# Supplementary material for: Measuring Site-specific Glycosylation Similarity between Influenza a Virus Variants with Statistical Certainty
Source: Mol Cell Proteomics. 2020 Nov 25;19(9):1533–45. doi: 10.1074/mcp.RA120.002031 (PMC8143645; doi:10.1074/mcp.RA120.002031)
Supplement: Supplementary file 2 [file mmc2.zip › mmc2/159250_2_supp_555327_qcjl6z.pdf]

Measuring site-specific glycosylation similarity between influenza A virus variants with statistical certainty

Authors: Deborah Chang, William E. Hackett, Lei Zhong, Xiu-Feng Wan, Joseph Zaia

List of materials:

Files

- Supplemental File 1. All peptides identified by Peaks Studio search
    - Submitted as an Excel file (File1_peaks_studio_protein-peptides_output.xlsx)
- Supplemental File 2. All proteins identified in each sample
    - Submitted as an Excel file (File2_identified_proteins.xlsx)
- Supplemental File 3. Glycopeptide abundances by replicate for each sample
    - Submitted as an Excel file (File3_glycopeptide_abundances_combined_replicates.xlsx)
- Supplemental File 4. Glycopeptide identifications and annotated spectra for AGP chymo samples
    - Submitted as three HTML files zipped into one file (File4_AGP_chymo_glycresoft_identifications.zip)
- Supplemental File 5. Glycopeptide identifications and annotated spectra for AGPgal chymo samples
    - Submitted as three HTML files zipped into one file (File5_AGPgal_chymo_glycresoft_identifications.zip)

- Supplemental File 6. Glycopeptide identifications and annotated spectra for AGP tryp samples
    - Submitted as three HTML files zipped into one file (File6_AGP_tryp_glycresoft_identifications.zip)
- Supplemental File 7. Glycopeptide identifications and annotated spectra for AGPgal tryp samples
    - Submitted as three HTML files zipped into one file (File7_AGPgal_tryp_glycresoft_identifications.zip)
- Supplemental File 8. Glycopeptide identifications and annotated spectra for Phil82 and Phil82gal chymo samples
    - Submitted as six HTML files zipped into one file (File8_Phil82_Phil82gal_chymo_glycresoft_identifications.zip)
- Supplemental File 9. Glycopeptide identifications and annotated spectra for Phil82 and Phil82gal tryp samples
    - Submitted as six HTML files zipped into one file (File9_Phil82_Phil82gal_tryp_glycresoft_identifications.zip)
- Supplemental File 10. Glycopeptide identifications and annotated spectra for mutant 5B8 and WT SWZ HA chymo samples
    - Submitted as six HTML files zipped into one file (File10_mutant5B8_WTSWZ_chymo_glycresoft_identifications.zip)
- Supplemental File 11. Glycopeptide identifications and annotated spectra for mutant 5B8 HA tryp samples

  - Submitted as three HTML files zipped into one file (File11_mutant5B8_tryp_glycresoft_identifications.zip)
- Supplemental File 12. Glycopeptide identifications and annotated spectra for WT SWZ HA tryp samples
  - Submitted as four HTML files zipped into one file (File12_WTSWZ_tryp_glycresoft_identifications.zip)

Figures

- Figure S1. WT and 5B8 mutant hemagglutinin (HA) sequences, and neuraminidase (NA) sequence of A/Switzerland/9715293/2013 virus.
- Figure S2. Hemagglutinin (HA) and neuraminidase (NA) sequences of A/Philippines/2/1982 virus.
- Figure S3. Quantification of glycopeptide abundances using GlycReSoft.
- Figure S4. Internal quality plots for example Tanimoto distributions.
- Figure S5. Internal quality plots for whole protein comparisons.
- Figure S6. Internal quality plots for site-specific comparisons of chymotryptic AGP vs. AGPgal.
- Figure S7. Site-specific Tanimoto distribution plots of tryptic AGP vs. AGPgal.
- Figure S8. Internal quality plots for site-specific comparisons of tryptic AGP vs. AGPgal.
- Figure S9. Total ion chromatograms of AGP and AGP replicates.
- Figure S10. Total ion chromatograms of Phil82 and Phil82 replicates.
- Figure S11. Total ion chromatograms of mutant 5B8 and WT SWZ13 HA replicates.

- Figure S12. Internal quality plots for site-specific comparisons of tryptic Phil82 HA vs. Phil82gal HA.
- Figure S13. Internal quality plots for site-specific comparisons of tryptic mutant 5B8 HA vs. WT SWZ13 HA.

Tables

- Table S1. Sample names and replicates.
- Table S2. Internal distribution heights and variances, test and null distribution overlap values, and confidence scores for all comparisons.

>cus|SWZHA|A/Switzerland/9715293/2013 HA SWZ 132Q, 219Y, 225D 20190316

QKLPGNDNSTATLCLGHHAVPNGTIVKTITNDRIEVTNATELVQNSSIGEICDSPHQILDGENC
TLIDALLGDPQCDGFQNKKWDLFVERSKAYSNCYPYDVPDYASLRSLVASSGTLEFNNESFNWA
GVTQNGTSSSCRRGSNSSFFSRLNWLTHLNSKYPALNVTMPNNEQFDKLYIWGVHHPVTDKDQI
FLYAQSSGRITVSTKRSQQAVIPNIGYRPRIRDIPSRISIYWTIVKPGDILLINSTGNLIAPRG
YFKIRSGKSSIMRSDAPIGKCKSECITPNGSIPNDKPFQNVNRITYGACPRYVKQSTLKLATGM
RNVPERQTRGIFGAIAGFIENGWEGMVDGWYGFRHQNSEGRGQAADLKSTQAAIDQINGKLNRL
IGKTNEKFHQIEKEFSEVEGRIQDLEKYVEDTKIDLWSYNAELLVALENQHTIDLTDSEMNKLF
EKTKKQLRENAEDMGNGCFKIYHKCDNACIGSIRNGTYDHDVYRDEALNNRFQIKGVELKSGYK
DWILWISFAISCFLLCVALLGFIMWACQKGNIRCNICI

>cus|SWZHA_5B8|A/Switzerland/9715293/2013 HA 5B8 132H, 219S, 225N 20190316

QKLPGNDNSTATLCLGHHAVPNGTIVKTITNDRIEVTNATELVQNSSIGEICDSPHQILDGENC
TLIDALLGDPQCDGFQNKKWDLFVERSKAYSNCYPYDVPDYASLRSLVASSGTLEFNNESFNWA
GVTHNGTSSSCRRGSNSSFFSRLNWLTHLNSKYPALNVTMPNNEQFDKLYIWGVHHPVTDKDQI
FLYAQSSGRITVSTKRSQQAVIPNIGSRPRIRNIPSRISIYWTIVKPGDILLINSTGNLIAPRG
YFKIRSGKSSIMRSDAPIGKCKSECITPNGSIPNDKPFQNVNRITYGACPRYVKQSTLKLATGM
RNVPERQTRGIFGAIAGFIENGWEGMVDGWYGFRHQNSEGRGQAADLKSTQAAIDQINGKLNRL
IGKTNEKFHQIEKEFSEVEGRIQDLEKYVEDTKIDLWSYNAELLVALENQHTIDLTDSEMNKLF
EKTKKQLRENAEDMGNGCFKIYHKCDNACIGSIRNGTYDHDVYRDEALNNRFQIKGVELKSGYK
DWILWISFAISCFLLCVALLGFIMWACQKGNIRCNICI

>cus|SWZNA|A/Switzerland/9715293/2013 NA protein

MNPNQKIITIGSVSLTISTICFFMQIAILITTVTLHFKQYEFNSPPNNQVMLCEPTIIERNITE
IVYLTNTTIEKEICPKPAEYRNWSKPQCGITGFAPFSKDNSIRLSAGGDIWVTREPYVSCDPDK
CYQFALGQGTTLNNVHSNNTVRDRTPYRTLLMNELGVPFHLGTKQVCIAWSSSSCHDGKAWLHV
CITGDDKNATASFIYNGRLVDSVVSWSKDILRTQESECVCINGTCTVVMTDGSASGKADTKILF
IEEGKIVHTSTLSGSAQHVEECSCYPRYPGVRCVCRDNWKGSNRPIVDINIKDHSIVSSYVCSG
LVGDTPRKNDSSSSSHCLDPNNEEGGHGVKGWAFDDGNDVWMGRTINETSRLGYETFKVIEGWS
NPKSKLQTNRQVIVDRGDRSGYSGIFSVEGKSCINRCFYVELIRGRKEETEVLWTSNSIVVFCG
TSGTYGTGSWPDGADLNLMPI

Figure S1. WT and 5B8 mutant hemagglutinin (HA) sequences, and neuraminidase (NA) sequence of A/Switzerland/9715293/2013 virus.

```
>iav|AFG99160|A/Phil/2/1982_HA A/Philippines/2/1982 1982// HA

MKTIIALSYMFCLVFAQNLPGNDNSTATLCLGHHAVPNGTLVKTITNDQIEVTNATELVQSSST
GRICDSPHRILDGKNCTLIDALLGDPHCDGFQNEKWDLFVERSKAFSNCYPYDVPDYASLRSLV
ASSGTLEFINEGFNWTGVTQSGGSSTCKRGSNNSFFSRLNWLYESESKYPVLNVTMPNNGKFDK
LYIWGIHHPSTDKEQTNLYIRASGRVTVSTKRSQQTVIPNIGSRPWVRGLSSRISIYWTIVKPG
DILLINSTGNLIAPRGYFKIRTGKSSIMRSDAPIGTCSSECITPNGSIPNDKPFQNVNKITYGA
CPRYVKQNTLKLATGMRNVPEKQTRGIFGAIAGFIENGWEGMVDGWYGFRHQNSEGTGQAADLK
STQAAIDQINGKLNRVIEKTNEKFHQIEKEFSEVEGRIQDLEKYVEDTKIDLWSYNAELLVALE
NQHTIDLTDSEMNKLFEKTRKQLRENAEDMGNGCFKIYHKCDNACIGSIRNGTYDHDVYRDEAL
NNRFQIKGVELKSGYKDWILWISFAISCFLLCVVLLGFIMWACQKGNIRCNICI

>iav|ADJ41819|A/Phil/2-MA/1982_NA A/Philippines/2-MA/1982
1982/06/01 NA

MNPNQKIITIGSVSLTIATICFLMQIAILVTTVTLHFKQYECSSPPNNQVVPCEPIIIERNITE
IVYLTNTTIEKEICPKLVEYRNWSKPQCKITGFAPFSKDNSIRLSAGGDIWVTREPYVSCDPGK
CYQFALGQGTTLDNKHSNDTIHDRTPYRTLLMNELGVPFHLGTRQVCIAWSSSSCHDGKAWLHV
CITGYDKNATASFIYDGRLVDSIGSWSKNILRTQESECVCINGTCTVVMTDGSASERADTKILF
IEEGKIVHISPLSGSAQHVEECSCYPRYPGVRCVCRDNWKGSNRPVVDINVKDYSIVSSYVCSG
LVGDTPRKNDRSSSSYCRNPNNEKGNHGVKGWAFDDGNDVWMGRTISEESRSGYETFKVIGGWS
TPNSKLQINRQVIVDSGNRSGYSGIFSVEGKSCINRCFYVELIRGREQETRVWWTSNSIVVFCG
TSGTYGTGSWPDGADINLMPI
```

Figure S2. Hemagglutinin (HA) and neuraminidase (NA) sequences of A/Philippines/2/1982 virus.

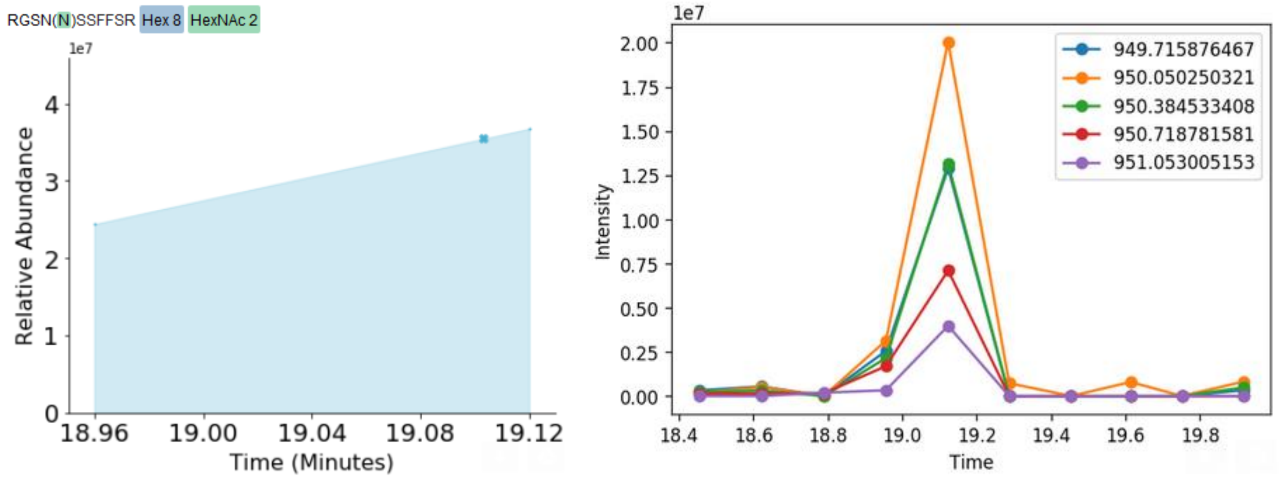

Figure S3. Quantification of glycopeptide abundances using GlycReSoft. The chromatogram plots in the GlycReSoft HTML files (supplemental files 4-12) were made from the same underlying data on which the quantification was done. Both rely on accurate processing by the deconvoluter. In this example shown here, the plot on the left is a deconvoluted chromatogram of a glycopeptide shown in the GlycReSoft results. Extracting the raw chromatograms for each of the isotopic peaks of this ion produces the plot on the right. The two points covered in the deconvoluted chromatogram (left) are the two points where most of the isotopic peaks (on the right) are non-zero. Due to the lack of MS1 scans spanning the chromatographic peak between 19.12 and 19.28 minutes, only the points at 18.96 and 19.12 minutes were used for quantification.

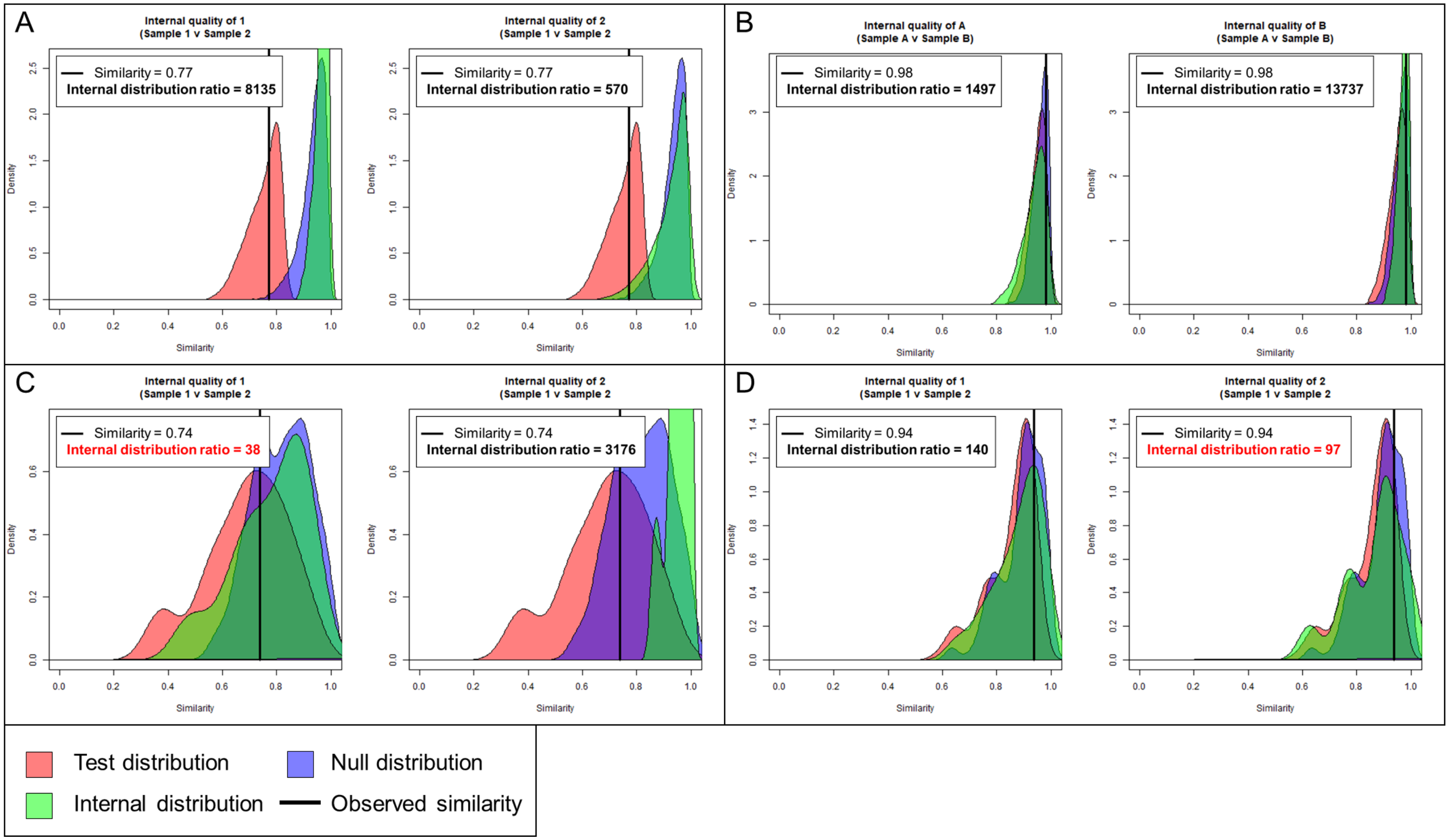

Figure S4. Internal quality plots for example Tanimoto distributions corresponding to the plots in Figure 1. An internal distribution (green) is drawn for each experimental group in each comparison (i.e. one for sample 1 and one for sample 2). The internal distribution ratio is quantifed by the height of the distribution divided by the variance. An internal distribution ratio of ≤100 is indicative of poor data quality. (A) Ideal plot with narrow null and test distributions and high internal distribution ratios. (B) Poorly resolved null and test distributions, but the internal distribution ratios are high, indicating good data quality. (C) Poorly resolved null and test distributions, but one internal distribution ratio is high, indicating poor data quality. (D) Inconsistencies among sample replicates results in multimodal distributions.

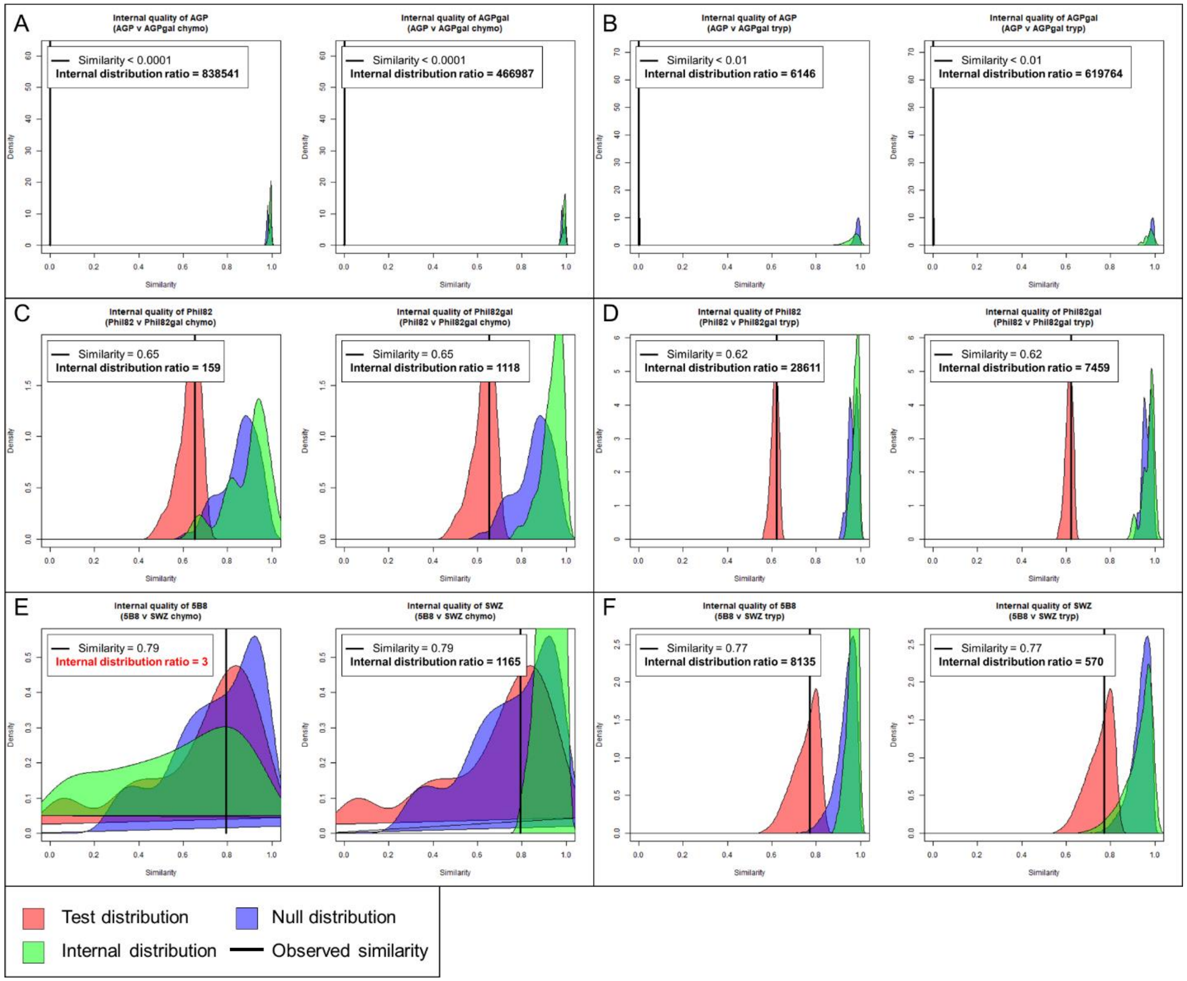

Figure S5. Internal quality plots for whole protein comparisons corresponding to the plots in Figure 2. An internal distribution (green) is drawn for each experimental group in each comparison. Internal distribution ratio is quantified by the height of the distribution divided by the variance. An internal distribution ratio of ≤100 is indicative of poor data quality. (A) chymotryptic AGP vs. AGP, (B) tryptic AGP vs. AGPgal, (C) chymotryptic Phil82 HA vs. Phil82gal HA, (D) tryptic Phil82 HA vs. Phil82gal HA, (E) chymotryptic mutant 5B8 HA vs. WT SWZ13 HA, and (F) tryptic mutant 5B8 HA vs. WT SWZ13 HA.

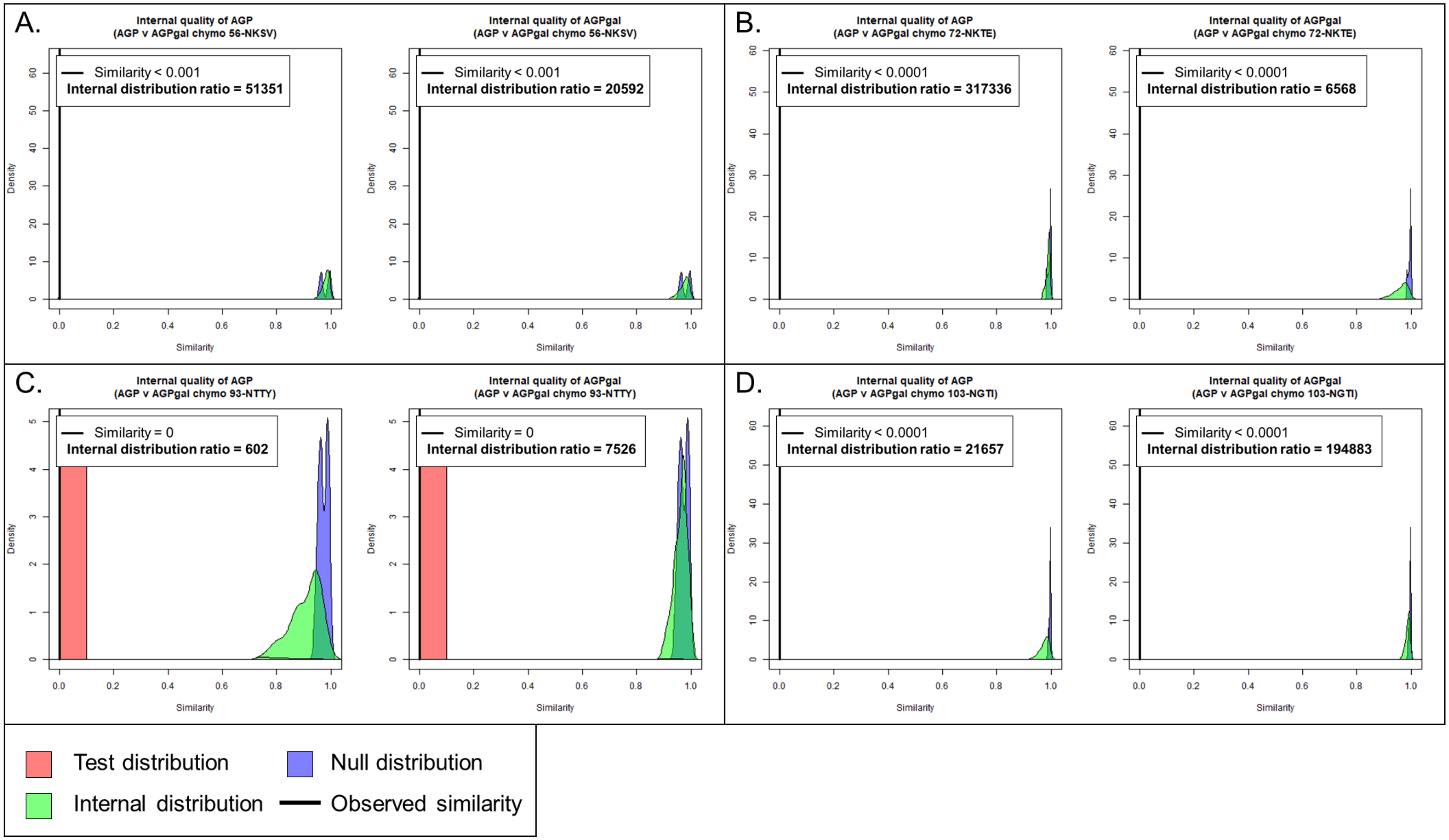

Figure S6. Internal quality plots for site-specific comparisons of chymotryptic glycopeptides of AGP and AGPgal corresponding to the plots in Figure 3. Site 33-NATL was not found for chymotrypsin. An internal distribution (green) is drawn for each experimental group in each comparison. Internal distribution ratio is quantified by the height of the distribution divided by the variance. An internal distribution ratio of ≤100 is indicative of poor data quality. (A) site 56-NKSV, (B) site 72-NKTE, (C) site 93-NTTY, and (D) site 103-NGTI).

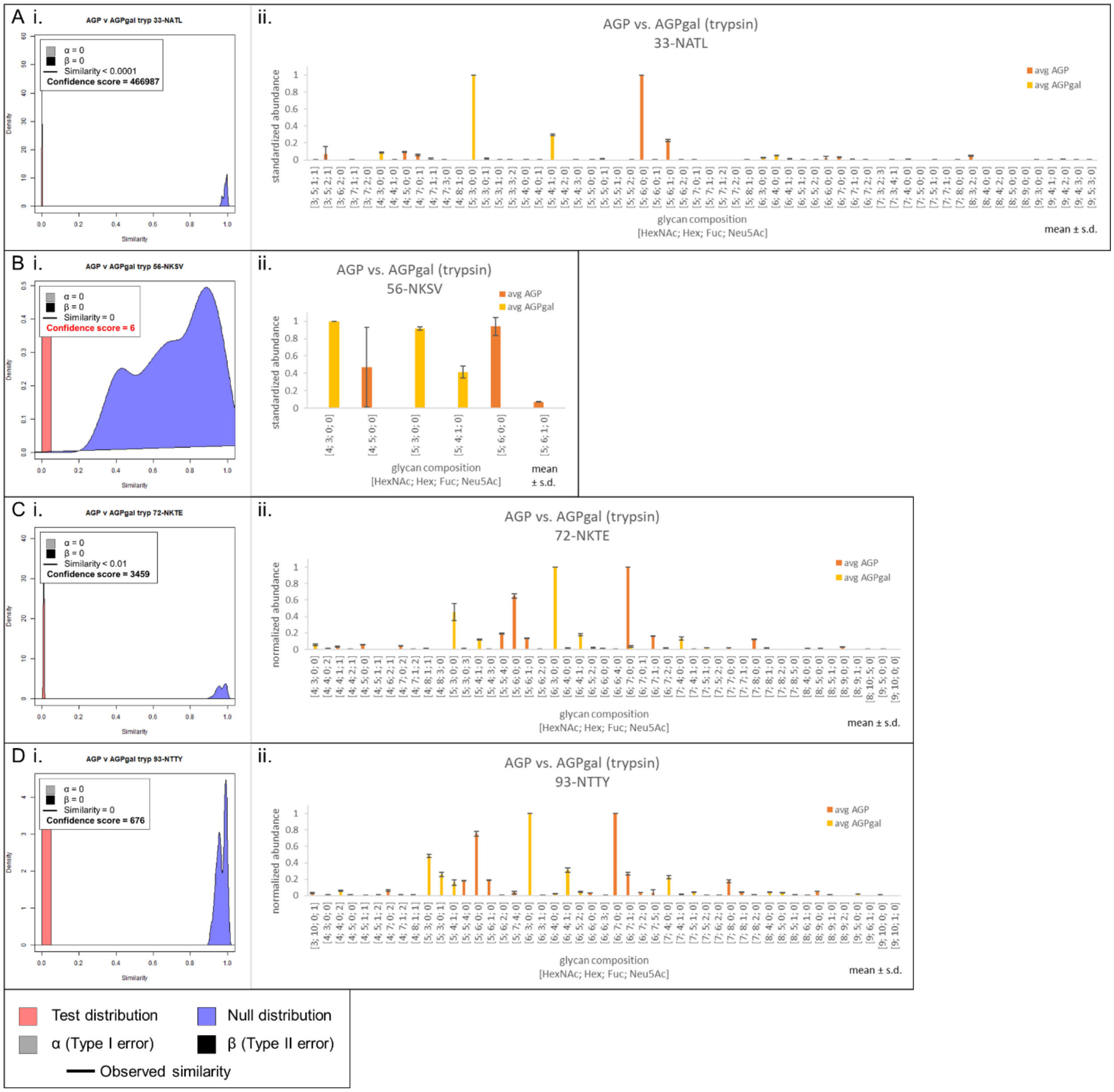

Figure S7. Site-specific comparisons for tryptic glycopeptides of AGP and AGPgal. Site 103-NGTI was not found for trypsin. (A) Tanimoto distribution plot (i) and standardized abundance bar plots (ii) for AGP vs. AGPgal at site 33-NATL, (B) site 56-NKSV, (C) site 72-NKTE, and (D) site 93-NTTY. In the Tanimoto distribution plots, the observed similarity is represented by the vertical black line, α and β are the Type I and II errors, respectively. A confidence score of ≥77 indicates good confidence. In the bar plots, the glycopeptide abundances were standardized to be in range [0,1], and averaged across technical replicates, not including missing values. Error bars show ± standard deviation.

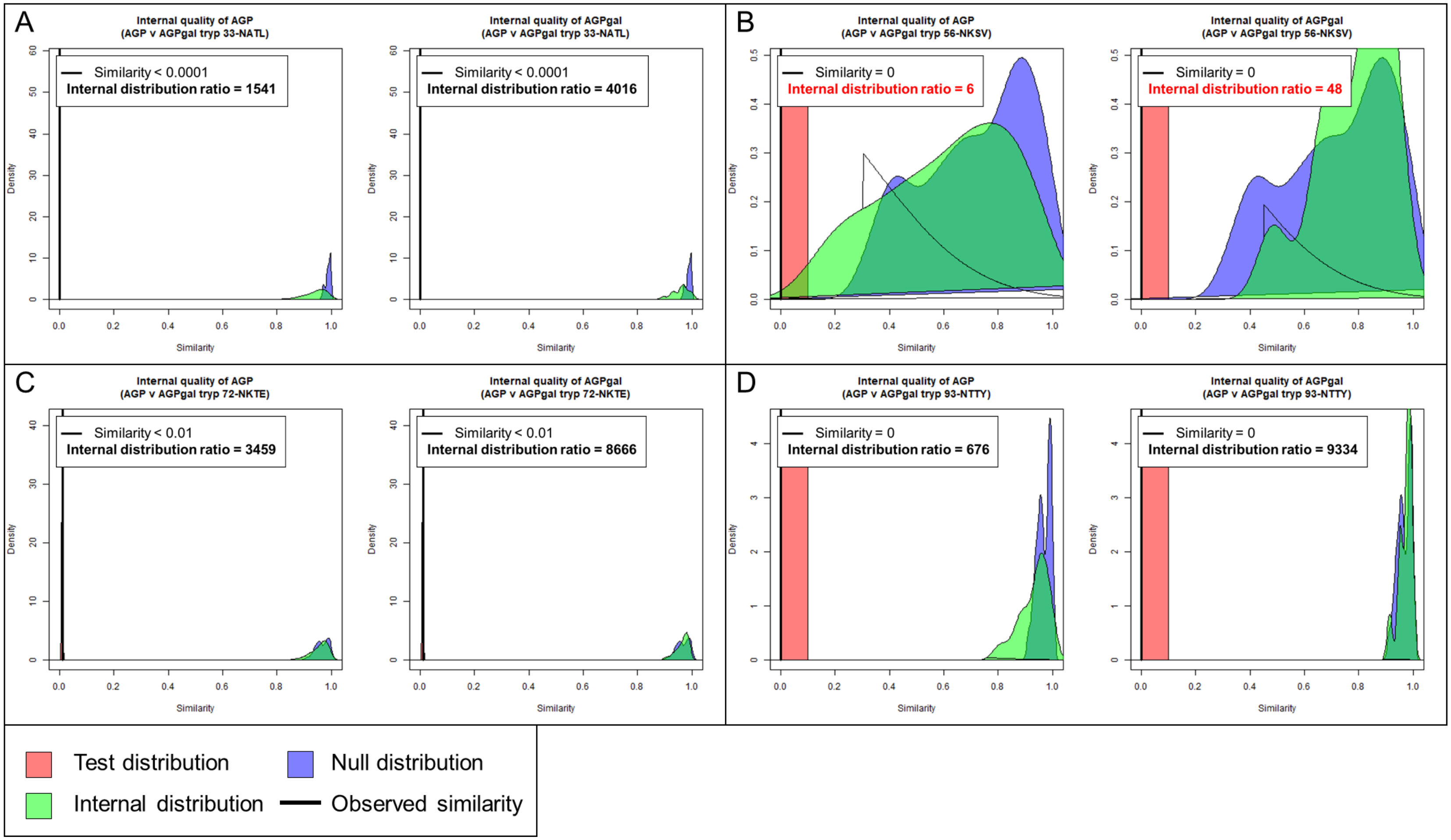

Figure S8. Internal quality plots for site-specific comparisons of tryptic glycopeptides of AGP and AGPgal corresponding to the plots in Figure S6. Site 103-NGTI was not found for trypsin. An internal distribution (green) is drawn for each experimental group in each comparison. Internal distribution ratio is quantified by the height of the distribution divided by the variance. An internal distribution ratio of ≤100 is indicative of poor data quality. (A) site 33-NATL, (B) site 56-NKSV, (C) site 72-NKTE, and (D) site 93-NTTY.

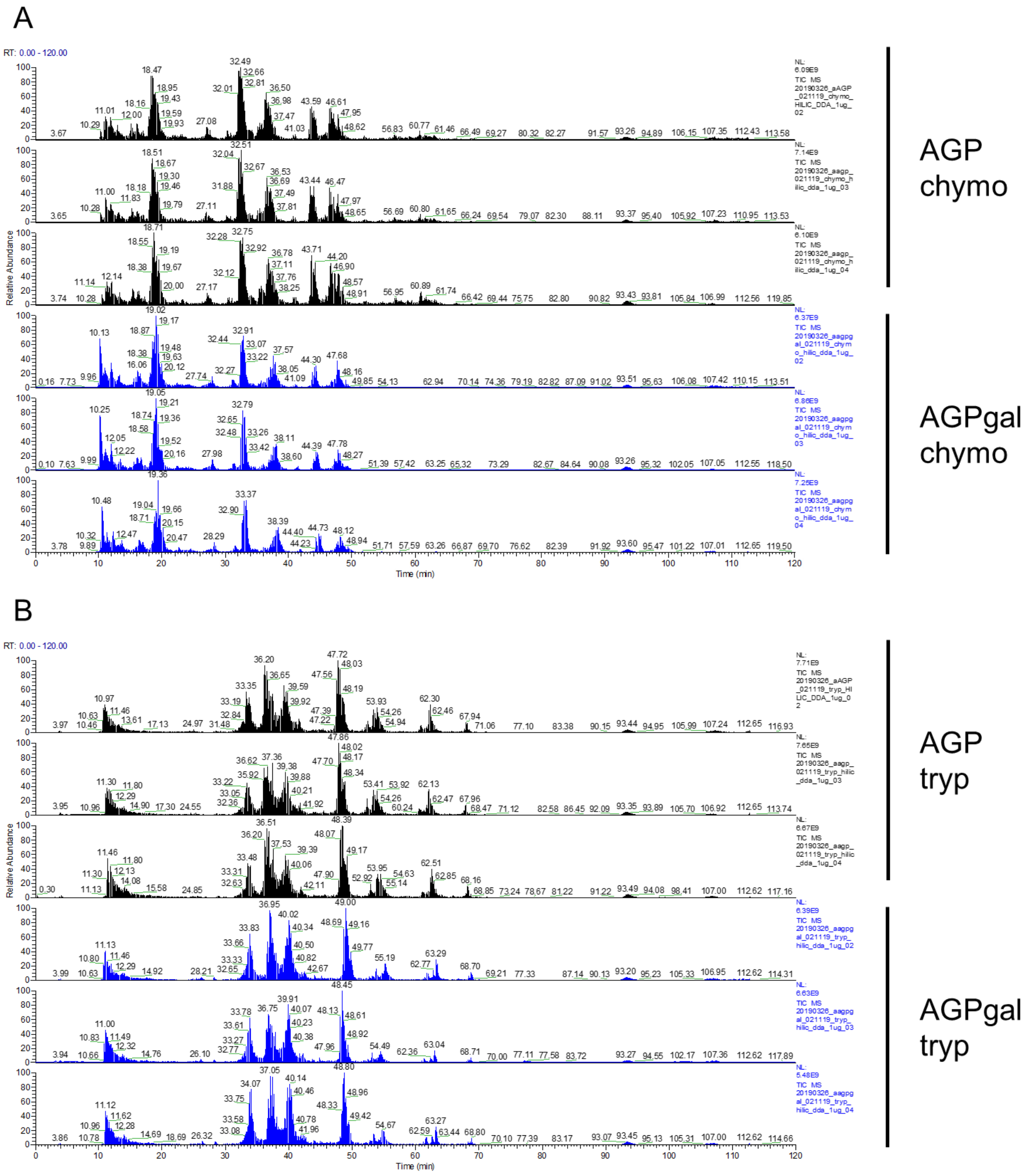

Figure S9. Total ion chromatograms of AGP and AGPgal replicates.

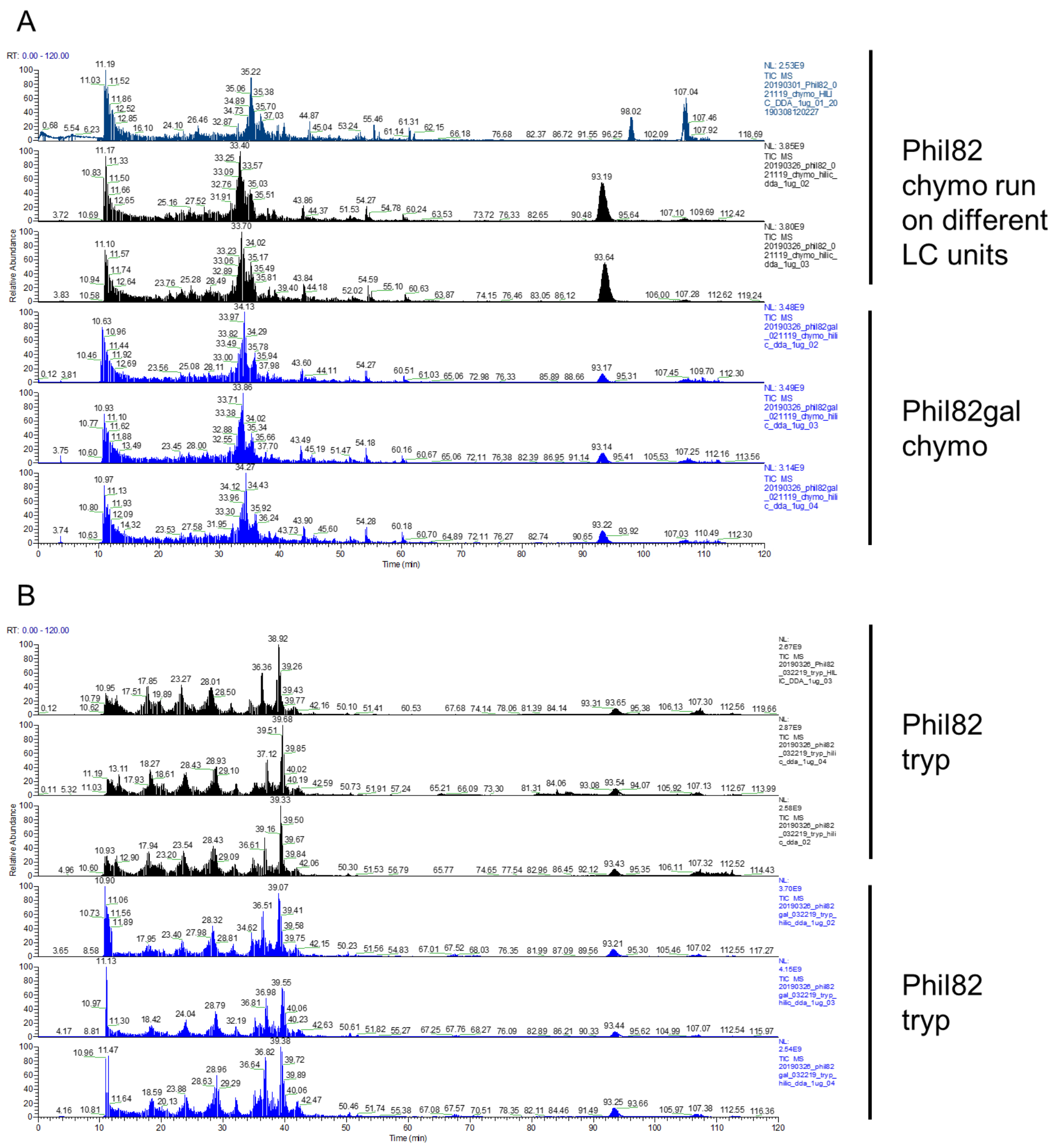

Figure S10. Total ion chromatograms of Phil82 and Phil82gal replicates. Note that one replicate for Phil82 chymo was run using a different LC unit.

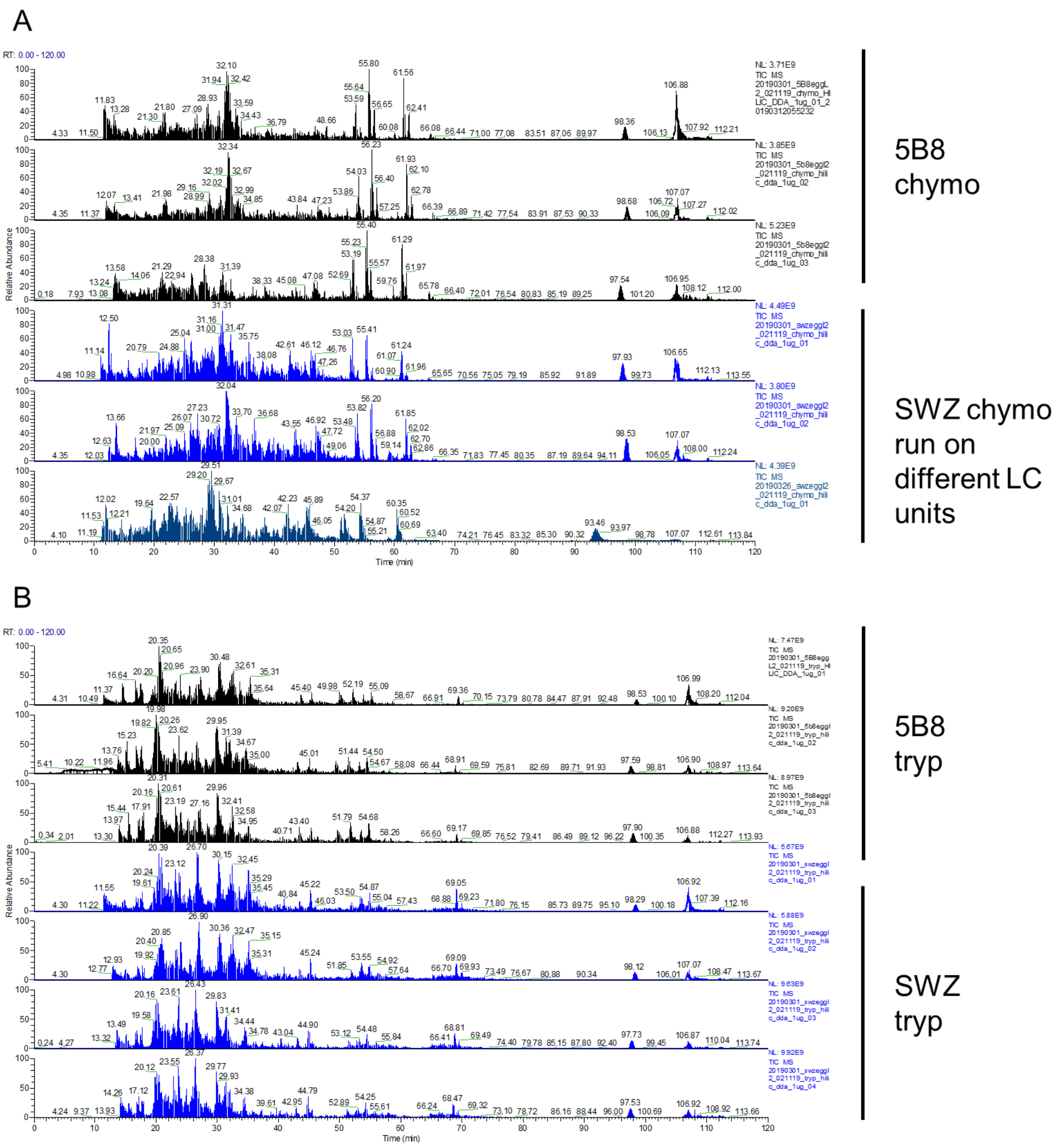

Figure S4. Total ion chromatograms of mutant 5B8 and WT SWZ13 HA replicates. Note that one replicate for WT SWZ13 HA chymo was run using a different LC unit.

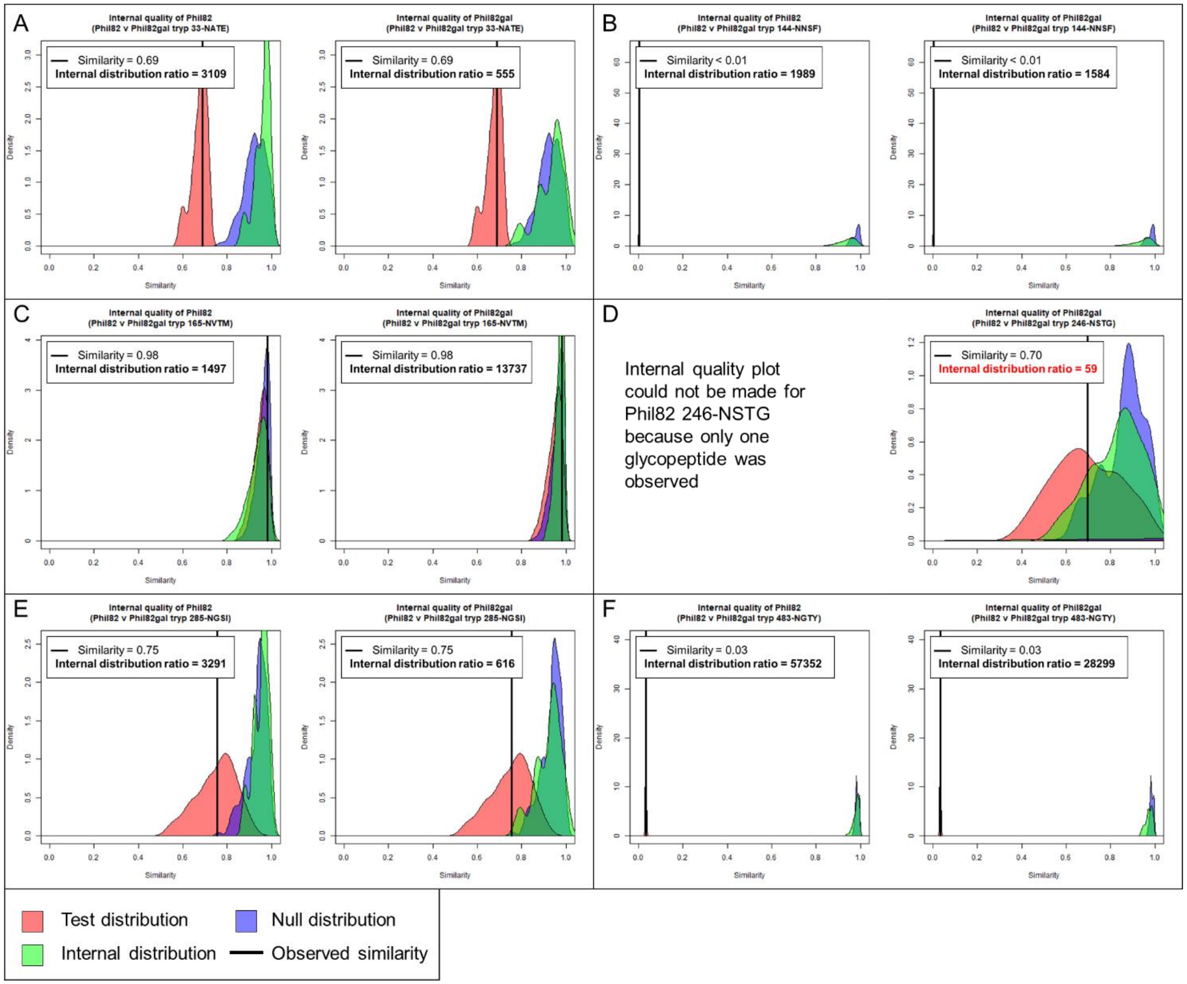

Figure S12. Internal quality plots for site-specific comparisons of tryptic glycopeptides of Phil82 HA and Phil82gal HA corresponding to the plots in Figure 4. An internal distribution (green) is drawn for each experimental group in each comparison. Internal distribution ratio is quantified by the height of the distribution divided by the variance. An internal distribution ratio of ≤100 is indicative of poor data quality. (A) site 38-NATE, (B) site 144-NNSF, (C) site 165-NVTM, (D) site 246-NSTG, (E) site 285-NGSI, and (F) site 483-NGTY.

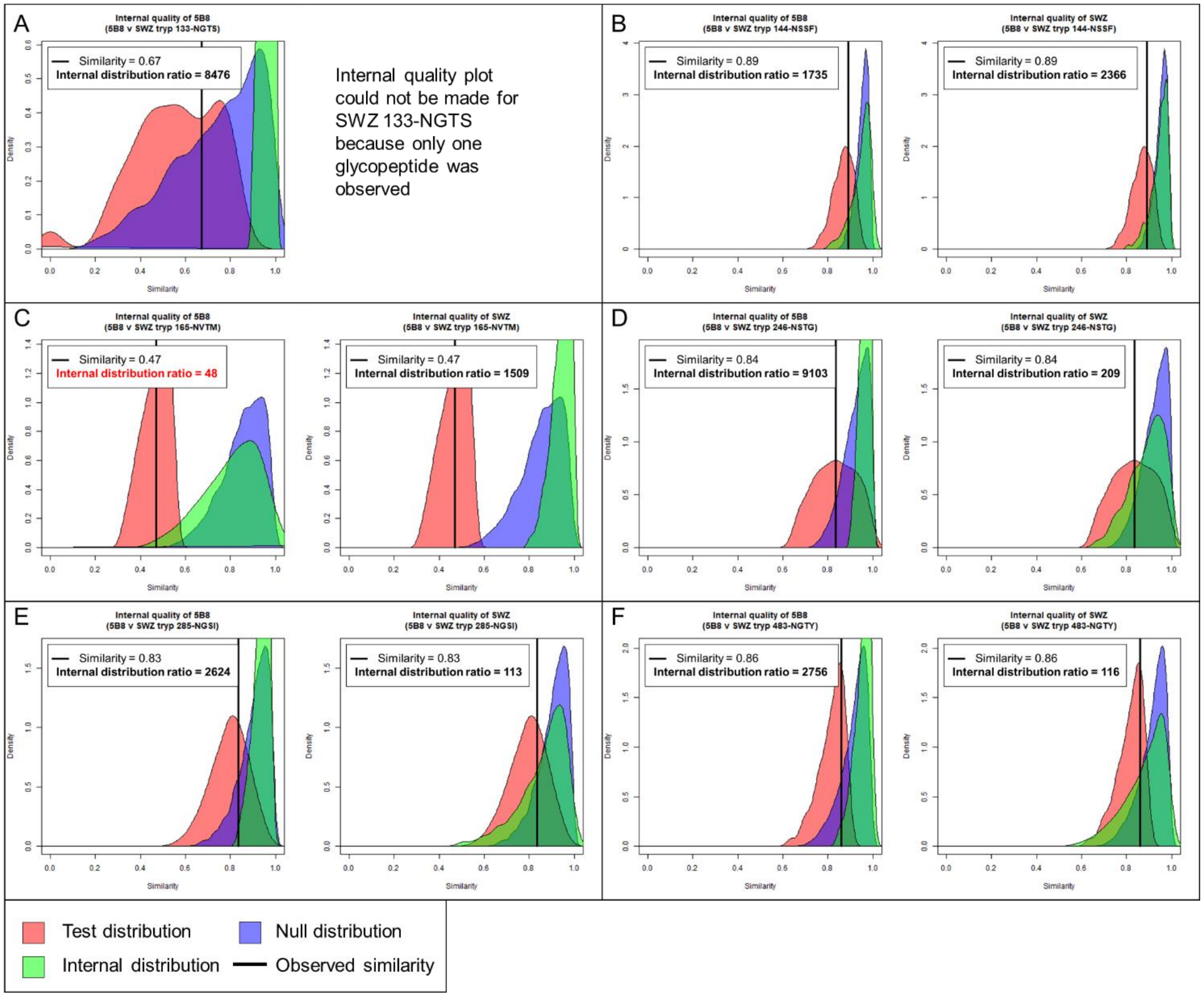

Figure S13. Internal quality plots for site-specific comparisons of tryptic glycopeptides of mutant 5B8 HA and WT SWZ13 HA corresponding to the plots in Figure 5. An internal distribution (green) is drawn for each experimental group in each comparison. Internal distribution ratio is quantified by the height of the distribution divided by the variance. An internal distribution ratio of ≤100 is indicative of poor data quality. (A) site 133-NGTS, (B) site 144-NSSF, (C) site 165-NVTM, (D) site 246-NSTG, (E) site 285-NGSI, and (F) site 483-NGTY.

Table S1. Sample names and replicates.

Sample type	Enzyme	# replicates
WT SWZ13 HA[a]	Chymotrypsin	3[c]
	Trypsin	4
mutant 5B8HA[b]	Chymotrypsin	3
	Trypsin	3
AGP	Chymotrypsin	3
	Trypsin	3
AGPgal	Chymotrypsin	3
	Trypsin	3
Phil82	Chymotrypsin	3[c]
	Trypsin	3
Phil82gal	Chymotrypsin	3
	Trypsin	3

[a]Wild-type IAV strain A/Switzerland/9715293/2013, expressed in egg.
[b]Mutant IAV strain A/Switzerland/9715293/2013, expressed in egg.
[c]One replicate was run using a different LC unit, but with the same LC parameters.

Table S2. Internal distribution heights and variances, test and null distribution overlap values, and confidence scores for all comparisons. The internal distribution ratio is defined as the internal distribution height divided by the internal distribution variance. The confidence score is defined as exp(-test/null overlap) multiplied by the lower of the two internal distribution ratios. A confidence score greater than the threshold of 77 is considered to be high confidence.

	**Sample 1 vs. Sample 2 comparison**	**Sample 1 height**	**Sample 2 height**	**Sample 1 internal variance**	**Sample 2 internal variance**	**Sample 1 internal distribution ratio**	**Sample 2 internal distribution ratio**	**Test/Null overlap**	**Confidence score**
**Fig. S3A**	Example A sample 1 vs. 2	2.599323	1.686711	0.00131135	0.004466707	1982	377	0.04	362
**Fig. S3B**	Example B sample 1 vs. 2	2.465665	5.071588	0.001646896	0.000369176	1497	13737	0.73	721
**Fig. S3C**	Example C sample 1 vs. 2	0.7181442	4.020666	0.01854203	0.001265741	38	3176	0.46	24
**Fig. S3D**	Example D sample 1 vs. 2	1.159522	1.093535	0.008238444	0.01126046	140	97	0.62	52
**Fig. S4A**	AGP v AGPgal chymo	20.44306	16.32354	2.44E-05	3.50E-05	838541	466987	0	466987
**Fig. S4B**	AGP v AGPgal tryp	4.188773	6.267085	0.000681454	0.000317091	6146	19764	0	6146
**Fig. S4C**	Phil82 v Phil82gal chymo	1.36992	2.508202	0.008562392	0.002243407	159	1118	0.04	153
**Fig. S4D**	Phil82 v Phil82gal tryp	6.435834	5.086898	0.000224937	0.000681956	28611	7459	0	7459
**Fig. S4E**	5B8 v SWZegg chymo	0.3026141	2.24111	0.08649666	0.00192244	3	1165	0.84	1
**Fig. S4F**	5B8 v SWZegg tryp	4.367964	2.241262	0.000536919	0.003930222	8135	570	0.03	553
**Fig. S5A**	AGP v AGPgal chymo NKSV	7.792945	6.023129	0.000151757	0.00029249	51351	20592	0	20592
**Fig. S5B**	AGP v AGPgal chymo NKTE	16.16169	4.089344	5.09E-05	0.000622563	317336	6568	0	6568
**Fig. S5C**	AGP v AGPgal chymo NTTY	1.877094	4.296286	0.003115758	0.000570787	602	7526	0	602

**Fig. S5D**	AGP v AGPgal chymo NGTI	5.919991	12.33261	0.000273348	6.33E-05	21657	194883	0	21657
**Fig. S7A**	AGP v AGPgal tryp NATL	2.46599	3.602016	0.001600071	0.000896896	3109	555	0	1541
**Fig. S7B**	AGP v AGPgal tryp NKSV	0.3611666	0.8522918	0.05490119	0.01766811	1989	1584	0	6
**Fig. S7C**	AGP v AGPgal tryp NKTE	3.306592	4.720276	0.00095585	0.000544668	1497	13737	0	3459
**Fig. S7D**	AGP v AGPgal tryp NTTY	1.975219	5.306906	0.002919273	0.000568546	NA	59	0	676
**Fig. S11A**	Phil82 v Phil82gal tryp NATE	3.614017	1.990508	0.001162099	0.003583734	3291	616	0	555
**Fig. S11B**	Phil82 v Phil82gal tryp NNSF	2.596542	2.674772	0.001304984	0.00168812	57352	28299	0	1584
**Fig. S11C**	Phil82 v Phil82gal tryp NVTM	2.465665	5.071588	0.001646896	0.000369176	3109	555	0.67	766
**Fig. S11D**	Phil82 v Phil82gal tryp NSTG	NA	0.8047484	NA	0.01344303	1989	1584	0.34	0
**Fig. S11E**	Phil82 v Phil82gal tryp NGSI	3.525224	1.994157	0.001071086	0.003234842	1497	13737	0.09	563
**Fig. S11F**	Phil82 v Phil82gal tryp NGTY	8.679483	6.142075	0.000151335	0.000217035	NA	59	0	28299
**Fig. S12A**	5B8 v SWZegg tryp NGTS	4.815018	NA	0.000568057	NA	8476	NA	0.46	0
**Fig. S12B**	5B8 v SWZegg tryp NSSF	2.853098	3.301326	0.001643503	0.001394915	1735	2366	0.12	1539
**Fig. S12C**	5B8 v SWZegg tryp NVTM	0.7357899	2.641839	0.01507687	0.001750124	48	1509	0	48
**Fig. S12D**	5B8 v SWZegg tryp NSTG	4.299102	1.253444	0.000472261	0.005969777	9103	209	0.36	146

**Fig. S12E**	5B8 v SWZegg tryp NGSI	3.021659	1.19003	0.001151345	0.01044038	2624	113	0.28	86
**Fig. S12F**	5B8 v SWZegg tryp NGTY	3.146439	1.340741	0.001141408	0.008075678	2756	166	0.19	137
