# Supplementary material for: Measuring Site-specific Glycosylation Similarity between Influenza a Virus Variants with Statistical Certainty
Source: Mol Cell Proteomics. 2020 Nov 25;19(9):1533–45. doi: 10.1074/mcp.RA120.002031 (PMC8143645; doi:10.1074/mcp.RA120.002031)
Supplement: Supplementary file 1 [file mmc1.zip › mmc1/159250_2_supp_555285_qcjklg.zip!/20190326_aAGP_021119_chymo_HILIC_DDA_1ug_02_glycopeptides.html]

## 20190326\_aAGP\_021119\_chymo\_HILIC\_DDA\_1ug\_02 @ GlycopeptideHypothesis-d20c1bda9db24ee0835dee5880292bd6

**MS^1^ Mass Accuracy:** 10.0 PPM  
**Grouping Tolerance:** 15.0 PPM  
**MS^n^ Mass Accuracy:** 20.0 PPM  
**Minimum `q`-Value For Clustering:** 0.05

**Glycopeptide Hypothesis:**
GlycopeptideHypothesis-d20c1bda9db24ee0835dee5880292bd6  
**Sample Searched:**
20190326\_aAGP\_021119\_chymo\_HILIC\_DDA\_1ug\_02

| Protein | Matched Glycopeptides |
| --- | --- |
| P02763\|A1AG1\_HUMAN | 140 |
| P19652\|A1AG2\_HUMAN | 120 |
| P13473\|LAMP2\_HUMAN | 3 |
| P00738\|HPT\_HUMAN | 2 |
| P05155\|IC1\_HUMAN | 2 |
| P13598\|ICAM2\_HUMAN | 1 |

### P02763|A1AG1\_HUMAN

**>P02763|A1AG1\_HUMAN**  
 `MALSWVLTVLSLLPLLEAQIPLCANLVPVPITNATLDQITGKWFYIASAFRNEEYNKSVQEIQATFFYFTPNKTEDTIFL  
REYQTRQDQCIYNTTYLNVQRENGTISRYVGGQEHFAHLLILRDTKTYMLAFDVNDEKNWGLSVYADKPETTKEQLGEFY  
EALDCLRIPKSDVVYTDWKKDKCEPLEKQHEKERKQEEGES`

| Site | Type | Count |
| --- | --- | --- |
| 33 | N-linked | 2 |
| 56 | N-linked | 37 |
| 93 | N-linked | 3 |
| 103 | N-linked | 36 |
| 72 | N-linked | 62 |

| Mass | Sequence | MS_2_ Score | `q`-value | MS_1_ Score | Signal | Protein Coordinates | Tandem Scans | Start Time | Apex Time | End Time |
| --- | --- | --- | --- | --- | --- | --- | --- | --- | --- | --- |
| 3152.2835 | TPN(N)KTEDTIF Hex 6 HexNAc 5 | 185.885 | 0.0 | 15.444 | 2.048e+08 | 69 - 79 | 8 | 21.97 | 23.67 | 24.69 |
| 3517.4168 | TPN(N)KTEDTIF Hex 7 HexNAc 6 | 184.472 | 0.0 | 15.148 | 3.352e+08 | 69 - 79 | 13 | 21.29 | 23.16 | 24.69 |
| 3462.4173 | YFTPN(N)KTEDTIF Hex 6 HexNAc 5 | 182.209 | 0.0 | 14.396 | 2.934e+09 | 67 - 79 | 56 | 35.99 | 37.15 | 77.85 |
| 3298.3425 | TPN(N)KTEDTIF Fuc 1 Hex 6 HexNAc 5 | 181.204 | 0.0 | 13.802 | 3.722e+07 | 69 - 79 | 4 | 23.16 | 23.50 | 24.01 |
| 3663.4752 | TPN(N)KTEDTIF Fuc 1 Hex 7 HexNAc 6 | 180.410 | 0.0 | 14.600 | 5.016e+07 | 69 - 79 | 4 | 22.65 | 22.99 | 23.33 |
| 2886.2325 | N(N)KSVQEIQATF Hex 5 HexNAc 4 | 179.877 | 0.0 | -3.245 | 2.823e+07 | 55 - 66 | 1 | 26.91 | 27.08 | 27.42 |
| 3827.5491 | YFTPN(N)KTEDTIF Hex 7 HexNAc 6 | 176.947 | 0.0 | 15.170 | 4.780e+09 | 67 - 79 | 45 | 35.48 | 36.50 | 43.25 |
| 3882.5493 | TPN(N)KTEDTIF Hex 8 HexNAc 7 | 175.217 | 0.0 | -4.321 | 2.690e+07 | 69 - 79 | 4 | 22.31 | 22.48 | 22.82 |
| 3973.6074 | YFTPN(N)KTEDTIF Fuc 1 Hex 7 HexNAc 6 | 174.484 | 0.0 | 15.054 | 9.933e+08 | 67 - 79 | 9 | 35.31 | 36.33 | 37.31 |
| 3609.4838 | FYFTPN(N)KTEDTIF Hex 6 HexNAc 5 | 173.970 | 0.0 | 14.789 | 2.352e+09 | 66 - 79 | 48 | 45.94 | 47.11 | 56.66 |
| 3974.6185 | FYFTPN(N)KTEDTIF Hex 7 HexNAc 6 | 172.785 | 0.0 | 15.465 | 4.479e+09 | 66 - 79 | 79 | 45.26 | 46.61 | 59.74 |
| 3299.3512 | FTPN(N)KTEDTIF Hex 6 HexNAc 5 | 172.640 | 0.0 | 15.470 | 4.351e+08 | 68 - 79 | 13 | 29.29 | 30.82 | 31.84 |
| 2787.1524 | TPN(N)KTEDTIF Hex 5 HexNAc 4 | 170.670 | 0.0 | 13.844 | 2.503e+07 | 69 - 79 | 3 | 23.67 | 24.18 | 25.03 |
| 3608.4742 | YFTPN(N)KTEDTIF Fuc 1 Hex 6 HexNAc 5 | 167.991 | 0.0 | 13.652 | 6.524e+08 | 67 - 79 | 12 | 35.82 | 36.98 | 38.81 |
| 3097.2824 | YFTPN(N)KTEDTIF Hex 5 HexNAc 4 | 167.567 | 0.0 | 14.444 | 3.297e+08 | 67 - 79 | 8 | 37.63 | 37.79 | 38.64 |
| 3664.4851 | FTPN(N)KTEDTIF Hex 7 HexNAc 6 | 166.271 | 0.0 | 16.123 | 6.929e+08 | 68 - 79 | 14 | 28.95 | 30.31 | 31.67 |
| 4120.6772 | FYFTPN(N)KTEDTIF Fuc 1 Hex 7 HexNAc 6 | 166.065 | 0.0 | 14.554 | 8.359e+08 | 66 - 79 | 13 | 45.60 | 46.45 | 47.61 |
| 4557.8158 | YFTPN(N)KTEDTIF Hex 9 HexNAc 8 | 165.209 | 0.0 | 15.550 | 2.393e+08 | 67 - 79 | 5 | 34.63 | 35.48 | 36.16 |
| 3755.5435 | FYFTPN(N)KTEDTIF Fuc 1 Hex 6 HexNAc 5 | 164.956 | 0.0 | 14.057 | 5.974e+08 | 66 - 79 | 14 | 45.94 | 46.94 | 50.84 |
| 3243.3395 | YFTPN(N)KTEDTIF Fuc 1 Hex 5 HexNAc 4 | 162.392 | 0.0 | -20.122 | 7.222e+06 | 67 - 79 | 1 | 37.47 | 37.63 | 37.63 |
| 4192.6838 | YFTPN(N)KTEDTIF Hex 8 HexNAc 7 | 161.342 | 0.0 | 14.997 | 8.710e+08 | 67 - 79 | 11 | 34.63 | 36.16 | 36.65 |
| 4119.6666 | YFTPN(N)KTEDTIF Fuc 2 Hex 7 HexNAc 6 | 161.108 | 0.0 | -3.916 | 1.512e+08 | 67 - 79 | 2 | 36.16 | 36.33 | 36.65 |
| 4266.7358 | FYFTPN(N)KTEDTIF Fuc 2 Hex 7 HexNAc 6 | 161.073 | 0.0 | 13.178 | 1.359e+08 | 66 - 79 | 3 | 46.11 | 46.28 | 46.78 |
| 3244.3509 | FYFTPN(N)KTEDTIF Hex 5 HexNAc 4 | 160.382 | 0.0 | 11.901 | 1.960e+08 | 66 - 79 | 4 | 47.61 | 47.78 | 48.45 |
| 3724.5922 | RNEEYN(N)KSVQEIQATFF Hex 5 HexNAc 4 | 160.097 | 0.0 | 16.834 | 3.167e+09 | 50 - 67 | 18 | 42.23 | 44.08 | 45.43 |
| 3672.5114 | EEYN(N)KSVQEIQATF Hex 6 HexNAc 5 | 159.708 | 0.0 | -3.608 | 7.869e+07 | 52 - 66 | 3 | 34.97 | 35.31 | 35.48 |
| 4338.7402 | YFTPN(N)KTEDTIF Fuc 1 Hex 8 HexNAc 7 | 158.954 | 0.0 | 12.504 | 1.618e+08 | 67 - 79 | 4 | 35.48 | 35.99 | 36.16 |
| 4339.7524 | FYFTPN(N)KTEDTIF Hex 8 HexNAc 7 | 158.054 | 0.0 | 14.872 | 6.139e+08 | 66 - 79 | 10 | 44.91 | 46.11 | 47.28 |
| 3881.5467 | TPN(N)KTEDTIF Fuc 3 Hex 6 HexNAc 5 Neu5Ac 1 | 158.010 | 0.0 | -19.167 | 6.064e+06 | 69 - 79 | 1 | 22.14 | 22.14 | 22.14 |
| 3577.5261 | RNEEYN(N)KSVQEIQATF Hex 5 HexNAc 4 | 157.941 | 0.0 | 15.563 | 8.583e+09 | 50 - 66 | 23 | 29.46 | 32.66 | 33.96 |
| 2934.2199 | FTPN(N)KTEDTIF Hex 5 HexNAc 4 | 156.667 | 0.0 | 14.362 | 4.611e+07 | 68 - 79 | 2 | 31.16 | 31.50 | 31.84 |
| 3942.6970 | RNEEYN(N)KSVQEIQATF Hex 6 HexNAc 5 | 156.622 | 0.0 | 14.015 | 6.717e+09 | 50 - 66 | 34 | 16.63 | 32.18 | 33.62 |
| 4235.7833 | RNEEYN(N)KSVQEIQATFF Fuc 1 Hex 6 HexNAc 5 | 156.104 | 0.0 | 15.723 | 1.521e+09 | 50 - 67 | 13 | 42.74 | 43.42 | 44.57 |
| 3810.5434 | FTPN(N)KTEDTIF Fuc 1 Hex 7 HexNAc 6 | 155.804 | 0.0 | 13.655 | 1.163e+08 | 68 - 79 | 6 | 29.80 | 30.14 | 30.82 |
| 3818.5718 | EEYN(N)KSVQEIQATF Fuc 1 Hex 6 HexNAc 5 | 155.065 | 0.0 | -3.564 | 4.109e+07 | 52 - 66 | 2 | 34.97 | 34.97 | 35.31 |
| 4089.7255 | RNEEYN(N)KSVQEIQATFF Hex 6 HexNAc 5 | 153.897 | 0.0 | 16.033 | 3.295e+09 | 50 - 67 | 23 | 41.37 | 43.59 | 45.09 |
| 3307.3789 | EEYN(N)KSVQEIQATF Hex 5 HexNAc 4 | 153.572 | 0.0 | -3.852 | 9.399e+07 | 52 - 66 | 4 | 35.48 | 35.65 | 35.99 |
| 3872.6601 | RNEEYN(N)KSVQEIQATFF Fuc 1 Hex 5 HexNAc 4 | 153.485 | 0.0 | 0.000 | 0.000e+00 | 50 - 67 | 1 | - | - | - |
| 4381.8436 | RNEEYN(N)KSVQEIQATFF Fuc 2 Hex 6 HexNAc 5 | 153.043 | 0.0 | -4.295 | 6.376e+07 | 50 - 67 | 3 | 43.25 | 43.25 | 43.75 |
| 3723.5844 | RNEEYN(N)KSVQEIQATF Fuc 1 Hex 5 HexNAc 4 | 152.641 | 0.0 | 9.051 | 3.263e+08 | 50 - 66 | 9 | 32.34 | 32.34 | 33.79 |
| 3870.6490 | RNEEYN(N)KSVQEIQATFF Fuc 1 Hex 5 HexNAc 4 | 152.601 | 0.0 | 10.216 | 1.241e+08 | 50 - 67 | 4 | 43.75 | 43.91 | 44.41 |
| 4088.8809 | RNEEYN(N)KSVQEIQATF Fuc 1 Hex 6 HexNAc 5 | 152.139 | 0.0 | 12.129 | 3.683e+09 | 50 - 66 | 89 | 23.67 | 32.01 | 36.16 |
| 4234.7764 | RNEEYN(N)KSVQEIQATF Fuc 2 Hex 6 HexNAc 5 | 151.504 | 0.0 | -3.890 | 1.194e+08 | 50 - 66 | 3 | 31.84 | 32.01 | 32.34 |
| 4703.8760 | YFTPN(N)KTEDTIF Fuc 1 Hex 9 HexNAc 8 | 151.485 | 0.0 | 11.396 | 3.816e+07 | 67 - 79 | 2 | 34.80 | 35.14 | 35.82 |
| 3251.3630 | N(N)KSVQEIQATF Hex 6 HexNAc 5 | 151.314 | 0.0 | -4.357 | 1.826e+07 | 55 - 66 | 2 | 26.39 | 26.56 | 26.91 |
| 4307.7909 | RNEEYN(N)KSVQEIQATF Hex 7 HexNAc 6 | 151.224 | 0.0 | -2.830 | 2.754e+08 | 50 - 66 | 3 | 31.84 | 32.01 | 32.34 |
| 3536.5187 | LNVQREN(N)GTISRY Hex 6 HexNAc 5 | 150.760 | 0.0 | 15.546 | 3.433e+09 | 96 - 109 | 12 | 18.16 | 18.95 | 20.10 |
| 3033.3009 | N(N)KSVQEIQATFF Hex 5 HexNAc 4 | 150.523 | 0.0 | -3.806 | 6.795e+06 | 55 - 67 | 1 | 41.03 | 41.20 | 41.54 |
| 3445.4091 | FTPN(N)KTEDTIF Fuc 1 Hex 6 HexNAc 5 | 149.127 | 0.0 | 13.377 | 7.836e+07 | 68 - 79 | 5 | 30.31 | 30.65 | 31.33 |
| 4003.5812 | YFTPN(N)KTEDTIF Hex 7 HexNAc 4 Neu5Ac 2 | 148.952 | 0.0 | 14.468 | 3.177e+08 | 67 - 79 | 7 | 37.63 | 37.79 | 38.81 |
| 3682.5771 | LNVQREN(N)GTISRY Fuc 1 Hex 6 HexNAc 5 | 148.057 | 0.004 | 14.268 | 1.251e+09 | 96 - 109 | 11 | 18.47 | 18.79 | 20.10 |
| 4150.6502 | FYFTPN(N)KTEDTIF Hex 7 HexNAc 4 Neu5Ac 2 | 146.963 | 0.004 | 12.962 | 2.219e+08 | 66 - 79 | 8 | 47.61 | 47.78 | 48.28 |
| 4454.8587 | RNEEYN(N)KSVQEIQATFF Hex 7 HexNAc 6 | 146.716 | 0.004 | 14.250 | 1.608e+08 | 50 - 67 | 5 | 42.91 | 43.25 | 43.75 |
| 3369.3489 | FYFTPN(N)KT Hex 7 HexNAc 6 | 146.166 | 0.0 | -4.298 | 1.358e+07 | 66 - 74 | 2 | 25.03 | 25.20 | 25.37 |
| 4704.8850 | FYFTPN(N)KTEDTIF Hex 9 HexNAc 8 | 146.054 | 0.004 | 15.705 | 1.574e+08 | 66 - 79 | 6 | 44.57 | 45.43 | 46.28 |
| 4029.6176 | FTPN(N)KTEDTIF Hex 8 HexNAc 7 | 145.728 | 0.004 | 14.824 | 5.933e+07 | 68 - 79 | 5 | 29.29 | 29.80 | 30.14 |
| 4296.7078 | FYFTPN(N)KTEDTIF Fuc 1 Hex 7 HexNAc 4 Neu5Ac 2 | 145.310 | 0.004 | -17.041 | 3.573e+07 | 66 - 79 | 2 | 47.44 | 47.61 | 47.61 |
| 4485.8101 | FYFTPN(N)KTEDTIF Fuc 1 Hex 8 HexNAc 7 | 144.153 | 0.004 | 15.113 | 1.269e+08 | 66 - 79 | 5 | 45.09 | 45.94 | 46.45 |
| 3795.5924 | RNEEYN(N)KSVQEIQAT Hex 6 HexNAc 5 | 142.425 | 0.008 | -19.788 | 2.323e+07 | 50 - 65 | 2 | 16.97 | 16.97 | 17.14 |
| 4600.9176 | RNEEYN(N)KSVQEIQATFF Fuc 1 Hex 7 HexNAc 6 | 142.117 | 0.008 | -3.066 | 6.145e+07 | 50 - 67 | 3 | 42.91 | 43.25 | 43.42 |
| 4453.8491 | RNEEYN(N)KSVQEIQATF Fuc 1 Hex 7 HexNAc 6 | 142.035 | 0.008 | -3.286 | 1.167e+08 | 50 - 66 | 2 | 31.67 | 32.01 | 32.18 |
| 3819.5784 | EEYN(N)KSVQEIQATFF Hex 6 HexNAc 5 | 141.620 | 0.008 | -4.099 | 2.581e+07 | 52 - 67 | 3 | 47.28 | 47.44 | 47.61 |
| 3213.4159 | RNEEYN(N)KSVQEIQATF Hex 4 HexNAc 3 | 141.387 | 0.008 | 0.000 | 0.000e+00 | 50 - 66 | 1 | - | - | - |
| 3171.3868 | LNVQREN(N)GTISRY Hex 5 HexNAc 4 | 141.268 | 0.008 | 13.573 | 2.403e+08 | 96 - 109 | 3 | 18.95 | 19.11 | 20.27 |
| 3575.5004 | YFTPN(N)KTEDTIFL Hex 6 HexNAc 5 | 140.103 | 0.010 | -3.288 | 2.478e+07 | 67 - 80 | 3 | 44.74 | 44.91 | 45.26 |
| 3754.5374 | YFTPN(N)KTEDTIF Fuc 2 Hex 6 HexNAc 5 | 140.093 | 0.010 | -6.403 | 7.285e+06 | 67 - 79 | 1 | 36.33 | 36.65 | 36.82 |
| 3693.4440 | TPN(N)KTEDTIF Hex 7 HexNAc 4 Neu5Ac 2 | 137.359 | 0.010 | -3.901 | 1.131e+07 | 69 - 79 | 4 | 24.01 | 24.18 | 24.35 |
| 2954.1863 | N(N)KTEDTIF Hex 6 HexNAc 5 | 137.076 | 0.0 | -20.552 | 2.922e+06 | 71 - 79 | 1 | 21.80 | 21.80 | 21.97 |
| 4922.9499 | YFTPN(N)KTEDTIF Hex 10 HexNAc 9 | 136.423 | 0.010 | -3.202 | 2.348e+07 | 67 - 79 | 1 | 34.63 | 34.97 | 34.97 |
| 3430.4557 | RNEEYN(N)KSVQEIQAT Hex 5 HexNAc 4 | 135.072 | 0.010 | -4.979 | 3.596e+07 | 50 - 65 | 3 | 16.97 | 17.14 | 17.47 |
| 3222.2832 | YFTPN(N)KT Hex 7 HexNAc 6 | 131.719 | 0.0 | -21.208 | 2.653e+06 | 67 - 74 | 1 | 14.00 | 14.00 | 14.00 |
| 4060.6035 | TPN(N)KTEDTIF Fuc 4 Hex 8 HexNAc 5 | 131.328 | 0.010 | 13.132 | 1.352e+08 | 69 - 79 | 4 | 37.63 | 37.79 | 38.47 |
| 3940.6327 | YFTPN(N)KTEDTIFL Hex 7 HexNAc 6 | 130.141 | 0.010 | -4.043 | 4.360e+07 | 67 - 80 | 3 | 44.24 | 44.41 | 44.74 |
| 4394.7532 | FTPN(N)KTEDTIF Hex 9 HexNAc 8 | 129.972 | 0.010 | 14.199 | 1.238e+07 | 68 - 79 | 2 | 28.78 | 29.12 | 29.80 |
| 4672.9231 | RNEEYN(N)KSVQEIQATF Hex 8 HexNAc 7 | 129.830 | 0.010 | -3.865 | 6.215e+07 | 50 - 66 | 2 | 31.50 | 31.67 | 32.01 |
| 4089.7153 | FRNEEYN(N)KSVQEIQATF Hex 6 HexNAc 5 | 129.747 | 0.010 | 11.283 | 4.398e+07 | 49 - 66 | 3 | 36.82 | 37.15 | 37.47 |
| 4193.7711 | LNVQREN(N)GTISRY Fuc 2 Hex 7 HexNAc 6 | 129.617 | 0.010 | 14.014 | 1.027e+09 | 96 - 109 | 7 | 17.82 | 18.32 | 19.76 |
| 3674.5208 | VQREN(N)GTISRY Hex 7 HexNAc 6 | 129.353 | 0.010 | -18.782 | 2.939e+07 | 98 - 109 | 3 | 12.50 | 12.50 | 12.50 |
| 3840.5163 | FTPN(N)KTEDTIF Hex 7 HexNAc 4 Neu5Ac 2 | 129.188 | 0.010 | -4.021 | 3.047e+07 | 68 - 79 | 4 | 31.16 | 31.33 | 31.67 |
| 3004.2150 | FYFTPN(N)KT Hex 6 HexNAc 5 | 128.436 | 0.0 | -3.564 | 6.282e+06 | 66 - 74 | 2 | 25.71 | 25.88 | 26.22 |
| 4425.7269 | N(N)KTEDTIF Fuc 5 Hex 5 HexNAc 8 Neu5Ac 1 | 128.291 | 0.0 | 0.000 | 0.000e+00 | 71 - 79 | 1 | - | - | - |
| 3569.4943 | NVQREN(N)GTISRY Fuc 1 Hex 6 HexNAc 5 | 126.126 | 0.010 | -4.889 | 4.377e+07 | 97 - 109 | 4 | 13.00 | 13.17 | 13.33 |
| 4631.9247 | LNVQREN(N)GTISRY Hex 9 HexNAc 8 | 126.122 | 0.010 | 14.912 | 1.005e+09 | 96 - 109 | 7 | 17.47 | 17.99 | 19.11 |
| 4042.5951 | QTRQDQC(C)IYN(N)TTY Hex 7 HexNAc 6 | 125.459 | 0.010 | 14.439 | 2.270e+08 | 83 - 96 | 8 | 20.78 | 21.80 | 22.65 |
| 3901.6506 | LNVQREN(N)GTISRY Hex 7 HexNAc 6 | 125.256 | 0.010 | 16.485 | 8.599e+09 | 96 - 109 | 13 | 17.82 | 18.63 | 19.76 |
| 4484.8008 | YFTPN(N)KTEDTIF Fuc 2 Hex 8 HexNAc 7 | 125.241 | 0.010 | 12.724 | 4.167e+07 | 67 - 79 | 2 | 35.31 | 35.82 | 35.99 |
| 4266.7901 | LNVQREN(N)GTISRY Hex 8 HexNAc 7 | 125.188 | 0.010 | 14.236 | 1.059e+09 | 96 - 109 | 6 | 17.65 | 18.16 | 19.11 |
| 3423.4343 | NVQREN(N)GTISRY Hex 6 HexNAc 5 | 125.035 | 0.010 | 13.246 | 1.583e+08 | 97 - 109 | 5 | 13.00 | 13.17 | 13.66 |
| 3788.5703 | NVQREN(N)GTISRY Hex 7 HexNAc 6 | 124.635 | 0.010 | 12.752 | 5.511e+08 | 97 - 109 | 6 | 12.83 | 13.00 | 13.66 |
| 3996.5976 | FYFTPN(N)KTEDTIF Hex 3 HexNAc 5 Neu5Ac 3 | 124.458 | 0.010 | 13.698 | 2.445e+08 | 66 - 79 | 3 | 45.77 | 46.61 | 47.11 |
| 4020.6075 | YFTPN(N)KTEDTIF Fuc 1 Hex 8 HexNAc 4 Neu5Ac 1 | 124.387 | 0.010 | -19.508 | 2.523e+07 | 67 - 79 | 2 | 37.79 | 37.79 | 37.96 |
| 3902.6430 | LN(D)VQREN(N)GTISRY Hex 7 HexNAc 6 | 123.940 | 0.010 | 8.332 | 4.492e+08 | 96 - 109 | 54 | 19.93 | 20.10 | 21.29 |
| 3578.5102 | RN(D)EEYN(N)KSVQEIQATF Hex 5 HexNAc 4 | 123.916 | 0.010 | 10.833 | 1.355e+09 | 50 - 66 | 30 | 34.13 | 35.48 | 36.50 |
| 3956.6051 | FTPN(N)KTEDTIF Fuc 2 Hex 7 HexNAc 6 | 123.669 | 0.010 | -4.494 | 1.500e+07 | 68 - 79 | 4 | 29.80 | 29.97 | 30.14 |
| 4631.8693 | FYFTPN(N)KTEDTIF Fuc 2 Hex 8 HexNAc 7 | 123.042 | 0.010 | -3.825 | 2.387e+07 | 66 - 79 | 2 | 45.43 | 45.77 | 45.94 |
| 4080.6908 | NVQREN(N)GTISRY Fuc 2 Hex 7 HexNAc 6 | 122.953 | 0.010 | -4.344 | 6.006e+07 | 97 - 109 | 3 | 12.83 | 12.83 | 13.17 |
| 3943.6432 | RN(D)EEYN(N)KSVQEIQATF Hex 6 HexNAc 5 | 121.638 | 0.010 | 10.764 | 1.210e+09 | 50 - 66 | 13 | 33.79 | 35.31 | 35.99 |
| 4207.6720 | TPN(N)KTEDTIF Fuc 3 Hex 3 HexNAc 9 Neu5Ac 1 | 121.517 | 0.010 | 9.487 | 1.090e+08 | 69 - 79 | 5 | 37.47 | 48.79 | 49.82 |
| 3771.5560 | FYFTPN(N)KTEDTIF Hex 7 HexNAc 5 | 121.206 | 0.010 | -20.320 | 1.376e+07 | 66 - 79 | 1 | 46.78 | 46.94 | 46.94 |
| 3934.6305 | NVQREN(N)GTISRY Fuc 1 Hex 7 HexNAc 6 | 120.757 | 0.010 | 12.442 | 1.794e+08 | 97 - 109 | 5 | 12.83 | 13.00 | 13.50 |
| 4137.7005 | FYFTPN(N)KTEDTIF Hex 8 HexNAc 6 | 120.686 | 0.010 | -20.565 | 9.074e+07 | 66 - 79 | 5 | 13.50 | 46.28 | 46.45 |
| 4339.8282 | LNVQREN(N)GTISRY Fuc 3 Hex 7 HexNAc 6 | 119.930 | 0.010 | 10.164 | 2.611e+08 | 96 - 109 | 3 | 18.16 | 18.32 | 18.79 |
| 4175.6764 | FTPN(N)KTEDTIF Fuc 1 Hex 8 HexNAc 7 | 119.589 | 0.010 | 10.714 | 1.042e+07 | 68 - 79 | 4 | 29.12 | 29.64 | 29.80 |
| 4047.7116 | LNVQREN(N)GTISRY Fuc 1 Hex 7 HexNAc 6 | 119.212 | 0.010 | 15.207 | 3.240e+09 | 96 - 109 | 10 | 17.82 | 18.32 | 19.76 |
| 4558.9101 | LNVQREN(N)GTISRY Fuc 2 Hex 8 HexNAc 7 | 119.056 | 0.010 | 15.302 | 2.082e+08 | 96 - 109 | 4 | 17.82 | 17.99 | 18.47 |
| 3677.4612 | QTRQDQC(C)IYN(N)TTY Hex 6 HexNAc 5 | 119.005 | 0.010 | 14.682 | 1.239e+08 | 83 - 96 | 7 | 21.12 | 22.14 | 22.65 |
| 3398.4319 | N(N)KSVQEIQATFF Hex 6 HexNAc 5 | 118.589 | 0.010 | -18.145 | 5.016e+06 | 55 - 67 | 2 | 40.35 | 40.52 | 40.52 |
| 3725.5782 | RN(D)EEYN(N)KSVQEIQATFF Hex 5 HexNAc 4 | 117.080 | 0.012 | 10.591 | 3.103e+08 | 50 - 67 | 10 | 45.60 | 47.11 | 47.28 |
| 5038.0545 | RNEEYN(N)KSVQEIQATF Hex 9 HexNAc 8 | 116.422 | 0.012 | -3.435 | 3.580e+07 | 50 - 66 | 2 | 31.16 | 31.50 | 31.67 |
| 4236.7703 | RN(D)EEYN(N)KSVQEIQATFF Fuc 1 Hex 6 HexNAc 5 | 116.336 | 0.014 | 9.723 | 1.687e+08 | 50 - 67 | 11 | 44.74 | 46.45 | 46.94 |
| 3124.3981 | LVPVPITN(N)ATL Hex 6 HexNAc 5 | 116.261 | 0.014 | -4.634 | 7.286e+06 | 25 - 36 | 2 | 45.60 | 45.60 | 45.94 |
| 4048.7008 | LN(D)VQREN(N)GTISRY Fuc 1 Hex 7 HexNAc 6 | 115.612 | 0.016 | 18.791 | 1.295e+08 | 96 - 109 | 8 | 19.76 | 19.93 | 20.61 |
| 4563.8441 | YFTPN(N)KT Fuc 5 Hex 7 HexNAc 9 | 112.144 | 0.0 | 0.000 | 0.000e+00 | 67 - 74 | 1 | - | - | - |
| 3828.6342 | LNVQREN(N)GTISRY Fuc 2 Hex 6 HexNAc 5 | 111.380 | 0.020 | 10.628 | 8.781e+07 | 96 - 109 | 2 | 18.32 | 18.47 | 18.95 |
| 4231.7186 | LNVQREN(N)GTISRY Fuc 1 Hex 4 HexNAc 5 Neu5Ac 3 | 110.997 | 0.020 | -4.201 | 6.062e+07 | 96 - 109 | 2 | 18.16 | 18.32 | 18.47 |
| 4090.7117 | RN(D)EEYN(N)KSVQEIQATFF Hex 6 HexNAc 5 | 110.503 | 0.021 | 9.926 | 3.272e+08 | 50 - 67 | 14 | 44.91 | 46.78 | 46.78 |
| 3454.4478 | EEYN(N)KSVQEIQATFF Hex 5 HexNAc 4 | 110.047 | 0.021 | -4.613 | 2.153e+07 | 52 - 67 | 3 | 47.78 | 47.95 | 48.28 |
| 4819.9920 | RNEEYN(N)KSVQEIQATFF Hex 8 HexNAc 7 | 109.655 | 0.021 | -2.920 | 1.690e+07 | 50 - 67 | 2 | 42.57 | 42.74 | 43.08 |
| 3613.4178 | FYFTPN(N)KTED Hex 7 HexNAc 6 | 109.648 | 0.021 | -4.778 | 5.253e+06 | 66 - 76 | 2 | 25.54 | 25.54 | 25.88 |
| 3823.5191 | QTRQDQC(C)IYN(N)TTY Fuc 1 Hex 6 HexNAc 5 | 109.438 | 0.021 | 13.941 | 3.556e+07 | 83 - 96 | 3 | 21.63 | 21.97 | 22.31 |
| 5070.0167 | FYFTPN(N)KTEDTIF Hex 10 HexNAc 9 | 108.645 | 0.021 | -3.908 | 1.775e+07 | 66 - 79 | 2 | 44.74 | 44.91 | 45.26 |
| 3901.6501 | LN(D)VQREN(N)GTISRY Fuc 3 Hex 5 HexNAc 4 Neu5Ac 1 | 108.471 | 0.023 | -21.356 | 1.204e+07 | 96 - 109 | 1 | 20.95 | 20.95 | 20.95 |
| 4518.8338 | NVQREN(N)GTISRY Hex 9 HexNAc 8 | 107.047 | 0.029 | -18.277 | 3.878e+07 | 97 - 109 | 1 | 12.67 | 12.67 | 12.83 |
| 4412.8473 | LNVQREN(N)GTISRY Fuc 1 Hex 8 HexNAc 7 | 106.757 | 0.029 | 15.218 | 4.255e+08 | 96 - 109 | 6 | 17.65 | 18.16 | 18.79 |
| 4777.9790 | LNVQREN(N)GTISRY Fuc 1 Hex 9 HexNAc 8 | 105.999 | 0.030 | 16.381 | 3.004e+08 | 96 - 109 | 3 | 17.47 | 17.99 | 18.32 |
| 4704.9621 | LNVQREN(N)GTISRY Fuc 3 Hex 8 HexNAc 7 | 103.380 | 0.033 | -3.170 | 8.344e+07 | 96 - 109 | 2 | 17.82 | 17.99 | 18.16 |
| 3981.6133 | LNVQREN(N)GTISRY Hex 10 HexNAc 4 | 103.369 | 0.033 | 12.317 | 3.535e+07 | 96 - 109 | 5 | 21.12 | 21.63 | 22.14 |
| 3638.4467 | TPN(N)KTEDTIF Hex 9 HexNAc 5 | 102.037 | 0.034 | 13.548 | 2.781e+07 | 69 - 79 | 4 | 38.13 | 38.30 | 38.98 |
| 4223.7449 | LNVQREN(N)GTISRY Fuc 1 Hex 7 HexNAc 4 Neu5Ac 2 | 101.793 | 0.034 | 13.503 | 1.119e+08 | 96 - 109 | 3 | 19.27 | 19.43 | 20.10 |
| 3469.5104 | C(C)ANLVPVPITN(N)ATL Hex 6 HexNAc 5 | 101.793 | 0.034 | 12.350 | 2.307e+07 | 22 - 36 | 6 | 50.16 | 50.50 | 51.18 |
| 3309.4022 | VQREN(N)GTISRY Hex 6 HexNAc 5 | 101.503 | 0.041 | -20.763 | 5.558e+06 | 98 - 109 | 1 | 12.67 | 12.67 | 12.67 |
| 4153.7036 | NVQREN(N)GTISRY Hex 8 HexNAc 7 | 101.262 | 0.041 | -19.053 | 4.353e+07 | 97 - 109 | 2 | 12.83 | 12.83 | 13.00 |
| 4997.0515 | LNVQREN(N)GTISRY Hex 10 HexNAc 9 | 101.194 | 0.041 | -2.794 | 1.461e+08 | 96 - 109 | 3 | 17.65 | 17.82 | 18.16 |
| 4924.0371 | LNVQREN(N)GTISRY Fuc 2 Hex 9 HexNAc 8 | 100.896 | 0.041 | -3.544 | 1.121e+08 | 96 - 109 | 3 | 17.65 | 17.82 | 18.16 |
| 3722.5529 | RNEEYN(N)KSVQEIQAT Fuc 2 Hex 5 HexNAc 4 | 99.957 | 0.046 | -19.545 | 2.088e+07 | 50 - 65 | 1 | 32.18 | 32.18 | 32.18 |
| 4077.6849 | LNVQREN(N)GTISRY Hex 7 HexNAc 4 Neu5Ac 2 | 99.574 | 0.046 | 14.024 | 3.720e+08 | 96 - 109 | 7 | 19.27 | 19.59 | 20.78 |
| 4807.9533 | LNVQREN(N)GTISRY Hex 9 HexNAc 6 Neu5Ac 2 | 99.258 | 0.047 | -19.541 | 3.097e+07 | 96 - 109 | 1 | 18.79 | 18.79 | 18.95 |
| 5143.1101 | LNVQREN(N)GTISRY Fuc 1 Hex 10 HexNAc 9 | 98.447 | 0.047 | -4.457 | 3.904e+07 | 96 - 109 | 2 | 17.65 | 17.82 | 17.99 |
| 3537.5110 | VQREN(N)GTISRY Fuc 1 Hex 4 HexNAc 7 | 98.422 | 0.047 | 9.485 | 1.122e+08 | 98 - 109 | 8 | 20.27 | 20.27 | 21.80 |
| 5070.0928 | LNVQREN(N)GTISRY Fuc 3 Hex 9 HexNAc 8 | 97.965 | 0.047 | -4.341 | 2.933e+07 | 96 - 109 | 1 | 17.65 | 17.82 | 18.16 |

### TPN(N)KTEDTIF Hex 6 HexNAc 5

**Mass:**  3152.2838

(1.218e-06)

**Charge States:** 3, 4

**Start Position**: 70

**End Position**: 80

| Profile Score | Tandem Score | TDA q-value | Volume | Spectrum Matches |
| --- | --- | --- | --- | --- |
| 15.4443 | 185.8852 | 0.0000 | 2.0477e+08 | 8 |

**Adduct Signal Fractions**

- **Unmodified**: 2.0477e+08

### TPN(N)KTEDTIF Hex 7 HexNAc 6

**Mass:**  3517.4163

(1.427e-06)

**Charge States:** 3, 4

**Start Position**: 70

**End Position**: 80

| Profile Score | Tandem Score | TDA q-value | Volume | Spectrum Matches |
| --- | --- | --- | --- | --- |
| 15.1480 | 184.4718 | 0.0000 | 3.3516e+08 | 13 |

**Adduct Signal Fractions**

- **Unmodified**: 3.3516e+08

### YFTPN(N)KTEDTIF Hex 6 HexNAc 5

**Mass:**  3462.4173

(1.477e-06)

**Charge States:** 2, 3, 4

**Start Position**: 68

**End Position**: 80

| Profile Score | Tandem Score | TDA q-value | Volume | Spectrum Matches |
| --- | --- | --- | --- | --- |
| 14.3961 | 182.2093 | 0.0000 | 2.9344e+09 | 56 |

**Adduct Signal Fractions**

- **Unmodified**: 2.9344e+09

### TPN(N)KTEDTIF Fuc 1 Hex 6 HexNAc 5

**Mass:**  3298.3423

(1.480e-06)

**Charge States:** 3, 4

**Start Position**: 70

**End Position**: 80

| Profile Score | Tandem Score | TDA q-value | Volume | Spectrum Matches |
| --- | --- | --- | --- | --- |
| 13.8017 | 181.2037 | 0.0000 | 3.7224e+07 | 4 |

**Adduct Signal Fractions**

- **Unmodified**: 3.7224e+07

### TPN(N)KTEDTIF Fuc 1 Hex 7 HexNAc 6

**Mass:**  3663.4738

(2.012e-06)

**Charge States:** 3, 4

**Start Position**: 70

**End Position**: 80

| Profile Score | Tandem Score | TDA q-value | Volume | Spectrum Matches |
| --- | --- | --- | --- | --- |
| 14.5996 | 180.4104 | 0.0000 | 5.0162e+07 | 4 |

**Adduct Signal Fractions**

- **Unmodified**: 5.0162e+07

### N(N)KSVQEIQATF Hex 5 HexNAc 4

**Mass:**  2886.2334

(2.069e-06)

**Charge States:** 3, 4

**Start Position**: 56

**End Position**: 67

| Profile Score | Tandem Score | TDA q-value | Volume | Spectrum Matches |
| --- | --- | --- | --- | --- |
| -3.2450 | 179.8773 | 0.0000 | 2.8226e+07 | 1 |

**Adduct Signal Fractions**

- **Unmodified**: 2.8226e+07

### YFTPN(N)KTEDTIF Hex 7 HexNAc 6

**Mass:**  3827.5502

(1.622e-06)

**Charge States:** 3, 4

**Start Position**: 68

**End Position**: 80

| Profile Score | Tandem Score | TDA q-value | Volume | Spectrum Matches |
| --- | --- | --- | --- | --- |
| 15.1699 | 176.9469 | 0.0000 | 4.7800e+09 | 45 |

**Adduct Signal Fractions**

- **Unmodified**: 4.7800e+09

### TPN(N)KTEDTIF Hex 8 HexNAc 7

**Mass:**  3882.5500

(1.443e-06)

**Charge States:** 3, 4

**Start Position**: 70

**End Position**: 80

| Profile Score | Tandem Score | TDA q-value | Volume | Spectrum Matches |
| --- | --- | --- | --- | --- |
| -4.3212 | 175.2173 | 0.0000 | 2.6897e+07 | 4 |

**Adduct Signal Fractions**

- **Unmodified**: 2.6897e+07

### YFTPN(N)KTEDTIF Fuc 1 Hex 7 HexNAc 6

**Mass:**  3973.6069

(2.368e-06)

**Charge States:** 3, 4

**Start Position**: 68

**End Position**: 80

| Profile Score | Tandem Score | TDA q-value | Volume | Spectrum Matches |
| --- | --- | --- | --- | --- |
| 15.0540 | 174.4842 | 0.0000 | 9.9334e+08 | 9 |

**Adduct Signal Fractions**

- **Unmodified**: 9.9334e+08

### FYFTPN(N)KTEDTIF Hex 6 HexNAc 5

**Mass:**  3609.4839

(9.855e-07)

**Charge States:** 3, 4

**Start Position**: 67

**End Position**: 80

| Profile Score | Tandem Score | TDA q-value | Volume | Spectrum Matches |
| --- | --- | --- | --- | --- |
| 14.7894 | 173.9697 | 0.0000 | 2.3522e+09 | 48 |

**Adduct Signal Fractions**

- **Unmodified**: 2.3522e+09

### FYFTPN(N)KTEDTIF Hex 7 HexNAc 6

**Mass:**  3974.6162

(7.113e-07)

**Charge States:** 3, 4

**Start Position**: 67

**End Position**: 80

| Profile Score | Tandem Score | TDA q-value | Volume | Spectrum Matches |
| --- | --- | --- | --- | --- |
| 15.4648 | 172.7852 | 0.0000 | 4.4793e+09 | 79 |

**Adduct Signal Fractions**

- **Unmodified**: 4.4793e+09

### FTPN(N)KTEDTIF Hex 6 HexNAc 5

**Mass:**  3299.3527

(1.237e-06)

**Charge States:** 3, 4

**Start Position**: 69

**End Position**: 80

| Profile Score | Tandem Score | TDA q-value | Volume | Spectrum Matches |
| --- | --- | --- | --- | --- |
| 15.4699 | 172.6405 | 0.0000 | 4.3512e+08 | 13 |

**Adduct Signal Fractions**

- **Unmodified**: 4.3512e+08

### TPN(N)KTEDTIF Hex 5 HexNAc 4

**Mass:**  2787.1521

(1.315e-06)

**Charge States:** 3

**Start Position**: 70

**End Position**: 80

| Profile Score | Tandem Score | TDA q-value | Volume | Spectrum Matches |
| --- | --- | --- | --- | --- |
| 13.8444 | 170.6700 | 0.0000 | 2.5032e+07 | 3 |

**Adduct Signal Fractions**

- **Unmodified**: 2.5032e+07

### YFTPN(N)KTEDTIF Fuc 1 Hex 6 HexNAc 5

**Mass:**  3608.4757

(1.745e-06)

**Charge States:** 3, 4

**Start Position**: 68

**End Position**: 80

| Profile Score | Tandem Score | TDA q-value | Volume | Spectrum Matches |
| --- | --- | --- | --- | --- |
| 13.6522 | 167.9915 | 0.0000 | 6.5243e+08 | 12 |

**Adduct Signal Fractions**

- **Unmodified**: 6.5243e+08

### YFTPN(N)KTEDTIF Hex 5 HexNAc 4

**Mass:**  3097.2822

(7.734e-07)

**Charge States:** 3, 4

**Start Position**: 68

**End Position**: 80

| Profile Score | Tandem Score | TDA q-value | Volume | Spectrum Matches |
| --- | --- | --- | --- | --- |
| 14.4442 | 167.5665 | 0.0000 | 3.2968e+08 | 8 |

**Adduct Signal Fractions**

- **Unmodified**: 3.2968e+08

### FTPN(N)KTEDTIF Hex 7 HexNAc 6

**Mass:**  3664.4866

(1.233e-06)

**Charge States:** 3, 4

**Start Position**: 69

**End Position**: 80

| Profile Score | Tandem Score | TDA q-value | Volume | Spectrum Matches |
| --- | --- | --- | --- | --- |
| 16.1226 | 166.2715 | 0.0000 | 6.9294e+08 | 14 |

**Adduct Signal Fractions**

- **Unmodified**: 6.9294e+08

### FYFTPN(N)KTEDTIF Fuc 1 Hex 7 HexNAc 6

**Mass:**  4120.6799

(9.779e-07)

**Charge States:** 3, 4

**Start Position**: 67

**End Position**: 80

| Profile Score | Tandem Score | TDA q-value | Volume | Spectrum Matches |
| --- | --- | --- | --- | --- |
| 14.5538 | 166.0649 | 0.0000 | 8.3594e+08 | 13 |

**Adduct Signal Fractions**

- **Unmodified**: 8.3594e+08

### YFTPN(N)KTEDTIF Hex 9 HexNAc 8

**Mass:**  4557.8171

(1.103e-06)

**Charge States:** 3, 4

**Start Position**: 68

**End Position**: 80

| Profile Score | Tandem Score | TDA q-value | Volume | Spectrum Matches |
| --- | --- | --- | --- | --- |
| 15.5500 | 165.2093 | 0.0000 | 2.3930e+08 | 5 |

**Adduct Signal Fractions**

- **Unmodified**: 2.3930e+08

### FYFTPN(N)KTEDTIF Fuc 1 Hex 6 HexNAc 5

**Mass:**  3755.5442

(7.090e-07)

**Charge States:** 3, 4

**Start Position**: 67

**End Position**: 80

| Profile Score | Tandem Score | TDA q-value | Volume | Spectrum Matches |
| --- | --- | --- | --- | --- |
| 14.0570 | 164.9562 | 0.0000 | 5.9737e+08 | 14 |

**Adduct Signal Fractions**

- **Unmodified**: 5.9737e+08

### YFTPN(N)KTEDTIF Fuc 1 Hex 5 HexNAc 4

**Mass:**  3243.3374

(1.278e-06)

**Charge States:** 3

**Start Position**: 68

**End Position**: 80

| Profile Score | Tandem Score | TDA q-value | Volume | Spectrum Matches |
| --- | --- | --- | --- | --- |
| -20.1219 | 162.3918 | 0.0000 | 7.2217e+06 | 1 |

**Adduct Signal Fractions**

- **Unmodified**: 7.2217e+06

### YFTPN(N)KTEDTIF Hex 8 HexNAc 7

**Mass:**  4192.6843

(1.584e-06)

**Charge States:** 3, 4

**Start Position**: 68

**End Position**: 80

| Profile Score | Tandem Score | TDA q-value | Volume | Spectrum Matches |
| --- | --- | --- | --- | --- |
| 14.9975 | 161.3422 | 0.0000 | 8.7096e+08 | 11 |

**Adduct Signal Fractions**

- **Unmodified**: 8.7096e+08

### YFTPN(N)KTEDTIF Fuc 2 Hex 7 HexNAc 6

**Mass:**  4119.6662

(2.510e-06)

**Charge States:** 3, 4

**Start Position**: 68

**End Position**: 80

| Profile Score | Tandem Score | TDA q-value | Volume | Spectrum Matches |
| --- | --- | --- | --- | --- |
| -3.9164 | 161.1085 | 0.0000 | 1.5125e+08 | 2 |

**Adduct Signal Fractions**

- **Unmodified**: 1.5125e+08

### FYFTPN(N)KTEDTIF Fuc 2 Hex 7 HexNAc 6

**Mass:**  4266.7365

(2.264e-06)

**Charge States:** 3, 4

**Start Position**: 67

**End Position**: 80

| Profile Score | Tandem Score | TDA q-value | Volume | Spectrum Matches |
| --- | --- | --- | --- | --- |
| 13.1778 | 161.0728 | 0.0000 | 1.3589e+08 | 3 |

**Adduct Signal Fractions**

- **Unmodified**: 1.3589e+08

### FYFTPN(N)KTEDTIF Hex 5 HexNAc 4

**Mass:**  3244.3500

(1.134e-06)

**Charge States:** 3, 4

**Start Position**: 67

**End Position**: 80

| Profile Score | Tandem Score | TDA q-value | Volume | Spectrum Matches |
| --- | --- | --- | --- | --- |
| 11.9005 | 160.3820 | 0.0000 | 1.9600e+08 | 4 |

**Adduct Signal Fractions**

- **Unmodified**: 1.9600e+08

### RNEEYN(N)KSVQEIQATFF Hex 5 HexNAc 4

**Mass:**  3724.5947

(9.100e-07)

**Charge States:** 3, 4

**Start Position**: 51

**End Position**: 68

| Profile Score | Tandem Score | TDA q-value | Volume | Spectrum Matches |
| --- | --- | --- | --- | --- |
| 16.8344 | 160.0969 | 0.0000 | 3.1670e+09 | 18 |

**Adduct Signal Fractions**

- **Unmodified**: 3.1670e+09

### EEYN(N)KSVQEIQATF Hex 6 HexNAc 5

**Mass:**  3672.5117

(1.241e-06)

**Charge States:** 3, 4

**Start Position**: 53

**End Position**: 67

| Profile Score | Tandem Score | TDA q-value | Volume | Spectrum Matches |
| --- | --- | --- | --- | --- |
| -3.6084 | 159.7083 | 0.0000 | 7.8689e+07 | 3 |

**Adduct Signal Fractions**

- **Unmodified**: 7.8689e+07

### YFTPN(N)KTEDTIF Fuc 1 Hex 8 HexNAc 7

**Mass:**  4338.7399

(1.610e-06)

**Charge States:** 3, 4

**Start Position**: 68

**End Position**: 80

| Profile Score | Tandem Score | TDA q-value | Volume | Spectrum Matches |
| --- | --- | --- | --- | --- |
| 12.5041 | 158.9535 | 0.0000 | 1.6184e+08 | 4 |

**Adduct Signal Fractions**

- **Unmodified**: 1.6184e+08

### FYFTPN(N)KTEDTIF Hex 8 HexNAc 7

**Mass:**  4339.7565

(1.729e-06)

**Charge States:** 3, 4

**Start Position**: 67

**End Position**: 80

| Profile Score | Tandem Score | TDA q-value | Volume | Spectrum Matches |
| --- | --- | --- | --- | --- |
| 14.8720 | 158.0543 | 0.0000 | 6.1386e+08 | 10 |

**Adduct Signal Fractions**

- **Unmodified**: 6.1386e+08

### TPN(N)KTEDTIF Fuc 3 Hex 6 HexNAc 5 Neu5Ac 1

**Mass:**  3881.5468

(-6.471e-07)

**Charge States:** 3, 4

**Start Position**: 70

**End Position**: 80

| Profile Score | Tandem Score | TDA q-value | Volume | Spectrum Matches |
| --- | --- | --- | --- | --- |
| -19.1671 | 158.0100 | 0.0000 | 6.0640e+06 | 1 |

**Adduct Signal Fractions**

- **Unmodified**: 6.0640e+06

### RNEEYN(N)KSVQEIQATF Hex 5 HexNAc 4

**Mass:**  3577.5268

(9.724e-07)

**Charge States:** 3, 4

**Start Position**: 51

**End Position**: 67

| Profile Score | Tandem Score | TDA q-value | Volume | Spectrum Matches |
| --- | --- | --- | --- | --- |
| 15.5628 | 157.9410 | 0.0000 | 8.5835e+09 | 23 |

**Adduct Signal Fractions**

- **Unmodified**: 8.5835e+09

### FTPN(N)KTEDTIF Hex 5 HexNAc 4

**Mass:**  2934.2202

(4.556e-07)

**Charge States:** 3

**Start Position**: 69

**End Position**: 80

| Profile Score | Tandem Score | TDA q-value | Volume | Spectrum Matches |
| --- | --- | --- | --- | --- |
| 14.3620 | 156.6674 | 0.0000 | 4.6114e+07 | 2 |

**Adduct Signal Fractions**

- **Unmodified**: 4.6114e+07

### RNEEYN(N)KSVQEIQATF Hex 6 HexNAc 5

**Mass:**  3942.6589

(1.833e-06)

**Charge States:** 3, 4

**Start Position**: 51

**End Position**: 67

| Profile Score | Tandem Score | TDA q-value | Volume | Spectrum Matches |
| --- | --- | --- | --- | --- |
| 14.0150 | 156.6215 | 0.0000 | 6.7169e+09 | 34 |

**Adduct Signal Fractions**

- **Unmodified**: 6.7169e+09

### RNEEYN(N)KSVQEIQATFF Fuc 1 Hex 6 HexNAc 5

**Mass:**  4235.7839

(1.018e-06)

**Charge States:** 3, 4

**Start Position**: 51

**End Position**: 68

| Profile Score | Tandem Score | TDA q-value | Volume | Spectrum Matches |
| --- | --- | --- | --- | --- |
| 15.7230 | 156.1039 | 0.0000 | 1.5208e+09 | 13 |

**Adduct Signal Fractions**

- **Unmodified**: 1.5208e+09

### FTPN(N)KTEDTIF Fuc 1 Hex 7 HexNAc 6

**Mass:**  3810.5432

(1.228e-06)

**Charge States:** 3, 4

**Start Position**: 69

**End Position**: 80

| Profile Score | Tandem Score | TDA q-value | Volume | Spectrum Matches |
| --- | --- | --- | --- | --- |
| 13.6550 | 155.8040 | 0.0000 | 1.1635e+08 | 6 |

**Adduct Signal Fractions**

- **Unmodified**: 1.1635e+08

### EEYN(N)KSVQEIQATF Fuc 1 Hex 6 HexNAc 5

**Mass:**  3818.5707

(1.381e-06)

**Charge States:** 3, 4

**Start Position**: 53

**End Position**: 67

| Profile Score | Tandem Score | TDA q-value | Volume | Spectrum Matches |
| --- | --- | --- | --- | --- |
| -3.5643 | 155.0650 | 0.0000 | 4.1085e+07 | 2 |

**Adduct Signal Fractions**

- **Unmodified**: 4.1085e+07

### RNEEYN(N)KSVQEIQATFF Hex 6 HexNAc 5

**Mass:**  4089.7248

(4.928e-07)

**Charge States:** 3, 4

**Start Position**: 51

**End Position**: 68

| Profile Score | Tandem Score | TDA q-value | Volume | Spectrum Matches |
| --- | --- | --- | --- | --- |
| 16.0327 | 153.8968 | 0.0000 | 3.2946e+09 | 23 |

**Adduct Signal Fractions**

- **Unmodified**: 3.2946e+09

### EEYN(N)KSVQEIQATF Hex 5 HexNAc 4

**Mass:**  3307.3778

(1.193e-06)

**Charge States:** 3, 4

**Start Position**: 53

**End Position**: 67

| Profile Score | Tandem Score | TDA q-value | Volume | Spectrum Matches |
| --- | --- | --- | --- | --- |
| -3.8519 | 153.5722 | 0.0000 | 9.3988e+07 | 4 |

**Adduct Signal Fractions**

- **Unmodified**: 9.3988e+07

### RNEEYN(N)KSVQEIQATFF Fuc 1 Hex 5 HexNAc 4

**Mass:**  3872.6601

(5.202e-04)

**Start Position**: 51

**End Position**: 68

| Profile Score | Tandem Score | TDA q-value | Volume | Spectrum Matches |
| --- | --- | --- | --- | --- |
| 0.0000 | 153.4854 | 0.0000 | 0.0000e+00 | 1 |

Adduct:

No Chromatogram Found

### RNEEYN(N)KSVQEIQATFF Fuc 2 Hex 6 HexNAc 5

**Mass:**  4381.8444

(1.799e-06)

**Charge States:** 4

**Start Position**: 51

**End Position**: 68

| Profile Score | Tandem Score | TDA q-value | Volume | Spectrum Matches |
| --- | --- | --- | --- | --- |
| -4.2945 | 153.0425 | 0.0000 | 6.3758e+07 | 3 |

**Adduct Signal Fractions**

- **Unmodified**: 6.3758e+07

### RNEEYN(N)KSVQEIQATF Fuc 1 Hex 5 HexNAc 4

**Mass:**  3723.5834

(2.049e-06)

**Charge States:** 3, 4

**Start Position**: 51

**End Position**: 67

| Profile Score | Tandem Score | TDA q-value | Volume | Spectrum Matches |
| --- | --- | --- | --- | --- |
| 9.0512 | 152.6414 | 0.0000 | 3.2629e+08 | 9 |

**Adduct Signal Fractions**

- **Unmodified**: 3.2629e+08

### RNEEYN(N)KSVQEIQATFF Fuc 1 Hex 5 HexNAc 4

**Mass:**  3870.6477

(3.232e-07)

**Charge States:** 3, 4

**Start Position**: 51

**End Position**: 68

| Profile Score | Tandem Score | TDA q-value | Volume | Spectrum Matches |
| --- | --- | --- | --- | --- |
| 10.2164 | 152.6006 | 0.0000 | 1.2408e+08 | 4 |

**Adduct Signal Fractions**

- **Unmodified**: 1.2408e+08

### RNEEYN(N)KSVQEIQATF Fuc 1 Hex 6 HexNAc 5

**Mass:**  4088.7192

(1.938e-06)

**Charge States:** 3, 4

**Start Position**: 51

**End Position**: 67

| Profile Score | Tandem Score | TDA q-value | Volume | Spectrum Matches |
| --- | --- | --- | --- | --- |
| 12.1294 | 152.1388 | 0.0000 | 3.6826e+09 | 89 |

**Adduct Signal Fractions**

- **Unmodified**: 3.6826e+09

### RNEEYN(N)KSVQEIQATF Fuc 2 Hex 6 HexNAc 5

**Mass:**  4234.7766

(1.025e-06)

**Charge States:** 3, 4

**Start Position**: 51

**End Position**: 67

| Profile Score | Tandem Score | TDA q-value | Volume | Spectrum Matches |
| --- | --- | --- | --- | --- |
| -3.8902 | 151.5035 | 0.0000 | 1.1945e+08 | 3 |

**Adduct Signal Fractions**

- **Unmodified**: 1.1945e+08

### YFTPN(N)KTEDTIF Fuc 1 Hex 9 HexNAc 8

**Mass:**  4703.8752

(1.932e-06)

**Charge States:** 4

**Start Position**: 68

**End Position**: 80

| Profile Score | Tandem Score | TDA q-value | Volume | Spectrum Matches |
| --- | --- | --- | --- | --- |
| 11.3959 | 151.4851 | 0.0000 | 3.8157e+07 | 2 |

**Adduct Signal Fractions**

- **Unmodified**: 3.8157e+07

### N(N)KSVQEIQATF Hex 6 HexNAc 5

**Mass:**  3251.3637

(1.812e-06)

**Charge States:** 3, 4

**Start Position**: 56

**End Position**: 67

| Profile Score | Tandem Score | TDA q-value | Volume | Spectrum Matches |
| --- | --- | --- | --- | --- |
| -4.3566 | 151.3142 | 0.0000 | 1.8263e+07 | 2 |

**Adduct Signal Fractions**

- **Unmodified**: 1.8263e+07

### RNEEYN(N)KSVQEIQATF Hex 7 HexNAc 6

**Mass:**  4307.7912

(1.295e-06)

**Charge States:** 4

**Start Position**: 51

**End Position**: 67

| Profile Score | Tandem Score | TDA q-value | Volume | Spectrum Matches |
| --- | --- | --- | --- | --- |
| -2.8303 | 151.2241 | 0.0000 | 2.7545e+08 | 3 |

**Adduct Signal Fractions**

- **Unmodified**: 2.7545e+08

### LNVQREN(N)GTISRY Hex 6 HexNAc 5

**Mass:**  3536.5197

(8.223e-07)

**Charge States:** 3, 4

**Start Position**: 97

**End Position**: 110

| Profile Score | Tandem Score | TDA q-value | Volume | Spectrum Matches |
| --- | --- | --- | --- | --- |
| 15.5459 | 150.7604 | 0.0000 | 3.4325e+09 | 12 |

**Adduct Signal Fractions**

- **Unmodified**: 3.4325e+09

### N(N)KSVQEIQATFF Hex 5 HexNAc 4

**Mass:**  3033.3023

(2.105e-06)

**Charge States:** 3

**Start Position**: 56

**End Position**: 68

| Profile Score | Tandem Score | TDA q-value | Volume | Spectrum Matches |
| --- | --- | --- | --- | --- |
| -3.8062 | 150.5228 | 0.0000 | 6.7947e+06 | 1 |

**Adduct Signal Fractions**

- **Unmodified**: 6.7947e+06

### FTPN(N)KTEDTIF Fuc 1 Hex 6 HexNAc 5

**Mass:**  3445.4093

(6.403e-07)

**Charge States:** 3, 4

**Start Position**: 69

**End Position**: 80

| Profile Score | Tandem Score | TDA q-value | Volume | Spectrum Matches |
| --- | --- | --- | --- | --- |
| 13.3767 | 149.1272 | 0.0000 | 7.8365e+07 | 5 |

**Adduct Signal Fractions**

- **Unmodified**: 7.8365e+07

### YFTPN(N)KTEDTIF Hex 7 HexNAc 4 Neu5Ac 2

**Mass:**  4003.5812

(1.178e-06)

**Charge States:** 3, 4

**Start Position**: 68

**End Position**: 80

| Profile Score | Tandem Score | TDA q-value | Volume | Spectrum Matches |
| --- | --- | --- | --- | --- |
| 14.4680 | 148.9524 | 0.0000 | 3.1771e+08 | 7 |

**Adduct Signal Fractions**

- **Unmodified**: 3.1771e+08

### LNVQREN(N)GTISRY Fuc 1 Hex 6 HexNAc 5

**Mass:**  3682.5773

(1.326e-06)

**Charge States:** 3, 4

**Start Position**: 97

**End Position**: 110

| Profile Score | Tandem Score | TDA q-value | Volume | Spectrum Matches |
| --- | --- | --- | --- | --- |
| 14.2685 | 148.0570 | 0.0040 | 1.2509e+09 | 11 |

**Adduct Signal Fractions**

- **Unmodified**: 1.2509e+09

### FYFTPN(N)KTEDTIF Hex 7 HexNAc 4 Neu5Ac 2

**Mass:**  4150.6486

(-2.130e-06)

**Charge States:** 3, 4

**Start Position**: 67

**End Position**: 80

| Profile Score | Tandem Score | TDA q-value | Volume | Spectrum Matches |
| --- | --- | --- | --- | --- |
| 12.9617 | 146.9630 | 0.0040 | 2.2186e+08 | 8 |

**Adduct Signal Fractions**

- **Unmodified**: 2.2186e+08

### RNEEYN(N)KSVQEIQATFF Hex 7 HexNAc 6

**Mass:**  4454.8596

(1.469e-06)

**Charge States:** 3, 4

**Start Position**: 51

**End Position**: 68

| Profile Score | Tandem Score | TDA q-value | Volume | Spectrum Matches |
| --- | --- | --- | --- | --- |
| 14.2504 | 146.7157 | 0.0040 | 1.6082e+08 | 5 |

**Adduct Signal Fractions**

- **Unmodified**: 1.6082e+08

### FYFTPN(N)KT Hex 7 HexNAc 6

**Mass:**  3369.3469

(1.209e-06)

**Charge States:** 3

**Start Position**: 67

**End Position**: 75

| Profile Score | Tandem Score | TDA q-value | Volume | Spectrum Matches |
| --- | --- | --- | --- | --- |
| -4.2977 | 146.1660 | 0.0000 | 1.3579e+07 | 2 |

**Adduct Signal Fractions**

- **Unmodified**: 1.3579e+07

### FYFTPN(N)KTEDTIF Hex 9 HexNAc 8

**Mass:**  4704.8840

(1.932e-06)

**Charge States:** 3, 4

**Start Position**: 67

**End Position**: 80

| Profile Score | Tandem Score | TDA q-value | Volume | Spectrum Matches |
| --- | --- | --- | --- | --- |
| 15.7048 | 146.0538 | 0.0040 | 1.5738e+08 | 6 |

**Adduct Signal Fractions**

- **Unmodified**: 1.5738e+08

### FTPN(N)KTEDTIF Hex 8 HexNAc 7

**Mass:**  4029.6181

(1.272e-06)

**Charge States:** 3, 4

**Start Position**: 69

**End Position**: 80

| Profile Score | Tandem Score | TDA q-value | Volume | Spectrum Matches |
| --- | --- | --- | --- | --- |
| 14.8242 | 145.7279 | 0.0040 | 5.9327e+07 | 5 |

**Adduct Signal Fractions**

- **Unmodified**: 5.9327e+07

### FYFTPN(N)KTEDTIF Fuc 1 Hex 7 HexNAc 4 Neu5Ac 2

**Mass:**  4296.7092

(1.034e-06)

**Charge States:** 3, 4

**Start Position**: 67

**End Position**: 80

| Profile Score | Tandem Score | TDA q-value | Volume | Spectrum Matches |
| --- | --- | --- | --- | --- |
| -17.0413 | 145.3099 | 0.0040 | 3.5729e+07 | 2 |

**Adduct Signal Fractions**

- **Unmodified**: 3.5729e+07

### FYFTPN(N)KTEDTIF Fuc 1 Hex 8 HexNAc 7

**Mass:**  4485.8102

(1.421e-06)

**Charge States:** 3, 4

**Start Position**: 67

**End Position**: 80

| Profile Score | Tandem Score | TDA q-value | Volume | Spectrum Matches |
| --- | --- | --- | --- | --- |
| 15.1126 | 144.1528 | 0.0040 | 1.2690e+08 | 5 |

**Adduct Signal Fractions**

- **Unmodified**: 1.2690e+08

### RNEEYN(N)KSVQEIQAT Hex 6 HexNAc 5

**Mass:**  3795.5905

(1.736e-06)

**Charge States:** 3, 4

**Start Position**: 51

**End Position**: 66

| Profile Score | Tandem Score | TDA q-value | Volume | Spectrum Matches |
| --- | --- | --- | --- | --- |
| -19.7881 | 142.4253 | 0.0077 | 2.3234e+07 | 2 |

**Adduct Signal Fractions**

- **Unmodified**: 2.3234e+07

### RNEEYN(N)KSVQEIQATFF Fuc 1 Hex 7 HexNAc 6

**Mass:**  4600.9191

(1.179e-06)

**Charge States:** 4

**Start Position**: 51

**End Position**: 68

| Profile Score | Tandem Score | TDA q-value | Volume | Spectrum Matches |
| --- | --- | --- | --- | --- |
| -3.0656 | 142.1167 | 0.0077 | 6.1450e+07 | 3 |

**Adduct Signal Fractions**

- **Unmodified**: 6.1450e+07

### RNEEYN(N)KSVQEIQATF Fuc 1 Hex 7 HexNAc 6

**Mass:**  4453.8503

(1.783e-06)

**Charge States:** 4

**Start Position**: 51

**End Position**: 67

| Profile Score | Tandem Score | TDA q-value | Volume | Spectrum Matches |
| --- | --- | --- | --- | --- |
| -3.2859 | 142.0348 | 0.0077 | 1.1665e+08 | 2 |

**Adduct Signal Fractions**

- **Unmodified**: 1.1665e+08

### EEYN(N)KSVQEIQATFF Hex 6 HexNAc 5

**Mass:**  3819.5824

(-1.418e-06)

**Charge States:** 3

**Start Position**: 53

**End Position**: 68

| Profile Score | Tandem Score | TDA q-value | Volume | Spectrum Matches |
| --- | --- | --- | --- | --- |
| -4.0990 | 141.6199 | 0.0077 | 2.5810e+07 | 3 |

**Adduct Signal Fractions**

- **Unmodified**: 2.5810e+07

### RNEEYN(N)KSVQEIQATF Hex 4 HexNAc 3

**Mass:**  3213.4159

(3.200e-04)

**Start Position**: 51

**End Position**: 67

| Profile Score | Tandem Score | TDA q-value | Volume | Spectrum Matches |
| --- | --- | --- | --- | --- |
| 0.0000 | 141.3875 | 0.0077 | 0.0000e+00 | 1 |

Adduct:

No Chromatogram Found

### LNVQREN(N)GTISRY Hex 5 HexNAc 4

**Mass:**  3171.3836

(3.462e-07)

**Charge States:** 3, 4

**Start Position**: 97

**End Position**: 110

| Profile Score | Tandem Score | TDA q-value | Volume | Spectrum Matches |
| --- | --- | --- | --- | --- |
| 13.5731 | 141.2681 | 0.0077 | 2.4027e+08 | 3 |

**Adduct Signal Fractions**

- **Unmodified**: 2.4027e+08

### YFTPN(N)KTEDTIFL Hex 6 HexNAc 5

**Mass:**  3575.5003

(2.305e-06)

**Charge States:** 3

**Start Position**: 68

**End Position**: 81

| Profile Score | Tandem Score | TDA q-value | Volume | Spectrum Matches |
| --- | --- | --- | --- | --- |
| -3.2875 | 140.1029 | 0.0099 | 2.4776e+07 | 3 |

**Adduct Signal Fractions**

- **Unmodified**: 2.4776e+07

### YFTPN(N)KTEDTIF Fuc 2 Hex 6 HexNAc 5

**Mass:**  3754.5364

(1.044e-06)

**Charge States:** 4

**Start Position**: 68

**End Position**: 80

| Profile Score | Tandem Score | TDA q-value | Volume | Spectrum Matches |
| --- | --- | --- | --- | --- |
| -6.4028 | 140.0932 | 0.0099 | 7.2855e+06 | 1 |

**Adduct Signal Fractions**

- **Unmodified**: 7.2855e+06

### TPN(N)KTEDTIF Hex 7 HexNAc 4 Neu5Ac 2

**Mass:**  3693.4462

(3.709e-07)

**Charge States:** 3, 4

**Start Position**: 70

**End Position**: 80

| Profile Score | Tandem Score | TDA q-value | Volume | Spectrum Matches |
| --- | --- | --- | --- | --- |
| -3.9009 | 137.3589 | 0.0099 | 1.1308e+07 | 4 |

**Adduct Signal Fractions**

- **Unmodified**: 1.1308e+07

### N(N)KTEDTIF Hex 6 HexNAc 5

**Mass:**  2954.1879

(2.777e-06)

**Charge States:** 3

**Start Position**: 72

**End Position**: 80

| Profile Score | Tandem Score | TDA q-value | Volume | Spectrum Matches |
| --- | --- | --- | --- | --- |
| -20.5515 | 137.0759 | 0.0000 | 2.9222e+06 | 1 |

**Adduct Signal Fractions**

- **Unmodified**: 2.9222e+06

### YFTPN(N)KTEDTIF Hex 10 HexNAc 9

**Mass:**  4922.9514

(1.512e-06)

**Charge States:** 4

**Start Position**: 68

**End Position**: 80

| Profile Score | Tandem Score | TDA q-value | Volume | Spectrum Matches |
| --- | --- | --- | --- | --- |
| -3.2018 | 136.4233 | 0.0099 | 2.3477e+07 | 1 |

**Adduct Signal Fractions**

- **Unmodified**: 2.3477e+07

### RNEEYN(N)KSVQEIQAT Hex 5 HexNAc 4

**Mass:**  3430.4562

(-2.197e-06)

**Charge States:** 3, 4

**Start Position**: 51

**End Position**: 66

| Profile Score | Tandem Score | TDA q-value | Volume | Spectrum Matches |
| --- | --- | --- | --- | --- |
| -4.9786 | 135.0718 | 0.0099 | 3.5961e+07 | 3 |

**Adduct Signal Fractions**

- **Unmodified**: 3.5961e+07

### YFTPN(N)KT Hex 7 HexNAc 6

**Mass:**  3222.2832

(2.722e-06)

**Charge States:** 3

**Start Position**: 68

**End Position**: 75

| Profile Score | Tandem Score | TDA q-value | Volume | Spectrum Matches |
| --- | --- | --- | --- | --- |
| -21.2077 | 131.7191 | 0.0000 | 2.6525e+06 | 1 |

**Adduct Signal Fractions**

- **Unmodified**: 2.6525e+06

### TPN(N)KTEDTIF Fuc 4 Hex 8 HexNAc 5

**Mass:**  4060.6040

(-3.406e-06)

**Charge States:** 3, 4

**Start Position**: 70

**End Position**: 80

| Profile Score | Tandem Score | TDA q-value | Volume | Spectrum Matches |
| --- | --- | --- | --- | --- |
| 13.1320 | 131.3275 | 0.0100 | 1.3515e+08 | 4 |

**Adduct Signal Fractions**

- **Unmodified**: 1.3515e+08

### YFTPN(N)KTEDTIFL Hex 7 HexNAc 6

**Mass:**  3940.6320

(1.698e-06)

**Charge States:** 3, 4

**Start Position**: 68

**End Position**: 81

| Profile Score | Tandem Score | TDA q-value | Volume | Spectrum Matches |
| --- | --- | --- | --- | --- |
| -4.0426 | 130.1405 | 0.0100 | 4.3605e+07 | 3 |

**Adduct Signal Fractions**

- **Unmodified**: 4.3605e+07

### FTPN(N)KTEDTIF Hex 9 HexNAc 8

**Mass:**  4394.7541

(2.616e-06)

**Charge States:** 4

**Start Position**: 69

**End Position**: 80

| Profile Score | Tandem Score | TDA q-value | Volume | Spectrum Matches |
| --- | --- | --- | --- | --- |
| 14.1987 | 129.9716 | 0.0100 | 1.2377e+07 | 2 |

**Adduct Signal Fractions**

- **Unmodified**: 1.2377e+07

### RNEEYN(N)KSVQEIQATF Hex 8 HexNAc 7

**Mass:**  4672.9226

(1.232e-06)

**Charge States:** 4

**Start Position**: 51

**End Position**: 67

| Profile Score | Tandem Score | TDA q-value | Volume | Spectrum Matches |
| --- | --- | --- | --- | --- |
| -3.8654 | 129.8300 | 0.0100 | 6.2149e+07 | 2 |

**Adduct Signal Fractions**

- **Unmodified**: 6.2149e+07

### FRNEEYN(N)KSVQEIQATF Hex 6 HexNAc 5

**Mass:**  4089.7270

(1.520e-06)

**Charge States:** 4

**Start Position**: 50

**End Position**: 67

| Profile Score | Tandem Score | TDA q-value | Volume | Spectrum Matches |
| --- | --- | --- | --- | --- |
| 11.2831 | 129.7472 | 0.0100 | 4.3981e+07 | 3 |

**Adduct Signal Fractions**

- **Unmodified**: 4.3981e+07

### LNVQREN(N)GTISRY Fuc 2 Hex 7 HexNAc 6

**Mass:**  4193.7717

(1.747e-06)

**Charge States:** 3, 4

**Start Position**: 97

**End Position**: 110

| Profile Score | Tandem Score | TDA q-value | Volume | Spectrum Matches |
| --- | --- | --- | --- | --- |
| 14.0135 | 129.6174 | 0.0100 | 1.0271e+09 | 7 |

**Adduct Signal Fractions**

- **Unmodified**: 1.0271e+09

### VQREN(N)GTISRY Hex 7 HexNAc 6

**Mass:**  3674.5222

(-2.706e-07)

**Charge States:** 3, 4

**Start Position**: 99

**End Position**: 110

| Profile Score | Tandem Score | TDA q-value | Volume | Spectrum Matches |
| --- | --- | --- | --- | --- |
| -18.7815 | 129.3528 | 0.0100 | 2.9394e+07 | 3 |

**Adduct Signal Fractions**

- **Unmodified**: 2.9394e+07

### FTPN(N)KTEDTIF Hex 7 HexNAc 4 Neu5Ac 2

**Mass:**  3840.5139

(1.415e-06)

**Charge States:** 3, 4

**Start Position**: 69

**End Position**: 80

| Profile Score | Tandem Score | TDA q-value | Volume | Spectrum Matches |
| --- | --- | --- | --- | --- |
| -4.0210 | 129.1882 | 0.0100 | 3.0471e+07 | 4 |

**Adduct Signal Fractions**

- **Unmodified**: 3.0471e+07

### FYFTPN(N)KT Hex 6 HexNAc 5

**Mass:**  3004.2147

(2.393e-06)

**Charge States:** 3

**Start Position**: 67

**End Position**: 75

| Profile Score | Tandem Score | TDA q-value | Volume | Spectrum Matches |
| --- | --- | --- | --- | --- |
| -3.5635 | 128.4357 | 0.0000 | 6.2823e+06 | 2 |

**Adduct Signal Fractions**

- **Unmodified**: 6.2823e+06

### N(N)KTEDTIF Fuc 5 Hex 5 HexNAc 8 Neu5Ac 1

**Mass:**  4425.7269

(6.731e-04)

**Start Position**: 72

**End Position**: 80

| Profile Score | Tandem Score | TDA q-value | Volume | Spectrum Matches |
| --- | --- | --- | --- | --- |
| 0.0000 | 128.2914 | 0.0000 | 0.0000e+00 | 1 |

Adduct:

No Chromatogram Found

### NVQREN(N)GTISRY Fuc 1 Hex 6 HexNAc 5

**Mass:**  3569.4904

(2.636e-06)

**Charge States:** 4

**Start Position**: 98

**End Position**: 110

| Profile Score | Tandem Score | TDA q-value | Volume | Spectrum Matches |
| --- | --- | --- | --- | --- |
| -4.8889 | 126.1260 | 0.0100 | 4.3766e+07 | 4 |

**Adduct Signal Fractions**

- **Unmodified**: 4.3766e+07

### LNVQREN(N)GTISRY Hex 9 HexNAc 8

**Mass:**  4631.9279

(1.323e-06)

**Charge States:** 3, 4

**Start Position**: 97

**End Position**: 110

| Profile Score | Tandem Score | TDA q-value | Volume | Spectrum Matches |
| --- | --- | --- | --- | --- |
| 14.9124 | 126.1221 | 0.0100 | 1.0048e+09 | 7 |

**Adduct Signal Fractions**

- **Unmodified**: 1.0048e+09

### QTRQDQC(C)IYN(N)TTY Hex 7 HexNAc 6

**Mass:**  4042.5954

(1.896e-06)

**Charge States:** 3, 4

**Start Position**: 84

**End Position**: 97

| Profile Score | Tandem Score | TDA q-value | Volume | Spectrum Matches |
| --- | --- | --- | --- | --- |
| 14.4387 | 125.4593 | 0.0100 | 2.2703e+08 | 8 |

**Adduct Signal Fractions**

- **Unmodified**: 2.2703e+08

### LNVQREN(N)GTISRY Hex 7 HexNAc 6

**Mass:**  3901.6484

(3.159e-06)

**Charge States:** 3, 4

**Start Position**: 97

**End Position**: 110

| Profile Score | Tandem Score | TDA q-value | Volume | Spectrum Matches |
| --- | --- | --- | --- | --- |
| 16.4852 | 125.2555 | 0.0100 | 8.5985e+09 | 13 |

**Adduct Signal Fractions**

- **Unmodified**: 8.5985e+09

### YFTPN(N)KTEDTIF Fuc 2 Hex 8 HexNAc 7

**Mass:**  4484.8034

(1.641e-06)

**Charge States:** 4

**Start Position**: 68

**End Position**: 80

| Profile Score | Tandem Score | TDA q-value | Volume | Spectrum Matches |
| --- | --- | --- | --- | --- |
| 12.7236 | 125.2408 | 0.0100 | 4.1671e+07 | 2 |

**Adduct Signal Fractions**

- **Unmodified**: 4.1671e+07

### LNVQREN(N)GTISRY Hex 8 HexNAc 7

**Mass:**  4266.7941

(1.090e-06)

**Charge States:** 3, 4

**Start Position**: 97

**End Position**: 110

| Profile Score | Tandem Score | TDA q-value | Volume | Spectrum Matches |
| --- | --- | --- | --- | --- |
| 14.2357 | 125.1878 | 0.0100 | 1.0594e+09 | 6 |

**Adduct Signal Fractions**

- **Unmodified**: 1.0594e+09

### NVQREN(N)GTISRY Hex 6 HexNAc 5

**Mass:**  3423.4341

(1.003e-06)

**Charge States:** 3

**Start Position**: 98

**End Position**: 110

| Profile Score | Tandem Score | TDA q-value | Volume | Spectrum Matches |
| --- | --- | --- | --- | --- |
| 13.2459 | 125.0345 | 0.0100 | 1.5826e+08 | 5 |

**Adduct Signal Fractions**

- **Unmodified**: 1.5826e+08

### NVQREN(N)GTISRY Hex 7 HexNAc 6

**Mass:**  3788.5717

(1.077e-06)

**Charge States:** 3, 4

**Start Position**: 98

**End Position**: 110

| Profile Score | Tandem Score | TDA q-value | Volume | Spectrum Matches |
| --- | --- | --- | --- | --- |
| 12.7518 | 124.6346 | 0.0100 | 5.5109e+08 | 6 |

**Adduct Signal Fractions**

- **Unmodified**: 5.5109e+08

### FYFTPN(N)KTEDTIF Hex 3 HexNAc 5 Neu5Ac 3

**Mass:**  3996.5952

(-2.641e-06)

**Charge States:** 3, 4

**Start Position**: 67

**End Position**: 80

| Profile Score | Tandem Score | TDA q-value | Volume | Spectrum Matches |
| --- | --- | --- | --- | --- |
| 13.6975 | 124.4576 | 0.0100 | 2.4449e+08 | 3 |

**Adduct Signal Fractions**

- **Unmodified**: 2.4449e+08

### YFTPN(N)KTEDTIF Fuc 1 Hex 8 HexNAc 4 Neu5Ac 1

**Mass:**  4020.6062

(3.669e-06)

**Charge States:** 3, 4

**Start Position**: 68

**End Position**: 80

| Profile Score | Tandem Score | TDA q-value | Volume | Spectrum Matches |
| --- | --- | --- | --- | --- |
| -19.5076 | 124.3871 | 0.0100 | 2.5230e+07 | 2 |

**Adduct Signal Fractions**

- **Unmodified**: 2.5230e+07

### LN(D)VQREN(N)GTISRY Hex 7 HexNAc 6

**Mass:**  3902.6435

(2.553e-04)

**Charge States:** 3, 4

**Start Position**: 97

**End Position**: 110

| Profile Score | Tandem Score | TDA q-value | Volume | Spectrum Matches |
| --- | --- | --- | --- | --- |
| 8.3320 | 123.9398 | 0.0100 | 4.4923e+08 | 54 |

**Adduct Signal Fractions**

- **Unmodified**: 4.4923e+08

### RN(D)EEYN(N)KSVQEIQATF Hex 5 HexNAc 4

**Mass:**  3578.5102

(2.764e-04)

**Charge States:** 3, 4

**Start Position**: 51

**End Position**: 67

| Profile Score | Tandem Score | TDA q-value | Volume | Spectrum Matches |
| --- | --- | --- | --- | --- |
| 10.8334 | 123.9156 | 0.0100 | 1.3552e+09 | 30 |

**Adduct Signal Fractions**

- **Unmodified**: 1.3552e+09

### FTPN(N)KTEDTIF Fuc 2 Hex 7 HexNAc 6

**Mass:**  3956.6020

(3.544e-06)

**Charge States:** 3, 4

**Start Position**: 69

**End Position**: 80

| Profile Score | Tandem Score | TDA q-value | Volume | Spectrum Matches |
| --- | --- | --- | --- | --- |
| -4.4939 | 123.6694 | 0.0100 | 1.5003e+07 | 4 |

**Adduct Signal Fractions**

- **Unmodified**: 1.5003e+07

### FYFTPN(N)KTEDTIF Fuc 2 Hex 8 HexNAc 7

**Mass:**  4631.8664

(2.524e-06)

**Charge States:** 4

**Start Position**: 67

**End Position**: 80

| Profile Score | Tandem Score | TDA q-value | Volume | Spectrum Matches |
| --- | --- | --- | --- | --- |
| -3.8249 | 123.0417 | 0.0100 | 2.3872e+07 | 2 |

**Adduct Signal Fractions**

- **Unmodified**: 2.3872e+07

### NVQREN(N)GTISRY Fuc 2 Hex 7 HexNAc 6

**Mass:**  4080.6909

(2.990e-06)

**Charge States:** 4

**Start Position**: 98

**End Position**: 110

| Profile Score | Tandem Score | TDA q-value | Volume | Spectrum Matches |
| --- | --- | --- | --- | --- |
| -4.3436 | 122.9535 | 0.0100 | 6.0061e+07 | 3 |

**Adduct Signal Fractions**

- **Unmodified**: 6.0061e+07

### RN(D)EEYN(N)KSVQEIQATF Hex 6 HexNAc 5

**Mass:**  3943.6415

(2.509e-04)

**Charge States:** 3, 4

**Start Position**: 51

**End Position**: 67

| Profile Score | Tandem Score | TDA q-value | Volume | Spectrum Matches |
| --- | --- | --- | --- | --- |
| 10.7640 | 121.6380 | 0.0100 | 1.2103e+09 | 13 |

**Adduct Signal Fractions**

- **Unmodified**: 1.2103e+09

### TPN(N)KTEDTIF Fuc 3 Hex 3 HexNAc 9 Neu5Ac 1

**Mass:**  4207.6721

(-9.919e-06)

**Charge States:** 3, 4

**Start Position**: 70

**End Position**: 80

| Profile Score | Tandem Score | TDA q-value | Volume | Spectrum Matches |
| --- | --- | --- | --- | --- |
| 9.4875 | 121.5172 | 0.0100 | 1.0897e+08 | 5 |

**Adduct Signal Fractions**

- **Unmodified**: 1.0897e+08

### FYFTPN(N)KTEDTIF Hex 7 HexNAc 5

**Mass:**  3771.5546

(5.733e-06)

**Charge States:** 4

**Start Position**: 67

**End Position**: 80

| Profile Score | Tandem Score | TDA q-value | Volume | Spectrum Matches |
| --- | --- | --- | --- | --- |
| -20.3197 | 121.2058 | 0.0100 | 1.3760e+07 | 1 |

**Adduct Signal Fractions**

- **Unmodified**: 1.3760e+07

### NVQREN(N)GTISRY Fuc 1 Hex 7 HexNAc 6

**Mass:**  3934.6308

(1.963e-06)

**Charge States:** 3, 4

**Start Position**: 98

**End Position**: 110

| Profile Score | Tandem Score | TDA q-value | Volume | Spectrum Matches |
| --- | --- | --- | --- | --- |
| 12.4420 | 120.7569 | 0.0100 | 1.7939e+08 | 5 |

**Adduct Signal Fractions**

- **Unmodified**: 1.7939e+08

### FYFTPN(N)KTEDTIF Hex 8 HexNAc 6

**Mass:**  4137.7014

(2.509e-04)

**Charge States:** 4

**Start Position**: 67

**End Position**: 80

| Profile Score | Tandem Score | TDA q-value | Volume | Spectrum Matches |
| --- | --- | --- | --- | --- |
| -20.5648 | 120.6863 | 0.0100 | 9.0743e+07 | 5 |

**Adduct Signal Fractions**

- **Unmodified**: 9.0743e+07

### LNVQREN(N)GTISRY Fuc 3 Hex 7 HexNAc 6

**Mass:**  4339.8298

(1.039e-06)

**Charge States:** 3, 4

**Start Position**: 97

**End Position**: 110

| Profile Score | Tandem Score | TDA q-value | Volume | Spectrum Matches |
| --- | --- | --- | --- | --- |
| 10.1645 | 119.9296 | 0.0100 | 2.6107e+08 | 3 |

**Adduct Signal Fractions**

- **Unmodified**: 2.6107e+08

### FTPN(N)KTEDTIF Fuc 1 Hex 8 HexNAc 7

**Mass:**  4175.6760

(1.723e-06)

**Charge States:** 3, 4

**Start Position**: 69

**End Position**: 80

| Profile Score | Tandem Score | TDA q-value | Volume | Spectrum Matches |
| --- | --- | --- | --- | --- |
| 10.7139 | 119.5888 | 0.0100 | 1.0416e+07 | 4 |

**Adduct Signal Fractions**

- **Unmodified**: 1.0416e+07

### LNVQREN(N)GTISRY Fuc 1 Hex 7 HexNAc 6

**Mass:**  4047.7153

(1.973e-06)

**Charge States:** 3, 4

**Start Position**: 97

**End Position**: 110

| Profile Score | Tandem Score | TDA q-value | Volume | Spectrum Matches |
| --- | --- | --- | --- | --- |
| 15.2073 | 119.2124 | 0.0100 | 3.2397e+09 | 10 |

**Adduct Signal Fractions**

- **Unmodified**: 3.2397e+09

### LNVQREN(N)GTISRY Fuc 2 Hex 8 HexNAc 7

**Mass:**  4558.9138

(1.521e-06)

**Charge States:** 4

**Start Position**: 97

**End Position**: 110

| Profile Score | Tandem Score | TDA q-value | Volume | Spectrum Matches |
| --- | --- | --- | --- | --- |
| 15.3023 | 119.0562 | 0.0100 | 2.0823e+08 | 4 |

**Adduct Signal Fractions**

- **Unmodified**: 2.0823e+08

### QTRQDQC(C)IYN(N)TTY Hex 6 HexNAc 5

**Mass:**  3677.4607

(1.451e-06)

**Charge States:** 3, 4

**Start Position**: 84

**End Position**: 97

| Profile Score | Tandem Score | TDA q-value | Volume | Spectrum Matches |
| --- | --- | --- | --- | --- |
| 14.6820 | 119.0051 | 0.0100 | 1.2395e+08 | 7 |

**Adduct Signal Fractions**

- **Unmodified**: 1.2395e+08

### N(N)KSVQEIQATFF Hex 6 HexNAc 5

**Mass:**  3398.4325

(4.159e-07)

**Charge States:** 3, 4

**Start Position**: 56

**End Position**: 68

| Profile Score | Tandem Score | TDA q-value | Volume | Spectrum Matches |
| --- | --- | --- | --- | --- |
| -18.1454 | 118.5895 | 0.0100 | 5.0156e+06 | 2 |

**Adduct Signal Fractions**

- **Unmodified**: 5.0156e+06

### RN(D)EEYN(N)KSVQEIQATFF Hex 5 HexNAc 4

**Mass:**  3725.5749

(2.640e-04)

**Charge States:** 3, 4

**Start Position**: 51

**End Position**: 68

| Profile Score | Tandem Score | TDA q-value | Volume | Spectrum Matches |
| --- | --- | --- | --- | --- |
| 10.5914 | 117.0803 | 0.0122 | 3.1034e+08 | 10 |

**Adduct Signal Fractions**

- **Unmodified**: 3.1034e+08

### RNEEYN(N)KSVQEIQATF Hex 9 HexNAc 8

**Mass:**  5038.0578

(1.809e-06)

**Charge States:** 4

**Start Position**: 51

**End Position**: 67

| Profile Score | Tandem Score | TDA q-value | Volume | Spectrum Matches |
| --- | --- | --- | --- | --- |
| -3.4352 | 116.4220 | 0.0122 | 3.5805e+07 | 2 |

**Adduct Signal Fractions**

- **Unmodified**: 3.5805e+07

### RN(D)EEYN(N)KSVQEIQATFF Fuc 1 Hex 6 HexNAc 5

**Mass:**  4236.7717

(2.359e-04)

**Charge States:** 3, 4

**Start Position**: 51

**End Position**: 68

| Profile Score | Tandem Score | TDA q-value | Volume | Spectrum Matches |
| --- | --- | --- | --- | --- |
| 9.7227 | 116.3355 | 0.0143 | 1.6873e+08 | 11 |

**Adduct Signal Fractions**

- **Unmodified**: 1.6873e+08

### LVPVPITN(N)ATL Hex 6 HexNAc 5

**Mass:**  3124.3972

(9.338e-07)

**Charge States:** 4

**Start Position**: 26

**End Position**: 37

| Profile Score | Tandem Score | TDA q-value | Volume | Spectrum Matches |
| --- | --- | --- | --- | --- |
| -4.6337 | 116.2614 | 0.0143 | 7.2858e+06 | 2 |

**Adduct Signal Fractions**

- **Unmodified**: 7.2858e+06

### LN(D)VQREN(N)GTISRY Fuc 1 Hex 7 HexNAc 6

**Mass:**  4048.7004

(2.462e-04)

**Charge States:** 3, 4

**Start Position**: 97

**End Position**: 110

| Profile Score | Tandem Score | TDA q-value | Volume | Spectrum Matches |
| --- | --- | --- | --- | --- |
| 18.7911 | 115.6119 | 0.0162 | 1.2947e+08 | 8 |

**Adduct Signal Fractions**

- **Unmodified**: 1.2947e+08

### YFTPN(N)KT Fuc 5 Hex 7 HexNAc 9

**Mass:**  4563.8441

(4.477e-04)

**Start Position**: 68

**End Position**: 75

| Profile Score | Tandem Score | TDA q-value | Volume | Spectrum Matches |
| --- | --- | --- | --- | --- |
| 0.0000 | 112.1439 | 0.0000 | 0.0000e+00 | 1 |

Adduct:

No Chromatogram Found

### LNVQREN(N)GTISRY Fuc 2 Hex 6 HexNAc 5

**Mass:**  3828.6391

(8.460e-07)

**Charge States:** 3, 4

**Start Position**: 97

**End Position**: 110

| Profile Score | Tandem Score | TDA q-value | Volume | Spectrum Matches |
| --- | --- | --- | --- | --- |
| 10.6283 | 111.3796 | 0.0199 | 8.7813e+07 | 2 |

**Adduct Signal Fractions**

- **Unmodified**: 8.7813e+07

### LNVQREN(N)GTISRY Fuc 1 Hex 4 HexNAc 5 Neu5Ac 3

**Mass:**  4231.7184

(-8.016e-06)

**Charge States:** 4, 5

**Start Position**: 97

**End Position**: 110

| Profile Score | Tandem Score | TDA q-value | Volume | Spectrum Matches |
| --- | --- | --- | --- | --- |
| -4.2012 | 110.9966 | 0.0199 | 6.0617e+07 | 2 |

**Adduct Signal Fractions**

- **Unmodified**: 6.0617e+07

### RN(D)EEYN(N)KSVQEIQATFF Hex 6 HexNAc 5

**Mass:**  4090.7089

(2.416e-04)

**Charge States:** 3, 4

**Start Position**: 51

**End Position**: 68

| Profile Score | Tandem Score | TDA q-value | Volume | Spectrum Matches |
| --- | --- | --- | --- | --- |
| 9.9262 | 110.5026 | 0.0211 | 3.2717e+08 | 14 |

**Adduct Signal Fractions**

- **Unmodified**: 3.2717e+08

### EEYN(N)KSVQEIQATFF Hex 5 HexNAc 4

**Mass:**  3454.4470

(7.192e-07)

**Charge States:** 3

**Start Position**: 53

**End Position**: 68

| Profile Score | Tandem Score | TDA q-value | Volume | Spectrum Matches |
| --- | --- | --- | --- | --- |
| -4.6133 | 110.0468 | 0.0211 | 2.1525e+07 | 3 |

**Adduct Signal Fractions**

- **Unmodified**: 2.1525e+07

### RNEEYN(N)KSVQEIQATFF Hex 8 HexNAc 7

**Mass:**  4819.9924

(1.890e-06)

**Charge States:** 4

**Start Position**: 51

**End Position**: 68

| Profile Score | Tandem Score | TDA q-value | Volume | Spectrum Matches |
| --- | --- | --- | --- | --- |
| -2.9200 | 109.6547 | 0.0211 | 1.6895e+07 | 2 |

**Adduct Signal Fractions**

- **Unmodified**: 1.6895e+07

### FYFTPN(N)KTED Hex 7 HexNAc 6

**Mass:**  3613.4170

(1.357e-06)

**Charge States:** 4

**Start Position**: 67

**End Position**: 77

| Profile Score | Tandem Score | TDA q-value | Volume | Spectrum Matches |
| --- | --- | --- | --- | --- |
| -4.7781 | 109.6483 | 0.0211 | 5.2527e+06 | 2 |

**Adduct Signal Fractions**

- **Unmodified**: 5.2527e+06

### QTRQDQC(C)IYN(N)TTY Fuc 1 Hex 6 HexNAc 5

**Mass:**  3823.5185

(1.817e-06)

**Charge States:** 3, 4

**Start Position**: 84

**End Position**: 97

| Profile Score | Tandem Score | TDA q-value | Volume | Spectrum Matches |
| --- | --- | --- | --- | --- |
| 13.9414 | 109.4381 | 0.0211 | 3.5560e+07 | 3 |

**Adduct Signal Fractions**

- **Unmodified**: 3.5560e+07

### FYFTPN(N)KTEDTIF Hex 10 HexNAc 9

**Mass:**  5070.0163

(1.545e-06)

**Charge States:** 4

**Start Position**: 67

**End Position**: 80

| Profile Score | Tandem Score | TDA q-value | Volume | Spectrum Matches |
| --- | --- | --- | --- | --- |
| -3.9078 | 108.6449 | 0.0211 | 1.7746e+07 | 2 |

**Adduct Signal Fractions**

- **Unmodified**: 1.7746e+07

### LN(D)VQREN(N)GTISRY Fuc 3 Hex 5 HexNAc 4 Neu5Ac 1

**Mass:**  3901.6501

(2.560e-04)

**Charge States:** 4

**Start Position**: 97

**End Position**: 110

| Profile Score | Tandem Score | TDA q-value | Volume | Spectrum Matches |
| --- | --- | --- | --- | --- |
| -21.3564 | 108.4713 | 0.0230 | 1.2041e+07 | 1 |

**Adduct Signal Fractions**

- **Unmodified**: 1.2041e+07

### NVQREN(N)GTISRY Hex 9 HexNAc 8

**Mass:**  4518.8303

(6.706e-07)

**Charge States:** 4

**Start Position**: 98

**End Position**: 110

| Profile Score | Tandem Score | TDA q-value | Volume | Spectrum Matches |
| --- | --- | --- | --- | --- |
| -18.2771 | 107.0466 | 0.0286 | 3.8784e+07 | 1 |

**Adduct Signal Fractions**

- **Unmodified**: 3.8784e+07

### LNVQREN(N)GTISRY Fuc 1 Hex 8 HexNAc 7

**Mass:**  4412.8478

(1.910e-06)

**Charge States:** 3, 4

**Start Position**: 97

**End Position**: 110

| Profile Score | Tandem Score | TDA q-value | Volume | Spectrum Matches |
| --- | --- | --- | --- | --- |
| 15.2185 | 106.7567 | 0.0286 | 4.2547e+08 | 6 |

**Adduct Signal Fractions**

- **Unmodified**: 4.2547e+08

### LNVQREN(N)GTISRY Fuc 1 Hex 9 HexNAc 8

**Mass:**  4777.9802

(2.029e-06)

**Charge States:** 3, 4

**Start Position**: 97

**End Position**: 110

| Profile Score | Tandem Score | TDA q-value | Volume | Spectrum Matches |
| --- | --- | --- | --- | --- |
| 16.3814 | 105.9988 | 0.0299 | 3.0042e+08 | 3 |

**Adduct Signal Fractions**

- **Unmodified**: 3.0042e+08

### LNVQREN(N)GTISRY Fuc 3 Hex 8 HexNAc 7

**Mass:**  4704.9597

(1.514e-06)

**Charge States:** 4

**Start Position**: 97

**End Position**: 110

| Profile Score | Tandem Score | TDA q-value | Volume | Spectrum Matches |
| --- | --- | --- | --- | --- |
| -3.1703 | 103.3802 | 0.0331 | 8.3439e+07 | 2 |

**Adduct Signal Fractions**

- **Unmodified**: 8.3439e+07

### LNVQREN(N)GTISRY Hex 10 HexNAc 4

**Mass:**  3981.6137

(-8.274e-06)

**Charge States:** 3, 4

**Start Position**: 97

**End Position**: 110

| Profile Score | Tandem Score | TDA q-value | Volume | Spectrum Matches |
| --- | --- | --- | --- | --- |
| 12.3169 | 103.3690 | 0.0331 | 3.5350e+07 | 5 |

**Adduct Signal Fractions**

- **Unmodified**: 3.5350e+07

### TPN(N)KTEDTIF Hex 9 HexNAc 5

**Mass:**  3638.4483

(2.691e-06)

**Charge States:** 3, 4

**Start Position**: 70

**End Position**: 80

| Profile Score | Tandem Score | TDA q-value | Volume | Spectrum Matches |
| --- | --- | --- | --- | --- |
| 13.5482 | 102.0374 | 0.0345 | 2.7809e+07 | 4 |

**Adduct Signal Fractions**

- **Unmodified**: 2.7809e+07

### LNVQREN(N)GTISRY Fuc 1 Hex 7 HexNAc 4 Neu5Ac 2

**Mass:**  4223.7458

(2.176e-06)

**Charge States:** 3, 4

**Start Position**: 97

**End Position**: 110

| Profile Score | Tandem Score | TDA q-value | Volume | Spectrum Matches |
| --- | --- | --- | --- | --- |
| 13.5026 | 101.7928 | 0.0345 | 1.1187e+08 | 3 |

**Adduct Signal Fractions**

- **Unmodified**: 1.1187e+08

### C(C)ANLVPVPITN(N)ATL Hex 6 HexNAc 5

**Mass:**  3469.5100

(1.431e-06)

**Charge States:** 3, 4

**Start Position**: 23

**End Position**: 37

| Profile Score | Tandem Score | TDA q-value | Volume | Spectrum Matches |
| --- | --- | --- | --- | --- |
| 12.3497 | 101.7927 | 0.0345 | 2.3070e+07 | 6 |

**Adduct Signal Fractions**

- **Unmodified**: 2.3070e+07

### VQREN(N)GTISRY Hex 6 HexNAc 5

**Mass:**  3309.4022

(4.376e-06)

**Charge States:** 3

**Start Position**: 99

**End Position**: 110

| Profile Score | Tandem Score | TDA q-value | Volume | Spectrum Matches |
| --- | --- | --- | --- | --- |
| -20.7635 | 101.5029 | 0.0414 | 5.5580e+06 | 1 |

**Adduct Signal Fractions**

- **Unmodified**: 5.5580e+06

### NVQREN(N)GTISRY Hex 8 HexNAc 7

**Mass:**  4153.7053

(1.408e-06)

**Charge States:** 4

**Start Position**: 98

**End Position**: 110

| Profile Score | Tandem Score | TDA q-value | Volume | Spectrum Matches |
| --- | --- | --- | --- | --- |
| -19.0530 | 101.2620 | 0.0414 | 4.3533e+07 | 2 |

**Adduct Signal Fractions**

- **Unmodified**: 4.3533e+07

### LNVQREN(N)GTISRY Hex 10 HexNAc 9

**Mass:**  4997.0500

(1.216e-06)

**Charge States:** 4

**Start Position**: 97

**End Position**: 110

| Profile Score | Tandem Score | TDA q-value | Volume | Spectrum Matches |
| --- | --- | --- | --- | --- |
| -2.7937 | 101.1939 | 0.0414 | 1.4608e+08 | 3 |

**Adduct Signal Fractions**

- **Unmodified**: 1.4608e+08

### LNVQREN(N)GTISRY Fuc 2 Hex 9 HexNAc 8

**Mass:**  4924.0363

(1.770e-06)

**Charge States:** 4

**Start Position**: 97

**End Position**: 110

| Profile Score | Tandem Score | TDA q-value | Volume | Spectrum Matches |
| --- | --- | --- | --- | --- |
| -3.5441 | 100.8961 | 0.0414 | 1.1214e+08 | 3 |

**Adduct Signal Fractions**

- **Unmodified**: 1.1214e+08

### RNEEYN(N)KSVQEIQAT Fuc 2 Hex 5 HexNAc 4

**Mass:**  3722.5687

(3.766e-07)

**Charge States:** 3, 4

**Start Position**: 51

**End Position**: 66

| Profile Score | Tandem Score | TDA q-value | Volume | Spectrum Matches |
| --- | --- | --- | --- | --- |
| -19.5450 | 99.9575 | 0.0463 | 2.0882e+07 | 1 |

**Adduct Signal Fractions**

- **Unmodified**: 2.0882e+07

### LNVQREN(N)GTISRY Hex 7 HexNAc 4 Neu5Ac 2

**Mass:**  4077.6843

(1.717e-06)

**Charge States:** 3, 4

**Start Position**: 97

**End Position**: 110

| Profile Score | Tandem Score | TDA q-value | Volume | Spectrum Matches |
| --- | --- | --- | --- | --- |
| 14.0243 | 99.5741 | 0.0463 | 3.7202e+08 | 7 |

**Adduct Signal Fractions**

- **Unmodified**: 3.7202e+08

### LNVQREN(N)GTISRY Hex 9 HexNAc 6 Neu5Ac 2

**Mass:**  4807.9538

(2.194e-06)

**Charge States:** 4

**Start Position**: 97

**End Position**: 110

| Profile Score | Tandem Score | TDA q-value | Volume | Spectrum Matches |
| --- | --- | --- | --- | --- |
| -19.5413 | 99.2583 | 0.0472 | 3.0973e+07 | 1 |

**Adduct Signal Fractions**

- **Unmodified**: 3.0973e+07

### LNVQREN(N)GTISRY Fuc 1 Hex 10 HexNAc 9

**Mass:**  5143.1076

(1.809e-06)

**Charge States:** 4

**Start Position**: 97

**End Position**: 110

| Profile Score | Tandem Score | TDA q-value | Volume | Spectrum Matches |
| --- | --- | --- | --- | --- |
| -4.4572 | 98.4465 | 0.0472 | 3.9040e+07 | 2 |

**Adduct Signal Fractions**

- **Unmodified**: 3.9040e+07

### VQREN(N)GTISRY Fuc 1 Hex 4 HexNAc 7

**Mass:**  3537.5153

(3.667e-06)

**Charge States:** 3, 4

**Start Position**: 99

**End Position**: 110

| Profile Score | Tandem Score | TDA q-value | Volume | Spectrum Matches |
| --- | --- | --- | --- | --- |
| 9.4853 | 98.4217 | 0.0472 | 1.1219e+08 | 8 |

**Adduct Signal Fractions**

- **Unmodified**: 1.1219e+08

### LNVQREN(N)GTISRY Fuc 3 Hex 9 HexNAc 8

**Mass:**  5070.0920

(1.696e-06)

**Charge States:** 4

**Start Position**: 97

**End Position**: 110

| Profile Score | Tandem Score | TDA q-value | Volume | Spectrum Matches |
| --- | --- | --- | --- | --- |
| -4.3406 | 97.9654 | 0.0472 | 2.9325e+07 | 1 |

**Adduct Signal Fractions**

- **Unmodified**: 2.9325e+07

### P19652|A1AG2\_HUMAN

**>P19652|A1AG2\_HUMAN**  
 `MALSWVLTVLSLLPLLEAQIPLCANLVPVPITNATLDRITGKWFYIASAFRNEEYNKSVQEIQATFFYFTPNKTEDTIFL  
REYQTRQNQCFYNSSYLNVQRENGTVSRYEGGREHVAHLLFLRDTKTLMFGSYLDDEKNWGLSFYADKPETTKEQLGEFY  
EALDCLCIPRSDVMYTDWKKDKCEPLEKQHEKERKQEEGES`

| Site | Type | Count |
| --- | --- | --- |
| 33 | N-linked | 4 |
| 56 | N-linked | 37 |
| 93 | N-linked | 2 |
| 103 | N-linked | 15 |
| 72 | N-linked | 62 |

| Mass | Sequence | MS_2_ Score | `q`-value | MS_1_ Score | Signal | Protein Coordinates | Tandem Scans | Start Time | Apex Time | End Time |
| --- | --- | --- | --- | --- | --- | --- | --- | --- | --- | --- |
| 3152.2835 | TPN(N)KTEDTIF Hex 6 HexNAc 5 | 185.885 | 0.0 | 15.444 | 2.048e+08 | 69 - 79 | 8 | 21.97 | 23.67 | 24.69 |
| 3517.4168 | TPN(N)KTEDTIF Hex 7 HexNAc 6 | 184.472 | 0.0 | 15.148 | 3.352e+08 | 69 - 79 | 13 | 21.29 | 23.16 | 24.69 |
| 3462.4173 | YFTPN(N)KTEDTIF Hex 6 HexNAc 5 | 182.209 | 0.0 | 14.396 | 2.934e+09 | 67 - 79 | 56 | 35.99 | 37.15 | 77.85 |
| 3298.3425 | TPN(N)KTEDTIF Fuc 1 Hex 6 HexNAc 5 | 181.204 | 0.0 | 13.802 | 3.722e+07 | 69 - 79 | 4 | 23.16 | 23.50 | 24.01 |
| 3663.4752 | TPN(N)KTEDTIF Fuc 1 Hex 7 HexNAc 6 | 180.410 | 0.0 | 14.600 | 5.016e+07 | 69 - 79 | 4 | 22.65 | 22.99 | 23.33 |
| 2886.2325 | N(N)KSVQEIQATF Hex 5 HexNAc 4 | 179.877 | 0.0 | -3.245 | 2.823e+07 | 55 - 66 | 1 | 26.91 | 27.08 | 27.42 |
| 3827.5491 | YFTPN(N)KTEDTIF Hex 7 HexNAc 6 | 176.947 | 0.0 | 15.170 | 4.780e+09 | 67 - 79 | 45 | 35.48 | 36.50 | 43.25 |
| 3882.5493 | TPN(N)KTEDTIF Hex 8 HexNAc 7 | 175.217 | 0.0 | -4.321 | 2.690e+07 | 69 - 79 | 4 | 22.31 | 22.48 | 22.82 |
| 3973.6074 | YFTPN(N)KTEDTIF Fuc 1 Hex 7 HexNAc 6 | 174.484 | 0.0 | 15.054 | 9.933e+08 | 67 - 79 | 9 | 35.31 | 36.33 | 37.31 |
| 3609.4838 | FYFTPN(N)KTEDTIF Hex 6 HexNAc 5 | 173.970 | 0.0 | 14.789 | 2.352e+09 | 66 - 79 | 48 | 45.94 | 47.11 | 56.66 |
| 3974.6185 | FYFTPN(N)KTEDTIF Hex 7 HexNAc 6 | 172.785 | 0.0 | 15.465 | 4.479e+09 | 66 - 79 | 79 | 45.26 | 46.61 | 59.74 |
| 3299.3512 | FTPN(N)KTEDTIF Hex 6 HexNAc 5 | 172.640 | 0.0 | 15.470 | 4.351e+08 | 68 - 79 | 13 | 29.29 | 30.82 | 31.84 |
| 2787.1524 | TPN(N)KTEDTIF Hex 5 HexNAc 4 | 170.670 | 0.0 | 13.844 | 2.503e+07 | 69 - 79 | 3 | 23.67 | 24.18 | 25.03 |
| 3608.4742 | YFTPN(N)KTEDTIF Fuc 1 Hex 6 HexNAc 5 | 167.991 | 0.0 | 13.652 | 6.524e+08 | 67 - 79 | 12 | 35.82 | 36.98 | 38.81 |
| 3097.2824 | YFTPN(N)KTEDTIF Hex 5 HexNAc 4 | 167.567 | 0.0 | 14.444 | 3.297e+08 | 67 - 79 | 8 | 37.63 | 37.79 | 38.64 |
| 3664.4851 | FTPN(N)KTEDTIF Hex 7 HexNAc 6 | 166.271 | 0.0 | 16.123 | 6.929e+08 | 68 - 79 | 14 | 28.95 | 30.31 | 31.67 |
| 4120.6772 | FYFTPN(N)KTEDTIF Fuc 1 Hex 7 HexNAc 6 | 166.065 | 0.0 | 14.554 | 8.359e+08 | 66 - 79 | 13 | 45.60 | 46.45 | 47.61 |
| 4557.8158 | YFTPN(N)KTEDTIF Hex 9 HexNAc 8 | 165.209 | 0.0 | 15.550 | 2.393e+08 | 67 - 79 | 5 | 34.63 | 35.48 | 36.16 |
| 3755.5435 | FYFTPN(N)KTEDTIF Fuc 1 Hex 6 HexNAc 5 | 164.956 | 0.0 | 14.057 | 5.974e+08 | 66 - 79 | 14 | 45.94 | 46.94 | 50.84 |
| 3243.3395 | YFTPN(N)KTEDTIF Fuc 1 Hex 5 HexNAc 4 | 162.392 | 0.0 | -20.122 | 7.222e+06 | 67 - 79 | 1 | 37.47 | 37.63 | 37.63 |
| 4192.6838 | YFTPN(N)KTEDTIF Hex 8 HexNAc 7 | 161.342 | 0.0 | 14.997 | 8.710e+08 | 67 - 79 | 11 | 34.63 | 36.16 | 36.65 |
| 4119.6666 | YFTPN(N)KTEDTIF Fuc 2 Hex 7 HexNAc 6 | 161.108 | 0.0 | -3.916 | 1.512e+08 | 67 - 79 | 2 | 36.16 | 36.33 | 36.65 |
| 4266.7358 | FYFTPN(N)KTEDTIF Fuc 2 Hex 7 HexNAc 6 | 161.073 | 0.0 | 13.178 | 1.359e+08 | 66 - 79 | 3 | 46.11 | 46.28 | 46.78 |
| 3244.3509 | FYFTPN(N)KTEDTIF Hex 5 HexNAc 4 | 160.382 | 0.0 | 11.901 | 1.960e+08 | 66 - 79 | 4 | 47.61 | 47.78 | 48.45 |
| 3724.5922 | RNEEYN(N)KSVQEIQATFF Hex 5 HexNAc 4 | 160.097 | 0.0 | 16.834 | 3.167e+09 | 50 - 67 | 18 | 42.23 | 44.08 | 45.43 |
| 3672.5114 | EEYN(N)KSVQEIQATF Hex 6 HexNAc 5 | 159.708 | 0.0 | -3.608 | 7.869e+07 | 52 - 66 | 3 | 34.97 | 35.31 | 35.48 |
| 4338.7402 | YFTPN(N)KTEDTIF Fuc 1 Hex 8 HexNAc 7 | 158.954 | 0.0 | 12.504 | 1.618e+08 | 67 - 79 | 4 | 35.48 | 35.99 | 36.16 |
| 4339.7524 | FYFTPN(N)KTEDTIF Hex 8 HexNAc 7 | 158.054 | 0.0 | 14.872 | 6.139e+08 | 66 - 79 | 10 | 44.91 | 46.11 | 47.28 |
| 3881.5467 | TPN(N)KTEDTIF Fuc 3 Hex 6 HexNAc 5 Neu5Ac 1 | 158.010 | 0.0 | -19.167 | 6.064e+06 | 69 - 79 | 1 | 22.14 | 22.14 | 22.14 |
| 3577.5261 | RNEEYN(N)KSVQEIQATF Hex 5 HexNAc 4 | 157.941 | 0.0 | 15.563 | 8.583e+09 | 50 - 66 | 23 | 29.46 | 32.66 | 33.96 |
| 2934.2199 | FTPN(N)KTEDTIF Hex 5 HexNAc 4 | 156.667 | 0.0 | 14.362 | 4.611e+07 | 68 - 79 | 2 | 31.16 | 31.50 | 31.84 |
| 3942.6970 | RNEEYN(N)KSVQEIQATF Hex 6 HexNAc 5 | 156.622 | 0.0 | 14.015 | 6.717e+09 | 50 - 66 | 34 | 16.63 | 32.18 | 33.62 |
| 4235.7833 | RNEEYN(N)KSVQEIQATFF Fuc 1 Hex 6 HexNAc 5 | 156.104 | 0.0 | 15.723 | 1.521e+09 | 50 - 67 | 13 | 42.74 | 43.42 | 44.57 |
| 3810.5434 | FTPN(N)KTEDTIF Fuc 1 Hex 7 HexNAc 6 | 155.804 | 0.0 | 13.655 | 1.163e+08 | 68 - 79 | 6 | 29.80 | 30.14 | 30.82 |
| 3818.5718 | EEYN(N)KSVQEIQATF Fuc 1 Hex 6 HexNAc 5 | 155.065 | 0.0 | -3.564 | 4.109e+07 | 52 - 66 | 2 | 34.97 | 34.97 | 35.31 |
| 3098.1779 | YN(N)SSYL Hex 7 HexNAc 6 | 154.620 | 0.0 | -18.895 | 3.496e+06 | 91 - 97 | 1 | 22.14 | 22.14 | 22.14 |
| 4089.7255 | RNEEYN(N)KSVQEIQATFF Hex 6 HexNAc 5 | 153.897 | 0.0 | 16.033 | 3.295e+09 | 50 - 67 | 23 | 41.37 | 43.59 | 45.09 |
| 3307.3789 | EEYN(N)KSVQEIQATF Hex 5 HexNAc 4 | 153.572 | 0.0 | -3.852 | 9.399e+07 | 52 - 66 | 4 | 35.48 | 35.65 | 35.99 |
| 3872.6601 | RNEEYN(N)KSVQEIQATFF Fuc 1 Hex 5 HexNAc 4 | 153.485 | 0.0 | 0.000 | 0.000e+00 | 50 - 67 | 1 | - | - | - |
| 4381.8436 | RNEEYN(N)KSVQEIQATFF Fuc 2 Hex 6 HexNAc 5 | 153.043 | 0.0 | -4.295 | 6.376e+07 | 50 - 67 | 3 | 43.25 | 43.25 | 43.75 |
| 3723.5844 | RNEEYN(N)KSVQEIQATF Fuc 1 Hex 5 HexNAc 4 | 152.641 | 0.0 | 9.051 | 3.263e+08 | 50 - 66 | 9 | 32.34 | 32.34 | 33.79 |
| 3870.6490 | RNEEYN(N)KSVQEIQATFF Fuc 1 Hex 5 HexNAc 4 | 152.601 | 0.0 | 10.216 | 1.241e+08 | 50 - 67 | 4 | 43.75 | 43.91 | 44.41 |
| 4088.8809 | RNEEYN(N)KSVQEIQATF Fuc 1 Hex 6 HexNAc 5 | 152.139 | 0.0 | 12.129 | 3.683e+09 | 50 - 66 | 89 | 23.67 | 32.01 | 36.16 |
| 4234.7764 | RNEEYN(N)KSVQEIQATF Fuc 2 Hex 6 HexNAc 5 | 151.504 | 0.0 | -3.890 | 1.194e+08 | 50 - 66 | 3 | 31.84 | 32.01 | 32.34 |
| 4703.8760 | YFTPN(N)KTEDTIF Fuc 1 Hex 9 HexNAc 8 | 151.485 | 0.0 | 11.396 | 3.816e+07 | 67 - 79 | 2 | 34.80 | 35.14 | 35.82 |
| 3251.3630 | N(N)KSVQEIQATF Hex 6 HexNAc 5 | 151.314 | 0.0 | -4.357 | 1.826e+07 | 55 - 66 | 2 | 26.39 | 26.56 | 26.91 |
| 3920.6046 | NVQREN(N)GTVSRY Fuc 1 Hex 7 HexNAc 6 | 151.254 | 0.0 | -3.017 | 3.327e+08 | 97 - 109 | 4 | 11.84 | 11.84 | 12.17 |
| 4307.7909 | RNEEYN(N)KSVQEIQATF Hex 7 HexNAc 6 | 151.224 | 0.0 | -2.830 | 2.754e+08 | 50 - 66 | 3 | 31.84 | 32.01 | 32.34 |
| 3033.3009 | N(N)KSVQEIQATFF Hex 5 HexNAc 4 | 150.523 | 0.0 | -3.806 | 6.795e+06 | 55 - 67 | 1 | 41.03 | 41.20 | 41.54 |
| 3445.4091 | FTPN(N)KTEDTIF Fuc 1 Hex 6 HexNAc 5 | 149.127 | 0.0 | 13.377 | 7.836e+07 | 68 - 79 | 5 | 30.31 | 30.65 | 31.33 |
| 4003.5812 | YFTPN(N)KTEDTIF Hex 7 HexNAc 4 Neu5Ac 2 | 148.952 | 0.0 | 14.468 | 3.177e+08 | 67 - 79 | 7 | 37.63 | 37.79 | 38.81 |
| 4150.6502 | FYFTPN(N)KTEDTIF Hex 7 HexNAc 4 Neu5Ac 2 | 146.963 | 0.004 | 12.962 | 2.219e+08 | 66 - 79 | 8 | 47.61 | 47.78 | 48.28 |
| 4454.8587 | RNEEYN(N)KSVQEIQATFF Hex 7 HexNAc 6 | 146.716 | 0.004 | 14.250 | 1.608e+08 | 50 - 67 | 5 | 42.91 | 43.25 | 43.75 |
| 3369.3489 | FYFTPN(N)KT Hex 7 HexNAc 6 | 146.166 | 0.0 | -4.298 | 1.358e+07 | 66 - 74 | 2 | 25.03 | 25.20 | 25.37 |
| 4704.8850 | FYFTPN(N)KTEDTIF Hex 9 HexNAc 8 | 146.054 | 0.004 | 15.705 | 1.574e+08 | 66 - 79 | 6 | 44.57 | 45.43 | 46.28 |
| 4029.6176 | FTPN(N)KTEDTIF Hex 8 HexNAc 7 | 145.728 | 0.004 | 14.824 | 5.933e+07 | 68 - 79 | 5 | 29.29 | 29.80 | 30.14 |
| 4296.7078 | FYFTPN(N)KTEDTIF Fuc 1 Hex 7 HexNAc 4 Neu5Ac 2 | 145.310 | 0.004 | -17.041 | 3.573e+07 | 66 - 79 | 2 | 47.44 | 47.61 | 47.61 |
| 4485.8101 | FYFTPN(N)KTEDTIF Fuc 1 Hex 8 HexNAc 7 | 144.153 | 0.004 | 15.113 | 1.269e+08 | 66 - 79 | 5 | 45.09 | 45.94 | 46.45 |
| 3795.5924 | RNEEYN(N)KSVQEIQAT Hex 6 HexNAc 5 | 142.425 | 0.008 | -19.788 | 2.323e+07 | 50 - 65 | 2 | 16.97 | 16.97 | 17.14 |
| 2733.0467 | YN(N)SSYL Hex 6 HexNAc 5 | 142.342 | 0.0 | -5.016 | 2.076e+06 | 91 - 97 | 1 | 22.65 | 22.82 | 22.99 |
| 4600.9176 | RNEEYN(N)KSVQEIQATFF Fuc 1 Hex 7 HexNAc 6 | 142.117 | 0.008 | -3.066 | 6.145e+07 | 50 - 67 | 3 | 42.91 | 43.25 | 43.42 |
| 4453.8491 | RNEEYN(N)KSVQEIQATF Fuc 1 Hex 7 HexNAc 6 | 142.035 | 0.008 | -3.286 | 1.167e+08 | 50 - 66 | 2 | 31.67 | 32.01 | 32.18 |
| 3819.5784 | EEYN(N)KSVQEIQATFF Hex 6 HexNAc 5 | 141.620 | 0.008 | -4.099 | 2.581e+07 | 52 - 67 | 3 | 47.28 | 47.44 | 47.61 |
| 3213.4159 | RNEEYN(N)KSVQEIQATF Hex 4 HexNAc 3 | 141.387 | 0.008 | 0.000 | 0.000e+00 | 50 - 66 | 1 | - | - | - |
| 3575.5004 | YFTPN(N)KTEDTIFL Hex 6 HexNAc 5 | 140.103 | 0.010 | -3.288 | 2.478e+07 | 67 - 80 | 3 | 44.74 | 44.91 | 45.26 |
| 3754.5374 | YFTPN(N)KTEDTIF Fuc 2 Hex 6 HexNAc 5 | 140.093 | 0.010 | -6.403 | 7.285e+06 | 67 - 79 | 1 | 36.33 | 36.65 | 36.82 |
| 3693.4440 | TPN(N)KTEDTIF Hex 7 HexNAc 4 Neu5Ac 2 | 137.359 | 0.010 | -3.901 | 1.131e+07 | 69 - 79 | 4 | 24.01 | 24.18 | 24.35 |
| 3555.4718 | NVQREN(N)GTVSRY Fuc 1 Hex 6 HexNAc 5 | 137.213 | 0.010 | -5.404 | 6.521e+07 | 97 - 109 | 3 | 11.84 | 12.00 | 12.17 |
| 2954.1863 | N(N)KTEDTIF Hex 6 HexNAc 5 | 137.076 | 0.0 | -20.552 | 2.922e+06 | 71 - 79 | 1 | 21.80 | 21.80 | 21.97 |
| 4922.9499 | YFTPN(N)KTEDTIF Hex 10 HexNAc 9 | 136.423 | 0.010 | -3.202 | 2.348e+07 | 67 - 79 | 1 | 34.63 | 34.97 | 34.97 |
| 3409.4139 | NVQREN(N)GTVSRY Hex 6 HexNAc 5 | 135.212 | 0.010 | -4.168 | 1.818e+08 | 97 - 109 | 4 | 11.84 | 12.00 | 12.34 |
| 3430.4557 | RNEEYN(N)KSVQEIQAT Hex 5 HexNAc 4 | 135.072 | 0.010 | -4.979 | 3.596e+07 | 50 - 65 | 3 | 16.97 | 17.14 | 17.47 |
| 4066.6606 | NVQREN(N)GTVSRY Fuc 2 Hex 7 HexNAc 6 | 133.443 | 0.010 | -18.634 | 7.740e+07 | 97 - 109 | 2 | 11.84 | 11.84 | 12.00 |
| 3222.2832 | YFTPN(N)KT Hex 7 HexNAc 6 | 131.719 | 0.0 | -21.208 | 2.653e+06 | 67 - 74 | 1 | 14.00 | 14.00 | 14.00 |
| 4060.6035 | TPN(N)KTEDTIF Fuc 4 Hex 8 HexNAc 5 | 131.328 | 0.010 | 13.132 | 1.352e+08 | 69 - 79 | 4 | 37.63 | 37.79 | 38.47 |
| 3940.6327 | YFTPN(N)KTEDTIFL Hex 7 HexNAc 6 | 130.141 | 0.010 | -4.043 | 4.360e+07 | 67 - 80 | 3 | 44.24 | 44.41 | 44.74 |
| 4394.7532 | FTPN(N)KTEDTIF Hex 9 HexNAc 8 | 129.972 | 0.010 | 14.199 | 1.238e+07 | 68 - 79 | 2 | 28.78 | 29.12 | 29.80 |
| 4672.9231 | RNEEYN(N)KSVQEIQATF Hex 8 HexNAc 7 | 129.830 | 0.010 | -3.865 | 6.215e+07 | 50 - 66 | 2 | 31.50 | 31.67 | 32.01 |
| 4089.7153 | FRNEEYN(N)KSVQEIQATF Hex 6 HexNAc 5 | 129.747 | 0.010 | 11.283 | 4.398e+07 | 49 - 66 | 3 | 36.82 | 37.15 | 37.47 |
| 3840.5163 | FTPN(N)KTEDTIF Hex 7 HexNAc 4 Neu5Ac 2 | 129.188 | 0.010 | -4.021 | 3.047e+07 | 68 - 79 | 4 | 31.16 | 31.33 | 31.67 |
| 3774.5463 | NVQREN(N)GTVSRY Hex 7 HexNAc 6 | 128.441 | 0.010 | 12.957 | 6.615e+08 | 97 - 109 | 6 | 11.84 | 11.84 | 12.67 |
| 3004.2150 | FYFTPN(N)KT Hex 6 HexNAc 5 | 128.436 | 0.0 | -3.564 | 6.282e+06 | 66 - 74 | 2 | 25.71 | 25.88 | 26.22 |
| 4425.7269 | N(N)KTEDTIF Fuc 5 Hex 5 HexNAc 8 Neu5Ac 1 | 128.291 | 0.0 | 0.000 | 0.000e+00 | 71 - 79 | 1 | - | - | - |
| 4484.8008 | YFTPN(N)KTEDTIF Fuc 2 Hex 8 HexNAc 7 | 125.241 | 0.010 | 12.724 | 4.167e+07 | 67 - 79 | 2 | 35.31 | 35.82 | 35.99 |
| 3996.5976 | FYFTPN(N)KTEDTIF Hex 3 HexNAc 5 Neu5Ac 3 | 124.458 | 0.010 | 13.698 | 2.445e+08 | 66 - 79 | 3 | 45.77 | 46.61 | 47.11 |
| 4020.6075 | YFTPN(N)KTEDTIF Fuc 1 Hex 8 HexNAc 4 Neu5Ac 1 | 124.387 | 0.010 | -19.508 | 2.523e+07 | 67 - 79 | 2 | 37.79 | 37.79 | 37.96 |
| 3578.5102 | RN(D)EEYN(N)KSVQEIQATF Hex 5 HexNAc 4 | 123.916 | 0.010 | 10.833 | 1.355e+09 | 50 - 66 | 30 | 34.13 | 35.48 | 36.50 |
| 3956.6051 | FTPN(N)KTEDTIF Fuc 2 Hex 7 HexNAc 6 | 123.669 | 0.010 | -4.494 | 1.500e+07 | 68 - 79 | 4 | 29.80 | 29.97 | 30.14 |
| 4631.8693 | FYFTPN(N)KTEDTIF Fuc 2 Hex 8 HexNAc 7 | 123.042 | 0.010 | -3.825 | 2.387e+07 | 66 - 79 | 2 | 45.43 | 45.77 | 45.94 |
| 3943.6432 | RN(D)EEYN(N)KSVQEIQATF Hex 6 HexNAc 5 | 121.638 | 0.010 | 10.764 | 1.210e+09 | 50 - 66 | 13 | 33.79 | 35.31 | 35.99 |
| 4207.6720 | TPN(N)KTEDTIF Fuc 3 Hex 3 HexNAc 9 Neu5Ac 1 | 121.517 | 0.010 | 9.487 | 1.090e+08 | 69 - 79 | 5 | 37.47 | 48.79 | 49.82 |
| 3771.5560 | FYFTPN(N)KTEDTIF Hex 7 HexNAc 5 | 121.206 | 0.010 | -20.320 | 1.376e+07 | 66 - 79 | 1 | 46.78 | 46.94 | 46.94 |
| 4033.6923 | LNVQREN(N)GTVSRY Fuc 1 Hex 7 HexNAc 6 | 120.817 | 0.010 | 13.799 | 2.984e+08 | 96 - 109 | 6 | 15.80 | 15.97 | 16.80 |
| 4137.7005 | FYFTPN(N)KTEDTIF Hex 8 HexNAc 6 | 120.686 | 0.010 | -20.565 | 9.074e+07 | 66 - 79 | 5 | 13.50 | 46.28 | 46.45 |
| 4175.6764 | FTPN(N)KTEDTIF Fuc 1 Hex 8 HexNAc 7 | 119.589 | 0.010 | 10.714 | 1.042e+07 | 68 - 79 | 4 | 29.12 | 29.64 | 29.80 |
| 3398.4319 | N(N)KSVQEIQATFF Hex 6 HexNAc 5 | 118.589 | 0.010 | -18.145 | 5.016e+06 | 55 - 67 | 2 | 40.35 | 40.52 | 40.52 |
| 3725.5782 | RN(D)EEYN(N)KSVQEIQATFF Hex 5 HexNAc 4 | 117.080 | 0.012 | 10.591 | 3.103e+08 | 50 - 67 | 10 | 45.60 | 47.11 | 47.28 |
| 5038.0545 | RNEEYN(N)KSVQEIQATF Hex 9 HexNAc 8 | 116.422 | 0.012 | -3.435 | 3.580e+07 | 50 - 66 | 2 | 31.16 | 31.50 | 31.67 |
| 4236.7703 | RN(D)EEYN(N)KSVQEIQATFF Fuc 1 Hex 6 HexNAc 5 | 116.336 | 0.014 | 9.723 | 1.687e+08 | 50 - 67 | 11 | 44.74 | 46.45 | 46.94 |
| 3124.3981 | LVPVPITN(N)ATL Hex 6 HexNAc 5 | 116.261 | 0.014 | -4.634 | 7.286e+06 | 25 - 36 | 2 | 45.60 | 45.60 | 45.94 |
| 4215.7273 | N(D)VQREN(N)GTVSRY Fuc 3 Hex 7 HexNAc 6 | 115.891 | 0.014 | 0.000 | 0.000e+00 | 97 - 109 | 1 | - | - | - |
| 3887.6366 | LNVQREN(N)GTVSRY Hex 7 HexNAc 6 | 115.511 | 0.016 | 13.893 | 5.672e+08 | 96 - 109 | 6 | 15.97 | 16.14 | 17.14 |
| 3362.4431 | TN(N)ATLDRITGKW Hex 6 HexNAc 5 | 114.978 | 0.016 | -4.183 | 2.074e+07 | 31 - 43 | 4 | 29.97 | 29.97 | 30.31 |
| 3668.5631 | LNVQREN(N)GTVSRY Fuc 1 Hex 6 HexNAc 5 | 114.087 | 0.018 | 12.943 | 6.715e+07 | 96 - 109 | 3 | 15.97 | 16.30 | 16.80 |
| 4563.8441 | YFTPN(N)KT Fuc 5 Hex 7 HexNAc 9 | 112.144 | 0.0 | 0.000 | 0.000e+00 | 67 - 74 | 1 | - | - | - |
| 3522.5023 | LNVQREN(N)GTVSRY Hex 6 HexNAc 5 | 111.471 | 0.020 | 14.906 | 1.196e+08 | 96 - 109 | 4 | 16.14 | 16.47 | 16.80 |
| 4090.7117 | RN(D)EEYN(N)KSVQEIQATFF Hex 6 HexNAc 5 | 110.503 | 0.021 | 9.926 | 3.272e+08 | 50 - 67 | 14 | 44.91 | 46.78 | 46.78 |
| 3454.4478 | EEYN(N)KSVQEIQATFF Hex 5 HexNAc 4 | 110.047 | 0.021 | -4.613 | 2.153e+07 | 52 - 67 | 3 | 47.78 | 47.95 | 48.28 |
| 4325.9635 | C(C)ANLVPVPITN(N)ATLDRITGKW Hex 6 HexNAc 5 | 109.695 | 0.021 | 9.668 | 1.023e+08 | 22 - 43 | 5 | 54.78 | 54.78 | 55.46 |
| 4819.9920 | RNEEYN(N)KSVQEIQATFF Hex 8 HexNAc 7 | 109.655 | 0.021 | -2.920 | 1.690e+07 | 50 - 67 | 2 | 42.57 | 42.74 | 43.08 |
| 3613.4178 | FYFTPN(N)KTED Hex 7 HexNAc 6 | 109.648 | 0.021 | -4.778 | 5.253e+06 | 66 - 76 | 2 | 25.54 | 25.54 | 25.88 |
| 5070.0167 | FYFTPN(N)KTEDTIF Hex 10 HexNAc 9 | 108.645 | 0.021 | -3.908 | 1.775e+07 | 66 - 79 | 2 | 44.74 | 44.91 | 45.26 |
| 4504.8128 | NVQREN(N)GTVSRY Hex 9 HexNAc 8 | 107.386 | 0.027 | -19.837 | 3.484e+07 | 97 - 109 | 1 | 11.67 | 11.67 | 11.84 |
| 4650.8679 | NVQREN(N)GTVSRY Fuc 1 Hex 9 HexNAc 8 | 105.854 | 0.030 | -20.705 | 1.327e+07 | 97 - 109 | 1 | 11.67 | 11.67 | 11.67 |
| 3638.4467 | TPN(N)KTEDTIF Hex 9 HexNAc 5 | 102.037 | 0.034 | 13.548 | 2.781e+07 | 69 - 79 | 4 | 38.13 | 38.30 | 38.98 |
| 3469.5104 | C(C)ANLVPVPITN(N)ATL Hex 6 HexNAc 5 | 101.793 | 0.034 | 12.350 | 2.307e+07 | 22 - 36 | 6 | 50.16 | 50.50 | 51.18 |
| 3669.5797 | LNVQREN(N)GTVSRY Fuc 1 Hex 6 HexNAc 5 | 100.914 | 0.041 | 0.000 | 0.000e+00 | 96 - 109 | 1 | - | - | - |
| 3722.5529 | RNEEYN(N)KSVQEIQAT Fuc 2 Hex 5 HexNAc 4 | 99.957 | 0.046 | -19.545 | 2.088e+07 | 50 - 65 | 1 | 32.18 | 32.18 | 32.18 |
| 4179.7517 | LNVQREN(N)GTVSRY Fuc 2 Hex 7 HexNAc 6 | 99.370 | 0.047 | -4.112 | 7.946e+07 | 96 - 109 | 3 | 15.80 | 15.97 | 16.30 |
| 4763.9601 | LNVQREN(N)GTVSRY Fuc 1 Hex 9 HexNAc 8 | 97.849 | 0.047 | -20.473 | 1.253e+07 | 96 - 109 | 1 | 15.47 | 15.47 | 15.64 |

### TPN(N)KTEDTIF Hex 6 HexNAc 5

**Mass:**  3152.2838

(1.218e-06)

**Charge States:** 3, 4

**Start Position**: 70

**End Position**: 80

| Profile Score | Tandem Score | TDA q-value | Volume | Spectrum Matches |
| --- | --- | --- | --- | --- |
| 15.4443 | 185.8852 | 0.0000 | 2.0477e+08 | 8 |

**Adduct Signal Fractions**

- **Unmodified**: 2.0477e+08

### TPN(N)KTEDTIF Hex 7 HexNAc 6

**Mass:**  3517.4163

(1.427e-06)

**Charge States:** 3, 4

**Start Position**: 70

**End Position**: 80

| Profile Score | Tandem Score | TDA q-value | Volume | Spectrum Matches |
| --- | --- | --- | --- | --- |
| 15.1480 | 184.4718 | 0.0000 | 3.3516e+08 | 13 |

**Adduct Signal Fractions**

- **Unmodified**: 3.3516e+08

### YFTPN(N)KTEDTIF Hex 6 HexNAc 5

**Mass:**  3462.4173

(1.477e-06)

**Charge States:** 2, 3, 4

**Start Position**: 68

**End Position**: 80

| Profile Score | Tandem Score | TDA q-value | Volume | Spectrum Matches |
| --- | --- | --- | --- | --- |
| 14.3961 | 182.2093 | 0.0000 | 2.9344e+09 | 56 |

**Adduct Signal Fractions**

- **Unmodified**: 2.9344e+09

### TPN(N)KTEDTIF Fuc 1 Hex 6 HexNAc 5

**Mass:**  3298.3423

(1.480e-06)

**Charge States:** 3, 4

**Start Position**: 70

**End Position**: 80

| Profile Score | Tandem Score | TDA q-value | Volume | Spectrum Matches |
| --- | --- | --- | --- | --- |
| 13.8017 | 181.2037 | 0.0000 | 3.7224e+07 | 4 |

**Adduct Signal Fractions**

- **Unmodified**: 3.7224e+07

### TPN(N)KTEDTIF Fuc 1 Hex 7 HexNAc 6

**Mass:**  3663.4738

(2.012e-06)

**Charge States:** 3, 4

**Start Position**: 70

**End Position**: 80

| Profile Score | Tandem Score | TDA q-value | Volume | Spectrum Matches |
| --- | --- | --- | --- | --- |
| 14.5996 | 180.4104 | 0.0000 | 5.0162e+07 | 4 |

**Adduct Signal Fractions**

- **Unmodified**: 5.0162e+07

### N(N)KSVQEIQATF Hex 5 HexNAc 4

**Mass:**  2886.2334

(2.069e-06)

**Charge States:** 3, 4

**Start Position**: 56

**End Position**: 67

| Profile Score | Tandem Score | TDA q-value | Volume | Spectrum Matches |
| --- | --- | --- | --- | --- |
| -3.2450 | 179.8773 | 0.0000 | 2.8226e+07 | 1 |

**Adduct Signal Fractions**

- **Unmodified**: 2.8226e+07

### YFTPN(N)KTEDTIF Hex 7 HexNAc 6

**Mass:**  3827.5502

(1.622e-06)

**Charge States:** 3, 4

**Start Position**: 68

**End Position**: 80

| Profile Score | Tandem Score | TDA q-value | Volume | Spectrum Matches |
| --- | --- | --- | --- | --- |
| 15.1699 | 176.9469 | 0.0000 | 4.7800e+09 | 45 |

**Adduct Signal Fractions**

- **Unmodified**: 4.7800e+09

### TPN(N)KTEDTIF Hex 8 HexNAc 7

**Mass:**  3882.5500

(1.443e-06)

**Charge States:** 3, 4

**Start Position**: 70

**End Position**: 80

| Profile Score | Tandem Score | TDA q-value | Volume | Spectrum Matches |
| --- | --- | --- | --- | --- |
| -4.3212 | 175.2173 | 0.0000 | 2.6897e+07 | 4 |

**Adduct Signal Fractions**

- **Unmodified**: 2.6897e+07

### YFTPN(N)KTEDTIF Fuc 1 Hex 7 HexNAc 6

**Mass:**  3973.6069

(2.368e-06)

**Charge States:** 3, 4

**Start Position**: 68

**End Position**: 80

| Profile Score | Tandem Score | TDA q-value | Volume | Spectrum Matches |
| --- | --- | --- | --- | --- |
| 15.0540 | 174.4842 | 0.0000 | 9.9334e+08 | 9 |

**Adduct Signal Fractions**

- **Unmodified**: 9.9334e+08

### FYFTPN(N)KTEDTIF Hex 6 HexNAc 5

**Mass:**  3609.4839

(9.855e-07)

**Charge States:** 3, 4

**Start Position**: 67

**End Position**: 80

| Profile Score | Tandem Score | TDA q-value | Volume | Spectrum Matches |
| --- | --- | --- | --- | --- |
| 14.7894 | 173.9697 | 0.0000 | 2.3522e+09 | 48 |

**Adduct Signal Fractions**

- **Unmodified**: 2.3522e+09

### FYFTPN(N)KTEDTIF Hex 7 HexNAc 6

**Mass:**  3974.6162

(7.113e-07)

**Charge States:** 3, 4

**Start Position**: 67

**End Position**: 80

| Profile Score | Tandem Score | TDA q-value | Volume | Spectrum Matches |
| --- | --- | --- | --- | --- |
| 15.4648 | 172.7852 | 0.0000 | 4.4793e+09 | 79 |

**Adduct Signal Fractions**

- **Unmodified**: 4.4793e+09

### FTPN(N)KTEDTIF Hex 6 HexNAc 5

**Mass:**  3299.3527

(1.237e-06)

**Charge States:** 3, 4

**Start Position**: 69

**End Position**: 80

| Profile Score | Tandem Score | TDA q-value | Volume | Spectrum Matches |
| --- | --- | --- | --- | --- |
| 15.4699 | 172.6405 | 0.0000 | 4.3512e+08 | 13 |

**Adduct Signal Fractions**

- **Unmodified**: 4.3512e+08

### TPN(N)KTEDTIF Hex 5 HexNAc 4

**Mass:**  2787.1521

(1.315e-06)

**Charge States:** 3

**Start Position**: 70

**End Position**: 80

| Profile Score | Tandem Score | TDA q-value | Volume | Spectrum Matches |
| --- | --- | --- | --- | --- |
| 13.8444 | 170.6700 | 0.0000 | 2.5032e+07 | 3 |

**Adduct Signal Fractions**

- **Unmodified**: 2.5032e+07

### YFTPN(N)KTEDTIF Fuc 1 Hex 6 HexNAc 5

**Mass:**  3608.4757

(1.745e-06)

**Charge States:** 3, 4

**Start Position**: 68

**End Position**: 80

| Profile Score | Tandem Score | TDA q-value | Volume | Spectrum Matches |
| --- | --- | --- | --- | --- |
| 13.6522 | 167.9915 | 0.0000 | 6.5243e+08 | 12 |

**Adduct Signal Fractions**

- **Unmodified**: 6.5243e+08

### YFTPN(N)KTEDTIF Hex 5 HexNAc 4

**Mass:**  3097.2822

(7.734e-07)

**Charge States:** 3, 4

**Start Position**: 68

**End Position**: 80

| Profile Score | Tandem Score | TDA q-value | Volume | Spectrum Matches |
| --- | --- | --- | --- | --- |
| 14.4442 | 167.5665 | 0.0000 | 3.2968e+08 | 8 |

**Adduct Signal Fractions**

- **Unmodified**: 3.2968e+08

### FTPN(N)KTEDTIF Hex 7 HexNAc 6

**Mass:**  3664.4866

(1.233e-06)

**Charge States:** 3, 4

**Start Position**: 69

**End Position**: 80

| Profile Score | Tandem Score | TDA q-value | Volume | Spectrum Matches |
| --- | --- | --- | --- | --- |
| 16.1226 | 166.2715 | 0.0000 | 6.9294e+08 | 14 |

**Adduct Signal Fractions**

- **Unmodified**: 6.9294e+08

### FYFTPN(N)KTEDTIF Fuc 1 Hex 7 HexNAc 6

**Mass:**  4120.6799

(9.779e-07)

**Charge States:** 3, 4

**Start Position**: 67

**End Position**: 80

| Profile Score | Tandem Score | TDA q-value | Volume | Spectrum Matches |
| --- | --- | --- | --- | --- |
| 14.5538 | 166.0649 | 0.0000 | 8.3594e+08 | 13 |

**Adduct Signal Fractions**

- **Unmodified**: 8.3594e+08

### YFTPN(N)KTEDTIF Hex 9 HexNAc 8

**Mass:**  4557.8171

(1.103e-06)

**Charge States:** 3, 4

**Start Position**: 68

**End Position**: 80

| Profile Score | Tandem Score | TDA q-value | Volume | Spectrum Matches |
| --- | --- | --- | --- | --- |
| 15.5500 | 165.2093 | 0.0000 | 2.3930e+08 | 5 |

**Adduct Signal Fractions**

- **Unmodified**: 2.3930e+08

### FYFTPN(N)KTEDTIF Fuc 1 Hex 6 HexNAc 5

**Mass:**  3755.5442

(7.090e-07)

**Charge States:** 3, 4

**Start Position**: 67

**End Position**: 80

| Profile Score | Tandem Score | TDA q-value | Volume | Spectrum Matches |
| --- | --- | --- | --- | --- |
| 14.0570 | 164.9562 | 0.0000 | 5.9737e+08 | 14 |

**Adduct Signal Fractions**

- **Unmodified**: 5.9737e+08

### YFTPN(N)KTEDTIF Fuc 1 Hex 5 HexNAc 4

**Mass:**  3243.3374

(1.278e-06)

**Charge States:** 3

**Start Position**: 68

**End Position**: 80

| Profile Score | Tandem Score | TDA q-value | Volume | Spectrum Matches |
| --- | --- | --- | --- | --- |
| -20.1219 | 162.3918 | 0.0000 | 7.2217e+06 | 1 |

**Adduct Signal Fractions**

- **Unmodified**: 7.2217e+06

### YFTPN(N)KTEDTIF Hex 8 HexNAc 7

**Mass:**  4192.6843

(1.584e-06)

**Charge States:** 3, 4

**Start Position**: 68

**End Position**: 80

| Profile Score | Tandem Score | TDA q-value | Volume | Spectrum Matches |
| --- | --- | --- | --- | --- |
| 14.9975 | 161.3422 | 0.0000 | 8.7096e+08 | 11 |

**Adduct Signal Fractions**

- **Unmodified**: 8.7096e+08

### YFTPN(N)KTEDTIF Fuc 2 Hex 7 HexNAc 6

**Mass:**  4119.6662

(2.510e-06)

**Charge States:** 3, 4

**Start Position**: 68

**End Position**: 80

| Profile Score | Tandem Score | TDA q-value | Volume | Spectrum Matches |
| --- | --- | --- | --- | --- |
| -3.9164 | 161.1085 | 0.0000 | 1.5125e+08 | 2 |

**Adduct Signal Fractions**

- **Unmodified**: 1.5125e+08

### FYFTPN(N)KTEDTIF Fuc 2 Hex 7 HexNAc 6

**Mass:**  4266.7365

(2.264e-06)

**Charge States:** 3, 4

**Start Position**: 67

**End Position**: 80

| Profile Score | Tandem Score | TDA q-value | Volume | Spectrum Matches |
| --- | --- | --- | --- | --- |
| 13.1778 | 161.0728 | 0.0000 | 1.3589e+08 | 3 |

**Adduct Signal Fractions**

- **Unmodified**: 1.3589e+08

### FYFTPN(N)KTEDTIF Hex 5 HexNAc 4

**Mass:**  3244.3500

(1.134e-06)

**Charge States:** 3, 4

**Start Position**: 67

**End Position**: 80

| Profile Score | Tandem Score | TDA q-value | Volume | Spectrum Matches |
| --- | --- | --- | --- | --- |
| 11.9005 | 160.3820 | 0.0000 | 1.9600e+08 | 4 |

**Adduct Signal Fractions**

- **Unmodified**: 1.9600e+08

### RNEEYN(N)KSVQEIQATFF Hex 5 HexNAc 4

**Mass:**  3724.5947

(9.100e-07)

**Charge States:** 3, 4

**Start Position**: 51

**End Position**: 68

| Profile Score | Tandem Score | TDA q-value | Volume | Spectrum Matches |
| --- | --- | --- | --- | --- |
| 16.8344 | 160.0969 | 0.0000 | 3.1670e+09 | 18 |

**Adduct Signal Fractions**

- **Unmodified**: 3.1670e+09

### EEYN(N)KSVQEIQATF Hex 6 HexNAc 5

**Mass:**  3672.5117

(1.241e-06)

**Charge States:** 3, 4

**Start Position**: 53

**End Position**: 67

| Profile Score | Tandem Score | TDA q-value | Volume | Spectrum Matches |
| --- | --- | --- | --- | --- |
| -3.6084 | 159.7083 | 0.0000 | 7.8689e+07 | 3 |

**Adduct Signal Fractions**

- **Unmodified**: 7.8689e+07

### YFTPN(N)KTEDTIF Fuc 1 Hex 8 HexNAc 7

**Mass:**  4338.7399

(1.610e-06)

**Charge States:** 3, 4

**Start Position**: 68

**End Position**: 80

| Profile Score | Tandem Score | TDA q-value | Volume | Spectrum Matches |
| --- | --- | --- | --- | --- |
| 12.5041 | 158.9535 | 0.0000 | 1.6184e+08 | 4 |

**Adduct Signal Fractions**

- **Unmodified**: 1.6184e+08

### FYFTPN(N)KTEDTIF Hex 8 HexNAc 7

**Mass:**  4339.7565

(1.729e-06)

**Charge States:** 3, 4

**Start Position**: 67

**End Position**: 80

| Profile Score | Tandem Score | TDA q-value | Volume | Spectrum Matches |
| --- | --- | --- | --- | --- |
| 14.8720 | 158.0543 | 0.0000 | 6.1386e+08 | 10 |

**Adduct Signal Fractions**

- **Unmodified**: 6.1386e+08

### TPN(N)KTEDTIF Fuc 3 Hex 6 HexNAc 5 Neu5Ac 1

**Mass:**  3881.5468

(-6.471e-07)

**Charge States:** 3, 4

**Start Position**: 70

**End Position**: 80

| Profile Score | Tandem Score | TDA q-value | Volume | Spectrum Matches |
| --- | --- | --- | --- | --- |
| -19.1671 | 158.0100 | 0.0000 | 6.0640e+06 | 1 |

**Adduct Signal Fractions**

- **Unmodified**: 6.0640e+06

### RNEEYN(N)KSVQEIQATF Hex 5 HexNAc 4

**Mass:**  3577.5268

(9.724e-07)

**Charge States:** 3, 4

**Start Position**: 51

**End Position**: 67

| Profile Score | Tandem Score | TDA q-value | Volume | Spectrum Matches |
| --- | --- | --- | --- | --- |
| 15.5628 | 157.9410 | 0.0000 | 8.5835e+09 | 23 |

**Adduct Signal Fractions**

- **Unmodified**: 8.5835e+09

### FTPN(N)KTEDTIF Hex 5 HexNAc 4

**Mass:**  2934.2202

(4.556e-07)

**Charge States:** 3

**Start Position**: 69

**End Position**: 80

| Profile Score | Tandem Score | TDA q-value | Volume | Spectrum Matches |
| --- | --- | --- | --- | --- |
| 14.3620 | 156.6674 | 0.0000 | 4.6114e+07 | 2 |

**Adduct Signal Fractions**

- **Unmodified**: 4.6114e+07

### RNEEYN(N)KSVQEIQATF Hex 6 HexNAc 5

**Mass:**  3942.6589

(1.833e-06)

**Charge States:** 3, 4

**Start Position**: 51

**End Position**: 67

| Profile Score | Tandem Score | TDA q-value | Volume | Spectrum Matches |
| --- | --- | --- | --- | --- |
| 14.0150 | 156.6215 | 0.0000 | 6.7169e+09 | 34 |

**Adduct Signal Fractions**

- **Unmodified**: 6.7169e+09

### RNEEYN(N)KSVQEIQATFF Fuc 1 Hex 6 HexNAc 5

**Mass:**  4235.7839

(1.018e-06)

**Charge States:** 3, 4

**Start Position**: 51

**End Position**: 68

| Profile Score | Tandem Score | TDA q-value | Volume | Spectrum Matches |
| --- | --- | --- | --- | --- |
| 15.7230 | 156.1039 | 0.0000 | 1.5208e+09 | 13 |

**Adduct Signal Fractions**

- **Unmodified**: 1.5208e+09

### FTPN(N)KTEDTIF Fuc 1 Hex 7 HexNAc 6

**Mass:**  3810.5432

(1.228e-06)

**Charge States:** 3, 4

**Start Position**: 69

**End Position**: 80

| Profile Score | Tandem Score | TDA q-value | Volume | Spectrum Matches |
| --- | --- | --- | --- | --- |
| 13.6550 | 155.8040 | 0.0000 | 1.1635e+08 | 6 |

**Adduct Signal Fractions**

- **Unmodified**: 1.1635e+08

### EEYN(N)KSVQEIQATF Fuc 1 Hex 6 HexNAc 5

**Mass:**  3818.5707

(1.381e-06)

**Charge States:** 3, 4

**Start Position**: 53

**End Position**: 67

| Profile Score | Tandem Score | TDA q-value | Volume | Spectrum Matches |
| --- | --- | --- | --- | --- |
| -3.5643 | 155.0650 | 0.0000 | 4.1085e+07 | 2 |

**Adduct Signal Fractions**

- **Unmodified**: 4.1085e+07

### YN(N)SSYL Hex 7 HexNAc 6

**Mass:**  3098.1776

(1.122e-06)

**Charge States:** 3, 4

**Start Position**: 92

**End Position**: 98

| Profile Score | Tandem Score | TDA q-value | Volume | Spectrum Matches |
| --- | --- | --- | --- | --- |
| -18.8950 | 154.6198 | 0.0000 | 3.4962e+06 | 1 |

**Adduct Signal Fractions**

- **Unmodified**: 3.4962e+06

### RNEEYN(N)KSVQEIQATFF Hex 6 HexNAc 5

**Mass:**  4089.7248

(4.928e-07)

**Charge States:** 3, 4

**Start Position**: 51

**End Position**: 68

| Profile Score | Tandem Score | TDA q-value | Volume | Spectrum Matches |
| --- | --- | --- | --- | --- |
| 16.0327 | 153.8968 | 0.0000 | 3.2946e+09 | 23 |

**Adduct Signal Fractions**

- **Unmodified**: 3.2946e+09

### EEYN(N)KSVQEIQATF Hex 5 HexNAc 4

**Mass:**  3307.3778

(1.193e-06)

**Charge States:** 3, 4

**Start Position**: 53

**End Position**: 67

| Profile Score | Tandem Score | TDA q-value | Volume | Spectrum Matches |
| --- | --- | --- | --- | --- |
| -3.8519 | 153.5722 | 0.0000 | 9.3988e+07 | 4 |

**Adduct Signal Fractions**

- **Unmodified**: 9.3988e+07

### RNEEYN(N)KSVQEIQATFF Fuc 1 Hex 5 HexNAc 4

**Mass:**  3872.6601

(5.202e-04)

**Start Position**: 51

**End Position**: 68

| Profile Score | Tandem Score | TDA q-value | Volume | Spectrum Matches |
| --- | --- | --- | --- | --- |
| 0.0000 | 153.4854 | 0.0000 | 0.0000e+00 | 1 |

Adduct:

No Chromatogram Found

### RNEEYN(N)KSVQEIQATFF Fuc 2 Hex 6 HexNAc 5

**Mass:**  4381.8444

(1.799e-06)

**Charge States:** 4

**Start Position**: 51

**End Position**: 68

| Profile Score | Tandem Score | TDA q-value | Volume | Spectrum Matches |
| --- | --- | --- | --- | --- |
| -4.2945 | 153.0425 | 0.0000 | 6.3758e+07 | 3 |

**Adduct Signal Fractions**

- **Unmodified**: 6.3758e+07

### RNEEYN(N)KSVQEIQATF Fuc 1 Hex 5 HexNAc 4

**Mass:**  3723.5834

(2.049e-06)

**Charge States:** 3, 4

**Start Position**: 51

**End Position**: 67

| Profile Score | Tandem Score | TDA q-value | Volume | Spectrum Matches |
| --- | --- | --- | --- | --- |
| 9.0512 | 152.6414 | 0.0000 | 3.2629e+08 | 9 |

**Adduct Signal Fractions**

- **Unmodified**: 3.2629e+08

### RNEEYN(N)KSVQEIQATFF Fuc 1 Hex 5 HexNAc 4

**Mass:**  3870.6477

(3.232e-07)

**Charge States:** 3, 4

**Start Position**: 51

**End Position**: 68

| Profile Score | Tandem Score | TDA q-value | Volume | Spectrum Matches |
| --- | --- | --- | --- | --- |
| 10.2164 | 152.6006 | 0.0000 | 1.2408e+08 | 4 |

**Adduct Signal Fractions**

- **Unmodified**: 1.2408e+08

### RNEEYN(N)KSVQEIQATF Fuc 1 Hex 6 HexNAc 5

**Mass:**  4088.7192

(1.938e-06)

**Charge States:** 3, 4

**Start Position**: 51

**End Position**: 67

| Profile Score | Tandem Score | TDA q-value | Volume | Spectrum Matches |
| --- | --- | --- | --- | --- |
| 12.1294 | 152.1388 | 0.0000 | 3.6826e+09 | 89 |

**Adduct Signal Fractions**

- **Unmodified**: 3.6826e+09

### RNEEYN(N)KSVQEIQATF Fuc 2 Hex 6 HexNAc 5

**Mass:**  4234.7766

(1.025e-06)

**Charge States:** 3, 4

**Start Position**: 51

**End Position**: 67

| Profile Score | Tandem Score | TDA q-value | Volume | Spectrum Matches |
| --- | --- | --- | --- | --- |
| -3.8902 | 151.5035 | 0.0000 | 1.1945e+08 | 3 |

**Adduct Signal Fractions**

- **Unmodified**: 1.1945e+08

### YFTPN(N)KTEDTIF Fuc 1 Hex 9 HexNAc 8

**Mass:**  4703.8752

(1.932e-06)

**Charge States:** 4

**Start Position**: 68

**End Position**: 80

| Profile Score | Tandem Score | TDA q-value | Volume | Spectrum Matches |
| --- | --- | --- | --- | --- |
| 11.3959 | 151.4851 | 0.0000 | 3.8157e+07 | 2 |

**Adduct Signal Fractions**

- **Unmodified**: 3.8157e+07

### N(N)KSVQEIQATF Hex 6 HexNAc 5

**Mass:**  3251.3637

(1.812e-06)

**Charge States:** 3, 4

**Start Position**: 56

**End Position**: 67

| Profile Score | Tandem Score | TDA q-value | Volume | Spectrum Matches |
| --- | --- | --- | --- | --- |
| -4.3566 | 151.3142 | 0.0000 | 1.8263e+07 | 2 |

**Adduct Signal Fractions**

- **Unmodified**: 1.8263e+07

### NVQREN(N)GTVSRY Fuc 1 Hex 7 HexNAc 6

**Mass:**  3920.6035

(-2.878e-07)

**Charge States:** 3, 4

**Start Position**: 98

**End Position**: 110

| Profile Score | Tandem Score | TDA q-value | Volume | Spectrum Matches |
| --- | --- | --- | --- | --- |
| -3.0170 | 151.2543 | 0.0000 | 3.3271e+08 | 4 |

**Adduct Signal Fractions**

- **Unmodified**: 3.3271e+08

### RNEEYN(N)KSVQEIQATF Hex 7 HexNAc 6

**Mass:**  4307.7912

(1.295e-06)

**Charge States:** 4

**Start Position**: 51

**End Position**: 67

| Profile Score | Tandem Score | TDA q-value | Volume | Spectrum Matches |
| --- | --- | --- | --- | --- |
| -2.8303 | 151.2241 | 0.0000 | 2.7545e+08 | 3 |

**Adduct Signal Fractions**

- **Unmodified**: 2.7545e+08

### N(N)KSVQEIQATFF Hex 5 HexNAc 4

**Mass:**  3033.3023

(2.105e-06)

**Charge States:** 3

**Start Position**: 56

**End Position**: 68

| Profile Score | Tandem Score | TDA q-value | Volume | Spectrum Matches |
| --- | --- | --- | --- | --- |
| -3.8062 | 150.5228 | 0.0000 | 6.7947e+06 | 1 |

**Adduct Signal Fractions**

- **Unmodified**: 6.7947e+06

### FTPN(N)KTEDTIF Fuc 1 Hex 6 HexNAc 5

**Mass:**  3445.4093

(6.403e-07)

**Charge States:** 3, 4

**Start Position**: 69

**End Position**: 80

| Profile Score | Tandem Score | TDA q-value | Volume | Spectrum Matches |
| --- | --- | --- | --- | --- |
| 13.3767 | 149.1272 | 0.0000 | 7.8365e+07 | 5 |

**Adduct Signal Fractions**

- **Unmodified**: 7.8365e+07

### YFTPN(N)KTEDTIF Hex 7 HexNAc 4 Neu5Ac 2

**Mass:**  4003.5812

(1.178e-06)

**Charge States:** 3, 4

**Start Position**: 68

**End Position**: 80

| Profile Score | Tandem Score | TDA q-value | Volume | Spectrum Matches |
| --- | --- | --- | --- | --- |
| 14.4680 | 148.9524 | 0.0000 | 3.1771e+08 | 7 |

**Adduct Signal Fractions**

- **Unmodified**: 3.1771e+08

### FYFTPN(N)KTEDTIF Hex 7 HexNAc 4 Neu5Ac 2

**Mass:**  4150.6486

(-2.130e-06)

**Charge States:** 3, 4

**Start Position**: 67

**End Position**: 80

| Profile Score | Tandem Score | TDA q-value | Volume | Spectrum Matches |
| --- | --- | --- | --- | --- |
| 12.9617 | 146.9630 | 0.0040 | 2.2186e+08 | 8 |

**Adduct Signal Fractions**

- **Unmodified**: 2.2186e+08

### RNEEYN(N)KSVQEIQATFF Hex 7 HexNAc 6

**Mass:**  4454.8596

(1.469e-06)

**Charge States:** 3, 4

**Start Position**: 51

**End Position**: 68

| Profile Score | Tandem Score | TDA q-value | Volume | Spectrum Matches |
| --- | --- | --- | --- | --- |
| 14.2504 | 146.7157 | 0.0040 | 1.6082e+08 | 5 |

**Adduct Signal Fractions**

- **Unmodified**: 1.6082e+08

### FYFTPN(N)KT Hex 7 HexNAc 6

**Mass:**  3369.3469

(1.209e-06)

**Charge States:** 3

**Start Position**: 67

**End Position**: 75

| Profile Score | Tandem Score | TDA q-value | Volume | Spectrum Matches |
| --- | --- | --- | --- | --- |
| -4.2977 | 146.1660 | 0.0000 | 1.3579e+07 | 2 |

**Adduct Signal Fractions**

- **Unmodified**: 1.3579e+07

### FYFTPN(N)KTEDTIF Hex 9 HexNAc 8

**Mass:**  4704.8840

(1.932e-06)

**Charge States:** 3, 4

**Start Position**: 67

**End Position**: 80

| Profile Score | Tandem Score | TDA q-value | Volume | Spectrum Matches |
| --- | --- | --- | --- | --- |
| 15.7048 | 146.0538 | 0.0040 | 1.5738e+08 | 6 |

**Adduct Signal Fractions**

- **Unmodified**: 1.5738e+08

### FTPN(N)KTEDTIF Hex 8 HexNAc 7

**Mass:**  4029.6181

(1.272e-06)

**Charge States:** 3, 4

**Start Position**: 69

**End Position**: 80

| Profile Score | Tandem Score | TDA q-value | Volume | Spectrum Matches |
| --- | --- | --- | --- | --- |
| 14.8242 | 145.7279 | 0.0040 | 5.9327e+07 | 5 |

**Adduct Signal Fractions**

- **Unmodified**: 5.9327e+07

### FYFTPN(N)KTEDTIF Fuc 1 Hex 7 HexNAc 4 Neu5Ac 2

**Mass:**  4296.7092

(1.034e-06)

**Charge States:** 3, 4

**Start Position**: 67

**End Position**: 80

| Profile Score | Tandem Score | TDA q-value | Volume | Spectrum Matches |
| --- | --- | --- | --- | --- |
| -17.0413 | 145.3099 | 0.0040 | 3.5729e+07 | 2 |

**Adduct Signal Fractions**

- **Unmodified**: 3.5729e+07

### FYFTPN(N)KTEDTIF Fuc 1 Hex 8 HexNAc 7

**Mass:**  4485.8102

(1.421e-06)

**Charge States:** 3, 4

**Start Position**: 67

**End Position**: 80

| Profile Score | Tandem Score | TDA q-value | Volume | Spectrum Matches |
| --- | --- | --- | --- | --- |
| 15.1126 | 144.1528 | 0.0040 | 1.2690e+08 | 5 |

**Adduct Signal Fractions**

- **Unmodified**: 1.2690e+08

### RNEEYN(N)KSVQEIQAT Hex 6 HexNAc 5

**Mass:**  3795.5905

(1.736e-06)

**Charge States:** 3, 4

**Start Position**: 51

**End Position**: 66

| Profile Score | Tandem Score | TDA q-value | Volume | Spectrum Matches |
| --- | --- | --- | --- | --- |
| -19.7881 | 142.4253 | 0.0077 | 2.3234e+07 | 2 |

**Adduct Signal Fractions**

- **Unmodified**: 2.3234e+07

### YN(N)SSYL Hex 6 HexNAc 5

**Mass:**  2733.0499

(2.860e-06)

**Charge States:** 3

**Start Position**: 92

**End Position**: 98

| Profile Score | Tandem Score | TDA q-value | Volume | Spectrum Matches |
| --- | --- | --- | --- | --- |
| -5.0162 | 142.3415 | 0.0000 | 2.0758e+06 | 1 |

**Adduct Signal Fractions**

- **Unmodified**: 2.0758e+06

### RNEEYN(N)KSVQEIQATFF Fuc 1 Hex 7 HexNAc 6

**Mass:**  4600.9191

(1.179e-06)

**Charge States:** 4

**Start Position**: 51

**End Position**: 68

| Profile Score | Tandem Score | TDA q-value | Volume | Spectrum Matches |
| --- | --- | --- | --- | --- |
| -3.0656 | 142.1167 | 0.0077 | 6.1450e+07 | 3 |

**Adduct Signal Fractions**

- **Unmodified**: 6.1450e+07

### RNEEYN(N)KSVQEIQATF Fuc 1 Hex 7 HexNAc 6

**Mass:**  4453.8503

(1.783e-06)

**Charge States:** 4

**Start Position**: 51

**End Position**: 67

| Profile Score | Tandem Score | TDA q-value | Volume | Spectrum Matches |
| --- | --- | --- | --- | --- |
| -3.2859 | 142.0348 | 0.0077 | 1.1665e+08 | 2 |

**Adduct Signal Fractions**

- **Unmodified**: 1.1665e+08

### EEYN(N)KSVQEIQATFF Hex 6 HexNAc 5

**Mass:**  3819.5824

(-1.418e-06)

**Charge States:** 3

**Start Position**: 53

**End Position**: 68

| Profile Score | Tandem Score | TDA q-value | Volume | Spectrum Matches |
| --- | --- | --- | --- | --- |
| -4.0990 | 141.6199 | 0.0077 | 2.5810e+07 | 3 |

**Adduct Signal Fractions**

- **Unmodified**: 2.5810e+07

### RNEEYN(N)KSVQEIQATF Hex 4 HexNAc 3

**Mass:**  3213.4159

(3.200e-04)

**Start Position**: 51

**End Position**: 67

| Profile Score | Tandem Score | TDA q-value | Volume | Spectrum Matches |
| --- | --- | --- | --- | --- |
| 0.0000 | 141.3875 | 0.0077 | 0.0000e+00 | 1 |

Adduct:

No Chromatogram Found

### YFTPN(N)KTEDTIFL Hex 6 HexNAc 5

**Mass:**  3575.5003

(2.305e-06)

**Charge States:** 3

**Start Position**: 68

**End Position**: 81

| Profile Score | Tandem Score | TDA q-value | Volume | Spectrum Matches |
| --- | --- | --- | --- | --- |
| -3.2875 | 140.1029 | 0.0099 | 2.4776e+07 | 3 |

**Adduct Signal Fractions**

- **Unmodified**: 2.4776e+07

### YFTPN(N)KTEDTIF Fuc 2 Hex 6 HexNAc 5

**Mass:**  3754.5364

(1.044e-06)

**Charge States:** 4

**Start Position**: 68

**End Position**: 80

| Profile Score | Tandem Score | TDA q-value | Volume | Spectrum Matches |
| --- | --- | --- | --- | --- |
| -6.4028 | 140.0932 | 0.0099 | 7.2855e+06 | 1 |

**Adduct Signal Fractions**

- **Unmodified**: 7.2855e+06

### TPN(N)KTEDTIF Hex 7 HexNAc 4 Neu5Ac 2

**Mass:**  3693.4462

(3.709e-07)

**Charge States:** 3, 4

**Start Position**: 70

**End Position**: 80

| Profile Score | Tandem Score | TDA q-value | Volume | Spectrum Matches |
| --- | --- | --- | --- | --- |
| -3.9009 | 137.3589 | 0.0099 | 1.1308e+07 | 4 |

**Adduct Signal Fractions**

- **Unmodified**: 1.1308e+07

### NVQREN(N)GTVSRY Fuc 1 Hex 6 HexNAc 5

**Mass:**  3555.4726

(-7.966e-07)

**Charge States:** 3

**Start Position**: 98

**End Position**: 110

| Profile Score | Tandem Score | TDA q-value | Volume | Spectrum Matches |
| --- | --- | --- | --- | --- |
| -5.4035 | 137.2126 | 0.0099 | 6.5206e+07 | 3 |

**Adduct Signal Fractions**

- **Unmodified**: 6.5206e+07

### N(N)KTEDTIF Hex 6 HexNAc 5

**Mass:**  2954.1879

(2.777e-06)

**Charge States:** 3

**Start Position**: 72

**End Position**: 80

| Profile Score | Tandem Score | TDA q-value | Volume | Spectrum Matches |
| --- | --- | --- | --- | --- |
| -20.5515 | 137.0759 | 0.0000 | 2.9222e+06 | 1 |

**Adduct Signal Fractions**

- **Unmodified**: 2.9222e+06

### YFTPN(N)KTEDTIF Hex 10 HexNAc 9

**Mass:**  4922.9514

(1.512e-06)

**Charge States:** 4

**Start Position**: 68

**End Position**: 80

| Profile Score | Tandem Score | TDA q-value | Volume | Spectrum Matches |
| --- | --- | --- | --- | --- |
| -3.2018 | 136.4233 | 0.0099 | 2.3477e+07 | 1 |

**Adduct Signal Fractions**

- **Unmodified**: 2.3477e+07

### NVQREN(N)GTVSRY Hex 6 HexNAc 5

**Mass:**  3409.4130

(-4.144e-06)

**Charge States:** 3

**Start Position**: 98

**End Position**: 110

| Profile Score | Tandem Score | TDA q-value | Volume | Spectrum Matches |
| --- | --- | --- | --- | --- |
| -4.1681 | 135.2118 | 0.0099 | 1.8175e+08 | 4 |

**Adduct Signal Fractions**

- **Unmodified**: 1.8175e+08

### RNEEYN(N)KSVQEIQAT Hex 5 HexNAc 4

**Mass:**  3430.4562

(-2.197e-06)

**Charge States:** 3, 4

**Start Position**: 51

**End Position**: 66

| Profile Score | Tandem Score | TDA q-value | Volume | Spectrum Matches |
| --- | --- | --- | --- | --- |
| -4.9786 | 135.0718 | 0.0099 | 3.5961e+07 | 3 |

**Adduct Signal Fractions**

- **Unmodified**: 3.5961e+07

### NVQREN(N)GTVSRY Fuc 2 Hex 7 HexNAc 6

**Mass:**  4066.6613

(-8.868e-07)

**Charge States:** 4

**Start Position**: 98

**End Position**: 110

| Profile Score | Tandem Score | TDA q-value | Volume | Spectrum Matches |
| --- | --- | --- | --- | --- |
| -18.6344 | 133.4432 | 0.0099 | 7.7397e+07 | 2 |

**Adduct Signal Fractions**

- **Unmodified**: 7.7397e+07

### YFTPN(N)KT Hex 7 HexNAc 6

**Mass:**  3222.2832

(2.722e-06)

**Charge States:** 3

**Start Position**: 68

**End Position**: 75

| Profile Score | Tandem Score | TDA q-value | Volume | Spectrum Matches |
| --- | --- | --- | --- | --- |
| -21.2077 | 131.7191 | 0.0000 | 2.6525e+06 | 1 |

**Adduct Signal Fractions**

- **Unmodified**: 2.6525e+06

### TPN(N)KTEDTIF Fuc 4 Hex 8 HexNAc 5

**Mass:**  4060.6040

(-3.406e-06)

**Charge States:** 3, 4

**Start Position**: 70

**End Position**: 80

| Profile Score | Tandem Score | TDA q-value | Volume | Spectrum Matches |
| --- | --- | --- | --- | --- |
| 13.1320 | 131.3275 | 0.0100 | 1.3515e+08 | 4 |

**Adduct Signal Fractions**

- **Unmodified**: 1.3515e+08

### YFTPN(N)KTEDTIFL Hex 7 HexNAc 6

**Mass:**  3940.6320

(1.698e-06)

**Charge States:** 3, 4

**Start Position**: 68

**End Position**: 81

| Profile Score | Tandem Score | TDA q-value | Volume | Spectrum Matches |
| --- | --- | --- | --- | --- |
| -4.0426 | 130.1405 | 0.0100 | 4.3605e+07 | 3 |

**Adduct Signal Fractions**

- **Unmodified**: 4.3605e+07

### FTPN(N)KTEDTIF Hex 9 HexNAc 8

**Mass:**  4394.7541

(2.616e-06)

**Charge States:** 4

**Start Position**: 69

**End Position**: 80

| Profile Score | Tandem Score | TDA q-value | Volume | Spectrum Matches |
| --- | --- | --- | --- | --- |
| 14.1987 | 129.9716 | 0.0100 | 1.2377e+07 | 2 |

**Adduct Signal Fractions**

- **Unmodified**: 1.2377e+07

### RNEEYN(N)KSVQEIQATF Hex 8 HexNAc 7

**Mass:**  4672.9226

(1.232e-06)

**Charge States:** 4

**Start Position**: 51

**End Position**: 67

| Profile Score | Tandem Score | TDA q-value | Volume | Spectrum Matches |
| --- | --- | --- | --- | --- |
| -3.8654 | 129.8300 | 0.0100 | 6.2149e+07 | 2 |

**Adduct Signal Fractions**

- **Unmodified**: 6.2149e+07

### FRNEEYN(N)KSVQEIQATF Hex 6 HexNAc 5

**Mass:**  4089.7270

(1.520e-06)

**Charge States:** 4

**Start Position**: 50

**End Position**: 67

| Profile Score | Tandem Score | TDA q-value | Volume | Spectrum Matches |
| --- | --- | --- | --- | --- |
| 11.2831 | 129.7472 | 0.0100 | 4.3981e+07 | 3 |

**Adduct Signal Fractions**

- **Unmodified**: 4.3981e+07

### FTPN(N)KTEDTIF Hex 7 HexNAc 4 Neu5Ac 2

**Mass:**  3840.5139

(1.415e-06)

**Charge States:** 3, 4

**Start Position**: 69

**End Position**: 80

| Profile Score | Tandem Score | TDA q-value | Volume | Spectrum Matches |
| --- | --- | --- | --- | --- |
| -4.0210 | 129.1882 | 0.0100 | 3.0471e+07 | 4 |

**Adduct Signal Fractions**

- **Unmodified**: 3.0471e+07

### NVQREN(N)GTVSRY Hex 7 HexNAc 6

**Mass:**  3774.5456

(-5.790e-07)

**Charge States:** 3, 4

**Start Position**: 98

**End Position**: 110

| Profile Score | Tandem Score | TDA q-value | Volume | Spectrum Matches |
| --- | --- | --- | --- | --- |
| 12.9574 | 128.4414 | 0.0100 | 6.6150e+08 | 6 |

**Adduct Signal Fractions**

- **Unmodified**: 6.6150e+08

### FYFTPN(N)KT Hex 6 HexNAc 5

**Mass:**  3004.2147

(2.393e-06)

**Charge States:** 3

**Start Position**: 67

**End Position**: 75

| Profile Score | Tandem Score | TDA q-value | Volume | Spectrum Matches |
| --- | --- | --- | --- | --- |
| -3.5635 | 128.4357 | 0.0000 | 6.2823e+06 | 2 |

**Adduct Signal Fractions**

- **Unmodified**: 6.2823e+06

### N(N)KTEDTIF Fuc 5 Hex 5 HexNAc 8 Neu5Ac 1

**Mass:**  4425.7269

(6.731e-04)

**Start Position**: 72

**End Position**: 80

| Profile Score | Tandem Score | TDA q-value | Volume | Spectrum Matches |
| --- | --- | --- | --- | --- |
| 0.0000 | 128.2914 | 0.0000 | 0.0000e+00 | 1 |

Adduct:

No Chromatogram Found

### YFTPN(N)KTEDTIF Fuc 2 Hex 8 HexNAc 7

**Mass:**  4484.8034

(1.641e-06)

**Charge States:** 4

**Start Position**: 68

**End Position**: 80

| Profile Score | Tandem Score | TDA q-value | Volume | Spectrum Matches |
| --- | --- | --- | --- | --- |
| 12.7236 | 125.2408 | 0.0100 | 4.1671e+07 | 2 |

**Adduct Signal Fractions**

- **Unmodified**: 4.1671e+07

### FYFTPN(N)KTEDTIF Hex 3 HexNAc 5 Neu5Ac 3

**Mass:**  3996.5952

(-2.641e-06)

**Charge States:** 3, 4

**Start Position**: 67

**End Position**: 80

| Profile Score | Tandem Score | TDA q-value | Volume | Spectrum Matches |
| --- | --- | --- | --- | --- |
| 13.6975 | 124.4576 | 0.0100 | 2.4449e+08 | 3 |

**Adduct Signal Fractions**

- **Unmodified**: 2.4449e+08

### YFTPN(N)KTEDTIF Fuc 1 Hex 8 HexNAc 4 Neu5Ac 1

**Mass:**  4020.6062

(3.669e-06)

**Charge States:** 3, 4

**Start Position**: 68

**End Position**: 80

| Profile Score | Tandem Score | TDA q-value | Volume | Spectrum Matches |
| --- | --- | --- | --- | --- |
| -19.5076 | 124.3871 | 0.0100 | 2.5230e+07 | 2 |

**Adduct Signal Fractions**

- **Unmodified**: 2.5230e+07

### RN(D)EEYN(N)KSVQEIQATF Hex 5 HexNAc 4

**Mass:**  3578.5102

(2.764e-04)

**Charge States:** 3, 4

**Start Position**: 51

**End Position**: 67

| Profile Score | Tandem Score | TDA q-value | Volume | Spectrum Matches |
| --- | --- | --- | --- | --- |
| 10.8334 | 123.9156 | 0.0100 | 1.3552e+09 | 30 |

**Adduct Signal Fractions**

- **Unmodified**: 1.3552e+09

### FTPN(N)KTEDTIF Fuc 2 Hex 7 HexNAc 6

**Mass:**  3956.6020

(3.544e-06)

**Charge States:** 3, 4

**Start Position**: 69

**End Position**: 80

| Profile Score | Tandem Score | TDA q-value | Volume | Spectrum Matches |
| --- | --- | --- | --- | --- |
| -4.4939 | 123.6694 | 0.0100 | 1.5003e+07 | 4 |

**Adduct Signal Fractions**

- **Unmodified**: 1.5003e+07

### FYFTPN(N)KTEDTIF Fuc 2 Hex 8 HexNAc 7

**Mass:**  4631.8664

(2.524e-06)

**Charge States:** 4

**Start Position**: 67

**End Position**: 80

| Profile Score | Tandem Score | TDA q-value | Volume | Spectrum Matches |
| --- | --- | --- | --- | --- |
| -3.8249 | 123.0417 | 0.0100 | 2.3872e+07 | 2 |

**Adduct Signal Fractions**

- **Unmodified**: 2.3872e+07

### RN(D)EEYN(N)KSVQEIQATF Hex 6 HexNAc 5

**Mass:**  3943.6415

(2.509e-04)

**Charge States:** 3, 4

**Start Position**: 51

**End Position**: 67

| Profile Score | Tandem Score | TDA q-value | Volume | Spectrum Matches |
| --- | --- | --- | --- | --- |
| 10.7640 | 121.6380 | 0.0100 | 1.2103e+09 | 13 |

**Adduct Signal Fractions**

- **Unmodified**: 1.2103e+09

### TPN(N)KTEDTIF Fuc 3 Hex 3 HexNAc 9 Neu5Ac 1

**Mass:**  4207.6721

(-9.919e-06)

**Charge States:** 3, 4

**Start Position**: 70

**End Position**: 80

| Profile Score | Tandem Score | TDA q-value | Volume | Spectrum Matches |
| --- | --- | --- | --- | --- |
| 9.4875 | 121.5172 | 0.0100 | 1.0897e+08 | 5 |

**Adduct Signal Fractions**

- **Unmodified**: 1.0897e+08

### FYFTPN(N)KTEDTIF Hex 7 HexNAc 5

**Mass:**  3771.5546

(5.733e-06)

**Charge States:** 4

**Start Position**: 67

**End Position**: 80

| Profile Score | Tandem Score | TDA q-value | Volume | Spectrum Matches |
| --- | --- | --- | --- | --- |
| -20.3197 | 121.2058 | 0.0100 | 1.3760e+07 | 1 |

**Adduct Signal Fractions**

- **Unmodified**: 1.3760e+07

### LNVQREN(N)GTVSRY Fuc 1 Hex 7 HexNAc 6

**Mass:**  4033.6901

(4.384e-07)

**Charge States:** 3, 4

**Start Position**: 97

**End Position**: 110

| Profile Score | Tandem Score | TDA q-value | Volume | Spectrum Matches |
| --- | --- | --- | --- | --- |
| 13.7994 | 120.8172 | 0.0100 | 2.9839e+08 | 6 |

**Adduct Signal Fractions**

- **Unmodified**: 2.9839e+08

### FYFTPN(N)KTEDTIF Hex 8 HexNAc 6

**Mass:**  4137.7014

(2.509e-04)

**Charge States:** 4

**Start Position**: 67

**End Position**: 80

| Profile Score | Tandem Score | TDA q-value | Volume | Spectrum Matches |
| --- | --- | --- | --- | --- |
| -20.5648 | 120.6863 | 0.0100 | 9.0743e+07 | 5 |

**Adduct Signal Fractions**

- **Unmodified**: 9.0743e+07

### FTPN(N)KTEDTIF Fuc 1 Hex 8 HexNAc 7

**Mass:**  4175.6760

(1.723e-06)

**Charge States:** 3, 4

**Start Position**: 69

**End Position**: 80

| Profile Score | Tandem Score | TDA q-value | Volume | Spectrum Matches |
| --- | --- | --- | --- | --- |
| 10.7139 | 119.5888 | 0.0100 | 1.0416e+07 | 4 |

**Adduct Signal Fractions**

- **Unmodified**: 1.0416e+07

### N(N)KSVQEIQATFF Hex 6 HexNAc 5

**Mass:**  3398.4325

(4.159e-07)

**Charge States:** 3, 4

**Start Position**: 56

**End Position**: 68

| Profile Score | Tandem Score | TDA q-value | Volume | Spectrum Matches |
| --- | --- | --- | --- | --- |
| -18.1454 | 118.5895 | 0.0100 | 5.0156e+06 | 2 |

**Adduct Signal Fractions**

- **Unmodified**: 5.0156e+06

### RN(D)EEYN(N)KSVQEIQATFF Hex 5 HexNAc 4

**Mass:**  3725.5749

(2.640e-04)

**Charge States:** 3, 4

**Start Position**: 51

**End Position**: 68

| Profile Score | Tandem Score | TDA q-value | Volume | Spectrum Matches |
| --- | --- | --- | --- | --- |
| 10.5914 | 117.0803 | 0.0122 | 3.1034e+08 | 10 |

**Adduct Signal Fractions**

- **Unmodified**: 3.1034e+08

### RNEEYN(N)KSVQEIQATF Hex 9 HexNAc 8

**Mass:**  5038.0578

(1.809e-06)

**Charge States:** 4

**Start Position**: 51

**End Position**: 67

| Profile Score | Tandem Score | TDA q-value | Volume | Spectrum Matches |
| --- | --- | --- | --- | --- |
| -3.4352 | 116.4220 | 0.0122 | 3.5805e+07 | 2 |

**Adduct Signal Fractions**

- **Unmodified**: 3.5805e+07

### RN(D)EEYN(N)KSVQEIQATFF Fuc 1 Hex 6 HexNAc 5

**Mass:**  4236.7717

(2.359e-04)

**Charge States:** 3, 4

**Start Position**: 51

**End Position**: 68

| Profile Score | Tandem Score | TDA q-value | Volume | Spectrum Matches |
| --- | --- | --- | --- | --- |
| 9.7227 | 116.3355 | 0.0143 | 1.6873e+08 | 11 |

**Adduct Signal Fractions**

- **Unmodified**: 1.6873e+08

### LVPVPITN(N)ATL Hex 6 HexNAc 5

**Mass:**  3124.3972

(9.338e-07)

**Charge States:** 4

**Start Position**: 26

**End Position**: 37

| Profile Score | Tandem Score | TDA q-value | Volume | Spectrum Matches |
| --- | --- | --- | --- | --- |
| -4.6337 | 116.2614 | 0.0143 | 7.2858e+06 | 2 |

**Adduct Signal Fractions**

- **Unmodified**: 7.2858e+06

### N(D)VQREN(N)GTVSRY Fuc 3 Hex 7 HexNAc 6

**Mass:**  4215.7273

(7.137e-04)

**Start Position**: 98

**End Position**: 110

| Profile Score | Tandem Score | TDA q-value | Volume | Spectrum Matches |
| --- | --- | --- | --- | --- |
| 0.0000 | 115.8912 | 0.0143 | 0.0000e+00 | 1 |

Adduct:

No Chromatogram Found

### LNVQREN(N)GTVSRY Hex 7 HexNAc 6

**Mass:**  3887.6362

(1.262e-06)

**Charge States:** 3, 4

**Start Position**: 97

**End Position**: 110

| Profile Score | Tandem Score | TDA q-value | Volume | Spectrum Matches |
| --- | --- | --- | --- | --- |
| 13.8935 | 115.5114 | 0.0162 | 5.6722e+08 | 6 |

**Adduct Signal Fractions**

- **Unmodified**: 5.6722e+08

### TN(N)ATLDRITGKW Hex 6 HexNAc 5

**Mass:**  3362.4417

(6.719e-07)

**Charge States:** 3, 4

**Start Position**: 32

**End Position**: 44

| Profile Score | Tandem Score | TDA q-value | Volume | Spectrum Matches |
| --- | --- | --- | --- | --- |
| -4.1832 | 114.9778 | 0.0162 | 2.0738e+07 | 4 |

**Adduct Signal Fractions**

- **Unmodified**: 2.0738e+07

### LNVQREN(N)GTVSRY Fuc 1 Hex 6 HexNAc 5

**Mass:**  3668.5651

(2.109e-06)

**Charge States:** 3, 4

**Start Position**: 97

**End Position**: 110

| Profile Score | Tandem Score | TDA q-value | Volume | Spectrum Matches |
| --- | --- | --- | --- | --- |
| 12.9426 | 114.0873 | 0.0183 | 6.7148e+07 | 3 |

**Adduct Signal Fractions**

- **Unmodified**: 6.7148e+07

### YFTPN(N)KT Fuc 5 Hex 7 HexNAc 9

**Mass:**  4563.8441

(4.477e-04)

**Start Position**: 68

**End Position**: 75

| Profile Score | Tandem Score | TDA q-value | Volume | Spectrum Matches |
| --- | --- | --- | --- | --- |
| 0.0000 | 112.1439 | 0.0000 | 0.0000e+00 | 1 |

Adduct:

No Chromatogram Found

### LNVQREN(N)GTVSRY Hex 6 HexNAc 5

**Mass:**  3522.5004

(1.915e-06)

**Charge States:** 3, 4

**Start Position**: 97

**End Position**: 110

| Profile Score | Tandem Score | TDA q-value | Volume | Spectrum Matches |
| --- | --- | --- | --- | --- |
| 14.9055 | 111.4710 | 0.0199 | 1.1957e+08 | 4 |

**Adduct Signal Fractions**

- **Unmodified**: 1.1957e+08

### RN(D)EEYN(N)KSVQEIQATFF Hex 6 HexNAc 5

**Mass:**  4090.7089

(2.416e-04)

**Charge States:** 3, 4

**Start Position**: 51

**End Position**: 68

| Profile Score | Tandem Score | TDA q-value | Volume | Spectrum Matches |
| --- | --- | --- | --- | --- |
| 9.9262 | 110.5026 | 0.0211 | 3.2717e+08 | 14 |

**Adduct Signal Fractions**

- **Unmodified**: 3.2717e+08

### EEYN(N)KSVQEIQATFF Hex 5 HexNAc 4

**Mass:**  3454.4470

(7.192e-07)

**Charge States:** 3

**Start Position**: 53

**End Position**: 68

| Profile Score | Tandem Score | TDA q-value | Volume | Spectrum Matches |
| --- | --- | --- | --- | --- |
| -4.6133 | 110.0468 | 0.0211 | 2.1525e+07 | 3 |

**Adduct Signal Fractions**

- **Unmodified**: 2.1525e+07

### C(C)ANLVPVPITN(N)ATLDRITGKW Hex 6 HexNAc 5

**Mass:**  4325.9641

(9.200e-07)

**Charge States:** 3, 4

**Start Position**: 23

**End Position**: 44

| Profile Score | Tandem Score | TDA q-value | Volume | Spectrum Matches |
| --- | --- | --- | --- | --- |
| 9.6676 | 109.6946 | 0.0211 | 1.0234e+08 | 5 |

**Adduct Signal Fractions**

- **Unmodified**: 1.0234e+08

### RNEEYN(N)KSVQEIQATFF Hex 8 HexNAc 7

**Mass:**  4819.9924

(1.890e-06)

**Charge States:** 4

**Start Position**: 51

**End Position**: 68

| Profile Score | Tandem Score | TDA q-value | Volume | Spectrum Matches |
| --- | --- | --- | --- | --- |
| -2.9200 | 109.6547 | 0.0211 | 1.6895e+07 | 2 |

**Adduct Signal Fractions**

- **Unmodified**: 1.6895e+07

### FYFTPN(N)KTED Hex 7 HexNAc 6

**Mass:**  3613.4170

(1.357e-06)

**Charge States:** 4

**Start Position**: 67

**End Position**: 77

| Profile Score | Tandem Score | TDA q-value | Volume | Spectrum Matches |
| --- | --- | --- | --- | --- |
| -4.7781 | 109.6483 | 0.0211 | 5.2527e+06 | 2 |

**Adduct Signal Fractions**

- **Unmodified**: 5.2527e+06

### FYFTPN(N)KTEDTIF Hex 10 HexNAc 9

**Mass:**  5070.0163

(1.545e-06)

**Charge States:** 4

**Start Position**: 67

**End Position**: 80

| Profile Score | Tandem Score | TDA q-value | Volume | Spectrum Matches |
| --- | --- | --- | --- | --- |
| -3.9078 | 108.6449 | 0.0211 | 1.7746e+07 | 2 |

**Adduct Signal Fractions**

- **Unmodified**: 1.7746e+07

### NVQREN(N)GTVSRY Hex 9 HexNAc 8

**Mass:**  4504.8137

(4.394e-07)

**Charge States:** 4

**Start Position**: 98

**End Position**: 110

| Profile Score | Tandem Score | TDA q-value | Volume | Spectrum Matches |
| --- | --- | --- | --- | --- |
| -19.8371 | 107.3858 | 0.0269 | 3.4845e+07 | 1 |

**Adduct Signal Fractions**

- **Unmodified**: 3.4845e+07

### NVQREN(N)GTVSRY Fuc 1 Hex 9 HexNAc 8

**Mass:**  4650.8679

(-3.810e-07)

**Charge States:** 4

**Start Position**: 98

**End Position**: 110

| Profile Score | Tandem Score | TDA q-value | Volume | Spectrum Matches |
| --- | --- | --- | --- | --- |
| -20.7045 | 105.8542 | 0.0299 | 1.3266e+07 | 1 |

**Adduct Signal Fractions**

- **Unmodified**: 1.3266e+07

### TPN(N)KTEDTIF Hex 9 HexNAc 5

**Mass:**  3638.4483

(2.691e-06)

**Charge States:** 3, 4

**Start Position**: 70

**End Position**: 80

| Profile Score | Tandem Score | TDA q-value | Volume | Spectrum Matches |
| --- | --- | --- | --- | --- |
| 13.5482 | 102.0374 | 0.0345 | 2.7809e+07 | 4 |

**Adduct Signal Fractions**

- **Unmodified**: 2.7809e+07

### C(C)ANLVPVPITN(N)ATL Hex 6 HexNAc 5

**Mass:**  3469.5100

(1.431e-06)

**Charge States:** 3, 4

**Start Position**: 23

**End Position**: 37

| Profile Score | Tandem Score | TDA q-value | Volume | Spectrum Matches |
| --- | --- | --- | --- | --- |
| 12.3497 | 101.7927 | 0.0345 | 2.3070e+07 | 6 |

**Adduct Signal Fractions**

- **Unmodified**: 2.3070e+07

### LNVQREN(N)GTVSRY Fuc 1 Hex 6 HexNAc 5

**Mass:**  3669.5797

(2.788e-04)

**Start Position**: 97

**End Position**: 110

| Profile Score | Tandem Score | TDA q-value | Volume | Spectrum Matches |
| --- | --- | --- | --- | --- |
| 0.0000 | 100.9142 | 0.0414 | 0.0000e+00 | 1 |

Adduct:

No Chromatogram Found

### RNEEYN(N)KSVQEIQAT Fuc 2 Hex 5 HexNAc 4

**Mass:**  3722.5687

(3.766e-07)

**Charge States:** 3, 4

**Start Position**: 51

**End Position**: 66

| Profile Score | Tandem Score | TDA q-value | Volume | Spectrum Matches |
| --- | --- | --- | --- | --- |
| -19.5450 | 99.9575 | 0.0463 | 2.0882e+07 | 1 |

**Adduct Signal Fractions**

- **Unmodified**: 2.0882e+07

### LNVQREN(N)GTVSRY Fuc 2 Hex 7 HexNAc 6

**Mass:**  4179.7497

(6.143e-07)

**Charge States:** 3, 4

**Start Position**: 97

**End Position**: 110

| Profile Score | Tandem Score | TDA q-value | Volume | Spectrum Matches |
| --- | --- | --- | --- | --- |
| -4.1125 | 99.3696 | 0.0472 | 7.9458e+07 | 3 |

**Adduct Signal Fractions**

- **Unmodified**: 7.9458e+07

### LNVQREN(N)GTVSRY Fuc 1 Hex 9 HexNAc 8

**Mass:**  4763.9597

(1.298e-06)

**Charge States:** 4

**Start Position**: 97

**End Position**: 110

| Profile Score | Tandem Score | TDA q-value | Volume | Spectrum Matches |
| --- | --- | --- | --- | --- |
| -20.4728 | 97.8488 | 0.0472 | 1.2534e+07 | 1 |

**Adduct Signal Fractions**

- **Unmodified**: 1.2534e+07

### P13473|LAMP2\_HUMAN

**>P13473|LAMP2\_HUMAN**  
 `MVCFRLFPVPGSGLVLVCLVLGAVRSYALELNLTDSENATCLYAKWQMNFTVRYETTNKTYKTVTISDHGTVTYNGSICG  
DDQNGPKIAVQFGPGFSWIANFTKAASTYSIDSVSFSYNTGDNTTFPDAEDKGILTVDELLAIRIPLNDLFRCNSLSTLE  
KNDVVQHYWDVLVQAFVQNGTVSTNEFLCDKDKTSTVAPTIHTTVPSPTTTPTPKEKPEAGTYSVNNGNDTCLLATMGLQ  
LNITQDKVASVININPNTTHSTGSCRSHTALLRLNSSTIKYLDFVFAVKNENRFYLKEVNISMYLVNGSVFSIANNNLSY  
WDAPLGSSYMCNKEQTVSVSGAFQINTFDLRVQPFNVTQGKYSTAQDCSADDDNFLVPIAVGAALAGVLILVLLAYFIGL  
KHHHAGYEQF`

| Site | Type | Count |
| --- | --- | --- |
| 307 | N-linked | 3 |

| Mass | Sequence | MS_2_ Score | `q`-value | MS_1_ Score | Signal | Protein Coordinates | Tandem Scans | Start Time | Apex Time | End Time |
| --- | --- | --- | --- | --- | --- | --- | --- | --- | --- | --- |
| 2722.1141 | LVN(N)GSVF Hex 6 HexNAc 5 | 176.281 | 0.0 | -3.982 | 7.893e+06 | 304 - 311 | 2 | 29.46 | 29.64 | 29.97 |
| 2868.1726 | LVN(N)GSVF Fuc 1 Hex 6 HexNAc 5 | 155.691 | 0.0 | -4.247 | 2.463e+06 | 304 - 311 | 2 | 29.12 | 29.29 | 29.46 |
| 2356.9805 | LVN(N)GSVF Hex 5 HexNAc 4 | 134.653 | 0.0 | -5.437 | 4.403e+06 | 304 - 311 | 1 | 29.97 | 30.14 | 30.48 |

### LVN(N)GSVF Hex 6 HexNAc 5

**Mass:**  2722.1136

(1.017e-06)

**Charge States:** 3

**Start Position**: 305

**End Position**: 312

| Profile Score | Tandem Score | TDA q-value | Volume | Spectrum Matches |
| --- | --- | --- | --- | --- |
| -3.9817 | 176.2810 | 0.0000 | 7.8927e+06 | 2 |

**Adduct Signal Fractions**

- **Unmodified**: 7.8927e+06

### LVN(N)GSVF Fuc 1 Hex 6 HexNAc 5

**Mass:**  2868.1726

(2.442e-06)

**Charge States:** 3

**Start Position**: 305

**End Position**: 312

| Profile Score | Tandem Score | TDA q-value | Volume | Spectrum Matches |
| --- | --- | --- | --- | --- |
| -4.2468 | 155.6913 | 0.0000 | 2.4633e+06 | 2 |

**Adduct Signal Fractions**

- **Unmodified**: 2.4633e+06

### LVN(N)GSVF Hex 5 HexNAc 4

**Mass:**  2356.9810

(7.446e-07)

**Charge States:** 3

**Start Position**: 305

**End Position**: 312

| Profile Score | Tandem Score | TDA q-value | Volume | Spectrum Matches |
| --- | --- | --- | --- | --- |
| -5.4370 | 134.6526 | 0.0000 | 4.4034e+06 | 1 |

**Adduct Signal Fractions**

- **Unmodified**: 4.4034e+06

### P00738|HPT\_HUMAN

**>P00738|HPT\_HUMAN**  
 `MSALGAVIALLLWGQLFAVDSGNDVTDIADDGCPKPPEIAHGYVEHSVRYQCKNYYKLRTEGDGVYTLNDKKQWINKAVG  
DKLPECEADDGCPKPPEIAHGYVEHSVRYQCKNYYKLRTEGDGVYTLNNEKQWINKAVGDKLPECEAVCGKPKNPANPVQ  
RILGGHLDAKGSFPWQAKMVSHHNLTTGATLINEQWLLTTAKNLFLNHSENATAKDIAPTLTLYVGKKQLVEIEKVVLHP  
NYSQVDIGLIKLKQKVSVNERVMPICLPSKDYAEVGRVGYVSGWGRNANFKFTDHLKYVMLPVADQDQCIRHYEGSTVPE  
KKTPKSPVGVQPILNEHTFCAGMSKYQEDTCYGDAGSAFAVHDLEEDTWYATGILSFDKSCAVAEYGVYVKVTSIQDWVQ  
KTIAEN`

| Site | Type | Count |
| --- | --- | --- |
| 184 | N-linked | 2 |

| Mass | Sequence | MS_2_ Score | `q`-value | MS_1_ Score | Signal | Protein Coordinates | Tandem Scans | Start Time | Apex Time | End Time |
| --- | --- | --- | --- | --- | --- | --- | --- | --- | --- | --- |
| 2872.2279 | VSHHN(N)LTTGATL Hex 5 HexNAc 4 | 157.430 | 0.0 | -4.251 | 2.594e+07 | 179 - 191 | 2 | 16.97 | 17.14 | 17.47 |
| 3237.3590 | VSHHN(N)LTTGATL Hex 6 HexNAc 5 | 157.365 | 0.0 | -3.535 | 1.087e+07 | 179 - 191 | 1 | 16.80 | 16.97 | 17.14 |

### VSHHN(N)LTTGATL Hex 5 HexNAc 4

**Mass:**  2872.2280

(1.684e-06)

**Charge States:** 3

**Start Position**: 180

**End Position**: 192

| Profile Score | Tandem Score | TDA q-value | Volume | Spectrum Matches |
| --- | --- | --- | --- | --- |
| -4.2513 | 157.4301 | 0.0000 | 2.5939e+07 | 2 |

**Adduct Signal Fractions**

- **Unmodified**: 2.5939e+07

### VSHHN(N)LTTGATL Hex 6 HexNAc 5

**Mass:**  3237.3590

(1.760e-06)

**Charge States:** 3

**Start Position**: 180

**End Position**: 192

| Profile Score | Tandem Score | TDA q-value | Volume | Spectrum Matches |
| --- | --- | --- | --- | --- |
| -3.5348 | 157.3648 | 0.0000 | 1.0869e+07 | 1 |

**Adduct Signal Fractions**

- **Unmodified**: 1.0869e+07

### P05155|IC1\_HUMAN

**>P05155|IC1\_HUMAN**  
 `MASRLTLLTLLLLLLAGDRASSNPNATSSSSQDPESLQDRGEGKVATTVISKMLFVEPILEVSSLPTTNSTTNSATKITA  
NTTDEPTTQPTTEPTTQPTIQPTQPTTQLPTDSPTQPTTGSFCPGPVTLCSDLESHSTEAVLGDALVDFSLKLYHAFSAM  
KKVETNMAFSPFSIASLLTQVLLGAGENTKTNLESILSYPKDFTCVHQALKGFTTKGVTSVSQIFHSPDLAIRDTFVNAS  
RTLYSSSPRVLSNNSDANLELINTWVAKNTNNKISRLLDSLPSDTRLVLLNAIYLSAKWKTTFDPKKTRMEPFHFKNSVI  
KVPMMNSKKYPVAHFIDQTLKAKVGQLQLSHNLSLVILVPQNLKHRLEDMEQALSPSVFKAIMEKLEMSKFQPTLLTLPR  
IKVTTSQDMLSIMEKLEFFDFSYDLNLCGLTEDPDLQVSAMQHQTVLELTETGVEAAAASAISVARTLLVFEVQQPFLFV  
LWDQQHKFPVFMGRVYDPRA`

| Site | Type | Count |
| --- | --- | --- |
| 238 | N-linked | 1 |
| 352 | N-linked | 1 |

| Mass | Sequence | MS_2_ Score | `q`-value | MS_1_ Score | Signal | Protein Coordinates | Tandem Scans | Start Time | Apex Time | End Time |
| --- | --- | --- | --- | --- | --- | --- | --- | --- | --- | --- |
| 3826.5165 | VN(N)ASRTL Fuc 1 Hex 8 HexNAc 8 | 120.099 | 0.0 | -21.098 | 1.765e+07 | 236 - 243 | 1 | 13.00 | 13.00 | 13.00 |
| 4139.6756 | QLSHN(N)LSL Fuc 1 Hex 9 HexNAc 8 | 105.369 | 0.0 | -5.608 | 4.637e+07 | 347 - 355 | 2 | 11.84 | 11.84 | 12.17 |

### VN(N)ASRTL Fuc 1 Hex 8 HexNAc 8

**Mass:**  3826.5148

(-5.451e-06)

**Charge States:** 4, 5

**Start Position**: 237

**End Position**: 244

| Profile Score | Tandem Score | TDA q-value | Volume | Spectrum Matches |
| --- | --- | --- | --- | --- |
| -21.0982 | 120.0992 | 0.0000 | 1.7645e+07 | 1 |

**Adduct Signal Fractions**

- **Unmodified**: 1.7645e+07

### QLSHN(N)LSL Fuc 1 Hex 9 HexNAc 8

**Mass:**  4139.6750

(3.872e-06)

**Charge States:** 4

**Start Position**: 348

**End Position**: 356

| Profile Score | Tandem Score | TDA q-value | Volume | Spectrum Matches |
| --- | --- | --- | --- | --- |
| -5.6080 | 105.3692 | 0.0000 | 4.6372e+07 | 2 |

**Adduct Signal Fractions**

- **Unmodified**: 4.6372e+07

### P13598|ICAM2\_HUMAN

**>P13598|ICAM2\_HUMAN**  
 `MSSFGYRTLTVALFTLICCPGSDEKVFEVHVRPKKLAVEPKGSLEVNCSTTCNQPEVGGLETSLDKILLDEQAQWKHYLV  
SNISHDTVLQCHFTCSGKQESMNSNVSVYQPPRQVILTLQPTLVAVGKSFTIECRVPTVEPLDSLTLFLFRGNETLHYET  
FGKAAPAPQEATATFNSTADREDGHRNFSCLAVLDLMSRGGNIFHKHSAPKMLEIYEPVSDSQMVIIVTVVSVLLSLFVT  
SVLLCFIFGQHLRQQRMGTYGVRAAWRRLPQAFRP`

| Site | Type | Count |
| --- | --- | --- |
| 153 | N-linked | 1 |

| Mass | Sequence | MS_2_ Score | `q`-value | MS_1_ Score | Signal | Protein Coordinates | Tandem Scans | Start Time | Apex Time | End Time |
| --- | --- | --- | --- | --- | --- | --- | --- | --- | --- | --- |
| 3814.4945 | RGN(N)ETL Hex 8 HexNAc 9 | 108.030 | 0.0 | 0.000 | 0.000e+00 | 150 - 156 | 1 | - | - | - |

### RGN(N)ETL Hex 8 HexNAc 9

**Mass:**  3814.4945

(5.265e-04)

**Start Position**: 151

**End Position**: 157

| Profile Score | Tandem Score | TDA q-value | Volume | Spectrum Matches |
| --- | --- | --- | --- | --- |
| 0.0000 | 108.0302 | 0.0000 | 0.0000e+00 | 1 |

Adduct:

No Chromatogram Found
