# Supplementary material for: Measuring Site-specific Glycosylation Similarity between Influenza a Virus Variants with Statistical Certainty
Source: Mol Cell Proteomics. 2020 Nov 25;19(9):1533–45. doi: 10.1074/mcp.RA120.002031 (PMC8143645; doi:10.1074/mcp.RA120.002031)
Supplement: Supplementary file 1 [file mmc1.zip › mmc1/159250_2_supp_555285_qcjklg.zip!/20190326_aAGP_021119_chymo_HILIC_DDA_1ug_03_glycopeptides.html]

## 20190326\_aAGP\_021119\_chymo\_HILIC\_DDA\_1ug\_03 @ GlycopeptideHypothesis-9354426a55b045d78004cc9c45aa74b8

**MS^1^ Mass Accuracy:** 10.0 PPM  
**Grouping Tolerance:** 15.0 PPM  
**MS^n^ Mass Accuracy:** 20.0 PPM  
**Minimum `q`-Value For Clustering:** 0.05

**Glycopeptide Hypothesis:**
GlycopeptideHypothesis-9354426a55b045d78004cc9c45aa74b8  
**Sample Searched:**
20190326\_aAGP\_021119\_chymo\_HILIC\_DDA\_1ug\_03

| Protein | Matched Glycopeptides |
| --- | --- |
| P02763\|A1AG1\_HUMAN | 142 |
| P19652\|A1AG2\_HUMAN | 122 |
| P13473\|LAMP2\_HUMAN | 3 |
| P00738\|HPT\_HUMAN | 2 |
| O95497\|VNN1\_HUMAN | 1 |

### P02763|A1AG1\_HUMAN

**>P02763|A1AG1\_HUMAN**  
 `MALSWVLTVLSLLPLLEAQIPLCANLVPVPITNATLDQITGKWFYIASAFRNEEYNKSVQEIQATFFYFTPNKTEDTIFL  
REYQTRQDQCIYNTTYLNVQRENGTISRYVGGQEHFAHLLILRDTKTYMLAFDVNDEKNWGLSVYADKPETTKEQLGEFY  
EALDCLRIPKSDVVYTDWKKDKCEPLEKQHEKERKQEEGES`

| Site | Type | Count |
| --- | --- | --- |
| 33 | N-linked | 2 |
| 56 | N-linked | 38 |
| 93 | N-linked | 4 |
| 103 | N-linked | 35 |
| 72 | N-linked | 63 |

| Mass | Sequence | MS_2_ Score | `q`-value | MS_1_ Score | Signal | Protein Coordinates | Tandem Scans | Start Time | Apex Time | End Time |
| --- | --- | --- | --- | --- | --- | --- | --- | --- | --- | --- |
| 3153.1487 | TPN(N)KTEDTIF Hex 6 HexNAc 5 | 184.031 | 0.0 | -2.681 | 3.687e+08 | 69 - 79 | 13 | 21.83 | 23.70 | 39.00 |
| 3299.3516 | FTPN(N)KTEDTIF Hex 6 HexNAc 5 | 183.315 | 0.0 | 14.798 | 4.834e+08 | 68 - 79 | 13 | 29.33 | 30.86 | 31.88 |
| 3517.4169 | TPN(N)KTEDTIF Hex 7 HexNAc 6 | 183.128 | 0.0 | 15.884 | 3.241e+08 | 69 - 79 | 9 | 22.34 | 23.02 | 24.55 |
| 2886.2312 | N(N)KSVQEIQATF Hex 5 HexNAc 4 | 181.122 | 0.0 | 11.996 | 2.953e+07 | 55 - 66 | 2 | 26.94 | 27.11 | 27.79 |
| 3251.3641 | N(N)KSVQEIQATF Hex 6 HexNAc 5 | 180.094 | 0.0 | -3.575 | 2.313e+07 | 55 - 66 | 2 | 26.26 | 26.43 | 26.77 |
| 2787.1524 | TPN(N)KTEDTIF Hex 5 HexNAc 4 | 178.291 | 0.0 | -2.534 | 2.611e+07 | 69 - 79 | 2 | 24.04 | 24.21 | 24.55 |
| 3097.2812 | YFTPN(N)KTEDTIF Hex 5 HexNAc 4 | 177.951 | 0.0 | 14.991 | 3.350e+08 | 67 - 79 | 7 | 37.49 | 37.81 | 38.66 |
| 3827.5475 | YFTPN(N)KTEDTIF Hex 7 HexNAc 6 | 175.335 | 0.0 | 15.245 | 4.940e+09 | 67 - 79 | 46 | 35.52 | 36.53 | 43.10 |
| 4192.6835 | YFTPN(N)KTEDTIF Hex 8 HexNAc 7 | 174.602 | 0.0 | 15.528 | 7.328e+08 | 67 - 79 | 10 | 34.66 | 36.02 | 36.69 |
| 3462.4150 | YFTPN(N)KTEDTIF Hex 6 HexNAc 5 | 172.053 | 0.0 | 15.105 | 3.435e+09 | 67 - 79 | 39 | 36.02 | 37.17 | 42.93 |
| 3663.4758 | TPN(N)KTEDTIF Fuc 1 Hex 7 HexNAc 6 | 170.725 | 0.0 | 14.389 | 5.083e+07 | 69 - 79 | 5 | 22.68 | 22.85 | 23.53 |
| 3973.6071 | YFTPN(N)KTEDTIF Fuc 1 Hex 7 HexNAc 6 | 170.569 | 0.0 | 14.265 | 8.375e+08 | 67 - 79 | 27 | 35.35 | 36.36 | 37.17 |
| 3608.4783 | YFTPN(N)KTEDTIF Fuc 1 Hex 6 HexNAc 5 | 168.110 | 0.0 | 15.408 | 6.641e+08 | 67 - 79 | 33 | 35.85 | 36.85 | 42.08 |
| 3609.4852 | FYFTPN(N)KTEDTIF Hex 6 HexNAc 5 | 167.738 | 0.0 | 14.728 | 2.499e+09 | 66 - 79 | 49 | 46.13 | 47.14 | 56.34 |
| 3755.5437 | FYFTPN(N)KTEDTIF Fuc 1 Hex 6 HexNAc 5 | 167.391 | 0.0 | 13.332 | 5.432e+08 | 66 - 79 | 9 | 45.96 | 46.97 | 48.14 |
| 3298.3430 | TPN(N)KTEDTIF Fuc 1 Hex 6 HexNAc 5 | 166.994 | 0.0 | 13.696 | 3.732e+07 | 69 - 79 | 4 | 23.02 | 23.36 | 23.87 |
| 3882.5504 | TPN(N)KTEDTIF Hex 8 HexNAc 7 | 166.932 | 0.0 | 14.673 | 2.999e+07 | 69 - 79 | 4 | 22.17 | 22.51 | 22.85 |
| 4120.6768 | FYFTPN(N)KTEDTIF Fuc 1 Hex 7 HexNAc 6 | 166.695 | 0.0 | 14.618 | 8.475e+08 | 66 - 79 | 12 | 45.62 | 46.30 | 47.64 |
| 4323.7536 | FYFTPN(N)KTEDTIF Fuc 1 Hex 7 HexNAc 7 | 166.147 | 0.0 | -3.642 | 3.927e+07 | 66 - 79 | 3 | 47.31 | 47.47 | 47.64 |
| 3398.4356 | N(N)KSVQEIQATFF Hex 6 HexNAc 5 | 166.058 | 0.0 | -4.529 | 7.991e+06 | 55 - 67 | 3 | 40.37 | 40.37 | 40.71 |
| 3974.6191 | FYFTPN(N)KTEDTIF Hex 7 HexNAc 6 | 165.663 | 0.0 | 15.300 | 4.672e+09 | 66 - 79 | 77 | 44.94 | 46.47 | 60.80 |
| 3664.4842 | FTPN(N)KTEDTIF Hex 7 HexNAc 6 | 165.492 | 0.0 | 15.444 | 6.673e+08 | 68 - 79 | 18 | 28.82 | 30.18 | 31.71 |
| 4266.7338 | FYFTPN(N)KTEDTIF Fuc 2 Hex 7 HexNAc 6 | 165.417 | 0.0 | 13.291 | 1.181e+08 | 66 - 79 | 3 | 46.13 | 46.30 | 46.81 |
| 4003.5787 | YFTPN(N)KTEDTIF Hex 7 HexNAc 4 Neu5Ac 2 | 165.145 | 0.0 | 15.302 | 3.124e+08 | 67 - 79 | 7 | 37.65 | 37.81 | 39.00 |
| 4338.7408 | YFTPN(N)KTEDTIF Fuc 1 Hex 8 HexNAc 7 | 164.640 | 0.0 | 15.733 | 1.472e+08 | 67 - 79 | 5 | 35.35 | 36.02 | 36.19 |
| 3307.3794 | EEYN(N)KSVQEIQATF Hex 5 HexNAc 4 | 162.548 | 0.0 | -3.483 | 7.724e+07 | 52 - 66 | 3 | 35.52 | 35.52 | 35.85 |
| 4119.6660 | YFTPN(N)KTEDTIF Fuc 2 Hex 7 HexNAc 6 | 162.437 | 0.0 | -3.420 | 1.237e+08 | 67 - 79 | 3 | 36.19 | 36.19 | 36.69 |
| 3672.5124 | EEYN(N)KSVQEIQATF Hex 6 HexNAc 5 | 162.212 | 0.0 | -3.460 | 7.690e+07 | 52 - 66 | 2 | 35.00 | 35.18 | 35.52 |
| 4557.8166 | YFTPN(N)KTEDTIF Hex 9 HexNAc 8 | 161.641 | 0.0 | 15.581 | 2.105e+08 | 67 - 79 | 7 | 34.33 | 35.18 | 36.36 |
| 3906.5500 | TPN(N)KTEDTIF Fuc 4 Hex 4 HexNAc 6 Neu5Ac 1 | 160.247 | 0.0 | -20.443 | 6.096e+07 | 69 - 79 | 1 | 36.69 | 36.85 | 36.85 |
| 3244.3520 | FYFTPN(N)KTEDTIF Hex 5 HexNAc 4 | 159.280 | 0.0 | 13.025 | 2.171e+08 | 66 - 79 | 4 | 47.64 | 47.64 | 48.48 |
| 3577.5284 | RNEEYN(N)KSVQEIQATF Hex 5 HexNAc 4 | 157.728 | 0.0 | 16.199 | 9.426e+09 | 50 - 66 | 19 | 30.69 | 32.51 | 33.99 |
| 4089.7278 | RNEEYN(N)KSVQEIQATFF Hex 6 HexNAc 5 | 157.676 | 0.0 | 16.880 | 3.593e+09 | 50 - 67 | 19 | 41.39 | 43.60 | 44.77 |
| 3724.5919 | RNEEYN(N)KSVQEIQATFF Hex 5 HexNAc 4 | 156.821 | 0.0 | 17.096 | 4.017e+09 | 50 - 67 | 18 | 39.68 | 43.93 | 45.28 |
| 4235.7851 | RNEEYN(N)KSVQEIQATFF Fuc 1 Hex 6 HexNAc 5 | 156.180 | 0.0 | 16.112 | 1.718e+09 | 50 - 67 | 16 | 41.56 | 43.44 | 44.77 |
| 3445.4093 | FTPN(N)KTEDTIF Fuc 1 Hex 6 HexNAc 5 | 155.855 | 0.0 | 13.048 | 9.308e+07 | 68 - 79 | 7 | 30.35 | 30.52 | 31.03 |
| 4704.8852 | FYFTPN(N)KTEDTIF Hex 9 HexNAc 8 | 154.381 | 0.0 | 15.125 | 1.681e+08 | 66 - 79 | 6 | 44.43 | 45.11 | 46.30 |
| 3942.6591 | RNEEYN(N)KSVQEIQATF Hex 6 HexNAc 5 | 154.356 | 0.0 | 15.791 | 7.022e+09 | 50 - 66 | 16 | 30.35 | 32.20 | 33.48 |
| 3810.5435 | FTPN(N)KTEDTIF Fuc 1 Hex 7 HexNAc 6 | 153.547 | 0.003 | 14.544 | 1.154e+08 | 68 - 79 | 6 | 29.67 | 30.01 | 30.69 |
| 4088.7161 | RNEEYN(N)KSVQEIQATF Fuc 1 Hex 6 HexNAc 5 | 153.401 | 0.003 | 14.863 | 3.442e+09 | 50 - 66 | 17 | 30.18 | 32.04 | 33.48 |
| 3243.3422 | YFTPN(N)KTEDTIF Fuc 1 Hex 5 HexNAc 4 | 153.019 | 0.003 | -20.002 | 7.317e+06 | 67 - 79 | 1 | 37.49 | 37.49 | 37.65 |
| 4600.9155 | RNEEYN(N)KSVQEIQATFF Fuc 1 Hex 7 HexNAc 6 | 152.949 | 0.003 | 14.099 | 6.472e+07 | 50 - 67 | 4 | 42.76 | 43.28 | 43.60 |
| 3870.6505 | RNEEYN(N)KSVQEIQATFF Fuc 1 Hex 5 HexNAc 4 | 152.421 | 0.003 | 13.538 | 1.605e+08 | 50 - 67 | 7 | 43.60 | 43.77 | 44.60 |
| 4922.9470 | YFTPN(N)KTEDTIF Hex 10 HexNAc 9 | 152.186 | 0.003 | -4.352 | 2.099e+07 | 67 - 79 | 2 | 34.66 | 34.83 | 35.00 |
| 3723.5877 | RNEEYN(N)KSVQEIQATF Fuc 1 Hex 5 HexNAc 4 | 152.112 | 0.003 | 11.712 | 3.585e+08 | 50 - 66 | 10 | 32.20 | 32.36 | 33.99 |
| 3430.4558 | RNEEYN(N)KSVQEIQAT Hex 5 HexNAc 4 | 151.886 | 0.003 | -3.873 | 4.255e+07 | 50 - 65 | 3 | 17.17 | 17.17 | 17.51 |
| 2934.2206 | FTPN(N)KTEDTIF Hex 5 HexNAc 4 | 151.459 | 0.003 | -2.826 | 5.043e+07 | 68 - 79 | 3 | 31.20 | 31.37 | 31.71 |
| 3004.2166 | FYFTPN(N)KT Hex 6 HexNAc 5 | 150.681 | 0.0 | -3.258 | 7.808e+06 | 66 - 74 | 2 | 25.57 | 25.74 | 26.09 |
| 4453.8501 | RNEEYN(N)KSVQEIQATF Fuc 1 Hex 7 HexNAc 6 | 150.559 | 0.003 | 14.298 | 1.576e+08 | 50 - 66 | 2 | 31.54 | 31.88 | 32.20 |
| 4339.7521 | FYFTPN(N)KTEDTIF Hex 8 HexNAc 7 | 150.401 | 0.003 | 14.467 | 6.185e+08 | 66 - 79 | 11 | 44.77 | 46.13 | 47.31 |
| 4060.6019 | TPN(N)KTEDTIF Fuc 4 Hex 8 HexNAc 5 | 150.313 | 0.003 | 13.202 | 1.360e+08 | 69 - 79 | 4 | 37.65 | 37.81 | 38.49 |
| 3033.3004 | N(N)KSVQEIQATFF Hex 5 HexNAc 4 | 149.181 | 0.003 | -4.431 | 8.091e+06 | 55 - 67 | 1 | 41.05 | 41.22 | 41.39 |
| 4307.7914 | RNEEYN(N)KSVQEIQATF Hex 7 HexNAc 6 | 148.584 | 0.003 | 14.425 | 3.599e+08 | 50 - 66 | 3 | 31.71 | 31.88 | 32.36 |
| 4381.8437 | RNEEYN(N)KSVQEIQATFF Fuc 2 Hex 6 HexNAc 5 | 148.351 | 0.003 | -3.987 | 6.808e+07 | 50 - 67 | 3 | 43.28 | 43.28 | 43.60 |
| 4484.7975 | YFTPN(N)KTEDTIF Fuc 2 Hex 8 HexNAc 7 | 148.164 | 0.003 | 9.828 | 3.204e+07 | 67 - 79 | 2 | 35.35 | 35.68 | 36.02 |
| 3940.6321 | YFTPN(N)KTEDTIFL Hex 7 HexNAc 6 | 145.734 | 0.003 | 14.054 | 5.170e+07 | 67 - 80 | 3 | 44.26 | 44.26 | 44.94 |
| 4150.6526 | FYFTPN(N)KTEDTIF Hex 7 HexNAc 4 Neu5Ac 2 | 145.492 | 0.003 | 13.524 | 2.610e+08 | 66 - 79 | 6 | 47.47 | 47.64 | 48.99 |
| 4020.6044 | YFTPN(N)KTEDTIF Fuc 1 Hex 8 HexNAc 4 Neu5Ac 1 | 145.240 | 0.003 | -4.496 | 2.866e+07 | 67 - 79 | 2 | 37.65 | 37.81 | 37.98 |
| 4234.7747 | RNEEYN(N)KSVQEIQATF Fuc 2 Hex 6 HexNAc 5 | 144.900 | 0.003 | -3.109 | 1.283e+08 | 50 - 66 | 2 | 31.88 | 31.88 | 32.20 |
| 4029.6176 | FTPN(N)KTEDTIF Hex 8 HexNAc 7 | 144.778 | 0.003 | 16.224 | 6.373e+07 | 68 - 79 | 6 | 29.16 | 29.67 | 30.01 |
| 4454.8597 | RNEEYN(N)KSVQEIQATFF Hex 7 HexNAc 6 | 144.230 | 0.003 | 13.828 | 1.706e+08 | 50 - 67 | 4 | 42.93 | 43.28 | 43.77 |
| 3996.5976 | FYFTPN(N)KTEDTIF Hex 3 HexNAc 5 Neu5Ac 3 | 143.320 | 0.003 | 13.714 | 2.504e+08 | 66 - 79 | 6 | 45.79 | 46.64 | 46.97 |
| 3818.5727 | EEYN(N)KSVQEIQATF Fuc 1 Hex 6 HexNAc 5 | 142.914 | 0.003 | -3.815 | 3.311e+07 | 52 - 66 | 3 | 34.83 | 35.00 | 35.18 |
| 3693.4441 | TPN(N)KTEDTIF Hex 7 HexNAc 4 Neu5Ac 2 | 142.767 | 0.003 | -18.452 | 9.759e+06 | 69 - 79 | 2 | 24.04 | 24.21 | 24.21 |
| 4672.9240 | RNEEYN(N)KSVQEIQATF Hex 8 HexNAc 7 | 141.382 | 0.003 | -3.636 | 6.603e+07 | 50 - 66 | 2 | 31.37 | 31.71 | 31.88 |
| 4485.8099 | FYFTPN(N)KTEDTIF Fuc 1 Hex 8 HexNAc 7 | 140.586 | 0.003 | 15.963 | 1.226e+08 | 66 - 79 | 5 | 45.28 | 45.96 | 46.30 |
| 3222.2816 | YFTPN(N)KT Hex 7 HexNAc 6 | 140.336 | 0.0 | -19.699 | 4.956e+06 | 67 - 74 | 1 | 13.99 | 14.15 | 14.15 |
| 3575.5009 | YFTPN(N)KTEDTIFL Hex 6 HexNAc 5 | 140.107 | 0.003 | -3.690 | 2.548e+07 | 67 - 80 | 4 | 44.60 | 44.94 | 45.11 |
| 3901.6506 | LNVQREN(N)GTISRY Hex 7 HexNAc 6 | 139.856 | 0.003 | 16.272 | 9.580e+09 | 96 - 109 | 12 | 17.85 | 18.51 | 19.79 |
| 4412.8462 | LNVQREN(N)GTISRY Fuc 1 Hex 8 HexNAc 7 | 139.618 | 0.003 | 14.076 | 4.122e+08 | 96 - 109 | 4 | 17.85 | 18.18 | 18.82 |
| 3536.5221 | LNVQREN(N)GTISRY Hex 6 HexNAc 5 | 139.075 | 0.003 | 15.499 | 3.694e+09 | 96 - 109 | 13 | 18.18 | 18.98 | 20.13 |
| 3795.5889 | RNEEYN(N)KSVQEIQAT Hex 6 HexNAc 5 | 136.327 | 0.003 | -20.075 | 2.638e+07 | 50 - 65 | 2 | 17.00 | 17.00 | 17.17 |
| 3934.6589 | NVQREN(N)GTISRY Fuc 1 Hex 7 HexNAc 6 | 135.713 | 0.003 | -9.361 | 1.876e+08 | 97 - 109 | 5 | 12.99 | 12.99 | 23.02 |
| 3677.4610 | QTRQDQC(C)IYN(N)TTY Hex 6 HexNAc 5 | 135.118 | 0.003 | 15.343 | 1.338e+08 | 83 - 96 | 6 | 21.49 | 22.00 | 22.51 |
| 4296.7078 | FYFTPN(N)KTEDTIF Fuc 1 Hex 7 HexNAc 4 Neu5Ac 2 | 134.778 | 0.003 | -4.498 | 4.221e+07 | 66 - 79 | 3 | 47.31 | 47.47 | 47.81 |
| 3682.5798 | LNVQREN(N)GTISRY Fuc 1 Hex 6 HexNAc 5 | 134.571 | 0.003 | 14.672 | 1.420e+09 | 96 - 109 | 11 | 18.18 | 18.82 | 20.13 |
| 3454.4501 | EEYN(N)KSVQEIQATFF Hex 5 HexNAc 4 | 134.292 | 0.003 | -4.566 | 2.523e+07 | 52 - 67 | 3 | 47.64 | 47.81 | 48.14 |
| 4149.6386 | YFTPN(N)KTEDTIF Fuc 1 Hex 7 HexNAc 4 Neu5Ac 2 | 133.045 | 0.003 | -4.720 | 5.568e+07 | 67 - 79 | 2 | 37.49 | 37.65 | 37.98 |
| 4089.7142 | FRNEEYN(N)KSVQEIQATF Hex 6 HexNAc 5 | 132.824 | 0.003 | 12.252 | 5.437e+07 | 49 - 66 | 2 | 36.85 | 37.17 | 37.49 |
| 3578.5103 | RN(D)EEYN(N)KSVQEIQATF Hex 5 HexNAc 4 | 132.670 | 0.003 | 10.988 | 1.247e+09 | 50 - 66 | 12 | 33.99 | 35.52 | 36.36 |
| 3171.3903 | LNVQREN(N)GTISRY Hex 5 HexNAc 4 | 131.749 | 0.003 | 14.716 | 2.534e+08 | 96 - 109 | 3 | 18.82 | 19.14 | 20.30 |
| 4425.7517 | N(N)KTEDTIF Fuc 5 Hex 5 HexNAc 8 Neu5Ac 1 | 131.647 | 0.0 | 0.000 | 0.000e+00 | 71 - 79 | 1 | - | - | - |
| 3943.6442 | RN(D)EEYN(N)KSVQEIQATF Hex 6 HexNAc 5 | 130.230 | 0.006 | 10.211 | 1.233e+09 | 50 - 66 | 14 | 33.65 | 35.18 | 36.19 |
| 3823.5195 | QTRQDQC(C)IYN(N)TTY Fuc 1 Hex 6 HexNAc 5 | 128.705 | 0.006 | 13.759 | 3.630e+07 | 83 - 96 | 4 | 21.49 | 21.83 | 22.34 |
| 3569.4905 | NVQREN(N)GTISRY Fuc 1 Hex 6 HexNAc 5 | 128.642 | 0.006 | -18.963 | 4.943e+07 | 97 - 109 | 2 | 13.16 | 13.33 | 13.33 |
| 3788.5650 | NVQREN(N)GTISRY Hex 7 HexNAc 6 | 128.549 | 0.006 | 12.718 | 4.999e+08 | 97 - 109 | 6 | 12.99 | 13.16 | 13.82 |
| 3819.5831 | EEYN(N)KSVQEIQATFF Hex 6 HexNAc 5 | 128.290 | 0.006 | -4.611 | 2.557e+07 | 52 - 67 | 3 | 47.14 | 47.31 | 47.64 |
| 3369.3492 | FYFTPN(N)KT Hex 7 HexNAc 6 | 128.278 | 0.0 | -3.667 | 1.266e+07 | 66 - 74 | 2 | 24.89 | 25.23 | 25.23 |
| 3674.5213 | VQREN(N)GTISRY Hex 7 HexNAc 6 | 128.200 | 0.006 | -20.771 | 2.216e+07 | 98 - 109 | 4 | 12.50 | 12.66 | 12.66 |
| 4193.7692 | LNVQREN(N)GTISRY Fuc 2 Hex 7 HexNAc 6 | 126.623 | 0.006 | 14.154 | 1.047e+09 | 96 - 109 | 7 | 17.85 | 18.35 | 19.63 |
| 4047.7095 | LNVQREN(N)GTISRY Fuc 1 Hex 7 HexNAc 6 | 125.910 | 0.006 | 14.921 | 3.354e+09 | 96 - 109 | 11 | 17.85 | 18.35 | 19.63 |
| 4042.5954 | QTRQDQC(C)IYN(N)TTY Hex 7 HexNAc 6 | 125.292 | 0.006 | 14.386 | 2.502e+08 | 83 - 96 | 7 | 20.64 | 21.66 | 22.34 |
| 4703.8747 | YFTPN(N)KTEDTIF Fuc 1 Hex 9 HexNAc 8 | 124.571 | 0.006 | 10.367 | 3.229e+07 | 67 - 79 | 2 | 34.83 | 35.18 | 35.68 |
| 4207.6720 | TPN(N)KTEDTIF Fuc 3 Hex 3 HexNAc 9 Neu5Ac 1 | 123.772 | 0.006 | 13.680 | 9.862e+07 | 69 - 79 | 4 | 48.65 | 48.82 | 49.50 |
| 3828.6350 | LNVQREN(N)GTISRY Fuc 2 Hex 6 HexNAc 5 | 122.225 | 0.013 | 10.171 | 9.833e+07 | 96 - 109 | 2 | 18.35 | 18.51 | 18.98 |
| 3840.5169 | FTPN(N)KTEDTIF Hex 7 HexNAc 4 Neu5Ac 2 | 122.174 | 0.013 | -4.317 | 3.237e+07 | 68 - 79 | 4 | 31.20 | 31.37 | 31.71 |
| 4631.8698 | FYFTPN(N)KTEDTIF Fuc 2 Hex 8 HexNAc 7 | 121.618 | 0.013 | 11.460 | 2.605e+07 | 66 - 79 | 3 | 45.28 | 45.79 | 45.96 |
| 3724.5929 | FRNEEYN(N)KSVQEIQATF Hex 5 HexNAc 4 | 121.101 | 0.013 | -4.903 | 3.489e+07 | 49 - 66 | 1 | 37.33 | 37.49 | 37.65 |
| 3423.4323 | NVQREN(N)GTISRY Hex 6 HexNAc 5 | 120.666 | 0.013 | 11.552 | 1.653e+08 | 97 - 109 | 4 | 13.16 | 13.33 | 13.99 |
| 3613.4181 | FYFTPN(N)KTED Hex 7 HexNAc 6 | 119.938 | 0.013 | -4.136 | 6.638e+06 | 66 - 76 | 2 | 25.40 | 25.40 | 25.74 |
| 4819.9928 | RNEEYN(N)KSVQEIQATFF Hex 8 HexNAc 7 | 119.881 | 0.013 | -3.454 | 1.929e+07 | 50 - 67 | 2 | 42.59 | 42.59 | 43.10 |
| 4089.7019 | RN(D)EEYN(N)KSVQEIQATF Fuc 1 Hex 6 HexNAc 5 | 119.718 | 0.015 | 11.197 | 5.640e+08 | 50 - 66 | 11 | 33.48 | 35.00 | 35.85 |
| 3124.3997 | LVPVPITN(N)ATL Hex 6 HexNAc 5 | 119.605 | 0.015 | -18.938 | 6.550e+06 | 25 - 36 | 1 | 45.62 | 45.62 | 45.79 |
| 3725.5802 | RN(D)EEYN(N)KSVQEIQATFF Hex 5 HexNAc 4 | 119.015 | 0.015 | 10.217 | 3.651e+08 | 50 - 67 | 10 | 45.28 | 46.97 | 47.31 |
| 4188.6547 | QTRQDQC(C)IYN(N)TTY Fuc 1 Hex 7 HexNAc 6 | 118.465 | 0.015 | 12.806 | 6.913e+07 | 83 - 96 | 4 | 21.15 | 21.49 | 22.00 |
| 4090.7129 | RN(D)EEYN(N)KSVQEIQATFF Hex 6 HexNAc 5 | 118.019 | 0.015 | 10.507 | 3.810e+08 | 50 - 67 | 30 | 44.94 | 46.64 | 46.81 |
| 4704.9621 | LNVQREN(N)GTISRY Fuc 3 Hex 8 HexNAc 7 | 117.307 | 0.017 | -4.025 | 7.473e+07 | 96 - 109 | 1 | 17.68 | 17.85 | 18.18 |
| 3309.3793 | VQREN(N)GTISRY Hex 6 HexNAc 5 | 117.200 | 0.017 | 0.000 | 0.000e+00 | 98 - 109 | 1 | - | - | - |
| 3956.6037 | FTPN(N)KTEDTIF Fuc 2 Hex 7 HexNAc 6 | 117.147 | 0.017 | -17.500 | 1.393e+07 | 68 - 79 | 2 | 29.84 | 29.84 | 30.01 |
| 5038.0541 | RNEEYN(N)KSVQEIQATF Hex 9 HexNAc 8 | 117.141 | 0.017 | -3.844 | 3.330e+07 | 50 - 66 | 3 | 31.20 | 31.37 | 31.54 |
| 4266.7882 | LNVQREN(N)GTISRY Hex 8 HexNAc 7 | 116.686 | 0.017 | 13.953 | 1.027e+09 | 96 - 109 | 6 | 17.85 | 18.18 | 19.46 |
| 4048.7001 | LN(D)VQREN(N)GTISRY Fuc 1 Hex 7 HexNAc 6 | 115.192 | 0.019 | 9.259 | 1.366e+08 | 96 - 109 | 8 | 19.79 | 20.30 | 20.47 |
| 4080.6810 | NVQREN(N)GTISRY Fuc 2 Hex 7 HexNAc 6 | 115.053 | 0.019 | -18.740 | 4.830e+07 | 97 - 109 | 2 | 12.99 | 12.99 | 13.16 |
| 3809.5355 | TPN(N)KTEDTIF Fuc 2 Hex 7 HexNAc 6 | 114.753 | 0.019 | -19.577 | 6.305e+06 | 69 - 79 | 1 | 22.68 | 22.68 | 22.85 |
| 4631.9252 | LNVQREN(N)GTISRY Hex 9 HexNAc 8 | 114.106 | 0.020 | 14.551 | 9.607e+08 | 96 - 109 | 6 | 17.51 | 18.01 | 19.30 |
| 4206.6664 | TPN(N)KTEDTIF Hex 10 HexNAc 7 | 113.141 | 0.020 | -4.578 | 1.487e+07 | 69 - 79 | 1 | 37.49 | 37.49 | 37.81 |
| 4118.6489 | YFTPN(N)KTEDTIF Hex 7 HexNAc 6 Neu5Ac 1 | 112.320 | 0.022 | 12.080 | 9.228e+06 | 67 - 79 | 3 | 39.34 | 39.68 | 40.20 |
| 4077.6856 | LNVQREN(N)GTISRY Hex 7 HexNAc 4 Neu5Ac 2 | 112.268 | 0.022 | 14.571 | 4.433e+08 | 96 - 109 | 6 | 19.30 | 19.63 | 20.64 |
| 4339.8257 | LNVQREN(N)GTISRY Fuc 3 Hex 7 HexNAc 6 | 111.919 | 0.024 | 9.319 | 2.689e+08 | 96 - 109 | 4 | 18.18 | 18.18 | 18.82 |
| 4777.9778 | LNVQREN(N)GTISRY Fuc 1 Hex 9 HexNAc 8 | 111.775 | 0.024 | 15.759 | 2.908e+08 | 96 - 109 | 3 | 17.51 | 18.01 | 18.35 |
| 4394.7532 | FTPN(N)KTEDTIF Hex 9 HexNAc 8 | 111.573 | 0.024 | 12.817 | 1.336e+07 | 68 - 79 | 2 | 28.65 | 28.82 | 29.67 |
| 4353.7267 | TPN(N)KTEDTIF Fuc 4 Hex 3 HexNAc 9 Neu5Ac 1 | 111.128 | 0.026 | -5.082 | 1.562e+07 | 69 - 79 | 1 | 48.48 | 48.65 | 48.82 |
| 3902.6427 | LN(D)VQREN(N)GTISRY Hex 7 HexNAc 6 | 110.774 | 0.029 | 10.084 | 3.594e+08 | 96 - 109 | 30 | 19.96 | 20.47 | 20.64 |
| 3537.5104 | VQREN(N)GTISRY Fuc 1 Hex 4 HexNAc 7 | 110.141 | 0.029 | 8.424 | 1.104e+08 | 98 - 109 | 6 | 20.30 | 20.47 | 21.32 |
| 4193.6893 | YFTPN(N)KTEDTIF Hex 8 HexNAc 7 | 109.893 | 0.031 | 0.000 | 0.000e+00 | 67 - 79 | 1 | - | - | - |
| 4818.9813 | RNEEYN(N)KSVQEIQATF Fuc 1 Hex 8 HexNAc 7 | 108.790 | 0.033 | -5.225 | 1.545e+07 | 50 - 66 | 2 | 31.37 | 31.54 | 31.71 |
| 4664.9069 | NVQREN(N)GTISRY Fuc 1 Hex 9 HexNAc 8 | 106.767 | 0.037 | -20.868 | 5.592e+06 | 97 - 109 | 1 | 12.66 | 12.66 | 12.66 |
| 4518.8289 | NVQREN(N)GTISRY Hex 9 HexNAc 8 | 106.597 | 0.037 | -19.025 | 3.204e+07 | 97 - 109 | 1 | 12.83 | 12.83 | 12.99 |
| 4924.0350 | LNVQREN(N)GTISRY Fuc 2 Hex 9 HexNAc 8 | 106.115 | 0.037 | -3.437 | 1.055e+08 | 96 - 109 | 3 | 17.68 | 17.85 | 18.18 |
| 4236.7691 | RN(D)EEYN(N)KSVQEIQATFF Fuc 1 Hex 6 HexNAc 5 | 105.865 | 0.037 | 10.632 | 1.832e+08 | 50 - 67 | 8 | 44.77 | 46.47 | 46.81 |
| 4246.6794 | TPN(N)KTEDTIF Fuc 3 Hex 7 HexNAc 6 Neu5Ac 1 | 105.662 | 0.037 | -18.554 | 4.396e+06 | 69 - 79 | 1 | 21.66 | 21.66 | 21.66 |
| 4283.8093 | LN(D)VQREN(N)GTISRY Fuc 4 Hex 7 HexNAc 5 | 105.401 | 0.037 | -20.737 | 3.622e+07 | 96 - 109 | 1 | 18.18 | 18.18 | 18.18 |
| 3058.2980 | NVQREN(N)GTISRY Hex 5 HexNAc 4 | 105.059 | 0.039 | -20.209 | 9.251e+06 | 97 - 109 | 1 | 13.33 | 13.49 | 13.49 |
| 3397.4247 | N(N)KSVQEIQATF Fuc 1 Hex 6 HexNAc 5 | 104.739 | 0.041 | -19.310 | 7.861e+06 | 55 - 66 | 1 | 26.09 | 26.26 | 26.26 |
| 4223.7431 | LNVQREN(N)GTISRY Fuc 1 Hex 7 HexNAc 4 Neu5Ac 2 | 104.003 | 0.044 | 14.194 | 1.384e+08 | 96 - 109 | 3 | 19.30 | 19.46 | 19.96 |
| 4192.7530 | LNVQREN(N)GTISRY Hex 7 HexNAc 6 Neu5Ac 1 | 103.427 | 0.044 | -18.496 | 9.747e+06 | 96 - 109 | 2 | 20.64 | 20.64 | 20.64 |
| 4558.9092 | LNVQREN(N)GTISRY Fuc 2 Hex 8 HexNAc 7 | 103.247 | 0.044 | 15.004 | 1.969e+08 | 96 - 109 | 3 | 17.68 | 18.01 | 18.51 |
| 5070.0924 | LNVQREN(N)GTISRY Fuc 3 Hex 9 HexNAc 8 | 102.527 | 0.044 | -3.497 | 2.935e+07 | 96 - 109 | 1 | 17.68 | 17.85 | 18.01 |
| 5289.1657 | LNVQREN(N)GTISRY Fuc 2 Hex 10 HexNAc 9 | 102.074 | 0.045 | -19.105 | 1.127e+07 | 96 - 109 | 1 | 17.51 | 17.68 | 17.68 |
| 4028.6073 | TPN(N)KTEDTIF Fuc 1 Hex 8 HexNAc 7 | 101.650 | 0.045 | -20.728 | 2.306e+06 | 69 - 79 | 1 | 22.34 | 22.34 | 22.51 |
| 4851.0199 | LNVQREN(N)GTISRY Fuc 4 Hex 8 HexNAc 7 | 100.833 | 0.048 | -4.533 | 1.812e+07 | 96 - 109 | 1 | 17.68 | 17.85 | 18.01 |
| 3469.5100 | C(C)ANLVPVPITN(N)ATL Hex 6 HexNAc 5 | 100.422 | 0.048 | 12.301 | 2.296e+07 | 22 - 36 | 5 | 50.19 | 50.36 | 51.21 |

### TPN(N)KTEDTIF Hex 6 HexNAc 5

**Mass:**  3152.2827

(1.363e-06)

**Charge States:** 3, 4

**Start Position**: 70

**End Position**: 80

| Profile Score | Tandem Score | TDA q-value | Volume | Spectrum Matches |
| --- | --- | --- | --- | --- |
| -2.6813 | 184.0308 | 0.0000 | 3.6872e+08 | 13 |

**Adduct Signal Fractions**

- **Unmodified**: 3.6872e+08

### FTPN(N)KTEDTIF Hex 6 HexNAc 5

**Mass:**  3299.3523

(8.094e-07)

**Charge States:** 3, 4

**Start Position**: 69

**End Position**: 80

| Profile Score | Tandem Score | TDA q-value | Volume | Spectrum Matches |
| --- | --- | --- | --- | --- |
| 14.7979 | 183.3149 | 0.0000 | 4.8339e+08 | 13 |

**Adduct Signal Fractions**

- **Unmodified**: 4.8339e+08

### TPN(N)KTEDTIF Hex 7 HexNAc 6

**Mass:**  3517.4163

(1.884e-06)

**Charge States:** 3, 4

**Start Position**: 70

**End Position**: 80

| Profile Score | Tandem Score | TDA q-value | Volume | Spectrum Matches |
| --- | --- | --- | --- | --- |
| 15.8842 | 183.1281 | 0.0000 | 3.2408e+08 | 9 |

**Adduct Signal Fractions**

- **Unmodified**: 3.2408e+08

### N(N)KSVQEIQATF Hex 5 HexNAc 4

**Mass:**  2886.2310

(2.074e-06)

**Charge States:** 3

**Start Position**: 56

**End Position**: 67

| Profile Score | Tandem Score | TDA q-value | Volume | Spectrum Matches |
| --- | --- | --- | --- | --- |
| 11.9963 | 181.1222 | 0.0000 | 2.9528e+07 | 2 |

**Adduct Signal Fractions**

- **Unmodified**: 2.9528e+07

### N(N)KSVQEIQATF Hex 6 HexNAc 5

**Mass:**  3251.3644

(1.475e-06)

**Charge States:** 3, 4

**Start Position**: 56

**End Position**: 67

| Profile Score | Tandem Score | TDA q-value | Volume | Spectrum Matches |
| --- | --- | --- | --- | --- |
| -3.5752 | 180.0941 | 0.0000 | 2.3126e+07 | 2 |

**Adduct Signal Fractions**

- **Unmodified**: 2.3126e+07

### TPN(N)KTEDTIF Hex 5 HexNAc 4

**Mass:**  2787.1523

(1.511e-06)

**Charge States:** 3

**Start Position**: 70

**End Position**: 80

| Profile Score | Tandem Score | TDA q-value | Volume | Spectrum Matches |
| --- | --- | --- | --- | --- |
| -2.5343 | 178.2909 | 0.0000 | 2.6107e+07 | 2 |

**Adduct Signal Fractions**

- **Unmodified**: 2.6107e+07

### YFTPN(N)KTEDTIF Hex 5 HexNAc 4

**Mass:**  3097.2793

(9.860e-07)

**Charge States:** 3, 4

**Start Position**: 68

**End Position**: 80

| Profile Score | Tandem Score | TDA q-value | Volume | Spectrum Matches |
| --- | --- | --- | --- | --- |
| 14.9911 | 177.9511 | 0.0000 | 3.3495e+08 | 7 |

**Adduct Signal Fractions**

- **Unmodified**: 3.3495e+08

### YFTPN(N)KTEDTIF Hex 7 HexNAc 6

**Mass:**  3827.5427

(7.937e-07)

**Charge States:** 3, 4

**Start Position**: 68

**End Position**: 80

| Profile Score | Tandem Score | TDA q-value | Volume | Spectrum Matches |
| --- | --- | --- | --- | --- |
| 15.2446 | 175.3346 | 0.0000 | 4.9403e+09 | 46 |

**Adduct Signal Fractions**

- **Unmodified**: 4.9403e+09

### YFTPN(N)KTEDTIF Hex 8 HexNAc 7

**Mass:**  4192.6862

(1.868e-06)

**Charge States:** 3, 4

**Start Position**: 68

**End Position**: 80

| Profile Score | Tandem Score | TDA q-value | Volume | Spectrum Matches |
| --- | --- | --- | --- | --- |
| 15.5279 | 174.6023 | 0.0000 | 7.3282e+08 | 10 |

**Adduct Signal Fractions**

- **Unmodified**: 7.3282e+08

### YFTPN(N)KTEDTIF Hex 6 HexNAc 5

**Mass:**  3462.4140

(1.738e-06)

**Charge States:** 2, 3, 4

**Start Position**: 68

**End Position**: 80

| Profile Score | Tandem Score | TDA q-value | Volume | Spectrum Matches |
| --- | --- | --- | --- | --- |
| 15.1046 | 172.0526 | 0.0000 | 3.4353e+09 | 39 |

**Adduct Signal Fractions**

- **Unmodified**: 3.4353e+09

### TPN(N)KTEDTIF Fuc 1 Hex 7 HexNAc 6

**Mass:**  3663.4768

(1.539e-06)

**Charge States:** 3, 4

**Start Position**: 70

**End Position**: 80

| Profile Score | Tandem Score | TDA q-value | Volume | Spectrum Matches |
| --- | --- | --- | --- | --- |
| 14.3886 | 170.7249 | 0.0000 | 5.0832e+07 | 5 |

**Adduct Signal Fractions**

- **Unmodified**: 5.0832e+07

### YFTPN(N)KTEDTIF Fuc 1 Hex 7 HexNAc 6

**Mass:**  3973.6081

(1.558e-06)

**Charge States:** 3, 4

**Start Position**: 68

**End Position**: 80

| Profile Score | Tandem Score | TDA q-value | Volume | Spectrum Matches |
| --- | --- | --- | --- | --- |
| 14.2654 | 170.5691 | 0.0000 | 8.3754e+08 | 27 |

**Adduct Signal Fractions**

- **Unmodified**: 8.3754e+08

### YFTPN(N)KTEDTIF Fuc 1 Hex 6 HexNAc 5

**Mass:**  3608.4717

(1.528e-06)

**Charge States:** 3, 4

**Start Position**: 68

**End Position**: 80

| Profile Score | Tandem Score | TDA q-value | Volume | Spectrum Matches |
| --- | --- | --- | --- | --- |
| 15.4076 | 168.1096 | 0.0000 | 6.6405e+08 | 33 |

**Adduct Signal Fractions**

- **Unmodified**: 6.6405e+08

### FYFTPN(N)KTEDTIF Hex 6 HexNAc 5

**Mass:**  3609.4854

(5.457e-07)

**Charge States:** 3, 4

**Start Position**: 67

**End Position**: 80

| Profile Score | Tandem Score | TDA q-value | Volume | Spectrum Matches |
| --- | --- | --- | --- | --- |
| 14.7279 | 167.7381 | 0.0000 | 2.4989e+09 | 49 |

**Adduct Signal Fractions**

- **Unmodified**: 2.4989e+09

### FYFTPN(N)KTEDTIF Fuc 1 Hex 6 HexNAc 5

**Mass:**  3755.5442

(1.078e-06)

**Charge States:** 3, 4

**Start Position**: 67

**End Position**: 80

| Profile Score | Tandem Score | TDA q-value | Volume | Spectrum Matches |
| --- | --- | --- | --- | --- |
| 13.3316 | 167.3913 | 0.0000 | 5.4319e+08 | 9 |

**Adduct Signal Fractions**

- **Unmodified**: 5.4319e+08

### TPN(N)KTEDTIF Fuc 1 Hex 6 HexNAc 5

**Mass:**  3298.3423

(2.218e-06)

**Charge States:** 3, 4

**Start Position**: 70

**End Position**: 80

| Profile Score | Tandem Score | TDA q-value | Volume | Spectrum Matches |
| --- | --- | --- | --- | --- |
| 13.6962 | 166.9940 | 0.0000 | 3.7323e+07 | 4 |

**Adduct Signal Fractions**

- **Unmodified**: 3.7323e+07

### TPN(N)KTEDTIF Hex 8 HexNAc 7

**Mass:**  3882.5485

(1.020e-06)

**Charge States:** 3, 4

**Start Position**: 70

**End Position**: 80

| Profile Score | Tandem Score | TDA q-value | Volume | Spectrum Matches |
| --- | --- | --- | --- | --- |
| 14.6735 | 166.9320 | 0.0000 | 2.9993e+07 | 4 |

**Adduct Signal Fractions**

- **Unmodified**: 2.9993e+07

### FYFTPN(N)KTEDTIF Fuc 1 Hex 7 HexNAc 6

**Mass:**  4120.6789

(1.855e-06)

**Charge States:** 3, 4

**Start Position**: 67

**End Position**: 80

| Profile Score | Tandem Score | TDA q-value | Volume | Spectrum Matches |
| --- | --- | --- | --- | --- |
| 14.6183 | 166.6950 | 0.0000 | 8.4755e+08 | 12 |

**Adduct Signal Fractions**

- **Unmodified**: 8.4755e+08

### FYFTPN(N)KTEDTIF Fuc 1 Hex 7 HexNAc 7

**Mass:**  4323.7536

(9.327e-07)

**Charge States:** 3, 4

**Start Position**: 67

**End Position**: 80

| Profile Score | Tandem Score | TDA q-value | Volume | Spectrum Matches |
| --- | --- | --- | --- | --- |
| -3.6418 | 166.1472 | 0.0000 | 3.9266e+07 | 3 |

**Adduct Signal Fractions**

- **Unmodified**: 3.9266e+07

### N(N)KSVQEIQATFF Hex 6 HexNAc 5

**Mass:**  3398.4365

(2.443e-06)

**Charge States:** 3

**Start Position**: 56

**End Position**: 68

| Profile Score | Tandem Score | TDA q-value | Volume | Spectrum Matches |
| --- | --- | --- | --- | --- |
| -4.5289 | 166.0576 | 0.0000 | 7.9908e+06 | 3 |

**Adduct Signal Fractions**

- **Unmodified**: 7.9908e+06

### FYFTPN(N)KTEDTIF Hex 7 HexNAc 6

**Mass:**  3974.6186

(1.835e-06)

**Charge States:** 3, 4

**Start Position**: 67

**End Position**: 80

| Profile Score | Tandem Score | TDA q-value | Volume | Spectrum Matches |
| --- | --- | --- | --- | --- |
| 15.3001 | 165.6626 | 0.0000 | 4.6720e+09 | 77 |

**Adduct Signal Fractions**

- **Unmodified**: 4.6720e+09

### FTPN(N)KTEDTIF Hex 7 HexNAc 6

**Mass:**  3664.4848

(1.077e-06)

**Charge States:** 3, 4

**Start Position**: 69

**End Position**: 80

| Profile Score | Tandem Score | TDA q-value | Volume | Spectrum Matches |
| --- | --- | --- | --- | --- |
| 15.4441 | 165.4918 | 0.0000 | 6.6726e+08 | 18 |

**Adduct Signal Fractions**

- **Unmodified**: 6.6726e+08

### FYFTPN(N)KTEDTIF Fuc 2 Hex 7 HexNAc 6

**Mass:**  4266.7341

(5.814e-07)

**Charge States:** 3, 4

**Start Position**: 67

**End Position**: 80

| Profile Score | Tandem Score | TDA q-value | Volume | Spectrum Matches |
| --- | --- | --- | --- | --- |
| 13.2913 | 165.4168 | 0.0000 | 1.1807e+08 | 3 |

**Adduct Signal Fractions**

- **Unmodified**: 1.1807e+08

### YFTPN(N)KTEDTIF Hex 7 HexNAc 4 Neu5Ac 2

**Mass:**  4003.5769

(9.294e-07)

**Charge States:** 3, 4

**Start Position**: 68

**End Position**: 80

| Profile Score | Tandem Score | TDA q-value | Volume | Spectrum Matches |
| --- | --- | --- | --- | --- |
| 15.3017 | 165.1453 | 0.0000 | 3.1237e+08 | 7 |

**Adduct Signal Fractions**

- **Unmodified**: 3.1237e+08

### YFTPN(N)KTEDTIF Fuc 1 Hex 8 HexNAc 7

**Mass:**  4338.7429

(1.306e-06)

**Charge States:** 3, 4

**Start Position**: 68

**End Position**: 80

| Profile Score | Tandem Score | TDA q-value | Volume | Spectrum Matches |
| --- | --- | --- | --- | --- |
| 15.7326 | 164.6403 | 0.0000 | 1.4719e+08 | 5 |

**Adduct Signal Fractions**

- **Unmodified**: 1.4719e+08

### EEYN(N)KSVQEIQATF Hex 5 HexNAc 4

**Mass:**  3307.3789

(9.130e-07)

**Charge States:** 3, 4

**Start Position**: 53

**End Position**: 67

| Profile Score | Tandem Score | TDA q-value | Volume | Spectrum Matches |
| --- | --- | --- | --- | --- |
| -3.4825 | 162.5484 | 0.0000 | 7.7241e+07 | 3 |

**Adduct Signal Fractions**

- **Unmodified**: 7.7241e+07

### YFTPN(N)KTEDTIF Fuc 2 Hex 7 HexNAc 6

**Mass:**  4119.6662

(1.685e-06)

**Charge States:** 3, 4

**Start Position**: 68

**End Position**: 80

| Profile Score | Tandem Score | TDA q-value | Volume | Spectrum Matches |
| --- | --- | --- | --- | --- |
| -3.4203 | 162.4366 | 0.0000 | 1.2368e+08 | 3 |

**Adduct Signal Fractions**

- **Unmodified**: 1.2368e+08

### EEYN(N)KSVQEIQATF Hex 6 HexNAc 5

**Mass:**  3672.5139

(1.546e-06)

**Charge States:** 3, 4

**Start Position**: 53

**End Position**: 67

| Profile Score | Tandem Score | TDA q-value | Volume | Spectrum Matches |
| --- | --- | --- | --- | --- |
| -3.4604 | 162.2122 | 0.0000 | 7.6897e+07 | 2 |

**Adduct Signal Fractions**

- **Unmodified**: 7.6897e+07

### YFTPN(N)KTEDTIF Hex 9 HexNAc 8

**Mass:**  4557.8176

(1.660e-06)

**Charge States:** 3, 4

**Start Position**: 68

**End Position**: 80

| Profile Score | Tandem Score | TDA q-value | Volume | Spectrum Matches |
| --- | --- | --- | --- | --- |
| 15.5811 | 161.6406 | 0.0000 | 2.1047e+08 | 7 |

**Adduct Signal Fractions**

- **Unmodified**: 2.1047e+08

### TPN(N)KTEDTIF Fuc 4 Hex 4 HexNAc 6 Neu5Ac 1

**Mass:**  3906.5463

(-8.017e-06)

**Charge States:** 3, 4

**Start Position**: 70

**End Position**: 80

| Profile Score | Tandem Score | TDA q-value | Volume | Spectrum Matches |
| --- | --- | --- | --- | --- |
| -20.4435 | 160.2474 | 0.0000 | 6.0955e+07 | 1 |

**Adduct Signal Fractions**

- **Unmodified**: 6.0955e+07

### FYFTPN(N)KTEDTIF Hex 5 HexNAc 4

**Mass:**  3244.3515

(1.058e-06)

**Charge States:** 3

**Start Position**: 67

**End Position**: 80

| Profile Score | Tandem Score | TDA q-value | Volume | Spectrum Matches |
| --- | --- | --- | --- | --- |
| 13.0253 | 159.2802 | 0.0000 | 2.1713e+08 | 4 |

**Adduct Signal Fractions**

- **Unmodified**: 2.1713e+08

### RNEEYN(N)KSVQEIQATF Hex 5 HexNAc 4

**Mass:**  3577.5291

(-4.646e-07)

**Charge States:** 3, 4

**Start Position**: 51

**End Position**: 67

| Profile Score | Tandem Score | TDA q-value | Volume | Spectrum Matches |
| --- | --- | --- | --- | --- |
| 16.1988 | 157.7283 | 0.0000 | 9.4257e+09 | 19 |

**Adduct Signal Fractions**

- **Unmodified**: 9.4257e+09

### RNEEYN(N)KSVQEIQATFF Hex 6 HexNAc 5

**Mass:**  4089.7297

(1.217e-06)

**Charge States:** 3, 4

**Start Position**: 51

**End Position**: 68

| Profile Score | Tandem Score | TDA q-value | Volume | Spectrum Matches |
| --- | --- | --- | --- | --- |
| 16.8803 | 157.6757 | 0.0000 | 3.5932e+09 | 19 |

**Adduct Signal Fractions**

- **Unmodified**: 3.5932e+09

### RNEEYN(N)KSVQEIQATFF Hex 5 HexNAc 4

**Mass:**  3724.5913

(8.720e-07)

**Charge States:** 3, 4

**Start Position**: 51

**End Position**: 68

| Profile Score | Tandem Score | TDA q-value | Volume | Spectrum Matches |
| --- | --- | --- | --- | --- |
| 17.0965 | 156.8207 | 0.0000 | 4.0165e+09 | 18 |

**Adduct Signal Fractions**

- **Unmodified**: 4.0165e+09

### RNEEYN(N)KSVQEIQATFF Fuc 1 Hex 6 HexNAc 5

**Mass:**  4235.7853

(8.776e-07)

**Charge States:** 3, 4

**Start Position**: 51

**End Position**: 68

| Profile Score | Tandem Score | TDA q-value | Volume | Spectrum Matches |
| --- | --- | --- | --- | --- |
| 16.1118 | 156.1799 | 0.0000 | 1.7181e+09 | 16 |

**Adduct Signal Fractions**

- **Unmodified**: 1.7181e+09

### FTPN(N)KTEDTIF Fuc 1 Hex 6 HexNAc 5

**Mass:**  3445.4093

(5.064e-07)

**Charge States:** 3, 4

**Start Position**: 69

**End Position**: 80

| Profile Score | Tandem Score | TDA q-value | Volume | Spectrum Matches |
| --- | --- | --- | --- | --- |
| 13.0481 | 155.8549 | 0.0000 | 9.3085e+07 | 7 |

**Adduct Signal Fractions**

- **Unmodified**: 9.3085e+07

### FYFTPN(N)KTEDTIF Hex 9 HexNAc 8

**Mass:**  4704.8859

(1.897e-06)

**Charge States:** 3, 4

**Start Position**: 67

**End Position**: 80

| Profile Score | Tandem Score | TDA q-value | Volume | Spectrum Matches |
| --- | --- | --- | --- | --- |
| 15.1254 | 154.3806 | 0.0000 | 1.6809e+08 | 6 |

**Adduct Signal Fractions**

- **Unmodified**: 1.6809e+08

### RNEEYN(N)KSVQEIQATF Hex 6 HexNAc 5

**Mass:**  3942.6596

(2.825e-06)

**Charge States:** 3, 4

**Start Position**: 51

**End Position**: 67

| Profile Score | Tandem Score | TDA q-value | Volume | Spectrum Matches |
| --- | --- | --- | --- | --- |
| 15.7912 | 154.3564 | 0.0000 | 7.0218e+09 | 16 |

**Adduct Signal Fractions**

- **Unmodified**: 7.0218e+09

### FTPN(N)KTEDTIF Fuc 1 Hex 7 HexNAc 6

**Mass:**  3810.5446

(1.496e-06)

**Charge States:** 3, 4

**Start Position**: 69

**End Position**: 80

| Profile Score | Tandem Score | TDA q-value | Volume | Spectrum Matches |
| --- | --- | --- | --- | --- |
| 14.5444 | 153.5470 | 0.0031 | 1.1535e+08 | 6 |

**Adduct Signal Fractions**

- **Unmodified**: 1.1535e+08

### RNEEYN(N)KSVQEIQATF Fuc 1 Hex 6 HexNAc 5

**Mass:**  4088.7126

(2.077e-06)

**Charge States:** 3, 4

**Start Position**: 51

**End Position**: 67

| Profile Score | Tandem Score | TDA q-value | Volume | Spectrum Matches |
| --- | --- | --- | --- | --- |
| 14.8626 | 153.4012 | 0.0031 | 3.4417e+09 | 17 |

**Adduct Signal Fractions**

- **Unmodified**: 3.4417e+09

### YFTPN(N)KTEDTIF Fuc 1 Hex 5 HexNAc 4

**Mass:**  3243.3451

(2.360e-06)

**Charge States:** 3

**Start Position**: 68

**End Position**: 80

| Profile Score | Tandem Score | TDA q-value | Volume | Spectrum Matches |
| --- | --- | --- | --- | --- |
| -20.0017 | 153.0186 | 0.0031 | 7.3169e+06 | 1 |

**Adduct Signal Fractions**

- **Unmodified**: 7.3169e+06

### RNEEYN(N)KSVQEIQATFF Fuc 1 Hex 7 HexNAc 6

**Mass:**  4600.9167

(1.620e-06)

**Charge States:** 3, 4

**Start Position**: 51

**End Position**: 68

| Profile Score | Tandem Score | TDA q-value | Volume | Spectrum Matches |
| --- | --- | --- | --- | --- |
| 14.0988 | 152.9488 | 0.0031 | 6.4718e+07 | 4 |

**Adduct Signal Fractions**

- **Unmodified**: 6.4718e+07

### RNEEYN(N)KSVQEIQATFF Fuc 1 Hex 5 HexNAc 4

**Mass:**  3870.6498

(1.523e-06)

**Charge States:** 3, 4

**Start Position**: 51

**End Position**: 68

| Profile Score | Tandem Score | TDA q-value | Volume | Spectrum Matches |
| --- | --- | --- | --- | --- |
| 13.5383 | 152.4214 | 0.0031 | 1.6054e+08 | 7 |

**Adduct Signal Fractions**

- **Unmodified**: 1.6054e+08

### YFTPN(N)KTEDTIF Hex 10 HexNAc 9

**Mass:**  4922.9455

(9.886e-07)

**Charge States:** 4

**Start Position**: 68

**End Position**: 80

| Profile Score | Tandem Score | TDA q-value | Volume | Spectrum Matches |
| --- | --- | --- | --- | --- |
| -4.3520 | 152.1862 | 0.0031 | 2.0988e+07 | 2 |

**Adduct Signal Fractions**

- **Unmodified**: 2.0988e+07

### RNEEYN(N)KSVQEIQATF Fuc 1 Hex 5 HexNAc 4

**Mass:**  3723.5873

(1.717e-06)

**Charge States:** 3, 4

**Start Position**: 51

**End Position**: 67

| Profile Score | Tandem Score | TDA q-value | Volume | Spectrum Matches |
| --- | --- | --- | --- | --- |
| 11.7119 | 152.1116 | 0.0031 | 3.5847e+08 | 10 |

**Adduct Signal Fractions**

- **Unmodified**: 3.5847e+08

### RNEEYN(N)KSVQEIQAT Hex 5 HexNAc 4

**Mass:**  3430.4547

(8.004e-07)

**Charge States:** 3

**Start Position**: 51

**End Position**: 66

| Profile Score | Tandem Score | TDA q-value | Volume | Spectrum Matches |
| --- | --- | --- | --- | --- |
| -3.8734 | 151.8857 | 0.0031 | 4.2548e+07 | 3 |

**Adduct Signal Fractions**

- **Unmodified**: 4.2548e+07

### FTPN(N)KTEDTIF Hex 5 HexNAc 4

**Mass:**  2934.2201

(6.688e-07)

**Charge States:** 3

**Start Position**: 69

**End Position**: 80

| Profile Score | Tandem Score | TDA q-value | Volume | Spectrum Matches |
| --- | --- | --- | --- | --- |
| -2.8260 | 151.4588 | 0.0031 | 5.0434e+07 | 3 |

**Adduct Signal Fractions**

- **Unmodified**: 5.0434e+07

### FYFTPN(N)KT Hex 6 HexNAc 5

**Mass:**  3004.2165

(2.933e-06)

**Charge States:** 3

**Start Position**: 67

**End Position**: 75

| Profile Score | Tandem Score | TDA q-value | Volume | Spectrum Matches |
| --- | --- | --- | --- | --- |
| -3.2583 | 150.6813 | 0.0000 | 7.8080e+06 | 2 |

**Adduct Signal Fractions**

- **Unmodified**: 7.8080e+06

### RNEEYN(N)KSVQEIQATF Fuc 1 Hex 7 HexNAc 6

**Mass:**  4453.8508

(1.808e-06)

**Charge States:** 4

**Start Position**: 51

**End Position**: 67

| Profile Score | Tandem Score | TDA q-value | Volume | Spectrum Matches |
| --- | --- | --- | --- | --- |
| 14.2984 | 150.5590 | 0.0031 | 1.5762e+08 | 2 |

**Adduct Signal Fractions**

- **Unmodified**: 1.5762e+08

### FYFTPN(N)KTEDTIF Hex 8 HexNAc 7

**Mass:**  4339.7551

(1.577e-06)

**Charge States:** 3, 4

**Start Position**: 67

**End Position**: 80

| Profile Score | Tandem Score | TDA q-value | Volume | Spectrum Matches |
| --- | --- | --- | --- | --- |
| 14.4672 | 150.4008 | 0.0031 | 6.1852e+08 | 11 |

**Adduct Signal Fractions**

- **Unmodified**: 6.1852e+08

### TPN(N)KTEDTIF Fuc 4 Hex 8 HexNAc 5

**Mass:**  4060.6000

(-3.102e-06)

**Charge States:** 3, 4

**Start Position**: 70

**End Position**: 80

| Profile Score | Tandem Score | TDA q-value | Volume | Spectrum Matches |
| --- | --- | --- | --- | --- |
| 13.2016 | 150.3132 | 0.0031 | 1.3595e+08 | 4 |

**Adduct Signal Fractions**

- **Unmodified**: 1.3595e+08

### N(N)KSVQEIQATFF Hex 5 HexNAc 4

**Mass:**  3033.3003

(1.440e-06)

**Charge States:** 3

**Start Position**: 56

**End Position**: 68

| Profile Score | Tandem Score | TDA q-value | Volume | Spectrum Matches |
| --- | --- | --- | --- | --- |
| -4.4311 | 149.1811 | 0.0031 | 8.0912e+06 | 1 |

**Adduct Signal Fractions**

- **Unmodified**: 8.0912e+06

### RNEEYN(N)KSVQEIQATF Hex 7 HexNAc 6

**Mass:**  4307.7922

(2.141e-06)

**Charge States:** 3, 4

**Start Position**: 51

**End Position**: 67

| Profile Score | Tandem Score | TDA q-value | Volume | Spectrum Matches |
| --- | --- | --- | --- | --- |
| 14.4255 | 148.5843 | 0.0031 | 3.5992e+08 | 3 |

**Adduct Signal Fractions**

- **Unmodified**: 3.5992e+08

### RNEEYN(N)KSVQEIQATFF Fuc 2 Hex 6 HexNAc 5

**Mass:**  4381.8435

(1.534e-06)

**Charge States:** 4

**Start Position**: 51

**End Position**: 68

| Profile Score | Tandem Score | TDA q-value | Volume | Spectrum Matches |
| --- | --- | --- | --- | --- |
| -3.9871 | 148.3505 | 0.0031 | 6.8085e+07 | 3 |

**Adduct Signal Fractions**

- **Unmodified**: 6.8085e+07

### YFTPN(N)KTEDTIF Fuc 2 Hex 8 HexNAc 7

**Mass:**  4484.7971

(1.152e-06)

**Charge States:** 3, 4

**Start Position**: 68

**End Position**: 80

| Profile Score | Tandem Score | TDA q-value | Volume | Spectrum Matches |
| --- | --- | --- | --- | --- |
| 9.8279 | 148.1639 | 0.0031 | 3.2036e+07 | 2 |

**Adduct Signal Fractions**

- **Unmodified**: 3.2036e+07

### YFTPN(N)KTEDTIFL Hex 7 HexNAc 6

**Mass:**  3940.6309

(7.348e-07)

**Charge States:** 3, 4

**Start Position**: 68

**End Position**: 81

| Profile Score | Tandem Score | TDA q-value | Volume | Spectrum Matches |
| --- | --- | --- | --- | --- |
| 14.0536 | 145.7341 | 0.0031 | 5.1702e+07 | 3 |

**Adduct Signal Fractions**

- **Unmodified**: 5.1702e+07

### FYFTPN(N)KTEDTIF Hex 7 HexNAc 4 Neu5Ac 2

**Mass:**  4150.6530

(2.170e-06)

**Charge States:** 3, 4

**Start Position**: 67

**End Position**: 80

| Profile Score | Tandem Score | TDA q-value | Volume | Spectrum Matches |
| --- | --- | --- | --- | --- |
| 13.5238 | 145.4916 | 0.0031 | 2.6097e+08 | 6 |

**Adduct Signal Fractions**

- **Unmodified**: 2.6097e+08

### YFTPN(N)KTEDTIF Fuc 1 Hex 8 HexNAc 4 Neu5Ac 1

**Mass:**  4020.6022

(2.685e-06)

**Charge States:** 3

**Start Position**: 68

**End Position**: 80

| Profile Score | Tandem Score | TDA q-value | Volume | Spectrum Matches |
| --- | --- | --- | --- | --- |
| -4.4960 | 145.2400 | 0.0031 | 2.8657e+07 | 2 |

**Adduct Signal Fractions**

- **Unmodified**: 2.8657e+07

### RNEEYN(N)KSVQEIQATF Fuc 2 Hex 6 HexNAc 5

**Mass:**  4234.7785

(2.446e-06)

**Charge States:** 4

**Start Position**: 51

**End Position**: 67

| Profile Score | Tandem Score | TDA q-value | Volume | Spectrum Matches |
| --- | --- | --- | --- | --- |
| -3.1088 | 144.8998 | 0.0031 | 1.2832e+08 | 2 |

**Adduct Signal Fractions**

- **Unmodified**: 1.2832e+08

### FTPN(N)KTEDTIF Hex 8 HexNAc 7

**Mass:**  4029.6181

(1.336e-06)

**Charge States:** 3, 4

**Start Position**: 69

**End Position**: 80

| Profile Score | Tandem Score | TDA q-value | Volume | Spectrum Matches |
| --- | --- | --- | --- | --- |
| 16.2241 | 144.7784 | 0.0031 | 6.3725e+07 | 6 |

**Adduct Signal Fractions**

- **Unmodified**: 6.3725e+07

### RNEEYN(N)KSVQEIQATFF Hex 7 HexNAc 6

**Mass:**  4454.8591

(1.367e-06)

**Charge States:** 3, 4

**Start Position**: 51

**End Position**: 68

| Profile Score | Tandem Score | TDA q-value | Volume | Spectrum Matches |
| --- | --- | --- | --- | --- |
| 13.8275 | 144.2299 | 0.0031 | 1.7057e+08 | 4 |

**Adduct Signal Fractions**

- **Unmodified**: 1.7057e+08

### FYFTPN(N)KTEDTIF Hex 3 HexNAc 5 Neu5Ac 3

**Mass:**  3996.5947

(-3.357e-06)

**Charge States:** 3, 4

**Start Position**: 67

**End Position**: 80

| Profile Score | Tandem Score | TDA q-value | Volume | Spectrum Matches |
| --- | --- | --- | --- | --- |
| 13.7141 | 143.3201 | 0.0031 | 2.5036e+08 | 6 |

**Adduct Signal Fractions**

- **Unmodified**: 2.5036e+08

### EEYN(N)KSVQEIQATF Fuc 1 Hex 6 HexNAc 5

**Mass:**  3818.5729

(1.590e-06)

**Charge States:** 3, 4

**Start Position**: 53

**End Position**: 67

| Profile Score | Tandem Score | TDA q-value | Volume | Spectrum Matches |
| --- | --- | --- | --- | --- |
| -3.8155 | 142.9135 | 0.0031 | 3.3111e+07 | 3 |

**Adduct Signal Fractions**

- **Unmodified**: 3.3111e+07

### TPN(N)KTEDTIF Hex 7 HexNAc 4 Neu5Ac 2

**Mass:**  3693.4462

(-5.159e-07)

**Charge States:** 3, 4

**Start Position**: 70

**End Position**: 80

| Profile Score | Tandem Score | TDA q-value | Volume | Spectrum Matches |
| --- | --- | --- | --- | --- |
| -18.4520 | 142.7669 | 0.0031 | 9.7591e+06 | 2 |

**Adduct Signal Fractions**

- **Unmodified**: 9.7591e+06

### RNEEYN(N)KSVQEIQATF Hex 8 HexNAc 7

**Mass:**  4672.9260

(2.011e-06)

**Charge States:** 4

**Start Position**: 51

**End Position**: 67

| Profile Score | Tandem Score | TDA q-value | Volume | Spectrum Matches |
| --- | --- | --- | --- | --- |
| -3.6365 | 141.3815 | 0.0031 | 6.6030e+07 | 2 |

**Adduct Signal Fractions**

- **Unmodified**: 6.6030e+07

### FYFTPN(N)KTEDTIF Fuc 1 Hex 8 HexNAc 7

**Mass:**  4485.8102

(1.694e-06)

**Charge States:** 3, 4

**Start Position**: 67

**End Position**: 80

| Profile Score | Tandem Score | TDA q-value | Volume | Spectrum Matches |
| --- | --- | --- | --- | --- |
| 15.9635 | 140.5857 | 0.0031 | 1.2259e+08 | 5 |

**Adduct Signal Fractions**

- **Unmodified**: 1.2259e+08

### YFTPN(N)KT Hex 7 HexNAc 6

**Mass:**  3222.2821

(2.448e-06)

**Charge States:** 3

**Start Position**: 68

**End Position**: 75

| Profile Score | Tandem Score | TDA q-value | Volume | Spectrum Matches |
| --- | --- | --- | --- | --- |
| -19.6987 | 140.3356 | 0.0000 | 4.9556e+06 | 1 |

**Adduct Signal Fractions**

- **Unmodified**: 4.9556e+06

### YFTPN(N)KTEDTIFL Hex 6 HexNAc 5

**Mass:**  3575.5010

(1.399e-06)

**Charge States:** 3

**Start Position**: 68

**End Position**: 81

| Profile Score | Tandem Score | TDA q-value | Volume | Spectrum Matches |
| --- | --- | --- | --- | --- |
| -3.6900 | 140.1073 | 0.0031 | 2.5477e+07 | 4 |

**Adduct Signal Fractions**

- **Unmodified**: 2.5477e+07

### LNVQREN(N)GTISRY Hex 7 HexNAc 6

**Mass:**  3901.6455

(1.392e-06)

**Charge States:** 3, 4

**Start Position**: 97

**End Position**: 110

| Profile Score | Tandem Score | TDA q-value | Volume | Spectrum Matches |
| --- | --- | --- | --- | --- |
| 16.2723 | 139.8563 | 0.0031 | 9.5805e+09 | 12 |

**Adduct Signal Fractions**

- **Unmodified**: 9.5805e+09

### LNVQREN(N)GTISRY Fuc 1 Hex 8 HexNAc 7

**Mass:**  4412.8439

(8.848e-07)

**Charge States:** 3, 4

**Start Position**: 97

**End Position**: 110

| Profile Score | Tandem Score | TDA q-value | Volume | Spectrum Matches |
| --- | --- | --- | --- | --- |
| 14.0761 | 139.6176 | 0.0031 | 4.1216e+08 | 4 |

**Adduct Signal Fractions**

- **Unmodified**: 4.1216e+08

### LNVQREN(N)GTISRY Hex 6 HexNAc 5

**Mass:**  3536.5222

(1.840e-06)

**Charge States:** 3, 4

**Start Position**: 97

**End Position**: 110

| Profile Score | Tandem Score | TDA q-value | Volume | Spectrum Matches |
| --- | --- | --- | --- | --- |
| 15.4990 | 139.0754 | 0.0031 | 3.6937e+09 | 13 |

**Adduct Signal Fractions**

- **Unmodified**: 3.6937e+09

### RNEEYN(N)KSVQEIQAT Hex 6 HexNAc 5

**Mass:**  3795.5898

(1.555e-06)

**Charge States:** 3, 4

**Start Position**: 51

**End Position**: 66

| Profile Score | Tandem Score | TDA q-value | Volume | Spectrum Matches |
| --- | --- | --- | --- | --- |
| -20.0752 | 136.3270 | 0.0031 | 2.6376e+07 | 2 |

**Adduct Signal Fractions**

- **Unmodified**: 2.6376e+07

### NVQREN(N)GTISRY Fuc 1 Hex 7 HexNAc 6

**Mass:**  3934.6225

(1.849e-07)

**Charge States:** 3, 4

**Start Position**: 98

**End Position**: 110

| Profile Score | Tandem Score | TDA q-value | Volume | Spectrum Matches |
| --- | --- | --- | --- | --- |
| -9.3609 | 135.7133 | 0.0031 | 1.8764e+08 | 5 |

**Adduct Signal Fractions**

- **Unmodified**: 1.8764e+08

### QTRQDQC(C)IYN(N)TTY Hex 6 HexNAc 5

**Mass:**  3677.4607

(1.382e-06)

**Charge States:** 3, 4

**Start Position**: 84

**End Position**: 97

| Profile Score | Tandem Score | TDA q-value | Volume | Spectrum Matches |
| --- | --- | --- | --- | --- |
| 15.3432 | 135.1185 | 0.0031 | 1.3375e+08 | 6 |

**Adduct Signal Fractions**

- **Unmodified**: 1.3375e+08

### FYFTPN(N)KTEDTIF Fuc 1 Hex 7 HexNAc 4 Neu5Ac 2

**Mass:**  4296.7062

(8.483e-07)

**Charge States:** 4

**Start Position**: 67

**End Position**: 80

| Profile Score | Tandem Score | TDA q-value | Volume | Spectrum Matches |
| --- | --- | --- | --- | --- |
| -4.4983 | 134.7783 | 0.0031 | 4.2210e+07 | 3 |

**Adduct Signal Fractions**

- **Unmodified**: 4.2210e+07

### LNVQREN(N)GTISRY Fuc 1 Hex 6 HexNAc 5

**Mass:**  3682.5795

(1.891e-06)

**Charge States:** 3, 4

**Start Position**: 97

**End Position**: 110

| Profile Score | Tandem Score | TDA q-value | Volume | Spectrum Matches |
| --- | --- | --- | --- | --- |
| 14.6724 | 134.5709 | 0.0031 | 1.4195e+09 | 11 |

**Adduct Signal Fractions**

- **Unmodified**: 1.4195e+09

### EEYN(N)KSVQEIQATFF Hex 5 HexNAc 4

**Mass:**  3454.4503

(9.792e-08)

**Charge States:** 3

**Start Position**: 53

**End Position**: 68

| Profile Score | Tandem Score | TDA q-value | Volume | Spectrum Matches |
| --- | --- | --- | --- | --- |
| -4.5663 | 134.2915 | 0.0031 | 2.5232e+07 | 3 |

**Adduct Signal Fractions**

- **Unmodified**: 2.5232e+07

### YFTPN(N)KTEDTIF Fuc 1 Hex 7 HexNAc 4 Neu5Ac 2

**Mass:**  4149.6364

(1.900e-06)

**Charge States:** 3, 4

**Start Position**: 68

**End Position**: 80

| Profile Score | Tandem Score | TDA q-value | Volume | Spectrum Matches |
| --- | --- | --- | --- | --- |
| -4.7205 | 133.0449 | 0.0031 | 5.5682e+07 | 2 |

**Adduct Signal Fractions**

- **Unmodified**: 5.5682e+07

### FRNEEYN(N)KSVQEIQATF Hex 6 HexNAc 5

**Mass:**  4089.7246

(9.506e-07)

**Charge States:** 4

**Start Position**: 50

**End Position**: 67

| Profile Score | Tandem Score | TDA q-value | Volume | Spectrum Matches |
| --- | --- | --- | --- | --- |
| 12.2524 | 132.8241 | 0.0031 | 5.4374e+07 | 2 |

**Adduct Signal Fractions**

- **Unmodified**: 5.4374e+07

### RN(D)EEYN(N)KSVQEIQATF Hex 5 HexNAc 4

**Mass:**  3578.5080

(2.761e-04)

**Charge States:** 3, 4

**Start Position**: 51

**End Position**: 67

| Profile Score | Tandem Score | TDA q-value | Volume | Spectrum Matches |
| --- | --- | --- | --- | --- |
| 10.9878 | 132.6702 | 0.0031 | 1.2475e+09 | 12 |

**Adduct Signal Fractions**

- **Unmodified**: 1.2475e+09

### LNVQREN(N)GTISRY Hex 5 HexNAc 4

**Mass:**  3171.3916

(2.186e-06)

**Charge States:** 3

**Start Position**: 97

**End Position**: 110

| Profile Score | Tandem Score | TDA q-value | Volume | Spectrum Matches |
| --- | --- | --- | --- | --- |
| 14.7158 | 131.7492 | 0.0031 | 2.5335e+08 | 3 |

**Adduct Signal Fractions**

- **Unmodified**: 2.5335e+08

### N(N)KTEDTIF Fuc 5 Hex 5 HexNAc 8 Neu5Ac 1

**Mass:**  4425.7517

(6.787e-04)

**Start Position**: 72

**End Position**: 80

| Profile Score | Tandem Score | TDA q-value | Volume | Spectrum Matches |
| --- | --- | --- | --- | --- |
| 0.0000 | 131.6471 | 0.0000 | 0.0000e+00 | 1 |

Adduct:

No Chromatogram Found

### RN(D)EEYN(N)KSVQEIQATF Hex 6 HexNAc 5

**Mass:**  3943.6447

(2.517e-04)

**Charge States:** 3, 4

**Start Position**: 51

**End Position**: 67

| Profile Score | Tandem Score | TDA q-value | Volume | Spectrum Matches |
| --- | --- | --- | --- | --- |
| 10.2105 | 130.2300 | 0.0056 | 1.2329e+09 | 14 |

**Adduct Signal Fractions**

- **Unmodified**: 1.2329e+09

### QTRQDQC(C)IYN(N)TTY Fuc 1 Hex 6 HexNAc 5

**Mass:**  3823.5177

(1.708e-06)

**Charge States:** 3, 4

**Start Position**: 84

**End Position**: 97

| Profile Score | Tandem Score | TDA q-value | Volume | Spectrum Matches |
| --- | --- | --- | --- | --- |
| 13.7590 | 128.7055 | 0.0056 | 3.6303e+07 | 4 |

**Adduct Signal Fractions**

- **Unmodified**: 3.6303e+07

### NVQREN(N)GTISRY Fuc 1 Hex 6 HexNAc 5

**Mass:**  3569.4922

(1.082e-06)

**Charge States:** 3, 4

**Start Position**: 98

**End Position**: 110

| Profile Score | Tandem Score | TDA q-value | Volume | Spectrum Matches |
| --- | --- | --- | --- | --- |
| -18.9628 | 128.6416 | 0.0056 | 4.9426e+07 | 2 |

**Adduct Signal Fractions**

- **Unmodified**: 4.9426e+07

### NVQREN(N)GTISRY Hex 7 HexNAc 6

**Mass:**  3788.5646

(2.543e-07)

**Charge States:** 3, 4

**Start Position**: 98

**End Position**: 110

| Profile Score | Tandem Score | TDA q-value | Volume | Spectrum Matches |
| --- | --- | --- | --- | --- |
| 12.7183 | 128.5495 | 0.0056 | 4.9995e+08 | 6 |

**Adduct Signal Fractions**

- **Unmodified**: 4.9995e+08

### EEYN(N)KSVQEIQATFF Hex 6 HexNAc 5

**Mass:**  3819.5821

(1.561e-06)

**Charge States:** 3, 4

**Start Position**: 53

**End Position**: 68

| Profile Score | Tandem Score | TDA q-value | Volume | Spectrum Matches |
| --- | --- | --- | --- | --- |
| -4.6109 | 128.2901 | 0.0056 | 2.5571e+07 | 3 |

**Adduct Signal Fractions**

- **Unmodified**: 2.5571e+07

### FYFTPN(N)KT Hex 7 HexNAc 6

**Mass:**  3369.3488

(1.900e-06)

**Charge States:** 3

**Start Position**: 67

**End Position**: 75

| Profile Score | Tandem Score | TDA q-value | Volume | Spectrum Matches |
| --- | --- | --- | --- | --- |
| -3.6667 | 128.2778 | 0.0000 | 1.2661e+07 | 2 |

**Adduct Signal Fractions**

- **Unmodified**: 1.2661e+07

### VQREN(N)GTISRY Hex 7 HexNAc 6

**Mass:**  3674.5236

(-1.013e-06)

**Charge States:** 3

**Start Position**: 99

**End Position**: 110

| Profile Score | Tandem Score | TDA q-value | Volume | Spectrum Matches |
| --- | --- | --- | --- | --- |
| -20.7708 | 128.2004 | 0.0056 | 2.2157e+07 | 4 |

**Adduct Signal Fractions**

- **Unmodified**: 2.2157e+07

### LNVQREN(N)GTISRY Fuc 2 Hex 7 HexNAc 6

**Mass:**  4193.7673

(1.611e-07)

**Charge States:** 3, 4

**Start Position**: 97

**End Position**: 110

| Profile Score | Tandem Score | TDA q-value | Volume | Spectrum Matches |
| --- | --- | --- | --- | --- |
| 14.1541 | 126.6225 | 0.0056 | 1.0466e+09 | 7 |

**Adduct Signal Fractions**

- **Unmodified**: 1.0466e+09

### LNVQREN(N)GTISRY Fuc 1 Hex 7 HexNAc 6

**Mass:**  4047.7104

(-2.032e-07)

**Charge States:** 3, 4

**Start Position**: 97

**End Position**: 110

| Profile Score | Tandem Score | TDA q-value | Volume | Spectrum Matches |
| --- | --- | --- | --- | --- |
| 14.9213 | 125.9097 | 0.0056 | 3.3545e+09 | 11 |

**Adduct Signal Fractions**

- **Unmodified**: 3.3545e+09

### QTRQDQC(C)IYN(N)TTY Hex 7 HexNAc 6

**Mass:**  4042.5954

(1.882e-06)

**Charge States:** 3, 4

**Start Position**: 84

**End Position**: 97

| Profile Score | Tandem Score | TDA q-value | Volume | Spectrum Matches |
| --- | --- | --- | --- | --- |
| 14.3859 | 125.2922 | 0.0056 | 2.5015e+08 | 7 |

**Adduct Signal Fractions**

- **Unmodified**: 2.5015e+08

### YFTPN(N)KTEDTIF Fuc 1 Hex 9 HexNAc 8

**Mass:**  4703.8747

(1.275e-06)

**Charge States:** 4

**Start Position**: 68

**End Position**: 80

| Profile Score | Tandem Score | TDA q-value | Volume | Spectrum Matches |
| --- | --- | --- | --- | --- |
| 10.3672 | 124.5711 | 0.0056 | 3.2291e+07 | 2 |

**Adduct Signal Fractions**

- **Unmodified**: 3.2291e+07

### TPN(N)KTEDTIF Fuc 3 Hex 3 HexNAc 9 Neu5Ac 1

**Mass:**  4207.6730

(-8.561e-06)

**Charge States:** 3, 4

**Start Position**: 70

**End Position**: 80

| Profile Score | Tandem Score | TDA q-value | Volume | Spectrum Matches |
| --- | --- | --- | --- | --- |
| 13.6800 | 123.7718 | 0.0056 | 9.8621e+07 | 4 |

**Adduct Signal Fractions**

- **Unmodified**: 9.8621e+07

### LNVQREN(N)GTISRY Fuc 2 Hex 6 HexNAc 5

**Mass:**  3828.6275

(-7.628e-07)

**Charge States:** 3, 4

**Start Position**: 97

**End Position**: 110

| Profile Score | Tandem Score | TDA q-value | Volume | Spectrum Matches |
| --- | --- | --- | --- | --- |
| 10.1712 | 122.2248 | 0.0129 | 9.8329e+07 | 2 |

**Adduct Signal Fractions**

- **Unmodified**: 9.8329e+07

### FTPN(N)KTEDTIF Hex 7 HexNAc 4 Neu5Ac 2

**Mass:**  3840.5173

(3.963e-07)

**Charge States:** 3, 4

**Start Position**: 69

**End Position**: 80

| Profile Score | Tandem Score | TDA q-value | Volume | Spectrum Matches |
| --- | --- | --- | --- | --- |
| -4.3167 | 122.1745 | 0.0129 | 3.2369e+07 | 4 |

**Adduct Signal Fractions**

- **Unmodified**: 3.2369e+07

### FYFTPN(N)KTEDTIF Fuc 2 Hex 8 HexNAc 7

**Mass:**  4631.8679

(1.667e-06)

**Charge States:** 4

**Start Position**: 67

**End Position**: 80

| Profile Score | Tandem Score | TDA q-value | Volume | Spectrum Matches |
| --- | --- | --- | --- | --- |
| 11.4599 | 121.6180 | 0.0129 | 2.6048e+07 | 3 |

**Adduct Signal Fractions**

- **Unmodified**: 2.6048e+07

### FRNEEYN(N)KSVQEIQATF Hex 5 HexNAc 4

**Mass:**  3724.5949

(7.750e-07)

**Charge States:** 4

**Start Position**: 50

**End Position**: 67

| Profile Score | Tandem Score | TDA q-value | Volume | Spectrum Matches |
| --- | --- | --- | --- | --- |
| -4.9031 | 121.1006 | 0.0129 | 3.4886e+07 | 1 |

**Adduct Signal Fractions**

- **Unmodified**: 3.4886e+07

### NVQREN(N)GTISRY Hex 6 HexNAc 5

**Mass:**  3423.4330

(6.622e-07)

**Charge States:** 3

**Start Position**: 98

**End Position**: 110

| Profile Score | Tandem Score | TDA q-value | Volume | Spectrum Matches |
| --- | --- | --- | --- | --- |
| 11.5521 | 120.6664 | 0.0129 | 1.6527e+08 | 4 |

**Adduct Signal Fractions**

- **Unmodified**: 1.6527e+08

### FYFTPN(N)KTED Hex 7 HexNAc 6

**Mass:**  3613.4196

(2.005e-06)

**Charge States:** 3

**Start Position**: 67

**End Position**: 77

| Profile Score | Tandem Score | TDA q-value | Volume | Spectrum Matches |
| --- | --- | --- | --- | --- |
| -4.1361 | 119.9380 | 0.0129 | 6.6385e+06 | 2 |

**Adduct Signal Fractions**

- **Unmodified**: 6.6385e+06

### RNEEYN(N)KSVQEIQATFF Hex 8 HexNAc 7

**Mass:**  4819.9934

(1.765e-06)

**Charge States:** 4

**Start Position**: 51

**End Position**: 68

| Profile Score | Tandem Score | TDA q-value | Volume | Spectrum Matches |
| --- | --- | --- | --- | --- |
| -3.4544 | 119.8810 | 0.0129 | 1.9293e+07 | 2 |

**Adduct Signal Fractions**

- **Unmodified**: 1.9293e+07

### RN(D)EEYN(N)KSVQEIQATF Fuc 1 Hex 6 HexNAc 5

**Mass:**  4089.7011

(2.429e-04)

**Charge States:** 3, 4

**Start Position**: 51

**End Position**: 67

| Profile Score | Tandem Score | TDA q-value | Volume | Spectrum Matches |
| --- | --- | --- | --- | --- |
| 11.1965 | 119.7176 | 0.0149 | 5.6400e+08 | 11 |

**Adduct Signal Fractions**

- **Unmodified**: 5.6400e+08

### LVPVPITN(N)ATL Hex 6 HexNAc 5

**Mass:**  3124.3991

(1.492e-06)

**Charge States:** 3, 4

**Start Position**: 26

**End Position**: 37

| Profile Score | Tandem Score | TDA q-value | Volume | Spectrum Matches |
| --- | --- | --- | --- | --- |
| -18.9381 | 119.6055 | 0.0149 | 6.5499e+06 | 1 |

**Adduct Signal Fractions**

- **Unmodified**: 6.5499e+06

### RN(D)EEYN(N)KSVQEIQATFF Hex 5 HexNAc 4

**Mass:**  3725.5797

(2.675e-04)

**Charge States:** 3, 4

**Start Position**: 51

**End Position**: 68

| Profile Score | Tandem Score | TDA q-value | Volume | Spectrum Matches |
| --- | --- | --- | --- | --- |
| 10.2173 | 119.0148 | 0.0149 | 3.6506e+08 | 10 |

**Adduct Signal Fractions**

- **Unmodified**: 3.6506e+08

### QTRQDQC(C)IYN(N)TTY Fuc 1 Hex 7 HexNAc 6

**Mass:**  4188.6560

(2.290e-06)

**Charge States:** 3, 4

**Start Position**: 84

**End Position**: 97

| Profile Score | Tandem Score | TDA q-value | Volume | Spectrum Matches |
| --- | --- | --- | --- | --- |
| 12.8056 | 118.4650 | 0.0149 | 6.9127e+07 | 4 |

**Adduct Signal Fractions**

- **Unmodified**: 6.9127e+07

### RN(D)EEYN(N)KSVQEIQATFF Hex 6 HexNAc 5

**Mass:**  4090.7082

(2.423e-04)

**Charge States:** 3, 4

**Start Position**: 51

**End Position**: 68

| Profile Score | Tandem Score | TDA q-value | Volume | Spectrum Matches |
| --- | --- | --- | --- | --- |
| 10.5069 | 118.0186 | 0.0149 | 3.8097e+08 | 30 |

**Adduct Signal Fractions**

- **Unmodified**: 3.8097e+08

### LNVQREN(N)GTISRY Fuc 3 Hex 8 HexNAc 7

**Mass:**  4704.9587

(1.296e-06)

**Charge States:** 4

**Start Position**: 97

**End Position**: 110

| Profile Score | Tandem Score | TDA q-value | Volume | Spectrum Matches |
| --- | --- | --- | --- | --- |
| -4.0246 | 117.3065 | 0.0165 | 7.4725e+07 | 1 |

**Adduct Signal Fractions**

- **Unmodified**: 7.4725e+07

### VQREN(N)GTISRY Hex 6 HexNAc 5

**Mass:**  3309.3793

(-2.537e-06)

**Start Position**: 99

**End Position**: 110

| Profile Score | Tandem Score | TDA q-value | Volume | Spectrum Matches |
| --- | --- | --- | --- | --- |
| 0.0000 | 117.2002 | 0.0165 | 0.0000e+00 | 1 |

Adduct:

No Chromatogram Found

### FTPN(N)KTEDTIF Fuc 2 Hex 7 HexNAc 6

**Mass:**  3956.6040

(1.905e-06)

**Charge States:** 3, 4

**Start Position**: 69

**End Position**: 80

| Profile Score | Tandem Score | TDA q-value | Volume | Spectrum Matches |
| --- | --- | --- | --- | --- |
| -17.5002 | 117.1472 | 0.0165 | 1.3930e+07 | 2 |

**Adduct Signal Fractions**

- **Unmodified**: 1.3930e+07

### RNEEYN(N)KSVQEIQATF Hex 9 HexNAc 8

**Mass:**  5038.0554

(-3.499e-07)

**Charge States:** 4

**Start Position**: 51

**End Position**: 67

| Profile Score | Tandem Score | TDA q-value | Volume | Spectrum Matches |
| --- | --- | --- | --- | --- |
| -3.8440 | 117.1415 | 0.0165 | 3.3303e+07 | 3 |

**Adduct Signal Fractions**

- **Unmodified**: 3.3303e+07

### LNVQREN(N)GTISRY Hex 8 HexNAc 7

**Mass:**  4266.7907

(6.923e-07)

**Charge States:** 3, 4

**Start Position**: 97

**End Position**: 110

| Profile Score | Tandem Score | TDA q-value | Volume | Spectrum Matches |
| --- | --- | --- | --- | --- |
| 13.9528 | 116.6863 | 0.0165 | 1.0268e+09 | 6 |

**Adduct Signal Fractions**

- **Unmodified**: 1.0268e+09

### LN(D)VQREN(N)GTISRY Fuc 1 Hex 7 HexNAc 6

**Mass:**  4048.7009

(2.470e-04)

**Charge States:** 3, 4

**Start Position**: 97

**End Position**: 110

| Profile Score | Tandem Score | TDA q-value | Volume | Spectrum Matches |
| --- | --- | --- | --- | --- |
| 9.2595 | 115.1916 | 0.0187 | 1.3655e+08 | 8 |

**Adduct Signal Fractions**

- **Unmodified**: 1.3655e+08

### NVQREN(N)GTISRY Fuc 2 Hex 7 HexNAc 6

**Mass:**  4080.6811

(6.191e-07)

**Charge States:** 4

**Start Position**: 98

**End Position**: 110

| Profile Score | Tandem Score | TDA q-value | Volume | Spectrum Matches |
| --- | --- | --- | --- | --- |
| -18.7396 | 115.0528 | 0.0187 | 4.8296e+07 | 2 |

**Adduct Signal Fractions**

- **Unmodified**: 4.8296e+07

### TPN(N)KTEDTIF Fuc 2 Hex 7 HexNAc 6

**Mass:**  3809.5353

(1.900e-06)

**Charge States:** 3, 4

**Start Position**: 70

**End Position**: 80

| Profile Score | Tandem Score | TDA q-value | Volume | Spectrum Matches |
| --- | --- | --- | --- | --- |
| -19.5768 | 114.7525 | 0.0187 | 6.3051e+06 | 1 |

**Adduct Signal Fractions**

- **Unmodified**: 6.3051e+06

### LNVQREN(N)GTISRY Hex 9 HexNAc 8

**Mass:**  4631.9304

(1.754e-06)

**Charge States:** 3, 4

**Start Position**: 97

**End Position**: 110

| Profile Score | Tandem Score | TDA q-value | Volume | Spectrum Matches |
| --- | --- | --- | --- | --- |
| 14.5506 | 114.1059 | 0.0201 | 9.6067e+08 | 6 |

**Adduct Signal Fractions**

- **Unmodified**: 9.6067e+08

### TPN(N)KTEDTIF Hex 10 HexNAc 7

**Mass:**  4206.6638

(3.238e-06)

**Charge States:** 3, 4

**Start Position**: 70

**End Position**: 80

| Profile Score | Tandem Score | TDA q-value | Volume | Spectrum Matches |
| --- | --- | --- | --- | --- |
| -4.5780 | 113.1410 | 0.0201 | 1.4872e+07 | 1 |

**Adduct Signal Fractions**

- **Unmodified**: 1.4872e+07

### YFTPN(N)KTEDTIF Hex 7 HexNAc 6 Neu5Ac 1

**Mass:**  4118.6486

(2.165e-06)

**Charge States:** 3, 4

**Start Position**: 68

**End Position**: 80

| Profile Score | Tandem Score | TDA q-value | Volume | Spectrum Matches |
| --- | --- | --- | --- | --- |
| 12.0796 | 112.3197 | 0.0222 | 9.2279e+06 | 3 |

**Adduct Signal Fractions**

- **Unmodified**: 9.2279e+06

### LNVQREN(N)GTISRY Hex 7 HexNAc 4 Neu5Ac 2

**Mass:**  4077.6852

(2.171e-06)

**Charge States:** 3, 4

**Start Position**: 97

**End Position**: 110

| Profile Score | Tandem Score | TDA q-value | Volume | Spectrum Matches |
| --- | --- | --- | --- | --- |
| 14.5709 | 112.2678 | 0.0222 | 4.4333e+08 | 6 |

**Adduct Signal Fractions**

- **Unmodified**: 4.4333e+08

### LNVQREN(N)GTISRY Fuc 3 Hex 7 HexNAc 6

**Mass:**  4339.8288

(1.853e-06)

**Charge States:** 3, 4

**Start Position**: 97

**End Position**: 110

| Profile Score | Tandem Score | TDA q-value | Volume | Spectrum Matches |
| --- | --- | --- | --- | --- |
| 9.3189 | 111.9194 | 0.0239 | 2.6888e+08 | 4 |

**Adduct Signal Fractions**

- **Unmodified**: 2.6888e+08

### LNVQREN(N)GTISRY Fuc 1 Hex 9 HexNAc 8

**Mass:**  4777.9811

(2.474e-06)

**Charge States:** 3, 4

**Start Position**: 97

**End Position**: 110

| Profile Score | Tandem Score | TDA q-value | Volume | Spectrum Matches |
| --- | --- | --- | --- | --- |
| 15.7586 | 111.7747 | 0.0239 | 2.9075e+08 | 3 |

**Adduct Signal Fractions**

- **Unmodified**: 2.9075e+08

### FTPN(N)KTEDTIF Hex 9 HexNAc 8

**Mass:**  4394.7512

(1.815e-06)

**Charge States:** 4

**Start Position**: 69

**End Position**: 80

| Profile Score | Tandem Score | TDA q-value | Volume | Spectrum Matches |
| --- | --- | --- | --- | --- |
| 12.8169 | 111.5732 | 0.0239 | 1.3360e+07 | 2 |

**Adduct Signal Fractions**

- **Unmodified**: 1.3360e+07

### TPN(N)KTEDTIF Fuc 4 Hex 3 HexNAc 9 Neu5Ac 1

**Mass:**  4353.7282

(-8.819e-06)

**Charge States:** 3, 4

**Start Position**: 70

**End Position**: 80

| Profile Score | Tandem Score | TDA q-value | Volume | Spectrum Matches |
| --- | --- | --- | --- | --- |
| -5.0824 | 111.1276 | 0.0259 | 1.5620e+07 | 1 |

**Adduct Signal Fractions**

- **Unmodified**: 1.5620e+07

### LN(D)VQREN(N)GTISRY Hex 7 HexNAc 6

**Mass:**  3902.6389

(2.551e-04)

**Charge States:** 3, 4

**Start Position**: 97

**End Position**: 110

| Profile Score | Tandem Score | TDA q-value | Volume | Spectrum Matches |
| --- | --- | --- | --- | --- |
| 10.0842 | 110.7736 | 0.0294 | 3.5937e+08 | 30 |

**Adduct Signal Fractions**

- **Unmodified**: 3.5937e+08

### VQREN(N)GTISRY Fuc 1 Hex 4 HexNAc 7

**Mass:**  3537.5070

(3.344e-06)

**Charge States:** 3, 4

**Start Position**: 99

**End Position**: 110

| Profile Score | Tandem Score | TDA q-value | Volume | Spectrum Matches |
| --- | --- | --- | --- | --- |
| 8.4239 | 110.1406 | 0.0294 | 1.1043e+08 | 6 |

**Adduct Signal Fractions**

- **Unmodified**: 1.1043e+08

### YFTPN(N)KTEDTIF Hex 8 HexNAc 7

**Mass:**  4193.6893

(2.416e-04)

**Start Position**: 68

**End Position**: 80

| Profile Score | Tandem Score | TDA q-value | Volume | Spectrum Matches |
| --- | --- | --- | --- | --- |
| 0.0000 | 109.8932 | 0.0312 | 0.0000e+00 | 1 |

Adduct:

No Chromatogram Found

### RNEEYN(N)KSVQEIQATF Fuc 1 Hex 8 HexNAc 7

**Mass:**  4818.9797

(1.253e-06)

**Charge States:** 4

**Start Position**: 51

**End Position**: 67

| Profile Score | Tandem Score | TDA q-value | Volume | Spectrum Matches |
| --- | --- | --- | --- | --- |
| -5.2246 | 108.7899 | 0.0328 | 1.5445e+07 | 2 |

**Adduct Signal Fractions**

- **Unmodified**: 1.5445e+07

### NVQREN(N)GTISRY Fuc 1 Hex 9 HexNAc 8

**Mass:**  4664.9069

(4.689e-06)

**Charge States:** 4

**Start Position**: 98

**End Position**: 110

| Profile Score | Tandem Score | TDA q-value | Volume | Spectrum Matches |
| --- | --- | --- | --- | --- |
| -20.8675 | 106.7674 | 0.0371 | 5.5922e+06 | 1 |

**Adduct Signal Fractions**

- **Unmodified**: 5.5922e+06

### NVQREN(N)GTISRY Hex 9 HexNAc 8

**Mass:**  4518.8283

(1.988e-07)

**Charge States:** 4

**Start Position**: 98

**End Position**: 110

| Profile Score | Tandem Score | TDA q-value | Volume | Spectrum Matches |
| --- | --- | --- | --- | --- |
| -19.0245 | 106.5971 | 0.0371 | 3.2044e+07 | 1 |

**Adduct Signal Fractions**

- **Unmodified**: 3.2044e+07

### LNVQREN(N)GTISRY Fuc 2 Hex 9 HexNAc 8

**Mass:**  4924.0339

(2.262e-06)

**Charge States:** 4

**Start Position**: 97

**End Position**: 110

| Profile Score | Tandem Score | TDA q-value | Volume | Spectrum Matches |
| --- | --- | --- | --- | --- |
| -3.4373 | 106.1153 | 0.0371 | 1.0551e+08 | 3 |

**Adduct Signal Fractions**

- **Unmodified**: 1.0551e+08

### RN(D)EEYN(N)KSVQEIQATFF Fuc 1 Hex 6 HexNAc 5

**Mass:**  4236.7658

(2.344e-04)

**Charge States:** 3, 4

**Start Position**: 51

**End Position**: 68

| Profile Score | Tandem Score | TDA q-value | Volume | Spectrum Matches |
| --- | --- | --- | --- | --- |
| 10.6321 | 105.8651 | 0.0371 | 1.8319e+08 | 8 |

**Adduct Signal Fractions**

- **Unmodified**: 1.8319e+08

### TPN(N)KTEDTIF Fuc 3 Hex 7 HexNAc 6 Neu5Ac 1

**Mass:**  4246.6794

(-4.427e-07)

**Charge States:** 4

**Start Position**: 70

**End Position**: 80

| Profile Score | Tandem Score | TDA q-value | Volume | Spectrum Matches |
| --- | --- | --- | --- | --- |
| -18.5537 | 105.6615 | 0.0371 | 4.3956e+06 | 1 |

**Adduct Signal Fractions**

- **Unmodified**: 4.3956e+06

### LN(D)VQREN(N)GTISRY Fuc 4 Hex 7 HexNAc 5

**Mass:**  4283.8093

(2.359e-04)

**Charge States:** 4

**Start Position**: 97

**End Position**: 110

| Profile Score | Tandem Score | TDA q-value | Volume | Spectrum Matches |
| --- | --- | --- | --- | --- |
| -20.7369 | 105.4013 | 0.0371 | 3.6225e+07 | 1 |

**Adduct Signal Fractions**

- **Unmodified**: 3.6225e+07

### NVQREN(N)GTISRY Hex 5 HexNAc 4

**Mass:**  3058.2949

(1.094e-06)

**Charge States:** 3

**Start Position**: 98

**End Position**: 110

| Profile Score | Tandem Score | TDA q-value | Volume | Spectrum Matches |
| --- | --- | --- | --- | --- |
| -20.2090 | 105.0586 | 0.0391 | 9.2509e+06 | 1 |

**Adduct Signal Fractions**

- **Unmodified**: 9.2509e+06

### N(N)KSVQEIQATF Fuc 1 Hex 6 HexNAc 5

**Mass:**  3397.4240

(1.326e-06)

**Charge States:** 3, 4

**Start Position**: 56

**End Position**: 67

| Profile Score | Tandem Score | TDA q-value | Volume | Spectrum Matches |
| --- | --- | --- | --- | --- |
| -19.3103 | 104.7389 | 0.0408 | 7.8611e+06 | 1 |

**Adduct Signal Fractions**

- **Unmodified**: 7.8611e+06

### LNVQREN(N)GTISRY Fuc 1 Hex 7 HexNAc 4 Neu5Ac 2

**Mass:**  4223.7443

(1.744e-06)

**Charge States:** 3, 4

**Start Position**: 97

**End Position**: 110

| Profile Score | Tandem Score | TDA q-value | Volume | Spectrum Matches |
| --- | --- | --- | --- | --- |
| 14.1936 | 104.0028 | 0.0438 | 1.3842e+08 | 3 |

**Adduct Signal Fractions**

- **Unmodified**: 1.3842e+08

### LNVQREN(N)GTISRY Hex 7 HexNAc 6 Neu5Ac 1

**Mass:**  4192.7526

(2.509e-06)

**Charge States:** 3, 4

**Start Position**: 97

**End Position**: 110

| Profile Score | Tandem Score | TDA q-value | Volume | Spectrum Matches |
| --- | --- | --- | --- | --- |
| -18.4958 | 103.4265 | 0.0438 | 9.7471e+06 | 2 |

**Adduct Signal Fractions**

- **Unmodified**: 9.7471e+06

### LNVQREN(N)GTISRY Fuc 2 Hex 8 HexNAc 7

**Mass:**  4558.9167

(1.364e-06)

**Charge States:** 3, 4

**Start Position**: 97

**End Position**: 110

| Profile Score | Tandem Score | TDA q-value | Volume | Spectrum Matches |
| --- | --- | --- | --- | --- |
| 15.0042 | 103.2475 | 0.0438 | 1.9686e+08 | 3 |

**Adduct Signal Fractions**

- **Unmodified**: 1.9686e+08

### LNVQREN(N)GTISRY Fuc 3 Hex 9 HexNAc 8

**Mass:**  5070.0920

(1.191e-06)

**Charge States:** 4

**Start Position**: 97

**End Position**: 110

| Profile Score | Tandem Score | TDA q-value | Volume | Spectrum Matches |
| --- | --- | --- | --- | --- |
| -3.4965 | 102.5270 | 0.0438 | 2.9351e+07 | 1 |

**Adduct Signal Fractions**

- **Unmodified**: 2.9351e+07

### LNVQREN(N)GTISRY Fuc 2 Hex 10 HexNAc 9

**Mass:**  5289.1647

(1.021e-06)

**Charge States:** 4

**Start Position**: 97

**End Position**: 110

| Profile Score | Tandem Score | TDA q-value | Volume | Spectrum Matches |
| --- | --- | --- | --- | --- |
| -19.1049 | 102.0735 | 0.0455 | 1.1271e+07 | 1 |

**Adduct Signal Fractions**

- **Unmodified**: 1.1271e+07

### TPN(N)KTEDTIF Fuc 1 Hex 8 HexNAc 7

**Mass:**  4028.6066

(1.050e-06)

**Charge States:** 4

**Start Position**: 70

**End Position**: 80

| Profile Score | Tandem Score | TDA q-value | Volume | Spectrum Matches |
| --- | --- | --- | --- | --- |
| -20.7277 | 101.6502 | 0.0455 | 2.3060e+06 | 1 |

**Adduct Signal Fractions**

- **Unmodified**: 2.3060e+06

### LNVQREN(N)GTISRY Fuc 4 Hex 8 HexNAc 7

**Mass:**  4851.0187

(1.664e-06)

**Charge States:** 4

**Start Position**: 97

**End Position**: 110

| Profile Score | Tandem Score | TDA q-value | Volume | Spectrum Matches |
| --- | --- | --- | --- | --- |
| -4.5331 | 100.8328 | 0.0484 | 1.8120e+07 | 1 |

**Adduct Signal Fractions**

- **Unmodified**: 1.8120e+07

### C(C)ANLVPVPITN(N)ATL Hex 6 HexNAc 5

**Mass:**  3469.5097

(1.540e-06)

**Charge States:** 3, 4

**Start Position**: 23

**End Position**: 37

| Profile Score | Tandem Score | TDA q-value | Volume | Spectrum Matches |
| --- | --- | --- | --- | --- |
| 12.3011 | 100.4223 | 0.0484 | 2.2961e+07 | 5 |

**Adduct Signal Fractions**

- **Unmodified**: 2.2961e+07

### P19652|A1AG2\_HUMAN

**>P19652|A1AG2\_HUMAN**  
 `MALSWVLTVLSLLPLLEAQIPLCANLVPVPITNATLDRITGKWFYIASAFRNEEYNKSVQEIQATFFYFTPNKTEDTIFL  
REYQTRQNQCFYNSSYLNVQRENGTVSRYEGGREHVAHLLFLRDTKTLMFGSYLDDEKNWGLSFYADKPETTKEQLGEFY  
EALDCLCIPRSDVMYTDWKKDKCEPLEKQHEKERKQEEGES`

| Site | Type | Count |
| --- | --- | --- |
| 33 | N-linked | 3 |
| 56 | N-linked | 38 |
| 93 | N-linked | 1 |
| 103 | N-linked | 17 |
| 72 | N-linked | 63 |

| Mass | Sequence | MS_2_ Score | `q`-value | MS_1_ Score | Signal | Protein Coordinates | Tandem Scans | Start Time | Apex Time | End Time |
| --- | --- | --- | --- | --- | --- | --- | --- | --- | --- | --- |
| 3153.1487 | TPN(N)KTEDTIF Hex 6 HexNAc 5 | 184.031 | 0.0 | -2.681 | 3.687e+08 | 69 - 79 | 13 | 21.83 | 23.70 | 39.00 |
| 3299.3516 | FTPN(N)KTEDTIF Hex 6 HexNAc 5 | 183.315 | 0.0 | 14.798 | 4.834e+08 | 68 - 79 | 13 | 29.33 | 30.86 | 31.88 |
| 3517.4169 | TPN(N)KTEDTIF Hex 7 HexNAc 6 | 183.128 | 0.0 | 15.884 | 3.241e+08 | 69 - 79 | 9 | 22.34 | 23.02 | 24.55 |
| 2886.2312 | N(N)KSVQEIQATF Hex 5 HexNAc 4 | 181.122 | 0.0 | 11.996 | 2.953e+07 | 55 - 66 | 2 | 26.94 | 27.11 | 27.79 |
| 3251.3641 | N(N)KSVQEIQATF Hex 6 HexNAc 5 | 180.094 | 0.0 | -3.575 | 2.313e+07 | 55 - 66 | 2 | 26.26 | 26.43 | 26.77 |
| 2787.1524 | TPN(N)KTEDTIF Hex 5 HexNAc 4 | 178.291 | 0.0 | -2.534 | 2.611e+07 | 69 - 79 | 2 | 24.04 | 24.21 | 24.55 |
| 3097.2812 | YFTPN(N)KTEDTIF Hex 5 HexNAc 4 | 177.951 | 0.0 | 14.991 | 3.350e+08 | 67 - 79 | 7 | 37.49 | 37.81 | 38.66 |
| 3827.5475 | YFTPN(N)KTEDTIF Hex 7 HexNAc 6 | 175.335 | 0.0 | 15.245 | 4.940e+09 | 67 - 79 | 46 | 35.52 | 36.53 | 43.10 |
| 4192.6835 | YFTPN(N)KTEDTIF Hex 8 HexNAc 7 | 174.602 | 0.0 | 15.528 | 7.328e+08 | 67 - 79 | 10 | 34.66 | 36.02 | 36.69 |
| 3462.4150 | YFTPN(N)KTEDTIF Hex 6 HexNAc 5 | 172.053 | 0.0 | 15.105 | 3.435e+09 | 67 - 79 | 39 | 36.02 | 37.17 | 42.93 |
| 3663.4758 | TPN(N)KTEDTIF Fuc 1 Hex 7 HexNAc 6 | 170.725 | 0.0 | 14.389 | 5.083e+07 | 69 - 79 | 5 | 22.68 | 22.85 | 23.53 |
| 3973.6071 | YFTPN(N)KTEDTIF Fuc 1 Hex 7 HexNAc 6 | 170.569 | 0.0 | 14.265 | 8.375e+08 | 67 - 79 | 27 | 35.35 | 36.36 | 37.17 |
| 3608.4783 | YFTPN(N)KTEDTIF Fuc 1 Hex 6 HexNAc 5 | 168.110 | 0.0 | 15.408 | 6.641e+08 | 67 - 79 | 33 | 35.85 | 36.85 | 42.08 |
| 3609.4852 | FYFTPN(N)KTEDTIF Hex 6 HexNAc 5 | 167.738 | 0.0 | 14.728 | 2.499e+09 | 66 - 79 | 49 | 46.13 | 47.14 | 56.34 |
| 3755.5437 | FYFTPN(N)KTEDTIF Fuc 1 Hex 6 HexNAc 5 | 167.391 | 0.0 | 13.332 | 5.432e+08 | 66 - 79 | 9 | 45.96 | 46.97 | 48.14 |
| 3298.3430 | TPN(N)KTEDTIF Fuc 1 Hex 6 HexNAc 5 | 166.994 | 0.0 | 13.696 | 3.732e+07 | 69 - 79 | 4 | 23.02 | 23.36 | 23.87 |
| 3882.5504 | TPN(N)KTEDTIF Hex 8 HexNAc 7 | 166.932 | 0.0 | 14.673 | 2.999e+07 | 69 - 79 | 4 | 22.17 | 22.51 | 22.85 |
| 4120.6768 | FYFTPN(N)KTEDTIF Fuc 1 Hex 7 HexNAc 6 | 166.695 | 0.0 | 14.618 | 8.475e+08 | 66 - 79 | 12 | 45.62 | 46.30 | 47.64 |
| 4323.7536 | FYFTPN(N)KTEDTIF Fuc 1 Hex 7 HexNAc 7 | 166.147 | 0.0 | -3.642 | 3.927e+07 | 66 - 79 | 3 | 47.31 | 47.47 | 47.64 |
| 3398.4356 | N(N)KSVQEIQATFF Hex 6 HexNAc 5 | 166.058 | 0.0 | -4.529 | 7.991e+06 | 55 - 67 | 3 | 40.37 | 40.37 | 40.71 |
| 3974.6191 | FYFTPN(N)KTEDTIF Hex 7 HexNAc 6 | 165.663 | 0.0 | 15.300 | 4.672e+09 | 66 - 79 | 77 | 44.94 | 46.47 | 60.80 |
| 3664.4842 | FTPN(N)KTEDTIF Hex 7 HexNAc 6 | 165.492 | 0.0 | 15.444 | 6.673e+08 | 68 - 79 | 18 | 28.82 | 30.18 | 31.71 |
| 4266.7338 | FYFTPN(N)KTEDTIF Fuc 2 Hex 7 HexNAc 6 | 165.417 | 0.0 | 13.291 | 1.181e+08 | 66 - 79 | 3 | 46.13 | 46.30 | 46.81 |
| 4003.5787 | YFTPN(N)KTEDTIF Hex 7 HexNAc 4 Neu5Ac 2 | 165.145 | 0.0 | 15.302 | 3.124e+08 | 67 - 79 | 7 | 37.65 | 37.81 | 39.00 |
| 4338.7408 | YFTPN(N)KTEDTIF Fuc 1 Hex 8 HexNAc 7 | 164.640 | 0.0 | 15.733 | 1.472e+08 | 67 - 79 | 5 | 35.35 | 36.02 | 36.19 |
| 3307.3794 | EEYN(N)KSVQEIQATF Hex 5 HexNAc 4 | 162.548 | 0.0 | -3.483 | 7.724e+07 | 52 - 66 | 3 | 35.52 | 35.52 | 35.85 |
| 4119.6660 | YFTPN(N)KTEDTIF Fuc 2 Hex 7 HexNAc 6 | 162.437 | 0.0 | -3.420 | 1.237e+08 | 67 - 79 | 3 | 36.19 | 36.19 | 36.69 |
| 3672.5124 | EEYN(N)KSVQEIQATF Hex 6 HexNAc 5 | 162.212 | 0.0 | -3.460 | 7.690e+07 | 52 - 66 | 2 | 35.00 | 35.18 | 35.52 |
| 4557.8166 | YFTPN(N)KTEDTIF Hex 9 HexNAc 8 | 161.641 | 0.0 | 15.581 | 2.105e+08 | 67 - 79 | 7 | 34.33 | 35.18 | 36.36 |
| 3906.5500 | TPN(N)KTEDTIF Fuc 4 Hex 4 HexNAc 6 Neu5Ac 1 | 160.247 | 0.0 | -20.443 | 6.096e+07 | 69 - 79 | 1 | 36.69 | 36.85 | 36.85 |
| 3244.3520 | FYFTPN(N)KTEDTIF Hex 5 HexNAc 4 | 159.280 | 0.0 | 13.025 | 2.171e+08 | 66 - 79 | 4 | 47.64 | 47.64 | 48.48 |
| 3577.5284 | RNEEYN(N)KSVQEIQATF Hex 5 HexNAc 4 | 157.728 | 0.0 | 16.199 | 9.426e+09 | 50 - 66 | 19 | 30.69 | 32.51 | 33.99 |
| 4089.7278 | RNEEYN(N)KSVQEIQATFF Hex 6 HexNAc 5 | 157.676 | 0.0 | 16.880 | 3.593e+09 | 50 - 67 | 19 | 41.39 | 43.60 | 44.77 |
| 3724.5919 | RNEEYN(N)KSVQEIQATFF Hex 5 HexNAc 4 | 156.821 | 0.0 | 17.096 | 4.017e+09 | 50 - 67 | 18 | 39.68 | 43.93 | 45.28 |
| 4235.7851 | RNEEYN(N)KSVQEIQATFF Fuc 1 Hex 6 HexNAc 5 | 156.180 | 0.0 | 16.112 | 1.718e+09 | 50 - 67 | 16 | 41.56 | 43.44 | 44.77 |
| 3445.4093 | FTPN(N)KTEDTIF Fuc 1 Hex 6 HexNAc 5 | 155.855 | 0.0 | 13.048 | 9.308e+07 | 68 - 79 | 7 | 30.35 | 30.52 | 31.03 |
| 3098.1802 | YN(N)SSYL Hex 7 HexNAc 6 | 155.213 | 0.0 | -19.179 | 5.535e+06 | 91 - 97 | 1 | 22.00 | 22.00 | 22.17 |
| 4704.8852 | FYFTPN(N)KTEDTIF Hex 9 HexNAc 8 | 154.381 | 0.0 | 15.125 | 1.681e+08 | 66 - 79 | 6 | 44.43 | 45.11 | 46.30 |
| 3942.6591 | RNEEYN(N)KSVQEIQATF Hex 6 HexNAc 5 | 154.356 | 0.0 | 15.791 | 7.022e+09 | 50 - 66 | 16 | 30.35 | 32.20 | 33.48 |
| 3810.5435 | FTPN(N)KTEDTIF Fuc 1 Hex 7 HexNAc 6 | 153.547 | 0.003 | 14.544 | 1.154e+08 | 68 - 79 | 6 | 29.67 | 30.01 | 30.69 |
| 4088.7161 | RNEEYN(N)KSVQEIQATF Fuc 1 Hex 6 HexNAc 5 | 153.401 | 0.003 | 14.863 | 3.442e+09 | 50 - 66 | 17 | 30.18 | 32.04 | 33.48 |
| 3243.3422 | YFTPN(N)KTEDTIF Fuc 1 Hex 5 HexNAc 4 | 153.019 | 0.003 | -20.002 | 7.317e+06 | 67 - 79 | 1 | 37.49 | 37.49 | 37.65 |
| 4600.9155 | RNEEYN(N)KSVQEIQATFF Fuc 1 Hex 7 HexNAc 6 | 152.949 | 0.003 | 14.099 | 6.472e+07 | 50 - 67 | 4 | 42.76 | 43.28 | 43.60 |
| 3870.6505 | RNEEYN(N)KSVQEIQATFF Fuc 1 Hex 5 HexNAc 4 | 152.421 | 0.003 | 13.538 | 1.605e+08 | 50 - 67 | 7 | 43.60 | 43.77 | 44.60 |
| 4922.9470 | YFTPN(N)KTEDTIF Hex 10 HexNAc 9 | 152.186 | 0.003 | -4.352 | 2.099e+07 | 67 - 79 | 2 | 34.66 | 34.83 | 35.00 |
| 3723.5877 | RNEEYN(N)KSVQEIQATF Fuc 1 Hex 5 HexNAc 4 | 152.112 | 0.003 | 11.712 | 3.585e+08 | 50 - 66 | 10 | 32.20 | 32.36 | 33.99 |
| 3430.4558 | RNEEYN(N)KSVQEIQAT Hex 5 HexNAc 4 | 151.886 | 0.003 | -3.873 | 4.255e+07 | 50 - 65 | 3 | 17.17 | 17.17 | 17.51 |
| 2934.2206 | FTPN(N)KTEDTIF Hex 5 HexNAc 4 | 151.459 | 0.003 | -2.826 | 5.043e+07 | 68 - 79 | 3 | 31.20 | 31.37 | 31.71 |
| 3004.2166 | FYFTPN(N)KT Hex 6 HexNAc 5 | 150.681 | 0.0 | -3.258 | 7.808e+06 | 66 - 74 | 2 | 25.57 | 25.74 | 26.09 |
| 4453.8501 | RNEEYN(N)KSVQEIQATF Fuc 1 Hex 7 HexNAc 6 | 150.559 | 0.003 | 14.298 | 1.576e+08 | 50 - 66 | 2 | 31.54 | 31.88 | 32.20 |
| 4339.7521 | FYFTPN(N)KTEDTIF Hex 8 HexNAc 7 | 150.401 | 0.003 | 14.467 | 6.185e+08 | 66 - 79 | 11 | 44.77 | 46.13 | 47.31 |
| 4060.6019 | TPN(N)KTEDTIF Fuc 4 Hex 8 HexNAc 5 | 150.313 | 0.003 | 13.202 | 1.360e+08 | 69 - 79 | 4 | 37.65 | 37.81 | 38.49 |
| 3033.3004 | N(N)KSVQEIQATFF Hex 5 HexNAc 4 | 149.181 | 0.003 | -4.431 | 8.091e+06 | 55 - 67 | 1 | 41.05 | 41.22 | 41.39 |
| 4307.7914 | RNEEYN(N)KSVQEIQATF Hex 7 HexNAc 6 | 148.584 | 0.003 | 14.425 | 3.599e+08 | 50 - 66 | 3 | 31.71 | 31.88 | 32.36 |
| 4381.8437 | RNEEYN(N)KSVQEIQATFF Fuc 2 Hex 6 HexNAc 5 | 148.351 | 0.003 | -3.987 | 6.808e+07 | 50 - 67 | 3 | 43.28 | 43.28 | 43.60 |
| 4484.7975 | YFTPN(N)KTEDTIF Fuc 2 Hex 8 HexNAc 7 | 148.164 | 0.003 | 9.828 | 3.204e+07 | 67 - 79 | 2 | 35.35 | 35.68 | 36.02 |
| 3940.6321 | YFTPN(N)KTEDTIFL Hex 7 HexNAc 6 | 145.734 | 0.003 | 14.054 | 5.170e+07 | 67 - 80 | 3 | 44.26 | 44.26 | 44.94 |
| 4150.6526 | FYFTPN(N)KTEDTIF Hex 7 HexNAc 4 Neu5Ac 2 | 145.492 | 0.003 | 13.524 | 2.610e+08 | 66 - 79 | 6 | 47.47 | 47.64 | 48.99 |
| 4020.6044 | YFTPN(N)KTEDTIF Fuc 1 Hex 8 HexNAc 4 Neu5Ac 1 | 145.240 | 0.003 | -4.496 | 2.866e+07 | 67 - 79 | 2 | 37.65 | 37.81 | 37.98 |
| 4234.7747 | RNEEYN(N)KSVQEIQATF Fuc 2 Hex 6 HexNAc 5 | 144.900 | 0.003 | -3.109 | 1.283e+08 | 50 - 66 | 2 | 31.88 | 31.88 | 32.20 |
| 4029.6176 | FTPN(N)KTEDTIF Hex 8 HexNAc 7 | 144.778 | 0.003 | 16.224 | 6.373e+07 | 68 - 79 | 6 | 29.16 | 29.67 | 30.01 |
| 4454.8597 | RNEEYN(N)KSVQEIQATFF Hex 7 HexNAc 6 | 144.230 | 0.003 | 13.828 | 1.706e+08 | 50 - 67 | 4 | 42.93 | 43.28 | 43.77 |
| 3996.5976 | FYFTPN(N)KTEDTIF Hex 3 HexNAc 5 Neu5Ac 3 | 143.320 | 0.003 | 13.714 | 2.504e+08 | 66 - 79 | 6 | 45.79 | 46.64 | 46.97 |
| 3818.5727 | EEYN(N)KSVQEIQATF Fuc 1 Hex 6 HexNAc 5 | 142.914 | 0.003 | -3.815 | 3.311e+07 | 52 - 66 | 3 | 34.83 | 35.00 | 35.18 |
| 3693.4441 | TPN(N)KTEDTIF Hex 7 HexNAc 4 Neu5Ac 2 | 142.767 | 0.003 | -18.452 | 9.759e+06 | 69 - 79 | 2 | 24.04 | 24.21 | 24.21 |
| 3920.6064 | NVQREN(N)GTVSRY Fuc 1 Hex 7 HexNAc 6 | 141.605 | 0.003 | -3.511 | 3.617e+08 | 97 - 109 | 4 | 11.83 | 11.83 | 12.33 |
| 4672.9240 | RNEEYN(N)KSVQEIQATF Hex 8 HexNAc 7 | 141.382 | 0.003 | -3.636 | 6.603e+07 | 50 - 66 | 2 | 31.37 | 31.71 | 31.88 |
| 4485.8099 | FYFTPN(N)KTEDTIF Fuc 1 Hex 8 HexNAc 7 | 140.586 | 0.003 | 15.963 | 1.226e+08 | 66 - 79 | 5 | 45.28 | 45.96 | 46.30 |
| 3222.2816 | YFTPN(N)KT Hex 7 HexNAc 6 | 140.336 | 0.0 | -19.699 | 4.956e+06 | 67 - 74 | 1 | 13.99 | 14.15 | 14.15 |
| 3575.5009 | YFTPN(N)KTEDTIFL Hex 6 HexNAc 5 | 140.107 | 0.003 | -3.690 | 2.548e+07 | 67 - 80 | 4 | 44.60 | 44.94 | 45.11 |
| 3795.5889 | RNEEYN(N)KSVQEIQAT Hex 6 HexNAc 5 | 136.327 | 0.003 | -20.075 | 2.638e+07 | 50 - 65 | 2 | 17.00 | 17.00 | 17.17 |
| 4296.7078 | FYFTPN(N)KTEDTIF Fuc 1 Hex 7 HexNAc 4 Neu5Ac 2 | 134.778 | 0.003 | -4.498 | 4.221e+07 | 66 - 79 | 3 | 47.31 | 47.47 | 47.81 |
| 3454.4501 | EEYN(N)KSVQEIQATFF Hex 5 HexNAc 4 | 134.292 | 0.003 | -4.566 | 2.523e+07 | 52 - 67 | 3 | 47.64 | 47.81 | 48.14 |
| 4149.6386 | YFTPN(N)KTEDTIF Fuc 1 Hex 7 HexNAc 4 Neu5Ac 2 | 133.045 | 0.003 | -4.720 | 5.568e+07 | 67 - 79 | 2 | 37.49 | 37.65 | 37.98 |
| 4089.7142 | FRNEEYN(N)KSVQEIQATF Hex 6 HexNAc 5 | 132.824 | 0.003 | 12.252 | 5.437e+07 | 49 - 66 | 2 | 36.85 | 37.17 | 37.49 |
| 3774.5482 | NVQREN(N)GTVSRY Hex 7 HexNAc 6 | 132.761 | 0.003 | 13.488 | 6.285e+08 | 97 - 109 | 6 | 11.83 | 12.00 | 12.50 |
| 3578.5103 | RN(D)EEYN(N)KSVQEIQATF Hex 5 HexNAc 4 | 132.670 | 0.003 | 10.988 | 1.247e+09 | 50 - 66 | 12 | 33.99 | 35.52 | 36.36 |
| 3555.4737 | NVQREN(N)GTVSRY Fuc 1 Hex 6 HexNAc 5 | 131.775 | 0.003 | -5.129 | 7.778e+07 | 97 - 109 | 3 | 12.00 | 12.00 | 12.33 |
| 4425.7517 | N(N)KTEDTIF Fuc 5 Hex 5 HexNAc 8 Neu5Ac 1 | 131.647 | 0.0 | 0.000 | 0.000e+00 | 71 - 79 | 1 | - | - | - |
| 3943.6442 | RN(D)EEYN(N)KSVQEIQATF Hex 6 HexNAc 5 | 130.230 | 0.006 | 10.211 | 1.233e+09 | 50 - 66 | 14 | 33.65 | 35.18 | 36.19 |
| 3409.4160 | NVQREN(N)GTVSRY Hex 6 HexNAc 5 | 128.502 | 0.006 | -4.597 | 1.537e+08 | 97 - 109 | 3 | 12.00 | 12.16 | 12.50 |
| 3819.5831 | EEYN(N)KSVQEIQATFF Hex 6 HexNAc 5 | 128.290 | 0.006 | -4.611 | 2.557e+07 | 52 - 67 | 3 | 47.14 | 47.31 | 47.64 |
| 3369.3492 | FYFTPN(N)KT Hex 7 HexNAc 6 | 128.278 | 0.0 | -3.667 | 1.266e+07 | 66 - 74 | 2 | 24.89 | 25.23 | 25.23 |
| 4703.8747 | YFTPN(N)KTEDTIF Fuc 1 Hex 9 HexNAc 8 | 124.571 | 0.006 | 10.367 | 3.229e+07 | 67 - 79 | 2 | 34.83 | 35.18 | 35.68 |
| 4207.6720 | TPN(N)KTEDTIF Fuc 3 Hex 3 HexNAc 9 Neu5Ac 1 | 123.772 | 0.006 | 13.680 | 9.862e+07 | 69 - 79 | 4 | 48.65 | 48.82 | 49.50 |
| 3522.5040 | LNVQREN(N)GTVSRY Hex 6 HexNAc 5 | 123.528 | 0.011 | 14.173 | 1.146e+08 | 96 - 109 | 3 | 16.33 | 16.50 | 17.00 |
| 3840.5169 | FTPN(N)KTEDTIF Hex 7 HexNAc 4 Neu5Ac 2 | 122.174 | 0.013 | -4.317 | 3.237e+07 | 68 - 79 | 4 | 31.20 | 31.37 | 31.71 |
| 4631.8698 | FYFTPN(N)KTEDTIF Fuc 2 Hex 8 HexNAc 7 | 121.618 | 0.013 | 11.460 | 2.605e+07 | 66 - 79 | 3 | 45.28 | 45.79 | 45.96 |
| 3724.5929 | FRNEEYN(N)KSVQEIQATF Hex 5 HexNAc 4 | 121.101 | 0.013 | -4.903 | 3.489e+07 | 49 - 66 | 1 | 37.33 | 37.49 | 37.65 |
| 4033.6910 | LNVQREN(N)GTVSRY Fuc 1 Hex 7 HexNAc 6 | 120.381 | 0.013 | 12.373 | 2.614e+08 | 96 - 109 | 5 | 16.01 | 16.16 | 17.17 |
| 4139.6801 | NVQREN(N)GTVSRY Hex 8 HexNAc 7 | 120.360 | 0.013 | -18.763 | 4.977e+07 | 97 - 109 | 2 | 11.83 | 11.83 | 11.83 |
| 3613.4181 | FYFTPN(N)KTED Hex 7 HexNAc 6 | 119.938 | 0.013 | -4.136 | 6.638e+06 | 66 - 76 | 2 | 25.40 | 25.40 | 25.74 |
| 4819.9928 | RNEEYN(N)KSVQEIQATFF Hex 8 HexNAc 7 | 119.881 | 0.013 | -3.454 | 1.929e+07 | 50 - 67 | 2 | 42.59 | 42.59 | 43.10 |
| 3887.6346 | LNVQREN(N)GTVSRY Hex 7 HexNAc 6 | 119.810 | 0.013 | 12.975 | 4.676e+08 | 96 - 109 | 7 | 16.16 | 16.33 | 17.17 |
| 4089.7019 | RN(D)EEYN(N)KSVQEIQATF Fuc 1 Hex 6 HexNAc 5 | 119.718 | 0.015 | 11.197 | 5.640e+08 | 50 - 66 | 11 | 33.48 | 35.00 | 35.85 |
| 3124.3997 | LVPVPITN(N)ATL Hex 6 HexNAc 5 | 119.605 | 0.015 | -18.938 | 6.550e+06 | 25 - 36 | 1 | 45.62 | 45.62 | 45.79 |
| 3725.5802 | RN(D)EEYN(N)KSVQEIQATFF Hex 5 HexNAc 4 | 119.015 | 0.015 | 10.217 | 3.651e+08 | 50 - 67 | 10 | 45.28 | 46.97 | 47.31 |
| 4035.6928 | LNVQREN(N)GTVSRY Fuc 1 Hex 7 HexNAc 6 | 118.617 | 0.015 | 0.000 | 0.000e+00 | 96 - 109 | 1 | - | - | - |
| 4066.6648 | NVQREN(N)GTVSRY Fuc 2 Hex 7 HexNAc 6 | 118.127 | 0.015 | -18.581 | 1.108e+08 | 97 - 109 | 2 | 11.83 | 11.83 | 12.00 |
| 4090.7129 | RN(D)EEYN(N)KSVQEIQATFF Hex 6 HexNAc 5 | 118.019 | 0.015 | 10.507 | 3.810e+08 | 50 - 67 | 30 | 44.94 | 46.64 | 46.81 |
| 3956.6037 | FTPN(N)KTEDTIF Fuc 2 Hex 7 HexNAc 6 | 117.147 | 0.017 | -17.500 | 1.393e+07 | 68 - 79 | 2 | 29.84 | 29.84 | 30.01 |
| 5038.0541 | RNEEYN(N)KSVQEIQATF Hex 9 HexNAc 8 | 117.141 | 0.017 | -3.844 | 3.330e+07 | 50 - 66 | 3 | 31.20 | 31.37 | 31.54 |
| 3660.5147 | VQREN(N)GTVSRY Hex 7 HexNAc 6 | 116.344 | 0.017 | -21.395 | 5.372e+06 | 98 - 109 | 1 | 11.67 | 11.67 | 11.67 |
| 3809.5355 | TPN(N)KTEDTIF Fuc 2 Hex 7 HexNAc 6 | 114.753 | 0.019 | -19.577 | 6.305e+06 | 69 - 79 | 1 | 22.68 | 22.68 | 22.85 |
| 3668.5626 | LNVQREN(N)GTVSRY Fuc 1 Hex 6 HexNAc 5 | 113.248 | 0.020 | -4.193 | 6.250e+07 | 96 - 109 | 4 | 16.33 | 16.50 | 16.84 |
| 4206.6664 | TPN(N)KTEDTIF Hex 10 HexNAc 7 | 113.141 | 0.020 | -4.578 | 1.487e+07 | 69 - 79 | 1 | 37.49 | 37.49 | 37.81 |
| 3523.5139 | LNVQREN(N)GTVSRY Hex 6 HexNAc 5 | 113.043 | 0.020 | 0.000 | 0.000e+00 | 96 - 109 | 1 | - | - | - |
| 4118.6489 | YFTPN(N)KTEDTIF Hex 7 HexNAc 6 Neu5Ac 1 | 112.320 | 0.022 | 12.080 | 9.228e+06 | 67 - 79 | 3 | 39.34 | 39.68 | 40.20 |
| 4394.7532 | FTPN(N)KTEDTIF Hex 9 HexNAc 8 | 111.573 | 0.024 | 12.817 | 1.336e+07 | 68 - 79 | 2 | 28.65 | 28.82 | 29.67 |
| 4353.7267 | TPN(N)KTEDTIF Fuc 4 Hex 3 HexNAc 9 Neu5Ac 1 | 111.128 | 0.026 | -5.082 | 1.562e+07 | 69 - 79 | 1 | 48.48 | 48.65 | 48.82 |
| 4193.6893 | YFTPN(N)KTEDTIF Hex 8 HexNAc 7 | 109.893 | 0.031 | 0.000 | 0.000e+00 | 67 - 79 | 1 | - | - | - |
| 3362.4427 | TN(N)ATLDRITGKW Hex 6 HexNAc 5 | 109.728 | 0.031 | -3.793 | 2.302e+07 | 31 - 43 | 4 | 29.84 | 30.01 | 30.18 |
| 4818.9813 | RNEEYN(N)KSVQEIQATF Fuc 1 Hex 8 HexNAc 7 | 108.790 | 0.033 | -5.225 | 1.545e+07 | 50 - 66 | 2 | 31.37 | 31.54 | 31.71 |
| 4236.7691 | RN(D)EEYN(N)KSVQEIQATFF Fuc 1 Hex 6 HexNAc 5 | 105.865 | 0.037 | 10.632 | 1.832e+08 | 50 - 67 | 8 | 44.77 | 46.47 | 46.81 |
| 4246.6794 | TPN(N)KTEDTIF Fuc 3 Hex 7 HexNAc 6 Neu5Ac 1 | 105.662 | 0.037 | -18.554 | 4.396e+06 | 69 - 79 | 1 | 21.66 | 21.66 | 21.66 |
| 4651.8753 | NVQREN(N)GTVSRY Fuc 1 Hex 9 HexNAc 8 | 105.513 | 0.037 | 0.000 | 0.000e+00 | 97 - 109 | 1 | - | - | - |
| 3397.4247 | N(N)KSVQEIQATF Fuc 1 Hex 6 HexNAc 5 | 104.739 | 0.041 | -19.310 | 7.861e+06 | 55 - 66 | 1 | 26.09 | 26.26 | 26.26 |
| 4179.7499 | LNVQREN(N)GTVSRY Fuc 2 Hex 7 HexNAc 6 | 103.589 | 0.044 | -4.098 | 7.064e+07 | 96 - 109 | 3 | 16.01 | 16.16 | 16.33 |
| 4028.6073 | TPN(N)KTEDTIF Fuc 1 Hex 8 HexNAc 7 | 101.650 | 0.045 | -20.728 | 2.306e+06 | 69 - 79 | 1 | 22.34 | 22.34 | 22.51 |
| 4212.7226 | N(D)VQREN(N)GTVSRY Fuc 1 Hex 7 HexNAc 6 Neu5Ac 1 | 101.302 | 0.047 | -20.877 | 2.805e+07 | 97 - 109 | 1 | 11.83 | 11.83 | 11.83 |
| 4617.9001 | LNVQREN(N)GTVSRY Hex 9 HexNAc 8 | 101.115 | 0.048 | -4.399 | 3.320e+07 | 96 - 109 | 3 | 15.67 | 15.84 | 16.01 |
| 3469.5100 | C(C)ANLVPVPITN(N)ATL Hex 6 HexNAc 5 | 100.422 | 0.048 | 12.301 | 2.296e+07 | 22 - 36 | 5 | 50.19 | 50.36 | 51.21 |

### TPN(N)KTEDTIF Hex 6 HexNAc 5

**Mass:**  3152.2827

(1.363e-06)

**Charge States:** 3, 4

**Start Position**: 70

**End Position**: 80

| Profile Score | Tandem Score | TDA q-value | Volume | Spectrum Matches |
| --- | --- | --- | --- | --- |
| -2.6813 | 184.0308 | 0.0000 | 3.6872e+08 | 13 |

**Adduct Signal Fractions**

- **Unmodified**: 3.6872e+08

### FTPN(N)KTEDTIF Hex 6 HexNAc 5

**Mass:**  3299.3523

(8.094e-07)

**Charge States:** 3, 4

**Start Position**: 69

**End Position**: 80

| Profile Score | Tandem Score | TDA q-value | Volume | Spectrum Matches |
| --- | --- | --- | --- | --- |
| 14.7979 | 183.3149 | 0.0000 | 4.8339e+08 | 13 |

**Adduct Signal Fractions**

- **Unmodified**: 4.8339e+08

### TPN(N)KTEDTIF Hex 7 HexNAc 6

**Mass:**  3517.4163

(1.884e-06)

**Charge States:** 3, 4

**Start Position**: 70

**End Position**: 80

| Profile Score | Tandem Score | TDA q-value | Volume | Spectrum Matches |
| --- | --- | --- | --- | --- |
| 15.8842 | 183.1281 | 0.0000 | 3.2408e+08 | 9 |

**Adduct Signal Fractions**

- **Unmodified**: 3.2408e+08

### N(N)KSVQEIQATF Hex 5 HexNAc 4

**Mass:**  2886.2310

(2.074e-06)

**Charge States:** 3

**Start Position**: 56

**End Position**: 67

| Profile Score | Tandem Score | TDA q-value | Volume | Spectrum Matches |
| --- | --- | --- | --- | --- |
| 11.9963 | 181.1222 | 0.0000 | 2.9528e+07 | 2 |

**Adduct Signal Fractions**

- **Unmodified**: 2.9528e+07

### N(N)KSVQEIQATF Hex 6 HexNAc 5

**Mass:**  3251.3644

(1.475e-06)

**Charge States:** 3, 4

**Start Position**: 56

**End Position**: 67

| Profile Score | Tandem Score | TDA q-value | Volume | Spectrum Matches |
| --- | --- | --- | --- | --- |
| -3.5752 | 180.0941 | 0.0000 | 2.3126e+07 | 2 |

**Adduct Signal Fractions**

- **Unmodified**: 2.3126e+07

### TPN(N)KTEDTIF Hex 5 HexNAc 4

**Mass:**  2787.1523

(1.511e-06)

**Charge States:** 3

**Start Position**: 70

**End Position**: 80

| Profile Score | Tandem Score | TDA q-value | Volume | Spectrum Matches |
| --- | --- | --- | --- | --- |
| -2.5343 | 178.2909 | 0.0000 | 2.6107e+07 | 2 |

**Adduct Signal Fractions**

- **Unmodified**: 2.6107e+07

### YFTPN(N)KTEDTIF Hex 5 HexNAc 4

**Mass:**  3097.2793

(9.860e-07)

**Charge States:** 3, 4

**Start Position**: 68

**End Position**: 80

| Profile Score | Tandem Score | TDA q-value | Volume | Spectrum Matches |
| --- | --- | --- | --- | --- |
| 14.9911 | 177.9511 | 0.0000 | 3.3495e+08 | 7 |

**Adduct Signal Fractions**

- **Unmodified**: 3.3495e+08

### YFTPN(N)KTEDTIF Hex 7 HexNAc 6

**Mass:**  3827.5427

(7.937e-07)

**Charge States:** 3, 4

**Start Position**: 68

**End Position**: 80

| Profile Score | Tandem Score | TDA q-value | Volume | Spectrum Matches |
| --- | --- | --- | --- | --- |
| 15.2446 | 175.3346 | 0.0000 | 4.9403e+09 | 46 |

**Adduct Signal Fractions**

- **Unmodified**: 4.9403e+09

### YFTPN(N)KTEDTIF Hex 8 HexNAc 7

**Mass:**  4192.6862

(1.868e-06)

**Charge States:** 3, 4

**Start Position**: 68

**End Position**: 80

| Profile Score | Tandem Score | TDA q-value | Volume | Spectrum Matches |
| --- | --- | --- | --- | --- |
| 15.5279 | 174.6023 | 0.0000 | 7.3282e+08 | 10 |

**Adduct Signal Fractions**

- **Unmodified**: 7.3282e+08

### YFTPN(N)KTEDTIF Hex 6 HexNAc 5

**Mass:**  3462.4140

(1.738e-06)

**Charge States:** 2, 3, 4

**Start Position**: 68

**End Position**: 80

| Profile Score | Tandem Score | TDA q-value | Volume | Spectrum Matches |
| --- | --- | --- | --- | --- |
| 15.1046 | 172.0526 | 0.0000 | 3.4353e+09 | 39 |

**Adduct Signal Fractions**

- **Unmodified**: 3.4353e+09

### TPN(N)KTEDTIF Fuc 1 Hex 7 HexNAc 6

**Mass:**  3663.4768

(1.539e-06)

**Charge States:** 3, 4

**Start Position**: 70

**End Position**: 80

| Profile Score | Tandem Score | TDA q-value | Volume | Spectrum Matches |
| --- | --- | --- | --- | --- |
| 14.3886 | 170.7249 | 0.0000 | 5.0832e+07 | 5 |

**Adduct Signal Fractions**

- **Unmodified**: 5.0832e+07

### YFTPN(N)KTEDTIF Fuc 1 Hex 7 HexNAc 6

**Mass:**  3973.6081

(1.558e-06)

**Charge States:** 3, 4

**Start Position**: 68

**End Position**: 80

| Profile Score | Tandem Score | TDA q-value | Volume | Spectrum Matches |
| --- | --- | --- | --- | --- |
| 14.2654 | 170.5691 | 0.0000 | 8.3754e+08 | 27 |

**Adduct Signal Fractions**

- **Unmodified**: 8.3754e+08

### YFTPN(N)KTEDTIF Fuc 1 Hex 6 HexNAc 5

**Mass:**  3608.4717

(1.528e-06)

**Charge States:** 3, 4

**Start Position**: 68

**End Position**: 80

| Profile Score | Tandem Score | TDA q-value | Volume | Spectrum Matches |
| --- | --- | --- | --- | --- |
| 15.4076 | 168.1096 | 0.0000 | 6.6405e+08 | 33 |

**Adduct Signal Fractions**

- **Unmodified**: 6.6405e+08

### FYFTPN(N)KTEDTIF Hex 6 HexNAc 5

**Mass:**  3609.4854

(5.457e-07)

**Charge States:** 3, 4

**Start Position**: 67

**End Position**: 80

| Profile Score | Tandem Score | TDA q-value | Volume | Spectrum Matches |
| --- | --- | --- | --- | --- |
| 14.7279 | 167.7381 | 0.0000 | 2.4989e+09 | 49 |

**Adduct Signal Fractions**

- **Unmodified**: 2.4989e+09

### FYFTPN(N)KTEDTIF Fuc 1 Hex 6 HexNAc 5

**Mass:**  3755.5442

(1.078e-06)

**Charge States:** 3, 4

**Start Position**: 67

**End Position**: 80

| Profile Score | Tandem Score | TDA q-value | Volume | Spectrum Matches |
| --- | --- | --- | --- | --- |
| 13.3316 | 167.3913 | 0.0000 | 5.4319e+08 | 9 |

**Adduct Signal Fractions**

- **Unmodified**: 5.4319e+08

### TPN(N)KTEDTIF Fuc 1 Hex 6 HexNAc 5

**Mass:**  3298.3423

(2.218e-06)

**Charge States:** 3, 4

**Start Position**: 70

**End Position**: 80

| Profile Score | Tandem Score | TDA q-value | Volume | Spectrum Matches |
| --- | --- | --- | --- | --- |
| 13.6962 | 166.9940 | 0.0000 | 3.7323e+07 | 4 |

**Adduct Signal Fractions**

- **Unmodified**: 3.7323e+07

### TPN(N)KTEDTIF Hex 8 HexNAc 7

**Mass:**  3882.5485

(1.020e-06)

**Charge States:** 3, 4

**Start Position**: 70

**End Position**: 80

| Profile Score | Tandem Score | TDA q-value | Volume | Spectrum Matches |
| --- | --- | --- | --- | --- |
| 14.6735 | 166.9320 | 0.0000 | 2.9993e+07 | 4 |

**Adduct Signal Fractions**

- **Unmodified**: 2.9993e+07

### FYFTPN(N)KTEDTIF Fuc 1 Hex 7 HexNAc 6

**Mass:**  4120.6789

(1.855e-06)

**Charge States:** 3, 4

**Start Position**: 67

**End Position**: 80

| Profile Score | Tandem Score | TDA q-value | Volume | Spectrum Matches |
| --- | --- | --- | --- | --- |
| 14.6183 | 166.6950 | 0.0000 | 8.4755e+08 | 12 |

**Adduct Signal Fractions**

- **Unmodified**: 8.4755e+08

### FYFTPN(N)KTEDTIF Fuc 1 Hex 7 HexNAc 7

**Mass:**  4323.7536

(9.327e-07)

**Charge States:** 3, 4

**Start Position**: 67

**End Position**: 80

| Profile Score | Tandem Score | TDA q-value | Volume | Spectrum Matches |
| --- | --- | --- | --- | --- |
| -3.6418 | 166.1472 | 0.0000 | 3.9266e+07 | 3 |

**Adduct Signal Fractions**

- **Unmodified**: 3.9266e+07

### N(N)KSVQEIQATFF Hex 6 HexNAc 5

**Mass:**  3398.4365

(2.443e-06)

**Charge States:** 3

**Start Position**: 56

**End Position**: 68

| Profile Score | Tandem Score | TDA q-value | Volume | Spectrum Matches |
| --- | --- | --- | --- | --- |
| -4.5289 | 166.0576 | 0.0000 | 7.9908e+06 | 3 |

**Adduct Signal Fractions**

- **Unmodified**: 7.9908e+06

### FYFTPN(N)KTEDTIF Hex 7 HexNAc 6

**Mass:**  3974.6186

(1.835e-06)

**Charge States:** 3, 4

**Start Position**: 67

**End Position**: 80

| Profile Score | Tandem Score | TDA q-value | Volume | Spectrum Matches |
| --- | --- | --- | --- | --- |
| 15.3001 | 165.6626 | 0.0000 | 4.6720e+09 | 77 |

**Adduct Signal Fractions**

- **Unmodified**: 4.6720e+09

### FTPN(N)KTEDTIF Hex 7 HexNAc 6

**Mass:**  3664.4848

(1.077e-06)

**Charge States:** 3, 4

**Start Position**: 69

**End Position**: 80

| Profile Score | Tandem Score | TDA q-value | Volume | Spectrum Matches |
| --- | --- | --- | --- | --- |
| 15.4441 | 165.4918 | 0.0000 | 6.6726e+08 | 18 |

**Adduct Signal Fractions**

- **Unmodified**: 6.6726e+08

### FYFTPN(N)KTEDTIF Fuc 2 Hex 7 HexNAc 6

**Mass:**  4266.7341

(5.814e-07)

**Charge States:** 3, 4

**Start Position**: 67

**End Position**: 80

| Profile Score | Tandem Score | TDA q-value | Volume | Spectrum Matches |
| --- | --- | --- | --- | --- |
| 13.2913 | 165.4168 | 0.0000 | 1.1807e+08 | 3 |

**Adduct Signal Fractions**

- **Unmodified**: 1.1807e+08

### YFTPN(N)KTEDTIF Hex 7 HexNAc 4 Neu5Ac 2

**Mass:**  4003.5769

(9.294e-07)

**Charge States:** 3, 4

**Start Position**: 68

**End Position**: 80

| Profile Score | Tandem Score | TDA q-value | Volume | Spectrum Matches |
| --- | --- | --- | --- | --- |
| 15.3017 | 165.1453 | 0.0000 | 3.1237e+08 | 7 |

**Adduct Signal Fractions**

- **Unmodified**: 3.1237e+08

### YFTPN(N)KTEDTIF Fuc 1 Hex 8 HexNAc 7

**Mass:**  4338.7429

(1.306e-06)

**Charge States:** 3, 4

**Start Position**: 68

**End Position**: 80

| Profile Score | Tandem Score | TDA q-value | Volume | Spectrum Matches |
| --- | --- | --- | --- | --- |
| 15.7326 | 164.6403 | 0.0000 | 1.4719e+08 | 5 |

**Adduct Signal Fractions**

- **Unmodified**: 1.4719e+08

### EEYN(N)KSVQEIQATF Hex 5 HexNAc 4

**Mass:**  3307.3789

(9.130e-07)

**Charge States:** 3, 4

**Start Position**: 53

**End Position**: 67

| Profile Score | Tandem Score | TDA q-value | Volume | Spectrum Matches |
| --- | --- | --- | --- | --- |
| -3.4825 | 162.5484 | 0.0000 | 7.7241e+07 | 3 |

**Adduct Signal Fractions**

- **Unmodified**: 7.7241e+07

### YFTPN(N)KTEDTIF Fuc 2 Hex 7 HexNAc 6

**Mass:**  4119.6662

(1.685e-06)

**Charge States:** 3, 4

**Start Position**: 68

**End Position**: 80

| Profile Score | Tandem Score | TDA q-value | Volume | Spectrum Matches |
| --- | --- | --- | --- | --- |
| -3.4203 | 162.4366 | 0.0000 | 1.2368e+08 | 3 |

**Adduct Signal Fractions**

- **Unmodified**: 1.2368e+08

### EEYN(N)KSVQEIQATF Hex 6 HexNAc 5

**Mass:**  3672.5139

(1.546e-06)

**Charge States:** 3, 4

**Start Position**: 53

**End Position**: 67

| Profile Score | Tandem Score | TDA q-value | Volume | Spectrum Matches |
| --- | --- | --- | --- | --- |
| -3.4604 | 162.2122 | 0.0000 | 7.6897e+07 | 2 |

**Adduct Signal Fractions**

- **Unmodified**: 7.6897e+07

### YFTPN(N)KTEDTIF Hex 9 HexNAc 8

**Mass:**  4557.8176

(1.660e-06)

**Charge States:** 3, 4

**Start Position**: 68

**End Position**: 80

| Profile Score | Tandem Score | TDA q-value | Volume | Spectrum Matches |
| --- | --- | --- | --- | --- |
| 15.5811 | 161.6406 | 0.0000 | 2.1047e+08 | 7 |

**Adduct Signal Fractions**

- **Unmodified**: 2.1047e+08

### TPN(N)KTEDTIF Fuc 4 Hex 4 HexNAc 6 Neu5Ac 1

**Mass:**  3906.5463

(-8.017e-06)

**Charge States:** 3, 4

**Start Position**: 70

**End Position**: 80

| Profile Score | Tandem Score | TDA q-value | Volume | Spectrum Matches |
| --- | --- | --- | --- | --- |
| -20.4435 | 160.2474 | 0.0000 | 6.0955e+07 | 1 |

**Adduct Signal Fractions**

- **Unmodified**: 6.0955e+07

### FYFTPN(N)KTEDTIF Hex 5 HexNAc 4

**Mass:**  3244.3515

(1.058e-06)

**Charge States:** 3

**Start Position**: 67

**End Position**: 80

| Profile Score | Tandem Score | TDA q-value | Volume | Spectrum Matches |
| --- | --- | --- | --- | --- |
| 13.0253 | 159.2802 | 0.0000 | 2.1713e+08 | 4 |

**Adduct Signal Fractions**

- **Unmodified**: 2.1713e+08

### RNEEYN(N)KSVQEIQATF Hex 5 HexNAc 4

**Mass:**  3577.5291

(-4.646e-07)

**Charge States:** 3, 4

**Start Position**: 51

**End Position**: 67

| Profile Score | Tandem Score | TDA q-value | Volume | Spectrum Matches |
| --- | --- | --- | --- | --- |
| 16.1988 | 157.7283 | 0.0000 | 9.4257e+09 | 19 |

**Adduct Signal Fractions**

- **Unmodified**: 9.4257e+09

### RNEEYN(N)KSVQEIQATFF Hex 6 HexNAc 5

**Mass:**  4089.7297

(1.217e-06)

**Charge States:** 3, 4

**Start Position**: 51

**End Position**: 68

| Profile Score | Tandem Score | TDA q-value | Volume | Spectrum Matches |
| --- | --- | --- | --- | --- |
| 16.8803 | 157.6757 | 0.0000 | 3.5932e+09 | 19 |

**Adduct Signal Fractions**

- **Unmodified**: 3.5932e+09

### RNEEYN(N)KSVQEIQATFF Hex 5 HexNAc 4

**Mass:**  3724.5913

(8.720e-07)

**Charge States:** 3, 4

**Start Position**: 51

**End Position**: 68

| Profile Score | Tandem Score | TDA q-value | Volume | Spectrum Matches |
| --- | --- | --- | --- | --- |
| 17.0965 | 156.8207 | 0.0000 | 4.0165e+09 | 18 |

**Adduct Signal Fractions**

- **Unmodified**: 4.0165e+09

### RNEEYN(N)KSVQEIQATFF Fuc 1 Hex 6 HexNAc 5

**Mass:**  4235.7853

(8.776e-07)

**Charge States:** 3, 4

**Start Position**: 51

**End Position**: 68

| Profile Score | Tandem Score | TDA q-value | Volume | Spectrum Matches |
| --- | --- | --- | --- | --- |
| 16.1118 | 156.1799 | 0.0000 | 1.7181e+09 | 16 |

**Adduct Signal Fractions**

- **Unmodified**: 1.7181e+09

### FTPN(N)KTEDTIF Fuc 1 Hex 6 HexNAc 5

**Mass:**  3445.4093

(5.064e-07)

**Charge States:** 3, 4

**Start Position**: 69

**End Position**: 80

| Profile Score | Tandem Score | TDA q-value | Volume | Spectrum Matches |
| --- | --- | --- | --- | --- |
| 13.0481 | 155.8549 | 0.0000 | 9.3085e+07 | 7 |

**Adduct Signal Fractions**

- **Unmodified**: 9.3085e+07

### YN(N)SSYL Hex 7 HexNAc 6

**Mass:**  3098.1795

(1.729e-06)

**Charge States:** 3

**Start Position**: 92

**End Position**: 98

| Profile Score | Tandem Score | TDA q-value | Volume | Spectrum Matches |
| --- | --- | --- | --- | --- |
| -19.1789 | 155.2132 | 0.0000 | 5.5352e+06 | 1 |

**Adduct Signal Fractions**

- **Unmodified**: 5.5352e+06

### FYFTPN(N)KTEDTIF Hex 9 HexNAc 8

**Mass:**  4704.8859

(1.897e-06)

**Charge States:** 3, 4

**Start Position**: 67

**End Position**: 80

| Profile Score | Tandem Score | TDA q-value | Volume | Spectrum Matches |
| --- | --- | --- | --- | --- |
| 15.1254 | 154.3806 | 0.0000 | 1.6809e+08 | 6 |

**Adduct Signal Fractions**

- **Unmodified**: 1.6809e+08

### RNEEYN(N)KSVQEIQATF Hex 6 HexNAc 5

**Mass:**  3942.6596

(2.825e-06)

**Charge States:** 3, 4

**Start Position**: 51

**End Position**: 67

| Profile Score | Tandem Score | TDA q-value | Volume | Spectrum Matches |
| --- | --- | --- | --- | --- |
| 15.7912 | 154.3564 | 0.0000 | 7.0218e+09 | 16 |

**Adduct Signal Fractions**

- **Unmodified**: 7.0218e+09

### FTPN(N)KTEDTIF Fuc 1 Hex 7 HexNAc 6

**Mass:**  3810.5446

(1.496e-06)

**Charge States:** 3, 4

**Start Position**: 69

**End Position**: 80

| Profile Score | Tandem Score | TDA q-value | Volume | Spectrum Matches |
| --- | --- | --- | --- | --- |
| 14.5444 | 153.5470 | 0.0031 | 1.1535e+08 | 6 |

**Adduct Signal Fractions**

- **Unmodified**: 1.1535e+08

### RNEEYN(N)KSVQEIQATF Fuc 1 Hex 6 HexNAc 5

**Mass:**  4088.7126

(2.077e-06)

**Charge States:** 3, 4

**Start Position**: 51

**End Position**: 67

| Profile Score | Tandem Score | TDA q-value | Volume | Spectrum Matches |
| --- | --- | --- | --- | --- |
| 14.8626 | 153.4012 | 0.0031 | 3.4417e+09 | 17 |

**Adduct Signal Fractions**

- **Unmodified**: 3.4417e+09

### YFTPN(N)KTEDTIF Fuc 1 Hex 5 HexNAc 4

**Mass:**  3243.3451

(2.360e-06)

**Charge States:** 3

**Start Position**: 68

**End Position**: 80

| Profile Score | Tandem Score | TDA q-value | Volume | Spectrum Matches |
| --- | --- | --- | --- | --- |
| -20.0017 | 153.0186 | 0.0031 | 7.3169e+06 | 1 |

**Adduct Signal Fractions**

- **Unmodified**: 7.3169e+06

### RNEEYN(N)KSVQEIQATFF Fuc 1 Hex 7 HexNAc 6

**Mass:**  4600.9167

(1.620e-06)

**Charge States:** 3, 4

**Start Position**: 51

**End Position**: 68

| Profile Score | Tandem Score | TDA q-value | Volume | Spectrum Matches |
| --- | --- | --- | --- | --- |
| 14.0988 | 152.9488 | 0.0031 | 6.4718e+07 | 4 |

**Adduct Signal Fractions**

- **Unmodified**: 6.4718e+07

### RNEEYN(N)KSVQEIQATFF Fuc 1 Hex 5 HexNAc 4

**Mass:**  3870.6498

(1.523e-06)

**Charge States:** 3, 4

**Start Position**: 51

**End Position**: 68

| Profile Score | Tandem Score | TDA q-value | Volume | Spectrum Matches |
| --- | --- | --- | --- | --- |
| 13.5383 | 152.4214 | 0.0031 | 1.6054e+08 | 7 |

**Adduct Signal Fractions**

- **Unmodified**: 1.6054e+08

### YFTPN(N)KTEDTIF Hex 10 HexNAc 9

**Mass:**  4922.9455

(9.886e-07)

**Charge States:** 4

**Start Position**: 68

**End Position**: 80

| Profile Score | Tandem Score | TDA q-value | Volume | Spectrum Matches |
| --- | --- | --- | --- | --- |
| -4.3520 | 152.1862 | 0.0031 | 2.0988e+07 | 2 |

**Adduct Signal Fractions**

- **Unmodified**: 2.0988e+07

### RNEEYN(N)KSVQEIQATF Fuc 1 Hex 5 HexNAc 4

**Mass:**  3723.5873

(1.717e-06)

**Charge States:** 3, 4

**Start Position**: 51

**End Position**: 67

| Profile Score | Tandem Score | TDA q-value | Volume | Spectrum Matches |
| --- | --- | --- | --- | --- |
| 11.7119 | 152.1116 | 0.0031 | 3.5847e+08 | 10 |

**Adduct Signal Fractions**

- **Unmodified**: 3.5847e+08

### RNEEYN(N)KSVQEIQAT Hex 5 HexNAc 4

**Mass:**  3430.4547

(8.004e-07)

**Charge States:** 3

**Start Position**: 51

**End Position**: 66

| Profile Score | Tandem Score | TDA q-value | Volume | Spectrum Matches |
| --- | --- | --- | --- | --- |
| -3.8734 | 151.8857 | 0.0031 | 4.2548e+07 | 3 |

**Adduct Signal Fractions**

- **Unmodified**: 4.2548e+07

### FTPN(N)KTEDTIF Hex 5 HexNAc 4

**Mass:**  2934.2201

(6.688e-07)

**Charge States:** 3

**Start Position**: 69

**End Position**: 80

| Profile Score | Tandem Score | TDA q-value | Volume | Spectrum Matches |
| --- | --- | --- | --- | --- |
| -2.8260 | 151.4588 | 0.0031 | 5.0434e+07 | 3 |

**Adduct Signal Fractions**

- **Unmodified**: 5.0434e+07

### FYFTPN(N)KT Hex 6 HexNAc 5

**Mass:**  3004.2165

(2.933e-06)

**Charge States:** 3

**Start Position**: 67

**End Position**: 75

| Profile Score | Tandem Score | TDA q-value | Volume | Spectrum Matches |
| --- | --- | --- | --- | --- |
| -3.2583 | 150.6813 | 0.0000 | 7.8080e+06 | 2 |

**Adduct Signal Fractions**

- **Unmodified**: 7.8080e+06

### RNEEYN(N)KSVQEIQATF Fuc 1 Hex 7 HexNAc 6

**Mass:**  4453.8508

(1.808e-06)

**Charge States:** 4

**Start Position**: 51

**End Position**: 67

| Profile Score | Tandem Score | TDA q-value | Volume | Spectrum Matches |
| --- | --- | --- | --- | --- |
| 14.2984 | 150.5590 | 0.0031 | 1.5762e+08 | 2 |

**Adduct Signal Fractions**

- **Unmodified**: 1.5762e+08

### FYFTPN(N)KTEDTIF Hex 8 HexNAc 7

**Mass:**  4339.7551

(1.577e-06)

**Charge States:** 3, 4

**Start Position**: 67

**End Position**: 80

| Profile Score | Tandem Score | TDA q-value | Volume | Spectrum Matches |
| --- | --- | --- | --- | --- |
| 14.4672 | 150.4008 | 0.0031 | 6.1852e+08 | 11 |

**Adduct Signal Fractions**

- **Unmodified**: 6.1852e+08

### TPN(N)KTEDTIF Fuc 4 Hex 8 HexNAc 5

**Mass:**  4060.6000

(-3.102e-06)

**Charge States:** 3, 4

**Start Position**: 70

**End Position**: 80

| Profile Score | Tandem Score | TDA q-value | Volume | Spectrum Matches |
| --- | --- | --- | --- | --- |
| 13.2016 | 150.3132 | 0.0031 | 1.3595e+08 | 4 |

**Adduct Signal Fractions**

- **Unmodified**: 1.3595e+08

### N(N)KSVQEIQATFF Hex 5 HexNAc 4

**Mass:**  3033.3003

(1.440e-06)

**Charge States:** 3

**Start Position**: 56

**End Position**: 68

| Profile Score | Tandem Score | TDA q-value | Volume | Spectrum Matches |
| --- | --- | --- | --- | --- |
| -4.4311 | 149.1811 | 0.0031 | 8.0912e+06 | 1 |

**Adduct Signal Fractions**

- **Unmodified**: 8.0912e+06

### RNEEYN(N)KSVQEIQATF Hex 7 HexNAc 6

**Mass:**  4307.7922

(2.141e-06)

**Charge States:** 3, 4

**Start Position**: 51

**End Position**: 67

| Profile Score | Tandem Score | TDA q-value | Volume | Spectrum Matches |
| --- | --- | --- | --- | --- |
| 14.4255 | 148.5843 | 0.0031 | 3.5992e+08 | 3 |

**Adduct Signal Fractions**

- **Unmodified**: 3.5992e+08

### RNEEYN(N)KSVQEIQATFF Fuc 2 Hex 6 HexNAc 5

**Mass:**  4381.8435

(1.534e-06)

**Charge States:** 4

**Start Position**: 51

**End Position**: 68

| Profile Score | Tandem Score | TDA q-value | Volume | Spectrum Matches |
| --- | --- | --- | --- | --- |
| -3.9871 | 148.3505 | 0.0031 | 6.8085e+07 | 3 |

**Adduct Signal Fractions**

- **Unmodified**: 6.8085e+07

### YFTPN(N)KTEDTIF Fuc 2 Hex 8 HexNAc 7

**Mass:**  4484.7971

(1.152e-06)

**Charge States:** 3, 4

**Start Position**: 68

**End Position**: 80

| Profile Score | Tandem Score | TDA q-value | Volume | Spectrum Matches |
| --- | --- | --- | --- | --- |
| 9.8279 | 148.1639 | 0.0031 | 3.2036e+07 | 2 |

**Adduct Signal Fractions**

- **Unmodified**: 3.2036e+07

### YFTPN(N)KTEDTIFL Hex 7 HexNAc 6

**Mass:**  3940.6309

(7.348e-07)

**Charge States:** 3, 4

**Start Position**: 68

**End Position**: 81

| Profile Score | Tandem Score | TDA q-value | Volume | Spectrum Matches |
| --- | --- | --- | --- | --- |
| 14.0536 | 145.7341 | 0.0031 | 5.1702e+07 | 3 |

**Adduct Signal Fractions**

- **Unmodified**: 5.1702e+07

### FYFTPN(N)KTEDTIF Hex 7 HexNAc 4 Neu5Ac 2

**Mass:**  4150.6530

(2.170e-06)

**Charge States:** 3, 4

**Start Position**: 67

**End Position**: 80

| Profile Score | Tandem Score | TDA q-value | Volume | Spectrum Matches |
| --- | --- | --- | --- | --- |
| 13.5238 | 145.4916 | 0.0031 | 2.6097e+08 | 6 |

**Adduct Signal Fractions**

- **Unmodified**: 2.6097e+08

### YFTPN(N)KTEDTIF Fuc 1 Hex 8 HexNAc 4 Neu5Ac 1

**Mass:**  4020.6022

(2.685e-06)

**Charge States:** 3

**Start Position**: 68

**End Position**: 80

| Profile Score | Tandem Score | TDA q-value | Volume | Spectrum Matches |
| --- | --- | --- | --- | --- |
| -4.4960 | 145.2400 | 0.0031 | 2.8657e+07 | 2 |

**Adduct Signal Fractions**

- **Unmodified**: 2.8657e+07

### RNEEYN(N)KSVQEIQATF Fuc 2 Hex 6 HexNAc 5

**Mass:**  4234.7785

(2.446e-06)

**Charge States:** 4

**Start Position**: 51

**End Position**: 67

| Profile Score | Tandem Score | TDA q-value | Volume | Spectrum Matches |
| --- | --- | --- | --- | --- |
| -3.1088 | 144.8998 | 0.0031 | 1.2832e+08 | 2 |

**Adduct Signal Fractions**

- **Unmodified**: 1.2832e+08

### FTPN(N)KTEDTIF Hex 8 HexNAc 7

**Mass:**  4029.6181

(1.336e-06)

**Charge States:** 3, 4

**Start Position**: 69

**End Position**: 80

| Profile Score | Tandem Score | TDA q-value | Volume | Spectrum Matches |
| --- | --- | --- | --- | --- |
| 16.2241 | 144.7784 | 0.0031 | 6.3725e+07 | 6 |

**Adduct Signal Fractions**

- **Unmodified**: 6.3725e+07

### RNEEYN(N)KSVQEIQATFF Hex 7 HexNAc 6

**Mass:**  4454.8591

(1.367e-06)

**Charge States:** 3, 4

**Start Position**: 51

**End Position**: 68

| Profile Score | Tandem Score | TDA q-value | Volume | Spectrum Matches |
| --- | --- | --- | --- | --- |
| 13.8275 | 144.2299 | 0.0031 | 1.7057e+08 | 4 |

**Adduct Signal Fractions**

- **Unmodified**: 1.7057e+08

### FYFTPN(N)KTEDTIF Hex 3 HexNAc 5 Neu5Ac 3

**Mass:**  3996.5947

(-3.357e-06)

**Charge States:** 3, 4

**Start Position**: 67

**End Position**: 80

| Profile Score | Tandem Score | TDA q-value | Volume | Spectrum Matches |
| --- | --- | --- | --- | --- |
| 13.7141 | 143.3201 | 0.0031 | 2.5036e+08 | 6 |

**Adduct Signal Fractions**

- **Unmodified**: 2.5036e+08

### EEYN(N)KSVQEIQATF Fuc 1 Hex 6 HexNAc 5

**Mass:**  3818.5729

(1.590e-06)

**Charge States:** 3, 4

**Start Position**: 53

**End Position**: 67

| Profile Score | Tandem Score | TDA q-value | Volume | Spectrum Matches |
| --- | --- | --- | --- | --- |
| -3.8155 | 142.9135 | 0.0031 | 3.3111e+07 | 3 |

**Adduct Signal Fractions**

- **Unmodified**: 3.3111e+07

### TPN(N)KTEDTIF Hex 7 HexNAc 4 Neu5Ac 2

**Mass:**  3693.4462

(-5.159e-07)

**Charge States:** 3, 4

**Start Position**: 70

**End Position**: 80

| Profile Score | Tandem Score | TDA q-value | Volume | Spectrum Matches |
| --- | --- | --- | --- | --- |
| -18.4520 | 142.7669 | 0.0031 | 9.7591e+06 | 2 |

**Adduct Signal Fractions**

- **Unmodified**: 9.7591e+06

### NVQREN(N)GTVSRY Fuc 1 Hex 7 HexNAc 6

**Mass:**  3920.6071

(2.177e-07)

**Charge States:** 3, 4

**Start Position**: 98

**End Position**: 110

| Profile Score | Tandem Score | TDA q-value | Volume | Spectrum Matches |
| --- | --- | --- | --- | --- |
| -3.5113 | 141.6054 | 0.0031 | 3.6170e+08 | 4 |

**Adduct Signal Fractions**

- **Unmodified**: 3.6170e+08

### RNEEYN(N)KSVQEIQATF Hex 8 HexNAc 7

**Mass:**  4672.9260

(2.011e-06)

**Charge States:** 4

**Start Position**: 51

**End Position**: 67

| Profile Score | Tandem Score | TDA q-value | Volume | Spectrum Matches |
| --- | --- | --- | --- | --- |
| -3.6365 | 141.3815 | 0.0031 | 6.6030e+07 | 2 |

**Adduct Signal Fractions**

- **Unmodified**: 6.6030e+07

### FYFTPN(N)KTEDTIF Fuc 1 Hex 8 HexNAc 7

**Mass:**  4485.8102

(1.694e-06)

**Charge States:** 3, 4

**Start Position**: 67

**End Position**: 80

| Profile Score | Tandem Score | TDA q-value | Volume | Spectrum Matches |
| --- | --- | --- | --- | --- |
| 15.9635 | 140.5857 | 0.0031 | 1.2259e+08 | 5 |

**Adduct Signal Fractions**

- **Unmodified**: 1.2259e+08

### YFTPN(N)KT Hex 7 HexNAc 6

**Mass:**  3222.2821

(2.448e-06)

**Charge States:** 3

**Start Position**: 68

**End Position**: 75

| Profile Score | Tandem Score | TDA q-value | Volume | Spectrum Matches |
| --- | --- | --- | --- | --- |
| -19.6987 | 140.3356 | 0.0000 | 4.9556e+06 | 1 |

**Adduct Signal Fractions**

- **Unmodified**: 4.9556e+06

### YFTPN(N)KTEDTIFL Hex 6 HexNAc 5

**Mass:**  3575.5010

(1.399e-06)

**Charge States:** 3

**Start Position**: 68

**End Position**: 81

| Profile Score | Tandem Score | TDA q-value | Volume | Spectrum Matches |
| --- | --- | --- | --- | --- |
| -3.6900 | 140.1073 | 0.0031 | 2.5477e+07 | 4 |

**Adduct Signal Fractions**

- **Unmodified**: 2.5477e+07

### RNEEYN(N)KSVQEIQAT Hex 6 HexNAc 5

**Mass:**  3795.5898

(1.555e-06)

**Charge States:** 3, 4

**Start Position**: 51

**End Position**: 66

| Profile Score | Tandem Score | TDA q-value | Volume | Spectrum Matches |
| --- | --- | --- | --- | --- |
| -20.0752 | 136.3270 | 0.0031 | 2.6376e+07 | 2 |

**Adduct Signal Fractions**

- **Unmodified**: 2.6376e+07

### FYFTPN(N)KTEDTIF Fuc 1 Hex 7 HexNAc 4 Neu5Ac 2

**Mass:**  4296.7062

(8.483e-07)

**Charge States:** 4

**Start Position**: 67

**End Position**: 80

| Profile Score | Tandem Score | TDA q-value | Volume | Spectrum Matches |
| --- | --- | --- | --- | --- |
| -4.4983 | 134.7783 | 0.0031 | 4.2210e+07 | 3 |

**Adduct Signal Fractions**

- **Unmodified**: 4.2210e+07

### EEYN(N)KSVQEIQATFF Hex 5 HexNAc 4

**Mass:**  3454.4503

(9.792e-08)

**Charge States:** 3

**Start Position**: 53

**End Position**: 68

| Profile Score | Tandem Score | TDA q-value | Volume | Spectrum Matches |
| --- | --- | --- | --- | --- |
| -4.5663 | 134.2915 | 0.0031 | 2.5232e+07 | 3 |

**Adduct Signal Fractions**

- **Unmodified**: 2.5232e+07

### YFTPN(N)KTEDTIF Fuc 1 Hex 7 HexNAc 4 Neu5Ac 2

**Mass:**  4149.6364

(1.900e-06)

**Charge States:** 3, 4

**Start Position**: 68

**End Position**: 80

| Profile Score | Tandem Score | TDA q-value | Volume | Spectrum Matches |
| --- | --- | --- | --- | --- |
| -4.7205 | 133.0449 | 0.0031 | 5.5682e+07 | 2 |

**Adduct Signal Fractions**

- **Unmodified**: 5.5682e+07

### FRNEEYN(N)KSVQEIQATF Hex 6 HexNAc 5

**Mass:**  4089.7246

(9.506e-07)

**Charge States:** 4

**Start Position**: 50

**End Position**: 67

| Profile Score | Tandem Score | TDA q-value | Volume | Spectrum Matches |
| --- | --- | --- | --- | --- |
| 12.2524 | 132.8241 | 0.0031 | 5.4374e+07 | 2 |

**Adduct Signal Fractions**

- **Unmodified**: 5.4374e+07

### NVQREN(N)GTVSRY Hex 7 HexNAc 6

**Mass:**  3774.5485

(-7.454e-09)

**Charge States:** 3, 4

**Start Position**: 98

**End Position**: 110

| Profile Score | Tandem Score | TDA q-value | Volume | Spectrum Matches |
| --- | --- | --- | --- | --- |
| 13.4877 | 132.7612 | 0.0031 | 6.2846e+08 | 6 |

**Adduct Signal Fractions**

- **Unmodified**: 6.2846e+08

### RN(D)EEYN(N)KSVQEIQATF Hex 5 HexNAc 4

**Mass:**  3578.5080

(2.761e-04)

**Charge States:** 3, 4

**Start Position**: 51

**End Position**: 67

| Profile Score | Tandem Score | TDA q-value | Volume | Spectrum Matches |
| --- | --- | --- | --- | --- |
| 10.9878 | 132.6702 | 0.0031 | 1.2475e+09 | 12 |

**Adduct Signal Fractions**

- **Unmodified**: 1.2475e+09

### NVQREN(N)GTVSRY Fuc 1 Hex 6 HexNAc 5

**Mass:**  3555.4737

(2.650e-07)

**Charge States:** 3

**Start Position**: 98

**End Position**: 110

| Profile Score | Tandem Score | TDA q-value | Volume | Spectrum Matches |
| --- | --- | --- | --- | --- |
| -5.1294 | 131.7750 | 0.0031 | 7.7783e+07 | 3 |

**Adduct Signal Fractions**

- **Unmodified**: 7.7783e+07

### N(N)KTEDTIF Fuc 5 Hex 5 HexNAc 8 Neu5Ac 1

**Mass:**  4425.7517

(6.787e-04)

**Start Position**: 72

**End Position**: 80

| Profile Score | Tandem Score | TDA q-value | Volume | Spectrum Matches |
| --- | --- | --- | --- | --- |
| 0.0000 | 131.6471 | 0.0000 | 0.0000e+00 | 1 |

Adduct:

No Chromatogram Found

### RN(D)EEYN(N)KSVQEIQATF Hex 6 HexNAc 5

**Mass:**  3943.6447

(2.517e-04)

**Charge States:** 3, 4

**Start Position**: 51

**End Position**: 67

| Profile Score | Tandem Score | TDA q-value | Volume | Spectrum Matches |
| --- | --- | --- | --- | --- |
| 10.2105 | 130.2300 | 0.0056 | 1.2329e+09 | 14 |

**Adduct Signal Fractions**

- **Unmodified**: 1.2329e+09

### NVQREN(N)GTVSRY Hex 6 HexNAc 5

**Mass:**  3409.4170

(5.383e-08)

**Charge States:** 3

**Start Position**: 98

**End Position**: 110

| Profile Score | Tandem Score | TDA q-value | Volume | Spectrum Matches |
| --- | --- | --- | --- | --- |
| -4.5966 | 128.5022 | 0.0056 | 1.5372e+08 | 3 |

**Adduct Signal Fractions**

- **Unmodified**: 1.5372e+08

### EEYN(N)KSVQEIQATFF Hex 6 HexNAc 5

**Mass:**  3819.5821

(1.561e-06)

**Charge States:** 3, 4

**Start Position**: 53

**End Position**: 68

| Profile Score | Tandem Score | TDA q-value | Volume | Spectrum Matches |
| --- | --- | --- | --- | --- |
| -4.6109 | 128.2901 | 0.0056 | 2.5571e+07 | 3 |

**Adduct Signal Fractions**

- **Unmodified**: 2.5571e+07

### FYFTPN(N)KT Hex 7 HexNAc 6

**Mass:**  3369.3488

(1.900e-06)

**Charge States:** 3

**Start Position**: 67

**End Position**: 75

| Profile Score | Tandem Score | TDA q-value | Volume | Spectrum Matches |
| --- | --- | --- | --- | --- |
| -3.6667 | 128.2778 | 0.0000 | 1.2661e+07 | 2 |

**Adduct Signal Fractions**

- **Unmodified**: 1.2661e+07

### YFTPN(N)KTEDTIF Fuc 1 Hex 9 HexNAc 8

**Mass:**  4703.8747

(1.275e-06)

**Charge States:** 4

**Start Position**: 68

**End Position**: 80

| Profile Score | Tandem Score | TDA q-value | Volume | Spectrum Matches |
| --- | --- | --- | --- | --- |
| 10.3672 | 124.5711 | 0.0056 | 3.2291e+07 | 2 |

**Adduct Signal Fractions**

- **Unmodified**: 3.2291e+07

### TPN(N)KTEDTIF Fuc 3 Hex 3 HexNAc 9 Neu5Ac 1

**Mass:**  4207.6730

(-8.561e-06)

**Charge States:** 3, 4

**Start Position**: 70

**End Position**: 80

| Profile Score | Tandem Score | TDA q-value | Volume | Spectrum Matches |
| --- | --- | --- | --- | --- |
| 13.6800 | 123.7718 | 0.0056 | 9.8621e+07 | 4 |

**Adduct Signal Fractions**

- **Unmodified**: 9.8621e+07

### LNVQREN(N)GTVSRY Hex 6 HexNAc 5

**Mass:**  3522.5033

(1.814e-06)

**Charge States:** 3, 4

**Start Position**: 97

**End Position**: 110

| Profile Score | Tandem Score | TDA q-value | Volume | Spectrum Matches |
| --- | --- | --- | --- | --- |
| 14.1729 | 123.5283 | 0.0110 | 1.1459e+08 | 3 |

**Adduct Signal Fractions**

- **Unmodified**: 1.1459e+08

### FTPN(N)KTEDTIF Hex 7 HexNAc 4 Neu5Ac 2

**Mass:**  3840.5173

(3.963e-07)

**Charge States:** 3, 4

**Start Position**: 69

**End Position**: 80

| Profile Score | Tandem Score | TDA q-value | Volume | Spectrum Matches |
| --- | --- | --- | --- | --- |
| -4.3167 | 122.1745 | 0.0129 | 3.2369e+07 | 4 |

**Adduct Signal Fractions**

- **Unmodified**: 3.2369e+07

### FYFTPN(N)KTEDTIF Fuc 2 Hex 8 HexNAc 7

**Mass:**  4631.8679

(1.667e-06)

**Charge States:** 4

**Start Position**: 67

**End Position**: 80

| Profile Score | Tandem Score | TDA q-value | Volume | Spectrum Matches |
| --- | --- | --- | --- | --- |
| 11.4599 | 121.6180 | 0.0129 | 2.6048e+07 | 3 |

**Adduct Signal Fractions**

- **Unmodified**: 2.6048e+07

### FRNEEYN(N)KSVQEIQATF Hex 5 HexNAc 4

**Mass:**  3724.5949

(7.750e-07)

**Charge States:** 4

**Start Position**: 50

**End Position**: 67

| Profile Score | Tandem Score | TDA q-value | Volume | Spectrum Matches |
| --- | --- | --- | --- | --- |
| -4.9031 | 121.1006 | 0.0129 | 3.4886e+07 | 1 |

**Adduct Signal Fractions**

- **Unmodified**: 3.4886e+07

### LNVQREN(N)GTVSRY Fuc 1 Hex 7 HexNAc 6

**Mass:**  4033.6894

(1.097e-06)

**Charge States:** 3, 4

**Start Position**: 97

**End Position**: 110

| Profile Score | Tandem Score | TDA q-value | Volume | Spectrum Matches |
| --- | --- | --- | --- | --- |
| 12.3729 | 120.3810 | 0.0129 | 2.6143e+08 | 5 |

**Adduct Signal Fractions**

- **Unmodified**: 2.6143e+08

### NVQREN(N)GTVSRY Hex 8 HexNAc 7

**Mass:**  4139.6784

(1.636e-06)

**Charge States:** 3, 4

**Start Position**: 98

**End Position**: 110

| Profile Score | Tandem Score | TDA q-value | Volume | Spectrum Matches |
| --- | --- | --- | --- | --- |
| -18.7627 | 120.3603 | 0.0129 | 4.9766e+07 | 2 |

**Adduct Signal Fractions**

- **Unmodified**: 4.9766e+07

### FYFTPN(N)KTED Hex 7 HexNAc 6

**Mass:**  3613.4196

(2.005e-06)

**Charge States:** 3

**Start Position**: 67

**End Position**: 77

| Profile Score | Tandem Score | TDA q-value | Volume | Spectrum Matches |
| --- | --- | --- | --- | --- |
| -4.1361 | 119.9380 | 0.0129 | 6.6385e+06 | 2 |

**Adduct Signal Fractions**

- **Unmodified**: 6.6385e+06

### RNEEYN(N)KSVQEIQATFF Hex 8 HexNAc 7

**Mass:**  4819.9934

(1.765e-06)

**Charge States:** 4

**Start Position**: 51

**End Position**: 68

| Profile Score | Tandem Score | TDA q-value | Volume | Spectrum Matches |
| --- | --- | --- | --- | --- |
| -3.4544 | 119.8810 | 0.0129 | 1.9293e+07 | 2 |

**Adduct Signal Fractions**

- **Unmodified**: 1.9293e+07

### LNVQREN(N)GTVSRY Hex 7 HexNAc 6

**Mass:**  3887.6342

(4.346e-08)

**Charge States:** 3, 4

**Start Position**: 97

**End Position**: 110

| Profile Score | Tandem Score | TDA q-value | Volume | Spectrum Matches |
| --- | --- | --- | --- | --- |
| 12.9754 | 119.8099 | 0.0129 | 4.6765e+08 | 7 |

**Adduct Signal Fractions**

- **Unmodified**: 4.6765e+08

### RN(D)EEYN(N)KSVQEIQATF Fuc 1 Hex 6 HexNAc 5

**Mass:**  4089.7011

(2.429e-04)

**Charge States:** 3, 4

**Start Position**: 51

**End Position**: 67

| Profile Score | Tandem Score | TDA q-value | Volume | Spectrum Matches |
| --- | --- | --- | --- | --- |
| 11.1965 | 119.7176 | 0.0149 | 5.6400e+08 | 11 |

**Adduct Signal Fractions**

- **Unmodified**: 5.6400e+08

### LVPVPITN(N)ATL Hex 6 HexNAc 5

**Mass:**  3124.3991

(1.492e-06)

**Charge States:** 3, 4

**Start Position**: 26

**End Position**: 37

| Profile Score | Tandem Score | TDA q-value | Volume | Spectrum Matches |
| --- | --- | --- | --- | --- |
| -18.9381 | 119.6055 | 0.0149 | 6.5499e+06 | 1 |

**Adduct Signal Fractions**

- **Unmodified**: 6.5499e+06

### RN(D)EEYN(N)KSVQEIQATFF Hex 5 HexNAc 4

**Mass:**  3725.5797

(2.675e-04)

**Charge States:** 3, 4

**Start Position**: 51

**End Position**: 68

| Profile Score | Tandem Score | TDA q-value | Volume | Spectrum Matches |
| --- | --- | --- | --- | --- |
| 10.2173 | 119.0148 | 0.0149 | 3.6506e+08 | 10 |

**Adduct Signal Fractions**

- **Unmodified**: 3.6506e+08

### LNVQREN(N)GTVSRY Fuc 1 Hex 7 HexNAc 6

**Mass:**  4035.6928

(4.967e-04)

**Start Position**: 97

**End Position**: 110

| Profile Score | Tandem Score | TDA q-value | Volume | Spectrum Matches |
| --- | --- | --- | --- | --- |
| 0.0000 | 118.6173 | 0.0149 | 0.0000e+00 | 1 |

Adduct:

No Chromatogram Found

### NVQREN(N)GTVSRY Fuc 2 Hex 7 HexNAc 6

**Mass:**  4066.6645

(3.969e-07)

**Charge States:** 3, 4

**Start Position**: 98

**End Position**: 110

| Profile Score | Tandem Score | TDA q-value | Volume | Spectrum Matches |
| --- | --- | --- | --- | --- |
| -18.5805 | 118.1275 | 0.0149 | 1.1077e+08 | 2 |

**Adduct Signal Fractions**

- **Unmodified**: 1.1077e+08

### RN(D)EEYN(N)KSVQEIQATFF Hex 6 HexNAc 5

**Mass:**  4090.7082

(2.423e-04)

**Charge States:** 3, 4

**Start Position**: 51

**End Position**: 68

| Profile Score | Tandem Score | TDA q-value | Volume | Spectrum Matches |
| --- | --- | --- | --- | --- |
| 10.5069 | 118.0186 | 0.0149 | 3.8097e+08 | 30 |

**Adduct Signal Fractions**

- **Unmodified**: 3.8097e+08

### FTPN(N)KTEDTIF Fuc 2 Hex 7 HexNAc 6

**Mass:**  3956.6040

(1.905e-06)

**Charge States:** 3, 4

**Start Position**: 69

**End Position**: 80

| Profile Score | Tandem Score | TDA q-value | Volume | Spectrum Matches |
| --- | --- | --- | --- | --- |
| -17.5002 | 117.1472 | 0.0165 | 1.3930e+07 | 2 |

**Adduct Signal Fractions**

- **Unmodified**: 1.3930e+07

### RNEEYN(N)KSVQEIQATF Hex 9 HexNAc 8

**Mass:**  5038.0554

(-3.499e-07)

**Charge States:** 4

**Start Position**: 51

**End Position**: 67

| Profile Score | Tandem Score | TDA q-value | Volume | Spectrum Matches |
| --- | --- | --- | --- | --- |
| -3.8440 | 117.1415 | 0.0165 | 3.3303e+07 | 3 |

**Adduct Signal Fractions**

- **Unmodified**: 3.3303e+07

### VQREN(N)GTVSRY Hex 7 HexNAc 6

**Mass:**  3660.5147

(2.868e-06)

**Charge States:** 3

**Start Position**: 99

**End Position**: 110

| Profile Score | Tandem Score | TDA q-value | Volume | Spectrum Matches |
| --- | --- | --- | --- | --- |
| -21.3947 | 116.3442 | 0.0165 | 5.3719e+06 | 1 |

**Adduct Signal Fractions**

- **Unmodified**: 5.3719e+06

### TPN(N)KTEDTIF Fuc 2 Hex 7 HexNAc 6

**Mass:**  3809.5353

(1.900e-06)

**Charge States:** 3, 4

**Start Position**: 70

**End Position**: 80

| Profile Score | Tandem Score | TDA q-value | Volume | Spectrum Matches |
| --- | --- | --- | --- | --- |
| -19.5768 | 114.7525 | 0.0187 | 6.3051e+06 | 1 |

**Adduct Signal Fractions**

- **Unmodified**: 6.3051e+06

### LNVQREN(N)GTVSRY Fuc 1 Hex 6 HexNAc 5

**Mass:**  3668.5625

(1.301e-06)

**Charge States:** 3, 4

**Start Position**: 97

**End Position**: 110

| Profile Score | Tandem Score | TDA q-value | Volume | Spectrum Matches |
| --- | --- | --- | --- | --- |
| -4.1930 | 113.2479 | 0.0201 | 6.2496e+07 | 4 |

**Adduct Signal Fractions**

- **Unmodified**: 6.2496e+07

### TPN(N)KTEDTIF Hex 10 HexNAc 7

**Mass:**  4206.6638

(3.238e-06)

**Charge States:** 3, 4

**Start Position**: 70

**End Position**: 80

| Profile Score | Tandem Score | TDA q-value | Volume | Spectrum Matches |
| --- | --- | --- | --- | --- |
| -4.5780 | 113.1410 | 0.0201 | 1.4872e+07 | 1 |

**Adduct Signal Fractions**

- **Unmodified**: 1.4872e+07

### LNVQREN(N)GTVSRY Hex 6 HexNAc 5

**Mass:**  3523.5139

(2.881e-04)

**Start Position**: 97

**End Position**: 110

| Profile Score | Tandem Score | TDA q-value | Volume | Spectrum Matches |
| --- | --- | --- | --- | --- |
| 0.0000 | 113.0425 | 0.0201 | 0.0000e+00 | 1 |

Adduct:

No Chromatogram Found

### YFTPN(N)KTEDTIF Hex 7 HexNAc 6 Neu5Ac 1

**Mass:**  4118.6486

(2.165e-06)

**Charge States:** 3, 4

**Start Position**: 68

**End Position**: 80

| Profile Score | Tandem Score | TDA q-value | Volume | Spectrum Matches |
| --- | --- | --- | --- | --- |
| 12.0796 | 112.3197 | 0.0222 | 9.2279e+06 | 3 |

**Adduct Signal Fractions**

- **Unmodified**: 9.2279e+06

### FTPN(N)KTEDTIF Hex 9 HexNAc 8

**Mass:**  4394.7512

(1.815e-06)

**Charge States:** 4

**Start Position**: 69

**End Position**: 80

| Profile Score | Tandem Score | TDA q-value | Volume | Spectrum Matches |
| --- | --- | --- | --- | --- |
| 12.8169 | 111.5732 | 0.0239 | 1.3360e+07 | 2 |

**Adduct Signal Fractions**

- **Unmodified**: 1.3360e+07

### TPN(N)KTEDTIF Fuc 4 Hex 3 HexNAc 9 Neu5Ac 1

**Mass:**  4353.7282

(-8.819e-06)

**Charge States:** 3, 4

**Start Position**: 70

**End Position**: 80

| Profile Score | Tandem Score | TDA q-value | Volume | Spectrum Matches |
| --- | --- | --- | --- | --- |
| -5.0824 | 111.1276 | 0.0259 | 1.5620e+07 | 1 |

**Adduct Signal Fractions**

- **Unmodified**: 1.5620e+07

### YFTPN(N)KTEDTIF Hex 8 HexNAc 7

**Mass:**  4193.6893

(2.416e-04)

**Start Position**: 68

**End Position**: 80

| Profile Score | Tandem Score | TDA q-value | Volume | Spectrum Matches |
| --- | --- | --- | --- | --- |
| 0.0000 | 109.8932 | 0.0312 | 0.0000e+00 | 1 |

Adduct:

No Chromatogram Found

### TN(N)ATLDRITGKW Hex 6 HexNAc 5

**Mass:**  3362.4420

(1.061e-06)

**Charge States:** 3, 4

**Start Position**: 32

**End Position**: 44

| Profile Score | Tandem Score | TDA q-value | Volume | Spectrum Matches |
| --- | --- | --- | --- | --- |
| -3.7931 | 109.7285 | 0.0312 | 2.3024e+07 | 4 |

**Adduct Signal Fractions**

- **Unmodified**: 2.3024e+07

### RNEEYN(N)KSVQEIQATF Fuc 1 Hex 8 HexNAc 7

**Mass:**  4818.9797

(1.253e-06)

**Charge States:** 4

**Start Position**: 51

**End Position**: 67

| Profile Score | Tandem Score | TDA q-value | Volume | Spectrum Matches |
| --- | --- | --- | --- | --- |
| -5.2246 | 108.7899 | 0.0328 | 1.5445e+07 | 2 |

**Adduct Signal Fractions**

- **Unmodified**: 1.5445e+07

### RN(D)EEYN(N)KSVQEIQATFF Fuc 1 Hex 6 HexNAc 5

**Mass:**  4236.7658

(2.344e-04)

**Charge States:** 3, 4

**Start Position**: 51

**End Position**: 68

| Profile Score | Tandem Score | TDA q-value | Volume | Spectrum Matches |
| --- | --- | --- | --- | --- |
| 10.6321 | 105.8651 | 0.0371 | 1.8319e+08 | 8 |

**Adduct Signal Fractions**

- **Unmodified**: 1.8319e+08

### TPN(N)KTEDTIF Fuc 3 Hex 7 HexNAc 6 Neu5Ac 1

**Mass:**  4246.6794

(-4.427e-07)

**Charge States:** 4

**Start Position**: 70

**End Position**: 80

| Profile Score | Tandem Score | TDA q-value | Volume | Spectrum Matches |
| --- | --- | --- | --- | --- |
| -18.5537 | 105.6615 | 0.0371 | 4.3956e+06 | 1 |

**Adduct Signal Fractions**

- **Unmodified**: 4.3956e+06

### NVQREN(N)GTVSRY Fuc 1 Hex 9 HexNAc 8

**Mass:**  4651.8753

(2.163e-04)

**Start Position**: 98

**End Position**: 110

| Profile Score | Tandem Score | TDA q-value | Volume | Spectrum Matches |
| --- | --- | --- | --- | --- |
| 0.0000 | 105.5127 | 0.0371 | 0.0000e+00 | 1 |

Adduct:

No Chromatogram Found

### N(N)KSVQEIQATF Fuc 1 Hex 6 HexNAc 5

**Mass:**  3397.4240

(1.326e-06)

**Charge States:** 3, 4

**Start Position**: 56

**End Position**: 67

| Profile Score | Tandem Score | TDA q-value | Volume | Spectrum Matches |
| --- | --- | --- | --- | --- |
| -19.3103 | 104.7389 | 0.0408 | 7.8611e+06 | 1 |

**Adduct Signal Fractions**

- **Unmodified**: 7.8611e+06

### LNVQREN(N)GTVSRY Fuc 2 Hex 7 HexNAc 6

**Mass:**  4179.7473

(1.278e-06)

**Charge States:** 4

**Start Position**: 97

**End Position**: 110

| Profile Score | Tandem Score | TDA q-value | Volume | Spectrum Matches |
| --- | --- | --- | --- | --- |
| -4.0979 | 103.5888 | 0.0438 | 7.0644e+07 | 3 |

**Adduct Signal Fractions**

- **Unmodified**: 7.0644e+07

### TPN(N)KTEDTIF Fuc 1 Hex 8 HexNAc 7

**Mass:**  4028.6066

(1.050e-06)

**Charge States:** 4

**Start Position**: 70

**End Position**: 80

| Profile Score | Tandem Score | TDA q-value | Volume | Spectrum Matches |
| --- | --- | --- | --- | --- |
| -20.7277 | 101.6502 | 0.0455 | 2.3060e+06 | 1 |

**Adduct Signal Fractions**

- **Unmodified**: 2.3060e+06

### N(D)VQREN(N)GTVSRY Fuc 1 Hex 7 HexNAc 6 Neu5Ac 1

**Mass:**  4212.7258

(2.435e-04)

**Charge States:** 3, 4

**Start Position**: 98

**End Position**: 110

| Profile Score | Tandem Score | TDA q-value | Volume | Spectrum Matches |
| --- | --- | --- | --- | --- |
| -20.8770 | 101.3020 | 0.0472 | 2.8049e+07 | 1 |

**Adduct Signal Fractions**

- **Unmodified**: 2.8049e+07

### LNVQREN(N)GTVSRY Hex 9 HexNAc 8

**Mass:**  4617.9025

(-3.366e-08)

**Charge States:** 4

**Start Position**: 97

**End Position**: 110

| Profile Score | Tandem Score | TDA q-value | Volume | Spectrum Matches |
| --- | --- | --- | --- | --- |
| -4.3989 | 101.1152 | 0.0484 | 3.3198e+07 | 3 |

**Adduct Signal Fractions**

- **Unmodified**: 3.3198e+07

### C(C)ANLVPVPITN(N)ATL Hex 6 HexNAc 5

**Mass:**  3469.5097

(1.540e-06)

**Charge States:** 3, 4

**Start Position**: 23

**End Position**: 37

| Profile Score | Tandem Score | TDA q-value | Volume | Spectrum Matches |
| --- | --- | --- | --- | --- |
| 12.3011 | 100.4223 | 0.0484 | 2.2961e+07 | 5 |

**Adduct Signal Fractions**

- **Unmodified**: 2.2961e+07

### P13473|LAMP2\_HUMAN

**>P13473|LAMP2\_HUMAN**  
 `MVCFRLFPVPGSGLVLVCLVLGAVRSYALELNLTDSENATCLYAKWQMNFTVRYETTNKTYKTVTISDHGTVTYNGSICG  
DDQNGPKIAVQFGPGFSWIANFTKAASTYSIDSVSFSYNTGDNTTFPDAEDKGILTVDELLAIRIPLNDLFRCNSLSTLE  
KNDVVQHYWDVLVQAFVQNGTVSTNEFLCDKDKTSTVAPTIHTTVPSPTTTPTPKEKPEAGTYSVNNGNDTCLLATMGLQ  
LNITQDKVASVININPNTTHSTGSCRSHTALLRLNSSTIKYLDFVFAVKNENRFYLKEVNISMYLVNGSVFSIANNNLSY  
WDAPLGSSYMCNKEQTVSVSGAFQINTFDLRVQPFNVTQGKYSTAQDCSADDDNFLVPIAVGAALAGVLILVLLAYFIGL  
KHHHAGYEQF`

| Site | Type | Count |
| --- | --- | --- |
| 307 | N-linked | 2 |
| 32 | N-linked | 1 |

| Mass | Sequence | MS_2_ Score | `q`-value | MS_1_ Score | Signal | Protein Coordinates | Tandem Scans | Start Time | Apex Time | End Time |
| --- | --- | --- | --- | --- | --- | --- | --- | --- | --- | --- |
| 2722.1136 | LVN(N)GSVF Hex 6 HexNAc 5 | 176.719 | 0.0 | 13.246 | 9.689e+06 | 304 - 311 | 2 | 29.33 | 29.50 | 30.01 |
| 3675.5269 | ALELN(N)L Fuc 5 Hex 4 HexNAc 8 | 139.879 | 0.0 | 0.000 | 0.000e+00 | 27 - 33 | 1 | - | - | - |
| 2869.1698 | LVN(N)GSVF Fuc 1 Hex 6 HexNAc 5 | 130.033 | 0.0 | 0.000 | 0.000e+00 | 304 - 311 | 1 | - | - | - |

### LVN(N)GSVF Hex 6 HexNAc 5

**Mass:**  2722.1129

(9.875e-07)

**Charge States:** 3

**Start Position**: 305

**End Position**: 312

| Profile Score | Tandem Score | TDA q-value | Volume | Spectrum Matches |
| --- | --- | --- | --- | --- |
| 13.2462 | 176.7193 | 0.0000 | 9.6888e+06 | 2 |

**Adduct Signal Fractions**

- **Unmodified**: 9.6888e+06

### ALELN(N)L Fuc 5 Hex 4 HexNAc 8

**Mass:**  3675.5269

(2.737e-04)

**Start Position**: 28

**End Position**: 34

| Profile Score | Tandem Score | TDA q-value | Volume | Spectrum Matches |
| --- | --- | --- | --- | --- |
| 0.0000 | 139.8792 | 0.0000 | 0.0000e+00 | 1 |

Adduct:

No Chromatogram Found

### LVN(N)GSVF Fuc 1 Hex 6 HexNAc 5

**Mass:**  2869.1698

(3.493e-04)

**Start Position**: 305

**End Position**: 312

| Profile Score | Tandem Score | TDA q-value | Volume | Spectrum Matches |
| --- | --- | --- | --- | --- |
| 0.0000 | 130.0331 | 0.0000 | 0.0000e+00 | 1 |

Adduct:

No Chromatogram Found

### P00738|HPT\_HUMAN

**>P00738|HPT\_HUMAN**  
 `MSALGAVIALLLWGQLFAVDSGNDVTDIADDGCPKPPEIAHGYVEHSVRYQCKNYYKLRTEGDGVYTLNDKKQWINKAVG  
DKLPECEADDGCPKPPEIAHGYVEHSVRYQCKNYYKLRTEGDGVYTLNNEKQWINKAVGDKLPECEAVCGKPKNPANPVQ  
RILGGHLDAKGSFPWQAKMVSHHNLTTGATLINEQWLLTTAKNLFLNHSENATAKDIAPTLTLYVGKKQLVEIEKVVLHP  
NYSQVDIGLIKLKQKVSVNERVMPICLPSKDYAEVGRVGYVSGWGRNANFKFTDHLKYVMLPVADQDQCIRHYEGSTVPE  
KKTPKSPVGVQPILNEHTFCAGMSKYQEDTCYGDAGSAFAVHDLEEDTWYATGILSFDKSCAVAEYGVYVKVTSIQDWVQ  
KTIAEN`

| Site | Type | Count |
| --- | --- | --- |
| 184 | N-linked | 2 |

| Mass | Sequence | MS_2_ Score | `q`-value | MS_1_ Score | Signal | Protein Coordinates | Tandem Scans | Start Time | Apex Time | End Time |
| --- | --- | --- | --- | --- | --- | --- | --- | --- | --- | --- |
| 2872.2269 | VSHHN(N)LTTGATL Hex 5 HexNAc 4 | 165.158 | 0.0 | -4.010 | 3.057e+07 | 179 - 191 | 2 | 17.17 | 17.17 | 17.51 |
| 3237.3572 | VSHHN(N)LTTGATL Hex 6 HexNAc 5 | 161.127 | 0.0 | -5.003 | 1.487e+07 | 179 - 191 | 1 | 16.84 | 17.00 | 17.17 |

### VSHHN(N)LTTGATL Hex 5 HexNAc 4

**Mass:**  2872.2266

(1.204e-06)

**Charge States:** 3

**Start Position**: 180

**End Position**: 192

| Profile Score | Tandem Score | TDA q-value | Volume | Spectrum Matches |
| --- | --- | --- | --- | --- |
| -4.0101 | 165.1576 | 0.0000 | 3.0571e+07 | 2 |

**Adduct Signal Fractions**

- **Unmodified**: 3.0571e+07

### VSHHN(N)LTTGATL Hex 6 HexNAc 5

**Mass:**  3237.3598

(1.406e-06)

**Charge States:** 3

**Start Position**: 180

**End Position**: 192

| Profile Score | Tandem Score | TDA q-value | Volume | Spectrum Matches |
| --- | --- | --- | --- | --- |
| -5.0033 | 161.1275 | 0.0000 | 1.4869e+07 | 1 |

**Adduct Signal Fractions**

- **Unmodified**: 1.4869e+07

### O95497|VNN1\_HUMAN

**>O95497|VNN1\_HUMAN**  
 `MTTQLPAYVAILLFYVSRASCQDTFTAAVYEHAAILPNATLTPVSREEALALMNRNLDILEGAITSAADQGAHIIVTPED  
AIYGWNFNRDSLYPYLEDIPDPEVNWIPCNNRNRFGQTPVQERLSCLAKNNSIYVVANIGDKKPCDTSDPQCPPDGRYQY  
NTDVVFDSQGKLVARYHKQNLFMGENQFNVPKEPEIVTFNTTFGSFGIFTCFDILFHDPAVTLVKDFHVDTIVFPTAWMN  
VLPHLSAVEFHSAWAMGMRVNFLASNIHYPSKKMTGSGIYAPNSSRAFHYDMKTEEGKLLLSQLDSHPSHSAVVNWTSYA  
SSIEALSSGNKEFKGTVFFDEFTFVKLTGVAGNYTVCQKDLCCHLSYKMSENIPNEVYALGAFDGLHTVEGRYYLQICTL  
LKCKTTNLNTCGDSAETASTRFEMFSLSGTFGTQYVFPEVLLSENQLAPGEFQVSTDGRLFSLKPTSGPVLTVTLFGRLY  
EKDWASNASSGLTAQARIIMLIVIAPIVCSLSW`

| Site | Type | Count |
| --- | --- | --- |
| 200 | N-linked | 1 |

| Mass | Sequence | MS_2_ Score | `q`-value | MS_1_ Score | Signal | Protein Coordinates | Tandem Scans | Start Time | Apex Time | End Time |
| --- | --- | --- | --- | --- | --- | --- | --- | --- | --- | --- |
| 3109.2031 | N(N)TTFGSF Fuc 1 Hex 6 HexNAc 6 | 126.520 | 0.0 | 12.967 | 1.083e+08 | 199 - 206 | 4 | 16.33 | 16.67 | 17.51 |

### N(N)TTFGSF Fuc 1 Hex 6 HexNAc 6

**Mass:**  3109.2050

(4.761e-06)

**Charge States:** 3

**Start Position**: 200

**End Position**: 207

| Profile Score | Tandem Score | TDA q-value | Volume | Spectrum Matches |
| --- | --- | --- | --- | --- |
| 12.9671 | 126.5197 | 0.0000 | 1.0835e+08 | 4 |

**Adduct Signal Fractions**

- **Unmodified**: 1.0835e+08
