# Supplementary material for: Measuring Site-specific Glycosylation Similarity between Influenza a Virus Variants with Statistical Certainty
Source: Mol Cell Proteomics. 2020 Nov 25;19(9):1533–45. doi: 10.1074/mcp.RA120.002031 (PMC8143645; doi:10.1074/mcp.RA120.002031)
Supplement: Supplementary file 1 [file mmc1.zip › mmc1/159250_2_supp_555285_qcjklg.zip!/20190326_aAGP_021119_chymo_HILIC_DDA_1ug_04_glycopeptides.html]

## 20190326\_aAGP\_021119\_chymo\_HILIC\_DDA\_1ug\_04 @ GlycopeptideHypothesis-f5496f1c75c44146beeabb7629ee9b5b

**MS^1^ Mass Accuracy:** 10.0 PPM  
**Grouping Tolerance:** 15.0 PPM  
**MS^n^ Mass Accuracy:** 20.0 PPM  
**Minimum `q`-Value For Clustering:** 0.05

**Glycopeptide Hypothesis:**
GlycopeptideHypothesis-f5496f1c75c44146beeabb7629ee9b5b  
**Sample Searched:**
20190326\_aAGP\_021119\_chymo\_HILIC\_DDA\_1ug\_04

| Protein | Matched Glycopeptides |
| --- | --- |
| P02763\|A1AG1\_HUMAN | 140 |
| P19652\|A1AG2\_HUMAN | 121 |
| P00738\|HPT\_HUMAN | 2 |
| P13473\|LAMP2\_HUMAN | 1 |

### P02763|A1AG1\_HUMAN

**>P02763|A1AG1\_HUMAN**  
 `MALSWVLTVLSLLPLLEAQIPLCANLVPVPITNATLDQITGKWFYIASAFRNEEYNKSVQEIQATFFYFTPNKTEDTIFL  
REYQTRQDQCIYNTTYLNVQRENGTISRYVGGQEHFAHLLILRDTKTYMLAFDVNDEKNWGLSVYADKPETTKEQLGEFY  
EALDCLRIPKSDVVYTDWKKDKCEPLEKQHEKERKQEEGES`

| Site | Type | Count |
| --- | --- | --- |
| 33 | N-linked | 2 |
| 56 | N-linked | 36 |
| 93 | N-linked | 4 |
| 103 | N-linked | 36 |
| 72 | N-linked | 62 |

| Mass | Sequence | MS_2_ Score | `q`-value | MS_1_ Score | Signal | Protein Coordinates | Tandem Scans | Start Time | Apex Time | End Time |
| --- | --- | --- | --- | --- | --- | --- | --- | --- | --- | --- |
| 3152.2829 | TPN(N)KTEDTIF Hex 6 HexNAc 5 | 182.079 | 0.0 | 15.206 | 1.948e+08 | 69 - 79 | 9 | 22.05 | 23.92 | 25.29 |
| 3517.4167 | TPN(N)KTEDTIF Hex 7 HexNAc 6 | 181.792 | 0.0 | 15.626 | 2.810e+08 | 69 - 79 | 10 | 21.36 | 23.24 | 24.77 |
| 2787.1525 | TPN(N)KTEDTIF Hex 5 HexNAc 4 | 181.778 | 0.0 | 14.263 | 2.212e+07 | 69 - 79 | 3 | 24.09 | 24.43 | 24.77 |
| 2886.2319 | N(N)KSVQEIQATF Hex 5 HexNAc 4 | 178.684 | 0.0 | 14.082 | 2.514e+07 | 55 - 66 | 2 | 27.00 | 27.34 | 27.68 |
| 3827.5488 | YFTPN(N)KTEDTIF Hex 7 HexNAc 6 | 177.218 | 0.0 | 14.918 | 4.580e+09 | 67 - 79 | 52 | 35.76 | 36.78 | 43.37 |
| 3973.6063 | YFTPN(N)KTEDTIF Fuc 1 Hex 7 HexNAc 6 | 176.426 | 0.0 | 14.804 | 8.900e+08 | 67 - 79 | 8 | 35.59 | 36.61 | 37.60 |
| 3097.2819 | YFTPN(N)KTEDTIF Hex 5 HexNAc 4 | 174.557 | 0.0 | 15.238 | 3.785e+08 | 67 - 79 | 5 | 37.76 | 38.08 | 38.93 |
| 3462.4150 | YFTPN(N)KTEDTIF Hex 6 HexNAc 5 | 174.243 | 0.0 | 14.173 | 2.895e+09 | 67 - 79 | 28 | 36.27 | 37.43 | 43.03 |
| 3299.3503 | FTPN(N)KTEDTIF Hex 6 HexNAc 5 | 173.039 | 0.0 | 15.036 | 4.095e+08 | 68 - 79 | 12 | 29.57 | 31.10 | 32.12 |
| 3307.3795 | EEYN(N)KSVQEIQATF Hex 5 HexNAc 4 | 170.890 | 0.0 | 12.169 | 8.537e+07 | 52 - 66 | 2 | 35.76 | 35.93 | 36.44 |
| 3298.3431 | TPN(N)KTEDTIF Fuc 1 Hex 6 HexNAc 5 | 170.587 | 0.0 | 13.408 | 3.114e+07 | 69 - 79 | 4 | 23.24 | 23.58 | 24.09 |
| 4614.8359 | YFTPN(N)KTEDTIF Fuc 4 Hex 7 HexNAc 7 | 169.379 | 0.0 | 15.607 | 8.189e+07 | 67 - 79 | 3 | 35.25 | 35.76 | 36.27 |
| 3609.4857 | FYFTPN(N)KTEDTIF Hex 6 HexNAc 5 | 168.667 | 0.0 | 14.759 | 2.786e+09 | 66 - 79 | 49 | 46.40 | 47.40 | 58.32 |
| 3664.4846 | FTPN(N)KTEDTIF Hex 7 HexNAc 6 | 168.062 | 0.0 | 16.265 | 6.629e+08 | 68 - 79 | 13 | 29.05 | 30.59 | 31.95 |
| 3608.4750 | YFTPN(N)KTEDTIF Fuc 1 Hex 6 HexNAc 5 | 167.922 | 0.0 | 14.317 | 5.999e+08 | 67 - 79 | 7 | 36.10 | 37.27 | 38.42 |
| 3755.5449 | FYFTPN(N)KTEDTIF Fuc 1 Hex 6 HexNAc 5 | 167.386 | 0.0 | 14.548 | 5.689e+08 | 66 - 79 | 11 | 46.06 | 47.06 | 48.40 |
| 3663.4750 | TPN(N)KTEDTIF Fuc 1 Hex 7 HexNAc 6 | 167.357 | 0.0 | 13.749 | 4.404e+07 | 69 - 79 | 4 | 22.90 | 23.07 | 23.76 |
| 4120.6790 | FYFTPN(N)KTEDTIF Fuc 1 Hex 7 HexNAc 6 | 166.335 | 0.0 | 14.398 | 8.777e+08 | 66 - 79 | 12 | 45.72 | 46.57 | 48.40 |
| 4192.6833 | YFTPN(N)KTEDTIF Hex 8 HexNAc 7 | 165.829 | 0.0 | 15.245 | 7.627e+08 | 67 - 79 | 11 | 34.91 | 36.44 | 37.27 |
| 3974.6213 | FYFTPN(N)KTEDTIF Hex 7 HexNAc 6 | 165.654 | 0.0 | 15.289 | 4.717e+09 | 66 - 79 | 82 | 45.55 | 46.73 | 60.72 |
| 4339.7521 | FYFTPN(N)KTEDTIF Hex 8 HexNAc 7 | 165.280 | 0.0 | 14.780 | 6.386e+08 | 66 - 79 | 9 | 45.03 | 46.23 | 46.90 |
| 4266.7362 | FYFTPN(N)KTEDTIF Fuc 2 Hex 7 HexNAc 6 | 164.176 | 0.0 | -16.199 | 1.187e+08 | 66 - 79 | 2 | 46.40 | 46.40 | 46.57 |
| 3882.5492 | TPN(N)KTEDTIF Hex 8 HexNAc 7 | 163.940 | 0.0 | -4.523 | 2.119e+07 | 69 - 79 | 4 | 22.39 | 22.73 | 22.90 |
| 4235.7881 | RNEEYN(N)KSVQEIQATFF Fuc 1 Hex 6 HexNAc 5 | 162.034 | 0.0 | 15.671 | 1.928e+09 | 50 - 67 | 16 | 41.83 | 43.54 | 44.86 |
| 3244.3524 | FYFTPN(N)KTEDTIF Hex 5 HexNAc 4 | 161.981 | 0.0 | 10.767 | 2.224e+08 | 66 - 79 | 4 | 47.06 | 47.90 | 48.74 |
| 3319.3141 | N(N)KTEDTIF Hex 7 HexNAc 6 | 161.405 | 0.0 | -20.235 | 3.217e+06 | 71 - 79 | 1 | 21.36 | 21.36 | 21.54 |
| 4089.7286 | RNEEYN(N)KSVQEIQATFF Hex 6 HexNAc 5 | 161.030 | 0.0 | 16.690 | 3.948e+09 | 50 - 67 | 19 | 41.49 | 43.71 | 44.86 |
| 4119.6668 | YFTPN(N)KTEDTIF Fuc 2 Hex 7 HexNAc 6 | 160.245 | 0.0 | -4.104 | 1.272e+08 | 67 - 79 | 2 | 36.44 | 36.44 | 36.94 |
| 4557.8147 | YFTPN(N)KTEDTIF Hex 9 HexNAc 8 | 159.947 | 0.0 | 15.398 | 2.304e+08 | 67 - 79 | 6 | 34.74 | 35.76 | 36.44 |
| 3672.5120 | EEYN(N)KSVQEIQATF Hex 6 HexNAc 5 | 158.828 | 0.0 | -3.199 | 8.124e+07 | 52 - 66 | 3 | 35.25 | 35.42 | 35.76 |
| 3723.5853 | RNEEYN(N)KSVQEIQATF Fuc 1 Hex 5 HexNAc 4 | 158.251 | 0.0 | 11.221 | 3.347e+08 | 50 - 66 | 9 | 32.44 | 32.61 | 34.06 |
| 3445.4094 | FTPN(N)KTEDTIF Fuc 1 Hex 6 HexNAc 5 | 158.247 | 0.0 | 13.429 | 7.412e+07 | 68 - 79 | 5 | 30.59 | 30.76 | 31.27 |
| 3004.2181 | FYFTPN(N)KT Hex 6 HexNAc 5 | 156.822 | 0.0 | -4.004 | 6.724e+06 | 66 - 74 | 2 | 25.80 | 25.97 | 26.32 |
| 3577.5255 | RNEEYN(N)KSVQEIQATF Hex 5 HexNAc 4 | 156.559 | 0.0 | 16.177 | 8.420e+09 | 50 - 66 | 22 | 24.77 | 32.75 | 34.23 |
| 4485.8103 | FYFTPN(N)KTEDTIF Fuc 1 Hex 8 HexNAc 7 | 156.496 | 0.0 | 14.851 | 1.309e+08 | 66 - 79 | 6 | 45.55 | 46.06 | 46.57 |
| 3724.5958 | RNEEYN(N)KSVQEIQATFF Hex 5 HexNAc 4 | 156.361 | 0.0 | 15.992 | 4.177e+09 | 50 - 67 | 17 | 42.35 | 44.20 | 45.55 |
| 3818.5708 | EEYN(N)KSVQEIQATF Fuc 1 Hex 6 HexNAc 5 | 155.479 | 0.0 | -4.046 | 3.410e+07 | 52 - 66 | 2 | 35.08 | 35.25 | 35.59 |
| 3454.4484 | EEYN(N)KSVQEIQATFF Hex 5 HexNAc 4 | 155.236 | 0.0 | -4.981 | 2.731e+07 | 52 - 67 | 1 | 47.90 | 47.90 | 48.40 |
| 3819.5820 | EEYN(N)KSVQEIQATFF Hex 6 HexNAc 5 | 154.973 | 0.0 | -5.626 | 2.607e+07 | 52 - 67 | 2 | 47.40 | 47.40 | 47.73 |
| 4381.8438 | RNEEYN(N)KSVQEIQATFF Fuc 2 Hex 6 HexNAc 5 | 153.767 | 0.0 | -3.714 | 7.341e+07 | 50 - 67 | 3 | 43.37 | 43.54 | 43.71 |
| 3942.6579 | RNEEYN(N)KSVQEIQATF Hex 6 HexNAc 5 | 153.511 | 0.0 | 15.010 | 6.008e+09 | 50 - 66 | 20 | 30.59 | 32.44 | 33.89 |
| 3870.6575 | RNEEYN(N)KSVQEIQATFF Fuc 1 Hex 5 HexNAc 4 | 153.010 | 0.0 | 13.132 | 1.534e+08 | 50 - 67 | 5 | 43.71 | 44.04 | 44.69 |
| 4020.6053 | YFTPN(N)KTEDTIF Fuc 1 Hex 8 HexNAc 4 Neu5Ac 1 | 152.837 | 0.0 | -4.526 | 5.374e+07 | 67 - 79 | 3 | 38.08 | 38.08 | 38.42 |
| 4338.7401 | YFTPN(N)KTEDTIF Fuc 1 Hex 8 HexNAc 7 | 152.427 | 0.0 | 14.683 | 1.449e+08 | 67 - 79 | 5 | 35.59 | 36.27 | 36.61 |
| 4003.5800 | YFTPN(N)KTEDTIF Hex 7 HexNAc 4 Neu5Ac 2 | 152.347 | 0.0 | 14.550 | 3.336e+08 | 67 - 79 | 6 | 37.92 | 38.08 | 38.93 |
| 4088.7160 | RNEEYN(N)KSVQEIQATF Fuc 1 Hex 6 HexNAc 5 | 152.329 | 0.0 | 14.587 | 2.951e+09 | 50 - 66 | 16 | 30.42 | 32.28 | 33.72 |
| 3810.5428 | FTPN(N)KTEDTIF Fuc 1 Hex 7 HexNAc 6 | 151.215 | 0.0 | 13.624 | 1.191e+08 | 68 - 79 | 6 | 30.08 | 30.25 | 30.93 |
| 4454.8604 | RNEEYN(N)KSVQEIQATFF Hex 7 HexNAc 6 | 150.949 | 0.0 | 14.902 | 1.854e+08 | 50 - 67 | 4 | 43.03 | 43.37 | 43.87 |
| 4307.7912 | RNEEYN(N)KSVQEIQATF Hex 7 HexNAc 6 | 150.565 | 0.0 | -2.648 | 2.895e+08 | 50 - 66 | 3 | 31.95 | 32.12 | 32.44 |
| 4234.7738 | RNEEYN(N)KSVQEIQATF Fuc 2 Hex 6 HexNAc 5 | 150.457 | 0.0 | -3.600 | 9.725e+07 | 50 - 66 | 3 | 32.12 | 32.12 | 32.44 |
| 3222.2737 | YFTPN(N)KT Hex 7 HexNAc 6 | 150.011 | 0.0 | -20.675 | 3.354e+06 | 67 - 74 | 1 | 14.28 | 14.28 | 14.28 |
| 4600.9167 | RNEEYN(N)KSVQEIQATFF Fuc 1 Hex 7 HexNAc 6 | 149.997 | 0.0 | 14.810 | 6.810e+07 | 50 - 67 | 3 | 42.86 | 43.37 | 43.54 |
| 4453.8502 | RNEEYN(N)KSVQEIQATF Fuc 1 Hex 7 HexNAc 6 | 149.738 | 0.0 | -3.481 | 1.288e+08 | 50 - 66 | 2 | 31.95 | 32.12 | 32.44 |
| 2934.2202 | FTPN(N)KTEDTIF Hex 5 HexNAc 4 | 149.602 | 0.0 | -3.493 | 4.012e+07 | 68 - 79 | 3 | 31.44 | 31.60 | 31.95 |
| 3682.5774 | LNVQREN(N)GTISRY Fuc 1 Hex 6 HexNAc 5 | 149.372 | 0.0 | 14.571 | 1.207e+09 | 96 - 109 | 11 | 18.38 | 19.03 | 20.34 |
| 4060.6029 | TPN(N)KTEDTIF Fuc 4 Hex 8 HexNAc 5 | 148.123 | 0.0 | 13.080 | 1.376e+08 | 69 - 79 | 4 | 37.92 | 38.08 | 38.59 |
| 4704.8852 | FYFTPN(N)KTEDTIF Hex 9 HexNAc 8 | 147.390 | 0.0 | 16.793 | 1.722e+08 | 66 - 79 | 6 | 44.86 | 45.55 | 46.40 |
| 4150.6523 | FYFTPN(N)KTEDTIF Hex 7 HexNAc 4 Neu5Ac 2 | 146.167 | 0.0 | 13.490 | 2.521e+08 | 66 - 79 | 5 | 47.73 | 47.90 | 48.91 |
| 3369.3485 | FYFTPN(N)KT Hex 7 HexNAc 6 | 145.901 | 0.0 | 12.889 | 1.334e+07 | 66 - 74 | 2 | 25.12 | 25.46 | 25.97 |
| 3613.4174 | FYFTPN(N)KTED Hex 7 HexNAc 6 | 144.957 | 0.0 | -18.071 | 5.227e+06 | 66 - 76 | 1 | 25.63 | 25.63 | 25.80 |
| 3536.5205 | LNVQREN(N)GTISRY Hex 6 HexNAc 5 | 143.326 | 0.0 | 15.808 | 3.297e+09 | 96 - 109 | 11 | 18.38 | 19.19 | 20.34 |
| 3430.4549 | RNEEYN(N)KSVQEIQAT Hex 5 HexNAc 4 | 142.590 | 0.0 | -4.607 | 3.418e+07 | 50 - 65 | 3 | 17.20 | 17.36 | 17.70 |
| 4207.6717 | TPN(N)KTEDTIF Fuc 3 Hex 3 HexNAc 9 Neu5Ac 1 | 140.943 | 0.0 | 13.939 | 1.021e+08 | 69 - 79 | 4 | 48.74 | 48.91 | 49.93 |
| 3693.4452 | TPN(N)KTEDTIF Hex 7 HexNAc 4 Neu5Ac 2 | 138.675 | 0.0 | -4.280 | 9.871e+06 | 69 - 79 | 3 | 24.26 | 24.43 | 24.60 |
| 4089.7123 | FRNEEYN(N)KSVQEIQATF Hex 6 HexNAc 5 | 138.033 | 0.0 | -5.395 | 4.085e+07 | 49 - 66 | 1 | 37.11 | 37.43 | 37.60 |
| 3849.5295 | YFTPN(N)KTEDTIF Hex 3 HexNAc 5 Neu5Ac 3 | 138.021 | 0.0 | 12.513 | 3.088e+08 | 67 - 79 | 2 | 36.61 | 36.78 | 37.43 |
| 4672.9234 | RNEEYN(N)KSVQEIQATF Hex 8 HexNAc 7 | 137.544 | 0.0 | -3.104 | 5.047e+07 | 50 - 66 | 2 | 31.60 | 31.77 | 32.12 |
| 3943.6431 | RN(D)EEYN(N)KSVQEIQATF Hex 6 HexNAc 5 | 135.678 | 0.0 | 11.626 | 1.211e+09 | 50 - 66 | 25 | 34.06 | 35.42 | 36.27 |
| 4149.6400 | YFTPN(N)KTEDTIF Fuc 1 Hex 7 HexNAc 4 Neu5Ac 2 | 134.694 | 0.0 | -5.481 | 3.971e+07 | 67 - 79 | 2 | 37.76 | 37.92 | 38.25 |
| 3934.6325 | NVQREN(N)GTISRY Fuc 1 Hex 7 HexNAc 6 | 133.692 | 0.0 | -3.770 | 1.478e+08 | 97 - 109 | 4 | 13.12 | 13.28 | 13.45 |
| 3578.5098 | RN(D)EEYN(N)KSVQEIQATF Hex 5 HexNAc 4 | 132.369 | 0.0 | 11.490 | 1.291e+09 | 50 - 66 | 12 | 34.40 | 35.76 | 36.94 |
| 3724.5889 | FRNEEYN(N)KSVQEIQATF Hex 5 HexNAc 4 | 131.880 | 0.0 | -5.113 | 3.104e+07 | 49 - 66 | 8 | 37.76 | 37.76 | 38.08 |
| 4029.6181 | FTPN(N)KTEDTIF Hex 8 HexNAc 7 | 129.670 | 0.003 | 15.805 | 5.182e+07 | 68 - 79 | 5 | 29.39 | 29.91 | 30.25 |
| 4296.7082 | FYFTPN(N)KTEDTIF Fuc 1 Hex 7 HexNAc 4 Neu5Ac 2 | 129.468 | 0.003 | -3.628 | 4.049e+07 | 66 - 79 | 2 | 47.56 | 47.73 | 47.90 |
| 3638.4464 | TPN(N)KTEDTIF Hex 9 HexNAc 5 | 128.680 | 0.003 | 12.545 | 3.402e+07 | 69 - 79 | 3 | 38.42 | 38.59 | 39.10 |
| 4703.8761 | YFTPN(N)KTEDTIF Fuc 1 Hex 9 HexNAc 8 | 128.357 | 0.003 | 14.003 | 3.907e+07 | 67 - 79 | 3 | 35.08 | 35.42 | 36.10 |
| 3569.4953 | NVQREN(N)GTISRY Fuc 1 Hex 6 HexNAc 5 | 127.042 | 0.003 | -4.235 | 3.770e+07 | 97 - 109 | 2 | 13.28 | 13.45 | 13.61 |
| 3171.3878 | LNVQREN(N)GTISRY Hex 5 HexNAc 4 | 126.352 | 0.003 | 13.699 | 2.451e+08 | 96 - 109 | 3 | 19.03 | 19.34 | 20.51 |
| 3788.5725 | NVQREN(N)GTISRY Hex 7 HexNAc 6 | 125.589 | 0.006 | 12.760 | 4.672e+08 | 97 - 109 | 6 | 13.12 | 13.28 | 13.95 |
| 4631.8691 | FYFTPN(N)KTEDTIF Fuc 2 Hex 8 HexNAc 7 | 125.362 | 0.006 | -3.794 | 2.302e+07 | 66 - 79 | 4 | 45.55 | 45.89 | 46.06 |
| 4042.5950 | QTRQDQC(C)IYN(N)TTY Hex 7 HexNAc 6 | 125.030 | 0.006 | 14.054 | 1.731e+08 | 83 - 96 | 7 | 20.85 | 21.88 | 22.73 |
| 4047.7097 | LNVQREN(N)GTISRY Fuc 1 Hex 7 HexNAc 6 | 124.900 | 0.006 | 15.267 | 2.832e+09 | 96 - 109 | 10 | 18.04 | 18.55 | 19.84 |
| 4048.7029 | LN(D)VQREN(N)GTISRY Fuc 1 Hex 7 HexNAc 6 | 124.209 | 0.006 | 10.399 | 1.240e+08 | 96 - 109 | 13 | 20.00 | 20.51 | 20.68 |
| 3423.4394 | NVQREN(N)GTISRY Hex 6 HexNAc 5 | 124.100 | 0.006 | 12.921 | 1.350e+08 | 97 - 109 | 4 | 13.28 | 13.45 | 13.95 |
| 3624.4945 | YFTPN(N)KTEDTIF Hex 7 HexNAc 5 | 123.299 | 0.008 | -21.586 | 1.083e+06 | 67 - 79 | 3 | 36.10 | 36.10 | 36.10 |
| 3956.6054 | FTPN(N)KTEDTIF Fuc 2 Hex 7 HexNAc 6 | 123.025 | 0.008 | -3.673 | 1.294e+07 | 68 - 79 | 3 | 29.91 | 30.08 | 30.25 |
| 3309.3989 | VQREN(N)GTISRY Hex 6 HexNAc 5 | 122.099 | 0.010 | -19.268 | 9.013e+06 | 98 - 109 | 1 | 12.95 | 12.95 | 12.95 |
| 3902.6434 | LN(D)VQREN(N)GTISRY Hex 7 HexNAc 6 | 122.084 | 0.010 | 8.322 | 3.656e+08 | 96 - 109 | 19 | 20.17 | 20.34 | 21.36 |
| 4167.6783 | FYFTPN(N)KTEDTIF Fuc 1 Hex 8 HexNAc 4 Neu5Ac 1 | 121.933 | 0.010 | -4.567 | 5.258e+07 | 66 - 79 | 2 | 47.73 | 47.90 | 48.23 |
| 4193.7691 | LNVQREN(N)GTISRY Fuc 2 Hex 7 HexNAc 6 | 121.050 | 0.010 | 13.377 | 8.984e+08 | 96 - 109 | 7 | 18.21 | 18.55 | 19.84 |
| 3901.6507 | LNVQREN(N)GTISRY Hex 7 HexNAc 6 | 120.411 | 0.010 | 16.287 | 8.336e+09 | 96 - 109 | 12 | 18.04 | 18.71 | 20.00 |
| 3795.5886 | RNEEYN(N)KSVQEIQAT Hex 6 HexNAc 5 | 120.338 | 0.010 | -5.617 | 2.247e+07 | 50 - 65 | 2 | 17.20 | 17.20 | 17.53 |
| 3676.5349 | VQREN(N)GTISRY Hex 7 HexNAc 6 | 120.094 | 0.010 | 0.000 | 0.000e+00 | 98 - 109 | 1 | - | - | - |
| 4353.7292 | TPN(N)KTEDTIF Fuc 4 Hex 3 HexNAc 9 Neu5Ac 1 | 119.860 | 0.010 | -4.741 | 1.909e+07 | 69 - 79 | 1 | 48.57 | 48.74 | 48.91 |
| 3828.6367 | LNVQREN(N)GTISRY Fuc 2 Hex 6 HexNAc 5 | 118.327 | 0.010 | 11.131 | 8.862e+07 | 96 - 109 | 2 | 18.38 | 18.71 | 19.19 |
| 3725.5810 | RN(D)EEYN(N)KSVQEIQATFF Hex 5 HexNAc 4 | 118.205 | 0.010 | 10.371 | 3.845e+08 | 50 - 67 | 9 | 45.55 | 47.23 | 47.56 |
| 4089.7020 | RN(D)EEYN(N)KSVQEIQATF Fuc 1 Hex 6 HexNAc 5 | 118.032 | 0.010 | 11.470 | 5.898e+08 | 50 - 66 | 15 | 33.72 | 35.42 | 36.27 |
| 4153.7036 | NVQREN(N)GTISRY Hex 8 HexNAc 7 | 117.398 | 0.012 | -5.765 | 4.105e+07 | 97 - 109 | 3 | 12.95 | 13.12 | 13.28 |
| 4090.7140 | RN(D)EEYN(N)KSVQEIQATFF Hex 6 HexNAc 5 | 117.100 | 0.012 | 10.655 | 4.124e+08 | 50 - 67 | 16 | 45.03 | 45.38 | 46.90 |
| 4412.8473 | LNVQREN(N)GTISRY Fuc 1 Hex 8 HexNAc 7 | 116.510 | 0.017 | 15.424 | 3.858e+08 | 96 - 109 | 5 | 17.87 | 18.38 | 19.03 |
| 4028.6028 | FTPN(N)KTEDTIF Fuc 3 Hex 6 HexNAc 5 Neu5Ac 1 | 116.371 | 0.017 | -19.030 | 7.691e+05 | 68 - 79 | 1 | 29.39 | 29.39 | 29.39 |
| 4484.8013 | YFTPN(N)KTEDTIF Fuc 2 Hex 8 HexNAc 7 | 114.681 | 0.018 | 9.916 | 3.093e+07 | 67 - 79 | 2 | 35.59 | 36.10 | 36.27 |
| 4266.7895 | LNVQREN(N)GTISRY Hex 8 HexNAc 7 | 114.646 | 0.018 | 15.519 | 9.595e+08 | 96 - 109 | 6 | 17.87 | 18.38 | 19.34 |
| 4922.9502 | YFTPN(N)KTEDTIF Hex 10 HexNAc 9 | 114.185 | 0.018 | -4.466 | 1.916e+07 | 67 - 79 | 1 | 34.91 | 35.08 | 35.25 |
| 4617.9012 | RNEEYN(N)KSVQEIQATFF Hex 8 HexNAc 6 | 113.143 | 0.018 | -6.011 | 2.724e+07 | 50 - 67 | 4 | 15.87 | 15.87 | 16.20 |
| 3840.5165 | FTPN(N)KTEDTIF Hex 7 HexNAc 4 Neu5Ac 2 | 111.825 | 0.021 | 13.873 | 2.418e+07 | 68 - 79 | 4 | 31.27 | 31.60 | 31.95 |
| 4339.8270 | LNVQREN(N)GTISRY Fuc 3 Hex 7 HexNAc 6 | 111.702 | 0.021 | 10.707 | 2.263e+08 | 96 - 109 | 4 | 18.38 | 18.55 | 19.03 |
| 3655.4711 | YFTPN(N)KTEDTIF Fuc 1 Hex 7 HexNAc 3 Neu5Ac 1 | 110.546 | 0.021 | -19.572 | 4.211e+06 | 67 - 79 | 1 | 38.59 | 38.59 | 38.76 |
| 4080.6931 | NVQREN(N)GTISRY Fuc 2 Hex 7 HexNAc 6 | 109.705 | 0.021 | -18.750 | 4.672e+07 | 97 - 109 | 2 | 13.12 | 13.12 | 13.28 |
| 4997.0528 | LNVQREN(N)GTISRY Hex 10 HexNAc 9 | 109.081 | 0.021 | -2.811 | 1.344e+08 | 96 - 109 | 3 | 17.87 | 18.04 | 18.38 |
| 3677.4608 | QTRQDQC(C)IYN(N)TTY Hex 6 HexNAc 5 | 108.720 | 0.021 | 14.584 | 1.023e+08 | 83 - 96 | 5 | 21.88 | 22.22 | 22.73 |
| 4558.9106 | LNVQREN(N)GTISRY Fuc 2 Hex 8 HexNAc 7 | 106.976 | 0.022 | -2.978 | 1.829e+08 | 96 - 109 | 3 | 18.04 | 18.21 | 18.55 |
| 3820.5899 | VQREN(N)GTISRY Fuc 1 Hex 7 HexNAc 6 | 106.292 | 0.022 | -21.350 | 4.415e+06 | 98 - 109 | 1 | 12.63 | 12.63 | 12.63 |
| 4777.9806 | LNVQREN(N)GTISRY Fuc 1 Hex 9 HexNAc 8 | 105.826 | 0.022 | 14.974 | 2.649e+08 | 96 - 109 | 2 | 17.87 | 18.21 | 18.55 |
| 3823.5199 | QTRQDQC(C)IYN(N)TTY Fuc 1 Hex 6 HexNAc 5 | 105.644 | 0.022 | 13.797 | 2.905e+07 | 83 - 96 | 3 | 21.54 | 22.05 | 22.56 |
| 4631.9261 | LNVQREN(N)GTISRY Hex 9 HexNAc 8 | 105.265 | 0.022 | 14.502 | 8.532e+08 | 96 - 109 | 5 | 17.70 | 18.21 | 19.34 |
| 4236.7714 | RN(D)EEYN(N)KSVQEIQATFF Fuc 1 Hex 6 HexNAc 5 | 105.015 | 0.024 | 10.324 | 2.039e+08 | 50 - 67 | 17 | 45.03 | 46.57 | 47.06 |
| 3674.5309 | VQREN(N)GTISRY Hex 7 HexNAc 6 | 104.820 | 0.025 | -19.877 | 2.213e+07 | 98 - 109 | 3 | 12.78 | 12.78 | 12.78 |
| 4518.8383 | NVQREN(N)GTISRY Hex 9 HexNAc 8 | 104.610 | 0.025 | -5.357 | 4.037e+07 | 97 - 109 | 3 | 12.95 | 12.95 | 13.28 |
| 4418.7712 | TPN(N)KTEDTIF Fuc 2 Hex 7 HexNAc 9 | 104.155 | 0.025 | -19.800 | 3.669e+06 | 69 - 79 | 1 | 46.90 | 46.90 | 46.90 |
| 4188.6543 | QTRQDQC(C)IYN(N)TTY Fuc 1 Hex 7 HexNAc 6 | 103.875 | 0.025 | 14.311 | 4.489e+07 | 83 - 96 | 4 | 21.36 | 21.71 | 22.22 |
| 4224.6964 | TPN(N)KTEDTIF Fuc 4 Hex 4 HexNAc 9 | 103.299 | 0.025 | -4.308 | 2.342e+07 | 69 - 79 | 2 | 48.91 | 48.91 | 49.42 |
| 4283.8118 | LN(D)VQREN(N)GTISRY Fuc 4 Hex 7 HexNAc 5 | 103.257 | 0.025 | -5.722 | 7.503e+07 | 96 - 109 | 1 | 18.21 | 18.38 | 18.71 |
| 4252.8088 | RNEEYN(N)KSVQEIQATFFY Hex 6 HexNAc 5 | 103.041 | 0.025 | -3.874 | 1.854e+08 | 50 - 68 | 2 | 43.37 | 43.54 | 43.87 |
| 3481.4479 | TPN(N)KTEDTIF Hex 3 HexNAc 9 | 102.328 | 0.026 | 0.000 | 0.000e+00 | 69 - 79 | 1 | - | - | - |
| 3960.6709 | RNEEYN(N)KSVQEIQATF Fuc 2 Hex 5 HexNAc 3 Neu5Ac 1 | 101.805 | 0.026 | -8.805 | 7.100e+06 | 50 - 66 | 5 | 34.23 | 34.23 | 34.74 |
| 3124.4019 | LVPVPITN(N)ATL Hex 6 HexNAc 5 | 101.594 | 0.028 | -20.717 | 3.473e+06 | 25 - 36 | 1 | 45.72 | 45.72 | 45.89 |
| 3537.5112 | VQREN(N)GTISRY Fuc 1 Hex 4 HexNAc 7 | 101.161 | 0.028 | 9.031 | 8.962e+07 | 98 - 109 | 21 | 20.51 | 20.51 | 21.54 |
| 4077.6301 | TPN(N)KTEDTIF Fuc 1 Hex 4 HexNAc 9 Neu5Ac 1 | 100.998 | 0.028 | -19.595 | 1.420e+07 | 69 - 79 | 2 | 38.08 | 38.08 | 38.25 |
| 4704.9647 | LNVQREN(N)GTISRY Fuc 3 Hex 8 HexNAc 7 | 100.387 | 0.031 | -4.446 | 7.019e+07 | 96 - 109 | 2 | 18.04 | 18.04 | 18.38 |
| 3469.5107 | C(C)ANLVPVPITN(N)ATL Hex 6 HexNAc 5 | 99.187 | 0.031 | 13.430 | 2.916e+07 | 22 - 36 | 5 | 50.28 | 50.45 | 51.13 |
| 4041.6757 | N(N)KSVQEIQATFF Fuc 3 Hex 6 HexNAc 6 | 98.319 | 0.032 | 0.000 | 0.000e+00 | 55 - 67 | 1 | - | - | - |
| 3965.6250 | NVQREN(N)GTISRY Fuc 2 Hex 7 HexNAc 4 Neu5Ac 1 | 98.238 | 0.034 | -5.127 | 1.954e+07 | 97 - 109 | 4 | 34.06 | 34.23 | 34.57 |
| 4818.9792 | RNEEYN(N)KSVQEIQATF Fuc 1 Hex 8 HexNAc 7 | 97.275 | 0.038 | -3.754 | 1.301e+07 | 50 - 66 | 2 | 31.60 | 31.77 | 31.95 |
| 4924.0378 | LNVQREN(N)GTISRY Fuc 2 Hex 9 HexNAc 8 | 96.989 | 0.039 | -3.018 | 9.744e+07 | 96 - 109 | 2 | 17.87 | 18.04 | 18.38 |
| 4326.6825 | TPN(N)KTEDTIF Fuc 3 Hex 10 HexNAc 4 Neu5Ac 1 | 95.989 | 0.042 | -4.278 | 1.974e+07 | 69 - 79 | 3 | 48.74 | 48.91 | 49.08 |
| 4795.0068 | LN(D)VQREN(N)GTISRY Fuc 5 Hex 8 HexNAc 6 | 95.794 | 0.042 | -6.535 | 4.218e+07 | 96 - 109 | 1 | 18.04 | 18.21 | 18.38 |
| 4077.6873 | LNVQREN(N)GTISRY Hex 7 HexNAc 4 Neu5Ac 2 | 95.356 | 0.042 | 14.422 | 3.889e+08 | 96 - 109 | 7 | 19.34 | 19.84 | 21.19 |
| 3981.6146 | LNVQREN(N)GTISRY Hex 10 HexNAc 4 | 93.880 | 0.043 | 11.201 | 2.776e+07 | 96 - 109 | 5 | 21.19 | 21.71 | 22.05 |
| 4226.7487 | NVQREN(N)GTISRY Fuc 3 Hex 7 HexNAc 6 | 91.767 | 0.048 | -20.996 | 6.002e+06 | 97 - 109 | 1 | 13.12 | 13.12 | 13.12 |

### TPN(N)KTEDTIF Hex 6 HexNAc 5

**Mass:**  3152.2820

(1.085e-06)

**Charge States:** 3, 4

**Start Position**: 70

**End Position**: 80

| Profile Score | Tandem Score | TDA q-value | Volume | Spectrum Matches |
| --- | --- | --- | --- | --- |
| 15.2062 | 182.0794 | 0.0000 | 1.9479e+08 | 9 |

**Adduct Signal Fractions**

- **Unmodified**: 1.9479e+08

### TPN(N)KTEDTIF Hex 7 HexNAc 6

**Mass:**  3517.4170

(9.486e-07)

**Charge States:** 3, 4

**Start Position**: 70

**End Position**: 80

| Profile Score | Tandem Score | TDA q-value | Volume | Spectrum Matches |
| --- | --- | --- | --- | --- |
| 15.6256 | 181.7918 | 0.0000 | 2.8104e+08 | 10 |

**Adduct Signal Fractions**

- **Unmodified**: 2.8104e+08

### TPN(N)KTEDTIF Hex 5 HexNAc 4

**Mass:**  2787.1527

(1.697e-06)

**Charge States:** 3

**Start Position**: 70

**End Position**: 80

| Profile Score | Tandem Score | TDA q-value | Volume | Spectrum Matches |
| --- | --- | --- | --- | --- |
| 14.2635 | 181.7775 | 0.0000 | 2.2118e+07 | 3 |

**Adduct Signal Fractions**

- **Unmodified**: 2.2118e+07

### N(N)KSVQEIQATF Hex 5 HexNAc 4

**Mass:**  2886.2318

(1.879e-06)

**Charge States:** 3

**Start Position**: 56

**End Position**: 67

| Profile Score | Tandem Score | TDA q-value | Volume | Spectrum Matches |
| --- | --- | --- | --- | --- |
| 14.0823 | 178.6837 | 0.0000 | 2.5141e+07 | 2 |

**Adduct Signal Fractions**

- **Unmodified**: 2.5141e+07

### YFTPN(N)KTEDTIF Hex 7 HexNAc 6

**Mass:**  3827.5453

(2.124e-06)

**Charge States:** 3, 4

**Start Position**: 68

**End Position**: 80

| Profile Score | Tandem Score | TDA q-value | Volume | Spectrum Matches |
| --- | --- | --- | --- | --- |
| 14.9178 | 177.2185 | 0.0000 | 4.5798e+09 | 52 |

**Adduct Signal Fractions**

- **Unmodified**: 4.5798e+09

### YFTPN(N)KTEDTIF Fuc 1 Hex 7 HexNAc 6

**Mass:**  3973.6052

(2.025e-06)

**Charge States:** 3, 4

**Start Position**: 68

**End Position**: 80

| Profile Score | Tandem Score | TDA q-value | Volume | Spectrum Matches |
| --- | --- | --- | --- | --- |
| 14.8042 | 176.4260 | 0.0000 | 8.8997e+08 | 8 |

**Adduct Signal Fractions**

- **Unmodified**: 8.8997e+08

### YFTPN(N)KTEDTIF Hex 5 HexNAc 4

**Mass:**  3097.2819

(2.442e-07)

**Charge States:** 2, 3

**Start Position**: 68

**End Position**: 80

| Profile Score | Tandem Score | TDA q-value | Volume | Spectrum Matches |
| --- | --- | --- | --- | --- |
| 15.2378 | 174.5574 | 0.0000 | 3.7855e+08 | 5 |

**Adduct Signal Fractions**

- **Unmodified**: 3.7855e+08

### YFTPN(N)KTEDTIF Hex 6 HexNAc 5

**Mass:**  3462.4143

(8.840e-07)

**Charge States:** 2, 3, 4

**Start Position**: 68

**End Position**: 80

| Profile Score | Tandem Score | TDA q-value | Volume | Spectrum Matches |
| --- | --- | --- | --- | --- |
| 14.1726 | 174.2428 | 0.0000 | 2.8950e+09 | 28 |

**Adduct Signal Fractions**

- **Unmodified**: 2.8950e+09

### FTPN(N)KTEDTIF Hex 6 HexNAc 5

**Mass:**  3299.3498

(6.490e-07)

**Charge States:** 3, 4

**Start Position**: 69

**End Position**: 80

| Profile Score | Tandem Score | TDA q-value | Volume | Spectrum Matches |
| --- | --- | --- | --- | --- |
| 15.0362 | 173.0391 | 0.0000 | 4.0949e+08 | 12 |

**Adduct Signal Fractions**

- **Unmodified**: 4.0949e+08

### EEYN(N)KSVQEIQATF Hex 5 HexNAc 4

**Mass:**  3307.3804

(4.293e-07)

**Charge States:** 3

**Start Position**: 53

**End Position**: 67

| Profile Score | Tandem Score | TDA q-value | Volume | Spectrum Matches |
| --- | --- | --- | --- | --- |
| 12.1693 | 170.8901 | 0.0000 | 8.5366e+07 | 2 |

**Adduct Signal Fractions**

- **Unmodified**: 8.5366e+07

### TPN(N)KTEDTIF Fuc 1 Hex 6 HexNAc 5

**Mass:**  3298.3430

(1.570e-06)

**Charge States:** 3, 4

**Start Position**: 70

**End Position**: 80

| Profile Score | Tandem Score | TDA q-value | Volume | Spectrum Matches |
| --- | --- | --- | --- | --- |
| 13.4078 | 170.5870 | 0.0000 | 3.1141e+07 | 4 |

**Adduct Signal Fractions**

- **Unmodified**: 3.1141e+07

### YFTPN(N)KTEDTIF Fuc 4 Hex 7 HexNAc 7

**Mass:**  4614.8342

(-3.773e-06)

**Charge States:** 3, 4

**Start Position**: 68

**End Position**: 80

| Profile Score | Tandem Score | TDA q-value | Volume | Spectrum Matches |
| --- | --- | --- | --- | --- |
| 15.6074 | 169.3788 | 0.0000 | 8.1892e+07 | 3 |

**Adduct Signal Fractions**

- **Unmodified**: 8.1892e+07

### FYFTPN(N)KTEDTIF Hex 6 HexNAc 5

**Mass:**  3609.4843

(1.898e-06)

**Charge States:** 3, 4

**Start Position**: 67

**End Position**: 80

| Profile Score | Tandem Score | TDA q-value | Volume | Spectrum Matches |
| --- | --- | --- | --- | --- |
| 14.7594 | 168.6670 | 0.0000 | 2.7860e+09 | 49 |

**Adduct Signal Fractions**

- **Unmodified**: 2.7860e+09

### FTPN(N)KTEDTIF Hex 7 HexNAc 6

**Mass:**  3664.4848

(1.249e-06)

**Charge States:** 3, 4

**Start Position**: 69

**End Position**: 80

| Profile Score | Tandem Score | TDA q-value | Volume | Spectrum Matches |
| --- | --- | --- | --- | --- |
| 16.2646 | 168.0623 | 0.0000 | 6.6287e+08 | 13 |

**Adduct Signal Fractions**

- **Unmodified**: 6.6287e+08

### YFTPN(N)KTEDTIF Fuc 1 Hex 6 HexNAc 5

**Mass:**  3608.4746

(1.906e-06)

**Charge States:** 3, 4

**Start Position**: 68

**End Position**: 80

| Profile Score | Tandem Score | TDA q-value | Volume | Spectrum Matches |
| --- | --- | --- | --- | --- |
| 14.3167 | 167.9220 | 0.0000 | 5.9993e+08 | 7 |

**Adduct Signal Fractions**

- **Unmodified**: 5.9993e+08

### FYFTPN(N)KTEDTIF Fuc 1 Hex 6 HexNAc 5

**Mass:**  3755.5453

(1.912e-06)

**Charge States:** 3, 4

**Start Position**: 67

**End Position**: 80

| Profile Score | Tandem Score | TDA q-value | Volume | Spectrum Matches |
| --- | --- | --- | --- | --- |
| 14.5482 | 167.3858 | 0.0000 | 5.6886e+08 | 11 |

**Adduct Signal Fractions**

- **Unmodified**: 5.6886e+08

### TPN(N)KTEDTIF Fuc 1 Hex 7 HexNAc 6

**Mass:**  3663.4755

(1.464e-06)

**Charge States:** 3, 4

**Start Position**: 70

**End Position**: 80

| Profile Score | Tandem Score | TDA q-value | Volume | Spectrum Matches |
| --- | --- | --- | --- | --- |
| 13.7494 | 167.3572 | 0.0000 | 4.4044e+07 | 4 |

**Adduct Signal Fractions**

- **Unmodified**: 4.4044e+07

### FYFTPN(N)KTEDTIF Fuc 1 Hex 7 HexNAc 6

**Mass:**  4120.6809

(2.247e-06)

**Charge States:** 3, 4

**Start Position**: 67

**End Position**: 80

| Profile Score | Tandem Score | TDA q-value | Volume | Spectrum Matches |
| --- | --- | --- | --- | --- |
| 14.3982 | 166.3353 | 0.0000 | 8.7767e+08 | 12 |

**Adduct Signal Fractions**

- **Unmodified**: 8.7767e+08

### YFTPN(N)KTEDTIF Hex 8 HexNAc 7

**Mass:**  4192.6833

(1.274e-06)

**Charge States:** 3, 4

**Start Position**: 68

**End Position**: 80

| Profile Score | Tandem Score | TDA q-value | Volume | Spectrum Matches |
| --- | --- | --- | --- | --- |
| 15.2448 | 165.8290 | 0.0000 | 7.6266e+08 | 11 |

**Adduct Signal Fractions**

- **Unmodified**: 7.6266e+08

### FYFTPN(N)KTEDTIF Hex 7 HexNAc 6

**Mass:**  3974.6226

(2.317e-06)

**Charge States:** 3, 4

**Start Position**: 67

**End Position**: 80

| Profile Score | Tandem Score | TDA q-value | Volume | Spectrum Matches |
| --- | --- | --- | --- | --- |
| 15.2894 | 165.6542 | 0.0000 | 4.7166e+09 | 82 |

**Adduct Signal Fractions**

- **Unmodified**: 4.7166e+09

### FYFTPN(N)KTEDTIF Hex 8 HexNAc 7

**Mass:**  4339.7546

(1.751e-06)

**Charge States:** 3, 4

**Start Position**: 67

**End Position**: 80

| Profile Score | Tandem Score | TDA q-value | Volume | Spectrum Matches |
| --- | --- | --- | --- | --- |
| 14.7801 | 165.2795 | 0.0000 | 6.3863e+08 | 9 |

**Adduct Signal Fractions**

- **Unmodified**: 6.3863e+08

### FYFTPN(N)KTEDTIF Fuc 2 Hex 7 HexNAc 6

**Mass:**  4266.7375

(2.138e-06)

**Charge States:** 4

**Start Position**: 67

**End Position**: 80

| Profile Score | Tandem Score | TDA q-value | Volume | Spectrum Matches |
| --- | --- | --- | --- | --- |
| -16.1994 | 164.1765 | 0.0000 | 1.1875e+08 | 2 |

**Adduct Signal Fractions**

- **Unmodified**: 1.1875e+08

### TPN(N)KTEDTIF Hex 8 HexNAc 7

**Mass:**  3882.5473

(1.107e-06)

**Charge States:** 3, 4

**Start Position**: 70

**End Position**: 80

| Profile Score | Tandem Score | TDA q-value | Volume | Spectrum Matches |
| --- | --- | --- | --- | --- |
| -4.5229 | 163.9402 | 0.0000 | 2.1186e+07 | 4 |

**Adduct Signal Fractions**

- **Unmodified**: 2.1186e+07

### RNEEYN(N)KSVQEIQATFF Fuc 1 Hex 6 HexNAc 5

**Mass:**  4235.7936

(7.762e-07)

**Charge States:** 3, 4

**Start Position**: 51

**End Position**: 68

| Profile Score | Tandem Score | TDA q-value | Volume | Spectrum Matches |
| --- | --- | --- | --- | --- |
| 15.6710 | 162.0343 | 0.0000 | 1.9278e+09 | 16 |

**Adduct Signal Fractions**

- **Unmodified**: 1.9278e+09

### FYFTPN(N)KTEDTIF Hex 5 HexNAc 4

**Mass:**  3244.3511

(9.271e-07)

**Charge States:** 3

**Start Position**: 67

**End Position**: 80

| Profile Score | Tandem Score | TDA q-value | Volume | Spectrum Matches |
| --- | --- | --- | --- | --- |
| 10.7671 | 161.9809 | 0.0000 | 2.2235e+08 | 4 |

**Adduct Signal Fractions**

- **Unmodified**: 2.2235e+08

### N(N)KTEDTIF Hex 7 HexNAc 6

**Mass:**  3319.3123

(1.737e-07)

**Charge States:** 3

**Start Position**: 72

**End Position**: 80

| Profile Score | Tandem Score | TDA q-value | Volume | Spectrum Matches |
| --- | --- | --- | --- | --- |
| -20.2355 | 161.4047 | 0.0000 | 3.2172e+06 | 1 |

**Adduct Signal Fractions**

- **Unmodified**: 3.2172e+06

### RNEEYN(N)KSVQEIQATFF Hex 6 HexNAc 5

**Mass:**  4089.7255

(2.978e-06)

**Charge States:** 3, 4

**Start Position**: 51

**End Position**: 68

| Profile Score | Tandem Score | TDA q-value | Volume | Spectrum Matches |
| --- | --- | --- | --- | --- |
| 16.6898 | 161.0298 | 0.0000 | 3.9484e+09 | 19 |

**Adduct Signal Fractions**

- **Unmodified**: 3.9484e+09

### YFTPN(N)KTEDTIF Fuc 2 Hex 7 HexNAc 6

**Mass:**  4119.6682

(1.994e-06)

**Charge States:** 3, 4

**Start Position**: 68

**End Position**: 80

| Profile Score | Tandem Score | TDA q-value | Volume | Spectrum Matches |
| --- | --- | --- | --- | --- |
| -4.1040 | 160.2445 | 0.0000 | 1.2720e+08 | 2 |

**Adduct Signal Fractions**

- **Unmodified**: 1.2720e+08

### YFTPN(N)KTEDTIF Hex 9 HexNAc 8

**Mass:**  4557.8166

(1.831e-06)

**Charge States:** 3, 4

**Start Position**: 68

**End Position**: 80

| Profile Score | Tandem Score | TDA q-value | Volume | Spectrum Matches |
| --- | --- | --- | --- | --- |
| 15.3983 | 159.9471 | 0.0000 | 2.3039e+08 | 6 |

**Adduct Signal Fractions**

- **Unmodified**: 2.3039e+08

### EEYN(N)KSVQEIQATF Hex 6 HexNAc 5

**Mass:**  3672.5121

(1.397e-06)

**Charge States:** 3

**Start Position**: 53

**End Position**: 67

| Profile Score | Tandem Score | TDA q-value | Volume | Spectrum Matches |
| --- | --- | --- | --- | --- |
| -3.1992 | 158.8277 | 0.0000 | 8.1237e+07 | 3 |

**Adduct Signal Fractions**

- **Unmodified**: 8.1237e+07

### RNEEYN(N)KSVQEIQATF Fuc 1 Hex 5 HexNAc 4

**Mass:**  3723.5857

(1.928e-06)

**Charge States:** 3, 4

**Start Position**: 51

**End Position**: 67

| Profile Score | Tandem Score | TDA q-value | Volume | Spectrum Matches |
| --- | --- | --- | --- | --- |
| 11.2212 | 158.2511 | 0.0000 | 3.3473e+08 | 9 |

**Adduct Signal Fractions**

- **Unmodified**: 3.3473e+08

### FTPN(N)KTEDTIF Fuc 1 Hex 6 HexNAc 5

**Mass:**  3445.4101

(3.906e-07)

**Charge States:** 3, 4

**Start Position**: 69

**End Position**: 80

| Profile Score | Tandem Score | TDA q-value | Volume | Spectrum Matches |
| --- | --- | --- | --- | --- |
| 13.4290 | 158.2472 | 0.0000 | 7.4120e+07 | 5 |

**Adduct Signal Fractions**

- **Unmodified**: 7.4120e+07

### FYFTPN(N)KT Hex 6 HexNAc 5

**Mass:**  3004.2171

(2.479e-06)

**Charge States:** 3

**Start Position**: 67

**End Position**: 75

| Profile Score | Tandem Score | TDA q-value | Volume | Spectrum Matches |
| --- | --- | --- | --- | --- |
| -4.0041 | 156.8222 | 0.0000 | 6.7236e+06 | 2 |

**Adduct Signal Fractions**

- **Unmodified**: 6.7236e+06

### RNEEYN(N)KSVQEIQATF Hex 5 HexNAc 4

**Mass:**  3577.5243

(1.699e-06)

**Charge States:** 3, 4

**Start Position**: 51

**End Position**: 67

| Profile Score | Tandem Score | TDA q-value | Volume | Spectrum Matches |
| --- | --- | --- | --- | --- |
| 16.1774 | 156.5592 | 0.0000 | 8.4197e+09 | 22 |

**Adduct Signal Fractions**

- **Unmodified**: 8.4197e+09

### FYFTPN(N)KTEDTIF Fuc 1 Hex 8 HexNAc 7

**Mass:**  4485.8098

(1.898e-06)

**Charge States:** 3, 4

**Start Position**: 67

**End Position**: 80

| Profile Score | Tandem Score | TDA q-value | Volume | Spectrum Matches |
| --- | --- | --- | --- | --- |
| 14.8512 | 156.4959 | 0.0000 | 1.3093e+08 | 6 |

**Adduct Signal Fractions**

- **Unmodified**: 1.3093e+08

### RNEEYN(N)KSVQEIQATFF Hex 5 HexNAc 4

**Mass:**  3724.5928

(8.245e-07)

**Charge States:** 3, 4

**Start Position**: 51

**End Position**: 68

| Profile Score | Tandem Score | TDA q-value | Volume | Spectrum Matches |
| --- | --- | --- | --- | --- |
| 15.9918 | 156.3606 | 0.0000 | 4.1767e+09 | 17 |

**Adduct Signal Fractions**

- **Unmodified**: 4.1767e+09

### EEYN(N)KSVQEIQATF Fuc 1 Hex 6 HexNAc 5

**Mass:**  3818.5717

(1.417e-06)

**Charge States:** 3

**Start Position**: 53

**End Position**: 67

| Profile Score | Tandem Score | TDA q-value | Volume | Spectrum Matches |
| --- | --- | --- | --- | --- |
| -4.0463 | 155.4792 | 0.0000 | 3.4096e+07 | 2 |

**Adduct Signal Fractions**

- **Unmodified**: 3.4096e+07

### EEYN(N)KSVQEIQATFF Hex 5 HexNAc 4

**Mass:**  3454.4448

(6.809e-08)

**Charge States:** 3

**Start Position**: 53

**End Position**: 68

| Profile Score | Tandem Score | TDA q-value | Volume | Spectrum Matches |
| --- | --- | --- | --- | --- |
| -4.9809 | 155.2356 | 0.0000 | 2.7311e+07 | 1 |

**Adduct Signal Fractions**

- **Unmodified**: 2.7311e+07

### EEYN(N)KSVQEIQATFF Hex 6 HexNAc 5

**Mass:**  3819.5806

(2.012e-06)

**Charge States:** 3, 4

**Start Position**: 53

**End Position**: 68

| Profile Score | Tandem Score | TDA q-value | Volume | Spectrum Matches |
| --- | --- | --- | --- | --- |
| -5.6259 | 154.9731 | 0.0000 | 2.6073e+07 | 2 |

**Adduct Signal Fractions**

- **Unmodified**: 2.6073e+07

### RNEEYN(N)KSVQEIQATFF Fuc 2 Hex 6 HexNAc 5

**Mass:**  4381.8478

(1.511e-06)

**Charge States:** 4

**Start Position**: 51

**End Position**: 68

| Profile Score | Tandem Score | TDA q-value | Volume | Spectrum Matches |
| --- | --- | --- | --- | --- |
| -3.7138 | 153.7672 | 0.0000 | 7.3407e+07 | 3 |

**Adduct Signal Fractions**

- **Unmodified**: 7.3407e+07

### RNEEYN(N)KSVQEIQATF Hex 6 HexNAc 5

**Mass:**  3942.6577

(1.717e-06)

**Charge States:** 3, 4

**Start Position**: 51

**End Position**: 67

| Profile Score | Tandem Score | TDA q-value | Volume | Spectrum Matches |
| --- | --- | --- | --- | --- |
| 15.0098 | 153.5112 | 0.0000 | 6.0077e+09 | 20 |

**Adduct Signal Fractions**

- **Unmodified**: 6.0077e+09

### RNEEYN(N)KSVQEIQATFF Fuc 1 Hex 5 HexNAc 4

**Mass:**  3870.6619

(2.368e-06)

**Charge States:** 3, 4

**Start Position**: 51

**End Position**: 68

| Profile Score | Tandem Score | TDA q-value | Volume | Spectrum Matches |
| --- | --- | --- | --- | --- |
| 13.1317 | 153.0097 | 0.0000 | 1.5337e+08 | 5 |

**Adduct Signal Fractions**

- **Unmodified**: 1.5337e+08

### YFTPN(N)KTEDTIF Fuc 1 Hex 8 HexNAc 4 Neu5Ac 1

**Mass:**  4020.6059

(2.924e-06)

**Charge States:** 3, 4

**Start Position**: 68

**End Position**: 80

| Profile Score | Tandem Score | TDA q-value | Volume | Spectrum Matches |
| --- | --- | --- | --- | --- |
| -4.5260 | 152.8367 | 0.0000 | 5.3739e+07 | 3 |

**Adduct Signal Fractions**

- **Unmodified**: 5.3739e+07

### YFTPN(N)KTEDTIF Fuc 1 Hex 8 HexNAc 7

**Mass:**  4338.7419

(7.933e-07)

**Charge States:** 3, 4

**Start Position**: 68

**End Position**: 80

| Profile Score | Tandem Score | TDA q-value | Volume | Spectrum Matches |
| --- | --- | --- | --- | --- |
| 14.6827 | 152.4270 | 0.0000 | 1.4490e+08 | 5 |

**Adduct Signal Fractions**

- **Unmodified**: 1.4490e+08

### YFTPN(N)KTEDTIF Hex 7 HexNAc 4 Neu5Ac 2

**Mass:**  4003.5807

(9.676e-07)

**Charge States:** 3, 4

**Start Position**: 68

**End Position**: 80

| Profile Score | Tandem Score | TDA q-value | Volume | Spectrum Matches |
| --- | --- | --- | --- | --- |
| 14.5502 | 152.3472 | 0.0000 | 3.3362e+08 | 6 |

**Adduct Signal Fractions**

- **Unmodified**: 3.3362e+08

### RNEEYN(N)KSVQEIQATF Fuc 1 Hex 6 HexNAc 5

**Mass:**  4088.7153

(1.681e-06)

**Charge States:** 3, 4

**Start Position**: 51

**End Position**: 67

| Profile Score | Tandem Score | TDA q-value | Volume | Spectrum Matches |
| --- | --- | --- | --- | --- |
| 14.5867 | 152.3287 | 0.0000 | 2.9510e+09 | 16 |

**Adduct Signal Fractions**

- **Unmodified**: 2.9510e+09

### FTPN(N)KTEDTIF Fuc 1 Hex 7 HexNAc 6

**Mass:**  3810.5421

(1.297e-06)

**Charge States:** 3, 4

**Start Position**: 69

**End Position**: 80

| Profile Score | Tandem Score | TDA q-value | Volume | Spectrum Matches |
| --- | --- | --- | --- | --- |
| 13.6241 | 151.2150 | 0.0000 | 1.1906e+08 | 6 |

**Adduct Signal Fractions**

- **Unmodified**: 1.1906e+08

### RNEEYN(N)KSVQEIQATFF Hex 7 HexNAc 6

**Mass:**  4454.8581

(1.209e-06)

**Charge States:** 3, 4

**Start Position**: 51

**End Position**: 68

| Profile Score | Tandem Score | TDA q-value | Volume | Spectrum Matches |
| --- | --- | --- | --- | --- |
| 14.9018 | 150.9494 | 0.0000 | 1.8544e+08 | 4 |

**Adduct Signal Fractions**

- **Unmodified**: 1.8544e+08

### RNEEYN(N)KSVQEIQATF Hex 7 HexNAc 6

**Mass:**  4307.7927

(1.279e-06)

**Charge States:** 3, 4

**Start Position**: 51

**End Position**: 67

| Profile Score | Tandem Score | TDA q-value | Volume | Spectrum Matches |
| --- | --- | --- | --- | --- |
| -2.6482 | 150.5649 | 0.0000 | 2.8955e+08 | 3 |

**Adduct Signal Fractions**

- **Unmodified**: 2.8955e+08

### RNEEYN(N)KSVQEIQATF Fuc 2 Hex 6 HexNAc 5

**Mass:**  4234.7780

(2.383e-06)

**Charge States:** 4

**Start Position**: 51

**End Position**: 67

| Profile Score | Tandem Score | TDA q-value | Volume | Spectrum Matches |
| --- | --- | --- | --- | --- |
| -3.6005 | 150.4575 | 0.0000 | 9.7249e+07 | 3 |

**Adduct Signal Fractions**

- **Unmodified**: 9.7249e+07

### YFTPN(N)KT Hex 7 HexNAc 6

**Mass:**  3222.2737

(-2.187e-07)

**Charge States:** 3

**Start Position**: 68

**End Position**: 75

| Profile Score | Tandem Score | TDA q-value | Volume | Spectrum Matches |
| --- | --- | --- | --- | --- |
| -20.6750 | 150.0115 | 0.0000 | 3.3539e+06 | 1 |

**Adduct Signal Fractions**

- **Unmodified**: 3.3539e+06

### RNEEYN(N)KSVQEIQATFF Fuc 1 Hex 7 HexNAc 6

**Mass:**  4600.9162

(1.143e-06)

**Charge States:** 3, 4

**Start Position**: 51

**End Position**: 68

| Profile Score | Tandem Score | TDA q-value | Volume | Spectrum Matches |
| --- | --- | --- | --- | --- |
| 14.8105 | 149.9970 | 0.0000 | 6.8100e+07 | 3 |

**Adduct Signal Fractions**

- **Unmodified**: 6.8100e+07

### RNEEYN(N)KSVQEIQATF Fuc 1 Hex 7 HexNAc 6

**Mass:**  4453.8513

(1.544e-06)

**Charge States:** 4

**Start Position**: 51

**End Position**: 67

| Profile Score | Tandem Score | TDA q-value | Volume | Spectrum Matches |
| --- | --- | --- | --- | --- |
| -3.4806 | 149.7379 | 0.0000 | 1.2881e+08 | 2 |

**Adduct Signal Fractions**

- **Unmodified**: 1.2881e+08

### FTPN(N)KTEDTIF Hex 5 HexNAc 4

**Mass:**  2934.2190

(1.669e-06)

**Charge States:** 3

**Start Position**: 69

**End Position**: 80

| Profile Score | Tandem Score | TDA q-value | Volume | Spectrum Matches |
| --- | --- | --- | --- | --- |
| -3.4927 | 149.6019 | 0.0000 | 4.0119e+07 | 3 |

**Adduct Signal Fractions**

- **Unmodified**: 4.0119e+07

### LNVQREN(N)GTISRY Fuc 1 Hex 6 HexNAc 5

**Mass:**  3682.5756

(1.021e-06)

**Charge States:** 3, 4

**Start Position**: 97

**End Position**: 110

| Profile Score | Tandem Score | TDA q-value | Volume | Spectrum Matches |
| --- | --- | --- | --- | --- |
| 14.5706 | 149.3716 | 0.0000 | 1.2066e+09 | 11 |

**Adduct Signal Fractions**

- **Unmodified**: 1.2066e+09

### TPN(N)KTEDTIF Fuc 4 Hex 8 HexNAc 5

**Mass:**  4060.6027

(-2.955e-06)

**Charge States:** 3, 4

**Start Position**: 70

**End Position**: 80

| Profile Score | Tandem Score | TDA q-value | Volume | Spectrum Matches |
| --- | --- | --- | --- | --- |
| 13.0799 | 148.1229 | 0.0000 | 1.3763e+08 | 4 |

**Adduct Signal Fractions**

- **Unmodified**: 1.3763e+08

### FYFTPN(N)KTEDTIF Hex 9 HexNAc 8

**Mass:**  4704.8850

(2.005e-06)

**Charge States:** 3, 4

**Start Position**: 67

**End Position**: 80

| Profile Score | Tandem Score | TDA q-value | Volume | Spectrum Matches |
| --- | --- | --- | --- | --- |
| 16.7934 | 147.3900 | 0.0000 | 1.7216e+08 | 6 |

**Adduct Signal Fractions**

- **Unmodified**: 1.7216e+08

### FYFTPN(N)KTEDTIF Hex 7 HexNAc 4 Neu5Ac 2

**Mass:**  4150.6501

(2.291e-06)

**Charge States:** 3, 4

**Start Position**: 67

**End Position**: 80

| Profile Score | Tandem Score | TDA q-value | Volume | Spectrum Matches |
| --- | --- | --- | --- | --- |
| 13.4904 | 146.1667 | 0.0000 | 2.5214e+08 | 5 |

**Adduct Signal Fractions**

- **Unmodified**: 2.5214e+08

### FYFTPN(N)KT Hex 7 HexNAc 6

**Mass:**  3369.3477

(2.075e-06)

**Charge States:** 3

**Start Position**: 67

**End Position**: 75

| Profile Score | Tandem Score | TDA q-value | Volume | Spectrum Matches |
| --- | --- | --- | --- | --- |
| 12.8887 | 145.9008 | 0.0000 | 1.3344e+07 | 2 |

**Adduct Signal Fractions**

- **Unmodified**: 1.3344e+07

### FYFTPN(N)KTED Hex 7 HexNAc 6

**Mass:**  3613.4177

(1.538e-06)

**Charge States:** 3

**Start Position**: 67

**End Position**: 77

| Profile Score | Tandem Score | TDA q-value | Volume | Spectrum Matches |
| --- | --- | --- | --- | --- |
| -18.0710 | 144.9568 | 0.0000 | 5.2266e+06 | 1 |

**Adduct Signal Fractions**

- **Unmodified**: 5.2266e+06

### LNVQREN(N)GTISRY Hex 6 HexNAc 5

**Mass:**  3536.5204

(1.580e-06)

**Charge States:** 3, 4

**Start Position**: 97

**End Position**: 110

| Profile Score | Tandem Score | TDA q-value | Volume | Spectrum Matches |
| --- | --- | --- | --- | --- |
| 15.8084 | 143.3262 | 0.0000 | 3.2971e+09 | 11 |

**Adduct Signal Fractions**

- **Unmodified**: 3.2971e+09

### RNEEYN(N)KSVQEIQAT Hex 5 HexNAc 4

**Mass:**  3430.4562

(-1.357e-06)

**Charge States:** 3

**Start Position**: 51

**End Position**: 66

| Profile Score | Tandem Score | TDA q-value | Volume | Spectrum Matches |
| --- | --- | --- | --- | --- |
| -4.6074 | 142.5895 | 0.0000 | 3.4182e+07 | 3 |

**Adduct Signal Fractions**

- **Unmodified**: 3.4182e+07

### TPN(N)KTEDTIF Fuc 3 Hex 3 HexNAc 9 Neu5Ac 1

**Mass:**  4207.6711

(-9.137e-06)

**Charge States:** 3, 4

**Start Position**: 70

**End Position**: 80

| Profile Score | Tandem Score | TDA q-value | Volume | Spectrum Matches |
| --- | --- | --- | --- | --- |
| 13.9395 | 140.9425 | 0.0000 | 1.0207e+08 | 4 |

**Adduct Signal Fractions**

- **Unmodified**: 1.0207e+08

### TPN(N)KTEDTIF Hex 7 HexNAc 4 Neu5Ac 2

**Mass:**  3693.4473

(2.864e-07)

**Charge States:** 3, 4

**Start Position**: 70

**End Position**: 80

| Profile Score | Tandem Score | TDA q-value | Volume | Spectrum Matches |
| --- | --- | --- | --- | --- |
| -4.2797 | 138.6748 | 0.0000 | 9.8711e+06 | 3 |

**Adduct Signal Fractions**

- **Unmodified**: 9.8711e+06

### FRNEEYN(N)KSVQEIQATF Hex 6 HexNAc 5

**Mass:**  4089.7226

(4.392e-07)

**Charge States:** 3, 4

**Start Position**: 50

**End Position**: 67

| Profile Score | Tandem Score | TDA q-value | Volume | Spectrum Matches |
| --- | --- | --- | --- | --- |
| -5.3951 | 138.0329 | 0.0000 | 4.0851e+07 | 1 |

**Adduct Signal Fractions**

- **Unmodified**: 4.0851e+07

### YFTPN(N)KTEDTIF Hex 3 HexNAc 5 Neu5Ac 3

**Mass:**  3849.5238

(-2.743e-06)

**Charge States:** 3, 4

**Start Position**: 68

**End Position**: 80

| Profile Score | Tandem Score | TDA q-value | Volume | Spectrum Matches |
| --- | --- | --- | --- | --- |
| 12.5131 | 138.0208 | 0.0000 | 3.0882e+08 | 2 |

**Adduct Signal Fractions**

- **Unmodified**: 3.0882e+08

### RNEEYN(N)KSVQEIQATF Hex 8 HexNAc 7

**Mass:**  4672.9230

(1.458e-06)

**Charge States:** 4

**Start Position**: 51

**End Position**: 67

| Profile Score | Tandem Score | TDA q-value | Volume | Spectrum Matches |
| --- | --- | --- | --- | --- |
| -3.1043 | 137.5435 | 0.0000 | 5.0466e+07 | 2 |

**Adduct Signal Fractions**

- **Unmodified**: 5.0466e+07

### RN(D)EEYN(N)KSVQEIQATF Hex 6 HexNAc 5

**Mass:**  3943.6440

(2.510e-04)

**Charge States:** 3, 4

**Start Position**: 51

**End Position**: 67

| Profile Score | Tandem Score | TDA q-value | Volume | Spectrum Matches |
| --- | --- | --- | --- | --- |
| 11.6261 | 135.6777 | 0.0000 | 1.2109e+09 | 25 |

**Adduct Signal Fractions**

- **Unmodified**: 1.2109e+09

### YFTPN(N)KTEDTIF Fuc 1 Hex 7 HexNAc 4 Neu5Ac 2

**Mass:**  4149.6414

(1.799e-06)

**Charge States:** 3

**Start Position**: 68

**End Position**: 80

| Profile Score | Tandem Score | TDA q-value | Volume | Spectrum Matches |
| --- | --- | --- | --- | --- |
| -5.4808 | 134.6943 | 0.0000 | 3.9710e+07 | 2 |

**Adduct Signal Fractions**

- **Unmodified**: 3.9710e+07

### NVQREN(N)GTISRY Fuc 1 Hex 7 HexNAc 6

**Mass:**  3934.6334

(3.668e-06)

**Charge States:** 3, 4

**Start Position**: 98

**End Position**: 110

| Profile Score | Tandem Score | TDA q-value | Volume | Spectrum Matches |
| --- | --- | --- | --- | --- |
| -3.7698 | 133.6917 | 0.0000 | 1.4783e+08 | 4 |

**Adduct Signal Fractions**

- **Unmodified**: 1.4783e+08

### RN(D)EEYN(N)KSVQEIQATF Hex 5 HexNAc 4

**Mass:**  3578.5072

(2.769e-04)

**Charge States:** 3, 4

**Start Position**: 51

**End Position**: 67

| Profile Score | Tandem Score | TDA q-value | Volume | Spectrum Matches |
| --- | --- | --- | --- | --- |
| 11.4898 | 132.3689 | 0.0000 | 1.2915e+09 | 12 |

**Adduct Signal Fractions**

- **Unmodified**: 1.2915e+09

### FRNEEYN(N)KSVQEIQATF Hex 5 HexNAc 4

**Mass:**  3724.5877

(-2.436e-07)

**Charge States:** 4

**Start Position**: 50

**End Position**: 67

| Profile Score | Tandem Score | TDA q-value | Volume | Spectrum Matches |
| --- | --- | --- | --- | --- |
| -5.1128 | 131.8802 | 0.0000 | 3.1037e+07 | 8 |

**Adduct Signal Fractions**

- **Unmodified**: 3.1037e+07

### FTPN(N)KTEDTIF Hex 8 HexNAc 7

**Mass:**  4029.6188

(1.096e-06)

**Charge States:** 3, 4

**Start Position**: 69

**End Position**: 80

| Profile Score | Tandem Score | TDA q-value | Volume | Spectrum Matches |
| --- | --- | --- | --- | --- |
| 15.8046 | 129.6700 | 0.0030 | 5.1817e+07 | 5 |

**Adduct Signal Fractions**

- **Unmodified**: 5.1817e+07

### FYFTPN(N)KTEDTIF Fuc 1 Hex 7 HexNAc 4 Neu5Ac 2

**Mass:**  4296.7121

(1.010e-06)

**Charge States:** 3

**Start Position**: 67

**End Position**: 80

| Profile Score | Tandem Score | TDA q-value | Volume | Spectrum Matches |
| --- | --- | --- | --- | --- |
| -3.6277 | 129.4679 | 0.0030 | 4.0485e+07 | 2 |

**Adduct Signal Fractions**

- **Unmodified**: 4.0485e+07

### TPN(N)KTEDTIF Hex 9 HexNAc 5

**Mass:**  3638.4468

(2.270e-06)

**Charge States:** 3

**Start Position**: 70

**End Position**: 80

| Profile Score | Tandem Score | TDA q-value | Volume | Spectrum Matches |
| --- | --- | --- | --- | --- |
| 12.5452 | 128.6801 | 0.0030 | 3.4018e+07 | 3 |

**Adduct Signal Fractions**

- **Unmodified**: 3.4018e+07

### YFTPN(N)KTEDTIF Fuc 1 Hex 9 HexNAc 8

**Mass:**  4703.8747

(1.771e-06)

**Charge States:** 4

**Start Position**: 68

**End Position**: 80

| Profile Score | Tandem Score | TDA q-value | Volume | Spectrum Matches |
| --- | --- | --- | --- | --- |
| 14.0031 | 128.3574 | 0.0030 | 3.9069e+07 | 3 |

**Adduct Signal Fractions**

- **Unmodified**: 3.9069e+07

### NVQREN(N)GTISRY Fuc 1 Hex 6 HexNAc 5

**Mass:**  3569.4955

(5.546e-07)

**Charge States:** 3

**Start Position**: 98

**End Position**: 110

| Profile Score | Tandem Score | TDA q-value | Volume | Spectrum Matches |
| --- | --- | --- | --- | --- |
| -4.2350 | 127.0415 | 0.0030 | 3.7699e+07 | 2 |

**Adduct Signal Fractions**

- **Unmodified**: 3.7699e+07

### LNVQREN(N)GTISRY Hex 5 HexNAc 4

**Mass:**  3171.3887

(1.987e-06)

**Charge States:** 3

**Start Position**: 97

**End Position**: 110

| Profile Score | Tandem Score | TDA q-value | Volume | Spectrum Matches |
| --- | --- | --- | --- | --- |
| 13.6991 | 126.3516 | 0.0030 | 2.4509e+08 | 3 |

**Adduct Signal Fractions**

- **Unmodified**: 2.4509e+08

### NVQREN(N)GTISRY Hex 7 HexNAc 6

**Mass:**  3788.5731

(1.787e-06)

**Charge States:** 3, 4

**Start Position**: 98

**End Position**: 110

| Profile Score | Tandem Score | TDA q-value | Volume | Spectrum Matches |
| --- | --- | --- | --- | --- |
| 12.7602 | 125.5888 | 0.0057 | 4.6717e+08 | 6 |

**Adduct Signal Fractions**

- **Unmodified**: 4.6717e+08

### FYFTPN(N)KTEDTIF Fuc 2 Hex 8 HexNAc 7

**Mass:**  4631.8693

(1.937e-06)

**Charge States:** 4

**Start Position**: 67

**End Position**: 80

| Profile Score | Tandem Score | TDA q-value | Volume | Spectrum Matches |
| --- | --- | --- | --- | --- |
| -3.7944 | 125.3620 | 0.0057 | 2.3016e+07 | 4 |

**Adduct Signal Fractions**

- **Unmodified**: 2.3016e+07

### QTRQDQC(C)IYN(N)TTY Hex 7 HexNAc 6

**Mass:**  4042.5946

(2.489e-06)

**Charge States:** 3, 4

**Start Position**: 84

**End Position**: 97

| Profile Score | Tandem Score | TDA q-value | Volume | Spectrum Matches |
| --- | --- | --- | --- | --- |
| 14.0536 | 125.0304 | 0.0057 | 1.7308e+08 | 7 |

**Adduct Signal Fractions**

- **Unmodified**: 1.7308e+08

### LNVQREN(N)GTISRY Fuc 1 Hex 7 HexNAc 6

**Mass:**  4047.7114

(1.135e-06)

**Charge States:** 3, 4

**Start Position**: 97

**End Position**: 110

| Profile Score | Tandem Score | TDA q-value | Volume | Spectrum Matches |
| --- | --- | --- | --- | --- |
| 15.2674 | 124.9005 | 0.0057 | 2.8317e+09 | 10 |

**Adduct Signal Fractions**

- **Unmodified**: 2.8317e+09

### LN(D)VQREN(N)GTISRY Fuc 1 Hex 7 HexNAc 6

**Mass:**  4048.7016

(2.467e-04)

**Charge States:** 3, 4

**Start Position**: 97

**End Position**: 110

| Profile Score | Tandem Score | TDA q-value | Volume | Spectrum Matches |
| --- | --- | --- | --- | --- |
| 10.3986 | 124.2085 | 0.0057 | 1.2401e+08 | 13 |

**Adduct Signal Fractions**

- **Unmodified**: 1.2401e+08

### NVQREN(N)GTISRY Hex 6 HexNAc 5

**Mass:**  3423.4381

(2.635e-06)

**Charge States:** 3

**Start Position**: 98

**End Position**: 110

| Profile Score | Tandem Score | TDA q-value | Volume | Spectrum Matches |
| --- | --- | --- | --- | --- |
| 12.9207 | 124.0997 | 0.0057 | 1.3501e+08 | 4 |

**Adduct Signal Fractions**

- **Unmodified**: 1.3501e+08

### YFTPN(N)KTEDTIF Hex 7 HexNAc 5

**Mass:**  3624.4945

(8.198e-06)

**Charge States:** 3

**Start Position**: 68

**End Position**: 80

| Profile Score | Tandem Score | TDA q-value | Volume | Spectrum Matches |
| --- | --- | --- | --- | --- |
| -21.5864 | 123.2991 | 0.0083 | 1.0830e+06 | 3 |

**Adduct Signal Fractions**

- **Unmodified**: 1.0830e+06

### FTPN(N)KTEDTIF Fuc 2 Hex 7 HexNAc 6

**Mass:**  3956.6043

(2.500e-06)

**Charge States:** 3

**Start Position**: 69

**End Position**: 80

| Profile Score | Tandem Score | TDA q-value | Volume | Spectrum Matches |
| --- | --- | --- | --- | --- |
| -3.6726 | 123.0247 | 0.0083 | 1.2937e+07 | 3 |

**Adduct Signal Fractions**

- **Unmodified**: 1.2937e+07

### VQREN(N)GTISRY Hex 6 HexNAc 5

**Mass:**  3309.3989

(3.461e-06)

**Charge States:** 3

**Start Position**: 99

**End Position**: 110

| Profile Score | Tandem Score | TDA q-value | Volume | Spectrum Matches |
| --- | --- | --- | --- | --- |
| -19.2685 | 122.0993 | 0.0100 | 9.0126e+06 | 1 |

**Adduct Signal Fractions**

- **Unmodified**: 9.0126e+06

### LN(D)VQREN(N)GTISRY Hex 7 HexNAc 6

**Mass:**  3902.6447

(2.562e-04)

**Charge States:** 3, 4

**Start Position**: 97

**End Position**: 110

| Profile Score | Tandem Score | TDA q-value | Volume | Spectrum Matches |
| --- | --- | --- | --- | --- |
| 8.3224 | 122.0843 | 0.0100 | 3.6562e+08 | 19 |

**Adduct Signal Fractions**

- **Unmodified**: 3.6562e+08

### FYFTPN(N)KTEDTIF Fuc 1 Hex 8 HexNAc 4 Neu5Ac 1

**Mass:**  4167.6809

(4.146e-06)

**Charge States:** 3, 4

**Start Position**: 67

**End Position**: 80

| Profile Score | Tandem Score | TDA q-value | Volume | Spectrum Matches |
| --- | --- | --- | --- | --- |
| -4.5674 | 121.9326 | 0.0100 | 5.2575e+07 | 2 |

**Adduct Signal Fractions**

- **Unmodified**: 5.2575e+07

### LNVQREN(N)GTISRY Fuc 2 Hex 7 HexNAc 6

**Mass:**  4193.7683

(2.094e-06)

**Charge States:** 3, 4

**Start Position**: 97

**End Position**: 110

| Profile Score | Tandem Score | TDA q-value | Volume | Spectrum Matches |
| --- | --- | --- | --- | --- |
| 13.3766 | 121.0498 | 0.0100 | 8.9840e+08 | 7 |

**Adduct Signal Fractions**

- **Unmodified**: 8.9840e+08

### LNVQREN(N)GTISRY Hex 7 HexNAc 6

**Mass:**  3901.6488

(2.036e-06)

**Charge States:** 3, 4

**Start Position**: 97

**End Position**: 110

| Profile Score | Tandem Score | TDA q-value | Volume | Spectrum Matches |
| --- | --- | --- | --- | --- |
| 16.2872 | 120.4114 | 0.0100 | 8.3360e+09 | 12 |

**Adduct Signal Fractions**

- **Unmodified**: 8.3360e+09

### RNEEYN(N)KSVQEIQAT Hex 6 HexNAc 5

**Mass:**  3795.5868

(1.815e-06)

**Charge States:** 3

**Start Position**: 51

**End Position**: 66

| Profile Score | Tandem Score | TDA q-value | Volume | Spectrum Matches |
| --- | --- | --- | --- | --- |
| -5.6170 | 120.3382 | 0.0100 | 2.2467e+07 | 2 |

**Adduct Signal Fractions**

- **Unmodified**: 2.2467e+07

### VQREN(N)GTISRY Hex 7 HexNAc 6

**Mass:**  3676.5349

(5.484e-04)

**Start Position**: 99

**End Position**: 110

| Profile Score | Tandem Score | TDA q-value | Volume | Spectrum Matches |
| --- | --- | --- | --- | --- |
| 0.0000 | 120.0936 | 0.0100 | 0.0000e+00 | 1 |

Adduct:

No Chromatogram Found

### TPN(N)KTEDTIF Fuc 4 Hex 3 HexNAc 9 Neu5Ac 1

**Mass:**  4353.7297

(-6.751e-06)

**Charge States:** 4

**Start Position**: 70

**End Position**: 80

| Profile Score | Tandem Score | TDA q-value | Volume | Spectrum Matches |
| --- | --- | --- | --- | --- |
| -4.7415 | 119.8604 | 0.0100 | 1.9090e+07 | 1 |

**Adduct Signal Fractions**

- **Unmodified**: 1.9090e+07

### LNVQREN(N)GTISRY Fuc 2 Hex 6 HexNAc 5

**Mass:**  3828.6337

(8.184e-07)

**Charge States:** 3, 4

**Start Position**: 97

**End Position**: 110

| Profile Score | Tandem Score | TDA q-value | Volume | Spectrum Matches |
| --- | --- | --- | --- | --- |
| 11.1314 | 118.3269 | 0.0100 | 8.8620e+07 | 2 |

**Adduct Signal Fractions**

- **Unmodified**: 8.8620e+07

### RN(D)EEYN(N)KSVQEIQATFF Hex 5 HexNAc 4

**Mass:**  3725.5801

(2.667e-04)

**Charge States:** 3, 4

**Start Position**: 51

**End Position**: 68

| Profile Score | Tandem Score | TDA q-value | Volume | Spectrum Matches |
| --- | --- | --- | --- | --- |
| 10.3707 | 118.2049 | 0.0100 | 3.8447e+08 | 9 |

**Adduct Signal Fractions**

- **Unmodified**: 3.8447e+08

### RN(D)EEYN(N)KSVQEIQATF Fuc 1 Hex 6 HexNAc 5

**Mass:**  4089.7006

(2.423e-04)

**Charge States:** 3, 4

**Start Position**: 51

**End Position**: 67

| Profile Score | Tandem Score | TDA q-value | Volume | Spectrum Matches |
| --- | --- | --- | --- | --- |
| 11.4698 | 118.0321 | 0.0100 | 5.8980e+08 | 15 |

**Adduct Signal Fractions**

- **Unmodified**: 5.8980e+08

### NVQREN(N)GTISRY Hex 8 HexNAc 7

**Mass:**  4153.7048

(1.581e-06)

**Charge States:** 4

**Start Position**: 98

**End Position**: 110

| Profile Score | Tandem Score | TDA q-value | Volume | Spectrum Matches |
| --- | --- | --- | --- | --- |
| -5.7648 | 117.3978 | 0.0123 | 4.1050e+07 | 3 |

**Adduct Signal Fractions**

- **Unmodified**: 4.1050e+07

### RN(D)EEYN(N)KSVQEIQATFF Hex 6 HexNAc 5

**Mass:**  4090.7143

(2.459e-04)

**Charge States:** 3, 4

**Start Position**: 51

**End Position**: 68

| Profile Score | Tandem Score | TDA q-value | Volume | Spectrum Matches |
| --- | --- | --- | --- | --- |
| 10.6548 | 117.0995 | 0.0123 | 4.1235e+08 | 16 |

**Adduct Signal Fractions**

- **Unmodified**: 4.1235e+08

### LNVQREN(N)GTISRY Fuc 1 Hex 8 HexNAc 7

**Mass:**  4412.8488

(1.738e-06)

**Charge States:** 3, 4

**Start Position**: 97

**End Position**: 110

| Profile Score | Tandem Score | TDA q-value | Volume | Spectrum Matches |
| --- | --- | --- | --- | --- |
| 15.4236 | 116.5095 | 0.0167 | 3.8583e+08 | 5 |

**Adduct Signal Fractions**

- **Unmodified**: 3.8583e+08

### FTPN(N)KTEDTIF Fuc 3 Hex 6 HexNAc 5 Neu5Ac 1

**Mass:**  4028.6028

(-3.657e-06)

**Charge States:** 3

**Start Position**: 69

**End Position**: 80

| Profile Score | Tandem Score | TDA q-value | Volume | Spectrum Matches |
| --- | --- | --- | --- | --- |
| -19.0298 | 116.3709 | 0.0167 | 7.6910e+05 | 1 |

**Adduct Signal Fractions**

- **Unmodified**: 7.6910e+05

### YFTPN(N)KTEDTIF Fuc 2 Hex 8 HexNAc 7

**Mass:**  4484.7995

(1.774e-06)

**Charge States:** 3, 4

**Start Position**: 68

**End Position**: 80

| Profile Score | Tandem Score | TDA q-value | Volume | Spectrum Matches |
| --- | --- | --- | --- | --- |
| 9.9161 | 114.6808 | 0.0184 | 3.0933e+07 | 2 |

**Adduct Signal Fractions**

- **Unmodified**: 3.0933e+07

### LNVQREN(N)GTISRY Hex 8 HexNAc 7

**Mass:**  4266.7951

(1.498e-06)

**Charge States:** 3, 4

**Start Position**: 97

**End Position**: 110

| Profile Score | Tandem Score | TDA q-value | Volume | Spectrum Matches |
| --- | --- | --- | --- | --- |
| 15.5190 | 114.6460 | 0.0184 | 9.5946e+08 | 6 |

**Adduct Signal Fractions**

- **Unmodified**: 9.5946e+08

### YFTPN(N)KTEDTIF Hex 10 HexNAc 9

**Mass:**  4922.9489

(1.646e-06)

**Charge States:** 4

**Start Position**: 68

**End Position**: 80

| Profile Score | Tandem Score | TDA q-value | Volume | Spectrum Matches |
| --- | --- | --- | --- | --- |
| -4.4665 | 114.1851 | 0.0184 | 1.9158e+07 | 1 |

**Adduct Signal Fractions**

- **Unmodified**: 1.9158e+07

### RNEEYN(N)KSVQEIQATFF Hex 8 HexNAc 6

**Mass:**  4617.9060

(2.185e-04)

**Charge States:** 4

**Start Position**: 51

**End Position**: 68

| Profile Score | Tandem Score | TDA q-value | Volume | Spectrum Matches |
| --- | --- | --- | --- | --- |
| -6.0109 | 113.1431 | 0.0184 | 2.7242e+07 | 4 |

**Adduct Signal Fractions**

- **Unmodified**: 2.7242e+07

### FTPN(N)KTEDTIF Hex 7 HexNAc 4 Neu5Ac 2

**Mass:**  3840.5154

(1.531e-06)

**Charge States:** 3, 4

**Start Position**: 69

**End Position**: 80

| Profile Score | Tandem Score | TDA q-value | Volume | Spectrum Matches |
| --- | --- | --- | --- | --- |
| 13.8731 | 111.8247 | 0.0210 | 2.4177e+07 | 4 |

**Adduct Signal Fractions**

- **Unmodified**: 2.4177e+07

### LNVQREN(N)GTISRY Fuc 3 Hex 7 HexNAc 6

**Mass:**  4339.8273

(1.102e-08)

**Charge States:** 3, 4

**Start Position**: 97

**End Position**: 110

| Profile Score | Tandem Score | TDA q-value | Volume | Spectrum Matches |
| --- | --- | --- | --- | --- |
| 10.7067 | 111.7017 | 0.0210 | 2.2627e+08 | 4 |

**Adduct Signal Fractions**

- **Unmodified**: 2.2627e+08

### YFTPN(N)KTEDTIF Fuc 1 Hex 7 HexNAc 3 Neu5Ac 1

**Mass:**  3655.4712

(3.258e-06)

**Charge States:** 3

**Start Position**: 68

**End Position**: 80

| Profile Score | Tandem Score | TDA q-value | Volume | Spectrum Matches |
| --- | --- | --- | --- | --- |
| -19.5717 | 110.5456 | 0.0210 | 4.2108e+06 | 1 |

**Adduct Signal Fractions**

- **Unmodified**: 4.2108e+06

### NVQREN(N)GTISRY Fuc 2 Hex 7 HexNAc 6

**Mass:**  4080.6928

(2.780e-06)

**Charge States:** 4

**Start Position**: 98

**End Position**: 110

| Profile Score | Tandem Score | TDA q-value | Volume | Spectrum Matches |
| --- | --- | --- | --- | --- |
| -18.7504 | 109.7049 | 0.0210 | 4.6719e+07 | 2 |

**Adduct Signal Fractions**

- **Unmodified**: 4.6719e+07

### LNVQREN(N)GTISRY Hex 10 HexNAc 9

**Mass:**  4997.0505

(1.623e-06)

**Charge States:** 4

**Start Position**: 97

**End Position**: 110

| Profile Score | Tandem Score | TDA q-value | Volume | Spectrum Matches |
| --- | --- | --- | --- | --- |
| -2.8111 | 109.0809 | 0.0210 | 1.3441e+08 | 3 |

**Adduct Signal Fractions**

- **Unmodified**: 1.3441e+08

### QTRQDQC(C)IYN(N)TTY Hex 6 HexNAc 5

**Mass:**  3677.4607

(1.569e-06)

**Charge States:** 3, 4

**Start Position**: 84

**End Position**: 97

| Profile Score | Tandem Score | TDA q-value | Volume | Spectrum Matches |
| --- | --- | --- | --- | --- |
| 14.5843 | 108.7199 | 0.0210 | 1.0227e+08 | 5 |

**Adduct Signal Fractions**

- **Unmodified**: 1.0227e+08

### LNVQREN(N)GTISRY Fuc 2 Hex 8 HexNAc 7

**Mass:**  4558.9167

(1.662e-06)

**Charge States:** 3, 4

**Start Position**: 97

**End Position**: 110

| Profile Score | Tandem Score | TDA q-value | Volume | Spectrum Matches |
| --- | --- | --- | --- | --- |
| -2.9777 | 106.9761 | 0.0221 | 1.8294e+08 | 3 |

**Adduct Signal Fractions**

- **Unmodified**: 1.8294e+08

### VQREN(N)GTISRY Fuc 1 Hex 7 HexNAc 6

**Mass:**  3820.5899

(3.194e-06)

**Charge States:** 3

**Start Position**: 99

**End Position**: 110

| Profile Score | Tandem Score | TDA q-value | Volume | Spectrum Matches |
| --- | --- | --- | --- | --- |
| -21.3499 | 106.2922 | 0.0221 | 4.4153e+06 | 1 |

**Adduct Signal Fractions**

- **Unmodified**: 4.4153e+06

### LNVQREN(N)GTISRY Fuc 1 Hex 9 HexNAc 8

**Mass:**  4777.9831

(1.926e-06)

**Charge States:** 3, 4

**Start Position**: 97

**End Position**: 110

| Profile Score | Tandem Score | TDA q-value | Volume | Spectrum Matches |
| --- | --- | --- | --- | --- |
| 14.9742 | 105.8258 | 0.0221 | 2.6491e+08 | 2 |

**Adduct Signal Fractions**

- **Unmodified**: 2.6491e+08

### QTRQDQC(C)IYN(N)TTY Fuc 1 Hex 6 HexNAc 5

**Mass:**  3823.5192

(3.301e-06)

**Charge States:** 3, 4

**Start Position**: 84

**End Position**: 97

| Profile Score | Tandem Score | TDA q-value | Volume | Spectrum Matches |
| --- | --- | --- | --- | --- |
| 13.7969 | 105.6444 | 0.0221 | 2.9050e+07 | 3 |

**Adduct Signal Fractions**

- **Unmodified**: 2.9050e+07

### LNVQREN(N)GTISRY Hex 9 HexNAc 8

**Mass:**  4631.9304

(1.728e-06)

**Charge States:** 3, 4

**Start Position**: 97

**End Position**: 110

| Profile Score | Tandem Score | TDA q-value | Volume | Spectrum Matches |
| --- | --- | --- | --- | --- |
| 14.5018 | 105.2649 | 0.0221 | 8.5323e+08 | 5 |

**Adduct Signal Fractions**

- **Unmodified**: 8.5323e+08

### RN(D)EEYN(N)KSVQEIQATFF Fuc 1 Hex 6 HexNAc 5

**Mass:**  4236.7712

(2.355e-04)

**Charge States:** 3, 4

**Start Position**: 51

**End Position**: 68

| Profile Score | Tandem Score | TDA q-value | Volume | Spectrum Matches |
| --- | --- | --- | --- | --- |
| 10.3240 | 105.0147 | 0.0240 | 2.0393e+08 | 17 |

**Adduct Signal Fractions**

- **Unmodified**: 2.0393e+08

### VQREN(N)GTISRY Hex 7 HexNAc 6

**Mass:**  3674.5307

(3.092e-06)

**Charge States:** 3, 4

**Start Position**: 99

**End Position**: 110

| Profile Score | Tandem Score | TDA q-value | Volume | Spectrum Matches |
| --- | --- | --- | --- | --- |
| -19.8768 | 104.8198 | 0.0248 | 2.2131e+07 | 3 |

**Adduct Signal Fractions**

- **Unmodified**: 2.2131e+07

### NVQREN(N)GTISRY Hex 9 HexNAc 8

**Mass:**  4518.8391

(2.605e-06)

**Charge States:** 4

**Start Position**: 98

**End Position**: 110

| Profile Score | Tandem Score | TDA q-value | Volume | Spectrum Matches |
| --- | --- | --- | --- | --- |
| -5.3570 | 104.6097 | 0.0248 | 4.0369e+07 | 3 |

**Adduct Signal Fractions**

- **Unmodified**: 4.0369e+07

### TPN(N)KTEDTIF Fuc 2 Hex 7 HexNAc 9

**Mass:**  4418.7712

(1.099e-06)

**Charge States:** 4

**Start Position**: 70

**End Position**: 80

| Profile Score | Tandem Score | TDA q-value | Volume | Spectrum Matches |
| --- | --- | --- | --- | --- |
| -19.8000 | 104.1555 | 0.0248 | 3.6689e+06 | 1 |

**Adduct Signal Fractions**

- **Unmodified**: 3.6689e+06

### QTRQDQC(C)IYN(N)TTY Fuc 1 Hex 7 HexNAc 6

**Mass:**  4188.6545

(1.864e-06)

**Charge States:** 3, 4

**Start Position**: 84

**End Position**: 97

| Profile Score | Tandem Score | TDA q-value | Volume | Spectrum Matches |
| --- | --- | --- | --- | --- |
| 14.3107 | 103.8753 | 0.0248 | 4.4890e+07 | 4 |

**Adduct Signal Fractions**

- **Unmodified**: 4.4890e+07

### TPN(N)KTEDTIF Fuc 4 Hex 4 HexNAc 9

**Mass:**  4224.6979

(-6.075e-06)

**Charge States:** 3

**Start Position**: 70

**End Position**: 80

| Profile Score | Tandem Score | TDA q-value | Volume | Spectrum Matches |
| --- | --- | --- | --- | --- |
| -4.3076 | 103.2985 | 0.0248 | 2.3417e+07 | 2 |

**Adduct Signal Fractions**

- **Unmodified**: 2.3417e+07

### LN(D)VQREN(N)GTISRY Fuc 4 Hex 7 HexNAc 5

**Mass:**  4283.8137

(2.368e-04)

**Charge States:** 4

**Start Position**: 97

**End Position**: 110

| Profile Score | Tandem Score | TDA q-value | Volume | Spectrum Matches |
| --- | --- | --- | --- | --- |
| -5.7218 | 103.2569 | 0.0248 | 7.5028e+07 | 1 |

**Adduct Signal Fractions**

- **Unmodified**: 7.5028e+07

### RNEEYN(N)KSVQEIQATFFY Hex 6 HexNAc 5

**Mass:**  4252.8088

(5.362e-06)

**Charge States:** 4

**Start Position**: 51

**End Position**: 69

| Profile Score | Tandem Score | TDA q-value | Volume | Spectrum Matches |
| --- | --- | --- | --- | --- |
| -3.8741 | 103.0406 | 0.0248 | 1.8541e+08 | 2 |

**Adduct Signal Fractions**

- **Unmodified**: 1.8541e+08

### TPN(N)KTEDTIF Hex 3 HexNAc 9

**Mass:**  3481.4479

(8.650e-04)

**Start Position**: 70

**End Position**: 80

| Profile Score | Tandem Score | TDA q-value | Volume | Spectrum Matches |
| --- | --- | --- | --- | --- |
| 0.0000 | 102.3279 | 0.0263 | 0.0000e+00 | 1 |

Adduct:

No Chromatogram Found

### RNEEYN(N)KSVQEIQATF Fuc 2 Hex 5 HexNAc 3 Neu5Ac 1

**Mass:**  3960.6701

(7.675e-04)

**Charge States:** 4

**Start Position**: 51

**End Position**: 67

| Profile Score | Tandem Score | TDA q-value | Volume | Spectrum Matches |
| --- | --- | --- | --- | --- |
| -8.8049 | 101.8048 | 0.0263 | 7.0998e+06 | 5 |

**Adduct Signal Fractions**

- **Unmodified**: 7.0998e+06

### LVPVPITN(N)ATL Hex 6 HexNAc 5

**Mass:**  3124.4046

(1.562e-06)

**Charge States:** 3

**Start Position**: 26

**End Position**: 37

| Profile Score | Tandem Score | TDA q-value | Volume | Spectrum Matches |
| --- | --- | --- | --- | --- |
| -20.7171 | 101.5941 | 0.0276 | 3.4732e+06 | 1 |

**Adduct Signal Fractions**

- **Unmodified**: 3.4732e+06

### VQREN(N)GTISRY Fuc 1 Hex 4 HexNAc 7

**Mass:**  3537.5117

(3.961e-06)

**Charge States:** 3, 4

**Start Position**: 99

**End Position**: 110

| Profile Score | Tandem Score | TDA q-value | Volume | Spectrum Matches |
| --- | --- | --- | --- | --- |
| 9.0306 | 101.1611 | 0.0276 | 8.9615e+07 | 21 |

**Adduct Signal Fractions**

- **Unmodified**: 8.9615e+07

### TPN(N)KTEDTIF Fuc 1 Hex 4 HexNAc 9 Neu5Ac 1

**Mass:**  4077.6275

(-4.375e-06)

**Charge States:** 3

**Start Position**: 70

**End Position**: 80

| Profile Score | Tandem Score | TDA q-value | Volume | Spectrum Matches |
| --- | --- | --- | --- | --- |
| -19.5951 | 100.9978 | 0.0276 | 1.4198e+07 | 2 |

**Adduct Signal Fractions**

- **Unmodified**: 1.4198e+07

### LNVQREN(N)GTISRY Fuc 3 Hex 8 HexNAc 7

**Mass:**  4704.9641

(2.438e-06)

**Charge States:** 4

**Start Position**: 97

**End Position**: 110

| Profile Score | Tandem Score | TDA q-value | Volume | Spectrum Matches |
| --- | --- | --- | --- | --- |
| -4.4463 | 100.3870 | 0.0308 | 7.0192e+07 | 2 |

**Adduct Signal Fractions**

- **Unmodified**: 7.0192e+07

### C(C)ANLVPVPITN(N)ATL Hex 6 HexNAc 5

**Mass:**  3469.5111

(1.682e-06)

**Charge States:** 3, 4

**Start Position**: 23

**End Position**: 37

| Profile Score | Tandem Score | TDA q-value | Volume | Spectrum Matches |
| --- | --- | --- | --- | --- |
| 13.4299 | 99.1874 | 0.0308 | 2.9159e+07 | 5 |

**Adduct Signal Fractions**

- **Unmodified**: 2.9159e+07

### N(N)KSVQEIQATFF Fuc 3 Hex 6 HexNAc 6

**Mass:**  4041.6757

(4.937e-04)

**Start Position**: 56

**End Position**: 68

| Profile Score | Tandem Score | TDA q-value | Volume | Spectrum Matches |
| --- | --- | --- | --- | --- |
| 0.0000 | 98.3194 | 0.0325 | 0.0000e+00 | 1 |

Adduct:

No Chromatogram Found

### NVQREN(N)GTISRY Fuc 2 Hex 7 HexNAc 4 Neu5Ac 1

**Mass:**  3965.6230

(6.539e-07)

**Charge States:** 4

**Start Position**: 98

**End Position**: 110

| Profile Score | Tandem Score | TDA q-value | Volume | Spectrum Matches |
| --- | --- | --- | --- | --- |
| -5.1271 | 98.2377 | 0.0341 | 1.9538e+07 | 4 |

**Adduct Signal Fractions**

- **Unmodified**: 1.9538e+07

### RNEEYN(N)KSVQEIQATF Fuc 1 Hex 8 HexNAc 7

**Mass:**  4818.9802

(1.118e-06)

**Charge States:** 4

**Start Position**: 51

**End Position**: 67

| Profile Score | Tandem Score | TDA q-value | Volume | Spectrum Matches |
| --- | --- | --- | --- | --- |
| -3.7536 | 97.2745 | 0.0376 | 1.3008e+07 | 2 |

**Adduct Signal Fractions**

- **Unmodified**: 1.3008e+07

### LNVQREN(N)GTISRY Fuc 2 Hex 9 HexNAc 8

**Mass:**  4924.0368

(1.264e-06)

**Charge States:** 4

**Start Position**: 97

**End Position**: 110

| Profile Score | Tandem Score | TDA q-value | Volume | Spectrum Matches |
| --- | --- | --- | --- | --- |
| -3.0182 | 96.9889 | 0.0393 | 9.7435e+07 | 2 |

**Adduct Signal Fractions**

- **Unmodified**: 9.7435e+07

### TPN(N)KTEDTIF Fuc 3 Hex 10 HexNAc 4 Neu5Ac 1

**Mass:**  4326.6838

(1.874e-06)

**Charge States:** 3, 4

**Start Position**: 70

**End Position**: 80

| Profile Score | Tandem Score | TDA q-value | Volume | Spectrum Matches |
| --- | --- | --- | --- | --- |
| -4.2780 | 95.9893 | 0.0420 | 1.9743e+07 | 3 |

**Adduct Signal Fractions**

- **Unmodified**: 1.9743e+07

### LN(D)VQREN(N)GTISRY Fuc 5 Hex 8 HexNAc 6

**Mass:**  4795.0090

(2.116e-04)

**Charge States:** 4

**Start Position**: 97

**End Position**: 110

| Profile Score | Tandem Score | TDA q-value | Volume | Spectrum Matches |
| --- | --- | --- | --- | --- |
| -6.5346 | 95.7939 | 0.0420 | 4.2176e+07 | 1 |

**Adduct Signal Fractions**

- **Unmodified**: 4.2176e+07

### LNVQREN(N)GTISRY Hex 7 HexNAc 4 Neu5Ac 2

**Mass:**  4077.6887

(1.364e-06)

**Charge States:** 3, 4

**Start Position**: 97

**End Position**: 110

| Profile Score | Tandem Score | TDA q-value | Volume | Spectrum Matches |
| --- | --- | --- | --- | --- |
| 14.4217 | 95.3562 | 0.0420 | 3.8893e+08 | 7 |

**Adduct Signal Fractions**

- **Unmodified**: 3.8893e+08

### LNVQREN(N)GTISRY Hex 10 HexNAc 4

**Mass:**  3981.6144

(-6.851e-06)

**Charge States:** 3, 4

**Start Position**: 97

**End Position**: 110

| Profile Score | Tandem Score | TDA q-value | Volume | Spectrum Matches |
| --- | --- | --- | --- | --- |
| 11.2012 | 93.8804 | 0.0432 | 2.7759e+07 | 5 |

**Adduct Signal Fractions**

- **Unmodified**: 2.7759e+07

### NVQREN(N)GTISRY Fuc 3 Hex 7 HexNAc 6

**Mass:**  4226.7487

(2.866e-06)

**Charge States:** 4

**Start Position**: 98

**End Position**: 110

| Profile Score | Tandem Score | TDA q-value | Volume | Spectrum Matches |
| --- | --- | --- | --- | --- |
| -20.9957 | 91.7674 | 0.0479 | 6.0020e+06 | 1 |

**Adduct Signal Fractions**

- **Unmodified**: 6.0020e+06

### P19652|A1AG2\_HUMAN

**>P19652|A1AG2\_HUMAN**  
 `MALSWVLTVLSLLPLLEAQIPLCANLVPVPITNATLDRITGKWFYIASAFRNEEYNKSVQEIQATFFYFTPNKTEDTIFL  
REYQTRQNQCFYNSSYLNVQRENGTVSRYEGGREHVAHLLFLRDTKTLMFGSYLDDEKNWGLSFYADKPETTKEQLGEFY  
EALDCLCIPRSDVMYTDWKKDKCEPLEKQHEKERKQEEGES`

| Site | Type | Count |
| --- | --- | --- |
| 33 | N-linked | 3 |
| 56 | N-linked | 36 |
| 93 | N-linked | 1 |
| 103 | N-linked | 19 |
| 72 | N-linked | 62 |

| Mass | Sequence | MS_2_ Score | `q`-value | MS_1_ Score | Signal | Protein Coordinates | Tandem Scans | Start Time | Apex Time | End Time |
| --- | --- | --- | --- | --- | --- | --- | --- | --- | --- | --- |
| 3152.2829 | TPN(N)KTEDTIF Hex 6 HexNAc 5 | 182.079 | 0.0 | 15.206 | 1.948e+08 | 69 - 79 | 9 | 22.05 | 23.92 | 25.29 |
| 3517.4167 | TPN(N)KTEDTIF Hex 7 HexNAc 6 | 181.792 | 0.0 | 15.626 | 2.810e+08 | 69 - 79 | 10 | 21.36 | 23.24 | 24.77 |
| 2787.1525 | TPN(N)KTEDTIF Hex 5 HexNAc 4 | 181.778 | 0.0 | 14.263 | 2.212e+07 | 69 - 79 | 3 | 24.09 | 24.43 | 24.77 |
| 2886.2319 | N(N)KSVQEIQATF Hex 5 HexNAc 4 | 178.684 | 0.0 | 14.082 | 2.514e+07 | 55 - 66 | 2 | 27.00 | 27.34 | 27.68 |
| 3827.5488 | YFTPN(N)KTEDTIF Hex 7 HexNAc 6 | 177.218 | 0.0 | 14.918 | 4.580e+09 | 67 - 79 | 52 | 35.76 | 36.78 | 43.37 |
| 3973.6063 | YFTPN(N)KTEDTIF Fuc 1 Hex 7 HexNAc 6 | 176.426 | 0.0 | 14.804 | 8.900e+08 | 67 - 79 | 8 | 35.59 | 36.61 | 37.60 |
| 3097.2819 | YFTPN(N)KTEDTIF Hex 5 HexNAc 4 | 174.557 | 0.0 | 15.238 | 3.785e+08 | 67 - 79 | 5 | 37.76 | 38.08 | 38.93 |
| 3462.4150 | YFTPN(N)KTEDTIF Hex 6 HexNAc 5 | 174.243 | 0.0 | 14.173 | 2.895e+09 | 67 - 79 | 28 | 36.27 | 37.43 | 43.03 |
| 3299.3503 | FTPN(N)KTEDTIF Hex 6 HexNAc 5 | 173.039 | 0.0 | 15.036 | 4.095e+08 | 68 - 79 | 12 | 29.57 | 31.10 | 32.12 |
| 3307.3795 | EEYN(N)KSVQEIQATF Hex 5 HexNAc 4 | 170.890 | 0.0 | 12.169 | 8.537e+07 | 52 - 66 | 2 | 35.76 | 35.93 | 36.44 |
| 3298.3431 | TPN(N)KTEDTIF Fuc 1 Hex 6 HexNAc 5 | 170.587 | 0.0 | 13.408 | 3.114e+07 | 69 - 79 | 4 | 23.24 | 23.58 | 24.09 |
| 4614.8359 | YFTPN(N)KTEDTIF Fuc 4 Hex 7 HexNAc 7 | 169.379 | 0.0 | 15.607 | 8.189e+07 | 67 - 79 | 3 | 35.25 | 35.76 | 36.27 |
| 3609.4857 | FYFTPN(N)KTEDTIF Hex 6 HexNAc 5 | 168.667 | 0.0 | 14.759 | 2.786e+09 | 66 - 79 | 49 | 46.40 | 47.40 | 58.32 |
| 3664.4846 | FTPN(N)KTEDTIF Hex 7 HexNAc 6 | 168.062 | 0.0 | 16.265 | 6.629e+08 | 68 - 79 | 13 | 29.05 | 30.59 | 31.95 |
| 3608.4750 | YFTPN(N)KTEDTIF Fuc 1 Hex 6 HexNAc 5 | 167.922 | 0.0 | 14.317 | 5.999e+08 | 67 - 79 | 7 | 36.10 | 37.27 | 38.42 |
| 3755.5449 | FYFTPN(N)KTEDTIF Fuc 1 Hex 6 HexNAc 5 | 167.386 | 0.0 | 14.548 | 5.689e+08 | 66 - 79 | 11 | 46.06 | 47.06 | 48.40 |
| 3663.4750 | TPN(N)KTEDTIF Fuc 1 Hex 7 HexNAc 6 | 167.357 | 0.0 | 13.749 | 4.404e+07 | 69 - 79 | 4 | 22.90 | 23.07 | 23.76 |
| 4120.6790 | FYFTPN(N)KTEDTIF Fuc 1 Hex 7 HexNAc 6 | 166.335 | 0.0 | 14.398 | 8.777e+08 | 66 - 79 | 12 | 45.72 | 46.57 | 48.40 |
| 4192.6833 | YFTPN(N)KTEDTIF Hex 8 HexNAc 7 | 165.829 | 0.0 | 15.245 | 7.627e+08 | 67 - 79 | 11 | 34.91 | 36.44 | 37.27 |
| 3974.6213 | FYFTPN(N)KTEDTIF Hex 7 HexNAc 6 | 165.654 | 0.0 | 15.289 | 4.717e+09 | 66 - 79 | 82 | 45.55 | 46.73 | 60.72 |
| 4339.7521 | FYFTPN(N)KTEDTIF Hex 8 HexNAc 7 | 165.280 | 0.0 | 14.780 | 6.386e+08 | 66 - 79 | 9 | 45.03 | 46.23 | 46.90 |
| 4266.7362 | FYFTPN(N)KTEDTIF Fuc 2 Hex 7 HexNAc 6 | 164.176 | 0.0 | -16.199 | 1.187e+08 | 66 - 79 | 2 | 46.40 | 46.40 | 46.57 |
| 3882.5492 | TPN(N)KTEDTIF Hex 8 HexNAc 7 | 163.940 | 0.0 | -4.523 | 2.119e+07 | 69 - 79 | 4 | 22.39 | 22.73 | 22.90 |
| 4235.7881 | RNEEYN(N)KSVQEIQATFF Fuc 1 Hex 6 HexNAc 5 | 162.034 | 0.0 | 15.671 | 1.928e+09 | 50 - 67 | 16 | 41.83 | 43.54 | 44.86 |
| 3244.3524 | FYFTPN(N)KTEDTIF Hex 5 HexNAc 4 | 161.981 | 0.0 | 10.767 | 2.224e+08 | 66 - 79 | 4 | 47.06 | 47.90 | 48.74 |
| 3319.3141 | N(N)KTEDTIF Hex 7 HexNAc 6 | 161.405 | 0.0 | -20.235 | 3.217e+06 | 71 - 79 | 1 | 21.36 | 21.36 | 21.54 |
| 4089.7286 | RNEEYN(N)KSVQEIQATFF Hex 6 HexNAc 5 | 161.030 | 0.0 | 16.690 | 3.948e+09 | 50 - 67 | 19 | 41.49 | 43.71 | 44.86 |
| 4119.6668 | YFTPN(N)KTEDTIF Fuc 2 Hex 7 HexNAc 6 | 160.245 | 0.0 | -4.104 | 1.272e+08 | 67 - 79 | 2 | 36.44 | 36.44 | 36.94 |
| 4557.8147 | YFTPN(N)KTEDTIF Hex 9 HexNAc 8 | 159.947 | 0.0 | 15.398 | 2.304e+08 | 67 - 79 | 6 | 34.74 | 35.76 | 36.44 |
| 3672.5120 | EEYN(N)KSVQEIQATF Hex 6 HexNAc 5 | 158.828 | 0.0 | -3.199 | 8.124e+07 | 52 - 66 | 3 | 35.25 | 35.42 | 35.76 |
| 3723.5853 | RNEEYN(N)KSVQEIQATF Fuc 1 Hex 5 HexNAc 4 | 158.251 | 0.0 | 11.221 | 3.347e+08 | 50 - 66 | 9 | 32.44 | 32.61 | 34.06 |
| 3445.4094 | FTPN(N)KTEDTIF Fuc 1 Hex 6 HexNAc 5 | 158.247 | 0.0 | 13.429 | 7.412e+07 | 68 - 79 | 5 | 30.59 | 30.76 | 31.27 |
| 3004.2181 | FYFTPN(N)KT Hex 6 HexNAc 5 | 156.822 | 0.0 | -4.004 | 6.724e+06 | 66 - 74 | 2 | 25.80 | 25.97 | 26.32 |
| 3577.5255 | RNEEYN(N)KSVQEIQATF Hex 5 HexNAc 4 | 156.559 | 0.0 | 16.177 | 8.420e+09 | 50 - 66 | 22 | 24.77 | 32.75 | 34.23 |
| 4485.8103 | FYFTPN(N)KTEDTIF Fuc 1 Hex 8 HexNAc 7 | 156.496 | 0.0 | 14.851 | 1.309e+08 | 66 - 79 | 6 | 45.55 | 46.06 | 46.57 |
| 3724.5958 | RNEEYN(N)KSVQEIQATFF Hex 5 HexNAc 4 | 156.361 | 0.0 | 15.992 | 4.177e+09 | 50 - 67 | 17 | 42.35 | 44.20 | 45.55 |
| 3818.5708 | EEYN(N)KSVQEIQATF Fuc 1 Hex 6 HexNAc 5 | 155.479 | 0.0 | -4.046 | 3.410e+07 | 52 - 66 | 2 | 35.08 | 35.25 | 35.59 |
| 3454.4484 | EEYN(N)KSVQEIQATFF Hex 5 HexNAc 4 | 155.236 | 0.0 | -4.981 | 2.731e+07 | 52 - 67 | 1 | 47.90 | 47.90 | 48.40 |
| 3819.5820 | EEYN(N)KSVQEIQATFF Hex 6 HexNAc 5 | 154.973 | 0.0 | -5.626 | 2.607e+07 | 52 - 67 | 2 | 47.40 | 47.40 | 47.73 |
| 3098.1790 | YN(N)SSYL Hex 7 HexNAc 6 | 154.912 | 0.0 | -19.798 | 3.223e+06 | 91 - 97 | 1 | 22.22 | 22.22 | 22.39 |
| 4381.8438 | RNEEYN(N)KSVQEIQATFF Fuc 2 Hex 6 HexNAc 5 | 153.767 | 0.0 | -3.714 | 7.341e+07 | 50 - 67 | 3 | 43.37 | 43.54 | 43.71 |
| 3942.6579 | RNEEYN(N)KSVQEIQATF Hex 6 HexNAc 5 | 153.511 | 0.0 | 15.010 | 6.008e+09 | 50 - 66 | 20 | 30.59 | 32.44 | 33.89 |
| 3870.6575 | RNEEYN(N)KSVQEIQATFF Fuc 1 Hex 5 HexNAc 4 | 153.010 | 0.0 | 13.132 | 1.534e+08 | 50 - 67 | 5 | 43.71 | 44.04 | 44.69 |
| 4020.6053 | YFTPN(N)KTEDTIF Fuc 1 Hex 8 HexNAc 4 Neu5Ac 1 | 152.837 | 0.0 | -4.526 | 5.374e+07 | 67 - 79 | 3 | 38.08 | 38.08 | 38.42 |
| 4338.7401 | YFTPN(N)KTEDTIF Fuc 1 Hex 8 HexNAc 7 | 152.427 | 0.0 | 14.683 | 1.449e+08 | 67 - 79 | 5 | 35.59 | 36.27 | 36.61 |
| 4003.5800 | YFTPN(N)KTEDTIF Hex 7 HexNAc 4 Neu5Ac 2 | 152.347 | 0.0 | 14.550 | 3.336e+08 | 67 - 79 | 6 | 37.92 | 38.08 | 38.93 |
| 4088.7160 | RNEEYN(N)KSVQEIQATF Fuc 1 Hex 6 HexNAc 5 | 152.329 | 0.0 | 14.587 | 2.951e+09 | 50 - 66 | 16 | 30.42 | 32.28 | 33.72 |
| 3810.5428 | FTPN(N)KTEDTIF Fuc 1 Hex 7 HexNAc 6 | 151.215 | 0.0 | 13.624 | 1.191e+08 | 68 - 79 | 6 | 30.08 | 30.25 | 30.93 |
| 4454.8604 | RNEEYN(N)KSVQEIQATFF Hex 7 HexNAc 6 | 150.949 | 0.0 | 14.902 | 1.854e+08 | 50 - 67 | 4 | 43.03 | 43.37 | 43.87 |
| 4307.7912 | RNEEYN(N)KSVQEIQATF Hex 7 HexNAc 6 | 150.565 | 0.0 | -2.648 | 2.895e+08 | 50 - 66 | 3 | 31.95 | 32.12 | 32.44 |
| 4234.7738 | RNEEYN(N)KSVQEIQATF Fuc 2 Hex 6 HexNAc 5 | 150.457 | 0.0 | -3.600 | 9.725e+07 | 50 - 66 | 3 | 32.12 | 32.12 | 32.44 |
| 3222.2737 | YFTPN(N)KT Hex 7 HexNAc 6 | 150.011 | 0.0 | -20.675 | 3.354e+06 | 67 - 74 | 1 | 14.28 | 14.28 | 14.28 |
| 4600.9167 | RNEEYN(N)KSVQEIQATFF Fuc 1 Hex 7 HexNAc 6 | 149.997 | 0.0 | 14.810 | 6.810e+07 | 50 - 67 | 3 | 42.86 | 43.37 | 43.54 |
| 4453.8502 | RNEEYN(N)KSVQEIQATF Fuc 1 Hex 7 HexNAc 6 | 149.738 | 0.0 | -3.481 | 1.288e+08 | 50 - 66 | 2 | 31.95 | 32.12 | 32.44 |
| 2934.2202 | FTPN(N)KTEDTIF Hex 5 HexNAc 4 | 149.602 | 0.0 | -3.493 | 4.012e+07 | 68 - 79 | 3 | 31.44 | 31.60 | 31.95 |
| 4060.6029 | TPN(N)KTEDTIF Fuc 4 Hex 8 HexNAc 5 | 148.123 | 0.0 | 13.080 | 1.376e+08 | 69 - 79 | 4 | 37.92 | 38.08 | 38.59 |
| 4704.8852 | FYFTPN(N)KTEDTIF Hex 9 HexNAc 8 | 147.390 | 0.0 | 16.793 | 1.722e+08 | 66 - 79 | 6 | 44.86 | 45.55 | 46.40 |
| 4150.6523 | FYFTPN(N)KTEDTIF Hex 7 HexNAc 4 Neu5Ac 2 | 146.167 | 0.0 | 13.490 | 2.521e+08 | 66 - 79 | 5 | 47.73 | 47.90 | 48.91 |
| 3369.3485 | FYFTPN(N)KT Hex 7 HexNAc 6 | 145.901 | 0.0 | 12.889 | 1.334e+07 | 66 - 74 | 2 | 25.12 | 25.46 | 25.97 |
| 3613.4174 | FYFTPN(N)KTED Hex 7 HexNAc 6 | 144.957 | 0.0 | -18.071 | 5.227e+06 | 66 - 76 | 1 | 25.63 | 25.63 | 25.80 |
| 4066.6753 | NVQREN(N)GTVSRY Fuc 2 Hex 7 HexNAc 6 | 144.115 | 0.0 | -19.452 | 8.060e+07 | 97 - 109 | 2 | 11.97 | 11.97 | 12.14 |
| 3920.6155 | NVQREN(N)GTVSRY Fuc 1 Hex 7 HexNAc 6 | 143.891 | 0.0 | -3.926 | 3.193e+08 | 97 - 109 | 4 | 11.97 | 11.97 | 12.47 |
| 3430.4549 | RNEEYN(N)KSVQEIQAT Hex 5 HexNAc 4 | 142.590 | 0.0 | -4.607 | 3.418e+07 | 50 - 65 | 3 | 17.20 | 17.36 | 17.70 |
| 3774.5571 | NVQREN(N)GTVSRY Hex 7 HexNAc 6 | 141.660 | 0.0 | 12.753 | 6.178e+08 | 97 - 109 | 7 | 11.97 | 12.14 | 12.95 |
| 4207.6717 | TPN(N)KTEDTIF Fuc 3 Hex 3 HexNAc 9 Neu5Ac 1 | 140.943 | 0.0 | 13.939 | 1.021e+08 | 69 - 79 | 4 | 48.74 | 48.91 | 49.93 |
| 3693.4452 | TPN(N)KTEDTIF Hex 7 HexNAc 4 Neu5Ac 2 | 138.675 | 0.0 | -4.280 | 9.871e+06 | 69 - 79 | 3 | 24.26 | 24.43 | 24.60 |
| 4089.7123 | FRNEEYN(N)KSVQEIQATF Hex 6 HexNAc 5 | 138.033 | 0.0 | -5.395 | 4.085e+07 | 49 - 66 | 1 | 37.11 | 37.43 | 37.60 |
| 3849.5295 | YFTPN(N)KTEDTIF Hex 3 HexNAc 5 Neu5Ac 3 | 138.021 | 0.0 | 12.513 | 3.088e+08 | 67 - 79 | 2 | 36.61 | 36.78 | 37.43 |
| 4672.9234 | RNEEYN(N)KSVQEIQATF Hex 8 HexNAc 7 | 137.544 | 0.0 | -3.104 | 5.047e+07 | 50 - 66 | 2 | 31.60 | 31.77 | 32.12 |
| 3943.6431 | RN(D)EEYN(N)KSVQEIQATF Hex 6 HexNAc 5 | 135.678 | 0.0 | 11.626 | 1.211e+09 | 50 - 66 | 25 | 34.06 | 35.42 | 36.27 |
| 4149.6400 | YFTPN(N)KTEDTIF Fuc 1 Hex 7 HexNAc 4 Neu5Ac 2 | 134.694 | 0.0 | -5.481 | 3.971e+07 | 67 - 79 | 2 | 37.76 | 37.92 | 38.25 |
| 3578.5098 | RN(D)EEYN(N)KSVQEIQATF Hex 5 HexNAc 4 | 132.369 | 0.0 | 11.490 | 1.291e+09 | 50 - 66 | 12 | 34.40 | 35.76 | 36.94 |
| 3409.4231 | NVQREN(N)GTVSRY Hex 6 HexNAc 5 | 132.247 | 0.0 | 10.552 | 1.745e+08 | 97 - 109 | 4 | 12.14 | 12.30 | 12.95 |
| 3724.5889 | FRNEEYN(N)KSVQEIQATF Hex 5 HexNAc 4 | 131.880 | 0.0 | -5.113 | 3.104e+07 | 49 - 66 | 8 | 37.76 | 37.76 | 38.08 |
| 4029.6181 | FTPN(N)KTEDTIF Hex 8 HexNAc 7 | 129.670 | 0.003 | 15.805 | 5.182e+07 | 68 - 79 | 5 | 29.39 | 29.91 | 30.25 |
| 4296.7082 | FYFTPN(N)KTEDTIF Fuc 1 Hex 7 HexNAc 4 Neu5Ac 2 | 129.468 | 0.003 | -3.628 | 4.049e+07 | 66 - 79 | 2 | 47.56 | 47.73 | 47.90 |
| 3555.4832 | NVQREN(N)GTVSRY Fuc 1 Hex 6 HexNAc 5 | 129.265 | 0.003 | -4.580 | 7.368e+07 | 97 - 109 | 2 | 12.14 | 12.14 | 12.47 |
| 3638.4464 | TPN(N)KTEDTIF Hex 9 HexNAc 5 | 128.680 | 0.003 | 12.545 | 3.402e+07 | 69 - 79 | 3 | 38.42 | 38.59 | 39.10 |
| 4703.8761 | YFTPN(N)KTEDTIF Fuc 1 Hex 9 HexNAc 8 | 128.357 | 0.003 | 14.003 | 3.907e+07 | 67 - 79 | 3 | 35.08 | 35.42 | 36.10 |
| 3887.6542 | LNVQREN(N)GTVSRY Hex 7 HexNAc 6 | 127.333 | 0.003 | 13.804 | 4.950e+08 | 96 - 109 | 10 | 16.36 | 16.53 | 17.70 |
| 4631.8691 | FYFTPN(N)KTEDTIF Fuc 2 Hex 8 HexNAc 7 | 125.362 | 0.006 | -3.794 | 2.302e+07 | 66 - 79 | 4 | 45.55 | 45.89 | 46.06 |
| 4033.6931 | LNVQREN(N)GTVSRY Fuc 1 Hex 7 HexNAc 6 | 124.567 | 0.006 | 13.429 | 2.614e+08 | 96 - 109 | 5 | 16.20 | 16.36 | 17.03 |
| 3624.4945 | YFTPN(N)KTEDTIF Hex 7 HexNAc 5 | 123.299 | 0.008 | -21.586 | 1.083e+06 | 67 - 79 | 3 | 36.10 | 36.10 | 36.10 |
| 3956.6054 | FTPN(N)KTEDTIF Fuc 2 Hex 7 HexNAc 6 | 123.025 | 0.008 | -3.673 | 1.294e+07 | 68 - 79 | 3 | 29.91 | 30.08 | 30.25 |
| 4167.6783 | FYFTPN(N)KTEDTIF Fuc 1 Hex 8 HexNAc 4 Neu5Ac 1 | 121.933 | 0.010 | -4.567 | 5.258e+07 | 66 - 79 | 2 | 47.73 | 47.90 | 48.23 |
| 4139.6907 | NVQREN(N)GTVSRY Hex 8 HexNAc 7 | 120.954 | 0.010 | -18.597 | 3.564e+07 | 97 - 109 | 2 | 11.97 | 11.97 | 11.97 |
| 3795.5886 | RNEEYN(N)KSVQEIQAT Hex 6 HexNAc 5 | 120.338 | 0.010 | -5.617 | 2.247e+07 | 50 - 65 | 2 | 17.20 | 17.20 | 17.53 |
| 4353.7292 | TPN(N)KTEDTIF Fuc 4 Hex 3 HexNAc 9 Neu5Ac 1 | 119.860 | 0.010 | -4.741 | 1.909e+07 | 69 - 79 | 1 | 48.57 | 48.74 | 48.91 |
| 3725.5810 | RN(D)EEYN(N)KSVQEIQATFF Hex 5 HexNAc 4 | 118.205 | 0.010 | 10.371 | 3.845e+08 | 50 - 67 | 9 | 45.55 | 47.23 | 47.56 |
| 4089.7020 | RN(D)EEYN(N)KSVQEIQATF Fuc 1 Hex 6 HexNAc 5 | 118.032 | 0.010 | 11.470 | 5.898e+08 | 50 - 66 | 15 | 33.72 | 35.42 | 36.27 |
| 4090.7140 | RN(D)EEYN(N)KSVQEIQATFF Hex 6 HexNAc 5 | 117.100 | 0.012 | 10.655 | 4.124e+08 | 50 - 67 | 16 | 45.03 | 45.38 | 46.90 |
| 4028.6028 | FTPN(N)KTEDTIF Fuc 3 Hex 6 HexNAc 5 Neu5Ac 1 | 116.371 | 0.017 | -19.030 | 7.691e+05 | 68 - 79 | 1 | 29.39 | 29.39 | 29.39 |
| 3522.5050 | LNVQREN(N)GTVSRY Hex 6 HexNAc 5 | 115.631 | 0.017 | -3.668 | 1.095e+08 | 96 - 109 | 4 | 16.53 | 16.69 | 17.03 |
| 3921.6040 | N(D)VQREN(N)GTVSRY Fuc 1 Hex 7 HexNAc 6 | 114.783 | 0.018 | -21.556 | 5.395e+06 | 97 - 109 | 3 | 13.61 | 13.61 | 13.61 |
| 4484.8013 | YFTPN(N)KTEDTIF Fuc 2 Hex 8 HexNAc 7 | 114.681 | 0.018 | 9.916 | 3.093e+07 | 67 - 79 | 2 | 35.59 | 36.10 | 36.27 |
| 4922.9502 | YFTPN(N)KTEDTIF Hex 10 HexNAc 9 | 114.185 | 0.018 | -4.466 | 1.916e+07 | 67 - 79 | 1 | 34.91 | 35.08 | 35.25 |
| 4617.9012 | RNEEYN(N)KSVQEIQATFF Hex 8 HexNAc 6 | 113.143 | 0.018 | -6.011 | 2.724e+07 | 50 - 67 | 4 | 15.87 | 15.87 | 16.20 |
| 3840.5165 | FTPN(N)KTEDTIF Hex 7 HexNAc 4 Neu5Ac 2 | 111.825 | 0.021 | 13.873 | 2.418e+07 | 68 - 79 | 4 | 31.27 | 31.60 | 31.95 |
| 3661.5178 | VQREN(N)GTVSRY Hex 7 HexNAc 6 | 111.509 | 0.021 | 0.000 | 0.000e+00 | 98 - 109 | 1 | - | - | - |
| 3655.4711 | YFTPN(N)KTEDTIF Fuc 1 Hex 7 HexNAc 3 Neu5Ac 1 | 110.546 | 0.021 | -19.572 | 4.211e+06 | 67 - 79 | 1 | 38.59 | 38.59 | 38.76 |
| 4504.8259 | NVQREN(N)GTVSRY Fuc 5 Hex 7 HexNAc 6 | 108.326 | 0.021 | -18.774 | 2.586e+07 | 97 - 109 | 1 | 11.97 | 11.97 | 11.97 |
| 4236.7714 | RN(D)EEYN(N)KSVQEIQATFF Fuc 1 Hex 6 HexNAc 5 | 105.015 | 0.024 | 10.324 | 2.039e+08 | 50 - 67 | 17 | 45.03 | 46.57 | 47.06 |
| 4418.7712 | TPN(N)KTEDTIF Fuc 2 Hex 7 HexNAc 9 | 104.155 | 0.025 | -19.800 | 3.669e+06 | 69 - 79 | 1 | 46.90 | 46.90 | 46.90 |
| 4224.6964 | TPN(N)KTEDTIF Fuc 4 Hex 4 HexNAc 9 | 103.299 | 0.025 | -4.308 | 2.342e+07 | 69 - 79 | 2 | 48.91 | 48.91 | 49.42 |
| 4252.8088 | RNEEYN(N)KSVQEIQATFFY Hex 6 HexNAc 5 | 103.041 | 0.025 | -3.874 | 1.854e+08 | 50 - 68 | 2 | 43.37 | 43.54 | 43.87 |
| 3668.5631 | LNVQREN(N)GTVSRY Fuc 1 Hex 6 HexNAc 5 | 102.965 | 0.025 | -4.247 | 5.978e+07 | 96 - 109 | 4 | 16.53 | 16.53 | 16.86 |
| 3481.4479 | TPN(N)KTEDTIF Hex 3 HexNAc 9 | 102.328 | 0.026 | 0.000 | 0.000e+00 | 69 - 79 | 1 | - | - | - |
| 3960.6709 | RNEEYN(N)KSVQEIQATF Fuc 2 Hex 5 HexNAc 3 Neu5Ac 1 | 101.805 | 0.026 | -8.805 | 7.100e+06 | 50 - 66 | 5 | 34.23 | 34.23 | 34.74 |
| 3124.4019 | LVPVPITN(N)ATL Hex 6 HexNAc 5 | 101.594 | 0.028 | -20.717 | 3.473e+06 | 25 - 36 | 1 | 45.72 | 45.72 | 45.89 |
| 3044.2875 | NVQREN(N)GTVSRY Hex 5 HexNAc 4 | 101.380 | 0.028 | -19.211 | 1.116e+07 | 97 - 109 | 1 | 12.30 | 12.30 | 12.30 |
| 4077.6301 | TPN(N)KTEDTIF Fuc 1 Hex 4 HexNAc 9 Neu5Ac 1 | 100.998 | 0.028 | -19.595 | 1.420e+07 | 69 - 79 | 2 | 38.08 | 38.08 | 38.25 |
| 4328.8281 | LNVQREN(N)GTVSRY Fuc 3 Hex 7 HexNAc 6 | 100.749 | 0.028 | 0.000 | 0.000e+00 | 96 - 109 | 1 | - | - | - |
| 3469.5107 | C(C)ANLVPVPITN(N)ATL Hex 6 HexNAc 5 | 99.187 | 0.031 | 13.430 | 2.916e+07 | 22 - 36 | 5 | 50.28 | 50.45 | 51.13 |
| 4041.6757 | N(N)KSVQEIQATFF Fuc 3 Hex 6 HexNAc 6 | 98.319 | 0.032 | 0.000 | 0.000e+00 | 55 - 67 | 1 | - | - | - |
| 4818.9792 | RNEEYN(N)KSVQEIQATF Fuc 1 Hex 8 HexNAc 7 | 97.275 | 0.038 | -3.754 | 1.301e+07 | 50 - 66 | 2 | 31.60 | 31.77 | 31.95 |
| 3362.4427 | TN(N)ATLDRITGKW Hex 6 HexNAc 5 | 96.329 | 0.041 | -4.637 | 2.438e+07 | 31 - 43 | 3 | 30.08 | 30.25 | 30.59 |
| 4326.6825 | TPN(N)KTEDTIF Fuc 3 Hex 10 HexNAc 4 Neu5Ac 1 | 95.989 | 0.042 | -4.278 | 1.974e+07 | 69 - 79 | 3 | 48.74 | 48.91 | 49.08 |
| 4179.7505 | LNVQREN(N)GTVSRY Fuc 2 Hex 7 HexNAc 6 | 95.887 | 0.042 | -4.437 | 7.068e+07 | 96 - 109 | 3 | 16.20 | 16.20 | 16.53 |
| 4214.7355 | NVQREN(N)GTVSRY Fuc 3 Hex 7 HexNAc 6 | 92.499 | 0.046 | 0.000 | 0.000e+00 | 97 - 109 | 1 | - | - | - |
| 4252.7644 | LNVQREN(N)GTVSRY Hex 8 HexNAc 7 | 92.428 | 0.046 | -20.188 | 3.308e+07 | 96 - 109 | 3 | 16.03 | 16.20 | 16.20 |
| 3776.5396 | VQREN(N)GTVSRY Fuc 1 Hex 5 HexNAc 6 Neu5Ac 1 | 92.026 | 0.048 | 0.000 | 0.000e+00 | 98 - 109 | 1 | - | - | - |

### TPN(N)KTEDTIF Hex 6 HexNAc 5

**Mass:**  3152.2820

(1.085e-06)

**Charge States:** 3, 4

**Start Position**: 70

**End Position**: 80

| Profile Score | Tandem Score | TDA q-value | Volume | Spectrum Matches |
| --- | --- | --- | --- | --- |
| 15.2062 | 182.0794 | 0.0000 | 1.9479e+08 | 9 |

**Adduct Signal Fractions**

- **Unmodified**: 1.9479e+08

### TPN(N)KTEDTIF Hex 7 HexNAc 6

**Mass:**  3517.4170

(9.486e-07)

**Charge States:** 3, 4

**Start Position**: 70

**End Position**: 80

| Profile Score | Tandem Score | TDA q-value | Volume | Spectrum Matches |
| --- | --- | --- | --- | --- |
| 15.6256 | 181.7918 | 0.0000 | 2.8104e+08 | 10 |

**Adduct Signal Fractions**

- **Unmodified**: 2.8104e+08

### TPN(N)KTEDTIF Hex 5 HexNAc 4

**Mass:**  2787.1527

(1.697e-06)

**Charge States:** 3

**Start Position**: 70

**End Position**: 80

| Profile Score | Tandem Score | TDA q-value | Volume | Spectrum Matches |
| --- | --- | --- | --- | --- |
| 14.2635 | 181.7775 | 0.0000 | 2.2118e+07 | 3 |

**Adduct Signal Fractions**

- **Unmodified**: 2.2118e+07

### N(N)KSVQEIQATF Hex 5 HexNAc 4

**Mass:**  2886.2318

(1.879e-06)

**Charge States:** 3

**Start Position**: 56

**End Position**: 67

| Profile Score | Tandem Score | TDA q-value | Volume | Spectrum Matches |
| --- | --- | --- | --- | --- |
| 14.0823 | 178.6837 | 0.0000 | 2.5141e+07 | 2 |

**Adduct Signal Fractions**

- **Unmodified**: 2.5141e+07

### YFTPN(N)KTEDTIF Hex 7 HexNAc 6

**Mass:**  3827.5453

(2.124e-06)

**Charge States:** 3, 4

**Start Position**: 68

**End Position**: 80

| Profile Score | Tandem Score | TDA q-value | Volume | Spectrum Matches |
| --- | --- | --- | --- | --- |
| 14.9178 | 177.2185 | 0.0000 | 4.5798e+09 | 52 |

**Adduct Signal Fractions**

- **Unmodified**: 4.5798e+09

### YFTPN(N)KTEDTIF Fuc 1 Hex 7 HexNAc 6

**Mass:**  3973.6052

(2.025e-06)

**Charge States:** 3, 4

**Start Position**: 68

**End Position**: 80

| Profile Score | Tandem Score | TDA q-value | Volume | Spectrum Matches |
| --- | --- | --- | --- | --- |
| 14.8042 | 176.4260 | 0.0000 | 8.8997e+08 | 8 |

**Adduct Signal Fractions**

- **Unmodified**: 8.8997e+08

### YFTPN(N)KTEDTIF Hex 5 HexNAc 4

**Mass:**  3097.2819

(2.442e-07)

**Charge States:** 2, 3

**Start Position**: 68

**End Position**: 80

| Profile Score | Tandem Score | TDA q-value | Volume | Spectrum Matches |
| --- | --- | --- | --- | --- |
| 15.2378 | 174.5574 | 0.0000 | 3.7855e+08 | 5 |

**Adduct Signal Fractions**

- **Unmodified**: 3.7855e+08

### YFTPN(N)KTEDTIF Hex 6 HexNAc 5

**Mass:**  3462.4143

(8.840e-07)

**Charge States:** 2, 3, 4

**Start Position**: 68

**End Position**: 80

| Profile Score | Tandem Score | TDA q-value | Volume | Spectrum Matches |
| --- | --- | --- | --- | --- |
| 14.1726 | 174.2428 | 0.0000 | 2.8950e+09 | 28 |

**Adduct Signal Fractions**

- **Unmodified**: 2.8950e+09

### FTPN(N)KTEDTIF Hex 6 HexNAc 5

**Mass:**  3299.3498

(6.490e-07)

**Charge States:** 3, 4

**Start Position**: 69

**End Position**: 80

| Profile Score | Tandem Score | TDA q-value | Volume | Spectrum Matches |
| --- | --- | --- | --- | --- |
| 15.0362 | 173.0391 | 0.0000 | 4.0949e+08 | 12 |

**Adduct Signal Fractions**

- **Unmodified**: 4.0949e+08

### EEYN(N)KSVQEIQATF Hex 5 HexNAc 4

**Mass:**  3307.3804

(4.293e-07)

**Charge States:** 3

**Start Position**: 53

**End Position**: 67

| Profile Score | Tandem Score | TDA q-value | Volume | Spectrum Matches |
| --- | --- | --- | --- | --- |
| 12.1693 | 170.8901 | 0.0000 | 8.5366e+07 | 2 |

**Adduct Signal Fractions**

- **Unmodified**: 8.5366e+07

### TPN(N)KTEDTIF Fuc 1 Hex 6 HexNAc 5

**Mass:**  3298.3430

(1.570e-06)

**Charge States:** 3, 4

**Start Position**: 70

**End Position**: 80

| Profile Score | Tandem Score | TDA q-value | Volume | Spectrum Matches |
| --- | --- | --- | --- | --- |
| 13.4078 | 170.5870 | 0.0000 | 3.1141e+07 | 4 |

**Adduct Signal Fractions**

- **Unmodified**: 3.1141e+07

### YFTPN(N)KTEDTIF Fuc 4 Hex 7 HexNAc 7

**Mass:**  4614.8342

(-3.773e-06)

**Charge States:** 3, 4

**Start Position**: 68

**End Position**: 80

| Profile Score | Tandem Score | TDA q-value | Volume | Spectrum Matches |
| --- | --- | --- | --- | --- |
| 15.6074 | 169.3788 | 0.0000 | 8.1892e+07 | 3 |

**Adduct Signal Fractions**

- **Unmodified**: 8.1892e+07

### FYFTPN(N)KTEDTIF Hex 6 HexNAc 5

**Mass:**  3609.4843

(1.898e-06)

**Charge States:** 3, 4

**Start Position**: 67

**End Position**: 80

| Profile Score | Tandem Score | TDA q-value | Volume | Spectrum Matches |
| --- | --- | --- | --- | --- |
| 14.7594 | 168.6670 | 0.0000 | 2.7860e+09 | 49 |

**Adduct Signal Fractions**

- **Unmodified**: 2.7860e+09

### FTPN(N)KTEDTIF Hex 7 HexNAc 6

**Mass:**  3664.4848

(1.249e-06)

**Charge States:** 3, 4

**Start Position**: 69

**End Position**: 80

| Profile Score | Tandem Score | TDA q-value | Volume | Spectrum Matches |
| --- | --- | --- | --- | --- |
| 16.2646 | 168.0623 | 0.0000 | 6.6287e+08 | 13 |

**Adduct Signal Fractions**

- **Unmodified**: 6.6287e+08

### YFTPN(N)KTEDTIF Fuc 1 Hex 6 HexNAc 5

**Mass:**  3608.4746

(1.906e-06)

**Charge States:** 3, 4

**Start Position**: 68

**End Position**: 80

| Profile Score | Tandem Score | TDA q-value | Volume | Spectrum Matches |
| --- | --- | --- | --- | --- |
| 14.3167 | 167.9220 | 0.0000 | 5.9993e+08 | 7 |

**Adduct Signal Fractions**

- **Unmodified**: 5.9993e+08

### FYFTPN(N)KTEDTIF Fuc 1 Hex 6 HexNAc 5

**Mass:**  3755.5453

(1.912e-06)

**Charge States:** 3, 4

**Start Position**: 67

**End Position**: 80

| Profile Score | Tandem Score | TDA q-value | Volume | Spectrum Matches |
| --- | --- | --- | --- | --- |
| 14.5482 | 167.3858 | 0.0000 | 5.6886e+08 | 11 |

**Adduct Signal Fractions**

- **Unmodified**: 5.6886e+08

### TPN(N)KTEDTIF Fuc 1 Hex 7 HexNAc 6

**Mass:**  3663.4755

(1.464e-06)

**Charge States:** 3, 4

**Start Position**: 70

**End Position**: 80

| Profile Score | Tandem Score | TDA q-value | Volume | Spectrum Matches |
| --- | --- | --- | --- | --- |
| 13.7494 | 167.3572 | 0.0000 | 4.4044e+07 | 4 |

**Adduct Signal Fractions**

- **Unmodified**: 4.4044e+07

### FYFTPN(N)KTEDTIF Fuc 1 Hex 7 HexNAc 6

**Mass:**  4120.6809

(2.247e-06)

**Charge States:** 3, 4

**Start Position**: 67

**End Position**: 80

| Profile Score | Tandem Score | TDA q-value | Volume | Spectrum Matches |
| --- | --- | --- | --- | --- |
| 14.3982 | 166.3353 | 0.0000 | 8.7767e+08 | 12 |

**Adduct Signal Fractions**

- **Unmodified**: 8.7767e+08

### YFTPN(N)KTEDTIF Hex 8 HexNAc 7

**Mass:**  4192.6833

(1.274e-06)

**Charge States:** 3, 4

**Start Position**: 68

**End Position**: 80

| Profile Score | Tandem Score | TDA q-value | Volume | Spectrum Matches |
| --- | --- | --- | --- | --- |
| 15.2448 | 165.8290 | 0.0000 | 7.6266e+08 | 11 |

**Adduct Signal Fractions**

- **Unmodified**: 7.6266e+08

### FYFTPN(N)KTEDTIF Hex 7 HexNAc 6

**Mass:**  3974.6226

(2.317e-06)

**Charge States:** 3, 4

**Start Position**: 67

**End Position**: 80

| Profile Score | Tandem Score | TDA q-value | Volume | Spectrum Matches |
| --- | --- | --- | --- | --- |
| 15.2894 | 165.6542 | 0.0000 | 4.7166e+09 | 82 |

**Adduct Signal Fractions**

- **Unmodified**: 4.7166e+09

### FYFTPN(N)KTEDTIF Hex 8 HexNAc 7

**Mass:**  4339.7546

(1.751e-06)

**Charge States:** 3, 4

**Start Position**: 67

**End Position**: 80

| Profile Score | Tandem Score | TDA q-value | Volume | Spectrum Matches |
| --- | --- | --- | --- | --- |
| 14.7801 | 165.2795 | 0.0000 | 6.3863e+08 | 9 |

**Adduct Signal Fractions**

- **Unmodified**: 6.3863e+08

### FYFTPN(N)KTEDTIF Fuc 2 Hex 7 HexNAc 6

**Mass:**  4266.7375

(2.138e-06)

**Charge States:** 4

**Start Position**: 67

**End Position**: 80

| Profile Score | Tandem Score | TDA q-value | Volume | Spectrum Matches |
| --- | --- | --- | --- | --- |
| -16.1994 | 164.1765 | 0.0000 | 1.1875e+08 | 2 |

**Adduct Signal Fractions**

- **Unmodified**: 1.1875e+08

### TPN(N)KTEDTIF Hex 8 HexNAc 7

**Mass:**  3882.5473

(1.107e-06)

**Charge States:** 3, 4

**Start Position**: 70

**End Position**: 80

| Profile Score | Tandem Score | TDA q-value | Volume | Spectrum Matches |
| --- | --- | --- | --- | --- |
| -4.5229 | 163.9402 | 0.0000 | 2.1186e+07 | 4 |

**Adduct Signal Fractions**

- **Unmodified**: 2.1186e+07

### RNEEYN(N)KSVQEIQATFF Fuc 1 Hex 6 HexNAc 5

**Mass:**  4235.7936

(7.762e-07)

**Charge States:** 3, 4

**Start Position**: 51

**End Position**: 68

| Profile Score | Tandem Score | TDA q-value | Volume | Spectrum Matches |
| --- | --- | --- | --- | --- |
| 15.6710 | 162.0343 | 0.0000 | 1.9278e+09 | 16 |

**Adduct Signal Fractions**

- **Unmodified**: 1.9278e+09

### FYFTPN(N)KTEDTIF Hex 5 HexNAc 4

**Mass:**  3244.3511

(9.271e-07)

**Charge States:** 3

**Start Position**: 67

**End Position**: 80

| Profile Score | Tandem Score | TDA q-value | Volume | Spectrum Matches |
| --- | --- | --- | --- | --- |
| 10.7671 | 161.9809 | 0.0000 | 2.2235e+08 | 4 |

**Adduct Signal Fractions**

- **Unmodified**: 2.2235e+08

### N(N)KTEDTIF Hex 7 HexNAc 6

**Mass:**  3319.3123

(1.737e-07)

**Charge States:** 3

**Start Position**: 72

**End Position**: 80

| Profile Score | Tandem Score | TDA q-value | Volume | Spectrum Matches |
| --- | --- | --- | --- | --- |
| -20.2355 | 161.4047 | 0.0000 | 3.2172e+06 | 1 |

**Adduct Signal Fractions**

- **Unmodified**: 3.2172e+06

### RNEEYN(N)KSVQEIQATFF Hex 6 HexNAc 5

**Mass:**  4089.7255

(2.978e-06)

**Charge States:** 3, 4

**Start Position**: 51

**End Position**: 68

| Profile Score | Tandem Score | TDA q-value | Volume | Spectrum Matches |
| --- | --- | --- | --- | --- |
| 16.6898 | 161.0298 | 0.0000 | 3.9484e+09 | 19 |

**Adduct Signal Fractions**

- **Unmodified**: 3.9484e+09

### YFTPN(N)KTEDTIF Fuc 2 Hex 7 HexNAc 6

**Mass:**  4119.6682

(1.994e-06)

**Charge States:** 3, 4

**Start Position**: 68

**End Position**: 80

| Profile Score | Tandem Score | TDA q-value | Volume | Spectrum Matches |
| --- | --- | --- | --- | --- |
| -4.1040 | 160.2445 | 0.0000 | 1.2720e+08 | 2 |

**Adduct Signal Fractions**

- **Unmodified**: 1.2720e+08

### YFTPN(N)KTEDTIF Hex 9 HexNAc 8

**Mass:**  4557.8166

(1.831e-06)

**Charge States:** 3, 4

**Start Position**: 68

**End Position**: 80

| Profile Score | Tandem Score | TDA q-value | Volume | Spectrum Matches |
| --- | --- | --- | --- | --- |
| 15.3983 | 159.9471 | 0.0000 | 2.3039e+08 | 6 |

**Adduct Signal Fractions**

- **Unmodified**: 2.3039e+08

### EEYN(N)KSVQEIQATF Hex 6 HexNAc 5

**Mass:**  3672.5121

(1.397e-06)

**Charge States:** 3

**Start Position**: 53

**End Position**: 67

| Profile Score | Tandem Score | TDA q-value | Volume | Spectrum Matches |
| --- | --- | --- | --- | --- |
| -3.1992 | 158.8277 | 0.0000 | 8.1237e+07 | 3 |

**Adduct Signal Fractions**

- **Unmodified**: 8.1237e+07

### RNEEYN(N)KSVQEIQATF Fuc 1 Hex 5 HexNAc 4

**Mass:**  3723.5857

(1.928e-06)

**Charge States:** 3, 4

**Start Position**: 51

**End Position**: 67

| Profile Score | Tandem Score | TDA q-value | Volume | Spectrum Matches |
| --- | --- | --- | --- | --- |
| 11.2212 | 158.2511 | 0.0000 | 3.3473e+08 | 9 |

**Adduct Signal Fractions**

- **Unmodified**: 3.3473e+08

### FTPN(N)KTEDTIF Fuc 1 Hex 6 HexNAc 5

**Mass:**  3445.4101

(3.906e-07)

**Charge States:** 3, 4

**Start Position**: 69

**End Position**: 80

| Profile Score | Tandem Score | TDA q-value | Volume | Spectrum Matches |
| --- | --- | --- | --- | --- |
| 13.4290 | 158.2472 | 0.0000 | 7.4120e+07 | 5 |

**Adduct Signal Fractions**

- **Unmodified**: 7.4120e+07

### FYFTPN(N)KT Hex 6 HexNAc 5

**Mass:**  3004.2171

(2.479e-06)

**Charge States:** 3

**Start Position**: 67

**End Position**: 75

| Profile Score | Tandem Score | TDA q-value | Volume | Spectrum Matches |
| --- | --- | --- | --- | --- |
| -4.0041 | 156.8222 | 0.0000 | 6.7236e+06 | 2 |

**Adduct Signal Fractions**

- **Unmodified**: 6.7236e+06

### RNEEYN(N)KSVQEIQATF Hex 5 HexNAc 4

**Mass:**  3577.5243

(1.699e-06)

**Charge States:** 3, 4

**Start Position**: 51

**End Position**: 67

| Profile Score | Tandem Score | TDA q-value | Volume | Spectrum Matches |
| --- | --- | --- | --- | --- |
| 16.1774 | 156.5592 | 0.0000 | 8.4197e+09 | 22 |

**Adduct Signal Fractions**

- **Unmodified**: 8.4197e+09

### FYFTPN(N)KTEDTIF Fuc 1 Hex 8 HexNAc 7

**Mass:**  4485.8098

(1.898e-06)

**Charge States:** 3, 4

**Start Position**: 67

**End Position**: 80

| Profile Score | Tandem Score | TDA q-value | Volume | Spectrum Matches |
| --- | --- | --- | --- | --- |
| 14.8512 | 156.4959 | 0.0000 | 1.3093e+08 | 6 |

**Adduct Signal Fractions**

- **Unmodified**: 1.3093e+08

### RNEEYN(N)KSVQEIQATFF Hex 5 HexNAc 4

**Mass:**  3724.5928

(8.245e-07)

**Charge States:** 3, 4

**Start Position**: 51

**End Position**: 68

| Profile Score | Tandem Score | TDA q-value | Volume | Spectrum Matches |
| --- | --- | --- | --- | --- |
| 15.9918 | 156.3606 | 0.0000 | 4.1767e+09 | 17 |

**Adduct Signal Fractions**

- **Unmodified**: 4.1767e+09

### EEYN(N)KSVQEIQATF Fuc 1 Hex 6 HexNAc 5

**Mass:**  3818.5717

(1.417e-06)

**Charge States:** 3

**Start Position**: 53

**End Position**: 67

| Profile Score | Tandem Score | TDA q-value | Volume | Spectrum Matches |
| --- | --- | --- | --- | --- |
| -4.0463 | 155.4792 | 0.0000 | 3.4096e+07 | 2 |

**Adduct Signal Fractions**

- **Unmodified**: 3.4096e+07

### EEYN(N)KSVQEIQATFF Hex 5 HexNAc 4

**Mass:**  3454.4448

(6.809e-08)

**Charge States:** 3

**Start Position**: 53

**End Position**: 68

| Profile Score | Tandem Score | TDA q-value | Volume | Spectrum Matches |
| --- | --- | --- | --- | --- |
| -4.9809 | 155.2356 | 0.0000 | 2.7311e+07 | 1 |

**Adduct Signal Fractions**

- **Unmodified**: 2.7311e+07

### EEYN(N)KSVQEIQATFF Hex 6 HexNAc 5

**Mass:**  3819.5806

(2.012e-06)

**Charge States:** 3, 4

**Start Position**: 53

**End Position**: 68

| Profile Score | Tandem Score | TDA q-value | Volume | Spectrum Matches |
| --- | --- | --- | --- | --- |
| -5.6259 | 154.9731 | 0.0000 | 2.6073e+07 | 2 |

**Adduct Signal Fractions**

- **Unmodified**: 2.6073e+07

### YN(N)SSYL Hex 7 HexNAc 6

**Mass:**  3098.1795

(1.636e-06)

**Charge States:** 3

**Start Position**: 92

**End Position**: 98

| Profile Score | Tandem Score | TDA q-value | Volume | Spectrum Matches |
| --- | --- | --- | --- | --- |
| -19.7981 | 154.9115 | 0.0000 | 3.2232e+06 | 1 |

**Adduct Signal Fractions**

- **Unmodified**: 3.2232e+06

### RNEEYN(N)KSVQEIQATFF Fuc 2 Hex 6 HexNAc 5

**Mass:**  4381.8478

(1.511e-06)

**Charge States:** 4

**Start Position**: 51

**End Position**: 68

| Profile Score | Tandem Score | TDA q-value | Volume | Spectrum Matches |
| --- | --- | --- | --- | --- |
| -3.7138 | 153.7672 | 0.0000 | 7.3407e+07 | 3 |

**Adduct Signal Fractions**

- **Unmodified**: 7.3407e+07

### RNEEYN(N)KSVQEIQATF Hex 6 HexNAc 5

**Mass:**  3942.6577

(1.717e-06)

**Charge States:** 3, 4

**Start Position**: 51

**End Position**: 67

| Profile Score | Tandem Score | TDA q-value | Volume | Spectrum Matches |
| --- | --- | --- | --- | --- |
| 15.0098 | 153.5112 | 0.0000 | 6.0077e+09 | 20 |

**Adduct Signal Fractions**

- **Unmodified**: 6.0077e+09

### RNEEYN(N)KSVQEIQATFF Fuc 1 Hex 5 HexNAc 4

**Mass:**  3870.6619

(2.368e-06)

**Charge States:** 3, 4

**Start Position**: 51

**End Position**: 68

| Profile Score | Tandem Score | TDA q-value | Volume | Spectrum Matches |
| --- | --- | --- | --- | --- |
| 13.1317 | 153.0097 | 0.0000 | 1.5337e+08 | 5 |

**Adduct Signal Fractions**

- **Unmodified**: 1.5337e+08

### YFTPN(N)KTEDTIF Fuc 1 Hex 8 HexNAc 4 Neu5Ac 1

**Mass:**  4020.6059

(2.924e-06)

**Charge States:** 3, 4

**Start Position**: 68

**End Position**: 80

| Profile Score | Tandem Score | TDA q-value | Volume | Spectrum Matches |
| --- | --- | --- | --- | --- |
| -4.5260 | 152.8367 | 0.0000 | 5.3739e+07 | 3 |

**Adduct Signal Fractions**

- **Unmodified**: 5.3739e+07

### YFTPN(N)KTEDTIF Fuc 1 Hex 8 HexNAc 7

**Mass:**  4338.7419

(7.933e-07)

**Charge States:** 3, 4

**Start Position**: 68

**End Position**: 80

| Profile Score | Tandem Score | TDA q-value | Volume | Spectrum Matches |
| --- | --- | --- | --- | --- |
| 14.6827 | 152.4270 | 0.0000 | 1.4490e+08 | 5 |

**Adduct Signal Fractions**

- **Unmodified**: 1.4490e+08

### YFTPN(N)KTEDTIF Hex 7 HexNAc 4 Neu5Ac 2

**Mass:**  4003.5807

(9.676e-07)

**Charge States:** 3, 4

**Start Position**: 68

**End Position**: 80

| Profile Score | Tandem Score | TDA q-value | Volume | Spectrum Matches |
| --- | --- | --- | --- | --- |
| 14.5502 | 152.3472 | 0.0000 | 3.3362e+08 | 6 |

**Adduct Signal Fractions**

- **Unmodified**: 3.3362e+08

### RNEEYN(N)KSVQEIQATF Fuc 1 Hex 6 HexNAc 5

**Mass:**  4088.7153

(1.681e-06)

**Charge States:** 3, 4

**Start Position**: 51

**End Position**: 67

| Profile Score | Tandem Score | TDA q-value | Volume | Spectrum Matches |
| --- | --- | --- | --- | --- |
| 14.5867 | 152.3287 | 0.0000 | 2.9510e+09 | 16 |

**Adduct Signal Fractions**

- **Unmodified**: 2.9510e+09

### FTPN(N)KTEDTIF Fuc 1 Hex 7 HexNAc 6

**Mass:**  3810.5421

(1.297e-06)

**Charge States:** 3, 4

**Start Position**: 69

**End Position**: 80

| Profile Score | Tandem Score | TDA q-value | Volume | Spectrum Matches |
| --- | --- | --- | --- | --- |
| 13.6241 | 151.2150 | 0.0000 | 1.1906e+08 | 6 |

**Adduct Signal Fractions**

- **Unmodified**: 1.1906e+08

### RNEEYN(N)KSVQEIQATFF Hex 7 HexNAc 6

**Mass:**  4454.8581

(1.209e-06)

**Charge States:** 3, 4

**Start Position**: 51

**End Position**: 68

| Profile Score | Tandem Score | TDA q-value | Volume | Spectrum Matches |
| --- | --- | --- | --- | --- |
| 14.9018 | 150.9494 | 0.0000 | 1.8544e+08 | 4 |

**Adduct Signal Fractions**

- **Unmodified**: 1.8544e+08

### RNEEYN(N)KSVQEIQATF Hex 7 HexNAc 6

**Mass:**  4307.7927

(1.279e-06)

**Charge States:** 3, 4

**Start Position**: 51

**End Position**: 67

| Profile Score | Tandem Score | TDA q-value | Volume | Spectrum Matches |
| --- | --- | --- | --- | --- |
| -2.6482 | 150.5649 | 0.0000 | 2.8955e+08 | 3 |

**Adduct Signal Fractions**

- **Unmodified**: 2.8955e+08

### RNEEYN(N)KSVQEIQATF Fuc 2 Hex 6 HexNAc 5

**Mass:**  4234.7780

(2.383e-06)

**Charge States:** 4

**Start Position**: 51

**End Position**: 67

| Profile Score | Tandem Score | TDA q-value | Volume | Spectrum Matches |
| --- | --- | --- | --- | --- |
| -3.6005 | 150.4575 | 0.0000 | 9.7249e+07 | 3 |

**Adduct Signal Fractions**

- **Unmodified**: 9.7249e+07

### YFTPN(N)KT Hex 7 HexNAc 6

**Mass:**  3222.2737

(-2.187e-07)

**Charge States:** 3

**Start Position**: 68

**End Position**: 75

| Profile Score | Tandem Score | TDA q-value | Volume | Spectrum Matches |
| --- | --- | --- | --- | --- |
| -20.6750 | 150.0115 | 0.0000 | 3.3539e+06 | 1 |

**Adduct Signal Fractions**

- **Unmodified**: 3.3539e+06

### RNEEYN(N)KSVQEIQATFF Fuc 1 Hex 7 HexNAc 6

**Mass:**  4600.9162

(1.143e-06)

**Charge States:** 3, 4

**Start Position**: 51

**End Position**: 68

| Profile Score | Tandem Score | TDA q-value | Volume | Spectrum Matches |
| --- | --- | --- | --- | --- |
| 14.8105 | 149.9970 | 0.0000 | 6.8100e+07 | 3 |

**Adduct Signal Fractions**

- **Unmodified**: 6.8100e+07

### RNEEYN(N)KSVQEIQATF Fuc 1 Hex 7 HexNAc 6

**Mass:**  4453.8513

(1.544e-06)

**Charge States:** 4

**Start Position**: 51

**End Position**: 67

| Profile Score | Tandem Score | TDA q-value | Volume | Spectrum Matches |
| --- | --- | --- | --- | --- |
| -3.4806 | 149.7379 | 0.0000 | 1.2881e+08 | 2 |

**Adduct Signal Fractions**

- **Unmodified**: 1.2881e+08

### FTPN(N)KTEDTIF Hex 5 HexNAc 4

**Mass:**  2934.2190

(1.669e-06)

**Charge States:** 3

**Start Position**: 69

**End Position**: 80

| Profile Score | Tandem Score | TDA q-value | Volume | Spectrum Matches |
| --- | --- | --- | --- | --- |
| -3.4927 | 149.6019 | 0.0000 | 4.0119e+07 | 3 |

**Adduct Signal Fractions**

- **Unmodified**: 4.0119e+07

### TPN(N)KTEDTIF Fuc 4 Hex 8 HexNAc 5

**Mass:**  4060.6027

(-2.955e-06)

**Charge States:** 3, 4

**Start Position**: 70

**End Position**: 80

| Profile Score | Tandem Score | TDA q-value | Volume | Spectrum Matches |
| --- | --- | --- | --- | --- |
| 13.0799 | 148.1229 | 0.0000 | 1.3763e+08 | 4 |

**Adduct Signal Fractions**

- **Unmodified**: 1.3763e+08

### FYFTPN(N)KTEDTIF Hex 9 HexNAc 8

**Mass:**  4704.8850

(2.005e-06)

**Charge States:** 3, 4

**Start Position**: 67

**End Position**: 80

| Profile Score | Tandem Score | TDA q-value | Volume | Spectrum Matches |
| --- | --- | --- | --- | --- |
| 16.7934 | 147.3900 | 0.0000 | 1.7216e+08 | 6 |

**Adduct Signal Fractions**

- **Unmodified**: 1.7216e+08

### FYFTPN(N)KTEDTIF Hex 7 HexNAc 4 Neu5Ac 2

**Mass:**  4150.6501

(2.291e-06)

**Charge States:** 3, 4

**Start Position**: 67

**End Position**: 80

| Profile Score | Tandem Score | TDA q-value | Volume | Spectrum Matches |
| --- | --- | --- | --- | --- |
| 13.4904 | 146.1667 | 0.0000 | 2.5214e+08 | 5 |

**Adduct Signal Fractions**

- **Unmodified**: 2.5214e+08

### FYFTPN(N)KT Hex 7 HexNAc 6

**Mass:**  3369.3477

(2.075e-06)

**Charge States:** 3

**Start Position**: 67

**End Position**: 75

| Profile Score | Tandem Score | TDA q-value | Volume | Spectrum Matches |
| --- | --- | --- | --- | --- |
| 12.8887 | 145.9008 | 0.0000 | 1.3344e+07 | 2 |

**Adduct Signal Fractions**

- **Unmodified**: 1.3344e+07

### FYFTPN(N)KTED Hex 7 HexNAc 6

**Mass:**  3613.4177

(1.538e-06)

**Charge States:** 3

**Start Position**: 67

**End Position**: 77

| Profile Score | Tandem Score | TDA q-value | Volume | Spectrum Matches |
| --- | --- | --- | --- | --- |
| -18.0710 | 144.9568 | 0.0000 | 5.2266e+06 | 1 |

**Adduct Signal Fractions**

- **Unmodified**: 5.2266e+06

### NVQREN(N)GTVSRY Fuc 2 Hex 7 HexNAc 6

**Mass:**  4066.6774

(3.575e-06)

**Charge States:** 3, 4

**Start Position**: 98

**End Position**: 110

| Profile Score | Tandem Score | TDA q-value | Volume | Spectrum Matches |
| --- | --- | --- | --- | --- |
| -19.4524 | 144.1146 | 0.0000 | 8.0604e+07 | 2 |

**Adduct Signal Fractions**

- **Unmodified**: 8.0604e+07

### NVQREN(N)GTVSRY Fuc 1 Hex 7 HexNAc 6

**Mass:**  3920.6171

(3.105e-06)

**Charge States:** 3, 4

**Start Position**: 98

**End Position**: 110

| Profile Score | Tandem Score | TDA q-value | Volume | Spectrum Matches |
| --- | --- | --- | --- | --- |
| -3.9257 | 143.8907 | 0.0000 | 3.1934e+08 | 4 |

**Adduct Signal Fractions**

- **Unmodified**: 3.1934e+08

### RNEEYN(N)KSVQEIQAT Hex 5 HexNAc 4

**Mass:**  3430.4562

(-1.357e-06)

**Charge States:** 3

**Start Position**: 51

**End Position**: 66

| Profile Score | Tandem Score | TDA q-value | Volume | Spectrum Matches |
| --- | --- | --- | --- | --- |
| -4.6074 | 142.5895 | 0.0000 | 3.4182e+07 | 3 |

**Adduct Signal Fractions**

- **Unmodified**: 3.4182e+07

### NVQREN(N)GTVSRY Hex 7 HexNAc 6

**Mass:**  3774.5572

(2.988e-06)

**Charge States:** 3, 4

**Start Position**: 98

**End Position**: 110

| Profile Score | Tandem Score | TDA q-value | Volume | Spectrum Matches |
| --- | --- | --- | --- | --- |
| 12.7526 | 141.6599 | 0.0000 | 6.1783e+08 | 7 |

**Adduct Signal Fractions**

- **Unmodified**: 6.1783e+08

### TPN(N)KTEDTIF Fuc 3 Hex 3 HexNAc 9 Neu5Ac 1

**Mass:**  4207.6711

(-9.137e-06)

**Charge States:** 3, 4

**Start Position**: 70

**End Position**: 80

| Profile Score | Tandem Score | TDA q-value | Volume | Spectrum Matches |
| --- | --- | --- | --- | --- |
| 13.9395 | 140.9425 | 0.0000 | 1.0207e+08 | 4 |

**Adduct Signal Fractions**

- **Unmodified**: 1.0207e+08

### TPN(N)KTEDTIF Hex 7 HexNAc 4 Neu5Ac 2

**Mass:**  3693.4473

(2.864e-07)

**Charge States:** 3, 4

**Start Position**: 70

**End Position**: 80

| Profile Score | Tandem Score | TDA q-value | Volume | Spectrum Matches |
| --- | --- | --- | --- | --- |
| -4.2797 | 138.6748 | 0.0000 | 9.8711e+06 | 3 |

**Adduct Signal Fractions**

- **Unmodified**: 9.8711e+06

### FRNEEYN(N)KSVQEIQATF Hex 6 HexNAc 5

**Mass:**  4089.7226

(4.392e-07)

**Charge States:** 3, 4

**Start Position**: 50

**End Position**: 67

| Profile Score | Tandem Score | TDA q-value | Volume | Spectrum Matches |
| --- | --- | --- | --- | --- |
| -5.3951 | 138.0329 | 0.0000 | 4.0851e+07 | 1 |

**Adduct Signal Fractions**

- **Unmodified**: 4.0851e+07

### YFTPN(N)KTEDTIF Hex 3 HexNAc 5 Neu5Ac 3

**Mass:**  3849.5238

(-2.743e-06)

**Charge States:** 3, 4

**Start Position**: 68

**End Position**: 80

| Profile Score | Tandem Score | TDA q-value | Volume | Spectrum Matches |
| --- | --- | --- | --- | --- |
| 12.5131 | 138.0208 | 0.0000 | 3.0882e+08 | 2 |

**Adduct Signal Fractions**

- **Unmodified**: 3.0882e+08

### RNEEYN(N)KSVQEIQATF Hex 8 HexNAc 7

**Mass:**  4672.9230

(1.458e-06)

**Charge States:** 4

**Start Position**: 51

**End Position**: 67

| Profile Score | Tandem Score | TDA q-value | Volume | Spectrum Matches |
| --- | --- | --- | --- | --- |
| -3.1043 | 137.5435 | 0.0000 | 5.0466e+07 | 2 |

**Adduct Signal Fractions**

- **Unmodified**: 5.0466e+07

### RN(D)EEYN(N)KSVQEIQATF Hex 6 HexNAc 5

**Mass:**  3943.6440

(2.510e-04)

**Charge States:** 3, 4

**Start Position**: 51

**End Position**: 67

| Profile Score | Tandem Score | TDA q-value | Volume | Spectrum Matches |
| --- | --- | --- | --- | --- |
| 11.6261 | 135.6777 | 0.0000 | 1.2109e+09 | 25 |

**Adduct Signal Fractions**

- **Unmodified**: 1.2109e+09

### YFTPN(N)KTEDTIF Fuc 1 Hex 7 HexNAc 4 Neu5Ac 2

**Mass:**  4149.6414

(1.799e-06)

**Charge States:** 3

**Start Position**: 68

**End Position**: 80

| Profile Score | Tandem Score | TDA q-value | Volume | Spectrum Matches |
| --- | --- | --- | --- | --- |
| -5.4808 | 134.6943 | 0.0000 | 3.9710e+07 | 2 |

**Adduct Signal Fractions**

- **Unmodified**: 3.9710e+07

### RN(D)EEYN(N)KSVQEIQATF Hex 5 HexNAc 4

**Mass:**  3578.5072

(2.769e-04)

**Charge States:** 3, 4

**Start Position**: 51

**End Position**: 67

| Profile Score | Tandem Score | TDA q-value | Volume | Spectrum Matches |
| --- | --- | --- | --- | --- |
| 11.4898 | 132.3689 | 0.0000 | 1.2915e+09 | 12 |

**Adduct Signal Fractions**

- **Unmodified**: 1.2915e+09

### NVQREN(N)GTVSRY Hex 6 HexNAc 5

**Mass:**  3409.4243

(2.088e-06)

**Charge States:** 3

**Start Position**: 98

**End Position**: 110

| Profile Score | Tandem Score | TDA q-value | Volume | Spectrum Matches |
| --- | --- | --- | --- | --- |
| 10.5516 | 132.2475 | 0.0000 | 1.7450e+08 | 4 |

**Adduct Signal Fractions**

- **Unmodified**: 1.7450e+08

### FRNEEYN(N)KSVQEIQATF Hex 5 HexNAc 4

**Mass:**  3724.5877

(-2.436e-07)

**Charge States:** 4

**Start Position**: 50

**End Position**: 67

| Profile Score | Tandem Score | TDA q-value | Volume | Spectrum Matches |
| --- | --- | --- | --- | --- |
| -5.1128 | 131.8802 | 0.0000 | 3.1037e+07 | 8 |

**Adduct Signal Fractions**

- **Unmodified**: 3.1037e+07

### FTPN(N)KTEDTIF Hex 8 HexNAc 7

**Mass:**  4029.6188

(1.096e-06)

**Charge States:** 3, 4

**Start Position**: 69

**End Position**: 80

| Profile Score | Tandem Score | TDA q-value | Volume | Spectrum Matches |
| --- | --- | --- | --- | --- |
| 15.8046 | 129.6700 | 0.0030 | 5.1817e+07 | 5 |

**Adduct Signal Fractions**

- **Unmodified**: 5.1817e+07

### FYFTPN(N)KTEDTIF Fuc 1 Hex 7 HexNAc 4 Neu5Ac 2

**Mass:**  4296.7121

(1.010e-06)

**Charge States:** 3

**Start Position**: 67

**End Position**: 80

| Profile Score | Tandem Score | TDA q-value | Volume | Spectrum Matches |
| --- | --- | --- | --- | --- |
| -3.6277 | 129.4679 | 0.0030 | 4.0485e+07 | 2 |

**Adduct Signal Fractions**

- **Unmodified**: 4.0485e+07

### NVQREN(N)GTVSRY Fuc 1 Hex 6 HexNAc 5

**Mass:**  3555.4813

(2.370e-06)

**Charge States:** 3

**Start Position**: 98

**End Position**: 110

| Profile Score | Tandem Score | TDA q-value | Volume | Spectrum Matches |
| --- | --- | --- | --- | --- |
| -4.5802 | 129.2646 | 0.0030 | 7.3681e+07 | 2 |

**Adduct Signal Fractions**

- **Unmodified**: 7.3681e+07

### TPN(N)KTEDTIF Hex 9 HexNAc 5

**Mass:**  3638.4468

(2.270e-06)

**Charge States:** 3

**Start Position**: 70

**End Position**: 80

| Profile Score | Tandem Score | TDA q-value | Volume | Spectrum Matches |
| --- | --- | --- | --- | --- |
| 12.5452 | 128.6801 | 0.0030 | 3.4018e+07 | 3 |

**Adduct Signal Fractions**

- **Unmodified**: 3.4018e+07

### YFTPN(N)KTEDTIF Fuc 1 Hex 9 HexNAc 8

**Mass:**  4703.8747

(1.771e-06)

**Charge States:** 4

**Start Position**: 68

**End Position**: 80

| Profile Score | Tandem Score | TDA q-value | Volume | Spectrum Matches |
| --- | --- | --- | --- | --- |
| 14.0031 | 128.3574 | 0.0030 | 3.9069e+07 | 3 |

**Adduct Signal Fractions**

- **Unmodified**: 3.9069e+07

### LNVQREN(N)GTVSRY Hex 7 HexNAc 6

**Mass:**  3887.6367

(1.240e-06)

**Charge States:** 3, 4

**Start Position**: 97

**End Position**: 110

| Profile Score | Tandem Score | TDA q-value | Volume | Spectrum Matches |
| --- | --- | --- | --- | --- |
| 13.8043 | 127.3331 | 0.0030 | 4.9500e+08 | 10 |

**Adduct Signal Fractions**

- **Unmodified**: 4.9500e+08

### FYFTPN(N)KTEDTIF Fuc 2 Hex 8 HexNAc 7

**Mass:**  4631.8693

(1.937e-06)

**Charge States:** 4

**Start Position**: 67

**End Position**: 80

| Profile Score | Tandem Score | TDA q-value | Volume | Spectrum Matches |
| --- | --- | --- | --- | --- |
| -3.7944 | 125.3620 | 0.0057 | 2.3016e+07 | 4 |

**Adduct Signal Fractions**

- **Unmodified**: 2.3016e+07

### LNVQREN(N)GTVSRY Fuc 1 Hex 7 HexNAc 6

**Mass:**  4033.6936

(9.999e-08)

**Charge States:** 3, 4

**Start Position**: 97

**End Position**: 110

| Profile Score | Tandem Score | TDA q-value | Volume | Spectrum Matches |
| --- | --- | --- | --- | --- |
| 13.4288 | 124.5668 | 0.0057 | 2.6142e+08 | 5 |

**Adduct Signal Fractions**

- **Unmodified**: 2.6142e+08

### YFTPN(N)KTEDTIF Hex 7 HexNAc 5

**Mass:**  3624.4945

(8.198e-06)

**Charge States:** 3

**Start Position**: 68

**End Position**: 80

| Profile Score | Tandem Score | TDA q-value | Volume | Spectrum Matches |
| --- | --- | --- | --- | --- |
| -21.5864 | 123.2991 | 0.0083 | 1.0830e+06 | 3 |

**Adduct Signal Fractions**

- **Unmodified**: 1.0830e+06

### FTPN(N)KTEDTIF Fuc 2 Hex 7 HexNAc 6

**Mass:**  3956.6043

(2.500e-06)

**Charge States:** 3

**Start Position**: 69

**End Position**: 80

| Profile Score | Tandem Score | TDA q-value | Volume | Spectrum Matches |
| --- | --- | --- | --- | --- |
| -3.6726 | 123.0247 | 0.0083 | 1.2937e+07 | 3 |

**Adduct Signal Fractions**

- **Unmodified**: 1.2937e+07

### FYFTPN(N)KTEDTIF Fuc 1 Hex 8 HexNAc 4 Neu5Ac 1

**Mass:**  4167.6809

(4.146e-06)

**Charge States:** 3, 4

**Start Position**: 67

**End Position**: 80

| Profile Score | Tandem Score | TDA q-value | Volume | Spectrum Matches |
| --- | --- | --- | --- | --- |
| -4.5674 | 121.9326 | 0.0100 | 5.2575e+07 | 2 |

**Adduct Signal Fractions**

- **Unmodified**: 5.2575e+07

### NVQREN(N)GTVSRY Hex 8 HexNAc 7

**Mass:**  4139.6877

(3.830e-06)

**Charge States:** 3, 4

**Start Position**: 98

**End Position**: 110

| Profile Score | Tandem Score | TDA q-value | Volume | Spectrum Matches |
| --- | --- | --- | --- | --- |
| -18.5975 | 120.9538 | 0.0100 | 3.5636e+07 | 2 |

**Adduct Signal Fractions**

- **Unmodified**: 3.5636e+07

### RNEEYN(N)KSVQEIQAT Hex 6 HexNAc 5

**Mass:**  3795.5868

(1.815e-06)

**Charge States:** 3

**Start Position**: 51

**End Position**: 66

| Profile Score | Tandem Score | TDA q-value | Volume | Spectrum Matches |
| --- | --- | --- | --- | --- |
| -5.6170 | 120.3382 | 0.0100 | 2.2467e+07 | 2 |

**Adduct Signal Fractions**

- **Unmodified**: 2.2467e+07

### TPN(N)KTEDTIF Fuc 4 Hex 3 HexNAc 9 Neu5Ac 1

**Mass:**  4353.7297

(-6.751e-06)

**Charge States:** 4

**Start Position**: 70

**End Position**: 80

| Profile Score | Tandem Score | TDA q-value | Volume | Spectrum Matches |
| --- | --- | --- | --- | --- |
| -4.7415 | 119.8604 | 0.0100 | 1.9090e+07 | 1 |

**Adduct Signal Fractions**

- **Unmodified**: 1.9090e+07

### RN(D)EEYN(N)KSVQEIQATFF Hex 5 HexNAc 4

**Mass:**  3725.5801

(2.667e-04)

**Charge States:** 3, 4

**Start Position**: 51

**End Position**: 68

| Profile Score | Tandem Score | TDA q-value | Volume | Spectrum Matches |
| --- | --- | --- | --- | --- |
| 10.3707 | 118.2049 | 0.0100 | 3.8447e+08 | 9 |

**Adduct Signal Fractions**

- **Unmodified**: 3.8447e+08

### RN(D)EEYN(N)KSVQEIQATF Fuc 1 Hex 6 HexNAc 5

**Mass:**  4089.7006

(2.423e-04)

**Charge States:** 3, 4

**Start Position**: 51

**End Position**: 67

| Profile Score | Tandem Score | TDA q-value | Volume | Spectrum Matches |
| --- | --- | --- | --- | --- |
| 11.4698 | 118.0321 | 0.0100 | 5.8980e+08 | 15 |

**Adduct Signal Fractions**

- **Unmodified**: 5.8980e+08

### RN(D)EEYN(N)KSVQEIQATFF Hex 6 HexNAc 5

**Mass:**  4090.7143

(2.459e-04)

**Charge States:** 3, 4

**Start Position**: 51

**End Position**: 68

| Profile Score | Tandem Score | TDA q-value | Volume | Spectrum Matches |
| --- | --- | --- | --- | --- |
| 10.6548 | 117.0995 | 0.0123 | 4.1235e+08 | 16 |

**Adduct Signal Fractions**

- **Unmodified**: 4.1235e+08

### FTPN(N)KTEDTIF Fuc 3 Hex 6 HexNAc 5 Neu5Ac 1

**Mass:**  4028.6028

(-3.657e-06)

**Charge States:** 3

**Start Position**: 69

**End Position**: 80

| Profile Score | Tandem Score | TDA q-value | Volume | Spectrum Matches |
| --- | --- | --- | --- | --- |
| -19.0298 | 116.3709 | 0.0167 | 7.6910e+05 | 1 |

**Adduct Signal Fractions**

- **Unmodified**: 7.6910e+05

### LNVQREN(N)GTVSRY Hex 6 HexNAc 5

**Mass:**  3522.5055

(1.417e-06)

**Charge States:** 3

**Start Position**: 97

**End Position**: 110

| Profile Score | Tandem Score | TDA q-value | Volume | Spectrum Matches |
| --- | --- | --- | --- | --- |
| -3.6679 | 115.6314 | 0.0167 | 1.0947e+08 | 4 |

**Adduct Signal Fractions**

- **Unmodified**: 1.0947e+08

### N(D)VQREN(N)GTVSRY Fuc 1 Hex 7 HexNAc 6

**Mass:**  3921.6040

(2.558e-04)

**Charge States:** 3

**Start Position**: 98

**End Position**: 110

| Profile Score | Tandem Score | TDA q-value | Volume | Spectrum Matches |
| --- | --- | --- | --- | --- |
| -21.5562 | 114.7835 | 0.0184 | 5.3950e+06 | 3 |

**Adduct Signal Fractions**

- **Unmodified**: 5.3950e+06

### YFTPN(N)KTEDTIF Fuc 2 Hex 8 HexNAc 7

**Mass:**  4484.7995

(1.774e-06)

**Charge States:** 3, 4

**Start Position**: 68

**End Position**: 80

| Profile Score | Tandem Score | TDA q-value | Volume | Spectrum Matches |
| --- | --- | --- | --- | --- |
| 9.9161 | 114.6808 | 0.0184 | 3.0933e+07 | 2 |

**Adduct Signal Fractions**

- **Unmodified**: 3.0933e+07

### YFTPN(N)KTEDTIF Hex 10 HexNAc 9

**Mass:**  4922.9489

(1.646e-06)

**Charge States:** 4

**Start Position**: 68

**End Position**: 80

| Profile Score | Tandem Score | TDA q-value | Volume | Spectrum Matches |
| --- | --- | --- | --- | --- |
| -4.4665 | 114.1851 | 0.0184 | 1.9158e+07 | 1 |

**Adduct Signal Fractions**

- **Unmodified**: 1.9158e+07

### RNEEYN(N)KSVQEIQATFF Hex 8 HexNAc 6

**Mass:**  4617.9060

(2.185e-04)

**Charge States:** 4

**Start Position**: 51

**End Position**: 68

| Profile Score | Tandem Score | TDA q-value | Volume | Spectrum Matches |
| --- | --- | --- | --- | --- |
| -6.0109 | 113.1431 | 0.0184 | 2.7242e+07 | 4 |

**Adduct Signal Fractions**

- **Unmodified**: 2.7242e+07

### FTPN(N)KTEDTIF Hex 7 HexNAc 4 Neu5Ac 2

**Mass:**  3840.5154

(1.531e-06)

**Charge States:** 3, 4

**Start Position**: 69

**End Position**: 80

| Profile Score | Tandem Score | TDA q-value | Volume | Spectrum Matches |
| --- | --- | --- | --- | --- |
| 13.8731 | 111.8247 | 0.0210 | 2.4177e+07 | 4 |

**Adduct Signal Fractions**

- **Unmodified**: 2.4177e+07

### VQREN(N)GTVSRY Hex 7 HexNAc 6

**Mass:**  3661.5178

(2.769e-04)

**Start Position**: 99

**End Position**: 110

| Profile Score | Tandem Score | TDA q-value | Volume | Spectrum Matches |
| --- | --- | --- | --- | --- |
| 0.0000 | 111.5089 | 0.0210 | 0.0000e+00 | 1 |

Adduct:

No Chromatogram Found

### YFTPN(N)KTEDTIF Fuc 1 Hex 7 HexNAc 3 Neu5Ac 1

**Mass:**  3655.4712

(3.258e-06)

**Charge States:** 3

**Start Position**: 68

**End Position**: 80

| Profile Score | Tandem Score | TDA q-value | Volume | Spectrum Matches |
| --- | --- | --- | --- | --- |
| -19.5717 | 110.5456 | 0.0210 | 4.2108e+06 | 1 |

**Adduct Signal Fractions**

- **Unmodified**: 4.2108e+06

### NVQREN(N)GTVSRY Fuc 5 Hex 7 HexNAc 6

**Mass:**  4504.8259

(-2.433e-06)

**Charge States:** 4

**Start Position**: 98

**End Position**: 110

| Profile Score | Tandem Score | TDA q-value | Volume | Spectrum Matches |
| --- | --- | --- | --- | --- |
| -18.7735 | 108.3263 | 0.0210 | 2.5863e+07 | 1 |

**Adduct Signal Fractions**

- **Unmodified**: 2.5863e+07

### RN(D)EEYN(N)KSVQEIQATFF Fuc 1 Hex 6 HexNAc 5

**Mass:**  4236.7712

(2.355e-04)

**Charge States:** 3, 4

**Start Position**: 51

**End Position**: 68

| Profile Score | Tandem Score | TDA q-value | Volume | Spectrum Matches |
| --- | --- | --- | --- | --- |
| 10.3240 | 105.0147 | 0.0240 | 2.0393e+08 | 17 |

**Adduct Signal Fractions**

- **Unmodified**: 2.0393e+08

### TPN(N)KTEDTIF Fuc 2 Hex 7 HexNAc 9

**Mass:**  4418.7712

(1.099e-06)

**Charge States:** 4

**Start Position**: 70

**End Position**: 80

| Profile Score | Tandem Score | TDA q-value | Volume | Spectrum Matches |
| --- | --- | --- | --- | --- |
| -19.8000 | 104.1555 | 0.0248 | 3.6689e+06 | 1 |

**Adduct Signal Fractions**

- **Unmodified**: 3.6689e+06

### TPN(N)KTEDTIF Fuc 4 Hex 4 HexNAc 9

**Mass:**  4224.6979

(-6.075e-06)

**Charge States:** 3

**Start Position**: 70

**End Position**: 80

| Profile Score | Tandem Score | TDA q-value | Volume | Spectrum Matches |
| --- | --- | --- | --- | --- |
| -4.3076 | 103.2985 | 0.0248 | 2.3417e+07 | 2 |

**Adduct Signal Fractions**

- **Unmodified**: 2.3417e+07

### RNEEYN(N)KSVQEIQATFFY Hex 6 HexNAc 5

**Mass:**  4252.8088

(5.362e-06)

**Charge States:** 4

**Start Position**: 51

**End Position**: 69

| Profile Score | Tandem Score | TDA q-value | Volume | Spectrum Matches |
| --- | --- | --- | --- | --- |
| -3.8741 | 103.0406 | 0.0248 | 1.8541e+08 | 2 |

**Adduct Signal Fractions**

- **Unmodified**: 1.8541e+08

### LNVQREN(N)GTVSRY Fuc 1 Hex 6 HexNAc 5

**Mass:**  3668.5629

(1.658e-06)

**Charge States:** 3, 4

**Start Position**: 97

**End Position**: 110

| Profile Score | Tandem Score | TDA q-value | Volume | Spectrum Matches |
| --- | --- | --- | --- | --- |
| -4.2470 | 102.9654 | 0.0248 | 5.9775e+07 | 4 |

**Adduct Signal Fractions**

- **Unmodified**: 5.9775e+07

### TPN(N)KTEDTIF Hex 3 HexNAc 9

**Mass:**  3481.4479

(8.650e-04)

**Start Position**: 70

**End Position**: 80

| Profile Score | Tandem Score | TDA q-value | Volume | Spectrum Matches |
| --- | --- | --- | --- | --- |
| 0.0000 | 102.3279 | 0.0263 | 0.0000e+00 | 1 |

Adduct:

No Chromatogram Found

### RNEEYN(N)KSVQEIQATF Fuc 2 Hex 5 HexNAc 3 Neu5Ac 1

**Mass:**  3960.6701

(7.675e-04)

**Charge States:** 4

**Start Position**: 51

**End Position**: 67

| Profile Score | Tandem Score | TDA q-value | Volume | Spectrum Matches |
| --- | --- | --- | --- | --- |
| -8.8049 | 101.8048 | 0.0263 | 7.0998e+06 | 5 |

**Adduct Signal Fractions**

- **Unmodified**: 7.0998e+06

### LVPVPITN(N)ATL Hex 6 HexNAc 5

**Mass:**  3124.4046

(1.562e-06)

**Charge States:** 3

**Start Position**: 26

**End Position**: 37

| Profile Score | Tandem Score | TDA q-value | Volume | Spectrum Matches |
| --- | --- | --- | --- | --- |
| -20.7171 | 101.5941 | 0.0276 | 3.4732e+06 | 1 |

**Adduct Signal Fractions**

- **Unmodified**: 3.4732e+06

### NVQREN(N)GTVSRY Hex 5 HexNAc 4

**Mass:**  3044.2875

(1.536e-06)

**Charge States:** 3

**Start Position**: 98

**End Position**: 110

| Profile Score | Tandem Score | TDA q-value | Volume | Spectrum Matches |
| --- | --- | --- | --- | --- |
| -19.2111 | 101.3803 | 0.0276 | 1.1164e+07 | 1 |

**Adduct Signal Fractions**

- **Unmodified**: 1.1164e+07

### TPN(N)KTEDTIF Fuc 1 Hex 4 HexNAc 9 Neu5Ac 1

**Mass:**  4077.6275

(-4.375e-06)

**Charge States:** 3

**Start Position**: 70

**End Position**: 80

| Profile Score | Tandem Score | TDA q-value | Volume | Spectrum Matches |
| --- | --- | --- | --- | --- |
| -19.5951 | 100.9978 | 0.0276 | 1.4198e+07 | 2 |

**Adduct Signal Fractions**

- **Unmodified**: 1.4198e+07

### LNVQREN(N)GTVSRY Fuc 3 Hex 7 HexNAc 6

**Mass:**  4328.8281

(6.989e-04)

**Start Position**: 97

**End Position**: 110

| Profile Score | Tandem Score | TDA q-value | Volume | Spectrum Matches |
| --- | --- | --- | --- | --- |
| 0.0000 | 100.7492 | 0.0276 | 0.0000e+00 | 1 |

Adduct:

No Chromatogram Found

### C(C)ANLVPVPITN(N)ATL Hex 6 HexNAc 5

**Mass:**  3469.5111

(1.682e-06)

**Charge States:** 3, 4

**Start Position**: 23

**End Position**: 37

| Profile Score | Tandem Score | TDA q-value | Volume | Spectrum Matches |
| --- | --- | --- | --- | --- |
| 13.4299 | 99.1874 | 0.0308 | 2.9159e+07 | 5 |

**Adduct Signal Fractions**

- **Unmodified**: 2.9159e+07

### N(N)KSVQEIQATFF Fuc 3 Hex 6 HexNAc 6

**Mass:**  4041.6757

(4.937e-04)

**Start Position**: 56

**End Position**: 68

| Profile Score | Tandem Score | TDA q-value | Volume | Spectrum Matches |
| --- | --- | --- | --- | --- |
| 0.0000 | 98.3194 | 0.0325 | 0.0000e+00 | 1 |

Adduct:

No Chromatogram Found

### RNEEYN(N)KSVQEIQATF Fuc 1 Hex 8 HexNAc 7

**Mass:**  4818.9802

(1.118e-06)

**Charge States:** 4

**Start Position**: 51

**End Position**: 67

| Profile Score | Tandem Score | TDA q-value | Volume | Spectrum Matches |
| --- | --- | --- | --- | --- |
| -3.7536 | 97.2745 | 0.0376 | 1.3008e+07 | 2 |

**Adduct Signal Fractions**

- **Unmodified**: 1.3008e+07

### TN(N)ATLDRITGKW Hex 6 HexNAc 5

**Mass:**  3362.4420

(1.168e-06)

**Charge States:** 3, 4

**Start Position**: 32

**End Position**: 44

| Profile Score | Tandem Score | TDA q-value | Volume | Spectrum Matches |
| --- | --- | --- | --- | --- |
| -4.6371 | 96.3291 | 0.0408 | 2.4380e+07 | 3 |

**Adduct Signal Fractions**

- **Unmodified**: 2.4380e+07

### TPN(N)KTEDTIF Fuc 3 Hex 10 HexNAc 4 Neu5Ac 1

**Mass:**  4326.6838

(1.874e-06)

**Charge States:** 3, 4

**Start Position**: 70

**End Position**: 80

| Profile Score | Tandem Score | TDA q-value | Volume | Spectrum Matches |
| --- | --- | --- | --- | --- |
| -4.2780 | 95.9893 | 0.0420 | 1.9743e+07 | 3 |

**Adduct Signal Fractions**

- **Unmodified**: 1.9743e+07

### LNVQREN(N)GTVSRY Fuc 2 Hex 7 HexNAc 6

**Mass:**  4179.7492

(5.721e-07)

**Charge States:** 3, 4

**Start Position**: 97

**End Position**: 110

| Profile Score | Tandem Score | TDA q-value | Volume | Spectrum Matches |
| --- | --- | --- | --- | --- |
| -4.4372 | 95.8868 | 0.0420 | 7.0679e+07 | 3 |

**Adduct Signal Fractions**

- **Unmodified**: 7.0679e+07

### NVQREN(N)GTVSRY Fuc 3 Hex 7 HexNAc 6

**Mass:**  4214.7355

(4.782e-04)

**Start Position**: 98

**End Position**: 110

| Profile Score | Tandem Score | TDA q-value | Volume | Spectrum Matches |
| --- | --- | --- | --- | --- |
| 0.0000 | 92.4995 | 0.0463 | 0.0000e+00 | 1 |

Adduct:

No Chromatogram Found

### LNVQREN(N)GTVSRY Hex 8 HexNAc 7

**Mass:**  4252.7624

(7.224e-07)

**Charge States:** 3, 4

**Start Position**: 97

**End Position**: 110

| Profile Score | Tandem Score | TDA q-value | Volume | Spectrum Matches |
| --- | --- | --- | --- | --- |
| -20.1876 | 92.4284 | 0.0463 | 3.3084e+07 | 3 |

**Adduct Signal Fractions**

- **Unmodified**: 3.3084e+07

### VQREN(N)GTVSRY Fuc 1 Hex 5 HexNAc 6 Neu5Ac 1

**Mass:**  3776.5396

(7.917e-04)

**Start Position**: 99

**End Position**: 110

| Profile Score | Tandem Score | TDA q-value | Volume | Spectrum Matches |
| --- | --- | --- | --- | --- |
| 0.0000 | 92.0257 | 0.0479 | 0.0000e+00 | 1 |

Adduct:

No Chromatogram Found

### P00738|HPT\_HUMAN

**>P00738|HPT\_HUMAN**  
 `MSALGAVIALLLWGQLFAVDSGNDVTDIADDGCPKPPEIAHGYVEHSVRYQCKNYYKLRTEGDGVYTLNDKKQWINKAVG  
DKLPECEADDGCPKPPEIAHGYVEHSVRYQCKNYYKLRTEGDGVYTLNNEKQWINKAVGDKLPECEAVCGKPKNPANPVQ  
RILGGHLDAKGSFPWQAKMVSHHNLTTGATLINEQWLLTTAKNLFLNHSENATAKDIAPTLTLYVGKKQLVEIEKVVLHP  
NYSQVDIGLIKLKQKVSVNERVMPICLPSKDYAEVGRVGYVSGWGRNANFKFTDHLKYVMLPVADQDQCIRHYEGSTVPE  
KKTPKSPVGVQPILNEHTFCAGMSKYQEDTCYGDAGSAFAVHDLEEDTWYATGILSFDKSCAVAEYGVYVKVTSIQDWVQ  
KTIAEN`

| Site | Type | Count |
| --- | --- | --- |
| 184 | N-linked | 2 |

| Mass | Sequence | MS_2_ Score | `q`-value | MS_1_ Score | Signal | Protein Coordinates | Tandem Scans | Start Time | Apex Time | End Time |
| --- | --- | --- | --- | --- | --- | --- | --- | --- | --- | --- |
| 2872.2265 | VSHHN(N)LTTGATL Hex 5 HexNAc 4 | 157.888 | 0.0 | -4.531 | 2.421e+07 | 179 - 191 | 2 | 17.20 | 17.36 | 17.70 |
| 3237.3612 | VSHHN(N)LTTGATL Hex 6 HexNAc 5 | 151.742 | 0.0 | -5.057 | 1.393e+07 | 179 - 191 | 2 | 17.03 | 17.20 | 17.53 |

### VSHHN(N)LTTGATL Hex 5 HexNAc 4

**Mass:**  2872.2277

(-1.099e-06)

**Charge States:** 3

**Start Position**: 180

**End Position**: 192

| Profile Score | Tandem Score | TDA q-value | Volume | Spectrum Matches |
| --- | --- | --- | --- | --- |
| -4.5311 | 157.8875 | 0.0000 | 2.4208e+07 | 2 |

**Adduct Signal Fractions**

- **Unmodified**: 2.4208e+07

### VSHHN(N)LTTGATL Hex 6 HexNAc 5

**Mass:**  3237.3612

(1.921e-06)

**Charge States:** 3

**Start Position**: 180

**End Position**: 192

| Profile Score | Tandem Score | TDA q-value | Volume | Spectrum Matches |
| --- | --- | --- | --- | --- |
| -5.0568 | 151.7422 | 0.0000 | 1.3932e+07 | 2 |

**Adduct Signal Fractions**

- **Unmodified**: 1.3932e+07

### P13473|LAMP2\_HUMAN

**>P13473|LAMP2\_HUMAN**  
 `MVCFRLFPVPGSGLVLVCLVLGAVRSYALELNLTDSENATCLYAKWQMNFTVRYETTNKTYKTVTISDHGTVTYNGSICG  
DDQNGPKIAVQFGPGFSWIANFTKAASTYSIDSVSFSYNTGDNTTFPDAEDKGILTVDELLAIRIPLNDLFRCNSLSTLE  
KNDVVQHYWDVLVQAFVQNGTVSTNEFLCDKDKTSTVAPTIHTTVPSPTTTPTPKEKPEAGTYSVNNGNDTCLLATMGLQ  
LNITQDKVASVININPNTTHSTGSCRSHTALLRLNSSTIKYLDFVFAVKNENRFYLKEVNISMYLVNGSVFSIANNNLSY  
WDAPLGSSYMCNKEQTVSVSGAFQINTFDLRVQPFNVTQGKYSTAQDCSADDDNFLVPIAVGAALAGVLILVLLAYFIGL  
KHHHAGYEQF`

| Site | Type | Count |
| --- | --- | --- |
| 307 | N-linked | 1 |

| Mass | Sequence | MS_2_ Score | `q`-value | MS_1_ Score | Signal | Protein Coordinates | Tandem Scans | Start Time | Apex Time | End Time |
| --- | --- | --- | --- | --- | --- | --- | --- | --- | --- | --- |
| 2722.1133 | LVN(N)GSVF Hex 6 HexNAc 5 | 176.255 | 0.0 | -3.852 | 8.129e+06 | 304 - 311 | 2 | 29.57 | 29.74 | 30.08 |

### LVN(N)GSVF Hex 6 HexNAc 5

**Mass:**  2722.1131

(1.303e-06)

**Charge States:** 3

**Start Position**: 305

**End Position**: 312

| Profile Score | Tandem Score | TDA q-value | Volume | Spectrum Matches |
| --- | --- | --- | --- | --- |
| -3.8521 | 176.2547 | 0.0000 | 8.1293e+06 | 2 |

**Adduct Signal Fractions**

- **Unmodified**: 8.1293e+06
