# Supplementary material for: Measuring Site-specific Glycosylation Similarity between Influenza a Virus Variants with Statistical Certainty
Source: Mol Cell Proteomics. 2020 Nov 25;19(9):1533–45. doi: 10.1074/mcp.RA120.002031 (PMC8143645; doi:10.1074/mcp.RA120.002031)
Supplement: Supplementary file 1 [file mmc1.zip › mmc1/159250_2_supp_555286_qcjklc.zip!/20190326_aAGPgal_021119_chymo_HILIC_DDA_1ug_02_glycopeptides.html]

## 20190326\_aAGPgal\_021119\_chymo\_HILIC\_DDA\_1ug\_02 @ GlycopeptideHypothesis-46ca10c57ea04e26a77f8c5a4f8bd5b2

**MS^1^ Mass Accuracy:** 10.0 PPM  
**Grouping Tolerance:** 15.0 PPM  
**MS^n^ Mass Accuracy:** 20.0 PPM  
**Minimum `q`-Value For Clustering:** 0.05

**Glycopeptide Hypothesis:**
GlycopeptideHypothesis-46ca10c57ea04e26a77f8c5a4f8bd5b2  
**Sample Searched:**
20190326\_aAGPgal\_021119\_chymo\_HILIC\_DDA\_1ug\_02

| Protein | Matched Glycopeptides |
| --- | --- |
| P02763\|A1AG1\_HUMAN | 159 |
| P19652\|A1AG2\_HUMAN | 139 |
| P00738\|HPT\_HUMAN | 2 |
| P13473\|LAMP2\_HUMAN | 2 |

### P02763|A1AG1\_HUMAN

**>P02763|A1AG1\_HUMAN**  
 `MALSWVLTVLSLLPLLEAQIPLCANLVPVPITNATLDQITGKWFYIASAFRNEEYNKSVQEIQATFFYFTPNKTEDTIFL  
REYQTRQDQCIYNTTYLNVQRENGTISRYVGGQEHFAHLLILRDTKTYMLAFDVNDEKNWGLSVYADKPETTKEQLGEFY  
EALDCLRIPKSDVVYTDWKKDKCEPLEKQHEKERKQEEGES`

| Site | Type | Count |
| --- | --- | --- |
| 33 | N-linked | 1 |
| 72 | N-linked | 67 |
| 93 | N-linked | 3 |
| 103 | N-linked | 41 |
| 56 | N-linked | 47 |

| Mass | Sequence | MS_2_ Score | `q`-value | MS_1_ Score | Signal | Protein Coordinates | Tandem Scans | Start Time | Apex Time | End Time |
| --- | --- | --- | --- | --- | --- | --- | --- | --- | --- | --- |
| 2869.2034 | TPN(N)KTEDTIF Hex 3 HexNAc 6 | 184.066 | 0.0 | 16.418 | 3.299e+08 | 69 - 79 | 11 | 22.31 | 24.17 | 25.52 |
| 3177.3146 | TPN(N)KTEDTIF Fuc 1 Hex 4 HexNAc 6 | 181.311 | 0.0 | 14.498 | 6.651e+07 | 69 - 79 | 5 | 22.99 | 23.67 | 24.33 |
| 2666.1249 | TPN(N)KTEDTIF Hex 3 HexNAc 5 | 180.990 | 0.0 | 15.638 | 1.735e+08 | 69 - 79 | 8 | 23.67 | 24.50 | 25.69 |
| 2974.2352 | TPN(N)KTEDTIF Fuc 1 Hex 4 HexNAc 5 | 180.842 | 0.0 | 14.098 | 4.180e+07 | 69 - 79 | 4 | 23.33 | 24.00 | 24.50 |
| 2463.0458 | TPN(N)KTEDTIF Hex 3 HexNAc 4 | 180.398 | 0.0 | 13.126 | 2.345e+07 | 69 - 79 | 4 | 24.50 | 24.84 | 25.35 |
| 3691.5393 | FYFTPN(N)KTEDTIF Hex 4 HexNAc 7 | 176.886 | 0.0 | 14.067 | 5.059e+08 | 66 - 79 | 12 | 45.81 | 46.84 | 47.84 |
| 3179.3357 | YFTPN(N)KTEDTIF Hex 3 HexNAc 6 | 176.033 | 0.0 | 14.835 | 3.536e+09 | 67 - 79 | 43 | 36.42 | 37.57 | 44.13 |
| 2773.1758 | YFTPN(N)KTEDTIF Hex 3 HexNAc 4 | 175.519 | 0.0 | 11.873 | 2.990e+08 | 67 - 79 | 9 | 37.09 | 38.21 | 40.75 |
| 3431.4350 | FYFTPN(N)KTEDTIF Fuc 1 Hex 4 HexNAc 5 | 175.209 | 0.0 | 12.800 | 4.204e+08 | 66 - 79 | 11 | 46.32 | 47.51 | 49.17 |
| 3284.3660 | YFTPN(N)KTEDTIF Fuc 1 Hex 4 HexNAc 5 | 174.854 | 0.0 | 13.992 | 5.773e+08 | 67 - 79 | 16 | 36.08 | 37.26 | 38.21 |
| 3634.5159 | FYFTPN(N)KTEDTIF Fuc 1 Hex 4 HexNAc 6 | 173.273 | 0.0 | 13.985 | 6.709e+08 | 66 - 79 | 16 | 46.15 | 47.18 | 48.83 |
| 3016.2721 | FTPN(N)KTEDTIF Hex 3 HexNAc 6 | 172.980 | 0.0 | 15.796 | 7.329e+08 | 68 - 79 | 12 | 29.76 | 31.34 | 32.27 |
| 3795.5585 | YFTPN(N)KTEDTIF Fuc 2 Hex 5 HexNAc 6 | 172.918 | 0.0 | 16.247 | 1.907e+08 | 67 - 79 | 4 | 36.08 | 36.59 | 37.09 |
| 2976.2553 | YFTPN(N)KTEDTIF Hex 3 HexNAc 5 | 171.977 | 0.0 | 15.024 | 1.936e+09 | 67 - 79 | 38 | 36.59 | 37.89 | 43.12 |
| 3487.4455 | YFTPN(N)KTEDTIF Fuc 1 Hex 4 HexNAc 6 | 171.166 | 0.0 | 16.078 | 1.075e+09 | 67 - 79 | 11 | 35.91 | 37.09 | 37.89 |
| 2775.1840 | YFTPN(N)KTEDTIF Hex 3 HexNAc 4 | 170.535 | 0.0 | 0.000 | 0.000e+00 | 67 - 79 | 1 | - | - | - |
| 3341.3902 | YFTPN(N)KTEDTIF Hex 4 HexNAc 6 | 169.623 | 0.0 | 16.272 | 1.761e+08 | 67 - 79 | 6 | 35.91 | 37.42 | 37.57 |
| 3326.4032 | FYFTPN(N)KTEDTIF Hex 3 HexNAc 6 | 169.167 | 0.0 | 15.446 | 2.824e+09 | 66 - 79 | 23 | 46.49 | 47.68 | 50.53 |
| 3544.4684 | YFTPN(N)KTEDTIF Hex 4 HexNAc 7 | 168.705 | 0.0 | 15.368 | 9.367e+08 | 67 - 79 | 13 | 35.57 | 37.09 | 37.89 |
| 3123.3269 | FYFTPN(N)KTEDTIF Hex 3 HexNAc 5 | 168.566 | 0.0 | 13.636 | 2.137e+09 | 66 - 79 | 132 | 47.01 | 48.00 | 98.34 |
| 3234.3378 | TPN(N)KTEDTIF Hex 4 HexNAc 7 | 167.753 | 0.0 | 12.460 | 4.364e+07 | 69 - 79 | 5 | 23.33 | 23.50 | 24.17 |
| 3942.6282 | FYFTPN(N)KTEDTIF Fuc 2 Hex 5 HexNAc 6 | 167.062 | 0.0 | 14.619 | 1.566e+08 | 66 - 79 | 7 | 45.81 | 46.84 | 47.34 |
| 3285.3789 | FYFTPN(N)KTEDTIF Hex 4 HexNAc 5 | 166.958 | 0.0 | 11.622 | 2.138e+07 | 66 - 79 | 4 | 47.18 | 47.68 | 47.84 |
| 2920.2445 | FYFTPN(N)KTEDTIF Hex 3 HexNAc 4 | 165.722 | 0.0 | 14.779 | 1.853e+08 | 66 - 79 | 11 | 48.16 | 48.32 | 51.88 |
| 2813.1922 | FTPN(N)KTEDTIF Hex 3 HexNAc 5 | 165.123 | 0.0 | 14.924 | 2.696e+08 | 68 - 79 | 11 | 30.10 | 31.59 | 32.27 |
| 3909.6045 | YFTPN(N)KTEDTIF Hex 5 HexNAc 8 | 164.975 | 0.0 | 14.199 | 1.596e+08 | 67 - 79 | 6 | 35.74 | 36.25 | 36.92 |
| 3747.5493 | YFTPN(N)KTEDTIF Hex 4 HexNAc 8 | 164.128 | 0.0 | 13.527 | 1.216e+08 | 67 - 79 | 6 | 36.25 | 36.59 | 37.09 |
| 3852.5810 | YFTPN(N)KTEDTIF Fuc 1 Hex 5 HexNAc 7 | 163.744 | 0.0 | 14.977 | 1.676e+08 | 67 - 79 | 6 | 36.08 | 36.59 | 37.09 |
| 3186.3524 | EEYN(N)KSVQEIQATF Hex 3 HexNAc 5 | 163.579 | 0.0 | 13.296 | 3.916e+07 | 52 - 66 | 3 | 35.57 | 35.74 | 36.25 |
| 3690.5265 | YFTPN(N)KTEDTIF Fuc 1 Hex 4 HexNAc 7 | 163.080 | 0.0 | 12.667 | 4.099e+07 | 67 - 79 | 3 | 36.42 | 36.92 | 37.09 |
| 3324.3820 | FTPN(N)KTEDTIF Fuc 1 Hex 4 HexNAc 6 | 162.293 | 0.0 | 14.451 | 1.214e+08 | 68 - 79 | 5 | 30.62 | 30.78 | 31.34 |
| 3121.3033 | FTPN(N)KTEDTIF Fuc 1 Hex 4 HexNAc 5 | 161.527 | 0.0 | 12.224 | 8.791e+07 | 68 - 79 | 5 | 30.78 | 31.12 | 31.76 |
| 3400.4853 | RNEEYN(N)KSVQEIQATFF Hex 3 HexNAc 4 | 159.834 | 0.0 | 16.134 | 1.941e+09 | 50 - 67 | 11 | 43.97 | 44.30 | 45.81 |
| 3031.2567 | TPN(N)KTEDTIF Hex 4 HexNAc 6 | 158.987 | 0.0 | 11.057 | 7.817e+06 | 69 - 79 | 3 | 23.67 | 23.83 | 24.33 |
| 3140.3849 | NVQREN(N)GTISRY Hex 3 HexNAc 6 | 158.446 | 0.0 | 14.350 | 7.931e+08 | 97 - 109 | 14 | 13.13 | 13.29 | 16.22 |
| 3603.5650 | RNEEYN(N)KSVQEIQATFF Hex 3 HexNAc 5 | 157.933 | 0.0 | 15.193 | 1.693e+09 | 50 - 67 | 10 | 43.97 | 44.13 | 45.47 |
| 3894.6191 | FYFTPN(N)KTEDTIF Hex 4 HexNAc 8 | 157.157 | 0.0 | 16.340 | 9.632e+07 | 66 - 79 | 5 | 46.15 | 46.66 | 47.34 |
| 3592.4804 | YFTPN(N)KTEDTIF Fuc 2 Hex 5 HexNAc 5 | 156.062 | 0.0 | -5.245 | 1.823e+07 | 67 - 79 | 3 | 36.42 | 36.59 | 36.92 |
| 3254.4040 | RN(D)EEYN(N)KSVQEIQATF Hex 3 HexNAc 4 | 155.960 | 0.0 | 10.136 | 5.852e+08 | 50 - 66 | 24 | 34.38 | 35.74 | 36.42 |
| 3138.3104 | YFTPN(N)KTEDTIF Hex 4 HexNAc 5 | 155.524 | 0.0 | -4.558 | 2.192e+07 | 67 - 79 | 1 | 37.09 | 37.42 | 37.57 |
| 3999.6512 | FYFTPN(N)KTEDTIF Fuc 1 Hex 5 HexNAc 7 | 155.455 | 0.0 | 16.282 | 1.046e+08 | 66 - 79 | 8 | 45.64 | 46.66 | 47.01 |
| 3253.4185 | RNEEYN(N)KSVQEIQATF Hex 3 HexNAc 4 | 155.349 | 0.0 | 15.943 | 5.852e+09 | 50 - 66 | 33 | 28.23 | 32.91 | 34.21 |
| 3488.4577 | FYFTPN(N)KTEDTIF Hex 4 HexNAc 6 | 155.287 | 0.0 | 10.367 | 5.102e+07 | 66 - 79 | 7 | 46.32 | 47.18 | 47.68 |
| 3911.6777 | RNEEYN(N)KSVQEIQATFF Fuc 1 Hex 4 HexNAc 5 | 155.166 | 0.0 | 15.167 | 1.296e+09 | 50 - 67 | 12 | 43.46 | 43.80 | 45.13 |
| 3764.6091 | RNEEYN(N)KSVQEIQATF Fuc 1 Hex 4 HexNAc 5 | 155.134 | 0.0 | 15.704 | 3.262e+09 | 50 - 66 | 15 | 30.45 | 32.44 | 33.71 |
| 3561.5484 | LNVQREN(N)GTISRY Fuc 1 Hex 4 HexNAc 6 | 154.949 | 0.0 | 14.369 | 4.029e+09 | 96 - 109 | 12 | 18.21 | 18.87 | 20.27 |
| 3456.4986 | RNEEYN(N)KSVQEIQATF Hex 3 HexNAc 5 | 154.615 | 0.0 | 15.494 | 4.640e+09 | 50 - 66 | 16 | 30.95 | 32.76 | 34.04 |
| 3517.4207 | YFTPN(N)KTEDTIF Hex 4 HexNAc 4 Neu5Ac 2 | 153.977 | 0.0 | 14.829 | 2.935e+08 | 67 - 79 | 9 | 38.21 | 38.37 | 39.56 |
| 3659.5774 | RNEEYN(N)KSVQEIQATF Hex 3 HexNAc 6 | 153.044 | 0.0 | -3.822 | 1.919e+08 | 50 - 66 | 3 | 32.44 | 32.61 | 32.76 |
| 4112.6822 | YFTPN(N)KTEDTIF Hex 5 HexNAc 9 | 152.247 | 0.0 | 13.597 | 4.815e+07 | 67 - 79 | 4 | 35.74 | 36.08 | 36.59 |
| 3399.4767 | RNEEYN(N)KSVQEIQATF Fuc 1 Hex 3 HexNAc 4 | 152.109 | 0.0 | 12.547 | 1.160e+08 | 50 - 66 | 5 | 32.91 | 33.07 | 33.71 |
| 4056.6724 | FYFTPN(N)KTEDTIF Hex 5 HexNAc 8 | 151.794 | 0.0 | 13.091 | 9.488e+07 | 66 - 79 | 6 | 45.81 | 46.15 | 47.18 |
| 3561.5300 | RNEEYN(N)KSVQEIQATF Fuc 1 Hex 4 HexNAc 4 | 151.665 | 0.0 | -3.925 | 1.369e+08 | 50 - 66 | 4 | 32.27 | 32.44 | 32.61 |
| 3708.5981 | RNEEYN(N)KSVQEIQATFF Fuc 1 Hex 4 HexNAc 4 | 151.585 | 0.0 | -3.648 | 5.779e+07 | 50 - 67 | 4 | 43.63 | 43.80 | 44.13 |
| 4259.7508 | FYFTPN(N)KTEDTIF Hex 5 HexNAc 9 | 151.533 | 0.0 | 12.305 | 3.204e+07 | 66 - 79 | 4 | 45.81 | 45.98 | 46.49 |
| 3806.6445 | RNEEYN(N)KSVQEIQATFF Hex 3 HexNAc 6 | 151.211 | 0.0 | -4.024 | 8.414e+07 | 50 - 67 | 7 | 43.80 | 43.97 | 44.13 |
| 3910.6731 | RNEEYN(N)KSVQEIQATF Fuc 2 Hex 4 HexNAc 5 | 151.111 | 0.0 | -20.008 | 2.670e+07 | 50 - 66 | 1 | 32.44 | 32.61 | 32.61 |
| 4055.6613 | YFTPN(N)KTEDTIF Fuc 1 Hex 5 HexNAc 8 | 151.070 | 0.0 | 12.206 | 4.037e+07 | 67 - 79 | 4 | 35.91 | 36.08 | 36.76 |
| 3967.6896 | RNEEYN(N)KSVQEIQATF Fuc 1 Hex 4 HexNAc 6 | 150.898 | 0.0 | -3.015 | 1.487e+08 | 50 - 66 | 3 | 32.10 | 32.44 | 32.61 |
| 2610.1140 | FTPN(N)KTEDTIF Hex 3 HexNAc 4 | 150.445 | 0.0 | -3.325 | 3.179e+07 | 68 - 79 | 4 | 31.76 | 31.93 | 32.27 |
| 3485.4283 | TPN(N)KTEDTIF Fuc 2 Hex 5 HexNAc 6 | 150.012 | 0.0 | -5.211 | 9.889e+06 | 69 - 79 | 2 | 22.99 | 23.16 | 23.50 |
| 2983.2727 | EEYN(N)KSVQEIQATF Hex 3 HexNAc 4 | 149.856 | 0.0 | -3.055 | 4.142e+07 | 52 - 66 | 15 | 35.74 | 35.91 | 36.25 |
| 3602.5576 | RNEEYN(N)KSVQEIQATF Fuc 1 Hex 3 HexNAc 5 | 149.408 | 0.0 | 9.996 | 1.093e+08 | 50 - 66 | 5 | 32.76 | 32.91 | 33.54 |
| 3381.4055 | FTPN(N)KTEDTIF Hex 4 HexNAc 7 | 149.312 | 0.0 | 14.761 | 7.337e+07 | 68 - 79 | 6 | 30.27 | 30.78 | 31.12 |
| 3534.4463 | YFTPN(N)KTEDTIF Fuc 1 Hex 5 HexNAc 4 Neu5Ac 1 | 148.859 | 0.0 | -5.130 | 1.627e+07 | 67 - 79 | 2 | 38.21 | 38.37 | 38.71 |
| 3618.5529 | RNEEYN(N)KSVQEIQATF Hex 4 HexNAc 5 | 148.600 | 0.0 | -4.755 | 5.227e+07 | 50 - 66 | 3 | 32.27 | 32.44 | 32.61 |
| 3089.3418 | YFTPN(N)KTEDTIFL Hex 3 HexNAc 5 | 148.453 | 0.0 | 13.444 | 1.179e+07 | 67 - 80 | 3 | 45.30 | 45.64 | 45.98 |
| 3494.4620 | EEYN(N)KSVQEIQATF Fuc 1 Hex 4 HexNAc 5 | 148.413 | 0.0 | -3.210 | 3.022e+07 | 52 - 66 | 3 | 35.06 | 35.23 | 35.40 |
| 4227.7902 | RNEEYN(N)KSVQEIQATF Hex 4 HexNAc 8 | 148.264 | 0.0 | -3.987 | 3.255e+07 | 50 - 66 | 1 | 31.93 | 32.10 | 32.27 |
| 3664.4895 | FYFTPN(N)KTEDTIF Hex 4 HexNAc 4 Neu5Ac 2 | 147.040 | 0.0 | 14.208 | 1.934e+08 | 66 - 79 | 12 | 48.32 | 48.49 | 50.70 |
| 3821.6338 | RNEEYN(N)KSVQEIQATF Hex 4 HexNAc 6 | 145.543 | 0.0 | 13.841 | 5.407e+08 | 50 - 66 | 9 | 31.59 | 32.61 | 33.71 |
| 3018.2777 | FTPN(N)KTEDTIF Hex 3 HexNAc 6 | 145.332 | 0.0 | 0.000 | 0.000e+00 | 68 - 79 | 1 | - | - | - |
| 3724.5905 | RNEEYN(N)KSVQEIQATFF Hex 5 HexNAc 4 | 144.376 | 0.0 | -19.309 | 1.319e+07 | 50 - 67 | 2 | 43.80 | 43.80 | 43.97 |
| 4057.7380 | RNEEYN(N)KSVQEIQATFF Fuc 2 Hex 4 HexNAc 5 | 144.068 | 0.0 | -18.948 | 1.810e+07 | 50 - 67 | 2 | 43.63 | 43.80 | 43.80 |
| 3765.6221 | RNEEYN(N)KSVQEIQATFF Hex 4 HexNAc 5 | 143.991 | 0.0 | -5.145 | 2.422e+07 | 50 - 67 | 3 | 43.63 | 43.97 | 43.97 |
| 3749.6229 | RNEEYN(N)KSVQEIQATFF Fuc 1 Hex 3 HexNAc 5 | 143.603 | 0.0 | -4.535 | 3.891e+07 | 50 - 67 | 3 | 43.97 | 43.97 | 44.30 |
| 4024.7126 | RNEEYN(N)KSVQEIQATF Hex 4 HexNAc 7 | 143.594 | 0.0 | -3.961 | 8.695e+07 | 50 - 66 | 3 | 32.10 | 32.27 | 32.61 |
| 3207.2907 | TPN(N)KTEDTIF Hex 4 HexNAc 4 Neu5Ac 2 | 142.821 | 0.0 | 14.336 | 1.626e+07 | 69 - 79 | 5 | 24.67 | 25.01 | 25.35 |
| 4332.8170 | RNEEYN(N)KSVQEIQATF Fuc 1 Hex 5 HexNAc 7 | 142.787 | 0.0 | -5.160 | 1.754e+07 | 50 - 66 | 2 | 31.76 | 31.93 | 32.27 |
| 3448.4626 | NVQREN(N)GTISRY Fuc 1 Hex 4 HexNAc 6 | 142.416 | 0.0 | 12.293 | 3.311e+08 | 97 - 109 | 7 | 12.97 | 13.13 | 13.62 |
| 3825.5283 | YFTPN(N)KTEDTIF Fuc 1 Hex 5 HexNAc 4 Neu5Ac 2 | 142.255 | 0.0 | -5.571 | 4.155e+07 | 67 - 79 | 2 | 37.74 | 37.89 | 38.21 |
| 3073.3167 | N(N)KSVQEIQATF Fuc 1 Hex 4 HexNAc 5 | 141.566 | 0.0 | -18.332 | 8.535e+06 | 55 - 66 | 1 | 26.55 | 26.72 | 26.72 |
| 3106.3489 | RNEEYN(N)KSVQEIQAT Hex 3 HexNAc 4 | 140.729 | 0.0 | -3.715 | 4.108e+07 | 50 - 65 | 3 | 17.21 | 17.37 | 17.54 |
| 3292.4209 | YFTPN(N)KTEDTIFL Hex 3 HexNAc 6 | 140.565 | 0.0 | 14.667 | 2.876e+07 | 67 - 80 | 5 | 45.13 | 45.30 | 45.81 |
| 3253.4369 | LNVQREN(N)GTISRY Hex 3 HexNAc 6 | 140.099 | 0.0 | 13.815 | 1.027e+10 | 96 - 109 | 39 | 18.38 | 19.02 | 27.74 |
| 3618.5691 | LNVQREN(N)GTISRY Hex 4 HexNAc 7 | 139.835 | 0.0 | 14.334 | 2.302e+09 | 96 - 109 | 17 | 18.21 | 18.72 | 21.29 |
| 4114.7570 | RNEEYN(N)KSVQEIQATFF Fuc 1 Hex 4 HexNAc 6 | 139.754 | 0.0 | 13.600 | 5.408e+07 | 50 - 67 | 3 | 43.12 | 43.63 | 43.97 |
| 3358.4681 | LNVQREN(N)GTISRY Fuc 1 Hex 4 HexNAc 5 | 138.840 | 0.0 | 13.993 | 1.050e+09 | 96 - 109 | 10 | 18.38 | 19.02 | 20.78 |
| 3394.3801 | QTRQDQC(C)IYN(N)TTY Hex 3 HexNAc 6 | 137.454 | 0.0 | 15.631 | 2.867e+08 | 83 - 96 | 10 | 21.29 | 22.48 | 22.99 |
| 3457.4837 | RN(D)EEYN(N)KSVQEIQATF Hex 3 HexNAc 5 | 136.467 | 0.0 | 11.513 | 5.144e+08 | 50 - 66 | 12 | 34.21 | 35.57 | 36.59 |
| 3192.3016 | TPN(N)KTEDTIF Fuc 3 Hex 3 HexNAc 4 Neu5Ac 1 | 136.379 | 0.0 | -19.407 | 3.164e+06 | 69 - 79 | 1 | 23.33 | 23.50 | 23.50 |
| 3309.4290 | RNEEYN(N)KSVQEIQAT Hex 3 HexNAc 5 | 135.817 | 0.0 | -4.686 | 3.259e+07 | 50 - 65 | 3 | 17.21 | 17.21 | 17.54 |
| 2937.2726 | NVQREN(N)GTISRY Hex 3 HexNAc 5 | 134.998 | 0.0 | 13.326 | 1.825e+08 | 97 - 109 | 4 | 13.13 | 13.29 | 13.95 |
| 2562.1234 | N(N)KSVQEIQATF Hex 3 HexNAc 4 | 132.465 | 0.0 | -3.328 | 2.040e+07 | 55 - 66 | 2 | 27.40 | 27.57 | 27.74 |
| 3764.6275 | LNVQREN(N)GTISRY Fuc 1 Hex 4 HexNAc 7 | 132.252 | 0.003 | -3.851 | 2.523e+08 | 96 - 109 | 3 | 18.38 | 18.55 | 18.87 |
| 3050.3568 | LNVQREN(N)GTISRY Hex 3 HexNAc 5 | 132.024 | 0.003 | 15.702 | 2.989e+09 | 96 - 109 | 12 | 18.55 | 19.32 | 20.61 |
| 3254.4308 | LN(D)VQREN(N)GTISRY Hex 3 HexNAc 6 | 131.897 | 0.003 | 7.047 | 2.988e+08 | 96 - 109 | 47 | 20.44 | 20.61 | 21.80 |
| 3756.5743 | NVQREN(N)GTISRY Fuc 2 Hex 5 HexNAc 6 | 131.836 | 0.003 | -5.175 | 8.328e+07 | 97 - 109 | 2 | 12.80 | 12.97 | 13.13 |
| 3666.5794 | LNVQREN(N)GTISRY Fuc 2 Hex 5 HexNAc 5 | 131.483 | 0.003 | -4.074 | 8.824e+07 | 96 - 109 | 2 | 18.38 | 18.55 | 18.72 |
| 3869.6585 | LNVQREN(N)GTISRY Fuc 2 Hex 5 HexNAc 6 | 131.220 | 0.003 | 14.479 | 1.535e+09 | 96 - 109 | 7 | 18.05 | 18.55 | 19.80 |
| 3546.5424 | RNEEYN(N)KSVQEIQATFF Fuc 1 Hex 3 HexNAc 4 | 130.977 | 0.003 | -5.130 | 2.678e+07 | 50 - 67 | 3 | 44.13 | 44.30 | 44.62 |
| 3998.6396 | YFTPN(N)KTEDTIF Fuc 2 Hex 5 HexNAc 7 | 130.672 | 0.003 | 12.465 | 2.551e+07 | 67 - 79 | 2 | 35.91 | 36.42 | 36.59 |
| 3026.3103 | VQREN(N)GTISRY Hex 3 HexNAc 6 | 130.614 | 0.003 | -3.325 | 4.447e+07 | 98 - 109 | 3 | 12.63 | 12.80 | 12.97 |
| 3505.4848 | NVQREN(N)GTISRY Hex 4 HexNAc 7 | 130.496 | 0.003 | -4.745 | 8.761e+07 | 97 - 109 | 3 | 12.97 | 13.13 | 13.29 |
| 3821.6495 | LNVQREN(N)GTISRY Hex 4 HexNAc 8 | 130.447 | 0.003 | 13.817 | 1.564e+09 | 96 - 109 | 10 | 18.05 | 18.55 | 19.80 |
| 4177.7733 | LNVQREN(N)GTISRY Fuc 3 Hex 6 HexNAc 6 | 130.201 | 0.003 | 15.879 | 4.470e+08 | 96 - 109 | 4 | 17.88 | 18.38 | 18.87 |
| 2828.1785 | TPN(N)KTEDTIF Hex 4 HexNAc 5 | 130.038 | 0.003 | -17.826 | 2.370e+06 | 69 - 79 | 1 | 24.00 | 24.00 | 24.17 |
| 3399.5221 | LNVQREN(N)GTISRY Fuc 1 Hex 3 HexNAc 6 | 129.661 | 0.003 | 10.732 | 1.592e+08 | 96 - 109 | 17 | 19.17 | 19.32 | 34.89 |
| 4072.7205 | RNEEYN(N)KSVQEIQATF Fuc 2 Hex 5 HexNAc 5 | 129.556 | 0.005 | -3.324 | 1.033e+08 | 50 - 66 | 3 | 31.93 | 32.10 | 32.44 |
| 3632.4958 | FTPN(N)KTEDTIF Fuc 2 Hex 5 HexNAc 6 | 129.511 | 0.005 | 12.459 | 1.669e+07 | 68 - 79 | 4 | 30.10 | 30.45 | 30.78 |
| 4089.7245 | RNEEYN(N)KSVQEIQATFF Hex 6 HexNAc 5 | 128.800 | 0.007 | -18.124 | 9.093e+06 | 50 - 67 | 2 | 43.46 | 43.63 | 43.63 |
| 3574.4461 | TPN(N)KTEDTIF Fuc 4 Hex 5 HexNAc 5 | 128.355 | 0.007 | 12.710 | 5.038e+07 | 69 - 79 | 4 | 38.21 | 38.37 | 38.88 |
| 2847.2774 | LNVQREN(N)GTISRY Hex 3 HexNAc 4 | 128.225 | 0.007 | 13.292 | 2.273e+08 | 96 - 109 | 5 | 19.32 | 19.48 | 20.61 |
| 3926.6823 | LNVQREN(N)GTISRY Fuc 1 Hex 5 HexNAc 7 | 128.064 | 0.007 | 11.594 | 6.439e+08 | 96 - 109 | 10 | 18.05 | 18.38 | 20.27 |
| 3681.5151 | FYFTPN(N)KTEDTIF Fuc 1 Hex 5 HexNAc 4 Neu5Ac 1 | 127.729 | 0.007 | -4.862 | 9.477e+06 | 66 - 79 | 1 | 48.49 | 48.49 | 48.83 |
| 4275.8008 | RNEEYN(N)KSVQEIQATF Fuc 2 Hex 5 HexNAc 6 | 127.484 | 0.007 | 10.474 | 2.979e+07 | 50 - 66 | 1 | 31.76 | 32.27 | 32.44 |
| 4186.7835 | LNVQREN(N)GTISRY Hex 5 HexNAc 9 | 126.537 | 0.007 | 14.851 | 2.791e+08 | 96 - 109 | 3 | 18.05 | 18.38 | 18.72 |
| 2709.1947 | N(N)KSVQEIQATFF Hex 3 HexNAc 4 | 125.684 | 0.007 | 11.641 | 4.752e+06 | 55 - 67 | 2 | 41.43 | 41.60 | 42.12 |
| 3837.5960 | FYFTPN(N)KTEDTIF Fuc 1 Hex 4 HexNAc 7 | 125.493 | 0.007 | 13.205 | 2.909e+07 | 66 - 79 | 4 | 46.49 | 47.01 | 47.18 |
| 2721.1356 | FYFTPN(N)KT Hex 3 HexNAc 6 | 125.488 | 0.0 | -2.622 | 1.271e+07 | 66 - 74 | 2 | 26.20 | 26.37 | 26.72 |
| 3604.5515 | RN(D)EEYN(N)KSVQEIQATFF Hex 3 HexNAc 5 | 124.980 | 0.009 | -5.944 | 4.305e+07 | 50 - 67 | 4 | 47.01 | 47.34 | 47.51 |
| 3354.3590 | FTPN(N)KTEDTIF Hex 4 HexNAc 4 Neu5Ac 2 | 124.776 | 0.009 | -3.893 | 2.500e+07 | 68 - 79 | 4 | 31.93 | 32.10 | 32.27 |
| 3702.4923 | QTRQDQC(C)IYN(N)TTY Fuc 1 Hex 4 HexNAc 6 | 124.724 | 0.009 | 14.611 | 7.224e+07 | 83 - 96 | 4 | 20.95 | 21.97 | 22.48 |
| 3245.3831 | NVQREN(N)GTISRY Fuc 1 Hex 4 HexNAc 5 | 124.190 | 0.009 | -4.158 | 6.171e+07 | 97 - 109 | 2 | 13.13 | 13.13 | 13.46 |
| 4217.7155 | YFTPN(N)KTEDTIF Fuc 1 Hex 6 HexNAc 8 | 123.604 | 0.011 | 10.071 | 1.883e+07 | 67 - 79 | 2 | 34.89 | 35.57 | 36.25 |
| 3708.5635 | NVQREN(N)GTISRY Hex 4 HexNAc 8 | 122.572 | 0.012 | -5.015 | 7.631e+07 | 97 - 109 | 3 | 12.97 | 12.97 | 13.29 |
| 3191.3001 | QTRQDQC(C)IYN(N)TTY Hex 3 HexNAc 5 | 122.396 | 0.012 | 14.160 | 1.198e+08 | 83 - 96 | 6 | 21.46 | 22.65 | 23.16 |
| 2912.2736 | N(N)KSVQEIQATFF Hex 3 HexNAc 5 | 122.103 | 0.012 | -4.055 | 5.645e+06 | 55 - 67 | 2 | 41.09 | 41.26 | 41.43 |
| 3495.4287 | RNEEYN(N)KSVQEIQATF Hex 7 HexNAc 2 | 122.036 | 0.012 | 10.607 | 3.788e+07 | 50 - 66 | 5 | 35.40 | 35.57 | 36.25 |
| 4171.7803 | RNEEYN(N)KSVQEIQATFF Hex 4 HexNAc 7 | 120.617 | 0.012 | 13.121 | 3.098e+07 | 50 - 67 | 4 | 43.12 | 43.29 | 43.97 |
| 3196.4145 | LNVQREN(N)GTISRY Fuc 1 Hex 3 HexNAc 5 | 120.547 | 0.012 | -4.904 | 6.325e+07 | 96 - 109 | 2 | 19.32 | 19.48 | 19.80 |
| 4219.7897 | RNEEYN(N)KSVQEIQATFF Fuc 2 Hex 5 HexNAc 5 | 120.521 | 0.012 | 13.673 | 3.541e+07 | 50 - 67 | 4 | 42.97 | 43.29 | 43.80 |
| 3562.5414 | LN(D)VQREN(N)GTISRY Fuc 1 Hex 4 HexNAc 6 | 120.468 | 0.012 | 8.649 | 1.064e+08 | 96 - 109 | 17 | 20.44 | 20.44 | 21.46 |
| 2518.0560 | FYFTPN(N)KT Hex 3 HexNAc 5 | 118.534 | 0.0 | -18.351 | 4.491e+06 | 66 - 74 | 1 | 26.55 | 26.72 | 26.72 |
| 3765.5951 | RN(D)EEYN(N)KSVQEIQATF Fuc 1 Hex 4 HexNAc 5 | 118.370 | 0.014 | 12.338 | 3.414e+08 | 50 - 66 | 13 | 33.87 | 35.23 | 35.91 |
| 4072.7430 | LNVQREN(N)GTISRY Fuc 2 Hex 5 HexNAc 7 | 118.100 | 0.014 | 15.429 | 2.360e+08 | 96 - 109 | 4 | 18.05 | 18.38 | 18.72 |
| 4129.7642 | LNVQREN(N)GTISRY Fuc 1 Hex 5 HexNAc 8 | 116.160 | 0.016 | 13.684 | 5.656e+08 | 96 - 109 | 6 | 17.88 | 18.38 | 19.32 |
| 3617.5399 | RNEEYN(N)KSVQEIQAT Fuc 1 Hex 4 HexNAc 5 | 115.325 | 0.016 | -18.877 | 1.550e+07 | 50 - 65 | 2 | 17.04 | 17.04 | 17.21 |
| 3220.3863 | N(N)KSVQEIQATFF Fuc 1 Hex 4 HexNAc 5 | 114.781 | 0.017 | -5.105 | 2.803e+06 | 55 - 67 | 2 | 40.41 | 40.41 | 40.75 |
| 3746.5399 | FTPN(N)KTEDTIF Hex 5 HexNAc 8 | 114.698 | 0.017 | 9.813 | 8.413e+06 | 68 - 79 | 3 | 29.76 | 29.93 | 30.62 |
| 3401.4690 | RN(D)EEYN(N)KSVQEIQATFF Hex 3 HexNAc 4 | 114.228 | 0.017 | -5.978 | 6.028e+07 | 50 - 67 | 5 | 47.34 | 47.51 | 47.68 |
| 3983.7058 | LNVQREN(N)GTISRY Hex 5 HexNAc 8 | 114.124 | 0.019 | -3.237 | 1.432e+08 | 96 - 109 | 2 | 18.21 | 18.38 | 18.72 |
| 2671.1012 | N(N)KTEDTIF Hex 3 HexNAc 6 | 114.122 | 0.0 | -19.903 | 3.891e+06 | 71 - 79 | 1 | 22.31 | 22.48 | 22.48 |
| 4016.6733 | NVQREN(N)GTISRY Fuc 1 Hex 5 HexNAc 8 | 113.899 | 0.019 | -19.458 | 2.551e+07 | 97 - 109 | 1 | 12.80 | 12.80 | 12.97 |
| 3415.4920 | LNVQREN(N)GTISRY Hex 4 HexNAc 6 | 113.462 | 0.019 | 11.310 | 2.626e+08 | 96 - 109 | 9 | 18.72 | 19.80 | 20.95 |
| 3314.3409 | TPN(N)KTEDTIF Hex 7 HexNAc 5 | 113.386 | 0.019 | 12.517 | 2.878e+07 | 69 - 79 | 3 | 38.37 | 38.71 | 39.22 |
| 4437.8723 | LNVQREN(N)GTISRY Fuc 2 Hex 6 HexNAc 8 | 111.941 | 0.022 | 12.916 | 2.204e+08 | 96 - 109 | 3 | 17.88 | 18.21 | 18.72 |
| 4380.8534 | LNVQREN(N)GTISRY Fuc 3 Hex 6 HexNAc 7 | 110.913 | 0.024 | -3.757 | 1.305e+08 | 96 - 109 | 3 | 17.88 | 18.21 | 18.38 |
| 3813.5947 | NVQREN(N)GTISRY Fuc 1 Hex 5 HexNAc 7 | 109.068 | 0.027 | -21.231 | 1.460e+07 | 97 - 109 | 2 | 12.80 | 12.97 | 12.97 |
| 3335.3837 | VQREN(N)GTISRY Fuc 1 Hex 4 HexNAc 6 | 109.049 | 0.027 | -5.165 | 6.337e+05 | 98 - 109 | 2 | 62.10 | 62.11 | 62.26 |
| 4234.7933 | LNVQREN(N)GTISRY Fuc 2 Hex 6 HexNAc 7 | 107.781 | 0.034 | 6.173 | 1.451e+08 | 96 - 109 | 4 | 18.05 | 18.21 | 19.02 |
| 3591.5262 | LNVQREN(N)GTISRY Hex 4 HexNAc 4 Neu5Ac 2 | 106.070 | 0.035 | 14.357 | 4.819e+08 | 96 - 109 | 10 | 19.48 | 19.96 | 21.29 |
| 3707.6054 | LNVQREN(N)GTISRY Fuc 2 Hex 4 HexNAc 6 | 106.021 | 0.035 | -19.787 | 2.134e+07 | 96 - 109 | 1 | 18.87 | 18.87 | 19.02 |
| 4145.7082 | FYFTPN(N)KTEDTIF Fuc 2 Hex 5 HexNAc 7 | 105.306 | 0.035 | 12.133 | 2.247e+07 | 66 - 79 | 4 | 45.98 | 46.49 | 46.84 |
| 3891.6391 | LNVQREN(N)GTISRY Fuc 1 Hex 8 HexNAc 3 Neu5Ac 1 | 105.246 | 0.035 | -18.639 | 4.884e+07 | 96 - 109 | 1 | 18.55 | 18.55 | 18.72 |
| 3051.3518 | LN(D)VQREN(N)GTISRY Hex 3 HexNAc 5 | 104.207 | 0.040 | 6.826 | 5.968e+07 | 96 - 109 | 6 | 20.61 | 20.78 | 21.46 |
| 4064.6836 | NVQREN(N)GTISRY Fuc 3 Hex 6 HexNAc 6 | 102.005 | 0.046 | -19.928 | 2.386e+07 | 97 - 109 | 2 | 12.80 | 12.80 | 12.80 |
| 4745.9855 | LNVQREN(N)GTISRY Fuc 3 Hex 7 HexNAc 8 | 101.696 | 0.046 | -3.280 | 4.562e+07 | 96 - 109 | 2 | 17.71 | 18.05 | 18.05 |
| 2983.3503 | C(C)ANLVPVPITN(N)ATL Hex 3 HexNAc 5 | 101.516 | 0.047 | 14.453 | 3.545e+07 | 22 - 36 | 7 | 50.95 | 51.16 | 52.07 |

### TPN(N)KTEDTIF Hex 3 HexNAc 6

**Mass:**  2869.2028

(9.350e-07)

**Charge States:** 3, 4

**Start Position**: 70

**End Position**: 80

| Profile Score | Tandem Score | TDA q-value | Volume | Spectrum Matches |
| --- | --- | --- | --- | --- |
| 16.4184 | 184.0663 | 0.0000 | 3.2987e+08 | 11 |

**Adduct Signal Fractions**

- **Unmodified**: 3.2987e+08

### TPN(N)KTEDTIF Fuc 1 Hex 4 HexNAc 6

**Mass:**  3177.3144

(9.528e-07)

**Charge States:** 3, 4

**Start Position**: 70

**End Position**: 80

| Profile Score | Tandem Score | TDA q-value | Volume | Spectrum Matches |
| --- | --- | --- | --- | --- |
| 14.4979 | 181.3112 | 0.0000 | 6.6510e+07 | 5 |

**Adduct Signal Fractions**

- **Unmodified**: 6.6510e+07

### TPN(N)KTEDTIF Hex 3 HexNAc 5

**Mass:**  2666.1247

(1.277e-06)

**Charge States:** 2, 3

**Start Position**: 70

**End Position**: 80

| Profile Score | Tandem Score | TDA q-value | Volume | Spectrum Matches |
| --- | --- | --- | --- | --- |
| 15.6377 | 180.9897 | 0.0000 | 1.7349e+08 | 8 |

**Adduct Signal Fractions**

- **Unmodified**: 1.7349e+08

### TPN(N)KTEDTIF Fuc 1 Hex 4 HexNAc 5

**Mass:**  2974.2357

(1.158e-06)

**Charge States:** 3

**Start Position**: 70

**End Position**: 80

| Profile Score | Tandem Score | TDA q-value | Volume | Spectrum Matches |
| --- | --- | --- | --- | --- |
| 14.0982 | 180.8416 | 0.0000 | 4.1800e+07 | 4 |

**Adduct Signal Fractions**

- **Unmodified**: 4.1800e+07

### TPN(N)KTEDTIF Hex 3 HexNAc 4

**Mass:**  2463.0459

(1.521e-06)

**Charge States:** 2, 3

**Start Position**: 70

**End Position**: 80

| Profile Score | Tandem Score | TDA q-value | Volume | Spectrum Matches |
| --- | --- | --- | --- | --- |
| 13.1256 | 180.3981 | 0.0000 | 2.3448e+07 | 4 |

**Adduct Signal Fractions**

- **Unmodified**: 2.3448e+07

### FYFTPN(N)KTEDTIF Hex 4 HexNAc 7

**Mass:**  3691.5393

(1.216e-06)

**Charge States:** 3, 4

**Start Position**: 67

**End Position**: 80

| Profile Score | Tandem Score | TDA q-value | Volume | Spectrum Matches |
| --- | --- | --- | --- | --- |
| 14.0667 | 176.8856 | 0.0000 | 5.0588e+08 | 12 |

**Adduct Signal Fractions**

- **Unmodified**: 5.0588e+08

### YFTPN(N)KTEDTIF Hex 3 HexNAc 6

**Mass:**  3179.3366

(5.983e-07)

**Charge States:** 2, 3, 4

**Start Position**: 68

**End Position**: 80

| Profile Score | Tandem Score | TDA q-value | Volume | Spectrum Matches |
| --- | --- | --- | --- | --- |
| 14.8353 | 176.0330 | 0.0000 | 3.5358e+09 | 43 |

**Adduct Signal Fractions**

- **Unmodified**: 3.5358e+09

### YFTPN(N)KTEDTIF Hex 3 HexNAc 4

**Mass:**  2773.1771

(1.116e-06)

**Charge States:** 2, 3

**Start Position**: 68

**End Position**: 80

| Profile Score | Tandem Score | TDA q-value | Volume | Spectrum Matches |
| --- | --- | --- | --- | --- |
| 11.8731 | 175.5189 | 0.0000 | 2.9904e+08 | 9 |

**Adduct Signal Fractions**

- **Unmodified**: 2.9904e+08

### FYFTPN(N)KTEDTIF Fuc 1 Hex 4 HexNAc 5

**Mass:**  3431.4347

(6.942e-07)

**Charge States:** 3, 4

**Start Position**: 67

**End Position**: 80

| Profile Score | Tandem Score | TDA q-value | Volume | Spectrum Matches |
| --- | --- | --- | --- | --- |
| 12.8003 | 175.2093 | 0.0000 | 4.2043e+08 | 11 |

**Adduct Signal Fractions**

- **Unmodified**: 4.2043e+08

### YFTPN(N)KTEDTIF Fuc 1 Hex 4 HexNAc 5

**Mass:**  3284.3651

(1.160e-06)

**Charge States:** 3, 4

**Start Position**: 68

**End Position**: 80

| Profile Score | Tandem Score | TDA q-value | Volume | Spectrum Matches |
| --- | --- | --- | --- | --- |
| 13.9918 | 174.8541 | 0.0000 | 5.7733e+08 | 16 |

**Adduct Signal Fractions**

- **Unmodified**: 5.7733e+08

### FYFTPN(N)KTEDTIF Fuc 1 Hex 4 HexNAc 6

**Mass:**  3634.5163

(1.100e-06)

**Charge States:** 3, 4

**Start Position**: 67

**End Position**: 80

| Profile Score | Tandem Score | TDA q-value | Volume | Spectrum Matches |
| --- | --- | --- | --- | --- |
| 13.9846 | 173.2734 | 0.0000 | 6.7092e+08 | 16 |

**Adduct Signal Fractions**

- **Unmodified**: 6.7092e+08

### FTPN(N)KTEDTIF Hex 3 HexNAc 6

**Mass:**  3016.2740

(4.736e-07)

**Charge States:** 3, 4

**Start Position**: 69

**End Position**: 80

| Profile Score | Tandem Score | TDA q-value | Volume | Spectrum Matches |
| --- | --- | --- | --- | --- |
| 15.7964 | 172.9800 | 0.0000 | 7.3287e+08 | 12 |

**Adduct Signal Fractions**

- **Unmodified**: 7.3287e+08

### YFTPN(N)KTEDTIF Fuc 2 Hex 5 HexNAc 6

**Mass:**  3795.5593

(1.988e-06)

**Charge States:** 3, 4

**Start Position**: 68

**End Position**: 80

| Profile Score | Tandem Score | TDA q-value | Volume | Spectrum Matches |
| --- | --- | --- | --- | --- |
| 16.2475 | 172.9183 | 0.0000 | 1.9067e+08 | 4 |

**Adduct Signal Fractions**

- **Unmodified**: 1.9067e+08

### YFTPN(N)KTEDTIF Hex 3 HexNAc 5

**Mass:**  2976.2558

(7.356e-07)

**Charge States:** 2, 3, 4

**Start Position**: 68

**End Position**: 80

| Profile Score | Tandem Score | TDA q-value | Volume | Spectrum Matches |
| --- | --- | --- | --- | --- |
| 15.0244 | 171.9773 | 0.0000 | 1.9356e+09 | 38 |

**Adduct Signal Fractions**

- **Unmodified**: 1.9356e+09

### YFTPN(N)KTEDTIF Fuc 1 Hex 4 HexNAc 6

**Mass:**  3487.4448

(1.068e-06)

**Charge States:** 3, 4

**Start Position**: 68

**End Position**: 80

| Profile Score | Tandem Score | TDA q-value | Volume | Spectrum Matches |
| --- | --- | --- | --- | --- |
| 16.0778 | 171.1660 | 0.0000 | 1.0747e+09 | 11 |

**Adduct Signal Fractions**

- **Unmodified**: 1.0747e+09

### YFTPN(N)KTEDTIF Hex 3 HexNAc 4

**Mass:**  2775.1840

(7.248e-04)

**Start Position**: 68

**End Position**: 80

| Profile Score | Tandem Score | TDA q-value | Volume | Spectrum Matches |
| --- | --- | --- | --- | --- |
| 0.0000 | 170.5346 | 0.0000 | 0.0000e+00 | 1 |

Adduct:

No Chromatogram Found

### YFTPN(N)KTEDTIF Hex 4 HexNAc 6

**Mass:**  3341.3897

(7.472e-07)

**Charge States:** 3, 4

**Start Position**: 68

**End Position**: 80

| Profile Score | Tandem Score | TDA q-value | Volume | Spectrum Matches |
| --- | --- | --- | --- | --- |
| 16.2720 | 169.6232 | 0.0000 | 1.7608e+08 | 6 |

**Adduct Signal Fractions**

- **Unmodified**: 1.7608e+08

### FYFTPN(N)KTEDTIF Hex 3 HexNAc 6

**Mass:**  3326.4036

(1.156e-06)

**Charge States:** 3, 4

**Start Position**: 67

**End Position**: 80

| Profile Score | Tandem Score | TDA q-value | Volume | Spectrum Matches |
| --- | --- | --- | --- | --- |
| 15.4463 | 169.1672 | 0.0000 | 2.8237e+09 | 23 |

**Adduct Signal Fractions**

- **Unmodified**: 2.8237e+09

### YFTPN(N)KTEDTIF Hex 4 HexNAc 7

**Mass:**  3544.4665

(8.170e-07)

**Charge States:** 3, 4

**Start Position**: 68

**End Position**: 80

| Profile Score | Tandem Score | TDA q-value | Volume | Spectrum Matches |
| --- | --- | --- | --- | --- |
| 15.3675 | 168.7046 | 0.0000 | 9.3672e+08 | 13 |

**Adduct Signal Fractions**

- **Unmodified**: 9.3672e+08

### FYFTPN(N)KTEDTIF Hex 3 HexNAc 5

**Mass:**  3123.3224

(1.480e-07)

**Charge States:** 2, 3, 4

**Start Position**: 67

**End Position**: 80

| Profile Score | Tandem Score | TDA q-value | Volume | Spectrum Matches |
| --- | --- | --- | --- | --- |
| 13.6359 | 168.5658 | 0.0000 | 2.1366e+09 | 132 |

**Adduct Signal Fractions**

- **Unmodified**: 2.1366e+09

### TPN(N)KTEDTIF Hex 4 HexNAc 7

**Mass:**  3234.3371

(9.267e-07)

**Charge States:** 3, 4

**Start Position**: 70

**End Position**: 80

| Profile Score | Tandem Score | TDA q-value | Volume | Spectrum Matches |
| --- | --- | --- | --- | --- |
| 12.4603 | 167.7525 | 0.0000 | 4.3638e+07 | 5 |

**Adduct Signal Fractions**

- **Unmodified**: 4.3638e+07

### FYFTPN(N)KTEDTIF Fuc 2 Hex 5 HexNAc 6

**Mass:**  3942.6276

(1.555e-06)

**Charge States:** 3, 4

**Start Position**: 67

**End Position**: 80

| Profile Score | Tandem Score | TDA q-value | Volume | Spectrum Matches |
| --- | --- | --- | --- | --- |
| 14.6190 | 167.0620 | 0.0000 | 1.5663e+08 | 7 |

**Adduct Signal Fractions**

- **Unmodified**: 1.5663e+08

### FYFTPN(N)KTEDTIF Hex 4 HexNAc 5

**Mass:**  3285.3784

(9.716e-07)

**Charge States:** 3

**Start Position**: 67

**End Position**: 80

| Profile Score | Tandem Score | TDA q-value | Volume | Spectrum Matches |
| --- | --- | --- | --- | --- |
| 11.6223 | 166.9576 | 0.0000 | 2.1383e+07 | 4 |

**Adduct Signal Fractions**

- **Unmodified**: 2.1383e+07

### FYFTPN(N)KTEDTIF Hex 3 HexNAc 4

**Mass:**  2920.2445

(6.469e-07)

**Charge States:** 2, 3, 4

**Start Position**: 67

**End Position**: 80

| Profile Score | Tandem Score | TDA q-value | Volume | Spectrum Matches |
| --- | --- | --- | --- | --- |
| 14.7787 | 165.7220 | 0.0000 | 1.8529e+08 | 11 |

**Adduct Signal Fractions**

- **Unmodified**: 1.8529e+08

### FTPN(N)KTEDTIF Hex 3 HexNAc 5

**Mass:**  2813.1915

(5.684e-07)

**Charge States:** 2, 3, 4

**Start Position**: 69

**End Position**: 80

| Profile Score | Tandem Score | TDA q-value | Volume | Spectrum Matches |
| --- | --- | --- | --- | --- |
| 14.9242 | 165.1230 | 0.0000 | 2.6963e+08 | 11 |

**Adduct Signal Fractions**

- **Unmodified**: 2.6963e+08

### YFTPN(N)KTEDTIF Hex 5 HexNAc 8

**Mass:**  3909.6042

(2.509e-06)

**Charge States:** 3, 4

**Start Position**: 68

**End Position**: 80

| Profile Score | Tandem Score | TDA q-value | Volume | Spectrum Matches |
| --- | --- | --- | --- | --- |
| 14.1988 | 164.9749 | 0.0000 | 1.5964e+08 | 6 |

**Adduct Signal Fractions**

- **Unmodified**: 1.5964e+08

### YFTPN(N)KTEDTIF Hex 4 HexNAc 8

**Mass:**  3747.5505

(1.368e-06)

**Charge States:** 3, 4

**Start Position**: 68

**End Position**: 80

| Profile Score | Tandem Score | TDA q-value | Volume | Spectrum Matches |
| --- | --- | --- | --- | --- |
| 13.5269 | 164.1276 | 0.0000 | 1.2160e+08 | 6 |

**Adduct Signal Fractions**

- **Unmodified**: 1.2160e+08

### YFTPN(N)KTEDTIF Fuc 1 Hex 5 HexNAc 7

**Mass:**  3852.5815

(1.641e-06)

**Charge States:** 3, 4

**Start Position**: 68

**End Position**: 80

| Profile Score | Tandem Score | TDA q-value | Volume | Spectrum Matches |
| --- | --- | --- | --- | --- |
| 14.9774 | 163.7442 | 0.0000 | 1.6759e+08 | 6 |

**Adduct Signal Fractions**

- **Unmodified**: 1.6759e+08

### EEYN(N)KSVQEIQATF Hex 3 HexNAc 5

**Mass:**  3186.3524

(7.725e-07)

**Charge States:** 3, 4

**Start Position**: 53

**End Position**: 67

| Profile Score | Tandem Score | TDA q-value | Volume | Spectrum Matches |
| --- | --- | --- | --- | --- |
| 13.2963 | 163.5790 | 0.0000 | 3.9162e+07 | 3 |

**Adduct Signal Fractions**

- **Unmodified**: 3.9162e+07

### YFTPN(N)KTEDTIF Fuc 1 Hex 4 HexNAc 7

**Mass:**  3690.5257

(1.313e-06)

**Charge States:** 3, 4

**Start Position**: 68

**End Position**: 80

| Profile Score | Tandem Score | TDA q-value | Volume | Spectrum Matches |
| --- | --- | --- | --- | --- |
| 12.6674 | 163.0801 | 0.0000 | 4.0987e+07 | 3 |

**Adduct Signal Fractions**

- **Unmodified**: 4.0987e+07

### FTPN(N)KTEDTIF Fuc 1 Hex 4 HexNAc 6

**Mass:**  3324.3817

(8.709e-07)

**Charge States:** 3, 4

**Start Position**: 69

**End Position**: 80

| Profile Score | Tandem Score | TDA q-value | Volume | Spectrum Matches |
| --- | --- | --- | --- | --- |
| 14.4512 | 162.2935 | 0.0000 | 1.2140e+08 | 5 |

**Adduct Signal Fractions**

- **Unmodified**: 1.2140e+08

### FTPN(N)KTEDTIF Fuc 1 Hex 4 HexNAc 5

**Mass:**  3121.3042

(1.079e-06)

**Charge States:** 3

**Start Position**: 69

**End Position**: 80

| Profile Score | Tandem Score | TDA q-value | Volume | Spectrum Matches |
| --- | --- | --- | --- | --- |
| 12.2238 | 161.5274 | 0.0000 | 8.7914e+07 | 5 |

**Adduct Signal Fractions**

- **Unmodified**: 8.7914e+07

### RNEEYN(N)KSVQEIQATFF Hex 3 HexNAc 4

**Mass:**  3400.4844

(1.075e-06)

**Charge States:** 3, 4

**Start Position**: 51

**End Position**: 68

| Profile Score | Tandem Score | TDA q-value | Volume | Spectrum Matches |
| --- | --- | --- | --- | --- |
| 16.1344 | 159.8344 | 0.0000 | 1.9407e+09 | 11 |

**Adduct Signal Fractions**

- **Unmodified**: 1.9407e+09

### TPN(N)KTEDTIF Hex 4 HexNAc 6

**Mass:**  3031.2583

(3.514e-08)

**Charge States:** 3

**Start Position**: 70

**End Position**: 80

| Profile Score | Tandem Score | TDA q-value | Volume | Spectrum Matches |
| --- | --- | --- | --- | --- |
| 11.0571 | 158.9871 | 0.0000 | 7.8173e+06 | 3 |

**Adduct Signal Fractions**

- **Unmodified**: 7.8173e+06

### NVQREN(N)GTISRY Hex 3 HexNAc 6

**Mass:**  3140.3490

(2.616e-07)

**Charge States:** 3, 4

**Start Position**: 98

**End Position**: 110

| Profile Score | Tandem Score | TDA q-value | Volume | Spectrum Matches |
| --- | --- | --- | --- | --- |
| 14.3496 | 158.4460 | 0.0000 | 7.9312e+08 | 14 |

**Adduct Signal Fractions**

- **Unmodified**: 7.9312e+08

### RNEEYN(N)KSVQEIQATFF Hex 3 HexNAc 5

**Mass:**  3603.5639

(1.190e-06)

**Charge States:** 3, 4

**Start Position**: 51

**End Position**: 68

| Profile Score | Tandem Score | TDA q-value | Volume | Spectrum Matches |
| --- | --- | --- | --- | --- |
| 15.1927 | 157.9330 | 0.0000 | 1.6925e+09 | 10 |

**Adduct Signal Fractions**

- **Unmodified**: 1.6925e+09

### FYFTPN(N)KTEDTIF Hex 4 HexNAc 8

**Mass:**  3894.6198

(2.036e-06)

**Charge States:** 3, 4

**Start Position**: 67

**End Position**: 80

| Profile Score | Tandem Score | TDA q-value | Volume | Spectrum Matches |
| --- | --- | --- | --- | --- |
| 16.3400 | 157.1573 | 0.0000 | 9.6316e+07 | 5 |

**Adduct Signal Fractions**

- **Unmodified**: 9.6316e+07

### YFTPN(N)KTEDTIF Fuc 2 Hex 5 HexNAc 5

**Mass:**  3592.4785

(1.018e-06)

**Charge States:** 3

**Start Position**: 68

**End Position**: 80

| Profile Score | Tandem Score | TDA q-value | Volume | Spectrum Matches |
| --- | --- | --- | --- | --- |
| -5.2453 | 156.0617 | 0.0000 | 1.8233e+07 | 3 |

**Adduct Signal Fractions**

- **Unmodified**: 1.8233e+07

### RN(D)EEYN(N)KSVQEIQATF Hex 3 HexNAc 4

**Mass:**  3254.4007

(3.047e-04)

**Charge States:** 3, 4

**Start Position**: 51

**End Position**: 67

| Profile Score | Tandem Score | TDA q-value | Volume | Spectrum Matches |
| --- | --- | --- | --- | --- |
| 10.1359 | 155.9603 | 0.0000 | 5.8519e+08 | 24 |

**Adduct Signal Fractions**

- **Unmodified**: 5.8519e+08

### YFTPN(N)KTEDTIF Hex 4 HexNAc 5

**Mass:**  3138.3122

(1.903e-06)

**Charge States:** 3

**Start Position**: 68

**End Position**: 80

| Profile Score | Tandem Score | TDA q-value | Volume | Spectrum Matches |
| --- | --- | --- | --- | --- |
| -4.5582 | 155.5236 | 0.0000 | 2.1919e+07 | 1 |

**Adduct Signal Fractions**

- **Unmodified**: 2.1919e+07

### FYFTPN(N)KTEDTIF Fuc 1 Hex 5 HexNAc 7

**Mass:**  3999.6508

(1.927e-06)

**Charge States:** 3, 4

**Start Position**: 67

**End Position**: 80

| Profile Score | Tandem Score | TDA q-value | Volume | Spectrum Matches |
| --- | --- | --- | --- | --- |
| 16.2824 | 155.4555 | 0.0000 | 1.0465e+08 | 8 |

**Adduct Signal Fractions**

- **Unmodified**: 1.0465e+08

### RNEEYN(N)KSVQEIQATF Hex 3 HexNAc 4

**Mass:**  3253.4192

(1.649e-06)

**Charge States:** 3, 4

**Start Position**: 51

**End Position**: 67

| Profile Score | Tandem Score | TDA q-value | Volume | Spectrum Matches |
| --- | --- | --- | --- | --- |
| 15.9433 | 155.3491 | 0.0000 | 5.8519e+09 | 33 |

**Adduct Signal Fractions**

- **Unmodified**: 5.8519e+09

### FYFTPN(N)KTEDTIF Hex 4 HexNAc 6

**Mass:**  3488.4589

(5.403e-07)

**Charge States:** 3, 4

**Start Position**: 67

**End Position**: 80

| Profile Score | Tandem Score | TDA q-value | Volume | Spectrum Matches |
| --- | --- | --- | --- | --- |
| 10.3674 | 155.2866 | 0.0000 | 5.1020e+07 | 7 |

**Adduct Signal Fractions**

- **Unmodified**: 5.1020e+07

### RNEEYN(N)KSVQEIQATFF Fuc 1 Hex 4 HexNAc 5

**Mass:**  3911.6782

(1.337e-06)

**Charge States:** 3, 4

**Start Position**: 51

**End Position**: 68

| Profile Score | Tandem Score | TDA q-value | Volume | Spectrum Matches |
| --- | --- | --- | --- | --- |
| 15.1672 | 155.1655 | 0.0000 | 1.2960e+09 | 12 |

**Adduct Signal Fractions**

- **Unmodified**: 1.2960e+09

### RNEEYN(N)KSVQEIQATF Fuc 1 Hex 4 HexNAc 5

**Mass:**  3764.6083

(1.990e-06)

**Charge States:** 3, 4

**Start Position**: 51

**End Position**: 67

| Profile Score | Tandem Score | TDA q-value | Volume | Spectrum Matches |
| --- | --- | --- | --- | --- |
| 15.7042 | 155.1342 | 0.0000 | 3.2617e+09 | 15 |

**Adduct Signal Fractions**

- **Unmodified**: 3.2617e+09

### LNVQREN(N)GTISRY Fuc 1 Hex 4 HexNAc 6

**Mass:**  3561.5484

(9.714e-07)

**Charge States:** 3, 4

**Start Position**: 97

**End Position**: 110

| Profile Score | Tandem Score | TDA q-value | Volume | Spectrum Matches |
| --- | --- | --- | --- | --- |
| 14.3690 | 154.9486 | 0.0000 | 4.0290e+09 | 12 |

**Adduct Signal Fractions**

- **Unmodified**: 4.0290e+09

### RNEEYN(N)KSVQEIQATF Hex 3 HexNAc 5

**Mass:**  3456.4993

(4.456e-07)

**Charge States:** 3, 4

**Start Position**: 51

**End Position**: 67

| Profile Score | Tandem Score | TDA q-value | Volume | Spectrum Matches |
| --- | --- | --- | --- | --- |
| 15.4944 | 154.6152 | 0.0000 | 4.6401e+09 | 16 |

**Adduct Signal Fractions**

- **Unmodified**: 4.6401e+09

### YFTPN(N)KTEDTIF Hex 4 HexNAc 4 Neu5Ac 2

**Mass:**  3517.4203

(7.796e-07)

**Charge States:** 3, 4

**Start Position**: 68

**End Position**: 80

| Profile Score | Tandem Score | TDA q-value | Volume | Spectrum Matches |
| --- | --- | --- | --- | --- |
| 14.8291 | 153.9767 | 0.0000 | 2.9345e+08 | 9 |

**Adduct Signal Fractions**

- **Unmodified**: 2.9345e+08

### RNEEYN(N)KSVQEIQATF Hex 3 HexNAc 6

**Mass:**  3659.5776

(7.467e-07)

**Charge States:** 3, 4

**Start Position**: 51

**End Position**: 67

| Profile Score | Tandem Score | TDA q-value | Volume | Spectrum Matches |
| --- | --- | --- | --- | --- |
| -3.8222 | 153.0441 | 0.0000 | 1.9185e+08 | 3 |

**Adduct Signal Fractions**

- **Unmodified**: 1.9185e+08

### YFTPN(N)KTEDTIF Hex 5 HexNAc 9

**Mass:**  4112.6823

(6.108e-07)

**Charge States:** 3, 4

**Start Position**: 68

**End Position**: 80

| Profile Score | Tandem Score | TDA q-value | Volume | Spectrum Matches |
| --- | --- | --- | --- | --- |
| 13.5966 | 152.2467 | 0.0000 | 4.8146e+07 | 4 |

**Adduct Signal Fractions**

- **Unmodified**: 4.8146e+07

### RNEEYN(N)KSVQEIQATF Fuc 1 Hex 3 HexNAc 4

**Mass:**  3399.4773

(1.498e-06)

**Charge States:** 3, 4

**Start Position**: 51

**End Position**: 67

| Profile Score | Tandem Score | TDA q-value | Volume | Spectrum Matches |
| --- | --- | --- | --- | --- |
| 12.5471 | 152.1094 | 0.0000 | 1.1596e+08 | 5 |

**Adduct Signal Fractions**

- **Unmodified**: 1.1596e+08

### FYFTPN(N)KTEDTIF Hex 5 HexNAc 8

**Mass:**  4056.6723

(1.675e-06)

**Charge States:** 3, 4

**Start Position**: 67

**End Position**: 80

| Profile Score | Tandem Score | TDA q-value | Volume | Spectrum Matches |
| --- | --- | --- | --- | --- |
| 13.0912 | 151.7944 | 0.0000 | 9.4876e+07 | 6 |

**Adduct Signal Fractions**

- **Unmodified**: 9.4876e+07

### RNEEYN(N)KSVQEIQATF Fuc 1 Hex 4 HexNAc 4

**Mass:**  3561.5290

(1.090e-06)

**Charge States:** 3, 4

**Start Position**: 51

**End Position**: 67

| Profile Score | Tandem Score | TDA q-value | Volume | Spectrum Matches |
| --- | --- | --- | --- | --- |
| -3.9248 | 151.6652 | 0.0000 | 1.3689e+08 | 4 |

**Adduct Signal Fractions**

- **Unmodified**: 1.3689e+08

### RNEEYN(N)KSVQEIQATFF Fuc 1 Hex 4 HexNAc 4

**Mass:**  3708.5986

(5.505e-07)

**Charge States:** 3, 4

**Start Position**: 51

**End Position**: 68

| Profile Score | Tandem Score | TDA q-value | Volume | Spectrum Matches |
| --- | --- | --- | --- | --- |
| -3.6481 | 151.5849 | 0.0000 | 5.7792e+07 | 4 |

**Adduct Signal Fractions**

- **Unmodified**: 5.7792e+07

### FYFTPN(N)KTEDTIF Hex 5 HexNAc 9

**Mass:**  4259.7512

(1.236e-06)

**Charge States:** 3, 4

**Start Position**: 67

**End Position**: 80

| Profile Score | Tandem Score | TDA q-value | Volume | Spectrum Matches |
| --- | --- | --- | --- | --- |
| 12.3054 | 151.5328 | 0.0000 | 3.2040e+07 | 4 |

**Adduct Signal Fractions**

- **Unmodified**: 3.2040e+07

### RNEEYN(N)KSVQEIQATFF Hex 3 HexNAc 6

**Mass:**  3806.6447

(2.119e-06)

**Charge States:** 3, 4

**Start Position**: 51

**End Position**: 68

| Profile Score | Tandem Score | TDA q-value | Volume | Spectrum Matches |
| --- | --- | --- | --- | --- |
| -4.0244 | 151.2112 | 0.0000 | 8.4142e+07 | 7 |

**Adduct Signal Fractions**

- **Unmodified**: 8.4142e+07

### RNEEYN(N)KSVQEIQATF Fuc 2 Hex 4 HexNAc 5

**Mass:**  3910.6699

(4.085e-06)

**Charge States:** 4

**Start Position**: 51

**End Position**: 67

| Profile Score | Tandem Score | TDA q-value | Volume | Spectrum Matches |
| --- | --- | --- | --- | --- |
| -20.0079 | 151.1112 | 0.0000 | 2.6698e+07 | 1 |

**Adduct Signal Fractions**

- **Unmodified**: 2.6698e+07

### YFTPN(N)KTEDTIF Fuc 1 Hex 5 HexNAc 8

**Mass:**  4055.6611

(7.357e-08)

**Charge States:** 3, 4

**Start Position**: 68

**End Position**: 80

| Profile Score | Tandem Score | TDA q-value | Volume | Spectrum Matches |
| --- | --- | --- | --- | --- |
| 12.2058 | 151.0703 | 0.0000 | 4.0367e+07 | 4 |

**Adduct Signal Fractions**

- **Unmodified**: 4.0367e+07

### RNEEYN(N)KSVQEIQATF Fuc 1 Hex 4 HexNAc 6

**Mass:**  3967.6906

(8.756e-07)

**Charge States:** 3, 4

**Start Position**: 51

**End Position**: 67

| Profile Score | Tandem Score | TDA q-value | Volume | Spectrum Matches |
| --- | --- | --- | --- | --- |
| -3.0149 | 150.8980 | 0.0000 | 1.4871e+08 | 3 |

**Adduct Signal Fractions**

- **Unmodified**: 1.4871e+08

### FTPN(N)KTEDTIF Hex 3 HexNAc 4

**Mass:**  2610.1137

(1.416e-06)

**Charge States:** 2, 3

**Start Position**: 69

**End Position**: 80

| Profile Score | Tandem Score | TDA q-value | Volume | Spectrum Matches |
| --- | --- | --- | --- | --- |
| -3.3245 | 150.4452 | 0.0000 | 3.1786e+07 | 4 |

**Adduct Signal Fractions**

- **Unmodified**: 3.1786e+07

### TPN(N)KTEDTIF Fuc 2 Hex 5 HexNAc 6

**Mass:**  3485.4285

(1.886e-06)

**Charge States:** 3, 4

**Start Position**: 70

**End Position**: 80

| Profile Score | Tandem Score | TDA q-value | Volume | Spectrum Matches |
| --- | --- | --- | --- | --- |
| -5.2114 | 150.0121 | 0.0000 | 9.8888e+06 | 2 |

**Adduct Signal Fractions**

- **Unmodified**: 9.8888e+06

### EEYN(N)KSVQEIQATF Hex 3 HexNAc 4

**Mass:**  2983.2725

(4.676e-07)

**Charge States:** 3

**Start Position**: 53

**End Position**: 67

| Profile Score | Tandem Score | TDA q-value | Volume | Spectrum Matches |
| --- | --- | --- | --- | --- |
| -3.0546 | 149.8556 | 0.0000 | 4.1422e+07 | 15 |

**Adduct Signal Fractions**

- **Unmodified**: 4.1422e+07

### RNEEYN(N)KSVQEIQATF Fuc 1 Hex 3 HexNAc 5

**Mass:**  3602.5576

(1.359e-06)

**Charge States:** 3, 4

**Start Position**: 51

**End Position**: 67

| Profile Score | Tandem Score | TDA q-value | Volume | Spectrum Matches |
| --- | --- | --- | --- | --- |
| 9.9961 | 149.4082 | 0.0000 | 1.0926e+08 | 5 |

**Adduct Signal Fractions**

- **Unmodified**: 1.0926e+08

### FTPN(N)KTEDTIF Hex 4 HexNAc 7

**Mass:**  3381.4037

(2.724e-06)

**Charge States:** 3, 4

**Start Position**: 69

**End Position**: 80

| Profile Score | Tandem Score | TDA q-value | Volume | Spectrum Matches |
| --- | --- | --- | --- | --- |
| 14.7612 | 149.3123 | 0.0000 | 7.3375e+07 | 6 |

**Adduct Signal Fractions**

- **Unmodified**: 7.3375e+07

### YFTPN(N)KTEDTIF Fuc 1 Hex 5 HexNAc 4 Neu5Ac 1

**Mass:**  3534.4455

(3.499e-06)

**Charge States:** 3

**Start Position**: 68

**End Position**: 80

| Profile Score | Tandem Score | TDA q-value | Volume | Spectrum Matches |
| --- | --- | --- | --- | --- |
| -5.1301 | 148.8592 | 0.0000 | 1.6270e+07 | 2 |

**Adduct Signal Fractions**

- **Unmodified**: 1.6270e+07

### RNEEYN(N)KSVQEIQATF Hex 4 HexNAc 5

**Mass:**  3618.5576

(2.781e-06)

**Charge States:** 3, 4

**Start Position**: 51

**End Position**: 67

| Profile Score | Tandem Score | TDA q-value | Volume | Spectrum Matches |
| --- | --- | --- | --- | --- |
| -4.7545 | 148.6004 | 0.0000 | 5.2266e+07 | 3 |

**Adduct Signal Fractions**

- **Unmodified**: 5.2266e+07

### YFTPN(N)KTEDTIFL Hex 3 HexNAc 5

**Mass:**  3089.3421

(1.500e-06)

**Charge States:** 3, 4

**Start Position**: 68

**End Position**: 81

| Profile Score | Tandem Score | TDA q-value | Volume | Spectrum Matches |
| --- | --- | --- | --- | --- |
| 13.4435 | 148.4529 | 0.0000 | 1.1792e+07 | 3 |

**Adduct Signal Fractions**

- **Unmodified**: 1.1792e+07

### EEYN(N)KSVQEIQATF Fuc 1 Hex 4 HexNAc 5

**Mass:**  3494.4644

(1.143e-06)

**Charge States:** 3, 4

**Start Position**: 53

**End Position**: 67

| Profile Score | Tandem Score | TDA q-value | Volume | Spectrum Matches |
| --- | --- | --- | --- | --- |
| -3.2100 | 148.4127 | 0.0000 | 3.0219e+07 | 3 |

**Adduct Signal Fractions**

- **Unmodified**: 3.0219e+07

### RNEEYN(N)KSVQEIQATF Hex 4 HexNAc 8

**Mass:**  4227.7902

(1.014e-06)

**Charge States:** 3, 4

**Start Position**: 51

**End Position**: 67

| Profile Score | Tandem Score | TDA q-value | Volume | Spectrum Matches |
| --- | --- | --- | --- | --- |
| -3.9872 | 148.2636 | 0.0000 | 3.2546e+07 | 1 |

**Adduct Signal Fractions**

- **Unmodified**: 3.2546e+07

### FYFTPN(N)KTEDTIF Hex 4 HexNAc 4 Neu5Ac 2

**Mass:**  3664.4892

(1.043e-06)

**Charge States:** 3, 4

**Start Position**: 67

**End Position**: 80

| Profile Score | Tandem Score | TDA q-value | Volume | Spectrum Matches |
| --- | --- | --- | --- | --- |
| 14.2084 | 147.0396 | 0.0000 | 1.9345e+08 | 12 |

**Adduct Signal Fractions**

- **Unmodified**: 1.9345e+08

### RNEEYN(N)KSVQEIQATF Hex 4 HexNAc 6

**Mass:**  3821.6350

(1.922e-06)

**Charge States:** 3, 4

**Start Position**: 51

**End Position**: 67

| Profile Score | Tandem Score | TDA q-value | Volume | Spectrum Matches |
| --- | --- | --- | --- | --- |
| 13.8411 | 145.5434 | 0.0000 | 5.4072e+08 | 9 |

**Adduct Signal Fractions**

- **Unmodified**: 5.4072e+08

### FTPN(N)KTEDTIF Hex 3 HexNAc 6

**Mass:**  3018.2777

(6.658e-04)

**Start Position**: 69

**End Position**: 80

| Profile Score | Tandem Score | TDA q-value | Volume | Spectrum Matches |
| --- | --- | --- | --- | --- |
| 0.0000 | 145.3323 | 0.0000 | 0.0000e+00 | 1 |

Adduct:

No Chromatogram Found

### RNEEYN(N)KSVQEIQATFF Hex 5 HexNAc 4

**Mass:**  3724.5942

(1.525e-06)

**Charge States:** 3, 4

**Start Position**: 51

**End Position**: 68

| Profile Score | Tandem Score | TDA q-value | Volume | Spectrum Matches |
| --- | --- | --- | --- | --- |
| -19.3085 | 144.3757 | 0.0000 | 1.3185e+07 | 2 |

**Adduct Signal Fractions**

- **Unmodified**: 1.3185e+07

### RNEEYN(N)KSVQEIQATFF Fuc 2 Hex 4 HexNAc 5

**Mass:**  4057.7360

(1.735e-06)

**Charge States:** 3, 4

**Start Position**: 51

**End Position**: 68

| Profile Score | Tandem Score | TDA q-value | Volume | Spectrum Matches |
| --- | --- | --- | --- | --- |
| -18.9484 | 144.0681 | 0.0000 | 1.8099e+07 | 2 |

**Adduct Signal Fractions**

- **Unmodified**: 1.8099e+07

### RNEEYN(N)KSVQEIQATFF Hex 4 HexNAc 5

**Mass:**  3765.6210

(1.976e-06)

**Charge States:** 3, 4

**Start Position**: 51

**End Position**: 68

| Profile Score | Tandem Score | TDA q-value | Volume | Spectrum Matches |
| --- | --- | --- | --- | --- |
| -5.1455 | 143.9905 | 0.0000 | 2.4222e+07 | 3 |

**Adduct Signal Fractions**

- **Unmodified**: 2.4222e+07

### RNEEYN(N)KSVQEIQATFF Fuc 1 Hex 3 HexNAc 5

**Mass:**  3749.6215

(3.872e-07)

**Charge States:** 4

**Start Position**: 51

**End Position**: 68

| Profile Score | Tandem Score | TDA q-value | Volume | Spectrum Matches |
| --- | --- | --- | --- | --- |
| -4.5353 | 143.6027 | 0.0000 | 3.8905e+07 | 3 |

**Adduct Signal Fractions**

- **Unmodified**: 3.8905e+07

### RNEEYN(N)KSVQEIQATF Hex 4 HexNAc 7

**Mass:**  4024.7123

(1.545e-06)

**Charge States:** 3, 4

**Start Position**: 51

**End Position**: 67

| Profile Score | Tandem Score | TDA q-value | Volume | Spectrum Matches |
| --- | --- | --- | --- | --- |
| -3.9612 | 143.5938 | 0.0000 | 8.6952e+07 | 3 |

**Adduct Signal Fractions**

- **Unmodified**: 8.6952e+07

### TPN(N)KTEDTIF Hex 4 HexNAc 4 Neu5Ac 2

**Mass:**  3207.2898

(1.982e-06)

**Charge States:** 3, 4

**Start Position**: 70

**End Position**: 80

| Profile Score | Tandem Score | TDA q-value | Volume | Spectrum Matches |
| --- | --- | --- | --- | --- |
| 14.3360 | 142.8214 | 0.0000 | 1.6260e+07 | 5 |

**Adduct Signal Fractions**

- **Unmodified**: 1.6260e+07

### RNEEYN(N)KSVQEIQATF Fuc 1 Hex 5 HexNAc 7

**Mass:**  4332.8210

(1.212e-06)

**Charge States:** 4

**Start Position**: 51

**End Position**: 67

| Profile Score | Tandem Score | TDA q-value | Volume | Spectrum Matches |
| --- | --- | --- | --- | --- |
| -5.1597 | 142.7871 | 0.0000 | 1.7537e+07 | 2 |

**Adduct Signal Fractions**

- **Unmodified**: 1.7537e+07

### NVQREN(N)GTISRY Fuc 1 Hex 4 HexNAc 6

**Mass:**  3448.4628

(-5.969e-07)

**Charge States:** 3, 4

**Start Position**: 98

**End Position**: 110

| Profile Score | Tandem Score | TDA q-value | Volume | Spectrum Matches |
| --- | --- | --- | --- | --- |
| 12.2934 | 142.4162 | 0.0000 | 3.3112e+08 | 7 |

**Adduct Signal Fractions**

- **Unmodified**: 3.3112e+08

### YFTPN(N)KTEDTIF Fuc 1 Hex 5 HexNAc 4 Neu5Ac 2

**Mass:**  3825.5322

(5.994e-07)

**Charge States:** 3, 4

**Start Position**: 68

**End Position**: 80

| Profile Score | Tandem Score | TDA q-value | Volume | Spectrum Matches |
| --- | --- | --- | --- | --- |
| -5.5711 | 142.2546 | 0.0000 | 4.1549e+07 | 2 |

**Adduct Signal Fractions**

- **Unmodified**: 4.1549e+07

### N(N)KSVQEIQATF Fuc 1 Hex 4 HexNAc 5

**Mass:**  3073.3163

(1.360e-06)

**Charge States:** 3, 4

**Start Position**: 56

**End Position**: 67

| Profile Score | Tandem Score | TDA q-value | Volume | Spectrum Matches |
| --- | --- | --- | --- | --- |
| -18.3319 | 141.5659 | 0.0000 | 8.5355e+06 | 1 |

**Adduct Signal Fractions**

- **Unmodified**: 8.5355e+06

### RNEEYN(N)KSVQEIQAT Hex 3 HexNAc 4

**Mass:**  3106.3478

(1.427e-06)

**Charge States:** 3

**Start Position**: 51

**End Position**: 66

| Profile Score | Tandem Score | TDA q-value | Volume | Spectrum Matches |
| --- | --- | --- | --- | --- |
| -3.7146 | 140.7289 | 0.0000 | 4.1084e+07 | 3 |

**Adduct Signal Fractions**

- **Unmodified**: 4.1084e+07

### YFTPN(N)KTEDTIFL Hex 3 HexNAc 6

**Mass:**  3292.4207

(1.320e-06)

**Charge States:** 3, 4

**Start Position**: 68

**End Position**: 81

| Profile Score | Tandem Score | TDA q-value | Volume | Spectrum Matches |
| --- | --- | --- | --- | --- |
| 14.6668 | 140.5652 | 0.0000 | 2.8760e+07 | 5 |

**Adduct Signal Fractions**

- **Unmodified**: 2.8760e+07

### LNVQREN(N)GTISRY Hex 3 HexNAc 6

**Mass:**  3253.4368

(2.292e-07)

**Charge States:** 2, 3, 4

**Start Position**: 97

**End Position**: 110

| Profile Score | Tandem Score | TDA q-value | Volume | Spectrum Matches |
| --- | --- | --- | --- | --- |
| 13.8145 | 140.0991 | 0.0000 | 1.0267e+10 | 39 |

**Adduct Signal Fractions**

- **Unmodified**: 1.0267e+10

### LNVQREN(N)GTISRY Hex 4 HexNAc 7

**Mass:**  3618.5695

(6.766e-07)

**Charge States:** 3, 4

**Start Position**: 97

**End Position**: 110

| Profile Score | Tandem Score | TDA q-value | Volume | Spectrum Matches |
| --- | --- | --- | --- | --- |
| 14.3336 | 139.8350 | 0.0000 | 2.3017e+09 | 17 |

**Adduct Signal Fractions**

- **Unmodified**: 2.3017e+09

### RNEEYN(N)KSVQEIQATFF Fuc 1 Hex 4 HexNAc 6

**Mass:**  4114.7570

(1.101e-06)

**Charge States:** 3, 4

**Start Position**: 51

**End Position**: 68

| Profile Score | Tandem Score | TDA q-value | Volume | Spectrum Matches |
| --- | --- | --- | --- | --- |
| 13.6002 | 139.7538 | 0.0000 | 5.4075e+07 | 3 |

**Adduct Signal Fractions**

- **Unmodified**: 5.4075e+07

### LNVQREN(N)GTISRY Fuc 1 Hex 4 HexNAc 5

**Mass:**  3358.4683

(2.680e-07)

**Charge States:** 3, 4

**Start Position**: 97

**End Position**: 110

| Profile Score | Tandem Score | TDA q-value | Volume | Spectrum Matches |
| --- | --- | --- | --- | --- |
| 13.9926 | 138.8404 | 0.0000 | 1.0497e+09 | 10 |

**Adduct Signal Fractions**

- **Unmodified**: 1.0497e+09

### QTRQDQC(C)IYN(N)TTY Hex 3 HexNAc 6

**Mass:**  3394.3804

(1.083e-06)

**Charge States:** 3, 4

**Start Position**: 84

**End Position**: 97

| Profile Score | Tandem Score | TDA q-value | Volume | Spectrum Matches |
| --- | --- | --- | --- | --- |
| 15.6308 | 137.4540 | 0.0000 | 2.8665e+08 | 10 |

**Adduct Signal Fractions**

- **Unmodified**: 2.8665e+08

### RN(D)EEYN(N)KSVQEIQATF Hex 3 HexNAc 5

**Mass:**  3457.4815

(2.857e-04)

**Charge States:** 3, 4

**Start Position**: 51

**End Position**: 67

| Profile Score | Tandem Score | TDA q-value | Volume | Spectrum Matches |
| --- | --- | --- | --- | --- |
| 11.5130 | 136.4674 | 0.0000 | 5.1442e+08 | 12 |

**Adduct Signal Fractions**

- **Unmodified**: 5.1442e+08

### TPN(N)KTEDTIF Fuc 3 Hex 3 HexNAc 4 Neu5Ac 1

**Mass:**  3192.3045

(-2.183e-06)

**Charge States:** 3

**Start Position**: 70

**End Position**: 80

| Profile Score | Tandem Score | TDA q-value | Volume | Spectrum Matches |
| --- | --- | --- | --- | --- |
| -19.4068 | 136.3787 | 0.0000 | 3.1639e+06 | 1 |

**Adduct Signal Fractions**

- **Unmodified**: 3.1639e+06

### RNEEYN(N)KSVQEIQAT Hex 3 HexNAc 5

**Mass:**  3309.4279

(7.137e-07)

**Charge States:** 3, 4

**Start Position**: 51

**End Position**: 66

| Profile Score | Tandem Score | TDA q-value | Volume | Spectrum Matches |
| --- | --- | --- | --- | --- |
| -4.6859 | 135.8166 | 0.0000 | 3.2591e+07 | 3 |

**Adduct Signal Fractions**

- **Unmodified**: 3.2591e+07

### NVQREN(N)GTISRY Hex 3 HexNAc 5

**Mass:**  2937.2704

(8.140e-07)

**Charge States:** 3

**Start Position**: 98

**End Position**: 110

| Profile Score | Tandem Score | TDA q-value | Volume | Spectrum Matches |
| --- | --- | --- | --- | --- |
| 13.3262 | 134.9985 | 0.0000 | 1.8251e+08 | 4 |

**Adduct Signal Fractions**

- **Unmodified**: 1.8251e+08

### N(N)KSVQEIQATF Hex 3 HexNAc 4

**Mass:**  2562.1239

(8.354e-07)

**Charge States:** 3

**Start Position**: 56

**End Position**: 67

| Profile Score | Tandem Score | TDA q-value | Volume | Spectrum Matches |
| --- | --- | --- | --- | --- |
| -3.3281 | 132.4650 | 0.0000 | 2.0401e+07 | 2 |

**Adduct Signal Fractions**

- **Unmodified**: 2.0401e+07

### LNVQREN(N)GTISRY Fuc 1 Hex 4 HexNAc 7

**Mass:**  3764.6281

(4.262e-08)

**Charge States:** 3, 4

**Start Position**: 97

**End Position**: 110

| Profile Score | Tandem Score | TDA q-value | Volume | Spectrum Matches |
| --- | --- | --- | --- | --- |
| -3.8508 | 132.2517 | 0.0027 | 2.5234e+08 | 3 |

**Adduct Signal Fractions**

- **Unmodified**: 2.5234e+08

### LNVQREN(N)GTISRY Hex 3 HexNAc 5

**Mass:**  3050.3558

(1.417e-07)

**Charge States:** 3, 4

**Start Position**: 97

**End Position**: 110

| Profile Score | Tandem Score | TDA q-value | Volume | Spectrum Matches |
| --- | --- | --- | --- | --- |
| 15.7023 | 132.0240 | 0.0027 | 2.9894e+09 | 12 |

**Adduct Signal Fractions**

- **Unmodified**: 2.9894e+09

### LN(D)VQREN(N)GTISRY Hex 3 HexNAc 6

**Mass:**  3254.4300

(3.068e-04)

**Charge States:** 3, 4

**Start Position**: 97

**End Position**: 110

| Profile Score | Tandem Score | TDA q-value | Volume | Spectrum Matches |
| --- | --- | --- | --- | --- |
| 7.0465 | 131.8971 | 0.0027 | 2.9883e+08 | 47 |

**Adduct Signal Fractions**

- **Unmodified**: 2.9883e+08

### NVQREN(N)GTISRY Fuc 2 Hex 5 HexNAc 6

**Mass:**  3756.5747

(7.198e-07)

**Charge States:** 4

**Start Position**: 98

**End Position**: 110

| Profile Score | Tandem Score | TDA q-value | Volume | Spectrum Matches |
| --- | --- | --- | --- | --- |
| -5.1754 | 131.8363 | 0.0027 | 8.3278e+07 | 2 |

**Adduct Signal Fractions**

- **Unmodified**: 8.3278e+07

### LNVQREN(N)GTISRY Fuc 2 Hex 5 HexNAc 5

**Mass:**  3666.5773

(3.664e-07)

**Charge States:** 3

**Start Position**: 97

**End Position**: 110

| Profile Score | Tandem Score | TDA q-value | Volume | Spectrum Matches |
| --- | --- | --- | --- | --- |
| -4.0742 | 131.4831 | 0.0027 | 8.8242e+07 | 2 |

**Adduct Signal Fractions**

- **Unmodified**: 8.8242e+07

### LNVQREN(N)GTISRY Fuc 2 Hex 5 HexNAc 6

**Mass:**  3869.6579

(3.616e-07)

**Charge States:** 3, 4

**Start Position**: 97

**End Position**: 110

| Profile Score | Tandem Score | TDA q-value | Volume | Spectrum Matches |
| --- | --- | --- | --- | --- |
| 14.4791 | 131.2197 | 0.0027 | 1.5349e+09 | 7 |

**Adduct Signal Fractions**

- **Unmodified**: 1.5349e+09

### RNEEYN(N)KSVQEIQATFF Fuc 1 Hex 3 HexNAc 4

**Mass:**  3546.5414

(1.182e-06)

**Charge States:** 3, 4

**Start Position**: 51

**End Position**: 68

| Profile Score | Tandem Score | TDA q-value | Volume | Spectrum Matches |
| --- | --- | --- | --- | --- |
| -5.1299 | 130.9766 | 0.0027 | 2.6778e+07 | 3 |

**Adduct Signal Fractions**

- **Unmodified**: 2.6778e+07

### YFTPN(N)KTEDTIF Fuc 2 Hex 5 HexNAc 7

**Mass:**  3998.6406

(1.727e-06)

**Charge States:** 3, 4

**Start Position**: 68

**End Position**: 80

| Profile Score | Tandem Score | TDA q-value | Volume | Spectrum Matches |
| --- | --- | --- | --- | --- |
| 12.4652 | 130.6718 | 0.0027 | 2.5514e+07 | 2 |

**Adduct Signal Fractions**

- **Unmodified**: 2.5514e+07

### VQREN(N)GTISRY Hex 3 HexNAc 6

**Mass:**  3026.3100

(1.732e-06)

**Charge States:** 3

**Start Position**: 99

**End Position**: 110

| Profile Score | Tandem Score | TDA q-value | Volume | Spectrum Matches |
| --- | --- | --- | --- | --- |
| -3.3247 | 130.6142 | 0.0027 | 4.4468e+07 | 3 |

**Adduct Signal Fractions**

- **Unmodified**: 4.4468e+07

### NVQREN(N)GTISRY Hex 4 HexNAc 7

**Mass:**  3505.4844

(-2.494e-07)

**Charge States:** 4

**Start Position**: 98

**End Position**: 110

| Profile Score | Tandem Score | TDA q-value | Volume | Spectrum Matches |
| --- | --- | --- | --- | --- |
| -4.7454 | 130.4958 | 0.0027 | 8.7610e+07 | 3 |

**Adduct Signal Fractions**

- **Unmodified**: 8.7610e+07

### LNVQREN(N)GTISRY Hex 4 HexNAc 8

**Mass:**  3821.6486

(5.570e-07)

**Charge States:** 3, 4

**Start Position**: 97

**End Position**: 110

| Profile Score | Tandem Score | TDA q-value | Volume | Spectrum Matches |
| --- | --- | --- | --- | --- |
| 13.8170 | 130.4474 | 0.0027 | 1.5643e+09 | 10 |

**Adduct Signal Fractions**

- **Unmodified**: 1.5643e+09

### LNVQREN(N)GTISRY Fuc 3 Hex 6 HexNAc 6

**Mass:**  4177.7751

(1.749e-06)

**Charge States:** 3, 4

**Start Position**: 97

**End Position**: 110

| Profile Score | Tandem Score | TDA q-value | Volume | Spectrum Matches |
| --- | --- | --- | --- | --- |
| 15.8792 | 130.2009 | 0.0027 | 4.4696e+08 | 4 |

**Adduct Signal Fractions**

- **Unmodified**: 4.4696e+08

### TPN(N)KTEDTIF Hex 4 HexNAc 5

**Mass:**  2828.1785

(1.485e-06)

**Charge States:** 3

**Start Position**: 70

**End Position**: 80

| Profile Score | Tandem Score | TDA q-value | Volume | Spectrum Matches |
| --- | --- | --- | --- | --- |
| -17.8264 | 130.0375 | 0.0027 | 2.3701e+06 | 1 |

**Adduct Signal Fractions**

- **Unmodified**: 2.3701e+06

### LNVQREN(N)GTISRY Fuc 1 Hex 3 HexNAc 6

**Mass:**  3399.4938

(2.946e-04)

**Charge States:** 3, 4

**Start Position**: 97

**End Position**: 110

| Profile Score | Tandem Score | TDA q-value | Volume | Spectrum Matches |
| --- | --- | --- | --- | --- |
| 10.7321 | 129.6613 | 0.0027 | 1.5921e+08 | 17 |

**Adduct Signal Fractions**

- **Unmodified**: 1.5921e+08

### RNEEYN(N)KSVQEIQATF Fuc 2 Hex 5 HexNAc 5

**Mass:**  4072.7211

(1.594e-06)

**Charge States:** 3, 4

**Start Position**: 51

**End Position**: 67

| Profile Score | Tandem Score | TDA q-value | Volume | Spectrum Matches |
| --- | --- | --- | --- | --- |
| -3.3237 | 129.5564 | 0.0052 | 1.0326e+08 | 3 |

**Adduct Signal Fractions**

- **Unmodified**: 1.0326e+08

### FTPN(N)KTEDTIF Fuc 2 Hex 5 HexNAc 6

**Mass:**  3632.4944

(1.023e-06)

**Charge States:** 3, 4

**Start Position**: 69

**End Position**: 80

| Profile Score | Tandem Score | TDA q-value | Volume | Spectrum Matches |
| --- | --- | --- | --- | --- |
| 12.4587 | 129.5111 | 0.0052 | 1.6687e+07 | 4 |

**Adduct Signal Fractions**

- **Unmodified**: 1.6687e+07

### RNEEYN(N)KSVQEIQATFF Hex 6 HexNAc 5

**Mass:**  4089.7260

(1.317e-06)

**Charge States:** 3, 4

**Start Position**: 51

**End Position**: 68

| Profile Score | Tandem Score | TDA q-value | Volume | Spectrum Matches |
| --- | --- | --- | --- | --- |
| -18.1236 | 128.8004 | 0.0071 | 9.0926e+06 | 2 |

**Adduct Signal Fractions**

- **Unmodified**: 9.0926e+06

### TPN(N)KTEDTIF Fuc 4 Hex 5 HexNAc 5

**Mass:**  3574.4430

(-4.815e-06)

**Charge States:** 3, 4

**Start Position**: 70

**End Position**: 80

| Profile Score | Tandem Score | TDA q-value | Volume | Spectrum Matches |
| --- | --- | --- | --- | --- |
| 12.7097 | 128.3550 | 0.0071 | 5.0377e+07 | 4 |

**Adduct Signal Fractions**

- **Unmodified**: 5.0377e+07

### LNVQREN(N)GTISRY Hex 3 HexNAc 4

**Mass:**  2847.2786

(1.904e-07)

**Charge States:** 3, 4

**Start Position**: 97

**End Position**: 110

| Profile Score | Tandem Score | TDA q-value | Volume | Spectrum Matches |
| --- | --- | --- | --- | --- |
| 13.2917 | 128.2246 | 0.0071 | 2.2731e+08 | 5 |

**Adduct Signal Fractions**

- **Unmodified**: 2.2731e+08

### LNVQREN(N)GTISRY Fuc 1 Hex 5 HexNAc 7

**Mass:**  3926.6874

(1.186e-06)

**Charge States:** 3, 4

**Start Position**: 97

**End Position**: 110

| Profile Score | Tandem Score | TDA q-value | Volume | Spectrum Matches |
| --- | --- | --- | --- | --- |
| 11.5945 | 128.0636 | 0.0071 | 6.4390e+08 | 10 |

**Adduct Signal Fractions**

- **Unmodified**: 6.4390e+08

### FYFTPN(N)KTEDTIF Fuc 1 Hex 5 HexNAc 4 Neu5Ac 1

**Mass:**  3681.5150

(3.724e-06)

**Charge States:** 3

**Start Position**: 67

**End Position**: 80

| Profile Score | Tandem Score | TDA q-value | Volume | Spectrum Matches |
| --- | --- | --- | --- | --- |
| -4.8619 | 127.7292 | 0.0071 | 9.4770e+06 | 1 |

**Adduct Signal Fractions**

- **Unmodified**: 9.4770e+06

### RNEEYN(N)KSVQEIQATF Fuc 2 Hex 5 HexNAc 6

**Mass:**  4275.8034

(5.389e-07)

**Charge States:** 3, 4

**Start Position**: 51

**End Position**: 67

| Profile Score | Tandem Score | TDA q-value | Volume | Spectrum Matches |
| --- | --- | --- | --- | --- |
| 10.4735 | 127.4842 | 0.0071 | 2.9790e+07 | 1 |

**Adduct Signal Fractions**

- **Unmodified**: 2.9790e+07

### LNVQREN(N)GTISRY Hex 5 HexNAc 9

**Mass:**  4186.7819

(1.399e-06)

**Charge States:** 3, 4

**Start Position**: 97

**End Position**: 110

| Profile Score | Tandem Score | TDA q-value | Volume | Spectrum Matches |
| --- | --- | --- | --- | --- |
| 14.8507 | 126.5366 | 0.0071 | 2.7914e+08 | 3 |

**Adduct Signal Fractions**

- **Unmodified**: 2.7914e+08

### N(N)KSVQEIQATFF Hex 3 HexNAc 4

**Mass:**  2709.1944

(1.870e-06)

**Charge States:** 3

**Start Position**: 56

**End Position**: 68

| Profile Score | Tandem Score | TDA q-value | Volume | Spectrum Matches |
| --- | --- | --- | --- | --- |
| 11.6412 | 125.6842 | 0.0071 | 4.7520e+06 | 2 |

**Adduct Signal Fractions**

- **Unmodified**: 4.7520e+06

### FYFTPN(N)KTEDTIF Fuc 1 Hex 4 HexNAc 7

**Mass:**  3837.5947

(8.754e-07)

**Charge States:** 3, 4

**Start Position**: 67

**End Position**: 80

| Profile Score | Tandem Score | TDA q-value | Volume | Spectrum Matches |
| --- | --- | --- | --- | --- |
| 13.2052 | 125.4935 | 0.0071 | 2.9094e+07 | 4 |

**Adduct Signal Fractions**

- **Unmodified**: 2.9094e+07

### FYFTPN(N)KT Hex 3 HexNAc 6

**Mass:**  2721.1358

(1.697e-06)

**Charge States:** 3

**Start Position**: 67

**End Position**: 75

| Profile Score | Tandem Score | TDA q-value | Volume | Spectrum Matches |
| --- | --- | --- | --- | --- |
| -2.6225 | 125.4881 | 0.0000 | 1.2710e+07 | 2 |

**Adduct Signal Fractions**

- **Unmodified**: 1.2710e+07

### RN(D)EEYN(N)KSVQEIQATFF Hex 3 HexNAc 5

**Mass:**  3604.5517

(2.747e-04)

**Charge States:** 3, 4

**Start Position**: 51

**End Position**: 68

| Profile Score | Tandem Score | TDA q-value | Volume | Spectrum Matches |
| --- | --- | --- | --- | --- |
| -5.9443 | 124.9799 | 0.0093 | 4.3055e+07 | 4 |

**Adduct Signal Fractions**

- **Unmodified**: 4.3055e+07

### FTPN(N)KTEDTIF Hex 4 HexNAc 4 Neu5Ac 2

**Mass:**  3354.3590

(1.258e-06)

**Charge States:** 3, 4

**Start Position**: 69

**End Position**: 80

| Profile Score | Tandem Score | TDA q-value | Volume | Spectrum Matches |
| --- | --- | --- | --- | --- |
| -3.8931 | 124.7760 | 0.0093 | 2.5000e+07 | 4 |

**Adduct Signal Fractions**

- **Unmodified**: 2.5000e+07

### QTRQDQC(C)IYN(N)TTY Fuc 1 Hex 4 HexNAc 6

**Mass:**  3702.4920

(1.097e-06)

**Charge States:** 3, 4

**Start Position**: 84

**End Position**: 97

| Profile Score | Tandem Score | TDA q-value | Volume | Spectrum Matches |
| --- | --- | --- | --- | --- |
| 14.6110 | 124.7244 | 0.0093 | 7.2239e+07 | 4 |

**Adduct Signal Fractions**

- **Unmodified**: 7.2239e+07

### NVQREN(N)GTISRY Fuc 1 Hex 4 HexNAc 5

**Mass:**  3245.3853

(7.440e-07)

**Charge States:** 3

**Start Position**: 98

**End Position**: 110

| Profile Score | Tandem Score | TDA q-value | Volume | Spectrum Matches |
| --- | --- | --- | --- | --- |
| -4.1581 | 124.1904 | 0.0093 | 6.1715e+07 | 2 |

**Adduct Signal Fractions**

- **Unmodified**: 6.1715e+07

### YFTPN(N)KTEDTIF Fuc 1 Hex 6 HexNAc 8

**Mass:**  4217.7141

(3.865e-07)

**Charge States:** 4

**Start Position**: 68

**End Position**: 80

| Profile Score | Tandem Score | TDA q-value | Volume | Spectrum Matches |
| --- | --- | --- | --- | --- |
| 10.0712 | 123.6036 | 0.0111 | 1.8835e+07 | 2 |

**Adduct Signal Fractions**

- **Unmodified**: 1.8835e+07

### NVQREN(N)GTISRY Hex 4 HexNAc 8

**Mass:**  3708.5632

(2.167e-07)

**Charge States:** 4

**Start Position**: 98

**End Position**: 110

| Profile Score | Tandem Score | TDA q-value | Volume | Spectrum Matches |
| --- | --- | --- | --- | --- |
| -5.0148 | 122.5719 | 0.0124 | 7.6306e+07 | 3 |

**Adduct Signal Fractions**

- **Unmodified**: 7.6306e+07

### QTRQDQC(C)IYN(N)TTY Hex 3 HexNAc 5

**Mass:**  3191.3003

(8.303e-07)

**Charge States:** 3, 4

**Start Position**: 84

**End Position**: 97

| Profile Score | Tandem Score | TDA q-value | Volume | Spectrum Matches |
| --- | --- | --- | --- | --- |
| 14.1603 | 122.3964 | 0.0124 | 1.1982e+08 | 6 |

**Adduct Signal Fractions**

- **Unmodified**: 1.1982e+08

### N(N)KSVQEIQATFF Hex 3 HexNAc 5

**Mass:**  2912.2734

(1.518e-06)

**Charge States:** 3

**Start Position**: 56

**End Position**: 68

| Profile Score | Tandem Score | TDA q-value | Volume | Spectrum Matches |
| --- | --- | --- | --- | --- |
| -4.0551 | 122.1026 | 0.0124 | 5.6445e+06 | 2 |

**Adduct Signal Fractions**

- **Unmodified**: 5.6445e+06

### RNEEYN(N)KSVQEIQATF Hex 7 HexNAc 2

**Mass:**  3495.4277

(-9.757e-06)

**Charge States:** 3, 4

**Start Position**: 51

**End Position**: 67

| Profile Score | Tandem Score | TDA q-value | Volume | Spectrum Matches |
| --- | --- | --- | --- | --- |
| 10.6074 | 122.0360 | 0.0124 | 3.7877e+07 | 5 |

**Adduct Signal Fractions**

- **Unmodified**: 3.7877e+07

### RNEEYN(N)KSVQEIQATFF Hex 4 HexNAc 7

**Mass:**  4171.7814

(1.483e-06)

**Charge States:** 3, 4

**Start Position**: 51

**End Position**: 68

| Profile Score | Tandem Score | TDA q-value | Volume | Spectrum Matches |
| --- | --- | --- | --- | --- |
| 13.1210 | 120.6175 | 0.0124 | 3.0980e+07 | 4 |

**Adduct Signal Fractions**

- **Unmodified**: 3.0980e+07

### LNVQREN(N)GTISRY Fuc 1 Hex 3 HexNAc 5

**Mass:**  3196.4177

(-1.788e-07)

**Charge States:** 3

**Start Position**: 97

**End Position**: 110

| Profile Score | Tandem Score | TDA q-value | Volume | Spectrum Matches |
| --- | --- | --- | --- | --- |
| -4.9043 | 120.5475 | 0.0124 | 6.3253e+07 | 2 |

**Adduct Signal Fractions**

- **Unmodified**: 6.3253e+07

### RNEEYN(N)KSVQEIQATFF Fuc 2 Hex 5 HexNAc 5

**Mass:**  4219.7912

(1.345e-06)

**Charge States:** 3, 4

**Start Position**: 51

**End Position**: 68

| Profile Score | Tandem Score | TDA q-value | Volume | Spectrum Matches |
| --- | --- | --- | --- | --- |
| 13.6728 | 120.5209 | 0.0124 | 3.5407e+07 | 4 |

**Adduct Signal Fractions**

- **Unmodified**: 3.5407e+07

### LN(D)VQREN(N)GTISRY Fuc 1 Hex 4 HexNAc 6

**Mass:**  3562.5410

(2.811e-04)

**Charge States:** 3, 4

**Start Position**: 97

**End Position**: 110

| Profile Score | Tandem Score | TDA q-value | Volume | Spectrum Matches |
| --- | --- | --- | --- | --- |
| 8.6492 | 120.4680 | 0.0124 | 1.0639e+08 | 17 |

**Adduct Signal Fractions**

- **Unmodified**: 1.0639e+08

### FYFTPN(N)KT Hex 3 HexNAc 5

**Mass:**  2518.0563

(1.175e-06)

**Charge States:** 3

**Start Position**: 67

**End Position**: 75

| Profile Score | Tandem Score | TDA q-value | Volume | Spectrum Matches |
| --- | --- | --- | --- | --- |
| -18.3507 | 118.5336 | 0.0000 | 4.4906e+06 | 1 |

**Adduct Signal Fractions**

- **Unmodified**: 4.4906e+06

### RN(D)EEYN(N)KSVQEIQATF Fuc 1 Hex 4 HexNAc 5

**Mass:**  3765.5944

(2.630e-04)

**Charge States:** 3, 4

**Start Position**: 51

**End Position**: 67

| Profile Score | Tandem Score | TDA q-value | Volume | Spectrum Matches |
| --- | --- | --- | --- | --- |
| 12.3379 | 118.3704 | 0.0140 | 3.4137e+08 | 13 |

**Adduct Signal Fractions**

- **Unmodified**: 3.4137e+08

### LNVQREN(N)GTISRY Fuc 2 Hex 5 HexNAc 7

**Mass:**  4072.7470

(1.283e-06)

**Charge States:** 3, 4

**Start Position**: 97

**End Position**: 110

| Profile Score | Tandem Score | TDA q-value | Volume | Spectrum Matches |
| --- | --- | --- | --- | --- |
| 15.4292 | 118.1002 | 0.0140 | 2.3602e+08 | 4 |

**Adduct Signal Fractions**

- **Unmodified**: 2.3602e+08

### LNVQREN(N)GTISRY Fuc 1 Hex 5 HexNAc 8

**Mass:**  4129.7678

(1.418e-06)

**Charge States:** 3, 4

**Start Position**: 97

**End Position**: 110

| Profile Score | Tandem Score | TDA q-value | Volume | Spectrum Matches |
| --- | --- | --- | --- | --- |
| 13.6836 | 116.1603 | 0.0157 | 5.6558e+08 | 6 |

**Adduct Signal Fractions**

- **Unmodified**: 5.6558e+08

### RNEEYN(N)KSVQEIQAT Fuc 1 Hex 4 HexNAc 5

**Mass:**  3617.5415

(1.456e-06)

**Charge States:** 3

**Start Position**: 51

**End Position**: 66

| Profile Score | Tandem Score | TDA q-value | Volume | Spectrum Matches |
| --- | --- | --- | --- | --- |
| -18.8770 | 115.3248 | 0.0157 | 1.5498e+07 | 2 |

**Adduct Signal Fractions**

- **Unmodified**: 1.5498e+07

### N(N)KSVQEIQATFF Fuc 1 Hex 4 HexNAc 5

**Mass:**  3220.3822

(5.659e-07)

**Charge States:** 3

**Start Position**: 56

**End Position**: 68

| Profile Score | Tandem Score | TDA q-value | Volume | Spectrum Matches |
| --- | --- | --- | --- | --- |
| -5.1051 | 114.7810 | 0.0174 | 2.8029e+06 | 2 |

**Adduct Signal Fractions**

- **Unmodified**: 2.8029e+06

### FTPN(N)KTEDTIF Hex 5 HexNAc 8

**Mass:**  3746.5393

(8.960e-07)

**Charge States:** 3, 4

**Start Position**: 69

**End Position**: 80

| Profile Score | Tandem Score | TDA q-value | Volume | Spectrum Matches |
| --- | --- | --- | --- | --- |
| 9.8128 | 114.6980 | 0.0174 | 8.4135e+06 | 3 |

**Adduct Signal Fractions**

- **Unmodified**: 8.4135e+06

### RN(D)EEYN(N)KSVQEIQATFF Hex 3 HexNAc 4

**Mass:**  3401.4680

(2.905e-04)

**Charge States:** 3, 4

**Start Position**: 51

**End Position**: 68

| Profile Score | Tandem Score | TDA q-value | Volume | Spectrum Matches |
| --- | --- | --- | --- | --- |
| -5.9781 | 114.2285 | 0.0174 | 6.0285e+07 | 5 |

**Adduct Signal Fractions**

- **Unmodified**: 6.0285e+07

### LNVQREN(N)GTISRY Hex 5 HexNAc 8

**Mass:**  3983.7075

(1.355e-06)

**Charge States:** 4

**Start Position**: 97

**End Position**: 110

| Profile Score | Tandem Score | TDA q-value | Volume | Spectrum Matches |
| --- | --- | --- | --- | --- |
| -3.2375 | 114.1241 | 0.0187 | 1.4318e+08 | 2 |

**Adduct Signal Fractions**

- **Unmodified**: 1.4318e+08

### N(N)KTEDTIF Hex 3 HexNAc 6

**Mass:**  2671.1012

(2.155e-07)

**Charge States:** 3

**Start Position**: 72

**End Position**: 80

| Profile Score | Tandem Score | TDA q-value | Volume | Spectrum Matches |
| --- | --- | --- | --- | --- |
| -19.9027 | 114.1219 | 0.0000 | 3.8911e+06 | 1 |

**Adduct Signal Fractions**

- **Unmodified**: 3.8911e+06

### NVQREN(N)GTISRY Fuc 1 Hex 5 HexNAc 8

**Mass:**  4016.6743

(1.260e-07)

**Charge States:** 4

**Start Position**: 98

**End Position**: 110

| Profile Score | Tandem Score | TDA q-value | Volume | Spectrum Matches |
| --- | --- | --- | --- | --- |
| -19.4578 | 113.8994 | 0.0187 | 2.5508e+07 | 1 |

**Adduct Signal Fractions**

- **Unmodified**: 2.5508e+07

### LNVQREN(N)GTISRY Hex 4 HexNAc 6

**Mass:**  3415.4954

(-2.156e-06)

**Charge States:** 3, 4

**Start Position**: 97

**End Position**: 110

| Profile Score | Tandem Score | TDA q-value | Volume | Spectrum Matches |
| --- | --- | --- | --- | --- |
| 11.3103 | 113.4624 | 0.0187 | 2.6258e+08 | 9 |

**Adduct Signal Fractions**

- **Unmodified**: 2.6258e+08

### TPN(N)KTEDTIF Hex 7 HexNAc 5

**Mass:**  3314.3417

(1.947e-06)

**Charge States:** 3, 4

**Start Position**: 70

**End Position**: 80

| Profile Score | Tandem Score | TDA q-value | Volume | Spectrum Matches |
| --- | --- | --- | --- | --- |
| 12.5170 | 113.3862 | 0.0187 | 2.8778e+07 | 3 |

**Adduct Signal Fractions**

- **Unmodified**: 2.8778e+07

### LNVQREN(N)GTISRY Fuc 2 Hex 6 HexNAc 8

**Mass:**  4437.8718

(7.651e-07)

**Charge States:** 4

**Start Position**: 97

**End Position**: 110

| Profile Score | Tandem Score | TDA q-value | Volume | Spectrum Matches |
| --- | --- | --- | --- | --- |
| 12.9161 | 111.9411 | 0.0220 | 2.2040e+08 | 3 |

**Adduct Signal Fractions**

- **Unmodified**: 2.2040e+08

### LNVQREN(N)GTISRY Fuc 3 Hex 6 HexNAc 7

**Mass:**  4380.8532

(8.516e-07)

**Charge States:** 3, 4

**Start Position**: 97

**End Position**: 110

| Profile Score | Tandem Score | TDA q-value | Volume | Spectrum Matches |
| --- | --- | --- | --- | --- |
| -3.7573 | 110.9133 | 0.0236 | 1.3047e+08 | 3 |

**Adduct Signal Fractions**

- **Unmodified**: 1.3047e+08

### NVQREN(N)GTISRY Fuc 1 Hex 5 HexNAc 7

**Mass:**  3813.6013

(-7.193e-07)

**Charge States:** 4

**Start Position**: 98

**End Position**: 110

| Profile Score | Tandem Score | TDA q-value | Volume | Spectrum Matches |
| --- | --- | --- | --- | --- |
| -21.2313 | 109.0676 | 0.0266 | 1.4599e+07 | 2 |

**Adduct Signal Fractions**

- **Unmodified**: 1.4599e+07

### VQREN(N)GTISRY Fuc 1 Hex 4 HexNAc 6

**Mass:**  3335.3798

(2.992e-04)

**Charge States:** 3

**Start Position**: 99

**End Position**: 110

| Profile Score | Tandem Score | TDA q-value | Volume | Spectrum Matches |
| --- | --- | --- | --- | --- |
| -5.1655 | 109.0486 | 0.0266 | 6.3375e+05 | 2 |

**Adduct Signal Fractions**

- **Unmodified**: 6.3375e+05

### LNVQREN(N)GTISRY Fuc 2 Hex 6 HexNAc 7

**Mass:**  4234.7941

(1.495e-06)

**Charge States:** 4

**Start Position**: 97

**End Position**: 110

| Profile Score | Tandem Score | TDA q-value | Volume | Spectrum Matches |
| --- | --- | --- | --- | --- |
| 6.1733 | 107.7808 | 0.0335 | 1.4511e+08 | 4 |

**Adduct Signal Fractions**

- **Unmodified**: 1.4511e+08

### LNVQREN(N)GTISRY Hex 4 HexNAc 4 Neu5Ac 2

**Mass:**  3591.5268

(2.617e-06)

**Charge States:** 3, 4

**Start Position**: 97

**End Position**: 110

| Profile Score | Tandem Score | TDA q-value | Volume | Spectrum Matches |
| --- | --- | --- | --- | --- |
| 14.3566 | 106.0697 | 0.0352 | 4.8194e+08 | 10 |

**Adduct Signal Fractions**

- **Unmodified**: 4.8194e+08

### LNVQREN(N)GTISRY Fuc 2 Hex 4 HexNAc 6

**Mass:**  3707.6079

(9.962e-07)

**Charge States:** 3, 4

**Start Position**: 97

**End Position**: 110

| Profile Score | Tandem Score | TDA q-value | Volume | Spectrum Matches |
| --- | --- | --- | --- | --- |
| -19.7871 | 106.0210 | 0.0352 | 2.1337e+07 | 1 |

**Adduct Signal Fractions**

- **Unmodified**: 2.1337e+07

### FYFTPN(N)KTEDTIF Fuc 2 Hex 5 HexNAc 7

**Mass:**  4145.7067

(1.716e-06)

**Charge States:** 3, 4

**Start Position**: 67

**End Position**: 80

| Profile Score | Tandem Score | TDA q-value | Volume | Spectrum Matches |
| --- | --- | --- | --- | --- |
| 12.1326 | 105.3056 | 0.0352 | 2.2466e+07 | 4 |

**Adduct Signal Fractions**

- **Unmodified**: 2.2466e+07

### LNVQREN(N)GTISRY Fuc 1 Hex 8 HexNAc 3 Neu5Ac 1

**Mass:**  3891.6369

(5.666e-06)

**Charge States:** 4

**Start Position**: 97

**End Position**: 110

| Profile Score | Tandem Score | TDA q-value | Volume | Spectrum Matches |
| --- | --- | --- | --- | --- |
| -18.6392 | 105.2464 | 0.0352 | 4.8844e+07 | 1 |

**Adduct Signal Fractions**

- **Unmodified**: 4.8844e+07

### LN(D)VQREN(N)GTISRY Hex 3 HexNAc 5

**Mass:**  3051.3563

(3.279e-04)

**Charge States:** 3, 4

**Start Position**: 97

**End Position**: 110

| Profile Score | Tandem Score | TDA q-value | Volume | Spectrum Matches |
| --- | --- | --- | --- | --- |
| 6.8263 | 104.2070 | 0.0401 | 5.9684e+07 | 6 |

**Adduct Signal Fractions**

- **Unmodified**: 5.9684e+07

### NVQREN(N)GTISRY Fuc 3 Hex 6 HexNAc 6

**Mass:**  4064.6840

(9.304e-08)

**Charge States:** 3, 4

**Start Position**: 98

**End Position**: 110

| Profile Score | Tandem Score | TDA q-value | Volume | Spectrum Matches |
| --- | --- | --- | --- | --- |
| -19.9278 | 102.0050 | 0.0456 | 2.3863e+07 | 2 |

**Adduct Signal Fractions**

- **Unmodified**: 2.3863e+07

### LNVQREN(N)GTISRY Fuc 3 Hex 7 HexNAc 8

**Mass:**  4745.9851

(1.171e-06)

**Charge States:** 4

**Start Position**: 97

**End Position**: 110

| Profile Score | Tandem Score | TDA q-value | Volume | Spectrum Matches |
| --- | --- | --- | --- | --- |
| -3.2802 | 101.6961 | 0.0456 | 4.5621e+07 | 2 |

**Adduct Signal Fractions**

- **Unmodified**: 4.5621e+07

### C(C)ANLVPVPITN(N)ATL Hex 3 HexNAc 5

**Mass:**  2983.3509

(1.450e-06)

**Charge States:** 3, 4

**Start Position**: 23

**End Position**: 37

| Profile Score | Tandem Score | TDA q-value | Volume | Spectrum Matches |
| --- | --- | --- | --- | --- |
| 14.4529 | 101.5163 | 0.0470 | 3.5452e+07 | 7 |

**Adduct Signal Fractions**

- **Unmodified**: 3.5452e+07

### P19652|A1AG2\_HUMAN

**>P19652|A1AG2\_HUMAN**  
 `MALSWVLTVLSLLPLLEAQIPLCANLVPVPITNATLDRITGKWFYIASAFRNEEYNKSVQEIQATFFYFTPNKTEDTIFL  
REYQTRQNQCFYNSSYLNVQRENGTVSRYEGGREHVAHLLFLRDTKTLMFGSYLDDEKNWGLSFYADKPETTKEQLGEFY  
EALDCLCIPRSDVMYTDWKKDKCEPLEKQHEKERKQEEGES`

| Site | Type | Count |
| --- | --- | --- |
| 33 | N-linked | 2 |
| 72 | N-linked | 67 |
| 93 | N-linked | 1 |
| 103 | N-linked | 22 |
| 56 | N-linked | 47 |

| Mass | Sequence | MS_2_ Score | `q`-value | MS_1_ Score | Signal | Protein Coordinates | Tandem Scans | Start Time | Apex Time | End Time |
| --- | --- | --- | --- | --- | --- | --- | --- | --- | --- | --- |
| 2869.2034 | TPN(N)KTEDTIF Hex 3 HexNAc 6 | 184.066 | 0.0 | 16.418 | 3.299e+08 | 69 - 79 | 11 | 22.31 | 24.17 | 25.52 |
| 3177.3146 | TPN(N)KTEDTIF Fuc 1 Hex 4 HexNAc 6 | 181.311 | 0.0 | 14.498 | 6.651e+07 | 69 - 79 | 5 | 22.99 | 23.67 | 24.33 |
| 2666.1249 | TPN(N)KTEDTIF Hex 3 HexNAc 5 | 180.990 | 0.0 | 15.638 | 1.735e+08 | 69 - 79 | 8 | 23.67 | 24.50 | 25.69 |
| 2974.2352 | TPN(N)KTEDTIF Fuc 1 Hex 4 HexNAc 5 | 180.842 | 0.0 | 14.098 | 4.180e+07 | 69 - 79 | 4 | 23.33 | 24.00 | 24.50 |
| 2463.0458 | TPN(N)KTEDTIF Hex 3 HexNAc 4 | 180.398 | 0.0 | 13.126 | 2.345e+07 | 69 - 79 | 4 | 24.50 | 24.84 | 25.35 |
| 3691.5393 | FYFTPN(N)KTEDTIF Hex 4 HexNAc 7 | 176.886 | 0.0 | 14.067 | 5.059e+08 | 66 - 79 | 12 | 45.81 | 46.84 | 47.84 |
| 3179.3357 | YFTPN(N)KTEDTIF Hex 3 HexNAc 6 | 176.033 | 0.0 | 14.835 | 3.536e+09 | 67 - 79 | 43 | 36.42 | 37.57 | 44.13 |
| 2773.1758 | YFTPN(N)KTEDTIF Hex 3 HexNAc 4 | 175.519 | 0.0 | 11.873 | 2.990e+08 | 67 - 79 | 9 | 37.09 | 38.21 | 40.75 |
| 3431.4350 | FYFTPN(N)KTEDTIF Fuc 1 Hex 4 HexNAc 5 | 175.209 | 0.0 | 12.800 | 4.204e+08 | 66 - 79 | 11 | 46.32 | 47.51 | 49.17 |
| 3284.3660 | YFTPN(N)KTEDTIF Fuc 1 Hex 4 HexNAc 5 | 174.854 | 0.0 | 13.992 | 5.773e+08 | 67 - 79 | 16 | 36.08 | 37.26 | 38.21 |
| 3634.5159 | FYFTPN(N)KTEDTIF Fuc 1 Hex 4 HexNAc 6 | 173.273 | 0.0 | 13.985 | 6.709e+08 | 66 - 79 | 16 | 46.15 | 47.18 | 48.83 |
| 3016.2721 | FTPN(N)KTEDTIF Hex 3 HexNAc 6 | 172.980 | 0.0 | 15.796 | 7.329e+08 | 68 - 79 | 12 | 29.76 | 31.34 | 32.27 |
| 3795.5585 | YFTPN(N)KTEDTIF Fuc 2 Hex 5 HexNAc 6 | 172.918 | 0.0 | 16.247 | 1.907e+08 | 67 - 79 | 4 | 36.08 | 36.59 | 37.09 |
| 2976.2553 | YFTPN(N)KTEDTIF Hex 3 HexNAc 5 | 171.977 | 0.0 | 15.024 | 1.936e+09 | 67 - 79 | 38 | 36.59 | 37.89 | 43.12 |
| 3487.4455 | YFTPN(N)KTEDTIF Fuc 1 Hex 4 HexNAc 6 | 171.166 | 0.0 | 16.078 | 1.075e+09 | 67 - 79 | 11 | 35.91 | 37.09 | 37.89 |
| 2775.1840 | YFTPN(N)KTEDTIF Hex 3 HexNAc 4 | 170.535 | 0.0 | 0.000 | 0.000e+00 | 67 - 79 | 1 | - | - | - |
| 3341.3902 | YFTPN(N)KTEDTIF Hex 4 HexNAc 6 | 169.623 | 0.0 | 16.272 | 1.761e+08 | 67 - 79 | 6 | 35.91 | 37.42 | 37.57 |
| 3326.4032 | FYFTPN(N)KTEDTIF Hex 3 HexNAc 6 | 169.167 | 0.0 | 15.446 | 2.824e+09 | 66 - 79 | 23 | 46.49 | 47.68 | 50.53 |
| 3544.4684 | YFTPN(N)KTEDTIF Hex 4 HexNAc 7 | 168.705 | 0.0 | 15.368 | 9.367e+08 | 67 - 79 | 13 | 35.57 | 37.09 | 37.89 |
| 3123.3269 | FYFTPN(N)KTEDTIF Hex 3 HexNAc 5 | 168.566 | 0.0 | 13.636 | 2.137e+09 | 66 - 79 | 132 | 47.01 | 48.00 | 98.34 |
| 3234.3378 | TPN(N)KTEDTIF Hex 4 HexNAc 7 | 167.753 | 0.0 | 12.460 | 4.364e+07 | 69 - 79 | 5 | 23.33 | 23.50 | 24.17 |
| 3942.6282 | FYFTPN(N)KTEDTIF Fuc 2 Hex 5 HexNAc 6 | 167.062 | 0.0 | 14.619 | 1.566e+08 | 66 - 79 | 7 | 45.81 | 46.84 | 47.34 |
| 3285.3789 | FYFTPN(N)KTEDTIF Hex 4 HexNAc 5 | 166.958 | 0.0 | 11.622 | 2.138e+07 | 66 - 79 | 4 | 47.18 | 47.68 | 47.84 |
| 2920.2445 | FYFTPN(N)KTEDTIF Hex 3 HexNAc 4 | 165.722 | 0.0 | 14.779 | 1.853e+08 | 66 - 79 | 11 | 48.16 | 48.32 | 51.88 |
| 2813.1922 | FTPN(N)KTEDTIF Hex 3 HexNAc 5 | 165.123 | 0.0 | 14.924 | 2.696e+08 | 68 - 79 | 11 | 30.10 | 31.59 | 32.27 |
| 3909.6045 | YFTPN(N)KTEDTIF Hex 5 HexNAc 8 | 164.975 | 0.0 | 14.199 | 1.596e+08 | 67 - 79 | 6 | 35.74 | 36.25 | 36.92 |
| 3747.5493 | YFTPN(N)KTEDTIF Hex 4 HexNAc 8 | 164.128 | 0.0 | 13.527 | 1.216e+08 | 67 - 79 | 6 | 36.25 | 36.59 | 37.09 |
| 3852.5810 | YFTPN(N)KTEDTIF Fuc 1 Hex 5 HexNAc 7 | 163.744 | 0.0 | 14.977 | 1.676e+08 | 67 - 79 | 6 | 36.08 | 36.59 | 37.09 |
| 3186.3524 | EEYN(N)KSVQEIQATF Hex 3 HexNAc 5 | 163.579 | 0.0 | 13.296 | 3.916e+07 | 52 - 66 | 3 | 35.57 | 35.74 | 36.25 |
| 3690.5265 | YFTPN(N)KTEDTIF Fuc 1 Hex 4 HexNAc 7 | 163.080 | 0.0 | 12.667 | 4.099e+07 | 67 - 79 | 3 | 36.42 | 36.92 | 37.09 |
| 3324.3820 | FTPN(N)KTEDTIF Fuc 1 Hex 4 HexNAc 6 | 162.293 | 0.0 | 14.451 | 1.214e+08 | 68 - 79 | 5 | 30.62 | 30.78 | 31.34 |
| 3121.3033 | FTPN(N)KTEDTIF Fuc 1 Hex 4 HexNAc 5 | 161.527 | 0.0 | 12.224 | 8.791e+07 | 68 - 79 | 5 | 30.78 | 31.12 | 31.76 |
| 3400.4853 | RNEEYN(N)KSVQEIQATFF Hex 3 HexNAc 4 | 159.834 | 0.0 | 16.134 | 1.941e+09 | 50 - 67 | 11 | 43.97 | 44.30 | 45.81 |
| 3031.2567 | TPN(N)KTEDTIF Hex 4 HexNAc 6 | 158.987 | 0.0 | 11.057 | 7.817e+06 | 69 - 79 | 3 | 23.67 | 23.83 | 24.33 |
| 3603.5650 | RNEEYN(N)KSVQEIQATFF Hex 3 HexNAc 5 | 157.933 | 0.0 | 15.193 | 1.693e+09 | 50 - 67 | 10 | 43.97 | 44.13 | 45.47 |
| 3894.6191 | FYFTPN(N)KTEDTIF Hex 4 HexNAc 8 | 157.157 | 0.0 | 16.340 | 9.632e+07 | 66 - 79 | 5 | 46.15 | 46.66 | 47.34 |
| 3592.4804 | YFTPN(N)KTEDTIF Fuc 2 Hex 5 HexNAc 5 | 156.062 | 0.0 | -5.245 | 1.823e+07 | 67 - 79 | 3 | 36.42 | 36.59 | 36.92 |
| 3254.4040 | RN(D)EEYN(N)KSVQEIQATF Hex 3 HexNAc 4 | 155.960 | 0.0 | 10.136 | 5.852e+08 | 50 - 66 | 24 | 34.38 | 35.74 | 36.42 |
| 3138.3104 | YFTPN(N)KTEDTIF Hex 4 HexNAc 5 | 155.524 | 0.0 | -4.558 | 2.192e+07 | 67 - 79 | 1 | 37.09 | 37.42 | 37.57 |
| 3999.6512 | FYFTPN(N)KTEDTIF Fuc 1 Hex 5 HexNAc 7 | 155.455 | 0.0 | 16.282 | 1.046e+08 | 66 - 79 | 8 | 45.64 | 46.66 | 47.01 |
| 3253.4185 | RNEEYN(N)KSVQEIQATF Hex 3 HexNAc 4 | 155.349 | 0.0 | 15.943 | 5.852e+09 | 50 - 66 | 33 | 28.23 | 32.91 | 34.21 |
| 3488.4577 | FYFTPN(N)KTEDTIF Hex 4 HexNAc 6 | 155.287 | 0.0 | 10.367 | 5.102e+07 | 66 - 79 | 7 | 46.32 | 47.18 | 47.68 |
| 3911.6777 | RNEEYN(N)KSVQEIQATFF Fuc 1 Hex 4 HexNAc 5 | 155.166 | 0.0 | 15.167 | 1.296e+09 | 50 - 67 | 12 | 43.46 | 43.80 | 45.13 |
| 3764.6091 | RNEEYN(N)KSVQEIQATF Fuc 1 Hex 4 HexNAc 5 | 155.134 | 0.0 | 15.704 | 3.262e+09 | 50 - 66 | 15 | 30.45 | 32.44 | 33.71 |
| 3456.4986 | RNEEYN(N)KSVQEIQATF Hex 3 HexNAc 5 | 154.615 | 0.0 | 15.494 | 4.640e+09 | 50 - 66 | 16 | 30.95 | 32.76 | 34.04 |
| 3517.4207 | YFTPN(N)KTEDTIF Hex 4 HexNAc 4 Neu5Ac 2 | 153.977 | 0.0 | 14.829 | 2.935e+08 | 67 - 79 | 9 | 38.21 | 38.37 | 39.56 |
| 3659.5774 | RNEEYN(N)KSVQEIQATF Hex 3 HexNAc 6 | 153.044 | 0.0 | -3.822 | 1.919e+08 | 50 - 66 | 3 | 32.44 | 32.61 | 32.76 |
| 4112.6822 | YFTPN(N)KTEDTIF Hex 5 HexNAc 9 | 152.247 | 0.0 | 13.597 | 4.815e+07 | 67 - 79 | 4 | 35.74 | 36.08 | 36.59 |
| 3399.4767 | RNEEYN(N)KSVQEIQATF Fuc 1 Hex 3 HexNAc 4 | 152.109 | 0.0 | 12.547 | 1.160e+08 | 50 - 66 | 5 | 32.91 | 33.07 | 33.71 |
| 4056.6724 | FYFTPN(N)KTEDTIF Hex 5 HexNAc 8 | 151.794 | 0.0 | 13.091 | 9.488e+07 | 66 - 79 | 6 | 45.81 | 46.15 | 47.18 |
| 3561.5300 | RNEEYN(N)KSVQEIQATF Fuc 1 Hex 4 HexNAc 4 | 151.665 | 0.0 | -3.925 | 1.369e+08 | 50 - 66 | 4 | 32.27 | 32.44 | 32.61 |
| 3708.5981 | RNEEYN(N)KSVQEIQATFF Fuc 1 Hex 4 HexNAc 4 | 151.585 | 0.0 | -3.648 | 5.779e+07 | 50 - 67 | 4 | 43.63 | 43.80 | 44.13 |
| 4259.7508 | FYFTPN(N)KTEDTIF Hex 5 HexNAc 9 | 151.533 | 0.0 | 12.305 | 3.204e+07 | 66 - 79 | 4 | 45.81 | 45.98 | 46.49 |
| 3806.6445 | RNEEYN(N)KSVQEIQATFF Hex 3 HexNAc 6 | 151.211 | 0.0 | -4.024 | 8.414e+07 | 50 - 67 | 7 | 43.80 | 43.97 | 44.13 |
| 3910.6731 | RNEEYN(N)KSVQEIQATF Fuc 2 Hex 4 HexNAc 5 | 151.111 | 0.0 | -20.008 | 2.670e+07 | 50 - 66 | 1 | 32.44 | 32.61 | 32.61 |
| 4055.6613 | YFTPN(N)KTEDTIF Fuc 1 Hex 5 HexNAc 8 | 151.070 | 0.0 | 12.206 | 4.037e+07 | 67 - 79 | 4 | 35.91 | 36.08 | 36.76 |
| 3967.6896 | RNEEYN(N)KSVQEIQATF Fuc 1 Hex 4 HexNAc 6 | 150.898 | 0.0 | -3.015 | 1.487e+08 | 50 - 66 | 3 | 32.10 | 32.44 | 32.61 |
| 2610.1140 | FTPN(N)KTEDTIF Hex 3 HexNAc 4 | 150.445 | 0.0 | -3.325 | 3.179e+07 | 68 - 79 | 4 | 31.76 | 31.93 | 32.27 |
| 3485.4283 | TPN(N)KTEDTIF Fuc 2 Hex 5 HexNAc 6 | 150.012 | 0.0 | -5.211 | 9.889e+06 | 69 - 79 | 2 | 22.99 | 23.16 | 23.50 |
| 2983.2727 | EEYN(N)KSVQEIQATF Hex 3 HexNAc 4 | 149.856 | 0.0 | -3.055 | 4.142e+07 | 52 - 66 | 15 | 35.74 | 35.91 | 36.25 |
| 3602.5576 | RNEEYN(N)KSVQEIQATF Fuc 1 Hex 3 HexNAc 5 | 149.408 | 0.0 | 9.996 | 1.093e+08 | 50 - 66 | 5 | 32.76 | 32.91 | 33.54 |
| 3381.4055 | FTPN(N)KTEDTIF Hex 4 HexNAc 7 | 149.312 | 0.0 | 14.761 | 7.337e+07 | 68 - 79 | 6 | 30.27 | 30.78 | 31.12 |
| 3534.4463 | YFTPN(N)KTEDTIF Fuc 1 Hex 5 HexNAc 4 Neu5Ac 1 | 148.859 | 0.0 | -5.130 | 1.627e+07 | 67 - 79 | 2 | 38.21 | 38.37 | 38.71 |
| 3618.5529 | RNEEYN(N)KSVQEIQATF Hex 4 HexNAc 5 | 148.600 | 0.0 | -4.755 | 5.227e+07 | 50 - 66 | 3 | 32.27 | 32.44 | 32.61 |
| 3089.3418 | YFTPN(N)KTEDTIFL Hex 3 HexNAc 5 | 148.453 | 0.0 | 13.444 | 1.179e+07 | 67 - 80 | 3 | 45.30 | 45.64 | 45.98 |
| 3494.4620 | EEYN(N)KSVQEIQATF Fuc 1 Hex 4 HexNAc 5 | 148.413 | 0.0 | -3.210 | 3.022e+07 | 52 - 66 | 3 | 35.06 | 35.23 | 35.40 |
| 4227.7902 | RNEEYN(N)KSVQEIQATF Hex 4 HexNAc 8 | 148.264 | 0.0 | -3.987 | 3.255e+07 | 50 - 66 | 1 | 31.93 | 32.10 | 32.27 |
| 3126.3381 | NVQREN(N)GTVSRY Hex 3 HexNAc 6 | 147.673 | 0.0 | 13.560 | 1.169e+09 | 97 - 109 | 8 | 11.82 | 11.98 | 13.13 |
| 3664.4895 | FYFTPN(N)KTEDTIF Hex 4 HexNAc 4 Neu5Ac 2 | 147.040 | 0.0 | 14.208 | 1.934e+08 | 66 - 79 | 12 | 48.32 | 48.49 | 50.70 |
| 2449.9655 | YN(N)SSYL Hex 3 HexNAc 6 | 146.925 | 0.0 | -4.795 | 4.470e+06 | 91 - 97 | 1 | 23.16 | 23.16 | 23.50 |
| 3239.4213 | LNVQREN(N)GTVSRY Hex 3 HexNAc 6 | 145.869 | 0.0 | 14.327 | 6.007e+08 | 96 - 109 | 9 | 16.39 | 16.55 | 17.88 |
| 3821.6338 | RNEEYN(N)KSVQEIQATF Hex 4 HexNAc 6 | 145.543 | 0.0 | 13.841 | 5.407e+08 | 50 - 66 | 9 | 31.59 | 32.61 | 33.71 |
| 3018.2777 | FTPN(N)KTEDTIF Hex 3 HexNAc 6 | 145.332 | 0.0 | 0.000 | 0.000e+00 | 68 - 79 | 1 | - | - | - |
| 3491.4722 | NVQREN(N)GTVSRY Hex 4 HexNAc 7 | 145.050 | 0.0 | -6.645 | 1.690e+08 | 97 - 109 | 6 | 11.82 | 11.98 | 12.63 |
| 3724.5905 | RNEEYN(N)KSVQEIQATFF Hex 5 HexNAc 4 | 144.376 | 0.0 | -19.309 | 1.319e+07 | 50 - 67 | 2 | 43.80 | 43.80 | 43.97 |
| 4057.7380 | RNEEYN(N)KSVQEIQATFF Fuc 2 Hex 4 HexNAc 5 | 144.068 | 0.0 | -18.948 | 1.810e+07 | 50 - 67 | 2 | 43.63 | 43.80 | 43.80 |
| 3765.6221 | RNEEYN(N)KSVQEIQATFF Hex 4 HexNAc 5 | 143.991 | 0.0 | -5.145 | 2.422e+07 | 50 - 67 | 3 | 43.63 | 43.97 | 43.97 |
| 2923.2597 | NVQREN(N)GTVSRY Hex 3 HexNAc 5 | 143.771 | 0.0 | -3.927 | 2.301e+08 | 97 - 109 | 3 | 11.98 | 12.14 | 12.47 |
| 3749.6229 | RNEEYN(N)KSVQEIQATFF Fuc 1 Hex 3 HexNAc 5 | 143.603 | 0.0 | -4.535 | 3.891e+07 | 50 - 67 | 3 | 43.97 | 43.97 | 44.30 |
| 4024.7126 | RNEEYN(N)KSVQEIQATF Hex 4 HexNAc 7 | 143.594 | 0.0 | -3.961 | 8.695e+07 | 50 - 66 | 3 | 32.10 | 32.27 | 32.61 |
| 3207.2907 | TPN(N)KTEDTIF Hex 4 HexNAc 4 Neu5Ac 2 | 142.821 | 0.0 | 14.336 | 1.626e+07 | 69 - 79 | 5 | 24.67 | 25.01 | 25.35 |
| 4332.8170 | RNEEYN(N)KSVQEIQATF Fuc 1 Hex 5 HexNAc 7 | 142.787 | 0.0 | -5.160 | 1.754e+07 | 50 - 66 | 2 | 31.76 | 31.93 | 32.27 |
| 3825.5283 | YFTPN(N)KTEDTIF Fuc 1 Hex 5 HexNAc 4 Neu5Ac 2 | 142.255 | 0.0 | -5.571 | 4.155e+07 | 67 - 79 | 2 | 37.74 | 37.89 | 38.21 |
| 3073.3167 | N(N)KSVQEIQATF Fuc 1 Hex 4 HexNAc 5 | 141.566 | 0.0 | -18.332 | 8.535e+06 | 55 - 66 | 1 | 26.55 | 26.72 | 26.72 |
| 3106.3489 | RNEEYN(N)KSVQEIQAT Hex 3 HexNAc 4 | 140.729 | 0.0 | -3.715 | 4.108e+07 | 50 - 65 | 3 | 17.21 | 17.37 | 17.54 |
| 3292.4209 | YFTPN(N)KTEDTIFL Hex 3 HexNAc 6 | 140.565 | 0.0 | 14.667 | 2.876e+07 | 67 - 80 | 5 | 45.13 | 45.30 | 45.81 |
| 4114.7570 | RNEEYN(N)KSVQEIQATFF Fuc 1 Hex 4 HexNAc 6 | 139.754 | 0.0 | 13.600 | 5.408e+07 | 50 - 67 | 3 | 43.12 | 43.63 | 43.97 |
| 3037.3489 | LNVQREN(N)GTVSRY Hex 3 HexNAc 5 | 138.983 | 0.0 | 0.000 | 0.000e+00 | 96 - 109 | 1 | - | - | - |
| 3742.5621 | NVQREN(N)GTVSRY Fuc 2 Hex 5 HexNAc 6 | 137.626 | 0.0 | -4.451 | 1.906e+08 | 97 - 109 | 3 | 11.82 | 11.82 | 12.14 |
| 3457.4837 | RN(D)EEYN(N)KSVQEIQATF Hex 3 HexNAc 5 | 136.467 | 0.0 | 11.513 | 5.144e+08 | 50 - 66 | 12 | 34.21 | 35.57 | 36.59 |
| 3192.3016 | TPN(N)KTEDTIF Fuc 3 Hex 3 HexNAc 4 Neu5Ac 1 | 136.379 | 0.0 | -19.407 | 3.164e+06 | 69 - 79 | 1 | 23.33 | 23.50 | 23.50 |
| 3434.4496 | NVQREN(N)GTVSRY Fuc 1 Hex 4 HexNAc 6 | 135.971 | 0.0 | 12.022 | 6.248e+08 | 97 - 109 | 7 | 11.82 | 11.98 | 12.80 |
| 3309.4290 | RNEEYN(N)KSVQEIQAT Hex 3 HexNAc 5 | 135.817 | 0.0 | -4.686 | 3.259e+07 | 50 - 65 | 3 | 17.21 | 17.21 | 17.54 |
| 2876.2836 | TN(N)ATLDRITGKW Hex 3 HexNAc 5 | 135.212 | 0.0 | -3.769 | 7.453e+06 | 31 - 43 | 1 | 30.45 | 30.62 | 30.95 |
| 2562.1234 | N(N)KSVQEIQATF Hex 3 HexNAc 4 | 132.465 | 0.0 | -3.328 | 2.040e+07 | 55 - 66 | 2 | 27.40 | 27.57 | 27.74 |
| 3547.5327 | LNVQREN(N)GTVSRY Fuc 1 Hex 4 HexNAc 6 | 132.029 | 0.003 | 13.405 | 4.289e+08 | 96 - 109 | 7 | 16.22 | 16.39 | 17.21 |
| 3546.5424 | RNEEYN(N)KSVQEIQATFF Fuc 1 Hex 3 HexNAc 4 | 130.977 | 0.003 | -5.130 | 2.678e+07 | 50 - 67 | 3 | 44.13 | 44.30 | 44.62 |
| 3998.6396 | YFTPN(N)KTEDTIF Fuc 2 Hex 5 HexNAc 7 | 130.672 | 0.003 | 12.465 | 2.551e+07 | 67 - 79 | 2 | 35.91 | 36.42 | 36.59 |
| 2828.1785 | TPN(N)KTEDTIF Hex 4 HexNAc 5 | 130.038 | 0.003 | -17.826 | 2.370e+06 | 69 - 79 | 1 | 24.00 | 24.00 | 24.17 |
| 4072.7205 | RNEEYN(N)KSVQEIQATF Fuc 2 Hex 5 HexNAc 5 | 129.556 | 0.005 | -3.324 | 1.033e+08 | 50 - 66 | 3 | 31.93 | 32.10 | 32.44 |
| 3632.4958 | FTPN(N)KTEDTIF Fuc 2 Hex 5 HexNAc 6 | 129.511 | 0.005 | 12.459 | 1.669e+07 | 68 - 79 | 4 | 30.10 | 30.45 | 30.78 |
| 4089.7245 | RNEEYN(N)KSVQEIQATFF Hex 6 HexNAc 5 | 128.800 | 0.007 | -18.124 | 9.093e+06 | 50 - 67 | 2 | 43.46 | 43.63 | 43.63 |
| 3574.4461 | TPN(N)KTEDTIF Fuc 4 Hex 5 HexNAc 5 | 128.355 | 0.007 | 12.710 | 5.038e+07 | 69 - 79 | 4 | 38.21 | 38.37 | 38.88 |
| 3231.3703 | NVQREN(N)GTVSRY Fuc 1 Hex 4 HexNAc 5 | 127.950 | 0.007 | -4.267 | 1.432e+08 | 97 - 109 | 1 | 11.98 | 11.98 | 12.31 |
| 3681.5151 | FYFTPN(N)KTEDTIF Fuc 1 Hex 5 HexNAc 4 Neu5Ac 1 | 127.729 | 0.007 | -4.862 | 9.477e+06 | 66 - 79 | 1 | 48.49 | 48.49 | 48.83 |
| 4275.8008 | RNEEYN(N)KSVQEIQATF Fuc 2 Hex 5 HexNAc 6 | 127.484 | 0.007 | 10.474 | 2.979e+07 | 50 - 66 | 1 | 31.76 | 32.27 | 32.44 |
| 2709.1947 | N(N)KSVQEIQATFF Hex 3 HexNAc 4 | 125.684 | 0.007 | 11.641 | 4.752e+06 | 55 - 67 | 2 | 41.43 | 41.60 | 42.12 |
| 3837.5960 | FYFTPN(N)KTEDTIF Fuc 1 Hex 4 HexNAc 7 | 125.493 | 0.007 | 13.205 | 2.909e+07 | 66 - 79 | 4 | 46.49 | 47.01 | 47.18 |
| 2721.1356 | FYFTPN(N)KT Hex 3 HexNAc 6 | 125.488 | 0.0 | -2.622 | 1.271e+07 | 66 - 74 | 2 | 26.20 | 26.37 | 26.72 |
| 3604.5515 | RN(D)EEYN(N)KSVQEIQATFF Hex 3 HexNAc 5 | 124.980 | 0.009 | -5.944 | 4.305e+07 | 50 - 67 | 4 | 47.01 | 47.34 | 47.51 |
| 3354.3590 | FTPN(N)KTEDTIF Hex 4 HexNAc 4 Neu5Ac 2 | 124.776 | 0.009 | -3.893 | 2.500e+07 | 68 - 79 | 4 | 31.93 | 32.10 | 32.27 |
| 3855.6454 | LNVQREN(N)GTVSRY Fuc 2 Hex 5 HexNAc 6 | 124.436 | 0.009 | -4.205 | 1.264e+08 | 96 - 109 | 3 | 15.90 | 16.22 | 16.39 |
| 3344.4529 | LNVQREN(N)GTVSRY Fuc 1 Hex 4 HexNAc 5 | 123.747 | 0.011 | 10.890 | 9.005e+07 | 96 - 109 | 3 | 16.39 | 16.55 | 17.21 |
| 4217.7155 | YFTPN(N)KTEDTIF Fuc 1 Hex 6 HexNAc 8 | 123.604 | 0.011 | 10.071 | 1.883e+07 | 67 - 79 | 2 | 34.89 | 35.57 | 36.25 |
| 2912.2736 | N(N)KSVQEIQATFF Hex 3 HexNAc 5 | 122.103 | 0.012 | -4.055 | 5.645e+06 | 55 - 67 | 2 | 41.09 | 41.26 | 41.43 |
| 3495.4287 | RNEEYN(N)KSVQEIQATF Hex 7 HexNAc 2 | 122.036 | 0.012 | 10.607 | 3.788e+07 | 50 - 66 | 5 | 35.40 | 35.57 | 36.25 |
| 4171.7803 | RNEEYN(N)KSVQEIQATFF Hex 4 HexNAc 7 | 120.617 | 0.012 | 13.121 | 3.098e+07 | 50 - 67 | 4 | 43.12 | 43.29 | 43.97 |
| 4219.7897 | RNEEYN(N)KSVQEIQATFF Fuc 2 Hex 5 HexNAc 5 | 120.521 | 0.012 | 13.673 | 3.541e+07 | 50 - 67 | 4 | 42.97 | 43.29 | 43.80 |
| 3036.3436 | LNVQREN(N)GTVSRY Hex 3 HexNAc 5 | 119.788 | 0.012 | 13.372 | 1.337e+08 | 96 - 109 | 3 | 16.71 | 16.71 | 17.37 |
| 2518.0560 | FYFTPN(N)KT Hex 3 HexNAc 5 | 118.534 | 0.0 | -18.351 | 4.491e+06 | 66 - 74 | 1 | 26.55 | 26.72 | 26.72 |
| 3765.5951 | RN(D)EEYN(N)KSVQEIQATF Fuc 1 Hex 4 HexNAc 5 | 118.370 | 0.014 | 12.338 | 3.414e+08 | 50 - 66 | 13 | 33.87 | 35.23 | 35.91 |
| 3617.5399 | RNEEYN(N)KSVQEIQAT Fuc 1 Hex 4 HexNAc 5 | 115.325 | 0.016 | -18.877 | 1.550e+07 | 50 - 65 | 2 | 17.04 | 17.04 | 17.21 |
| 3220.3863 | N(N)KSVQEIQATFF Fuc 1 Hex 4 HexNAc 5 | 114.781 | 0.017 | -5.105 | 2.803e+06 | 55 - 67 | 2 | 40.41 | 40.41 | 40.75 |
| 3746.5399 | FTPN(N)KTEDTIF Hex 5 HexNAc 8 | 114.698 | 0.017 | 9.813 | 8.413e+06 | 68 - 79 | 3 | 29.76 | 29.93 | 30.62 |
| 3401.4690 | RN(D)EEYN(N)KSVQEIQATFF Hex 3 HexNAc 4 | 114.228 | 0.017 | -5.978 | 6.028e+07 | 50 - 67 | 5 | 47.34 | 47.51 | 47.68 |
| 2671.1012 | N(N)KTEDTIF Hex 3 HexNAc 6 | 114.122 | 0.0 | -19.903 | 3.891e+06 | 71 - 79 | 1 | 22.31 | 22.48 | 22.48 |
| 3314.3409 | TPN(N)KTEDTIF Hex 7 HexNAc 5 | 113.386 | 0.019 | 12.517 | 2.878e+07 | 69 - 79 | 3 | 38.37 | 38.71 | 39.22 |
| 3694.5527 | NVQREN(N)GTVSRY Hex 4 HexNAc 8 | 113.385 | 0.019 | -19.549 | 9.682e+07 | 97 - 109 | 4 | 11.82 | 11.82 | 11.98 |
| 3464.4214 | NVQREN(N)GTVSRY Hex 4 HexNAc 4 Neu5Ac 2 | 111.451 | 0.022 | -20.471 | 2.594e+07 | 97 - 109 | 2 | 12.14 | 12.14 | 12.31 |
| 3807.6367 | LNVQREN(N)GTVSRY Hex 4 HexNAc 8 | 109.247 | 0.027 | -4.600 | 6.762e+07 | 96 - 109 | 2 | 16.06 | 16.22 | 16.39 |
| 3799.5864 | NVQREN(N)GTVSRY Fuc 1 Hex 5 HexNAc 7 | 109.147 | 0.027 | -18.880 | 3.307e+07 | 97 - 109 | 2 | 11.82 | 11.82 | 11.82 |
| 3604.5574 | LNVQREN(N)GTVSRY Hex 4 HexNAc 7 | 109.054 | 0.027 | -18.625 | 6.627e+07 | 96 - 109 | 3 | 16.22 | 16.22 | 16.39 |
| 4050.6744 | NVQREN(N)GTVSRY Fuc 3 Hex 6 HexNAc 6 | 108.459 | 0.030 | -20.145 | 3.366e+07 | 97 - 109 | 2 | 11.65 | 11.82 | 11.82 |
| 3012.2982 | VQREN(N)GTVSRY Hex 3 HexNAc 6 | 107.163 | 0.035 | -18.779 | 1.720e+07 | 98 - 109 | 2 | 11.65 | 11.65 | 11.65 |
| 4059.6879 | NVQREN(N)GTVSRY Hex 5 HexNAc 9 | 105.962 | 0.035 | -20.162 | 1.145e+07 | 97 - 109 | 1 | 11.82 | 11.82 | 11.82 |
| 4108.7161 | NVQREN(N)GTVSRY Fuc 2 Hex 6 HexNAc 7 | 105.641 | 0.035 | 0.000 | 0.000e+00 | 97 - 109 | 1 | - | - | - |
| 4145.7082 | FYFTPN(N)KTEDTIF Fuc 2 Hex 5 HexNAc 7 | 105.306 | 0.035 | 12.133 | 2.247e+07 | 66 - 79 | 4 | 45.98 | 46.49 | 46.84 |
| 4163.7546 | LNVQREN(N)GTVSRY Fuc 3 Hex 6 HexNAc 6 | 104.086 | 0.042 | -20.939 | 2.096e+07 | 96 - 109 | 2 | 15.90 | 15.90 | 15.90 |
| 2983.3503 | C(C)ANLVPVPITN(N)ATL Hex 3 HexNAc 5 | 101.516 | 0.047 | 14.453 | 3.545e+07 | 22 - 36 | 7 | 50.95 | 51.16 | 52.07 |

### TPN(N)KTEDTIF Hex 3 HexNAc 6

**Mass:**  2869.2028

(9.350e-07)

**Charge States:** 3, 4

**Start Position**: 70

**End Position**: 80

| Profile Score | Tandem Score | TDA q-value | Volume | Spectrum Matches |
| --- | --- | --- | --- | --- |
| 16.4184 | 184.0663 | 0.0000 | 3.2987e+08 | 11 |

**Adduct Signal Fractions**

- **Unmodified**: 3.2987e+08

### TPN(N)KTEDTIF Fuc 1 Hex 4 HexNAc 6

**Mass:**  3177.3144

(9.528e-07)

**Charge States:** 3, 4

**Start Position**: 70

**End Position**: 80

| Profile Score | Tandem Score | TDA q-value | Volume | Spectrum Matches |
| --- | --- | --- | --- | --- |
| 14.4979 | 181.3112 | 0.0000 | 6.6510e+07 | 5 |

**Adduct Signal Fractions**

- **Unmodified**: 6.6510e+07

### TPN(N)KTEDTIF Hex 3 HexNAc 5

**Mass:**  2666.1247

(1.277e-06)

**Charge States:** 2, 3

**Start Position**: 70

**End Position**: 80

| Profile Score | Tandem Score | TDA q-value | Volume | Spectrum Matches |
| --- | --- | --- | --- | --- |
| 15.6377 | 180.9897 | 0.0000 | 1.7349e+08 | 8 |

**Adduct Signal Fractions**

- **Unmodified**: 1.7349e+08

### TPN(N)KTEDTIF Fuc 1 Hex 4 HexNAc 5

**Mass:**  2974.2357

(1.158e-06)

**Charge States:** 3

**Start Position**: 70

**End Position**: 80

| Profile Score | Tandem Score | TDA q-value | Volume | Spectrum Matches |
| --- | --- | --- | --- | --- |
| 14.0982 | 180.8416 | 0.0000 | 4.1800e+07 | 4 |

**Adduct Signal Fractions**

- **Unmodified**: 4.1800e+07

### TPN(N)KTEDTIF Hex 3 HexNAc 4

**Mass:**  2463.0459

(1.521e-06)

**Charge States:** 2, 3

**Start Position**: 70

**End Position**: 80

| Profile Score | Tandem Score | TDA q-value | Volume | Spectrum Matches |
| --- | --- | --- | --- | --- |
| 13.1256 | 180.3981 | 0.0000 | 2.3448e+07 | 4 |

**Adduct Signal Fractions**

- **Unmodified**: 2.3448e+07

### FYFTPN(N)KTEDTIF Hex 4 HexNAc 7

**Mass:**  3691.5393

(1.216e-06)

**Charge States:** 3, 4

**Start Position**: 67

**End Position**: 80

| Profile Score | Tandem Score | TDA q-value | Volume | Spectrum Matches |
| --- | --- | --- | --- | --- |
| 14.0667 | 176.8856 | 0.0000 | 5.0588e+08 | 12 |

**Adduct Signal Fractions**

- **Unmodified**: 5.0588e+08

### YFTPN(N)KTEDTIF Hex 3 HexNAc 6

**Mass:**  3179.3366

(5.983e-07)

**Charge States:** 2, 3, 4

**Start Position**: 68

**End Position**: 80

| Profile Score | Tandem Score | TDA q-value | Volume | Spectrum Matches |
| --- | --- | --- | --- | --- |
| 14.8353 | 176.0330 | 0.0000 | 3.5358e+09 | 43 |

**Adduct Signal Fractions**

- **Unmodified**: 3.5358e+09

### YFTPN(N)KTEDTIF Hex 3 HexNAc 4

**Mass:**  2773.1771

(1.116e-06)

**Charge States:** 2, 3

**Start Position**: 68

**End Position**: 80

| Profile Score | Tandem Score | TDA q-value | Volume | Spectrum Matches |
| --- | --- | --- | --- | --- |
| 11.8731 | 175.5189 | 0.0000 | 2.9904e+08 | 9 |

**Adduct Signal Fractions**

- **Unmodified**: 2.9904e+08

### FYFTPN(N)KTEDTIF Fuc 1 Hex 4 HexNAc 5

**Mass:**  3431.4347

(6.942e-07)

**Charge States:** 3, 4

**Start Position**: 67

**End Position**: 80

| Profile Score | Tandem Score | TDA q-value | Volume | Spectrum Matches |
| --- | --- | --- | --- | --- |
| 12.8003 | 175.2093 | 0.0000 | 4.2043e+08 | 11 |

**Adduct Signal Fractions**

- **Unmodified**: 4.2043e+08

### YFTPN(N)KTEDTIF Fuc 1 Hex 4 HexNAc 5

**Mass:**  3284.3651

(1.160e-06)

**Charge States:** 3, 4

**Start Position**: 68

**End Position**: 80

| Profile Score | Tandem Score | TDA q-value | Volume | Spectrum Matches |
| --- | --- | --- | --- | --- |
| 13.9918 | 174.8541 | 0.0000 | 5.7733e+08 | 16 |

**Adduct Signal Fractions**

- **Unmodified**: 5.7733e+08

### FYFTPN(N)KTEDTIF Fuc 1 Hex 4 HexNAc 6

**Mass:**  3634.5163

(1.100e-06)

**Charge States:** 3, 4

**Start Position**: 67

**End Position**: 80

| Profile Score | Tandem Score | TDA q-value | Volume | Spectrum Matches |
| --- | --- | --- | --- | --- |
| 13.9846 | 173.2734 | 0.0000 | 6.7092e+08 | 16 |

**Adduct Signal Fractions**

- **Unmodified**: 6.7092e+08

### FTPN(N)KTEDTIF Hex 3 HexNAc 6

**Mass:**  3016.2740

(4.736e-07)

**Charge States:** 3, 4

**Start Position**: 69

**End Position**: 80

| Profile Score | Tandem Score | TDA q-value | Volume | Spectrum Matches |
| --- | --- | --- | --- | --- |
| 15.7964 | 172.9800 | 0.0000 | 7.3287e+08 | 12 |

**Adduct Signal Fractions**

- **Unmodified**: 7.3287e+08

### YFTPN(N)KTEDTIF Fuc 2 Hex 5 HexNAc 6

**Mass:**  3795.5593

(1.988e-06)

**Charge States:** 3, 4

**Start Position**: 68

**End Position**: 80

| Profile Score | Tandem Score | TDA q-value | Volume | Spectrum Matches |
| --- | --- | --- | --- | --- |
| 16.2475 | 172.9183 | 0.0000 | 1.9067e+08 | 4 |

**Adduct Signal Fractions**

- **Unmodified**: 1.9067e+08

### YFTPN(N)KTEDTIF Hex 3 HexNAc 5

**Mass:**  2976.2558

(7.356e-07)

**Charge States:** 2, 3, 4

**Start Position**: 68

**End Position**: 80

| Profile Score | Tandem Score | TDA q-value | Volume | Spectrum Matches |
| --- | --- | --- | --- | --- |
| 15.0244 | 171.9773 | 0.0000 | 1.9356e+09 | 38 |

**Adduct Signal Fractions**

- **Unmodified**: 1.9356e+09

### YFTPN(N)KTEDTIF Fuc 1 Hex 4 HexNAc 6

**Mass:**  3487.4448

(1.068e-06)

**Charge States:** 3, 4

**Start Position**: 68

**End Position**: 80

| Profile Score | Tandem Score | TDA q-value | Volume | Spectrum Matches |
| --- | --- | --- | --- | --- |
| 16.0778 | 171.1660 | 0.0000 | 1.0747e+09 | 11 |

**Adduct Signal Fractions**

- **Unmodified**: 1.0747e+09

### YFTPN(N)KTEDTIF Hex 3 HexNAc 4

**Mass:**  2775.1840

(7.248e-04)

**Start Position**: 68

**End Position**: 80

| Profile Score | Tandem Score | TDA q-value | Volume | Spectrum Matches |
| --- | --- | --- | --- | --- |
| 0.0000 | 170.5346 | 0.0000 | 0.0000e+00 | 1 |

Adduct:

No Chromatogram Found

### YFTPN(N)KTEDTIF Hex 4 HexNAc 6

**Mass:**  3341.3897

(7.472e-07)

**Charge States:** 3, 4

**Start Position**: 68

**End Position**: 80

| Profile Score | Tandem Score | TDA q-value | Volume | Spectrum Matches |
| --- | --- | --- | --- | --- |
| 16.2720 | 169.6232 | 0.0000 | 1.7608e+08 | 6 |

**Adduct Signal Fractions**

- **Unmodified**: 1.7608e+08

### FYFTPN(N)KTEDTIF Hex 3 HexNAc 6

**Mass:**  3326.4036

(1.156e-06)

**Charge States:** 3, 4

**Start Position**: 67

**End Position**: 80

| Profile Score | Tandem Score | TDA q-value | Volume | Spectrum Matches |
| --- | --- | --- | --- | --- |
| 15.4463 | 169.1672 | 0.0000 | 2.8237e+09 | 23 |

**Adduct Signal Fractions**

- **Unmodified**: 2.8237e+09

### YFTPN(N)KTEDTIF Hex 4 HexNAc 7

**Mass:**  3544.4665

(8.170e-07)

**Charge States:** 3, 4

**Start Position**: 68

**End Position**: 80

| Profile Score | Tandem Score | TDA q-value | Volume | Spectrum Matches |
| --- | --- | --- | --- | --- |
| 15.3675 | 168.7046 | 0.0000 | 9.3672e+08 | 13 |

**Adduct Signal Fractions**

- **Unmodified**: 9.3672e+08

### FYFTPN(N)KTEDTIF Hex 3 HexNAc 5

**Mass:**  3123.3224

(1.480e-07)

**Charge States:** 2, 3, 4

**Start Position**: 67

**End Position**: 80

| Profile Score | Tandem Score | TDA q-value | Volume | Spectrum Matches |
| --- | --- | --- | --- | --- |
| 13.6359 | 168.5658 | 0.0000 | 2.1366e+09 | 132 |

**Adduct Signal Fractions**

- **Unmodified**: 2.1366e+09

### TPN(N)KTEDTIF Hex 4 HexNAc 7

**Mass:**  3234.3371

(9.267e-07)

**Charge States:** 3, 4

**Start Position**: 70

**End Position**: 80

| Profile Score | Tandem Score | TDA q-value | Volume | Spectrum Matches |
| --- | --- | --- | --- | --- |
| 12.4603 | 167.7525 | 0.0000 | 4.3638e+07 | 5 |

**Adduct Signal Fractions**

- **Unmodified**: 4.3638e+07

### FYFTPN(N)KTEDTIF Fuc 2 Hex 5 HexNAc 6

**Mass:**  3942.6276

(1.555e-06)

**Charge States:** 3, 4

**Start Position**: 67

**End Position**: 80

| Profile Score | Tandem Score | TDA q-value | Volume | Spectrum Matches |
| --- | --- | --- | --- | --- |
| 14.6190 | 167.0620 | 0.0000 | 1.5663e+08 | 7 |

**Adduct Signal Fractions**

- **Unmodified**: 1.5663e+08

### FYFTPN(N)KTEDTIF Hex 4 HexNAc 5

**Mass:**  3285.3784

(9.716e-07)

**Charge States:** 3

**Start Position**: 67

**End Position**: 80

| Profile Score | Tandem Score | TDA q-value | Volume | Spectrum Matches |
| --- | --- | --- | --- | --- |
| 11.6223 | 166.9576 | 0.0000 | 2.1383e+07 | 4 |

**Adduct Signal Fractions**

- **Unmodified**: 2.1383e+07

### FYFTPN(N)KTEDTIF Hex 3 HexNAc 4

**Mass:**  2920.2445

(6.469e-07)

**Charge States:** 2, 3, 4

**Start Position**: 67

**End Position**: 80

| Profile Score | Tandem Score | TDA q-value | Volume | Spectrum Matches |
| --- | --- | --- | --- | --- |
| 14.7787 | 165.7220 | 0.0000 | 1.8529e+08 | 11 |

**Adduct Signal Fractions**

- **Unmodified**: 1.8529e+08

### FTPN(N)KTEDTIF Hex 3 HexNAc 5

**Mass:**  2813.1915

(5.684e-07)

**Charge States:** 2, 3, 4

**Start Position**: 69

**End Position**: 80

| Profile Score | Tandem Score | TDA q-value | Volume | Spectrum Matches |
| --- | --- | --- | --- | --- |
| 14.9242 | 165.1230 | 0.0000 | 2.6963e+08 | 11 |

**Adduct Signal Fractions**

- **Unmodified**: 2.6963e+08

### YFTPN(N)KTEDTIF Hex 5 HexNAc 8

**Mass:**  3909.6042

(2.509e-06)

**Charge States:** 3, 4

**Start Position**: 68

**End Position**: 80

| Profile Score | Tandem Score | TDA q-value | Volume | Spectrum Matches |
| --- | --- | --- | --- | --- |
| 14.1988 | 164.9749 | 0.0000 | 1.5964e+08 | 6 |

**Adduct Signal Fractions**

- **Unmodified**: 1.5964e+08

### YFTPN(N)KTEDTIF Hex 4 HexNAc 8

**Mass:**  3747.5505

(1.368e-06)

**Charge States:** 3, 4

**Start Position**: 68

**End Position**: 80

| Profile Score | Tandem Score | TDA q-value | Volume | Spectrum Matches |
| --- | --- | --- | --- | --- |
| 13.5269 | 164.1276 | 0.0000 | 1.2160e+08 | 6 |

**Adduct Signal Fractions**

- **Unmodified**: 1.2160e+08

### YFTPN(N)KTEDTIF Fuc 1 Hex 5 HexNAc 7

**Mass:**  3852.5815

(1.641e-06)

**Charge States:** 3, 4

**Start Position**: 68

**End Position**: 80

| Profile Score | Tandem Score | TDA q-value | Volume | Spectrum Matches |
| --- | --- | --- | --- | --- |
| 14.9774 | 163.7442 | 0.0000 | 1.6759e+08 | 6 |

**Adduct Signal Fractions**

- **Unmodified**: 1.6759e+08

### EEYN(N)KSVQEIQATF Hex 3 HexNAc 5

**Mass:**  3186.3524

(7.725e-07)

**Charge States:** 3, 4

**Start Position**: 53

**End Position**: 67

| Profile Score | Tandem Score | TDA q-value | Volume | Spectrum Matches |
| --- | --- | --- | --- | --- |
| 13.2963 | 163.5790 | 0.0000 | 3.9162e+07 | 3 |

**Adduct Signal Fractions**

- **Unmodified**: 3.9162e+07

### YFTPN(N)KTEDTIF Fuc 1 Hex 4 HexNAc 7

**Mass:**  3690.5257

(1.313e-06)

**Charge States:** 3, 4

**Start Position**: 68

**End Position**: 80

| Profile Score | Tandem Score | TDA q-value | Volume | Spectrum Matches |
| --- | --- | --- | --- | --- |
| 12.6674 | 163.0801 | 0.0000 | 4.0987e+07 | 3 |

**Adduct Signal Fractions**

- **Unmodified**: 4.0987e+07

### FTPN(N)KTEDTIF Fuc 1 Hex 4 HexNAc 6

**Mass:**  3324.3817

(8.709e-07)

**Charge States:** 3, 4

**Start Position**: 69

**End Position**: 80

| Profile Score | Tandem Score | TDA q-value | Volume | Spectrum Matches |
| --- | --- | --- | --- | --- |
| 14.4512 | 162.2935 | 0.0000 | 1.2140e+08 | 5 |

**Adduct Signal Fractions**

- **Unmodified**: 1.2140e+08

### FTPN(N)KTEDTIF Fuc 1 Hex 4 HexNAc 5

**Mass:**  3121.3042

(1.079e-06)

**Charge States:** 3

**Start Position**: 69

**End Position**: 80

| Profile Score | Tandem Score | TDA q-value | Volume | Spectrum Matches |
| --- | --- | --- | --- | --- |
| 12.2238 | 161.5274 | 0.0000 | 8.7914e+07 | 5 |

**Adduct Signal Fractions**

- **Unmodified**: 8.7914e+07

### RNEEYN(N)KSVQEIQATFF Hex 3 HexNAc 4

**Mass:**  3400.4844

(1.075e-06)

**Charge States:** 3, 4

**Start Position**: 51

**End Position**: 68

| Profile Score | Tandem Score | TDA q-value | Volume | Spectrum Matches |
| --- | --- | --- | --- | --- |
| 16.1344 | 159.8344 | 0.0000 | 1.9407e+09 | 11 |

**Adduct Signal Fractions**

- **Unmodified**: 1.9407e+09

### TPN(N)KTEDTIF Hex 4 HexNAc 6

**Mass:**  3031.2583

(3.514e-08)

**Charge States:** 3

**Start Position**: 70

**End Position**: 80

| Profile Score | Tandem Score | TDA q-value | Volume | Spectrum Matches |
| --- | --- | --- | --- | --- |
| 11.0571 | 158.9871 | 0.0000 | 7.8173e+06 | 3 |

**Adduct Signal Fractions**

- **Unmodified**: 7.8173e+06

### RNEEYN(N)KSVQEIQATFF Hex 3 HexNAc 5

**Mass:**  3603.5639

(1.190e-06)

**Charge States:** 3, 4

**Start Position**: 51

**End Position**: 68

| Profile Score | Tandem Score | TDA q-value | Volume | Spectrum Matches |
| --- | --- | --- | --- | --- |
| 15.1927 | 157.9330 | 0.0000 | 1.6925e+09 | 10 |

**Adduct Signal Fractions**

- **Unmodified**: 1.6925e+09

### FYFTPN(N)KTEDTIF Hex 4 HexNAc 8

**Mass:**  3894.6198

(2.036e-06)

**Charge States:** 3, 4

**Start Position**: 67

**End Position**: 80

| Profile Score | Tandem Score | TDA q-value | Volume | Spectrum Matches |
| --- | --- | --- | --- | --- |
| 16.3400 | 157.1573 | 0.0000 | 9.6316e+07 | 5 |

**Adduct Signal Fractions**

- **Unmodified**: 9.6316e+07

### YFTPN(N)KTEDTIF Fuc 2 Hex 5 HexNAc 5

**Mass:**  3592.4785

(1.018e-06)

**Charge States:** 3

**Start Position**: 68

**End Position**: 80

| Profile Score | Tandem Score | TDA q-value | Volume | Spectrum Matches |
| --- | --- | --- | --- | --- |
| -5.2453 | 156.0617 | 0.0000 | 1.8233e+07 | 3 |

**Adduct Signal Fractions**

- **Unmodified**: 1.8233e+07

### RN(D)EEYN(N)KSVQEIQATF Hex 3 HexNAc 4

**Mass:**  3254.4007

(3.047e-04)

**Charge States:** 3, 4

**Start Position**: 51

**End Position**: 67

| Profile Score | Tandem Score | TDA q-value | Volume | Spectrum Matches |
| --- | --- | --- | --- | --- |
| 10.1359 | 155.9603 | 0.0000 | 5.8519e+08 | 24 |

**Adduct Signal Fractions**

- **Unmodified**: 5.8519e+08

### YFTPN(N)KTEDTIF Hex 4 HexNAc 5

**Mass:**  3138.3122

(1.903e-06)

**Charge States:** 3

**Start Position**: 68

**End Position**: 80

| Profile Score | Tandem Score | TDA q-value | Volume | Spectrum Matches |
| --- | --- | --- | --- | --- |
| -4.5582 | 155.5236 | 0.0000 | 2.1919e+07 | 1 |

**Adduct Signal Fractions**

- **Unmodified**: 2.1919e+07

### FYFTPN(N)KTEDTIF Fuc 1 Hex 5 HexNAc 7

**Mass:**  3999.6508

(1.927e-06)

**Charge States:** 3, 4

**Start Position**: 67

**End Position**: 80

| Profile Score | Tandem Score | TDA q-value | Volume | Spectrum Matches |
| --- | --- | --- | --- | --- |
| 16.2824 | 155.4555 | 0.0000 | 1.0465e+08 | 8 |

**Adduct Signal Fractions**

- **Unmodified**: 1.0465e+08

### RNEEYN(N)KSVQEIQATF Hex 3 HexNAc 4

**Mass:**  3253.4192

(1.649e-06)

**Charge States:** 3, 4

**Start Position**: 51

**End Position**: 67

| Profile Score | Tandem Score | TDA q-value | Volume | Spectrum Matches |
| --- | --- | --- | --- | --- |
| 15.9433 | 155.3491 | 0.0000 | 5.8519e+09 | 33 |

**Adduct Signal Fractions**

- **Unmodified**: 5.8519e+09

### FYFTPN(N)KTEDTIF Hex 4 HexNAc 6

**Mass:**  3488.4589

(5.403e-07)

**Charge States:** 3, 4

**Start Position**: 67

**End Position**: 80

| Profile Score | Tandem Score | TDA q-value | Volume | Spectrum Matches |
| --- | --- | --- | --- | --- |
| 10.3674 | 155.2866 | 0.0000 | 5.1020e+07 | 7 |

**Adduct Signal Fractions**

- **Unmodified**: 5.1020e+07

### RNEEYN(N)KSVQEIQATFF Fuc 1 Hex 4 HexNAc 5

**Mass:**  3911.6782

(1.337e-06)

**Charge States:** 3, 4

**Start Position**: 51

**End Position**: 68

| Profile Score | Tandem Score | TDA q-value | Volume | Spectrum Matches |
| --- | --- | --- | --- | --- |
| 15.1672 | 155.1655 | 0.0000 | 1.2960e+09 | 12 |

**Adduct Signal Fractions**

- **Unmodified**: 1.2960e+09

### RNEEYN(N)KSVQEIQATF Fuc 1 Hex 4 HexNAc 5

**Mass:**  3764.6083

(1.990e-06)

**Charge States:** 3, 4

**Start Position**: 51

**End Position**: 67

| Profile Score | Tandem Score | TDA q-value | Volume | Spectrum Matches |
| --- | --- | --- | --- | --- |
| 15.7042 | 155.1342 | 0.0000 | 3.2617e+09 | 15 |

**Adduct Signal Fractions**

- **Unmodified**: 3.2617e+09

### RNEEYN(N)KSVQEIQATF Hex 3 HexNAc 5

**Mass:**  3456.4993

(4.456e-07)

**Charge States:** 3, 4

**Start Position**: 51

**End Position**: 67

| Profile Score | Tandem Score | TDA q-value | Volume | Spectrum Matches |
| --- | --- | --- | --- | --- |
| 15.4944 | 154.6152 | 0.0000 | 4.6401e+09 | 16 |

**Adduct Signal Fractions**

- **Unmodified**: 4.6401e+09

### YFTPN(N)KTEDTIF Hex 4 HexNAc 4 Neu5Ac 2

**Mass:**  3517.4203

(7.796e-07)

**Charge States:** 3, 4

**Start Position**: 68

**End Position**: 80

| Profile Score | Tandem Score | TDA q-value | Volume | Spectrum Matches |
| --- | --- | --- | --- | --- |
| 14.8291 | 153.9767 | 0.0000 | 2.9345e+08 | 9 |

**Adduct Signal Fractions**

- **Unmodified**: 2.9345e+08

### RNEEYN(N)KSVQEIQATF Hex 3 HexNAc 6

**Mass:**  3659.5776

(7.467e-07)

**Charge States:** 3, 4

**Start Position**: 51

**End Position**: 67

| Profile Score | Tandem Score | TDA q-value | Volume | Spectrum Matches |
| --- | --- | --- | --- | --- |
| -3.8222 | 153.0441 | 0.0000 | 1.9185e+08 | 3 |

**Adduct Signal Fractions**

- **Unmodified**: 1.9185e+08

### YFTPN(N)KTEDTIF Hex 5 HexNAc 9

**Mass:**  4112.6823

(6.108e-07)

**Charge States:** 3, 4

**Start Position**: 68

**End Position**: 80

| Profile Score | Tandem Score | TDA q-value | Volume | Spectrum Matches |
| --- | --- | --- | --- | --- |
| 13.5966 | 152.2467 | 0.0000 | 4.8146e+07 | 4 |

**Adduct Signal Fractions**

- **Unmodified**: 4.8146e+07

### RNEEYN(N)KSVQEIQATF Fuc 1 Hex 3 HexNAc 4

**Mass:**  3399.4773

(1.498e-06)

**Charge States:** 3, 4

**Start Position**: 51

**End Position**: 67

| Profile Score | Tandem Score | TDA q-value | Volume | Spectrum Matches |
| --- | --- | --- | --- | --- |
| 12.5471 | 152.1094 | 0.0000 | 1.1596e+08 | 5 |

**Adduct Signal Fractions**

- **Unmodified**: 1.1596e+08

### FYFTPN(N)KTEDTIF Hex 5 HexNAc 8

**Mass:**  4056.6723

(1.675e-06)

**Charge States:** 3, 4

**Start Position**: 67

**End Position**: 80

| Profile Score | Tandem Score | TDA q-value | Volume | Spectrum Matches |
| --- | --- | --- | --- | --- |
| 13.0912 | 151.7944 | 0.0000 | 9.4876e+07 | 6 |

**Adduct Signal Fractions**

- **Unmodified**: 9.4876e+07

### RNEEYN(N)KSVQEIQATF Fuc 1 Hex 4 HexNAc 4

**Mass:**  3561.5290

(1.090e-06)

**Charge States:** 3, 4

**Start Position**: 51

**End Position**: 67

| Profile Score | Tandem Score | TDA q-value | Volume | Spectrum Matches |
| --- | --- | --- | --- | --- |
| -3.9248 | 151.6652 | 0.0000 | 1.3689e+08 | 4 |

**Adduct Signal Fractions**

- **Unmodified**: 1.3689e+08

### RNEEYN(N)KSVQEIQATFF Fuc 1 Hex 4 HexNAc 4

**Mass:**  3708.5986

(5.505e-07)

**Charge States:** 3, 4

**Start Position**: 51

**End Position**: 68

| Profile Score | Tandem Score | TDA q-value | Volume | Spectrum Matches |
| --- | --- | --- | --- | --- |
| -3.6481 | 151.5849 | 0.0000 | 5.7792e+07 | 4 |

**Adduct Signal Fractions**

- **Unmodified**: 5.7792e+07

### FYFTPN(N)KTEDTIF Hex 5 HexNAc 9

**Mass:**  4259.7512

(1.236e-06)

**Charge States:** 3, 4

**Start Position**: 67

**End Position**: 80

| Profile Score | Tandem Score | TDA q-value | Volume | Spectrum Matches |
| --- | --- | --- | --- | --- |
| 12.3054 | 151.5328 | 0.0000 | 3.2040e+07 | 4 |

**Adduct Signal Fractions**

- **Unmodified**: 3.2040e+07

### RNEEYN(N)KSVQEIQATFF Hex 3 HexNAc 6

**Mass:**  3806.6447

(2.119e-06)

**Charge States:** 3, 4

**Start Position**: 51

**End Position**: 68

| Profile Score | Tandem Score | TDA q-value | Volume | Spectrum Matches |
| --- | --- | --- | --- | --- |
| -4.0244 | 151.2112 | 0.0000 | 8.4142e+07 | 7 |

**Adduct Signal Fractions**

- **Unmodified**: 8.4142e+07

### RNEEYN(N)KSVQEIQATF Fuc 2 Hex 4 HexNAc 5

**Mass:**  3910.6699

(4.085e-06)

**Charge States:** 4

**Start Position**: 51

**End Position**: 67

| Profile Score | Tandem Score | TDA q-value | Volume | Spectrum Matches |
| --- | --- | --- | --- | --- |
| -20.0079 | 151.1112 | 0.0000 | 2.6698e+07 | 1 |

**Adduct Signal Fractions**

- **Unmodified**: 2.6698e+07

### YFTPN(N)KTEDTIF Fuc 1 Hex 5 HexNAc 8

**Mass:**  4055.6611

(7.357e-08)

**Charge States:** 3, 4

**Start Position**: 68

**End Position**: 80

| Profile Score | Tandem Score | TDA q-value | Volume | Spectrum Matches |
| --- | --- | --- | --- | --- |
| 12.2058 | 151.0703 | 0.0000 | 4.0367e+07 | 4 |

**Adduct Signal Fractions**

- **Unmodified**: 4.0367e+07

### RNEEYN(N)KSVQEIQATF Fuc 1 Hex 4 HexNAc 6

**Mass:**  3967.6906

(8.756e-07)

**Charge States:** 3, 4

**Start Position**: 51

**End Position**: 67

| Profile Score | Tandem Score | TDA q-value | Volume | Spectrum Matches |
| --- | --- | --- | --- | --- |
| -3.0149 | 150.8980 | 0.0000 | 1.4871e+08 | 3 |

**Adduct Signal Fractions**

- **Unmodified**: 1.4871e+08

### FTPN(N)KTEDTIF Hex 3 HexNAc 4

**Mass:**  2610.1137

(1.416e-06)

**Charge States:** 2, 3

**Start Position**: 69

**End Position**: 80

| Profile Score | Tandem Score | TDA q-value | Volume | Spectrum Matches |
| --- | --- | --- | --- | --- |
| -3.3245 | 150.4452 | 0.0000 | 3.1786e+07 | 4 |

**Adduct Signal Fractions**

- **Unmodified**: 3.1786e+07

### TPN(N)KTEDTIF Fuc 2 Hex 5 HexNAc 6

**Mass:**  3485.4285

(1.886e-06)

**Charge States:** 3, 4

**Start Position**: 70

**End Position**: 80

| Profile Score | Tandem Score | TDA q-value | Volume | Spectrum Matches |
| --- | --- | --- | --- | --- |
| -5.2114 | 150.0121 | 0.0000 | 9.8888e+06 | 2 |

**Adduct Signal Fractions**

- **Unmodified**: 9.8888e+06

### EEYN(N)KSVQEIQATF Hex 3 HexNAc 4

**Mass:**  2983.2725

(4.676e-07)

**Charge States:** 3

**Start Position**: 53

**End Position**: 67

| Profile Score | Tandem Score | TDA q-value | Volume | Spectrum Matches |
| --- | --- | --- | --- | --- |
| -3.0546 | 149.8556 | 0.0000 | 4.1422e+07 | 15 |

**Adduct Signal Fractions**

- **Unmodified**: 4.1422e+07

### RNEEYN(N)KSVQEIQATF Fuc 1 Hex 3 HexNAc 5

**Mass:**  3602.5576

(1.359e-06)

**Charge States:** 3, 4

**Start Position**: 51

**End Position**: 67

| Profile Score | Tandem Score | TDA q-value | Volume | Spectrum Matches |
| --- | --- | --- | --- | --- |
| 9.9961 | 149.4082 | 0.0000 | 1.0926e+08 | 5 |

**Adduct Signal Fractions**

- **Unmodified**: 1.0926e+08

### FTPN(N)KTEDTIF Hex 4 HexNAc 7

**Mass:**  3381.4037

(2.724e-06)

**Charge States:** 3, 4

**Start Position**: 69

**End Position**: 80

| Profile Score | Tandem Score | TDA q-value | Volume | Spectrum Matches |
| --- | --- | --- | --- | --- |
| 14.7612 | 149.3123 | 0.0000 | 7.3375e+07 | 6 |

**Adduct Signal Fractions**

- **Unmodified**: 7.3375e+07

### YFTPN(N)KTEDTIF Fuc 1 Hex 5 HexNAc 4 Neu5Ac 1

**Mass:**  3534.4455

(3.499e-06)

**Charge States:** 3

**Start Position**: 68

**End Position**: 80

| Profile Score | Tandem Score | TDA q-value | Volume | Spectrum Matches |
| --- | --- | --- | --- | --- |
| -5.1301 | 148.8592 | 0.0000 | 1.6270e+07 | 2 |

**Adduct Signal Fractions**

- **Unmodified**: 1.6270e+07

### RNEEYN(N)KSVQEIQATF Hex 4 HexNAc 5

**Mass:**  3618.5576

(2.781e-06)

**Charge States:** 3, 4

**Start Position**: 51

**End Position**: 67

| Profile Score | Tandem Score | TDA q-value | Volume | Spectrum Matches |
| --- | --- | --- | --- | --- |
| -4.7545 | 148.6004 | 0.0000 | 5.2266e+07 | 3 |

**Adduct Signal Fractions**

- **Unmodified**: 5.2266e+07

### YFTPN(N)KTEDTIFL Hex 3 HexNAc 5

**Mass:**  3089.3421

(1.500e-06)

**Charge States:** 3, 4

**Start Position**: 68

**End Position**: 81

| Profile Score | Tandem Score | TDA q-value | Volume | Spectrum Matches |
| --- | --- | --- | --- | --- |
| 13.4435 | 148.4529 | 0.0000 | 1.1792e+07 | 3 |

**Adduct Signal Fractions**

- **Unmodified**: 1.1792e+07

### EEYN(N)KSVQEIQATF Fuc 1 Hex 4 HexNAc 5

**Mass:**  3494.4644

(1.143e-06)

**Charge States:** 3, 4

**Start Position**: 53

**End Position**: 67

| Profile Score | Tandem Score | TDA q-value | Volume | Spectrum Matches |
| --- | --- | --- | --- | --- |
| -3.2100 | 148.4127 | 0.0000 | 3.0219e+07 | 3 |

**Adduct Signal Fractions**

- **Unmodified**: 3.0219e+07

### RNEEYN(N)KSVQEIQATF Hex 4 HexNAc 8

**Mass:**  4227.7902

(1.014e-06)

**Charge States:** 3, 4

**Start Position**: 51

**End Position**: 67

| Profile Score | Tandem Score | TDA q-value | Volume | Spectrum Matches |
| --- | --- | --- | --- | --- |
| -3.9872 | 148.2636 | 0.0000 | 3.2546e+07 | 1 |

**Adduct Signal Fractions**

- **Unmodified**: 3.2546e+07

### NVQREN(N)GTVSRY Hex 3 HexNAc 6

**Mass:**  3126.3378

(2.887e-07)

**Charge States:** 3, 4

**Start Position**: 98

**End Position**: 110

| Profile Score | Tandem Score | TDA q-value | Volume | Spectrum Matches |
| --- | --- | --- | --- | --- |
| 13.5600 | 147.6733 | 0.0000 | 1.1685e+09 | 8 |

**Adduct Signal Fractions**

- **Unmodified**: 1.1685e+09

### FYFTPN(N)KTEDTIF Hex 4 HexNAc 4 Neu5Ac 2

**Mass:**  3664.4892

(1.043e-06)

**Charge States:** 3, 4

**Start Position**: 67

**End Position**: 80

| Profile Score | Tandem Score | TDA q-value | Volume | Spectrum Matches |
| --- | --- | --- | --- | --- |
| 14.2084 | 147.0396 | 0.0000 | 1.9345e+08 | 12 |

**Adduct Signal Fractions**

- **Unmodified**: 1.9345e+08

### YN(N)SSYL Hex 3 HexNAc 6

**Mass:**  2449.9658

(1.138e-06)

**Charge States:** 3

**Start Position**: 92

**End Position**: 98

| Profile Score | Tandem Score | TDA q-value | Volume | Spectrum Matches |
| --- | --- | --- | --- | --- |
| -4.7952 | 146.9254 | 0.0000 | 4.4699e+06 | 1 |

**Adduct Signal Fractions**

- **Unmodified**: 4.4699e+06

### LNVQREN(N)GTVSRY Hex 3 HexNAc 6

**Mass:**  3239.4204

(8.681e-08)

**Charge States:** 3, 4

**Start Position**: 97

**End Position**: 110

| Profile Score | Tandem Score | TDA q-value | Volume | Spectrum Matches |
| --- | --- | --- | --- | --- |
| 14.3266 | 145.8687 | 0.0000 | 6.0068e+08 | 9 |

**Adduct Signal Fractions**

- **Unmodified**: 6.0068e+08

### RNEEYN(N)KSVQEIQATF Hex 4 HexNAc 6

**Mass:**  3821.6350

(1.922e-06)

**Charge States:** 3, 4

**Start Position**: 51

**End Position**: 67

| Profile Score | Tandem Score | TDA q-value | Volume | Spectrum Matches |
| --- | --- | --- | --- | --- |
| 13.8411 | 145.5434 | 0.0000 | 5.4072e+08 | 9 |

**Adduct Signal Fractions**

- **Unmodified**: 5.4072e+08

### FTPN(N)KTEDTIF Hex 3 HexNAc 6

**Mass:**  3018.2777

(6.658e-04)

**Start Position**: 69

**End Position**: 80

| Profile Score | Tandem Score | TDA q-value | Volume | Spectrum Matches |
| --- | --- | --- | --- | --- |
| 0.0000 | 145.3323 | 0.0000 | 0.0000e+00 | 1 |

Adduct:

No Chromatogram Found

### NVQREN(N)GTVSRY Hex 4 HexNAc 7

**Mass:**  3491.4691

(1.539e-06)

**Charge States:** 3, 4

**Start Position**: 98

**End Position**: 110

| Profile Score | Tandem Score | TDA q-value | Volume | Spectrum Matches |
| --- | --- | --- | --- | --- |
| -6.6447 | 145.0498 | 0.0000 | 1.6896e+08 | 6 |

**Adduct Signal Fractions**

- **Unmodified**: 1.6896e+08

### RNEEYN(N)KSVQEIQATFF Hex 5 HexNAc 4

**Mass:**  3724.5942

(1.525e-06)

**Charge States:** 3, 4

**Start Position**: 51

**End Position**: 68

| Profile Score | Tandem Score | TDA q-value | Volume | Spectrum Matches |
| --- | --- | --- | --- | --- |
| -19.3085 | 144.3757 | 0.0000 | 1.3185e+07 | 2 |

**Adduct Signal Fractions**

- **Unmodified**: 1.3185e+07

### RNEEYN(N)KSVQEIQATFF Fuc 2 Hex 4 HexNAc 5

**Mass:**  4057.7360

(1.735e-06)

**Charge States:** 3, 4

**Start Position**: 51

**End Position**: 68

| Profile Score | Tandem Score | TDA q-value | Volume | Spectrum Matches |
| --- | --- | --- | --- | --- |
| -18.9484 | 144.0681 | 0.0000 | 1.8099e+07 | 2 |

**Adduct Signal Fractions**

- **Unmodified**: 1.8099e+07

### RNEEYN(N)KSVQEIQATFF Hex 4 HexNAc 5

**Mass:**  3765.6210

(1.976e-06)

**Charge States:** 3, 4

**Start Position**: 51

**End Position**: 68

| Profile Score | Tandem Score | TDA q-value | Volume | Spectrum Matches |
| --- | --- | --- | --- | --- |
| -5.1455 | 143.9905 | 0.0000 | 2.4222e+07 | 3 |

**Adduct Signal Fractions**

- **Unmodified**: 2.4222e+07

### NVQREN(N)GTVSRY Hex 3 HexNAc 5

**Mass:**  2923.2599

(7.420e-07)

**Charge States:** 3

**Start Position**: 98

**End Position**: 110

| Profile Score | Tandem Score | TDA q-value | Volume | Spectrum Matches |
| --- | --- | --- | --- | --- |
| -3.9266 | 143.7711 | 0.0000 | 2.3013e+08 | 3 |

**Adduct Signal Fractions**

- **Unmodified**: 2.3013e+08

### RNEEYN(N)KSVQEIQATFF Fuc 1 Hex 3 HexNAc 5

**Mass:**  3749.6215

(3.872e-07)

**Charge States:** 4

**Start Position**: 51

**End Position**: 68

| Profile Score | Tandem Score | TDA q-value | Volume | Spectrum Matches |
| --- | --- | --- | --- | --- |
| -4.5353 | 143.6027 | 0.0000 | 3.8905e+07 | 3 |

**Adduct Signal Fractions**

- **Unmodified**: 3.8905e+07

### RNEEYN(N)KSVQEIQATF Hex 4 HexNAc 7

**Mass:**  4024.7123

(1.545e-06)

**Charge States:** 3, 4

**Start Position**: 51

**End Position**: 67

| Profile Score | Tandem Score | TDA q-value | Volume | Spectrum Matches |
| --- | --- | --- | --- | --- |
| -3.9612 | 143.5938 | 0.0000 | 8.6952e+07 | 3 |

**Adduct Signal Fractions**

- **Unmodified**: 8.6952e+07

### TPN(N)KTEDTIF Hex 4 HexNAc 4 Neu5Ac 2

**Mass:**  3207.2898

(1.982e-06)

**Charge States:** 3, 4

**Start Position**: 70

**End Position**: 80

| Profile Score | Tandem Score | TDA q-value | Volume | Spectrum Matches |
| --- | --- | --- | --- | --- |
| 14.3360 | 142.8214 | 0.0000 | 1.6260e+07 | 5 |

**Adduct Signal Fractions**

- **Unmodified**: 1.6260e+07

### RNEEYN(N)KSVQEIQATF Fuc 1 Hex 5 HexNAc 7

**Mass:**  4332.8210

(1.212e-06)

**Charge States:** 4

**Start Position**: 51

**End Position**: 67

| Profile Score | Tandem Score | TDA q-value | Volume | Spectrum Matches |
| --- | --- | --- | --- | --- |
| -5.1597 | 142.7871 | 0.0000 | 1.7537e+07 | 2 |

**Adduct Signal Fractions**

- **Unmodified**: 1.7537e+07

### YFTPN(N)KTEDTIF Fuc 1 Hex 5 HexNAc 4 Neu5Ac 2

**Mass:**  3825.5322

(5.994e-07)

**Charge States:** 3, 4

**Start Position**: 68

**End Position**: 80

| Profile Score | Tandem Score | TDA q-value | Volume | Spectrum Matches |
| --- | --- | --- | --- | --- |
| -5.5711 | 142.2546 | 0.0000 | 4.1549e+07 | 2 |

**Adduct Signal Fractions**

- **Unmodified**: 4.1549e+07

### N(N)KSVQEIQATF Fuc 1 Hex 4 HexNAc 5

**Mass:**  3073.3163

(1.360e-06)

**Charge States:** 3, 4

**Start Position**: 56

**End Position**: 67

| Profile Score | Tandem Score | TDA q-value | Volume | Spectrum Matches |
| --- | --- | --- | --- | --- |
| -18.3319 | 141.5659 | 0.0000 | 8.5355e+06 | 1 |

**Adduct Signal Fractions**

- **Unmodified**: 8.5355e+06

### RNEEYN(N)KSVQEIQAT Hex 3 HexNAc 4

**Mass:**  3106.3478

(1.427e-06)

**Charge States:** 3

**Start Position**: 51

**End Position**: 66

| Profile Score | Tandem Score | TDA q-value | Volume | Spectrum Matches |
| --- | --- | --- | --- | --- |
| -3.7146 | 140.7289 | 0.0000 | 4.1084e+07 | 3 |

**Adduct Signal Fractions**

- **Unmodified**: 4.1084e+07

### YFTPN(N)KTEDTIFL Hex 3 HexNAc 6

**Mass:**  3292.4207

(1.320e-06)

**Charge States:** 3, 4

**Start Position**: 68

**End Position**: 81

| Profile Score | Tandem Score | TDA q-value | Volume | Spectrum Matches |
| --- | --- | --- | --- | --- |
| 14.6668 | 140.5652 | 0.0000 | 2.8760e+07 | 5 |

**Adduct Signal Fractions**

- **Unmodified**: 2.8760e+07

### RNEEYN(N)KSVQEIQATFF Fuc 1 Hex 4 HexNAc 6

**Mass:**  4114.7570

(1.101e-06)

**Charge States:** 3, 4

**Start Position**: 51

**End Position**: 68

| Profile Score | Tandem Score | TDA q-value | Volume | Spectrum Matches |
| --- | --- | --- | --- | --- |
| 13.6002 | 139.7538 | 0.0000 | 5.4075e+07 | 3 |

**Adduct Signal Fractions**

- **Unmodified**: 5.4075e+07

### LNVQREN(N)GTVSRY Hex 3 HexNAc 5

**Mass:**  3037.3489

(3.321e-04)

**Start Position**: 97

**End Position**: 110

| Profile Score | Tandem Score | TDA q-value | Volume | Spectrum Matches |
| --- | --- | --- | --- | --- |
| 0.0000 | 138.9832 | 0.0000 | 0.0000e+00 | 1 |

Adduct:

No Chromatogram Found

### NVQREN(N)GTVSRY Fuc 2 Hex 5 HexNAc 6

**Mass:**  3742.5627

(1.399e-06)

**Charge States:** 3

**Start Position**: 98

**End Position**: 110

| Profile Score | Tandem Score | TDA q-value | Volume | Spectrum Matches |
| --- | --- | --- | --- | --- |
| -4.4511 | 137.6257 | 0.0000 | 1.9062e+08 | 3 |

**Adduct Signal Fractions**

- **Unmodified**: 1.9062e+08

### RN(D)EEYN(N)KSVQEIQATF Hex 3 HexNAc 5

**Mass:**  3457.4815

(2.857e-04)

**Charge States:** 3, 4

**Start Position**: 51

**End Position**: 67

| Profile Score | Tandem Score | TDA q-value | Volume | Spectrum Matches |
| --- | --- | --- | --- | --- |
| 11.5130 | 136.4674 | 0.0000 | 5.1442e+08 | 12 |

**Adduct Signal Fractions**

- **Unmodified**: 5.1442e+08

### TPN(N)KTEDTIF Fuc 3 Hex 3 HexNAc 4 Neu5Ac 1

**Mass:**  3192.3045

(-2.183e-06)

**Charge States:** 3

**Start Position**: 70

**End Position**: 80

| Profile Score | Tandem Score | TDA q-value | Volume | Spectrum Matches |
| --- | --- | --- | --- | --- |
| -19.4068 | 136.3787 | 0.0000 | 3.1639e+06 | 1 |

**Adduct Signal Fractions**

- **Unmodified**: 3.1639e+06

### NVQREN(N)GTVSRY Fuc 1 Hex 4 HexNAc 6

**Mass:**  3434.4490

(1.463e-06)

**Charge States:** 3, 4

**Start Position**: 98

**End Position**: 110

| Profile Score | Tandem Score | TDA q-value | Volume | Spectrum Matches |
| --- | --- | --- | --- | --- |
| 12.0218 | 135.9705 | 0.0000 | 6.2482e+08 | 7 |

**Adduct Signal Fractions**

- **Unmodified**: 6.2482e+08

### RNEEYN(N)KSVQEIQAT Hex 3 HexNAc 5

**Mass:**  3309.4279

(7.137e-07)

**Charge States:** 3, 4

**Start Position**: 51

**End Position**: 66

| Profile Score | Tandem Score | TDA q-value | Volume | Spectrum Matches |
| --- | --- | --- | --- | --- |
| -4.6859 | 135.8166 | 0.0000 | 3.2591e+07 | 3 |

**Adduct Signal Fractions**

- **Unmodified**: 3.2591e+07

### TN(N)ATLDRITGKW Hex 3 HexNAc 5

**Mass:**  2876.2831

(7.456e-07)

**Charge States:** 3

**Start Position**: 32

**End Position**: 44

| Profile Score | Tandem Score | TDA q-value | Volume | Spectrum Matches |
| --- | --- | --- | --- | --- |
| -3.7694 | 135.2116 | 0.0000 | 7.4528e+06 | 1 |

**Adduct Signal Fractions**

- **Unmodified**: 7.4528e+06

### N(N)KSVQEIQATF Hex 3 HexNAc 4

**Mass:**  2562.1239

(8.354e-07)

**Charge States:** 3

**Start Position**: 56

**End Position**: 67

| Profile Score | Tandem Score | TDA q-value | Volume | Spectrum Matches |
| --- | --- | --- | --- | --- |
| -3.3281 | 132.4650 | 0.0000 | 2.0401e+07 | 2 |

**Adduct Signal Fractions**

- **Unmodified**: 2.0401e+07

### LNVQREN(N)GTVSRY Fuc 1 Hex 4 HexNAc 6

**Mass:**  3547.5331

(8.735e-07)

**Charge States:** 3, 4

**Start Position**: 97

**End Position**: 110

| Profile Score | Tandem Score | TDA q-value | Volume | Spectrum Matches |
| --- | --- | --- | --- | --- |
| 13.4050 | 132.0286 | 0.0027 | 4.2894e+08 | 7 |

**Adduct Signal Fractions**

- **Unmodified**: 4.2894e+08

### RNEEYN(N)KSVQEIQATFF Fuc 1 Hex 3 HexNAc 4

**Mass:**  3546.5414

(1.182e-06)

**Charge States:** 3, 4

**Start Position**: 51

**End Position**: 68

| Profile Score | Tandem Score | TDA q-value | Volume | Spectrum Matches |
| --- | --- | --- | --- | --- |
| -5.1299 | 130.9766 | 0.0027 | 2.6778e+07 | 3 |

**Adduct Signal Fractions**

- **Unmodified**: 2.6778e+07

### YFTPN(N)KTEDTIF Fuc 2 Hex 5 HexNAc 7

**Mass:**  3998.6406

(1.727e-06)

**Charge States:** 3, 4

**Start Position**: 68

**End Position**: 80

| Profile Score | Tandem Score | TDA q-value | Volume | Spectrum Matches |
| --- | --- | --- | --- | --- |
| 12.4652 | 130.6718 | 0.0027 | 2.5514e+07 | 2 |

**Adduct Signal Fractions**

- **Unmodified**: 2.5514e+07

### TPN(N)KTEDTIF Hex 4 HexNAc 5

**Mass:**  2828.1785

(1.485e-06)

**Charge States:** 3

**Start Position**: 70

**End Position**: 80

| Profile Score | Tandem Score | TDA q-value | Volume | Spectrum Matches |
| --- | --- | --- | --- | --- |
| -17.8264 | 130.0375 | 0.0027 | 2.3701e+06 | 1 |

**Adduct Signal Fractions**

- **Unmodified**: 2.3701e+06

### RNEEYN(N)KSVQEIQATF Fuc 2 Hex 5 HexNAc 5

**Mass:**  4072.7211

(1.594e-06)

**Charge States:** 3, 4

**Start Position**: 51

**End Position**: 67

| Profile Score | Tandem Score | TDA q-value | Volume | Spectrum Matches |
| --- | --- | --- | --- | --- |
| -3.3237 | 129.5564 | 0.0052 | 1.0326e+08 | 3 |

**Adduct Signal Fractions**

- **Unmodified**: 1.0326e+08

### FTPN(N)KTEDTIF Fuc 2 Hex 5 HexNAc 6

**Mass:**  3632.4944

(1.023e-06)

**Charge States:** 3, 4

**Start Position**: 69

**End Position**: 80

| Profile Score | Tandem Score | TDA q-value | Volume | Spectrum Matches |
| --- | --- | --- | --- | --- |
| 12.4587 | 129.5111 | 0.0052 | 1.6687e+07 | 4 |

**Adduct Signal Fractions**

- **Unmodified**: 1.6687e+07

### RNEEYN(N)KSVQEIQATFF Hex 6 HexNAc 5

**Mass:**  4089.7260

(1.317e-06)

**Charge States:** 3, 4

**Start Position**: 51

**End Position**: 68

| Profile Score | Tandem Score | TDA q-value | Volume | Spectrum Matches |
| --- | --- | --- | --- | --- |
| -18.1236 | 128.8004 | 0.0071 | 9.0926e+06 | 2 |

**Adduct Signal Fractions**

- **Unmodified**: 9.0926e+06

### TPN(N)KTEDTIF Fuc 4 Hex 5 HexNAc 5

**Mass:**  3574.4430

(-4.815e-06)

**Charge States:** 3, 4

**Start Position**: 70

**End Position**: 80

| Profile Score | Tandem Score | TDA q-value | Volume | Spectrum Matches |
| --- | --- | --- | --- | --- |
| 12.7097 | 128.3550 | 0.0071 | 5.0377e+07 | 4 |

**Adduct Signal Fractions**

- **Unmodified**: 5.0377e+07

### NVQREN(N)GTVSRY Fuc 1 Hex 4 HexNAc 5

**Mass:**  3231.3700

(9.196e-07)

**Charge States:** 3

**Start Position**: 98

**End Position**: 110

| Profile Score | Tandem Score | TDA q-value | Volume | Spectrum Matches |
| --- | --- | --- | --- | --- |
| -4.2672 | 127.9501 | 0.0071 | 1.4317e+08 | 1 |

**Adduct Signal Fractions**

- **Unmodified**: 1.4317e+08

### FYFTPN(N)KTEDTIF Fuc 1 Hex 5 HexNAc 4 Neu5Ac 1

**Mass:**  3681.5150

(3.724e-06)

**Charge States:** 3

**Start Position**: 67

**End Position**: 80

| Profile Score | Tandem Score | TDA q-value | Volume | Spectrum Matches |
| --- | --- | --- | --- | --- |
| -4.8619 | 127.7292 | 0.0071 | 9.4770e+06 | 1 |

**Adduct Signal Fractions**

- **Unmodified**: 9.4770e+06

### RNEEYN(N)KSVQEIQATF Fuc 2 Hex 5 HexNAc 6

**Mass:**  4275.8034

(5.389e-07)

**Charge States:** 3, 4

**Start Position**: 51

**End Position**: 67

| Profile Score | Tandem Score | TDA q-value | Volume | Spectrum Matches |
| --- | --- | --- | --- | --- |
| 10.4735 | 127.4842 | 0.0071 | 2.9790e+07 | 1 |

**Adduct Signal Fractions**

- **Unmodified**: 2.9790e+07

### N(N)KSVQEIQATFF Hex 3 HexNAc 4

**Mass:**  2709.1944

(1.870e-06)

**Charge States:** 3

**Start Position**: 56

**End Position**: 68

| Profile Score | Tandem Score | TDA q-value | Volume | Spectrum Matches |
| --- | --- | --- | --- | --- |
| 11.6412 | 125.6842 | 0.0071 | 4.7520e+06 | 2 |

**Adduct Signal Fractions**

- **Unmodified**: 4.7520e+06

### FYFTPN(N)KTEDTIF Fuc 1 Hex 4 HexNAc 7

**Mass:**  3837.5947

(8.754e-07)

**Charge States:** 3, 4

**Start Position**: 67

**End Position**: 80

| Profile Score | Tandem Score | TDA q-value | Volume | Spectrum Matches |
| --- | --- | --- | --- | --- |
| 13.2052 | 125.4935 | 0.0071 | 2.9094e+07 | 4 |

**Adduct Signal Fractions**

- **Unmodified**: 2.9094e+07

### FYFTPN(N)KT Hex 3 HexNAc 6

**Mass:**  2721.1358

(1.697e-06)

**Charge States:** 3

**Start Position**: 67

**End Position**: 75

| Profile Score | Tandem Score | TDA q-value | Volume | Spectrum Matches |
| --- | --- | --- | --- | --- |
| -2.6225 | 125.4881 | 0.0000 | 1.2710e+07 | 2 |

**Adduct Signal Fractions**

- **Unmodified**: 1.2710e+07

### RN(D)EEYN(N)KSVQEIQATFF Hex 3 HexNAc 5

**Mass:**  3604.5517

(2.747e-04)

**Charge States:** 3, 4

**Start Position**: 51

**End Position**: 68

| Profile Score | Tandem Score | TDA q-value | Volume | Spectrum Matches |
| --- | --- | --- | --- | --- |
| -5.9443 | 124.9799 | 0.0093 | 4.3055e+07 | 4 |

**Adduct Signal Fractions**

- **Unmodified**: 4.3055e+07

### FTPN(N)KTEDTIF Hex 4 HexNAc 4 Neu5Ac 2

**Mass:**  3354.3590

(1.258e-06)

**Charge States:** 3, 4

**Start Position**: 69

**End Position**: 80

| Profile Score | Tandem Score | TDA q-value | Volume | Spectrum Matches |
| --- | --- | --- | --- | --- |
| -3.8931 | 124.7760 | 0.0093 | 2.5000e+07 | 4 |

**Adduct Signal Fractions**

- **Unmodified**: 2.5000e+07

### LNVQREN(N)GTVSRY Fuc 2 Hex 5 HexNAc 6

**Mass:**  3855.6455

(5.641e-07)

**Charge States:** 3, 4

**Start Position**: 97

**End Position**: 110

| Profile Score | Tandem Score | TDA q-value | Volume | Spectrum Matches |
| --- | --- | --- | --- | --- |
| -4.2053 | 124.4364 | 0.0093 | 1.2638e+08 | 3 |

**Adduct Signal Fractions**

- **Unmodified**: 1.2638e+08

### LNVQREN(N)GTVSRY Fuc 1 Hex 4 HexNAc 5

**Mass:**  3344.4516

(2.697e-08)

**Charge States:** 3, 4

**Start Position**: 97

**End Position**: 110

| Profile Score | Tandem Score | TDA q-value | Volume | Spectrum Matches |
| --- | --- | --- | --- | --- |
| 10.8902 | 123.7468 | 0.0111 | 9.0051e+07 | 3 |

**Adduct Signal Fractions**

- **Unmodified**: 9.0051e+07

### YFTPN(N)KTEDTIF Fuc 1 Hex 6 HexNAc 8

**Mass:**  4217.7141

(3.865e-07)

**Charge States:** 4

**Start Position**: 68

**End Position**: 80

| Profile Score | Tandem Score | TDA q-value | Volume | Spectrum Matches |
| --- | --- | --- | --- | --- |
| 10.0712 | 123.6036 | 0.0111 | 1.8835e+07 | 2 |

**Adduct Signal Fractions**

- **Unmodified**: 1.8835e+07

### N(N)KSVQEIQATFF Hex 3 HexNAc 5

**Mass:**  2912.2734

(1.518e-06)

**Charge States:** 3

**Start Position**: 56

**End Position**: 68

| Profile Score | Tandem Score | TDA q-value | Volume | Spectrum Matches |
| --- | --- | --- | --- | --- |
| -4.0551 | 122.1026 | 0.0124 | 5.6445e+06 | 2 |

**Adduct Signal Fractions**

- **Unmodified**: 5.6445e+06

### RNEEYN(N)KSVQEIQATF Hex 7 HexNAc 2

**Mass:**  3495.4277

(-9.757e-06)

**Charge States:** 3, 4

**Start Position**: 51

**End Position**: 67

| Profile Score | Tandem Score | TDA q-value | Volume | Spectrum Matches |
| --- | --- | --- | --- | --- |
| 10.6074 | 122.0360 | 0.0124 | 3.7877e+07 | 5 |

**Adduct Signal Fractions**

- **Unmodified**: 3.7877e+07

### RNEEYN(N)KSVQEIQATFF Hex 4 HexNAc 7

**Mass:**  4171.7814

(1.483e-06)

**Charge States:** 3, 4

**Start Position**: 51

**End Position**: 68

| Profile Score | Tandem Score | TDA q-value | Volume | Spectrum Matches |
| --- | --- | --- | --- | --- |
| 13.1210 | 120.6175 | 0.0124 | 3.0980e+07 | 4 |

**Adduct Signal Fractions**

- **Unmodified**: 3.0980e+07

### RNEEYN(N)KSVQEIQATFF Fuc 2 Hex 5 HexNAc 5

**Mass:**  4219.7912

(1.345e-06)

**Charge States:** 3, 4

**Start Position**: 51

**End Position**: 68

| Profile Score | Tandem Score | TDA q-value | Volume | Spectrum Matches |
| --- | --- | --- | --- | --- |
| 13.6728 | 120.5209 | 0.0124 | 3.5407e+07 | 4 |

**Adduct Signal Fractions**

- **Unmodified**: 3.5407e+07

### LNVQREN(N)GTVSRY Hex 3 HexNAc 5

**Mass:**  3036.3420

(1.662e-06)

**Charge States:** 3

**Start Position**: 97

**End Position**: 110

| Profile Score | Tandem Score | TDA q-value | Volume | Spectrum Matches |
| --- | --- | --- | --- | --- |
| 13.3716 | 119.7884 | 0.0124 | 1.3369e+08 | 3 |

**Adduct Signal Fractions**

- **Unmodified**: 1.3369e+08

### FYFTPN(N)KT Hex 3 HexNAc 5

**Mass:**  2518.0563

(1.175e-06)

**Charge States:** 3

**Start Position**: 67

**End Position**: 75

| Profile Score | Tandem Score | TDA q-value | Volume | Spectrum Matches |
| --- | --- | --- | --- | --- |
| -18.3507 | 118.5336 | 0.0000 | 4.4906e+06 | 1 |

**Adduct Signal Fractions**

- **Unmodified**: 4.4906e+06

### RN(D)EEYN(N)KSVQEIQATF Fuc 1 Hex 4 HexNAc 5

**Mass:**  3765.5944

(2.630e-04)

**Charge States:** 3, 4

**Start Position**: 51

**End Position**: 67

| Profile Score | Tandem Score | TDA q-value | Volume | Spectrum Matches |
| --- | --- | --- | --- | --- |
| 12.3379 | 118.3704 | 0.0140 | 3.4137e+08 | 13 |

**Adduct Signal Fractions**

- **Unmodified**: 3.4137e+08

### RNEEYN(N)KSVQEIQAT Fuc 1 Hex 4 HexNAc 5

**Mass:**  3617.5415

(1.456e-06)

**Charge States:** 3

**Start Position**: 51

**End Position**: 66

| Profile Score | Tandem Score | TDA q-value | Volume | Spectrum Matches |
| --- | --- | --- | --- | --- |
| -18.8770 | 115.3248 | 0.0157 | 1.5498e+07 | 2 |

**Adduct Signal Fractions**

- **Unmodified**: 1.5498e+07

### N(N)KSVQEIQATFF Fuc 1 Hex 4 HexNAc 5

**Mass:**  3220.3822

(5.659e-07)

**Charge States:** 3

**Start Position**: 56

**End Position**: 68

| Profile Score | Tandem Score | TDA q-value | Volume | Spectrum Matches |
| --- | --- | --- | --- | --- |
| -5.1051 | 114.7810 | 0.0174 | 2.8029e+06 | 2 |

**Adduct Signal Fractions**

- **Unmodified**: 2.8029e+06

### FTPN(N)KTEDTIF Hex 5 HexNAc 8

**Mass:**  3746.5393

(8.960e-07)

**Charge States:** 3, 4

**Start Position**: 69

**End Position**: 80

| Profile Score | Tandem Score | TDA q-value | Volume | Spectrum Matches |
| --- | --- | --- | --- | --- |
| 9.8128 | 114.6980 | 0.0174 | 8.4135e+06 | 3 |

**Adduct Signal Fractions**

- **Unmodified**: 8.4135e+06

### RN(D)EEYN(N)KSVQEIQATFF Hex 3 HexNAc 4

**Mass:**  3401.4680

(2.905e-04)

**Charge States:** 3, 4

**Start Position**: 51

**End Position**: 68

| Profile Score | Tandem Score | TDA q-value | Volume | Spectrum Matches |
| --- | --- | --- | --- | --- |
| -5.9781 | 114.2285 | 0.0174 | 6.0285e+07 | 5 |

**Adduct Signal Fractions**

- **Unmodified**: 6.0285e+07

### N(N)KTEDTIF Hex 3 HexNAc 6

**Mass:**  2671.1012

(2.155e-07)

**Charge States:** 3

**Start Position**: 72

**End Position**: 80

| Profile Score | Tandem Score | TDA q-value | Volume | Spectrum Matches |
| --- | --- | --- | --- | --- |
| -19.9027 | 114.1219 | 0.0000 | 3.8911e+06 | 1 |

**Adduct Signal Fractions**

- **Unmodified**: 3.8911e+06

### TPN(N)KTEDTIF Hex 7 HexNAc 5

**Mass:**  3314.3417

(1.947e-06)

**Charge States:** 3, 4

**Start Position**: 70

**End Position**: 80

| Profile Score | Tandem Score | TDA q-value | Volume | Spectrum Matches |
| --- | --- | --- | --- | --- |
| 12.5170 | 113.3862 | 0.0187 | 2.8778e+07 | 3 |

**Adduct Signal Fractions**

- **Unmodified**: 2.8778e+07

### NVQREN(N)GTVSRY Hex 4 HexNAc 8

**Mass:**  3694.5537

(1.532e-06)

**Charge States:** 3, 4

**Start Position**: 98

**End Position**: 110

| Profile Score | Tandem Score | TDA q-value | Volume | Spectrum Matches |
| --- | --- | --- | --- | --- |
| -19.5494 | 113.3846 | 0.0187 | 9.6823e+07 | 4 |

**Adduct Signal Fractions**

- **Unmodified**: 9.6823e+07

### NVQREN(N)GTVSRY Hex 4 HexNAc 4 Neu5Ac 2

**Mass:**  3464.4208

(1.587e-08)

**Charge States:** 3

**Start Position**: 98

**End Position**: 110

| Profile Score | Tandem Score | TDA q-value | Volume | Spectrum Matches |
| --- | --- | --- | --- | --- |
| -20.4710 | 111.4512 | 0.0220 | 2.5938e+07 | 2 |

**Adduct Signal Fractions**

- **Unmodified**: 2.5938e+07

### LNVQREN(N)GTVSRY Hex 4 HexNAc 8

**Mass:**  3807.6376

(1.534e-06)

**Charge States:** 3, 4

**Start Position**: 97

**End Position**: 110

| Profile Score | Tandem Score | TDA q-value | Volume | Spectrum Matches |
| --- | --- | --- | --- | --- |
| -4.5999 | 109.2472 | 0.0266 | 6.7616e+07 | 2 |

**Adduct Signal Fractions**

- **Unmodified**: 6.7616e+07

### NVQREN(N)GTVSRY Fuc 1 Hex 5 HexNAc 7

**Mass:**  3799.5891

(7.594e-07)

**Charge States:** 3, 4

**Start Position**: 98

**End Position**: 110

| Profile Score | Tandem Score | TDA q-value | Volume | Spectrum Matches |
| --- | --- | --- | --- | --- |
| -18.8799 | 109.1474 | 0.0266 | 3.3075e+07 | 2 |

**Adduct Signal Fractions**

- **Unmodified**: 3.3075e+07

### LNVQREN(N)GTVSRY Hex 4 HexNAc 7

**Mass:**  3604.5578

(1.320e-07)

**Charge States:** 3, 4

**Start Position**: 97

**End Position**: 110

| Profile Score | Tandem Score | TDA q-value | Volume | Spectrum Matches |
| --- | --- | --- | --- | --- |
| -18.6246 | 109.0541 | 0.0266 | 6.6266e+07 | 3 |

**Adduct Signal Fractions**

- **Unmodified**: 6.6266e+07

### NVQREN(N)GTVSRY Fuc 3 Hex 6 HexNAc 6

**Mass:**  4050.6750

(9.296e-07)

**Charge States:** 4

**Start Position**: 98

**End Position**: 110

| Profile Score | Tandem Score | TDA q-value | Volume | Spectrum Matches |
| --- | --- | --- | --- | --- |
| -20.1454 | 108.4588 | 0.0302 | 3.3656e+07 | 2 |

**Adduct Signal Fractions**

- **Unmodified**: 3.3656e+07

### VQREN(N)GTVSRY Hex 3 HexNAc 6

**Mass:**  3012.2982

(1.763e-06)

**Charge States:** 3

**Start Position**: 99

**End Position**: 110

| Profile Score | Tandem Score | TDA q-value | Volume | Spectrum Matches |
| --- | --- | --- | --- | --- |
| -18.7792 | 107.1631 | 0.0350 | 1.7198e+07 | 2 |

**Adduct Signal Fractions**

- **Unmodified**: 1.7198e+07

### NVQREN(N)GTVSRY Hex 5 HexNAc 9

**Mass:**  4059.6879

(2.063e-06)

**Charge States:** 4

**Start Position**: 98

**End Position**: 110

| Profile Score | Tandem Score | TDA q-value | Volume | Spectrum Matches |
| --- | --- | --- | --- | --- |
| -20.1623 | 105.9618 | 0.0352 | 1.1453e+07 | 1 |

**Adduct Signal Fractions**

- **Unmodified**: 1.1453e+07

### NVQREN(N)GTVSRY Fuc 2 Hex 6 HexNAc 7

**Mass:**  4108.7161

(2.499e-04)

**Start Position**: 98

**End Position**: 110

| Profile Score | Tandem Score | TDA q-value | Volume | Spectrum Matches |
| --- | --- | --- | --- | --- |
| 0.0000 | 105.6414 | 0.0352 | 0.0000e+00 | 1 |

Adduct:

No Chromatogram Found

### FYFTPN(N)KTEDTIF Fuc 2 Hex 5 HexNAc 7

**Mass:**  4145.7067

(1.716e-06)

**Charge States:** 3, 4

**Start Position**: 67

**End Position**: 80

| Profile Score | Tandem Score | TDA q-value | Volume | Spectrum Matches |
| --- | --- | --- | --- | --- |
| 12.1326 | 105.3056 | 0.0352 | 2.2466e+07 | 4 |

**Adduct Signal Fractions**

- **Unmodified**: 2.2466e+07

### LNVQREN(N)GTVSRY Fuc 3 Hex 6 HexNAc 6

**Mass:**  4163.7551

(6.962e-07)

**Charge States:** 3, 4

**Start Position**: 97

**End Position**: 110

| Profile Score | Tandem Score | TDA q-value | Volume | Spectrum Matches |
| --- | --- | --- | --- | --- |
| -20.9395 | 104.0857 | 0.0416 | 2.0963e+07 | 2 |

**Adduct Signal Fractions**

- **Unmodified**: 2.0963e+07

### C(C)ANLVPVPITN(N)ATL Hex 3 HexNAc 5

**Mass:**  2983.3509

(1.450e-06)

**Charge States:** 3, 4

**Start Position**: 23

**End Position**: 37

| Profile Score | Tandem Score | TDA q-value | Volume | Spectrum Matches |
| --- | --- | --- | --- | --- |
| 14.4529 | 101.5163 | 0.0470 | 3.5452e+07 | 7 |

**Adduct Signal Fractions**

- **Unmodified**: 3.5452e+07

### P00738|HPT\_HUMAN

**>P00738|HPT\_HUMAN**  
 `MSALGAVIALLLWGQLFAVDSGNDVTDIADDGCPKPPEIAHGYVEHSVRYQCKNYYKLRTEGDGVYTLNDKKQWINKAVG  
DKLPECEADDGCPKPPEIAHGYVEHSVRYQCKNYYKLRTEGDGVYTLNNEKQWINKAVGDKLPECEAVCGKPKNPANPVQ  
RILGGHLDAKGSFPWQAKMVSHHNLTTGATLINEQWLLTTAKNLFLNHSENATAKDIAPTLTLYVGKKQLVEIEKVVLHP  
NYSQVDIGLIKLKQKVSVNERVMPICLPSKDYAEVGRVGYVSGWGRNANFKFTDHLKYVMLPVADQDQCIRHYEGSTVPE  
KKTPKSPVGVQPILNEHTFCAGMSKYQEDTCYGDAGSAFAVHDLEEDTWYATGILSFDKSCAVAEYGVYVKVTSIQDWVQ  
KTIAEN`

| Site | Type | Count |
| --- | --- | --- |
| 184 | N-linked | 2 |

| Mass | Sequence | MS_2_ Score | `q`-value | MS_1_ Score | Signal | Protein Coordinates | Tandem Scans | Start Time | Apex Time | End Time |
| --- | --- | --- | --- | --- | --- | --- | --- | --- | --- | --- |
| 2751.1992 | VSHHN(N)LTTGATL Hex 3 HexNAc 5 | 165.620 | 0.0 | -18.651 | 1.283e+07 | 179 - 191 | 1 | 17.37 | 17.37 | 17.54 |
| 2548.1203 | VSHHN(N)LTTGATL Hex 3 HexNAc 4 | 164.892 | 0.0 | 10.750 | 3.559e+07 | 179 - 191 | 2 | 17.37 | 17.54 | 18.05 |

### VSHHN(N)LTTGATL Hex 3 HexNAc 5

**Mass:**  2751.1984

(6.058e-07)

**Charge States:** 3

**Start Position**: 180

**End Position**: 192

| Profile Score | Tandem Score | TDA q-value | Volume | Spectrum Matches |
| --- | --- | --- | --- | --- |
| -18.6508 | 165.6200 | 0.0000 | 1.2835e+07 | 1 |

**Adduct Signal Fractions**

- **Unmodified**: 1.2835e+07

### VSHHN(N)LTTGATL Hex 3 HexNAc 4

**Mass:**  2548.1207

(3.521e-07)

**Charge States:** 3

**Start Position**: 180

**End Position**: 192

| Profile Score | Tandem Score | TDA q-value | Volume | Spectrum Matches |
| --- | --- | --- | --- | --- |
| 10.7500 | 164.8918 | 0.0000 | 3.5586e+07 | 2 |

**Adduct Signal Fractions**

- **Unmodified**: 3.5586e+07

### P13473|LAMP2\_HUMAN

**>P13473|LAMP2\_HUMAN**  
 `MVCFRLFPVPGSGLVLVCLVLGAVRSYALELNLTDSENATCLYAKWQMNFTVRYETTNKTYKTVTISDHGTVTYNGSICG  
DDQNGPKIAVQFGPGFSWIANFTKAASTYSIDSVSFSYNTGDNTTFPDAEDKGILTVDELLAIRIPLNDLFRCNSLSTLE  
KNDVVQHYWDVLVQAFVQNGTVSTNEFLCDKDKTSTVAPTIHTTVPSPTTTPTPKEKPEAGTYSVNNGNDTCLLATMGLQ  
LNITQDKVASVININPNTTHSTGSCRSHTALLRLNSSTIKYLDFVFAVKNENRFYLKEVNISMYLVNGSVFSIANNNLSY  
WDAPLGSSYMCNKEQTVSVSGAFQINTFDLRVQPFNVTQGKYSTAQDCSADDDNFLVPIAVGAALAGVLILVLLAYFIGL  
KHHHAGYEQF`

| Site | Type | Count |
| --- | --- | --- |
| 179 | N-linked | 1 |
| 307 | N-linked | 1 |

| Mass | Sequence | MS_2_ Score | `q`-value | MS_1_ Score | Signal | Protein Coordinates | Tandem Scans | Start Time | Apex Time | End Time |
| --- | --- | --- | --- | --- | --- | --- | --- | --- | --- | --- |
| 2235.9523 | LVN(N)GSVF Hex 3 HexNAc 5 | 106.679 | 0.0 | -4.350 | 5.060e+06 | 304 - 311 | 3 | 30.45 | 30.62 | 30.78 |
| 2696.1127 | VQN(N)GTVSTNEF Hex 3 HexNAc 5 | 103.794 | 0.043 | -20.326 | 2.451e+06 | 176 - 187 | 1 | 23.16 | 23.16 | 23.33 |

### LVN(N)GSVF Hex 3 HexNAc 5

**Mass:**  2235.9538

(-1.322e-07)

**Charge States:** 3

**Start Position**: 305

**End Position**: 312

| Profile Score | Tandem Score | TDA q-value | Volume | Spectrum Matches |
| --- | --- | --- | --- | --- |
| -4.3501 | 106.6792 | 0.0000 | 5.0596e+06 | 3 |

**Adduct Signal Fractions**

- **Unmodified**: 5.0596e+06

### VQN(N)GTVSTNEF Hex 3 HexNAc 5

**Mass:**  2696.1115

(1.649e-06)

**Charge States:** 3

**Start Position**: 177

**End Position**: 188

| Profile Score | Tandem Score | TDA q-value | Volume | Spectrum Matches |
| --- | --- | --- | --- | --- |
| -20.3261 | 103.7943 | 0.0430 | 2.4512e+06 | 1 |

**Adduct Signal Fractions**

- **Unmodified**: 2.4512e+06
