# Supplementary material for: Measuring Site-specific Glycosylation Similarity between Influenza a Virus Variants with Statistical Certainty
Source: Mol Cell Proteomics. 2020 Nov 25;19(9):1533–45. doi: 10.1074/mcp.RA120.002031 (PMC8143645; doi:10.1074/mcp.RA120.002031)
Supplement: Supplementary file 1 [file mmc1.zip › mmc1/159250_2_supp_555286_qcjklc.zip!/20190326_aAGPgal_021119_chymo_HILIC_DDA_1ug_03_glycopeptides.html]

## 20190326\_aAGPgal\_021119\_chymo\_HILIC\_DDA\_1ug\_03 @ GlycopeptideHypothesis-2c3e584c13e14270ad4dc26024936e9b

**MS^1^ Mass Accuracy:** 10.0 PPM  
**Grouping Tolerance:** 15.0 PPM  
**MS^n^ Mass Accuracy:** 20.0 PPM  
**Minimum `q`-Value For Clustering:** 0.05

**Glycopeptide Hypothesis:**
GlycopeptideHypothesis-2c3e584c13e14270ad4dc26024936e9b  
**Sample Searched:**
20190326\_aAGPgal\_021119\_chymo\_HILIC\_DDA\_1ug\_03

| Protein | Matched Glycopeptides |
| --- | --- |
| P02763\|A1AG1\_HUMAN | 165 |
| P19652\|A1AG2\_HUMAN | 142 |
| P00738\|HPT\_HUMAN | 2 |
| P13473\|LAMP2\_HUMAN | 1 |

### P02763|A1AG1\_HUMAN

**>P02763|A1AG1\_HUMAN**  
 `MALSWVLTVLSLLPLLEAQIPLCANLVPVPITNATLDQITGKWFYIASAFRNEEYNKSVQEIQATFFYFTPNKTEDTIFL  
REYQTRQDQCIYNTTYLNVQRENGTISRYVGGQEHFAHLLILRDTKTYMLAFDVNDEKNWGLSVYADKPETTKEQLGEFY  
EALDCLRIPKSDVVYTDWKKDKCEPLEKQHEKERKQEEGES`

| Site | Type | Count |
| --- | --- | --- |
| 33 | N-linked | 1 |
| 72 | N-linked | 71 |
| 93 | N-linked | 5 |
| 103 | N-linked | 41 |
| 56 | N-linked | 47 |

| Mass | Sequence | MS_2_ Score | `q`-value | MS_1_ Score | Signal | Protein Coordinates | Tandem Scans | Start Time | Apex Time | End Time |
| --- | --- | --- | --- | --- | --- | --- | --- | --- | --- | --- |
| 2974.2350 | TPN(N)KTEDTIF Fuc 1 Hex 4 HexNAc 5 | 182.175 | 0.0 | 14.351 | 3.699e+07 | 69 - 79 | 3 | 23.56 | 24.06 | 24.57 |
| 2666.1250 | TPN(N)KTEDTIF Hex 3 HexNAc 5 | 181.471 | 0.0 | 15.599 | 1.613e+08 | 69 - 79 | 8 | 22.71 | 24.57 | 25.59 |
| 2869.2038 | TPN(N)KTEDTIF Hex 3 HexNAc 6 | 180.669 | 0.0 | 16.310 | 2.943e+08 | 69 - 79 | 11 | 22.37 | 24.23 | 25.59 |
| 3177.3140 | TPN(N)KTEDTIF Fuc 1 Hex 4 HexNAc 6 | 180.571 | 0.0 | 14.474 | 6.442e+07 | 69 - 79 | 4 | 23.22 | 23.73 | 24.40 |
| 2976.2606 | YFTPN(N)KTEDTIF Hex 3 HexNAc 5 | 178.871 | 0.0 | 15.488 | 1.800e+09 | 67 - 79 | 134 | 36.97 | 38.11 | 100.43 |
| 2562.1235 | N(N)KSVQEIQATF Hex 3 HexNAc 4 | 178.578 | 0.0 | -4.396 | 2.278e+07 | 55 - 66 | 1 | 27.47 | 27.64 | 27.98 |
| 3544.4699 | YFTPN(N)KTEDTIF Hex 4 HexNAc 7 | 178.290 | 0.0 | 15.131 | 8.357e+08 | 67 - 79 | 11 | 35.78 | 36.97 | 38.27 |
| 3179.3357 | YFTPN(N)KTEDTIF Hex 3 HexNAc 6 | 176.919 | 0.0 | 14.916 | 3.132e+09 | 67 - 79 | 31 | 36.63 | 37.80 | 44.22 |
| 3284.3680 | YFTPN(N)KTEDTIF Fuc 1 Hex 4 HexNAc 5 | 175.084 | 0.0 | 14.429 | 4.647e+08 | 67 - 79 | 5 | 36.29 | 37.47 | 38.11 |
| 2463.0463 | TPN(N)KTEDTIF Hex 3 HexNAc 4 | 174.520 | 0.0 | 13.890 | 2.188e+07 | 69 - 79 | 4 | 24.57 | 24.91 | 25.42 |
| 3691.5384 | FYFTPN(N)KTEDTIF Hex 4 HexNAc 7 | 172.734 | 0.0 | 15.135 | 4.549e+08 | 66 - 79 | 10 | 46.08 | 47.27 | 47.78 |
| 3487.4473 | YFTPN(N)KTEDTIF Fuc 1 Hex 4 HexNAc 6 | 171.681 | 0.0 | 15.893 | 8.484e+08 | 67 - 79 | 10 | 36.12 | 37.31 | 38.11 |
| 2773.1769 | YFTPN(N)KTEDTIF Hex 3 HexNAc 4 | 170.818 | 0.0 | 14.105 | 2.592e+08 | 67 - 79 | 15 | 37.31 | 38.43 | 40.81 |
| 3326.4043 | FYFTPN(N)KTEDTIF Hex 3 HexNAc 6 | 169.855 | 0.0 | 13.657 | 5.052e+09 | 66 - 79 | 224 | 46.76 | 47.78 | 92.81 |
| 3234.3373 | TPN(N)KTEDTIF Hex 4 HexNAc 7 | 169.110 | 0.0 | 12.654 | 4.478e+07 | 69 - 79 | 4 | 23.22 | 23.56 | 24.23 |
| 3634.5164 | FYFTPN(N)KTEDTIF Fuc 1 Hex 4 HexNAc 6 | 168.539 | 0.0 | 14.079 | 7.851e+08 | 66 - 79 | 44 | 46.42 | 47.27 | 63.05 |
| 3431.4352 | FYFTPN(N)KTEDTIF Fuc 1 Hex 4 HexNAc 5 | 168.486 | 0.0 | 14.044 | 3.449e+08 | 66 - 79 | 8 | 46.76 | 47.61 | 48.94 |
| 2920.2448 | FYFTPN(N)KTEDTIF Hex 3 HexNAc 4 | 167.778 | 0.0 | 14.993 | 1.796e+08 | 66 - 79 | 13 | 48.27 | 48.43 | 52.08 |
| 2765.2045 | N(N)KSVQEIQATF Hex 3 HexNAc 5 | 167.731 | 0.0 | -3.717 | 1.639e+07 | 55 - 66 | 1 | 27.29 | 27.47 | 27.64 |
| 3341.3887 | YFTPN(N)KTEDTIF Hex 4 HexNAc 6 | 167.523 | 0.0 | 13.398 | 1.441e+08 | 67 - 79 | 5 | 36.29 | 37.64 | 37.96 |
| 3123.3270 | FYFTPN(N)KTEDTIF Hex 3 HexNAc 5 | 167.444 | 0.0 | 13.869 | 2.332e+09 | 66 - 79 | 150 | 47.10 | 48.10 | 99.92 |
| 3747.5514 | YFTPN(N)KTEDTIF Hex 4 HexNAc 8 | 166.350 | 0.0 | 15.086 | 1.200e+08 | 67 - 79 | 5 | 36.63 | 36.80 | 37.47 |
| 3852.5823 | YFTPN(N)KTEDTIF Fuc 1 Hex 5 HexNAc 7 | 166.138 | 0.0 | 15.215 | 1.350e+08 | 67 - 79 | 6 | 36.12 | 36.80 | 37.14 |
| 3186.3526 | EEYN(N)KSVQEIQATF Hex 3 HexNAc 5 | 165.637 | 0.0 | -3.245 | 3.409e+07 | 52 - 66 | 4 | 35.78 | 35.95 | 36.29 |
| 3016.2701 | FTPN(N)KTEDTIF Hex 3 HexNAc 6 | 165.125 | 0.0 | 14.830 | 6.212e+08 | 68 - 79 | 10 | 29.84 | 31.48 | 31.97 |
| 2813.1931 | FTPN(N)KTEDTIF Hex 3 HexNAc 5 | 164.276 | 0.0 | -0.517 | 2.157e+08 | 68 - 79 | 9 | 30.19 | 31.63 | 113.96 |
| 3324.3845 | FTPN(N)KTEDTIF Fuc 1 Hex 4 HexNAc 6 | 163.733 | 0.0 | 13.661 | 1.114e+08 | 68 - 79 | 7 | 30.70 | 30.87 | 64.24 |
| 3909.6045 | YFTPN(N)KTEDTIF Hex 5 HexNAc 8 | 163.064 | 0.0 | 12.850 | 1.196e+08 | 67 - 79 | 5 | 36.12 | 36.46 | 37.14 |
| 3121.3011 | FTPN(N)KTEDTIF Fuc 1 Hex 4 HexNAc 5 | 162.647 | 0.0 | 12.914 | 7.392e+07 | 68 - 79 | 4 | 30.87 | 31.20 | 31.80 |
| 3837.5975 | FYFTPN(N)KTEDTIF Fuc 1 Hex 4 HexNAc 7 | 162.403 | 0.0 | 12.051 | 2.682e+07 | 66 - 79 | 4 | 46.76 | 47.27 | 47.44 |
| 3073.3152 | N(N)KSVQEIQATF Fuc 1 Hex 4 HexNAc 5 | 160.716 | 0.0 | -4.343 | 8.613e+06 | 55 - 66 | 1 | 26.61 | 26.78 | 27.12 |
| 3795.5594 | YFTPN(N)KTEDTIF Fuc 2 Hex 5 HexNAc 6 | 159.579 | 0.0 | 14.529 | 1.692e+08 | 67 - 79 | 3 | 36.63 | 36.97 | 37.47 |
| 3967.6922 | RNEEYN(N)KSVQEIQATF Fuc 1 Hex 4 HexNAc 6 | 158.901 | 0.0 | 14.855 | 1.369e+08 | 50 - 66 | 2 | 32.14 | 32.48 | 32.79 |
| 3456.5000 | RNEEYN(N)KSVQEIQATF Hex 3 HexNAc 5 | 158.475 | 0.0 | 15.989 | 5.690e+09 | 50 - 66 | 15 | 30.87 | 32.79 | 34.25 |
| 3494.4645 | EEYN(N)KSVQEIQATF Fuc 1 Hex 4 HexNAc 5 | 158.123 | 0.0 | -3.575 | 2.242e+07 | 52 - 66 | 2 | 35.27 | 35.44 | 35.61 |
| 3942.6283 | FYFTPN(N)KTEDTIF Fuc 2 Hex 5 HexNAc 6 | 157.966 | 0.0 | 15.204 | 1.018e+08 | 66 - 79 | 6 | 46.08 | 46.93 | 47.44 |
| 3400.4853 | RNEEYN(N)KSVQEIQATFF Hex 3 HexNAc 4 | 157.371 | 0.0 | 17.174 | 1.915e+09 | 50 - 67 | 12 | 43.54 | 44.56 | 45.91 |
| 3911.6766 | RNEEYN(N)KSVQEIQATFF Fuc 1 Hex 4 HexNAc 5 | 157.057 | 0.0 | 16.072 | 1.050e+09 | 50 - 67 | 18 | 41.66 | 44.06 | 45.39 |
| 3999.6508 | FYFTPN(N)KTEDTIF Fuc 1 Hex 5 HexNAc 7 | 156.863 | 0.0 | 14.148 | 6.746e+07 | 66 - 79 | 5 | 45.74 | 46.76 | 47.61 |
| 3546.5431 | RNEEYN(N)KSVQEIQATFF Fuc 1 Hex 3 HexNAc 4 | 156.238 | 0.0 | -4.828 | 3.189e+07 | 50 - 67 | 3 | 44.39 | 44.56 | 44.72 |
| 3592.4795 | YFTPN(N)KTEDTIF Fuc 2 Hex 5 HexNAc 5 | 156.124 | 0.0 | -19.569 | 1.440e+07 | 67 - 79 | 1 | 36.80 | 36.80 | 36.97 |
| 3690.5295 | YFTPN(N)KTEDTIF Fuc 1 Hex 4 HexNAc 7 | 155.624 | 0.0 | -4.547 | 3.546e+07 | 67 - 79 | 3 | 36.80 | 37.14 | 37.31 |
| 3517.4202 | YFTPN(N)KTEDTIF Hex 4 HexNAc 4 Neu5Ac 2 | 154.595 | 0.0 | 13.990 | 2.468e+08 | 67 - 79 | 7 | 38.43 | 38.60 | 39.44 |
| 3534.4462 | YFTPN(N)KTEDTIF Fuc 1 Hex 5 HexNAc 4 Neu5Ac 1 | 154.545 | 0.0 | 12.400 | 4.689e+07 | 67 - 79 | 3 | 38.43 | 38.60 | 39.44 |
| 3603.5642 | RNEEYN(N)KSVQEIQATFF Hex 3 HexNAc 5 | 154.400 | 0.0 | 15.126 | 1.568e+09 | 50 - 67 | 10 | 44.22 | 44.39 | 45.74 |
| 3253.4180 | RNEEYN(N)KSVQEIQATF Hex 3 HexNAc 4 | 153.831 | 0.0 | 16.706 | 7.172e+09 | 50 - 66 | 29 | 28.31 | 33.11 | 34.42 |
| 3764.6131 | RNEEYN(N)KSVQEIQATF Fuc 1 Hex 4 HexNAc 5 | 152.643 | 0.0 | 16.006 | 3.295e+09 | 50 - 66 | 15 | 31.48 | 32.48 | 33.91 |
| 3106.3488 | RNEEYN(N)KSVQEIQAT Hex 3 HexNAc 4 | 152.610 | 0.0 | -16.940 | 3.542e+07 | 50 - 65 | 1 | 17.40 | 17.40 | 17.57 |
| 3681.5171 | FYFTPN(N)KTEDTIF Fuc 1 Hex 5 HexNAc 4 Neu5Ac 1 | 152.049 | 0.0 | 12.706 | 2.715e+07 | 66 - 79 | 3 | 48.43 | 48.60 | 49.11 |
| 3561.5334 | RNEEYN(N)KSVQEIQATF Fuc 1 Hex 4 HexNAc 4 | 151.976 | 0.0 | 15.587 | 1.658e+08 | 50 - 66 | 3 | 31.80 | 32.65 | 32.79 |
| 3399.4751 | RNEEYN(N)KSVQEIQATF Fuc 1 Hex 3 HexNAc 4 | 151.845 | 0.0 | 11.599 | 1.441e+08 | 50 - 66 | 8 | 33.11 | 33.26 | 34.25 |
| 4112.6827 | YFTPN(N)KTEDTIF Hex 5 HexNAc 9 | 151.183 | 0.0 | -3.896 | 4.027e+07 | 67 - 79 | 3 | 36.12 | 36.12 | 36.80 |
| 3602.5561 | RNEEYN(N)KSVQEIQATF Fuc 1 Hex 3 HexNAc 5 | 150.936 | 0.0 | 11.921 | 1.503e+08 | 50 - 66 | 7 | 32.79 | 33.11 | 34.08 |
| 3659.5806 | RNEEYN(N)KSVQEIQATF Hex 3 HexNAc 6 | 150.906 | 0.0 | 10.848 | 3.130e+08 | 50 - 66 | 7 | 32.48 | 32.65 | 34.08 |
| 2610.1144 | FTPN(N)KTEDTIF Hex 3 HexNAc 4 | 150.786 | 0.0 | -3.327 | 3.430e+07 | 68 - 79 | 3 | 31.80 | 31.97 | 32.31 |
| 3381.4057 | FTPN(N)KTEDTIF Hex 4 HexNAc 7 | 150.780 | 0.0 | 13.062 | 7.057e+07 | 68 - 79 | 4 | 30.53 | 30.87 | 31.20 |
| 3485.4276 | TPN(N)KTEDTIF Fuc 2 Hex 5 HexNAc 6 | 150.482 | 0.0 | -4.545 | 8.469e+06 | 69 - 79 | 1 | 23.05 | 23.22 | 23.56 |
| 3894.6200 | FYFTPN(N)KTEDTIF Hex 4 HexNAc 8 | 149.980 | 0.0 | 16.024 | 6.870e+07 | 66 - 79 | 5 | 46.59 | 46.76 | 47.61 |
| 4072.7217 | RNEEYN(N)KSVQEIQATF Fuc 2 Hex 5 HexNAc 5 | 149.745 | 0.0 | -2.029 | 9.374e+07 | 50 - 66 | 3 | 32.14 | 32.14 | 32.48 |
| 2983.2742 | EEYN(N)KSVQEIQATF Hex 3 HexNAc 4 | 148.230 | 0.0 | -3.387 | 4.319e+07 | 52 - 66 | 2 | 35.95 | 36.12 | 36.46 |
| 3254.4040 | RN(D)EEYN(N)KSVQEIQATF Hex 3 HexNAc 4 | 147.778 | 0.0 | 9.866 | 6.573e+08 | 50 - 66 | 94 | 34.59 | 35.95 | 36.63 |
| 3542.4487 | TPN(N)KTEDTIF Fuc 1 Hex 5 HexNAc 7 | 147.408 | 0.0 | -19.106 | 4.001e+06 | 69 - 79 | 1 | 23.05 | 23.05 | 23.22 |
| 3708.5963 | RNEEYN(N)KSVQEIQATFF Fuc 1 Hex 4 HexNAc 4 | 146.543 | 0.0 | -4.665 | 4.734e+07 | 50 - 67 | 3 | 43.89 | 44.06 | 44.39 |
| 4024.7142 | RNEEYN(N)KSVQEIQATF Hex 4 HexNAc 7 | 146.132 | 0.0 | -4.400 | 8.394e+07 | 50 - 66 | 4 | 32.14 | 32.31 | 32.65 |
| 4056.6732 | FYFTPN(N)KTEDTIF Hex 5 HexNAc 8 | 146.007 | 0.0 | 12.897 | 6.528e+07 | 66 - 79 | 6 | 46.08 | 46.42 | 47.10 |
| 3664.4897 | FYFTPN(N)KTEDTIF Hex 4 HexNAc 4 Neu5Ac 2 | 146.007 | 0.0 | 13.584 | 1.495e+08 | 66 - 79 | 10 | 48.43 | 48.60 | 50.30 |
| 3618.5552 | RNEEYN(N)KSVQEIQATF Hex 4 HexNAc 5 | 145.888 | 0.0 | -5.041 | 7.276e+07 | 50 - 66 | 3 | 32.31 | 32.65 | 32.79 |
| 3138.3123 | YFTPN(N)KTEDTIF Hex 4 HexNAc 5 | 145.005 | 0.0 | -19.918 | 1.569e+07 | 67 - 79 | 1 | 37.64 | 37.64 | 37.80 |
| 3253.4399 | LNVQREN(N)GTISRY Hex 3 HexNAc 6 | 144.824 | 0.0 | 13.808 | 1.013e+10 | 96 - 109 | 12 | 18.41 | 19.05 | 20.50 |
| 3292.4203 | YFTPN(N)KTEDTIFL Hex 3 HexNAc 6 | 144.797 | 0.0 | 14.547 | 2.405e+07 | 67 - 80 | 5 | 45.39 | 45.56 | 46.08 |
| 3765.6180 | RNEEYN(N)KSVQEIQATFF Hex 4 HexNAc 5 | 144.744 | 0.0 | -4.418 | 2.721e+07 | 50 - 67 | 3 | 43.89 | 44.22 | 44.39 |
| 3749.6239 | RNEEYN(N)KSVQEIQATFF Fuc 1 Hex 3 HexNAc 5 | 144.527 | 0.0 | -17.940 | 3.282e+07 | 50 - 67 | 2 | 44.22 | 44.39 | 44.39 |
| 3561.5513 | LNVQREN(N)GTISRY Fuc 1 Hex 4 HexNAc 6 | 144.289 | 0.0 | 14.458 | 3.718e+09 | 96 - 109 | 13 | 18.41 | 18.90 | 21.35 |
| 3488.4592 | FYFTPN(N)KTEDTIF Hex 4 HexNAc 6 | 143.952 | 0.0 | 11.821 | 4.842e+07 | 66 - 79 | 5 | 46.59 | 47.27 | 47.78 |
| 2575.0711 | YFTPN(N)KT Hex 3 HexNAc 6 | 143.357 | 0.0 | 0.000 | 0.000e+00 | 67 - 74 | 1 | - | - | - |
| 3140.3527 | NVQREN(N)GTISRY Hex 3 HexNAc 6 | 142.874 | 0.0 | 13.517 | 7.880e+08 | 97 - 109 | 8 | 13.21 | 13.37 | 14.78 |
| 4275.8012 | RNEEYN(N)KSVQEIQATF Fuc 2 Hex 5 HexNAc 6 | 141.600 | 0.003 | 12.994 | 2.634e+07 | 50 - 66 | 3 | 31.80 | 32.31 | 32.48 |
| 3050.3603 | LNVQREN(N)GTISRY Hex 3 HexNAc 5 | 141.511 | 0.003 | 15.317 | 2.736e+09 | 96 - 109 | 10 | 18.74 | 19.36 | 20.67 |
| 4219.7882 | RNEEYN(N)KSVQEIQATFF Fuc 2 Hex 5 HexNAc 5 | 141.499 | 0.003 | 12.889 | 3.428e+07 | 50 - 67 | 4 | 43.37 | 43.54 | 44.06 |
| 3207.2916 | TPN(N)KTEDTIF Hex 4 HexNAc 4 Neu5Ac 2 | 141.267 | 0.003 | 13.116 | 1.343e+07 | 69 - 79 | 2 | 24.91 | 25.08 | 25.59 |
| 3358.4720 | LNVQREN(N)GTISRY Fuc 1 Hex 4 HexNAc 5 | 140.970 | 0.003 | 13.903 | 1.060e+09 | 96 - 109 | 8 | 18.58 | 19.05 | 20.50 |
| 4055.6636 | YFTPN(N)KTEDTIF Fuc 1 Hex 5 HexNAc 8 | 140.701 | 0.003 | 7.856 | 3.175e+07 | 67 - 79 | 4 | 36.12 | 36.29 | 36.80 |
| 4259.7504 | FYFTPN(N)KTEDTIF Hex 5 HexNAc 9 | 140.483 | 0.003 | 11.610 | 2.295e+07 | 66 - 79 | 4 | 46.08 | 46.25 | 46.76 |
| 4114.7572 | RNEEYN(N)KSVQEIQATFF Fuc 1 Hex 4 HexNAc 6 | 140.037 | 0.003 | 13.253 | 5.724e+07 | 50 - 67 | 4 | 43.54 | 43.89 | 44.22 |
| 3285.3767 | FYFTPN(N)KTEDTIF Hex 4 HexNAc 5 | 139.815 | 0.003 | -4.937 | 1.327e+07 | 66 - 79 | 2 | 47.44 | 47.78 | 47.78 |
| 3191.3003 | QTRQDQC(C)IYN(N)TTY Hex 3 HexNAc 5 | 139.783 | 0.003 | 14.934 | 9.513e+07 | 83 - 96 | 4 | 22.20 | 22.71 | 23.39 |
| 3089.3417 | YFTPN(N)KTEDTIFL Hex 3 HexNAc 5 | 139.776 | 0.003 | 13.944 | 1.192e+07 | 67 - 80 | 3 | 45.56 | 45.91 | 46.25 |
| 3764.6311 | LNVQREN(N)GTISRY Fuc 1 Hex 4 HexNAc 7 | 138.130 | 0.003 | -3.755 | 2.382e+08 | 96 - 109 | 3 | 18.41 | 18.58 | 18.90 |
| 4227.7897 | RNEEYN(N)KSVQEIQATF Hex 4 HexNAc 8 | 138.071 | 0.003 | -3.783 | 3.867e+07 | 50 - 66 | 3 | 31.97 | 32.14 | 32.48 |
| 3806.6439 | RNEEYN(N)KSVQEIQATFF Hex 3 HexNAc 6 | 138.001 | 0.003 | -4.282 | 7.432e+07 | 50 - 67 | 4 | 44.06 | 44.22 | 44.56 |
| 3617.5442 | RNEEYN(N)KSVQEIQAT Fuc 1 Hex 4 HexNAc 5 | 137.920 | 0.003 | -19.318 | 1.159e+07 | 50 - 65 | 1 | 17.07 | 17.07 | 17.23 |
| 3399.5500 | LNVQREN(N)GTISRY Fuc 1 Hex 3 HexNAc 6 | 137.581 | 0.003 | 11.772 | 1.573e+08 | 96 - 109 | 18 | 19.21 | 19.36 | 35.27 |
| 3708.5671 | NVQREN(N)GTISRY Hex 4 HexNAc 8 | 137.347 | 0.003 | -19.221 | 7.729e+07 | 97 - 109 | 2 | 13.04 | 13.04 | 13.21 |
| 2709.1939 | N(N)KSVQEIQATFF Hex 3 HexNAc 4 | 137.200 | 0.003 | -2.982 | 4.634e+06 | 55 - 67 | 2 | 41.66 | 41.83 | 42.17 |
| 2937.2736 | NVQREN(N)GTISRY Hex 3 HexNAc 5 | 136.947 | 0.003 | 12.761 | 2.035e+08 | 97 - 109 | 3 | 13.37 | 13.54 | 14.19 |
| 2721.1355 | FYFTPN(N)KT Hex 3 HexNAc 6 | 135.851 | 0.0 | -3.670 | 1.249e+07 | 66 - 74 | 2 | 26.27 | 26.44 | 26.78 |
| 3649.5046 | YFTPN(N)KTEDTIF Fuc 1 Hex 5 HexNAc 6 | 135.229 | 0.003 | -4.994 | 7.825e+06 | 67 - 79 | 2 | 36.63 | 36.97 | 36.97 |
| 3825.5309 | YFTPN(N)KTEDTIF Fuc 1 Hex 5 HexNAc 4 Neu5Ac 2 | 134.822 | 0.003 | -5.534 | 2.996e+07 | 67 - 79 | 2 | 37.96 | 38.11 | 38.43 |
| 3354.3586 | FTPN(N)KTEDTIF Hex 4 HexNAc 4 Neu5Ac 2 | 134.255 | 0.003 | -4.270 | 2.287e+07 | 68 - 79 | 2 | 31.97 | 32.14 | 32.31 |
| 4057.7338 | RNEEYN(N)KSVQEIQATFF Fuc 2 Hex 4 HexNAc 5 | 133.505 | 0.003 | -4.411 | 1.537e+07 | 50 - 67 | 2 | 43.89 | 44.06 | 44.22 |
| 3394.3803 | QTRQDQC(C)IYN(N)TTY Hex 3 HexNAc 6 | 133.362 | 0.003 | 15.480 | 2.565e+08 | 83 - 96 | 8 | 21.35 | 22.37 | 23.22 |
| 3910.6723 | RNEEYN(N)KSVQEIQATF Fuc 2 Hex 4 HexNAc 5 | 131.928 | 0.003 | -4.216 | 4.335e+07 | 50 - 66 | 2 | 32.48 | 32.65 | 32.79 |
| 2847.2810 | LNVQREN(N)GTISRY Hex 3 HexNAc 4 | 131.013 | 0.005 | 13.787 | 2.004e+08 | 96 - 109 | 4 | 19.36 | 19.52 | 20.84 |
| 3505.4850 | NVQREN(N)GTISRY Hex 4 HexNAc 7 | 130.397 | 0.005 | -18.397 | 6.579e+07 | 97 - 109 | 1 | 13.04 | 13.21 | 13.21 |
| 3334.4207 | VQREN(N)GTISRY Fuc 1 Hex 4 HexNAc 6 | 130.295 | 0.005 | -19.840 | 1.362e+07 | 98 - 109 | 1 | 12.71 | 12.71 | 12.71 |
| 3604.5517 | RN(D)EEYN(N)KSVQEIQATFF Hex 3 HexNAc 5 | 129.668 | 0.005 | -5.790 | 4.619e+07 | 50 - 67 | 12 | 47.27 | 47.44 | 47.61 |
| 3448.4642 | NVQREN(N)GTISRY Fuc 1 Hex 4 HexNAc 6 | 128.935 | 0.005 | 13.053 | 3.293e+08 | 97 - 109 | 5 | 13.04 | 13.21 | 13.86 |
| 3765.5948 | RN(D)EEYN(N)KSVQEIQATF Fuc 1 Hex 4 HexNAc 5 | 128.492 | 0.005 | 11.442 | 3.215e+08 | 50 - 66 | 11 | 34.08 | 35.44 | 36.29 |
| 2965.2034 | FYFTPN(N)KTED Hex 3 HexNAc 6 | 128.317 | 0.005 | -18.309 | 3.707e+06 | 66 - 76 | 1 | 26.61 | 26.78 | 26.78 |
| 3821.6370 | RNEEYN(N)KSVQEIQATF Hex 4 HexNAc 6 | 127.125 | 0.008 | 12.189 | 5.712e+08 | 50 - 66 | 7 | 32.31 | 32.65 | 33.91 |
| 3309.4293 | RNEEYN(N)KSVQEIQAT Hex 3 HexNAc 5 | 127.034 | 0.008 | 11.922 | 3.505e+07 | 50 - 65 | 3 | 17.23 | 17.40 | 17.91 |
| 4129.7673 | LNVQREN(N)GTISRY Fuc 1 Hex 5 HexNAc 8 | 126.651 | 0.008 | 13.544 | 5.209e+08 | 96 - 109 | 4 | 18.24 | 18.41 | 19.05 |
| 3926.6862 | LNVQREN(N)GTISRY Fuc 1 Hex 5 HexNAc 7 | 126.156 | 0.010 | 11.099 | 5.989e+08 | 96 - 109 | 9 | 18.24 | 18.58 | 20.00 |
| 2982.2892 | TPN(N)KTEDTIFL Hex 3 HexNAc 6 | 125.982 | 0.010 | -17.336 | 2.330e+06 | 69 - 80 | 3 | 34.93 | 35.10 | 35.10 |
| 4186.7859 | LNVQREN(N)GTISRY Hex 5 HexNAc 9 | 125.683 | 0.010 | 10.364 | 3.109e+08 | 96 - 109 | 2 | 18.24 | 18.41 | 19.05 |
| 3457.4832 | RN(D)EEYN(N)KSVQEIQATF Hex 3 HexNAc 5 | 125.420 | 0.012 | 9.503 | 5.503e+08 | 50 - 66 | 14 | 34.25 | 35.78 | 37.64 |
| 3618.5709 | LNVQREN(N)GTISRY Hex 4 HexNAc 7 | 125.197 | 0.012 | 14.632 | 2.206e+09 | 96 - 109 | 14 | 18.24 | 18.74 | 20.67 |
| 3499.4139 | QTRQDQC(C)IYN(N)TTY Fuc 1 Hex 4 HexNAc 5 | 124.916 | 0.012 | -3.600 | 2.845e+07 | 83 - 96 | 2 | 22.20 | 22.20 | 22.54 |
| 3400.4796 | FRNEEYN(N)KSVQEIQATF Hex 3 HexNAc 4 | 124.913 | 0.012 | -19.878 | 8.259e+06 | 49 - 66 | 1 | 37.80 | 37.80 | 37.80 |
| 3415.4935 | LNVQREN(N)GTISRY Hex 4 HexNAc 6 | 124.499 | 0.012 | -18.203 | 5.443e+07 | 96 - 109 | 1 | 18.74 | 18.74 | 18.90 |
| 3756.5763 | NVQREN(N)GTISRY Fuc 2 Hex 5 HexNAc 6 | 123.823 | 0.012 | -19.046 | 8.005e+07 | 97 - 109 | 2 | 13.04 | 13.04 | 13.21 |
| 2671.1033 | N(N)KTEDTIF Hex 3 HexNAc 6 | 123.563 | 0.0 | -19.164 | 4.933e+06 | 71 - 79 | 1 | 22.37 | 22.54 | 22.54 |
| 3254.4305 | LN(D)VQREN(N)GTISRY Hex 3 HexNAc 6 | 123.203 | 0.012 | 7.850 | 3.025e+08 | 96 - 109 | 81 | 20.50 | 21.18 | 21.86 |
| 3632.4949 | FTPN(N)KTEDTIF Fuc 2 Hex 5 HexNAc 6 | 122.973 | 0.012 | 12.885 | 1.359e+07 | 68 - 79 | 3 | 30.19 | 30.53 | 30.87 |
| 3821.6532 | LNVQREN(N)GTISRY Hex 4 HexNAc 8 | 122.942 | 0.012 | 13.707 | 1.459e+09 | 96 - 109 | 9 | 18.08 | 18.58 | 20.00 |
| 4177.7762 | LNVQREN(N)GTISRY Fuc 3 Hex 6 HexNAc 6 | 122.732 | 0.012 | -2.990 | 4.132e+08 | 96 - 109 | 3 | 18.24 | 18.41 | 18.74 |
| 3220.3817 | N(N)KSVQEIQATFF Fuc 1 Hex 4 HexNAc 5 | 122.687 | 0.012 | -3.672 | 2.771e+06 | 55 - 67 | 2 | 40.64 | 40.81 | 41.15 |
| 3429.4090 | YFTPN(N)KTEDTIF Hex 4 HexNAc 5 Neu5Ac 1 | 122.506 | 0.012 | -5.351 | 3.225e+06 | 67 - 79 | 2 | 40.81 | 40.98 | 41.32 |
| 3574.4426 | TPN(N)KTEDTIF Fuc 4 Hex 5 HexNAc 5 | 119.857 | 0.016 | -4.376 | 4.041e+07 | 69 - 79 | 2 | 38.43 | 38.60 | 38.93 |
| 3031.2579 | TPN(N)KTEDTIF Hex 4 HexNAc 6 | 119.520 | 0.016 | 13.024 | 8.384e+06 | 69 - 79 | 2 | 23.73 | 23.89 | 24.57 |
| 4202.7307 | FYFTPN(N)KTEDTIF Fuc 1 Hex 5 HexNAc 8 | 119.464 | 0.016 | 12.732 | 1.705e+07 | 66 - 79 | 3 | 46.08 | 46.25 | 46.76 |
| 3702.4913 | QTRQDQC(C)IYN(N)TTY Fuc 1 Hex 4 HexNAc 6 | 118.497 | 0.016 | 14.029 | 5.955e+07 | 83 - 96 | 5 | 21.86 | 22.03 | 22.71 |
| 2518.0523 | FYFTPN(N)KT Hex 3 HexNAc 5 | 118.432 | 0.0 | -18.723 | 5.309e+06 | 66 - 74 | 2 | 26.78 | 26.78 | 26.95 |
| 4332.8209 | RNEEYN(N)KSVQEIQATF Fuc 1 Hex 5 HexNAc 7 | 117.602 | 0.018 | -4.471 | 1.596e+07 | 50 - 66 | 1 | 31.97 | 31.97 | 32.31 |
| 3331.3660 | YFTPN(N)KTEDTIF Fuc 1 Hex 5 HexNAc 3 Neu5Ac 1 | 117.501 | 0.018 | -4.948 | 5.309e+06 | 67 - 79 | 2 | 38.77 | 38.77 | 39.10 |
| 3026.3145 | VQREN(N)GTISRY Hex 3 HexNAc 6 | 117.437 | 0.018 | -4.033 | 4.931e+07 | 98 - 109 | 2 | 12.71 | 12.87 | 13.04 |
| 3314.3407 | TPN(N)KTEDTIF Hex 7 HexNAc 5 | 117.026 | 0.018 | 11.467 | 2.448e+07 | 69 - 79 | 2 | 38.77 | 38.93 | 39.44 |
| 3196.4181 | LNVQREN(N)GTISRY Fuc 1 Hex 3 HexNAc 5 | 116.544 | 0.019 | -4.532 | 5.827e+07 | 96 - 109 | 2 | 19.36 | 19.52 | 19.84 |
| 4072.7455 | LNVQREN(N)GTISRY Fuc 2 Hex 5 HexNAc 7 | 116.318 | 0.019 | -3.301 | 2.327e+08 | 96 - 109 | 3 | 18.24 | 18.41 | 18.74 |
| 3869.6617 | LNVQREN(N)GTISRY Fuc 2 Hex 5 HexNAc 6 | 115.366 | 0.019 | 14.501 | 1.429e+09 | 96 - 109 | 6 | 18.24 | 18.74 | 19.68 |
| 4745.9846 | LNVQREN(N)GTISRY Fuc 3 Hex 7 HexNAc 8 | 114.722 | 0.019 | -4.871 | 4.358e+07 | 96 - 109 | 3 | 17.91 | 18.08 | 18.41 |
| 2934.2659 | RNEEYN(N)KSVQEIQ Hex 3 HexNAc 4 | 114.146 | 0.020 | -19.803 | 6.454e+06 | 50 - 63 | 1 | 14.95 | 14.95 | 15.12 |
| 3415.4751 | RNEEYN(N)KSVQEIQATF Hex 4 HexNAc 4 | 113.169 | 0.020 | -20.221 | 2.286e+07 | 50 - 66 | 1 | 32.65 | 32.65 | 32.79 |
| 3392.4535 | VQREN(N)GTISRY Hex 4 HexNAc 7 | 112.430 | 0.020 | 0.000 | 0.000e+00 | 98 - 109 | 1 | - | - | - |
| 4437.8757 | LNVQREN(N)GTISRY Fuc 2 Hex 6 HexNAc 8 | 111.424 | 0.020 | 13.508 | 1.748e+08 | 96 - 109 | 3 | 18.08 | 18.24 | 18.74 |
| 3707.6104 | LNVQREN(N)GTISRY Fuc 2 Hex 4 HexNAc 6 | 111.280 | 0.020 | -5.492 | 2.421e+07 | 96 - 109 | 1 | 18.74 | 18.90 | 19.05 |
| 3666.5810 | LNVQREN(N)GTISRY Fuc 2 Hex 5 HexNAc 5 | 111.241 | 0.020 | -19.138 | 7.467e+07 | 96 - 109 | 1 | 18.58 | 18.74 | 18.74 |
| 3178.3248 | FTPN(N)KTEDTIF Hex 4 HexNAc 6 | 111.200 | 0.020 | -5.095 | 8.899e+06 | 68 - 79 | 2 | 30.87 | 31.04 | 31.20 |
| 3689.5168 | FTPN(N)KTEDTIF Fuc 1 Hex 5 HexNAc 7 | 111.187 | 0.020 | 12.370 | 8.721e+06 | 68 - 79 | 4 | 30.01 | 30.36 | 30.70 |
| 3540.4381 | QTRQDQC(C)IYN(N)TTY Fuc 1 Hex 3 HexNAc 6 | 111.102 | 0.020 | 12.470 | 1.005e+07 | 83 - 96 | 2 | 22.03 | 22.37 | 22.71 |
| 3983.7074 | LNVQREN(N)GTISRY Hex 5 HexNAc 8 | 110.530 | 0.022 | -3.880 | 1.320e+08 | 96 - 109 | 2 | 18.41 | 18.41 | 18.74 |
| 3591.5209 | LNVQREN(N)GTISRY Hex 4 HexNAc 4 Neu5Ac 2 | 110.156 | 0.022 | 14.286 | 4.365e+08 | 96 - 109 | 8 | 19.68 | 20.00 | 21.35 |
| 3515.4076 | TPN(N)KTEDTIF Fuc 1 Hex 5 HexNAc 4 Neu5Ac 2 | 109.602 | 0.022 | 0.000 | 0.000e+00 | 69 - 79 | 1 | - | - | - |
| 2823.2354 | VQREN(N)GTISRY Hex 3 HexNAc 5 | 109.423 | 0.022 | -19.884 | 7.099e+06 | 98 - 109 | 1 | 12.87 | 12.87 | 12.87 |
| 4016.6773 | NVQREN(N)GTISRY Fuc 1 Hex 5 HexNAc 8 | 109.036 | 0.024 | -20.552 | 2.455e+07 | 97 - 109 | 2 | 12.87 | 12.87 | 13.04 |
| 4064.6975 | NVQREN(N)GTISRY Fuc 3 Hex 6 HexNAc 6 | 108.837 | 0.026 | -20.276 | 1.701e+07 | 97 - 109 | 2 | 12.87 | 12.87 | 12.87 |
| 3926.6701 | RNEEYN(N)KSVQEIQATF Fuc 1 Hex 5 HexNAc 5 | 107.560 | 0.027 | -6.225 | 8.641e+06 | 50 - 66 | 1 | 32.14 | 32.48 | 32.48 |
| 2983.3502 | C(C)ANLVPVPITN(N)ATL Hex 3 HexNAc 5 | 107.165 | 0.027 | 13.320 | 2.241e+07 | 22 - 36 | 7 | 51.15 | 51.39 | 52.23 |
| 3401.4755 | RN(D)EEYN(N)KSVQEIQATFF Hex 3 HexNAc 4 | 105.416 | 0.038 | 7.036 | 5.696e+07 | 50 - 67 | 6 | 46.08 | 46.25 | 47.10 |
| 3245.3839 | NVQREN(N)GTISRY Fuc 1 Hex 4 HexNAc 5 | 104.327 | 0.042 | 10.552 | 7.889e+07 | 97 - 109 | 3 | 13.21 | 13.37 | 13.86 |
| 4075.7065 | NVQREN(N)GTISRY Hex 5 HexNAc 9 | 102.957 | 0.045 | 0.000 | 0.000e+00 | 97 - 109 | 1 | - | - | - |
| 4380.8543 | LNVQREN(N)GTISRY Fuc 3 Hex 6 HexNAc 7 | 102.256 | 0.046 | -3.518 | 1.132e+08 | 96 - 109 | 3 | 18.08 | 18.24 | 18.58 |
| 3972.6016 | FYFTPN(N)KTEDTIF Fuc 1 Hex 5 HexNAc 4 Neu5Ac 2 | 100.754 | 0.050 | -5.447 | 1.924e+07 | 66 - 79 | 2 | 47.95 | 48.10 | 48.27 |
| 3495.4501 | LNVQREN(N)GTISRY Hex 7 HexNAc 4 | 100.703 | 0.050 | 12.486 | 3.406e+07 | 96 - 109 | 4 | 21.52 | 22.03 | 22.20 |
| 2849.2873 | LNVQREN(N)GTISRY Hex 3 HexNAc 4 | 100.199 | 0.050 | 0.000 | 0.000e+00 | 96 - 109 | 1 | - | - | - |

### TPN(N)KTEDTIF Fuc 1 Hex 4 HexNAc 5

**Mass:**  2974.2339

(5.382e-07)

**Charge States:** 3

**Start Position**: 70

**End Position**: 80

| Profile Score | Tandem Score | TDA q-value | Volume | Spectrum Matches |
| --- | --- | --- | --- | --- |
| 14.3506 | 182.1750 | 0.0000 | 3.6992e+07 | 3 |

**Adduct Signal Fractions**

- **Unmodified**: 3.6992e+07

### TPN(N)KTEDTIF Hex 3 HexNAc 5

**Mass:**  2666.1247

(1.424e-06)

**Charge States:** 2, 3

**Start Position**: 70

**End Position**: 80

| Profile Score | Tandem Score | TDA q-value | Volume | Spectrum Matches |
| --- | --- | --- | --- | --- |
| 15.5991 | 181.4711 | 0.0000 | 1.6133e+08 | 8 |

**Adduct Signal Fractions**

- **Unmodified**: 1.6133e+08

### TPN(N)KTEDTIF Hex 3 HexNAc 6

**Mass:**  2869.2037

(1.013e-06)

**Charge States:** 3, 4

**Start Position**: 70

**End Position**: 80

| Profile Score | Tandem Score | TDA q-value | Volume | Spectrum Matches |
| --- | --- | --- | --- | --- |
| 16.3100 | 180.6692 | 0.0000 | 2.9431e+08 | 11 |

**Adduct Signal Fractions**

- **Unmodified**: 2.9431e+08

### TPN(N)KTEDTIF Fuc 1 Hex 4 HexNAc 6

**Mass:**  3177.3136

(5.581e-07)

**Charge States:** 3, 4

**Start Position**: 70

**End Position**: 80

| Profile Score | Tandem Score | TDA q-value | Volume | Spectrum Matches |
| --- | --- | --- | --- | --- |
| 14.4737 | 180.5706 | 0.0000 | 6.4424e+07 | 4 |

**Adduct Signal Fractions**

- **Unmodified**: 6.4424e+07

### YFTPN(N)KTEDTIF Hex 3 HexNAc 5

**Mass:**  2976.2569

(8.199e-07)

**Charge States:** 2, 3, 4

**Start Position**: 68

**End Position**: 80

| Profile Score | Tandem Score | TDA q-value | Volume | Spectrum Matches |
| --- | --- | --- | --- | --- |
| 15.4880 | 178.8706 | 0.0000 | 1.7999e+09 | 134 |

**Adduct Signal Fractions**

- **Unmodified**: 1.7999e+09

### N(N)KSVQEIQATF Hex 3 HexNAc 4

**Mass:**  2562.1245

(1.041e-06)

**Charge States:** 3

**Start Position**: 56

**End Position**: 67

| Profile Score | Tandem Score | TDA q-value | Volume | Spectrum Matches |
| --- | --- | --- | --- | --- |
| -4.3955 | 178.5779 | 0.0000 | 2.2775e+07 | 1 |

**Adduct Signal Fractions**

- **Unmodified**: 2.2775e+07

### YFTPN(N)KTEDTIF Hex 4 HexNAc 7

**Mass:**  3544.4679

(1.659e-06)

**Charge States:** 3, 4

**Start Position**: 68

**End Position**: 80

| Profile Score | Tandem Score | TDA q-value | Volume | Spectrum Matches |
| --- | --- | --- | --- | --- |
| 15.1306 | 178.2902 | 0.0000 | 8.3569e+08 | 11 |

**Adduct Signal Fractions**

- **Unmodified**: 8.3569e+08

### YFTPN(N)KTEDTIF Hex 3 HexNAc 6

**Mass:**  3179.3351

(4.864e-07)

**Charge States:** 2, 3, 4

**Start Position**: 68

**End Position**: 80

| Profile Score | Tandem Score | TDA q-value | Volume | Spectrum Matches |
| --- | --- | --- | --- | --- |
| 14.9162 | 176.9188 | 0.0000 | 3.1320e+09 | 31 |

**Adduct Signal Fractions**

- **Unmodified**: 3.1320e+09

### YFTPN(N)KTEDTIF Fuc 1 Hex 4 HexNAc 5

**Mass:**  3284.3684

(9.639e-07)

**Charge States:** 3, 4

**Start Position**: 68

**End Position**: 80

| Profile Score | Tandem Score | TDA q-value | Volume | Spectrum Matches |
| --- | --- | --- | --- | --- |
| 14.4294 | 175.0841 | 0.0000 | 4.6468e+08 | 5 |

**Adduct Signal Fractions**

- **Unmodified**: 4.6468e+08

### TPN(N)KTEDTIF Hex 3 HexNAc 4

**Mass:**  2463.0459

(2.075e-06)

**Charge States:** 2, 3

**Start Position**: 70

**End Position**: 80

| Profile Score | Tandem Score | TDA q-value | Volume | Spectrum Matches |
| --- | --- | --- | --- | --- |
| 13.8903 | 174.5203 | 0.0000 | 2.1883e+07 | 4 |

**Adduct Signal Fractions**

- **Unmodified**: 2.1883e+07

### FYFTPN(N)KTEDTIF Hex 4 HexNAc 7

**Mass:**  3691.5379

(1.657e-06)

**Charge States:** 3, 4

**Start Position**: 67

**End Position**: 80

| Profile Score | Tandem Score | TDA q-value | Volume | Spectrum Matches |
| --- | --- | --- | --- | --- |
| 15.1352 | 172.7342 | 0.0000 | 4.5490e+08 | 10 |

**Adduct Signal Fractions**

- **Unmodified**: 4.5490e+08

### YFTPN(N)KTEDTIF Fuc 1 Hex 4 HexNAc 6

**Mass:**  3487.4463

(1.210e-06)

**Charge States:** 3, 4

**Start Position**: 68

**End Position**: 80

| Profile Score | Tandem Score | TDA q-value | Volume | Spectrum Matches |
| --- | --- | --- | --- | --- |
| 15.8934 | 171.6813 | 0.0000 | 8.4840e+08 | 10 |

**Adduct Signal Fractions**

- **Unmodified**: 8.4840e+08

### YFTPN(N)KTEDTIF Hex 3 HexNAc 4

**Mass:**  2773.1773

(9.537e-07)

**Charge States:** 2, 3

**Start Position**: 68

**End Position**: 80

| Profile Score | Tandem Score | TDA q-value | Volume | Spectrum Matches |
| --- | --- | --- | --- | --- |
| 14.1046 | 170.8177 | 0.0000 | 2.5925e+08 | 15 |

**Adduct Signal Fractions**

- **Unmodified**: 2.5925e+08

### FYFTPN(N)KTEDTIF Hex 3 HexNAc 6

**Mass:**  3326.4054

(1.003e-06)

**Charge States:** 2, 3, 4

**Start Position**: 67

**End Position**: 80

| Profile Score | Tandem Score | TDA q-value | Volume | Spectrum Matches |
| --- | --- | --- | --- | --- |
| 13.6566 | 169.8548 | 0.0000 | 5.0523e+09 | 224 |

**Adduct Signal Fractions**

- **Unmodified**: 5.0523e+09

### TPN(N)KTEDTIF Hex 4 HexNAc 7

**Mass:**  3234.3367

(1.360e-06)

**Charge States:** 3

**Start Position**: 70

**End Position**: 80

| Profile Score | Tandem Score | TDA q-value | Volume | Spectrum Matches |
| --- | --- | --- | --- | --- |
| 12.6538 | 169.1100 | 0.0000 | 4.4781e+07 | 4 |

**Adduct Signal Fractions**

- **Unmodified**: 4.4781e+07

### FYFTPN(N)KTEDTIF Fuc 1 Hex 4 HexNAc 6

**Mass:**  3634.5177

(1.168e-06)

**Charge States:** 3, 4

**Start Position**: 67

**End Position**: 80

| Profile Score | Tandem Score | TDA q-value | Volume | Spectrum Matches |
| --- | --- | --- | --- | --- |
| 14.0793 | 168.5392 | 0.0000 | 7.8515e+08 | 44 |

**Adduct Signal Fractions**

- **Unmodified**: 7.8515e+08

### FYFTPN(N)KTEDTIF Fuc 1 Hex 4 HexNAc 5

**Mass:**  3431.4347

(6.905e-07)

**Charge States:** 3, 4

**Start Position**: 67

**End Position**: 80

| Profile Score | Tandem Score | TDA q-value | Volume | Spectrum Matches |
| --- | --- | --- | --- | --- |
| 14.0443 | 168.4857 | 0.0000 | 3.4488e+08 | 8 |

**Adduct Signal Fractions**

- **Unmodified**: 3.4488e+08

### FYFTPN(N)KTEDTIF Hex 3 HexNAc 4

**Mass:**  2920.2447

(8.629e-07)

**Charge States:** 2, 3

**Start Position**: 67

**End Position**: 80

| Profile Score | Tandem Score | TDA q-value | Volume | Spectrum Matches |
| --- | --- | --- | --- | --- |
| 14.9930 | 167.7783 | 0.0000 | 1.7957e+08 | 13 |

**Adduct Signal Fractions**

- **Unmodified**: 1.7957e+08

### N(N)KSVQEIQATF Hex 3 HexNAc 5

**Mass:**  2765.2040

(1.958e-06)

**Charge States:** 3

**Start Position**: 56

**End Position**: 67

| Profile Score | Tandem Score | TDA q-value | Volume | Spectrum Matches |
| --- | --- | --- | --- | --- |
| -3.7172 | 167.7310 | 0.0000 | 1.6395e+07 | 1 |

**Adduct Signal Fractions**

- **Unmodified**: 1.6395e+07

### YFTPN(N)KTEDTIF Hex 4 HexNAc 6

**Mass:**  3341.3886

(7.641e-07)

**Charge States:** 3

**Start Position**: 68

**End Position**: 80

| Profile Score | Tandem Score | TDA q-value | Volume | Spectrum Matches |
| --- | --- | --- | --- | --- |
| 13.3975 | 167.5230 | 0.0000 | 1.4413e+08 | 5 |

**Adduct Signal Fractions**

- **Unmodified**: 1.4413e+08

### FYFTPN(N)KTEDTIF Hex 3 HexNAc 5

**Mass:**  3123.3246

(6.453e-07)

**Charge States:** 2, 3, 4

**Start Position**: 67

**End Position**: 80

| Profile Score | Tandem Score | TDA q-value | Volume | Spectrum Matches |
| --- | --- | --- | --- | --- |
| 13.8687 | 167.4439 | 0.0000 | 2.3323e+09 | 150 |

**Adduct Signal Fractions**

- **Unmodified**: 2.3323e+09

### YFTPN(N)KTEDTIF Hex 4 HexNAc 8

**Mass:**  3747.5520

(2.080e-06)

**Charge States:** 3, 4

**Start Position**: 68

**End Position**: 80

| Profile Score | Tandem Score | TDA q-value | Volume | Spectrum Matches |
| --- | --- | --- | --- | --- |
| 15.0864 | 166.3502 | 0.0000 | 1.2002e+08 | 5 |

**Adduct Signal Fractions**

- **Unmodified**: 1.2002e+08

### YFTPN(N)KTEDTIF Fuc 1 Hex 5 HexNAc 7

**Mass:**  3852.5828

(8.580e-07)

**Charge States:** 3, 4

**Start Position**: 68

**End Position**: 80

| Profile Score | Tandem Score | TDA q-value | Volume | Spectrum Matches |
| --- | --- | --- | --- | --- |
| 15.2152 | 166.1384 | 0.0000 | 1.3500e+08 | 6 |

**Adduct Signal Fractions**

- **Unmodified**: 1.3500e+08

### EEYN(N)KSVQEIQATF Hex 3 HexNAc 5

**Mass:**  3186.3532

(5.244e-07)

**Charge States:** 3

**Start Position**: 53

**End Position**: 67

| Profile Score | Tandem Score | TDA q-value | Volume | Spectrum Matches |
| --- | --- | --- | --- | --- |
| -3.2449 | 165.6371 | 0.0000 | 3.4091e+07 | 4 |

**Adduct Signal Fractions**

- **Unmodified**: 3.4091e+07

### FTPN(N)KTEDTIF Hex 3 HexNAc 6

**Mass:**  3016.2724

(1.016e-06)

**Charge States:** 2, 3, 4

**Start Position**: 69

**End Position**: 80

| Profile Score | Tandem Score | TDA q-value | Volume | Spectrum Matches |
| --- | --- | --- | --- | --- |
| 14.8299 | 165.1247 | 0.0000 | 6.2117e+08 | 10 |

**Adduct Signal Fractions**

- **Unmodified**: 6.2117e+08

### FTPN(N)KTEDTIF Hex 3 HexNAc 5

**Mass:**  2813.1919

(1.051e-06)

**Charge States:** 8, 2, 3

**Start Position**: 69

**End Position**: 80

| Profile Score | Tandem Score | TDA q-value | Volume | Spectrum Matches |
| --- | --- | --- | --- | --- |
| -0.5167 | 164.2759 | 0.0000 | 2.1572e+08 | 9 |

**Adduct Signal Fractions**

- **Unmodified**: 2.1572e+08

### FTPN(N)KTEDTIF Fuc 1 Hex 4 HexNAc 6

**Mass:**  3324.3836

(6.552e-07)

**Charge States:** 3, 4

**Start Position**: 69

**End Position**: 80

| Profile Score | Tandem Score | TDA q-value | Volume | Spectrum Matches |
| --- | --- | --- | --- | --- |
| 13.6611 | 163.7329 | 0.0000 | 1.1138e+08 | 7 |

**Adduct Signal Fractions**

- **Unmodified**: 1.1138e+08

### YFTPN(N)KTEDTIF Hex 5 HexNAc 8

**Mass:**  3909.6033

(1.834e-06)

**Charge States:** 3, 4

**Start Position**: 68

**End Position**: 80

| Profile Score | Tandem Score | TDA q-value | Volume | Spectrum Matches |
| --- | --- | --- | --- | --- |
| 12.8503 | 163.0644 | 0.0000 | 1.1960e+08 | 5 |

**Adduct Signal Fractions**

- **Unmodified**: 1.1960e+08

### FTPN(N)KTEDTIF Fuc 1 Hex 4 HexNAc 5

**Mass:**  3121.2998

(-3.228e-07)

**Charge States:** 3

**Start Position**: 69

**End Position**: 80

| Profile Score | Tandem Score | TDA q-value | Volume | Spectrum Matches |
| --- | --- | --- | --- | --- |
| 12.9140 | 162.6466 | 0.0000 | 7.3921e+07 | 4 |

**Adduct Signal Fractions**

- **Unmodified**: 7.3921e+07

### FYFTPN(N)KTEDTIF Fuc 1 Hex 4 HexNAc 7

**Mass:**  3837.5996

(1.570e-06)

**Charge States:** 3, 4

**Start Position**: 67

**End Position**: 80

| Profile Score | Tandem Score | TDA q-value | Volume | Spectrum Matches |
| --- | --- | --- | --- | --- |
| 12.0515 | 162.4026 | 0.0000 | 2.6818e+07 | 4 |

**Adduct Signal Fractions**

- **Unmodified**: 2.6818e+07

### N(N)KSVQEIQATF Fuc 1 Hex 4 HexNAc 5

**Mass:**  3073.3152

(1.097e-06)

**Charge States:** 3

**Start Position**: 56

**End Position**: 67

| Profile Score | Tandem Score | TDA q-value | Volume | Spectrum Matches |
| --- | --- | --- | --- | --- |
| -4.3429 | 160.7157 | 0.0000 | 8.6132e+06 | 1 |

**Adduct Signal Fractions**

- **Unmodified**: 8.6132e+06

### YFTPN(N)KTEDTIF Fuc 2 Hex 5 HexNAc 6

**Mass:**  3795.5579

(9.783e-07)

**Charge States:** 3, 4

**Start Position**: 68

**End Position**: 80

| Profile Score | Tandem Score | TDA q-value | Volume | Spectrum Matches |
| --- | --- | --- | --- | --- |
| 14.5285 | 159.5787 | 0.0000 | 1.6918e+08 | 3 |

**Adduct Signal Fractions**

- **Unmodified**: 1.6918e+08

### RNEEYN(N)KSVQEIQATF Fuc 1 Hex 4 HexNAc 6

**Mass:**  3967.6923

(1.785e-06)

**Charge States:** 3, 4

**Start Position**: 51

**End Position**: 67

| Profile Score | Tandem Score | TDA q-value | Volume | Spectrum Matches |
| --- | --- | --- | --- | --- |
| 14.8547 | 158.9014 | 0.0000 | 1.3687e+08 | 2 |

**Adduct Signal Fractions**

- **Unmodified**: 1.3687e+08

### RNEEYN(N)KSVQEIQATF Hex 3 HexNAc 5

**Mass:**  3456.4997

(-2.502e-07)

**Charge States:** 3, 4

**Start Position**: 51

**End Position**: 67

| Profile Score | Tandem Score | TDA q-value | Volume | Spectrum Matches |
| --- | --- | --- | --- | --- |
| 15.9894 | 158.4746 | 0.0000 | 5.6901e+09 | 15 |

**Adduct Signal Fractions**

- **Unmodified**: 5.6901e+09

### EEYN(N)KSVQEIQATF Fuc 1 Hex 4 HexNAc 5

**Mass:**  3494.4658

(1.492e-06)

**Charge States:** 3

**Start Position**: 53

**End Position**: 67

| Profile Score | Tandem Score | TDA q-value | Volume | Spectrum Matches |
| --- | --- | --- | --- | --- |
| -3.5750 | 158.1228 | 0.0000 | 2.2416e+07 | 2 |

**Adduct Signal Fractions**

- **Unmodified**: 2.2416e+07

### FYFTPN(N)KTEDTIF Fuc 2 Hex 5 HexNAc 6

**Mass:**  3942.6289

(1.476e-06)

**Charge States:** 3, 4

**Start Position**: 67

**End Position**: 80

| Profile Score | Tandem Score | TDA q-value | Volume | Spectrum Matches |
| --- | --- | --- | --- | --- |
| 15.2038 | 157.9657 | 0.0000 | 1.0178e+08 | 6 |

**Adduct Signal Fractions**

- **Unmodified**: 1.0178e+08

### RNEEYN(N)KSVQEIQATFF Hex 3 HexNAc 4

**Mass:**  3400.4844

(4.595e-07)

**Charge States:** 3, 4

**Start Position**: 51

**End Position**: 68

| Profile Score | Tandem Score | TDA q-value | Volume | Spectrum Matches |
| --- | --- | --- | --- | --- |
| 17.1740 | 157.3706 | 0.0000 | 1.9149e+09 | 12 |

**Adduct Signal Fractions**

- **Unmodified**: 1.9149e+09

### RNEEYN(N)KSVQEIQATFF Fuc 1 Hex 4 HexNAc 5

**Mass:**  3911.6784

(4.390e-08)

**Charge States:** 3, 4

**Start Position**: 51

**End Position**: 68

| Profile Score | Tandem Score | TDA q-value | Volume | Spectrum Matches |
| --- | --- | --- | --- | --- |
| 16.0720 | 157.0567 | 0.0000 | 1.0505e+09 | 18 |

**Adduct Signal Fractions**

- **Unmodified**: 1.0505e+09

### FYFTPN(N)KTEDTIF Fuc 1 Hex 5 HexNAc 7

**Mass:**  3999.6505

(1.601e-06)

**Charge States:** 3, 4

**Start Position**: 67

**End Position**: 80

| Profile Score | Tandem Score | TDA q-value | Volume | Spectrum Matches |
| --- | --- | --- | --- | --- |
| 14.1476 | 156.8633 | 0.0000 | 6.7462e+07 | 5 |

**Adduct Signal Fractions**

- **Unmodified**: 6.7462e+07

### RNEEYN(N)KSVQEIQATFF Fuc 1 Hex 3 HexNAc 4

**Mass:**  3546.5440

(1.344e-06)

**Charge States:** 3, 4

**Start Position**: 51

**End Position**: 68

| Profile Score | Tandem Score | TDA q-value | Volume | Spectrum Matches |
| --- | --- | --- | --- | --- |
| -4.8283 | 156.2382 | 0.0000 | 3.1887e+07 | 3 |

**Adduct Signal Fractions**

- **Unmodified**: 3.1887e+07

### YFTPN(N)KTEDTIF Fuc 2 Hex 5 HexNAc 5

**Mass:**  3592.4789

(1.104e-06)

**Charge States:** 3

**Start Position**: 68

**End Position**: 80

| Profile Score | Tandem Score | TDA q-value | Volume | Spectrum Matches |
| --- | --- | --- | --- | --- |
| -19.5689 | 156.1241 | 0.0000 | 1.4401e+07 | 1 |

**Adduct Signal Fractions**

- **Unmodified**: 1.4401e+07

### YFTPN(N)KTEDTIF Fuc 1 Hex 4 HexNAc 7

**Mass:**  3690.5308

(2.161e-06)

**Charge States:** 3, 4

**Start Position**: 68

**End Position**: 80

| Profile Score | Tandem Score | TDA q-value | Volume | Spectrum Matches |
| --- | --- | --- | --- | --- |
| -4.5467 | 155.6239 | 0.0000 | 3.5460e+07 | 3 |

**Adduct Signal Fractions**

- **Unmodified**: 3.5460e+07

### YFTPN(N)KTEDTIF Hex 4 HexNAc 4 Neu5Ac 2

**Mass:**  3517.4188

(1.224e-06)

**Charge States:** 3, 4

**Start Position**: 68

**End Position**: 80

| Profile Score | Tandem Score | TDA q-value | Volume | Spectrum Matches |
| --- | --- | --- | --- | --- |
| 13.9896 | 154.5954 | 0.0000 | 2.4680e+08 | 7 |

**Adduct Signal Fractions**

- **Unmodified**: 2.4680e+08

### YFTPN(N)KTEDTIF Fuc 1 Hex 5 HexNAc 4 Neu5Ac 1

**Mass:**  3534.4444

(4.319e-06)

**Charge States:** 3

**Start Position**: 68

**End Position**: 80

| Profile Score | Tandem Score | TDA q-value | Volume | Spectrum Matches |
| --- | --- | --- | --- | --- |
| 12.3999 | 154.5445 | 0.0000 | 4.6892e+07 | 3 |

**Adduct Signal Fractions**

- **Unmodified**: 4.6892e+07

### RNEEYN(N)KSVQEIQATFF Hex 3 HexNAc 5

**Mass:**  3603.5630

(6.553e-07)

**Charge States:** 3, 4

**Start Position**: 51

**End Position**: 68

| Profile Score | Tandem Score | TDA q-value | Volume | Spectrum Matches |
| --- | --- | --- | --- | --- |
| 15.1258 | 154.3998 | 0.0000 | 1.5677e+09 | 10 |

**Adduct Signal Fractions**

- **Unmodified**: 1.5677e+09

### RNEEYN(N)KSVQEIQATF Hex 3 HexNAc 4

**Mass:**  3253.4141

(9.969e-07)

**Charge States:** 3, 4

**Start Position**: 51

**End Position**: 67

| Profile Score | Tandem Score | TDA q-value | Volume | Spectrum Matches |
| --- | --- | --- | --- | --- |
| 16.7060 | 153.8311 | 0.0000 | 7.1716e+09 | 29 |

**Adduct Signal Fractions**

- **Unmodified**: 7.1716e+09

### RNEEYN(N)KSVQEIQATF Fuc 1 Hex 4 HexNAc 5

**Mass:**  3764.6123

(2.556e-06)

**Charge States:** 3, 4

**Start Position**: 51

**End Position**: 67

| Profile Score | Tandem Score | TDA q-value | Volume | Spectrum Matches |
| --- | --- | --- | --- | --- |
| 16.0058 | 152.6435 | 0.0000 | 3.2955e+09 | 15 |

**Adduct Signal Fractions**

- **Unmodified**: 3.2955e+09

### RNEEYN(N)KSVQEIQAT Hex 3 HexNAc 4

**Mass:**  3106.3482

(6.955e-07)

**Charge States:** 3

**Start Position**: 51

**End Position**: 66

| Profile Score | Tandem Score | TDA q-value | Volume | Spectrum Matches |
| --- | --- | --- | --- | --- |
| -16.9398 | 152.6099 | 0.0000 | 3.5419e+07 | 1 |

**Adduct Signal Fractions**

- **Unmodified**: 3.5419e+07

### FYFTPN(N)KTEDTIF Fuc 1 Hex 5 HexNAc 4 Neu5Ac 1

**Mass:**  3681.5161

(6.328e-06)

**Charge States:** 3

**Start Position**: 67

**End Position**: 80

| Profile Score | Tandem Score | TDA q-value | Volume | Spectrum Matches |
| --- | --- | --- | --- | --- |
| 12.7060 | 152.0488 | 0.0000 | 2.7146e+07 | 3 |

**Adduct Signal Fractions**

- **Unmodified**: 2.7146e+07

### RNEEYN(N)KSVQEIQATF Fuc 1 Hex 4 HexNAc 4

**Mass:**  3561.5344

(2.715e-06)

**Charge States:** 3, 4

**Start Position**: 51

**End Position**: 67

| Profile Score | Tandem Score | TDA q-value | Volume | Spectrum Matches |
| --- | --- | --- | --- | --- |
| 15.5872 | 151.9757 | 0.0000 | 1.6582e+08 | 3 |

**Adduct Signal Fractions**

- **Unmodified**: 1.6582e+08

### RNEEYN(N)KSVQEIQATF Fuc 1 Hex 3 HexNAc 4

**Mass:**  3399.4740

(2.442e-07)

**Charge States:** 3, 4

**Start Position**: 51

**End Position**: 67

| Profile Score | Tandem Score | TDA q-value | Volume | Spectrum Matches |
| --- | --- | --- | --- | --- |
| 11.5993 | 151.8453 | 0.0000 | 1.4407e+08 | 8 |

**Adduct Signal Fractions**

- **Unmodified**: 1.4407e+08

### YFTPN(N)KTEDTIF Hex 5 HexNAc 9

**Mass:**  4112.6813

(1.356e-06)

**Charge States:** 3, 4

**Start Position**: 68

**End Position**: 80

| Profile Score | Tandem Score | TDA q-value | Volume | Spectrum Matches |
| --- | --- | --- | --- | --- |
| -3.8965 | 151.1826 | 0.0000 | 4.0269e+07 | 3 |

**Adduct Signal Fractions**

- **Unmodified**: 4.0269e+07

### RNEEYN(N)KSVQEIQATF Fuc 1 Hex 3 HexNAc 5

**Mass:**  3602.5534

(9.268e-07)

**Charge States:** 3, 4

**Start Position**: 51

**End Position**: 67

| Profile Score | Tandem Score | TDA q-value | Volume | Spectrum Matches |
| --- | --- | --- | --- | --- |
| 11.9208 | 150.9363 | 0.0000 | 1.5030e+08 | 7 |

**Adduct Signal Fractions**

- **Unmodified**: 1.5030e+08

### RNEEYN(N)KSVQEIQATF Hex 3 HexNAc 6

**Mass:**  3659.5823

(2.539e-06)

**Charge States:** 3, 4

**Start Position**: 51

**End Position**: 67

| Profile Score | Tandem Score | TDA q-value | Volume | Spectrum Matches |
| --- | --- | --- | --- | --- |
| 10.8484 | 150.9055 | 0.0000 | 3.1299e+08 | 7 |

**Adduct Signal Fractions**

- **Unmodified**: 3.1299e+08

### FTPN(N)KTEDTIF Hex 3 HexNAc 4

**Mass:**  2610.1144

(8.446e-07)

**Charge States:** 2, 3

**Start Position**: 69

**End Position**: 80

| Profile Score | Tandem Score | TDA q-value | Volume | Spectrum Matches |
| --- | --- | --- | --- | --- |
| -3.3267 | 150.7862 | 0.0000 | 3.4297e+07 | 3 |

**Adduct Signal Fractions**

- **Unmodified**: 3.4297e+07

### FTPN(N)KTEDTIF Hex 4 HexNAc 7

**Mass:**  3381.4048

(8.557e-07)

**Charge States:** 3, 4

**Start Position**: 69

**End Position**: 80

| Profile Score | Tandem Score | TDA q-value | Volume | Spectrum Matches |
| --- | --- | --- | --- | --- |
| 13.0617 | 150.7796 | 0.0000 | 7.0572e+07 | 4 |

**Adduct Signal Fractions**

- **Unmodified**: 7.0572e+07

### TPN(N)KTEDTIF Fuc 2 Hex 5 HexNAc 6

**Mass:**  3485.4270

(1.344e-06)

**Charge States:** 3

**Start Position**: 70

**End Position**: 80

| Profile Score | Tandem Score | TDA q-value | Volume | Spectrum Matches |
| --- | --- | --- | --- | --- |
| -4.5449 | 150.4819 | 0.0000 | 8.4693e+06 | 1 |

**Adduct Signal Fractions**

- **Unmodified**: 8.4693e+06

### FYFTPN(N)KTEDTIF Hex 4 HexNAc 8

**Mass:**  3894.6198

(1.793e-06)

**Charge States:** 3, 4

**Start Position**: 67

**End Position**: 80

| Profile Score | Tandem Score | TDA q-value | Volume | Spectrum Matches |
| --- | --- | --- | --- | --- |
| 16.0244 | 149.9802 | 0.0000 | 6.8705e+07 | 5 |

**Adduct Signal Fractions**

- **Unmodified**: 6.8705e+07

### RNEEYN(N)KSVQEIQATF Fuc 2 Hex 5 HexNAc 5

**Mass:**  4072.7221

(1.347e-06)

**Charge States:** 3, 4

**Start Position**: 51

**End Position**: 67

| Profile Score | Tandem Score | TDA q-value | Volume | Spectrum Matches |
| --- | --- | --- | --- | --- |
| -2.0290 | 149.7450 | 0.0000 | 9.3738e+07 | 3 |

**Adduct Signal Fractions**

- **Unmodified**: 9.3738e+07

### EEYN(N)KSVQEIQATF Hex 3 HexNAc 4

**Mass:**  2983.2740

(1.254e-06)

**Charge States:** 3

**Start Position**: 53

**End Position**: 67

| Profile Score | Tandem Score | TDA q-value | Volume | Spectrum Matches |
| --- | --- | --- | --- | --- |
| -3.3868 | 148.2295 | 0.0000 | 4.3192e+07 | 2 |

**Adduct Signal Fractions**

- **Unmodified**: 4.3192e+07

### RN(D)EEYN(N)KSVQEIQATF Hex 3 HexNAc 4

**Mass:**  3254.4021

(3.038e-04)

**Charge States:** 3, 4

**Start Position**: 51

**End Position**: 67

| Profile Score | Tandem Score | TDA q-value | Volume | Spectrum Matches |
| --- | --- | --- | --- | --- |
| 9.8657 | 147.7783 | 0.0000 | 6.5733e+08 | 94 |

**Adduct Signal Fractions**

- **Unmodified**: 6.5733e+08

### TPN(N)KTEDTIF Fuc 1 Hex 5 HexNAc 7

**Mass:**  3542.4475

(1.076e-06)

**Charge States:** 3

**Start Position**: 70

**End Position**: 80

| Profile Score | Tandem Score | TDA q-value | Volume | Spectrum Matches |
| --- | --- | --- | --- | --- |
| -19.1065 | 147.4084 | 0.0000 | 4.0006e+06 | 1 |

**Adduct Signal Fractions**

- **Unmodified**: 4.0006e+06

### RNEEYN(N)KSVQEIQATFF Fuc 1 Hex 4 HexNAc 4

**Mass:**  3708.5952

(3.769e-06)

**Charge States:** 3, 4

**Start Position**: 51

**End Position**: 68

| Profile Score | Tandem Score | TDA q-value | Volume | Spectrum Matches |
| --- | --- | --- | --- | --- |
| -4.6651 | 146.5426 | 0.0000 | 4.7344e+07 | 3 |

**Adduct Signal Fractions**

- **Unmodified**: 4.7344e+07

### RNEEYN(N)KSVQEIQATF Hex 4 HexNAc 7

**Mass:**  4024.7121

(3.556e-06)

**Charge States:** 3, 4

**Start Position**: 51

**End Position**: 67

| Profile Score | Tandem Score | TDA q-value | Volume | Spectrum Matches |
| --- | --- | --- | --- | --- |
| -4.4004 | 146.1322 | 0.0000 | 8.3937e+07 | 4 |

**Adduct Signal Fractions**

- **Unmodified**: 8.3937e+07

### FYFTPN(N)KTEDTIF Hex 5 HexNAc 8

**Mass:**  4056.6726

(1.756e-06)

**Charge States:** 3, 4

**Start Position**: 67

**End Position**: 80

| Profile Score | Tandem Score | TDA q-value | Volume | Spectrum Matches |
| --- | --- | --- | --- | --- |
| 12.8967 | 146.0071 | 0.0000 | 6.5280e+07 | 6 |

**Adduct Signal Fractions**

- **Unmodified**: 6.5280e+07

### FYFTPN(N)KTEDTIF Hex 4 HexNAc 4 Neu5Ac 2

**Mass:**  3664.4899

(1.000e-06)

**Charge States:** 3, 4

**Start Position**: 67

**End Position**: 80

| Profile Score | Tandem Score | TDA q-value | Volume | Spectrum Matches |
| --- | --- | --- | --- | --- |
| 13.5840 | 146.0067 | 0.0000 | 1.4952e+08 | 10 |

**Adduct Signal Fractions**

- **Unmodified**: 1.4952e+08

### RNEEYN(N)KSVQEIQATF Hex 4 HexNAc 5

**Mass:**  3618.5542

(2.560e-06)

**Charge States:** 3, 4

**Start Position**: 51

**End Position**: 67

| Profile Score | Tandem Score | TDA q-value | Volume | Spectrum Matches |
| --- | --- | --- | --- | --- |
| -5.0413 | 145.8878 | 0.0000 | 7.2763e+07 | 3 |

**Adduct Signal Fractions**

- **Unmodified**: 7.2763e+07

### YFTPN(N)KTEDTIF Hex 4 HexNAc 5

**Mass:**  3138.3118

(2.225e-06)

**Charge States:** 3

**Start Position**: 68

**End Position**: 80

| Profile Score | Tandem Score | TDA q-value | Volume | Spectrum Matches |
| --- | --- | --- | --- | --- |
| -19.9178 | 145.0047 | 0.0000 | 1.5691e+07 | 1 |

**Adduct Signal Fractions**

- **Unmodified**: 1.5691e+07

### LNVQREN(N)GTISRY Hex 3 HexNAc 6

**Mass:**  3253.4390

(1.608e-06)

**Charge States:** 2, 3, 4

**Start Position**: 97

**End Position**: 110

| Profile Score | Tandem Score | TDA q-value | Volume | Spectrum Matches |
| --- | --- | --- | --- | --- |
| 13.8083 | 144.8243 | 0.0000 | 1.0130e+10 | 12 |

**Adduct Signal Fractions**

- **Unmodified**: 1.0130e+10

### YFTPN(N)KTEDTIFL Hex 3 HexNAc 6

**Mass:**  3292.4203

(1.114e-06)

**Charge States:** 3, 4

**Start Position**: 68

**End Position**: 81

| Profile Score | Tandem Score | TDA q-value | Volume | Spectrum Matches |
| --- | --- | --- | --- | --- |
| 14.5467 | 144.7965 | 0.0000 | 2.4047e+07 | 5 |

**Adduct Signal Fractions**

- **Unmodified**: 2.4047e+07

### RNEEYN(N)KSVQEIQATFF Hex 4 HexNAc 5

**Mass:**  3765.6168

(-5.867e-08)

**Charge States:** 3, 4

**Start Position**: 51

**End Position**: 68

| Profile Score | Tandem Score | TDA q-value | Volume | Spectrum Matches |
| --- | --- | --- | --- | --- |
| -4.4176 | 144.7441 | 0.0000 | 2.7205e+07 | 3 |

**Adduct Signal Fractions**

- **Unmodified**: 2.7205e+07

### RNEEYN(N)KSVQEIQATFF Fuc 1 Hex 3 HexNAc 5

**Mass:**  3749.6197

(9.711e-07)

**Charge States:** 3, 4

**Start Position**: 51

**End Position**: 68

| Profile Score | Tandem Score | TDA q-value | Volume | Spectrum Matches |
| --- | --- | --- | --- | --- |
| -17.9399 | 144.5266 | 0.0000 | 3.2818e+07 | 2 |

**Adduct Signal Fractions**

- **Unmodified**: 3.2818e+07

### LNVQREN(N)GTISRY Fuc 1 Hex 4 HexNAc 6

**Mass:**  3561.5520

(1.876e-06)

**Charge States:** 3, 4

**Start Position**: 97

**End Position**: 110

| Profile Score | Tandem Score | TDA q-value | Volume | Spectrum Matches |
| --- | --- | --- | --- | --- |
| 14.4578 | 144.2893 | 0.0000 | 3.7177e+09 | 13 |

**Adduct Signal Fractions**

- **Unmodified**: 3.7177e+09

### FYFTPN(N)KTEDTIF Hex 4 HexNAc 6

**Mass:**  3488.4596

(1.718e-06)

**Charge States:** 3

**Start Position**: 67

**End Position**: 80

| Profile Score | Tandem Score | TDA q-value | Volume | Spectrum Matches |
| --- | --- | --- | --- | --- |
| 11.8208 | 143.9516 | 0.0000 | 4.8425e+07 | 5 |

**Adduct Signal Fractions**

- **Unmodified**: 4.8425e+07

### YFTPN(N)KT Hex 3 HexNAc 6

**Mass:**  2575.0711

(3.916e-04)

**Start Position**: 68

**End Position**: 75

| Profile Score | Tandem Score | TDA q-value | Volume | Spectrum Matches |
| --- | --- | --- | --- | --- |
| 0.0000 | 143.3566 | 0.0000 | 0.0000e+00 | 1 |

Adduct:

No Chromatogram Found

### NVQREN(N)GTISRY Hex 3 HexNAc 6

**Mass:**  3140.3523

(7.192e-07)

**Charge States:** 3

**Start Position**: 98

**End Position**: 110

| Profile Score | Tandem Score | TDA q-value | Volume | Spectrum Matches |
| --- | --- | --- | --- | --- |
| 13.5173 | 142.8736 | 0.0000 | 7.8799e+08 | 8 |

**Adduct Signal Fractions**

- **Unmodified**: 7.8799e+08

### RNEEYN(N)KSVQEIQATF Fuc 2 Hex 5 HexNAc 6

**Mass:**  4275.8019

(1.490e-06)

**Charge States:** 3, 4

**Start Position**: 51

**End Position**: 67

| Profile Score | Tandem Score | TDA q-value | Volume | Spectrum Matches |
| --- | --- | --- | --- | --- |
| 12.9939 | 141.6000 | 0.0029 | 2.6339e+07 | 3 |

**Adduct Signal Fractions**

- **Unmodified**: 2.6339e+07

### LNVQREN(N)GTISRY Hex 3 HexNAc 5

**Mass:**  3050.3611

(1.632e-06)

**Charge States:** 3, 4

**Start Position**: 97

**End Position**: 110

| Profile Score | Tandem Score | TDA q-value | Volume | Spectrum Matches |
| --- | --- | --- | --- | --- |
| 15.3169 | 141.5113 | 0.0029 | 2.7361e+09 | 10 |

**Adduct Signal Fractions**

- **Unmodified**: 2.7361e+09

### RNEEYN(N)KSVQEIQATFF Fuc 2 Hex 5 HexNAc 5

**Mass:**  4219.7902

(1.597e-06)

**Charge States:** 3, 4

**Start Position**: 51

**End Position**: 68

| Profile Score | Tandem Score | TDA q-value | Volume | Spectrum Matches |
| --- | --- | --- | --- | --- |
| 12.8886 | 141.4993 | 0.0029 | 3.4275e+07 | 4 |

**Adduct Signal Fractions**

- **Unmodified**: 3.4275e+07

### TPN(N)KTEDTIF Hex 4 HexNAc 4 Neu5Ac 2

**Mass:**  3207.2913

(1.886e-06)

**Charge States:** 3, 4

**Start Position**: 70

**End Position**: 80

| Profile Score | Tandem Score | TDA q-value | Volume | Spectrum Matches |
| --- | --- | --- | --- | --- |
| 13.1162 | 141.2675 | 0.0029 | 1.3432e+07 | 2 |

**Adduct Signal Fractions**

- **Unmodified**: 1.3432e+07

### LNVQREN(N)GTISRY Fuc 1 Hex 4 HexNAc 5

**Mass:**  3358.4723

(1.573e-06)

**Charge States:** 3, 4

**Start Position**: 97

**End Position**: 110

| Profile Score | Tandem Score | TDA q-value | Volume | Spectrum Matches |
| --- | --- | --- | --- | --- |
| 13.9032 | 140.9696 | 0.0029 | 1.0605e+09 | 8 |

**Adduct Signal Fractions**

- **Unmodified**: 1.0605e+09

### YFTPN(N)KTEDTIF Fuc 1 Hex 5 HexNAc 8

**Mass:**  4055.6621

(2.537e-06)

**Charge States:** 3, 4

**Start Position**: 68

**End Position**: 80

| Profile Score | Tandem Score | TDA q-value | Volume | Spectrum Matches |
| --- | --- | --- | --- | --- |
| 7.8561 | 140.7007 | 0.0029 | 3.1750e+07 | 4 |

**Adduct Signal Fractions**

- **Unmodified**: 3.1750e+07

### FYFTPN(N)KTEDTIF Hex 5 HexNAc 9

**Mass:**  4259.7521

(1.879e-06)

**Charge States:** 3, 4

**Start Position**: 67

**End Position**: 80

| Profile Score | Tandem Score | TDA q-value | Volume | Spectrum Matches |
| --- | --- | --- | --- | --- |
| 11.6097 | 140.4833 | 0.0029 | 2.2955e+07 | 4 |

**Adduct Signal Fractions**

- **Unmodified**: 2.2955e+07

### RNEEYN(N)KSVQEIQATFF Fuc 1 Hex 4 HexNAc 6

**Mass:**  4114.7565

(1.052e-06)

**Charge States:** 3, 4

**Start Position**: 51

**End Position**: 68

| Profile Score | Tandem Score | TDA q-value | Volume | Spectrum Matches |
| --- | --- | --- | --- | --- |
| 13.2533 | 140.0369 | 0.0029 | 5.7241e+07 | 4 |

**Adduct Signal Fractions**

- **Unmodified**: 5.7241e+07

### FYFTPN(N)KTEDTIF Hex 4 HexNAc 5

**Mass:**  3285.3751

(1.851e-07)

**Charge States:** 3

**Start Position**: 67

**End Position**: 80

| Profile Score | Tandem Score | TDA q-value | Volume | Spectrum Matches |
| --- | --- | --- | --- | --- |
| -4.9367 | 139.8149 | 0.0029 | 1.3265e+07 | 2 |

**Adduct Signal Fractions**

- **Unmodified**: 1.3265e+07

### QTRQDQC(C)IYN(N)TTY Hex 3 HexNAc 5

**Mass:**  3191.3010

(1.260e-06)

**Charge States:** 3

**Start Position**: 84

**End Position**: 97

| Profile Score | Tandem Score | TDA q-value | Volume | Spectrum Matches |
| --- | --- | --- | --- | --- |
| 14.9342 | 139.7834 | 0.0029 | 9.5135e+07 | 4 |

**Adduct Signal Fractions**

- **Unmodified**: 9.5135e+07

### YFTPN(N)KTEDTIFL Hex 3 HexNAc 5

**Mass:**  3089.3417

(1.403e-06)

**Charge States:** 3

**Start Position**: 68

**End Position**: 81

| Profile Score | Tandem Score | TDA q-value | Volume | Spectrum Matches |
| --- | --- | --- | --- | --- |
| 13.9440 | 139.7763 | 0.0029 | 1.1916e+07 | 3 |

**Adduct Signal Fractions**

- **Unmodified**: 1.1916e+07

### LNVQREN(N)GTISRY Fuc 1 Hex 4 HexNAc 7

**Mass:**  3764.6336

(2.104e-06)

**Charge States:** 3, 4

**Start Position**: 97

**End Position**: 110

| Profile Score | Tandem Score | TDA q-value | Volume | Spectrum Matches |
| --- | --- | --- | --- | --- |
| -3.7549 | 138.1299 | 0.0029 | 2.3819e+08 | 3 |

**Adduct Signal Fractions**

- **Unmodified**: 2.3819e+08

### RNEEYN(N)KSVQEIQATF Hex 4 HexNAc 8

**Mass:**  4227.7897

(1.396e-06)

**Charge States:** 3, 4

**Start Position**: 51

**End Position**: 67

| Profile Score | Tandem Score | TDA q-value | Volume | Spectrum Matches |
| --- | --- | --- | --- | --- |
| -3.7831 | 138.0715 | 0.0029 | 3.8672e+07 | 3 |

**Adduct Signal Fractions**

- **Unmodified**: 3.8672e+07

### RNEEYN(N)KSVQEIQATFF Hex 3 HexNAc 6

**Mass:**  3806.6442

(2.460e-07)

**Charge States:** 3, 4

**Start Position**: 51

**End Position**: 68

| Profile Score | Tandem Score | TDA q-value | Volume | Spectrum Matches |
| --- | --- | --- | --- | --- |
| -4.2816 | 138.0007 | 0.0029 | 7.4318e+07 | 4 |

**Adduct Signal Fractions**

- **Unmodified**: 7.4318e+07

### RNEEYN(N)KSVQEIQAT Fuc 1 Hex 4 HexNAc 5

**Mass:**  3617.5438

(2.120e-06)

**Charge States:** 3

**Start Position**: 51

**End Position**: 66

| Profile Score | Tandem Score | TDA q-value | Volume | Spectrum Matches |
| --- | --- | --- | --- | --- |
| -19.3184 | 137.9198 | 0.0029 | 1.1593e+07 | 1 |

**Adduct Signal Fractions**

- **Unmodified**: 1.1593e+07

### LNVQREN(N)GTISRY Fuc 1 Hex 3 HexNAc 6

**Mass:**  3399.4975

(2.960e-04)

**Charge States:** 3, 4

**Start Position**: 97

**End Position**: 110

| Profile Score | Tandem Score | TDA q-value | Volume | Spectrum Matches |
| --- | --- | --- | --- | --- |
| 11.7716 | 137.5813 | 0.0029 | 1.5731e+08 | 18 |

**Adduct Signal Fractions**

- **Unmodified**: 1.5731e+08

### NVQREN(N)GTISRY Hex 4 HexNAc 8

**Mass:**  3708.5678

(1.251e-06)

**Charge States:** 3, 4

**Start Position**: 98

**End Position**: 110

| Profile Score | Tandem Score | TDA q-value | Volume | Spectrum Matches |
| --- | --- | --- | --- | --- |
| -19.2215 | 137.3470 | 0.0029 | 7.7288e+07 | 2 |

**Adduct Signal Fractions**

- **Unmodified**: 7.7288e+07

### N(N)KSVQEIQATFF Hex 3 HexNAc 4

**Mass:**  2709.1939

(1.417e-06)

**Charge States:** 3

**Start Position**: 56

**End Position**: 68

| Profile Score | Tandem Score | TDA q-value | Volume | Spectrum Matches |
| --- | --- | --- | --- | --- |
| -2.9819 | 137.1995 | 0.0029 | 4.6341e+06 | 2 |

**Adduct Signal Fractions**

- **Unmodified**: 4.6341e+06

### NVQREN(N)GTISRY Hex 3 HexNAc 5

**Mass:**  2937.2737

(5.191e-07)

**Charge States:** 3

**Start Position**: 98

**End Position**: 110

| Profile Score | Tandem Score | TDA q-value | Volume | Spectrum Matches |
| --- | --- | --- | --- | --- |
| 12.7613 | 136.9466 | 0.0029 | 2.0352e+08 | 3 |

**Adduct Signal Fractions**

- **Unmodified**: 2.0352e+08

### FYFTPN(N)KT Hex 3 HexNAc 6

**Mass:**  2721.1349

(1.639e-06)

**Charge States:** 3

**Start Position**: 67

**End Position**: 75

| Profile Score | Tandem Score | TDA q-value | Volume | Spectrum Matches |
| --- | --- | --- | --- | --- |
| -3.6704 | 135.8514 | 0.0000 | 1.2492e+07 | 2 |

**Adduct Signal Fractions**

- **Unmodified**: 1.2492e+07

### YFTPN(N)KTEDTIF Fuc 1 Hex 5 HexNAc 6

**Mass:**  3649.5053

(2.437e-06)

**Charge States:** 3

**Start Position**: 68

**End Position**: 80

| Profile Score | Tandem Score | TDA q-value | Volume | Spectrum Matches |
| --- | --- | --- | --- | --- |
| -4.9939 | 135.2286 | 0.0029 | 7.8248e+06 | 2 |

**Adduct Signal Fractions**

- **Unmodified**: 7.8248e+06

### YFTPN(N)KTEDTIF Fuc 1 Hex 5 HexNAc 4 Neu5Ac 2

**Mass:**  3825.5304

(5.496e-07)

**Charge States:** 3

**Start Position**: 68

**End Position**: 80

| Profile Score | Tandem Score | TDA q-value | Volume | Spectrum Matches |
| --- | --- | --- | --- | --- |
| -5.5335 | 134.8218 | 0.0029 | 2.9963e+07 | 2 |

**Adduct Signal Fractions**

- **Unmodified**: 2.9963e+07

### FTPN(N)KTEDTIF Hex 4 HexNAc 4 Neu5Ac 2

**Mass:**  3354.3583

(1.155e-06)

**Charge States:** 3, 4

**Start Position**: 69

**End Position**: 80

| Profile Score | Tandem Score | TDA q-value | Volume | Spectrum Matches |
| --- | --- | --- | --- | --- |
| -4.2697 | 134.2545 | 0.0029 | 2.2869e+07 | 2 |

**Adduct Signal Fractions**

- **Unmodified**: 2.2869e+07

### RNEEYN(N)KSVQEIQATFF Fuc 2 Hex 4 HexNAc 5

**Mass:**  4057.7326

(1.105e-06)

**Charge States:** 3

**Start Position**: 51

**End Position**: 68

| Profile Score | Tandem Score | TDA q-value | Volume | Spectrum Matches |
| --- | --- | --- | --- | --- |
| -4.4109 | 133.5045 | 0.0029 | 1.5369e+07 | 2 |

**Adduct Signal Fractions**

- **Unmodified**: 1.5369e+07

### QTRQDQC(C)IYN(N)TTY Hex 3 HexNAc 6

**Mass:**  3394.3819

(1.568e-06)

**Charge States:** 3, 4

**Start Position**: 84

**End Position**: 97

| Profile Score | Tandem Score | TDA q-value | Volume | Spectrum Matches |
| --- | --- | --- | --- | --- |
| 15.4802 | 133.3624 | 0.0029 | 2.5649e+08 | 8 |

**Adduct Signal Fractions**

- **Unmodified**: 2.5649e+08

### RNEEYN(N)KSVQEIQATF Fuc 2 Hex 4 HexNAc 5

**Mass:**  3910.6719

(2.395e-06)

**Charge States:** 3, 4

**Start Position**: 51

**End Position**: 67

| Profile Score | Tandem Score | TDA q-value | Volume | Spectrum Matches |
| --- | --- | --- | --- | --- |
| -4.2158 | 131.9282 | 0.0029 | 4.3351e+07 | 2 |

**Adduct Signal Fractions**

- **Unmodified**: 4.3351e+07

### LNVQREN(N)GTISRY Hex 3 HexNAc 4

**Mass:**  2847.2830

(2.195e-06)

**Charge States:** 3

**Start Position**: 97

**End Position**: 110

| Profile Score | Tandem Score | TDA q-value | Volume | Spectrum Matches |
| --- | --- | --- | --- | --- |
| 13.7867 | 131.0133 | 0.0055 | 2.0043e+08 | 4 |

**Adduct Signal Fractions**

- **Unmodified**: 2.0043e+08

### NVQREN(N)GTISRY Hex 4 HexNAc 7

**Mass:**  3505.4851

(4.283e-07)

**Charge States:** 3

**Start Position**: 98

**End Position**: 110

| Profile Score | Tandem Score | TDA q-value | Volume | Spectrum Matches |
| --- | --- | --- | --- | --- |
| -18.3974 | 130.3971 | 0.0055 | 6.5787e+07 | 1 |

**Adduct Signal Fractions**

- **Unmodified**: 6.5787e+07

### VQREN(N)GTISRY Fuc 1 Hex 4 HexNAc 6

**Mass:**  3334.4199

(1.989e-07)

**Charge States:** 3, 4

**Start Position**: 99

**End Position**: 110

| Profile Score | Tandem Score | TDA q-value | Volume | Spectrum Matches |
| --- | --- | --- | --- | --- |
| -19.8400 | 130.2946 | 0.0055 | 1.3624e+07 | 1 |

**Adduct Signal Fractions**

- **Unmodified**: 1.3624e+07

### RN(D)EEYN(N)KSVQEIQATFF Hex 3 HexNAc 5

**Mass:**  3604.5514

(2.751e-04)

**Charge States:** 3

**Start Position**: 51

**End Position**: 68

| Profile Score | Tandem Score | TDA q-value | Volume | Spectrum Matches |
| --- | --- | --- | --- | --- |
| -5.7903 | 129.6681 | 0.0055 | 4.6193e+07 | 12 |

**Adduct Signal Fractions**

- **Unmodified**: 4.6193e+07

### NVQREN(N)GTISRY Fuc 1 Hex 4 HexNAc 6

**Mass:**  3448.4646

(3.220e-07)

**Charge States:** 3

**Start Position**: 98

**End Position**: 110

| Profile Score | Tandem Score | TDA q-value | Volume | Spectrum Matches |
| --- | --- | --- | --- | --- |
| 13.0527 | 128.9347 | 0.0055 | 3.2931e+08 | 5 |

**Adduct Signal Fractions**

- **Unmodified**: 3.2931e+08

### RN(D)EEYN(N)KSVQEIQATF Fuc 1 Hex 4 HexNAc 5

**Mass:**  3765.5927

(2.633e-04)

**Charge States:** 3, 4

**Start Position**: 51

**End Position**: 67

| Profile Score | Tandem Score | TDA q-value | Volume | Spectrum Matches |
| --- | --- | --- | --- | --- |
| 11.4420 | 128.4925 | 0.0055 | 3.2149e+08 | 11 |

**Adduct Signal Fractions**

- **Unmodified**: 3.2149e+08

### FYFTPN(N)KTED Hex 3 HexNAc 6

**Mass:**  2965.2037

(3.641e-07)

**Charge States:** 3

**Start Position**: 67

**End Position**: 77

| Profile Score | Tandem Score | TDA q-value | Volume | Spectrum Matches |
| --- | --- | --- | --- | --- |
| -18.3085 | 128.3172 | 0.0055 | 3.7069e+06 | 1 |

**Adduct Signal Fractions**

- **Unmodified**: 3.7069e+06

### RNEEYN(N)KSVQEIQATF Hex 4 HexNAc 6

**Mass:**  3821.6358

(1.895e-06)

**Charge States:** 3, 4

**Start Position**: 51

**End Position**: 67

| Profile Score | Tandem Score | TDA q-value | Volume | Spectrum Matches |
| --- | --- | --- | --- | --- |
| 12.1893 | 127.1249 | 0.0079 | 5.7117e+08 | 7 |

**Adduct Signal Fractions**

- **Unmodified**: 5.7117e+08

### RNEEYN(N)KSVQEIQAT Hex 3 HexNAc 5

**Mass:**  3309.4286

(2.667e-06)

**Charge States:** 3

**Start Position**: 51

**End Position**: 66

| Profile Score | Tandem Score | TDA q-value | Volume | Spectrum Matches |
| --- | --- | --- | --- | --- |
| 11.9219 | 127.0341 | 0.0079 | 3.5050e+07 | 3 |

**Adduct Signal Fractions**

- **Unmodified**: 3.5050e+07

### LNVQREN(N)GTISRY Fuc 1 Hex 5 HexNAc 8

**Mass:**  4129.7702

(1.719e-06)

**Charge States:** 3, 4

**Start Position**: 97

**End Position**: 110

| Profile Score | Tandem Score | TDA q-value | Volume | Spectrum Matches |
| --- | --- | --- | --- | --- |
| 13.5439 | 126.6515 | 0.0079 | 5.2087e+08 | 4 |

**Adduct Signal Fractions**

- **Unmodified**: 5.2087e+08

### LNVQREN(N)GTISRY Fuc 1 Hex 5 HexNAc 7

**Mass:**  3926.6845

(2.741e-06)

**Charge States:** 3, 4

**Start Position**: 97

**End Position**: 110

| Profile Score | Tandem Score | TDA q-value | Volume | Spectrum Matches |
| --- | --- | --- | --- | --- |
| 11.0986 | 126.1564 | 0.0104 | 5.9894e+08 | 9 |

**Adduct Signal Fractions**

- **Unmodified**: 5.9894e+08

### TPN(N)KTEDTIFL Hex 3 HexNAc 6

**Mass:**  2982.2885

(1.887e-06)

**Charge States:** 3

**Start Position**: 70

**End Position**: 81

| Profile Score | Tandem Score | TDA q-value | Volume | Spectrum Matches |
| --- | --- | --- | --- | --- |
| -17.3359 | 125.9819 | 0.0104 | 2.3295e+06 | 3 |

**Adduct Signal Fractions**

- **Unmodified**: 2.3295e+06

### LNVQREN(N)GTISRY Hex 5 HexNAc 9

**Mass:**  4186.7849

(2.462e-06)

**Charge States:** 3, 4

**Start Position**: 97

**End Position**: 110

| Profile Score | Tandem Score | TDA q-value | Volume | Spectrum Matches |
| --- | --- | --- | --- | --- |
| 10.3636 | 125.6825 | 0.0104 | 3.1093e+08 | 2 |

**Adduct Signal Fractions**

- **Unmodified**: 3.1093e+08

### RN(D)EEYN(N)KSVQEIQATF Hex 3 HexNAc 5

**Mass:**  3457.4804

(2.864e-04)

**Charge States:** 3, 4

**Start Position**: 51

**End Position**: 67

| Profile Score | Tandem Score | TDA q-value | Volume | Spectrum Matches |
| --- | --- | --- | --- | --- |
| 9.5034 | 125.4199 | 0.0120 | 5.5031e+08 | 14 |

**Adduct Signal Fractions**

- **Unmodified**: 5.5031e+08

### LNVQREN(N)GTISRY Hex 4 HexNAc 7

**Mass:**  3618.5696

(1.313e-06)

**Charge States:** 3, 4

**Start Position**: 97

**End Position**: 110

| Profile Score | Tandem Score | TDA q-value | Volume | Spectrum Matches |
| --- | --- | --- | --- | --- |
| 14.6323 | 125.1969 | 0.0120 | 2.2065e+09 | 14 |

**Adduct Signal Fractions**

- **Unmodified**: 2.2065e+09

### QTRQDQC(C)IYN(N)TTY Fuc 1 Hex 4 HexNAc 5

**Mass:**  3499.4134

(1.602e-06)

**Charge States:** 3

**Start Position**: 84

**End Position**: 97

| Profile Score | Tandem Score | TDA q-value | Volume | Spectrum Matches |
| --- | --- | --- | --- | --- |
| -3.6002 | 124.9157 | 0.0120 | 2.8445e+07 | 2 |

**Adduct Signal Fractions**

- **Unmodified**: 2.8445e+07

### FRNEEYN(N)KSVQEIQATF Hex 3 HexNAc 4

**Mass:**  3400.4796

(-9.558e-07)

**Charge States:** 3

**Start Position**: 50

**End Position**: 67

| Profile Score | Tandem Score | TDA q-value | Volume | Spectrum Matches |
| --- | --- | --- | --- | --- |
| -19.8783 | 124.9134 | 0.0120 | 8.2594e+06 | 1 |

**Adduct Signal Fractions**

- **Unmodified**: 8.2594e+06

### LNVQREN(N)GTISRY Hex 4 HexNAc 6

**Mass:**  3415.4932

(1.355e-06)

**Charge States:** 3

**Start Position**: 97

**End Position**: 110

| Profile Score | Tandem Score | TDA q-value | Volume | Spectrum Matches |
| --- | --- | --- | --- | --- |
| -18.2033 | 124.4993 | 0.0120 | 5.4431e+07 | 1 |

**Adduct Signal Fractions**

- **Unmodified**: 5.4431e+07

### NVQREN(N)GTISRY Fuc 2 Hex 5 HexNAc 6

**Mass:**  3756.5777

(1.204e-06)

**Charge States:** 3, 4

**Start Position**: 98

**End Position**: 110

| Profile Score | Tandem Score | TDA q-value | Volume | Spectrum Matches |
| --- | --- | --- | --- | --- |
| -19.0458 | 123.8226 | 0.0120 | 8.0054e+07 | 2 |

**Adduct Signal Fractions**

- **Unmodified**: 8.0054e+07

### N(N)KTEDTIF Hex 3 HexNAc 6

**Mass:**  2671.1028

(8.310e-07)

**Charge States:** 3

**Start Position**: 72

**End Position**: 80

| Profile Score | Tandem Score | TDA q-value | Volume | Spectrum Matches |
| --- | --- | --- | --- | --- |
| -19.1641 | 123.5627 | 0.0000 | 4.9330e+06 | 1 |

**Adduct Signal Fractions**

- **Unmodified**: 4.9330e+06

### LN(D)VQREN(N)GTISRY Hex 3 HexNAc 6

**Mass:**  3254.4274

(3.058e-04)

**Charge States:** 3, 4

**Start Position**: 97

**End Position**: 110

| Profile Score | Tandem Score | TDA q-value | Volume | Spectrum Matches |
| --- | --- | --- | --- | --- |
| 7.8501 | 123.2025 | 0.0120 | 3.0250e+08 | 81 |

**Adduct Signal Fractions**

- **Unmodified**: 3.0250e+08

### FTPN(N)KTEDTIF Fuc 2 Hex 5 HexNAc 6

**Mass:**  3632.4951

(1.141e-06)

**Charge States:** 3, 4

**Start Position**: 69

**End Position**: 80

| Profile Score | Tandem Score | TDA q-value | Volume | Spectrum Matches |
| --- | --- | --- | --- | --- |
| 12.8846 | 122.9728 | 0.0120 | 1.3588e+07 | 3 |

**Adduct Signal Fractions**

- **Unmodified**: 1.3588e+07

### LNVQREN(N)GTISRY Hex 4 HexNAc 8

**Mass:**  3821.6537

(1.734e-06)

**Charge States:** 3, 4

**Start Position**: 97

**End Position**: 110

| Profile Score | Tandem Score | TDA q-value | Volume | Spectrum Matches |
| --- | --- | --- | --- | --- |
| 13.7068 | 122.9416 | 0.0120 | 1.4590e+09 | 9 |

**Adduct Signal Fractions**

- **Unmodified**: 1.4590e+09

### LNVQREN(N)GTISRY Fuc 3 Hex 6 HexNAc 6

**Mass:**  4177.7780

(2.449e-06)

**Charge States:** 3, 4

**Start Position**: 97

**End Position**: 110

| Profile Score | Tandem Score | TDA q-value | Volume | Spectrum Matches |
| --- | --- | --- | --- | --- |
| -2.9898 | 122.7318 | 0.0120 | 4.1317e+08 | 3 |

**Adduct Signal Fractions**

- **Unmodified**: 4.1317e+08

### N(N)KSVQEIQATFF Fuc 1 Hex 4 HexNAc 5

**Mass:**  3220.3841

(2.128e-07)

**Charge States:** 3

**Start Position**: 56

**End Position**: 68

| Profile Score | Tandem Score | TDA q-value | Volume | Spectrum Matches |
| --- | --- | --- | --- | --- |
| -3.6723 | 122.6868 | 0.0120 | 2.7710e+06 | 2 |

**Adduct Signal Fractions**

- **Unmodified**: 2.7710e+06

### YFTPN(N)KTEDTIF Hex 4 HexNAc 5 Neu5Ac 1

**Mass:**  3429.4052

(2.248e-06)

**Charge States:** 3

**Start Position**: 68

**End Position**: 80

| Profile Score | Tandem Score | TDA q-value | Volume | Spectrum Matches |
| --- | --- | --- | --- | --- |
| -5.3508 | 122.5059 | 0.0120 | 3.2249e+06 | 2 |

**Adduct Signal Fractions**

- **Unmodified**: 3.2249e+06

### TPN(N)KTEDTIF Fuc 4 Hex 5 HexNAc 5

**Mass:**  3574.4423

(-4.212e-06)

**Charge States:** 3

**Start Position**: 70

**End Position**: 80

| Profile Score | Tandem Score | TDA q-value | Volume | Spectrum Matches |
| --- | --- | --- | --- | --- |
| -4.3760 | 119.8574 | 0.0159 | 4.0415e+07 | 2 |

**Adduct Signal Fractions**

- **Unmodified**: 4.0415e+07

### TPN(N)KTEDTIF Hex 4 HexNAc 6

**Mass:**  3031.2616

(2.575e-06)

**Charge States:** 3

**Start Position**: 70

**End Position**: 80

| Profile Score | Tandem Score | TDA q-value | Volume | Spectrum Matches |
| --- | --- | --- | --- | --- |
| 13.0238 | 119.5198 | 0.0159 | 8.3843e+06 | 2 |

**Adduct Signal Fractions**

- **Unmodified**: 8.3843e+06

### FYFTPN(N)KTEDTIF Fuc 1 Hex 5 HexNAc 8

**Mass:**  4202.7302

(1.659e-06)

**Charge States:** 3, 4

**Start Position**: 67

**End Position**: 80

| Profile Score | Tandem Score | TDA q-value | Volume | Spectrum Matches |
| --- | --- | --- | --- | --- |
| 12.7323 | 119.4638 | 0.0159 | 1.7054e+07 | 3 |

**Adduct Signal Fractions**

- **Unmodified**: 1.7054e+07

### QTRQDQC(C)IYN(N)TTY Fuc 1 Hex 4 HexNAc 6

**Mass:**  3702.4912

(8.498e-07)

**Charge States:** 3, 4

**Start Position**: 84

**End Position**: 97

| Profile Score | Tandem Score | TDA q-value | Volume | Spectrum Matches |
| --- | --- | --- | --- | --- |
| 14.0285 | 118.4970 | 0.0159 | 5.9549e+07 | 5 |

**Adduct Signal Fractions**

- **Unmodified**: 5.9549e+07

### FYFTPN(N)KT Hex 3 HexNAc 5

**Mass:**  2518.0534

(5.519e-07)

**Charge States:** 3

**Start Position**: 67

**End Position**: 75

| Profile Score | Tandem Score | TDA q-value | Volume | Spectrum Matches |
| --- | --- | --- | --- | --- |
| -18.7231 | 118.4319 | 0.0000 | 5.3087e+06 | 2 |

**Adduct Signal Fractions**

- **Unmodified**: 5.3087e+06

### RNEEYN(N)KSVQEIQATF Fuc 1 Hex 5 HexNAc 7

**Mass:**  4332.8210

(1.124e-06)

**Charge States:** 3

**Start Position**: 51

**End Position**: 67

| Profile Score | Tandem Score | TDA q-value | Volume | Spectrum Matches |
| --- | --- | --- | --- | --- |
| -4.4712 | 117.6024 | 0.0178 | 1.5961e+07 | 1 |

**Adduct Signal Fractions**

- **Unmodified**: 1.5961e+07

### YFTPN(N)KTEDTIF Fuc 1 Hex 5 HexNAc 3 Neu5Ac 1

**Mass:**  3331.3657

(3.649e-06)

**Charge States:** 3

**Start Position**: 68

**End Position**: 80

| Profile Score | Tandem Score | TDA q-value | Volume | Spectrum Matches |
| --- | --- | --- | --- | --- |
| -4.9482 | 117.5013 | 0.0178 | 5.3090e+06 | 2 |

**Adduct Signal Fractions**

- **Unmodified**: 5.3090e+06

### VQREN(N)GTISRY Hex 3 HexNAc 6

**Mass:**  3026.3153

(2.184e-06)

**Charge States:** 3

**Start Position**: 99

**End Position**: 110

| Profile Score | Tandem Score | TDA q-value | Volume | Spectrum Matches |
| --- | --- | --- | --- | --- |
| -4.0328 | 117.4374 | 0.0178 | 4.9307e+07 | 2 |

**Adduct Signal Fractions**

- **Unmodified**: 4.9307e+07

### TPN(N)KTEDTIF Hex 7 HexNAc 5

**Mass:**  3314.3410

(2.162e-06)

**Charge States:** 3

**Start Position**: 70

**End Position**: 80

| Profile Score | Tandem Score | TDA q-value | Volume | Spectrum Matches |
| --- | --- | --- | --- | --- |
| 11.4672 | 117.0262 | 0.0178 | 2.4482e+07 | 2 |

**Adduct Signal Fractions**

- **Unmodified**: 2.4482e+07

### LNVQREN(N)GTISRY Fuc 1 Hex 3 HexNAc 5

**Mass:**  3196.4203

(8.613e-07)

**Charge States:** 3

**Start Position**: 97

**End Position**: 110

| Profile Score | Tandem Score | TDA q-value | Volume | Spectrum Matches |
| --- | --- | --- | --- | --- |
| -4.5315 | 116.5436 | 0.0194 | 5.8269e+07 | 2 |

**Adduct Signal Fractions**

- **Unmodified**: 5.8269e+07

### LNVQREN(N)GTISRY Fuc 2 Hex 5 HexNAc 7

**Mass:**  4072.7497

(1.836e-06)

**Charge States:** 3, 4

**Start Position**: 97

**End Position**: 110

| Profile Score | Tandem Score | TDA q-value | Volume | Spectrum Matches |
| --- | --- | --- | --- | --- |
| -3.3005 | 116.3180 | 0.0194 | 2.3265e+08 | 3 |

**Adduct Signal Fractions**

- **Unmodified**: 2.3265e+08

### LNVQREN(N)GTISRY Fuc 2 Hex 5 HexNAc 6

**Mass:**  3869.6596

(1.693e-06)

**Charge States:** 3, 4

**Start Position**: 97

**End Position**: 110

| Profile Score | Tandem Score | TDA q-value | Volume | Spectrum Matches |
| --- | --- | --- | --- | --- |
| 14.5015 | 115.3661 | 0.0194 | 1.4287e+09 | 6 |

**Adduct Signal Fractions**

- **Unmodified**: 1.4287e+09

### LNVQREN(N)GTISRY Fuc 3 Hex 7 HexNAc 8

**Mass:**  4745.9851

(2.148e-06)

**Charge States:** 4

**Start Position**: 97

**End Position**: 110

| Profile Score | Tandem Score | TDA q-value | Volume | Spectrum Matches |
| --- | --- | --- | --- | --- |
| -4.8709 | 114.7222 | 0.0194 | 4.3579e+07 | 3 |

**Adduct Signal Fractions**

- **Unmodified**: 4.3579e+07

### RNEEYN(N)KSVQEIQ Hex 3 HexNAc 4

**Mass:**  2934.2656

(1.502e-06)

**Charge States:** 3

**Start Position**: 51

**End Position**: 64

| Profile Score | Tandem Score | TDA q-value | Volume | Spectrum Matches |
| --- | --- | --- | --- | --- |
| -19.8033 | 114.1459 | 0.0205 | 6.4542e+06 | 1 |

**Adduct Signal Fractions**

- **Unmodified**: 6.4542e+06

### RNEEYN(N)KSVQEIQATF Hex 4 HexNAc 4

**Mass:**  3415.4741

(2.638e-06)

**Charge States:** 3

**Start Position**: 51

**End Position**: 67

| Profile Score | Tandem Score | TDA q-value | Volume | Spectrum Matches |
| --- | --- | --- | --- | --- |
| -20.2214 | 113.1694 | 0.0205 | 2.2862e+07 | 1 |

**Adduct Signal Fractions**

- **Unmodified**: 2.2862e+07

### VQREN(N)GTISRY Hex 4 HexNAc 7

**Mass:**  3392.4535

(2.986e-04)

**Start Position**: 99

**End Position**: 110

| Profile Score | Tandem Score | TDA q-value | Volume | Spectrum Matches |
| --- | --- | --- | --- | --- |
| 0.0000 | 112.4303 | 0.0205 | 0.0000e+00 | 1 |

Adduct:

No Chromatogram Found

### LNVQREN(N)GTISRY Fuc 2 Hex 6 HexNAc 8

**Mass:**  4437.8762

(1.377e-06)

**Charge States:** 3, 4

**Start Position**: 97

**End Position**: 110

| Profile Score | Tandem Score | TDA q-value | Volume | Spectrum Matches |
| --- | --- | --- | --- | --- |
| 13.5082 | 111.4237 | 0.0205 | 1.7484e+08 | 3 |

**Adduct Signal Fractions**

- **Unmodified**: 1.7484e+08

### LNVQREN(N)GTISRY Fuc 2 Hex 4 HexNAc 6

**Mass:**  3707.6127

(2.250e-06)

**Charge States:** 3, 4

**Start Position**: 97

**End Position**: 110

| Profile Score | Tandem Score | TDA q-value | Volume | Spectrum Matches |
| --- | --- | --- | --- | --- |
| -5.4922 | 111.2799 | 0.0205 | 2.4213e+07 | 1 |

**Adduct Signal Fractions**

- **Unmodified**: 2.4213e+07

### LNVQREN(N)GTISRY Fuc 2 Hex 5 HexNAc 5

**Mass:**  3666.5799

(1.536e-06)

**Charge States:** 3, 4

**Start Position**: 97

**End Position**: 110

| Profile Score | Tandem Score | TDA q-value | Volume | Spectrum Matches |
| --- | --- | --- | --- | --- |
| -19.1376 | 111.2413 | 0.0205 | 7.4672e+07 | 1 |

**Adduct Signal Fractions**

- **Unmodified**: 7.4672e+07

### FTPN(N)KTEDTIF Hex 4 HexNAc 6

**Mass:**  3178.3251

(1.460e-06)

**Charge States:** 3

**Start Position**: 69

**End Position**: 80

| Profile Score | Tandem Score | TDA q-value | Volume | Spectrum Matches |
| --- | --- | --- | --- | --- |
| -5.0952 | 111.1996 | 0.0205 | 8.8989e+06 | 2 |

**Adduct Signal Fractions**

- **Unmodified**: 8.8989e+06

### FTPN(N)KTEDTIF Fuc 1 Hex 5 HexNAc 7

**Mass:**  3689.5193

(1.866e-06)

**Charge States:** 3, 4

**Start Position**: 69

**End Position**: 80

| Profile Score | Tandem Score | TDA q-value | Volume | Spectrum Matches |
| --- | --- | --- | --- | --- |
| 12.3695 | 111.1868 | 0.0205 | 8.7210e+06 | 4 |

**Adduct Signal Fractions**

- **Unmodified**: 8.7210e+06

### QTRQDQC(C)IYN(N)TTY Fuc 1 Hex 3 HexNAc 6

**Mass:**  3540.4389

(1.755e-06)

**Charge States:** 3

**Start Position**: 84

**End Position**: 97

| Profile Score | Tandem Score | TDA q-value | Volume | Spectrum Matches |
| --- | --- | --- | --- | --- |
| 12.4697 | 111.1018 | 0.0205 | 1.0052e+07 | 2 |

**Adduct Signal Fractions**

- **Unmodified**: 1.0052e+07

### LNVQREN(N)GTISRY Hex 5 HexNAc 8

**Mass:**  3983.7111

(2.791e-06)

**Charge States:** 3, 4

**Start Position**: 97

**End Position**: 110

| Profile Score | Tandem Score | TDA q-value | Volume | Spectrum Matches |
| --- | --- | --- | --- | --- |
| -3.8799 | 110.5298 | 0.0220 | 1.3203e+08 | 2 |

**Adduct Signal Fractions**

- **Unmodified**: 1.3203e+08

### LNVQREN(N)GTISRY Hex 4 HexNAc 4 Neu5Ac 2

**Mass:**  3591.5202

(3.658e-07)

**Charge States:** 3, 4

**Start Position**: 97

**End Position**: 110

| Profile Score | Tandem Score | TDA q-value | Volume | Spectrum Matches |
| --- | --- | --- | --- | --- |
| 14.2856 | 110.1559 | 0.0220 | 4.3650e+08 | 8 |

**Adduct Signal Fractions**

- **Unmodified**: 4.3650e+08

### TPN(N)KTEDTIF Fuc 1 Hex 5 HexNAc 4 Neu5Ac 2

**Mass:**  3515.4076

(3.116e-06)

**Start Position**: 70

**End Position**: 80

| Profile Score | Tandem Score | TDA q-value | Volume | Spectrum Matches |
| --- | --- | --- | --- | --- |
| 0.0000 | 109.6019 | 0.0220 | 0.0000e+00 | 1 |

Adduct:

No Chromatogram Found

### VQREN(N)GTISRY Hex 3 HexNAc 5

**Mass:**  2823.2354

(2.220e-06)

**Charge States:** 3

**Start Position**: 99

**End Position**: 110

| Profile Score | Tandem Score | TDA q-value | Volume | Spectrum Matches |
| --- | --- | --- | --- | --- |
| -19.8835 | 109.4234 | 0.0220 | 7.0989e+06 | 1 |

**Adduct Signal Fractions**

- **Unmodified**: 7.0989e+06

### NVQREN(N)GTISRY Fuc 1 Hex 5 HexNAc 8

**Mass:**  4016.6796

(1.465e-06)

**Charge States:** 3, 4

**Start Position**: 98

**End Position**: 110

| Profile Score | Tandem Score | TDA q-value | Volume | Spectrum Matches |
| --- | --- | --- | --- | --- |
| -20.5517 | 109.0357 | 0.0240 | 2.4546e+07 | 2 |

**Adduct Signal Fractions**

- **Unmodified**: 2.4546e+07

### NVQREN(N)GTISRY Fuc 3 Hex 6 HexNAc 6

**Mass:**  4064.6982

(3.563e-06)

**Charge States:** 3, 4

**Start Position**: 98

**End Position**: 110

| Profile Score | Tandem Score | TDA q-value | Volume | Spectrum Matches |
| --- | --- | --- | --- | --- |
| -20.2760 | 108.8373 | 0.0257 | 1.7007e+07 | 2 |

**Adduct Signal Fractions**

- **Unmodified**: 1.7007e+07

### RNEEYN(N)KSVQEIQATF Fuc 1 Hex 5 HexNAc 5

**Mass:**  3926.6658

(3.327e-06)

**Charge States:** 4

**Start Position**: 51

**End Position**: 67

| Profile Score | Tandem Score | TDA q-value | Volume | Spectrum Matches |
| --- | --- | --- | --- | --- |
| -6.2254 | 107.5597 | 0.0271 | 8.6414e+06 | 1 |

**Adduct Signal Fractions**

- **Unmodified**: 8.6414e+06

### C(C)ANLVPVPITN(N)ATL Hex 3 HexNAc 5

**Mass:**  2983.3505

(1.332e-06)

**Charge States:** 3, 4

**Start Position**: 23

**End Position**: 37

| Profile Score | Tandem Score | TDA q-value | Volume | Spectrum Matches |
| --- | --- | --- | --- | --- |
| 13.3198 | 107.1652 | 0.0271 | 2.2413e+07 | 7 |

**Adduct Signal Fractions**

- **Unmodified**: 2.2413e+07

### RN(D)EEYN(N)KSVQEIQATFF Hex 3 HexNAc 4

**Mass:**  3401.4739

(2.925e-04)

**Charge States:** 3, 4

**Start Position**: 51

**End Position**: 68

| Profile Score | Tandem Score | TDA q-value | Volume | Spectrum Matches |
| --- | --- | --- | --- | --- |
| 7.0357 | 105.4158 | 0.0382 | 5.6958e+07 | 6 |

**Adduct Signal Fractions**

- **Unmodified**: 5.6958e+07

### NVQREN(N)GTISRY Fuc 1 Hex 4 HexNAc 5

**Mass:**  3245.3834

(4.126e-07)

**Charge States:** 3

**Start Position**: 98

**End Position**: 110

| Profile Score | Tandem Score | TDA q-value | Volume | Spectrum Matches |
| --- | --- | --- | --- | --- |
| 10.5523 | 104.3265 | 0.0418 | 7.8887e+07 | 3 |

**Adduct Signal Fractions**

- **Unmodified**: 7.8887e+07

### NVQREN(N)GTISRY Hex 5 HexNAc 9

**Mass:**  4075.7065

(4.937e-04)

**Start Position**: 98

**End Position**: 110

| Profile Score | Tandem Score | TDA q-value | Volume | Spectrum Matches |
| --- | --- | --- | --- | --- |
| 0.0000 | 102.9575 | 0.0447 | 0.0000e+00 | 1 |

Adduct:

No Chromatogram Found

### LNVQREN(N)GTISRY Fuc 3 Hex 6 HexNAc 7

**Mass:**  4380.8566

(7.568e-07)

**Charge States:** 3, 4

**Start Position**: 97

**End Position**: 110

| Profile Score | Tandem Score | TDA q-value | Volume | Spectrum Matches |
| --- | --- | --- | --- | --- |
| -3.5183 | 102.2563 | 0.0457 | 1.1317e+08 | 3 |

**Adduct Signal Fractions**

- **Unmodified**: 1.1317e+08

### FYFTPN(N)KTEDTIF Fuc 1 Hex 5 HexNAc 4 Neu5Ac 2

**Mass:**  3972.5996

(1.575e-06)

**Charge States:** 3, 4

**Start Position**: 67

**End Position**: 80

| Profile Score | Tandem Score | TDA q-value | Volume | Spectrum Matches |
| --- | --- | --- | --- | --- |
| -5.4468 | 100.7539 | 0.0497 | 1.9241e+07 | 2 |

**Adduct Signal Fractions**

- **Unmodified**: 1.9241e+07

### LNVQREN(N)GTISRY Hex 7 HexNAc 4

**Mass:**  3495.4495

(-8.038e-06)

**Charge States:** 3, 4

**Start Position**: 97

**End Position**: 110

| Profile Score | Tandem Score | TDA q-value | Volume | Spectrum Matches |
| --- | --- | --- | --- | --- |
| 12.4863 | 100.7029 | 0.0497 | 3.4065e+07 | 4 |

**Adduct Signal Fractions**

- **Unmodified**: 3.4065e+07

### LNVQREN(N)GTISRY Hex 3 HexNAc 4

**Mass:**  2849.2873

(7.061e-04)

**Start Position**: 97

**End Position**: 110

| Profile Score | Tandem Score | TDA q-value | Volume | Spectrum Matches |
| --- | --- | --- | --- | --- |
| 0.0000 | 100.1990 | 0.0497 | 0.0000e+00 | 1 |

Adduct:

No Chromatogram Found

### P19652|A1AG2\_HUMAN

**>P19652|A1AG2\_HUMAN**  
 `MALSWVLTVLSLLPLLEAQIPLCANLVPVPITNATLDRITGKWFYIASAFRNEEYNKSVQEIQATFFYFTPNKTEDTIFL  
REYQTRQNQCFYNSSYLNVQRENGTVSRYEGGREHVAHLLFLRDTKTLMFGSYLDDEKNWGLSFYADKPETTKEQLGEFY  
EALDCLCIPRSDVMYTDWKKDKCEPLEKQHEKERKQEEGES`

| Site | Type | Count |
| --- | --- | --- |
| 33 | N-linked | 2 |
| 72 | N-linked | 71 |
| 93 | N-linked | 1 |
| 103 | N-linked | 21 |
| 56 | N-linked | 47 |

| Mass | Sequence | MS_2_ Score | `q`-value | MS_1_ Score | Signal | Protein Coordinates | Tandem Scans | Start Time | Apex Time | End Time |
| --- | --- | --- | --- | --- | --- | --- | --- | --- | --- | --- |
| 2974.2350 | TPN(N)KTEDTIF Fuc 1 Hex 4 HexNAc 5 | 182.175 | 0.0 | 14.351 | 3.699e+07 | 69 - 79 | 3 | 23.56 | 24.06 | 24.57 |
| 2666.1250 | TPN(N)KTEDTIF Hex 3 HexNAc 5 | 181.471 | 0.0 | 15.599 | 1.613e+08 | 69 - 79 | 8 | 22.71 | 24.57 | 25.59 |
| 2869.2038 | TPN(N)KTEDTIF Hex 3 HexNAc 6 | 180.669 | 0.0 | 16.310 | 2.943e+08 | 69 - 79 | 11 | 22.37 | 24.23 | 25.59 |
| 3177.3140 | TPN(N)KTEDTIF Fuc 1 Hex 4 HexNAc 6 | 180.571 | 0.0 | 14.474 | 6.442e+07 | 69 - 79 | 4 | 23.22 | 23.73 | 24.40 |
| 2976.2606 | YFTPN(N)KTEDTIF Hex 3 HexNAc 5 | 178.871 | 0.0 | 15.488 | 1.800e+09 | 67 - 79 | 134 | 36.97 | 38.11 | 100.43 |
| 2562.1235 | N(N)KSVQEIQATF Hex 3 HexNAc 4 | 178.578 | 0.0 | -4.396 | 2.278e+07 | 55 - 66 | 1 | 27.47 | 27.64 | 27.98 |
| 3544.4699 | YFTPN(N)KTEDTIF Hex 4 HexNAc 7 | 178.290 | 0.0 | 15.131 | 8.357e+08 | 67 - 79 | 11 | 35.78 | 36.97 | 38.27 |
| 3179.3357 | YFTPN(N)KTEDTIF Hex 3 HexNAc 6 | 176.919 | 0.0 | 14.916 | 3.132e+09 | 67 - 79 | 31 | 36.63 | 37.80 | 44.22 |
| 3284.3680 | YFTPN(N)KTEDTIF Fuc 1 Hex 4 HexNAc 5 | 175.084 | 0.0 | 14.429 | 4.647e+08 | 67 - 79 | 5 | 36.29 | 37.47 | 38.11 |
| 2463.0463 | TPN(N)KTEDTIF Hex 3 HexNAc 4 | 174.520 | 0.0 | 13.890 | 2.188e+07 | 69 - 79 | 4 | 24.57 | 24.91 | 25.42 |
| 3691.5384 | FYFTPN(N)KTEDTIF Hex 4 HexNAc 7 | 172.734 | 0.0 | 15.135 | 4.549e+08 | 66 - 79 | 10 | 46.08 | 47.27 | 47.78 |
| 3487.4473 | YFTPN(N)KTEDTIF Fuc 1 Hex 4 HexNAc 6 | 171.681 | 0.0 | 15.893 | 8.484e+08 | 67 - 79 | 10 | 36.12 | 37.31 | 38.11 |
| 2773.1769 | YFTPN(N)KTEDTIF Hex 3 HexNAc 4 | 170.818 | 0.0 | 14.105 | 2.592e+08 | 67 - 79 | 15 | 37.31 | 38.43 | 40.81 |
| 3326.4043 | FYFTPN(N)KTEDTIF Hex 3 HexNAc 6 | 169.855 | 0.0 | 13.657 | 5.052e+09 | 66 - 79 | 224 | 46.76 | 47.78 | 92.81 |
| 3234.3373 | TPN(N)KTEDTIF Hex 4 HexNAc 7 | 169.110 | 0.0 | 12.654 | 4.478e+07 | 69 - 79 | 4 | 23.22 | 23.56 | 24.23 |
| 3634.5164 | FYFTPN(N)KTEDTIF Fuc 1 Hex 4 HexNAc 6 | 168.539 | 0.0 | 14.079 | 7.851e+08 | 66 - 79 | 44 | 46.42 | 47.27 | 63.05 |
| 3431.4352 | FYFTPN(N)KTEDTIF Fuc 1 Hex 4 HexNAc 5 | 168.486 | 0.0 | 14.044 | 3.449e+08 | 66 - 79 | 8 | 46.76 | 47.61 | 48.94 |
| 2920.2448 | FYFTPN(N)KTEDTIF Hex 3 HexNAc 4 | 167.778 | 0.0 | 14.993 | 1.796e+08 | 66 - 79 | 13 | 48.27 | 48.43 | 52.08 |
| 2765.2045 | N(N)KSVQEIQATF Hex 3 HexNAc 5 | 167.731 | 0.0 | -3.717 | 1.639e+07 | 55 - 66 | 1 | 27.29 | 27.47 | 27.64 |
| 3341.3887 | YFTPN(N)KTEDTIF Hex 4 HexNAc 6 | 167.523 | 0.0 | 13.398 | 1.441e+08 | 67 - 79 | 5 | 36.29 | 37.64 | 37.96 |
| 3123.3270 | FYFTPN(N)KTEDTIF Hex 3 HexNAc 5 | 167.444 | 0.0 | 13.869 | 2.332e+09 | 66 - 79 | 150 | 47.10 | 48.10 | 99.92 |
| 3747.5514 | YFTPN(N)KTEDTIF Hex 4 HexNAc 8 | 166.350 | 0.0 | 15.086 | 1.200e+08 | 67 - 79 | 5 | 36.63 | 36.80 | 37.47 |
| 3852.5823 | YFTPN(N)KTEDTIF Fuc 1 Hex 5 HexNAc 7 | 166.138 | 0.0 | 15.215 | 1.350e+08 | 67 - 79 | 6 | 36.12 | 36.80 | 37.14 |
| 3186.3526 | EEYN(N)KSVQEIQATF Hex 3 HexNAc 5 | 165.637 | 0.0 | -3.245 | 3.409e+07 | 52 - 66 | 4 | 35.78 | 35.95 | 36.29 |
| 3016.2701 | FTPN(N)KTEDTIF Hex 3 HexNAc 6 | 165.125 | 0.0 | 14.830 | 6.212e+08 | 68 - 79 | 10 | 29.84 | 31.48 | 31.97 |
| 2813.1931 | FTPN(N)KTEDTIF Hex 3 HexNAc 5 | 164.276 | 0.0 | -0.517 | 2.157e+08 | 68 - 79 | 9 | 30.19 | 31.63 | 113.96 |
| 3324.3845 | FTPN(N)KTEDTIF Fuc 1 Hex 4 HexNAc 6 | 163.733 | 0.0 | 13.661 | 1.114e+08 | 68 - 79 | 7 | 30.70 | 30.87 | 64.24 |
| 3909.6045 | YFTPN(N)KTEDTIF Hex 5 HexNAc 8 | 163.064 | 0.0 | 12.850 | 1.196e+08 | 67 - 79 | 5 | 36.12 | 36.46 | 37.14 |
| 3121.3011 | FTPN(N)KTEDTIF Fuc 1 Hex 4 HexNAc 5 | 162.647 | 0.0 | 12.914 | 7.392e+07 | 68 - 79 | 4 | 30.87 | 31.20 | 31.80 |
| 3837.5975 | FYFTPN(N)KTEDTIF Fuc 1 Hex 4 HexNAc 7 | 162.403 | 0.0 | 12.051 | 2.682e+07 | 66 - 79 | 4 | 46.76 | 47.27 | 47.44 |
| 3073.3152 | N(N)KSVQEIQATF Fuc 1 Hex 4 HexNAc 5 | 160.716 | 0.0 | -4.343 | 8.613e+06 | 55 - 66 | 1 | 26.61 | 26.78 | 27.12 |
| 3795.5594 | YFTPN(N)KTEDTIF Fuc 2 Hex 5 HexNAc 6 | 159.579 | 0.0 | 14.529 | 1.692e+08 | 67 - 79 | 3 | 36.63 | 36.97 | 37.47 |
| 3742.5598 | NVQREN(N)GTVSRY Fuc 2 Hex 5 HexNAc 6 | 159.404 | 0.0 | -4.529 | 1.930e+08 | 97 - 109 | 3 | 11.89 | 11.89 | 12.22 |
| 3126.3342 | NVQREN(N)GTVSRY Hex 3 HexNAc 6 | 159.281 | 0.0 | 13.538 | 1.233e+09 | 97 - 109 | 6 | 11.89 | 12.05 | 13.04 |
| 3967.6922 | RNEEYN(N)KSVQEIQATF Fuc 1 Hex 4 HexNAc 6 | 158.901 | 0.0 | 14.855 | 1.369e+08 | 50 - 66 | 2 | 32.14 | 32.48 | 32.79 |
| 3456.5000 | RNEEYN(N)KSVQEIQATF Hex 3 HexNAc 5 | 158.475 | 0.0 | 15.989 | 5.690e+09 | 50 - 66 | 15 | 30.87 | 32.79 | 34.25 |
| 3494.4645 | EEYN(N)KSVQEIQATF Fuc 1 Hex 4 HexNAc 5 | 158.123 | 0.0 | -3.575 | 2.242e+07 | 52 - 66 | 2 | 35.27 | 35.44 | 35.61 |
| 3942.6283 | FYFTPN(N)KTEDTIF Fuc 2 Hex 5 HexNAc 6 | 157.966 | 0.0 | 15.204 | 1.018e+08 | 66 - 79 | 6 | 46.08 | 46.93 | 47.44 |
| 3400.4853 | RNEEYN(N)KSVQEIQATFF Hex 3 HexNAc 4 | 157.371 | 0.0 | 17.174 | 1.915e+09 | 50 - 67 | 12 | 43.54 | 44.56 | 45.91 |
| 3911.6766 | RNEEYN(N)KSVQEIQATFF Fuc 1 Hex 4 HexNAc 5 | 157.057 | 0.0 | 16.072 | 1.050e+09 | 50 - 67 | 18 | 41.66 | 44.06 | 45.39 |
| 3999.6508 | FYFTPN(N)KTEDTIF Fuc 1 Hex 5 HexNAc 7 | 156.863 | 0.0 | 14.148 | 6.746e+07 | 66 - 79 | 5 | 45.74 | 46.76 | 47.61 |
| 3546.5431 | RNEEYN(N)KSVQEIQATFF Fuc 1 Hex 3 HexNAc 4 | 156.238 | 0.0 | -4.828 | 3.189e+07 | 50 - 67 | 3 | 44.39 | 44.56 | 44.72 |
| 3592.4795 | YFTPN(N)KTEDTIF Fuc 2 Hex 5 HexNAc 5 | 156.124 | 0.0 | -19.569 | 1.440e+07 | 67 - 79 | 1 | 36.80 | 36.80 | 36.97 |
| 3690.5295 | YFTPN(N)KTEDTIF Fuc 1 Hex 4 HexNAc 7 | 155.624 | 0.0 | -4.547 | 3.546e+07 | 67 - 79 | 3 | 36.80 | 37.14 | 37.31 |
| 3231.3652 | NVQREN(N)GTVSRY Fuc 1 Hex 4 HexNAc 5 | 154.924 | 0.0 | -3.975 | 1.504e+08 | 97 - 109 | 2 | 12.05 | 12.05 | 12.54 |
| 3517.4202 | YFTPN(N)KTEDTIF Hex 4 HexNAc 4 Neu5Ac 2 | 154.595 | 0.0 | 13.990 | 2.468e+08 | 67 - 79 | 7 | 38.43 | 38.60 | 39.44 |
| 3534.4462 | YFTPN(N)KTEDTIF Fuc 1 Hex 5 HexNAc 4 Neu5Ac 1 | 154.545 | 0.0 | 12.400 | 4.689e+07 | 67 - 79 | 3 | 38.43 | 38.60 | 39.44 |
| 3603.5642 | RNEEYN(N)KSVQEIQATFF Hex 3 HexNAc 5 | 154.400 | 0.0 | 15.126 | 1.568e+09 | 50 - 67 | 10 | 44.22 | 44.39 | 45.74 |
| 3253.4180 | RNEEYN(N)KSVQEIQATF Hex 3 HexNAc 4 | 153.831 | 0.0 | 16.706 | 7.172e+09 | 50 - 66 | 29 | 28.31 | 33.11 | 34.42 |
| 3764.6131 | RNEEYN(N)KSVQEIQATF Fuc 1 Hex 4 HexNAc 5 | 152.643 | 0.0 | 16.006 | 3.295e+09 | 50 - 66 | 15 | 31.48 | 32.48 | 33.91 |
| 3106.3488 | RNEEYN(N)KSVQEIQAT Hex 3 HexNAc 4 | 152.610 | 0.0 | -16.940 | 3.542e+07 | 50 - 65 | 1 | 17.40 | 17.40 | 17.57 |
| 3681.5171 | FYFTPN(N)KTEDTIF Fuc 1 Hex 5 HexNAc 4 Neu5Ac 1 | 152.049 | 0.0 | 12.706 | 2.715e+07 | 66 - 79 | 3 | 48.43 | 48.60 | 49.11 |
| 3561.5334 | RNEEYN(N)KSVQEIQATF Fuc 1 Hex 4 HexNAc 4 | 151.976 | 0.0 | 15.587 | 1.658e+08 | 50 - 66 | 3 | 31.80 | 32.65 | 32.79 |
| 3399.4751 | RNEEYN(N)KSVQEIQATF Fuc 1 Hex 3 HexNAc 4 | 151.845 | 0.0 | 11.599 | 1.441e+08 | 50 - 66 | 8 | 33.11 | 33.26 | 34.25 |
| 4112.6827 | YFTPN(N)KTEDTIF Hex 5 HexNAc 9 | 151.183 | 0.0 | -3.896 | 4.027e+07 | 67 - 79 | 3 | 36.12 | 36.12 | 36.80 |
| 3602.5561 | RNEEYN(N)KSVQEIQATF Fuc 1 Hex 3 HexNAc 5 | 150.936 | 0.0 | 11.921 | 1.503e+08 | 50 - 66 | 7 | 32.79 | 33.11 | 34.08 |
| 3659.5806 | RNEEYN(N)KSVQEIQATF Hex 3 HexNAc 6 | 150.906 | 0.0 | 10.848 | 3.130e+08 | 50 - 66 | 7 | 32.48 | 32.65 | 34.08 |
| 2610.1144 | FTPN(N)KTEDTIF Hex 3 HexNAc 4 | 150.786 | 0.0 | -3.327 | 3.430e+07 | 68 - 79 | 3 | 31.80 | 31.97 | 32.31 |
| 3381.4057 | FTPN(N)KTEDTIF Hex 4 HexNAc 7 | 150.780 | 0.0 | 13.062 | 7.057e+07 | 68 - 79 | 4 | 30.53 | 30.87 | 31.20 |
| 3485.4276 | TPN(N)KTEDTIF Fuc 2 Hex 5 HexNAc 6 | 150.482 | 0.0 | -4.545 | 8.469e+06 | 69 - 79 | 1 | 23.05 | 23.22 | 23.56 |
| 3894.6200 | FYFTPN(N)KTEDTIF Hex 4 HexNAc 8 | 149.980 | 0.0 | 16.024 | 6.870e+07 | 66 - 79 | 5 | 46.59 | 46.76 | 47.61 |
| 4072.7217 | RNEEYN(N)KSVQEIQATF Fuc 2 Hex 5 HexNAc 5 | 149.745 | 0.0 | -2.029 | 9.374e+07 | 50 - 66 | 3 | 32.14 | 32.14 | 32.48 |
| 2983.2742 | EEYN(N)KSVQEIQATF Hex 3 HexNAc 4 | 148.230 | 0.0 | -3.387 | 4.319e+07 | 52 - 66 | 2 | 35.95 | 36.12 | 36.46 |
| 3254.4040 | RN(D)EEYN(N)KSVQEIQATF Hex 3 HexNAc 4 | 147.778 | 0.0 | 9.866 | 6.573e+08 | 50 - 66 | 94 | 34.59 | 35.95 | 36.63 |
| 3542.4487 | TPN(N)KTEDTIF Fuc 1 Hex 5 HexNAc 7 | 147.408 | 0.0 | -19.106 | 4.001e+06 | 69 - 79 | 1 | 23.05 | 23.05 | 23.22 |
| 3708.5963 | RNEEYN(N)KSVQEIQATFF Fuc 1 Hex 4 HexNAc 4 | 146.543 | 0.0 | -4.665 | 4.734e+07 | 50 - 67 | 3 | 43.89 | 44.06 | 44.39 |
| 4024.7142 | RNEEYN(N)KSVQEIQATF Hex 4 HexNAc 7 | 146.132 | 0.0 | -4.400 | 8.394e+07 | 50 - 66 | 4 | 32.14 | 32.31 | 32.65 |
| 4056.6732 | FYFTPN(N)KTEDTIF Hex 5 HexNAc 8 | 146.007 | 0.0 | 12.897 | 6.528e+07 | 66 - 79 | 6 | 46.08 | 46.42 | 47.10 |
| 3664.4897 | FYFTPN(N)KTEDTIF Hex 4 HexNAc 4 Neu5Ac 2 | 146.007 | 0.0 | 13.584 | 1.495e+08 | 66 - 79 | 10 | 48.43 | 48.60 | 50.30 |
| 3618.5552 | RNEEYN(N)KSVQEIQATF Hex 4 HexNAc 5 | 145.888 | 0.0 | -5.041 | 7.276e+07 | 50 - 66 | 3 | 32.31 | 32.65 | 32.79 |
| 3138.3123 | YFTPN(N)KTEDTIF Hex 4 HexNAc 5 | 145.005 | 0.0 | -19.918 | 1.569e+07 | 67 - 79 | 1 | 37.64 | 37.64 | 37.80 |
| 3292.4203 | YFTPN(N)KTEDTIFL Hex 3 HexNAc 6 | 144.797 | 0.0 | 14.547 | 2.405e+07 | 67 - 80 | 5 | 45.39 | 45.56 | 46.08 |
| 3765.6180 | RNEEYN(N)KSVQEIQATFF Hex 4 HexNAc 5 | 144.744 | 0.0 | -4.418 | 2.721e+07 | 50 - 67 | 3 | 43.89 | 44.22 | 44.39 |
| 3749.6239 | RNEEYN(N)KSVQEIQATFF Fuc 1 Hex 3 HexNAc 5 | 144.527 | 0.0 | -17.940 | 3.282e+07 | 50 - 67 | 2 | 44.22 | 44.39 | 44.39 |
| 3694.5486 | NVQREN(N)GTVSRY Hex 4 HexNAc 8 | 144.195 | 0.0 | -5.597 | 1.084e+08 | 97 - 109 | 3 | 11.89 | 11.89 | 12.22 |
| 3488.4592 | FYFTPN(N)KTEDTIF Hex 4 HexNAc 6 | 143.952 | 0.0 | 11.821 | 4.842e+07 | 66 - 79 | 5 | 46.59 | 47.27 | 47.78 |
| 2575.0711 | YFTPN(N)KT Hex 3 HexNAc 6 | 143.357 | 0.0 | 0.000 | 0.000e+00 | 67 - 74 | 1 | - | - | - |
| 2449.9659 | YN(N)SSYL Hex 3 HexNAc 6 | 142.698 | 0.0 | -19.358 | 2.911e+06 | 91 - 97 | 1 | 23.22 | 23.22 | 23.39 |
| 3239.4217 | LNVQREN(N)GTVSRY Hex 3 HexNAc 6 | 142.237 | 0.003 | 14.004 | 5.989e+08 | 96 - 109 | 7 | 16.57 | 16.73 | 17.57 |
| 4275.8012 | RNEEYN(N)KSVQEIQATF Fuc 2 Hex 5 HexNAc 6 | 141.600 | 0.003 | 12.994 | 2.634e+07 | 50 - 66 | 3 | 31.80 | 32.31 | 32.48 |
| 4219.7882 | RNEEYN(N)KSVQEIQATFF Fuc 2 Hex 5 HexNAc 5 | 141.499 | 0.003 | 12.889 | 3.428e+07 | 50 - 67 | 4 | 43.37 | 43.54 | 44.06 |
| 3207.2916 | TPN(N)KTEDTIF Hex 4 HexNAc 4 Neu5Ac 2 | 141.267 | 0.003 | 13.116 | 1.343e+07 | 69 - 79 | 2 | 24.91 | 25.08 | 25.59 |
| 4055.6636 | YFTPN(N)KTEDTIF Fuc 1 Hex 5 HexNAc 8 | 140.701 | 0.003 | 7.856 | 3.175e+07 | 67 - 79 | 4 | 36.12 | 36.29 | 36.80 |
| 4259.7504 | FYFTPN(N)KTEDTIF Hex 5 HexNAc 9 | 140.483 | 0.003 | 11.610 | 2.295e+07 | 66 - 79 | 4 | 46.08 | 46.25 | 46.76 |
| 4114.7572 | RNEEYN(N)KSVQEIQATFF Fuc 1 Hex 4 HexNAc 6 | 140.037 | 0.003 | 13.253 | 5.724e+07 | 50 - 67 | 4 | 43.54 | 43.89 | 44.22 |
| 3285.3767 | FYFTPN(N)KTEDTIF Hex 4 HexNAc 5 | 139.815 | 0.003 | -4.937 | 1.327e+07 | 66 - 79 | 2 | 47.44 | 47.78 | 47.78 |
| 3089.3417 | YFTPN(N)KTEDTIFL Hex 3 HexNAc 5 | 139.776 | 0.003 | 13.944 | 1.192e+07 | 67 - 80 | 3 | 45.56 | 45.91 | 46.25 |
| 4227.7897 | RNEEYN(N)KSVQEIQATF Hex 4 HexNAc 8 | 138.071 | 0.003 | -3.783 | 3.867e+07 | 50 - 66 | 3 | 31.97 | 32.14 | 32.48 |
| 3806.6439 | RNEEYN(N)KSVQEIQATFF Hex 3 HexNAc 6 | 138.001 | 0.003 | -4.282 | 7.432e+07 | 50 - 67 | 4 | 44.06 | 44.22 | 44.56 |
| 3617.5442 | RNEEYN(N)KSVQEIQAT Fuc 1 Hex 4 HexNAc 5 | 137.920 | 0.003 | -19.318 | 1.159e+07 | 50 - 65 | 1 | 17.07 | 17.07 | 17.23 |
| 3434.4453 | NVQREN(N)GTVSRY Fuc 1 Hex 4 HexNAc 6 | 137.578 | 0.003 | 11.882 | 6.363e+08 | 97 - 109 | 5 | 11.89 | 12.05 | 12.71 |
| 2709.1939 | N(N)KSVQEIQATFF Hex 3 HexNAc 4 | 137.200 | 0.003 | -2.982 | 4.634e+06 | 55 - 67 | 2 | 41.66 | 41.83 | 42.17 |
| 2923.2551 | NVQREN(N)GTVSRY Hex 3 HexNAc 5 | 136.439 | 0.003 | -3.259 | 2.384e+08 | 97 - 109 | 2 | 12.05 | 12.22 | 12.54 |
| 2721.1355 | FYFTPN(N)KT Hex 3 HexNAc 6 | 135.851 | 0.0 | -3.670 | 1.249e+07 | 66 - 74 | 2 | 26.27 | 26.44 | 26.78 |
| 3649.5046 | YFTPN(N)KTEDTIF Fuc 1 Hex 5 HexNAc 6 | 135.229 | 0.003 | -4.994 | 7.825e+06 | 67 - 79 | 2 | 36.63 | 36.97 | 36.97 |
| 3825.5309 | YFTPN(N)KTEDTIF Fuc 1 Hex 5 HexNAc 4 Neu5Ac 2 | 134.822 | 0.003 | -5.534 | 2.996e+07 | 67 - 79 | 2 | 37.96 | 38.11 | 38.43 |
| 3354.3586 | FTPN(N)KTEDTIF Hex 4 HexNAc 4 Neu5Ac 2 | 134.255 | 0.003 | -4.270 | 2.287e+07 | 68 - 79 | 2 | 31.97 | 32.14 | 32.31 |
| 4057.7338 | RNEEYN(N)KSVQEIQATFF Fuc 2 Hex 4 HexNAc 5 | 133.505 | 0.003 | -4.411 | 1.537e+07 | 50 - 67 | 2 | 43.89 | 44.06 | 44.22 |
| 3855.6449 | LNVQREN(N)GTVSRY Fuc 2 Hex 5 HexNAc 6 | 132.623 | 0.003 | -4.344 | 8.212e+07 | 96 - 109 | 5 | 16.24 | 16.24 | 16.57 |
| 3910.6723 | RNEEYN(N)KSVQEIQATF Fuc 2 Hex 4 HexNAc 5 | 131.928 | 0.003 | -4.216 | 4.335e+07 | 50 - 66 | 2 | 32.48 | 32.65 | 32.79 |
| 3036.3411 | LNVQREN(N)GTVSRY Hex 3 HexNAc 5 | 130.821 | 0.005 | 13.065 | 1.229e+08 | 96 - 109 | 4 | 16.73 | 16.90 | 17.91 |
| 3547.5339 | LNVQREN(N)GTVSRY Fuc 1 Hex 4 HexNAc 6 | 130.792 | 0.005 | 13.330 | 3.344e+08 | 96 - 109 | 6 | 16.40 | 16.57 | 17.57 |
| 3604.5517 | RN(D)EEYN(N)KSVQEIQATFF Hex 3 HexNAc 5 | 129.668 | 0.005 | -5.790 | 4.619e+07 | 50 - 67 | 12 | 47.27 | 47.44 | 47.61 |
| 3765.5948 | RN(D)EEYN(N)KSVQEIQATF Fuc 1 Hex 4 HexNAc 5 | 128.492 | 0.005 | 11.442 | 3.215e+08 | 50 - 66 | 11 | 34.08 | 35.44 | 36.29 |
| 2965.2034 | FYFTPN(N)KTED Hex 3 HexNAc 6 | 128.317 | 0.005 | -18.309 | 3.707e+06 | 66 - 76 | 1 | 26.61 | 26.78 | 26.78 |
| 3821.6370 | RNEEYN(N)KSVQEIQATF Hex 4 HexNAc 6 | 127.125 | 0.008 | 12.189 | 5.712e+08 | 50 - 66 | 7 | 32.31 | 32.65 | 33.91 |
| 3309.4293 | RNEEYN(N)KSVQEIQAT Hex 3 HexNAc 5 | 127.034 | 0.008 | 11.922 | 3.505e+07 | 50 - 65 | 3 | 17.23 | 17.40 | 17.91 |
| 2982.2892 | TPN(N)KTEDTIFL Hex 3 HexNAc 6 | 125.982 | 0.010 | -17.336 | 2.330e+06 | 69 - 80 | 3 | 34.93 | 35.10 | 35.10 |
| 3457.4832 | RN(D)EEYN(N)KSVQEIQATF Hex 3 HexNAc 5 | 125.420 | 0.012 | 9.503 | 5.503e+08 | 50 - 66 | 14 | 34.25 | 35.78 | 37.64 |
| 3400.4796 | FRNEEYN(N)KSVQEIQATF Hex 3 HexNAc 4 | 124.913 | 0.012 | -19.878 | 8.259e+06 | 49 - 66 | 1 | 37.80 | 37.80 | 37.80 |
| 3012.2948 | VQREN(N)GTVSRY Hex 3 HexNAc 6 | 124.139 | 0.012 | -20.644 | 2.537e+07 | 98 - 109 | 1 | 11.72 | 11.72 | 11.89 |
| 3491.4681 | NVQREN(N)GTVSRY Hex 4 HexNAc 7 | 124.067 | 0.012 | 6.506 | 1.500e+08 | 97 - 109 | 4 | 11.89 | 12.05 | 12.87 |
| 2671.1033 | N(N)KTEDTIF Hex 3 HexNAc 6 | 123.563 | 0.0 | -19.164 | 4.933e+06 | 71 - 79 | 1 | 22.37 | 22.54 | 22.54 |
| 3632.4949 | FTPN(N)KTEDTIF Fuc 2 Hex 5 HexNAc 6 | 122.973 | 0.012 | 12.885 | 1.359e+07 | 68 - 79 | 3 | 30.19 | 30.53 | 30.87 |
| 3220.3817 | N(N)KSVQEIQATFF Fuc 1 Hex 4 HexNAc 5 | 122.687 | 0.012 | -3.672 | 2.771e+06 | 55 - 67 | 2 | 40.64 | 40.81 | 41.15 |
| 3429.4090 | YFTPN(N)KTEDTIF Hex 4 HexNAc 5 Neu5Ac 1 | 122.506 | 0.012 | -5.351 | 3.225e+06 | 67 - 79 | 2 | 40.81 | 40.98 | 41.32 |
| 3604.5571 | LNVQREN(N)GTVSRY Hex 4 HexNAc 7 | 122.292 | 0.012 | -4.611 | 4.557e+07 | 96 - 109 | 3 | 16.24 | 16.40 | 16.57 |
| 2876.2830 | TN(N)ATLDRITGKW Hex 3 HexNAc 5 | 120.426 | 0.016 | -3.692 | 6.969e+06 | 31 - 43 | 1 | 30.53 | 30.70 | 31.04 |
| 3574.4426 | TPN(N)KTEDTIF Fuc 4 Hex 5 HexNAc 5 | 119.857 | 0.016 | -4.376 | 4.041e+07 | 69 - 79 | 2 | 38.43 | 38.60 | 38.93 |
| 3031.2579 | TPN(N)KTEDTIF Hex 4 HexNAc 6 | 119.520 | 0.016 | 13.024 | 8.384e+06 | 69 - 79 | 2 | 23.73 | 23.89 | 24.57 |
| 4202.7307 | FYFTPN(N)KTEDTIF Fuc 1 Hex 5 HexNAc 8 | 119.464 | 0.016 | 12.732 | 1.705e+07 | 66 - 79 | 3 | 46.08 | 46.25 | 46.76 |
| 2518.0523 | FYFTPN(N)KT Hex 3 HexNAc 5 | 118.432 | 0.0 | -18.723 | 5.309e+06 | 66 - 74 | 2 | 26.78 | 26.78 | 26.95 |
| 4332.8209 | RNEEYN(N)KSVQEIQATF Fuc 1 Hex 5 HexNAc 7 | 117.602 | 0.018 | -4.471 | 1.596e+07 | 50 - 66 | 1 | 31.97 | 31.97 | 32.31 |
| 3807.6343 | LNVQREN(N)GTVSRY Hex 4 HexNAc 8 | 117.580 | 0.018 | -5.319 | 4.516e+07 | 96 - 109 | 3 | 16.24 | 16.24 | 16.57 |
| 3331.3660 | YFTPN(N)KTEDTIF Fuc 1 Hex 5 HexNAc 3 Neu5Ac 1 | 117.501 | 0.018 | -4.948 | 5.309e+06 | 67 - 79 | 2 | 38.77 | 38.77 | 39.10 |
| 3314.3407 | TPN(N)KTEDTIF Hex 7 HexNAc 5 | 117.026 | 0.018 | 11.467 | 2.448e+07 | 69 - 79 | 2 | 38.77 | 38.93 | 39.44 |
| 3344.4541 | LNVQREN(N)GTVSRY Fuc 1 Hex 4 HexNAc 5 | 114.806 | 0.019 | 11.597 | 8.257e+07 | 96 - 109 | 3 | 16.57 | 16.73 | 17.23 |
| 2934.2659 | RNEEYN(N)KSVQEIQ Hex 3 HexNAc 4 | 114.146 | 0.020 | -19.803 | 6.454e+06 | 50 - 63 | 1 | 14.95 | 14.95 | 15.12 |
| 3415.4751 | RNEEYN(N)KSVQEIQATF Hex 4 HexNAc 4 | 113.169 | 0.020 | -20.221 | 2.286e+07 | 50 - 66 | 1 | 32.65 | 32.65 | 32.79 |
| 3178.3248 | FTPN(N)KTEDTIF Hex 4 HexNAc 6 | 111.200 | 0.020 | -5.095 | 8.899e+06 | 68 - 79 | 2 | 30.87 | 31.04 | 31.20 |
| 3689.5168 | FTPN(N)KTEDTIF Fuc 1 Hex 5 HexNAc 7 | 111.187 | 0.020 | 12.370 | 8.721e+06 | 68 - 79 | 4 | 30.01 | 30.36 | 30.70 |
| 3515.4076 | TPN(N)KTEDTIF Fuc 1 Hex 5 HexNAc 4 Neu5Ac 2 | 109.602 | 0.022 | 0.000 | 0.000e+00 | 69 - 79 | 1 | - | - | - |
| 3320.3981 | VQREN(N)GTVSRY Fuc 1 Hex 4 HexNAc 6 | 108.745 | 0.026 | -19.402 | 9.690e+06 | 98 - 109 | 1 | 11.72 | 11.72 | 11.72 |
| 3947.6383 | NVQREN(N)GTVSRY Fuc 2 Hex 5 HexNAc 7 | 108.148 | 0.027 | 0.000 | 0.000e+00 | 97 - 109 | 1 | - | - | - |
| 3926.6701 | RNEEYN(N)KSVQEIQATF Fuc 1 Hex 5 HexNAc 5 | 107.560 | 0.027 | -6.225 | 8.641e+06 | 50 - 66 | 1 | 32.14 | 32.48 | 32.48 |
| 2983.3502 | C(C)ANLVPVPITN(N)ATL Hex 3 HexNAc 5 | 107.165 | 0.027 | 13.320 | 2.241e+07 | 22 - 36 | 7 | 51.15 | 51.39 | 52.23 |
| 3799.5781 | NVQREN(N)GTVSRY Fuc 1 Hex 5 HexNAc 7 | 105.481 | 0.037 | -19.230 | 1.839e+07 | 97 - 109 | 1 | 11.89 | 11.89 | 11.89 |
| 3401.4755 | RN(D)EEYN(N)KSVQEIQATFF Hex 3 HexNAc 4 | 105.416 | 0.038 | 7.036 | 5.696e+07 | 50 - 67 | 6 | 46.08 | 46.25 | 47.10 |
| 4002.6629 | NVQREN(N)GTVSRY Fuc 1 Hex 5 HexNAc 8 | 105.040 | 0.038 | -20.197 | 3.784e+07 | 97 - 109 | 2 | 11.89 | 11.89 | 11.89 |
| 3914.6525 | LNVQREN(N)GTVSRY Fuc 1 Hex 5 HexNAc 7 | 103.653 | 0.045 | 0.000 | 0.000e+00 | 96 - 109 | 1 | - | - | - |
| 3464.4228 | NVQREN(N)GTVSRY Hex 4 HexNAc 4 Neu5Ac 2 | 102.765 | 0.045 | -19.668 | 2.664e+07 | 97 - 109 | 2 | 12.22 | 12.22 | 12.38 |
| 3972.6016 | FYFTPN(N)KTEDTIF Fuc 1 Hex 5 HexNAc 4 Neu5Ac 2 | 100.754 | 0.050 | -5.447 | 1.924e+07 | 66 - 79 | 2 | 47.95 | 48.10 | 48.27 |

### TPN(N)KTEDTIF Fuc 1 Hex 4 HexNAc 5

**Mass:**  2974.2339

(5.382e-07)

**Charge States:** 3

**Start Position**: 70

**End Position**: 80

| Profile Score | Tandem Score | TDA q-value | Volume | Spectrum Matches |
| --- | --- | --- | --- | --- |
| 14.3506 | 182.1750 | 0.0000 | 3.6992e+07 | 3 |

**Adduct Signal Fractions**

- **Unmodified**: 3.6992e+07

### TPN(N)KTEDTIF Hex 3 HexNAc 5

**Mass:**  2666.1247

(1.424e-06)

**Charge States:** 2, 3

**Start Position**: 70

**End Position**: 80

| Profile Score | Tandem Score | TDA q-value | Volume | Spectrum Matches |
| --- | --- | --- | --- | --- |
| 15.5991 | 181.4711 | 0.0000 | 1.6133e+08 | 8 |

**Adduct Signal Fractions**

- **Unmodified**: 1.6133e+08

### TPN(N)KTEDTIF Hex 3 HexNAc 6

**Mass:**  2869.2037

(1.013e-06)

**Charge States:** 3, 4

**Start Position**: 70

**End Position**: 80

| Profile Score | Tandem Score | TDA q-value | Volume | Spectrum Matches |
| --- | --- | --- | --- | --- |
| 16.3100 | 180.6692 | 0.0000 | 2.9431e+08 | 11 |

**Adduct Signal Fractions**

- **Unmodified**: 2.9431e+08

### TPN(N)KTEDTIF Fuc 1 Hex 4 HexNAc 6

**Mass:**  3177.3136

(5.581e-07)

**Charge States:** 3, 4

**Start Position**: 70

**End Position**: 80

| Profile Score | Tandem Score | TDA q-value | Volume | Spectrum Matches |
| --- | --- | --- | --- | --- |
| 14.4737 | 180.5706 | 0.0000 | 6.4424e+07 | 4 |

**Adduct Signal Fractions**

- **Unmodified**: 6.4424e+07

### YFTPN(N)KTEDTIF Hex 3 HexNAc 5

**Mass:**  2976.2569

(8.199e-07)

**Charge States:** 2, 3, 4

**Start Position**: 68

**End Position**: 80

| Profile Score | Tandem Score | TDA q-value | Volume | Spectrum Matches |
| --- | --- | --- | --- | --- |
| 15.4880 | 178.8706 | 0.0000 | 1.7999e+09 | 134 |

**Adduct Signal Fractions**

- **Unmodified**: 1.7999e+09

### N(N)KSVQEIQATF Hex 3 HexNAc 4

**Mass:**  2562.1245

(1.041e-06)

**Charge States:** 3

**Start Position**: 56

**End Position**: 67

| Profile Score | Tandem Score | TDA q-value | Volume | Spectrum Matches |
| --- | --- | --- | --- | --- |
| -4.3955 | 178.5779 | 0.0000 | 2.2775e+07 | 1 |

**Adduct Signal Fractions**

- **Unmodified**: 2.2775e+07

### YFTPN(N)KTEDTIF Hex 4 HexNAc 7

**Mass:**  3544.4679

(1.659e-06)

**Charge States:** 3, 4

**Start Position**: 68

**End Position**: 80

| Profile Score | Tandem Score | TDA q-value | Volume | Spectrum Matches |
| --- | --- | --- | --- | --- |
| 15.1306 | 178.2902 | 0.0000 | 8.3569e+08 | 11 |

**Adduct Signal Fractions**

- **Unmodified**: 8.3569e+08

### YFTPN(N)KTEDTIF Hex 3 HexNAc 6

**Mass:**  3179.3351

(4.864e-07)

**Charge States:** 2, 3, 4

**Start Position**: 68

**End Position**: 80

| Profile Score | Tandem Score | TDA q-value | Volume | Spectrum Matches |
| --- | --- | --- | --- | --- |
| 14.9162 | 176.9188 | 0.0000 | 3.1320e+09 | 31 |

**Adduct Signal Fractions**

- **Unmodified**: 3.1320e+09

### YFTPN(N)KTEDTIF Fuc 1 Hex 4 HexNAc 5

**Mass:**  3284.3684

(9.639e-07)

**Charge States:** 3, 4

**Start Position**: 68

**End Position**: 80

| Profile Score | Tandem Score | TDA q-value | Volume | Spectrum Matches |
| --- | --- | --- | --- | --- |
| 14.4294 | 175.0841 | 0.0000 | 4.6468e+08 | 5 |

**Adduct Signal Fractions**

- **Unmodified**: 4.6468e+08

### TPN(N)KTEDTIF Hex 3 HexNAc 4

**Mass:**  2463.0459

(2.075e-06)

**Charge States:** 2, 3

**Start Position**: 70

**End Position**: 80

| Profile Score | Tandem Score | TDA q-value | Volume | Spectrum Matches |
| --- | --- | --- | --- | --- |
| 13.8903 | 174.5203 | 0.0000 | 2.1883e+07 | 4 |

**Adduct Signal Fractions**

- **Unmodified**: 2.1883e+07

### FYFTPN(N)KTEDTIF Hex 4 HexNAc 7

**Mass:**  3691.5379

(1.657e-06)

**Charge States:** 3, 4

**Start Position**: 67

**End Position**: 80

| Profile Score | Tandem Score | TDA q-value | Volume | Spectrum Matches |
| --- | --- | --- | --- | --- |
| 15.1352 | 172.7342 | 0.0000 | 4.5490e+08 | 10 |

**Adduct Signal Fractions**

- **Unmodified**: 4.5490e+08

### YFTPN(N)KTEDTIF Fuc 1 Hex 4 HexNAc 6

**Mass:**  3487.4463

(1.210e-06)

**Charge States:** 3, 4

**Start Position**: 68

**End Position**: 80

| Profile Score | Tandem Score | TDA q-value | Volume | Spectrum Matches |
| --- | --- | --- | --- | --- |
| 15.8934 | 171.6813 | 0.0000 | 8.4840e+08 | 10 |

**Adduct Signal Fractions**

- **Unmodified**: 8.4840e+08

### YFTPN(N)KTEDTIF Hex 3 HexNAc 4

**Mass:**  2773.1773

(9.537e-07)

**Charge States:** 2, 3

**Start Position**: 68

**End Position**: 80

| Profile Score | Tandem Score | TDA q-value | Volume | Spectrum Matches |
| --- | --- | --- | --- | --- |
| 14.1046 | 170.8177 | 0.0000 | 2.5925e+08 | 15 |

**Adduct Signal Fractions**

- **Unmodified**: 2.5925e+08

### FYFTPN(N)KTEDTIF Hex 3 HexNAc 6

**Mass:**  3326.4054

(1.003e-06)

**Charge States:** 2, 3, 4

**Start Position**: 67

**End Position**: 80

| Profile Score | Tandem Score | TDA q-value | Volume | Spectrum Matches |
| --- | --- | --- | --- | --- |
| 13.6566 | 169.8548 | 0.0000 | 5.0523e+09 | 224 |

**Adduct Signal Fractions**

- **Unmodified**: 5.0523e+09

### TPN(N)KTEDTIF Hex 4 HexNAc 7

**Mass:**  3234.3367

(1.360e-06)

**Charge States:** 3

**Start Position**: 70

**End Position**: 80

| Profile Score | Tandem Score | TDA q-value | Volume | Spectrum Matches |
| --- | --- | --- | --- | --- |
| 12.6538 | 169.1100 | 0.0000 | 4.4781e+07 | 4 |

**Adduct Signal Fractions**

- **Unmodified**: 4.4781e+07

### FYFTPN(N)KTEDTIF Fuc 1 Hex 4 HexNAc 6

**Mass:**  3634.5177

(1.168e-06)

**Charge States:** 3, 4

**Start Position**: 67

**End Position**: 80

| Profile Score | Tandem Score | TDA q-value | Volume | Spectrum Matches |
| --- | --- | --- | --- | --- |
| 14.0793 | 168.5392 | 0.0000 | 7.8515e+08 | 44 |

**Adduct Signal Fractions**

- **Unmodified**: 7.8515e+08

### FYFTPN(N)KTEDTIF Fuc 1 Hex 4 HexNAc 5

**Mass:**  3431.4347

(6.905e-07)

**Charge States:** 3, 4

**Start Position**: 67

**End Position**: 80

| Profile Score | Tandem Score | TDA q-value | Volume | Spectrum Matches |
| --- | --- | --- | --- | --- |
| 14.0443 | 168.4857 | 0.0000 | 3.4488e+08 | 8 |

**Adduct Signal Fractions**

- **Unmodified**: 3.4488e+08

### FYFTPN(N)KTEDTIF Hex 3 HexNAc 4

**Mass:**  2920.2447

(8.629e-07)

**Charge States:** 2, 3

**Start Position**: 67

**End Position**: 80

| Profile Score | Tandem Score | TDA q-value | Volume | Spectrum Matches |
| --- | --- | --- | --- | --- |
| 14.9930 | 167.7783 | 0.0000 | 1.7957e+08 | 13 |

**Adduct Signal Fractions**

- **Unmodified**: 1.7957e+08

### N(N)KSVQEIQATF Hex 3 HexNAc 5

**Mass:**  2765.2040

(1.958e-06)

**Charge States:** 3

**Start Position**: 56

**End Position**: 67

| Profile Score | Tandem Score | TDA q-value | Volume | Spectrum Matches |
| --- | --- | --- | --- | --- |
| -3.7172 | 167.7310 | 0.0000 | 1.6395e+07 | 1 |

**Adduct Signal Fractions**

- **Unmodified**: 1.6395e+07

### YFTPN(N)KTEDTIF Hex 4 HexNAc 6

**Mass:**  3341.3886

(7.641e-07)

**Charge States:** 3

**Start Position**: 68

**End Position**: 80

| Profile Score | Tandem Score | TDA q-value | Volume | Spectrum Matches |
| --- | --- | --- | --- | --- |
| 13.3975 | 167.5230 | 0.0000 | 1.4413e+08 | 5 |

**Adduct Signal Fractions**

- **Unmodified**: 1.4413e+08

### FYFTPN(N)KTEDTIF Hex 3 HexNAc 5

**Mass:**  3123.3246

(6.453e-07)

**Charge States:** 2, 3, 4

**Start Position**: 67

**End Position**: 80

| Profile Score | Tandem Score | TDA q-value | Volume | Spectrum Matches |
| --- | --- | --- | --- | --- |
| 13.8687 | 167.4439 | 0.0000 | 2.3323e+09 | 150 |

**Adduct Signal Fractions**

- **Unmodified**: 2.3323e+09

### YFTPN(N)KTEDTIF Hex 4 HexNAc 8

**Mass:**  3747.5520

(2.080e-06)

**Charge States:** 3, 4

**Start Position**: 68

**End Position**: 80

| Profile Score | Tandem Score | TDA q-value | Volume | Spectrum Matches |
| --- | --- | --- | --- | --- |
| 15.0864 | 166.3502 | 0.0000 | 1.2002e+08 | 5 |

**Adduct Signal Fractions**

- **Unmodified**: 1.2002e+08

### YFTPN(N)KTEDTIF Fuc 1 Hex 5 HexNAc 7

**Mass:**  3852.5828

(8.580e-07)

**Charge States:** 3, 4

**Start Position**: 68

**End Position**: 80

| Profile Score | Tandem Score | TDA q-value | Volume | Spectrum Matches |
| --- | --- | --- | --- | --- |
| 15.2152 | 166.1384 | 0.0000 | 1.3500e+08 | 6 |

**Adduct Signal Fractions**

- **Unmodified**: 1.3500e+08

### EEYN(N)KSVQEIQATF Hex 3 HexNAc 5

**Mass:**  3186.3532

(5.244e-07)

**Charge States:** 3

**Start Position**: 53

**End Position**: 67

| Profile Score | Tandem Score | TDA q-value | Volume | Spectrum Matches |
| --- | --- | --- | --- | --- |
| -3.2449 | 165.6371 | 0.0000 | 3.4091e+07 | 4 |

**Adduct Signal Fractions**

- **Unmodified**: 3.4091e+07

### FTPN(N)KTEDTIF Hex 3 HexNAc 6

**Mass:**  3016.2724

(1.016e-06)

**Charge States:** 2, 3, 4

**Start Position**: 69

**End Position**: 80

| Profile Score | Tandem Score | TDA q-value | Volume | Spectrum Matches |
| --- | --- | --- | --- | --- |
| 14.8299 | 165.1247 | 0.0000 | 6.2117e+08 | 10 |

**Adduct Signal Fractions**

- **Unmodified**: 6.2117e+08

### FTPN(N)KTEDTIF Hex 3 HexNAc 5

**Mass:**  2813.1919

(1.051e-06)

**Charge States:** 8, 2, 3

**Start Position**: 69

**End Position**: 80

| Profile Score | Tandem Score | TDA q-value | Volume | Spectrum Matches |
| --- | --- | --- | --- | --- |
| -0.5167 | 164.2759 | 0.0000 | 2.1572e+08 | 9 |

**Adduct Signal Fractions**

- **Unmodified**: 2.1572e+08

### FTPN(N)KTEDTIF Fuc 1 Hex 4 HexNAc 6

**Mass:**  3324.3836

(6.552e-07)

**Charge States:** 3, 4

**Start Position**: 69

**End Position**: 80

| Profile Score | Tandem Score | TDA q-value | Volume | Spectrum Matches |
| --- | --- | --- | --- | --- |
| 13.6611 | 163.7329 | 0.0000 | 1.1138e+08 | 7 |

**Adduct Signal Fractions**

- **Unmodified**: 1.1138e+08

### YFTPN(N)KTEDTIF Hex 5 HexNAc 8

**Mass:**  3909.6033

(1.834e-06)

**Charge States:** 3, 4

**Start Position**: 68

**End Position**: 80

| Profile Score | Tandem Score | TDA q-value | Volume | Spectrum Matches |
| --- | --- | --- | --- | --- |
| 12.8503 | 163.0644 | 0.0000 | 1.1960e+08 | 5 |

**Adduct Signal Fractions**

- **Unmodified**: 1.1960e+08

### FTPN(N)KTEDTIF Fuc 1 Hex 4 HexNAc 5

**Mass:**  3121.2998

(-3.228e-07)

**Charge States:** 3

**Start Position**: 69

**End Position**: 80

| Profile Score | Tandem Score | TDA q-value | Volume | Spectrum Matches |
| --- | --- | --- | --- | --- |
| 12.9140 | 162.6466 | 0.0000 | 7.3921e+07 | 4 |

**Adduct Signal Fractions**

- **Unmodified**: 7.3921e+07

### FYFTPN(N)KTEDTIF Fuc 1 Hex 4 HexNAc 7

**Mass:**  3837.5996

(1.570e-06)

**Charge States:** 3, 4

**Start Position**: 67

**End Position**: 80

| Profile Score | Tandem Score | TDA q-value | Volume | Spectrum Matches |
| --- | --- | --- | --- | --- |
| 12.0515 | 162.4026 | 0.0000 | 2.6818e+07 | 4 |

**Adduct Signal Fractions**

- **Unmodified**: 2.6818e+07

### N(N)KSVQEIQATF Fuc 1 Hex 4 HexNAc 5

**Mass:**  3073.3152

(1.097e-06)

**Charge States:** 3

**Start Position**: 56

**End Position**: 67

| Profile Score | Tandem Score | TDA q-value | Volume | Spectrum Matches |
| --- | --- | --- | --- | --- |
| -4.3429 | 160.7157 | 0.0000 | 8.6132e+06 | 1 |

**Adduct Signal Fractions**

- **Unmodified**: 8.6132e+06

### YFTPN(N)KTEDTIF Fuc 2 Hex 5 HexNAc 6

**Mass:**  3795.5579

(9.783e-07)

**Charge States:** 3, 4

**Start Position**: 68

**End Position**: 80

| Profile Score | Tandem Score | TDA q-value | Volume | Spectrum Matches |
| --- | --- | --- | --- | --- |
| 14.5285 | 159.5787 | 0.0000 | 1.6918e+08 | 3 |

**Adduct Signal Fractions**

- **Unmodified**: 1.6918e+08

### NVQREN(N)GTVSRY Fuc 2 Hex 5 HexNAc 6

**Mass:**  3742.5595

(5.581e-07)

**Charge States:** 3

**Start Position**: 98

**End Position**: 110

| Profile Score | Tandem Score | TDA q-value | Volume | Spectrum Matches |
| --- | --- | --- | --- | --- |
| -4.5289 | 159.4041 | 0.0000 | 1.9304e+08 | 3 |

**Adduct Signal Fractions**

- **Unmodified**: 1.9304e+08

### NVQREN(N)GTVSRY Hex 3 HexNAc 6

**Mass:**  3126.3338

(8.089e-07)

**Charge States:** 3, 4

**Start Position**: 98

**End Position**: 110

| Profile Score | Tandem Score | TDA q-value | Volume | Spectrum Matches |
| --- | --- | --- | --- | --- |
| 13.5382 | 159.2809 | 0.0000 | 1.2330e+09 | 6 |

**Adduct Signal Fractions**

- **Unmodified**: 1.2330e+09

### RNEEYN(N)KSVQEIQATF Fuc 1 Hex 4 HexNAc 6

**Mass:**  3967.6923

(1.785e-06)

**Charge States:** 3, 4

**Start Position**: 51

**End Position**: 67

| Profile Score | Tandem Score | TDA q-value | Volume | Spectrum Matches |
| --- | --- | --- | --- | --- |
| 14.8547 | 158.9014 | 0.0000 | 1.3687e+08 | 2 |

**Adduct Signal Fractions**

- **Unmodified**: 1.3687e+08

### RNEEYN(N)KSVQEIQATF Hex 3 HexNAc 5

**Mass:**  3456.4997

(-2.502e-07)

**Charge States:** 3, 4

**Start Position**: 51

**End Position**: 67

| Profile Score | Tandem Score | TDA q-value | Volume | Spectrum Matches |
| --- | --- | --- | --- | --- |
| 15.9894 | 158.4746 | 0.0000 | 5.6901e+09 | 15 |

**Adduct Signal Fractions**

- **Unmodified**: 5.6901e+09

### EEYN(N)KSVQEIQATF Fuc 1 Hex 4 HexNAc 5

**Mass:**  3494.4658

(1.492e-06)

**Charge States:** 3

**Start Position**: 53

**End Position**: 67

| Profile Score | Tandem Score | TDA q-value | Volume | Spectrum Matches |
| --- | --- | --- | --- | --- |
| -3.5750 | 158.1228 | 0.0000 | 2.2416e+07 | 2 |

**Adduct Signal Fractions**

- **Unmodified**: 2.2416e+07

### FYFTPN(N)KTEDTIF Fuc 2 Hex 5 HexNAc 6

**Mass:**  3942.6289

(1.476e-06)

**Charge States:** 3, 4

**Start Position**: 67

**End Position**: 80

| Profile Score | Tandem Score | TDA q-value | Volume | Spectrum Matches |
| --- | --- | --- | --- | --- |
| 15.2038 | 157.9657 | 0.0000 | 1.0178e+08 | 6 |

**Adduct Signal Fractions**

- **Unmodified**: 1.0178e+08

### RNEEYN(N)KSVQEIQATFF Hex 3 HexNAc 4

**Mass:**  3400.4844

(4.595e-07)

**Charge States:** 3, 4

**Start Position**: 51

**End Position**: 68

| Profile Score | Tandem Score | TDA q-value | Volume | Spectrum Matches |
| --- | --- | --- | --- | --- |
| 17.1740 | 157.3706 | 0.0000 | 1.9149e+09 | 12 |

**Adduct Signal Fractions**

- **Unmodified**: 1.9149e+09

### RNEEYN(N)KSVQEIQATFF Fuc 1 Hex 4 HexNAc 5

**Mass:**  3911.6784

(4.390e-08)

**Charge States:** 3, 4

**Start Position**: 51

**End Position**: 68

| Profile Score | Tandem Score | TDA q-value | Volume | Spectrum Matches |
| --- | --- | --- | --- | --- |
| 16.0720 | 157.0567 | 0.0000 | 1.0505e+09 | 18 |

**Adduct Signal Fractions**

- **Unmodified**: 1.0505e+09

### FYFTPN(N)KTEDTIF Fuc 1 Hex 5 HexNAc 7

**Mass:**  3999.6505

(1.601e-06)

**Charge States:** 3, 4

**Start Position**: 67

**End Position**: 80

| Profile Score | Tandem Score | TDA q-value | Volume | Spectrum Matches |
| --- | --- | --- | --- | --- |
| 14.1476 | 156.8633 | 0.0000 | 6.7462e+07 | 5 |

**Adduct Signal Fractions**

- **Unmodified**: 6.7462e+07

### RNEEYN(N)KSVQEIQATFF Fuc 1 Hex 3 HexNAc 4

**Mass:**  3546.5440

(1.344e-06)

**Charge States:** 3, 4

**Start Position**: 51

**End Position**: 68

| Profile Score | Tandem Score | TDA q-value | Volume | Spectrum Matches |
| --- | --- | --- | --- | --- |
| -4.8283 | 156.2382 | 0.0000 | 3.1887e+07 | 3 |

**Adduct Signal Fractions**

- **Unmodified**: 3.1887e+07

### YFTPN(N)KTEDTIF Fuc 2 Hex 5 HexNAc 5

**Mass:**  3592.4789

(1.104e-06)

**Charge States:** 3

**Start Position**: 68

**End Position**: 80

| Profile Score | Tandem Score | TDA q-value | Volume | Spectrum Matches |
| --- | --- | --- | --- | --- |
| -19.5689 | 156.1241 | 0.0000 | 1.4401e+07 | 1 |

**Adduct Signal Fractions**

- **Unmodified**: 1.4401e+07

### YFTPN(N)KTEDTIF Fuc 1 Hex 4 HexNAc 7

**Mass:**  3690.5308

(2.161e-06)

**Charge States:** 3, 4

**Start Position**: 68

**End Position**: 80

| Profile Score | Tandem Score | TDA q-value | Volume | Spectrum Matches |
| --- | --- | --- | --- | --- |
| -4.5467 | 155.6239 | 0.0000 | 3.5460e+07 | 3 |

**Adduct Signal Fractions**

- **Unmodified**: 3.5460e+07

### NVQREN(N)GTVSRY Fuc 1 Hex 4 HexNAc 5

**Mass:**  3231.3653

(-5.763e-07)

**Charge States:** 3

**Start Position**: 98

**End Position**: 110

| Profile Score | Tandem Score | TDA q-value | Volume | Spectrum Matches |
| --- | --- | --- | --- | --- |
| -3.9754 | 154.9245 | 0.0000 | 1.5039e+08 | 2 |

**Adduct Signal Fractions**

- **Unmodified**: 1.5039e+08

### YFTPN(N)KTEDTIF Hex 4 HexNAc 4 Neu5Ac 2

**Mass:**  3517.4188

(1.224e-06)

**Charge States:** 3, 4

**Start Position**: 68

**End Position**: 80

| Profile Score | Tandem Score | TDA q-value | Volume | Spectrum Matches |
| --- | --- | --- | --- | --- |
| 13.9896 | 154.5954 | 0.0000 | 2.4680e+08 | 7 |

**Adduct Signal Fractions**

- **Unmodified**: 2.4680e+08

### YFTPN(N)KTEDTIF Fuc 1 Hex 5 HexNAc 4 Neu5Ac 1

**Mass:**  3534.4444

(4.319e-06)

**Charge States:** 3

**Start Position**: 68

**End Position**: 80

| Profile Score | Tandem Score | TDA q-value | Volume | Spectrum Matches |
| --- | --- | --- | --- | --- |
| 12.3999 | 154.5445 | 0.0000 | 4.6892e+07 | 3 |

**Adduct Signal Fractions**

- **Unmodified**: 4.6892e+07

### RNEEYN(N)KSVQEIQATFF Hex 3 HexNAc 5

**Mass:**  3603.5630

(6.553e-07)

**Charge States:** 3, 4

**Start Position**: 51

**End Position**: 68

| Profile Score | Tandem Score | TDA q-value | Volume | Spectrum Matches |
| --- | --- | --- | --- | --- |
| 15.1258 | 154.3998 | 0.0000 | 1.5677e+09 | 10 |

**Adduct Signal Fractions**

- **Unmodified**: 1.5677e+09

### RNEEYN(N)KSVQEIQATF Hex 3 HexNAc 4

**Mass:**  3253.4141

(9.969e-07)

**Charge States:** 3, 4

**Start Position**: 51

**End Position**: 67

| Profile Score | Tandem Score | TDA q-value | Volume | Spectrum Matches |
| --- | --- | --- | --- | --- |
| 16.7060 | 153.8311 | 0.0000 | 7.1716e+09 | 29 |

**Adduct Signal Fractions**

- **Unmodified**: 7.1716e+09

### RNEEYN(N)KSVQEIQATF Fuc 1 Hex 4 HexNAc 5

**Mass:**  3764.6123

(2.556e-06)

**Charge States:** 3, 4

**Start Position**: 51

**End Position**: 67

| Profile Score | Tandem Score | TDA q-value | Volume | Spectrum Matches |
| --- | --- | --- | --- | --- |
| 16.0058 | 152.6435 | 0.0000 | 3.2955e+09 | 15 |

**Adduct Signal Fractions**

- **Unmodified**: 3.2955e+09

### RNEEYN(N)KSVQEIQAT Hex 3 HexNAc 4

**Mass:**  3106.3482

(6.955e-07)

**Charge States:** 3

**Start Position**: 51

**End Position**: 66

| Profile Score | Tandem Score | TDA q-value | Volume | Spectrum Matches |
| --- | --- | --- | --- | --- |
| -16.9398 | 152.6099 | 0.0000 | 3.5419e+07 | 1 |

**Adduct Signal Fractions**

- **Unmodified**: 3.5419e+07

### FYFTPN(N)KTEDTIF Fuc 1 Hex 5 HexNAc 4 Neu5Ac 1

**Mass:**  3681.5161

(6.328e-06)

**Charge States:** 3

**Start Position**: 67

**End Position**: 80

| Profile Score | Tandem Score | TDA q-value | Volume | Spectrum Matches |
| --- | --- | --- | --- | --- |
| 12.7060 | 152.0488 | 0.0000 | 2.7146e+07 | 3 |

**Adduct Signal Fractions**

- **Unmodified**: 2.7146e+07

### RNEEYN(N)KSVQEIQATF Fuc 1 Hex 4 HexNAc 4

**Mass:**  3561.5344

(2.715e-06)

**Charge States:** 3, 4

**Start Position**: 51

**End Position**: 67

| Profile Score | Tandem Score | TDA q-value | Volume | Spectrum Matches |
| --- | --- | --- | --- | --- |
| 15.5872 | 151.9757 | 0.0000 | 1.6582e+08 | 3 |

**Adduct Signal Fractions**

- **Unmodified**: 1.6582e+08

### RNEEYN(N)KSVQEIQATF Fuc 1 Hex 3 HexNAc 4

**Mass:**  3399.4740

(2.442e-07)

**Charge States:** 3, 4

**Start Position**: 51

**End Position**: 67

| Profile Score | Tandem Score | TDA q-value | Volume | Spectrum Matches |
| --- | --- | --- | --- | --- |
| 11.5993 | 151.8453 | 0.0000 | 1.4407e+08 | 8 |

**Adduct Signal Fractions**

- **Unmodified**: 1.4407e+08

### YFTPN(N)KTEDTIF Hex 5 HexNAc 9

**Mass:**  4112.6813

(1.356e-06)

**Charge States:** 3, 4

**Start Position**: 68

**End Position**: 80

| Profile Score | Tandem Score | TDA q-value | Volume | Spectrum Matches |
| --- | --- | --- | --- | --- |
| -3.8965 | 151.1826 | 0.0000 | 4.0269e+07 | 3 |

**Adduct Signal Fractions**

- **Unmodified**: 4.0269e+07

### RNEEYN(N)KSVQEIQATF Fuc 1 Hex 3 HexNAc 5

**Mass:**  3602.5534

(9.268e-07)

**Charge States:** 3, 4

**Start Position**: 51

**End Position**: 67

| Profile Score | Tandem Score | TDA q-value | Volume | Spectrum Matches |
| --- | --- | --- | --- | --- |
| 11.9208 | 150.9363 | 0.0000 | 1.5030e+08 | 7 |

**Adduct Signal Fractions**

- **Unmodified**: 1.5030e+08

### RNEEYN(N)KSVQEIQATF Hex 3 HexNAc 6

**Mass:**  3659.5823

(2.539e-06)

**Charge States:** 3, 4

**Start Position**: 51

**End Position**: 67

| Profile Score | Tandem Score | TDA q-value | Volume | Spectrum Matches |
| --- | --- | --- | --- | --- |
| 10.8484 | 150.9055 | 0.0000 | 3.1299e+08 | 7 |

**Adduct Signal Fractions**

- **Unmodified**: 3.1299e+08

### FTPN(N)KTEDTIF Hex 3 HexNAc 4

**Mass:**  2610.1144

(8.446e-07)

**Charge States:** 2, 3

**Start Position**: 69

**End Position**: 80

| Profile Score | Tandem Score | TDA q-value | Volume | Spectrum Matches |
| --- | --- | --- | --- | --- |
| -3.3267 | 150.7862 | 0.0000 | 3.4297e+07 | 3 |

**Adduct Signal Fractions**

- **Unmodified**: 3.4297e+07

### FTPN(N)KTEDTIF Hex 4 HexNAc 7

**Mass:**  3381.4048

(8.557e-07)

**Charge States:** 3, 4

**Start Position**: 69

**End Position**: 80

| Profile Score | Tandem Score | TDA q-value | Volume | Spectrum Matches |
| --- | --- | --- | --- | --- |
| 13.0617 | 150.7796 | 0.0000 | 7.0572e+07 | 4 |

**Adduct Signal Fractions**

- **Unmodified**: 7.0572e+07

### TPN(N)KTEDTIF Fuc 2 Hex 5 HexNAc 6

**Mass:**  3485.4270

(1.344e-06)

**Charge States:** 3

**Start Position**: 70

**End Position**: 80

| Profile Score | Tandem Score | TDA q-value | Volume | Spectrum Matches |
| --- | --- | --- | --- | --- |
| -4.5449 | 150.4819 | 0.0000 | 8.4693e+06 | 1 |

**Adduct Signal Fractions**

- **Unmodified**: 8.4693e+06

### FYFTPN(N)KTEDTIF Hex 4 HexNAc 8

**Mass:**  3894.6198

(1.793e-06)

**Charge States:** 3, 4

**Start Position**: 67

**End Position**: 80

| Profile Score | Tandem Score | TDA q-value | Volume | Spectrum Matches |
| --- | --- | --- | --- | --- |
| 16.0244 | 149.9802 | 0.0000 | 6.8705e+07 | 5 |

**Adduct Signal Fractions**

- **Unmodified**: 6.8705e+07

### RNEEYN(N)KSVQEIQATF Fuc 2 Hex 5 HexNAc 5

**Mass:**  4072.7221

(1.347e-06)

**Charge States:** 3, 4

**Start Position**: 51

**End Position**: 67

| Profile Score | Tandem Score | TDA q-value | Volume | Spectrum Matches |
| --- | --- | --- | --- | --- |
| -2.0290 | 149.7450 | 0.0000 | 9.3738e+07 | 3 |

**Adduct Signal Fractions**

- **Unmodified**: 9.3738e+07

### EEYN(N)KSVQEIQATF Hex 3 HexNAc 4

**Mass:**  2983.2740

(1.254e-06)

**Charge States:** 3

**Start Position**: 53

**End Position**: 67

| Profile Score | Tandem Score | TDA q-value | Volume | Spectrum Matches |
| --- | --- | --- | --- | --- |
| -3.3868 | 148.2295 | 0.0000 | 4.3192e+07 | 2 |

**Adduct Signal Fractions**

- **Unmodified**: 4.3192e+07

### RN(D)EEYN(N)KSVQEIQATF Hex 3 HexNAc 4

**Mass:**  3254.4021

(3.038e-04)

**Charge States:** 3, 4

**Start Position**: 51

**End Position**: 67

| Profile Score | Tandem Score | TDA q-value | Volume | Spectrum Matches |
| --- | --- | --- | --- | --- |
| 9.8657 | 147.7783 | 0.0000 | 6.5733e+08 | 94 |

**Adduct Signal Fractions**

- **Unmodified**: 6.5733e+08

### TPN(N)KTEDTIF Fuc 1 Hex 5 HexNAc 7

**Mass:**  3542.4475

(1.076e-06)

**Charge States:** 3

**Start Position**: 70

**End Position**: 80

| Profile Score | Tandem Score | TDA q-value | Volume | Spectrum Matches |
| --- | --- | --- | --- | --- |
| -19.1065 | 147.4084 | 0.0000 | 4.0006e+06 | 1 |

**Adduct Signal Fractions**

- **Unmodified**: 4.0006e+06

### RNEEYN(N)KSVQEIQATFF Fuc 1 Hex 4 HexNAc 4

**Mass:**  3708.5952

(3.769e-06)

**Charge States:** 3, 4

**Start Position**: 51

**End Position**: 68

| Profile Score | Tandem Score | TDA q-value | Volume | Spectrum Matches |
| --- | --- | --- | --- | --- |
| -4.6651 | 146.5426 | 0.0000 | 4.7344e+07 | 3 |

**Adduct Signal Fractions**

- **Unmodified**: 4.7344e+07

### RNEEYN(N)KSVQEIQATF Hex 4 HexNAc 7

**Mass:**  4024.7121

(3.556e-06)

**Charge States:** 3, 4

**Start Position**: 51

**End Position**: 67

| Profile Score | Tandem Score | TDA q-value | Volume | Spectrum Matches |
| --- | --- | --- | --- | --- |
| -4.4004 | 146.1322 | 0.0000 | 8.3937e+07 | 4 |

**Adduct Signal Fractions**

- **Unmodified**: 8.3937e+07

### FYFTPN(N)KTEDTIF Hex 5 HexNAc 8

**Mass:**  4056.6726

(1.756e-06)

**Charge States:** 3, 4

**Start Position**: 67

**End Position**: 80

| Profile Score | Tandem Score | TDA q-value | Volume | Spectrum Matches |
| --- | --- | --- | --- | --- |
| 12.8967 | 146.0071 | 0.0000 | 6.5280e+07 | 6 |

**Adduct Signal Fractions**

- **Unmodified**: 6.5280e+07

### FYFTPN(N)KTEDTIF Hex 4 HexNAc 4 Neu5Ac 2

**Mass:**  3664.4899

(1.000e-06)

**Charge States:** 3, 4

**Start Position**: 67

**End Position**: 80

| Profile Score | Tandem Score | TDA q-value | Volume | Spectrum Matches |
| --- | --- | --- | --- | --- |
| 13.5840 | 146.0067 | 0.0000 | 1.4952e+08 | 10 |

**Adduct Signal Fractions**

- **Unmodified**: 1.4952e+08

### RNEEYN(N)KSVQEIQATF Hex 4 HexNAc 5

**Mass:**  3618.5542

(2.560e-06)

**Charge States:** 3, 4

**Start Position**: 51

**End Position**: 67

| Profile Score | Tandem Score | TDA q-value | Volume | Spectrum Matches |
| --- | --- | --- | --- | --- |
| -5.0413 | 145.8878 | 0.0000 | 7.2763e+07 | 3 |

**Adduct Signal Fractions**

- **Unmodified**: 7.2763e+07

### YFTPN(N)KTEDTIF Hex 4 HexNAc 5

**Mass:**  3138.3118

(2.225e-06)

**Charge States:** 3

**Start Position**: 68

**End Position**: 80

| Profile Score | Tandem Score | TDA q-value | Volume | Spectrum Matches |
| --- | --- | --- | --- | --- |
| -19.9178 | 145.0047 | 0.0000 | 1.5691e+07 | 1 |

**Adduct Signal Fractions**

- **Unmodified**: 1.5691e+07

### YFTPN(N)KTEDTIFL Hex 3 HexNAc 6

**Mass:**  3292.4203

(1.114e-06)

**Charge States:** 3, 4

**Start Position**: 68

**End Position**: 81

| Profile Score | Tandem Score | TDA q-value | Volume | Spectrum Matches |
| --- | --- | --- | --- | --- |
| 14.5467 | 144.7965 | 0.0000 | 2.4047e+07 | 5 |

**Adduct Signal Fractions**

- **Unmodified**: 2.4047e+07

### RNEEYN(N)KSVQEIQATFF Hex 4 HexNAc 5

**Mass:**  3765.6168

(-5.867e-08)

**Charge States:** 3, 4

**Start Position**: 51

**End Position**: 68

| Profile Score | Tandem Score | TDA q-value | Volume | Spectrum Matches |
| --- | --- | --- | --- | --- |
| -4.4176 | 144.7441 | 0.0000 | 2.7205e+07 | 3 |

**Adduct Signal Fractions**

- **Unmodified**: 2.7205e+07

### RNEEYN(N)KSVQEIQATFF Fuc 1 Hex 3 HexNAc 5

**Mass:**  3749.6197

(9.711e-07)

**Charge States:** 3, 4

**Start Position**: 51

**End Position**: 68

| Profile Score | Tandem Score | TDA q-value | Volume | Spectrum Matches |
| --- | --- | --- | --- | --- |
| -17.9399 | 144.5266 | 0.0000 | 3.2818e+07 | 2 |

**Adduct Signal Fractions**

- **Unmodified**: 3.2818e+07

### NVQREN(N)GTVSRY Hex 4 HexNAc 8

**Mass:**  3694.5503

(7.800e-07)

**Charge States:** 3

**Start Position**: 98

**End Position**: 110

| Profile Score | Tandem Score | TDA q-value | Volume | Spectrum Matches |
| --- | --- | --- | --- | --- |
| -5.5970 | 144.1950 | 0.0000 | 1.0841e+08 | 3 |

**Adduct Signal Fractions**

- **Unmodified**: 1.0841e+08

### FYFTPN(N)KTEDTIF Hex 4 HexNAc 6

**Mass:**  3488.4596

(1.718e-06)

**Charge States:** 3

**Start Position**: 67

**End Position**: 80

| Profile Score | Tandem Score | TDA q-value | Volume | Spectrum Matches |
| --- | --- | --- | --- | --- |
| 11.8208 | 143.9516 | 0.0000 | 4.8425e+07 | 5 |

**Adduct Signal Fractions**

- **Unmodified**: 4.8425e+07

### YFTPN(N)KT Hex 3 HexNAc 6

**Mass:**  2575.0711

(3.916e-04)

**Start Position**: 68

**End Position**: 75

| Profile Score | Tandem Score | TDA q-value | Volume | Spectrum Matches |
| --- | --- | --- | --- | --- |
| 0.0000 | 143.3566 | 0.0000 | 0.0000e+00 | 1 |

Adduct:

No Chromatogram Found

### YN(N)SSYL Hex 3 HexNAc 6

**Mass:**  2449.9661

(1.330e-06)

**Charge States:** 3

**Start Position**: 92

**End Position**: 98

| Profile Score | Tandem Score | TDA q-value | Volume | Spectrum Matches |
| --- | --- | --- | --- | --- |
| -19.3581 | 142.6980 | 0.0000 | 2.9113e+06 | 1 |

**Adduct Signal Fractions**

- **Unmodified**: 2.9113e+06

### LNVQREN(N)GTVSRY Hex 3 HexNAc 6

**Mass:**  3239.4215

(9.294e-07)

**Charge States:** 3, 4

**Start Position**: 97

**End Position**: 110

| Profile Score | Tandem Score | TDA q-value | Volume | Spectrum Matches |
| --- | --- | --- | --- | --- |
| 14.0043 | 142.2373 | 0.0029 | 5.9893e+08 | 7 |

**Adduct Signal Fractions**

- **Unmodified**: 5.9893e+08

### RNEEYN(N)KSVQEIQATF Fuc 2 Hex 5 HexNAc 6

**Mass:**  4275.8019

(1.490e-06)

**Charge States:** 3, 4

**Start Position**: 51

**End Position**: 67

| Profile Score | Tandem Score | TDA q-value | Volume | Spectrum Matches |
| --- | --- | --- | --- | --- |
| 12.9939 | 141.6000 | 0.0029 | 2.6339e+07 | 3 |

**Adduct Signal Fractions**

- **Unmodified**: 2.6339e+07

### RNEEYN(N)KSVQEIQATFF Fuc 2 Hex 5 HexNAc 5

**Mass:**  4219.7902

(1.597e-06)

**Charge States:** 3, 4

**Start Position**: 51

**End Position**: 68

| Profile Score | Tandem Score | TDA q-value | Volume | Spectrum Matches |
| --- | --- | --- | --- | --- |
| 12.8886 | 141.4993 | 0.0029 | 3.4275e+07 | 4 |

**Adduct Signal Fractions**

- **Unmodified**: 3.4275e+07

### TPN(N)KTEDTIF Hex 4 HexNAc 4 Neu5Ac 2

**Mass:**  3207.2913

(1.886e-06)

**Charge States:** 3, 4

**Start Position**: 70

**End Position**: 80

| Profile Score | Tandem Score | TDA q-value | Volume | Spectrum Matches |
| --- | --- | --- | --- | --- |
| 13.1162 | 141.2675 | 0.0029 | 1.3432e+07 | 2 |

**Adduct Signal Fractions**

- **Unmodified**: 1.3432e+07

### YFTPN(N)KTEDTIF Fuc 1 Hex 5 HexNAc 8

**Mass:**  4055.6621

(2.537e-06)

**Charge States:** 3, 4

**Start Position**: 68

**End Position**: 80

| Profile Score | Tandem Score | TDA q-value | Volume | Spectrum Matches |
| --- | --- | --- | --- | --- |
| 7.8561 | 140.7007 | 0.0029 | 3.1750e+07 | 4 |

**Adduct Signal Fractions**

- **Unmodified**: 3.1750e+07

### FYFTPN(N)KTEDTIF Hex 5 HexNAc 9

**Mass:**  4259.7521

(1.879e-06)

**Charge States:** 3, 4

**Start Position**: 67

**End Position**: 80

| Profile Score | Tandem Score | TDA q-value | Volume | Spectrum Matches |
| --- | --- | --- | --- | --- |
| 11.6097 | 140.4833 | 0.0029 | 2.2955e+07 | 4 |

**Adduct Signal Fractions**

- **Unmodified**: 2.2955e+07

### RNEEYN(N)KSVQEIQATFF Fuc 1 Hex 4 HexNAc 6

**Mass:**  4114.7565

(1.052e-06)

**Charge States:** 3, 4

**Start Position**: 51

**End Position**: 68

| Profile Score | Tandem Score | TDA q-value | Volume | Spectrum Matches |
| --- | --- | --- | --- | --- |
| 13.2533 | 140.0369 | 0.0029 | 5.7241e+07 | 4 |

**Adduct Signal Fractions**

- **Unmodified**: 5.7241e+07

### FYFTPN(N)KTEDTIF Hex 4 HexNAc 5

**Mass:**  3285.3751

(1.851e-07)

**Charge States:** 3

**Start Position**: 67

**End Position**: 80

| Profile Score | Tandem Score | TDA q-value | Volume | Spectrum Matches |
| --- | --- | --- | --- | --- |
| -4.9367 | 139.8149 | 0.0029 | 1.3265e+07 | 2 |

**Adduct Signal Fractions**

- **Unmodified**: 1.3265e+07

### YFTPN(N)KTEDTIFL Hex 3 HexNAc 5

**Mass:**  3089.3417

(1.403e-06)

**Charge States:** 3

**Start Position**: 68

**End Position**: 81

| Profile Score | Tandem Score | TDA q-value | Volume | Spectrum Matches |
| --- | --- | --- | --- | --- |
| 13.9440 | 139.7763 | 0.0029 | 1.1916e+07 | 3 |

**Adduct Signal Fractions**

- **Unmodified**: 1.1916e+07

### RNEEYN(N)KSVQEIQATF Hex 4 HexNAc 8

**Mass:**  4227.7897

(1.396e-06)

**Charge States:** 3, 4

**Start Position**: 51

**End Position**: 67

| Profile Score | Tandem Score | TDA q-value | Volume | Spectrum Matches |
| --- | --- | --- | --- | --- |
| -3.7831 | 138.0715 | 0.0029 | 3.8672e+07 | 3 |

**Adduct Signal Fractions**

- **Unmodified**: 3.8672e+07

### RNEEYN(N)KSVQEIQATFF Hex 3 HexNAc 6

**Mass:**  3806.6442

(2.460e-07)

**Charge States:** 3, 4

**Start Position**: 51

**End Position**: 68

| Profile Score | Tandem Score | TDA q-value | Volume | Spectrum Matches |
| --- | --- | --- | --- | --- |
| -4.2816 | 138.0007 | 0.0029 | 7.4318e+07 | 4 |

**Adduct Signal Fractions**

- **Unmodified**: 7.4318e+07

### RNEEYN(N)KSVQEIQAT Fuc 1 Hex 4 HexNAc 5

**Mass:**  3617.5438

(2.120e-06)

**Charge States:** 3

**Start Position**: 51

**End Position**: 66

| Profile Score | Tandem Score | TDA q-value | Volume | Spectrum Matches |
| --- | --- | --- | --- | --- |
| -19.3184 | 137.9198 | 0.0029 | 1.1593e+07 | 1 |

**Adduct Signal Fractions**

- **Unmodified**: 1.1593e+07

### NVQREN(N)GTVSRY Fuc 1 Hex 4 HexNAc 6

**Mass:**  3434.4450

(-6.655e-07)

**Charge States:** 3, 4

**Start Position**: 98

**End Position**: 110

| Profile Score | Tandem Score | TDA q-value | Volume | Spectrum Matches |
| --- | --- | --- | --- | --- |
| 11.8818 | 137.5782 | 0.0029 | 6.3633e+08 | 5 |

**Adduct Signal Fractions**

- **Unmodified**: 6.3633e+08

### N(N)KSVQEIQATFF Hex 3 HexNAc 4

**Mass:**  2709.1939

(1.417e-06)

**Charge States:** 3

**Start Position**: 56

**End Position**: 68

| Profile Score | Tandem Score | TDA q-value | Volume | Spectrum Matches |
| --- | --- | --- | --- | --- |
| -2.9819 | 137.1995 | 0.0029 | 4.6341e+06 | 2 |

**Adduct Signal Fractions**

- **Unmodified**: 4.6341e+06

### NVQREN(N)GTVSRY Hex 3 HexNAc 5

**Mass:**  2923.2546

(-8.995e-07)

**Charge States:** 3

**Start Position**: 98

**End Position**: 110

| Profile Score | Tandem Score | TDA q-value | Volume | Spectrum Matches |
| --- | --- | --- | --- | --- |
| -3.2587 | 136.4392 | 0.0029 | 2.3839e+08 | 2 |

**Adduct Signal Fractions**

- **Unmodified**: 2.3839e+08

### FYFTPN(N)KT Hex 3 HexNAc 6

**Mass:**  2721.1349

(1.639e-06)

**Charge States:** 3

**Start Position**: 67

**End Position**: 75

| Profile Score | Tandem Score | TDA q-value | Volume | Spectrum Matches |
| --- | --- | --- | --- | --- |
| -3.6704 | 135.8514 | 0.0000 | 1.2492e+07 | 2 |

**Adduct Signal Fractions**

- **Unmodified**: 1.2492e+07

### YFTPN(N)KTEDTIF Fuc 1 Hex 5 HexNAc 6

**Mass:**  3649.5053

(2.437e-06)

**Charge States:** 3

**Start Position**: 68

**End Position**: 80

| Profile Score | Tandem Score | TDA q-value | Volume | Spectrum Matches |
| --- | --- | --- | --- | --- |
| -4.9939 | 135.2286 | 0.0029 | 7.8248e+06 | 2 |

**Adduct Signal Fractions**

- **Unmodified**: 7.8248e+06

### YFTPN(N)KTEDTIF Fuc 1 Hex 5 HexNAc 4 Neu5Ac 2

**Mass:**  3825.5304

(5.496e-07)

**Charge States:** 3

**Start Position**: 68

**End Position**: 80

| Profile Score | Tandem Score | TDA q-value | Volume | Spectrum Matches |
| --- | --- | --- | --- | --- |
| -5.5335 | 134.8218 | 0.0029 | 2.9963e+07 | 2 |

**Adduct Signal Fractions**

- **Unmodified**: 2.9963e+07

### FTPN(N)KTEDTIF Hex 4 HexNAc 4 Neu5Ac 2

**Mass:**  3354.3583

(1.155e-06)

**Charge States:** 3, 4

**Start Position**: 69

**End Position**: 80

| Profile Score | Tandem Score | TDA q-value | Volume | Spectrum Matches |
| --- | --- | --- | --- | --- |
| -4.2697 | 134.2545 | 0.0029 | 2.2869e+07 | 2 |

**Adduct Signal Fractions**

- **Unmodified**: 2.2869e+07

### RNEEYN(N)KSVQEIQATFF Fuc 2 Hex 4 HexNAc 5

**Mass:**  4057.7326

(1.105e-06)

**Charge States:** 3

**Start Position**: 51

**End Position**: 68

| Profile Score | Tandem Score | TDA q-value | Volume | Spectrum Matches |
| --- | --- | --- | --- | --- |
| -4.4109 | 133.5045 | 0.0029 | 1.5369e+07 | 2 |

**Adduct Signal Fractions**

- **Unmodified**: 1.5369e+07

### LNVQREN(N)GTVSRY Fuc 2 Hex 5 HexNAc 6

**Mass:**  3855.6443

(7.706e-07)

**Charge States:** 3, 4

**Start Position**: 97

**End Position**: 110

| Profile Score | Tandem Score | TDA q-value | Volume | Spectrum Matches |
| --- | --- | --- | --- | --- |
| -4.3442 | 132.6232 | 0.0029 | 8.2124e+07 | 5 |

**Adduct Signal Fractions**

- **Unmodified**: 8.2124e+07

### RNEEYN(N)KSVQEIQATF Fuc 2 Hex 4 HexNAc 5

**Mass:**  3910.6719

(2.395e-06)

**Charge States:** 3, 4

**Start Position**: 51

**End Position**: 67

| Profile Score | Tandem Score | TDA q-value | Volume | Spectrum Matches |
| --- | --- | --- | --- | --- |
| -4.2158 | 131.9282 | 0.0029 | 4.3351e+07 | 2 |

**Adduct Signal Fractions**

- **Unmodified**: 4.3351e+07

### LNVQREN(N)GTVSRY Hex 3 HexNAc 5

**Mass:**  3036.3407

(-5.160e-07)

**Charge States:** 3

**Start Position**: 97

**End Position**: 110

| Profile Score | Tandem Score | TDA q-value | Volume | Spectrum Matches |
| --- | --- | --- | --- | --- |
| 13.0651 | 130.8214 | 0.0055 | 1.2290e+08 | 4 |

**Adduct Signal Fractions**

- **Unmodified**: 1.2290e+08

### LNVQREN(N)GTVSRY Fuc 1 Hex 4 HexNAc 6

**Mass:**  3547.5346

(7.864e-07)

**Charge States:** 3, 4

**Start Position**: 97

**End Position**: 110

| Profile Score | Tandem Score | TDA q-value | Volume | Spectrum Matches |
| --- | --- | --- | --- | --- |
| 13.3297 | 130.7922 | 0.0055 | 3.3439e+08 | 6 |

**Adduct Signal Fractions**

- **Unmodified**: 3.3439e+08

### RN(D)EEYN(N)KSVQEIQATFF Hex 3 HexNAc 5

**Mass:**  3604.5514

(2.751e-04)

**Charge States:** 3

**Start Position**: 51

**End Position**: 68

| Profile Score | Tandem Score | TDA q-value | Volume | Spectrum Matches |
| --- | --- | --- | --- | --- |
| -5.7903 | 129.6681 | 0.0055 | 4.6193e+07 | 12 |

**Adduct Signal Fractions**

- **Unmodified**: 4.6193e+07

### RN(D)EEYN(N)KSVQEIQATF Fuc 1 Hex 4 HexNAc 5

**Mass:**  3765.5927

(2.633e-04)

**Charge States:** 3, 4

**Start Position**: 51

**End Position**: 67

| Profile Score | Tandem Score | TDA q-value | Volume | Spectrum Matches |
| --- | --- | --- | --- | --- |
| 11.4420 | 128.4925 | 0.0055 | 3.2149e+08 | 11 |

**Adduct Signal Fractions**

- **Unmodified**: 3.2149e+08

### FYFTPN(N)KTED Hex 3 HexNAc 6

**Mass:**  2965.2037

(3.641e-07)

**Charge States:** 3

**Start Position**: 67

**End Position**: 77

| Profile Score | Tandem Score | TDA q-value | Volume | Spectrum Matches |
| --- | --- | --- | --- | --- |
| -18.3085 | 128.3172 | 0.0055 | 3.7069e+06 | 1 |

**Adduct Signal Fractions**

- **Unmodified**: 3.7069e+06

### RNEEYN(N)KSVQEIQATF Hex 4 HexNAc 6

**Mass:**  3821.6358

(1.895e-06)

**Charge States:** 3, 4

**Start Position**: 51

**End Position**: 67

| Profile Score | Tandem Score | TDA q-value | Volume | Spectrum Matches |
| --- | --- | --- | --- | --- |
| 12.1893 | 127.1249 | 0.0079 | 5.7117e+08 | 7 |

**Adduct Signal Fractions**

- **Unmodified**: 5.7117e+08

### RNEEYN(N)KSVQEIQAT Hex 3 HexNAc 5

**Mass:**  3309.4286

(2.667e-06)

**Charge States:** 3

**Start Position**: 51

**End Position**: 66

| Profile Score | Tandem Score | TDA q-value | Volume | Spectrum Matches |
| --- | --- | --- | --- | --- |
| 11.9219 | 127.0341 | 0.0079 | 3.5050e+07 | 3 |

**Adduct Signal Fractions**

- **Unmodified**: 3.5050e+07

### TPN(N)KTEDTIFL Hex 3 HexNAc 6

**Mass:**  2982.2885

(1.887e-06)

**Charge States:** 3

**Start Position**: 70

**End Position**: 81

| Profile Score | Tandem Score | TDA q-value | Volume | Spectrum Matches |
| --- | --- | --- | --- | --- |
| -17.3359 | 125.9819 | 0.0104 | 2.3295e+06 | 3 |

**Adduct Signal Fractions**

- **Unmodified**: 2.3295e+06

### RN(D)EEYN(N)KSVQEIQATF Hex 3 HexNAc 5

**Mass:**  3457.4804

(2.864e-04)

**Charge States:** 3, 4

**Start Position**: 51

**End Position**: 67

| Profile Score | Tandem Score | TDA q-value | Volume | Spectrum Matches |
| --- | --- | --- | --- | --- |
| 9.5034 | 125.4199 | 0.0120 | 5.5031e+08 | 14 |

**Adduct Signal Fractions**

- **Unmodified**: 5.5031e+08

### FRNEEYN(N)KSVQEIQATF Hex 3 HexNAc 4

**Mass:**  3400.4796

(-9.558e-07)

**Charge States:** 3

**Start Position**: 50

**End Position**: 67

| Profile Score | Tandem Score | TDA q-value | Volume | Spectrum Matches |
| --- | --- | --- | --- | --- |
| -19.8783 | 124.9134 | 0.0120 | 8.2594e+06 | 1 |

**Adduct Signal Fractions**

- **Unmodified**: 8.2594e+06

### VQREN(N)GTVSRY Hex 3 HexNAc 6

**Mass:**  3012.2949

(6.710e-07)

**Charge States:** 3

**Start Position**: 99

**End Position**: 110

| Profile Score | Tandem Score | TDA q-value | Volume | Spectrum Matches |
| --- | --- | --- | --- | --- |
| -20.6441 | 124.1393 | 0.0120 | 2.5371e+07 | 1 |

**Adduct Signal Fractions**

- **Unmodified**: 2.5371e+07

### NVQREN(N)GTVSRY Hex 4 HexNAc 7

**Mass:**  3491.4666

(-2.034e-08)

**Charge States:** 3

**Start Position**: 98

**End Position**: 110

| Profile Score | Tandem Score | TDA q-value | Volume | Spectrum Matches |
| --- | --- | --- | --- | --- |
| 6.5063 | 124.0670 | 0.0120 | 1.5001e+08 | 4 |

**Adduct Signal Fractions**

- **Unmodified**: 1.5001e+08

### N(N)KTEDTIF Hex 3 HexNAc 6

**Mass:**  2671.1028

(8.310e-07)

**Charge States:** 3

**Start Position**: 72

**End Position**: 80

| Profile Score | Tandem Score | TDA q-value | Volume | Spectrum Matches |
| --- | --- | --- | --- | --- |
| -19.1641 | 123.5627 | 0.0000 | 4.9330e+06 | 1 |

**Adduct Signal Fractions**

- **Unmodified**: 4.9330e+06

### FTPN(N)KTEDTIF Fuc 2 Hex 5 HexNAc 6

**Mass:**  3632.4951

(1.141e-06)

**Charge States:** 3, 4

**Start Position**: 69

**End Position**: 80

| Profile Score | Tandem Score | TDA q-value | Volume | Spectrum Matches |
| --- | --- | --- | --- | --- |
| 12.8846 | 122.9728 | 0.0120 | 1.3588e+07 | 3 |

**Adduct Signal Fractions**

- **Unmodified**: 1.3588e+07

### N(N)KSVQEIQATFF Fuc 1 Hex 4 HexNAc 5

**Mass:**  3220.3841

(2.128e-07)

**Charge States:** 3

**Start Position**: 56

**End Position**: 68

| Profile Score | Tandem Score | TDA q-value | Volume | Spectrum Matches |
| --- | --- | --- | --- | --- |
| -3.6723 | 122.6868 | 0.0120 | 2.7710e+06 | 2 |

**Adduct Signal Fractions**

- **Unmodified**: 2.7710e+06

### YFTPN(N)KTEDTIF Hex 4 HexNAc 5 Neu5Ac 1

**Mass:**  3429.4052

(2.248e-06)

**Charge States:** 3

**Start Position**: 68

**End Position**: 80

| Profile Score | Tandem Score | TDA q-value | Volume | Spectrum Matches |
| --- | --- | --- | --- | --- |
| -5.3508 | 122.5059 | 0.0120 | 3.2249e+06 | 2 |

**Adduct Signal Fractions**

- **Unmodified**: 3.2249e+06

### LNVQREN(N)GTVSRY Hex 4 HexNAc 7

**Mass:**  3604.5547

(7.739e-07)

**Charge States:** 3

**Start Position**: 97

**End Position**: 110

| Profile Score | Tandem Score | TDA q-value | Volume | Spectrum Matches |
| --- | --- | --- | --- | --- |
| -4.6105 | 122.2916 | 0.0120 | 4.5572e+07 | 3 |

**Adduct Signal Fractions**

- **Unmodified**: 4.5572e+07

### TN(N)ATLDRITGKW Hex 3 HexNAc 5

**Mass:**  2876.2827

(6.306e-07)

**Charge States:** 3

**Start Position**: 32

**End Position**: 44

| Profile Score | Tandem Score | TDA q-value | Volume | Spectrum Matches |
| --- | --- | --- | --- | --- |
| -3.6925 | 120.4259 | 0.0159 | 6.9686e+06 | 1 |

**Adduct Signal Fractions**

- **Unmodified**: 6.9686e+06

### TPN(N)KTEDTIF Fuc 4 Hex 5 HexNAc 5

**Mass:**  3574.4423

(-4.212e-06)

**Charge States:** 3

**Start Position**: 70

**End Position**: 80

| Profile Score | Tandem Score | TDA q-value | Volume | Spectrum Matches |
| --- | --- | --- | --- | --- |
| -4.3760 | 119.8574 | 0.0159 | 4.0415e+07 | 2 |

**Adduct Signal Fractions**

- **Unmodified**: 4.0415e+07

### TPN(N)KTEDTIF Hex 4 HexNAc 6

**Mass:**  3031.2616

(2.575e-06)

**Charge States:** 3

**Start Position**: 70

**End Position**: 80

| Profile Score | Tandem Score | TDA q-value | Volume | Spectrum Matches |
| --- | --- | --- | --- | --- |
| 13.0238 | 119.5198 | 0.0159 | 8.3843e+06 | 2 |

**Adduct Signal Fractions**

- **Unmodified**: 8.3843e+06

### FYFTPN(N)KTEDTIF Fuc 1 Hex 5 HexNAc 8

**Mass:**  4202.7302

(1.659e-06)

**Charge States:** 3, 4

**Start Position**: 67

**End Position**: 80

| Profile Score | Tandem Score | TDA q-value | Volume | Spectrum Matches |
| --- | --- | --- | --- | --- |
| 12.7323 | 119.4638 | 0.0159 | 1.7054e+07 | 3 |

**Adduct Signal Fractions**

- **Unmodified**: 1.7054e+07

### FYFTPN(N)KT Hex 3 HexNAc 5

**Mass:**  2518.0534

(5.519e-07)

**Charge States:** 3

**Start Position**: 67

**End Position**: 75

| Profile Score | Tandem Score | TDA q-value | Volume | Spectrum Matches |
| --- | --- | --- | --- | --- |
| -18.7231 | 118.4319 | 0.0000 | 5.3087e+06 | 2 |

**Adduct Signal Fractions**

- **Unmodified**: 5.3087e+06

### RNEEYN(N)KSVQEIQATF Fuc 1 Hex 5 HexNAc 7

**Mass:**  4332.8210

(1.124e-06)

**Charge States:** 3

**Start Position**: 51

**End Position**: 67

| Profile Score | Tandem Score | TDA q-value | Volume | Spectrum Matches |
| --- | --- | --- | --- | --- |
| -4.4712 | 117.6024 | 0.0178 | 1.5961e+07 | 1 |

**Adduct Signal Fractions**

- **Unmodified**: 1.5961e+07

### LNVQREN(N)GTVSRY Hex 4 HexNAc 8

**Mass:**  3807.6335

(4.727e-07)

**Charge States:** 3, 4

**Start Position**: 97

**End Position**: 110

| Profile Score | Tandem Score | TDA q-value | Volume | Spectrum Matches |
| --- | --- | --- | --- | --- |
| -5.3187 | 117.5802 | 0.0178 | 4.5156e+07 | 3 |

**Adduct Signal Fractions**

- **Unmodified**: 4.5156e+07

### YFTPN(N)KTEDTIF Fuc 1 Hex 5 HexNAc 3 Neu5Ac 1

**Mass:**  3331.3657

(3.649e-06)

**Charge States:** 3

**Start Position**: 68

**End Position**: 80

| Profile Score | Tandem Score | TDA q-value | Volume | Spectrum Matches |
| --- | --- | --- | --- | --- |
| -4.9482 | 117.5013 | 0.0178 | 5.3090e+06 | 2 |

**Adduct Signal Fractions**

- **Unmodified**: 5.3090e+06

### TPN(N)KTEDTIF Hex 7 HexNAc 5

**Mass:**  3314.3410

(2.162e-06)

**Charge States:** 3

**Start Position**: 70

**End Position**: 80

| Profile Score | Tandem Score | TDA q-value | Volume | Spectrum Matches |
| --- | --- | --- | --- | --- |
| 11.4672 | 117.0262 | 0.0178 | 2.4482e+07 | 2 |

**Adduct Signal Fractions**

- **Unmodified**: 2.4482e+07

### LNVQREN(N)GTVSRY Fuc 1 Hex 4 HexNAc 5

**Mass:**  3344.4538

(1.028e-06)

**Charge States:** 3

**Start Position**: 97

**End Position**: 110

| Profile Score | Tandem Score | TDA q-value | Volume | Spectrum Matches |
| --- | --- | --- | --- | --- |
| 11.5966 | 114.8058 | 0.0194 | 8.2572e+07 | 3 |

**Adduct Signal Fractions**

- **Unmodified**: 8.2572e+07

### RNEEYN(N)KSVQEIQ Hex 3 HexNAc 4

**Mass:**  2934.2656

(1.502e-06)

**Charge States:** 3

**Start Position**: 51

**End Position**: 64

| Profile Score | Tandem Score | TDA q-value | Volume | Spectrum Matches |
| --- | --- | --- | --- | --- |
| -19.8033 | 114.1459 | 0.0205 | 6.4542e+06 | 1 |

**Adduct Signal Fractions**

- **Unmodified**: 6.4542e+06

### RNEEYN(N)KSVQEIQATF Hex 4 HexNAc 4

**Mass:**  3415.4741

(2.638e-06)

**Charge States:** 3

**Start Position**: 51

**End Position**: 67

| Profile Score | Tandem Score | TDA q-value | Volume | Spectrum Matches |
| --- | --- | --- | --- | --- |
| -20.2214 | 113.1694 | 0.0205 | 2.2862e+07 | 1 |

**Adduct Signal Fractions**

- **Unmodified**: 2.2862e+07

### FTPN(N)KTEDTIF Hex 4 HexNAc 6

**Mass:**  3178.3251

(1.460e-06)

**Charge States:** 3

**Start Position**: 69

**End Position**: 80

| Profile Score | Tandem Score | TDA q-value | Volume | Spectrum Matches |
| --- | --- | --- | --- | --- |
| -5.0952 | 111.1996 | 0.0205 | 8.8989e+06 | 2 |

**Adduct Signal Fractions**

- **Unmodified**: 8.8989e+06

### FTPN(N)KTEDTIF Fuc 1 Hex 5 HexNAc 7

**Mass:**  3689.5193

(1.866e-06)

**Charge States:** 3, 4

**Start Position**: 69

**End Position**: 80

| Profile Score | Tandem Score | TDA q-value | Volume | Spectrum Matches |
| --- | --- | --- | --- | --- |
| 12.3695 | 111.1868 | 0.0205 | 8.7210e+06 | 4 |

**Adduct Signal Fractions**

- **Unmodified**: 8.7210e+06

### TPN(N)KTEDTIF Fuc 1 Hex 5 HexNAc 4 Neu5Ac 2

**Mass:**  3515.4076

(3.116e-06)

**Start Position**: 70

**End Position**: 80

| Profile Score | Tandem Score | TDA q-value | Volume | Spectrum Matches |
| --- | --- | --- | --- | --- |
| 0.0000 | 109.6019 | 0.0220 | 0.0000e+00 | 1 |

Adduct:

No Chromatogram Found

### VQREN(N)GTVSRY Fuc 1 Hex 4 HexNAc 6

**Mass:**  3320.3981

(-1.662e-06)

**Charge States:** 3

**Start Position**: 99

**End Position**: 110

| Profile Score | Tandem Score | TDA q-value | Volume | Spectrum Matches |
| --- | --- | --- | --- | --- |
| -19.4024 | 108.7450 | 0.0257 | 9.6897e+06 | 1 |

**Adduct Signal Fractions**

- **Unmodified**: 9.6897e+06

### NVQREN(N)GTVSRY Fuc 2 Hex 5 HexNAc 7

**Mass:**  3947.6383

(5.073e-04)

**Start Position**: 98

**End Position**: 110

| Profile Score | Tandem Score | TDA q-value | Volume | Spectrum Matches |
| --- | --- | --- | --- | --- |
| 0.0000 | 108.1478 | 0.0271 | 0.0000e+00 | 1 |

Adduct:

No Chromatogram Found

### RNEEYN(N)KSVQEIQATF Fuc 1 Hex 5 HexNAc 5

**Mass:**  3926.6658

(3.327e-06)

**Charge States:** 4

**Start Position**: 51

**End Position**: 67

| Profile Score | Tandem Score | TDA q-value | Volume | Spectrum Matches |
| --- | --- | --- | --- | --- |
| -6.2254 | 107.5597 | 0.0271 | 8.6414e+06 | 1 |

**Adduct Signal Fractions**

- **Unmodified**: 8.6414e+06

### C(C)ANLVPVPITN(N)ATL Hex 3 HexNAc 5

**Mass:**  2983.3505

(1.332e-06)

**Charge States:** 3, 4

**Start Position**: 23

**End Position**: 37

| Profile Score | Tandem Score | TDA q-value | Volume | Spectrum Matches |
| --- | --- | --- | --- | --- |
| 13.3198 | 107.1652 | 0.0271 | 2.2413e+07 | 7 |

**Adduct Signal Fractions**

- **Unmodified**: 2.2413e+07

### NVQREN(N)GTVSRY Fuc 1 Hex 5 HexNAc 7

**Mass:**  3799.5781

(-1.762e-07)

**Charge States:** 3

**Start Position**: 98

**End Position**: 110

| Profile Score | Tandem Score | TDA q-value | Volume | Spectrum Matches |
| --- | --- | --- | --- | --- |
| -19.2300 | 105.4813 | 0.0366 | 1.8389e+07 | 1 |

**Adduct Signal Fractions**

- **Unmodified**: 1.8389e+07

### RN(D)EEYN(N)KSVQEIQATFF Hex 3 HexNAc 4

**Mass:**  3401.4739

(2.925e-04)

**Charge States:** 3, 4

**Start Position**: 51

**End Position**: 68

| Profile Score | Tandem Score | TDA q-value | Volume | Spectrum Matches |
| --- | --- | --- | --- | --- |
| 7.0357 | 105.4158 | 0.0382 | 5.6958e+07 | 6 |

**Adduct Signal Fractions**

- **Unmodified**: 5.6958e+07

### NVQREN(N)GTVSRY Fuc 1 Hex 5 HexNAc 8

**Mass:**  4002.6630

(1.158e-06)

**Charge States:** 3, 4

**Start Position**: 98

**End Position**: 110

| Profile Score | Tandem Score | TDA q-value | Volume | Spectrum Matches |
| --- | --- | --- | --- | --- |
| -20.1967 | 105.0400 | 0.0382 | 3.7843e+07 | 2 |

**Adduct Signal Fractions**

- **Unmodified**: 3.7843e+07

### LNVQREN(N)GTVSRY Fuc 1 Hex 5 HexNAc 7

**Mass:**  3914.6525

(5.085e-04)

**Start Position**: 97

**End Position**: 110

| Profile Score | Tandem Score | TDA q-value | Volume | Spectrum Matches |
| --- | --- | --- | --- | --- |
| 0.0000 | 103.6535 | 0.0447 | 0.0000e+00 | 1 |

Adduct:

No Chromatogram Found

### NVQREN(N)GTVSRY Hex 4 HexNAc 4 Neu5Ac 2

**Mass:**  3464.4223

(4.220e-07)

**Charge States:** 3

**Start Position**: 98

**End Position**: 110

| Profile Score | Tandem Score | TDA q-value | Volume | Spectrum Matches |
| --- | --- | --- | --- | --- |
| -19.6681 | 102.7647 | 0.0447 | 2.6638e+07 | 2 |

**Adduct Signal Fractions**

- **Unmodified**: 2.6638e+07

### FYFTPN(N)KTEDTIF Fuc 1 Hex 5 HexNAc 4 Neu5Ac 2

**Mass:**  3972.5996

(1.575e-06)

**Charge States:** 3, 4

**Start Position**: 67

**End Position**: 80

| Profile Score | Tandem Score | TDA q-value | Volume | Spectrum Matches |
| --- | --- | --- | --- | --- |
| -5.4468 | 100.7539 | 0.0497 | 1.9241e+07 | 2 |

**Adduct Signal Fractions**

- **Unmodified**: 1.9241e+07

### P00738|HPT\_HUMAN

**>P00738|HPT\_HUMAN**  
 `MSALGAVIALLLWGQLFAVDSGNDVTDIADDGCPKPPEIAHGYVEHSVRYQCKNYYKLRTEGDGVYTLNDKKQWINKAVG  
DKLPECEADDGCPKPPEIAHGYVEHSVRYQCKNYYKLRTEGDGVYTLNNEKQWINKAVGDKLPECEAVCGKPKNPANPVQ  
RILGGHLDAKGSFPWQAKMVSHHNLTTGATLINEQWLLTTAKNLFLNHSENATAKDIAPTLTLYVGKKQLVEIEKVVLHP  
NYSQVDIGLIKLKQKVSVNERVMPICLPSKDYAEVGRVGYVSGWGRNANFKFTDHLKYVMLPVADQDQCIRHYEGSTVPE  
KKTPKSPVGVQPILNEHTFCAGMSKYQEDTCYGDAGSAFAVHDLEEDTWYATGILSFDKSCAVAEYGVYVKVTSIQDWVQ  
KTIAEN`

| Site | Type | Count |
| --- | --- | --- |
| 184 | N-linked | 2 |

| Mass | Sequence | MS_2_ Score | `q`-value | MS_1_ Score | Signal | Protein Coordinates | Tandem Scans | Start Time | Apex Time | End Time |
| --- | --- | --- | --- | --- | --- | --- | --- | --- | --- | --- |
| 2548.1200 | VSHHN(N)LTTGATL Hex 3 HexNAc 4 | 168.737 | 0.0 | -3.559 | 2.928e+07 | 179 - 191 | 2 | 17.40 | 17.57 | 17.91 |
| 2751.1997 | VSHHN(N)LTTGATL Hex 3 HexNAc 5 | 160.041 | 0.0 | -4.506 | 1.299e+07 | 179 - 191 | 2 | 17.40 | 17.57 | 17.74 |

### VSHHN(N)LTTGATL Hex 3 HexNAc 4

**Mass:**  2548.1198

(9.461e-07)

**Charge States:** 3

**Start Position**: 180

**End Position**: 192

| Profile Score | Tandem Score | TDA q-value | Volume | Spectrum Matches |
| --- | --- | --- | --- | --- |
| -3.5585 | 168.7371 | 0.0000 | 2.9283e+07 | 2 |

**Adduct Signal Fractions**

- **Unmodified**: 2.9283e+07

### VSHHN(N)LTTGATL Hex 3 HexNAc 5

**Mass:**  2751.1994

(1.539e-06)

**Charge States:** 3

**Start Position**: 180

**End Position**: 192

| Profile Score | Tandem Score | TDA q-value | Volume | Spectrum Matches |
| --- | --- | --- | --- | --- |
| -4.5064 | 160.0406 | 0.0000 | 1.2991e+07 | 2 |

**Adduct Signal Fractions**

- **Unmodified**: 1.2991e+07

### P13473|LAMP2\_HUMAN

**>P13473|LAMP2\_HUMAN**  
 `MVCFRLFPVPGSGLVLVCLVLGAVRSYALELNLTDSENATCLYAKWQMNFTVRYETTNKTYKTVTISDHGTVTYNGSICG  
DDQNGPKIAVQFGPGFSWIANFTKAASTYSIDSVSFSYNTGDNTTFPDAEDKGILTVDELLAIRIPLNDLFRCNSLSTLE  
KNDVVQHYWDVLVQAFVQNGTVSTNEFLCDKDKTSTVAPTIHTTVPSPTTTPTPKEKPEAGTYSVNNGNDTCLLATMGLQ  
LNITQDKVASVININPNTTHSTGSCRSHTALLRLNSSTIKYLDFVFAVKNENRFYLKEVNISMYLVNGSVFSIANNNLSY  
WDAPLGSSYMCNKEQTVSVSGAFQINTFDLRVQPFNVTQGKYSTAQDCSADDDNFLVPIAVGAALAGVLILVLLAYFIGL  
KHHHAGYEQF`

| Site | Type | Count |
| --- | --- | --- |
| 307 | N-linked | 1 |

| Mass | Sequence | MS_2_ Score | `q`-value | MS_1_ Score | Signal | Protein Coordinates | Tandem Scans | Start Time | Apex Time | End Time |
| --- | --- | --- | --- | --- | --- | --- | --- | --- | --- | --- |
| 2235.9530 | LVN(N)GSVF Hex 3 HexNAc 5 | 143.604 | 0.0 | -5.512 | 3.269e+06 | 304 - 311 | 2 | 30.53 | 30.70 | 30.87 |

### LVN(N)GSVF Hex 3 HexNAc 5

**Mass:**  2235.9532

(7.454e-07)

**Charge States:** 3

**Start Position**: 305

**End Position**: 312

| Profile Score | Tandem Score | TDA q-value | Volume | Spectrum Matches |
| --- | --- | --- | --- | --- |
| -5.5116 | 143.6045 | 0.0000 | 3.2691e+06 | 2 |

**Adduct Signal Fractions**

- **Unmodified**: 3.2691e+06
