# Supplementary material for: Measuring Site-specific Glycosylation Similarity between Influenza a Virus Variants with Statistical Certainty
Source: Mol Cell Proteomics. 2020 Nov 25;19(9):1533–45. doi: 10.1074/mcp.RA120.002031 (PMC8143645; doi:10.1074/mcp.RA120.002031)
Supplement: Supplementary file 1 [file mmc1.zip › mmc1/159250_2_supp_555286_qcjklc.zip!/20190326_aAGPgal_021119_chymo_HILIC_DDA_1ug_04_glycopeptides.html]

## 20190326\_aAGPgal\_021119\_chymo\_HILIC\_DDA\_1ug\_04 @ GlycopeptideHypothesis-a0b3cb8241fa45a282fabd7643b59803

**MS^1^ Mass Accuracy:** 10.0 PPM  
**Grouping Tolerance:** 15.0 PPM  
**MS^n^ Mass Accuracy:** 20.0 PPM  
**Minimum `q`-Value For Clustering:** 0.05

**Glycopeptide Hypothesis:**
GlycopeptideHypothesis-a0b3cb8241fa45a282fabd7643b59803  
**Sample Searched:**
20190326\_aAGPgal\_021119\_chymo\_HILIC\_DDA\_1ug\_04

| Protein | Matched Glycopeptides |
| --- | --- |
| P02763\|A1AG1\_HUMAN | 170 |
| P19652\|A1AG2\_HUMAN | 150 |
| P00738\|HPT\_HUMAN | 2 |
| P13473\|LAMP2\_HUMAN | 1 |
| P14745\|CD44\_PAPHA | 1 |
| P16070\|CD44\_HUMAN | 1 |

### P02763|A1AG1\_HUMAN

**>P02763|A1AG1\_HUMAN**  
 `MALSWVLTVLSLLPLLEAQIPLCANLVPVPITNATLDQITGKWFYIASAFRNEEYNKSVQEIQATFFYFTPNKTEDTIFL  
REYQTRQDQCIYNTTYLNVQRENGTISRYVGGQEHFAHLLILRDTKTYMLAFDVNDEKNWGLSVYADKPETTKEQLGEFY  
EALDCLRIPKSDVVYTDWKKDKCEPLEKQHEKERKQEEGES`

| Site | Type | Count |
| --- | --- | --- |
| 33 | N-linked | 1 |
| 72 | N-linked | 71 |
| 93 | N-linked | 5 |
| 103 | N-linked | 47 |
| 56 | N-linked | 46 |

| Mass | Sequence | MS_2_ Score | `q`-value | MS_1_ Score | Signal | Protein Coordinates | Tandem Scans | Start Time | Apex Time | End Time |
| --- | --- | --- | --- | --- | --- | --- | --- | --- | --- | --- |
| 2666.1252 | TPN(N)KTEDTIF Hex 3 HexNAc 5 | 186.358 | 0.0 | 16.194 | 1.389e+08 | 69 - 79 | 7 | 24.37 | 24.88 | 25.90 |
| 2869.2039 | TPN(N)KTEDTIF Hex 3 HexNAc 6 | 181.114 | 0.0 | 16.293 | 3.123e+08 | 69 - 79 | 12 | 22.51 | 24.54 | 25.90 |
| 3177.3145 | TPN(N)KTEDTIF Fuc 1 Hex 4 HexNAc 6 | 180.178 | 0.0 | 15.076 | 5.672e+07 | 69 - 79 | 5 | 23.53 | 24.04 | 24.54 |
| 2976.2572 | YFTPN(N)KTEDTIF Hex 3 HexNAc 5 | 179.364 | 0.0 | 15.318 | 1.868e+09 | 67 - 79 | 24 | 37.08 | 38.39 | 43.88 |
| 2773.1769 | YFTPN(N)KTEDTIF Hex 3 HexNAc 4 | 177.685 | 0.0 | 15.290 | 2.768e+08 | 67 - 79 | 9 | 38.55 | 38.71 | 41.26 |
| 2765.2041 | N(N)KSVQEIQATF Hex 3 HexNAc 5 | 175.410 | 0.0 | -3.678 | 1.537e+07 | 55 - 66 | 1 | 27.43 | 27.60 | 27.77 |
| 3123.3240 | FYFTPN(N)KTEDTIF Hex 3 HexNAc 5 | 175.237 | 0.0 | 13.519 | 2.061e+09 | 66 - 79 | 114 | 47.44 | 48.61 | 92.55 |
| 3544.4720 | YFTPN(N)KTEDTIF Hex 4 HexNAc 7 | 175.202 | 0.0 | 13.707 | 8.793e+08 | 67 - 79 | 18 | 36.23 | 37.42 | 52.55 |
| 2974.2344 | TPN(N)KTEDTIF Fuc 1 Hex 4 HexNAc 5 | 175.093 | 0.0 | 14.845 | 3.612e+07 | 69 - 79 | 3 | 23.70 | 24.21 | 24.71 |
| 3284.3917 | YFTPN(N)KTEDTIF Fuc 1 Hex 4 HexNAc 5 | 174.901 | 0.0 | 12.597 | 5.422e+08 | 67 - 79 | 13 | 36.56 | 37.75 | 48.28 |
| 3326.4039 | FYFTPN(N)KTEDTIF Hex 3 HexNAc 6 | 174.829 | 0.0 | 13.231 | 4.532e+09 | 66 - 79 | 215 | 47.27 | 48.28 | 92.94 |
| 3179.3358 | YFTPN(N)KTEDTIF Hex 3 HexNAc 6 | 174.803 | 0.0 | 15.137 | 3.039e+09 | 67 - 79 | 37 | 36.91 | 38.07 | 44.56 |
| 3234.3375 | TPN(N)KTEDTIF Hex 4 HexNAc 7 | 174.585 | 0.0 | 12.506 | 3.608e+07 | 69 - 79 | 4 | 23.53 | 23.87 | 24.37 |
| 2562.1240 | N(N)KSVQEIQATF Hex 3 HexNAc 4 | 172.874 | 0.0 | -4.229 | 1.787e+07 | 55 - 66 | 1 | 27.60 | 27.94 | 28.12 |
| 3341.3896 | YFTPN(N)KTEDTIF Hex 4 HexNAc 6 | 170.695 | 0.0 | 12.756 | 1.528e+08 | 67 - 79 | 4 | 37.25 | 37.91 | 38.71 |
| 3121.3021 | FTPN(N)KTEDTIF Fuc 1 Hex 4 HexNAc 5 | 169.878 | 0.0 | 12.703 | 7.195e+07 | 68 - 79 | 4 | 31.00 | 31.34 | 32.23 |
| 3431.4360 | FYFTPN(N)KTEDTIF Fuc 1 Hex 4 HexNAc 5 | 169.777 | 0.0 | 14.231 | 3.382e+08 | 66 - 79 | 8 | 46.93 | 47.95 | 49.62 |
| 3691.5384 | FYFTPN(N)KTEDTIF Hex 4 HexNAc 7 | 169.516 | 0.0 | 15.540 | 4.649e+08 | 66 - 79 | 10 | 46.41 | 47.61 | 48.28 |
| 3073.3152 | N(N)KSVQEIQATF Fuc 1 Hex 4 HexNAc 5 | 169.070 | 0.0 | -18.219 | 7.275e+06 | 55 - 66 | 1 | 26.92 | 26.92 | 27.09 |
| 3795.5588 | YFTPN(N)KTEDTIF Fuc 2 Hex 5 HexNAc 6 | 168.722 | 0.0 | 15.613 | 1.791e+08 | 67 - 79 | 4 | 36.73 | 37.25 | 37.58 |
| 3634.5161 | FYFTPN(N)KTEDTIF Fuc 1 Hex 4 HexNAc 6 | 168.379 | 0.0 | 14.867 | 7.221e+08 | 66 - 79 | 36 | 46.75 | 47.78 | 54.59 |
| 3852.5815 | YFTPN(N)KTEDTIF Fuc 1 Hex 5 HexNAc 7 | 167.953 | 0.0 | 16.453 | 1.503e+08 | 67 - 79 | 6 | 36.56 | 37.08 | 37.58 |
| 3016.2722 | FTPN(N)KTEDTIF Hex 3 HexNAc 6 | 167.437 | 0.0 | 14.574 | 8.000e+08 | 68 - 79 | 11 | 30.15 | 31.65 | 32.23 |
| 3487.4475 | YFTPN(N)KTEDTIF Fuc 1 Hex 4 HexNAc 6 | 166.586 | 0.0 | 16.213 | 9.741e+08 | 67 - 79 | 10 | 36.39 | 37.58 | 38.55 |
| 2463.0459 | TPN(N)KTEDTIF Hex 3 HexNAc 4 | 165.798 | 0.0 | 14.343 | 2.135e+07 | 69 - 79 | 4 | 24.71 | 25.05 | 25.56 |
| 3909.6041 | YFTPN(N)KTEDTIF Hex 5 HexNAc 8 | 165.541 | 0.0 | 13.414 | 1.380e+08 | 67 - 79 | 6 | 36.39 | 36.73 | 37.42 |
| 2813.1919 | FTPN(N)KTEDTIF Hex 3 HexNAc 5 | 165.262 | 0.0 | 14.297 | 2.507e+08 | 68 - 79 | 10 | 30.49 | 31.89 | 32.74 |
| 3324.3825 | FTPN(N)KTEDTIF Fuc 1 Hex 4 HexNAc 6 | 164.097 | 0.0 | 12.798 | 1.036e+08 | 68 - 79 | 7 | 30.83 | 31.17 | 31.89 |
| 3138.3084 | YFTPN(N)KTEDTIF Hex 4 HexNAc 5 | 162.963 | 0.0 | -20.009 | 9.662e+06 | 67 - 79 | 2 | 37.75 | 38.07 | 38.07 |
| 3400.4864 | RNEEYN(N)KSVQEIQATFF Hex 3 HexNAc 4 | 162.591 | 0.0 | 17.268 | 1.855e+09 | 50 - 67 | 15 | 43.88 | 45.06 | 46.41 |
| 3292.4205 | YFTPN(N)KTEDTIFL Hex 3 HexNAc 6 | 162.055 | 0.0 | 15.975 | 2.374e+07 | 67 - 80 | 5 | 45.73 | 45.90 | 46.41 |
| 3494.4627 | EEYN(N)KSVQEIQATF Fuc 1 Hex 4 HexNAc 5 | 161.970 | 0.0 | 12.489 | 1.969e+07 | 52 - 66 | 2 | 35.54 | 35.71 | 36.23 |
| 3603.5642 | RNEEYN(N)KSVQEIQATFF Hex 3 HexNAc 5 | 161.915 | 0.0 | 15.756 | 1.664e+09 | 50 - 67 | 10 | 44.56 | 44.73 | 46.07 |
| 3747.5494 | YFTPN(N)KTEDTIF Hex 4 HexNAc 8 | 161.629 | 0.0 | 16.798 | 1.315e+08 | 67 - 79 | 5 | 36.91 | 37.08 | 37.75 |
| 3534.4466 | YFTPN(N)KTEDTIF Fuc 1 Hex 5 HexNAc 4 Neu5Ac 1 | 160.992 | 0.0 | 13.554 | 4.037e+07 | 67 - 79 | 2 | 38.71 | 38.87 | 39.38 |
| 2671.1026 | N(N)KTEDTIF Hex 3 HexNAc 6 | 160.533 | 0.0 | -5.165 | 4.598e+06 | 71 - 79 | 1 | 22.51 | 22.68 | 22.85 |
| 4114.7566 | RNEEYN(N)KSVQEIQATFF Fuc 1 Hex 4 HexNAc 6 | 159.456 | 0.0 | 14.250 | 5.007e+07 | 50 - 67 | 4 | 43.88 | 44.23 | 44.56 |
| 3381.4055 | FTPN(N)KTEDTIF Hex 4 HexNAc 7 | 158.368 | 0.0 | 14.116 | 6.204e+07 | 68 - 79 | 4 | 30.66 | 31.00 | 31.34 |
| 3690.5273 | YFTPN(N)KTEDTIF Fuc 1 Hex 4 HexNAc 7 | 158.257 | 0.0 | 14.071 | 4.583e+07 | 67 - 79 | 4 | 36.91 | 37.42 | 37.58 |
| 3999.6505 | FYFTPN(N)KTEDTIF Fuc 1 Hex 5 HexNAc 7 | 157.455 | 0.0 | 16.617 | 7.007e+07 | 66 - 79 | 5 | 46.41 | 46.93 | 47.61 |
| 2978.2550 | YFTPN(N)KTEDTIF Hex 3 HexNAc 5 | 157.236 | 0.0 | 0.000 | 0.000e+00 | 67 - 79 | 1 | - | - | - |
| 3485.4263 | TPN(N)KTEDTIF Fuc 2 Hex 5 HexNAc 6 | 157.030 | 0.0 | 11.356 | 7.660e+06 | 69 - 79 | 2 | 23.02 | 23.53 | 23.70 |
| 3764.6099 | RNEEYN(N)KSVQEIQATF Fuc 1 Hex 4 HexNAc 5 | 156.667 | 0.0 | 16.301 | 3.555e+09 | 50 - 66 | 14 | 31.72 | 32.74 | 34.18 |
| 3911.6802 | RNEEYN(N)KSVQEIQATFF Fuc 1 Hex 4 HexNAc 5 | 156.662 | 0.0 | 15.913 | 1.164e+09 | 50 - 67 | 12 | 44.05 | 44.40 | 45.73 |
| 3942.6281 | FYFTPN(N)KTEDTIF Fuc 2 Hex 5 HexNAc 6 | 156.381 | 0.0 | 14.359 | 1.002e+08 | 66 - 79 | 5 | 46.41 | 47.27 | 47.78 |
| 2920.2445 | FYFTPN(N)KTEDTIF Hex 3 HexNAc 4 | 155.981 | 0.0 | 14.938 | 1.740e+08 | 66 - 79 | 13 | 48.61 | 48.77 | 52.47 |
| 2983.2738 | EEYN(N)KSVQEIQATF Hex 3 HexNAc 4 | 155.872 | 0.0 | -3.373 | 4.018e+07 | 52 - 66 | 2 | 36.23 | 36.39 | 36.73 |
| 3254.4025 | RN(D)EEYN(N)KSVQEIQATF Hex 3 HexNAc 4 | 155.410 | 0.0 | 10.441 | 3.097e+08 | 50 - 66 | 20 | 36.06 | 36.23 | 36.91 |
| 3253.4178 | RNEEYN(N)KSVQEIQATF Hex 3 HexNAc 4 | 155.175 | 0.0 | 16.305 | 6.476e+09 | 50 - 66 | 25 | 28.62 | 33.37 | 34.69 |
| 4112.6820 | YFTPN(N)KTEDTIF Hex 5 HexNAc 9 | 154.777 | 0.0 | 9.875 | 4.142e+07 | 67 - 79 | 3 | 36.39 | 36.39 | 37.08 |
| 3517.4200 | YFTPN(N)KTEDTIF Hex 4 HexNAc 4 Neu5Ac 2 | 154.512 | 0.0 | 14.389 | 2.533e+08 | 67 - 79 | 8 | 38.71 | 38.87 | 40.06 |
| 2847.2830 | LNVQREN(N)GTISRY Hex 3 HexNAc 4 | 154.090 | 0.0 | 12.671 | 1.932e+08 | 96 - 109 | 3 | 19.66 | 19.83 | 20.81 |
| 3456.4980 | RNEEYN(N)KSVQEIQATF Hex 3 HexNAc 5 | 153.571 | 0.0 | 15.885 | 5.208e+09 | 50 - 66 | 17 | 31.00 | 33.05 | 34.52 |
| 3210.3506 | YFTPN(N)KTEDTIF Fuc 1 Hex 3 HexNAc 4 Neu5Ac 1 | 153.408 | 0.0 | 0.000 | 0.000e+00 | 67 - 79 | 1 | - | - | - |
| 3561.5537 | LNVQREN(N)GTISRY Fuc 1 Hex 4 HexNAc 6 | 153.361 | 0.0 | 14.403 | 3.575e+09 | 96 - 109 | 11 | 18.54 | 19.04 | 20.47 |
| 3186.3533 | EEYN(N)KSVQEIQATF Hex 3 HexNAc 5 | 153.257 | 0.0 | 14.206 | 3.560e+07 | 52 - 66 | 2 | 36.06 | 36.23 | 36.73 |
| 3618.5526 | RNEEYN(N)KSVQEIQATF Hex 4 HexNAc 5 | 153.247 | 0.0 | 17.397 | 7.745e+07 | 50 - 66 | 3 | 32.40 | 32.90 | 33.05 |
| 3253.4984 | LNVQREN(N)GTISRY Hex 3 HexNAc 6 | 152.864 | 0.0 | 11.851 | 9.801e+09 | 96 - 109 | 182 | 16.54 | 19.36 | 114.27 |
| 3399.4748 | RNEEYN(N)KSVQEIQATF Fuc 1 Hex 3 HexNAc 4 | 152.786 | 0.0 | 12.798 | 1.238e+08 | 50 - 66 | 6 | 33.37 | 33.52 | 34.86 |
| 3967.6901 | RNEEYN(N)KSVQEIQATF Fuc 1 Hex 4 HexNAc 6 | 151.913 | 0.0 | -3.267 | 1.466e+08 | 50 - 66 | 3 | 32.40 | 32.74 | 32.90 |
| 3561.5312 | RNEEYN(N)KSVQEIQATF Fuc 1 Hex 4 HexNAc 4 | 151.801 | 0.0 | 15.370 | 1.688e+08 | 50 - 66 | 3 | 32.23 | 32.90 | 33.05 |
| 4072.7212 | RNEEYN(N)KSVQEIQATF Fuc 2 Hex 5 HexNAc 5 | 151.413 | 0.0 | 14.232 | 9.414e+07 | 50 - 66 | 4 | 32.23 | 32.57 | 32.90 |
| 3592.4782 | YFTPN(N)KTEDTIF Fuc 2 Hex 5 HexNAc 5 | 151.162 | 0.0 | -5.321 | 1.748e+07 | 67 - 79 | 1 | 37.08 | 37.08 | 37.42 |
| 3659.5790 | RNEEYN(N)KSVQEIQATF Hex 3 HexNAc 6 | 150.966 | 0.0 | 11.924 | 2.928e+08 | 50 - 66 | 6 | 32.90 | 32.90 | 34.18 |
| 3602.5551 | RNEEYN(N)KSVQEIQATF Fuc 1 Hex 3 HexNAc 5 | 150.932 | 0.0 | 13.086 | 1.219e+08 | 50 - 66 | 3 | 33.05 | 33.37 | 33.85 |
| 3031.2589 | TPN(N)KTEDTIF Hex 4 HexNAc 6 | 150.828 | 0.0 | 11.829 | 8.029e+06 | 69 - 79 | 2 | 23.87 | 24.21 | 24.71 |
| 4024.7114 | RNEEYN(N)KSVQEIQATF Hex 4 HexNAc 7 | 150.521 | 0.0 | -4.781 | 8.387e+07 | 50 - 66 | 4 | 32.40 | 32.57 | 32.90 |
| 2610.1129 | FTPN(N)KTEDTIF Hex 3 HexNAc 4 | 150.235 | 0.0 | -3.744 | 3.153e+07 | 68 - 79 | 4 | 32.06 | 32.23 | 32.57 |
| 3207.2900 | TPN(N)KTEDTIF Hex 4 HexNAc 4 Neu5Ac 2 | 150.098 | 0.0 | 13.627 | 1.534e+07 | 69 - 79 | 3 | 24.88 | 25.22 | 25.90 |
| 4056.6726 | FYFTPN(N)KTEDTIF Hex 5 HexNAc 8 | 148.806 | 0.0 | 12.180 | 7.708e+07 | 66 - 79 | 6 | 46.41 | 46.75 | 47.61 |
| 3708.5980 | RNEEYN(N)KSVQEIQATFF Fuc 1 Hex 4 HexNAc 4 | 148.702 | 0.0 | -4.435 | 4.970e+07 | 50 - 67 | 2 | 44.23 | 44.40 | 44.73 |
| 3806.6446 | RNEEYN(N)KSVQEIQATFF Hex 3 HexNAc 6 | 148.546 | 0.0 | 11.810 | 7.417e+07 | 50 - 67 | 5 | 44.56 | 44.56 | 45.39 |
| 3106.3494 | RNEEYN(N)KSVQEIQAT Hex 3 HexNAc 4 | 148.390 | 0.0 | -3.133 | 4.065e+07 | 50 - 65 | 2 | 17.53 | 17.70 | 17.87 |
| 3192.3049 | TPN(N)KTEDTIF Fuc 3 Hex 3 HexNAc 4 Neu5Ac 1 | 147.835 | 0.0 | -18.904 | 2.814e+06 | 69 - 79 | 1 | 23.53 | 23.53 | 23.70 |
| 3089.3419 | YFTPN(N)KTEDTIFL Hex 3 HexNAc 5 | 146.360 | 0.0 | 14.011 | 1.038e+07 | 67 - 80 | 3 | 45.90 | 46.24 | 46.58 |
| 2965.2060 | FYFTPN(N)KTED Hex 3 HexNAc 6 | 144.930 | 0.0 | -4.732 | 3.635e+06 | 66 - 76 | 1 | 26.75 | 26.92 | 27.09 |
| 2937.2727 | NVQREN(N)GTISRY Hex 3 HexNAc 5 | 144.631 | 0.0 | 12.574 | 2.108e+08 | 97 - 109 | 3 | 13.62 | 13.79 | 14.45 |
| 3664.4897 | FYFTPN(N)KTEDTIF Hex 4 HexNAc 4 Neu5Ac 2 | 144.093 | 0.0 | 13.300 | 1.538e+08 | 66 - 79 | 9 | 48.77 | 48.94 | 49.78 |
| 4227.7907 | RNEEYN(N)KSVQEIQATF Hex 4 HexNAc 8 | 143.879 | 0.0 | -3.417 | 3.589e+07 | 50 - 66 | 3 | 32.23 | 32.40 | 32.74 |
| 3618.5943 | LNVQREN(N)GTISRY Hex 4 HexNAc 7 | 143.667 | 0.0 | 11.574 | 2.175e+09 | 96 - 109 | 14 | 18.37 | 19.04 | 44.90 |
| 3354.3583 | FTPN(N)KTEDTIF Hex 4 HexNAc 4 Neu5Ac 2 | 142.804 | 0.0 | -3.540 | 2.266e+07 | 68 - 79 | 2 | 32.23 | 32.40 | 32.57 |
| 3910.6657 | RNEEYN(N)KSVQEIQATF Fuc 2 Hex 4 HexNAc 5 | 142.777 | 0.0 | -4.947 | 4.099e+07 | 50 - 66 | 2 | 32.74 | 32.90 | 33.05 |
| 2912.2712 | N(N)KSVQEIQATFF Hex 3 HexNAc 5 | 142.686 | 0.0 | 13.954 | 6.269e+06 | 55 - 67 | 2 | 41.60 | 41.77 | 42.28 |
| 3894.6191 | FYFTPN(N)KTEDTIF Hex 4 HexNAc 8 | 142.297 | 0.0 | 15.718 | 7.074e+07 | 66 - 79 | 5 | 46.41 | 47.27 | 47.95 |
| 3415.4712 | RNEEYN(N)KSVQEIQATF Hex 4 HexNAc 4 | 142.221 | 0.0 | -20.488 | 2.155e+07 | 50 - 66 | 1 | 32.90 | 33.05 | 33.05 |
| 3546.5414 | RNEEYN(N)KSVQEIQATFF Fuc 1 Hex 3 HexNAc 4 | 141.318 | 0.0 | 11.884 | 3.377e+07 | 50 - 67 | 4 | 44.73 | 44.90 | 45.56 |
| 3708.5631 | NVQREN(N)GTISRY Hex 4 HexNAc 8 | 141.229 | 0.0 | -18.849 | 7.397e+07 | 97 - 109 | 2 | 13.29 | 13.29 | 13.46 |
| 3765.5953 | RN(D)EEYN(N)KSVQEIQATF Fuc 1 Hex 4 HexNAc 5 | 141.211 | 0.0 | 12.141 | 2.807e+08 | 50 - 66 | 11 | 34.35 | 35.71 | 36.91 |
| 3681.5157 | FYFTPN(N)KTEDTIF Fuc 1 Hex 5 HexNAc 4 Neu5Ac 1 | 140.808 | 0.0 | 13.043 | 2.573e+07 | 66 - 79 | 3 | 48.77 | 48.94 | 49.45 |
| 3488.4593 | FYFTPN(N)KTEDTIF Hex 4 HexNAc 6 | 140.492 | 0.0 | 11.579 | 5.377e+07 | 66 - 79 | 7 | 46.93 | 47.78 | 48.28 |
| 4219.7892 | RNEEYN(N)KSVQEIQATFF Fuc 2 Hex 5 HexNAc 5 | 140.488 | 0.0 | 14.276 | 2.810e+07 | 50 - 67 | 4 | 43.71 | 43.88 | 44.40 |
| 2721.1355 | FYFTPN(N)KT Hex 3 HexNAc 6 | 139.619 | 0.0 | -3.423 | 1.101e+07 | 66 - 74 | 1 | 26.58 | 26.58 | 26.92 |
| 3749.6214 | RNEEYN(N)KSVQEIQATFF Fuc 1 Hex 3 HexNAc 5 | 139.358 | 0.0 | -4.270 | 3.460e+07 | 50 - 67 | 2 | 44.56 | 44.73 | 44.90 |
| 3358.4742 | LNVQREN(N)GTISRY Fuc 1 Hex 4 HexNAc 5 | 138.903 | 0.0 | 13.177 | 9.925e+08 | 96 - 109 | 8 | 18.71 | 19.36 | 20.81 |
| 3399.5037 | LNVQREN(N)GTISRY Fuc 1 Hex 3 HexNAc 6 | 138.881 | 0.0 | 11.781 | 1.234e+08 | 96 - 109 | 2 | 19.36 | 19.66 | 19.99 |
| 3546.4606 | YFTPN(N)KTEDTIF Hex 4 HexNAc 7 | 136.664 | 0.0 | 0.000 | 0.000e+00 | 67 - 79 | 1 | - | - | - |
| 3309.4307 | RNEEYN(N)KSVQEIQAT Hex 3 HexNAc 5 | 136.433 | 0.0 | -4.448 | 2.996e+07 | 50 - 65 | 5 | 17.53 | 17.70 | 18.04 |
| 3457.4830 | RN(D)EEYN(N)KSVQEIQATF Hex 3 HexNAc 5 | 134.904 | 0.0 | 10.189 | 5.192e+08 | 50 - 66 | 12 | 34.69 | 36.06 | 36.91 |
| 3050.3632 | LNVQREN(N)GTISRY Hex 3 HexNAc 5 | 133.445 | 0.0 | 14.874 | 2.575e+09 | 96 - 109 | 9 | 19.04 | 19.66 | 20.81 |
| 4275.7999 | RNEEYN(N)KSVQEIQATF Fuc 2 Hex 5 HexNAc 6 | 132.675 | 0.0 | 11.703 | 2.555e+07 | 50 - 66 | 3 | 32.06 | 32.57 | 32.74 |
| 3448.4619 | NVQREN(N)GTISRY Fuc 1 Hex 4 HexNAc 6 | 131.824 | 0.0 | 13.716 | 3.465e+08 | 97 - 109 | 4 | 13.29 | 13.46 | 14.11 |
| 3303.3758 | YFTPN(N)KTEDTIF Hex 5 HexNAc 5 | 131.619 | 0.0 | -7.463 | 2.391e+06 | 67 - 79 | 3 | 29.47 | 29.64 | 29.81 |
| 3574.4415 | TPN(N)KTEDTIF Fuc 4 Hex 5 HexNAc 5 | 131.187 | 0.0 | 13.565 | 3.885e+07 | 69 - 79 | 3 | 38.71 | 39.04 | 39.55 |
| 3869.6633 | LNVQREN(N)GTISRY Fuc 2 Hex 5 HexNAc 6 | 129.878 | 0.0 | 14.173 | 1.313e+09 | 96 - 109 | 9 | 18.37 | 18.88 | 19.99 |
| 4177.7748 | LNVQREN(N)GTISRY Fuc 3 Hex 6 HexNAc 6 | 129.430 | 0.0 | 15.652 | 4.339e+08 | 96 - 109 | 4 | 18.20 | 18.71 | 19.04 |
| 3178.3229 | FTPN(N)KTEDTIF Hex 4 HexNAc 6 | 128.024 | 0.0 | -5.013 | 9.413e+06 | 68 - 79 | 2 | 31.00 | 31.34 | 31.47 |
| 4055.6618 | YFTPN(N)KTEDTIF Fuc 1 Hex 5 HexNAc 8 | 128.012 | 0.0 | 15.211 | 3.687e+07 | 67 - 79 | 4 | 36.39 | 36.56 | 37.25 |
| 3191.3009 | QTRQDQC(C)IYN(N)TTY Hex 3 HexNAc 5 | 127.998 | 0.0 | 15.237 | 9.755e+07 | 83 - 96 | 4 | 22.51 | 22.85 | 23.36 |
| 3756.5719 | NVQREN(N)GTISRY Fuc 2 Hex 5 HexNAc 6 | 127.978 | 0.0 | -18.775 | 8.406e+07 | 97 - 109 | 4 | 13.29 | 13.29 | 13.46 |
| 3702.4913 | QTRQDQC(C)IYN(N)TTY Fuc 1 Hex 4 HexNAc 6 | 127.978 | 0.0 | 13.850 | 6.171e+07 | 83 - 96 | 4 | 22.00 | 22.34 | 23.19 |
| 4057.7390 | RNEEYN(N)KSVQEIQATFF Fuc 2 Hex 4 HexNAc 5 | 127.768 | 0.0 | -4.716 | 1.619e+07 | 50 - 67 | 2 | 44.23 | 44.40 | 44.56 |
| 3617.5404 | RNEEYN(N)KSVQEIQAT Fuc 1 Hex 4 HexNAc 5 | 127.302 | 0.0 | -19.138 | 1.416e+07 | 50 - 65 | 2 | 17.36 | 17.36 | 17.53 |
| 3825.5369 | YFTPN(N)KTEDTIF Fuc 1 Hex 5 HexNAc 4 Neu5Ac 2 | 127.154 | 0.003 | 11.251 | 3.182e+07 | 67 - 79 | 2 | 38.24 | 38.39 | 38.87 |
| 3765.6195 | RNEEYN(N)KSVQEIQATFF Hex 4 HexNAc 5 | 127.131 | 0.003 | -5.202 | 2.659e+07 | 50 - 67 | 4 | 44.23 | 44.40 | 44.73 |
| 3140.3519 | NVQREN(N)GTISRY Hex 3 HexNAc 6 | 127.096 | 0.003 | 14.151 | 8.466e+08 | 97 - 109 | 6 | 13.46 | 13.62 | 14.78 |
| 4332.8213 | RNEEYN(N)KSVQEIQATF Fuc 1 Hex 5 HexNAc 7 | 126.458 | 0.003 | -4.103 | 1.401e+07 | 50 - 66 | 1 | 32.23 | 32.23 | 32.57 |
| 2709.1929 | N(N)KSVQEIQATFF Hex 3 HexNAc 4 | 125.262 | 0.005 | -4.730 | 4.587e+06 | 55 - 67 | 2 | 41.94 | 42.11 | 42.45 |
| 3926.6868 | LNVQREN(N)GTISRY Fuc 1 Hex 5 HexNAc 7 | 125.081 | 0.005 | 9.653 | 5.602e+08 | 96 - 109 | 10 | 18.54 | 18.71 | 20.64 |
| 4072.7442 | LNVQREN(N)GTISRY Fuc 2 Hex 5 HexNAc 7 | 124.707 | 0.005 | 14.041 | 2.186e+08 | 96 - 109 | 3 | 18.37 | 18.71 | 19.04 |
| 2574.0663 | YFTPN(N)KT Hex 3 HexNAc 6 | 124.304 | 0.0 | -21.164 | 4.715e+06 | 67 - 74 | 1 | 14.78 | 14.78 | 14.94 |
| 2982.2864 | TPN(N)KTEDTIFL Hex 3 HexNAc 6 | 123.807 | 0.005 | -19.702 | 1.623e+06 | 69 - 80 | 1 | 35.20 | 35.20 | 35.37 |
| 4129.7672 | LNVQREN(N)GTISRY Fuc 1 Hex 5 HexNAc 8 | 122.896 | 0.007 | 16.114 | 5.099e+08 | 96 - 109 | 4 | 18.20 | 18.71 | 19.04 |
| 4202.7307 | FYFTPN(N)KTEDTIF Fuc 1 Hex 5 HexNAc 8 | 122.390 | 0.007 | 12.943 | 2.032e+07 | 66 - 79 | 4 | 46.41 | 46.75 | 47.27 |
| 2518.0534 | FYFTPN(N)KT Hex 3 HexNAc 5 | 122.375 | 0.0 | -18.770 | 5.006e+06 | 66 - 74 | 1 | 26.92 | 26.92 | 27.09 |
| 2934.2653 | RNEEYN(N)KSVQEIQ Hex 3 HexNAc 4 | 122.309 | 0.007 | -20.236 | 2.891e+06 | 50 - 63 | 1 | 15.28 | 15.28 | 15.28 |
| 3821.6549 | LNVQREN(N)GTISRY Hex 4 HexNAc 8 | 121.662 | 0.007 | 13.488 | 1.351e+09 | 96 - 109 | 9 | 18.37 | 18.88 | 20.15 |
| 4065.7069 | NVQREN(N)GTISRY Fuc 3 Hex 6 HexNAc 6 | 121.427 | 0.007 | 0.000 | 0.000e+00 | 97 - 109 | 2 | - | - | - |
| 3394.3807 | QTRQDQC(C)IYN(N)TTY Hex 3 HexNAc 6 | 121.331 | 0.007 | 15.866 | 2.870e+08 | 83 - 96 | 7 | 21.48 | 22.68 | 23.19 |
| 3666.5855 | LNVQREN(N)GTISRY Fuc 2 Hex 5 HexNAc 5 | 121.093 | 0.007 | -19.091 | 8.143e+07 | 96 - 109 | 1 | 18.88 | 18.88 | 19.04 |
| 3499.4148 | QTRQDQC(C)IYN(N)TTY Fuc 1 Hex 4 HexNAc 5 | 120.677 | 0.007 | 12.092 | 3.594e+07 | 83 - 96 | 2 | 22.34 | 22.51 | 23.19 |
| 3707.6127 | LNVQREN(N)GTISRY Fuc 2 Hex 4 HexNAc 6 | 119.008 | 0.007 | -19.989 | 1.186e+07 | 96 - 109 | 1 | 19.21 | 19.21 | 19.21 |
| 3632.4941 | FTPN(N)KTEDTIF Fuc 2 Hex 5 HexNAc 6 | 118.924 | 0.007 | -4.257 | 1.248e+07 | 68 - 79 | 3 | 30.49 | 30.66 | 31.00 |
| 3591.5232 | LNVQREN(N)GTISRY Hex 4 HexNAc 4 Neu5Ac 2 | 118.488 | 0.009 | 14.505 | 4.692e+08 | 96 - 109 | 9 | 19.83 | 20.15 | 21.83 |
| 4145.7089 | FYFTPN(N)KTEDTIF Fuc 2 Hex 5 HexNAc 7 | 118.121 | 0.009 | 11.660 | 1.346e+07 | 66 - 79 | 4 | 46.58 | 47.10 | 47.44 |
| 3196.4208 | LNVQREN(N)GTISRY Fuc 1 Hex 3 HexNAc 5 | 118.081 | 0.009 | -4.218 | 5.516e+07 | 96 - 109 | 2 | 19.66 | 19.83 | 19.99 |
| 3334.4188 | VQREN(N)GTISRY Fuc 1 Hex 4 HexNAc 6 | 117.576 | 0.009 | -17.890 | 1.773e+07 | 98 - 109 | 1 | 12.96 | 12.96 | 12.96 |
| 3821.6338 | RNEEYN(N)KSVQEIQATF Hex 4 HexNAc 6 | 117.151 | 0.011 | 14.626 | 5.619e+08 | 50 - 66 | 9 | 31.89 | 32.90 | 34.18 |
| 3584.4877 | FTPN(N)KTEDTIF Hex 4 HexNAc 8 | 116.943 | 0.011 | -4.758 | 7.538e+06 | 68 - 79 | 4 | 30.49 | 30.83 | 31.00 |
| 3026.3385 | VQREN(N)GTISRY Hex 3 HexNAc 6 | 116.540 | 0.011 | -18.967 | 4.346e+07 | 98 - 109 | 2 | 13.13 | 13.13 | 40.74 |
| 4234.7954 | LNVQREN(N)GTISRY Fuc 2 Hex 6 HexNAc 7 | 115.693 | 0.011 | -3.892 | 9.600e+07 | 96 - 109 | 3 | 18.37 | 18.54 | 18.88 |
| 3415.6166 | LNVQREN(N)GTISRY Hex 4 HexNAc 6 | 115.259 | 0.012 | -8.413 | 6.283e+07 | 96 - 109 | 7 | 18.88 | 19.04 | 37.25 |
| 3437.4161 | TPN(N)KTEDTIF Hex 4 HexNAc 8 | 114.611 | 0.012 | -5.253 | 3.741e+06 | 69 - 79 | 2 | 23.36 | 23.53 | 23.87 |
| 3505.6839 | NVQREN(N)GTISRY Hex 4 HexNAc 7 | 114.471 | 0.012 | -5.131 | 1.440e+08 | 97 - 109 | 16 | 13.46 | 13.46 | 49.45 |
| 3764.6326 | LNVQREN(N)GTISRY Fuc 1 Hex 4 HexNAc 7 | 114.464 | 0.012 | 14.129 | 2.346e+08 | 96 - 109 | 4 | 18.71 | 18.88 | 19.36 |
| 2824.2379 | VQREN(N)GTISRY Hex 3 HexNAc 5 | 114.384 | 0.012 | 0.000 | 0.000e+00 | 98 - 109 | 1 | - | - | - |
| 4018.6717 | NVQREN(N)GTISRY Fuc 1 Hex 5 HexNAc 8 | 113.790 | 0.014 | 0.000 | 0.000e+00 | 97 - 109 | 2 | - | - | - |
| 3331.3667 | YFTPN(N)KTEDTIF Fuc 1 Hex 5 HexNAc 3 Neu5Ac 1 | 113.557 | 0.014 | -5.738 | 3.255e+06 | 67 - 79 | 1 | 39.04 | 39.21 | 39.38 |
| 4332.8450 | LNVQREN(N)GTISRY Fuc 1 Hex 5 HexNAc 9 | 111.173 | 0.016 | -18.542 | 1.672e+07 | 96 - 109 | 1 | 18.54 | 18.54 | 18.71 |
| 4089.7275 | RNEEYN(N)KSVQEIQATFF Hex 6 HexNAc 5 | 111.030 | 0.016 | -18.356 | 7.982e+06 | 50 - 67 | 2 | 44.05 | 44.23 | 44.23 |
| 3983.7076 | LNVQREN(N)GTISRY Hex 5 HexNAc 8 | 110.111 | 0.017 | -3.556 | 1.547e+08 | 96 - 109 | 2 | 18.54 | 18.71 | 19.04 |
| 3899.6352 | LNVQREN(N)GTISRY Fuc 1 Hex 5 HexNAc 4 Neu5Ac 2 | 108.004 | 0.023 | 11.319 | 1.372e+08 | 96 - 109 | 3 | 19.83 | 19.99 | 20.47 |
| 3371.3847 | FTPN(N)KTEDTIF Fuc 1 Hex 5 HexNAc 4 Neu5Ac 1 | 107.956 | 0.025 | -19.972 | 3.396e+06 | 68 - 79 | 1 | 32.23 | 32.40 | 32.40 |
| 4536.9123 | RNEEYN(N)KSVQEIQATF Fuc 1 Hex 5 HexNAc 8 | 107.437 | 0.027 | -21.162 | 4.088e+05 | 50 - 66 | 2 | 43.54 | 43.54 | 43.54 |
| 4437.8757 | LNVQREN(N)GTISRY Fuc 2 Hex 6 HexNAc 8 | 104.875 | 0.028 | 12.903 | 1.884e+08 | 96 - 109 | 3 | 18.20 | 18.54 | 19.04 |
| 4380.8534 | LNVQREN(N)GTISRY Fuc 3 Hex 6 HexNAc 7 | 104.762 | 0.029 | -3.212 | 1.157e+08 | 96 - 109 | 3 | 18.20 | 18.54 | 18.71 |
| 2983.3501 | C(C)ANLVPVPITN(N)ATL Hex 3 HexNAc 5 | 103.866 | 0.031 | 14.396 | 2.314e+07 | 22 - 36 | 5 | 51.50 | 51.71 | 52.42 |
| 3401.4697 | RN(D)EEYN(N)KSVQEIQATFF Hex 3 HexNAc 4 | 102.878 | 0.031 | -5.398 | 4.891e+07 | 50 - 67 | 11 | 47.78 | 47.95 | 48.12 |
| 3813.5969 | NVQREN(N)GTISRY Fuc 1 Hex 5 HexNAc 7 | 102.326 | 0.031 | -20.090 | 1.045e+07 | 97 - 109 | 2 | 13.29 | 13.29 | 13.29 |
| 3540.4379 | QTRQDQC(C)IYN(N)TTY Fuc 1 Hex 3 HexNAc 6 | 101.805 | 0.032 | -3.499 | 9.446e+06 | 83 - 96 | 1 | 22.51 | 22.51 | 22.85 |
| 4494.8962 | LNVQREN(N)GTISRY Fuc 1 Hex 6 HexNAc 9 | 100.937 | 0.034 | 10.392 | 7.224e+07 | 96 - 109 | 1 | 18.20 | 18.54 | 19.04 |
| 3643.5364 | VQREN(N)GTISRY Fuc 2 Hex 5 HexNAc 6 | 100.034 | 0.034 | 0.000 | 0.000e+00 | 98 - 109 | 1 | - | - | - |
| 4186.7860 | LNVQREN(N)GTISRY Hex 5 HexNAc 9 | 99.973 | 0.034 | 12.958 | 2.965e+08 | 96 - 109 | 4 | 18.20 | 18.71 | 19.21 |
| 3972.6001 | FYFTPN(N)KTEDTIF Fuc 1 Hex 5 HexNAc 4 Neu5Ac 2 | 99.643 | 0.034 | -20.074 | 1.354e+07 | 66 - 79 | 2 | 48.28 | 48.44 | 48.44 |
| 3245.3832 | NVQREN(N)GTISRY Fuc 1 Hex 4 HexNAc 5 | 99.547 | 0.034 | -5.390 | 8.453e+07 | 97 - 109 | 3 | 13.46 | 13.62 | 13.95 |
| 4745.9851 | LNVQREN(N)GTISRY Fuc 3 Hex 7 HexNAc 8 | 96.761 | 0.042 | -4.538 | 4.208e+07 | 96 - 109 | 2 | 18.04 | 18.37 | 18.37 |
| 3090.3738 | LNVQREN(N)GTISR Hex 3 HexNAc 6 | 94.844 | 0.045 | -20.392 | 5.677e+06 | 96 - 108 | 1 | 14.45 | 14.61 | 14.61 |
| 2734.1930 | NVQREN(N)GTISRY Hex 3 HexNAc 4 | 94.065 | 0.045 | -19.168 | 1.314e+07 | 97 - 109 | 1 | 13.79 | 13.79 | 13.95 |
| 4542.9053 | LNVQREN(N)GTISRY Fuc 3 Hex 7 HexNAc 7 | 93.155 | 0.048 | -19.170 | 1.365e+07 | 96 - 109 | 1 | 18.20 | 18.20 | 18.37 |
| 3478.4372 | NVQREN(N)GTISRY Hex 4 HexNAc 4 Neu5Ac 2 | 92.926 | 0.048 | -20.101 | 2.079e+07 | 97 - 109 | 2 | 13.95 | 13.95 | 14.11 |
| 4389.8671 | LNVQREN(N)GTISRY Fuc 5 Hex 3 HexNAc 8 | 92.922 | 0.048 | -19.525 | 2.865e+07 | 96 - 109 | 2 | 18.54 | 18.54 | 18.71 |

### TPN(N)KTEDTIF Hex 3 HexNAc 5

**Mass:**  2666.1251

(1.284e-06)

**Charge States:** 2, 3

**Start Position**: 70

**End Position**: 80

| Profile Score | Tandem Score | TDA q-value | Volume | Spectrum Matches |
| --- | --- | --- | --- | --- |
| 16.1935 | 186.3579 | 0.0000 | 1.3891e+08 | 7 |

**Adduct Signal Fractions**

- **Unmodified**: 1.3891e+08

### TPN(N)KTEDTIF Hex 3 HexNAc 6

**Mass:**  2869.2042

(1.151e-06)

**Charge States:** 2, 3, 4

**Start Position**: 70

**End Position**: 80

| Profile Score | Tandem Score | TDA q-value | Volume | Spectrum Matches |
| --- | --- | --- | --- | --- |
| 16.2927 | 181.1143 | 0.0000 | 3.1235e+08 | 12 |

**Adduct Signal Fractions**

- **Unmodified**: 3.1235e+08

### TPN(N)KTEDTIF Fuc 1 Hex 4 HexNAc 6

**Mass:**  3177.3155

(1.166e-06)

**Charge States:** 3

**Start Position**: 70

**End Position**: 80

| Profile Score | Tandem Score | TDA q-value | Volume | Spectrum Matches |
| --- | --- | --- | --- | --- |
| 15.0758 | 180.1785 | 0.0000 | 5.6725e+07 | 5 |

**Adduct Signal Fractions**

- **Unmodified**: 5.6725e+07

### YFTPN(N)KTEDTIF Hex 3 HexNAc 5

**Mass:**  2976.2576

(9.816e-07)

**Charge States:** 2, 3, 4

**Start Position**: 68

**End Position**: 80

| Profile Score | Tandem Score | TDA q-value | Volume | Spectrum Matches |
| --- | --- | --- | --- | --- |
| 15.3176 | 179.3644 | 0.0000 | 1.8681e+09 | 24 |

**Adduct Signal Fractions**

- **Unmodified**: 1.8681e+09

### YFTPN(N)KTEDTIF Hex 3 HexNAc 4

**Mass:**  2773.1766

(1.445e-06)

**Charge States:** 2, 3

**Start Position**: 68

**End Position**: 80

| Profile Score | Tandem Score | TDA q-value | Volume | Spectrum Matches |
| --- | --- | --- | --- | --- |
| 15.2900 | 177.6851 | 0.0000 | 2.7684e+08 | 9 |

**Adduct Signal Fractions**

- **Unmodified**: 2.7684e+08

### N(N)KSVQEIQATF Hex 3 HexNAc 5

**Mass:**  2765.2042

(1.760e-06)

**Charge States:** 3

**Start Position**: 56

**End Position**: 67

| Profile Score | Tandem Score | TDA q-value | Volume | Spectrum Matches |
| --- | --- | --- | --- | --- |
| -3.6782 | 175.4104 | 0.0000 | 1.5365e+07 | 1 |

**Adduct Signal Fractions**

- **Unmodified**: 1.5365e+07

### FYFTPN(N)KTEDTIF Hex 3 HexNAc 5

**Mass:**  3123.3228

(3.539e-07)

**Charge States:** 2, 3, 4

**Start Position**: 67

**End Position**: 80

| Profile Score | Tandem Score | TDA q-value | Volume | Spectrum Matches |
| --- | --- | --- | --- | --- |
| 13.5187 | 175.2373 | 0.0000 | 2.0608e+09 | 114 |

**Adduct Signal Fractions**

- **Unmodified**: 2.0608e+09

### YFTPN(N)KTEDTIF Hex 4 HexNAc 7

**Mass:**  3544.4686

(1.076e-06)

**Charge States:** 3, 4

**Start Position**: 68

**End Position**: 80

| Profile Score | Tandem Score | TDA q-value | Volume | Spectrum Matches |
| --- | --- | --- | --- | --- |
| 13.7067 | 175.2019 | 0.0000 | 8.7930e+08 | 18 |

**Adduct Signal Fractions**

- **Unmodified**: 8.7930e+08

### TPN(N)KTEDTIF Fuc 1 Hex 4 HexNAc 5

**Mass:**  2974.2326

(1.710e-06)

**Charge States:** 3

**Start Position**: 70

**End Position**: 80

| Profile Score | Tandem Score | TDA q-value | Volume | Spectrum Matches |
| --- | --- | --- | --- | --- |
| 14.8447 | 175.0932 | 0.0000 | 3.6115e+07 | 3 |

**Adduct Signal Fractions**

- **Unmodified**: 3.6115e+07

### YFTPN(N)KTEDTIF Fuc 1 Hex 4 HexNAc 5

**Mass:**  3284.3681

(8.133e-07)

**Charge States:** 3, 4

**Start Position**: 68

**End Position**: 80

| Profile Score | Tandem Score | TDA q-value | Volume | Spectrum Matches |
| --- | --- | --- | --- | --- |
| 12.5967 | 174.9015 | 0.0000 | 5.4224e+08 | 13 |

**Adduct Signal Fractions**

- **Unmodified**: 5.4224e+08

### FYFTPN(N)KTEDTIF Hex 3 HexNAc 6

**Mass:**  3326.4032

(1.596e-07)

**Charge States:** 3, 4

**Start Position**: 67

**End Position**: 80

| Profile Score | Tandem Score | TDA q-value | Volume | Spectrum Matches |
| --- | --- | --- | --- | --- |
| 13.2311 | 174.8285 | 0.0000 | 4.5324e+09 | 215 |

**Adduct Signal Fractions**

- **Unmodified**: 4.5324e+09

### YFTPN(N)KTEDTIF Hex 3 HexNAc 6

**Mass:**  3179.3358

(8.401e-07)

**Charge States:** 2, 3, 4

**Start Position**: 68

**End Position**: 80

| Profile Score | Tandem Score | TDA q-value | Volume | Spectrum Matches |
| --- | --- | --- | --- | --- |
| 15.1370 | 174.8034 | 0.0000 | 3.0392e+09 | 37 |

**Adduct Signal Fractions**

- **Unmodified**: 3.0392e+09

### TPN(N)KTEDTIF Hex 4 HexNAc 7

**Mass:**  3234.3371

(1.203e-06)

**Charge States:** 3

**Start Position**: 70

**End Position**: 80

| Profile Score | Tandem Score | TDA q-value | Volume | Spectrum Matches |
| --- | --- | --- | --- | --- |
| 12.5064 | 174.5851 | 0.0000 | 3.6080e+07 | 4 |

**Adduct Signal Fractions**

- **Unmodified**: 3.6080e+07

### N(N)KSVQEIQATF Hex 3 HexNAc 4

**Mass:**  2562.1236

(5.024e-07)

**Charge States:** 3

**Start Position**: 56

**End Position**: 67

| Profile Score | Tandem Score | TDA q-value | Volume | Spectrum Matches |
| --- | --- | --- | --- | --- |
| -4.2291 | 172.8736 | 0.0000 | 1.7870e+07 | 1 |

**Adduct Signal Fractions**

- **Unmodified**: 1.7870e+07

### YFTPN(N)KTEDTIF Hex 4 HexNAc 6

**Mass:**  3341.3900

(1.527e-06)

**Charge States:** 3

**Start Position**: 68

**End Position**: 80

| Profile Score | Tandem Score | TDA q-value | Volume | Spectrum Matches |
| --- | --- | --- | --- | --- |
| 12.7558 | 170.6947 | 0.0000 | 1.5281e+08 | 4 |

**Adduct Signal Fractions**

- **Unmodified**: 1.5281e+08

### FTPN(N)KTEDTIF Fuc 1 Hex 4 HexNAc 5

**Mass:**  3121.3024

(6.464e-07)

**Charge States:** 3

**Start Position**: 69

**End Position**: 80

| Profile Score | Tandem Score | TDA q-value | Volume | Spectrum Matches |
| --- | --- | --- | --- | --- |
| 12.7032 | 169.8782 | 0.0000 | 7.1951e+07 | 4 |

**Adduct Signal Fractions**

- **Unmodified**: 7.1951e+07

### FYFTPN(N)KTEDTIF Fuc 1 Hex 4 HexNAc 5

**Mass:**  3431.4358

(9.087e-07)

**Charge States:** 3, 4

**Start Position**: 67

**End Position**: 80

| Profile Score | Tandem Score | TDA q-value | Volume | Spectrum Matches |
| --- | --- | --- | --- | --- |
| 14.2311 | 169.7775 | 0.0000 | 3.3817e+08 | 8 |

**Adduct Signal Fractions**

- **Unmodified**: 3.3817e+08

### FYFTPN(N)KTEDTIF Hex 4 HexNAc 7

**Mass:**  3691.5375

(1.156e-06)

**Charge States:** 3, 4

**Start Position**: 67

**End Position**: 80

| Profile Score | Tandem Score | TDA q-value | Volume | Spectrum Matches |
| --- | --- | --- | --- | --- |
| 15.5398 | 169.5160 | 0.0000 | 4.6489e+08 | 10 |

**Adduct Signal Fractions**

- **Unmodified**: 4.6489e+08

### N(N)KSVQEIQATF Fuc 1 Hex 4 HexNAc 5

**Mass:**  3073.3159

(1.240e-06)

**Charge States:** 3

**Start Position**: 56

**End Position**: 67

| Profile Score | Tandem Score | TDA q-value | Volume | Spectrum Matches |
| --- | --- | --- | --- | --- |
| -18.2193 | 169.0696 | 0.0000 | 7.2745e+06 | 1 |

**Adduct Signal Fractions**

- **Unmodified**: 7.2745e+06

### YFTPN(N)KTEDTIF Fuc 2 Hex 5 HexNAc 6

**Mass:**  3795.5575

(9.129e-07)

**Charge States:** 3, 4

**Start Position**: 68

**End Position**: 80

| Profile Score | Tandem Score | TDA q-value | Volume | Spectrum Matches |
| --- | --- | --- | --- | --- |
| 15.6129 | 168.7221 | 0.0000 | 1.7905e+08 | 4 |

**Adduct Signal Fractions**

- **Unmodified**: 1.7905e+08

### FYFTPN(N)KTEDTIF Fuc 1 Hex 4 HexNAc 6

**Mass:**  3634.5163

(1.212e-06)

**Charge States:** 3, 4

**Start Position**: 67

**End Position**: 80

| Profile Score | Tandem Score | TDA q-value | Volume | Spectrum Matches |
| --- | --- | --- | --- | --- |
| 14.8673 | 168.3795 | 0.0000 | 7.2206e+08 | 36 |

**Adduct Signal Fractions**

- **Unmodified**: 7.2206e+08

### YFTPN(N)KTEDTIF Fuc 1 Hex 5 HexNAc 7

**Mass:**  3852.5795

(1.346e-06)

**Charge States:** 3, 4

**Start Position**: 68

**End Position**: 80

| Profile Score | Tandem Score | TDA q-value | Volume | Spectrum Matches |
| --- | --- | --- | --- | --- |
| 16.4528 | 167.9528 | 0.0000 | 1.5028e+08 | 6 |

**Adduct Signal Fractions**

- **Unmodified**: 1.5028e+08

### FTPN(N)KTEDTIF Hex 3 HexNAc 6

**Mass:**  3016.2722

(9.073e-07)

**Charge States:** 2, 3, 4

**Start Position**: 69

**End Position**: 80

| Profile Score | Tandem Score | TDA q-value | Volume | Spectrum Matches |
| --- | --- | --- | --- | --- |
| 14.5738 | 167.4370 | 0.0000 | 8.0001e+08 | 11 |

**Adduct Signal Fractions**

- **Unmodified**: 8.0001e+08

### YFTPN(N)KTEDTIF Fuc 1 Hex 4 HexNAc 6

**Mass:**  3487.4478

(1.259e-06)

**Charge States:** 3, 4

**Start Position**: 68

**End Position**: 80

| Profile Score | Tandem Score | TDA q-value | Volume | Spectrum Matches |
| --- | --- | --- | --- | --- |
| 16.2127 | 166.5862 | 0.0000 | 9.7413e+08 | 10 |

**Adduct Signal Fractions**

- **Unmodified**: 9.7413e+08

### TPN(N)KTEDTIF Hex 3 HexNAc 4

**Mass:**  2463.0461

(2.994e-06)

**Charge States:** 2, 3

**Start Position**: 70

**End Position**: 80

| Profile Score | Tandem Score | TDA q-value | Volume | Spectrum Matches |
| --- | --- | --- | --- | --- |
| 14.3433 | 165.7976 | 0.0000 | 2.1349e+07 | 4 |

**Adduct Signal Fractions**

- **Unmodified**: 2.1349e+07

### YFTPN(N)KTEDTIF Hex 5 HexNAc 8

**Mass:**  3909.6033

(1.595e-06)

**Charge States:** 3, 4

**Start Position**: 68

**End Position**: 80

| Profile Score | Tandem Score | TDA q-value | Volume | Spectrum Matches |
| --- | --- | --- | --- | --- |
| 13.4136 | 165.5412 | 0.0000 | 1.3798e+08 | 6 |

**Adduct Signal Fractions**

- **Unmodified**: 1.3798e+08

### FTPN(N)KTEDTIF Hex 3 HexNAc 5

**Mass:**  2813.1908

(2.581e-07)

**Charge States:** 2, 3, 4

**Start Position**: 69

**End Position**: 80

| Profile Score | Tandem Score | TDA q-value | Volume | Spectrum Matches |
| --- | --- | --- | --- | --- |
| 14.2970 | 165.2617 | 0.0000 | 2.5074e+08 | 10 |

**Adduct Signal Fractions**

- **Unmodified**: 2.5074e+08

### FTPN(N)KTEDTIF Fuc 1 Hex 4 HexNAc 6

**Mass:**  3324.3832

(9.570e-07)

**Charge States:** 3

**Start Position**: 69

**End Position**: 80

| Profile Score | Tandem Score | TDA q-value | Volume | Spectrum Matches |
| --- | --- | --- | --- | --- |
| 12.7977 | 164.0973 | 0.0000 | 1.0357e+08 | 7 |

**Adduct Signal Fractions**

- **Unmodified**: 1.0357e+08

### YFTPN(N)KTEDTIF Hex 4 HexNAc 5

**Mass:**  3138.3063

(1.469e-06)

**Charge States:** 3

**Start Position**: 68

**End Position**: 80

| Profile Score | Tandem Score | TDA q-value | Volume | Spectrum Matches |
| --- | --- | --- | --- | --- |
| -20.0087 | 162.9634 | 0.0000 | 9.6619e+06 | 2 |

**Adduct Signal Fractions**

- **Unmodified**: 9.6619e+06

### RNEEYN(N)KSVQEIQATFF Hex 3 HexNAc 4

**Mass:**  3400.4855

(1.033e-06)

**Charge States:** 3, 4

**Start Position**: 51

**End Position**: 68

| Profile Score | Tandem Score | TDA q-value | Volume | Spectrum Matches |
| --- | --- | --- | --- | --- |
| 17.2684 | 162.5909 | 0.0000 | 1.8552e+09 | 15 |

**Adduct Signal Fractions**

- **Unmodified**: 1.8552e+09

### YFTPN(N)KTEDTIFL Hex 3 HexNAc 6

**Mass:**  3292.4203

(1.312e-06)

**Charge States:** 3, 4

**Start Position**: 68

**End Position**: 81

| Profile Score | Tandem Score | TDA q-value | Volume | Spectrum Matches |
| --- | --- | --- | --- | --- |
| 15.9755 | 162.0550 | 0.0000 | 2.3738e+07 | 5 |

**Adduct Signal Fractions**

- **Unmodified**: 2.3738e+07

### EEYN(N)KSVQEIQATF Fuc 1 Hex 4 HexNAc 5

**Mass:**  3494.4644

(1.090e-06)

**Charge States:** 3

**Start Position**: 53

**End Position**: 67

| Profile Score | Tandem Score | TDA q-value | Volume | Spectrum Matches |
| --- | --- | --- | --- | --- |
| 12.4887 | 161.9701 | 0.0000 | 1.9690e+07 | 2 |

**Adduct Signal Fractions**

- **Unmodified**: 1.9690e+07

### RNEEYN(N)KSVQEIQATFF Hex 3 HexNAc 5

**Mass:**  3603.5623

(1.508e-06)

**Charge States:** 3, 4

**Start Position**: 51

**End Position**: 68

| Profile Score | Tandem Score | TDA q-value | Volume | Spectrum Matches |
| --- | --- | --- | --- | --- |
| 15.7557 | 161.9148 | 0.0000 | 1.6643e+09 | 10 |

**Adduct Signal Fractions**

- **Unmodified**: 1.6643e+09

### YFTPN(N)KTEDTIF Hex 4 HexNAc 8

**Mass:**  3747.5495

(9.606e-07)

**Charge States:** 3, 4

**Start Position**: 68

**End Position**: 80

| Profile Score | Tandem Score | TDA q-value | Volume | Spectrum Matches |
| --- | --- | --- | --- | --- |
| 16.7979 | 161.6290 | 0.0000 | 1.3149e+08 | 5 |

**Adduct Signal Fractions**

- **Unmodified**: 1.3149e+08

### YFTPN(N)KTEDTIF Fuc 1 Hex 5 HexNAc 4 Neu5Ac 1

**Mass:**  3534.4480

(4.260e-06)

**Charge States:** 3

**Start Position**: 68

**End Position**: 80

| Profile Score | Tandem Score | TDA q-value | Volume | Spectrum Matches |
| --- | --- | --- | --- | --- |
| 13.5537 | 160.9916 | 0.0000 | 4.0367e+07 | 2 |

**Adduct Signal Fractions**

- **Unmodified**: 4.0367e+07

### N(N)KTEDTIF Hex 3 HexNAc 6

**Mass:**  2671.1024

(7.347e-07)

**Charge States:** 3

**Start Position**: 72

**End Position**: 80

| Profile Score | Tandem Score | TDA q-value | Volume | Spectrum Matches |
| --- | --- | --- | --- | --- |
| -5.1647 | 160.5334 | 0.0000 | 4.5978e+06 | 1 |

**Adduct Signal Fractions**

- **Unmodified**: 4.5978e+06

### RNEEYN(N)KSVQEIQATFF Fuc 1 Hex 4 HexNAc 6

**Mass:**  4114.7575

(1.225e-06)

**Charge States:** 3, 4

**Start Position**: 51

**End Position**: 68

| Profile Score | Tandem Score | TDA q-value | Volume | Spectrum Matches |
| --- | --- | --- | --- | --- |
| 14.2499 | 159.4559 | 0.0000 | 5.0075e+07 | 4 |

**Adduct Signal Fractions**

- **Unmodified**: 5.0075e+07

### FTPN(N)KTEDTIF Hex 4 HexNAc 7

**Mass:**  3381.4052

(1.012e-06)

**Charge States:** 3, 4

**Start Position**: 69

**End Position**: 80

| Profile Score | Tandem Score | TDA q-value | Volume | Spectrum Matches |
| --- | --- | --- | --- | --- |
| 14.1165 | 158.3684 | 0.0000 | 6.2044e+07 | 4 |

**Adduct Signal Fractions**

- **Unmodified**: 6.2044e+07

### YFTPN(N)KTEDTIF Fuc 1 Hex 4 HexNAc 7

**Mass:**  3690.5253

(6.826e-07)

**Charge States:** 3

**Start Position**: 68

**End Position**: 80

| Profile Score | Tandem Score | TDA q-value | Volume | Spectrum Matches |
| --- | --- | --- | --- | --- |
| 14.0707 | 158.2566 | 0.0000 | 4.5829e+07 | 4 |

**Adduct Signal Fractions**

- **Unmodified**: 4.5829e+07

### FYFTPN(N)KTEDTIF Fuc 1 Hex 5 HexNAc 7

**Mass:**  3999.6513

(1.466e-06)

**Charge States:** 3, 4

**Start Position**: 67

**End Position**: 80

| Profile Score | Tandem Score | TDA q-value | Volume | Spectrum Matches |
| --- | --- | --- | --- | --- |
| 16.6167 | 157.4554 | 0.0000 | 7.0065e+07 | 5 |

**Adduct Signal Fractions**

- **Unmodified**: 7.0065e+07

### YFTPN(N)KTEDTIF Hex 3 HexNAc 5

**Mass:**  2978.2550

(6.726e-04)

**Start Position**: 68

**End Position**: 80

| Profile Score | Tandem Score | TDA q-value | Volume | Spectrum Matches |
| --- | --- | --- | --- | --- |
| 0.0000 | 157.2360 | 0.0000 | 0.0000e+00 | 1 |

Adduct:

No Chromatogram Found

### TPN(N)KTEDTIF Fuc 2 Hex 5 HexNAc 6

**Mass:**  3485.4248

(1.428e-06)

**Charge States:** 3

**Start Position**: 70

**End Position**: 80

| Profile Score | Tandem Score | TDA q-value | Volume | Spectrum Matches |
| --- | --- | --- | --- | --- |
| 11.3558 | 157.0296 | 0.0000 | 7.6605e+06 | 2 |

**Adduct Signal Fractions**

- **Unmodified**: 7.6605e+06

### RNEEYN(N)KSVQEIQATF Fuc 1 Hex 4 HexNAc 5

**Mass:**  3764.6090

(6.483e-07)

**Charge States:** 3, 4

**Start Position**: 51

**End Position**: 67

| Profile Score | Tandem Score | TDA q-value | Volume | Spectrum Matches |
| --- | --- | --- | --- | --- |
| 16.3005 | 156.6666 | 0.0000 | 3.5549e+09 | 14 |

**Adduct Signal Fractions**

- **Unmodified**: 3.5549e+09

### RNEEYN(N)KSVQEIQATFF Fuc 1 Hex 4 HexNAc 5

**Mass:**  3911.6867

(1.170e-06)

**Charge States:** 3, 4

**Start Position**: 51

**End Position**: 68

| Profile Score | Tandem Score | TDA q-value | Volume | Spectrum Matches |
| --- | --- | --- | --- | --- |
| 15.9133 | 156.6615 | 0.0000 | 1.1642e+09 | 12 |

**Adduct Signal Fractions**

- **Unmodified**: 1.1642e+09

### FYFTPN(N)KTEDTIF Fuc 2 Hex 5 HexNAc 6

**Mass:**  3942.6289

(1.562e-06)

**Charge States:** 3, 4

**Start Position**: 67

**End Position**: 80

| Profile Score | Tandem Score | TDA q-value | Volume | Spectrum Matches |
| --- | --- | --- | --- | --- |
| 14.3595 | 156.3812 | 0.0000 | 1.0021e+08 | 5 |

**Adduct Signal Fractions**

- **Unmodified**: 1.0021e+08

### FYFTPN(N)KTEDTIF Hex 3 HexNAc 4

**Mass:**  2920.2443

(7.180e-07)

**Charge States:** 2, 3

**Start Position**: 67

**End Position**: 80

| Profile Score | Tandem Score | TDA q-value | Volume | Spectrum Matches |
| --- | --- | --- | --- | --- |
| 14.9376 | 155.9814 | 0.0000 | 1.7404e+08 | 13 |

**Adduct Signal Fractions**

- **Unmodified**: 1.7404e+08

### EEYN(N)KSVQEIQATF Hex 3 HexNAc 4

**Mass:**  2983.2740

(1.004e-06)

**Charge States:** 3

**Start Position**: 53

**End Position**: 67

| Profile Score | Tandem Score | TDA q-value | Volume | Spectrum Matches |
| --- | --- | --- | --- | --- |
| -3.3733 | 155.8721 | 0.0000 | 4.0179e+07 | 2 |

**Adduct Signal Fractions**

- **Unmodified**: 4.0179e+07

### RN(D)EEYN(N)KSVQEIQATF Hex 3 HexNAc 4

**Mass:**  3254.4010

(3.041e-04)

**Charge States:** 3, 4

**Start Position**: 51

**End Position**: 67

| Profile Score | Tandem Score | TDA q-value | Volume | Spectrum Matches |
| --- | --- | --- | --- | --- |
| 10.4407 | 155.4102 | 0.0000 | 3.0965e+08 | 20 |

**Adduct Signal Fractions**

- **Unmodified**: 3.0965e+08

### RNEEYN(N)KSVQEIQATF Hex 3 HexNAc 4

**Mass:**  3253.4155

(1.423e-06)

**Charge States:** 3, 4

**Start Position**: 51

**End Position**: 67

| Profile Score | Tandem Score | TDA q-value | Volume | Spectrum Matches |
| --- | --- | --- | --- | --- |
| 16.3049 | 155.1749 | 0.0000 | 6.4758e+09 | 25 |

**Adduct Signal Fractions**

- **Unmodified**: 6.4758e+09

### YFTPN(N)KTEDTIF Hex 5 HexNAc 9

**Mass:**  4112.6813

(1.165e-06)

**Charge States:** 3, 4

**Start Position**: 68

**End Position**: 80

| Profile Score | Tandem Score | TDA q-value | Volume | Spectrum Matches |
| --- | --- | --- | --- | --- |
| 9.8745 | 154.7768 | 0.0000 | 4.1416e+07 | 3 |

**Adduct Signal Fractions**

- **Unmodified**: 4.1416e+07

### YFTPN(N)KTEDTIF Hex 4 HexNAc 4 Neu5Ac 2

**Mass:**  3517.4207

(4.402e-07)

**Charge States:** 3, 4

**Start Position**: 68

**End Position**: 80

| Profile Score | Tandem Score | TDA q-value | Volume | Spectrum Matches |
| --- | --- | --- | --- | --- |
| 14.3895 | 154.5119 | 0.0000 | 2.5334e+08 | 8 |

**Adduct Signal Fractions**

- **Unmodified**: 2.5334e+08

### LNVQREN(N)GTISRY Hex 3 HexNAc 4

**Mass:**  2847.2849

(2.474e-06)

**Charge States:** 3

**Start Position**: 97

**End Position**: 110

| Profile Score | Tandem Score | TDA q-value | Volume | Spectrum Matches |
| --- | --- | --- | --- | --- |
| 12.6706 | 154.0900 | 0.0000 | 1.9318e+08 | 3 |

**Adduct Signal Fractions**

- **Unmodified**: 1.9318e+08

### RNEEYN(N)KSVQEIQATF Hex 3 HexNAc 5

**Mass:**  3456.4971

(1.657e-06)

**Charge States:** 3, 4

**Start Position**: 51

**End Position**: 67

| Profile Score | Tandem Score | TDA q-value | Volume | Spectrum Matches |
| --- | --- | --- | --- | --- |
| 15.8854 | 153.5713 | 0.0000 | 5.2077e+09 | 17 |

**Adduct Signal Fractions**

- **Unmodified**: 5.2077e+09

### YFTPN(N)KTEDTIF Fuc 1 Hex 3 HexNAc 4 Neu5Ac 1

**Mass:**  3210.3506

(7.274e-06)

**Start Position**: 68

**End Position**: 80

| Profile Score | Tandem Score | TDA q-value | Volume | Spectrum Matches |
| --- | --- | --- | --- | --- |
| 0.0000 | 153.4085 | 0.0000 | 0.0000e+00 | 1 |

Adduct:

No Chromatogram Found

### LNVQREN(N)GTISRY Fuc 1 Hex 4 HexNAc 6

**Mass:**  3561.5528

(2.428e-06)

**Charge States:** 3, 4

**Start Position**: 97

**End Position**: 110

| Profile Score | Tandem Score | TDA q-value | Volume | Spectrum Matches |
| --- | --- | --- | --- | --- |
| 14.4029 | 153.3611 | 0.0000 | 3.5748e+09 | 11 |

**Adduct Signal Fractions**

- **Unmodified**: 3.5748e+09

### EEYN(N)KSVQEIQATF Hex 3 HexNAc 5

**Mass:**  3186.3532

(1.098e-06)

**Charge States:** 3

**Start Position**: 53

**End Position**: 67

| Profile Score | Tandem Score | TDA q-value | Volume | Spectrum Matches |
| --- | --- | --- | --- | --- |
| 14.2059 | 153.2567 | 0.0000 | 3.5602e+07 | 2 |

**Adduct Signal Fractions**

- **Unmodified**: 3.5602e+07

### RNEEYN(N)KSVQEIQATF Hex 4 HexNAc 5

**Mass:**  3618.5531

(8.739e-07)

**Charge States:** 3, 4

**Start Position**: 51

**End Position**: 67

| Profile Score | Tandem Score | TDA q-value | Volume | Spectrum Matches |
| --- | --- | --- | --- | --- |
| 17.3974 | 153.2472 | 0.0000 | 7.7454e+07 | 3 |

**Adduct Signal Fractions**

- **Unmodified**: 7.7454e+07

### LNVQREN(N)GTISRY Hex 3 HexNAc 6

**Mass:**  3253.4412

(2.433e-06)

**Charge States:** 3, 4

**Start Position**: 97

**End Position**: 110

| Profile Score | Tandem Score | TDA q-value | Volume | Spectrum Matches |
| --- | --- | --- | --- | --- |
| 11.8506 | 152.8639 | 0.0000 | 9.8009e+09 | 182 |

**Adduct Signal Fractions**

- **Unmodified**: 9.8009e+09

### RNEEYN(N)KSVQEIQATF Fuc 1 Hex 3 HexNAc 4

**Mass:**  3399.4733

(6.886e-07)

**Charge States:** 3

**Start Position**: 51

**End Position**: 67

| Profile Score | Tandem Score | TDA q-value | Volume | Spectrum Matches |
| --- | --- | --- | --- | --- |
| 12.7984 | 152.7860 | 0.0000 | 1.2378e+08 | 6 |

**Adduct Signal Fractions**

- **Unmodified**: 1.2378e+08

### RNEEYN(N)KSVQEIQATF Fuc 1 Hex 4 HexNAc 6

**Mass:**  3967.6896

(1.097e-06)

**Charge States:** 3, 4

**Start Position**: 51

**End Position**: 67

| Profile Score | Tandem Score | TDA q-value | Volume | Spectrum Matches |
| --- | --- | --- | --- | --- |
| -3.2671 | 151.9130 | 0.0000 | 1.4661e+08 | 3 |

**Adduct Signal Fractions**

- **Unmodified**: 1.4661e+08

### RNEEYN(N)KSVQEIQATF Fuc 1 Hex 4 HexNAc 4

**Mass:**  3561.5304

(1.491e-06)

**Charge States:** 3, 4

**Start Position**: 51

**End Position**: 67

| Profile Score | Tandem Score | TDA q-value | Volume | Spectrum Matches |
| --- | --- | --- | --- | --- |
| 15.3697 | 151.8008 | 0.0000 | 1.6879e+08 | 3 |

**Adduct Signal Fractions**

- **Unmodified**: 1.6879e+08

### RNEEYN(N)KSVQEIQATF Fuc 2 Hex 5 HexNAc 5

**Mass:**  4072.7206

(1.323e-06)

**Charge States:** 3, 4

**Start Position**: 51

**End Position**: 67

| Profile Score | Tandem Score | TDA q-value | Volume | Spectrum Matches |
| --- | --- | --- | --- | --- |
| 14.2320 | 151.4132 | 0.0000 | 9.4135e+07 | 4 |

**Adduct Signal Fractions**

- **Unmodified**: 9.4135e+07

### YFTPN(N)KTEDTIF Fuc 2 Hex 5 HexNAc 5

**Mass:**  3592.4793

(1.291e-06)

**Charge States:** 3

**Start Position**: 68

**End Position**: 80

| Profile Score | Tandem Score | TDA q-value | Volume | Spectrum Matches |
| --- | --- | --- | --- | --- |
| -5.3213 | 151.1621 | 0.0000 | 1.7479e+07 | 1 |

**Adduct Signal Fractions**

- **Unmodified**: 1.7479e+07

### RNEEYN(N)KSVQEIQATF Hex 3 HexNAc 6

**Mass:**  3659.5786

(1.522e-06)

**Charge States:** 3, 4

**Start Position**: 51

**End Position**: 67

| Profile Score | Tandem Score | TDA q-value | Volume | Spectrum Matches |
| --- | --- | --- | --- | --- |
| 11.9239 | 150.9656 | 0.0000 | 2.9284e+08 | 6 |

**Adduct Signal Fractions**

- **Unmodified**: 2.9284e+08

### RNEEYN(N)KSVQEIQATF Fuc 1 Hex 3 HexNAc 5

**Mass:**  3602.5552

(6.522e-07)

**Charge States:** 3, 4

**Start Position**: 51

**End Position**: 67

| Profile Score | Tandem Score | TDA q-value | Volume | Spectrum Matches |
| --- | --- | --- | --- | --- |
| 13.0862 | 150.9318 | 0.0000 | 1.2190e+08 | 3 |

**Adduct Signal Fractions**

- **Unmodified**: 1.2190e+08

### TPN(N)KTEDTIF Hex 4 HexNAc 6

**Mass:**  3031.2572

(1.235e-06)

**Charge States:** 3

**Start Position**: 70

**End Position**: 80

| Profile Score | Tandem Score | TDA q-value | Volume | Spectrum Matches |
| --- | --- | --- | --- | --- |
| 11.8294 | 150.8280 | 0.0000 | 8.0294e+06 | 2 |

**Adduct Signal Fractions**

- **Unmodified**: 8.0294e+06

### RNEEYN(N)KSVQEIQATF Hex 4 HexNAc 7

**Mass:**  4024.7114

(2.987e-06)

**Charge States:** 3, 4

**Start Position**: 51

**End Position**: 67

| Profile Score | Tandem Score | TDA q-value | Volume | Spectrum Matches |
| --- | --- | --- | --- | --- |
| -4.7807 | 150.5214 | 0.0000 | 8.3870e+07 | 4 |

**Adduct Signal Fractions**

- **Unmodified**: 8.3870e+07

### FTPN(N)KTEDTIF Hex 3 HexNAc 4

**Mass:**  2610.1131

(1.478e-06)

**Charge States:** 2, 3

**Start Position**: 69

**End Position**: 80

| Profile Score | Tandem Score | TDA q-value | Volume | Spectrum Matches |
| --- | --- | --- | --- | --- |
| -3.7443 | 150.2350 | 0.0000 | 3.1535e+07 | 4 |

**Adduct Signal Fractions**

- **Unmodified**: 3.1535e+07

### TPN(N)KTEDTIF Hex 4 HexNAc 4 Neu5Ac 2

**Mass:**  3207.2902

(6.518e-07)

**Charge States:** 3

**Start Position**: 70

**End Position**: 80

| Profile Score | Tandem Score | TDA q-value | Volume | Spectrum Matches |
| --- | --- | --- | --- | --- |
| 13.6269 | 150.0978 | 0.0000 | 1.5336e+07 | 3 |

**Adduct Signal Fractions**

- **Unmodified**: 1.5336e+07

### FYFTPN(N)KTEDTIF Hex 5 HexNAc 8

**Mass:**  4056.6716

(1.508e-06)

**Charge States:** 3, 4

**Start Position**: 67

**End Position**: 80

| Profile Score | Tandem Score | TDA q-value | Volume | Spectrum Matches |
| --- | --- | --- | --- | --- |
| 12.1802 | 148.8063 | 0.0000 | 7.7079e+07 | 6 |

**Adduct Signal Fractions**

- **Unmodified**: 7.7079e+07

### RNEEYN(N)KSVQEIQATFF Fuc 1 Hex 4 HexNAc 4

**Mass:**  3708.6015

(2.090e-06)

**Charge States:** 3, 4

**Start Position**: 51

**End Position**: 68

| Profile Score | Tandem Score | TDA q-value | Volume | Spectrum Matches |
| --- | --- | --- | --- | --- |
| -4.4355 | 148.7022 | 0.0000 | 4.9703e+07 | 2 |

**Adduct Signal Fractions**

- **Unmodified**: 4.9703e+07

### RNEEYN(N)KSVQEIQATFF Hex 3 HexNAc 6

**Mass:**  3806.6464

(1.245e-06)

**Charge States:** 3, 4

**Start Position**: 51

**End Position**: 68

| Profile Score | Tandem Score | TDA q-value | Volume | Spectrum Matches |
| --- | --- | --- | --- | --- |
| 11.8105 | 148.5456 | 0.0000 | 7.4175e+07 | 5 |

**Adduct Signal Fractions**

- **Unmodified**: 7.4175e+07

### RNEEYN(N)KSVQEIQAT Hex 3 HexNAc 4

**Mass:**  3106.3500

(1.211e-06)

**Charge States:** 3

**Start Position**: 51

**End Position**: 66

| Profile Score | Tandem Score | TDA q-value | Volume | Spectrum Matches |
| --- | --- | --- | --- | --- |
| -3.1328 | 148.3899 | 0.0000 | 4.0650e+07 | 2 |

**Adduct Signal Fractions**

- **Unmodified**: 4.0650e+07

### TPN(N)KTEDTIF Fuc 3 Hex 3 HexNAc 4 Neu5Ac 1

**Mass:**  3192.3026

(-1.058e-06)

**Charge States:** 3

**Start Position**: 70

**End Position**: 80

| Profile Score | Tandem Score | TDA q-value | Volume | Spectrum Matches |
| --- | --- | --- | --- | --- |
| -18.9043 | 147.8347 | 0.0000 | 2.8142e+06 | 1 |

**Adduct Signal Fractions**

- **Unmodified**: 2.8142e+06

### YFTPN(N)KTEDTIFL Hex 3 HexNAc 5

**Mass:**  3089.3424

(1.657e-06)

**Charge States:** 3

**Start Position**: 68

**End Position**: 81

| Profile Score | Tandem Score | TDA q-value | Volume | Spectrum Matches |
| --- | --- | --- | --- | --- |
| 14.0106 | 146.3595 | 0.0000 | 1.0382e+07 | 3 |

**Adduct Signal Fractions**

- **Unmodified**: 1.0382e+07

### FYFTPN(N)KTED Hex 3 HexNAc 6

**Mass:**  2965.2063

(1.757e-06)

**Charge States:** 3

**Start Position**: 67

**End Position**: 77

| Profile Score | Tandem Score | TDA q-value | Volume | Spectrum Matches |
| --- | --- | --- | --- | --- |
| -4.7318 | 144.9300 | 0.0000 | 3.6353e+06 | 1 |

**Adduct Signal Fractions**

- **Unmodified**: 3.6353e+06

### NVQREN(N)GTISRY Hex 3 HexNAc 5

**Mass:**  2937.2726

(-1.524e-07)

**Charge States:** 3

**Start Position**: 98

**End Position**: 110

| Profile Score | Tandem Score | TDA q-value | Volume | Spectrum Matches |
| --- | --- | --- | --- | --- |
| 12.5737 | 144.6309 | 0.0000 | 2.1082e+08 | 3 |

**Adduct Signal Fractions**

- **Unmodified**: 2.1082e+08

### FYFTPN(N)KTEDTIF Hex 4 HexNAc 4 Neu5Ac 2

**Mass:**  3664.4903

(7.204e-07)

**Charge States:** 3, 4

**Start Position**: 67

**End Position**: 80

| Profile Score | Tandem Score | TDA q-value | Volume | Spectrum Matches |
| --- | --- | --- | --- | --- |
| 13.3004 | 144.0927 | 0.0000 | 1.5378e+08 | 9 |

**Adduct Signal Fractions**

- **Unmodified**: 1.5378e+08

### RNEEYN(N)KSVQEIQATF Hex 4 HexNAc 8

**Mass:**  4227.7907

(8.152e-07)

**Charge States:** 3, 4

**Start Position**: 51

**End Position**: 67

| Profile Score | Tandem Score | TDA q-value | Volume | Spectrum Matches |
| --- | --- | --- | --- | --- |
| -3.4169 | 143.8792 | 0.0000 | 3.5887e+07 | 3 |

**Adduct Signal Fractions**

- **Unmodified**: 3.5887e+07

### LNVQREN(N)GTISRY Hex 4 HexNAc 7

**Mass:**  3618.5736

(1.556e-06)

**Charge States:** 3, 4

**Start Position**: 97

**End Position**: 110

| Profile Score | Tandem Score | TDA q-value | Volume | Spectrum Matches |
| --- | --- | --- | --- | --- |
| 11.5738 | 143.6666 | 0.0000 | 2.1753e+09 | 14 |

**Adduct Signal Fractions**

- **Unmodified**: 2.1753e+09

### FTPN(N)KTEDTIF Hex 4 HexNAc 4 Neu5Ac 2

**Mass:**  3354.3587

(8.804e-07)

**Charge States:** 3, 4

**Start Position**: 69

**End Position**: 80

| Profile Score | Tandem Score | TDA q-value | Volume | Spectrum Matches |
| --- | --- | --- | --- | --- |
| -3.5395 | 142.8038 | 0.0000 | 2.2656e+07 | 2 |

**Adduct Signal Fractions**

- **Unmodified**: 2.2656e+07

### RNEEYN(N)KSVQEIQATF Fuc 2 Hex 4 HexNAc 5

**Mass:**  3910.6660

(2.554e-06)

**Charge States:** 3

**Start Position**: 51

**End Position**: 67

| Profile Score | Tandem Score | TDA q-value | Volume | Spectrum Matches |
| --- | --- | --- | --- | --- |
| -4.9466 | 142.7765 | 0.0000 | 4.0989e+07 | 2 |

**Adduct Signal Fractions**

- **Unmodified**: 4.0989e+07

### N(N)KSVQEIQATFF Hex 3 HexNAc 5

**Mass:**  2912.2716

(6.644e-07)

**Charge States:** 3

**Start Position**: 56

**End Position**: 68

| Profile Score | Tandem Score | TDA q-value | Volume | Spectrum Matches |
| --- | --- | --- | --- | --- |
| 13.9539 | 142.6858 | 0.0000 | 6.2695e+06 | 2 |

**Adduct Signal Fractions**

- **Unmodified**: 6.2695e+06

### FYFTPN(N)KTEDTIF Hex 4 HexNAc 8

**Mass:**  3894.6190

(1.521e-06)

**Charge States:** 3, 4

**Start Position**: 67

**End Position**: 80

| Profile Score | Tandem Score | TDA q-value | Volume | Spectrum Matches |
| --- | --- | --- | --- | --- |
| 15.7177 | 142.2970 | 0.0000 | 7.0736e+07 | 5 |

**Adduct Signal Fractions**

- **Unmodified**: 7.0736e+07

### RNEEYN(N)KSVQEIQATF Hex 4 HexNAc 4

**Mass:**  3415.4708

(1.272e-06)

**Charge States:** 3

**Start Position**: 51

**End Position**: 67

| Profile Score | Tandem Score | TDA q-value | Volume | Spectrum Matches |
| --- | --- | --- | --- | --- |
| -20.4880 | 142.2208 | 0.0000 | 2.1547e+07 | 1 |

**Adduct Signal Fractions**

- **Unmodified**: 2.1547e+07

### RNEEYN(N)KSVQEIQATFF Fuc 1 Hex 3 HexNAc 4

**Mass:**  3546.5462

(-1.484e-06)

**Charge States:** 3

**Start Position**: 51

**End Position**: 68

| Profile Score | Tandem Score | TDA q-value | Volume | Spectrum Matches |
| --- | --- | --- | --- | --- |
| 11.8837 | 141.3180 | 0.0000 | 3.3775e+07 | 4 |

**Adduct Signal Fractions**

- **Unmodified**: 3.3775e+07

### NVQREN(N)GTISRY Hex 4 HexNAc 8

**Mass:**  3708.5612

(-5.329e-07)

**Charge States:** 3, 4

**Start Position**: 98

**End Position**: 110

| Profile Score | Tandem Score | TDA q-value | Volume | Spectrum Matches |
| --- | --- | --- | --- | --- |
| -18.8486 | 141.2285 | 0.0000 | 7.3973e+07 | 2 |

**Adduct Signal Fractions**

- **Unmodified**: 7.3973e+07

### RN(D)EEYN(N)KSVQEIQATF Fuc 1 Hex 4 HexNAc 5

**Mass:**  3765.5934

(2.629e-04)

**Charge States:** 3, 4

**Start Position**: 51

**End Position**: 67

| Profile Score | Tandem Score | TDA q-value | Volume | Spectrum Matches |
| --- | --- | --- | --- | --- |
| 12.1408 | 141.2106 | 0.0000 | 2.8068e+08 | 11 |

**Adduct Signal Fractions**

- **Unmodified**: 2.8068e+08

### FYFTPN(N)KTEDTIF Fuc 1 Hex 5 HexNAc 4 Neu5Ac 1

**Mass:**  3681.5169

(4.259e-06)

**Charge States:** 3

**Start Position**: 67

**End Position**: 80

| Profile Score | Tandem Score | TDA q-value | Volume | Spectrum Matches |
| --- | --- | --- | --- | --- |
| 13.0426 | 140.8075 | 0.0000 | 2.5728e+07 | 3 |

**Adduct Signal Fractions**

- **Unmodified**: 2.5728e+07

### FYFTPN(N)KTEDTIF Hex 4 HexNAc 6

**Mass:**  3488.4603

(1.885e-06)

**Charge States:** 3

**Start Position**: 67

**End Position**: 80

| Profile Score | Tandem Score | TDA q-value | Volume | Spectrum Matches |
| --- | --- | --- | --- | --- |
| 11.5792 | 140.4916 | 0.0000 | 5.3769e+07 | 7 |

**Adduct Signal Fractions**

- **Unmodified**: 5.3769e+07

### RNEEYN(N)KSVQEIQATFF Fuc 2 Hex 5 HexNAc 5

**Mass:**  4219.7912

(1.759e-06)

**Charge States:** 3, 4

**Start Position**: 51

**End Position**: 68

| Profile Score | Tandem Score | TDA q-value | Volume | Spectrum Matches |
| --- | --- | --- | --- | --- |
| 14.2758 | 140.4879 | 0.0000 | 2.8098e+07 | 4 |

**Adduct Signal Fractions**

- **Unmodified**: 2.8098e+07

### FYFTPN(N)KT Hex 3 HexNAc 6

**Mass:**  2721.1353

(1.382e-06)

**Charge States:** 3

**Start Position**: 67

**End Position**: 75

| Profile Score | Tandem Score | TDA q-value | Volume | Spectrum Matches |
| --- | --- | --- | --- | --- |
| -3.4228 | 139.6192 | 0.0000 | 1.1012e+07 | 1 |

**Adduct Signal Fractions**

- **Unmodified**: 1.1012e+07

### RNEEYN(N)KSVQEIQATFF Fuc 1 Hex 3 HexNAc 5

**Mass:**  3749.6211

(1.645e-06)

**Charge States:** 4

**Start Position**: 51

**End Position**: 68

| Profile Score | Tandem Score | TDA q-value | Volume | Spectrum Matches |
| --- | --- | --- | --- | --- |
| -4.2701 | 139.3584 | 0.0000 | 3.4603e+07 | 2 |

**Adduct Signal Fractions**

- **Unmodified**: 3.4603e+07

### LNVQREN(N)GTISRY Fuc 1 Hex 4 HexNAc 5

**Mass:**  3358.4741

(2.613e-06)

**Charge States:** 3, 4

**Start Position**: 97

**End Position**: 110

| Profile Score | Tandem Score | TDA q-value | Volume | Spectrum Matches |
| --- | --- | --- | --- | --- |
| 13.1774 | 138.9026 | 0.0000 | 9.9249e+08 | 8 |

**Adduct Signal Fractions**

- **Unmodified**: 9.9249e+08

### LNVQREN(N)GTISRY Fuc 1 Hex 3 HexNAc 6

**Mass:**  3399.5044

(2.385e-06)

**Charge States:** 3

**Start Position**: 97

**End Position**: 110

| Profile Score | Tandem Score | TDA q-value | Volume | Spectrum Matches |
| --- | --- | --- | --- | --- |
| 11.7812 | 138.8807 | 0.0000 | 1.2341e+08 | 2 |

**Adduct Signal Fractions**

- **Unmodified**: 1.2341e+08

### YFTPN(N)KTEDTIF Hex 4 HexNAc 7

**Mass:**  3546.4606

(5.630e-04)

**Start Position**: 68

**End Position**: 80

| Profile Score | Tandem Score | TDA q-value | Volume | Spectrum Matches |
| --- | --- | --- | --- | --- |
| 0.0000 | 136.6640 | 0.0000 | 0.0000e+00 | 1 |

Adduct:

No Chromatogram Found

### RNEEYN(N)KSVQEIQAT Hex 3 HexNAc 5

**Mass:**  3309.4293

(2.148e-06)

**Charge States:** 3

**Start Position**: 51

**End Position**: 66

| Profile Score | Tandem Score | TDA q-value | Volume | Spectrum Matches |
| --- | --- | --- | --- | --- |
| -4.4481 | 136.4326 | 0.0000 | 2.9960e+07 | 5 |

**Adduct Signal Fractions**

- **Unmodified**: 2.9960e+07

### RN(D)EEYN(N)KSVQEIQATF Hex 3 HexNAc 5

**Mass:**  3457.4811

(2.864e-04)

**Charge States:** 3, 4

**Start Position**: 51

**End Position**: 67

| Profile Score | Tandem Score | TDA q-value | Volume | Spectrum Matches |
| --- | --- | --- | --- | --- |
| 10.1888 | 134.9039 | 0.0000 | 5.1920e+08 | 12 |

**Adduct Signal Fractions**

- **Unmodified**: 5.1920e+08

### LNVQREN(N)GTISRY Hex 3 HexNAc 5

**Mass:**  3050.3642

(2.700e-06)

**Charge States:** 3, 4

**Start Position**: 97

**End Position**: 110

| Profile Score | Tandem Score | TDA q-value | Volume | Spectrum Matches |
| --- | --- | --- | --- | --- |
| 14.8740 | 133.4447 | 0.0000 | 2.5754e+09 | 9 |

**Adduct Signal Fractions**

- **Unmodified**: 2.5754e+09

### RNEEYN(N)KSVQEIQATF Fuc 2 Hex 5 HexNAc 6

**Mass:**  4275.8010

(1.287e-06)

**Charge States:** 3, 4

**Start Position**: 51

**End Position**: 67

| Profile Score | Tandem Score | TDA q-value | Volume | Spectrum Matches |
| --- | --- | --- | --- | --- |
| 11.7028 | 132.6749 | 0.0000 | 2.5548e+07 | 3 |

**Adduct Signal Fractions**

- **Unmodified**: 2.5548e+07

### NVQREN(N)GTISRY Fuc 1 Hex 4 HexNAc 6

**Mass:**  3448.4613

(2.519e-07)

**Charge States:** 3

**Start Position**: 98

**End Position**: 110

| Profile Score | Tandem Score | TDA q-value | Volume | Spectrum Matches |
| --- | --- | --- | --- | --- |
| 13.7159 | 131.8240 | 0.0000 | 3.4645e+08 | 4 |

**Adduct Signal Fractions**

- **Unmodified**: 3.4645e+08

### YFTPN(N)KTEDTIF Hex 5 HexNAc 5

**Mass:**  3303.3718

(9.205e-04)

**Charge States:** 3

**Start Position**: 68

**End Position**: 80

| Profile Score | Tandem Score | TDA q-value | Volume | Spectrum Matches |
| --- | --- | --- | --- | --- |
| -7.4625 | 131.6192 | 0.0000 | 2.3907e+06 | 3 |

**Adduct Signal Fractions**

- **Unmodified**: 2.3907e+06

### TPN(N)KTEDTIF Fuc 4 Hex 5 HexNAc 5

**Mass:**  3574.4419

(-4.697e-06)

**Charge States:** 3

**Start Position**: 70

**End Position**: 80

| Profile Score | Tandem Score | TDA q-value | Volume | Spectrum Matches |
| --- | --- | --- | --- | --- |
| 13.5653 | 131.1868 | 0.0000 | 3.8853e+07 | 3 |

**Adduct Signal Fractions**

- **Unmodified**: 3.8853e+07

### LNVQREN(N)GTISRY Fuc 2 Hex 5 HexNAc 6

**Mass:**  3869.6625

(1.865e-06)

**Charge States:** 3, 4

**Start Position**: 97

**End Position**: 110

| Profile Score | Tandem Score | TDA q-value | Volume | Spectrum Matches |
| --- | --- | --- | --- | --- |
| 14.1725 | 129.8783 | 0.0000 | 1.3129e+09 | 9 |

**Adduct Signal Fractions**

- **Unmodified**: 1.3129e+09

### LNVQREN(N)GTISRY Fuc 3 Hex 6 HexNAc 6

**Mass:**  4177.7756

(1.507e-06)

**Charge States:** 3, 4

**Start Position**: 97

**End Position**: 110

| Profile Score | Tandem Score | TDA q-value | Volume | Spectrum Matches |
| --- | --- | --- | --- | --- |
| 15.6515 | 129.4302 | 0.0000 | 4.3395e+08 | 4 |

**Adduct Signal Fractions**

- **Unmodified**: 4.3395e+08

### FTPN(N)KTEDTIF Hex 4 HexNAc 6

**Mass:**  3178.3218

(1.252e-06)

**Charge States:** 3

**Start Position**: 69

**End Position**: 80

| Profile Score | Tandem Score | TDA q-value | Volume | Spectrum Matches |
| --- | --- | --- | --- | --- |
| -5.0125 | 128.0242 | 0.0000 | 9.4129e+06 | 2 |

**Adduct Signal Fractions**

- **Unmodified**: 9.4129e+06

### YFTPN(N)KTEDTIF Fuc 1 Hex 5 HexNAc 8

**Mass:**  4055.6614

(1.763e-06)

**Charge States:** 3, 4

**Start Position**: 68

**End Position**: 80

| Profile Score | Tandem Score | TDA q-value | Volume | Spectrum Matches |
| --- | --- | --- | --- | --- |
| 15.2114 | 128.0118 | 0.0000 | 3.6873e+07 | 4 |

**Adduct Signal Fractions**

- **Unmodified**: 3.6873e+07

### QTRQDQC(C)IYN(N)TTY Hex 3 HexNAc 5

**Mass:**  3191.3007

(1.427e-06)

**Charge States:** 3, 4

**Start Position**: 84

**End Position**: 97

| Profile Score | Tandem Score | TDA q-value | Volume | Spectrum Matches |
| --- | --- | --- | --- | --- |
| 15.2369 | 127.9976 | 0.0000 | 9.7546e+07 | 4 |

**Adduct Signal Fractions**

- **Unmodified**: 9.7546e+07

### NVQREN(N)GTISRY Fuc 2 Hex 5 HexNAc 6

**Mass:**  3756.5729

(-4.879e-08)

**Charge States:** 3, 4

**Start Position**: 98

**End Position**: 110

| Profile Score | Tandem Score | TDA q-value | Volume | Spectrum Matches |
| --- | --- | --- | --- | --- |
| -18.7753 | 127.9780 | 0.0000 | 8.4063e+07 | 4 |

**Adduct Signal Fractions**

- **Unmodified**: 8.4063e+07

### QTRQDQC(C)IYN(N)TTY Fuc 1 Hex 4 HexNAc 6

**Mass:**  3702.4901

(1.343e-06)

**Charge States:** 3, 4

**Start Position**: 84

**End Position**: 97

| Profile Score | Tandem Score | TDA q-value | Volume | Spectrum Matches |
| --- | --- | --- | --- | --- |
| 13.8504 | 127.9778 | 0.0000 | 6.1707e+07 | 4 |

**Adduct Signal Fractions**

- **Unmodified**: 6.1707e+07

### RNEEYN(N)KSVQEIQATFF Fuc 2 Hex 4 HexNAc 5

**Mass:**  4057.7419

(2.126e-06)

**Charge States:** 3, 4

**Start Position**: 51

**End Position**: 68

| Profile Score | Tandem Score | TDA q-value | Volume | Spectrum Matches |
| --- | --- | --- | --- | --- |
| -4.7163 | 127.7678 | 0.0000 | 1.6191e+07 | 2 |

**Adduct Signal Fractions**

- **Unmodified**: 1.6191e+07

### RNEEYN(N)KSVQEIQAT Fuc 1 Hex 4 HexNAc 5

**Mass:**  3617.5416

(1.525e-06)

**Charge States:** 3

**Start Position**: 51

**End Position**: 66

| Profile Score | Tandem Score | TDA q-value | Volume | Spectrum Matches |
| --- | --- | --- | --- | --- |
| -19.1379 | 127.3018 | 0.0000 | 1.4164e+07 | 2 |

**Adduct Signal Fractions**

- **Unmodified**: 1.4164e+07

### YFTPN(N)KTEDTIF Fuc 1 Hex 5 HexNAc 4 Neu5Ac 2

**Mass:**  3825.5374

(2.360e-06)

**Charge States:** 3, 4

**Start Position**: 68

**End Position**: 80

| Profile Score | Tandem Score | TDA q-value | Volume | Spectrum Matches |
| --- | --- | --- | --- | --- |
| 11.2507 | 127.1535 | 0.0026 | 3.1824e+07 | 2 |

**Adduct Signal Fractions**

- **Unmodified**: 3.1824e+07

### RNEEYN(N)KSVQEIQATFF Hex 4 HexNAc 5

**Mass:**  3765.6212

(1.149e-06)

**Charge States:** 3, 4

**Start Position**: 51

**End Position**: 68

| Profile Score | Tandem Score | TDA q-value | Volume | Spectrum Matches |
| --- | --- | --- | --- | --- |
| -5.2024 | 127.1312 | 0.0026 | 2.6592e+07 | 4 |

**Adduct Signal Fractions**

- **Unmodified**: 2.6592e+07

### NVQREN(N)GTISRY Hex 3 HexNAc 6

**Mass:**  3140.3516

(4.132e-07)

**Charge States:** 3, 4

**Start Position**: 98

**End Position**: 110

| Profile Score | Tandem Score | TDA q-value | Volume | Spectrum Matches |
| --- | --- | --- | --- | --- |
| 14.1505 | 127.0956 | 0.0026 | 8.4663e+08 | 6 |

**Adduct Signal Fractions**

- **Unmodified**: 8.4663e+08

### RNEEYN(N)KSVQEIQATF Fuc 1 Hex 5 HexNAc 7

**Mass:**  4332.8215

(1.205e-06)

**Charge States:** 3

**Start Position**: 51

**End Position**: 67

| Profile Score | Tandem Score | TDA q-value | Volume | Spectrum Matches |
| --- | --- | --- | --- | --- |
| -4.1033 | 126.4582 | 0.0026 | 1.4014e+07 | 1 |

**Adduct Signal Fractions**

- **Unmodified**: 1.4014e+07

### N(N)KSVQEIQATFF Hex 3 HexNAc 4

**Mass:**  2709.1931

(4.707e-07)

**Charge States:** 3

**Start Position**: 56

**End Position**: 68

| Profile Score | Tandem Score | TDA q-value | Volume | Spectrum Matches |
| --- | --- | --- | --- | --- |
| -4.7297 | 125.2615 | 0.0050 | 4.5867e+06 | 2 |

**Adduct Signal Fractions**

- **Unmodified**: 4.5867e+06

### LNVQREN(N)GTISRY Fuc 1 Hex 5 HexNAc 7

**Mass:**  3926.6892

(7.366e-07)

**Charge States:** 3, 4

**Start Position**: 97

**End Position**: 110

| Profile Score | Tandem Score | TDA q-value | Volume | Spectrum Matches |
| --- | --- | --- | --- | --- |
| 9.6530 | 125.0806 | 0.0050 | 5.6017e+08 | 10 |

**Adduct Signal Fractions**

- **Unmodified**: 5.6017e+08

### LNVQREN(N)GTISRY Fuc 2 Hex 5 HexNAc 7

**Mass:**  4072.7475

(1.214e-06)

**Charge States:** 3, 4

**Start Position**: 97

**End Position**: 110

| Profile Score | Tandem Score | TDA q-value | Volume | Spectrum Matches |
| --- | --- | --- | --- | --- |
| 14.0406 | 124.7073 | 0.0050 | 2.1860e+08 | 3 |

**Adduct Signal Fractions**

- **Unmodified**: 2.1860e+08

### YFTPN(N)KT Hex 3 HexNAc 6

**Mass:**  2574.0666

(1.420e-06)

**Charge States:** 3

**Start Position**: 68

**End Position**: 75

| Profile Score | Tandem Score | TDA q-value | Volume | Spectrum Matches |
| --- | --- | --- | --- | --- |
| -21.1643 | 124.3038 | 0.0000 | 4.7148e+06 | 1 |

**Adduct Signal Fractions**

- **Unmodified**: 4.7148e+06

### TPN(N)KTEDTIFL Hex 3 HexNAc 6

**Mass:**  2982.2856

(1.648e-07)

**Charge States:** 3

**Start Position**: 70

**End Position**: 81

| Profile Score | Tandem Score | TDA q-value | Volume | Spectrum Matches |
| --- | --- | --- | --- | --- |
| -19.7015 | 123.8074 | 0.0050 | 1.6234e+06 | 1 |

**Adduct Signal Fractions**

- **Unmodified**: 1.6234e+06

### LNVQREN(N)GTISRY Fuc 1 Hex 5 HexNAc 8

**Mass:**  4129.7702

(2.147e-06)

**Charge States:** 3, 4

**Start Position**: 97

**End Position**: 110

| Profile Score | Tandem Score | TDA q-value | Volume | Spectrum Matches |
| --- | --- | --- | --- | --- |
| 16.1139 | 122.8964 | 0.0067 | 5.0989e+08 | 4 |

**Adduct Signal Fractions**

- **Unmodified**: 5.0989e+08

### FYFTPN(N)KTEDTIF Fuc 1 Hex 5 HexNAc 8

**Mass:**  4202.7302

(1.604e-06)

**Charge States:** 3, 4

**Start Position**: 67

**End Position**: 80

| Profile Score | Tandem Score | TDA q-value | Volume | Spectrum Matches |
| --- | --- | --- | --- | --- |
| 12.9433 | 122.3904 | 0.0067 | 2.0317e+07 | 4 |

**Adduct Signal Fractions**

- **Unmodified**: 2.0317e+07

### FYFTPN(N)KT Hex 3 HexNAc 5

**Mass:**  2518.0539

(7.184e-07)

**Charge States:** 3

**Start Position**: 67

**End Position**: 75

| Profile Score | Tandem Score | TDA q-value | Volume | Spectrum Matches |
| --- | --- | --- | --- | --- |
| -18.7700 | 122.3752 | 0.0000 | 5.0063e+06 | 1 |

**Adduct Signal Fractions**

- **Unmodified**: 5.0063e+06

### RNEEYN(N)KSVQEIQ Hex 3 HexNAc 4

**Mass:**  2934.2653

(1.392e-06)

**Charge States:** 3

**Start Position**: 51

**End Position**: 64

| Profile Score | Tandem Score | TDA q-value | Volume | Spectrum Matches |
| --- | --- | --- | --- | --- |
| -20.2358 | 122.3085 | 0.0067 | 2.8905e+06 | 1 |

**Adduct Signal Fractions**

- **Unmodified**: 2.8905e+06

### LNVQREN(N)GTISRY Hex 4 HexNAc 8

**Mass:**  3821.6550

(1.896e-06)

**Charge States:** 3, 4

**Start Position**: 97

**End Position**: 110

| Profile Score | Tandem Score | TDA q-value | Volume | Spectrum Matches |
| --- | --- | --- | --- | --- |
| 13.4877 | 121.6623 | 0.0067 | 1.3508e+09 | 9 |

**Adduct Signal Fractions**

- **Unmodified**: 1.3508e+09

### NVQREN(N)GTISRY Fuc 3 Hex 6 HexNAc 6

**Mass:**  4065.7069

(2.517e-04)

**Start Position**: 98

**End Position**: 110

| Profile Score | Tandem Score | TDA q-value | Volume | Spectrum Matches |
| --- | --- | --- | --- | --- |
| 0.0000 | 121.4265 | 0.0067 | 0.0000e+00 | 2 |

Adduct:

No Chromatogram Found

### QTRQDQC(C)IYN(N)TTY Hex 3 HexNAc 6

**Mass:**  3394.3811

(1.424e-06)

**Charge States:** 3, 4

**Start Position**: 84

**End Position**: 97

| Profile Score | Tandem Score | TDA q-value | Volume | Spectrum Matches |
| --- | --- | --- | --- | --- |
| 15.8659 | 121.3309 | 0.0067 | 2.8705e+08 | 7 |

**Adduct Signal Fractions**

- **Unmodified**: 2.8705e+08

### LNVQREN(N)GTISRY Fuc 2 Hex 5 HexNAc 5

**Mass:**  3666.5853

(2.110e-06)

**Charge States:** 3, 4

**Start Position**: 97

**End Position**: 110

| Profile Score | Tandem Score | TDA q-value | Volume | Spectrum Matches |
| --- | --- | --- | --- | --- |
| -19.0910 | 121.0929 | 0.0067 | 8.1435e+07 | 1 |

**Adduct Signal Fractions**

- **Unmodified**: 8.1435e+07

### QTRQDQC(C)IYN(N)TTY Fuc 1 Hex 4 HexNAc 5

**Mass:**  3499.4126

(1.352e-06)

**Charge States:** 3

**Start Position**: 84

**End Position**: 97

| Profile Score | Tandem Score | TDA q-value | Volume | Spectrum Matches |
| --- | --- | --- | --- | --- |
| 12.0920 | 120.6767 | 0.0067 | 3.5945e+07 | 2 |

**Adduct Signal Fractions**

- **Unmodified**: 3.5945e+07

### LNVQREN(N)GTISRY Fuc 2 Hex 4 HexNAc 6

**Mass:**  3707.6127

(2.277e-06)

**Charge States:** 3

**Start Position**: 97

**End Position**: 110

| Profile Score | Tandem Score | TDA q-value | Volume | Spectrum Matches |
| --- | --- | --- | --- | --- |
| -19.9887 | 119.0078 | 0.0067 | 1.1858e+07 | 1 |

**Adduct Signal Fractions**

- **Unmodified**: 1.1858e+07

### FTPN(N)KTEDTIF Fuc 2 Hex 5 HexNAc 6

**Mass:**  3632.4951

(1.239e-06)

**Charge States:** 3, 4

**Start Position**: 69

**End Position**: 80

| Profile Score | Tandem Score | TDA q-value | Volume | Spectrum Matches |
| --- | --- | --- | --- | --- |
| -4.2565 | 118.9238 | 0.0067 | 1.2483e+07 | 3 |

**Adduct Signal Fractions**

- **Unmodified**: 1.2483e+07

### LNVQREN(N)GTISRY Hex 4 HexNAc 4 Neu5Ac 2

**Mass:**  3591.5238

(3.465e-07)

**Charge States:** 3, 4

**Start Position**: 97

**End Position**: 110

| Profile Score | Tandem Score | TDA q-value | Volume | Spectrum Matches |
| --- | --- | --- | --- | --- |
| 14.5052 | 118.4885 | 0.0087 | 4.6919e+08 | 9 |

**Adduct Signal Fractions**

- **Unmodified**: 4.6919e+08

### FYFTPN(N)KTEDTIF Fuc 2 Hex 5 HexNAc 7

**Mass:**  4145.7092

(1.866e-06)

**Charge States:** 3, 4

**Start Position**: 67

**End Position**: 80

| Profile Score | Tandem Score | TDA q-value | Volume | Spectrum Matches |
| --- | --- | --- | --- | --- |
| 11.6597 | 118.1211 | 0.0087 | 1.3455e+07 | 4 |

**Adduct Signal Fractions**

- **Unmodified**: 1.3455e+07

### LNVQREN(N)GTISRY Fuc 1 Hex 3 HexNAc 5

**Mass:**  3196.4210

(2.419e-06)

**Charge States:** 3

**Start Position**: 97

**End Position**: 110

| Profile Score | Tandem Score | TDA q-value | Volume | Spectrum Matches |
| --- | --- | --- | --- | --- |
| -4.2176 | 118.0811 | 0.0087 | 5.5161e+07 | 2 |

**Adduct Signal Fractions**

- **Unmodified**: 5.5161e+07

### VQREN(N)GTISRY Fuc 1 Hex 4 HexNAc 6

**Mass:**  3334.4188

(-9.173e-08)

**Charge States:** 3

**Start Position**: 99

**End Position**: 110

| Profile Score | Tandem Score | TDA q-value | Volume | Spectrum Matches |
| --- | --- | --- | --- | --- |
| -17.8901 | 117.5764 | 0.0087 | 1.7731e+07 | 1 |

**Adduct Signal Fractions**

- **Unmodified**: 1.7731e+07

### RNEEYN(N)KSVQEIQATF Hex 4 HexNAc 6

**Mass:**  3821.6317

(1.513e-06)

**Charge States:** 3, 4

**Start Position**: 51

**End Position**: 67

| Profile Score | Tandem Score | TDA q-value | Volume | Spectrum Matches |
| --- | --- | --- | --- | --- |
| 14.6260 | 117.1512 | 0.0105 | 5.6186e+08 | 9 |

**Adduct Signal Fractions**

- **Unmodified**: 5.6186e+08

### FTPN(N)KTEDTIF Hex 4 HexNAc 8

**Mass:**  3584.4842

(1.997e-06)

**Charge States:** 3, 4

**Start Position**: 69

**End Position**: 80

| Profile Score | Tandem Score | TDA q-value | Volume | Spectrum Matches |
| --- | --- | --- | --- | --- |
| -4.7585 | 116.9429 | 0.0105 | 7.5378e+06 | 4 |

**Adduct Signal Fractions**

- **Unmodified**: 7.5378e+06

### VQREN(N)GTISRY Hex 3 HexNAc 6

**Mass:**  3026.3117

(3.333e-04)

**Charge States:** 3

**Start Position**: 99

**End Position**: 110

| Profile Score | Tandem Score | TDA q-value | Volume | Spectrum Matches |
| --- | --- | --- | --- | --- |
| -18.9670 | 116.5397 | 0.0105 | 4.3456e+07 | 2 |

**Adduct Signal Fractions**

- **Unmodified**: 4.3456e+07

### LNVQREN(N)GTISRY Fuc 2 Hex 6 HexNAc 7

**Mass:**  4234.7980

(2.585e-06)

**Charge States:** 3, 4

**Start Position**: 97

**End Position**: 110

| Profile Score | Tandem Score | TDA q-value | Volume | Spectrum Matches |
| --- | --- | --- | --- | --- |
| -3.8925 | 115.6931 | 0.0105 | 9.6001e+07 | 3 |

**Adduct Signal Fractions**

- **Unmodified**: 9.6001e+07

### LNVQREN(N)GTISRY Hex 4 HexNAc 6

**Mass:**  3415.4957

(2.119e-06)

**Charge States:** 3

**Start Position**: 97

**End Position**: 110

| Profile Score | Tandem Score | TDA q-value | Volume | Spectrum Matches |
| --- | --- | --- | --- | --- |
| -8.4129 | 115.2593 | 0.0124 | 6.2825e+07 | 7 |

**Adduct Signal Fractions**

- **Unmodified**: 6.2825e+07

### TPN(N)KTEDTIF Hex 4 HexNAc 8

**Mass:**  3437.4201

(2.146e-06)

**Charge States:** 3

**Start Position**: 70

**End Position**: 80

| Profile Score | Tandem Score | TDA q-value | Volume | Spectrum Matches |
| --- | --- | --- | --- | --- |
| -5.2528 | 114.6114 | 0.0124 | 3.7413e+06 | 2 |

**Adduct Signal Fractions**

- **Unmodified**: 3.7413e+06

### NVQREN(N)GTISRY Hex 4 HexNAc 7

**Mass:**  3505.4833

(5.727e-04)

**Charge States:** 3

**Start Position**: 98

**End Position**: 110

| Profile Score | Tandem Score | TDA q-value | Volume | Spectrum Matches |
| --- | --- | --- | --- | --- |
| -5.1314 | 114.4714 | 0.0124 | 1.4400e+08 | 16 |

**Adduct Signal Fractions**

- **Unmodified**: 1.4400e+08

### LNVQREN(N)GTISRY Fuc 1 Hex 4 HexNAc 7

**Mass:**  3764.6310

(1.900e-06)

**Charge States:** 3, 4

**Start Position**: 97

**End Position**: 110

| Profile Score | Tandem Score | TDA q-value | Volume | Spectrum Matches |
| --- | --- | --- | --- | --- |
| 14.1291 | 114.4638 | 0.0124 | 2.3458e+08 | 4 |

**Adduct Signal Fractions**

- **Unmodified**: 2.3458e+08

### VQREN(N)GTISRY Hex 3 HexNAc 5

**Mass:**  2824.2379

(3.573e-04)

**Start Position**: 99

**End Position**: 110

| Profile Score | Tandem Score | TDA q-value | Volume | Spectrum Matches |
| --- | --- | --- | --- | --- |
| 0.0000 | 114.3838 | 0.0124 | 0.0000e+00 | 1 |

Adduct:

No Chromatogram Found

### NVQREN(N)GTISRY Fuc 1 Hex 5 HexNAc 8

**Mass:**  4018.6717

(4.974e-04)

**Start Position**: 98

**End Position**: 110

| Profile Score | Tandem Score | TDA q-value | Volume | Spectrum Matches |
| --- | --- | --- | --- | --- |
| 0.0000 | 113.7897 | 0.0141 | 0.0000e+00 | 2 |

Adduct:

No Chromatogram Found

### YFTPN(N)KTEDTIF Fuc 1 Hex 5 HexNAc 3 Neu5Ac 1

**Mass:**  3331.3672

(4.111e-06)

**Charge States:** 3

**Start Position**: 68

**End Position**: 80

| Profile Score | Tandem Score | TDA q-value | Volume | Spectrum Matches |
| --- | --- | --- | --- | --- |
| -5.7385 | 113.5567 | 0.0141 | 3.2549e+06 | 1 |

**Adduct Signal Fractions**

- **Unmodified**: 3.2549e+06

### LNVQREN(N)GTISRY Fuc 1 Hex 5 HexNAc 9

**Mass:**  4332.8478

(2.413e-06)

**Charge States:** 3

**Start Position**: 97

**End Position**: 110

| Profile Score | Tandem Score | TDA q-value | Volume | Spectrum Matches |
| --- | --- | --- | --- | --- |
| -18.5416 | 111.1725 | 0.0159 | 1.6718e+07 | 1 |

**Adduct Signal Fractions**

- **Unmodified**: 1.6718e+07

### RNEEYN(N)KSVQEIQATFF Hex 6 HexNAc 5

**Mass:**  4089.7285

(6.495e-07)

**Charge States:** 3, 4

**Start Position**: 51

**End Position**: 68

| Profile Score | Tandem Score | TDA q-value | Volume | Spectrum Matches |
| --- | --- | --- | --- | --- |
| -18.3561 | 111.0300 | 0.0159 | 7.9823e+06 | 2 |

**Adduct Signal Fractions**

- **Unmodified**: 7.9823e+06

### LNVQREN(N)GTISRY Hex 5 HexNAc 8

**Mass:**  3983.7099

(1.700e-06)

**Charge States:** 3, 4

**Start Position**: 97

**End Position**: 110

| Profile Score | Tandem Score | TDA q-value | Volume | Spectrum Matches |
| --- | --- | --- | --- | --- |
| -3.5558 | 110.1114 | 0.0173 | 1.5471e+08 | 2 |

**Adduct Signal Fractions**

- **Unmodified**: 1.5471e+08

### LNVQREN(N)GTISRY Fuc 1 Hex 5 HexNAc 4 Neu5Ac 2

**Mass:**  3899.6328

(2.410e-06)

**Charge States:** 3, 4

**Start Position**: 97

**End Position**: 110

| Profile Score | Tandem Score | TDA q-value | Volume | Spectrum Matches |
| --- | --- | --- | --- | --- |
| 11.3194 | 108.0038 | 0.0230 | 1.3716e+08 | 3 |

**Adduct Signal Fractions**

- **Unmodified**: 1.3716e+08

### FTPN(N)KTEDTIF Fuc 1 Hex 5 HexNAc 4 Neu5Ac 1

**Mass:**  3371.3845

(4.649e-06)

**Charge States:** 3

**Start Position**: 69

**End Position**: 80

| Profile Score | Tandem Score | TDA q-value | Volume | Spectrum Matches |
| --- | --- | --- | --- | --- |
| -19.9719 | 107.9555 | 0.0248 | 3.3963e+06 | 1 |

**Adduct Signal Fractions**

- **Unmodified**: 3.3963e+06

### RNEEYN(N)KSVQEIQATF Fuc 1 Hex 5 HexNAc 8

**Mass:**  4536.9123

(2.204e-04)

**Charge States:** 4

**Start Position**: 51

**End Position**: 67

| Profile Score | Tandem Score | TDA q-value | Volume | Spectrum Matches |
| --- | --- | --- | --- | --- |
| -21.1623 | 107.4370 | 0.0265 | 4.0880e+05 | 2 |

**Adduct Signal Fractions**

- **Unmodified**: 4.0880e+05

### LNVQREN(N)GTISRY Fuc 2 Hex 6 HexNAc 8

**Mass:**  4437.8771

(7.325e-07)

**Charge States:** 3, 4

**Start Position**: 97

**End Position**: 110

| Profile Score | Tandem Score | TDA q-value | Volume | Spectrum Matches |
| --- | --- | --- | --- | --- |
| 12.9032 | 104.8749 | 0.0276 | 1.8839e+08 | 3 |

**Adduct Signal Fractions**

- **Unmodified**: 1.8839e+08

### LNVQREN(N)GTISRY Fuc 3 Hex 6 HexNAc 7

**Mass:**  4380.8547

(9.915e-07)

**Charge States:** 3, 4

**Start Position**: 97

**End Position**: 110

| Profile Score | Tandem Score | TDA q-value | Volume | Spectrum Matches |
| --- | --- | --- | --- | --- |
| -3.2119 | 104.7624 | 0.0293 | 1.1566e+08 | 3 |

**Adduct Signal Fractions**

- **Unmodified**: 1.1566e+08

### C(C)ANLVPVPITN(N)ATL Hex 3 HexNAc 5

**Mass:**  2983.3498

(1.074e-06)

**Charge States:** 3

**Start Position**: 23

**End Position**: 37

| Profile Score | Tandem Score | TDA q-value | Volume | Spectrum Matches |
| --- | --- | --- | --- | --- |
| 14.3961 | 103.8660 | 0.0307 | 2.3144e+07 | 5 |

**Adduct Signal Fractions**

- **Unmodified**: 2.3144e+07

### RN(D)EEYN(N)KSVQEIQATFF Hex 3 HexNAc 4

**Mass:**  3401.4702

(2.905e-04)

**Charge States:** 3

**Start Position**: 51

**End Position**: 68

| Profile Score | Tandem Score | TDA q-value | Volume | Spectrum Matches |
| --- | --- | --- | --- | --- |
| -5.3982 | 102.8776 | 0.0307 | 4.8906e+07 | 11 |

**Adduct Signal Fractions**

- **Unmodified**: 4.8906e+07

### NVQREN(N)GTISRY Fuc 1 Hex 5 HexNAc 7

**Mass:**  3813.5978

(8.652e-07)

**Charge States:** 3, 4

**Start Position**: 98

**End Position**: 110

| Profile Score | Tandem Score | TDA q-value | Volume | Spectrum Matches |
| --- | --- | --- | --- | --- |
| -20.0902 | 102.3265 | 0.0307 | 1.0450e+07 | 2 |

**Adduct Signal Fractions**

- **Unmodified**: 1.0450e+07

### QTRQDQC(C)IYN(N)TTY Fuc 1 Hex 3 HexNAc 6

**Mass:**  3540.4385

(1.136e-06)

**Charge States:** 3

**Start Position**: 84

**End Position**: 97

| Profile Score | Tandem Score | TDA q-value | Volume | Spectrum Matches |
| --- | --- | --- | --- | --- |
| -3.4985 | 101.8050 | 0.0322 | 9.4458e+06 | 1 |

**Adduct Signal Fractions**

- **Unmodified**: 9.4458e+06

### LNVQREN(N)GTISRY Fuc 1 Hex 6 HexNAc 9

**Mass:**  4494.8967

(1.600e-06)

**Charge States:** 4

**Start Position**: 97

**End Position**: 110

| Profile Score | Tandem Score | TDA q-value | Volume | Spectrum Matches |
| --- | --- | --- | --- | --- |
| 10.3920 | 100.9366 | 0.0336 | 7.2241e+07 | 1 |

**Adduct Signal Fractions**

- **Unmodified**: 7.2241e+07

### VQREN(N)GTISRY Fuc 2 Hex 5 HexNAc 6

**Mass:**  3643.5364

(2.763e-04)

**Start Position**: 99

**End Position**: 110

| Profile Score | Tandem Score | TDA q-value | Volume | Spectrum Matches |
| --- | --- | --- | --- | --- |
| 0.0000 | 100.0340 | 0.0342 | 0.0000e+00 | 1 |

Adduct:

No Chromatogram Found

### LNVQREN(N)GTISRY Hex 5 HexNAc 9

**Mass:**  4186.7849

(1.652e-06)

**Charge States:** 3, 4

**Start Position**: 97

**End Position**: 110

| Profile Score | Tandem Score | TDA q-value | Volume | Spectrum Matches |
| --- | --- | --- | --- | --- |
| 12.9582 | 99.9734 | 0.0342 | 2.9647e+08 | 4 |

**Adduct Signal Fractions**

- **Unmodified**: 2.9647e+08

### FYFTPN(N)KTEDTIF Fuc 1 Hex 5 HexNAc 4 Neu5Ac 2

**Mass:**  3972.6011

(9.906e-07)

**Charge States:** 3

**Start Position**: 67

**End Position**: 80

| Profile Score | Tandem Score | TDA q-value | Volume | Spectrum Matches |
| --- | --- | --- | --- | --- |
| -20.0742 | 99.6434 | 0.0342 | 1.3535e+07 | 2 |

**Adduct Signal Fractions**

- **Unmodified**: 1.3535e+07

### NVQREN(N)GTISRY Fuc 1 Hex 4 HexNAc 5

**Mass:**  3245.3823

(-6.994e-07)

**Charge States:** 3

**Start Position**: 98

**End Position**: 110

| Profile Score | Tandem Score | TDA q-value | Volume | Spectrum Matches |
| --- | --- | --- | --- | --- |
| -5.3895 | 99.5473 | 0.0342 | 8.4534e+07 | 3 |

**Adduct Signal Fractions**

- **Unmodified**: 8.4534e+07

### LNVQREN(N)GTISRY Fuc 3 Hex 7 HexNAc 8

**Mass:**  4745.9860

(1.396e-06)

**Charge States:** 4

**Start Position**: 97

**End Position**: 110

| Profile Score | Tandem Score | TDA q-value | Volume | Spectrum Matches |
| --- | --- | --- | --- | --- |
| -4.5379 | 96.7607 | 0.0422 | 4.2075e+07 | 2 |

**Adduct Signal Fractions**

- **Unmodified**: 4.2075e+07

### LNVQREN(N)GTISR Hex 3 HexNAc 6

**Mass:**  3090.3693

(2.182e-06)

**Charge States:** 3

**Start Position**: 97

**End Position**: 109

| Profile Score | Tandem Score | TDA q-value | Volume | Spectrum Matches |
| --- | --- | --- | --- | --- |
| -20.3915 | 94.8442 | 0.0446 | 5.6769e+06 | 1 |

**Adduct Signal Fractions**

- **Unmodified**: 5.6769e+06

### NVQREN(N)GTISRY Hex 3 HexNAc 4

**Mass:**  2734.1944

(5.917e-07)

**Charge States:** 3

**Start Position**: 98

**End Position**: 110

| Profile Score | Tandem Score | TDA q-value | Volume | Spectrum Matches |
| --- | --- | --- | --- | --- |
| -19.1684 | 94.0650 | 0.0446 | 1.3139e+07 | 1 |

**Adduct Signal Fractions**

- **Unmodified**: 1.3139e+07

### LNVQREN(N)GTISRY Fuc 3 Hex 7 HexNAc 7

**Mass:**  4542.9060

(1.336e-06)

**Charge States:** 4

**Start Position**: 97

**End Position**: 110

| Profile Score | Tandem Score | TDA q-value | Volume | Spectrum Matches |
| --- | --- | --- | --- | --- |
| -19.1701 | 93.1552 | 0.0483 | 1.3651e+07 | 1 |

**Adduct Signal Fractions**

- **Unmodified**: 1.3651e+07

### NVQREN(N)GTISRY Hex 4 HexNAc 4 Neu5Ac 2

**Mass:**  3478.4372

(2.039e-07)

**Charge States:** 3

**Start Position**: 98

**End Position**: 110

| Profile Score | Tandem Score | TDA q-value | Volume | Spectrum Matches |
| --- | --- | --- | --- | --- |
| -20.1011 | 92.9258 | 0.0483 | 2.0792e+07 | 2 |

**Adduct Signal Fractions**

- **Unmodified**: 2.0792e+07

### LNVQREN(N)GTISRY Fuc 5 Hex 3 HexNAc 8

**Mass:**  4389.8659

(-4.070e-06)

**Charge States:** 4

**Start Position**: 97

**End Position**: 110

| Profile Score | Tandem Score | TDA q-value | Volume | Spectrum Matches |
| --- | --- | --- | --- | --- |
| -19.5247 | 92.9222 | 0.0483 | 2.8647e+07 | 2 |

**Adduct Signal Fractions**

- **Unmodified**: 2.8647e+07

### P19652|A1AG2\_HUMAN

**>P19652|A1AG2\_HUMAN**  
 `MALSWVLTVLSLLPLLEAQIPLCANLVPVPITNATLDRITGKWFYIASAFRNEEYNKSVQEIQATFFYFTPNKTEDTIFL  
REYQTRQNQCFYNSSYLNVQRENGTVSRYEGGREHVAHLLFLRDTKTLMFGSYLDDEKNWGLSFYADKPETTKEQLGEFY  
EALDCLCIPRSDVMYTDWKKDKCEPLEKQHEKERKQEEGES`

| Site | Type | Count |
| --- | --- | --- |
| 33 | N-linked | 2 |
| 72 | N-linked | 71 |
| 93 | N-linked | 1 |
| 103 | N-linked | 30 |
| 56 | N-linked | 46 |

| Mass | Sequence | MS_2_ Score | `q`-value | MS_1_ Score | Signal | Protein Coordinates | Tandem Scans | Start Time | Apex Time | End Time |
| --- | --- | --- | --- | --- | --- | --- | --- | --- | --- | --- |
| 2666.1252 | TPN(N)KTEDTIF Hex 3 HexNAc 5 | 186.358 | 0.0 | 16.194 | 1.389e+08 | 69 - 79 | 7 | 24.37 | 24.88 | 25.90 |
| 2869.2039 | TPN(N)KTEDTIF Hex 3 HexNAc 6 | 181.114 | 0.0 | 16.293 | 3.123e+08 | 69 - 79 | 12 | 22.51 | 24.54 | 25.90 |
| 3177.3145 | TPN(N)KTEDTIF Fuc 1 Hex 4 HexNAc 6 | 180.178 | 0.0 | 15.076 | 5.672e+07 | 69 - 79 | 5 | 23.53 | 24.04 | 24.54 |
| 2976.2572 | YFTPN(N)KTEDTIF Hex 3 HexNAc 5 | 179.364 | 0.0 | 15.318 | 1.868e+09 | 67 - 79 | 24 | 37.08 | 38.39 | 43.88 |
| 2773.1769 | YFTPN(N)KTEDTIF Hex 3 HexNAc 4 | 177.685 | 0.0 | 15.290 | 2.768e+08 | 67 - 79 | 9 | 38.55 | 38.71 | 41.26 |
| 2765.2041 | N(N)KSVQEIQATF Hex 3 HexNAc 5 | 175.410 | 0.0 | -3.678 | 1.537e+07 | 55 - 66 | 1 | 27.43 | 27.60 | 27.77 |
| 3123.3240 | FYFTPN(N)KTEDTIF Hex 3 HexNAc 5 | 175.237 | 0.0 | 13.519 | 2.061e+09 | 66 - 79 | 114 | 47.44 | 48.61 | 92.55 |
| 3544.4720 | YFTPN(N)KTEDTIF Hex 4 HexNAc 7 | 175.202 | 0.0 | 13.707 | 8.793e+08 | 67 - 79 | 18 | 36.23 | 37.42 | 52.55 |
| 2974.2344 | TPN(N)KTEDTIF Fuc 1 Hex 4 HexNAc 5 | 175.093 | 0.0 | 14.845 | 3.612e+07 | 69 - 79 | 3 | 23.70 | 24.21 | 24.71 |
| 3284.3917 | YFTPN(N)KTEDTIF Fuc 1 Hex 4 HexNAc 5 | 174.901 | 0.0 | 12.597 | 5.422e+08 | 67 - 79 | 13 | 36.56 | 37.75 | 48.28 |
| 3326.4039 | FYFTPN(N)KTEDTIF Hex 3 HexNAc 6 | 174.829 | 0.0 | 13.231 | 4.532e+09 | 66 - 79 | 215 | 47.27 | 48.28 | 92.94 |
| 3179.3358 | YFTPN(N)KTEDTIF Hex 3 HexNAc 6 | 174.803 | 0.0 | 15.137 | 3.039e+09 | 67 - 79 | 37 | 36.91 | 38.07 | 44.56 |
| 3234.3375 | TPN(N)KTEDTIF Hex 4 HexNAc 7 | 174.585 | 0.0 | 12.506 | 3.608e+07 | 69 - 79 | 4 | 23.53 | 23.87 | 24.37 |
| 2562.1240 | N(N)KSVQEIQATF Hex 3 HexNAc 4 | 172.874 | 0.0 | -4.229 | 1.787e+07 | 55 - 66 | 1 | 27.60 | 27.94 | 28.12 |
| 3341.3896 | YFTPN(N)KTEDTIF Hex 4 HexNAc 6 | 170.695 | 0.0 | 12.756 | 1.528e+08 | 67 - 79 | 4 | 37.25 | 37.91 | 38.71 |
| 3121.3021 | FTPN(N)KTEDTIF Fuc 1 Hex 4 HexNAc 5 | 169.878 | 0.0 | 12.703 | 7.195e+07 | 68 - 79 | 4 | 31.00 | 31.34 | 32.23 |
| 3431.4360 | FYFTPN(N)KTEDTIF Fuc 1 Hex 4 HexNAc 5 | 169.777 | 0.0 | 14.231 | 3.382e+08 | 66 - 79 | 8 | 46.93 | 47.95 | 49.62 |
| 3691.5384 | FYFTPN(N)KTEDTIF Hex 4 HexNAc 7 | 169.516 | 0.0 | 15.540 | 4.649e+08 | 66 - 79 | 10 | 46.41 | 47.61 | 48.28 |
| 3073.3152 | N(N)KSVQEIQATF Fuc 1 Hex 4 HexNAc 5 | 169.070 | 0.0 | -18.219 | 7.275e+06 | 55 - 66 | 1 | 26.92 | 26.92 | 27.09 |
| 3795.5588 | YFTPN(N)KTEDTIF Fuc 2 Hex 5 HexNAc 6 | 168.722 | 0.0 | 15.613 | 1.791e+08 | 67 - 79 | 4 | 36.73 | 37.25 | 37.58 |
| 3634.5161 | FYFTPN(N)KTEDTIF Fuc 1 Hex 4 HexNAc 6 | 168.379 | 0.0 | 14.867 | 7.221e+08 | 66 - 79 | 36 | 46.75 | 47.78 | 54.59 |
| 3852.5815 | YFTPN(N)KTEDTIF Fuc 1 Hex 5 HexNAc 7 | 167.953 | 0.0 | 16.453 | 1.503e+08 | 67 - 79 | 6 | 36.56 | 37.08 | 37.58 |
| 3016.2722 | FTPN(N)KTEDTIF Hex 3 HexNAc 6 | 167.437 | 0.0 | 14.574 | 8.000e+08 | 68 - 79 | 11 | 30.15 | 31.65 | 32.23 |
| 3487.4475 | YFTPN(N)KTEDTIF Fuc 1 Hex 4 HexNAc 6 | 166.586 | 0.0 | 16.213 | 9.741e+08 | 67 - 79 | 10 | 36.39 | 37.58 | 38.55 |
| 2463.0459 | TPN(N)KTEDTIF Hex 3 HexNAc 4 | 165.798 | 0.0 | 14.343 | 2.135e+07 | 69 - 79 | 4 | 24.71 | 25.05 | 25.56 |
| 3909.6041 | YFTPN(N)KTEDTIF Hex 5 HexNAc 8 | 165.541 | 0.0 | 13.414 | 1.380e+08 | 67 - 79 | 6 | 36.39 | 36.73 | 37.42 |
| 2813.1919 | FTPN(N)KTEDTIF Hex 3 HexNAc 5 | 165.262 | 0.0 | 14.297 | 2.507e+08 | 68 - 79 | 10 | 30.49 | 31.89 | 32.74 |
| 3324.3825 | FTPN(N)KTEDTIF Fuc 1 Hex 4 HexNAc 6 | 164.097 | 0.0 | 12.798 | 1.036e+08 | 68 - 79 | 7 | 30.83 | 31.17 | 31.89 |
| 3138.3084 | YFTPN(N)KTEDTIF Hex 4 HexNAc 5 | 162.963 | 0.0 | -20.009 | 9.662e+06 | 67 - 79 | 2 | 37.75 | 38.07 | 38.07 |
| 3400.4864 | RNEEYN(N)KSVQEIQATFF Hex 3 HexNAc 4 | 162.591 | 0.0 | 17.268 | 1.855e+09 | 50 - 67 | 15 | 43.88 | 45.06 | 46.41 |
| 3292.4205 | YFTPN(N)KTEDTIFL Hex 3 HexNAc 6 | 162.055 | 0.0 | 15.975 | 2.374e+07 | 67 - 80 | 5 | 45.73 | 45.90 | 46.41 |
| 3494.4627 | EEYN(N)KSVQEIQATF Fuc 1 Hex 4 HexNAc 5 | 161.970 | 0.0 | 12.489 | 1.969e+07 | 52 - 66 | 2 | 35.54 | 35.71 | 36.23 |
| 3603.5642 | RNEEYN(N)KSVQEIQATFF Hex 3 HexNAc 5 | 161.915 | 0.0 | 15.756 | 1.664e+09 | 50 - 67 | 10 | 44.56 | 44.73 | 46.07 |
| 3747.5494 | YFTPN(N)KTEDTIF Hex 4 HexNAc 8 | 161.629 | 0.0 | 16.798 | 1.315e+08 | 67 - 79 | 5 | 36.91 | 37.08 | 37.75 |
| 3534.4466 | YFTPN(N)KTEDTIF Fuc 1 Hex 5 HexNAc 4 Neu5Ac 1 | 160.992 | 0.0 | 13.554 | 4.037e+07 | 67 - 79 | 2 | 38.71 | 38.87 | 39.38 |
| 2671.1026 | N(N)KTEDTIF Hex 3 HexNAc 6 | 160.533 | 0.0 | -5.165 | 4.598e+06 | 71 - 79 | 1 | 22.51 | 22.68 | 22.85 |
| 4114.7566 | RNEEYN(N)KSVQEIQATFF Fuc 1 Hex 4 HexNAc 6 | 159.456 | 0.0 | 14.250 | 5.007e+07 | 50 - 67 | 4 | 43.88 | 44.23 | 44.56 |
| 3744.5650 | NVQREN(N)GTVSRY Fuc 2 Hex 5 HexNAc 6 | 158.606 | 0.0 | 0.000 | 0.000e+00 | 97 - 109 | 1 | - | - | - |
| 3381.4055 | FTPN(N)KTEDTIF Hex 4 HexNAc 7 | 158.368 | 0.0 | 14.116 | 6.204e+07 | 68 - 79 | 4 | 30.66 | 31.00 | 31.34 |
| 3690.5273 | YFTPN(N)KTEDTIF Fuc 1 Hex 4 HexNAc 7 | 158.257 | 0.0 | 14.071 | 4.583e+07 | 67 - 79 | 4 | 36.91 | 37.42 | 37.58 |
| 3999.6505 | FYFTPN(N)KTEDTIF Fuc 1 Hex 5 HexNAc 7 | 157.455 | 0.0 | 16.617 | 7.007e+07 | 66 - 79 | 5 | 46.41 | 46.93 | 47.61 |
| 2978.2550 | YFTPN(N)KTEDTIF Hex 3 HexNAc 5 | 157.236 | 0.0 | 0.000 | 0.000e+00 | 67 - 79 | 1 | - | - | - |
| 3485.4263 | TPN(N)KTEDTIF Fuc 2 Hex 5 HexNAc 6 | 157.030 | 0.0 | 11.356 | 7.660e+06 | 69 - 79 | 2 | 23.02 | 23.53 | 23.70 |
| 3764.6099 | RNEEYN(N)KSVQEIQATF Fuc 1 Hex 4 HexNAc 5 | 156.667 | 0.0 | 16.301 | 3.555e+09 | 50 - 66 | 14 | 31.72 | 32.74 | 34.18 |
| 3911.6802 | RNEEYN(N)KSVQEIQATFF Fuc 1 Hex 4 HexNAc 5 | 156.662 | 0.0 | 15.913 | 1.164e+09 | 50 - 67 | 12 | 44.05 | 44.40 | 45.73 |
| 3942.6281 | FYFTPN(N)KTEDTIF Fuc 2 Hex 5 HexNAc 6 | 156.381 | 0.0 | 14.359 | 1.002e+08 | 66 - 79 | 5 | 46.41 | 47.27 | 47.78 |
| 2920.2445 | FYFTPN(N)KTEDTIF Hex 3 HexNAc 4 | 155.981 | 0.0 | 14.938 | 1.740e+08 | 66 - 79 | 13 | 48.61 | 48.77 | 52.47 |
| 2983.2738 | EEYN(N)KSVQEIQATF Hex 3 HexNAc 4 | 155.872 | 0.0 | -3.373 | 4.018e+07 | 52 - 66 | 2 | 36.23 | 36.39 | 36.73 |
| 3254.4025 | RN(D)EEYN(N)KSVQEIQATF Hex 3 HexNAc 4 | 155.410 | 0.0 | 10.441 | 3.097e+08 | 50 - 66 | 20 | 36.06 | 36.23 | 36.91 |
| 3253.4178 | RNEEYN(N)KSVQEIQATF Hex 3 HexNAc 4 | 155.175 | 0.0 | 16.305 | 6.476e+09 | 50 - 66 | 25 | 28.62 | 33.37 | 34.69 |
| 4112.6820 | YFTPN(N)KTEDTIF Hex 5 HexNAc 9 | 154.777 | 0.0 | 9.875 | 4.142e+07 | 67 - 79 | 3 | 36.39 | 36.39 | 37.08 |
| 3517.4200 | YFTPN(N)KTEDTIF Hex 4 HexNAc 4 Neu5Ac 2 | 154.512 | 0.0 | 14.389 | 2.533e+08 | 67 - 79 | 8 | 38.71 | 38.87 | 40.06 |
| 3126.3368 | NVQREN(N)GTVSRY Hex 3 HexNAc 6 | 154.480 | 0.0 | 13.698 | 1.164e+09 | 97 - 109 | 8 | 12.14 | 12.31 | 13.46 |
| 3456.4980 | RNEEYN(N)KSVQEIQATF Hex 3 HexNAc 5 | 153.571 | 0.0 | 15.885 | 5.208e+09 | 50 - 66 | 17 | 31.00 | 33.05 | 34.52 |
| 3210.3506 | YFTPN(N)KTEDTIF Fuc 1 Hex 3 HexNAc 4 Neu5Ac 1 | 153.408 | 0.0 | 0.000 | 0.000e+00 | 67 - 79 | 1 | - | - | - |
| 3186.3533 | EEYN(N)KSVQEIQATF Hex 3 HexNAc 5 | 153.257 | 0.0 | 14.206 | 3.560e+07 | 52 - 66 | 2 | 36.06 | 36.23 | 36.73 |
| 3618.5526 | RNEEYN(N)KSVQEIQATF Hex 4 HexNAc 5 | 153.247 | 0.0 | 17.397 | 7.745e+07 | 50 - 66 | 3 | 32.40 | 32.90 | 33.05 |
| 3399.4748 | RNEEYN(N)KSVQEIQATF Fuc 1 Hex 3 HexNAc 4 | 152.786 | 0.0 | 12.798 | 1.238e+08 | 50 - 66 | 6 | 33.37 | 33.52 | 34.86 |
| 3967.6901 | RNEEYN(N)KSVQEIQATF Fuc 1 Hex 4 HexNAc 6 | 151.913 | 0.0 | -3.267 | 1.466e+08 | 50 - 66 | 3 | 32.40 | 32.74 | 32.90 |
| 3561.5312 | RNEEYN(N)KSVQEIQATF Fuc 1 Hex 4 HexNAc 4 | 151.801 | 0.0 | 15.370 | 1.688e+08 | 50 - 66 | 3 | 32.23 | 32.90 | 33.05 |
| 4072.7212 | RNEEYN(N)KSVQEIQATF Fuc 2 Hex 5 HexNAc 5 | 151.413 | 0.0 | 14.232 | 9.414e+07 | 50 - 66 | 4 | 32.23 | 32.57 | 32.90 |
| 3592.4782 | YFTPN(N)KTEDTIF Fuc 2 Hex 5 HexNAc 5 | 151.162 | 0.0 | -5.321 | 1.748e+07 | 67 - 79 | 1 | 37.08 | 37.08 | 37.42 |
| 3659.5790 | RNEEYN(N)KSVQEIQATF Hex 3 HexNAc 6 | 150.966 | 0.0 | 11.924 | 2.928e+08 | 50 - 66 | 6 | 32.90 | 32.90 | 34.18 |
| 3602.5551 | RNEEYN(N)KSVQEIQATF Fuc 1 Hex 3 HexNAc 5 | 150.932 | 0.0 | 13.086 | 1.219e+08 | 50 - 66 | 3 | 33.05 | 33.37 | 33.85 |
| 3031.2589 | TPN(N)KTEDTIF Hex 4 HexNAc 6 | 150.828 | 0.0 | 11.829 | 8.029e+06 | 69 - 79 | 2 | 23.87 | 24.21 | 24.71 |
| 4024.7114 | RNEEYN(N)KSVQEIQATF Hex 4 HexNAc 7 | 150.521 | 0.0 | -4.781 | 8.387e+07 | 50 - 66 | 4 | 32.40 | 32.57 | 32.90 |
| 2610.1129 | FTPN(N)KTEDTIF Hex 3 HexNAc 4 | 150.235 | 0.0 | -3.744 | 3.153e+07 | 68 - 79 | 4 | 32.06 | 32.23 | 32.57 |
| 3207.2900 | TPN(N)KTEDTIF Hex 4 HexNAc 4 Neu5Ac 2 | 150.098 | 0.0 | 13.627 | 1.534e+07 | 69 - 79 | 3 | 24.88 | 25.22 | 25.90 |
| 4056.6726 | FYFTPN(N)KTEDTIF Hex 5 HexNAc 8 | 148.806 | 0.0 | 12.180 | 7.708e+07 | 66 - 79 | 6 | 46.41 | 46.75 | 47.61 |
| 3708.5980 | RNEEYN(N)KSVQEIQATFF Fuc 1 Hex 4 HexNAc 4 | 148.702 | 0.0 | -4.435 | 4.970e+07 | 50 - 67 | 2 | 44.23 | 44.40 | 44.73 |
| 3806.6446 | RNEEYN(N)KSVQEIQATFF Hex 3 HexNAc 6 | 148.546 | 0.0 | 11.810 | 7.417e+07 | 50 - 67 | 5 | 44.56 | 44.56 | 45.39 |
| 3106.3494 | RNEEYN(N)KSVQEIQAT Hex 3 HexNAc 4 | 148.390 | 0.0 | -3.133 | 4.065e+07 | 50 - 65 | 2 | 17.53 | 17.70 | 17.87 |
| 3192.3049 | TPN(N)KTEDTIF Fuc 3 Hex 3 HexNAc 4 Neu5Ac 1 | 147.835 | 0.0 | -18.904 | 2.814e+06 | 69 - 79 | 1 | 23.53 | 23.53 | 23.70 |
| 3089.3419 | YFTPN(N)KTEDTIFL Hex 3 HexNAc 5 | 146.360 | 0.0 | 14.011 | 1.038e+07 | 67 - 80 | 3 | 45.90 | 46.24 | 46.58 |
| 3239.4232 | LNVQREN(N)GTVSRY Hex 3 HexNAc 6 | 146.311 | 0.0 | 14.357 | 5.592e+08 | 96 - 109 | 6 | 16.71 | 17.03 | 18.04 |
| 2965.2060 | FYFTPN(N)KTED Hex 3 HexNAc 6 | 144.930 | 0.0 | -4.732 | 3.635e+06 | 66 - 76 | 1 | 26.75 | 26.92 | 27.09 |
| 3491.4710 | NVQREN(N)GTVSRY Hex 4 HexNAc 7 | 144.807 | 0.0 | 13.584 | 1.705e+08 | 97 - 109 | 5 | 12.14 | 12.14 | 13.13 |
| 3664.4897 | FYFTPN(N)KTEDTIF Hex 4 HexNAc 4 Neu5Ac 2 | 144.093 | 0.0 | 13.300 | 1.538e+08 | 66 - 79 | 9 | 48.77 | 48.94 | 49.78 |
| 4227.7907 | RNEEYN(N)KSVQEIQATF Hex 4 HexNAc 8 | 143.879 | 0.0 | -3.417 | 3.589e+07 | 50 - 66 | 3 | 32.23 | 32.40 | 32.74 |
| 3354.3583 | FTPN(N)KTEDTIF Hex 4 HexNAc 4 Neu5Ac 2 | 142.804 | 0.0 | -3.540 | 2.266e+07 | 68 - 79 | 2 | 32.23 | 32.40 | 32.57 |
| 3910.6657 | RNEEYN(N)KSVQEIQATF Fuc 2 Hex 4 HexNAc 5 | 142.777 | 0.0 | -4.947 | 4.099e+07 | 50 - 66 | 2 | 32.74 | 32.90 | 33.05 |
| 2912.2712 | N(N)KSVQEIQATFF Hex 3 HexNAc 5 | 142.686 | 0.0 | 13.954 | 6.269e+06 | 55 - 67 | 2 | 41.60 | 41.77 | 42.28 |
| 3894.6191 | FYFTPN(N)KTEDTIF Hex 4 HexNAc 8 | 142.297 | 0.0 | 15.718 | 7.074e+07 | 66 - 79 | 5 | 46.41 | 47.27 | 47.95 |
| 3415.4712 | RNEEYN(N)KSVQEIQATF Hex 4 HexNAc 4 | 142.221 | 0.0 | -20.488 | 2.155e+07 | 50 - 66 | 1 | 32.90 | 33.05 | 33.05 |
| 2449.9658 | YN(N)SSYL Hex 3 HexNAc 6 | 142.133 | 0.0 | -5.454 | 3.118e+06 | 91 - 97 | 1 | 23.36 | 23.53 | 23.87 |
| 3231.3680 | NVQREN(N)GTVSRY Fuc 1 Hex 4 HexNAc 5 | 141.884 | 0.0 | 12.816 | 1.546e+08 | 97 - 109 | 2 | 12.14 | 12.31 | 12.80 |
| 3546.5414 | RNEEYN(N)KSVQEIQATFF Fuc 1 Hex 3 HexNAc 4 | 141.318 | 0.0 | 11.884 | 3.377e+07 | 50 - 67 | 4 | 44.73 | 44.90 | 45.56 |
| 3765.5953 | RN(D)EEYN(N)KSVQEIQATF Fuc 1 Hex 4 HexNAc 5 | 141.211 | 0.0 | 12.141 | 2.807e+08 | 50 - 66 | 11 | 34.35 | 35.71 | 36.91 |
| 3681.5157 | FYFTPN(N)KTEDTIF Fuc 1 Hex 5 HexNAc 4 Neu5Ac 1 | 140.808 | 0.0 | 13.043 | 2.573e+07 | 66 - 79 | 3 | 48.77 | 48.94 | 49.45 |
| 3488.4593 | FYFTPN(N)KTEDTIF Hex 4 HexNAc 6 | 140.492 | 0.0 | 11.579 | 5.377e+07 | 66 - 79 | 7 | 46.93 | 47.78 | 48.28 |
| 4219.7892 | RNEEYN(N)KSVQEIQATFF Fuc 2 Hex 5 HexNAc 5 | 140.488 | 0.0 | 14.276 | 2.810e+07 | 50 - 67 | 4 | 43.71 | 43.88 | 44.40 |
| 2721.1355 | FYFTPN(N)KT Hex 3 HexNAc 6 | 139.619 | 0.0 | -3.423 | 1.101e+07 | 66 - 74 | 1 | 26.58 | 26.58 | 26.92 |
| 3749.6214 | RNEEYN(N)KSVQEIQATFF Fuc 1 Hex 3 HexNAc 5 | 139.358 | 0.0 | -4.270 | 3.460e+07 | 50 - 67 | 2 | 44.56 | 44.73 | 44.90 |
| 3546.4606 | YFTPN(N)KTEDTIF Hex 4 HexNAc 7 | 136.664 | 0.0 | 0.000 | 0.000e+00 | 67 - 79 | 1 | - | - | - |
| 3309.4307 | RNEEYN(N)KSVQEIQAT Hex 3 HexNAc 5 | 136.433 | 0.0 | -4.448 | 2.996e+07 | 50 - 65 | 5 | 17.53 | 17.70 | 18.04 |
| 3457.4830 | RN(D)EEYN(N)KSVQEIQATF Hex 3 HexNAc 5 | 134.904 | 0.0 | 10.189 | 5.192e+08 | 50 - 66 | 12 | 34.69 | 36.06 | 36.91 |
| 4050.6728 | NVQREN(N)GTVSRY Fuc 3 Hex 6 HexNAc 6 | 134.487 | 0.0 | -20.108 | 2.872e+07 | 97 - 109 | 2 | 11.98 | 11.98 | 12.14 |
| 2923.2584 | NVQREN(N)GTVSRY Hex 3 HexNAc 5 | 134.364 | 0.0 | 12.069 | 2.572e+08 | 97 - 109 | 3 | 12.31 | 12.47 | 13.13 |
| 4275.7999 | RNEEYN(N)KSVQEIQATF Fuc 2 Hex 5 HexNAc 6 | 132.675 | 0.0 | 11.703 | 2.555e+07 | 50 - 66 | 3 | 32.06 | 32.57 | 32.74 |
| 3434.4478 | NVQREN(N)GTVSRY Fuc 1 Hex 4 HexNAc 6 | 132.465 | 0.0 | 12.430 | 6.075e+08 | 97 - 109 | 5 | 12.14 | 12.31 | 13.13 |
| 3303.3758 | YFTPN(N)KTEDTIF Hex 5 HexNAc 5 | 131.619 | 0.0 | -7.463 | 2.391e+06 | 67 - 79 | 3 | 29.47 | 29.64 | 29.81 |
| 3574.4415 | TPN(N)KTEDTIF Fuc 4 Hex 5 HexNAc 5 | 131.187 | 0.0 | 13.565 | 3.885e+07 | 69 - 79 | 3 | 38.71 | 39.04 | 39.55 |
| 3178.3229 | FTPN(N)KTEDTIF Hex 4 HexNAc 6 | 128.024 | 0.0 | -5.013 | 9.413e+06 | 68 - 79 | 2 | 31.00 | 31.34 | 31.47 |
| 4055.6618 | YFTPN(N)KTEDTIF Fuc 1 Hex 5 HexNAc 8 | 128.012 | 0.0 | 15.211 | 3.687e+07 | 67 - 79 | 4 | 36.39 | 36.56 | 37.25 |
| 3547.5330 | LNVQREN(N)GTVSRY Fuc 1 Hex 4 HexNAc 6 | 127.867 | 0.0 | 13.939 | 3.771e+08 | 96 - 109 | 5 | 16.71 | 16.71 | 17.70 |
| 4057.7390 | RNEEYN(N)KSVQEIQATFF Fuc 2 Hex 4 HexNAc 5 | 127.768 | 0.0 | -4.716 | 1.619e+07 | 50 - 67 | 2 | 44.23 | 44.40 | 44.56 |
| 3617.5404 | RNEEYN(N)KSVQEIQAT Fuc 1 Hex 4 HexNAc 5 | 127.302 | 0.0 | -19.138 | 1.416e+07 | 50 - 65 | 2 | 17.36 | 17.36 | 17.53 |
| 3825.5369 | YFTPN(N)KTEDTIF Fuc 1 Hex 5 HexNAc 4 Neu5Ac 2 | 127.154 | 0.003 | 11.251 | 3.182e+07 | 67 - 79 | 2 | 38.24 | 38.39 | 38.87 |
| 3765.6195 | RNEEYN(N)KSVQEIQATFF Hex 4 HexNAc 5 | 127.131 | 0.003 | -5.202 | 2.659e+07 | 50 - 67 | 4 | 44.23 | 44.40 | 44.73 |
| 4332.8213 | RNEEYN(N)KSVQEIQATF Fuc 1 Hex 5 HexNAc 7 | 126.458 | 0.003 | -4.103 | 1.401e+07 | 50 - 66 | 1 | 32.23 | 32.23 | 32.57 |
| 2709.1929 | N(N)KSVQEIQATFF Hex 3 HexNAc 4 | 125.262 | 0.005 | -4.730 | 4.587e+06 | 55 - 67 | 2 | 41.94 | 42.11 | 42.45 |
| 3549.5443 | LNVQREN(N)GTVSRY Fuc 1 Hex 4 HexNAc 6 | 125.124 | 0.005 | 0.000 | 0.000e+00 | 96 - 109 | 1 | - | - | - |
| 2574.0663 | YFTPN(N)KT Hex 3 HexNAc 6 | 124.304 | 0.0 | -21.164 | 4.715e+06 | 67 - 74 | 1 | 14.78 | 14.78 | 14.94 |
| 2982.2864 | TPN(N)KTEDTIFL Hex 3 HexNAc 6 | 123.807 | 0.005 | -19.702 | 1.623e+06 | 69 - 80 | 1 | 35.20 | 35.20 | 35.37 |
| 4202.7307 | FYFTPN(N)KTEDTIF Fuc 1 Hex 5 HexNAc 8 | 122.390 | 0.007 | 12.943 | 2.032e+07 | 66 - 79 | 4 | 46.41 | 46.75 | 47.27 |
| 2518.0534 | FYFTPN(N)KT Hex 3 HexNAc 5 | 122.375 | 0.0 | -18.770 | 5.006e+06 | 66 - 74 | 1 | 26.92 | 26.92 | 27.09 |
| 2934.2653 | RNEEYN(N)KSVQEIQ Hex 3 HexNAc 4 | 122.309 | 0.007 | -20.236 | 2.891e+06 | 50 - 63 | 1 | 15.28 | 15.28 | 15.28 |
| 3604.5567 | LNVQREN(N)GTVSRY Hex 4 HexNAc 7 | 122.119 | 0.007 | -4.434 | 5.938e+07 | 96 - 109 | 3 | 16.54 | 16.71 | 16.87 |
| 3344.4555 | LNVQREN(N)GTVSRY Fuc 1 Hex 4 HexNAc 5 | 121.717 | 0.007 | 12.869 | 8.681e+07 | 96 - 109 | 3 | 16.71 | 16.87 | 17.53 |
| 3036.3437 | LNVQREN(N)GTVSRY Hex 3 HexNAc 5 | 121.704 | 0.007 | 14.234 | 1.200e+08 | 96 - 109 | 4 | 16.87 | 17.20 | 17.70 |
| 3694.5505 | NVQREN(N)GTVSRY Hex 4 HexNAc 8 | 121.106 | 0.007 | -19.028 | 1.010e+08 | 97 - 109 | 2 | 12.14 | 12.14 | 12.31 |
| 3742.5595 | NVQREN(N)GTVSRY Fuc 2 Hex 5 HexNAc 6 | 120.046 | 0.007 | -18.113 | 1.729e+08 | 97 - 109 | 2 | 12.14 | 12.14 | 12.31 |
| 3632.4941 | FTPN(N)KTEDTIF Fuc 2 Hex 5 HexNAc 6 | 118.924 | 0.007 | -4.257 | 1.248e+07 | 68 - 79 | 3 | 30.49 | 30.66 | 31.00 |
| 4145.7089 | FYFTPN(N)KTEDTIF Fuc 2 Hex 5 HexNAc 7 | 118.121 | 0.009 | 11.660 | 1.346e+07 | 66 - 79 | 4 | 46.58 | 47.10 | 47.44 |
| 3821.6338 | RNEEYN(N)KSVQEIQATF Hex 4 HexNAc 6 | 117.151 | 0.011 | 14.626 | 5.619e+08 | 50 - 66 | 9 | 31.89 | 32.90 | 34.18 |
| 3584.4877 | FTPN(N)KTEDTIF Hex 4 HexNAc 8 | 116.943 | 0.011 | -4.758 | 7.538e+06 | 68 - 79 | 4 | 30.49 | 30.83 | 31.00 |
| 3855.6443 | LNVQREN(N)GTVSRY Fuc 2 Hex 5 HexNAc 6 | 116.942 | 0.011 | -4.307 | 9.475e+07 | 96 - 109 | 4 | 16.38 | 16.54 | 16.87 |
| 4002.6630 | NVQREN(N)GTVSRY Fuc 1 Hex 5 HexNAc 8 | 114.618 | 0.012 | -19.873 | 3.966e+07 | 97 - 109 | 1 | 11.98 | 12.14 | 12.14 |
| 3437.4161 | TPN(N)KTEDTIF Hex 4 HexNAc 8 | 114.611 | 0.012 | -5.253 | 3.741e+06 | 69 - 79 | 2 | 23.36 | 23.53 | 23.87 |
| 3331.3667 | YFTPN(N)KTEDTIF Fuc 1 Hex 5 HexNAc 3 Neu5Ac 1 | 113.557 | 0.014 | -5.738 | 3.255e+06 | 67 - 79 | 1 | 39.04 | 39.21 | 39.38 |
| 3012.2968 | VQREN(N)GTVSRY Hex 3 HexNAc 6 | 112.308 | 0.014 | -18.357 | 1.826e+07 | 98 - 109 | 1 | 11.98 | 11.98 | 11.98 |
| 3321.4087 | VQREN(N)GTVSRY Fuc 1 Hex 4 HexNAc 6 | 112.122 | 0.014 | 0.000 | 0.000e+00 | 98 - 109 | 1 | - | - | - |
| 4089.7275 | RNEEYN(N)KSVQEIQATFF Hex 6 HexNAc 5 | 111.030 | 0.016 | -18.356 | 7.982e+06 | 50 - 67 | 2 | 44.05 | 44.23 | 44.23 |
| 3371.3847 | FTPN(N)KTEDTIF Fuc 1 Hex 5 HexNAc 4 Neu5Ac 1 | 107.956 | 0.025 | -19.972 | 3.396e+06 | 68 - 79 | 1 | 32.23 | 32.40 | 32.40 |
| 4536.9123 | RNEEYN(N)KSVQEIQATF Fuc 1 Hex 5 HexNAc 8 | 107.437 | 0.027 | -21.162 | 4.088e+05 | 50 - 66 | 2 | 43.54 | 43.54 | 43.54 |
| 3808.6303 | LNVQREN(N)GTVSRY Hex 4 HexNAc 8 | 107.425 | 0.027 | 0.000 | 0.000e+00 | 96 - 109 | 1 | - | - | - |
| 3799.5803 | NVQREN(N)GTVSRY Fuc 1 Hex 5 HexNAc 7 | 105.161 | 0.028 | -18.397 | 1.572e+07 | 97 - 109 | 2 | 12.14 | 12.14 | 12.14 |
| 4310.7722 | NVQREN(N)GTVSRY Fuc 2 Hex 6 HexNAc 8 | 104.106 | 0.031 | -20.195 | 1.038e+07 | 97 - 109 | 1 | 11.98 | 11.98 | 11.98 |
| 2983.3501 | C(C)ANLVPVPITN(N)ATL Hex 3 HexNAc 5 | 103.866 | 0.031 | 14.396 | 2.314e+07 | 22 - 36 | 5 | 51.50 | 51.71 | 52.42 |
| 3401.4697 | RN(D)EEYN(N)KSVQEIQATFF Hex 3 HexNAc 4 | 102.878 | 0.031 | -5.398 | 4.891e+07 | 50 - 67 | 11 | 47.78 | 47.95 | 48.12 |
| 3637.5269 | NVQREN(N)GTVSRY Fuc 1 Hex 4 HexNAc 7 | 100.848 | 0.034 | -21.054 | 8.144e+06 | 97 - 109 | 2 | 12.14 | 12.14 | 12.14 |
| 4004.6674 | N(D)VQREN(N)GTVSRY Fuc 1 Hex 5 HexNAc 8 | 100.413 | 0.034 | 0.000 | 0.000e+00 | 97 - 109 | 1 | - | - | - |
| 3972.6001 | FYFTPN(N)KTEDTIF Fuc 1 Hex 5 HexNAc 4 Neu5Ac 2 | 99.643 | 0.034 | -20.074 | 1.354e+07 | 66 - 79 | 2 | 48.28 | 48.44 | 48.44 |
| 3744.5815 | N(D)VQREN(N)GTVSRY Fuc 2 Hex 5 HexNAc 6 | 99.290 | 0.034 | 0.000 | 0.000e+00 | 97 - 109 | 1 | - | - | - |
| 2876.2841 | TN(N)ATLDRITGKW Hex 3 HexNAc 5 | 96.560 | 0.042 | -4.315 | 5.198e+06 | 31 - 43 | 2 | 30.83 | 31.00 | 31.17 |
| 3464.4216 | NVQREN(N)GTVSRY Hex 4 HexNAc 4 Neu5Ac 2 | 94.129 | 0.045 | -19.644 | 2.923e+07 | 97 - 109 | 1 | 12.47 | 12.63 | 12.63 |
| 4003.6709 | NVQREN(N)GTVSRY Fuc 1 Hex 5 HexNAc 8 | 93.994 | 0.046 | 0.000 | 0.000e+00 | 97 - 109 | 1 | - | - | - |
| 3695.5699 | NVQREN(N)GTVSRY Hex 4 HexNAc 8 | 93.939 | 0.046 | 0.000 | 0.000e+00 | 97 - 109 | 1 | - | - | - |
| 3773.5396 | NVQREN(N)GTVSRY Fuc 3 Hex 5 HexNAc 4 Neu5Ac 1 | 93.905 | 0.046 | 0.000 | 0.000e+00 | 97 - 109 | 1 | - | - | - |
| 4163.7604 | LNVQREN(N)GTVSRY Fuc 3 Hex 6 HexNAc 6 | 93.349 | 0.047 | -21.153 | 1.612e+07 | 96 - 109 | 2 | 16.21 | 16.21 | 16.38 |

### TPN(N)KTEDTIF Hex 3 HexNAc 5

**Mass:**  2666.1251

(1.284e-06)

**Charge States:** 2, 3

**Start Position**: 70

**End Position**: 80

| Profile Score | Tandem Score | TDA q-value | Volume | Spectrum Matches |
| --- | --- | --- | --- | --- |
| 16.1935 | 186.3579 | 0.0000 | 1.3891e+08 | 7 |

**Adduct Signal Fractions**

- **Unmodified**: 1.3891e+08

### TPN(N)KTEDTIF Hex 3 HexNAc 6

**Mass:**  2869.2042

(1.151e-06)

**Charge States:** 2, 3, 4

**Start Position**: 70

**End Position**: 80

| Profile Score | Tandem Score | TDA q-value | Volume | Spectrum Matches |
| --- | --- | --- | --- | --- |
| 16.2927 | 181.1143 | 0.0000 | 3.1235e+08 | 12 |

**Adduct Signal Fractions**

- **Unmodified**: 3.1235e+08

### TPN(N)KTEDTIF Fuc 1 Hex 4 HexNAc 6

**Mass:**  3177.3155

(1.166e-06)

**Charge States:** 3

**Start Position**: 70

**End Position**: 80

| Profile Score | Tandem Score | TDA q-value | Volume | Spectrum Matches |
| --- | --- | --- | --- | --- |
| 15.0758 | 180.1785 | 0.0000 | 5.6725e+07 | 5 |

**Adduct Signal Fractions**

- **Unmodified**: 5.6725e+07

### YFTPN(N)KTEDTIF Hex 3 HexNAc 5

**Mass:**  2976.2576

(9.816e-07)

**Charge States:** 2, 3, 4

**Start Position**: 68

**End Position**: 80

| Profile Score | Tandem Score | TDA q-value | Volume | Spectrum Matches |
| --- | --- | --- | --- | --- |
| 15.3176 | 179.3644 | 0.0000 | 1.8681e+09 | 24 |

**Adduct Signal Fractions**

- **Unmodified**: 1.8681e+09

### YFTPN(N)KTEDTIF Hex 3 HexNAc 4

**Mass:**  2773.1766

(1.445e-06)

**Charge States:** 2, 3

**Start Position**: 68

**End Position**: 80

| Profile Score | Tandem Score | TDA q-value | Volume | Spectrum Matches |
| --- | --- | --- | --- | --- |
| 15.2900 | 177.6851 | 0.0000 | 2.7684e+08 | 9 |

**Adduct Signal Fractions**

- **Unmodified**: 2.7684e+08

### N(N)KSVQEIQATF Hex 3 HexNAc 5

**Mass:**  2765.2042

(1.760e-06)

**Charge States:** 3

**Start Position**: 56

**End Position**: 67

| Profile Score | Tandem Score | TDA q-value | Volume | Spectrum Matches |
| --- | --- | --- | --- | --- |
| -3.6782 | 175.4104 | 0.0000 | 1.5365e+07 | 1 |

**Adduct Signal Fractions**

- **Unmodified**: 1.5365e+07

### FYFTPN(N)KTEDTIF Hex 3 HexNAc 5

**Mass:**  3123.3228

(3.539e-07)

**Charge States:** 2, 3, 4

**Start Position**: 67

**End Position**: 80

| Profile Score | Tandem Score | TDA q-value | Volume | Spectrum Matches |
| --- | --- | --- | --- | --- |
| 13.5187 | 175.2373 | 0.0000 | 2.0608e+09 | 114 |

**Adduct Signal Fractions**

- **Unmodified**: 2.0608e+09

### YFTPN(N)KTEDTIF Hex 4 HexNAc 7

**Mass:**  3544.4686

(1.076e-06)

**Charge States:** 3, 4

**Start Position**: 68

**End Position**: 80

| Profile Score | Tandem Score | TDA q-value | Volume | Spectrum Matches |
| --- | --- | --- | --- | --- |
| 13.7067 | 175.2019 | 0.0000 | 8.7930e+08 | 18 |

**Adduct Signal Fractions**

- **Unmodified**: 8.7930e+08

### TPN(N)KTEDTIF Fuc 1 Hex 4 HexNAc 5

**Mass:**  2974.2326

(1.710e-06)

**Charge States:** 3

**Start Position**: 70

**End Position**: 80

| Profile Score | Tandem Score | TDA q-value | Volume | Spectrum Matches |
| --- | --- | --- | --- | --- |
| 14.8447 | 175.0932 | 0.0000 | 3.6115e+07 | 3 |

**Adduct Signal Fractions**

- **Unmodified**: 3.6115e+07

### YFTPN(N)KTEDTIF Fuc 1 Hex 4 HexNAc 5

**Mass:**  3284.3681

(8.133e-07)

**Charge States:** 3, 4

**Start Position**: 68

**End Position**: 80

| Profile Score | Tandem Score | TDA q-value | Volume | Spectrum Matches |
| --- | --- | --- | --- | --- |
| 12.5967 | 174.9015 | 0.0000 | 5.4224e+08 | 13 |

**Adduct Signal Fractions**

- **Unmodified**: 5.4224e+08

### FYFTPN(N)KTEDTIF Hex 3 HexNAc 6

**Mass:**  3326.4032

(1.596e-07)

**Charge States:** 3, 4

**Start Position**: 67

**End Position**: 80

| Profile Score | Tandem Score | TDA q-value | Volume | Spectrum Matches |
| --- | --- | --- | --- | --- |
| 13.2311 | 174.8285 | 0.0000 | 4.5324e+09 | 215 |

**Adduct Signal Fractions**

- **Unmodified**: 4.5324e+09

### YFTPN(N)KTEDTIF Hex 3 HexNAc 6

**Mass:**  3179.3358

(8.401e-07)

**Charge States:** 2, 3, 4

**Start Position**: 68

**End Position**: 80

| Profile Score | Tandem Score | TDA q-value | Volume | Spectrum Matches |
| --- | --- | --- | --- | --- |
| 15.1370 | 174.8034 | 0.0000 | 3.0392e+09 | 37 |

**Adduct Signal Fractions**

- **Unmodified**: 3.0392e+09

### TPN(N)KTEDTIF Hex 4 HexNAc 7

**Mass:**  3234.3371

(1.203e-06)

**Charge States:** 3

**Start Position**: 70

**End Position**: 80

| Profile Score | Tandem Score | TDA q-value | Volume | Spectrum Matches |
| --- | --- | --- | --- | --- |
| 12.5064 | 174.5851 | 0.0000 | 3.6080e+07 | 4 |

**Adduct Signal Fractions**

- **Unmodified**: 3.6080e+07

### N(N)KSVQEIQATF Hex 3 HexNAc 4

**Mass:**  2562.1236

(5.024e-07)

**Charge States:** 3

**Start Position**: 56

**End Position**: 67

| Profile Score | Tandem Score | TDA q-value | Volume | Spectrum Matches |
| --- | --- | --- | --- | --- |
| -4.2291 | 172.8736 | 0.0000 | 1.7870e+07 | 1 |

**Adduct Signal Fractions**

- **Unmodified**: 1.7870e+07

### YFTPN(N)KTEDTIF Hex 4 HexNAc 6

**Mass:**  3341.3900

(1.527e-06)

**Charge States:** 3

**Start Position**: 68

**End Position**: 80

| Profile Score | Tandem Score | TDA q-value | Volume | Spectrum Matches |
| --- | --- | --- | --- | --- |
| 12.7558 | 170.6947 | 0.0000 | 1.5281e+08 | 4 |

**Adduct Signal Fractions**

- **Unmodified**: 1.5281e+08

### FTPN(N)KTEDTIF Fuc 1 Hex 4 HexNAc 5

**Mass:**  3121.3024

(6.464e-07)

**Charge States:** 3

**Start Position**: 69

**End Position**: 80

| Profile Score | Tandem Score | TDA q-value | Volume | Spectrum Matches |
| --- | --- | --- | --- | --- |
| 12.7032 | 169.8782 | 0.0000 | 7.1951e+07 | 4 |

**Adduct Signal Fractions**

- **Unmodified**: 7.1951e+07

### FYFTPN(N)KTEDTIF Fuc 1 Hex 4 HexNAc 5

**Mass:**  3431.4358

(9.087e-07)

**Charge States:** 3, 4

**Start Position**: 67

**End Position**: 80

| Profile Score | Tandem Score | TDA q-value | Volume | Spectrum Matches |
| --- | --- | --- | --- | --- |
| 14.2311 | 169.7775 | 0.0000 | 3.3817e+08 | 8 |

**Adduct Signal Fractions**

- **Unmodified**: 3.3817e+08

### FYFTPN(N)KTEDTIF Hex 4 HexNAc 7

**Mass:**  3691.5375

(1.156e-06)

**Charge States:** 3, 4

**Start Position**: 67

**End Position**: 80

| Profile Score | Tandem Score | TDA q-value | Volume | Spectrum Matches |
| --- | --- | --- | --- | --- |
| 15.5398 | 169.5160 | 0.0000 | 4.6489e+08 | 10 |

**Adduct Signal Fractions**

- **Unmodified**: 4.6489e+08

### N(N)KSVQEIQATF Fuc 1 Hex 4 HexNAc 5

**Mass:**  3073.3159

(1.240e-06)

**Charge States:** 3

**Start Position**: 56

**End Position**: 67

| Profile Score | Tandem Score | TDA q-value | Volume | Spectrum Matches |
| --- | --- | --- | --- | --- |
| -18.2193 | 169.0696 | 0.0000 | 7.2745e+06 | 1 |

**Adduct Signal Fractions**

- **Unmodified**: 7.2745e+06

### YFTPN(N)KTEDTIF Fuc 2 Hex 5 HexNAc 6

**Mass:**  3795.5575

(9.129e-07)

**Charge States:** 3, 4

**Start Position**: 68

**End Position**: 80

| Profile Score | Tandem Score | TDA q-value | Volume | Spectrum Matches |
| --- | --- | --- | --- | --- |
| 15.6129 | 168.7221 | 0.0000 | 1.7905e+08 | 4 |

**Adduct Signal Fractions**

- **Unmodified**: 1.7905e+08

### FYFTPN(N)KTEDTIF Fuc 1 Hex 4 HexNAc 6

**Mass:**  3634.5163

(1.212e-06)

**Charge States:** 3, 4

**Start Position**: 67

**End Position**: 80

| Profile Score | Tandem Score | TDA q-value | Volume | Spectrum Matches |
| --- | --- | --- | --- | --- |
| 14.8673 | 168.3795 | 0.0000 | 7.2206e+08 | 36 |

**Adduct Signal Fractions**

- **Unmodified**: 7.2206e+08

### YFTPN(N)KTEDTIF Fuc 1 Hex 5 HexNAc 7

**Mass:**  3852.5795

(1.346e-06)

**Charge States:** 3, 4

**Start Position**: 68

**End Position**: 80

| Profile Score | Tandem Score | TDA q-value | Volume | Spectrum Matches |
| --- | --- | --- | --- | --- |
| 16.4528 | 167.9528 | 0.0000 | 1.5028e+08 | 6 |

**Adduct Signal Fractions**

- **Unmodified**: 1.5028e+08

### FTPN(N)KTEDTIF Hex 3 HexNAc 6

**Mass:**  3016.2722

(9.073e-07)

**Charge States:** 2, 3, 4

**Start Position**: 69

**End Position**: 80

| Profile Score | Tandem Score | TDA q-value | Volume | Spectrum Matches |
| --- | --- | --- | --- | --- |
| 14.5738 | 167.4370 | 0.0000 | 8.0001e+08 | 11 |

**Adduct Signal Fractions**

- **Unmodified**: 8.0001e+08

### YFTPN(N)KTEDTIF Fuc 1 Hex 4 HexNAc 6

**Mass:**  3487.4478

(1.259e-06)

**Charge States:** 3, 4

**Start Position**: 68

**End Position**: 80

| Profile Score | Tandem Score | TDA q-value | Volume | Spectrum Matches |
| --- | --- | --- | --- | --- |
| 16.2127 | 166.5862 | 0.0000 | 9.7413e+08 | 10 |

**Adduct Signal Fractions**

- **Unmodified**: 9.7413e+08

### TPN(N)KTEDTIF Hex 3 HexNAc 4

**Mass:**  2463.0461

(2.994e-06)

**Charge States:** 2, 3

**Start Position**: 70

**End Position**: 80

| Profile Score | Tandem Score | TDA q-value | Volume | Spectrum Matches |
| --- | --- | --- | --- | --- |
| 14.3433 | 165.7976 | 0.0000 | 2.1349e+07 | 4 |

**Adduct Signal Fractions**

- **Unmodified**: 2.1349e+07

### YFTPN(N)KTEDTIF Hex 5 HexNAc 8

**Mass:**  3909.6033

(1.595e-06)

**Charge States:** 3, 4

**Start Position**: 68

**End Position**: 80

| Profile Score | Tandem Score | TDA q-value | Volume | Spectrum Matches |
| --- | --- | --- | --- | --- |
| 13.4136 | 165.5412 | 0.0000 | 1.3798e+08 | 6 |

**Adduct Signal Fractions**

- **Unmodified**: 1.3798e+08

### FTPN(N)KTEDTIF Hex 3 HexNAc 5

**Mass:**  2813.1908

(2.581e-07)

**Charge States:** 2, 3, 4

**Start Position**: 69

**End Position**: 80

| Profile Score | Tandem Score | TDA q-value | Volume | Spectrum Matches |
| --- | --- | --- | --- | --- |
| 14.2970 | 165.2617 | 0.0000 | 2.5074e+08 | 10 |

**Adduct Signal Fractions**

- **Unmodified**: 2.5074e+08

### FTPN(N)KTEDTIF Fuc 1 Hex 4 HexNAc 6

**Mass:**  3324.3832

(9.570e-07)

**Charge States:** 3

**Start Position**: 69

**End Position**: 80

| Profile Score | Tandem Score | TDA q-value | Volume | Spectrum Matches |
| --- | --- | --- | --- | --- |
| 12.7977 | 164.0973 | 0.0000 | 1.0357e+08 | 7 |

**Adduct Signal Fractions**

- **Unmodified**: 1.0357e+08

### YFTPN(N)KTEDTIF Hex 4 HexNAc 5

**Mass:**  3138.3063

(1.469e-06)

**Charge States:** 3

**Start Position**: 68

**End Position**: 80

| Profile Score | Tandem Score | TDA q-value | Volume | Spectrum Matches |
| --- | --- | --- | --- | --- |
| -20.0087 | 162.9634 | 0.0000 | 9.6619e+06 | 2 |

**Adduct Signal Fractions**

- **Unmodified**: 9.6619e+06

### RNEEYN(N)KSVQEIQATFF Hex 3 HexNAc 4

**Mass:**  3400.4855

(1.033e-06)

**Charge States:** 3, 4

**Start Position**: 51

**End Position**: 68

| Profile Score | Tandem Score | TDA q-value | Volume | Spectrum Matches |
| --- | --- | --- | --- | --- |
| 17.2684 | 162.5909 | 0.0000 | 1.8552e+09 | 15 |

**Adduct Signal Fractions**

- **Unmodified**: 1.8552e+09

### YFTPN(N)KTEDTIFL Hex 3 HexNAc 6

**Mass:**  3292.4203

(1.312e-06)

**Charge States:** 3, 4

**Start Position**: 68

**End Position**: 81

| Profile Score | Tandem Score | TDA q-value | Volume | Spectrum Matches |
| --- | --- | --- | --- | --- |
| 15.9755 | 162.0550 | 0.0000 | 2.3738e+07 | 5 |

**Adduct Signal Fractions**

- **Unmodified**: 2.3738e+07

### EEYN(N)KSVQEIQATF Fuc 1 Hex 4 HexNAc 5

**Mass:**  3494.4644

(1.090e-06)

**Charge States:** 3

**Start Position**: 53

**End Position**: 67

| Profile Score | Tandem Score | TDA q-value | Volume | Spectrum Matches |
| --- | --- | --- | --- | --- |
| 12.4887 | 161.9701 | 0.0000 | 1.9690e+07 | 2 |

**Adduct Signal Fractions**

- **Unmodified**: 1.9690e+07

### RNEEYN(N)KSVQEIQATFF Hex 3 HexNAc 5

**Mass:**  3603.5623

(1.508e-06)

**Charge States:** 3, 4

**Start Position**: 51

**End Position**: 68

| Profile Score | Tandem Score | TDA q-value | Volume | Spectrum Matches |
| --- | --- | --- | --- | --- |
| 15.7557 | 161.9148 | 0.0000 | 1.6643e+09 | 10 |

**Adduct Signal Fractions**

- **Unmodified**: 1.6643e+09

### YFTPN(N)KTEDTIF Hex 4 HexNAc 8

**Mass:**  3747.5495

(9.606e-07)

**Charge States:** 3, 4

**Start Position**: 68

**End Position**: 80

| Profile Score | Tandem Score | TDA q-value | Volume | Spectrum Matches |
| --- | --- | --- | --- | --- |
| 16.7979 | 161.6290 | 0.0000 | 1.3149e+08 | 5 |

**Adduct Signal Fractions**

- **Unmodified**: 1.3149e+08

### YFTPN(N)KTEDTIF Fuc 1 Hex 5 HexNAc 4 Neu5Ac 1

**Mass:**  3534.4480

(4.260e-06)

**Charge States:** 3

**Start Position**: 68

**End Position**: 80

| Profile Score | Tandem Score | TDA q-value | Volume | Spectrum Matches |
| --- | --- | --- | --- | --- |
| 13.5537 | 160.9916 | 0.0000 | 4.0367e+07 | 2 |

**Adduct Signal Fractions**

- **Unmodified**: 4.0367e+07

### N(N)KTEDTIF Hex 3 HexNAc 6

**Mass:**  2671.1024

(7.347e-07)

**Charge States:** 3

**Start Position**: 72

**End Position**: 80

| Profile Score | Tandem Score | TDA q-value | Volume | Spectrum Matches |
| --- | --- | --- | --- | --- |
| -5.1647 | 160.5334 | 0.0000 | 4.5978e+06 | 1 |

**Adduct Signal Fractions**

- **Unmodified**: 4.5978e+06

### RNEEYN(N)KSVQEIQATFF Fuc 1 Hex 4 HexNAc 6

**Mass:**  4114.7575

(1.225e-06)

**Charge States:** 3, 4

**Start Position**: 51

**End Position**: 68

| Profile Score | Tandem Score | TDA q-value | Volume | Spectrum Matches |
| --- | --- | --- | --- | --- |
| 14.2499 | 159.4559 | 0.0000 | 5.0075e+07 | 4 |

**Adduct Signal Fractions**

- **Unmodified**: 5.0075e+07

### NVQREN(N)GTVSRY Fuc 2 Hex 5 HexNAc 6

**Mass:**  3744.5650

(5.364e-04)

**Start Position**: 98

**End Position**: 110

| Profile Score | Tandem Score | TDA q-value | Volume | Spectrum Matches |
| --- | --- | --- | --- | --- |
| 0.0000 | 158.6058 | 0.0000 | 0.0000e+00 | 1 |

Adduct:

No Chromatogram Found

### FTPN(N)KTEDTIF Hex 4 HexNAc 7

**Mass:**  3381.4052

(1.012e-06)

**Charge States:** 3, 4

**Start Position**: 69

**End Position**: 80

| Profile Score | Tandem Score | TDA q-value | Volume | Spectrum Matches |
| --- | --- | --- | --- | --- |
| 14.1165 | 158.3684 | 0.0000 | 6.2044e+07 | 4 |

**Adduct Signal Fractions**

- **Unmodified**: 6.2044e+07

### YFTPN(N)KTEDTIF Fuc 1 Hex 4 HexNAc 7

**Mass:**  3690.5253

(6.826e-07)

**Charge States:** 3

**Start Position**: 68

**End Position**: 80

| Profile Score | Tandem Score | TDA q-value | Volume | Spectrum Matches |
| --- | --- | --- | --- | --- |
| 14.0707 | 158.2566 | 0.0000 | 4.5829e+07 | 4 |

**Adduct Signal Fractions**

- **Unmodified**: 4.5829e+07

### FYFTPN(N)KTEDTIF Fuc 1 Hex 5 HexNAc 7

**Mass:**  3999.6513

(1.466e-06)

**Charge States:** 3, 4

**Start Position**: 67

**End Position**: 80

| Profile Score | Tandem Score | TDA q-value | Volume | Spectrum Matches |
| --- | --- | --- | --- | --- |
| 16.6167 | 157.4554 | 0.0000 | 7.0065e+07 | 5 |

**Adduct Signal Fractions**

- **Unmodified**: 7.0065e+07

### YFTPN(N)KTEDTIF Hex 3 HexNAc 5

**Mass:**  2978.2550

(6.726e-04)

**Start Position**: 68

**End Position**: 80

| Profile Score | Tandem Score | TDA q-value | Volume | Spectrum Matches |
| --- | --- | --- | --- | --- |
| 0.0000 | 157.2360 | 0.0000 | 0.0000e+00 | 1 |

Adduct:

No Chromatogram Found

### TPN(N)KTEDTIF Fuc 2 Hex 5 HexNAc 6

**Mass:**  3485.4248

(1.428e-06)

**Charge States:** 3

**Start Position**: 70

**End Position**: 80

| Profile Score | Tandem Score | TDA q-value | Volume | Spectrum Matches |
| --- | --- | --- | --- | --- |
| 11.3558 | 157.0296 | 0.0000 | 7.6605e+06 | 2 |

**Adduct Signal Fractions**

- **Unmodified**: 7.6605e+06

### RNEEYN(N)KSVQEIQATF Fuc 1 Hex 4 HexNAc 5

**Mass:**  3764.6090

(6.483e-07)

**Charge States:** 3, 4

**Start Position**: 51

**End Position**: 67

| Profile Score | Tandem Score | TDA q-value | Volume | Spectrum Matches |
| --- | --- | --- | --- | --- |
| 16.3005 | 156.6666 | 0.0000 | 3.5549e+09 | 14 |

**Adduct Signal Fractions**

- **Unmodified**: 3.5549e+09

### RNEEYN(N)KSVQEIQATFF Fuc 1 Hex 4 HexNAc 5

**Mass:**  3911.6867

(1.170e-06)

**Charge States:** 3, 4

**Start Position**: 51

**End Position**: 68

| Profile Score | Tandem Score | TDA q-value | Volume | Spectrum Matches |
| --- | --- | --- | --- | --- |
| 15.9133 | 156.6615 | 0.0000 | 1.1642e+09 | 12 |

**Adduct Signal Fractions**

- **Unmodified**: 1.1642e+09

### FYFTPN(N)KTEDTIF Fuc 2 Hex 5 HexNAc 6

**Mass:**  3942.6289

(1.562e-06)

**Charge States:** 3, 4

**Start Position**: 67

**End Position**: 80

| Profile Score | Tandem Score | TDA q-value | Volume | Spectrum Matches |
| --- | --- | --- | --- | --- |
| 14.3595 | 156.3812 | 0.0000 | 1.0021e+08 | 5 |

**Adduct Signal Fractions**

- **Unmodified**: 1.0021e+08

### FYFTPN(N)KTEDTIF Hex 3 HexNAc 4

**Mass:**  2920.2443

(7.180e-07)

**Charge States:** 2, 3

**Start Position**: 67

**End Position**: 80

| Profile Score | Tandem Score | TDA q-value | Volume | Spectrum Matches |
| --- | --- | --- | --- | --- |
| 14.9376 | 155.9814 | 0.0000 | 1.7404e+08 | 13 |

**Adduct Signal Fractions**

- **Unmodified**: 1.7404e+08

### EEYN(N)KSVQEIQATF Hex 3 HexNAc 4

**Mass:**  2983.2740

(1.004e-06)

**Charge States:** 3

**Start Position**: 53

**End Position**: 67

| Profile Score | Tandem Score | TDA q-value | Volume | Spectrum Matches |
| --- | --- | --- | --- | --- |
| -3.3733 | 155.8721 | 0.0000 | 4.0179e+07 | 2 |

**Adduct Signal Fractions**

- **Unmodified**: 4.0179e+07

### RN(D)EEYN(N)KSVQEIQATF Hex 3 HexNAc 4

**Mass:**  3254.4010

(3.041e-04)

**Charge States:** 3, 4

**Start Position**: 51

**End Position**: 67

| Profile Score | Tandem Score | TDA q-value | Volume | Spectrum Matches |
| --- | --- | --- | --- | --- |
| 10.4407 | 155.4102 | 0.0000 | 3.0965e+08 | 20 |

**Adduct Signal Fractions**

- **Unmodified**: 3.0965e+08

### RNEEYN(N)KSVQEIQATF Hex 3 HexNAc 4

**Mass:**  3253.4155

(1.423e-06)

**Charge States:** 3, 4

**Start Position**: 51

**End Position**: 67

| Profile Score | Tandem Score | TDA q-value | Volume | Spectrum Matches |
| --- | --- | --- | --- | --- |
| 16.3049 | 155.1749 | 0.0000 | 6.4758e+09 | 25 |

**Adduct Signal Fractions**

- **Unmodified**: 6.4758e+09

### YFTPN(N)KTEDTIF Hex 5 HexNAc 9

**Mass:**  4112.6813

(1.165e-06)

**Charge States:** 3, 4

**Start Position**: 68

**End Position**: 80

| Profile Score | Tandem Score | TDA q-value | Volume | Spectrum Matches |
| --- | --- | --- | --- | --- |
| 9.8745 | 154.7768 | 0.0000 | 4.1416e+07 | 3 |

**Adduct Signal Fractions**

- **Unmodified**: 4.1416e+07

### YFTPN(N)KTEDTIF Hex 4 HexNAc 4 Neu5Ac 2

**Mass:**  3517.4207

(4.402e-07)

**Charge States:** 3, 4

**Start Position**: 68

**End Position**: 80

| Profile Score | Tandem Score | TDA q-value | Volume | Spectrum Matches |
| --- | --- | --- | --- | --- |
| 14.3895 | 154.5119 | 0.0000 | 2.5334e+08 | 8 |

**Adduct Signal Fractions**

- **Unmodified**: 2.5334e+08

### NVQREN(N)GTVSRY Hex 3 HexNAc 6

**Mass:**  3126.3363

(-1.126e-06)

**Charge States:** 3

**Start Position**: 98

**End Position**: 110

| Profile Score | Tandem Score | TDA q-value | Volume | Spectrum Matches |
| --- | --- | --- | --- | --- |
| 13.6977 | 154.4795 | 0.0000 | 1.1640e+09 | 8 |

**Adduct Signal Fractions**

- **Unmodified**: 1.1640e+09

### RNEEYN(N)KSVQEIQATF Hex 3 HexNAc 5

**Mass:**  3456.4971

(1.657e-06)

**Charge States:** 3, 4

**Start Position**: 51

**End Position**: 67

| Profile Score | Tandem Score | TDA q-value | Volume | Spectrum Matches |
| --- | --- | --- | --- | --- |
| 15.8854 | 153.5713 | 0.0000 | 5.2077e+09 | 17 |

**Adduct Signal Fractions**

- **Unmodified**: 5.2077e+09

### YFTPN(N)KTEDTIF Fuc 1 Hex 3 HexNAc 4 Neu5Ac 1

**Mass:**  3210.3506

(7.274e-06)

**Start Position**: 68

**End Position**: 80

| Profile Score | Tandem Score | TDA q-value | Volume | Spectrum Matches |
| --- | --- | --- | --- | --- |
| 0.0000 | 153.4085 | 0.0000 | 0.0000e+00 | 1 |

Adduct:

No Chromatogram Found

### EEYN(N)KSVQEIQATF Hex 3 HexNAc 5

**Mass:**  3186.3532

(1.098e-06)

**Charge States:** 3

**Start Position**: 53

**End Position**: 67

| Profile Score | Tandem Score | TDA q-value | Volume | Spectrum Matches |
| --- | --- | --- | --- | --- |
| 14.2059 | 153.2567 | 0.0000 | 3.5602e+07 | 2 |

**Adduct Signal Fractions**

- **Unmodified**: 3.5602e+07

### RNEEYN(N)KSVQEIQATF Hex 4 HexNAc 5

**Mass:**  3618.5531

(8.739e-07)

**Charge States:** 3, 4

**Start Position**: 51

**End Position**: 67

| Profile Score | Tandem Score | TDA q-value | Volume | Spectrum Matches |
| --- | --- | --- | --- | --- |
| 17.3974 | 153.2472 | 0.0000 | 7.7454e+07 | 3 |

**Adduct Signal Fractions**

- **Unmodified**: 7.7454e+07

### RNEEYN(N)KSVQEIQATF Fuc 1 Hex 3 HexNAc 4

**Mass:**  3399.4733

(6.886e-07)

**Charge States:** 3

**Start Position**: 51

**End Position**: 67

| Profile Score | Tandem Score | TDA q-value | Volume | Spectrum Matches |
| --- | --- | --- | --- | --- |
| 12.7984 | 152.7860 | 0.0000 | 1.2378e+08 | 6 |

**Adduct Signal Fractions**

- **Unmodified**: 1.2378e+08

### RNEEYN(N)KSVQEIQATF Fuc 1 Hex 4 HexNAc 6

**Mass:**  3967.6896

(1.097e-06)

**Charge States:** 3, 4

**Start Position**: 51

**End Position**: 67

| Profile Score | Tandem Score | TDA q-value | Volume | Spectrum Matches |
| --- | --- | --- | --- | --- |
| -3.2671 | 151.9130 | 0.0000 | 1.4661e+08 | 3 |

**Adduct Signal Fractions**

- **Unmodified**: 1.4661e+08

### RNEEYN(N)KSVQEIQATF Fuc 1 Hex 4 HexNAc 4

**Mass:**  3561.5304

(1.491e-06)

**Charge States:** 3, 4

**Start Position**: 51

**End Position**: 67

| Profile Score | Tandem Score | TDA q-value | Volume | Spectrum Matches |
| --- | --- | --- | --- | --- |
| 15.3697 | 151.8008 | 0.0000 | 1.6879e+08 | 3 |

**Adduct Signal Fractions**

- **Unmodified**: 1.6879e+08

### RNEEYN(N)KSVQEIQATF Fuc 2 Hex 5 HexNAc 5

**Mass:**  4072.7206

(1.323e-06)

**Charge States:** 3, 4

**Start Position**: 51

**End Position**: 67

| Profile Score | Tandem Score | TDA q-value | Volume | Spectrum Matches |
| --- | --- | --- | --- | --- |
| 14.2320 | 151.4132 | 0.0000 | 9.4135e+07 | 4 |

**Adduct Signal Fractions**

- **Unmodified**: 9.4135e+07

### YFTPN(N)KTEDTIF Fuc 2 Hex 5 HexNAc 5

**Mass:**  3592.4793

(1.291e-06)

**Charge States:** 3

**Start Position**: 68

**End Position**: 80

| Profile Score | Tandem Score | TDA q-value | Volume | Spectrum Matches |
| --- | --- | --- | --- | --- |
| -5.3213 | 151.1621 | 0.0000 | 1.7479e+07 | 1 |

**Adduct Signal Fractions**

- **Unmodified**: 1.7479e+07

### RNEEYN(N)KSVQEIQATF Hex 3 HexNAc 6

**Mass:**  3659.5786

(1.522e-06)

**Charge States:** 3, 4

**Start Position**: 51

**End Position**: 67

| Profile Score | Tandem Score | TDA q-value | Volume | Spectrum Matches |
| --- | --- | --- | --- | --- |
| 11.9239 | 150.9656 | 0.0000 | 2.9284e+08 | 6 |

**Adduct Signal Fractions**

- **Unmodified**: 2.9284e+08

### RNEEYN(N)KSVQEIQATF Fuc 1 Hex 3 HexNAc 5

**Mass:**  3602.5552

(6.522e-07)

**Charge States:** 3, 4

**Start Position**: 51

**End Position**: 67

| Profile Score | Tandem Score | TDA q-value | Volume | Spectrum Matches |
| --- | --- | --- | --- | --- |
| 13.0862 | 150.9318 | 0.0000 | 1.2190e+08 | 3 |

**Adduct Signal Fractions**

- **Unmodified**: 1.2190e+08

### TPN(N)KTEDTIF Hex 4 HexNAc 6

**Mass:**  3031.2572

(1.235e-06)

**Charge States:** 3

**Start Position**: 70

**End Position**: 80

| Profile Score | Tandem Score | TDA q-value | Volume | Spectrum Matches |
| --- | --- | --- | --- | --- |
| 11.8294 | 150.8280 | 0.0000 | 8.0294e+06 | 2 |

**Adduct Signal Fractions**

- **Unmodified**: 8.0294e+06

### RNEEYN(N)KSVQEIQATF Hex 4 HexNAc 7

**Mass:**  4024.7114

(2.987e-06)

**Charge States:** 3, 4

**Start Position**: 51

**End Position**: 67

| Profile Score | Tandem Score | TDA q-value | Volume | Spectrum Matches |
| --- | --- | --- | --- | --- |
| -4.7807 | 150.5214 | 0.0000 | 8.3870e+07 | 4 |

**Adduct Signal Fractions**

- **Unmodified**: 8.3870e+07

### FTPN(N)KTEDTIF Hex 3 HexNAc 4

**Mass:**  2610.1131

(1.478e-06)

**Charge States:** 2, 3

**Start Position**: 69

**End Position**: 80

| Profile Score | Tandem Score | TDA q-value | Volume | Spectrum Matches |
| --- | --- | --- | --- | --- |
| -3.7443 | 150.2350 | 0.0000 | 3.1535e+07 | 4 |

**Adduct Signal Fractions**

- **Unmodified**: 3.1535e+07

### TPN(N)KTEDTIF Hex 4 HexNAc 4 Neu5Ac 2

**Mass:**  3207.2902

(6.518e-07)

**Charge States:** 3

**Start Position**: 70

**End Position**: 80

| Profile Score | Tandem Score | TDA q-value | Volume | Spectrum Matches |
| --- | --- | --- | --- | --- |
| 13.6269 | 150.0978 | 0.0000 | 1.5336e+07 | 3 |

**Adduct Signal Fractions**

- **Unmodified**: 1.5336e+07

### FYFTPN(N)KTEDTIF Hex 5 HexNAc 8

**Mass:**  4056.6716

(1.508e-06)

**Charge States:** 3, 4

**Start Position**: 67

**End Position**: 80

| Profile Score | Tandem Score | TDA q-value | Volume | Spectrum Matches |
| --- | --- | --- | --- | --- |
| 12.1802 | 148.8063 | 0.0000 | 7.7079e+07 | 6 |

**Adduct Signal Fractions**

- **Unmodified**: 7.7079e+07

### RNEEYN(N)KSVQEIQATFF Fuc 1 Hex 4 HexNAc 4

**Mass:**  3708.6015

(2.090e-06)

**Charge States:** 3, 4

**Start Position**: 51

**End Position**: 68

| Profile Score | Tandem Score | TDA q-value | Volume | Spectrum Matches |
| --- | --- | --- | --- | --- |
| -4.4355 | 148.7022 | 0.0000 | 4.9703e+07 | 2 |

**Adduct Signal Fractions**

- **Unmodified**: 4.9703e+07

### RNEEYN(N)KSVQEIQATFF Hex 3 HexNAc 6

**Mass:**  3806.6464

(1.245e-06)

**Charge States:** 3, 4

**Start Position**: 51

**End Position**: 68

| Profile Score | Tandem Score | TDA q-value | Volume | Spectrum Matches |
| --- | --- | --- | --- | --- |
| 11.8105 | 148.5456 | 0.0000 | 7.4175e+07 | 5 |

**Adduct Signal Fractions**

- **Unmodified**: 7.4175e+07

### RNEEYN(N)KSVQEIQAT Hex 3 HexNAc 4

**Mass:**  3106.3500

(1.211e-06)

**Charge States:** 3

**Start Position**: 51

**End Position**: 66

| Profile Score | Tandem Score | TDA q-value | Volume | Spectrum Matches |
| --- | --- | --- | --- | --- |
| -3.1328 | 148.3899 | 0.0000 | 4.0650e+07 | 2 |

**Adduct Signal Fractions**

- **Unmodified**: 4.0650e+07

### TPN(N)KTEDTIF Fuc 3 Hex 3 HexNAc 4 Neu5Ac 1

**Mass:**  3192.3026

(-1.058e-06)

**Charge States:** 3

**Start Position**: 70

**End Position**: 80

| Profile Score | Tandem Score | TDA q-value | Volume | Spectrum Matches |
| --- | --- | --- | --- | --- |
| -18.9043 | 147.8347 | 0.0000 | 2.8142e+06 | 1 |

**Adduct Signal Fractions**

- **Unmodified**: 2.8142e+06

### YFTPN(N)KTEDTIFL Hex 3 HexNAc 5

**Mass:**  3089.3424

(1.657e-06)

**Charge States:** 3

**Start Position**: 68

**End Position**: 81

| Profile Score | Tandem Score | TDA q-value | Volume | Spectrum Matches |
| --- | --- | --- | --- | --- |
| 14.0106 | 146.3595 | 0.0000 | 1.0382e+07 | 3 |

**Adduct Signal Fractions**

- **Unmodified**: 1.0382e+07

### LNVQREN(N)GTVSRY Hex 3 HexNAc 6

**Mass:**  3239.4237

(9.220e-07)

**Charge States:** 3, 4

**Start Position**: 97

**End Position**: 110

| Profile Score | Tandem Score | TDA q-value | Volume | Spectrum Matches |
| --- | --- | --- | --- | --- |
| 14.3567 | 146.3108 | 0.0000 | 5.5915e+08 | 6 |

**Adduct Signal Fractions**

- **Unmodified**: 5.5915e+08

### FYFTPN(N)KTED Hex 3 HexNAc 6

**Mass:**  2965.2063

(1.757e-06)

**Charge States:** 3

**Start Position**: 67

**End Position**: 77

| Profile Score | Tandem Score | TDA q-value | Volume | Spectrum Matches |
| --- | --- | --- | --- | --- |
| -4.7318 | 144.9300 | 0.0000 | 3.6353e+06 | 1 |

**Adduct Signal Fractions**

- **Unmodified**: 3.6353e+06

### NVQREN(N)GTVSRY Hex 4 HexNAc 7

**Mass:**  3491.4724

(1.273e-06)

**Charge States:** 3

**Start Position**: 98

**End Position**: 110

| Profile Score | Tandem Score | TDA q-value | Volume | Spectrum Matches |
| --- | --- | --- | --- | --- |
| 13.5837 | 144.8075 | 0.0000 | 1.7055e+08 | 5 |

**Adduct Signal Fractions**

- **Unmodified**: 1.7055e+08

### FYFTPN(N)KTEDTIF Hex 4 HexNAc 4 Neu5Ac 2

**Mass:**  3664.4903

(7.204e-07)

**Charge States:** 3, 4

**Start Position**: 67

**End Position**: 80

| Profile Score | Tandem Score | TDA q-value | Volume | Spectrum Matches |
| --- | --- | --- | --- | --- |
| 13.3004 | 144.0927 | 0.0000 | 1.5378e+08 | 9 |

**Adduct Signal Fractions**

- **Unmodified**: 1.5378e+08

### RNEEYN(N)KSVQEIQATF Hex 4 HexNAc 8

**Mass:**  4227.7907

(8.152e-07)

**Charge States:** 3, 4

**Start Position**: 51

**End Position**: 67

| Profile Score | Tandem Score | TDA q-value | Volume | Spectrum Matches |
| --- | --- | --- | --- | --- |
| -3.4169 | 143.8792 | 0.0000 | 3.5887e+07 | 3 |

**Adduct Signal Fractions**

- **Unmodified**: 3.5887e+07

### FTPN(N)KTEDTIF Hex 4 HexNAc 4 Neu5Ac 2

**Mass:**  3354.3587

(8.804e-07)

**Charge States:** 3, 4

**Start Position**: 69

**End Position**: 80

| Profile Score | Tandem Score | TDA q-value | Volume | Spectrum Matches |
| --- | --- | --- | --- | --- |
| -3.5395 | 142.8038 | 0.0000 | 2.2656e+07 | 2 |

**Adduct Signal Fractions**

- **Unmodified**: 2.2656e+07

### RNEEYN(N)KSVQEIQATF Fuc 2 Hex 4 HexNAc 5

**Mass:**  3910.6660

(2.554e-06)

**Charge States:** 3

**Start Position**: 51

**End Position**: 67

| Profile Score | Tandem Score | TDA q-value | Volume | Spectrum Matches |
| --- | --- | --- | --- | --- |
| -4.9466 | 142.7765 | 0.0000 | 4.0989e+07 | 2 |

**Adduct Signal Fractions**

- **Unmodified**: 4.0989e+07

### N(N)KSVQEIQATFF Hex 3 HexNAc 5

**Mass:**  2912.2716

(6.644e-07)

**Charge States:** 3

**Start Position**: 56

**End Position**: 68

| Profile Score | Tandem Score | TDA q-value | Volume | Spectrum Matches |
| --- | --- | --- | --- | --- |
| 13.9539 | 142.6858 | 0.0000 | 6.2695e+06 | 2 |

**Adduct Signal Fractions**

- **Unmodified**: 6.2695e+06

### FYFTPN(N)KTEDTIF Hex 4 HexNAc 8

**Mass:**  3894.6190

(1.521e-06)

**Charge States:** 3, 4

**Start Position**: 67

**End Position**: 80

| Profile Score | Tandem Score | TDA q-value | Volume | Spectrum Matches |
| --- | --- | --- | --- | --- |
| 15.7177 | 142.2970 | 0.0000 | 7.0736e+07 | 5 |

**Adduct Signal Fractions**

- **Unmodified**: 7.0736e+07

### RNEEYN(N)KSVQEIQATF Hex 4 HexNAc 4

**Mass:**  3415.4708

(1.272e-06)

**Charge States:** 3

**Start Position**: 51

**End Position**: 67

| Profile Score | Tandem Score | TDA q-value | Volume | Spectrum Matches |
| --- | --- | --- | --- | --- |
| -20.4880 | 142.2208 | 0.0000 | 2.1547e+07 | 1 |

**Adduct Signal Fractions**

- **Unmodified**: 2.1547e+07

### YN(N)SSYL Hex 3 HexNAc 6

**Mass:**  2449.9656

(1.051e-06)

**Charge States:** 3

**Start Position**: 92

**End Position**: 98

| Profile Score | Tandem Score | TDA q-value | Volume | Spectrum Matches |
| --- | --- | --- | --- | --- |
| -5.4543 | 142.1331 | 0.0000 | 3.1179e+06 | 1 |

**Adduct Signal Fractions**

- **Unmodified**: 3.1179e+06

### NVQREN(N)GTVSRY Fuc 1 Hex 4 HexNAc 5

**Mass:**  3231.3682

(2.973e-07)

**Charge States:** 3

**Start Position**: 98

**End Position**: 110

| Profile Score | Tandem Score | TDA q-value | Volume | Spectrum Matches |
| --- | --- | --- | --- | --- |
| 12.8159 | 141.8843 | 0.0000 | 1.5459e+08 | 2 |

**Adduct Signal Fractions**

- **Unmodified**: 1.5459e+08

### RNEEYN(N)KSVQEIQATFF Fuc 1 Hex 3 HexNAc 4

**Mass:**  3546.5462

(-1.484e-06)

**Charge States:** 3

**Start Position**: 51

**End Position**: 68

| Profile Score | Tandem Score | TDA q-value | Volume | Spectrum Matches |
| --- | --- | --- | --- | --- |
| 11.8837 | 141.3180 | 0.0000 | 3.3775e+07 | 4 |

**Adduct Signal Fractions**

- **Unmodified**: 3.3775e+07

### RN(D)EEYN(N)KSVQEIQATF Fuc 1 Hex 4 HexNAc 5

**Mass:**  3765.5934

(2.629e-04)

**Charge States:** 3, 4

**Start Position**: 51

**End Position**: 67

| Profile Score | Tandem Score | TDA q-value | Volume | Spectrum Matches |
| --- | --- | --- | --- | --- |
| 12.1408 | 141.2106 | 0.0000 | 2.8068e+08 | 11 |

**Adduct Signal Fractions**

- **Unmodified**: 2.8068e+08

### FYFTPN(N)KTEDTIF Fuc 1 Hex 5 HexNAc 4 Neu5Ac 1

**Mass:**  3681.5169

(4.259e-06)

**Charge States:** 3

**Start Position**: 67

**End Position**: 80

| Profile Score | Tandem Score | TDA q-value | Volume | Spectrum Matches |
| --- | --- | --- | --- | --- |
| 13.0426 | 140.8075 | 0.0000 | 2.5728e+07 | 3 |

**Adduct Signal Fractions**

- **Unmodified**: 2.5728e+07

### FYFTPN(N)KTEDTIF Hex 4 HexNAc 6

**Mass:**  3488.4603

(1.885e-06)

**Charge States:** 3

**Start Position**: 67

**End Position**: 80

| Profile Score | Tandem Score | TDA q-value | Volume | Spectrum Matches |
| --- | --- | --- | --- | --- |
| 11.5792 | 140.4916 | 0.0000 | 5.3769e+07 | 7 |

**Adduct Signal Fractions**

- **Unmodified**: 5.3769e+07

### RNEEYN(N)KSVQEIQATFF Fuc 2 Hex 5 HexNAc 5

**Mass:**  4219.7912

(1.759e-06)

**Charge States:** 3, 4

**Start Position**: 51

**End Position**: 68

| Profile Score | Tandem Score | TDA q-value | Volume | Spectrum Matches |
| --- | --- | --- | --- | --- |
| 14.2758 | 140.4879 | 0.0000 | 2.8098e+07 | 4 |

**Adduct Signal Fractions**

- **Unmodified**: 2.8098e+07

### FYFTPN(N)KT Hex 3 HexNAc 6

**Mass:**  2721.1353

(1.382e-06)

**Charge States:** 3

**Start Position**: 67

**End Position**: 75

| Profile Score | Tandem Score | TDA q-value | Volume | Spectrum Matches |
| --- | --- | --- | --- | --- |
| -3.4228 | 139.6192 | 0.0000 | 1.1012e+07 | 1 |

**Adduct Signal Fractions**

- **Unmodified**: 1.1012e+07

### RNEEYN(N)KSVQEIQATFF Fuc 1 Hex 3 HexNAc 5

**Mass:**  3749.6211

(1.645e-06)

**Charge States:** 4

**Start Position**: 51

**End Position**: 68

| Profile Score | Tandem Score | TDA q-value | Volume | Spectrum Matches |
| --- | --- | --- | --- | --- |
| -4.2701 | 139.3584 | 0.0000 | 3.4603e+07 | 2 |

**Adduct Signal Fractions**

- **Unmodified**: 3.4603e+07

### YFTPN(N)KTEDTIF Hex 4 HexNAc 7

**Mass:**  3546.4606

(5.630e-04)

**Start Position**: 68

**End Position**: 80

| Profile Score | Tandem Score | TDA q-value | Volume | Spectrum Matches |
| --- | --- | --- | --- | --- |
| 0.0000 | 136.6640 | 0.0000 | 0.0000e+00 | 1 |

Adduct:

No Chromatogram Found

### RNEEYN(N)KSVQEIQAT Hex 3 HexNAc 5

**Mass:**  3309.4293

(2.148e-06)

**Charge States:** 3

**Start Position**: 51

**End Position**: 66

| Profile Score | Tandem Score | TDA q-value | Volume | Spectrum Matches |
| --- | --- | --- | --- | --- |
| -4.4481 | 136.4326 | 0.0000 | 2.9960e+07 | 5 |

**Adduct Signal Fractions**

- **Unmodified**: 2.9960e+07

### RN(D)EEYN(N)KSVQEIQATF Hex 3 HexNAc 5

**Mass:**  3457.4811

(2.864e-04)

**Charge States:** 3, 4

**Start Position**: 51

**End Position**: 67

| Profile Score | Tandem Score | TDA q-value | Volume | Spectrum Matches |
| --- | --- | --- | --- | --- |
| 10.1888 | 134.9039 | 0.0000 | 5.1920e+08 | 12 |

**Adduct Signal Fractions**

- **Unmodified**: 5.1920e+08

### NVQREN(N)GTVSRY Fuc 3 Hex 6 HexNAc 6

**Mass:**  4050.6666

(-3.148e-07)

**Charge States:** 3

**Start Position**: 98

**End Position**: 110

| Profile Score | Tandem Score | TDA q-value | Volume | Spectrum Matches |
| --- | --- | --- | --- | --- |
| -20.1083 | 134.4868 | 0.0000 | 2.8723e+07 | 2 |

**Adduct Signal Fractions**

- **Unmodified**: 2.8723e+07

### NVQREN(N)GTVSRY Hex 3 HexNAc 5

**Mass:**  2923.2588

(-1.170e-07)

**Charge States:** 3

**Start Position**: 98

**End Position**: 110

| Profile Score | Tandem Score | TDA q-value | Volume | Spectrum Matches |
| --- | --- | --- | --- | --- |
| 12.0685 | 134.3643 | 0.0000 | 2.5725e+08 | 3 |

**Adduct Signal Fractions**

- **Unmodified**: 2.5725e+08

### RNEEYN(N)KSVQEIQATF Fuc 2 Hex 5 HexNAc 6

**Mass:**  4275.8010

(1.287e-06)

**Charge States:** 3, 4

**Start Position**: 51

**End Position**: 67

| Profile Score | Tandem Score | TDA q-value | Volume | Spectrum Matches |
| --- | --- | --- | --- | --- |
| 11.7028 | 132.6749 | 0.0000 | 2.5548e+07 | 3 |

**Adduct Signal Fractions**

- **Unmodified**: 2.5548e+07

### NVQREN(N)GTVSRY Fuc 1 Hex 4 HexNAc 6

**Mass:**  3434.4472

(6.330e-07)

**Charge States:** 3

**Start Position**: 98

**End Position**: 110

| Profile Score | Tandem Score | TDA q-value | Volume | Spectrum Matches |
| --- | --- | --- | --- | --- |
| 12.4303 | 132.4647 | 0.0000 | 6.0751e+08 | 5 |

**Adduct Signal Fractions**

- **Unmodified**: 6.0751e+08

### YFTPN(N)KTEDTIF Hex 5 HexNAc 5

**Mass:**  3303.3718

(9.205e-04)

**Charge States:** 3

**Start Position**: 68

**End Position**: 80

| Profile Score | Tandem Score | TDA q-value | Volume | Spectrum Matches |
| --- | --- | --- | --- | --- |
| -7.4625 | 131.6192 | 0.0000 | 2.3907e+06 | 3 |

**Adduct Signal Fractions**

- **Unmodified**: 2.3907e+06

### TPN(N)KTEDTIF Fuc 4 Hex 5 HexNAc 5

**Mass:**  3574.4419

(-4.697e-06)

**Charge States:** 3

**Start Position**: 70

**End Position**: 80

| Profile Score | Tandem Score | TDA q-value | Volume | Spectrum Matches |
| --- | --- | --- | --- | --- |
| 13.5653 | 131.1868 | 0.0000 | 3.8853e+07 | 3 |

**Adduct Signal Fractions**

- **Unmodified**: 3.8853e+07

### FTPN(N)KTEDTIF Hex 4 HexNAc 6

**Mass:**  3178.3218

(1.252e-06)

**Charge States:** 3

**Start Position**: 69

**End Position**: 80

| Profile Score | Tandem Score | TDA q-value | Volume | Spectrum Matches |
| --- | --- | --- | --- | --- |
| -5.0125 | 128.0242 | 0.0000 | 9.4129e+06 | 2 |

**Adduct Signal Fractions**

- **Unmodified**: 9.4129e+06

### YFTPN(N)KTEDTIF Fuc 1 Hex 5 HexNAc 8

**Mass:**  4055.6614

(1.763e-06)

**Charge States:** 3, 4

**Start Position**: 68

**End Position**: 80

| Profile Score | Tandem Score | TDA q-value | Volume | Spectrum Matches |
| --- | --- | --- | --- | --- |
| 15.2114 | 128.0118 | 0.0000 | 3.6873e+07 | 4 |

**Adduct Signal Fractions**

- **Unmodified**: 3.6873e+07

### LNVQREN(N)GTVSRY Fuc 1 Hex 4 HexNAc 6

**Mass:**  3547.5338

(9.042e-07)

**Charge States:** 3, 4

**Start Position**: 97

**End Position**: 110

| Profile Score | Tandem Score | TDA q-value | Volume | Spectrum Matches |
| --- | --- | --- | --- | --- |
| 13.9393 | 127.8666 | 0.0000 | 3.7710e+08 | 5 |

**Adduct Signal Fractions**

- **Unmodified**: 3.7710e+08

### RNEEYN(N)KSVQEIQATFF Fuc 2 Hex 4 HexNAc 5

**Mass:**  4057.7419

(2.126e-06)

**Charge States:** 3, 4

**Start Position**: 51

**End Position**: 68

| Profile Score | Tandem Score | TDA q-value | Volume | Spectrum Matches |
| --- | --- | --- | --- | --- |
| -4.7163 | 127.7678 | 0.0000 | 1.6191e+07 | 2 |

**Adduct Signal Fractions**

- **Unmodified**: 1.6191e+07

### RNEEYN(N)KSVQEIQAT Fuc 1 Hex 4 HexNAc 5

**Mass:**  3617.5416

(1.525e-06)

**Charge States:** 3

**Start Position**: 51

**End Position**: 66

| Profile Score | Tandem Score | TDA q-value | Volume | Spectrum Matches |
| --- | --- | --- | --- | --- |
| -19.1379 | 127.3018 | 0.0000 | 1.4164e+07 | 2 |

**Adduct Signal Fractions**

- **Unmodified**: 1.4164e+07

### YFTPN(N)KTEDTIF Fuc 1 Hex 5 HexNAc 4 Neu5Ac 2

**Mass:**  3825.5374

(2.360e-06)

**Charge States:** 3, 4

**Start Position**: 68

**End Position**: 80

| Profile Score | Tandem Score | TDA q-value | Volume | Spectrum Matches |
| --- | --- | --- | --- | --- |
| 11.2507 | 127.1535 | 0.0026 | 3.1824e+07 | 2 |

**Adduct Signal Fractions**

- **Unmodified**: 3.1824e+07

### RNEEYN(N)KSVQEIQATFF Hex 4 HexNAc 5

**Mass:**  3765.6212

(1.149e-06)

**Charge States:** 3, 4

**Start Position**: 51

**End Position**: 68

| Profile Score | Tandem Score | TDA q-value | Volume | Spectrum Matches |
| --- | --- | --- | --- | --- |
| -5.2024 | 127.1312 | 0.0026 | 2.6592e+07 | 4 |

**Adduct Signal Fractions**

- **Unmodified**: 2.6592e+07

### RNEEYN(N)KSVQEIQATF Fuc 1 Hex 5 HexNAc 7

**Mass:**  4332.8215

(1.205e-06)

**Charge States:** 3

**Start Position**: 51

**End Position**: 67

| Profile Score | Tandem Score | TDA q-value | Volume | Spectrum Matches |
| --- | --- | --- | --- | --- |
| -4.1033 | 126.4582 | 0.0026 | 1.4014e+07 | 1 |

**Adduct Signal Fractions**

- **Unmodified**: 1.4014e+07

### N(N)KSVQEIQATFF Hex 3 HexNAc 4

**Mass:**  2709.1931

(4.707e-07)

**Charge States:** 3

**Start Position**: 56

**End Position**: 68

| Profile Score | Tandem Score | TDA q-value | Volume | Spectrum Matches |
| --- | --- | --- | --- | --- |
| -4.7297 | 125.2615 | 0.0050 | 4.5867e+06 | 2 |

**Adduct Signal Fractions**

- **Unmodified**: 4.5867e+06

### LNVQREN(N)GTVSRY Fuc 1 Hex 4 HexNAc 6

**Mass:**  3549.5443

(5.676e-04)

**Start Position**: 97

**End Position**: 110

| Profile Score | Tandem Score | TDA q-value | Volume | Spectrum Matches |
| --- | --- | --- | --- | --- |
| 0.0000 | 125.1245 | 0.0050 | 0.0000e+00 | 1 |

Adduct:

No Chromatogram Found

### YFTPN(N)KT Hex 3 HexNAc 6

**Mass:**  2574.0666

(1.420e-06)

**Charge States:** 3

**Start Position**: 68

**End Position**: 75

| Profile Score | Tandem Score | TDA q-value | Volume | Spectrum Matches |
| --- | --- | --- | --- | --- |
| -21.1643 | 124.3038 | 0.0000 | 4.7148e+06 | 1 |

**Adduct Signal Fractions**

- **Unmodified**: 4.7148e+06

### TPN(N)KTEDTIFL Hex 3 HexNAc 6

**Mass:**  2982.2856

(1.648e-07)

**Charge States:** 3

**Start Position**: 70

**End Position**: 81

| Profile Score | Tandem Score | TDA q-value | Volume | Spectrum Matches |
| --- | --- | --- | --- | --- |
| -19.7015 | 123.8074 | 0.0050 | 1.6234e+06 | 1 |

**Adduct Signal Fractions**

- **Unmodified**: 1.6234e+06

### FYFTPN(N)KTEDTIF Fuc 1 Hex 5 HexNAc 8

**Mass:**  4202.7302

(1.604e-06)

**Charge States:** 3, 4

**Start Position**: 67

**End Position**: 80

| Profile Score | Tandem Score | TDA q-value | Volume | Spectrum Matches |
| --- | --- | --- | --- | --- |
| 12.9433 | 122.3904 | 0.0067 | 2.0317e+07 | 4 |

**Adduct Signal Fractions**

- **Unmodified**: 2.0317e+07

### FYFTPN(N)KT Hex 3 HexNAc 5

**Mass:**  2518.0539

(7.184e-07)

**Charge States:** 3

**Start Position**: 67

**End Position**: 75

| Profile Score | Tandem Score | TDA q-value | Volume | Spectrum Matches |
| --- | --- | --- | --- | --- |
| -18.7700 | 122.3752 | 0.0000 | 5.0063e+06 | 1 |

**Adduct Signal Fractions**

- **Unmodified**: 5.0063e+06

### RNEEYN(N)KSVQEIQ Hex 3 HexNAc 4

**Mass:**  2934.2653

(1.392e-06)

**Charge States:** 3

**Start Position**: 51

**End Position**: 64

| Profile Score | Tandem Score | TDA q-value | Volume | Spectrum Matches |
| --- | --- | --- | --- | --- |
| -20.2358 | 122.3085 | 0.0067 | 2.8905e+06 | 1 |

**Adduct Signal Fractions**

- **Unmodified**: 2.8905e+06

### LNVQREN(N)GTVSRY Hex 4 HexNAc 7

**Mass:**  3604.5540

(1.548e-06)

**Charge States:** 3, 4

**Start Position**: 97

**End Position**: 110

| Profile Score | Tandem Score | TDA q-value | Volume | Spectrum Matches |
| --- | --- | --- | --- | --- |
| -4.4335 | 122.1193 | 0.0067 | 5.9377e+07 | 3 |

**Adduct Signal Fractions**

- **Unmodified**: 5.9377e+07

### LNVQREN(N)GTVSRY Fuc 1 Hex 4 HexNAc 5

**Mass:**  3344.4556

(9.137e-07)

**Charge States:** 3

**Start Position**: 97

**End Position**: 110

| Profile Score | Tandem Score | TDA q-value | Volume | Spectrum Matches |
| --- | --- | --- | --- | --- |
| 12.8687 | 121.7168 | 0.0067 | 8.6807e+07 | 3 |

**Adduct Signal Fractions**

- **Unmodified**: 8.6807e+07

### LNVQREN(N)GTVSRY Hex 3 HexNAc 5

**Mass:**  3036.3424

(1.191e-06)

**Charge States:** 3

**Start Position**: 97

**End Position**: 110

| Profile Score | Tandem Score | TDA q-value | Volume | Spectrum Matches |
| --- | --- | --- | --- | --- |
| 14.2336 | 121.7038 | 0.0067 | 1.1998e+08 | 4 |

**Adduct Signal Fractions**

- **Unmodified**: 1.1998e+08

### NVQREN(N)GTVSRY Hex 4 HexNAc 8

**Mass:**  3694.5496

(5.537e-07)

**Charge States:** 3, 4

**Start Position**: 98

**End Position**: 110

| Profile Score | Tandem Score | TDA q-value | Volume | Spectrum Matches |
| --- | --- | --- | --- | --- |
| -19.0281 | 121.1062 | 0.0067 | 1.0103e+08 | 2 |

**Adduct Signal Fractions**

- **Unmodified**: 1.0103e+08

### NVQREN(N)GTVSRY Fuc 2 Hex 5 HexNAc 6

**Mass:**  3742.5613

(-7.980e-07)

**Charge States:** 3, 4

**Start Position**: 98

**End Position**: 110

| Profile Score | Tandem Score | TDA q-value | Volume | Spectrum Matches |
| --- | --- | --- | --- | --- |
| -18.1126 | 120.0460 | 0.0067 | 1.7292e+08 | 2 |

**Adduct Signal Fractions**

- **Unmodified**: 1.7292e+08

### FTPN(N)KTEDTIF Fuc 2 Hex 5 HexNAc 6

**Mass:**  3632.4951

(1.239e-06)

**Charge States:** 3, 4

**Start Position**: 69

**End Position**: 80

| Profile Score | Tandem Score | TDA q-value | Volume | Spectrum Matches |
| --- | --- | --- | --- | --- |
| -4.2565 | 118.9238 | 0.0067 | 1.2483e+07 | 3 |

**Adduct Signal Fractions**

- **Unmodified**: 1.2483e+07

### FYFTPN(N)KTEDTIF Fuc 2 Hex 5 HexNAc 7

**Mass:**  4145.7092

(1.866e-06)

**Charge States:** 3, 4

**Start Position**: 67

**End Position**: 80

| Profile Score | Tandem Score | TDA q-value | Volume | Spectrum Matches |
| --- | --- | --- | --- | --- |
| 11.6597 | 118.1211 | 0.0087 | 1.3455e+07 | 4 |

**Adduct Signal Fractions**

- **Unmodified**: 1.3455e+07

### RNEEYN(N)KSVQEIQATF Hex 4 HexNAc 6

**Mass:**  3821.6317

(1.513e-06)

**Charge States:** 3, 4

**Start Position**: 51

**End Position**: 67

| Profile Score | Tandem Score | TDA q-value | Volume | Spectrum Matches |
| --- | --- | --- | --- | --- |
| 14.6260 | 117.1512 | 0.0105 | 5.6186e+08 | 9 |

**Adduct Signal Fractions**

- **Unmodified**: 5.6186e+08

### FTPN(N)KTEDTIF Hex 4 HexNAc 8

**Mass:**  3584.4842

(1.997e-06)

**Charge States:** 3, 4

**Start Position**: 69

**End Position**: 80

| Profile Score | Tandem Score | TDA q-value | Volume | Spectrum Matches |
| --- | --- | --- | --- | --- |
| -4.7585 | 116.9429 | 0.0105 | 7.5378e+06 | 4 |

**Adduct Signal Fractions**

- **Unmodified**: 7.5378e+06

### LNVQREN(N)GTVSRY Fuc 2 Hex 5 HexNAc 6

**Mass:**  3855.6439

(8.925e-07)

**Charge States:** 3, 4

**Start Position**: 97

**End Position**: 110

| Profile Score | Tandem Score | TDA q-value | Volume | Spectrum Matches |
| --- | --- | --- | --- | --- |
| -4.3068 | 116.9418 | 0.0105 | 9.4747e+07 | 4 |

**Adduct Signal Fractions**

- **Unmodified**: 9.4747e+07

### NVQREN(N)GTVSRY Fuc 1 Hex 5 HexNAc 8

**Mass:**  4002.6640

(1.650e-06)

**Charge States:** 3

**Start Position**: 98

**End Position**: 110

| Profile Score | Tandem Score | TDA q-value | Volume | Spectrum Matches |
| --- | --- | --- | --- | --- |
| -19.8735 | 114.6185 | 0.0124 | 3.9663e+07 | 1 |

**Adduct Signal Fractions**

- **Unmodified**: 3.9663e+07

### TPN(N)KTEDTIF Hex 4 HexNAc 8

**Mass:**  3437.4201

(2.146e-06)

**Charge States:** 3

**Start Position**: 70

**End Position**: 80

| Profile Score | Tandem Score | TDA q-value | Volume | Spectrum Matches |
| --- | --- | --- | --- | --- |
| -5.2528 | 114.6114 | 0.0124 | 3.7413e+06 | 2 |

**Adduct Signal Fractions**

- **Unmodified**: 3.7413e+06

### YFTPN(N)KTEDTIF Fuc 1 Hex 5 HexNAc 3 Neu5Ac 1

**Mass:**  3331.3672

(4.111e-06)

**Charge States:** 3

**Start Position**: 68

**End Position**: 80

| Profile Score | Tandem Score | TDA q-value | Volume | Spectrum Matches |
| --- | --- | --- | --- | --- |
| -5.7385 | 113.5567 | 0.0141 | 3.2549e+06 | 1 |

**Adduct Signal Fractions**

- **Unmodified**: 3.2549e+06

### VQREN(N)GTVSRY Hex 3 HexNAc 6

**Mass:**  3012.2968

(1.254e-06)

**Charge States:** 3

**Start Position**: 99

**End Position**: 110

| Profile Score | Tandem Score | TDA q-value | Volume | Spectrum Matches |
| --- | --- | --- | --- | --- |
| -18.3569 | 112.3084 | 0.0141 | 1.8265e+07 | 1 |

**Adduct Signal Fractions**

- **Unmodified**: 1.8265e+07

### VQREN(N)GTVSRY Fuc 1 Hex 4 HexNAc 6

**Mass:**  3321.4087

(3.027e-04)

**Start Position**: 99

**End Position**: 110

| Profile Score | Tandem Score | TDA q-value | Volume | Spectrum Matches |
| --- | --- | --- | --- | --- |
| 0.0000 | 112.1221 | 0.0141 | 0.0000e+00 | 1 |

Adduct:

No Chromatogram Found

### RNEEYN(N)KSVQEIQATFF Hex 6 HexNAc 5

**Mass:**  4089.7285

(6.495e-07)

**Charge States:** 3, 4

**Start Position**: 51

**End Position**: 68

| Profile Score | Tandem Score | TDA q-value | Volume | Spectrum Matches |
| --- | --- | --- | --- | --- |
| -18.3561 | 111.0300 | 0.0159 | 7.9823e+06 | 2 |

**Adduct Signal Fractions**

- **Unmodified**: 7.9823e+06

### FTPN(N)KTEDTIF Fuc 1 Hex 5 HexNAc 4 Neu5Ac 1

**Mass:**  3371.3845

(4.649e-06)

**Charge States:** 3

**Start Position**: 69

**End Position**: 80

| Profile Score | Tandem Score | TDA q-value | Volume | Spectrum Matches |
| --- | --- | --- | --- | --- |
| -19.9719 | 107.9555 | 0.0248 | 3.3963e+06 | 1 |

**Adduct Signal Fractions**

- **Unmodified**: 3.3963e+06

### RNEEYN(N)KSVQEIQATF Fuc 1 Hex 5 HexNAc 8

**Mass:**  4536.9123

(2.204e-04)

**Charge States:** 4

**Start Position**: 51

**End Position**: 67

| Profile Score | Tandem Score | TDA q-value | Volume | Spectrum Matches |
| --- | --- | --- | --- | --- |
| -21.1623 | 107.4370 | 0.0265 | 4.0880e+05 | 2 |

**Adduct Signal Fractions**

- **Unmodified**: 4.0880e+05

### LNVQREN(N)GTVSRY Hex 4 HexNAc 8

**Mass:**  3808.6303

(2.623e-04)

**Start Position**: 97

**End Position**: 110

| Profile Score | Tandem Score | TDA q-value | Volume | Spectrum Matches |
| --- | --- | --- | --- | --- |
| 0.0000 | 107.4247 | 0.0265 | 0.0000e+00 | 1 |

Adduct:

No Chromatogram Found

### NVQREN(N)GTVSRY Fuc 1 Hex 5 HexNAc 7

**Mass:**  3799.5803

(4.289e-07)

**Charge States:** 3

**Start Position**: 98

**End Position**: 110

| Profile Score | Tandem Score | TDA q-value | Volume | Spectrum Matches |
| --- | --- | --- | --- | --- |
| -18.3971 | 105.1612 | 0.0276 | 1.5722e+07 | 2 |

**Adduct Signal Fractions**

- **Unmodified**: 1.5722e+07

### NVQREN(N)GTVSRY Fuc 2 Hex 6 HexNAc 8

**Mass:**  4310.7722

(7.287e-07)

**Charge States:** 4

**Start Position**: 98

**End Position**: 110

| Profile Score | Tandem Score | TDA q-value | Volume | Spectrum Matches |
| --- | --- | --- | --- | --- |
| -20.1947 | 104.1056 | 0.0307 | 1.0384e+07 | 1 |

**Adduct Signal Fractions**

- **Unmodified**: 1.0384e+07

### C(C)ANLVPVPITN(N)ATL Hex 3 HexNAc 5

**Mass:**  2983.3498

(1.074e-06)

**Charge States:** 3

**Start Position**: 23

**End Position**: 37

| Profile Score | Tandem Score | TDA q-value | Volume | Spectrum Matches |
| --- | --- | --- | --- | --- |
| 14.3961 | 103.8660 | 0.0307 | 2.3144e+07 | 5 |

**Adduct Signal Fractions**

- **Unmodified**: 2.3144e+07

### RN(D)EEYN(N)KSVQEIQATFF Hex 3 HexNAc 4

**Mass:**  3401.4702

(2.905e-04)

**Charge States:** 3

**Start Position**: 51

**End Position**: 68

| Profile Score | Tandem Score | TDA q-value | Volume | Spectrum Matches |
| --- | --- | --- | --- | --- |
| -5.3982 | 102.8776 | 0.0307 | 4.8906e+07 | 11 |

**Adduct Signal Fractions**

- **Unmodified**: 4.8906e+07

### NVQREN(N)GTVSRY Fuc 1 Hex 4 HexNAc 7

**Mass:**  3637.5269

(2.303e-07)

**Charge States:** 3

**Start Position**: 98

**End Position**: 110

| Profile Score | Tandem Score | TDA q-value | Volume | Spectrum Matches |
| --- | --- | --- | --- | --- |
| -21.0544 | 100.8481 | 0.0336 | 8.1439e+06 | 2 |

**Adduct Signal Fractions**

- **Unmodified**: 8.1439e+06

### N(D)VQREN(N)GTVSRY Fuc 1 Hex 5 HexNAc 8

**Mass:**  4004.6674

(5.020e-04)

**Start Position**: 98

**End Position**: 110

| Profile Score | Tandem Score | TDA q-value | Volume | Spectrum Matches |
| --- | --- | --- | --- | --- |
| 0.0000 | 100.4128 | 0.0342 | 0.0000e+00 | 1 |

Adduct:

No Chromatogram Found

### FYFTPN(N)KTEDTIF Fuc 1 Hex 5 HexNAc 4 Neu5Ac 2

**Mass:**  3972.6011

(9.906e-07)

**Charge States:** 3

**Start Position**: 67

**End Position**: 80

| Profile Score | Tandem Score | TDA q-value | Volume | Spectrum Matches |
| --- | --- | --- | --- | --- |
| -20.0742 | 99.6434 | 0.0342 | 1.3535e+07 | 2 |

**Adduct Signal Fractions**

- **Unmodified**: 1.3535e+07

### N(D)VQREN(N)GTVSRY Fuc 2 Hex 5 HexNAc 6

**Mass:**  3744.5815

(5.408e-04)

**Start Position**: 98

**End Position**: 110

| Profile Score | Tandem Score | TDA q-value | Volume | Spectrum Matches |
| --- | --- | --- | --- | --- |
| 0.0000 | 99.2902 | 0.0342 | 0.0000e+00 | 1 |

Adduct:

No Chromatogram Found

### TN(N)ATLDRITGKW Hex 3 HexNAc 5

**Mass:**  2876.2860

(9.711e-07)

**Charge States:** 3

**Start Position**: 32

**End Position**: 44

| Profile Score | Tandem Score | TDA q-value | Volume | Spectrum Matches |
| --- | --- | --- | --- | --- |
| -4.3150 | 96.5602 | 0.0422 | 5.1981e+06 | 2 |

**Adduct Signal Fractions**

- **Unmodified**: 5.1981e+06

### NVQREN(N)GTVSRY Hex 4 HexNAc 4 Neu5Ac 2

**Mass:**  3464.4237

(-4.930e-07)

**Charge States:** 3

**Start Position**: 98

**End Position**: 110

| Profile Score | Tandem Score | TDA q-value | Volume | Spectrum Matches |
| --- | --- | --- | --- | --- |
| -19.6442 | 94.1290 | 0.0446 | 2.9234e+07 | 1 |

**Adduct Signal Fractions**

- **Unmodified**: 2.9234e+07

### NVQREN(N)GTVSRY Fuc 1 Hex 5 HexNAc 8

**Mass:**  4003.6709

(2.530e-04)

**Start Position**: 98

**End Position**: 110

| Profile Score | Tandem Score | TDA q-value | Volume | Spectrum Matches |
| --- | --- | --- | --- | --- |
| 0.0000 | 93.9939 | 0.0458 | 0.0000e+00 | 1 |

Adduct:

No Chromatogram Found

### NVQREN(N)GTVSRY Hex 4 HexNAc 8

**Mass:**  3695.5699

(2.767e-04)

**Start Position**: 98

**End Position**: 110

| Profile Score | Tandem Score | TDA q-value | Volume | Spectrum Matches |
| --- | --- | --- | --- | --- |
| 0.0000 | 93.9390 | 0.0458 | 0.0000e+00 | 1 |

Adduct:

No Chromatogram Found

### NVQREN(N)GTVSRY Fuc 3 Hex 5 HexNAc 4 Neu5Ac 1

**Mass:**  3773.5396

(-3.264e-06)

**Start Position**: 98

**End Position**: 110

| Profile Score | Tandem Score | TDA q-value | Volume | Spectrum Matches |
| --- | --- | --- | --- | --- |
| 0.0000 | 93.9054 | 0.0458 | 0.0000e+00 | 1 |

Adduct:

No Chromatogram Found

### LNVQREN(N)GTVSRY Fuc 3 Hex 6 HexNAc 6

**Mass:**  4163.7590

(-3.540e-07)

**Charge States:** 4

**Start Position**: 97

**End Position**: 110

| Profile Score | Tandem Score | TDA q-value | Volume | Spectrum Matches |
| --- | --- | --- | --- | --- |
| -21.1534 | 93.3486 | 0.0475 | 1.6118e+07 | 2 |

**Adduct Signal Fractions**

- **Unmodified**: 1.6118e+07

### P00738|HPT\_HUMAN

**>P00738|HPT\_HUMAN**  
 `MSALGAVIALLLWGQLFAVDSGNDVTDIADDGCPKPPEIAHGYVEHSVRYQCKNYYKLRTEGDGVYTLNDKKQWINKAVG  
DKLPECEADDGCPKPPEIAHGYVEHSVRYQCKNYYKLRTEGDGVYTLNNEKQWINKAVGDKLPECEAVCGKPKNPANPVQ  
RILGGHLDAKGSFPWQAKMVSHHNLTTGATLINEQWLLTTAKNLFLNHSENATAKDIAPTLTLYVGKKQLVEIEKVVLHP  
NYSQVDIGLIKLKQKVSVNERVMPICLPSKDYAEVGRVGYVSGWGRNANFKFTDHLKYVMLPVADQDQCIRHYEGSTVPE  
KKTPKSPVGVQPILNEHTFCAGMSKYQEDTCYGDAGSAFAVHDLEEDTWYATGILSFDKSCAVAEYGVYVKVTSIQDWVQ  
KTIAEN`

| Site | Type | Count |
| --- | --- | --- |
| 184 | N-linked | 2 |

| Mass | Sequence | MS_2_ Score | `q`-value | MS_1_ Score | Signal | Protein Coordinates | Tandem Scans | Start Time | Apex Time | End Time |
| --- | --- | --- | --- | --- | --- | --- | --- | --- | --- | --- |
| 2548.1196 | VSHHN(N)LTTGATL Hex 3 HexNAc 4 | 165.663 | 0.0 | -3.039 | 2.609e+07 | 179 - 191 | 2 | 17.70 | 17.87 | 18.04 |
| 2751.1997 | VSHHN(N)LTTGATL Hex 3 HexNAc 5 | 163.882 | 0.0 | -18.602 | 1.270e+07 | 179 - 191 | 1 | 17.70 | 17.70 | 17.87 |

### VSHHN(N)LTTGATL Hex 3 HexNAc 4

**Mass:**  2548.1191

(1.199e-06)

**Charge States:** 3

**Start Position**: 180

**End Position**: 192

| Profile Score | Tandem Score | TDA q-value | Volume | Spectrum Matches |
| --- | --- | --- | --- | --- |
| -3.0388 | 165.6625 | 0.0000 | 2.6087e+07 | 2 |

**Adduct Signal Fractions**

- **Unmodified**: 2.6087e+07

### VSHHN(N)LTTGATL Hex 3 HexNAc 5

**Mass:**  2751.1995

(9.581e-07)

**Charge States:** 3

**Start Position**: 180

**End Position**: 192

| Profile Score | Tandem Score | TDA q-value | Volume | Spectrum Matches |
| --- | --- | --- | --- | --- |
| -18.6020 | 163.8822 | 0.0000 | 1.2702e+07 | 1 |

**Adduct Signal Fractions**

- **Unmodified**: 1.2702e+07

### P13473|LAMP2\_HUMAN

**>P13473|LAMP2\_HUMAN**  
 `MVCFRLFPVPGSGLVLVCLVLGAVRSYALELNLTDSENATCLYAKWQMNFTVRYETTNKTYKTVTISDHGTVTYNGSICG  
DDQNGPKIAVQFGPGFSWIANFTKAASTYSIDSVSFSYNTGDNTTFPDAEDKGILTVDELLAIRIPLNDLFRCNSLSTLE  
KNDVVQHYWDVLVQAFVQNGTVSTNEFLCDKDKTSTVAPTIHTTVPSPTTTPTPKEKPEAGTYSVNNGNDTCLLATMGLQ  
LNITQDKVASVININPNTTHSTGSCRSHTALLRLNSSTIKYLDFVFAVKNENRFYLKEVNISMYLVNGSVFSIANNNLSY  
WDAPLGSSYMCNKEQTVSVSGAFQINTFDLRVQPFNVTQGKYSTAQDCSADDDNFLVPIAVGAALAGVLILVLLAYFIGL  
KHHHAGYEQF`

| Site | Type | Count |
| --- | --- | --- |
| 307 | N-linked | 1 |

| Mass | Sequence | MS_2_ Score | `q`-value | MS_1_ Score | Signal | Protein Coordinates | Tandem Scans | Start Time | Apex Time | End Time |
| --- | --- | --- | --- | --- | --- | --- | --- | --- | --- | --- |
| 2235.9522 | LVN(N)GSVF Hex 3 HexNAc 5 | 144.109 | 0.0 | -19.781 | 2.325e+06 | 304 - 311 | 2 | 30.83 | 30.83 | 31.00 |

### LVN(N)GSVF Hex 3 HexNAc 5

**Mass:**  2235.9519

(7.914e-07)

**Charge States:** 3

**Start Position**: 305

**End Position**: 312

| Profile Score | Tandem Score | TDA q-value | Volume | Spectrum Matches |
| --- | --- | --- | --- | --- |
| -19.7807 | 144.1095 | 0.0000 | 2.3249e+06 | 2 |

**Adduct Signal Fractions**

- **Unmodified**: 2.3249e+06

### P14745|CD44\_PAPHA

**>P14745|CD44\_PAPHA**  
 `MDKFWWRAAWGLCLVQLSLAQIDLNITCRFEGIYHVEKNGRYSISRTEAADLCKAFNSTLPTMAQMEKALSIGFETCRYG  
FIEGHVVIPRIHPNSICAANNTGVYILTSNTSQYDTYCFNASAPPGEDCTSVTDLPNAFDGPITITIVNRDGTRYVKKGE  
YRTNPEDINPSSPTDDDVSSGSSSERSSTLGGYIFYNHFSTSPPIPDEDGPWITDSTDRTPATRDQGAFDPSGGSHTTHG  
SESAGHSHGSREGGANTTSGPLRTPQIPEWLIILASLLALALILAVCIAVNSRRRCGQKKKLVINNGNGAVEDRKSSGLN  
GEASKSQEMVHLVNKESSETPDQFMTADETRNLQNVDMKIGV`

| Site | Type | Count |
| --- | --- | --- |
| 25 | N-linked | 1 |

| Mass | Sequence | MS_2_ Score | `q`-value | MS_1_ Score | Signal | Protein Coordinates | Tandem Scans | Start Time | Apex Time | End Time |
| --- | --- | --- | --- | --- | --- | --- | --- | --- | --- | --- |
| 3907.6101 | QIDLN(N)ITC(C)RF Fuc 3 Hex 6 HexNAc 6 | 93.197 | 0.048 | -4.904 | 7.955e+07 | 20 - 30 | 2 | 18.71 | 18.88 | 19.04 |

### QIDLN(N)ITC(C)RF Fuc 3 Hex 6 HexNAc 6

**Mass:**  3907.6086

(1.073e-06)

**Charge States:** 4, 5

**Start Position**: 21

**End Position**: 31

| Profile Score | Tandem Score | TDA q-value | Volume | Spectrum Matches |
| --- | --- | --- | --- | --- |
| -4.9040 | 93.1972 | 0.0483 | 7.9547e+07 | 2 |

**Adduct Signal Fractions**

- **Unmodified**: 7.9547e+07

### P16070|CD44\_HUMAN

**>P16070|CD44\_HUMAN**  
 `MDKFWWHAAWGLCLVPLSLAQIDLNITCRFAGVFHVEKNGRYSISRTEAADLCKAFNSTLPTMAQMEKALSIGFETCRYG  
FIEGHVVIPRIHPNSICAANNTGVYILTSNTSQYDTYCFNASAPPEEDCTSVTDLPNAFDGPITITIVNRDGTRYVQKGE  
YRTNPEDIYPSNPTDDDVSSGSSSERSSTSGGYIFYTFSTVHPIPDEDSPWITDSTDRIPATTLMSTSATATETATKRQE  
TWDWFSWLFLPSESKNHLHTTTQMAGTSSNTISAGWEPNEENEDERDRHLSFSGSGIDDDEDFISSTISTTPRAFDHTKQ  
NQDWTQWNPSHSNPEVLLQTTTRMTDVDRNGTTAYEGNWNPEAHPPLIHHEHHEEEETPHSTSTIQATPSSTTEETATQK  
EQWFGNRWHEGYRQTPKEDSHSTTGTAAASAHTSHPMQGRTTPSPEDSSWTDFFNPISHPMGRGHQAGRRMDMDSSHSIT  
LQPTANPNTGLVEDLDRTGPLSMTTQQSNSQSFSTSHEGLEEDKDHPTTSTLTSSNRNDVTGGRRDPNHSEGSTTLLEGY  
TSHYPHTKESRTFIPVTSAKTGSFGVTAVTVGDSNSNVNRSLSGDQDTFHPSGGSHTTHGSESDGHSHGSQEGGANTTSG  
PIRTPQIPEWLIILASLLALALILAVCIAVNSRRRCGQKKKLVINSGNGAVEDRKPSGLNGEASKSQEMVHLVNKESSET  
PDQFMTADETRNLQNVDMKIGV`

| Site | Type | Count |
| --- | --- | --- |
| 25 | N-linked | 1 |

| Mass | Sequence | MS_2_ Score | `q`-value | MS_1_ Score | Signal | Protein Coordinates | Tandem Scans | Start Time | Apex Time | End Time |
| --- | --- | --- | --- | --- | --- | --- | --- | --- | --- | --- |
| 3907.6101 | QIDLN(N)ITC(C)RF Fuc 3 Hex 6 HexNAc 6 | 93.197 | 0.048 | -4.904 | 7.955e+07 | 20 - 30 | 2 | 18.71 | 18.88 | 19.04 |

### QIDLN(N)ITC(C)RF Fuc 3 Hex 6 HexNAc 6

**Mass:**  3907.6086

(1.073e-06)

**Charge States:** 4, 5

**Start Position**: 21

**End Position**: 31

| Profile Score | Tandem Score | TDA q-value | Volume | Spectrum Matches |
| --- | --- | --- | --- | --- |
| -4.9040 | 93.1972 | 0.0483 | 7.9547e+07 | 2 |

**Adduct Signal Fractions**

- **Unmodified**: 7.9547e+07
