# Supplementary material for: Measuring Site-specific Glycosylation Similarity between Influenza a Virus Variants with Statistical Certainty
Source: Mol Cell Proteomics. 2020 Nov 25;19(9):1533–45. doi: 10.1074/mcp.RA120.002031 (PMC8143645; doi:10.1074/mcp.RA120.002031)
Supplement: Supplementary file 1 [file mmc1.zip › mmc1/159250_2_supp_555287_qcjklk.zip!/20190326_aAGP_021119_tryp_HILIC_DDA_1ug_03_glycopeptides.html]

## 20190326\_aAGP\_021119\_tryp\_HILIC\_DDA\_1ug\_03 @ GlycopeptideHypothesis-1e2c1a7fd9654d69bd41ffd619161ab3

**MS^1^ Mass Accuracy:** 10.0 PPM  
**Grouping Tolerance:** 15.0 PPM  
**MS^n^ Mass Accuracy:** 20.0 PPM  
**Minimum `q`-Value For Clustering:** 0.05

**Glycopeptide Hypothesis:**
GlycopeptideHypothesis-1e2c1a7fd9654d69bd41ffd619161ab3  
**Sample Searched:**
20190326\_aAGP\_021119\_tryp\_HILIC\_DDA\_1ug\_03

| Protein | Matched Glycopeptides |
| --- | --- |
| P02763\|A1AG1\_HUMAN | 177 |
| P19652\|A1AG2\_HUMAN | 170 |
| P00738\|HPT\_HUMAN | 8 |
| Q9Y5Y7\|LYVE1\_HUMAN | 8 |
| P05155\|IC1\_HUMAN | 7 |
| Q12913\|PTPRJ\_HUMAN | 4 |
| O95497\|VNN1\_HUMAN | 4 |
| P02790\|HEMO\_HUMAN | 4 |
| Q5R543\|HEMO\_PONAB | 4 |
| P11279\|LAMP1\_HUMAN | 3 |
| O75144\|ICOSL\_HUMAN | 3 |

### P02763|A1AG1\_HUMAN

**>P02763|A1AG1\_HUMAN**  
 `MALSWVLTVLSLLPLLEAQIPLCANLVPVPITNATLDQITGKWFYIASAFRNEEYNKSVQEIQATFFYFTPNKTEDTIFL  
REYQTRQDQCIYNTTYLNVQRENGTISRYVGGQEHFAHLLILRDTKTYMLAFDVNDEKNWGLSVYADKPETTKEQLGEFY  
EALDCLRIPKSDVVYTDWKKDKCEPLEKQHEKERKQEEGES`

| Site | Type | Count |
| --- | --- | --- |
| 33 | N-linked | 57 |
| 56 | N-linked | 2 |
| 93 | N-linked | 31 |
| 72 | N-linked | 87 |

| Mass | Sequence | MS_2_ Score | `q`-value | MS_1_ Score | Signal | Protein Coordinates | Tandem Scans | Start Time | Apex Time | End Time |
| --- | --- | --- | --- | --- | --- | --- | --- | --- | --- | --- |
| 2390.9659 | YFTPN(N)K Hex 5 HexNAc 4 | 188.015 | 0.0 | -5.902 | 2.572e+07 | 67 - 73 | 3 | 14.08 | 14.08 | 14.40 |
| 2757.0946 | YFTPN(N)K Hex 6 HexNAc 5 | 187.448 | 0.0 | 0.000 | 0.000e+00 | 67 - 73 | 1 | - | - | - |
| 2756.0978 | YFTPN(N)K Hex 6 HexNAc 5 | 185.448 | 0.0 | 14.112 | 3.130e+08 | 67 - 73 | 5 | 13.79 | 14.08 | 14.57 |
| 3268.2989 | FYFTPN(N)K Hex 7 HexNAc 6 | 184.028 | 0.0 | 15.013 | 1.597e+08 | 66 - 73 | 8 | 23.93 | 24.55 | 25.74 |
| 2903.1661 | FYFTPN(N)K Hex 6 HexNAc 5 | 183.752 | 0.0 | 14.804 | 1.202e+08 | 66 - 73 | 12 | 24.55 | 25.24 | 27.73 |
| 2417.9212 | NEEYN(N)K Hex 5 HexNAc 4 | 181.265 | 0.0 | 11.722 | 7.881e+07 | 51 - 57 | 8 | 3.78 | 3.95 | 5.14 |
| 2783.0539 | NEEYN(N)K Hex 6 HexNAc 5 | 181.199 | 0.0 | 11.501 | 6.456e+07 | 51 - 57 | 7 | 3.78 | 3.95 | 5.03 |
| 3906.6677 | SVQEIQATFFYFTPN(N)K Hex 6 HexNAc 5 | 175.216 | 0.0 | 14.922 | 2.357e+09 | 57 - 73 | 54 | 50.16 | 53.92 | 61.62 |
| 4052.7252 | SVQEIQATFFYFTPN(N)K Fuc 1 Hex 6 HexNAc 5 | 175.061 | 0.0 | 13.796 | 4.928e+08 | 57 - 73 | 17 | 53.07 | 53.75 | 57.68 |
| 4271.8037 | SVQEIQATFFYFTPN(N)K Hex 7 HexNAc 6 | 173.546 | 0.0 | 14.194 | 2.926e+09 | 57 - 73 | 55 | 49.65 | 53.41 | 113.34 |
| 4417.8722 | SVQEIQATFFYFTPN(N)K Fuc 1 Hex 7 HexNAc 6 | 166.205 | 0.0 | 13.778 | 5.800e+08 | 57 - 73 | 17 | 53.07 | 53.24 | 58.19 |
| 4636.9331 | SVQEIQATFFYFTPN(N)K Hex 8 HexNAc 7 | 164.673 | 0.0 | 14.168 | 3.987e+08 | 57 - 73 | 9 | 52.39 | 52.90 | 53.75 |
| 4563.9202 | SVQEIQATFFYFTPN(N)K Fuc 2 Hex 7 HexNAc 6 | 164.123 | 0.0 | -3.890 | 8.526e+07 | 57 - 73 | 2 | 53.07 | 53.07 | 53.58 |
| 3912.7903 | LVPVPITN(N)ATLDQITGK Fuc 1 Hex 6 HexNAc 5 | 163.384 | 0.0 | 15.013 | 3.654e+09 | 25 - 42 | 66 | 41.41 | 47.70 | 113.39 |
| 3541.5338 | SVQEIQATFFYFTPN(N)K Hex 5 HexNAc 4 | 161.196 | 0.0 | 13.435 | 2.793e+08 | 57 - 73 | 15 | 54.26 | 54.60 | 58.87 |
| 3766.7416 | LVPVPITN(N)ATLDQITGK Hex 6 HexNAc 5 | 159.947 | 0.0 | 14.766 | 1.686e+10 | 25 - 42 | 594 | 41.41 | 48.02 | 113.54 |
| 3969.8107 | PVPITN(N)ATLDQITGK Fuc 2 Hex 3 HexNAc 8 | 158.914 | 0.0 | 14.714 | 7.778e+08 | 27 - 42 | 7 | 48.34 | 48.49 | 49.82 |
| 3222.3220 | ATFFYFTPN(N)K Hex 6 HexNAc 5 | 156.715 | 0.0 | 14.807 | 4.694e+07 | 63 - 73 | 2 | 40.72 | 40.89 | 41.41 |
| 3587.4555 | ATFFYFTPN(N)K Hex 7 HexNAc 6 | 155.053 | 0.0 | 13.115 | 7.577e+07 | 63 - 73 | 3 | 40.21 | 40.38 | 40.89 |
| 3401.5988 | LVPVPITN(N)ATLDQITGK Hex 5 HexNAc 4 | 153.831 | 0.0 | 14.572 | 1.647e+09 | 25 - 42 | 16 | 41.92 | 48.49 | 50.16 |
| 4464.8545 | SVQEIQATFFYFTPN(N)K Fuc 1 Hex 8 HexNAc 4 Neu5Ac 1 | 153.781 | 0.0 | 12.606 | 5.258e+07 | 57 - 73 | 4 | 54.26 | 54.43 | 55.11 |
| 4782.9907 | SVQEIQATFFYFTPN(N)K Fuc 1 Hex 8 HexNAc 7 | 150.195 | 0.0 | 12.204 | 7.721e+07 | 57 - 73 | 3 | 52.39 | 52.73 | 53.24 |
| 5028.2309 | SVQEIQATFFYFTPN(N)KTEDTIFLR Fuc 1 Hex 6 HexNAc 5 | 146.640 | 0.0 | 12.452 | 1.422e+08 | 57 - 81 | 5 | 61.96 | 62.30 | 63.84 |
| 4882.1739 | SVQEIQATFFYFTPN(N)KTEDTIFLR Hex 6 HexNAc 5 | 145.935 | 0.0 | 14.519 | 5.916e+08 | 57 - 81 | 19 | 59.90 | 62.47 | 65.38 |
| 5002.0649 | SVQEIQATFFYFTPN(N)K Hex 9 HexNAc 8 | 144.329 | 0.0 | 13.666 | 8.196e+07 | 57 - 73 | 4 | 51.87 | 52.04 | 52.90 |
| 3369.3751 | ATFFYFTPN(N)K Fuc 1 Hex 6 HexNAc 5 | 142.068 | 0.0 | 0.000 | 0.000e+00 | 63 - 73 | 1 | - | - | - |
| 4929.0559 | SVQEIQATFFYFTPN(N)K Fuc 2 Hex 8 HexNAc 7 | 141.885 | 0.0 | 12.440 | 1.670e+07 | 57 - 73 | 3 | 52.21 | 52.73 | 52.90 |
| 4131.8685 | LVPVPITN(N)ATLDQITGK Hex 7 HexNAc 6 | 141.604 | 0.0 | 15.029 | 5.216e+08 | 25 - 42 | 6 | 41.24 | 47.53 | 47.86 |
| 4447.8313 | SVQEIQATFFYFTPN(N)K Hex 7 HexNAc 4 Neu5Ac 2 | 140.464 | 0.0 | 12.183 | 1.349e+08 | 57 - 73 | 6 | 54.26 | 54.43 | 55.28 |
| 4028.8285 | PVPITN(N)ATLDQITGK Fuc 1 Hex 3 HexNAc 9 | 140.130 | 0.0 | 0.000 | 0.000e+00 | 27 - 42 | 1 | - | - | - |
| 5247.3098 | SVQEIQATFFYFTPN(N)KTEDTIFLR Hex 7 HexNAc 6 | 139.398 | 0.0 | 13.334 | 1.220e+09 | 57 - 81 | 24 | 60.41 | 61.96 | 67.44 |
| 4242.7898 | YFTPN(N)KTEDTIFLR Fuc 1 Hex 7 HexNAc 6 | 138.288 | 0.0 | 9.772 | 2.376e+08 | 67 - 81 | 4 | 36.36 | 36.43 | 36.90 |
| 3786.6016 | TPN(N)KTEDTIFLR Hex 7 HexNAc 6 | 137.885 | 0.0 | 14.490 | 6.767e+07 | 69 - 81 | 6 | 24.90 | 25.24 | 26.06 |
| 3902.6099 | QDQC(C)IYN(N)TTYLNVQR Hex 6 HexNAc 5 | 135.070 | 0.0 | 11.094 | 1.902e+09 | 86 - 101 | 8 | 31.34 | 33.72 | 34.73 |
| 4188.8876 | PVPITN(N)ATLDQITGK Fuc 1 Hex 4 HexNAc 9 | 134.155 | 0.0 | -4.505 | 8.523e+07 | 27 - 42 | 3 | 48.17 | 48.34 | 48.49 |
| 3731.6034 | YFTPN(N)KTEDTIFLR Hex 6 HexNAc 5 | 133.207 | 0.0 | 10.647 | 1.091e+09 | 67 - 81 | 10 | 35.24 | 37.21 | 39.04 |
| 4020.7149 | SVQEIQATFFYFTPN(N)K Fuc 3 Hex 4 HexNAc 5 | 131.836 | 0.0 | -19.965 | 1.237e+07 | 57 - 73 | 1 | 53.58 | 53.58 | 53.75 |
| 4267.7478 | QDQC(C)IYN(N)TTYLNVQR Hex 7 HexNAc 6 | 131.778 | 0.0 | 13.826 | 2.550e+09 | 86 - 101 | 8 | 31.00 | 33.39 | 33.88 |
| 4096.7358 | YFTPN(N)KTEDTIFLR Hex 7 HexNAc 6 | 131.659 | 0.0 | 11.228 | 2.145e+09 | 67 - 81 | 123 | 34.90 | 36.74 | 91.78 |
| 3366.4682 | YFTPN(N)KTEDTIFLR Hex 5 HexNAc 4 | 131.047 | 0.0 | 14.973 | 5.973e+07 | 67 - 81 | 1 | 37.68 | 37.68 | 38.36 |
| 4048.6687 | QDQC(C)IYN(N)TTYLNVQR Fuc 1 Hex 6 HexNAc 5 | 128.758 | 0.0 | 12.826 | 5.017e+08 | 86 - 101 | 5 | 33.22 | 33.55 | 34.56 |
| 3877.6605 | YFTPN(N)KTEDTIFLR Fuc 1 Hex 6 HexNAc 5 | 127.666 | 0.0 | 13.358 | 2.778e+08 | 67 - 81 | 3 | 36.74 | 37.05 | 37.36 |
| 4277.9269 | LVPVPITN(N)ATLDQITGK Fuc 1 Hex 7 HexNAc 6 | 127.300 | 0.0 | 13.947 | 1.858e+08 | 25 - 42 | 4 | 47.02 | 47.53 | 47.70 |
| 4632.8779 | QDQC(C)IYN(N)TTYLNVQR Hex 8 HexNAc 7 | 126.869 | 0.0 | 13.578 | 4.787e+08 | 86 - 101 | 6 | 32.19 | 32.70 | 33.72 |
| 4109.7466 | SVQEIQATFFYFTPN(N)K Hex 6 HexNAc 6 | 126.323 | 0.0 | 12.424 | 6.664e+07 | 57 - 73 | 7 | 54.26 | 54.43 | 54.94 |
| 4328.8205 | SVQEIQATFFYFTPN(N)K Fuc 4 Hex 5 HexNAc 5 | 126.295 | 0.0 | 12.214 | 6.150e+08 | 57 - 73 | 26 | 52.56 | 54.09 | 56.99 |
| 4593.8893 | SVQEIQATFFYFTPN(N)K Fuc 1 Hex 7 HexNAc 4 Neu5Ac 2 | 125.373 | 0.0 | -19.392 | 1.248e+07 | 57 - 73 | 3 | 54.09 | 54.26 | 54.26 |
| 4413.8033 | QDQC(C)IYN(N)TTYLNVQR Fuc 1 Hex 7 HexNAc 6 | 125.357 | 0.0 | 12.194 | 6.695e+08 | 86 - 101 | 5 | 32.88 | 33.22 | 34.22 |
| 3878.6703 | FYFTPN(N)KTEDTIFLR Hex 6 HexNAc 5 | 124.304 | 0.0 | 14.986 | 8.360e+07 | 66 - 81 | 6 | 45.49 | 45.83 | 46.51 |
| 3537.4758 | QDQC(C)IYN(N)TTYLNVQR Hex 5 HexNAc 4 | 123.055 | 0.0 | -4.073 | 4.017e+07 | 86 - 101 | 2 | 33.88 | 34.05 | 34.39 |
| 4436.8889 | SVQEIQATFFYFTPN(N)K Hex 8 HexNAc 6 | 121.744 | 0.0 | 0.000 | 0.000e+00 | 57 - 73 | 1 | - | - | - |
| 4778.9336 | QDQC(C)IYN(N)TTYLNVQR Fuc 1 Hex 8 HexNAc 7 | 121.449 | 0.0 | 13.526 | 1.136e+08 | 86 - 101 | 4 | 32.36 | 32.88 | 33.39 |
| 3840.6777 | PVPITN(N)ATLDQITGK Hex 9 HexNAc 4 | 120.949 | 0.0 | 0.000 | 0.000e+00 | 27 - 42 | 1 | - | - | - |
| 3421.4693 | TPN(N)KTEDTIFLR Hex 6 HexNAc 5 | 120.776 | 0.0 | 14.705 | 3.361e+07 | 69 - 81 | 7 | 25.41 | 25.74 | 26.29 |
| 4243.8039 | FYFTPN(N)KTEDTIFLR Hex 7 HexNAc 6 | 120.562 | 0.0 | 14.544 | 2.775e+08 | 66 - 81 | 8 | 43.97 | 45.49 | 46.85 |
| 3568.5392 | FTPN(N)KTEDTIFLR Hex 6 HexNAc 5 | 120.194 | 0.0 | 14.757 | 8.049e+07 | 68 - 81 | 5 | 31.68 | 32.02 | 32.70 |
| 4057.8296 | PVPITN(N)ATLDQITGK Fuc 2 Hex 3 HexNAc 7 Neu5Ac 1 | 118.705 | 0.0 | 11.500 | 2.613e+07 | 27 - 42 | 6 | 50.16 | 50.68 | 51.19 |
| 4559.8618 | QDQC(C)IYN(N)TTYLNVQR Fuc 2 Hex 7 HexNAc 6 | 118.557 | 0.0 | 13.897 | 1.080e+08 | 86 - 101 | 3 | 32.88 | 33.05 | 33.55 |
| 4998.0103 | QDQC(C)IYN(N)TTYLNVQR Hex 9 HexNAc 8 | 117.267 | 0.0 | 13.417 | 1.466e+08 | 86 - 101 | 3 | 32.02 | 32.36 | 32.70 |
| 4497.0007 | LVPVPITN(N)ATLDQITGK Hex 8 HexNAc 7 | 116.633 | 0.0 | 13.358 | 8.593e+07 | 25 - 42 | 6 | 46.85 | 47.36 | 47.53 |
| 4198.7915 | SVQEIQATFFYFTPN(N)K Fuc 2 Hex 6 HexNAc 5 | 115.325 | 0.0 | -19.521 | 1.105e+07 | 57 - 73 | 1 | 53.41 | 53.58 | 53.58 |
| 4074.8617 | LVPVPITN(N)ATLDQITGK Fuc 1 Hex 7 HexNAc 5 | 115.038 | 0.0 | 3.560 | 9.838e+06 | 25 - 42 | 3 | 47.53 | 47.53 | 51.02 |
| 4133.8648 | PVPITN(N)ATLDQITGK Fuc 2 Hex 4 HexNAc 8 | 114.780 | 0.0 | 0.000 | 0.000e+00 | 27 - 42 | 1 | - | - | - |
| 4098.7433 | YFTPN(N)KTEDTIFLR Hex 7 HexNAc 6 | 114.366 | 0.0 | 0.000 | 0.000e+00 | 67 - 81 | 2 | - | - | - |
| 4461.8636 | YFTPN(N)KTEDTIFLR Hex 8 HexNAc 7 | 114.279 | 0.0 | 12.900 | 2.030e+08 | 67 - 81 | 4 | 34.56 | 35.75 | 36.07 |
| 3784.7485 | LVPVPITN(N)ATLDQITGK Fuc 2 Hex 5 HexNAc 3 Neu5Ac 1 | 113.396 | 0.0 | -9.867 | 6.719e+07 | 25 - 42 | 40 | 49.99 | 50.16 | 74.16 |
| 4058.8522 | PVPITN(N)ATLDQITGK Fuc 4 Hex 3 HexNAc 7 | 112.459 | 0.0 | -5.076 | 3.414e+07 | 27 - 42 | 1 | 47.53 | 47.70 | 47.86 |
| 3971.8150 | PVPITN(N)ATLDQITGK Fuc 2 Hex 3 HexNAc 8 | 110.172 | 0.0 | 0.000 | 0.000e+00 | 27 - 42 | 1 | - | - | - |
| 3959.7939 | LVPVPITN(N)ATLDQITGK Fuc 1 Hex 7 HexNAc 3 Neu5Ac 1 | 109.301 | 0.0 | -18.733 | 3.289e+07 | 25 - 42 | 2 | 48.97 | 49.14 | 49.14 |
| 4493.9133 | SVQEIQATFFYFTPN(N)K Fuc 4 Hex 6 HexNAc 5 | 108.614 | 0.0 | 0.000 | 0.000e+00 | 57 - 73 | 1 | - | - | - |
| 4024.7291 | FYFTPN(N)KTEDTIFLR Fuc 1 Hex 6 HexNAc 5 | 108.483 | 0.0 | 13.683 | 2.095e+07 | 66 - 81 | 3 | 45.49 | 45.66 | 46.17 |
| 4024.7275 | PVPITN(N)ATLDQITGK Fuc 1 Hex 8 HexNAc 5 | 108.483 | 0.0 | -5.215 | 1.793e+07 | 27 - 42 | 3 | 52.21 | 52.21 | 52.56 |
| 4589.8302 | QDQC(C)IYN(N)TTYLNVQR Fuc 1 Hex 7 HexNAc 4 Neu5Ac 2 | 108.477 | 0.0 | -5.179 | 3.793e+07 | 86 - 101 | 2 | 34.05 | 34.22 | 34.56 |
| 4299.8165 | YFTPN(N)KTEDTIFLR Hex 7 HexNAc 7 | 108.306 | 0.0 | -5.140 | 7.868e+07 | 67 - 81 | 1 | 36.90 | 37.05 | 37.36 |
| 3968.6736 | PVPITN(N)ATLDQITGK Fuc 2 Hex 8 HexNAc 4 | 106.927 | 0.0 | -21.295 | 1.065e+06 | 27 - 42 | 3 | 24.22 | 24.22 | 24.38 |
| 4389.8615 | FYFTPN(N)KTEDTIFLR Fuc 1 Hex 7 HexNAc 6 | 106.149 | 0.0 | 14.078 | 5.220e+07 | 66 - 81 | 5 | 45.15 | 45.32 | 45.83 |
| 4443.7724 | QDQC(C)IYN(N)TTYLNVQR Hex 7 HexNAc 4 Neu5Ac 2 | 104.861 | 0.0 | 12.383 | 2.021e+08 | 86 - 101 | 4 | 34.05 | 34.39 | 35.24 |
| 3933.6711 | FTPN(N)KTEDTIFLR Hex 7 HexNAc 6 | 104.466 | 0.0 | 13.689 | 1.566e+08 | 68 - 81 | 6 | 31.34 | 31.51 | 32.19 |
| 3602.5945 | PVPITN(N)ATLDQITGK Fuc 2 Hex 7 HexNAc 3 | 101.698 | 0.0 | -19.404 | 5.973e+06 | 27 - 42 | 1 | 51.87 | 52.04 | 52.04 |
| 4693.9535 | SVQEIQATFFYFTPN(N)K Fuc 4 Hex 6 HexNAc 6 | 100.107 | 0.0 | 10.249 | 3.866e+07 | 57 - 73 | 2 | 53.24 | 53.58 | 54.09 |
| 3567.5270 | TPN(N)KTEDTIFLR Fuc 1 Hex 6 HexNAc 5 | 98.674 | 0.0 | -5.233 | 5.711e+06 | 69 - 81 | 3 | 25.41 | 25.41 | 25.74 |
| 3736.5652 | IYN(N)TTYLNVQR Hex 7 HexNAc 6 | 97.243 | 0.0 | 15.917 | 2.907e+07 | 90 - 101 | 4 | 27.95 | 28.18 | 28.41 |
| 4826.9992 | YFTPN(N)KTEDTIFLR Hex 9 HexNAc 8 | 97.064 | 0.0 | -3.362 | 6.335e+07 | 67 - 81 | 2 | 35.24 | 35.41 | 35.75 |
| 4533.0581 | LVPVPITN(N)ATLDQITGK Fuc 3 Hex 3 HexNAc 9 | 96.550 | 0.0 | 0.000 | 0.000e+00 | 25 - 42 | 1 | - | - | - |
| 4088.8248 | PVPITN(N)ATLDQITGK Fuc 3 Hex 3 HexNAc 5 Neu5Ac 2 | 96.173 | 0.0 | -4.897 | 3.331e+07 | 27 - 42 | 2 | 48.82 | 48.82 | 49.14 |
| 3714.5961 | FTPN(N)KTEDTIFLR Fuc 1 Hex 6 HexNAc 5 | 96.093 | 0.0 | -3.998 | 1.697e+07 | 68 - 81 | 3 | 31.51 | 31.68 | 32.02 |
| 4272.7522 | YFTPN(N)KTEDTIFLR Hex 7 HexNAc 4 Neu5Ac 2 | 95.677 | 0.0 | -3.742 | 1.312e+08 | 67 - 81 | 5 | 37.68 | 37.85 | 38.02 |
| 4643.0587 | PVPITN(N)ATLDQITGK Fuc 3 Hex 5 HexNAc 9 | 93.668 | 0.0 | 13.132 | 1.448e+07 | 27 - 42 | 2 | 46.68 | 47.19 | 47.36 |
| 4517.0385 | SVQEIQATFFYFTPN(N)KTEDTIFLR Hex 5 HexNAc 4 | 91.884 | 0.0 | -19.076 | 2.030e+07 | 57 - 81 | 2 | 62.81 | 62.81 | 62.98 |
| 5393.3658 | SVQEIQATFFYFTPN(N)KTEDTIFLR Fuc 1 Hex 7 HexNAc 6 | 91.208 | 0.0 | 12.612 | 2.404e+08 | 57 - 81 | 6 | 61.62 | 61.96 | 63.50 |
| 5612.4369 | SVQEIQATFFYFTPN(N)KTEDTIFLR Hex 8 HexNAc 7 | 90.282 | 0.0 | 14.428 | 2.101e+08 | 57 - 81 | 5 | 61.27 | 61.62 | 62.13 |
| 3882.7759 | PVPITN(N)ATLDQITGK Hex 3 HexNAc 9 | 89.088 | 0.0 | 0.000 | 0.000e+00 | 27 - 42 | 1 | - | - | - |
| 3932.6600 | TPN(N)KTEDTIFLR Fuc 1 Hex 7 HexNAc 6 | 87.478 | 0.0 | -18.890 | 7.631e+06 | 69 - 81 | 2 | 24.90 | 25.07 | 25.07 |
| 4298.8018 | FTPN(N)KTEDTIFLR Hex 8 HexNAc 7 | 86.373 | 0.0 | -4.363 | 6.986e+06 | 68 - 81 | 7 | 30.66 | 31.00 | 31.00 |
| 4079.7258 | FTPN(N)KTEDTIFLR Fuc 1 Hex 7 HexNAc 6 | 85.313 | 0.0 | -4.281 | 2.494e+07 | 68 - 81 | 3 | 31.17 | 31.34 | 31.68 |
| 3056.3362 | TPN(N)KTEDTIFLR Hex 5 HexNAc 4 | 84.932 | 0.0 | -3.822 | 3.968e+06 | 69 - 81 | 3 | 25.91 | 26.06 | 26.29 |
| 4608.9339 | FYFTPN(N)KTEDTIFLR Hex 8 HexNAc 7 | 84.883 | 0.0 | 14.259 | 2.380e+07 | 66 - 81 | 3 | 44.47 | 44.98 | 45.15 |
| 5192.1323 | YFTPN(N)KTEDTIFLR Hex 10 HexNAc 9 | 84.277 | 0.0 | -19.547 | 6.145e+06 | 67 - 81 | 1 | 35.07 | 35.07 | 35.24 |
| 4118.7146 | YFTPN(N)KTEDTIFLR Hex 3 HexNAc 5 Neu5Ac 3 | 84.169 | 0.0 | -4.685 | 7.787e+07 | 67 - 81 | 1 | 36.62 | 36.74 | 36.90 |
| 3371.4294 | IYN(N)TTYLNVQR Hex 6 HexNAc 5 | 79.869 | 0.002 | 11.731 | 4.549e+07 | 90 - 101 | 4 | 28.41 | 28.57 | 28.90 |
| 3951.7360 | FTPN(N)KTEDTIFLR Fuc 2 Hex 6 HexNAc 4 Neu5Ac 1 | 79.768 | 0.002 | -6.358 | 5.406e+07 | 68 - 81 | 4 | 48.02 | 48.02 | 48.34 |
| 3788.6234 | FYFTPN(N)KTEDTIFLR Fuc 1 Hex 4 HexNAc 4 Neu5Ac 1 | 75.836 | 0.006 | 14.922 | 2.409e+08 | 66 - 81 | 5 | 37.68 | 37.85 | 38.70 |
| 5539.4226 | SVQEIQATFFYFTPN(N)KTEDTIFLR Fuc 2 Hex 7 HexNAc 6 | 75.015 | 0.006 | -19.391 | 2.255e+07 | 57 - 81 | 1 | 61.79 | 61.79 | 61.96 |
| 4116.7043 | TPN(N)KTEDTIFLR Fuc 1 Hex 4 HexNAc 5 Neu5Ac 3 | 73.194 | 0.006 | -19.666 | 1.218e+07 | 69 - 81 | 1 | 36.53 | 36.53 | 36.53 |
| 4419.8388 | FYFTPN(N)KTEDTIFLR Hex 7 HexNAc 4 Neu5Ac 2 | 73.167 | 0.006 | -19.154 | 8.152e+06 | 66 - 81 | 3 | 46.34 | 46.34 | 46.51 |
| 4111.8475 | C(C)ANLVPVPITN(N)ATLDQITGK Hex 6 HexNAc 5 | 68.409 | 0.007 | 12.322 | 1.157e+08 | 22 - 42 | 4 | 51.36 | 51.53 | 52.39 |
| 5367.1972 | SVQEIQATFFYFTPN(N)K Hex 10 HexNAc 9 | 66.902 | 0.007 | -5.012 | 1.080e+07 | 57 - 73 | 1 | 51.53 | 51.87 | 52.04 |
| 4474.8791 | SVQEIQATFFYFTPN(N)K Hex 7 HexNAc 7 | 59.983 | 0.007 | 11.694 | 7.569e+07 | 57 - 73 | 4 | 53.75 | 53.92 | 54.60 |
| 4924.9943 | QDQC(C)IYN(N)TTYLNVQR Fuc 2 Hex 8 HexNAc 7 | 58.998 | 0.007 | 13.740 | 2.738e+07 | 86 - 101 | 2 | 32.19 | 32.70 | 32.88 |
| 4257.9041 | C(C)ANLVPVPITN(N)ATLDQITGK Fuc 1 Hex 6 HexNAc 5 | 57.997 | 0.007 | 12.891 | 3.128e+07 | 22 - 42 | 3 | 51.19 | 51.36 | 51.87 |
| 5148.1311 | SVQEIQATFFYFTPN(N)K Fuc 1 Hex 9 HexNAc 8 | 56.095 | 0.007 | -6.242 | 6.067e+06 | 57 - 73 | 1 | 51.87 | 52.04 | 52.21 |
| 4640.0180 | PVPITN(N)ATLDQITGK Fuc 2 Hex 3 HexNAc 7 Neu5Ac 3 | 52.732 | 0.007 | -5.224 | 7.798e+06 | 27 - 42 | 1 | 56.99 | 57.16 | 57.33 |
| 4316.8880 | PVPITN(N)ATLDQITGK Fuc 3 Hex 8 HexNAc 5 | 52.308 | 0.007 | -4.907 | 2.928e+07 | 27 - 42 | 2 | 47.02 | 47.53 | 47.53 |
| 4460.8009 | QDQC(C)IYN(N)TTYLNVQR Fuc 1 Hex 8 HexNAc 4 Neu5Ac 1 | 50.382 | 0.007 | -5.751 | 4.046e+07 | 86 - 101 | 2 | 34.22 | 34.39 | 34.73 |
| 5977.5676 | SVQEIQATFFYFTPN(N)KTEDTIFLR Hex 9 HexNAc 8 | 48.646 | 0.007 | 14.198 | 7.338e+07 | 57 - 81 | 6 | 60.76 | 60.93 | 61.79 |
| 4289.7255 | QDQC(C)IYN(N)TTYLNVQR Hex 3 HexNAc 5 Neu5Ac 3 | 48.295 | 0.007 | 9.848 | 3.077e+08 | 86 - 101 | 4 | 33.05 | 33.39 | 34.22 |
| 3880.7834 | LVPVPITN(N)ATLDQITGK Fuc 3 Hex 4 HexNAc 5 | 47.348 | 0.007 | -5.973 | 9.541e+07 | 25 - 42 | 6 | 47.53 | 47.53 | 51.19 |
| 5144.0668 | QDQC(C)IYN(N)TTYLNVQR Fuc 1 Hex 9 HexNAc 8 | 47.015 | 0.007 | -3.919 | 2.463e+07 | 86 - 101 | 2 | 32.02 | 32.19 | 32.36 |
| 5363.1402 | QDQC(C)IYN(N)TTYLNVQR Hex 10 HexNAc 9 | 45.663 | 0.007 | -4.128 | 2.786e+07 | 86 - 101 | 2 | 31.85 | 32.02 | 32.19 |
| 4562.8962 | SVQEIQATFFYFTPN(N)K Hex 7 HexNAc 6 Neu5Ac 1 | 45.115 | 0.007 | -20.745 | 5.306e+05 | 57 - 73 | 1 | 56.14 | 56.14 | 56.14 |
| 5423.3358 | SVQEIQATFFYFTPN(N)KTEDTIFLR Hex 7 HexNAc 4 Neu5Ac 2 | 44.728 | 0.007 | -3.903 | 3.148e+07 | 57 - 81 | 3 | 62.81 | 62.98 | 63.33 |
| 3931.8197 | LVPVPITN(N)ATLDQITGK Hex 7 HexNAc 5 | 44.340 | 0.007 | 0.000 | 0.000e+00 | 25 - 42 | 1 | - | - | - |
| 4243.8595 | PVPITN(N)ATLDQITGK Fuc 3 Hex 7 HexNAc 4 Neu5Ac 1 | 43.973 | 0.007 | 0.000 | 0.000e+00 | 27 - 42 | 1 | - | - | - |
| 3950.7348 | PVPITN(N)ATLDQITGK Fuc 1 Hex 7 HexNAc 4 Neu5Ac 1 | 43.767 | 0.007 | -3.576 | 2.596e+08 | 27 - 42 | 3 | 47.53 | 47.70 | 47.86 |
| 3846.7006 | PVPITN(N)ATLDQITGK Fuc 2 Hex 6 HexNAc 5 | 42.963 | 0.007 | -5.575 | 6.012e+06 | 27 - 42 | 2 | 51.36 | 51.70 | 51.87 |
| 4862.1356 | LVPVPITN(N)ATLDQITGK Hex 9 HexNAc 8 | 42.899 | 0.007 | -4.598 | 5.978e+06 | 25 - 42 | 1 | 46.34 | 46.68 | 46.85 |
| 3841.7728 | LVPVPITN(N)ATLDQITGK Fuc 3 Hex 5 HexNAc 4 | 42.524 | 0.007 | 3.936 | 2.048e+07 | 25 - 42 | 5 | 49.82 | 50.68 | 51.53 |
| 5758.4931 | SVQEIQATFFYFTPN(N)KTEDTIFLR Fuc 1 Hex 8 HexNAc 7 | 42.164 | 0.007 | 15.737 | 3.805e+07 | 57 - 81 | 3 | 61.10 | 61.62 | 61.79 |
| 3513.5363 | FYFTPN(N)KTEDTIFLR Hex 5 HexNAc 4 | 41.630 | 0.007 | -5.615 | 2.818e+06 | 66 - 81 | 1 | 46.17 | 46.34 | 46.51 |
| 4273.6961 | QDQC(C)IYN(N)TTYLNVQR Hex 9 HexNAc 3 Neu5Ac 1 | 41.543 | 0.007 | 10.013 | 2.307e+08 | 86 - 101 | 3 | 34.05 | 34.22 | 35.07 |
| 4105.6901 | QDQC(C)IYN(N)TTYLNVQR Hex 6 HexNAc 6 | 41.331 | 0.007 | 13.847 | 8.980e+07 | 86 - 101 | 3 | 33.55 | 33.72 | 34.56 |
| 3789.6109 | TPN(N)KTEDTIFLR Hex 7 HexNAc 6 | 40.343 | 0.007 | 0.000 | 0.000e+00 | 69 - 81 | 1 | - | - | - |
| 5290.1191 | QDQC(C)IYN(N)TTYLNVQR Fuc 2 Hex 9 HexNAc 8 | 38.203 | 0.007 | -5.153 | 4.706e+06 | 86 - 101 | 1 | 31.85 | 32.02 | 32.19 |
| 4060.8574 | LVPVPITN(N)ATLDQITGK Fuc 2 Hex 6 HexNAc 5 | 38.135 | 0.007 | 0.000 | 0.000e+00 | 25 - 42 | 1 | - | - | - |
| 4998.0114 | QDQC(C)IYN(N)TTYLNVQR Fuc 5 Hex 7 HexNAc 6 | 37.920 | 0.007 | -19.838 | 3.636e+06 | 86 - 101 | 1 | 33.05 | 33.05 | 33.05 |
| 4638.9945 | PVPITN(N)ATLDQITGK Hex 3 HexNAc 7 Neu5Ac 4 | 37.608 | 0.007 | -19.182 | 2.504e+06 | 27 - 42 | 2 | 57.51 | 57.51 | 57.68 |
| 4423.9853 | LVPVPITN(N)ATLDQITGK Fuc 2 Hex 7 HexNAc 6 | 37.377 | 0.007 | -4.964 | 1.926e+07 | 25 - 42 | 1 | 47.02 | 47.53 | 47.53 |
| 3754.5859 | YFTPN(N)KTEDTIFLR Fuc 1 Hex 9 HexNAc 2 | 37.127 | 0.007 | -18.945 | 1.465e+07 | 67 - 81 | 2 | 37.21 | 37.21 | 37.21 |
| 3913.7861 | PVPITN(N)ATLDQITGK Fuc 3 Hex 3 HexNAc 7 | 36.212 | 0.008 | -8.800 | 6.107e+07 | 27 - 42 | 25 | 48.65 | 49.99 | 92.64 |
| 4975.0717 | FYFTPN(N)KTEDTIFLR Hex 9 HexNAc 8 | 35.821 | 0.008 | 0.000 | 0.000e+00 | 66 - 81 | 1 | - | - | - |
| 3934.6793 | YFTPN(N)KTEDTIFLR Hex 6 HexNAc 6 | 35.624 | 0.008 | -4.484 | 5.309e+07 | 67 - 81 | 4 | 37.53 | 37.53 | 37.85 |
| 4153.7556 | YFTPN(N)KTEDTIFLR Fuc 4 Hex 5 HexNAc 5 | 35.408 | 0.009 | 13.726 | 5.029e+08 | 67 - 81 | 3 | 36.90 | 37.36 | 38.19 |
| 4640.9905 | LVPVPITN(N)ATLDQITGK Hex 6 HexNAc 5 Neu5Ac 3 | 35.399 | 0.009 | 0.000 | 0.000e+00 | 25 - 42 | 1 | - | - | - |
| 4436.7741 | QDQC(C)IYN(N)TTYLNVQR Fuc 2 Hex 10 HexNAc 3 | 34.884 | 0.009 | -19.847 | 8.426e+06 | 86 - 101 | 1 | 34.05 | 34.05 | 34.22 |
| 4456.8132 | ATFFYFTPN(N)K Fuc 4 Hex 5 HexNAc 9 | 34.522 | 0.011 | -19.983 | 1.053e+07 | 63 - 73 | 1 | 53.41 | 53.41 | 53.41 |
| 4268.7265 | QDQC(C)IYN(N)TTYLN(D)VQR Hex 7 HexNAc 6 | 33.859 | 0.015 | 8.633 | 6.018e+07 | 86 - 101 | 8 | 31.85 | 32.02 | 32.53 |
| 4476.9786 | C(C)ANLVPVPITN(N)ATLDQITGK Hex 7 HexNAc 6 | 33.735 | 0.015 | -19.170 | 2.146e+06 | 22 - 42 | 1 | 51.19 | 51.19 | 51.19 |
| 5304.3238 | SVQEIQATFFYFTPN(N)KTEDTIFLR Fuc 4 Hex 5 HexNAc 5 | 33.377 | 0.016 | 13.313 | 2.606e+08 | 57 - 81 | 9 | 62.30 | 62.64 | 64.36 |
| 3884.5124 | QDQC(C)IYN(N)TTYLN Hex 7 HexNAc 6 | 33.020 | 0.018 | -18.425 | 8.040e+06 | 86 - 98 | 1 | 31.85 | 32.02 | 32.02 |
| 3969.8099 | LVPVPITN(N)ATLDQITGK Hex 6 HexNAc 6 | 33.013 | 0.018 | 4.616 | 1.376e+08 | 25 - 42 | 5 | 41.92 | 47.53 | 47.70 |
| 5509.1964 | QDQC(C)IYN(N)TTYLNVQR Fuc 1 Hex 10 HexNAc 9 | 32.674 | 0.023 | -19.268 | 5.873e+06 | 86 - 101 | 1 | 31.85 | 31.85 | 32.02 |
| 4607.8617 | QDQC(C)IYN(N)TTYLNVQR Fuc 2 Hex 8 HexNAc 4 Neu5Ac 1 | 32.434 | 0.025 | 0.000 | 0.000e+00 | 86 - 101 | 1 | - | - | - |
| 4547.0821 | LVPVPITN(N)ATLDQITGK Fuc 2 Hex 4 HexNAc 9 | 32.015 | 0.028 | 10.691 | 9.460e+07 | 25 - 42 | 11 | 68.99 | 69.28 | 70.56 |
| 3737.5705 | IYN(N)TTYLNVQR Hex 7 HexNAc 6 | 31.862 | 0.029 | 0.000 | 0.000e+00 | 90 - 101 | 1 | - | - | - |
| 5440.3599 | SVQEIQATFFYFTPN(N)KTEDTIFLR Fuc 1 Hex 8 HexNAc 4 Neu5Ac 1 | 31.387 | 0.031 | -4.937 | 1.006e+07 | 57 - 81 | 1 | 62.81 | 62.98 | 63.15 |
| 3987.8308 | LVPVPITN(N)ATLDQITGK Fuc 2 Hex 5 HexNAc 4 Neu5Ac 1 | 30.823 | 0.031 | -22.232 | 1.186e+06 | 25 - 42 | 3 | 50.50 | 50.50 | 50.68 |
| 4659.0856 | LVPVPITN(N)ATLDQITGK Fuc 3 Hex 7 HexNAc 5 Neu5Ac 1 | 30.460 | 0.031 | -20.110 | 1.805e+06 | 25 - 42 | 1 | 67.62 | 67.62 | 67.62 |
| 3768.7393 | LVPVPITN(N)ATLDQITGK Hex 6 HexNAc 5 | 30.279 | 0.032 | 0.000 | 0.000e+00 | 25 - 42 | 1 | - | - | - |
| 4008.7512 | PVPITN(N)ATLDQITGK Fuc 2 Hex 7 HexNAc 5 | 30.262 | 0.032 | -19.848 | 1.208e+06 | 27 - 42 | 1 | 51.19 | 51.19 | 51.36 |
| 4199.7697 | SVQEIQATFFYFTPN(N)K Hex 6 HexNAc 5 Neu5Ac 1 | 30.029 | 0.032 | 0.000 | 0.000e+00 | 57 - 73 | 1 | - | - | - |
| 4692.1595 | QIPLC(C)ANLVPVPITN(N)ATLDQITGK Hex 5 HexNAc 5 Neu5Ac 1 | 29.837 | 0.032 | 16.190 | 3.095e+08 | 18 - 42 | 7 | 67.10 | 67.62 | 68.65 |
| 3880.7752 | PVPITN(N)ATLDQITGK Hex 3 HexNAc 9 | 29.261 | 0.035 | -18.454 | 7.669e+07 | 27 - 42 | 2 | 48.34 | 48.49 | 48.49 |
| 4620.9416 | SVQEIQATFFYFTPN(N)K Fuc 1 Hex 7 HexNAc 7 | 29.130 | 0.036 | -19.632 | 3.047e+06 | 57 - 73 | 1 | 53.75 | 53.75 | 53.75 |
| 4563.1199 | QIPLC(C)ANLVPVPITN(N)ATLDQITGK Hex 6 HexNAc 5 | 28.797 | 0.037 | 12.090 | 4.030e+08 | 18 - 42 | 10 | 67.27 | 67.96 | 68.82 |
| 4324.7637 | QDQC(C)IYN(N)TTYLNVQR Fuc 4 Hex 5 HexNAc 5 | 28.446 | 0.037 | 12.916 | 4.791e+08 | 86 - 101 | 5 | 33.55 | 33.55 | 34.22 |
| 4131.8657 | PVPITN(N)ATLDQITGK Fuc 2 Hex 4 HexNAc 8 | 28.428 | 0.037 | -5.808 | 3.998e+06 | 27 - 42 | 2 | 46.00 | 46.17 | 46.51 |
| 3882.6243 | IYN(N)TTYLNVQR Fuc 1 Hex 7 HexNAc 6 | 27.857 | 0.039 | 12.432 | 9.730e+06 | 90 - 101 | 3 | 27.77 | 28.02 | 28.02 |
| 7813.3374 | WFYIASAFRNEEYNKSVQEIQATFFYFTPN(N)K Fuc 5 Hex 10 HexNAc 8 | 27.627 | 0.039 | -18.452 | 3.423e+07 | 42 - 73 | 1 | 53.92 | 53.92 | 54.09 |
| 3049.2877 | N(N)ATLDQITGK Hex 6 HexNAc 5 | 27.563 | 0.041 | 0.000 | 0.000e+00 | 32 - 42 | 1 | - | - | - |
| 4709.1776 | QIPLC(C)ANLVPVPITN(N)ATLDQITGK Fuc 1 Hex 6 HexNAc 5 | 27.553 | 0.041 | 12.586 | 1.301e+08 | 18 - 42 | 6 | 67.10 | 67.62 | 68.65 |
| 3961.8028 | LVPVPITN(N)ATLDQITGK Fuc 1 Hex 7 HexNAc 3 Neu5Ac 1 | 27.541 | 0.041 | 0.000 | 0.000e+00 | 25 - 42 | 1 | - | - | - |
| 4730.1090 | C(C)ANLVPVPITN(N)ATLDQITGK Fuc 2 Hex 3 HexNAc 9 | 27.503 | 0.041 | -4.058 | 4.616e+07 | 22 - 42 | 5 | 67.44 | 67.62 | 67.96 |
| 4689.8946 | QDQC(C)IYN(N)TTYLNVQR Fuc 4 Hex 6 HexNAc 6 | 27.064 | 0.043 | 12.630 | 7.563e+07 | 86 - 101 | 4 | 32.70 | 32.88 | 33.39 |
| 3804.6749 | PVPITN(N)ATLDQITGK Hex 7 HexNAc 4 Neu5Ac 1 | 27.020 | 0.043 | 15.726 | 1.015e+09 | 27 - 42 | 15 | 47.36 | 48.17 | 48.65 |
| 4151.7405 | TPN(N)KTEDTIFLR Hex 8 HexNAc 7 | 26.648 | 0.045 | -5.587 | 4.262e+06 | 69 - 81 | 2 | 24.38 | 24.55 | 24.90 |
| 4974.0668 | FYFTPN(N)KTEDTIFLR Hex 9 HexNAc 8 | 26.565 | 0.045 | -20.985 | 2.814e+06 | 66 - 81 | 1 | 44.30 | 44.30 | 44.47 |
| 4693.1425 | LVPVPITN(N)ATLDQITGK Fuc 3 Hex 4 HexNAc 9 | 26.353 | 0.047 | 10.228 | 2.348e+07 | 25 - 42 | 9 | 68.65 | 68.99 | 70.29 |

### YFTPN(N)K Hex 5 HexNAc 4

**Mass:**  2390.9613

(3.245e-06)

**Charge States:** 2

**Start Position**: 68

**End Position**: 74

| Profile Score | Tandem Score | TDA q-value | Volume | Spectrum Matches |
| --- | --- | --- | --- | --- |
| -5.9023 | 188.0146 | 0.0000 | 2.5716e+07 | 3 |

**Adduct Signal Fractions**

- **Unmodified**: 2.5716e+07

### YFTPN(N)K Hex 6 HexNAc 5

**Mass:**  2757.0946

(3.629e-04)

**Start Position**: 68

**End Position**: 74

| Profile Score | Tandem Score | TDA q-value | Volume | Spectrum Matches |
| --- | --- | --- | --- | --- |
| 0.0000 | 187.4481 | 0.0000 | 0.0000e+00 | 1 |

Adduct:

No Chromatogram Found

### YFTPN(N)K Hex 6 HexNAc 5

**Mass:**  2756.0980

(1.251e-06)

**Charge States:** 2, 3

**Start Position**: 68

**End Position**: 74

| Profile Score | Tandem Score | TDA q-value | Volume | Spectrum Matches |
| --- | --- | --- | --- | --- |
| 14.1125 | 185.4478 | 0.0000 | 3.1298e+08 | 5 |

**Adduct Signal Fractions**

- **Unmodified**: 3.1298e+08

### FYFTPN(N)K Hex 7 HexNAc 6

**Mass:**  3268.2991

(1.201e-06)

**Charge States:** 3, 4

**Start Position**: 67

**End Position**: 74

| Profile Score | Tandem Score | TDA q-value | Volume | Spectrum Matches |
| --- | --- | --- | --- | --- |
| 15.0132 | 184.0277 | 0.0000 | 1.5971e+08 | 8 |

**Adduct Signal Fractions**

- **Unmodified**: 1.5971e+08

### FYFTPN(N)K Hex 6 HexNAc 5

**Mass:**  2903.1654

(8.364e-07)

**Charge States:** 2, 3

**Start Position**: 67

**End Position**: 74

| Profile Score | Tandem Score | TDA q-value | Volume | Spectrum Matches |
| --- | --- | --- | --- | --- |
| 14.8043 | 183.7520 | 0.0000 | 1.2021e+08 | 12 |

**Adduct Signal Fractions**

- **Unmodified**: 1.2021e+08

### NEEYN(N)K Hex 5 HexNAc 4

**Mass:**  2417.9220

(3.145e-07)

**Charge States:** 2, 3

**Start Position**: 52

**End Position**: 58

| Profile Score | Tandem Score | TDA q-value | Volume | Spectrum Matches |
| --- | --- | --- | --- | --- |
| 11.7217 | 181.2652 | 0.0000 | 7.8808e+07 | 8 |

**Adduct Signal Fractions**

- **Unmodified**: 7.8808e+07

### NEEYN(N)K Hex 6 HexNAc 5

**Mass:**  2783.0550

(6.045e-07)

**Charge States:** 3

**Start Position**: 52

**End Position**: 58

| Profile Score | Tandem Score | TDA q-value | Volume | Spectrum Matches |
| --- | --- | --- | --- | --- |
| 11.5010 | 181.1988 | 0.0000 | 6.4556e+07 | 7 |

**Adduct Signal Fractions**

- **Unmodified**: 6.4556e+07

### SVQEIQATFFYFTPN(N)K Hex 6 HexNAc 5

**Mass:**  3906.6691

(2.258e-06)

**Charge States:** 3, 4, 5

**Start Position**: 58

**End Position**: 74

| Profile Score | Tandem Score | TDA q-value | Volume | Spectrum Matches |
| --- | --- | --- | --- | --- |
| 14.9224 | 175.2159 | 0.0000 | 2.3571e+09 | 54 |

**Adduct Signal Fractions**

- **Unmodified**: 2.3571e+09

### SVQEIQATFFYFTPN(N)K Fuc 1 Hex 6 HexNAc 5

**Mass:**  4052.7248

(2.304e-06)

**Charge States:** 3, 4

**Start Position**: 58

**End Position**: 74

| Profile Score | Tandem Score | TDA q-value | Volume | Spectrum Matches |
| --- | --- | --- | --- | --- |
| 13.7957 | 175.0613 | 0.0000 | 4.9285e+08 | 17 |

**Adduct Signal Fractions**

- **Unmodified**: 4.9285e+08

### SVQEIQATFFYFTPN(N)K Hex 7 HexNAc 6

**Mass:**  4271.8083

(2.156e-06)

**Charge States:** 3, 4, 5

**Start Position**: 58

**End Position**: 74

| Profile Score | Tandem Score | TDA q-value | Volume | Spectrum Matches |
| --- | --- | --- | --- | --- |
| 14.1936 | 173.5464 | 0.0000 | 2.9258e+09 | 55 |

**Adduct Signal Fractions**

- **Unmodified**: 2.9258e+09

### SVQEIQATFFYFTPN(N)K Fuc 1 Hex 7 HexNAc 6

**Mass:**  4417.8635

(2.282e-04)

**Charge States:** 3, 4, 5

**Start Position**: 58

**End Position**: 74

| Profile Score | Tandem Score | TDA q-value | Volume | Spectrum Matches |
| --- | --- | --- | --- | --- |
| 13.7776 | 166.2051 | 0.0000 | 5.7995e+08 | 17 |

**Adduct Signal Fractions**

- **Unmodified**: 5.7995e+08

### SVQEIQATFFYFTPN(N)K Hex 8 HexNAc 7

**Mass:**  4636.9318

(7.010e-07)

**Charge States:** 3, 4, 5

**Start Position**: 58

**End Position**: 74

| Profile Score | Tandem Score | TDA q-value | Volume | Spectrum Matches |
| --- | --- | --- | --- | --- |
| 14.1683 | 164.6732 | 0.0000 | 3.9873e+08 | 9 |

**Adduct Signal Fractions**

- **Unmodified**: 3.9873e+08

### SVQEIQATFFYFTPN(N)K Fuc 2 Hex 7 HexNAc 6

**Mass:**  4563.9216

(2.855e-06)

**Charge States:** 3, 4

**Start Position**: 58

**End Position**: 74

| Profile Score | Tandem Score | TDA q-value | Volume | Spectrum Matches |
| --- | --- | --- | --- | --- |
| -3.8901 | 164.1234 | 0.0000 | 8.5262e+07 | 2 |

**Adduct Signal Fractions**

- **Unmodified**: 8.5262e+07

### LVPVPITN(N)ATLDQITGK Fuc 1 Hex 6 HexNAc 5

**Mass:**  3912.7944

(-5.584e-07)

**Charge States:** 3, 4

**Start Position**: 26

**End Position**: 43

| Profile Score | Tandem Score | TDA q-value | Volume | Spectrum Matches |
| --- | --- | --- | --- | --- |
| 15.0126 | 163.3836 | 0.0000 | 3.6543e+09 | 66 |

**Adduct Signal Fractions**

- **Unmodified**: 3.6543e+09

### SVQEIQATFFYFTPN(N)K Hex 5 HexNAc 4

**Mass:**  3541.5324

(1.144e-06)

**Charge States:** 3, 4

**Start Position**: 58

**End Position**: 74

| Profile Score | Tandem Score | TDA q-value | Volume | Spectrum Matches |
| --- | --- | --- | --- | --- |
| 13.4348 | 161.1955 | 0.0000 | 2.7931e+08 | 15 |

**Adduct Signal Fractions**

- **Unmodified**: 2.7931e+08

### LVPVPITN(N)ATLDQITGK Hex 6 HexNAc 5

**Mass:**  3766.7287

(4.111e-07)

**Charge States:** 3, 4

**Start Position**: 26

**End Position**: 43

| Profile Score | Tandem Score | TDA q-value | Volume | Spectrum Matches |
| --- | --- | --- | --- | --- |
| 14.7661 | 159.9473 | 0.0000 | 1.6861e+10 | 594 |

**Adduct Signal Fractions**

- **Unmodified**: 1.6861e+10

### PVPITN(N)ATLDQITGK Fuc 2 Hex 3 HexNAc 8

**Mass:**  3969.8115

(9.665e-06)

**Charge States:** 3, 4

**Start Position**: 28

**End Position**: 43

| Profile Score | Tandem Score | TDA q-value | Volume | Spectrum Matches |
| --- | --- | --- | --- | --- |
| 14.7142 | 158.9144 | 0.0000 | 7.7784e+08 | 7 |

**Adduct Signal Fractions**

- **Unmodified**: 7.7784e+08

### ATFFYFTPN(N)K Hex 6 HexNAc 5

**Mass:**  3222.3213

(2.110e-06)

**Charge States:** 3

**Start Position**: 64

**End Position**: 74

| Profile Score | Tandem Score | TDA q-value | Volume | Spectrum Matches |
| --- | --- | --- | --- | --- |
| 14.8071 | 156.7150 | 0.0000 | 4.6937e+07 | 2 |

**Adduct Signal Fractions**

- **Unmodified**: 4.6937e+07

### ATFFYFTPN(N)K Hex 7 HexNAc 6

**Mass:**  3587.4563

(2.840e-06)

**Charge States:** 3, 4

**Start Position**: 64

**End Position**: 74

| Profile Score | Tandem Score | TDA q-value | Volume | Spectrum Matches |
| --- | --- | --- | --- | --- |
| 13.1155 | 155.0526 | 0.0000 | 7.5767e+07 | 3 |

**Adduct Signal Fractions**

- **Unmodified**: 7.5767e+07

### LVPVPITN(N)ATLDQITGK Hex 5 HexNAc 4

**Mass:**  3401.5988

(6.823e-07)

**Charge States:** 3, 4

**Start Position**: 26

**End Position**: 43

| Profile Score | Tandem Score | TDA q-value | Volume | Spectrum Matches |
| --- | --- | --- | --- | --- |
| 14.5715 | 153.8310 | 0.0000 | 1.6465e+09 | 16 |

**Adduct Signal Fractions**

- **Unmodified**: 1.6465e+09

### SVQEIQATFFYFTPN(N)K Fuc 1 Hex 8 HexNAc 4 Neu5Ac 1

**Mass:**  4464.8566

(3.716e-06)

**Charge States:** 3, 4

**Start Position**: 58

**End Position**: 74

| Profile Score | Tandem Score | TDA q-value | Volume | Spectrum Matches |
| --- | --- | --- | --- | --- |
| 12.6064 | 153.7809 | 0.0000 | 5.2580e+07 | 4 |

**Adduct Signal Fractions**

- **Unmodified**: 5.2580e+07

### SVQEIQATFFYFTPN(N)K Fuc 1 Hex 8 HexNAc 7

**Mass:**  4782.9924

(2.055e-06)

**Charge States:** 3, 4

**Start Position**: 58

**End Position**: 74

| Profile Score | Tandem Score | TDA q-value | Volume | Spectrum Matches |
| --- | --- | --- | --- | --- |
| 12.2037 | 150.1947 | 0.0000 | 7.7208e+07 | 3 |

**Adduct Signal Fractions**

- **Unmodified**: 7.7208e+07

### SVQEIQATFFYFTPN(N)KTEDTIFLR Fuc 1 Hex 6 HexNAc 5

**Mass:**  5028.2297

(1.853e-06)

**Charge States:** 4, 5

**Start Position**: 58

**End Position**: 82

| Profile Score | Tandem Score | TDA q-value | Volume | Spectrum Matches |
| --- | --- | --- | --- | --- |
| 12.4524 | 146.6399 | 0.0000 | 1.4224e+08 | 5 |

**Adduct Signal Fractions**

- **Unmodified**: 1.4224e+08

### SVQEIQATFFYFTPN(N)KTEDTIFLR Hex 6 HexNAc 5

**Mass:**  4882.1765

(2.142e-06)

**Charge States:** 3, 4, 5

**Start Position**: 58

**End Position**: 82

| Profile Score | Tandem Score | TDA q-value | Volume | Spectrum Matches |
| --- | --- | --- | --- | --- |
| 14.5191 | 145.9353 | 0.0000 | 5.9164e+08 | 19 |

**Adduct Signal Fractions**

- **Unmodified**: 5.9164e+08

### SVQEIQATFFYFTPN(N)K Hex 9 HexNAc 8

**Mass:**  5002.0686

(2.249e-06)

**Charge States:** 4, 5

**Start Position**: 58

**End Position**: 74

| Profile Score | Tandem Score | TDA q-value | Volume | Spectrum Matches |
| --- | --- | --- | --- | --- |
| 13.6658 | 144.3287 | 0.0000 | 8.1956e+07 | 4 |

**Adduct Signal Fractions**

- **Unmodified**: 8.1956e+07

### ATFFYFTPN(N)K Fuc 1 Hex 6 HexNAc 5

**Mass:**  3369.3751

(2.972e-04)

**Start Position**: 64

**End Position**: 74

| Profile Score | Tandem Score | TDA q-value | Volume | Spectrum Matches |
| --- | --- | --- | --- | --- |
| 0.0000 | 142.0677 | 0.0000 | 0.0000e+00 | 1 |

Adduct:

No Chromatogram Found

### SVQEIQATFFYFTPN(N)K Fuc 2 Hex 8 HexNAc 7

**Mass:**  4929.0529

(2.804e-06)

**Charge States:** 4

**Start Position**: 58

**End Position**: 74

| Profile Score | Tandem Score | TDA q-value | Volume | Spectrum Matches |
| --- | --- | --- | --- | --- |
| 12.4397 | 141.8855 | 0.0000 | 1.6702e+07 | 3 |

**Adduct Signal Fractions**

- **Unmodified**: 1.6702e+07

### LVPVPITN(N)ATLDQITGK Hex 7 HexNAc 6

**Mass:**  4131.8732

(1.606e-06)

**Charge States:** 3, 4

**Start Position**: 26

**End Position**: 43

| Profile Score | Tandem Score | TDA q-value | Volume | Spectrum Matches |
| --- | --- | --- | --- | --- |
| 15.0291 | 141.6039 | 0.0000 | 5.2164e+08 | 6 |

**Adduct Signal Fractions**

- **Unmodified**: 5.2164e+08

### SVQEIQATFFYFTPN(N)K Hex 7 HexNAc 4 Neu5Ac 2

**Mass:**  4447.8327

(2.078e-06)

**Charge States:** 3, 4

**Start Position**: 58

**End Position**: 74

| Profile Score | Tandem Score | TDA q-value | Volume | Spectrum Matches |
| --- | --- | --- | --- | --- |
| 12.1826 | 140.4643 | 0.0000 | 1.3486e+08 | 6 |

**Adduct Signal Fractions**

- **Unmodified**: 1.3486e+08

### PVPITN(N)ATLDQITGK Fuc 1 Hex 3 HexNAc 9

**Mass:**  4028.8285

(5.056e-04)

**Start Position**: 28

**End Position**: 43

| Profile Score | Tandem Score | TDA q-value | Volume | Spectrum Matches |
| --- | --- | --- | --- | --- |
| 0.0000 | 140.1296 | 0.0000 | 0.0000e+00 | 1 |

Adduct:

No Chromatogram Found

### SVQEIQATFFYFTPN(N)KTEDTIFLR Hex 7 HexNAc 6

**Mass:**  5247.3186

(3.440e-06)

**Charge States:** 3, 4, 5

**Start Position**: 58

**End Position**: 82

| Profile Score | Tandem Score | TDA q-value | Volume | Spectrum Matches |
| --- | --- | --- | --- | --- |
| 13.3341 | 139.3981 | 0.0000 | 1.2201e+09 | 24 |

**Adduct Signal Fractions**

- **Unmodified**: 1.2201e+09

### YFTPN(N)KTEDTIFLR Fuc 1 Hex 7 HexNAc 6

**Mass:**  4242.7907

(3.852e-07)

**Charge States:** 3, 4

**Start Position**: 68

**End Position**: 82

| Profile Score | Tandem Score | TDA q-value | Volume | Spectrum Matches |
| --- | --- | --- | --- | --- |
| 9.7723 | 138.2884 | 0.0000 | 2.3763e+08 | 4 |

**Adduct Signal Fractions**

- **Unmodified**: 2.3763e+08

### TPN(N)KTEDTIFLR Hex 7 HexNAc 6

**Mass:**  3786.6015

(1.054e-06)

**Charge States:** 3, 4

**Start Position**: 70

**End Position**: 82

| Profile Score | Tandem Score | TDA q-value | Volume | Spectrum Matches |
| --- | --- | --- | --- | --- |
| 14.4896 | 137.8852 | 0.0000 | 6.7669e+07 | 6 |

**Adduct Signal Fractions**

- **Unmodified**: 6.7669e+07

### QDQC(C)IYN(N)TTYLNVQR Hex 6 HexNAc 5

**Mass:**  3902.6109

(1.858e-06)

**Charge States:** 3, 4

**Start Position**: 87

**End Position**: 102

| Profile Score | Tandem Score | TDA q-value | Volume | Spectrum Matches |
| --- | --- | --- | --- | --- |
| 11.0945 | 135.0701 | 0.0000 | 1.9022e+09 | 8 |

**Adduct Signal Fractions**

- **Unmodified**: 1.9022e+09

### PVPITN(N)ATLDQITGK Fuc 1 Hex 4 HexNAc 9

**Mass:**  4188.8850

(9.770e-06)

**Charge States:** 3, 4

**Start Position**: 28

**End Position**: 43

| Profile Score | Tandem Score | TDA q-value | Volume | Spectrum Matches |
| --- | --- | --- | --- | --- |
| -4.5055 | 134.1553 | 0.0000 | 8.5230e+07 | 3 |

**Adduct Signal Fractions**

- **Unmodified**: 8.5230e+07

### YFTPN(N)KTEDTIFLR Hex 6 HexNAc 5

**Mass:**  3731.6054

(1.393e-06)

**Charge States:** 3, 4

**Start Position**: 68

**End Position**: 82

| Profile Score | Tandem Score | TDA q-value | Volume | Spectrum Matches |
| --- | --- | --- | --- | --- |
| 10.6470 | 133.2073 | 0.0000 | 1.0905e+09 | 10 |

**Adduct Signal Fractions**

- **Unmodified**: 1.0905e+09

### SVQEIQATFFYFTPN(N)K Fuc 3 Hex 4 HexNAc 5

**Mass:**  4020.7202

(-2.050e-06)

**Charge States:** 3, 4

**Start Position**: 58

**End Position**: 74

| Profile Score | Tandem Score | TDA q-value | Volume | Spectrum Matches |
| --- | --- | --- | --- | --- |
| -19.9652 | 131.8360 | 0.0000 | 1.2370e+07 | 1 |

**Adduct Signal Fractions**

- **Unmodified**: 1.2370e+07

### QDQC(C)IYN(N)TTYLNVQR Hex 7 HexNAc 6

**Mass:**  4267.7512

(1.320e-06)

**Charge States:** 3, 4, 5

**Start Position**: 87

**End Position**: 102

| Profile Score | Tandem Score | TDA q-value | Volume | Spectrum Matches |
| --- | --- | --- | --- | --- |
| 13.8260 | 131.7780 | 0.0000 | 2.5503e+09 | 8 |

**Adduct Signal Fractions**

- **Unmodified**: 2.5503e+09

### YFTPN(N)KTEDTIFLR Hex 7 HexNAc 6

**Mass:**  4096.7331

(2.062e-06)

**Charge States:** 3, 4

**Start Position**: 68

**End Position**: 82

| Profile Score | Tandem Score | TDA q-value | Volume | Spectrum Matches |
| --- | --- | --- | --- | --- |
| 11.2278 | 131.6591 | 0.0000 | 2.1452e+09 | 123 |

**Adduct Signal Fractions**

- **Unmodified**: 2.1452e+09

### YFTPN(N)KTEDTIFLR Hex 5 HexNAc 4

**Mass:**  3366.4678

(9.407e-07)

**Charge States:** 3, 4

**Start Position**: 68

**End Position**: 82

| Profile Score | Tandem Score | TDA q-value | Volume | Spectrum Matches |
| --- | --- | --- | --- | --- |
| 14.9733 | 131.0474 | 0.0000 | 5.9734e+07 | 1 |

**Adduct Signal Fractions**

- **Unmodified**: 5.9734e+07

### QDQC(C)IYN(N)TTYLNVQR Fuc 1 Hex 6 HexNAc 5

**Mass:**  4048.6701

(2.483e-06)

**Charge States:** 3, 4

**Start Position**: 87

**End Position**: 102

| Profile Score | Tandem Score | TDA q-value | Volume | Spectrum Matches |
| --- | --- | --- | --- | --- |
| 12.8258 | 128.7576 | 0.0000 | 5.0174e+08 | 5 |

**Adduct Signal Fractions**

- **Unmodified**: 5.0174e+08

### YFTPN(N)KTEDTIFLR Fuc 1 Hex 6 HexNAc 5

**Mass:**  3877.6569

(7.826e-07)

**Charge States:** 3, 4

**Start Position**: 68

**End Position**: 82

| Profile Score | Tandem Score | TDA q-value | Volume | Spectrum Matches |
| --- | --- | --- | --- | --- |
| 13.3577 | 127.6661 | 0.0000 | 2.7783e+08 | 3 |

**Adduct Signal Fractions**

- **Unmodified**: 2.7783e+08

### LVPVPITN(N)ATLDQITGK Fuc 1 Hex 7 HexNAc 6

**Mass:**  4277.9294

(1.160e-06)

**Charge States:** 3, 4

**Start Position**: 26

**End Position**: 43

| Profile Score | Tandem Score | TDA q-value | Volume | Spectrum Matches |
| --- | --- | --- | --- | --- |
| 13.9474 | 127.2997 | 0.0000 | 1.8580e+08 | 4 |

**Adduct Signal Fractions**

- **Unmodified**: 1.8580e+08

### QDQC(C)IYN(N)TTYLNVQR Hex 8 HexNAc 7

**Mass:**  4632.8791

(1.450e-06)

**Charge States:** 3, 4

**Start Position**: 87

**End Position**: 102

| Profile Score | Tandem Score | TDA q-value | Volume | Spectrum Matches |
| --- | --- | --- | --- | --- |
| 13.5779 | 126.8689 | 0.0000 | 4.7870e+08 | 6 |

**Adduct Signal Fractions**

- **Unmodified**: 4.7870e+08

### SVQEIQATFFYFTPN(N)K Hex 6 HexNAc 6

**Mass:**  4109.7453

(2.459e-06)

**Charge States:** 3, 4

**Start Position**: 58

**End Position**: 74

| Profile Score | Tandem Score | TDA q-value | Volume | Spectrum Matches |
| --- | --- | --- | --- | --- |
| 12.4238 | 126.3226 | 0.0000 | 6.6639e+07 | 7 |

**Adduct Signal Fractions**

- **Unmodified**: 6.6639e+07

### SVQEIQATFFYFTPN(N)K Fuc 4 Hex 5 HexNAc 5

**Mass:**  4328.8234

(-3.425e-06)

**Charge States:** 3, 4, 5

**Start Position**: 58

**End Position**: 74

| Profile Score | Tandem Score | TDA q-value | Volume | Spectrum Matches |
| --- | --- | --- | --- | --- |
| 12.2136 | 126.2955 | 0.0000 | 6.1505e+08 | 26 |

**Adduct Signal Fractions**

- **Unmodified**: 6.1505e+08

### SVQEIQATFFYFTPN(N)K Fuc 1 Hex 7 HexNAc 4 Neu5Ac 2

**Mass:**  4593.8884

(1.587e-06)

**Charge States:** 4

**Start Position**: 58

**End Position**: 74

| Profile Score | Tandem Score | TDA q-value | Volume | Spectrum Matches |
| --- | --- | --- | --- | --- |
| -19.3917 | 125.3733 | 0.0000 | 1.2482e+07 | 3 |

**Adduct Signal Fractions**

- **Unmodified**: 1.2482e+07

### QDQC(C)IYN(N)TTYLNVQR Fuc 1 Hex 7 HexNAc 6

**Mass:**  4413.8063

(2.931e-06)

**Charge States:** 3, 4

**Start Position**: 87

**End Position**: 102

| Profile Score | Tandem Score | TDA q-value | Volume | Spectrum Matches |
| --- | --- | --- | --- | --- |
| 12.1944 | 125.3570 | 0.0000 | 6.6950e+08 | 5 |

**Adduct Signal Fractions**

- **Unmodified**: 6.6950e+08

### FYFTPN(N)KTEDTIFLR Hex 6 HexNAc 5

**Mass:**  3878.6717

(4.876e-07)

**Charge States:** 3, 4

**Start Position**: 67

**End Position**: 82

| Profile Score | Tandem Score | TDA q-value | Volume | Spectrum Matches |
| --- | --- | --- | --- | --- |
| 14.9860 | 124.3039 | 0.0000 | 8.3599e+07 | 6 |

**Adduct Signal Fractions**

- **Unmodified**: 8.3599e+07

### QDQC(C)IYN(N)TTYLNVQR Hex 5 HexNAc 4

**Mass:**  3537.4777

(1.877e-06)

**Charge States:** 3

**Start Position**: 87

**End Position**: 102

| Profile Score | Tandem Score | TDA q-value | Volume | Spectrum Matches |
| --- | --- | --- | --- | --- |
| -4.0728 | 123.0550 | 0.0000 | 4.0167e+07 | 2 |

**Adduct Signal Fractions**

- **Unmodified**: 4.0167e+07

### SVQEIQATFFYFTPN(N)K Hex 8 HexNAc 6

**Mass:**  4436.8889

(6.864e-04)

**Start Position**: 58

**End Position**: 74

| Profile Score | Tandem Score | TDA q-value | Volume | Spectrum Matches |
| --- | --- | --- | --- | --- |
| 0.0000 | 121.7438 | 0.0000 | 0.0000e+00 | 1 |

Adduct:

No Chromatogram Found

### QDQC(C)IYN(N)TTYLNVQR Fuc 1 Hex 8 HexNAc 7

**Mass:**  4778.9338

(2.072e-06)

**Charge States:** 3, 4

**Start Position**: 87

**End Position**: 102

| Profile Score | Tandem Score | TDA q-value | Volume | Spectrum Matches |
| --- | --- | --- | --- | --- |
| 13.5259 | 121.4492 | 0.0000 | 1.1360e+08 | 4 |

**Adduct Signal Fractions**

- **Unmodified**: 1.1360e+08

### PVPITN(N)ATLDQITGK Hex 9 HexNAc 4

**Mass:**  3840.6777

(7.877e-04)

**Start Position**: 28

**End Position**: 43

| Profile Score | Tandem Score | TDA q-value | Volume | Spectrum Matches |
| --- | --- | --- | --- | --- |
| 0.0000 | 120.9494 | 0.0000 | 0.0000e+00 | 1 |

Adduct:

No Chromatogram Found

### TPN(N)KTEDTIFLR Hex 6 HexNAc 5

**Mass:**  3421.4690

(1.273e-06)

**Charge States:** 3, 4

**Start Position**: 70

**End Position**: 82

| Profile Score | Tandem Score | TDA q-value | Volume | Spectrum Matches |
| --- | --- | --- | --- | --- |
| 14.7048 | 120.7758 | 0.0000 | 3.3612e+07 | 7 |

**Adduct Signal Fractions**

- **Unmodified**: 3.3612e+07

### FYFTPN(N)KTEDTIFLR Hex 7 HexNAc 6

**Mass:**  4243.8059

(8.887e-07)

**Charge States:** 3, 4

**Start Position**: 67

**End Position**: 82

| Profile Score | Tandem Score | TDA q-value | Volume | Spectrum Matches |
| --- | --- | --- | --- | --- |
| 14.5437 | 120.5617 | 0.0000 | 2.7752e+08 | 8 |

**Adduct Signal Fractions**

- **Unmodified**: 2.7752e+08

### FTPN(N)KTEDTIFLR Hex 6 HexNAc 5

**Mass:**  3568.5393

(1.308e-06)

**Charge States:** 3, 4

**Start Position**: 69

**End Position**: 82

| Profile Score | Tandem Score | TDA q-value | Volume | Spectrum Matches |
| --- | --- | --- | --- | --- |
| 14.7569 | 120.1939 | 0.0000 | 8.0490e+07 | 5 |

**Adduct Signal Fractions**

- **Unmodified**: 8.0490e+07

### PVPITN(N)ATLDQITGK Fuc 2 Hex 3 HexNAc 7 Neu5Ac 1

**Mass:**  4057.8305

(9.695e-06)

**Charge States:** 3, 4

**Start Position**: 28

**End Position**: 43

| Profile Score | Tandem Score | TDA q-value | Volume | Spectrum Matches |
| --- | --- | --- | --- | --- |
| 11.5001 | 118.7046 | 0.0000 | 2.6128e+07 | 6 |

**Adduct Signal Fractions**

- **Unmodified**: 2.6128e+07

### QDQC(C)IYN(N)TTYLNVQR Fuc 2 Hex 7 HexNAc 6

**Mass:**  4559.8635

(2.731e-06)

**Charge States:** 3, 4

**Start Position**: 87

**End Position**: 102

| Profile Score | Tandem Score | TDA q-value | Volume | Spectrum Matches |
| --- | --- | --- | --- | --- |
| 13.8968 | 118.5571 | 0.0000 | 1.0800e+08 | 3 |

**Adduct Signal Fractions**

- **Unmodified**: 1.0800e+08

### QDQC(C)IYN(N)TTYLNVQR Hex 9 HexNAc 8

**Mass:**  4998.0114

(2.279e-06)

**Charge States:** 3, 4

**Start Position**: 87

**End Position**: 102

| Profile Score | Tandem Score | TDA q-value | Volume | Spectrum Matches |
| --- | --- | --- | --- | --- |
| 13.4169 | 117.2665 | 0.0000 | 1.4663e+08 | 3 |

**Adduct Signal Fractions**

- **Unmodified**: 1.4663e+08

### LVPVPITN(N)ATLDQITGK Hex 8 HexNAc 7

**Mass:**  4497.0007

(1.876e-06)

**Charge States:** 3, 4, 5

**Start Position**: 26

**End Position**: 43

| Profile Score | Tandem Score | TDA q-value | Volume | Spectrum Matches |
| --- | --- | --- | --- | --- |
| 13.3583 | 116.6329 | 0.0000 | 8.5928e+07 | 6 |

**Adduct Signal Fractions**

- **Unmodified**: 8.5928e+07

### SVQEIQATFFYFTPN(N)K Fuc 2 Hex 6 HexNAc 5

**Mass:**  4198.7907

(3.446e-06)

**Charge States:** 3, 4

**Start Position**: 58

**End Position**: 74

| Profile Score | Tandem Score | TDA q-value | Volume | Spectrum Matches |
| --- | --- | --- | --- | --- |
| -19.5214 | 115.3254 | 0.0000 | 1.1054e+07 | 1 |

**Adduct Signal Fractions**

- **Unmodified**: 1.1054e+07

### LVPVPITN(N)ATLDQITGK Fuc 1 Hex 7 HexNAc 5

**Mass:**  4074.8718

(8.094e-06)

**Charge States:** 3, 4

**Start Position**: 26

**End Position**: 43

| Profile Score | Tandem Score | TDA q-value | Volume | Spectrum Matches |
| --- | --- | --- | --- | --- |
| 3.5601 | 115.0381 | 0.0000 | 9.8379e+06 | 3 |

**Adduct Signal Fractions**

- **Unmodified**: 9.8379e+06

### PVPITN(N)ATLDQITGK Fuc 2 Hex 4 HexNAc 8

**Mass:**  4133.8648

(4.940e-04)

**Start Position**: 28

**End Position**: 43

| Profile Score | Tandem Score | TDA q-value | Volume | Spectrum Matches |
| --- | --- | --- | --- | --- |
| 0.0000 | 114.7802 | 0.0000 | 0.0000e+00 | 1 |

Adduct:

No Chromatogram Found

### YFTPN(N)KTEDTIFLR Hex 7 HexNAc 6

**Mass:**  4098.7433

(7.375e-04)

**Start Position**: 68

**End Position**: 82

| Profile Score | Tandem Score | TDA q-value | Volume | Spectrum Matches |
| --- | --- | --- | --- | --- |
| 0.0000 | 114.3662 | 0.0000 | 0.0000e+00 | 2 |

Adduct:

No Chromatogram Found

### YFTPN(N)KTEDTIFLR Hex 8 HexNAc 7

**Mass:**  4461.8669

(9.227e-07)

**Charge States:** 3, 4

**Start Position**: 68

**End Position**: 82

| Profile Score | Tandem Score | TDA q-value | Volume | Spectrum Matches |
| --- | --- | --- | --- | --- |
| 12.8998 | 114.2786 | 0.0000 | 2.0303e+08 | 4 |

**Adduct Signal Fractions**

- **Unmodified**: 2.0303e+08

### LVPVPITN(N)ATLDQITGK Fuc 2 Hex 5 HexNAc 3 Neu5Ac 1

**Mass:**  3784.7466

(8.018e-04)

**Charge States:** 3, 4

**Start Position**: 26

**End Position**: 43

| Profile Score | Tandem Score | TDA q-value | Volume | Spectrum Matches |
| --- | --- | --- | --- | --- |
| -9.8667 | 113.3962 | 0.0000 | 6.7186e+07 | 40 |

**Adduct Signal Fractions**

- **Unmodified**: 6.7186e+07

### PVPITN(N)ATLDQITGK Fuc 4 Hex 3 HexNAc 7

**Mass:**  4058.8515

(6.130e-06)

**Charge States:** 4

**Start Position**: 28

**End Position**: 43

| Profile Score | Tandem Score | TDA q-value | Volume | Spectrum Matches |
| --- | --- | --- | --- | --- |
| -5.0759 | 112.4590 | 0.0000 | 3.4141e+07 | 1 |

**Adduct Signal Fractions**

- **Unmodified**: 3.4141e+07

### PVPITN(N)ATLDQITGK Fuc 2 Hex 3 HexNAc 8

**Mass:**  3971.8150

(5.149e-04)

**Start Position**: 28

**End Position**: 43

| Profile Score | Tandem Score | TDA q-value | Volume | Spectrum Matches |
| --- | --- | --- | --- | --- |
| 0.0000 | 110.1717 | 0.0000 | 0.0000e+00 | 1 |

Adduct:

No Chromatogram Found

### LVPVPITN(N)ATLDQITGK Fuc 1 Hex 7 HexNAc 3 Neu5Ac 1

**Mass:**  3959.7932

(4.647e-06)

**Charge States:** 3, 4

**Start Position**: 26

**End Position**: 43

| Profile Score | Tandem Score | TDA q-value | Volume | Spectrum Matches |
| --- | --- | --- | --- | --- |
| -18.7328 | 109.3006 | 0.0000 | 3.2888e+07 | 2 |

**Adduct Signal Fractions**

- **Unmodified**: 3.2888e+07

### SVQEIQATFFYFTPN(N)K Fuc 4 Hex 6 HexNAc 5

**Mass:**  4493.9133

(6.728e-04)

**Start Position**: 58

**End Position**: 74

| Profile Score | Tandem Score | TDA q-value | Volume | Spectrum Matches |
| --- | --- | --- | --- | --- |
| 0.0000 | 108.6138 | 0.0000 | 0.0000e+00 | 1 |

Adduct:

No Chromatogram Found

### FYFTPN(N)KTEDTIFLR Fuc 1 Hex 6 HexNAc 5

**Mass:**  4024.7294

(2.450e-06)

**Charge States:** 3, 4

**Start Position**: 67

**End Position**: 82

| Profile Score | Tandem Score | TDA q-value | Volume | Spectrum Matches |
| --- | --- | --- | --- | --- |
| 13.6825 | 108.4833 | 0.0000 | 2.0955e+07 | 3 |

**Adduct Signal Fractions**

- **Unmodified**: 2.0955e+07

### PVPITN(N)ATLDQITGK Fuc 1 Hex 8 HexNAc 5

**Mass:**  4024.7297

(-1.468e-06)

**Charge States:** 4

**Start Position**: 28

**End Position**: 43

| Profile Score | Tandem Score | TDA q-value | Volume | Spectrum Matches |
| --- | --- | --- | --- | --- |
| -5.2148 | 108.4833 | 0.0000 | 1.7934e+07 | 3 |

**Adduct Signal Fractions**

- **Unmodified**: 1.7934e+07

### QDQC(C)IYN(N)TTYLNVQR Fuc 1 Hex 7 HexNAc 4 Neu5Ac 2

**Mass:**  4589.8298

(1.794e-06)

**Charge States:** 3, 4

**Start Position**: 87

**End Position**: 102

| Profile Score | Tandem Score | TDA q-value | Volume | Spectrum Matches |
| --- | --- | --- | --- | --- |
| -5.1785 | 108.4775 | 0.0000 | 3.7930e+07 | 2 |

**Adduct Signal Fractions**

- **Unmodified**: 3.7930e+07

### YFTPN(N)KTEDTIFLR Hex 7 HexNAc 7

**Mass:**  4299.8176

(2.108e-06)

**Charge States:** 4

**Start Position**: 68

**End Position**: 82

| Profile Score | Tandem Score | TDA q-value | Volume | Spectrum Matches |
| --- | --- | --- | --- | --- |
| -5.1401 | 108.3059 | 0.0000 | 7.8683e+07 | 1 |

**Adduct Signal Fractions**

- **Unmodified**: 7.8683e+07

### PVPITN(N)ATLDQITGK Fuc 2 Hex 8 HexNAc 4

**Mass:**  3968.6694

(2.468e-04)

**Charge States:** 4

**Start Position**: 28

**End Position**: 43

| Profile Score | Tandem Score | TDA q-value | Volume | Spectrum Matches |
| --- | --- | --- | --- | --- |
| -21.2953 | 106.9270 | 0.0000 | 1.0648e+06 | 3 |

**Adduct Signal Fractions**

- **Unmodified**: 1.0648e+06

### FYFTPN(N)KTEDTIFLR Fuc 1 Hex 7 HexNAc 6

**Mass:**  4389.8620

(1.053e-06)

**Charge States:** 3, 4

**Start Position**: 67

**End Position**: 82

| Profile Score | Tandem Score | TDA q-value | Volume | Spectrum Matches |
| --- | --- | --- | --- | --- |
| 14.0782 | 106.1492 | 0.0000 | 5.2201e+07 | 5 |

**Adduct Signal Fractions**

- **Unmodified**: 5.2201e+07

### QDQC(C)IYN(N)TTYLNVQR Hex 7 HexNAc 4 Neu5Ac 2

**Mass:**  4443.7736

(1.390e-06)

**Charge States:** 3, 4

**Start Position**: 87

**End Position**: 102

| Profile Score | Tandem Score | TDA q-value | Volume | Spectrum Matches |
| --- | --- | --- | --- | --- |
| 12.3826 | 104.8612 | 0.0000 | 2.0206e+08 | 4 |

**Adduct Signal Fractions**

- **Unmodified**: 2.0206e+08

### FTPN(N)KTEDTIFLR Hex 7 HexNAc 6

**Mass:**  3933.6713

(7.723e-07)

**Charge States:** 3, 4

**Start Position**: 69

**End Position**: 82

| Profile Score | Tandem Score | TDA q-value | Volume | Spectrum Matches |
| --- | --- | --- | --- | --- |
| 13.6888 | 104.4659 | 0.0000 | 1.5662e+08 | 6 |

**Adduct Signal Fractions**

- **Unmodified**: 1.5662e+08

### PVPITN(N)ATLDQITGK Fuc 2 Hex 7 HexNAc 3

**Mass:**  3602.5940

(2.288e-06)

**Charge States:** 3, 4

**Start Position**: 28

**End Position**: 43

| Profile Score | Tandem Score | TDA q-value | Volume | Spectrum Matches |
| --- | --- | --- | --- | --- |
| -19.4042 | 101.6982 | 0.0000 | 5.9725e+06 | 1 |

**Adduct Signal Fractions**

- **Unmodified**: 5.9725e+06

### SVQEIQATFFYFTPN(N)K Fuc 4 Hex 6 HexNAc 6

**Mass:**  4693.9543

(-3.658e-06)

**Charge States:** 4

**Start Position**: 58

**End Position**: 74

| Profile Score | Tandem Score | TDA q-value | Volume | Spectrum Matches |
| --- | --- | --- | --- | --- |
| 10.2485 | 100.1067 | 0.0000 | 3.8658e+07 | 2 |

**Adduct Signal Fractions**

- **Unmodified**: 3.8658e+07

### TPN(N)KTEDTIFLR Fuc 1 Hex 6 HexNAc 5

**Mass:**  3567.5268

(1.013e-06)

**Charge States:** 3

**Start Position**: 70

**End Position**: 82

| Profile Score | Tandem Score | TDA q-value | Volume | Spectrum Matches |
| --- | --- | --- | --- | --- |
| -5.2334 | 98.6742 | 0.0000 | 5.7110e+06 | 3 |

**Adduct Signal Fractions**

- **Unmodified**: 5.7110e+06

### IYN(N)TTYLNVQR Hex 7 HexNAc 6

**Mass:**  3736.5664

(1.521e-06)

**Charge States:** 3, 4

**Start Position**: 91

**End Position**: 102

| Profile Score | Tandem Score | TDA q-value | Volume | Spectrum Matches |
| --- | --- | --- | --- | --- |
| 15.9167 | 97.2431 | 0.0000 | 2.9066e+07 | 4 |

**Adduct Signal Fractions**

- **Unmodified**: 2.9066e+07

### YFTPN(N)KTEDTIFLR Hex 9 HexNAc 8

**Mass:**  4826.9953

(2.349e-06)

**Charge States:** 4

**Start Position**: 68

**End Position**: 82

| Profile Score | Tandem Score | TDA q-value | Volume | Spectrum Matches |
| --- | --- | --- | --- | --- |
| -3.3621 | 97.0643 | 0.0000 | 6.3353e+07 | 2 |

**Adduct Signal Fractions**

- **Unmodified**: 6.3353e+07

### LVPVPITN(N)ATLDQITGK Fuc 3 Hex 3 HexNAc 9

**Mass:**  4533.0581

(4.408e-04)

**Start Position**: 26

**End Position**: 43

| Profile Score | Tandem Score | TDA q-value | Volume | Spectrum Matches |
| --- | --- | --- | --- | --- |
| 0.0000 | 96.5504 | 0.0000 | 0.0000e+00 | 1 |

Adduct:

No Chromatogram Found

### PVPITN(N)ATLDQITGK Fuc 3 Hex 3 HexNAc 5 Neu5Ac 2

**Mass:**  4088.8195

(9.252e-06)

**Charge States:** 3, 4

**Start Position**: 28

**End Position**: 43

| Profile Score | Tandem Score | TDA q-value | Volume | Spectrum Matches |
| --- | --- | --- | --- | --- |
| -4.8966 | 96.1734 | 0.0000 | 3.3313e+07 | 2 |

**Adduct Signal Fractions**

- **Unmodified**: 3.3313e+07

### FTPN(N)KTEDTIFLR Fuc 1 Hex 6 HexNAc 5

**Mass:**  3714.5963

(-1.507e-06)

**Charge States:** 3, 4

**Start Position**: 69

**End Position**: 82

| Profile Score | Tandem Score | TDA q-value | Volume | Spectrum Matches |
| --- | --- | --- | --- | --- |
| -3.9985 | 96.0927 | 0.0000 | 1.6967e+07 | 3 |

**Adduct Signal Fractions**

- **Unmodified**: 1.6967e+07

### YFTPN(N)KTEDTIFLR Hex 7 HexNAc 4 Neu5Ac 2

**Mass:**  4272.7644

(-4.454e-06)

**Charge States:** 4

**Start Position**: 68

**End Position**: 82

| Profile Score | Tandem Score | TDA q-value | Volume | Spectrum Matches |
| --- | --- | --- | --- | --- |
| -3.7424 | 95.6772 | 0.0000 | 1.3120e+08 | 5 |

**Adduct Signal Fractions**

- **Unmodified**: 1.3120e+08

### PVPITN(N)ATLDQITGK Fuc 3 Hex 5 HexNAc 9

**Mass:**  4643.0593

(9.737e-06)

**Charge States:** 4

**Start Position**: 28

**End Position**: 43

| Profile Score | Tandem Score | TDA q-value | Volume | Spectrum Matches |
| --- | --- | --- | --- | --- |
| 13.1320 | 93.6678 | 0.0000 | 1.4479e+07 | 2 |

**Adduct Signal Fractions**

- **Unmodified**: 1.4479e+07

### SVQEIQATFFYFTPN(N)KTEDTIFLR Hex 5 HexNAc 4

**Mass:**  4517.0388

(1.748e-06)

**Charge States:** 3, 4, 5

**Start Position**: 58

**End Position**: 82

| Profile Score | Tandem Score | TDA q-value | Volume | Spectrum Matches |
| --- | --- | --- | --- | --- |
| -19.0764 | 91.8838 | 0.0000 | 2.0297e+07 | 2 |

**Adduct Signal Fractions**

- **Unmodified**: 2.0297e+07

### SVQEIQATFFYFTPN(N)KTEDTIFLR Fuc 1 Hex 7 HexNAc 6

**Mass:**  5393.3654

(1.797e-06)

**Charge States:** 4, 5

**Start Position**: 58

**End Position**: 82

| Profile Score | Tandem Score | TDA q-value | Volume | Spectrum Matches |
| --- | --- | --- | --- | --- |
| 12.6122 | 91.2079 | 0.0000 | 2.4041e+08 | 6 |

**Adduct Signal Fractions**

- **Unmodified**: 2.4041e+08

### SVQEIQATFFYFTPN(N)KTEDTIFLR Hex 8 HexNAc 7

**Mass:**  5612.4362

(2.149e-06)

**Charge States:** 4, 5

**Start Position**: 58

**End Position**: 82

| Profile Score | Tandem Score | TDA q-value | Volume | Spectrum Matches |
| --- | --- | --- | --- | --- |
| 14.4282 | 90.2821 | 0.0000 | 2.1011e+08 | 5 |

**Adduct Signal Fractions**

- **Unmodified**: 2.1011e+08

### PVPITN(N)ATLDQITGK Hex 3 HexNAc 9

**Mass:**  3882.7759

(5.260e-04)

**Start Position**: 28

**End Position**: 43

| Profile Score | Tandem Score | TDA q-value | Volume | Spectrum Matches |
| --- | --- | --- | --- | --- |
| 0.0000 | 89.0884 | 0.0000 | 0.0000e+00 | 1 |

Adduct:

No Chromatogram Found

### TPN(N)KTEDTIFLR Fuc 1 Hex 7 HexNAc 6

**Mass:**  3932.6596

(1.082e-06)

**Charge States:** 3, 4

**Start Position**: 70

**End Position**: 82

| Profile Score | Tandem Score | TDA q-value | Volume | Spectrum Matches |
| --- | --- | --- | --- | --- |
| -18.8900 | 87.4782 | 0.0000 | 7.6306e+06 | 2 |

**Adduct Signal Fractions**

- **Unmodified**: 7.6306e+06

### FTPN(N)KTEDTIFLR Hex 8 HexNAc 7

**Mass:**  4298.7995

(3.602e-07)

**Charge States:** 3, 4

**Start Position**: 69

**End Position**: 82

| Profile Score | Tandem Score | TDA q-value | Volume | Spectrum Matches |
| --- | --- | --- | --- | --- |
| -4.3632 | 86.3729 | 0.0000 | 6.9860e+06 | 7 |

**Adduct Signal Fractions**

- **Unmodified**: 6.9860e+06

### FTPN(N)KTEDTIFLR Fuc 1 Hex 7 HexNAc 6

**Mass:**  4079.7268

(-5.028e-07)

**Charge States:** 3, 4

**Start Position**: 69

**End Position**: 82

| Profile Score | Tandem Score | TDA q-value | Volume | Spectrum Matches |
| --- | --- | --- | --- | --- |
| -4.2813 | 85.3134 | 0.0000 | 2.4940e+07 | 3 |

**Adduct Signal Fractions**

- **Unmodified**: 2.4940e+07

### TPN(N)KTEDTIFLR Hex 5 HexNAc 4

**Mass:**  3056.3333

(1.868e-06)

**Charge States:** 3

**Start Position**: 70

**End Position**: 82

| Profile Score | Tandem Score | TDA q-value | Volume | Spectrum Matches |
| --- | --- | --- | --- | --- |
| -3.8223 | 84.9324 | 0.0000 | 3.9675e+06 | 3 |

**Adduct Signal Fractions**

- **Unmodified**: 3.9675e+06

### FYFTPN(N)KTEDTIFLR Hex 8 HexNAc 7

**Mass:**  4608.9299

(5.185e-08)

**Charge States:** 3, 4

**Start Position**: 67

**End Position**: 82

| Profile Score | Tandem Score | TDA q-value | Volume | Spectrum Matches |
| --- | --- | --- | --- | --- |
| 14.2593 | 84.8825 | 0.0000 | 2.3804e+07 | 3 |

**Adduct Signal Fractions**

- **Unmodified**: 2.3804e+07

### YFTPN(N)KTEDTIFLR Hex 10 HexNAc 9

**Mass:**  5192.1296

(7.659e-07)

**Charge States:** 4

**Start Position**: 68

**End Position**: 82

| Profile Score | Tandem Score | TDA q-value | Volume | Spectrum Matches |
| --- | --- | --- | --- | --- |
| -19.5474 | 84.2767 | 0.0000 | 6.1450e+06 | 1 |

**Adduct Signal Fractions**

- **Unmodified**: 6.1450e+06

### YFTPN(N)KTEDTIFLR Hex 3 HexNAc 5 Neu5Ac 3

**Mass:**  4118.7175

(-2.123e-06)

**Charge States:** 4

**Start Position**: 68

**End Position**: 82

| Profile Score | Tandem Score | TDA q-value | Volume | Spectrum Matches |
| --- | --- | --- | --- | --- |
| -4.6846 | 84.1685 | 0.0000 | 7.7869e+07 | 1 |

**Adduct Signal Fractions**

- **Unmodified**: 7.7869e+07

### IYN(N)TTYLNVQR Hex 6 HexNAc 5

**Mass:**  3371.4300

(2.475e-07)

**Charge States:** 3, 4

**Start Position**: 91

**End Position**: 102

| Profile Score | Tandem Score | TDA q-value | Volume | Spectrum Matches |
| --- | --- | --- | --- | --- |
| 11.7311 | 79.8690 | 0.0020 | 4.5488e+07 | 4 |

**Adduct Signal Fractions**

- **Unmodified**: 4.5488e+07

### FTPN(N)KTEDTIFLR Fuc 2 Hex 6 HexNAc 4 Neu5Ac 1

**Mass:**  3951.7258

(7.691e-04)

**Charge States:** 4

**Start Position**: 69

**End Position**: 82

| Profile Score | Tandem Score | TDA q-value | Volume | Spectrum Matches |
| --- | --- | --- | --- | --- |
| -6.3583 | 79.7678 | 0.0020 | 5.4058e+07 | 4 |

**Adduct Signal Fractions**

- **Unmodified**: 5.4058e+07

### FYFTPN(N)KTEDTIFLR Fuc 1 Hex 4 HexNAc 4 Neu5Ac 1

**Mass:**  3788.6229

(-2.683e-06)

**Charge States:** 3, 4

**Start Position**: 67

**End Position**: 82

| Profile Score | Tandem Score | TDA q-value | Volume | Spectrum Matches |
| --- | --- | --- | --- | --- |
| 14.9221 | 75.8358 | 0.0060 | 2.4086e+08 | 5 |

**Adduct Signal Fractions**

- **Unmodified**: 2.4086e+08

### SVQEIQATFFYFTPN(N)KTEDTIFLR Fuc 2 Hex 7 HexNAc 6

**Mass:**  5539.4235

(2.300e-06)

**Charge States:** 4

**Start Position**: 58

**End Position**: 82

| Profile Score | Tandem Score | TDA q-value | Volume | Spectrum Matches |
| --- | --- | --- | --- | --- |
| -19.3911 | 75.0154 | 0.0060 | 2.2546e+07 | 1 |

**Adduct Signal Fractions**

- **Unmodified**: 2.2546e+07

### TPN(N)KTEDTIFLR Fuc 1 Hex 4 HexNAc 5 Neu5Ac 3

**Mass:**  4116.7043

(1.169e-07)

**Charge States:** 4

**Start Position**: 70

**End Position**: 82

| Profile Score | Tandem Score | TDA q-value | Volume | Spectrum Matches |
| --- | --- | --- | --- | --- |
| -19.6665 | 73.1945 | 0.0060 | 1.2180e+07 | 1 |

**Adduct Signal Fractions**

- **Unmodified**: 1.2180e+07

### FYFTPN(N)KTEDTIFLR Hex 7 HexNAc 4 Neu5Ac 2

**Mass:**  4419.8391

(2.110e-06)

**Charge States:** 3, 4

**Start Position**: 67

**End Position**: 82

| Profile Score | Tandem Score | TDA q-value | Volume | Spectrum Matches |
| --- | --- | --- | --- | --- |
| -19.1542 | 73.1673 | 0.0060 | 8.1523e+06 | 3 |

**Adduct Signal Fractions**

- **Unmodified**: 8.1523e+06

### C(C)ANLVPVPITN(N)ATLDQITGK Hex 6 HexNAc 5

**Mass:**  4111.8469

(2.183e-06)

**Charge States:** 3, 4

**Start Position**: 23

**End Position**: 43

| Profile Score | Tandem Score | TDA q-value | Volume | Spectrum Matches |
| --- | --- | --- | --- | --- |
| 12.3224 | 68.4089 | 0.0066 | 1.1565e+08 | 4 |

**Adduct Signal Fractions**

- **Unmodified**: 1.1565e+08

### SVQEIQATFFYFTPN(N)K Hex 10 HexNAc 9

**Mass:**  5367.1970

(1.076e-06)

**Charge States:** 4

**Start Position**: 58

**End Position**: 74

| Profile Score | Tandem Score | TDA q-value | Volume | Spectrum Matches |
| --- | --- | --- | --- | --- |
| -5.0119 | 66.9025 | 0.0066 | 1.0802e+07 | 1 |

**Adduct Signal Fractions**

- **Unmodified**: 1.0802e+07

### SVQEIQATFFYFTPN(N)K Hex 7 HexNAc 7

**Mass:**  4474.8796

(1.353e-06)

**Charge States:** 3, 4

**Start Position**: 58

**End Position**: 74

| Profile Score | Tandem Score | TDA q-value | Volume | Spectrum Matches |
| --- | --- | --- | --- | --- |
| 11.6939 | 59.9832 | 0.0066 | 7.5687e+07 | 4 |

**Adduct Signal Fractions**

- **Unmodified**: 7.5687e+07

### QDQC(C)IYN(N)TTYLNVQR Fuc 2 Hex 8 HexNAc 7

**Mass:**  4924.9934

(2.041e-06)

**Charge States:** 4

**Start Position**: 87

**End Position**: 102

| Profile Score | Tandem Score | TDA q-value | Volume | Spectrum Matches |
| --- | --- | --- | --- | --- |
| 13.7399 | 58.9980 | 0.0066 | 2.7379e+07 | 2 |

**Adduct Signal Fractions**

- **Unmodified**: 2.7379e+07

### C(C)ANLVPVPITN(N)ATLDQITGK Fuc 1 Hex 6 HexNAc 5

**Mass:**  4257.9045

(2.237e-06)

**Charge States:** 3, 4

**Start Position**: 23

**End Position**: 43

| Profile Score | Tandem Score | TDA q-value | Volume | Spectrum Matches |
| --- | --- | --- | --- | --- |
| 12.8909 | 57.9967 | 0.0066 | 3.1280e+07 | 3 |

**Adduct Signal Fractions**

- **Unmodified**: 3.1280e+07

### SVQEIQATFFYFTPN(N)K Fuc 1 Hex 9 HexNAc 8

**Mass:**  5148.1369

(4.272e-06)

**Charge States:** 4

**Start Position**: 58

**End Position**: 74

| Profile Score | Tandem Score | TDA q-value | Volume | Spectrum Matches |
| --- | --- | --- | --- | --- |
| -6.2418 | 56.0952 | 0.0066 | 6.0671e+06 | 1 |

**Adduct Signal Fractions**

- **Unmodified**: 6.0671e+06

### PVPITN(N)ATLDQITGK Fuc 2 Hex 3 HexNAc 7 Neu5Ac 3

**Mass:**  4640.0207

(9.252e-06)

**Charge States:** 4

**Start Position**: 28

**End Position**: 43

| Profile Score | Tandem Score | TDA q-value | Volume | Spectrum Matches |
| --- | --- | --- | --- | --- |
| -5.2244 | 52.7321 | 0.0066 | 7.7976e+06 | 1 |

**Adduct Signal Fractions**

- **Unmodified**: 7.7976e+06

### PVPITN(N)ATLDQITGK Fuc 3 Hex 8 HexNAc 5

**Mass:**  4316.8859

(7.150e-06)

**Charge States:** 4

**Start Position**: 28

**End Position**: 43

| Profile Score | Tandem Score | TDA q-value | Volume | Spectrum Matches |
| --- | --- | --- | --- | --- |
| -4.9070 | 52.3083 | 0.0066 | 2.9278e+07 | 2 |

**Adduct Signal Fractions**

- **Unmodified**: 2.9278e+07

### QDQC(C)IYN(N)TTYLNVQR Fuc 1 Hex 8 HexNAc 4 Neu5Ac 1

**Mass:**  4460.8019

(3.040e-06)

**Charge States:** 3, 4

**Start Position**: 87

**End Position**: 102

| Profile Score | Tandem Score | TDA q-value | Volume | Spectrum Matches |
| --- | --- | --- | --- | --- |
| -5.7512 | 50.3817 | 0.0066 | 4.0464e+07 | 2 |

**Adduct Signal Fractions**

- **Unmodified**: 4.0464e+07

### SVQEIQATFFYFTPN(N)KTEDTIFLR Hex 9 HexNAc 8

**Mass:**  5977.5646

(9.288e-07)

**Charge States:** 4, 5

**Start Position**: 58

**End Position**: 82

| Profile Score | Tandem Score | TDA q-value | Volume | Spectrum Matches |
| --- | --- | --- | --- | --- |
| 14.1984 | 48.6462 | 0.0066 | 7.3384e+07 | 6 |

**Adduct Signal Fractions**

- **Unmodified**: 7.3384e+07

### QDQC(C)IYN(N)TTYLNVQR Hex 3 HexNAc 5 Neu5Ac 3

**Mass:**  4289.7209

(-3.784e-06)

**Charge States:** 3, 4

**Start Position**: 87

**End Position**: 102

| Profile Score | Tandem Score | TDA q-value | Volume | Spectrum Matches |
| --- | --- | --- | --- | --- |
| 9.8480 | 48.2946 | 0.0066 | 3.0769e+08 | 4 |

**Adduct Signal Fractions**

- **Unmodified**: 3.0769e+08

### LVPVPITN(N)ATLDQITGK Fuc 3 Hex 4 HexNAc 5

**Mass:**  3880.7858

(-5.686e-06)

**Charge States:** 3, 4

**Start Position**: 26

**End Position**: 43

| Profile Score | Tandem Score | TDA q-value | Volume | Spectrum Matches |
| --- | --- | --- | --- | --- |
| -5.9735 | 47.3483 | 0.0066 | 9.5408e+07 | 6 |

**Adduct Signal Fractions**

- **Unmodified**: 9.5408e+07

### QDQC(C)IYN(N)TTYLNVQR Fuc 1 Hex 9 HexNAc 8

**Mass:**  5144.0661

(1.787e-06)

**Charge States:** 4

**Start Position**: 87

**End Position**: 102

| Profile Score | Tandem Score | TDA q-value | Volume | Spectrum Matches |
| --- | --- | --- | --- | --- |
| -3.9194 | 47.0148 | 0.0066 | 2.4635e+07 | 2 |

**Adduct Signal Fractions**

- **Unmodified**: 2.4635e+07

### QDQC(C)IYN(N)TTYLNVQR Hex 10 HexNAc 9

**Mass:**  5363.1403

(1.441e-06)

**Charge States:** 4

**Start Position**: 87

**End Position**: 102

| Profile Score | Tandem Score | TDA q-value | Volume | Spectrum Matches |
| --- | --- | --- | --- | --- |
| -4.1277 | 45.6633 | 0.0066 | 2.7855e+07 | 2 |

**Adduct Signal Fractions**

- **Unmodified**: 2.7855e+07

### SVQEIQATFFYFTPN(N)K Hex 7 HexNAc 6 Neu5Ac 1

**Mass:**  4562.8962

(1.787e-06)

**Charge States:** 4

**Start Position**: 58

**End Position**: 74

| Profile Score | Tandem Score | TDA q-value | Volume | Spectrum Matches |
| --- | --- | --- | --- | --- |
| -20.7451 | 45.1151 | 0.0066 | 5.3062e+05 | 1 |

**Adduct Signal Fractions**

- **Unmodified**: 5.3062e+05

### SVQEIQATFFYFTPN(N)KTEDTIFLR Hex 7 HexNAc 4 Neu5Ac 2

**Mass:**  5423.3361

(1.697e-06)

**Charge States:** 4

**Start Position**: 58

**End Position**: 82

| Profile Score | Tandem Score | TDA q-value | Volume | Spectrum Matches |
| --- | --- | --- | --- | --- |
| -3.9029 | 44.7278 | 0.0066 | 3.1480e+07 | 3 |

**Adduct Signal Fractions**

- **Unmodified**: 3.1480e+07

### LVPVPITN(N)ATLDQITGK Hex 7 HexNAc 5

**Mass:**  3931.8197

(7.735e-04)

**Start Position**: 26

**End Position**: 43

| Profile Score | Tandem Score | TDA q-value | Volume | Spectrum Matches |
| --- | --- | --- | --- | --- |
| 0.0000 | 44.3404 | 0.0066 | 0.0000e+00 | 1 |

Adduct:

No Chromatogram Found

### PVPITN(N)ATLDQITGK Fuc 3 Hex 7 HexNAc 4 Neu5Ac 1

**Mass:**  4243.8595

(2.454e-04)

**Start Position**: 28

**End Position**: 43

| Profile Score | Tandem Score | TDA q-value | Volume | Spectrum Matches |
| --- | --- | --- | --- | --- |
| 0.0000 | 43.9729 | 0.0066 | 0.0000e+00 | 1 |

Adduct:

No Chromatogram Found

### PVPITN(N)ATLDQITGK Fuc 1 Hex 7 HexNAc 4 Neu5Ac 1

**Mass:**  3950.7358

(8.458e-06)

**Charge States:** 4

**Start Position**: 28

**End Position**: 43

| Profile Score | Tandem Score | TDA q-value | Volume | Spectrum Matches |
| --- | --- | --- | --- | --- |
| -3.5757 | 43.7666 | 0.0066 | 2.5963e+08 | 3 |

**Adduct Signal Fractions**

- **Unmodified**: 2.5963e+08

### PVPITN(N)ATLDQITGK Fuc 2 Hex 6 HexNAc 5

**Mass:**  3846.6918

(1.507e-07)

**Charge States:** 3, 4

**Start Position**: 28

**End Position**: 43

| Profile Score | Tandem Score | TDA q-value | Volume | Spectrum Matches |
| --- | --- | --- | --- | --- |
| -5.5751 | 42.9628 | 0.0066 | 6.0123e+06 | 2 |

**Adduct Signal Fractions**

- **Unmodified**: 6.0123e+06

### LVPVPITN(N)ATLDQITGK Hex 9 HexNAc 8

**Mass:**  4862.1354

(2.378e-06)

**Charge States:** 4

**Start Position**: 26

**End Position**: 43

| Profile Score | Tandem Score | TDA q-value | Volume | Spectrum Matches |
| --- | --- | --- | --- | --- |
| -4.5977 | 42.8995 | 0.0066 | 5.9781e+06 | 1 |

**Adduct Signal Fractions**

- **Unmodified**: 5.9781e+06

### LVPVPITN(N)ATLDQITGK Fuc 3 Hex 5 HexNAc 4

**Mass:**  3841.7686

(5.256e-04)

**Charge States:** 3, 4

**Start Position**: 26

**End Position**: 43

| Profile Score | Tandem Score | TDA q-value | Volume | Spectrum Matches |
| --- | --- | --- | --- | --- |
| 3.9355 | 42.5240 | 0.0066 | 2.0478e+07 | 5 |

**Adduct Signal Fractions**

- **Unmodified**: 2.0478e+07

### SVQEIQATFFYFTPN(N)KTEDTIFLR Fuc 1 Hex 8 HexNAc 7

**Mass:**  5758.4948

(8.526e-07)

**Charge States:** 4, 5

**Start Position**: 58

**End Position**: 82

| Profile Score | Tandem Score | TDA q-value | Volume | Spectrum Matches |
| --- | --- | --- | --- | --- |
| 15.7365 | 42.1645 | 0.0066 | 3.8055e+07 | 3 |

**Adduct Signal Fractions**

- **Unmodified**: 3.8055e+07

### FYFTPN(N)KTEDTIFLR Hex 5 HexNAc 4

**Mass:**  3513.5370

(5.991e-07)

**Charge States:** 3

**Start Position**: 67

**End Position**: 82

| Profile Score | Tandem Score | TDA q-value | Volume | Spectrum Matches |
| --- | --- | --- | --- | --- |
| -5.6148 | 41.6298 | 0.0066 | 2.8176e+06 | 1 |

**Adduct Signal Fractions**

- **Unmodified**: 2.8176e+06

### QDQC(C)IYN(N)TTYLNVQR Hex 9 HexNAc 3 Neu5Ac 1

**Mass:**  4273.6960

(-1.820e-06)

**Charge States:** 3, 4

**Start Position**: 87

**End Position**: 102

| Profile Score | Tandem Score | TDA q-value | Volume | Spectrum Matches |
| --- | --- | --- | --- | --- |
| 10.0129 | 41.5431 | 0.0066 | 2.3073e+08 | 3 |

**Adduct Signal Fractions**

- **Unmodified**: 2.3073e+08

### QDQC(C)IYN(N)TTYLNVQR Hex 6 HexNAc 6

**Mass:**  4105.6926

(2.457e-06)

**Charge States:** 3, 4

**Start Position**: 87

**End Position**: 102

| Profile Score | Tandem Score | TDA q-value | Volume | Spectrum Matches |
| --- | --- | --- | --- | --- |
| 13.8469 | 41.3308 | 0.0066 | 8.9796e+07 | 3 |

**Adduct Signal Fractions**

- **Unmodified**: 8.9796e+07

### TPN(N)KTEDTIFLR Hex 7 HexNAc 6

**Mass:**  3789.6109

(7.958e-04)

**Start Position**: 70

**End Position**: 82

| Profile Score | Tandem Score | TDA q-value | Volume | Spectrum Matches |
| --- | --- | --- | --- | --- |
| 0.0000 | 40.3430 | 0.0066 | 0.0000e+00 | 1 |

Adduct:

No Chromatogram Found

### QDQC(C)IYN(N)TTYLNVQR Fuc 2 Hex 9 HexNAc 8

**Mass:**  5290.1140

(-2.829e-07)

**Charge States:** 4

**Start Position**: 87

**End Position**: 102

| Profile Score | Tandem Score | TDA q-value | Volume | Spectrum Matches |
| --- | --- | --- | --- | --- |
| -5.1535 | 38.2034 | 0.0066 | 4.7059e+06 | 1 |

**Adduct Signal Fractions**

- **Unmodified**: 4.7059e+06

### LVPVPITN(N)ATLDQITGK Fuc 2 Hex 6 HexNAc 5

**Mass:**  4060.8574

(4.961e-04)

**Start Position**: 26

**End Position**: 43

| Profile Score | Tandem Score | TDA q-value | Volume | Spectrum Matches |
| --- | --- | --- | --- | --- |
| 0.0000 | 38.1346 | 0.0066 | 0.0000e+00 | 1 |

Adduct:

No Chromatogram Found

### QDQC(C)IYN(N)TTYLNVQR Fuc 5 Hex 7 HexNAc 6

**Mass:**  4998.0114

(-2.749e-06)

**Charge States:** 4

**Start Position**: 87

**End Position**: 102

| Profile Score | Tandem Score | TDA q-value | Volume | Spectrum Matches |
| --- | --- | --- | --- | --- |
| -19.8385 | 37.9204 | 0.0066 | 3.6355e+06 | 1 |

**Adduct Signal Fractions**

- **Unmodified**: 3.6355e+06

### PVPITN(N)ATLDQITGK Hex 3 HexNAc 7 Neu5Ac 4

**Mass:**  4638.9899

(6.999e-06)

**Charge States:** 4

**Start Position**: 28

**End Position**: 43

| Profile Score | Tandem Score | TDA q-value | Volume | Spectrum Matches |
| --- | --- | --- | --- | --- |
| -19.1821 | 37.6076 | 0.0066 | 2.5037e+06 | 2 |

**Adduct Signal Fractions**

- **Unmodified**: 2.5037e+06

### LVPVPITN(N)ATLDQITGK Fuc 2 Hex 7 HexNAc 6

**Mass:**  4423.9875

(2.730e-06)

**Charge States:** 3, 4

**Start Position**: 26

**End Position**: 43

| Profile Score | Tandem Score | TDA q-value | Volume | Spectrum Matches |
| --- | --- | --- | --- | --- |
| -4.9641 | 37.3768 | 0.0066 | 1.9263e+07 | 1 |

**Adduct Signal Fractions**

- **Unmodified**: 1.9263e+07

### YFTPN(N)KTEDTIFLR Fuc 1 Hex 9 HexNAc 2

**Mass:**  3754.5859

(2.856e-06)

**Charge States:** 4

**Start Position**: 68

**End Position**: 82

| Profile Score | Tandem Score | TDA q-value | Volume | Spectrum Matches |
| --- | --- | --- | --- | --- |
| -18.9447 | 37.1271 | 0.0066 | 1.4647e+07 | 2 |

**Adduct Signal Fractions**

- **Unmodified**: 1.4647e+07

### PVPITN(N)ATLDQITGK Fuc 3 Hex 3 HexNAc 7

**Mass:**  3913.7814

(2.660e-04)

**Charge States:** 3, 4

**Start Position**: 28

**End Position**: 43

| Profile Score | Tandem Score | TDA q-value | Volume | Spectrum Matches |
| --- | --- | --- | --- | --- |
| -8.8003 | 36.2115 | 0.0077 | 6.1071e+07 | 25 |

**Adduct Signal Fractions**

- **Unmodified**: 6.1071e+07

### FYFTPN(N)KTEDTIFLR Hex 9 HexNAc 8

**Mass:**  4975.0717

(2.030e-04)

**Start Position**: 67

**End Position**: 82

| Profile Score | Tandem Score | TDA q-value | Volume | Spectrum Matches |
| --- | --- | --- | --- | --- |
| 0.0000 | 35.8215 | 0.0077 | 0.0000e+00 | 1 |

Adduct:

No Chromatogram Found

### YFTPN(N)KTEDTIFLR Hex 6 HexNAc 6

**Mass:**  3934.6800

(9.215e-07)

**Charge States:** 4

**Start Position**: 68

**End Position**: 82

| Profile Score | Tandem Score | TDA q-value | Volume | Spectrum Matches |
| --- | --- | --- | --- | --- |
| -4.4843 | 35.6241 | 0.0077 | 5.3090e+07 | 4 |

**Adduct Signal Fractions**

- **Unmodified**: 5.3090e+07

### YFTPN(N)KTEDTIFLR Fuc 4 Hex 5 HexNAc 5

**Mass:**  4153.7565

(-3.986e-06)

**Charge States:** 3, 4

**Start Position**: 68

**End Position**: 82

| Profile Score | Tandem Score | TDA q-value | Volume | Spectrum Matches |
| --- | --- | --- | --- | --- |
| 13.7262 | 35.4078 | 0.0089 | 5.0294e+08 | 3 |

**Adduct Signal Fractions**

- **Unmodified**: 5.0294e+08

### LVPVPITN(N)ATLDQITGK Hex 6 HexNAc 5 Neu5Ac 3

**Mass:**  4640.9905

(2.104e-04)

**Start Position**: 26

**End Position**: 43

| Profile Score | Tandem Score | TDA q-value | Volume | Spectrum Matches |
| --- | --- | --- | --- | --- |
| 0.0000 | 35.3991 | 0.0089 | 0.0000e+00 | 1 |

Adduct:

No Chromatogram Found

### QDQC(C)IYN(N)TTYLNVQR Fuc 2 Hex 10 HexNAc 3

**Mass:**  4436.7766

(1.082e-06)

**Charge States:** 3

**Start Position**: 87

**End Position**: 102

| Profile Score | Tandem Score | TDA q-value | Volume | Spectrum Matches |
| --- | --- | --- | --- | --- |
| -19.8466 | 34.8839 | 0.0089 | 8.4263e+06 | 1 |

**Adduct Signal Fractions**

- **Unmodified**: 8.4263e+06

### ATFFYFTPN(N)K Fuc 4 Hex 5 HexNAc 9

**Mass:**  4456.8132

(2.227e-07)

**Charge States:** 4

**Start Position**: 64

**End Position**: 74

| Profile Score | Tandem Score | TDA q-value | Volume | Spectrum Matches |
| --- | --- | --- | --- | --- |
| -19.9834 | 34.5223 | 0.0113 | 1.0531e+07 | 1 |

**Adduct Signal Fractions**

- **Unmodified**: 1.0531e+07

### QDQC(C)IYN(N)TTYLN(D)VQR Hex 7 HexNAc 6

**Mass:**  4268.7272

(2.324e-04)

**Charge States:** 3, 4

**Start Position**: 87

**End Position**: 102

| Profile Score | Tandem Score | TDA q-value | Volume | Spectrum Matches |
| --- | --- | --- | --- | --- |
| 8.6334 | 33.8593 | 0.0148 | 6.0178e+07 | 8 |

**Adduct Signal Fractions**

- **Unmodified**: 6.0178e+07

### C(C)ANLVPVPITN(N)ATLDQITGK Hex 7 HexNAc 6

**Mass:**  4476.9816

(2.398e-06)

**Charge States:** 3, 4

**Start Position**: 23

**End Position**: 43

| Profile Score | Tandem Score | TDA q-value | Volume | Spectrum Matches |
| --- | --- | --- | --- | --- |
| -19.1696 | 33.7347 | 0.0148 | 2.1460e+06 | 1 |

**Adduct Signal Fractions**

- **Unmodified**: 2.1460e+06

### SVQEIQATFFYFTPN(N)KTEDTIFLR Fuc 4 Hex 5 HexNAc 5

**Mass:**  5304.3254

(-3.134e-06)

**Charge States:** 3, 4, 5

**Start Position**: 58

**End Position**: 82

| Profile Score | Tandem Score | TDA q-value | Volume | Spectrum Matches |
| --- | --- | --- | --- | --- |
| 13.3132 | 33.3774 | 0.0159 | 2.6062e+08 | 9 |

**Adduct Signal Fractions**

- **Unmodified**: 2.6062e+08

### QDQC(C)IYN(N)TTYLN Hex 7 HexNAc 6

**Mass:**  3884.5116

(1.132e-06)

**Charge States:** 3

**Start Position**: 87

**End Position**: 99

| Profile Score | Tandem Score | TDA q-value | Volume | Spectrum Matches |
| --- | --- | --- | --- | --- |
| -18.4245 | 33.0201 | 0.0181 | 8.0398e+06 | 1 |

**Adduct Signal Fractions**

- **Unmodified**: 8.0398e+06

### LVPVPITN(N)ATLDQITGK Hex 6 HexNAc 6

**Mass:**  3969.8195

(2.123e-06)

**Charge States:** 3, 4

**Start Position**: 26

**End Position**: 43

| Profile Score | Tandem Score | TDA q-value | Volume | Spectrum Matches |
| --- | --- | --- | --- | --- |
| 4.6158 | 33.0133 | 0.0181 | 1.3755e+08 | 5 |

**Adduct Signal Fractions**

- **Unmodified**: 1.3755e+08

### QDQC(C)IYN(N)TTYLNVQR Fuc 1 Hex 10 HexNAc 9

**Mass:**  5509.1940

(7.078e-07)

**Charge States:** 4

**Start Position**: 87

**End Position**: 102

| Profile Score | Tandem Score | TDA q-value | Volume | Spectrum Matches |
| --- | --- | --- | --- | --- |
| -19.2677 | 32.6735 | 0.0228 | 5.8732e+06 | 1 |

**Adduct Signal Fractions**

- **Unmodified**: 5.8732e+06

### QDQC(C)IYN(N)TTYLNVQR Fuc 2 Hex 8 HexNAc 4 Neu5Ac 1

**Mass:**  4607.8617

(2.216e-04)

**Start Position**: 87

**End Position**: 102

| Profile Score | Tandem Score | TDA q-value | Volume | Spectrum Matches |
| --- | --- | --- | --- | --- |
| 0.0000 | 32.4336 | 0.0250 | 0.0000e+00 | 1 |

Adduct:

No Chromatogram Found

### LVPVPITN(N)ATLDQITGK Fuc 2 Hex 4 HexNAc 9

**Mass:**  4547.0817

(6.151e-06)

**Charge States:** 3, 4

**Start Position**: 26

**End Position**: 43

| Profile Score | Tandem Score | TDA q-value | Volume | Spectrum Matches |
| --- | --- | --- | --- | --- |
| 10.6912 | 32.0146 | 0.0280 | 9.4600e+07 | 11 |

**Adduct Signal Fractions**

- **Unmodified**: 9.4600e+07

### IYN(N)TTYLNVQR Hex 7 HexNAc 6

**Mass:**  3737.5705

(2.703e-04)

**Start Position**: 91

**End Position**: 102

| Profile Score | Tandem Score | TDA q-value | Volume | Spectrum Matches |
| --- | --- | --- | --- | --- |
| 0.0000 | 31.8615 | 0.0291 | 0.0000e+00 | 1 |

Adduct:

No Chromatogram Found

### SVQEIQATFFYFTPN(N)KTEDTIFLR Fuc 1 Hex 8 HexNAc 4 Neu5Ac 1

**Mass:**  5440.3605

(3.344e-06)

**Charge States:** 4

**Start Position**: 58

**End Position**: 82

| Profile Score | Tandem Score | TDA q-value | Volume | Spectrum Matches |
| --- | --- | --- | --- | --- |
| -4.9368 | 31.3865 | 0.0312 | 1.0060e+07 | 1 |

**Adduct Signal Fractions**

- **Unmodified**: 1.0060e+07

### LVPVPITN(N)ATLDQITGK Fuc 2 Hex 5 HexNAc 4 Neu5Ac 1

**Mass:**  3987.8267

(7.627e-04)

**Charge States:** 3

**Start Position**: 26

**End Position**: 43

| Profile Score | Tandem Score | TDA q-value | Volume | Spectrum Matches |
| --- | --- | --- | --- | --- |
| -22.2320 | 30.8229 | 0.0312 | 1.1857e+06 | 3 |

**Adduct Signal Fractions**

- **Unmodified**: 1.1857e+06

### LVPVPITN(N)ATLDQITGK Fuc 3 Hex 7 HexNAc 5 Neu5Ac 1

**Mass:**  4659.0856

(2.192e-04)

**Charge States:** 4

**Start Position**: 26

**End Position**: 43

| Profile Score | Tandem Score | TDA q-value | Volume | Spectrum Matches |
| --- | --- | --- | --- | --- |
| -20.1096 | 30.4601 | 0.0312 | 1.8051e+06 | 1 |

**Adduct Signal Fractions**

- **Unmodified**: 1.8051e+06

### LVPVPITN(N)ATLDQITGK Hex 6 HexNAc 5

**Mass:**  3768.7393

(5.340e-04)

**Start Position**: 26

**End Position**: 43

| Profile Score | Tandem Score | TDA q-value | Volume | Spectrum Matches |
| --- | --- | --- | --- | --- |
| 0.0000 | 30.2785 | 0.0319 | 0.0000e+00 | 1 |

Adduct:

No Chromatogram Found

### PVPITN(N)ATLDQITGK Fuc 2 Hex 7 HexNAc 5

**Mass:**  4008.7419

(-5.807e-07)

**Charge States:** 4

**Start Position**: 28

**End Position**: 43

| Profile Score | Tandem Score | TDA q-value | Volume | Spectrum Matches |
| --- | --- | --- | --- | --- |
| -19.8477 | 30.2617 | 0.0319 | 1.2084e+06 | 1 |

**Adduct Signal Fractions**

- **Unmodified**: 1.2084e+06

### SVQEIQATFFYFTPN(N)K Hex 6 HexNAc 5 Neu5Ac 1

**Mass:**  4199.7697

(4.798e-04)

**Start Position**: 58

**End Position**: 74

| Profile Score | Tandem Score | TDA q-value | Volume | Spectrum Matches |
| --- | --- | --- | --- | --- |
| 0.0000 | 30.0293 | 0.0324 | 0.0000e+00 | 1 |

Adduct:

No Chromatogram Found

### QIPLC(C)ANLVPVPITN(N)ATLDQITGK Hex 5 HexNAc 5 Neu5Ac 1

**Mass:**  4692.1672

(1.356e-06)

**Charge States:** 3, 4

**Start Position**: 19

**End Position**: 43

| Profile Score | Tandem Score | TDA q-value | Volume | Spectrum Matches |
| --- | --- | --- | --- | --- |
| 16.1904 | 29.8372 | 0.0324 | 3.0951e+08 | 7 |

**Adduct Signal Fractions**

- **Unmodified**: 3.0951e+08

### PVPITN(N)ATLDQITGK Hex 3 HexNAc 9

**Mass:**  3880.7763

(9.377e-06)

**Charge States:** 3, 4

**Start Position**: 28

**End Position**: 43

| Profile Score | Tandem Score | TDA q-value | Volume | Spectrum Matches |
| --- | --- | --- | --- | --- |
| -18.4541 | 29.2608 | 0.0352 | 7.6690e+07 | 2 |

**Adduct Signal Fractions**

- **Unmodified**: 7.6690e+07

### SVQEIQATFFYFTPN(N)K Fuc 1 Hex 7 HexNAc 7

**Mass:**  4620.9416

(2.525e-06)

**Charge States:** 4

**Start Position**: 58

**End Position**: 74

| Profile Score | Tandem Score | TDA q-value | Volume | Spectrum Matches |
| --- | --- | --- | --- | --- |
| -19.6321 | 29.1296 | 0.0361 | 3.0465e+06 | 1 |

**Adduct Signal Fractions**

- **Unmodified**: 3.0465e+06

### QIPLC(C)ANLVPVPITN(N)ATLDQITGK Hex 6 HexNAc 5

**Mass:**  4563.1223

(8.840e-07)

**Charge States:** 3, 4

**Start Position**: 19

**End Position**: 43

| Profile Score | Tandem Score | TDA q-value | Volume | Spectrum Matches |
| --- | --- | --- | --- | --- |
| 12.0904 | 28.7966 | 0.0368 | 4.0296e+08 | 10 |

**Adduct Signal Fractions**

- **Unmodified**: 4.0296e+08

### QDQC(C)IYN(N)TTYLNVQR Fuc 4 Hex 5 HexNAc 5

**Mass:**  4324.7644

(-5.021e-06)

**Charge States:** 3, 4

**Start Position**: 87

**End Position**: 102

| Profile Score | Tandem Score | TDA q-value | Volume | Spectrum Matches |
| --- | --- | --- | --- | --- |
| 12.9164 | 28.4457 | 0.0370 | 4.7913e+08 | 5 |

**Adduct Signal Fractions**

- **Unmodified**: 4.7913e+08

### PVPITN(N)ATLDQITGK Fuc 2 Hex 4 HexNAc 8

**Mass:**  4131.8669

(9.980e-06)

**Charge States:** 4

**Start Position**: 28

**End Position**: 43

| Profile Score | Tandem Score | TDA q-value | Volume | Spectrum Matches |
| --- | --- | --- | --- | --- |
| -5.8083 | 28.4281 | 0.0370 | 3.9977e+06 | 2 |

**Adduct Signal Fractions**

- **Unmodified**: 3.9977e+06

### IYN(N)TTYLNVQR Fuc 1 Hex 7 HexNAc 6

**Mass:**  3882.6232

(1.215e-06)

**Charge States:** 3, 4

**Start Position**: 91

**End Position**: 102

| Profile Score | Tandem Score | TDA q-value | Volume | Spectrum Matches |
| --- | --- | --- | --- | --- |
| 12.4316 | 27.8570 | 0.0385 | 9.7296e+06 | 3 |

**Adduct Signal Fractions**

- **Unmodified**: 9.7296e+06

### WFYIASAFRNEEYNKSVQEIQATFFYFTPN(N)K Fuc 5 Hex 10 HexNAc 8

**Mass:**  7813.3413

(5.329e-06)

**Charge States:** 5

**Start Position**: 43

**End Position**: 74

| Profile Score | Tandem Score | TDA q-value | Volume | Spectrum Matches |
| --- | --- | --- | --- | --- |
| -18.4517 | 27.6269 | 0.0392 | 3.4232e+07 | 1 |

**Adduct Signal Fractions**

- **Unmodified**: 3.4232e+07

### N(N)ATLDQITGK Hex 6 HexNAc 5

**Mass:**  3049.2877

(6.622e-04)

**Start Position**: 33

**End Position**: 43

| Profile Score | Tandem Score | TDA q-value | Volume | Spectrum Matches |
| --- | --- | --- | --- | --- |
| 0.0000 | 27.5633 | 0.0406 | 0.0000e+00 | 1 |

Adduct:

No Chromatogram Found

### QIPLC(C)ANLVPVPITN(N)ATLDQITGK Fuc 1 Hex 6 HexNAc 5

**Mass:**  4709.1804

(9.163e-07)

**Charge States:** 3, 4

**Start Position**: 19

**End Position**: 43

| Profile Score | Tandem Score | TDA q-value | Volume | Spectrum Matches |
| --- | --- | --- | --- | --- |
| 12.5861 | 27.5529 | 0.0406 | 1.3014e+08 | 6 |

**Adduct Signal Fractions**

- **Unmodified**: 1.3014e+08

### LVPVPITN(N)ATLDQITGK Fuc 1 Hex 7 HexNAc 3 Neu5Ac 1

**Mass:**  3961.8028

(5.120e-04)

**Start Position**: 26

**End Position**: 43

| Profile Score | Tandem Score | TDA q-value | Volume | Spectrum Matches |
| --- | --- | --- | --- | --- |
| 0.0000 | 27.5414 | 0.0406 | 0.0000e+00 | 1 |

Adduct:

No Chromatogram Found

### C(C)ANLVPVPITN(N)ATLDQITGK Fuc 2 Hex 3 HexNAc 9

**Mass:**  4730.1052

(1.225e-06)

**Charge States:** 4

**Start Position**: 23

**End Position**: 43

| Profile Score | Tandem Score | TDA q-value | Volume | Spectrum Matches |
| --- | --- | --- | --- | --- |
| -4.0584 | 27.5035 | 0.0406 | 4.6156e+07 | 5 |

**Adduct Signal Fractions**

- **Unmodified**: 4.6156e+07

### QDQC(C)IYN(N)TTYLNVQR Fuc 4 Hex 6 HexNAc 6

**Mass:**  4689.8986

(-7.830e-06)

**Charge States:** 3, 4

**Start Position**: 87

**End Position**: 102

| Profile Score | Tandem Score | TDA q-value | Volume | Spectrum Matches |
| --- | --- | --- | --- | --- |
| 12.6304 | 27.0642 | 0.0431 | 7.5632e+07 | 4 |

**Adduct Signal Fractions**

- **Unmodified**: 7.5632e+07

### PVPITN(N)ATLDQITGK Hex 7 HexNAc 4 Neu5Ac 1

**Mass:**  3804.6804

(7.876e-06)

**Charge States:** 3, 4, 5

**Start Position**: 28

**End Position**: 43

| Profile Score | Tandem Score | TDA q-value | Volume | Spectrum Matches |
| --- | --- | --- | --- | --- |
| 15.7257 | 27.0204 | 0.0431 | 1.0153e+09 | 15 |

**Adduct Signal Fractions**

- **Unmodified**: 1.0153e+09

### TPN(N)KTEDTIFLR Hex 8 HexNAc 7

**Mass:**  4151.7375

(1.102e-06)

**Charge States:** 4

**Start Position**: 70

**End Position**: 82

| Profile Score | Tandem Score | TDA q-value | Volume | Spectrum Matches |
| --- | --- | --- | --- | --- |
| -5.5874 | 26.6481 | 0.0455 | 4.2620e+06 | 2 |

**Adduct Signal Fractions**

- **Unmodified**: 4.2620e+06

### FYFTPN(N)KTEDTIFLR Hex 9 HexNAc 8

**Mass:**  4974.0617

(2.504e-06)

**Charge States:** 4

**Start Position**: 67

**End Position**: 82

| Profile Score | Tandem Score | TDA q-value | Volume | Spectrum Matches |
| --- | --- | --- | --- | --- |
| -20.9849 | 26.5648 | 0.0455 | 2.8136e+06 | 1 |

**Adduct Signal Fractions**

- **Unmodified**: 2.8136e+06

### LVPVPITN(N)ATLDQITGK Fuc 3 Hex 4 HexNAc 9

**Mass:**  4693.1418

(5.999e-06)

**Charge States:** 3, 4

**Start Position**: 26

**End Position**: 43

| Profile Score | Tandem Score | TDA q-value | Volume | Spectrum Matches |
| --- | --- | --- | --- | --- |
| 10.2281 | 26.3526 | 0.0469 | 2.3482e+07 | 9 |

**Adduct Signal Fractions**

- **Unmodified**: 2.3482e+07

### P19652|A1AG2\_HUMAN

**>P19652|A1AG2\_HUMAN**  
 `MALSWVLTVLSLLPLLEAQIPLCANLVPVPITNATLDRITGKWFYIASAFRNEEYNKSVQEIQATFFYFTPNKTEDTIFL  
REYQTRQNQCFYNSSYLNVQRENGTVSRYEGGREHVAHLLFLRDTKTLMFGSYLDDEKNWGLSFYADKPETTKEQLGEFY  
EALDCLCIPRSDVMYTDWKKDKCEPLEKQHEKERKQEEGES`

| Site | Type | Count |
| --- | --- | --- |
| 33 | N-linked | 66 |
| 56 | N-linked | 2 |
| 93 | N-linked | 15 |
| 72 | N-linked | 87 |

| Mass | Sequence | MS_2_ Score | `q`-value | MS_1_ Score | Signal | Protein Coordinates | Tandem Scans | Start Time | Apex Time | End Time |
| --- | --- | --- | --- | --- | --- | --- | --- | --- | --- | --- |
| 2390.9659 | YFTPN(N)K Hex 5 HexNAc 4 | 188.015 | 0.0 | -5.902 | 2.572e+07 | 67 - 73 | 3 | 14.08 | 14.08 | 14.40 |
| 2757.0946 | YFTPN(N)K Hex 6 HexNAc 5 | 187.448 | 0.0 | 0.000 | 0.000e+00 | 67 - 73 | 1 | - | - | - |
| 2756.0978 | YFTPN(N)K Hex 6 HexNAc 5 | 185.448 | 0.0 | 14.112 | 3.130e+08 | 67 - 73 | 5 | 13.79 | 14.08 | 14.57 |
| 3268.2989 | FYFTPN(N)K Hex 7 HexNAc 6 | 184.028 | 0.0 | 15.013 | 1.597e+08 | 66 - 73 | 8 | 23.93 | 24.55 | 25.74 |
| 2903.1661 | FYFTPN(N)K Hex 6 HexNAc 5 | 183.752 | 0.0 | 14.804 | 1.202e+08 | 66 - 73 | 12 | 24.55 | 25.24 | 27.73 |
| 2417.9212 | NEEYN(N)K Hex 5 HexNAc 4 | 181.265 | 0.0 | 11.722 | 7.881e+07 | 51 - 57 | 8 | 3.78 | 3.95 | 5.14 |
| 2783.0539 | NEEYN(N)K Hex 6 HexNAc 5 | 181.199 | 0.0 | 11.501 | 6.456e+07 | 51 - 57 | 7 | 3.78 | 3.95 | 5.03 |
| 3906.6677 | SVQEIQATFFYFTPN(N)K Hex 6 HexNAc 5 | 175.216 | 0.0 | 14.922 | 2.357e+09 | 57 - 73 | 54 | 50.16 | 53.92 | 61.62 |
| 4052.7252 | SVQEIQATFFYFTPN(N)K Fuc 1 Hex 6 HexNAc 5 | 175.061 | 0.0 | 13.796 | 4.928e+08 | 57 - 73 | 17 | 53.07 | 53.75 | 57.68 |
| 4271.8037 | SVQEIQATFFYFTPN(N)K Hex 7 HexNAc 6 | 173.546 | 0.0 | 14.194 | 2.926e+09 | 57 - 73 | 55 | 49.65 | 53.41 | 113.34 |
| 4417.8722 | SVQEIQATFFYFTPN(N)K Fuc 1 Hex 7 HexNAc 6 | 166.205 | 0.0 | 13.778 | 5.800e+08 | 57 - 73 | 17 | 53.07 | 53.24 | 58.19 |
| 4636.9331 | SVQEIQATFFYFTPN(N)K Hex 8 HexNAc 7 | 164.673 | 0.0 | 14.168 | 3.987e+08 | 57 - 73 | 9 | 52.39 | 52.90 | 53.75 |
| 4563.9202 | SVQEIQATFFYFTPN(N)K Fuc 2 Hex 7 HexNAc 6 | 164.123 | 0.0 | -3.890 | 8.526e+07 | 57 - 73 | 2 | 53.07 | 53.07 | 53.58 |
| 3541.5338 | SVQEIQATFFYFTPN(N)K Hex 5 HexNAc 4 | 161.196 | 0.0 | 13.435 | 2.793e+08 | 57 - 73 | 15 | 54.26 | 54.60 | 58.87 |
| 3395.5248 | LVPVPITN(N)ATLDR Hex 6 HexNAc 5 | 158.327 | 0.0 | 11.580 | 1.770e+10 | 25 - 38 | 615 | 29.71 | 36.62 | 113.85 |
| 3030.3956 | LVPVPITN(N)ATLDR Hex 5 HexNAc 4 | 157.121 | 0.0 | 14.523 | 1.132e+09 | 25 - 38 | 8 | 36.74 | 37.05 | 38.53 |
| 3222.3220 | ATFFYFTPN(N)K Hex 6 HexNAc 5 | 156.715 | 0.0 | 14.807 | 4.694e+07 | 63 - 73 | 2 | 40.72 | 40.89 | 41.41 |
| 3760.6579 | LVPVPITN(N)ATLDR Hex 7 HexNAc 6 | 155.717 | 0.0 | -2.584 | 8.010e+08 | 25 - 38 | 4 | 35.75 | 35.92 | 36.07 |
| 3541.5821 | LVPVPITN(N)ATLDR Fuc 1 Hex 6 HexNAc 5 | 155.067 | 0.0 | -2.529 | 3.963e+09 | 25 - 38 | 29 | 29.54 | 36.07 | 113.23 |
| 3587.4555 | ATFFYFTPN(N)K Hex 7 HexNAc 6 | 155.053 | 0.0 | 13.115 | 7.577e+07 | 63 - 73 | 3 | 40.21 | 40.38 | 40.89 |
| 4464.8545 | SVQEIQATFFYFTPN(N)K Fuc 1 Hex 8 HexNAc 4 Neu5Ac 1 | 153.781 | 0.0 | 12.606 | 5.258e+07 | 57 - 73 | 4 | 54.26 | 54.43 | 55.11 |
| 4782.9907 | SVQEIQATFFYFTPN(N)K Fuc 1 Hex 8 HexNAc 7 | 150.195 | 0.0 | 12.204 | 7.721e+07 | 57 - 73 | 3 | 52.39 | 52.73 | 53.24 |
| 3740.6386 | C(C)ANLVPVPITN(N)ATLDR Hex 6 HexNAc 5 | 147.096 | 0.0 | 13.298 | 2.396e+08 | 22 - 38 | 6 | 41.58 | 41.92 | 42.43 |
| 5028.2309 | SVQEIQATFFYFTPN(N)KTEDTIFLR Fuc 1 Hex 6 HexNAc 5 | 146.640 | 0.0 | 12.452 | 1.422e+08 | 57 - 81 | 5 | 61.96 | 62.30 | 63.84 |
| 4882.1739 | SVQEIQATFFYFTPN(N)KTEDTIFLR Hex 6 HexNAc 5 | 145.935 | 0.0 | 14.519 | 5.916e+08 | 57 - 81 | 19 | 59.90 | 62.47 | 65.38 |
| 5002.0649 | SVQEIQATFFYFTPN(N)K Hex 9 HexNAc 8 | 144.329 | 0.0 | 13.666 | 8.196e+07 | 57 - 73 | 4 | 51.87 | 52.04 | 52.90 |
| 3369.3751 | ATFFYFTPN(N)K Fuc 1 Hex 6 HexNAc 5 | 142.068 | 0.0 | 0.000 | 0.000e+00 | 63 - 73 | 1 | - | - | - |
| 4929.0559 | SVQEIQATFFYFTPN(N)K Fuc 2 Hex 8 HexNAc 7 | 141.885 | 0.0 | 12.440 | 1.670e+07 | 57 - 73 | 3 | 52.21 | 52.73 | 52.90 |
| 4447.8313 | SVQEIQATFFYFTPN(N)K Hex 7 HexNAc 4 Neu5Ac 2 | 140.464 | 0.0 | 12.183 | 1.349e+08 | 57 - 73 | 6 | 54.26 | 54.43 | 55.28 |
| 4125.7927 | LVPVPITN(N)ATLDR Hex 8 HexNAc 7 | 140.453 | 0.0 | 12.259 | 1.440e+08 | 25 - 38 | 5 | 35.24 | 35.75 | 35.92 |
| 3906.7185 | LVPVPITN(N)ATLDR Fuc 1 Hex 7 HexNAc 6 | 139.552 | 0.0 | -4.258 | 2.934e+08 | 25 - 38 | 4 | 35.58 | 35.92 | 36.07 |
| 5247.3098 | SVQEIQATFFYFTPN(N)KTEDTIFLR Hex 7 HexNAc 6 | 139.398 | 0.0 | 13.334 | 1.220e+09 | 57 - 81 | 24 | 60.41 | 61.96 | 67.44 |
| 3819.6399 | PVPITN(N)ATLDR Fuc 1 Hex 4 HexNAc 9 | 138.719 | 0.0 | -8.956 | 1.450e+08 | 27 - 38 | 15 | 41.58 | 47.86 | 72.25 |
| 3687.6410 | LVPVPITN(N)ATLDR Fuc 2 Hex 6 HexNAc 5 | 138.625 | 0.0 | -17.878 | 3.862e+07 | 25 - 38 | 1 | 35.92 | 35.92 | 35.92 |
| 4242.7898 | YFTPN(N)KTEDTIFLR Fuc 1 Hex 7 HexNAc 6 | 138.288 | 0.0 | 9.772 | 2.376e+08 | 67 - 81 | 4 | 36.36 | 36.43 | 36.90 |
| 3786.6016 | TPN(N)KTEDTIFLR Hex 7 HexNAc 6 | 137.885 | 0.0 | 14.490 | 6.767e+07 | 69 - 81 | 6 | 24.90 | 25.24 | 26.06 |
| 3560.6105 | LVPVPITN(N)ATLDR Hex 7 HexNAc 5 | 133.891 | 0.0 | 0.000 | 0.000e+00 | 25 - 38 | 1 | - | - | - |
| 3731.6034 | YFTPN(N)KTEDTIFLR Hex 6 HexNAc 5 | 133.207 | 0.0 | 10.647 | 1.091e+09 | 67 - 81 | 10 | 35.24 | 37.21 | 39.04 |
| 4020.7149 | SVQEIQATFFYFTPN(N)K Fuc 3 Hex 4 HexNAc 5 | 131.836 | 0.0 | -19.965 | 1.237e+07 | 57 - 73 | 1 | 53.58 | 53.58 | 53.75 |
| 4096.7358 | YFTPN(N)KTEDTIFLR Hex 7 HexNAc 6 | 131.659 | 0.0 | 11.228 | 2.145e+09 | 67 - 81 | 123 | 34.90 | 36.74 | 91.78 |
| 3031.3939 | LVPVPITN(N)ATLDR Hex 5 HexNAc 4 | 131.216 | 0.0 | 0.000 | 0.000e+00 | 25 - 38 | 1 | - | - | - |
| 3366.4682 | YFTPN(N)KTEDTIFLR Hex 5 HexNAc 4 | 131.047 | 0.0 | 14.973 | 5.973e+07 | 67 - 81 | 1 | 37.68 | 37.68 | 38.36 |
| 4272.7087 | QNQC(C)FYN(N)SSYLNVQR Hex 7 HexNAc 6 | 128.649 | 0.0 | 9.790 | 1.119e+09 | 86 - 101 | 5 | 33.05 | 33.22 | 40.04 |
| 3282.4424 | VPVPITN(N)ATLDR Hex 6 HexNAc 5 | 127.959 | 0.0 | -3.812 | 1.016e+07 | 26 - 38 | 3 | 29.88 | 30.05 | 30.32 |
| 3877.6605 | YFTPN(N)KTEDTIFLR Fuc 1 Hex 6 HexNAc 5 | 127.666 | 0.0 | 13.358 | 2.778e+08 | 67 - 81 | 3 | 36.74 | 37.05 | 37.36 |
| 4109.7466 | SVQEIQATFFYFTPN(N)K Hex 6 HexNAc 6 | 126.323 | 0.0 | 12.424 | 6.664e+07 | 57 - 73 | 7 | 54.26 | 54.43 | 54.94 |
| 4328.8205 | SVQEIQATFFYFTPN(N)K Fuc 4 Hex 5 HexNAc 5 | 126.295 | 0.0 | 12.214 | 6.150e+08 | 57 - 73 | 26 | 52.56 | 54.09 | 56.99 |
| 4593.8893 | SVQEIQATFFYFTPN(N)K Fuc 1 Hex 7 HexNAc 4 Neu5Ac 2 | 125.373 | 0.0 | -19.392 | 1.248e+07 | 57 - 73 | 3 | 54.09 | 54.26 | 54.26 |
| 3878.6703 | FYFTPN(N)KTEDTIFLR Hex 6 HexNAc 5 | 124.304 | 0.0 | 14.986 | 8.360e+07 | 66 - 81 | 6 | 45.49 | 45.83 | 46.51 |
| 3509.5717 | NLVPVPITN(N)ATLDR Hex 6 HexNAc 5 | 123.531 | 0.0 | -4.331 | 1.712e+07 | 24 - 38 | 3 | 40.72 | 40.72 | 41.07 |
| 3598.6087 | LVPVPITN(N)ATLDR Hex 6 HexNAc 6 | 123.334 | 0.0 | 14.511 | 9.744e+08 | 25 - 38 | 7 | 36.90 | 37.21 | 38.36 |
| 3907.5775 | QNQC(C)FYN(N)SSYLNVQR Hex 6 HexNAc 5 | 122.890 | 0.0 | 10.380 | 7.295e+08 | 86 - 101 | 5 | 33.39 | 33.55 | 40.38 |
| 3452.5485 | NLVPVPITN(N)ATLDR Fuc 1 Hex 6 HexNAc 4 | 122.765 | 0.0 | 15.268 | 3.697e+09 | 24 - 38 | 9 | 37.05 | 37.36 | 38.87 |
| 4436.8889 | SVQEIQATFFYFTPN(N)K Hex 8 HexNAc 6 | 121.744 | 0.0 | 0.000 | 0.000e+00 | 57 - 73 | 1 | - | - | - |
| 3588.5853 | LVPVPITN(N)ATLDR Fuc 1 Hex 7 HexNAc 3 Neu5Ac 1 | 121.374 | 0.0 | -19.312 | 4.017e+07 | 25 - 38 | 1 | 37.53 | 37.53 | 37.68 |
| 3421.4693 | TPN(N)KTEDTIFLR Hex 6 HexNAc 5 | 120.776 | 0.0 | 14.705 | 3.361e+07 | 69 - 81 | 7 | 25.41 | 25.74 | 26.29 |
| 4243.8039 | FYFTPN(N)KTEDTIFLR Hex 7 HexNAc 6 | 120.562 | 0.0 | 14.544 | 2.775e+08 | 66 - 81 | 8 | 43.97 | 45.49 | 46.85 |
| 3568.5392 | FTPN(N)KTEDTIFLR Hex 6 HexNAc 5 | 120.194 | 0.0 | 14.757 | 8.049e+07 | 68 - 81 | 5 | 31.68 | 32.02 | 32.70 |
| 3559.6042 | LVPVPITN(N)ATLDR Hex 7 HexNAc 5 | 119.571 | 0.0 | -11.795 | 1.107e+06 | 25 - 38 | 40 | 72.02 | 72.23 | 87.95 |
| 3762.6652 | LVPVPITN(N)ATLDR Hex 7 HexNAc 6 | 119.011 | 0.0 | 0.000 | 0.000e+00 | 25 - 38 | 2 | - | - | - |
| 4637.8536 | QNQC(C)FYN(N)SSYLNVQR Hex 8 HexNAc 7 | 117.326 | 0.0 | 12.821 | 7.905e+07 | 86 - 101 | 1 | 32.36 | 32.53 | 33.22 |
| 4198.7915 | SVQEIQATFFYFTPN(N)K Fuc 2 Hex 6 HexNAc 5 | 115.325 | 0.0 | -19.521 | 1.105e+07 | 57 - 73 | 1 | 53.41 | 53.58 | 53.58 |
| 4098.7433 | YFTPN(N)KTEDTIFLR Hex 7 HexNAc 6 | 114.366 | 0.0 | 0.000 | 0.000e+00 | 67 - 81 | 2 | - | - | - |
| 4461.8636 | YFTPN(N)KTEDTIFLR Hex 8 HexNAc 7 | 114.279 | 0.0 | 12.900 | 2.030e+08 | 67 - 81 | 4 | 34.56 | 35.75 | 36.07 |
| 4418.7710 | QNQC(C)FYN(N)SSYLNVQR Fuc 1 Hex 7 HexNAc 6 | 114.154 | 0.0 | -8.495 | 3.199e+08 | 86 - 101 | 3 | 32.88 | 33.05 | 39.21 |
| 3375.5030 | C(C)ANLVPVPITN(N)ATLDR Hex 5 HexNAc 4 | 112.699 | 0.0 | -4.740 | 1.259e+07 | 22 - 38 | 1 | 42.09 | 42.26 | 42.43 |
| 4053.6366 | QNQC(C)FYN(N)SSYLNVQR Fuc 1 Hex 6 HexNAc 5 | 109.274 | 0.0 | -6.148 | 1.380e+08 | 86 - 101 | 3 | 33.22 | 33.39 | 39.88 |
| 4493.9133 | SVQEIQATFFYFTPN(N)K Fuc 4 Hex 6 HexNAc 5 | 108.614 | 0.0 | 0.000 | 0.000e+00 | 57 - 73 | 1 | - | - | - |
| 4024.7291 | FYFTPN(N)KTEDTIFLR Fuc 1 Hex 6 HexNAc 5 | 108.483 | 0.0 | 13.683 | 2.095e+07 | 66 - 81 | 3 | 45.49 | 45.66 | 46.17 |
| 4299.8165 | YFTPN(N)KTEDTIFLR Hex 7 HexNAc 7 | 108.306 | 0.0 | -5.140 | 7.868e+07 | 67 - 81 | 1 | 36.90 | 37.05 | 37.36 |
| 4389.8615 | FYFTPN(N)KTEDTIFLR Fuc 1 Hex 7 HexNAc 6 | 106.149 | 0.0 | 14.078 | 5.220e+07 | 66 - 81 | 5 | 45.15 | 45.32 | 45.83 |
| 3580.6082 | ANLVPVPITN(N)ATLDR Hex 6 HexNAc 5 | 105.110 | 0.0 | -4.760 | 2.143e+07 | 23 - 38 | 1 | 41.07 | 41.24 | 41.58 |
| 3933.6711 | FTPN(N)KTEDTIFLR Hex 7 HexNAc 6 | 104.466 | 0.0 | 13.689 | 1.566e+08 | 68 - 81 | 6 | 31.34 | 31.51 | 32.19 |
| 4175.8767 | LVPVPITN(N)ATLDR Fuc 2 Hex 4 HexNAc 9 | 101.196 | 0.0 | 12.525 | 1.315e+08 | 25 - 38 | 11 | 63.50 | 63.67 | 65.21 |
| 4693.9535 | SVQEIQATFFYFTPN(N)K Fuc 4 Hex 6 HexNAc 6 | 100.107 | 0.0 | 10.249 | 3.866e+07 | 57 - 73 | 2 | 53.24 | 53.58 | 54.09 |
| 3509.5712 | LVPVPITN(N)ATLDR Fuc 3 Hex 4 HexNAc 5 | 99.507 | 0.0 | -17.595 | 2.649e+07 | 25 - 38 | 2 | 47.36 | 47.53 | 47.53 |
| 3567.5270 | TPN(N)KTEDTIFLR Fuc 1 Hex 6 HexNAc 5 | 98.674 | 0.0 | -5.233 | 5.711e+06 | 69 - 81 | 3 | 25.41 | 25.41 | 25.74 |
| 4826.9992 | YFTPN(N)KTEDTIFLR Hex 9 HexNAc 8 | 97.064 | 0.0 | -3.362 | 6.335e+07 | 67 - 81 | 2 | 35.24 | 35.41 | 35.75 |
| 3714.5961 | FTPN(N)KTEDTIFLR Fuc 1 Hex 6 HexNAc 5 | 96.093 | 0.0 | -3.998 | 1.697e+07 | 68 - 81 | 3 | 31.51 | 31.68 | 32.02 |
| 4272.7522 | YFTPN(N)KTEDTIFLR Hex 7 HexNAc 4 Neu5Ac 2 | 95.677 | 0.0 | -3.742 | 1.312e+08 | 67 - 81 | 5 | 37.68 | 37.85 | 38.02 |
| 3690.6445 | LVPVPITN(N)ATLDR Fuc 2 Hex 6 HexNAc 5 | 95.587 | 0.0 | 0.000 | 0.000e+00 | 25 - 38 | 1 | - | - | - |
| 4190.8883 | LVPVPITN(N)ATLDR Fuc 4 Hex 3 HexNAc 7 Neu5Ac 1 | 95.407 | 0.0 | 11.714 | 1.832e+08 | 25 - 38 | 12 | 59.38 | 60.93 | 61.27 |
| 3903.5973 | PVPITN(N)ATLDR Fuc 1 Hex 9 HexNAc 4 Neu5Ac 1 | 92.869 | 0.0 | -4.487 | 6.154e+07 | 27 - 38 | 4 | 34.90 | 34.90 | 35.41 |
| 4517.0385 | SVQEIQATFFYFTPN(N)KTEDTIFLR Hex 5 HexNAc 4 | 91.884 | 0.0 | -19.076 | 2.030e+07 | 57 - 81 | 2 | 62.81 | 62.81 | 62.98 |
| 5393.3658 | SVQEIQATFFYFTPN(N)KTEDTIFLR Fuc 1 Hex 7 HexNAc 6 | 91.208 | 0.0 | 12.612 | 2.404e+08 | 57 - 81 | 6 | 61.62 | 61.96 | 63.50 |
| 5612.4369 | SVQEIQATFFYFTPN(N)KTEDTIFLR Hex 8 HexNAc 7 | 90.282 | 0.0 | 14.428 | 2.101e+08 | 57 - 81 | 5 | 61.27 | 61.62 | 62.13 |
| 3932.6600 | TPN(N)KTEDTIFLR Fuc 1 Hex 7 HexNAc 6 | 87.478 | 0.0 | -18.890 | 7.631e+06 | 69 - 81 | 2 | 24.90 | 25.07 | 25.07 |
| 4272.8757 | LVPVPITN(N)ATLDR Fuc 1 Hex 8 HexNAc 7 | 86.731 | 0.0 | 0.000 | 0.000e+00 | 25 - 38 | 1 | - | - | - |
| 4298.8018 | FTPN(N)KTEDTIFLR Hex 8 HexNAc 7 | 86.373 | 0.0 | -4.363 | 6.986e+06 | 68 - 81 | 7 | 30.66 | 31.00 | 31.00 |
| 4079.7258 | FTPN(N)KTEDTIFLR Fuc 1 Hex 7 HexNAc 6 | 85.313 | 0.0 | -4.281 | 2.494e+07 | 68 - 81 | 3 | 31.17 | 31.34 | 31.68 |
| 3056.3362 | TPN(N)KTEDTIFLR Hex 5 HexNAc 4 | 84.932 | 0.0 | -3.822 | 3.968e+06 | 69 - 81 | 3 | 25.91 | 26.06 | 26.29 |
| 4608.9339 | FYFTPN(N)KTEDTIFLR Hex 8 HexNAc 7 | 84.883 | 0.0 | 14.259 | 2.380e+07 | 66 - 81 | 3 | 44.47 | 44.98 | 45.15 |
| 5192.1323 | YFTPN(N)KTEDTIFLR Hex 10 HexNAc 9 | 84.277 | 0.0 | -19.547 | 6.145e+06 | 67 - 81 | 1 | 35.07 | 35.07 | 35.24 |
| 4118.7146 | YFTPN(N)KTEDTIFLR Hex 3 HexNAc 5 Neu5Ac 3 | 84.169 | 0.0 | -4.685 | 7.787e+07 | 67 - 81 | 1 | 36.62 | 36.74 | 36.90 |
| 3685.5719 | PVPITN(N)ATLDR Hex 3 HexNAc 7 Neu5Ac 2 | 80.705 | 0.002 | -5.344 | 1.045e+07 | 27 - 38 | 1 | 40.55 | 40.72 | 40.89 |
| 3704.6654 | LVPVPITN(N)ATLDR Fuc 1 Hex 7 HexNAc 5 | 80.365 | 0.002 | -19.695 | 2.093e+07 | 25 - 38 | 1 | 35.92 | 35.92 | 35.92 |
| 3951.7360 | FTPN(N)KTEDTIFLR Fuc 2 Hex 6 HexNAc 4 Neu5Ac 1 | 79.768 | 0.002 | -6.358 | 5.406e+07 | 68 - 81 | 4 | 48.02 | 48.02 | 48.34 |
| 3742.5186 | FYN(N)SSYLNVQR Hex 7 HexNAc 6 | 78.051 | 0.004 | 14.154 | 1.346e+07 | 90 - 101 | 3 | 29.03 | 29.20 | 29.71 |
| 3230.3177 | YN(N)SSYLNVQR Hex 6 HexNAc 5 | 76.323 | 0.006 | 11.969 | 5.967e+06 | 91 - 101 | 2 | 22.54 | 22.62 | 22.86 |
| 3788.6234 | FYFTPN(N)KTEDTIFLR Fuc 1 Hex 4 HexNAc 4 Neu5Ac 1 | 75.836 | 0.006 | 14.922 | 2.409e+08 | 66 - 81 | 5 | 37.68 | 37.85 | 38.70 |
| 5539.4226 | SVQEIQATFFYFTPN(N)KTEDTIFLR Fuc 2 Hex 7 HexNAc 6 | 75.015 | 0.006 | -19.391 | 2.255e+07 | 57 - 81 | 1 | 61.79 | 61.79 | 61.96 |
| 3453.5389 | VPVPITN(N)ATLDR Fuc 2 Hex 4 HexNAc 6 | 73.457 | 0.006 | -4.302 | 4.062e+07 | 26 - 38 | 27 | 40.04 | 40.21 | 40.38 |
| 4116.7043 | TPN(N)KTEDTIFLR Fuc 1 Hex 4 HexNAc 5 Neu5Ac 3 | 73.194 | 0.006 | -19.666 | 1.218e+07 | 69 - 81 | 1 | 36.53 | 36.53 | 36.53 |
| 4419.8388 | FYFTPN(N)KTEDTIFLR Hex 7 HexNAc 4 Neu5Ac 2 | 73.167 | 0.006 | -19.154 | 8.152e+06 | 66 - 81 | 3 | 46.34 | 46.34 | 46.51 |
| 3428.5020 | VPVPITN(N)ATLDR Fuc 1 Hex 6 HexNAc 5 | 72.907 | 0.006 | -5.057 | 1.902e+06 | 26 - 38 | 1 | 29.71 | 29.71 | 30.05 |
| 5367.1972 | SVQEIQATFFYFTPN(N)K Hex 10 HexNAc 9 | 66.902 | 0.007 | -5.012 | 1.080e+07 | 57 - 73 | 1 | 51.53 | 51.87 | 52.04 |
| 3923.7440 | NLVPVPITN(N)ATLDR Hex 3 HexNAc 8 Neu5Ac 1 | 65.438 | 0.007 | -3.980 | 1.077e+08 | 24 - 38 | 3 | 35.58 | 35.92 | 35.92 |
| 3617.6374 | NLVPVPITN(N)ATLDR Fuc 1 Hex 7 HexNAc 4 | 61.392 | 0.007 | 0.000 | 0.000e+00 | 24 - 38 | 1 | - | - | - |
| 4474.8791 | SVQEIQATFFYFTPN(N)K Hex 7 HexNAc 7 | 59.983 | 0.007 | 11.694 | 7.569e+07 | 57 - 73 | 4 | 53.75 | 53.92 | 54.60 |
| 3886.6967 | C(C)ANLVPVPITN(N)ATLDR Fuc 1 Hex 6 HexNAc 5 | 56.315 | 0.007 | -3.537 | 1.370e+08 | 22 - 38 | 6 | 41.58 | 52.04 | 52.73 |
| 5148.1311 | SVQEIQATFFYFTPN(N)K Fuc 1 Hex 9 HexNAc 8 | 56.095 | 0.007 | -6.242 | 6.067e+06 | 57 - 73 | 1 | 51.87 | 52.04 | 52.21 |
| 4212.8417 | ANLVPVPITN(N)ATLDR Fuc 1 Hex 9 HexNAc 5 | 55.076 | 0.007 | -6.185 | 1.570e+08 | 23 - 38 | 4 | 61.44 | 62.13 | 62.30 |
| 4191.9172 | QIPLC(C)ANLVPVPITN(N)ATLDR Hex 6 HexNAc 5 | 55.018 | 0.007 | 12.748 | 6.118e+08 | 18 - 38 | 10 | 61.44 | 62.13 | 63.33 |
| 4374.8705 | ANLVPVPITN(N)ATLDR Fuc 1 Hex 10 HexNAc 5 | 53.859 | 0.007 | -4.760 | 2.789e+07 | 23 - 38 | 1 | 61.79 | 61.79 | 62.13 |
| 3817.6811 | NLVPVPITN(N)ATLDR Fuc 1 Hex 7 HexNAc 5 | 48.951 | 0.007 | -4.599 | 1.123e+08 | 24 - 38 | 3 | 36.90 | 36.90 | 37.21 |
| 5977.5676 | SVQEIQATFFYFTPN(N)KTEDTIFLR Hex 9 HexNAc 8 | 48.646 | 0.007 | 14.198 | 7.338e+07 | 57 - 81 | 6 | 60.76 | 60.93 | 61.79 |
| 3087.4171 | NLVPVPITN(N)ATLDR Fuc 1 Hex 5 HexNAc 3 | 47.288 | 0.007 | 15.172 | 2.038e+08 | 24 - 38 | 3 | 37.68 | 37.85 | 38.70 |
| 3887.6827 | C(C)AN(D)LVPVPITN(N)ATLDR Fuc 1 Hex 6 HexNAc 5 | 45.479 | 0.007 | 8.015 | 4.745e+07 | 22 - 38 | 8 | 43.46 | 43.63 | 44.47 |
| 4562.8962 | SVQEIQATFFYFTPN(N)K Hex 7 HexNAc 6 Neu5Ac 1 | 45.115 | 0.007 | -20.745 | 5.306e+05 | 57 - 73 | 1 | 56.14 | 56.14 | 56.14 |
| 5423.3358 | SVQEIQATFFYFTPN(N)KTEDTIFLR Hex 7 HexNAc 4 Neu5Ac 2 | 44.728 | 0.007 | -3.903 | 3.148e+07 | 57 - 81 | 3 | 62.81 | 62.98 | 63.33 |
| 3944.6621 | PVPITN(N)ATLDR Fuc 1 Hex 8 HexNAc 5 Neu5Ac 1 | 43.545 | 0.007 | -18.144 | 3.131e+07 | 27 - 38 | 1 | 35.75 | 35.92 | 35.92 |
| 3543.5821 | LVPVPITN(N)ATLDR Fuc 1 Hex 6 HexNAc 5 | 42.861 | 0.007 | 0.000 | 0.000e+00 | 25 - 38 | 1 | - | - | - |
| 5758.4931 | SVQEIQATFFYFTPN(N)KTEDTIFLR Fuc 1 Hex 8 HexNAc 7 | 42.164 | 0.007 | 15.737 | 3.805e+07 | 57 - 81 | 3 | 61.10 | 61.62 | 61.79 |
| 3405.5365 | LVPVPITN(N)ATLDR Fuc 2 Hex 3 HexNAc 6 | 42.089 | 0.007 | 0.000 | 0.000e+00 | 25 - 38 | 1 | - | - | - |
| 3513.5363 | FYFTPN(N)KTEDTIFLR Hex 5 HexNAc 4 | 41.630 | 0.007 | -5.615 | 2.818e+06 | 66 - 81 | 1 | 46.17 | 46.34 | 46.51 |
| 4320.9528 | QIPLC(C)ANLVPVPITN(N)ATLDR Hex 5 HexNAc 5 Neu5Ac 1 | 41.101 | 0.007 | 15.125 | 4.189e+08 | 18 - 38 | 7 | 60.93 | 61.96 | 62.98 |
| 3741.6210 | C(C)AN(D)LVPVPITN(N)ATLDR Hex 6 HexNAc 5 | 40.403 | 0.007 | 9.951 | 2.170e+08 | 22 - 38 | 13 | 42.60 | 43.80 | 44.98 |
| 3789.6109 | TPN(N)KTEDTIFLR Hex 7 HexNAc 6 | 40.343 | 0.007 | 0.000 | 0.000e+00 | 69 - 81 | 1 | - | - | - |
| 3580.5400 | LVPVPITN(N)ATLDR Fuc 1 Hex 10 HexNAc 2 | 37.458 | 0.007 | -20.661 | 6.784e+06 | 25 - 38 | 2 | 36.74 | 36.74 | 36.74 |
| 3398.5365 | LVPVPITN(N)ATLDR Hex 6 HexNAc 5 | 37.333 | 0.007 | 0.000 | 0.000e+00 | 25 - 38 | 1 | - | - | - |
| 3754.5859 | YFTPN(N)KTEDTIFLR Fuc 1 Hex 9 HexNAc 2 | 37.127 | 0.007 | -18.945 | 1.465e+07 | 67 - 81 | 2 | 37.21 | 37.21 | 37.21 |
| 4052.7772 | LVPVPITN(N)ATLDR Fuc 2 Hex 7 HexNAc 6 | 36.704 | 0.007 | -18.738 | 3.082e+07 | 25 - 38 | 1 | 35.75 | 35.92 | 35.92 |
| 3797.6619 | C(C)AN(D)LVPVPITN(N)ATLDR Fuc 2 Hex 4 HexNAc 4 Neu5Ac 1 | 36.330 | 0.008 | 10.804 | 1.054e+08 | 22 - 38 | 5 | 42.60 | 42.95 | 43.46 |
| 4975.0717 | FYFTPN(N)KTEDTIFLR Hex 9 HexNAc 8 | 35.821 | 0.008 | 0.000 | 0.000e+00 | 66 - 81 | 1 | - | - | - |
| 3934.6793 | YFTPN(N)KTEDTIFLR Hex 6 HexNAc 6 | 35.624 | 0.008 | -4.484 | 5.309e+07 | 67 - 81 | 4 | 37.53 | 37.53 | 37.85 |
| 4153.7556 | YFTPN(N)KTEDTIFLR Fuc 4 Hex 5 HexNAc 5 | 35.408 | 0.009 | 13.726 | 5.029e+08 | 67 - 81 | 3 | 36.90 | 37.36 | 38.19 |
| 3475.4948 | LVPVPITN(N)ATLDR Hex 9 HexNAc 3 | 34.746 | 0.010 | -20.713 | 2.385e+06 | 25 - 38 | 1 | 40.04 | 40.04 | 40.04 |
| 3595.4973 | PVPITN(N)ATLDR Hex 8 HexNAc 4 Neu5Ac 1 | 34.707 | 0.010 | 0.000 | 0.000e+00 | 27 - 38 | 1 | - | - | - |
| 4456.8132 | ATFFYFTPN(N)K Fuc 4 Hex 5 HexNAc 9 | 34.522 | 0.011 | -19.983 | 1.053e+07 | 63 - 73 | 1 | 53.41 | 53.41 | 53.41 |
| 4337.9746 | QIPLC(C)ANLVPVPITN(N)ATLDR Fuc 1 Hex 6 HexNAc 5 | 34.032 | 0.015 | 14.303 | 1.653e+08 | 18 - 38 | 4 | 61.62 | 61.96 | 62.30 |
| 4434.7196 | QN(D)QC(C)FYN(N)SSYLNVQR Fuc 3 Hex 6 HexNAc 4 Neu5Ac 1 | 33.497 | 0.015 | -6.505 | 7.708e+07 | 86 - 101 | 2 | 39.04 | 39.04 | 39.38 |
| 5304.3238 | SVQEIQATFFYFTPN(N)KTEDTIFLR Fuc 4 Hex 5 HexNAc 5 | 33.377 | 0.016 | 13.313 | 2.606e+08 | 57 - 81 | 9 | 62.30 | 62.64 | 64.36 |
| 5285.2516 | ANLVPVPITN(N)ATLDR Fuc 5 Hex 7 HexNAc 9 | 33.197 | 0.016 | 9.288 | 1.260e+08 | 23 - 38 | 3 | 61.79 | 61.96 | 62.64 |
| 4490.9313 | LVPVPITN(N)ATLDR Hex 9 HexNAc 8 | 33.105 | 0.017 | -7.077 | 4.888e+06 | 25 - 38 | 1 | 35.07 | 35.07 | 35.41 |
| 4358.9062 | ANLVPVPITN(N)ATLDR Fuc 2 Hex 9 HexNAc 5 | 32.305 | 0.026 | -4.763 | 4.134e+07 | 23 - 38 | 2 | 61.62 | 61.96 | 61.96 |
| 3977.7205 | LVPVPITN(N)ATLDR Hex 6 HexNAc 5 Neu5Ac 2 | 32.071 | 0.028 | -4.710 | 8.838e+06 | 25 - 38 | 2 | 42.09 | 42.26 | 42.43 |
| 3239.4253 | PVPITN(N)ATLDR Fuc 2 Hex 4 HexNAc 4 Neu5Ac 1 | 31.998 | 0.028 | 14.863 | 5.230e+07 | 27 - 38 | 4 | 42.26 | 42.43 | 43.29 |
| 4336.9450 | LVPVPITN(N)ATLDR Fuc 5 Hex 3 HexNAc 7 Neu5Ac 1 | 31.726 | 0.031 | 10.720 | 3.855e+07 | 25 - 38 | 6 | 59.38 | 60.93 | 61.10 |
| 5440.3599 | SVQEIQATFFYFTPN(N)KTEDTIFLR Fuc 1 Hex 8 HexNAc 4 Neu5Ac 1 | 31.387 | 0.031 | -4.937 | 1.006e+07 | 57 - 81 | 1 | 62.81 | 62.98 | 63.15 |
| 3869.6715 | C(C)ANLVPVPITN(N)ATLDR Hex 5 HexNAc 5 Neu5Ac 1 | 30.863 | 0.031 | 15.461 | 2.809e+08 | 22 - 38 | 5 | 51.87 | 52.04 | 52.90 |
| 3256.4522 | LVPVPITN(N)ATLDR Fuc 1 Hex 8 HexNAc 2 | 30.559 | 0.031 | -3.617 | 3.311e+07 | 25 - 38 | 2 | 42.26 | 42.43 | 42.77 |
| 3434.7841 | PVPITN(N)ATLDR Fuc 2 Hex 7 HexNAc 4 | 30.503 | 0.031 | -4.747 | 3.448e+07 | 27 - 38 | 63 | 17.46 | 36.74 | 82.92 |
| 3417.5064 | C(C)AN(D)LVPVPITN(N)ATLDR Hex 4 HexNAc 5 | 30.331 | 0.032 | 10.282 | 1.162e+09 | 22 - 38 | 1 | 36.07 | 36.62 | 38.36 |
| 4268.8098 | LVPVPITN(N)ATLDR Hex 6 HexNAc 5 Neu5Ac 3 | 30.099 | 0.032 | 11.609 | 1.259e+07 | 25 - 38 | 2 | 45.66 | 45.83 | 46.34 |
| 4199.7697 | SVQEIQATFFYFTPN(N)K Hex 6 HexNAc 5 Neu5Ac 1 | 30.029 | 0.032 | 0.000 | 0.000e+00 | 57 - 73 | 1 | - | - | - |
| 4054.6230 | QN(D)QC(C)FYN(N)SSYLNVQR Fuc 1 Hex 6 HexNAc 5 | 29.967 | 0.032 | -6.139 | 2.246e+07 | 86 - 101 | 2 | 34.39 | 34.39 | 34.73 |
| 3377.3867 | FYN(N)SSYLNVQR Hex 6 HexNAc 5 | 29.902 | 0.032 | -4.724 | 4.441e+06 | 90 - 101 | 3 | 29.37 | 29.54 | 29.71 |
| 3595.4507 | YN(N)SSYLNVQR Hex 7 HexNAc 6 | 29.704 | 0.032 | -4.159 | 5.672e+06 | 91 - 101 | 2 | 22.18 | 22.30 | 22.48 |
| 5002.9782 | QNQC(C)FYN(N)SSYLNVQR Hex 9 HexNAc 8 | 29.383 | 0.034 | -19.534 | 8.496e+06 | 86 - 101 | 1 | 37.68 | 37.68 | 37.68 |
| 4564.8367 | QNQC(C)FYN(N)SSYLNVQR Fuc 2 Hex 7 HexNAc 6 | 29.175 | 0.036 | -5.278 | 1.951e+07 | 86 - 101 | 1 | 38.53 | 38.70 | 39.04 |
| 4620.9416 | SVQEIQATFFYFTPN(N)K Fuc 1 Hex 7 HexNAc 7 | 29.130 | 0.036 | -19.632 | 3.047e+06 | 57 - 73 | 1 | 53.75 | 53.75 | 53.75 |
| 3744.5116 | FYN(N)SSYLNVQR Hex 7 HexNAc 6 | 27.983 | 0.038 | 0.000 | 0.000e+00 | 90 - 101 | 1 | - | - | - |
| 3742.6909 | ANLVPVPITN(N)ATLDR Hex 7 HexNAc 5 | 27.632 | 0.039 | -19.214 | 1.652e+06 | 23 - 38 | 1 | 41.07 | 41.07 | 41.07 |
| 7813.3374 | WFYIASAFRNEEYNKSVQEIQATFFYFTPN(N)K Fuc 5 Hex 10 HexNAc 8 | 27.627 | 0.039 | -18.452 | 3.423e+07 | 42 - 73 | 1 | 53.92 | 53.92 | 54.09 |
| 4321.9519 | QIPLC(C)ANLVPVPITN(N)ATLDR Fuc 2 Hex 5 HexNAc 5 | 27.497 | 0.041 | -19.744 | 1.338e+07 | 18 - 38 | 1 | 62.30 | 62.30 | 62.30 |
| 3654.5293 | PVPITN(N)ATLDR Fuc 1 Hex 8 HexNAc 5 | 27.263 | 0.041 | 0.000 | 0.000e+00 | 27 - 38 | 1 | - | - | - |
| 4321.9334 | LVPVPITN(N)ATLDR Fuc 3 Hex 4 HexNAc 9 | 27.107 | 0.042 | 12.664 | 3.235e+07 | 25 - 38 | 9 | 62.98 | 63.33 | 64.70 |
| 4151.7405 | TPN(N)KTEDTIFLR Hex 8 HexNAc 7 | 26.648 | 0.045 | -5.587 | 4.262e+06 | 69 - 81 | 2 | 24.38 | 24.55 | 24.90 |
| 4974.0668 | FYFTPN(N)KTEDTIFLR Hex 9 HexNAc 8 | 26.565 | 0.045 | -20.985 | 2.814e+06 | 66 - 81 | 1 | 44.30 | 44.30 | 44.47 |
| 4653.8104 | QN(D)QC(C)FYN(N)SSYLNVQR Fuc 2 Hex 7 HexNAc 5 Neu5Ac 1 | 26.188 | 0.048 | -6.428 | 7.717e+07 | 86 - 101 | 2 | 38.36 | 38.53 | 38.87 |

### YFTPN(N)K Hex 5 HexNAc 4

**Mass:**  2390.9613

(3.245e-06)

**Charge States:** 2

**Start Position**: 68

**End Position**: 74

| Profile Score | Tandem Score | TDA q-value | Volume | Spectrum Matches |
| --- | --- | --- | --- | --- |
| -5.9023 | 188.0146 | 0.0000 | 2.5716e+07 | 3 |

**Adduct Signal Fractions**

- **Unmodified**: 2.5716e+07

### YFTPN(N)K Hex 6 HexNAc 5

**Mass:**  2757.0946

(3.629e-04)

**Start Position**: 68

**End Position**: 74

| Profile Score | Tandem Score | TDA q-value | Volume | Spectrum Matches |
| --- | --- | --- | --- | --- |
| 0.0000 | 187.4481 | 0.0000 | 0.0000e+00 | 1 |

Adduct:

No Chromatogram Found

### YFTPN(N)K Hex 6 HexNAc 5

**Mass:**  2756.0980

(1.251e-06)

**Charge States:** 2, 3

**Start Position**: 68

**End Position**: 74

| Profile Score | Tandem Score | TDA q-value | Volume | Spectrum Matches |
| --- | --- | --- | --- | --- |
| 14.1125 | 185.4478 | 0.0000 | 3.1298e+08 | 5 |

**Adduct Signal Fractions**

- **Unmodified**: 3.1298e+08

### FYFTPN(N)K Hex 7 HexNAc 6

**Mass:**  3268.2991

(1.201e-06)

**Charge States:** 3, 4

**Start Position**: 67

**End Position**: 74

| Profile Score | Tandem Score | TDA q-value | Volume | Spectrum Matches |
| --- | --- | --- | --- | --- |
| 15.0132 | 184.0277 | 0.0000 | 1.5971e+08 | 8 |

**Adduct Signal Fractions**

- **Unmodified**: 1.5971e+08

### FYFTPN(N)K Hex 6 HexNAc 5

**Mass:**  2903.1654

(8.364e-07)

**Charge States:** 2, 3

**Start Position**: 67

**End Position**: 74

| Profile Score | Tandem Score | TDA q-value | Volume | Spectrum Matches |
| --- | --- | --- | --- | --- |
| 14.8043 | 183.7520 | 0.0000 | 1.2021e+08 | 12 |

**Adduct Signal Fractions**

- **Unmodified**: 1.2021e+08

### NEEYN(N)K Hex 5 HexNAc 4

**Mass:**  2417.9220

(3.145e-07)

**Charge States:** 2, 3

**Start Position**: 52

**End Position**: 58

| Profile Score | Tandem Score | TDA q-value | Volume | Spectrum Matches |
| --- | --- | --- | --- | --- |
| 11.7217 | 181.2652 | 0.0000 | 7.8808e+07 | 8 |

**Adduct Signal Fractions**

- **Unmodified**: 7.8808e+07

### NEEYN(N)K Hex 6 HexNAc 5

**Mass:**  2783.0550

(6.045e-07)

**Charge States:** 3

**Start Position**: 52

**End Position**: 58

| Profile Score | Tandem Score | TDA q-value | Volume | Spectrum Matches |
| --- | --- | --- | --- | --- |
| 11.5010 | 181.1988 | 0.0000 | 6.4556e+07 | 7 |

**Adduct Signal Fractions**

- **Unmodified**: 6.4556e+07

### SVQEIQATFFYFTPN(N)K Hex 6 HexNAc 5

**Mass:**  3906.6691

(2.258e-06)

**Charge States:** 3, 4, 5

**Start Position**: 58

**End Position**: 74

| Profile Score | Tandem Score | TDA q-value | Volume | Spectrum Matches |
| --- | --- | --- | --- | --- |
| 14.9224 | 175.2159 | 0.0000 | 2.3571e+09 | 54 |

**Adduct Signal Fractions**

- **Unmodified**: 2.3571e+09

### SVQEIQATFFYFTPN(N)K Fuc 1 Hex 6 HexNAc 5

**Mass:**  4052.7248

(2.304e-06)

**Charge States:** 3, 4

**Start Position**: 58

**End Position**: 74

| Profile Score | Tandem Score | TDA q-value | Volume | Spectrum Matches |
| --- | --- | --- | --- | --- |
| 13.7957 | 175.0613 | 0.0000 | 4.9285e+08 | 17 |

**Adduct Signal Fractions**

- **Unmodified**: 4.9285e+08

### SVQEIQATFFYFTPN(N)K Hex 7 HexNAc 6

**Mass:**  4271.8083

(2.156e-06)

**Charge States:** 3, 4, 5

**Start Position**: 58

**End Position**: 74

| Profile Score | Tandem Score | TDA q-value | Volume | Spectrum Matches |
| --- | --- | --- | --- | --- |
| 14.1936 | 173.5464 | 0.0000 | 2.9258e+09 | 55 |

**Adduct Signal Fractions**

- **Unmodified**: 2.9258e+09

### SVQEIQATFFYFTPN(N)K Fuc 1 Hex 7 HexNAc 6

**Mass:**  4417.8635

(2.282e-04)

**Charge States:** 3, 4, 5

**Start Position**: 58

**End Position**: 74

| Profile Score | Tandem Score | TDA q-value | Volume | Spectrum Matches |
| --- | --- | --- | --- | --- |
| 13.7776 | 166.2051 | 0.0000 | 5.7995e+08 | 17 |

**Adduct Signal Fractions**

- **Unmodified**: 5.7995e+08

### SVQEIQATFFYFTPN(N)K Hex 8 HexNAc 7

**Mass:**  4636.9318

(7.010e-07)

**Charge States:** 3, 4, 5

**Start Position**: 58

**End Position**: 74

| Profile Score | Tandem Score | TDA q-value | Volume | Spectrum Matches |
| --- | --- | --- | --- | --- |
| 14.1683 | 164.6732 | 0.0000 | 3.9873e+08 | 9 |

**Adduct Signal Fractions**

- **Unmodified**: 3.9873e+08

### SVQEIQATFFYFTPN(N)K Fuc 2 Hex 7 HexNAc 6

**Mass:**  4563.9216

(2.855e-06)

**Charge States:** 3, 4

**Start Position**: 58

**End Position**: 74

| Profile Score | Tandem Score | TDA q-value | Volume | Spectrum Matches |
| --- | --- | --- | --- | --- |
| -3.8901 | 164.1234 | 0.0000 | 8.5262e+07 | 2 |

**Adduct Signal Fractions**

- **Unmodified**: 8.5262e+07

### SVQEIQATFFYFTPN(N)K Hex 5 HexNAc 4

**Mass:**  3541.5324

(1.144e-06)

**Charge States:** 3, 4

**Start Position**: 58

**End Position**: 74

| Profile Score | Tandem Score | TDA q-value | Volume | Spectrum Matches |
| --- | --- | --- | --- | --- |
| 13.4348 | 161.1955 | 0.0000 | 2.7931e+08 | 15 |

**Adduct Signal Fractions**

- **Unmodified**: 2.7931e+08

### LVPVPITN(N)ATLDR Hex 6 HexNAc 5

**Mass:**  3395.5244

(3.980e-07)

**Charge States:** 2, 3, 4

**Start Position**: 26

**End Position**: 39

| Profile Score | Tandem Score | TDA q-value | Volume | Spectrum Matches |
| --- | --- | --- | --- | --- |
| 11.5801 | 158.3266 | 0.0000 | 1.7703e+10 | 615 |

**Adduct Signal Fractions**

- **Unmodified**: 1.7703e+10

### LVPVPITN(N)ATLDR Hex 5 HexNAc 4

**Mass:**  3030.3973

(2.334e-06)

**Charge States:** 2, 3, 4

**Start Position**: 26

**End Position**: 39

| Profile Score | Tandem Score | TDA q-value | Volume | Spectrum Matches |
| --- | --- | --- | --- | --- |
| 14.5231 | 157.1212 | 0.0000 | 1.1318e+09 | 8 |

**Adduct Signal Fractions**

- **Unmodified**: 1.1318e+09

### ATFFYFTPN(N)K Hex 6 HexNAc 5

**Mass:**  3222.3213

(2.110e-06)

**Charge States:** 3

**Start Position**: 64

**End Position**: 74

| Profile Score | Tandem Score | TDA q-value | Volume | Spectrum Matches |
| --- | --- | --- | --- | --- |
| 14.8071 | 156.7150 | 0.0000 | 4.6937e+07 | 2 |

**Adduct Signal Fractions**

- **Unmodified**: 4.6937e+07

### LVPVPITN(N)ATLDR Hex 7 HexNAc 6

**Mass:**  3760.6587

(1.262e-06)

**Charge States:** 3, 4

**Start Position**: 26

**End Position**: 39

| Profile Score | Tandem Score | TDA q-value | Volume | Spectrum Matches |
| --- | --- | --- | --- | --- |
| -2.5842 | 155.7173 | 0.0000 | 8.0100e+08 | 4 |

**Adduct Signal Fractions**

- **Unmodified**: 8.0100e+08

### LVPVPITN(N)ATLDR Fuc 1 Hex 6 HexNAc 5

**Mass:**  3541.5807

(1.372e-06)

**Charge States:** 3, 4

**Start Position**: 26

**End Position**: 39

| Profile Score | Tandem Score | TDA q-value | Volume | Spectrum Matches |
| --- | --- | --- | --- | --- |
| -2.5291 | 155.0668 | 0.0000 | 3.9631e+09 | 29 |

**Adduct Signal Fractions**

- **Unmodified**: 3.9631e+09

### ATFFYFTPN(N)K Hex 7 HexNAc 6

**Mass:**  3587.4563

(2.840e-06)

**Charge States:** 3, 4

**Start Position**: 64

**End Position**: 74

| Profile Score | Tandem Score | TDA q-value | Volume | Spectrum Matches |
| --- | --- | --- | --- | --- |
| 13.1155 | 155.0526 | 0.0000 | 7.5767e+07 | 3 |

**Adduct Signal Fractions**

- **Unmodified**: 7.5767e+07

### SVQEIQATFFYFTPN(N)K Fuc 1 Hex 8 HexNAc 4 Neu5Ac 1

**Mass:**  4464.8566

(3.716e-06)

**Charge States:** 3, 4

**Start Position**: 58

**End Position**: 74

| Profile Score | Tandem Score | TDA q-value | Volume | Spectrum Matches |
| --- | --- | --- | --- | --- |
| 12.6064 | 153.7809 | 0.0000 | 5.2580e+07 | 4 |

**Adduct Signal Fractions**

- **Unmodified**: 5.2580e+07

### SVQEIQATFFYFTPN(N)K Fuc 1 Hex 8 HexNAc 7

**Mass:**  4782.9924

(2.055e-06)

**Charge States:** 3, 4

**Start Position**: 58

**End Position**: 74

| Profile Score | Tandem Score | TDA q-value | Volume | Spectrum Matches |
| --- | --- | --- | --- | --- |
| 12.2037 | 150.1947 | 0.0000 | 7.7208e+07 | 3 |

**Adduct Signal Fractions**

- **Unmodified**: 7.7208e+07

### C(C)ANLVPVPITN(N)ATLDR Hex 6 HexNAc 5

**Mass:**  3740.6394

(2.404e-06)

**Charge States:** 3, 4

**Start Position**: 23

**End Position**: 39

| Profile Score | Tandem Score | TDA q-value | Volume | Spectrum Matches |
| --- | --- | --- | --- | --- |
| 13.2980 | 147.0962 | 0.0000 | 2.3962e+08 | 6 |

**Adduct Signal Fractions**

- **Unmodified**: 2.3962e+08

### SVQEIQATFFYFTPN(N)KTEDTIFLR Fuc 1 Hex 6 HexNAc 5

**Mass:**  5028.2297

(1.853e-06)

**Charge States:** 4, 5

**Start Position**: 58

**End Position**: 82

| Profile Score | Tandem Score | TDA q-value | Volume | Spectrum Matches |
| --- | --- | --- | --- | --- |
| 12.4524 | 146.6399 | 0.0000 | 1.4224e+08 | 5 |

**Adduct Signal Fractions**

- **Unmodified**: 1.4224e+08

### SVQEIQATFFYFTPN(N)KTEDTIFLR Hex 6 HexNAc 5

**Mass:**  4882.1765

(2.142e-06)

**Charge States:** 3, 4, 5

**Start Position**: 58

**End Position**: 82

| Profile Score | Tandem Score | TDA q-value | Volume | Spectrum Matches |
| --- | --- | --- | --- | --- |
| 14.5191 | 145.9353 | 0.0000 | 5.9164e+08 | 19 |

**Adduct Signal Fractions**

- **Unmodified**: 5.9164e+08

### SVQEIQATFFYFTPN(N)K Hex 9 HexNAc 8

**Mass:**  5002.0686

(2.249e-06)

**Charge States:** 4, 5

**Start Position**: 58

**End Position**: 74

| Profile Score | Tandem Score | TDA q-value | Volume | Spectrum Matches |
| --- | --- | --- | --- | --- |
| 13.6658 | 144.3287 | 0.0000 | 8.1956e+07 | 4 |

**Adduct Signal Fractions**

- **Unmodified**: 8.1956e+07

### ATFFYFTPN(N)K Fuc 1 Hex 6 HexNAc 5

**Mass:**  3369.3751

(2.972e-04)

**Start Position**: 64

**End Position**: 74

| Profile Score | Tandem Score | TDA q-value | Volume | Spectrum Matches |
| --- | --- | --- | --- | --- |
| 0.0000 | 142.0677 | 0.0000 | 0.0000e+00 | 1 |

Adduct:

No Chromatogram Found

### SVQEIQATFFYFTPN(N)K Fuc 2 Hex 8 HexNAc 7

**Mass:**  4929.0529

(2.804e-06)

**Charge States:** 4

**Start Position**: 58

**End Position**: 74

| Profile Score | Tandem Score | TDA q-value | Volume | Spectrum Matches |
| --- | --- | --- | --- | --- |
| 12.4397 | 141.8855 | 0.0000 | 1.6702e+07 | 3 |

**Adduct Signal Fractions**

- **Unmodified**: 1.6702e+07

### SVQEIQATFFYFTPN(N)K Hex 7 HexNAc 4 Neu5Ac 2

**Mass:**  4447.8327

(2.078e-06)

**Charge States:** 3, 4

**Start Position**: 58

**End Position**: 74

| Profile Score | Tandem Score | TDA q-value | Volume | Spectrum Matches |
| --- | --- | --- | --- | --- |
| 12.1826 | 140.4643 | 0.0000 | 1.3486e+08 | 6 |

**Adduct Signal Fractions**

- **Unmodified**: 1.3486e+08

### LVPVPITN(N)ATLDR Hex 8 HexNAc 7

**Mass:**  4125.7922

(1.258e-06)

**Charge States:** 3, 4

**Start Position**: 26

**End Position**: 39

| Profile Score | Tandem Score | TDA q-value | Volume | Spectrum Matches |
| --- | --- | --- | --- | --- |
| 12.2589 | 140.4532 | 0.0000 | 1.4403e+08 | 5 |

**Adduct Signal Fractions**

- **Unmodified**: 1.4403e+08

### LVPVPITN(N)ATLDR Fuc 1 Hex 7 HexNAc 6

**Mass:**  3906.7175

(1.256e-06)

**Charge States:** 3, 4

**Start Position**: 26

**End Position**: 39

| Profile Score | Tandem Score | TDA q-value | Volume | Spectrum Matches |
| --- | --- | --- | --- | --- |
| -4.2585 | 139.5523 | 0.0000 | 2.9336e+08 | 4 |

**Adduct Signal Fractions**

- **Unmodified**: 2.9336e+08

### SVQEIQATFFYFTPN(N)KTEDTIFLR Hex 7 HexNAc 6

**Mass:**  5247.3186

(3.440e-06)

**Charge States:** 3, 4, 5

**Start Position**: 58

**End Position**: 82

| Profile Score | Tandem Score | TDA q-value | Volume | Spectrum Matches |
| --- | --- | --- | --- | --- |
| 13.3341 | 139.3981 | 0.0000 | 1.2201e+09 | 24 |

**Adduct Signal Fractions**

- **Unmodified**: 1.2201e+09

### PVPITN(N)ATLDR Fuc 1 Hex 4 HexNAc 9

**Mass:**  3819.6364

(5.307e-04)

**Charge States:** 3, 4

**Start Position**: 28

**End Position**: 39

| Profile Score | Tandem Score | TDA q-value | Volume | Spectrum Matches |
| --- | --- | --- | --- | --- |
| -8.9565 | 138.7193 | 0.0000 | 1.4502e+08 | 15 |

**Adduct Signal Fractions**

- **Unmodified**: 1.4502e+08

### LVPVPITN(N)ATLDR Fuc 2 Hex 6 HexNAc 5

**Mass:**  3687.6414

(8.727e-07)

**Charge States:** 3, 4

**Start Position**: 26

**End Position**: 39

| Profile Score | Tandem Score | TDA q-value | Volume | Spectrum Matches |
| --- | --- | --- | --- | --- |
| -17.8777 | 138.6254 | 0.0000 | 3.8620e+07 | 1 |

**Adduct Signal Fractions**

- **Unmodified**: 3.8620e+07

### YFTPN(N)KTEDTIFLR Fuc 1 Hex 7 HexNAc 6

**Mass:**  4242.7907

(3.852e-07)

**Charge States:** 3, 4

**Start Position**: 68

**End Position**: 82

| Profile Score | Tandem Score | TDA q-value | Volume | Spectrum Matches |
| --- | --- | --- | --- | --- |
| 9.7723 | 138.2884 | 0.0000 | 2.3763e+08 | 4 |

**Adduct Signal Fractions**

- **Unmodified**: 2.3763e+08

### TPN(N)KTEDTIFLR Hex 7 HexNAc 6

**Mass:**  3786.6015

(1.054e-06)

**Charge States:** 3, 4

**Start Position**: 70

**End Position**: 82

| Profile Score | Tandem Score | TDA q-value | Volume | Spectrum Matches |
| --- | --- | --- | --- | --- |
| 14.4896 | 137.8852 | 0.0000 | 6.7669e+07 | 6 |

**Adduct Signal Fractions**

- **Unmodified**: 6.7669e+07

### LVPVPITN(N)ATLDR Hex 7 HexNAc 5

**Mass:**  3560.6105

(8.532e-04)

**Start Position**: 26

**End Position**: 39

| Profile Score | Tandem Score | TDA q-value | Volume | Spectrum Matches |
| --- | --- | --- | --- | --- |
| 0.0000 | 133.8911 | 0.0000 | 0.0000e+00 | 1 |

Adduct:

No Chromatogram Found

### YFTPN(N)KTEDTIFLR Hex 6 HexNAc 5

**Mass:**  3731.6054

(1.393e-06)

**Charge States:** 3, 4

**Start Position**: 68

**End Position**: 82

| Profile Score | Tandem Score | TDA q-value | Volume | Spectrum Matches |
| --- | --- | --- | --- | --- |
| 10.6470 | 133.2073 | 0.0000 | 1.0905e+09 | 10 |

**Adduct Signal Fractions**

- **Unmodified**: 1.0905e+09

### SVQEIQATFFYFTPN(N)K Fuc 3 Hex 4 HexNAc 5

**Mass:**  4020.7202

(-2.050e-06)

**Charge States:** 3, 4

**Start Position**: 58

**End Position**: 74

| Profile Score | Tandem Score | TDA q-value | Volume | Spectrum Matches |
| --- | --- | --- | --- | --- |
| -19.9652 | 131.8360 | 0.0000 | 1.2370e+07 | 1 |

**Adduct Signal Fractions**

- **Unmodified**: 1.2370e+07

### YFTPN(N)KTEDTIFLR Hex 7 HexNAc 6

**Mass:**  4096.7331

(2.062e-06)

**Charge States:** 3, 4

**Start Position**: 68

**End Position**: 82

| Profile Score | Tandem Score | TDA q-value | Volume | Spectrum Matches |
| --- | --- | --- | --- | --- |
| 11.2278 | 131.6591 | 0.0000 | 2.1452e+09 | 123 |

**Adduct Signal Fractions**

- **Unmodified**: 2.1452e+09

### LVPVPITN(N)ATLDR Hex 5 HexNAc 4

**Mass:**  3031.3939

(3.312e-04)

**Start Position**: 26

**End Position**: 39

| Profile Score | Tandem Score | TDA q-value | Volume | Spectrum Matches |
| --- | --- | --- | --- | --- |
| 0.0000 | 131.2162 | 0.0000 | 0.0000e+00 | 1 |

Adduct:

No Chromatogram Found

### YFTPN(N)KTEDTIFLR Hex 5 HexNAc 4

**Mass:**  3366.4678

(9.407e-07)

**Charge States:** 3, 4

**Start Position**: 68

**End Position**: 82

| Profile Score | Tandem Score | TDA q-value | Volume | Spectrum Matches |
| --- | --- | --- | --- | --- |
| 14.9733 | 131.0474 | 0.0000 | 5.9734e+07 | 1 |

**Adduct Signal Fractions**

- **Unmodified**: 5.9734e+07

### QNQC(C)FYN(N)SSYLNVQR Hex 7 HexNAc 6

**Mass:**  4272.7116

(1.279e-06)

**Charge States:** 3, 4

**Start Position**: 87

**End Position**: 102

| Profile Score | Tandem Score | TDA q-value | Volume | Spectrum Matches |
| --- | --- | --- | --- | --- |
| 9.7900 | 128.6487 | 0.0000 | 1.1192e+09 | 5 |

**Adduct Signal Fractions**

- **Unmodified**: 1.1192e+09

### VPVPITN(N)ATLDR Hex 6 HexNAc 5

**Mass:**  3282.4436

(1.035e-06)

**Charge States:** 3

**Start Position**: 27

**End Position**: 39

| Profile Score | Tandem Score | TDA q-value | Volume | Spectrum Matches |
| --- | --- | --- | --- | --- |
| -3.8123 | 127.9587 | 0.0000 | 1.0155e+07 | 3 |

**Adduct Signal Fractions**

- **Unmodified**: 1.0155e+07

### YFTPN(N)KTEDTIFLR Fuc 1 Hex 6 HexNAc 5

**Mass:**  3877.6569

(7.826e-07)

**Charge States:** 3, 4

**Start Position**: 68

**End Position**: 82

| Profile Score | Tandem Score | TDA q-value | Volume | Spectrum Matches |
| --- | --- | --- | --- | --- |
| 13.3577 | 127.6661 | 0.0000 | 2.7783e+08 | 3 |

**Adduct Signal Fractions**

- **Unmodified**: 2.7783e+08

### SVQEIQATFFYFTPN(N)K Hex 6 HexNAc 6

**Mass:**  4109.7453

(2.459e-06)

**Charge States:** 3, 4

**Start Position**: 58

**End Position**: 74

| Profile Score | Tandem Score | TDA q-value | Volume | Spectrum Matches |
| --- | --- | --- | --- | --- |
| 12.4238 | 126.3226 | 0.0000 | 6.6639e+07 | 7 |

**Adduct Signal Fractions**

- **Unmodified**: 6.6639e+07

### SVQEIQATFFYFTPN(N)K Fuc 4 Hex 5 HexNAc 5

**Mass:**  4328.8234

(-3.425e-06)

**Charge States:** 3, 4, 5

**Start Position**: 58

**End Position**: 74

| Profile Score | Tandem Score | TDA q-value | Volume | Spectrum Matches |
| --- | --- | --- | --- | --- |
| 12.2136 | 126.2955 | 0.0000 | 6.1505e+08 | 26 |

**Adduct Signal Fractions**

- **Unmodified**: 6.1505e+08

### SVQEIQATFFYFTPN(N)K Fuc 1 Hex 7 HexNAc 4 Neu5Ac 2

**Mass:**  4593.8884

(1.587e-06)

**Charge States:** 4

**Start Position**: 58

**End Position**: 74

| Profile Score | Tandem Score | TDA q-value | Volume | Spectrum Matches |
| --- | --- | --- | --- | --- |
| -19.3917 | 125.3733 | 0.0000 | 1.2482e+07 | 3 |

**Adduct Signal Fractions**

- **Unmodified**: 1.2482e+07

### FYFTPN(N)KTEDTIFLR Hex 6 HexNAc 5

**Mass:**  3878.6717

(4.876e-07)

**Charge States:** 3, 4

**Start Position**: 67

**End Position**: 82

| Profile Score | Tandem Score | TDA q-value | Volume | Spectrum Matches |
| --- | --- | --- | --- | --- |
| 14.9860 | 124.3039 | 0.0000 | 8.3599e+07 | 6 |

**Adduct Signal Fractions**

- **Unmodified**: 8.3599e+07

### NLVPVPITN(N)ATLDR Hex 6 HexNAc 5

**Mass:**  3509.5728

(2.179e-06)

**Charge States:** 3

**Start Position**: 25

**End Position**: 39

| Profile Score | Tandem Score | TDA q-value | Volume | Spectrum Matches |
| --- | --- | --- | --- | --- |
| -4.3314 | 123.5313 | 0.0000 | 1.7120e+07 | 3 |

**Adduct Signal Fractions**

- **Unmodified**: 1.7120e+07

### LVPVPITN(N)ATLDR Hex 6 HexNAc 6

**Mass:**  3598.6100

(2.268e-06)

**Charge States:** 3, 4

**Start Position**: 26

**End Position**: 39

| Profile Score | Tandem Score | TDA q-value | Volume | Spectrum Matches |
| --- | --- | --- | --- | --- |
| 14.5115 | 123.3341 | 0.0000 | 9.7437e+08 | 7 |

**Adduct Signal Fractions**

- **Unmodified**: 9.7437e+08

### QNQC(C)FYN(N)SSYLNVQR Hex 6 HexNAc 5

**Mass:**  3907.5811

(2.021e-06)

**Charge States:** 3, 4

**Start Position**: 87

**End Position**: 102

| Profile Score | Tandem Score | TDA q-value | Volume | Spectrum Matches |
| --- | --- | --- | --- | --- |
| 10.3799 | 122.8905 | 0.0000 | 7.2951e+08 | 5 |

**Adduct Signal Fractions**

- **Unmodified**: 7.2951e+08

### NLVPVPITN(N)ATLDR Fuc 1 Hex 6 HexNAc 4

**Mass:**  3452.5482

(7.010e-07)

**Charge States:** 2, 3, 4

**Start Position**: 25

**End Position**: 39

| Profile Score | Tandem Score | TDA q-value | Volume | Spectrum Matches |
| --- | --- | --- | --- | --- |
| 15.2684 | 122.7651 | 0.0000 | 3.6966e+09 | 9 |

**Adduct Signal Fractions**

- **Unmodified**: 3.6966e+09

### SVQEIQATFFYFTPN(N)K Hex 8 HexNAc 6

**Mass:**  4436.8889

(6.864e-04)

**Start Position**: 58

**End Position**: 74

| Profile Score | Tandem Score | TDA q-value | Volume | Spectrum Matches |
| --- | --- | --- | --- | --- |
| 0.0000 | 121.7438 | 0.0000 | 0.0000e+00 | 1 |

Adduct:

No Chromatogram Found

### LVPVPITN(N)ATLDR Fuc 1 Hex 7 HexNAc 3 Neu5Ac 1

**Mass:**  3588.5861

(4.538e-06)

**Charge States:** 3

**Start Position**: 26

**End Position**: 39

| Profile Score | Tandem Score | TDA q-value | Volume | Spectrum Matches |
| --- | --- | --- | --- | --- |
| -19.3118 | 121.3741 | 0.0000 | 4.0165e+07 | 1 |

**Adduct Signal Fractions**

- **Unmodified**: 4.0165e+07

### TPN(N)KTEDTIFLR Hex 6 HexNAc 5

**Mass:**  3421.4690

(1.273e-06)

**Charge States:** 3, 4

**Start Position**: 70

**End Position**: 82

| Profile Score | Tandem Score | TDA q-value | Volume | Spectrum Matches |
| --- | --- | --- | --- | --- |
| 14.7048 | 120.7758 | 0.0000 | 3.3612e+07 | 7 |

**Adduct Signal Fractions**

- **Unmodified**: 3.3612e+07

### FYFTPN(N)KTEDTIFLR Hex 7 HexNAc 6

**Mass:**  4243.8059

(8.887e-07)

**Charge States:** 3, 4

**Start Position**: 67

**End Position**: 82

| Profile Score | Tandem Score | TDA q-value | Volume | Spectrum Matches |
| --- | --- | --- | --- | --- |
| 14.5437 | 120.5617 | 0.0000 | 2.7752e+08 | 8 |

**Adduct Signal Fractions**

- **Unmodified**: 2.7752e+08

### FTPN(N)KTEDTIFLR Hex 6 HexNAc 5

**Mass:**  3568.5393

(1.308e-06)

**Charge States:** 3, 4

**Start Position**: 69

**End Position**: 82

| Profile Score | Tandem Score | TDA q-value | Volume | Spectrum Matches |
| --- | --- | --- | --- | --- |
| 14.7569 | 120.1939 | 0.0000 | 8.0490e+07 | 5 |

**Adduct Signal Fractions**

- **Unmodified**: 8.0490e+07

### LVPVPITN(N)ATLDR Hex 7 HexNAc 5

**Mass:**  3559.6034

(5.720e-04)

**Charge States:** 3

**Start Position**: 26

**End Position**: 39

| Profile Score | Tandem Score | TDA q-value | Volume | Spectrum Matches |
| --- | --- | --- | --- | --- |
| -11.7947 | 119.5707 | 0.0000 | 1.1074e+06 | 40 |

**Adduct Signal Fractions**

- **Unmodified**: 1.1074e+06

### LVPVPITN(N)ATLDR Hex 7 HexNAc 6

**Mass:**  3762.6652

(5.346e-04)

**Start Position**: 26

**End Position**: 39

| Profile Score | Tandem Score | TDA q-value | Volume | Spectrum Matches |
| --- | --- | --- | --- | --- |
| 0.0000 | 119.0108 | 0.0000 | 0.0000e+00 | 2 |

Adduct:

No Chromatogram Found

### QNQC(C)FYN(N)SSYLNVQR Hex 8 HexNAc 7

**Mass:**  4637.8522

(2.490e-06)

**Charge States:** 3, 4

**Start Position**: 87

**End Position**: 102

| Profile Score | Tandem Score | TDA q-value | Volume | Spectrum Matches |
| --- | --- | --- | --- | --- |
| 12.8206 | 117.3265 | 0.0000 | 7.9053e+07 | 1 |

**Adduct Signal Fractions**

- **Unmodified**: 7.9053e+07

### SVQEIQATFFYFTPN(N)K Fuc 2 Hex 6 HexNAc 5

**Mass:**  4198.7907

(3.446e-06)

**Charge States:** 3, 4

**Start Position**: 58

**End Position**: 74

| Profile Score | Tandem Score | TDA q-value | Volume | Spectrum Matches |
| --- | --- | --- | --- | --- |
| -19.5214 | 115.3254 | 0.0000 | 1.1054e+07 | 1 |

**Adduct Signal Fractions**

- **Unmodified**: 1.1054e+07

### YFTPN(N)KTEDTIFLR Hex 7 HexNAc 6

**Mass:**  4098.7433

(7.375e-04)

**Start Position**: 68

**End Position**: 82

| Profile Score | Tandem Score | TDA q-value | Volume | Spectrum Matches |
| --- | --- | --- | --- | --- |
| 0.0000 | 114.3662 | 0.0000 | 0.0000e+00 | 2 |

Adduct:

No Chromatogram Found

### YFTPN(N)KTEDTIFLR Hex 8 HexNAc 7

**Mass:**  4461.8669

(9.227e-07)

**Charge States:** 3, 4

**Start Position**: 68

**End Position**: 82

| Profile Score | Tandem Score | TDA q-value | Volume | Spectrum Matches |
| --- | --- | --- | --- | --- |
| 12.8998 | 114.2786 | 0.0000 | 2.0303e+08 | 4 |

**Adduct Signal Fractions**

- **Unmodified**: 2.0303e+08

### QNQC(C)FYN(N)SSYLNVQR Fuc 1 Hex 7 HexNAc 6

**Mass:**  4418.7741

(1.515e-06)

**Charge States:** 3, 4

**Start Position**: 87

**End Position**: 102

| Profile Score | Tandem Score | TDA q-value | Volume | Spectrum Matches |
| --- | --- | --- | --- | --- |
| -8.4948 | 114.1542 | 0.0000 | 3.1995e+08 | 3 |

**Adduct Signal Fractions**

- **Unmodified**: 3.1995e+08

### C(C)ANLVPVPITN(N)ATLDR Hex 5 HexNAc 4

**Mass:**  3375.5030

(6.044e-07)

**Charge States:** 3

**Start Position**: 23

**End Position**: 39

| Profile Score | Tandem Score | TDA q-value | Volume | Spectrum Matches |
| --- | --- | --- | --- | --- |
| -4.7399 | 112.6995 | 0.0000 | 1.2586e+07 | 1 |

**Adduct Signal Fractions**

- **Unmodified**: 1.2586e+07

### QNQC(C)FYN(N)SSYLNVQR Fuc 1 Hex 6 HexNAc 5

**Mass:**  4053.6406

(2.452e-06)

**Charge States:** 3, 4

**Start Position**: 87

**End Position**: 102

| Profile Score | Tandem Score | TDA q-value | Volume | Spectrum Matches |
| --- | --- | --- | --- | --- |
| -6.1478 | 109.2741 | 0.0000 | 1.3797e+08 | 3 |

**Adduct Signal Fractions**

- **Unmodified**: 1.3797e+08

### SVQEIQATFFYFTPN(N)K Fuc 4 Hex 6 HexNAc 5

**Mass:**  4493.9133

(6.728e-04)

**Start Position**: 58

**End Position**: 74

| Profile Score | Tandem Score | TDA q-value | Volume | Spectrum Matches |
| --- | --- | --- | --- | --- |
| 0.0000 | 108.6138 | 0.0000 | 0.0000e+00 | 1 |

Adduct:

No Chromatogram Found

### FYFTPN(N)KTEDTIFLR Fuc 1 Hex 6 HexNAc 5

**Mass:**  4024.7294

(2.450e-06)

**Charge States:** 3, 4

**Start Position**: 67

**End Position**: 82

| Profile Score | Tandem Score | TDA q-value | Volume | Spectrum Matches |
| --- | --- | --- | --- | --- |
| 13.6825 | 108.4833 | 0.0000 | 2.0955e+07 | 3 |

**Adduct Signal Fractions**

- **Unmodified**: 2.0955e+07

### YFTPN(N)KTEDTIFLR Hex 7 HexNAc 7

**Mass:**  4299.8176

(2.108e-06)

**Charge States:** 4

**Start Position**: 68

**End Position**: 82

| Profile Score | Tandem Score | TDA q-value | Volume | Spectrum Matches |
| --- | --- | --- | --- | --- |
| -5.1401 | 108.3059 | 0.0000 | 7.8683e+07 | 1 |

**Adduct Signal Fractions**

- **Unmodified**: 7.8683e+07

### FYFTPN(N)KTEDTIFLR Fuc 1 Hex 7 HexNAc 6

**Mass:**  4389.8620

(1.053e-06)

**Charge States:** 3, 4

**Start Position**: 67

**End Position**: 82

| Profile Score | Tandem Score | TDA q-value | Volume | Spectrum Matches |
| --- | --- | --- | --- | --- |
| 14.0782 | 106.1492 | 0.0000 | 5.2201e+07 | 5 |

**Adduct Signal Fractions**

- **Unmodified**: 5.2201e+07

### ANLVPVPITN(N)ATLDR Hex 6 HexNAc 5

**Mass:**  3580.6089

(1.768e-06)

**Charge States:** 3

**Start Position**: 24

**End Position**: 39

| Profile Score | Tandem Score | TDA q-value | Volume | Spectrum Matches |
| --- | --- | --- | --- | --- |
| -4.7605 | 105.1104 | 0.0000 | 2.1428e+07 | 1 |

**Adduct Signal Fractions**

- **Unmodified**: 2.1428e+07

### FTPN(N)KTEDTIFLR Hex 7 HexNAc 6

**Mass:**  3933.6713

(7.723e-07)

**Charge States:** 3, 4

**Start Position**: 69

**End Position**: 82

| Profile Score | Tandem Score | TDA q-value | Volume | Spectrum Matches |
| --- | --- | --- | --- | --- |
| 13.6888 | 104.4659 | 0.0000 | 1.5662e+08 | 6 |

**Adduct Signal Fractions**

- **Unmodified**: 1.5662e+08

### LVPVPITN(N)ATLDR Fuc 2 Hex 4 HexNAc 9

**Mass:**  4175.8762

(6.301e-06)

**Charge States:** 3, 4

**Start Position**: 26

**End Position**: 39

| Profile Score | Tandem Score | TDA q-value | Volume | Spectrum Matches |
| --- | --- | --- | --- | --- |
| 12.5253 | 101.1962 | 0.0000 | 1.3153e+08 | 11 |

**Adduct Signal Fractions**

- **Unmodified**: 1.3153e+08

### SVQEIQATFFYFTPN(N)K Fuc 4 Hex 6 HexNAc 6

**Mass:**  4693.9543

(-3.658e-06)

**Charge States:** 4

**Start Position**: 58

**End Position**: 74

| Profile Score | Tandem Score | TDA q-value | Volume | Spectrum Matches |
| --- | --- | --- | --- | --- |
| 10.2485 | 100.1067 | 0.0000 | 3.8658e+07 | 2 |

**Adduct Signal Fractions**

- **Unmodified**: 3.8658e+07

### LVPVPITN(N)ATLDR Fuc 3 Hex 4 HexNAc 5

**Mass:**  3509.5717

(5.481e-07)

**Charge States:** 3, 4

**Start Position**: 26

**End Position**: 39

| Profile Score | Tandem Score | TDA q-value | Volume | Spectrum Matches |
| --- | --- | --- | --- | --- |
| -17.5950 | 99.5071 | 0.0000 | 2.6487e+07 | 2 |

**Adduct Signal Fractions**

- **Unmodified**: 2.6487e+07

### TPN(N)KTEDTIFLR Fuc 1 Hex 6 HexNAc 5

**Mass:**  3567.5268

(1.013e-06)

**Charge States:** 3

**Start Position**: 70

**End Position**: 82

| Profile Score | Tandem Score | TDA q-value | Volume | Spectrum Matches |
| --- | --- | --- | --- | --- |
| -5.2334 | 98.6742 | 0.0000 | 5.7110e+06 | 3 |

**Adduct Signal Fractions**

- **Unmodified**: 5.7110e+06

### YFTPN(N)KTEDTIFLR Hex 9 HexNAc 8

**Mass:**  4826.9953

(2.349e-06)

**Charge States:** 4

**Start Position**: 68

**End Position**: 82

| Profile Score | Tandem Score | TDA q-value | Volume | Spectrum Matches |
| --- | --- | --- | --- | --- |
| -3.3621 | 97.0643 | 0.0000 | 6.3353e+07 | 2 |

**Adduct Signal Fractions**

- **Unmodified**: 6.3353e+07

### FTPN(N)KTEDTIFLR Fuc 1 Hex 6 HexNAc 5

**Mass:**  3714.5963

(-1.507e-06)

**Charge States:** 3, 4

**Start Position**: 69

**End Position**: 82

| Profile Score | Tandem Score | TDA q-value | Volume | Spectrum Matches |
| --- | --- | --- | --- | --- |
| -3.9985 | 96.0927 | 0.0000 | 1.6967e+07 | 3 |

**Adduct Signal Fractions**

- **Unmodified**: 1.6967e+07

### YFTPN(N)KTEDTIFLR Hex 7 HexNAc 4 Neu5Ac 2

**Mass:**  4272.7644

(-4.454e-06)

**Charge States:** 4

**Start Position**: 68

**End Position**: 82

| Profile Score | Tandem Score | TDA q-value | Volume | Spectrum Matches |
| --- | --- | --- | --- | --- |
| -3.7424 | 95.6772 | 0.0000 | 1.3120e+08 | 5 |

**Adduct Signal Fractions**

- **Unmodified**: 1.3120e+08

### LVPVPITN(N)ATLDR Fuc 2 Hex 6 HexNAc 5

**Mass:**  3690.6445

(8.152e-04)

**Start Position**: 26

**End Position**: 39

| Profile Score | Tandem Score | TDA q-value | Volume | Spectrum Matches |
| --- | --- | --- | --- | --- |
| 0.0000 | 95.5865 | 0.0000 | 0.0000e+00 | 1 |

Adduct:

No Chromatogram Found

### LVPVPITN(N)ATLDR Fuc 4 Hex 3 HexNAc 7 Neu5Ac 1

**Mass:**  4190.8898

(9.595e-06)

**Charge States:** 3, 4

**Start Position**: 26

**End Position**: 39

| Profile Score | Tandem Score | TDA q-value | Volume | Spectrum Matches |
| --- | --- | --- | --- | --- |
| 11.7143 | 95.4069 | 0.0000 | 1.8320e+08 | 12 |

**Adduct Signal Fractions**

- **Unmodified**: 1.8320e+08

### PVPITN(N)ATLDR Fuc 1 Hex 9 HexNAc 4 Neu5Ac 1

**Mass:**  3903.5982

(-2.730e-06)

**Charge States:** 3, 4

**Start Position**: 28

**End Position**: 39

| Profile Score | Tandem Score | TDA q-value | Volume | Spectrum Matches |
| --- | --- | --- | --- | --- |
| -4.4870 | 92.8692 | 0.0000 | 6.1544e+07 | 4 |

**Adduct Signal Fractions**

- **Unmodified**: 6.1544e+07

### SVQEIQATFFYFTPN(N)KTEDTIFLR Hex 5 HexNAc 4

**Mass:**  4517.0388

(1.748e-06)

**Charge States:** 3, 4, 5

**Start Position**: 58

**End Position**: 82

| Profile Score | Tandem Score | TDA q-value | Volume | Spectrum Matches |
| --- | --- | --- | --- | --- |
| -19.0764 | 91.8838 | 0.0000 | 2.0297e+07 | 2 |

**Adduct Signal Fractions**

- **Unmodified**: 2.0297e+07

### SVQEIQATFFYFTPN(N)KTEDTIFLR Fuc 1 Hex 7 HexNAc 6

**Mass:**  5393.3654

(1.797e-06)

**Charge States:** 4, 5

**Start Position**: 58

**End Position**: 82

| Profile Score | Tandem Score | TDA q-value | Volume | Spectrum Matches |
| --- | --- | --- | --- | --- |
| 12.6122 | 91.2079 | 0.0000 | 2.4041e+08 | 6 |

**Adduct Signal Fractions**

- **Unmodified**: 2.4041e+08

### SVQEIQATFFYFTPN(N)KTEDTIFLR Hex 8 HexNAc 7

**Mass:**  5612.4362

(2.149e-06)

**Charge States:** 4, 5

**Start Position**: 58

**End Position**: 82

| Profile Score | Tandem Score | TDA q-value | Volume | Spectrum Matches |
| --- | --- | --- | --- | --- |
| 14.4282 | 90.2821 | 0.0000 | 2.1011e+08 | 5 |

**Adduct Signal Fractions**

- **Unmodified**: 2.1011e+08

### TPN(N)KTEDTIFLR Fuc 1 Hex 7 HexNAc 6

**Mass:**  3932.6596

(1.082e-06)

**Charge States:** 3, 4

**Start Position**: 70

**End Position**: 82

| Profile Score | Tandem Score | TDA q-value | Volume | Spectrum Matches |
| --- | --- | --- | --- | --- |
| -18.8900 | 87.4782 | 0.0000 | 7.6306e+06 | 2 |

**Adduct Signal Fractions**

- **Unmodified**: 7.6306e+06

### LVPVPITN(N)ATLDR Fuc 1 Hex 8 HexNAc 7

**Mass:**  4272.8757

(2.414e-04)

**Start Position**: 26

**End Position**: 39

| Profile Score | Tandem Score | TDA q-value | Volume | Spectrum Matches |
| --- | --- | --- | --- | --- |
| 0.0000 | 86.7315 | 0.0000 | 0.0000e+00 | 1 |

Adduct:

No Chromatogram Found

### FTPN(N)KTEDTIFLR Hex 8 HexNAc 7

**Mass:**  4298.7995

(3.602e-07)

**Charge States:** 3, 4

**Start Position**: 69

**End Position**: 82

| Profile Score | Tandem Score | TDA q-value | Volume | Spectrum Matches |
| --- | --- | --- | --- | --- |
| -4.3632 | 86.3729 | 0.0000 | 6.9860e+06 | 7 |

**Adduct Signal Fractions**

- **Unmodified**: 6.9860e+06

### FTPN(N)KTEDTIFLR Fuc 1 Hex 7 HexNAc 6

**Mass:**  4079.7268

(-5.028e-07)

**Charge States:** 3, 4

**Start Position**: 69

**End Position**: 82

| Profile Score | Tandem Score | TDA q-value | Volume | Spectrum Matches |
| --- | --- | --- | --- | --- |
| -4.2813 | 85.3134 | 0.0000 | 2.4940e+07 | 3 |

**Adduct Signal Fractions**

- **Unmodified**: 2.4940e+07

### TPN(N)KTEDTIFLR Hex 5 HexNAc 4

**Mass:**  3056.3333

(1.868e-06)

**Charge States:** 3

**Start Position**: 70

**End Position**: 82

| Profile Score | Tandem Score | TDA q-value | Volume | Spectrum Matches |
| --- | --- | --- | --- | --- |
| -3.8223 | 84.9324 | 0.0000 | 3.9675e+06 | 3 |

**Adduct Signal Fractions**

- **Unmodified**: 3.9675e+06

### FYFTPN(N)KTEDTIFLR Hex 8 HexNAc 7

**Mass:**  4608.9299

(5.185e-08)

**Charge States:** 3, 4

**Start Position**: 67

**End Position**: 82

| Profile Score | Tandem Score | TDA q-value | Volume | Spectrum Matches |
| --- | --- | --- | --- | --- |
| 14.2593 | 84.8825 | 0.0000 | 2.3804e+07 | 3 |

**Adduct Signal Fractions**

- **Unmodified**: 2.3804e+07

### YFTPN(N)KTEDTIFLR Hex 10 HexNAc 9

**Mass:**  5192.1296

(7.659e-07)

**Charge States:** 4

**Start Position**: 68

**End Position**: 82

| Profile Score | Tandem Score | TDA q-value | Volume | Spectrum Matches |
| --- | --- | --- | --- | --- |
| -19.5474 | 84.2767 | 0.0000 | 6.1450e+06 | 1 |

**Adduct Signal Fractions**

- **Unmodified**: 6.1450e+06

### YFTPN(N)KTEDTIFLR Hex 3 HexNAc 5 Neu5Ac 3

**Mass:**  4118.7175

(-2.123e-06)

**Charge States:** 4

**Start Position**: 68

**End Position**: 82

| Profile Score | Tandem Score | TDA q-value | Volume | Spectrum Matches |
| --- | --- | --- | --- | --- |
| -4.6846 | 84.1685 | 0.0000 | 7.7869e+07 | 1 |

**Adduct Signal Fractions**

- **Unmodified**: 7.7869e+07

### PVPITN(N)ATLDR Hex 3 HexNAc 7 Neu5Ac 2

**Mass:**  3685.5741

(3.599e-06)

**Charge States:** 3

**Start Position**: 28

**End Position**: 39

| Profile Score | Tandem Score | TDA q-value | Volume | Spectrum Matches |
| --- | --- | --- | --- | --- |
| -5.3445 | 80.7047 | 0.0020 | 1.0447e+07 | 1 |

**Adduct Signal Fractions**

- **Unmodified**: 1.0447e+07

### LVPVPITN(N)ATLDR Fuc 1 Hex 7 HexNAc 5

**Mass:**  3704.6654

(2.778e-04)

**Charge States:** 3

**Start Position**: 26

**End Position**: 39

| Profile Score | Tandem Score | TDA q-value | Volume | Spectrum Matches |
| --- | --- | --- | --- | --- |
| -19.6945 | 80.3645 | 0.0020 | 2.0927e+07 | 1 |

**Adduct Signal Fractions**

- **Unmodified**: 2.0927e+07

### FTPN(N)KTEDTIFLR Fuc 2 Hex 6 HexNAc 4 Neu5Ac 1

**Mass:**  3951.7258

(7.691e-04)

**Charge States:** 4

**Start Position**: 69

**End Position**: 82

| Profile Score | Tandem Score | TDA q-value | Volume | Spectrum Matches |
| --- | --- | --- | --- | --- |
| -6.3583 | 79.7678 | 0.0020 | 5.4058e+07 | 4 |

**Adduct Signal Fractions**

- **Unmodified**: 5.4058e+07

### FYN(N)SSYLNVQR Hex 7 HexNAc 6

**Mass:**  3742.5196

(1.069e-06)

**Charge States:** 3, 4

**Start Position**: 91

**End Position**: 102

| Profile Score | Tandem Score | TDA q-value | Volume | Spectrum Matches |
| --- | --- | --- | --- | --- |
| 14.1538 | 78.0511 | 0.0041 | 1.3458e+07 | 3 |

**Adduct Signal Fractions**

- **Unmodified**: 1.3458e+07

### YN(N)SSYLNVQR Hex 6 HexNAc 5

**Mass:**  3230.3172

(1.391e-06)

**Charge States:** 3

**Start Position**: 92

**End Position**: 102

| Profile Score | Tandem Score | TDA q-value | Volume | Spectrum Matches |
| --- | --- | --- | --- | --- |
| 11.9690 | 76.3232 | 0.0060 | 5.9669e+06 | 2 |

**Adduct Signal Fractions**

- **Unmodified**: 5.9669e+06

### FYFTPN(N)KTEDTIFLR Fuc 1 Hex 4 HexNAc 4 Neu5Ac 1

**Mass:**  3788.6229

(-2.683e-06)

**Charge States:** 3, 4

**Start Position**: 67

**End Position**: 82

| Profile Score | Tandem Score | TDA q-value | Volume | Spectrum Matches |
| --- | --- | --- | --- | --- |
| 14.9221 | 75.8358 | 0.0060 | 2.4086e+08 | 5 |

**Adduct Signal Fractions**

- **Unmodified**: 2.4086e+08

### SVQEIQATFFYFTPN(N)KTEDTIFLR Fuc 2 Hex 7 HexNAc 6

**Mass:**  5539.4235

(2.300e-06)

**Charge States:** 4

**Start Position**: 58

**End Position**: 82

| Profile Score | Tandem Score | TDA q-value | Volume | Spectrum Matches |
| --- | --- | --- | --- | --- |
| -19.3911 | 75.0154 | 0.0060 | 2.2546e+07 | 1 |

**Adduct Signal Fractions**

- **Unmodified**: 2.2546e+07

### VPVPITN(N)ATLDR Fuc 2 Hex 4 HexNAc 6

**Mass:**  3453.5374

(3.395e-06)

**Charge States:** 3, 4

**Start Position**: 27

**End Position**: 39

| Profile Score | Tandem Score | TDA q-value | Volume | Spectrum Matches |
| --- | --- | --- | --- | --- |
| -4.3022 | 73.4572 | 0.0060 | 4.0619e+07 | 27 |

**Adduct Signal Fractions**

- **Unmodified**: 4.0619e+07

### TPN(N)KTEDTIFLR Fuc 1 Hex 4 HexNAc 5 Neu5Ac 3

**Mass:**  4116.7043

(1.169e-07)

**Charge States:** 4

**Start Position**: 70

**End Position**: 82

| Profile Score | Tandem Score | TDA q-value | Volume | Spectrum Matches |
| --- | --- | --- | --- | --- |
| -19.6665 | 73.1945 | 0.0060 | 1.2180e+07 | 1 |

**Adduct Signal Fractions**

- **Unmodified**: 1.2180e+07

### FYFTPN(N)KTEDTIFLR Hex 7 HexNAc 4 Neu5Ac 2

**Mass:**  4419.8391

(2.110e-06)

**Charge States:** 3, 4

**Start Position**: 67

**End Position**: 82

| Profile Score | Tandem Score | TDA q-value | Volume | Spectrum Matches |
| --- | --- | --- | --- | --- |
| -19.1542 | 73.1673 | 0.0060 | 8.1523e+06 | 3 |

**Adduct Signal Fractions**

- **Unmodified**: 8.1523e+06

### VPVPITN(N)ATLDR Fuc 1 Hex 6 HexNAc 5

**Mass:**  3428.5003

(1.178e-06)

**Charge States:** 3

**Start Position**: 27

**End Position**: 39

| Profile Score | Tandem Score | TDA q-value | Volume | Spectrum Matches |
| --- | --- | --- | --- | --- |
| -5.0568 | 72.9072 | 0.0060 | 1.9017e+06 | 1 |

**Adduct Signal Fractions**

- **Unmodified**: 1.9017e+06

### SVQEIQATFFYFTPN(N)K Hex 10 HexNAc 9

**Mass:**  5367.1970

(1.076e-06)

**Charge States:** 4

**Start Position**: 58

**End Position**: 74

| Profile Score | Tandem Score | TDA q-value | Volume | Spectrum Matches |
| --- | --- | --- | --- | --- |
| -5.0119 | 66.9025 | 0.0066 | 1.0802e+07 | 1 |

**Adduct Signal Fractions**

- **Unmodified**: 1.0802e+07

### NLVPVPITN(N)ATLDR Hex 3 HexNAc 8 Neu5Ac 1

**Mass:**  3923.7427

(6.055e-07)

**Charge States:** 3

**Start Position**: 25

**End Position**: 39

| Profile Score | Tandem Score | TDA q-value | Volume | Spectrum Matches |
| --- | --- | --- | --- | --- |
| -3.9804 | 65.4381 | 0.0066 | 1.0766e+08 | 3 |

**Adduct Signal Fractions**

- **Unmodified**: 1.0766e+08

### NLVPVPITN(N)ATLDR Fuc 1 Hex 7 HexNAc 4

**Mass:**  3617.6374

(8.412e-04)

**Start Position**: 25

**End Position**: 39

| Profile Score | Tandem Score | TDA q-value | Volume | Spectrum Matches |
| --- | --- | --- | --- | --- |
| 0.0000 | 61.3923 | 0.0066 | 0.0000e+00 | 1 |

Adduct:

No Chromatogram Found

### SVQEIQATFFYFTPN(N)K Hex 7 HexNAc 7

**Mass:**  4474.8796

(1.353e-06)

**Charge States:** 3, 4

**Start Position**: 58

**End Position**: 74

| Profile Score | Tandem Score | TDA q-value | Volume | Spectrum Matches |
| --- | --- | --- | --- | --- |
| 11.6939 | 59.9832 | 0.0066 | 7.5687e+07 | 4 |

**Adduct Signal Fractions**

- **Unmodified**: 7.5687e+07

### C(C)ANLVPVPITN(N)ATLDR Fuc 1 Hex 6 HexNAc 5

**Mass:**  3886.6972

(-1.108e-06)

**Charge States:** 3, 4

**Start Position**: 23

**End Position**: 39

| Profile Score | Tandem Score | TDA q-value | Volume | Spectrum Matches |
| --- | --- | --- | --- | --- |
| -3.5368 | 56.3146 | 0.0066 | 1.3695e+08 | 6 |

**Adduct Signal Fractions**

- **Unmodified**: 1.3695e+08

### SVQEIQATFFYFTPN(N)K Fuc 1 Hex 9 HexNAc 8

**Mass:**  5148.1369

(4.272e-06)

**Charge States:** 4

**Start Position**: 58

**End Position**: 74

| Profile Score | Tandem Score | TDA q-value | Volume | Spectrum Matches |
| --- | --- | --- | --- | --- |
| -6.2418 | 56.0952 | 0.0066 | 6.0671e+06 | 1 |

**Adduct Signal Fractions**

- **Unmodified**: 6.0671e+06

### ANLVPVPITN(N)ATLDR Fuc 1 Hex 9 HexNAc 5

**Mass:**  4212.8395

(8.068e-06)

**Charge States:** 4

**Start Position**: 24

**End Position**: 39

| Profile Score | Tandem Score | TDA q-value | Volume | Spectrum Matches |
| --- | --- | --- | --- | --- |
| -6.1852 | 55.0762 | 0.0066 | 1.5697e+08 | 4 |

**Adduct Signal Fractions**

- **Unmodified**: 1.5697e+08

### QIPLC(C)ANLVPVPITN(N)ATLDR Hex 6 HexNAc 5

**Mass:**  4191.9193

(1.507e-06)

**Charge States:** 3, 4

**Start Position**: 19

**End Position**: 39

| Profile Score | Tandem Score | TDA q-value | Volume | Spectrum Matches |
| --- | --- | --- | --- | --- |
| 12.7477 | 55.0178 | 0.0066 | 6.1181e+08 | 10 |

**Adduct Signal Fractions**

- **Unmodified**: 6.1181e+08

### ANLVPVPITN(N)ATLDR Fuc 1 Hex 10 HexNAc 5

**Mass:**  4374.8684

(-2.651e-08)

**Charge States:** 3

**Start Position**: 24

**End Position**: 39

| Profile Score | Tandem Score | TDA q-value | Volume | Spectrum Matches |
| --- | --- | --- | --- | --- |
| -4.7597 | 53.8590 | 0.0066 | 2.7885e+07 | 1 |

**Adduct Signal Fractions**

- **Unmodified**: 2.7885e+07

### NLVPVPITN(N)ATLDR Fuc 1 Hex 7 HexNAc 5

**Mass:**  3817.6770

(1.233e-06)

**Charge States:** 3, 4

**Start Position**: 25

**End Position**: 39

| Profile Score | Tandem Score | TDA q-value | Volume | Spectrum Matches |
| --- | --- | --- | --- | --- |
| -4.5989 | 48.9514 | 0.0066 | 1.1231e+08 | 3 |

**Adduct Signal Fractions**

- **Unmodified**: 1.1231e+08

### SVQEIQATFFYFTPN(N)KTEDTIFLR Hex 9 HexNAc 8

**Mass:**  5977.5646

(9.288e-07)

**Charge States:** 4, 5

**Start Position**: 58

**End Position**: 82

| Profile Score | Tandem Score | TDA q-value | Volume | Spectrum Matches |
| --- | --- | --- | --- | --- |
| 14.1984 | 48.6462 | 0.0066 | 7.3384e+07 | 6 |

**Adduct Signal Fractions**

- **Unmodified**: 7.3384e+07

### NLVPVPITN(N)ATLDR Fuc 1 Hex 5 HexNAc 3

**Mass:**  3087.4162

(1.537e-06)

**Charge States:** 3, 4

**Start Position**: 25

**End Position**: 39

| Profile Score | Tandem Score | TDA q-value | Volume | Spectrum Matches |
| --- | --- | --- | --- | --- |
| 15.1717 | 47.2881 | 0.0066 | 2.0380e+08 | 3 |

**Adduct Signal Fractions**

- **Unmodified**: 2.0380e+08

### C(C)AN(D)LVPVPITN(N)ATLDR Fuc 1 Hex 6 HexNAc 5

**Mass:**  3887.6808

(2.551e-04)

**Charge States:** 3, 4

**Start Position**: 23

**End Position**: 39

| Profile Score | Tandem Score | TDA q-value | Volume | Spectrum Matches |
| --- | --- | --- | --- | --- |
| 8.0150 | 45.4793 | 0.0066 | 4.7453e+07 | 8 |

**Adduct Signal Fractions**

- **Unmodified**: 4.7453e+07

### SVQEIQATFFYFTPN(N)K Hex 7 HexNAc 6 Neu5Ac 1

**Mass:**  4562.8962

(1.787e-06)

**Charge States:** 4

**Start Position**: 58

**End Position**: 74

| Profile Score | Tandem Score | TDA q-value | Volume | Spectrum Matches |
| --- | --- | --- | --- | --- |
| -20.7451 | 45.1151 | 0.0066 | 5.3062e+05 | 1 |

**Adduct Signal Fractions**

- **Unmodified**: 5.3062e+05

### SVQEIQATFFYFTPN(N)KTEDTIFLR Hex 7 HexNAc 4 Neu5Ac 2

**Mass:**  5423.3361

(1.697e-06)

**Charge States:** 4

**Start Position**: 58

**End Position**: 82

| Profile Score | Tandem Score | TDA q-value | Volume | Spectrum Matches |
| --- | --- | --- | --- | --- |
| -3.9029 | 44.7278 | 0.0066 | 3.1480e+07 | 3 |

**Adduct Signal Fractions**

- **Unmodified**: 3.1480e+07

### PVPITN(N)ATLDR Fuc 1 Hex 8 HexNAc 5 Neu5Ac 1

**Mass:**  3944.6628

(8.610e-06)

**Charge States:** 4

**Start Position**: 28

**End Position**: 39

| Profile Score | Tandem Score | TDA q-value | Volume | Spectrum Matches |
| --- | --- | --- | --- | --- |
| -18.1439 | 43.5452 | 0.0066 | 3.1308e+07 | 1 |

**Adduct Signal Fractions**

- **Unmodified**: 3.1308e+07

### LVPVPITN(N)ATLDR Fuc 1 Hex 6 HexNAc 5

**Mass:**  3543.5821

(5.652e-04)

**Start Position**: 26

**End Position**: 39

| Profile Score | Tandem Score | TDA q-value | Volume | Spectrum Matches |
| --- | --- | --- | --- | --- |
| 0.0000 | 42.8614 | 0.0066 | 0.0000e+00 | 1 |

Adduct:

No Chromatogram Found

### SVQEIQATFFYFTPN(N)KTEDTIFLR Fuc 1 Hex 8 HexNAc 7

**Mass:**  5758.4948

(8.526e-07)

**Charge States:** 4, 5

**Start Position**: 58

**End Position**: 82

| Profile Score | Tandem Score | TDA q-value | Volume | Spectrum Matches |
| --- | --- | --- | --- | --- |
| 15.7365 | 42.1645 | 0.0066 | 3.8055e+07 | 3 |

**Adduct Signal Fractions**

- **Unmodified**: 3.8055e+07

### LVPVPITN(N)ATLDR Fuc 2 Hex 3 HexNAc 6

**Mass:**  3405.5365

(2.871e-04)

**Start Position**: 26

**End Position**: 39

| Profile Score | Tandem Score | TDA q-value | Volume | Spectrum Matches |
| --- | --- | --- | --- | --- |
| 0.0000 | 42.0887 | 0.0066 | 0.0000e+00 | 1 |

Adduct:

No Chromatogram Found

### FYFTPN(N)KTEDTIFLR Hex 5 HexNAc 4

**Mass:**  3513.5370

(5.991e-07)

**Charge States:** 3

**Start Position**: 67

**End Position**: 82

| Profile Score | Tandem Score | TDA q-value | Volume | Spectrum Matches |
| --- | --- | --- | --- | --- |
| -5.6148 | 41.6298 | 0.0066 | 2.8176e+06 | 1 |

**Adduct Signal Fractions**

- **Unmodified**: 2.8176e+06

### QIPLC(C)ANLVPVPITN(N)ATLDR Hex 5 HexNAc 5 Neu5Ac 1

**Mass:**  4320.9587

(8.150e-07)

**Charge States:** 3, 4

**Start Position**: 19

**End Position**: 39

| Profile Score | Tandem Score | TDA q-value | Volume | Spectrum Matches |
| --- | --- | --- | --- | --- |
| 15.1245 | 41.1007 | 0.0066 | 4.1891e+08 | 7 |

**Adduct Signal Fractions**

- **Unmodified**: 4.1891e+08

### C(C)AN(D)LVPVPITN(N)ATLDR Hex 6 HexNAc 5

**Mass:**  3741.6212

(2.644e-04)

**Charge States:** 3, 4

**Start Position**: 23

**End Position**: 39

| Profile Score | Tandem Score | TDA q-value | Volume | Spectrum Matches |
| --- | --- | --- | --- | --- |
| 9.9512 | 40.4030 | 0.0066 | 2.1697e+08 | 13 |

**Adduct Signal Fractions**

- **Unmodified**: 2.1697e+08

### TPN(N)KTEDTIFLR Hex 7 HexNAc 6

**Mass:**  3789.6109

(7.958e-04)

**Start Position**: 70

**End Position**: 82

| Profile Score | Tandem Score | TDA q-value | Volume | Spectrum Matches |
| --- | --- | --- | --- | --- |
| 0.0000 | 40.3430 | 0.0066 | 0.0000e+00 | 1 |

Adduct:

No Chromatogram Found

### LVPVPITN(N)ATLDR Fuc 1 Hex 10 HexNAc 2

**Mass:**  3580.5400

(-6.300e-06)

**Charge States:** 4

**Start Position**: 26

**End Position**: 39

| Profile Score | Tandem Score | TDA q-value | Volume | Spectrum Matches |
| --- | --- | --- | --- | --- |
| -20.6610 | 37.4581 | 0.0066 | 6.7840e+06 | 2 |

**Adduct Signal Fractions**

- **Unmodified**: 6.7840e+06

### LVPVPITN(N)ATLDR Hex 6 HexNAc 5

**Mass:**  3398.5365

(8.877e-04)

**Start Position**: 26

**End Position**: 39

| Profile Score | Tandem Score | TDA q-value | Volume | Spectrum Matches |
| --- | --- | --- | --- | --- |
| 0.0000 | 37.3327 | 0.0066 | 0.0000e+00 | 1 |

Adduct:

No Chromatogram Found

### YFTPN(N)KTEDTIFLR Fuc 1 Hex 9 HexNAc 2

**Mass:**  3754.5859

(2.856e-06)

**Charge States:** 4

**Start Position**: 68

**End Position**: 82

| Profile Score | Tandem Score | TDA q-value | Volume | Spectrum Matches |
| --- | --- | --- | --- | --- |
| -18.9447 | 37.1271 | 0.0066 | 1.4647e+07 | 2 |

**Adduct Signal Fractions**

- **Unmodified**: 1.4647e+07

### LVPVPITN(N)ATLDR Fuc 2 Hex 7 HexNAc 6

**Mass:**  4052.7807

(2.551e-06)

**Charge States:** 3, 4

**Start Position**: 26

**End Position**: 39

| Profile Score | Tandem Score | TDA q-value | Volume | Spectrum Matches |
| --- | --- | --- | --- | --- |
| -18.7376 | 36.7038 | 0.0066 | 3.0820e+07 | 1 |

**Adduct Signal Fractions**

- **Unmodified**: 3.0820e+07

### C(C)AN(D)LVPVPITN(N)ATLDR Fuc 2 Hex 4 HexNAc 4 Neu5Ac 1

**Mass:**  3797.6603

(2.643e-04)

**Charge States:** 3, 4

**Start Position**: 23

**End Position**: 39

| Profile Score | Tandem Score | TDA q-value | Volume | Spectrum Matches |
| --- | --- | --- | --- | --- |
| 10.8042 | 36.3303 | 0.0077 | 1.0538e+08 | 5 |

**Adduct Signal Fractions**

- **Unmodified**: 1.0538e+08

### FYFTPN(N)KTEDTIFLR Hex 9 HexNAc 8

**Mass:**  4975.0717

(2.030e-04)

**Start Position**: 67

**End Position**: 82

| Profile Score | Tandem Score | TDA q-value | Volume | Spectrum Matches |
| --- | --- | --- | --- | --- |
| 0.0000 | 35.8215 | 0.0077 | 0.0000e+00 | 1 |

Adduct:

No Chromatogram Found

### YFTPN(N)KTEDTIFLR Hex 6 HexNAc 6

**Mass:**  3934.6800

(9.215e-07)

**Charge States:** 4

**Start Position**: 68

**End Position**: 82

| Profile Score | Tandem Score | TDA q-value | Volume | Spectrum Matches |
| --- | --- | --- | --- | --- |
| -4.4843 | 35.6241 | 0.0077 | 5.3090e+07 | 4 |

**Adduct Signal Fractions**

- **Unmodified**: 5.3090e+07

### YFTPN(N)KTEDTIFLR Fuc 4 Hex 5 HexNAc 5

**Mass:**  4153.7565

(-3.986e-06)

**Charge States:** 3, 4

**Start Position**: 68

**End Position**: 82

| Profile Score | Tandem Score | TDA q-value | Volume | Spectrum Matches |
| --- | --- | --- | --- | --- |
| 13.7262 | 35.4078 | 0.0089 | 5.0294e+08 | 3 |

**Adduct Signal Fractions**

- **Unmodified**: 5.0294e+08

### LVPVPITN(N)ATLDR Hex 9 HexNAc 3

**Mass:**  3475.4954

(-8.163e-06)

**Charge States:** 3, 4

**Start Position**: 26

**End Position**: 39

| Profile Score | Tandem Score | TDA q-value | Volume | Spectrum Matches |
| --- | --- | --- | --- | --- |
| -20.7127 | 34.7457 | 0.0101 | 2.3847e+06 | 1 |

**Adduct Signal Fractions**

- **Unmodified**: 2.3847e+06

### PVPITN(N)ATLDR Hex 8 HexNAc 4 Neu5Ac 1

**Mass:**  3595.4973

(1.589e-06)

**Start Position**: 28

**End Position**: 39

| Profile Score | Tandem Score | TDA q-value | Volume | Spectrum Matches |
| --- | --- | --- | --- | --- |
| 0.0000 | 34.7075 | 0.0101 | 0.0000e+00 | 1 |

Adduct:

No Chromatogram Found

### ATFFYFTPN(N)K Fuc 4 Hex 5 HexNAc 9

**Mass:**  4456.8132

(2.227e-07)

**Charge States:** 4

**Start Position**: 64

**End Position**: 74

| Profile Score | Tandem Score | TDA q-value | Volume | Spectrum Matches |
| --- | --- | --- | --- | --- |
| -19.9834 | 34.5223 | 0.0113 | 1.0531e+07 | 1 |

**Adduct Signal Fractions**

- **Unmodified**: 1.0531e+07

### QIPLC(C)ANLVPVPITN(N)ATLDR Fuc 1 Hex 6 HexNAc 5

**Mass:**  4337.9782

(1.509e-06)

**Charge States:** 3, 4

**Start Position**: 19

**End Position**: 39

| Profile Score | Tandem Score | TDA q-value | Volume | Spectrum Matches |
| --- | --- | --- | --- | --- |
| 14.3028 | 34.0321 | 0.0148 | 1.6528e+08 | 4 |

**Adduct Signal Fractions**

- **Unmodified**: 1.6528e+08

### QN(D)QC(C)FYN(N)SSYLNVQR Fuc 3 Hex 6 HexNAc 4 Neu5Ac 1

**Mass:**  4434.7116

(2.142e-04)

**Charge States:** 4

**Start Position**: 87

**End Position**: 102

| Profile Score | Tandem Score | TDA q-value | Volume | Spectrum Matches |
| --- | --- | --- | --- | --- |
| -6.5054 | 33.4966 | 0.0148 | 7.7081e+07 | 2 |

**Adduct Signal Fractions**

- **Unmodified**: 7.7081e+07

### SVQEIQATFFYFTPN(N)KTEDTIFLR Fuc 4 Hex 5 HexNAc 5

**Mass:**  5304.3254

(-3.134e-06)

**Charge States:** 3, 4, 5

**Start Position**: 58

**End Position**: 82

| Profile Score | Tandem Score | TDA q-value | Volume | Spectrum Matches |
| --- | --- | --- | --- | --- |
| 13.3132 | 33.3774 | 0.0159 | 2.6062e+08 | 9 |

**Adduct Signal Fractions**

- **Unmodified**: 2.6062e+08

### ANLVPVPITN(N)ATLDR Fuc 5 Hex 7 HexNAc 9

**Mass:**  5285.2474

(-5.702e-07)

**Charge States:** 4, 5

**Start Position**: 24

**End Position**: 39

| Profile Score | Tandem Score | TDA q-value | Volume | Spectrum Matches |
| --- | --- | --- | --- | --- |
| 9.2882 | 33.1967 | 0.0159 | 1.2599e+08 | 3 |

**Adduct Signal Fractions**

- **Unmodified**: 1.2599e+08

### LVPVPITN(N)ATLDR Hex 9 HexNAc 8

**Mass:**  4490.9260

(1.563e-06)

**Charge States:** 4

**Start Position**: 26

**End Position**: 39

| Profile Score | Tandem Score | TDA q-value | Volume | Spectrum Matches |
| --- | --- | --- | --- | --- |
| -7.0775 | 33.1053 | 0.0170 | 4.8881e+06 | 1 |

**Adduct Signal Fractions**

- **Unmodified**: 4.8881e+06

### ANLVPVPITN(N)ATLDR Fuc 2 Hex 9 HexNAc 5

**Mass:**  4358.9040

(6.272e-06)

**Charge States:** 3, 4

**Start Position**: 24

**End Position**: 39

| Profile Score | Tandem Score | TDA q-value | Volume | Spectrum Matches |
| --- | --- | --- | --- | --- |
| -4.7635 | 32.3050 | 0.0260 | 4.1344e+07 | 2 |

**Adduct Signal Fractions**

- **Unmodified**: 4.1344e+07

### LVPVPITN(N)ATLDR Hex 6 HexNAc 5 Neu5Ac 2

**Mass:**  3977.7194

(1.551e-06)

**Charge States:** 3

**Start Position**: 26

**End Position**: 39

| Profile Score | Tandem Score | TDA q-value | Volume | Spectrum Matches |
| --- | --- | --- | --- | --- |
| -4.7097 | 32.0706 | 0.0280 | 8.8380e+06 | 2 |

**Adduct Signal Fractions**

- **Unmodified**: 8.8380e+06

### PVPITN(N)ATLDR Fuc 2 Hex 4 HexNAc 4 Neu5Ac 1

**Mass:**  3239.4259

(9.163e-06)

**Charge States:** 3, 4

**Start Position**: 28

**End Position**: 39

| Profile Score | Tandem Score | TDA q-value | Volume | Spectrum Matches |
| --- | --- | --- | --- | --- |
| 14.8634 | 31.9980 | 0.0280 | 5.2298e+07 | 4 |

**Adduct Signal Fractions**

- **Unmodified**: 5.2298e+07

### LVPVPITN(N)ATLDR Fuc 5 Hex 3 HexNAc 7 Neu5Ac 1

**Mass:**  4336.9460

(8.280e-06)

**Charge States:** 3, 4

**Start Position**: 26

**End Position**: 39

| Profile Score | Tandem Score | TDA q-value | Volume | Spectrum Matches |
| --- | --- | --- | --- | --- |
| 10.7203 | 31.7263 | 0.0312 | 3.8553e+07 | 6 |

**Adduct Signal Fractions**

- **Unmodified**: 3.8553e+07

### SVQEIQATFFYFTPN(N)KTEDTIFLR Fuc 1 Hex 8 HexNAc 4 Neu5Ac 1

**Mass:**  5440.3605

(3.344e-06)

**Charge States:** 4

**Start Position**: 58

**End Position**: 82

| Profile Score | Tandem Score | TDA q-value | Volume | Spectrum Matches |
| --- | --- | --- | --- | --- |
| -4.9368 | 31.3865 | 0.0312 | 1.0060e+07 | 1 |

**Adduct Signal Fractions**

- **Unmodified**: 1.0060e+07

### C(C)ANLVPVPITN(N)ATLDR Hex 5 HexNAc 5 Neu5Ac 1

**Mass:**  3869.6731

(-1.600e-06)

**Charge States:** 3, 4

**Start Position**: 23

**End Position**: 39

| Profile Score | Tandem Score | TDA q-value | Volume | Spectrum Matches |
| --- | --- | --- | --- | --- |
| 15.4609 | 30.8633 | 0.0312 | 2.8089e+08 | 5 |

**Adduct Signal Fractions**

- **Unmodified**: 2.8089e+08

### LVPVPITN(N)ATLDR Fuc 1 Hex 8 HexNAc 2

**Mass:**  3256.4525

(1.445e-06)

**Charge States:** 3

**Start Position**: 26

**End Position**: 39

| Profile Score | Tandem Score | TDA q-value | Volume | Spectrum Matches |
| --- | --- | --- | --- | --- |
| -3.6174 | 30.5594 | 0.0312 | 3.3111e+07 | 2 |

**Adduct Signal Fractions**

- **Unmodified**: 3.3111e+07

### PVPITN(N)ATLDR Fuc 2 Hex 7 HexNAc 4

**Mass:**  3434.4787

(3.482e-06)

**Charge States:** 3, 4

**Start Position**: 28

**End Position**: 39

| Profile Score | Tandem Score | TDA q-value | Volume | Spectrum Matches |
| --- | --- | --- | --- | --- |
| -4.7472 | 30.5029 | 0.0312 | 3.4484e+07 | 63 |

**Adduct Signal Fractions**

- **Unmodified**: 3.4484e+07

### C(C)AN(D)LVPVPITN(N)ATLDR Hex 4 HexNAc 5

**Mass:**  3417.5037

(2.867e-04)

**Charge States:** 3, 4

**Start Position**: 23

**End Position**: 39

| Profile Score | Tandem Score | TDA q-value | Volume | Spectrum Matches |
| --- | --- | --- | --- | --- |
| 10.2823 | 30.3307 | 0.0319 | 1.1625e+09 | 1 |

**Adduct Signal Fractions**

- **Unmodified**: 1.1625e+09

### LVPVPITN(N)ATLDR Hex 6 HexNAc 5 Neu5Ac 3

**Mass:**  4268.8127

(-6.030e-07)

**Charge States:** 3, 4

**Start Position**: 26

**End Position**: 39

| Profile Score | Tandem Score | TDA q-value | Volume | Spectrum Matches |
| --- | --- | --- | --- | --- |
| 11.6090 | 30.0992 | 0.0324 | 1.2585e+07 | 2 |

**Adduct Signal Fractions**

- **Unmodified**: 1.2585e+07

### SVQEIQATFFYFTPN(N)K Hex 6 HexNAc 5 Neu5Ac 1

**Mass:**  4199.7697

(4.798e-04)

**Start Position**: 58

**End Position**: 74

| Profile Score | Tandem Score | TDA q-value | Volume | Spectrum Matches |
| --- | --- | --- | --- | --- |
| 0.0000 | 30.0293 | 0.0324 | 0.0000e+00 | 1 |

Adduct:

No Chromatogram Found

### QN(D)QC(C)FYN(N)SSYLNVQR Fuc 1 Hex 6 HexNAc 5

**Mass:**  4054.6199

(2.442e-04)

**Charge States:** 3, 4

**Start Position**: 87

**End Position**: 102

| Profile Score | Tandem Score | TDA q-value | Volume | Spectrum Matches |
| --- | --- | --- | --- | --- |
| -6.1388 | 29.9674 | 0.0324 | 2.2463e+07 | 2 |

**Adduct Signal Fractions**

- **Unmodified**: 2.2463e+07

### FYN(N)SSYLNVQR Hex 6 HexNAc 5

**Mass:**  3377.3871

(1.642e-06)

**Charge States:** 3

**Start Position**: 91

**End Position**: 102

| Profile Score | Tandem Score | TDA q-value | Volume | Spectrum Matches |
| --- | --- | --- | --- | --- |
| -4.7245 | 29.9018 | 0.0324 | 4.4407e+06 | 3 |

**Adduct Signal Fractions**

- **Unmodified**: 4.4407e+06

### YN(N)SSYLNVQR Hex 7 HexNAc 6

**Mass:**  3595.4536

(2.275e-06)

**Charge States:** 3

**Start Position**: 92

**End Position**: 102

| Profile Score | Tandem Score | TDA q-value | Volume | Spectrum Matches |
| --- | --- | --- | --- | --- |
| -4.1594 | 29.7038 | 0.0324 | 5.6717e+06 | 2 |

**Adduct Signal Fractions**

- **Unmodified**: 5.6717e+06

### QNQC(C)FYN(N)SSYLNVQR Hex 9 HexNAc 8

**Mass:**  5002.9782

(1.844e-06)

**Charge States:** 4

**Start Position**: 87

**End Position**: 102

| Profile Score | Tandem Score | TDA q-value | Volume | Spectrum Matches |
| --- | --- | --- | --- | --- |
| -19.5338 | 29.3835 | 0.0343 | 8.4960e+06 | 1 |

**Adduct Signal Fractions**

- **Unmodified**: 8.4960e+06

### QNQC(C)FYN(N)SSYLNVQR Fuc 2 Hex 7 HexNAc 6

**Mass:**  4564.8376

(3.787e-06)

**Charge States:** 3, 4

**Start Position**: 87

**End Position**: 102

| Profile Score | Tandem Score | TDA q-value | Volume | Spectrum Matches |
| --- | --- | --- | --- | --- |
| -5.2783 | 29.1750 | 0.0361 | 1.9508e+07 | 1 |

**Adduct Signal Fractions**

- **Unmodified**: 1.9508e+07

### SVQEIQATFFYFTPN(N)K Fuc 1 Hex 7 HexNAc 7

**Mass:**  4620.9416

(2.525e-06)

**Charge States:** 4

**Start Position**: 58

**End Position**: 74

| Profile Score | Tandem Score | TDA q-value | Volume | Spectrum Matches |
| --- | --- | --- | --- | --- |
| -19.6321 | 29.1296 | 0.0361 | 3.0465e+06 | 1 |

**Adduct Signal Fractions**

- **Unmodified**: 3.0465e+06

### FYN(N)SSYLNVQR Hex 7 HexNAc 6

**Mass:**  3744.5116

(5.338e-04)

**Start Position**: 91

**End Position**: 102

| Profile Score | Tandem Score | TDA q-value | Volume | Spectrum Matches |
| --- | --- | --- | --- | --- |
| 0.0000 | 27.9829 | 0.0379 | 0.0000e+00 | 1 |

Adduct:

No Chromatogram Found

### ANLVPVPITN(N)ATLDR Hex 7 HexNAc 5

**Mass:**  3742.6909

(9.554e-06)

**Charge States:** 3

**Start Position**: 24

**End Position**: 39

| Profile Score | Tandem Score | TDA q-value | Volume | Spectrum Matches |
| --- | --- | --- | --- | --- |
| -19.2144 | 27.6319 | 0.0392 | 1.6517e+06 | 1 |

**Adduct Signal Fractions**

- **Unmodified**: 1.6517e+06

### WFYIASAFRNEEYNKSVQEIQATFFYFTPN(N)K Fuc 5 Hex 10 HexNAc 8

**Mass:**  7813.3413

(5.329e-06)

**Charge States:** 5

**Start Position**: 43

**End Position**: 74

| Profile Score | Tandem Score | TDA q-value | Volume | Spectrum Matches |
| --- | --- | --- | --- | --- |
| -18.4517 | 27.6269 | 0.0392 | 3.4232e+07 | 1 |

**Adduct Signal Fractions**

- **Unmodified**: 3.4232e+07

### QIPLC(C)ANLVPVPITN(N)ATLDR Fuc 2 Hex 5 HexNAc 5

**Mass:**  4321.9519

(-5.432e-06)

**Charge States:** 4

**Start Position**: 19

**End Position**: 39

| Profile Score | Tandem Score | TDA q-value | Volume | Spectrum Matches |
| --- | --- | --- | --- | --- |
| -19.7435 | 27.4967 | 0.0406 | 1.3380e+07 | 1 |

**Adduct Signal Fractions**

- **Unmodified**: 1.3380e+07

### PVPITN(N)ATLDR Fuc 1 Hex 8 HexNAc 5

**Mass:**  3654.5293

(2.726e-04)

**Start Position**: 28

**End Position**: 39

| Profile Score | Tandem Score | TDA q-value | Volume | Spectrum Matches |
| --- | --- | --- | --- | --- |
| 0.0000 | 27.2630 | 0.0406 | 0.0000e+00 | 1 |

Adduct:

No Chromatogram Found

### LVPVPITN(N)ATLDR Fuc 3 Hex 4 HexNAc 9

**Mass:**  4321.9348

(6.227e-06)

**Charge States:** 3, 4

**Start Position**: 26

**End Position**: 39

| Profile Score | Tandem Score | TDA q-value | Volume | Spectrum Matches |
| --- | --- | --- | --- | --- |
| 12.6639 | 27.1073 | 0.0424 | 3.2353e+07 | 9 |

**Adduct Signal Fractions**

- **Unmodified**: 3.2353e+07

### TPN(N)KTEDTIFLR Hex 8 HexNAc 7

**Mass:**  4151.7375

(1.102e-06)

**Charge States:** 4

**Start Position**: 70

**End Position**: 82

| Profile Score | Tandem Score | TDA q-value | Volume | Spectrum Matches |
| --- | --- | --- | --- | --- |
| -5.5874 | 26.6481 | 0.0455 | 4.2620e+06 | 2 |

**Adduct Signal Fractions**

- **Unmodified**: 4.2620e+06

### FYFTPN(N)KTEDTIFLR Hex 9 HexNAc 8

**Mass:**  4974.0617

(2.504e-06)

**Charge States:** 4

**Start Position**: 67

**End Position**: 82

| Profile Score | Tandem Score | TDA q-value | Volume | Spectrum Matches |
| --- | --- | --- | --- | --- |
| -20.9849 | 26.5648 | 0.0455 | 2.8136e+06 | 1 |

**Adduct Signal Fractions**

- **Unmodified**: 2.8136e+06

### QN(D)QC(C)FYN(N)SSYLNVQR Fuc 2 Hex 7 HexNAc 5 Neu5Ac 1

**Mass:**  4653.8102

(2.097e-04)

**Charge States:** 4

**Start Position**: 87

**End Position**: 102

| Profile Score | Tandem Score | TDA q-value | Volume | Spectrum Matches |
| --- | --- | --- | --- | --- |
| -6.4277 | 26.1880 | 0.0482 | 7.7170e+07 | 2 |

**Adduct Signal Fractions**

- **Unmodified**: 7.7170e+07

### P00738|HPT\_HUMAN

**>P00738|HPT\_HUMAN**  
 `MSALGAVIALLLWGQLFAVDSGNDVTDIADDGCPKPPEIAHGYVEHSVRYQCKNYYKLRTEGDGVYTLNDKKQWINKAVG  
DKLPECEADDGCPKPPEIAHGYVEHSVRYQCKNYYKLRTEGDGVYTLNNEKQWINKAVGDKLPECEAVCGKPKNPANPVQ  
RILGGHLDAKGSFPWQAKMVSHHNLTTGATLINEQWLLTTAKNLFLNHSENATAKDIAPTLTLYVGKKQLVEIEKVVLHP  
NYSQVDIGLIKLKQKVSVNERVMPICLPSKDYAEVGRVGYVSGWGRNANFKFTDHLKYVMLPVADQDQCIRHYEGSTVPE  
KKTPKSPVGVQPILNEHTFCAGMSKYQEDTCYGDAGSAFAVHDLEEDTWYATGILSFDKSCAVAEYGVYVKVTSIQDWVQ  
KTIAEN`

| Site | Type | Count |
| --- | --- | --- |
| 241 | N-linked | 5 |
| 211 | N-linked | 3 |

| Mass | Sequence | MS_2_ Score | `q`-value | MS_1_ Score | Signal | Protein Coordinates | Tandem Scans | Start Time | Apex Time | End Time |
| --- | --- | --- | --- | --- | --- | --- | --- | --- | --- | --- |
| 3416.5915 | VVLHPN(N)YSQVDIGLIK Hex 5 HexNAc 4 | 154.991 | 0.0 | -2.938 | 4.281e+07 | 235 - 251 | 3 | 42.43 | 42.43 | 42.77 |
| 3781.7317 | VVLHPN(N)YSQVDIGLIK Hex 6 HexNAc 5 | 153.072 | 0.0 | -18.110 | 2.302e+07 | 235 - 251 | 2 | 42.26 | 42.26 | 42.43 |
| 4146.8577 | VVLHPN(N)YSQVDIGLIK Hex 7 HexNAc 6 | 147.445 | 0.0 | -18.365 | 1.309e+07 | 235 - 251 | 2 | 41.92 | 41.92 | 42.09 |
| 2892.2230 | VVLHPN(N)YSQVD Hex 5 HexNAc 4 | 112.227 | 0.0 | -4.014 | 4.434e+06 | 235 - 246 | 2 | 23.93 | 24.07 | 24.22 |
| 3257.3531 | VVLHPN(N)YSQVD Hex 6 HexNAc 5 | 106.380 | 0.0 | -4.210 | 3.680e+06 | 235 - 246 | 1 | 23.60 | 23.76 | 23.93 |
| 5433.1619 | NLFLNHSEN(N)ATAK Fuc 5 Hex 10 HexNAc 8 | 105.666 | 0.0 | -5.181 | 1.671e+07 | 202 - 215 | 2 | 17.63 | 17.77 | 17.93 |
| 5068.0303 | NLFLNHSEN(N)ATAK Fuc 5 Hex 9 HexNAc 7 | 101.812 | 0.0 | -4.243 | 2.144e+07 | 202 - 215 | 2 | 17.77 | 18.04 | 18.15 |
| 4702.9018 | NLFLNHSEN(N)ATAK Hex 10 HexNAc 8 | 90.523 | 0.0 | -20.680 | 4.685e+06 | 202 - 215 | 1 | 18.15 | 18.15 | 18.31 |

### VVLHPN(N)YSQVDIGLIK Hex 5 HexNAc 4

**Mass:**  3416.5918

(1.104e-06)

**Charge States:** 3, 4

**Start Position**: 236

**End Position**: 252

| Profile Score | Tandem Score | TDA q-value | Volume | Spectrum Matches |
| --- | --- | --- | --- | --- |
| -2.9375 | 154.9913 | 0.0000 | 4.2809e+07 | 3 |

**Adduct Signal Fractions**

- **Unmodified**: 4.2809e+07

### VVLHPN(N)YSQVDIGLIK Hex 6 HexNAc 5

**Mass:**  3781.7301

(3.239e-06)

**Charge States:** 4

**Start Position**: 236

**End Position**: 252

| Profile Score | Tandem Score | TDA q-value | Volume | Spectrum Matches |
| --- | --- | --- | --- | --- |
| -18.1099 | 153.0723 | 0.0000 | 2.3024e+07 | 2 |

**Adduct Signal Fractions**

- **Unmodified**: 2.3024e+07

### VVLHPN(N)YSQVDIGLIK Hex 7 HexNAc 6

**Mass:**  4146.8566

(1.621e-06)

**Charge States:** 3, 4

**Start Position**: 236

**End Position**: 252

| Profile Score | Tandem Score | TDA q-value | Volume | Spectrum Matches |
| --- | --- | --- | --- | --- |
| -18.3650 | 147.4448 | 0.0000 | 1.3088e+07 | 2 |

**Adduct Signal Fractions**

- **Unmodified**: 1.3088e+07

### VVLHPN(N)YSQVD Hex 5 HexNAc 4

**Mass:**  2892.2230

(2.074e-06)

**Charge States:** 3

**Start Position**: 236

**End Position**: 247

| Profile Score | Tandem Score | TDA q-value | Volume | Spectrum Matches |
| --- | --- | --- | --- | --- |
| -4.0143 | 112.2269 | 0.0000 | 4.4336e+06 | 2 |

**Adduct Signal Fractions**

- **Unmodified**: 4.4336e+06

### VVLHPN(N)YSQVD Hex 6 HexNAc 5

**Mass:**  3257.3541

(1.528e-06)

**Charge States:** 3

**Start Position**: 236

**End Position**: 247

| Profile Score | Tandem Score | TDA q-value | Volume | Spectrum Matches |
| --- | --- | --- | --- | --- |
| -4.2104 | 106.3799 | 0.0000 | 3.6796e+06 | 1 |

**Adduct Signal Fractions**

- **Unmodified**: 3.6796e+06

### NLFLNHSEN(N)ATAK Fuc 5 Hex 10 HexNAc 8

**Mass:**  5433.1608

(-3.363e-06)

**Charge States:** 4

**Start Position**: 203

**End Position**: 216

| Profile Score | Tandem Score | TDA q-value | Volume | Spectrum Matches |
| --- | --- | --- | --- | --- |
| -5.1814 | 105.6659 | 0.0000 | 1.6713e+07 | 2 |

**Adduct Signal Fractions**

- **Unmodified**: 1.6713e+07

### NLFLNHSEN(N)ATAK Fuc 5 Hex 9 HexNAc 7

**Mass:**  5068.0270

(-3.882e-06)

**Charge States:** 4

**Start Position**: 203

**End Position**: 216

| Profile Score | Tandem Score | TDA q-value | Volume | Spectrum Matches |
| --- | --- | --- | --- | --- |
| -4.2428 | 101.8121 | 0.0000 | 2.1439e+07 | 2 |

**Adduct Signal Fractions**

- **Unmodified**: 2.1439e+07

### NLFLNHSEN(N)ATAK Hex 10 HexNAc 8

**Mass:**  4702.9001

(2.206e-06)

**Charge States:** 4

**Start Position**: 203

**End Position**: 216

| Profile Score | Tandem Score | TDA q-value | Volume | Spectrum Matches |
| --- | --- | --- | --- | --- |
| -20.6796 | 90.5233 | 0.0000 | 4.6848e+06 | 1 |

**Adduct Signal Fractions**

- **Unmodified**: 4.6848e+06

### Q9Y5Y7|LYVE1\_HUMAN

**>Q9Y5Y7|LYVE1\_HUMAN**  
 `MARCFSLVLLLTSIWTTRLLVQGSLRAEELSIQVSCRIMGITLVSKKANQQLNFTEAKEACRLLGLSLAGKDQVETALKA  
SFETCSYGWVGDGFVVISRISPNPKCGKNGVGVLIWKVPVSRQFAAYCYNSSDTWTNSCIPEIITTKDPIFNTQTATQTT  
EFIVSDSTYSVASPYSTIPAPTTTPPAPASTSIPRRKKLICVTEVFMETSTMSTETEPFVENKAAFKNEAAGFGGVPTAL  
LVLALLFFGAAAGLGFCYVKRYVKAFPFTNKNQQKEMIETKVVKEEKANDSNPNEESKKTDKNPEESKSPSKTTVRCLEA  
EV`

| Site | Type | Count |
| --- | --- | --- |
| 53 | N-linked | 8 |

| Mass | Sequence | MS_2_ Score | `q`-value | MS_1_ Score | Signal | Protein Coordinates | Tandem Scans | Start Time | Apex Time | End Time |
| --- | --- | --- | --- | --- | --- | --- | --- | --- | --- | --- |
| 3013.3043 | KANQQLN(N)FTEAK Hex 5 HexNAc 4 | 151.214 | 0.0 | -19.057 | 6.773e+06 | 46 - 58 | 1 | 17.63 | 17.63 | 17.77 |
| 3378.4363 | KANQQLN(N)FTEAK Hex 6 HexNAc 5 | 138.935 | 0.0 | -3.870 | 2.009e+07 | 46 - 58 | 2 | 17.46 | 17.63 | 17.77 |
| 3250.3463 | ANQQLN(N)FTEAK Hex 6 HexNAc 5 | 130.917 | 0.0 | 11.671 | 1.655e+07 | 47 - 58 | 4 | 20.78 | 21.29 | 21.61 |
| 2885.2184 | ANQQLN(N)FTEAK Hex 5 HexNAc 4 | 115.902 | 0.0 | -5.432 | 4.640e+06 | 47 - 58 | 2 | 21.17 | 21.45 | 21.61 |
| 3615.4798 | ANQQLN(N)FTEAK Hex 7 HexNAc 6 | 102.624 | 0.0 | -5.396 | 2.988e+06 | 47 - 58 | 2 | 21.00 | 21.00 | 21.29 |
| 3743.5783 | KANQQLN(N)FTEAK Hex 7 HexNAc 6 | 94.872 | 0.0 | -20.163 | 2.662e+06 | 46 - 58 | 1 | 17.46 | 17.46 | 17.46 |
| 3380.4409 | KANQQLN(N)FTEAK Hex 6 HexNAc 5 | 92.075 | 0.0 | 0.000 | 0.000e+00 | 46 - 58 | 1 | - | - | - |
| 3379.4282 | KAN(D)QQLN(N)FTEAK Hex 6 HexNAc 5 | 34.084 | 0.014 | -6.905 | 3.708e+06 | 46 - 58 | 2 | 18.31 | 18.37 | 18.57 |

### KANQQLN(N)FTEAK Hex 5 HexNAc 4

**Mass:**  3013.3033

(4.330e-07)

**Charge States:** 3

**Start Position**: 47

**End Position**: 59

| Profile Score | Tandem Score | TDA q-value | Volume | Spectrum Matches |
| --- | --- | --- | --- | --- |
| -19.0575 | 151.2137 | 0.0000 | 6.7734e+06 | 1 |

**Adduct Signal Fractions**

- **Unmodified**: 6.7734e+06

### KANQQLN(N)FTEAK Hex 6 HexNAc 5

**Mass:**  3378.4370

(4.410e-07)

**Charge States:** 3

**Start Position**: 47

**End Position**: 59

| Profile Score | Tandem Score | TDA q-value | Volume | Spectrum Matches |
| --- | --- | --- | --- | --- |
| -3.8699 | 138.9347 | 0.0000 | 2.0090e+07 | 2 |

**Adduct Signal Fractions**

- **Unmodified**: 2.0090e+07

### ANQQLN(N)FTEAK Hex 6 HexNAc 5

**Mass:**  3250.3471

(2.358e-06)

**Charge States:** 3

**Start Position**: 48

**End Position**: 59

| Profile Score | Tandem Score | TDA q-value | Volume | Spectrum Matches |
| --- | --- | --- | --- | --- |
| 11.6706 | 130.9167 | 0.0000 | 1.6551e+07 | 4 |

**Adduct Signal Fractions**

- **Unmodified**: 1.6551e+07

### ANQQLN(N)FTEAK Hex 5 HexNAc 4

**Mass:**  2885.2124

(1.860e-06)

**Charge States:** 3

**Start Position**: 48

**End Position**: 59

| Profile Score | Tandem Score | TDA q-value | Volume | Spectrum Matches |
| --- | --- | --- | --- | --- |
| -5.4316 | 115.9019 | 0.0000 | 4.6401e+06 | 2 |

**Adduct Signal Fractions**

- **Unmodified**: 4.6401e+06

### ANQQLN(N)FTEAK Hex 7 HexNAc 6

**Mass:**  3615.4795

(2.242e-06)

**Charge States:** 3

**Start Position**: 48

**End Position**: 59

| Profile Score | Tandem Score | TDA q-value | Volume | Spectrum Matches |
| --- | --- | --- | --- | --- |
| -5.3963 | 102.6242 | 0.0000 | 2.9879e+06 | 2 |

**Adduct Signal Fractions**

- **Unmodified**: 2.9879e+06

### KANQQLN(N)FTEAK Hex 7 HexNAc 6

**Mass:**  3743.5783

(3.102e-06)

**Charge States:** 3

**Start Position**: 47

**End Position**: 59

| Profile Score | Tandem Score | TDA q-value | Volume | Spectrum Matches |
| --- | --- | --- | --- | --- |
| -20.1634 | 94.8723 | 0.0000 | 2.6616e+06 | 1 |

**Adduct Signal Fractions**

- **Unmodified**: 2.6616e+06

### KANQQLN(N)FTEAK Hex 6 HexNAc 5

**Mass:**  3380.4409

(5.939e-04)

**Start Position**: 47

**End Position**: 59

| Profile Score | Tandem Score | TDA q-value | Volume | Spectrum Matches |
| --- | --- | --- | --- | --- |
| 0.0000 | 92.0749 | 0.0000 | 0.0000e+00 | 1 |

Adduct:

No Chromatogram Found

### KAN(D)QQLN(N)FTEAK Hex 6 HexNAc 5

**Mass:**  3379.4295

(2.924e-04)

**Charge States:** 3

**Start Position**: 47

**End Position**: 59

| Profile Score | Tandem Score | TDA q-value | Volume | Spectrum Matches |
| --- | --- | --- | --- | --- |
| -6.9051 | 34.0844 | 0.0138 | 3.7083e+06 | 2 |

**Adduct Signal Fractions**

- **Unmodified**: 3.7083e+06

### P05155|IC1\_HUMAN

**>P05155|IC1\_HUMAN**  
 `MASRLTLLTLLLLLLAGDRASSNPNATSSSSQDPESLQDRGEGKVATTVISKMLFVEPILEVSSLPTTNSTTNSATKITA  
NTTDEPTTQPTTEPTTQPTIQPTQPTTQLPTDSPTQPTTGSFCPGPVTLCSDLESHSTEAVLGDALVDFSLKLYHAFSAM  
KKVETNMAFSPFSIASLLTQVLLGAGENTKTNLESILSYPKDFTCVHQALKGFTTKGVTSVSQIFHSPDLAIRDTFVNAS  
RTLYSSSPRVLSNNSDANLELINTWVAKNTNNKISRLLDSLPSDTRLVLLNAIYLSAKWKTTFDPKKTRMEPFHFKNSVI  
KVPMMNSKKYPVAHFIDQTLKAKVGQLQLSHNLSLVILVPQNLKHRLEDMEQALSPSVFKAIMEKLEMSKFQPTLLTLPR  
IKVTTSQDMLSIMEKLEFFDFSYDLNLCGLTEDPDLQVSAMQHQTVLELTETGVEAAAASAISVARTLLVFEVQQPFLFV  
LWDQQHKFPVFMGRVYDPRA`

| Site | Type | Count |
| --- | --- | --- |
| 25 | N-linked | 7 |

| Mass | Sequence | MS_2_ Score | `q`-value | MS_1_ Score | Signal | Protein Coordinates | Tandem Scans | Start Time | Apex Time | End Time |
| --- | --- | --- | --- | --- | --- | --- | --- | --- | --- | --- |
| 3554.4329 | NPN(N)ATSSSSQDPESLQDR Hex 5 HexNAc 4 | 77.830 | 0.004 | -19.342 | 1.678e+07 | 22 - 40 | 1 | 15.27 | 15.27 | 15.44 |
| 3919.5643 | NPN(N)ATSSSSQDPESLQDR Hex 6 HexNAc 5 | 71.807 | 0.007 | -20.238 | 3.609e+07 | 22 - 40 | 4 | 14.08 | 14.24 | 15.27 |
| 4284.6947 | NPN(N)ATSSSSQDPESLQDR Hex 7 HexNAc 6 | 56.105 | 0.007 | -19.940 | 1.108e+07 | 22 - 40 | 1 | 13.95 | 14.08 | 14.08 |
| 4067.6365 | NPN(N)ATSSSSQDPESLQDR Fuc 1 Hex 6 HexNAc 5 | 44.280 | 0.007 | 0.000 | 0.000e+00 | 22 - 40 | 1 | - | - | - |
| 3702.5017 | NPN(N)ATSSSSQDPESLQDR Fuc 1 Hex 5 HexNAc 4 | 43.690 | 0.007 | 0.000 | 0.000e+00 | 22 - 40 | 2 | - | - | - |
| 4431.7705 | NPN(N)ATSSSSQDPESLQDR Fuc 1 Hex 7 HexNAc 6 | 36.592 | 0.007 | 0.000 | 0.000e+00 | 22 - 40 | 1 | - | - | - |
| 3556.4434 | NPN(N)ATSSSSQDPESLQDR Hex 5 HexNAc 4 | 32.509 | 0.024 | 0.000 | 0.000e+00 | 22 - 40 | 2 | - | - | - |

### NPN(N)ATSSSSQDPESLQDR Hex 5 HexNAc 4

**Mass:**  3554.4307

(9.505e-07)

**Charge States:** 3

**Start Position**: 23

**End Position**: 41

| Profile Score | Tandem Score | TDA q-value | Volume | Spectrum Matches |
| --- | --- | --- | --- | --- |
| -19.3417 | 77.8302 | 0.0041 | 1.6785e+07 | 1 |

**Adduct Signal Fractions**

- **Unmodified**: 1.6785e+07

### NPN(N)ATSSSSQDPESLQDR Hex 6 HexNAc 5

**Mass:**  3919.5591

(2.056e-06)

**Charge States:** 3, 4

**Start Position**: 23

**End Position**: 41

| Profile Score | Tandem Score | TDA q-value | Volume | Spectrum Matches |
| --- | --- | --- | --- | --- |
| -20.2380 | 71.8068 | 0.0066 | 3.6088e+07 | 4 |

**Adduct Signal Fractions**

- **Unmodified**: 3.6088e+07

### NPN(N)ATSSSSQDPESLQDR Hex 7 HexNAc 6

**Mass:**  4284.6936

(1.047e-06)

**Charge States:** 4

**Start Position**: 23

**End Position**: 41

| Profile Score | Tandem Score | TDA q-value | Volume | Spectrum Matches |
| --- | --- | --- | --- | --- |
| -19.9396 | 56.1054 | 0.0066 | 1.1084e+07 | 1 |

**Adduct Signal Fractions**

- **Unmodified**: 1.1084e+07

### NPN(N)ATSSSSQDPESLQDR Fuc 1 Hex 6 HexNAc 5

**Mass:**  4067.6365

(4.966e-04)

**Start Position**: 23

**End Position**: 41

| Profile Score | Tandem Score | TDA q-value | Volume | Spectrum Matches |
| --- | --- | --- | --- | --- |
| 0.0000 | 44.2798 | 0.0066 | 0.0000e+00 | 1 |

Adduct:

No Chromatogram Found

### NPN(N)ATSSSSQDPESLQDR Fuc 1 Hex 5 HexNAc 4

**Mass:**  3702.5017

(5.449e-04)

**Start Position**: 23

**End Position**: 41

| Profile Score | Tandem Score | TDA q-value | Volume | Spectrum Matches |
| --- | --- | --- | --- | --- |
| 0.0000 | 43.6899 | 0.0066 | 0.0000e+00 | 2 |

Adduct:

No Chromatogram Found

### NPN(N)ATSSSSQDPESLQDR Fuc 1 Hex 7 HexNAc 6

**Mass:**  4431.7705

(2.304e-04)

**Start Position**: 23

**End Position**: 41

| Profile Score | Tandem Score | TDA q-value | Volume | Spectrum Matches |
| --- | --- | --- | --- | --- |
| 0.0000 | 36.5923 | 0.0066 | 0.0000e+00 | 1 |

Adduct:

No Chromatogram Found

### NPN(N)ATSSSSQDPESLQDR Hex 5 HexNAc 4

**Mass:**  3556.4434

(5.672e-04)

**Start Position**: 23

**End Position**: 41

| Profile Score | Tandem Score | TDA q-value | Volume | Spectrum Matches |
| --- | --- | --- | --- | --- |
| 0.0000 | 32.5091 | 0.0238 | 0.0000e+00 | 2 |

Adduct:

No Chromatogram Found

### Q12913|PTPRJ\_HUMAN

**>Q12913|PTPRJ\_HUMAN**  
 `MKPAAREARLPPRSPGLRWALPLLLLLLRLGQILCAGGTPSPIPDPSVATVATGENGITQISSTAESFHKQNGTGTPQVE  
TNTSEDGESSGANDSLRTPEQGSNGTDGASQKTPSSTGPSPVFDIKAVSISPTNVILTWKSNDTAASEYKYVVKHKMENE  
KTITVVHQPWCNITGLRPATSYVFSITPGIGNETWGDPRVIKVITEPIPVSDLRVALTGVRKAALSWSNGNGTASCRVLL  
ESIGSHEELTQDSRLQVNISGLKPGVQYNINPYLLQSNKTKGDPLGTEGGLDASNTERSRAGSPTAPVHDESLVGPVDPS  
SGQQSRDTEVLLVGLEPGTRYNATVYSQAANGTEGQPQAIEFRTNAIQVFDVTAVNISATSLTLIWKVSDNESSSNYTYK  
IHVAGETDSSNLNVSEPRAVIPGLRSSTFYNITVCPVLGDIEGTPGFLQVHTPPVPVSDFRVTVVSTTEIGLAWSSHDAE  
SFQMHITQEGAGNSRVEITTNQSIIIGGLFPGTKYCFEIVPKGPNGTEGASRTVCNRTVPSAVFDIHVVYVTTTEMWLDW  
KSPDGASEYVYHLVIESKHGSNHTSTYDKAITLQGLIPGTLYNITISPEVDHVWGDPNSTAQYTRPSNVSNIDVSTNTTA  
ATLSWQNFDDASPTYSYCLLIEKAGNSSNATQVVTDIGITDATVTELIPGSSYTVEIFAQVGDGIKSLEPGRKSFCTDPA  
SMASFDCEVVPKEPALVLKWTCPPGANAGFELEVSSGAWNNATHLESCSSENGTEYRTEVTYLNFSTSYNISITTVSCGK  
MAAPTRNTCTTGITDPPPPDGSPNITSVSHNSVKVKFSGFEASHGPIKAYAVILTTGEAGHPSADVLKYTYEDFKKGASD  
TYVTYLIRTEEKGRSQSLSEVLKYEIDVGNESTTLGYYNGKLEPLGSYRACVAGFTNITFHPQNKGLIDGAESYVSFSRY  
SDAVSLPQDPGVICGAVFGCIFGALVIVTVGGFIFWRKKRKDAKNNEVSFSQIKPKKSKLIRVENFEAYFKKQQADSNCG  
FAEEYEDLKLVGISQPKYAAELAENRGKNRYNNVLPYDISRVKLSVQTHSTDDYINANYMPGYHSKKDFIATQGPLPNTL  
KDFWRMVWEKNVYAIIMLTKCVEQGRTKCEEYWPSKQAQDYGDITVAMTSEIVLPEWTIRDFTVKNIQTSESHPLRQFHF  
TSWPDHGVPDTTDLLINFRYLVRDYMKQSPPESPILVHCSAGVGRTGTFIAIDRLIYQIENENTVDVYGIVYDLRMHRPL  
MVQTEDQYVFLNQCVLDIVRSQKDSKVDLIYQNTTAMTIYENLAPVTTFGKTNGYIA`

| Site | Type | Count |
| --- | --- | --- |
| 413 | N-linked | 4 |

| Mass | Sequence | MS_2_ Score | `q`-value | MS_1_ Score | Signal | Protein Coordinates | Tandem Scans | Start Time | Apex Time | End Time |
| --- | --- | --- | --- | --- | --- | --- | --- | --- | --- | --- |
| 3911.6707 | IHVAGETDSSNLN(N)VSEPR Hex 6 HexNAc 5 | 155.666 | 0.0 | 15.496 | 1.270e+07 | 400 - 418 | 5 | 23.39 | 23.60 | 23.93 |
| 4057.7082 | IHVAGETDSSNLN(N)VSEPR Fuc 1 Hex 6 HexNAc 5 | 29.716 | 0.032 | -3.942 | 3.615e+06 | 400 - 418 | 2 | 23.34 | 23.39 | 23.49 |
| 4422.8437 | IHVAGETDSSNLN(N)VSEPR Fuc 1 Hex 7 HexNAc 6 | 28.780 | 0.037 | -4.159 | 2.848e+06 | 400 - 418 | 1 | 23.05 | 23.24 | 23.24 |
| 3546.5138 | IHVAGETDSSNLN(N)VSEPR Hex 5 HexNAc 4 | 26.737 | 0.045 | -20.042 | 1.230e+06 | 400 - 418 | 2 | 23.76 | 23.76 | 23.76 |

### IHVAGETDSSNLN(N)VSEPR Hex 6 HexNAc 5

**Mass:**  3911.6477

(1.303e-06)

**Charge States:** 3, 4

**Start Position**: 401

**End Position**: 419

| Profile Score | Tandem Score | TDA q-value | Volume | Spectrum Matches |
| --- | --- | --- | --- | --- |
| 15.4957 | 155.6656 | 0.0000 | 1.2700e+07 | 5 |

**Adduct Signal Fractions**

- **Unmodified**: 1.2700e+07

### IHVAGETDSSNLN(N)VSEPR Fuc 1 Hex 6 HexNAc 5

**Mass:**  4057.7094

(2.823e-06)

**Charge States:** 4

**Start Position**: 401

**End Position**: 419

| Profile Score | Tandem Score | TDA q-value | Volume | Spectrum Matches |
| --- | --- | --- | --- | --- |
| -3.9422 | 29.7158 | 0.0324 | 3.6146e+06 | 2 |

**Adduct Signal Fractions**

- **Unmodified**: 3.6146e+06

### IHVAGETDSSNLN(N)VSEPR Fuc 1 Hex 7 HexNAc 6

**Mass:**  4422.8478

(3.443e-06)

**Charge States:** 4

**Start Position**: 401

**End Position**: 419

| Profile Score | Tandem Score | TDA q-value | Volume | Spectrum Matches |
| --- | --- | --- | --- | --- |
| -4.1593 | 28.7800 | 0.0370 | 2.8483e+06 | 1 |

**Adduct Signal Fractions**

- **Unmodified**: 2.8483e+06

### IHVAGETDSSNLN(N)VSEPR Hex 5 HexNAc 4

**Mass:**  3546.5147

(1.234e-06)

**Charge States:** 3, 4

**Start Position**: 401

**End Position**: 419

| Profile Score | Tandem Score | TDA q-value | Volume | Spectrum Matches |
| --- | --- | --- | --- | --- |
| -20.0415 | 26.7369 | 0.0447 | 1.2303e+06 | 2 |

**Adduct Signal Fractions**

- **Unmodified**: 1.2303e+06

### O95497|VNN1\_HUMAN

**>O95497|VNN1\_HUMAN**  
 `MTTQLPAYVAILLFYVSRASCQDTFTAAVYEHAAILPNATLTPVSREEALALMNRNLDILEGAITSAADQGAHIIVTPED  
AIYGWNFNRDSLYPYLEDIPDPEVNWIPCNNRNRFGQTPVQERLSCLAKNNSIYVVANIGDKKPCDTSDPQCPPDGRYQY  
NTDVVFDSQGKLVARYHKQNLFMGENQFNVPKEPEIVTFNTTFGSFGIFTCFDILFHDPAVTLVKDFHVDTIVFPTAWMN  
VLPHLSAVEFHSAWAMGMRVNFLASNIHYPSKKMTGSGIYAPNSSRAFHYDMKTEEGKLLLSQLDSHPSHSAVVNWTSYA  
SSIEALSSGNKEFKGTVFFDEFTFVKLTGVAGNYTVCQKDLCCHLSYKMSENIPNEVYALGAFDGLHTVEGRYYLQICTL  
LKCKTTNLNTCGDSAETASTRFEMFSLSGTFGTQYVFPEVLLSENQLAPGEFQVSTDGRLFSLKPTSGPVLTVTLFGRLY  
EKDWASNASSGLTAQARIIMLIVIAPIVCSLSW`

| Site | Type | Count |
| --- | --- | --- |
| 353 | N-linked | 4 |

| Mass | Sequence | MS_2_ Score | `q`-value | MS_1_ Score | Signal | Protein Coordinates | Tandem Scans | Start Time | Apex Time | End Time |
| --- | --- | --- | --- | --- | --- | --- | --- | --- | --- | --- |
| 3397.4165 | LTGVAGN(N)YTVC(C)QK Hex 6 HexNAc 5 | 127.439 | 0.0 | -3.759 | 6.877e+06 | 346 - 359 | 2 | 21.94 | 22.07 | 22.30 |
| 3032.2837 | LTGVAGN(N)YTVC(C)QK Hex 5 HexNAc 4 | 32.878 | 0.018 | 13.764 | 4.455e+06 | 346 - 359 | 3 | 22.18 | 22.30 | 22.54 |
| 3543.4739 | LTGVAGN(N)YTVC(C)QK Fuc 1 Hex 6 HexNAc 5 | 31.732 | 0.031 | -5.675 | 1.655e+06 | 346 - 359 | 2 | 21.78 | 21.78 | 22.07 |
| 3398.4160 | LTGVAGN(N)YTVC(C)QK Hex 6 HexNAc 5 | 30.468 | 0.031 | 0.000 | 0.000e+00 | 346 - 359 | 1 | - | - | - |

### LTGVAGN(N)YTVC(C)QK Hex 6 HexNAc 5

**Mass:**  3397.4148

(1.906e-06)

**Charge States:** 3

**Start Position**: 347

**End Position**: 360

| Profile Score | Tandem Score | TDA q-value | Volume | Spectrum Matches |
| --- | --- | --- | --- | --- |
| -3.7593 | 127.4387 | 0.0000 | 6.8775e+06 | 2 |

**Adduct Signal Fractions**

- **Unmodified**: 6.8775e+06

### LTGVAGN(N)YTVC(C)QK Hex 5 HexNAc 4

**Mass:**  3032.2827

(2.502e-06)

**Charge States:** 3

**Start Position**: 347

**End Position**: 360

| Profile Score | Tandem Score | TDA q-value | Volume | Spectrum Matches |
| --- | --- | --- | --- | --- |
| 13.7641 | 32.8775 | 0.0181 | 4.4548e+06 | 3 |

**Adduct Signal Fractions**

- **Unmodified**: 4.4548e+06

### LTGVAGN(N)YTVC(C)QK Fuc 1 Hex 6 HexNAc 5

**Mass:**  3543.4751

(1.763e-06)

**Charge States:** 3

**Start Position**: 347

**End Position**: 360

| Profile Score | Tandem Score | TDA q-value | Volume | Spectrum Matches |
| --- | --- | --- | --- | --- |
| -5.6750 | 31.7315 | 0.0312 | 1.6554e+06 | 2 |

**Adduct Signal Fractions**

- **Unmodified**: 1.6554e+06

### LTGVAGN(N)YTVC(C)QK Hex 6 HexNAc 5

**Mass:**  3398.4160

(2.958e-04)

**Start Position**: 347

**End Position**: 360

| Profile Score | Tandem Score | TDA q-value | Volume | Spectrum Matches |
| --- | --- | --- | --- | --- |
| 0.0000 | 30.4684 | 0.0312 | 0.0000e+00 | 1 |

Adduct:

No Chromatogram Found

### P02790|HEMO\_HUMAN

**>P02790|HEMO\_HUMAN**  
 `MARVLGAPVALGLWSLCWSLAIATPLPPTSAHGNVAEGETKPDPDVTERCSDGWSFDATTLDDNGTMLFFKGEFVWKSHK  
WDRELISERWKNFPSPVDAAFRQGHNSVFLIKGDKVWVYPPEKKEKGYPKLLQDEFPGIPSPLDAAVECHRGECQAEGVL  
FFQGDREWFWDLATGTMKERSWPAVGNCSSALRWLGRYYCFQGNQFLRFDPVRGEVPPRYPRDVRDYFMPCPGRGHGHRN  
GTGHGNSTHHGPEYMRCSPHLVLSALTSDNHGATYAFSGTHYWRLDTSRDGWHSWPIAHQWPQGPSAVDAAFSWEEKLYL  
VQGTQVYVFLTKGGYTLVSGYPKRLEKEVGTPHGIILDSVDAAFICPGSSRLHIMAGRRLWWLDLKSGAQATWTELPWPH  
EKVDGALCMEKSLGPNSCSANGPGLYLIHGPNLYCYSDVEKLNAAKALPQPQNVTSLLGCTH`

| Site | Type | Count |
| --- | --- | --- |
| 187 | N-linked | 3 |
| 453 | N-linked | 1 |

| Mass | Sequence | MS_2_ Score | `q`-value | MS_1_ Score | Signal | Protein Coordinates | Tandem Scans | Start Time | Apex Time | End Time |
| --- | --- | --- | --- | --- | --- | --- | --- | --- | --- | --- |
| 3026.2440 | SWPAVGN(N)C(C)SSALR Hex 5 HexNAc 4 | 134.477 | 0.0 | 14.081 | 1.054e+07 | 180 - 193 | 3 | 30.83 | 31.17 | 31.51 |
| 3357.4564 | ALPQPQN(N)VTSLLGC(C)TH Hex 5 HexNAc 4 | 123.202 | 0.0 | 13.786 | 3.848e+07 | 446 - 462 | 3 | 43.97 | 44.30 | 44.81 |
| 3391.3796 | SWPAVGN(N)C(C)SSALR Hex 6 HexNAc 5 | 38.984 | 0.007 | -5.664 | 2.277e+06 | 180 - 193 | 2 | 30.66 | 30.83 | 31.00 |
| 3172.3072 | SWPAVGN(N)C(C)SSALR Fuc 1 Hex 5 HexNAc 4 | 38.102 | 0.007 | 0.000 | 0.000e+00 | 180 - 193 | 1 | - | - | - |

### SWPAVGN(N)C(C)SSALR Hex 5 HexNAc 4

**Mass:**  3026.2426

(-1.898e-07)

**Charge States:** 3

**Start Position**: 181

**End Position**: 194

| Profile Score | Tandem Score | TDA q-value | Volume | Spectrum Matches |
| --- | --- | --- | --- | --- |
| 14.0810 | 134.4768 | 0.0000 | 1.0543e+07 | 3 |

**Adduct Signal Fractions**

- **Unmodified**: 1.0543e+07

### ALPQPQN(N)VTSLLGC(C)TH Hex 5 HexNAc 4

**Mass:**  3357.4561

(6.594e-07)

**Charge States:** 3

**Start Position**: 447

**End Position**: 463

| Profile Score | Tandem Score | TDA q-value | Volume | Spectrum Matches |
| --- | --- | --- | --- | --- |
| 13.7862 | 123.2023 | 0.0000 | 3.8484e+07 | 3 |

**Adduct Signal Fractions**

- **Unmodified**: 3.8484e+07

### SWPAVGN(N)C(C)SSALR Hex 6 HexNAc 5

**Mass:**  3391.3793

(2.224e-06)

**Charge States:** 3

**Start Position**: 181

**End Position**: 194

| Profile Score | Tandem Score | TDA q-value | Volume | Spectrum Matches |
| --- | --- | --- | --- | --- |
| -5.6639 | 38.9835 | 0.0066 | 2.2771e+06 | 2 |

**Adduct Signal Fractions**

- **Unmodified**: 2.2771e+06

### SWPAVGN(N)C(C)SSALR Fuc 1 Hex 5 HexNAc 4

**Mass:**  3172.3072

(1.914e-06)

**Start Position**: 181

**End Position**: 194

| Profile Score | Tandem Score | TDA q-value | Volume | Spectrum Matches |
| --- | --- | --- | --- | --- |
| 0.0000 | 38.1022 | 0.0066 | 0.0000e+00 | 1 |

Adduct:

No Chromatogram Found

### Q5R543|HEMO\_PONAB

**>Q5R543|HEMO\_PONAB**  
 `MARALGAPIALGLWSLCWSLAIATPLPPTSAHGNVAEGETKPDPDVTERCSDGWSFDATTLDDNGTMLFFKGEFVWKSHK  
WDRELISERWKNFPSPVDAAFRQGHNSVFLIKGDKVWVYPPEKKEKGYPKLLQDEFPGIPSPLDAAVECHRGECQAEGVL  
FFQGDREWFWDLATGTMKERSWPAVGNCSSALRWLGRYYCFQGNQFLRFDPVRGEVPPRYPRDVRDYFMPCPGRGHGHRN  
GTGHGNGTHHGPEYMRCSPHLVLSALTSDNHGATYAFSGTHYWRLDTSRDGWHSWPIAHQWPQGPSTVDAAFSWEEKLYL  
VQGTQVYVFLTKGGYTLVSGYPKQLEKEVGTPHGIILDSVDAAFICPGSSRLHIMAGRRLWWLDLKSGAQALWTELPWPH  
EKVDGALCVEKSLGPNSCSANGPGLYLIHGPNLYCYSDVEKLNAAKALPQPQNVTSLLGCTH`

| Site | Type | Count |
| --- | --- | --- |
| 187 | N-linked | 3 |
| 453 | N-linked | 1 |

| Mass | Sequence | MS_2_ Score | `q`-value | MS_1_ Score | Signal | Protein Coordinates | Tandem Scans | Start Time | Apex Time | End Time |
| --- | --- | --- | --- | --- | --- | --- | --- | --- | --- | --- |
| 3026.2440 | SWPAVGN(N)C(C)SSALR Hex 5 HexNAc 4 | 134.477 | 0.0 | 14.081 | 1.054e+07 | 180 - 193 | 3 | 30.83 | 31.17 | 31.51 |
| 3357.4564 | ALPQPQN(N)VTSLLGC(C)TH Hex 5 HexNAc 4 | 123.202 | 0.0 | 13.786 | 3.848e+07 | 446 - 462 | 3 | 43.97 | 44.30 | 44.81 |
| 3391.3796 | SWPAVGN(N)C(C)SSALR Hex 6 HexNAc 5 | 38.984 | 0.007 | -5.664 | 2.277e+06 | 180 - 193 | 2 | 30.66 | 30.83 | 31.00 |
| 3172.3072 | SWPAVGN(N)C(C)SSALR Fuc 1 Hex 5 HexNAc 4 | 38.102 | 0.007 | 0.000 | 0.000e+00 | 180 - 193 | 1 | - | - | - |

### SWPAVGN(N)C(C)SSALR Hex 5 HexNAc 4

**Mass:**  3026.2426

(-1.898e-07)

**Charge States:** 3

**Start Position**: 181

**End Position**: 194

| Profile Score | Tandem Score | TDA q-value | Volume | Spectrum Matches |
| --- | --- | --- | --- | --- |
| 14.0810 | 134.4768 | 0.0000 | 1.0543e+07 | 3 |

**Adduct Signal Fractions**

- **Unmodified**: 1.0543e+07

### ALPQPQN(N)VTSLLGC(C)TH Hex 5 HexNAc 4

**Mass:**  3357.4561

(6.594e-07)

**Charge States:** 3

**Start Position**: 447

**End Position**: 463

| Profile Score | Tandem Score | TDA q-value | Volume | Spectrum Matches |
| --- | --- | --- | --- | --- |
| 13.7862 | 123.2023 | 0.0000 | 3.8484e+07 | 3 |

**Adduct Signal Fractions**

- **Unmodified**: 3.8484e+07

### SWPAVGN(N)C(C)SSALR Hex 6 HexNAc 5

**Mass:**  3391.3793

(2.224e-06)

**Charge States:** 3

**Start Position**: 181

**End Position**: 194

| Profile Score | Tandem Score | TDA q-value | Volume | Spectrum Matches |
| --- | --- | --- | --- | --- |
| -5.6639 | 38.9835 | 0.0066 | 2.2771e+06 | 2 |

**Adduct Signal Fractions**

- **Unmodified**: 2.2771e+06

### SWPAVGN(N)C(C)SSALR Fuc 1 Hex 5 HexNAc 4

**Mass:**  3172.3072

(1.914e-06)

**Start Position**: 181

**End Position**: 194

| Profile Score | Tandem Score | TDA q-value | Volume | Spectrum Matches |
| --- | --- | --- | --- | --- |
| 0.0000 | 38.1022 | 0.0066 | 0.0000e+00 | 1 |

Adduct:

No Chromatogram Found

### P11279|LAMP1\_HUMAN

**>P11279|LAMP1\_HUMAN**  
 `MAAPGSARRPLLLLLLLLLLGLMHCASAAMFMVKNGNGTACIMANFSAAFSVNYDTKSGPKNMTFDLPSDATVVLNRSSC  
GKENTSDPSLVIAFGRGHTLTLNFTRNATRYSVQLMSFVYNLSDTHLFPNASSKEIKTVESITDIRADIDKKYRCVSGTQ  
VHMNNVTVTLHDATIQAYLSNSSFSRGETRCEQDRPSPTTAPPAPPSPSPSPVPKSPSVDKYNVSGTNGTCLLASMGLQL  
NLTYERKDNTTVTRLLNINPNKTSASGSCGAHLVTLELHSEGTTVLLFQFGMNASSSRFFLQGIQLNTILPDARDPAFKA  
ANGSLRALQATVGNSYKCNAEEHVRVTKAFSVNIFKVWVQAFKVEGGQFGSVEECLLDENSMLIPIAVGGALAGLVLIVL  
IAYLVGRKRSHAGYQTI`

| Site | Type | Count |
| --- | --- | --- |
| 103 | N-linked | 3 |

| Mass | Sequence | MS_2_ Score | `q`-value | MS_1_ Score | Signal | Protein Coordinates | Tandem Scans | Start Time | Apex Time | End Time |
| --- | --- | --- | --- | --- | --- | --- | --- | --- | --- | --- |
| 2740.1734 | GHTLTLN(N)FTR Hex 6 HexNAc 3 | 111.445 | 0.0 | -4.415 | 1.999e+06 | 96 - 106 | 4 | 30.05 | 30.19 | 30.49 |
| 2579.1295 | GHTLTLN(N)FTR Hex 5 HexNAc 3 | 30.052 | 0.032 | 0.000 | 0.000e+00 | 96 - 106 | 1 | - | - | - |
| 2781.1989 | GHTLTLN(N)FTR Hex 5 HexNAc 4 | 29.793 | 0.032 | -4.311 | 1.232e+06 | 96 - 106 | 2 | 30.05 | 30.19 | 30.49 |

### GHTLTLN(N)FTR Hex 6 HexNAc 3

**Mass:**  2740.1731

(-1.296e-06)

**Charge States:** 3

**Start Position**: 97

**End Position**: 107

| Profile Score | Tandem Score | TDA q-value | Volume | Spectrum Matches |
| --- | --- | --- | --- | --- |
| -4.4148 | 111.4447 | 0.0000 | 1.9988e+06 | 4 |

**Adduct Signal Fractions**

- **Unmodified**: 1.9988e+06

### GHTLTLN(N)FTR Hex 5 HexNAc 3

**Mass:**  2579.1295

(3.928e-04)

**Start Position**: 97

**End Position**: 107

| Profile Score | Tandem Score | TDA q-value | Volume | Spectrum Matches |
| --- | --- | --- | --- | --- |
| 0.0000 | 30.0525 | 0.0324 | 0.0000e+00 | 1 |

Adduct:

No Chromatogram Found

### GHTLTLN(N)FTR Hex 5 HexNAc 4

**Mass:**  2781.1988

(9.499e-07)

**Charge States:** 3

**Start Position**: 97

**End Position**: 107

| Profile Score | Tandem Score | TDA q-value | Volume | Spectrum Matches |
| --- | --- | --- | --- | --- |
| -4.3114 | 29.7932 | 0.0324 | 1.2317e+06 | 2 |

**Adduct Signal Fractions**

- **Unmodified**: 1.2317e+06

### O75144|ICOSL\_HUMAN

**>O75144|ICOSL\_HUMAN**  
 `MRLGSPGLLFLLFSSLRADTQEKEVRAMVGSDVELSCACPEGSRFDLNDVYVYWQTSESKTVVTYHIPQNSSLENVDSRY  
RNRALMSPAGMLRGDFSLRLFNVTPQDEQKFHCLVLSQSLGFQEVLSVEVTLHVAANFSVPVVSAPHSPSQDELTFTCTS  
INGYPRPNVYWINKTDNSLLDQALQNDTVFLNMRGLYDVVSVLRIARTPSVNIGCCIENVLLQQNLTVGSQTGNDIGERD  
KITENPVSTGEKNAATWSILAVLCLLVVVAVAIGWVCRDRCLQHSYAGAWAVSPETELTGHV`

| Site | Type | Count |
| --- | --- | --- |
| 70 | N-linked | 2 |
| 102 | N-linked | 1 |

| Mass | Sequence | MS_2_ Score | `q`-value | MS_1_ Score | Signal | Protein Coordinates | Tandem Scans | Start Time | Apex Time | End Time |
| --- | --- | --- | --- | --- | --- | --- | --- | --- | --- | --- |
| 4291.8565 | TVVTYHIPQN(N)SSLENVDSR Fuc 1 Hex 6 HexNAc 5 | 32.804 | 0.020 | -20.661 | 1.375e+06 | 60 - 79 | 3 | 30.49 | 30.66 | 30.66 |
| 2940.2467 | LFN(N)VTPQDEQK Hex 5 HexNAc 4 | 30.384 | 0.032 | -20.483 | 9.401e+05 | 99 - 110 | 1 | 25.41 | 25.41 | 25.58 |
| 4145.7885 | TVVTYHIPQN(N)SSLENVDSR Hex 6 HexNAc 5 | 27.399 | 0.041 | -19.240 | 1.344e+06 | 60 - 79 | 2 | 30.66 | 30.83 | 30.83 |

### TVVTYHIPQN(N)SSLENVDSR Fuc 1 Hex 6 HexNAc 5

**Mass:**  4291.8586

(4.034e-06)

**Charge States:** 4

**Start Position**: 61

**End Position**: 80

| Profile Score | Tandem Score | TDA q-value | Volume | Spectrum Matches |
| --- | --- | --- | --- | --- |
| -20.6615 | 32.8042 | 0.0205 | 1.3751e+06 | 3 |

**Adduct Signal Fractions**

- **Unmodified**: 1.3751e+06

### LFN(N)VTPQDEQK Hex 5 HexNAc 4

**Mass:**  2940.2472

(3.055e-06)

**Charge States:** 3

**Start Position**: 100

**End Position**: 111

| Profile Score | Tandem Score | TDA q-value | Volume | Spectrum Matches |
| --- | --- | --- | --- | --- |
| -20.4833 | 30.3839 | 0.0319 | 9.4007e+05 | 1 |

**Adduct Signal Fractions**

- **Unmodified**: 9.4007e+05

### TVVTYHIPQN(N)SSLENVDSR Hex 6 HexNAc 5

**Mass:**  4145.7878

(2.690e-06)

**Charge States:** 4

**Start Position**: 61

**End Position**: 80

| Profile Score | Tandem Score | TDA q-value | Volume | Spectrum Matches |
| --- | --- | --- | --- | --- |
| -19.2396 | 27.3994 | 0.0406 | 1.3436e+06 | 2 |

**Adduct Signal Fractions**

- **Unmodified**: 1.3436e+06
