# Supplementary material for: Measuring Site-specific Glycosylation Similarity between Influenza a Virus Variants with Statistical Certainty
Source: Mol Cell Proteomics. 2020 Nov 25;19(9):1533–45. doi: 10.1074/mcp.RA120.002031 (PMC8143645; doi:10.1074/mcp.RA120.002031)
Supplement: Supplementary file 1 [file mmc1.zip › mmc1/159250_2_supp_555287_qcjklk.zip!/20190326_aAGP_021119_tryp_HILIC_DDA_1ug_04_glycopeptides.html]

## 20190326\_aAGP\_021119\_tryp\_HILIC\_DDA\_1ug\_04 @ GlycopeptideHypothesis-30b8a4d69f3940c38f79a25b688d8c39

**MS^1^ Mass Accuracy:** 10.0 PPM  
**Grouping Tolerance:** 15.0 PPM  
**MS^n^ Mass Accuracy:** 20.0 PPM  
**Minimum `q`-Value For Clustering:** 0.05

**Glycopeptide Hypothesis:**
GlycopeptideHypothesis-30b8a4d69f3940c38f79a25b688d8c39  
**Sample Searched:**
20190326\_aAGP\_021119\_tryp\_HILIC\_DDA\_1ug\_04

| Protein | Matched Glycopeptides |
| --- | --- |
| P19652\|A1AG2\_HUMAN | 189 |
| P02763\|A1AG1\_HUMAN | 186 |
| P00738\|HPT\_HUMAN | 8 |
| P05155\|IC1\_HUMAN | 8 |
| Q9Y5Y7\|LYVE1\_HUMAN | 8 |
| O75144\|ICOSL\_HUMAN | 5 |
| P02790\|HEMO\_HUMAN | 4 |
| Q5R543\|HEMO\_PONAB | 4 |
| Q12913\|PTPRJ\_HUMAN | 3 |
| O95497\|VNN1\_HUMAN | 2 |
| P11279\|LAMP1\_HUMAN | 1 |

### P19652|A1AG2\_HUMAN

**>P19652|A1AG2\_HUMAN**  
 `MALSWVLTVLSLLPLLEAQIPLCANLVPVPITNATLDRITGKWFYIASAFRNEEYNKSVQEIQATFFYFTPNKTEDTIFL  
REYQTRQNQCFYNSSYLNVQRENGTVSRYEGGREHVAHLLFLRDTKTLMFGSYLDDEKNWGLSFYADKPETTKEQLGEFY  
EALDCLCIPRSDVMYTDWKKDKCEPLEKQHEKERKQEEGES`

| Site | Type | Count |
| --- | --- | --- |
| 33 | N-linked | 64 |
| 56 | N-linked | 3 |
| 93 | N-linked | 23 |
| 72 | N-linked | 99 |

| Mass | Sequence | MS_2_ Score | `q`-value | MS_1_ Score | Signal | Protein Coordinates | Tandem Scans | Start Time | Apex Time | End Time |
| --- | --- | --- | --- | --- | --- | --- | --- | --- | --- | --- |
| 2756.0974 | YFTPN(N)K Hex 6 HexNAc 5 | 187.498 | 0.0 | 13.791 | 2.565e+08 | 67 - 73 | 3 | 14.25 | 14.41 | 14.91 |
| 3121.2318 | YFTPN(N)K Hex 7 HexNAc 6 | 187.178 | 0.0 | 13.782 | 3.649e+08 | 67 - 73 | 4 | 13.94 | 14.08 | 14.74 |
| 3268.2995 | FYFTPN(N)K Hex 7 HexNAc 6 | 184.423 | 0.0 | 15.291 | 1.154e+08 | 66 - 73 | 8 | 24.42 | 24.85 | 26.18 |
| 2903.1667 | FYFTPN(N)K Hex 6 HexNAc 5 | 183.305 | 0.0 | 13.636 | 8.964e+07 | 66 - 73 | 14 | 25.19 | 25.53 | 28.37 |
| 2417.9227 | NEEYN(N)K Hex 5 HexNAc 4 | 182.356 | 0.0 | 11.263 | 5.950e+07 | 51 - 57 | 7 | 3.97 | 4.14 | 5.37 |
| 2929.1116 | NEEYN(N)K Fuc 1 Hex 6 HexNAc 5 | 181.805 | 0.0 | -3.515 | 2.091e+07 | 51 - 57 | 7 | 3.97 | 4.14 | 10.79 |
| 2783.0555 | NEEYN(N)K Hex 6 HexNAc 5 | 181.465 | 0.0 | 13.061 | 2.804e+08 | 51 - 57 | 20 | 3.97 | 4.14 | 10.79 |
| 3906.6665 | SVQEIQATFFYFTPN(N)K Hex 6 HexNAc 5 | 174.243 | 0.0 | 15.076 | 1.932e+09 | 57 - 73 | 41 | 50.53 | 54.46 | 62.33 |
| 2902.1548 | YFTPN(N)K Fuc 1 Hex 6 HexNAc 5 | 174.140 | 0.0 | -18.632 | 3.791e+07 | 67 - 73 | 1 | 14.08 | 14.25 | 14.25 |
| 3267.2876 | YFTPN(N)K Fuc 1 Hex 7 HexNAc 6 | 173.371 | 0.0 | -4.635 | 6.048e+07 | 67 - 73 | 2 | 13.78 | 13.94 | 14.25 |
| 3486.3637 | YFTPN(N)K Hex 8 HexNAc 7 | 172.606 | 0.0 | -20.311 | 1.935e+07 | 67 - 73 | 1 | 13.78 | 13.94 | 13.94 |
| 4636.9346 | SVQEIQATFFYFTPN(N)K Hex 8 HexNAc 7 | 172.360 | 0.0 | 13.532 | 3.064e+08 | 57 - 73 | 7 | 53.09 | 53.61 | 54.29 |
| 2392.9588 | YFTPN(N)K Hex 5 HexNAc 4 | 167.873 | 0.0 | 0.000 | 0.000e+00 | 67 - 73 | 1 | - | - | - |
| 4417.8622 | SVQEIQATFFYFTPN(N)K Fuc 1 Hex 7 HexNAc 6 | 167.604 | 0.0 | 13.761 | 4.974e+08 | 57 - 73 | 13 | 53.61 | 53.78 | 57.02 |
| 4052.7261 | SVQEIQATFFYFTPN(N)K Fuc 1 Hex 6 HexNAc 5 | 166.895 | 0.0 | 13.683 | 4.173e+08 | 57 - 73 | 13 | 53.95 | 54.29 | 57.36 |
| 2757.1032 | YFTPN(N)K Hex 6 HexNAc 5 | 166.705 | 0.0 | 0.000 | 0.000e+00 | 67 - 73 | 1 | - | - | - |
| 2390.9674 | YFTPN(N)K Hex 5 HexNAc 4 | 166.049 | 0.0 | -20.584 | 1.828e+07 | 67 - 73 | 2 | 14.57 | 14.57 | 14.74 |
| 3541.5339 | SVQEIQATFFYFTPN(N)K Hex 5 HexNAc 4 | 165.700 | 0.0 | 13.872 | 2.413e+08 | 57 - 73 | 12 | 54.97 | 55.14 | 58.05 |
| 3222.3222 | ATFFYFTPN(N)K Hex 6 HexNAc 5 | 164.263 | 0.0 | -3.140 | 3.783e+07 | 63 - 73 | 2 | 41.08 | 41.25 | 41.59 |
| 3049.2255 | FYFTPN(N)K Fuc 1 Hex 6 HexNAc 5 | 163.976 | 0.034 | 14.121 | 1.590e+07 | 66 - 73 | 3 | 25.02 | 25.36 | 25.84 |
| 4271.8036 | SVQEIQATFFYFTPN(N)K Hex 7 HexNAc 6 | 163.209 | 0.0 | 15.017 | 2.306e+09 | 57 - 73 | 48 | 50.35 | 53.95 | 113.29 |
| 3050.2342 | FFYFTPN(N)K Hex 6 HexNAc 5 | 163.027 | 0.034 | -19.052 | 2.092e+07 | 65 - 73 | 2 | 36.03 | 36.03 | 36.20 |
| 3415.3697 | FFYFTPN(N)K Hex 7 HexNAc 6 | 161.026 | 0.034 | -2.619 | 3.575e+07 | 65 - 73 | 2 | 35.35 | 35.52 | 35.69 |
| 3395.5308 | LVPVPITN(N)ATLDR Hex 6 HexNAc 5 | 158.535 | 0.0 | 13.305 | 1.541e+10 | 25 - 38 | 542 | 30.01 | 36.51 | 114.09 |
| 3030.3946 | LVPVPITN(N)ATLDR Hex 5 HexNAc 4 | 157.425 | 0.0 | 15.231 | 1.051e+09 | 25 - 38 | 8 | 36.80 | 37.21 | 38.88 |
| 3587.4552 | ATFFYFTPN(N)K Hex 7 HexNAc 6 | 156.181 | 0.0 | 13.199 | 6.601e+07 | 63 - 73 | 3 | 40.39 | 40.56 | 41.25 |
| 4782.9933 | SVQEIQATFFYFTPN(N)K Fuc 1 Hex 8 HexNAc 7 | 156.146 | 0.0 | 12.333 | 6.298e+07 | 57 - 73 | 5 | 52.92 | 53.44 | 53.95 |
| 4464.8569 | SVQEIQATFFYFTPN(N)K Fuc 1 Hex 8 HexNAc 4 Neu5Ac 1 | 154.264 | 0.0 | 13.712 | 5.406e+07 | 57 - 73 | 4 | 54.80 | 54.97 | 55.65 |
| 4563.9198 | SVQEIQATFFYFTPN(N)K Fuc 2 Hex 7 HexNAc 6 | 153.860 | 0.0 | -3.572 | 7.004e+07 | 57 - 73 | 4 | 53.61 | 53.78 | 54.12 |
| 3541.5902 | LVPVPITN(N)ATLDR Fuc 1 Hex 6 HexNAc 5 | 152.733 | 0.0 | 13.020 | 3.612e+09 | 25 - 38 | 23 | 29.84 | 36.20 | 43.82 |
| 4882.1738 | SVQEIQATFFYFTPN(N)KTEDTIFLR Hex 6 HexNAc 5 | 143.239 | 0.0 | 12.839 | 5.186e+08 | 57 - 81 | 17 | 60.45 | 62.85 | 66.10 |
| 3368.3746 | ATFFYFTPN(N)K Fuc 1 Hex 6 HexNAc 5 | 142.391 | 0.0 | -20.161 | 5.229e+06 | 63 - 73 | 1 | 40.74 | 40.91 | 40.91 |
| 5002.0667 | SVQEIQATFFYFTPN(N)K Hex 9 HexNAc 8 | 141.343 | 0.0 | 11.885 | 5.875e+07 | 57 - 73 | 2 | 52.58 | 52.92 | 53.44 |
| 5002.0667 | SVQEIQATFFYFTPN(N)K Fuc 5 Hex 7 HexNAc 6 | 141.343 | 0.0 | 11.885 | 5.875e+07 | 57 - 73 | 2 | 52.58 | 52.92 | 53.44 |
| 3588.5885 | LVPVPITN(N)ATLDR Fuc 1 Hex 7 HexNAc 3 Neu5Ac 1 | 140.327 | 0.0 | -4.320 | 5.020e+07 | 25 - 38 | 1 | 37.70 | 37.70 | 38.03 |
| 5028.2314 | SVQEIQATFFYFTPN(N)KTEDTIFLR Fuc 1 Hex 6 HexNAc 5 | 140.061 | 0.0 | 12.570 | 1.191e+08 | 57 - 81 | 7 | 62.51 | 62.68 | 64.73 |
| 4447.8321 | SVQEIQATFFYFTPN(N)K Hex 7 HexNAc 4 Neu5Ac 2 | 139.277 | 0.0 | 13.461 | 1.197e+08 | 57 - 73 | 7 | 54.80 | 54.97 | 56.68 |
| 3687.6513 | LVPVPITN(N)ATLDR Fuc 2 Hex 6 HexNAc 5 | 137.254 | 0.0 | -18.373 | 4.999e+07 | 25 - 38 | 2 | 36.03 | 36.20 | 36.20 |
| 4243.8033 | FYFTPN(N)KTEDTIFLR Hex 7 HexNAc 6 | 136.485 | 0.0 | 13.345 | 1.961e+08 | 66 - 81 | 11 | 44.16 | 45.68 | 47.56 |
| 3731.6018 | YFTPN(N)KTEDTIFLR Hex 6 HexNAc 5 | 135.460 | 0.0 | 10.888 | 1.048e+09 | 67 - 81 | 8 | 35.35 | 37.38 | 39.22 |
| 3760.6641 | LVPVPITN(N)ATLDR Hex 7 HexNAc 6 | 135.240 | 0.0 | 15.697 | 6.908e+08 | 25 - 38 | 6 | 35.86 | 36.20 | 37.06 |
| 5247.3084 | SVQEIQATFFYFTPN(N)KTEDTIFLR Hex 7 HexNAc 6 | 134.393 | 0.0 | 13.409 | 1.235e+09 | 57 - 81 | 25 | 60.10 | 62.51 | 68.16 |
| 3786.6025 | TPN(N)KTEDTIFLR Hex 7 HexNAc 6 | 133.715 | 0.0 | 13.452 | 5.229e+07 | 69 - 81 | 8 | 25.36 | 25.70 | 26.30 |
| 3282.4427 | VPVPITN(N)ATLDR Hex 6 HexNAc 5 | 132.243 | 0.0 | 19.656 | 1.046e+07 | 26 - 38 | 4 | 30.18 | 30.35 | 30.74 |
| 3877.6623 | YFTPN(N)KTEDTIFLR Fuc 1 Hex 6 HexNAc 5 | 132.092 | 0.0 | -3.782 | 2.463e+08 | 67 - 81 | 4 | 35.35 | 37.21 | 37.53 |
| 3906.7208 | LVPVPITN(N)ATLDR Fuc 1 Hex 7 HexNAc 6 | 132.034 | 0.0 | -3.522 | 2.130e+08 | 25 - 38 | 4 | 35.86 | 36.03 | 36.20 |
| 4125.7913 | LVPVPITN(N)ATLDR Hex 8 HexNAc 7 | 130.494 | 0.0 | -3.100 | 1.112e+08 | 25 - 38 | 4 | 35.52 | 36.03 | 36.03 |
| 4690.8519 | ATFFYFTPN(N)K Fuc 5 Hex 5 HexNAc 8 Neu5Ac 1 | 130.132 | 0.0 | -5.370 | 1.315e+07 | 63 - 73 | 2 | 53.27 | 53.27 | 53.61 |
| 3421.4699 | TPN(N)KTEDTIFLR Hex 6 HexNAc 5 | 127.492 | 0.0 | 13.344 | 3.001e+07 | 69 - 81 | 6 | 26.01 | 26.18 | 27.02 |
| 4328.8209 | SVQEIQATFFYFTPN(N)K Fuc 4 Hex 5 HexNAc 5 | 127.198 | 0.0 | 11.789 | 5.138e+08 | 57 - 73 | 25 | 53.09 | 54.80 | 57.53 |
| 4593.8936 | SVQEIQATFFYFTPN(N)K Fuc 1 Hex 7 HexNAc 4 Neu5Ac 2 | 126.229 | 0.0 | -20.071 | 1.231e+07 | 57 - 73 | 1 | 54.80 | 54.80 | 54.97 |
| 4096.7404 | YFTPN(N)KTEDTIFLR Hex 7 HexNAc 6 | 125.693 | 0.0 | 13.184 | 2.069e+09 | 67 - 81 | 21 | 35.18 | 36.80 | 44.16 |
| 4242.8246 | YFTPN(N)KTEDTIFLR Fuc 1 Hex 7 HexNAc 6 | 124.688 | 0.0 | -6.381 | 2.167e+08 | 67 - 81 | 6 | 36.51 | 36.65 | 61.65 |
| 3879.6742 | YFTPN(N)KTEDTIFLR Fuc 1 Hex 6 HexNAc 5 | 124.296 | 0.0 | 0.000 | 0.000e+00 | 67 - 81 | 1 | - | - | - |
| 4109.7460 | SVQEIQATFFYFTPN(N)K Hex 6 HexNAc 6 | 124.206 | 0.0 | 12.244 | 5.282e+07 | 57 - 73 | 3 | 54.97 | 54.97 | 55.82 |
| 3740.6402 | C(C)ANLVPVPITN(N)ATLDR Hex 6 HexNAc 5 | 124.162 | 0.0 | 15.113 | 1.815e+08 | 22 - 38 | 5 | 41.93 | 42.11 | 42.62 |
| 3366.4691 | YFTPN(N)KTEDTIFLR Hex 5 HexNAc 4 | 124.024 | 0.0 | -4.454 | 7.378e+07 | 67 - 81 | 1 | 37.70 | 37.86 | 38.19 |
| 3907.5768 | QNQC(C)FYN(N)SSYLNVQR Hex 6 HexNAc 5 | 123.805 | 0.0 | 10.297 | 6.463e+08 | 86 - 101 | 5 | 33.48 | 33.82 | 40.39 |
| 3509.5723 | NLVPVPITN(N)ATLDR Hex 6 HexNAc 5 | 123.744 | 0.0 | -6.305 | 3.474e+07 | 24 - 38 | 3 | 40.91 | 47.90 | 47.90 |
| 4299.8120 | YFTPN(N)KTEDTIFLR Hex 7 HexNAc 7 | 123.178 | 0.0 | -5.038 | 7.567e+07 | 67 - 81 | 3 | 37.06 | 37.21 | 37.53 |
| 4053.6395 | QNQC(C)FYN(N)SSYLNVQR Fuc 1 Hex 6 HexNAc 5 | 121.167 | 0.0 | -5.292 | 5.013e+07 | 86 - 101 | 2 | 33.31 | 33.48 | 33.65 |
| 3470.5978 | LVPVPITN(N)ATLDR Fuc 3 Hex 5 HexNAc 4 | 121.084 | 0.0 | 0.000 | 0.000e+00 | 25 - 38 | 1 | - | - | - |
| 4418.7713 | QNQC(C)FYN(N)SSYLNVQR Fuc 1 Hex 7 HexNAc 6 | 120.905 | 0.0 | 7.393 | 3.183e+08 | 86 - 101 | 5 | 33.14 | 33.31 | 39.89 |
| 3686.6283 | LVPVPITN(N)ATLDR Hex 6 HexNAc 5 Neu5Ac 1 | 120.592 | 0.0 | 10.773 | 2.633e+07 | 25 - 38 | 1 | 39.05 | 39.56 | 39.89 |
| 3445.5298 | LVPVPITN(N)ATLDR Fuc 2 Hex 7 HexNAc 3 | 118.946 | 0.0 | -23.124 | 4.043e+05 | 25 - 38 | 3 | 30.01 | 30.01 | 30.01 |
| 3733.5127 | ATFFYFTPN(N)K Fuc 1 Hex 7 HexNAc 6 | 118.626 | 0.0 | -18.182 | 6.893e+06 | 63 - 73 | 1 | 40.22 | 40.22 | 40.39 |
| 4973.0651 | YFTPN(N)KTEDTIFLR Fuc 1 Hex 9 HexNAc 8 | 117.566 | 0.0 | -19.116 | 2.395e+06 | 67 - 81 | 1 | 35.35 | 35.35 | 35.35 |
| 4461.8686 | YFTPN(N)KTEDTIFLR Hex 8 HexNAc 7 | 116.516 | 0.0 | 13.542 | 1.775e+08 | 67 - 81 | 3 | 35.69 | 36.03 | 36.36 |
| 4389.8616 | YFTPN(N)KTEDTIFLR Fuc 2 Hex 7 HexNAc 6 | 115.730 | 0.0 | -6.169 | 3.407e+07 | 67 - 81 | 8 | 45.51 | 45.68 | 46.02 |
| 3452.5487 | NLVPVPITN(N)ATLDR Fuc 1 Hex 6 HexNAc 4 | 114.836 | 0.0 | 16.147 | 3.078e+09 | 24 - 38 | 12 | 37.06 | 37.53 | 39.05 |
| 3598.6055 | NLVPVPITN(N)ATLDR Fuc 2 Hex 6 HexNAc 4 | 114.261 | 0.0 | 13.912 | 8.853e+08 | 24 - 38 | 5 | 37.06 | 37.38 | 38.19 |
| 4637.8553 | QNQC(C)FYN(N)SSYLNVQR Hex 8 HexNAc 7 | 112.114 | 0.0 | -5.942 | 4.906e+07 | 86 - 101 | 1 | 32.63 | 33.14 | 33.14 |
| 4474.8765 | SVQEIQATFFYFTPN(N)K Hex 7 HexNAc 7 | 110.321 | 0.0 | 10.974 | 5.677e+07 | 57 - 73 | 3 | 54.29 | 54.46 | 55.48 |
| 3031.4194 | LVPVPITN(N)ATLDR Hex 5 HexNAc 4 | 109.662 | 0.0 | 0.000 | 0.000e+00 | 25 - 38 | 1 | - | - | - |
| 3878.6697 | FYFTPN(N)KTEDTIFLR Hex 6 HexNAc 5 | 108.994 | 0.0 | 13.111 | 6.677e+07 | 66 - 81 | 5 | 46.02 | 46.19 | 47.21 |
| 3886.5724 | PVPITN(N)ATLDR Hex 8 HexNAc 4 Neu5Ac 2 | 108.405 | 0.0 | 7.622 | 1.785e+08 | 27 - 38 | 8 | 40.91 | 41.08 | 41.76 |
| 4272.7112 | QNQC(C)FYN(N)SSYLNVQR Hex 7 HexNAc 6 | 108.210 | 0.0 | 8.883 | 1.064e+09 | 86 - 101 | 4 | 33.14 | 33.31 | 39.56 |
| 3895.6905 | YFTPN(N)KTEDTIFLR Hex 7 HexNAc 5 | 107.735 | 0.0 | 0.000 | 0.000e+00 | 67 - 81 | 1 | - | - | - |
| 3580.6088 | ANLVPVPITN(N)ATLDR Hex 6 HexNAc 5 | 105.154 | 0.0 | -17.077 | 1.490e+07 | 23 - 38 | 1 | 41.42 | 41.42 | 41.59 |
| 3817.6838 | NLVPVPITN(N)ATLDR Fuc 1 Hex 7 HexNAc 5 | 104.347 | 0.0 | -4.444 | 8.972e+07 | 24 - 38 | 2 | 37.06 | 37.21 | 37.38 |
| 3541.5832 | PVPITN(N)ATLDR Fuc 3 Hex 3 HexNAc 7 | 103.622 | 0.0 | 7.413 | 2.091e+07 | 27 - 38 | 6 | 44.49 | 44.66 | 47.38 |
| 4020.7100 | SVQEIQATFFYFTPN(N)K Fuc 3 Hex 4 HexNAc 5 | 103.619 | 0.0 | -19.131 | 9.858e+06 | 57 - 73 | 2 | 54.12 | 54.29 | 54.29 |
| 3903.5977 | PVPITN(N)ATLDR Fuc 1 Hex 9 HexNAc 4 Neu5Ac 1 | 103.055 | 0.0 | -18.214 | 9.790e+06 | 27 - 38 | 2 | 41.59 | 41.59 | 41.76 |
| 4517.0370 | SVQEIQATFFYFTPN(N)KTEDTIFLR Hex 5 HexNAc 4 | 101.817 | 0.0 | -3.930 | 1.974e+07 | 57 - 81 | 2 | 63.19 | 63.19 | 63.53 |
| 3568.5397 | FTPN(N)KTEDTIFLR Hex 6 HexNAc 5 | 97.357 | 0.0 | -2.894 | 6.942e+07 | 68 - 81 | 4 | 32.12 | 32.29 | 32.63 |
| 3428.5021 | VPVPITN(N)ATLDR Fuc 1 Hex 6 HexNAc 5 | 96.831 | 0.0 | -4.678 | 2.215e+06 | 26 - 38 | 2 | 30.01 | 30.18 | 30.35 |
| 5393.3643 | SVQEIQATFFYFTPN(N)KTEDTIFLR Fuc 1 Hex 7 HexNAc 6 | 96.551 | 0.0 | 13.059 | 2.553e+08 | 57 - 81 | 10 | 61.30 | 62.33 | 64.73 |
| 3567.5293 | TPN(N)KTEDTIFLR Fuc 1 Hex 6 HexNAc 5 | 96.307 | 0.0 | -4.430 | 4.815e+06 | 69 - 81 | 3 | 25.84 | 25.84 | 26.18 |
| 4826.9981 | YFTPN(N)KTEDTIFLR Hex 9 HexNAc 8 | 95.055 | 0.0 | 11.679 | 4.966e+07 | 67 - 81 | 2 | 35.35 | 35.69 | 36.03 |
| 3933.6716 | FTPN(N)KTEDTIFLR Hex 7 HexNAc 6 | 94.046 | 0.0 | 14.313 | 1.310e+08 | 68 - 81 | 6 | 31.61 | 31.77 | 32.63 |
| 3422.4748 | TPN(N)KTEDTIFLR Hex 6 HexNAc 5 | 93.876 | 0.0 | 0.000 | 0.000e+00 | 69 - 81 | 1 | - | - | - |
| 4608.9343 | FYFTPN(N)KTEDTIFLR Hex 8 HexNAc 7 | 92.701 | 0.0 | 12.809 | 2.289e+07 | 66 - 81 | 4 | 44.83 | 45.17 | 45.51 |
| 3597.5217 | PVPITN(N)ATLDR Fuc 2 Hex 8 HexNAc 4 | 88.929 | 0.0 | 0.000 | 0.000e+00 | 27 - 38 | 1 | - | - | - |
| 3453.5379 | VPVPITN(N)ATLDR Fuc 2 Hex 4 HexNAc 6 | 88.587 | 0.0 | -4.635 | 3.698e+07 | 26 - 38 | 12 | 40.22 | 40.39 | 40.56 |
| 4272.7549 | YFTPN(N)KTEDTIFLR Hex 7 HexNAc 4 Neu5Ac 2 | 88.062 | 0.0 | -4.075 | 1.374e+08 | 67 - 81 | 10 | 37.86 | 38.03 | 38.19 |
| 3714.5966 | FTPN(N)KTEDTIFLR Fuc 1 Hex 6 HexNAc 5 | 86.031 | 0.0 | -3.821 | 1.409e+07 | 68 - 81 | 2 | 31.94 | 32.12 | 32.29 |
| 4609.8993 | YFTPN(N)KTEDTIFLR Fuc 1 Hex 8 HexNAc 7 | 84.859 | 0.0 | 0.000 | 0.000e+00 | 67 - 81 | 1 | - | - | - |
| 4268.8120 | PVPITN(N)ATLDR Fuc 2 Hex 3 HexNAc 7 Neu5Ac 3 | 84.650 | 0.0 | -18.682 | 8.533e+06 | 27 - 38 | 2 | 46.02 | 46.02 | 46.19 |
| 4079.7259 | FTPN(N)KTEDTIFLR Fuc 1 Hex 7 HexNAc 6 | 84.231 | 0.0 | -3.376 | 2.935e+07 | 68 - 81 | 5 | 31.53 | 31.61 | 31.94 |
| 3249.4129 | PVPITN(N)ATLDR Fuc 1 Hex 3 HexNAc 7 | 83.776 | 0.0 | 0.000 | 0.000e+00 | 27 - 38 | 1 | - | - | - |
| 4300.5882 | FTPN(N)KTEDTIFLR Hex 8 HexNAc 7 | 82.657 | 0.0 | 10.113 | 6.518e+07 | 68 - 81 | 10 | 31.02 | 47.04 | 47.38 |
| 3932.6586 | TPN(N)KTEDTIFLR Fuc 1 Hex 7 HexNAc 6 | 81.957 | 0.0 | -4.692 | 7.704e+06 | 69 - 81 | 3 | 25.36 | 25.53 | 25.70 |
| 5539.4244 | SVQEIQATFFYFTPN(N)KTEDTIFLR Fuc 2 Hex 7 HexNAc 6 | 81.287 | 0.002 | -20.156 | 2.407e+07 | 57 - 81 | 1 | 62.16 | 62.33 | 62.33 |
| 3470.5630 | LVPVPITN(N)ATLDR Fuc 3 Hex 5 HexNAc 4 | 81.092 | 0.002 | -21.173 | 1.241e+07 | 25 - 38 | 2 | 40.22 | 40.39 | 40.39 |
| 3508.5942 | LVPVPITN(N)ATLDR Fuc 1 Hex 4 HexNAc 5 Neu5Ac 1 | 77.297 | 0.002 | -18.887 | 1.995e+07 | 25 - 38 | 1 | 36.03 | 36.20 | 36.20 |
| 3056.3377 | TPN(N)KTEDTIFLR Hex 5 HexNAc 4 | 76.332 | 0.004 | 10.230 | 6.463e+06 | 69 - 81 | 2 | 26.43 | 26.52 | 26.82 |
| 4191.9175 | QIPLC(C)ANLVPVPITN(N)ATLDR Hex 6 HexNAc 5 | 75.827 | 0.004 | 13.346 | 6.189e+08 | 18 - 38 | 9 | 61.82 | 62.51 | 63.70 |
| 3685.5768 | PVPITN(N)ATLDR Hex 3 HexNAc 7 Neu5Ac 2 | 75.630 | 0.004 | 7.185 | 1.452e+07 | 27 - 38 | 1 | 40.74 | 40.91 | 41.59 |
| 3741.6226 | C(C)AN(D)LVPVPITN(N)ATLDR Hex 6 HexNAc 5 | 75.059 | 0.004 | 8.925 | 1.714e+08 | 22 - 38 | 11 | 42.62 | 43.99 | 45.00 |
| 3788.6236 | FYFTPN(N)KTEDTIFLR Fuc 1 Hex 4 HexNAc 4 Neu5Ac 1 | 72.960 | 0.005 | 14.074 | 2.608e+08 | 66 - 81 | 5 | 37.86 | 38.03 | 38.53 |
| 4929.0581 | SVQEIQATFFYFTPN(N)K Fuc 2 Hex 8 HexNAc 7 | 69.103 | 0.005 | 11.354 | 1.280e+07 | 57 - 73 | 2 | 52.92 | 53.27 | 53.61 |
| 5367.1969 | SVQEIQATFFYFTPN(N)K Hex 10 HexNAc 9 | 65.611 | 0.005 | -4.491 | 6.331e+06 | 57 - 73 | 1 | 52.24 | 52.41 | 52.58 |
| 3923.7486 | NLVPVPITN(N)ATLDR Hex 3 HexNAc 8 Neu5Ac 1 | 65.396 | 0.005 | -3.987 | 7.461e+07 | 24 - 38 | 3 | 35.86 | 36.20 | 36.20 |
| 4470.8186 | PVPITN(N)ATLDR Hex 3 HexNAc 8 Neu5Ac 4 | 65.072 | 0.005 | -4.674 | 9.712e+07 | 27 - 38 | 5 | 33.48 | 33.65 | 33.99 |
| 4113.7672 | FYFTPN(N)KTEDTIFLR Fuc 3 Hex 6 HexNAc 4 | 63.898 | 0.005 | -4.629 | 9.844e+07 | 66 - 81 | 2 | 36.80 | 36.80 | 37.06 |
| 5148.1262 | SVQEIQATFFYFTPN(N)K Fuc 1 Hex 9 HexNAc 8 | 61.775 | 0.005 | -21.179 | 2.927e+06 | 57 - 73 | 1 | 52.41 | 52.58 | 52.58 |
| 4693.9566 | SVQEIQATFFYFTPN(N)K Fuc 4 Hex 6 HexNAc 6 | 60.976 | 0.005 | 8.134 | 3.673e+07 | 57 - 73 | 2 | 53.78 | 53.78 | 54.46 |
| 5612.4365 | SVQEIQATFFYFTPN(N)KTEDTIFLR Hex 8 HexNAc 7 | 60.030 | 0.005 | 16.381 | 1.901e+08 | 57 - 81 | 4 | 61.65 | 61.82 | 62.51 |
| 3886.7009 | C(C)ANLVPVPITN(N)ATLDR Fuc 1 Hex 6 HexNAc 5 | 57.062 | 0.005 | 11.543 | 1.213e+08 | 22 - 38 | 6 | 41.76 | 52.58 | 53.27 |
| 4492.9081 | SVQEIQATFFYFTPN(N)K Fuc 4 Hex 6 HexNAc 5 | 54.697 | 0.005 | 0.000 | 0.000e+00 | 57 - 73 | 1 | - | - | - |
| 4024.7276 | FYFTPN(N)KTEDTIFLR Fuc 1 Hex 6 HexNAc 5 | 53.074 | 0.005 | 12.860 | 1.710e+07 | 66 - 81 | 4 | 45.85 | 46.02 | 46.53 |
| 4564.8360 | QNQC(C)FYN(N)SSYLNVQR Fuc 2 Hex 7 HexNAc 6 | 48.519 | 0.005 | -5.747 | 2.182e+07 | 86 - 101 | 1 | 33.14 | 33.31 | 33.48 |
| 3375.5038 | C(C)ANLVPVPITN(N)ATLDR Hex 5 HexNAc 4 | 47.961 | 0.005 | -4.560 | 1.277e+07 | 22 - 38 | 6 | 42.45 | 42.45 | 43.82 |
| 5440.3622 | SVQEIQATFFYFTPN(N)KTEDTIFLR Fuc 1 Hex 8 HexNAc 4 Neu5Ac 1 | 47.657 | 0.005 | -4.309 | 1.049e+07 | 57 - 81 | 1 | 63.36 | 63.36 | 63.70 |
| 4337.9734 | QIPLC(C)ANLVPVPITN(N)ATLDR Fuc 1 Hex 6 HexNAc 5 | 45.304 | 0.005 | 13.106 | 1.847e+08 | 18 - 38 | 6 | 61.65 | 62.33 | 63.02 |
| 4374.8687 | ANLVPVPITN(N)ATLDR Fuc 1 Hex 10 HexNAc 5 | 44.916 | 0.005 | -19.414 | 1.590e+07 | 23 - 38 | 2 | 62.33 | 62.33 | 62.33 |
| 5977.5682 | SVQEIQATFFYFTPN(N)KTEDTIFLR Hex 9 HexNAc 8 | 44.143 | 0.005 | 14.835 | 6.254e+07 | 57 - 81 | 4 | 61.13 | 61.48 | 62.33 |
| 4620.9440 | SVQEIQATFFYFTPN(N)K Fuc 1 Hex 7 HexNAc 7 | 42.796 | 0.005 | -20.438 | 3.818e+06 | 57 - 73 | 1 | 54.29 | 54.29 | 54.29 |
| 4190.8879 | LVPVPITN(N)ATLDR Fuc 4 Hex 3 HexNAc 7 Neu5Ac 1 | 41.024 | 0.005 | 12.998 | 1.464e+08 | 25 - 38 | 9 | 60.10 | 61.30 | 61.82 |
| 5150.1185 | SVQEIQATFFYFTPN(N)K Fuc 1 Hex 9 HexNAc 8 | 40.953 | 0.005 | 0.000 | 0.000e+00 | 57 - 73 | 1 | - | - | - |
| 3087.4168 | NLVPVPITN(N)ATLDR Fuc 1 Hex 5 HexNAc 3 | 40.535 | 0.006 | 14.306 | 2.164e+08 | 24 - 38 | 2 | 37.86 | 38.03 | 39.05 |
| 5758.4931 | SVQEIQATFFYFTPN(N)KTEDTIFLR Fuc 1 Hex 8 HexNAc 7 | 40.211 | 0.006 | -4.734 | 3.399e+07 | 57 - 81 | 3 | 61.65 | 61.99 | 61.99 |
| 4289.7286 | QC(C)FYN(N)SSYLNVQR Fuc 2 Hex 5 HexNAc 6 Neu5Ac 1 | 39.111 | 0.006 | 13.256 | 3.272e+08 | 88 - 101 | 1 | 33.31 | 33.48 | 33.99 |
| 4448.7451 | QNQC(C)FYN(N)SSYLNVQR Hex 7 HexNAc 4 Neu5Ac 2 | 38.939 | 0.006 | -18.483 | 1.401e+07 | 86 - 101 | 2 | 34.33 | 34.33 | 34.33 |
| 3924.5967 | QC(C)FYN(N)SSYLNVQR Fuc 2 Hex 4 HexNAc 5 Neu5Ac 1 | 38.925 | 0.006 | 13.448 | 2.095e+08 | 88 - 101 | 4 | 33.65 | 33.99 | 34.33 |
| 3450.4543 | PVPITN(N)ATLDR Fuc 1 Hex 8 HexNAc 4 | 38.532 | 0.006 | 0.000 | 0.000e+00 | 27 - 38 | 1 | - | - | - |
| 4175.8774 | LVPVPITN(N)ATLDR Fuc 2 Hex 4 HexNAc 9 | 38.071 | 0.009 | 13.465 | 1.137e+08 | 25 - 38 | 11 | 63.70 | 64.05 | 65.59 |
| 4212.8415 | ANLVPVPITN(N)ATLDR Fuc 1 Hex 9 HexNAc 5 | 38.014 | 0.009 | -5.857 | 9.182e+07 | 23 - 38 | 2 | 62.51 | 62.51 | 63.02 |
| 3513.5370 | FYFTPN(N)KTEDTIFLR Hex 5 HexNAc 4 | 37.117 | 0.010 | -19.577 | 2.749e+06 | 66 - 81 | 1 | 46.53 | 46.70 | 46.70 |
| 3797.6625 | C(C)AN(D)LVPVPITN(N)ATLDR Fuc 2 Hex 4 HexNAc 4 Neu5Ac 1 | 36.934 | 0.010 | -5.961 | 8.394e+07 | 22 - 38 | 4 | 42.96 | 43.13 | 43.47 |
| 4273.6972 | QC(C)FYN(N)SSYLNVQR Fuc 3 Hex 4 HexNAc 6 Neu5Ac 1 | 36.777 | 0.010 | 11.504 | 1.858e+08 | 88 - 101 | 1 | 34.16 | 34.50 | 35.35 |
| 4153.7553 | YFTPN(N)KTEDTIFLR Fuc 4 Hex 5 HexNAc 5 | 36.663 | 0.010 | 13.118 | 4.645e+08 | 67 - 81 | 4 | 37.21 | 37.38 | 38.53 |
| 3557.6006 | LVPVPITN(N)ATLDR Hex 7 HexNAc 5 | 36.241 | 0.010 | -19.443 | 1.224e+06 | 25 - 38 | 1 | 44.49 | 44.49 | 44.49 |
| 4977.0741 | FYFTPN(N)KTEDTIFLR Hex 9 HexNAc 8 | 33.578 | 0.013 | 0.000 | 0.000e+00 | 66 - 81 | 1 | - | - | - |
| 4273.6947 | QN(D)QC(C)FYN(N)SSYLNVQR Hex 7 HexNAc 6 | 33.333 | 0.013 | -6.785 | 2.146e+08 | 86 - 101 | 2 | 39.72 | 39.72 | 40.91 |
| 5285.2519 | ANLVPVPITN(N)ATLDR Fuc 5 Hex 7 HexNAc 9 | 33.060 | 0.013 | 10.258 | 1.478e+08 | 23 - 38 | 4 | 62.33 | 62.51 | 63.87 |
| 5614.4453 | SVQEIQATFFYFTPN(N)KTEDTIFLR Hex 8 HexNAc 7 | 32.862 | 0.014 | 0.000 | 0.000e+00 | 57 - 81 | 1 | - | - | - |
| 4419.7582 | QN(D)QC(C)FYN(N)SSYLNVQR Fuc 1 Hex 7 HexNAc 6 | 31.971 | 0.019 | -6.045 | 2.763e+07 | 86 - 101 | 1 | 34.16 | 34.33 | 34.50 |
| 4285.8360 | LVPVPITN(N)ATLDR Fuc 1 Hex 7 HexNAc 5 Neu5Ac 2 | 31.855 | 0.019 | -4.825 | 4.578e+06 | 25 - 38 | 1 | 45.68 | 46.02 | 46.19 |
| 4320.9509 | QIPLC(C)ANLVPVPITN(N)ATLDR Hex 5 HexNAc 5 Neu5Ac 1 | 31.538 | 0.019 | 15.483 | 3.620e+08 | 18 - 38 | 7 | 61.48 | 62.33 | 63.36 |
| 5192.1403 | YFTPN(N)KTEDTIFLR Fuc 5 Hex 8 HexNAc 7 | 31.375 | 0.019 | -4.590 | 5.292e+06 | 67 - 81 | 1 | 35.18 | 35.35 | 35.52 |
| 5002.9932 | QNQC(C)FYN(N)SSYLNVQR Fuc 5 Hex 7 HexNAc 6 | 31.134 | 0.020 | -5.768 | 2.219e+07 | 86 - 101 | 1 | 32.29 | 32.63 | 32.63 |
| 3887.6822 | C(C)AN(D)LVPVPITN(N)ATLDR Fuc 1 Hex 6 HexNAc 5 | 31.073 | 0.020 | 7.791 | 3.581e+07 | 22 - 38 | 5 | 43.64 | 43.82 | 44.66 |
| 4101.7126 | FTPN(N)KTEDTIFLR Hex 10 HexNAc 3 Neu5Ac 1 | 30.438 | 0.021 | -20.571 | 1.042e+06 | 68 - 81 | 1 | 31.61 | 31.77 | 31.77 |
| 3742.5177 | FYN(N)SSYLNVQR Hex 7 HexNAc 6 | 29.788 | 0.024 | -4.050 | 8.816e+06 | 90 - 101 | 3 | 29.43 | 29.55 | 29.84 |
| 4170.7834 | FYFTPN(N)KTEDTIFLR Fuc 2 Hex 6 HexNAc 5 | 29.641 | 0.024 | -19.569 | 2.292e+07 | 66 - 81 | 1 | 37.38 | 37.38 | 37.53 |
| 3434.4733 | PVPITN(N)ATLDR Fuc 2 Hex 7 HexNAc 4 | 29.447 | 0.024 | -4.142 | 6.979e+05 | 27 - 38 | 15 | 73.69 | 73.92 | 85.45 |
| 3726.6696 | ANLVPVPITN(N)ATLDR Fuc 1 Hex 6 HexNAc 5 | 29.426 | 0.024 | -18.606 | 4.678e+06 | 23 - 38 | 1 | 41.25 | 41.25 | 41.42 |
| 3423.4966 | FYFTPN(N)KTEDTIFLR Fuc 1 Hex 3 HexNAc 3 Neu5Ac 1 | 28.817 | 0.025 | -19.037 | 1.523e+07 | 66 - 81 | 2 | 38.36 | 38.36 | 38.53 |
| 7813.3370 | WFYIASAFRNEEYNKSVQEIQATFFYFTPN(N)K Fuc 5 Hex 10 HexNAc 8 | 28.593 | 0.028 | -4.675 | 2.605e+07 | 42 - 73 | 1 | 54.46 | 54.46 | 54.80 |
| 3977.7207 | LVPVPITN(N)ATLDR Hex 6 HexNAc 5 Neu5Ac 2 | 28.388 | 0.028 | -4.809 | 9.736e+06 | 25 - 38 | 2 | 42.11 | 42.45 | 42.62 |
| 5002.9701 | QNQC(C)FYN(N)SSYLNVQR Hex 9 HexNAc 8 | 28.136 | 0.029 | -20.942 | 1.006e+07 | 86 - 101 | 1 | 37.70 | 37.70 | 37.86 |
| 3256.4532 | LVPVPITN(N)ATLDR Fuc 1 Hex 8 HexNAc 2 | 27.843 | 0.031 | 14.551 | 3.432e+07 | 25 - 38 | 2 | 42.45 | 42.79 | 43.13 |
| 3905.6872 | PVPITN(N)ATLDR Fuc 1 Hex 4 HexNAc 8 Neu5Ac 1 | 27.779 | 0.031 | -19.579 | 1.893e+06 | 27 - 38 | 2 | 43.47 | 43.47 | 43.64 |
| 3612.5861 | LVPVPITN(N)ATLDR Hex 5 HexNAc 4 Neu5Ac 2 | 27.642 | 0.031 | -20.134 | 2.717e+06 | 25 - 38 | 2 | 42.79 | 42.79 | 42.96 |
| 3934.6802 | FYFTPN(N)KTEDTIFLR Fuc 2 Hex 4 HexNAc 4 Neu5Ac 1 | 27.574 | 0.033 | -18.612 | 5.093e+07 | 66 - 81 | 1 | 37.70 | 37.70 | 37.86 |
| 5368.1179 | QNQC(C)FYN(N)SSYLNVQR Fuc 5 Hex 8 HexNAc 7 | 27.158 | 0.037 | -19.637 | 3.416e+06 | 86 - 101 | 1 | 32.12 | 32.12 | 32.12 |
| 3798.6099 | PVPITN(N)ATLDR Hex 8 HexNAc 5 Neu5Ac 1 | 27.078 | 0.037 | -18.819 | 6.146e+07 | 27 - 38 | 1 | 36.03 | 36.20 | 36.20 |
| 4321.9328 | LVPVPITN(N)ATLDR Fuc 3 Hex 4 HexNAc 9 | 26.841 | 0.037 | 15.403 | 2.693e+07 | 25 - 38 | 7 | 63.70 | 63.87 | 65.07 |
| 5904.5400 | SVQEIQATFFYFTPN(N)KTEDTIFLR Fuc 2 Hex 8 HexNAc 7 | 26.688 | 0.037 | -4.533 | 6.113e+06 | 57 - 81 | 1 | 61.65 | 61.82 | 61.99 |
| 4288.6693 | QN(D)QC(C)FYN(N)SSYLNVQR Fuc 2 Hex 6 HexNAc 4 Neu5Ac 1 | 26.569 | 0.037 | 8.082 | 1.590e+08 | 86 - 101 | 2 | 38.71 | 39.56 | 39.72 |
| 3595.4522 | YN(N)SSYLNVQR Hex 7 HexNAc 6 | 26.201 | 0.037 | -4.684 | 4.699e+06 | 91 - 101 | 2 | 22.52 | 22.61 | 22.83 |
| 4336.9448 | LVPVPITN(N)ATLDR Fuc 5 Hex 3 HexNAc 7 Neu5Ac 1 | 26.085 | 0.037 | 9.911 | 3.156e+07 | 25 - 38 | 7 | 59.93 | 61.30 | 61.48 |
| 4164.6779 | FYN(N)SSYLNVQR Fuc 4 Hex 6 HexNAc 6 | 26.042 | 0.038 | -3.000 | 7.095e+07 | 90 - 101 | 3 | 41.25 | 41.25 | 41.76 |
| 3826.7857 | QIPLC(C)ANLVPVPITN(N)ATLDR Hex 5 HexNAc 4 | 26.006 | 0.038 | 13.624 | 5.640e+07 | 18 - 38 | 3 | 62.85 | 63.19 | 63.70 |
| 3869.6711 | C(C)ANLVPVPITN(N)ATLDR Hex 5 HexNAc 5 Neu5Ac 1 | 25.660 | 0.039 | 15.201 | 1.749e+08 | 22 - 38 | 5 | 52.41 | 52.58 | 53.27 |
| 6342.6973 | SVQEIQATFFYFTPN(N)KTEDTIFLR Hex 10 HexNAc 9 | 25.513 | 0.042 | -5.815 | 6.204e+06 | 57 - 81 | 1 | 61.13 | 61.30 | 61.48 |
| 3432.3889 | ATFFYFTPN(N)K Fuc 2 Hex 8 HexNAc 3 | 25.196 | 0.045 | -18.346 | 8.769e+06 | 63 - 73 | 1 | 35.35 | 35.52 | 35.52 |
| 4478.8941 | FYFTPN(N)KTEDTIFLR Fuc 3 Hex 7 HexNAc 5 | 25.171 | 0.045 | -5.679 | 2.657e+07 | 66 - 81 | 1 | 35.86 | 36.03 | 36.20 |
| 4435.7480 | QN(D)QC(C)FYN(N)SSYLNVQR Hex 8 HexNAc 6 | 24.396 | 0.045 | 9.844 | 7.844e+07 | 86 - 101 | 2 | 38.71 | 39.22 | 39.72 |
| 3239.4261 | PVPITN(N)ATLDR Fuc 2 Hex 4 HexNAc 4 Neu5Ac 1 | 24.155 | 0.045 | 13.539 | 4.230e+07 | 27 - 38 | 4 | 42.62 | 42.79 | 43.30 |
| 3757.6658 | ANLVPVPITN(N)ATLDR Fuc 2 Hex 6 HexNAc 3 Neu5Ac 1 | 24.134 | 0.045 | 12.121 | 8.654e+07 | 23 - 38 | 3 | 41.93 | 42.11 | 42.62 |
| 4465.7619 | QNQC(C)FYN(N)SSYLNVQR Fuc 1 Hex 8 HexNAc 4 Neu5Ac 1 | 24.013 | 0.045 | -6.000 | 1.331e+07 | 86 - 101 | 1 | 34.33 | 34.33 | 34.67 |
| 5304.3235 | SVQEIQATFFYFTPN(N)KTEDTIFLR Fuc 4 Hex 5 HexNAc 5 | 23.744 | 0.046 | 12.858 | 2.236e+08 | 57 - 81 | 12 | 62.85 | 63.19 | 65.59 |
| 4213.8580 | C(C)AN(D)LVPVPITN(N)ATLDR Fuc 1 Hex 3 HexNAc 9 | 23.736 | 0.046 | 11.222 | 1.329e+08 | 22 - 38 | 4 | 62.51 | 62.68 | 63.19 |
| 5423.3371 | SVQEIQATFFYFTPN(N)KTEDTIFLR Hex 7 HexNAc 4 Neu5Ac 2 | 23.640 | 0.049 | -4.042 | 2.579e+07 | 57 - 81 | 1 | 63.36 | 63.36 | 63.70 |
| 3230.3156 | YN(N)SSYLNVQR Hex 6 HexNAc 5 | 23.478 | 0.049 | 12.209 | 7.831e+06 | 91 - 101 | 3 | 22.97 | 23.05 | 23.42 |
| 4163.7386 | PVPITN(N)ATLDR Hex 9 HexNAc 6 Neu5Ac 1 | 23.440 | 0.049 | -5.280 | 1.084e+07 | 27 - 38 | 1 | 35.69 | 35.86 | 36.03 |
| 3799.5460 | FYN(N)SSYLNVQR Fuc 4 Hex 5 HexNAc 5 | 23.255 | 0.049 | -19.140 | 2.580e+07 | 90 - 101 | 1 | 41.76 | 41.76 | 41.93 |

### YFTPN(N)K Hex 6 HexNAc 5

**Mass:**  2756.0958

(2.698e-06)

**Charge States:** 2, 3

**Start Position**: 68

**End Position**: 74

| Profile Score | Tandem Score | TDA q-value | Volume | Spectrum Matches |
| --- | --- | --- | --- | --- |
| 13.7913 | 187.4984 | 0.0000 | 2.5647e+08 | 3 |

**Adduct Signal Fractions**

- **Unmodified**: 2.5647e+08

### YFTPN(N)K Hex 7 HexNAc 6

**Mass:**  3121.2328

(9.366e-07)

**Charge States:** 3

**Start Position**: 68

**End Position**: 74

| Profile Score | Tandem Score | TDA q-value | Volume | Spectrum Matches |
| --- | --- | --- | --- | --- |
| 13.7821 | 187.1777 | 0.0000 | 3.6487e+08 | 4 |

**Adduct Signal Fractions**

- **Unmodified**: 3.6487e+08

### FYFTPN(N)K Hex 7 HexNAc 6

**Mass:**  3268.2991

(1.211e-06)

**Charge States:** 3

**Start Position**: 67

**End Position**: 74

| Profile Score | Tandem Score | TDA q-value | Volume | Spectrum Matches |
| --- | --- | --- | --- | --- |
| 15.2914 | 184.4226 | 0.0000 | 1.1539e+08 | 8 |

**Adduct Signal Fractions**

- **Unmodified**: 1.1539e+08

### FYFTPN(N)K Hex 6 HexNAc 5

**Mass:**  2903.1666

(1.323e-06)

**Charge States:** 2, 3

**Start Position**: 67

**End Position**: 74

| Profile Score | Tandem Score | TDA q-value | Volume | Spectrum Matches |
| --- | --- | --- | --- | --- |
| 13.6363 | 183.3051 | 0.0000 | 8.9641e+07 | 14 |

**Adduct Signal Fractions**

- **Unmodified**: 8.9641e+07

### NEEYN(N)K Hex 5 HexNAc 4

**Mass:**  2417.9227

(9.089e-07)

**Charge States:** 2, 3

**Start Position**: 52

**End Position**: 58

| Profile Score | Tandem Score | TDA q-value | Volume | Spectrum Matches |
| --- | --- | --- | --- | --- |
| 11.2627 | 182.3559 | 0.0000 | 5.9499e+07 | 7 |

**Adduct Signal Fractions**

- **Unmodified**: 5.9499e+07

### NEEYN(N)K Fuc 1 Hex 6 HexNAc 5

**Mass:**  2929.1109

(9.414e-07)

**Charge States:** 3

**Start Position**: 52

**End Position**: 58

| Profile Score | Tandem Score | TDA q-value | Volume | Spectrum Matches |
| --- | --- | --- | --- | --- |
| -3.5151 | 181.8048 | 0.0000 | 2.0912e+07 | 7 |

**Adduct Signal Fractions**

- **Unmodified**: 2.0912e+07

### NEEYN(N)K Hex 6 HexNAc 5

**Mass:**  2783.0537

(6.698e-07)

**Charge States:** 2, 3

**Start Position**: 52

**End Position**: 58

| Profile Score | Tandem Score | TDA q-value | Volume | Spectrum Matches |
| --- | --- | --- | --- | --- |
| 13.0609 | 181.4648 | 0.0000 | 2.8035e+08 | 20 |

**Adduct Signal Fractions**

- **Unmodified**: 2.8035e+08

### SVQEIQATFFYFTPN(N)K Hex 6 HexNAc 5

**Mass:**  3906.6663

(1.457e-06)

**Charge States:** 3, 4, 5

**Start Position**: 58

**End Position**: 74

| Profile Score | Tandem Score | TDA q-value | Volume | Spectrum Matches |
| --- | --- | --- | --- | --- |
| 15.0761 | 174.2427 | 0.0000 | 1.9316e+09 | 41 |

**Adduct Signal Fractions**

- **Unmodified**: 1.9316e+09

### YFTPN(N)K Fuc 1 Hex 6 HexNAc 5

**Mass:**  2902.1555

(-5.692e-07)

**Charge States:** 3

**Start Position**: 68

**End Position**: 74

| Profile Score | Tandem Score | TDA q-value | Volume | Spectrum Matches |
| --- | --- | --- | --- | --- |
| -18.6323 | 174.1396 | 0.0000 | 3.7915e+07 | 1 |

**Adduct Signal Fractions**

- **Unmodified**: 3.7915e+07

### YFTPN(N)K Fuc 1 Hex 7 HexNAc 6

**Mass:**  3267.2884

(1.490e-06)

**Charge States:** 3

**Start Position**: 68

**End Position**: 74

| Profile Score | Tandem Score | TDA q-value | Volume | Spectrum Matches |
| --- | --- | --- | --- | --- |
| -4.6348 | 173.3706 | 0.0000 | 6.0483e+07 | 2 |

**Adduct Signal Fractions**

- **Unmodified**: 6.0483e+07

### YFTPN(N)K Hex 8 HexNAc 7

**Mass:**  3486.3642

(1.170e-06)

**Charge States:** 3

**Start Position**: 68

**End Position**: 74

| Profile Score | Tandem Score | TDA q-value | Volume | Spectrum Matches |
| --- | --- | --- | --- | --- |
| -20.3105 | 172.6064 | 0.0000 | 1.9350e+07 | 1 |

**Adduct Signal Fractions**

- **Unmodified**: 1.9350e+07

### SVQEIQATFFYFTPN(N)K Hex 8 HexNAc 7

**Mass:**  4636.9357

(2.165e-06)

**Charge States:** 3, 4, 5

**Start Position**: 58

**End Position**: 74

| Profile Score | Tandem Score | TDA q-value | Volume | Spectrum Matches |
| --- | --- | --- | --- | --- |
| 13.5319 | 172.3604 | 0.0000 | 3.0643e+08 | 7 |

**Adduct Signal Fractions**

- **Unmodified**: 3.0643e+08

### YFTPN(N)K Hex 5 HexNAc 4

**Mass:**  2392.9588

(8.351e-04)

**Start Position**: 68

**End Position**: 74

| Profile Score | Tandem Score | TDA q-value | Volume | Spectrum Matches |
| --- | --- | --- | --- | --- |
| 0.0000 | 167.8728 | 0.0000 | 0.0000e+00 | 1 |

Adduct:

No Chromatogram Found

### SVQEIQATFFYFTPN(N)K Fuc 1 Hex 7 HexNAc 6

**Mass:**  4417.8684

(1.140e-06)

**Charge States:** 3, 4

**Start Position**: 58

**End Position**: 74

| Profile Score | Tandem Score | TDA q-value | Volume | Spectrum Matches |
| --- | --- | --- | --- | --- |
| 13.7613 | 167.6039 | 0.0000 | 4.9742e+08 | 13 |

**Adduct Signal Fractions**

- **Unmodified**: 4.9742e+08

### SVQEIQATFFYFTPN(N)K Fuc 1 Hex 6 HexNAc 5

**Mass:**  4052.7266

(1.801e-06)

**Charge States:** 3, 4

**Start Position**: 58

**End Position**: 74

| Profile Score | Tandem Score | TDA q-value | Volume | Spectrum Matches |
| --- | --- | --- | --- | --- |
| 13.6826 | 166.8947 | 0.0000 | 4.1727e+08 | 13 |

**Adduct Signal Fractions**

- **Unmodified**: 4.1727e+08

### YFTPN(N)K Hex 6 HexNAc 5

**Mass:**  2757.1032

(3.660e-04)

**Start Position**: 68

**End Position**: 74

| Profile Score | Tandem Score | TDA q-value | Volume | Spectrum Matches |
| --- | --- | --- | --- | --- |
| 0.0000 | 166.7052 | 0.0000 | 0.0000e+00 | 1 |

Adduct:

No Chromatogram Found

### YFTPN(N)K Hex 5 HexNAc 4

**Mass:**  2390.9698

(-1.680e-06)

**Charge States:** 2

**Start Position**: 68

**End Position**: 74

| Profile Score | Tandem Score | TDA q-value | Volume | Spectrum Matches |
| --- | --- | --- | --- | --- |
| -20.5840 | 166.0494 | 0.0000 | 1.8277e+07 | 2 |

**Adduct Signal Fractions**

- **Unmodified**: 1.8277e+07

### SVQEIQATFFYFTPN(N)K Hex 5 HexNAc 4

**Mass:**  3541.5331

(1.562e-06)

**Charge States:** 3, 4

**Start Position**: 58

**End Position**: 74

| Profile Score | Tandem Score | TDA q-value | Volume | Spectrum Matches |
| --- | --- | --- | --- | --- |
| 13.8719 | 165.6998 | 0.0000 | 2.4128e+08 | 12 |

**Adduct Signal Fractions**

- **Unmodified**: 2.4128e+08

### ATFFYFTPN(N)K Hex 6 HexNAc 5

**Mass:**  3222.3231

(1.682e-06)

**Charge States:** 3

**Start Position**: 64

**End Position**: 74

| Profile Score | Tandem Score | TDA q-value | Volume | Spectrum Matches |
| --- | --- | --- | --- | --- |
| -3.1395 | 164.2631 | 0.0000 | 3.7834e+07 | 2 |

**Adduct Signal Fractions**

- **Unmodified**: 3.7834e+07

### FYFTPN(N)K Fuc 1 Hex 6 HexNAc 5

**Mass:**  3049.2259

(1.966e-06)

**Charge States:** 3

**Start Position**: 67

**End Position**: 74

| Profile Score | Tandem Score | TDA q-value | Volume | Spectrum Matches |
| --- | --- | --- | --- | --- |
| 14.1209 | 163.9755 | 0.0345 | 1.5895e+07 | 3 |

**Adduct Signal Fractions**

- **Unmodified**: 1.5895e+07

### SVQEIQATFFYFTPN(N)K Hex 7 HexNAc 6

**Mass:**  4271.8107

(1.996e-06)

**Charge States:** 3, 4, 5

**Start Position**: 58

**End Position**: 74

| Profile Score | Tandem Score | TDA q-value | Volume | Spectrum Matches |
| --- | --- | --- | --- | --- |
| 15.0169 | 163.2087 | 0.0000 | 2.3057e+09 | 48 |

**Adduct Signal Fractions**

- **Unmodified**: 2.3057e+09

### FFYFTPN(N)K Hex 6 HexNAc 5

**Mass:**  3050.2337

(7.933e-07)

**Charge States:** 3

**Start Position**: 66

**End Position**: 74

| Profile Score | Tandem Score | TDA q-value | Volume | Spectrum Matches |
| --- | --- | --- | --- | --- |
| -19.0523 | 163.0275 | 0.0345 | 2.0923e+07 | 2 |

**Adduct Signal Fractions**

- **Unmodified**: 2.0923e+07

### FFYFTPN(N)K Hex 7 HexNAc 6

**Mass:**  3415.3694

(2.247e-06)

**Charge States:** 3

**Start Position**: 66

**End Position**: 74

| Profile Score | Tandem Score | TDA q-value | Volume | Spectrum Matches |
| --- | --- | --- | --- | --- |
| -2.6190 | 161.0261 | 0.0345 | 3.5746e+07 | 2 |

**Adduct Signal Fractions**

- **Unmodified**: 3.5746e+07

### LVPVPITN(N)ATLDR Hex 6 HexNAc 5

**Mass:**  3395.5310

(9.790e-07)

**Charge States:** 2, 3, 4

**Start Position**: 26

**End Position**: 39

| Profile Score | Tandem Score | TDA q-value | Volume | Spectrum Matches |
| --- | --- | --- | --- | --- |
| 13.3053 | 158.5346 | 0.0000 | 1.5415e+10 | 542 |

**Adduct Signal Fractions**

- **Unmodified**: 1.5415e+10

### LVPVPITN(N)ATLDR Hex 5 HexNAc 4

**Mass:**  3030.3944

(1.378e-06)

**Charge States:** 2, 3, 4

**Start Position**: 26

**End Position**: 39

| Profile Score | Tandem Score | TDA q-value | Volume | Spectrum Matches |
| --- | --- | --- | --- | --- |
| 15.2315 | 157.4253 | 0.0000 | 1.0508e+09 | 8 |

**Adduct Signal Fractions**

- **Unmodified**: 1.0508e+09

### ATFFYFTPN(N)K Hex 7 HexNAc 6

**Mass:**  3587.4560

(1.980e-06)

**Charge States:** 3

**Start Position**: 64

**End Position**: 74

| Profile Score | Tandem Score | TDA q-value | Volume | Spectrum Matches |
| --- | --- | --- | --- | --- |
| 13.1990 | 156.1807 | 0.0000 | 6.6008e+07 | 3 |

**Adduct Signal Fractions**

- **Unmodified**: 6.6008e+07

### SVQEIQATFFYFTPN(N)K Fuc 1 Hex 8 HexNAc 7

**Mass:**  4782.9919

(1.913e-06)

**Charge States:** 3, 4

**Start Position**: 58

**End Position**: 74

| Profile Score | Tandem Score | TDA q-value | Volume | Spectrum Matches |
| --- | --- | --- | --- | --- |
| 12.3328 | 156.1457 | 0.0000 | 6.2981e+07 | 5 |

**Adduct Signal Fractions**

- **Unmodified**: 6.2981e+07

### SVQEIQATFFYFTPN(N)K Fuc 1 Hex 8 HexNAc 4 Neu5Ac 1

**Mass:**  4464.8591

(4.341e-06)

**Charge States:** 3, 4

**Start Position**: 58

**End Position**: 74

| Profile Score | Tandem Score | TDA q-value | Volume | Spectrum Matches |
| --- | --- | --- | --- | --- |
| 13.7119 | 154.2645 | 0.0000 | 5.4061e+07 | 4 |

**Adduct Signal Fractions**

- **Unmodified**: 5.4061e+07

### SVQEIQATFFYFTPN(N)K Fuc 2 Hex 7 HexNAc 6

**Mass:**  4563.9211

(2.633e-06)

**Charge States:** 3, 4

**Start Position**: 58

**End Position**: 74

| Profile Score | Tandem Score | TDA q-value | Volume | Spectrum Matches |
| --- | --- | --- | --- | --- |
| -3.5722 | 153.8604 | 0.0000 | 7.0037e+07 | 4 |

**Adduct Signal Fractions**

- **Unmodified**: 7.0037e+07

### LVPVPITN(N)ATLDR Fuc 1 Hex 6 HexNAc 5

**Mass:**  3541.5906

(3.243e-06)

**Charge States:** 3, 4

**Start Position**: 26

**End Position**: 39

| Profile Score | Tandem Score | TDA q-value | Volume | Spectrum Matches |
| --- | --- | --- | --- | --- |
| 13.0203 | 152.7327 | 0.0000 | 3.6118e+09 | 23 |

**Adduct Signal Fractions**

- **Unmodified**: 3.6118e+09

### SVQEIQATFFYFTPN(N)KTEDTIFLR Hex 6 HexNAc 5

**Mass:**  4882.1769

(1.804e-06)

**Charge States:** 3, 4, 5

**Start Position**: 58

**End Position**: 82

| Profile Score | Tandem Score | TDA q-value | Volume | Spectrum Matches |
| --- | --- | --- | --- | --- |
| 12.8385 | 143.2387 | 0.0000 | 5.1862e+08 | 17 |

**Adduct Signal Fractions**

- **Unmodified**: 5.1862e+08

### ATFFYFTPN(N)K Fuc 1 Hex 6 HexNAc 5

**Mass:**  3368.3750

(3.047e-07)

**Charge States:** 3

**Start Position**: 64

**End Position**: 74

| Profile Score | Tandem Score | TDA q-value | Volume | Spectrum Matches |
| --- | --- | --- | --- | --- |
| -20.1613 | 142.3912 | 0.0000 | 5.2294e+06 | 1 |

**Adduct Signal Fractions**

- **Unmodified**: 5.2294e+06

### SVQEIQATFFYFTPN(N)K Hex 9 HexNAc 8

**Mass:**  5002.0646

(2.520e-06)

**Charge States:** 3, 4, 5

**Start Position**: 58

**End Position**: 74

| Profile Score | Tandem Score | TDA q-value | Volume | Spectrum Matches |
| --- | --- | --- | --- | --- |
| 11.8853 | 141.3428 | 0.0000 | 5.8746e+07 | 2 |

**Adduct Signal Fractions**

- **Unmodified**: 5.8746e+07

### SVQEIQATFFYFTPN(N)K Fuc 5 Hex 7 HexNAc 6

**Mass:**  5002.0646

(-2.508e-06)

**Charge States:** 3, 4, 5

**Start Position**: 58

**End Position**: 74

| Profile Score | Tandem Score | TDA q-value | Volume | Spectrum Matches |
| --- | --- | --- | --- | --- |
| 11.8853 | 141.3428 | 0.0000 | 5.8746e+07 | 2 |

**Adduct Signal Fractions**

- **Unmodified**: 5.8746e+07

### LVPVPITN(N)ATLDR Fuc 1 Hex 7 HexNAc 3 Neu5Ac 1

**Mass:**  3588.5864

(4.618e-06)

**Charge States:** 3

**Start Position**: 26

**End Position**: 39

| Profile Score | Tandem Score | TDA q-value | Volume | Spectrum Matches |
| --- | --- | --- | --- | --- |
| -4.3205 | 140.3273 | 0.0000 | 5.0204e+07 | 1 |

**Adduct Signal Fractions**

- **Unmodified**: 5.0204e+07

### SVQEIQATFFYFTPN(N)KTEDTIFLR Fuc 1 Hex 6 HexNAc 5

**Mass:**  5028.2321

(2.224e-06)

**Charge States:** 4, 5

**Start Position**: 58

**End Position**: 82

| Profile Score | Tandem Score | TDA q-value | Volume | Spectrum Matches |
| --- | --- | --- | --- | --- |
| 12.5696 | 140.0613 | 0.0000 | 1.1908e+08 | 7 |

**Adduct Signal Fractions**

- **Unmodified**: 1.1908e+08

### SVQEIQATFFYFTPN(N)K Hex 7 HexNAc 4 Neu5Ac 2

**Mass:**  4447.8332

(3.076e-06)

**Charge States:** 3, 4

**Start Position**: 58

**End Position**: 74

| Profile Score | Tandem Score | TDA q-value | Volume | Spectrum Matches |
| --- | --- | --- | --- | --- |
| 13.4609 | 139.2767 | 0.0000 | 1.1966e+08 | 7 |

**Adduct Signal Fractions**

- **Unmodified**: 1.1966e+08

### LVPVPITN(N)ATLDR Fuc 2 Hex 6 HexNAc 5

**Mass:**  3687.6531

(4.047e-06)

**Charge States:** 3, 4

**Start Position**: 26

**End Position**: 39

| Profile Score | Tandem Score | TDA q-value | Volume | Spectrum Matches |
| --- | --- | --- | --- | --- |
| -18.3729 | 137.2544 | 0.0000 | 4.9995e+07 | 2 |

**Adduct Signal Fractions**

- **Unmodified**: 4.9995e+07

### FYFTPN(N)KTEDTIFLR Hex 7 HexNAc 6

**Mass:**  4243.8044

(1.801e-06)

**Charge States:** 3, 4

**Start Position**: 67

**End Position**: 82

| Profile Score | Tandem Score | TDA q-value | Volume | Spectrum Matches |
| --- | --- | --- | --- | --- |
| 13.3454 | 136.4847 | 0.0000 | 1.9613e+08 | 11 |

**Adduct Signal Fractions**

- **Unmodified**: 1.9613e+08

### YFTPN(N)KTEDTIFLR Hex 6 HexNAc 5

**Mass:**  3731.6010

(1.028e-06)

**Charge States:** 3, 4

**Start Position**: 68

**End Position**: 82

| Profile Score | Tandem Score | TDA q-value | Volume | Spectrum Matches |
| --- | --- | --- | --- | --- |
| 10.8882 | 135.4603 | 0.0000 | 1.0478e+09 | 8 |

**Adduct Signal Fractions**

- **Unmodified**: 1.0478e+09

### LVPVPITN(N)ATLDR Hex 7 HexNAc 6

**Mass:**  3760.6657

(1.465e-06)

**Charge States:** 3, 4

**Start Position**: 26

**End Position**: 39

| Profile Score | Tandem Score | TDA q-value | Volume | Spectrum Matches |
| --- | --- | --- | --- | --- |
| 15.6965 | 135.2403 | 0.0000 | 6.9078e+08 | 6 |

**Adduct Signal Fractions**

- **Unmodified**: 6.9078e+08

### SVQEIQATFFYFTPN(N)KTEDTIFLR Hex 7 HexNAc 6

**Mass:**  5247.3112

(2.347e-06)

**Charge States:** 3, 4, 5

**Start Position**: 58

**End Position**: 82

| Profile Score | Tandem Score | TDA q-value | Volume | Spectrum Matches |
| --- | --- | --- | --- | --- |
| 13.4092 | 134.3931 | 0.0000 | 1.2351e+09 | 25 |

**Adduct Signal Fractions**

- **Unmodified**: 1.2351e+09

### TPN(N)KTEDTIFLR Hex 7 HexNAc 6

**Mass:**  3786.6015

(1.251e-06)

**Charge States:** 3, 4

**Start Position**: 70

**End Position**: 82

| Profile Score | Tandem Score | TDA q-value | Volume | Spectrum Matches |
| --- | --- | --- | --- | --- |
| 13.4523 | 133.7153 | 0.0000 | 5.2289e+07 | 8 |

**Adduct Signal Fractions**

- **Unmodified**: 5.2289e+07

### VPVPITN(N)ATLDR Hex 6 HexNAc 5

**Mass:**  3282.4425

(1.445e-06)

**Charge States:** 3

**Start Position**: 27

**End Position**: 39

| Profile Score | Tandem Score | TDA q-value | Volume | Spectrum Matches |
| --- | --- | --- | --- | --- |
| 19.6560 | 132.2431 | 0.0000 | 1.0457e+07 | 4 |

**Adduct Signal Fractions**

- **Unmodified**: 1.0457e+07

### YFTPN(N)KTEDTIFLR Fuc 1 Hex 6 HexNAc 5

**Mass:**  3877.6587

(3.851e-06)

**Charge States:** 3, 4

**Start Position**: 68

**End Position**: 82

| Profile Score | Tandem Score | TDA q-value | Volume | Spectrum Matches |
| --- | --- | --- | --- | --- |
| -3.7825 | 132.0920 | 0.0000 | 2.4626e+08 | 4 |

**Adduct Signal Fractions**

- **Unmodified**: 2.4626e+08

### LVPVPITN(N)ATLDR Fuc 1 Hex 7 HexNAc 6

**Mass:**  3906.7176

(3.040e-06)

**Charge States:** 3, 4

**Start Position**: 26

**End Position**: 39

| Profile Score | Tandem Score | TDA q-value | Volume | Spectrum Matches |
| --- | --- | --- | --- | --- |
| -3.5221 | 132.0344 | 0.0000 | 2.1300e+08 | 4 |

**Adduct Signal Fractions**

- **Unmodified**: 2.1300e+08

### LVPVPITN(N)ATLDR Hex 8 HexNAc 7

**Mass:**  4125.7908

(7.594e-07)

**Charge States:** 3, 4

**Start Position**: 26

**End Position**: 39

| Profile Score | Tandem Score | TDA q-value | Volume | Spectrum Matches |
| --- | --- | --- | --- | --- |
| -3.1000 | 130.4938 | 0.0000 | 1.1122e+08 | 4 |

**Adduct Signal Fractions**

- **Unmodified**: 1.1122e+08

### ATFFYFTPN(N)K Fuc 5 Hex 5 HexNAc 8 Neu5Ac 1

**Mass:**  4690.8508

(-7.222e-06)

**Charge States:** 4

**Start Position**: 64

**End Position**: 74

| Profile Score | Tandem Score | TDA q-value | Volume | Spectrum Matches |
| --- | --- | --- | --- | --- |
| -5.3701 | 130.1318 | 0.0000 | 1.3145e+07 | 2 |

**Adduct Signal Fractions**

- **Unmodified**: 1.3145e+07

### TPN(N)KTEDTIFLR Hex 6 HexNAc 5

**Mass:**  3421.4694

(1.188e-06)

**Charge States:** 3, 4

**Start Position**: 70

**End Position**: 82

| Profile Score | Tandem Score | TDA q-value | Volume | Spectrum Matches |
| --- | --- | --- | --- | --- |
| 13.3440 | 127.4916 | 0.0000 | 3.0012e+07 | 6 |

**Adduct Signal Fractions**

- **Unmodified**: 3.0012e+07

### SVQEIQATFFYFTPN(N)K Fuc 4 Hex 5 HexNAc 5

**Mass:**  4328.8259

(-3.097e-06)

**Charge States:** 3, 4

**Start Position**: 58

**End Position**: 74

| Profile Score | Tandem Score | TDA q-value | Volume | Spectrum Matches |
| --- | --- | --- | --- | --- |
| 11.7894 | 127.1977 | 0.0000 | 5.1379e+08 | 25 |

**Adduct Signal Fractions**

- **Unmodified**: 5.1379e+08

### SVQEIQATFFYFTPN(N)K Fuc 1 Hex 7 HexNAc 4 Neu5Ac 2

**Mass:**  4593.8942

(2.518e-06)

**Charge States:** 3, 4

**Start Position**: 58

**End Position**: 74

| Profile Score | Tandem Score | TDA q-value | Volume | Spectrum Matches |
| --- | --- | --- | --- | --- |
| -20.0711 | 126.2293 | 0.0000 | 1.2312e+07 | 1 |

**Adduct Signal Fractions**

- **Unmodified**: 1.2312e+07

### YFTPN(N)KTEDTIFLR Hex 7 HexNAc 6

**Mass:**  4096.7429

(1.398e-06)

**Charge States:** 3, 4

**Start Position**: 68

**End Position**: 82

| Profile Score | Tandem Score | TDA q-value | Volume | Spectrum Matches |
| --- | --- | --- | --- | --- |
| 13.1841 | 125.6926 | 0.0000 | 2.0690e+09 | 21 |

**Adduct Signal Fractions**

- **Unmodified**: 2.0690e+09

### YFTPN(N)KTEDTIFLR Fuc 1 Hex 7 HexNAc 6

**Mass:**  4242.7945

(3.127e-06)

**Charge States:** 3, 4

**Start Position**: 68

**End Position**: 82

| Profile Score | Tandem Score | TDA q-value | Volume | Spectrum Matches |
| --- | --- | --- | --- | --- |
| -6.3814 | 124.6881 | 0.0000 | 2.1671e+08 | 6 |

**Adduct Signal Fractions**

- **Unmodified**: 2.1671e+08

### YFTPN(N)KTEDTIFLR Fuc 1 Hex 6 HexNAc 5

**Mass:**  3879.6742

(5.207e-04)

**Start Position**: 68

**End Position**: 82

| Profile Score | Tandem Score | TDA q-value | Volume | Spectrum Matches |
| --- | --- | --- | --- | --- |
| 0.0000 | 124.2960 | 0.0000 | 0.0000e+00 | 1 |

Adduct:

No Chromatogram Found

### SVQEIQATFFYFTPN(N)K Hex 6 HexNAc 6

**Mass:**  4109.7471

(1.630e-06)

**Charge States:** 3, 4

**Start Position**: 58

**End Position**: 74

| Profile Score | Tandem Score | TDA q-value | Volume | Spectrum Matches |
| --- | --- | --- | --- | --- |
| 12.2444 | 124.2056 | 0.0000 | 5.2821e+07 | 3 |

**Adduct Signal Fractions**

- **Unmodified**: 5.2821e+07

### C(C)ANLVPVPITN(N)ATLDR Hex 6 HexNAc 5

**Mass:**  3740.6409

(2.044e-06)

**Charge States:** 3, 4

**Start Position**: 23

**End Position**: 39

| Profile Score | Tandem Score | TDA q-value | Volume | Spectrum Matches |
| --- | --- | --- | --- | --- |
| 15.1132 | 124.1624 | 0.0000 | 1.8146e+08 | 5 |

**Adduct Signal Fractions**

- **Unmodified**: 1.8146e+08

### YFTPN(N)KTEDTIFLR Hex 5 HexNAc 4

**Mass:**  3366.4682

(9.788e-07)

**Charge States:** 3, 4

**Start Position**: 68

**End Position**: 82

| Profile Score | Tandem Score | TDA q-value | Volume | Spectrum Matches |
| --- | --- | --- | --- | --- |
| -4.4539 | 124.0236 | 0.0000 | 7.3776e+07 | 1 |

**Adduct Signal Fractions**

- **Unmodified**: 7.3776e+07

### QNQC(C)FYN(N)SSYLNVQR Hex 6 HexNAc 5

**Mass:**  3907.5825

(1.506e-06)

**Charge States:** 3, 4

**Start Position**: 87

**End Position**: 102

| Profile Score | Tandem Score | TDA q-value | Volume | Spectrum Matches |
| --- | --- | --- | --- | --- |
| 10.2967 | 123.8054 | 0.0000 | 6.4631e+08 | 5 |

**Adduct Signal Fractions**

- **Unmodified**: 6.4631e+08

### NLVPVPITN(N)ATLDR Hex 6 HexNAc 5

**Mass:**  3509.5735

(1.876e-06)

**Charge States:** 3

**Start Position**: 25

**End Position**: 39

| Profile Score | Tandem Score | TDA q-value | Volume | Spectrum Matches |
| --- | --- | --- | --- | --- |
| -6.3055 | 123.7438 | 0.0000 | 3.4737e+07 | 3 |

**Adduct Signal Fractions**

- **Unmodified**: 3.4737e+07

### YFTPN(N)KTEDTIFLR Hex 7 HexNAc 7

**Mass:**  4299.8137

(3.730e-06)

**Charge States:** 4

**Start Position**: 68

**End Position**: 82

| Profile Score | Tandem Score | TDA q-value | Volume | Spectrum Matches |
| --- | --- | --- | --- | --- |
| -5.0385 | 123.1778 | 0.0000 | 7.5670e+07 | 3 |

**Adduct Signal Fractions**

- **Unmodified**: 7.5670e+07

### QNQC(C)FYN(N)SSYLNVQR Fuc 1 Hex 6 HexNAc 5

**Mass:**  4053.6381

(1.911e-06)

**Charge States:** 3

**Start Position**: 87

**End Position**: 102

| Profile Score | Tandem Score | TDA q-value | Volume | Spectrum Matches |
| --- | --- | --- | --- | --- |
| -5.2916 | 121.1673 | 0.0000 | 5.0127e+07 | 2 |

**Adduct Signal Fractions**

- **Unmodified**: 5.0127e+07

### LVPVPITN(N)ATLDR Fuc 3 Hex 5 HexNAc 4

**Mass:**  3470.5978

(5.864e-04)

**Start Position**: 26

**End Position**: 39

| Profile Score | Tandem Score | TDA q-value | Volume | Spectrum Matches |
| --- | --- | --- | --- | --- |
| 0.0000 | 121.0841 | 0.0000 | 0.0000e+00 | 1 |

Adduct:

No Chromatogram Found

### QNQC(C)FYN(N)SSYLNVQR Fuc 1 Hex 7 HexNAc 6

**Mass:**  4418.7741

(1.064e-06)

**Charge States:** 3, 4

**Start Position**: 87

**End Position**: 102

| Profile Score | Tandem Score | TDA q-value | Volume | Spectrum Matches |
| --- | --- | --- | --- | --- |
| 7.3929 | 120.9048 | 0.0000 | 3.1826e+08 | 5 |

**Adduct Signal Fractions**

- **Unmodified**: 3.1826e+08

### LVPVPITN(N)ATLDR Hex 6 HexNAc 5 Neu5Ac 1

**Mass:**  3686.6281

(2.747e-06)

**Charge States:** 3, 4

**Start Position**: 26

**End Position**: 39

| Profile Score | Tandem Score | TDA q-value | Volume | Spectrum Matches |
| --- | --- | --- | --- | --- |
| 10.7733 | 120.5916 | 0.0000 | 2.6334e+07 | 1 |

**Adduct Signal Fractions**

- **Unmodified**: 2.6334e+07

### LVPVPITN(N)ATLDR Fuc 2 Hex 7 HexNAc 3

**Mass:**  3445.5298

(5.890e-04)

**Charge States:** 3

**Start Position**: 26

**End Position**: 39

| Profile Score | Tandem Score | TDA q-value | Volume | Spectrum Matches |
| --- | --- | --- | --- | --- |
| -23.1244 | 118.9456 | 0.0000 | 4.0434e+05 | 3 |

**Adduct Signal Fractions**

- **Unmodified**: 4.0434e+05

### ATFFYFTPN(N)K Fuc 1 Hex 7 HexNAc 6

**Mass:**  3733.5075

(3.254e-07)

**Charge States:** 3

**Start Position**: 64

**End Position**: 74

| Profile Score | Tandem Score | TDA q-value | Volume | Spectrum Matches |
| --- | --- | --- | --- | --- |
| -18.1818 | 118.6259 | 0.0000 | 6.8934e+06 | 1 |

**Adduct Signal Fractions**

- **Unmodified**: 6.8934e+06

### YFTPN(N)KTEDTIFLR Fuc 1 Hex 9 HexNAc 8

**Mass:**  4973.0651

(2.780e-06)

**Charge States:** 4

**Start Position**: 68

**End Position**: 82

| Profile Score | Tandem Score | TDA q-value | Volume | Spectrum Matches |
| --- | --- | --- | --- | --- |
| -19.1159 | 117.5660 | 0.0000 | 2.3952e+06 | 1 |

**Adduct Signal Fractions**

- **Unmodified**: 2.3952e+06

### YFTPN(N)KTEDTIFLR Hex 8 HexNAc 7

**Mass:**  4461.8659

(4.125e-06)

**Charge States:** 3, 4

**Start Position**: 68

**End Position**: 82

| Profile Score | Tandem Score | TDA q-value | Volume | Spectrum Matches |
| --- | --- | --- | --- | --- |
| 13.5425 | 116.5158 | 0.0000 | 1.7748e+08 | 3 |

**Adduct Signal Fractions**

- **Unmodified**: 1.7748e+08

### YFTPN(N)KTEDTIFLR Fuc 2 Hex 7 HexNAc 6

**Mass:**  4389.8630

(2.307e-04)

**Charge States:** 3, 4

**Start Position**: 68

**End Position**: 82

| Profile Score | Tandem Score | TDA q-value | Volume | Spectrum Matches |
| --- | --- | --- | --- | --- |
| -6.1687 | 115.7301 | 0.0000 | 3.4075e+07 | 8 |

**Adduct Signal Fractions**

- **Unmodified**: 3.4075e+07

### NLVPVPITN(N)ATLDR Fuc 1 Hex 6 HexNAc 4

**Mass:**  3452.5493

(1.158e-06)

**Charge States:** 3, 4

**Start Position**: 25

**End Position**: 39

| Profile Score | Tandem Score | TDA q-value | Volume | Spectrum Matches |
| --- | --- | --- | --- | --- |
| 16.1472 | 114.8358 | 0.0000 | 3.0777e+09 | 12 |

**Adduct Signal Fractions**

- **Unmodified**: 3.0777e+09

### NLVPVPITN(N)ATLDR Fuc 2 Hex 6 HexNAc 4

**Mass:**  3598.6045

(1.714e-06)

**Charge States:** 3, 4

**Start Position**: 25

**End Position**: 39

| Profile Score | Tandem Score | TDA q-value | Volume | Spectrum Matches |
| --- | --- | --- | --- | --- |
| 13.9118 | 114.2609 | 0.0000 | 8.8527e+08 | 5 |

**Adduct Signal Fractions**

- **Unmodified**: 8.8527e+08

### QNQC(C)FYN(N)SSYLNVQR Hex 8 HexNAc 7

**Mass:**  4637.8552

(3.327e-06)

**Charge States:** 3, 4

**Start Position**: 87

**End Position**: 102

| Profile Score | Tandem Score | TDA q-value | Volume | Spectrum Matches |
| --- | --- | --- | --- | --- |
| -5.9422 | 112.1135 | 0.0000 | 4.9061e+07 | 1 |

**Adduct Signal Fractions**

- **Unmodified**: 4.9061e+07

### SVQEIQATFFYFTPN(N)K Hex 7 HexNAc 7

**Mass:**  4474.8781

(2.386e-06)

**Charge States:** 3, 4

**Start Position**: 58

**End Position**: 74

| Profile Score | Tandem Score | TDA q-value | Volume | Spectrum Matches |
| --- | --- | --- | --- | --- |
| 10.9741 | 110.3211 | 0.0000 | 5.6775e+07 | 3 |

**Adduct Signal Fractions**

- **Unmodified**: 5.6775e+07

### LVPVPITN(N)ATLDR Hex 5 HexNAc 4

**Mass:**  3031.4194

(3.396e-04)

**Start Position**: 26

**End Position**: 39

| Profile Score | Tandem Score | TDA q-value | Volume | Spectrum Matches |
| --- | --- | --- | --- | --- |
| 0.0000 | 109.6621 | 0.0000 | 0.0000e+00 | 1 |

Adduct:

No Chromatogram Found

### FYFTPN(N)KTEDTIFLR Hex 6 HexNAc 5

**Mass:**  3878.6702

(1.690e-06)

**Charge States:** 3, 4

**Start Position**: 67

**End Position**: 82

| Profile Score | Tandem Score | TDA q-value | Volume | Spectrum Matches |
| --- | --- | --- | --- | --- |
| 13.1108 | 108.9941 | 0.0000 | 6.6773e+07 | 5 |

**Adduct Signal Fractions**

- **Unmodified**: 6.6773e+07

### PVPITN(N)ATLDR Hex 8 HexNAc 4 Neu5Ac 2

**Mass:**  3886.5697

(-2.281e-06)

**Charge States:** 3, 4

**Start Position**: 28

**End Position**: 39

| Profile Score | Tandem Score | TDA q-value | Volume | Spectrum Matches |
| --- | --- | --- | --- | --- |
| 7.6223 | 108.4049 | 0.0000 | 1.7847e+08 | 8 |

**Adduct Signal Fractions**

- **Unmodified**: 1.7847e+08

### QNQC(C)FYN(N)SSYLNVQR Hex 7 HexNAc 6

**Mass:**  4272.7110

(1.565e-06)

**Charge States:** 3, 4

**Start Position**: 87

**End Position**: 102

| Profile Score | Tandem Score | TDA q-value | Volume | Spectrum Matches |
| --- | --- | --- | --- | --- |
| 8.8828 | 108.2098 | 0.0000 | 1.0638e+09 | 4 |

**Adduct Signal Fractions**

- **Unmodified**: 1.0638e+09

### YFTPN(N)KTEDTIFLR Hex 7 HexNAc 5

**Mass:**  3895.6905

(5.241e-04)

**Start Position**: 68

**End Position**: 82

| Profile Score | Tandem Score | TDA q-value | Volume | Spectrum Matches |
| --- | --- | --- | --- | --- |
| 0.0000 | 107.7345 | 0.0000 | 0.0000e+00 | 1 |

Adduct:

No Chromatogram Found

### ANLVPVPITN(N)ATLDR Hex 6 HexNAc 5

**Mass:**  3580.6085

(1.909e-06)

**Charge States:** 3

**Start Position**: 24

**End Position**: 39

| Profile Score | Tandem Score | TDA q-value | Volume | Spectrum Matches |
| --- | --- | --- | --- | --- |
| -17.0772 | 105.1541 | 0.0000 | 1.4903e+07 | 1 |

**Adduct Signal Fractions**

- **Unmodified**: 1.4903e+07

### NLVPVPITN(N)ATLDR Fuc 1 Hex 7 HexNAc 5

**Mass:**  3817.6811

(3.566e-06)

**Charge States:** 3

**Start Position**: 25

**End Position**: 39

| Profile Score | Tandem Score | TDA q-value | Volume | Spectrum Matches |
| --- | --- | --- | --- | --- |
| -4.4440 | 104.3472 | 0.0000 | 8.9716e+07 | 2 |

**Adduct Signal Fractions**

- **Unmodified**: 8.9716e+07

### PVPITN(N)ATLDR Fuc 3 Hex 3 HexNAc 7

**Mass:**  3541.5844

(9.977e-06)

**Charge States:** 3, 4

**Start Position**: 28

**End Position**: 39

| Profile Score | Tandem Score | TDA q-value | Volume | Spectrum Matches |
| --- | --- | --- | --- | --- |
| 7.4128 | 103.6217 | 0.0000 | 2.0906e+07 | 6 |

**Adduct Signal Fractions**

- **Unmodified**: 2.0906e+07

### SVQEIQATFFYFTPN(N)K Fuc 3 Hex 4 HexNAc 5

**Mass:**  4020.7095

(-3.970e-06)

**Charge States:** 3, 4

**Start Position**: 58

**End Position**: 74

| Profile Score | Tandem Score | TDA q-value | Volume | Spectrum Matches |
| --- | --- | --- | --- | --- |
| -19.1308 | 103.6187 | 0.0000 | 9.8584e+06 | 2 |

**Adduct Signal Fractions**

- **Unmodified**: 9.8584e+06

### PVPITN(N)ATLDR Fuc 1 Hex 9 HexNAc 4 Neu5Ac 1

**Mass:**  3903.5916

(4.400e-07)

**Charge States:** 3

**Start Position**: 28

**End Position**: 39

| Profile Score | Tandem Score | TDA q-value | Volume | Spectrum Matches |
| --- | --- | --- | --- | --- |
| -18.2145 | 103.0546 | 0.0000 | 9.7902e+06 | 2 |

**Adduct Signal Fractions**

- **Unmodified**: 9.7902e+06

### SVQEIQATFFYFTPN(N)KTEDTIFLR Hex 5 HexNAc 4

**Mass:**  4517.0373

(1.424e-06)

**Charge States:** 4

**Start Position**: 58

**End Position**: 82

| Profile Score | Tandem Score | TDA q-value | Volume | Spectrum Matches |
| --- | --- | --- | --- | --- |
| -3.9298 | 101.8173 | 0.0000 | 1.9743e+07 | 2 |

**Adduct Signal Fractions**

- **Unmodified**: 1.9743e+07

### FTPN(N)KTEDTIFLR Hex 6 HexNAc 5

**Mass:**  3568.5393

(1.590e-06)

**Charge States:** 3, 4

**Start Position**: 69

**End Position**: 82

| Profile Score | Tandem Score | TDA q-value | Volume | Spectrum Matches |
| --- | --- | --- | --- | --- |
| -2.8936 | 97.3570 | 0.0000 | 6.9422e+07 | 4 |

**Adduct Signal Fractions**

- **Unmodified**: 6.9422e+07

### VPVPITN(N)ATLDR Fuc 1 Hex 6 HexNAc 5

**Mass:**  3428.5014

(1.393e-06)

**Charge States:** 3

**Start Position**: 27

**End Position**: 39

| Profile Score | Tandem Score | TDA q-value | Volume | Spectrum Matches |
| --- | --- | --- | --- | --- |
| -4.6777 | 96.8313 | 0.0000 | 2.2148e+06 | 2 |

**Adduct Signal Fractions**

- **Unmodified**: 2.2148e+06

### SVQEIQATFFYFTPN(N)KTEDTIFLR Fuc 1 Hex 7 HexNAc 6

**Mass:**  5393.3644

(1.445e-06)

**Charge States:** 4, 5

**Start Position**: 58

**End Position**: 82

| Profile Score | Tandem Score | TDA q-value | Volume | Spectrum Matches |
| --- | --- | --- | --- | --- |
| 13.0593 | 96.5508 | 0.0000 | 2.5527e+08 | 10 |

**Adduct Signal Fractions**

- **Unmodified**: 2.5527e+08

### TPN(N)KTEDTIFLR Fuc 1 Hex 6 HexNAc 5

**Mass:**  3567.5286

(1.573e-06)

**Charge States:** 3

**Start Position**: 70

**End Position**: 82

| Profile Score | Tandem Score | TDA q-value | Volume | Spectrum Matches |
| --- | --- | --- | --- | --- |
| -4.4299 | 96.3067 | 0.0000 | 4.8148e+06 | 3 |

**Adduct Signal Fractions**

- **Unmodified**: 4.8148e+06

### YFTPN(N)KTEDTIFLR Hex 9 HexNAc 8

**Mass:**  4826.9973

(2.824e-06)

**Charge States:** 3, 4

**Start Position**: 68

**End Position**: 82

| Profile Score | Tandem Score | TDA q-value | Volume | Spectrum Matches |
| --- | --- | --- | --- | --- |
| 11.6786 | 95.0554 | 0.0000 | 4.9663e+07 | 2 |

**Adduct Signal Fractions**

- **Unmodified**: 4.9663e+07

### FTPN(N)KTEDTIFLR Hex 7 HexNAc 6

**Mass:**  3933.6699

(1.832e-06)

**Charge States:** 3, 4

**Start Position**: 69

**End Position**: 82

| Profile Score | Tandem Score | TDA q-value | Volume | Spectrum Matches |
| --- | --- | --- | --- | --- |
| 14.3132 | 94.0461 | 0.0000 | 1.3098e+08 | 6 |

**Adduct Signal Fractions**

- **Unmodified**: 1.3098e+08

### TPN(N)KTEDTIFLR Hex 6 HexNAc 5

**Mass:**  3422.4748

(2.951e-04)

**Start Position**: 70

**End Position**: 82

| Profile Score | Tandem Score | TDA q-value | Volume | Spectrum Matches |
| --- | --- | --- | --- | --- |
| 0.0000 | 93.8757 | 0.0000 | 0.0000e+00 | 1 |

Adduct:

No Chromatogram Found

### FYFTPN(N)KTEDTIFLR Hex 8 HexNAc 7

**Mass:**  4608.9318

(4.053e-07)

**Charge States:** 3, 4

**Start Position**: 67

**End Position**: 82

| Profile Score | Tandem Score | TDA q-value | Volume | Spectrum Matches |
| --- | --- | --- | --- | --- |
| 12.8087 | 92.7007 | 0.0000 | 2.2892e+07 | 4 |

**Adduct Signal Fractions**

- **Unmodified**: 2.2892e+07

### PVPITN(N)ATLDR Fuc 2 Hex 8 HexNAc 4

**Mass:**  3597.5217

(2.807e-04)

**Start Position**: 28

**End Position**: 39

| Profile Score | Tandem Score | TDA q-value | Volume | Spectrum Matches |
| --- | --- | --- | --- | --- |
| 0.0000 | 88.9288 | 0.0000 | 0.0000e+00 | 1 |

Adduct:

No Chromatogram Found

### VPVPITN(N)ATLDR Fuc 2 Hex 4 HexNAc 6

**Mass:**  3453.5377

(2.751e-06)

**Charge States:** 3, 4

**Start Position**: 27

**End Position**: 39

| Profile Score | Tandem Score | TDA q-value | Volume | Spectrum Matches |
| --- | --- | --- | --- | --- |
| -4.6351 | 88.5865 | 0.0000 | 3.6983e+07 | 12 |

**Adduct Signal Fractions**

- **Unmodified**: 3.6983e+07

### YFTPN(N)KTEDTIFLR Hex 7 HexNAc 4 Neu5Ac 2

**Mass:**  4272.7614

(8.079e-07)

**Charge States:** 4

**Start Position**: 68

**End Position**: 82

| Profile Score | Tandem Score | TDA q-value | Volume | Spectrum Matches |
| --- | --- | --- | --- | --- |
| -4.0754 | 88.0616 | 0.0000 | 1.3738e+08 | 10 |

**Adduct Signal Fractions**

- **Unmodified**: 1.3738e+08

### FTPN(N)KTEDTIFLR Fuc 1 Hex 6 HexNAc 5

**Mass:**  3714.5960

(1.221e-06)

**Charge States:** 3

**Start Position**: 69

**End Position**: 82

| Profile Score | Tandem Score | TDA q-value | Volume | Spectrum Matches |
| --- | --- | --- | --- | --- |
| -3.8206 | 86.0311 | 0.0000 | 1.4091e+07 | 2 |

**Adduct Signal Fractions**

- **Unmodified**: 1.4091e+07

### YFTPN(N)KTEDTIFLR Fuc 1 Hex 8 HexNAc 7

**Mass:**  4609.8993

(4.297e-04)

**Start Position**: 68

**End Position**: 82

| Profile Score | Tandem Score | TDA q-value | Volume | Spectrum Matches |
| --- | --- | --- | --- | --- |
| 0.0000 | 84.8593 | 0.0000 | 0.0000e+00 | 1 |

Adduct:

No Chromatogram Found

### PVPITN(N)ATLDR Fuc 2 Hex 3 HexNAc 7 Neu5Ac 3

**Mass:**  4268.8122

(9.374e-06)

**Charge States:** 3, 4

**Start Position**: 28

**End Position**: 39

| Profile Score | Tandem Score | TDA q-value | Volume | Spectrum Matches |
| --- | --- | --- | --- | --- |
| -18.6818 | 84.6501 | 0.0000 | 8.5333e+06 | 2 |

**Adduct Signal Fractions**

- **Unmodified**: 8.5333e+06

### FTPN(N)KTEDTIFLR Fuc 1 Hex 7 HexNAc 6

**Mass:**  4079.7277

(-1.130e-06)

**Charge States:** 3, 4

**Start Position**: 69

**End Position**: 82

| Profile Score | Tandem Score | TDA q-value | Volume | Spectrum Matches |
| --- | --- | --- | --- | --- |
| -3.3763 | 84.2312 | 0.0000 | 2.9353e+07 | 5 |

**Adduct Signal Fractions**

- **Unmodified**: 2.9353e+07

### PVPITN(N)ATLDR Fuc 1 Hex 3 HexNAc 7

**Mass:**  3249.4129

(-4.679e-06)

**Start Position**: 28

**End Position**: 39

| Profile Score | Tandem Score | TDA q-value | Volume | Spectrum Matches |
| --- | --- | --- | --- | --- |
| 0.0000 | 83.7764 | 0.0000 | 0.0000e+00 | 1 |

Adduct:

No Chromatogram Found

### FTPN(N)KTEDTIFLR Hex 8 HexNAc 7

**Mass:**  4300.8269

(1.341e-06)

**Charge States:** 3, 4

**Start Position**: 69

**End Position**: 82

| Profile Score | Tandem Score | TDA q-value | Volume | Spectrum Matches |
| --- | --- | --- | --- | --- |
| 10.1134 | 82.6567 | 0.0000 | 6.5176e+07 | 10 |

**Adduct Signal Fractions**

- **Unmodified**: 6.5176e+07

### TPN(N)KTEDTIFLR Fuc 1 Hex 7 HexNAc 6

**Mass:**  3932.6606

(7.810e-07)

**Charge States:** 3

**Start Position**: 70

**End Position**: 82

| Profile Score | Tandem Score | TDA q-value | Volume | Spectrum Matches |
| --- | --- | --- | --- | --- |
| -4.6920 | 81.9569 | 0.0000 | 7.7042e+06 | 3 |

**Adduct Signal Fractions**

- **Unmodified**: 7.7042e+06

### SVQEIQATFFYFTPN(N)KTEDTIFLR Fuc 2 Hex 7 HexNAc 6

**Mass:**  5539.4255

(1.864e-06)

**Charge States:** 4

**Start Position**: 58

**End Position**: 82

| Profile Score | Tandem Score | TDA q-value | Volume | Spectrum Matches |
| --- | --- | --- | --- | --- |
| -20.1555 | 81.2866 | 0.0022 | 2.4066e+07 | 1 |

**Adduct Signal Fractions**

- **Unmodified**: 2.4066e+07

### LVPVPITN(N)ATLDR Fuc 3 Hex 5 HexNAc 4

**Mass:**  3470.5636

(5.804e-04)

**Charge States:** 3

**Start Position**: 26

**End Position**: 39

| Profile Score | Tandem Score | TDA q-value | Volume | Spectrum Matches |
| --- | --- | --- | --- | --- |
| -21.1725 | 81.0919 | 0.0022 | 1.2411e+07 | 2 |

**Adduct Signal Fractions**

- **Unmodified**: 1.2411e+07

### LVPVPITN(N)ATLDR Fuc 1 Hex 4 HexNAc 5 Neu5Ac 1

**Mass:**  3508.5946

(6.995e-06)

**Charge States:** 3

**Start Position**: 26

**End Position**: 39

| Profile Score | Tandem Score | TDA q-value | Volume | Spectrum Matches |
| --- | --- | --- | --- | --- |
| -18.8869 | 77.2969 | 0.0022 | 1.9947e+07 | 1 |

**Adduct Signal Fractions**

- **Unmodified**: 1.9947e+07

### TPN(N)KTEDTIFLR Hex 5 HexNAc 4

**Mass:**  3056.3380

(1.557e-06)

**Charge States:** 3

**Start Position**: 70

**End Position**: 82

| Profile Score | Tandem Score | TDA q-value | Volume | Spectrum Matches |
| --- | --- | --- | --- | --- |
| 10.2303 | 76.3319 | 0.0044 | 6.4634e+06 | 2 |

**Adduct Signal Fractions**

- **Unmodified**: 6.4634e+06

### QIPLC(C)ANLVPVPITN(N)ATLDR Hex 6 HexNAc 5

**Mass:**  4191.9193

(2.299e-06)

**Charge States:** 3, 4

**Start Position**: 19

**End Position**: 39

| Profile Score | Tandem Score | TDA q-value | Volume | Spectrum Matches |
| --- | --- | --- | --- | --- |
| 13.3457 | 75.8269 | 0.0044 | 6.1894e+08 | 9 |

**Adduct Signal Fractions**

- **Unmodified**: 6.1894e+08

### PVPITN(N)ATLDR Hex 3 HexNAc 7 Neu5Ac 2

**Mass:**  3685.5734

(3.357e-06)

**Charge States:** 3

**Start Position**: 28

**End Position**: 39

| Profile Score | Tandem Score | TDA q-value | Volume | Spectrum Matches |
| --- | --- | --- | --- | --- |
| 7.1846 | 75.6299 | 0.0044 | 1.4516e+07 | 1 |

**Adduct Signal Fractions**

- **Unmodified**: 1.4516e+07

### C(C)AN(D)LVPVPITN(N)ATLDR Hex 6 HexNAc 5

**Mass:**  3741.6227

(2.646e-04)

**Charge States:** 3, 4

**Start Position**: 23

**End Position**: 39

| Profile Score | Tandem Score | TDA q-value | Volume | Spectrum Matches |
| --- | --- | --- | --- | --- |
| 8.9246 | 75.0590 | 0.0044 | 1.7140e+08 | 11 |

**Adduct Signal Fractions**

- **Unmodified**: 1.7140e+08

### FYFTPN(N)KTEDTIFLR Fuc 1 Hex 4 HexNAc 4 Neu5Ac 1

**Mass:**  3788.6244

(-2.988e-06)

**Charge States:** 3, 4

**Start Position**: 67

**End Position**: 82

| Profile Score | Tandem Score | TDA q-value | Volume | Spectrum Matches |
| --- | --- | --- | --- | --- |
| 14.0736 | 72.9602 | 0.0048 | 2.6075e+08 | 5 |

**Adduct Signal Fractions**

- **Unmodified**: 2.6075e+08

### SVQEIQATFFYFTPN(N)K Fuc 2 Hex 8 HexNAc 7

**Mass:**  4929.0529

(2.502e-06)

**Charge States:** 4

**Start Position**: 58

**End Position**: 74

| Profile Score | Tandem Score | TDA q-value | Volume | Spectrum Matches |
| --- | --- | --- | --- | --- |
| 11.3535 | 69.1029 | 0.0048 | 1.2795e+07 | 2 |

**Adduct Signal Fractions**

- **Unmodified**: 1.2795e+07

### SVQEIQATFFYFTPN(N)K Hex 10 HexNAc 9

**Mass:**  5367.2004

(6.658e-07)

**Charge States:** 4

**Start Position**: 58

**End Position**: 74

| Profile Score | Tandem Score | TDA q-value | Volume | Spectrum Matches |
| --- | --- | --- | --- | --- |
| -4.4911 | 65.6114 | 0.0048 | 6.3307e+06 | 1 |

**Adduct Signal Fractions**

- **Unmodified**: 6.3307e+06

### NLVPVPITN(N)ATLDR Hex 3 HexNAc 8 Neu5Ac 1

**Mass:**  3923.7515

(1.638e-06)

**Charge States:** 3, 4

**Start Position**: 25

**End Position**: 39

| Profile Score | Tandem Score | TDA q-value | Volume | Spectrum Matches |
| --- | --- | --- | --- | --- |
| -3.9870 | 65.3958 | 0.0048 | 7.4612e+07 | 3 |

**Adduct Signal Fractions**

- **Unmodified**: 7.4612e+07

### PVPITN(N)ATLDR Hex 3 HexNAc 8 Neu5Ac 4

**Mass:**  4470.8190

(-6.346e-06)

**Charge States:** 3, 4

**Start Position**: 28

**End Position**: 39

| Profile Score | Tandem Score | TDA q-value | Volume | Spectrum Matches |
| --- | --- | --- | --- | --- |
| -4.6741 | 65.0720 | 0.0048 | 9.7123e+07 | 5 |

**Adduct Signal Fractions**

- **Unmodified**: 9.7123e+07

### FYFTPN(N)KTEDTIFLR Fuc 3 Hex 6 HexNAc 4

**Mass:**  4113.7663

(1.605e-06)

**Charge States:** 4

**Start Position**: 67

**End Position**: 82

| Profile Score | Tandem Score | TDA q-value | Volume | Spectrum Matches |
| --- | --- | --- | --- | --- |
| -4.6290 | 63.8984 | 0.0048 | 9.8440e+07 | 2 |

**Adduct Signal Fractions**

- **Unmodified**: 9.8440e+07

### SVQEIQATFFYFTPN(N)K Fuc 1 Hex 9 HexNAc 8

**Mass:**  5148.1271

(1.880e-06)

**Charge States:** 4

**Start Position**: 58

**End Position**: 74

| Profile Score | Tandem Score | TDA q-value | Volume | Spectrum Matches |
| --- | --- | --- | --- | --- |
| -21.1788 | 61.7755 | 0.0048 | 2.9274e+06 | 1 |

**Adduct Signal Fractions**

- **Unmodified**: 2.9274e+06

### SVQEIQATFFYFTPN(N)K Fuc 4 Hex 6 HexNAc 6

**Mass:**  4693.9572

(-3.363e-06)

**Charge States:** 3, 4

**Start Position**: 58

**End Position**: 74

| Profile Score | Tandem Score | TDA q-value | Volume | Spectrum Matches |
| --- | --- | --- | --- | --- |
| 8.1335 | 60.9756 | 0.0048 | 3.6728e+07 | 2 |

**Adduct Signal Fractions**

- **Unmodified**: 3.6728e+07

### SVQEIQATFFYFTPN(N)KTEDTIFLR Hex 8 HexNAc 7

**Mass:**  5612.4367

(9.255e-07)

**Charge States:** 4, 5

**Start Position**: 58

**End Position**: 82

| Profile Score | Tandem Score | TDA q-value | Volume | Spectrum Matches |
| --- | --- | --- | --- | --- |
| 16.3812 | 60.0297 | 0.0048 | 1.9014e+08 | 4 |

**Adduct Signal Fractions**

- **Unmodified**: 1.9014e+08

### C(C)ANLVPVPITN(N)ATLDR Fuc 1 Hex 6 HexNAc 5

**Mass:**  3886.6961

(7.821e-06)

**Charge States:** 3, 4

**Start Position**: 23

**End Position**: 39

| Profile Score | Tandem Score | TDA q-value | Volume | Spectrum Matches |
| --- | --- | --- | --- | --- |
| 11.5434 | 57.0620 | 0.0048 | 1.2130e+08 | 6 |

**Adduct Signal Fractions**

- **Unmodified**: 1.2130e+08

### SVQEIQATFFYFTPN(N)K Fuc 4 Hex 6 HexNAc 5

**Mass:**  4492.9081

(4.489e-04)

**Start Position**: 58

**End Position**: 74

| Profile Score | Tandem Score | TDA q-value | Volume | Spectrum Matches |
| --- | --- | --- | --- | --- |
| 0.0000 | 54.6974 | 0.0048 | 0.0000e+00 | 1 |

Adduct:

No Chromatogram Found

### FYFTPN(N)KTEDTIFLR Fuc 1 Hex 6 HexNAc 5

**Mass:**  4024.7294

(1.092e-06)

**Charge States:** 3, 4

**Start Position**: 67

**End Position**: 82

| Profile Score | Tandem Score | TDA q-value | Volume | Spectrum Matches |
| --- | --- | --- | --- | --- |
| 12.8602 | 53.0742 | 0.0048 | 1.7095e+07 | 4 |

**Adduct Signal Fractions**

- **Unmodified**: 1.7095e+07

### QNQC(C)FYN(N)SSYLNVQR Fuc 2 Hex 7 HexNAc 6

**Mass:**  4564.8391

(3.127e-06)

**Charge States:** 3

**Start Position**: 87

**End Position**: 102

| Profile Score | Tandem Score | TDA q-value | Volume | Spectrum Matches |
| --- | --- | --- | --- | --- |
| -5.7469 | 48.5188 | 0.0048 | 2.1821e+07 | 1 |

**Adduct Signal Fractions**

- **Unmodified**: 2.1821e+07

### C(C)ANLVPVPITN(N)ATLDR Hex 5 HexNAc 4

**Mass:**  3375.5026

(2.142e-06)

**Charge States:** 3

**Start Position**: 23

**End Position**: 39

| Profile Score | Tandem Score | TDA q-value | Volume | Spectrum Matches |
| --- | --- | --- | --- | --- |
| -4.5596 | 47.9606 | 0.0048 | 1.2773e+07 | 6 |

**Adduct Signal Fractions**

- **Unmodified**: 1.2773e+07

### SVQEIQATFFYFTPN(N)KTEDTIFLR Fuc 1 Hex 8 HexNAc 4 Neu5Ac 1

**Mass:**  5440.3620

(3.607e-06)

**Charge States:** 4

**Start Position**: 58

**End Position**: 82

| Profile Score | Tandem Score | TDA q-value | Volume | Spectrum Matches |
| --- | --- | --- | --- | --- |
| -4.3088 | 47.6568 | 0.0048 | 1.0485e+07 | 1 |

**Adduct Signal Fractions**

- **Unmodified**: 1.0485e+07

### QIPLC(C)ANLVPVPITN(N)ATLDR Fuc 1 Hex 6 HexNAc 5

**Mass:**  4337.9787

(1.899e-06)

**Charge States:** 3, 4

**Start Position**: 19

**End Position**: 39

| Profile Score | Tandem Score | TDA q-value | Volume | Spectrum Matches |
| --- | --- | --- | --- | --- |
| 13.1059 | 45.3037 | 0.0048 | 1.8466e+08 | 6 |

**Adduct Signal Fractions**

- **Unmodified**: 1.8466e+08

### ANLVPVPITN(N)ATLDR Fuc 1 Hex 10 HexNAc 5

**Mass:**  4374.8688

(-6.338e-07)

**Charge States:** 3, 4

**Start Position**: 24

**End Position**: 39

| Profile Score | Tandem Score | TDA q-value | Volume | Spectrum Matches |
| --- | --- | --- | --- | --- |
| -19.4138 | 44.9158 | 0.0048 | 1.5895e+07 | 2 |

**Adduct Signal Fractions**

- **Unmodified**: 1.5895e+07

### SVQEIQATFFYFTPN(N)KTEDTIFLR Hex 9 HexNAc 8

**Mass:**  5977.5661

(9.978e-07)

**Charge States:** 4, 5

**Start Position**: 58

**End Position**: 82

| Profile Score | Tandem Score | TDA q-value | Volume | Spectrum Matches |
| --- | --- | --- | --- | --- |
| 14.8349 | 44.1430 | 0.0048 | 6.2540e+07 | 4 |

**Adduct Signal Fractions**

- **Unmodified**: 6.2540e+07

### SVQEIQATFFYFTPN(N)K Fuc 1 Hex 7 HexNAc 7

**Mass:**  4620.9436

(2.953e-06)

**Charge States:** 3, 4

**Start Position**: 58

**End Position**: 74

| Profile Score | Tandem Score | TDA q-value | Volume | Spectrum Matches |
| --- | --- | --- | --- | --- |
| -20.4382 | 42.7957 | 0.0048 | 3.8179e+06 | 1 |

**Adduct Signal Fractions**

- **Unmodified**: 3.8179e+06

### LVPVPITN(N)ATLDR Fuc 4 Hex 3 HexNAc 7 Neu5Ac 1

**Mass:**  4190.8864

(9.070e-06)

**Charge States:** 3, 4

**Start Position**: 26

**End Position**: 39

| Profile Score | Tandem Score | TDA q-value | Volume | Spectrum Matches |
| --- | --- | --- | --- | --- |
| 12.9984 | 41.0240 | 0.0048 | 1.4642e+08 | 9 |

**Adduct Signal Fractions**

- **Unmodified**: 1.4642e+08

### SVQEIQATFFYFTPN(N)K Fuc 1 Hex 9 HexNAc 8

**Mass:**  5150.1185

(3.892e-04)

**Start Position**: 58

**End Position**: 74

| Profile Score | Tandem Score | TDA q-value | Volume | Spectrum Matches |
| --- | --- | --- | --- | --- |
| 0.0000 | 40.9527 | 0.0048 | 0.0000e+00 | 1 |

Adduct:

No Chromatogram Found

### NLVPVPITN(N)ATLDR Fuc 1 Hex 5 HexNAc 3

**Mass:**  3087.4162

(1.859e-06)

**Charge States:** 3

**Start Position**: 25

**End Position**: 39

| Profile Score | Tandem Score | TDA q-value | Volume | Spectrum Matches |
| --- | --- | --- | --- | --- |
| 14.3059 | 40.5347 | 0.0060 | 2.1644e+08 | 2 |

**Adduct Signal Fractions**

- **Unmodified**: 2.1644e+08

### SVQEIQATFFYFTPN(N)KTEDTIFLR Fuc 1 Hex 8 HexNAc 7

**Mass:**  5758.4929

(1.338e-06)

**Charge States:** 4, 5

**Start Position**: 58

**End Position**: 82

| Profile Score | Tandem Score | TDA q-value | Volume | Spectrum Matches |
| --- | --- | --- | --- | --- |
| -4.7344 | 40.2115 | 0.0060 | 3.3992e+07 | 3 |

**Adduct Signal Fractions**

- **Unmodified**: 3.3992e+07

### QC(C)FYN(N)SSYLNVQR Fuc 2 Hex 5 HexNAc 6 Neu5Ac 1

**Mass:**  4289.7266

(5.963e-06)

**Charge States:** 3, 4

**Start Position**: 89

**End Position**: 102

| Profile Score | Tandem Score | TDA q-value | Volume | Spectrum Matches |
| --- | --- | --- | --- | --- |
| 13.2557 | 39.1110 | 0.0060 | 3.2722e+08 | 1 |

**Adduct Signal Fractions**

- **Unmodified**: 3.2722e+08

### QNQC(C)FYN(N)SSYLNVQR Hex 7 HexNAc 4 Neu5Ac 2

**Mass:**  4448.7468

(2.266e-06)

**Charge States:** 3, 4

**Start Position**: 87

**End Position**: 102

| Profile Score | Tandem Score | TDA q-value | Volume | Spectrum Matches |
| --- | --- | --- | --- | --- |
| -18.4827 | 38.9386 | 0.0060 | 1.4005e+07 | 2 |

**Adduct Signal Fractions**

- **Unmodified**: 1.4005e+07

### QC(C)FYN(N)SSYLNVQR Fuc 2 Hex 4 HexNAc 5 Neu5Ac 1

**Mass:**  3924.5949

(6.391e-06)

**Charge States:** 3, 4

**Start Position**: 89

**End Position**: 102

| Profile Score | Tandem Score | TDA q-value | Volume | Spectrum Matches |
| --- | --- | --- | --- | --- |
| 13.4480 | 38.9254 | 0.0060 | 2.0946e+08 | 4 |

**Adduct Signal Fractions**

- **Unmodified**: 2.0946e+08

### PVPITN(N)ATLDR Fuc 1 Hex 8 HexNAc 4

**Mass:**  3450.4543

(5.736e-08)

**Start Position**: 28

**End Position**: 39

| Profile Score | Tandem Score | TDA q-value | Volume | Spectrum Matches |
| --- | --- | --- | --- | --- |
| 0.0000 | 38.5318 | 0.0060 | 0.0000e+00 | 1 |

Adduct:

No Chromatogram Found

### LVPVPITN(N)ATLDR Fuc 2 Hex 4 HexNAc 9

**Mass:**  4175.8757

(6.112e-06)

**Charge States:** 3, 4

**Start Position**: 26

**End Position**: 39

| Profile Score | Tandem Score | TDA q-value | Volume | Spectrum Matches |
| --- | --- | --- | --- | --- |
| 13.4650 | 38.0709 | 0.0088 | 1.1366e+08 | 11 |

**Adduct Signal Fractions**

- **Unmodified**: 1.1366e+08

### ANLVPVPITN(N)ATLDR Fuc 1 Hex 9 HexNAc 5

**Mass:**  4212.8449

(4.452e-06)

**Charge States:** 4

**Start Position**: 24

**End Position**: 39

| Profile Score | Tandem Score | TDA q-value | Volume | Spectrum Matches |
| --- | --- | --- | --- | --- |
| -5.8574 | 38.0142 | 0.0088 | 9.1824e+07 | 2 |

**Adduct Signal Fractions**

- **Unmodified**: 9.1824e+07

### FYFTPN(N)KTEDTIFLR Hex 5 HexNAc 4

**Mass:**  3513.5366

(1.818e-06)

**Charge States:** 3

**Start Position**: 67

**End Position**: 82

| Profile Score | Tandem Score | TDA q-value | Volume | Spectrum Matches |
| --- | --- | --- | --- | --- |
| -19.5768 | 37.1165 | 0.0097 | 2.7490e+06 | 1 |

**Adduct Signal Fractions**

- **Unmodified**: 2.7490e+06

### C(C)AN(D)LVPVPITN(N)ATLDR Fuc 2 Hex 4 HexNAc 4 Neu5Ac 1

**Mass:**  3797.6618

(2.650e-04)

**Charge States:** 3, 4

**Start Position**: 23

**End Position**: 39

| Profile Score | Tandem Score | TDA q-value | Volume | Spectrum Matches |
| --- | --- | --- | --- | --- |
| -5.9612 | 36.9341 | 0.0097 | 8.3937e+07 | 4 |

**Adduct Signal Fractions**

- **Unmodified**: 8.3937e+07

### QC(C)FYN(N)SSYLNVQR Fuc 3 Hex 4 HexNAc 6 Neu5Ac 1

**Mass:**  4273.6979

(-3.643e-06)

**Charge States:** 3, 4

**Start Position**: 89

**End Position**: 102

| Profile Score | Tandem Score | TDA q-value | Volume | Spectrum Matches |
| --- | --- | --- | --- | --- |
| 11.5037 | 36.7770 | 0.0097 | 1.8576e+08 | 1 |

**Adduct Signal Fractions**

- **Unmodified**: 1.8576e+08

### YFTPN(N)KTEDTIFLR Fuc 4 Hex 5 HexNAc 5

**Mass:**  4153.7585

(-4.708e-06)

**Charge States:** 3, 4

**Start Position**: 68

**End Position**: 82

| Profile Score | Tandem Score | TDA q-value | Volume | Spectrum Matches |
| --- | --- | --- | --- | --- |
| 13.1177 | 36.6632 | 0.0097 | 4.6450e+08 | 4 |

**Adduct Signal Fractions**

- **Unmodified**: 4.6450e+08

### LVPVPITN(N)ATLDR Hex 7 HexNAc 5

**Mass:**  3557.6006

(7.101e-06)

**Charge States:** 3

**Start Position**: 26

**End Position**: 39

| Profile Score | Tandem Score | TDA q-value | Volume | Spectrum Matches |
| --- | --- | --- | --- | --- |
| -19.4432 | 36.2408 | 0.0097 | 1.2244e+06 | 1 |

**Adduct Signal Fractions**

- **Unmodified**: 1.2244e+06

### FYFTPN(N)KTEDTIFLR Hex 9 HexNAc 8

**Mass:**  4977.0741

(6.056e-04)

**Start Position**: 67

**End Position**: 82

| Profile Score | Tandem Score | TDA q-value | Volume | Spectrum Matches |
| --- | --- | --- | --- | --- |
| 0.0000 | 33.5778 | 0.0132 | 0.0000e+00 | 1 |

Adduct:

No Chromatogram Found

### QN(D)QC(C)FYN(N)SSYLNVQR Hex 7 HexNAc 6

**Mass:**  4273.6935

(2.322e-04)

**Charge States:** 3, 4

**Start Position**: 87

**End Position**: 102

| Profile Score | Tandem Score | TDA q-value | Volume | Spectrum Matches |
| --- | --- | --- | --- | --- |
| -6.7852 | 33.3334 | 0.0132 | 2.1456e+08 | 2 |

**Adduct Signal Fractions**

- **Unmodified**: 2.1456e+08

### ANLVPVPITN(N)ATLDR Fuc 5 Hex 7 HexNAc 9

**Mass:**  5285.2487

(-1.620e-06)

**Charge States:** 4, 5

**Start Position**: 24

**End Position**: 39

| Profile Score | Tandem Score | TDA q-value | Volume | Spectrum Matches |
| --- | --- | --- | --- | --- |
| 10.2575 | 33.0600 | 0.0132 | 1.4777e+08 | 4 |

**Adduct Signal Fractions**

- **Unmodified**: 1.4777e+08

### SVQEIQATFFYFTPN(N)KTEDTIFLR Hex 8 HexNAc 7

**Mass:**  5614.4453

(3.596e-04)

**Start Position**: 58

**End Position**: 82

| Profile Score | Tandem Score | TDA q-value | Volume | Spectrum Matches |
| --- | --- | --- | --- | --- |
| 0.0000 | 32.8616 | 0.0144 | 0.0000e+00 | 1 |

Adduct:

No Chromatogram Found

### QN(D)QC(C)FYN(N)SSYLNVQR Fuc 1 Hex 7 HexNAc 6

**Mass:**  4419.7590

(2.244e-04)

**Charge States:** 3

**Start Position**: 87

**End Position**: 102

| Profile Score | Tandem Score | TDA q-value | Volume | Spectrum Matches |
| --- | --- | --- | --- | --- |
| -6.0447 | 31.9712 | 0.0187 | 2.7629e+07 | 1 |

**Adduct Signal Fractions**

- **Unmodified**: 2.7629e+07

### LVPVPITN(N)ATLDR Fuc 1 Hex 7 HexNAc 5 Neu5Ac 2

**Mass:**  4285.8411

(2.573e-06)

**Charge States:** 3

**Start Position**: 26

**End Position**: 39

| Profile Score | Tandem Score | TDA q-value | Volume | Spectrum Matches |
| --- | --- | --- | --- | --- |
| -4.8247 | 31.8553 | 0.0187 | 4.5776e+06 | 1 |

**Adduct Signal Fractions**

- **Unmodified**: 4.5776e+06

### QIPLC(C)ANLVPVPITN(N)ATLDR Hex 5 HexNAc 5 Neu5Ac 1

**Mass:**  4320.9558

(-1.338e-06)

**Charge States:** 3, 4

**Start Position**: 19

**End Position**: 39

| Profile Score | Tandem Score | TDA q-value | Volume | Spectrum Matches |
| --- | --- | --- | --- | --- |
| 15.4827 | 31.5385 | 0.0187 | 3.6196e+08 | 7 |

**Adduct Signal Fractions**

- **Unmodified**: 3.6196e+08

### YFTPN(N)KTEDTIFLR Fuc 5 Hex 8 HexNAc 7

**Mass:**  5192.1384

(-2.401e-06)

**Charge States:** 4

**Start Position**: 68

**End Position**: 82

| Profile Score | Tandem Score | TDA q-value | Volume | Spectrum Matches |
| --- | --- | --- | --- | --- |
| -4.5899 | 31.3749 | 0.0187 | 5.2917e+06 | 1 |

**Adduct Signal Fractions**

- **Unmodified**: 5.2917e+06

### QNQC(C)FYN(N)SSYLNVQR Fuc 5 Hex 7 HexNAc 6

**Mass:**  5002.9948

(-1.480e-06)

**Charge States:** 4

**Start Position**: 87

**End Position**: 102

| Profile Score | Tandem Score | TDA q-value | Volume | Spectrum Matches |
| --- | --- | --- | --- | --- |
| -5.7676 | 31.1339 | 0.0199 | 2.2188e+07 | 1 |

**Adduct Signal Fractions**

- **Unmodified**: 2.2188e+07

### C(C)AN(D)LVPVPITN(N)ATLDR Fuc 1 Hex 6 HexNAc 5

**Mass:**  3887.6823

(2.552e-04)

**Charge States:** 3, 4

**Start Position**: 23

**End Position**: 39

| Profile Score | Tandem Score | TDA q-value | Volume | Spectrum Matches |
| --- | --- | --- | --- | --- |
| 7.7906 | 31.0726 | 0.0199 | 3.5809e+07 | 5 |

**Adduct Signal Fractions**

- **Unmodified**: 3.5809e+07

### FTPN(N)KTEDTIFLR Hex 10 HexNAc 3 Neu5Ac 1

**Mass:**  4101.7106

(8.201e-06)

**Charge States:** 4

**Start Position**: 69

**End Position**: 82

| Profile Score | Tandem Score | TDA q-value | Volume | Spectrum Matches |
| --- | --- | --- | --- | --- |
| -20.5708 | 30.4379 | 0.0208 | 1.0423e+06 | 1 |

**Adduct Signal Fractions**

- **Unmodified**: 1.0423e+06

### FYN(N)SSYLNVQR Hex 7 HexNAc 6

**Mass:**  3742.5188

(4.420e-07)

**Charge States:** 3, 4

**Start Position**: 91

**End Position**: 102

| Profile Score | Tandem Score | TDA q-value | Volume | Spectrum Matches |
| --- | --- | --- | --- | --- |
| -4.0496 | 29.7878 | 0.0236 | 8.8158e+06 | 3 |

**Adduct Signal Fractions**

- **Unmodified**: 8.8158e+06

### FYFTPN(N)KTEDTIFLR Fuc 2 Hex 6 HexNAc 5

**Mass:**  4170.7819

(1.769e-07)

**Charge States:** 4

**Start Position**: 67

**End Position**: 82

| Profile Score | Tandem Score | TDA q-value | Volume | Spectrum Matches |
| --- | --- | --- | --- | --- |
| -19.5686 | 29.6412 | 0.0236 | 2.2917e+07 | 1 |

**Adduct Signal Fractions**

- **Unmodified**: 2.2917e+07

### PVPITN(N)ATLDR Fuc 2 Hex 7 HexNAc 4

**Mass:**  3434.4706

(6.248e-06)

**Charge States:** 4

**Start Position**: 28

**End Position**: 39

| Profile Score | Tandem Score | TDA q-value | Volume | Spectrum Matches |
| --- | --- | --- | --- | --- |
| -4.1422 | 29.4475 | 0.0236 | 6.9788e+05 | 15 |

**Adduct Signal Fractions**

- **Unmodified**: 6.9788e+05

### ANLVPVPITN(N)ATLDR Fuc 1 Hex 6 HexNAc 5

**Mass:**  3726.6644

(1.139e-06)

**Charge States:** 3

**Start Position**: 24

**End Position**: 39

| Profile Score | Tandem Score | TDA q-value | Volume | Spectrum Matches |
| --- | --- | --- | --- | --- |
| -18.6063 | 29.4260 | 0.0236 | 4.6778e+06 | 1 |

**Adduct Signal Fractions**

- **Unmodified**: 4.6778e+06

### FYFTPN(N)KTEDTIFLR Fuc 1 Hex 3 HexNAc 3 Neu5Ac 1

**Mass:**  3423.4960

(-1.610e-06)

**Charge States:** 3, 4

**Start Position**: 67

**End Position**: 82

| Profile Score | Tandem Score | TDA q-value | Volume | Spectrum Matches |
| --- | --- | --- | --- | --- |
| -19.0367 | 28.8173 | 0.0253 | 1.5234e+07 | 2 |

**Adduct Signal Fractions**

- **Unmodified**: 1.5234e+07

### WFYIASAFRNEEYNKSVQEIQATFFYFTPN(N)K Fuc 5 Hex 10 HexNAc 8

**Mass:**  7813.3462

(5.975e-06)

**Charge States:** 5

**Start Position**: 43

**End Position**: 74

| Profile Score | Tandem Score | TDA q-value | Volume | Spectrum Matches |
| --- | --- | --- | --- | --- |
| -4.6751 | 28.5925 | 0.0284 | 2.6046e+07 | 1 |

**Adduct Signal Fractions**

- **Unmodified**: 2.6046e+07

### LVPVPITN(N)ATLDR Hex 6 HexNAc 5 Neu5Ac 2

**Mass:**  3977.7302

(4.246e-06)

**Charge States:** 3, 4

**Start Position**: 26

**End Position**: 39

| Profile Score | Tandem Score | TDA q-value | Volume | Spectrum Matches |
| --- | --- | --- | --- | --- |
| -4.8088 | 28.3877 | 0.0284 | 9.7359e+06 | 2 |

**Adduct Signal Fractions**

- **Unmodified**: 9.7359e+06

### QNQC(C)FYN(N)SSYLNVQR Hex 9 HexNAc 8

**Mass:**  5002.9689

(4.849e-07)

**Charge States:** 4

**Start Position**: 87

**End Position**: 102

| Profile Score | Tandem Score | TDA q-value | Volume | Spectrum Matches |
| --- | --- | --- | --- | --- |
| -20.9424 | 28.1364 | 0.0293 | 1.0058e+07 | 1 |

**Adduct Signal Fractions**

- **Unmodified**: 1.0058e+07

### LVPVPITN(N)ATLDR Fuc 1 Hex 8 HexNAc 2

**Mass:**  3256.4533

(1.630e-06)

**Charge States:** 3

**Start Position**: 26

**End Position**: 39

| Profile Score | Tandem Score | TDA q-value | Volume | Spectrum Matches |
| --- | --- | --- | --- | --- |
| 14.5506 | 27.8429 | 0.0308 | 3.4318e+07 | 2 |

**Adduct Signal Fractions**

- **Unmodified**: 3.4318e+07

### PVPITN(N)ATLDR Fuc 1 Hex 4 HexNAc 8 Neu5Ac 1

**Mass:**  3905.6852

(9.397e-06)

**Charge States:** 4

**Start Position**: 28

**End Position**: 39

| Profile Score | Tandem Score | TDA q-value | Volume | Spectrum Matches |
| --- | --- | --- | --- | --- |
| -19.5791 | 27.7789 | 0.0308 | 1.8932e+06 | 2 |

**Adduct Signal Fractions**

- **Unmodified**: 1.8932e+06

### LVPVPITN(N)ATLDR Hex 5 HexNAc 4 Neu5Ac 2

**Mass:**  3612.5879

(1.957e-06)

**Charge States:** 3, 4

**Start Position**: 26

**End Position**: 39

| Profile Score | Tandem Score | TDA q-value | Volume | Spectrum Matches |
| --- | --- | --- | --- | --- |
| -20.1342 | 27.6420 | 0.0308 | 2.7172e+06 | 2 |

**Adduct Signal Fractions**

- **Unmodified**: 2.7172e+06

### FYFTPN(N)KTEDTIFLR Fuc 2 Hex 4 HexNAc 4 Neu5Ac 1

**Mass:**  3934.6807

(-2.780e-06)

**Charge States:** 3, 4

**Start Position**: 67

**End Position**: 82

| Profile Score | Tandem Score | TDA q-value | Volume | Spectrum Matches |
| --- | --- | --- | --- | --- |
| -18.6121 | 27.5739 | 0.0328 | 5.0932e+07 | 1 |

**Adduct Signal Fractions**

- **Unmodified**: 5.0932e+07

### QNQC(C)FYN(N)SSYLNVQR Fuc 5 Hex 8 HexNAc 7

**Mass:**  5368.1179

(-1.516e-06)

**Charge States:** 4

**Start Position**: 87

**End Position**: 102

| Profile Score | Tandem Score | TDA q-value | Volume | Spectrum Matches |
| --- | --- | --- | --- | --- |
| -19.6366 | 27.1580 | 0.0370 | 3.4165e+06 | 1 |

**Adduct Signal Fractions**

- **Unmodified**: 3.4165e+06

### PVPITN(N)ATLDR Hex 8 HexNAc 5 Neu5Ac 1

**Mass:**  3798.6113

(9.468e-06)

**Charge States:** 3, 4

**Start Position**: 28

**End Position**: 39

| Profile Score | Tandem Score | TDA q-value | Volume | Spectrum Matches |
| --- | --- | --- | --- | --- |
| -18.8193 | 27.0780 | 0.0373 | 6.1460e+07 | 1 |

**Adduct Signal Fractions**

- **Unmodified**: 6.1460e+07

### LVPVPITN(N)ATLDR Fuc 3 Hex 4 HexNAc 9

**Mass:**  4321.9348

(6.351e-06)

**Charge States:** 3, 4

**Start Position**: 26

**End Position**: 39

| Profile Score | Tandem Score | TDA q-value | Volume | Spectrum Matches |
| --- | --- | --- | --- | --- |
| 15.4032 | 26.8412 | 0.0373 | 2.6928e+07 | 7 |

**Adduct Signal Fractions**

- **Unmodified**: 2.6928e+07

### SVQEIQATFFYFTPN(N)KTEDTIFLR Fuc 2 Hex 8 HexNAc 7

**Mass:**  5904.5476

(7.260e-07)

**Charge States:** 4

**Start Position**: 58

**End Position**: 82

| Profile Score | Tandem Score | TDA q-value | Volume | Spectrum Matches |
| --- | --- | --- | --- | --- |
| -4.5329 | 26.6879 | 0.0373 | 6.1134e+06 | 1 |

**Adduct Signal Fractions**

- **Unmodified**: 6.1134e+06

### QN(D)QC(C)FYN(N)SSYLNVQR Fuc 2 Hex 6 HexNAc 4 Neu5Ac 1

**Mass:**  4288.6584

(2.274e-04)

**Charge States:** 4

**Start Position**: 87

**End Position**: 102

| Profile Score | Tandem Score | TDA q-value | Volume | Spectrum Matches |
| --- | --- | --- | --- | --- |
| 8.0822 | 26.5689 | 0.0373 | 1.5903e+08 | 2 |

**Adduct Signal Fractions**

- **Unmodified**: 1.5903e+08

### YN(N)SSYLNVQR Hex 7 HexNAc 6

**Mass:**  3595.4518

(2.605e-06)

**Charge States:** 3

**Start Position**: 92

**End Position**: 102

| Profile Score | Tandem Score | TDA q-value | Volume | Spectrum Matches |
| --- | --- | --- | --- | --- |
| -4.6839 | 26.2005 | 0.0373 | 4.6988e+06 | 2 |

**Adduct Signal Fractions**

- **Unmodified**: 4.6988e+06

### LVPVPITN(N)ATLDR Fuc 5 Hex 3 HexNAc 7 Neu5Ac 1

**Mass:**  4336.9403

(9.594e-06)

**Charge States:** 3, 4

**Start Position**: 26

**End Position**: 39

| Profile Score | Tandem Score | TDA q-value | Volume | Spectrum Matches |
| --- | --- | --- | --- | --- |
| 9.9110 | 26.0846 | 0.0373 | 3.1556e+07 | 7 |

**Adduct Signal Fractions**

- **Unmodified**: 3.1556e+07

### FYN(N)SSYLNVQR Fuc 4 Hex 6 HexNAc 6

**Mass:**  4164.6776

(-3.616e-06)

**Charge States:** 3

**Start Position**: 91

**End Position**: 102

| Profile Score | Tandem Score | TDA q-value | Volume | Spectrum Matches |
| --- | --- | --- | --- | --- |
| -3.0000 | 26.0420 | 0.0380 | 7.0955e+07 | 3 |

**Adduct Signal Fractions**

- **Unmodified**: 7.0955e+07

### QIPLC(C)ANLVPVPITN(N)ATLDR Hex 5 HexNAc 4

**Mass:**  3826.7869

(1.335e-06)

**Charge States:** 3, 4

**Start Position**: 19

**End Position**: 39

| Profile Score | Tandem Score | TDA q-value | Volume | Spectrum Matches |
| --- | --- | --- | --- | --- |
| 13.6237 | 26.0062 | 0.0380 | 5.6401e+07 | 3 |

**Adduct Signal Fractions**

- **Unmodified**: 5.6401e+07

### C(C)ANLVPVPITN(N)ATLDR Hex 5 HexNAc 5 Neu5Ac 1

**Mass:**  3869.6728

(-7.630e-07)

**Charge States:** 3, 4

**Start Position**: 23

**End Position**: 39

| Profile Score | Tandem Score | TDA q-value | Volume | Spectrum Matches |
| --- | --- | --- | --- | --- |
| 15.2007 | 25.6596 | 0.0395 | 1.7489e+08 | 5 |

**Adduct Signal Fractions**

- **Unmodified**: 1.7489e+08

### SVQEIQATFFYFTPN(N)KTEDTIFLR Hex 10 HexNAc 9

**Mass:**  6342.6970

(8.763e-07)

**Charge States:** 4

**Start Position**: 58

**End Position**: 82

| Profile Score | Tandem Score | TDA q-value | Volume | Spectrum Matches |
| --- | --- | --- | --- | --- |
| -5.8151 | 25.5133 | 0.0421 | 6.2037e+06 | 1 |

**Adduct Signal Fractions**

- **Unmodified**: 6.2037e+06

### ATFFYFTPN(N)K Fuc 2 Hex 8 HexNAc 3

**Mass:**  3432.3869

(4.225e-06)

**Charge States:** 3

**Start Position**: 64

**End Position**: 74

| Profile Score | Tandem Score | TDA q-value | Volume | Spectrum Matches |
| --- | --- | --- | --- | --- |
| -18.3456 | 25.1956 | 0.0445 | 8.7695e+06 | 1 |

**Adduct Signal Fractions**

- **Unmodified**: 8.7695e+06

### FYFTPN(N)KTEDTIFLR Fuc 3 Hex 7 HexNAc 5

**Mass:**  4478.8942

(2.336e-07)

**Charge States:** 4

**Start Position**: 67

**End Position**: 82

| Profile Score | Tandem Score | TDA q-value | Volume | Spectrum Matches |
| --- | --- | --- | --- | --- |
| -5.6786 | 25.1710 | 0.0445 | 2.6567e+07 | 1 |

**Adduct Signal Fractions**

- **Unmodified**: 2.6567e+07

### QN(D)QC(C)FYN(N)SSYLNVQR Hex 8 HexNAc 6

**Mass:**  4435.7331

(2.200e-04)

**Charge States:** 3, 4

**Start Position**: 87

**End Position**: 102

| Profile Score | Tandem Score | TDA q-value | Volume | Spectrum Matches |
| --- | --- | --- | --- | --- |
| 9.8444 | 24.3965 | 0.0445 | 7.8442e+07 | 2 |

**Adduct Signal Fractions**

- **Unmodified**: 7.8442e+07

### PVPITN(N)ATLDR Fuc 2 Hex 4 HexNAc 4 Neu5Ac 1

**Mass:**  3239.4259

(9.398e-06)

**Charge States:** 3, 4

**Start Position**: 28

**End Position**: 39

| Profile Score | Tandem Score | TDA q-value | Volume | Spectrum Matches |
| --- | --- | --- | --- | --- |
| 13.5395 | 24.1548 | 0.0445 | 4.2297e+07 | 4 |

**Adduct Signal Fractions**

- **Unmodified**: 4.2297e+07

### ANLVPVPITN(N)ATLDR Fuc 2 Hex 6 HexNAc 3 Neu5Ac 1

**Mass:**  3757.6660

(3.386e-06)

**Charge States:** 3, 4

**Start Position**: 24

**End Position**: 39

| Profile Score | Tandem Score | TDA q-value | Volume | Spectrum Matches |
| --- | --- | --- | --- | --- |
| 12.1210 | 24.1339 | 0.0449 | 8.6544e+07 | 3 |

**Adduct Signal Fractions**

- **Unmodified**: 8.6544e+07

### QNQC(C)FYN(N)SSYLNVQR Fuc 1 Hex 8 HexNAc 4 Neu5Ac 1

**Mass:**  4465.7561

(3.123e-06)

**Charge States:** 3, 4

**Start Position**: 87

**End Position**: 102

| Profile Score | Tandem Score | TDA q-value | Volume | Spectrum Matches |
| --- | --- | --- | --- | --- |
| -5.9995 | 24.0130 | 0.0449 | 1.3305e+07 | 1 |

**Adduct Signal Fractions**

- **Unmodified**: 1.3305e+07

### SVQEIQATFFYFTPN(N)KTEDTIFLR Fuc 4 Hex 5 HexNAc 5

**Mass:**  5304.3254

(-3.953e-06)

**Charge States:** 3, 4, 5

**Start Position**: 58

**End Position**: 82

| Profile Score | Tandem Score | TDA q-value | Volume | Spectrum Matches |
| --- | --- | --- | --- | --- |
| 12.8581 | 23.7441 | 0.0464 | 2.2358e+08 | 12 |

**Adduct Signal Fractions**

- **Unmodified**: 2.2358e+08

### C(C)AN(D)LVPVPITN(N)ATLDR Fuc 1 Hex 3 HexNAc 9

**Mass:**  4213.8620

(2.387e-04)

**Charge States:** 3

**Start Position**: 23

**End Position**: 39

| Profile Score | Tandem Score | TDA q-value | Volume | Spectrum Matches |
| --- | --- | --- | --- | --- |
| 11.2222 | 23.7364 | 0.0464 | 1.3294e+08 | 4 |

**Adduct Signal Fractions**

- **Unmodified**: 1.3294e+08

### SVQEIQATFFYFTPN(N)KTEDTIFLR Hex 7 HexNAc 4 Neu5Ac 2

**Mass:**  5423.3371

(1.638e-06)

**Charge States:** 4

**Start Position**: 58

**End Position**: 82

| Profile Score | Tandem Score | TDA q-value | Volume | Spectrum Matches |
| --- | --- | --- | --- | --- |
| -4.0422 | 23.6397 | 0.0487 | 2.5786e+07 | 1 |

**Adduct Signal Fractions**

- **Unmodified**: 2.5786e+07

### YN(N)SSYLNVQR Hex 6 HexNAc 5

**Mass:**  3230.3179

(1.984e-06)

**Charge States:** 3

**Start Position**: 92

**End Position**: 102

| Profile Score | Tandem Score | TDA q-value | Volume | Spectrum Matches |
| --- | --- | --- | --- | --- |
| 12.2091 | 23.4781 | 0.0491 | 7.8309e+06 | 3 |

**Adduct Signal Fractions**

- **Unmodified**: 7.8309e+06

### PVPITN(N)ATLDR Hex 9 HexNAc 6 Neu5Ac 1

**Mass:**  4163.7326

(7.027e-06)

**Charge States:** 4

**Start Position**: 28

**End Position**: 39

| Profile Score | Tandem Score | TDA q-value | Volume | Spectrum Matches |
| --- | --- | --- | --- | --- |
| -5.2798 | 23.4398 | 0.0491 | 1.0840e+07 | 1 |

**Adduct Signal Fractions**

- **Unmodified**: 1.0840e+07

### FYN(N)SSYLNVQR Fuc 4 Hex 5 HexNAc 5

**Mass:**  3799.5470

(-3.486e-06)

**Charge States:** 3

**Start Position**: 91

**End Position**: 102

| Profile Score | Tandem Score | TDA q-value | Volume | Spectrum Matches |
| --- | --- | --- | --- | --- |
| -19.1397 | 23.2552 | 0.0491 | 2.5796e+07 | 1 |

**Adduct Signal Fractions**

- **Unmodified**: 2.5796e+07

### P02763|A1AG1\_HUMAN

**>P02763|A1AG1\_HUMAN**  
 `MALSWVLTVLSLLPLLEAQIPLCANLVPVPITNATLDQITGKWFYIASAFRNEEYNKSVQEIQATFFYFTPNKTEDTIFL  
REYQTRQDQCIYNTTYLNVQRENGTISRYVGGQEHFAHLLILRDTKTYMLAFDVNDEKNWGLSVYADKPETTKEQLGEFY  
EALDCLRIPKSDVVYTDWKKDKCEPLEKQHEKERKQEEGES`

| Site | Type | Count |
| --- | --- | --- |
| 33 | N-linked | 52 |
| 56 | N-linked | 3 |
| 93 | N-linked | 32 |
| 72 | N-linked | 99 |

| Mass | Sequence | MS_2_ Score | `q`-value | MS_1_ Score | Signal | Protein Coordinates | Tandem Scans | Start Time | Apex Time | End Time |
| --- | --- | --- | --- | --- | --- | --- | --- | --- | --- | --- |
| 2756.0974 | YFTPN(N)K Hex 6 HexNAc 5 | 187.498 | 0.0 | 13.791 | 2.565e+08 | 67 - 73 | 3 | 14.25 | 14.41 | 14.91 |
| 3121.2318 | YFTPN(N)K Hex 7 HexNAc 6 | 187.178 | 0.0 | 13.782 | 3.649e+08 | 67 - 73 | 4 | 13.94 | 14.08 | 14.74 |
| 3268.2995 | FYFTPN(N)K Hex 7 HexNAc 6 | 184.423 | 0.0 | 15.291 | 1.154e+08 | 66 - 73 | 8 | 24.42 | 24.85 | 26.18 |
| 2903.1667 | FYFTPN(N)K Hex 6 HexNAc 5 | 183.305 | 0.0 | 13.636 | 8.964e+07 | 66 - 73 | 14 | 25.19 | 25.53 | 28.37 |
| 2417.9227 | NEEYN(N)K Hex 5 HexNAc 4 | 182.356 | 0.0 | 11.263 | 5.950e+07 | 51 - 57 | 7 | 3.97 | 4.14 | 5.37 |
| 2929.1116 | NEEYN(N)K Fuc 1 Hex 6 HexNAc 5 | 181.805 | 0.0 | -3.515 | 2.091e+07 | 51 - 57 | 7 | 3.97 | 4.14 | 10.79 |
| 2783.0555 | NEEYN(N)K Hex 6 HexNAc 5 | 181.465 | 0.0 | 13.061 | 2.804e+08 | 51 - 57 | 20 | 3.97 | 4.14 | 10.79 |
| 3906.6665 | SVQEIQATFFYFTPN(N)K Hex 6 HexNAc 5 | 174.243 | 0.0 | 15.076 | 1.932e+09 | 57 - 73 | 41 | 50.53 | 54.46 | 62.33 |
| 2902.1548 | YFTPN(N)K Fuc 1 Hex 6 HexNAc 5 | 174.140 | 0.0 | -18.632 | 3.791e+07 | 67 - 73 | 1 | 14.08 | 14.25 | 14.25 |
| 3267.2876 | YFTPN(N)K Fuc 1 Hex 7 HexNAc 6 | 173.371 | 0.0 | -4.635 | 6.048e+07 | 67 - 73 | 2 | 13.78 | 13.94 | 14.25 |
| 3486.3637 | YFTPN(N)K Hex 8 HexNAc 7 | 172.606 | 0.0 | -20.311 | 1.935e+07 | 67 - 73 | 1 | 13.78 | 13.94 | 13.94 |
| 4636.9346 | SVQEIQATFFYFTPN(N)K Hex 8 HexNAc 7 | 172.360 | 0.0 | 13.532 | 3.064e+08 | 57 - 73 | 7 | 53.09 | 53.61 | 54.29 |
| 2392.9588 | YFTPN(N)K Hex 5 HexNAc 4 | 167.873 | 0.0 | 0.000 | 0.000e+00 | 67 - 73 | 1 | - | - | - |
| 4417.8622 | SVQEIQATFFYFTPN(N)K Fuc 1 Hex 7 HexNAc 6 | 167.604 | 0.0 | 13.761 | 4.974e+08 | 57 - 73 | 13 | 53.61 | 53.78 | 57.02 |
| 4052.7261 | SVQEIQATFFYFTPN(N)K Fuc 1 Hex 6 HexNAc 5 | 166.895 | 0.0 | 13.683 | 4.173e+08 | 57 - 73 | 13 | 53.95 | 54.29 | 57.36 |
| 2757.1032 | YFTPN(N)K Hex 6 HexNAc 5 | 166.705 | 0.0 | 0.000 | 0.000e+00 | 67 - 73 | 1 | - | - | - |
| 2390.9674 | YFTPN(N)K Hex 5 HexNAc 4 | 166.049 | 0.0 | -20.584 | 1.828e+07 | 67 - 73 | 2 | 14.57 | 14.57 | 14.74 |
| 3541.5339 | SVQEIQATFFYFTPN(N)K Hex 5 HexNAc 4 | 165.700 | 0.0 | 13.872 | 2.413e+08 | 57 - 73 | 12 | 54.97 | 55.14 | 58.05 |
| 3222.3222 | ATFFYFTPN(N)K Hex 6 HexNAc 5 | 164.263 | 0.0 | -3.140 | 3.783e+07 | 63 - 73 | 2 | 41.08 | 41.25 | 41.59 |
| 3049.2255 | FYFTPN(N)K Fuc 1 Hex 6 HexNAc 5 | 163.976 | 0.034 | 14.121 | 1.590e+07 | 66 - 73 | 3 | 25.02 | 25.36 | 25.84 |
| 4271.8036 | SVQEIQATFFYFTPN(N)K Hex 7 HexNAc 6 | 163.209 | 0.0 | 15.017 | 2.306e+09 | 57 - 73 | 48 | 50.35 | 53.95 | 113.29 |
| 3050.2342 | FFYFTPN(N)K Hex 6 HexNAc 5 | 163.027 | 0.034 | -19.052 | 2.092e+07 | 65 - 73 | 2 | 36.03 | 36.03 | 36.20 |
| 3415.3697 | FFYFTPN(N)K Hex 7 HexNAc 6 | 161.026 | 0.034 | -2.619 | 3.575e+07 | 65 - 73 | 2 | 35.35 | 35.52 | 35.69 |
| 3912.7937 | LVPVPITN(N)ATLDQITGK Fuc 1 Hex 6 HexNAc 5 | 158.046 | 0.0 | 14.681 | 3.525e+09 | 25 - 42 | 35 | 41.59 | 48.07 | 52.41 |
| 3587.4552 | ATFFYFTPN(N)K Hex 7 HexNAc 6 | 156.181 | 0.0 | 13.199 | 6.601e+07 | 63 - 73 | 3 | 40.39 | 40.56 | 41.25 |
| 4782.9933 | SVQEIQATFFYFTPN(N)K Fuc 1 Hex 8 HexNAc 7 | 156.146 | 0.0 | 12.333 | 6.298e+07 | 57 - 73 | 5 | 52.92 | 53.44 | 53.95 |
| 4464.8569 | SVQEIQATFFYFTPN(N)K Fuc 1 Hex 8 HexNAc 4 Neu5Ac 1 | 154.264 | 0.0 | 13.712 | 5.406e+07 | 57 - 73 | 4 | 54.80 | 54.97 | 55.65 |
| 4563.9198 | SVQEIQATFFYFTPN(N)K Fuc 2 Hex 7 HexNAc 6 | 153.860 | 0.0 | -3.572 | 7.004e+07 | 57 - 73 | 4 | 53.61 | 53.78 | 54.12 |
| 3766.7434 | LVPVPITN(N)ATLDQITGK Hex 6 HexNAc 5 | 152.056 | 0.0 | 14.739 | 1.537e+10 | 25 - 42 | 556 | 41.76 | 48.39 | 113.85 |
| 3401.6004 | LVPVPITN(N)ATLDQITGK Hex 5 HexNAc 4 | 150.805 | 0.0 | 15.211 | 1.455e+09 | 25 - 42 | 17 | 42.11 | 48.85 | 50.87 |
| 4277.9262 | LVPVPITN(N)ATLDQITGK Fuc 1 Hex 7 HexNAc 6 | 144.666 | 0.0 | 14.791 | 1.877e+08 | 25 - 42 | 5 | 47.38 | 47.90 | 48.22 |
| 4882.1738 | SVQEIQATFFYFTPN(N)KTEDTIFLR Hex 6 HexNAc 5 | 143.239 | 0.0 | 12.839 | 5.186e+08 | 57 - 81 | 17 | 60.45 | 62.85 | 66.10 |
| 3368.3746 | ATFFYFTPN(N)K Fuc 1 Hex 6 HexNAc 5 | 142.391 | 0.0 | -20.161 | 5.229e+06 | 63 - 73 | 1 | 40.74 | 40.91 | 40.91 |
| 5002.0667 | SVQEIQATFFYFTPN(N)K Hex 9 HexNAc 8 | 141.343 | 0.0 | 11.885 | 5.875e+07 | 57 - 73 | 2 | 52.58 | 52.92 | 53.44 |
| 5002.0667 | SVQEIQATFFYFTPN(N)K Fuc 5 Hex 7 HexNAc 6 | 141.343 | 0.0 | 11.885 | 5.875e+07 | 57 - 73 | 2 | 52.58 | 52.92 | 53.44 |
| 5028.2314 | SVQEIQATFFYFTPN(N)KTEDTIFLR Fuc 1 Hex 6 HexNAc 5 | 140.061 | 0.0 | 12.570 | 1.191e+08 | 57 - 81 | 7 | 62.51 | 62.68 | 64.73 |
| 4447.8321 | SVQEIQATFFYFTPN(N)K Hex 7 HexNAc 4 Neu5Ac 2 | 139.277 | 0.0 | 13.461 | 1.197e+08 | 57 - 73 | 7 | 54.80 | 54.97 | 56.68 |
| 4243.8033 | FYFTPN(N)KTEDTIFLR Hex 7 HexNAc 6 | 136.485 | 0.0 | 13.345 | 1.961e+08 | 66 - 81 | 11 | 44.16 | 45.68 | 47.56 |
| 4131.8688 | LVPVPITN(N)ATLDQITGK Hex 7 HexNAc 6 | 136.092 | 0.0 | 14.734 | 5.604e+08 | 25 - 42 | 11 | 41.08 | 48.07 | 48.70 |
| 3731.6018 | YFTPN(N)KTEDTIFLR Hex 6 HexNAc 5 | 135.460 | 0.0 | 10.888 | 1.048e+09 | 67 - 81 | 8 | 35.35 | 37.38 | 39.22 |
| 5247.3084 | SVQEIQATFFYFTPN(N)KTEDTIFLR Hex 7 HexNAc 6 | 134.393 | 0.0 | 13.409 | 1.235e+09 | 57 - 81 | 25 | 60.10 | 62.51 | 68.16 |
| 3786.6025 | TPN(N)KTEDTIFLR Hex 7 HexNAc 6 | 133.715 | 0.0 | 13.452 | 5.229e+07 | 69 - 81 | 8 | 25.36 | 25.70 | 26.30 |
| 3877.6623 | YFTPN(N)KTEDTIFLR Fuc 1 Hex 6 HexNAc 5 | 132.092 | 0.0 | -3.782 | 2.463e+08 | 67 - 81 | 4 | 35.35 | 37.21 | 37.53 |
| 4048.6690 | QDQC(C)IYN(N)TTYLNVQR Fuc 1 Hex 6 HexNAc 5 | 131.430 | 0.0 | -2.218 | 4.239e+08 | 86 - 101 | 4 | 33.65 | 33.82 | 34.16 |
| 3902.6098 | QDQC(C)IYN(N)TTYLNVQR Hex 6 HexNAc 5 | 131.350 | 0.0 | 9.314 | 1.786e+09 | 86 - 101 | 8 | 31.61 | 33.99 | 34.84 |
| 4188.8887 | PVPITN(N)ATLDQITGK Fuc 1 Hex 4 HexNAc 9 | 130.343 | 0.0 | 7.921 | 9.062e+07 | 27 - 42 | 5 | 47.21 | 48.70 | 48.85 |
| 4690.8519 | ATFFYFTPN(N)K Fuc 5 Hex 5 HexNAc 8 Neu5Ac 1 | 130.132 | 0.0 | -5.370 | 1.315e+07 | 63 - 73 | 2 | 53.27 | 53.27 | 53.61 |
| 4632.8794 | QDQC(C)IYN(N)TTYLNVQR Hex 8 HexNAc 7 | 128.484 | 0.0 | 15.268 | 3.816e+08 | 86 - 101 | 4 | 32.80 | 32.97 | 33.65 |
| 3421.4699 | TPN(N)KTEDTIFLR Hex 6 HexNAc 5 | 127.492 | 0.0 | 13.344 | 3.001e+07 | 69 - 81 | 6 | 26.01 | 26.18 | 27.02 |
| 4328.8209 | SVQEIQATFFYFTPN(N)K Fuc 4 Hex 5 HexNAc 5 | 127.198 | 0.0 | 11.789 | 5.138e+08 | 57 - 73 | 25 | 53.09 | 54.80 | 57.53 |
| 4413.8001 | QDQC(C)IYN(N)TTYLNVQR Fuc 1 Hex 7 HexNAc 6 | 126.973 | 0.0 | 12.235 | 5.941e+08 | 86 - 101 | 6 | 33.14 | 33.48 | 34.33 |
| 4267.7532 | QDQC(C)IYN(N)TTYLNVQR Hex 7 HexNAc 6 | 126.613 | 0.0 | -1.398 | 2.311e+09 | 86 - 101 | 44 | 31.36 | 33.65 | 100.12 |
| 4593.8936 | SVQEIQATFFYFTPN(N)K Fuc 1 Hex 7 HexNAc 4 Neu5Ac 2 | 126.229 | 0.0 | -20.071 | 1.231e+07 | 57 - 73 | 1 | 54.80 | 54.80 | 54.97 |
| 4096.7404 | YFTPN(N)KTEDTIFLR Hex 7 HexNAc 6 | 125.693 | 0.0 | 13.184 | 2.069e+09 | 67 - 81 | 21 | 35.18 | 36.80 | 44.16 |
| 4242.8246 | YFTPN(N)KTEDTIFLR Fuc 1 Hex 7 HexNAc 6 | 124.688 | 0.0 | -6.381 | 2.167e+08 | 67 - 81 | 6 | 36.51 | 36.65 | 61.65 |
| 3879.6742 | YFTPN(N)KTEDTIFLR Fuc 1 Hex 6 HexNAc 5 | 124.296 | 0.0 | 0.000 | 0.000e+00 | 67 - 81 | 1 | - | - | - |
| 4109.7460 | SVQEIQATFFYFTPN(N)K Hex 6 HexNAc 6 | 124.206 | 0.0 | 12.244 | 5.282e+07 | 57 - 73 | 3 | 54.97 | 54.97 | 55.82 |
| 3366.4691 | YFTPN(N)KTEDTIFLR Hex 5 HexNAc 4 | 124.024 | 0.0 | -4.454 | 7.378e+07 | 67 - 81 | 1 | 37.70 | 37.86 | 38.19 |
| 4299.8120 | YFTPN(N)KTEDTIFLR Hex 7 HexNAc 7 | 123.178 | 0.0 | -5.038 | 7.567e+07 | 67 - 81 | 3 | 37.06 | 37.21 | 37.53 |
| 4435.7895 | QDQC(C)IYN(N)TTYLNVQR Hex 10 HexNAc 3 Neu5Ac 1 | 119.256 | 0.0 | -5.442 | 9.558e+07 | 86 - 101 | 1 | 33.14 | 33.31 | 33.65 |
| 3733.5127 | ATFFYFTPN(N)K Fuc 1 Hex 7 HexNAc 6 | 118.626 | 0.0 | -18.182 | 6.893e+06 | 63 - 73 | 1 | 40.22 | 40.22 | 40.39 |
| 4973.0651 | YFTPN(N)KTEDTIFLR Fuc 1 Hex 9 HexNAc 8 | 117.566 | 0.0 | -19.116 | 2.395e+06 | 67 - 81 | 1 | 35.35 | 35.35 | 35.35 |
| 4559.8585 | QDQC(C)IYN(N)TTYLNVQR Fuc 2 Hex 7 HexNAc 6 | 116.888 | 0.0 | 10.589 | 9.771e+07 | 86 - 101 | 2 | 33.31 | 33.31 | 33.99 |
| 4497.0003 | LVPVPITN(N)ATLDQITGK Hex 8 HexNAc 7 | 116.845 | 0.0 | 8.880 | 7.321e+07 | 25 - 42 | 4 | 47.21 | 47.73 | 47.90 |
| 4461.8686 | YFTPN(N)KTEDTIFLR Hex 8 HexNAc 7 | 116.516 | 0.0 | 13.542 | 1.775e+08 | 67 - 81 | 3 | 35.69 | 36.03 | 36.36 |
| 4389.8616 | YFTPN(N)KTEDTIFLR Fuc 2 Hex 7 HexNAc 6 | 115.730 | 0.0 | -6.169 | 3.407e+07 | 67 - 81 | 8 | 45.51 | 45.68 | 46.02 |
| 4778.9346 | QDQC(C)IYN(N)TTYLNVQR Fuc 1 Hex 8 HexNAc 7 | 115.387 | 0.0 | 13.422 | 9.870e+07 | 86 - 101 | 4 | 32.63 | 33.14 | 33.31 |
| 3959.7924 | LVPVPITN(N)ATLDQITGK Fuc 1 Hex 7 HexNAc 3 Neu5Ac 1 | 114.637 | 0.0 | -3.894 | 3.837e+07 | 25 - 42 | 3 | 49.50 | 49.50 | 50.01 |
| 3967.7137 | PVPITN(N)ATLDQITGK Fuc 2 Hex 8 HexNAc 4 | 113.223 | 0.0 | 0.000 | 0.000e+00 | 27 - 42 | 2 | - | - | - |
| 3950.7367 | PVPITN(N)ATLDQITGK Fuc 1 Hex 7 HexNAc 4 Neu5Ac 1 | 110.973 | 0.0 | -3.889 | 3.228e+08 | 27 - 42 | 3 | 47.90 | 48.07 | 48.39 |
| 4474.8765 | SVQEIQATFFYFTPN(N)K Hex 7 HexNAc 7 | 110.321 | 0.0 | 10.974 | 5.677e+07 | 57 - 73 | 3 | 54.29 | 54.46 | 55.48 |
| 4090.8337 | PVPITN(N)ATLDQITGK Fuc 2 Hex 5 HexNAc 7 | 109.913 | 0.0 | 0.000 | 0.000e+00 | 27 - 42 | 1 | - | - | - |
| 3878.6697 | FYFTPN(N)KTEDTIFLR Hex 6 HexNAc 5 | 108.994 | 0.0 | 13.111 | 6.677e+07 | 66 - 81 | 5 | 46.02 | 46.19 | 47.21 |
| 3895.6905 | YFTPN(N)KTEDTIFLR Hex 7 HexNAc 5 | 107.735 | 0.0 | 0.000 | 0.000e+00 | 67 - 81 | 1 | - | - | - |
| 3838.6665 | PVPITN(N)ATLDQITGK Hex 9 HexNAc 4 | 106.398 | 0.0 | 0.000 | 0.000e+00 | 27 - 42 | 1 | - | - | - |
| 4060.8457 | PVPITN(N)ATLDQITGK Fuc 4 Hex 3 HexNAc 7 | 106.002 | 0.0 | 0.000 | 0.000e+00 | 27 - 42 | 1 | - | - | - |
| 4643.0586 | PVPITN(N)ATLDQITGK Fuc 3 Hex 5 HexNAc 9 | 105.215 | 0.0 | 9.496 | 9.966e+06 | 27 - 42 | 3 | 47.04 | 47.38 | 47.73 |
| 4020.7100 | SVQEIQATFFYFTPN(N)K Fuc 3 Hex 4 HexNAc 5 | 103.619 | 0.0 | -19.131 | 9.858e+06 | 57 - 73 | 2 | 54.12 | 54.29 | 54.29 |
| 4517.0370 | SVQEIQATFFYFTPN(N)KTEDTIFLR Hex 5 HexNAc 4 | 101.817 | 0.0 | -3.930 | 1.974e+07 | 57 - 81 | 2 | 63.19 | 63.19 | 63.53 |
| 3602.5917 | PVPITN(N)ATLDQITGK Fuc 2 Hex 7 HexNAc 3 | 100.953 | 0.0 | -18.914 | 3.862e+06 | 27 - 42 | 1 | 52.41 | 52.58 | 52.58 |
| 3568.5397 | FTPN(N)KTEDTIFLR Hex 6 HexNAc 5 | 97.357 | 0.0 | -2.894 | 6.942e+07 | 68 - 81 | 4 | 32.12 | 32.29 | 32.63 |
| 4640.0216 | PVPITN(N)ATLDQITGK Fuc 2 Hex 3 HexNAc 7 Neu5Ac 3 | 97.036 | 0.0 | -4.762 | 6.757e+06 | 27 - 42 | 3 | 57.53 | 57.71 | 58.05 |
| 4057.8312 | LVPVPITN(N)ATLDQITGK Hex 6 HexNAc 5 Neu5Ac 1 | 96.926 | 0.0 | 12.326 | 1.967e+07 | 25 - 42 | 6 | 50.70 | 51.21 | 51.72 |
| 5393.3643 | SVQEIQATFFYFTPN(N)KTEDTIFLR Fuc 1 Hex 7 HexNAc 6 | 96.551 | 0.0 | 13.059 | 2.553e+08 | 57 - 81 | 10 | 61.30 | 62.33 | 64.73 |
| 3567.5293 | TPN(N)KTEDTIFLR Fuc 1 Hex 6 HexNAc 5 | 96.307 | 0.0 | -4.430 | 4.815e+06 | 69 - 81 | 3 | 25.84 | 25.84 | 26.18 |
| 4826.9981 | YFTPN(N)KTEDTIFLR Hex 9 HexNAc 8 | 95.055 | 0.0 | 11.679 | 4.966e+07 | 67 - 81 | 2 | 35.35 | 35.69 | 36.03 |
| 3371.4304 | IYN(N)TTYLNVQR Hex 6 HexNAc 5 | 94.309 | 0.0 | 13.036 | 4.055e+07 | 90 - 101 | 4 | 28.74 | 28.92 | 29.33 |
| 3933.6716 | FTPN(N)KTEDTIFLR Hex 7 HexNAc 6 | 94.046 | 0.0 | 14.313 | 1.310e+08 | 68 - 81 | 6 | 31.61 | 31.77 | 32.63 |
| 3422.4748 | TPN(N)KTEDTIFLR Hex 6 HexNAc 5 | 93.876 | 0.0 | 0.000 | 0.000e+00 | 69 - 81 | 1 | - | - | - |
| 4088.8254 | PVPITN(N)ATLDQITGK Fuc 3 Hex 3 HexNAc 5 Neu5Ac 2 | 92.787 | 0.0 | -5.114 | 2.608e+07 | 27 - 42 | 1 | 49.17 | 49.33 | 49.67 |
| 4608.9343 | FYFTPN(N)KTEDTIFLR Hex 8 HexNAc 7 | 92.701 | 0.0 | 12.809 | 2.289e+07 | 66 - 81 | 4 | 44.83 | 45.17 | 45.51 |
| 4443.7724 | QDQC(C)IYN(N)TTYLNVQR Hex 7 HexNAc 4 Neu5Ac 2 | 88.944 | 0.0 | 12.784 | 1.380e+08 | 86 - 101 | 3 | 34.33 | 34.50 | 35.18 |
| 4272.7549 | YFTPN(N)KTEDTIFLR Hex 7 HexNAc 4 Neu5Ac 2 | 88.062 | 0.0 | -4.075 | 1.374e+08 | 67 - 81 | 10 | 37.86 | 38.03 | 38.19 |
| 4058.8536 | LVPVPITN(N)ATLDQITGK Fuc 2 Hex 6 HexNAc 5 | 86.425 | 0.0 | -5.379 | 3.512e+07 | 25 - 42 | 1 | 47.90 | 48.07 | 48.22 |
| 3714.5966 | FTPN(N)KTEDTIFLR Fuc 1 Hex 6 HexNAc 5 | 86.031 | 0.0 | -3.821 | 1.409e+07 | 68 - 81 | 2 | 31.94 | 32.12 | 32.29 |
| 4609.8993 | YFTPN(N)KTEDTIFLR Fuc 1 Hex 8 HexNAc 7 | 84.859 | 0.0 | 0.000 | 0.000e+00 | 67 - 81 | 1 | - | - | - |
| 4079.7259 | FTPN(N)KTEDTIFLR Fuc 1 Hex 7 HexNAc 6 | 84.231 | 0.0 | -3.376 | 2.935e+07 | 68 - 81 | 5 | 31.53 | 31.61 | 31.94 |
| 4300.5882 | FTPN(N)KTEDTIFLR Hex 8 HexNAc 7 | 82.657 | 0.0 | 10.113 | 6.518e+07 | 68 - 81 | 10 | 31.02 | 47.04 | 47.38 |
| 3932.6586 | TPN(N)KTEDTIFLR Fuc 1 Hex 7 HexNAc 6 | 81.957 | 0.0 | -4.692 | 7.704e+06 | 69 - 81 | 3 | 25.36 | 25.53 | 25.70 |
| 5539.4244 | SVQEIQATFFYFTPN(N)KTEDTIFLR Fuc 2 Hex 7 HexNAc 6 | 81.287 | 0.002 | -20.156 | 2.407e+07 | 57 - 81 | 1 | 62.16 | 62.33 | 62.33 |
| 3056.3377 | TPN(N)KTEDTIFLR Hex 5 HexNAc 4 | 76.332 | 0.004 | 10.230 | 6.463e+06 | 69 - 81 | 2 | 26.43 | 26.52 | 26.82 |
| 3788.6236 | FYFTPN(N)KTEDTIFLR Fuc 1 Hex 4 HexNAc 4 Neu5Ac 1 | 72.960 | 0.005 | 14.074 | 2.608e+08 | 66 - 81 | 5 | 37.86 | 38.03 | 38.53 |
| 4929.0581 | SVQEIQATFFYFTPN(N)K Fuc 2 Hex 8 HexNAc 7 | 69.103 | 0.005 | 11.354 | 1.280e+07 | 57 - 73 | 2 | 52.92 | 53.27 | 53.61 |
| 5367.1969 | SVQEIQATFFYFTPN(N)K Hex 10 HexNAc 9 | 65.611 | 0.005 | -4.491 | 6.331e+06 | 57 - 73 | 1 | 52.24 | 52.41 | 52.58 |
| 4113.7672 | FYFTPN(N)KTEDTIFLR Fuc 3 Hex 6 HexNAc 4 | 63.898 | 0.005 | -4.629 | 9.844e+07 | 66 - 81 | 2 | 36.80 | 36.80 | 37.06 |
| 3931.8249 | LVPVPITN(N)ATLDQITGK Hex 7 HexNAc 5 | 61.892 | 0.005 | 0.000 | 0.000e+00 | 25 - 42 | 1 | - | - | - |
| 5148.1262 | SVQEIQATFFYFTPN(N)K Fuc 1 Hex 9 HexNAc 8 | 61.775 | 0.005 | -21.179 | 2.927e+06 | 57 - 73 | 1 | 52.41 | 52.58 | 52.58 |
| 4693.9566 | SVQEIQATFFYFTPN(N)K Fuc 4 Hex 6 HexNAc 6 | 60.976 | 0.005 | 8.134 | 3.673e+07 | 57 - 73 | 2 | 53.78 | 53.78 | 54.46 |
| 5612.4365 | SVQEIQATFFYFTPN(N)KTEDTIFLR Hex 8 HexNAc 7 | 60.030 | 0.005 | 16.381 | 1.901e+08 | 57 - 81 | 4 | 61.65 | 61.82 | 62.51 |
| 4111.8450 | C(C)ANLVPVPITN(N)ATLDQITGK Hex 6 HexNAc 5 | 58.537 | 0.005 | 15.155 | 2.141e+08 | 22 - 42 | 9 | 51.89 | 61.13 | 61.65 |
| 4998.0113 | QDQC(C)IYN(N)TTYLNVQR Hex 9 HexNAc 8 | 57.561 | 0.005 | 11.808 | 1.351e+08 | 86 - 101 | 3 | 32.29 | 32.63 | 33.14 |
| 4492.9081 | SVQEIQATFFYFTPN(N)K Fuc 4 Hex 6 HexNAc 5 | 54.697 | 0.005 | 0.000 | 0.000e+00 | 57 - 73 | 1 | - | - | - |
| 4024.7276 | FYFTPN(N)KTEDTIFLR Fuc 1 Hex 6 HexNAc 5 | 53.074 | 0.005 | 12.860 | 1.710e+07 | 66 - 81 | 4 | 45.85 | 46.02 | 46.53 |
| 4257.9026 | C(C)ANLVPVPITN(N)ATLDQITGK Fuc 1 Hex 6 HexNAc 5 | 50.510 | 0.005 | 14.021 | 1.929e+07 | 22 - 42 | 2 | 51.72 | 51.89 | 52.41 |
| 4924.9937 | QDQC(C)IYN(N)TTYLNVQR Fuc 2 Hex 8 HexNAc 7 | 49.924 | 0.005 | 13.536 | 2.182e+07 | 86 - 101 | 2 | 32.46 | 32.97 | 33.14 |
| 3537.4758 | QDQC(C)IYN(N)TTYLNVQR Hex 5 HexNAc 4 | 48.288 | 0.005 | -4.482 | 3.249e+07 | 86 - 101 | 2 | 34.16 | 34.33 | 34.67 |
| 3969.8132 | LVPVPITN(N)ATLDQITGK Hex 6 HexNAc 6 | 47.749 | 0.005 | 11.070 | 7.468e+08 | 25 - 42 | 12 | 42.11 | 49.01 | 50.01 |
| 5440.3622 | SVQEIQATFFYFTPN(N)KTEDTIFLR Fuc 1 Hex 8 HexNAc 4 Neu5Ac 1 | 47.657 | 0.005 | -4.309 | 1.049e+07 | 57 - 81 | 1 | 63.36 | 63.36 | 63.70 |
| 5144.0682 | QDQC(C)IYN(N)TTYLNVQR Fuc 1 Hex 9 HexNAc 8 | 46.478 | 0.005 | -4.658 | 1.890e+07 | 86 - 101 | 2 | 32.29 | 32.46 | 32.80 |
| 3882.7937 | LVPVPITN(N)ATLDQITGK Fuc 3 Hex 4 HexNAc 5 | 45.838 | 0.005 | 0.000 | 0.000e+00 | 25 - 42 | 1 | - | - | - |
| 4460.8037 | QDQC(C)IYN(N)TTYLNVQR Fuc 1 Hex 8 HexNAc 4 Neu5Ac 1 | 45.242 | 0.005 | 11.400 | 3.018e+07 | 86 - 101 | 1 | 34.33 | 34.50 | 35.01 |
| 5977.5682 | SVQEIQATFFYFTPN(N)KTEDTIFLR Hex 9 HexNAc 8 | 44.143 | 0.005 | 14.835 | 6.254e+07 | 57 - 81 | 4 | 61.13 | 61.48 | 62.33 |
| 4461.8159 | QDQC(C)IYN(N)TTYLNVQR Fuc 3 Hex 8 HexNAc 4 | 43.594 | 0.005 | -5.507 | 8.025e+06 | 86 - 101 | 2 | 34.67 | 34.67 | 35.01 |
| 4620.9440 | SVQEIQATFFYFTPN(N)K Fuc 1 Hex 7 HexNAc 7 | 42.796 | 0.005 | -20.438 | 3.818e+06 | 57 - 73 | 1 | 54.29 | 54.29 | 54.29 |
| 5150.1185 | SVQEIQATFFYFTPN(N)K Fuc 1 Hex 9 HexNAc 8 | 40.953 | 0.005 | 0.000 | 0.000e+00 | 57 - 73 | 1 | - | - | - |
| 5758.4931 | SVQEIQATFFYFTPN(N)KTEDTIFLR Fuc 1 Hex 8 HexNAc 7 | 40.211 | 0.006 | -4.734 | 3.399e+07 | 57 - 81 | 3 | 61.65 | 61.99 | 61.99 |
| 4709.1775 | QIPLC(C)ANLVPVPITN(N)ATLDQITGK Fuc 1 Hex 6 HexNAc 5 | 39.976 | 0.006 | 12.447 | 1.105e+08 | 18 - 42 | 7 | 67.30 | 67.99 | 68.68 |
| 3822.6870 | PVPITN(N)ATLDQITGK Fuc 1 Hex 8 HexNAc 4 | 38.967 | 0.006 | -6.681 | 2.320e+05 | 27 - 42 | 1 | 79.41 | 79.47 | 79.70 |
| 4105.6906 | QDQC(C)IYN(N)TTYLNVQR Hex 6 HexNAc 6 | 38.383 | 0.006 | 12.437 | 8.493e+07 | 86 - 101 | 1 | 33.82 | 33.99 | 34.67 |
| 3966.7056 | PVPITN(N)ATLDQITGK Hex 8 HexNAc 4 Neu5Ac 1 | 38.350 | 0.006 | -19.210 | 1.076e+08 | 27 - 42 | 1 | 48.07 | 48.07 | 48.22 |
| 3884.5161 | QDQC(C)IYN(N)TTYLN Hex 7 HexNAc 6 | 38.123 | 0.007 | -19.524 | 3.864e+06 | 86 - 98 | 1 | 32.12 | 32.29 | 32.29 |
| 3903.5979 | QDQC(C)IYN(N)TTYLN(D)VQR Hex 6 HexNAc 5 | 37.921 | 0.009 | -4.396 | 9.208e+07 | 86 - 101 | 11 | 32.46 | 35.01 | 35.69 |
| 3513.5370 | FYFTPN(N)KTEDTIFLR Hex 5 HexNAc 4 | 37.117 | 0.010 | -19.577 | 2.749e+06 | 66 - 81 | 1 | 46.53 | 46.70 | 46.70 |
| 4153.7553 | YFTPN(N)KTEDTIFLR Fuc 4 Hex 5 HexNAc 5 | 36.663 | 0.010 | 13.118 | 4.645e+08 | 67 - 81 | 4 | 37.21 | 37.38 | 38.53 |
| 3951.7380 | PVPITN(N)ATLDQITGK Fuc 3 Hex 7 HexNAc 4 | 36.446 | 0.010 | -4.666 | 4.012e+07 | 27 - 42 | 1 | 48.39 | 48.53 | 48.70 |
| 4334.9433 | PVPITN(N)ATLDQITGK Fuc 2 Hex 4 HexNAc 9 | 35.559 | 0.010 | -20.462 | 3.694e+06 | 27 - 42 | 1 | 47.73 | 47.73 | 47.90 |
| 4074.8590 | LVPVPITN(N)ATLDQITGK Fuc 1 Hex 7 HexNAc 5 | 34.138 | 0.012 | 11.495 | 6.247e+06 | 25 - 42 | 2 | 50.87 | 51.21 | 51.55 |
| 4705.9173 | QDQC(C)IYN(N)TTYLNVQR Fuc 3 Hex 7 HexNAc 6 | 33.827 | 0.013 | -20.177 | 4.872e+06 | 86 - 101 | 2 | 33.31 | 33.31 | 33.48 |
| 4194.7357 | QDQC(C)IYN(N)TTYLNVQR Fuc 2 Hex 6 HexNAc 5 | 33.815 | 0.013 | -5.228 | 7.979e+06 | 86 - 101 | 2 | 33.31 | 33.65 | 33.65 |
| 4977.0741 | FYFTPN(N)KTEDTIFLR Hex 9 HexNAc 8 | 33.578 | 0.013 | 0.000 | 0.000e+00 | 66 - 81 | 1 | - | - | - |
| 5614.4453 | SVQEIQATFFYFTPN(N)KTEDTIFLR Hex 8 HexNAc 7 | 32.862 | 0.014 | 0.000 | 0.000e+00 | 57 - 81 | 1 | - | - | - |
| 4589.8338 | QDQC(C)IYN(N)TTYLNVQR Fuc 1 Hex 7 HexNAc 4 Neu5Ac 2 | 32.735 | 0.016 | -4.567 | 2.497e+07 | 86 - 101 | 2 | 34.33 | 34.33 | 34.67 |
| 3736.5651 | IYN(N)TTYLNVQR Hex 7 HexNAc 6 | 32.258 | 0.018 | 13.279 | 2.852e+07 | 90 - 101 | 5 | 28.33 | 28.52 | 28.80 |
| 5192.1403 | YFTPN(N)KTEDTIFLR Fuc 5 Hex 8 HexNAc 7 | 31.375 | 0.019 | -4.590 | 5.292e+06 | 67 - 81 | 1 | 35.18 | 35.35 | 35.52 |
| 4131.8443 | PVPITN(N)ATLDQITGK Fuc 2 Hex 4 HexNAc 8 | 31.117 | 0.020 | -18.972 | 7.476e+05 | 27 - 42 | 1 | 58.90 | 58.90 | 58.90 |
| 4657.0424 | LVPVPITN(N)ATLDQITGK Fuc 1 Hex 7 HexNAc 5 Neu5Ac 2 | 30.844 | 0.021 | -5.532 | 2.076e+06 | 25 - 42 | 1 | 57.71 | 57.71 | 58.05 |
| 4101.7126 | FTPN(N)KTEDTIFLR Hex 10 HexNAc 3 Neu5Ac 1 | 30.438 | 0.021 | -20.571 | 1.042e+06 | 68 - 81 | 1 | 31.61 | 31.77 | 31.77 |
| 4423.9869 | LVPVPITN(N)ATLDQITGK Fuc 2 Hex 7 HexNAc 6 | 30.311 | 0.022 | 7.629 | 1.899e+07 | 25 - 42 | 2 | 47.21 | 47.90 | 47.90 |
| 4563.1219 | QIPLC(C)ANLVPVPITN(N)ATLDQITGK Hex 6 HexNAc 5 | 30.176 | 0.022 | 13.433 | 4.108e+08 | 18 - 42 | 9 | 67.65 | 68.34 | 69.19 |
| 4170.7834 | FYFTPN(N)KTEDTIFLR Fuc 2 Hex 6 HexNAc 5 | 29.641 | 0.024 | -19.569 | 2.292e+07 | 66 - 81 | 1 | 37.38 | 37.38 | 37.53 |
| 4547.0821 | LVPVPITN(N)ATLDQITGK Fuc 2 Hex 4 HexNAc 9 | 29.366 | 0.024 | 8.620 | 6.992e+07 | 25 - 42 | 11 | 64.05 | 69.64 | 70.73 |
| 4649.9020 | QDQC(C)IYN(N)TTYLN(D)VQR Fuc 4 Hex 7 HexNAc 5 | 29.173 | 0.024 | 13.006 | 1.206e+08 | 86 - 101 | 4 | 32.80 | 32.97 | 33.48 |
| 3517.4876 | IYN(N)TTYLNVQR Fuc 1 Hex 6 HexNAc 5 | 29.119 | 0.024 | -4.001 | 3.786e+06 | 90 - 101 | 1 | 28.61 | 28.67 | 28.80 |
| 3761.5649 | N(N)ATLDQITGK Fuc 1 Hex 7 HexNAc 7 | 28.910 | 0.025 | -4.957 | 1.022e+08 | 32 - 42 | 4 | 52.75 | 52.92 | 53.09 |
| 3423.4966 | FYFTPN(N)KTEDTIFLR Fuc 1 Hex 3 HexNAc 3 Neu5Ac 1 | 28.817 | 0.025 | -19.037 | 1.523e+07 | 66 - 81 | 2 | 38.36 | 38.36 | 38.53 |
| 7813.3370 | WFYIASAFRNEEYNKSVQEIQATFFYFTPN(N)K Fuc 5 Hex 10 HexNAc 8 | 28.593 | 0.028 | -4.675 | 2.605e+07 | 42 - 73 | 1 | 54.46 | 54.46 | 54.80 |
| 3822.7695 | LVPVPITN(N)ATLDQITGK Fuc 2 Hex 4 HexNAc 4 Neu5Ac 1 | 28.550 | 0.028 | -17.980 | 6.855e+07 | 25 - 42 | 2 | 47.56 | 47.56 | 47.73 |
| 4324.7649 | QDQC(C)IYN(N)TTYLNVQR Fuc 4 Hex 5 HexNAc 5 | 28.152 | 0.029 | 13.011 | 4.485e+08 | 86 - 101 | 6 | 33.65 | 33.82 | 35.01 |
| 4606.8649 | QDQC(C)IYN(N)TTYLNVQR Fuc 2 Hex 8 HexNAc 4 Neu5Ac 1 | 28.132 | 0.029 | -20.506 | 7.056e+06 | 86 - 101 | 1 | 34.33 | 34.33 | 34.50 |
| 3810.7363 | LVPVPITN(N)ATLDQITGK Hex 5 HexNAc 6 | 27.927 | 0.030 | -24.147 | 1.746e+06 | 25 - 42 | 9 | 64.56 | 65.59 | 98.05 |
| 4268.7296 | QDQC(C)IYN(N)TTYLN(D)VQR Hex 7 HexNAc 6 | 27.607 | 0.033 | -20.467 | 8.676e+07 | 86 - 101 | 2 | 34.67 | 34.67 | 34.84 |
| 3934.6802 | FYFTPN(N)KTEDTIFLR Fuc 2 Hex 4 HexNAc 4 Neu5Ac 1 | 27.574 | 0.033 | -18.612 | 5.093e+07 | 66 - 81 | 1 | 37.70 | 37.70 | 37.86 |
| 4414.7862 | QDQC(C)IYN(N)TTYLN(D)VQR Fuc 1 Hex 7 HexNAc 6 | 26.847 | 0.037 | -20.944 | 2.602e+07 | 86 - 101 | 1 | 34.50 | 34.50 | 34.67 |
| 3914.7904 | LVPVPITN(N)ATLDQITGK Fuc 1 Hex 6 HexNAc 5 | 26.788 | 0.037 | 0.000 | 0.000e+00 | 25 - 42 | 1 | - | - | - |
| 5904.5400 | SVQEIQATFFYFTPN(N)KTEDTIFLR Fuc 2 Hex 8 HexNAc 7 | 26.688 | 0.037 | -4.533 | 6.113e+06 | 57 - 81 | 1 | 61.65 | 61.82 | 61.99 |
| 4242.8512 | PVPITN(N)ATLDQITGK Fuc 3 Hex 7 HexNAc 4 Neu5Ac 1 | 25.915 | 0.039 | -21.607 | 1.382e+06 | 27 - 42 | 1 | 61.82 | 61.82 | 61.82 |
| 3804.6769 | PVPITN(N)ATLDQITGK Hex 7 HexNAc 4 Neu5Ac 1 | 25.797 | 0.039 | 14.107 | 1.148e+09 | 27 - 42 | 11 | 48.07 | 48.53 | 50.35 |
| 3847.7581 | LVPVPITN(N)ATLDQITGK Hex 9 HexNAc 3 | 25.705 | 0.039 | 0.000 | 0.000e+00 | 25 - 42 | 1 | - | - | - |
| 4007.7564 | PVPITN(N)ATLDQITGK Hex 7 HexNAc 5 Neu5Ac 1 | 25.700 | 0.039 | 12.821 | 1.025e+08 | 27 - 42 | 3 | 48.85 | 49.01 | 49.84 |
| 4692.1564 | QIPLC(C)ANLVPVPITN(N)ATLDQITGK Hex 5 HexNAc 5 Neu5Ac 1 | 25.673 | 0.039 | 15.424 | 2.074e+08 | 18 - 42 | 9 | 67.30 | 67.99 | 69.02 |
| 3925.5873 | QDQC(C)IYN(N)TTYLNVQR Fuc 1 Hex 9 HexNAc 2 | 25.551 | 0.042 | 9.562 | 3.493e+07 | 86 - 101 | 1 | 34.50 | 34.84 | 35.69 |
| 6342.6973 | SVQEIQATFFYFTPN(N)KTEDTIFLR Hex 10 HexNAc 9 | 25.513 | 0.042 | -5.815 | 6.204e+06 | 57 - 81 | 1 | 61.13 | 61.30 | 61.48 |
| 4693.1395 | LVPVPITN(N)ATLDQITGK Fuc 3 Hex 4 HexNAc 9 | 25.420 | 0.042 | 3.743 | 1.671e+07 | 25 - 42 | 6 | 69.19 | 69.36 | 70.08 |
| 3432.3889 | ATFFYFTPN(N)K Fuc 2 Hex 8 HexNAc 3 | 25.196 | 0.045 | -18.346 | 8.769e+06 | 63 - 73 | 1 | 35.35 | 35.52 | 35.52 |
| 4478.8941 | FYFTPN(N)KTEDTIFLR Fuc 3 Hex 7 HexNAc 5 | 25.171 | 0.045 | -5.679 | 2.657e+07 | 66 - 81 | 1 | 35.86 | 36.03 | 36.20 |
| 4862.1339 | LVPVPITN(N)ATLDQITGK Hex 9 HexNAc 8 | 25.034 | 0.045 | 8.121 | 7.801e+06 | 25 - 42 | 3 | 46.70 | 47.04 | 47.56 |
| 3769.7345 | LVPVPITN(N)ATLDQITGK Hex 6 HexNAc 5 | 25.017 | 0.045 | 0.000 | 0.000e+00 | 25 - 42 | 1 | - | - | - |
| 4562.0934 | LVPVPITN(N)ATLDQITGK Fuc 4 Hex 3 HexNAc 7 Neu5Ac 1 | 24.998 | 0.045 | 11.042 | 7.482e+07 | 25 - 42 | 8 | 66.10 | 67.48 | 67.65 |
| 3765.7119 | PVPITN(N)ATLDQITGK Hex 3 HexNAc 7 Neu5Ac 1 | 24.921 | 0.045 | -2.900 | 3.765e+06 | 27 - 42 | 1 | 72.29 | 72.42 | 72.42 |
| 3460.6237 | LVPVPITN(N)ATLDQITGK Fuc 2 Hex 3 HexNAc 3 Neu5Ac 1 | 24.857 | 0.045 | 0.000 | 0.000e+00 | 25 - 42 | 1 | - | - | - |
| 4314.9261 | C(C)ANLVPVPITN(N)ATLDQITGK Hex 6 HexNAc 6 | 24.735 | 0.045 | -18.879 | 1.247e+07 | 22 - 42 | 2 | 52.75 | 52.75 | 52.92 |
| 3882.6240 | IYN(N)TTYLNVQR Fuc 1 Hex 7 HexNAc 6 | 24.596 | 0.045 | 11.693 | 9.159e+06 | 90 - 101 | 2 | 28.18 | 28.37 | 28.46 |
| 3846.6966 | PVPITN(N)ATLDQITGK Fuc 2 Hex 6 HexNAc 5 | 24.272 | 0.045 | -5.218 | 3.772e+06 | 27 - 42 | 2 | 52.07 | 52.24 | 52.41 |
| 4049.6579 | QDQC(C)IYN(N)TTYLN(D)VQR Fuc 1 Hex 6 HexNAc 5 | 23.835 | 0.046 | -5.505 | 1.470e+07 | 86 - 101 | 1 | 34.84 | 34.84 | 35.35 |
| 5304.3235 | SVQEIQATFFYFTPN(N)KTEDTIFLR Fuc 4 Hex 5 HexNAc 5 | 23.744 | 0.046 | 12.858 | 2.236e+08 | 57 - 81 | 12 | 62.85 | 63.19 | 65.59 |
| 5423.3371 | SVQEIQATFFYFTPN(N)KTEDTIFLR Hex 7 HexNAc 4 Neu5Ac 2 | 23.640 | 0.049 | -4.042 | 2.579e+07 | 57 - 81 | 1 | 63.36 | 63.36 | 63.70 |

### YFTPN(N)K Hex 6 HexNAc 5

**Mass:**  2756.0958

(2.698e-06)

**Charge States:** 2, 3

**Start Position**: 68

**End Position**: 74

| Profile Score | Tandem Score | TDA q-value | Volume | Spectrum Matches |
| --- | --- | --- | --- | --- |
| 13.7913 | 187.4984 | 0.0000 | 2.5647e+08 | 3 |

**Adduct Signal Fractions**

- **Unmodified**: 2.5647e+08

### YFTPN(N)K Hex 7 HexNAc 6

**Mass:**  3121.2328

(9.366e-07)

**Charge States:** 3

**Start Position**: 68

**End Position**: 74

| Profile Score | Tandem Score | TDA q-value | Volume | Spectrum Matches |
| --- | --- | --- | --- | --- |
| 13.7821 | 187.1777 | 0.0000 | 3.6487e+08 | 4 |

**Adduct Signal Fractions**

- **Unmodified**: 3.6487e+08

### FYFTPN(N)K Hex 7 HexNAc 6

**Mass:**  3268.2991

(1.211e-06)

**Charge States:** 3

**Start Position**: 67

**End Position**: 74

| Profile Score | Tandem Score | TDA q-value | Volume | Spectrum Matches |
| --- | --- | --- | --- | --- |
| 15.2914 | 184.4226 | 0.0000 | 1.1539e+08 | 8 |

**Adduct Signal Fractions**

- **Unmodified**: 1.1539e+08

### FYFTPN(N)K Hex 6 HexNAc 5

**Mass:**  2903.1666

(1.323e-06)

**Charge States:** 2, 3

**Start Position**: 67

**End Position**: 74

| Profile Score | Tandem Score | TDA q-value | Volume | Spectrum Matches |
| --- | --- | --- | --- | --- |
| 13.6363 | 183.3051 | 0.0000 | 8.9641e+07 | 14 |

**Adduct Signal Fractions**

- **Unmodified**: 8.9641e+07

### NEEYN(N)K Hex 5 HexNAc 4

**Mass:**  2417.9227

(9.089e-07)

**Charge States:** 2, 3

**Start Position**: 52

**End Position**: 58

| Profile Score | Tandem Score | TDA q-value | Volume | Spectrum Matches |
| --- | --- | --- | --- | --- |
| 11.2627 | 182.3559 | 0.0000 | 5.9499e+07 | 7 |

**Adduct Signal Fractions**

- **Unmodified**: 5.9499e+07

### NEEYN(N)K Fuc 1 Hex 6 HexNAc 5

**Mass:**  2929.1109

(9.414e-07)

**Charge States:** 3

**Start Position**: 52

**End Position**: 58

| Profile Score | Tandem Score | TDA q-value | Volume | Spectrum Matches |
| --- | --- | --- | --- | --- |
| -3.5151 | 181.8048 | 0.0000 | 2.0912e+07 | 7 |

**Adduct Signal Fractions**

- **Unmodified**: 2.0912e+07

### NEEYN(N)K Hex 6 HexNAc 5

**Mass:**  2783.0537

(6.698e-07)

**Charge States:** 2, 3

**Start Position**: 52

**End Position**: 58

| Profile Score | Tandem Score | TDA q-value | Volume | Spectrum Matches |
| --- | --- | --- | --- | --- |
| 13.0609 | 181.4648 | 0.0000 | 2.8035e+08 | 20 |

**Adduct Signal Fractions**

- **Unmodified**: 2.8035e+08

### SVQEIQATFFYFTPN(N)K Hex 6 HexNAc 5

**Mass:**  3906.6663

(1.457e-06)

**Charge States:** 3, 4, 5

**Start Position**: 58

**End Position**: 74

| Profile Score | Tandem Score | TDA q-value | Volume | Spectrum Matches |
| --- | --- | --- | --- | --- |
| 15.0761 | 174.2427 | 0.0000 | 1.9316e+09 | 41 |

**Adduct Signal Fractions**

- **Unmodified**: 1.9316e+09

### YFTPN(N)K Fuc 1 Hex 6 HexNAc 5

**Mass:**  2902.1555

(-5.692e-07)

**Charge States:** 3

**Start Position**: 68

**End Position**: 74

| Profile Score | Tandem Score | TDA q-value | Volume | Spectrum Matches |
| --- | --- | --- | --- | --- |
| -18.6323 | 174.1396 | 0.0000 | 3.7915e+07 | 1 |

**Adduct Signal Fractions**

- **Unmodified**: 3.7915e+07

### YFTPN(N)K Fuc 1 Hex 7 HexNAc 6

**Mass:**  3267.2884

(1.490e-06)

**Charge States:** 3

**Start Position**: 68

**End Position**: 74

| Profile Score | Tandem Score | TDA q-value | Volume | Spectrum Matches |
| --- | --- | --- | --- | --- |
| -4.6348 | 173.3706 | 0.0000 | 6.0483e+07 | 2 |

**Adduct Signal Fractions**

- **Unmodified**: 6.0483e+07

### YFTPN(N)K Hex 8 HexNAc 7

**Mass:**  3486.3642

(1.170e-06)

**Charge States:** 3

**Start Position**: 68

**End Position**: 74

| Profile Score | Tandem Score | TDA q-value | Volume | Spectrum Matches |
| --- | --- | --- | --- | --- |
| -20.3105 | 172.6064 | 0.0000 | 1.9350e+07 | 1 |

**Adduct Signal Fractions**

- **Unmodified**: 1.9350e+07

### SVQEIQATFFYFTPN(N)K Hex 8 HexNAc 7

**Mass:**  4636.9357

(2.165e-06)

**Charge States:** 3, 4, 5

**Start Position**: 58

**End Position**: 74

| Profile Score | Tandem Score | TDA q-value | Volume | Spectrum Matches |
| --- | --- | --- | --- | --- |
| 13.5319 | 172.3604 | 0.0000 | 3.0643e+08 | 7 |

**Adduct Signal Fractions**

- **Unmodified**: 3.0643e+08

### YFTPN(N)K Hex 5 HexNAc 4

**Mass:**  2392.9588

(8.351e-04)

**Start Position**: 68

**End Position**: 74

| Profile Score | Tandem Score | TDA q-value | Volume | Spectrum Matches |
| --- | --- | --- | --- | --- |
| 0.0000 | 167.8728 | 0.0000 | 0.0000e+00 | 1 |

Adduct:

No Chromatogram Found

### SVQEIQATFFYFTPN(N)K Fuc 1 Hex 7 HexNAc 6

**Mass:**  4417.8684

(1.140e-06)

**Charge States:** 3, 4

**Start Position**: 58

**End Position**: 74

| Profile Score | Tandem Score | TDA q-value | Volume | Spectrum Matches |
| --- | --- | --- | --- | --- |
| 13.7613 | 167.6039 | 0.0000 | 4.9742e+08 | 13 |

**Adduct Signal Fractions**

- **Unmodified**: 4.9742e+08

### SVQEIQATFFYFTPN(N)K Fuc 1 Hex 6 HexNAc 5

**Mass:**  4052.7266

(1.801e-06)

**Charge States:** 3, 4

**Start Position**: 58

**End Position**: 74

| Profile Score | Tandem Score | TDA q-value | Volume | Spectrum Matches |
| --- | --- | --- | --- | --- |
| 13.6826 | 166.8947 | 0.0000 | 4.1727e+08 | 13 |

**Adduct Signal Fractions**

- **Unmodified**: 4.1727e+08

### YFTPN(N)K Hex 6 HexNAc 5

**Mass:**  2757.1032

(3.660e-04)

**Start Position**: 68

**End Position**: 74

| Profile Score | Tandem Score | TDA q-value | Volume | Spectrum Matches |
| --- | --- | --- | --- | --- |
| 0.0000 | 166.7052 | 0.0000 | 0.0000e+00 | 1 |

Adduct:

No Chromatogram Found

### YFTPN(N)K Hex 5 HexNAc 4

**Mass:**  2390.9698

(-1.680e-06)

**Charge States:** 2

**Start Position**: 68

**End Position**: 74

| Profile Score | Tandem Score | TDA q-value | Volume | Spectrum Matches |
| --- | --- | --- | --- | --- |
| -20.5840 | 166.0494 | 0.0000 | 1.8277e+07 | 2 |

**Adduct Signal Fractions**

- **Unmodified**: 1.8277e+07

### SVQEIQATFFYFTPN(N)K Hex 5 HexNAc 4

**Mass:**  3541.5331

(1.562e-06)

**Charge States:** 3, 4

**Start Position**: 58

**End Position**: 74

| Profile Score | Tandem Score | TDA q-value | Volume | Spectrum Matches |
| --- | --- | --- | --- | --- |
| 13.8719 | 165.6998 | 0.0000 | 2.4128e+08 | 12 |

**Adduct Signal Fractions**

- **Unmodified**: 2.4128e+08

### ATFFYFTPN(N)K Hex 6 HexNAc 5

**Mass:**  3222.3231

(1.682e-06)

**Charge States:** 3

**Start Position**: 64

**End Position**: 74

| Profile Score | Tandem Score | TDA q-value | Volume | Spectrum Matches |
| --- | --- | --- | --- | --- |
| -3.1395 | 164.2631 | 0.0000 | 3.7834e+07 | 2 |

**Adduct Signal Fractions**

- **Unmodified**: 3.7834e+07

### FYFTPN(N)K Fuc 1 Hex 6 HexNAc 5

**Mass:**  3049.2259

(1.966e-06)

**Charge States:** 3

**Start Position**: 67

**End Position**: 74

| Profile Score | Tandem Score | TDA q-value | Volume | Spectrum Matches |
| --- | --- | --- | --- | --- |
| 14.1209 | 163.9755 | 0.0345 | 1.5895e+07 | 3 |

**Adduct Signal Fractions**

- **Unmodified**: 1.5895e+07

### SVQEIQATFFYFTPN(N)K Hex 7 HexNAc 6

**Mass:**  4271.8107

(1.996e-06)

**Charge States:** 3, 4, 5

**Start Position**: 58

**End Position**: 74

| Profile Score | Tandem Score | TDA q-value | Volume | Spectrum Matches |
| --- | --- | --- | --- | --- |
| 15.0169 | 163.2087 | 0.0000 | 2.3057e+09 | 48 |

**Adduct Signal Fractions**

- **Unmodified**: 2.3057e+09

### FFYFTPN(N)K Hex 6 HexNAc 5

**Mass:**  3050.2337

(7.933e-07)

**Charge States:** 3

**Start Position**: 66

**End Position**: 74

| Profile Score | Tandem Score | TDA q-value | Volume | Spectrum Matches |
| --- | --- | --- | --- | --- |
| -19.0523 | 163.0275 | 0.0345 | 2.0923e+07 | 2 |

**Adduct Signal Fractions**

- **Unmodified**: 2.0923e+07

### FFYFTPN(N)K Hex 7 HexNAc 6

**Mass:**  3415.3694

(2.247e-06)

**Charge States:** 3

**Start Position**: 66

**End Position**: 74

| Profile Score | Tandem Score | TDA q-value | Volume | Spectrum Matches |
| --- | --- | --- | --- | --- |
| -2.6190 | 161.0261 | 0.0345 | 3.5746e+07 | 2 |

**Adduct Signal Fractions**

- **Unmodified**: 3.5746e+07

### LVPVPITN(N)ATLDQITGK Fuc 1 Hex 6 HexNAc 5

**Mass:**  3912.7926

(8.460e-07)

**Charge States:** 3, 4

**Start Position**: 26

**End Position**: 43

| Profile Score | Tandem Score | TDA q-value | Volume | Spectrum Matches |
| --- | --- | --- | --- | --- |
| 14.6811 | 158.0456 | 0.0000 | 3.5253e+09 | 35 |

**Adduct Signal Fractions**

- **Unmodified**: 3.5253e+09

### ATFFYFTPN(N)K Hex 7 HexNAc 6

**Mass:**  3587.4560

(1.980e-06)

**Charge States:** 3

**Start Position**: 64

**End Position**: 74

| Profile Score | Tandem Score | TDA q-value | Volume | Spectrum Matches |
| --- | --- | --- | --- | --- |
| 13.1990 | 156.1807 | 0.0000 | 6.6008e+07 | 3 |

**Adduct Signal Fractions**

- **Unmodified**: 6.6008e+07

### SVQEIQATFFYFTPN(N)K Fuc 1 Hex 8 HexNAc 7

**Mass:**  4782.9919

(1.913e-06)

**Charge States:** 3, 4

**Start Position**: 58

**End Position**: 74

| Profile Score | Tandem Score | TDA q-value | Volume | Spectrum Matches |
| --- | --- | --- | --- | --- |
| 12.3328 | 156.1457 | 0.0000 | 6.2981e+07 | 5 |

**Adduct Signal Fractions**

- **Unmodified**: 6.2981e+07

### SVQEIQATFFYFTPN(N)K Fuc 1 Hex 8 HexNAc 4 Neu5Ac 1

**Mass:**  4464.8591

(4.341e-06)

**Charge States:** 3, 4

**Start Position**: 58

**End Position**: 74

| Profile Score | Tandem Score | TDA q-value | Volume | Spectrum Matches |
| --- | --- | --- | --- | --- |
| 13.7119 | 154.2645 | 0.0000 | 5.4061e+07 | 4 |

**Adduct Signal Fractions**

- **Unmodified**: 5.4061e+07

### SVQEIQATFFYFTPN(N)K Fuc 2 Hex 7 HexNAc 6

**Mass:**  4563.9211

(2.633e-06)

**Charge States:** 3, 4

**Start Position**: 58

**End Position**: 74

| Profile Score | Tandem Score | TDA q-value | Volume | Spectrum Matches |
| --- | --- | --- | --- | --- |
| -3.5722 | 153.8604 | 0.0000 | 7.0037e+07 | 4 |

**Adduct Signal Fractions**

- **Unmodified**: 7.0037e+07

### LVPVPITN(N)ATLDQITGK Hex 6 HexNAc 5

**Mass:**  3766.7316

(9.611e-07)

**Charge States:** 3, 4

**Start Position**: 26

**End Position**: 43

| Profile Score | Tandem Score | TDA q-value | Volume | Spectrum Matches |
| --- | --- | --- | --- | --- |
| 14.7387 | 152.0562 | 0.0000 | 1.5369e+10 | 556 |

**Adduct Signal Fractions**

- **Unmodified**: 1.5369e+10

### LVPVPITN(N)ATLDQITGK Hex 5 HexNAc 4

**Mass:**  3401.6013

(9.536e-07)

**Charge States:** 3, 4

**Start Position**: 26

**End Position**: 43

| Profile Score | Tandem Score | TDA q-value | Volume | Spectrum Matches |
| --- | --- | --- | --- | --- |
| 15.2107 | 150.8047 | 0.0000 | 1.4546e+09 | 17 |

**Adduct Signal Fractions**

- **Unmodified**: 1.4546e+09

### LVPVPITN(N)ATLDQITGK Fuc 1 Hex 7 HexNAc 6

**Mass:**  4277.9289

(1.321e-06)

**Charge States:** 3, 4

**Start Position**: 26

**End Position**: 43

| Profile Score | Tandem Score | TDA q-value | Volume | Spectrum Matches |
| --- | --- | --- | --- | --- |
| 14.7912 | 144.6660 | 0.0000 | 1.8769e+08 | 5 |

**Adduct Signal Fractions**

- **Unmodified**: 1.8769e+08

### SVQEIQATFFYFTPN(N)KTEDTIFLR Hex 6 HexNAc 5

**Mass:**  4882.1769

(1.804e-06)

**Charge States:** 3, 4, 5

**Start Position**: 58

**End Position**: 82

| Profile Score | Tandem Score | TDA q-value | Volume | Spectrum Matches |
| --- | --- | --- | --- | --- |
| 12.8385 | 143.2387 | 0.0000 | 5.1862e+08 | 17 |

**Adduct Signal Fractions**

- **Unmodified**: 5.1862e+08

### ATFFYFTPN(N)K Fuc 1 Hex 6 HexNAc 5

**Mass:**  3368.3750

(3.047e-07)

**Charge States:** 3

**Start Position**: 64

**End Position**: 74

| Profile Score | Tandem Score | TDA q-value | Volume | Spectrum Matches |
| --- | --- | --- | --- | --- |
| -20.1613 | 142.3912 | 0.0000 | 5.2294e+06 | 1 |

**Adduct Signal Fractions**

- **Unmodified**: 5.2294e+06

### SVQEIQATFFYFTPN(N)K Hex 9 HexNAc 8

**Mass:**  5002.0646

(2.520e-06)

**Charge States:** 3, 4, 5

**Start Position**: 58

**End Position**: 74

| Profile Score | Tandem Score | TDA q-value | Volume | Spectrum Matches |
| --- | --- | --- | --- | --- |
| 11.8853 | 141.3428 | 0.0000 | 5.8746e+07 | 2 |

**Adduct Signal Fractions**

- **Unmodified**: 5.8746e+07

### SVQEIQATFFYFTPN(N)K Fuc 5 Hex 7 HexNAc 6

**Mass:**  5002.0646

(-2.508e-06)

**Charge States:** 3, 4, 5

**Start Position**: 58

**End Position**: 74

| Profile Score | Tandem Score | TDA q-value | Volume | Spectrum Matches |
| --- | --- | --- | --- | --- |
| 11.8853 | 141.3428 | 0.0000 | 5.8746e+07 | 2 |

**Adduct Signal Fractions**

- **Unmodified**: 5.8746e+07

### SVQEIQATFFYFTPN(N)KTEDTIFLR Fuc 1 Hex 6 HexNAc 5

**Mass:**  5028.2321

(2.224e-06)

**Charge States:** 4, 5

**Start Position**: 58

**End Position**: 82

| Profile Score | Tandem Score | TDA q-value | Volume | Spectrum Matches |
| --- | --- | --- | --- | --- |
| 12.5696 | 140.0613 | 0.0000 | 1.1908e+08 | 7 |

**Adduct Signal Fractions**

- **Unmodified**: 1.1908e+08

### SVQEIQATFFYFTPN(N)K Hex 7 HexNAc 4 Neu5Ac 2

**Mass:**  4447.8332

(3.076e-06)

**Charge States:** 3, 4

**Start Position**: 58

**End Position**: 74

| Profile Score | Tandem Score | TDA q-value | Volume | Spectrum Matches |
| --- | --- | --- | --- | --- |
| 13.4609 | 139.2767 | 0.0000 | 1.1966e+08 | 7 |

**Adduct Signal Fractions**

- **Unmodified**: 1.1966e+08

### FYFTPN(N)KTEDTIFLR Hex 7 HexNAc 6

**Mass:**  4243.8044

(1.801e-06)

**Charge States:** 3, 4

**Start Position**: 67

**End Position**: 82

| Profile Score | Tandem Score | TDA q-value | Volume | Spectrum Matches |
| --- | --- | --- | --- | --- |
| 13.3454 | 136.4847 | 0.0000 | 1.9613e+08 | 11 |

**Adduct Signal Fractions**

- **Unmodified**: 1.9613e+08

### LVPVPITN(N)ATLDQITGK Hex 7 HexNAc 6

**Mass:**  4131.8674

(2.069e-06)

**Charge States:** 3, 4

**Start Position**: 26

**End Position**: 43

| Profile Score | Tandem Score | TDA q-value | Volume | Spectrum Matches |
| --- | --- | --- | --- | --- |
| 14.7342 | 136.0920 | 0.0000 | 5.6040e+08 | 11 |

**Adduct Signal Fractions**

- **Unmodified**: 5.6040e+08

### YFTPN(N)KTEDTIFLR Hex 6 HexNAc 5

**Mass:**  3731.6010

(1.028e-06)

**Charge States:** 3, 4

**Start Position**: 68

**End Position**: 82

| Profile Score | Tandem Score | TDA q-value | Volume | Spectrum Matches |
| --- | --- | --- | --- | --- |
| 10.8882 | 135.4603 | 0.0000 | 1.0478e+09 | 8 |

**Adduct Signal Fractions**

- **Unmodified**: 1.0478e+09

### SVQEIQATFFYFTPN(N)KTEDTIFLR Hex 7 HexNAc 6

**Mass:**  5247.3112

(2.347e-06)

**Charge States:** 3, 4, 5

**Start Position**: 58

**End Position**: 82

| Profile Score | Tandem Score | TDA q-value | Volume | Spectrum Matches |
| --- | --- | --- | --- | --- |
| 13.4092 | 134.3931 | 0.0000 | 1.2351e+09 | 25 |

**Adduct Signal Fractions**

- **Unmodified**: 1.2351e+09

### TPN(N)KTEDTIFLR Hex 7 HexNAc 6

**Mass:**  3786.6015

(1.251e-06)

**Charge States:** 3, 4

**Start Position**: 70

**End Position**: 82

| Profile Score | Tandem Score | TDA q-value | Volume | Spectrum Matches |
| --- | --- | --- | --- | --- |
| 13.4523 | 133.7153 | 0.0000 | 5.2289e+07 | 8 |

**Adduct Signal Fractions**

- **Unmodified**: 5.2289e+07

### YFTPN(N)KTEDTIFLR Fuc 1 Hex 6 HexNAc 5

**Mass:**  3877.6587

(3.851e-06)

**Charge States:** 3, 4

**Start Position**: 68

**End Position**: 82

| Profile Score | Tandem Score | TDA q-value | Volume | Spectrum Matches |
| --- | --- | --- | --- | --- |
| -3.7825 | 132.0920 | 0.0000 | 2.4626e+08 | 4 |

**Adduct Signal Fractions**

- **Unmodified**: 2.4626e+08

### QDQC(C)IYN(N)TTYLNVQR Fuc 1 Hex 6 HexNAc 5

**Mass:**  4048.6693

(1.763e-06)

**Charge States:** 3, 4

**Start Position**: 87

**End Position**: 102

| Profile Score | Tandem Score | TDA q-value | Volume | Spectrum Matches |
| --- | --- | --- | --- | --- |
| -2.2175 | 131.4300 | 0.0000 | 4.2389e+08 | 4 |

**Adduct Signal Fractions**

- **Unmodified**: 4.2389e+08

### QDQC(C)IYN(N)TTYLNVQR Hex 6 HexNAc 5

**Mass:**  3902.6090

(2.221e-06)

**Charge States:** 3, 4

**Start Position**: 87

**End Position**: 102

| Profile Score | Tandem Score | TDA q-value | Volume | Spectrum Matches |
| --- | --- | --- | --- | --- |
| 9.3135 | 131.3497 | 0.0000 | 1.7862e+09 | 8 |

**Adduct Signal Fractions**

- **Unmodified**: 1.7862e+09

### PVPITN(N)ATLDQITGK Fuc 1 Hex 4 HexNAc 9

**Mass:**  4188.8884

(9.064e-06)

**Charge States:** 3, 4

**Start Position**: 28

**End Position**: 43

| Profile Score | Tandem Score | TDA q-value | Volume | Spectrum Matches |
| --- | --- | --- | --- | --- |
| 7.9209 | 130.3427 | 0.0000 | 9.0617e+07 | 5 |

**Adduct Signal Fractions**

- **Unmodified**: 9.0617e+07

### ATFFYFTPN(N)K Fuc 5 Hex 5 HexNAc 8 Neu5Ac 1

**Mass:**  4690.8508

(-7.222e-06)

**Charge States:** 4

**Start Position**: 64

**End Position**: 74

| Profile Score | Tandem Score | TDA q-value | Volume | Spectrum Matches |
| --- | --- | --- | --- | --- |
| -5.3701 | 130.1318 | 0.0000 | 1.3145e+07 | 2 |

**Adduct Signal Fractions**

- **Unmodified**: 1.3145e+07

### QDQC(C)IYN(N)TTYLNVQR Hex 8 HexNAc 7

**Mass:**  4632.8820

(3.117e-06)

**Charge States:** 3, 4

**Start Position**: 87

**End Position**: 102

| Profile Score | Tandem Score | TDA q-value | Volume | Spectrum Matches |
| --- | --- | --- | --- | --- |
| 15.2676 | 128.4842 | 0.0000 | 3.8158e+08 | 4 |

**Adduct Signal Fractions**

- **Unmodified**: 3.8158e+08

### TPN(N)KTEDTIFLR Hex 6 HexNAc 5

**Mass:**  3421.4694

(1.188e-06)

**Charge States:** 3, 4

**Start Position**: 70

**End Position**: 82

| Profile Score | Tandem Score | TDA q-value | Volume | Spectrum Matches |
| --- | --- | --- | --- | --- |
| 13.3440 | 127.4916 | 0.0000 | 3.0012e+07 | 6 |

**Adduct Signal Fractions**

- **Unmodified**: 3.0012e+07

### SVQEIQATFFYFTPN(N)K Fuc 4 Hex 5 HexNAc 5

**Mass:**  4328.8259

(-3.097e-06)

**Charge States:** 3, 4

**Start Position**: 58

**End Position**: 74

| Profile Score | Tandem Score | TDA q-value | Volume | Spectrum Matches |
| --- | --- | --- | --- | --- |
| 11.7894 | 127.1977 | 0.0000 | 5.1379e+08 | 25 |

**Adduct Signal Fractions**

- **Unmodified**: 5.1379e+08

### QDQC(C)IYN(N)TTYLNVQR Fuc 1 Hex 7 HexNAc 6

**Mass:**  4413.8010

(1.706e-06)

**Charge States:** 3, 4

**Start Position**: 87

**End Position**: 102

| Profile Score | Tandem Score | TDA q-value | Volume | Spectrum Matches |
| --- | --- | --- | --- | --- |
| 12.2347 | 126.9733 | 0.0000 | 5.9409e+08 | 6 |

**Adduct Signal Fractions**

- **Unmodified**: 5.9409e+08

### QDQC(C)IYN(N)TTYLNVQR Hex 7 HexNAc 6

**Mass:**  4267.7411

(3.060e-06)

**Charge States:** 3, 4

**Start Position**: 87

**End Position**: 102

| Profile Score | Tandem Score | TDA q-value | Volume | Spectrum Matches |
| --- | --- | --- | --- | --- |
| -1.3983 | 126.6127 | 0.0000 | 2.3113e+09 | 44 |

**Adduct Signal Fractions**

- **Unmodified**: 2.3113e+09

### SVQEIQATFFYFTPN(N)K Fuc 1 Hex 7 HexNAc 4 Neu5Ac 2

**Mass:**  4593.8942

(2.518e-06)

**Charge States:** 3, 4

**Start Position**: 58

**End Position**: 74

| Profile Score | Tandem Score | TDA q-value | Volume | Spectrum Matches |
| --- | --- | --- | --- | --- |
| -20.0711 | 126.2293 | 0.0000 | 1.2312e+07 | 1 |

**Adduct Signal Fractions**

- **Unmodified**: 1.2312e+07

### YFTPN(N)KTEDTIFLR Hex 7 HexNAc 6

**Mass:**  4096.7429

(1.398e-06)

**Charge States:** 3, 4

**Start Position**: 68

**End Position**: 82

| Profile Score | Tandem Score | TDA q-value | Volume | Spectrum Matches |
| --- | --- | --- | --- | --- |
| 13.1841 | 125.6926 | 0.0000 | 2.0690e+09 | 21 |

**Adduct Signal Fractions**

- **Unmodified**: 2.0690e+09

### YFTPN(N)KTEDTIFLR Fuc 1 Hex 7 HexNAc 6

**Mass:**  4242.7945

(3.127e-06)

**Charge States:** 3, 4

**Start Position**: 68

**End Position**: 82

| Profile Score | Tandem Score | TDA q-value | Volume | Spectrum Matches |
| --- | --- | --- | --- | --- |
| -6.3814 | 124.6881 | 0.0000 | 2.1671e+08 | 6 |

**Adduct Signal Fractions**

- **Unmodified**: 2.1671e+08

### YFTPN(N)KTEDTIFLR Fuc 1 Hex 6 HexNAc 5

**Mass:**  3879.6742

(5.207e-04)

**Start Position**: 68

**End Position**: 82

| Profile Score | Tandem Score | TDA q-value | Volume | Spectrum Matches |
| --- | --- | --- | --- | --- |
| 0.0000 | 124.2960 | 0.0000 | 0.0000e+00 | 1 |

Adduct:

No Chromatogram Found

### SVQEIQATFFYFTPN(N)K Hex 6 HexNAc 6

**Mass:**  4109.7471

(1.630e-06)

**Charge States:** 3, 4

**Start Position**: 58

**End Position**: 74

| Profile Score | Tandem Score | TDA q-value | Volume | Spectrum Matches |
| --- | --- | --- | --- | --- |
| 12.2444 | 124.2056 | 0.0000 | 5.2821e+07 | 3 |

**Adduct Signal Fractions**

- **Unmodified**: 5.2821e+07

### YFTPN(N)KTEDTIFLR Hex 5 HexNAc 4

**Mass:**  3366.4682

(9.788e-07)

**Charge States:** 3, 4

**Start Position**: 68

**End Position**: 82

| Profile Score | Tandem Score | TDA q-value | Volume | Spectrum Matches |
| --- | --- | --- | --- | --- |
| -4.4539 | 124.0236 | 0.0000 | 7.3776e+07 | 1 |

**Adduct Signal Fractions**

- **Unmodified**: 7.3776e+07

### YFTPN(N)KTEDTIFLR Hex 7 HexNAc 7

**Mass:**  4299.8137

(3.730e-06)

**Charge States:** 4

**Start Position**: 68

**End Position**: 82

| Profile Score | Tandem Score | TDA q-value | Volume | Spectrum Matches |
| --- | --- | --- | --- | --- |
| -5.0385 | 123.1778 | 0.0000 | 7.5670e+07 | 3 |

**Adduct Signal Fractions**

- **Unmodified**: 7.5670e+07

### QDQC(C)IYN(N)TTYLNVQR Hex 10 HexNAc 3 Neu5Ac 1

**Mass:**  4435.7927

(9.309e-06)

**Charge States:** 3, 4

**Start Position**: 87

**End Position**: 102

| Profile Score | Tandem Score | TDA q-value | Volume | Spectrum Matches |
| --- | --- | --- | --- | --- |
| -5.4415 | 119.2563 | 0.0000 | 9.5578e+07 | 1 |

**Adduct Signal Fractions**

- **Unmodified**: 9.5578e+07

### ATFFYFTPN(N)K Fuc 1 Hex 7 HexNAc 6

**Mass:**  3733.5075

(3.254e-07)

**Charge States:** 3

**Start Position**: 64

**End Position**: 74

| Profile Score | Tandem Score | TDA q-value | Volume | Spectrum Matches |
| --- | --- | --- | --- | --- |
| -18.1818 | 118.6259 | 0.0000 | 6.8934e+06 | 1 |

**Adduct Signal Fractions**

- **Unmodified**: 6.8934e+06

### YFTPN(N)KTEDTIFLR Fuc 1 Hex 9 HexNAc 8

**Mass:**  4973.0651

(2.780e-06)

**Charge States:** 4

**Start Position**: 68

**End Position**: 82

| Profile Score | Tandem Score | TDA q-value | Volume | Spectrum Matches |
| --- | --- | --- | --- | --- |
| -19.1159 | 117.5660 | 0.0000 | 2.3952e+06 | 1 |

**Adduct Signal Fractions**

- **Unmodified**: 2.3952e+06

### QDQC(C)IYN(N)TTYLNVQR Fuc 2 Hex 7 HexNAc 6

**Mass:**  4559.8630

(2.554e-06)

**Charge States:** 3, 4

**Start Position**: 87

**End Position**: 102

| Profile Score | Tandem Score | TDA q-value | Volume | Spectrum Matches |
| --- | --- | --- | --- | --- |
| 10.5890 | 116.8877 | 0.0000 | 9.7707e+07 | 2 |

**Adduct Signal Fractions**

- **Unmodified**: 9.7707e+07

### LVPVPITN(N)ATLDQITGK Hex 8 HexNAc 7

**Mass:**  4497.0012

(1.918e-06)

**Charge States:** 3, 4

**Start Position**: 26

**End Position**: 43

| Profile Score | Tandem Score | TDA q-value | Volume | Spectrum Matches |
| --- | --- | --- | --- | --- |
| 8.8803 | 116.8445 | 0.0000 | 7.3206e+07 | 4 |

**Adduct Signal Fractions**

- **Unmodified**: 7.3206e+07

### YFTPN(N)KTEDTIFLR Hex 8 HexNAc 7

**Mass:**  4461.8659

(4.125e-06)

**Charge States:** 3, 4

**Start Position**: 68

**End Position**: 82

| Profile Score | Tandem Score | TDA q-value | Volume | Spectrum Matches |
| --- | --- | --- | --- | --- |
| 13.5425 | 116.5158 | 0.0000 | 1.7748e+08 | 3 |

**Adduct Signal Fractions**

- **Unmodified**: 1.7748e+08

### YFTPN(N)KTEDTIFLR Fuc 2 Hex 7 HexNAc 6

**Mass:**  4389.8630

(2.307e-04)

**Charge States:** 3, 4

**Start Position**: 68

**End Position**: 82

| Profile Score | Tandem Score | TDA q-value | Volume | Spectrum Matches |
| --- | --- | --- | --- | --- |
| -6.1687 | 115.7301 | 0.0000 | 3.4075e+07 | 8 |

**Adduct Signal Fractions**

- **Unmodified**: 3.4075e+07

### QDQC(C)IYN(N)TTYLNVQR Fuc 1 Hex 8 HexNAc 7

**Mass:**  4778.9348

(2.332e-06)

**Charge States:** 3, 4

**Start Position**: 87

**End Position**: 102

| Profile Score | Tandem Score | TDA q-value | Volume | Spectrum Matches |
| --- | --- | --- | --- | --- |
| 13.4217 | 115.3868 | 0.0000 | 9.8698e+07 | 4 |

**Adduct Signal Fractions**

- **Unmodified**: 9.8698e+07

### LVPVPITN(N)ATLDQITGK Fuc 1 Hex 7 HexNAc 3 Neu5Ac 1

**Mass:**  3959.7921

(4.218e-06)

**Charge States:** 3, 4

**Start Position**: 26

**End Position**: 43

| Profile Score | Tandem Score | TDA q-value | Volume | Spectrum Matches |
| --- | --- | --- | --- | --- |
| -3.8935 | 114.6367 | 0.0000 | 3.8368e+07 | 3 |

**Adduct Signal Fractions**

- **Unmodified**: 3.8368e+07

### PVPITN(N)ATLDQITGK Fuc 2 Hex 8 HexNAc 4

**Mass:**  3967.7137

(2.508e-04)

**Start Position**: 28

**End Position**: 43

| Profile Score | Tandem Score | TDA q-value | Volume | Spectrum Matches |
| --- | --- | --- | --- | --- |
| 0.0000 | 113.2231 | 0.0000 | 0.0000e+00 | 2 |

Adduct:

No Chromatogram Found

### PVPITN(N)ATLDQITGK Fuc 1 Hex 7 HexNAc 4 Neu5Ac 1

**Mass:**  3950.7363

(5.926e-06)

**Charge States:** 4

**Start Position**: 28

**End Position**: 43

| Profile Score | Tandem Score | TDA q-value | Volume | Spectrum Matches |
| --- | --- | --- | --- | --- |
| -3.8893 | 110.9727 | 0.0000 | 3.2284e+08 | 3 |

**Adduct Signal Fractions**

- **Unmodified**: 3.2284e+08

### SVQEIQATFFYFTPN(N)K Hex 7 HexNAc 7

**Mass:**  4474.8781

(2.386e-06)

**Charge States:** 3, 4

**Start Position**: 58

**End Position**: 74

| Profile Score | Tandem Score | TDA q-value | Volume | Spectrum Matches |
| --- | --- | --- | --- | --- |
| 10.9741 | 110.3211 | 0.0000 | 5.6775e+07 | 3 |

**Adduct Signal Fractions**

- **Unmodified**: 5.6775e+07

### PVPITN(N)ATLDQITGK Fuc 2 Hex 5 HexNAc 7

**Mass:**  4090.8337

(8.905e-06)

**Start Position**: 28

**End Position**: 43

| Profile Score | Tandem Score | TDA q-value | Volume | Spectrum Matches |
| --- | --- | --- | --- | --- |
| 0.0000 | 109.9131 | 0.0000 | 0.0000e+00 | 1 |

Adduct:

No Chromatogram Found

### FYFTPN(N)KTEDTIFLR Hex 6 HexNAc 5

**Mass:**  3878.6702

(1.690e-06)

**Charge States:** 3, 4

**Start Position**: 67

**End Position**: 82

| Profile Score | Tandem Score | TDA q-value | Volume | Spectrum Matches |
| --- | --- | --- | --- | --- |
| 13.1108 | 108.9941 | 0.0000 | 6.6773e+07 | 5 |

**Adduct Signal Fractions**

- **Unmodified**: 6.6773e+07

### YFTPN(N)KTEDTIFLR Hex 7 HexNAc 5

**Mass:**  3895.6905

(5.241e-04)

**Start Position**: 68

**End Position**: 82

| Profile Score | Tandem Score | TDA q-value | Volume | Spectrum Matches |
| --- | --- | --- | --- | --- |
| 0.0000 | 107.7345 | 0.0000 | 0.0000e+00 | 1 |

Adduct:

No Chromatogram Found

### PVPITN(N)ATLDQITGK Hex 9 HexNAc 4

**Mass:**  3838.6665

(2.637e-04)

**Start Position**: 28

**End Position**: 43

| Profile Score | Tandem Score | TDA q-value | Volume | Spectrum Matches |
| --- | --- | --- | --- | --- |
| 0.0000 | 106.3978 | 0.0000 | 0.0000e+00 | 1 |

Adduct:

No Chromatogram Found

### PVPITN(N)ATLDQITGK Fuc 4 Hex 3 HexNAc 7

**Mass:**  4060.8457

(5.022e-04)

**Start Position**: 28

**End Position**: 43

| Profile Score | Tandem Score | TDA q-value | Volume | Spectrum Matches |
| --- | --- | --- | --- | --- |
| 0.0000 | 106.0019 | 0.0000 | 0.0000e+00 | 1 |

Adduct:

No Chromatogram Found

### PVPITN(N)ATLDQITGK Fuc 3 Hex 5 HexNAc 9

**Mass:**  4643.0578

(9.260e-06)

**Charge States:** 4

**Start Position**: 28

**End Position**: 43

| Profile Score | Tandem Score | TDA q-value | Volume | Spectrum Matches |
| --- | --- | --- | --- | --- |
| 9.4955 | 105.2149 | 0.0000 | 9.9663e+06 | 3 |

**Adduct Signal Fractions**

- **Unmodified**: 9.9663e+06

### SVQEIQATFFYFTPN(N)K Fuc 3 Hex 4 HexNAc 5

**Mass:**  4020.7095

(-3.970e-06)

**Charge States:** 3, 4

**Start Position**: 58

**End Position**: 74

| Profile Score | Tandem Score | TDA q-value | Volume | Spectrum Matches |
| --- | --- | --- | --- | --- |
| -19.1308 | 103.6187 | 0.0000 | 9.8584e+06 | 2 |

**Adduct Signal Fractions**

- **Unmodified**: 9.8584e+06

### SVQEIQATFFYFTPN(N)KTEDTIFLR Hex 5 HexNAc 4

**Mass:**  4517.0373

(1.424e-06)

**Charge States:** 4

**Start Position**: 58

**End Position**: 82

| Profile Score | Tandem Score | TDA q-value | Volume | Spectrum Matches |
| --- | --- | --- | --- | --- |
| -3.9298 | 101.8173 | 0.0000 | 1.9743e+07 | 2 |

**Adduct Signal Fractions**

- **Unmodified**: 1.9743e+07

### PVPITN(N)ATLDQITGK Fuc 2 Hex 7 HexNAc 3

**Mass:**  3602.5936

(1.896e-06)

**Charge States:** 3, 4

**Start Position**: 28

**End Position**: 43

| Profile Score | Tandem Score | TDA q-value | Volume | Spectrum Matches |
| --- | --- | --- | --- | --- |
| -18.9144 | 100.9532 | 0.0000 | 3.8619e+06 | 1 |

**Adduct Signal Fractions**

- **Unmodified**: 3.8619e+06

### FTPN(N)KTEDTIFLR Hex 6 HexNAc 5

**Mass:**  3568.5393

(1.590e-06)

**Charge States:** 3, 4

**Start Position**: 69

**End Position**: 82

| Profile Score | Tandem Score | TDA q-value | Volume | Spectrum Matches |
| --- | --- | --- | --- | --- |
| -2.8936 | 97.3570 | 0.0000 | 6.9422e+07 | 4 |

**Adduct Signal Fractions**

- **Unmodified**: 6.9422e+07

### PVPITN(N)ATLDQITGK Fuc 2 Hex 3 HexNAc 7 Neu5Ac 3

**Mass:**  4640.0187

(9.324e-06)

**Charge States:** 4

**Start Position**: 28

**End Position**: 43

| Profile Score | Tandem Score | TDA q-value | Volume | Spectrum Matches |
| --- | --- | --- | --- | --- |
| -4.7617 | 97.0355 | 0.0000 | 6.7571e+06 | 3 |

**Adduct Signal Fractions**

- **Unmodified**: 6.7571e+06

### LVPVPITN(N)ATLDQITGK Hex 6 HexNAc 5 Neu5Ac 1

**Mass:**  4057.8312

(1.954e-06)

**Charge States:** 3, 4

**Start Position**: 26

**End Position**: 43

| Profile Score | Tandem Score | TDA q-value | Volume | Spectrum Matches |
| --- | --- | --- | --- | --- |
| 12.3264 | 96.9261 | 0.0000 | 1.9667e+07 | 6 |

**Adduct Signal Fractions**

- **Unmodified**: 1.9667e+07

### SVQEIQATFFYFTPN(N)KTEDTIFLR Fuc 1 Hex 7 HexNAc 6

**Mass:**  5393.3644

(1.445e-06)

**Charge States:** 4, 5

**Start Position**: 58

**End Position**: 82

| Profile Score | Tandem Score | TDA q-value | Volume | Spectrum Matches |
| --- | --- | --- | --- | --- |
| 13.0593 | 96.5508 | 0.0000 | 2.5527e+08 | 10 |

**Adduct Signal Fractions**

- **Unmodified**: 2.5527e+08

### TPN(N)KTEDTIFLR Fuc 1 Hex 6 HexNAc 5

**Mass:**  3567.5286

(1.573e-06)

**Charge States:** 3

**Start Position**: 70

**End Position**: 82

| Profile Score | Tandem Score | TDA q-value | Volume | Spectrum Matches |
| --- | --- | --- | --- | --- |
| -4.4299 | 96.3067 | 0.0000 | 4.8148e+06 | 3 |

**Adduct Signal Fractions**

- **Unmodified**: 4.8148e+06

### YFTPN(N)KTEDTIFLR Hex 9 HexNAc 8

**Mass:**  4826.9973

(2.824e-06)

**Charge States:** 3, 4

**Start Position**: 68

**End Position**: 82

| Profile Score | Tandem Score | TDA q-value | Volume | Spectrum Matches |
| --- | --- | --- | --- | --- |
| 11.6786 | 95.0554 | 0.0000 | 4.9663e+07 | 2 |

**Adduct Signal Fractions**

- **Unmodified**: 4.9663e+07

### IYN(N)TTYLNVQR Hex 6 HexNAc 5

**Mass:**  3371.4285

(6.850e-07)

**Charge States:** 3

**Start Position**: 91

**End Position**: 102

| Profile Score | Tandem Score | TDA q-value | Volume | Spectrum Matches |
| --- | --- | --- | --- | --- |
| 13.0355 | 94.3085 | 0.0000 | 4.0554e+07 | 4 |

**Adduct Signal Fractions**

- **Unmodified**: 4.0554e+07

### FTPN(N)KTEDTIFLR Hex 7 HexNAc 6

**Mass:**  3933.6699

(1.832e-06)

**Charge States:** 3, 4

**Start Position**: 69

**End Position**: 82

| Profile Score | Tandem Score | TDA q-value | Volume | Spectrum Matches |
| --- | --- | --- | --- | --- |
| 14.3132 | 94.0461 | 0.0000 | 1.3098e+08 | 6 |

**Adduct Signal Fractions**

- **Unmodified**: 1.3098e+08

### TPN(N)KTEDTIFLR Hex 6 HexNAc 5

**Mass:**  3422.4748

(2.951e-04)

**Start Position**: 70

**End Position**: 82

| Profile Score | Tandem Score | TDA q-value | Volume | Spectrum Matches |
| --- | --- | --- | --- | --- |
| 0.0000 | 93.8757 | 0.0000 | 0.0000e+00 | 1 |

Adduct:

No Chromatogram Found

### PVPITN(N)ATLDQITGK Fuc 3 Hex 3 HexNAc 5 Neu5Ac 2

**Mass:**  4088.8269

(9.827e-06)

**Charge States:** 3

**Start Position**: 28

**End Position**: 43

| Profile Score | Tandem Score | TDA q-value | Volume | Spectrum Matches |
| --- | --- | --- | --- | --- |
| -5.1135 | 92.7874 | 0.0000 | 2.6080e+07 | 1 |

**Adduct Signal Fractions**

- **Unmodified**: 2.6080e+07

### FYFTPN(N)KTEDTIFLR Hex 8 HexNAc 7

**Mass:**  4608.9318

(4.053e-07)

**Charge States:** 3, 4

**Start Position**: 67

**End Position**: 82

| Profile Score | Tandem Score | TDA q-value | Volume | Spectrum Matches |
| --- | --- | --- | --- | --- |
| 12.8087 | 92.7007 | 0.0000 | 2.2892e+07 | 4 |

**Adduct Signal Fractions**

- **Unmodified**: 2.2892e+07

### QDQC(C)IYN(N)TTYLNVQR Hex 7 HexNAc 4 Neu5Ac 2

**Mass:**  4443.7741

(1.079e-06)

**Charge States:** 3, 4

**Start Position**: 87

**End Position**: 102

| Profile Score | Tandem Score | TDA q-value | Volume | Spectrum Matches |
| --- | --- | --- | --- | --- |
| 12.7843 | 88.9436 | 0.0000 | 1.3804e+08 | 3 |

**Adduct Signal Fractions**

- **Unmodified**: 1.3804e+08

### YFTPN(N)KTEDTIFLR Hex 7 HexNAc 4 Neu5Ac 2

**Mass:**  4272.7614

(8.079e-07)

**Charge States:** 4

**Start Position**: 68

**End Position**: 82

| Profile Score | Tandem Score | TDA q-value | Volume | Spectrum Matches |
| --- | --- | --- | --- | --- |
| -4.0754 | 88.0616 | 0.0000 | 1.3738e+08 | 10 |

**Adduct Signal Fractions**

- **Unmodified**: 1.3738e+08

### LVPVPITN(N)ATLDQITGK Fuc 2 Hex 6 HexNAc 5

**Mass:**  4058.8557

(3.331e-06)

**Charge States:** 3, 4

**Start Position**: 26

**End Position**: 43

| Profile Score | Tandem Score | TDA q-value | Volume | Spectrum Matches |
| --- | --- | --- | --- | --- |
| -5.3789 | 86.4250 | 0.0000 | 3.5123e+07 | 1 |

**Adduct Signal Fractions**

- **Unmodified**: 3.5123e+07

### FTPN(N)KTEDTIFLR Fuc 1 Hex 6 HexNAc 5

**Mass:**  3714.5960

(1.221e-06)

**Charge States:** 3

**Start Position**: 69

**End Position**: 82

| Profile Score | Tandem Score | TDA q-value | Volume | Spectrum Matches |
| --- | --- | --- | --- | --- |
| -3.8206 | 86.0311 | 0.0000 | 1.4091e+07 | 2 |

**Adduct Signal Fractions**

- **Unmodified**: 1.4091e+07

### YFTPN(N)KTEDTIFLR Fuc 1 Hex 8 HexNAc 7

**Mass:**  4609.8993

(4.297e-04)

**Start Position**: 68

**End Position**: 82

| Profile Score | Tandem Score | TDA q-value | Volume | Spectrum Matches |
| --- | --- | --- | --- | --- |
| 0.0000 | 84.8593 | 0.0000 | 0.0000e+00 | 1 |

Adduct:

No Chromatogram Found

### FTPN(N)KTEDTIFLR Fuc 1 Hex 7 HexNAc 6

**Mass:**  4079.7277

(-1.130e-06)

**Charge States:** 3, 4

**Start Position**: 69

**End Position**: 82

| Profile Score | Tandem Score | TDA q-value | Volume | Spectrum Matches |
| --- | --- | --- | --- | --- |
| -3.3763 | 84.2312 | 0.0000 | 2.9353e+07 | 5 |

**Adduct Signal Fractions**

- **Unmodified**: 2.9353e+07

### FTPN(N)KTEDTIFLR Hex 8 HexNAc 7

**Mass:**  4300.8269

(1.341e-06)

**Charge States:** 3, 4

**Start Position**: 69

**End Position**: 82

| Profile Score | Tandem Score | TDA q-value | Volume | Spectrum Matches |
| --- | --- | --- | --- | --- |
| 10.1134 | 82.6567 | 0.0000 | 6.5176e+07 | 10 |

**Adduct Signal Fractions**

- **Unmodified**: 6.5176e+07

### TPN(N)KTEDTIFLR Fuc 1 Hex 7 HexNAc 6

**Mass:**  3932.6606

(7.810e-07)

**Charge States:** 3

**Start Position**: 70

**End Position**: 82

| Profile Score | Tandem Score | TDA q-value | Volume | Spectrum Matches |
| --- | --- | --- | --- | --- |
| -4.6920 | 81.9569 | 0.0000 | 7.7042e+06 | 3 |

**Adduct Signal Fractions**

- **Unmodified**: 7.7042e+06

### SVQEIQATFFYFTPN(N)KTEDTIFLR Fuc 2 Hex 7 HexNAc 6

**Mass:**  5539.4255

(1.864e-06)

**Charge States:** 4

**Start Position**: 58

**End Position**: 82

| Profile Score | Tandem Score | TDA q-value | Volume | Spectrum Matches |
| --- | --- | --- | --- | --- |
| -20.1555 | 81.2866 | 0.0022 | 2.4066e+07 | 1 |

**Adduct Signal Fractions**

- **Unmodified**: 2.4066e+07

### TPN(N)KTEDTIFLR Hex 5 HexNAc 4

**Mass:**  3056.3380

(1.557e-06)

**Charge States:** 3

**Start Position**: 70

**End Position**: 82

| Profile Score | Tandem Score | TDA q-value | Volume | Spectrum Matches |
| --- | --- | --- | --- | --- |
| 10.2303 | 76.3319 | 0.0044 | 6.4634e+06 | 2 |

**Adduct Signal Fractions**

- **Unmodified**: 6.4634e+06

### FYFTPN(N)KTEDTIFLR Fuc 1 Hex 4 HexNAc 4 Neu5Ac 1

**Mass:**  3788.6244

(-2.988e-06)

**Charge States:** 3, 4

**Start Position**: 67

**End Position**: 82

| Profile Score | Tandem Score | TDA q-value | Volume | Spectrum Matches |
| --- | --- | --- | --- | --- |
| 14.0736 | 72.9602 | 0.0048 | 2.6075e+08 | 5 |

**Adduct Signal Fractions**

- **Unmodified**: 2.6075e+08

### SVQEIQATFFYFTPN(N)K Fuc 2 Hex 8 HexNAc 7

**Mass:**  4929.0529

(2.502e-06)

**Charge States:** 4

**Start Position**: 58

**End Position**: 74

| Profile Score | Tandem Score | TDA q-value | Volume | Spectrum Matches |
| --- | --- | --- | --- | --- |
| 11.3535 | 69.1029 | 0.0048 | 1.2795e+07 | 2 |

**Adduct Signal Fractions**

- **Unmodified**: 1.2795e+07

### SVQEIQATFFYFTPN(N)K Hex 10 HexNAc 9

**Mass:**  5367.2004

(6.658e-07)

**Charge States:** 4

**Start Position**: 58

**End Position**: 74

| Profile Score | Tandem Score | TDA q-value | Volume | Spectrum Matches |
| --- | --- | --- | --- | --- |
| -4.4911 | 65.6114 | 0.0048 | 6.3307e+06 | 1 |

**Adduct Signal Fractions**

- **Unmodified**: 6.3307e+06

### FYFTPN(N)KTEDTIFLR Fuc 3 Hex 6 HexNAc 4

**Mass:**  4113.7663

(1.605e-06)

**Charge States:** 4

**Start Position**: 67

**End Position**: 82

| Profile Score | Tandem Score | TDA q-value | Volume | Spectrum Matches |
| --- | --- | --- | --- | --- |
| -4.6290 | 63.8984 | 0.0048 | 9.8440e+07 | 2 |

**Adduct Signal Fractions**

- **Unmodified**: 9.8440e+07

### LVPVPITN(N)ATLDQITGK Hex 7 HexNAc 5

**Mass:**  3931.8249

(7.748e-04)

**Start Position**: 26

**End Position**: 43

| Profile Score | Tandem Score | TDA q-value | Volume | Spectrum Matches |
| --- | --- | --- | --- | --- |
| 0.0000 | 61.8922 | 0.0048 | 0.0000e+00 | 1 |

Adduct:

No Chromatogram Found

### SVQEIQATFFYFTPN(N)K Fuc 1 Hex 9 HexNAc 8

**Mass:**  5148.1271

(1.880e-06)

**Charge States:** 4

**Start Position**: 58

**End Position**: 74

| Profile Score | Tandem Score | TDA q-value | Volume | Spectrum Matches |
| --- | --- | --- | --- | --- |
| -21.1788 | 61.7755 | 0.0048 | 2.9274e+06 | 1 |

**Adduct Signal Fractions**

- **Unmodified**: 2.9274e+06

### SVQEIQATFFYFTPN(N)K Fuc 4 Hex 6 HexNAc 6

**Mass:**  4693.9572

(-3.363e-06)

**Charge States:** 3, 4

**Start Position**: 58

**End Position**: 74

| Profile Score | Tandem Score | TDA q-value | Volume | Spectrum Matches |
| --- | --- | --- | --- | --- |
| 8.1335 | 60.9756 | 0.0048 | 3.6728e+07 | 2 |

**Adduct Signal Fractions**

- **Unmodified**: 3.6728e+07

### SVQEIQATFFYFTPN(N)KTEDTIFLR Hex 8 HexNAc 7

**Mass:**  5612.4367

(9.255e-07)

**Charge States:** 4, 5

**Start Position**: 58

**End Position**: 82

| Profile Score | Tandem Score | TDA q-value | Volume | Spectrum Matches |
| --- | --- | --- | --- | --- |
| 16.3812 | 60.0297 | 0.0048 | 1.9014e+08 | 4 |

**Adduct Signal Fractions**

- **Unmodified**: 1.9014e+08

### C(C)ANLVPVPITN(N)ATLDQITGK Hex 6 HexNAc 5

**Mass:**  4111.8458

(2.214e-06)

**Charge States:** 3, 4

**Start Position**: 23

**End Position**: 43

| Profile Score | Tandem Score | TDA q-value | Volume | Spectrum Matches |
| --- | --- | --- | --- | --- |
| 15.1551 | 58.5372 | 0.0048 | 2.1412e+08 | 9 |

**Adduct Signal Fractions**

- **Unmodified**: 2.1412e+08

### QDQC(C)IYN(N)TTYLNVQR Hex 9 HexNAc 8

**Mass:**  4998.0129

(2.596e-06)

**Charge States:** 3, 4

**Start Position**: 87

**End Position**: 102

| Profile Score | Tandem Score | TDA q-value | Volume | Spectrum Matches |
| --- | --- | --- | --- | --- |
| 11.8079 | 57.5615 | 0.0048 | 1.3510e+08 | 3 |

**Adduct Signal Fractions**

- **Unmodified**: 1.3510e+08

### SVQEIQATFFYFTPN(N)K Fuc 4 Hex 6 HexNAc 5

**Mass:**  4492.9081

(4.489e-04)

**Start Position**: 58

**End Position**: 74

| Profile Score | Tandem Score | TDA q-value | Volume | Spectrum Matches |
| --- | --- | --- | --- | --- |
| 0.0000 | 54.6974 | 0.0048 | 0.0000e+00 | 1 |

Adduct:

No Chromatogram Found

### FYFTPN(N)KTEDTIFLR Fuc 1 Hex 6 HexNAc 5

**Mass:**  4024.7294

(1.092e-06)

**Charge States:** 3, 4

**Start Position**: 67

**End Position**: 82

| Profile Score | Tandem Score | TDA q-value | Volume | Spectrum Matches |
| --- | --- | --- | --- | --- |
| 12.8602 | 53.0742 | 0.0048 | 1.7095e+07 | 4 |

**Adduct Signal Fractions**

- **Unmodified**: 1.7095e+07

### C(C)ANLVPVPITN(N)ATLDQITGK Fuc 1 Hex 6 HexNAc 5

**Mass:**  4257.9010

(1.043e-06)

**Charge States:** 3, 4

**Start Position**: 23

**End Position**: 43

| Profile Score | Tandem Score | TDA q-value | Volume | Spectrum Matches |
| --- | --- | --- | --- | --- |
| 14.0213 | 50.5103 | 0.0048 | 1.9291e+07 | 2 |

**Adduct Signal Fractions**

- **Unmodified**: 1.9291e+07

### QDQC(C)IYN(N)TTYLNVQR Fuc 2 Hex 8 HexNAc 7

**Mass:**  4924.9938

(2.108e-06)

**Charge States:** 4

**Start Position**: 87

**End Position**: 102

| Profile Score | Tandem Score | TDA q-value | Volume | Spectrum Matches |
| --- | --- | --- | --- | --- |
| 13.5359 | 49.9245 | 0.0048 | 2.1825e+07 | 2 |

**Adduct Signal Fractions**

- **Unmodified**: 2.1825e+07

### QDQC(C)IYN(N)TTYLNVQR Hex 5 HexNAc 4

**Mass:**  3537.4769

(1.569e-06)

**Charge States:** 3

**Start Position**: 87

**End Position**: 102

| Profile Score | Tandem Score | TDA q-value | Volume | Spectrum Matches |
| --- | --- | --- | --- | --- |
| -4.4825 | 48.2879 | 0.0048 | 3.2492e+07 | 2 |

**Adduct Signal Fractions**

- **Unmodified**: 3.2492e+07

### LVPVPITN(N)ATLDQITGK Hex 6 HexNAc 6

**Mass:**  3969.8120

(1.466e-06)

**Charge States:** 3, 4

**Start Position**: 26

**End Position**: 43

| Profile Score | Tandem Score | TDA q-value | Volume | Spectrum Matches |
| --- | --- | --- | --- | --- |
| 11.0695 | 47.7494 | 0.0048 | 7.4676e+08 | 12 |

**Adduct Signal Fractions**

- **Unmodified**: 7.4676e+08

### SVQEIQATFFYFTPN(N)KTEDTIFLR Fuc 1 Hex 8 HexNAc 4 Neu5Ac 1

**Mass:**  5440.3620

(3.607e-06)

**Charge States:** 4

**Start Position**: 58

**End Position**: 82

| Profile Score | Tandem Score | TDA q-value | Volume | Spectrum Matches |
| --- | --- | --- | --- | --- |
| -4.3088 | 47.6568 | 0.0048 | 1.0485e+07 | 1 |

**Adduct Signal Fractions**

- **Unmodified**: 1.0485e+07

### QDQC(C)IYN(N)TTYLNVQR Fuc 1 Hex 9 HexNAc 8

**Mass:**  5144.0690

(7.813e-07)

**Charge States:** 4

**Start Position**: 87

**End Position**: 102

| Profile Score | Tandem Score | TDA q-value | Volume | Spectrum Matches |
| --- | --- | --- | --- | --- |
| -4.6576 | 46.4777 | 0.0048 | 1.8903e+07 | 2 |

**Adduct Signal Fractions**

- **Unmodified**: 1.8903e+07

### LVPVPITN(N)ATLDQITGK Fuc 3 Hex 4 HexNAc 5

**Mass:**  3882.7937

(5.147e-04)

**Start Position**: 26

**End Position**: 43

| Profile Score | Tandem Score | TDA q-value | Volume | Spectrum Matches |
| --- | --- | --- | --- | --- |
| 0.0000 | 45.8379 | 0.0048 | 0.0000e+00 | 1 |

Adduct:

No Chromatogram Found

### QDQC(C)IYN(N)TTYLNVQR Fuc 1 Hex 8 HexNAc 4 Neu5Ac 1

**Mass:**  4460.7991

(3.636e-06)

**Charge States:** 3

**Start Position**: 87

**End Position**: 102

| Profile Score | Tandem Score | TDA q-value | Volume | Spectrum Matches |
| --- | --- | --- | --- | --- |
| 11.3995 | 45.2419 | 0.0048 | 3.0178e+07 | 1 |

**Adduct Signal Fractions**

- **Unmodified**: 3.0178e+07

### SVQEIQATFFYFTPN(N)KTEDTIFLR Hex 9 HexNAc 8

**Mass:**  5977.5661

(9.978e-07)

**Charge States:** 4, 5

**Start Position**: 58

**End Position**: 82

| Profile Score | Tandem Score | TDA q-value | Volume | Spectrum Matches |
| --- | --- | --- | --- | --- |
| 14.8349 | 44.1430 | 0.0048 | 6.2540e+07 | 4 |

**Adduct Signal Fractions**

- **Unmodified**: 6.2540e+07

### QDQC(C)IYN(N)TTYLNVQR Fuc 3 Hex 8 HexNAc 4

**Mass:**  4461.8102

(1.560e-06)

**Charge States:** 3, 4

**Start Position**: 87

**End Position**: 102

| Profile Score | Tandem Score | TDA q-value | Volume | Spectrum Matches |
| --- | --- | --- | --- | --- |
| -5.5069 | 43.5939 | 0.0048 | 8.0247e+06 | 2 |

**Adduct Signal Fractions**

- **Unmodified**: 8.0247e+06

### SVQEIQATFFYFTPN(N)K Fuc 1 Hex 7 HexNAc 7

**Mass:**  4620.9436

(2.953e-06)

**Charge States:** 3, 4

**Start Position**: 58

**End Position**: 74

| Profile Score | Tandem Score | TDA q-value | Volume | Spectrum Matches |
| --- | --- | --- | --- | --- |
| -20.4382 | 42.7957 | 0.0048 | 3.8179e+06 | 1 |

**Adduct Signal Fractions**

- **Unmodified**: 3.8179e+06

### SVQEIQATFFYFTPN(N)K Fuc 1 Hex 9 HexNAc 8

**Mass:**  5150.1185

(3.892e-04)

**Start Position**: 58

**End Position**: 74

| Profile Score | Tandem Score | TDA q-value | Volume | Spectrum Matches |
| --- | --- | --- | --- | --- |
| 0.0000 | 40.9527 | 0.0048 | 0.0000e+00 | 1 |

Adduct:

No Chromatogram Found

### SVQEIQATFFYFTPN(N)KTEDTIFLR Fuc 1 Hex 8 HexNAc 7

**Mass:**  5758.4929

(1.338e-06)

**Charge States:** 4, 5

**Start Position**: 58

**End Position**: 82

| Profile Score | Tandem Score | TDA q-value | Volume | Spectrum Matches |
| --- | --- | --- | --- | --- |
| -4.7344 | 40.2115 | 0.0060 | 3.3992e+07 | 3 |

**Adduct Signal Fractions**

- **Unmodified**: 3.3992e+07

### QIPLC(C)ANLVPVPITN(N)ATLDQITGK Fuc 1 Hex 6 HexNAc 5

**Mass:**  4709.1799

(8.300e-07)

**Charge States:** 3, 4

**Start Position**: 19

**End Position**: 43

| Profile Score | Tandem Score | TDA q-value | Volume | Spectrum Matches |
| --- | --- | --- | --- | --- |
| 12.4467 | 39.9757 | 0.0060 | 1.1045e+08 | 7 |

**Adduct Signal Fractions**

- **Unmodified**: 1.1045e+08

### PVPITN(N)ATLDQITGK Fuc 1 Hex 8 HexNAc 4

**Mass:**  3822.6782

(2.603e-04)

**Charge States:** 4

**Start Position**: 28

**End Position**: 43

| Profile Score | Tandem Score | TDA q-value | Volume | Spectrum Matches |
| --- | --- | --- | --- | --- |
| -6.6806 | 38.9672 | 0.0060 | 2.3197e+05 | 1 |

**Adduct Signal Fractions**

- **Unmodified**: 2.3197e+05

### QDQC(C)IYN(N)TTYLNVQR Hex 6 HexNAc 6

**Mass:**  4105.6884

(1.373e-06)

**Charge States:** 3, 4

**Start Position**: 87

**End Position**: 102

| Profile Score | Tandem Score | TDA q-value | Volume | Spectrum Matches |
| --- | --- | --- | --- | --- |
| 12.4368 | 38.3827 | 0.0060 | 8.4929e+07 | 1 |

**Adduct Signal Fractions**

- **Unmodified**: 8.4929e+07

### PVPITN(N)ATLDQITGK Hex 8 HexNAc 4 Neu5Ac 1

**Mass:**  3966.7050

(1.982e-06)

**Charge States:** 4

**Start Position**: 28

**End Position**: 43

| Profile Score | Tandem Score | TDA q-value | Volume | Spectrum Matches |
| --- | --- | --- | --- | --- |
| -19.2099 | 38.3502 | 0.0060 | 1.0761e+08 | 1 |

**Adduct Signal Fractions**

- **Unmodified**: 1.0761e+08

### QDQC(C)IYN(N)TTYLN Hex 7 HexNAc 6

**Mass:**  3884.5167

(1.348e-06)

**Charge States:** 3

**Start Position**: 87

**End Position**: 99

| Profile Score | Tandem Score | TDA q-value | Volume | Spectrum Matches |
| --- | --- | --- | --- | --- |
| -19.5241 | 38.1230 | 0.0075 | 3.8644e+06 | 1 |

**Adduct Signal Fractions**

- **Unmodified**: 3.8644e+06

### QDQC(C)IYN(N)TTYLN(D)VQR Hex 6 HexNAc 5

**Mass:**  3903.5996

(2.548e-04)

**Charge States:** 3, 4

**Start Position**: 87

**End Position**: 102

| Profile Score | Tandem Score | TDA q-value | Volume | Spectrum Matches |
| --- | --- | --- | --- | --- |
| -4.3956 | 37.9208 | 0.0088 | 9.2078e+07 | 11 |

**Adduct Signal Fractions**

- **Unmodified**: 9.2078e+07

### FYFTPN(N)KTEDTIFLR Hex 5 HexNAc 4

**Mass:**  3513.5366

(1.818e-06)

**Charge States:** 3

**Start Position**: 67

**End Position**: 82

| Profile Score | Tandem Score | TDA q-value | Volume | Spectrum Matches |
| --- | --- | --- | --- | --- |
| -19.5768 | 37.1165 | 0.0097 | 2.7490e+06 | 1 |

**Adduct Signal Fractions**

- **Unmodified**: 2.7490e+06

### YFTPN(N)KTEDTIFLR Fuc 4 Hex 5 HexNAc 5

**Mass:**  4153.7585

(-4.708e-06)

**Charge States:** 3, 4

**Start Position**: 68

**End Position**: 82

| Profile Score | Tandem Score | TDA q-value | Volume | Spectrum Matches |
| --- | --- | --- | --- | --- |
| 13.1177 | 36.6632 | 0.0097 | 4.6450e+08 | 4 |

**Adduct Signal Fractions**

- **Unmodified**: 4.6450e+08

### PVPITN(N)ATLDQITGK Fuc 3 Hex 7 HexNAc 4

**Mass:**  3951.7365

(3.654e-06)

**Charge States:** 3, 4

**Start Position**: 28

**End Position**: 43

| Profile Score | Tandem Score | TDA q-value | Volume | Spectrum Matches |
| --- | --- | --- | --- | --- |
| -4.6660 | 36.4456 | 0.0097 | 4.0118e+07 | 1 |

**Adduct Signal Fractions**

- **Unmodified**: 4.0118e+07

### PVPITN(N)ATLDQITGK Fuc 2 Hex 4 HexNAc 9

**Mass:**  4334.9450

(9.676e-06)

**Charge States:** 3

**Start Position**: 28

**End Position**: 43

| Profile Score | Tandem Score | TDA q-value | Volume | Spectrum Matches |
| --- | --- | --- | --- | --- |
| -20.4619 | 35.5586 | 0.0097 | 3.6944e+06 | 1 |

**Adduct Signal Fractions**

- **Unmodified**: 3.6944e+06

### LVPVPITN(N)ATLDQITGK Fuc 1 Hex 7 HexNAc 5

**Mass:**  4074.8596

(4.423e-06)

**Charge States:** 3, 4

**Start Position**: 26

**End Position**: 43

| Profile Score | Tandem Score | TDA q-value | Volume | Spectrum Matches |
| --- | --- | --- | --- | --- |
| 11.4949 | 34.1375 | 0.0122 | 6.2469e+06 | 2 |

**Adduct Signal Fractions**

- **Unmodified**: 6.2469e+06

### QDQC(C)IYN(N)TTYLNVQR Fuc 3 Hex 7 HexNAc 6

**Mass:**  4705.9202

(2.371e-06)

**Charge States:** 3

**Start Position**: 87

**End Position**: 102

| Profile Score | Tandem Score | TDA q-value | Volume | Spectrum Matches |
| --- | --- | --- | --- | --- |
| -20.1771 | 33.8272 | 0.0132 | 4.8717e+06 | 2 |

**Adduct Signal Fractions**

- **Unmodified**: 4.8717e+06

### QDQC(C)IYN(N)TTYLNVQR Fuc 2 Hex 6 HexNAc 5

**Mass:**  4194.7355

(3.947e-06)

**Charge States:** 3

**Start Position**: 87

**End Position**: 102

| Profile Score | Tandem Score | TDA q-value | Volume | Spectrum Matches |
| --- | --- | --- | --- | --- |
| -5.2282 | 33.8149 | 0.0132 | 7.9792e+06 | 2 |

**Adduct Signal Fractions**

- **Unmodified**: 7.9792e+06

### FYFTPN(N)KTEDTIFLR Hex 9 HexNAc 8

**Mass:**  4977.0741

(6.056e-04)

**Start Position**: 67

**End Position**: 82

| Profile Score | Tandem Score | TDA q-value | Volume | Spectrum Matches |
| --- | --- | --- | --- | --- |
| 0.0000 | 33.5778 | 0.0132 | 0.0000e+00 | 1 |

Adduct:

No Chromatogram Found

### SVQEIQATFFYFTPN(N)KTEDTIFLR Hex 8 HexNAc 7

**Mass:**  5614.4453

(3.596e-04)

**Start Position**: 58

**End Position**: 82

| Profile Score | Tandem Score | TDA q-value | Volume | Spectrum Matches |
| --- | --- | --- | --- | --- |
| 0.0000 | 32.8616 | 0.0144 | 0.0000e+00 | 1 |

Adduct:

No Chromatogram Found

### QDQC(C)IYN(N)TTYLNVQR Fuc 1 Hex 7 HexNAc 4 Neu5Ac 2

**Mass:**  4589.8366

(1.481e-06)

**Charge States:** 3

**Start Position**: 87

**End Position**: 102

| Profile Score | Tandem Score | TDA q-value | Volume | Spectrum Matches |
| --- | --- | --- | --- | --- |
| -4.5670 | 32.7350 | 0.0157 | 2.4966e+07 | 2 |

**Adduct Signal Fractions**

- **Unmodified**: 2.4966e+07

### IYN(N)TTYLNVQR Hex 7 HexNAc 6

**Mass:**  3736.5657

(1.455e-06)

**Charge States:** 3, 4

**Start Position**: 91

**End Position**: 102

| Profile Score | Tandem Score | TDA q-value | Volume | Spectrum Matches |
| --- | --- | --- | --- | --- |
| 13.2795 | 32.2583 | 0.0181 | 2.8524e+07 | 5 |

**Adduct Signal Fractions**

- **Unmodified**: 2.8524e+07

### YFTPN(N)KTEDTIFLR Fuc 5 Hex 8 HexNAc 7

**Mass:**  5192.1384

(-2.401e-06)

**Charge States:** 4

**Start Position**: 68

**End Position**: 82

| Profile Score | Tandem Score | TDA q-value | Volume | Spectrum Matches |
| --- | --- | --- | --- | --- |
| -4.5899 | 31.3749 | 0.0187 | 5.2917e+06 | 1 |

**Adduct Signal Fractions**

- **Unmodified**: 5.2917e+06

### PVPITN(N)ATLDQITGK Fuc 2 Hex 4 HexNAc 8

**Mass:**  4131.8443

(4.980e-06)

**Charge States:** 3

**Start Position**: 28

**End Position**: 43

| Profile Score | Tandem Score | TDA q-value | Volume | Spectrum Matches |
| --- | --- | --- | --- | --- |
| -18.9715 | 31.1168 | 0.0199 | 7.4759e+05 | 1 |

**Adduct Signal Fractions**

- **Unmodified**: 7.4759e+05

### LVPVPITN(N)ATLDQITGK Fuc 1 Hex 7 HexNAc 5 Neu5Ac 2

**Mass:**  4657.0485

(4.038e-06)

**Charge States:** 4

**Start Position**: 26

**End Position**: 43

| Profile Score | Tandem Score | TDA q-value | Volume | Spectrum Matches |
| --- | --- | --- | --- | --- |
| -5.5317 | 30.8435 | 0.0208 | 2.0756e+06 | 1 |

**Adduct Signal Fractions**

- **Unmodified**: 2.0756e+06

### FTPN(N)KTEDTIFLR Hex 10 HexNAc 3 Neu5Ac 1

**Mass:**  4101.7106

(8.201e-06)

**Charge States:** 4

**Start Position**: 69

**End Position**: 82

| Profile Score | Tandem Score | TDA q-value | Volume | Spectrum Matches |
| --- | --- | --- | --- | --- |
| -20.5708 | 30.4379 | 0.0208 | 1.0423e+06 | 1 |

**Adduct Signal Fractions**

- **Unmodified**: 1.0423e+06

### LVPVPITN(N)ATLDQITGK Fuc 2 Hex 7 HexNAc 6

**Mass:**  4423.9885

(2.229e-06)

**Charge States:** 3, 4

**Start Position**: 26

**End Position**: 43

| Profile Score | Tandem Score | TDA q-value | Volume | Spectrum Matches |
| --- | --- | --- | --- | --- |
| 7.6286 | 30.3107 | 0.0217 | 1.8994e+07 | 2 |

**Adduct Signal Fractions**

- **Unmodified**: 1.8994e+07

### QIPLC(C)ANLVPVPITN(N)ATLDQITGK Hex 6 HexNAc 5

**Mass:**  4563.1227

(1.348e-06)

**Charge States:** 3, 4

**Start Position**: 19

**End Position**: 43

| Profile Score | Tandem Score | TDA q-value | Volume | Spectrum Matches |
| --- | --- | --- | --- | --- |
| 13.4334 | 30.1765 | 0.0217 | 4.1078e+08 | 9 |

**Adduct Signal Fractions**

- **Unmodified**: 4.1078e+08

### FYFTPN(N)KTEDTIFLR Fuc 2 Hex 6 HexNAc 5

**Mass:**  4170.7819

(1.769e-07)

**Charge States:** 4

**Start Position**: 67

**End Position**: 82

| Profile Score | Tandem Score | TDA q-value | Volume | Spectrum Matches |
| --- | --- | --- | --- | --- |
| -19.5686 | 29.6412 | 0.0236 | 2.2917e+07 | 1 |

**Adduct Signal Fractions**

- **Unmodified**: 2.2917e+07

### LVPVPITN(N)ATLDQITGK Fuc 2 Hex 4 HexNAc 9

**Mass:**  4547.0817

(5.053e-06)

**Charge States:** 3, 4

**Start Position**: 26

**End Position**: 43

| Profile Score | Tandem Score | TDA q-value | Volume | Spectrum Matches |
| --- | --- | --- | --- | --- |
| 8.6203 | 29.3657 | 0.0236 | 6.9924e+07 | 11 |

**Adduct Signal Fractions**

- **Unmodified**: 6.9924e+07

### QDQC(C)IYN(N)TTYLN(D)VQR Fuc 4 Hex 7 HexNAc 5

**Mass:**  4649.9035

(2.182e-04)

**Charge States:** 3, 4

**Start Position**: 87

**End Position**: 102

| Profile Score | Tandem Score | TDA q-value | Volume | Spectrum Matches |
| --- | --- | --- | --- | --- |
| 13.0060 | 29.1731 | 0.0244 | 1.2056e+08 | 4 |

**Adduct Signal Fractions**

- **Unmodified**: 1.2056e+08

### IYN(N)TTYLNVQR Fuc 1 Hex 6 HexNAc 5

**Mass:**  3517.4877

(2.993e-07)

**Charge States:** 3

**Start Position**: 91

**End Position**: 102

| Profile Score | Tandem Score | TDA q-value | Volume | Spectrum Matches |
| --- | --- | --- | --- | --- |
| -4.0012 | 29.1193 | 0.0244 | 3.7863e+06 | 1 |

**Adduct Signal Fractions**

- **Unmodified**: 3.7863e+06

### N(N)ATLDQITGK Fuc 1 Hex 7 HexNAc 7

**Mass:**  3761.5665

(5.602e-06)

**Charge States:** 3, 4

**Start Position**: 33

**End Position**: 43

| Profile Score | Tandem Score | TDA q-value | Volume | Spectrum Matches |
| --- | --- | --- | --- | --- |
| -4.9572 | 28.9096 | 0.0253 | 1.0222e+08 | 4 |

**Adduct Signal Fractions**

- **Unmodified**: 1.0222e+08

### FYFTPN(N)KTEDTIFLR Fuc 1 Hex 3 HexNAc 3 Neu5Ac 1

**Mass:**  3423.4960

(-1.610e-06)

**Charge States:** 3, 4

**Start Position**: 67

**End Position**: 82

| Profile Score | Tandem Score | TDA q-value | Volume | Spectrum Matches |
| --- | --- | --- | --- | --- |
| -19.0367 | 28.8173 | 0.0253 | 1.5234e+07 | 2 |

**Adduct Signal Fractions**

- **Unmodified**: 1.5234e+07

### WFYIASAFRNEEYNKSVQEIQATFFYFTPN(N)K Fuc 5 Hex 10 HexNAc 8

**Mass:**  7813.3462

(5.975e-06)

**Charge States:** 5

**Start Position**: 43

**End Position**: 74

| Profile Score | Tandem Score | TDA q-value | Volume | Spectrum Matches |
| --- | --- | --- | --- | --- |
| -4.6751 | 28.5925 | 0.0284 | 2.6046e+07 | 1 |

**Adduct Signal Fractions**

- **Unmodified**: 2.6046e+07

### LVPVPITN(N)ATLDQITGK Fuc 2 Hex 4 HexNAc 4 Neu5Ac 1

**Mass:**  3822.7696

(4.021e-06)

**Charge States:** 4

**Start Position**: 26

**End Position**: 43

| Profile Score | Tandem Score | TDA q-value | Volume | Spectrum Matches |
| --- | --- | --- | --- | --- |
| -17.9798 | 28.5496 | 0.0284 | 6.8551e+07 | 2 |

**Adduct Signal Fractions**

- **Unmodified**: 6.8551e+07

### QDQC(C)IYN(N)TTYLNVQR Fuc 4 Hex 5 HexNAc 5

**Mass:**  4324.7658

(-3.777e-06)

**Charge States:** 3, 4

**Start Position**: 87

**End Position**: 102

| Profile Score | Tandem Score | TDA q-value | Volume | Spectrum Matches |
| --- | --- | --- | --- | --- |
| 13.0107 | 28.1517 | 0.0293 | 4.4846e+08 | 6 |

**Adduct Signal Fractions**

- **Unmodified**: 4.4846e+08

### QDQC(C)IYN(N)TTYLNVQR Fuc 2 Hex 8 HexNAc 4 Neu5Ac 1

**Mass:**  4606.8678

(4.647e-06)

**Charge States:** 3, 4

**Start Position**: 87

**End Position**: 102

| Profile Score | Tandem Score | TDA q-value | Volume | Spectrum Matches |
| --- | --- | --- | --- | --- |
| -20.5062 | 28.1322 | 0.0293 | 7.0559e+06 | 1 |

**Adduct Signal Fractions**

- **Unmodified**: 7.0559e+06

### LVPVPITN(N)ATLDQITGK Hex 5 HexNAc 6

**Mass:**  3810.7388

(7.834e-04)

**Charge States:** 3, 4

**Start Position**: 26

**End Position**: 43

| Profile Score | Tandem Score | TDA q-value | Volume | Spectrum Matches |
| --- | --- | --- | --- | --- |
| -24.1471 | 27.9270 | 0.0301 | 1.7460e+06 | 9 |

**Adduct Signal Fractions**

- **Unmodified**: 1.7460e+06

### QDQC(C)IYN(N)TTYLN(D)VQR Hex 7 HexNAc 6

**Mass:**  4268.7273

(2.335e-04)

**Charge States:** 3, 4

**Start Position**: 87

**End Position**: 102

| Profile Score | Tandem Score | TDA q-value | Volume | Spectrum Matches |
| --- | --- | --- | --- | --- |
| -20.4665 | 27.6068 | 0.0328 | 8.6756e+07 | 2 |

**Adduct Signal Fractions**

- **Unmodified**: 8.6756e+07

### FYFTPN(N)KTEDTIFLR Fuc 2 Hex 4 HexNAc 4 Neu5Ac 1

**Mass:**  3934.6807

(-2.780e-06)

**Charge States:** 3, 4

**Start Position**: 67

**End Position**: 82

| Profile Score | Tandem Score | TDA q-value | Volume | Spectrum Matches |
| --- | --- | --- | --- | --- |
| -18.6121 | 27.5739 | 0.0328 | 5.0932e+07 | 1 |

**Adduct Signal Fractions**

- **Unmodified**: 5.0932e+07

### QDQC(C)IYN(N)TTYLN(D)VQR Fuc 1 Hex 7 HexNAc 6

**Mass:**  4414.7897

(2.258e-04)

**Charge States:** 3, 4

**Start Position**: 87

**End Position**: 102

| Profile Score | Tandem Score | TDA q-value | Volume | Spectrum Matches |
| --- | --- | --- | --- | --- |
| -20.9437 | 26.8472 | 0.0373 | 2.6021e+07 | 1 |

**Adduct Signal Fractions**

- **Unmodified**: 2.6021e+07

### LVPVPITN(N)ATLDQITGK Fuc 1 Hex 6 HexNAc 5

**Mass:**  3914.7904

(5.123e-04)

**Start Position**: 26

**End Position**: 43

| Profile Score | Tandem Score | TDA q-value | Volume | Spectrum Matches |
| --- | --- | --- | --- | --- |
| 0.0000 | 26.7876 | 0.0373 | 0.0000e+00 | 1 |

Adduct:

No Chromatogram Found

### SVQEIQATFFYFTPN(N)KTEDTIFLR Fuc 2 Hex 8 HexNAc 7

**Mass:**  5904.5476

(7.260e-07)

**Charge States:** 4

**Start Position**: 58

**End Position**: 82

| Profile Score | Tandem Score | TDA q-value | Volume | Spectrum Matches |
| --- | --- | --- | --- | --- |
| -4.5329 | 26.6879 | 0.0373 | 6.1134e+06 | 1 |

**Adduct Signal Fractions**

- **Unmodified**: 6.1134e+06

### PVPITN(N)ATLDQITGK Fuc 3 Hex 7 HexNAc 4 Neu5Ac 1

**Mass:**  4242.8512

(7.821e-06)

**Charge States:** 3

**Start Position**: 28

**End Position**: 43

| Profile Score | Tandem Score | TDA q-value | Volume | Spectrum Matches |
| --- | --- | --- | --- | --- |
| -21.6071 | 25.9146 | 0.0395 | 1.3818e+06 | 1 |

**Adduct Signal Fractions**

- **Unmodified**: 1.3818e+06

### PVPITN(N)ATLDQITGK Hex 7 HexNAc 4 Neu5Ac 1

**Mass:**  3804.6743

(9.562e-06)

**Charge States:** 3, 4, 5

**Start Position**: 28

**End Position**: 43

| Profile Score | Tandem Score | TDA q-value | Volume | Spectrum Matches |
| --- | --- | --- | --- | --- |
| 14.1065 | 25.7973 | 0.0395 | 1.1479e+09 | 11 |

**Adduct Signal Fractions**

- **Unmodified**: 1.1479e+09

### LVPVPITN(N)ATLDQITGK Hex 9 HexNAc 3

**Mass:**  3847.7581

(2.679e-04)

**Start Position**: 26

**End Position**: 43

| Profile Score | Tandem Score | TDA q-value | Volume | Spectrum Matches |
| --- | --- | --- | --- | --- |
| 0.0000 | 25.7051 | 0.0395 | 0.0000e+00 | 1 |

Adduct:

No Chromatogram Found

### PVPITN(N)ATLDQITGK Hex 7 HexNAc 5 Neu5Ac 1

**Mass:**  4007.7561

(8.171e-06)

**Charge States:** 4

**Start Position**: 28

**End Position**: 43

| Profile Score | Tandem Score | TDA q-value | Volume | Spectrum Matches |
| --- | --- | --- | --- | --- |
| 12.8207 | 25.7001 | 0.0395 | 1.0250e+08 | 3 |

**Adduct Signal Fractions**

- **Unmodified**: 1.0250e+08

### QIPLC(C)ANLVPVPITN(N)ATLDQITGK Hex 5 HexNAc 5 Neu5Ac 1

**Mass:**  4692.1613

(7.979e-08)

**Charge States:** 3, 4

**Start Position**: 19

**End Position**: 43

| Profile Score | Tandem Score | TDA q-value | Volume | Spectrum Matches |
| --- | --- | --- | --- | --- |
| 15.4243 | 25.6727 | 0.0395 | 2.0738e+08 | 9 |

**Adduct Signal Fractions**

- **Unmodified**: 2.0738e+08

### QDQC(C)IYN(N)TTYLNVQR Fuc 1 Hex 9 HexNAc 2

**Mass:**  3925.5928

(-1.159e-07)

**Charge States:** 3, 4

**Start Position**: 87

**End Position**: 102

| Profile Score | Tandem Score | TDA q-value | Volume | Spectrum Matches |
| --- | --- | --- | --- | --- |
| 9.5616 | 25.5513 | 0.0421 | 3.4929e+07 | 1 |

**Adduct Signal Fractions**

- **Unmodified**: 3.4929e+07

### SVQEIQATFFYFTPN(N)KTEDTIFLR Hex 10 HexNAc 9

**Mass:**  6342.6970

(8.763e-07)

**Charge States:** 4

**Start Position**: 58

**End Position**: 82

| Profile Score | Tandem Score | TDA q-value | Volume | Spectrum Matches |
| --- | --- | --- | --- | --- |
| -5.8151 | 25.5133 | 0.0421 | 6.2037e+06 | 1 |

**Adduct Signal Fractions**

- **Unmodified**: 6.2037e+06

### LVPVPITN(N)ATLDQITGK Fuc 3 Hex 4 HexNAc 9

**Mass:**  4693.1408

(5.804e-06)

**Charge States:** 3, 4

**Start Position**: 26

**End Position**: 43

| Profile Score | Tandem Score | TDA q-value | Volume | Spectrum Matches |
| --- | --- | --- | --- | --- |
| 3.7428 | 25.4204 | 0.0421 | 1.6711e+07 | 6 |

**Adduct Signal Fractions**

- **Unmodified**: 1.6711e+07

### ATFFYFTPN(N)K Fuc 2 Hex 8 HexNAc 3

**Mass:**  3432.3869

(4.225e-06)

**Charge States:** 3

**Start Position**: 64

**End Position**: 74

| Profile Score | Tandem Score | TDA q-value | Volume | Spectrum Matches |
| --- | --- | --- | --- | --- |
| -18.3456 | 25.1956 | 0.0445 | 8.7695e+06 | 1 |

**Adduct Signal Fractions**

- **Unmodified**: 8.7695e+06

### FYFTPN(N)KTEDTIFLR Fuc 3 Hex 7 HexNAc 5

**Mass:**  4478.8942

(2.336e-07)

**Charge States:** 4

**Start Position**: 67

**End Position**: 82

| Profile Score | Tandem Score | TDA q-value | Volume | Spectrum Matches |
| --- | --- | --- | --- | --- |
| -5.6786 | 25.1710 | 0.0445 | 2.6567e+07 | 1 |

**Adduct Signal Fractions**

- **Unmodified**: 2.6567e+07

### LVPVPITN(N)ATLDQITGK Hex 9 HexNAc 8

**Mass:**  4862.1325

(2.328e-06)

**Charge States:** 4

**Start Position**: 26

**End Position**: 43

| Profile Score | Tandem Score | TDA q-value | Volume | Spectrum Matches |
| --- | --- | --- | --- | --- |
| 8.1213 | 25.0344 | 0.0445 | 7.8013e+06 | 3 |

**Adduct Signal Fractions**

- **Unmodified**: 7.8013e+06

### LVPVPITN(N)ATLDQITGK Hex 6 HexNAc 5

**Mass:**  3769.7345

(7.982e-04)

**Start Position**: 26

**End Position**: 43

| Profile Score | Tandem Score | TDA q-value | Volume | Spectrum Matches |
| --- | --- | --- | --- | --- |
| 0.0000 | 25.0174 | 0.0445 | 0.0000e+00 | 1 |

Adduct:

No Chromatogram Found

### LVPVPITN(N)ATLDQITGK Fuc 4 Hex 3 HexNAc 7 Neu5Ac 1

**Mass:**  4562.0935

(8.348e-06)

**Charge States:** 3, 4

**Start Position**: 26

**End Position**: 43

| Profile Score | Tandem Score | TDA q-value | Volume | Spectrum Matches |
| --- | --- | --- | --- | --- |
| 11.0418 | 24.9978 | 0.0445 | 7.4817e+07 | 8 |

**Adduct Signal Fractions**

- **Unmodified**: 7.4817e+07

### PVPITN(N)ATLDQITGK Hex 3 HexNAc 7 Neu5Ac 1

**Mass:**  3765.7132

(8.767e-06)

**Charge States:** 3

**Start Position**: 28

**End Position**: 43

| Profile Score | Tandem Score | TDA q-value | Volume | Spectrum Matches |
| --- | --- | --- | --- | --- |
| -2.9004 | 24.9213 | 0.0445 | 3.7655e+06 | 1 |

**Adduct Signal Fractions**

- **Unmodified**: 3.7655e+06

### LVPVPITN(N)ATLDQITGK Fuc 2 Hex 3 HexNAc 3 Neu5Ac 1

**Mass:**  3460.6237

(8.681e-04)

**Start Position**: 26

**End Position**: 43

| Profile Score | Tandem Score | TDA q-value | Volume | Spectrum Matches |
| --- | --- | --- | --- | --- |
| 0.0000 | 24.8575 | 0.0445 | 0.0000e+00 | 1 |

Adduct:

No Chromatogram Found

### C(C)ANLVPVPITN(N)ATLDQITGK Hex 6 HexNAc 6

**Mass:**  4314.9204

(1.724e-06)

**Charge States:** 3, 4

**Start Position**: 23

**End Position**: 43

| Profile Score | Tandem Score | TDA q-value | Volume | Spectrum Matches |
| --- | --- | --- | --- | --- |
| -18.8786 | 24.7346 | 0.0445 | 1.2475e+07 | 2 |

**Adduct Signal Fractions**

- **Unmodified**: 1.2475e+07

### IYN(N)TTYLNVQR Fuc 1 Hex 7 HexNAc 6

**Mass:**  3882.6267

(1.611e-06)

**Charge States:** 3, 4

**Start Position**: 91

**End Position**: 102

| Profile Score | Tandem Score | TDA q-value | Volume | Spectrum Matches |
| --- | --- | --- | --- | --- |
| 11.6927 | 24.5964 | 0.0445 | 9.1593e+06 | 2 |

**Adduct Signal Fractions**

- **Unmodified**: 9.1593e+06

### PVPITN(N)ATLDQITGK Fuc 2 Hex 6 HexNAc 5

**Mass:**  3846.6934

(-3.545e-07)

**Charge States:** 3

**Start Position**: 28

**End Position**: 43

| Profile Score | Tandem Score | TDA q-value | Volume | Spectrum Matches |
| --- | --- | --- | --- | --- |
| -5.2177 | 24.2725 | 0.0445 | 3.7717e+06 | 2 |

**Adduct Signal Fractions**

- **Unmodified**: 3.7717e+06

### QDQC(C)IYN(N)TTYLN(D)VQR Fuc 1 Hex 6 HexNAc 5

**Mass:**  4049.6577

(2.461e-04)

**Charge States:** 3, 4

**Start Position**: 87

**End Position**: 102

| Profile Score | Tandem Score | TDA q-value | Volume | Spectrum Matches |
| --- | --- | --- | --- | --- |
| -5.5047 | 23.8348 | 0.0455 | 1.4696e+07 | 1 |

**Adduct Signal Fractions**

- **Unmodified**: 1.4696e+07

### SVQEIQATFFYFTPN(N)KTEDTIFLR Fuc 4 Hex 5 HexNAc 5

**Mass:**  5304.3254

(-3.953e-06)

**Charge States:** 3, 4, 5

**Start Position**: 58

**End Position**: 82

| Profile Score | Tandem Score | TDA q-value | Volume | Spectrum Matches |
| --- | --- | --- | --- | --- |
| 12.8581 | 23.7441 | 0.0464 | 2.2358e+08 | 12 |

**Adduct Signal Fractions**

- **Unmodified**: 2.2358e+08

### SVQEIQATFFYFTPN(N)KTEDTIFLR Hex 7 HexNAc 4 Neu5Ac 2

**Mass:**  5423.3371

(1.638e-06)

**Charge States:** 4

**Start Position**: 58

**End Position**: 82

| Profile Score | Tandem Score | TDA q-value | Volume | Spectrum Matches |
| --- | --- | --- | --- | --- |
| -4.0422 | 23.6397 | 0.0487 | 2.5786e+07 | 1 |

**Adduct Signal Fractions**

- **Unmodified**: 2.5786e+07

### P00738|HPT\_HUMAN

**>P00738|HPT\_HUMAN**  
 `MSALGAVIALLLWGQLFAVDSGNDVTDIADDGCPKPPEIAHGYVEHSVRYQCKNYYKLRTEGDGVYTLNDKKQWINKAVG  
DKLPECEADDGCPKPPEIAHGYVEHSVRYQCKNYYKLRTEGDGVYTLNNEKQWINKAVGDKLPECEAVCGKPKNPANPVQ  
RILGGHLDAKGSFPWQAKMVSHHNLTTGATLINEQWLLTTAKNLFLNHSENATAKDIAPTLTLYVGKKQLVEIEKVVLHP  
NYSQVDIGLIKLKQKVSVNERVMPICLPSKDYAEVGRVGYVSGWGRNANFKFTDHLKYVMLPVADQDQCIRHYEGSTVPE  
KKTPKSPVGVQPILNEHTFCAGMSKYQEDTCYGDAGSAFAVHDLEEDTWYATGILSFDKSCAVAEYGVYVKVTSIQDWVQ  
KTIAEN`

| Site | Type | Count |
| --- | --- | --- |
| 241 | N-linked | 5 |
| 211 | N-linked | 2 |
| 207 | N-linked | 1 |

| Mass | Sequence | MS_2_ Score | `q`-value | MS_1_ Score | Signal | Protein Coordinates | Tandem Scans | Start Time | Apex Time | End Time |
| --- | --- | --- | --- | --- | --- | --- | --- | --- | --- | --- |
| 3416.5921 | VVLHPN(N)YSQVDIGLIK Hex 5 HexNAc 4 | 166.089 | 0.0 | -3.589 | 3.901e+07 | 235 - 251 | 3 | 42.62 | 42.79 | 42.96 |
| 3781.7304 | VVLHPN(N)YSQVDIGLIK Hex 6 HexNAc 5 | 157.746 | 0.0 | -18.094 | 2.622e+07 | 235 - 251 | 2 | 42.45 | 42.45 | 42.62 |
| 4146.8558 | VVLHPN(N)YSQVDIGLIK Hex 7 HexNAc 6 | 134.675 | 0.0 | -4.494 | 1.489e+07 | 235 - 251 | 2 | 42.11 | 42.11 | 42.45 |
| 3257.3554 | VVLHPN(N)YSQVD Hex 6 HexNAc 5 | 106.543 | 0.0 | 13.643 | 4.340e+06 | 235 - 246 | 2 | 23.96 | 24.19 | 24.27 |
| 5068.0320 | NLFLN(N)HSENATAK Fuc 5 Hex 9 HexNAc 7 | 97.259 | 0.0 | -18.510 | 1.131e+07 | 202 - 215 | 1 | 18.45 | 18.45 | 18.61 |
| 5433.1618 | NLFLNHSEN(N)ATAK Fuc 5 Hex 10 HexNAc 8 | 93.737 | 0.0 | -19.536 | 1.264e+07 | 202 - 215 | 1 | 18.16 | 18.29 | 18.29 |
| 5581.2001 | NLFLNHSEN(N)ATAK Fuc 4 Hex 10 HexNAc 8 Neu5Ac 1 | 36.274 | 0.010 | 0.000 | 0.000e+00 | 202 - 215 | 1 | - | - | - |
| 2892.2234 | VVLHPN(N)YSQVD Hex 5 HexNAc 4 | 34.603 | 0.012 | -3.159 | 5.160e+06 | 235 - 246 | 2 | 24.27 | 24.42 | 24.55 |

### VVLHPN(N)YSQVDIGLIK Hex 5 HexNAc 4

**Mass:**  3416.5918

(1.661e-06)

**Charge States:** 3, 4

**Start Position**: 236

**End Position**: 252

| Profile Score | Tandem Score | TDA q-value | Volume | Spectrum Matches |
| --- | --- | --- | --- | --- |
| -3.5892 | 166.0893 | 0.0000 | 3.9008e+07 | 3 |

**Adduct Signal Fractions**

- **Unmodified**: 3.9008e+07

### VVLHPN(N)YSQVDIGLIK Hex 6 HexNAc 5

**Mass:**  3781.7294

(3.011e-06)

**Charge States:** 4

**Start Position**: 236

**End Position**: 252

| Profile Score | Tandem Score | TDA q-value | Volume | Spectrum Matches |
| --- | --- | --- | --- | --- |
| -18.0935 | 157.7456 | 0.0000 | 2.6215e+07 | 2 |

**Adduct Signal Fractions**

- **Unmodified**: 2.6215e+07

### VVLHPN(N)YSQVDIGLIK Hex 7 HexNAc 6

**Mass:**  4146.8571

(1.004e-06)

**Charge States:** 3

**Start Position**: 236

**End Position**: 252

| Profile Score | Tandem Score | TDA q-value | Volume | Spectrum Matches |
| --- | --- | --- | --- | --- |
| -4.4944 | 134.6746 | 0.0000 | 1.4890e+07 | 2 |

**Adduct Signal Fractions**

- **Unmodified**: 1.4890e+07

### VVLHPN(N)YSQVD Hex 6 HexNAc 5

**Mass:**  3257.3556

(2.024e-06)

**Charge States:** 3

**Start Position**: 236

**End Position**: 247

| Profile Score | Tandem Score | TDA q-value | Volume | Spectrum Matches |
| --- | --- | --- | --- | --- |
| 13.6428 | 106.5432 | 0.0000 | 4.3402e+06 | 2 |

**Adduct Signal Fractions**

- **Unmodified**: 4.3402e+06

### NLFLN(N)HSENATAK Fuc 5 Hex 9 HexNAc 7

**Mass:**  5068.0344

(-2.426e-06)

**Charge States:** 4

**Start Position**: 203

**End Position**: 216

| Profile Score | Tandem Score | TDA q-value | Volume | Spectrum Matches |
| --- | --- | --- | --- | --- |
| -18.5100 | 97.2594 | 0.0000 | 1.1311e+07 | 1 |

**Adduct Signal Fractions**

- **Unmodified**: 1.1311e+07

### NLFLNHSEN(N)ATAK Fuc 5 Hex 10 HexNAc 8

**Mass:**  5433.1662

(-4.046e-06)

**Charge States:** 4

**Start Position**: 203

**End Position**: 216

| Profile Score | Tandem Score | TDA q-value | Volume | Spectrum Matches |
| --- | --- | --- | --- | --- |
| -19.5360 | 93.7368 | 0.0000 | 1.2636e+07 | 1 |

**Adduct Signal Fractions**

- **Unmodified**: 1.2636e+07

### NLFLNHSEN(N)ATAK Fuc 4 Hex 10 HexNAc 8 Neu5Ac 1

**Mass:**  5581.2001

(5.349e-04)

**Start Position**: 203

**End Position**: 216

| Profile Score | Tandem Score | TDA q-value | Volume | Spectrum Matches |
| --- | --- | --- | --- | --- |
| 0.0000 | 36.2740 | 0.0097 | 0.0000e+00 | 1 |

Adduct:

No Chromatogram Found

### VVLHPN(N)YSQVD Hex 5 HexNAc 4

**Mass:**  2892.2235

(2.554e-06)

**Charge States:** 3

**Start Position**: 236

**End Position**: 247

| Profile Score | Tandem Score | TDA q-value | Volume | Spectrum Matches |
| --- | --- | --- | --- | --- |
| -3.1586 | 34.6031 | 0.0122 | 5.1601e+06 | 2 |

**Adduct Signal Fractions**

- **Unmodified**: 5.1601e+06

### P05155|IC1\_HUMAN

**>P05155|IC1\_HUMAN**  
 `MASRLTLLTLLLLLLAGDRASSNPNATSSSSQDPESLQDRGEGKVATTVISKMLFVEPILEVSSLPTTNSTTNSATKITA  
NTTDEPTTQPTTEPTTQPTIQPTQPTTQLPTDSPTQPTTGSFCPGPVTLCSDLESHSTEAVLGDALVDFSLKLYHAFSAM  
KKVETNMAFSPFSIASLLTQVLLGAGENTKTNLESILSYPKDFTCVHQALKGFTTKGVTSVSQIFHSPDLAIRDTFVNAS  
RTLYSSSPRVLSNNSDANLELINTWVAKNTNNKISRLLDSLPSDTRLVLLNAIYLSAKWKTTFDPKKTRMEPFHFKNSVI  
KVPMMNSKKYPVAHFIDQTLKAKVGQLQLSHNLSLVILVPQNLKHRLEDMEQALSPSVFKAIMEKLEMSKFQPTLLTLPR  
IKVTTSQDMLSIMEKLEFFDFSYDLNLCGLTEDPDLQVSAMQHQTVLELTETGVEAAAASAISVARTLLVFEVQQPFLFV  
LWDQQHKFPVFMGRVYDPRA`

| Site | Type | Count |
| --- | --- | --- |
| 25 | N-linked | 8 |

| Mass | Sequence | MS_2_ Score | `q`-value | MS_1_ Score | Signal | Protein Coordinates | Tandem Scans | Start Time | Apex Time | End Time |
| --- | --- | --- | --- | --- | --- | --- | --- | --- | --- | --- |
| 3919.6120 | NPN(N)ATSSSSQDPESLQDR Hex 6 HexNAc 5 | 135.391 | 0.0 | -19.389 | 1.961e+07 | 22 - 40 | 3 | 14.57 | 14.57 | 28.37 |
| 3554.4328 | NPN(N)ATSSSSQDPESLQDR Hex 5 HexNAc 4 | 118.200 | 0.0 | -18.887 | 1.654e+07 | 22 - 40 | 1 | 15.70 | 15.84 | 15.84 |
| 3922.5811 | NPN(N)ATSSSSQDPESLQDR Hex 6 HexNAc 5 | 67.881 | 0.005 | 0.000 | 0.000e+00 | 22 - 40 | 3 | - | - | - |
| 3555.4357 | NPN(N)ATSSSSQDPESLQDR Hex 5 HexNAc 4 | 50.769 | 0.005 | 0.000 | 0.000e+00 | 22 - 40 | 1 | - | - | - |
| 4066.6192 | NPN(N)ATSSSSQDPESLQDR Fuc 1 Hex 6 HexNAc 5 | 50.544 | 0.005 | 0.000 | 0.000e+00 | 22 - 40 | 1 | - | - | - |
| 3701.4955 | NPN(N)ATSSSSQDPESLQDR Fuc 1 Hex 5 HexNAc 4 | 50.274 | 0.005 | 0.000 | 0.000e+00 | 22 - 40 | 1 | - | - | - |
| 4284.6892 | NPN(N)ATSSSSQDPESLQDR Hex 7 HexNAc 6 | 49.288 | 0.005 | -18.887 | 5.961e+06 | 22 - 40 | 1 | 14.41 | 14.41 | 14.41 |
| 3700.4865 | NPN(N)ATSSSSQDPESLQDR Fuc 1 Hex 5 HexNAc 4 | 34.170 | 0.012 | -19.927 | 8.839e+06 | 22 - 40 | 1 | 15.70 | 15.70 | 15.84 |

### NPN(N)ATSSSSQDPESLQDR Hex 6 HexNAc 5

**Mass:**  3919.5694

(2.496e-06)

**Charge States:** 4

**Start Position**: 23

**End Position**: 41

| Profile Score | Tandem Score | TDA q-value | Volume | Spectrum Matches |
| --- | --- | --- | --- | --- |
| -19.3891 | 135.3908 | 0.0000 | 1.9608e+07 | 3 |

**Adduct Signal Fractions**

- **Unmodified**: 1.9608e+07

### NPN(N)ATSSSSQDPESLQDR Hex 5 HexNAc 4

**Mass:**  3554.4318

(1.982e-06)

**Charge States:** 3

**Start Position**: 23

**End Position**: 41

| Profile Score | Tandem Score | TDA q-value | Volume | Spectrum Matches |
| --- | --- | --- | --- | --- |
| -18.8867 | 118.1998 | 0.0000 | 1.6536e+07 | 1 |

**Adduct Signal Fractions**

- **Unmodified**: 1.6536e+07

### NPN(N)ATSSSSQDPESLQDR Hex 6 HexNAc 5

**Mass:**  3922.5811

(5.147e-04)

**Start Position**: 23

**End Position**: 41

| Profile Score | Tandem Score | TDA q-value | Volume | Spectrum Matches |
| --- | --- | --- | --- | --- |
| 0.0000 | 67.8813 | 0.0048 | 0.0000e+00 | 3 |

Adduct:

No Chromatogram Found

### NPN(N)ATSSSSQDPESLQDR Hex 5 HexNAc 4

**Mass:**  3555.4357

(2.837e-04)

**Start Position**: 23

**End Position**: 41

| Profile Score | Tandem Score | TDA q-value | Volume | Spectrum Matches |
| --- | --- | --- | --- | --- |
| 0.0000 | 50.7693 | 0.0048 | 0.0000e+00 | 1 |

Adduct:

No Chromatogram Found

### NPN(N)ATSSSSQDPESLQDR Fuc 1 Hex 6 HexNAc 5

**Mass:**  4066.6192

(2.464e-04)

**Start Position**: 23

**End Position**: 41

| Profile Score | Tandem Score | TDA q-value | Volume | Spectrum Matches |
| --- | --- | --- | --- | --- |
| 0.0000 | 50.5441 | 0.0048 | 0.0000e+00 | 1 |

Adduct:

No Chromatogram Found

### NPN(N)ATSSSSQDPESLQDR Fuc 1 Hex 5 HexNAc 4

**Mass:**  3701.4955

(2.730e-04)

**Start Position**: 23

**End Position**: 41

| Profile Score | Tandem Score | TDA q-value | Volume | Spectrum Matches |
| --- | --- | --- | --- | --- |
| 0.0000 | 50.2743 | 0.0048 | 0.0000e+00 | 1 |

Adduct:

No Chromatogram Found

### NPN(N)ATSSSSQDPESLQDR Hex 7 HexNAc 6

**Mass:**  4284.6892

(-5.575e-07)

**Charge States:** 4

**Start Position**: 23

**End Position**: 41

| Profile Score | Tandem Score | TDA q-value | Volume | Spectrum Matches |
| --- | --- | --- | --- | --- |
| -18.8874 | 49.2880 | 0.0048 | 5.9605e+06 | 1 |

**Adduct Signal Fractions**

- **Unmodified**: 5.9605e+06

### NPN(N)ATSSSSQDPESLQDR Fuc 1 Hex 5 HexNAc 4

**Mass:**  3700.4899

(-2.229e-06)

**Charge States:** 3

**Start Position**: 23

**End Position**: 41

| Profile Score | Tandem Score | TDA q-value | Volume | Spectrum Matches |
| --- | --- | --- | --- | --- |
| -19.9267 | 34.1704 | 0.0122 | 8.8387e+06 | 1 |

**Adduct Signal Fractions**

- **Unmodified**: 8.8387e+06

### Q9Y5Y7|LYVE1\_HUMAN

**>Q9Y5Y7|LYVE1\_HUMAN**  
 `MARCFSLVLLLTSIWTTRLLVQGSLRAEELSIQVSCRIMGITLVSKKANQQLNFTEAKEACRLLGLSLAGKDQVETALKA  
SFETCSYGWVGDGFVVISRISPNPKCGKNGVGVLIWKVPVSRQFAAYCYNSSDTWTNSCIPEIITTKDPIFNTQTATQTT  
EFIVSDSTYSVASPYSTIPAPTTTPPAPASTSIPRRKKLICVTEVFMETSTMSTETEPFVENKAAFKNEAAGFGGVPTAL  
LVLALLFFGAAAGLGFCYVKRYVKAFPFTNKNQQKEMIETKVVKEEKANDSNPNEESKKTDKNPEESKSPSKTTVRCLEA  
EV`

| Site | Type | Count |
| --- | --- | --- |
| 53 | N-linked | 8 |

| Mass | Sequence | MS_2_ Score | `q`-value | MS_1_ Score | Signal | Protein Coordinates | Tandem Scans | Start Time | Apex Time | End Time |
| --- | --- | --- | --- | --- | --- | --- | --- | --- | --- | --- |
| 3378.4398 | KANQQLN(N)FTEAK Hex 6 HexNAc 5 | 159.233 | 0.0 | -3.983 | 2.366e+07 | 46 - 58 | 2 | 17.99 | 17.99 | 18.45 |
| 3013.3079 | KANQQLN(N)FTEAK Hex 5 HexNAc 4 | 150.620 | 0.0 | -19.220 | 7.786e+06 | 46 - 58 | 1 | 18.16 | 18.16 | 18.29 |
| 3743.5660 | KANQQLN(N)FTEAK Hex 7 HexNAc 6 | 122.453 | 0.0 | -20.061 | 4.048e+06 | 46 - 58 | 1 | 17.83 | 17.99 | 17.99 |
| 3250.3475 | ANQQLN(N)FTEAK Hex 6 HexNAc 5 | 108.523 | 0.0 | -4.472 | 1.316e+07 | 47 - 58 | 2 | 21.57 | 21.73 | 22.05 |
| 3615.4750 | ANQQLN(N)FTEAK Hex 7 HexNAc 6 | 103.279 | 0.0 | -4.978 | 3.549e+06 | 47 - 58 | 2 | 21.35 | 21.57 | 21.57 |
| 3268.3813 | ANQQLN(N)FTEAK Fuc 2 Hex 5 HexNAc 3 Neu5Ac 1 | 91.035 | 0.0 | 0.000 | 0.000e+00 | 47 - 58 | 1 | - | - | - |
| 3379.4267 | KAN(D)QQLN(N)FTEAK Hex 6 HexNAc 5 | 31.242 | 0.019 | -5.812 | 3.837e+06 | 46 - 58 | 2 | 18.78 | 18.78 | 18.95 |
| 2885.2174 | ANQQLN(N)FTEAK Hex 5 HexNAc 4 | 30.033 | 0.023 | -5.327 | 3.917e+06 | 47 - 58 | 2 | 21.57 | 21.90 | 22.05 |

### KANQQLN(N)FTEAK Hex 6 HexNAc 5

**Mass:**  3378.4407

(1.947e-06)

**Charge States:** 3

**Start Position**: 47

**End Position**: 59

| Profile Score | Tandem Score | TDA q-value | Volume | Spectrum Matches |
| --- | --- | --- | --- | --- |
| -3.9828 | 159.2331 | 0.0000 | 2.3657e+07 | 2 |

**Adduct Signal Fractions**

- **Unmodified**: 2.3657e+07

### KANQQLN(N)FTEAK Hex 5 HexNAc 4

**Mass:**  3013.3068

(1.581e-06)

**Charge States:** 3

**Start Position**: 47

**End Position**: 59

| Profile Score | Tandem Score | TDA q-value | Volume | Spectrum Matches |
| --- | --- | --- | --- | --- |
| -19.2199 | 150.6198 | 0.0000 | 7.7862e+06 | 1 |

**Adduct Signal Fractions**

- **Unmodified**: 7.7862e+06

### KANQQLN(N)FTEAK Hex 7 HexNAc 6

**Mass:**  3743.5684

(-9.211e-07)

**Charge States:** 3

**Start Position**: 47

**End Position**: 59

| Profile Score | Tandem Score | TDA q-value | Volume | Spectrum Matches |
| --- | --- | --- | --- | --- |
| -20.0611 | 122.4531 | 0.0000 | 4.0485e+06 | 1 |

**Adduct Signal Fractions**

- **Unmodified**: 4.0485e+06

### ANQQLN(N)FTEAK Hex 6 HexNAc 5

**Mass:**  3250.3456

(3.358e-06)

**Charge States:** 3

**Start Position**: 48

**End Position**: 59

| Profile Score | Tandem Score | TDA q-value | Volume | Spectrum Matches |
| --- | --- | --- | --- | --- |
| -4.4720 | 108.5227 | 0.0000 | 1.3165e+07 | 2 |

**Adduct Signal Fractions**

- **Unmodified**: 1.3165e+07

### ANQQLN(N)FTEAK Hex 7 HexNAc 6

**Mass:**  3615.4744

(7.805e-07)

**Charge States:** 3

**Start Position**: 48

**End Position**: 59

| Profile Score | Tandem Score | TDA q-value | Volume | Spectrum Matches |
| --- | --- | --- | --- | --- |
| -4.9783 | 103.2790 | 0.0000 | 3.5486e+06 | 2 |

**Adduct Signal Fractions**

- **Unmodified**: 3.5486e+06

### ANQQLN(N)FTEAK Fuc 2 Hex 5 HexNAc 3 Neu5Ac 1

**Mass:**  3268.3813

(9.317e-04)

**Start Position**: 48

**End Position**: 59

| Profile Score | Tandem Score | TDA q-value | Volume | Spectrum Matches |
| --- | --- | --- | --- | --- |
| 0.0000 | 91.0348 | 0.0000 | 0.0000e+00 | 1 |

Adduct:

No Chromatogram Found

### KAN(D)QQLN(N)FTEAK Hex 6 HexNAc 5

**Mass:**  3379.4284

(2.943e-04)

**Charge States:** 3

**Start Position**: 47

**End Position**: 59

| Profile Score | Tandem Score | TDA q-value | Volume | Spectrum Matches |
| --- | --- | --- | --- | --- |
| -5.8121 | 31.2419 | 0.0187 | 3.8371e+06 | 2 |

**Adduct Signal Fractions**

- **Unmodified**: 3.8371e+06

### ANQQLN(N)FTEAK Hex 5 HexNAc 4

**Mass:**  2885.2150

(6.148e-06)

**Charge States:** 3

**Start Position**: 48

**End Position**: 59

| Profile Score | Tandem Score | TDA q-value | Volume | Spectrum Matches |
| --- | --- | --- | --- | --- |
| -5.3272 | 30.0333 | 0.0229 | 3.9167e+06 | 2 |

**Adduct Signal Fractions**

- **Unmodified**: 3.9167e+06

### O75144|ICOSL\_HUMAN

**>O75144|ICOSL\_HUMAN**  
 `MRLGSPGLLFLLFSSLRADTQEKEVRAMVGSDVELSCACPEGSRFDLNDVYVYWQTSESKTVVTYHIPQNSSLENVDSRY  
RNRALMSPAGMLRGDFSLRLFNVTPQDEQKFHCLVLSQSLGFQEVLSVEVTLHVAANFSVPVVSAPHSPSQDELTFTCTS  
INGYPRPNVYWINKTDNSLLDQALQNDTVFLNMRGLYDVVSVLRIARTPSVNIGCCIENVLLQQNLTVGSQTGNDIGERD  
KITENPVSTGEKNAATWSILAVLCLLVVVAVAIGWVCRDRCLQHSYAGAWAVSPETELTGHV`

| Site | Type | Count |
| --- | --- | --- |
| 70 | N-linked | 2 |
| 102 | N-linked | 3 |

| Mass | Sequence | MS_2_ Score | `q`-value | MS_1_ Score | Signal | Protein Coordinates | Tandem Scans | Start Time | Apex Time | End Time |
| --- | --- | --- | --- | --- | --- | --- | --- | --- | --- | --- |
| 3305.3812 | LFN(N)VTPQDEQK Hex 6 HexNAc 5 | 28.258 | 0.028 | -21.001 | 4.796e+05 | 99 - 110 | 1 | 25.36 | 25.36 | 25.36 |
| 4145.7832 | TVVTYHIPQN(N)SSLENVDSR Hex 6 HexNAc 5 | 27.001 | 0.037 | -20.851 | 6.443e+05 | 60 - 79 | 2 | 31.02 | 31.02 | 31.02 |
| 2940.2473 | LFN(N)VTPQDEQK Hex 5 HexNAc 4 | 26.188 | 0.037 | -20.337 | 4.324e+05 | 99 - 110 | 1 | 25.84 | 25.84 | 25.84 |
| 4291.8516 | TVVTYHIPQN(N)SSLENVDSR Fuc 1 Hex 6 HexNAc 5 | 25.580 | 0.042 | -20.591 | 1.496e+06 | 60 - 79 | 5 | 30.90 | 31.02 | 31.02 |
| 3452.4439 | LFN(N)VTPQDEQK Fuc 1 Hex 6 HexNAc 5 | 24.621 | 0.045 | 0.000 | 0.000e+00 | 99 - 110 | 1 | - | - | - |

### LFN(N)VTPQDEQK Hex 6 HexNAc 5

**Mass:**  3305.3812

(3.326e-06)

**Charge States:** 3

**Start Position**: 100

**End Position**: 111

| Profile Score | Tandem Score | TDA q-value | Volume | Spectrum Matches |
| --- | --- | --- | --- | --- |
| -21.0010 | 28.2578 | 0.0284 | 4.7956e+05 | 1 |

**Adduct Signal Fractions**

- **Unmodified**: 4.7956e+05

### TVVTYHIPQN(N)SSLENVDSR Hex 6 HexNAc 5

**Mass:**  4145.7863

(1.695e-06)

**Charge States:** 3, 4

**Start Position**: 61

**End Position**: 80

| Profile Score | Tandem Score | TDA q-value | Volume | Spectrum Matches |
| --- | --- | --- | --- | --- |
| -20.8506 | 27.0012 | 0.0373 | 6.4430e+05 | 2 |

**Adduct Signal Fractions**

- **Unmodified**: 6.4430e+05

### LFN(N)VTPQDEQK Hex 5 HexNAc 4

**Mass:**  2940.2473

(3.145e-06)

**Charge States:** 3

**Start Position**: 100

**End Position**: 111

| Profile Score | Tandem Score | TDA q-value | Volume | Spectrum Matches |
| --- | --- | --- | --- | --- |
| -20.3371 | 26.1881 | 0.0373 | 4.3237e+05 | 1 |

**Adduct Signal Fractions**

- **Unmodified**: 4.3237e+05

### TVVTYHIPQN(N)SSLENVDSR Fuc 1 Hex 6 HexNAc 5

**Mass:**  4291.8522

(3.746e-06)

**Charge States:** 3, 4

**Start Position**: 61

**End Position**: 80

| Profile Score | Tandem Score | TDA q-value | Volume | Spectrum Matches |
| --- | --- | --- | --- | --- |
| -20.5908 | 25.5800 | 0.0421 | 1.4956e+06 | 5 |

**Adduct Signal Fractions**

- **Unmodified**: 1.4956e+06

### LFN(N)VTPQDEQK Fuc 1 Hex 6 HexNAc 5

**Mass:**  3452.4439

(2.943e-04)

**Start Position**: 100

**End Position**: 111

| Profile Score | Tandem Score | TDA q-value | Volume | Spectrum Matches |
| --- | --- | --- | --- | --- |
| 0.0000 | 24.6209 | 0.0445 | 0.0000e+00 | 1 |

Adduct:

No Chromatogram Found

### P02790|HEMO\_HUMAN

**>P02790|HEMO\_HUMAN**  
 `MARVLGAPVALGLWSLCWSLAIATPLPPTSAHGNVAEGETKPDPDVTERCSDGWSFDATTLDDNGTMLFFKGEFVWKSHK  
WDRELISERWKNFPSPVDAAFRQGHNSVFLIKGDKVWVYPPEKKEKGYPKLLQDEFPGIPSPLDAAVECHRGECQAEGVL  
FFQGDREWFWDLATGTMKERSWPAVGNCSSALRWLGRYYCFQGNQFLRFDPVRGEVPPRYPRDVRDYFMPCPGRGHGHRN  
GTGHGNSTHHGPEYMRCSPHLVLSALTSDNHGATYAFSGTHYWRLDTSRDGWHSWPIAHQWPQGPSAVDAAFSWEEKLYL  
VQGTQVYVFLTKGGYTLVSGYPKRLEKEVGTPHGIILDSVDAAFICPGSSRLHIMAGRRLWWLDLKSGAQATWTELPWPH  
EKVDGALCMEKSLGPNSCSANGPGLYLIHGPNLYCYSDVEKLNAAKALPQPQNVTSLLGCTH`

| Site | Type | Count |
| --- | --- | --- |
| 187 | N-linked | 3 |
| 453 | N-linked | 1 |

| Mass | Sequence | MS_2_ Score | `q`-value | MS_1_ Score | Signal | Protein Coordinates | Tandem Scans | Start Time | Apex Time | End Time |
| --- | --- | --- | --- | --- | --- | --- | --- | --- | --- | --- |
| 3026.2459 | SWPAVGN(N)C(C)SSALR Hex 5 HexNAc 4 | 117.486 | 0.0 | 12.596 | 1.165e+07 | 180 - 193 | 2 | 31.19 | 31.53 | 31.94 |
| 3357.4563 | ALPQPQN(N)VTSLLGC(C)TH Hex 5 HexNAc 4 | 92.336 | 0.0 | 13.741 | 3.050e+07 | 446 - 462 | 3 | 44.16 | 44.66 | 45.17 |
| 3172.3064 | SWPAVGN(N)C(C)SSALR Fuc 1 Hex 5 HexNAc 4 | 35.935 | 0.010 | -20.313 | 1.279e+06 | 180 - 193 | 1 | 31.36 | 31.36 | 31.61 |
| 3391.3812 | SWPAVGN(N)C(C)SSALR Hex 6 HexNAc 5 | 28.201 | 0.029 | -5.961 | 2.175e+06 | 180 - 193 | 2 | 31.02 | 31.19 | 31.36 |

### SWPAVGN(N)C(C)SSALR Hex 5 HexNAc 4

**Mass:**  3026.2465

(4.332e-07)

**Charge States:** 3

**Start Position**: 181

**End Position**: 194

| Profile Score | Tandem Score | TDA q-value | Volume | Spectrum Matches |
| --- | --- | --- | --- | --- |
| 12.5962 | 117.4861 | 0.0000 | 1.1649e+07 | 2 |

**Adduct Signal Fractions**

- **Unmodified**: 1.1649e+07

### ALPQPQN(N)VTSLLGC(C)TH Hex 5 HexNAc 4

**Mass:**  3357.4568

(6.730e-07)

**Charge States:** 3

**Start Position**: 447

**End Position**: 463

| Profile Score | Tandem Score | TDA q-value | Volume | Spectrum Matches |
| --- | --- | --- | --- | --- |
| 13.7406 | 92.3355 | 0.0000 | 3.0504e+07 | 3 |

**Adduct Signal Fractions**

- **Unmodified**: 3.0504e+07

### SWPAVGN(N)C(C)SSALR Fuc 1 Hex 5 HexNAc 4

**Mass:**  3172.3061

(1.588e-06)

**Charge States:** 3

**Start Position**: 181

**End Position**: 194

| Profile Score | Tandem Score | TDA q-value | Volume | Spectrum Matches |
| --- | --- | --- | --- | --- |
| -20.3131 | 35.9350 | 0.0097 | 1.2793e+06 | 1 |

**Adduct Signal Fractions**

- **Unmodified**: 1.2793e+06

### SWPAVGN(N)C(C)SSALR Hex 6 HexNAc 5

**Mass:**  3391.3804

(1.921e-06)

**Charge States:** 3

**Start Position**: 181

**End Position**: 194

| Profile Score | Tandem Score | TDA q-value | Volume | Spectrum Matches |
| --- | --- | --- | --- | --- |
| -5.9613 | 28.2014 | 0.0293 | 2.1755e+06 | 2 |

**Adduct Signal Fractions**

- **Unmodified**: 2.1755e+06

### Q5R543|HEMO\_PONAB

**>Q5R543|HEMO\_PONAB**  
 `MARALGAPIALGLWSLCWSLAIATPLPPTSAHGNVAEGETKPDPDVTERCSDGWSFDATTLDDNGTMLFFKGEFVWKSHK  
WDRELISERWKNFPSPVDAAFRQGHNSVFLIKGDKVWVYPPEKKEKGYPKLLQDEFPGIPSPLDAAVECHRGECQAEGVL  
FFQGDREWFWDLATGTMKERSWPAVGNCSSALRWLGRYYCFQGNQFLRFDPVRGEVPPRYPRDVRDYFMPCPGRGHGHRN  
GTGHGNGTHHGPEYMRCSPHLVLSALTSDNHGATYAFSGTHYWRLDTSRDGWHSWPIAHQWPQGPSTVDAAFSWEEKLYL  
VQGTQVYVFLTKGGYTLVSGYPKQLEKEVGTPHGIILDSVDAAFICPGSSRLHIMAGRRLWWLDLKSGAQALWTELPWPH  
EKVDGALCVEKSLGPNSCSANGPGLYLIHGPNLYCYSDVEKLNAAKALPQPQNVTSLLGCTH`

| Site | Type | Count |
| --- | --- | --- |
| 187 | N-linked | 3 |
| 453 | N-linked | 1 |

| Mass | Sequence | MS_2_ Score | `q`-value | MS_1_ Score | Signal | Protein Coordinates | Tandem Scans | Start Time | Apex Time | End Time |
| --- | --- | --- | --- | --- | --- | --- | --- | --- | --- | --- |
| 3026.2459 | SWPAVGN(N)C(C)SSALR Hex 5 HexNAc 4 | 117.486 | 0.0 | 12.596 | 1.165e+07 | 180 - 193 | 2 | 31.19 | 31.53 | 31.94 |
| 3357.4563 | ALPQPQN(N)VTSLLGC(C)TH Hex 5 HexNAc 4 | 92.336 | 0.0 | 13.741 | 3.050e+07 | 446 - 462 | 3 | 44.16 | 44.66 | 45.17 |
| 3172.3064 | SWPAVGN(N)C(C)SSALR Fuc 1 Hex 5 HexNAc 4 | 35.935 | 0.010 | -20.313 | 1.279e+06 | 180 - 193 | 1 | 31.36 | 31.36 | 31.61 |
| 3391.3812 | SWPAVGN(N)C(C)SSALR Hex 6 HexNAc 5 | 28.201 | 0.029 | -5.961 | 2.175e+06 | 180 - 193 | 2 | 31.02 | 31.19 | 31.36 |

### SWPAVGN(N)C(C)SSALR Hex 5 HexNAc 4

**Mass:**  3026.2465

(4.332e-07)

**Charge States:** 3

**Start Position**: 181

**End Position**: 194

| Profile Score | Tandem Score | TDA q-value | Volume | Spectrum Matches |
| --- | --- | --- | --- | --- |
| 12.5962 | 117.4861 | 0.0000 | 1.1649e+07 | 2 |

**Adduct Signal Fractions**

- **Unmodified**: 1.1649e+07

### ALPQPQN(N)VTSLLGC(C)TH Hex 5 HexNAc 4

**Mass:**  3357.4568

(6.730e-07)

**Charge States:** 3

**Start Position**: 447

**End Position**: 463

| Profile Score | Tandem Score | TDA q-value | Volume | Spectrum Matches |
| --- | --- | --- | --- | --- |
| 13.7406 | 92.3355 | 0.0000 | 3.0504e+07 | 3 |

**Adduct Signal Fractions**

- **Unmodified**: 3.0504e+07

### SWPAVGN(N)C(C)SSALR Fuc 1 Hex 5 HexNAc 4

**Mass:**  3172.3061

(1.588e-06)

**Charge States:** 3

**Start Position**: 181

**End Position**: 194

| Profile Score | Tandem Score | TDA q-value | Volume | Spectrum Matches |
| --- | --- | --- | --- | --- |
| -20.3131 | 35.9350 | 0.0097 | 1.2793e+06 | 1 |

**Adduct Signal Fractions**

- **Unmodified**: 1.2793e+06

### SWPAVGN(N)C(C)SSALR Hex 6 HexNAc 5

**Mass:**  3391.3804

(1.921e-06)

**Charge States:** 3

**Start Position**: 181

**End Position**: 194

| Profile Score | Tandem Score | TDA q-value | Volume | Spectrum Matches |
| --- | --- | --- | --- | --- |
| -5.9613 | 28.2014 | 0.0293 | 2.1755e+06 | 2 |

**Adduct Signal Fractions**

- **Unmodified**: 2.1755e+06

### Q12913|PTPRJ\_HUMAN

**>Q12913|PTPRJ\_HUMAN**  
 `MKPAAREARLPPRSPGLRWALPLLLLLLRLGQILCAGGTPSPIPDPSVATVATGENGITQISSTAESFHKQNGTGTPQVE  
TNTSEDGESSGANDSLRTPEQGSNGTDGASQKTPSSTGPSPVFDIKAVSISPTNVILTWKSNDTAASEYKYVVKHKMENE  
KTITVVHQPWCNITGLRPATSYVFSITPGIGNETWGDPRVIKVITEPIPVSDLRVALTGVRKAALSWSNGNGTASCRVLL  
ESIGSHEELTQDSRLQVNISGLKPGVQYNINPYLLQSNKTKGDPLGTEGGLDASNTERSRAGSPTAPVHDESLVGPVDPS  
SGQQSRDTEVLLVGLEPGTRYNATVYSQAANGTEGQPQAIEFRTNAIQVFDVTAVNISATSLTLIWKVSDNESSSNYTYK  
IHVAGETDSSNLNVSEPRAVIPGLRSSTFYNITVCPVLGDIEGTPGFLQVHTPPVPVSDFRVTVVSTTEIGLAWSSHDAE  
SFQMHITQEGAGNSRVEITTNQSIIIGGLFPGTKYCFEIVPKGPNGTEGASRTVCNRTVPSAVFDIHVVYVTTTEMWLDW  
KSPDGASEYVYHLVIESKHGSNHTSTYDKAITLQGLIPGTLYNITISPEVDHVWGDPNSTAQYTRPSNVSNIDVSTNTTA  
ATLSWQNFDDASPTYSYCLLIEKAGNSSNATQVVTDIGITDATVTELIPGSSYTVEIFAQVGDGIKSLEPGRKSFCTDPA  
SMASFDCEVVPKEPALVLKWTCPPGANAGFELEVSSGAWNNATHLESCSSENGTEYRTEVTYLNFSTSYNISITTVSCGK  
MAAPTRNTCTTGITDPPPPDGSPNITSVSHNSVKVKFSGFEASHGPIKAYAVILTTGEAGHPSADVLKYTYEDFKKGASD  
TYVTYLIRTEEKGRSQSLSEVLKYEIDVGNESTTLGYYNGKLEPLGSYRACVAGFTNITFHPQNKGLIDGAESYVSFSRY  
SDAVSLPQDPGVICGAVFGCIFGALVIVTVGGFIFWRKKRKDAKNNEVSFSQIKPKKSKLIRVENFEAYFKKQQADSNCG  
FAEEYEDLKLVGISQPKYAAELAENRGKNRYNNVLPYDISRVKLSVQTHSTDDYINANYMPGYHSKKDFIATQGPLPNTL  
KDFWRMVWEKNVYAIIMLTKCVEQGRTKCEEYWPSKQAQDYGDITVAMTSEIVLPEWTIRDFTVKNIQTSESHPLRQFHF  
TSWPDHGVPDTTDLLINFRYLVRDYMKQSPPESPILVHCSAGVGRTGTFIAIDRLIYQIENENTVDVYGIVYDLRMHRPL  
MVQTEDQYVFLNQCVLDIVRSQKDSKVDLIYQNTTAMTIYENLAPVTTFGKTNGYIA`

| Site | Type | Count |
| --- | --- | --- |
| 413 | N-linked | 3 |

| Mass | Sequence | MS_2_ Score | `q`-value | MS_1_ Score | Signal | Protein Coordinates | Tandem Scans | Start Time | Apex Time | End Time |
| --- | --- | --- | --- | --- | --- | --- | --- | --- | --- | --- |
| 3911.6485 | IHVAGETDSSNLN(N)VSEPR Hex 6 HexNAc 5 | 141.806 | 0.0 | 13.868 | 2.076e+07 | 400 - 418 | 6 | 23.83 | 23.96 | 24.27 |
| 4057.7076 | IHVAGETDSSNLN(N)VSEPR Fuc 1 Hex 6 HexNAc 5 | 26.852 | 0.037 | 10.395 | 5.323e+06 | 400 - 418 | 2 | 23.77 | 23.77 | 23.96 |
| 3546.5130 | IHVAGETDSSNLN(N)VSEPR Hex 5 HexNAc 4 | 26.271 | 0.037 | -4.237 | 2.649e+06 | 400 - 418 | 2 | 24.11 | 24.19 | 24.34 |

### IHVAGETDSSNLN(N)VSEPR Hex 6 HexNAc 5

**Mass:**  3911.6497

(1.940e-06)

**Charge States:** 3, 4

**Start Position**: 401

**End Position**: 419

| Profile Score | Tandem Score | TDA q-value | Volume | Spectrum Matches |
| --- | --- | --- | --- | --- |
| 13.8678 | 141.8063 | 0.0000 | 2.0763e+07 | 6 |

**Adduct Signal Fractions**

- **Unmodified**: 2.0763e+07

### IHVAGETDSSNLN(N)VSEPR Fuc 1 Hex 6 HexNAc 5

**Mass:**  4057.6990

(4.430e-07)

**Charge States:** 3, 4

**Start Position**: 401

**End Position**: 419

| Profile Score | Tandem Score | TDA q-value | Volume | Spectrum Matches |
| --- | --- | --- | --- | --- |
| 10.3955 | 26.8517 | 0.0373 | 5.3235e+06 | 2 |

**Adduct Signal Fractions**

- **Unmodified**: 5.3235e+06

### IHVAGETDSSNLN(N)VSEPR Hex 5 HexNAc 4

**Mass:**  3546.5117

(2.189e-06)

**Charge States:** 3

**Start Position**: 401

**End Position**: 419

| Profile Score | Tandem Score | TDA q-value | Volume | Spectrum Matches |
| --- | --- | --- | --- | --- |
| -4.2375 | 26.2711 | 0.0373 | 2.6487e+06 | 2 |

**Adduct Signal Fractions**

- **Unmodified**: 2.6487e+06

### O95497|VNN1\_HUMAN

**>O95497|VNN1\_HUMAN**  
 `MTTQLPAYVAILLFYVSRASCQDTFTAAVYEHAAILPNATLTPVSREEALALMNRNLDILEGAITSAADQGAHIIVTPED  
AIYGWNFNRDSLYPYLEDIPDPEVNWIPCNNRNRFGQTPVQERLSCLAKNNSIYVVANIGDKKPCDTSDPQCPPDGRYQY  
NTDVVFDSQGKLVARYHKQNLFMGENQFNVPKEPEIVTFNTTFGSFGIFTCFDILFHDPAVTLVKDFHVDTIVFPTAWMN  
VLPHLSAVEFHSAWAMGMRVNFLASNIHYPSKKMTGSGIYAPNSSRAFHYDMKTEEGKLLLSQLDSHPSHSAVVNWTSYA  
SSIEALSSGNKEFKGTVFFDEFTFVKLTGVAGNYTVCQKDLCCHLSYKMSENIPNEVYALGAFDGLHTVEGRYYLQICTL  
LKCKTTNLNTCGDSAETASTRFEMFSLSGTFGTQYVFPEVLLSENQLAPGEFQVSTDGRLFSLKPTSGPVLTVTLFGRLY  
EKDWASNASSGLTAQARIIMLIVIAPIVCSLSW`

| Site | Type | Count |
| --- | --- | --- |
| 353 | N-linked | 2 |

| Mass | Sequence | MS_2_ Score | `q`-value | MS_1_ Score | Signal | Protein Coordinates | Tandem Scans | Start Time | Apex Time | End Time |
| --- | --- | --- | --- | --- | --- | --- | --- | --- | --- | --- |
| 3397.4169 | LTGVAGN(N)YTVC(C)QK Hex 6 HexNAc 5 | 124.102 | 0.0 | -2.677 | 9.214e+06 | 346 - 359 | 2 | 22.35 | 22.43 | 22.61 |
| 3032.2795 | LTGVAGN(N)YTVC(C)QK Hex 5 HexNAc 4 | 30.353 | 0.022 | -18.846 | 1.789e+06 | 346 - 359 | 2 | 22.61 | 22.61 | 22.83 |

### LTGVAGN(N)YTVC(C)QK Hex 6 HexNAc 5

**Mass:**  3397.4181

(1.713e-06)

**Charge States:** 3

**Start Position**: 347

**End Position**: 360

| Profile Score | Tandem Score | TDA q-value | Volume | Spectrum Matches |
| --- | --- | --- | --- | --- |
| -2.6769 | 124.1016 | 0.0000 | 9.2143e+06 | 2 |

**Adduct Signal Fractions**

- **Unmodified**: 9.2143e+06

### LTGVAGN(N)YTVC(C)QK Hex 5 HexNAc 4

**Mass:**  3032.2796

(2.559e-07)

**Charge States:** 3

**Start Position**: 347

**End Position**: 360

| Profile Score | Tandem Score | TDA q-value | Volume | Spectrum Matches |
| --- | --- | --- | --- | --- |
| -18.8464 | 30.3533 | 0.0217 | 1.7891e+06 | 2 |

**Adduct Signal Fractions**

- **Unmodified**: 1.7891e+06

### P11279|LAMP1\_HUMAN

**>P11279|LAMP1\_HUMAN**  
 `MAAPGSARRPLLLLLLLLLLGLMHCASAAMFMVKNGNGTACIMANFSAAFSVNYDTKSGPKNMTFDLPSDATVVLNRSSC  
GKENTSDPSLVIAFGRGHTLTLNFTRNATRYSVQLMSFVYNLSDTHLFPNASSKEIKTVESITDIRADIDKKYRCVSGTQ  
VHMNNVTVTLHDATIQAYLSNSSFSRGETRCEQDRPSPTTAPPAPPSPSPSPVPKSPSVDKYNVSGTNGTCLLASMGLQL  
NLTYERKDNTTVTRLLNINPNKTSASGSCGAHLVTLELHSEGTTVLLFQFGMNASSSRFFLQGIQLNTILPDARDPAFKA  
ANGSLRALQATVGNSYKCNAEEHVRVTKAFSVNIFKVWVQAFKVEGGQFGSVEECLLDENSMLIPIAVGGALAGLVLIVL  
IAYLVGRKRSHAGYQTI`

| Site | Type | Count |
| --- | --- | --- |
| 103 | N-linked | 1 |

| Mass | Sequence | MS_2_ Score | `q`-value | MS_1_ Score | Signal | Protein Coordinates | Tandem Scans | Start Time | Apex Time | End Time |
| --- | --- | --- | --- | --- | --- | --- | --- | --- | --- | --- |
| 2740.1720 | GHTLTLN(N)FTR Hex 6 HexNAc 3 | 137.118 | 0.0 | -3.848 | 2.002e+06 | 96 - 106 | 2 | 30.62 | 30.62 | 30.90 |

### GHTLTLN(N)FTR Hex 6 HexNAc 3

**Mass:**  2740.1722

(9.334e-07)

**Charge States:** 3

**Start Position**: 97

**End Position**: 107

| Profile Score | Tandem Score | TDA q-value | Volume | Spectrum Matches |
| --- | --- | --- | --- | --- |
| -3.8477 | 137.1178 | 0.0000 | 2.0018e+06 | 2 |

**Adduct Signal Fractions**

- **Unmodified**: 2.0018e+06
