# Supplementary material for: Measuring Site-specific Glycosylation Similarity between Influenza a Virus Variants with Statistical Certainty
Source: Mol Cell Proteomics. 2020 Nov 25;19(9):1533–45. doi: 10.1074/mcp.RA120.002031 (PMC8143645; doi:10.1074/mcp.RA120.002031)
Supplement: Supplementary file 1 [file mmc1.zip › mmc1/159250_2_supp_555287_qcjklk.zip!/20190326_aAGP_021119_tryp_HILIC_DDA_1ug_02_glycopeptides.html]

## 20190326\_aAGP\_021119\_tryp\_HILIC\_DDA\_1ug\_02 @ GlycopeptideHypothesis-b5ba9801025a462782ecb0926aef4030

**MS^1^ Mass Accuracy:** 10.0 PPM  
**Grouping Tolerance:** 15.0 PPM  
**MS^n^ Mass Accuracy:** 20.0 PPM  
**Minimum `q`-Value For Clustering:** 0.05

**Glycopeptide Hypothesis:**
GlycopeptideHypothesis-b5ba9801025a462782ecb0926aef4030  
**Sample Searched:**
20190326\_aAGP\_021119\_tryp\_HILIC\_DDA\_1ug\_02

| Protein | Matched Glycopeptides |
| --- | --- |
| P02763\|A1AG1\_HUMAN | 213 |
| P19652\|A1AG2\_HUMAN | 209 |
| P00738\|HPT\_HUMAN | 11 |
| P05155\|IC1\_HUMAN | 10 |
| Q9Y5Y7\|LYVE1\_HUMAN | 9 |
| Q12913\|PTPRJ\_HUMAN | 4 |
| P02790\|HEMO\_HUMAN | 4 |
| Q5R543\|HEMO\_PONAB | 4 |
| O95497\|VNN1\_HUMAN | 3 |
| P11279\|LAMP1\_HUMAN | 3 |
| O75144\|ICOSL\_HUMAN | 3 |
| Q13201\|MMRN1\_HUMAN | 2 |

### P02763|A1AG1\_HUMAN

**>P02763|A1AG1\_HUMAN**  
 `MALSWVLTVLSLLPLLEAQIPLCANLVPVPITNATLDQITGKWFYIASAFRNEEYNKSVQEIQATFFYFTPNKTEDTIFL  
REYQTRQDQCIYNTTYLNVQRENGTISRYVGGQEHFAHLLILRDTKTYMLAFDVNDEKNWGLSVYADKPETTKEQLGEFY  
EALDCLRIPKSDVVYTDWKKDKCEPLEKQHEKERKQEEGES`

| Site | Type | Count |
| --- | --- | --- |
| 33 | N-linked | 72 |
| 56 | N-linked | 3 |
| 93 | N-linked | 38 |
| 72 | N-linked | 100 |

| Mass | Sequence | MS_2_ Score | `q`-value | MS_1_ Score | Signal | Protein Coordinates | Tandem Scans | Start Time | Apex Time | End Time |
| --- | --- | --- | --- | --- | --- | --- | --- | --- | --- | --- |
| 2390.9639 | YFTPN(N)K Hex 5 HexNAc 4 | 187.938 | 0.0 | -20.299 | 2.877e+07 | 67 - 73 | 2 | 13.61 | 13.61 | 13.78 |
| 2756.0988 | YFTPN(N)K Hex 6 HexNAc 5 | 187.465 | 0.0 | 15.094 | 2.770e+08 | 67 - 73 | 5 | 13.28 | 13.61 | 14.11 |
| 3268.2988 | FYFTPN(N)K Hex 7 HexNAc 6 | 183.308 | 0.0 | 14.523 | 1.750e+08 | 66 - 73 | 10 | 23.79 | 24.29 | 25.82 |
| 2783.0554 | NEEYN(N)K Hex 6 HexNAc 5 | 182.563 | 0.0 | 14.190 | 4.182e+08 | 51 - 57 | 15 | 3.80 | 3.80 | 9.50 |
| 2417.9215 | NEEYN(N)K Hex 5 HexNAc 4 | 181.312 | 0.0 | 12.527 | 8.418e+07 | 51 - 57 | 7 | 3.80 | 3.80 | 5.41 |
| 3121.2294 | YFTPN(N)K Hex 7 HexNAc 6 | 180.694 | 0.0 | 13.287 | 3.179e+08 | 67 - 73 | 5 | 12.95 | 13.12 | 13.94 |
| 2929.1119 | NEEYN(N)K Fuc 1 Hex 6 HexNAc 5 | 180.467 | 0.0 | 12.006 | 2.903e+07 | 51 - 57 | 3 | 3.80 | 3.80 | 4.96 |
| 3906.6667 | SVQEIQATFFYFTPN(N)K Hex 6 HexNAc 5 | 175.203 | 0.0 | 14.728 | 2.969e+09 | 57 - 73 | 49 | 50.18 | 53.93 | 113.13 |
| 4052.7252 | SVQEIQATFFYFTPN(N)K Fuc 1 Hex 6 HexNAc 5 | 174.617 | 0.0 | 14.247 | 6.065e+08 | 57 - 73 | 18 | 53.42 | 53.76 | 58.36 |
| 2902.1548 | YFTPN(N)K Fuc 1 Hex 6 HexNAc 5 | 173.938 | 0.0 | -4.622 | 4.384e+07 | 67 - 73 | 3 | 13.12 | 13.28 | 13.45 |
| 4271.8026 | SVQEIQATFFYFTPN(N)K Hex 7 HexNAc 6 | 173.876 | 0.0 | 13.287 | 3.690e+09 | 57 - 73 | 129 | 49.66 | 53.42 | 113.24 |
| 3486.3624 | YFTPN(N)K Hex 8 HexNAc 7 | 172.730 | 0.0 | -19.746 | 1.855e+07 | 67 - 73 | 1 | 12.95 | 12.95 | 13.12 |
| 3267.2908 | YFTPN(N)K Fuc 1 Hex 7 HexNAc 6 | 171.645 | 0.0 | -4.772 | 4.415e+07 | 67 - 73 | 2 | 12.95 | 12.95 | 13.28 |
| 2903.1657 | FYFTPN(N)K Hex 6 HexNAc 5 | 169.843 | 0.0 | 14.484 | 1.431e+08 | 66 - 73 | 13 | 24.46 | 24.97 | 27.29 |
| 4417.8609 | SVQEIQATFFYFTPN(N)K Fuc 1 Hex 7 HexNAc 6 | 167.701 | 0.0 | 13.458 | 6.737e+08 | 57 - 73 | 17 | 53.08 | 53.25 | 57.33 |
| 4636.9345 | SVQEIQATFFYFTPN(N)K Hex 8 HexNAc 7 | 166.705 | 0.0 | 13.810 | 4.789e+08 | 57 - 73 | 10 | 52.40 | 52.91 | 54.10 |
| 4563.9203 | SVQEIQATFFYFTPN(N)K Fuc 2 Hex 7 HexNAc 6 | 166.687 | 0.0 | 10.977 | 9.026e+07 | 57 - 73 | 2 | 53.08 | 53.25 | 53.76 |
| 3541.5340 | SVQEIQATFFYFTPN(N)K Hex 5 HexNAc 4 | 164.842 | 0.0 | 13.935 | 3.082e+08 | 57 - 73 | 18 | 54.43 | 54.60 | 58.02 |
| 3912.7910 | LVPVPITN(N)ATLDQITGK Fuc 1 Hex 6 HexNAc 5 | 161.575 | 0.0 | 14.922 | 4.605e+09 | 25 - 42 | 41 | 41.28 | 47.56 | 113.09 |
| 3969.8113 | PVPITN(N)ATLDQITGK Fuc 2 Hex 3 HexNAc 8 | 161.436 | 0.0 | 11.881 | 1.073e+09 | 27 - 42 | 15 | 41.79 | 48.50 | 49.83 |
| 4782.9916 | SVQEIQATFFYFTPN(N)K Fuc 1 Hex 8 HexNAc 7 | 156.863 | 0.0 | 13.146 | 9.007e+07 | 57 - 73 | 5 | 52.40 | 52.91 | 53.25 |
| 3222.3235 | ATFFYFTPN(N)K Hex 6 HexNAc 5 | 156.352 | 0.0 | -3.497 | 4.873e+07 | 63 - 73 | 2 | 40.77 | 40.94 | 41.28 |
| 3587.4538 | ATFFYFTPN(N)K Hex 7 HexNAc 6 | 156.157 | 0.0 | 13.054 | 7.305e+07 | 63 - 73 | 2 | 40.09 | 40.26 | 41.11 |
| 3766.7323 | LVPVPITN(N)ATLDQITGK Hex 6 HexNAc 5 | 155.110 | 0.0 | 15.103 | 1.891e+10 | 25 - 42 | 524 | 41.45 | 47.87 | 113.54 |
| 3401.5981 | LVPVPITN(N)ATLDQITGK Hex 5 HexNAc 4 | 154.559 | 0.0 | 15.267 | 1.971e+09 | 25 - 42 | 18 | 41.96 | 48.35 | 50.18 |
| 4464.8571 | SVQEIQATFFYFTPN(N)K Fuc 1 Hex 8 HexNAc 4 Neu5Ac 1 | 153.576 | 0.0 | -4.800 | 3.414e+07 | 57 - 73 | 2 | 54.43 | 54.43 | 54.94 |
| 3823.7535 | PVPITN(N)ATLDQITGK Fuc 1 Hex 3 HexNAc 8 | 149.239 | 0.0 | 15.995 | 2.982e+09 | 27 - 42 | 5 | 48.35 | 48.66 | 49.66 |
| 4882.1720 | SVQEIQATFFYFTPN(N)KTEDTIFLR Hex 6 HexNAc 5 | 148.881 | 0.0 | 14.196 | 6.601e+08 | 57 - 81 | 24 | 60.07 | 62.63 | 67.43 |
| 5002.0652 | SVQEIQATFFYFTPN(N)K Hex 9 HexNAc 8 | 147.971 | 0.0 | 12.690 | 9.985e+07 | 57 - 73 | 7 | 51.89 | 52.06 | 53.08 |
| 3731.6011 | YFTPN(N)KTEDTIFLR Hex 6 HexNAc 5 | 147.234 | 0.0 | 13.361 | 1.028e+09 | 67 - 81 | 22 | 35.21 | 37.27 | 43.32 |
| 3902.6098 | QDQC(C)IYN(N)TTYLNVQR Hex 6 HexNAc 5 | 143.310 | 0.0 | 15.001 | 2.490e+09 | 86 - 101 | 11 | 31.31 | 33.85 | 35.21 |
| 3368.3790 | ATFFYFTPN(N)K Fuc 1 Hex 6 HexNAc 5 | 142.003 | 0.0 | -20.135 | 3.762e+06 | 63 - 73 | 1 | 40.60 | 40.60 | 40.60 |
| 4447.8376 | SVQEIQATFFYFTPN(N)K Hex 7 HexNAc 4 Neu5Ac 2 | 140.087 | 0.0 | 12.329 | 1.844e+08 | 57 - 73 | 6 | 53.76 | 54.43 | 55.79 |
| 4242.7938 | YFTPN(N)KTEDTIFLR Fuc 1 Hex 7 HexNAc 6 | 137.525 | 0.0 | 8.644 | 2.314e+08 | 67 - 81 | 5 | 34.86 | 36.50 | 37.11 |
| 5028.2311 | SVQEIQATFFYFTPN(N)KTEDTIFLR Fuc 1 Hex 6 HexNAc 5 | 136.844 | 0.0 | 12.186 | 1.523e+08 | 57 - 81 | 8 | 62.30 | 62.46 | 64.17 |
| 3786.6011 | TPN(N)KTEDTIFLR Hex 7 HexNAc 6 | 136.622 | 0.0 | 14.378 | 7.909e+07 | 69 - 81 | 7 | 24.80 | 24.97 | 25.82 |
| 4277.9253 | LVPVPITN(N)ATLDQITGK Fuc 1 Hex 7 HexNAc 6 | 135.695 | 0.0 | 13.996 | 2.528e+08 | 25 - 42 | 5 | 47.05 | 47.39 | 47.87 |
| 4048.6677 | QDQC(C)IYN(N)TTYLNVQR Fuc 1 Hex 6 HexNAc 5 | 132.562 | 0.0 | 13.543 | 6.389e+08 | 86 - 101 | 6 | 33.35 | 33.69 | 34.52 |
| 4328.8215 | SVQEIQATFFYFTPN(N)K Fuc 4 Hex 5 HexNAc 5 | 130.567 | 0.0 | 13.972 | 7.752e+08 | 57 - 73 | 25 | 52.40 | 54.26 | 59.04 |
| 4131.8675 | LVPVPITN(N)ATLDQITGK Hex 7 HexNAc 6 | 130.122 | 0.0 | 14.484 | 7.059e+08 | 25 - 42 | 10 | 41.11 | 47.56 | 48.19 |
| 4188.8856 | PVPITN(N)ATLDQITGK Fuc 1 Hex 4 HexNAc 9 | 129.935 | 0.0 | -4.782 | 1.003e+08 | 27 - 42 | 3 | 48.03 | 48.19 | 48.50 |
| 3537.4745 | QDQC(C)IYN(N)TTYLNVQR Hex 5 HexNAc 4 | 128.448 | 0.0 | -4.150 | 4.524e+07 | 86 - 101 | 3 | 34.02 | 34.19 | 34.36 |
| 4929.0530 | SVQEIQATFFYFTPN(N)K Fuc 2 Hex 8 HexNAc 7 | 128.386 | 0.0 | 11.356 | 2.290e+07 | 57 - 73 | 2 | 52.23 | 52.74 | 53.08 |
| 4267.7478 | QDQC(C)IYN(N)TTYLNVQR Hex 7 HexNAc 6 | 127.593 | 0.0 | 14.269 | 3.258e+09 | 86 - 101 | 9 | 30.97 | 33.35 | 34.36 |
| 3839.6689 | PVPITN(N)ATLDQITGK Hex 9 HexNAc 4 | 127.544 | 0.0 | 0.000 | 0.000e+00 | 27 - 42 | 1 | - | - | - |
| 3877.6588 | YFTPN(N)KTEDTIFLR Fuc 1 Hex 6 HexNAc 5 | 127.249 | 0.0 | 8.368 | 2.547e+08 | 67 - 81 | 4 | 35.21 | 36.96 | 37.27 |
| 4497.0006 | LVPVPITN(N)ATLDQITGK Hex 8 HexNAc 7 | 126.811 | 0.0 | 12.377 | 1.156e+08 | 25 - 42 | 6 | 46.71 | 47.22 | 47.56 |
| 4096.7345 | YFTPN(N)KTEDTIFLR Hex 7 HexNAc 6 | 126.700 | 0.0 | 12.520 | 1.953e+09 | 67 - 81 | 21 | 35.04 | 36.80 | 46.37 |
| 4413.8040 | QDQC(C)IYN(N)TTYLNVQR Fuc 1 Hex 7 HexNAc 6 | 125.894 | 0.0 | 11.714 | 9.875e+08 | 86 - 101 | 6 | 30.97 | 33.19 | 34.19 |
| 4109.7466 | SVQEIQATFFYFTPN(N)K Hex 6 HexNAc 6 | 125.792 | 0.0 | 9.705 | 7.623e+07 | 57 - 73 | 4 | 54.43 | 54.43 | 55.11 |
| 3882.7577 | PVPITN(N)ATLDQITGK Hex 3 HexNAc 9 | 125.083 | 0.0 | 0.000 | 0.000e+00 | 27 - 42 | 1 | - | - | - |
| 4243.8037 | FYFTPN(N)KTEDTIFLR Hex 7 HexNAc 6 | 124.809 | 0.0 | 12.199 | 2.539e+08 | 66 - 81 | 8 | 43.83 | 45.35 | 46.37 |
| 4632.8792 | QDQC(C)IYN(N)TTYLNVQR Hex 8 HexNAc 7 | 124.598 | 0.0 | 16.118 | 6.792e+08 | 86 - 101 | 6 | 32.50 | 32.84 | 33.52 |
| 5247.3065 | SVQEIQATFFYFTPN(N)KTEDTIFLR Hex 7 HexNAc 6 | 123.744 | 0.0 | 13.127 | 1.457e+09 | 57 - 81 | 30 | 59.38 | 62.30 | 67.94 |
| 3878.6702 | FYFTPN(N)KTEDTIFLR Hex 6 HexNAc 5 | 120.953 | 0.0 | 13.441 | 7.915e+07 | 66 - 81 | 5 | 45.52 | 45.69 | 46.54 |
| 4593.8929 | SVQEIQATFFYFTPN(N)K Fuc 1 Hex 7 HexNAc 4 Neu5Ac 2 | 120.813 | 0.0 | -5.102 | 2.192e+07 | 57 - 73 | 1 | 54.26 | 54.26 | 54.60 |
| 4026.8315 | PVPITN(N)ATLDQITGK Fuc 1 Hex 3 HexNAc 9 | 119.201 | 0.0 | -21.587 | 5.373e+06 | 27 - 42 | 1 | 48.03 | 48.03 | 48.03 |
| 4198.7898 | SVQEIQATFFYFTPN(N)K Fuc 2 Hex 6 HexNAc 5 | 119.044 | 0.0 | -5.173 | 1.086e+07 | 57 - 73 | 1 | 53.42 | 53.59 | 53.76 |
| 3959.7940 | LVPVPITN(N)ATLDQITGK Fuc 1 Hex 7 HexNAc 3 Neu5Ac 1 | 118.852 | 0.0 | -4.390 | 2.573e+07 | 25 - 42 | 2 | 48.82 | 48.99 | 49.15 |
| 4998.0095 | QDQC(C)IYN(N)TTYLNVQR Fuc 5 Hex 7 HexNAc 6 | 118.094 | 0.0 | 12.041 | 2.148e+08 | 86 - 101 | 4 | 32.16 | 32.50 | 33.01 |
| 4436.8925 | SVQEIQATFFYFTPN(N)K Hex 8 HexNAc 6 | 117.113 | 0.0 | 0.000 | 0.000e+00 | 57 - 73 | 1 | - | - | - |
| 3366.4689 | YFTPN(N)KTEDTIFLR Hex 5 HexNAc 4 | 116.679 | 0.0 | -18.735 | 5.128e+07 | 67 - 81 | 1 | 37.58 | 37.75 | 37.75 |
| 4058.8525 | LVPVPITN(N)ATLDQITGK Fuc 2 Hex 6 HexNAc 5 | 116.327 | 0.0 | -5.416 | 5.636e+07 | 25 - 42 | 5 | 47.39 | 47.56 | 47.87 |
| 3421.4689 | TPN(N)KTEDTIFLR Hex 6 HexNAc 5 | 116.239 | 0.0 | 13.961 | 3.927e+07 | 69 - 81 | 7 | 25.31 | 25.48 | 26.29 |
| 4461.8677 | YFTPN(N)KTEDTIFLR Hex 8 HexNAc 7 | 115.367 | 0.0 | 12.733 | 2.286e+08 | 67 - 81 | 4 | 35.71 | 35.88 | 36.34 |
| 4778.9343 | QDQC(C)IYN(N)TTYLNVQR Fuc 1 Hex 8 HexNAc 7 | 114.791 | 0.0 | 15.003 | 1.619e+08 | 86 - 101 | 3 | 32.50 | 32.67 | 33.19 |
| 4024.7287 | PVPITN(N)ATLDQITGK Fuc 1 Hex 8 HexNAc 5 | 114.254 | 0.0 | -18.453 | 5.913e+06 | 27 - 42 | 3 | 52.40 | 52.40 | 52.40 |
| 3736.5645 | IYN(N)TTYLNVQR Hex 7 HexNAc 6 | 113.024 | 0.0 | 15.356 | 3.695e+07 | 90 - 101 | 4 | 27.94 | 28.14 | 28.42 |
| 3928.6446 | ATFFYFTPN(N)K Fuc 2 Hex 3 HexNAc 8 Neu5Ac 1 | 112.388 | 0.0 | 12.499 | 2.276e+08 | 63 - 73 | 1 | 53.76 | 53.93 | 55.79 |
| 4559.8609 | QDQC(C)IYN(N)TTYLNVQR Fuc 2 Hex 7 HexNAc 6 | 111.185 | 0.0 | -4.079 | 1.349e+08 | 86 - 101 | 2 | 33.01 | 33.19 | 33.52 |
| 3933.6712 | FTPN(N)KTEDTIFLR Hex 7 HexNAc 6 | 111.140 | 0.0 | 13.537 | 1.740e+08 | 68 - 81 | 6 | 29.62 | 31.65 | 32.33 |
| 4474.8782 | SVQEIQATFFYFTPN(N)K Hex 7 HexNAc 7 | 110.771 | 0.0 | 12.159 | 1.034e+08 | 57 - 73 | 3 | 53.76 | 53.93 | 54.77 |
| 3568.5387 | FTPN(N)KTEDTIFLR Hex 6 HexNAc 5 | 109.822 | 0.0 | 14.240 | 9.332e+07 | 68 - 81 | 5 | 31.65 | 31.99 | 32.84 |
| 4423.9851 | LVPVPITN(N)ATLDQITGK Fuc 2 Hex 7 HexNAc 6 | 109.533 | 0.0 | 7.093 | 2.932e+07 | 25 - 42 | 2 | 46.88 | 47.39 | 47.56 |
| 4443.7722 | QDQC(C)IYN(N)TTYLNVQR Hex 7 HexNAc 4 Neu5Ac 2 | 106.453 | 0.0 | 12.281 | 2.082e+08 | 86 - 101 | 2 | 34.19 | 34.36 | 35.21 |
| 4607.9113 | YFTPN(N)KTEDTIFLR Fuc 1 Hex 8 HexNAc 7 | 105.924 | 0.0 | -20.109 | 1.240e+07 | 67 - 81 | 1 | 35.88 | 35.88 | 35.88 |
| 4074.8552 | LVPVPITN(N)ATLDQITGK Fuc 1 Hex 7 HexNAc 5 | 105.814 | 0.0 | -5.333 | 3.846e+06 | 25 - 42 | 2 | 50.69 | 50.69 | 51.03 |
| 3821.6552 | PVPITN(N)ATLDQITGK Fuc 1 Hex 8 HexNAc 4 | 104.114 | 0.0 | -20.419 | 4.959e+06 | 27 - 42 | 3 | 47.72 | 47.72 | 47.72 |
| 4517.0353 | SVQEIQATFFYFTPN(N)KTEDTIFLR Hex 5 HexNAc 4 | 103.953 | 0.0 | -4.286 | 1.825e+07 | 57 - 81 | 2 | 62.97 | 63.15 | 63.32 |
| 3932.6608 | TPN(N)KTEDTIFLR Fuc 1 Hex 7 HexNAc 6 | 103.560 | 0.0 | -4.610 | 1.002e+07 | 69 - 81 | 2 | 24.80 | 24.97 | 25.14 |
| 4693.9511 | SVQEIQATFFYFTPN(N)K Fuc 4 Hex 6 HexNAc 6 | 103.303 | 0.0 | 13.512 | 5.681e+07 | 57 - 73 | 3 | 53.08 | 53.59 | 53.93 |
| 5612.4363 | SVQEIQATFFYFTPN(N)KTEDTIFLR Hex 8 HexNAc 7 | 102.956 | 0.0 | 14.470 | 2.530e+08 | 57 - 81 | 5 | 61.27 | 61.78 | 62.80 |
| 4118.7167 | YFTPN(N)KTEDTIFLR Hex 3 HexNAc 5 Neu5Ac 3 | 102.303 | 0.0 | -4.828 | 7.155e+07 | 67 - 81 | 3 | 36.65 | 36.80 | 36.96 |
| 4827.0011 | YFTPN(N)KTEDTIFLR Hex 9 HexNAc 8 | 101.948 | 0.0 | 12.443 | 6.497e+07 | 67 - 81 | 2 | 35.21 | 35.55 | 35.88 |
| 4299.8141 | YFTPN(N)KTEDTIFLR Hex 7 HexNAc 7 | 100.926 | 0.0 | -5.145 | 7.040e+07 | 67 - 81 | 2 | 36.96 | 37.11 | 37.27 |
| 3602.5933 | PVPITN(N)ATLDQITGK Fuc 2 Hex 7 HexNAc 3 | 98.242 | 0.0 | -4.216 | 7.116e+06 | 27 - 42 | 2 | 51.89 | 52.06 | 52.23 |
| 4337.9702 | PVPITN(N)ATLDQITGK Fuc 2 Hex 4 HexNAc 9 | 97.948 | 0.0 | 7.886 | 1.245e+08 | 27 - 42 | 6 | 61.44 | 62.13 | 62.46 |
| 3548.6539 | LVPVPITN(N)ATLDQITGK Fuc 1 Hex 5 HexNAc 4 | 96.598 | 0.0 | 0.000 | 0.000e+00 | 25 - 42 | 1 | - | - | - |
| 4020.7094 | SVQEIQATFFYFTPN(N)K Fuc 3 Hex 4 HexNAc 5 | 96.544 | 0.0 | 7.330 | 2.791e+07 | 57 - 73 | 2 | 53.59 | 54.43 | 54.60 |
| 4589.8298 | QDQC(C)IYN(N)TTYLNVQR Fuc 1 Hex 7 HexNAc 4 Neu5Ac 2 | 96.449 | 0.0 | -5.116 | 3.472e+07 | 86 - 101 | 1 | 34.02 | 34.36 | 34.36 |
| 4024.7283 | FYFTPN(N)KTEDTIFLR Fuc 1 Hex 6 HexNAc 5 | 95.781 | 0.0 | -4.069 | 2.237e+07 | 66 - 81 | 3 | 45.35 | 45.52 | 45.86 |
| 4640.0212 | PVPITN(N)ATLDQITGK Fuc 2 Hex 3 HexNAc 7 Neu5Ac 3 | 94.900 | 0.0 | -5.027 | 1.034e+07 | 27 - 42 | 1 | 57.16 | 57.33 | 57.68 |
| 4391.8577 | YFTPN(N)KTEDTIFLR Fuc 2 Hex 7 HexNAc 6 | 94.684 | 0.0 | 0.000 | 0.000e+00 | 67 - 81 | 1 | - | - | - |
| 3732.6097 | YFTPN(N)KTEDTIFLR Hex 6 HexNAc 5 | 94.613 | 0.0 | 0.000 | 0.000e+00 | 67 - 81 | 1 | - | - | - |
| 5393.3665 | SVQEIQATFFYFTPN(N)KTEDTIFLR Fuc 1 Hex 7 HexNAc 6 | 93.713 | 0.0 | 12.769 | 2.825e+08 | 57 - 81 | 6 | 60.93 | 62.13 | 63.49 |
| 4643.0608 | PVPITN(N)ATLDQITGK Fuc 3 Hex 5 HexNAc 9 | 93.698 | 0.0 | 14.922 | 1.891e+07 | 27 - 42 | 3 | 46.54 | 47.22 | 47.39 |
| 4272.7523 | YFTPN(N)KTEDTIFLR Hex 7 HexNAc 4 Neu5Ac 2 | 92.819 | 0.0 | -4.087 | 1.404e+08 | 67 - 81 | 4 | 37.43 | 37.91 | 37.91 |
| 4057.8301 | LVPVPITN(N)ATLDQITGK Hex 6 HexNAc 5 Neu5Ac 1 | 92.720 | 0.0 | 13.009 | 3.285e+07 | 25 - 42 | 6 | 50.01 | 50.69 | 51.37 |
| 4608.9354 | FYFTPN(N)KTEDTIFLR Hex 8 HexNAc 7 | 86.115 | 0.0 | 19.425 | 2.814e+07 | 66 - 81 | 2 | 44.34 | 44.84 | 45.01 |
| 4079.7272 | FTPN(N)KTEDTIFLR Fuc 1 Hex 7 HexNAc 6 | 85.165 | 0.0 | -4.048 | 3.168e+07 | 68 - 81 | 3 | 31.14 | 31.31 | 31.65 |
| 4080.7221 | FTPN(N)KTEDTIFLR Fuc 1 Hex 7 HexNAc 6 | 83.277 | 0.0 | 0.000 | 0.000e+00 | 68 - 81 | 1 | - | - | - |
| 3714.5973 | FTPN(N)KTEDTIFLR Fuc 1 Hex 6 HexNAc 5 | 83.218 | 0.0 | -3.736 | 1.861e+07 | 68 - 81 | 3 | 31.65 | 31.82 | 32.16 |
| 3056.3385 | TPN(N)KTEDTIFLR Hex 5 HexNAc 4 | 81.908 | 0.0 | 11.703 | 5.285e+06 | 69 - 81 | 3 | 25.82 | 25.99 | 26.22 |
| 4298.8896 | FTPN(N)KTEDTIFLR Hex 8 HexNAc 7 | 81.236 | 0.0 | -5.336 | 1.257e+07 | 68 - 81 | 7 | 30.63 | 31.14 | 31.14 |
| 5539.4241 | SVQEIQATFFYFTPN(N)KTEDTIFLR Fuc 2 Hex 7 HexNAc 6 | 75.530 | 0.003 | -19.839 | 3.115e+07 | 57 - 81 | 2 | 61.95 | 61.95 | 62.13 |
| 5367.2000 | SVQEIQATFFYFTPN(N)K Hex 10 HexNAc 9 | 74.236 | 0.003 | -4.917 | 1.116e+07 | 57 - 73 | 1 | 51.72 | 51.89 | 52.06 |
| 4419.8344 | FYFTPN(N)KTEDTIFLR Hex 7 HexNAc 4 Neu5Ac 2 | 73.976 | 0.003 | -4.737 | 7.641e+06 | 66 - 81 | 4 | 46.03 | 46.20 | 46.37 |
| 4802.0249 | SVQEIQATFFYFTPN(N)K Hex 9 HexNAc 7 | 69.303 | 0.003 | 0.000 | 0.000e+00 | 57 - 73 | 1 | - | - | - |
| 4153.7559 | YFTPN(N)KTEDTIFLR Fuc 4 Hex 5 HexNAc 5 | 67.535 | 0.003 | 13.695 | 4.810e+08 | 67 - 81 | 6 | 34.86 | 37.27 | 38.42 |
| 5977.5676 | SVQEIQATFFYFTPN(N)KTEDTIFLR Hex 9 HexNAc 8 | 66.621 | 0.003 | 13.178 | 8.205e+07 | 57 - 81 | 6 | 60.93 | 61.44 | 62.13 |
| 4111.8447 | C(C)ANLVPVPITN(N)ATLDQITGK Hex 6 HexNAc 5 | 65.138 | 0.003 | -2.740 | 2.398e+08 | 22 - 42 | 12 | 45.52 | 51.54 | 61.61 |
| 4257.9050 | C(C)ANLVPVPITN(N)ATLDQITGK Fuc 1 Hex 6 HexNAc 5 | 64.103 | 0.003 | 13.847 | 3.277e+07 | 22 - 42 | 3 | 51.20 | 51.37 | 51.89 |
| 3783.7504 | LVPVPITN(N)ATLDQITGK Fuc 2 Hex 5 HexNAc 3 Neu5Ac 1 | 62.162 | 0.003 | 10.844 | 2.555e+09 | 25 - 42 | 99 | 42.13 | 48.03 | 50.35 |
| 5758.4946 | SVQEIQATFFYFTPN(N)KTEDTIFLR Fuc 1 Hex 8 HexNAc 7 | 60.207 | 0.003 | 13.980 | 4.880e+07 | 57 - 81 | 4 | 61.27 | 61.78 | 62.13 |
| 5144.0652 | QDQC(C)IYN(N)TTYLNVQR Fuc 1 Hex 9 HexNAc 8 | 56.691 | 0.003 | 11.482 | 3.839e+07 | 86 - 101 | 3 | 31.99 | 32.33 | 32.84 |
| 5148.1241 | SVQEIQATFFYFTPN(N)K Fuc 1 Hex 9 HexNAc 8 | 56.267 | 0.003 | 7.373 | 1.196e+07 | 57 - 73 | 2 | 51.89 | 52.06 | 52.57 |
| 3930.8070 | LVPVPITN(N)ATLDQITGK Hex 7 HexNAc 5 | 54.399 | 0.003 | -6.615 | 1.746e+07 | 25 - 42 | 6 | 49.83 | 49.83 | 50.35 |
| 3846.7013 | PVPITN(N)ATLDQITGK Fuc 2 Hex 6 HexNAc 5 | 51.666 | 0.003 | -5.077 | 7.248e+06 | 27 - 42 | 2 | 51.37 | 51.72 | 51.89 |
| 4389.8608 | FYFTPN(N)KTEDTIFLR Fuc 1 Hex 7 HexNAc 6 | 51.409 | 0.003 | 12.675 | 4.580e+07 | 66 - 81 | 4 | 45.01 | 45.18 | 45.69 |
| 3456.5104 | PVPITN(N)ATLDQITGK Fuc 1 Hex 7 HexNAc 3 | 48.561 | 0.003 | 0.000 | 0.000e+00 | 27 - 42 | 1 | - | - | - |
| 5423.3359 | SVQEIQATFFYFTPN(N)KTEDTIFLR Hex 7 HexNAc 4 Neu5Ac 2 | 48.106 | 0.003 | -3.332 | 3.354e+07 | 57 - 81 | 3 | 63.15 | 63.32 | 63.66 |
| 4194.7320 | QDQC(C)IYN(N)TTYLNVQR Fuc 2 Hex 6 HexNAc 5 | 47.721 | 0.003 | -5.313 | 9.825e+06 | 86 - 101 | 2 | 33.19 | 33.35 | 33.52 |
| 4105.6893 | QDQC(C)IYN(N)TTYLNVQR Hex 6 HexNAc 6 | 47.140 | 0.003 | -3.869 | 1.249e+08 | 86 - 101 | 4 | 33.69 | 33.85 | 34.19 |
| 3950.7378 | PVPITN(N)ATLDQITGK Fuc 1 Hex 7 HexNAc 4 Neu5Ac 1 | 46.238 | 0.003 | -19.620 | 3.175e+07 | 27 - 42 | 1 | 48.19 | 48.19 | 48.35 |
| 4188.8924 | LVPVPITN(N)ATLDQITGK Fuc 4 Hex 5 HexNAc 5 | 45.580 | 0.003 | 8.822 | 3.294e+07 | 25 - 42 | 3 | 46.71 | 47.39 | 47.39 |
| 3951.7367 | PVPITN(N)ATLDQITGK Fuc 3 Hex 7 HexNAc 4 | 45.213 | 0.003 | -19.783 | 4.034e+07 | 27 - 42 | 2 | 47.87 | 47.87 | 48.03 |
| 4562.8958 | SVQEIQATFFYFTPN(N)K Hex 7 HexNAc 6 Neu5Ac 1 | 45.141 | 0.003 | -5.509 | 3.610e+06 | 57 - 73 | 2 | 56.31 | 56.48 | 56.82 |
| 4924.9933 | QDQC(C)IYN(N)TTYLNVQR Fuc 2 Hex 8 HexNAc 7 | 45.131 | 0.003 | -4.117 | 3.684e+07 | 86 - 101 | 2 | 32.50 | 32.84 | 33.01 |
| 4608.8649 | QDQC(C)IYN(N)TTYLNVQR Fuc 2 Hex 8 HexNAc 4 Neu5Ac 1 | 43.919 | 0.003 | 0.000 | 0.000e+00 | 86 - 101 | 1 | - | - | - |
| 5363.1391 | QDQC(C)IYN(N)TTYLNVQR Hex 10 HexNAc 9 | 43.260 | 0.003 | -3.836 | 3.543e+07 | 86 - 101 | 1 | 31.82 | 31.99 | 32.33 |
| 3567.5263 | TPN(N)KTEDTIFLR Fuc 1 Hex 6 HexNAc 5 | 42.131 | 0.003 | -5.418 | 7.785e+06 | 69 - 81 | 4 | 25.14 | 25.31 | 25.48 |
| 3931.8241 | LVPVPITN(N)ATLDQITGK Hex 7 HexNAc 5 | 42.018 | 0.003 | 0.000 | 0.000e+00 | 25 - 42 | 1 | - | - | - |
| 4470.8245 | QDQC(C)IYN(N)TTYLNVQR Hex 7 HexNAc 7 | 41.833 | 0.003 | -3.555 | 1.243e+08 | 86 - 101 | 3 | 33.35 | 33.52 | 33.69 |
| 4862.1337 | LVPVPITN(N)ATLDQITGK Hex 9 HexNAc 8 | 40.484 | 0.003 | 8.987 | 9.753e+06 | 25 - 42 | 2 | 46.20 | 46.54 | 47.22 |
| 4535.9191 | FYFTPN(N)KTEDTIFLR Fuc 2 Hex 7 HexNAc 6 | 40.372 | 0.003 | -20.987 | 2.079e+06 | 66 - 81 | 1 | 45.01 | 45.01 | 45.01 |
| 3880.7843 | LVPVPITN(N)ATLDQITGK Fuc 3 Hex 4 HexNAc 5 | 39.932 | 0.004 | -18.292 | 1.031e+08 | 25 - 42 | 5 | 47.39 | 47.56 | 51.20 |
| 5304.3241 | SVQEIQATFFYFTPN(N)KTEDTIFLR Fuc 4 Hex 5 HexNAc 5 | 39.848 | 0.005 | 13.683 | 2.705e+08 | 57 - 81 | 12 | 62.46 | 62.97 | 65.20 |
| 4344.8583 | SVQEIQATFFYFTPN(N)K Fuc 3 Hex 6 HexNAc 5 | 39.645 | 0.005 | -19.429 | 8.762e+06 | 57 - 73 | 1 | 54.60 | 54.77 | 54.77 |
| 3855.7893 | LVPVPITN(N)ATLDQITGK Fuc 2 Hex 6 HexNAc 4 | 38.932 | 0.005 | 0.000 | 0.000e+00 | 25 - 42 | 1 | - | - | - |
| 3788.6233 | FYFTPN(N)KTEDTIFLR Fuc 1 Hex 4 HexNAc 4 Neu5Ac 1 | 37.745 | 0.006 | 14.593 | 2.292e+08 | 66 - 81 | 4 | 37.58 | 37.75 | 38.59 |
| 3367.4815 | YFTPN(N)KTEDTIFLR Hex 5 HexNAc 4 | 37.365 | 0.006 | 0.000 | 0.000e+00 | 67 - 81 | 1 | - | - | - |
| 4547.0810 | LVPVPITN(N)ATLDQITGK Fuc 2 Hex 4 HexNAc 9 | 37.090 | 0.006 | 13.243 | 1.087e+08 | 25 - 42 | 12 | 69.14 | 69.43 | 70.71 |
| 4279.9221 | PVPITN(N)ATLDQITGK Fuc 3 Hex 4 HexNAc 8 | 36.962 | 0.007 | 0.000 | 0.000e+00 | 27 - 42 | 1 | - | - | - |
| 3971.8301 | LVPVPITN(N)ATLDQITGK Hex 6 HexNAc 6 | 36.604 | 0.007 | 0.000 | 0.000e+00 | 25 - 42 | 2 | - | - | - |
| 4268.7331 | QDQC(C)IYN(N)TTYLN(D)VQR Hex 7 HexNAc 6 | 35.914 | 0.007 | -4.070 | 2.328e+08 | 86 - 101 | 29 | 31.99 | 34.52 | 106.18 |
| 4460.8011 | QDQC(C)IYN(N)TTYLNVQR Fuc 1 Hex 8 HexNAc 4 Neu5Ac 1 | 35.735 | 0.007 | -5.354 | 2.660e+07 | 86 - 101 | 1 | 34.36 | 34.36 | 34.69 |
| 4325.7127 | ATFFYFTPN(N)K Hex 6 HexNAc 9 Neu5Ac 1 | 35.720 | 0.007 | -4.356 | 1.328e+08 | 63 - 73 | 3 | 53.42 | 53.42 | 53.76 |
| 4590.8321 | QDQC(C)IYN(N)TTYLNVQR Fuc 3 Hex 7 HexNAc 4 Neu5Ac 1 | 35.370 | 0.007 | -19.697 | 6.380e+06 | 86 - 101 | 1 | 34.52 | 34.52 | 34.69 |
| 4227.8019 | FTPN(N)KTEDTIFLR Fuc 2 Hex 7 HexNAc 6 | 35.011 | 0.010 | -22.670 | 4.651e+06 | 68 - 81 | 2 | 62.80 | 62.80 | 62.80 |
| 4432.8341 | QDQC(C)IYN(N)TTYLNVQR Hex 8 HexNAc 6 | 34.882 | 0.010 | 0.000 | 0.000e+00 | 86 - 101 | 1 | - | - | - |
| 3884.5128 | QDQC(C)IYN(N)TTYLN Hex 7 HexNAc 6 | 34.836 | 0.010 | -19.062 | 1.484e+07 | 86 - 98 | 2 | 31.99 | 32.16 | 32.16 |
| 4435.7841 | QDQC(C)IYN(N)TTYLNVQR Hex 10 HexNAc 3 Neu5Ac 1 | 33.944 | 0.010 | -4.806 | 9.461e+07 | 86 - 101 | 1 | 33.01 | 33.19 | 33.35 |
| 3903.5985 | QDQC(C)IYN(N)TTYLN(D)VQR Hex 6 HexNAc 5 | 33.718 | 0.011 | -7.698 | 8.178e+07 | 86 - 101 | 5 | 32.33 | 34.52 | 34.52 |
| 4564.9021 | SVQEIQATFFYFTPN(N)K Fuc 3 Hex 5 HexNAc 4 Neu5Ac 2 | 33.648 | 0.011 | 0.000 | 0.000e+00 | 57 - 73 | 1 | - | - | - |
| 3805.6898 | PVPITN(N)ATLDQITGK Fuc 2 Hex 7 HexNAc 4 | 33.439 | 0.011 | -19.810 | 1.020e+07 | 27 - 42 | 1 | 48.35 | 48.35 | 48.35 |
| 3746.7150 | C(C)ANLVPVPITN(N)ATLDQITGK Hex 5 HexNAc 4 | 32.799 | 0.014 | -18.611 | 7.610e+06 | 22 - 42 | 2 | 51.72 | 51.89 | 51.89 |
| 5440.3608 | SVQEIQATFFYFTPN(N)KTEDTIFLR Fuc 1 Hex 8 HexNAc 4 Neu5Ac 1 | 32.349 | 0.016 | -4.560 | 5.913e+06 | 57 - 81 | 1 | 63.15 | 63.32 | 63.49 |
| 3882.6232 | IYN(N)TTYLNVQR Fuc 1 Hex 7 HexNAc 6 | 32.344 | 0.016 | 11.905 | 1.710e+07 | 90 - 101 | 3 | 27.75 | 27.94 | 28.01 |
| 3934.6797 | FYFTPN(N)KTEDTIFLR Fuc 2 Hex 4 HexNAc 4 Neu5Ac 1 | 32.341 | 0.016 | -18.609 | 4.168e+07 | 66 - 81 | 4 | 37.58 | 37.58 | 37.75 |
| 3371.4297 | IYN(N)TTYLNVQR Hex 6 HexNAc 5 | 32.268 | 0.016 | 10.827 | 2.618e+07 | 90 - 101 | 3 | 28.42 | 28.51 | 28.95 |
| 7813.3406 | WFYIASAFRNEEYNKSVQEIQATFFYFTPN(N)K Fuc 5 Hex 10 HexNAc 8 | 32.122 | 0.016 | -4.185 | 3.861e+07 | 42 - 73 | 1 | 53.93 | 53.93 | 54.26 |
| 3519.3788 | QDQC(C)IYN(N)TTYLN Hex 6 HexNAc 5 | 31.955 | 0.017 | -18.488 | 4.580e+06 | 86 - 98 | 1 | 32.50 | 32.50 | 32.50 |
| 4245.8625 | PVPITN(N)ATLDQITGK Hex 9 HexNAc 6 | 31.216 | 0.018 | 0.000 | 0.000e+00 | 27 - 42 | 1 | - | - | - |
| 3481.5632 | PVPITN(N)ATLDQITGK Fuc 2 Hex 5 HexNAc 4 | 30.873 | 0.018 | -19.149 | 2.696e+06 | 27 - 42 | 1 | 51.89 | 52.06 | 52.06 |
| 4693.1428 | LVPVPITN(N)ATLDQITGK Fuc 3 Hex 4 HexNAc 9 | 30.301 | 0.022 | 10.538 | 2.816e+07 | 25 - 42 | 7 | 68.97 | 69.29 | 70.36 |
| 4169.7891 | PVPITN(N)ATLDQITGK Hex 8 HexNAc 5 Neu5Ac 1 | 30.076 | 0.022 | -19.808 | 8.634e+06 | 27 - 42 | 1 | 52.06 | 52.06 | 52.23 |
| 5509.2011 | QDQC(C)IYN(N)TTYLNVQR Fuc 1 Hex 10 HexNAc 9 | 30.014 | 0.022 | -19.461 | 6.770e+06 | 86 - 101 | 1 | 31.82 | 31.99 | 31.99 |
| 4049.6578 | QDQC(C)IYN(N)TTYLN(D)VQR Fuc 1 Hex 6 HexNAc 5 | 29.762 | 0.022 | -19.735 | 2.223e+07 | 86 - 101 | 2 | 34.69 | 34.69 | 34.86 |
| 4562.0930 | LVPVPITN(N)ATLDQITGK Fuc 4 Hex 3 HexNAc 7 Neu5Ac 1 | 29.596 | 0.023 | 12.189 | 9.847e+07 | 25 - 42 | 9 | 65.89 | 67.09 | 67.43 |
| 4259.9197 | C(C)ANLVPVPITN(N)ATLDQITGK Fuc 1 Hex 6 HexNAc 5 | 29.542 | 0.023 | 0.000 | 0.000e+00 | 22 - 42 | 1 | - | - | - |
| 4692.1567 | QIPLC(C)ANLVPVPITN(N)ATLDQITGK Hex 5 HexNAc 5 Neu5Ac 1 | 29.402 | 0.024 | 15.949 | 3.579e+08 | 18 - 42 | 12 | 66.06 | 67.94 | 68.80 |
| 3880.7763 | PVPITN(N)ATLDQITGK Hex 3 HexNAc 9 | 29.141 | 0.025 | -19.528 | 7.163e+07 | 27 - 42 | 2 | 48.19 | 48.35 | 48.35 |
| 4151.7314 | TPN(N)KTEDTIFLR Hex 8 HexNAc 7 | 29.014 | 0.027 | -4.874 | 4.745e+06 | 69 - 81 | 1 | 24.29 | 24.46 | 24.63 |
| 4657.0559 | LVPVPITN(N)ATLDQITGK Fuc 1 Hex 7 HexNAc 5 Neu5Ac 2 | 28.607 | 0.030 | -20.503 | 6.987e+05 | 25 - 42 | 1 | 57.33 | 57.33 | 57.33 |
| 4563.1188 | QIPLC(C)ANLVPVPITN(N)ATLDQITGK Hex 6 HexNAc 5 | 28.142 | 0.031 | 11.861 | 2.819e+08 | 18 - 42 | 10 | 67.60 | 68.11 | 69.14 |
| 4541.0122 | C(C)ANLVPVPITN(N)ATLDQITGK Fuc 1 Hex 9 HexNAc 4 | 28.087 | 0.031 | -20.529 | 2.137e+06 | 22 - 42 | 1 | 63.32 | 63.32 | 63.32 |
| 4273.8113 | SVQEIQATFFYFTPN(N)K Hex 7 HexNAc 6 | 27.775 | 0.035 | 0.000 | 0.000e+00 | 57 - 73 | 1 | - | - | - |
| 4786.0777 | LVPVPITN(N)ATLDQITGK Fuc 1 Hex 6 HexNAc 5 Neu5Ac 3 | 27.579 | 0.035 | -5.106 | 2.741e+06 | 25 - 42 | 2 | 56.99 | 57.16 | 57.33 |
| 3048.2845 | N(N)ATLDQITGK Hex 6 HexNAc 5 | 26.811 | 0.036 | 0.000 | 0.000e+00 | 32 - 42 | 1 | - | - | - |
| 3804.6747 | PVPITN(N)ATLDQITGK Hex 7 HexNAc 4 Neu5Ac 1 | 26.739 | 0.036 | 14.043 | 1.047e+09 | 27 - 42 | 7 | 47.72 | 48.03 | 48.50 |
| 4041.7549 | PVPITN(N)ATLDQITGK Hex 9 HexNAc 5 | 26.662 | 0.036 | 0.000 | 0.000e+00 | 27 - 42 | 1 | - | - | - |
| 4324.7627 | QDQC(C)IYN(N)TTYLNVQR Fuc 4 Hex 5 HexNAc 5 | 26.653 | 0.036 | 14.795 | 6.647e+08 | 86 - 101 | 4 | 33.52 | 33.69 | 34.36 |
| 4709.1788 | QIPLC(C)ANLVPVPITN(N)ATLDQITGK Fuc 1 Hex 6 HexNAc 5 | 26.069 | 0.036 | 13.145 | 7.945e+07 | 18 - 42 | 3 | 67.26 | 67.94 | 68.11 |
| 4418.8018 | YFTPN(N)KTEDTIFLR Fuc 1 Hex 7 HexNAc 4 Neu5Ac 2 | 26.048 | 0.036 | -20.228 | 1.764e+07 | 67 - 81 | 1 | 37.58 | 37.75 | 37.75 |
| 3737.5732 | IYN(N)TTYLNVQR Hex 7 HexNAc 6 | 26.019 | 0.036 | 0.000 | 0.000e+00 | 90 - 101 | 1 | - | - | - |
| 5192.1384 | YFTPN(N)KTEDTIFLR Hex 10 HexNAc 9 | 25.878 | 0.036 | -19.649 | 3.337e+06 | 67 - 81 | 1 | 35.21 | 35.21 | 35.21 |
| 5192.1384 | YFTPN(N)KTEDTIFLR Fuc 5 Hex 8 HexNAc 7 | 25.878 | 0.036 | -19.649 | 3.337e+06 | 67 - 81 | 1 | 35.21 | 35.21 | 35.21 |
| 5290.1242 | QDQC(C)IYN(N)TTYLNVQR Fuc 2 Hex 9 HexNAc 8 | 25.812 | 0.036 | -4.759 | 8.379e+06 | 86 - 101 | 1 | 31.99 | 32.16 | 32.33 |
| 4649.9022 | QDQC(C)IYN(N)TTYLN(D)VQR Fuc 4 Hex 7 HexNAc 5 | 25.308 | 0.036 | -6.381 | 8.129e+07 | 86 - 101 | 2 | 32.67 | 32.67 | 33.01 |
| 4266.7917 | FYFTPN(N)KTEDTIFLR Fuc 2 Hex 3 HexNAc 5 Neu5Ac 2 | 25.264 | 0.036 | -19.267 | 1.604e+07 | 66 - 81 | 1 | 45.18 | 45.35 | 45.35 |
| 3761.5597 | N(N)ATLDQITGK Fuc 1 Hex 7 HexNAc 7 | 24.721 | 0.039 | 13.764 | 1.262e+08 | 32 - 42 | 4 | 52.06 | 52.40 | 52.74 |
| 4476.9843 | C(C)ANLVPVPITN(N)ATLDQITGK Hex 7 HexNAc 6 | 24.620 | 0.039 | -5.392 | 3.760e+06 | 22 - 42 | 1 | 51.03 | 51.20 | 51.37 |
| 6126.6276 | SVQEIQATFFYFTPN(N)KTEDTIFLR Fuc 1 Hex 9 HexNAc 8 | 24.611 | 0.039 | 0.000 | 0.000e+00 | 57 - 81 | 1 | - | - | - |
| 4007.7563 | PVPITN(N)ATLDQITGK Hex 7 HexNAc 5 Neu5Ac 1 | 24.491 | 0.039 | -4.369 | 1.242e+08 | 27 - 42 | 4 | 48.35 | 48.35 | 48.82 |
| 3725.7302 | LVPVPITN(N)ATLDQITGK Hex 7 HexNAc 4 | 24.361 | 0.040 | -19.385 | 1.710e+06 | 25 - 42 | 1 | 44.84 | 44.84 | 44.84 |
| 4334.9193 | LVPVPITN(N)ATLDQITGK Hex 7 HexNAc 7 | 23.892 | 0.040 | -19.482 | 1.061e+07 | 25 - 42 | 1 | 47.22 | 47.39 | 47.39 |
| 3958.6558 | QDQC(C)IYN(N)TTYLNVQR Fuc 2 Hex 4 HexNAc 4 Neu5Ac 1 | 23.860 | 0.040 | -18.400 | 9.989e+06 | 86 - 101 | 2 | 34.86 | 34.86 | 34.86 |
| 3750.7158 | LVPVPITN(N)ATLDQITGK Fuc 1 Hex 5 HexNAc 5 | 23.738 | 0.041 | -20.675 | 7.069e+06 | 25 - 42 | 1 | 47.72 | 47.72 | 47.72 |
| 4203.8977 | LVPVPITN(N)ATLDQITGK Fuc 1 Hex 6 HexNAc 5 Neu5Ac 1 | 23.478 | 0.042 | -20.227 | 1.712e+06 | 25 - 42 | 1 | 50.52 | 50.52 | 50.69 |
| 4314.9269 | C(C)ANLVPVPITN(N)ATLDQITGK Hex 6 HexNAc 6 | 23.395 | 0.044 | -18.795 | 1.594e+07 | 22 - 42 | 2 | 52.23 | 52.23 | 52.40 |
| 4008.7751 | PVPITN(N)ATLDQITGK Fuc 2 Hex 7 HexNAc 5 | 23.381 | 0.044 | -5.472 | 1.870e+06 | 27 - 42 | 1 | 51.03 | 51.20 | 51.37 |
| 4300.8264 | FYFTPN(N)KTEDTIFLR Fuc 4 Hex 5 HexNAc 5 | 23.310 | 0.044 | -3.124 | 5.316e+07 | 66 - 81 | 3 | 46.37 | 46.54 | 46.88 |
| 4026.8422 | LVPVPITN(N)ATLDQITGK Fuc 4 Hex 4 HexNAc 5 | 23.283 | 0.044 | -4.867 | 3.510e+07 | 25 - 42 | 1 | 47.22 | 47.39 | 47.56 |
| 4128.6821 | N(N)ATLDQITGK Fuc 1 Hex 8 HexNAc 8 | 23.222 | 0.045 | 0.000 | 0.000e+00 | 32 - 42 | 1 | - | - | - |
| 4294.7720 | SVQEIQATFFYFTPN(N)K Fuc 1 Hex 10 HexNAc 3 | 23.194 | 0.046 | -19.268 | 3.311e+06 | 57 - 73 | 1 | 53.59 | 53.59 | 53.59 |
| 6342.7030 | SVQEIQATFFYFTPN(N)KTEDTIFLR Hex 10 HexNAc 9 | 23.185 | 0.046 | -5.556 | 1.088e+07 | 57 - 81 | 2 | 60.75 | 60.93 | 61.10 |
| 5062.0905 | SVQEIQATFFYFTPN(N)K Fuc 4 Hex 7 HexNAc 7 | 23.016 | 0.046 | 0.000 | 0.000e+00 | 57 - 73 | 1 | - | - | - |
| 4795.9579 | QDQC(C)IYN(N)TTYLN(D)VQR Hex 9 HexNAc 7 | 22.978 | 0.046 | -6.747 | 2.129e+07 | 86 - 101 | 1 | 32.50 | 33.01 | 33.01 |
| 4289.8136 | SVQEIQATFFYFTPN(N)K Fuc 2 Hex 6 HexNAc 4 Neu5Ac 1 | 22.936 | 0.046 | -22.120 | 4.933e+06 | 57 - 73 | 11 | 58.19 | 58.36 | 58.36 |
| 4095.8055 | C(C)ANLVPVPITN(N)ATLDQITGK Fuc 1 Hex 5 HexNAc 5 | 22.924 | 0.046 | -3.526 | 5.148e+07 | 22 - 42 | 4 | 61.61 | 61.78 | 62.13 |
| 4187.8993 | LVPVPITN(N)ATLDQITGK Fuc 2 Hex 5 HexNAc 5 Neu5Ac 1 | 22.758 | 0.046 | 0.000 | 0.000e+00 | 25 - 42 | 1 | - | - | - |
| 3968.8251 | LVPVPITN(N)ATLDQITGK Fuc 3 Hex 4 HexNAc 4 Neu5Ac 1 | 22.379 | 0.048 | -18.195 | 3.645e+07 | 25 - 42 | 2 | 46.88 | 47.05 | 47.05 |
| 4429.7965 | QDQC(C)IYN(N)TTYLNVQR Hex 8 HexNAc 6 | 22.169 | 0.050 | 10.212 | 4.480e+07 | 86 - 101 | 4 | 32.16 | 32.50 | 32.84 |

### YFTPN(N)K Hex 5 HexNAc 4

**Mass:**  2390.9646

(-9.445e-07)

**Charge States:** 2, 3

**Start Position**: 68

**End Position**: 74

| Profile Score | Tandem Score | TDA q-value | Volume | Spectrum Matches |
| --- | --- | --- | --- | --- |
| -20.2986 | 187.9382 | 0.0000 | 2.8771e+07 | 2 |

**Adduct Signal Fractions**

- **Unmodified**: 2.8771e+07

### YFTPN(N)K Hex 6 HexNAc 5

**Mass:**  2756.0989

(1.115e-06)

**Charge States:** 3

**Start Position**: 68

**End Position**: 74

| Profile Score | Tandem Score | TDA q-value | Volume | Spectrum Matches |
| --- | --- | --- | --- | --- |
| 15.0940 | 187.4647 | 0.0000 | 2.7702e+08 | 5 |

**Adduct Signal Fractions**

- **Unmodified**: 2.7702e+08

### FYFTPN(N)K Hex 7 HexNAc 6

**Mass:**  3268.2987

(1.290e-06)

**Charge States:** 3, 4

**Start Position**: 67

**End Position**: 74

| Profile Score | Tandem Score | TDA q-value | Volume | Spectrum Matches |
| --- | --- | --- | --- | --- |
| 14.5230 | 183.3084 | 0.0000 | 1.7500e+08 | 10 |

**Adduct Signal Fractions**

- **Unmodified**: 1.7500e+08

### NEEYN(N)K Hex 6 HexNAc 5

**Mass:**  2783.0553

(5.679e-07)

**Charge States:** 2, 3

**Start Position**: 52

**End Position**: 58

| Profile Score | Tandem Score | TDA q-value | Volume | Spectrum Matches |
| --- | --- | --- | --- | --- |
| 14.1904 | 182.5628 | 0.0000 | 4.1822e+08 | 15 |

**Adduct Signal Fractions**

- **Unmodified**: 4.1822e+08

### NEEYN(N)K Hex 5 HexNAc 4

**Mass:**  2417.9232

(7.168e-07)

**Charge States:** 2, 3

**Start Position**: 52

**End Position**: 58

| Profile Score | Tandem Score | TDA q-value | Volume | Spectrum Matches |
| --- | --- | --- | --- | --- |
| 12.5270 | 181.3118 | 0.0000 | 8.4181e+07 | 7 |

**Adduct Signal Fractions**

- **Unmodified**: 8.4181e+07

### YFTPN(N)K Hex 7 HexNAc 6

**Mass:**  3121.2306

(1.280e-06)

**Charge States:** 3

**Start Position**: 68

**End Position**: 74

| Profile Score | Tandem Score | TDA q-value | Volume | Spectrum Matches |
| --- | --- | --- | --- | --- |
| 13.2871 | 180.6938 | 0.0000 | 3.1787e+08 | 5 |

**Adduct Signal Fractions**

- **Unmodified**: 3.1787e+08

### NEEYN(N)K Fuc 1 Hex 6 HexNAc 5

**Mass:**  2929.1145

(9.957e-07)

**Charge States:** 3

**Start Position**: 52

**End Position**: 58

| Profile Score | Tandem Score | TDA q-value | Volume | Spectrum Matches |
| --- | --- | --- | --- | --- |
| 12.0063 | 180.4669 | 0.0000 | 2.9033e+07 | 3 |

**Adduct Signal Fractions**

- **Unmodified**: 2.9033e+07

### SVQEIQATFFYFTPN(N)K Hex 6 HexNAc 5

**Mass:**  3906.6660

(1.810e-06)

**Charge States:** 3, 4, 5

**Start Position**: 58

**End Position**: 74

| Profile Score | Tandem Score | TDA q-value | Volume | Spectrum Matches |
| --- | --- | --- | --- | --- |
| 14.7280 | 175.2034 | 0.0000 | 2.9688e+09 | 49 |

**Adduct Signal Fractions**

- **Unmodified**: 2.9688e+09

### SVQEIQATFFYFTPN(N)K Fuc 1 Hex 6 HexNAc 5

**Mass:**  4052.7270

(1.127e-06)

**Charge States:** 3, 4, 5

**Start Position**: 58

**End Position**: 74

| Profile Score | Tandem Score | TDA q-value | Volume | Spectrum Matches |
| --- | --- | --- | --- | --- |
| 14.2474 | 174.6170 | 0.0000 | 6.0646e+08 | 18 |

**Adduct Signal Fractions**

- **Unmodified**: 6.0646e+08

### YFTPN(N)K Fuc 1 Hex 6 HexNAc 5

**Mass:**  2902.1537

(4.886e-07)

**Charge States:** 3

**Start Position**: 68

**End Position**: 74

| Profile Score | Tandem Score | TDA q-value | Volume | Spectrum Matches |
| --- | --- | --- | --- | --- |
| -4.6216 | 173.9382 | 0.0000 | 4.3836e+07 | 3 |

**Adduct Signal Fractions**

- **Unmodified**: 4.3836e+07

### SVQEIQATFFYFTPN(N)K Hex 7 HexNAc 6

**Mass:**  4271.8093

(2.038e-06)

**Charge States:** 3, 4, 5

**Start Position**: 58

**End Position**: 74

| Profile Score | Tandem Score | TDA q-value | Volume | Spectrum Matches |
| --- | --- | --- | --- | --- |
| 13.2866 | 173.8761 | 0.0000 | 3.6903e+09 | 129 |

**Adduct Signal Fractions**

- **Unmodified**: 3.6903e+09

### YFTPN(N)K Hex 8 HexNAc 7

**Mass:**  3486.3623

(1.027e-06)

**Charge States:** 3

**Start Position**: 68

**End Position**: 74

| Profile Score | Tandem Score | TDA q-value | Volume | Spectrum Matches |
| --- | --- | --- | --- | --- |
| -19.7459 | 172.7300 | 0.0000 | 1.8550e+07 | 1 |

**Adduct Signal Fractions**

- **Unmodified**: 1.8550e+07

### YFTPN(N)K Fuc 1 Hex 7 HexNAc 6

**Mass:**  3267.2902

(1.767e-06)

**Charge States:** 3

**Start Position**: 68

**End Position**: 74

| Profile Score | Tandem Score | TDA q-value | Volume | Spectrum Matches |
| --- | --- | --- | --- | --- |
| -4.7720 | 171.6449 | 0.0000 | 4.4153e+07 | 2 |

**Adduct Signal Fractions**

- **Unmodified**: 4.4153e+07

### FYFTPN(N)K Hex 6 HexNAc 5

**Mass:**  2903.1655

(9.097e-07)

**Charge States:** 2, 3, 4

**Start Position**: 67

**End Position**: 74

| Profile Score | Tandem Score | TDA q-value | Volume | Spectrum Matches |
| --- | --- | --- | --- | --- |
| 14.4837 | 169.8429 | 0.0000 | 1.4314e+08 | 13 |

**Adduct Signal Fractions**

- **Unmodified**: 1.4314e+08

### SVQEIQATFFYFTPN(N)K Fuc 1 Hex 7 HexNAc 6

**Mass:**  4417.8664

(1.478e-06)

**Charge States:** 3, 4, 5

**Start Position**: 58

**End Position**: 74

| Profile Score | Tandem Score | TDA q-value | Volume | Spectrum Matches |
| --- | --- | --- | --- | --- |
| 13.4584 | 167.7006 | 0.0000 | 6.7371e+08 | 17 |

**Adduct Signal Fractions**

- **Unmodified**: 6.7371e+08

### SVQEIQATFFYFTPN(N)K Hex 8 HexNAc 7

**Mass:**  4636.9338

(2.973e-06)

**Charge States:** 3, 4, 5

**Start Position**: 58

**End Position**: 74

| Profile Score | Tandem Score | TDA q-value | Volume | Spectrum Matches |
| --- | --- | --- | --- | --- |
| 13.8104 | 166.7048 | 0.0000 | 4.7888e+08 | 10 |

**Adduct Signal Fractions**

- **Unmodified**: 4.7888e+08

### SVQEIQATFFYFTPN(N)K Fuc 2 Hex 7 HexNAc 6

**Mass:**  4563.9221

(2.084e-06)

**Charge States:** 4

**Start Position**: 58

**End Position**: 74

| Profile Score | Tandem Score | TDA q-value | Volume | Spectrum Matches |
| --- | --- | --- | --- | --- |
| 10.9767 | 166.6869 | 0.0000 | 9.0257e+07 | 2 |

**Adduct Signal Fractions**

- **Unmodified**: 9.0257e+07

### SVQEIQATFFYFTPN(N)K Hex 5 HexNAc 4

**Mass:**  3541.5327

(1.822e-06)

**Charge States:** 3, 4

**Start Position**: 58

**End Position**: 74

| Profile Score | Tandem Score | TDA q-value | Volume | Spectrum Matches |
| --- | --- | --- | --- | --- |
| 13.9355 | 164.8419 | 0.0000 | 3.0824e+08 | 18 |

**Adduct Signal Fractions**

- **Unmodified**: 3.0824e+08

### LVPVPITN(N)ATLDQITGK Fuc 1 Hex 6 HexNAc 5

**Mass:**  3912.7922

(1.838e-06)

**Charge States:** 3, 4

**Start Position**: 26

**End Position**: 43

| Profile Score | Tandem Score | TDA q-value | Volume | Spectrum Matches |
| --- | --- | --- | --- | --- |
| 14.9220 | 161.5752 | 0.0000 | 4.6054e+09 | 41 |

**Adduct Signal Fractions**

- **Unmodified**: 4.6054e+09

### PVPITN(N)ATLDQITGK Fuc 2 Hex 3 HexNAc 8

**Mass:**  3969.8083

(9.411e-06)

**Charge States:** 3, 4

**Start Position**: 28

**End Position**: 43

| Profile Score | Tandem Score | TDA q-value | Volume | Spectrum Matches |
| --- | --- | --- | --- | --- |
| 11.8811 | 161.4356 | 0.0000 | 1.0731e+09 | 15 |

**Adduct Signal Fractions**

- **Unmodified**: 1.0731e+09

### SVQEIQATFFYFTPN(N)K Fuc 1 Hex 8 HexNAc 7

**Mass:**  4782.9914

(2.325e-06)

**Charge States:** 3, 4

**Start Position**: 58

**End Position**: 74

| Profile Score | Tandem Score | TDA q-value | Volume | Spectrum Matches |
| --- | --- | --- | --- | --- |
| 13.1461 | 156.8631 | 0.0000 | 9.0067e+07 | 5 |

**Adduct Signal Fractions**

- **Unmodified**: 9.0067e+07

### ATFFYFTPN(N)K Hex 6 HexNAc 5

**Mass:**  3222.3239

(2.008e-06)

**Charge States:** 3, 4

**Start Position**: 64

**End Position**: 74

| Profile Score | Tandem Score | TDA q-value | Volume | Spectrum Matches |
| --- | --- | --- | --- | --- |
| -3.4965 | 156.3519 | 0.0000 | 4.8726e+07 | 2 |

**Adduct Signal Fractions**

- **Unmodified**: 4.8726e+07

### ATFFYFTPN(N)K Hex 7 HexNAc 6

**Mass:**  3587.4541

(1.607e-06)

**Charge States:** 3, 4

**Start Position**: 64

**End Position**: 74

| Profile Score | Tandem Score | TDA q-value | Volume | Spectrum Matches |
| --- | --- | --- | --- | --- |
| 13.0535 | 156.1570 | 0.0000 | 7.3053e+07 | 2 |

**Adduct Signal Fractions**

- **Unmodified**: 7.3053e+07

### LVPVPITN(N)ATLDQITGK Hex 6 HexNAc 5

**Mass:**  3766.7326

(1.202e-06)

**Charge States:** 3, 4

**Start Position**: 26

**End Position**: 43

| Profile Score | Tandem Score | TDA q-value | Volume | Spectrum Matches |
| --- | --- | --- | --- | --- |
| 15.1031 | 155.1102 | 0.0000 | 1.8908e+10 | 524 |

**Adduct Signal Fractions**

- **Unmodified**: 1.8908e+10

### LVPVPITN(N)ATLDQITGK Hex 5 HexNAc 4

**Mass:**  3401.5966

(1.792e-07)

**Charge States:** 3, 4

**Start Position**: 26

**End Position**: 43

| Profile Score | Tandem Score | TDA q-value | Volume | Spectrum Matches |
| --- | --- | --- | --- | --- |
| 15.2672 | 154.5588 | 0.0000 | 1.9709e+09 | 18 |

**Adduct Signal Fractions**

- **Unmodified**: 1.9709e+09

### SVQEIQATFFYFTPN(N)K Fuc 1 Hex 8 HexNAc 4 Neu5Ac 1

**Mass:**  4464.8610

(4.770e-06)

**Charge States:** 4

**Start Position**: 58

**End Position**: 74

| Profile Score | Tandem Score | TDA q-value | Volume | Spectrum Matches |
| --- | --- | --- | --- | --- |
| -4.8004 | 153.5758 | 0.0000 | 3.4141e+07 | 2 |

**Adduct Signal Fractions**

- **Unmodified**: 3.4141e+07

### PVPITN(N)ATLDQITGK Fuc 1 Hex 3 HexNAc 8

**Mass:**  3823.7521

(3.168e-06)

**Charge States:** 3, 4

**Start Position**: 28

**End Position**: 43

| Profile Score | Tandem Score | TDA q-value | Volume | Spectrum Matches |
| --- | --- | --- | --- | --- |
| 15.9947 | 149.2387 | 0.0000 | 2.9816e+09 | 5 |

**Adduct Signal Fractions**

- **Unmodified**: 2.9816e+09

### SVQEIQATFFYFTPN(N)KTEDTIFLR Hex 6 HexNAc 5

**Mass:**  4882.1730

(1.867e-06)

**Charge States:** 3, 4, 5

**Start Position**: 58

**End Position**: 82

| Profile Score | Tandem Score | TDA q-value | Volume | Spectrum Matches |
| --- | --- | --- | --- | --- |
| 14.1963 | 148.8807 | 0.0000 | 6.6008e+08 | 24 |

**Adduct Signal Fractions**

- **Unmodified**: 6.6008e+08

### SVQEIQATFFYFTPN(N)K Hex 9 HexNAc 8

**Mass:**  5002.0676

(1.459e-06)

**Charge States:** 4, 5

**Start Position**: 58

**End Position**: 74

| Profile Score | Tandem Score | TDA q-value | Volume | Spectrum Matches |
| --- | --- | --- | --- | --- |
| 12.6904 | 147.9710 | 0.0000 | 9.9854e+07 | 7 |

**Adduct Signal Fractions**

- **Unmodified**: 9.9854e+07

### YFTPN(N)KTEDTIFLR Hex 6 HexNAc 5

**Mass:**  3731.6005

(7.536e-07)

**Charge States:** 3, 4

**Start Position**: 68

**End Position**: 82

| Profile Score | Tandem Score | TDA q-value | Volume | Spectrum Matches |
| --- | --- | --- | --- | --- |
| 13.3612 | 147.2339 | 0.0000 | 1.0284e+09 | 22 |

**Adduct Signal Fractions**

- **Unmodified**: 1.0284e+09

### QDQC(C)IYN(N)TTYLNVQR Hex 6 HexNAc 5

**Mass:**  3902.6110

(1.158e-06)

**Charge States:** 3, 4, 5

**Start Position**: 87

**End Position**: 102

| Profile Score | Tandem Score | TDA q-value | Volume | Spectrum Matches |
| --- | --- | --- | --- | --- |
| 15.0008 | 143.3101 | 0.0000 | 2.4903e+09 | 11 |

**Adduct Signal Fractions**

- **Unmodified**: 2.4903e+09

### ATFFYFTPN(N)K Fuc 1 Hex 6 HexNAc 5

**Mass:**  3368.3790

(1.462e-06)

**Charge States:** 3

**Start Position**: 64

**End Position**: 74

| Profile Score | Tandem Score | TDA q-value | Volume | Spectrum Matches |
| --- | --- | --- | --- | --- |
| -20.1352 | 142.0029 | 0.0000 | 3.7619e+06 | 1 |

**Adduct Signal Fractions**

- **Unmodified**: 3.7619e+06

### SVQEIQATFFYFTPN(N)K Hex 7 HexNAc 4 Neu5Ac 2

**Mass:**  4447.8342

(3.477e-06)

**Charge States:** 3, 4

**Start Position**: 58

**End Position**: 74

| Profile Score | Tandem Score | TDA q-value | Volume | Spectrum Matches |
| --- | --- | --- | --- | --- |
| 12.3286 | 140.0869 | 0.0000 | 1.8440e+08 | 6 |

**Adduct Signal Fractions**

- **Unmodified**: 1.8440e+08

### YFTPN(N)KTEDTIFLR Fuc 1 Hex 7 HexNAc 6

**Mass:**  4242.7941

(4.729e-10)

**Charge States:** 4

**Start Position**: 68

**End Position**: 82

| Profile Score | Tandem Score | TDA q-value | Volume | Spectrum Matches |
| --- | --- | --- | --- | --- |
| 8.6435 | 137.5249 | 0.0000 | 2.3144e+08 | 5 |

**Adduct Signal Fractions**

- **Unmodified**: 2.3144e+08

### SVQEIQATFFYFTPN(N)KTEDTIFLR Fuc 1 Hex 6 HexNAc 5

**Mass:**  5028.2321

(2.478e-06)

**Charge States:** 4, 5

**Start Position**: 58

**End Position**: 82

| Profile Score | Tandem Score | TDA q-value | Volume | Spectrum Matches |
| --- | --- | --- | --- | --- |
| 12.1861 | 136.8445 | 0.0000 | 1.5229e+08 | 8 |

**Adduct Signal Fractions**

- **Unmodified**: 1.5229e+08

### TPN(N)KTEDTIFLR Hex 7 HexNAc 6

**Mass:**  3786.6013

(9.784e-07)

**Charge States:** 3, 4

**Start Position**: 70

**End Position**: 82

| Profile Score | Tandem Score | TDA q-value | Volume | Spectrum Matches |
| --- | --- | --- | --- | --- |
| 14.3781 | 136.6219 | 0.0000 | 7.9095e+07 | 7 |

**Adduct Signal Fractions**

- **Unmodified**: 7.9095e+07

### LVPVPITN(N)ATLDQITGK Fuc 1 Hex 7 HexNAc 6

**Mass:**  4277.9279

(2.259e-06)

**Charge States:** 3, 4

**Start Position**: 26

**End Position**: 43

| Profile Score | Tandem Score | TDA q-value | Volume | Spectrum Matches |
| --- | --- | --- | --- | --- |
| 13.9961 | 135.6951 | 0.0000 | 2.5280e+08 | 5 |

**Adduct Signal Fractions**

- **Unmodified**: 2.5280e+08

### QDQC(C)IYN(N)TTYLNVQR Fuc 1 Hex 6 HexNAc 5

**Mass:**  4048.6679

(1.613e-06)

**Charge States:** 3, 4

**Start Position**: 87

**End Position**: 102

| Profile Score | Tandem Score | TDA q-value | Volume | Spectrum Matches |
| --- | --- | --- | --- | --- |
| 13.5432 | 132.5621 | 0.0000 | 6.3887e+08 | 6 |

**Adduct Signal Fractions**

- **Unmodified**: 6.3887e+08

### SVQEIQATFFYFTPN(N)K Fuc 4 Hex 5 HexNAc 5

**Mass:**  4328.8239

(-4.039e-06)

**Charge States:** 3, 4, 5

**Start Position**: 58

**End Position**: 74

| Profile Score | Tandem Score | TDA q-value | Volume | Spectrum Matches |
| --- | --- | --- | --- | --- |
| 13.9719 | 130.5669 | 0.0000 | 7.7517e+08 | 25 |

**Adduct Signal Fractions**

- **Unmodified**: 7.7517e+08

### LVPVPITN(N)ATLDQITGK Hex 7 HexNAc 6

**Mass:**  4131.8654

(2.299e-06)

**Charge States:** 3, 4

**Start Position**: 26

**End Position**: 43

| Profile Score | Tandem Score | TDA q-value | Volume | Spectrum Matches |
| --- | --- | --- | --- | --- |
| 14.4839 | 130.1220 | 0.0000 | 7.0585e+08 | 10 |

**Adduct Signal Fractions**

- **Unmodified**: 7.0585e+08

### PVPITN(N)ATLDQITGK Fuc 1 Hex 4 HexNAc 9

**Mass:**  4188.8874

(9.051e-06)

**Charge States:** 4

**Start Position**: 28

**End Position**: 43

| Profile Score | Tandem Score | TDA q-value | Volume | Spectrum Matches |
| --- | --- | --- | --- | --- |
| -4.7816 | 129.9347 | 0.0000 | 1.0029e+08 | 3 |

**Adduct Signal Fractions**

- **Unmodified**: 1.0029e+08

### QDQC(C)IYN(N)TTYLNVQR Hex 5 HexNAc 4

**Mass:**  3537.4748

(6.906e-07)

**Charge States:** 3, 4

**Start Position**: 87

**End Position**: 102

| Profile Score | Tandem Score | TDA q-value | Volume | Spectrum Matches |
| --- | --- | --- | --- | --- |
| -4.1503 | 128.4477 | 0.0000 | 4.5244e+07 | 3 |

**Adduct Signal Fractions**

- **Unmodified**: 4.5244e+07

### SVQEIQATFFYFTPN(N)K Fuc 2 Hex 8 HexNAc 7

**Mass:**  4929.0539

(2.681e-06)

**Charge States:** 4

**Start Position**: 58

**End Position**: 74

| Profile Score | Tandem Score | TDA q-value | Volume | Spectrum Matches |
| --- | --- | --- | --- | --- |
| 11.3556 | 128.3865 | 0.0000 | 2.2904e+07 | 2 |

**Adduct Signal Fractions**

- **Unmodified**: 2.2904e+07

### QDQC(C)IYN(N)TTYLNVQR Hex 7 HexNAc 6

**Mass:**  4267.7512

(1.730e-06)

**Charge States:** 3, 4

**Start Position**: 87

**End Position**: 102

| Profile Score | Tandem Score | TDA q-value | Volume | Spectrum Matches |
| --- | --- | --- | --- | --- |
| 14.2689 | 127.5930 | 0.0000 | 3.2579e+09 | 9 |

**Adduct Signal Fractions**

- **Unmodified**: 3.2579e+09

### PVPITN(N)ATLDQITGK Hex 9 HexNAc 4

**Mass:**  3839.6689

(5.249e-04)

**Start Position**: 28

**End Position**: 43

| Profile Score | Tandem Score | TDA q-value | Volume | Spectrum Matches |
| --- | --- | --- | --- | --- |
| 0.0000 | 127.5435 | 0.0000 | 0.0000e+00 | 1 |

Adduct:

No Chromatogram Found

### YFTPN(N)KTEDTIFLR Fuc 1 Hex 6 HexNAc 5

**Mass:**  3877.6572

(1.430e-06)

**Charge States:** 3, 4

**Start Position**: 68

**End Position**: 82

| Profile Score | Tandem Score | TDA q-value | Volume | Spectrum Matches |
| --- | --- | --- | --- | --- |
| 8.3685 | 127.2492 | 0.0000 | 2.5470e+08 | 4 |

**Adduct Signal Fractions**

- **Unmodified**: 2.5470e+08

### LVPVPITN(N)ATLDQITGK Hex 8 HexNAc 7

**Mass:**  4497.0002

(2.250e-06)

**Charge States:** 3, 4, 5

**Start Position**: 26

**End Position**: 43

| Profile Score | Tandem Score | TDA q-value | Volume | Spectrum Matches |
| --- | --- | --- | --- | --- |
| 12.3775 | 126.8106 | 0.0000 | 1.1562e+08 | 6 |

**Adduct Signal Fractions**

- **Unmodified**: 1.1562e+08

### YFTPN(N)KTEDTIFLR Hex 7 HexNAc 6

**Mass:**  4096.7326

(-1.236e-06)

**Charge States:** 3, 4

**Start Position**: 68

**End Position**: 82

| Profile Score | Tandem Score | TDA q-value | Volume | Spectrum Matches |
| --- | --- | --- | --- | --- |
| 12.5200 | 126.6999 | 0.0000 | 1.9531e+09 | 21 |

**Adduct Signal Fractions**

- **Unmodified**: 1.9531e+09

### QDQC(C)IYN(N)TTYLNVQR Fuc 1 Hex 7 HexNAc 6

**Mass:**  4413.8127

(8.965e-07)

**Charge States:** 3, 4

**Start Position**: 87

**End Position**: 102

| Profile Score | Tandem Score | TDA q-value | Volume | Spectrum Matches |
| --- | --- | --- | --- | --- |
| 11.7142 | 125.8940 | 0.0000 | 9.8745e+08 | 6 |

**Adduct Signal Fractions**

- **Unmodified**: 9.8745e+08

### SVQEIQATFFYFTPN(N)K Hex 6 HexNAc 6

**Mass:**  4109.7477

(2.020e-06)

**Charge States:** 3, 4

**Start Position**: 58

**End Position**: 74

| Profile Score | Tandem Score | TDA q-value | Volume | Spectrum Matches |
| --- | --- | --- | --- | --- |
| 9.7049 | 125.7924 | 0.0000 | 7.6235e+07 | 4 |

**Adduct Signal Fractions**

- **Unmodified**: 7.6235e+07

### PVPITN(N)ATLDQITGK Hex 3 HexNAc 9

**Mass:**  3882.7577

(5.213e-04)

**Start Position**: 28

**End Position**: 43

| Profile Score | Tandem Score | TDA q-value | Volume | Spectrum Matches |
| --- | --- | --- | --- | --- |
| 0.0000 | 125.0831 | 0.0000 | 0.0000e+00 | 1 |

Adduct:

No Chromatogram Found

### FYFTPN(N)KTEDTIFLR Hex 7 HexNAc 6

**Mass:**  4243.8044

(1.732e-06)

**Charge States:** 3, 4

**Start Position**: 67

**End Position**: 82

| Profile Score | Tandem Score | TDA q-value | Volume | Spectrum Matches |
| --- | --- | --- | --- | --- |
| 12.1988 | 124.8086 | 0.0000 | 2.5394e+08 | 8 |

**Adduct Signal Fractions**

- **Unmodified**: 2.5394e+08

### QDQC(C)IYN(N)TTYLNVQR Hex 8 HexNAc 7

**Mass:**  4632.8806

(1.819e-06)

**Charge States:** 3, 4, 5

**Start Position**: 87

**End Position**: 102

| Profile Score | Tandem Score | TDA q-value | Volume | Spectrum Matches |
| --- | --- | --- | --- | --- |
| 16.1182 | 124.5981 | 0.0000 | 6.7920e+08 | 6 |

**Adduct Signal Fractions**

- **Unmodified**: 6.7920e+08

### SVQEIQATFFYFTPN(N)KTEDTIFLR Hex 7 HexNAc 6

**Mass:**  5247.3063

(2.611e-06)

**Charge States:** 3, 4, 5, 6

**Start Position**: 58

**End Position**: 82

| Profile Score | Tandem Score | TDA q-value | Volume | Spectrum Matches |
| --- | --- | --- | --- | --- |
| 13.1269 | 123.7439 | 0.0000 | 1.4568e+09 | 30 |

**Adduct Signal Fractions**

- **Unmodified**: 1.4568e+09

### FYFTPN(N)KTEDTIFLR Hex 6 HexNAc 5

**Mass:**  3878.6711

(1.201e-06)

**Charge States:** 3, 4

**Start Position**: 67

**End Position**: 82

| Profile Score | Tandem Score | TDA q-value | Volume | Spectrum Matches |
| --- | --- | --- | --- | --- |
| 13.4413 | 120.9525 | 0.0000 | 7.9152e+07 | 5 |

**Adduct Signal Fractions**

- **Unmodified**: 7.9152e+07

### SVQEIQATFFYFTPN(N)K Fuc 1 Hex 7 HexNAc 4 Neu5Ac 2

**Mass:**  4593.8933

(2.363e-06)

**Charge States:** 4

**Start Position**: 58

**End Position**: 74

| Profile Score | Tandem Score | TDA q-value | Volume | Spectrum Matches |
| --- | --- | --- | --- | --- |
| -5.1024 | 120.8128 | 0.0000 | 2.1924e+07 | 1 |

**Adduct Signal Fractions**

- **Unmodified**: 2.1924e+07

### PVPITN(N)ATLDQITGK Fuc 1 Hex 3 HexNAc 9

**Mass:**  4026.8315

(9.715e-06)

**Charge States:** 4

**Start Position**: 28

**End Position**: 43

| Profile Score | Tandem Score | TDA q-value | Volume | Spectrum Matches |
| --- | --- | --- | --- | --- |
| -21.5869 | 119.2012 | 0.0000 | 5.3733e+06 | 1 |

**Adduct Signal Fractions**

- **Unmodified**: 5.3733e+06

### SVQEIQATFFYFTPN(N)K Fuc 2 Hex 6 HexNAc 5

**Mass:**  4198.7902

(3.378e-06)

**Charge States:** 4

**Start Position**: 58

**End Position**: 74

| Profile Score | Tandem Score | TDA q-value | Volume | Spectrum Matches |
| --- | --- | --- | --- | --- |
| -5.1730 | 119.0445 | 0.0000 | 1.0857e+07 | 1 |

**Adduct Signal Fractions**

- **Unmodified**: 1.0857e+07

### LVPVPITN(N)ATLDQITGK Fuc 1 Hex 7 HexNAc 3 Neu5Ac 1

**Mass:**  3959.7951

(4.360e-06)

**Charge States:** 3

**Start Position**: 26

**End Position**: 43

| Profile Score | Tandem Score | TDA q-value | Volume | Spectrum Matches |
| --- | --- | --- | --- | --- |
| -4.3904 | 118.8516 | 0.0000 | 2.5730e+07 | 2 |

**Adduct Signal Fractions**

- **Unmodified**: 2.5730e+07

### QDQC(C)IYN(N)TTYLNVQR Fuc 5 Hex 7 HexNAc 6

**Mass:**  4998.0090

(-3.225e-06)

**Charge States:** 3, 4

**Start Position**: 87

**End Position**: 102

| Profile Score | Tandem Score | TDA q-value | Volume | Spectrum Matches |
| --- | --- | --- | --- | --- |
| 12.0406 | 118.0937 | 0.0000 | 2.1477e+08 | 4 |

**Adduct Signal Fractions**

- **Unmodified**: 2.1477e+08

### SVQEIQATFFYFTPN(N)K Hex 8 HexNAc 6

**Mass:**  4436.8925

(6.872e-04)

**Start Position**: 58

**End Position**: 74

| Profile Score | Tandem Score | TDA q-value | Volume | Spectrum Matches |
| --- | --- | --- | --- | --- |
| 0.0000 | 117.1125 | 0.0000 | 0.0000e+00 | 1 |

Adduct:

No Chromatogram Found

### YFTPN(N)KTEDTIFLR Hex 5 HexNAc 4

**Mass:**  3366.4689

(6.018e-07)

**Charge States:** 3, 4

**Start Position**: 68

**End Position**: 82

| Profile Score | Tandem Score | TDA q-value | Volume | Spectrum Matches |
| --- | --- | --- | --- | --- |
| -18.7353 | 116.6787 | 0.0000 | 5.1285e+07 | 1 |

**Adduct Signal Fractions**

- **Unmodified**: 5.1285e+07

### LVPVPITN(N)ATLDQITGK Fuc 2 Hex 6 HexNAc 5

**Mass:**  4058.8498

(8.491e-07)

**Charge States:** 3, 4

**Start Position**: 26

**End Position**: 43

| Profile Score | Tandem Score | TDA q-value | Volume | Spectrum Matches |
| --- | --- | --- | --- | --- |
| -5.4161 | 116.3274 | 0.0000 | 5.6359e+07 | 5 |

**Adduct Signal Fractions**

- **Unmodified**: 5.6359e+07

### TPN(N)KTEDTIFLR Hex 6 HexNAc 5

**Mass:**  3421.4683

(1.102e-06)

**Charge States:** 3, 4

**Start Position**: 70

**End Position**: 82

| Profile Score | Tandem Score | TDA q-value | Volume | Spectrum Matches |
| --- | --- | --- | --- | --- |
| 13.9611 | 116.2390 | 0.0000 | 3.9272e+07 | 7 |

**Adduct Signal Fractions**

- **Unmodified**: 3.9272e+07

### YFTPN(N)KTEDTIFLR Hex 8 HexNAc 7

**Mass:**  4461.8669

(8.782e-07)

**Charge States:** 4

**Start Position**: 68

**End Position**: 82

| Profile Score | Tandem Score | TDA q-value | Volume | Spectrum Matches |
| --- | --- | --- | --- | --- |
| 12.7335 | 115.3675 | 0.0000 | 2.2856e+08 | 4 |

**Adduct Signal Fractions**

- **Unmodified**: 2.2856e+08

### QDQC(C)IYN(N)TTYLNVQR Fuc 1 Hex 8 HexNAc 7

**Mass:**  4778.9348

(1.806e-06)

**Charge States:** 3, 4

**Start Position**: 87

**End Position**: 102

| Profile Score | Tandem Score | TDA q-value | Volume | Spectrum Matches |
| --- | --- | --- | --- | --- |
| 15.0032 | 114.7909 | 0.0000 | 1.6192e+08 | 3 |

**Adduct Signal Fractions**

- **Unmodified**: 1.6192e+08

### PVPITN(N)ATLDQITGK Fuc 1 Hex 8 HexNAc 5

**Mass:**  4024.7277

(-2.139e-06)

**Charge States:** 3, 4

**Start Position**: 28

**End Position**: 43

| Profile Score | Tandem Score | TDA q-value | Volume | Spectrum Matches |
| --- | --- | --- | --- | --- |
| -18.4528 | 114.2542 | 0.0000 | 5.9129e+06 | 3 |

**Adduct Signal Fractions**

- **Unmodified**: 5.9129e+06

### IYN(N)TTYLNVQR Hex 7 HexNAc 6

**Mass:**  3736.5646

(1.035e-06)

**Charge States:** 3, 4

**Start Position**: 91

**End Position**: 102

| Profile Score | Tandem Score | TDA q-value | Volume | Spectrum Matches |
| --- | --- | --- | --- | --- |
| 15.3562 | 113.0240 | 0.0000 | 3.6946e+07 | 4 |

**Adduct Signal Fractions**

- **Unmodified**: 3.6946e+07

### ATFFYFTPN(N)K Fuc 2 Hex 3 HexNAc 8 Neu5Ac 1

**Mass:**  3928.6430

(7.945e-06)

**Charge States:** 3, 4

**Start Position**: 64

**End Position**: 74

| Profile Score | Tandem Score | TDA q-value | Volume | Spectrum Matches |
| --- | --- | --- | --- | --- |
| 12.4987 | 112.3875 | 0.0000 | 2.2765e+08 | 1 |

**Adduct Signal Fractions**

- **Unmodified**: 2.2765e+08

### QDQC(C)IYN(N)TTYLNVQR Fuc 2 Hex 7 HexNAc 6

**Mass:**  4559.8630

(1.393e-06)

**Charge States:** 4

**Start Position**: 87

**End Position**: 102

| Profile Score | Tandem Score | TDA q-value | Volume | Spectrum Matches |
| --- | --- | --- | --- | --- |
| -4.0785 | 111.1855 | 0.0000 | 1.3493e+08 | 2 |

**Adduct Signal Fractions**

- **Unmodified**: 1.3493e+08

### FTPN(N)KTEDTIFLR Hex 7 HexNAc 6

**Mass:**  3933.6708

(1.257e-06)

**Charge States:** 3, 4

**Start Position**: 69

**End Position**: 82

| Profile Score | Tandem Score | TDA q-value | Volume | Spectrum Matches |
| --- | --- | --- | --- | --- |
| 13.5369 | 111.1400 | 0.0000 | 1.7397e+08 | 6 |

**Adduct Signal Fractions**

- **Unmodified**: 1.7397e+08

### SVQEIQATFFYFTPN(N)K Hex 7 HexNAc 7

**Mass:**  4474.8781

(1.809e-06)

**Charge States:** 3, 4

**Start Position**: 58

**End Position**: 74

| Profile Score | Tandem Score | TDA q-value | Volume | Spectrum Matches |
| --- | --- | --- | --- | --- |
| 12.1592 | 110.7709 | 0.0000 | 1.0339e+08 | 3 |

**Adduct Signal Fractions**

- **Unmodified**: 1.0339e+08

### FTPN(N)KTEDTIFLR Hex 6 HexNAc 5

**Mass:**  3568.5385

(9.490e-07)

**Charge States:** 3, 4

**Start Position**: 69

**End Position**: 82

| Profile Score | Tandem Score | TDA q-value | Volume | Spectrum Matches |
| --- | --- | --- | --- | --- |
| 14.2403 | 109.8215 | 0.0000 | 9.3317e+07 | 5 |

**Adduct Signal Fractions**

- **Unmodified**: 9.3317e+07

### LVPVPITN(N)ATLDQITGK Fuc 2 Hex 7 HexNAc 6

**Mass:**  4423.9880

(2.693e-06)

**Charge States:** 4

**Start Position**: 26

**End Position**: 43

| Profile Score | Tandem Score | TDA q-value | Volume | Spectrum Matches |
| --- | --- | --- | --- | --- |
| 7.0926 | 109.5331 | 0.0000 | 2.9323e+07 | 2 |

**Adduct Signal Fractions**

- **Unmodified**: 2.9323e+07

### QDQC(C)IYN(N)TTYLNVQR Hex 7 HexNAc 4 Neu5Ac 2

**Mass:**  4443.7717

(1.465e-06)

**Charge States:** 3, 4

**Start Position**: 87

**End Position**: 102

| Profile Score | Tandem Score | TDA q-value | Volume | Spectrum Matches |
| --- | --- | --- | --- | --- |
| 12.2811 | 106.4529 | 0.0000 | 2.0824e+08 | 2 |

**Adduct Signal Fractions**

- **Unmodified**: 2.0824e+08

### YFTPN(N)KTEDTIFLR Fuc 1 Hex 8 HexNAc 7

**Mass:**  4607.9113

(-1.746e-06)

**Charge States:** 4

**Start Position**: 68

**End Position**: 82

| Profile Score | Tandem Score | TDA q-value | Volume | Spectrum Matches |
| --- | --- | --- | --- | --- |
| -20.1090 | 105.9241 | 0.0000 | 1.2398e+07 | 1 |

**Adduct Signal Fractions**

- **Unmodified**: 1.2398e+07

### LVPVPITN(N)ATLDQITGK Fuc 1 Hex 7 HexNAc 5

**Mass:**  4074.8571

(6.180e-06)

**Charge States:** 4

**Start Position**: 26

**End Position**: 43

| Profile Score | Tandem Score | TDA q-value | Volume | Spectrum Matches |
| --- | --- | --- | --- | --- |
| -5.3334 | 105.8139 | 0.0000 | 3.8463e+06 | 2 |

**Adduct Signal Fractions**

- **Unmodified**: 3.8463e+06

### PVPITN(N)ATLDQITGK Fuc 1 Hex 8 HexNAc 4

**Mass:**  3821.6552

(-9.780e-07)

**Charge States:** 5

**Start Position**: 28

**End Position**: 43

| Profile Score | Tandem Score | TDA q-value | Volume | Spectrum Matches |
| --- | --- | --- | --- | --- |
| -20.4190 | 104.1141 | 0.0000 | 4.9588e+06 | 3 |

**Adduct Signal Fractions**

- **Unmodified**: 4.9588e+06

### SVQEIQATFFYFTPN(N)KTEDTIFLR Hex 5 HexNAc 4

**Mass:**  4517.0368

(1.392e-06)

**Charge States:** 4

**Start Position**: 58

**End Position**: 82

| Profile Score | Tandem Score | TDA q-value | Volume | Spectrum Matches |
| --- | --- | --- | --- | --- |
| -4.2861 | 103.9531 | 0.0000 | 1.8247e+07 | 2 |

**Adduct Signal Fractions**

- **Unmodified**: 1.8247e+07

### TPN(N)KTEDTIFLR Fuc 1 Hex 7 HexNAc 6

**Mass:**  3932.6606

(1.447e-06)

**Charge States:** 4

**Start Position**: 70

**End Position**: 82

| Profile Score | Tandem Score | TDA q-value | Volume | Spectrum Matches |
| --- | --- | --- | --- | --- |
| -4.6100 | 103.5604 | 0.0000 | 1.0023e+07 | 2 |

**Adduct Signal Fractions**

- **Unmodified**: 1.0023e+07

### SVQEIQATFFYFTPN(N)K Fuc 4 Hex 6 HexNAc 6

**Mass:**  4693.9562

(-3.940e-06)

**Charge States:** 4

**Start Position**: 58

**End Position**: 74

| Profile Score | Tandem Score | TDA q-value | Volume | Spectrum Matches |
| --- | --- | --- | --- | --- |
| 13.5122 | 103.3033 | 0.0000 | 5.6809e+07 | 3 |

**Adduct Signal Fractions**

- **Unmodified**: 5.6809e+07

### SVQEIQATFFYFTPN(N)KTEDTIFLR Hex 8 HexNAc 7

**Mass:**  5612.4362

(1.786e-06)

**Charge States:** 4, 5

**Start Position**: 58

**End Position**: 82

| Profile Score | Tandem Score | TDA q-value | Volume | Spectrum Matches |
| --- | --- | --- | --- | --- |
| 14.4699 | 102.9564 | 0.0000 | 2.5303e+08 | 5 |

**Adduct Signal Fractions**

- **Unmodified**: 2.5303e+08

### YFTPN(N)KTEDTIFLR Hex 3 HexNAc 5 Neu5Ac 3

**Mass:**  4118.7155

(-1.234e-06)

**Charge States:** 4

**Start Position**: 68

**End Position**: 82

| Profile Score | Tandem Score | TDA q-value | Volume | Spectrum Matches |
| --- | --- | --- | --- | --- |
| -4.8281 | 102.3033 | 0.0000 | 7.1547e+07 | 3 |

**Adduct Signal Fractions**

- **Unmodified**: 7.1547e+07

### YFTPN(N)KTEDTIFLR Hex 9 HexNAc 8

**Mass:**  4826.9982

(9.451e-07)

**Charge States:** 4

**Start Position**: 68

**End Position**: 82

| Profile Score | Tandem Score | TDA q-value | Volume | Spectrum Matches |
| --- | --- | --- | --- | --- |
| 12.4434 | 101.9477 | 0.0000 | 6.4966e+07 | 2 |

**Adduct Signal Fractions**

- **Unmodified**: 6.4966e+07

### YFTPN(N)KTEDTIFLR Hex 7 HexNAc 7

**Mass:**  4299.8127

(7.697e-07)

**Charge States:** 4

**Start Position**: 68

**End Position**: 82

| Profile Score | Tandem Score | TDA q-value | Volume | Spectrum Matches |
| --- | --- | --- | --- | --- |
| -5.1445 | 100.9264 | 0.0000 | 7.0400e+07 | 2 |

**Adduct Signal Fractions**

- **Unmodified**: 7.0400e+07

### PVPITN(N)ATLDQITGK Fuc 2 Hex 7 HexNAc 3

**Mass:**  3602.5947

(2.039e-06)

**Charge States:** 3

**Start Position**: 28

**End Position**: 43

| Profile Score | Tandem Score | TDA q-value | Volume | Spectrum Matches |
| --- | --- | --- | --- | --- |
| -4.2157 | 98.2417 | 0.0000 | 7.1156e+06 | 2 |

**Adduct Signal Fractions**

- **Unmodified**: 7.1156e+06

### PVPITN(N)ATLDQITGK Fuc 2 Hex 4 HexNAc 9

**Mass:**  4337.9719

(7.039e-04)

**Charge States:** 3, 4

**Start Position**: 28

**End Position**: 43

| Profile Score | Tandem Score | TDA q-value | Volume | Spectrum Matches |
| --- | --- | --- | --- | --- |
| 7.8863 | 97.9477 | 0.0000 | 1.2446e+08 | 6 |

**Adduct Signal Fractions**

- **Unmodified**: 1.2446e+08

### LVPVPITN(N)ATLDQITGK Fuc 1 Hex 5 HexNAc 4

**Mass:**  3548.6539

(2.819e-04)

**Start Position**: 26

**End Position**: 43

| Profile Score | Tandem Score | TDA q-value | Volume | Spectrum Matches |
| --- | --- | --- | --- | --- |
| 0.0000 | 96.5984 | 0.0000 | 0.0000e+00 | 1 |

Adduct:

No Chromatogram Found

### SVQEIQATFFYFTPN(N)K Fuc 3 Hex 4 HexNAc 5

**Mass:**  4020.7092

(-2.024e-06)

**Charge States:** 3, 4

**Start Position**: 58

**End Position**: 74

| Profile Score | Tandem Score | TDA q-value | Volume | Spectrum Matches |
| --- | --- | --- | --- | --- |
| 7.3298 | 96.5438 | 0.0000 | 2.7910e+07 | 2 |

**Adduct Signal Fractions**

- **Unmodified**: 2.7910e+07

### QDQC(C)IYN(N)TTYLNVQR Fuc 1 Hex 7 HexNAc 4 Neu5Ac 2

**Mass:**  4589.8298

(9.368e-07)

**Charge States:** 4

**Start Position**: 87

**End Position**: 102

| Profile Score | Tandem Score | TDA q-value | Volume | Spectrum Matches |
| --- | --- | --- | --- | --- |
| -5.1160 | 96.4488 | 0.0000 | 3.4719e+07 | 1 |

**Adduct Signal Fractions**

- **Unmodified**: 3.4719e+07

### FYFTPN(N)KTEDTIFLR Fuc 1 Hex 6 HexNAc 5

**Mass:**  4024.7292

(9.248e-07)

**Charge States:** 4

**Start Position**: 67

**End Position**: 82

| Profile Score | Tandem Score | TDA q-value | Volume | Spectrum Matches |
| --- | --- | --- | --- | --- |
| -4.0690 | 95.7808 | 0.0000 | 2.2372e+07 | 3 |

**Adduct Signal Fractions**

- **Unmodified**: 2.2372e+07

### PVPITN(N)ATLDQITGK Fuc 2 Hex 3 HexNAc 7 Neu5Ac 3

**Mass:**  4640.0212

(9.381e-06)

**Charge States:** 4, 5

**Start Position**: 28

**End Position**: 43

| Profile Score | Tandem Score | TDA q-value | Volume | Spectrum Matches |
| --- | --- | --- | --- | --- |
| -5.0274 | 94.8998 | 0.0000 | 1.0342e+07 | 1 |

**Adduct Signal Fractions**

- **Unmodified**: 1.0342e+07

### YFTPN(N)KTEDTIFLR Fuc 2 Hex 7 HexNAc 6

**Mass:**  4391.8577

(6.864e-04)

**Start Position**: 68

**End Position**: 82

| Profile Score | Tandem Score | TDA q-value | Volume | Spectrum Matches |
| --- | --- | --- | --- | --- |
| 0.0000 | 94.6840 | 0.0000 | 0.0000e+00 | 1 |

Adduct:

No Chromatogram Found

### YFTPN(N)KTEDTIFLR Hex 6 HexNAc 5

**Mass:**  3732.6097

(2.714e-04)

**Start Position**: 68

**End Position**: 82

| Profile Score | Tandem Score | TDA q-value | Volume | Spectrum Matches |
| --- | --- | --- | --- | --- |
| 0.0000 | 94.6126 | 0.0000 | 0.0000e+00 | 1 |

Adduct:

No Chromatogram Found

### SVQEIQATFFYFTPN(N)KTEDTIFLR Fuc 1 Hex 7 HexNAc 6

**Mass:**  5393.3644

(2.397e-06)

**Charge States:** 4, 5

**Start Position**: 58

**End Position**: 82

| Profile Score | Tandem Score | TDA q-value | Volume | Spectrum Matches |
| --- | --- | --- | --- | --- |
| 12.7694 | 93.7131 | 0.0000 | 2.8246e+08 | 6 |

**Adduct Signal Fractions**

- **Unmodified**: 2.8246e+08

### PVPITN(N)ATLDQITGK Fuc 3 Hex 5 HexNAc 9

**Mass:**  4643.0617

(9.707e-06)

**Charge States:** 4

**Start Position**: 28

**End Position**: 43

| Profile Score | Tandem Score | TDA q-value | Volume | Spectrum Matches |
| --- | --- | --- | --- | --- |
| 14.9222 | 93.6978 | 0.0000 | 1.8914e+07 | 3 |

**Adduct Signal Fractions**

- **Unmodified**: 1.8914e+07

### YFTPN(N)KTEDTIFLR Hex 7 HexNAc 4 Neu5Ac 2

**Mass:**  4272.7614

(-5.884e-07)

**Charge States:** 4

**Start Position**: 68

**End Position**: 82

| Profile Score | Tandem Score | TDA q-value | Volume | Spectrum Matches |
| --- | --- | --- | --- | --- |
| -4.0872 | 92.8185 | 0.0000 | 1.4040e+08 | 4 |

**Adduct Signal Fractions**

- **Unmodified**: 1.4040e+08

### LVPVPITN(N)ATLDQITGK Hex 6 HexNAc 5 Neu5Ac 1

**Mass:**  4057.8290

(1.777e-06)

**Charge States:** 3, 4

**Start Position**: 26

**End Position**: 43

| Profile Score | Tandem Score | TDA q-value | Volume | Spectrum Matches |
| --- | --- | --- | --- | --- |
| 13.0089 | 92.7202 | 0.0000 | 3.2851e+07 | 6 |

**Adduct Signal Fractions**

- **Unmodified**: 3.2851e+07

### FYFTPN(N)KTEDTIFLR Hex 8 HexNAc 7

**Mass:**  4608.9348

(1.556e-06)

**Charge States:** 4

**Start Position**: 67

**End Position**: 82

| Profile Score | Tandem Score | TDA q-value | Volume | Spectrum Matches |
| --- | --- | --- | --- | --- |
| 19.4247 | 86.1148 | 0.0000 | 2.8137e+07 | 2 |

**Adduct Signal Fractions**

- **Unmodified**: 2.8137e+07

### FTPN(N)KTEDTIFLR Fuc 1 Hex 7 HexNAc 6

**Mass:**  4079.7275

(1.047e-06)

**Charge States:** 3, 4

**Start Position**: 69

**End Position**: 82

| Profile Score | Tandem Score | TDA q-value | Volume | Spectrum Matches |
| --- | --- | --- | --- | --- |
| -4.0476 | 85.1653 | 0.0000 | 3.1681e+07 | 3 |

**Adduct Signal Fractions**

- **Unmodified**: 3.1681e+07

### FTPN(N)KTEDTIFLR Fuc 1 Hex 7 HexNAc 6

**Mass:**  4080.7221

(2.447e-04)

**Start Position**: 69

**End Position**: 82

| Profile Score | Tandem Score | TDA q-value | Volume | Spectrum Matches |
| --- | --- | --- | --- | --- |
| 0.0000 | 83.2774 | 0.0000 | 0.0000e+00 | 1 |

Adduct:

No Chromatogram Found

### FTPN(N)KTEDTIFLR Fuc 1 Hex 6 HexNAc 5

**Mass:**  3714.5966

(1.515e-06)

**Charge States:** 3, 4

**Start Position**: 69

**End Position**: 82

| Profile Score | Tandem Score | TDA q-value | Volume | Spectrum Matches |
| --- | --- | --- | --- | --- |
| -3.7361 | 83.2181 | 0.0000 | 1.8606e+07 | 3 |

**Adduct Signal Fractions**

- **Unmodified**: 1.8606e+07

### TPN(N)KTEDTIFLR Hex 5 HexNAc 4

**Mass:**  3056.3388

(2.207e-06)

**Charge States:** 3

**Start Position**: 70

**End Position**: 82

| Profile Score | Tandem Score | TDA q-value | Volume | Spectrum Matches |
| --- | --- | --- | --- | --- |
| 11.7029 | 81.9076 | 0.0000 | 5.2848e+06 | 3 |

**Adduct Signal Fractions**

- **Unmodified**: 5.2848e+06

### FTPN(N)KTEDTIFLR Hex 8 HexNAc 7

**Mass:**  4298.7995

(1.715e-06)

**Charge States:** 3, 4

**Start Position**: 69

**End Position**: 82

| Profile Score | Tandem Score | TDA q-value | Volume | Spectrum Matches |
| --- | --- | --- | --- | --- |
| -5.3360 | 81.2359 | 0.0000 | 1.2572e+07 | 7 |

**Adduct Signal Fractions**

- **Unmodified**: 1.2572e+07

### SVQEIQATFFYFTPN(N)KTEDTIFLR Fuc 2 Hex 7 HexNAc 6

**Mass:**  5539.4221

(2.052e-06)

**Charge States:** 4, 5

**Start Position**: 58

**End Position**: 82

| Profile Score | Tandem Score | TDA q-value | Volume | Spectrum Matches |
| --- | --- | --- | --- | --- |
| -19.8386 | 75.5299 | 0.0026 | 3.1151e+07 | 2 |

**Adduct Signal Fractions**

- **Unmodified**: 3.1151e+07

### SVQEIQATFFYFTPN(N)K Hex 10 HexNAc 9

**Mass:**  5367.2014

(1.795e-06)

**Charge States:** 4

**Start Position**: 58

**End Position**: 74

| Profile Score | Tandem Score | TDA q-value | Volume | Spectrum Matches |
| --- | --- | --- | --- | --- |
| -4.9170 | 74.2359 | 0.0026 | 1.1156e+07 | 1 |

**Adduct Signal Fractions**

- **Unmodified**: 1.1156e+07

### FYFTPN(N)KTEDTIFLR Hex 7 HexNAc 4 Neu5Ac 2

**Mass:**  4419.8342

(1.058e-06)

**Charge States:** 4

**Start Position**: 67

**End Position**: 82

| Profile Score | Tandem Score | TDA q-value | Volume | Spectrum Matches |
| --- | --- | --- | --- | --- |
| -4.7372 | 73.9759 | 0.0026 | 7.6413e+06 | 4 |

**Adduct Signal Fractions**

- **Unmodified**: 7.6413e+06

### SVQEIQATFFYFTPN(N)K Hex 9 HexNAc 7

**Mass:**  4802.0249

(6.350e-04)

**Start Position**: 58

**End Position**: 74

| Profile Score | Tandem Score | TDA q-value | Volume | Spectrum Matches |
| --- | --- | --- | --- | --- |
| 0.0000 | 69.3034 | 0.0026 | 0.0000e+00 | 1 |

Adduct:

No Chromatogram Found

### YFTPN(N)KTEDTIFLR Fuc 4 Hex 5 HexNAc 5

**Mass:**  4153.7546

(-5.154e-06)

**Charge States:** 3, 4

**Start Position**: 68

**End Position**: 82

| Profile Score | Tandem Score | TDA q-value | Volume | Spectrum Matches |
| --- | --- | --- | --- | --- |
| 13.6947 | 67.5354 | 0.0026 | 4.8101e+08 | 6 |

**Adduct Signal Fractions**

- **Unmodified**: 4.8101e+08

### SVQEIQATFFYFTPN(N)KTEDTIFLR Hex 9 HexNAc 8

**Mass:**  5977.5674

(1.284e-06)

**Charge States:** 4, 5

**Start Position**: 58

**End Position**: 82

| Profile Score | Tandem Score | TDA q-value | Volume | Spectrum Matches |
| --- | --- | --- | --- | --- |
| 13.1785 | 66.6207 | 0.0026 | 8.2052e+07 | 6 |

**Adduct Signal Fractions**

- **Unmodified**: 8.2052e+07

### C(C)ANLVPVPITN(N)ATLDQITGK Hex 6 HexNAc 5

**Mass:**  4111.8464

(1.997e-06)

**Charge States:** 3, 4

**Start Position**: 23

**End Position**: 43

| Profile Score | Tandem Score | TDA q-value | Volume | Spectrum Matches |
| --- | --- | --- | --- | --- |
| -2.7398 | 65.1382 | 0.0026 | 2.3980e+08 | 12 |

**Adduct Signal Fractions**

- **Unmodified**: 2.3980e+08

### C(C)ANLVPVPITN(N)ATLDQITGK Fuc 1 Hex 6 HexNAc 5

**Mass:**  4257.9050

(1.958e-06)

**Charge States:** 3, 4

**Start Position**: 23

**End Position**: 43

| Profile Score | Tandem Score | TDA q-value | Volume | Spectrum Matches |
| --- | --- | --- | --- | --- |
| 13.8469 | 64.1030 | 0.0026 | 3.2766e+07 | 3 |

**Adduct Signal Fractions**

- **Unmodified**: 3.2766e+07

### LVPVPITN(N)ATLDQITGK Fuc 2 Hex 5 HexNAc 3 Neu5Ac 1

**Mass:**  3783.7553

(5.364e-04)

**Charge States:** 3, 4

**Start Position**: 26

**End Position**: 43

| Profile Score | Tandem Score | TDA q-value | Volume | Spectrum Matches |
| --- | --- | --- | --- | --- |
| 10.8440 | 62.1622 | 0.0026 | 2.5550e+09 | 99 |

**Adduct Signal Fractions**

- **Unmodified**: 2.5550e+09

### SVQEIQATFFYFTPN(N)KTEDTIFLR Fuc 1 Hex 8 HexNAc 7

**Mass:**  5758.4909

(2.355e-06)

**Charge States:** 4, 5

**Start Position**: 58

**End Position**: 82

| Profile Score | Tandem Score | TDA q-value | Volume | Spectrum Matches |
| --- | --- | --- | --- | --- |
| 13.9799 | 60.2073 | 0.0026 | 4.8796e+07 | 4 |

**Adduct Signal Fractions**

- **Unmodified**: 4.8796e+07

### QDQC(C)IYN(N)TTYLNVQR Fuc 1 Hex 9 HexNAc 8

**Mass:**  5144.0676

(1.896e-06)

**Charge States:** 4

**Start Position**: 87

**End Position**: 102

| Profile Score | Tandem Score | TDA q-value | Volume | Spectrum Matches |
| --- | --- | --- | --- | --- |
| 11.4820 | 56.6908 | 0.0026 | 3.8394e+07 | 3 |

**Adduct Signal Fractions**

- **Unmodified**: 3.8394e+07

### SVQEIQATFFYFTPN(N)K Fuc 1 Hex 9 HexNAc 8

**Mass:**  5148.1262

(1.585e-06)

**Charge States:** 4

**Start Position**: 58

**End Position**: 74

| Profile Score | Tandem Score | TDA q-value | Volume | Spectrum Matches |
| --- | --- | --- | --- | --- |
| 7.3730 | 56.2667 | 0.0026 | 1.1962e+07 | 2 |

**Adduct Signal Fractions**

- **Unmodified**: 1.1962e+07

### LVPVPITN(N)ATLDQITGK Hex 7 HexNAc 5

**Mass:**  3930.8044

(5.200e-04)

**Charge States:** 3

**Start Position**: 26

**End Position**: 43

| Profile Score | Tandem Score | TDA q-value | Volume | Spectrum Matches |
| --- | --- | --- | --- | --- |
| -6.6148 | 54.3994 | 0.0026 | 1.7461e+07 | 6 |

**Adduct Signal Fractions**

- **Unmodified**: 1.7461e+07

### PVPITN(N)ATLDQITGK Fuc 2 Hex 6 HexNAc 5

**Mass:**  3846.6921

(1.931e-07)

**Charge States:** 3, 4

**Start Position**: 28

**End Position**: 43

| Profile Score | Tandem Score | TDA q-value | Volume | Spectrum Matches |
| --- | --- | --- | --- | --- |
| -5.0774 | 51.6661 | 0.0026 | 7.2483e+06 | 2 |

**Adduct Signal Fractions**

- **Unmodified**: 7.2483e+06

### FYFTPN(N)KTEDTIFLR Fuc 1 Hex 7 HexNAc 6

**Mass:**  4389.8605

(9.806e-07)

**Charge States:** 3, 4

**Start Position**: 67

**End Position**: 82

| Profile Score | Tandem Score | TDA q-value | Volume | Spectrum Matches |
| --- | --- | --- | --- | --- |
| 12.6751 | 51.4086 | 0.0026 | 4.5801e+07 | 4 |

**Adduct Signal Fractions**

- **Unmodified**: 4.5801e+07

### PVPITN(N)ATLDQITGK Fuc 1 Hex 7 HexNAc 3

**Mass:**  3456.5104

(-4.959e-06)

**Start Position**: 28

**End Position**: 43

| Profile Score | Tandem Score | TDA q-value | Volume | Spectrum Matches |
| --- | --- | --- | --- | --- |
| 0.0000 | 48.5614 | 0.0026 | 0.0000e+00 | 1 |

Adduct:

No Chromatogram Found

### SVQEIQATFFYFTPN(N)KTEDTIFLR Hex 7 HexNAc 4 Neu5Ac 2

**Mass:**  5423.3352

(1.886e-06)

**Charge States:** 4

**Start Position**: 58

**End Position**: 82

| Profile Score | Tandem Score | TDA q-value | Volume | Spectrum Matches |
| --- | --- | --- | --- | --- |
| -3.3321 | 48.1059 | 0.0026 | 3.3537e+07 | 3 |

**Adduct Signal Fractions**

- **Unmodified**: 3.3537e+07

### QDQC(C)IYN(N)TTYLNVQR Fuc 2 Hex 6 HexNAc 5

**Mass:**  4194.7346

(3.721e-06)

**Charge States:** 3, 4

**Start Position**: 87

**End Position**: 102

| Profile Score | Tandem Score | TDA q-value | Volume | Spectrum Matches |
| --- | --- | --- | --- | --- |
| -5.3128 | 47.7208 | 0.0026 | 9.8251e+06 | 2 |

**Adduct Signal Fractions**

- **Unmodified**: 9.8251e+06

### QDQC(C)IYN(N)TTYLNVQR Hex 6 HexNAc 6

**Mass:**  4105.6906

(9.161e-07)

**Charge States:** 3, 4

**Start Position**: 87

**End Position**: 102

| Profile Score | Tandem Score | TDA q-value | Volume | Spectrum Matches |
| --- | --- | --- | --- | --- |
| -3.8689 | 47.1400 | 0.0026 | 1.2486e+08 | 4 |

**Adduct Signal Fractions**

- **Unmodified**: 1.2486e+08

### PVPITN(N)ATLDQITGK Fuc 1 Hex 7 HexNAc 4 Neu5Ac 1

**Mass:**  3950.7375

(8.911e-06)

**Charge States:** 4

**Start Position**: 28

**End Position**: 43

| Profile Score | Tandem Score | TDA q-value | Volume | Spectrum Matches |
| --- | --- | --- | --- | --- |
| -19.6197 | 46.2383 | 0.0026 | 3.1749e+07 | 1 |

**Adduct Signal Fractions**

- **Unmodified**: 3.1749e+07

### LVPVPITN(N)ATLDQITGK Fuc 4 Hex 5 HexNAc 5

**Mass:**  4188.8918

(-3.592e-06)

**Charge States:** 3, 4

**Start Position**: 26

**End Position**: 43

| Profile Score | Tandem Score | TDA q-value | Volume | Spectrum Matches |
| --- | --- | --- | --- | --- |
| 8.8218 | 45.5797 | 0.0026 | 3.2936e+07 | 3 |

**Adduct Signal Fractions**

- **Unmodified**: 3.2936e+07

### PVPITN(N)ATLDQITGK Fuc 3 Hex 7 HexNAc 4

**Mass:**  3951.7390

(4.124e-06)

**Charge States:** 4

**Start Position**: 28

**End Position**: 43

| Profile Score | Tandem Score | TDA q-value | Volume | Spectrum Matches |
| --- | --- | --- | --- | --- |
| -19.7834 | 45.2127 | 0.0026 | 4.0336e+07 | 2 |

**Adduct Signal Fractions**

- **Unmodified**: 4.0336e+07

### SVQEIQATFFYFTPN(N)K Hex 7 HexNAc 6 Neu5Ac 1

**Mass:**  4562.8952

(1.640e-06)

**Charge States:** 4

**Start Position**: 58

**End Position**: 74

| Profile Score | Tandem Score | TDA q-value | Volume | Spectrum Matches |
| --- | --- | --- | --- | --- |
| -5.5090 | 45.1411 | 0.0026 | 3.6096e+06 | 2 |

**Adduct Signal Fractions**

- **Unmodified**: 3.6096e+06

### QDQC(C)IYN(N)TTYLNVQR Fuc 2 Hex 8 HexNAc 7

**Mass:**  4924.9919

(2.717e-06)

**Charge States:** 4

**Start Position**: 87

**End Position**: 102

| Profile Score | Tandem Score | TDA q-value | Volume | Spectrum Matches |
| --- | --- | --- | --- | --- |
| -4.1171 | 45.1311 | 0.0026 | 3.6837e+07 | 2 |

**Adduct Signal Fractions**

- **Unmodified**: 3.6837e+07

### QDQC(C)IYN(N)TTYLNVQR Fuc 2 Hex 8 HexNAc 4 Neu5Ac 1

**Mass:**  4608.8649

(4.394e-04)

**Start Position**: 87

**End Position**: 102

| Profile Score | Tandem Score | TDA q-value | Volume | Spectrum Matches |
| --- | --- | --- | --- | --- |
| 0.0000 | 43.9189 | 0.0026 | 0.0000e+00 | 1 |

Adduct:

No Chromatogram Found

### QDQC(C)IYN(N)TTYLNVQR Hex 10 HexNAc 9

**Mass:**  5363.1398

(1.162e-06)

**Charge States:** 4

**Start Position**: 87

**End Position**: 102

| Profile Score | Tandem Score | TDA q-value | Volume | Spectrum Matches |
| --- | --- | --- | --- | --- |
| -3.8363 | 43.2598 | 0.0026 | 3.5428e+07 | 1 |

**Adduct Signal Fractions**

- **Unmodified**: 3.5428e+07

### TPN(N)KTEDTIFLR Fuc 1 Hex 6 HexNAc 5

**Mass:**  3567.5256

(-2.205e-07)

**Charge States:** 3, 4

**Start Position**: 70

**End Position**: 82

| Profile Score | Tandem Score | TDA q-value | Volume | Spectrum Matches |
| --- | --- | --- | --- | --- |
| -5.4179 | 42.1310 | 0.0026 | 7.7850e+06 | 4 |

**Adduct Signal Fractions**

- **Unmodified**: 7.7850e+06

### LVPVPITN(N)ATLDQITGK Hex 7 HexNAc 5

**Mass:**  3931.8241

(7.746e-04)

**Start Position**: 26

**End Position**: 43

| Profile Score | Tandem Score | TDA q-value | Volume | Spectrum Matches |
| --- | --- | --- | --- | --- |
| 0.0000 | 42.0178 | 0.0026 | 0.0000e+00 | 1 |

Adduct:

No Chromatogram Found

### QDQC(C)IYN(N)TTYLNVQR Hex 7 HexNAc 7

**Mass:**  4470.8249

(2.255e-06)

**Charge States:** 4

**Start Position**: 87

**End Position**: 102

| Profile Score | Tandem Score | TDA q-value | Volume | Spectrum Matches |
| --- | --- | --- | --- | --- |
| -3.5553 | 41.8332 | 0.0026 | 1.2427e+08 | 3 |

**Adduct Signal Fractions**

- **Unmodified**: 1.2427e+08

### LVPVPITN(N)ATLDQITGK Hex 9 HexNAc 8

**Mass:**  4862.1320

(1.780e-06)

**Charge States:** 4

**Start Position**: 26

**End Position**: 43

| Profile Score | Tandem Score | TDA q-value | Volume | Spectrum Matches |
| --- | --- | --- | --- | --- |
| 8.9875 | 40.4839 | 0.0026 | 9.7528e+06 | 2 |

**Adduct Signal Fractions**

- **Unmodified**: 9.7528e+06

### FYFTPN(N)KTEDTIFLR Fuc 2 Hex 7 HexNAc 6

**Mass:**  4535.9191

(1.314e-06)

**Charge States:** 4

**Start Position**: 67

**End Position**: 82

| Profile Score | Tandem Score | TDA q-value | Volume | Spectrum Matches |
| --- | --- | --- | --- | --- |
| -20.9870 | 40.3722 | 0.0026 | 2.0786e+06 | 1 |

**Adduct Signal Fractions**

- **Unmodified**: 2.0786e+06

### LVPVPITN(N)ATLDQITGK Fuc 3 Hex 4 HexNAc 5

**Mass:**  3880.7885

(-4.212e-06)

**Charge States:** 3, 4

**Start Position**: 26

**End Position**: 43

| Profile Score | Tandem Score | TDA q-value | Volume | Spectrum Matches |
| --- | --- | --- | --- | --- |
| -18.2923 | 39.9318 | 0.0038 | 1.0315e+08 | 5 |

**Adduct Signal Fractions**

- **Unmodified**: 1.0315e+08

### SVQEIQATFFYFTPN(N)KTEDTIFLR Fuc 4 Hex 5 HexNAc 5

**Mass:**  5304.3249

(-3.138e-06)

**Charge States:** 4, 5

**Start Position**: 58

**End Position**: 82

| Profile Score | Tandem Score | TDA q-value | Volume | Spectrum Matches |
| --- | --- | --- | --- | --- |
| 13.6832 | 39.8478 | 0.0049 | 2.7053e+08 | 12 |

**Adduct Signal Fractions**

- **Unmodified**: 2.7053e+08

### SVQEIQATFFYFTPN(N)K Fuc 3 Hex 6 HexNAc 5

**Mass:**  4344.8581

(5.568e-06)

**Charge States:** 3

**Start Position**: 58

**End Position**: 74

| Profile Score | Tandem Score | TDA q-value | Volume | Spectrum Matches |
| --- | --- | --- | --- | --- |
| -19.4294 | 39.6451 | 0.0049 | 8.7621e+06 | 1 |

**Adduct Signal Fractions**

- **Unmodified**: 8.7621e+06

### LVPVPITN(N)ATLDQITGK Fuc 2 Hex 6 HexNAc 4

**Mass:**  3855.7893

(6.444e-06)

**Start Position**: 26

**End Position**: 43

| Profile Score | Tandem Score | TDA q-value | Volume | Spectrum Matches |
| --- | --- | --- | --- | --- |
| 0.0000 | 38.9322 | 0.0049 | 0.0000e+00 | 1 |

Adduct:

No Chromatogram Found

### FYFTPN(N)KTEDTIFLR Fuc 1 Hex 4 HexNAc 4 Neu5Ac 1

**Mass:**  3788.6230

(-3.217e-06)

**Charge States:** 4

**Start Position**: 67

**End Position**: 82

| Profile Score | Tandem Score | TDA q-value | Volume | Spectrum Matches |
| --- | --- | --- | --- | --- |
| 14.5926 | 37.7448 | 0.0060 | 2.2921e+08 | 4 |

**Adduct Signal Fractions**

- **Unmodified**: 2.2921e+08

### YFTPN(N)KTEDTIFLR Hex 5 HexNAc 4

**Mass:**  3367.4815

(3.020e-04)

**Start Position**: 68

**End Position**: 82

| Profile Score | Tandem Score | TDA q-value | Volume | Spectrum Matches |
| --- | --- | --- | --- | --- |
| 0.0000 | 37.3655 | 0.0060 | 0.0000e+00 | 1 |

Adduct:

No Chromatogram Found

### LVPVPITN(N)ATLDQITGK Fuc 2 Hex 4 HexNAc 9

**Mass:**  4547.0803

(5.847e-06)

**Charge States:** 3, 4

**Start Position**: 26

**End Position**: 43

| Profile Score | Tandem Score | TDA q-value | Volume | Spectrum Matches |
| --- | --- | --- | --- | --- |
| 13.2428 | 37.0903 | 0.0060 | 1.0870e+08 | 12 |

**Adduct Signal Fractions**

- **Unmodified**: 1.0870e+08

### PVPITN(N)ATLDQITGK Fuc 3 Hex 4 HexNAc 8

**Mass:**  4279.9221

(4.770e-04)

**Start Position**: 28

**End Position**: 43

| Profile Score | Tandem Score | TDA q-value | Volume | Spectrum Matches |
| --- | --- | --- | --- | --- |
| 0.0000 | 36.9622 | 0.0069 | 0.0000e+00 | 1 |

Adduct:

No Chromatogram Found

### LVPVPITN(N)ATLDQITGK Hex 6 HexNAc 6

**Mass:**  3971.8301

(5.075e-04)

**Start Position**: 26

**End Position**: 43

| Profile Score | Tandem Score | TDA q-value | Volume | Spectrum Matches |
| --- | --- | --- | --- | --- |
| 0.0000 | 36.6036 | 0.0069 | 0.0000e+00 | 2 |

Adduct:

No Chromatogram Found

### QDQC(C)IYN(N)TTYLN(D)VQR Hex 7 HexNAc 6

**Mass:**  4268.7331

(2.334e-04)

**Charge States:** 3, 4

**Start Position**: 87

**End Position**: 102

| Profile Score | Tandem Score | TDA q-value | Volume | Spectrum Matches |
| --- | --- | --- | --- | --- |
| -4.0697 | 35.9139 | 0.0069 | 2.3280e+08 | 29 |

**Adduct Signal Fractions**

- **Unmodified**: 2.3280e+08

### QDQC(C)IYN(N)TTYLNVQR Fuc 1 Hex 8 HexNAc 4 Neu5Ac 1

**Mass:**  4460.7990

(5.104e-06)

**Charge States:** 3, 4

**Start Position**: 87

**End Position**: 102

| Profile Score | Tandem Score | TDA q-value | Volume | Spectrum Matches |
| --- | --- | --- | --- | --- |
| -5.3543 | 35.7348 | 0.0069 | 2.6602e+07 | 1 |

**Adduct Signal Fractions**

- **Unmodified**: 2.6602e+07

### ATFFYFTPN(N)K Hex 6 HexNAc 9 Neu5Ac 1

**Mass:**  4325.7092

(-3.499e-06)

**Charge States:** 4, 5

**Start Position**: 64

**End Position**: 74

| Profile Score | Tandem Score | TDA q-value | Volume | Spectrum Matches |
| --- | --- | --- | --- | --- |
| -4.3562 | 35.7205 | 0.0069 | 1.3279e+08 | 3 |

**Adduct Signal Fractions**

- **Unmodified**: 1.3279e+08

### QDQC(C)IYN(N)TTYLNVQR Fuc 3 Hex 7 HexNAc 4 Neu5Ac 1

**Mass:**  4590.8327

(-2.875e-06)

**Charge States:** 4

**Start Position**: 87

**End Position**: 102

| Profile Score | Tandem Score | TDA q-value | Volume | Spectrum Matches |
| --- | --- | --- | --- | --- |
| -19.6971 | 35.3697 | 0.0069 | 6.3804e+06 | 1 |

**Adduct Signal Fractions**

- **Unmodified**: 6.3804e+06

### FTPN(N)KTEDTIFLR Fuc 2 Hex 7 HexNAc 6

**Mass:**  4227.8019

(4.764e-04)

**Charge States:** 4

**Start Position**: 69

**End Position**: 82

| Profile Score | Tandem Score | TDA q-value | Volume | Spectrum Matches |
| --- | --- | --- | --- | --- |
| -22.6701 | 35.0108 | 0.0100 | 4.6510e+06 | 2 |

**Adduct Signal Fractions**

- **Unmodified**: 4.6510e+06

### QDQC(C)IYN(N)TTYLNVQR Hex 8 HexNAc 6

**Mass:**  4432.8341

(6.876e-04)

**Start Position**: 87

**End Position**: 102

| Profile Score | Tandem Score | TDA q-value | Volume | Spectrum Matches |
| --- | --- | --- | --- | --- |
| 0.0000 | 34.8817 | 0.0100 | 0.0000e+00 | 1 |

Adduct:

No Chromatogram Found

### QDQC(C)IYN(N)TTYLN Hex 7 HexNAc 6

**Mass:**  3884.5151

(9.429e-07)

**Charge States:** 3, 4

**Start Position**: 87

**End Position**: 99

| Profile Score | Tandem Score | TDA q-value | Volume | Spectrum Matches |
| --- | --- | --- | --- | --- |
| -19.0622 | 34.8357 | 0.0100 | 1.4842e+07 | 2 |

**Adduct Signal Fractions**

- **Unmodified**: 1.4842e+07

### QDQC(C)IYN(N)TTYLNVQR Hex 10 HexNAc 3 Neu5Ac 1

**Mass:**  4435.7834

(7.253e-06)

**Charge States:** 3, 4

**Start Position**: 87

**End Position**: 102

| Profile Score | Tandem Score | TDA q-value | Volume | Spectrum Matches |
| --- | --- | --- | --- | --- |
| -4.8060 | 33.9442 | 0.0100 | 9.4608e+07 | 1 |

**Adduct Signal Fractions**

- **Unmodified**: 9.4608e+07

### QDQC(C)IYN(N)TTYLN(D)VQR Hex 6 HexNAc 5

**Mass:**  3903.6054

(2.548e-04)

**Charge States:** 3, 4

**Start Position**: 87

**End Position**: 102

| Profile Score | Tandem Score | TDA q-value | Volume | Spectrum Matches |
| --- | --- | --- | --- | --- |
| -7.6976 | 33.7183 | 0.0108 | 8.1784e+07 | 5 |

**Adduct Signal Fractions**

- **Unmodified**: 8.1784e+07

### SVQEIQATFFYFTPN(N)K Fuc 3 Hex 5 HexNAc 4 Neu5Ac 2

**Mass:**  4564.9021

(6.597e-04)

**Start Position**: 58

**End Position**: 74

| Profile Score | Tandem Score | TDA q-value | Volume | Spectrum Matches |
| --- | --- | --- | --- | --- |
| 0.0000 | 33.6481 | 0.0108 | 0.0000e+00 | 1 |

Adduct:

No Chromatogram Found

### PVPITN(N)ATLDQITGK Fuc 2 Hex 7 HexNAc 4

**Mass:**  3805.6898

(6.536e-06)

**Charge States:** 3

**Start Position**: 28

**End Position**: 43

| Profile Score | Tandem Score | TDA q-value | Volume | Spectrum Matches |
| --- | --- | --- | --- | --- |
| -19.8103 | 33.4388 | 0.0108 | 1.0203e+07 | 1 |

**Adduct Signal Fractions**

- **Unmodified**: 1.0203e+07

### C(C)ANLVPVPITN(N)ATLDQITGK Hex 5 HexNAc 4

**Mass:**  3746.7152

(2.342e-06)

**Charge States:** 3, 4

**Start Position**: 23

**End Position**: 43

| Profile Score | Tandem Score | TDA q-value | Volume | Spectrum Matches |
| --- | --- | --- | --- | --- |
| -18.6108 | 32.7986 | 0.0137 | 7.6098e+06 | 2 |

**Adduct Signal Fractions**

- **Unmodified**: 7.6098e+06

### SVQEIQATFFYFTPN(N)KTEDTIFLR Fuc 1 Hex 8 HexNAc 4 Neu5Ac 1

**Mass:**  5440.3586

(4.099e-06)

**Charge States:** 4

**Start Position**: 58

**End Position**: 82

| Profile Score | Tandem Score | TDA q-value | Volume | Spectrum Matches |
| --- | --- | --- | --- | --- |
| -4.5598 | 32.3491 | 0.0155 | 5.9131e+06 | 1 |

**Adduct Signal Fractions**

- **Unmodified**: 5.9131e+06

### IYN(N)TTYLNVQR Fuc 1 Hex 7 HexNAc 6

**Mass:**  3882.6242

(1.480e-06)

**Charge States:** 3, 4

**Start Position**: 91

**End Position**: 102

| Profile Score | Tandem Score | TDA q-value | Volume | Spectrum Matches |
| --- | --- | --- | --- | --- |
| 11.9051 | 32.3439 | 0.0155 | 1.7104e+07 | 3 |

**Adduct Signal Fractions**

- **Unmodified**: 1.7104e+07

### FYFTPN(N)KTEDTIFLR Fuc 2 Hex 4 HexNAc 4 Neu5Ac 1

**Mass:**  3934.6823

(-2.348e-06)

**Charge States:** 4

**Start Position**: 67

**End Position**: 82

| Profile Score | Tandem Score | TDA q-value | Volume | Spectrum Matches |
| --- | --- | --- | --- | --- |
| -18.6091 | 32.3406 | 0.0155 | 4.1679e+07 | 4 |

**Adduct Signal Fractions**

- **Unmodified**: 4.1679e+07

### IYN(N)TTYLNVQR Hex 6 HexNAc 5

**Mass:**  3371.4278

(7.285e-07)

**Charge States:** 3, 4

**Start Position**: 91

**End Position**: 102

| Profile Score | Tandem Score | TDA q-value | Volume | Spectrum Matches |
| --- | --- | --- | --- | --- |
| 10.8270 | 32.2679 | 0.0155 | 2.6185e+07 | 3 |

**Adduct Signal Fractions**

- **Unmodified**: 2.6185e+07

### WFYIASAFRNEEYNKSVQEIQATFFYFTPN(N)K Fuc 5 Hex 10 HexNAc 8

**Mass:**  7813.3346

(4.511e-06)

**Charge States:** 5

**Start Position**: 43

**End Position**: 74

| Profile Score | Tandem Score | TDA q-value | Volume | Spectrum Matches |
| --- | --- | --- | --- | --- |
| -4.1854 | 32.1221 | 0.0165 | 3.8610e+07 | 1 |

**Adduct Signal Fractions**

- **Unmodified**: 3.8610e+07

### QDQC(C)IYN(N)TTYLN Hex 6 HexNAc 5

**Mass:**  3519.3788

(1.069e-06)

**Charge States:** 3

**Start Position**: 87

**End Position**: 99

| Profile Score | Tandem Score | TDA q-value | Volume | Spectrum Matches |
| --- | --- | --- | --- | --- |
| -18.4883 | 31.9555 | 0.0174 | 4.5805e+06 | 1 |

**Adduct Signal Fractions**

- **Unmodified**: 4.5805e+06

### PVPITN(N)ATLDQITGK Hex 9 HexNAc 6

**Mass:**  4245.8625

(4.829e-04)

**Start Position**: 28

**End Position**: 43

| Profile Score | Tandem Score | TDA q-value | Volume | Spectrum Matches |
| --- | --- | --- | --- | --- |
| 0.0000 | 31.2158 | 0.0180 | 0.0000e+00 | 1 |

Adduct:

No Chromatogram Found

### PVPITN(N)ATLDQITGK Fuc 2 Hex 5 HexNAc 4

**Mass:**  3481.5602

(2.496e-06)

**Charge States:** 3

**Start Position**: 28

**End Position**: 43

| Profile Score | Tandem Score | TDA q-value | Volume | Spectrum Matches |
| --- | --- | --- | --- | --- |
| -19.1489 | 30.8731 | 0.0180 | 2.6963e+06 | 1 |

**Adduct Signal Fractions**

- **Unmodified**: 2.6963e+06

### LVPVPITN(N)ATLDQITGK Fuc 3 Hex 4 HexNAc 9

**Mass:**  4693.1428

(5.259e-06)

**Charge States:** 3, 4

**Start Position**: 26

**End Position**: 43

| Profile Score | Tandem Score | TDA q-value | Volume | Spectrum Matches |
| --- | --- | --- | --- | --- |
| 10.5380 | 30.3007 | 0.0222 | 2.8157e+07 | 7 |

**Adduct Signal Fractions**

- **Unmodified**: 2.8157e+07

### PVPITN(N)ATLDQITGK Hex 8 HexNAc 5 Neu5Ac 1

**Mass:**  4169.7858

(2.372e-06)

**Charge States:** 3

**Start Position**: 28

**End Position**: 43

| Profile Score | Tandem Score | TDA q-value | Volume | Spectrum Matches |
| --- | --- | --- | --- | --- |
| -19.8085 | 30.0758 | 0.0222 | 8.6341e+06 | 1 |

**Adduct Signal Fractions**

- **Unmodified**: 8.6341e+06

### QDQC(C)IYN(N)TTYLNVQR Fuc 1 Hex 10 HexNAc 9

**Mass:**  5509.2023

(1.678e-06)

**Charge States:** 4

**Start Position**: 87

**End Position**: 102

| Profile Score | Tandem Score | TDA q-value | Volume | Spectrum Matches |
| --- | --- | --- | --- | --- |
| -19.4607 | 30.0137 | 0.0222 | 6.7704e+06 | 1 |

**Adduct Signal Fractions**

- **Unmodified**: 6.7704e+06

### QDQC(C)IYN(N)TTYLN(D)VQR Fuc 1 Hex 6 HexNAc 5

**Mass:**  4049.6589

(2.465e-04)

**Charge States:** 3, 4

**Start Position**: 87

**End Position**: 102

| Profile Score | Tandem Score | TDA q-value | Volume | Spectrum Matches |
| --- | --- | --- | --- | --- |
| -19.7354 | 29.7617 | 0.0222 | 2.2227e+07 | 2 |

**Adduct Signal Fractions**

- **Unmodified**: 2.2227e+07

### LVPVPITN(N)ATLDQITGK Fuc 4 Hex 3 HexNAc 7 Neu5Ac 1

**Mass:**  4562.0935

(8.452e-06)

**Charge States:** 3, 4

**Start Position**: 26

**End Position**: 43

| Profile Score | Tandem Score | TDA q-value | Volume | Spectrum Matches |
| --- | --- | --- | --- | --- |
| 12.1895 | 29.5962 | 0.0230 | 9.8473e+07 | 9 |

**Adduct Signal Fractions**

- **Unmodified**: 9.8473e+07

### C(C)ANLVPVPITN(N)ATLDQITGK Fuc 1 Hex 6 HexNAc 5

**Mass:**  4259.9197

(4.751e-04)

**Start Position**: 23

**End Position**: 43

| Profile Score | Tandem Score | TDA q-value | Volume | Spectrum Matches |
| --- | --- | --- | --- | --- |
| 0.0000 | 29.5420 | 0.0230 | 0.0000e+00 | 1 |

Adduct:

No Chromatogram Found

### QIPLC(C)ANLVPVPITN(N)ATLDQITGK Hex 5 HexNAc 5 Neu5Ac 1

**Mass:**  4692.1574

(-7.986e-07)

**Charge States:** 3, 4

**Start Position**: 19

**End Position**: 43

| Profile Score | Tandem Score | TDA q-value | Volume | Spectrum Matches |
| --- | --- | --- | --- | --- |
| 15.9486 | 29.4017 | 0.0239 | 3.5791e+08 | 12 |

**Adduct Signal Fractions**

- **Unmodified**: 3.5791e+08

### PVPITN(N)ATLDQITGK Hex 3 HexNAc 9

**Mass:**  3880.7748

(9.409e-06)

**Charge States:** 3, 4

**Start Position**: 28

**End Position**: 43

| Profile Score | Tandem Score | TDA q-value | Volume | Spectrum Matches |
| --- | --- | --- | --- | --- |
| -19.5281 | 29.1411 | 0.0247 | 7.1632e+07 | 2 |

**Adduct Signal Fractions**

- **Unmodified**: 7.1632e+07

### TPN(N)KTEDTIFLR Hex 8 HexNAc 7

**Mass:**  4151.7331

(7.937e-07)

**Charge States:** 4

**Start Position**: 70

**End Position**: 82

| Profile Score | Tandem Score | TDA q-value | Volume | Spectrum Matches |
| --- | --- | --- | --- | --- |
| -4.8740 | 29.0144 | 0.0273 | 4.7446e+06 | 1 |

**Adduct Signal Fractions**

- **Unmodified**: 4.7446e+06

### LVPVPITN(N)ATLDQITGK Fuc 1 Hex 7 HexNAc 5 Neu5Ac 2

**Mass:**  4657.0559

(5.618e-06)

**Charge States:** 4

**Start Position**: 26

**End Position**: 43

| Profile Score | Tandem Score | TDA q-value | Volume | Spectrum Matches |
| --- | --- | --- | --- | --- |
| -20.5025 | 28.6069 | 0.0298 | 6.9868e+05 | 1 |

**Adduct Signal Fractions**

- **Unmodified**: 6.9868e+05

### QIPLC(C)ANLVPVPITN(N)ATLDQITGK Hex 6 HexNAc 5

**Mass:**  4563.1198

(9.899e-07)

**Charge States:** 3, 4

**Start Position**: 19

**End Position**: 43

| Profile Score | Tandem Score | TDA q-value | Volume | Spectrum Matches |
| --- | --- | --- | --- | --- |
| 11.8607 | 28.1420 | 0.0311 | 2.8191e+08 | 10 |

**Adduct Signal Fractions**

- **Unmodified**: 2.8191e+08

### C(C)ANLVPVPITN(N)ATLDQITGK Fuc 1 Hex 9 HexNAc 4

**Mass:**  4541.0148

(8.618e-06)

**Charge States:** 3, 4

**Start Position**: 23

**End Position**: 43

| Profile Score | Tandem Score | TDA q-value | Volume | Spectrum Matches |
| --- | --- | --- | --- | --- |
| -20.5294 | 28.0871 | 0.0311 | 2.1373e+06 | 1 |

**Adduct Signal Fractions**

- **Unmodified**: 2.1373e+06

### SVQEIQATFFYFTPN(N)K Hex 7 HexNAc 6

**Mass:**  4273.8113

(4.726e-04)

**Start Position**: 58

**End Position**: 74

| Profile Score | Tandem Score | TDA q-value | Volume | Spectrum Matches |
| --- | --- | --- | --- | --- |
| 0.0000 | 27.7755 | 0.0349 | 0.0000e+00 | 1 |

Adduct:

No Chromatogram Found

### LVPVPITN(N)ATLDQITGK Fuc 1 Hex 6 HexNAc 5 Neu5Ac 3

**Mass:**  4786.0788

(1.393e-06)

**Charge States:** 4

**Start Position**: 26

**End Position**: 43

| Profile Score | Tandem Score | TDA q-value | Volume | Spectrum Matches |
| --- | --- | --- | --- | --- |
| -5.1057 | 27.5787 | 0.0352 | 2.7408e+06 | 2 |

**Adduct Signal Fractions**

- **Unmodified**: 2.7408e+06

### N(N)ATLDQITGK Hex 6 HexNAc 5

**Mass:**  3048.2845

(3.330e-04)

**Start Position**: 33

**End Position**: 43

| Profile Score | Tandem Score | TDA q-value | Volume | Spectrum Matches |
| --- | --- | --- | --- | --- |
| 0.0000 | 26.8112 | 0.0360 | 0.0000e+00 | 1 |

Adduct:

No Chromatogram Found

### PVPITN(N)ATLDQITGK Hex 7 HexNAc 4 Neu5Ac 1

**Mass:**  3804.6721

(9.466e-06)

**Charge States:** 3, 4, 5

**Start Position**: 28

**End Position**: 43

| Profile Score | Tandem Score | TDA q-value | Volume | Spectrum Matches |
| --- | --- | --- | --- | --- |
| 14.0428 | 26.7387 | 0.0360 | 1.0468e+09 | 7 |

**Adduct Signal Fractions**

- **Unmodified**: 1.0468e+09

### PVPITN(N)ATLDQITGK Hex 9 HexNAc 5

**Mass:**  4041.7549

(2.527e-04)

**Start Position**: 28

**End Position**: 43

| Profile Score | Tandem Score | TDA q-value | Volume | Spectrum Matches |
| --- | --- | --- | --- | --- |
| 0.0000 | 26.6618 | 0.0360 | 0.0000e+00 | 1 |

Adduct:

No Chromatogram Found

### QDQC(C)IYN(N)TTYLNVQR Fuc 4 Hex 5 HexNAc 5

**Mass:**  4324.7619

(-3.634e-06)

**Charge States:** 3, 4

**Start Position**: 87

**End Position**: 102

| Profile Score | Tandem Score | TDA q-value | Volume | Spectrum Matches |
| --- | --- | --- | --- | --- |
| 14.7949 | 26.6529 | 0.0360 | 6.6473e+08 | 4 |

**Adduct Signal Fractions**

- **Unmodified**: 6.6473e+08

### QIPLC(C)ANLVPVPITN(N)ATLDQITGK Fuc 1 Hex 6 HexNAc 5

**Mass:**  4709.1804

(7.564e-07)

**Charge States:** 3, 4

**Start Position**: 19

**End Position**: 43

| Profile Score | Tandem Score | TDA q-value | Volume | Spectrum Matches |
| --- | --- | --- | --- | --- |
| 13.1445 | 26.0695 | 0.0360 | 7.9447e+07 | 3 |

**Adduct Signal Fractions**

- **Unmodified**: 7.9447e+07

### YFTPN(N)KTEDTIFLR Fuc 1 Hex 7 HexNAc 4 Neu5Ac 2

**Mass:**  4418.7917

(-6.199e-06)

**Charge States:** 4

**Start Position**: 68

**End Position**: 82

| Profile Score | Tandem Score | TDA q-value | Volume | Spectrum Matches |
| --- | --- | --- | --- | --- |
| -20.2277 | 26.0476 | 0.0360 | 1.7642e+07 | 1 |

**Adduct Signal Fractions**

- **Unmodified**: 1.7642e+07

### IYN(N)TTYLNVQR Hex 7 HexNAc 6

**Mass:**  3737.5732

(2.710e-04)

**Start Position**: 91

**End Position**: 102

| Profile Score | Tandem Score | TDA q-value | Volume | Spectrum Matches |
| --- | --- | --- | --- | --- |
| 0.0000 | 26.0192 | 0.0360 | 0.0000e+00 | 1 |

Adduct:

No Chromatogram Found

### YFTPN(N)KTEDTIFLR Hex 10 HexNAc 9

**Mass:**  5192.1384

(2.416e-06)

**Charge States:** 4

**Start Position**: 68

**End Position**: 82

| Profile Score | Tandem Score | TDA q-value | Volume | Spectrum Matches |
| --- | --- | --- | --- | --- |
| -19.6486 | 25.8778 | 0.0360 | 3.3367e+06 | 1 |

**Adduct Signal Fractions**

- **Unmodified**: 3.3367e+06

### YFTPN(N)KTEDTIFLR Fuc 5 Hex 8 HexNAc 7

**Mass:**  5192.1384

(-2.428e-06)

**Charge States:** 4

**Start Position**: 68

**End Position**: 82

| Profile Score | Tandem Score | TDA q-value | Volume | Spectrum Matches |
| --- | --- | --- | --- | --- |
| -19.6486 | 25.8778 | 0.0360 | 3.3367e+06 | 1 |

**Adduct Signal Fractions**

- **Unmodified**: 3.3367e+06

### QDQC(C)IYN(N)TTYLNVQR Fuc 2 Hex 9 HexNAc 8

**Mass:**  5290.1203

(3.327e-06)

**Charge States:** 4

**Start Position**: 87

**End Position**: 102

| Profile Score | Tandem Score | TDA q-value | Volume | Spectrum Matches |
| --- | --- | --- | --- | --- |
| -4.7593 | 25.8116 | 0.0360 | 8.3793e+06 | 1 |

**Adduct Signal Fractions**

- **Unmodified**: 8.3793e+06

### QDQC(C)IYN(N)TTYLN(D)VQR Fuc 4 Hex 7 HexNAc 5

**Mass:**  4649.9020

(2.182e-04)

**Charge States:** 3, 4

**Start Position**: 87

**End Position**: 102

| Profile Score | Tandem Score | TDA q-value | Volume | Spectrum Matches |
| --- | --- | --- | --- | --- |
| -6.3814 | 25.3080 | 0.0362 | 8.1292e+07 | 2 |

**Adduct Signal Fractions**

- **Unmodified**: 8.1292e+07

### FYFTPN(N)KTEDTIFLR Fuc 2 Hex 3 HexNAc 5 Neu5Ac 2

**Mass:**  4266.7922

(-4.979e-06)

**Charge States:** 4

**Start Position**: 67

**End Position**: 82

| Profile Score | Tandem Score | TDA q-value | Volume | Spectrum Matches |
| --- | --- | --- | --- | --- |
| -19.2674 | 25.2643 | 0.0362 | 1.6044e+07 | 1 |

**Adduct Signal Fractions**

- **Unmodified**: 1.6044e+07

### N(N)ATLDQITGK Fuc 1 Hex 7 HexNAc 7

**Mass:**  3761.5662

(5.121e-06)

**Charge States:** 3, 4

**Start Position**: 33

**End Position**: 43

| Profile Score | Tandem Score | TDA q-value | Volume | Spectrum Matches |
| --- | --- | --- | --- | --- |
| 13.7636 | 24.7213 | 0.0395 | 1.2620e+08 | 4 |

**Adduct Signal Fractions**

- **Unmodified**: 1.2620e+08

### C(C)ANLVPVPITN(N)ATLDQITGK Hex 7 HexNAc 6

**Mass:**  4476.9831

(7.123e-06)

**Charge States:** 4

**Start Position**: 23

**End Position**: 43

| Profile Score | Tandem Score | TDA q-value | Volume | Spectrum Matches |
| --- | --- | --- | --- | --- |
| -5.3924 | 24.6204 | 0.0395 | 3.7604e+06 | 1 |

**Adduct Signal Fractions**

- **Unmodified**: 3.7604e+06

### SVQEIQATFFYFTPN(N)KTEDTIFLR Fuc 1 Hex 9 HexNAc 8

**Mass:**  6126.6276

(4.916e-04)

**Start Position**: 58

**End Position**: 82

| Profile Score | Tandem Score | TDA q-value | Volume | Spectrum Matches |
| --- | --- | --- | --- | --- |
| 0.0000 | 24.6108 | 0.0395 | 0.0000e+00 | 1 |

Adduct:

No Chromatogram Found

### PVPITN(N)ATLDQITGK Hex 7 HexNAc 5 Neu5Ac 1

**Mass:**  4007.7556

(8.156e-06)

**Charge States:** 4

**Start Position**: 28

**End Position**: 43

| Profile Score | Tandem Score | TDA q-value | Volume | Spectrum Matches |
| --- | --- | --- | --- | --- |
| -4.3689 | 24.4913 | 0.0395 | 1.2421e+08 | 4 |

**Adduct Signal Fractions**

- **Unmodified**: 1.2421e+08

### LVPVPITN(N)ATLDQITGK Hex 7 HexNAc 4

**Mass:**  3725.7302

(7.740e-06)

**Charge States:** 4

**Start Position**: 26

**End Position**: 43

| Profile Score | Tandem Score | TDA q-value | Volume | Spectrum Matches |
| --- | --- | --- | --- | --- |
| -19.3854 | 24.3607 | 0.0399 | 1.7096e+06 | 1 |

**Adduct Signal Fractions**

- **Unmodified**: 1.7096e+06

### LVPVPITN(N)ATLDQITGK Hex 7 HexNAc 7

**Mass:**  4334.8937

(2.763e-06)

**Charge States:** 4

**Start Position**: 26

**End Position**: 43

| Profile Score | Tandem Score | TDA q-value | Volume | Spectrum Matches |
| --- | --- | --- | --- | --- |
| -19.4824 | 23.8924 | 0.0404 | 1.0608e+07 | 1 |

**Adduct Signal Fractions**

- **Unmodified**: 1.0608e+07

### QDQC(C)IYN(N)TTYLNVQR Fuc 2 Hex 4 HexNAc 4 Neu5Ac 1

**Mass:**  3958.6611

(8.013e-06)

**Charge States:** 3, 4

**Start Position**: 87

**End Position**: 102

| Profile Score | Tandem Score | TDA q-value | Volume | Spectrum Matches |
| --- | --- | --- | --- | --- |
| -18.4001 | 23.8596 | 0.0404 | 9.9885e+06 | 2 |

**Adduct Signal Fractions**

- **Unmodified**: 9.9885e+06

### LVPVPITN(N)ATLDQITGK Fuc 1 Hex 5 HexNAc 5

**Mass:**  3750.7158

(-4.612e-06)

**Charge States:** 4

**Start Position**: 26

**End Position**: 43

| Profile Score | Tandem Score | TDA q-value | Volume | Spectrum Matches |
| --- | --- | --- | --- | --- |
| -20.6755 | 23.7384 | 0.0410 | 7.0687e+06 | 1 |

**Adduct Signal Fractions**

- **Unmodified**: 7.0687e+06

### LVPVPITN(N)ATLDQITGK Fuc 1 Hex 6 HexNAc 5 Neu5Ac 1

**Mass:**  4203.8977

(3.918e-06)

**Charge States:** 4

**Start Position**: 26

**End Position**: 43

| Profile Score | Tandem Score | TDA q-value | Volume | Spectrum Matches |
| --- | --- | --- | --- | --- |
| -20.2274 | 23.4777 | 0.0416 | 1.7117e+06 | 1 |

**Adduct Signal Fractions**

- **Unmodified**: 1.7117e+06

### C(C)ANLVPVPITN(N)ATLDQITGK Hex 6 HexNAc 6

**Mass:**  4314.9260

(1.827e-06)

**Charge States:** 3, 4

**Start Position**: 23

**End Position**: 43

| Profile Score | Tandem Score | TDA q-value | Volume | Spectrum Matches |
| --- | --- | --- | --- | --- |
| -18.7947 | 23.3946 | 0.0437 | 1.5942e+07 | 2 |

**Adduct Signal Fractions**

- **Unmodified**: 1.5942e+07

### PVPITN(N)ATLDQITGK Fuc 2 Hex 7 HexNAc 5

**Mass:**  4008.7397

(-1.095e-06)

**Charge States:** 4

**Start Position**: 28

**End Position**: 43

| Profile Score | Tandem Score | TDA q-value | Volume | Spectrum Matches |
| --- | --- | --- | --- | --- |
| -5.4725 | 23.3807 | 0.0437 | 1.8700e+06 | 1 |

**Adduct Signal Fractions**

- **Unmodified**: 1.8700e+06

### FYFTPN(N)KTEDTIFLR Fuc 4 Hex 5 HexNAc 5

**Mass:**  4300.8239

(-4.722e-06)

**Charge States:** 4

**Start Position**: 67

**End Position**: 82

| Profile Score | Tandem Score | TDA q-value | Volume | Spectrum Matches |
| --- | --- | --- | --- | --- |
| -3.1241 | 23.3099 | 0.0441 | 5.3155e+07 | 3 |

**Adduct Signal Fractions**

- **Unmodified**: 5.3155e+07

### LVPVPITN(N)ATLDQITGK Fuc 4 Hex 4 HexNAc 5

**Mass:**  4026.8430

(-2.721e-06)

**Charge States:** 4

**Start Position**: 26

**End Position**: 43

| Profile Score | Tandem Score | TDA q-value | Volume | Spectrum Matches |
| --- | --- | --- | --- | --- |
| -4.8675 | 23.2833 | 0.0441 | 3.5096e+07 | 1 |

**Adduct Signal Fractions**

- **Unmodified**: 3.5096e+07

### N(N)ATLDQITGK Fuc 1 Hex 8 HexNAc 8

**Mass:**  4128.6821

(4.872e-04)

**Start Position**: 33

**End Position**: 43

| Profile Score | Tandem Score | TDA q-value | Volume | Spectrum Matches |
| --- | --- | --- | --- | --- |
| 0.0000 | 23.2220 | 0.0455 | 0.0000e+00 | 1 |

Adduct:

No Chromatogram Found

### SVQEIQATFFYFTPN(N)K Fuc 1 Hex 10 HexNAc 3

**Mass:**  4294.7720

(2.691e-07)

**Charge States:** 5

**Start Position**: 58

**End Position**: 74

| Profile Score | Tandem Score | TDA q-value | Volume | Spectrum Matches |
| --- | --- | --- | --- | --- |
| -19.2679 | 23.1942 | 0.0456 | 3.3107e+06 | 1 |

**Adduct Signal Fractions**

- **Unmodified**: 3.3107e+06

### SVQEIQATFFYFTPN(N)KTEDTIFLR Hex 10 HexNAc 9

**Mass:**  6342.6907

(-1.840e-07)

**Charge States:** 5

**Start Position**: 58

**End Position**: 82

| Profile Score | Tandem Score | TDA q-value | Volume | Spectrum Matches |
| --- | --- | --- | --- | --- |
| -5.5563 | 23.1848 | 0.0456 | 1.0882e+07 | 2 |

**Adduct Signal Fractions**

- **Unmodified**: 1.0882e+07

### SVQEIQATFFYFTPN(N)K Fuc 4 Hex 7 HexNAc 7

**Mass:**  5062.0905

(5.904e-04)

**Start Position**: 58

**End Position**: 74

| Profile Score | Tandem Score | TDA q-value | Volume | Spectrum Matches |
| --- | --- | --- | --- | --- |
| 0.0000 | 23.0156 | 0.0456 | 0.0000e+00 | 1 |

Adduct:

No Chromatogram Found

### QDQC(C)IYN(N)TTYLN(D)VQR Hex 9 HexNAc 7

**Mass:**  4795.9631

(2.146e-04)

**Charge States:** 4

**Start Position**: 87

**End Position**: 102

| Profile Score | Tandem Score | TDA q-value | Volume | Spectrum Matches |
| --- | --- | --- | --- | --- |
| -6.7470 | 22.9781 | 0.0456 | 2.1295e+07 | 1 |

**Adduct Signal Fractions**

- **Unmodified**: 2.1295e+07

### SVQEIQATFFYFTPN(N)K Fuc 2 Hex 6 HexNAc 4 Neu5Ac 1

**Mass:**  4289.8127

(7.072e-04)

**Charge States:** 3, 4

**Start Position**: 58

**End Position**: 74

| Profile Score | Tandem Score | TDA q-value | Volume | Spectrum Matches |
| --- | --- | --- | --- | --- |
| -22.1197 | 22.9363 | 0.0456 | 4.9330e+06 | 11 |

**Adduct Signal Fractions**

- **Unmodified**: 4.9330e+06

### C(C)ANLVPVPITN(N)ATLDQITGK Fuc 1 Hex 5 HexNAc 5

**Mass:**  4095.8054

(-7.886e-06)

**Charge States:** 3, 4

**Start Position**: 23

**End Position**: 43

| Profile Score | Tandem Score | TDA q-value | Volume | Spectrum Matches |
| --- | --- | --- | --- | --- |
| -3.5261 | 22.9244 | 0.0456 | 5.1485e+07 | 4 |

**Adduct Signal Fractions**

- **Unmodified**: 5.1485e+07

### LVPVPITN(N)ATLDQITGK Fuc 2 Hex 5 HexNAc 5 Neu5Ac 1

**Mass:**  4187.8993

(3.076e-06)

**Start Position**: 26

**End Position**: 43

| Profile Score | Tandem Score | TDA q-value | Volume | Spectrum Matches |
| --- | --- | --- | --- | --- |
| 0.0000 | 22.7583 | 0.0462 | 0.0000e+00 | 1 |

Adduct:

No Chromatogram Found

### LVPVPITN(N)ATLDQITGK Fuc 3 Hex 4 HexNAc 4 Neu5Ac 1

**Mass:**  3968.8232

(3.978e-06)

**Charge States:** 3, 4

**Start Position**: 26

**End Position**: 43

| Profile Score | Tandem Score | TDA q-value | Volume | Spectrum Matches |
| --- | --- | --- | --- | --- |
| -18.1950 | 22.3792 | 0.0479 | 3.6447e+07 | 2 |

**Adduct Signal Fractions**

- **Unmodified**: 3.6447e+07

### QDQC(C)IYN(N)TTYLNVQR Hex 8 HexNAc 6

**Mass:**  4429.7946

(2.657e-06)

**Charge States:** 3, 4

**Start Position**: 87

**End Position**: 102

| Profile Score | Tandem Score | TDA q-value | Volume | Spectrum Matches |
| --- | --- | --- | --- | --- |
| 10.2118 | 22.1689 | 0.0497 | 4.4798e+07 | 4 |

**Adduct Signal Fractions**

- **Unmodified**: 4.4798e+07

### P19652|A1AG2\_HUMAN

**>P19652|A1AG2\_HUMAN**  
 `MALSWVLTVLSLLPLLEAQIPLCANLVPVPITNATLDRITGKWFYIASAFRNEEYNKSVQEIQATFFYFTPNKTEDTIFL  
REYQTRQNQCFYNSSYLNVQRENGTVSRYEGGREHVAHLLFLRDTKTLMFGSYLDDEKNWGLSFYADKPETTKEQLGEFY  
EALDCLCIPRSDVMYTDWKKDKCEPLEKQHEKERKQEEGES`

| Site | Type | Count |
| --- | --- | --- |
| 33 | N-linked | 78 |
| 56 | N-linked | 3 |
| 93 | N-linked | 28 |
| 72 | N-linked | 100 |

| Mass | Sequence | MS_2_ Score | `q`-value | MS_1_ Score | Signal | Protein Coordinates | Tandem Scans | Start Time | Apex Time | End Time |
| --- | --- | --- | --- | --- | --- | --- | --- | --- | --- | --- |
| 2390.9639 | YFTPN(N)K Hex 5 HexNAc 4 | 187.938 | 0.0 | -20.299 | 2.877e+07 | 67 - 73 | 2 | 13.61 | 13.61 | 13.78 |
| 2756.0988 | YFTPN(N)K Hex 6 HexNAc 5 | 187.465 | 0.0 | 15.094 | 2.770e+08 | 67 - 73 | 5 | 13.28 | 13.61 | 14.11 |
| 3268.2988 | FYFTPN(N)K Hex 7 HexNAc 6 | 183.308 | 0.0 | 14.523 | 1.750e+08 | 66 - 73 | 10 | 23.79 | 24.29 | 25.82 |
| 2783.0554 | NEEYN(N)K Hex 6 HexNAc 5 | 182.563 | 0.0 | 14.190 | 4.182e+08 | 51 - 57 | 15 | 3.80 | 3.80 | 9.50 |
| 2417.9215 | NEEYN(N)K Hex 5 HexNAc 4 | 181.312 | 0.0 | 12.527 | 8.418e+07 | 51 - 57 | 7 | 3.80 | 3.80 | 5.41 |
| 3121.2294 | YFTPN(N)K Hex 7 HexNAc 6 | 180.694 | 0.0 | 13.287 | 3.179e+08 | 67 - 73 | 5 | 12.95 | 13.12 | 13.94 |
| 2929.1119 | NEEYN(N)K Fuc 1 Hex 6 HexNAc 5 | 180.467 | 0.0 | 12.006 | 2.903e+07 | 51 - 57 | 3 | 3.80 | 3.80 | 4.96 |
| 3906.6667 | SVQEIQATFFYFTPN(N)K Hex 6 HexNAc 5 | 175.203 | 0.0 | 14.728 | 2.969e+09 | 57 - 73 | 49 | 50.18 | 53.93 | 113.13 |
| 4052.7252 | SVQEIQATFFYFTPN(N)K Fuc 1 Hex 6 HexNAc 5 | 174.617 | 0.0 | 14.247 | 6.065e+08 | 57 - 73 | 18 | 53.42 | 53.76 | 58.36 |
| 2902.1548 | YFTPN(N)K Fuc 1 Hex 6 HexNAc 5 | 173.938 | 0.0 | -4.622 | 4.384e+07 | 67 - 73 | 3 | 13.12 | 13.28 | 13.45 |
| 4271.8026 | SVQEIQATFFYFTPN(N)K Hex 7 HexNAc 6 | 173.876 | 0.0 | 13.287 | 3.690e+09 | 57 - 73 | 129 | 49.66 | 53.42 | 113.24 |
| 3486.3624 | YFTPN(N)K Hex 8 HexNAc 7 | 172.730 | 0.0 | -19.746 | 1.855e+07 | 67 - 73 | 1 | 12.95 | 12.95 | 13.12 |
| 3267.2908 | YFTPN(N)K Fuc 1 Hex 7 HexNAc 6 | 171.645 | 0.0 | -4.772 | 4.415e+07 | 67 - 73 | 2 | 12.95 | 12.95 | 13.28 |
| 3395.5270 | LVPVPITN(N)ATLDR Hex 6 HexNAc 5 | 170.471 | 0.0 | 13.130 | 1.924e+10 | 25 - 38 | 718 | 29.79 | 36.50 | 113.72 |
| 2903.1657 | FYFTPN(N)K Hex 6 HexNAc 5 | 169.843 | 0.0 | 14.484 | 1.431e+08 | 66 - 73 | 13 | 24.46 | 24.97 | 27.29 |
| 4417.8609 | SVQEIQATFFYFTPN(N)K Fuc 1 Hex 7 HexNAc 6 | 167.701 | 0.0 | 13.458 | 6.737e+08 | 57 - 73 | 17 | 53.08 | 53.25 | 57.33 |
| 4636.9345 | SVQEIQATFFYFTPN(N)K Hex 8 HexNAc 7 | 166.705 | 0.0 | 13.810 | 4.789e+08 | 57 - 73 | 10 | 52.40 | 52.91 | 54.10 |
| 4563.9203 | SVQEIQATFFYFTPN(N)K Fuc 2 Hex 7 HexNAc 6 | 166.687 | 0.0 | 10.977 | 9.026e+07 | 57 - 73 | 2 | 53.08 | 53.25 | 53.76 |
| 3030.3928 | LVPVPITN(N)ATLDR Hex 5 HexNAc 4 | 166.227 | 0.0 | 13.582 | 1.160e+09 | 25 - 38 | 7 | 36.80 | 37.11 | 38.59 |
| 3541.5340 | SVQEIQATFFYFTPN(N)K Hex 5 HexNAc 4 | 164.842 | 0.0 | 13.935 | 3.082e+08 | 57 - 73 | 18 | 54.43 | 54.60 | 58.02 |
| 4782.9916 | SVQEIQATFFYFTPN(N)K Fuc 1 Hex 8 HexNAc 7 | 156.863 | 0.0 | 13.146 | 9.007e+07 | 57 - 73 | 5 | 52.40 | 52.91 | 53.25 |
| 3541.5843 | LVPVPITN(N)ATLDR Fuc 1 Hex 6 HexNAc 5 | 156.719 | 0.0 | 13.397 | 5.628e+09 | 25 - 38 | 231 | 29.62 | 36.05 | 113.31 |
| 3222.3235 | ATFFYFTPN(N)K Hex 6 HexNAc 5 | 156.352 | 0.0 | -3.497 | 4.873e+07 | 63 - 73 | 2 | 40.77 | 40.94 | 41.28 |
| 3587.4538 | ATFFYFTPN(N)K Hex 7 HexNAc 6 | 156.157 | 0.0 | 13.054 | 7.305e+07 | 63 - 73 | 2 | 40.09 | 40.26 | 41.11 |
| 3740.6383 | C(C)ANLVPVPITN(N)ATLDR Hex 6 HexNAc 5 | 155.846 | 0.0 | 10.829 | 4.884e+08 | 22 - 38 | 10 | 41.62 | 41.79 | 52.91 |
| 4464.8571 | SVQEIQATFFYFTPN(N)K Fuc 1 Hex 8 HexNAc 4 Neu5Ac 1 | 153.576 | 0.0 | -4.800 | 3.414e+07 | 57 - 73 | 2 | 54.43 | 54.43 | 54.94 |
| 3760.6572 | LVPVPITN(N)ATLDR Hex 7 HexNAc 6 | 151.908 | 0.0 | 14.538 | 9.791e+08 | 25 - 38 | 6 | 35.71 | 36.05 | 36.50 |
| 3906.7163 | PVPITN(N)ATLDR Fuc 3 Hex 4 HexNAc 8 | 149.324 | 0.0 | -3.027 | 2.926e+08 | 27 - 38 | 2 | 35.88 | 36.05 | 36.20 |
| 4882.1720 | SVQEIQATFFYFTPN(N)KTEDTIFLR Hex 6 HexNAc 5 | 148.881 | 0.0 | 14.196 | 6.601e+08 | 57 - 81 | 24 | 60.07 | 62.63 | 67.43 |
| 5002.0652 | SVQEIQATFFYFTPN(N)K Hex 9 HexNAc 8 | 147.971 | 0.0 | 12.690 | 9.985e+07 | 57 - 73 | 7 | 51.89 | 52.06 | 53.08 |
| 3731.6011 | YFTPN(N)KTEDTIFLR Hex 6 HexNAc 5 | 147.234 | 0.0 | 13.361 | 1.028e+09 | 67 - 81 | 22 | 35.21 | 37.27 | 43.32 |
| 3572.5629 | LVPVPITN(N)ATLDR Fuc 2 Hex 6 HexNAc 3 Neu5Ac 1 | 147.141 | 0.0 | 0.000 | 0.000e+00 | 25 - 38 | 1 | - | - | - |
| 3231.3903 | PVPITN(N)ATLDR Fuc 2 Hex 7 HexNAc 3 | 142.786 | 0.0 | -18.347 | 3.220e+06 | 27 - 38 | 1 | 40.43 | 40.43 | 40.60 |
| 3282.4416 | VPVPITN(N)ATLDR Hex 6 HexNAc 5 | 142.096 | 0.0 | -3.188 | 1.314e+07 | 26 - 38 | 3 | 29.97 | 29.97 | 30.30 |
| 3368.3790 | ATFFYFTPN(N)K Fuc 1 Hex 6 HexNAc 5 | 142.003 | 0.0 | -20.135 | 3.762e+06 | 63 - 73 | 1 | 40.60 | 40.60 | 40.60 |
| 4447.8376 | SVQEIQATFFYFTPN(N)K Hex 7 HexNAc 4 Neu5Ac 2 | 140.087 | 0.0 | 12.329 | 1.844e+08 | 57 - 73 | 6 | 53.76 | 54.43 | 55.79 |
| 3817.6796 | PVPITN(N)ATLDR Fuc 1 Hex 4 HexNAc 9 | 138.428 | 0.0 | -4.517 | 1.037e+08 | 27 - 38 | 2 | 36.80 | 36.96 | 37.11 |
| 4242.7938 | YFTPN(N)KTEDTIFLR Fuc 1 Hex 7 HexNAc 6 | 137.525 | 0.0 | 8.644 | 2.314e+08 | 67 - 81 | 5 | 34.86 | 36.50 | 37.11 |
| 5028.2311 | SVQEIQATFFYFTPN(N)KTEDTIFLR Fuc 1 Hex 6 HexNAc 5 | 136.844 | 0.0 | 12.186 | 1.523e+08 | 57 - 81 | 8 | 62.30 | 62.46 | 64.17 |
| 3786.6011 | TPN(N)KTEDTIFLR Hex 7 HexNAc 6 | 136.622 | 0.0 | 14.378 | 7.909e+07 | 69 - 81 | 7 | 24.80 | 24.97 | 25.82 |
| 3032.4151 | LVPVPITN(N)ATLDR Hex 5 HexNAc 4 | 136.204 | 0.0 | 0.000 | 0.000e+00 | 25 - 38 | 1 | - | - | - |
| 3687.6428 | LVPVPITN(N)ATLDR Fuc 2 Hex 6 HexNAc 5 | 134.594 | 0.0 | -4.699 | 8.814e+07 | 25 - 38 | 3 | 35.88 | 36.05 | 36.20 |
| 3903.5969 | PVPITN(N)ATLDR Fuc 1 Hex 9 HexNAc 4 Neu5Ac 1 | 133.807 | 0.0 | -4.474 | 1.107e+08 | 27 - 38 | 4 | 34.86 | 34.86 | 35.55 |
| 3907.5743 | QNQC(C)FYN(N)SSYLNVQR Hex 6 HexNAc 5 | 132.930 | 0.0 | 10.242 | 7.943e+08 | 86 - 101 | 9 | 33.35 | 33.69 | 40.60 |
| 4125.7923 | LVPVPITN(N)ATLDR Hex 8 HexNAc 7 | 131.657 | 0.0 | 12.303 | 1.617e+08 | 25 - 38 | 3 | 35.38 | 35.88 | 36.05 |
| 4328.8215 | SVQEIQATFFYFTPN(N)K Fuc 4 Hex 5 HexNAc 5 | 130.567 | 0.0 | 13.972 | 7.752e+08 | 57 - 73 | 25 | 52.40 | 54.26 | 59.04 |
| 4929.0530 | SVQEIQATFFYFTPN(N)K Fuc 2 Hex 8 HexNAc 7 | 128.386 | 0.0 | 11.356 | 2.290e+07 | 57 - 73 | 2 | 52.23 | 52.74 | 53.08 |
| 3877.6588 | YFTPN(N)KTEDTIFLR Fuc 1 Hex 6 HexNAc 5 | 127.249 | 0.0 | 8.368 | 2.547e+08 | 67 - 81 | 4 | 35.21 | 36.96 | 37.27 |
| 4096.7345 | YFTPN(N)KTEDTIFLR Hex 7 HexNAc 6 | 126.700 | 0.0 | 12.520 | 1.953e+09 | 67 - 81 | 21 | 35.04 | 36.80 | 46.37 |
| 4109.7466 | SVQEIQATFFYFTPN(N)K Hex 6 HexNAc 6 | 125.792 | 0.0 | 9.705 | 7.623e+07 | 57 - 73 | 4 | 54.43 | 54.43 | 55.11 |
| 4243.8037 | FYFTPN(N)KTEDTIFLR Hex 7 HexNAc 6 | 124.809 | 0.0 | 12.199 | 2.539e+08 | 66 - 81 | 8 | 43.83 | 45.35 | 46.37 |
| 3598.6056 | NLVPVPITN(N)ATLDR Fuc 2 Hex 6 HexNAc 4 | 124.305 | 0.0 | 13.476 | 1.020e+09 | 24 - 38 | 6 | 36.96 | 37.11 | 38.42 |
| 5247.3065 | SVQEIQATFFYFTPN(N)KTEDTIFLR Hex 7 HexNAc 6 | 123.744 | 0.0 | 13.127 | 1.457e+09 | 57 - 81 | 30 | 59.38 | 62.30 | 67.94 |
| 4418.7682 | QNQC(C)FYN(N)SSYLNVQR Fuc 1 Hex 7 HexNAc 6 | 123.570 | 0.0 | 6.273 | 3.684e+08 | 86 - 101 | 3 | 32.84 | 33.19 | 39.59 |
| 4053.6388 | QNQC(C)FYN(N)SSYLNVQR Fuc 1 Hex 6 HexNAc 5 | 123.441 | 0.0 | -5.239 | 8.906e+07 | 86 - 101 | 2 | 33.19 | 33.35 | 33.52 |
| 3878.6702 | FYFTPN(N)KTEDTIFLR Hex 6 HexNAc 5 | 120.953 | 0.0 | 13.441 | 7.915e+07 | 66 - 81 | 5 | 45.52 | 45.69 | 46.54 |
| 4593.8929 | SVQEIQATFFYFTPN(N)K Fuc 1 Hex 7 HexNAc 4 Neu5Ac 2 | 120.813 | 0.0 | -5.102 | 2.192e+07 | 57 - 73 | 1 | 54.26 | 54.26 | 54.60 |
| 4198.7898 | SVQEIQATFFYFTPN(N)K Fuc 2 Hex 6 HexNAc 5 | 119.044 | 0.0 | -5.173 | 1.086e+07 | 57 - 73 | 1 | 53.42 | 53.59 | 53.76 |
| 3588.5877 | LVPVPITN(N)ATLDR Fuc 1 Hex 7 HexNAc 3 Neu5Ac 1 | 117.335 | 0.0 | -19.353 | 2.565e+07 | 25 - 38 | 1 | 37.58 | 37.58 | 37.75 |
| 4436.8925 | SVQEIQATFFYFTPN(N)K Hex 8 HexNAc 6 | 117.113 | 0.0 | 0.000 | 0.000e+00 | 57 - 73 | 1 | - | - | - |
| 3686.6264 | LVPVPITN(N)ATLDR Hex 6 HexNAc 5 Neu5Ac 1 | 116.761 | 0.0 | 11.032 | 2.903e+07 | 25 - 38 | 1 | 38.76 | 39.26 | 39.43 |
| 3366.4689 | YFTPN(N)KTEDTIFLR Hex 5 HexNAc 4 | 116.679 | 0.0 | -18.735 | 5.128e+07 | 67 - 81 | 1 | 37.58 | 37.75 | 37.75 |
| 3421.4689 | TPN(N)KTEDTIFLR Hex 6 HexNAc 5 | 116.239 | 0.0 | 13.961 | 3.927e+07 | 69 - 81 | 7 | 25.31 | 25.48 | 26.29 |
| 3906.7252 | LVPVPITN(N)ATLDR Fuc 1 Hex 7 HexNAc 6 | 115.696 | 0.0 | -18.714 | 2.224e+07 | 25 - 38 | 2 | 35.71 | 35.71 | 35.71 |
| 4461.8677 | YFTPN(N)KTEDTIFLR Hex 8 HexNAc 7 | 115.367 | 0.0 | 12.733 | 2.286e+08 | 67 - 81 | 4 | 35.71 | 35.88 | 36.34 |
| 3509.5700 | LVPVPITN(N)ATLDR Fuc 3 Hex 4 HexNAc 5 | 115.018 | 0.0 | -5.002 | 1.712e+07 | 25 - 38 | 3 | 40.60 | 40.77 | 40.94 |
| 4272.7083 | QNQC(C)FYN(N)SSYLNVQR Hex 7 HexNAc 6 | 112.602 | 0.0 | 10.176 | 1.321e+09 | 86 - 101 | 15 | 30.80 | 33.19 | 40.26 |
| 3928.6446 | ATFFYFTPN(N)K Fuc 2 Hex 3 HexNAc 8 Neu5Ac 1 | 112.388 | 0.0 | 12.499 | 2.276e+08 | 63 - 73 | 1 | 53.76 | 53.93 | 55.79 |
| 3580.6100 | ANLVPVPITN(N)ATLDR Hex 6 HexNAc 5 | 112.261 | 0.0 | -3.649 | 2.822e+07 | 23 - 38 | 3 | 41.11 | 41.28 | 41.45 |
| 3933.6712 | FTPN(N)KTEDTIFLR Hex 7 HexNAc 6 | 111.140 | 0.0 | 13.537 | 1.740e+08 | 68 - 81 | 6 | 29.62 | 31.65 | 32.33 |
| 4474.8782 | SVQEIQATFFYFTPN(N)K Hex 7 HexNAc 7 | 110.771 | 0.0 | 12.159 | 1.034e+08 | 57 - 73 | 3 | 53.76 | 53.93 | 54.77 |
| 3568.5387 | FTPN(N)KTEDTIFLR Hex 6 HexNAc 5 | 109.822 | 0.0 | 14.240 | 9.332e+07 | 68 - 81 | 5 | 31.65 | 31.99 | 32.84 |
| 3559.6029 | LVPVPITN(N)ATLDR Hex 7 HexNAc 5 | 108.319 | 0.0 | 0.000 | 0.000e+00 | 25 - 38 | 1 | - | - | - |
| 4637.8542 | QNQC(C)FYN(N)SSYLNVQR Hex 8 HexNAc 7 | 106.752 | 0.0 | -5.784 | 1.145e+08 | 86 - 101 | 1 | 32.50 | 32.67 | 33.01 |
| 4564.8364 | QNQC(C)FYN(N)SSYLNVQR Fuc 2 Hex 7 HexNAc 6 | 106.653 | 0.0 | -19.754 | 3.186e+07 | 86 - 101 | 1 | 33.01 | 33.01 | 33.19 |
| 4607.9113 | YFTPN(N)KTEDTIFLR Fuc 1 Hex 8 HexNAc 7 | 105.924 | 0.0 | -20.109 | 1.240e+07 | 67 - 81 | 1 | 35.88 | 35.88 | 35.88 |
| 3452.5500 | NLVPVPITN(N)ATLDR Fuc 1 Hex 6 HexNAc 4 | 105.365 | 0.0 | 15.486 | 3.770e+09 | 24 - 38 | 11 | 37.11 | 37.43 | 38.93 |
| 4052.7764 | LVPVPITN(N)ATLDR Fuc 2 Hex 7 HexNAc 6 | 104.327 | 0.0 | -18.660 | 3.810e+07 | 25 - 38 | 1 | 35.88 | 35.88 | 36.05 |
| 4517.0353 | SVQEIQATFFYFTPN(N)KTEDTIFLR Hex 5 HexNAc 4 | 103.953 | 0.0 | -4.286 | 1.825e+07 | 57 - 81 | 2 | 62.97 | 63.15 | 63.32 |
| 3932.6608 | TPN(N)KTEDTIFLR Fuc 1 Hex 7 HexNAc 6 | 103.560 | 0.0 | -4.610 | 1.002e+07 | 69 - 81 | 2 | 24.80 | 24.97 | 25.14 |
| 4693.9511 | SVQEIQATFFYFTPN(N)K Fuc 4 Hex 6 HexNAc 6 | 103.303 | 0.0 | 13.512 | 5.681e+07 | 57 - 73 | 3 | 53.08 | 53.59 | 53.93 |
| 3886.5704 | PVPITN(N)ATLDR Hex 8 HexNAc 4 Neu5Ac 2 | 103.170 | 0.0 | 7.215 | 3.364e+08 | 27 - 38 | 8 | 40.77 | 40.77 | 41.45 |
| 5612.4363 | SVQEIQATFFYFTPN(N)KTEDTIFLR Hex 8 HexNAc 7 | 102.956 | 0.0 | 14.470 | 2.530e+08 | 57 - 81 | 5 | 61.27 | 61.78 | 62.80 |
| 4118.7167 | YFTPN(N)KTEDTIFLR Hex 3 HexNAc 5 Neu5Ac 3 | 102.303 | 0.0 | -4.828 | 7.155e+07 | 67 - 81 | 3 | 36.65 | 36.80 | 36.96 |
| 4268.8113 | PVPITN(N)ATLDR Fuc 2 Hex 3 HexNAc 7 Neu5Ac 3 | 102.074 | 0.0 | -4.056 | 2.322e+07 | 27 - 38 | 3 | 45.35 | 45.52 | 45.86 |
| 4827.0011 | YFTPN(N)KTEDTIFLR Hex 9 HexNAc 8 | 101.948 | 0.0 | 12.443 | 6.497e+07 | 67 - 81 | 2 | 35.21 | 35.55 | 35.88 |
| 4299.8141 | YFTPN(N)KTEDTIFLR Hex 7 HexNAc 7 | 100.926 | 0.0 | -5.145 | 7.040e+07 | 67 - 81 | 2 | 36.96 | 37.11 | 37.27 |
| 3595.4956 | PVPITN(N)ATLDR Hex 8 HexNAc 4 Neu5Ac 1 | 100.589 | 0.0 | -6.390 | 1.329e+08 | 27 - 38 | 2 | 36.05 | 36.05 | 36.65 |
| 3433.4718 | PVPITN(N)ATLDR Hex 7 HexNAc 4 Neu5Ac 1 | 99.668 | 0.0 | 14.246 | 9.684e+08 | 27 - 38 | 9 | 36.20 | 36.50 | 37.27 |
| 3743.6317 | PVPITN(N)ATLDR Fuc 1 Hex 3 HexNAc 8 Neu5Ac 1 | 97.943 | 0.0 | 0.000 | 0.000e+00 | 27 - 38 | 1 | - | - | - |
| 3689.6443 | LVPVPITN(N)ATLDR Fuc 2 Hex 6 HexNAc 5 | 97.607 | 0.0 | 0.000 | 0.000e+00 | 25 - 38 | 1 | - | - | - |
| 4020.7094 | SVQEIQATFFYFTPN(N)K Fuc 3 Hex 4 HexNAc 5 | 96.544 | 0.0 | 7.330 | 2.791e+07 | 57 - 73 | 2 | 53.59 | 54.43 | 54.60 |
| 4024.7283 | FYFTPN(N)KTEDTIFLR Fuc 1 Hex 6 HexNAc 5 | 95.781 | 0.0 | -4.069 | 2.237e+07 | 66 - 81 | 3 | 45.35 | 45.52 | 45.86 |
| 4190.8880 | LVPVPITN(N)ATLDR Fuc 4 Hex 3 HexNAc 7 Neu5Ac 1 | 95.410 | 0.0 | 13.625 | 1.968e+08 | 25 - 38 | 12 | 59.55 | 61.10 | 61.61 |
| 3741.6215 | C(C)AN(D)LVPVPITN(N)ATLDR Hex 6 HexNAc 5 | 95.008 | 0.0 | 9.366 | 2.460e+08 | 22 - 38 | 13 | 42.47 | 43.66 | 44.68 |
| 4391.8577 | YFTPN(N)KTEDTIFLR Fuc 2 Hex 7 HexNAc 6 | 94.684 | 0.0 | 0.000 | 0.000e+00 | 67 - 81 | 1 | - | - | - |
| 3732.6097 | YFTPN(N)KTEDTIFLR Hex 6 HexNAc 5 | 94.613 | 0.0 | 0.000 | 0.000e+00 | 67 - 81 | 1 | - | - | - |
| 4448.7423 | QNQC(C)FYN(N)SSYLNVQR Hex 7 HexNAc 4 Neu5Ac 2 | 93.909 | 0.0 | -20.020 | 3.749e+07 | 86 - 101 | 2 | 34.19 | 34.19 | 34.36 |
| 5393.3665 | SVQEIQATFFYFTPN(N)KTEDTIFLR Fuc 1 Hex 7 HexNAc 6 | 93.713 | 0.0 | 12.769 | 2.825e+08 | 57 - 81 | 6 | 60.93 | 62.13 | 63.49 |
| 4272.7523 | YFTPN(N)KTEDTIFLR Hex 7 HexNAc 4 Neu5Ac 2 | 92.819 | 0.0 | -4.087 | 1.404e+08 | 67 - 81 | 4 | 37.43 | 37.91 | 37.91 |
| 4175.8777 | NLVPVPITN(N)ATLDR Fuc 4 Hex 4 HexNAc 7 | 91.884 | 0.0 | 13.379 | 1.300e+08 | 24 - 38 | 11 | 63.66 | 63.83 | 65.37 |
| 3778.6909 | LVPVPITN(N)ATLDR Fuc 2 Hex 6 HexNAc 4 Neu5Ac 1 | 90.279 | 0.0 | 0.000 | 0.000e+00 | 25 - 38 | 1 | - | - | - |
| 3428.4987 | VPVPITN(N)ATLDR Fuc 1 Hex 6 HexNAc 5 | 89.013 | 0.0 | -5.435 | 2.895e+06 | 26 - 38 | 4 | 29.62 | 29.79 | 29.97 |
| 4608.9354 | FYFTPN(N)KTEDTIFLR Hex 8 HexNAc 7 | 86.115 | 0.0 | 19.425 | 2.814e+07 | 66 - 81 | 2 | 44.34 | 44.84 | 45.01 |
| 4490.9345 | LVPVPITN(N)ATLDR Fuc 5 Hex 7 HexNAc 6 | 86.026 | 0.0 | -20.487 | 5.458e+06 | 25 - 38 | 1 | 35.04 | 35.21 | 35.21 |
| 3412.5526 | LVPVPITN(N)ATLDR Fuc 2 Hex 5 HexNAc 3 Neu5Ac 1 | 85.957 | 0.0 | 10.011 | 2.449e+09 | 25 - 38 | 1 | 36.05 | 36.50 | 39.10 |
| 3977.7200 | LVPVPITN(N)ATLDR Hex 6 HexNAc 5 Neu5Ac 2 | 85.803 | 0.0 | -4.963 | 1.084e+07 | 25 - 38 | 3 | 41.96 | 42.13 | 42.30 |
| 4079.7272 | FTPN(N)KTEDTIFLR Fuc 1 Hex 7 HexNAc 6 | 85.165 | 0.0 | -4.048 | 3.168e+07 | 68 - 81 | 3 | 31.14 | 31.31 | 31.65 |
| 3597.5145 | PVPITN(N)ATLDR Fuc 2 Hex 8 HexNAc 4 | 84.767 | 0.0 | 0.000 | 0.000e+00 | 27 - 38 | 1 | - | - | - |
| 4080.7221 | FTPN(N)KTEDTIFLR Fuc 1 Hex 7 HexNAc 6 | 83.277 | 0.0 | 0.000 | 0.000e+00 | 68 - 81 | 1 | - | - | - |
| 3714.5973 | FTPN(N)KTEDTIFLR Fuc 1 Hex 6 HexNAc 5 | 83.218 | 0.0 | -3.736 | 1.861e+07 | 68 - 81 | 3 | 31.65 | 31.82 | 32.16 |
| 3056.3385 | TPN(N)KTEDTIFLR Hex 5 HexNAc 4 | 81.908 | 0.0 | 11.703 | 5.285e+06 | 69 - 81 | 3 | 25.82 | 25.99 | 26.22 |
| 3378.3910 | FYN(N)SSYLNVQR Hex 6 HexNAc 5 | 81.739 | 0.0 | 0.000 | 0.000e+00 | 90 - 101 | 1 | - | - | - |
| 4298.8896 | FTPN(N)KTEDTIFLR Hex 8 HexNAc 7 | 81.236 | 0.0 | -5.336 | 1.257e+07 | 68 - 81 | 7 | 30.63 | 31.14 | 31.14 |
| 3453.5381 | VPVPITN(N)ATLDR Fuc 2 Hex 4 HexNAc 6 | 76.820 | 0.003 | -4.141 | 4.278e+07 | 26 - 38 | 5 | 39.92 | 40.09 | 40.43 |
| 3230.3148 | YN(N)SSYLNVQR Hex 6 HexNAc 5 | 76.571 | 0.003 | 10.467 | 8.669e+06 | 91 - 101 | 3 | 22.26 | 22.42 | 22.71 |
| 5539.4241 | SVQEIQATFFYFTPN(N)KTEDTIFLR Fuc 2 Hex 7 HexNAc 6 | 75.530 | 0.003 | -19.839 | 3.115e+07 | 57 - 81 | 2 | 61.95 | 61.95 | 62.13 |
| 3596.4601 | YN(N)SSYLNVQR Hex 7 HexNAc 6 | 75.224 | 0.003 | 0.000 | 0.000e+00 | 91 - 101 | 1 | - | - | - |
| 3742.5177 | FYN(N)SSYLNVQR Hex 7 HexNAc 6 | 75.047 | 0.003 | 12.915 | 1.599e+07 | 90 - 101 | 5 | 29.11 | 29.28 | 29.79 |
| 5367.2000 | SVQEIQATFFYFTPN(N)K Hex 10 HexNAc 9 | 74.236 | 0.003 | -4.917 | 1.116e+07 | 57 - 73 | 1 | 51.72 | 51.89 | 52.06 |
| 4419.8344 | FYFTPN(N)KTEDTIFLR Hex 7 HexNAc 4 Neu5Ac 2 | 73.976 | 0.003 | -4.737 | 7.641e+06 | 66 - 81 | 4 | 46.03 | 46.20 | 46.37 |
| 4267.7477 | PVPITN(N)ATLDR Hex 3 HexNAc 7 Neu5Ac 4 | 72.791 | 0.003 | -21.370 | 2.966e+06 | 27 - 38 | 1 | 35.55 | 35.55 | 35.55 |
| 4802.0249 | SVQEIQATFFYFTPN(N)K Hex 9 HexNAc 7 | 69.303 | 0.003 | 0.000 | 0.000e+00 | 57 - 73 | 1 | - | - | - |
| 4153.7559 | YFTPN(N)KTEDTIFLR Fuc 4 Hex 5 HexNAc 5 | 67.535 | 0.003 | 13.695 | 4.810e+08 | 67 - 81 | 6 | 34.86 | 37.27 | 38.42 |
| 5977.5676 | SVQEIQATFFYFTPN(N)KTEDTIFLR Hex 9 HexNAc 8 | 66.621 | 0.003 | 13.178 | 8.205e+07 | 57 - 81 | 6 | 60.93 | 61.44 | 62.13 |
| 4191.9157 | QIPLC(C)ANLVPVPITN(N)ATLDR Hex 6 HexNAc 5 | 66.476 | 0.003 | 12.471 | 4.331e+08 | 18 - 38 | 8 | 61.61 | 62.30 | 63.66 |
| 5758.4946 | SVQEIQATFFYFTPN(N)KTEDTIFLR Fuc 1 Hex 8 HexNAc 7 | 60.207 | 0.003 | 13.980 | 4.880e+07 | 57 - 81 | 4 | 61.27 | 61.78 | 62.13 |
| 5148.1241 | SVQEIQATFFYFTPN(N)K Fuc 1 Hex 9 HexNAc 8 | 56.267 | 0.003 | 7.373 | 1.196e+07 | 57 - 73 | 2 | 51.89 | 52.06 | 52.57 |
| 3375.5047 | C(C)ANLVPVPITN(N)ATLDR Hex 5 HexNAc 4 | 56.206 | 0.003 | -4.857 | 1.294e+07 | 22 - 38 | 2 | 42.13 | 42.30 | 42.47 |
| 3887.6820 | C(C)AN(D)LVPVPITN(N)ATLDR Fuc 1 Hex 6 HexNAc 5 | 54.507 | 0.003 | 8.197 | 5.463e+07 | 22 - 38 | 6 | 43.32 | 43.49 | 44.34 |
| 4389.8608 | FYFTPN(N)KTEDTIFLR Fuc 1 Hex 7 HexNAc 6 | 51.409 | 0.003 | 12.675 | 4.580e+07 | 66 - 81 | 4 | 45.01 | 45.18 | 45.69 |
| 3886.7076 | C(C)ANLVPVPITN(N)ATLDR Fuc 1 Hex 6 HexNAc 5 | 50.444 | 0.003 | -19.220 | 4.594e+07 | 22 - 38 | 2 | 41.62 | 41.62 | 41.79 |
| 4320.9506 | QIPLC(C)ANLVPVPITN(N)ATLDR Hex 5 HexNAc 5 Neu5Ac 1 | 49.967 | 0.003 | 15.038 | 5.410e+08 | 18 - 38 | 9 | 61.27 | 62.13 | 62.97 |
| 3563.5639 | C(C)AN(D)LVPVPITN(N)ATLDR Fuc 1 Hex 4 HexNAc 5 | 49.162 | 0.003 | 10.976 | 3.450e+08 | 22 - 38 | 2 | 35.88 | 36.05 | 36.50 |
| 5423.3359 | SVQEIQATFFYFTPN(N)KTEDTIFLR Hex 7 HexNAc 4 Neu5Ac 2 | 48.106 | 0.003 | -3.332 | 3.354e+07 | 57 - 81 | 3 | 63.15 | 63.32 | 63.66 |
| 4374.8689 | ANLVPVPITN(N)ATLDR Fuc 1 Hex 10 HexNAc 5 | 47.384 | 0.003 | -4.663 | 3.024e+07 | 23 - 38 | 1 | 61.95 | 62.13 | 62.30 |
| 4562.8958 | SVQEIQATFFYFTPN(N)K Hex 7 HexNAc 6 Neu5Ac 1 | 45.141 | 0.003 | -5.509 | 3.610e+06 | 57 - 73 | 2 | 56.31 | 56.48 | 56.82 |
| 3509.5712 | NLVPVPITN(N)ATLDR Hex 6 HexNAc 5 | 43.635 | 0.003 | -4.631 | 3.403e+07 | 24 - 38 | 1 | 47.22 | 47.39 | 47.56 |
| 5285.2530 | ANLVPVPITN(N)ATLDR Fuc 5 Hex 7 HexNAc 9 | 42.962 | 0.003 | -4.990 | 1.026e+08 | 23 - 38 | 2 | 62.13 | 62.30 | 62.46 |
| 3087.4162 | NLVPVPITN(N)ATLDR Fuc 1 Hex 5 HexNAc 3 | 42.892 | 0.003 | 14.362 | 2.034e+08 | 24 - 38 | 3 | 37.75 | 37.91 | 38.59 |
| 3567.5263 | TPN(N)KTEDTIFLR Fuc 1 Hex 6 HexNAc 5 | 42.131 | 0.003 | -5.418 | 7.785e+06 | 69 - 81 | 4 | 25.14 | 25.31 | 25.48 |
| 3417.5083 | C(C)AN(D)LVPVPITN(N)ATLDR Hex 4 HexNAc 5 | 41.957 | 0.003 | 10.217 | 1.169e+09 | 22 - 38 | 3 | 36.20 | 36.50 | 38.93 |
| 4535.9191 | FYFTPN(N)KTEDTIFLR Fuc 2 Hex 7 HexNAc 6 | 40.372 | 0.003 | -20.987 | 2.079e+06 | 66 - 81 | 1 | 45.01 | 45.01 | 45.01 |
| 4467.7150 | QN(D)QC(C)FYN(N)SSYLNVQR Fuc 3 Hex 8 HexNAc 4 | 40.118 | 0.004 | -19.954 | 1.971e+07 | 86 - 101 | 2 | 33.19 | 33.19 | 33.19 |
| 4783.9077 | QNQC(C)FYN(N)SSYLNVQR Fuc 1 Hex 8 HexNAc 7 | 40.076 | 0.004 | -20.639 | 8.176e+06 | 86 - 101 | 1 | 32.33 | 32.50 | 32.50 |
| 5304.3241 | SVQEIQATFFYFTPN(N)KTEDTIFLR Fuc 4 Hex 5 HexNAc 5 | 39.848 | 0.005 | 13.683 | 2.705e+08 | 57 - 81 | 12 | 62.46 | 62.97 | 65.20 |
| 4344.8583 | SVQEIQATFFYFTPN(N)K Fuc 3 Hex 6 HexNAc 5 | 39.645 | 0.005 | -19.429 | 8.762e+06 | 57 - 73 | 1 | 54.60 | 54.77 | 54.77 |
| 3963.7379 | NLVPVPITN(N)ATLDR Fuc 2 Hex 7 HexNAc 5 | 39.638 | 0.005 | -21.080 | 1.727e+07 | 24 - 38 | 1 | 36.80 | 36.96 | 36.96 |
| 4321.9353 | NLVPVPITN(N)ATLDR Fuc 5 Hex 4 HexNAc 7 | 38.984 | 0.005 | 11.378 | 3.231e+07 | 24 - 38 | 8 | 63.49 | 63.66 | 64.69 |
| 4273.7103 | QN(D)QC(C)FYN(N)SSYLNVQR Hex 7 HexNAc 6 | 37.809 | 0.006 | 8.820 | 3.718e+08 | 86 - 101 | 6 | 34.02 | 34.36 | 40.60 |
| 3788.6233 | FYFTPN(N)KTEDTIFLR Fuc 1 Hex 4 HexNAc 4 Neu5Ac 1 | 37.745 | 0.006 | 14.593 | 2.292e+08 | 66 - 81 | 4 | 37.58 | 37.75 | 38.59 |
| 3367.4815 | YFTPN(N)KTEDTIFLR Hex 5 HexNAc 4 | 37.365 | 0.006 | 0.000 | 0.000e+00 | 67 - 81 | 1 | - | - | - |
| 3797.6629 | C(C)AN(D)LVPVPITN(N)ATLDR Fuc 2 Hex 4 HexNAc 4 Neu5Ac 1 | 36.822 | 0.007 | -5.825 | 1.148e+08 | 22 - 38 | 4 | 42.64 | 42.81 | 43.15 |
| 3581.5289 | LVPVPITN(N)ATLDR Fuc 1 Hex 10 HexNAc 2 | 36.703 | 0.007 | 0.000 | 0.000e+00 | 25 - 38 | 2 | - | - | - |
| 4325.7127 | ATFFYFTPN(N)K Hex 6 HexNAc 9 Neu5Ac 1 | 35.720 | 0.007 | -4.356 | 1.328e+08 | 63 - 73 | 3 | 53.42 | 53.42 | 53.76 |
| 3757.6657 | ANLVPVPITN(N)ATLDR Fuc 2 Hex 6 HexNAc 3 Neu5Ac 1 | 35.385 | 0.007 | 12.355 | 5.454e+07 | 23 - 38 | 2 | 41.62 | 41.79 | 42.30 |
| 4227.8019 | FTPN(N)KTEDTIFLR Fuc 2 Hex 7 HexNAc 6 | 35.011 | 0.010 | -22.670 | 4.651e+06 | 68 - 81 | 2 | 62.80 | 62.80 | 62.80 |
| 4375.8532 | PVPITN(N)ATLDR Fuc 2 Hex 6 HexNAc 8 Neu5Ac 1 | 33.960 | 0.010 | -21.067 | 1.750e+05 | 27 - 38 | 1 | 64.52 | 64.52 | 64.52 |
| 4330.7179 | QC(C)FYN(N)SSYLNVQR Fuc 2 Hex 4 HexNAc 7 Neu5Ac 1 | 33.707 | 0.011 | -6.343 | 2.269e+07 | 88 - 101 | 1 | 34.36 | 34.52 | 34.86 |
| 4564.9021 | SVQEIQATFFYFTPN(N)K Fuc 3 Hex 5 HexNAc 4 Neu5Ac 2 | 33.648 | 0.011 | 0.000 | 0.000e+00 | 57 - 73 | 1 | - | - | - |
| 4212.8421 | ANLVPVPITN(N)ATLDR Fuc 1 Hex 9 HexNAc 5 | 33.327 | 0.011 | 11.482 | 1.875e+08 | 23 - 38 | 5 | 60.93 | 62.30 | 63.15 |
| 4336.9434 | LVPVPITN(N)ATLDR Fuc 5 Hex 3 HexNAc 7 Neu5Ac 1 | 33.103 | 0.012 | 11.919 | 4.632e+07 | 25 - 38 | 6 | 59.73 | 60.93 | 61.27 |
| 4580.8557 | QNQC(C)FYN(N)SSYLNVQR Fuc 1 Hex 8 HexNAc 6 | 32.769 | 0.014 | -20.527 | 2.332e+06 | 86 - 101 | 1 | 33.01 | 33.01 | 33.01 |
| 3869.6710 | C(C)ANLVPVPITN(N)ATLDR Hex 5 HexNAc 5 Neu5Ac 1 | 32.558 | 0.015 | 15.988 | 3.117e+08 | 22 - 38 | 6 | 51.72 | 52.06 | 52.74 |
| 5440.3608 | SVQEIQATFFYFTPN(N)KTEDTIFLR Fuc 1 Hex 8 HexNAc 4 Neu5Ac 1 | 32.349 | 0.016 | -4.560 | 5.913e+06 | 57 - 81 | 1 | 63.15 | 63.32 | 63.49 |
| 3934.6797 | FYFTPN(N)KTEDTIFLR Fuc 2 Hex 4 HexNAc 4 Neu5Ac 1 | 32.341 | 0.016 | -18.609 | 4.168e+07 | 66 - 81 | 4 | 37.58 | 37.58 | 37.75 |
| 7813.3406 | WFYIASAFRNEEYNKSVQEIQATFFYFTPN(N)K Fuc 5 Hex 10 HexNAc 8 | 32.122 | 0.016 | -4.185 | 3.861e+07 | 42 - 73 | 1 | 53.93 | 53.93 | 54.26 |
| 3377.3860 | FYN(N)SSYLNVQR Hex 6 HexNAc 5 | 31.876 | 0.017 | -4.445 | 5.577e+06 | 90 - 101 | 2 | 29.62 | 29.62 | 29.97 |
| 5002.9675 | QNQC(C)FYN(N)SSYLNVQR Hex 9 HexNAc 8 | 31.456 | 0.018 | -19.383 | 9.779e+06 | 86 - 101 | 1 | 37.75 | 37.75 | 37.75 |
| 3491.4880 | PVPITN(N)ATLDR Fuc 1 Hex 7 HexNAc 5 | 31.188 | 0.018 | -20.123 | 2.559e+06 | 27 - 38 | 1 | 40.09 | 40.09 | 40.09 |
| 3595.4488 | YN(N)SSYLNVQR Hex 7 HexNAc 6 | 30.299 | 0.022 | -4.053 | 8.569e+06 | 91 - 101 | 2 | 21.93 | 21.93 | 22.18 |
| 3256.4517 | LVPVPITN(N)ATLDR Fuc 1 Hex 8 HexNAc 2 | 29.616 | 0.023 | -4.107 | 2.163e+07 | 25 - 38 | 2 | 42.30 | 42.47 | 42.81 |
| 4151.7314 | TPN(N)KTEDTIFLR Hex 8 HexNAc 7 | 29.014 | 0.027 | -4.874 | 4.745e+06 | 69 - 81 | 1 | 24.29 | 24.46 | 24.63 |
| 3239.4249 | PVPITN(N)ATLDR Fuc 2 Hex 4 HexNAc 4 Neu5Ac 1 | 28.560 | 0.031 | 13.039 | 6.002e+07 | 27 - 38 | 5 | 42.30 | 42.47 | 42.98 |
| 3935.6824 | ANLVPVPITN(N)ATLDR Fuc 1 Hex 8 HexNAc 3 Neu5Ac 1 | 28.325 | 0.031 | -20.467 | 2.040e+07 | 23 - 38 | 5 | 36.96 | 36.96 | 37.11 |
| 4540.0238 | LVPVPITN(N)ATLDR Fuc 5 Hex 3 HexNAc 8 Neu5Ac 1 | 27.992 | 0.032 | 11.510 | 7.714e+07 | 25 - 38 | 4 | 61.27 | 61.78 | 62.13 |
| 3826.7849 | QIPLC(C)ANLVPVPITN(N)ATLDR Hex 5 HexNAc 4 | 27.817 | 0.034 | -3.583 | 3.918e+07 | 18 - 38 | 3 | 62.80 | 62.80 | 63.15 |
| 4434.7303 | QN(D)QC(C)FYN(N)SSYLNVQR Fuc 3 Hex 6 HexNAc 4 Neu5Ac 1 | 27.796 | 0.035 | 9.443 | 8.980e+07 | 86 - 101 | 3 | 38.59 | 39.10 | 39.43 |
| 4273.8113 | SVQEIQATFFYFTPN(N)K Hex 7 HexNAc 6 | 27.775 | 0.035 | 0.000 | 0.000e+00 | 57 - 73 | 1 | - | - | - |
| 3435.4477 | PVPITN(N)ATLDR Fuc 2 Hex 7 HexNAc 4 | 27.135 | 0.036 | -21.426 | 2.185e+06 | 27 - 38 | 3 | 17.13 | 17.13 | 17.13 |
| 3579.5283 | PVPITN(N)ATLDR Fuc 1 Hex 7 HexNAc 4 Neu5Ac 1 | 27.110 | 0.036 | 12.477 | 3.661e+08 | 27 - 38 | 3 | 35.88 | 36.05 | 36.50 |
| 3888.5765 | FYN(N)SSYLNVQR Fuc 1 Hex 7 HexNAc 6 | 26.953 | 0.036 | -18.910 | 3.195e+06 | 90 - 101 | 3 | 28.95 | 29.11 | 29.11 |
| 3543.5889 | LVPVPITN(N)ATLDR Fuc 1 Hex 6 HexNAc 5 | 26.854 | 0.036 | 0.000 | 0.000e+00 | 25 - 38 | 1 | - | - | - |
| 3965.5862 | QC(C)FYN(N)SSYLNVQR Fuc 2 Hex 3 HexNAc 6 Neu5Ac 1 | 26.244 | 0.036 | -5.223 | 1.696e+07 | 88 - 101 | 2 | 34.69 | 34.86 | 35.21 |
| 3943.7207 | C(C)ANLVPVPITN(N)ATLDR Hex 6 HexNAc 6 | 26.197 | 0.036 | 10.000 | 3.175e+07 | 22 - 38 | 4 | 42.47 | 42.64 | 43.15 |
| 4521.8777 | LVPVPITN(N)ATLDR Fuc 1 Hex 9 HexNAc 6 Neu5Ac 1 | 26.091 | 0.036 | -20.303 | 2.687e+06 | 25 - 38 | 1 | 55.11 | 55.11 | 55.28 |
| 4418.8018 | YFTPN(N)KTEDTIFLR Fuc 1 Hex 7 HexNAc 4 Neu5Ac 2 | 26.048 | 0.036 | -20.228 | 1.764e+07 | 67 - 81 | 1 | 37.58 | 37.75 | 37.75 |
| 3923.7423 | NLVPVPITN(N)ATLDR Hex 3 HexNAc 8 Neu5Ac 1 | 25.973 | 0.036 | -5.002 | 7.564e+07 | 24 - 38 | 2 | 35.71 | 35.88 | 36.05 |
| 5368.1125 | QNQC(C)FYN(N)SSYLNVQR Hex 10 HexNAc 9 | 25.954 | 0.036 | -19.987 | 6.482e+06 | 86 - 101 | 1 | 31.82 | 31.99 | 31.99 |
| 4577.9738 | ANLVPVPITN(N)ATLDR Fuc 1 Hex 10 HexNAc 6 | 25.925 | 0.036 | -20.230 | 3.000e+06 | 23 - 38 | 1 | 61.78 | 61.78 | 61.78 |
| 5192.1384 | YFTPN(N)KTEDTIFLR Hex 10 HexNAc 9 | 25.878 | 0.036 | -19.649 | 3.337e+06 | 67 - 81 | 1 | 35.21 | 35.21 | 35.21 |
| 5192.1384 | YFTPN(N)KTEDTIFLR Fuc 5 Hex 8 HexNAc 7 | 25.878 | 0.036 | -19.649 | 3.337e+06 | 67 - 81 | 1 | 35.21 | 35.21 | 35.21 |
| 2917.3144 | VPVPITN(N)ATLDR Hex 5 HexNAc 4 | 25.271 | 0.036 | -20.388 | 3.247e+05 | 26 - 38 | 1 | 30.30 | 30.30 | 30.46 |
| 4256.6707 | QC(C)FYN(N)SSYLNVQR Fuc 1 Hex 10 HexNAc 4 | 25.265 | 0.036 | 9.832 | 7.522e+08 | 88 - 101 | 1 | 39.59 | 39.76 | 41.28 |
| 4266.7917 | FYFTPN(N)KTEDTIFLR Fuc 2 Hex 3 HexNAc 5 Neu5Ac 2 | 25.264 | 0.036 | -19.267 | 1.604e+07 | 66 - 81 | 1 | 45.18 | 45.35 | 45.35 |
| 3964.5968 | QN(D)QC(C)FYN(N)SSYLNVQR Fuc 2 Hex 4 HexNAc 4 Neu5Ac 1 | 25.045 | 0.036 | -20.035 | 5.213e+07 | 86 - 101 | 2 | 33.69 | 33.85 | 33.85 |
| 3434.3902 | YN(N)SSYLNVQR Hex 6 HexNAc 6 | 25.016 | 0.036 | 0.000 | 0.000e+00 | 91 - 101 | 1 | - | - | - |
| 6126.6276 | SVQEIQATFFYFTPN(N)KTEDTIFLR Fuc 1 Hex 9 HexNAc 8 | 24.611 | 0.039 | 0.000 | 0.000e+00 | 57 - 81 | 1 | - | - | - |
| 4070.6120 | QN(D)QC(C)FYN(N)SSYLNVQR Hex 7 HexNAc 5 | 24.055 | 0.040 | -19.919 | 1.292e+07 | 86 - 101 | 1 | 39.26 | 39.43 | 39.43 |
| 3766.6111 | LVPVPITN(N)ATLDR Hex 9 HexNAc 3 Neu5Ac 1 | 23.984 | 0.040 | 0.000 | 0.000e+00 | 25 - 38 | 1 | - | - | - |
| 4300.8264 | FYFTPN(N)KTEDTIFLR Fuc 4 Hex 5 HexNAc 5 | 23.310 | 0.044 | -3.124 | 5.316e+07 | 66 - 81 | 3 | 46.37 | 46.54 | 46.88 |
| 4294.7720 | SVQEIQATFFYFTPN(N)K Fuc 1 Hex 10 HexNAc 3 | 23.194 | 0.046 | -19.268 | 3.311e+06 | 57 - 73 | 1 | 53.59 | 53.59 | 53.59 |
| 6342.7030 | SVQEIQATFFYFTPN(N)KTEDTIFLR Hex 10 HexNAc 9 | 23.185 | 0.046 | -5.556 | 1.088e+07 | 57 - 81 | 2 | 60.75 | 60.93 | 61.10 |
| 4358.9008 | ANLVPVPITN(N)ATLDR Fuc 2 Hex 9 HexNAc 5 | 23.145 | 0.046 | -18.335 | 4.935e+07 | 23 - 38 | 2 | 61.95 | 62.13 | 62.13 |
| 4283.7458 | QC(C)FYN(N)SSYLNVQR Fuc 2 Hex 3 HexNAc 9 | 23.115 | 0.046 | 3.250 | 3.367e+08 | 88 - 101 | 6 | 31.65 | 33.35 | 33.35 |
| 5062.0905 | SVQEIQATFFYFTPN(N)K Fuc 4 Hex 7 HexNAc 7 | 23.016 | 0.046 | 0.000 | 0.000e+00 | 57 - 73 | 1 | - | - | - |
| 3496.5379 | VPVPITN(N)ATLDR Fuc 2 Hex 3 HexNAc 7 | 23.014 | 0.046 | -21.375 | 1.315e+06 | 26 - 38 | 2 | 50.86 | 50.86 | 50.86 |
| 4289.8136 | SVQEIQATFFYFTPN(N)K Fuc 2 Hex 6 HexNAc 4 Neu5Ac 1 | 22.936 | 0.046 | -22.120 | 4.933e+06 | 57 - 73 | 11 | 58.19 | 58.36 | 58.36 |
| 3655.6295 | NLVPVPITN(N)ATLDR Fuc 1 Hex 6 HexNAc 5 | 22.585 | 0.048 | -4.746 | 1.063e+07 | 24 - 38 | 2 | 47.05 | 47.05 | 47.39 |

### YFTPN(N)K Hex 5 HexNAc 4

**Mass:**  2390.9646

(-9.445e-07)

**Charge States:** 2, 3

**Start Position**: 68

**End Position**: 74

| Profile Score | Tandem Score | TDA q-value | Volume | Spectrum Matches |
| --- | --- | --- | --- | --- |
| -20.2986 | 187.9382 | 0.0000 | 2.8771e+07 | 2 |

**Adduct Signal Fractions**

- **Unmodified**: 2.8771e+07

### YFTPN(N)K Hex 6 HexNAc 5

**Mass:**  2756.0989

(1.115e-06)

**Charge States:** 3

**Start Position**: 68

**End Position**: 74

| Profile Score | Tandem Score | TDA q-value | Volume | Spectrum Matches |
| --- | --- | --- | --- | --- |
| 15.0940 | 187.4647 | 0.0000 | 2.7702e+08 | 5 |

**Adduct Signal Fractions**

- **Unmodified**: 2.7702e+08

### FYFTPN(N)K Hex 7 HexNAc 6

**Mass:**  3268.2987

(1.290e-06)

**Charge States:** 3, 4

**Start Position**: 67

**End Position**: 74

| Profile Score | Tandem Score | TDA q-value | Volume | Spectrum Matches |
| --- | --- | --- | --- | --- |
| 14.5230 | 183.3084 | 0.0000 | 1.7500e+08 | 10 |

**Adduct Signal Fractions**

- **Unmodified**: 1.7500e+08

### NEEYN(N)K Hex 6 HexNAc 5

**Mass:**  2783.0553

(5.679e-07)

**Charge States:** 2, 3

**Start Position**: 52

**End Position**: 58

| Profile Score | Tandem Score | TDA q-value | Volume | Spectrum Matches |
| --- | --- | --- | --- | --- |
| 14.1904 | 182.5628 | 0.0000 | 4.1822e+08 | 15 |

**Adduct Signal Fractions**

- **Unmodified**: 4.1822e+08

### NEEYN(N)K Hex 5 HexNAc 4

**Mass:**  2417.9232

(7.168e-07)

**Charge States:** 2, 3

**Start Position**: 52

**End Position**: 58

| Profile Score | Tandem Score | TDA q-value | Volume | Spectrum Matches |
| --- | --- | --- | --- | --- |
| 12.5270 | 181.3118 | 0.0000 | 8.4181e+07 | 7 |

**Adduct Signal Fractions**

- **Unmodified**: 8.4181e+07

### YFTPN(N)K Hex 7 HexNAc 6

**Mass:**  3121.2306

(1.280e-06)

**Charge States:** 3

**Start Position**: 68

**End Position**: 74

| Profile Score | Tandem Score | TDA q-value | Volume | Spectrum Matches |
| --- | --- | --- | --- | --- |
| 13.2871 | 180.6938 | 0.0000 | 3.1787e+08 | 5 |

**Adduct Signal Fractions**

- **Unmodified**: 3.1787e+08

### NEEYN(N)K Fuc 1 Hex 6 HexNAc 5

**Mass:**  2929.1145

(9.957e-07)

**Charge States:** 3

**Start Position**: 52

**End Position**: 58

| Profile Score | Tandem Score | TDA q-value | Volume | Spectrum Matches |
| --- | --- | --- | --- | --- |
| 12.0063 | 180.4669 | 0.0000 | 2.9033e+07 | 3 |

**Adduct Signal Fractions**

- **Unmodified**: 2.9033e+07

### SVQEIQATFFYFTPN(N)K Hex 6 HexNAc 5

**Mass:**  3906.6660

(1.810e-06)

**Charge States:** 3, 4, 5

**Start Position**: 58

**End Position**: 74

| Profile Score | Tandem Score | TDA q-value | Volume | Spectrum Matches |
| --- | --- | --- | --- | --- |
| 14.7280 | 175.2034 | 0.0000 | 2.9688e+09 | 49 |

**Adduct Signal Fractions**

- **Unmodified**: 2.9688e+09

### SVQEIQATFFYFTPN(N)K Fuc 1 Hex 6 HexNAc 5

**Mass:**  4052.7270

(1.127e-06)

**Charge States:** 3, 4, 5

**Start Position**: 58

**End Position**: 74

| Profile Score | Tandem Score | TDA q-value | Volume | Spectrum Matches |
| --- | --- | --- | --- | --- |
| 14.2474 | 174.6170 | 0.0000 | 6.0646e+08 | 18 |

**Adduct Signal Fractions**

- **Unmodified**: 6.0646e+08

### YFTPN(N)K Fuc 1 Hex 6 HexNAc 5

**Mass:**  2902.1537

(4.886e-07)

**Charge States:** 3

**Start Position**: 68

**End Position**: 74

| Profile Score | Tandem Score | TDA q-value | Volume | Spectrum Matches |
| --- | --- | --- | --- | --- |
| -4.6216 | 173.9382 | 0.0000 | 4.3836e+07 | 3 |

**Adduct Signal Fractions**

- **Unmodified**: 4.3836e+07

### SVQEIQATFFYFTPN(N)K Hex 7 HexNAc 6

**Mass:**  4271.8093

(2.038e-06)

**Charge States:** 3, 4, 5

**Start Position**: 58

**End Position**: 74

| Profile Score | Tandem Score | TDA q-value | Volume | Spectrum Matches |
| --- | --- | --- | --- | --- |
| 13.2866 | 173.8761 | 0.0000 | 3.6903e+09 | 129 |

**Adduct Signal Fractions**

- **Unmodified**: 3.6903e+09

### YFTPN(N)K Hex 8 HexNAc 7

**Mass:**  3486.3623

(1.027e-06)

**Charge States:** 3

**Start Position**: 68

**End Position**: 74

| Profile Score | Tandem Score | TDA q-value | Volume | Spectrum Matches |
| --- | --- | --- | --- | --- |
| -19.7459 | 172.7300 | 0.0000 | 1.8550e+07 | 1 |

**Adduct Signal Fractions**

- **Unmodified**: 1.8550e+07

### YFTPN(N)K Fuc 1 Hex 7 HexNAc 6

**Mass:**  3267.2902

(1.767e-06)

**Charge States:** 3

**Start Position**: 68

**End Position**: 74

| Profile Score | Tandem Score | TDA q-value | Volume | Spectrum Matches |
| --- | --- | --- | --- | --- |
| -4.7720 | 171.6449 | 0.0000 | 4.4153e+07 | 2 |

**Adduct Signal Fractions**

- **Unmodified**: 4.4153e+07

### LVPVPITN(N)ATLDR Hex 6 HexNAc 5

**Mass:**  3395.5274

(1.312e-06)

**Charge States:** 2, 3, 4

**Start Position**: 26

**End Position**: 39

| Profile Score | Tandem Score | TDA q-value | Volume | Spectrum Matches |
| --- | --- | --- | --- | --- |
| 13.1301 | 170.4707 | 0.0000 | 1.9245e+10 | 718 |

**Adduct Signal Fractions**

- **Unmodified**: 1.9245e+10

### FYFTPN(N)K Hex 6 HexNAc 5

**Mass:**  2903.1655

(9.097e-07)

**Charge States:** 2, 3, 4

**Start Position**: 67

**End Position**: 74

| Profile Score | Tandem Score | TDA q-value | Volume | Spectrum Matches |
| --- | --- | --- | --- | --- |
| 14.4837 | 169.8429 | 0.0000 | 1.4314e+08 | 13 |

**Adduct Signal Fractions**

- **Unmodified**: 1.4314e+08

### SVQEIQATFFYFTPN(N)K Fuc 1 Hex 7 HexNAc 6

**Mass:**  4417.8664

(1.478e-06)

**Charge States:** 3, 4, 5

**Start Position**: 58

**End Position**: 74

| Profile Score | Tandem Score | TDA q-value | Volume | Spectrum Matches |
| --- | --- | --- | --- | --- |
| 13.4584 | 167.7006 | 0.0000 | 6.7371e+08 | 17 |

**Adduct Signal Fractions**

- **Unmodified**: 6.7371e+08

### SVQEIQATFFYFTPN(N)K Hex 8 HexNAc 7

**Mass:**  4636.9338

(2.973e-06)

**Charge States:** 3, 4, 5

**Start Position**: 58

**End Position**: 74

| Profile Score | Tandem Score | TDA q-value | Volume | Spectrum Matches |
| --- | --- | --- | --- | --- |
| 13.8104 | 166.7048 | 0.0000 | 4.7888e+08 | 10 |

**Adduct Signal Fractions**

- **Unmodified**: 4.7888e+08

### SVQEIQATFFYFTPN(N)K Fuc 2 Hex 7 HexNAc 6

**Mass:**  4563.9221

(2.084e-06)

**Charge States:** 4

**Start Position**: 58

**End Position**: 74

| Profile Score | Tandem Score | TDA q-value | Volume | Spectrum Matches |
| --- | --- | --- | --- | --- |
| 10.9767 | 166.6869 | 0.0000 | 9.0257e+07 | 2 |

**Adduct Signal Fractions**

- **Unmodified**: 9.0257e+07

### LVPVPITN(N)ATLDR Hex 5 HexNAc 4

**Mass:**  3030.3929

(9.050e-07)

**Charge States:** 2, 3, 4

**Start Position**: 26

**End Position**: 39

| Profile Score | Tandem Score | TDA q-value | Volume | Spectrum Matches |
| --- | --- | --- | --- | --- |
| 13.5823 | 166.2269 | 0.0000 | 1.1604e+09 | 7 |

**Adduct Signal Fractions**

- **Unmodified**: 1.1604e+09

### SVQEIQATFFYFTPN(N)K Hex 5 HexNAc 4

**Mass:**  3541.5327

(1.822e-06)

**Charge States:** 3, 4

**Start Position**: 58

**End Position**: 74

| Profile Score | Tandem Score | TDA q-value | Volume | Spectrum Matches |
| --- | --- | --- | --- | --- |
| 13.9355 | 164.8419 | 0.0000 | 3.0824e+08 | 18 |

**Adduct Signal Fractions**

- **Unmodified**: 3.0824e+08

### SVQEIQATFFYFTPN(N)K Fuc 1 Hex 8 HexNAc 7

**Mass:**  4782.9914

(2.325e-06)

**Charge States:** 3, 4

**Start Position**: 58

**End Position**: 74

| Profile Score | Tandem Score | TDA q-value | Volume | Spectrum Matches |
| --- | --- | --- | --- | --- |
| 13.1461 | 156.8631 | 0.0000 | 9.0067e+07 | 5 |

**Adduct Signal Fractions**

- **Unmodified**: 9.0067e+07

### LVPVPITN(N)ATLDR Fuc 1 Hex 6 HexNAc 5

**Mass:**  3541.5818

(9.940e-07)

**Charge States:** 3, 4

**Start Position**: 26

**End Position**: 39

| Profile Score | Tandem Score | TDA q-value | Volume | Spectrum Matches |
| --- | --- | --- | --- | --- |
| 13.3967 | 156.7186 | 0.0000 | 5.6278e+09 | 231 |

**Adduct Signal Fractions**

- **Unmodified**: 5.6278e+09

### ATFFYFTPN(N)K Hex 6 HexNAc 5

**Mass:**  3222.3239

(2.008e-06)

**Charge States:** 3, 4

**Start Position**: 64

**End Position**: 74

| Profile Score | Tandem Score | TDA q-value | Volume | Spectrum Matches |
| --- | --- | --- | --- | --- |
| -3.4965 | 156.3519 | 0.0000 | 4.8726e+07 | 2 |

**Adduct Signal Fractions**

- **Unmodified**: 4.8726e+07

### ATFFYFTPN(N)K Hex 7 HexNAc 6

**Mass:**  3587.4541

(1.607e-06)

**Charge States:** 3, 4

**Start Position**: 64

**End Position**: 74

| Profile Score | Tandem Score | TDA q-value | Volume | Spectrum Matches |
| --- | --- | --- | --- | --- |
| 13.0535 | 156.1570 | 0.0000 | 7.3053e+07 | 2 |

**Adduct Signal Fractions**

- **Unmodified**: 7.3053e+07

### C(C)ANLVPVPITN(N)ATLDR Hex 6 HexNAc 5

**Mass:**  3740.6365

(2.453e-06)

**Charge States:** 3, 4

**Start Position**: 23

**End Position**: 39

| Profile Score | Tandem Score | TDA q-value | Volume | Spectrum Matches |
| --- | --- | --- | --- | --- |
| 10.8286 | 155.8465 | 0.0000 | 4.8845e+08 | 10 |

**Adduct Signal Fractions**

- **Unmodified**: 4.8845e+08

### SVQEIQATFFYFTPN(N)K Fuc 1 Hex 8 HexNAc 4 Neu5Ac 1

**Mass:**  4464.8610

(4.770e-06)

**Charge States:** 4

**Start Position**: 58

**End Position**: 74

| Profile Score | Tandem Score | TDA q-value | Volume | Spectrum Matches |
| --- | --- | --- | --- | --- |
| -4.8004 | 153.5758 | 0.0000 | 3.4141e+07 | 2 |

**Adduct Signal Fractions**

- **Unmodified**: 3.4141e+07

### LVPVPITN(N)ATLDR Hex 7 HexNAc 6

**Mass:**  3760.6545

(-1.922e-08)

**Charge States:** 3, 4

**Start Position**: 26

**End Position**: 39

| Profile Score | Tandem Score | TDA q-value | Volume | Spectrum Matches |
| --- | --- | --- | --- | --- |
| 14.5382 | 151.9082 | 0.0000 | 9.7911e+08 | 6 |

**Adduct Signal Fractions**

- **Unmodified**: 9.7911e+08

### PVPITN(N)ATLDR Fuc 3 Hex 4 HexNAc 8

**Mass:**  3906.7133

(9.519e-06)

**Charge States:** 4

**Start Position**: 28

**End Position**: 39

| Profile Score | Tandem Score | TDA q-value | Volume | Spectrum Matches |
| --- | --- | --- | --- | --- |
| -3.0274 | 149.3241 | 0.0000 | 2.9258e+08 | 2 |

**Adduct Signal Fractions**

- **Unmodified**: 2.9258e+08

### SVQEIQATFFYFTPN(N)KTEDTIFLR Hex 6 HexNAc 5

**Mass:**  4882.1730

(1.867e-06)

**Charge States:** 3, 4, 5

**Start Position**: 58

**End Position**: 82

| Profile Score | Tandem Score | TDA q-value | Volume | Spectrum Matches |
| --- | --- | --- | --- | --- |
| 14.1963 | 148.8807 | 0.0000 | 6.6008e+08 | 24 |

**Adduct Signal Fractions**

- **Unmodified**: 6.6008e+08

### SVQEIQATFFYFTPN(N)K Hex 9 HexNAc 8

**Mass:**  5002.0676

(1.459e-06)

**Charge States:** 4, 5

**Start Position**: 58

**End Position**: 74

| Profile Score | Tandem Score | TDA q-value | Volume | Spectrum Matches |
| --- | --- | --- | --- | --- |
| 12.6904 | 147.9710 | 0.0000 | 9.9854e+07 | 7 |

**Adduct Signal Fractions**

- **Unmodified**: 9.9854e+07

### YFTPN(N)KTEDTIFLR Hex 6 HexNAc 5

**Mass:**  3731.6005

(7.536e-07)

**Charge States:** 3, 4

**Start Position**: 68

**End Position**: 82

| Profile Score | Tandem Score | TDA q-value | Volume | Spectrum Matches |
| --- | --- | --- | --- | --- |
| 13.3612 | 147.2339 | 0.0000 | 1.0284e+09 | 22 |

**Adduct Signal Fractions**

- **Unmodified**: 1.0284e+09

### LVPVPITN(N)ATLDR Fuc 2 Hex 6 HexNAc 3 Neu5Ac 1

**Mass:**  3572.5629

(-3.348e-06)

**Start Position**: 26

**End Position**: 39

| Profile Score | Tandem Score | TDA q-value | Volume | Spectrum Matches |
| --- | --- | --- | --- | --- |
| 0.0000 | 147.1408 | 0.0000 | 0.0000e+00 | 1 |

Adduct:

No Chromatogram Found

### PVPITN(N)ATLDR Fuc 2 Hex 7 HexNAc 3

**Mass:**  3231.3903

(3.251e-06)

**Charge States:** 4

**Start Position**: 28

**End Position**: 39

| Profile Score | Tandem Score | TDA q-value | Volume | Spectrum Matches |
| --- | --- | --- | --- | --- |
| -18.3473 | 142.7859 | 0.0000 | 3.2196e+06 | 1 |

**Adduct Signal Fractions**

- **Unmodified**: 3.2196e+06

### VPVPITN(N)ATLDR Hex 6 HexNAc 5

**Mass:**  3282.4411

(8.612e-07)

**Charge States:** 3

**Start Position**: 27

**End Position**: 39

| Profile Score | Tandem Score | TDA q-value | Volume | Spectrum Matches |
| --- | --- | --- | --- | --- |
| -3.1877 | 142.0961 | 0.0000 | 1.3137e+07 | 3 |

**Adduct Signal Fractions**

- **Unmodified**: 1.3137e+07

### ATFFYFTPN(N)K Fuc 1 Hex 6 HexNAc 5

**Mass:**  3368.3790

(1.462e-06)

**Charge States:** 3

**Start Position**: 64

**End Position**: 74

| Profile Score | Tandem Score | TDA q-value | Volume | Spectrum Matches |
| --- | --- | --- | --- | --- |
| -20.1352 | 142.0029 | 0.0000 | 3.7619e+06 | 1 |

**Adduct Signal Fractions**

- **Unmodified**: 3.7619e+06

### SVQEIQATFFYFTPN(N)K Hex 7 HexNAc 4 Neu5Ac 2

**Mass:**  4447.8342

(3.477e-06)

**Charge States:** 3, 4

**Start Position**: 58

**End Position**: 74

| Profile Score | Tandem Score | TDA q-value | Volume | Spectrum Matches |
| --- | --- | --- | --- | --- |
| 12.3286 | 140.0869 | 0.0000 | 1.8440e+08 | 6 |

**Adduct Signal Fractions**

- **Unmodified**: 1.8440e+08

### PVPITN(N)ATLDR Fuc 1 Hex 4 HexNAc 9

**Mass:**  3817.6818

(9.895e-06)

**Charge States:** 3, 4

**Start Position**: 28

**End Position**: 39

| Profile Score | Tandem Score | TDA q-value | Volume | Spectrum Matches |
| --- | --- | --- | --- | --- |
| -4.5168 | 138.4279 | 0.0000 | 1.0372e+08 | 2 |

**Adduct Signal Fractions**

- **Unmodified**: 1.0372e+08

### YFTPN(N)KTEDTIFLR Fuc 1 Hex 7 HexNAc 6

**Mass:**  4242.7941

(4.729e-10)

**Charge States:** 4

**Start Position**: 68

**End Position**: 82

| Profile Score | Tandem Score | TDA q-value | Volume | Spectrum Matches |
| --- | --- | --- | --- | --- |
| 8.6435 | 137.5249 | 0.0000 | 2.3144e+08 | 5 |

**Adduct Signal Fractions**

- **Unmodified**: 2.3144e+08

### SVQEIQATFFYFTPN(N)KTEDTIFLR Fuc 1 Hex 6 HexNAc 5

**Mass:**  5028.2321

(2.478e-06)

**Charge States:** 4, 5

**Start Position**: 58

**End Position**: 82

| Profile Score | Tandem Score | TDA q-value | Volume | Spectrum Matches |
| --- | --- | --- | --- | --- |
| 12.1861 | 136.8445 | 0.0000 | 1.5229e+08 | 8 |

**Adduct Signal Fractions**

- **Unmodified**: 1.5229e+08

### TPN(N)KTEDTIFLR Hex 7 HexNAc 6

**Mass:**  3786.6013

(9.784e-07)

**Charge States:** 3, 4

**Start Position**: 70

**End Position**: 82

| Profile Score | Tandem Score | TDA q-value | Volume | Spectrum Matches |
| --- | --- | --- | --- | --- |
| 14.3781 | 136.6219 | 0.0000 | 7.9095e+07 | 7 |

**Adduct Signal Fractions**

- **Unmodified**: 7.9095e+07

### LVPVPITN(N)ATLDR Hex 5 HexNAc 4

**Mass:**  3032.4151

(6.682e-04)

**Start Position**: 26

**End Position**: 39

| Profile Score | Tandem Score | TDA q-value | Volume | Spectrum Matches |
| --- | --- | --- | --- | --- |
| 0.0000 | 136.2041 | 0.0000 | 0.0000e+00 | 1 |

Adduct:

No Chromatogram Found

### LVPVPITN(N)ATLDR Fuc 2 Hex 6 HexNAc 5

**Mass:**  3687.6408

(1.423e-06)

**Charge States:** 3, 4

**Start Position**: 26

**End Position**: 39

| Profile Score | Tandem Score | TDA q-value | Volume | Spectrum Matches |
| --- | --- | --- | --- | --- |
| -4.6986 | 134.5944 | 0.0000 | 8.8145e+07 | 3 |

**Adduct Signal Fractions**

- **Unmodified**: 8.8145e+07

### PVPITN(N)ATLDR Fuc 1 Hex 9 HexNAc 4 Neu5Ac 1

**Mass:**  3903.6000

(-3.203e-06)

**Charge States:** 3, 4

**Start Position**: 28

**End Position**: 39

| Profile Score | Tandem Score | TDA q-value | Volume | Spectrum Matches |
| --- | --- | --- | --- | --- |
| -4.4738 | 133.8065 | 0.0000 | 1.1074e+08 | 4 |

**Adduct Signal Fractions**

- **Unmodified**: 1.1074e+08

### QNQC(C)FYN(N)SSYLNVQR Hex 6 HexNAc 5

**Mass:**  3907.5783

(2.169e-06)

**Charge States:** 3, 4

**Start Position**: 87

**End Position**: 102

| Profile Score | Tandem Score | TDA q-value | Volume | Spectrum Matches |
| --- | --- | --- | --- | --- |
| 10.2422 | 132.9302 | 0.0000 | 7.9428e+08 | 9 |

**Adduct Signal Fractions**

- **Unmodified**: 7.9428e+08

### LVPVPITN(N)ATLDR Hex 8 HexNAc 7

**Mass:**  4125.7932

(3.219e-07)

**Charge States:** 3, 4

**Start Position**: 26

**End Position**: 39

| Profile Score | Tandem Score | TDA q-value | Volume | Spectrum Matches |
| --- | --- | --- | --- | --- |
| 12.3031 | 131.6567 | 0.0000 | 1.6170e+08 | 3 |

**Adduct Signal Fractions**

- **Unmodified**: 1.6170e+08

### SVQEIQATFFYFTPN(N)K Fuc 4 Hex 5 HexNAc 5

**Mass:**  4328.8239

(-4.039e-06)

**Charge States:** 3, 4, 5

**Start Position**: 58

**End Position**: 74

| Profile Score | Tandem Score | TDA q-value | Volume | Spectrum Matches |
| --- | --- | --- | --- | --- |
| 13.9719 | 130.5669 | 0.0000 | 7.7517e+08 | 25 |

**Adduct Signal Fractions**

- **Unmodified**: 7.7517e+08

### SVQEIQATFFYFTPN(N)K Fuc 2 Hex 8 HexNAc 7

**Mass:**  4929.0539

(2.681e-06)

**Charge States:** 4

**Start Position**: 58

**End Position**: 74

| Profile Score | Tandem Score | TDA q-value | Volume | Spectrum Matches |
| --- | --- | --- | --- | --- |
| 11.3556 | 128.3865 | 0.0000 | 2.2904e+07 | 2 |

**Adduct Signal Fractions**

- **Unmodified**: 2.2904e+07

### YFTPN(N)KTEDTIFLR Fuc 1 Hex 6 HexNAc 5

**Mass:**  3877.6572

(1.430e-06)

**Charge States:** 3, 4

**Start Position**: 68

**End Position**: 82

| Profile Score | Tandem Score | TDA q-value | Volume | Spectrum Matches |
| --- | --- | --- | --- | --- |
| 8.3685 | 127.2492 | 0.0000 | 2.5470e+08 | 4 |

**Adduct Signal Fractions**

- **Unmodified**: 2.5470e+08

### YFTPN(N)KTEDTIFLR Hex 7 HexNAc 6

**Mass:**  4096.7326

(-1.236e-06)

**Charge States:** 3, 4

**Start Position**: 68

**End Position**: 82

| Profile Score | Tandem Score | TDA q-value | Volume | Spectrum Matches |
| --- | --- | --- | --- | --- |
| 12.5200 | 126.6999 | 0.0000 | 1.9531e+09 | 21 |

**Adduct Signal Fractions**

- **Unmodified**: 1.9531e+09

### SVQEIQATFFYFTPN(N)K Hex 6 HexNAc 6

**Mass:**  4109.7477

(2.020e-06)

**Charge States:** 3, 4

**Start Position**: 58

**End Position**: 74

| Profile Score | Tandem Score | TDA q-value | Volume | Spectrum Matches |
| --- | --- | --- | --- | --- |
| 9.7049 | 125.7924 | 0.0000 | 7.6235e+07 | 4 |

**Adduct Signal Fractions**

- **Unmodified**: 7.6235e+07

### FYFTPN(N)KTEDTIFLR Hex 7 HexNAc 6

**Mass:**  4243.8044

(1.732e-06)

**Charge States:** 3, 4

**Start Position**: 67

**End Position**: 82

| Profile Score | Tandem Score | TDA q-value | Volume | Spectrum Matches |
| --- | --- | --- | --- | --- |
| 12.1988 | 124.8086 | 0.0000 | 2.5394e+08 | 8 |

**Adduct Signal Fractions**

- **Unmodified**: 2.5394e+08

### NLVPVPITN(N)ATLDR Fuc 2 Hex 6 HexNAc 4

**Mass:**  3598.6049

(9.759e-07)

**Charge States:** 3, 4

**Start Position**: 25

**End Position**: 39

| Profile Score | Tandem Score | TDA q-value | Volume | Spectrum Matches |
| --- | --- | --- | --- | --- |
| 13.4761 | 124.3054 | 0.0000 | 1.0202e+09 | 6 |

**Adduct Signal Fractions**

- **Unmodified**: 1.0202e+09

### SVQEIQATFFYFTPN(N)KTEDTIFLR Hex 7 HexNAc 6

**Mass:**  5247.3063

(2.611e-06)

**Charge States:** 3, 4, 5, 6

**Start Position**: 58

**End Position**: 82

| Profile Score | Tandem Score | TDA q-value | Volume | Spectrum Matches |
| --- | --- | --- | --- | --- |
| 13.1269 | 123.7439 | 0.0000 | 1.4568e+09 | 30 |

**Adduct Signal Fractions**

- **Unmodified**: 1.4568e+09

### QNQC(C)FYN(N)SSYLNVQR Fuc 1 Hex 7 HexNAc 6

**Mass:**  4418.7692

(2.258e-06)

**Charge States:** 3, 4

**Start Position**: 87

**End Position**: 102

| Profile Score | Tandem Score | TDA q-value | Volume | Spectrum Matches |
| --- | --- | --- | --- | --- |
| 6.2733 | 123.5698 | 0.0000 | 3.6840e+08 | 3 |

**Adduct Signal Fractions**

- **Unmodified**: 3.6840e+08

### QNQC(C)FYN(N)SSYLNVQR Fuc 1 Hex 6 HexNAc 5

**Mass:**  4053.6376

(1.825e-06)

**Charge States:** 3, 4

**Start Position**: 87

**End Position**: 102

| Profile Score | Tandem Score | TDA q-value | Volume | Spectrum Matches |
| --- | --- | --- | --- | --- |
| -5.2388 | 123.4413 | 0.0000 | 8.9057e+07 | 2 |

**Adduct Signal Fractions**

- **Unmodified**: 8.9057e+07

### FYFTPN(N)KTEDTIFLR Hex 6 HexNAc 5

**Mass:**  3878.6711

(1.201e-06)

**Charge States:** 3, 4

**Start Position**: 67

**End Position**: 82

| Profile Score | Tandem Score | TDA q-value | Volume | Spectrum Matches |
| --- | --- | --- | --- | --- |
| 13.4413 | 120.9525 | 0.0000 | 7.9152e+07 | 5 |

**Adduct Signal Fractions**

- **Unmodified**: 7.9152e+07

### SVQEIQATFFYFTPN(N)K Fuc 1 Hex 7 HexNAc 4 Neu5Ac 2

**Mass:**  4593.8933

(2.363e-06)

**Charge States:** 4

**Start Position**: 58

**End Position**: 74

| Profile Score | Tandem Score | TDA q-value | Volume | Spectrum Matches |
| --- | --- | --- | --- | --- |
| -5.1024 | 120.8128 | 0.0000 | 2.1924e+07 | 1 |

**Adduct Signal Fractions**

- **Unmodified**: 2.1924e+07

### SVQEIQATFFYFTPN(N)K Fuc 2 Hex 6 HexNAc 5

**Mass:**  4198.7902

(3.378e-06)

**Charge States:** 4

**Start Position**: 58

**End Position**: 74

| Profile Score | Tandem Score | TDA q-value | Volume | Spectrum Matches |
| --- | --- | --- | --- | --- |
| -5.1730 | 119.0445 | 0.0000 | 1.0857e+07 | 1 |

**Adduct Signal Fractions**

- **Unmodified**: 1.0857e+07

### LVPVPITN(N)ATLDR Fuc 1 Hex 7 HexNAc 3 Neu5Ac 1

**Mass:**  3588.5879

(5.009e-06)

**Charge States:** 3

**Start Position**: 26

**End Position**: 39

| Profile Score | Tandem Score | TDA q-value | Volume | Spectrum Matches |
| --- | --- | --- | --- | --- |
| -19.3527 | 117.3347 | 0.0000 | 2.5655e+07 | 1 |

**Adduct Signal Fractions**

- **Unmodified**: 2.5655e+07

### SVQEIQATFFYFTPN(N)K Hex 8 HexNAc 6

**Mass:**  4436.8925

(6.872e-04)

**Start Position**: 58

**End Position**: 74

| Profile Score | Tandem Score | TDA q-value | Volume | Spectrum Matches |
| --- | --- | --- | --- | --- |
| 0.0000 | 117.1125 | 0.0000 | 0.0000e+00 | 1 |

Adduct:

No Chromatogram Found

### LVPVPITN(N)ATLDR Hex 6 HexNAc 5 Neu5Ac 1

**Mass:**  3686.6269

(2.446e-06)

**Charge States:** 3, 4

**Start Position**: 26

**End Position**: 39

| Profile Score | Tandem Score | TDA q-value | Volume | Spectrum Matches |
| --- | --- | --- | --- | --- |
| 11.0319 | 116.7606 | 0.0000 | 2.9030e+07 | 1 |

**Adduct Signal Fractions**

- **Unmodified**: 2.9030e+07

### YFTPN(N)KTEDTIFLR Hex 5 HexNAc 4

**Mass:**  3366.4689

(6.018e-07)

**Charge States:** 3, 4

**Start Position**: 68

**End Position**: 82

| Profile Score | Tandem Score | TDA q-value | Volume | Spectrum Matches |
| --- | --- | --- | --- | --- |
| -18.7353 | 116.6787 | 0.0000 | 5.1285e+07 | 1 |

**Adduct Signal Fractions**

- **Unmodified**: 5.1285e+07

### TPN(N)KTEDTIFLR Hex 6 HexNAc 5

**Mass:**  3421.4683

(1.102e-06)

**Charge States:** 3, 4

**Start Position**: 70

**End Position**: 82

| Profile Score | Tandem Score | TDA q-value | Volume | Spectrum Matches |
| --- | --- | --- | --- | --- |
| 13.9611 | 116.2390 | 0.0000 | 3.9272e+07 | 7 |

**Adduct Signal Fractions**

- **Unmodified**: 3.9272e+07

### LVPVPITN(N)ATLDR Fuc 1 Hex 7 HexNAc 6

**Mass:**  3906.7268

(3.657e-06)

**Charge States:** 3, 4

**Start Position**: 26

**End Position**: 39

| Profile Score | Tandem Score | TDA q-value | Volume | Spectrum Matches |
| --- | --- | --- | --- | --- |
| -18.7139 | 115.6960 | 0.0000 | 2.2245e+07 | 2 |

**Adduct Signal Fractions**

- **Unmodified**: 2.2245e+07

### YFTPN(N)KTEDTIFLR Hex 8 HexNAc 7

**Mass:**  4461.8669

(8.782e-07)

**Charge States:** 4

**Start Position**: 68

**End Position**: 82

| Profile Score | Tandem Score | TDA q-value | Volume | Spectrum Matches |
| --- | --- | --- | --- | --- |
| 12.7335 | 115.3675 | 0.0000 | 2.2856e+08 | 4 |

**Adduct Signal Fractions**

- **Unmodified**: 2.2856e+08

### LVPVPITN(N)ATLDR Fuc 3 Hex 4 HexNAc 5

**Mass:**  3509.5706

(-5.649e-06)

**Charge States:** 3

**Start Position**: 26

**End Position**: 39

| Profile Score | Tandem Score | TDA q-value | Volume | Spectrum Matches |
| --- | --- | --- | --- | --- |
| -5.0019 | 115.0183 | 0.0000 | 1.7116e+07 | 3 |

**Adduct Signal Fractions**

- **Unmodified**: 1.7116e+07

### QNQC(C)FYN(N)SSYLNVQR Hex 7 HexNAc 6

**Mass:**  4272.7189

(1.251e-06)

**Charge States:** 3, 4

**Start Position**: 87

**End Position**: 102

| Profile Score | Tandem Score | TDA q-value | Volume | Spectrum Matches |
| --- | --- | --- | --- | --- |
| 10.1759 | 112.6024 | 0.0000 | 1.3208e+09 | 15 |

**Adduct Signal Fractions**

- **Unmodified**: 1.3208e+09

### ATFFYFTPN(N)K Fuc 2 Hex 3 HexNAc 8 Neu5Ac 1

**Mass:**  3928.6430

(7.945e-06)

**Charge States:** 3, 4

**Start Position**: 64

**End Position**: 74

| Profile Score | Tandem Score | TDA q-value | Volume | Spectrum Matches |
| --- | --- | --- | --- | --- |
| 12.4987 | 112.3875 | 0.0000 | 2.2765e+08 | 1 |

**Adduct Signal Fractions**

- **Unmodified**: 2.2765e+08

### ANLVPVPITN(N)ATLDR Hex 6 HexNAc 5

**Mass:**  3580.6100

(1.639e-06)

**Charge States:** 3

**Start Position**: 24

**End Position**: 39

| Profile Score | Tandem Score | TDA q-value | Volume | Spectrum Matches |
| --- | --- | --- | --- | --- |
| -3.6495 | 112.2614 | 0.0000 | 2.8221e+07 | 3 |

**Adduct Signal Fractions**

- **Unmodified**: 2.8221e+07

### FTPN(N)KTEDTIFLR Hex 7 HexNAc 6

**Mass:**  3933.6708

(1.257e-06)

**Charge States:** 3, 4

**Start Position**: 69

**End Position**: 82

| Profile Score | Tandem Score | TDA q-value | Volume | Spectrum Matches |
| --- | --- | --- | --- | --- |
| 13.5369 | 111.1400 | 0.0000 | 1.7397e+08 | 6 |

**Adduct Signal Fractions**

- **Unmodified**: 1.7397e+08

### SVQEIQATFFYFTPN(N)K Hex 7 HexNAc 7

**Mass:**  4474.8781

(1.809e-06)

**Charge States:** 3, 4

**Start Position**: 58

**End Position**: 74

| Profile Score | Tandem Score | TDA q-value | Volume | Spectrum Matches |
| --- | --- | --- | --- | --- |
| 12.1592 | 110.7709 | 0.0000 | 1.0339e+08 | 3 |

**Adduct Signal Fractions**

- **Unmodified**: 1.0339e+08

### FTPN(N)KTEDTIFLR Hex 6 HexNAc 5

**Mass:**  3568.5385

(9.490e-07)

**Charge States:** 3, 4

**Start Position**: 69

**End Position**: 82

| Profile Score | Tandem Score | TDA q-value | Volume | Spectrum Matches |
| --- | --- | --- | --- | --- |
| 14.2403 | 109.8215 | 0.0000 | 9.3317e+07 | 5 |

**Adduct Signal Fractions**

- **Unmodified**: 9.3317e+07

### LVPVPITN(N)ATLDR Hex 7 HexNAc 5

**Mass:**  3559.6029

(5.700e-04)

**Start Position**: 26

**End Position**: 39

| Profile Score | Tandem Score | TDA q-value | Volume | Spectrum Matches |
| --- | --- | --- | --- | --- |
| 0.0000 | 108.3191 | 0.0000 | 0.0000e+00 | 1 |

Adduct:

No Chromatogram Found

### QNQC(C)FYN(N)SSYLNVQR Hex 8 HexNAc 7

**Mass:**  4637.8542

(1.935e-06)

**Charge States:** 3, 4

**Start Position**: 87

**End Position**: 102

| Profile Score | Tandem Score | TDA q-value | Volume | Spectrum Matches |
| --- | --- | --- | --- | --- |
| -5.7845 | 106.7519 | 0.0000 | 1.1450e+08 | 1 |

**Adduct Signal Fractions**

- **Unmodified**: 1.1450e+08

### QNQC(C)FYN(N)SSYLNVQR Fuc 2 Hex 7 HexNAc 6

**Mass:**  4564.8342

(3.081e-06)

**Charge States:** 4

**Start Position**: 87

**End Position**: 102

| Profile Score | Tandem Score | TDA q-value | Volume | Spectrum Matches |
| --- | --- | --- | --- | --- |
| -19.7542 | 106.6529 | 0.0000 | 3.1858e+07 | 1 |

**Adduct Signal Fractions**

- **Unmodified**: 3.1858e+07

### YFTPN(N)KTEDTIFLR Fuc 1 Hex 8 HexNAc 7

**Mass:**  4607.9113

(-1.746e-06)

**Charge States:** 4

**Start Position**: 68

**End Position**: 82

| Profile Score | Tandem Score | TDA q-value | Volume | Spectrum Matches |
| --- | --- | --- | --- | --- |
| -20.1090 | 105.9241 | 0.0000 | 1.2398e+07 | 1 |

**Adduct Signal Fractions**

- **Unmodified**: 1.2398e+07

### NLVPVPITN(N)ATLDR Fuc 1 Hex 6 HexNAc 4

**Mass:**  3452.5519

(9.421e-07)

**Charge States:** 3, 4

**Start Position**: 25

**End Position**: 39

| Profile Score | Tandem Score | TDA q-value | Volume | Spectrum Matches |
| --- | --- | --- | --- | --- |
| 15.4858 | 105.3648 | 0.0000 | 3.7699e+09 | 11 |

**Adduct Signal Fractions**

- **Unmodified**: 3.7699e+09

### LVPVPITN(N)ATLDR Fuc 2 Hex 7 HexNAc 6

**Mass:**  4052.7766

(1.550e-06)

**Charge States:** 4

**Start Position**: 26

**End Position**: 39

| Profile Score | Tandem Score | TDA q-value | Volume | Spectrum Matches |
| --- | --- | --- | --- | --- |
| -18.6597 | 104.3272 | 0.0000 | 3.8097e+07 | 1 |

**Adduct Signal Fractions**

- **Unmodified**: 3.8097e+07

### SVQEIQATFFYFTPN(N)KTEDTIFLR Hex 5 HexNAc 4

**Mass:**  4517.0368

(1.392e-06)

**Charge States:** 4

**Start Position**: 58

**End Position**: 82

| Profile Score | Tandem Score | TDA q-value | Volume | Spectrum Matches |
| --- | --- | --- | --- | --- |
| -4.2861 | 103.9531 | 0.0000 | 1.8247e+07 | 2 |

**Adduct Signal Fractions**

- **Unmodified**: 1.8247e+07

### TPN(N)KTEDTIFLR Fuc 1 Hex 7 HexNAc 6

**Mass:**  3932.6606

(1.447e-06)

**Charge States:** 4

**Start Position**: 70

**End Position**: 82

| Profile Score | Tandem Score | TDA q-value | Volume | Spectrum Matches |
| --- | --- | --- | --- | --- |
| -4.6100 | 103.5604 | 0.0000 | 1.0023e+07 | 2 |

**Adduct Signal Fractions**

- **Unmodified**: 1.0023e+07

### SVQEIQATFFYFTPN(N)K Fuc 4 Hex 6 HexNAc 6

**Mass:**  4693.9562

(-3.940e-06)

**Charge States:** 4

**Start Position**: 58

**End Position**: 74

| Profile Score | Tandem Score | TDA q-value | Volume | Spectrum Matches |
| --- | --- | --- | --- | --- |
| 13.5122 | 103.3033 | 0.0000 | 5.6809e+07 | 3 |

**Adduct Signal Fractions**

- **Unmodified**: 5.6809e+07

### PVPITN(N)ATLDR Hex 8 HexNAc 4 Neu5Ac 2

**Mass:**  3886.5722

(-7.583e-06)

**Charge States:** 3, 4

**Start Position**: 28

**End Position**: 39

| Profile Score | Tandem Score | TDA q-value | Volume | Spectrum Matches |
| --- | --- | --- | --- | --- |
| 7.2147 | 103.1703 | 0.0000 | 3.3640e+08 | 8 |

**Adduct Signal Fractions**

- **Unmodified**: 3.3640e+08

### SVQEIQATFFYFTPN(N)KTEDTIFLR Hex 8 HexNAc 7

**Mass:**  5612.4362

(1.786e-06)

**Charge States:** 4, 5

**Start Position**: 58

**End Position**: 82

| Profile Score | Tandem Score | TDA q-value | Volume | Spectrum Matches |
| --- | --- | --- | --- | --- |
| 14.4699 | 102.9564 | 0.0000 | 2.5303e+08 | 5 |

**Adduct Signal Fractions**

- **Unmodified**: 2.5303e+08

### YFTPN(N)KTEDTIFLR Hex 3 HexNAc 5 Neu5Ac 3

**Mass:**  4118.7155

(-1.234e-06)

**Charge States:** 4

**Start Position**: 68

**End Position**: 82

| Profile Score | Tandem Score | TDA q-value | Volume | Spectrum Matches |
| --- | --- | --- | --- | --- |
| -4.8281 | 102.3033 | 0.0000 | 7.1547e+07 | 3 |

**Adduct Signal Fractions**

- **Unmodified**: 7.1547e+07

### PVPITN(N)ATLDR Fuc 2 Hex 3 HexNAc 7 Neu5Ac 3

**Mass:**  4268.8132

(9.545e-06)

**Charge States:** 3, 4

**Start Position**: 28

**End Position**: 39

| Profile Score | Tandem Score | TDA q-value | Volume | Spectrum Matches |
| --- | --- | --- | --- | --- |
| -4.0556 | 102.0735 | 0.0000 | 2.3219e+07 | 3 |

**Adduct Signal Fractions**

- **Unmodified**: 2.3219e+07

### YFTPN(N)KTEDTIFLR Hex 9 HexNAc 8

**Mass:**  4826.9982

(9.451e-07)

**Charge States:** 4

**Start Position**: 68

**End Position**: 82

| Profile Score | Tandem Score | TDA q-value | Volume | Spectrum Matches |
| --- | --- | --- | --- | --- |
| 12.4434 | 101.9477 | 0.0000 | 6.4966e+07 | 2 |

**Adduct Signal Fractions**

- **Unmodified**: 6.4966e+07

### YFTPN(N)KTEDTIFLR Hex 7 HexNAc 7

**Mass:**  4299.8127

(7.697e-07)

**Charge States:** 4

**Start Position**: 68

**End Position**: 82

| Profile Score | Tandem Score | TDA q-value | Volume | Spectrum Matches |
| --- | --- | --- | --- | --- |
| -5.1445 | 100.9264 | 0.0000 | 7.0400e+07 | 2 |

**Adduct Signal Fractions**

- **Unmodified**: 7.0400e+07

### PVPITN(N)ATLDR Hex 8 HexNAc 4 Neu5Ac 1

**Mass:**  3595.4943

(7.621e-07)

**Charge States:** 4

**Start Position**: 28

**End Position**: 39

| Profile Score | Tandem Score | TDA q-value | Volume | Spectrum Matches |
| --- | --- | --- | --- | --- |
| -6.3895 | 100.5889 | 0.0000 | 1.3295e+08 | 2 |

**Adduct Signal Fractions**

- **Unmodified**: 1.3295e+08

### PVPITN(N)ATLDR Hex 7 HexNAc 4 Neu5Ac 1

**Mass:**  3433.4719

(8.859e-06)

**Charge States:** 3, 4, 5

**Start Position**: 28

**End Position**: 39

| Profile Score | Tandem Score | TDA q-value | Volume | Spectrum Matches |
| --- | --- | --- | --- | --- |
| 14.2457 | 99.6676 | 0.0000 | 9.6841e+08 | 9 |

**Adduct Signal Fractions**

- **Unmodified**: 9.6841e+08

### PVPITN(N)ATLDR Fuc 1 Hex 3 HexNAc 8 Neu5Ac 1

**Mass:**  3743.6317

(7.702e-06)

**Start Position**: 28

**End Position**: 39

| Profile Score | Tandem Score | TDA q-value | Volume | Spectrum Matches |
| --- | --- | --- | --- | --- |
| 0.0000 | 97.9426 | 0.0000 | 0.0000e+00 | 1 |

Adduct:

No Chromatogram Found

### LVPVPITN(N)ATLDR Fuc 2 Hex 6 HexNAc 5

**Mass:**  3689.6443

(5.440e-04)

**Start Position**: 26

**End Position**: 39

| Profile Score | Tandem Score | TDA q-value | Volume | Spectrum Matches |
| --- | --- | --- | --- | --- |
| 0.0000 | 97.6075 | 0.0000 | 0.0000e+00 | 1 |

Adduct:

No Chromatogram Found

### SVQEIQATFFYFTPN(N)K Fuc 3 Hex 4 HexNAc 5

**Mass:**  4020.7092

(-2.024e-06)

**Charge States:** 3, 4

**Start Position**: 58

**End Position**: 74

| Profile Score | Tandem Score | TDA q-value | Volume | Spectrum Matches |
| --- | --- | --- | --- | --- |
| 7.3298 | 96.5438 | 0.0000 | 2.7910e+07 | 2 |

**Adduct Signal Fractions**

- **Unmodified**: 2.7910e+07

### FYFTPN(N)KTEDTIFLR Fuc 1 Hex 6 HexNAc 5

**Mass:**  4024.7292

(9.248e-07)

**Charge States:** 4

**Start Position**: 67

**End Position**: 82

| Profile Score | Tandem Score | TDA q-value | Volume | Spectrum Matches |
| --- | --- | --- | --- | --- |
| -4.0690 | 95.7808 | 0.0000 | 2.2372e+07 | 3 |

**Adduct Signal Fractions**

- **Unmodified**: 2.2372e+07

### LVPVPITN(N)ATLDR Fuc 4 Hex 3 HexNAc 7 Neu5Ac 1

**Mass:**  4190.8859

(9.439e-06)

**Charge States:** 3, 4

**Start Position**: 26

**End Position**: 39

| Profile Score | Tandem Score | TDA q-value | Volume | Spectrum Matches |
| --- | --- | --- | --- | --- |
| 13.6249 | 95.4103 | 0.0000 | 1.9681e+08 | 12 |

**Adduct Signal Fractions**

- **Unmodified**: 1.9681e+08

### C(C)AN(D)LVPVPITN(N)ATLDR Hex 6 HexNAc 5

**Mass:**  3741.6212

(2.646e-04)

**Charge States:** 3, 4

**Start Position**: 23

**End Position**: 39

| Profile Score | Tandem Score | TDA q-value | Volume | Spectrum Matches |
| --- | --- | --- | --- | --- |
| 9.3656 | 95.0082 | 0.0000 | 2.4604e+08 | 13 |

**Adduct Signal Fractions**

- **Unmodified**: 2.4604e+08

### YFTPN(N)KTEDTIFLR Fuc 2 Hex 7 HexNAc 6

**Mass:**  4391.8577

(6.864e-04)

**Start Position**: 68

**End Position**: 82

| Profile Score | Tandem Score | TDA q-value | Volume | Spectrum Matches |
| --- | --- | --- | --- | --- |
| 0.0000 | 94.6840 | 0.0000 | 0.0000e+00 | 1 |

Adduct:

No Chromatogram Found

### YFTPN(N)KTEDTIFLR Hex 6 HexNAc 5

**Mass:**  3732.6097

(2.714e-04)

**Start Position**: 68

**End Position**: 82

| Profile Score | Tandem Score | TDA q-value | Volume | Spectrum Matches |
| --- | --- | --- | --- | --- |
| 0.0000 | 94.6126 | 0.0000 | 0.0000e+00 | 1 |

Adduct:

No Chromatogram Found

### QNQC(C)FYN(N)SSYLNVQR Hex 7 HexNAc 4 Neu5Ac 2

**Mass:**  4448.7394

(6.399e-07)

**Charge States:** 4

**Start Position**: 87

**End Position**: 102

| Profile Score | Tandem Score | TDA q-value | Volume | Spectrum Matches |
| --- | --- | --- | --- | --- |
| -20.0198 | 93.9094 | 0.0000 | 3.7493e+07 | 2 |

**Adduct Signal Fractions**

- **Unmodified**: 3.7493e+07

### SVQEIQATFFYFTPN(N)KTEDTIFLR Fuc 1 Hex 7 HexNAc 6

**Mass:**  5393.3644

(2.397e-06)

**Charge States:** 4, 5

**Start Position**: 58

**End Position**: 82

| Profile Score | Tandem Score | TDA q-value | Volume | Spectrum Matches |
| --- | --- | --- | --- | --- |
| 12.7694 | 93.7131 | 0.0000 | 2.8246e+08 | 6 |

**Adduct Signal Fractions**

- **Unmodified**: 2.8246e+08

### YFTPN(N)KTEDTIFLR Hex 7 HexNAc 4 Neu5Ac 2

**Mass:**  4272.7614

(-5.884e-07)

**Charge States:** 4

**Start Position**: 68

**End Position**: 82

| Profile Score | Tandem Score | TDA q-value | Volume | Spectrum Matches |
| --- | --- | --- | --- | --- |
| -4.0872 | 92.8185 | 0.0000 | 1.4040e+08 | 4 |

**Adduct Signal Fractions**

- **Unmodified**: 1.4040e+08

### NLVPVPITN(N)ATLDR Fuc 4 Hex 4 HexNAc 7

**Mass:**  4175.8776

(6.275e-06)

**Charge States:** 3, 4

**Start Position**: 25

**End Position**: 39

| Profile Score | Tandem Score | TDA q-value | Volume | Spectrum Matches |
| --- | --- | --- | --- | --- |
| 13.3792 | 91.8838 | 0.0000 | 1.2997e+08 | 11 |

**Adduct Signal Fractions**

- **Unmodified**: 1.2997e+08

### LVPVPITN(N)ATLDR Fuc 2 Hex 6 HexNAc 4 Neu5Ac 1

**Mass:**  3778.6909

(8.043e-04)

**Start Position**: 26

**End Position**: 39

| Profile Score | Tandem Score | TDA q-value | Volume | Spectrum Matches |
| --- | --- | --- | --- | --- |
| 0.0000 | 90.2792 | 0.0000 | 0.0000e+00 | 1 |

Adduct:

No Chromatogram Found

### VPVPITN(N)ATLDR Fuc 1 Hex 6 HexNAc 5

**Mass:**  3428.4995

(-2.138e-07)

**Charge States:** 3

**Start Position**: 27

**End Position**: 39

| Profile Score | Tandem Score | TDA q-value | Volume | Spectrum Matches |
| --- | --- | --- | --- | --- |
| -5.4350 | 89.0134 | 0.0000 | 2.8954e+06 | 4 |

**Adduct Signal Fractions**

- **Unmodified**: 2.8954e+06

### FYFTPN(N)KTEDTIFLR Hex 8 HexNAc 7

**Mass:**  4608.9348

(1.556e-06)

**Charge States:** 4

**Start Position**: 67

**End Position**: 82

| Profile Score | Tandem Score | TDA q-value | Volume | Spectrum Matches |
| --- | --- | --- | --- | --- |
| 19.4247 | 86.1148 | 0.0000 | 2.8137e+07 | 2 |

**Adduct Signal Fractions**

- **Unmodified**: 2.8137e+07

### LVPVPITN(N)ATLDR Fuc 5 Hex 7 HexNAc 6

**Mass:**  4490.9338

(-1.904e-06)

**Charge States:** 4

**Start Position**: 26

**End Position**: 39

| Profile Score | Tandem Score | TDA q-value | Volume | Spectrum Matches |
| --- | --- | --- | --- | --- |
| -20.4873 | 86.0265 | 0.0000 | 5.4581e+06 | 1 |

**Adduct Signal Fractions**

- **Unmodified**: 5.4581e+06

### LVPVPITN(N)ATLDR Fuc 2 Hex 5 HexNAc 3 Neu5Ac 1

**Mass:**  3412.5525

(5.966e-04)

**Charge States:** 3, 4

**Start Position**: 26

**End Position**: 39

| Profile Score | Tandem Score | TDA q-value | Volume | Spectrum Matches |
| --- | --- | --- | --- | --- |
| 10.0108 | 85.9570 | 0.0000 | 2.4486e+09 | 1 |

**Adduct Signal Fractions**

- **Unmodified**: 2.4486e+09

### LVPVPITN(N)ATLDR Hex 6 HexNAc 5 Neu5Ac 2

**Mass:**  3977.7214

(2.077e-06)

**Charge States:** 3, 4

**Start Position**: 26

**End Position**: 39

| Profile Score | Tandem Score | TDA q-value | Volume | Spectrum Matches |
| --- | --- | --- | --- | --- |
| -4.9633 | 85.8025 | 0.0000 | 1.0840e+07 | 3 |

**Adduct Signal Fractions**

- **Unmodified**: 1.0840e+07

### FTPN(N)KTEDTIFLR Fuc 1 Hex 7 HexNAc 6

**Mass:**  4079.7275

(1.047e-06)

**Charge States:** 3, 4

**Start Position**: 69

**End Position**: 82

| Profile Score | Tandem Score | TDA q-value | Volume | Spectrum Matches |
| --- | --- | --- | --- | --- |
| -4.0476 | 85.1653 | 0.0000 | 3.1681e+07 | 3 |

**Adduct Signal Fractions**

- **Unmodified**: 3.1681e+07

### PVPITN(N)ATLDR Fuc 2 Hex 8 HexNAc 4

**Mass:**  3597.5145

(2.787e-04)

**Start Position**: 28

**End Position**: 39

| Profile Score | Tandem Score | TDA q-value | Volume | Spectrum Matches |
| --- | --- | --- | --- | --- |
| 0.0000 | 84.7673 | 0.0000 | 0.0000e+00 | 1 |

Adduct:

No Chromatogram Found

### FTPN(N)KTEDTIFLR Fuc 1 Hex 7 HexNAc 6

**Mass:**  4080.7221

(2.447e-04)

**Start Position**: 69

**End Position**: 82

| Profile Score | Tandem Score | TDA q-value | Volume | Spectrum Matches |
| --- | --- | --- | --- | --- |
| 0.0000 | 83.2774 | 0.0000 | 0.0000e+00 | 1 |

Adduct:

No Chromatogram Found

### FTPN(N)KTEDTIFLR Fuc 1 Hex 6 HexNAc 5

**Mass:**  3714.5966

(1.515e-06)

**Charge States:** 3, 4

**Start Position**: 69

**End Position**: 82

| Profile Score | Tandem Score | TDA q-value | Volume | Spectrum Matches |
| --- | --- | --- | --- | --- |
| -3.7361 | 83.2181 | 0.0000 | 1.8606e+07 | 3 |

**Adduct Signal Fractions**

- **Unmodified**: 1.8606e+07

### TPN(N)KTEDTIFLR Hex 5 HexNAc 4

**Mass:**  3056.3388

(2.207e-06)

**Charge States:** 3

**Start Position**: 70

**End Position**: 82

| Profile Score | Tandem Score | TDA q-value | Volume | Spectrum Matches |
| --- | --- | --- | --- | --- |
| 11.7029 | 81.9076 | 0.0000 | 5.2848e+06 | 3 |

**Adduct Signal Fractions**

- **Unmodified**: 5.2848e+06

### FYN(N)SSYLNVQR Hex 6 HexNAc 5

**Mass:**  3378.3910

(2.989e-04)

**Start Position**: 91

**End Position**: 102

| Profile Score | Tandem Score | TDA q-value | Volume | Spectrum Matches |
| --- | --- | --- | --- | --- |
| 0.0000 | 81.7388 | 0.0000 | 0.0000e+00 | 1 |

Adduct:

No Chromatogram Found

### FTPN(N)KTEDTIFLR Hex 8 HexNAc 7

**Mass:**  4298.7995

(1.715e-06)

**Charge States:** 3, 4

**Start Position**: 69

**End Position**: 82

| Profile Score | Tandem Score | TDA q-value | Volume | Spectrum Matches |
| --- | --- | --- | --- | --- |
| -5.3360 | 81.2359 | 0.0000 | 1.2572e+07 | 7 |

**Adduct Signal Fractions**

- **Unmodified**: 1.2572e+07

### VPVPITN(N)ATLDR Fuc 2 Hex 4 HexNAc 6

**Mass:**  3453.5370

(2.621e-06)

**Charge States:** 4

**Start Position**: 27

**End Position**: 39

| Profile Score | Tandem Score | TDA q-value | Volume | Spectrum Matches |
| --- | --- | --- | --- | --- |
| -4.1406 | 76.8196 | 0.0026 | 4.2782e+07 | 5 |

**Adduct Signal Fractions**

- **Unmodified**: 4.2782e+07

### YN(N)SSYLNVQR Hex 6 HexNAc 5

**Mass:**  3230.3142

(1.614e-06)

**Charge States:** 3

**Start Position**: 92

**End Position**: 102

| Profile Score | Tandem Score | TDA q-value | Volume | Spectrum Matches |
| --- | --- | --- | --- | --- |
| 10.4667 | 76.5706 | 0.0026 | 8.6688e+06 | 3 |

**Adduct Signal Fractions**

- **Unmodified**: 8.6688e+06

### SVQEIQATFFYFTPN(N)KTEDTIFLR Fuc 2 Hex 7 HexNAc 6

**Mass:**  5539.4221

(2.052e-06)

**Charge States:** 4, 5

**Start Position**: 58

**End Position**: 82

| Profile Score | Tandem Score | TDA q-value | Volume | Spectrum Matches |
| --- | --- | --- | --- | --- |
| -19.8386 | 75.5299 | 0.0026 | 3.1151e+07 | 2 |

**Adduct Signal Fractions**

- **Unmodified**: 3.1151e+07

### YN(N)SSYLNVQR Hex 7 HexNAc 6

**Mass:**  3596.4601

(2.822e-04)

**Start Position**: 92

**End Position**: 102

| Profile Score | Tandem Score | TDA q-value | Volume | Spectrum Matches |
| --- | --- | --- | --- | --- |
| 0.0000 | 75.2244 | 0.0026 | 0.0000e+00 | 1 |

Adduct:

No Chromatogram Found

### FYN(N)SSYLNVQR Hex 7 HexNAc 6

**Mass:**  3742.5168

(1.130e-06)

**Charge States:** 3, 4

**Start Position**: 91

**End Position**: 102

| Profile Score | Tandem Score | TDA q-value | Volume | Spectrum Matches |
| --- | --- | --- | --- | --- |
| 12.9146 | 75.0465 | 0.0026 | 1.5990e+07 | 5 |

**Adduct Signal Fractions**

- **Unmodified**: 1.5990e+07

### SVQEIQATFFYFTPN(N)K Hex 10 HexNAc 9

**Mass:**  5367.2014

(1.795e-06)

**Charge States:** 4

**Start Position**: 58

**End Position**: 74

| Profile Score | Tandem Score | TDA q-value | Volume | Spectrum Matches |
| --- | --- | --- | --- | --- |
| -4.9170 | 74.2359 | 0.0026 | 1.1156e+07 | 1 |

**Adduct Signal Fractions**

- **Unmodified**: 1.1156e+07

### FYFTPN(N)KTEDTIFLR Hex 7 HexNAc 4 Neu5Ac 2

**Mass:**  4419.8342

(1.058e-06)

**Charge States:** 4

**Start Position**: 67

**End Position**: 82

| Profile Score | Tandem Score | TDA q-value | Volume | Spectrum Matches |
| --- | --- | --- | --- | --- |
| -4.7372 | 73.9759 | 0.0026 | 7.6413e+06 | 4 |

**Adduct Signal Fractions**

- **Unmodified**: 7.6413e+06

### PVPITN(N)ATLDR Hex 3 HexNAc 7 Neu5Ac 4

**Mass:**  4267.7477

(-9.242e-07)

**Charge States:** 4

**Start Position**: 28

**End Position**: 39

| Profile Score | Tandem Score | TDA q-value | Volume | Spectrum Matches |
| --- | --- | --- | --- | --- |
| -21.3699 | 72.7914 | 0.0026 | 2.9664e+06 | 1 |

**Adduct Signal Fractions**

- **Unmodified**: 2.9664e+06

### SVQEIQATFFYFTPN(N)K Hex 9 HexNAc 7

**Mass:**  4802.0249

(6.350e-04)

**Start Position**: 58

**End Position**: 74

| Profile Score | Tandem Score | TDA q-value | Volume | Spectrum Matches |
| --- | --- | --- | --- | --- |
| 0.0000 | 69.3034 | 0.0026 | 0.0000e+00 | 1 |

Adduct:

No Chromatogram Found

### YFTPN(N)KTEDTIFLR Fuc 4 Hex 5 HexNAc 5

**Mass:**  4153.7546

(-5.154e-06)

**Charge States:** 3, 4

**Start Position**: 68

**End Position**: 82

| Profile Score | Tandem Score | TDA q-value | Volume | Spectrum Matches |
| --- | --- | --- | --- | --- |
| 13.6947 | 67.5354 | 0.0026 | 4.8101e+08 | 6 |

**Adduct Signal Fractions**

- **Unmodified**: 4.8101e+08

### SVQEIQATFFYFTPN(N)KTEDTIFLR Hex 9 HexNAc 8

**Mass:**  5977.5674

(1.284e-06)

**Charge States:** 4, 5

**Start Position**: 58

**End Position**: 82

| Profile Score | Tandem Score | TDA q-value | Volume | Spectrum Matches |
| --- | --- | --- | --- | --- |
| 13.1785 | 66.6207 | 0.0026 | 8.2052e+07 | 6 |

**Adduct Signal Fractions**

- **Unmodified**: 8.2052e+07

### QIPLC(C)ANLVPVPITN(N)ATLDR Hex 6 HexNAc 5

**Mass:**  4191.9186

(1.415e-06)

**Charge States:** 3, 4

**Start Position**: 19

**End Position**: 39

| Profile Score | Tandem Score | TDA q-value | Volume | Spectrum Matches |
| --- | --- | --- | --- | --- |
| 12.4707 | 66.4761 | 0.0026 | 4.3313e+08 | 8 |

**Adduct Signal Fractions**

- **Unmodified**: 4.3313e+08

### SVQEIQATFFYFTPN(N)KTEDTIFLR Fuc 1 Hex 8 HexNAc 7

**Mass:**  5758.4909

(2.355e-06)

**Charge States:** 4, 5

**Start Position**: 58

**End Position**: 82

| Profile Score | Tandem Score | TDA q-value | Volume | Spectrum Matches |
| --- | --- | --- | --- | --- |
| 13.9799 | 60.2073 | 0.0026 | 4.8796e+07 | 4 |

**Adduct Signal Fractions**

- **Unmodified**: 4.8796e+07

### SVQEIQATFFYFTPN(N)K Fuc 1 Hex 9 HexNAc 8

**Mass:**  5148.1262

(1.585e-06)

**Charge States:** 4

**Start Position**: 58

**End Position**: 74

| Profile Score | Tandem Score | TDA q-value | Volume | Spectrum Matches |
| --- | --- | --- | --- | --- |
| 7.3730 | 56.2667 | 0.0026 | 1.1962e+07 | 2 |

**Adduct Signal Fractions**

- **Unmodified**: 1.1962e+07

### C(C)ANLVPVPITN(N)ATLDR Hex 5 HexNAc 4

**Mass:**  3375.5044

(1.352e-06)

**Charge States:** 3

**Start Position**: 23

**End Position**: 39

| Profile Score | Tandem Score | TDA q-value | Volume | Spectrum Matches |
| --- | --- | --- | --- | --- |
| -4.8575 | 56.2064 | 0.0026 | 1.2941e+07 | 2 |

**Adduct Signal Fractions**

- **Unmodified**: 1.2941e+07

### C(C)AN(D)LVPVPITN(N)ATLDR Fuc 1 Hex 6 HexNAc 5

**Mass:**  3887.6812

(2.553e-04)

**Charge States:** 3, 4

**Start Position**: 23

**End Position**: 39

| Profile Score | Tandem Score | TDA q-value | Volume | Spectrum Matches |
| --- | --- | --- | --- | --- |
| 8.1972 | 54.5072 | 0.0026 | 5.4634e+07 | 6 |

**Adduct Signal Fractions**

- **Unmodified**: 5.4634e+07

### FYFTPN(N)KTEDTIFLR Fuc 1 Hex 7 HexNAc 6

**Mass:**  4389.8605

(9.806e-07)

**Charge States:** 3, 4

**Start Position**: 67

**End Position**: 82

| Profile Score | Tandem Score | TDA q-value | Volume | Spectrum Matches |
| --- | --- | --- | --- | --- |
| 12.6751 | 51.4086 | 0.0026 | 4.5801e+07 | 4 |

**Adduct Signal Fractions**

- **Unmodified**: 4.5801e+07

### C(C)ANLVPVPITN(N)ATLDR Fuc 1 Hex 6 HexNAc 5

**Mass:**  3886.7321

(3.757e-07)

**Charge States:** 3, 4

**Start Position**: 23

**End Position**: 39

| Profile Score | Tandem Score | TDA q-value | Volume | Spectrum Matches |
| --- | --- | --- | --- | --- |
| -19.2200 | 50.4439 | 0.0026 | 4.5935e+07 | 2 |

**Adduct Signal Fractions**

- **Unmodified**: 4.5935e+07

### QIPLC(C)ANLVPVPITN(N)ATLDR Hex 5 HexNAc 5 Neu5Ac 1

**Mass:**  4320.9538

(-2.358e-06)

**Charge States:** 3, 4

**Start Position**: 19

**End Position**: 39

| Profile Score | Tandem Score | TDA q-value | Volume | Spectrum Matches |
| --- | --- | --- | --- | --- |
| 15.0384 | 49.9667 | 0.0026 | 5.4105e+08 | 9 |

**Adduct Signal Fractions**

- **Unmodified**: 5.4105e+08

### C(C)AN(D)LVPVPITN(N)ATLDR Fuc 1 Hex 4 HexNAc 5

**Mass:**  3563.5598

(2.767e-04)

**Charge States:** 3, 4

**Start Position**: 23

**End Position**: 39

| Profile Score | Tandem Score | TDA q-value | Volume | Spectrum Matches |
| --- | --- | --- | --- | --- |
| 10.9757 | 49.1620 | 0.0026 | 3.4497e+08 | 2 |

**Adduct Signal Fractions**

- **Unmodified**: 3.4497e+08

### SVQEIQATFFYFTPN(N)KTEDTIFLR Hex 7 HexNAc 4 Neu5Ac 2

**Mass:**  5423.3352

(1.886e-06)

**Charge States:** 4

**Start Position**: 58

**End Position**: 82

| Profile Score | Tandem Score | TDA q-value | Volume | Spectrum Matches |
| --- | --- | --- | --- | --- |
| -3.3321 | 48.1059 | 0.0026 | 3.3537e+07 | 3 |

**Adduct Signal Fractions**

- **Unmodified**: 3.3537e+07

### ANLVPVPITN(N)ATLDR Fuc 1 Hex 10 HexNAc 5

**Mass:**  4374.8674

(-2.579e-08)

**Charge States:** 4

**Start Position**: 24

**End Position**: 39

| Profile Score | Tandem Score | TDA q-value | Volume | Spectrum Matches |
| --- | --- | --- | --- | --- |
| -4.6626 | 47.3836 | 0.0026 | 3.0237e+07 | 1 |

**Adduct Signal Fractions**

- **Unmodified**: 3.0237e+07

### SVQEIQATFFYFTPN(N)K Hex 7 HexNAc 6 Neu5Ac 1

**Mass:**  4562.8952

(1.640e-06)

**Charge States:** 4

**Start Position**: 58

**End Position**: 74

| Profile Score | Tandem Score | TDA q-value | Volume | Spectrum Matches |
| --- | --- | --- | --- | --- |
| -5.5090 | 45.1411 | 0.0026 | 3.6096e+06 | 2 |

**Adduct Signal Fractions**

- **Unmodified**: 3.6096e+06

### NLVPVPITN(N)ATLDR Hex 6 HexNAc 5

**Mass:**  3509.5720

(1.762e-06)

**Charge States:** 3

**Start Position**: 25

**End Position**: 39

| Profile Score | Tandem Score | TDA q-value | Volume | Spectrum Matches |
| --- | --- | --- | --- | --- |
| -4.6315 | 43.6352 | 0.0026 | 3.4026e+07 | 1 |

**Adduct Signal Fractions**

- **Unmodified**: 3.4026e+07

### ANLVPVPITN(N)ATLDR Fuc 5 Hex 7 HexNAc 9

**Mass:**  5285.2548

(-1.467e-06)

**Charge States:** 5

**Start Position**: 24

**End Position**: 39

| Profile Score | Tandem Score | TDA q-value | Volume | Spectrum Matches |
| --- | --- | --- | --- | --- |
| -4.9899 | 42.9620 | 0.0026 | 1.0259e+08 | 2 |

**Adduct Signal Fractions**

- **Unmodified**: 1.0259e+08

### NLVPVPITN(N)ATLDR Fuc 1 Hex 5 HexNAc 3

**Mass:**  3087.4151

(1.330e-06)

**Charge States:** 3, 4

**Start Position**: 25

**End Position**: 39

| Profile Score | Tandem Score | TDA q-value | Volume | Spectrum Matches |
| --- | --- | --- | --- | --- |
| 14.3616 | 42.8924 | 0.0026 | 2.0339e+08 | 3 |

**Adduct Signal Fractions**

- **Unmodified**: 2.0339e+08

### TPN(N)KTEDTIFLR Fuc 1 Hex 6 HexNAc 5

**Mass:**  3567.5256

(-2.205e-07)

**Charge States:** 3, 4

**Start Position**: 70

**End Position**: 82

| Profile Score | Tandem Score | TDA q-value | Volume | Spectrum Matches |
| --- | --- | --- | --- | --- |
| -5.4179 | 42.1310 | 0.0026 | 7.7850e+06 | 4 |

**Adduct Signal Fractions**

- **Unmodified**: 7.7850e+06

### C(C)AN(D)LVPVPITN(N)ATLDR Hex 4 HexNAc 5

**Mass:**  3417.5084

(2.873e-04)

**Charge States:** 3, 4

**Start Position**: 23

**End Position**: 39

| Profile Score | Tandem Score | TDA q-value | Volume | Spectrum Matches |
| --- | --- | --- | --- | --- |
| 10.2168 | 41.9573 | 0.0026 | 1.1691e+09 | 3 |

**Adduct Signal Fractions**

- **Unmodified**: 1.1691e+09

### FYFTPN(N)KTEDTIFLR Fuc 2 Hex 7 HexNAc 6

**Mass:**  4535.9191

(1.314e-06)

**Charge States:** 4

**Start Position**: 67

**End Position**: 82

| Profile Score | Tandem Score | TDA q-value | Volume | Spectrum Matches |
| --- | --- | --- | --- | --- |
| -20.9870 | 40.3722 | 0.0026 | 2.0786e+06 | 1 |

**Adduct Signal Fractions**

- **Unmodified**: 2.0786e+06

### QN(D)QC(C)FYN(N)SSYLNVQR Fuc 3 Hex 8 HexNAc 4

**Mass:**  4467.7150

(2.189e-04)

**Charge States:** 4

**Start Position**: 87

**End Position**: 102

| Profile Score | Tandem Score | TDA q-value | Volume | Spectrum Matches |
| --- | --- | --- | --- | --- |
| -19.9544 | 40.1181 | 0.0038 | 1.9708e+07 | 2 |

**Adduct Signal Fractions**

- **Unmodified**: 1.9708e+07

### QNQC(C)FYN(N)SSYLNVQR Fuc 1 Hex 8 HexNAc 7

**Mass:**  4783.9089

(2.077e-06)

**Charge States:** 4

**Start Position**: 87

**End Position**: 102

| Profile Score | Tandem Score | TDA q-value | Volume | Spectrum Matches |
| --- | --- | --- | --- | --- |
| -20.6395 | 40.0761 | 0.0038 | 8.1760e+06 | 1 |

**Adduct Signal Fractions**

- **Unmodified**: 8.1760e+06

### SVQEIQATFFYFTPN(N)KTEDTIFLR Fuc 4 Hex 5 HexNAc 5

**Mass:**  5304.3249

(-3.138e-06)

**Charge States:** 4, 5

**Start Position**: 58

**End Position**: 82

| Profile Score | Tandem Score | TDA q-value | Volume | Spectrum Matches |
| --- | --- | --- | --- | --- |
| 13.6832 | 39.8478 | 0.0049 | 2.7053e+08 | 12 |

**Adduct Signal Fractions**

- **Unmodified**: 2.7053e+08

### SVQEIQATFFYFTPN(N)K Fuc 3 Hex 6 HexNAc 5

**Mass:**  4344.8581

(5.568e-06)

**Charge States:** 3

**Start Position**: 58

**End Position**: 74

| Profile Score | Tandem Score | TDA q-value | Volume | Spectrum Matches |
| --- | --- | --- | --- | --- |
| -19.4294 | 39.6451 | 0.0049 | 8.7621e+06 | 1 |

**Adduct Signal Fractions**

- **Unmodified**: 8.7621e+06

### NLVPVPITN(N)ATLDR Fuc 2 Hex 7 HexNAc 5

**Mass:**  3963.7380

(9.781e-07)

**Charge States:** 4

**Start Position**: 25

**End Position**: 39

| Profile Score | Tandem Score | TDA q-value | Volume | Spectrum Matches |
| --- | --- | --- | --- | --- |
| -21.0804 | 39.6378 | 0.0049 | 1.7274e+07 | 1 |

**Adduct Signal Fractions**

- **Unmodified**: 1.7274e+07

### NLVPVPITN(N)ATLDR Fuc 5 Hex 4 HexNAc 7

**Mass:**  4321.9352

(6.889e-06)

**Charge States:** 3, 4

**Start Position**: 25

**End Position**: 39

| Profile Score | Tandem Score | TDA q-value | Volume | Spectrum Matches |
| --- | --- | --- | --- | --- |
| 11.3781 | 38.9837 | 0.0049 | 3.2311e+07 | 8 |

**Adduct Signal Fractions**

- **Unmodified**: 3.2311e+07

### QN(D)QC(C)FYN(N)SSYLNVQR Hex 7 HexNAc 6

**Mass:**  4273.6940

(2.325e-04)

**Charge States:** 3, 4

**Start Position**: 87

**End Position**: 102

| Profile Score | Tandem Score | TDA q-value | Volume | Spectrum Matches |
| --- | --- | --- | --- | --- |
| 8.8205 | 37.8093 | 0.0060 | 3.7179e+08 | 6 |

**Adduct Signal Fractions**

- **Unmodified**: 3.7179e+08

### FYFTPN(N)KTEDTIFLR Fuc 1 Hex 4 HexNAc 4 Neu5Ac 1

**Mass:**  3788.6230

(-3.217e-06)

**Charge States:** 4

**Start Position**: 67

**End Position**: 82

| Profile Score | Tandem Score | TDA q-value | Volume | Spectrum Matches |
| --- | --- | --- | --- | --- |
| 14.5926 | 37.7448 | 0.0060 | 2.2921e+08 | 4 |

**Adduct Signal Fractions**

- **Unmodified**: 2.2921e+08

### YFTPN(N)KTEDTIFLR Hex 5 HexNAc 4

**Mass:**  3367.4815

(3.020e-04)

**Start Position**: 68

**End Position**: 82

| Profile Score | Tandem Score | TDA q-value | Volume | Spectrum Matches |
| --- | --- | --- | --- | --- |
| 0.0000 | 37.3655 | 0.0060 | 0.0000e+00 | 1 |

Adduct:

No Chromatogram Found

### C(C)AN(D)LVPVPITN(N)ATLDR Fuc 2 Hex 4 HexNAc 4 Neu5Ac 1

**Mass:**  3797.6625

(2.643e-04)

**Charge States:** 3, 4

**Start Position**: 23

**End Position**: 39

| Profile Score | Tandem Score | TDA q-value | Volume | Spectrum Matches |
| --- | --- | --- | --- | --- |
| -5.8246 | 36.8222 | 0.0069 | 1.1479e+08 | 4 |

**Adduct Signal Fractions**

- **Unmodified**: 1.1479e+08

### LVPVPITN(N)ATLDR Fuc 1 Hex 10 HexNAc 2

**Mass:**  3581.5289

(2.724e-04)

**Start Position**: 26

**End Position**: 39

| Profile Score | Tandem Score | TDA q-value | Volume | Spectrum Matches |
| --- | --- | --- | --- | --- |
| 0.0000 | 36.7026 | 0.0069 | 0.0000e+00 | 2 |

Adduct:

No Chromatogram Found

### ATFFYFTPN(N)K Hex 6 HexNAc 9 Neu5Ac 1

**Mass:**  4325.7092

(-3.499e-06)

**Charge States:** 4, 5

**Start Position**: 64

**End Position**: 74

| Profile Score | Tandem Score | TDA q-value | Volume | Spectrum Matches |
| --- | --- | --- | --- | --- |
| -4.3562 | 35.7205 | 0.0069 | 1.3279e+08 | 3 |

**Adduct Signal Fractions**

- **Unmodified**: 1.3279e+08

### ANLVPVPITN(N)ATLDR Fuc 2 Hex 6 HexNAc 3 Neu5Ac 1

**Mass:**  3757.6642

(2.510e-06)

**Charge States:** 3, 4

**Start Position**: 24

**End Position**: 39

| Profile Score | Tandem Score | TDA q-value | Volume | Spectrum Matches |
| --- | --- | --- | --- | --- |
| 12.3554 | 35.3850 | 0.0069 | 5.4543e+07 | 2 |

**Adduct Signal Fractions**

- **Unmodified**: 5.4543e+07

### FTPN(N)KTEDTIFLR Fuc 2 Hex 7 HexNAc 6

**Mass:**  4227.8019

(4.764e-04)

**Charge States:** 4

**Start Position**: 69

**End Position**: 82

| Profile Score | Tandem Score | TDA q-value | Volume | Spectrum Matches |
| --- | --- | --- | --- | --- |
| -22.6701 | 35.0108 | 0.0100 | 4.6510e+06 | 2 |

**Adduct Signal Fractions**

- **Unmodified**: 4.6510e+06

### PVPITN(N)ATLDR Fuc 2 Hex 6 HexNAc 8 Neu5Ac 1

**Mass:**  4375.8532

(7.814e-06)

**Charge States:** 4

**Start Position**: 28

**End Position**: 39

| Profile Score | Tandem Score | TDA q-value | Volume | Spectrum Matches |
| --- | --- | --- | --- | --- |
| -21.0674 | 33.9603 | 0.0100 | 1.7498e+05 | 1 |

**Adduct Signal Fractions**

- **Unmodified**: 1.7498e+05

### QC(C)FYN(N)SSYLNVQR Fuc 2 Hex 4 HexNAc 7 Neu5Ac 1

**Mass:**  4330.7155

(-3.422e-06)

**Charge States:** 4

**Start Position**: 89

**End Position**: 102

| Profile Score | Tandem Score | TDA q-value | Volume | Spectrum Matches |
| --- | --- | --- | --- | --- |
| -6.3428 | 33.7067 | 0.0108 | 2.2695e+07 | 1 |

**Adduct Signal Fractions**

- **Unmodified**: 2.2695e+07

### SVQEIQATFFYFTPN(N)K Fuc 3 Hex 5 HexNAc 4 Neu5Ac 2

**Mass:**  4564.9021

(6.597e-04)

**Start Position**: 58

**End Position**: 74

| Profile Score | Tandem Score | TDA q-value | Volume | Spectrum Matches |
| --- | --- | --- | --- | --- |
| 0.0000 | 33.6481 | 0.0108 | 0.0000e+00 | 1 |

Adduct:

No Chromatogram Found

### ANLVPVPITN(N)ATLDR Fuc 1 Hex 9 HexNAc 5

**Mass:**  4212.8381

(4.818e-06)

**Charge States:** 4

**Start Position**: 24

**End Position**: 39

| Profile Score | Tandem Score | TDA q-value | Volume | Spectrum Matches |
| --- | --- | --- | --- | --- |
| 11.4821 | 33.3268 | 0.0108 | 1.8749e+08 | 5 |

**Adduct Signal Fractions**

- **Unmodified**: 1.8749e+08

### LVPVPITN(N)ATLDR Fuc 5 Hex 3 HexNAc 7 Neu5Ac 1

**Mass:**  4336.9421

(8.709e-06)

**Charge States:** 3, 4

**Start Position**: 26

**End Position**: 39

| Profile Score | Tandem Score | TDA q-value | Volume | Spectrum Matches |
| --- | --- | --- | --- | --- |
| 11.9187 | 33.1030 | 0.0118 | 4.6316e+07 | 6 |

**Adduct Signal Fractions**

- **Unmodified**: 4.6316e+07

### QNQC(C)FYN(N)SSYLNVQR Fuc 1 Hex 8 HexNAc 6

**Mass:**  4580.8557

(8.855e-06)

**Charge States:** 4

**Start Position**: 87

**End Position**: 102

| Profile Score | Tandem Score | TDA q-value | Volume | Spectrum Matches |
| --- | --- | --- | --- | --- |
| -20.5272 | 32.7690 | 0.0137 | 2.3322e+06 | 1 |

**Adduct Signal Fractions**

- **Unmodified**: 2.3322e+06

### C(C)ANLVPVPITN(N)ATLDR Hex 5 HexNAc 5 Neu5Ac 1

**Mass:**  3869.6702

(-3.672e-07)

**Charge States:** 3, 4

**Start Position**: 23

**End Position**: 39

| Profile Score | Tandem Score | TDA q-value | Volume | Spectrum Matches |
| --- | --- | --- | --- | --- |
| 15.9877 | 32.5576 | 0.0146 | 3.1172e+08 | 6 |

**Adduct Signal Fractions**

- **Unmodified**: 3.1172e+08

### SVQEIQATFFYFTPN(N)KTEDTIFLR Fuc 1 Hex 8 HexNAc 4 Neu5Ac 1

**Mass:**  5440.3586

(4.099e-06)

**Charge States:** 4

**Start Position**: 58

**End Position**: 82

| Profile Score | Tandem Score | TDA q-value | Volume | Spectrum Matches |
| --- | --- | --- | --- | --- |
| -4.5598 | 32.3491 | 0.0155 | 5.9131e+06 | 1 |

**Adduct Signal Fractions**

- **Unmodified**: 5.9131e+06

### FYFTPN(N)KTEDTIFLR Fuc 2 Hex 4 HexNAc 4 Neu5Ac 1

**Mass:**  3934.6823

(-2.348e-06)

**Charge States:** 4

**Start Position**: 67

**End Position**: 82

| Profile Score | Tandem Score | TDA q-value | Volume | Spectrum Matches |
| --- | --- | --- | --- | --- |
| -18.6091 | 32.3406 | 0.0155 | 4.1679e+07 | 4 |

**Adduct Signal Fractions**

- **Unmodified**: 4.1679e+07

### WFYIASAFRNEEYNKSVQEIQATFFYFTPN(N)K Fuc 5 Hex 10 HexNAc 8

**Mass:**  7813.3346

(4.511e-06)

**Charge States:** 5

**Start Position**: 43

**End Position**: 74

| Profile Score | Tandem Score | TDA q-value | Volume | Spectrum Matches |
| --- | --- | --- | --- | --- |
| -4.1854 | 32.1221 | 0.0165 | 3.8610e+07 | 1 |

**Adduct Signal Fractions**

- **Unmodified**: 3.8610e+07

### FYN(N)SSYLNVQR Hex 6 HexNAc 5

**Mass:**  3377.3853

(2.018e-06)

**Charge States:** 3

**Start Position**: 91

**End Position**: 102

| Profile Score | Tandem Score | TDA q-value | Volume | Spectrum Matches |
| --- | --- | --- | --- | --- |
| -4.4452 | 31.8758 | 0.0174 | 5.5775e+06 | 2 |

**Adduct Signal Fractions**

- **Unmodified**: 5.5775e+06

### QNQC(C)FYN(N)SSYLNVQR Hex 9 HexNAc 8

**Mass:**  5002.9675

(-2.979e-07)

**Charge States:** 4

**Start Position**: 87

**End Position**: 102

| Profile Score | Tandem Score | TDA q-value | Volume | Spectrum Matches |
| --- | --- | --- | --- | --- |
| -19.3827 | 31.4562 | 0.0180 | 9.7788e+06 | 1 |

**Adduct Signal Fractions**

- **Unmodified**: 9.7788e+06

### PVPITN(N)ATLDR Fuc 1 Hex 7 HexNAc 5

**Mass:**  3491.4880

(2.084e-06)

**Charge States:** 4

**Start Position**: 28

**End Position**: 39

| Profile Score | Tandem Score | TDA q-value | Volume | Spectrum Matches |
| --- | --- | --- | --- | --- |
| -20.1229 | 31.1881 | 0.0180 | 2.5593e+06 | 1 |

**Adduct Signal Fractions**

- **Unmodified**: 2.5593e+06

### YN(N)SSYLNVQR Hex 7 HexNAc 6

**Mass:**  3595.4459

(6.802e-07)

**Charge States:** 3, 4

**Start Position**: 92

**End Position**: 102

| Profile Score | Tandem Score | TDA q-value | Volume | Spectrum Matches |
| --- | --- | --- | --- | --- |
| -4.0533 | 30.2989 | 0.0222 | 8.5687e+06 | 2 |

**Adduct Signal Fractions**

- **Unmodified**: 8.5687e+06

### LVPVPITN(N)ATLDR Fuc 1 Hex 8 HexNAc 2

**Mass:**  3256.4518

(1.152e-06)

**Charge States:** 3

**Start Position**: 26

**End Position**: 39

| Profile Score | Tandem Score | TDA q-value | Volume | Spectrum Matches |
| --- | --- | --- | --- | --- |
| -4.1069 | 29.6161 | 0.0230 | 2.1631e+07 | 2 |

**Adduct Signal Fractions**

- **Unmodified**: 2.1631e+07

### TPN(N)KTEDTIFLR Hex 8 HexNAc 7

**Mass:**  4151.7331

(7.937e-07)

**Charge States:** 4

**Start Position**: 70

**End Position**: 82

| Profile Score | Tandem Score | TDA q-value | Volume | Spectrum Matches |
| --- | --- | --- | --- | --- |
| -4.8740 | 29.0144 | 0.0273 | 4.7446e+06 | 1 |

**Adduct Signal Fractions**

- **Unmodified**: 4.7446e+06

### PVPITN(N)ATLDR Fuc 2 Hex 4 HexNAc 4 Neu5Ac 1

**Mass:**  3239.4248

(8.827e-06)

**Charge States:** 3, 4

**Start Position**: 28

**End Position**: 39

| Profile Score | Tandem Score | TDA q-value | Volume | Spectrum Matches |
| --- | --- | --- | --- | --- |
| 13.0391 | 28.5601 | 0.0306 | 6.0017e+07 | 5 |

**Adduct Signal Fractions**

- **Unmodified**: 6.0017e+07

### ANLVPVPITN(N)ATLDR Fuc 1 Hex 8 HexNAc 3 Neu5Ac 1

**Mass:**  3935.6835

(-4.874e-06)

**Charge States:** 4

**Start Position**: 24

**End Position**: 39

| Profile Score | Tandem Score | TDA q-value | Volume | Spectrum Matches |
| --- | --- | --- | --- | --- |
| -20.4674 | 28.3249 | 0.0311 | 2.0400e+07 | 5 |

**Adduct Signal Fractions**

- **Unmodified**: 2.0400e+07

### LVPVPITN(N)ATLDR Fuc 5 Hex 3 HexNAc 8 Neu5Ac 1

**Mass:**  4540.0256

(8.092e-06)

**Charge States:** 3, 4

**Start Position**: 26

**End Position**: 39

| Profile Score | Tandem Score | TDA q-value | Volume | Spectrum Matches |
| --- | --- | --- | --- | --- |
| 11.5097 | 27.9918 | 0.0318 | 7.7137e+07 | 4 |

**Adduct Signal Fractions**

- **Unmodified**: 7.7137e+07

### QIPLC(C)ANLVPVPITN(N)ATLDR Hex 5 HexNAc 4

**Mass:**  3826.7851

(1.409e-06)

**Charge States:** 3, 4

**Start Position**: 19

**End Position**: 39

| Profile Score | Tandem Score | TDA q-value | Volume | Spectrum Matches |
| --- | --- | --- | --- | --- |
| -3.5828 | 27.8167 | 0.0343 | 3.9183e+07 | 3 |

**Adduct Signal Fractions**

- **Unmodified**: 3.9183e+07

### QN(D)QC(C)FYN(N)SSYLNVQR Fuc 3 Hex 6 HexNAc 4 Neu5Ac 1

**Mass:**  4434.7243

(2.179e-04)

**Charge States:** 4

**Start Position**: 87

**End Position**: 102

| Profile Score | Tandem Score | TDA q-value | Volume | Spectrum Matches |
| --- | --- | --- | --- | --- |
| 9.4429 | 27.7957 | 0.0349 | 8.9799e+07 | 3 |

**Adduct Signal Fractions**

- **Unmodified**: 8.9799e+07

### SVQEIQATFFYFTPN(N)K Hex 7 HexNAc 6

**Mass:**  4273.8113

(4.726e-04)

**Start Position**: 58

**End Position**: 74

| Profile Score | Tandem Score | TDA q-value | Volume | Spectrum Matches |
| --- | --- | --- | --- | --- |
| 0.0000 | 27.7755 | 0.0349 | 0.0000e+00 | 1 |

Adduct:

No Chromatogram Found

### PVPITN(N)ATLDR Fuc 2 Hex 7 HexNAc 4

**Mass:**  3435.4477

(2.959e-04)

**Charge States:** 3

**Start Position**: 28

**End Position**: 39

| Profile Score | Tandem Score | TDA q-value | Volume | Spectrum Matches |
| --- | --- | --- | --- | --- |
| -21.4259 | 27.1346 | 0.0360 | 2.1849e+06 | 3 |

**Adduct Signal Fractions**

- **Unmodified**: 2.1849e+06

### PVPITN(N)ATLDR Fuc 1 Hex 7 HexNAc 4 Neu5Ac 1

**Mass:**  3579.5248

(9.023e-06)

**Charge States:** 4

**Start Position**: 28

**End Position**: 39

| Profile Score | Tandem Score | TDA q-value | Volume | Spectrum Matches |
| --- | --- | --- | --- | --- |
| 12.4768 | 27.1102 | 0.0360 | 3.6608e+08 | 3 |

**Adduct Signal Fractions**

- **Unmodified**: 3.6608e+08

### FYN(N)SSYLNVQR Fuc 1 Hex 7 HexNAc 6

**Mass:**  3888.5754

(1.725e-06)

**Charge States:** 3, 4

**Start Position**: 91

**End Position**: 102

| Profile Score | Tandem Score | TDA q-value | Volume | Spectrum Matches |
| --- | --- | --- | --- | --- |
| -18.9102 | 26.9531 | 0.0360 | 3.1953e+06 | 3 |

**Adduct Signal Fractions**

- **Unmodified**: 3.1953e+06

### LVPVPITN(N)ATLDR Fuc 1 Hex 6 HexNAc 5

**Mass:**  3543.5889

(5.672e-04)

**Start Position**: 26

**End Position**: 39

| Profile Score | Tandem Score | TDA q-value | Volume | Spectrum Matches |
| --- | --- | --- | --- | --- |
| 0.0000 | 26.8536 | 0.0360 | 0.0000e+00 | 1 |

Adduct:

No Chromatogram Found

### QC(C)FYN(N)SSYLNVQR Fuc 2 Hex 3 HexNAc 6 Neu5Ac 1

**Mass:**  3965.5876

(-4.362e-06)

**Charge States:** 4

**Start Position**: 89

**End Position**: 102

| Profile Score | Tandem Score | TDA q-value | Volume | Spectrum Matches |
| --- | --- | --- | --- | --- |
| -5.2232 | 26.2439 | 0.0360 | 1.6960e+07 | 2 |

**Adduct Signal Fractions**

- **Unmodified**: 1.6960e+07

### C(C)ANLVPVPITN(N)ATLDR Hex 6 HexNAc 6

**Mass:**  3943.7177

(3.629e-06)

**Charge States:** 3, 4

**Start Position**: 23

**End Position**: 39

| Profile Score | Tandem Score | TDA q-value | Volume | Spectrum Matches |
| --- | --- | --- | --- | --- |
| 10.0001 | 26.1970 | 0.0360 | 3.1752e+07 | 4 |

**Adduct Signal Fractions**

- **Unmodified**: 3.1752e+07

### LVPVPITN(N)ATLDR Fuc 1 Hex 9 HexNAc 6 Neu5Ac 1

**Mass:**  4521.8771

(-8.048e-06)

**Charge States:** 4

**Start Position**: 26

**End Position**: 39

| Profile Score | Tandem Score | TDA q-value | Volume | Spectrum Matches |
| --- | --- | --- | --- | --- |
| -20.3028 | 26.0905 | 0.0360 | 2.6869e+06 | 1 |

**Adduct Signal Fractions**

- **Unmodified**: 2.6869e+06

### YFTPN(N)KTEDTIFLR Fuc 1 Hex 7 HexNAc 4 Neu5Ac 2

**Mass:**  4418.7917

(-6.199e-06)

**Charge States:** 4

**Start Position**: 68

**End Position**: 82

| Profile Score | Tandem Score | TDA q-value | Volume | Spectrum Matches |
| --- | --- | --- | --- | --- |
| -20.2277 | 26.0476 | 0.0360 | 1.7642e+07 | 1 |

**Adduct Signal Fractions**

- **Unmodified**: 1.7642e+07

### NLVPVPITN(N)ATLDR Hex 3 HexNAc 8 Neu5Ac 1

**Mass:**  3923.7434

(1.351e-06)

**Charge States:** 3

**Start Position**: 25

**End Position**: 39

| Profile Score | Tandem Score | TDA q-value | Volume | Spectrum Matches |
| --- | --- | --- | --- | --- |
| -5.0016 | 25.9728 | 0.0360 | 7.5635e+07 | 2 |

**Adduct Signal Fractions**

- **Unmodified**: 7.5635e+07

### QNQC(C)FYN(N)SSYLNVQR Hex 10 HexNAc 9

**Mass:**  5368.1125

(2.103e-06)

**Charge States:** 4

**Start Position**: 87

**End Position**: 102

| Profile Score | Tandem Score | TDA q-value | Volume | Spectrum Matches |
| --- | --- | --- | --- | --- |
| -19.9866 | 25.9539 | 0.0360 | 6.4822e+06 | 1 |

**Adduct Signal Fractions**

- **Unmodified**: 6.4822e+06

### ANLVPVPITN(N)ATLDR Fuc 1 Hex 10 HexNAc 6

**Mass:**  4577.9738

(5.042e-06)

**Charge States:** 4

**Start Position**: 24

**End Position**: 39

| Profile Score | Tandem Score | TDA q-value | Volume | Spectrum Matches |
| --- | --- | --- | --- | --- |
| -20.2296 | 25.9253 | 0.0360 | 2.9999e+06 | 1 |

**Adduct Signal Fractions**

- **Unmodified**: 2.9999e+06

### YFTPN(N)KTEDTIFLR Hex 10 HexNAc 9

**Mass:**  5192.1384

(2.416e-06)

**Charge States:** 4

**Start Position**: 68

**End Position**: 82

| Profile Score | Tandem Score | TDA q-value | Volume | Spectrum Matches |
| --- | --- | --- | --- | --- |
| -19.6486 | 25.8778 | 0.0360 | 3.3367e+06 | 1 |

**Adduct Signal Fractions**

- **Unmodified**: 3.3367e+06

### YFTPN(N)KTEDTIFLR Fuc 5 Hex 8 HexNAc 7

**Mass:**  5192.1384

(-2.428e-06)

**Charge States:** 4

**Start Position**: 68

**End Position**: 82

| Profile Score | Tandem Score | TDA q-value | Volume | Spectrum Matches |
| --- | --- | --- | --- | --- |
| -19.6486 | 25.8778 | 0.0360 | 3.3367e+06 | 1 |

**Adduct Signal Fractions**

- **Unmodified**: 3.3367e+06

### VPVPITN(N)ATLDR Hex 5 HexNAc 4

**Mass:**  2917.3117

(1.936e-06)

**Charge States:** 3

**Start Position**: 27

**End Position**: 39

| Profile Score | Tandem Score | TDA q-value | Volume | Spectrum Matches |
| --- | --- | --- | --- | --- |
| -20.3884 | 25.2712 | 0.0362 | 3.2475e+05 | 1 |

**Adduct Signal Fractions**

- **Unmodified**: 3.2475e+05

### QC(C)FYN(N)SSYLNVQR Fuc 1 Hex 10 HexNAc 4

**Mass:**  4256.6677

(2.800e-06)

**Charge States:** 3, 4

**Start Position**: 89

**End Position**: 102

| Profile Score | Tandem Score | TDA q-value | Volume | Spectrum Matches |
| --- | --- | --- | --- | --- |
| 9.8319 | 25.2649 | 0.0362 | 7.5216e+08 | 1 |

**Adduct Signal Fractions**

- **Unmodified**: 7.5216e+08

### FYFTPN(N)KTEDTIFLR Fuc 2 Hex 3 HexNAc 5 Neu5Ac 2

**Mass:**  4266.7922

(-4.979e-06)

**Charge States:** 4

**Start Position**: 67

**End Position**: 82

| Profile Score | Tandem Score | TDA q-value | Volume | Spectrum Matches |
| --- | --- | --- | --- | --- |
| -19.2674 | 25.2643 | 0.0362 | 1.6044e+07 | 1 |

**Adduct Signal Fractions**

- **Unmodified**: 1.6044e+07

### QN(D)QC(C)FYN(N)SSYLNVQR Fuc 2 Hex 4 HexNAc 4 Neu5Ac 1

**Mass:**  3964.5949

(2.524e-04)

**Charge States:** 3, 4

**Start Position**: 87

**End Position**: 102

| Profile Score | Tandem Score | TDA q-value | Volume | Spectrum Matches |
| --- | --- | --- | --- | --- |
| -20.0353 | 25.0452 | 0.0362 | 5.2132e+07 | 2 |

**Adduct Signal Fractions**

- **Unmodified**: 5.2132e+07

### YN(N)SSYLNVQR Hex 6 HexNAc 6

**Mass:**  3434.3902

(2.906e-04)

**Start Position**: 92

**End Position**: 102

| Profile Score | Tandem Score | TDA q-value | Volume | Spectrum Matches |
| --- | --- | --- | --- | --- |
| 0.0000 | 25.0164 | 0.0362 | 0.0000e+00 | 1 |

Adduct:

No Chromatogram Found

### SVQEIQATFFYFTPN(N)KTEDTIFLR Fuc 1 Hex 9 HexNAc 8

**Mass:**  6126.6276

(4.916e-04)

**Start Position**: 58

**End Position**: 82

| Profile Score | Tandem Score | TDA q-value | Volume | Spectrum Matches |
| --- | --- | --- | --- | --- |
| 0.0000 | 24.6108 | 0.0395 | 0.0000e+00 | 1 |

Adduct:

No Chromatogram Found

### QN(D)QC(C)FYN(N)SSYLNVQR Hex 7 HexNAc 5

**Mass:**  4070.6043

(2.406e-04)

**Charge States:** 3

**Start Position**: 87

**End Position**: 102

| Profile Score | Tandem Score | TDA q-value | Volume | Spectrum Matches |
| --- | --- | --- | --- | --- |
| -19.9192 | 24.0546 | 0.0399 | 1.2916e+07 | 1 |

**Adduct Signal Fractions**

- **Unmodified**: 1.2916e+07

### LVPVPITN(N)ATLDR Hex 9 HexNAc 3 Neu5Ac 1

**Mass:**  3766.6111

(-1.709e-06)

**Start Position**: 26

**End Position**: 39

| Profile Score | Tandem Score | TDA q-value | Volume | Spectrum Matches |
| --- | --- | --- | --- | --- |
| 0.0000 | 23.9843 | 0.0399 | 0.0000e+00 | 1 |

Adduct:

No Chromatogram Found

### FYFTPN(N)KTEDTIFLR Fuc 4 Hex 5 HexNAc 5

**Mass:**  4300.8239

(-4.722e-06)

**Charge States:** 4

**Start Position**: 67

**End Position**: 82

| Profile Score | Tandem Score | TDA q-value | Volume | Spectrum Matches |
| --- | --- | --- | --- | --- |
| -3.1241 | 23.3099 | 0.0441 | 5.3155e+07 | 3 |

**Adduct Signal Fractions**

- **Unmodified**: 5.3155e+07

### SVQEIQATFFYFTPN(N)K Fuc 1 Hex 10 HexNAc 3

**Mass:**  4294.7720

(2.691e-07)

**Charge States:** 5

**Start Position**: 58

**End Position**: 74

| Profile Score | Tandem Score | TDA q-value | Volume | Spectrum Matches |
| --- | --- | --- | --- | --- |
| -19.2679 | 23.1942 | 0.0456 | 3.3107e+06 | 1 |

**Adduct Signal Fractions**

- **Unmodified**: 3.3107e+06

### SVQEIQATFFYFTPN(N)KTEDTIFLR Hex 10 HexNAc 9

**Mass:**  6342.6907

(-1.840e-07)

**Charge States:** 5

**Start Position**: 58

**End Position**: 82

| Profile Score | Tandem Score | TDA q-value | Volume | Spectrum Matches |
| --- | --- | --- | --- | --- |
| -5.5563 | 23.1848 | 0.0456 | 1.0882e+07 | 2 |

**Adduct Signal Fractions**

- **Unmodified**: 1.0882e+07

### ANLVPVPITN(N)ATLDR Fuc 2 Hex 9 HexNAc 5

**Mass:**  4358.8991

(5.528e-06)

**Charge States:** 4

**Start Position**: 24

**End Position**: 39

| Profile Score | Tandem Score | TDA q-value | Volume | Spectrum Matches |
| --- | --- | --- | --- | --- |
| -18.3348 | 23.1445 | 0.0456 | 4.9350e+07 | 2 |

**Adduct Signal Fractions**

- **Unmodified**: 4.9350e+07

### QC(C)FYN(N)SSYLNVQR Fuc 2 Hex 3 HexNAc 9

**Mass:**  4283.7570

(-6.752e-07)

**Charge States:** 3, 4

**Start Position**: 89

**End Position**: 102

| Profile Score | Tandem Score | TDA q-value | Volume | Spectrum Matches |
| --- | --- | --- | --- | --- |
| 3.2500 | 23.1153 | 0.0456 | 3.3672e+08 | 6 |

**Adduct Signal Fractions**

- **Unmodified**: 3.3672e+08

### SVQEIQATFFYFTPN(N)K Fuc 4 Hex 7 HexNAc 7

**Mass:**  5062.0905

(5.904e-04)

**Start Position**: 58

**End Position**: 74

| Profile Score | Tandem Score | TDA q-value | Volume | Spectrum Matches |
| --- | --- | --- | --- | --- |
| 0.0000 | 23.0156 | 0.0456 | 0.0000e+00 | 1 |

Adduct:

No Chromatogram Found

### VPVPITN(N)ATLDR Fuc 2 Hex 3 HexNAc 7

**Mass:**  3496.5379

(5.656e-04)

**Charge States:** 3

**Start Position**: 27

**End Position**: 39

| Profile Score | Tandem Score | TDA q-value | Volume | Spectrum Matches |
| --- | --- | --- | --- | --- |
| -21.3754 | 23.0140 | 0.0456 | 1.3146e+06 | 2 |

**Adduct Signal Fractions**

- **Unmodified**: 1.3146e+06

### SVQEIQATFFYFTPN(N)K Fuc 2 Hex 6 HexNAc 4 Neu5Ac 1

**Mass:**  4289.8127

(7.072e-04)

**Charge States:** 3, 4

**Start Position**: 58

**End Position**: 74

| Profile Score | Tandem Score | TDA q-value | Volume | Spectrum Matches |
| --- | --- | --- | --- | --- |
| -22.1197 | 22.9363 | 0.0456 | 4.9330e+06 | 11 |

**Adduct Signal Fractions**

- **Unmodified**: 4.9330e+06

### NLVPVPITN(N)ATLDR Fuc 1 Hex 6 HexNAc 5

**Mass:**  3655.6283

(1.387e-06)

**Charge States:** 3

**Start Position**: 25

**End Position**: 39

| Profile Score | Tandem Score | TDA q-value | Volume | Spectrum Matches |
| --- | --- | --- | --- | --- |
| -4.7455 | 22.5850 | 0.0479 | 1.0628e+07 | 2 |

**Adduct Signal Fractions**

- **Unmodified**: 1.0628e+07

### P00738|HPT\_HUMAN

**>P00738|HPT\_HUMAN**  
 `MSALGAVIALLLWGQLFAVDSGNDVTDIADDGCPKPPEIAHGYVEHSVRYQCKNYYKLRTEGDGVYTLNDKKQWINKAVG  
DKLPECEADDGCPKPPEIAHGYVEHSVRYQCKNYYKLRTEGDGVYTLNNEKQWINKAVGDKLPECEAVCGKPKNPANPVQ  
RILGGHLDAKGSFPWQAKMVSHHNLTTGATLINEQWLLTTAKNLFLNHSENATAKDIAPTLTLYVGKKQLVEIEKVVLHP  
NYSQVDIGLIKLKQKVSVNERVMPICLPSKDYAEVGRVGYVSGWGRNANFKFTDHLKYVMLPVADQDQCIRHYEGSTVPE  
KKTPKSPVGVQPILNEHTFCAGMSKYQEDTCYGDAGSAFAVHDLEEDTWYATGILSFDKSCAVAEYGVYVKVTSIQDWVQ  
KTIAEN`

| Site | Type | Count |
| --- | --- | --- |
| 241 | N-linked | 6 |
| 211 | N-linked | 4 |
| 207 | N-linked | 1 |

| Mass | Sequence | MS_2_ Score | `q`-value | MS_1_ Score | Signal | Protein Coordinates | Tandem Scans | Start Time | Apex Time | End Time |
| --- | --- | --- | --- | --- | --- | --- | --- | --- | --- | --- |
| 3416.5915 | VVLHPN(N)YSQVDIGLIK Hex 5 HexNAc 4 | 155.865 | 0.0 | -3.696 | 3.796e+07 | 235 - 251 | 4 | 42.30 | 42.47 | 42.81 |
| 4146.8577 | VVLHPN(N)YSQVDIGLIK Hex 7 HexNAc 6 | 155.006 | 0.0 | -19.559 | 1.473e+07 | 235 - 251 | 1 | 41.79 | 41.79 | 41.96 |
| 2892.2216 | VVLHPN(N)YSQVD Hex 5 HexNAc 4 | 121.934 | 0.0 | -18.192 | 4.372e+06 | 235 - 246 | 2 | 23.79 | 23.79 | 23.95 |
| 5068.0329 | NLFLNHSEN(N)ATAK Fuc 5 Hex 9 HexNAc 7 | 93.771 | 0.0 | -4.054 | 1.714e+07 | 202 - 215 | 2 | 17.45 | 17.56 | 17.72 |
| 5433.1650 | NLFLNHSEN(N)ATAK Fuc 5 Hex 10 HexNAc 8 | 42.833 | 0.003 | -4.421 | 1.853e+07 | 202 - 215 | 3 | 17.13 | 17.28 | 17.45 |
| 2893.2217 | VVLHPN(N)YSQVD Hex 5 HexNAc 4 | 42.251 | 0.003 | 0.000 | 0.000e+00 | 235 - 246 | 1 | - | - | - |
| 4702.8976 | NLFLN(N)HSENATAK Hex 10 HexNAc 8 | 34.681 | 0.010 | -19.571 | 5.422e+06 | 202 - 215 | 1 | 17.72 | 17.72 | 17.85 |
| 3257.3531 | VVLHPN(N)YSQVD Hex 6 HexNAc 5 | 32.135 | 0.016 | -5.002 | 3.697e+06 | 235 - 246 | 2 | 23.35 | 23.64 | 23.64 |
| 5580.2225 | NLFLNHSEN(N)ATAK Fuc 4 Hex 10 HexNAc 8 Neu5Ac 1 | 27.516 | 0.035 | 0.000 | 0.000e+00 | 202 - 215 | 1 | - | - | - |
| 5216.0861 | NLFLNHSEN(N)ATAK Fuc 4 Hex 9 HexNAc 7 Neu5Ac 1 | 26.458 | 0.036 | 0.000 | 0.000e+00 | 202 - 215 | 1 | - | - | - |
| 3929.8149 | VVLHPN(N)YSQVDIGLIK Fuc 1 Hex 6 HexNAc 5 | 22.915 | 0.046 | 9.639 | 8.767e+08 | 235 - 251 | 13 | 41.28 | 47.56 | 48.50 |

### VVLHPN(N)YSQVDIGLIK Hex 5 HexNAc 4

**Mass:**  3416.5904

(2.782e-06)

**Charge States:** 3, 4

**Start Position**: 236

**End Position**: 252

| Profile Score | Tandem Score | TDA q-value | Volume | Spectrum Matches |
| --- | --- | --- | --- | --- |
| -3.6964 | 155.8651 | 0.0000 | 3.7957e+07 | 4 |

**Adduct Signal Fractions**

- **Unmodified**: 3.7957e+07

### VVLHPN(N)YSQVDIGLIK Hex 7 HexNAc 6

**Mass:**  4146.8581

(2.005e-06)

**Charge States:** 3, 4

**Start Position**: 236

**End Position**: 252

| Profile Score | Tandem Score | TDA q-value | Volume | Spectrum Matches |
| --- | --- | --- | --- | --- |
| -19.5588 | 155.0060 | 0.0000 | 1.4725e+07 | 1 |

**Adduct Signal Fractions**

- **Unmodified**: 1.4725e+07

### VVLHPN(N)YSQVD Hex 5 HexNAc 4

**Mass:**  2892.2210

(1.856e-06)

**Charge States:** 3

**Start Position**: 236

**End Position**: 247

| Profile Score | Tandem Score | TDA q-value | Volume | Spectrum Matches |
| --- | --- | --- | --- | --- |
| -18.1919 | 121.9341 | 0.0000 | 4.3715e+06 | 2 |

**Adduct Signal Fractions**

- **Unmodified**: 4.3715e+06

### NLFLNHSEN(N)ATAK Fuc 5 Hex 9 HexNAc 7

**Mass:**  5068.0310

(-1.860e-06)

**Charge States:** 4

**Start Position**: 203

**End Position**: 216

| Profile Score | Tandem Score | TDA q-value | Volume | Spectrum Matches |
| --- | --- | --- | --- | --- |
| -4.0535 | 93.7710 | 0.0000 | 1.7135e+07 | 2 |

**Adduct Signal Fractions**

- **Unmodified**: 1.7135e+07

### NLFLNHSEN(N)ATAK Fuc 5 Hex 10 HexNAc 8

**Mass:**  5433.1638

(-2.838e-06)

**Charge States:** 4

**Start Position**: 203

**End Position**: 216

| Profile Score | Tandem Score | TDA q-value | Volume | Spectrum Matches |
| --- | --- | --- | --- | --- |
| -4.4206 | 42.8330 | 0.0026 | 1.8527e+07 | 3 |

**Adduct Signal Fractions**

- **Unmodified**: 1.8527e+07

### VVLHPN(N)YSQVD Hex 5 HexNAc 4

**Mass:**  2893.2217

(3.474e-04)

**Start Position**: 236

**End Position**: 247

| Profile Score | Tandem Score | TDA q-value | Volume | Spectrum Matches |
| --- | --- | --- | --- | --- |
| 0.0000 | 42.2507 | 0.0026 | 0.0000e+00 | 1 |

Adduct:

No Chromatogram Found

### NLFLN(N)HSENATAK Hex 10 HexNAc 8

**Mass:**  4702.8967

(1.501e-06)

**Charge States:** 4

**Start Position**: 203

**End Position**: 216

| Profile Score | Tandem Score | TDA q-value | Volume | Spectrum Matches |
| --- | --- | --- | --- | --- |
| -19.5714 | 34.6814 | 0.0100 | 5.4220e+06 | 1 |

**Adduct Signal Fractions**

- **Unmodified**: 5.4220e+06

### VVLHPN(N)YSQVD Hex 6 HexNAc 5

**Mass:**  3257.3560

(2.089e-06)

**Charge States:** 3

**Start Position**: 236

**End Position**: 247

| Profile Score | Tandem Score | TDA q-value | Volume | Spectrum Matches |
| --- | --- | --- | --- | --- |
| -5.0018 | 32.1349 | 0.0155 | 3.6969e+06 | 2 |

**Adduct Signal Fractions**

- **Unmodified**: 3.6969e+06

### NLFLNHSEN(N)ATAK Fuc 4 Hex 10 HexNAc 8 Neu5Ac 1

**Mass:**  5580.2225

(3.596e-04)

**Start Position**: 203

**End Position**: 216

| Profile Score | Tandem Score | TDA q-value | Volume | Spectrum Matches |
| --- | --- | --- | --- | --- |
| 0.0000 | 27.5157 | 0.0352 | 0.0000e+00 | 1 |

Adduct:

No Chromatogram Found

### NLFLNHSEN(N)ATAK Fuc 4 Hex 9 HexNAc 7 Neu5Ac 1

**Mass:**  5216.0861

(5.758e-04)

**Start Position**: 203

**End Position**: 216

| Profile Score | Tandem Score | TDA q-value | Volume | Spectrum Matches |
| --- | --- | --- | --- | --- |
| 0.0000 | 26.4575 | 0.0360 | 0.0000e+00 | 1 |

Adduct:

No Chromatogram Found

### VVLHPN(N)YSQVDIGLIK Fuc 1 Hex 6 HexNAc 5

**Mass:**  3929.8134

(5.134e-04)

**Charge States:** 3, 4

**Start Position**: 236

**End Position**: 252

| Profile Score | Tandem Score | TDA q-value | Volume | Spectrum Matches |
| --- | --- | --- | --- | --- |
| 9.6390 | 22.9151 | 0.0456 | 8.7669e+08 | 13 |

**Adduct Signal Fractions**

- **Unmodified**: 8.7669e+08

### P05155|IC1\_HUMAN

**>P05155|IC1\_HUMAN**  
 `MASRLTLLTLLLLLLAGDRASSNPNATSSSSQDPESLQDRGEGKVATTVISKMLFVEPILEVSSLPTTNSTTNSATKITA  
NTTDEPTTQPTTEPTTQPTIQPTQPTTQLPTDSPTQPTTGSFCPGPVTLCSDLESHSTEAVLGDALVDFSLKLYHAFSAM  
KKVETNMAFSPFSIASLLTQVLLGAGENTKTNLESILSYPKDFTCVHQALKGFTTKGVTSVSQIFHSPDLAIRDTFVNAS  
RTLYSSSPRVLSNNSDANLELINTWVAKNTNNKISRLLDSLPSDTRLVLLNAIYLSAKWKTTFDPKKTRMEPFHFKNSVI  
KVPMMNSKKYPVAHFIDQTLKAKVGQLQLSHNLSLVILVPQNLKHRLEDMEQALSPSVFKAIMEKLEMSKFQPTLLTLPR  
IKVTTSQDMLSIMEKLEFFDFSYDLNLCGLTEDPDLQVSAMQHQTVLELTETGVEAAAASAISVARTLLVFEVQQPFLFV  
LWDQQHKFPVFMGRVYDPRA`

| Site | Type | Count |
| --- | --- | --- |
| 25 | N-linked | 9 |
| 253 | N-linked | 1 |

| Mass | Sequence | MS_2_ Score | `q`-value | MS_1_ Score | Signal | Protein Coordinates | Tandem Scans | Start Time | Apex Time | End Time |
| --- | --- | --- | --- | --- | --- | --- | --- | --- | --- | --- |
| 3919.5644 | NPN(N)ATSSSSQDPESLQDR Hex 6 HexNAc 5 | 140.891 | 0.0 | -5.110 | 4.951e+07 | 22 - 40 | 3 | 13.45 | 13.61 | 13.94 |
| 3554.4316 | NPN(N)ATSSSSQDPESLQDR Hex 5 HexNAc 4 | 73.593 | 0.003 | -5.722 | 1.874e+07 | 22 - 40 | 3 | 14.72 | 14.88 | 15.03 |
| 4284.6975 | NPN(N)ATSSSSQDPESLQDR Hex 7 HexNAc 6 | 64.824 | 0.003 | -18.903 | 1.212e+07 | 22 - 40 | 2 | 13.45 | 13.45 | 13.61 |
| 3701.5069 | NPN(N)ATSSSSQDPESLQDR Fuc 1 Hex 5 HexNAc 4 | 51.737 | 0.003 | 0.000 | 0.000e+00 | 22 - 40 | 1 | - | - | - |
| 3920.5615 | NPN(N)ATSSSSQDPESLQDR Hex 6 HexNAc 5 | 49.220 | 0.003 | 0.000 | 0.000e+00 | 22 - 40 | 2 | - | - | - |
| 3700.4888 | NPN(N)ATSSSSQDPESLQDR Fuc 1 Hex 5 HexNAc 4 | 46.126 | 0.003 | -20.215 | 4.684e+06 | 22 - 40 | 1 | 14.72 | 14.72 | 14.72 |
| 4431.7669 | NPN(N)ATSSSSQDPESLQDR Fuc 1 Hex 7 HexNAc 6 | 39.783 | 0.005 | 0.000 | 0.000e+00 | 22 - 40 | 1 | - | - | - |
| 4067.6413 | NPN(N)ATSSSSQDPESLQDR Fuc 1 Hex 6 HexNAc 5 | 35.767 | 0.007 | 0.000 | 0.000e+00 | 22 - 40 | 1 | - | - | - |
| 4233.8500 | VLSN(N)NSDANLELINTWVAK Fuc 1 Hex 6 HexNAc 5 | 22.979 | 0.046 | -19.701 | 1.960e+06 | 249 - 268 | 1 | 51.03 | 51.03 | 51.20 |
| 4065.6142 | NPN(N)ATSSSSQDPESLQDR Fuc 1 Hex 6 HexNAc 5 | 22.721 | 0.046 | -19.584 | 4.733e+06 | 22 - 40 | 1 | 13.61 | 13.61 | 13.61 |

### NPN(N)ATSSSSQDPESLQDR Hex 6 HexNAc 5

**Mass:**  3919.5646

(1.132e-06)

**Charge States:** 4

**Start Position**: 23

**End Position**: 41

| Profile Score | Tandem Score | TDA q-value | Volume | Spectrum Matches |
| --- | --- | --- | --- | --- |
| -5.1098 | 140.8909 | 0.0000 | 4.9508e+07 | 3 |

**Adduct Signal Fractions**

- **Unmodified**: 4.9508e+07

### NPN(N)ATSSSSQDPESLQDR Hex 5 HexNAc 4

**Mass:**  3554.4354

(-1.031e-06)

**Charge States:** 3

**Start Position**: 23

**End Position**: 41

| Profile Score | Tandem Score | TDA q-value | Volume | Spectrum Matches |
| --- | --- | --- | --- | --- |
| -5.7219 | 73.5930 | 0.0026 | 1.8741e+07 | 3 |

**Adduct Signal Fractions**

- **Unmodified**: 1.8741e+07

### NPN(N)ATSSSSQDPESLQDR Hex 7 HexNAc 6

**Mass:**  4284.6975

(1.322e-06)

**Charge States:** 4

**Start Position**: 23

**End Position**: 41

| Profile Score | Tandem Score | TDA q-value | Volume | Spectrum Matches |
| --- | --- | --- | --- | --- |
| -18.9028 | 64.8239 | 0.0026 | 1.2123e+07 | 2 |

**Adduct Signal Fractions**

- **Unmodified**: 1.2123e+07

### NPN(N)ATSSSSQDPESLQDR Fuc 1 Hex 5 HexNAc 4

**Mass:**  3701.5069

(2.761e-04)

**Start Position**: 23

**End Position**: 41

| Profile Score | Tandem Score | TDA q-value | Volume | Spectrum Matches |
| --- | --- | --- | --- | --- |
| 0.0000 | 51.7370 | 0.0026 | 0.0000e+00 | 1 |

Adduct:

No Chromatogram Found

### NPN(N)ATSSSSQDPESLQDR Hex 6 HexNAc 5

**Mass:**  3920.5615

(2.556e-04)

**Start Position**: 23

**End Position**: 41

| Profile Score | Tandem Score | TDA q-value | Volume | Spectrum Matches |
| --- | --- | --- | --- | --- |
| 0.0000 | 49.2200 | 0.0026 | 0.0000e+00 | 2 |

Adduct:

No Chromatogram Found

### NPN(N)ATSSSSQDPESLQDR Fuc 1 Hex 5 HexNAc 4

**Mass:**  3700.4888

(9.834e-07)

**Charge States:** 3

**Start Position**: 23

**End Position**: 41

| Profile Score | Tandem Score | TDA q-value | Volume | Spectrum Matches |
| --- | --- | --- | --- | --- |
| -20.2149 | 46.1258 | 0.0026 | 4.6837e+06 | 1 |

**Adduct Signal Fractions**

- **Unmodified**: 4.6837e+06

### NPN(N)ATSSSSQDPESLQDR Fuc 1 Hex 7 HexNAc 6

**Mass:**  4431.7669

(2.296e-04)

**Start Position**: 23

**End Position**: 41

| Profile Score | Tandem Score | TDA q-value | Volume | Spectrum Matches |
| --- | --- | --- | --- | --- |
| 0.0000 | 39.7828 | 0.0049 | 0.0000e+00 | 1 |

Adduct:

No Chromatogram Found

### NPN(N)ATSSSSQDPESLQDR Fuc 1 Hex 6 HexNAc 5

**Mass:**  4067.6413

(4.978e-04)

**Start Position**: 23

**End Position**: 41

| Profile Score | Tandem Score | TDA q-value | Volume | Spectrum Matches |
| --- | --- | --- | --- | --- |
| 0.0000 | 35.7674 | 0.0069 | 0.0000e+00 | 1 |

Adduct:

No Chromatogram Found

### VLSN(N)NSDANLELINTWVAK Fuc 1 Hex 6 HexNAc 5

**Mass:**  4233.8493

(-1.766e-06)

**Charge States:** 4

**Start Position**: 250

**End Position**: 269

| Profile Score | Tandem Score | TDA q-value | Volume | Spectrum Matches |
| --- | --- | --- | --- | --- |
| -19.7014 | 22.9794 | 0.0456 | 1.9597e+06 | 1 |

**Adduct Signal Fractions**

- **Unmodified**: 1.9597e+06

### NPN(N)ATSSSSQDPESLQDR Fuc 1 Hex 6 HexNAc 5

**Mass:**  4065.6142

(-7.619e-07)

**Charge States:** 4

**Start Position**: 23

**End Position**: 41

| Profile Score | Tandem Score | TDA q-value | Volume | Spectrum Matches |
| --- | --- | --- | --- | --- |
| -19.5841 | 22.7207 | 0.0462 | 4.7334e+06 | 1 |

**Adduct Signal Fractions**

- **Unmodified**: 4.7334e+06

### Q9Y5Y7|LYVE1\_HUMAN

**>Q9Y5Y7|LYVE1\_HUMAN**  
 `MARCFSLVLLLTSIWTTRLLVQGSLRAEELSIQVSCRIMGITLVSKKANQQLNFTEAKEACRLLGLSLAGKDQVETALKA  
SFETCSYGWVGDGFVVISRISPNPKCGKNGVGVLIWKVPVSRQFAAYCYNSSDTWTNSCIPEIITTKDPIFNTQTATQTT  
EFIVSDSTYSVASPYSTIPAPTTTPPAPASTSIPRRKKLICVTEVFMETSTMSTETEPFVENKAAFKNEAAGFGGVPTAL  
LVLALLFFGAAAGLGFCYVKRYVKAFPFTNKNQQKEMIETKVVKEEKANDSNPNEESKKTDKNPEESKSPSKTTVRCLEA  
EV`

| Site | Type | Count |
| --- | --- | --- |
| 53 | N-linked | 9 |

| Mass | Sequence | MS_2_ Score | `q`-value | MS_1_ Score | Signal | Protein Coordinates | Tandem Scans | Start Time | Apex Time | End Time |
| --- | --- | --- | --- | --- | --- | --- | --- | --- | --- | --- |
| 3013.3068 | KANQQLN(N)FTEAK Hex 5 HexNAc 4 | 155.507 | 0.0 | -4.436 | 8.236e+06 | 46 - 58 | 1 | 17.13 | 17.28 | 17.45 |
| 3378.4394 | KANQQLN(N)FTEAK Hex 6 HexNAc 5 | 137.441 | 0.0 | -5.035 | 2.605e+07 | 46 - 58 | 3 | 16.96 | 17.13 | 17.45 |
| 3250.4232 | ANQQLN(N)FTEAK Hex 6 HexNAc 5 | 130.635 | 0.0 | 10.480 | 2.378e+07 | 47 - 58 | 7 | 20.65 | 21.02 | 21.93 |
| 3743.5747 | KANQQLN(N)FTEAK Hex 7 HexNAc 6 | 118.704 | 0.0 | -20.389 | 4.208e+06 | 46 - 58 | 2 | 16.96 | 16.96 | 16.96 |
| 3615.4796 | ANQQLN(N)FTEAK Hex 7 HexNAc 6 | 100.506 | 0.0 | -4.783 | 3.752e+06 | 47 - 58 | 2 | 20.51 | 20.65 | 20.87 |
| 2885.2166 | ANQQLN(N)FTEAK Hex 5 HexNAc 4 | 94.106 | 0.0 | -4.558 | 8.162e+06 | 47 - 58 | 2 | 20.87 | 21.15 | 21.29 |
| 3267.3781 | ANQQLN(N)FTEAK Fuc 2 Hex 5 HexNAc 3 Neu5Ac 1 | 32.854 | 0.013 | 0.000 | 0.000e+00 | 47 - 58 | 1 | - | - | - |
| 3617.4841 | ANQQLN(N)FTEAK Hex 7 HexNAc 6 | 28.203 | 0.031 | 0.000 | 0.000e+00 | 47 - 58 | 1 | - | - | - |
| 3379.4329 | KAN(D)QQLN(N)FTEAK Hex 6 HexNAc 5 | 23.028 | 0.046 | -21.048 | 2.384e+06 | 46 - 58 | 1 | 17.72 | 17.85 | 17.85 |

### KANQQLN(N)FTEAK Hex 5 HexNAc 4

**Mass:**  3013.3077

(1.867e-06)

**Charge States:** 3

**Start Position**: 47

**End Position**: 59

| Profile Score | Tandem Score | TDA q-value | Volume | Spectrum Matches |
| --- | --- | --- | --- | --- |
| -4.4356 | 155.5067 | 0.0000 | 8.2356e+06 | 1 |

**Adduct Signal Fractions**

- **Unmodified**: 8.2356e+06

### KANQQLN(N)FTEAK Hex 6 HexNAc 5

**Mass:**  3378.4381

(1.498e-06)

**Charge States:** 3

**Start Position**: 47

**End Position**: 59

| Profile Score | Tandem Score | TDA q-value | Volume | Spectrum Matches |
| --- | --- | --- | --- | --- |
| -5.0347 | 137.4415 | 0.0000 | 2.6053e+07 | 3 |

**Adduct Signal Fractions**

- **Unmodified**: 2.6053e+07

### ANQQLN(N)FTEAK Hex 6 HexNAc 5

**Mass:**  3250.3456

(1.890e-06)

**Charge States:** 3

**Start Position**: 48

**End Position**: 59

| Profile Score | Tandem Score | TDA q-value | Volume | Spectrum Matches |
| --- | --- | --- | --- | --- |
| 10.4800 | 130.6352 | 0.0000 | 2.3776e+07 | 7 |

**Adduct Signal Fractions**

- **Unmodified**: 2.3776e+07

### KANQQLN(N)FTEAK Hex 7 HexNAc 6

**Mass:**  3743.5772

(2.809e-06)

**Charge States:** 3, 4

**Start Position**: 47

**End Position**: 59

| Profile Score | Tandem Score | TDA q-value | Volume | Spectrum Matches |
| --- | --- | --- | --- | --- |
| -20.3886 | 118.7039 | 0.0000 | 4.2083e+06 | 2 |

**Adduct Signal Fractions**

- **Unmodified**: 4.2083e+06

### ANQQLN(N)FTEAK Hex 7 HexNAc 6

**Mass:**  3615.4806

(2.472e-06)

**Charge States:** 3

**Start Position**: 48

**End Position**: 59

| Profile Score | Tandem Score | TDA q-value | Volume | Spectrum Matches |
| --- | --- | --- | --- | --- |
| -4.7829 | 100.5060 | 0.0000 | 3.7521e+06 | 2 |

**Adduct Signal Fractions**

- **Unmodified**: 3.7521e+06

### ANQQLN(N)FTEAK Hex 5 HexNAc 4

**Mass:**  2885.2132

(5.494e-06)

**Charge States:** 3

**Start Position**: 48

**End Position**: 59

| Profile Score | Tandem Score | TDA q-value | Volume | Spectrum Matches |
| --- | --- | --- | --- | --- |
| -4.5583 | 94.1055 | 0.0000 | 8.1620e+06 | 2 |

**Adduct Signal Fractions**

- **Unmodified**: 8.1620e+06

### ANQQLN(N)FTEAK Fuc 2 Hex 5 HexNAc 3 Neu5Ac 1

**Mass:**  3267.3781

(6.245e-04)

**Start Position**: 48

**End Position**: 59

| Profile Score | Tandem Score | TDA q-value | Volume | Spectrum Matches |
| --- | --- | --- | --- | --- |
| 0.0000 | 32.8539 | 0.0128 | 0.0000e+00 | 1 |

Adduct:

No Chromatogram Found

### ANQQLN(N)FTEAK Hex 7 HexNAc 6

**Mass:**  3617.4841

(5.567e-04)

**Start Position**: 48

**End Position**: 59

| Profile Score | Tandem Score | TDA q-value | Volume | Spectrum Matches |
| --- | --- | --- | --- | --- |
| 0.0000 | 28.2026 | 0.0311 | 0.0000e+00 | 1 |

Adduct:

No Chromatogram Found

### KAN(D)QQLN(N)FTEAK Hex 6 HexNAc 5

**Mass:**  3379.4295

(2.968e-04)

**Charge States:** 3

**Start Position**: 47

**End Position**: 59

| Profile Score | Tandem Score | TDA q-value | Volume | Spectrum Matches |
| --- | --- | --- | --- | --- |
| -21.0480 | 23.0278 | 0.0456 | 2.3840e+06 | 1 |

**Adduct Signal Fractions**

- **Unmodified**: 2.3840e+06

### Q12913|PTPRJ\_HUMAN

**>Q12913|PTPRJ\_HUMAN**  
 `MKPAAREARLPPRSPGLRWALPLLLLLLRLGQILCAGGTPSPIPDPSVATVATGENGITQISSTAESFHKQNGTGTPQVE  
TNTSEDGESSGANDSLRTPEQGSNGTDGASQKTPSSTGPSPVFDIKAVSISPTNVILTWKSNDTAASEYKYVVKHKMENE  
KTITVVHQPWCNITGLRPATSYVFSITPGIGNETWGDPRVIKVITEPIPVSDLRVALTGVRKAALSWSNGNGTASCRVLL  
ESIGSHEELTQDSRLQVNISGLKPGVQYNINPYLLQSNKTKGDPLGTEGGLDASNTERSRAGSPTAPVHDESLVGPVDPS  
SGQQSRDTEVLLVGLEPGTRYNATVYSQAANGTEGQPQAIEFRTNAIQVFDVTAVNISATSLTLIWKVSDNESSSNYTYK  
IHVAGETDSSNLNVSEPRAVIPGLRSSTFYNITVCPVLGDIEGTPGFLQVHTPPVPVSDFRVTVVSTTEIGLAWSSHDAE  
SFQMHITQEGAGNSRVEITTNQSIIIGGLFPGTKYCFEIVPKGPNGTEGASRTVCNRTVPSAVFDIHVVYVTTTEMWLDW  
KSPDGASEYVYHLVIESKHGSNHTSTYDKAITLQGLIPGTLYNITISPEVDHVWGDPNSTAQYTRPSNVSNIDVSTNTTA  
ATLSWQNFDDASPTYSYCLLIEKAGNSSNATQVVTDIGITDATVTELIPGSSYTVEIFAQVGDGIKSLEPGRKSFCTDPA  
SMASFDCEVVPKEPALVLKWTCPPGANAGFELEVSSGAWNNATHLESCSSENGTEYRTEVTYLNFSTSYNISITTVSCGK  
MAAPTRNTCTTGITDPPPPDGSPNITSVSHNSVKVKFSGFEASHGPIKAYAVILTTGEAGHPSADVLKYTYEDFKKGASD  
TYVTYLIRTEEKGRSQSLSEVLKYEIDVGNESTTLGYYNGKLEPLGSYRACVAGFTNITFHPQNKGLIDGAESYVSFSRY  
SDAVSLPQDPGVICGAVFGCIFGALVIVTVGGFIFWRKKRKDAKNNEVSFSQIKPKKSKLIRVENFEAYFKKQQADSNCG  
FAEEYEDLKLVGISQPKYAAELAENRGKNRYNNVLPYDISRVKLSVQTHSTDDYINANYMPGYHSKKDFIATQGPLPNTL  
KDFWRMVWEKNVYAIIMLTKCVEQGRTKCEEYWPSKQAQDYGDITVAMTSEIVLPEWTIRDFTVKNIQTSESHPLRQFHF  
TSWPDHGVPDTTDLLINFRYLVRDYMKQSPPESPILVHCSAGVGRTGTFIAIDRLIYQIENENTVDVYGIVYDLRMHRPL  
MVQTEDQYVFLNQCVLDIVRSQKDSKVDLIYQNTTAMTIYENLAPVTTFGKTNGYIA`

| Site | Type | Count |
| --- | --- | --- |
| 413 | N-linked | 4 |

| Mass | Sequence | MS_2_ Score | `q`-value | MS_1_ Score | Signal | Protein Coordinates | Tandem Scans | Start Time | Apex Time | End Time |
| --- | --- | --- | --- | --- | --- | --- | --- | --- | --- | --- |
| 3911.6471 | IHVAGETDSSNLN(N)VSEPR Hex 6 HexNAc 5 | 76.481 | 0.003 | 14.245 | 1.449e+07 | 400 - 418 | 5 | 23.25 | 23.35 | 23.79 |
| 3547.0775 | IHVAGETDSSNLN(N)VSEPR Hex 5 HexNAc 4 | 53.629 | 0.003 | -20.437 | 4.716e+06 | 400 - 418 | 3 | 23.64 | 47.39 | 47.39 |
| 4057.7030 | IHVAGETDSSNLN(N)VSEPR Fuc 1 Hex 6 HexNAc 5 | 42.690 | 0.003 | -3.031 | 4.306e+06 | 400 - 418 | 2 | 23.15 | 23.25 | 23.35 |
| 4422.8365 | IHVAGETDSSNLN(N)VSEPR Fuc 1 Hex 7 HexNAc 6 | 27.924 | 0.032 | 10.744 | 5.766e+06 | 400 - 418 | 2 | 22.86 | 22.99 | 23.15 |

### IHVAGETDSSNLN(N)VSEPR Hex 6 HexNAc 5

**Mass:**  3911.6477

(9.740e-07)

**Charge States:** 3, 4

**Start Position**: 401

**End Position**: 419

| Profile Score | Tandem Score | TDA q-value | Volume | Spectrum Matches |
| --- | --- | --- | --- | --- |
| 14.2450 | 76.4807 | 0.0026 | 1.4493e+07 | 5 |

**Adduct Signal Fractions**

- **Unmodified**: 1.4493e+07

### IHVAGETDSSNLN(N)VSEPR Hex 5 HexNAc 4

**Mass:**  3547.5144

(2.826e-04)

**Charge States:** 4

**Start Position**: 401

**End Position**: 419

| Profile Score | Tandem Score | TDA q-value | Volume | Spectrum Matches |
| --- | --- | --- | --- | --- |
| -20.4372 | 53.6293 | 0.0026 | 4.7158e+06 | 3 |

**Adduct Signal Fractions**

- **Unmodified**: 4.7158e+06

### IHVAGETDSSNLN(N)VSEPR Fuc 1 Hex 6 HexNAc 5

**Mass:**  4057.7043

(8.772e-07)

**Charge States:** 4

**Start Position**: 401

**End Position**: 419

| Profile Score | Tandem Score | TDA q-value | Volume | Spectrum Matches |
| --- | --- | --- | --- | --- |
| -3.0309 | 42.6897 | 0.0026 | 4.3058e+06 | 2 |

**Adduct Signal Fractions**

- **Unmodified**: 4.3058e+06

### IHVAGETDSSNLN(N)VSEPR Fuc 1 Hex 7 HexNAc 6

**Mass:**  4422.8366

(2.800e-06)

**Charge States:** 4

**Start Position**: 401

**End Position**: 419

| Profile Score | Tandem Score | TDA q-value | Volume | Spectrum Matches |
| --- | --- | --- | --- | --- |
| 10.7443 | 27.9238 | 0.0318 | 5.7655e+06 | 2 |

**Adduct Signal Fractions**

- **Unmodified**: 5.7655e+06

### P02790|HEMO\_HUMAN

**>P02790|HEMO\_HUMAN**  
 `MARVLGAPVALGLWSLCWSLAIATPLPPTSAHGNVAEGETKPDPDVTERCSDGWSFDATTLDDNGTMLFFKGEFVWKSHK  
WDRELISERWKNFPSPVDAAFRQGHNSVFLIKGDKVWVYPPEKKEKGYPKLLQDEFPGIPSPLDAAVECHRGECQAEGVL  
FFQGDREWFWDLATGTMKERSWPAVGNCSSALRWLGRYYCFQGNQFLRFDPVRGEVPPRYPRDVRDYFMPCPGRGHGHRN  
GTGHGNSTHHGPEYMRCSPHLVLSALTSDNHGATYAFSGTHYWRLDTSRDGWHSWPIAHQWPQGPSAVDAAFSWEEKLYL  
VQGTQVYVFLTKGGYTLVSGYPKRLEKEVGTPHGIILDSVDAAFICPGSSRLHIMAGRRLWWLDLKSGAQATWTELPWPH  
EKVDGALCMEKSLGPNSCSANGPGLYLIHGPNLYCYSDVEKLNAAKALPQPQNVTSLLGCTH`

| Site | Type | Count |
| --- | --- | --- |
| 187 | N-linked | 3 |
| 453 | N-linked | 1 |

| Mass | Sequence | MS_2_ Score | `q`-value | MS_1_ Score | Signal | Protein Coordinates | Tandem Scans | Start Time | Apex Time | End Time |
| --- | --- | --- | --- | --- | --- | --- | --- | --- | --- | --- |
| 3026.2462 | SWPAVGN(N)C(C)SSALR Hex 5 HexNAc 4 | 120.361 | 0.0 | 14.999 | 1.702e+07 | 180 - 193 | 2 | 30.80 | 31.14 | 31.48 |
| 3357.4569 | ALPQPQN(N)VTSLLGC(C)TH Hex 5 HexNAc 4 | 106.138 | 0.0 | 13.976 | 3.939e+07 | 446 - 462 | 4 | 43.83 | 44.17 | 44.68 |
| 3391.3800 | SWPAVGN(N)C(C)SSALR Hex 6 HexNAc 5 | 102.402 | 0.0 | -5.078 | 3.450e+06 | 180 - 193 | 1 | 30.63 | 30.97 | 30.97 |
| 3172.3076 | SWPAVGN(N)C(C)SSALR Fuc 1 Hex 5 HexNAc 4 | 31.249 | 0.018 | -19.283 | 2.212e+06 | 180 - 193 | 1 | 30.97 | 31.14 | 31.14 |

### SWPAVGN(N)C(C)SSALR Hex 5 HexNAc 4

**Mass:**  3026.2461

(6.936e-07)

**Charge States:** 3

**Start Position**: 181

**End Position**: 194

| Profile Score | Tandem Score | TDA q-value | Volume | Spectrum Matches |
| --- | --- | --- | --- | --- |
| 14.9993 | 120.3612 | 0.0000 | 1.7024e+07 | 2 |

**Adduct Signal Fractions**

- **Unmodified**: 1.7024e+07

### ALPQPQN(N)VTSLLGC(C)TH Hex 5 HexNAc 4

**Mass:**  3357.4568

(8.902e-07)

**Charge States:** 3, 4

**Start Position**: 447

**End Position**: 463

| Profile Score | Tandem Score | TDA q-value | Volume | Spectrum Matches |
| --- | --- | --- | --- | --- |
| 13.9764 | 106.1382 | 0.0000 | 3.9389e+07 | 4 |

**Adduct Signal Fractions**

- **Unmodified**: 3.9389e+07

### SWPAVGN(N)C(C)SSALR Hex 6 HexNAc 5

**Mass:**  3391.3797

(1.154e-06)

**Charge States:** 3

**Start Position**: 181

**End Position**: 194

| Profile Score | Tandem Score | TDA q-value | Volume | Spectrum Matches |
| --- | --- | --- | --- | --- |
| -5.0784 | 102.4018 | 0.0000 | 3.4496e+06 | 1 |

**Adduct Signal Fractions**

- **Unmodified**: 3.4496e+06

### SWPAVGN(N)C(C)SSALR Fuc 1 Hex 5 HexNAc 4

**Mass:**  3172.3079

(1.864e-06)

**Charge States:** 3

**Start Position**: 181

**End Position**: 194

| Profile Score | Tandem Score | TDA q-value | Volume | Spectrum Matches |
| --- | --- | --- | --- | --- |
| -19.2834 | 31.2488 | 0.0180 | 2.2125e+06 | 1 |

**Adduct Signal Fractions**

- **Unmodified**: 2.2125e+06

### Q5R543|HEMO\_PONAB

**>Q5R543|HEMO\_PONAB**  
 `MARALGAPIALGLWSLCWSLAIATPLPPTSAHGNVAEGETKPDPDVTERCSDGWSFDATTLDDNGTMLFFKGEFVWKSHK  
WDRELISERWKNFPSPVDAAFRQGHNSVFLIKGDKVWVYPPEKKEKGYPKLLQDEFPGIPSPLDAAVECHRGECQAEGVL  
FFQGDREWFWDLATGTMKERSWPAVGNCSSALRWLGRYYCFQGNQFLRFDPVRGEVPPRYPRDVRDYFMPCPGRGHGHRN  
GTGHGNGTHHGPEYMRCSPHLVLSALTSDNHGATYAFSGTHYWRLDTSRDGWHSWPIAHQWPQGPSTVDAAFSWEEKLYL  
VQGTQVYVFLTKGGYTLVSGYPKQLEKEVGTPHGIILDSVDAAFICPGSSRLHIMAGRRLWWLDLKSGAQALWTELPWPH  
EKVDGALCVEKSLGPNSCSANGPGLYLIHGPNLYCYSDVEKLNAAKALPQPQNVTSLLGCTH`

| Site | Type | Count |
| --- | --- | --- |
| 187 | N-linked | 3 |
| 453 | N-linked | 1 |

| Mass | Sequence | MS_2_ Score | `q`-value | MS_1_ Score | Signal | Protein Coordinates | Tandem Scans | Start Time | Apex Time | End Time |
| --- | --- | --- | --- | --- | --- | --- | --- | --- | --- | --- |
| 3026.2462 | SWPAVGN(N)C(C)SSALR Hex 5 HexNAc 4 | 120.361 | 0.0 | 14.999 | 1.702e+07 | 180 - 193 | 2 | 30.80 | 31.14 | 31.48 |
| 3357.4569 | ALPQPQN(N)VTSLLGC(C)TH Hex 5 HexNAc 4 | 106.138 | 0.0 | 13.976 | 3.939e+07 | 446 - 462 | 4 | 43.83 | 44.17 | 44.68 |
| 3391.3800 | SWPAVGN(N)C(C)SSALR Hex 6 HexNAc 5 | 102.402 | 0.0 | -5.078 | 3.450e+06 | 180 - 193 | 1 | 30.63 | 30.97 | 30.97 |
| 3172.3076 | SWPAVGN(N)C(C)SSALR Fuc 1 Hex 5 HexNAc 4 | 31.249 | 0.018 | -19.283 | 2.212e+06 | 180 - 193 | 1 | 30.97 | 31.14 | 31.14 |

### SWPAVGN(N)C(C)SSALR Hex 5 HexNAc 4

**Mass:**  3026.2461

(6.936e-07)

**Charge States:** 3

**Start Position**: 181

**End Position**: 194

| Profile Score | Tandem Score | TDA q-value | Volume | Spectrum Matches |
| --- | --- | --- | --- | --- |
| 14.9993 | 120.3612 | 0.0000 | 1.7024e+07 | 2 |

**Adduct Signal Fractions**

- **Unmodified**: 1.7024e+07

### ALPQPQN(N)VTSLLGC(C)TH Hex 5 HexNAc 4

**Mass:**  3357.4568

(8.902e-07)

**Charge States:** 3, 4

**Start Position**: 447

**End Position**: 463

| Profile Score | Tandem Score | TDA q-value | Volume | Spectrum Matches |
| --- | --- | --- | --- | --- |
| 13.9764 | 106.1382 | 0.0000 | 3.9389e+07 | 4 |

**Adduct Signal Fractions**

- **Unmodified**: 3.9389e+07

### SWPAVGN(N)C(C)SSALR Hex 6 HexNAc 5

**Mass:**  3391.3797

(1.154e-06)

**Charge States:** 3

**Start Position**: 181

**End Position**: 194

| Profile Score | Tandem Score | TDA q-value | Volume | Spectrum Matches |
| --- | --- | --- | --- | --- |
| -5.0784 | 102.4018 | 0.0000 | 3.4496e+06 | 1 |

**Adduct Signal Fractions**

- **Unmodified**: 3.4496e+06

### SWPAVGN(N)C(C)SSALR Fuc 1 Hex 5 HexNAc 4

**Mass:**  3172.3079

(1.864e-06)

**Charge States:** 3

**Start Position**: 181

**End Position**: 194

| Profile Score | Tandem Score | TDA q-value | Volume | Spectrum Matches |
| --- | --- | --- | --- | --- |
| -19.2834 | 31.2488 | 0.0180 | 2.2125e+06 | 1 |

**Adduct Signal Fractions**

- **Unmodified**: 2.2125e+06

### O95497|VNN1\_HUMAN

**>O95497|VNN1\_HUMAN**  
 `MTTQLPAYVAILLFYVSRASCQDTFTAAVYEHAAILPNATLTPVSREEALALMNRNLDILEGAITSAADQGAHIIVTPED  
AIYGWNFNRDSLYPYLEDIPDPEVNWIPCNNRNRFGQTPVQERLSCLAKNNSIYVVANIGDKKPCDTSDPQCPPDGRYQY  
NTDVVFDSQGKLVARYHKQNLFMGENQFNVPKEPEIVTFNTTFGSFGIFTCFDILFHDPAVTLVKDFHVDTIVFPTAWMN  
VLPHLSAVEFHSAWAMGMRVNFLASNIHYPSKKMTGSGIYAPNSSRAFHYDMKTEEGKLLLSQLDSHPSHSAVVNWTSYA  
SSIEALSSGNKEFKGTVFFDEFTFVKLTGVAGNYTVCQKDLCCHLSYKMSENIPNEVYALGAFDGLHTVEGRYYLQICTL  
LKCKTTNLNTCGDSAETASTRFEMFSLSGTFGTQYVFPEVLLSENQLAPGEFQVSTDGRLFSLKPTSGPVLTVTLFGRLY  
EKDWASNASSGLTAQARIIMLIVIAPIVCSLSW`

| Site | Type | Count |
| --- | --- | --- |
| 353 | N-linked | 3 |

| Mass | Sequence | MS_2_ Score | `q`-value | MS_1_ Score | Signal | Protein Coordinates | Tandem Scans | Start Time | Apex Time | End Time |
| --- | --- | --- | --- | --- | --- | --- | --- | --- | --- | --- |
| 3397.4155 | LTGVAGN(N)YTVC(C)QK Hex 6 HexNAc 5 | 41.433 | 0.003 | -4.305 | 7.312e+06 | 346 - 359 | 2 | 21.62 | 21.79 | 22.05 |
| 3033.2869 | LTGVAGN(N)YTVC(C)QK Hex 5 HexNAc 4 | 32.829 | 0.014 | 0.000 | 0.000e+00 | 346 - 359 | 1 | - | - | - |
| 3032.2819 | LTGVAGN(N)YTVC(C)QK Hex 5 HexNAc 4 | 31.441 | 0.018 | 11.147 | 5.806e+06 | 346 - 359 | 1 | 21.93 | 22.05 | 22.26 |

### LTGVAGN(N)YTVC(C)QK Hex 6 HexNAc 5

**Mass:**  3397.4159

(7.898e-07)

**Charge States:** 3

**Start Position**: 347

**End Position**: 360

| Profile Score | Tandem Score | TDA q-value | Volume | Spectrum Matches |
| --- | --- | --- | --- | --- |
| -4.3047 | 41.4331 | 0.0026 | 7.3116e+06 | 2 |

**Adduct Signal Fractions**

- **Unmodified**: 7.3116e+06

### LTGVAGN(N)YTVC(C)QK Hex 5 HexNAc 4

**Mass:**  3033.2869

(3.324e-04)

**Start Position**: 347

**End Position**: 360

| Profile Score | Tandem Score | TDA q-value | Volume | Spectrum Matches |
| --- | --- | --- | --- | --- |
| 0.0000 | 32.8287 | 0.0137 | 0.0000e+00 | 1 |

Adduct:

No Chromatogram Found

### LTGVAGN(N)YTVC(C)QK Hex 5 HexNAc 4

**Mass:**  3032.2835

(4.736e-07)

**Charge States:** 3

**Start Position**: 347

**End Position**: 360

| Profile Score | Tandem Score | TDA q-value | Volume | Spectrum Matches |
| --- | --- | --- | --- | --- |
| 11.1467 | 31.4412 | 0.0180 | 5.8056e+06 | 1 |

**Adduct Signal Fractions**

- **Unmodified**: 5.8056e+06

### P11279|LAMP1\_HUMAN

**>P11279|LAMP1\_HUMAN**  
 `MAAPGSARRPLLLLLLLLLLGLMHCASAAMFMVKNGNGTACIMANFSAAFSVNYDTKSGPKNMTFDLPSDATVVLNRSSC  
GKENTSDPSLVIAFGRGHTLTLNFTRNATRYSVQLMSFVYNLSDTHLFPNASSKEIKTVESITDIRADIDKKYRCVSGTQ  
VHMNNVTVTLHDATIQAYLSNSSFSRGETRCEQDRPSPTTAPPAPPSPSPSPVPKSPSVDKYNVSGTNGTCLLASMGLQL  
NLTYERKDNTTVTRLLNINPNKTSASGSCGAHLVTLELHSEGTTVLLFQFGMNASSSRFFLQGIQLNTILPDARDPAFKA  
ANGSLRALQATVGNSYKCNAEEHVRVTKAFSVNIFKVWVQAFKVEGGQFGSVEECLLDENSMLIPIAVGGALAGLVLIVL  
IAYLVGRKRSHAGYQTI`

| Site | Type | Count |
| --- | --- | --- |
| 103 | N-linked | 3 |

| Mass | Sequence | MS_2_ Score | `q`-value | MS_1_ Score | Signal | Protein Coordinates | Tandem Scans | Start Time | Apex Time | End Time |
| --- | --- | --- | --- | --- | --- | --- | --- | --- | --- | --- |
| 2740.1732 | GHTLTLN(N)FTR Hex 6 HexNAc 3 | 122.765 | 0.0 | -3.769 | 2.222e+06 | 96 - 106 | 2 | 30.14 | 30.30 | 30.46 |
| 2578.1200 | GHTLTLN(N)FTR Hex 5 HexNAc 3 | 113.578 | 0.0 | -4.874 | 1.154e+06 | 96 - 106 | 1 | 30.30 | 30.46 | 30.63 |
| 2781.1972 | GHTLTLN(N)FTR Hex 5 HexNAc 4 | 33.635 | 0.011 | -5.348 | 1.149e+06 | 96 - 106 | 1 | 30.14 | 30.14 | 30.46 |

### GHTLTLN(N)FTR Hex 6 HexNAc 3

**Mass:**  2740.1724

(1.294e-06)

**Charge States:** 3

**Start Position**: 97

**End Position**: 107

| Profile Score | Tandem Score | TDA q-value | Volume | Spectrum Matches |
| --- | --- | --- | --- | --- |
| -3.7694 | 122.7654 | 0.0000 | 2.2220e+06 | 2 |

**Adduct Signal Fractions**

- **Unmodified**: 2.2220e+06

### GHTLTLN(N)FTR Hex 5 HexNAc 3

**Mass:**  2578.1206

(1.443e-06)

**Charge States:** 3

**Start Position**: 97

**End Position**: 107

| Profile Score | Tandem Score | TDA q-value | Volume | Spectrum Matches |
| --- | --- | --- | --- | --- |
| -4.8740 | 113.5778 | 0.0000 | 1.1542e+06 | 1 |

**Adduct Signal Fractions**

- **Unmodified**: 1.1542e+06

### GHTLTLN(N)FTR Hex 5 HexNAc 4

**Mass:**  2781.1975

(5.207e-07)

**Charge States:** 3

**Start Position**: 97

**End Position**: 107

| Profile Score | Tandem Score | TDA q-value | Volume | Spectrum Matches |
| --- | --- | --- | --- | --- |
| -5.3483 | 33.6350 | 0.0108 | 1.1486e+06 | 1 |

**Adduct Signal Fractions**

- **Unmodified**: 1.1486e+06

### O75144|ICOSL\_HUMAN

**>O75144|ICOSL\_HUMAN**  
 `MRLGSPGLLFLLFSSLRADTQEKEVRAMVGSDVELSCACPEGSRFDLNDVYVYWQTSESKTVVTYHIPQNSSLENVDSRY  
RNRALMSPAGMLRGDFSLRLFNVTPQDEQKFHCLVLSQSLGFQEVLSVEVTLHVAANFSVPVVSAPHSPSQDELTFTCTS  
INGYPRPNVYWINKTDNSLLDQALQNDTVFLNMRGLYDVVSVLRIARTPSVNIGCCIENVLLQQNLTVGSQTGNDIGERD  
KITENPVSTGEKNAATWSILAVLCLLVVVAVAIGWVCRDRCLQHSYAGAWAVSPETELTGHV`

| Site | Type | Count |
| --- | --- | --- |
| 70 | N-linked | 2 |
| 102 | N-linked | 1 |

| Mass | Sequence | MS_2_ Score | `q`-value | MS_1_ Score | Signal | Protein Coordinates | Tandem Scans | Start Time | Apex Time | End Time |
| --- | --- | --- | --- | --- | --- | --- | --- | --- | --- | --- |
| 4145.7873 | TVVTYHIPQN(N)SSLENVDSR Hex 6 HexNAc 5 | 43.516 | 0.003 | -19.648 | 1.222e+06 | 60 - 79 | 1 | 30.80 | 30.80 | 30.80 |
| 4291.8526 | TVVTYHIPQN(N)SSLENVDSR Fuc 1 Hex 6 HexNAc 5 | 39.766 | 0.005 | -20.123 | 2.152e+06 | 60 - 79 | 3 | 30.63 | 30.63 | 30.80 |
| 2940.2463 | LFN(N)VTPQDEQK Hex 5 HexNAc 4 | 38.312 | 0.005 | -20.789 | 1.380e+06 | 99 - 110 | 1 | 25.14 | 25.31 | 25.31 |

### TVVTYHIPQN(N)SSLENVDSR Hex 6 HexNAc 5

**Mass:**  4145.7863

(1.691e-06)

**Charge States:** 3, 4

**Start Position**: 61

**End Position**: 80

| Profile Score | Tandem Score | TDA q-value | Volume | Spectrum Matches |
| --- | --- | --- | --- | --- |
| -19.6477 | 43.5161 | 0.0026 | 1.2223e+06 | 1 |

**Adduct Signal Fractions**

- **Unmodified**: 1.2223e+06

### TVVTYHIPQN(N)SSLENVDSR Fuc 1 Hex 6 HexNAc 5

**Mass:**  4291.8522

(3.462e-06)

**Charge States:** 4

**Start Position**: 61

**End Position**: 80

| Profile Score | Tandem Score | TDA q-value | Volume | Spectrum Matches |
| --- | --- | --- | --- | --- |
| -20.1228 | 39.7656 | 0.0049 | 2.1525e+06 | 3 |

**Adduct Signal Fractions**

- **Unmodified**: 2.1525e+06

### LFN(N)VTPQDEQK Hex 5 HexNAc 4

**Mass:**  2940.2433

(1.787e-06)

**Charge States:** 3

**Start Position**: 100

**End Position**: 111

| Profile Score | Tandem Score | TDA q-value | Volume | Spectrum Matches |
| --- | --- | --- | --- | --- |
| -20.7889 | 38.3117 | 0.0049 | 1.3801e+06 | 1 |

**Adduct Signal Fractions**

- **Unmodified**: 1.3801e+06

### Q13201|MMRN1\_HUMAN

**>Q13201|MMRN1\_HUMAN**  
 `MKGARLFVLLSSLWSGGIGLNNSKHSWTIPEDGNSQKTMPSASVPPNKIQSLQILPTTRVMSAEIATTPEARTSEDSLLK  
STLPPSETSAPAEGVRNQTLTSTEKAEGVVKLQNLTLPTNASIKFNPGAESVVLSNSTLKFLQSFARKSNEQATSLNTVG  
GTGGIGGVGGTGGVGNRAPRETYLSRGDSSSSQRTDYQKSNFETTRGKNWCAYVHTRLSPTVILDNQVTYVPGGKGPCGW  
TGGSCPQRSQKISNPVYRMQHKIVTSLDWRCCPGYSGPKCQLRAQEQQSLIHTNQAESHTAVGRGVAEQQQQQGCGDPEV  
MQKMTDQVNYQAMKLTLLQKKIDNISLTVNDVRNTYSSLEGKVSEDKSREFQSLLKGLKSKSINVLIRDIVREQFKIFQN  
DMQETVAQLFKTVSSLSEDLESTRQIIQKVNESVVSIAAQQKFVLVQENRPTLTDIVELRNHIVNVRQEMTLTCEKPIKE  
LEVKQTHLEGALEQEHSRSILYYESLNKTLSKLKEVHEQLLSTEQVSDQKNAPAAESVSNNVTEYMSTLHENIKKQSLMM  
LQMFEDLHIQESKINNLTVSLEMEKESLRGECEDMLSKCRNDFKFQLKDTEENLHVLNQTLAEVLFPMDNKMDKMSEQLN  
DLTYDMEILQPLLEQGASLRQTMTYEQPKEAIVIRKKIENLTSAVNSLNFIIKELTKRHNLLRNEVQGRDDALERRINEY  
ALEMEDGLNKTMTIINNAIDFIQDNYALKETLSTIKDNSEIHHKCTSDMETILTFIPQFHRLNDSIQTLVNDNQRYNFVL  
QVAKTLAGIPRDEKLNQSNFQKMYQMFNETTSQVRKYQQNMSHLEEKLLLTTKISKNFETRLQDIESKVTQTLIPYYISV  
KKGSVVTNERDQALQLQVLNSRFKALEAKSIHLSINFFSLNKTLHEVLTMCHNASTSVSELNATIPKWIKHSLPDIQLLQ  
KGLTEFVEPIIQIKTQAALSNLTCCIDRSLPGSLANVVKSQKQVKSLPKKINALKKPTVNLTTVLIGRTQRNTDNIIYPE  
EYSSCSRHPCQNGGTCINGRTSFTCACRHPFTGDNCTIKLVEENALAPDFSKGSYRYAPMVAFFASHTYGMTIPGPILFN  
NLDVNYGASYTPRTGKFRIPYLGVYVFKYTIESFSAHISGFLVVDGIDKLAFESENINSEIHCDRVLTGDALLELNYGQE  
VWLRLAKGTIPAKFPPVTTFSGYLLYRT`

| Site | Type | Count |
| --- | --- | --- |
| 507 | N-linked | 1 |
| 828 | N-linked | 1 |

| Mass | Sequence | MS_2_ Score | `q`-value | MS_1_ Score | Signal | Protein Coordinates | Tandem Scans | Start Time | Apex Time | End Time |
| --- | --- | --- | --- | --- | --- | --- | --- | --- | --- | --- |
| 4456.7251 | MYQMFN(N)ETTSQVR Fuc 1 Hex 9 HexNAc 6 | 27.097 | 0.036 | -20.441 | 1.855e+07 | 822 - 835 | 1 | 33.01 | 33.19 | 33.19 |
| 3923.6909 | SILYYESLN(N)K Fuc 4 Hex 3 HexNAc 8 | 22.214 | 0.050 | 12.900 | 4.841e+08 | 498 - 508 | 16 | 53.25 | 53.93 | 57.68 |

### MYQMFN(N)ETTSQVR Fuc 1 Hex 9 HexNAc 6

**Mass:**  4456.7258

(-2.285e-06)

**Charge States:** 4

**Start Position**: 823

**End Position**: 836

| Profile Score | Tandem Score | TDA q-value | Volume | Spectrum Matches |
| --- | --- | --- | --- | --- |
| -20.4414 | 27.0971 | 0.0360 | 1.8553e+07 | 1 |

**Adduct Signal Fractions**

- **Unmodified**: 1.8553e+07

### SILYYESLN(N)K Fuc 4 Hex 3 HexNAc 8

**Mass:**  3923.6889

(9.150e-06)

**Charge States:** 3, 4

**Start Position**: 499

**End Position**: 509

| Profile Score | Tandem Score | TDA q-value | Volume | Spectrum Matches |
| --- | --- | --- | --- | --- |
| 12.9002 | 22.2145 | 0.0497 | 4.8405e+08 | 16 |

**Adduct Signal Fractions**

- **Unmodified**: 4.8405e+08
