# Supplementary material for: Measuring Site-specific Glycosylation Similarity between Influenza a Virus Variants with Statistical Certainty
Source: Mol Cell Proteomics. 2020 Nov 25;19(9):1533–45. doi: 10.1074/mcp.RA120.002031 (PMC8143645; doi:10.1074/mcp.RA120.002031)
Supplement: Supplementary file 1 [file mmc1.zip › mmc1/159250_2_supp_555288_qcjkln.zip!/20190326_aAGPgal_021119_tryp_HILIC_DDA_1ug_03_glycopeptides.html]

## 20190326\_aAGPgal\_021119\_tryp\_HILIC\_DDA\_1ug\_03 @ GlycopeptideHypothesis-400b854021f949b0b58c30c4553a853e

**MS^1^ Mass Accuracy:** 10.0 PPM  
**Grouping Tolerance:** 15.0 PPM  
**MS^n^ Mass Accuracy:** 20.0 PPM  
**Minimum `q`-Value For Clustering:** 0.05

**Glycopeptide Hypothesis:**
GlycopeptideHypothesis-400b854021f949b0b58c30c4553a853e  
**Sample Searched:**
20190326\_aAGPgal\_021119\_tryp\_HILIC\_DDA\_1ug\_03

| Protein | Matched Glycopeptides |
| --- | --- |
| P02763\|A1AG1\_HUMAN | 160 |
| P19652\|A1AG2\_HUMAN | 158 |
| P00738\|HPT\_HUMAN | 14 |
| Q9Y5Y7\|LYVE1\_HUMAN | 9 |
| P05155\|IC1\_HUMAN | 7 |
| Q12913\|PTPRJ\_HUMAN | 6 |
| O95497\|VNN1\_HUMAN | 3 |
| P02790\|HEMO\_HUMAN | 3 |
| Q5R543\|HEMO\_PONAB | 3 |
| P11279\|LAMP1\_HUMAN | 3 |
| O75144\|ICOSL\_HUMAN | 1 |

### P02763|A1AG1\_HUMAN

**>P02763|A1AG1\_HUMAN**  
 `MALSWVLTVLSLLPLLEAQIPLCANLVPVPITNATLDQITGKWFYIASAFRNEEYNKSVQEIQATFFYFTPNKTEDTIFL  
REYQTRQDQCIYNTTYLNVQRENGTISRYVGGQEHFAHLLILRDTKTYMLAFDVNDEKNWGLSVYADKPETTKEQLGEFY  
EALDCLRIPKSDVVYTDWKKDKCEPLEKQHEKERKQEEGES`

| Site | Type | Count |
| --- | --- | --- |
| 33 | N-linked | 42 |
| 56 | N-linked | 3 |
| 93 | N-linked | 38 |
| 72 | N-linked | 77 |

| Mass | Sequence | MS_2_ Score | `q`-value | MS_1_ Score | Signal | Protein Coordinates | Tandem Scans | Start Time | Apex Time | End Time |
| --- | --- | --- | --- | --- | --- | --- | --- | --- | --- | --- |
| 2473.0179 | YFTPN(N)K Hex 3 HexNAc 6 | 188.898 | 0.0 | 14.137 | 3.107e+08 | 67 - 73 | 5 | 13.65 | 13.81 | 14.29 |
| 2781.1281 | YFTPN(N)K Fuc 1 Hex 4 HexNAc 6 | 186.556 | 0.0 | -4.323 | 4.931e+07 | 67 - 73 | 3 | 13.48 | 13.48 | 13.81 |
| 2620.0856 | FYFTPN(N)K Hex 3 HexNAc 6 | 183.035 | 0.0 | 13.158 | 1.155e+08 | 66 - 73 | 11 | 25.02 | 25.36 | 27.92 |
| 2578.0496 | YFTPN(N)K Fuc 1 Hex 4 HexNAc 5 | 181.096 | 0.0 | -19.295 | 3.412e+07 | 67 - 73 | 2 | 13.65 | 13.65 | 13.81 |
| 2296.8955 | NEEYN(N)K Hex 3 HexNAc 5 | 180.688 | 0.0 | 10.718 | 3.117e+07 | 51 - 57 | 7 | 3.77 | 3.94 | 4.99 |
| 2605.0069 | NEEYN(N)K Fuc 1 Hex 4 HexNAc 5 | 178.950 | 0.0 | 11.035 | 1.259e+07 | 51 - 57 | 4 | 3.77 | 3.94 | 4.82 |
| 3988.7196 | SVQEIQATFFYFTPN(N)K Hex 4 HexNAc 7 | 173.301 | 0.0 | 10.801 | 2.305e+08 | 57 - 73 | 10 | 53.47 | 53.64 | 55.33 |
| 2838.1508 | YFTPN(N)K Hex 4 HexNAc 7 | 173.030 | 0.0 | -5.133 | 2.478e+07 | 67 - 73 | 1 | 13.48 | 13.48 | 13.81 |
| 2269.9384 | YFTPN(N)K Hex 3 HexNAc 5 | 172.718 | 0.0 | 12.486 | 1.952e+08 | 67 - 73 | 5 | 13.81 | 13.98 | 14.45 |
| 2066.8589 | YFTPN(N)K Hex 3 HexNAc 4 | 172.517 | 0.0 | -19.096 | 2.513e+07 | 67 - 73 | 3 | 13.98 | 13.98 | 14.15 |
| 2939.2404 | ATFFYFTPN(N)K Hex 3 HexNAc 6 | 172.154 | 0.0 | -3.409 | 3.969e+07 | 63 - 73 | 3 | 41.22 | 41.39 | 41.73 |
| 3728.6183 | SVQEIQATFFYFTPN(N)K Fuc 1 Hex 4 HexNAc 5 | 171.606 | 0.0 | 13.093 | 1.770e+08 | 57 - 73 | 8 | 53.98 | 54.15 | 55.50 |
| 2417.0063 | FYFTPN(N)K Hex 3 HexNAc 5 | 170.522 | 0.0 | 13.059 | 6.659e+07 | 66 - 73 | 11 | 25.36 | 25.82 | 27.71 |
| 3420.5054 | SVQEIQATFFYFTPN(N)K Hex 3 HexNAc 5 | 168.319 | 0.0 | 14.862 | 6.027e+08 | 57 - 73 | 29 | 50.92 | 54.65 | 59.16 |
| 3623.5857 | SVQEIQATFFYFTPN(N)K Hex 3 HexNAc 6 | 167.708 | 0.0 | 14.624 | 1.060e+09 | 57 - 73 | 25 | 54.15 | 54.49 | 57.95 |
| 3931.6989 | SVQEIQATFFYFTPN(N)K Fuc 1 Hex 4 HexNAc 6 | 165.633 | 0.0 | 13.253 | 2.884e+08 | 57 - 73 | 12 | 53.64 | 53.98 | 56.18 |
| 2093.8160 | NEEYN(N)K Hex 3 HexNAc 4 | 163.328 | 0.0 | 9.567 | 3.463e+07 | 51 - 57 | 10 | 3.77 | 3.94 | 10.66 |
| 2840.1601 | YFTPN(N)K Hex 4 HexNAc 7 | 160.539 | 0.0 | 0.000 | 0.000e+00 | 67 - 73 | 1 | - | - | - |
| 4239.8071 | SVQEIQATFFYFTPN(N)K Fuc 2 Hex 5 HexNAc 6 | 157.904 | 0.0 | 13.499 | 4.557e+07 | 57 - 73 | 4 | 53.30 | 53.64 | 54.15 |
| 2736.1615 | ATFFYFTPN(N)K Hex 3 HexNAc 5 | 156.464 | 0.0 | -4.085 | 2.291e+07 | 63 - 73 | 3 | 41.56 | 41.73 | 41.90 |
| 3217.4261 | SVQEIQATFFYFTPN(N)K Hex 3 HexNAc 4 | 153.432 | 0.0 | 12.807 | 7.656e+07 | 57 - 73 | 8 | 54.82 | 54.99 | 56.79 |
| 4271.7980 | SVQEIQATFFYFTPN(N)K Hex 7 HexNAc 6 | 153.210 | 0.0 | 12.622 | 4.845e+07 | 57 - 73 | 1 | 53.30 | 53.30 | 54.15 |
| 3280.5713 | LVPVPITN(N)ATLDQITGK Hex 3 HexNAc 5 | 152.872 | 0.0 | 14.275 | 1.348e+10 | 25 - 42 | 514 | 41.90 | 48.45 | 113.36 |
| 3791.7687 | LVPVPITN(N)ATLDQITGK Fuc 1 Hex 4 HexNAc 6 | 152.184 | 0.0 | 14.069 | 1.712e+08 | 25 - 42 | 7 | 41.56 | 48.13 | 50.07 |
| 3484.0787 | LVPVPITN(N)ATLDQITGK Hex 3 HexNAc 6 | 151.894 | 0.0 | 8.524 | 4.407e+08 | 25 - 42 | 15 | 41.73 | 48.30 | 64.34 |
| 3906.6679 | SVQEIQATFFYFTPN(N)K Hex 6 HexNAc 5 | 148.554 | 0.0 | 13.102 | 3.737e+07 | 57 - 73 | 4 | 53.47 | 53.81 | 54.32 |
| 4191.7990 | SVQEIQATFFYFTPN(N)K Hex 4 HexNAc 8 | 147.421 | 0.0 | 13.144 | 2.603e+07 | 57 - 73 | 4 | 53.30 | 53.47 | 54.15 |
| 3077.4921 | LVPVPITN(N)ATLDQITGK Hex 3 HexNAc 4 | 146.432 | 0.0 | 14.266 | 1.151e+09 | 25 - 42 | 23 | 42.07 | 48.92 | 52.79 |
| 3138.3886 | TPN(N)KTEDTIFLR Hex 3 HexNAc 6 | 146.253 | 0.0 | 12.390 | 4.965e+07 | 69 - 81 | 6 | 25.65 | 25.82 | 26.36 |
| 3588.6841 | LVPVPITN(N)ATLDQITGK Fuc 1 Hex 4 HexNAc 5 | 145.300 | 0.0 | 15.218 | 3.845e+09 | 25 - 42 | 30 | 41.56 | 48.13 | 52.11 |
| 3426.6309 | LVPVPITN(N)ATLDQITGK Fuc 1 Hex 3 HexNAc 5 | 144.109 | 0.0 | -4.566 | 6.382e+07 | 25 - 42 | 2 | 48.76 | 48.76 | 49.07 |
| 4134.7761 | SVQEIQATFFYFTPN(N)K Fuc 1 Hex 4 HexNAc 7 | 143.609 | 0.0 | -4.673 | 1.116e+07 | 57 - 73 | 3 | 53.47 | 53.81 | 53.98 |
| 4556.9335 | SVQEIQATFFYFTPN(N)K Hex 5 HexNAc 9 | 143.334 | 0.0 | -20.405 | 2.019e+06 | 57 - 73 | 1 | 52.79 | 52.79 | 52.79 |
| 3619.5278 | QDQC(C)IYN(N)TTYLNVQR Hex 3 HexNAc 6 | 141.514 | 0.0 | 10.735 | 3.111e+09 | 86 - 101 | 8 | 31.41 | 33.78 | 34.93 |
| 3044.2653 | ATFFYFTPN(N)K Fuc 1 Hex 4 HexNAc 5 | 140.360 | 0.0 | -17.444 | 5.646e+06 | 63 - 73 | 1 | 41.05 | 41.05 | 41.22 |
| 4296.8291 | SVQEIQATFFYFTPN(N)K Fuc 1 Hex 5 HexNAc 7 | 139.286 | 0.0 | 10.080 | 3.223e+07 | 57 - 73 | 3 | 52.96 | 53.30 | 54.15 |
| 3785.6418 | SVQEIQATFFYFTPN(N)K Hex 4 HexNAc 6 | 137.591 | 0.0 | 11.500 | 3.327e+07 | 57 - 73 | 5 | 53.81 | 54.82 | 55.50 |
| 3756.6324 | YFTPN(N)KTEDTIFLR Fuc 1 Hex 4 HexNAc 6 | 137.425 | 0.0 | -3.746 | 2.114e+08 | 67 - 81 | 8 | 35.27 | 36.90 | 37.59 |
| 3896.7960 | LVPVPITN(N)ATLDQITGK Fuc 2 Hex 5 HexNAc 5 | 136.975 | 0.0 | -4.151 | 3.432e+07 | 25 - 42 | 2 | 47.63 | 47.63 | 47.96 |
| 3442.6281 | LVPVPITN(N)ATLDQITGK Hex 4 HexNAc 5 | 136.831 | 0.0 | -5.452 | 6.583e+07 | 25 - 42 | 2 | 47.96 | 48.13 | 48.30 |
| 3416.4477 | QDQC(C)IYN(N)TTYLNVQR Hex 3 HexNAc 5 | 136.636 | 0.0 | 12.995 | 1.526e+09 | 86 - 101 | 8 | 33.61 | 33.93 | 34.93 |
| 3961.6701 | SVQEIQATFFYFTPN(N)K Hex 4 HexNAc 4 Neu5Ac 2 | 136.180 | 0.0 | 12.825 | 3.857e+07 | 57 - 73 | 4 | 54.99 | 55.16 | 56.18 |
| 3962.6862 | SVQEIQATFFYFTPN(N)K Fuc 2 Hex 4 HexNAc 4 Neu5Ac 1 | 134.851 | 0.0 | -19.860 | 2.049e+06 | 57 - 73 | 2 | 54.82 | 54.82 | 54.82 |
| 3984.6631 | QDQC(C)IYN(N)TTYLNVQR Hex 4 HexNAc 7 | 134.185 | 0.0 | 15.754 | 7.630e+08 | 86 - 101 | 6 | 32.94 | 33.44 | 34.25 |
| 4235.7497 | QDQC(C)IYN(N)TTYLNVQR Fuc 2 Hex 5 HexNAc 6 | 133.129 | 0.0 | -3.000 | 1.599e+08 | 86 - 101 | 2 | 33.11 | 33.27 | 33.44 |
| 3448.5201 | YFTPN(N)KTEDTIFLR Hex 3 HexNAc 6 | 132.896 | 0.0 | 13.175 | 1.560e+09 | 67 - 81 | 18 | 35.60 | 37.29 | 39.41 |
| 3042.3631 | YFTPN(N)KTEDTIFLR Hex 3 HexNAc 4 | 131.009 | 0.0 | 12.940 | 1.085e+08 | 67 - 81 | 3 | 37.59 | 37.91 | 38.91 |
| 3247.3479 | ATFFYFTPN(N)K Fuc 1 Hex 4 HexNAc 6 | 130.528 | 0.0 | -21.185 | 4.150e+06 | 63 - 73 | 1 | 40.71 | 40.71 | 40.88 |
| 3645.7068 | LVPVPITN(N)ATLDQITGK Hex 4 HexNAc 6 | 129.907 | 0.0 | 13.143 | 6.238e+08 | 25 - 42 | 12 | 47.46 | 48.92 | 50.58 |
| 3724.5588 | QDQC(C)IYN(N)TTYLNVQR Fuc 1 Hex 4 HexNAc 5 | 129.835 | 0.0 | 10.343 | 4.293e+08 | 86 - 101 | 7 | 33.44 | 33.61 | 34.59 |
| 3553.5530 | YFTPN(N)KTEDTIFLR Fuc 1 Hex 4 HexNAc 5 | 126.450 | 0.0 | 8.546 | 1.435e+08 | 67 - 81 | 4 | 37.06 | 37.16 | 37.76 |
| 3428.6393 | LVPVPITN(N)ATLDQITGK Fuc 1 Hex 3 HexNAc 5 | 125.865 | 0.0 | 0.000 | 0.000e+00 | 25 - 42 | 2 | - | - | - |
| 3245.4419 | YFTPN(N)KTEDTIFLR Hex 3 HexNAc 5 | 125.497 | 0.0 | 9.524 | 7.132e+08 | 67 - 81 | 8 | 35.77 | 37.59 | 41.90 |
| 3392.5103 | FYFTPN(N)KTEDTIFLR Hex 3 HexNAc 5 | 124.704 | 0.0 | 13.538 | 4.977e+07 | 66 - 81 | 6 | 46.10 | 46.27 | 47.12 |
| 2935.3088 | TPN(N)KTEDTIFLR Hex 3 HexNAc 5 | 124.529 | 0.0 | 12.823 | 3.948e+07 | 69 - 81 | 6 | 25.82 | 26.10 | 26.83 |
| 3927.6392 | QDQC(C)IYN(N)TTYLNVQR Fuc 1 Hex 4 HexNAc 6 | 124.151 | 0.0 | 13.596 | 1.092e+09 | 86 - 101 | 5 | 33.11 | 33.44 | 34.42 |
| 3848.7887 | LVPVPITN(N)ATLDQITGK Hex 4 HexNAc 7 | 124.104 | 0.0 | 10.828 | 8.118e+07 | 25 - 42 | 4 | 47.46 | 47.96 | 48.13 |
| 4130.7193 | QDQC(C)IYN(N)TTYLNVQR Fuc 1 Hex 4 HexNAc 7 | 123.498 | 0.0 | 12.472 | 5.590e+07 | 86 - 101 | 2 | 32.94 | 33.27 | 33.61 |
| 3213.3683 | QDQC(C)IYN(N)TTYLNVQR Hex 3 HexNAc 4 | 121.727 | 0.0 | -5.520 | 2.316e+07 | 86 - 101 | 2 | 34.10 | 34.25 | 34.42 |
| 4099.8781 | LVPVPITN(N)ATLDQITGK Fuc 2 Hex 5 HexNAc 6 | 121.193 | 0.0 | -4.873 | 1.987e+07 | 25 - 42 | 2 | 47.29 | 47.96 | 47.96 |
| 3595.5904 | FYFTPN(N)KTEDTIFLR Hex 3 HexNAc 6 | 121.079 | 0.0 | 13.985 | 1.089e+08 | 66 - 81 | 5 | 45.93 | 46.10 | 47.29 |
| 3606.7016 | LVPVPITN(N)ATLDQITGK Hex 5 HexNAc 5 | 119.100 | 0.0 | -20.769 | 1.231e+07 | 25 - 42 | 2 | 48.92 | 48.92 | 50.41 |
| 4349.7958 | QDQC(C)IYN(N)TTYLNVQR Hex 5 HexNAc 8 | 117.060 | 0.0 | 8.332 | 1.207e+08 | 86 - 101 | 3 | 32.60 | 32.77 | 33.44 |
| 3957.6126 | QDQC(C)IYN(N)TTYLNVQR Hex 4 HexNAc 4 Neu5Ac 2 | 116.991 | 0.0 | 13.081 | 2.088e+08 | 86 - 101 | 5 | 34.25 | 34.59 | 35.43 |
| 3766.7345 | LVPVPITN(N)ATLDQITGK Hex 6 HexNAc 5 | 116.355 | 0.0 | 8.945 | 7.285e+07 | 25 - 42 | 12 | 45.09 | 47.96 | 48.13 |
| 3813.6537 | YFTPN(N)KTEDTIFLR Hex 4 HexNAc 7 | 116.115 | 0.0 | 9.582 | 1.721e+08 | 67 - 81 | 3 | 36.27 | 36.75 | 37.76 |
| 3786.6077 | TPN(N)KTEDTIFLR Hex 7 HexNAc 6 | 115.917 | 0.0 | 14.446 | 9.777e+07 | 69 - 81 | 2 | 37.91 | 38.07 | 38.74 |
| 4064.7425 | YFTPN(N)KTEDTIFLR Fuc 2 Hex 5 HexNAc 6 | 114.294 | 0.0 | -5.323 | 3.190e+07 | 67 - 81 | 1 | 36.27 | 36.60 | 36.60 |
| 3555.5597 | YFTPN(N)KTEDTIFLR Fuc 1 Hex 4 HexNAc 5 | 113.186 | 0.0 | 0.000 | 0.000e+00 | 67 - 81 | 1 | - | - | - |
| 3444.6565 | LVPVPITN(N)ATLDQITGK Hex 4 HexNAc 5 | 110.456 | 0.0 | 0.000 | 0.000e+00 | 25 - 42 | 3 | - | - | - |
| 3781.5291 | ATFFYFTPN(N)K Fuc 3 Hex 8 HexNAc 4 | 110.230 | 0.0 | -20.654 | 4.479e+06 | 63 - 73 | 1 | 53.98 | 54.15 | 54.15 |
| 4121.7664 | YFTPN(N)KTEDTIFLR Fuc 1 Hex 5 HexNAc 7 | 108.074 | 0.0 | -3.427 | 2.608e+07 | 67 - 81 | 2 | 35.94 | 36.10 | 36.27 |
| 4292.7739 | QDQC(C)IYN(N)TTYLNVQR Fuc 1 Hex 5 HexNAc 7 | 107.684 | 0.0 | 14.805 | 1.364e+08 | 86 - 101 | 5 | 32.43 | 32.94 | 33.44 |
| 3781.5807 | QDQC(C)IYN(N)TTYLNVQR Hex 4 HexNAc 6 | 105.822 | 0.0 | 9.653 | 8.860e+07 | 86 - 101 | 4 | 33.11 | 33.78 | 33.93 |
| 4178.7887 | YFTPN(N)KTEDTIFLR Hex 5 HexNAc 8 | 105.656 | 0.0 | -3.217 | 3.654e+07 | 67 - 81 | 2 | 35.77 | 35.94 | 36.10 |
| 3088.3524 | IYN(N)TTYLNVQR Hex 3 HexNAc 6 | 105.632 | 0.0 | 13.379 | 3.546e+07 | 90 - 101 | 6 | 28.43 | 28.61 | 29.16 |
| 4274.8009 | SVQEIQATFFYFTPN(N)K Hex 7 HexNAc 6 | 104.581 | 0.0 | 0.000 | 0.000e+00 | 57 - 73 | 1 | - | - | - |
| 4381.8639 | YFTPN(N)KTEDTIFLR Hex 5 HexNAc 9 | 103.919 | 0.0 | -5.018 | 1.273e+07 | 67 - 81 | 1 | 35.77 | 35.94 | 36.10 |
| 3285.4575 | FTPN(N)KTEDTIFLR Hex 3 HexNAc 6 | 103.814 | 0.0 | 14.055 | 9.863e+07 | 68 - 81 | 4 | 31.92 | 32.09 | 32.94 |
| 3845.6759 | PVPITN(N)ATLDQITGK Hex 6 HexNAc 5 Neu5Ac 1 | 101.853 | 0.0 | -4.833 | 6.787e+06 | 27 - 42 | 1 | 52.28 | 52.45 | 52.62 |
| 3960.7244 | FYFTPN(N)KTEDTIFLR Hex 4 HexNAc 7 | 100.297 | 0.0 | 13.567 | 1.734e+07 | 66 - 81 | 4 | 45.25 | 45.42 | 45.93 |
| 4156.8976 | LVPVPITN(N)ATLDQITGK Fuc 1 Hex 5 HexNAc 7 | 96.021 | 0.0 | 11.481 | 1.443e+07 | 25 - 42 | 2 | 47.29 | 47.63 | 47.96 |
| 3903.7025 | FYFTPN(N)KTEDTIFLR Fuc 1 Hex 4 HexNAc 6 | 95.915 | 0.0 | -2.725 | 2.459e+07 | 66 - 81 | 4 | 45.59 | 45.76 | 46.10 |
| 3390.4887 | FTPN(N)KTEDTIFLR Fuc 1 Hex 4 HexNAc 5 | 94.923 | 0.0 | -4.668 | 1.341e+07 | 68 - 81 | 3 | 31.75 | 31.75 | 32.26 |
| 3700.6209 | FYFTPN(N)KTEDTIFLR Fuc 1 Hex 4 HexNAc 5 | 94.612 | 0.0 | 12.623 | 1.606e+07 | 66 - 81 | 5 | 45.76 | 45.93 | 46.44 |
| 2885.2728 | IYN(N)TTYLNVQR Hex 3 HexNAc 5 | 94.590 | 0.0 | 13.430 | 2.556e+07 | 90 - 101 | 3 | 28.61 | 28.81 | 29.25 |
| 3243.4232 | TPN(N)KTEDTIFLR Fuc 1 Hex 4 HexNAc 5 | 93.120 | 0.0 | -4.903 | 6.750e+06 | 69 - 81 | 2 | 25.53 | 25.53 | 25.82 |
| 3082.3781 | FTPN(N)KTEDTIFLR Hex 3 HexNAc 5 | 92.793 | 0.0 | 13.789 | 4.580e+07 | 68 - 81 | 4 | 32.09 | 32.26 | 32.77 |
| 3446.9585 | TPN(N)KTEDTIFLR Fuc 1 Hex 4 HexNAc 6 | 87.840 | 0.0 | 5.010 | 1.331e+07 | 69 - 81 | 22 | 25.19 | 25.36 | 28.94 |
| 3593.5694 | FTPN(N)KTEDTIFLR Fuc 1 Hex 4 HexNAc 6 | 79.178 | 0.0 | -3.999 | 1.919e+07 | 68 - 81 | 4 | 31.41 | 31.58 | 31.92 |
| 4599.0894 | SVQEIQATFFYFTPN(N)KTEDTIFLR Hex 3 HexNAc 6 | 70.206 | 0.0 | -18.853 | 1.963e+07 | 57 - 81 | 2 | 62.70 | 62.87 | 62.87 |
| 4052.7234 | SVQEIQATFFYFTPN(N)K Fuc 1 Hex 6 HexNAc 5 | 61.267 | 0.0 | -5.060 | 7.248e+06 | 57 - 73 | 2 | 53.47 | 53.64 | 53.81 |
| 4187.7411 | QDQC(C)IYN(N)TTYLNVQR Hex 4 HexNAc 8 | 60.807 | 0.0 | 17.576 | 1.334e+08 | 86 - 101 | 3 | 32.77 | 32.94 | 33.61 |
| 4395.9988 | SVQEIQATFFYFTPN(N)KTEDTIFLR Hex 3 HexNAc 5 | 59.720 | 0.0 | -5.168 | 9.126e+06 | 57 - 81 | 2 | 63.04 | 63.04 | 63.35 |
| 3978.6970 | SVQEIQATFFYFTPN(N)K Fuc 1 Hex 5 HexNAc 4 Neu5Ac 1 | 59.282 | 0.0 | -4.613 | 8.019e+06 | 57 - 73 | 2 | 54.99 | 55.16 | 55.33 |
| 3953.8180 | LVPVPITN(N)ATLDQITGK Fuc 1 Hex 5 HexNAc 6 | 56.240 | 0.0 | 8.835 | 3.496e+07 | 25 - 42 | 4 | 47.29 | 47.96 | 48.13 |
| 3625.6849 | C(C)ANLVPVPITN(N)ATLDQITGK Hex 3 HexNAc 5 | 54.614 | 0.0 | 14.409 | 1.005e+08 | 22 - 42 | 5 | 51.77 | 51.94 | 52.45 |
| 4353.8470 | SVQEIQATFFYFTPN(N)K Hex 5 HexNAc 8 | 54.409 | 0.0 | 10.857 | 1.624e+07 | 57 - 73 | 1 | 52.79 | 52.96 | 53.47 |
| 3726.5575 | QDQC(C)IYN(N)TTYLNVQR Fuc 1 Hex 4 HexNAc 5 | 54.290 | 0.0 | 0.000 | 0.000e+00 | 86 - 101 | 1 | - | - | - |
| 3522.5844 | PVPITN(N)ATLDQITGK Fuc 2 Hex 4 HexNAc 5 | 53.981 | 0.0 | -5.561 | 4.287e+06 | 27 - 42 | 2 | 51.94 | 52.11 | 52.28 |
| 3189.4301 | FYFTPN(N)KTEDTIFLR Hex 3 HexNAc 4 | 51.734 | 0.0 | -3.878 | 6.693e+06 | 66 - 81 | 1 | 46.44 | 46.61 | 46.95 |
| 3385.6054 | LVPVPITN(N)ATLDQITGK Fuc 1 Hex 4 HexNAc 4 | 50.094 | 0.0 | -20.744 | 1.324e+07 | 25 - 42 | 1 | 48.13 | 48.13 | 48.30 |
| 3933.7971 | C(C)ANLVPVPITN(N)ATLDQITGK Fuc 1 Hex 4 HexNAc 5 | 49.063 | 0.0 | 12.901 | 2.723e+07 | 22 - 42 | 4 | 51.43 | 51.60 | 52.11 |
| 4325.8623 | FYFTPN(N)KTEDTIFLR Fuc 5 Hex 3 HexNAc 6 | 48.127 | 0.0 | -21.295 | 2.419e+06 | 66 - 81 | 1 | 44.76 | 44.76 | 44.92 |
| 3422.6051 | C(C)ANLVPVPITN(N)ATLDQITGK Hex 3 HexNAc 4 | 47.973 | 0.0 | -3.826 | 1.134e+07 | 22 - 42 | 3 | 51.94 | 52.11 | 52.45 |
| 4328.8204 | SVQEIQATFFYFTPN(N)K Fuc 4 Hex 5 HexNAc 5 | 47.851 | 0.0 | -18.399 | 7.249e+06 | 57 - 73 | 1 | 53.98 | 53.98 | 54.15 |
| 3974.6379 | QDQC(C)IYN(N)TTYLNVQR Fuc 1 Hex 5 HexNAc 4 Neu5Ac 1 | 47.421 | 0.0 | -4.909 | 2.027e+07 | 86 - 101 | 2 | 34.42 | 34.59 | 34.76 |
| 3733.7225 | LVPVPITN(N)ATLDQITGK Hex 4 HexNAc 5 Neu5Ac 1 | 47.142 | 0.0 | 12.011 | 1.613e+07 | 25 - 42 | 5 | 50.58 | 51.09 | 51.43 |
| 3788.6589 | SVQEIQATFFYFTPN(N)K Hex 4 HexNAc 6 | 46.690 | 0.0 | 0.000 | 0.000e+00 | 57 - 73 | 1 | - | - | - |
| 3765.5827 | QDQC(C)IYN(N)TTYLNVQR Fuc 1 Hex 3 HexNAc 6 | 46.646 | 0.0 | -19.864 | 1.404e+07 | 86 - 101 | 1 | 33.61 | 33.61 | 33.78 |
| 3440.5399 | PVPITN(N)ATLDQITGK Fuc 2 Hex 6 HexNAc 3 | 45.979 | 0.0 | -19.835 | 2.454e+06 | 27 - 42 | 2 | 51.94 | 51.94 | 51.94 |
| 4497.8645 | QDQC(C)IYN(N)TTYLNVQR Fuc 1 Hex 5 HexNAc 8 | 45.775 | 0.0 | 0.000 | 0.000e+00 | 86 - 101 | 1 | - | - | - |
| 4327.8112 | SVQEIQATFFYFTPN(N)K Fuc 2 Hex 5 HexNAc 5 Neu5Ac 1 | 45.558 | 0.0 | -19.510 | 2.078e+06 | 57 - 73 | 1 | 54.32 | 54.32 | 54.32 |
| 3562.5082 | QDQC(C)IYN(N)TTYLNVQR Fuc 1 Hex 3 HexNAc 5 | 45.407 | 0.0 | -19.853 | 6.868e+06 | 86 - 101 | 1 | 33.78 | 33.78 | 33.78 |
| 4640.0203 | PVPITN(N)ATLDQITGK Fuc 2 Hex 3 HexNAc 7 Neu5Ac 3 | 44.621 | 0.0 | 12.730 | 4.854e+06 | 27 - 42 | 4 | 56.79 | 56.96 | 57.39 |
| 3636.0457 | LVPVPITN(N)ATLDQITGK Fuc 1 Hex 5 HexNAc 3 Neu5Ac 1 | 43.713 | 0.0 | -2.404 | 1.127e+07 | 25 - 42 | 2 | 49.40 | 49.40 | 66.74 |
| 4438.8310 | QDQC(C)IYN(N)TTYLNVQR Fuc 2 Hex 5 HexNAc 7 | 43.542 | 0.0 | 12.425 | 2.727e+07 | 86 - 101 | 2 | 32.60 | 33.11 | 33.27 |
| 4552.8744 | QDQC(C)IYN(N)TTYLNVQR Hex 5 HexNAc 9 | 43.478 | 0.0 | 12.495 | 6.314e+07 | 86 - 101 | 2 | 32.43 | 32.60 | 33.11 |
| 3912.7940 | LVPVPITN(N)ATLDQITGK Fuc 1 Hex 6 HexNAc 5 | 42.274 | 0.0 | 8.279 | 8.495e+06 | 25 - 42 | 3 | 46.61 | 47.29 | 47.46 |
| 4442.8876 | SVQEIQATFFYFTPN(N)K Fuc 2 Hex 5 HexNAc 7 | 41.302 | 0.0 | -5.122 | 3.696e+06 | 57 - 73 | 1 | 53.30 | 53.47 | 53.64 |
| 5247.3021 | SVQEIQATFFYFTPN(N)KTEDTIFLR Hex 7 HexNAc 6 | 40.389 | 0.0 | 11.495 | 1.280e+07 | 57 - 81 | 3 | 61.67 | 62.02 | 62.70 |
| 3417.4379 | QDQC(C)IYN(N)TTYLN(D)VQR Hex 3 HexNAc 5 | 40.156 | 0.0 | -19.644 | 3.567e+07 | 86 - 101 | 2 | 35.10 | 35.10 | 35.27 |
| 2879.3004 | FTPN(N)KTEDTIFLR Hex 3 HexNAc 4 | 40.095 | 0.0 | -4.747 | 5.004e+06 | 68 - 81 | 1 | 32.43 | 32.43 | 32.77 |
| 4805.9857 | QDQC(C)IYN(N)TTYLNVQR Fuc 2 Hex 6 HexNAc 8 | 39.866 | 0.0 | 0.000 | 0.000e+00 | 86 - 101 | 1 | - | - | - |
| 4495.8544 | QDQC(C)IYN(N)TTYLNVQR Fuc 1 Hex 5 HexNAc 8 | 39.860 | 0.0 | -4.418 | 3.389e+07 | 86 - 101 | 1 | 32.43 | 32.77 | 32.94 |
| 3849.7809 | LVPVPITN(N)ATLDQITGK Hex 4 HexNAc 7 | 38.687 | 0.0 | 0.000 | 0.000e+00 | 25 - 42 | 1 | - | - | - |
| 3238.3102 | QDQC(C)IYN(N)TTYLN Hex 3 HexNAc 6 | 38.328 | 0.0 | 0.000 | 0.000e+00 | 86 - 98 | 1 | - | - | - |
| 4010.8432 | LVPVPITN(N)ATLDQITGK Hex 5 HexNAc 7 | 38.208 | 0.0 | -5.780 | 8.356e+06 | 25 - 42 | 1 | 47.46 | 47.63 | 47.96 |
| 3663.7329 | LVPVPITN(N)ATLDQITGK Fuc 2 Hex 3 HexNAc 4 Neu5Ac 1 | 37.938 | 0.0 | 0.000 | 0.000e+00 | 25 - 42 | 1 | - | - | - |
| 3650.5919 | FTPN(N)KTEDTIFLR Hex 4 HexNAc 7 | 37.830 | 0.0 | -4.996 | 1.107e+07 | 68 - 81 | 4 | 31.24 | 31.41 | 31.75 |
| 4657.9058 | QDQC(C)IYN(N)TTYLNVQR Fuc 1 Hex 6 HexNAc 8 | 36.659 | 0.0 | -19.031 | 8.175e+06 | 86 - 101 | 1 | 32.26 | 32.43 | 32.43 |
| 3620.5157 | QDQC(C)IYN(N)TTYLN(D)VQR Hex 3 HexNAc 6 | 36.402 | 0.0 | -19.937 | 6.032e+07 | 86 - 101 | 6 | 32.43 | 35.10 | 35.10 |
| 3610.5771 | YFTPN(N)KTEDTIFLR Hex 4 HexNAc 6 | 36.078 | 0.0 | -19.837 | 2.829e+07 | 67 - 81 | 1 | 37.59 | 37.59 | 37.76 |
| 4514.8885 | QDQC(C)IYN(N)TTYLNVQR Fuc 5 Hex 4 HexNAc 6 | 36.061 | 0.0 | 0.000 | 0.000e+00 | 86 - 101 | 1 | - | - | - |
| 4499.9108 | SVQEIQATFFYFTPN(N)K Fuc 1 Hex 5 HexNAc 8 | 35.954 | 0.0 | -21.316 | 7.234e+05 | 57 - 73 | 1 | 52.79 | 52.79 | 52.79 |
| 3590.6905 | LVPVPITN(N)ATLDQITGK Fuc 1 Hex 4 HexNAc 5 | 34.554 | 0.003 | 0.000 | 0.000e+00 | 25 - 42 | 2 | - | - | - |
| 3139.3882 | TPN(N)KTEDTIFLR Hex 3 HexNAc 6 | 34.480 | 0.003 | 0.000 | 0.000e+00 | 69 - 81 | 1 | - | - | - |
| 4265.7239 | QDQC(C)IYN(N)TTYLNVQR Fuc 1 Hex 5 HexNAc 4 Neu5Ac 2 | 34.380 | 0.003 | -5.871 | 2.237e+07 | 86 - 101 | 2 | 34.10 | 34.25 | 34.42 |
| 4659.9189 | QDQC(C)IYN(N)TTYLNVQR Fuc 1 Hex 6 HexNAc 8 | 33.521 | 0.005 | 0.000 | 0.000e+00 | 86 - 101 | 1 | - | - | - |
| 4907.1965 | SVQEIQATFFYFTPN(N)KTEDTIFLR Fuc 1 Hex 4 HexNAc 6 | 33.288 | 0.005 | -21.355 | 1.949e+06 | 57 - 81 | 1 | 62.53 | 62.53 | 62.53 |
| 4788.0809 | PVPITN(N)ATLDQITGK Fuc 5 Hex 3 HexNAc 7 Neu5Ac 2 | 32.742 | 0.005 | 0.000 | 0.000e+00 | 27 - 42 | 1 | - | - | - |
| 3122.2564 | QDQC(C)IYN(N)TTYL Hex 3 HexNAc 6 | 32.202 | 0.007 | -19.581 | 5.657e+06 | 86 - 97 | 1 | 37.59 | 37.59 | 37.59 |
| 4600.8882 | QDQC(C)IYN(N)TTYLNVQR Fuc 2 Hex 6 HexNAc 7 | 31.909 | 0.009 | -5.519 | 1.092e+07 | 86 - 101 | 1 | 32.26 | 32.77 | 32.77 |
| 4860.9875 | QDQC(C)IYN(N)TTYLNVQR Fuc 1 Hex 6 HexNAc 9 | 31.387 | 0.012 | -5.569 | 6.095e+06 | 86 - 101 | 1 | 32.26 | 32.26 | 32.60 |
| 3607.7155 | LVPVPITN(N)ATLDQITGK Hex 5 HexNAc 5 | 31.057 | 0.012 | 0.000 | 0.000e+00 | 25 - 42 | 1 | - | - | - |
| 2732.2302 | TPN(N)KTEDTIFLR Hex 3 HexNAc 4 | 30.885 | 0.013 | 9.749 | 5.844e+06 | 69 - 81 | 2 | 26.21 | 26.21 | 26.70 |
| 3193.3840 | IYN(N)TTYLNVQR Fuc 1 Hex 4 HexNAc 5 | 30.740 | 0.013 | -4.122 | 4.050e+06 | 90 - 101 | 2 | 28.26 | 28.33 | 28.52 |
| 4213.9168 | LVPVPITN(N)ATLDQITGK Hex 5 HexNAc 8 | 30.563 | 0.013 | -5.217 | 4.921e+06 | 25 - 42 | 1 | 46.95 | 47.12 | 47.46 |
| 3314.3578 | ATFFYFTPN(N)K Fuc 2 Hex 6 HexNAc 4 | 30.549 | 0.013 | 0.000 | 0.000e+00 | 63 - 73 | 1 | - | - | - |
| 4385.0710 | QIPLC(C)ANLVPVPITN(N)ATLDQITGK Fuc 1 Hex 4 HexNAc 5 | 29.974 | 0.017 | 11.364 | 4.303e+07 | 18 - 42 | 4 | 67.72 | 68.19 | 68.54 |
| 4543.8578 | QDQC(C)IYN(N)TTYLNVQR Fuc 3 Hex 6 HexNAc 6 | 29.678 | 0.019 | -20.463 | 1.085e+07 | 86 - 101 | 1 | 32.77 | 32.94 | 32.94 |
| 4051.8652 | LVPVPITN(N)ATLDQITGK Hex 4 HexNAc 8 | 29.435 | 0.020 | -5.514 | 4.105e+06 | 25 - 42 | 1 | 47.46 | 47.46 | 47.79 |
| 3647.7201 | LVPVPITN(N)ATLDQITGK Hex 4 HexNAc 6 | 28.399 | 0.025 | 0.000 | 0.000e+00 | 25 - 42 | 1 | - | - | - |
| 4368.0503 | QIPLC(C)ANLVPVPITN(N)ATLDQITGK Hex 3 HexNAc 5 Neu5Ac 1 | 28.041 | 0.027 | 14.084 | 1.678e+08 | 18 - 42 | 6 | 68.06 | 68.19 | 68.85 |
| 3990.8185 | C(C)ANLVPVPITN(N)ATLDQITGK Hex 4 HexNAc 6 | 26.511 | 0.039 | -3.818 | 9.452e+06 | 22 - 42 | 3 | 52.45 | 52.45 | 52.79 |
| 3626.6293 | PVPITN(N)ATLDQITGK Fuc 1 Hex 5 HexNAc 4 Neu5Ac 1 | 25.836 | 0.039 | -3.538 | 3.134e+08 | 27 - 42 | 3 | 47.96 | 48.13 | 48.45 |
| 3821.7384 | LVPVPITN(N)ATLDQITGK Hex 4 HexNAc 4 Neu5Ac 2 | 25.743 | 0.040 | -20.132 | 5.468e+06 | 25 - 42 | 1 | 49.07 | 49.07 | 49.07 |
| 3901.6800 | FTPN(N)KTEDTIFLR Fuc 2 Hex 5 HexNAc 6 | 25.316 | 0.042 | -19.092 | 2.793e+06 | 68 - 81 | 2 | 31.07 | 31.24 | 31.24 |
| 3699.5530 | QDQC(C)IYN(N)TTYLNVQR Hex 6 HexNAc 4 | 25.276 | 0.043 | 0.000 | 0.000e+00 | 86 - 101 | 1 | - | - | - |
| 3398.4688 | IYN(N)TTYLNVQR Fuc 1 Hex 4 HexNAc 6 | 24.862 | 0.049 | 0.000 | 0.000e+00 | 90 - 101 | 1 | - | - | - |

### YFTPN(N)K Hex 3 HexNAc 6

**Mass:**  2473.0180

(1.102e-06)

**Charge States:** 2, 3

**Start Position**: 68

**End Position**: 74

| Profile Score | Tandem Score | TDA q-value | Volume | Spectrum Matches |
| --- | --- | --- | --- | --- |
| 14.1375 | 188.8985 | 0.0000 | 3.1074e+08 | 5 |

**Adduct Signal Fractions**

- **Unmodified**: 3.1074e+08

### YFTPN(N)K Fuc 1 Hex 4 HexNAc 6

**Mass:**  2781.1280

(9.012e-07)

**Charge States:** 3

**Start Position**: 68

**End Position**: 74

| Profile Score | Tandem Score | TDA q-value | Volume | Spectrum Matches |
| --- | --- | --- | --- | --- |
| -4.3227 | 186.5559 | 0.0000 | 4.9309e+07 | 3 |

**Adduct Signal Fractions**

- **Unmodified**: 4.9309e+07

### FYFTPN(N)K Hex 3 HexNAc 6

**Mass:**  2620.0854

(6.577e-07)

**Charge States:** 2, 3

**Start Position**: 67

**End Position**: 74

| Profile Score | Tandem Score | TDA q-value | Volume | Spectrum Matches |
| --- | --- | --- | --- | --- |
| 13.1581 | 183.0354 | 0.0000 | 1.1546e+08 | 11 |

**Adduct Signal Fractions**

- **Unmodified**: 1.1546e+08

### YFTPN(N)K Fuc 1 Hex 4 HexNAc 5

**Mass:**  2578.0492

(2.518e-06)

**Charge States:** 3

**Start Position**: 68

**End Position**: 74

| Profile Score | Tandem Score | TDA q-value | Volume | Spectrum Matches |
| --- | --- | --- | --- | --- |
| -19.2955 | 181.0960 | 0.0000 | 3.4123e+07 | 2 |

**Adduct Signal Fractions**

- **Unmodified**: 3.4123e+07

### NEEYN(N)K Hex 3 HexNAc 5

**Mass:**  2296.8960

(-2.573e-07)

**Charge States:** 2, 3

**Start Position**: 52

**End Position**: 58

| Profile Score | Tandem Score | TDA q-value | Volume | Spectrum Matches |
| --- | --- | --- | --- | --- |
| 10.7175 | 180.6881 | 0.0000 | 3.1170e+07 | 7 |

**Adduct Signal Fractions**

- **Unmodified**: 3.1170e+07

### NEEYN(N)K Fuc 1 Hex 4 HexNAc 5

**Mass:**  2605.0080

(1.805e-06)

**Charge States:** 3

**Start Position**: 52

**End Position**: 58

| Profile Score | Tandem Score | TDA q-value | Volume | Spectrum Matches |
| --- | --- | --- | --- | --- |
| 11.0349 | 178.9503 | 0.0000 | 1.2594e+07 | 4 |

**Adduct Signal Fractions**

- **Unmodified**: 1.2594e+07

### SVQEIQATFFYFTPN(N)K Hex 4 HexNAc 7

**Mass:**  3988.7204

(1.798e-06)

**Charge States:** 3, 4

**Start Position**: 58

**End Position**: 74

| Profile Score | Tandem Score | TDA q-value | Volume | Spectrum Matches |
| --- | --- | --- | --- | --- |
| 10.8008 | 173.3012 | 0.0000 | 2.3053e+08 | 10 |

**Adduct Signal Fractions**

- **Unmodified**: 2.3053e+08

### YFTPN(N)K Hex 4 HexNAc 7

**Mass:**  2838.1501

(1.098e-06)

**Charge States:** 3

**Start Position**: 68

**End Position**: 74

| Profile Score | Tandem Score | TDA q-value | Volume | Spectrum Matches |
| --- | --- | --- | --- | --- |
| -5.1332 | 173.0297 | 0.0000 | 2.4780e+07 | 1 |

**Adduct Signal Fractions**

- **Unmodified**: 2.4780e+07

### YFTPN(N)K Hex 3 HexNAc 5

**Mass:**  2269.9379

(6.187e-07)

**Charge States:** 2, 3

**Start Position**: 68

**End Position**: 74

| Profile Score | Tandem Score | TDA q-value | Volume | Spectrum Matches |
| --- | --- | --- | --- | --- |
| 12.4864 | 172.7184 | 0.0000 | 1.9518e+08 | 5 |

**Adduct Signal Fractions**

- **Unmodified**: 1.9518e+08

### YFTPN(N)K Hex 3 HexNAc 4

**Mass:**  2066.8585

(9.695e-07)

**Charge States:** 2, 3

**Start Position**: 68

**End Position**: 74

| Profile Score | Tandem Score | TDA q-value | Volume | Spectrum Matches |
| --- | --- | --- | --- | --- |
| -19.0961 | 172.5173 | 0.0000 | 2.5133e+07 | 3 |

**Adduct Signal Fractions**

- **Unmodified**: 2.5133e+07

### ATFFYFTPN(N)K Hex 3 HexNAc 6

**Mass:**  2939.2408

(1.068e-06)

**Charge States:** 3

**Start Position**: 64

**End Position**: 74

| Profile Score | Tandem Score | TDA q-value | Volume | Spectrum Matches |
| --- | --- | --- | --- | --- |
| -3.4085 | 172.1543 | 0.0000 | 3.9688e+07 | 3 |

**Adduct Signal Fractions**

- **Unmodified**: 3.9688e+07

### SVQEIQATFFYFTPN(N)K Fuc 1 Hex 4 HexNAc 5

**Mass:**  3728.6182

(1.723e-06)

**Charge States:** 3, 4

**Start Position**: 58

**End Position**: 74

| Profile Score | Tandem Score | TDA q-value | Volume | Spectrum Matches |
| --- | --- | --- | --- | --- |
| 13.0932 | 171.6062 | 0.0000 | 1.7703e+08 | 8 |

**Adduct Signal Fractions**

- **Unmodified**: 1.7703e+08

### FYFTPN(N)K Hex 3 HexNAc 5

**Mass:**  2417.0062

(7.675e-07)

**Charge States:** 2, 3

**Start Position**: 67

**End Position**: 74

| Profile Score | Tandem Score | TDA q-value | Volume | Spectrum Matches |
| --- | --- | --- | --- | --- |
| 13.0585 | 170.5224 | 0.0000 | 6.6595e+07 | 11 |

**Adduct Signal Fractions**

- **Unmodified**: 6.6595e+07

### SVQEIQATFFYFTPN(N)K Hex 3 HexNAc 5

**Mass:**  3420.5055

(1.649e-06)

**Charge States:** 2, 3, 4

**Start Position**: 58

**End Position**: 74

| Profile Score | Tandem Score | TDA q-value | Volume | Spectrum Matches |
| --- | --- | --- | --- | --- |
| 14.8623 | 168.3189 | 0.0000 | 6.0272e+08 | 29 |

**Adduct Signal Fractions**

- **Unmodified**: 6.0272e+08

### SVQEIQATFFYFTPN(N)K Hex 3 HexNAc 6

**Mass:**  3623.5856

(2.220e-06)

**Charge States:** 3, 4, 5

**Start Position**: 58

**End Position**: 74

| Profile Score | Tandem Score | TDA q-value | Volume | Spectrum Matches |
| --- | --- | --- | --- | --- |
| 14.6236 | 167.7075 | 0.0000 | 1.0604e+09 | 25 |

**Adduct Signal Fractions**

- **Unmodified**: 1.0604e+09

### SVQEIQATFFYFTPN(N)K Fuc 1 Hex 4 HexNAc 6

**Mass:**  3931.7004

(1.412e-06)

**Charge States:** 3, 4, 5

**Start Position**: 58

**End Position**: 74

| Profile Score | Tandem Score | TDA q-value | Volume | Spectrum Matches |
| --- | --- | --- | --- | --- |
| 13.2533 | 165.6330 | 0.0000 | 2.8839e+08 | 12 |

**Adduct Signal Fractions**

- **Unmodified**: 2.8839e+08

### NEEYN(N)K Hex 3 HexNAc 4

**Mass:**  2093.8172

(4.759e-07)

**Charge States:** 2, 3

**Start Position**: 52

**End Position**: 58

| Profile Score | Tandem Score | TDA q-value | Volume | Spectrum Matches |
| --- | --- | --- | --- | --- |
| 9.5670 | 163.3281 | 0.0000 | 3.4634e+07 | 10 |

**Adduct Signal Fractions**

- **Unmodified**: 3.4634e+07

### YFTPN(N)K Hex 4 HexNAc 7

**Mass:**  2840.1601

(7.091e-04)

**Start Position**: 68

**End Position**: 74

| Profile Score | Tandem Score | TDA q-value | Volume | Spectrum Matches |
| --- | --- | --- | --- | --- |
| 0.0000 | 160.5392 | 0.0000 | 0.0000e+00 | 1 |

Adduct:

No Chromatogram Found

### SVQEIQATFFYFTPN(N)K Fuc 2 Hex 5 HexNAc 6

**Mass:**  4239.8078

(1.255e-06)

**Charge States:** 3, 4

**Start Position**: 58

**End Position**: 74

| Profile Score | Tandem Score | TDA q-value | Volume | Spectrum Matches |
| --- | --- | --- | --- | --- |
| 13.4989 | 157.9036 | 0.0000 | 4.5572e+07 | 4 |

**Adduct Signal Fractions**

- **Unmodified**: 4.5572e+07

### ATFFYFTPN(N)K Hex 3 HexNAc 5

**Mass:**  2736.1615

(1.732e-06)

**Charge States:** 2, 3

**Start Position**: 64

**End Position**: 74

| Profile Score | Tandem Score | TDA q-value | Volume | Spectrum Matches |
| --- | --- | --- | --- | --- |
| -4.0848 | 156.4640 | 0.0000 | 2.2914e+07 | 3 |

**Adduct Signal Fractions**

- **Unmodified**: 2.2914e+07

### SVQEIQATFFYFTPN(N)K Hex 3 HexNAc 4

**Mass:**  3217.4265

(9.472e-07)

**Charge States:** 3, 4

**Start Position**: 58

**End Position**: 74

| Profile Score | Tandem Score | TDA q-value | Volume | Spectrum Matches |
| --- | --- | --- | --- | --- |
| 12.8068 | 153.4321 | 0.0000 | 7.6558e+07 | 8 |

**Adduct Signal Fractions**

- **Unmodified**: 7.6558e+07

### SVQEIQATFFYFTPN(N)K Hex 7 HexNAc 6

**Mass:**  4271.7985

(1.674e-06)

**Charge States:** 3, 4

**Start Position**: 58

**End Position**: 74

| Profile Score | Tandem Score | TDA q-value | Volume | Spectrum Matches |
| --- | --- | --- | --- | --- |
| 12.6216 | 153.2095 | 0.0000 | 4.8446e+07 | 1 |

**Adduct Signal Fractions**

- **Unmodified**: 4.8446e+07

### LVPVPITN(N)ATLDQITGK Hex 3 HexNAc 5

**Mass:**  3280.5719

(7.546e-07)

**Charge States:** 2, 3, 4

**Start Position**: 26

**End Position**: 43

| Profile Score | Tandem Score | TDA q-value | Volume | Spectrum Matches |
| --- | --- | --- | --- | --- |
| 14.2752 | 152.8725 | 0.0000 | 1.3483e+10 | 514 |

**Adduct Signal Fractions**

- **Unmodified**: 1.3483e+10

### LVPVPITN(N)ATLDQITGK Fuc 1 Hex 4 HexNAc 6

**Mass:**  3791.7653

(1.528e-06)

**Charge States:** 3, 4

**Start Position**: 26

**End Position**: 43

| Profile Score | Tandem Score | TDA q-value | Volume | Spectrum Matches |
| --- | --- | --- | --- | --- |
| 14.0692 | 152.1836 | 0.0000 | 1.7123e+08 | 7 |

**Adduct Signal Fractions**

- **Unmodified**: 1.7123e+08

### LVPVPITN(N)ATLDQITGK Hex 3 HexNAc 6

**Mass:**  3483.6523

(1.100e-06)

**Charge States:** 3, 4

**Start Position**: 26

**End Position**: 43

| Profile Score | Tandem Score | TDA q-value | Volume | Spectrum Matches |
| --- | --- | --- | --- | --- |
| 8.5242 | 151.8936 | 0.0000 | 4.4068e+08 | 15 |

**Adduct Signal Fractions**

- **Unmodified**: 4.4068e+08

### SVQEIQATFFYFTPN(N)K Hex 6 HexNAc 5

**Mass:**  3906.6689

(1.090e-06)

**Charge States:** 3, 4

**Start Position**: 58

**End Position**: 74

| Profile Score | Tandem Score | TDA q-value | Volume | Spectrum Matches |
| --- | --- | --- | --- | --- |
| 13.1017 | 148.5536 | 0.0000 | 3.7369e+07 | 4 |

**Adduct Signal Fractions**

- **Unmodified**: 3.7369e+07

### SVQEIQATFFYFTPN(N)K Hex 4 HexNAc 8

**Mass:**  4191.7995

(1.848e-06)

**Charge States:** 3, 4

**Start Position**: 58

**End Position**: 74

| Profile Score | Tandem Score | TDA q-value | Volume | Spectrum Matches |
| --- | --- | --- | --- | --- |
| 13.1443 | 147.4205 | 0.0000 | 2.6034e+07 | 4 |

**Adduct Signal Fractions**

- **Unmodified**: 2.6034e+07

### LVPVPITN(N)ATLDQITGK Hex 3 HexNAc 4

**Mass:**  3077.4933

(-1.236e-06)

**Charge States:** 2, 3, 4

**Start Position**: 26

**End Position**: 43

| Profile Score | Tandem Score | TDA q-value | Volume | Spectrum Matches |
| --- | --- | --- | --- | --- |
| 14.2662 | 146.4321 | 0.0000 | 1.1511e+09 | 23 |

**Adduct Signal Fractions**

- **Unmodified**: 1.1511e+09

### TPN(N)KTEDTIFLR Hex 3 HexNAc 6

**Mass:**  3138.3883

(6.401e-07)

**Charge States:** 3, 4

**Start Position**: 70

**End Position**: 82

| Profile Score | Tandem Score | TDA q-value | Volume | Spectrum Matches |
| --- | --- | --- | --- | --- |
| 12.3900 | 146.2525 | 0.0000 | 4.9653e+07 | 6 |

**Adduct Signal Fractions**

- **Unmodified**: 4.9653e+07

### LVPVPITN(N)ATLDQITGK Fuc 1 Hex 4 HexNAc 5

**Mass:**  3588.6840

(1.108e-06)

**Charge States:** 3, 4

**Start Position**: 26

**End Position**: 43

| Profile Score | Tandem Score | TDA q-value | Volume | Spectrum Matches |
| --- | --- | --- | --- | --- |
| 15.2184 | 145.3004 | 0.0000 | 3.8455e+09 | 30 |

**Adduct Signal Fractions**

- **Unmodified**: 3.8455e+09

### LVPVPITN(N)ATLDQITGK Fuc 1 Hex 3 HexNAc 5

**Mass:**  3426.6320

(5.937e-07)

**Charge States:** 4

**Start Position**: 26

**End Position**: 43

| Profile Score | Tandem Score | TDA q-value | Volume | Spectrum Matches |
| --- | --- | --- | --- | --- |
| -4.5663 | 144.1091 | 0.0000 | 6.3820e+07 | 2 |

**Adduct Signal Fractions**

- **Unmodified**: 6.3820e+07

### SVQEIQATFFYFTPN(N)K Fuc 1 Hex 4 HexNAc 7

**Mass:**  4134.7770

(1.372e-06)

**Charge States:** 3, 4

**Start Position**: 58

**End Position**: 74

| Profile Score | Tandem Score | TDA q-value | Volume | Spectrum Matches |
| --- | --- | --- | --- | --- |
| -4.6729 | 143.6086 | 0.0000 | 1.1160e+07 | 3 |

**Adduct Signal Fractions**

- **Unmodified**: 1.1160e+07

### SVQEIQATFFYFTPN(N)K Hex 5 HexNAc 9

**Mass:**  4556.9333

(1.810e-06)

**Charge States:** 3, 4

**Start Position**: 58

**End Position**: 74

| Profile Score | Tandem Score | TDA q-value | Volume | Spectrum Matches |
| --- | --- | --- | --- | --- |
| -20.4051 | 143.3341 | 0.0000 | 2.0191e+06 | 1 |

**Adduct Signal Fractions**

- **Unmodified**: 2.0191e+06

### QDQC(C)IYN(N)TTYLNVQR Hex 3 HexNAc 6

**Mass:**  3619.5280

(8.362e-07)

**Charge States:** 3, 4

**Start Position**: 87

**End Position**: 102

| Profile Score | Tandem Score | TDA q-value | Volume | Spectrum Matches |
| --- | --- | --- | --- | --- |
| 10.7354 | 141.5136 | 0.0000 | 3.1108e+09 | 8 |

**Adduct Signal Fractions**

- **Unmodified**: 3.1108e+09

### ATFFYFTPN(N)K Fuc 1 Hex 4 HexNAc 5

**Mass:**  3044.2692

(2.877e-07)

**Charge States:** 3

**Start Position**: 64

**End Position**: 74

| Profile Score | Tandem Score | TDA q-value | Volume | Spectrum Matches |
| --- | --- | --- | --- | --- |
| -17.4436 | 140.3602 | 0.0000 | 5.6464e+06 | 1 |

**Adduct Signal Fractions**

- **Unmodified**: 5.6464e+06

### SVQEIQATFFYFTPN(N)K Fuc 1 Hex 5 HexNAc 7

**Mass:**  4296.8303

(5.712e-07)

**Charge States:** 3, 4

**Start Position**: 58

**End Position**: 74

| Profile Score | Tandem Score | TDA q-value | Volume | Spectrum Matches |
| --- | --- | --- | --- | --- |
| 10.0804 | 139.2859 | 0.0000 | 3.2234e+07 | 3 |

**Adduct Signal Fractions**

- **Unmodified**: 3.2234e+07

### SVQEIQATFFYFTPN(N)K Hex 4 HexNAc 6

**Mass:**  3785.6372

(1.634e-06)

**Charge States:** 3, 4

**Start Position**: 58

**End Position**: 74

| Profile Score | Tandem Score | TDA q-value | Volume | Spectrum Matches |
| --- | --- | --- | --- | --- |
| 11.4996 | 137.5905 | 0.0000 | 3.3274e+07 | 5 |

**Adduct Signal Fractions**

- **Unmodified**: 3.3274e+07

### YFTPN(N)KTEDTIFLR Fuc 1 Hex 4 HexNAc 6

**Mass:**  3756.6308

(1.358e-06)

**Charge States:** 3, 4

**Start Position**: 68

**End Position**: 82

| Profile Score | Tandem Score | TDA q-value | Volume | Spectrum Matches |
| --- | --- | --- | --- | --- |
| -3.7464 | 137.4253 | 0.0000 | 2.1143e+08 | 8 |

**Adduct Signal Fractions**

- **Unmodified**: 2.1143e+08

### LVPVPITN(N)ATLDQITGK Fuc 2 Hex 5 HexNAc 5

**Mass:**  3896.7976

(1.688e-06)

**Charge States:** 3, 4

**Start Position**: 26

**End Position**: 43

| Profile Score | Tandem Score | TDA q-value | Volume | Spectrum Matches |
| --- | --- | --- | --- | --- |
| -4.1506 | 136.9747 | 0.0000 | 3.4318e+07 | 2 |

**Adduct Signal Fractions**

- **Unmodified**: 3.4318e+07

### LVPVPITN(N)ATLDQITGK Hex 4 HexNAc 5

**Mass:**  3442.6267

(1.218e-06)

**Charge States:** 3, 4

**Start Position**: 26

**End Position**: 43

| Profile Score | Tandem Score | TDA q-value | Volume | Spectrum Matches |
| --- | --- | --- | --- | --- |
| -5.4518 | 136.8305 | 0.0000 | 6.5826e+07 | 2 |

**Adduct Signal Fractions**

- **Unmodified**: 6.5826e+07

### QDQC(C)IYN(N)TTYLNVQR Hex 3 HexNAc 5

**Mass:**  3416.4479

(7.736e-07)

**Charge States:** 3, 4

**Start Position**: 87

**End Position**: 102

| Profile Score | Tandem Score | TDA q-value | Volume | Spectrum Matches |
| --- | --- | --- | --- | --- |
| 12.9951 | 136.6355 | 0.0000 | 1.5264e+09 | 8 |

**Adduct Signal Fractions**

- **Unmodified**: 1.5264e+09

### SVQEIQATFFYFTPN(N)K Hex 4 HexNAc 4 Neu5Ac 2

**Mass:**  3961.6699

(9.364e-07)

**Charge States:** 3, 4

**Start Position**: 58

**End Position**: 74

| Profile Score | Tandem Score | TDA q-value | Volume | Spectrum Matches |
| --- | --- | --- | --- | --- |
| 12.8255 | 136.1798 | 0.0000 | 3.8574e+07 | 4 |

**Adduct Signal Fractions**

- **Unmodified**: 3.8574e+07

### SVQEIQATFFYFTPN(N)K Fuc 2 Hex 4 HexNAc 4 Neu5Ac 1

**Mass:**  3962.6862

(-6.470e-08)

**Charge States:** 4

**Start Position**: 58

**End Position**: 74

| Profile Score | Tandem Score | TDA q-value | Volume | Spectrum Matches |
| --- | --- | --- | --- | --- |
| -19.8603 | 134.8513 | 0.0000 | 2.0486e+06 | 2 |

**Adduct Signal Fractions**

- **Unmodified**: 2.0486e+06

### QDQC(C)IYN(N)TTYLNVQR Hex 4 HexNAc 7

**Mass:**  3984.6657

(1.546e-06)

**Charge States:** 3, 4

**Start Position**: 87

**End Position**: 102

| Profile Score | Tandem Score | TDA q-value | Volume | Spectrum Matches |
| --- | --- | --- | --- | --- |
| 15.7537 | 134.1848 | 0.0000 | 7.6298e+08 | 6 |

**Adduct Signal Fractions**

- **Unmodified**: 7.6298e+08

### QDQC(C)IYN(N)TTYLNVQR Fuc 2 Hex 5 HexNAc 6

**Mass:**  4235.7497

(9.945e-07)

**Charge States:** 3, 4

**Start Position**: 87

**End Position**: 102

| Profile Score | Tandem Score | TDA q-value | Volume | Spectrum Matches |
| --- | --- | --- | --- | --- |
| -2.9996 | 133.1287 | 0.0000 | 1.5994e+08 | 2 |

**Adduct Signal Fractions**

- **Unmodified**: 1.5994e+08

### YFTPN(N)KTEDTIFLR Hex 3 HexNAc 6

**Mass:**  3448.5185

(7.302e-07)

**Charge States:** 3, 4

**Start Position**: 68

**End Position**: 82

| Profile Score | Tandem Score | TDA q-value | Volume | Spectrum Matches |
| --- | --- | --- | --- | --- |
| 13.1748 | 132.8957 | 0.0000 | 1.5597e+09 | 18 |

**Adduct Signal Fractions**

- **Unmodified**: 1.5597e+09

### YFTPN(N)KTEDTIFLR Hex 3 HexNAc 4

**Mass:**  3042.3625

(8.936e-07)

**Charge States:** 3

**Start Position**: 68

**End Position**: 82

| Profile Score | Tandem Score | TDA q-value | Volume | Spectrum Matches |
| --- | --- | --- | --- | --- |
| 12.9403 | 131.0090 | 0.0000 | 1.0851e+08 | 3 |

**Adduct Signal Fractions**

- **Unmodified**: 1.0851e+08

### ATFFYFTPN(N)K Fuc 1 Hex 4 HexNAc 6

**Mass:**  3247.3456

(-6.629e-07)

**Charge States:** 3

**Start Position**: 64

**End Position**: 74

| Profile Score | Tandem Score | TDA q-value | Volume | Spectrum Matches |
| --- | --- | --- | --- | --- |
| -21.1847 | 130.5278 | 0.0000 | 4.1502e+06 | 1 |

**Adduct Signal Fractions**

- **Unmodified**: 4.1502e+06

### LVPVPITN(N)ATLDQITGK Hex 4 HexNAc 6

**Mass:**  3645.7062

(1.561e-06)

**Charge States:** 3, 4

**Start Position**: 26

**End Position**: 43

| Profile Score | Tandem Score | TDA q-value | Volume | Spectrum Matches |
| --- | --- | --- | --- | --- |
| 13.1431 | 129.9071 | 0.0000 | 6.2379e+08 | 12 |

**Adduct Signal Fractions**

- **Unmodified**: 6.2379e+08

### QDQC(C)IYN(N)TTYLNVQR Fuc 1 Hex 4 HexNAc 5

**Mass:**  3724.5591

(1.324e-06)

**Charge States:** 3, 4

**Start Position**: 87

**End Position**: 102

| Profile Score | Tandem Score | TDA q-value | Volume | Spectrum Matches |
| --- | --- | --- | --- | --- |
| 10.3431 | 129.8347 | 0.0000 | 4.2928e+08 | 7 |

**Adduct Signal Fractions**

- **Unmodified**: 4.2928e+08

### YFTPN(N)KTEDTIFLR Fuc 1 Hex 4 HexNAc 5

**Mass:**  3553.5525

(1.607e-06)

**Charge States:** 3, 4

**Start Position**: 68

**End Position**: 82

| Profile Score | Tandem Score | TDA q-value | Volume | Spectrum Matches |
| --- | --- | --- | --- | --- |
| 8.5456 | 126.4495 | 0.0000 | 1.4353e+08 | 4 |

**Adduct Signal Fractions**

- **Unmodified**: 1.4353e+08

### LVPVPITN(N)ATLDQITGK Fuc 1 Hex 3 HexNAc 5

**Mass:**  3428.6393

(5.871e-04)

**Start Position**: 26

**End Position**: 43

| Profile Score | Tandem Score | TDA q-value | Volume | Spectrum Matches |
| --- | --- | --- | --- | --- |
| 0.0000 | 125.8647 | 0.0000 | 0.0000e+00 | 2 |

Adduct:

No Chromatogram Found

### YFTPN(N)KTEDTIFLR Hex 3 HexNAc 5

**Mass:**  3245.4417

(6.439e-07)

**Charge States:** 3, 4

**Start Position**: 68

**End Position**: 82

| Profile Score | Tandem Score | TDA q-value | Volume | Spectrum Matches |
| --- | --- | --- | --- | --- |
| 9.5237 | 125.4966 | 0.0000 | 7.1318e+08 | 8 |

**Adduct Signal Fractions**

- **Unmodified**: 7.1318e+08

### FYFTPN(N)KTEDTIFLR Hex 3 HexNAc 5

**Mass:**  3392.5102

(9.952e-07)

**Charge States:** 3, 4

**Start Position**: 67

**End Position**: 82

| Profile Score | Tandem Score | TDA q-value | Volume | Spectrum Matches |
| --- | --- | --- | --- | --- |
| 13.5385 | 124.7043 | 0.0000 | 4.9772e+07 | 6 |

**Adduct Signal Fractions**

- **Unmodified**: 4.9772e+07

### TPN(N)KTEDTIFLR Hex 3 HexNAc 5

**Mass:**  2935.3093

(9.055e-07)

**Charge States:** 3, 4

**Start Position**: 70

**End Position**: 82

| Profile Score | Tandem Score | TDA q-value | Volume | Spectrum Matches |
| --- | --- | --- | --- | --- |
| 12.8228 | 124.5287 | 0.0000 | 3.9479e+07 | 6 |

**Adduct Signal Fractions**

- **Unmodified**: 3.9479e+07

### QDQC(C)IYN(N)TTYLNVQR Fuc 1 Hex 4 HexNAc 6

**Mass:**  3927.6394

(2.878e-07)

**Charge States:** 3, 4

**Start Position**: 87

**End Position**: 102

| Profile Score | Tandem Score | TDA q-value | Volume | Spectrum Matches |
| --- | --- | --- | --- | --- |
| 13.5955 | 124.1514 | 0.0000 | 1.0920e+09 | 5 |

**Adduct Signal Fractions**

- **Unmodified**: 1.0920e+09

### LVPVPITN(N)ATLDQITGK Hex 4 HexNAc 7

**Mass:**  3848.7897

(2.225e-06)

**Charge States:** 3, 4

**Start Position**: 26

**End Position**: 43

| Profile Score | Tandem Score | TDA q-value | Volume | Spectrum Matches |
| --- | --- | --- | --- | --- |
| 10.8280 | 124.1036 | 0.0000 | 8.1181e+07 | 4 |

**Adduct Signal Fractions**

- **Unmodified**: 8.1181e+07

### QDQC(C)IYN(N)TTYLNVQR Fuc 1 Hex 4 HexNAc 7

**Mass:**  4130.7209

(1.714e-07)

**Charge States:** 3, 4

**Start Position**: 87

**End Position**: 102

| Profile Score | Tandem Score | TDA q-value | Volume | Spectrum Matches |
| --- | --- | --- | --- | --- |
| 12.4720 | 123.4985 | 0.0000 | 5.5904e+07 | 2 |

**Adduct Signal Fractions**

- **Unmodified**: 5.5904e+07

### QDQC(C)IYN(N)TTYLNVQR Hex 3 HexNAc 4

**Mass:**  3213.3697

(9.397e-07)

**Charge States:** 3

**Start Position**: 87

**End Position**: 102

| Profile Score | Tandem Score | TDA q-value | Volume | Spectrum Matches |
| --- | --- | --- | --- | --- |
| -5.5204 | 121.7267 | 0.0000 | 2.3157e+07 | 2 |

**Adduct Signal Fractions**

- **Unmodified**: 2.3157e+07

### LVPVPITN(N)ATLDQITGK Fuc 2 Hex 5 HexNAc 6

**Mass:**  4099.8811

(1.347e-06)

**Charge States:** 3, 4

**Start Position**: 26

**End Position**: 43

| Profile Score | Tandem Score | TDA q-value | Volume | Spectrum Matches |
| --- | --- | --- | --- | --- |
| -4.8727 | 121.1930 | 0.0000 | 1.9867e+07 | 2 |

**Adduct Signal Fractions**

- **Unmodified**: 1.9867e+07

### FYFTPN(N)KTEDTIFLR Hex 3 HexNAc 6

**Mass:**  3595.5899

(1.189e-06)

**Charge States:** 3, 4

**Start Position**: 67

**End Position**: 82

| Profile Score | Tandem Score | TDA q-value | Volume | Spectrum Matches |
| --- | --- | --- | --- | --- |
| 13.9848 | 121.0792 | 0.0000 | 1.0894e+08 | 5 |

**Adduct Signal Fractions**

- **Unmodified**: 1.0894e+08

### LVPVPITN(N)ATLDQITGK Hex 5 HexNAc 5

**Mass:**  3606.7033

(5.627e-04)

**Charge States:** 3

**Start Position**: 26

**End Position**: 43

| Profile Score | Tandem Score | TDA q-value | Volume | Spectrum Matches |
| --- | --- | --- | --- | --- |
| -20.7695 | 119.1003 | 0.0000 | 1.2309e+07 | 2 |

**Adduct Signal Fractions**

- **Unmodified**: 1.2309e+07

### QDQC(C)IYN(N)TTYLNVQR Hex 5 HexNAc 8

**Mass:**  4349.7966

(1.887e-06)

**Charge States:** 3, 4

**Start Position**: 87

**End Position**: 102

| Profile Score | Tandem Score | TDA q-value | Volume | Spectrum Matches |
| --- | --- | --- | --- | --- |
| 8.3322 | 117.0596 | 0.0000 | 1.2068e+08 | 3 |

**Adduct Signal Fractions**

- **Unmodified**: 1.2068e+08

### QDQC(C)IYN(N)TTYLNVQR Hex 4 HexNAc 4 Neu5Ac 2

**Mass:**  3957.6140

(5.966e-07)

**Charge States:** 3, 4

**Start Position**: 87

**End Position**: 102

| Profile Score | Tandem Score | TDA q-value | Volume | Spectrum Matches |
| --- | --- | --- | --- | --- |
| 13.0812 | 116.9915 | 0.0000 | 2.0883e+08 | 5 |

**Adduct Signal Fractions**

- **Unmodified**: 2.0883e+08

### LVPVPITN(N)ATLDQITGK Hex 6 HexNAc 5

**Mass:**  3766.7394

(1.760e-06)

**Charge States:** 3, 4

**Start Position**: 26

**End Position**: 43

| Profile Score | Tandem Score | TDA q-value | Volume | Spectrum Matches |
| --- | --- | --- | --- | --- |
| 8.9448 | 116.3547 | 0.0000 | 7.2845e+07 | 12 |

**Adduct Signal Fractions**

- **Unmodified**: 7.2845e+07

### YFTPN(N)KTEDTIFLR Hex 4 HexNAc 7

**Mass:**  3813.6535

(4.950e-07)

**Charge States:** 3, 4

**Start Position**: 68

**End Position**: 82

| Profile Score | Tandem Score | TDA q-value | Volume | Spectrum Matches |
| --- | --- | --- | --- | --- |
| 9.5819 | 116.1153 | 0.0000 | 1.7214e+08 | 3 |

**Adduct Signal Fractions**

- **Unmodified**: 1.7214e+08

### TPN(N)KTEDTIFLR Hex 7 HexNAc 6

**Mass:**  3786.6081

(2.747e-06)

**Charge States:** 3, 4

**Start Position**: 70

**End Position**: 82

| Profile Score | Tandem Score | TDA q-value | Volume | Spectrum Matches |
| --- | --- | --- | --- | --- |
| 14.4463 | 115.9167 | 0.0000 | 9.7774e+07 | 2 |

**Adduct Signal Fractions**

- **Unmodified**: 9.7774e+07

### YFTPN(N)KTEDTIFLR Fuc 2 Hex 5 HexNAc 6

**Mass:**  4064.7424

(1.509e-07)

**Charge States:** 4

**Start Position**: 68

**End Position**: 82

| Profile Score | Tandem Score | TDA q-value | Volume | Spectrum Matches |
| --- | --- | --- | --- | --- |
| -5.3229 | 114.2941 | 0.0000 | 3.1904e+07 | 1 |

**Adduct Signal Fractions**

- **Unmodified**: 3.1904e+07

### YFTPN(N)KTEDTIFLR Fuc 1 Hex 4 HexNAc 5

**Mass:**  3555.5597

(5.658e-04)

**Start Position**: 68

**End Position**: 82

| Profile Score | Tandem Score | TDA q-value | Volume | Spectrum Matches |
| --- | --- | --- | --- | --- |
| 0.0000 | 113.1860 | 0.0000 | 0.0000e+00 | 1 |

Adduct:

No Chromatogram Found

### LVPVPITN(N)ATLDQITGK Hex 4 HexNAc 5

**Mass:**  3444.6565

(2.934e-04)

**Start Position**: 26

**End Position**: 43

| Profile Score | Tandem Score | TDA q-value | Volume | Spectrum Matches |
| --- | --- | --- | --- | --- |
| 0.0000 | 110.4565 | 0.0000 | 0.0000e+00 | 3 |

Adduct:

No Chromatogram Found

### ATFFYFTPN(N)K Fuc 3 Hex 8 HexNAc 4

**Mass:**  3781.5297

(3.593e-06)

**Charge States:** 4

**Start Position**: 64

**End Position**: 74

| Profile Score | Tandem Score | TDA q-value | Volume | Spectrum Matches |
| --- | --- | --- | --- | --- |
| -20.6538 | 110.2303 | 0.0000 | 4.4793e+06 | 1 |

**Adduct Signal Fractions**

- **Unmodified**: 4.4793e+06

### YFTPN(N)KTEDTIFLR Fuc 1 Hex 5 HexNAc 7

**Mass:**  4121.7658

(1.824e-06)

**Charge States:** 4

**Start Position**: 68

**End Position**: 82

| Profile Score | Tandem Score | TDA q-value | Volume | Spectrum Matches |
| --- | --- | --- | --- | --- |
| -3.4273 | 108.0735 | 0.0000 | 2.6080e+07 | 2 |

**Adduct Signal Fractions**

- **Unmodified**: 2.6080e+07

### QDQC(C)IYN(N)TTYLNVQR Fuc 1 Hex 5 HexNAc 7

**Mass:**  4292.7751

(1.899e-06)

**Charge States:** 3, 4

**Start Position**: 87

**End Position**: 102

| Profile Score | Tandem Score | TDA q-value | Volume | Spectrum Matches |
| --- | --- | --- | --- | --- |
| 14.8054 | 107.6845 | 0.0000 | 1.3640e+08 | 5 |

**Adduct Signal Fractions**

- **Unmodified**: 1.3640e+08

### QDQC(C)IYN(N)TTYLNVQR Hex 4 HexNAc 6

**Mass:**  3781.5796

(2.311e-06)

**Charge States:** 3, 4

**Start Position**: 87

**End Position**: 102

| Profile Score | Tandem Score | TDA q-value | Volume | Spectrum Matches |
| --- | --- | --- | --- | --- |
| 9.6528 | 105.8219 | 0.0000 | 8.8599e+07 | 4 |

**Adduct Signal Fractions**

- **Unmodified**: 8.8599e+07

### YFTPN(N)KTEDTIFLR Hex 5 HexNAc 8

**Mass:**  4178.7873

(1.213e-06)

**Charge States:** 4

**Start Position**: 68

**End Position**: 82

| Profile Score | Tandem Score | TDA q-value | Volume | Spectrum Matches |
| --- | --- | --- | --- | --- |
| -3.2171 | 105.6556 | 0.0000 | 3.6544e+07 | 2 |

**Adduct Signal Fractions**

- **Unmodified**: 3.6544e+07

### IYN(N)TTYLNVQR Hex 3 HexNAc 6

**Mass:**  3088.3522

(9.865e-07)

**Charge States:** 3, 4

**Start Position**: 91

**End Position**: 102

| Profile Score | Tandem Score | TDA q-value | Volume | Spectrum Matches |
| --- | --- | --- | --- | --- |
| 13.3785 | 105.6324 | 0.0000 | 3.5460e+07 | 6 |

**Adduct Signal Fractions**

- **Unmodified**: 3.5460e+07

### SVQEIQATFFYFTPN(N)K Hex 7 HexNAc 6

**Mass:**  4274.8009

(7.042e-04)

**Start Position**: 58

**End Position**: 74

| Profile Score | Tandem Score | TDA q-value | Volume | Spectrum Matches |
| --- | --- | --- | --- | --- |
| 0.0000 | 104.5811 | 0.0000 | 0.0000e+00 | 1 |

Adduct:

No Chromatogram Found

### YFTPN(N)KTEDTIFLR Hex 5 HexNAc 9

**Mass:**  4381.8649

(3.430e-07)

**Charge States:** 4

**Start Position**: 68

**End Position**: 82

| Profile Score | Tandem Score | TDA q-value | Volume | Spectrum Matches |
| --- | --- | --- | --- | --- |
| -5.0183 | 103.9188 | 0.0000 | 1.2732e+07 | 1 |

**Adduct Signal Fractions**

- **Unmodified**: 1.2732e+07

### FTPN(N)KTEDTIFLR Hex 3 HexNAc 6

**Mass:**  3285.4572

(6.883e-07)

**Charge States:** 3, 4

**Start Position**: 69

**End Position**: 82

| Profile Score | Tandem Score | TDA q-value | Volume | Spectrum Matches |
| --- | --- | --- | --- | --- |
| 14.0549 | 103.8139 | 0.0000 | 9.8634e+07 | 4 |

**Adduct Signal Fractions**

- **Unmodified**: 9.8634e+07

### PVPITN(N)ATLDQITGK Hex 6 HexNAc 5 Neu5Ac 1

**Mass:**  3845.6767

(1.522e-06)

**Charge States:** 4

**Start Position**: 28

**End Position**: 43

| Profile Score | Tandem Score | TDA q-value | Volume | Spectrum Matches |
| --- | --- | --- | --- | --- |
| -4.8325 | 101.8531 | 0.0000 | 6.7867e+06 | 1 |

**Adduct Signal Fractions**

- **Unmodified**: 6.7867e+06

### FYFTPN(N)KTEDTIFLR Hex 4 HexNAc 7

**Mass:**  3960.7238

(1.842e-06)

**Charge States:** 3, 4

**Start Position**: 67

**End Position**: 82

| Profile Score | Tandem Score | TDA q-value | Volume | Spectrum Matches |
| --- | --- | --- | --- | --- |
| 13.5667 | 100.2973 | 0.0000 | 1.7339e+07 | 4 |

**Adduct Signal Fractions**

- **Unmodified**: 1.7339e+07

### LVPVPITN(N)ATLDQITGK Fuc 1 Hex 5 HexNAc 7

**Mass:**  4156.8977

(1.382e-06)

**Charge States:** 3, 4

**Start Position**: 26

**End Position**: 43

| Profile Score | Tandem Score | TDA q-value | Volume | Spectrum Matches |
| --- | --- | --- | --- | --- |
| 11.4814 | 96.0215 | 0.0000 | 1.4429e+07 | 2 |

**Adduct Signal Fractions**

- **Unmodified**: 1.4429e+07

### FYFTPN(N)KTEDTIFLR Fuc 1 Hex 4 HexNAc 6

**Mass:**  3903.7036

(1.386e-06)

**Charge States:** 3, 4

**Start Position**: 67

**End Position**: 82

| Profile Score | Tandem Score | TDA q-value | Volume | Spectrum Matches |
| --- | --- | --- | --- | --- |
| -2.7250 | 95.9147 | 0.0000 | 2.4591e+07 | 4 |

**Adduct Signal Fractions**

- **Unmodified**: 2.4591e+07

### FTPN(N)KTEDTIFLR Fuc 1 Hex 4 HexNAc 5

**Mass:**  3390.4883

(7.421e-07)

**Charge States:** 3, 4

**Start Position**: 69

**End Position**: 82

| Profile Score | Tandem Score | TDA q-value | Volume | Spectrum Matches |
| --- | --- | --- | --- | --- |
| -4.6680 | 94.9232 | 0.0000 | 1.3411e+07 | 3 |

**Adduct Signal Fractions**

- **Unmodified**: 1.3411e+07

### FYFTPN(N)KTEDTIFLR Fuc 1 Hex 4 HexNAc 5

**Mass:**  3700.6203

(8.418e-07)

**Charge States:** 3, 4

**Start Position**: 67

**End Position**: 82

| Profile Score | Tandem Score | TDA q-value | Volume | Spectrum Matches |
| --- | --- | --- | --- | --- |
| 12.6229 | 94.6121 | 0.0000 | 1.6060e+07 | 5 |

**Adduct Signal Fractions**

- **Unmodified**: 1.6060e+07

### IYN(N)TTYLNVQR Hex 3 HexNAc 5

**Mass:**  2885.2723

(7.963e-07)

**Charge States:** 3

**Start Position**: 91

**End Position**: 102

| Profile Score | Tandem Score | TDA q-value | Volume | Spectrum Matches |
| --- | --- | --- | --- | --- |
| 13.4303 | 94.5901 | 0.0000 | 2.5557e+07 | 3 |

**Adduct Signal Fractions**

- **Unmodified**: 2.5557e+07

### TPN(N)KTEDTIFLR Fuc 1 Hex 4 HexNAc 5

**Mass:**  3243.4224

(1.511e-06)

**Charge States:** 3

**Start Position**: 70

**End Position**: 82

| Profile Score | Tandem Score | TDA q-value | Volume | Spectrum Matches |
| --- | --- | --- | --- | --- |
| -4.9033 | 93.1202 | 0.0000 | 6.7496e+06 | 2 |

**Adduct Signal Fractions**

- **Unmodified**: 6.7496e+06

### FTPN(N)KTEDTIFLR Hex 3 HexNAc 5

**Mass:**  3082.3782

(1.183e-06)

**Charge States:** 3, 4

**Start Position**: 69

**End Position**: 82

| Profile Score | Tandem Score | TDA q-value | Volume | Spectrum Matches |
| --- | --- | --- | --- | --- |
| 13.7894 | 92.7930 | 0.0000 | 4.5800e+07 | 4 |

**Adduct Signal Fractions**

- **Unmodified**: 4.5800e+07

### TPN(N)KTEDTIFLR Fuc 1 Hex 4 HexNAc 6

**Mass:**  3446.5006

(5.822e-04)

**Charge States:** 3, 4

**Start Position**: 70

**End Position**: 82

| Profile Score | Tandem Score | TDA q-value | Volume | Spectrum Matches |
| --- | --- | --- | --- | --- |
| 5.0099 | 87.8404 | 0.0000 | 1.3308e+07 | 22 |

**Adduct Signal Fractions**

- **Unmodified**: 1.3308e+07

### FTPN(N)KTEDTIFLR Fuc 1 Hex 4 HexNAc 6

**Mass:**  3593.5695

(1.400e-06)

**Charge States:** 3, 4

**Start Position**: 69

**End Position**: 82

| Profile Score | Tandem Score | TDA q-value | Volume | Spectrum Matches |
| --- | --- | --- | --- | --- |
| -3.9994 | 79.1777 | 0.0000 | 1.9190e+07 | 4 |

**Adduct Signal Fractions**

- **Unmodified**: 1.9190e+07

### SVQEIQATFFYFTPN(N)KTEDTIFLR Hex 3 HexNAc 6

**Mass:**  4599.0886

(1.075e-06)

**Charge States:** 3, 4, 5

**Start Position**: 58

**End Position**: 82

| Profile Score | Tandem Score | TDA q-value | Volume | Spectrum Matches |
| --- | --- | --- | --- | --- |
| -18.8530 | 70.2060 | 0.0000 | 1.9632e+07 | 2 |

**Adduct Signal Fractions**

- **Unmodified**: 1.9632e+07

### SVQEIQATFFYFTPN(N)K Fuc 1 Hex 6 HexNAc 5

**Mass:**  4052.7206

(6.206e-07)

**Charge States:** 3

**Start Position**: 58

**End Position**: 74

| Profile Score | Tandem Score | TDA q-value | Volume | Spectrum Matches |
| --- | --- | --- | --- | --- |
| -5.0605 | 61.2669 | 0.0000 | 7.2481e+06 | 2 |

**Adduct Signal Fractions**

- **Unmodified**: 7.2481e+06

### QDQC(C)IYN(N)TTYLNVQR Hex 4 HexNAc 8

**Mass:**  4187.7409

(1.198e-06)

**Charge States:** 3, 4

**Start Position**: 87

**End Position**: 102

| Profile Score | Tandem Score | TDA q-value | Volume | Spectrum Matches |
| --- | --- | --- | --- | --- |
| 17.5762 | 60.8069 | 0.0000 | 1.3344e+08 | 3 |

**Adduct Signal Fractions**

- **Unmodified**: 1.3344e+08

### SVQEIQATFFYFTPN(N)KTEDTIFLR Hex 3 HexNAc 5

**Mass:**  4395.9997

(-1.064e-06)

**Charge States:** 4

**Start Position**: 58

**End Position**: 82

| Profile Score | Tandem Score | TDA q-value | Volume | Spectrum Matches |
| --- | --- | --- | --- | --- |
| -5.1680 | 59.7198 | 0.0000 | 9.1259e+06 | 2 |

**Adduct Signal Fractions**

- **Unmodified**: 9.1259e+06

### SVQEIQATFFYFTPN(N)K Fuc 1 Hex 5 HexNAc 4 Neu5Ac 1

**Mass:**  3978.6996

(3.689e-06)

**Charge States:** 3

**Start Position**: 58

**End Position**: 74

| Profile Score | Tandem Score | TDA q-value | Volume | Spectrum Matches |
| --- | --- | --- | --- | --- |
| -4.6128 | 59.2824 | 0.0000 | 8.0188e+06 | 2 |

**Adduct Signal Fractions**

- **Unmodified**: 8.0188e+06

### LVPVPITN(N)ATLDQITGK Fuc 1 Hex 5 HexNAc 6

**Mass:**  3953.8098

(2.068e-06)

**Charge States:** 3, 4

**Start Position**: 26

**End Position**: 43

| Profile Score | Tandem Score | TDA q-value | Volume | Spectrum Matches |
| --- | --- | --- | --- | --- |
| 8.8351 | 56.2403 | 0.0000 | 3.4956e+07 | 4 |

**Adduct Signal Fractions**

- **Unmodified**: 3.4956e+07

### C(C)ANLVPVPITN(N)ATLDQITGK Hex 3 HexNAc 5

**Mass:**  3625.6851

(1.281e-06)

**Charge States:** 3, 4

**Start Position**: 23

**End Position**: 43

| Profile Score | Tandem Score | TDA q-value | Volume | Spectrum Matches |
| --- | --- | --- | --- | --- |
| 14.4086 | 54.6144 | 0.0000 | 1.0051e+08 | 5 |

**Adduct Signal Fractions**

- **Unmodified**: 1.0051e+08

### SVQEIQATFFYFTPN(N)K Hex 5 HexNAc 8

**Mass:**  4353.8464

(3.282e-07)

**Charge States:** 4

**Start Position**: 58

**End Position**: 74

| Profile Score | Tandem Score | TDA q-value | Volume | Spectrum Matches |
| --- | --- | --- | --- | --- |
| 10.8572 | 54.4092 | 0.0000 | 1.6239e+07 | 1 |

**Adduct Signal Fractions**

- **Unmodified**: 1.6239e+07

### QDQC(C)IYN(N)TTYLNVQR Fuc 1 Hex 4 HexNAc 5

**Mass:**  3726.5575

(5.375e-04)

**Start Position**: 87

**End Position**: 102

| Profile Score | Tandem Score | TDA q-value | Volume | Spectrum Matches |
| --- | --- | --- | --- | --- |
| 0.0000 | 54.2900 | 0.0000 | 0.0000e+00 | 1 |

Adduct:

No Chromatogram Found

### PVPITN(N)ATLDQITGK Fuc 2 Hex 4 HexNAc 5

**Mass:**  3522.5879

(-2.989e-06)

**Charge States:** 4

**Start Position**: 28

**End Position**: 43

| Profile Score | Tandem Score | TDA q-value | Volume | Spectrum Matches |
| --- | --- | --- | --- | --- |
| -5.5614 | 53.9808 | 0.0000 | 4.2866e+06 | 2 |

**Adduct Signal Fractions**

- **Unmodified**: 4.2866e+06

### FYFTPN(N)KTEDTIFLR Hex 3 HexNAc 4

**Mass:**  3189.4312

(6.460e-07)

**Charge States:** 3

**Start Position**: 67

**End Position**: 82

| Profile Score | Tandem Score | TDA q-value | Volume | Spectrum Matches |
| --- | --- | --- | --- | --- |
| -3.8780 | 51.7342 | 0.0000 | 6.6925e+06 | 1 |

**Adduct Signal Fractions**

- **Unmodified**: 6.6925e+06

### LVPVPITN(N)ATLDQITGK Fuc 1 Hex 4 HexNAc 4

**Mass:**  3385.5998

(4.360e-06)

**Charge States:** 3

**Start Position**: 26

**End Position**: 43

| Profile Score | Tandem Score | TDA q-value | Volume | Spectrum Matches |
| --- | --- | --- | --- | --- |
| -20.7435 | 50.0942 | 0.0000 | 1.3241e+07 | 1 |

**Adduct Signal Fractions**

- **Unmodified**: 1.3241e+07

### C(C)ANLVPVPITN(N)ATLDQITGK Fuc 1 Hex 4 HexNAc 5

**Mass:**  3933.7968

(1.967e-06)

**Charge States:** 3, 4

**Start Position**: 23

**End Position**: 43

| Profile Score | Tandem Score | TDA q-value | Volume | Spectrum Matches |
| --- | --- | --- | --- | --- |
| 12.9007 | 49.0629 | 0.0000 | 2.7231e+07 | 4 |

**Adduct Signal Fractions**

- **Unmodified**: 2.7231e+07

### FYFTPN(N)KTEDTIFLR Fuc 5 Hex 3 HexNAc 6

**Mass:**  4325.8649

(-2.545e-06)

**Charge States:** 4

**Start Position**: 67

**End Position**: 82

| Profile Score | Tandem Score | TDA q-value | Volume | Spectrum Matches |
| --- | --- | --- | --- | --- |
| -21.2946 | 48.1275 | 0.0000 | 2.4195e+06 | 1 |

**Adduct Signal Fractions**

- **Unmodified**: 2.4195e+06

### C(C)ANLVPVPITN(N)ATLDQITGK Hex 3 HexNAc 4

**Mass:**  3422.6046

(1.053e-06)

**Charge States:** 3

**Start Position**: 23

**End Position**: 43

| Profile Score | Tandem Score | TDA q-value | Volume | Spectrum Matches |
| --- | --- | --- | --- | --- |
| -3.8256 | 47.9726 | 0.0000 | 1.1339e+07 | 3 |

**Adduct Signal Fractions**

- **Unmodified**: 1.1339e+07

### SVQEIQATFFYFTPN(N)K Fuc 4 Hex 5 HexNAc 5

**Mass:**  4328.8229

(-3.762e-06)

**Charge States:** 4

**Start Position**: 58

**End Position**: 74

| Profile Score | Tandem Score | TDA q-value | Volume | Spectrum Matches |
| --- | --- | --- | --- | --- |
| -18.3993 | 47.8507 | 0.0000 | 7.2487e+06 | 1 |

**Adduct Signal Fractions**

- **Unmodified**: 7.2487e+06

### QDQC(C)IYN(N)TTYLNVQR Fuc 1 Hex 5 HexNAc 4 Neu5Ac 1

**Mass:**  3974.6369

(3.449e-06)

**Charge States:** 3

**Start Position**: 87

**End Position**: 102

| Profile Score | Tandem Score | TDA q-value | Volume | Spectrum Matches |
| --- | --- | --- | --- | --- |
| -4.9094 | 47.4207 | 0.0000 | 2.0266e+07 | 2 |

**Adduct Signal Fractions**

- **Unmodified**: 2.0266e+07

### LVPVPITN(N)ATLDQITGK Hex 4 HexNAc 5 Neu5Ac 1

**Mass:**  3733.7226

(9.641e-07)

**Charge States:** 3, 4

**Start Position**: 26

**End Position**: 43

| Profile Score | Tandem Score | TDA q-value | Volume | Spectrum Matches |
| --- | --- | --- | --- | --- |
| 12.0108 | 47.1421 | 0.0000 | 1.6125e+07 | 5 |

**Adduct Signal Fractions**

- **Unmodified**: 1.6125e+07

### SVQEIQATFFYFTPN(N)K Hex 4 HexNAc 6

**Mass:**  3788.6589

(7.990e-04)

**Start Position**: 58

**End Position**: 74

| Profile Score | Tandem Score | TDA q-value | Volume | Spectrum Matches |
| --- | --- | --- | --- | --- |
| 0.0000 | 46.6902 | 0.0000 | 0.0000e+00 | 1 |

Adduct:

No Chromatogram Found

### QDQC(C)IYN(N)TTYLNVQR Fuc 1 Hex 3 HexNAc 6

**Mass:**  3765.5817

(-1.328e-07)

**Charge States:** 3

**Start Position**: 87

**End Position**: 102

| Profile Score | Tandem Score | TDA q-value | Volume | Spectrum Matches |
| --- | --- | --- | --- | --- |
| -19.8635 | 46.6463 | 0.0000 | 1.4042e+07 | 1 |

**Adduct Signal Fractions**

- **Unmodified**: 1.4042e+07

### PVPITN(N)ATLDQITGK Fuc 2 Hex 6 HexNAc 3

**Mass:**  3440.5394

(1.978e-06)

**Charge States:** 3, 4

**Start Position**: 28

**End Position**: 43

| Profile Score | Tandem Score | TDA q-value | Volume | Spectrum Matches |
| --- | --- | --- | --- | --- |
| -19.8350 | 45.9786 | 0.0000 | 2.4541e+06 | 2 |

**Adduct Signal Fractions**

- **Unmodified**: 2.4541e+06

### QDQC(C)IYN(N)TTYLNVQR Fuc 1 Hex 5 HexNAc 8

**Mass:**  4497.8645

(4.489e-04)

**Start Position**: 87

**End Position**: 102

| Profile Score | Tandem Score | TDA q-value | Volume | Spectrum Matches |
| --- | --- | --- | --- | --- |
| 0.0000 | 45.7748 | 0.0000 | 0.0000e+00 | 1 |

Adduct:

No Chromatogram Found

### SVQEIQATFFYFTPN(N)K Fuc 2 Hex 5 HexNAc 5 Neu5Ac 1

**Mass:**  4327.8112

(-1.699e-06)

**Charge States:** 4

**Start Position**: 58

**End Position**: 74

| Profile Score | Tandem Score | TDA q-value | Volume | Spectrum Matches |
| --- | --- | --- | --- | --- |
| -19.5104 | 45.5583 | 0.0000 | 2.0784e+06 | 1 |

**Adduct Signal Fractions**

- **Unmodified**: 2.0784e+06

### QDQC(C)IYN(N)TTYLNVQR Fuc 1 Hex 3 HexNAc 5

**Mass:**  3562.5082

(1.572e-06)

**Charge States:** 3

**Start Position**: 87

**End Position**: 102

| Profile Score | Tandem Score | TDA q-value | Volume | Spectrum Matches |
| --- | --- | --- | --- | --- |
| -19.8530 | 45.4067 | 0.0000 | 6.8679e+06 | 1 |

**Adduct Signal Fractions**

- **Unmodified**: 6.8679e+06

### PVPITN(N)ATLDQITGK Fuc 2 Hex 3 HexNAc 7 Neu5Ac 3

**Mass:**  4640.0241

(8.564e-06)

**Charge States:** 4

**Start Position**: 28

**End Position**: 43

| Profile Score | Tandem Score | TDA q-value | Volume | Spectrum Matches |
| --- | --- | --- | --- | --- |
| 12.7296 | 44.6213 | 0.0000 | 4.8539e+06 | 4 |

**Adduct Signal Fractions**

- **Unmodified**: 4.8539e+06

### LVPVPITN(N)ATLDQITGK Fuc 1 Hex 5 HexNAc 3 Neu5Ac 1

**Mass:**  3635.6808

(3.085e-06)

**Charge States:** 3

**Start Position**: 26

**End Position**: 43

| Profile Score | Tandem Score | TDA q-value | Volume | Spectrum Matches |
| --- | --- | --- | --- | --- |
| -2.4035 | 43.7129 | 0.0000 | 1.1270e+07 | 2 |

**Adduct Signal Fractions**

- **Unmodified**: 1.1270e+07

### QDQC(C)IYN(N)TTYLNVQR Fuc 2 Hex 5 HexNAc 7

**Mass:**  4438.8273

(1.589e-06)

**Charge States:** 3, 4

**Start Position**: 87

**End Position**: 102

| Profile Score | Tandem Score | TDA q-value | Volume | Spectrum Matches |
| --- | --- | --- | --- | --- |
| 12.4246 | 43.5425 | 0.0000 | 2.7267e+07 | 2 |

**Adduct Signal Fractions**

- **Unmodified**: 2.7267e+07

### QDQC(C)IYN(N)TTYLNVQR Hex 5 HexNAc 9

**Mass:**  4552.8747

(4.381e-08)

**Charge States:** 3, 4

**Start Position**: 87

**End Position**: 102

| Profile Score | Tandem Score | TDA q-value | Volume | Spectrum Matches |
| --- | --- | --- | --- | --- |
| 12.4949 | 43.4777 | 0.0000 | 6.3138e+07 | 2 |

**Adduct Signal Fractions**

- **Unmodified**: 6.3138e+07

### LVPVPITN(N)ATLDQITGK Fuc 1 Hex 6 HexNAc 5

**Mass:**  3912.7927

(1.742e-06)

**Charge States:** 3, 4

**Start Position**: 26

**End Position**: 43

| Profile Score | Tandem Score | TDA q-value | Volume | Spectrum Matches |
| --- | --- | --- | --- | --- |
| 8.2786 | 42.2739 | 0.0000 | 8.4949e+06 | 3 |

**Adduct Signal Fractions**

- **Unmodified**: 8.4949e+06

### SVQEIQATFFYFTPN(N)K Fuc 2 Hex 5 HexNAc 7

**Mass:**  4442.8859

(7.825e-07)

**Charge States:** 4

**Start Position**: 58

**End Position**: 74

| Profile Score | Tandem Score | TDA q-value | Volume | Spectrum Matches |
| --- | --- | --- | --- | --- |
| -5.1218 | 41.3017 | 0.0000 | 3.6955e+06 | 1 |

**Adduct Signal Fractions**

- **Unmodified**: 3.6955e+06

### SVQEIQATFFYFTPN(N)KTEDTIFLR Hex 7 HexNAc 6

**Mass:**  5247.3039

(6.689e-07)

**Charge States:** 4

**Start Position**: 58

**End Position**: 82

| Profile Score | Tandem Score | TDA q-value | Volume | Spectrum Matches |
| --- | --- | --- | --- | --- |
| 11.4949 | 40.3894 | 0.0000 | 1.2795e+07 | 3 |

**Adduct Signal Fractions**

- **Unmodified**: 1.2795e+07

### QDQC(C)IYN(N)TTYLN(D)VQR Hex 3 HexNAc 5

**Mass:**  3417.4389

(2.910e-04)

**Charge States:** 3, 4

**Start Position**: 87

**End Position**: 102

| Profile Score | Tandem Score | TDA q-value | Volume | Spectrum Matches |
| --- | --- | --- | --- | --- |
| -19.6441 | 40.1565 | 0.0000 | 3.5672e+07 | 2 |

**Adduct Signal Fractions**

- **Unmodified**: 3.5672e+07

### FTPN(N)KTEDTIFLR Hex 3 HexNAc 4

**Mass:**  2879.2994

(1.239e-06)

**Charge States:** 3

**Start Position**: 69

**End Position**: 82

| Profile Score | Tandem Score | TDA q-value | Volume | Spectrum Matches |
| --- | --- | --- | --- | --- |
| -4.7469 | 40.0948 | 0.0000 | 5.0038e+06 | 1 |

**Adduct Signal Fractions**

- **Unmodified**: 5.0038e+06

### QDQC(C)IYN(N)TTYLNVQR Fuc 2 Hex 6 HexNAc 8

**Mass:**  4805.9857

(4.223e-04)

**Start Position**: 87

**End Position**: 102

| Profile Score | Tandem Score | TDA q-value | Volume | Spectrum Matches |
| --- | --- | --- | --- | --- |
| 0.0000 | 39.8659 | 0.0000 | 0.0000e+00 | 1 |

Adduct:

No Chromatogram Found

### QDQC(C)IYN(N)TTYLNVQR Fuc 1 Hex 5 HexNAc 8

**Mass:**  4495.8547

(2.091e-06)

**Charge States:** 4

**Start Position**: 87

**End Position**: 102

| Profile Score | Tandem Score | TDA q-value | Volume | Spectrum Matches |
| --- | --- | --- | --- | --- |
| -4.4178 | 39.8602 | 0.0000 | 3.3887e+07 | 1 |

**Adduct Signal Fractions**

- **Unmodified**: 3.3887e+07

### LVPVPITN(N)ATLDQITGK Hex 4 HexNAc 7

**Mass:**  3849.7809

(2.598e-04)

**Start Position**: 26

**End Position**: 43

| Profile Score | Tandem Score | TDA q-value | Volume | Spectrum Matches |
| --- | --- | --- | --- | --- |
| 0.0000 | 38.6871 | 0.0000 | 0.0000e+00 | 1 |

Adduct:

No Chromatogram Found

### QDQC(C)IYN(N)TTYLN Hex 3 HexNAc 6

**Mass:**  3238.3102

(6.224e-04)

**Start Position**: 87

**End Position**: 99

| Profile Score | Tandem Score | TDA q-value | Volume | Spectrum Matches |
| --- | --- | --- | --- | --- |
| 0.0000 | 38.3280 | 0.0000 | 0.0000e+00 | 1 |

Adduct:

No Chromatogram Found

### LVPVPITN(N)ATLDQITGK Hex 5 HexNAc 7

**Mass:**  4010.8435

(2.381e-06)

**Charge States:** 3, 4

**Start Position**: 26

**End Position**: 43

| Profile Score | Tandem Score | TDA q-value | Volume | Spectrum Matches |
| --- | --- | --- | --- | --- |
| -5.7801 | 38.2080 | 0.0000 | 8.3559e+06 | 1 |

**Adduct Signal Fractions**

- **Unmodified**: 8.3559e+06

### LVPVPITN(N)ATLDQITGK Fuc 2 Hex 3 HexNAc 4 Neu5Ac 1

**Mass:**  3663.7329

(8.281e-04)

**Start Position**: 26

**End Position**: 43

| Profile Score | Tandem Score | TDA q-value | Volume | Spectrum Matches |
| --- | --- | --- | --- | --- |
| 0.0000 | 37.9381 | 0.0000 | 0.0000e+00 | 1 |

Adduct:

No Chromatogram Found

### FTPN(N)KTEDTIFLR Hex 4 HexNAc 7

**Mass:**  3650.5891

(1.514e-06)

**Charge States:** 3, 4

**Start Position**: 69

**End Position**: 82

| Profile Score | Tandem Score | TDA q-value | Volume | Spectrum Matches |
| --- | --- | --- | --- | --- |
| -4.9957 | 37.8298 | 0.0000 | 1.1066e+07 | 4 |

**Adduct Signal Fractions**

- **Unmodified**: 1.1066e+07

### QDQC(C)IYN(N)TTYLNVQR Fuc 1 Hex 6 HexNAc 8

**Mass:**  4657.9074

(1.727e-06)

**Charge States:** 4

**Start Position**: 87

**End Position**: 102

| Profile Score | Tandem Score | TDA q-value | Volume | Spectrum Matches |
| --- | --- | --- | --- | --- |
| -19.0308 | 36.6593 | 0.0000 | 8.1745e+06 | 1 |

**Adduct Signal Fractions**

- **Unmodified**: 8.1745e+06

### QDQC(C)IYN(N)TTYLN(D)VQR Hex 3 HexNAc 6

**Mass:**  3620.5178

(2.731e-04)

**Charge States:** 3

**Start Position**: 87

**End Position**: 102

| Profile Score | Tandem Score | TDA q-value | Volume | Spectrum Matches |
| --- | --- | --- | --- | --- |
| -19.9374 | 36.4022 | 0.0000 | 6.0319e+07 | 6 |

**Adduct Signal Fractions**

- **Unmodified**: 6.0319e+07

### YFTPN(N)KTEDTIFLR Hex 4 HexNAc 6

**Mass:**  3610.5738

(8.381e-07)

**Charge States:** 3, 4

**Start Position**: 68

**End Position**: 82

| Profile Score | Tandem Score | TDA q-value | Volume | Spectrum Matches |
| --- | --- | --- | --- | --- |
| -19.8366 | 36.0782 | 0.0000 | 2.8288e+07 | 1 |

**Adduct Signal Fractions**

- **Unmodified**: 2.8288e+07

### QDQC(C)IYN(N)TTYLNVQR Fuc 5 Hex 4 HexNAc 6

**Mass:**  4514.8885

(6.698e-04)

**Start Position**: 87

**End Position**: 102

| Profile Score | Tandem Score | TDA q-value | Volume | Spectrum Matches |
| --- | --- | --- | --- | --- |
| 0.0000 | 36.0608 | 0.0000 | 0.0000e+00 | 1 |

Adduct:

No Chromatogram Found

### SVQEIQATFFYFTPN(N)K Fuc 1 Hex 5 HexNAc 8

**Mass:**  4499.9108

(1.619e-06)

**Charge States:** 4

**Start Position**: 58

**End Position**: 74

| Profile Score | Tandem Score | TDA q-value | Volume | Spectrum Matches |
| --- | --- | --- | --- | --- |
| -21.3156 | 35.9541 | 0.0000 | 7.2343e+05 | 1 |

**Adduct Signal Fractions**

- **Unmodified**: 7.2343e+05

### LVPVPITN(N)ATLDQITGK Fuc 1 Hex 4 HexNAc 5

**Mass:**  3590.6905

(5.603e-04)

**Start Position**: 26

**End Position**: 43

| Profile Score | Tandem Score | TDA q-value | Volume | Spectrum Matches |
| --- | --- | --- | --- | --- |
| 0.0000 | 34.5544 | 0.0028 | 0.0000e+00 | 2 |

Adduct:

No Chromatogram Found

### TPN(N)KTEDTIFLR Hex 3 HexNAc 6

**Mass:**  3139.3882

(3.193e-04)

**Start Position**: 70

**End Position**: 82

| Profile Score | Tandem Score | TDA q-value | Volume | Spectrum Matches |
| --- | --- | --- | --- | --- |
| 0.0000 | 34.4797 | 0.0028 | 0.0000e+00 | 1 |

Adduct:

No Chromatogram Found

### QDQC(C)IYN(N)TTYLNVQR Fuc 1 Hex 5 HexNAc 4 Neu5Ac 2

**Mass:**  4265.7253

(-4.663e-07)

**Charge States:** 4

**Start Position**: 87

**End Position**: 102

| Profile Score | Tandem Score | TDA q-value | Volume | Spectrum Matches |
| --- | --- | --- | --- | --- |
| -5.8712 | 34.3803 | 0.0028 | 2.2373e+07 | 2 |

**Adduct Signal Fractions**

- **Unmodified**: 2.2373e+07

### QDQC(C)IYN(N)TTYLNVQR Fuc 1 Hex 6 HexNAc 8

**Mass:**  4659.9189

(4.336e-04)

**Start Position**: 87

**End Position**: 102

| Profile Score | Tandem Score | TDA q-value | Volume | Spectrum Matches |
| --- | --- | --- | --- | --- |
| 0.0000 | 33.5210 | 0.0053 | 0.0000e+00 | 1 |

Adduct:

No Chromatogram Found

### SVQEIQATFFYFTPN(N)KTEDTIFLR Fuc 1 Hex 4 HexNAc 6

**Mass:**  4907.1965

(4.451e-07)

**Charge States:** 4

**Start Position**: 58

**End Position**: 82

| Profile Score | Tandem Score | TDA q-value | Volume | Spectrum Matches |
| --- | --- | --- | --- | --- |
| -21.3553 | 33.2882 | 0.0053 | 1.9487e+06 | 1 |

**Adduct Signal Fractions**

- **Unmodified**: 1.9487e+06

### PVPITN(N)ATLDQITGK Fuc 5 Hex 3 HexNAc 7 Neu5Ac 2

**Mass:**  4788.0809

(2.141e-04)

**Start Position**: 28

**End Position**: 43

| Profile Score | Tandem Score | TDA q-value | Volume | Spectrum Matches |
| --- | --- | --- | --- | --- |
| 0.0000 | 32.7423 | 0.0053 | 0.0000e+00 | 1 |

Adduct:

No Chromatogram Found

### QDQC(C)IYN(N)TTYL Hex 3 HexNAc 6

**Mass:**  3122.2564

(1.083e-06)

**Charge States:** 3

**Start Position**: 87

**End Position**: 98

| Profile Score | Tandem Score | TDA q-value | Volume | Spectrum Matches |
| --- | --- | --- | --- | --- |
| -19.5814 | 32.2018 | 0.0066 | 5.6568e+06 | 1 |

**Adduct Signal Fractions**

- **Unmodified**: 5.6568e+06

### QDQC(C)IYN(N)TTYLNVQR Fuc 2 Hex 6 HexNAc 7

**Mass:**  4600.8913

(2.041e-06)

**Charge States:** 4

**Start Position**: 87

**End Position**: 102

| Profile Score | Tandem Score | TDA q-value | Volume | Spectrum Matches |
| --- | --- | --- | --- | --- |
| -5.5187 | 31.9086 | 0.0092 | 1.0921e+07 | 1 |

**Adduct Signal Fractions**

- **Unmodified**: 1.0921e+07

### QDQC(C)IYN(N)TTYLNVQR Fuc 1 Hex 6 HexNAc 9

**Mass:**  4860.9880

(1.915e-06)

**Charge States:** 4

**Start Position**: 87

**End Position**: 102

| Profile Score | Tandem Score | TDA q-value | Volume | Spectrum Matches |
| --- | --- | --- | --- | --- |
| -5.5687 | 31.3867 | 0.0116 | 6.0949e+06 | 1 |

**Adduct Signal Fractions**

- **Unmodified**: 6.0949e+06

### LVPVPITN(N)ATLDQITGK Hex 5 HexNAc 5

**Mass:**  3607.7155

(8.434e-04)

**Start Position**: 26

**End Position**: 43

| Profile Score | Tandem Score | TDA q-value | Volume | Spectrum Matches |
| --- | --- | --- | --- | --- |
| 0.0000 | 31.0571 | 0.0116 | 0.0000e+00 | 1 |

Adduct:

No Chromatogram Found

### TPN(N)KTEDTIFLR Hex 3 HexNAc 4

**Mass:**  2732.2296

(5.044e-07)

**Charge States:** 3

**Start Position**: 70

**End Position**: 82

| Profile Score | Tandem Score | TDA q-value | Volume | Spectrum Matches |
| --- | --- | --- | --- | --- |
| 9.7492 | 30.8847 | 0.0126 | 5.8435e+06 | 2 |

**Adduct Signal Fractions**

- **Unmodified**: 5.8435e+06

### IYN(N)TTYLNVQR Fuc 1 Hex 4 HexNAc 5

**Mass:**  3193.3837

(2.322e-06)

**Charge States:** 3

**Start Position**: 91

**End Position**: 102

| Profile Score | Tandem Score | TDA q-value | Volume | Spectrum Matches |
| --- | --- | --- | --- | --- |
| -4.1218 | 30.7404 | 0.0126 | 4.0500e+06 | 2 |

**Adduct Signal Fractions**

- **Unmodified**: 4.0500e+06

### LVPVPITN(N)ATLDQITGK Hex 5 HexNAc 8

**Mass:**  4213.9167

(8.580e-07)

**Charge States:** 4

**Start Position**: 26

**End Position**: 43

| Profile Score | Tandem Score | TDA q-value | Volume | Spectrum Matches |
| --- | --- | --- | --- | --- |
| -5.2173 | 30.5627 | 0.0126 | 4.9213e+06 | 1 |

**Adduct Signal Fractions**

- **Unmodified**: 4.9213e+06

### ATFFYFTPN(N)K Fuc 2 Hex 6 HexNAc 4

**Mass:**  3314.3578

(9.076e-04)

**Start Position**: 64

**End Position**: 74

| Profile Score | Tandem Score | TDA q-value | Volume | Spectrum Matches |
| --- | --- | --- | --- | --- |
| 0.0000 | 30.5487 | 0.0126 | 0.0000e+00 | 1 |

Adduct:

No Chromatogram Found

### QIPLC(C)ANLVPVPITN(N)ATLDQITGK Fuc 1 Hex 4 HexNAc 5

**Mass:**  4385.0729

(5.909e-07)

**Charge States:** 3, 4

**Start Position**: 19

**End Position**: 43

| Profile Score | Tandem Score | TDA q-value | Volume | Spectrum Matches |
| --- | --- | --- | --- | --- |
| 11.3639 | 29.9739 | 0.0174 | 4.3029e+07 | 4 |

**Adduct Signal Fractions**

- **Unmodified**: 4.3029e+07

### QDQC(C)IYN(N)TTYLNVQR Fuc 3 Hex 6 HexNAc 6

**Mass:**  4543.8586

(5.122e-07)

**Charge States:** 3, 4

**Start Position**: 87

**End Position**: 102

| Profile Score | Tandem Score | TDA q-value | Volume | Spectrum Matches |
| --- | --- | --- | --- | --- |
| -20.4630 | 29.6783 | 0.0186 | 1.0849e+07 | 1 |

**Adduct Signal Fractions**

- **Unmodified**: 1.0849e+07

### LVPVPITN(N)ATLDQITGK Hex 4 HexNAc 8

**Mass:**  4051.8657

(1.295e-06)

**Charge States:** 4

**Start Position**: 26

**End Position**: 43

| Profile Score | Tandem Score | TDA q-value | Volume | Spectrum Matches |
| --- | --- | --- | --- | --- |
| -5.5144 | 29.4353 | 0.0195 | 4.1048e+06 | 1 |

**Adduct Signal Fractions**

- **Unmodified**: 4.1048e+06

### LVPVPITN(N)ATLDQITGK Hex 4 HexNAc 6

**Mass:**  3647.7201

(5.536e-04)

**Start Position**: 26

**End Position**: 43

| Profile Score | Tandem Score | TDA q-value | Volume | Spectrum Matches |
| --- | --- | --- | --- | --- |
| 0.0000 | 28.3987 | 0.0250 | 0.0000e+00 | 1 |

Adduct:

No Chromatogram Found

### QIPLC(C)ANLVPVPITN(N)ATLDQITGK Hex 3 HexNAc 5 Neu5Ac 1

**Mass:**  4368.0529

(-5.530e-07)

**Charge States:** 3, 4

**Start Position**: 19

**End Position**: 43

| Profile Score | Tandem Score | TDA q-value | Volume | Spectrum Matches |
| --- | --- | --- | --- | --- |
| 14.0841 | 28.0412 | 0.0271 | 1.6784e+08 | 6 |

**Adduct Signal Fractions**

- **Unmodified**: 1.6784e+08

### C(C)ANLVPVPITN(N)ATLDQITGK Hex 4 HexNAc 6

**Mass:**  3990.8161

(9.548e-07)

**Charge States:** 4

**Start Position**: 23

**End Position**: 43

| Profile Score | Tandem Score | TDA q-value | Volume | Spectrum Matches |
| --- | --- | --- | --- | --- |
| -3.8177 | 26.5111 | 0.0388 | 9.4519e+06 | 3 |

**Adduct Signal Fractions**

- **Unmodified**: 9.4519e+06

### PVPITN(N)ATLDQITGK Fuc 1 Hex 5 HexNAc 4 Neu5Ac 1

**Mass:**  3626.6301

(8.345e-06)

**Charge States:** 4

**Start Position**: 28

**End Position**: 43

| Profile Score | Tandem Score | TDA q-value | Volume | Spectrum Matches |
| --- | --- | --- | --- | --- |
| -3.5384 | 25.8364 | 0.0391 | 3.1342e+08 | 3 |

**Adduct Signal Fractions**

- **Unmodified**: 3.1342e+08

### LVPVPITN(N)ATLDQITGK Hex 4 HexNAc 4 Neu5Ac 2

**Mass:**  3821.7351

(3.161e-07)

**Charge States:** 3, 4

**Start Position**: 26

**End Position**: 43

| Profile Score | Tandem Score | TDA q-value | Volume | Spectrum Matches |
| --- | --- | --- | --- | --- |
| -20.1320 | 25.7434 | 0.0401 | 5.4683e+06 | 1 |

**Adduct Signal Fractions**

- **Unmodified**: 5.4683e+06

### FTPN(N)KTEDTIFLR Fuc 2 Hex 5 HexNAc 6

**Mass:**  3901.6809

(1.210e-06)

**Charge States:** 4

**Start Position**: 69

**End Position**: 82

| Profile Score | Tandem Score | TDA q-value | Volume | Spectrum Matches |
| --- | --- | --- | --- | --- |
| -19.0922 | 25.3162 | 0.0417 | 2.7929e+06 | 2 |

**Adduct Signal Fractions**

- **Unmodified**: 2.7929e+06

### QDQC(C)IYN(N)TTYLNVQR Hex 6 HexNAc 4

**Mass:**  3699.5530

(7.874e-06)

**Start Position**: 87

**End Position**: 102

| Profile Score | Tandem Score | TDA q-value | Volume | Spectrum Matches |
| --- | --- | --- | --- | --- |
| 0.0000 | 25.2755 | 0.0427 | 0.0000e+00 | 1 |

Adduct:

No Chromatogram Found

### IYN(N)TTYLNVQR Fuc 1 Hex 4 HexNAc 6

**Mass:**  3398.4688

(5.914e-04)

**Start Position**: 91

**End Position**: 102

| Profile Score | Tandem Score | TDA q-value | Volume | Spectrum Matches |
| --- | --- | --- | --- | --- |
| 0.0000 | 24.8619 | 0.0495 | 0.0000e+00 | 1 |

Adduct:

No Chromatogram Found

### P19652|A1AG2\_HUMAN

**>P19652|A1AG2\_HUMAN**  
 `MALSWVLTVLSLLPLLEAQIPLCANLVPVPITNATLDRITGKWFYIASAFRNEEYNKSVQEIQATFFYFTPNKTEDTIFL  
REYQTRQNQCFYNSSYLNVQRENGTVSRYEGGREHVAHLLFLRDTKTLMFGSYLDDEKNWGLSFYADKPETTKEQLGEFY  
EALDCLCIPRSDVMYTDWKKDKCEPLEKQHEKERKQEEGES`

| Site | Type | Count |
| --- | --- | --- |
| 33 | N-linked | 53 |
| 56 | N-linked | 3 |
| 93 | N-linked | 25 |
| 72 | N-linked | 77 |

| Mass | Sequence | MS_2_ Score | `q`-value | MS_1_ Score | Signal | Protein Coordinates | Tandem Scans | Start Time | Apex Time | End Time |
| --- | --- | --- | --- | --- | --- | --- | --- | --- | --- | --- |
| 2473.0179 | YFTPN(N)K Hex 3 HexNAc 6 | 188.898 | 0.0 | 14.137 | 3.107e+08 | 67 - 73 | 5 | 13.65 | 13.81 | 14.29 |
| 2781.1281 | YFTPN(N)K Fuc 1 Hex 4 HexNAc 6 | 186.556 | 0.0 | -4.323 | 4.931e+07 | 67 - 73 | 3 | 13.48 | 13.48 | 13.81 |
| 2620.0856 | FYFTPN(N)K Hex 3 HexNAc 6 | 183.035 | 0.0 | 13.158 | 1.155e+08 | 66 - 73 | 11 | 25.02 | 25.36 | 27.92 |
| 2578.0496 | YFTPN(N)K Fuc 1 Hex 4 HexNAc 5 | 181.096 | 0.0 | -19.295 | 3.412e+07 | 67 - 73 | 2 | 13.65 | 13.65 | 13.81 |
| 2296.8955 | NEEYN(N)K Hex 3 HexNAc 5 | 180.688 | 0.0 | 10.718 | 3.117e+07 | 51 - 57 | 7 | 3.77 | 3.94 | 4.99 |
| 2605.0069 | NEEYN(N)K Fuc 1 Hex 4 HexNAc 5 | 178.950 | 0.0 | 11.035 | 1.259e+07 | 51 - 57 | 4 | 3.77 | 3.94 | 4.82 |
| 3988.7196 | SVQEIQATFFYFTPN(N)K Hex 4 HexNAc 7 | 173.301 | 0.0 | 10.801 | 2.305e+08 | 57 - 73 | 10 | 53.47 | 53.64 | 55.33 |
| 2838.1508 | YFTPN(N)K Hex 4 HexNAc 7 | 173.030 | 0.0 | -5.133 | 2.478e+07 | 67 - 73 | 1 | 13.48 | 13.48 | 13.81 |
| 2269.9384 | YFTPN(N)K Hex 3 HexNAc 5 | 172.718 | 0.0 | 12.486 | 1.952e+08 | 67 - 73 | 5 | 13.81 | 13.98 | 14.45 |
| 2066.8589 | YFTPN(N)K Hex 3 HexNAc 4 | 172.517 | 0.0 | -19.096 | 2.513e+07 | 67 - 73 | 3 | 13.98 | 13.98 | 14.15 |
| 2939.2404 | ATFFYFTPN(N)K Hex 3 HexNAc 6 | 172.154 | 0.0 | -3.409 | 3.969e+07 | 63 - 73 | 3 | 41.22 | 41.39 | 41.73 |
| 3728.6183 | SVQEIQATFFYFTPN(N)K Fuc 1 Hex 4 HexNAc 5 | 171.606 | 0.0 | 13.093 | 1.770e+08 | 57 - 73 | 8 | 53.98 | 54.15 | 55.50 |
| 2417.0063 | FYFTPN(N)K Hex 3 HexNAc 5 | 170.522 | 0.0 | 13.059 | 6.659e+07 | 66 - 73 | 11 | 25.36 | 25.82 | 27.71 |
| 3420.5054 | SVQEIQATFFYFTPN(N)K Hex 3 HexNAc 5 | 168.319 | 0.0 | 14.862 | 6.027e+08 | 57 - 73 | 29 | 50.92 | 54.65 | 59.16 |
| 3623.5857 | SVQEIQATFFYFTPN(N)K Hex 3 HexNAc 6 | 167.708 | 0.0 | 14.624 | 1.060e+09 | 57 - 73 | 25 | 54.15 | 54.49 | 57.95 |
| 3931.6989 | SVQEIQATFFYFTPN(N)K Fuc 1 Hex 4 HexNAc 6 | 165.633 | 0.0 | 13.253 | 2.884e+08 | 57 - 73 | 12 | 53.64 | 53.98 | 56.18 |
| 2093.8160 | NEEYN(N)K Hex 3 HexNAc 4 | 163.328 | 0.0 | 9.567 | 3.463e+07 | 51 - 57 | 10 | 3.77 | 3.94 | 10.66 |
| 2909.3673 | LVPVPITN(N)ATLDR Hex 3 HexNAc 5 | 161.792 | 0.0 | 10.901 | 1.271e+10 | 25 - 38 | 426 | 29.91 | 36.90 | 113.53 |
| 2840.1601 | YFTPN(N)K Hex 4 HexNAc 7 | 160.539 | 0.0 | 0.000 | 0.000e+00 | 67 - 73 | 1 | - | - | - |
| 2706.2909 | LVPVPITN(N)ATLDR Hex 3 HexNAc 4 | 159.315 | 0.0 | 14.181 | 1.117e+09 | 25 - 38 | 11 | 36.60 | 37.29 | 70.87 |
| 3217.4772 | LVPVPITN(N)ATLDR Fuc 1 Hex 4 HexNAc 5 | 158.688 | 0.0 | 14.979 | 3.602e+09 | 25 - 38 | 20 | 36.27 | 36.60 | 113.20 |
| 4239.8071 | SVQEIQATFFYFTPN(N)K Fuc 2 Hex 5 HexNAc 6 | 157.904 | 0.0 | 13.499 | 4.557e+07 | 57 - 73 | 4 | 53.30 | 53.64 | 54.15 |
| 2736.1615 | ATFFYFTPN(N)K Hex 3 HexNAc 5 | 156.464 | 0.0 | -4.085 | 2.291e+07 | 63 - 73 | 3 | 41.56 | 41.73 | 41.90 |
| 3072.4264 | LVPVPITN(N)ATLDR Hex 4 HexNAc 5 | 155.854 | 0.0 | 0.000 | 0.000e+00 | 25 - 38 | 2 | - | - | - |
| 3217.4261 | SVQEIQATFFYFTPN(N)K Hex 3 HexNAc 4 | 153.432 | 0.0 | 12.807 | 7.656e+07 | 57 - 73 | 8 | 54.82 | 54.99 | 56.79 |
| 4271.7980 | SVQEIQATFFYFTPN(N)K Hex 7 HexNAc 6 | 153.210 | 0.0 | 12.622 | 4.845e+07 | 57 - 73 | 1 | 53.30 | 53.30 | 54.15 |
| 3071.4180 | LVPVPITN(N)ATLDR Hex 4 HexNAc 5 | 150.102 | 0.0 | -3.861 | 8.052e+07 | 25 - 38 | 3 | 36.27 | 36.75 | 36.75 |
| 3906.6679 | SVQEIQATFFYFTPN(N)K Hex 6 HexNAc 5 | 148.554 | 0.0 | 13.102 | 3.737e+07 | 57 - 73 | 4 | 53.47 | 53.81 | 54.32 |
| 4191.7990 | SVQEIQATFFYFTPN(N)K Hex 4 HexNAc 8 | 147.421 | 0.0 | 13.144 | 2.603e+07 | 57 - 73 | 4 | 53.30 | 53.47 | 54.15 |
| 3274.5005 | LVPVPITN(N)ATLDR Hex 4 HexNAc 6 | 146.655 | 0.0 | 9.672 | 7.546e+08 | 25 - 38 | 12 | 30.59 | 37.45 | 38.23 |
| 3138.3886 | TPN(N)KTEDTIFLR Hex 3 HexNAc 6 | 146.253 | 0.0 | 12.390 | 4.965e+07 | 69 - 81 | 6 | 25.65 | 25.82 | 26.36 |
| 3112.4460 | LVPVPITN(N)ATLDR Hex 3 HexNAc 6 | 145.977 | 0.0 | 10.888 | 2.901e+08 | 25 - 38 | 4 | 36.60 | 36.75 | 38.07 |
| 3056.4280 | LVPVPITN(N)ATLDR Fuc 1 Hex 3 HexNAc 5 | 145.029 | 0.0 | 0.000 | 0.000e+00 | 25 - 38 | 1 | - | - | - |
| 4134.7761 | SVQEIQATFFYFTPN(N)K Fuc 1 Hex 4 HexNAc 7 | 143.609 | 0.0 | -4.673 | 1.116e+07 | 57 - 73 | 3 | 53.47 | 53.81 | 53.98 |
| 4556.9335 | SVQEIQATFFYFTPN(N)K Hex 5 HexNAc 9 | 143.334 | 0.0 | -20.405 | 2.019e+06 | 57 - 73 | 1 | 52.79 | 52.79 | 52.79 |
| 3055.4281 | LVPVPITN(N)ATLDR Fuc 1 Hex 3 HexNAc 5 | 142.726 | 0.0 | -19.232 | 2.337e+07 | 25 - 38 | 2 | 37.16 | 37.16 | 37.16 |
| 3114.4529 | LVPVPITN(N)ATLDR Hex 3 HexNAc 6 | 142.705 | 0.0 | 0.000 | 0.000e+00 | 25 - 38 | 1 | - | - | - |
| 3420.5577 | LVPVPITN(N)ATLDR Fuc 1 Hex 4 HexNAc 6 | 142.203 | 0.0 | 13.231 | 1.118e+08 | 25 - 38 | 4 | 36.10 | 36.44 | 36.75 |
| 3562.5901 | C(C)ANLVPVPITN(N)ATLDR Fuc 1 Hex 4 HexNAc 5 | 140.925 | 0.0 | -4.587 | 5.430e+07 | 22 - 38 | 4 | 41.73 | 41.90 | 42.07 |
| 3044.2653 | ATFFYFTPN(N)K Fuc 1 Hex 4 HexNAc 5 | 140.360 | 0.0 | -17.444 | 5.646e+06 | 63 - 73 | 1 | 41.05 | 41.05 | 41.22 |
| 4296.8291 | SVQEIQATFFYFTPN(N)K Fuc 1 Hex 5 HexNAc 7 | 139.286 | 0.0 | 10.080 | 3.223e+07 | 57 - 73 | 3 | 52.96 | 53.30 | 54.15 |
| 3785.6418 | SVQEIQATFFYFTPN(N)K Hex 4 HexNAc 6 | 137.591 | 0.0 | 11.500 | 3.327e+07 | 57 - 73 | 5 | 53.81 | 54.82 | 55.50 |
| 3756.6324 | YFTPN(N)KTEDTIFLR Fuc 1 Hex 4 HexNAc 6 | 137.425 | 0.0 | -3.746 | 2.114e+08 | 67 - 81 | 8 | 35.27 | 36.90 | 37.59 |
| 2796.2822 | VPVPITN(N)ATLDR Hex 3 HexNAc 5 | 136.884 | 0.0 | -4.126 | 7.824e+06 | 26 - 38 | 2 | 30.25 | 30.25 | 30.76 |
| 3961.6701 | SVQEIQATFFYFTPN(N)K Hex 4 HexNAc 4 Neu5Ac 2 | 136.180 | 0.0 | 12.825 | 3.857e+07 | 57 - 73 | 4 | 54.99 | 55.16 | 56.18 |
| 3962.6862 | SVQEIQATFFYFTPN(N)K Fuc 2 Hex 4 HexNAc 4 Neu5Ac 1 | 134.851 | 0.0 | -19.860 | 2.049e+06 | 57 - 73 | 2 | 54.82 | 54.82 | 54.82 |
| 3254.5600 | C(C)ANLVPVPITN(N)ATLDR Hex 3 HexNAc 5 | 134.399 | 0.0 | 12.578 | 4.899e+08 | 22 - 38 | 23 | 42.07 | 42.24 | 53.81 |
| 3448.5201 | YFTPN(N)KTEDTIFLR Hex 3 HexNAc 6 | 132.896 | 0.0 | 13.175 | 1.560e+09 | 67 - 81 | 18 | 35.60 | 37.29 | 39.41 |
| 3292.5262 | LVPVPITN(N)ATLDR Fuc 2 Hex 3 HexNAc 4 Neu5Ac 1 | 131.513 | 0.0 | 0.000 | 0.000e+00 | 25 - 38 | 2 | - | - | - |
| 3042.3631 | YFTPN(N)KTEDTIFLR Hex 3 HexNAc 4 | 131.009 | 0.0 | 12.940 | 1.085e+08 | 67 - 81 | 3 | 37.59 | 37.91 | 38.91 |
| 3247.3479 | ATFFYFTPN(N)K Fuc 1 Hex 4 HexNAc 6 | 130.528 | 0.0 | -21.185 | 4.150e+06 | 63 - 73 | 1 | 40.71 | 40.71 | 40.88 |
| 3264.4768 | LVPVPITN(N)ATLDR Fuc 1 Hex 5 HexNAc 3 Neu5Ac 1 | 130.198 | 0.0 | -20.403 | 2.645e+07 | 25 - 38 | 1 | 37.59 | 37.76 | 37.76 |
| 3364.5394 | LVPVPITN(N)ATLDR Fuc 2 Hex 4 HexNAc 5 | 130.032 | 0.0 | 0.000 | 0.000e+00 | 25 - 38 | 3 | - | - | - |
| 3728.6702 | LVPVPITN(N)ATLDR Fuc 2 Hex 5 HexNAc 6 | 127.387 | 0.0 | -18.531 | 2.139e+07 | 25 - 38 | 2 | 36.10 | 36.27 | 36.27 |
| 3553.5530 | YFTPN(N)KTEDTIFLR Fuc 1 Hex 4 HexNAc 5 | 126.450 | 0.0 | 8.546 | 1.435e+08 | 67 - 81 | 4 | 37.06 | 37.16 | 37.76 |
| 3624.4939 | QNQC(C)FYN(N)SSYLNVQR Hex 3 HexNAc 6 | 125.899 | 0.0 | 12.080 | 1.216e+09 | 86 - 101 | 6 | 33.44 | 33.61 | 40.38 |
| 3245.4419 | YFTPN(N)KTEDTIFLR Hex 3 HexNAc 5 | 125.497 | 0.0 | 9.524 | 7.132e+08 | 67 - 81 | 8 | 35.77 | 37.59 | 41.90 |
| 3392.5103 | FYFTPN(N)KTEDTIFLR Hex 3 HexNAc 5 | 124.704 | 0.0 | 13.538 | 4.977e+07 | 66 - 81 | 6 | 46.10 | 46.27 | 47.12 |
| 3477.5795 | LVPVPITN(N)ATLDR Hex 4 HexNAc 7 | 124.569 | 0.0 | -4.877 | 6.677e+07 | 25 - 38 | 3 | 35.94 | 36.27 | 36.44 |
| 2935.3088 | TPN(N)KTEDTIFLR Hex 3 HexNAc 5 | 124.529 | 0.0 | 12.823 | 3.948e+07 | 69 - 81 | 6 | 25.82 | 26.10 | 26.83 |
| 2966.3893 | NLVPVPITN(N)ATLDR Fuc 1 Hex 3 HexNAc 4 | 123.991 | 0.0 | 15.690 | 1.625e+09 | 24 - 38 | 8 | 37.59 | 37.91 | 38.74 |
| 3525.5898 | LVPVPITN(N)ATLDR Fuc 2 Hex 5 HexNAc 5 | 123.163 | 0.0 | -4.588 | 4.601e+07 | 25 - 38 | 2 | 35.94 | 36.10 | 36.27 |
| 3421.4135 | QNQC(C)FYN(N)SSYLNVQR Hex 3 HexNAc 5 | 123.067 | 0.0 | 8.428 | 5.091e+08 | 86 - 101 | 4 | 33.61 | 33.78 | 40.38 |
| 3932.6107 | QNQC(C)FYN(N)SSYLNVQR Fuc 1 Hex 4 HexNAc 6 | 122.839 | 0.0 | 12.649 | 4.772e+08 | 86 - 101 | 5 | 33.11 | 33.27 | 39.75 |
| 3595.5904 | FYFTPN(N)KTEDTIFLR Hex 3 HexNAc 6 | 121.079 | 0.0 | 13.985 | 1.089e+08 | 66 - 81 | 5 | 45.93 | 46.10 | 47.29 |
| 3094.4466 | ANLVPVPITN(N)ATLDR Hex 3 HexNAc 5 | 119.548 | 0.0 | -3.634 | 1.695e+07 | 23 - 38 | 1 | 41.56 | 41.73 | 41.90 |
| 3422.5569 | LVPVPITN(N)ATLDR Fuc 1 Hex 4 HexNAc 6 | 117.625 | 0.0 | 0.000 | 0.000e+00 | 25 - 38 | 1 | - | - | - |
| 3813.6537 | YFTPN(N)KTEDTIFLR Hex 4 HexNAc 7 | 116.115 | 0.0 | 9.582 | 1.721e+08 | 67 - 81 | 3 | 36.27 | 36.75 | 37.76 |
| 3786.6077 | TPN(N)KTEDTIFLR Hex 7 HexNAc 6 | 115.917 | 0.0 | 14.446 | 9.777e+07 | 69 - 81 | 2 | 37.91 | 38.07 | 38.74 |
| 4064.7425 | YFTPN(N)KTEDTIFLR Fuc 2 Hex 5 HexNAc 6 | 114.294 | 0.0 | -5.323 | 3.190e+07 | 67 - 81 | 1 | 36.27 | 36.60 | 36.60 |
| 3555.5597 | YFTPN(N)KTEDTIFLR Fuc 1 Hex 4 HexNAc 5 | 113.186 | 0.0 | 0.000 | 0.000e+00 | 67 - 81 | 1 | - | - | - |
| 3395.5300 | LVPVPITN(N)ATLDR Hex 6 HexNAc 5 | 111.698 | 0.0 | 7.360 | 4.708e+07 | 25 - 38 | 5 | 35.27 | 35.77 | 36.44 |
| 3729.5293 | QNQC(C)FYN(N)SSYLNVQR Fuc 1 Hex 4 HexNAc 5 | 110.269 | 0.0 | -7.305 | 1.649e+08 | 86 - 101 | 3 | 33.27 | 33.44 | 39.75 |
| 3781.5291 | ATFFYFTPN(N)K Fuc 3 Hex 8 HexNAc 4 | 110.230 | 0.0 | -20.654 | 4.479e+06 | 63 - 73 | 1 | 53.98 | 54.15 | 54.15 |
| 3439.5892 | LVPVPITN(N)ATLDR Hex 5 HexNAc 6 | 109.967 | 0.0 | 0.000 | 0.000e+00 | 25 - 38 | 2 | - | - | - |
| 3989.6338 | QNQC(C)FYN(N)SSYLNVQR Hex 4 HexNAc 7 | 108.853 | 0.0 | 12.352 | 1.770e+08 | 86 - 101 | 2 | 32.77 | 33.44 | 33.61 |
| 3582.6173 | LVPVPITN(N)ATLDR Fuc 1 Hex 5 HexNAc 6 | 108.108 | 0.0 | -5.306 | 2.868e+07 | 25 - 38 | 1 | 35.77 | 35.94 | 36.27 |
| 4121.7664 | YFTPN(N)KTEDTIFLR Fuc 1 Hex 5 HexNAc 7 | 108.074 | 0.0 | -3.427 | 2.608e+07 | 67 - 81 | 2 | 35.94 | 36.10 | 36.27 |
| 3584.6201 | LVPVPITN(N)ATLDR Fuc 1 Hex 5 HexNAc 6 | 107.240 | 0.0 | 0.000 | 0.000e+00 | 25 - 38 | 2 | - | - | - |
| 3293.5300 | NLVPVPITN(N)ATLDR Fuc 1 Hex 5 HexNAc 4 | 106.985 | 0.0 | 0.000 | 0.000e+00 | 24 - 38 | 1 | - | - | - |
| 4178.7887 | YFTPN(N)KTEDTIFLR Hex 5 HexNAc 8 | 105.656 | 0.0 | -3.217 | 3.654e+07 | 67 - 81 | 2 | 35.77 | 35.94 | 36.10 |
| 4274.8009 | SVQEIQATFFYFTPN(N)K Hex 7 HexNAc 6 | 104.581 | 0.0 | 0.000 | 0.000e+00 | 57 - 73 | 1 | - | - | - |
| 4381.8639 | YFTPN(N)KTEDTIFLR Hex 5 HexNAc 9 | 103.919 | 0.0 | -5.018 | 1.273e+07 | 67 - 81 | 1 | 35.77 | 35.94 | 36.10 |
| 3285.4575 | FTPN(N)KTEDTIFLR Hex 3 HexNAc 6 | 103.814 | 0.0 | 14.055 | 9.863e+07 | 68 - 81 | 4 | 31.92 | 32.09 | 32.94 |
| 4192.7144 | QNQC(C)FYN(N)SSYLNVQR Hex 4 HexNAc 8 | 101.018 | 0.0 | -6.134 | 3.830e+07 | 86 - 101 | 2 | 32.77 | 32.77 | 33.27 |
| 3960.7244 | FYFTPN(N)KTEDTIFLR Hex 4 HexNAc 7 | 100.297 | 0.0 | 13.567 | 1.734e+07 | 66 - 81 | 4 | 45.25 | 45.42 | 45.93 |
| 2763.3087 | NLVPVPITN(N)ATLDR Fuc 1 Hex 3 HexNAc 3 | 98.954 | 0.0 | 14.667 | 1.029e+08 | 24 - 38 | 2 | 37.91 | 38.07 | 38.74 |
| 3023.4104 | NLVPVPITN(N)ATLDR Hex 3 HexNAc 5 | 97.567 | 0.0 | -17.981 | 6.950e+06 | 24 - 38 | 1 | 41.22 | 41.22 | 41.39 |
| 3903.7025 | FYFTPN(N)KTEDTIFLR Fuc 1 Hex 4 HexNAc 6 | 95.915 | 0.0 | -2.725 | 2.459e+07 | 66 - 81 | 4 | 45.59 | 45.76 | 46.10 |
| 3390.4887 | FTPN(N)KTEDTIFLR Fuc 1 Hex 4 HexNAc 5 | 94.923 | 0.0 | -4.668 | 1.341e+07 | 68 - 81 | 3 | 31.75 | 31.75 | 32.26 |
| 4018.7377 | LVPVPITN(N)ATLDR Hex 5 HexNAc 6 Neu5Ac 2 | 94.850 | 0.0 | 0.000 | 0.000e+00 | 25 - 38 | 1 | - | - | - |
| 3700.6209 | FYFTPN(N)KTEDTIFLR Fuc 1 Hex 4 HexNAc 5 | 94.612 | 0.0 | 12.623 | 1.606e+07 | 66 - 81 | 5 | 45.76 | 45.93 | 46.44 |
| 3151.3776 | PVPITN(N)ATLDR Fuc 2 Hex 4 HexNAc 5 | 93.807 | 0.0 | -20.261 | 5.946e+06 | 27 - 38 | 2 | 40.55 | 40.55 | 40.71 |
| 3243.4232 | TPN(N)KTEDTIFLR Fuc 1 Hex 4 HexNAc 5 | 93.120 | 0.0 | -4.903 | 6.750e+06 | 69 - 81 | 2 | 25.53 | 25.53 | 25.82 |
| 3082.3781 | FTPN(N)KTEDTIFLR Hex 3 HexNAc 5 | 92.793 | 0.0 | 13.789 | 4.580e+07 | 68 - 81 | 4 | 32.09 | 32.26 | 32.77 |
| 3094.3068 | FYN(N)SSYLNVQR Hex 3 HexNAc 6 | 91.913 | 0.0 | -3.668 | 1.001e+07 | 90 - 101 | 3 | 29.57 | 29.74 | 30.08 |
| 3446.9585 | TPN(N)KTEDTIFLR Fuc 1 Hex 4 HexNAc 6 | 87.840 | 0.0 | 5.010 | 1.331e+07 | 69 - 81 | 22 | 25.19 | 25.36 | 28.94 |
| 3785.6918 | LVPVPITN(N)ATLDR Fuc 1 Hex 5 HexNAc 7 | 86.117 | 0.0 | -5.551 | 1.907e+07 | 25 - 38 | 2 | 35.77 | 36.10 | 36.27 |
| 3104.3946 | VPVPITN(N)ATLDR Fuc 1 Hex 4 HexNAc 5 | 81.676 | 0.0 | -4.929 | 2.225e+06 | 26 - 38 | 1 | 29.74 | 29.91 | 30.08 |
| 3962.5855 | QNQC(C)FYN(N)SSYLNVQR Hex 4 HexNAc 4 Neu5Ac 2 | 80.271 | 0.0 | -4.449 | 5.631e+07 | 86 - 101 | 1 | 34.25 | 34.25 | 34.59 |
| 2947.2367 | YN(N)SSYLNVQR Hex 3 HexNAc 6 | 79.472 | 0.0 | 11.889 | 1.718e+07 | 91 - 101 | 4 | 22.49 | 22.64 | 22.88 |
| 3593.5694 | FTPN(N)KTEDTIFLR Fuc 1 Hex 4 HexNAc 6 | 79.178 | 0.0 | -3.999 | 1.919e+07 | 68 - 81 | 4 | 31.41 | 31.58 | 31.92 |
| 4599.0894 | SVQEIQATFFYFTPN(N)KTEDTIFLR Hex 3 HexNAc 6 | 70.206 | 0.0 | -18.853 | 1.963e+07 | 57 - 81 | 2 | 62.70 | 62.87 | 62.87 |
| 3545.5646 | C(C)ANLVPVPITN(N)ATLDR Hex 3 HexNAc 5 Neu5Ac 1 | 66.620 | 0.0 | 15.853 | 2.076e+08 | 22 - 38 | 7 | 52.11 | 52.45 | 53.13 |
| 4052.7234 | SVQEIQATFFYFTPN(N)K Fuc 1 Hex 6 HexNAc 5 | 61.267 | 0.0 | -5.060 | 7.248e+06 | 57 - 73 | 2 | 53.47 | 53.64 | 53.81 |
| 4395.9988 | SVQEIQATFFYFTPN(N)KTEDTIFLR Hex 3 HexNAc 5 | 59.720 | 0.0 | -5.168 | 9.126e+06 | 57 - 81 | 2 | 63.04 | 63.04 | 63.35 |
| 3978.6970 | SVQEIQATFFYFTPN(N)K Fuc 1 Hex 5 HexNAc 4 Neu5Ac 1 | 59.282 | 0.0 | -4.613 | 8.019e+06 | 57 - 73 | 2 | 54.99 | 55.16 | 55.33 |
| 3051.3983 | C(C)ANLVPVPITN(N)ATLDR Hex 3 HexNAc 4 | 58.582 | 0.0 | -4.527 | 1.166e+07 | 22 - 38 | 2 | 42.40 | 42.57 | 42.74 |
| 4353.8470 | SVQEIQATFFYFTPN(N)K Hex 5 HexNAc 8 | 54.409 | 0.0 | 10.857 | 1.624e+07 | 57 - 73 | 1 | 52.79 | 52.96 | 53.47 |
| 3189.4301 | FYFTPN(N)KTEDTIFLR Hex 3 HexNAc 4 | 51.734 | 0.0 | -3.878 | 6.693e+06 | 66 - 81 | 1 | 46.44 | 46.61 | 46.95 |
| 4325.8623 | FYFTPN(N)KTEDTIFLR Fuc 5 Hex 3 HexNAc 6 | 48.127 | 0.0 | -21.295 | 2.419e+06 | 66 - 81 | 1 | 44.76 | 44.76 | 44.92 |
| 3996.8439 | QIPLC(C)ANLVPVPITN(N)ATLDR Hex 3 HexNAc 5 Neu5Ac 1 | 48.002 | 0.0 | 15.924 | 3.765e+08 | 18 - 38 | 13 | 61.50 | 62.36 | 63.70 |
| 4328.8204 | SVQEIQATFFYFTPN(N)K Fuc 4 Hex 5 HexNAc 5 | 47.851 | 0.0 | -18.399 | 7.249e+06 | 57 - 73 | 1 | 53.98 | 53.98 | 54.15 |
| 3788.6589 | SVQEIQATFFYFTPN(N)K Hex 4 HexNAc 6 | 46.690 | 0.0 | 0.000 | 0.000e+00 | 57 - 73 | 1 | - | - | - |
| 4922.9747 | QNQC(C)FYN(N)SSYLNVQR Fuc 5 Hex 4 HexNAc 8 | 45.810 | 0.0 | 12.549 | 2.911e+07 | 86 - 101 | 5 | 30.08 | 30.25 | 30.76 |
| 4327.8112 | SVQEIQATFFYFTPN(N)K Fuc 2 Hex 5 HexNAc 5 Neu5Ac 1 | 45.558 | 0.0 | -19.510 | 2.078e+06 | 57 - 73 | 1 | 54.32 | 54.32 | 54.32 |
| 4240.7120 | QNQC(C)FYN(N)SSYLNVQR Fuc 2 Hex 5 HexNAc 6 | 44.281 | 0.0 | -17.948 | 1.531e+07 | 86 - 101 | 2 | 32.94 | 32.94 | 32.94 |
| 3169.4671 | NLVPVPITN(N)ATLDR Fuc 1 Hex 3 HexNAc 5 | 43.932 | 0.0 | -18.232 | 3.757e+07 | 24 - 38 | 1 | 37.59 | 37.59 | 37.76 |
| 3625.4824 | QN(D)QC(C)FYN(N)SSYLNVQR Hex 3 HexNAc 6 | 43.048 | 0.0 | -4.578 | 3.667e+08 | 86 - 101 | 7 | 34.25 | 34.76 | 41.39 |
| 2964.2785 | PVPITN(N)ATLDR Fuc 1 Hex 5 HexNAc 4 | 42.913 | 0.0 | -18.162 | 1.673e+07 | 27 - 38 | 1 | 37.29 | 37.29 | 37.29 |
| 3331.5307 | NLVPVPITN(N)ATLDR Fuc 1 Hex 4 HexNAc 5 | 42.844 | 0.0 | -19.647 | 1.742e+07 | 24 - 38 | 1 | 37.45 | 37.45 | 37.59 |
| 4354.7686 | QNQC(C)FYN(N)SSYLNVQR Hex 5 HexNAc 8 | 42.001 | 0.0 | -20.724 | 9.768e+06 | 86 - 101 | 1 | 32.43 | 32.60 | 32.60 |
| 3705.7541 | QIPLC(C)ANLVPVPITN(N)ATLDR Hex 3 HexNAc 5 | 42.000 | 0.0 | 13.423 | 3.603e+08 | 18 - 38 | 10 | 62.36 | 63.04 | 64.34 |
| 4442.8876 | SVQEIQATFFYFTPN(N)K Fuc 2 Hex 5 HexNAc 7 | 41.302 | 0.0 | -5.122 | 3.696e+06 | 57 - 73 | 1 | 53.30 | 53.47 | 53.64 |
| 3933.5943 | QN(D)QC(C)FYN(N)SSYLNVQR Fuc 1 Hex 4 HexNAc 6 | 40.442 | 0.0 | -5.799 | 4.400e+07 | 86 - 101 | 3 | 34.25 | 34.42 | 34.59 |
| 5247.3021 | SVQEIQATFFYFTPN(N)KTEDTIFLR Hex 7 HexNAc 6 | 40.389 | 0.0 | 11.495 | 1.280e+07 | 57 - 81 | 3 | 61.67 | 62.02 | 62.70 |
| 2879.3004 | FTPN(N)KTEDTIFLR Hex 3 HexNAc 4 | 40.095 | 0.0 | -4.747 | 5.004e+06 | 68 - 81 | 1 | 32.43 | 32.43 | 32.77 |
| 3816.6742 | LVPVPITN(N)ATLDR Fuc 2 Hex 5 HexNAc 5 Neu5Ac 1 | 40.073 | 0.0 | 0.000 | 0.000e+00 | 25 - 38 | 1 | - | - | - |
| 4500.8286 | QNQC(C)FYN(N)SSYLNVQR Fuc 1 Hex 5 HexNAc 8 | 39.930 | 0.0 | -20.997 | 4.676e+06 | 86 - 101 | 1 | 32.43 | 32.60 | 32.60 |
| 4268.8159 | LVPVPITN(N)ATLDR Hex 6 HexNAc 5 Neu5Ac 3 | 39.736 | 0.0 | 10.305 | 8.451e+06 | 25 - 38 | 3 | 44.92 | 45.42 | 45.59 |
| 3650.5919 | FTPN(N)KTEDTIFLR Hex 4 HexNAc 7 | 37.830 | 0.0 | -4.996 | 1.107e+07 | 68 - 81 | 4 | 31.24 | 31.41 | 31.75 |
| 3266.4784 | LVPVPITN(N)ATLDR Fuc 1 Hex 5 HexNAc 3 Neu5Ac 1 | 37.675 | 0.0 | 0.000 | 0.000e+00 | 25 - 38 | 1 | - | - | - |
| 5126.0518 | QNQC(C)FYN(N)SSYLNVQR Fuc 5 Hex 4 HexNAc 9 | 36.363 | 0.0 | -3.671 | 1.868e+07 | 86 - 101 | 3 | 29.91 | 29.91 | 30.25 |
| 3610.5771 | YFTPN(N)KTEDTIFLR Hex 4 HexNAc 6 | 36.078 | 0.0 | -19.837 | 2.829e+07 | 67 - 81 | 1 | 37.59 | 37.59 | 37.76 |
| 4499.9108 | SVQEIQATFFYFTPN(N)K Fuc 1 Hex 5 HexNAc 8 | 35.954 | 0.0 | -21.316 | 7.234e+05 | 57 - 73 | 1 | 52.79 | 52.79 | 52.79 |
| 3271.5036 | ANLVPVPITN(N)ATLDR Fuc 2 Hex 3 HexNAc 3 Neu5Ac 1 | 34.695 | 0.003 | 11.681 | 7.739e+07 | 23 - 38 | 3 | 42.07 | 42.24 | 42.91 |
| 3139.3882 | TPN(N)KTEDTIFLR Hex 3 HexNAc 6 | 34.480 | 0.003 | 0.000 | 0.000e+00 | 69 - 81 | 1 | - | - | - |
| 4013.8668 | QIPLC(C)ANLVPVPITN(N)ATLDR Fuc 1 Hex 4 HexNAc 5 | 34.207 | 0.003 | 13.817 | 1.036e+08 | 18 - 38 | 8 | 61.67 | 62.36 | 63.18 |
| 4907.1965 | SVQEIQATFFYFTPN(N)KTEDTIFLR Fuc 1 Hex 4 HexNAc 6 | 33.288 | 0.005 | -21.355 | 1.949e+06 | 57 - 81 | 1 | 62.53 | 62.53 | 62.53 |
| 4006.6476 | QN(D)QC(C)FYN(N)SSYLNVQR Fuc 4 Hex 3 HexNAc 5 | 32.151 | 0.007 | 12.979 | 6.169e+07 | 86 - 101 | 2 | 32.94 | 33.11 | 33.61 |
| 3563.5725 | C(C)AN(D)LVPVPITN(N)ATLDR Fuc 1 Hex 4 HexNAc 5 | 31.351 | 0.012 | 10.994 | 1.838e+07 | 22 - 38 | 3 | 43.59 | 43.76 | 44.25 |
| 3404.4929 | PVPITN(N)ATLDR Fuc 2 Hex 5 HexNAc 4 Neu5Ac 1 | 31.032 | 0.012 | 0.000 | 0.000e+00 | 27 - 38 | 1 | - | - | - |
| 2732.2302 | TPN(N)KTEDTIFLR Hex 3 HexNAc 4 | 30.885 | 0.013 | 9.749 | 5.844e+06 | 69 - 81 | 2 | 26.21 | 26.21 | 26.70 |
| 3746.5021 | QN(D)QC(C)FYN(N)SSYLNVQR Hex 5 HexNAc 5 | 30.679 | 0.013 | -20.018 | 2.247e+07 | 86 - 101 | 1 | 39.58 | 39.58 | 39.58 |
| 3314.3578 | ATFFYFTPN(N)K Fuc 2 Hex 6 HexNAc 4 | 30.549 | 0.013 | 0.000 | 0.000e+00 | 63 - 73 | 1 | - | - | - |
| 4719.8938 | QNQC(C)FYN(N)SSYLNVQR Hex 6 HexNAc 9 | 30.136 | 0.017 | -4.102 | 7.286e+06 | 86 - 101 | 3 | 30.25 | 30.42 | 30.59 |
| 2744.1583 | YN(N)SSYLNVQR Hex 3 HexNAc 5 | 29.624 | 0.020 | 11.137 | 7.261e+06 | 91 - 101 | 2 | 22.67 | 22.83 | 22.88 |
| 3255.4610 | C(C)AN(D)LVPVPITN(N)ATLDR Hex 3 HexNAc 5 | 28.659 | 0.022 | -20.279 | 3.395e+07 | 22 - 38 | 1 | 44.25 | 44.25 | 44.42 |
| 3608.4572 | QC(C)FYN(N)SSYLNVQR Fuc 1 Hex 6 HexNAc 4 | 28.130 | 0.027 | 13.816 | 5.638e+08 | 88 - 101 | 3 | 40.23 | 40.38 | 41.90 |
| 3402.4172 | FYN(N)SSYLNVQR Fuc 1 Hex 4 HexNAc 6 | 27.746 | 0.029 | 13.271 | 4.676e+06 | 90 - 101 | 3 | 29.08 | 29.25 | 29.41 |
| 3255.4228 | PVPITN(N)ATLDR Fuc 1 Hex 5 HexNAc 4 Neu5Ac 1 | 26.646 | 0.037 | 13.701 | 4.140e+08 | 27 - 38 | 5 | 36.27 | 36.60 | 36.90 |
| 3219.4840 | LVPVPITN(N)ATLDR Fuc 1 Hex 4 HexNAc 5 | 26.548 | 0.038 | 0.000 | 0.000e+00 | 25 - 38 | 2 | - | - | - |
| 4034.7931 | ANLVPVPITN(N)ATLDR Fuc 2 Hex 7 HexNAc 5 | 26.470 | 0.039 | -3.881 | 4.622e+07 | 23 - 38 | 4 | 62.19 | 62.36 | 62.70 |
| 3541.5832 | LVPVPITN(N)ATLDR Fuc 1 Hex 6 HexNAc 5 | 26.343 | 0.039 | 0.000 | 0.000e+00 | 25 - 38 | 1 | - | - | - |
| 3620.5069 | YN(N)SSYLNVQR Fuc 1 Hex 5 HexNAc 7 | 26.309 | 0.039 | -19.149 | 1.203e+07 | 91 - 101 | 3 | 41.39 | 41.39 | 41.39 |
| 4557.8493 | QNQC(C)FYN(N)SSYLNVQR Fuc 5 Hex 3 HexNAc 7 | 25.997 | 0.039 | -19.546 | 8.253e+06 | 86 - 101 | 1 | 32.26 | 32.43 | 32.43 |
| 3948.5661 | QNQC(C)FYN(N)SSYLNVQR Hex 5 HexNAc 6 | 25.391 | 0.042 | -4.088 | 6.450e+07 | 86 - 101 | 3 | 39.24 | 39.41 | 39.58 |
| 3901.6800 | FTPN(N)KTEDTIFLR Fuc 2 Hex 5 HexNAc 6 | 25.316 | 0.042 | -19.092 | 2.793e+06 | 68 - 81 | 2 | 31.07 | 31.24 | 31.24 |

### YFTPN(N)K Hex 3 HexNAc 6

**Mass:**  2473.0180

(1.102e-06)

**Charge States:** 2, 3

**Start Position**: 68

**End Position**: 74

| Profile Score | Tandem Score | TDA q-value | Volume | Spectrum Matches |
| --- | --- | --- | --- | --- |
| 14.1375 | 188.8985 | 0.0000 | 3.1074e+08 | 5 |

**Adduct Signal Fractions**

- **Unmodified**: 3.1074e+08

### YFTPN(N)K Fuc 1 Hex 4 HexNAc 6

**Mass:**  2781.1280

(9.012e-07)

**Charge States:** 3

**Start Position**: 68

**End Position**: 74

| Profile Score | Tandem Score | TDA q-value | Volume | Spectrum Matches |
| --- | --- | --- | --- | --- |
| -4.3227 | 186.5559 | 0.0000 | 4.9309e+07 | 3 |

**Adduct Signal Fractions**

- **Unmodified**: 4.9309e+07

### FYFTPN(N)K Hex 3 HexNAc 6

**Mass:**  2620.0854

(6.577e-07)

**Charge States:** 2, 3

**Start Position**: 67

**End Position**: 74

| Profile Score | Tandem Score | TDA q-value | Volume | Spectrum Matches |
| --- | --- | --- | --- | --- |
| 13.1581 | 183.0354 | 0.0000 | 1.1546e+08 | 11 |

**Adduct Signal Fractions**

- **Unmodified**: 1.1546e+08

### YFTPN(N)K Fuc 1 Hex 4 HexNAc 5

**Mass:**  2578.0492

(2.518e-06)

**Charge States:** 3

**Start Position**: 68

**End Position**: 74

| Profile Score | Tandem Score | TDA q-value | Volume | Spectrum Matches |
| --- | --- | --- | --- | --- |
| -19.2955 | 181.0960 | 0.0000 | 3.4123e+07 | 2 |

**Adduct Signal Fractions**

- **Unmodified**: 3.4123e+07

### NEEYN(N)K Hex 3 HexNAc 5

**Mass:**  2296.8960

(-2.573e-07)

**Charge States:** 2, 3

**Start Position**: 52

**End Position**: 58

| Profile Score | Tandem Score | TDA q-value | Volume | Spectrum Matches |
| --- | --- | --- | --- | --- |
| 10.7175 | 180.6881 | 0.0000 | 3.1170e+07 | 7 |

**Adduct Signal Fractions**

- **Unmodified**: 3.1170e+07

### NEEYN(N)K Fuc 1 Hex 4 HexNAc 5

**Mass:**  2605.0080

(1.805e-06)

**Charge States:** 3

**Start Position**: 52

**End Position**: 58

| Profile Score | Tandem Score | TDA q-value | Volume | Spectrum Matches |
| --- | --- | --- | --- | --- |
| 11.0349 | 178.9503 | 0.0000 | 1.2594e+07 | 4 |

**Adduct Signal Fractions**

- **Unmodified**: 1.2594e+07

### SVQEIQATFFYFTPN(N)K Hex 4 HexNAc 7

**Mass:**  3988.7204

(1.798e-06)

**Charge States:** 3, 4

**Start Position**: 58

**End Position**: 74

| Profile Score | Tandem Score | TDA q-value | Volume | Spectrum Matches |
| --- | --- | --- | --- | --- |
| 10.8008 | 173.3012 | 0.0000 | 2.3053e+08 | 10 |

**Adduct Signal Fractions**

- **Unmodified**: 2.3053e+08

### YFTPN(N)K Hex 4 HexNAc 7

**Mass:**  2838.1501

(1.098e-06)

**Charge States:** 3

**Start Position**: 68

**End Position**: 74

| Profile Score | Tandem Score | TDA q-value | Volume | Spectrum Matches |
| --- | --- | --- | --- | --- |
| -5.1332 | 173.0297 | 0.0000 | 2.4780e+07 | 1 |

**Adduct Signal Fractions**

- **Unmodified**: 2.4780e+07

### YFTPN(N)K Hex 3 HexNAc 5

**Mass:**  2269.9379

(6.187e-07)

**Charge States:** 2, 3

**Start Position**: 68

**End Position**: 74

| Profile Score | Tandem Score | TDA q-value | Volume | Spectrum Matches |
| --- | --- | --- | --- | --- |
| 12.4864 | 172.7184 | 0.0000 | 1.9518e+08 | 5 |

**Adduct Signal Fractions**

- **Unmodified**: 1.9518e+08

### YFTPN(N)K Hex 3 HexNAc 4

**Mass:**  2066.8585

(9.695e-07)

**Charge States:** 2, 3

**Start Position**: 68

**End Position**: 74

| Profile Score | Tandem Score | TDA q-value | Volume | Spectrum Matches |
| --- | --- | --- | --- | --- |
| -19.0961 | 172.5173 | 0.0000 | 2.5133e+07 | 3 |

**Adduct Signal Fractions**

- **Unmodified**: 2.5133e+07

### ATFFYFTPN(N)K Hex 3 HexNAc 6

**Mass:**  2939.2408

(1.068e-06)

**Charge States:** 3

**Start Position**: 64

**End Position**: 74

| Profile Score | Tandem Score | TDA q-value | Volume | Spectrum Matches |
| --- | --- | --- | --- | --- |
| -3.4085 | 172.1543 | 0.0000 | 3.9688e+07 | 3 |

**Adduct Signal Fractions**

- **Unmodified**: 3.9688e+07

### SVQEIQATFFYFTPN(N)K Fuc 1 Hex 4 HexNAc 5

**Mass:**  3728.6182

(1.723e-06)

**Charge States:** 3, 4

**Start Position**: 58

**End Position**: 74

| Profile Score | Tandem Score | TDA q-value | Volume | Spectrum Matches |
| --- | --- | --- | --- | --- |
| 13.0932 | 171.6062 | 0.0000 | 1.7703e+08 | 8 |

**Adduct Signal Fractions**

- **Unmodified**: 1.7703e+08

### FYFTPN(N)K Hex 3 HexNAc 5

**Mass:**  2417.0062

(7.675e-07)

**Charge States:** 2, 3

**Start Position**: 67

**End Position**: 74

| Profile Score | Tandem Score | TDA q-value | Volume | Spectrum Matches |
| --- | --- | --- | --- | --- |
| 13.0585 | 170.5224 | 0.0000 | 6.6595e+07 | 11 |

**Adduct Signal Fractions**

- **Unmodified**: 6.6595e+07

### SVQEIQATFFYFTPN(N)K Hex 3 HexNAc 5

**Mass:**  3420.5055

(1.649e-06)

**Charge States:** 2, 3, 4

**Start Position**: 58

**End Position**: 74

| Profile Score | Tandem Score | TDA q-value | Volume | Spectrum Matches |
| --- | --- | --- | --- | --- |
| 14.8623 | 168.3189 | 0.0000 | 6.0272e+08 | 29 |

**Adduct Signal Fractions**

- **Unmodified**: 6.0272e+08

### SVQEIQATFFYFTPN(N)K Hex 3 HexNAc 6

**Mass:**  3623.5856

(2.220e-06)

**Charge States:** 3, 4, 5

**Start Position**: 58

**End Position**: 74

| Profile Score | Tandem Score | TDA q-value | Volume | Spectrum Matches |
| --- | --- | --- | --- | --- |
| 14.6236 | 167.7075 | 0.0000 | 1.0604e+09 | 25 |

**Adduct Signal Fractions**

- **Unmodified**: 1.0604e+09

### SVQEIQATFFYFTPN(N)K Fuc 1 Hex 4 HexNAc 6

**Mass:**  3931.7004

(1.412e-06)

**Charge States:** 3, 4, 5

**Start Position**: 58

**End Position**: 74

| Profile Score | Tandem Score | TDA q-value | Volume | Spectrum Matches |
| --- | --- | --- | --- | --- |
| 13.2533 | 165.6330 | 0.0000 | 2.8839e+08 | 12 |

**Adduct Signal Fractions**

- **Unmodified**: 2.8839e+08

### NEEYN(N)K Hex 3 HexNAc 4

**Mass:**  2093.8172

(4.759e-07)

**Charge States:** 2, 3

**Start Position**: 52

**End Position**: 58

| Profile Score | Tandem Score | TDA q-value | Volume | Spectrum Matches |
| --- | --- | --- | --- | --- |
| 9.5670 | 163.3281 | 0.0000 | 3.4634e+07 | 10 |

**Adduct Signal Fractions**

- **Unmodified**: 3.4634e+07

### LVPVPITN(N)ATLDR Hex 3 HexNAc 5

**Mass:**  2909.3670

(1.843e-06)

**Charge States:** 2, 3, 4

**Start Position**: 26

**End Position**: 39

| Profile Score | Tandem Score | TDA q-value | Volume | Spectrum Matches |
| --- | --- | --- | --- | --- |
| 10.9014 | 161.7919 | 0.0000 | 1.2706e+10 | 426 |

**Adduct Signal Fractions**

- **Unmodified**: 1.2706e+10

### YFTPN(N)K Hex 4 HexNAc 7

**Mass:**  2840.1601

(7.091e-04)

**Start Position**: 68

**End Position**: 74

| Profile Score | Tandem Score | TDA q-value | Volume | Spectrum Matches |
| --- | --- | --- | --- | --- |
| 0.0000 | 160.5392 | 0.0000 | 0.0000e+00 | 1 |

Adduct:

No Chromatogram Found

### LVPVPITN(N)ATLDR Hex 3 HexNAc 4

**Mass:**  2706.2854

(3.722e-04)

**Charge States:** 2, 3, 4

**Start Position**: 26

**End Position**: 39

| Profile Score | Tandem Score | TDA q-value | Volume | Spectrum Matches |
| --- | --- | --- | --- | --- |
| 14.1807 | 159.3150 | 0.0000 | 1.1172e+09 | 11 |

**Adduct Signal Fractions**

- **Unmodified**: 1.1172e+09

### LVPVPITN(N)ATLDR Fuc 1 Hex 4 HexNAc 5

**Mass:**  3217.4771

(3.030e-07)

**Charge States:** 2, 3, 4

**Start Position**: 26

**End Position**: 39

| Profile Score | Tandem Score | TDA q-value | Volume | Spectrum Matches |
| --- | --- | --- | --- | --- |
| 14.9787 | 158.6879 | 0.0000 | 3.6019e+09 | 20 |

**Adduct Signal Fractions**

- **Unmodified**: 3.6019e+09

### SVQEIQATFFYFTPN(N)K Fuc 2 Hex 5 HexNAc 6

**Mass:**  4239.8078

(1.255e-06)

**Charge States:** 3, 4

**Start Position**: 58

**End Position**: 74

| Profile Score | Tandem Score | TDA q-value | Volume | Spectrum Matches |
| --- | --- | --- | --- | --- |
| 13.4989 | 157.9036 | 0.0000 | 4.5572e+07 | 4 |

**Adduct Signal Fractions**

- **Unmodified**: 4.5572e+07

### ATFFYFTPN(N)K Hex 3 HexNAc 5

**Mass:**  2736.1615

(1.732e-06)

**Charge States:** 2, 3

**Start Position**: 64

**End Position**: 74

| Profile Score | Tandem Score | TDA q-value | Volume | Spectrum Matches |
| --- | --- | --- | --- | --- |
| -4.0848 | 156.4640 | 0.0000 | 2.2914e+07 | 3 |

**Adduct Signal Fractions**

- **Unmodified**: 2.2914e+07

### LVPVPITN(N)ATLDR Hex 4 HexNAc 5

**Mass:**  3072.4264

(6.558e-04)

**Start Position**: 26

**End Position**: 39

| Profile Score | Tandem Score | TDA q-value | Volume | Spectrum Matches |
| --- | --- | --- | --- | --- |
| 0.0000 | 155.8538 | 0.0000 | 0.0000e+00 | 2 |

Adduct:

No Chromatogram Found

### SVQEIQATFFYFTPN(N)K Hex 3 HexNAc 4

**Mass:**  3217.4265

(9.472e-07)

**Charge States:** 3, 4

**Start Position**: 58

**End Position**: 74

| Profile Score | Tandem Score | TDA q-value | Volume | Spectrum Matches |
| --- | --- | --- | --- | --- |
| 12.8068 | 153.4321 | 0.0000 | 7.6558e+07 | 8 |

**Adduct Signal Fractions**

- **Unmodified**: 7.6558e+07

### SVQEIQATFFYFTPN(N)K Hex 7 HexNAc 6

**Mass:**  4271.7985

(1.674e-06)

**Charge States:** 3, 4

**Start Position**: 58

**End Position**: 74

| Profile Score | Tandem Score | TDA q-value | Volume | Spectrum Matches |
| --- | --- | --- | --- | --- |
| 12.6216 | 153.2095 | 0.0000 | 4.8446e+07 | 1 |

**Adduct Signal Fractions**

- **Unmodified**: 4.8446e+07

### LVPVPITN(N)ATLDR Hex 4 HexNAc 5

**Mass:**  3071.4219

(-5.939e-08)

**Charge States:** 3

**Start Position**: 26

**End Position**: 39

| Profile Score | Tandem Score | TDA q-value | Volume | Spectrum Matches |
| --- | --- | --- | --- | --- |
| -3.8613 | 150.1016 | 0.0000 | 8.0522e+07 | 3 |

**Adduct Signal Fractions**

- **Unmodified**: 8.0522e+07

### SVQEIQATFFYFTPN(N)K Hex 6 HexNAc 5

**Mass:**  3906.6689

(1.090e-06)

**Charge States:** 3, 4

**Start Position**: 58

**End Position**: 74

| Profile Score | Tandem Score | TDA q-value | Volume | Spectrum Matches |
| --- | --- | --- | --- | --- |
| 13.1017 | 148.5536 | 0.0000 | 3.7369e+07 | 4 |

**Adduct Signal Fractions**

- **Unmodified**: 3.7369e+07

### SVQEIQATFFYFTPN(N)K Hex 4 HexNAc 8

**Mass:**  4191.7995

(1.848e-06)

**Charge States:** 3, 4

**Start Position**: 58

**End Position**: 74

| Profile Score | Tandem Score | TDA q-value | Volume | Spectrum Matches |
| --- | --- | --- | --- | --- |
| 13.1443 | 147.4205 | 0.0000 | 2.6034e+07 | 4 |

**Adduct Signal Fractions**

- **Unmodified**: 2.6034e+07

### LVPVPITN(N)ATLDR Hex 4 HexNAc 6

**Mass:**  3274.4990

(1.531e-06)

**Charge States:** 3, 4

**Start Position**: 26

**End Position**: 39

| Profile Score | Tandem Score | TDA q-value | Volume | Spectrum Matches |
| --- | --- | --- | --- | --- |
| 9.6723 | 146.6547 | 0.0000 | 7.5457e+08 | 12 |

**Adduct Signal Fractions**

- **Unmodified**: 7.5457e+08

### TPN(N)KTEDTIFLR Hex 3 HexNAc 6

**Mass:**  3138.3883

(6.401e-07)

**Charge States:** 3, 4

**Start Position**: 70

**End Position**: 82

| Profile Score | Tandem Score | TDA q-value | Volume | Spectrum Matches |
| --- | --- | --- | --- | --- |
| 12.3900 | 146.2525 | 0.0000 | 4.9653e+07 | 6 |

**Adduct Signal Fractions**

- **Unmodified**: 4.9653e+07

### LVPVPITN(N)ATLDR Hex 3 HexNAc 6

**Mass:**  3112.4463

(-1.774e-07)

**Charge States:** 3, 4

**Start Position**: 26

**End Position**: 39

| Profile Score | Tandem Score | TDA q-value | Volume | Spectrum Matches |
| --- | --- | --- | --- | --- |
| 10.8885 | 145.9769 | 0.0000 | 2.9009e+08 | 4 |

**Adduct Signal Fractions**

- **Unmodified**: 2.9009e+08

### LVPVPITN(N)ATLDR Fuc 1 Hex 3 HexNAc 5

**Mass:**  3056.4280

(3.293e-04)

**Start Position**: 26

**End Position**: 39

| Profile Score | Tandem Score | TDA q-value | Volume | Spectrum Matches |
| --- | --- | --- | --- | --- |
| 0.0000 | 145.0290 | 0.0000 | 0.0000e+00 | 1 |

Adduct:

No Chromatogram Found

### SVQEIQATFFYFTPN(N)K Fuc 1 Hex 4 HexNAc 7

**Mass:**  4134.7770

(1.372e-06)

**Charge States:** 3, 4

**Start Position**: 58

**End Position**: 74

| Profile Score | Tandem Score | TDA q-value | Volume | Spectrum Matches |
| --- | --- | --- | --- | --- |
| -4.6729 | 143.6086 | 0.0000 | 1.1160e+07 | 3 |

**Adduct Signal Fractions**

- **Unmodified**: 1.1160e+07

### SVQEIQATFFYFTPN(N)K Hex 5 HexNAc 9

**Mass:**  4556.9333

(1.810e-06)

**Charge States:** 3, 4

**Start Position**: 58

**End Position**: 74

| Profile Score | Tandem Score | TDA q-value | Volume | Spectrum Matches |
| --- | --- | --- | --- | --- |
| -20.4051 | 143.3341 | 0.0000 | 2.0191e+06 | 1 |

**Adduct Signal Fractions**

- **Unmodified**: 2.0191e+06

### LVPVPITN(N)ATLDR Fuc 1 Hex 3 HexNAc 5

**Mass:**  3055.4275

(1.833e-06)

**Charge States:** 3, 4

**Start Position**: 26

**End Position**: 39

| Profile Score | Tandem Score | TDA q-value | Volume | Spectrum Matches |
| --- | --- | --- | --- | --- |
| -19.2322 | 142.7256 | 0.0000 | 2.3370e+07 | 2 |

**Adduct Signal Fractions**

- **Unmodified**: 2.3370e+07

### LVPVPITN(N)ATLDR Hex 3 HexNAc 6

**Mass:**  3114.4529

(6.457e-04)

**Start Position**: 26

**End Position**: 39

| Profile Score | Tandem Score | TDA q-value | Volume | Spectrum Matches |
| --- | --- | --- | --- | --- |
| 0.0000 | 142.7055 | 0.0000 | 0.0000e+00 | 1 |

Adduct:

No Chromatogram Found

### LVPVPITN(N)ATLDR Fuc 1 Hex 4 HexNAc 6

**Mass:**  3420.5571

(1.739e-06)

**Charge States:** 3, 4

**Start Position**: 26

**End Position**: 39

| Profile Score | Tandem Score | TDA q-value | Volume | Spectrum Matches |
| --- | --- | --- | --- | --- |
| 13.2307 | 142.2031 | 0.0000 | 1.1184e+08 | 4 |

**Adduct Signal Fractions**

- **Unmodified**: 1.1184e+08

### C(C)ANLVPVPITN(N)ATLDR Fuc 1 Hex 4 HexNAc 5

**Mass:**  3562.5895

(1.876e-06)

**Charge States:** 3, 4

**Start Position**: 23

**End Position**: 39

| Profile Score | Tandem Score | TDA q-value | Volume | Spectrum Matches |
| --- | --- | --- | --- | --- |
| -4.5866 | 140.9250 | 0.0000 | 5.4304e+07 | 4 |

**Adduct Signal Fractions**

- **Unmodified**: 5.4304e+07

### ATFFYFTPN(N)K Fuc 1 Hex 4 HexNAc 5

**Mass:**  3044.2692

(2.877e-07)

**Charge States:** 3

**Start Position**: 64

**End Position**: 74

| Profile Score | Tandem Score | TDA q-value | Volume | Spectrum Matches |
| --- | --- | --- | --- | --- |
| -17.4436 | 140.3602 | 0.0000 | 5.6464e+06 | 1 |

**Adduct Signal Fractions**

- **Unmodified**: 5.6464e+06

### SVQEIQATFFYFTPN(N)K Fuc 1 Hex 5 HexNAc 7

**Mass:**  4296.8303

(5.712e-07)

**Charge States:** 3, 4

**Start Position**: 58

**End Position**: 74

| Profile Score | Tandem Score | TDA q-value | Volume | Spectrum Matches |
| --- | --- | --- | --- | --- |
| 10.0804 | 139.2859 | 0.0000 | 3.2234e+07 | 3 |

**Adduct Signal Fractions**

- **Unmodified**: 3.2234e+07

### SVQEIQATFFYFTPN(N)K Hex 4 HexNAc 6

**Mass:**  3785.6372

(1.634e-06)

**Charge States:** 3, 4

**Start Position**: 58

**End Position**: 74

| Profile Score | Tandem Score | TDA q-value | Volume | Spectrum Matches |
| --- | --- | --- | --- | --- |
| 11.4996 | 137.5905 | 0.0000 | 3.3274e+07 | 5 |

**Adduct Signal Fractions**

- **Unmodified**: 3.3274e+07

### YFTPN(N)KTEDTIFLR Fuc 1 Hex 4 HexNAc 6

**Mass:**  3756.6308

(1.358e-06)

**Charge States:** 3, 4

**Start Position**: 68

**End Position**: 82

| Profile Score | Tandem Score | TDA q-value | Volume | Spectrum Matches |
| --- | --- | --- | --- | --- |
| -3.7464 | 137.4253 | 0.0000 | 2.1143e+08 | 8 |

**Adduct Signal Fractions**

- **Unmodified**: 2.1143e+08

### VPVPITN(N)ATLDR Hex 3 HexNAc 5

**Mass:**  2796.2825

(9.422e-07)

**Charge States:** 3

**Start Position**: 27

**End Position**: 39

| Profile Score | Tandem Score | TDA q-value | Volume | Spectrum Matches |
| --- | --- | --- | --- | --- |
| -4.1256 | 136.8837 | 0.0000 | 7.8243e+06 | 2 |

**Adduct Signal Fractions**

- **Unmodified**: 7.8243e+06

### SVQEIQATFFYFTPN(N)K Hex 4 HexNAc 4 Neu5Ac 2

**Mass:**  3961.6699

(9.364e-07)

**Charge States:** 3, 4

**Start Position**: 58

**End Position**: 74

| Profile Score | Tandem Score | TDA q-value | Volume | Spectrum Matches |
| --- | --- | --- | --- | --- |
| 12.8255 | 136.1798 | 0.0000 | 3.8574e+07 | 4 |

**Adduct Signal Fractions**

- **Unmodified**: 3.8574e+07

### SVQEIQATFFYFTPN(N)K Fuc 2 Hex 4 HexNAc 4 Neu5Ac 1

**Mass:**  3962.6862

(-6.470e-08)

**Charge States:** 4

**Start Position**: 58

**End Position**: 74

| Profile Score | Tandem Score | TDA q-value | Volume | Spectrum Matches |
| --- | --- | --- | --- | --- |
| -19.8603 | 134.8513 | 0.0000 | 2.0486e+06 | 2 |

**Adduct Signal Fractions**

- **Unmodified**: 2.0486e+06

### C(C)ANLVPVPITN(N)ATLDR Hex 3 HexNAc 5

**Mass:**  3254.4761

(8.433e-07)

**Charge States:** 3, 4

**Start Position**: 23

**End Position**: 39

| Profile Score | Tandem Score | TDA q-value | Volume | Spectrum Matches |
| --- | --- | --- | --- | --- |
| 12.5782 | 134.3991 | 0.0000 | 4.8987e+08 | 23 |

**Adduct Signal Fractions**

- **Unmodified**: 4.8987e+08

### YFTPN(N)KTEDTIFLR Hex 3 HexNAc 6

**Mass:**  3448.5185

(7.302e-07)

**Charge States:** 3, 4

**Start Position**: 68

**End Position**: 82

| Profile Score | Tandem Score | TDA q-value | Volume | Spectrum Matches |
| --- | --- | --- | --- | --- |
| 13.1748 | 132.8957 | 0.0000 | 1.5597e+09 | 18 |

**Adduct Signal Fractions**

- **Unmodified**: 1.5597e+09

### LVPVPITN(N)ATLDR Fuc 2 Hex 3 HexNAc 4 Neu5Ac 1

**Mass:**  3292.5262

(9.212e-04)

**Start Position**: 26

**End Position**: 39

| Profile Score | Tandem Score | TDA q-value | Volume | Spectrum Matches |
| --- | --- | --- | --- | --- |
| 0.0000 | 131.5125 | 0.0000 | 0.0000e+00 | 2 |

Adduct:

No Chromatogram Found

### YFTPN(N)KTEDTIFLR Hex 3 HexNAc 4

**Mass:**  3042.3625

(8.936e-07)

**Charge States:** 3

**Start Position**: 68

**End Position**: 82

| Profile Score | Tandem Score | TDA q-value | Volume | Spectrum Matches |
| --- | --- | --- | --- | --- |
| 12.9403 | 131.0090 | 0.0000 | 1.0851e+08 | 3 |

**Adduct Signal Fractions**

- **Unmodified**: 1.0851e+08

### ATFFYFTPN(N)K Fuc 1 Hex 4 HexNAc 6

**Mass:**  3247.3456

(-6.629e-07)

**Charge States:** 3

**Start Position**: 64

**End Position**: 74

| Profile Score | Tandem Score | TDA q-value | Volume | Spectrum Matches |
| --- | --- | --- | --- | --- |
| -21.1847 | 130.5278 | 0.0000 | 4.1502e+06 | 1 |

**Adduct Signal Fractions**

- **Unmodified**: 4.1502e+06

### LVPVPITN(N)ATLDR Fuc 1 Hex 5 HexNAc 3 Neu5Ac 1

**Mass:**  3264.4762

(4.056e-06)

**Charge States:** 3

**Start Position**: 26

**End Position**: 39

| Profile Score | Tandem Score | TDA q-value | Volume | Spectrum Matches |
| --- | --- | --- | --- | --- |
| -20.4030 | 130.1980 | 0.0000 | 2.6447e+07 | 1 |

**Adduct Signal Fractions**

- **Unmodified**: 2.6447e+07

### LVPVPITN(N)ATLDR Fuc 2 Hex 4 HexNAc 5

**Mass:**  3364.5394

(-2.755e-06)

**Start Position**: 26

**End Position**: 39

| Profile Score | Tandem Score | TDA q-value | Volume | Spectrum Matches |
| --- | --- | --- | --- | --- |
| 0.0000 | 130.0318 | 0.0000 | 0.0000e+00 | 3 |

Adduct:

No Chromatogram Found

### LVPVPITN(N)ATLDR Fuc 2 Hex 5 HexNAc 6

**Mass:**  3728.6717

(3.668e-06)

**Charge States:** 4

**Start Position**: 26

**End Position**: 39

| Profile Score | Tandem Score | TDA q-value | Volume | Spectrum Matches |
| --- | --- | --- | --- | --- |
| -18.5309 | 127.3869 | 0.0000 | 2.1387e+07 | 2 |

**Adduct Signal Fractions**

- **Unmodified**: 2.1387e+07

### YFTPN(N)KTEDTIFLR Fuc 1 Hex 4 HexNAc 5

**Mass:**  3553.5525

(1.607e-06)

**Charge States:** 3, 4

**Start Position**: 68

**End Position**: 82

| Profile Score | Tandem Score | TDA q-value | Volume | Spectrum Matches |
| --- | --- | --- | --- | --- |
| 8.5456 | 126.4495 | 0.0000 | 1.4353e+08 | 4 |

**Adduct Signal Fractions**

- **Unmodified**: 1.4353e+08

### QNQC(C)FYN(N)SSYLNVQR Hex 3 HexNAc 6

**Mass:**  3624.4956

(1.219e-06)

**Charge States:** 3, 4

**Start Position**: 87

**End Position**: 102

| Profile Score | Tandem Score | TDA q-value | Volume | Spectrum Matches |
| --- | --- | --- | --- | --- |
| 12.0799 | 125.8989 | 0.0000 | 1.2161e+09 | 6 |

**Adduct Signal Fractions**

- **Unmodified**: 1.2161e+09

### YFTPN(N)KTEDTIFLR Hex 3 HexNAc 5

**Mass:**  3245.4417

(6.439e-07)

**Charge States:** 3, 4

**Start Position**: 68

**End Position**: 82

| Profile Score | Tandem Score | TDA q-value | Volume | Spectrum Matches |
| --- | --- | --- | --- | --- |
| 9.5237 | 125.4966 | 0.0000 | 7.1318e+08 | 8 |

**Adduct Signal Fractions**

- **Unmodified**: 7.1318e+08

### FYFTPN(N)KTEDTIFLR Hex 3 HexNAc 5

**Mass:**  3392.5102

(9.952e-07)

**Charge States:** 3, 4

**Start Position**: 67

**End Position**: 82

| Profile Score | Tandem Score | TDA q-value | Volume | Spectrum Matches |
| --- | --- | --- | --- | --- |
| 13.5385 | 124.7043 | 0.0000 | 4.9772e+07 | 6 |

**Adduct Signal Fractions**

- **Unmodified**: 4.9772e+07

### LVPVPITN(N)ATLDR Hex 4 HexNAc 7

**Mass:**  3477.5766

(2.810e-07)

**Charge States:** 3, 4

**Start Position**: 26

**End Position**: 39

| Profile Score | Tandem Score | TDA q-value | Volume | Spectrum Matches |
| --- | --- | --- | --- | --- |
| -4.8772 | 124.5690 | 0.0000 | 6.6766e+07 | 3 |

**Adduct Signal Fractions**

- **Unmodified**: 6.6766e+07

### TPN(N)KTEDTIFLR Hex 3 HexNAc 5

**Mass:**  2935.3093

(9.055e-07)

**Charge States:** 3, 4

**Start Position**: 70

**End Position**: 82

| Profile Score | Tandem Score | TDA q-value | Volume | Spectrum Matches |
| --- | --- | --- | --- | --- |
| 12.8228 | 124.5287 | 0.0000 | 3.9479e+07 | 6 |

**Adduct Signal Fractions**

- **Unmodified**: 3.9479e+07

### NLVPVPITN(N)ATLDR Fuc 1 Hex 3 HexNAc 4

**Mass:**  2966.3906

(4.900e-07)

**Charge States:** 2, 3, 4

**Start Position**: 25

**End Position**: 39

| Profile Score | Tandem Score | TDA q-value | Volume | Spectrum Matches |
| --- | --- | --- | --- | --- |
| 15.6904 | 123.9910 | 0.0000 | 1.6250e+09 | 8 |

**Adduct Signal Fractions**

- **Unmodified**: 1.6250e+09

### LVPVPITN(N)ATLDR Fuc 2 Hex 5 HexNAc 5

**Mass:**  3525.5901

(1.326e-06)

**Charge States:** 4

**Start Position**: 26

**End Position**: 39

| Profile Score | Tandem Score | TDA q-value | Volume | Spectrum Matches |
| --- | --- | --- | --- | --- |
| -4.5876 | 123.1635 | 0.0000 | 4.6013e+07 | 2 |

**Adduct Signal Fractions**

- **Unmodified**: 4.6013e+07

### QNQC(C)FYN(N)SSYLNVQR Hex 3 HexNAc 5

**Mass:**  3421.4192

(1.167e-06)

**Charge States:** 3, 4

**Start Position**: 87

**End Position**: 102

| Profile Score | Tandem Score | TDA q-value | Volume | Spectrum Matches |
| --- | --- | --- | --- | --- |
| 8.4283 | 123.0673 | 0.0000 | 5.0914e+08 | 4 |

**Adduct Signal Fractions**

- **Unmodified**: 5.0914e+08

### QNQC(C)FYN(N)SSYLNVQR Fuc 1 Hex 4 HexNAc 6

**Mass:**  3932.6098

(1.154e-06)

**Charge States:** 3, 4

**Start Position**: 87

**End Position**: 102

| Profile Score | Tandem Score | TDA q-value | Volume | Spectrum Matches |
| --- | --- | --- | --- | --- |
| 12.6491 | 122.8389 | 0.0000 | 4.7722e+08 | 5 |

**Adduct Signal Fractions**

- **Unmodified**: 4.7722e+08

### FYFTPN(N)KTEDTIFLR Hex 3 HexNAc 6

**Mass:**  3595.5899

(1.189e-06)

**Charge States:** 3, 4

**Start Position**: 67

**End Position**: 82

| Profile Score | Tandem Score | TDA q-value | Volume | Spectrum Matches |
| --- | --- | --- | --- | --- |
| 13.9848 | 121.0792 | 0.0000 | 1.0894e+08 | 5 |

**Adduct Signal Fractions**

- **Unmodified**: 1.0894e+08

### ANLVPVPITN(N)ATLDR Hex 3 HexNAc 5

**Mass:**  3094.4474

(6.833e-07)

**Charge States:** 3

**Start Position**: 24

**End Position**: 39

| Profile Score | Tandem Score | TDA q-value | Volume | Spectrum Matches |
| --- | --- | --- | --- | --- |
| -3.6342 | 119.5477 | 0.0000 | 1.6946e+07 | 1 |

**Adduct Signal Fractions**

- **Unmodified**: 1.6946e+07

### LVPVPITN(N)ATLDR Fuc 1 Hex 4 HexNAc 6

**Mass:**  3422.5569

(5.855e-04)

**Start Position**: 26

**End Position**: 39

| Profile Score | Tandem Score | TDA q-value | Volume | Spectrum Matches |
| --- | --- | --- | --- | --- |
| 0.0000 | 117.6254 | 0.0000 | 0.0000e+00 | 1 |

Adduct:

No Chromatogram Found

### YFTPN(N)KTEDTIFLR Hex 4 HexNAc 7

**Mass:**  3813.6535

(4.950e-07)

**Charge States:** 3, 4

**Start Position**: 68

**End Position**: 82

| Profile Score | Tandem Score | TDA q-value | Volume | Spectrum Matches |
| --- | --- | --- | --- | --- |
| 9.5819 | 116.1153 | 0.0000 | 1.7214e+08 | 3 |

**Adduct Signal Fractions**

- **Unmodified**: 1.7214e+08

### TPN(N)KTEDTIFLR Hex 7 HexNAc 6

**Mass:**  3786.6081

(2.747e-06)

**Charge States:** 3, 4

**Start Position**: 70

**End Position**: 82

| Profile Score | Tandem Score | TDA q-value | Volume | Spectrum Matches |
| --- | --- | --- | --- | --- |
| 14.4463 | 115.9167 | 0.0000 | 9.7774e+07 | 2 |

**Adduct Signal Fractions**

- **Unmodified**: 9.7774e+07

### YFTPN(N)KTEDTIFLR Fuc 2 Hex 5 HexNAc 6

**Mass:**  4064.7424

(1.509e-07)

**Charge States:** 4

**Start Position**: 68

**End Position**: 82

| Profile Score | Tandem Score | TDA q-value | Volume | Spectrum Matches |
| --- | --- | --- | --- | --- |
| -5.3229 | 114.2941 | 0.0000 | 3.1904e+07 | 1 |

**Adduct Signal Fractions**

- **Unmodified**: 3.1904e+07

### YFTPN(N)KTEDTIFLR Fuc 1 Hex 4 HexNAc 5

**Mass:**  3555.5597

(5.658e-04)

**Start Position**: 68

**End Position**: 82

| Profile Score | Tandem Score | TDA q-value | Volume | Spectrum Matches |
| --- | --- | --- | --- | --- |
| 0.0000 | 113.1860 | 0.0000 | 0.0000e+00 | 1 |

Adduct:

No Chromatogram Found

### LVPVPITN(N)ATLDR Hex 6 HexNAc 5

**Mass:**  3395.5310

(3.797e-06)

**Charge States:** 3, 4

**Start Position**: 26

**End Position**: 39

| Profile Score | Tandem Score | TDA q-value | Volume | Spectrum Matches |
| --- | --- | --- | --- | --- |
| 7.3602 | 111.6984 | 0.0000 | 4.7084e+07 | 5 |

**Adduct Signal Fractions**

- **Unmodified**: 4.7084e+07

### QNQC(C)FYN(N)SSYLNVQR Fuc 1 Hex 4 HexNAc 5

**Mass:**  3729.5304

(1.811e-06)

**Charge States:** 3, 4

**Start Position**: 87

**End Position**: 102

| Profile Score | Tandem Score | TDA q-value | Volume | Spectrum Matches |
| --- | --- | --- | --- | --- |
| -7.3054 | 110.2691 | 0.0000 | 1.6492e+08 | 3 |

**Adduct Signal Fractions**

- **Unmodified**: 1.6492e+08

### ATFFYFTPN(N)K Fuc 3 Hex 8 HexNAc 4

**Mass:**  3781.5297

(3.593e-06)

**Charge States:** 4

**Start Position**: 64

**End Position**: 74

| Profile Score | Tandem Score | TDA q-value | Volume | Spectrum Matches |
| --- | --- | --- | --- | --- |
| -20.6538 | 110.2303 | 0.0000 | 4.4793e+06 | 1 |

**Adduct Signal Fractions**

- **Unmodified**: 4.4793e+06

### LVPVPITN(N)ATLDR Hex 5 HexNAc 6

**Mass:**  3439.5892

(8.847e-04)

**Start Position**: 26

**End Position**: 39

| Profile Score | Tandem Score | TDA q-value | Volume | Spectrum Matches |
| --- | --- | --- | --- | --- |
| 0.0000 | 109.9666 | 0.0000 | 0.0000e+00 | 2 |

Adduct:

No Chromatogram Found

### QNQC(C)FYN(N)SSYLNVQR Hex 4 HexNAc 7

**Mass:**  3989.6318

(9.157e-07)

**Charge States:** 3, 4

**Start Position**: 87

**End Position**: 102

| Profile Score | Tandem Score | TDA q-value | Volume | Spectrum Matches |
| --- | --- | --- | --- | --- |
| 12.3525 | 108.8534 | 0.0000 | 1.7701e+08 | 2 |

**Adduct Signal Fractions**

- **Unmodified**: 1.7701e+08

### LVPVPITN(N)ATLDR Fuc 1 Hex 5 HexNAc 6

**Mass:**  3582.6120

(1.435e-06)

**Charge States:** 3, 4

**Start Position**: 26

**End Position**: 39

| Profile Score | Tandem Score | TDA q-value | Volume | Spectrum Matches |
| --- | --- | --- | --- | --- |
| -5.3058 | 108.1079 | 0.0000 | 2.8676e+07 | 1 |

**Adduct Signal Fractions**

- **Unmodified**: 2.8676e+07

### YFTPN(N)KTEDTIFLR Fuc 1 Hex 5 HexNAc 7

**Mass:**  4121.7658

(1.824e-06)

**Charge States:** 4

**Start Position**: 68

**End Position**: 82

| Profile Score | Tandem Score | TDA q-value | Volume | Spectrum Matches |
| --- | --- | --- | --- | --- |
| -3.4273 | 108.0735 | 0.0000 | 2.6080e+07 | 2 |

**Adduct Signal Fractions**

- **Unmodified**: 2.6080e+07

### LVPVPITN(N)ATLDR Fuc 1 Hex 5 HexNAc 6

**Mass:**  3584.6201

(8.398e-04)

**Start Position**: 26

**End Position**: 39

| Profile Score | Tandem Score | TDA q-value | Volume | Spectrum Matches |
| --- | --- | --- | --- | --- |
| 0.0000 | 107.2404 | 0.0000 | 0.0000e+00 | 2 |

Adduct:

No Chromatogram Found

### NLVPVPITN(N)ATLDR Fuc 1 Hex 5 HexNAc 4

**Mass:**  3293.5300

(9.236e-04)

**Start Position**: 25

**End Position**: 39

| Profile Score | Tandem Score | TDA q-value | Volume | Spectrum Matches |
| --- | --- | --- | --- | --- |
| 0.0000 | 106.9854 | 0.0000 | 0.0000e+00 | 1 |

Adduct:

No Chromatogram Found

### YFTPN(N)KTEDTIFLR Hex 5 HexNAc 8

**Mass:**  4178.7873

(1.213e-06)

**Charge States:** 4

**Start Position**: 68

**End Position**: 82

| Profile Score | Tandem Score | TDA q-value | Volume | Spectrum Matches |
| --- | --- | --- | --- | --- |
| -3.2171 | 105.6556 | 0.0000 | 3.6544e+07 | 2 |

**Adduct Signal Fractions**

- **Unmodified**: 3.6544e+07

### SVQEIQATFFYFTPN(N)K Hex 7 HexNAc 6

**Mass:**  4274.8009

(7.042e-04)

**Start Position**: 58

**End Position**: 74

| Profile Score | Tandem Score | TDA q-value | Volume | Spectrum Matches |
| --- | --- | --- | --- | --- |
| 0.0000 | 104.5811 | 0.0000 | 0.0000e+00 | 1 |

Adduct:

No Chromatogram Found

### YFTPN(N)KTEDTIFLR Hex 5 HexNAc 9

**Mass:**  4381.8649

(3.430e-07)

**Charge States:** 4

**Start Position**: 68

**End Position**: 82

| Profile Score | Tandem Score | TDA q-value | Volume | Spectrum Matches |
| --- | --- | --- | --- | --- |
| -5.0183 | 103.9188 | 0.0000 | 1.2732e+07 | 1 |

**Adduct Signal Fractions**

- **Unmodified**: 1.2732e+07

### FTPN(N)KTEDTIFLR Hex 3 HexNAc 6

**Mass:**  3285.4572

(6.883e-07)

**Charge States:** 3, 4

**Start Position**: 69

**End Position**: 82

| Profile Score | Tandem Score | TDA q-value | Volume | Spectrum Matches |
| --- | --- | --- | --- | --- |
| 14.0549 | 103.8139 | 0.0000 | 9.8634e+07 | 4 |

**Adduct Signal Fractions**

- **Unmodified**: 9.8634e+07

### QNQC(C)FYN(N)SSYLNVQR Hex 4 HexNAc 8

**Mass:**  4192.7141

(2.228e-06)

**Charge States:** 3, 4

**Start Position**: 87

**End Position**: 102

| Profile Score | Tandem Score | TDA q-value | Volume | Spectrum Matches |
| --- | --- | --- | --- | --- |
| -6.1339 | 101.0181 | 0.0000 | 3.8305e+07 | 2 |

**Adduct Signal Fractions**

- **Unmodified**: 3.8305e+07

### FYFTPN(N)KTEDTIFLR Hex 4 HexNAc 7

**Mass:**  3960.7238

(1.842e-06)

**Charge States:** 3, 4

**Start Position**: 67

**End Position**: 82

| Profile Score | Tandem Score | TDA q-value | Volume | Spectrum Matches |
| --- | --- | --- | --- | --- |
| 13.5667 | 100.2973 | 0.0000 | 1.7339e+07 | 4 |

**Adduct Signal Fractions**

- **Unmodified**: 1.7339e+07

### NLVPVPITN(N)ATLDR Fuc 1 Hex 3 HexNAc 3

**Mass:**  2763.3081

(4.755e-06)

**Charge States:** 2, 3

**Start Position**: 25

**End Position**: 39

| Profile Score | Tandem Score | TDA q-value | Volume | Spectrum Matches |
| --- | --- | --- | --- | --- |
| 14.6670 | 98.9540 | 0.0000 | 1.0290e+08 | 2 |

**Adduct Signal Fractions**

- **Unmodified**: 1.0290e+08

### NLVPVPITN(N)ATLDR Hex 3 HexNAc 5

**Mass:**  3023.4100

(1.048e-06)

**Charge States:** 3

**Start Position**: 25

**End Position**: 39

| Profile Score | Tandem Score | TDA q-value | Volume | Spectrum Matches |
| --- | --- | --- | --- | --- |
| -17.9807 | 97.5667 | 0.0000 | 6.9496e+06 | 1 |

**Adduct Signal Fractions**

- **Unmodified**: 6.9496e+06

### FYFTPN(N)KTEDTIFLR Fuc 1 Hex 4 HexNAc 6

**Mass:**  3903.7036

(1.386e-06)

**Charge States:** 3, 4

**Start Position**: 67

**End Position**: 82

| Profile Score | Tandem Score | TDA q-value | Volume | Spectrum Matches |
| --- | --- | --- | --- | --- |
| -2.7250 | 95.9147 | 0.0000 | 2.4591e+07 | 4 |

**Adduct Signal Fractions**

- **Unmodified**: 2.4591e+07

### FTPN(N)KTEDTIFLR Fuc 1 Hex 4 HexNAc 5

**Mass:**  3390.4883

(7.421e-07)

**Charge States:** 3, 4

**Start Position**: 69

**End Position**: 82

| Profile Score | Tandem Score | TDA q-value | Volume | Spectrum Matches |
| --- | --- | --- | --- | --- |
| -4.6680 | 94.9232 | 0.0000 | 1.3411e+07 | 3 |

**Adduct Signal Fractions**

- **Unmodified**: 1.3411e+07

### LVPVPITN(N)ATLDR Hex 5 HexNAc 6 Neu5Ac 2

**Mass:**  4018.7377

(-5.115e-07)

**Start Position**: 26

**End Position**: 39

| Profile Score | Tandem Score | TDA q-value | Volume | Spectrum Matches |
| --- | --- | --- | --- | --- |
| 0.0000 | 94.8500 | 0.0000 | 0.0000e+00 | 1 |

Adduct:

No Chromatogram Found

### FYFTPN(N)KTEDTIFLR Fuc 1 Hex 4 HexNAc 5

**Mass:**  3700.6203

(8.418e-07)

**Charge States:** 3, 4

**Start Position**: 67

**End Position**: 82

| Profile Score | Tandem Score | TDA q-value | Volume | Spectrum Matches |
| --- | --- | --- | --- | --- |
| 12.6229 | 94.6121 | 0.0000 | 1.6060e+07 | 5 |

**Adduct Signal Fractions**

- **Unmodified**: 1.6060e+07

### PVPITN(N)ATLDR Fuc 2 Hex 4 HexNAc 5

**Mass:**  3151.3793

(-2.962e-07)

**Charge States:** 3

**Start Position**: 28

**End Position**: 39

| Profile Score | Tandem Score | TDA q-value | Volume | Spectrum Matches |
| --- | --- | --- | --- | --- |
| -20.2615 | 93.8070 | 0.0000 | 5.9457e+06 | 2 |

**Adduct Signal Fractions**

- **Unmodified**: 5.9457e+06

### TPN(N)KTEDTIFLR Fuc 1 Hex 4 HexNAc 5

**Mass:**  3243.4224

(1.511e-06)

**Charge States:** 3

**Start Position**: 70

**End Position**: 82

| Profile Score | Tandem Score | TDA q-value | Volume | Spectrum Matches |
| --- | --- | --- | --- | --- |
| -4.9033 | 93.1202 | 0.0000 | 6.7496e+06 | 2 |

**Adduct Signal Fractions**

- **Unmodified**: 6.7496e+06

### FTPN(N)KTEDTIFLR Hex 3 HexNAc 5

**Mass:**  3082.3782

(1.183e-06)

**Charge States:** 3, 4

**Start Position**: 69

**End Position**: 82

| Profile Score | Tandem Score | TDA q-value | Volume | Spectrum Matches |
| --- | --- | --- | --- | --- |
| 13.7894 | 92.7930 | 0.0000 | 4.5800e+07 | 4 |

**Adduct Signal Fractions**

- **Unmodified**: 4.5800e+07

### FYN(N)SSYLNVQR Hex 3 HexNAc 6

**Mass:**  3094.3072

(1.408e-06)

**Charge States:** 3

**Start Position**: 91

**End Position**: 102

| Profile Score | Tandem Score | TDA q-value | Volume | Spectrum Matches |
| --- | --- | --- | --- | --- |
| -3.6678 | 91.9129 | 0.0000 | 1.0012e+07 | 3 |

**Adduct Signal Fractions**

- **Unmodified**: 1.0012e+07

### TPN(N)KTEDTIFLR Fuc 1 Hex 4 HexNAc 6

**Mass:**  3446.5006

(5.822e-04)

**Charge States:** 3, 4

**Start Position**: 70

**End Position**: 82

| Profile Score | Tandem Score | TDA q-value | Volume | Spectrum Matches |
| --- | --- | --- | --- | --- |
| 5.0099 | 87.8404 | 0.0000 | 1.3308e+07 | 22 |

**Adduct Signal Fractions**

- **Unmodified**: 1.3308e+07

### LVPVPITN(N)ATLDR Fuc 1 Hex 5 HexNAc 7

**Mass:**  3785.6953

(4.735e-07)

**Charge States:** 3, 4

**Start Position**: 26

**End Position**: 39

| Profile Score | Tandem Score | TDA q-value | Volume | Spectrum Matches |
| --- | --- | --- | --- | --- |
| -5.5506 | 86.1172 | 0.0000 | 1.9068e+07 | 2 |

**Adduct Signal Fractions**

- **Unmodified**: 1.9068e+07

### VPVPITN(N)ATLDR Fuc 1 Hex 4 HexNAc 5

**Mass:**  3104.3911

(2.093e-07)

**Charge States:** 3

**Start Position**: 27

**End Position**: 39

| Profile Score | Tandem Score | TDA q-value | Volume | Spectrum Matches |
| --- | --- | --- | --- | --- |
| -4.9288 | 81.6763 | 0.0000 | 2.2251e+06 | 1 |

**Adduct Signal Fractions**

- **Unmodified**: 2.2251e+06

### QNQC(C)FYN(N)SSYLNVQR Hex 4 HexNAc 4 Neu5Ac 2

**Mass:**  3962.5830

(1.698e-06)

**Charge States:** 3, 4

**Start Position**: 87

**End Position**: 102

| Profile Score | Tandem Score | TDA q-value | Volume | Spectrum Matches |
| --- | --- | --- | --- | --- |
| -4.4492 | 80.2706 | 0.0000 | 5.6311e+07 | 1 |

**Adduct Signal Fractions**

- **Unmodified**: 5.6311e+07

### YN(N)SSYLNVQR Hex 3 HexNAc 6

**Mass:**  2947.2376

(1.407e-06)

**Charge States:** 3

**Start Position**: 92

**End Position**: 102

| Profile Score | Tandem Score | TDA q-value | Volume | Spectrum Matches |
| --- | --- | --- | --- | --- |
| 11.8894 | 79.4717 | 0.0000 | 1.7182e+07 | 4 |

**Adduct Signal Fractions**

- **Unmodified**: 1.7182e+07

### FTPN(N)KTEDTIFLR Fuc 1 Hex 4 HexNAc 6

**Mass:**  3593.5695

(1.400e-06)

**Charge States:** 3, 4

**Start Position**: 69

**End Position**: 82

| Profile Score | Tandem Score | TDA q-value | Volume | Spectrum Matches |
| --- | --- | --- | --- | --- |
| -3.9994 | 79.1777 | 0.0000 | 1.9190e+07 | 4 |

**Adduct Signal Fractions**

- **Unmodified**: 1.9190e+07

### SVQEIQATFFYFTPN(N)KTEDTIFLR Hex 3 HexNAc 6

**Mass:**  4599.0886

(1.075e-06)

**Charge States:** 3, 4, 5

**Start Position**: 58

**End Position**: 82

| Profile Score | Tandem Score | TDA q-value | Volume | Spectrum Matches |
| --- | --- | --- | --- | --- |
| -18.8530 | 70.2060 | 0.0000 | 1.9632e+07 | 2 |

**Adduct Signal Fractions**

- **Unmodified**: 1.9632e+07

### C(C)ANLVPVPITN(N)ATLDR Hex 3 HexNAc 5 Neu5Ac 1

**Mass:**  3545.5658

(-1.204e-06)

**Charge States:** 3, 4

**Start Position**: 23

**End Position**: 39

| Profile Score | Tandem Score | TDA q-value | Volume | Spectrum Matches |
| --- | --- | --- | --- | --- |
| 15.8534 | 66.6200 | 0.0000 | 2.0763e+08 | 7 |

**Adduct Signal Fractions**

- **Unmodified**: 2.0763e+08

### SVQEIQATFFYFTPN(N)K Fuc 1 Hex 6 HexNAc 5

**Mass:**  4052.7206

(6.206e-07)

**Charge States:** 3

**Start Position**: 58

**End Position**: 74

| Profile Score | Tandem Score | TDA q-value | Volume | Spectrum Matches |
| --- | --- | --- | --- | --- |
| -5.0605 | 61.2669 | 0.0000 | 7.2481e+06 | 2 |

**Adduct Signal Fractions**

- **Unmodified**: 7.2481e+06

### SVQEIQATFFYFTPN(N)KTEDTIFLR Hex 3 HexNAc 5

**Mass:**  4395.9997

(-1.064e-06)

**Charge States:** 4

**Start Position**: 58

**End Position**: 82

| Profile Score | Tandem Score | TDA q-value | Volume | Spectrum Matches |
| --- | --- | --- | --- | --- |
| -5.1680 | 59.7198 | 0.0000 | 9.1259e+06 | 2 |

**Adduct Signal Fractions**

- **Unmodified**: 9.1259e+06

### SVQEIQATFFYFTPN(N)K Fuc 1 Hex 5 HexNAc 4 Neu5Ac 1

**Mass:**  3978.6996

(3.689e-06)

**Charge States:** 3

**Start Position**: 58

**End Position**: 74

| Profile Score | Tandem Score | TDA q-value | Volume | Spectrum Matches |
| --- | --- | --- | --- | --- |
| -4.6128 | 59.2824 | 0.0000 | 8.0188e+06 | 2 |

**Adduct Signal Fractions**

- **Unmodified**: 8.0188e+06

### C(C)ANLVPVPITN(N)ATLDR Hex 3 HexNAc 4

**Mass:**  3051.3967

(1.693e-06)

**Charge States:** 3

**Start Position**: 23

**End Position**: 39

| Profile Score | Tandem Score | TDA q-value | Volume | Spectrum Matches |
| --- | --- | --- | --- | --- |
| -4.5269 | 58.5818 | 0.0000 | 1.1656e+07 | 2 |

**Adduct Signal Fractions**

- **Unmodified**: 1.1656e+07

### SVQEIQATFFYFTPN(N)K Hex 5 HexNAc 8

**Mass:**  4353.8464

(3.282e-07)

**Charge States:** 4

**Start Position**: 58

**End Position**: 74

| Profile Score | Tandem Score | TDA q-value | Volume | Spectrum Matches |
| --- | --- | --- | --- | --- |
| 10.8572 | 54.4092 | 0.0000 | 1.6239e+07 | 1 |

**Adduct Signal Fractions**

- **Unmodified**: 1.6239e+07

### FYFTPN(N)KTEDTIFLR Hex 3 HexNAc 4

**Mass:**  3189.4312

(6.460e-07)

**Charge States:** 3

**Start Position**: 67

**End Position**: 82

| Profile Score | Tandem Score | TDA q-value | Volume | Spectrum Matches |
| --- | --- | --- | --- | --- |
| -3.8780 | 51.7342 | 0.0000 | 6.6925e+06 | 1 |

**Adduct Signal Fractions**

- **Unmodified**: 6.6925e+06

### FYFTPN(N)KTEDTIFLR Fuc 5 Hex 3 HexNAc 6

**Mass:**  4325.8649

(-2.545e-06)

**Charge States:** 4

**Start Position**: 67

**End Position**: 82

| Profile Score | Tandem Score | TDA q-value | Volume | Spectrum Matches |
| --- | --- | --- | --- | --- |
| -21.2946 | 48.1275 | 0.0000 | 2.4195e+06 | 1 |

**Adduct Signal Fractions**

- **Unmodified**: 2.4195e+06

### QIPLC(C)ANLVPVPITN(N)ATLDR Hex 3 HexNAc 5 Neu5Ac 1

**Mass:**  3996.8474

(-5.518e-07)

**Charge States:** 3, 4

**Start Position**: 19

**End Position**: 39

| Profile Score | Tandem Score | TDA q-value | Volume | Spectrum Matches |
| --- | --- | --- | --- | --- |
| 15.9242 | 48.0021 | 0.0000 | 3.7652e+08 | 13 |

**Adduct Signal Fractions**

- **Unmodified**: 3.7652e+08

### SVQEIQATFFYFTPN(N)K Fuc 4 Hex 5 HexNAc 5

**Mass:**  4328.8229

(-3.762e-06)

**Charge States:** 4

**Start Position**: 58

**End Position**: 74

| Profile Score | Tandem Score | TDA q-value | Volume | Spectrum Matches |
| --- | --- | --- | --- | --- |
| -18.3993 | 47.8507 | 0.0000 | 7.2487e+06 | 1 |

**Adduct Signal Fractions**

- **Unmodified**: 7.2487e+06

### SVQEIQATFFYFTPN(N)K Hex 4 HexNAc 6

**Mass:**  3788.6589

(7.990e-04)

**Start Position**: 58

**End Position**: 74

| Profile Score | Tandem Score | TDA q-value | Volume | Spectrum Matches |
| --- | --- | --- | --- | --- |
| 0.0000 | 46.6902 | 0.0000 | 0.0000e+00 | 1 |

Adduct:

No Chromatogram Found

### QNQC(C)FYN(N)SSYLNVQR Fuc 5 Hex 4 HexNAc 8

**Mass:**  4922.9743

(-3.959e-06)

**Charge States:** 4

**Start Position**: 87

**End Position**: 102

| Profile Score | Tandem Score | TDA q-value | Volume | Spectrum Matches |
| --- | --- | --- | --- | --- |
| 12.5489 | 45.8100 | 0.0000 | 2.9112e+07 | 5 |

**Adduct Signal Fractions**

- **Unmodified**: 2.9112e+07

### SVQEIQATFFYFTPN(N)K Fuc 2 Hex 5 HexNAc 5 Neu5Ac 1

**Mass:**  4327.8112

(-1.699e-06)

**Charge States:** 4

**Start Position**: 58

**End Position**: 74

| Profile Score | Tandem Score | TDA q-value | Volume | Spectrum Matches |
| --- | --- | --- | --- | --- |
| -19.5104 | 45.5583 | 0.0000 | 2.0784e+06 | 1 |

**Adduct Signal Fractions**

- **Unmodified**: 2.0784e+06

### QNQC(C)FYN(N)SSYLNVQR Fuc 2 Hex 5 HexNAc 6

**Mass:**  4240.7082

(-1.487e-06)

**Charge States:** 3, 4

**Start Position**: 87

**End Position**: 102

| Profile Score | Tandem Score | TDA q-value | Volume | Spectrum Matches |
| --- | --- | --- | --- | --- |
| -17.9481 | 44.2808 | 0.0000 | 1.5311e+07 | 2 |

**Adduct Signal Fractions**

- **Unmodified**: 1.5311e+07

### NLVPVPITN(N)ATLDR Fuc 1 Hex 3 HexNAc 5

**Mass:**  3169.4690

(1.397e-06)

**Charge States:** 3

**Start Position**: 25

**End Position**: 39

| Profile Score | Tandem Score | TDA q-value | Volume | Spectrum Matches |
| --- | --- | --- | --- | --- |
| -18.2320 | 43.9322 | 0.0000 | 3.7571e+07 | 1 |

**Adduct Signal Fractions**

- **Unmodified**: 3.7571e+07

### QN(D)QC(C)FYN(N)SSYLNVQR Hex 3 HexNAc 6

**Mass:**  3625.4807

(2.723e-04)

**Charge States:** 3, 4

**Start Position**: 87

**End Position**: 102

| Profile Score | Tandem Score | TDA q-value | Volume | Spectrum Matches |
| --- | --- | --- | --- | --- |
| -4.5780 | 43.0480 | 0.0000 | 3.6668e+08 | 7 |

**Adduct Signal Fractions**

- **Unmodified**: 3.6668e+08

### PVPITN(N)ATLDR Fuc 1 Hex 5 HexNAc 4

**Mass:**  2964.2785

(-5.767e-06)

**Charge States:** 4

**Start Position**: 28

**End Position**: 39

| Profile Score | Tandem Score | TDA q-value | Volume | Spectrum Matches |
| --- | --- | --- | --- | --- |
| -18.1621 | 42.9134 | 0.0000 | 1.6726e+07 | 1 |

**Adduct Signal Fractions**

- **Unmodified**: 1.6726e+07

### NLVPVPITN(N)ATLDR Fuc 1 Hex 4 HexNAc 5

**Mass:**  3331.5324

(3.409e-06)

**Charge States:** 3

**Start Position**: 25

**End Position**: 39

| Profile Score | Tandem Score | TDA q-value | Volume | Spectrum Matches |
| --- | --- | --- | --- | --- |
| -19.6471 | 42.8439 | 0.0000 | 1.7425e+07 | 1 |

**Adduct Signal Fractions**

- **Unmodified**: 1.7425e+07

### QNQC(C)FYN(N)SSYLNVQR Hex 5 HexNAc 8

**Mass:**  4354.7683

(2.282e-06)

**Charge States:** 3, 4

**Start Position**: 87

**End Position**: 102

| Profile Score | Tandem Score | TDA q-value | Volume | Spectrum Matches |
| --- | --- | --- | --- | --- |
| -20.7241 | 42.0008 | 0.0000 | 9.7678e+06 | 1 |

**Adduct Signal Fractions**

- **Unmodified**: 9.7678e+06

### QIPLC(C)ANLVPVPITN(N)ATLDR Hex 3 HexNAc 5

**Mass:**  3705.7549

(4.244e-07)

**Charge States:** 3, 4

**Start Position**: 19

**End Position**: 39

| Profile Score | Tandem Score | TDA q-value | Volume | Spectrum Matches |
| --- | --- | --- | --- | --- |
| 13.4229 | 41.9999 | 0.0000 | 3.6029e+08 | 10 |

**Adduct Signal Fractions**

- **Unmodified**: 3.6029e+08

### SVQEIQATFFYFTPN(N)K Fuc 2 Hex 5 HexNAc 7

**Mass:**  4442.8859

(7.825e-07)

**Charge States:** 4

**Start Position**: 58

**End Position**: 74

| Profile Score | Tandem Score | TDA q-value | Volume | Spectrum Matches |
| --- | --- | --- | --- | --- |
| -5.1218 | 41.3017 | 0.0000 | 3.6955e+06 | 1 |

**Adduct Signal Fractions**

- **Unmodified**: 3.6955e+06

### QN(D)QC(C)FYN(N)SSYLNVQR Fuc 1 Hex 4 HexNAc 6

**Mass:**  3933.5939

(2.520e-04)

**Charge States:** 4

**Start Position**: 87

**End Position**: 102

| Profile Score | Tandem Score | TDA q-value | Volume | Spectrum Matches |
| --- | --- | --- | --- | --- |
| -5.7989 | 40.4420 | 0.0000 | 4.3997e+07 | 3 |

**Adduct Signal Fractions**

- **Unmodified**: 4.3997e+07

### SVQEIQATFFYFTPN(N)KTEDTIFLR Hex 7 HexNAc 6

**Mass:**  5247.3039

(6.689e-07)

**Charge States:** 4

**Start Position**: 58

**End Position**: 82

| Profile Score | Tandem Score | TDA q-value | Volume | Spectrum Matches |
| --- | --- | --- | --- | --- |
| 11.4949 | 40.3894 | 0.0000 | 1.2795e+07 | 3 |

**Adduct Signal Fractions**

- **Unmodified**: 1.2795e+07

### FTPN(N)KTEDTIFLR Hex 3 HexNAc 4

**Mass:**  2879.2994

(1.239e-06)

**Charge States:** 3

**Start Position**: 69

**End Position**: 82

| Profile Score | Tandem Score | TDA q-value | Volume | Spectrum Matches |
| --- | --- | --- | --- | --- |
| -4.7469 | 40.0948 | 0.0000 | 5.0038e+06 | 1 |

**Adduct Signal Fractions**

- **Unmodified**: 5.0038e+06

### LVPVPITN(N)ATLDR Fuc 2 Hex 5 HexNAc 5 Neu5Ac 1

**Mass:**  3816.6742

(-1.731e-06)

**Start Position**: 26

**End Position**: 39

| Profile Score | Tandem Score | TDA q-value | Volume | Spectrum Matches |
| --- | --- | --- | --- | --- |
| 0.0000 | 40.0735 | 0.0000 | 0.0000e+00 | 1 |

Adduct:

No Chromatogram Found

### QNQC(C)FYN(N)SSYLNVQR Fuc 1 Hex 5 HexNAc 8

**Mass:**  4500.8308

(2.377e-06)

**Charge States:** 4

**Start Position**: 87

**End Position**: 102

| Profile Score | Tandem Score | TDA q-value | Volume | Spectrum Matches |
| --- | --- | --- | --- | --- |
| -20.9973 | 39.9303 | 0.0000 | 4.6762e+06 | 1 |

**Adduct Signal Fractions**

- **Unmodified**: 4.6762e+06

### LVPVPITN(N)ATLDR Hex 6 HexNAc 5 Neu5Ac 3

**Mass:**  4268.8117

(3.320e-06)

**Charge States:** 4

**Start Position**: 26

**End Position**: 39

| Profile Score | Tandem Score | TDA q-value | Volume | Spectrum Matches |
| --- | --- | --- | --- | --- |
| 10.3053 | 39.7360 | 0.0000 | 8.4510e+06 | 3 |

**Adduct Signal Fractions**

- **Unmodified**: 8.4510e+06

### FTPN(N)KTEDTIFLR Hex 4 HexNAc 7

**Mass:**  3650.5891

(1.514e-06)

**Charge States:** 3, 4

**Start Position**: 69

**End Position**: 82

| Profile Score | Tandem Score | TDA q-value | Volume | Spectrum Matches |
| --- | --- | --- | --- | --- |
| -4.9957 | 37.8298 | 0.0000 | 1.1066e+07 | 4 |

**Adduct Signal Fractions**

- **Unmodified**: 1.1066e+07

### LVPVPITN(N)ATLDR Fuc 1 Hex 5 HexNAc 3 Neu5Ac 1

**Mass:**  3266.4784

(6.170e-04)

**Start Position**: 26

**End Position**: 39

| Profile Score | Tandem Score | TDA q-value | Volume | Spectrum Matches |
| --- | --- | --- | --- | --- |
| 0.0000 | 37.6748 | 0.0000 | 0.0000e+00 | 1 |

Adduct:

No Chromatogram Found

### QNQC(C)FYN(N)SSYLNVQR Fuc 5 Hex 4 HexNAc 9

**Mass:**  5126.0529

(-4.041e-06)

**Charge States:** 4

**Start Position**: 87

**End Position**: 102

| Profile Score | Tandem Score | TDA q-value | Volume | Spectrum Matches |
| --- | --- | --- | --- | --- |
| -3.6708 | 36.3631 | 0.0000 | 1.8682e+07 | 3 |

**Adduct Signal Fractions**

- **Unmodified**: 1.8682e+07

### YFTPN(N)KTEDTIFLR Hex 4 HexNAc 6

**Mass:**  3610.5738

(8.381e-07)

**Charge States:** 3, 4

**Start Position**: 68

**End Position**: 82

| Profile Score | Tandem Score | TDA q-value | Volume | Spectrum Matches |
| --- | --- | --- | --- | --- |
| -19.8366 | 36.0782 | 0.0000 | 2.8288e+07 | 1 |

**Adduct Signal Fractions**

- **Unmodified**: 2.8288e+07

### SVQEIQATFFYFTPN(N)K Fuc 1 Hex 5 HexNAc 8

**Mass:**  4499.9108

(1.619e-06)

**Charge States:** 4

**Start Position**: 58

**End Position**: 74

| Profile Score | Tandem Score | TDA q-value | Volume | Spectrum Matches |
| --- | --- | --- | --- | --- |
| -21.3156 | 35.9541 | 0.0000 | 7.2343e+05 | 1 |

**Adduct Signal Fractions**

- **Unmodified**: 7.2343e+05

### ANLVPVPITN(N)ATLDR Fuc 2 Hex 3 HexNAc 3 Neu5Ac 1

**Mass:**  3271.5034

(2.250e-06)

**Charge States:** 3

**Start Position**: 24

**End Position**: 39

| Profile Score | Tandem Score | TDA q-value | Volume | Spectrum Matches |
| --- | --- | --- | --- | --- |
| 11.6807 | 34.6951 | 0.0028 | 7.7389e+07 | 3 |

**Adduct Signal Fractions**

- **Unmodified**: 7.7389e+07

### TPN(N)KTEDTIFLR Hex 3 HexNAc 6

**Mass:**  3139.3882

(3.193e-04)

**Start Position**: 70

**End Position**: 82

| Profile Score | Tandem Score | TDA q-value | Volume | Spectrum Matches |
| --- | --- | --- | --- | --- |
| 0.0000 | 34.4797 | 0.0028 | 0.0000e+00 | 1 |

Adduct:

No Chromatogram Found

### QIPLC(C)ANLVPVPITN(N)ATLDR Fuc 1 Hex 4 HexNAc 5

**Mass:**  4013.8694

(8.348e-07)

**Charge States:** 3, 4

**Start Position**: 19

**End Position**: 39

| Profile Score | Tandem Score | TDA q-value | Volume | Spectrum Matches |
| --- | --- | --- | --- | --- |
| 13.8166 | 34.2067 | 0.0028 | 1.0355e+08 | 8 |

**Adduct Signal Fractions**

- **Unmodified**: 1.0355e+08

### SVQEIQATFFYFTPN(N)KTEDTIFLR Fuc 1 Hex 4 HexNAc 6

**Mass:**  4907.1965

(4.451e-07)

**Charge States:** 4

**Start Position**: 58

**End Position**: 82

| Profile Score | Tandem Score | TDA q-value | Volume | Spectrum Matches |
| --- | --- | --- | --- | --- |
| -21.3553 | 33.2882 | 0.0053 | 1.9487e+06 | 1 |

**Adduct Signal Fractions**

- **Unmodified**: 1.9487e+06

### QN(D)QC(C)FYN(N)SSYLNVQR Fuc 4 Hex 3 HexNAc 5

**Mass:**  4006.6413

(2.487e-04)

**Charge States:** 3, 4

**Start Position**: 87

**End Position**: 102

| Profile Score | Tandem Score | TDA q-value | Volume | Spectrum Matches |
| --- | --- | --- | --- | --- |
| 12.9785 | 32.1511 | 0.0066 | 6.1695e+07 | 2 |

**Adduct Signal Fractions**

- **Unmodified**: 6.1695e+07

### C(C)AN(D)LVPVPITN(N)ATLDR Fuc 1 Hex 4 HexNAc 5

**Mass:**  3563.5757

(2.757e-04)

**Charge States:** 3, 4

**Start Position**: 23

**End Position**: 39

| Profile Score | Tandem Score | TDA q-value | Volume | Spectrum Matches |
| --- | --- | --- | --- | --- |
| 10.9937 | 31.3510 | 0.0116 | 1.8375e+07 | 3 |

**Adduct Signal Fractions**

- **Unmodified**: 1.8375e+07

### PVPITN(N)ATLDR Fuc 2 Hex 5 HexNAc 4 Neu5Ac 1

**Mass:**  3404.4929

(8.949e-04)

**Start Position**: 28

**End Position**: 39

| Profile Score | Tandem Score | TDA q-value | Volume | Spectrum Matches |
| --- | --- | --- | --- | --- |
| 0.0000 | 31.0319 | 0.0116 | 0.0000e+00 | 1 |

Adduct:

No Chromatogram Found

### TPN(N)KTEDTIFLR Hex 3 HexNAc 4

**Mass:**  2732.2296

(5.044e-07)

**Charge States:** 3

**Start Position**: 70

**End Position**: 82

| Profile Score | Tandem Score | TDA q-value | Volume | Spectrum Matches |
| --- | --- | --- | --- | --- |
| 9.7492 | 30.8847 | 0.0126 | 5.8435e+06 | 2 |

**Adduct Signal Fractions**

- **Unmodified**: 5.8435e+06

### QN(D)QC(C)FYN(N)SSYLNVQR Hex 5 HexNAc 5

**Mass:**  3746.5021

(2.623e-04)

**Charge States:** 3

**Start Position**: 87

**End Position**: 102

| Profile Score | Tandem Score | TDA q-value | Volume | Spectrum Matches |
| --- | --- | --- | --- | --- |
| -20.0184 | 30.6787 | 0.0126 | 2.2466e+07 | 1 |

**Adduct Signal Fractions**

- **Unmodified**: 2.2466e+07

### ATFFYFTPN(N)K Fuc 2 Hex 6 HexNAc 4

**Mass:**  3314.3578

(9.076e-04)

**Start Position**: 64

**End Position**: 74

| Profile Score | Tandem Score | TDA q-value | Volume | Spectrum Matches |
| --- | --- | --- | --- | --- |
| 0.0000 | 30.5487 | 0.0126 | 0.0000e+00 | 1 |

Adduct:

No Chromatogram Found

### QNQC(C)FYN(N)SSYLNVQR Hex 6 HexNAc 9

**Mass:**  4719.8962

(7.765e-08)

**Charge States:** 4

**Start Position**: 87

**End Position**: 102

| Profile Score | Tandem Score | TDA q-value | Volume | Spectrum Matches |
| --- | --- | --- | --- | --- |
| -4.1015 | 30.1359 | 0.0174 | 7.2864e+06 | 3 |

**Adduct Signal Fractions**

- **Unmodified**: 7.2864e+06

### YN(N)SSYLNVQR Hex 3 HexNAc 5

**Mass:**  2744.1579

(1.428e-06)

**Charge States:** 3

**Start Position**: 92

**End Position**: 102

| Profile Score | Tandem Score | TDA q-value | Volume | Spectrum Matches |
| --- | --- | --- | --- | --- |
| 11.1373 | 29.6239 | 0.0195 | 7.2608e+06 | 2 |

**Adduct Signal Fractions**

- **Unmodified**: 7.2608e+06

### C(C)AN(D)LVPVPITN(N)ATLDR Hex 3 HexNAc 5

**Mass:**  3255.4608

(3.033e-04)

**Charge States:** 3

**Start Position**: 23

**End Position**: 39

| Profile Score | Tandem Score | TDA q-value | Volume | Spectrum Matches |
| --- | --- | --- | --- | --- |
| -20.2789 | 28.6590 | 0.0215 | 3.3947e+07 | 1 |

**Adduct Signal Fractions**

- **Unmodified**: 3.3947e+07

### QC(C)FYN(N)SSYLNVQR Fuc 1 Hex 6 HexNAc 4

**Mass:**  3608.4574

(1.917e-06)

**Charge States:** 3, 4

**Start Position**: 89

**End Position**: 102

| Profile Score | Tandem Score | TDA q-value | Volume | Spectrum Matches |
| --- | --- | --- | --- | --- |
| 13.8159 | 28.1301 | 0.0271 | 5.6379e+08 | 3 |

**Adduct Signal Fractions**

- **Unmodified**: 5.6379e+08

### FYN(N)SSYLNVQR Fuc 1 Hex 4 HexNAc 6

**Mass:**  3402.4154

(1.195e-06)

**Charge States:** 3, 4

**Start Position**: 91

**End Position**: 102

| Profile Score | Tandem Score | TDA q-value | Volume | Spectrum Matches |
| --- | --- | --- | --- | --- |
| 13.2705 | 27.7460 | 0.0292 | 4.6760e+06 | 3 |

**Adduct Signal Fractions**

- **Unmodified**: 4.6760e+06

### PVPITN(N)ATLDR Fuc 1 Hex 5 HexNAc 4 Neu5Ac 1

**Mass:**  3255.4228

(8.593e-06)

**Charge States:** 3, 4

**Start Position**: 28

**End Position**: 39

| Profile Score | Tandem Score | TDA q-value | Volume | Spectrum Matches |
| --- | --- | --- | --- | --- |
| 13.7015 | 26.6456 | 0.0373 | 4.1396e+08 | 5 |

**Adduct Signal Fractions**

- **Unmodified**: 4.1396e+08

### LVPVPITN(N)ATLDR Fuc 1 Hex 4 HexNAc 5

**Mass:**  3219.4840

(6.245e-04)

**Start Position**: 26

**End Position**: 39

| Profile Score | Tandem Score | TDA q-value | Volume | Spectrum Matches |
| --- | --- | --- | --- | --- |
| 0.0000 | 26.5480 | 0.0383 | 0.0000e+00 | 2 |

Adduct:

No Chromatogram Found

### ANLVPVPITN(N)ATLDR Fuc 2 Hex 7 HexNAc 5

**Mass:**  4034.7895

(3.710e-06)

**Charge States:** 4

**Start Position**: 24

**End Position**: 39

| Profile Score | Tandem Score | TDA q-value | Volume | Spectrum Matches |
| --- | --- | --- | --- | --- |
| -3.8810 | 26.4699 | 0.0388 | 4.6224e+07 | 4 |

**Adduct Signal Fractions**

- **Unmodified**: 4.6224e+07

### LVPVPITN(N)ATLDR Fuc 1 Hex 6 HexNAc 5

**Mass:**  3541.5832

(8.188e-07)

**Start Position**: 26

**End Position**: 39

| Profile Score | Tandem Score | TDA q-value | Volume | Spectrum Matches |
| --- | --- | --- | --- | --- |
| 0.0000 | 26.3427 | 0.0388 | 0.0000e+00 | 1 |

Adduct:

No Chromatogram Found

### YN(N)SSYLNVQR Fuc 1 Hex 5 HexNAc 7

**Mass:**  3620.5069

(8.301e-06)

**Charge States:** 3

**Start Position**: 92

**End Position**: 102

| Profile Score | Tandem Score | TDA q-value | Volume | Spectrum Matches |
| --- | --- | --- | --- | --- |
| -19.1492 | 26.3094 | 0.0388 | 1.2030e+07 | 3 |

**Adduct Signal Fractions**

- **Unmodified**: 1.2030e+07

### QNQC(C)FYN(N)SSYLNVQR Fuc 5 Hex 3 HexNAc 7

**Mass:**  4557.8503

(-2.614e-06)

**Charge States:** 4

**Start Position**: 87

**End Position**: 102

| Profile Score | Tandem Score | TDA q-value | Volume | Spectrum Matches |
| --- | --- | --- | --- | --- |
| -19.5462 | 25.9972 | 0.0391 | 8.2530e+06 | 1 |

**Adduct Signal Fractions**

- **Unmodified**: 8.2530e+06

### QNQC(C)FYN(N)SSYLNVQR Hex 5 HexNAc 6

**Mass:**  3948.5651

(-8.055e-06)

**Charge States:** 3

**Start Position**: 87

**End Position**: 102

| Profile Score | Tandem Score | TDA q-value | Volume | Spectrum Matches |
| --- | --- | --- | --- | --- |
| -4.0883 | 25.3910 | 0.0417 | 6.4502e+07 | 3 |

**Adduct Signal Fractions**

- **Unmodified**: 6.4502e+07

### FTPN(N)KTEDTIFLR Fuc 2 Hex 5 HexNAc 6

**Mass:**  3901.6809

(1.210e-06)

**Charge States:** 4

**Start Position**: 69

**End Position**: 82

| Profile Score | Tandem Score | TDA q-value | Volume | Spectrum Matches |
| --- | --- | --- | --- | --- |
| -19.0922 | 25.3162 | 0.0417 | 2.7929e+06 | 2 |

**Adduct Signal Fractions**

- **Unmodified**: 2.7929e+06

### P00738|HPT\_HUMAN

**>P00738|HPT\_HUMAN**  
 `MSALGAVIALLLWGQLFAVDSGNDVTDIADDGCPKPPEIAHGYVEHSVRYQCKNYYKLRTEGDGVYTLNDKKQWINKAVG  
DKLPECEADDGCPKPPEIAHGYVEHSVRYQCKNYYKLRTEGDGVYTLNNEKQWINKAVGDKLPECEAVCGKPKNPANPVQ  
RILGGHLDAKGSFPWQAKMVSHHNLTTGATLINEQWLLTTAKNLFLNHSENATAKDIAPTLTLYVGKKQLVEIEKVVLHP  
NYSQVDIGLIKLKQKVSVNERVMPICLPSKDYAEVGRVGYVSGWGRNANFKFTDHLKYVMLPVADQDQCIRHYEGSTVPE  
KKTPKSPVGVQPILNEHTFCAGMSKYQEDTCYGDAGSAFAVHDLEEDTWYATGILSFDKSCAVAEYGVYVKVTSIQDWVQ  
KTIAEN`

| Site | Type | Count |
| --- | --- | --- |
| 241 | N-linked | 4 |
| 211 | N-linked | 8 |
| 207 | N-linked | 2 |

| Mass | Sequence | MS_2_ Score | `q`-value | MS_1_ Score | Signal | Protein Coordinates | Tandem Scans | Start Time | Apex Time | End Time |
| --- | --- | --- | --- | --- | --- | --- | --- | --- | --- | --- |
| 3092.4833 | VVLHPN(N)YSQVDIGLIK Hex 3 HexNAc 4 | 171.651 | 0.0 | 12.800 | 4.525e+07 | 235 - 251 | 4 | 42.57 | 42.57 | 43.25 |
| 3498.6456 | VVLHPN(N)YSQVDIGLIK Hex 3 HexNAc 6 | 141.698 | 0.0 | -19.968 | 1.051e+07 | 235 - 251 | 2 | 42.24 | 42.40 | 42.40 |
| 4460.8414 | NLFLNHSEN(N)ATAK Fuc 5 Hex 4 HexNAc 8 | 123.950 | 0.0 | -3.990 | 3.867e+07 | 202 - 215 | 4 | 18.27 | 18.27 | 18.70 |
| 4257.7628 | NLFLN(N)HSENATAK Hex 6 HexNAc 9 | 103.381 | 0.0 | 12.433 | 3.099e+07 | 202 - 215 | 2 | 18.27 | 18.42 | 18.86 |
| 4054.6820 | NLFLNHSEN(N)ATAK Hex 6 HexNAc 8 | 46.384 | 0.0 | -4.363 | 8.584e+06 | 202 - 215 | 3 | 18.56 | 18.56 | 18.77 |
| 4258.7501 | NLFLN(N)HSENATAK Fuc 5 Hex 4 HexNAc 7 | 45.147 | 0.0 | -20.922 | 1.685e+06 | 202 - 215 | 3 | 22.11 | 22.11 | 22.28 |
| 4565.8725 | NLFLNHSEN(N)ATAK Fuc 1 Hex 7 HexNAc 9 | 41.291 | 0.0 | -19.991 | 5.763e+06 | 202 - 215 | 2 | 18.10 | 18.27 | 18.27 |
| 4461.8415 | N(D)LFLNHSEN(N)ATAK Fuc 5 Hex 4 HexNAc 8 | 40.640 | 0.0 | -21.257 | 1.613e+06 | 202 - 215 | 1 | 18.10 | 18.10 | 18.10 |
| 4663.9211 | NLFLNHSEN(N)ATAK Fuc 5 Hex 4 HexNAc 9 | 32.224 | 0.005 | -4.624 | 1.502e+07 | 202 - 215 | 1 | 17.97 | 18.10 | 18.27 |
| 4478.8331 | NLFLNHSEN(N)ATAK Fuc 4 Hex 5 HexNAc 8 | 31.172 | 0.012 | 8.679 | 7.431e+07 | 202 - 215 | 3 | 38.40 | 38.57 | 39.07 |
| 4768.9522 | N(D)LFLNHSEN(N)ATAK Fuc 4 Hex 5 HexNAc 8 Neu5Ac 1 | 29.607 | 0.020 | -6.185 | 1.182e+07 | 202 - 215 | 1 | 17.90 | 17.97 | 18.10 |
| 2568.1144 | VVLHPN(N)YSQVD Hex 3 HexNAc 4 | 26.200 | 0.039 | 13.498 | 8.638e+06 | 235 - 246 | 3 | 23.91 | 24.03 | 24.31 |
| 2771.1928 | VVLHPN(N)YSQVD Hex 3 HexNAc 5 | 25.470 | 0.042 | 12.280 | 6.748e+06 | 235 - 246 | 2 | 23.76 | 23.91 | 24.03 |
| 4769.9589 | NLFLNHSEN(N)ATAK Fuc 4 Hex 5 HexNAc 8 Neu5Ac 1 | 25.318 | 0.042 | 0.000 | 0.000e+00 | 202 - 215 | 1 | - | - | - |

### VVLHPN(N)YSQVDIGLIK Hex 3 HexNAc 4

**Mass:**  3092.4834

(1.100e-06)

**Charge States:** 3, 4

**Start Position**: 236

**End Position**: 252

| Profile Score | Tandem Score | TDA q-value | Volume | Spectrum Matches |
| --- | --- | --- | --- | --- |
| 12.8000 | 171.6509 | 0.0000 | 4.5249e+07 | 4 |

**Adduct Signal Fractions**

- **Unmodified**: 4.5249e+07

### VVLHPN(N)YSQVDIGLIK Hex 3 HexNAc 6

**Mass:**  3498.6483

(2.731e-06)

**Charge States:** 3, 4

**Start Position**: 236

**End Position**: 252

| Profile Score | Tandem Score | TDA q-value | Volume | Spectrum Matches |
| --- | --- | --- | --- | --- |
| -19.9677 | 141.6979 | 0.0000 | 1.0510e+07 | 2 |

**Adduct Signal Fractions**

- **Unmodified**: 1.0510e+07

### NLFLNHSEN(N)ATAK Fuc 5 Hex 4 HexNAc 8

**Mass:**  4460.8410

(-4.585e-06)

**Charge States:** 4

**Start Position**: 203

**End Position**: 216

| Profile Score | Tandem Score | TDA q-value | Volume | Spectrum Matches |
| --- | --- | --- | --- | --- |
| -3.9899 | 123.9504 | 0.0000 | 3.8667e+07 | 4 |

**Adduct Signal Fractions**

- **Unmodified**: 3.8667e+07

### NLFLN(N)HSENATAK Hex 6 HexNAc 9

**Mass:**  4257.7639

(1.196e-06)

**Charge States:** 4

**Start Position**: 203

**End Position**: 216

| Profile Score | Tandem Score | TDA q-value | Volume | Spectrum Matches |
| --- | --- | --- | --- | --- |
| 12.4327 | 103.3805 | 0.0000 | 3.0991e+07 | 2 |

**Adduct Signal Fractions**

- **Unmodified**: 3.0991e+07

### NLFLNHSEN(N)ATAK Hex 6 HexNAc 8

**Mass:**  4054.6816

(8.217e-07)

**Charge States:** 4

**Start Position**: 203

**End Position**: 216

| Profile Score | Tandem Score | TDA q-value | Volume | Spectrum Matches |
| --- | --- | --- | --- | --- |
| -4.3628 | 46.3840 | 0.0000 | 8.5839e+06 | 3 |

**Adduct Signal Fractions**

- **Unmodified**: 8.5839e+06

### NLFLN(N)HSENATAK Fuc 5 Hex 4 HexNAc 7

**Mass:**  4258.7468

(2.331e-04)

**Charge States:** 4

**Start Position**: 203

**End Position**: 216

| Profile Score | Tandem Score | TDA q-value | Volume | Spectrum Matches |
| --- | --- | --- | --- | --- |
| -20.9222 | 45.1467 | 0.0000 | 1.6853e+06 | 3 |

**Adduct Signal Fractions**

- **Unmodified**: 1.6853e+06

### NLFLNHSEN(N)ATAK Fuc 1 Hex 7 HexNAc 9

**Mass:**  4565.8762

(3.865e-08)

**Charge States:** 4

**Start Position**: 203

**End Position**: 216

| Profile Score | Tandem Score | TDA q-value | Volume | Spectrum Matches |
| --- | --- | --- | --- | --- |
| -19.9907 | 41.2908 | 0.0000 | 5.7633e+06 | 2 |

**Adduct Signal Fractions**

- **Unmodified**: 5.7633e+06

### N(D)LFLNHSEN(N)ATAK Fuc 5 Hex 4 HexNAc 8

**Mass:**  4461.8415

(2.196e-04)

**Charge States:** 4

**Start Position**: 203

**End Position**: 216

| Profile Score | Tandem Score | TDA q-value | Volume | Spectrum Matches |
| --- | --- | --- | --- | --- |
| -21.2574 | 40.6399 | 0.0000 | 1.6126e+06 | 1 |

**Adduct Signal Fractions**

- **Unmodified**: 1.6126e+06

### NLFLNHSEN(N)ATAK Fuc 5 Hex 4 HexNAc 9

**Mass:**  4663.9201

(-3.972e-06)

**Charge States:** 4

**Start Position**: 203

**End Position**: 216

| Profile Score | Tandem Score | TDA q-value | Volume | Spectrum Matches |
| --- | --- | --- | --- | --- |
| -4.6240 | 32.2241 | 0.0053 | 1.5019e+07 | 1 |

**Adduct Signal Fractions**

- **Unmodified**: 1.5019e+07

### NLFLNHSEN(N)ATAK Fuc 4 Hex 5 HexNAc 8

**Mass:**  4478.8381

(4.475e-04)

**Charge States:** 3, 4

**Start Position**: 203

**End Position**: 216

| Profile Score | Tandem Score | TDA q-value | Volume | Spectrum Matches |
| --- | --- | --- | --- | --- |
| 8.6788 | 31.1722 | 0.0116 | 7.4314e+07 | 3 |

**Adduct Signal Fractions**

- **Unmodified**: 7.4314e+07

### N(D)LFLNHSEN(N)ATAK Fuc 4 Hex 5 HexNAc 8 Neu5Ac 1

**Mass:**  4768.9523

(2.086e-04)

**Charge States:** 4

**Start Position**: 203

**End Position**: 216

| Profile Score | Tandem Score | TDA q-value | Volume | Spectrum Matches |
| --- | --- | --- | --- | --- |
| -6.1851 | 29.6074 | 0.0195 | 1.1820e+07 | 1 |

**Adduct Signal Fractions**

- **Unmodified**: 1.1820e+07

### VVLHPN(N)YSQVD Hex 3 HexNAc 4

**Mass:**  2568.1135

(1.126e-06)

**Charge States:** 3

**Start Position**: 236

**End Position**: 247

| Profile Score | Tandem Score | TDA q-value | Volume | Spectrum Matches |
| --- | --- | --- | --- | --- |
| 13.4983 | 26.2003 | 0.0391 | 8.6384e+06 | 3 |

**Adduct Signal Fractions**

- **Unmodified**: 8.6384e+06

### VVLHPN(N)YSQVD Hex 3 HexNAc 5

**Mass:**  2771.1926

(1.791e-06)

**Charge States:** 3

**Start Position**: 236

**End Position**: 247

| Profile Score | Tandem Score | TDA q-value | Volume | Spectrum Matches |
| --- | --- | --- | --- | --- |
| 12.2800 | 25.4698 | 0.0417 | 6.7475e+06 | 2 |

**Adduct Signal Fractions**

- **Unmodified**: 6.7475e+06

### NLFLNHSEN(N)ATAK Fuc 4 Hex 5 HexNAc 8 Neu5Ac 1

**Mass:**  4769.9589

(4.208e-04)

**Start Position**: 203

**End Position**: 216

| Profile Score | Tandem Score | TDA q-value | Volume | Spectrum Matches |
| --- | --- | --- | --- | --- |
| 0.0000 | 25.3185 | 0.0417 | 0.0000e+00 | 1 |

Adduct:

No Chromatogram Found

### Q9Y5Y7|LYVE1\_HUMAN

**>Q9Y5Y7|LYVE1\_HUMAN**  
 `MARCFSLVLLLTSIWTTRLLVQGSLRAEELSIQVSCRIMGITLVSKKANQQLNFTEAKEACRLLGLSLAGKDQVETALKA  
SFETCSYGWVGDGFVVISRISPNPKCGKNGVGVLIWKVPVSRQFAAYCYNSSDTWTNSCIPEIITTKDPIFNTQTATQTT  
EFIVSDSTYSVASPYSTIPAPTTTPPAPASTSIPRRKKLICVTEVFMETSTMSTETEPFVENKAAFKNEAAGFGGVPTAL  
LVLALLFFGAAAGLGFCYVKRYVKAFPFTNKNQQKEMIETKVVKEEKANDSNPNEESKKTDKNPEESKSPSKTTVRCLEA  
EV`

| Site | Type | Count |
| --- | --- | --- |
| 53 | N-linked | 9 |

| Mass | Sequence | MS_2_ Score | `q`-value | MS_1_ Score | Signal | Protein Coordinates | Tandem Scans | Start Time | Apex Time | End Time |
| --- | --- | --- | --- | --- | --- | --- | --- | --- | --- | --- |
| 2892.2778 | KANQQLN(N)FTEAK Hex 3 HexNAc 5 | 182.929 | 0.0 | 12.199 | 4.686e+07 | 46 - 58 | 2 | 17.48 | 17.60 | 17.90 |
| 2689.1995 | KANQQLN(N)FTEAK Hex 3 HexNAc 4 | 151.469 | 0.0 | -3.762 | 1.714e+07 | 46 - 58 | 2 | 17.60 | 17.65 | 17.80 |
| 2764.1848 | ANQQLN(N)FTEAK Hex 3 HexNAc 5 | 135.960 | 0.0 | 12.949 | 1.629e+07 | 47 - 58 | 3 | 21.15 | 21.36 | 21.83 |
| 2561.1103 | ANQQLN(N)FTEAK Hex 3 HexNAc 4 | 116.803 | 0.0 | 11.048 | 5.197e+06 | 47 - 58 | 4 | 21.22 | 21.51 | 21.83 |
| 3129.3163 | ANQQLN(N)FTEAK Hex 4 HexNAc 6 | 113.571 | 0.0 | -2.888 | 5.524e+06 | 47 - 58 | 1 | 21.15 | 21.22 | 21.36 |
| 3623.5846 | KANQQLN(N)FTEAK Hex 5 HexNAc 7 | 97.804 | 0.0 | -6.908 | 1.441e+07 | 46 - 58 | 47 | 50.75 | 50.92 | 98.58 |
| 3257.4073 | KANQQLN(N)FTEAK Hex 4 HexNAc 6 | 92.229 | 0.0 | -20.102 | 8.603e+06 | 46 - 58 | 2 | 17.48 | 17.48 | 17.60 |
| 3495.4429 | ANQQLN(N)FTEAK Hex 5 HexNAc 7 | 35.343 | 0.003 | 0.000 | 0.000e+00 | 47 - 58 | 1 | - | - | - |
| 3258.4110 | KAN(D)QQLN(N)FTEAK Hex 4 HexNAc 6 | 28.661 | 0.022 | -21.564 | 1.218e+06 | 46 - 58 | 1 | 17.35 | 17.35 | 17.35 |

### KANQQLN(N)FTEAK Hex 3 HexNAc 5

**Mass:**  2892.2779

(4.688e-07)

**Charge States:** 3

**Start Position**: 47

**End Position**: 59

| Profile Score | Tandem Score | TDA q-value | Volume | Spectrum Matches |
| --- | --- | --- | --- | --- |
| 12.1991 | 182.9294 | 0.0000 | 4.6858e+07 | 2 |

**Adduct Signal Fractions**

- **Unmodified**: 4.6858e+07

### KANQQLN(N)FTEAK Hex 3 HexNAc 4

**Mass:**  2689.2000

(1.371e-06)

**Charge States:** 3

**Start Position**: 47

**End Position**: 59

| Profile Score | Tandem Score | TDA q-value | Volume | Spectrum Matches |
| --- | --- | --- | --- | --- |
| -3.7615 | 151.4688 | 0.0000 | 1.7137e+07 | 2 |

**Adduct Signal Fractions**

- **Unmodified**: 1.7137e+07

### ANQQLN(N)FTEAK Hex 3 HexNAc 5

**Mass:**  2764.1845

(6.108e-07)

**Charge States:** 3

**Start Position**: 48

**End Position**: 59

| Profile Score | Tandem Score | TDA q-value | Volume | Spectrum Matches |
| --- | --- | --- | --- | --- |
| 12.9487 | 135.9599 | 0.0000 | 1.6292e+07 | 3 |

**Adduct Signal Fractions**

- **Unmodified**: 1.6292e+07

### ANQQLN(N)FTEAK Hex 3 HexNAc 4

**Mass:**  2561.1064

(1.917e-06)

**Charge States:** 3

**Start Position**: 48

**End Position**: 59

| Profile Score | Tandem Score | TDA q-value | Volume | Spectrum Matches |
| --- | --- | --- | --- | --- |
| 11.0482 | 116.8027 | 0.0000 | 5.1975e+06 | 4 |

**Adduct Signal Fractions**

- **Unmodified**: 5.1975e+06

### ANQQLN(N)FTEAK Hex 4 HexNAc 6

**Mass:**  3129.3151

(6.512e-07)

**Charge States:** 3

**Start Position**: 48

**End Position**: 59

| Profile Score | Tandem Score | TDA q-value | Volume | Spectrum Matches |
| --- | --- | --- | --- | --- |
| -2.8881 | 113.5709 | 0.0000 | 5.5240e+06 | 1 |

**Adduct Signal Fractions**

- **Unmodified**: 5.5240e+06

### KANQQLN(N)FTEAK Hex 5 HexNAc 7

**Mass:**  3623.5860

(2.811e-04)

**Charge States:** 3, 4

**Start Position**: 47

**End Position**: 59

| Profile Score | Tandem Score | TDA q-value | Volume | Spectrum Matches |
| --- | --- | --- | --- | --- |
| -6.9078 | 97.8039 | 0.0000 | 1.4411e+07 | 47 |

**Adduct Signal Fractions**

- **Unmodified**: 1.4411e+07

### KANQQLN(N)FTEAK Hex 4 HexNAc 6

**Mass:**  3257.4054

(1.700e-07)

**Charge States:** 3

**Start Position**: 47

**End Position**: 59

| Profile Score | Tandem Score | TDA q-value | Volume | Spectrum Matches |
| --- | --- | --- | --- | --- |
| -20.1023 | 92.2288 | 0.0000 | 8.6029e+06 | 2 |

**Adduct Signal Fractions**

- **Unmodified**: 8.6029e+06

### ANQQLN(N)FTEAK Hex 5 HexNAc 7

**Mass:**  3495.4429

(2.855e-04)

**Start Position**: 48

**End Position**: 59

| Profile Score | Tandem Score | TDA q-value | Volume | Spectrum Matches |
| --- | --- | --- | --- | --- |
| 0.0000 | 35.3430 | 0.0028 | 0.0000e+00 | 1 |

Adduct:

No Chromatogram Found

### KAN(D)QQLN(N)FTEAK Hex 4 HexNAc 6

**Mass:**  3258.4110

(3.079e-04)

**Charge States:** 3

**Start Position**: 47

**End Position**: 59

| Profile Score | Tandem Score | TDA q-value | Volume | Spectrum Matches |
| --- | --- | --- | --- | --- |
| -21.5640 | 28.6608 | 0.0215 | 1.2179e+06 | 1 |

**Adduct Signal Fractions**

- **Unmodified**: 1.2179e+06

### P05155|IC1\_HUMAN

**>P05155|IC1\_HUMAN**  
 `MASRLTLLTLLLLLLAGDRASSNPNATSSSSQDPESLQDRGEGKVATTVISKMLFVEPILEVSSLPTTNSTTNSATKITA  
NTTDEPTTQPTTEPTTQPTIQPTQPTTQLPTDSPTQPTTGSFCPGPVTLCSDLESHSTEAVLGDALVDFSLKLYHAFSAM  
KKVETNMAFSPFSIASLLTQVLLGAGENTKTNLESILSYPKDFTCVHQALKGFTTKGVTSVSQIFHSPDLAIRDTFVNAS  
RTLYSSSPRVLSNNSDANLELINTWVAKNTNNKISRLLDSLPSDTRLVLLNAIYLSAKWKTTFDPKKTRMEPFHFKNSVI  
KVPMMNSKKYPVAHFIDQTLKAKVGQLQLSHNLSLVILVPQNLKHRLEDMEQALSPSVFKAIMEKLEMSKFQPTLLTLPR  
IKVTTSQDMLSIMEKLEFFDFSYDLNLCGLTEDPDLQVSAMQHQTVLELTETGVEAAAASAISVARTLLVFEVQQPFLFV  
LWDQQHKFPVFMGRVYDPRA`

| Site | Type | Count |
| --- | --- | --- |
| 25 | N-linked | 7 |

| Mass | Sequence | MS_2_ Score | `q`-value | MS_1_ Score | Signal | Protein Coordinates | Tandem Scans | Start Time | Apex Time | End Time |
| --- | --- | --- | --- | --- | --- | --- | --- | --- | --- | --- |
| 3595.4567 | NPN(N)ATSSSSQDPESLQDR Hex 4 HexNAc 5 | 125.152 | 0.0 | -5.204 | 4.576e+07 | 22 - 40 | 3 | 13.81 | 13.98 | 14.29 |
| 3798.5383 | NPN(N)ATSSSSQDPESLQDR Hex 4 HexNAc 6 | 104.750 | 0.0 | -20.814 | 1.425e+07 | 22 - 40 | 2 | 13.81 | 13.98 | 13.98 |
| 3433.4042 | NPN(N)ATSSSSQDPESLQDR Hex 3 HexNAc 5 | 62.428 | 0.0 | -5.763 | 1.300e+07 | 22 - 40 | 3 | 15.07 | 15.24 | 15.40 |
| 3377.3902 | NPN(N)ATSSSSQDPESLQDR Fuc 1 Hex 3 HexNAc 4 | 55.428 | 0.0 | 0.000 | 0.000e+00 | 22 - 40 | 1 | - | - | - |
| 3230.3262 | NPN(N)ATSSSSQDPESLQDR Hex 3 HexNAc 4 | 54.950 | 0.0 | -4.581 | 2.360e+07 | 22 - 40 | 2 | 15.07 | 15.24 | 15.40 |
| 3743.5342 | NPN(N)ATSSSSQDPESLQDR Fuc 1 Hex 4 HexNAc 5 | 45.287 | 0.0 | 0.000 | 0.000e+00 | 22 - 40 | 2 | - | - | - |
| 3376.3824 | NPN(N)ATSSSSQDPESLQDR Fuc 1 Hex 3 HexNAc 4 | 44.689 | 0.0 | -19.719 | 1.093e+07 | 22 - 40 | 2 | 15.07 | 15.07 | 15.24 |

### NPN(N)ATSSSSQDPESLQDR Hex 4 HexNAc 5

**Mass:**  3595.4573

(1.130e-06)

**Charge States:** 3

**Start Position**: 23

**End Position**: 41

| Profile Score | Tandem Score | TDA q-value | Volume | Spectrum Matches |
| --- | --- | --- | --- | --- |
| -5.2043 | 125.1517 | 0.0000 | 4.5758e+07 | 3 |

**Adduct Signal Fractions**

- **Unmodified**: 4.5758e+07

### NPN(N)ATSSSSQDPESLQDR Hex 4 HexNAc 6

**Mass:**  3798.5385

(3.142e-07)

**Charge States:** 3, 4

**Start Position**: 23

**End Position**: 41

| Profile Score | Tandem Score | TDA q-value | Volume | Spectrum Matches |
| --- | --- | --- | --- | --- |
| -20.8140 | 104.7495 | 0.0000 | 1.4255e+07 | 2 |

**Adduct Signal Fractions**

- **Unmodified**: 1.4255e+07

### NPN(N)ATSSSSQDPESLQDR Hex 3 HexNAc 5

**Mass:**  3433.4035

(5.313e-07)

**Charge States:** 3

**Start Position**: 23

**End Position**: 41

| Profile Score | Tandem Score | TDA q-value | Volume | Spectrum Matches |
| --- | --- | --- | --- | --- |
| -5.7625 | 62.4276 | 0.0000 | 1.3002e+07 | 3 |

**Adduct Signal Fractions**

- **Unmodified**: 1.3002e+07

### NPN(N)ATSSSSQDPESLQDR Fuc 1 Hex 3 HexNAc 4

**Mass:**  3377.3902

(2.993e-04)

**Start Position**: 23

**End Position**: 41

| Profile Score | Tandem Score | TDA q-value | Volume | Spectrum Matches |
| --- | --- | --- | --- | --- |
| 0.0000 | 55.4277 | 0.0000 | 0.0000e+00 | 1 |

Adduct:

No Chromatogram Found

### NPN(N)ATSSSSQDPESLQDR Hex 3 HexNAc 4

**Mass:**  3230.3263

(1.623e-06)

**Charge States:** 3

**Start Position**: 23

**End Position**: 41

| Profile Score | Tandem Score | TDA q-value | Volume | Spectrum Matches |
| --- | --- | --- | --- | --- |
| -4.5815 | 54.9504 | 0.0000 | 2.3605e+07 | 2 |

**Adduct Signal Fractions**

- **Unmodified**: 2.3605e+07

### NPN(N)ATSSSSQDPESLQDR Fuc 1 Hex 4 HexNAc 5

**Mass:**  3743.5342

(8.003e-04)

**Start Position**: 23

**End Position**: 41

| Profile Score | Tandem Score | TDA q-value | Volume | Spectrum Matches |
| --- | --- | --- | --- | --- |
| 0.0000 | 45.2874 | 0.0000 | 0.0000e+00 | 2 |

Adduct:

No Chromatogram Found

### NPN(N)ATSSSSQDPESLQDR Fuc 1 Hex 3 HexNAc 4

**Mass:**  3376.3841

(-2.114e-07)

**Charge States:** 3

**Start Position**: 23

**End Position**: 41

| Profile Score | Tandem Score | TDA q-value | Volume | Spectrum Matches |
| --- | --- | --- | --- | --- |
| -19.7194 | 44.6886 | 0.0000 | 1.0931e+07 | 2 |

**Adduct Signal Fractions**

- **Unmodified**: 1.0931e+07

### Q12913|PTPRJ\_HUMAN

**>Q12913|PTPRJ\_HUMAN**  
 `MKPAAREARLPPRSPGLRWALPLLLLLLRLGQILCAGGTPSPIPDPSVATVATGENGITQISSTAESFHKQNGTGTPQVE  
TNTSEDGESSGANDSLRTPEQGSNGTDGASQKTPSSTGPSPVFDIKAVSISPTNVILTWKSNDTAASEYKYVVKHKMENE  
KTITVVHQPWCNITGLRPATSYVFSITPGIGNETWGDPRVIKVITEPIPVSDLRVALTGVRKAALSWSNGNGTASCRVLL  
ESIGSHEELTQDSRLQVNISGLKPGVQYNINPYLLQSNKTKGDPLGTEGGLDASNTERSRAGSPTAPVHDESLVGPVDPS  
SGQQSRDTEVLLVGLEPGTRYNATVYSQAANGTEGQPQAIEFRTNAIQVFDVTAVNISATSLTLIWKVSDNESSSNYTYK  
IHVAGETDSSNLNVSEPRAVIPGLRSSTFYNITVCPVLGDIEGTPGFLQVHTPPVPVSDFRVTVVSTTEIGLAWSSHDAE  
SFQMHITQEGAGNSRVEITTNQSIIIGGLFPGTKYCFEIVPKGPNGTEGASRTVCNRTVPSAVFDIHVVYVTTTEMWLDW  
KSPDGASEYVYHLVIESKHGSNHTSTYDKAITLQGLIPGTLYNITISPEVDHVWGDPNSTAQYTRPSNVSNIDVSTNTTA  
ATLSWQNFDDASPTYSYCLLIEKAGNSSNATQVVTDIGITDATVTELIPGSSYTVEIFAQVGDGIKSLEPGRKSFCTDPA  
SMASFDCEVVPKEPALVLKWTCPPGANAGFELEVSSGAWNNATHLESCSSENGTEYRTEVTYLNFSTSYNISITTVSCGK  
MAAPTRNTCTTGITDPPPPDGSPNITSVSHNSVKVKFSGFEASHGPIKAYAVILTTGEAGHPSADVLKYTYEDFKKGASD  
TYVTYLIRTEEKGRSQSLSEVLKYEIDVGNESTTLGYYNGKLEPLGSYRACVAGFTNITFHPQNKGLIDGAESYVSFSRY  
SDAVSLPQDPGVICGAVFGCIFGALVIVTVGGFIFWRKKRKDAKNNEVSFSQIKPKKSKLIRVENFEAYFKKQQADSNCG  
FAEEYEDLKLVGISQPKYAAELAENRGKNRYNNVLPYDISRVKLSVQTHSTDDYINANYMPGYHSKKDFIATQGPLPNTL  
KDFWRMVWEKNVYAIIMLTKCVEQGRTKCEEYWPSKQAQDYGDITVAMTSEIVLPEWTIRDFTVKNIQTSESHPLRQFHF  
TSWPDHGVPDTTDLLINFRYLVRDYMKQSPPESPILVHCSAGVGRTGTFIAIDRLIYQIENENTVDVYGIVYDLRMHRPL  
MVQTEDQYVFLNQCVLDIVRSQKDSKVDLIYQNTTAMTIYENLAPVTTFGKTNGYIA`

| Site | Type | Count |
| --- | --- | --- |
| 525 | N-linked | 1 |
| 413 | N-linked | 4 |
| 231 | N-linked | 1 |

| Mass | Sequence | MS_2_ Score | `q`-value | MS_1_ Score | Signal | Protein Coordinates | Tandem Scans | Start Time | Apex Time | End Time |
| --- | --- | --- | --- | --- | --- | --- | --- | --- | --- | --- |
| 3425.4841 | IHVAGETDSSNLN(N)VSEPR Hex 3 HexNAc 5 | 75.789 | 0.0 | -2.249 | 2.153e+07 | 400 - 418 | 7 | 23.50 | 23.58 | 43.42 |
| 3970.5704 | GPN(N)GTEGASR Fuc 1 Hex 9 HexNAc 7 | 70.312 | 0.0 | -18.861 | 4.053e+07 | 522 - 532 | 1 | 33.27 | 33.27 | 33.44 |
| 3734.6029 | IHVAGETDSSNLN(N)VSEPR Fuc 1 Hex 4 HexNAc 5 | 34.936 | 0.003 | 0.000 | 0.000e+00 | 400 - 418 | 1 | - | - | - |
| 3733.5972 | IHVAGETDSSNLN(N)VSEPR Fuc 1 Hex 4 HexNAc 5 | 33.881 | 0.004 | 7.620 | 6.411e+06 | 400 - 418 | 2 | 23.33 | 23.33 | 23.44 |
| 3222.4039 | IHVAGETDSSNLN(N)VSEPR Hex 3 HexNAc 4 | 32.899 | 0.005 | -4.082 | 2.245e+06 | 400 - 418 | 2 | 23.65 | 23.76 | 23.88 |
| 2850.1518 | AALSWSN(D)GN(N)GTASC(C)R Hex 3 HexNAc 4 | 26.073 | 0.039 | 9.526 | 1.017e+07 | 222 - 237 | 3 | 22.74 | 22.86 | 23.41 |

### IHVAGETDSSNLN(N)VSEPR Hex 3 HexNAc 5

**Mass:**  3425.4871

(1.007e-06)

**Charge States:** 3, 4

**Start Position**: 401

**End Position**: 419

| Profile Score | Tandem Score | TDA q-value | Volume | Spectrum Matches |
| --- | --- | --- | --- | --- |
| -2.2494 | 75.7893 | 0.0000 | 2.1533e+07 | 7 |

**Adduct Signal Fractions**

- **Unmodified**: 2.1533e+07

### GPN(N)GTEGASR Fuc 1 Hex 9 HexNAc 7

**Mass:**  3970.5566

(9.186e-06)

**Charge States:** 4

**Start Position**: 523

**End Position**: 533

| Profile Score | Tandem Score | TDA q-value | Volume | Spectrum Matches |
| --- | --- | --- | --- | --- |
| -18.8614 | 70.3125 | 0.0000 | 4.0527e+07 | 1 |

**Adduct Signal Fractions**

- **Unmodified**: 4.0527e+07

### IHVAGETDSSNLN(N)VSEPR Fuc 1 Hex 4 HexNAc 5

**Mass:**  3734.6029

(2.700e-04)

**Start Position**: 401

**End Position**: 419

| Profile Score | Tandem Score | TDA q-value | Volume | Spectrum Matches |
| --- | --- | --- | --- | --- |
| 0.0000 | 34.9359 | 0.0028 | 0.0000e+00 | 1 |

Adduct:

No Chromatogram Found

### IHVAGETDSSNLN(N)VSEPR Fuc 1 Hex 4 HexNAc 5

**Mass:**  3733.5964

(1.931e-06)

**Charge States:** 3, 4

**Start Position**: 401

**End Position**: 419

| Profile Score | Tandem Score | TDA q-value | Volume | Spectrum Matches |
| --- | --- | --- | --- | --- |
| 7.6197 | 33.8809 | 0.0041 | 6.4107e+06 | 2 |

**Adduct Signal Fractions**

- **Unmodified**: 6.4107e+06

### IHVAGETDSSNLN(N)VSEPR Hex 3 HexNAc 4

**Mass:**  3222.4037

(3.422e-06)

**Charge States:** 3

**Start Position**: 401

**End Position**: 419

| Profile Score | Tandem Score | TDA q-value | Volume | Spectrum Matches |
| --- | --- | --- | --- | --- |
| -4.0824 | 32.8987 | 0.0053 | 2.2454e+06 | 2 |

**Adduct Signal Fractions**

- **Unmodified**: 2.2454e+06

### AALSWSN(D)GN(N)GTASC(C)R Hex 3 HexNAc 4

**Mass:**  2850.1507

(3.462e-04)

**Charge States:** 3

**Start Position**: 223

**End Position**: 238

| Profile Score | Tandem Score | TDA q-value | Volume | Spectrum Matches |
| --- | --- | --- | --- | --- |
| 9.5257 | 26.0734 | 0.0391 | 1.0168e+07 | 3 |

**Adduct Signal Fractions**

- **Unmodified**: 1.0168e+07

### O95497|VNN1\_HUMAN

**>O95497|VNN1\_HUMAN**  
 `MTTQLPAYVAILLFYVSRASCQDTFTAAVYEHAAILPNATLTPVSREEALALMNRNLDILEGAITSAADQGAHIIVTPED  
AIYGWNFNRDSLYPYLEDIPDPEVNWIPCNNRNRFGQTPVQERLSCLAKNNSIYVVANIGDKKPCDTSDPQCPPDGRYQY  
NTDVVFDSQGKLVARYHKQNLFMGENQFNVPKEPEIVTFNTTFGSFGIFTCFDILFHDPAVTLVKDFHVDTIVFPTAWMN  
VLPHLSAVEFHSAWAMGMRVNFLASNIHYPSKKMTGSGIYAPNSSRAFHYDMKTEEGKLLLSQLDSHPSHSAVVNWTSYA  
SSIEALSSGNKEFKGTVFFDEFTFVKLTGVAGNYTVCQKDLCCHLSYKMSENIPNEVYALGAFDGLHTVEGRYYLQICTL  
LKCKTTNLNTCGDSAETASTRFEMFSLSGTFGTQYVFPEVLLSENQLAPGEFQVSTDGRLFSLKPTSGPVLTVTLFGRLY  
EKDWASNASSGLTAQARIIMLIVIAPIVCSLSW`

| Site | Type | Count |
| --- | --- | --- |
| 353 | N-linked | 3 |

| Mass | Sequence | MS_2_ Score | `q`-value | MS_1_ Score | Signal | Protein Coordinates | Tandem Scans | Start Time | Apex Time | End Time |
| --- | --- | --- | --- | --- | --- | --- | --- | --- | --- | --- |
| 2911.2564 | LTGVAGN(N)YTVC(C)QK Hex 3 HexNAc 5 | 106.824 | 0.0 | -3.933 | 7.761e+06 | 346 - 359 | 2 | 21.96 | 22.11 | 22.39 |
| 3219.3683 | LTGVAGN(N)YTVC(C)QK Fuc 1 Hex 4 HexNAc 5 | 98.817 | 0.0 | -19.450 | 1.471e+06 | 346 - 359 | 1 | 21.66 | 21.83 | 21.83 |
| 2708.1737 | LTGVAGN(N)YTVC(C)QK Hex 3 HexNAc 4 | 28.623 | 0.022 | -5.186 | 3.056e+06 | 346 - 359 | 1 | 22.11 | 22.28 | 22.49 |

### LTGVAGN(N)YTVC(C)QK Hex 3 HexNAc 5

**Mass:**  2911.2561

(2.146e-08)

**Charge States:** 3

**Start Position**: 347

**End Position**: 360

| Profile Score | Tandem Score | TDA q-value | Volume | Spectrum Matches |
| --- | --- | --- | --- | --- |
| -3.9333 | 106.8243 | 0.0000 | 7.7606e+06 | 2 |

**Adduct Signal Fractions**

- **Unmodified**: 7.7606e+06

### LTGVAGN(N)YTVC(C)QK Fuc 1 Hex 4 HexNAc 5

**Mass:**  3219.3697

(1.938e-07)

**Charge States:** 3

**Start Position**: 347

**End Position**: 360

| Profile Score | Tandem Score | TDA q-value | Volume | Spectrum Matches |
| --- | --- | --- | --- | --- |
| -19.4499 | 98.8174 | 0.0000 | 1.4709e+06 | 1 |

**Adduct Signal Fractions**

- **Unmodified**: 1.4709e+06

### LTGVAGN(N)YTVC(C)QK Hex 3 HexNAc 4

**Mass:**  2708.1751

(6.469e-07)

**Charge States:** 3

**Start Position**: 347

**End Position**: 360

| Profile Score | Tandem Score | TDA q-value | Volume | Spectrum Matches |
| --- | --- | --- | --- | --- |
| -5.1859 | 28.6235 | 0.0215 | 3.0558e+06 | 1 |

**Adduct Signal Fractions**

- **Unmodified**: 3.0558e+06

### P02790|HEMO\_HUMAN

**>P02790|HEMO\_HUMAN**  
 `MARVLGAPVALGLWSLCWSLAIATPLPPTSAHGNVAEGETKPDPDVTERCSDGWSFDATTLDDNGTMLFFKGEFVWKSHK  
WDRELISERWKNFPSPVDAAFRQGHNSVFLIKGDKVWVYPPEKKEKGYPKLLQDEFPGIPSPLDAAVECHRGECQAEGVL  
FFQGDREWFWDLATGTMKERSWPAVGNCSSALRWLGRYYCFQGNQFLRFDPVRGEVPPRYPRDVRDYFMPCPGRGHGHRN  
GTGHGNSTHHGPEYMRCSPHLVLSALTSDNHGATYAFSGTHYWRLDTSRDGWHSWPIAHQWPQGPSAVDAAFSWEEKLYL  
VQGTQVYVFLTKGGYTLVSGYPKRLEKEVGTPHGIILDSVDAAFICPGSSRLHIMAGRRLWWLDLKSGAQATWTELPWPH  
EKVDGALCMEKSLGPNSCSANGPGLYLIHGPNLYCYSDVEKLNAAKALPQPQNVTSLLGCTH`

| Site | Type | Count |
| --- | --- | --- |
| 187 | N-linked | 2 |
| 453 | N-linked | 1 |

| Mass | Sequence | MS_2_ Score | `q`-value | MS_1_ Score | Signal | Protein Coordinates | Tandem Scans | Start Time | Apex Time | End Time |
| --- | --- | --- | --- | --- | --- | --- | --- | --- | --- | --- |
| 2702.1396 | SWPAVGN(N)C(C)SSALR Hex 3 HexNAc 4 | 115.231 | 0.0 | 12.724 | 8.850e+06 | 180 - 193 | 3 | 31.07 | 31.24 | 31.75 |
| 3033.3503 | ALPQPQN(N)VTSLLGC(C)TH Hex 3 HexNAc 4 | 92.513 | 0.0 | 15.247 | 2.297e+07 | 446 - 462 | 2 | 44.25 | 44.59 | 44.92 |
| 2905.2210 | SWPAVGN(N)C(C)SSALR Hex 3 HexNAc 5 | 41.884 | 0.0 | -4.801 | 3.104e+06 | 180 - 193 | 2 | 30.93 | 31.07 | 31.24 |

### SWPAVGN(N)C(C)SSALR Hex 3 HexNAc 4

**Mass:**  2702.1392

(2.872e-07)

**Charge States:** 3

**Start Position**: 181

**End Position**: 194

| Profile Score | Tandem Score | TDA q-value | Volume | Spectrum Matches |
| --- | --- | --- | --- | --- |
| 12.7237 | 115.2312 | 0.0000 | 8.8495e+06 | 3 |

**Adduct Signal Fractions**

- **Unmodified**: 8.8495e+06

### ALPQPQN(N)VTSLLGC(C)TH Hex 3 HexNAc 4

**Mass:**  3033.3497

(4.916e-07)

**Charge States:** 3

**Start Position**: 447

**End Position**: 463

| Profile Score | Tandem Score | TDA q-value | Volume | Spectrum Matches |
| --- | --- | --- | --- | --- |
| 15.2470 | 92.5128 | 0.0000 | 2.2967e+07 | 2 |

**Adduct Signal Fractions**

- **Unmodified**: 2.2967e+07

### SWPAVGN(N)C(C)SSALR Hex 3 HexNAc 5

**Mass:**  2905.2207

(1.074e-06)

**Charge States:** 3

**Start Position**: 181

**End Position**: 194

| Profile Score | Tandem Score | TDA q-value | Volume | Spectrum Matches |
| --- | --- | --- | --- | --- |
| -4.8007 | 41.8844 | 0.0000 | 3.1043e+06 | 2 |

**Adduct Signal Fractions**

- **Unmodified**: 3.1043e+06

### Q5R543|HEMO\_PONAB

**>Q5R543|HEMO\_PONAB**  
 `MARALGAPIALGLWSLCWSLAIATPLPPTSAHGNVAEGETKPDPDVTERCSDGWSFDATTLDDNGTMLFFKGEFVWKSHK  
WDRELISERWKNFPSPVDAAFRQGHNSVFLIKGDKVWVYPPEKKEKGYPKLLQDEFPGIPSPLDAAVECHRGECQAEGVL  
FFQGDREWFWDLATGTMKERSWPAVGNCSSALRWLGRYYCFQGNQFLRFDPVRGEVPPRYPRDVRDYFMPCPGRGHGHRN  
GTGHGNGTHHGPEYMRCSPHLVLSALTSDNHGATYAFSGTHYWRLDTSRDGWHSWPIAHQWPQGPSTVDAAFSWEEKLYL  
VQGTQVYVFLTKGGYTLVSGYPKQLEKEVGTPHGIILDSVDAAFICPGSSRLHIMAGRRLWWLDLKSGAQALWTELPWPH  
EKVDGALCVEKSLGPNSCSANGPGLYLIHGPNLYCYSDVEKLNAAKALPQPQNVTSLLGCTH`

| Site | Type | Count |
| --- | --- | --- |
| 187 | N-linked | 2 |
| 453 | N-linked | 1 |

| Mass | Sequence | MS_2_ Score | `q`-value | MS_1_ Score | Signal | Protein Coordinates | Tandem Scans | Start Time | Apex Time | End Time |
| --- | --- | --- | --- | --- | --- | --- | --- | --- | --- | --- |
| 2702.1396 | SWPAVGN(N)C(C)SSALR Hex 3 HexNAc 4 | 115.231 | 0.0 | 12.724 | 8.850e+06 | 180 - 193 | 3 | 31.07 | 31.24 | 31.75 |
| 3033.3503 | ALPQPQN(N)VTSLLGC(C)TH Hex 3 HexNAc 4 | 92.513 | 0.0 | 15.247 | 2.297e+07 | 446 - 462 | 2 | 44.25 | 44.59 | 44.92 |
| 2905.2210 | SWPAVGN(N)C(C)SSALR Hex 3 HexNAc 5 | 41.884 | 0.0 | -4.801 | 3.104e+06 | 180 - 193 | 2 | 30.93 | 31.07 | 31.24 |

### SWPAVGN(N)C(C)SSALR Hex 3 HexNAc 4

**Mass:**  2702.1392

(2.872e-07)

**Charge States:** 3

**Start Position**: 181

**End Position**: 194

| Profile Score | Tandem Score | TDA q-value | Volume | Spectrum Matches |
| --- | --- | --- | --- | --- |
| 12.7237 | 115.2312 | 0.0000 | 8.8495e+06 | 3 |

**Adduct Signal Fractions**

- **Unmodified**: 8.8495e+06

### ALPQPQN(N)VTSLLGC(C)TH Hex 3 HexNAc 4

**Mass:**  3033.3497

(4.916e-07)

**Charge States:** 3

**Start Position**: 447

**End Position**: 463

| Profile Score | Tandem Score | TDA q-value | Volume | Spectrum Matches |
| --- | --- | --- | --- | --- |
| 15.2470 | 92.5128 | 0.0000 | 2.2967e+07 | 2 |

**Adduct Signal Fractions**

- **Unmodified**: 2.2967e+07

### SWPAVGN(N)C(C)SSALR Hex 3 HexNAc 5

**Mass:**  2905.2207

(1.074e-06)

**Charge States:** 3

**Start Position**: 181

**End Position**: 194

| Profile Score | Tandem Score | TDA q-value | Volume | Spectrum Matches |
| --- | --- | --- | --- | --- |
| -4.8007 | 41.8844 | 0.0000 | 3.1043e+06 | 2 |

**Adduct Signal Fractions**

- **Unmodified**: 3.1043e+06

### P11279|LAMP1\_HUMAN

**>P11279|LAMP1\_HUMAN**  
 `MAAPGSARRPLLLLLLLLLLGLMHCASAAMFMVKNGNGTACIMANFSAAFSVNYDTKSGPKNMTFDLPSDATVVLNRSSC  
GKENTSDPSLVIAFGRGHTLTLNFTRNATRYSVQLMSFVYNLSDTHLFPNASSKEIKTVESITDIRADIDKKYRCVSGTQ  
VHMNNVTVTLHDATIQAYLSNSSFSRGETRCEQDRPSPTTAPPAPPSPSPSPVPKSPSVDKYNVSGTNGTCLLASMGLQL  
NLTYERKDNTTVTRLLNINPNKTSASGSCGAHLVTLELHSEGTTVLLFQFGMNASSSRFFLQGIQLNTILPDARDPAFKA  
ANGSLRALQATVGNSYKCNAEEHVRVTKAFSVNIFKVWVQAFKVEGGQFGSVEECLLDENSMLIPIAVGGALAGLVLIVL  
IAYLVGRKRSHAGYQTI`

| Site | Type | Count |
| --- | --- | --- |
| 103 | N-linked | 3 |

| Mass | Sequence | MS_2_ Score | `q`-value | MS_1_ Score | Signal | Protein Coordinates | Tandem Scans | Start Time | Apex Time | End Time |
| --- | --- | --- | --- | --- | --- | --- | --- | --- | --- | --- |
| 2578.1202 | GHTLTLN(N)FTR Hex 5 HexNAc 3 | 137.554 | 0.0 | -3.425 | 4.287e+06 | 96 - 106 | 2 | 29.91 | 30.08 | 30.42 |
| 2416.0654 | GHTLTLN(N)FTR Hex 4 HexNAc 3 | 137.364 | 0.0 | -18.508 | 2.541e+06 | 96 - 106 | 2 | 30.25 | 30.25 | 30.42 |
| 2457.0923 | GHTLTLN(N)FTR Hex 3 HexNAc 4 | 115.021 | 0.0 | -5.874 | 1.356e+06 | 96 - 106 | 1 | 30.25 | 30.25 | 30.59 |

### GHTLTLN(N)FTR Hex 5 HexNAc 3

**Mass:**  2578.1204

(1.139e-06)

**Charge States:** 3

**Start Position**: 97

**End Position**: 107

| Profile Score | Tandem Score | TDA q-value | Volume | Spectrum Matches |
| --- | --- | --- | --- | --- |
| -3.4254 | 137.5539 | 0.0000 | 4.2867e+06 | 2 |

**Adduct Signal Fractions**

- **Unmodified**: 4.2867e+06

### GHTLTLN(N)FTR Hex 4 HexNAc 3

**Mass:**  2416.0653

(5.183e-07)

**Charge States:** 3

**Start Position**: 97

**End Position**: 107

| Profile Score | Tandem Score | TDA q-value | Volume | Spectrum Matches |
| --- | --- | --- | --- | --- |
| -18.5080 | 137.3639 | 0.0000 | 2.5406e+06 | 2 |

**Adduct Signal Fractions**

- **Unmodified**: 2.5406e+06

### GHTLTLN(N)FTR Hex 3 HexNAc 4

**Mass:**  2457.0921

(6.294e-07)

**Charge States:** 3

**Start Position**: 97

**End Position**: 107

| Profile Score | Tandem Score | TDA q-value | Volume | Spectrum Matches |
| --- | --- | --- | --- | --- |
| -5.8737 | 115.0208 | 0.0000 | 1.3562e+06 | 1 |

**Adduct Signal Fractions**

- **Unmodified**: 1.3562e+06

### O75144|ICOSL\_HUMAN

**>O75144|ICOSL\_HUMAN**  
 `MRLGSPGLLFLLFSSLRADTQEKEVRAMVGSDVELSCACPEGSRFDLNDVYVYWQTSESKTVVTYHIPQNSSLENVDSRY  
RNRALMSPAGMLRGDFSLRLFNVTPQDEQKFHCLVLSQSLGFQEVLSVEVTLHVAANFSVPVVSAPHSPSQDELTFTCTS  
INGYPRPNVYWINKTDNSLLDQALQNDTVFLNMRGLYDVVSVLRIARTPSVNIGCCIENVLLQQNLTVGSQTGNDIGERD  
KITENPVSTGEKNAATWSILAVLCLLVVVAVAIGWVCRDRCLQHSYAGAWAVSPETELTGHV`

| Site | Type | Count |
| --- | --- | --- |
| 102 | N-linked | 1 |

| Mass | Sequence | MS_2_ Score | `q`-value | MS_1_ Score | Signal | Protein Coordinates | Tandem Scans | Start Time | Apex Time | End Time |
| --- | --- | --- | --- | --- | --- | --- | --- | --- | --- | --- |
| 2819.2190 | LFN(N)VTPQDEQK Hex 3 HexNAc 5 | 27.774 | 0.029 | -21.359 | 1.314e+06 | 99 - 110 | 1 | 25.36 | 25.53 | 25.53 |

### LFN(N)VTPQDEQK Hex 3 HexNAc 5

**Mass:**  2819.2203

(1.980e-06)

**Charge States:** 3

**Start Position**: 100

**End Position**: 111

| Profile Score | Tandem Score | TDA q-value | Volume | Spectrum Matches |
| --- | --- | --- | --- | --- |
| -21.3588 | 27.7740 | 0.0292 | 1.3142e+06 | 1 |

**Adduct Signal Fractions**

- **Unmodified**: 1.3142e+06
