# Supplementary material for: Measuring Site-specific Glycosylation Similarity between Influenza a Virus Variants with Statistical Certainty
Source: Mol Cell Proteomics. 2020 Nov 25;19(9):1533–45. doi: 10.1074/mcp.RA120.002031 (PMC8143645; doi:10.1074/mcp.RA120.002031)
Supplement: Supplementary file 1 [file mmc1.zip › mmc1/159250_2_supp_555288_qcjkln.zip!/20190326_aAGPgal_021119_tryp_HILIC_DDA_1ug_04_glycopeptides.html]

## 20190326\_aAGPgal\_021119\_tryp\_HILIC\_DDA\_1ug\_04 @ GlycopeptideHypothesis-23645d1e9717497c816618b3e3f4f827

**MS^1^ Mass Accuracy:** 10.0 PPM  
**Grouping Tolerance:** 15.0 PPM  
**MS^n^ Mass Accuracy:** 20.0 PPM  
**Minimum `q`-Value For Clustering:** 0.05

**Glycopeptide Hypothesis:**
GlycopeptideHypothesis-23645d1e9717497c816618b3e3f4f827  
**Sample Searched:**
20190326\_aAGPgal\_021119\_tryp\_HILIC\_DDA\_1ug\_04

| Protein | Matched Glycopeptides |
| --- | --- |
| P19652\|A1AG2\_HUMAN | 153 |
| P02763\|A1AG1\_HUMAN | 145 |
| P00738\|HPT\_HUMAN | 11 |
| P05155\|IC1\_HUMAN | 10 |
| Q9Y5Y7\|LYVE1\_HUMAN | 9 |
| Q12913\|PTPRJ\_HUMAN | 4 |
| O95497\|VNN1\_HUMAN | 3 |
| P11279\|LAMP1\_HUMAN | 3 |
| P21163\|PNGF\_ELIMR | 2 |
| Q13201\|MMRN1\_HUMAN | 2 |
| P02790\|HEMO\_HUMAN | 2 |
| Q5R543\|HEMO\_PONAB | 2 |

### P19652|A1AG2\_HUMAN

**>P19652|A1AG2\_HUMAN**  
 `MALSWVLTVLSLLPLLEAQIPLCANLVPVPITNATLDRITGKWFYIASAFRNEEYNKSVQEIQATFFYFTPNKTEDTIFL  
REYQTRQNQCFYNSSYLNVQRENGTVSRYEGGREHVAHLLFLRDTKTLMFGSYLDDEKNWGLSFYADKPETTKEQLGEFY  
EALDCLCIPRSDVMYTDWKKDKCEPLEKQHEKERKQEEGES`

| Site | Type | Count |
| --- | --- | --- |
| 33 | N-linked | 53 |
| 56 | N-linked | 1 |
| 93 | N-linked | 22 |
| 72 | N-linked | 77 |

| Mass | Sequence | MS_2_ Score | `q`-value | MS_1_ Score | Signal | Protein Coordinates | Tandem Scans | Start Time | Apex Time | End Time |
| --- | --- | --- | --- | --- | --- | --- | --- | --- | --- | --- |
| 2269.9383 | YFTPN(N)K Hex 3 HexNAc 5 | 187.370 | 0.0 | 13.524 | 2.080e+08 | 67 - 73 | 8 | 13.98 | 14.28 | 14.79 |
| 2473.0178 | YFTPN(N)K Hex 3 HexNAc 6 | 187.048 | 0.0 | 14.232 | 3.743e+08 | 67 - 73 | 6 | 13.85 | 13.98 | 14.69 |
| 2620.0865 | FYFTPN(N)K Hex 3 HexNAc 6 | 183.031 | 0.0 | 13.497 | 1.039e+08 | 66 - 73 | 11 | 25.20 | 25.55 | 28.15 |
| 2580.0490 | YFTPN(N)K Fuc 1 Hex 4 HexNAc 5 | 182.056 | 0.0 | 0.000 | 0.000e+00 | 67 - 73 | 1 | - | - | - |
| 2093.8166 | NEEYN(N)K Hex 3 HexNAc 4 | 181.316 | 0.0 | 11.811 | 5.132e+07 | 51 - 57 | 6 | 3.86 | 3.86 | 5.03 |
| 3988.7195 | SVQEIQATFFYFTPN(N)K Hex 4 HexNAc 7 | 175.474 | 0.0 | 11.370 | 2.228e+08 | 57 - 73 | 14 | 53.66 | 54.16 | 56.87 |
| 3623.5855 | SVQEIQATFFYFTPN(N)K Hex 3 HexNAc 6 | 174.109 | 0.0 | 14.863 | 1.198e+09 | 57 - 73 | 24 | 50.94 | 54.67 | 58.40 |
| 3931.6975 | SVQEIQATFFYFTPN(N)K Fuc 1 Hex 4 HexNAc 6 | 173.486 | 0.0 | 13.256 | 2.677e+08 | 57 - 73 | 9 | 53.99 | 54.33 | 56.37 |
| 3728.6173 | SVQEIQATFFYFTPN(N)K Fuc 1 Hex 4 HexNAc 5 | 165.419 | 0.0 | 13.586 | 2.024e+08 | 57 - 73 | 7 | 54.16 | 54.50 | 55.68 |
| 3420.5048 | SVQEIQATFFYFTPN(N)K Hex 3 HexNAc 5 | 165.182 | 0.0 | 15.343 | 7.045e+08 | 57 - 73 | 28 | 51.11 | 55.01 | 59.08 |
| 2909.3661 | LVPVPITN(N)ATLDR Hex 3 HexNAc 5 | 160.474 | 0.0 | 11.576 | 1.267e+10 | 25 - 38 | 333 | 30.23 | 37.36 | 113.87 |
| 3217.4776 | LVPVPITN(N)ATLDR Fuc 1 Hex 4 HexNAc 5 | 160.226 | 0.0 | 10.016 | 3.513e+09 | 25 - 38 | 108 | 29.73 | 36.74 | 113.23 |
| 3475.4241 | ATFFYFTPN(N)K Fuc 2 Hex 7 HexNAc 4 | 158.594 | 0.0 | 0.000 | 0.000e+00 | 63 - 73 | 1 | - | - | - |
| 2706.2867 | LVPVPITN(N)ATLDR Hex 3 HexNAc 4 | 157.765 | 0.0 | 13.906 | 1.089e+09 | 25 - 38 | 16 | 37.36 | 37.67 | 72.85 |
| 3138.3888 | TPN(N)KTEDTIFLR Hex 3 HexNAc 6 | 157.147 | 0.0 | 13.262 | 4.373e+07 | 69 - 81 | 5 | 25.84 | 26.01 | 27.22 |
| 3112.4447 | LVPVPITN(N)ATLDR Hex 3 HexNAc 6 | 156.657 | 0.0 | 9.642 | 2.880e+08 | 25 - 38 | 4 | 36.90 | 37.05 | 38.30 |
| 4191.8000 | SVQEIQATFFYFTPN(N)K Hex 4 HexNAc 8 | 153.685 | 0.0 | -3.706 | 2.317e+07 | 57 - 73 | 4 | 53.66 | 53.82 | 54.16 |
| 3071.4205 | LVPVPITN(N)ATLDR Hex 4 HexNAc 5 | 149.912 | 0.0 | 12.290 | 9.764e+07 | 25 - 38 | 4 | 36.57 | 37.05 | 37.36 |
| 4239.8077 | SVQEIQATFFYFTPN(N)K Fuc 2 Hex 5 HexNAc 6 | 149.415 | 0.0 | 13.540 | 4.168e+07 | 57 - 73 | 4 | 53.66 | 53.82 | 54.50 |
| 2736.1608 | ATFFYFTPN(N)K Hex 3 HexNAc 5 | 149.237 | 0.0 | -3.211 | 2.263e+07 | 63 - 73 | 2 | 41.79 | 41.96 | 42.13 |
| 2939.2405 | ATFFYFTPN(N)K Hex 3 HexNAc 6 | 147.421 | 0.0 | 14.396 | 4.502e+07 | 63 - 73 | 2 | 41.45 | 41.62 | 42.13 |
| 4296.8316 | SVQEIQATFFYFTPN(N)K Fuc 1 Hex 5 HexNAc 7 | 146.346 | 0.0 | 8.959 | 3.352e+07 | 57 - 73 | 6 | 53.32 | 53.49 | 54.50 |
| 3217.4274 | SVQEIQATFFYFTPN(N)K Hex 3 HexNAc 4 | 145.717 | 0.0 | 12.924 | 7.206e+07 | 57 - 73 | 6 | 55.17 | 55.34 | 57.04 |
| 3274.4998 | LVPVPITN(N)ATLDR Hex 4 HexNAc 6 | 144.796 | 0.0 | 11.753 | 7.669e+08 | 25 - 38 | 8 | 36.40 | 37.67 | 38.46 |
| 3055.4235 | LVPVPITN(N)ATLDR Fuc 1 Hex 3 HexNAc 5 | 143.607 | 0.0 | 10.747 | 6.662e+07 | 25 - 38 | 2 | 37.05 | 37.36 | 37.67 |
| 2708.2867 | LVPVPITN(N)ATLDR Hex 3 HexNAc 4 | 141.384 | 0.0 | 0.000 | 0.000e+00 | 25 - 38 | 1 | - | - | - |
| 3785.6414 | SVQEIQATFFYFTPN(N)K Hex 4 HexNAc 6 | 141.180 | 0.0 | 10.647 | 3.105e+07 | 57 - 73 | 5 | 53.99 | 55.17 | 55.34 |
| 3562.5902 | C(C)ANLVPVPITN(N)ATLDR Fuc 1 Hex 4 HexNAc 5 | 141.048 | 0.0 | 12.050 | 5.843e+07 | 22 - 38 | 4 | 41.96 | 42.13 | 42.64 |
| 3420.5580 | LVPVPITN(N)ATLDR Fuc 1 Hex 4 HexNAc 6 | 138.851 | 0.0 | 17.209 | 1.887e+08 | 25 - 38 | 3 | 36.24 | 36.57 | 36.90 |
| 3448.5209 | YFTPN(N)KTEDTIFLR Hex 3 HexNAc 6 | 138.139 | 0.0 | 11.339 | 1.568e+09 | 67 - 81 | 13 | 35.73 | 37.67 | 42.13 |
| 3962.6694 | SVQEIQATFFYFTPN(N)K Fuc 2 Hex 4 HexNAc 4 Neu5Ac 1 | 135.371 | 0.0 | -18.803 | 2.712e+06 | 57 - 73 | 1 | 55.17 | 55.17 | 55.17 |
| 3276.5056 | LVPVPITN(N)ATLDR Hex 4 HexNAc 6 | 133.867 | 0.0 | 0.000 | 0.000e+00 | 25 - 38 | 1 | - | - | - |
| 3247.3493 | ATFFYFTPN(N)K Fuc 1 Hex 4 HexNAc 6 | 133.482 | 0.0 | 0.000 | 0.000e+00 | 63 - 73 | 1 | - | - | - |
| 3813.6511 | YFTPN(N)KTEDTIFLR Hex 4 HexNAc 7 | 133.432 | 0.0 | 8.580 | 1.386e+08 | 67 - 81 | 5 | 36.74 | 37.05 | 37.83 |
| 3245.4421 | YFTPN(N)KTEDTIFLR Hex 3 HexNAc 5 | 132.939 | 0.0 | 10.945 | 7.353e+08 | 67 - 81 | 6 | 35.90 | 37.83 | 39.14 |
| 3477.5798 | LVPVPITN(N)ATLDR Hex 4 HexNAc 7 | 132.734 | 0.0 | -4.865 | 1.026e+08 | 25 - 38 | 4 | 36.24 | 36.57 | 36.74 |
| 3069.3307 | PVPITN(N)ATLDR Fuc 2 Hex 6 HexNAc 3 | 132.542 | 0.0 | -19.104 | 1.040e+07 | 27 - 38 | 1 | 40.46 | 40.46 | 40.62 |
| 3254.4765 | C(C)ANLVPVPITN(N)ATLDR Hex 3 HexNAc 5 | 132.184 | 0.0 | 12.168 | 4.901e+08 | 22 - 38 | 9 | 42.30 | 53.32 | 54.16 |
| 3421.4174 | QNQC(C)FYN(N)SSYLNVQR Hex 3 HexNAc 5 | 131.915 | 0.0 | -3.291 | 2.997e+08 | 86 - 101 | 4 | 33.75 | 33.91 | 34.07 |
| 3756.6305 | YFTPN(N)KTEDTIFLR Fuc 1 Hex 4 HexNAc 6 | 129.745 | 0.0 | -4.729 | 2.476e+08 | 67 - 81 | 8 | 35.39 | 37.21 | 37.83 |
| 3044.2652 | ATFFYFTPN(N)K Fuc 1 Hex 4 HexNAc 5 | 129.281 | 0.0 | -4.112 | 6.116e+06 | 63 - 73 | 1 | 41.28 | 41.45 | 41.62 |
| 2796.2828 | VPVPITN(N)ATLDR Hex 3 HexNAc 5 | 127.954 | 0.0 | -2.852 | 7.016e+06 | 26 - 38 | 3 | 30.40 | 30.57 | 30.88 |
| 3554.5465 | YFTPN(N)KTEDTIFLR Fuc 1 Hex 4 HexNAc 5 | 127.803 | 0.0 | 0.000 | 0.000e+00 | 67 - 81 | 1 | - | - | - |
| 3961.6704 | SVQEIQATFFYFTPN(N)K Hex 4 HexNAc 4 Neu5Ac 2 | 127.405 | 0.0 | 12.747 | 3.631e+07 | 57 - 73 | 4 | 55.17 | 55.51 | 56.37 |
| 4556.9323 | SVQEIQATFFYFTPN(N)K Hex 5 HexNAc 9 | 126.575 | 0.0 | -19.428 | 3.312e+06 | 57 - 73 | 1 | 53.15 | 53.15 | 53.15 |
| 3624.4951 | QNQC(C)FYN(N)SSYLNVQR Hex 3 HexNAc 6 | 125.711 | 0.0 | 6.611 | 1.400e+09 | 86 - 101 | 6 | 33.58 | 33.75 | 41.11 |
| 3553.5521 | YFTPN(N)KTEDTIFLR Fuc 1 Hex 4 HexNAc 5 | 125.602 | 0.0 | -4.600 | 1.685e+08 | 67 - 81 | 3 | 37.36 | 37.36 | 37.83 |
| 3264.4787 | LVPVPITN(N)ATLDR Fuc 1 Hex 5 HexNAc 3 Neu5Ac 1 | 125.455 | 0.0 | -20.219 | 2.147e+07 | 25 - 38 | 1 | 37.83 | 37.99 | 37.99 |
| 3595.5912 | FYFTPN(N)KTEDTIFLR Hex 3 HexNAc 6 | 125.187 | 0.0 | 13.427 | 1.067e+08 | 66 - 81 | 5 | 46.29 | 46.46 | 47.65 |
| 3023.4111 | NLVPVPITN(N)ATLDR Hex 3 HexNAc 5 | 124.127 | 0.0 | -4.385 | 1.008e+07 | 24 - 38 | 1 | 41.45 | 41.45 | 41.79 |
| 3932.6138 | QNQC(C)FYN(N)SSYLNVQR Fuc 1 Hex 4 HexNAc 6 | 123.313 | 0.0 | 11.076 | 3.525e+08 | 86 - 101 | 3 | 33.41 | 33.58 | 39.48 |
| 4065.7453 | YFTPN(N)KTEDTIFLR Fuc 2 Hex 5 HexNAc 6 | 122.822 | 0.0 | 0.000 | 0.000e+00 | 67 - 81 | 1 | - | - | - |
| 3728.6724 | LVPVPITN(N)ATLDR Fuc 2 Hex 5 HexNAc 6 | 122.816 | 0.0 | -5.078 | 1.141e+07 | 25 - 38 | 4 | 35.90 | 36.40 | 36.40 |
| 3525.5915 | LVPVPITN(N)ATLDR Fuc 2 Hex 5 HexNAc 5 | 121.533 | 0.0 | -4.163 | 4.677e+07 | 25 - 38 | 2 | 36.24 | 36.24 | 36.57 |
| 4134.7769 | SVQEIQATFFYFTPN(N)K Fuc 1 Hex 4 HexNAc 7 | 121.290 | 0.0 | -4.909 | 8.205e+06 | 57 - 73 | 2 | 53.82 | 54.16 | 54.33 |
| 3379.5342 | LVPVPITN(N)ATLDR Fuc 1 Hex 5 HexNAc 5 | 120.877 | 0.0 | -16.886 | 9.452e+06 | 25 - 38 | 1 | 36.57 | 36.57 | 36.57 |
| 3729.5266 | QNQC(C)FYN(N)SSYLNVQR Fuc 1 Hex 4 HexNAc 5 | 117.621 | 0.0 | -9.888 | 1.752e+08 | 86 - 101 | 4 | 33.58 | 33.58 | 39.97 |
| 2935.3081 | TPN(N)KTEDTIFLR Hex 3 HexNAc 5 | 117.605 | 0.0 | 12.345 | 4.101e+07 | 69 - 81 | 6 | 26.01 | 26.32 | 27.38 |
| 2966.3904 | NLVPVPITN(N)ATLDR Fuc 1 Hex 3 HexNAc 4 | 115.887 | 0.0 | 14.766 | 1.471e+09 | 24 - 38 | 18 | 37.99 | 38.14 | 39.14 |
| 3815.6605 | YFTPN(N)KTEDTIFLR Hex 4 HexNAc 7 | 115.664 | 0.0 | 0.000 | 0.000e+00 | 67 - 81 | 2 | - | - | - |
| 3104.3930 | VPVPITN(N)ATLDR Fuc 1 Hex 4 HexNAc 5 | 115.557 | 0.0 | -18.549 | 1.791e+06 | 26 - 38 | 1 | 30.06 | 30.06 | 30.23 |
| 3978.6950 | SVQEIQATFFYFTPN(N)K Fuc 1 Hex 5 HexNAc 4 Neu5Ac 1 | 112.640 | 0.0 | -4.866 | 6.645e+06 | 57 - 73 | 2 | 55.34 | 55.51 | 55.68 |
| 3051.3994 | C(C)ANLVPVPITN(N)ATLDR Hex 3 HexNAc 4 | 111.822 | 0.0 | 10.016 | 1.229e+07 | 22 - 38 | 2 | 42.64 | 42.80 | 43.48 |
| 3903.7030 | FYFTPN(N)KTEDTIFLR Fuc 1 Hex 4 HexNAc 6 | 111.819 | 0.0 | 13.833 | 2.760e+07 | 66 - 81 | 4 | 45.78 | 45.95 | 46.63 |
| 3304.3796 | ATFFYFTPN(N)K Hex 4 HexNAc 7 | 111.324 | 0.0 | -6.040 | 3.524e+06 | 63 - 73 | 1 | 40.78 | 40.95 | 41.11 |
| 3582.6173 | LVPVPITN(N)ATLDR Fuc 1 Hex 5 HexNAc 6 | 111.249 | 0.0 | -4.542 | 3.386e+07 | 25 - 38 | 2 | 36.07 | 36.57 | 36.57 |
| 3082.3782 | FTPN(N)KTEDTIFLR Hex 3 HexNAc 5 | 110.755 | 0.0 | 12.913 | 4.227e+07 | 68 - 81 | 3 | 32.39 | 32.56 | 33.08 |
| 3285.4573 | FTPN(N)KTEDTIFLR Hex 3 HexNAc 6 | 110.619 | 0.0 | 13.077 | 8.725e+07 | 68 - 81 | 7 | 30.40 | 32.22 | 32.91 |
| 2732.2283 | TPN(N)KTEDTIFLR Hex 3 HexNAc 4 | 109.753 | 0.0 | 10.027 | 7.822e+06 | 69 - 81 | 2 | 26.32 | 26.49 | 27.10 |
| 3073.4229 | LVPVPITN(N)ATLDR Hex 4 HexNAc 5 | 109.668 | 0.0 | 0.000 | 0.000e+00 | 25 - 38 | 1 | - | - | - |
| 3042.3627 | YFTPN(N)KTEDTIFLR Hex 3 HexNAc 4 | 109.273 | 0.0 | -1.040 | 7.902e+07 | 67 - 81 | 2 | 36.24 | 38.14 | 38.97 |
| 3392.5112 | FYFTPN(N)KTEDTIFLR Hex 3 HexNAc 5 | 109.203 | 0.0 | 12.062 | 5.054e+07 | 66 - 81 | 5 | 46.46 | 46.63 | 47.65 |
| 3113.4529 | LVPVPITN(N)ATLDR Hex 3 HexNAc 6 | 108.968 | 0.0 | 0.000 | 0.000e+00 | 25 - 38 | 3 | - | - | - |
| 3989.6356 | QNQC(C)FYN(N)SSYLNVQR Hex 4 HexNAc 7 | 108.951 | 0.0 | 13.517 | 1.548e+08 | 86 - 101 | 2 | 33.08 | 33.25 | 33.91 |
| 3189.4300 | FYFTPN(N)KTEDTIFLR Hex 3 HexNAc 4 | 108.757 | 0.0 | -3.702 | 6.028e+06 | 66 - 81 | 1 | 46.63 | 46.80 | 47.14 |
| 3151.3768 | PVPITN(N)ATLDR Fuc 2 Hex 4 HexNAc 5 | 107.802 | 0.0 | -5.854 | 6.250e+06 | 27 - 38 | 2 | 40.62 | 40.78 | 40.95 |
| 3786.6072 | TPN(N)KTEDTIFLR Hex 7 HexNAc 6 | 107.484 | 0.0 | 13.763 | 1.029e+08 | 69 - 81 | 4 | 38.30 | 38.46 | 38.97 |
| 3169.4671 | NLVPVPITN(N)ATLDR Fuc 1 Hex 3 HexNAc 5 | 105.234 | 0.0 | -4.385 | 4.343e+07 | 24 - 38 | 2 | 37.83 | 37.99 | 38.30 |
| 4178.7885 | YFTPN(N)KTEDTIFLR Hex 5 HexNAc 8 | 103.246 | 0.0 | -3.974 | 3.131e+07 | 67 - 81 | 2 | 36.07 | 36.24 | 36.57 |
| 3593.5700 | FTPN(N)KTEDTIFLR Fuc 1 Hex 4 HexNAc 6 | 102.488 | 0.0 | -3.671 | 2.019e+07 | 68 - 81 | 3 | 31.71 | 31.88 | 32.22 |
| 3140.3956 | TPN(N)KTEDTIFLR Hex 3 HexNAc 6 | 101.033 | 0.0 | 0.000 | 0.000e+00 | 69 - 81 | 1 | - | - | - |
| 3700.6215 | FYFTPN(N)KTEDTIFLR Fuc 1 Hex 4 HexNAc 5 | 100.560 | 0.0 | -3.937 | 1.433e+07 | 66 - 81 | 2 | 45.95 | 46.12 | 46.46 |
| 3364.5364 | LVPVPITN(N)ATLDR Fuc 2 Hex 4 HexNAc 5 | 98.751 | 0.0 | 0.000 | 0.000e+00 | 25 - 38 | 1 | - | - | - |
| 3496.6129 | LVPVPITN(N)ATLDR Fuc 4 Hex 3 HexNAc 5 | 97.942 | 0.0 | 0.000 | 0.000e+00 | 25 - 38 | 1 | - | - | - |
| 3292.5193 | LVPVPITN(N)ATLDR Fuc 2 Hex 3 HexNAc 4 Neu5Ac 1 | 96.718 | 0.0 | 0.000 | 0.000e+00 | 25 - 38 | 1 | - | - | - |
| 3255.4223 | PVPITN(N)ATLDR Fuc 1 Hex 5 HexNAc 4 Neu5Ac 1 | 96.095 | 0.0 | -3.961 | 3.906e+08 | 27 - 38 | 5 | 36.57 | 36.74 | 37.05 |
| 4381.8690 | YFTPN(N)KTEDTIFLR Hex 5 HexNAc 9 | 95.437 | 0.0 | -19.892 | 9.003e+06 | 67 - 81 | 1 | 36.07 | 36.07 | 36.24 |
| 4121.7656 | YFTPN(N)KTEDTIFLR Fuc 1 Hex 5 HexNAc 7 | 94.770 | 0.0 | -5.005 | 3.630e+07 | 67 - 81 | 3 | 36.24 | 36.57 | 36.74 |
| 2763.3089 | NLVPVPITN(N)ATLDR Fuc 1 Hex 3 HexNAc 3 | 90.268 | 0.0 | 14.133 | 1.089e+08 | 24 - 38 | 3 | 38.14 | 38.46 | 38.97 |
| 3293.5457 | NLVPVPITN(N)ATLDR Fuc 1 Hex 5 HexNAc 4 | 89.214 | 0.0 | -8.692 | 1.397e+05 | 24 - 38 | 1 | 72.40 | 72.50 | 72.50 |
| 3960.7256 | FYFTPN(N)KTEDTIFLR Hex 4 HexNAc 7 | 88.330 | 0.0 | -3.561 | 1.727e+07 | 66 - 81 | 5 | 45.61 | 45.95 | 46.12 |
| 3390.4913 | FTPN(N)KTEDTIFLR Fuc 1 Hex 4 HexNAc 5 | 88.300 | 0.0 | -3.288 | 1.163e+07 | 68 - 81 | 3 | 31.88 | 32.05 | 32.22 |
| 4268.8153 | LVPVPITN(N)ATLDR Hex 6 HexNAc 5 Neu5Ac 3 | 87.138 | 0.0 | 11.187 | 9.397e+06 | 25 - 38 | 4 | 45.27 | 45.61 | 45.95 |
| 4016.7357 | YFTPN(N)KTEDTIFLR Hex 4 HexNAc 8 | 87.137 | 0.0 | -19.459 | 1.430e+07 | 67 - 81 | 1 | 36.57 | 36.57 | 36.57 |
| 3650.5910 | FTPN(N)KTEDTIFLR Hex 4 HexNAc 7 | 83.719 | 0.0 | -4.761 | 1.085e+07 | 68 - 81 | 4 | 31.55 | 31.88 | 32.05 |
| 3446.4997 | TPN(N)KTEDTIFLR Fuc 1 Hex 4 HexNAc 6 | 82.878 | 0.0 | -4.250 | 8.504e+06 | 69 - 81 | 4 | 25.38 | 25.55 | 25.84 |
| 3243.4239 | TPN(N)KTEDTIFLR Fuc 1 Hex 4 HexNAc 5 | 81.837 | 0.0 | -4.718 | 3.919e+06 | 69 - 81 | 3 | 25.68 | 25.84 | 26.01 |
| 3255.4623 | C(C)AN(D)LVPVPITN(N)ATLDR Hex 3 HexNAc 5 | 77.965 | 0.002 | -2.520 | 1.595e+07 | 22 - 38 | 6 | 43.31 | 43.31 | 45.61 |
| 3787.6481 | SVQEIQATFFYFTPN(N)K Hex 4 HexNAc 6 | 73.078 | 0.002 | 0.000 | 0.000e+00 | 57 - 73 | 1 | - | - | - |
| 3582.5617 | SVQEIQATFFYFTPN(N)K Hex 4 HexNAc 5 | 70.064 | 0.002 | -5.860 | 8.368e+06 | 57 - 73 | 1 | 54.50 | 54.67 | 55.01 |
| 4094.7244 | YFTPN(N)KTEDTIFLR Fuc 1 Hex 5 HexNAc 4 Neu5Ac 2 | 67.457 | 0.002 | -20.675 | 9.138e+06 | 67 - 81 | 1 | 37.99 | 37.99 | 38.14 |
| 4004.7431 | SVQEIQATFFYFTPN(N)K Fuc 4 Hex 3 HexNAc 5 | 66.090 | 0.002 | -20.858 | 2.397e+06 | 57 - 73 | 1 | 54.84 | 54.84 | 54.84 |
| 4599.0866 | SVQEIQATFFYFTPN(N)KTEDTIFLR Hex 3 HexNAc 6 | 63.742 | 0.002 | -4.628 | 2.503e+07 | 57 - 81 | 2 | 62.93 | 63.10 | 63.44 |
| 3996.8436 | QIPLC(C)ANLVPVPITN(N)ATLDR Hex 3 HexNAc 5 Neu5Ac 1 | 62.155 | 0.002 | 15.967 | 4.036e+08 | 18 - 38 | 11 | 61.73 | 62.59 | 63.61 |
| 4396.0163 | SVQEIQATFFYFTPN(N)KTEDTIFLR Hex 3 HexNAc 5 | 47.215 | 0.003 | -19.684 | 6.018e+06 | 57 - 81 | 1 | 63.27 | 63.27 | 63.27 |
| 4557.8463 | QNQC(C)FYN(N)SSYLNVQR Hex 5 HexNAc 9 | 46.454 | 0.003 | -19.423 | 5.034e+06 | 86 - 101 | 1 | 32.56 | 32.56 | 32.73 |
| 4269.7850 | SVQEIQATFFYFTPN(N)K Fuc 1 Hex 5 HexNAc 4 Neu5Ac 2 | 46.044 | 0.003 | -20.889 | 3.262e+06 | 57 - 73 | 1 | 54.84 | 54.84 | 54.84 |
| 3382.3979 | QC(C)FYN(N)SSYLNVQR Hex 3 HexNAc 6 | 45.234 | 0.003 | -19.801 | 1.170e+07 | 88 - 101 | 1 | 33.91 | 34.07 | 34.07 |
| 4442.8908 | SVQEIQATFFYFTPN(N)K Fuc 2 Hex 5 HexNAc 7 | 44.180 | 0.003 | -20.013 | 3.658e+06 | 57 - 73 | 1 | 53.66 | 53.66 | 53.82 |
| 3730.6765 | LVPVPITN(N)ATLDR Fuc 2 Hex 5 HexNAc 6 | 43.506 | 0.003 | 0.000 | 0.000e+00 | 25 - 38 | 1 | - | - | - |
| 3094.4462 | ANLVPVPITN(N)ATLDR Hex 3 HexNAc 5 | 42.257 | 0.003 | -3.844 | 1.651e+07 | 23 - 38 | 2 | 41.79 | 41.96 | 42.30 |
| 3014.3992 | LVPVPITN(N)ATLDR Fuc 1 Hex 4 HexNAc 4 | 41.541 | 0.003 | 0.000 | 0.000e+00 | 25 - 38 | 1 | - | - | - |
| 3965.5885 | QNQC(C)FYN(N)SSYLNVQR Fuc 2 Hex 4 HexNAc 4 Neu5Ac 1 | 41.422 | 0.003 | -6.811 | 9.332e+07 | 86 - 101 | 1 | 33.58 | 33.75 | 33.91 |
| 4064.7412 | YFTPN(N)KTEDTIFLR Fuc 2 Hex 5 HexNAc 6 | 41.065 | 0.003 | -20.251 | 8.280e+06 | 67 - 81 | 1 | 36.90 | 36.90 | 36.90 |
| 4353.8481 | SVQEIQATFFYFTPN(N)K Hex 5 HexNAc 8 | 40.897 | 0.003 | 9.795 | 2.100e+07 | 57 - 73 | 1 | 53.15 | 53.32 | 53.99 |
| 4907.2009 | SVQEIQATFFYFTPN(N)KTEDTIFLR Fuc 1 Hex 4 HexNAc 6 | 38.827 | 0.003 | -19.715 | 2.335e+06 | 57 - 81 | 1 | 62.76 | 62.76 | 62.76 |
| 4354.7658 | QNQC(C)FYN(N)SSYLNVQR Hex 5 HexNAc 8 | 38.800 | 0.003 | -4.931 | 9.113e+06 | 86 - 101 | 1 | 32.56 | 32.73 | 32.91 |
| 4050.7623 | ANLVPVPITN(N)ATLDR Fuc 1 Hex 8 HexNAc 5 | 38.316 | 0.005 | -5.606 | 3.104e+07 | 23 - 38 | 3 | 62.59 | 62.59 | 62.93 |
| 3545.5647 | C(C)ANLVPVPITN(N)ATLDR Hex 3 HexNAc 5 Neu5Ac 1 | 37.666 | 0.005 | 16.758 | 2.156e+08 | 22 - 38 | 6 | 52.30 | 52.81 | 53.49 |
| 3639.6522 | LVPVPITN(N)ATLDR Hex 5 HexNAc 7 | 37.550 | 0.005 | -5.973 | 8.842e+06 | 25 - 38 | 1 | 36.24 | 36.24 | 36.57 |
| 3705.7552 | QIPLC(C)ANLVPVPITN(N)ATLDR Hex 3 HexNAc 5 | 37.543 | 0.005 | 13.470 | 4.906e+08 | 18 - 38 | 12 | 62.59 | 63.27 | 64.58 |
| 4922.9749 | QNQC(C)FYN(N)SSYLNVQR Fuc 5 Hex 4 HexNAc 8 | 37.002 | 0.006 | 14.670 | 2.671e+07 | 86 - 101 | 5 | 30.23 | 30.40 | 30.88 |
| 3625.4820 | QN(D)QC(C)FYN(N)SSYLNVQR Hex 3 HexNAc 6 | 36.979 | 0.006 | -4.517 | 2.823e+08 | 86 - 101 | 5 | 34.72 | 35.05 | 41.45 |
| 3271.5035 | ANLVPVPITN(N)ATLDR Fuc 2 Hex 3 HexNAc 3 Neu5Ac 1 | 36.667 | 0.006 | -4.072 | 6.610e+07 | 23 - 38 | 2 | 42.47 | 42.64 | 42.97 |
| 4211.8110 | FYFTPN(N)KTEDTIFLR Fuc 2 Hex 5 HexNAc 6 | 35.863 | 0.007 | -5.135 | 3.352e+06 | 66 - 81 | 1 | 45.44 | 45.61 | 45.78 |
| 4013.8671 | QIPLC(C)ANLVPVPITN(N)ATLDR Fuc 1 Hex 4 HexNAc 5 | 35.851 | 0.007 | 13.669 | 1.428e+08 | 18 - 38 | 8 | 62.08 | 62.59 | 63.78 |
| 3933.5960 | QN(D)QC(C)FYN(N)SSYLNVQR Fuc 1 Hex 4 HexNAc 6 | 35.291 | 0.007 | -5.873 | 4.412e+07 | 86 - 101 | 2 | 34.39 | 34.55 | 34.72 |
| 3782.5322 | ATFFYFTPN(N)K Fuc 1 Hex 3 HexNAc 8 Neu5Ac 1 | 34.719 | 0.007 | -20.291 | 9.196e+06 | 63 - 73 | 1 | 54.50 | 54.50 | 54.67 |
| 4240.7159 | QNQC(C)FYN(N)SSYLNVQR Fuc 2 Hex 5 HexNAc 6 | 34.645 | 0.007 | -18.343 | 3.196e+07 | 86 - 101 | 1 | 33.08 | 33.25 | 33.25 |
| 3503.5218 | TPN(N)KTEDTIFLR Hex 4 HexNAc 7 | 34.067 | 0.010 | -5.181 | 4.598e+06 | 69 - 81 | 2 | 25.38 | 25.55 | 25.84 |
| 4499.9082 | SVQEIQATFFYFTPN(N)K Fuc 1 Hex 5 HexNAc 8 | 33.430 | 0.011 | -20.361 | 3.372e+06 | 57 - 73 | 1 | 53.15 | 53.32 | 53.32 |
| 2879.2976 | FTPN(N)KTEDTIFLR Hex 3 HexNAc 4 | 33.418 | 0.011 | -4.903 | 5.428e+06 | 68 - 81 | 1 | 32.56 | 32.73 | 33.08 |
| 3502.6757 | QIPLC(C)ANLVPVPITN(N)ATLDR Hex 3 HexNAc 4 | 33.356 | 0.011 | 13.475 | 3.095e+07 | 18 - 38 | 3 | 63.27 | 63.61 | 64.27 |
| 3600.6403 | LVPVPITN(N)ATLDR Hex 6 HexNAc 6 | 32.839 | 0.011 | 0.000 | 0.000e+00 | 25 - 38 | 1 | - | - | - |
| 2891.2254 | FYN(N)SSYLNVQR Hex 3 HexNAc 5 | 31.904 | 0.013 | -4.174 | 2.703e+06 | 90 - 101 | 2 | 29.89 | 30.06 | 30.23 |
| 4034.7903 | ANLVPVPITN(N)ATLDR Fuc 2 Hex 7 HexNAc 5 | 31.069 | 0.017 | -3.934 | 3.334e+07 | 23 - 38 | 3 | 62.42 | 62.59 | 62.76 |
| 4192.7137 | QNQC(C)FYN(N)SSYLNVQR Hex 4 HexNAc 8 | 30.428 | 0.019 | -5.153 | 3.116e+07 | 86 - 101 | 2 | 32.91 | 33.08 | 33.41 |
| 3785.6931 | LVPVPITN(N)ATLDR Fuc 1 Hex 5 HexNAc 7 | 30.197 | 0.020 | 10.530 | 2.010e+07 | 25 - 38 | 1 | 35.90 | 36.24 | 36.57 |
| 3031.3885 | LVPVPITN(N)ATLDR Hex 5 HexNAc 4 | 29.791 | 0.021 | 0.000 | 0.000e+00 | 25 - 38 | 1 | - | - | - |
| 3331.5207 | NLVPVPITN(N)ATLDR Fuc 1 Hex 4 HexNAc 5 | 29.706 | 0.021 | -20.471 | 7.751e+06 | 24 - 38 | 1 | 37.83 | 37.83 | 37.83 |
| 3901.6733 | FTPN(N)KTEDTIFLR Fuc 2 Hex 5 HexNAc 6 | 29.081 | 0.023 | -5.476 | 2.487e+06 | 68 - 81 | 2 | 31.38 | 31.55 | 31.71 |
| 3094.3060 | FYN(N)SSYLNVQR Hex 3 HexNAc 6 | 29.050 | 0.024 | -3.327 | 7.198e+06 | 90 - 101 | 3 | 29.73 | 29.89 | 30.23 |
| 3610.5727 | YFTPN(N)KTEDTIFLR Hex 4 HexNAc 6 | 28.576 | 0.029 | -5.826 | 1.595e+07 | 67 - 81 | 1 | 37.83 | 37.99 | 38.14 |
| 3471.5230 | FTPN(N)KTEDTIFLR Fuc 1 Hex 7 HexNAc 3 | 28.524 | 0.032 | -6.368 | 1.807e+06 | 68 - 81 | 2 | 24.86 | 25.03 | 25.20 |
| 4704.1193 | SVQEIQATFFYFTPN(N)KTEDTIFLR Fuc 1 Hex 4 HexNAc 5 | 28.219 | 0.033 | -19.365 | 2.451e+06 | 57 - 81 | 1 | 62.76 | 62.93 | 62.93 |
| 3219.4738 | LVPVPITN(N)ATLDR Fuc 1 Hex 4 HexNAc 5 | 27.883 | 0.035 | 0.000 | 0.000e+00 | 25 - 38 | 1 | - | - | - |
| 2947.2377 | YN(N)SSYLNVQR Hex 3 HexNAc 6 | 27.371 | 0.035 | 12.024 | 1.404e+07 | 91 - 101 | 2 | 22.70 | 22.92 | 23.15 |
| 3730.4981 | QN(D)QC(C)FYN(N)SSYLNVQR Fuc 1 Hex 4 HexNAc 5 | 27.277 | 0.035 | -6.348 | 1.456e+07 | 86 - 101 | 4 | 34.55 | 34.72 | 34.88 |
| 3619.5029 | YN(N)SSYLNVQR Fuc 4 Hex 3 HexNAc 5 Neu5Ac 1 | 27.218 | 0.035 | -19.298 | 1.923e+07 | 91 - 101 | 2 | 41.62 | 41.62 | 41.62 |
| 3746.5006 | QN(D)QC(C)FYN(N)SSYLNVQR Hex 5 HexNAc 5 | 26.434 | 0.046 | -19.459 | 2.164e+07 | 86 - 101 | 1 | 39.81 | 39.81 | 39.81 |
| 4006.6490 | QN(D)QC(C)FYN(N)SSYLNVQR Fuc 4 Hex 3 HexNAc 5 | 25.735 | 0.046 | 12.813 | 5.918e+07 | 86 - 101 | 2 | 33.08 | 33.41 | 33.91 |
| 7247.1739 | ITGKWFYIASAFRNEEYNKSVQEIQATFFYFTPN(N)K Fuc 2 Hex 8 HexNAc 7 | 25.623 | 0.046 | -18.950 | 1.380e+07 | 38 - 73 | 1 | 54.67 | 54.84 | 54.84 |
| 3255.3456 | YN(N)SSYLNVQR Fuc 1 Hex 4 HexNAc 6 | 25.506 | 0.046 | -20.845 | 9.641e+05 | 91 - 101 | 1 | 22.44 | 22.44 | 22.56 |

### YFTPN(N)K Hex 3 HexNAc 5

**Mass:**  2269.9387

(1.331e-06)

**Charge States:** 2, 3

**Start Position**: 68

**End Position**: 74

| Profile Score | Tandem Score | TDA q-value | Volume | Spectrum Matches |
| --- | --- | --- | --- | --- |
| 13.5240 | 187.3703 | 0.0000 | 2.0802e+08 | 8 |

**Adduct Signal Fractions**

- **Unmodified**: 2.0802e+08

### YFTPN(N)K Hex 3 HexNAc 6

**Mass:**  2473.0177

(1.244e-06)

**Charge States:** 2, 3

**Start Position**: 68

**End Position**: 74

| Profile Score | Tandem Score | TDA q-value | Volume | Spectrum Matches |
| --- | --- | --- | --- | --- |
| 14.2318 | 187.0480 | 0.0000 | 3.7432e+08 | 6 |

**Adduct Signal Fractions**

- **Unmodified**: 3.7432e+08

### FYFTPN(N)K Hex 3 HexNAc 6

**Mass:**  2620.0865

(1.028e-06)

**Charge States:** 2, 3

**Start Position**: 67

**End Position**: 74

| Profile Score | Tandem Score | TDA q-value | Volume | Spectrum Matches |
| --- | --- | --- | --- | --- |
| 13.4967 | 183.0308 | 0.0000 | 1.0392e+08 | 11 |

**Adduct Signal Fractions**

- **Unmodified**: 1.0392e+08

### YFTPN(N)K Fuc 1 Hex 4 HexNAc 5

**Mass:**  2580.0490

(7.767e-04)

**Start Position**: 68

**End Position**: 74

| Profile Score | Tandem Score | TDA q-value | Volume | Spectrum Matches |
| --- | --- | --- | --- | --- |
| 0.0000 | 182.0556 | 0.0000 | 0.0000e+00 | 1 |

Adduct:

No Chromatogram Found

### NEEYN(N)K Hex 3 HexNAc 4

**Mass:**  2093.8160

(2.256e-08)

**Charge States:** 2, 3

**Start Position**: 52

**End Position**: 58

| Profile Score | Tandem Score | TDA q-value | Volume | Spectrum Matches |
| --- | --- | --- | --- | --- |
| 11.8107 | 181.3157 | 0.0000 | 5.1317e+07 | 6 |

**Adduct Signal Fractions**

- **Unmodified**: 5.1317e+07

### SVQEIQATFFYFTPN(N)K Hex 4 HexNAc 7

**Mass:**  3988.7204

(1.216e-06)

**Charge States:** 3, 4

**Start Position**: 58

**End Position**: 74

| Profile Score | Tandem Score | TDA q-value | Volume | Spectrum Matches |
| --- | --- | --- | --- | --- |
| 11.3700 | 175.4739 | 0.0000 | 2.2284e+08 | 14 |

**Adduct Signal Fractions**

- **Unmodified**: 2.2284e+08

### SVQEIQATFFYFTPN(N)K Hex 3 HexNAc 6

**Mass:**  3623.5860

(1.309e-06)

**Charge States:** 3, 4

**Start Position**: 58

**End Position**: 74

| Profile Score | Tandem Score | TDA q-value | Volume | Spectrum Matches |
| --- | --- | --- | --- | --- |
| 14.8625 | 174.1088 | 0.0000 | 1.1977e+09 | 24 |

**Adduct Signal Fractions**

- **Unmodified**: 1.1977e+09

### SVQEIQATFFYFTPN(N)K Fuc 1 Hex 4 HexNAc 6

**Mass:**  3931.6970

(2.111e-06)

**Charge States:** 3, 4

**Start Position**: 58

**End Position**: 74

| Profile Score | Tandem Score | TDA q-value | Volume | Spectrum Matches |
| --- | --- | --- | --- | --- |
| 13.2563 | 173.4863 | 0.0000 | 2.6774e+08 | 9 |

**Adduct Signal Fractions**

- **Unmodified**: 2.6774e+08

### SVQEIQATFFYFTPN(N)K Fuc 1 Hex 4 HexNAc 5

**Mass:**  3728.6175

(9.848e-07)

**Charge States:** 3, 4

**Start Position**: 58

**End Position**: 74

| Profile Score | Tandem Score | TDA q-value | Volume | Spectrum Matches |
| --- | --- | --- | --- | --- |
| 13.5860 | 165.4185 | 0.0000 | 2.0244e+08 | 7 |

**Adduct Signal Fractions**

- **Unmodified**: 2.0244e+08

### SVQEIQATFFYFTPN(N)K Hex 3 HexNAc 5

**Mass:**  3420.5037

(1.062e-06)

**Charge States:** 2, 3, 4

**Start Position**: 58

**End Position**: 74

| Profile Score | Tandem Score | TDA q-value | Volume | Spectrum Matches |
| --- | --- | --- | --- | --- |
| 15.3434 | 165.1817 | 0.0000 | 7.0452e+08 | 28 |

**Adduct Signal Fractions**

- **Unmodified**: 7.0452e+08

### LVPVPITN(N)ATLDR Hex 3 HexNAc 5

**Mass:**  2909.3670

(2.825e-07)

**Charge States:** 2, 3, 4

**Start Position**: 26

**End Position**: 39

| Profile Score | Tandem Score | TDA q-value | Volume | Spectrum Matches |
| --- | --- | --- | --- | --- |
| 11.5762 | 160.4743 | 0.0000 | 1.2670e+10 | 333 |

**Adduct Signal Fractions**

- **Unmodified**: 1.2670e+10

### LVPVPITN(N)ATLDR Fuc 1 Hex 4 HexNAc 5

**Mass:**  3217.4774

(5.701e-07)

**Charge States:** 2, 3, 4

**Start Position**: 26

**End Position**: 39

| Profile Score | Tandem Score | TDA q-value | Volume | Spectrum Matches |
| --- | --- | --- | --- | --- |
| 10.0157 | 160.2259 | 0.0000 | 3.5131e+09 | 108 |

**Adduct Signal Fractions**

- **Unmodified**: 3.5131e+09

### ATFFYFTPN(N)K Fuc 2 Hex 7 HexNAc 4

**Mass:**  3475.4241

(5.812e-04)

**Start Position**: 64

**End Position**: 74

| Profile Score | Tandem Score | TDA q-value | Volume | Spectrum Matches |
| --- | --- | --- | --- | --- |
| 0.0000 | 158.5941 | 0.0000 | 0.0000e+00 | 1 |

Adduct:

No Chromatogram Found

### LVPVPITN(N)ATLDR Hex 3 HexNAc 4

**Mass:**  2706.2869

(3.757e-04)

**Charge States:** 2, 3, 4

**Start Position**: 26

**End Position**: 39

| Profile Score | Tandem Score | TDA q-value | Volume | Spectrum Matches |
| --- | --- | --- | --- | --- |
| 13.9057 | 157.7649 | 0.0000 | 1.0890e+09 | 16 |

**Adduct Signal Fractions**

- **Unmodified**: 1.0890e+09

### TPN(N)KTEDTIFLR Hex 3 HexNAc 6

**Mass:**  3138.3894

(9.927e-07)

**Charge States:** 3, 4

**Start Position**: 70

**End Position**: 82

| Profile Score | Tandem Score | TDA q-value | Volume | Spectrum Matches |
| --- | --- | --- | --- | --- |
| 13.2619 | 157.1468 | 0.0000 | 4.3731e+07 | 5 |

**Adduct Signal Fractions**

- **Unmodified**: 4.3731e+07

### LVPVPITN(N)ATLDR Hex 3 HexNAc 6

**Mass:**  3112.4448

(2.338e-07)

**Charge States:** 3, 4

**Start Position**: 26

**End Position**: 39

| Profile Score | Tandem Score | TDA q-value | Volume | Spectrum Matches |
| --- | --- | --- | --- | --- |
| 9.6422 | 156.6570 | 0.0000 | 2.8801e+08 | 4 |

**Adduct Signal Fractions**

- **Unmodified**: 2.8801e+08

### SVQEIQATFFYFTPN(N)K Hex 4 HexNAc 8

**Mass:**  4191.7985

(8.251e-07)

**Charge States:** 3, 4

**Start Position**: 58

**End Position**: 74

| Profile Score | Tandem Score | TDA q-value | Volume | Spectrum Matches |
| --- | --- | --- | --- | --- |
| -3.7057 | 153.6847 | 0.0000 | 2.3167e+07 | 4 |

**Adduct Signal Fractions**

- **Unmodified**: 2.3167e+07

### LVPVPITN(N)ATLDR Hex 4 HexNAc 5

**Mass:**  3071.4201

(3.170e-07)

**Charge States:** 3, 4

**Start Position**: 26

**End Position**: 39

| Profile Score | Tandem Score | TDA q-value | Volume | Spectrum Matches |
| --- | --- | --- | --- | --- |
| 12.2903 | 149.9122 | 0.0000 | 9.7639e+07 | 4 |

**Adduct Signal Fractions**

- **Unmodified**: 9.7639e+07

### SVQEIQATFFYFTPN(N)K Fuc 2 Hex 5 HexNAc 6

**Mass:**  4239.8088

(1.121e-06)

**Charge States:** 3, 4

**Start Position**: 58

**End Position**: 74

| Profile Score | Tandem Score | TDA q-value | Volume | Spectrum Matches |
| --- | --- | --- | --- | --- |
| 13.5404 | 149.4146 | 0.0000 | 4.1684e+07 | 4 |

**Adduct Signal Fractions**

- **Unmodified**: 4.1684e+07

### ATFFYFTPN(N)K Hex 3 HexNAc 5

**Mass:**  2736.1604

(1.017e-06)

**Charge States:** 3

**Start Position**: 64

**End Position**: 74

| Profile Score | Tandem Score | TDA q-value | Volume | Spectrum Matches |
| --- | --- | --- | --- | --- |
| -3.2105 | 149.2368 | 0.0000 | 2.2634e+07 | 2 |

**Adduct Signal Fractions**

- **Unmodified**: 2.2634e+07

### ATFFYFTPN(N)K Hex 3 HexNAc 6

**Mass:**  2939.2410

(1.117e-06)

**Charge States:** 3

**Start Position**: 64

**End Position**: 74

| Profile Score | Tandem Score | TDA q-value | Volume | Spectrum Matches |
| --- | --- | --- | --- | --- |
| 14.3965 | 147.4212 | 0.0000 | 4.5021e+07 | 2 |

**Adduct Signal Fractions**

- **Unmodified**: 4.5021e+07

### SVQEIQATFFYFTPN(N)K Fuc 1 Hex 5 HexNAc 7

**Mass:**  4296.8376

(2.407e-06)

**Charge States:** 3, 4

**Start Position**: 58

**End Position**: 74

| Profile Score | Tandem Score | TDA q-value | Volume | Spectrum Matches |
| --- | --- | --- | --- | --- |
| 8.9586 | 146.3463 | 0.0000 | 3.3518e+07 | 6 |

**Adduct Signal Fractions**

- **Unmodified**: 3.3518e+07

### SVQEIQATFFYFTPN(N)K Hex 3 HexNAc 4

**Mass:**  3217.4287

(7.251e-07)

**Charge States:** 3, 4

**Start Position**: 58

**End Position**: 74

| Profile Score | Tandem Score | TDA q-value | Volume | Spectrum Matches |
| --- | --- | --- | --- | --- |
| 12.9241 | 145.7175 | 0.0000 | 7.2059e+07 | 6 |

**Adduct Signal Fractions**

- **Unmodified**: 7.2059e+07

### LVPVPITN(N)ATLDR Hex 4 HexNAc 6

**Mass:**  3274.4990

(1.732e-06)

**Charge States:** 3, 4

**Start Position**: 26

**End Position**: 39

| Profile Score | Tandem Score | TDA q-value | Volume | Spectrum Matches |
| --- | --- | --- | --- | --- |
| 11.7535 | 144.7958 | 0.0000 | 7.6695e+08 | 8 |

**Adduct Signal Fractions**

- **Unmodified**: 7.6695e+08

### LVPVPITN(N)ATLDR Fuc 1 Hex 3 HexNAc 5

**Mass:**  3055.4253

(-1.177e-06)

**Charge States:** 3

**Start Position**: 26

**End Position**: 39

| Profile Score | Tandem Score | TDA q-value | Volume | Spectrum Matches |
| --- | --- | --- | --- | --- |
| 10.7471 | 143.6068 | 0.0000 | 6.6620e+07 | 2 |

**Adduct Signal Fractions**

- **Unmodified**: 6.6620e+07

### LVPVPITN(N)ATLDR Hex 3 HexNAc 4

**Mass:**  2708.2867

(7.398e-04)

**Start Position**: 26

**End Position**: 39

| Profile Score | Tandem Score | TDA q-value | Volume | Spectrum Matches |
| --- | --- | --- | --- | --- |
| 0.0000 | 141.3835 | 0.0000 | 0.0000e+00 | 1 |

Adduct:

No Chromatogram Found

### SVQEIQATFFYFTPN(N)K Hex 4 HexNAc 6

**Mass:**  3785.6380

(8.799e-07)

**Charge States:** 3, 4

**Start Position**: 58

**End Position**: 74

| Profile Score | Tandem Score | TDA q-value | Volume | Spectrum Matches |
| --- | --- | --- | --- | --- |
| 10.6471 | 141.1802 | 0.0000 | 3.1053e+07 | 5 |

**Adduct Signal Fractions**

- **Unmodified**: 3.1053e+07

### C(C)ANLVPVPITN(N)ATLDR Fuc 1 Hex 4 HexNAc 5

**Mass:**  3562.5902

(2.822e-06)

**Charge States:** 3, 4

**Start Position**: 23

**End Position**: 39

| Profile Score | Tandem Score | TDA q-value | Volume | Spectrum Matches |
| --- | --- | --- | --- | --- |
| 12.0501 | 141.0477 | 0.0000 | 5.8433e+07 | 4 |

**Adduct Signal Fractions**

- **Unmodified**: 5.8433e+07

### LVPVPITN(N)ATLDR Fuc 1 Hex 4 HexNAc 6

**Mass:**  3420.5586

(9.001e-07)

**Charge States:** 3

**Start Position**: 26

**End Position**: 39

| Profile Score | Tandem Score | TDA q-value | Volume | Spectrum Matches |
| --- | --- | --- | --- | --- |
| 17.2092 | 138.8512 | 0.0000 | 1.8870e+08 | 3 |

**Adduct Signal Fractions**

- **Unmodified**: 1.8870e+08

### YFTPN(N)KTEDTIFLR Hex 3 HexNAc 6

**Mass:**  3448.5207

(1.053e-06)

**Charge States:** 3, 4

**Start Position**: 68

**End Position**: 82

| Profile Score | Tandem Score | TDA q-value | Volume | Spectrum Matches |
| --- | --- | --- | --- | --- |
| 11.3390 | 138.1390 | 0.0000 | 1.5676e+09 | 13 |

**Adduct Signal Fractions**

- **Unmodified**: 1.5676e+09

### SVQEIQATFFYFTPN(N)K Fuc 2 Hex 4 HexNAc 4 Neu5Ac 1

**Mass:**  3962.6694

(-4.327e-06)

**Charge States:** 4

**Start Position**: 58

**End Position**: 74

| Profile Score | Tandem Score | TDA q-value | Volume | Spectrum Matches |
| --- | --- | --- | --- | --- |
| -18.8027 | 135.3714 | 0.0000 | 2.7120e+06 | 1 |

**Adduct Signal Fractions**

- **Unmodified**: 2.7120e+06

### LVPVPITN(N)ATLDR Hex 4 HexNAc 6

**Mass:**  3276.5056

(6.137e-04)

**Start Position**: 26

**End Position**: 39

| Profile Score | Tandem Score | TDA q-value | Volume | Spectrum Matches |
| --- | --- | --- | --- | --- |
| 0.0000 | 133.8668 | 0.0000 | 0.0000e+00 | 1 |

Adduct:

No Chromatogram Found

### ATFFYFTPN(N)K Fuc 1 Hex 4 HexNAc 6

**Mass:**  3247.3493

(5.084e-07)

**Start Position**: 64

**End Position**: 74

| Profile Score | Tandem Score | TDA q-value | Volume | Spectrum Matches |
| --- | --- | --- | --- | --- |
| 0.0000 | 133.4824 | 0.0000 | 0.0000e+00 | 1 |

Adduct:

No Chromatogram Found

### YFTPN(N)KTEDTIFLR Hex 4 HexNAc 7

**Mass:**  3813.6535

(9.061e-07)

**Charge States:** 4

**Start Position**: 68

**End Position**: 82

| Profile Score | Tandem Score | TDA q-value | Volume | Spectrum Matches |
| --- | --- | --- | --- | --- |
| 8.5799 | 133.4317 | 0.0000 | 1.3858e+08 | 5 |

**Adduct Signal Fractions**

- **Unmodified**: 1.3858e+08

### YFTPN(N)KTEDTIFLR Hex 3 HexNAc 5

**Mass:**  3245.4417

(1.687e-06)

**Charge States:** 3, 4

**Start Position**: 68

**End Position**: 82

| Profile Score | Tandem Score | TDA q-value | Volume | Spectrum Matches |
| --- | --- | --- | --- | --- |
| 10.9449 | 132.9386 | 0.0000 | 7.3529e+08 | 6 |

**Adduct Signal Fractions**

- **Unmodified**: 7.3529e+08

### LVPVPITN(N)ATLDR Hex 4 HexNAc 7

**Mass:**  3477.5824

(2.000e-06)

**Charge States:** 3, 4

**Start Position**: 26

**End Position**: 39

| Profile Score | Tandem Score | TDA q-value | Volume | Spectrum Matches |
| --- | --- | --- | --- | --- |
| -4.8647 | 132.7335 | 0.0000 | 1.0264e+08 | 4 |

**Adduct Signal Fractions**

- **Unmodified**: 1.0264e+08

### PVPITN(N)ATLDR Fuc 2 Hex 6 HexNAc 3

**Mass:**  3069.3297

(8.452e-07)

**Charge States:** 3

**Start Position**: 28

**End Position**: 39

| Profile Score | Tandem Score | TDA q-value | Volume | Spectrum Matches |
| --- | --- | --- | --- | --- |
| -19.1041 | 132.5425 | 0.0000 | 1.0403e+07 | 1 |

**Adduct Signal Fractions**

- **Unmodified**: 1.0403e+07

### C(C)ANLVPVPITN(N)ATLDR Hex 3 HexNAc 5

**Mass:**  3254.4772

(7.988e-07)

**Charge States:** 3, 4

**Start Position**: 23

**End Position**: 39

| Profile Score | Tandem Score | TDA q-value | Volume | Spectrum Matches |
| --- | --- | --- | --- | --- |
| 12.1675 | 132.1842 | 0.0000 | 4.9007e+08 | 9 |

**Adduct Signal Fractions**

- **Unmodified**: 4.9007e+08

### QNQC(C)FYN(N)SSYLNVQR Hex 3 HexNAc 5

**Mass:**  3421.4185

(9.840e-07)

**Charge States:** 3, 4

**Start Position**: 87

**End Position**: 102

| Profile Score | Tandem Score | TDA q-value | Volume | Spectrum Matches |
| --- | --- | --- | --- | --- |
| -3.2913 | 131.9147 | 0.0000 | 2.9971e+08 | 4 |

**Adduct Signal Fractions**

- **Unmodified**: 2.9971e+08

### YFTPN(N)KTEDTIFLR Fuc 1 Hex 4 HexNAc 6

**Mass:**  3756.6330

(5.121e-07)

**Charge States:** 3, 4

**Start Position**: 68

**End Position**: 82

| Profile Score | Tandem Score | TDA q-value | Volume | Spectrum Matches |
| --- | --- | --- | --- | --- |
| -4.7286 | 129.7446 | 0.0000 | 2.4765e+08 | 8 |

**Adduct Signal Fractions**

- **Unmodified**: 2.4765e+08

### ATFFYFTPN(N)K Fuc 1 Hex 4 HexNAc 5

**Mass:**  3044.2624

(-1.118e-07)

**Charge States:** 3

**Start Position**: 64

**End Position**: 74

| Profile Score | Tandem Score | TDA q-value | Volume | Spectrum Matches |
| --- | --- | --- | --- | --- |
| -4.1116 | 129.2809 | 0.0000 | 6.1161e+06 | 1 |

**Adduct Signal Fractions**

- **Unmodified**: 6.1161e+06

### VPVPITN(N)ATLDR Hex 3 HexNAc 5

**Mass:**  2796.2829

(5.600e-07)

**Charge States:** 3

**Start Position**: 27

**End Position**: 39

| Profile Score | Tandem Score | TDA q-value | Volume | Spectrum Matches |
| --- | --- | --- | --- | --- |
| -2.8525 | 127.9542 | 0.0000 | 7.0157e+06 | 3 |

**Adduct Signal Fractions**

- **Unmodified**: 7.0157e+06

### YFTPN(N)KTEDTIFLR Fuc 1 Hex 4 HexNAc 5

**Mass:**  3554.5465

(2.806e-04)

**Start Position**: 68

**End Position**: 82

| Profile Score | Tandem Score | TDA q-value | Volume | Spectrum Matches |
| --- | --- | --- | --- | --- |
| 0.0000 | 127.8034 | 0.0000 | 0.0000e+00 | 1 |

Adduct:

No Chromatogram Found

### SVQEIQATFFYFTPN(N)K Hex 4 HexNAc 4 Neu5Ac 2

**Mass:**  3961.6728

(2.006e-06)

**Charge States:** 3, 4

**Start Position**: 58

**End Position**: 74

| Profile Score | Tandem Score | TDA q-value | Volume | Spectrum Matches |
| --- | --- | --- | --- | --- |
| 12.7470 | 127.4052 | 0.0000 | 3.6313e+07 | 4 |

**Adduct Signal Fractions**

- **Unmodified**: 3.6313e+07

### SVQEIQATFFYFTPN(N)K Hex 5 HexNAc 9

**Mass:**  4556.9323

(1.649e-06)

**Charge States:** 4

**Start Position**: 58

**End Position**: 74

| Profile Score | Tandem Score | TDA q-value | Volume | Spectrum Matches |
| --- | --- | --- | --- | --- |
| -19.4276 | 126.5748 | 0.0000 | 3.3121e+06 | 1 |

**Adduct Signal Fractions**

- **Unmodified**: 3.3121e+06

### QNQC(C)FYN(N)SSYLNVQR Hex 3 HexNAc 6

**Mass:**  3624.4989

(1.599e-06)

**Charge States:** 3, 4

**Start Position**: 87

**End Position**: 102

| Profile Score | Tandem Score | TDA q-value | Volume | Spectrum Matches |
| --- | --- | --- | --- | --- |
| 6.6109 | 125.7109 | 0.0000 | 1.4001e+09 | 6 |

**Adduct Signal Fractions**

- **Unmodified**: 1.4001e+09

### YFTPN(N)KTEDTIFLR Fuc 1 Hex 4 HexNAc 5

**Mass:**  3553.5510

(1.258e-06)

**Charge States:** 3, 4

**Start Position**: 68

**End Position**: 82

| Profile Score | Tandem Score | TDA q-value | Volume | Spectrum Matches |
| --- | --- | --- | --- | --- |
| -4.6002 | 125.6023 | 0.0000 | 1.6850e+08 | 3 |

**Adduct Signal Fractions**

- **Unmodified**: 1.6850e+08

### LVPVPITN(N)ATLDR Fuc 1 Hex 5 HexNAc 3 Neu5Ac 1

**Mass:**  3264.4791

(4.125e-06)

**Charge States:** 3

**Start Position**: 26

**End Position**: 39

| Profile Score | Tandem Score | TDA q-value | Volume | Spectrum Matches |
| --- | --- | --- | --- | --- |
| -20.2188 | 125.4555 | 0.0000 | 2.1474e+07 | 1 |

**Adduct Signal Fractions**

- **Unmodified**: 2.1474e+07

### FYFTPN(N)KTEDTIFLR Hex 3 HexNAc 6

**Mass:**  3595.5917

(1.614e-06)

**Charge States:** 3, 4

**Start Position**: 67

**End Position**: 82

| Profile Score | Tandem Score | TDA q-value | Volume | Spectrum Matches |
| --- | --- | --- | --- | --- |
| 13.4275 | 125.1874 | 0.0000 | 1.0669e+08 | 5 |

**Adduct Signal Fractions**

- **Unmodified**: 1.0669e+08

### NLVPVPITN(N)ATLDR Hex 3 HexNAc 5

**Mass:**  3023.4111

(1.394e-06)

**Charge States:** 3

**Start Position**: 25

**End Position**: 39

| Profile Score | Tandem Score | TDA q-value | Volume | Spectrum Matches |
| --- | --- | --- | --- | --- |
| -4.3854 | 124.1273 | 0.0000 | 1.0083e+07 | 1 |

**Adduct Signal Fractions**

- **Unmodified**: 1.0083e+07

### QNQC(C)FYN(N)SSYLNVQR Fuc 1 Hex 4 HexNAc 6

**Mass:**  3932.6109

(1.786e-06)

**Charge States:** 3, 4

**Start Position**: 87

**End Position**: 102

| Profile Score | Tandem Score | TDA q-value | Volume | Spectrum Matches |
| --- | --- | --- | --- | --- |
| 11.0760 | 123.3134 | 0.0000 | 3.5252e+08 | 3 |

**Adduct Signal Fractions**

- **Unmodified**: 3.5252e+08

### YFTPN(N)KTEDTIFLR Fuc 2 Hex 5 HexNAc 6

**Mass:**  4065.7453

(2.475e-04)

**Start Position**: 68

**End Position**: 82

| Profile Score | Tandem Score | TDA q-value | Volume | Spectrum Matches |
| --- | --- | --- | --- | --- |
| 0.0000 | 122.8224 | 0.0000 | 0.0000e+00 | 1 |

Adduct:

No Chromatogram Found

### LVPVPITN(N)ATLDR Fuc 2 Hex 5 HexNAc 6

**Mass:**  3728.6749

(2.742e-06)

**Charge States:** 4

**Start Position**: 26

**End Position**: 39

| Profile Score | Tandem Score | TDA q-value | Volume | Spectrum Matches |
| --- | --- | --- | --- | --- |
| -5.0781 | 122.8160 | 0.0000 | 1.1414e+07 | 4 |

**Adduct Signal Fractions**

- **Unmodified**: 1.1414e+07

### LVPVPITN(N)ATLDR Fuc 2 Hex 5 HexNAc 5

**Mass:**  3525.5930

(2.136e-06)

**Charge States:** 3

**Start Position**: 26

**End Position**: 39

| Profile Score | Tandem Score | TDA q-value | Volume | Spectrum Matches |
| --- | --- | --- | --- | --- |
| -4.1635 | 121.5332 | 0.0000 | 4.6772e+07 | 2 |

**Adduct Signal Fractions**

- **Unmodified**: 4.6772e+07

### SVQEIQATFFYFTPN(N)K Fuc 1 Hex 4 HexNAc 7

**Mass:**  4134.7795

(1.044e-06)

**Charge States:** 3, 4

**Start Position**: 58

**End Position**: 74

| Profile Score | Tandem Score | TDA q-value | Volume | Spectrum Matches |
| --- | --- | --- | --- | --- |
| -4.9093 | 121.2899 | 0.0000 | 8.2048e+06 | 2 |

**Adduct Signal Fractions**

- **Unmodified**: 8.2048e+06

### LVPVPITN(N)ATLDR Fuc 1 Hex 5 HexNAc 5

**Mass:**  3379.5342

(1.974e-06)

**Charge States:** 3

**Start Position**: 26

**End Position**: 39

| Profile Score | Tandem Score | TDA q-value | Volume | Spectrum Matches |
| --- | --- | --- | --- | --- |
| -16.8862 | 120.8769 | 0.0000 | 9.4521e+06 | 1 |

**Adduct Signal Fractions**

- **Unmodified**: 9.4521e+06

### QNQC(C)FYN(N)SSYLNVQR Fuc 1 Hex 4 HexNAc 5

**Mass:**  3729.5308

(1.713e-06)

**Charge States:** 3, 4

**Start Position**: 87

**End Position**: 102

| Profile Score | Tandem Score | TDA q-value | Volume | Spectrum Matches |
| --- | --- | --- | --- | --- |
| -9.8885 | 117.6211 | 0.0000 | 1.7518e+08 | 4 |

**Adduct Signal Fractions**

- **Unmodified**: 1.7518e+08

### TPN(N)KTEDTIFLR Hex 3 HexNAc 5

**Mass:**  2935.3077

(2.897e-07)

**Charge States:** 3

**Start Position**: 70

**End Position**: 82

| Profile Score | Tandem Score | TDA q-value | Volume | Spectrum Matches |
| --- | --- | --- | --- | --- |
| 12.3453 | 117.6051 | 0.0000 | 4.1006e+07 | 6 |

**Adduct Signal Fractions**

- **Unmodified**: 4.1006e+07

### NLVPVPITN(N)ATLDR Fuc 1 Hex 3 HexNAc 4

**Mass:**  2966.3917

(1.409e-06)

**Charge States:** 2, 3, 4

**Start Position**: 25

**End Position**: 39

| Profile Score | Tandem Score | TDA q-value | Volume | Spectrum Matches |
| --- | --- | --- | --- | --- |
| 14.7660 | 115.8871 | 0.0000 | 1.4705e+09 | 18 |

**Adduct Signal Fractions**

- **Unmodified**: 1.4705e+09

### YFTPN(N)KTEDTIFLR Hex 4 HexNAc 7

**Mass:**  3815.6605

(5.272e-04)

**Start Position**: 68

**End Position**: 82

| Profile Score | Tandem Score | TDA q-value | Volume | Spectrum Matches |
| --- | --- | --- | --- | --- |
| 0.0000 | 115.6638 | 0.0000 | 0.0000e+00 | 2 |

Adduct:

No Chromatogram Found

### VPVPITN(N)ATLDR Fuc 1 Hex 4 HexNAc 5

**Mass:**  3104.3937

(9.858e-07)

**Charge States:** 3

**Start Position**: 27

**End Position**: 39

| Profile Score | Tandem Score | TDA q-value | Volume | Spectrum Matches |
| --- | --- | --- | --- | --- |
| -18.5492 | 115.5570 | 0.0000 | 1.7906e+06 | 1 |

**Adduct Signal Fractions**

- **Unmodified**: 1.7906e+06

### SVQEIQATFFYFTPN(N)K Fuc 1 Hex 5 HexNAc 4 Neu5Ac 1

**Mass:**  3978.6897

(4.686e-06)

**Charge States:** 3

**Start Position**: 58

**End Position**: 74

| Profile Score | Tandem Score | TDA q-value | Volume | Spectrum Matches |
| --- | --- | --- | --- | --- |
| -4.8660 | 112.6396 | 0.0000 | 6.6452e+06 | 2 |

**Adduct Signal Fractions**

- **Unmodified**: 6.6452e+06

### C(C)ANLVPVPITN(N)ATLDR Hex 3 HexNAc 4

**Mass:**  3051.3982

(2.906e-06)

**Charge States:** 3

**Start Position**: 23

**End Position**: 39

| Profile Score | Tandem Score | TDA q-value | Volume | Spectrum Matches |
| --- | --- | --- | --- | --- |
| 10.0161 | 111.8217 | 0.0000 | 1.2288e+07 | 2 |

**Adduct Signal Fractions**

- **Unmodified**: 1.2288e+07

### FYFTPN(N)KTEDTIFLR Fuc 1 Hex 4 HexNAc 6

**Mass:**  3903.7036

(1.658e-06)

**Charge States:** 3, 4

**Start Position**: 67

**End Position**: 82

| Profile Score | Tandem Score | TDA q-value | Volume | Spectrum Matches |
| --- | --- | --- | --- | --- |
| 13.8328 | 111.8188 | 0.0000 | 2.7600e+07 | 4 |

**Adduct Signal Fractions**

- **Unmodified**: 2.7600e+07

### ATFFYFTPN(N)K Hex 4 HexNAc 7

**Mass:**  3304.3833

(-5.787e-07)

**Charge States:** 3

**Start Position**: 64

**End Position**: 74

| Profile Score | Tandem Score | TDA q-value | Volume | Spectrum Matches |
| --- | --- | --- | --- | --- |
| -6.0399 | 111.3241 | 0.0000 | 3.5241e+06 | 1 |

**Adduct Signal Fractions**

- **Unmodified**: 3.5241e+06

### LVPVPITN(N)ATLDR Fuc 1 Hex 5 HexNAc 6

**Mass:**  3582.6179

(3.114e-06)

**Charge States:** 3, 4

**Start Position**: 26

**End Position**: 39

| Profile Score | Tandem Score | TDA q-value | Volume | Spectrum Matches |
| --- | --- | --- | --- | --- |
| -4.5424 | 111.2493 | 0.0000 | 3.3857e+07 | 2 |

**Adduct Signal Fractions**

- **Unmodified**: 3.3857e+07

### FTPN(N)KTEDTIFLR Hex 3 HexNAc 5

**Mass:**  3082.3793

(3.931e-07)

**Charge States:** 3, 4

**Start Position**: 69

**End Position**: 82

| Profile Score | Tandem Score | TDA q-value | Volume | Spectrum Matches |
| --- | --- | --- | --- | --- |
| 12.9133 | 110.7548 | 0.0000 | 4.2271e+07 | 3 |

**Adduct Signal Fractions**

- **Unmodified**: 4.2271e+07

### FTPN(N)KTEDTIFLR Hex 3 HexNAc 6

**Mass:**  3285.4575

(8.236e-07)

**Charge States:** 3, 4

**Start Position**: 69

**End Position**: 82

| Profile Score | Tandem Score | TDA q-value | Volume | Spectrum Matches |
| --- | --- | --- | --- | --- |
| 13.0770 | 110.6195 | 0.0000 | 8.7251e+07 | 7 |

**Adduct Signal Fractions**

- **Unmodified**: 8.7251e+07

### TPN(N)KTEDTIFLR Hex 3 HexNAc 4

**Mass:**  2732.2252

(1.118e-06)

**Charge States:** 3

**Start Position**: 70

**End Position**: 82

| Profile Score | Tandem Score | TDA q-value | Volume | Spectrum Matches |
| --- | --- | --- | --- | --- |
| 10.0274 | 109.7526 | 0.0000 | 7.8218e+06 | 2 |

**Adduct Signal Fractions**

- **Unmodified**: 7.8218e+06

### LVPVPITN(N)ATLDR Hex 4 HexNAc 5

**Mass:**  3073.4229

(6.532e-04)

**Start Position**: 26

**End Position**: 39

| Profile Score | Tandem Score | TDA q-value | Volume | Spectrum Matches |
| --- | --- | --- | --- | --- |
| 0.0000 | 109.6677 | 0.0000 | 0.0000e+00 | 1 |

Adduct:

No Chromatogram Found

### YFTPN(N)KTEDTIFLR Hex 3 HexNAc 4

**Mass:**  3042.3631

(1.619e-06)

**Charge States:** 3

**Start Position**: 68

**End Position**: 82

| Profile Score | Tandem Score | TDA q-value | Volume | Spectrum Matches |
| --- | --- | --- | --- | --- |
| -1.0405 | 109.2734 | 0.0000 | 7.9021e+07 | 2 |

**Adduct Signal Fractions**

- **Unmodified**: 7.9021e+07

### FYFTPN(N)KTEDTIFLR Hex 3 HexNAc 5

**Mass:**  3392.5120

(1.273e-06)

**Charge States:** 3, 4

**Start Position**: 67

**End Position**: 82

| Profile Score | Tandem Score | TDA q-value | Volume | Spectrum Matches |
| --- | --- | --- | --- | --- |
| 12.0623 | 109.2030 | 0.0000 | 5.0537e+07 | 5 |

**Adduct Signal Fractions**

- **Unmodified**: 5.0537e+07

### LVPVPITN(N)ATLDR Hex 3 HexNAc 6

**Mass:**  3113.4529

(3.244e-04)

**Start Position**: 26

**End Position**: 39

| Profile Score | Tandem Score | TDA q-value | Volume | Spectrum Matches |
| --- | --- | --- | --- | --- |
| 0.0000 | 108.9679 | 0.0000 | 0.0000e+00 | 3 |

Adduct:

No Chromatogram Found

### QNQC(C)FYN(N)SSYLNVQR Hex 4 HexNAc 7

**Mass:**  3989.6311

(1.113e-06)

**Charge States:** 3, 4

**Start Position**: 87

**End Position**: 102

| Profile Score | Tandem Score | TDA q-value | Volume | Spectrum Matches |
| --- | --- | --- | --- | --- |
| 13.5170 | 108.9506 | 0.0000 | 1.5481e+08 | 2 |

**Adduct Signal Fractions**

- **Unmodified**: 1.5481e+08

### FYFTPN(N)KTEDTIFLR Hex 3 HexNAc 4

**Mass:**  3189.4319

(1.351e-06)

**Charge States:** 3

**Start Position**: 67

**End Position**: 82

| Profile Score | Tandem Score | TDA q-value | Volume | Spectrum Matches |
| --- | --- | --- | --- | --- |
| -3.7024 | 108.7568 | 0.0000 | 6.0280e+06 | 1 |

**Adduct Signal Fractions**

- **Unmodified**: 6.0280e+06

### PVPITN(N)ATLDR Fuc 2 Hex 4 HexNAc 5

**Mass:**  3151.3767

(-1.105e-06)

**Charge States:** 3

**Start Position**: 28

**End Position**: 39

| Profile Score | Tandem Score | TDA q-value | Volume | Spectrum Matches |
| --- | --- | --- | --- | --- |
| -5.8538 | 107.8024 | 0.0000 | 6.2501e+06 | 2 |

**Adduct Signal Fractions**

- **Unmodified**: 6.2501e+06

### TPN(N)KTEDTIFLR Hex 7 HexNAc 6

**Mass:**  3786.6055

(2.949e-06)

**Charge States:** 3, 4

**Start Position**: 70

**End Position**: 82

| Profile Score | Tandem Score | TDA q-value | Volume | Spectrum Matches |
| --- | --- | --- | --- | --- |
| 13.7635 | 107.4835 | 0.0000 | 1.0293e+08 | 4 |

**Adduct Signal Fractions**

- **Unmodified**: 1.0293e+08

### NLVPVPITN(N)ATLDR Fuc 1 Hex 3 HexNAc 5

**Mass:**  3169.4672

(3.206e-07)

**Charge States:** 3

**Start Position**: 25

**End Position**: 39

| Profile Score | Tandem Score | TDA q-value | Volume | Spectrum Matches |
| --- | --- | --- | --- | --- |
| -4.3854 | 105.2338 | 0.0000 | 4.3429e+07 | 2 |

**Adduct Signal Fractions**

- **Unmodified**: 4.3429e+07

### YFTPN(N)KTEDTIFLR Hex 5 HexNAc 8

**Mass:**  4178.7883

(1.387e-06)

**Charge States:** 3, 4

**Start Position**: 68

**End Position**: 82

| Profile Score | Tandem Score | TDA q-value | Volume | Spectrum Matches |
| --- | --- | --- | --- | --- |
| -3.9740 | 103.2455 | 0.0000 | 3.1309e+07 | 2 |

**Adduct Signal Fractions**

- **Unmodified**: 3.1309e+07

### FTPN(N)KTEDTIFLR Fuc 1 Hex 4 HexNAc 6

**Mass:**  3593.5702

(1.423e-06)

**Charge States:** 3, 4

**Start Position**: 69

**End Position**: 82

| Profile Score | Tandem Score | TDA q-value | Volume | Spectrum Matches |
| --- | --- | --- | --- | --- |
| -3.6705 | 102.4885 | 0.0000 | 2.0194e+07 | 3 |

**Adduct Signal Fractions**

- **Unmodified**: 2.0194e+07

### TPN(N)KTEDTIFLR Hex 3 HexNAc 6

**Mass:**  3140.3956

(6.403e-04)

**Start Position**: 70

**End Position**: 82

| Profile Score | Tandem Score | TDA q-value | Volume | Spectrum Matches |
| --- | --- | --- | --- | --- |
| 0.0000 | 101.0326 | 0.0000 | 0.0000e+00 | 1 |

Adduct:

No Chromatogram Found

### FYFTPN(N)KTEDTIFLR Fuc 1 Hex 4 HexNAc 5

**Mass:**  3700.6214

(9.506e-07)

**Charge States:** 3, 4

**Start Position**: 67

**End Position**: 82

| Profile Score | Tandem Score | TDA q-value | Volume | Spectrum Matches |
| --- | --- | --- | --- | --- |
| -3.9366 | 100.5597 | 0.0000 | 1.4332e+07 | 2 |

**Adduct Signal Fractions**

- **Unmodified**: 1.4332e+07

### LVPVPITN(N)ATLDR Fuc 2 Hex 4 HexNAc 5

**Mass:**  3364.5364

(2.984e-04)

**Start Position**: 26

**End Position**: 39

| Profile Score | Tandem Score | TDA q-value | Volume | Spectrum Matches |
| --- | --- | --- | --- | --- |
| 0.0000 | 98.7509 | 0.0000 | 0.0000e+00 | 1 |

Adduct:

No Chromatogram Found

### LVPVPITN(N)ATLDR Fuc 4 Hex 3 HexNAc 5

**Mass:**  3496.6129

(8.637e-04)

**Start Position**: 26

**End Position**: 39

| Profile Score | Tandem Score | TDA q-value | Volume | Spectrum Matches |
| --- | --- | --- | --- | --- |
| 0.0000 | 97.9419 | 0.0000 | 0.0000e+00 | 1 |

Adduct:

No Chromatogram Found

### LVPVPITN(N)ATLDR Fuc 2 Hex 3 HexNAc 4 Neu5Ac 1

**Mass:**  3292.5193

(9.191e-04)

**Start Position**: 26

**End Position**: 39

| Profile Score | Tandem Score | TDA q-value | Volume | Spectrum Matches |
| --- | --- | --- | --- | --- |
| 0.0000 | 96.7180 | 0.0000 | 0.0000e+00 | 1 |

Adduct:

No Chromatogram Found

### PVPITN(N)ATLDR Fuc 1 Hex 5 HexNAc 4 Neu5Ac 1

**Mass:**  3255.4226

(9.707e-06)

**Charge States:** 3, 4

**Start Position**: 28

**End Position**: 39

| Profile Score | Tandem Score | TDA q-value | Volume | Spectrum Matches |
| --- | --- | --- | --- | --- |
| -3.9612 | 96.0949 | 0.0000 | 3.9061e+08 | 5 |

**Adduct Signal Fractions**

- **Unmodified**: 3.9061e+08

### YFTPN(N)KTEDTIFLR Hex 5 HexNAc 9

**Mass:**  4381.8703

(2.003e-06)

**Charge States:** 3, 4

**Start Position**: 68

**End Position**: 82

| Profile Score | Tandem Score | TDA q-value | Volume | Spectrum Matches |
| --- | --- | --- | --- | --- |
| -19.8915 | 95.4370 | 0.0000 | 9.0030e+06 | 1 |

**Adduct Signal Fractions**

- **Unmodified**: 9.0030e+06

### YFTPN(N)KTEDTIFLR Fuc 1 Hex 5 HexNAc 7

**Mass:**  4121.7658

(1.237e-06)

**Charge States:** 3, 4

**Start Position**: 68

**End Position**: 82

| Profile Score | Tandem Score | TDA q-value | Volume | Spectrum Matches |
| --- | --- | --- | --- | --- |
| -5.0055 | 94.7699 | 0.0000 | 3.6302e+07 | 3 |

**Adduct Signal Fractions**

- **Unmodified**: 3.6302e+07

### NLVPVPITN(N)ATLDR Fuc 1 Hex 3 HexNAc 3

**Mass:**  2763.3074

(1.475e-06)

**Charge States:** 2, 3

**Start Position**: 25

**End Position**: 39

| Profile Score | Tandem Score | TDA q-value | Volume | Spectrum Matches |
| --- | --- | --- | --- | --- |
| 14.1333 | 90.2684 | 0.0000 | 1.0889e+08 | 3 |

**Adduct Signal Fractions**

- **Unmodified**: 1.0889e+08

### NLVPVPITN(N)ATLDR Fuc 1 Hex 5 HexNAc 4

**Mass:**  3293.5442

(9.247e-04)

**Charge States:** 3

**Start Position**: 25

**End Position**: 39

| Profile Score | Tandem Score | TDA q-value | Volume | Spectrum Matches |
| --- | --- | --- | --- | --- |
| -8.6920 | 89.2143 | 0.0000 | 1.3973e+05 | 1 |

**Adduct Signal Fractions**

- **Unmodified**: 1.3973e+05

### FYFTPN(N)KTEDTIFLR Hex 4 HexNAc 7

**Mass:**  3960.7263

(1.969e-06)

**Charge States:** 3, 4

**Start Position**: 67

**End Position**: 82

| Profile Score | Tandem Score | TDA q-value | Volume | Spectrum Matches |
| --- | --- | --- | --- | --- |
| -3.5609 | 88.3300 | 0.0000 | 1.7273e+07 | 5 |

**Adduct Signal Fractions**

- **Unmodified**: 1.7273e+07

### FTPN(N)KTEDTIFLR Fuc 1 Hex 4 HexNAc 5

**Mass:**  3390.4912

(1.809e-06)

**Charge States:** 3

**Start Position**: 69

**End Position**: 82

| Profile Score | Tandem Score | TDA q-value | Volume | Spectrum Matches |
| --- | --- | --- | --- | --- |
| -3.2879 | 88.2995 | 0.0000 | 1.1631e+07 | 3 |

**Adduct Signal Fractions**

- **Unmodified**: 1.1631e+07

### LVPVPITN(N)ATLDR Hex 6 HexNAc 5 Neu5Ac 3

**Mass:**  4268.8098

(2.099e-06)

**Charge States:** 3, 4

**Start Position**: 26

**End Position**: 39

| Profile Score | Tandem Score | TDA q-value | Volume | Spectrum Matches |
| --- | --- | --- | --- | --- |
| 11.1871 | 87.1377 | 0.0000 | 9.3973e+06 | 4 |

**Adduct Signal Fractions**

- **Unmodified**: 9.3973e+06

### YFTPN(N)KTEDTIFLR Hex 4 HexNAc 8

**Mass:**  4016.7373

(1.969e-06)

**Charge States:** 3, 4

**Start Position**: 68

**End Position**: 82

| Profile Score | Tandem Score | TDA q-value | Volume | Spectrum Matches |
| --- | --- | --- | --- | --- |
| -19.4592 | 87.1374 | 0.0000 | 1.4300e+07 | 1 |

**Adduct Signal Fractions**

- **Unmodified**: 1.4300e+07

### FTPN(N)KTEDTIFLR Hex 4 HexNAc 7

**Mass:**  3650.5915

(7.763e-07)

**Charge States:** 3, 4

**Start Position**: 69

**End Position**: 82

| Profile Score | Tandem Score | TDA q-value | Volume | Spectrum Matches |
| --- | --- | --- | --- | --- |
| -4.7605 | 83.7187 | 0.0000 | 1.0851e+07 | 4 |

**Adduct Signal Fractions**

- **Unmodified**: 1.0851e+07

### TPN(N)KTEDTIFLR Fuc 1 Hex 4 HexNAc 6

**Mass:**  3446.4973

(1.575e-06)

**Charge States:** 3, 4

**Start Position**: 70

**End Position**: 82

| Profile Score | Tandem Score | TDA q-value | Volume | Spectrum Matches |
| --- | --- | --- | --- | --- |
| -4.2505 | 82.8777 | 0.0000 | 8.5045e+06 | 4 |

**Adduct Signal Fractions**

- **Unmodified**: 8.5045e+06

### TPN(N)KTEDTIFLR Fuc 1 Hex 4 HexNAc 5

**Mass:**  3243.4235

(1.923e-06)

**Charge States:** 3

**Start Position**: 70

**End Position**: 82

| Profile Score | Tandem Score | TDA q-value | Volume | Spectrum Matches |
| --- | --- | --- | --- | --- |
| -4.7181 | 81.8374 | 0.0000 | 3.9189e+06 | 3 |

**Adduct Signal Fractions**

- **Unmodified**: 3.9189e+06

### C(C)AN(D)LVPVPITN(N)ATLDR Hex 3 HexNAc 5

**Mass:**  3255.4700

(3.029e-04)

**Charge States:** 3

**Start Position**: 23

**End Position**: 39

| Profile Score | Tandem Score | TDA q-value | Volume | Spectrum Matches |
| --- | --- | --- | --- | --- |
| -2.5200 | 77.9654 | 0.0020 | 1.5948e+07 | 6 |

**Adduct Signal Fractions**

- **Unmodified**: 1.5948e+07

### SVQEIQATFFYFTPN(N)K Hex 4 HexNAc 6

**Mass:**  3787.6481

(5.320e-04)

**Start Position**: 58

**End Position**: 74

| Profile Score | Tandem Score | TDA q-value | Volume | Spectrum Matches |
| --- | --- | --- | --- | --- |
| 0.0000 | 73.0777 | 0.0020 | 0.0000e+00 | 1 |

Adduct:

No Chromatogram Found

### SVQEIQATFFYFTPN(N)K Hex 4 HexNAc 5

**Mass:**  3582.5619

(2.063e-06)

**Charge States:** 3

**Start Position**: 58

**End Position**: 74

| Profile Score | Tandem Score | TDA q-value | Volume | Spectrum Matches |
| --- | --- | --- | --- | --- |
| -5.8596 | 70.0636 | 0.0020 | 8.3678e+06 | 1 |

**Adduct Signal Fractions**

- **Unmodified**: 8.3678e+06

### YFTPN(N)KTEDTIFLR Fuc 1 Hex 5 HexNAc 4 Neu5Ac 2

**Mass:**  4094.7219

(2.045e-06)

**Charge States:** 4

**Start Position**: 68

**End Position**: 82

| Profile Score | Tandem Score | TDA q-value | Volume | Spectrum Matches |
| --- | --- | --- | --- | --- |
| -20.6748 | 67.4565 | 0.0020 | 9.1378e+06 | 1 |

**Adduct Signal Fractions**

- **Unmodified**: 9.1378e+06

### SVQEIQATFFYFTPN(N)K Fuc 4 Hex 3 HexNAc 5

**Mass:**  4004.7431

(2.424e-06)

**Charge States:** 3

**Start Position**: 58

**End Position**: 74

| Profile Score | Tandem Score | TDA q-value | Volume | Spectrum Matches |
| --- | --- | --- | --- | --- |
| -20.8579 | 66.0905 | 0.0020 | 2.3969e+06 | 1 |

**Adduct Signal Fractions**

- **Unmodified**: 2.3969e+06

### SVQEIQATFFYFTPN(N)KTEDTIFLR Hex 3 HexNAc 6

**Mass:**  4599.0852

(6.531e-07)

**Charge States:** 4

**Start Position**: 58

**End Position**: 82

| Profile Score | Tandem Score | TDA q-value | Volume | Spectrum Matches |
| --- | --- | --- | --- | --- |
| -4.6283 | 63.7422 | 0.0020 | 2.5033e+07 | 2 |

**Adduct Signal Fractions**

- **Unmodified**: 2.5033e+07

### QIPLC(C)ANLVPVPITN(N)ATLDR Hex 3 HexNAc 5 Neu5Ac 1

**Mass:**  3996.8443

(-9.201e-07)

**Charge States:** 3, 4

**Start Position**: 19

**End Position**: 39

| Profile Score | Tandem Score | TDA q-value | Volume | Spectrum Matches |
| --- | --- | --- | --- | --- |
| 15.9668 | 62.1551 | 0.0020 | 4.0355e+08 | 11 |

**Adduct Signal Fractions**

- **Unmodified**: 4.0355e+08

### SVQEIQATFFYFTPN(N)KTEDTIFLR Hex 3 HexNAc 5

**Mass:**  4396.0163

(2.728e-06)

**Charge States:** 4

**Start Position**: 58

**End Position**: 82

| Profile Score | Tandem Score | TDA q-value | Volume | Spectrum Matches |
| --- | --- | --- | --- | --- |
| -19.6842 | 47.2154 | 0.0032 | 6.0176e+06 | 1 |

**Adduct Signal Fractions**

- **Unmodified**: 6.0176e+06

### QNQC(C)FYN(N)SSYLNVQR Hex 5 HexNAc 9

**Mass:**  4557.8449

(1.732e-06)

**Charge States:** 3

**Start Position**: 87

**End Position**: 102

| Profile Score | Tandem Score | TDA q-value | Volume | Spectrum Matches |
| --- | --- | --- | --- | --- |
| -19.4233 | 46.4543 | 0.0032 | 5.0344e+06 | 1 |

**Adduct Signal Fractions**

- **Unmodified**: 5.0344e+06

### SVQEIQATFFYFTPN(N)K Fuc 1 Hex 5 HexNAc 4 Neu5Ac 2

**Mass:**  4269.7853

(2.003e-06)

**Charge States:** 3, 4

**Start Position**: 58

**End Position**: 74

| Profile Score | Tandem Score | TDA q-value | Volume | Spectrum Matches |
| --- | --- | --- | --- | --- |
| -20.8885 | 46.0435 | 0.0032 | 3.2616e+06 | 1 |

**Adduct Signal Fractions**

- **Unmodified**: 3.2616e+06

### QC(C)FYN(N)SSYLNVQR Hex 3 HexNAc 6

**Mass:**  3382.3910

(-1.996e-07)

**Charge States:** 3

**Start Position**: 89

**End Position**: 102

| Profile Score | Tandem Score | TDA q-value | Volume | Spectrum Matches |
| --- | --- | --- | --- | --- |
| -19.8006 | 45.2345 | 0.0032 | 1.1698e+07 | 1 |

**Adduct Signal Fractions**

- **Unmodified**: 1.1698e+07

### SVQEIQATFFYFTPN(N)K Fuc 2 Hex 5 HexNAc 7

**Mass:**  4442.8908

(2.003e-06)

**Charge States:** 4

**Start Position**: 58

**End Position**: 74

| Profile Score | Tandem Score | TDA q-value | Volume | Spectrum Matches |
| --- | --- | --- | --- | --- |
| -20.0135 | 44.1798 | 0.0032 | 3.6576e+06 | 1 |

**Adduct Signal Fractions**

- **Unmodified**: 3.6576e+06

### LVPVPITN(N)ATLDR Fuc 2 Hex 5 HexNAc 6

**Mass:**  3730.6765

(5.395e-04)

**Start Position**: 26

**End Position**: 39

| Profile Score | Tandem Score | TDA q-value | Volume | Spectrum Matches |
| --- | --- | --- | --- | --- |
| 0.0000 | 43.5057 | 0.0032 | 0.0000e+00 | 1 |

Adduct:

No Chromatogram Found

### ANLVPVPITN(N)ATLDR Hex 3 HexNAc 5

**Mass:**  3094.4470

(4.978e-07)

**Charge States:** 3

**Start Position**: 24

**End Position**: 39

| Profile Score | Tandem Score | TDA q-value | Volume | Spectrum Matches |
| --- | --- | --- | --- | --- |
| -3.8438 | 42.2569 | 0.0032 | 1.6505e+07 | 2 |

**Adduct Signal Fractions**

- **Unmodified**: 1.6505e+07

### LVPVPITN(N)ATLDR Fuc 1 Hex 4 HexNAc 4

**Mass:**  3014.3992

(1.298e-06)

**Start Position**: 26

**End Position**: 39

| Profile Score | Tandem Score | TDA q-value | Volume | Spectrum Matches |
| --- | --- | --- | --- | --- |
| 0.0000 | 41.5406 | 0.0032 | 0.0000e+00 | 1 |

Adduct:

No Chromatogram Found

### QNQC(C)FYN(N)SSYLNVQR Fuc 2 Hex 4 HexNAc 4 Neu5Ac 1

**Mass:**  3965.5869

(4.996e-04)

**Charge States:** 4

**Start Position**: 87

**End Position**: 102

| Profile Score | Tandem Score | TDA q-value | Volume | Spectrum Matches |
| --- | --- | --- | --- | --- |
| -6.8112 | 41.4217 | 0.0032 | 9.3320e+07 | 1 |

**Adduct Signal Fractions**

- **Unmodified**: 9.3320e+07

### YFTPN(N)KTEDTIFLR Fuc 2 Hex 5 HexNAc 6

**Mass:**  4064.7412

(4.295e-07)

**Charge States:** 3

**Start Position**: 68

**End Position**: 82

| Profile Score | Tandem Score | TDA q-value | Volume | Spectrum Matches |
| --- | --- | --- | --- | --- |
| -20.2507 | 41.0645 | 0.0032 | 8.2796e+06 | 1 |

**Adduct Signal Fractions**

- **Unmodified**: 8.2796e+06

### SVQEIQATFFYFTPN(N)K Hex 5 HexNAc 8

**Mass:**  4353.8493

(-5.984e-07)

**Charge States:** 4

**Start Position**: 58

**End Position**: 74

| Profile Score | Tandem Score | TDA q-value | Volume | Spectrum Matches |
| --- | --- | --- | --- | --- |
| 9.7954 | 40.8973 | 0.0032 | 2.0998e+07 | 1 |

**Adduct Signal Fractions**

- **Unmodified**: 2.0998e+07

### SVQEIQATFFYFTPN(N)KTEDTIFLR Fuc 1 Hex 4 HexNAc 6

**Mass:**  4907.2009

(1.349e-06)

**Charge States:** 4

**Start Position**: 58

**End Position**: 82

| Profile Score | Tandem Score | TDA q-value | Volume | Spectrum Matches |
| --- | --- | --- | --- | --- |
| -19.7147 | 38.8266 | 0.0032 | 2.3349e+06 | 1 |

**Adduct Signal Fractions**

- **Unmodified**: 2.3349e+06

### QNQC(C)FYN(N)SSYLNVQR Hex 5 HexNAc 8

**Mass:**  4354.7634

(1.295e-06)

**Charge States:** 3, 4

**Start Position**: 87

**End Position**: 102

| Profile Score | Tandem Score | TDA q-value | Volume | Spectrum Matches |
| --- | --- | --- | --- | --- |
| -4.9309 | 38.8002 | 0.0032 | 9.1135e+06 | 1 |

**Adduct Signal Fractions**

- **Unmodified**: 9.1135e+06

### ANLVPVPITN(N)ATLDR Fuc 1 Hex 8 HexNAc 5

**Mass:**  4050.7656

(-8.065e-08)

**Charge States:** 3

**Start Position**: 24

**End Position**: 39

| Profile Score | Tandem Score | TDA q-value | Volume | Spectrum Matches |
| --- | --- | --- | --- | --- |
| -5.6063 | 38.3163 | 0.0047 | 3.1038e+07 | 3 |

**Adduct Signal Fractions**

- **Unmodified**: 3.1038e+07

### C(C)ANLVPVPITN(N)ATLDR Hex 3 HexNAc 5 Neu5Ac 1

**Mass:**  3545.5651

(-1.337e-06)

**Charge States:** 3, 4

**Start Position**: 23

**End Position**: 39

| Profile Score | Tandem Score | TDA q-value | Volume | Spectrum Matches |
| --- | --- | --- | --- | --- |
| 16.7583 | 37.6661 | 0.0047 | 2.1565e+08 | 6 |

**Adduct Signal Fractions**

- **Unmodified**: 2.1565e+08

### LVPVPITN(N)ATLDR Hex 5 HexNAc 7

**Mass:**  3639.6630

(6.712e-06)

**Charge States:** 3

**Start Position**: 26

**End Position**: 39

| Profile Score | Tandem Score | TDA q-value | Volume | Spectrum Matches |
| --- | --- | --- | --- | --- |
| -5.9726 | 37.5505 | 0.0047 | 8.8417e+06 | 1 |

**Adduct Signal Fractions**

- **Unmodified**: 8.8417e+06

### QIPLC(C)ANLVPVPITN(N)ATLDR Hex 3 HexNAc 5

**Mass:**  3705.7589

(1.290e-06)

**Charge States:** 3, 4

**Start Position**: 19

**End Position**: 39

| Profile Score | Tandem Score | TDA q-value | Volume | Spectrum Matches |
| --- | --- | --- | --- | --- |
| 13.4702 | 37.5425 | 0.0047 | 4.9059e+08 | 12 |

**Adduct Signal Fractions**

- **Unmodified**: 4.9059e+08

### QNQC(C)FYN(N)SSYLNVQR Fuc 5 Hex 4 HexNAc 8

**Mass:**  4922.9748

(-4.399e-06)

**Charge States:** 4

**Start Position**: 87

**End Position**: 102

| Profile Score | Tandem Score | TDA q-value | Volume | Spectrum Matches |
| --- | --- | --- | --- | --- |
| 14.6698 | 37.0020 | 0.0059 | 2.6705e+07 | 5 |

**Adduct Signal Fractions**

- **Unmodified**: 2.6705e+07

### QN(D)QC(C)FYN(N)SSYLNVQR Hex 3 HexNAc 6

**Mass:**  3625.4826

(2.732e-04)

**Charge States:** 3, 4

**Start Position**: 87

**End Position**: 102

| Profile Score | Tandem Score | TDA q-value | Volume | Spectrum Matches |
| --- | --- | --- | --- | --- |
| -4.5172 | 36.9788 | 0.0059 | 2.8229e+08 | 5 |

**Adduct Signal Fractions**

- **Unmodified**: 2.8229e+08

### ANLVPVPITN(N)ATLDR Fuc 2 Hex 3 HexNAc 3 Neu5Ac 1

**Mass:**  3271.5031

(2.613e-06)

**Charge States:** 3

**Start Position**: 24

**End Position**: 39

| Profile Score | Tandem Score | TDA q-value | Volume | Spectrum Matches |
| --- | --- | --- | --- | --- |
| -4.0720 | 36.6668 | 0.0059 | 6.6098e+07 | 2 |

**Adduct Signal Fractions**

- **Unmodified**: 6.6098e+07

### FYFTPN(N)KTEDTIFLR Fuc 2 Hex 5 HexNAc 6

**Mass:**  4211.8117

(9.324e-07)

**Charge States:** 4

**Start Position**: 67

**End Position**: 82

| Profile Score | Tandem Score | TDA q-value | Volume | Spectrum Matches |
| --- | --- | --- | --- | --- |
| -5.1354 | 35.8627 | 0.0071 | 3.3520e+06 | 1 |

**Adduct Signal Fractions**

- **Unmodified**: 3.3520e+06

### QIPLC(C)ANLVPVPITN(N)ATLDR Fuc 1 Hex 4 HexNAc 5

**Mass:**  4013.8687

(6.220e-07)

**Charge States:** 3, 4

**Start Position**: 19

**End Position**: 39

| Profile Score | Tandem Score | TDA q-value | Volume | Spectrum Matches |
| --- | --- | --- | --- | --- |
| 13.6687 | 35.8510 | 0.0071 | 1.4282e+08 | 8 |

**Adduct Signal Fractions**

- **Unmodified**: 1.4282e+08

### QN(D)QC(C)FYN(N)SSYLNVQR Fuc 1 Hex 4 HexNAc 6

**Mass:**  3933.5923

(2.515e-04)

**Charge States:** 3, 4

**Start Position**: 87

**End Position**: 102

| Profile Score | Tandem Score | TDA q-value | Volume | Spectrum Matches |
| --- | --- | --- | --- | --- |
| -5.8725 | 35.2913 | 0.0071 | 4.4121e+07 | 2 |

**Adduct Signal Fractions**

- **Unmodified**: 4.4121e+07

### ATFFYFTPN(N)K Fuc 1 Hex 3 HexNAc 8 Neu5Ac 1

**Mass:**  3782.5324

(-4.387e-06)

**Charge States:** 4

**Start Position**: 64

**End Position**: 74

| Profile Score | Tandem Score | TDA q-value | Volume | Spectrum Matches |
| --- | --- | --- | --- | --- |
| -20.2911 | 34.7192 | 0.0071 | 9.1957e+06 | 1 |

**Adduct Signal Fractions**

- **Unmodified**: 9.1957e+06

### QNQC(C)FYN(N)SSYLNVQR Fuc 2 Hex 5 HexNAc 6

**Mass:**  4240.7131

(2.320e-06)

**Charge States:** 3, 4

**Start Position**: 87

**End Position**: 102

| Profile Score | Tandem Score | TDA q-value | Volume | Spectrum Matches |
| --- | --- | --- | --- | --- |
| -18.3430 | 34.6452 | 0.0071 | 3.1961e+07 | 1 |

**Adduct Signal Fractions**

- **Unmodified**: 3.1961e+07

### TPN(N)KTEDTIFLR Hex 4 HexNAc 7

**Mass:**  3503.5193

(1.177e-06)

**Charge States:** 3, 4

**Start Position**: 70

**End Position**: 82

| Profile Score | Tandem Score | TDA q-value | Volume | Spectrum Matches |
| --- | --- | --- | --- | --- |
| -5.1810 | 34.0669 | 0.0097 | 4.5981e+06 | 2 |

**Adduct Signal Fractions**

- **Unmodified**: 4.5981e+06

### SVQEIQATFFYFTPN(N)K Fuc 1 Hex 5 HexNAc 8

**Mass:**  4499.9103

(1.525e-06)

**Charge States:** 4

**Start Position**: 58

**End Position**: 74

| Profile Score | Tandem Score | TDA q-value | Volume | Spectrum Matches |
| --- | --- | --- | --- | --- |
| -20.3606 | 33.4300 | 0.0105 | 3.3720e+06 | 1 |

**Adduct Signal Fractions**

- **Unmodified**: 3.3720e+06

### FTPN(N)KTEDTIFLR Hex 3 HexNAc 4

**Mass:**  2879.2976

(5.942e-07)

**Charge States:** 3

**Start Position**: 69

**End Position**: 82

| Profile Score | Tandem Score | TDA q-value | Volume | Spectrum Matches |
| --- | --- | --- | --- | --- |
| -4.9030 | 33.4177 | 0.0105 | 5.4284e+06 | 1 |

**Adduct Signal Fractions**

- **Unmodified**: 5.4284e+06

### QIPLC(C)ANLVPVPITN(N)ATLDR Hex 3 HexNAc 4

**Mass:**  3502.6756

(4.436e-07)

**Charge States:** 3

**Start Position**: 19

**End Position**: 39

| Profile Score | Tandem Score | TDA q-value | Volume | Spectrum Matches |
| --- | --- | --- | --- | --- |
| 13.4754 | 33.3564 | 0.0105 | 3.0953e+07 | 3 |

**Adduct Signal Fractions**

- **Unmodified**: 3.0953e+07

### LVPVPITN(N)ATLDR Hex 6 HexNAc 6

**Mass:**  3600.6403

(5.665e-04)

**Start Position**: 26

**End Position**: 39

| Profile Score | Tandem Score | TDA q-value | Volume | Spectrum Matches |
| --- | --- | --- | --- | --- |
| 0.0000 | 32.8394 | 0.0105 | 0.0000e+00 | 1 |

Adduct:

No Chromatogram Found

### FYN(N)SSYLNVQR Hex 3 HexNAc 5

**Mass:**  2891.2265

(-1.153e-06)

**Charge States:** 3

**Start Position**: 91

**End Position**: 102

| Profile Score | Tandem Score | TDA q-value | Volume | Spectrum Matches |
| --- | --- | --- | --- | --- |
| -4.1745 | 31.9037 | 0.0129 | 2.7028e+06 | 2 |

**Adduct Signal Fractions**

- **Unmodified**: 2.7028e+06

### ANLVPVPITN(N)ATLDR Fuc 2 Hex 7 HexNAc 5

**Mass:**  4034.7883

(4.869e-06)

**Charge States:** 4

**Start Position**: 24

**End Position**: 39

| Profile Score | Tandem Score | TDA q-value | Volume | Spectrum Matches |
| --- | --- | --- | --- | --- |
| -3.9345 | 31.0690 | 0.0166 | 3.3341e+07 | 3 |

**Adduct Signal Fractions**

- **Unmodified**: 3.3341e+07

### QNQC(C)FYN(N)SSYLNVQR Hex 4 HexNAc 8

**Mass:**  4192.7126

(1.904e-06)

**Charge States:** 3, 4

**Start Position**: 87

**End Position**: 102

| Profile Score | Tandem Score | TDA q-value | Volume | Spectrum Matches |
| --- | --- | --- | --- | --- |
| -5.1527 | 30.4281 | 0.0189 | 3.1164e+07 | 2 |

**Adduct Signal Fractions**

- **Unmodified**: 3.1164e+07

### LVPVPITN(N)ATLDR Fuc 1 Hex 5 HexNAc 7

**Mass:**  3785.6951

(2.878e-06)

**Charge States:** 3, 4

**Start Position**: 26

**End Position**: 39

| Profile Score | Tandem Score | TDA q-value | Volume | Spectrum Matches |
| --- | --- | --- | --- | --- |
| 10.5300 | 30.1973 | 0.0201 | 2.0101e+07 | 1 |

**Adduct Signal Fractions**

- **Unmodified**: 2.0101e+07

### LVPVPITN(N)ATLDR Hex 5 HexNAc 4

**Mass:**  3031.3885

(3.294e-04)

**Start Position**: 26

**End Position**: 39

| Profile Score | Tandem Score | TDA q-value | Volume | Spectrum Matches |
| --- | --- | --- | --- | --- |
| 0.0000 | 29.7912 | 0.0208 | 0.0000e+00 | 1 |

Adduct:

No Chromatogram Found

### NLVPVPITN(N)ATLDR Fuc 1 Hex 4 HexNAc 5

**Mass:**  3331.5207

(8.836e-07)

**Charge States:** 3

**Start Position**: 25

**End Position**: 39

| Profile Score | Tandem Score | TDA q-value | Volume | Spectrum Matches |
| --- | --- | --- | --- | --- |
| -20.4711 | 29.7064 | 0.0208 | 7.7505e+06 | 1 |

**Adduct Signal Fractions**

- **Unmodified**: 7.7505e+06

### FTPN(N)KTEDTIFLR Fuc 2 Hex 5 HexNAc 6

**Mass:**  3901.6721

(-1.173e-07)

**Charge States:** 3

**Start Position**: 69

**End Position**: 82

| Profile Score | Tandem Score | TDA q-value | Volume | Spectrum Matches |
| --- | --- | --- | --- | --- |
| -5.4757 | 29.0813 | 0.0231 | 2.4874e+06 | 2 |

**Adduct Signal Fractions**

- **Unmodified**: 2.4874e+06

### FYN(N)SSYLNVQR Hex 3 HexNAc 6

**Mass:**  3094.3057

(1.423e-06)

**Charge States:** 3

**Start Position**: 91

**End Position**: 102

| Profile Score | Tandem Score | TDA q-value | Volume | Spectrum Matches |
| --- | --- | --- | --- | --- |
| -3.3272 | 29.0503 | 0.0241 | 7.1980e+06 | 3 |

**Adduct Signal Fractions**

- **Unmodified**: 7.1980e+06

### YFTPN(N)KTEDTIFLR Hex 4 HexNAc 6

**Mass:**  3610.5756

(1.363e-06)

**Charge States:** 3, 4

**Start Position**: 68

**End Position**: 82

| Profile Score | Tandem Score | TDA q-value | Volume | Spectrum Matches |
| --- | --- | --- | --- | --- |
| -5.8263 | 28.5761 | 0.0287 | 1.5954e+07 | 1 |

**Adduct Signal Fractions**

- **Unmodified**: 1.5954e+07

### FTPN(N)KTEDTIFLR Fuc 1 Hex 7 HexNAc 3

**Mass:**  3471.5249

(2.942e-04)

**Charge States:** 3, 4

**Start Position**: 69

**End Position**: 82

| Profile Score | Tandem Score | TDA q-value | Volume | Spectrum Matches |
| --- | --- | --- | --- | --- |
| -6.3684 | 28.5242 | 0.0321 | 1.8071e+06 | 2 |

**Adduct Signal Fractions**

- **Unmodified**: 1.8071e+06

### SVQEIQATFFYFTPN(N)KTEDTIFLR Fuc 1 Hex 4 HexNAc 5

**Mass:**  4704.1174

(1.392e-06)

**Charge States:** 4

**Start Position**: 58

**End Position**: 82

| Profile Score | Tandem Score | TDA q-value | Volume | Spectrum Matches |
| --- | --- | --- | --- | --- |
| -19.3653 | 28.2192 | 0.0331 | 2.4507e+06 | 1 |

**Adduct Signal Fractions**

- **Unmodified**: 2.4507e+06

### LVPVPITN(N)ATLDR Fuc 1 Hex 4 HexNAc 5

**Mass:**  3219.4738

(6.213e-04)

**Start Position**: 26

**End Position**: 39

| Profile Score | Tandem Score | TDA q-value | Volume | Spectrum Matches |
| --- | --- | --- | --- | --- |
| 0.0000 | 27.8825 | 0.0346 | 0.0000e+00 | 1 |

Adduct:

No Chromatogram Found

### YN(N)SSYLNVQR Hex 3 HexNAc 6

**Mass:**  2947.2379

(1.289e-06)

**Charge States:** 3

**Start Position**: 92

**End Position**: 102

| Profile Score | Tandem Score | TDA q-value | Volume | Spectrum Matches |
| --- | --- | --- | --- | --- |
| 12.0236 | 27.3713 | 0.0353 | 1.4043e+07 | 2 |

**Adduct Signal Fractions**

- **Unmodified**: 1.4043e+07

### QN(D)QC(C)FYN(N)SSYLNVQR Fuc 1 Hex 4 HexNAc 5

**Mass:**  3730.5111

(2.645e-04)

**Charge States:** 3, 4

**Start Position**: 87

**End Position**: 102

| Profile Score | Tandem Score | TDA q-value | Volume | Spectrum Matches |
| --- | --- | --- | --- | --- |
| -6.3477 | 27.2771 | 0.0353 | 1.4559e+07 | 4 |

**Adduct Signal Fractions**

- **Unmodified**: 1.4559e+07

### YN(N)SSYLNVQR Fuc 4 Hex 3 HexNAc 5 Neu5Ac 1

**Mass:**  3619.5060

(6.766e-06)

**Charge States:** 3, 4

**Start Position**: 92

**End Position**: 102

| Profile Score | Tandem Score | TDA q-value | Volume | Spectrum Matches |
| --- | --- | --- | --- | --- |
| -19.2983 | 27.2180 | 0.0353 | 1.9232e+07 | 2 |

**Adduct Signal Fractions**

- **Unmodified**: 1.9232e+07

### QN(D)QC(C)FYN(N)SSYLNVQR Hex 5 HexNAc 5

**Mass:**  3746.5006

(2.619e-04)

**Charge States:** 3

**Start Position**: 87

**End Position**: 102

| Profile Score | Tandem Score | TDA q-value | Volume | Spectrum Matches |
| --- | --- | --- | --- | --- |
| -19.4592 | 26.4343 | 0.0464 | 2.1641e+07 | 1 |

**Adduct Signal Fractions**

- **Unmodified**: 2.1641e+07

### QN(D)QC(C)FYN(N)SSYLNVQR Fuc 4 Hex 3 HexNAc 5

**Mass:**  4006.6459

(2.499e-04)

**Charge States:** 3, 4

**Start Position**: 87

**End Position**: 102

| Profile Score | Tandem Score | TDA q-value | Volume | Spectrum Matches |
| --- | --- | --- | --- | --- |
| 12.8127 | 25.7345 | 0.0464 | 5.9183e+07 | 2 |

**Adduct Signal Fractions**

- **Unmodified**: 5.9183e+07

### ITGKWFYIASAFRNEEYNKSVQEIQATFFYFTPN(N)K Fuc 2 Hex 8 HexNAc 7

**Mass:**  7247.1688

(-1.173e-06)

**Charge States:** 5

**Start Position**: 39

**End Position**: 74

| Profile Score | Tandem Score | TDA q-value | Volume | Spectrum Matches |
| --- | --- | --- | --- | --- |
| -18.9501 | 25.6227 | 0.0464 | 1.3802e+07 | 1 |

**Adduct Signal Fractions**

- **Unmodified**: 1.3802e+07

### YN(N)SSYLNVQR Fuc 1 Hex 4 HexNAc 6

**Mass:**  3255.3407

(-1.268e-06)

**Charge States:** 3

**Start Position**: 92

**End Position**: 102

| Profile Score | Tandem Score | TDA q-value | Volume | Spectrum Matches |
| --- | --- | --- | --- | --- |
| -20.8445 | 25.5058 | 0.0464 | 9.6413e+05 | 1 |

**Adduct Signal Fractions**

- **Unmodified**: 9.6413e+05

### P02763|A1AG1\_HUMAN

**>P02763|A1AG1\_HUMAN**  
 `MALSWVLTVLSLLPLLEAQIPLCANLVPVPITNATLDQITGKWFYIASAFRNEEYNKSVQEIQATFFYFTPNKTEDTIFL  
REYQTRQDQCIYNTTYLNVQRENGTISRYVGGQEHFAHLLILRDTKTYMLAFDVNDEKNWGLSVYADKPETTKEQLGEFY  
EALDCLRIPKSDVVYTDWKKDKCEPLEKQHEKERKQEEGES`

| Site | Type | Count |
| --- | --- | --- |
| 33 | N-linked | 35 |
| 56 | N-linked | 1 |
| 93 | N-linked | 33 |
| 72 | N-linked | 76 |

| Mass | Sequence | MS_2_ Score | `q`-value | MS_1_ Score | Signal | Protein Coordinates | Tandem Scans | Start Time | Apex Time | End Time |
| --- | --- | --- | --- | --- | --- | --- | --- | --- | --- | --- |
| 2269.9383 | YFTPN(N)K Hex 3 HexNAc 5 | 187.370 | 0.0 | 13.524 | 2.080e+08 | 67 - 73 | 8 | 13.98 | 14.28 | 14.79 |
| 2473.0178 | YFTPN(N)K Hex 3 HexNAc 6 | 187.048 | 0.0 | 14.232 | 3.743e+08 | 67 - 73 | 6 | 13.85 | 13.98 | 14.69 |
| 2620.0865 | FYFTPN(N)K Hex 3 HexNAc 6 | 183.031 | 0.0 | 13.497 | 1.039e+08 | 66 - 73 | 11 | 25.20 | 25.55 | 28.15 |
| 2580.0490 | YFTPN(N)K Fuc 1 Hex 4 HexNAc 5 | 182.056 | 0.0 | 0.000 | 0.000e+00 | 67 - 73 | 1 | - | - | - |
| 2093.8166 | NEEYN(N)K Hex 3 HexNAc 4 | 181.316 | 0.0 | 11.811 | 5.132e+07 | 51 - 57 | 6 | 3.86 | 3.86 | 5.03 |
| 3988.7195 | SVQEIQATFFYFTPN(N)K Hex 4 HexNAc 7 | 175.474 | 0.0 | 11.370 | 2.228e+08 | 57 - 73 | 14 | 53.66 | 54.16 | 56.87 |
| 3623.5855 | SVQEIQATFFYFTPN(N)K Hex 3 HexNAc 6 | 174.109 | 0.0 | 14.863 | 1.198e+09 | 57 - 73 | 24 | 50.94 | 54.67 | 58.40 |
| 3931.6975 | SVQEIQATFFYFTPN(N)K Fuc 1 Hex 4 HexNAc 6 | 173.486 | 0.0 | 13.256 | 2.677e+08 | 57 - 73 | 9 | 53.99 | 54.33 | 56.37 |
| 3728.6173 | SVQEIQATFFYFTPN(N)K Fuc 1 Hex 4 HexNAc 5 | 165.419 | 0.0 | 13.586 | 2.024e+08 | 57 - 73 | 7 | 54.16 | 54.50 | 55.68 |
| 3420.5048 | SVQEIQATFFYFTPN(N)K Hex 3 HexNAc 5 | 165.182 | 0.0 | 15.343 | 7.045e+08 | 57 - 73 | 28 | 51.11 | 55.01 | 59.08 |
| 3280.5713 | LVPVPITN(N)ATLDQITGK Hex 3 HexNAc 5 | 164.314 | 0.0 | 14.323 | 1.334e+10 | 25 - 42 | 513 | 42.13 | 48.80 | 113.46 |
| 3475.4241 | ATFFYFTPN(N)K Fuc 2 Hex 7 HexNAc 4 | 158.594 | 0.0 | 0.000 | 0.000e+00 | 63 - 73 | 1 | - | - | - |
| 3138.3888 | TPN(N)KTEDTIFLR Hex 3 HexNAc 6 | 157.147 | 0.0 | 13.262 | 4.373e+07 | 69 - 81 | 5 | 25.84 | 26.01 | 27.22 |
| 4191.8000 | SVQEIQATFFYFTPN(N)K Hex 4 HexNAc 8 | 153.685 | 0.0 | -3.706 | 2.317e+07 | 57 - 73 | 4 | 53.66 | 53.82 | 54.16 |
| 3588.6810 | LVPVPITN(N)ATLDQITGK Fuc 1 Hex 4 HexNAc 5 | 152.113 | 0.0 | 15.711 | 4.060e+09 | 25 - 42 | 68 | 41.79 | 48.50 | 113.14 |
| 4239.8077 | SVQEIQATFFYFTPN(N)K Fuc 2 Hex 5 HexNAc 6 | 149.415 | 0.0 | 13.540 | 4.168e+07 | 57 - 73 | 4 | 53.66 | 53.82 | 54.50 |
| 2736.1608 | ATFFYFTPN(N)K Hex 3 HexNAc 5 | 149.237 | 0.0 | -3.211 | 2.263e+07 | 63 - 73 | 2 | 41.79 | 41.96 | 42.13 |
| 2939.2405 | ATFFYFTPN(N)K Hex 3 HexNAc 6 | 147.421 | 0.0 | 14.396 | 4.502e+07 | 63 - 73 | 2 | 41.45 | 41.62 | 42.13 |
| 3791.7609 | LVPVPITN(N)ATLDQITGK Fuc 1 Hex 4 HexNAc 6 | 147.295 | 0.0 | 13.370 | 1.973e+08 | 25 - 42 | 5 | 41.79 | 48.33 | 48.65 |
| 4296.8316 | SVQEIQATFFYFTPN(N)K Fuc 1 Hex 5 HexNAc 7 | 146.346 | 0.0 | 8.959 | 3.352e+07 | 57 - 73 | 6 | 53.32 | 53.49 | 54.50 |
| 3217.4274 | SVQEIQATFFYFTPN(N)K Hex 3 HexNAc 4 | 145.717 | 0.0 | 12.924 | 7.206e+07 | 57 - 73 | 6 | 55.17 | 55.34 | 57.04 |
| 3077.4945 | LVPVPITN(N)ATLDQITGK Hex 3 HexNAc 4 | 144.556 | 0.0 | 14.704 | 1.113e+09 | 25 - 42 | 28 | 42.30 | 49.11 | 53.15 |
| 3483.6489 | LVPVPITN(N)ATLDQITGK Hex 3 HexNAc 6 | 144.430 | 0.0 | -2.885 | 3.378e+08 | 25 - 42 | 9 | 42.13 | 48.65 | 50.43 |
| 3426.6304 | LVPVPITN(N)ATLDQITGK Fuc 1 Hex 3 HexNAc 5 | 143.650 | 0.0 | -4.799 | 6.883e+07 | 25 - 42 | 3 | 48.96 | 49.11 | 49.27 |
| 3619.5267 | QDQC(C)IYN(N)TTYLNVQR Hex 3 HexNAc 6 | 141.791 | 0.0 | 14.494 | 3.354e+09 | 86 - 101 | 10 | 31.55 | 34.07 | 35.05 |
| 3785.6414 | SVQEIQATFFYFTPN(N)K Hex 4 HexNAc 6 | 141.180 | 0.0 | 10.647 | 3.105e+07 | 57 - 73 | 5 | 53.99 | 55.17 | 55.34 |
| 3442.6246 | LVPVPITN(N)ATLDQITGK Hex 4 HexNAc 5 | 138.761 | 0.0 | -5.264 | 7.023e+07 | 25 - 42 | 3 | 48.16 | 48.65 | 48.65 |
| 3448.5209 | YFTPN(N)KTEDTIFLR Hex 3 HexNAc 6 | 138.139 | 0.0 | 11.339 | 1.568e+09 | 67 - 81 | 13 | 35.73 | 37.67 | 42.13 |
| 4292.7730 | QDQC(C)IYN(N)TTYLNVQR Fuc 1 Hex 5 HexNAc 7 | 136.623 | 0.0 | 12.790 | 1.327e+08 | 86 - 101 | 5 | 32.73 | 33.08 | 33.75 |
| 3984.6636 | QDQC(C)IYN(N)TTYLNVQR Hex 4 HexNAc 7 | 135.518 | 0.0 | 14.091 | 7.113e+08 | 86 - 101 | 6 | 33.25 | 33.41 | 34.88 |
| 3962.6694 | SVQEIQATFFYFTPN(N)K Fuc 2 Hex 4 HexNAc 4 Neu5Ac 1 | 135.371 | 0.0 | -18.803 | 2.712e+06 | 57 - 73 | 1 | 55.17 | 55.17 | 55.17 |
| 3416.4470 | QDQC(C)IYN(N)TTYLNVQR Hex 3 HexNAc 5 | 133.823 | 0.0 | 13.640 | 1.540e+09 | 86 - 101 | 9 | 31.71 | 34.23 | 35.22 |
| 4235.7502 | QDQC(C)IYN(N)TTYLNVQR Fuc 2 Hex 5 HexNAc 6 | 133.661 | 0.0 | 14.483 | 1.369e+08 | 86 - 101 | 3 | 33.08 | 33.41 | 33.91 |
| 3247.3493 | ATFFYFTPN(N)K Fuc 1 Hex 4 HexNAc 6 | 133.482 | 0.0 | 0.000 | 0.000e+00 | 63 - 73 | 1 | - | - | - |
| 3813.6511 | YFTPN(N)KTEDTIFLR Hex 4 HexNAc 7 | 133.432 | 0.0 | 8.580 | 1.386e+08 | 67 - 81 | 5 | 36.74 | 37.05 | 37.83 |
| 3245.4421 | YFTPN(N)KTEDTIFLR Hex 3 HexNAc 5 | 132.939 | 0.0 | 10.945 | 7.353e+08 | 67 - 81 | 6 | 35.90 | 37.83 | 39.14 |
| 3724.5599 | QDQC(C)IYN(N)TTYLNVQR Fuc 1 Hex 4 HexNAc 5 | 130.137 | 0.0 | -1.061 | 6.824e+08 | 86 - 101 | 8 | 33.58 | 33.75 | 40.14 |
| 3756.6305 | YFTPN(N)KTEDTIFLR Fuc 1 Hex 4 HexNAc 6 | 129.745 | 0.0 | -4.729 | 2.476e+08 | 67 - 81 | 8 | 35.39 | 37.21 | 37.83 |
| 3848.7866 | LVPVPITN(N)ATLDQITGK Hex 4 HexNAc 7 | 129.429 | 0.0 | 11.282 | 9.484e+07 | 25 - 42 | 5 | 47.82 | 48.33 | 48.50 |
| 3896.7967 | LVPVPITN(N)ATLDQITGK Fuc 2 Hex 5 HexNAc 5 | 129.313 | 0.0 | -4.151 | 3.297e+07 | 25 - 42 | 2 | 47.82 | 47.99 | 48.33 |
| 3044.2652 | ATFFYFTPN(N)K Fuc 1 Hex 4 HexNAc 5 | 129.281 | 0.0 | -4.112 | 6.116e+06 | 63 - 73 | 1 | 41.28 | 41.45 | 41.62 |
| 3554.5465 | YFTPN(N)KTEDTIFLR Fuc 1 Hex 4 HexNAc 5 | 127.803 | 0.0 | 0.000 | 0.000e+00 | 67 - 81 | 1 | - | - | - |
| 3961.6704 | SVQEIQATFFYFTPN(N)K Hex 4 HexNAc 4 Neu5Ac 2 | 127.405 | 0.0 | 12.747 | 3.631e+07 | 57 - 73 | 4 | 55.17 | 55.51 | 56.37 |
| 4556.9323 | SVQEIQATFFYFTPN(N)K Hex 5 HexNAc 9 | 126.575 | 0.0 | -19.428 | 3.312e+06 | 57 - 73 | 1 | 53.15 | 53.15 | 53.15 |
| 3645.7057 | LVPVPITN(N)ATLDQITGK Hex 4 HexNAc 6 | 125.704 | 0.0 | 12.639 | 7.009e+08 | 25 - 42 | 14 | 42.30 | 49.11 | 50.26 |
| 3553.5521 | YFTPN(N)KTEDTIFLR Fuc 1 Hex 4 HexNAc 5 | 125.602 | 0.0 | -4.600 | 1.685e+08 | 67 - 81 | 3 | 37.36 | 37.36 | 37.83 |
| 3595.5912 | FYFTPN(N)KTEDTIFLR Hex 3 HexNAc 6 | 125.187 | 0.0 | 13.427 | 1.067e+08 | 66 - 81 | 5 | 46.29 | 46.46 | 47.65 |
| 3927.6407 | QDQC(C)IYN(N)TTYLNVQR Fuc 1 Hex 4 HexNAc 6 | 123.767 | 0.0 | 12.911 | 9.763e+08 | 86 - 101 | 5 | 33.41 | 33.75 | 34.72 |
| 3213.3672 | QDQC(C)IYN(N)TTYLNVQR Hex 3 HexNAc 4 | 123.497 | 0.0 | -19.246 | 2.100e+07 | 86 - 101 | 1 | 34.39 | 34.39 | 34.55 |
| 4065.7453 | YFTPN(N)KTEDTIFLR Fuc 2 Hex 5 HexNAc 6 | 122.822 | 0.0 | 0.000 | 0.000e+00 | 67 - 81 | 1 | - | - | - |
| 4134.7769 | SVQEIQATFFYFTPN(N)K Fuc 1 Hex 4 HexNAc 7 | 121.290 | 0.0 | -4.909 | 8.205e+06 | 57 - 73 | 2 | 53.82 | 54.16 | 54.33 |
| 3606.7203 | LVPVPITN(N)ATLDQITGK Hex 5 HexNAc 5 | 117.753 | 0.0 | -22.757 | 8.664e+06 | 25 - 42 | 9 | 50.43 | 50.60 | 71.06 |
| 2935.3081 | TPN(N)KTEDTIFLR Hex 3 HexNAc 5 | 117.605 | 0.0 | 12.345 | 4.101e+07 | 69 - 81 | 6 | 26.01 | 26.32 | 27.38 |
| 3815.6605 | YFTPN(N)KTEDTIFLR Hex 4 HexNAc 7 | 115.664 | 0.0 | 0.000 | 0.000e+00 | 67 - 81 | 2 | - | - | - |
| 3444.6529 | LVPVPITN(N)ATLDQITGK Hex 4 HexNAc 5 | 115.249 | 0.0 | 0.000 | 0.000e+00 | 25 - 42 | 1 | - | - | - |
| 3088.3532 | IYN(N)TTYLNVQR Hex 3 HexNAc 6 | 113.282 | 0.0 | 13.895 | 5.941e+07 | 90 - 101 | 6 | 28.64 | 28.84 | 29.38 |
| 3978.6950 | SVQEIQATFFYFTPN(N)K Fuc 1 Hex 5 HexNAc 4 Neu5Ac 1 | 112.640 | 0.0 | -4.866 | 6.645e+06 | 57 - 73 | 2 | 55.34 | 55.51 | 55.68 |
| 3903.7030 | FYFTPN(N)KTEDTIFLR Fuc 1 Hex 4 HexNAc 6 | 111.819 | 0.0 | 13.833 | 2.760e+07 | 66 - 81 | 4 | 45.78 | 45.95 | 46.63 |
| 3304.3796 | ATFFYFTPN(N)K Hex 4 HexNAc 7 | 111.324 | 0.0 | -6.040 | 3.524e+06 | 63 - 73 | 1 | 40.78 | 40.95 | 41.11 |
| 3957.6125 | QDQC(C)IYN(N)TTYLNVQR Hex 4 HexNAc 4 Neu5Ac 2 | 111.010 | 0.0 | 12.893 | 1.901e+08 | 86 - 101 | 6 | 34.55 | 34.72 | 35.39 |
| 3082.3782 | FTPN(N)KTEDTIFLR Hex 3 HexNAc 5 | 110.755 | 0.0 | 12.913 | 4.227e+07 | 68 - 81 | 3 | 32.39 | 32.56 | 33.08 |
| 3285.4573 | FTPN(N)KTEDTIFLR Hex 3 HexNAc 6 | 110.619 | 0.0 | 13.077 | 8.725e+07 | 68 - 81 | 7 | 30.40 | 32.22 | 32.91 |
| 4349.7959 | QDQC(C)IYN(N)TTYLNVQR Hex 5 HexNAc 8 | 110.339 | 0.0 | 11.902 | 1.054e+08 | 86 - 101 | 3 | 32.73 | 32.91 | 33.58 |
| 3236.3018 | QDQC(C)IYN(N)TTYLN Hex 3 HexNAc 6 | 109.811 | 0.0 | -4.814 | 1.078e+07 | 86 - 98 | 1 | 32.73 | 32.91 | 33.08 |
| 2732.2283 | TPN(N)KTEDTIFLR Hex 3 HexNAc 4 | 109.753 | 0.0 | 10.027 | 7.822e+06 | 69 - 81 | 2 | 26.32 | 26.49 | 27.10 |
| 3440.5381 | PVPITN(N)ATLDQITGK Fuc 2 Hex 6 HexNAc 3 | 109.569 | 0.0 | -20.249 | 3.053e+06 | 27 - 42 | 2 | 52.13 | 52.30 | 52.30 |
| 3042.3627 | YFTPN(N)KTEDTIFLR Hex 3 HexNAc 4 | 109.273 | 0.0 | -1.040 | 7.902e+07 | 67 - 81 | 2 | 36.24 | 38.14 | 38.97 |
| 3392.5112 | FYFTPN(N)KTEDTIFLR Hex 3 HexNAc 5 | 109.203 | 0.0 | 12.062 | 5.054e+07 | 66 - 81 | 5 | 46.46 | 46.63 | 47.65 |
| 3189.4300 | FYFTPN(N)KTEDTIFLR Hex 3 HexNAc 4 | 108.757 | 0.0 | -3.702 | 6.028e+06 | 66 - 81 | 1 | 46.63 | 46.80 | 47.14 |
| 4266.7233 | QDQC(C)IYN(N)TTYLNVQR Fuc 3 Hex 5 HexNAc 4 Neu5Ac 1 | 108.014 | 0.0 | -20.629 | 9.402e+06 | 86 - 101 | 2 | 34.39 | 34.39 | 34.39 |
| 3786.6072 | TPN(N)KTEDTIFLR Hex 7 HexNAc 6 | 107.484 | 0.0 | 13.763 | 1.029e+08 | 69 - 81 | 4 | 38.30 | 38.46 | 38.97 |
| 3845.6789 | PVPITN(N)ATLDQITGK Hex 6 HexNAc 5 Neu5Ac 1 | 107.302 | 0.0 | -18.859 | 5.818e+06 | 27 - 42 | 2 | 52.64 | 52.64 | 52.64 |
| 4187.7403 | QDQC(C)IYN(N)TTYLNVQR Hex 4 HexNAc 8 | 106.778 | 0.0 | 11.992 | 1.335e+08 | 86 - 101 | 3 | 33.08 | 33.25 | 33.91 |
| 3781.5820 | QDQC(C)IYN(N)TTYLNVQR Hex 4 HexNAc 6 | 106.382 | 0.0 | -3.931 | 7.303e+07 | 86 - 101 | 3 | 33.91 | 34.07 | 34.23 |
| 4178.7885 | YFTPN(N)KTEDTIFLR Hex 5 HexNAc 8 | 103.246 | 0.0 | -3.974 | 3.131e+07 | 67 - 81 | 2 | 36.07 | 36.24 | 36.57 |
| 3593.5700 | FTPN(N)KTEDTIFLR Fuc 1 Hex 4 HexNAc 6 | 102.488 | 0.0 | -3.671 | 2.019e+07 | 68 - 81 | 3 | 31.71 | 31.88 | 32.22 |
| 3140.3956 | TPN(N)KTEDTIFLR Hex 3 HexNAc 6 | 101.033 | 0.0 | 0.000 | 0.000e+00 | 69 - 81 | 1 | - | - | - |
| 3700.6215 | FYFTPN(N)KTEDTIFLR Fuc 1 Hex 4 HexNAc 5 | 100.560 | 0.0 | -3.937 | 1.433e+07 | 66 - 81 | 2 | 45.95 | 46.12 | 46.46 |
| 4099.8767 | LVPVPITN(N)ATLDQITGK Fuc 2 Hex 5 HexNAc 6 | 99.863 | 0.0 | 7.026 | 2.320e+07 | 25 - 42 | 3 | 47.48 | 48.16 | 48.33 |
| 2885.2735 | IYN(N)TTYLNVQR Hex 3 HexNAc 5 | 99.660 | 0.0 | 15.787 | 4.986e+07 | 90 - 101 | 4 | 28.79 | 29.01 | 29.47 |
| 4381.8690 | YFTPN(N)KTEDTIFLR Hex 5 HexNAc 9 | 95.437 | 0.0 | -19.892 | 9.003e+06 | 67 - 81 | 1 | 36.07 | 36.07 | 36.24 |
| 3522.5848 | PVPITN(N)ATLDQITGK Fuc 2 Hex 4 HexNAc 5 | 94.866 | 0.0 | -4.597 | 3.975e+06 | 27 - 42 | 3 | 52.30 | 52.47 | 52.64 |
| 4121.7656 | YFTPN(N)KTEDTIFLR Fuc 1 Hex 5 HexNAc 7 | 94.770 | 0.0 | -5.005 | 3.630e+07 | 67 - 81 | 3 | 36.24 | 36.57 | 36.74 |
| 3718.6549 | PVPITN(N)ATLDQITGK Hex 7 HexNAc 5 | 93.546 | 0.0 | 0.000 | 0.000e+00 | 27 - 42 | 1 | - | - | - |
| 3960.7256 | FYFTPN(N)KTEDTIFLR Hex 4 HexNAc 7 | 88.330 | 0.0 | -3.561 | 1.727e+07 | 66 - 81 | 5 | 45.61 | 45.95 | 46.12 |
| 3390.4913 | FTPN(N)KTEDTIFLR Fuc 1 Hex 4 HexNAc 5 | 88.300 | 0.0 | -3.288 | 1.163e+07 | 68 - 81 | 3 | 31.88 | 32.05 | 32.22 |
| 4016.7357 | YFTPN(N)KTEDTIFLR Hex 4 HexNAc 8 | 87.137 | 0.0 | -19.459 | 1.430e+07 | 67 - 81 | 1 | 36.57 | 36.57 | 36.57 |
| 3650.5910 | FTPN(N)KTEDTIFLR Hex 4 HexNAc 7 | 83.719 | 0.0 | -4.761 | 1.085e+07 | 68 - 81 | 4 | 31.55 | 31.88 | 32.05 |
| 3446.4997 | TPN(N)KTEDTIFLR Fuc 1 Hex 4 HexNAc 6 | 82.878 | 0.0 | -4.250 | 8.504e+06 | 69 - 81 | 4 | 25.38 | 25.55 | 25.84 |
| 3243.4239 | TPN(N)KTEDTIFLR Fuc 1 Hex 4 HexNAc 5 | 81.837 | 0.0 | -4.718 | 3.919e+06 | 69 - 81 | 3 | 25.68 | 25.84 | 26.01 |
| 3787.6481 | SVQEIQATFFYFTPN(N)K Hex 4 HexNAc 6 | 73.078 | 0.002 | 0.000 | 0.000e+00 | 57 - 73 | 1 | - | - | - |
| 3582.5617 | SVQEIQATFFYFTPN(N)K Hex 4 HexNAc 5 | 70.064 | 0.002 | -5.860 | 8.368e+06 | 57 - 73 | 1 | 54.50 | 54.67 | 55.01 |
| 4094.7244 | YFTPN(N)KTEDTIFLR Fuc 1 Hex 5 HexNAc 4 Neu5Ac 2 | 67.457 | 0.002 | -20.675 | 9.138e+06 | 67 - 81 | 1 | 37.99 | 37.99 | 38.14 |
| 4004.7431 | SVQEIQATFFYFTPN(N)K Fuc 4 Hex 3 HexNAc 5 | 66.090 | 0.002 | -20.858 | 2.397e+06 | 57 - 73 | 1 | 54.84 | 54.84 | 54.84 |
| 3625.6853 | C(C)ANLVPVPITN(N)ATLDQITGK Hex 3 HexNAc 5 | 64.930 | 0.002 | 14.223 | 9.683e+07 | 22 - 42 | 6 | 52.13 | 52.30 | 52.81 |
| 4599.0866 | SVQEIQATFFYFTPN(N)KTEDTIFLR Hex 3 HexNAc 6 | 63.742 | 0.002 | -4.628 | 2.503e+07 | 57 - 81 | 2 | 62.93 | 63.10 | 63.44 |
| 3953.8183 | LVPVPITN(N)ATLDQITGK Fuc 1 Hex 5 HexNAc 6 | 63.246 | 0.002 | 6.491 | 3.183e+07 | 25 - 42 | 3 | 47.65 | 47.82 | 48.33 |
| 4156.8981 | LVPVPITN(N)ATLDQITGK Fuc 1 Hex 5 HexNAc 7 | 61.730 | 0.002 | 13.808 | 1.519e+07 | 25 - 42 | 2 | 47.31 | 47.99 | 48.16 |
| 4495.8528 | QDQC(C)IYN(N)TTYLNVQR Fuc 1 Hex 5 HexNAc 8 | 60.903 | 0.002 | -4.460 | 3.348e+07 | 86 - 101 | 3 | 32.73 | 32.91 | 33.25 |
| 3635.8038 | LVPVPITN(N)ATLDQITGK Fuc 1 Hex 5 HexNAc 3 Neu5Ac 1 | 56.968 | 0.002 | -3.572 | 2.051e+07 | 25 - 42 | 4 | 49.42 | 49.58 | 66.70 |
| 3933.7968 | C(C)ANLVPVPITN(N)ATLDQITGK Fuc 1 Hex 4 HexNAc 5 | 56.184 | 0.002 | 11.962 | 3.062e+07 | 22 - 42 | 3 | 51.79 | 51.96 | 52.47 |
| 4130.7190 | QDQC(C)IYN(N)TTYLNVQR Fuc 1 Hex 4 HexNAc 7 | 54.336 | 0.002 | 13.802 | 4.824e+07 | 86 - 101 | 3 | 33.25 | 33.58 | 33.91 |
| 3733.7236 | LVPVPITN(N)ATLDQITGK Hex 4 HexNAc 5 Neu5Ac 1 | 53.766 | 0.002 | 13.114 | 1.435e+07 | 25 - 42 | 6 | 50.77 | 51.28 | 51.79 |
| 4552.8730 | QDQC(C)IYN(N)TTYLNVQR Hex 5 HexNAc 9 | 53.676 | 0.002 | -4.960 | 5.944e+07 | 86 - 101 | 1 | 32.73 | 32.73 | 33.25 |
| 4438.8301 | QDQC(C)IYN(N)TTYLNVQR Fuc 2 Hex 5 HexNAc 7 | 53.398 | 0.002 | -5.187 | 2.936e+07 | 86 - 101 | 3 | 32.91 | 33.25 | 33.41 |
| 3765.5857 | QDQC(C)IYN(N)TTYLNVQR Fuc 1 Hex 3 HexNAc 6 | 49.999 | 0.003 | -19.838 | 1.311e+07 | 86 - 101 | 1 | 33.91 | 33.91 | 33.91 |
| 4010.8463 | LVPVPITN(N)ATLDQITGK Hex 5 HexNAc 7 | 48.469 | 0.003 | -4.910 | 7.844e+06 | 25 - 42 | 1 | 47.82 | 47.82 | 48.16 |
| 3750.7426 | LVPVPITN(N)ATLDQITGK Fuc 1 Hex 5 HexNAc 5 | 48.447 | 0.003 | -5.302 | 1.288e+07 | 25 - 42 | 3 | 47.82 | 48.33 | 51.62 |
| 4396.0163 | SVQEIQATFFYFTPN(N)KTEDTIFLR Hex 3 HexNAc 5 | 47.215 | 0.003 | -19.684 | 6.018e+06 | 57 - 81 | 1 | 63.27 | 63.27 | 63.27 |
| 4269.7850 | SVQEIQATFFYFTPN(N)K Fuc 1 Hex 5 HexNAc 4 Neu5Ac 2 | 46.044 | 0.003 | -20.889 | 3.262e+06 | 57 - 73 | 1 | 54.84 | 54.84 | 54.84 |
| 3830.7094 | PVPITN(N)ATLDQITGK Fuc 3 Hex 5 HexNAc 5 | 45.432 | 0.003 | -19.348 | 1.584e+07 | 27 - 42 | 1 | 48.33 | 48.33 | 48.33 |
| 3974.6375 | QDQC(C)IYN(N)TTYLNVQR Fuc 1 Hex 5 HexNAc 4 Neu5Ac 1 | 44.856 | 0.003 | -5.302 | 1.809e+07 | 86 - 101 | 2 | 34.55 | 34.88 | 35.05 |
| 4442.8908 | SVQEIQATFFYFTPN(N)K Fuc 2 Hex 5 HexNAc 7 | 44.180 | 0.003 | -20.013 | 3.658e+06 | 57 - 73 | 1 | 53.66 | 53.66 | 53.82 |
| 4640.0187 | PVPITN(N)ATLDQITGK Fuc 2 Hex 3 HexNAc 7 Neu5Ac 3 | 41.743 | 0.003 | 13.770 | 4.060e+06 | 27 - 42 | 3 | 57.19 | 57.46 | 57.75 |
| 4064.7412 | YFTPN(N)KTEDTIFLR Fuc 2 Hex 5 HexNAc 6 | 41.065 | 0.003 | -20.251 | 8.280e+06 | 67 - 81 | 1 | 36.90 | 36.90 | 36.90 |
| 4353.8481 | SVQEIQATFFYFTPN(N)K Hex 5 HexNAc 8 | 40.897 | 0.003 | 9.795 | 2.100e+07 | 57 - 73 | 1 | 53.15 | 53.32 | 53.99 |
| 4860.9770 | QDQC(C)IYN(N)TTYLNVQR Fuc 1 Hex 6 HexNAc 9 | 39.500 | 0.003 | -19.921 | 4.808e+06 | 86 - 101 | 1 | 32.39 | 32.56 | 32.56 |
| 4907.2009 | SVQEIQATFFYFTPN(N)KTEDTIFLR Fuc 1 Hex 4 HexNAc 6 | 38.827 | 0.003 | -19.715 | 2.335e+06 | 57 - 81 | 1 | 62.76 | 62.76 | 62.76 |
| 4657.9059 | QDQC(C)IYN(N)TTYLNVQR Fuc 1 Hex 6 HexNAc 8 | 37.942 | 0.005 | -4.727 | 9.998e+06 | 86 - 101 | 2 | 32.39 | 32.56 | 32.73 |
| 3422.6076 | C(C)ANLVPVPITN(N)ATLDQITGK Hex 3 HexNAc 4 | 36.648 | 0.006 | -3.700 | 1.088e+07 | 22 - 42 | 3 | 52.30 | 52.47 | 52.64 |
| 4600.8851 | QDQC(C)IYN(N)TTYLNVQR Fuc 2 Hex 6 HexNAc 7 | 36.554 | 0.006 | -5.724 | 1.052e+07 | 86 - 101 | 1 | 32.39 | 32.91 | 32.91 |
| 3417.4368 | QDQC(C)IYN(N)TTYLN(D)VQR Hex 3 HexNAc 5 | 36.222 | 0.006 | -19.765 | 2.723e+07 | 86 - 101 | 2 | 35.39 | 35.39 | 35.56 |
| 3735.7345 | LVPVPITN(N)ATLDQITGK Fuc 2 Hex 4 HexNAc 5 | 36.184 | 0.007 | 0.000 | 0.000e+00 | 25 - 42 | 1 | - | - | - |
| 4211.8110 | FYFTPN(N)KTEDTIFLR Fuc 2 Hex 5 HexNAc 6 | 35.863 | 0.007 | -5.135 | 3.352e+06 | 66 - 81 | 1 | 45.44 | 45.61 | 45.78 |
| 3782.5322 | ATFFYFTPN(N)K Fuc 1 Hex 3 HexNAc 8 Neu5Ac 1 | 34.719 | 0.007 | -20.291 | 9.196e+06 | 63 - 73 | 1 | 54.50 | 54.50 | 54.67 |
| 3503.5218 | TPN(N)KTEDTIFLR Hex 4 HexNAc 7 | 34.067 | 0.010 | -5.181 | 4.598e+06 | 69 - 81 | 2 | 25.38 | 25.55 | 25.84 |
| 3620.5162 | QDQC(C)IYN(N)TTYLN(D)VQR Hex 3 HexNAc 6 | 34.037 | 0.010 | -19.966 | 8.888e+07 | 86 - 101 | 4 | 32.56 | 35.22 | 35.39 |
| 3867.8213 | LVPVPITN(N)ATLDQITGK Fuc 4 Hex 3 HexNAc 5 | 33.653 | 0.011 | 0.000 | 0.000e+00 | 25 - 42 | 1 | - | - | - |
| 4499.9082 | SVQEIQATFFYFTPN(N)K Fuc 1 Hex 5 HexNAc 8 | 33.430 | 0.011 | -20.361 | 3.372e+06 | 57 - 73 | 1 | 53.15 | 53.32 | 53.32 |
| 2879.2976 | FTPN(N)KTEDTIFLR Hex 3 HexNAc 4 | 33.418 | 0.011 | -4.903 | 5.428e+06 | 68 - 81 | 1 | 32.56 | 32.73 | 33.08 |
| 4368.0489 | QIPLC(C)ANLVPVPITN(N)ATLDQITGK Hex 3 HexNAc 5 Neu5Ac 1 | 32.801 | 0.011 | 17.484 | 1.326e+08 | 18 - 42 | 6 | 67.78 | 68.29 | 68.80 |
| 4543.8612 | QDQC(C)IYN(N)TTYLNVQR Fuc 3 Hex 6 HexNAc 6 | 32.192 | 0.012 | -19.853 | 9.364e+06 | 86 - 101 | 1 | 33.08 | 33.08 | 33.25 |
| 3647.7165 | LVPVPITN(N)ATLDQITGK Hex 4 HexNAc 6 | 32.054 | 0.012 | 0.000 | 0.000e+00 | 25 - 42 | 1 | - | - | - |
| 4577.8865 | QDQC(C)IYN(N)TTYLNVQR Fuc 1 Hex 8 HexNAc 6 | 30.013 | 0.021 | 0.000 | 0.000e+00 | 86 - 101 | 1 | - | - | - |
| 4805.9881 | QDQC(C)IYN(N)TTYLNVQR Fuc 2 Hex 6 HexNAc 8 | 29.660 | 0.021 | 0.000 | 0.000e+00 | 86 - 101 | 1 | - | - | - |
| 3901.6733 | FTPN(N)KTEDTIFLR Fuc 2 Hex 5 HexNAc 6 | 29.081 | 0.023 | -5.476 | 2.487e+06 | 68 - 81 | 2 | 31.38 | 31.55 | 31.71 |
| 4213.9214 | LVPVPITN(N)ATLDQITGK Fuc 5 Hex 3 HexNAc 6 | 28.759 | 0.024 | -5.408 | 4.242e+06 | 25 - 42 | 1 | 47.31 | 47.48 | 47.65 |
| 3610.5727 | YFTPN(N)KTEDTIFLR Hex 4 HexNAc 6 | 28.576 | 0.029 | -5.826 | 1.595e+07 | 67 - 81 | 1 | 37.83 | 37.99 | 38.14 |
| 3471.5230 | FTPN(N)KTEDTIFLR Fuc 1 Hex 7 HexNAc 3 | 28.524 | 0.032 | -6.368 | 1.807e+06 | 68 - 81 | 2 | 24.86 | 25.03 | 25.20 |
| 4704.1193 | SVQEIQATFFYFTPN(N)KTEDTIFLR Fuc 1 Hex 4 HexNAc 5 | 28.219 | 0.033 | -19.365 | 2.451e+06 | 57 - 81 | 1 | 62.76 | 62.93 | 62.93 |
| 3194.3752 | IYN(N)TTYLNVQR Fuc 1 Hex 4 HexNAc 5 | 27.852 | 0.035 | 0.000 | 0.000e+00 | 90 - 101 | 1 | - | - | - |
| 3707.5336 | QDQC(C)IYN(N)TTYLNVQR Hex 3 HexNAc 5 Neu5Ac 1 | 27.784 | 0.035 | 14.210 | 8.468e+08 | 86 - 101 | 5 | 39.31 | 39.97 | 40.78 |
| 3396.4617 | IYN(N)TTYLNVQR Fuc 1 Hex 4 HexNAc 6 | 27.593 | 0.035 | 11.063 | 2.808e+07 | 90 - 101 | 2 | 28.33 | 28.40 | 28.64 |
| 3990.8160 | C(C)ANLVPVPITN(N)ATLDQITGK Hex 4 HexNAc 6 | 27.445 | 0.035 | -4.390 | 1.049e+07 | 22 - 42 | 2 | 52.64 | 52.81 | 53.15 |
| 3282.5764 | LVPVPITN(N)ATLDQITGK Hex 3 HexNAc 5 | 25.376 | 0.046 | 0.000 | 0.000e+00 | 25 - 42 | 1 | - | - | - |

### YFTPN(N)K Hex 3 HexNAc 5

**Mass:**  2269.9387

(1.331e-06)

**Charge States:** 2, 3

**Start Position**: 68

**End Position**: 74

| Profile Score | Tandem Score | TDA q-value | Volume | Spectrum Matches |
| --- | --- | --- | --- | --- |
| 13.5240 | 187.3703 | 0.0000 | 2.0802e+08 | 8 |

**Adduct Signal Fractions**

- **Unmodified**: 2.0802e+08

### YFTPN(N)K Hex 3 HexNAc 6

**Mass:**  2473.0177

(1.244e-06)

**Charge States:** 2, 3

**Start Position**: 68

**End Position**: 74

| Profile Score | Tandem Score | TDA q-value | Volume | Spectrum Matches |
| --- | --- | --- | --- | --- |
| 14.2318 | 187.0480 | 0.0000 | 3.7432e+08 | 6 |

**Adduct Signal Fractions**

- **Unmodified**: 3.7432e+08

### FYFTPN(N)K Hex 3 HexNAc 6

**Mass:**  2620.0865

(1.028e-06)

**Charge States:** 2, 3

**Start Position**: 67

**End Position**: 74

| Profile Score | Tandem Score | TDA q-value | Volume | Spectrum Matches |
| --- | --- | --- | --- | --- |
| 13.4967 | 183.0308 | 0.0000 | 1.0392e+08 | 11 |

**Adduct Signal Fractions**

- **Unmodified**: 1.0392e+08

### YFTPN(N)K Fuc 1 Hex 4 HexNAc 5

**Mass:**  2580.0490

(7.767e-04)

**Start Position**: 68

**End Position**: 74

| Profile Score | Tandem Score | TDA q-value | Volume | Spectrum Matches |
| --- | --- | --- | --- | --- |
| 0.0000 | 182.0556 | 0.0000 | 0.0000e+00 | 1 |

Adduct:

No Chromatogram Found

### NEEYN(N)K Hex 3 HexNAc 4

**Mass:**  2093.8160

(2.256e-08)

**Charge States:** 2, 3

**Start Position**: 52

**End Position**: 58

| Profile Score | Tandem Score | TDA q-value | Volume | Spectrum Matches |
| --- | --- | --- | --- | --- |
| 11.8107 | 181.3157 | 0.0000 | 5.1317e+07 | 6 |

**Adduct Signal Fractions**

- **Unmodified**: 5.1317e+07

### SVQEIQATFFYFTPN(N)K Hex 4 HexNAc 7

**Mass:**  3988.7204

(1.216e-06)

**Charge States:** 3, 4

**Start Position**: 58

**End Position**: 74

| Profile Score | Tandem Score | TDA q-value | Volume | Spectrum Matches |
| --- | --- | --- | --- | --- |
| 11.3700 | 175.4739 | 0.0000 | 2.2284e+08 | 14 |

**Adduct Signal Fractions**

- **Unmodified**: 2.2284e+08

### SVQEIQATFFYFTPN(N)K Hex 3 HexNAc 6

**Mass:**  3623.5860

(1.309e-06)

**Charge States:** 3, 4

**Start Position**: 58

**End Position**: 74

| Profile Score | Tandem Score | TDA q-value | Volume | Spectrum Matches |
| --- | --- | --- | --- | --- |
| 14.8625 | 174.1088 | 0.0000 | 1.1977e+09 | 24 |

**Adduct Signal Fractions**

- **Unmodified**: 1.1977e+09

### SVQEIQATFFYFTPN(N)K Fuc 1 Hex 4 HexNAc 6

**Mass:**  3931.6970

(2.111e-06)

**Charge States:** 3, 4

**Start Position**: 58

**End Position**: 74

| Profile Score | Tandem Score | TDA q-value | Volume | Spectrum Matches |
| --- | --- | --- | --- | --- |
| 13.2563 | 173.4863 | 0.0000 | 2.6774e+08 | 9 |

**Adduct Signal Fractions**

- **Unmodified**: 2.6774e+08

### SVQEIQATFFYFTPN(N)K Fuc 1 Hex 4 HexNAc 5

**Mass:**  3728.6175

(9.848e-07)

**Charge States:** 3, 4

**Start Position**: 58

**End Position**: 74

| Profile Score | Tandem Score | TDA q-value | Volume | Spectrum Matches |
| --- | --- | --- | --- | --- |
| 13.5860 | 165.4185 | 0.0000 | 2.0244e+08 | 7 |

**Adduct Signal Fractions**

- **Unmodified**: 2.0244e+08

### SVQEIQATFFYFTPN(N)K Hex 3 HexNAc 5

**Mass:**  3420.5037

(1.062e-06)

**Charge States:** 2, 3, 4

**Start Position**: 58

**End Position**: 74

| Profile Score | Tandem Score | TDA q-value | Volume | Spectrum Matches |
| --- | --- | --- | --- | --- |
| 15.3434 | 165.1817 | 0.0000 | 7.0452e+08 | 28 |

**Adduct Signal Fractions**

- **Unmodified**: 7.0452e+08

### LVPVPITN(N)ATLDQITGK Hex 3 HexNAc 5

**Mass:**  3280.5719

(2.360e-07)

**Charge States:** 2, 3, 4

**Start Position**: 26

**End Position**: 43

| Profile Score | Tandem Score | TDA q-value | Volume | Spectrum Matches |
| --- | --- | --- | --- | --- |
| 14.3227 | 164.3136 | 0.0000 | 1.3343e+10 | 513 |

**Adduct Signal Fractions**

- **Unmodified**: 1.3343e+10

### ATFFYFTPN(N)K Fuc 2 Hex 7 HexNAc 4

**Mass:**  3475.4241

(5.812e-04)

**Start Position**: 64

**End Position**: 74

| Profile Score | Tandem Score | TDA q-value | Volume | Spectrum Matches |
| --- | --- | --- | --- | --- |
| 0.0000 | 158.5941 | 0.0000 | 0.0000e+00 | 1 |

Adduct:

No Chromatogram Found

### TPN(N)KTEDTIFLR Hex 3 HexNAc 6

**Mass:**  3138.3894

(9.927e-07)

**Charge States:** 3, 4

**Start Position**: 70

**End Position**: 82

| Profile Score | Tandem Score | TDA q-value | Volume | Spectrum Matches |
| --- | --- | --- | --- | --- |
| 13.2619 | 157.1468 | 0.0000 | 4.3731e+07 | 5 |

**Adduct Signal Fractions**

- **Unmodified**: 4.3731e+07

### SVQEIQATFFYFTPN(N)K Hex 4 HexNAc 8

**Mass:**  4191.7985

(8.251e-07)

**Charge States:** 3, 4

**Start Position**: 58

**End Position**: 74

| Profile Score | Tandem Score | TDA q-value | Volume | Spectrum Matches |
| --- | --- | --- | --- | --- |
| -3.7057 | 153.6847 | 0.0000 | 2.3167e+07 | 4 |

**Adduct Signal Fractions**

- **Unmodified**: 2.3167e+07

### LVPVPITN(N)ATLDQITGK Fuc 1 Hex 4 HexNAc 5

**Mass:**  3588.6799

(2.105e-07)

**Charge States:** 3, 4

**Start Position**: 26

**End Position**: 43

| Profile Score | Tandem Score | TDA q-value | Volume | Spectrum Matches |
| --- | --- | --- | --- | --- |
| 15.7107 | 152.1133 | 0.0000 | 4.0600e+09 | 68 |

**Adduct Signal Fractions**

- **Unmodified**: 4.0600e+09

### SVQEIQATFFYFTPN(N)K Fuc 2 Hex 5 HexNAc 6

**Mass:**  4239.8088

(1.121e-06)

**Charge States:** 3, 4

**Start Position**: 58

**End Position**: 74

| Profile Score | Tandem Score | TDA q-value | Volume | Spectrum Matches |
| --- | --- | --- | --- | --- |
| 13.5404 | 149.4146 | 0.0000 | 4.1684e+07 | 4 |

**Adduct Signal Fractions**

- **Unmodified**: 4.1684e+07

### ATFFYFTPN(N)K Hex 3 HexNAc 5

**Mass:**  2736.1604

(1.017e-06)

**Charge States:** 3

**Start Position**: 64

**End Position**: 74

| Profile Score | Tandem Score | TDA q-value | Volume | Spectrum Matches |
| --- | --- | --- | --- | --- |
| -3.2105 | 149.2368 | 0.0000 | 2.2634e+07 | 2 |

**Adduct Signal Fractions**

- **Unmodified**: 2.2634e+07

### ATFFYFTPN(N)K Hex 3 HexNAc 6

**Mass:**  2939.2410

(1.117e-06)

**Charge States:** 3

**Start Position**: 64

**End Position**: 74

| Profile Score | Tandem Score | TDA q-value | Volume | Spectrum Matches |
| --- | --- | --- | --- | --- |
| 14.3965 | 147.4212 | 0.0000 | 4.5021e+07 | 2 |

**Adduct Signal Fractions**

- **Unmodified**: 4.5021e+07

### LVPVPITN(N)ATLDQITGK Fuc 1 Hex 4 HexNAc 6

**Mass:**  3791.7609

(1.075e-06)

**Charge States:** 3, 4

**Start Position**: 26

**End Position**: 43

| Profile Score | Tandem Score | TDA q-value | Volume | Spectrum Matches |
| --- | --- | --- | --- | --- |
| 13.3699 | 147.2950 | 0.0000 | 1.9733e+08 | 5 |

**Adduct Signal Fractions**

- **Unmodified**: 1.9733e+08

### SVQEIQATFFYFTPN(N)K Fuc 1 Hex 5 HexNAc 7

**Mass:**  4296.8376

(2.407e-06)

**Charge States:** 3, 4

**Start Position**: 58

**End Position**: 74

| Profile Score | Tandem Score | TDA q-value | Volume | Spectrum Matches |
| --- | --- | --- | --- | --- |
| 8.9586 | 146.3463 | 0.0000 | 3.3518e+07 | 6 |

**Adduct Signal Fractions**

- **Unmodified**: 3.3518e+07

### SVQEIQATFFYFTPN(N)K Hex 3 HexNAc 4

**Mass:**  3217.4287

(7.251e-07)

**Charge States:** 3, 4

**Start Position**: 58

**End Position**: 74

| Profile Score | Tandem Score | TDA q-value | Volume | Spectrum Matches |
| --- | --- | --- | --- | --- |
| 12.9241 | 145.7175 | 0.0000 | 7.2059e+07 | 6 |

**Adduct Signal Fractions**

- **Unmodified**: 7.2059e+07

### LVPVPITN(N)ATLDQITGK Hex 3 HexNAc 4

**Mass:**  3077.4907

(1.594e-07)

**Charge States:** 2, 3, 4

**Start Position**: 26

**End Position**: 43

| Profile Score | Tandem Score | TDA q-value | Volume | Spectrum Matches |
| --- | --- | --- | --- | --- |
| 14.7041 | 144.5559 | 0.0000 | 1.1131e+09 | 28 |

**Adduct Signal Fractions**

- **Unmodified**: 1.1131e+09

### LVPVPITN(N)ATLDQITGK Hex 3 HexNAc 6

**Mass:**  3483.6483

(1.660e-07)

**Charge States:** 3, 4

**Start Position**: 26

**End Position**: 43

| Profile Score | Tandem Score | TDA q-value | Volume | Spectrum Matches |
| --- | --- | --- | --- | --- |
| -2.8853 | 144.4304 | 0.0000 | 3.3782e+08 | 9 |

**Adduct Signal Fractions**

- **Unmodified**: 3.3782e+08

### LVPVPITN(N)ATLDQITGK Fuc 1 Hex 3 HexNAc 5

**Mass:**  3426.6297

(9.854e-07)

**Charge States:** 3, 4

**Start Position**: 26

**End Position**: 43

| Profile Score | Tandem Score | TDA q-value | Volume | Spectrum Matches |
| --- | --- | --- | --- | --- |
| -4.7989 | 143.6498 | 0.0000 | 6.8827e+07 | 3 |

**Adduct Signal Fractions**

- **Unmodified**: 6.8827e+07

### QDQC(C)IYN(N)TTYLNVQR Hex 3 HexNAc 6

**Mass:**  3619.5250

(7.395e-07)

**Charge States:** 3, 4

**Start Position**: 87

**End Position**: 102

| Profile Score | Tandem Score | TDA q-value | Volume | Spectrum Matches |
| --- | --- | --- | --- | --- |
| 14.4941 | 141.7909 | 0.0000 | 3.3542e+09 | 10 |

**Adduct Signal Fractions**

- **Unmodified**: 3.3542e+09

### SVQEIQATFFYFTPN(N)K Hex 4 HexNAc 6

**Mass:**  3785.6380

(8.799e-07)

**Charge States:** 3, 4

**Start Position**: 58

**End Position**: 74

| Profile Score | Tandem Score | TDA q-value | Volume | Spectrum Matches |
| --- | --- | --- | --- | --- |
| 10.6471 | 141.1802 | 0.0000 | 3.1053e+07 | 5 |

**Adduct Signal Fractions**

- **Unmodified**: 3.1053e+07

### LVPVPITN(N)ATLDQITGK Hex 4 HexNAc 5

**Mass:**  3442.6250

(2.181e-06)

**Charge States:** 3

**Start Position**: 26

**End Position**: 43

| Profile Score | Tandem Score | TDA q-value | Volume | Spectrum Matches |
| --- | --- | --- | --- | --- |
| -5.2636 | 138.7609 | 0.0000 | 7.0233e+07 | 3 |

**Adduct Signal Fractions**

- **Unmodified**: 7.0233e+07

### YFTPN(N)KTEDTIFLR Hex 3 HexNAc 6

**Mass:**  3448.5207

(1.053e-06)

**Charge States:** 3, 4

**Start Position**: 68

**End Position**: 82

| Profile Score | Tandem Score | TDA q-value | Volume | Spectrum Matches |
| --- | --- | --- | --- | --- |
| 11.3390 | 138.1390 | 0.0000 | 1.5676e+09 | 13 |

**Adduct Signal Fractions**

- **Unmodified**: 1.5676e+09

### QDQC(C)IYN(N)TTYLNVQR Fuc 1 Hex 5 HexNAc 7

**Mass:**  4292.7751

(1.855e-06)

**Charge States:** 3, 4

**Start Position**: 87

**End Position**: 102

| Profile Score | Tandem Score | TDA q-value | Volume | Spectrum Matches |
| --- | --- | --- | --- | --- |
| 12.7897 | 136.6233 | 0.0000 | 1.3268e+08 | 5 |

**Adduct Signal Fractions**

- **Unmodified**: 1.3268e+08

### QDQC(C)IYN(N)TTYLNVQR Hex 4 HexNAc 7

**Mass:**  3984.6643

(3.352e-06)

**Charge States:** 3, 4

**Start Position**: 87

**End Position**: 102

| Profile Score | Tandem Score | TDA q-value | Volume | Spectrum Matches |
| --- | --- | --- | --- | --- |
| 14.0908 | 135.5180 | 0.0000 | 7.1133e+08 | 6 |

**Adduct Signal Fractions**

- **Unmodified**: 7.1133e+08

### SVQEIQATFFYFTPN(N)K Fuc 2 Hex 4 HexNAc 4 Neu5Ac 1

**Mass:**  3962.6694

(-4.327e-06)

**Charge States:** 4

**Start Position**: 58

**End Position**: 74

| Profile Score | Tandem Score | TDA q-value | Volume | Spectrum Matches |
| --- | --- | --- | --- | --- |
| -18.8027 | 135.3714 | 0.0000 | 2.7120e+06 | 1 |

**Adduct Signal Fractions**

- **Unmodified**: 2.7120e+06

### QDQC(C)IYN(N)TTYLNVQR Hex 3 HexNAc 5

**Mass:**  3416.4479

(8.185e-07)

**Charge States:** 3, 4

**Start Position**: 87

**End Position**: 102

| Profile Score | Tandem Score | TDA q-value | Volume | Spectrum Matches |
| --- | --- | --- | --- | --- |
| 13.6397 | 133.8230 | 0.0000 | 1.5398e+09 | 9 |

**Adduct Signal Fractions**

- **Unmodified**: 1.5398e+09

### QDQC(C)IYN(N)TTYLNVQR Fuc 2 Hex 5 HexNAc 6

**Mass:**  4235.7512

(1.045e-06)

**Charge States:** 3, 4

**Start Position**: 87

**End Position**: 102

| Profile Score | Tandem Score | TDA q-value | Volume | Spectrum Matches |
| --- | --- | --- | --- | --- |
| 14.4833 | 133.6614 | 0.0000 | 1.3692e+08 | 3 |

**Adduct Signal Fractions**

- **Unmodified**: 1.3692e+08

### ATFFYFTPN(N)K Fuc 1 Hex 4 HexNAc 6

**Mass:**  3247.3493

(5.084e-07)

**Start Position**: 64

**End Position**: 74

| Profile Score | Tandem Score | TDA q-value | Volume | Spectrum Matches |
| --- | --- | --- | --- | --- |
| 0.0000 | 133.4824 | 0.0000 | 0.0000e+00 | 1 |

Adduct:

No Chromatogram Found

### YFTPN(N)KTEDTIFLR Hex 4 HexNAc 7

**Mass:**  3813.6535

(9.061e-07)

**Charge States:** 4

**Start Position**: 68

**End Position**: 82

| Profile Score | Tandem Score | TDA q-value | Volume | Spectrum Matches |
| --- | --- | --- | --- | --- |
| 8.5799 | 133.4317 | 0.0000 | 1.3858e+08 | 5 |

**Adduct Signal Fractions**

- **Unmodified**: 1.3858e+08

### YFTPN(N)KTEDTIFLR Hex 3 HexNAc 5

**Mass:**  3245.4417

(1.687e-06)

**Charge States:** 3, 4

**Start Position**: 68

**End Position**: 82

| Profile Score | Tandem Score | TDA q-value | Volume | Spectrum Matches |
| --- | --- | --- | --- | --- |
| 10.9449 | 132.9386 | 0.0000 | 7.3529e+08 | 6 |

**Adduct Signal Fractions**

- **Unmodified**: 7.3529e+08

### QDQC(C)IYN(N)TTYLNVQR Fuc 1 Hex 4 HexNAc 5

**Mass:**  3724.5617

(1.423e-06)

**Charge States:** 3, 4

**Start Position**: 87

**End Position**: 102

| Profile Score | Tandem Score | TDA q-value | Volume | Spectrum Matches |
| --- | --- | --- | --- | --- |
| -1.0614 | 130.1373 | 0.0000 | 6.8238e+08 | 8 |

**Adduct Signal Fractions**

- **Unmodified**: 6.8238e+08

### YFTPN(N)KTEDTIFLR Fuc 1 Hex 4 HexNAc 6

**Mass:**  3756.6330

(5.121e-07)

**Charge States:** 3, 4

**Start Position**: 68

**End Position**: 82

| Profile Score | Tandem Score | TDA q-value | Volume | Spectrum Matches |
| --- | --- | --- | --- | --- |
| -4.7286 | 129.7446 | 0.0000 | 2.4765e+08 | 8 |

**Adduct Signal Fractions**

- **Unmodified**: 2.4765e+08

### LVPVPITN(N)ATLDQITGK Hex 4 HexNAc 7

**Mass:**  3848.7875

(1.689e-06)

**Charge States:** 3, 4

**Start Position**: 26

**End Position**: 43

| Profile Score | Tandem Score | TDA q-value | Volume | Spectrum Matches |
| --- | --- | --- | --- | --- |
| 11.2822 | 129.4288 | 0.0000 | 9.4841e+07 | 5 |

**Adduct Signal Fractions**

- **Unmodified**: 9.4841e+07

### LVPVPITN(N)ATLDQITGK Fuc 2 Hex 5 HexNAc 5

**Mass:**  3896.7961

(2.065e-06)

**Charge States:** 3, 4

**Start Position**: 26

**End Position**: 43

| Profile Score | Tandem Score | TDA q-value | Volume | Spectrum Matches |
| --- | --- | --- | --- | --- |
| -4.1510 | 129.3131 | 0.0000 | 3.2966e+07 | 2 |

**Adduct Signal Fractions**

- **Unmodified**: 3.2966e+07

### ATFFYFTPN(N)K Fuc 1 Hex 4 HexNAc 5

**Mass:**  3044.2624

(-1.118e-07)

**Charge States:** 3

**Start Position**: 64

**End Position**: 74

| Profile Score | Tandem Score | TDA q-value | Volume | Spectrum Matches |
| --- | --- | --- | --- | --- |
| -4.1116 | 129.2809 | 0.0000 | 6.1161e+06 | 1 |

**Adduct Signal Fractions**

- **Unmodified**: 6.1161e+06

### YFTPN(N)KTEDTIFLR Fuc 1 Hex 4 HexNAc 5

**Mass:**  3554.5465

(2.806e-04)

**Start Position**: 68

**End Position**: 82

| Profile Score | Tandem Score | TDA q-value | Volume | Spectrum Matches |
| --- | --- | --- | --- | --- |
| 0.0000 | 127.8034 | 0.0000 | 0.0000e+00 | 1 |

Adduct:

No Chromatogram Found

### SVQEIQATFFYFTPN(N)K Hex 4 HexNAc 4 Neu5Ac 2

**Mass:**  3961.6728

(2.006e-06)

**Charge States:** 3, 4

**Start Position**: 58

**End Position**: 74

| Profile Score | Tandem Score | TDA q-value | Volume | Spectrum Matches |
| --- | --- | --- | --- | --- |
| 12.7470 | 127.4052 | 0.0000 | 3.6313e+07 | 4 |

**Adduct Signal Fractions**

- **Unmodified**: 3.6313e+07

### SVQEIQATFFYFTPN(N)K Hex 5 HexNAc 9

**Mass:**  4556.9323

(1.649e-06)

**Charge States:** 4

**Start Position**: 58

**End Position**: 74

| Profile Score | Tandem Score | TDA q-value | Volume | Spectrum Matches |
| --- | --- | --- | --- | --- |
| -19.4276 | 126.5748 | 0.0000 | 3.3121e+06 | 1 |

**Adduct Signal Fractions**

- **Unmodified**: 3.3121e+06

### LVPVPITN(N)ATLDQITGK Hex 4 HexNAc 6

**Mass:**  3645.7033

(1.444e-06)

**Charge States:** 3, 4

**Start Position**: 26

**End Position**: 43

| Profile Score | Tandem Score | TDA q-value | Volume | Spectrum Matches |
| --- | --- | --- | --- | --- |
| 12.6385 | 125.7044 | 0.0000 | 7.0089e+08 | 14 |

**Adduct Signal Fractions**

- **Unmodified**: 7.0089e+08

### YFTPN(N)KTEDTIFLR Fuc 1 Hex 4 HexNAc 5

**Mass:**  3553.5510

(1.258e-06)

**Charge States:** 3, 4

**Start Position**: 68

**End Position**: 82

| Profile Score | Tandem Score | TDA q-value | Volume | Spectrum Matches |
| --- | --- | --- | --- | --- |
| -4.6002 | 125.6023 | 0.0000 | 1.6850e+08 | 3 |

**Adduct Signal Fractions**

- **Unmodified**: 1.6850e+08

### FYFTPN(N)KTEDTIFLR Hex 3 HexNAc 6

**Mass:**  3595.5917

(1.614e-06)

**Charge States:** 3, 4

**Start Position**: 67

**End Position**: 82

| Profile Score | Tandem Score | TDA q-value | Volume | Spectrum Matches |
| --- | --- | --- | --- | --- |
| 13.4275 | 125.1874 | 0.0000 | 1.0669e+08 | 5 |

**Adduct Signal Fractions**

- **Unmodified**: 1.0669e+08

### QDQC(C)IYN(N)TTYLNVQR Fuc 1 Hex 4 HexNAc 6

**Mass:**  3927.6415

(1.208e-06)

**Charge States:** 3, 4

**Start Position**: 87

**End Position**: 102

| Profile Score | Tandem Score | TDA q-value | Volume | Spectrum Matches |
| --- | --- | --- | --- | --- |
| 12.9112 | 123.7672 | 0.0000 | 9.7630e+08 | 5 |

**Adduct Signal Fractions**

- **Unmodified**: 9.7630e+08

### QDQC(C)IYN(N)TTYLNVQR Hex 3 HexNAc 4

**Mass:**  3213.3678

(7.576e-07)

**Charge States:** 3

**Start Position**: 87

**End Position**: 102

| Profile Score | Tandem Score | TDA q-value | Volume | Spectrum Matches |
| --- | --- | --- | --- | --- |
| -19.2459 | 123.4965 | 0.0000 | 2.0995e+07 | 1 |

**Adduct Signal Fractions**

- **Unmodified**: 2.0995e+07

### YFTPN(N)KTEDTIFLR Fuc 2 Hex 5 HexNAc 6

**Mass:**  4065.7453

(2.475e-04)

**Start Position**: 68

**End Position**: 82

| Profile Score | Tandem Score | TDA q-value | Volume | Spectrum Matches |
| --- | --- | --- | --- | --- |
| 0.0000 | 122.8224 | 0.0000 | 0.0000e+00 | 1 |

Adduct:

No Chromatogram Found

### SVQEIQATFFYFTPN(N)K Fuc 1 Hex 4 HexNAc 7

**Mass:**  4134.7795

(1.044e-06)

**Charge States:** 3, 4

**Start Position**: 58

**End Position**: 74

| Profile Score | Tandem Score | TDA q-value | Volume | Spectrum Matches |
| --- | --- | --- | --- | --- |
| -4.9093 | 121.2899 | 0.0000 | 8.2048e+06 | 2 |

**Adduct Signal Fractions**

- **Unmodified**: 8.2048e+06

### LVPVPITN(N)ATLDQITGK Hex 5 HexNAc 5

**Mass:**  3606.7007

(5.652e-04)

**Charge States:** 3, 4

**Start Position**: 26

**End Position**: 43

| Profile Score | Tandem Score | TDA q-value | Volume | Spectrum Matches |
| --- | --- | --- | --- | --- |
| -22.7567 | 117.7530 | 0.0000 | 8.6644e+06 | 9 |

**Adduct Signal Fractions**

- **Unmodified**: 8.6644e+06

### TPN(N)KTEDTIFLR Hex 3 HexNAc 5

**Mass:**  2935.3077

(2.897e-07)

**Charge States:** 3

**Start Position**: 70

**End Position**: 82

| Profile Score | Tandem Score | TDA q-value | Volume | Spectrum Matches |
| --- | --- | --- | --- | --- |
| 12.3453 | 117.6051 | 0.0000 | 4.1006e+07 | 6 |

**Adduct Signal Fractions**

- **Unmodified**: 4.1006e+07

### YFTPN(N)KTEDTIFLR Hex 4 HexNAc 7

**Mass:**  3815.6605

(5.272e-04)

**Start Position**: 68

**End Position**: 82

| Profile Score | Tandem Score | TDA q-value | Volume | Spectrum Matches |
| --- | --- | --- | --- | --- |
| 0.0000 | 115.6638 | 0.0000 | 0.0000e+00 | 2 |

Adduct:

No Chromatogram Found

### LVPVPITN(N)ATLDQITGK Hex 4 HexNAc 5

**Mass:**  3444.6529

(5.898e-04)

**Start Position**: 26

**End Position**: 43

| Profile Score | Tandem Score | TDA q-value | Volume | Spectrum Matches |
| --- | --- | --- | --- | --- |
| 0.0000 | 115.2495 | 0.0000 | 0.0000e+00 | 1 |

Adduct:

No Chromatogram Found

### IYN(N)TTYLNVQR Hex 3 HexNAc 6

**Mass:**  3088.3529

(1.470e-06)

**Charge States:** 3, 4

**Start Position**: 91

**End Position**: 102

| Profile Score | Tandem Score | TDA q-value | Volume | Spectrum Matches |
| --- | --- | --- | --- | --- |
| 13.8953 | 113.2823 | 0.0000 | 5.9406e+07 | 6 |

**Adduct Signal Fractions**

- **Unmodified**: 5.9406e+07

### SVQEIQATFFYFTPN(N)K Fuc 1 Hex 5 HexNAc 4 Neu5Ac 1

**Mass:**  3978.6897

(4.686e-06)

**Charge States:** 3

**Start Position**: 58

**End Position**: 74

| Profile Score | Tandem Score | TDA q-value | Volume | Spectrum Matches |
| --- | --- | --- | --- | --- |
| -4.8660 | 112.6396 | 0.0000 | 6.6452e+06 | 2 |

**Adduct Signal Fractions**

- **Unmodified**: 6.6452e+06

### FYFTPN(N)KTEDTIFLR Fuc 1 Hex 4 HexNAc 6

**Mass:**  3903.7036

(1.658e-06)

**Charge States:** 3, 4

**Start Position**: 67

**End Position**: 82

| Profile Score | Tandem Score | TDA q-value | Volume | Spectrum Matches |
| --- | --- | --- | --- | --- |
| 13.8328 | 111.8188 | 0.0000 | 2.7600e+07 | 4 |

**Adduct Signal Fractions**

- **Unmodified**: 2.7600e+07

### ATFFYFTPN(N)K Hex 4 HexNAc 7

**Mass:**  3304.3833

(-5.787e-07)

**Charge States:** 3

**Start Position**: 64

**End Position**: 74

| Profile Score | Tandem Score | TDA q-value | Volume | Spectrum Matches |
| --- | --- | --- | --- | --- |
| -6.0399 | 111.3241 | 0.0000 | 3.5241e+06 | 1 |

**Adduct Signal Fractions**

- **Unmodified**: 3.5241e+06

### QDQC(C)IYN(N)TTYLNVQR Hex 4 HexNAc 4 Neu5Ac 2

**Mass:**  3957.6137

(5.539e-07)

**Charge States:** 3, 4

**Start Position**: 87

**End Position**: 102

| Profile Score | Tandem Score | TDA q-value | Volume | Spectrum Matches |
| --- | --- | --- | --- | --- |
| 12.8927 | 111.0105 | 0.0000 | 1.9005e+08 | 6 |

**Adduct Signal Fractions**

- **Unmodified**: 1.9005e+08

### FTPN(N)KTEDTIFLR Hex 3 HexNAc 5

**Mass:**  3082.3793

(3.931e-07)

**Charge States:** 3, 4

**Start Position**: 69

**End Position**: 82

| Profile Score | Tandem Score | TDA q-value | Volume | Spectrum Matches |
| --- | --- | --- | --- | --- |
| 12.9133 | 110.7548 | 0.0000 | 4.2271e+07 | 3 |

**Adduct Signal Fractions**

- **Unmodified**: 4.2271e+07

### FTPN(N)KTEDTIFLR Hex 3 HexNAc 6

**Mass:**  3285.4575

(8.236e-07)

**Charge States:** 3, 4

**Start Position**: 69

**End Position**: 82

| Profile Score | Tandem Score | TDA q-value | Volume | Spectrum Matches |
| --- | --- | --- | --- | --- |
| 13.0770 | 110.6195 | 0.0000 | 8.7251e+07 | 7 |

**Adduct Signal Fractions**

- **Unmodified**: 8.7251e+07

### QDQC(C)IYN(N)TTYLNVQR Hex 5 HexNAc 8

**Mass:**  4349.7966

(4.784e-07)

**Charge States:** 3, 4

**Start Position**: 87

**End Position**: 102

| Profile Score | Tandem Score | TDA q-value | Volume | Spectrum Matches |
| --- | --- | --- | --- | --- |
| 11.9024 | 110.3387 | 0.0000 | 1.0541e+08 | 3 |

**Adduct Signal Fractions**

- **Unmodified**: 1.0541e+08

### QDQC(C)IYN(N)TTYLN Hex 3 HexNAc 6

**Mass:**  3236.3018

(1.818e-06)

**Charge States:** 3

**Start Position**: 87

**End Position**: 99

| Profile Score | Tandem Score | TDA q-value | Volume | Spectrum Matches |
| --- | --- | --- | --- | --- |
| -4.8141 | 109.8109 | 0.0000 | 1.0778e+07 | 1 |

**Adduct Signal Fractions**

- **Unmodified**: 1.0778e+07

### TPN(N)KTEDTIFLR Hex 3 HexNAc 4

**Mass:**  2732.2252

(1.118e-06)

**Charge States:** 3

**Start Position**: 70

**End Position**: 82

| Profile Score | Tandem Score | TDA q-value | Volume | Spectrum Matches |
| --- | --- | --- | --- | --- |
| 10.0274 | 109.7526 | 0.0000 | 7.8218e+06 | 2 |

**Adduct Signal Fractions**

- **Unmodified**: 7.8218e+06

### PVPITN(N)ATLDQITGK Fuc 2 Hex 6 HexNAc 3

**Mass:**  3440.5354

(2.828e-06)

**Charge States:** 3, 4

**Start Position**: 28

**End Position**: 43

| Profile Score | Tandem Score | TDA q-value | Volume | Spectrum Matches |
| --- | --- | --- | --- | --- |
| -20.2492 | 109.5688 | 0.0000 | 3.0525e+06 | 2 |

**Adduct Signal Fractions**

- **Unmodified**: 3.0525e+06

### YFTPN(N)KTEDTIFLR Hex 3 HexNAc 4

**Mass:**  3042.3631

(1.619e-06)

**Charge States:** 3

**Start Position**: 68

**End Position**: 82

| Profile Score | Tandem Score | TDA q-value | Volume | Spectrum Matches |
| --- | --- | --- | --- | --- |
| -1.0405 | 109.2734 | 0.0000 | 7.9021e+07 | 2 |

**Adduct Signal Fractions**

- **Unmodified**: 7.9021e+07

### FYFTPN(N)KTEDTIFLR Hex 3 HexNAc 5

**Mass:**  3392.5120

(1.273e-06)

**Charge States:** 3, 4

**Start Position**: 67

**End Position**: 82

| Profile Score | Tandem Score | TDA q-value | Volume | Spectrum Matches |
| --- | --- | --- | --- | --- |
| 12.0623 | 109.2030 | 0.0000 | 5.0537e+07 | 5 |

**Adduct Signal Fractions**

- **Unmodified**: 5.0537e+07

### FYFTPN(N)KTEDTIFLR Hex 3 HexNAc 4

**Mass:**  3189.4319

(1.351e-06)

**Charge States:** 3

**Start Position**: 67

**End Position**: 82

| Profile Score | Tandem Score | TDA q-value | Volume | Spectrum Matches |
| --- | --- | --- | --- | --- |
| -3.7024 | 108.7568 | 0.0000 | 6.0280e+06 | 1 |

**Adduct Signal Fractions**

- **Unmodified**: 6.0280e+06

### QDQC(C)IYN(N)TTYLNVQR Fuc 3 Hex 5 HexNAc 4 Neu5Ac 1

**Mass:**  4266.7214

(-4.379e-06)

**Charge States:** 3, 4

**Start Position**: 87

**End Position**: 102

| Profile Score | Tandem Score | TDA q-value | Volume | Spectrum Matches |
| --- | --- | --- | --- | --- |
| -20.6291 | 108.0135 | 0.0000 | 9.4020e+06 | 2 |

**Adduct Signal Fractions**

- **Unmodified**: 9.4020e+06

### TPN(N)KTEDTIFLR Hex 7 HexNAc 6

**Mass:**  3786.6055

(2.949e-06)

**Charge States:** 3, 4

**Start Position**: 70

**End Position**: 82

| Profile Score | Tandem Score | TDA q-value | Volume | Spectrum Matches |
| --- | --- | --- | --- | --- |
| 13.7635 | 107.4835 | 0.0000 | 1.0293e+08 | 4 |

**Adduct Signal Fractions**

- **Unmodified**: 1.0293e+08

### PVPITN(N)ATLDQITGK Hex 6 HexNAc 5 Neu5Ac 1

**Mass:**  3845.6782

(2.276e-06)

**Charge States:** 3, 4

**Start Position**: 28

**End Position**: 43

| Profile Score | Tandem Score | TDA q-value | Volume | Spectrum Matches |
| --- | --- | --- | --- | --- |
| -18.8590 | 107.3016 | 0.0000 | 5.8176e+06 | 2 |

**Adduct Signal Fractions**

- **Unmodified**: 5.8176e+06

### QDQC(C)IYN(N)TTYLNVQR Hex 4 HexNAc 8

**Mass:**  4187.7394

(1.261e-06)

**Charge States:** 3, 4

**Start Position**: 87

**End Position**: 102

| Profile Score | Tandem Score | TDA q-value | Volume | Spectrum Matches |
| --- | --- | --- | --- | --- |
| 11.9922 | 106.7777 | 0.0000 | 1.3348e+08 | 3 |

**Adduct Signal Fractions**

- **Unmodified**: 1.3348e+08

### QDQC(C)IYN(N)TTYLNVQR Hex 4 HexNAc 6

**Mass:**  3781.5792

(2.304e-06)

**Charge States:** 3

**Start Position**: 87

**End Position**: 102

| Profile Score | Tandem Score | TDA q-value | Volume | Spectrum Matches |
| --- | --- | --- | --- | --- |
| -3.9312 | 106.3817 | 0.0000 | 7.3031e+07 | 3 |

**Adduct Signal Fractions**

- **Unmodified**: 7.3031e+07

### YFTPN(N)KTEDTIFLR Hex 5 HexNAc 8

**Mass:**  4178.7883

(1.387e-06)

**Charge States:** 3, 4

**Start Position**: 68

**End Position**: 82

| Profile Score | Tandem Score | TDA q-value | Volume | Spectrum Matches |
| --- | --- | --- | --- | --- |
| -3.9740 | 103.2455 | 0.0000 | 3.1309e+07 | 2 |

**Adduct Signal Fractions**

- **Unmodified**: 3.1309e+07

### FTPN(N)KTEDTIFLR Fuc 1 Hex 4 HexNAc 6

**Mass:**  3593.5702

(1.423e-06)

**Charge States:** 3, 4

**Start Position**: 69

**End Position**: 82

| Profile Score | Tandem Score | TDA q-value | Volume | Spectrum Matches |
| --- | --- | --- | --- | --- |
| -3.6705 | 102.4885 | 0.0000 | 2.0194e+07 | 3 |

**Adduct Signal Fractions**

- **Unmodified**: 2.0194e+07

### TPN(N)KTEDTIFLR Hex 3 HexNAc 6

**Mass:**  3140.3956

(6.403e-04)

**Start Position**: 70

**End Position**: 82

| Profile Score | Tandem Score | TDA q-value | Volume | Spectrum Matches |
| --- | --- | --- | --- | --- |
| 0.0000 | 101.0326 | 0.0000 | 0.0000e+00 | 1 |

Adduct:

No Chromatogram Found

### FYFTPN(N)KTEDTIFLR Fuc 1 Hex 4 HexNAc 5

**Mass:**  3700.6214

(9.506e-07)

**Charge States:** 3, 4

**Start Position**: 67

**End Position**: 82

| Profile Score | Tandem Score | TDA q-value | Volume | Spectrum Matches |
| --- | --- | --- | --- | --- |
| -3.9366 | 100.5597 | 0.0000 | 1.4332e+07 | 2 |

**Adduct Signal Fractions**

- **Unmodified**: 1.4332e+07

### LVPVPITN(N)ATLDQITGK Fuc 2 Hex 5 HexNAc 6

**Mass:**  4099.8762

(1.486e-06)

**Charge States:** 3, 4

**Start Position**: 26

**End Position**: 43

| Profile Score | Tandem Score | TDA q-value | Volume | Spectrum Matches |
| --- | --- | --- | --- | --- |
| 7.0258 | 99.8631 | 0.0000 | 2.3200e+07 | 3 |

**Adduct Signal Fractions**

- **Unmodified**: 2.3200e+07

### IYN(N)TTYLNVQR Hex 3 HexNAc 5

**Mass:**  2885.2741

(1.181e-06)

**Charge States:** 3

**Start Position**: 91

**End Position**: 102

| Profile Score | Tandem Score | TDA q-value | Volume | Spectrum Matches |
| --- | --- | --- | --- | --- |
| 15.7867 | 99.6601 | 0.0000 | 4.9858e+07 | 4 |

**Adduct Signal Fractions**

- **Unmodified**: 4.9858e+07

### YFTPN(N)KTEDTIFLR Hex 5 HexNAc 9

**Mass:**  4381.8703

(2.003e-06)

**Charge States:** 3, 4

**Start Position**: 68

**End Position**: 82

| Profile Score | Tandem Score | TDA q-value | Volume | Spectrum Matches |
| --- | --- | --- | --- | --- |
| -19.8915 | 95.4370 | 0.0000 | 9.0030e+06 | 1 |

**Adduct Signal Fractions**

- **Unmodified**: 9.0030e+06

### PVPITN(N)ATLDQITGK Fuc 2 Hex 4 HexNAc 5

**Mass:**  3522.5835

(-2.328e-07)

**Charge States:** 3

**Start Position**: 28

**End Position**: 43

| Profile Score | Tandem Score | TDA q-value | Volume | Spectrum Matches |
| --- | --- | --- | --- | --- |
| -4.5971 | 94.8660 | 0.0000 | 3.9746e+06 | 3 |

**Adduct Signal Fractions**

- **Unmodified**: 3.9746e+06

### YFTPN(N)KTEDTIFLR Fuc 1 Hex 5 HexNAc 7

**Mass:**  4121.7658

(1.237e-06)

**Charge States:** 3, 4

**Start Position**: 68

**End Position**: 82

| Profile Score | Tandem Score | TDA q-value | Volume | Spectrum Matches |
| --- | --- | --- | --- | --- |
| -5.0055 | 94.7699 | 0.0000 | 3.6302e+07 | 3 |

**Adduct Signal Fractions**

- **Unmodified**: 3.6302e+07

### PVPITN(N)ATLDQITGK Hex 7 HexNAc 5

**Mass:**  3718.6549

(5.453e-04)

**Start Position**: 28

**End Position**: 43

| Profile Score | Tandem Score | TDA q-value | Volume | Spectrum Matches |
| --- | --- | --- | --- | --- |
| 0.0000 | 93.5456 | 0.0000 | 0.0000e+00 | 1 |

Adduct:

No Chromatogram Found

### FYFTPN(N)KTEDTIFLR Hex 4 HexNAc 7

**Mass:**  3960.7263

(1.969e-06)

**Charge States:** 3, 4

**Start Position**: 67

**End Position**: 82

| Profile Score | Tandem Score | TDA q-value | Volume | Spectrum Matches |
| --- | --- | --- | --- | --- |
| -3.5609 | 88.3300 | 0.0000 | 1.7273e+07 | 5 |

**Adduct Signal Fractions**

- **Unmodified**: 1.7273e+07

### FTPN(N)KTEDTIFLR Fuc 1 Hex 4 HexNAc 5

**Mass:**  3390.4912

(1.809e-06)

**Charge States:** 3

**Start Position**: 69

**End Position**: 82

| Profile Score | Tandem Score | TDA q-value | Volume | Spectrum Matches |
| --- | --- | --- | --- | --- |
| -3.2879 | 88.2995 | 0.0000 | 1.1631e+07 | 3 |

**Adduct Signal Fractions**

- **Unmodified**: 1.1631e+07

### YFTPN(N)KTEDTIFLR Hex 4 HexNAc 8

**Mass:**  4016.7373

(1.969e-06)

**Charge States:** 3, 4

**Start Position**: 68

**End Position**: 82

| Profile Score | Tandem Score | TDA q-value | Volume | Spectrum Matches |
| --- | --- | --- | --- | --- |
| -19.4592 | 87.1374 | 0.0000 | 1.4300e+07 | 1 |

**Adduct Signal Fractions**

- **Unmodified**: 1.4300e+07

### FTPN(N)KTEDTIFLR Hex 4 HexNAc 7

**Mass:**  3650.5915

(7.763e-07)

**Charge States:** 3, 4

**Start Position**: 69

**End Position**: 82

| Profile Score | Tandem Score | TDA q-value | Volume | Spectrum Matches |
| --- | --- | --- | --- | --- |
| -4.7605 | 83.7187 | 0.0000 | 1.0851e+07 | 4 |

**Adduct Signal Fractions**

- **Unmodified**: 1.0851e+07

### TPN(N)KTEDTIFLR Fuc 1 Hex 4 HexNAc 6

**Mass:**  3446.4973

(1.575e-06)

**Charge States:** 3, 4

**Start Position**: 70

**End Position**: 82

| Profile Score | Tandem Score | TDA q-value | Volume | Spectrum Matches |
| --- | --- | --- | --- | --- |
| -4.2505 | 82.8777 | 0.0000 | 8.5045e+06 | 4 |

**Adduct Signal Fractions**

- **Unmodified**: 8.5045e+06

### TPN(N)KTEDTIFLR Fuc 1 Hex 4 HexNAc 5

**Mass:**  3243.4235

(1.923e-06)

**Charge States:** 3

**Start Position**: 70

**End Position**: 82

| Profile Score | Tandem Score | TDA q-value | Volume | Spectrum Matches |
| --- | --- | --- | --- | --- |
| -4.7181 | 81.8374 | 0.0000 | 3.9189e+06 | 3 |

**Adduct Signal Fractions**

- **Unmodified**: 3.9189e+06

### SVQEIQATFFYFTPN(N)K Hex 4 HexNAc 6

**Mass:**  3787.6481

(5.320e-04)

**Start Position**: 58

**End Position**: 74

| Profile Score | Tandem Score | TDA q-value | Volume | Spectrum Matches |
| --- | --- | --- | --- | --- |
| 0.0000 | 73.0777 | 0.0020 | 0.0000e+00 | 1 |

Adduct:

No Chromatogram Found

### SVQEIQATFFYFTPN(N)K Hex 4 HexNAc 5

**Mass:**  3582.5619

(2.063e-06)

**Charge States:** 3

**Start Position**: 58

**End Position**: 74

| Profile Score | Tandem Score | TDA q-value | Volume | Spectrum Matches |
| --- | --- | --- | --- | --- |
| -5.8596 | 70.0636 | 0.0020 | 8.3678e+06 | 1 |

**Adduct Signal Fractions**

- **Unmodified**: 8.3678e+06

### YFTPN(N)KTEDTIFLR Fuc 1 Hex 5 HexNAc 4 Neu5Ac 2

**Mass:**  4094.7219

(2.045e-06)

**Charge States:** 4

**Start Position**: 68

**End Position**: 82

| Profile Score | Tandem Score | TDA q-value | Volume | Spectrum Matches |
| --- | --- | --- | --- | --- |
| -20.6748 | 67.4565 | 0.0020 | 9.1378e+06 | 1 |

**Adduct Signal Fractions**

- **Unmodified**: 9.1378e+06

### SVQEIQATFFYFTPN(N)K Fuc 4 Hex 3 HexNAc 5

**Mass:**  4004.7431

(2.424e-06)

**Charge States:** 3

**Start Position**: 58

**End Position**: 74

| Profile Score | Tandem Score | TDA q-value | Volume | Spectrum Matches |
| --- | --- | --- | --- | --- |
| -20.8579 | 66.0905 | 0.0020 | 2.3969e+06 | 1 |

**Adduct Signal Fractions**

- **Unmodified**: 2.3969e+06

### C(C)ANLVPVPITN(N)ATLDQITGK Hex 3 HexNAc 5

**Mass:**  3625.6851

(1.127e-06)

**Charge States:** 3, 4

**Start Position**: 23

**End Position**: 43

| Profile Score | Tandem Score | TDA q-value | Volume | Spectrum Matches |
| --- | --- | --- | --- | --- |
| 14.2227 | 64.9298 | 0.0020 | 9.6831e+07 | 6 |

**Adduct Signal Fractions**

- **Unmodified**: 9.6831e+07

### SVQEIQATFFYFTPN(N)KTEDTIFLR Hex 3 HexNAc 6

**Mass:**  4599.0852

(6.531e-07)

**Charge States:** 4

**Start Position**: 58

**End Position**: 82

| Profile Score | Tandem Score | TDA q-value | Volume | Spectrum Matches |
| --- | --- | --- | --- | --- |
| -4.6283 | 63.7422 | 0.0020 | 2.5033e+07 | 2 |

**Adduct Signal Fractions**

- **Unmodified**: 2.5033e+07

### LVPVPITN(N)ATLDQITGK Fuc 1 Hex 5 HexNAc 6

**Mass:**  3953.8193

(1.776e-06)

**Charge States:** 3, 4

**Start Position**: 26

**End Position**: 43

| Profile Score | Tandem Score | TDA q-value | Volume | Spectrum Matches |
| --- | --- | --- | --- | --- |
| 6.4908 | 63.2465 | 0.0020 | 3.1835e+07 | 3 |

**Adduct Signal Fractions**

- **Unmodified**: 3.1835e+07

### LVPVPITN(N)ATLDQITGK Fuc 1 Hex 5 HexNAc 7

**Mass:**  4156.8962

(1.066e-06)

**Charge States:** 3, 4

**Start Position**: 26

**End Position**: 43

| Profile Score | Tandem Score | TDA q-value | Volume | Spectrum Matches |
| --- | --- | --- | --- | --- |
| 13.8084 | 61.7304 | 0.0020 | 1.5194e+07 | 2 |

**Adduct Signal Fractions**

- **Unmodified**: 1.5194e+07

### QDQC(C)IYN(N)TTYLNVQR Fuc 1 Hex 5 HexNAc 8

**Mass:**  4495.8547

(1.411e-06)

**Charge States:** 4

**Start Position**: 87

**End Position**: 102

| Profile Score | Tandem Score | TDA q-value | Volume | Spectrum Matches |
| --- | --- | --- | --- | --- |
| -4.4597 | 60.9028 | 0.0020 | 3.3483e+07 | 3 |

**Adduct Signal Fractions**

- **Unmodified**: 3.3483e+07

### LVPVPITN(N)ATLDQITGK Fuc 1 Hex 5 HexNAc 3 Neu5Ac 1

**Mass:**  3635.6856

(1.897e-06)

**Charge States:** 3

**Start Position**: 26

**End Position**: 43

| Profile Score | Tandem Score | TDA q-value | Volume | Spectrum Matches |
| --- | --- | --- | --- | --- |
| -3.5717 | 56.9684 | 0.0020 | 2.0512e+07 | 4 |

**Adduct Signal Fractions**

- **Unmodified**: 2.0512e+07

### C(C)ANLVPVPITN(N)ATLDQITGK Fuc 1 Hex 4 HexNAc 5

**Mass:**  3933.7967

(2.668e-06)

**Charge States:** 3, 4

**Start Position**: 23

**End Position**: 43

| Profile Score | Tandem Score | TDA q-value | Volume | Spectrum Matches |
| --- | --- | --- | --- | --- |
| 11.9622 | 56.1843 | 0.0020 | 3.0622e+07 | 3 |

**Adduct Signal Fractions**

- **Unmodified**: 3.0622e+07

### QDQC(C)IYN(N)TTYLNVQR Fuc 1 Hex 4 HexNAc 7

**Mass:**  4130.7189

(1.362e-06)

**Charge States:** 3, 4

**Start Position**: 87

**End Position**: 102

| Profile Score | Tandem Score | TDA q-value | Volume | Spectrum Matches |
| --- | --- | --- | --- | --- |
| 13.8019 | 54.3355 | 0.0020 | 4.8237e+07 | 3 |

**Adduct Signal Fractions**

- **Unmodified**: 4.8237e+07

### LVPVPITN(N)ATLDQITGK Hex 4 HexNAc 5 Neu5Ac 1

**Mass:**  3733.7231

(1.444e-06)

**Charge States:** 3, 4

**Start Position**: 26

**End Position**: 43

| Profile Score | Tandem Score | TDA q-value | Volume | Spectrum Matches |
| --- | --- | --- | --- | --- |
| 13.1138 | 53.7658 | 0.0020 | 1.4353e+07 | 6 |

**Adduct Signal Fractions**

- **Unmodified**: 1.4353e+07

### QDQC(C)IYN(N)TTYLNVQR Hex 5 HexNAc 9

**Mass:**  4552.8737

(1.133e-06)

**Charge States:** 3, 4

**Start Position**: 87

**End Position**: 102

| Profile Score | Tandem Score | TDA q-value | Volume | Spectrum Matches |
| --- | --- | --- | --- | --- |
| -4.9599 | 53.6764 | 0.0020 | 5.9440e+07 | 1 |

**Adduct Signal Fractions**

- **Unmodified**: 5.9440e+07

### QDQC(C)IYN(N)TTYLNVQR Fuc 2 Hex 5 HexNAc 7

**Mass:**  4438.8264

(3.580e-07)

**Charge States:** 3, 4

**Start Position**: 87

**End Position**: 102

| Profile Score | Tandem Score | TDA q-value | Volume | Spectrum Matches |
| --- | --- | --- | --- | --- |
| -5.1871 | 53.3983 | 0.0020 | 2.9357e+07 | 3 |

**Adduct Signal Fractions**

- **Unmodified**: 2.9357e+07

### QDQC(C)IYN(N)TTYLNVQR Fuc 1 Hex 3 HexNAc 6

**Mass:**  3765.5857

(9.701e-07)

**Charge States:** 3

**Start Position**: 87

**End Position**: 102

| Profile Score | Tandem Score | TDA q-value | Volume | Spectrum Matches |
| --- | --- | --- | --- | --- |
| -19.8381 | 49.9990 | 0.0032 | 1.3107e+07 | 1 |

**Adduct Signal Fractions**

- **Unmodified**: 1.3107e+07

### LVPVPITN(N)ATLDQITGK Hex 5 HexNAc 7

**Mass:**  4010.8496

(3.902e-06)

**Charge States:** 3, 4

**Start Position**: 26

**End Position**: 43

| Profile Score | Tandem Score | TDA q-value | Volume | Spectrum Matches |
| --- | --- | --- | --- | --- |
| -4.9103 | 48.4692 | 0.0032 | 7.8443e+06 | 1 |

**Adduct Signal Fractions**

- **Unmodified**: 7.8443e+06

### LVPVPITN(N)ATLDQITGK Fuc 1 Hex 5 HexNAc 5

**Mass:**  3750.7381

(2.105e-06)

**Charge States:** 3, 4

**Start Position**: 26

**End Position**: 43

| Profile Score | Tandem Score | TDA q-value | Volume | Spectrum Matches |
| --- | --- | --- | --- | --- |
| -5.3021 | 48.4467 | 0.0032 | 1.2883e+07 | 3 |

**Adduct Signal Fractions**

- **Unmodified**: 1.2883e+07

### SVQEIQATFFYFTPN(N)KTEDTIFLR Hex 3 HexNAc 5

**Mass:**  4396.0163

(2.728e-06)

**Charge States:** 4

**Start Position**: 58

**End Position**: 82

| Profile Score | Tandem Score | TDA q-value | Volume | Spectrum Matches |
| --- | --- | --- | --- | --- |
| -19.6842 | 47.2154 | 0.0032 | 6.0176e+06 | 1 |

**Adduct Signal Fractions**

- **Unmodified**: 6.0176e+06

### SVQEIQATFFYFTPN(N)K Fuc 1 Hex 5 HexNAc 4 Neu5Ac 2

**Mass:**  4269.7853

(2.003e-06)

**Charge States:** 3, 4

**Start Position**: 58

**End Position**: 74

| Profile Score | Tandem Score | TDA q-value | Volume | Spectrum Matches |
| --- | --- | --- | --- | --- |
| -20.8885 | 46.0435 | 0.0032 | 3.2616e+06 | 1 |

**Adduct Signal Fractions**

- **Unmodified**: 3.2616e+06

### PVPITN(N)ATLDQITGK Fuc 3 Hex 5 HexNAc 5

**Mass:**  3830.7094

(3.373e-06)

**Charge States:** 4

**Start Position**: 28

**End Position**: 43

| Profile Score | Tandem Score | TDA q-value | Volume | Spectrum Matches |
| --- | --- | --- | --- | --- |
| -19.3483 | 45.4318 | 0.0032 | 1.5837e+07 | 1 |

**Adduct Signal Fractions**

- **Unmodified**: 1.5837e+07

### QDQC(C)IYN(N)TTYLNVQR Fuc 1 Hex 5 HexNAc 4 Neu5Ac 1

**Mass:**  3974.6376

(3.724e-06)

**Charge States:** 3

**Start Position**: 87

**End Position**: 102

| Profile Score | Tandem Score | TDA q-value | Volume | Spectrum Matches |
| --- | --- | --- | --- | --- |
| -5.3022 | 44.8562 | 0.0032 | 1.8090e+07 | 2 |

**Adduct Signal Fractions**

- **Unmodified**: 1.8090e+07

### SVQEIQATFFYFTPN(N)K Fuc 2 Hex 5 HexNAc 7

**Mass:**  4442.8908

(2.003e-06)

**Charge States:** 4

**Start Position**: 58

**End Position**: 74

| Profile Score | Tandem Score | TDA q-value | Volume | Spectrum Matches |
| --- | --- | --- | --- | --- |
| -20.0135 | 44.1798 | 0.0032 | 3.6576e+06 | 1 |

**Adduct Signal Fractions**

- **Unmodified**: 3.6576e+06

### PVPITN(N)ATLDQITGK Fuc 2 Hex 3 HexNAc 7 Neu5Ac 3

**Mass:**  4640.0187

(8.872e-06)

**Charge States:** 4

**Start Position**: 28

**End Position**: 43

| Profile Score | Tandem Score | TDA q-value | Volume | Spectrum Matches |
| --- | --- | --- | --- | --- |
| 13.7699 | 41.7435 | 0.0032 | 4.0602e+06 | 3 |

**Adduct Signal Fractions**

- **Unmodified**: 4.0602e+06

### YFTPN(N)KTEDTIFLR Fuc 2 Hex 5 HexNAc 6

**Mass:**  4064.7412

(4.295e-07)

**Charge States:** 3

**Start Position**: 68

**End Position**: 82

| Profile Score | Tandem Score | TDA q-value | Volume | Spectrum Matches |
| --- | --- | --- | --- | --- |
| -20.2507 | 41.0645 | 0.0032 | 8.2796e+06 | 1 |

**Adduct Signal Fractions**

- **Unmodified**: 8.2796e+06

### SVQEIQATFFYFTPN(N)K Hex 5 HexNAc 8

**Mass:**  4353.8493

(-5.984e-07)

**Charge States:** 4

**Start Position**: 58

**End Position**: 74

| Profile Score | Tandem Score | TDA q-value | Volume | Spectrum Matches |
| --- | --- | --- | --- | --- |
| 9.7954 | 40.8973 | 0.0032 | 2.0998e+07 | 1 |

**Adduct Signal Fractions**

- **Unmodified**: 2.0998e+07

### QDQC(C)IYN(N)TTYLNVQR Fuc 1 Hex 6 HexNAc 9

**Mass:**  4860.9782

(-8.239e-07)

**Charge States:** 4

**Start Position**: 87

**End Position**: 102

| Profile Score | Tandem Score | TDA q-value | Volume | Spectrum Matches |
| --- | --- | --- | --- | --- |
| -19.9206 | 39.4999 | 0.0032 | 4.8078e+06 | 1 |

**Adduct Signal Fractions**

- **Unmodified**: 4.8078e+06

### SVQEIQATFFYFTPN(N)KTEDTIFLR Fuc 1 Hex 4 HexNAc 6

**Mass:**  4907.2009

(1.349e-06)

**Charge States:** 4

**Start Position**: 58

**End Position**: 82

| Profile Score | Tandem Score | TDA q-value | Volume | Spectrum Matches |
| --- | --- | --- | --- | --- |
| -19.7147 | 38.8266 | 0.0032 | 2.3349e+06 | 1 |

**Adduct Signal Fractions**

- **Unmodified**: 2.3349e+06

### QDQC(C)IYN(N)TTYLNVQR Fuc 1 Hex 6 HexNAc 8

**Mass:**  4657.9040

(2.428e-06)

**Charge States:** 4

**Start Position**: 87

**End Position**: 102

| Profile Score | Tandem Score | TDA q-value | Volume | Spectrum Matches |
| --- | --- | --- | --- | --- |
| -4.7267 | 37.9420 | 0.0047 | 9.9978e+06 | 2 |

**Adduct Signal Fractions**

- **Unmodified**: 9.9978e+06

### C(C)ANLVPVPITN(N)ATLDQITGK Hex 3 HexNAc 4

**Mass:**  3422.6072

(2.342e-06)

**Charge States:** 3, 4

**Start Position**: 23

**End Position**: 43

| Profile Score | Tandem Score | TDA q-value | Volume | Spectrum Matches |
| --- | --- | --- | --- | --- |
| -3.6995 | 36.6477 | 0.0059 | 1.0879e+07 | 3 |

**Adduct Signal Fractions**

- **Unmodified**: 1.0879e+07

### QDQC(C)IYN(N)TTYLNVQR Fuc 2 Hex 6 HexNAc 7

**Mass:**  4600.8884

(9.657e-07)

**Charge States:** 3, 4

**Start Position**: 87

**End Position**: 102

| Profile Score | Tandem Score | TDA q-value | Volume | Spectrum Matches |
| --- | --- | --- | --- | --- |
| -5.7240 | 36.5543 | 0.0059 | 1.0518e+07 | 1 |

**Adduct Signal Fractions**

- **Unmodified**: 1.0518e+07

### QDQC(C)IYN(N)TTYLN(D)VQR Hex 3 HexNAc 5

**Mass:**  3417.4356

(2.901e-04)

**Charge States:** 3

**Start Position**: 87

**End Position**: 102

| Profile Score | Tandem Score | TDA q-value | Volume | Spectrum Matches |
| --- | --- | --- | --- | --- |
| -19.7649 | 36.2220 | 0.0059 | 2.7232e+07 | 2 |

**Adduct Signal Fractions**

- **Unmodified**: 2.7232e+07

### LVPVPITN(N)ATLDQITGK Fuc 2 Hex 4 HexNAc 5

**Mass:**  3735.7345

(2.668e-04)

**Start Position**: 26

**End Position**: 43

| Profile Score | Tandem Score | TDA q-value | Volume | Spectrum Matches |
| --- | --- | --- | --- | --- |
| 0.0000 | 36.1837 | 0.0071 | 0.0000e+00 | 1 |

Adduct:

No Chromatogram Found

### FYFTPN(N)KTEDTIFLR Fuc 2 Hex 5 HexNAc 6

**Mass:**  4211.8117

(9.324e-07)

**Charge States:** 4

**Start Position**: 67

**End Position**: 82

| Profile Score | Tandem Score | TDA q-value | Volume | Spectrum Matches |
| --- | --- | --- | --- | --- |
| -5.1354 | 35.8627 | 0.0071 | 3.3520e+06 | 1 |

**Adduct Signal Fractions**

- **Unmodified**: 3.3520e+06

### ATFFYFTPN(N)K Fuc 1 Hex 3 HexNAc 8 Neu5Ac 1

**Mass:**  3782.5324

(-4.387e-06)

**Charge States:** 4

**Start Position**: 64

**End Position**: 74

| Profile Score | Tandem Score | TDA q-value | Volume | Spectrum Matches |
| --- | --- | --- | --- | --- |
| -20.2911 | 34.7192 | 0.0071 | 9.1957e+06 | 1 |

**Adduct Signal Fractions**

- **Unmodified**: 9.1957e+06

### TPN(N)KTEDTIFLR Hex 4 HexNAc 7

**Mass:**  3503.5193

(1.177e-06)

**Charge States:** 3, 4

**Start Position**: 70

**End Position**: 82

| Profile Score | Tandem Score | TDA q-value | Volume | Spectrum Matches |
| --- | --- | --- | --- | --- |
| -5.1810 | 34.0669 | 0.0097 | 4.5981e+06 | 2 |

**Adduct Signal Fractions**

- **Unmodified**: 4.5981e+06

### QDQC(C)IYN(N)TTYLN(D)VQR Hex 3 HexNAc 6

**Mass:**  3620.5175

(2.745e-04)

**Charge States:** 3, 4

**Start Position**: 87

**End Position**: 102

| Profile Score | Tandem Score | TDA q-value | Volume | Spectrum Matches |
| --- | --- | --- | --- | --- |
| -19.9655 | 34.0366 | 0.0097 | 8.8885e+07 | 4 |

**Adduct Signal Fractions**

- **Unmodified**: 8.8885e+07

### LVPVPITN(N)ATLDQITGK Fuc 4 Hex 3 HexNAc 5

**Mass:**  3867.8213

(7.814e-04)

**Start Position**: 26

**End Position**: 43

| Profile Score | Tandem Score | TDA q-value | Volume | Spectrum Matches |
| --- | --- | --- | --- | --- |
| 0.0000 | 33.6534 | 0.0105 | 0.0000e+00 | 1 |

Adduct:

No Chromatogram Found

### SVQEIQATFFYFTPN(N)K Fuc 1 Hex 5 HexNAc 8

**Mass:**  4499.9103

(1.525e-06)

**Charge States:** 4

**Start Position**: 58

**End Position**: 74

| Profile Score | Tandem Score | TDA q-value | Volume | Spectrum Matches |
| --- | --- | --- | --- | --- |
| -20.3606 | 33.4300 | 0.0105 | 3.3720e+06 | 1 |

**Adduct Signal Fractions**

- **Unmodified**: 3.3720e+06

### FTPN(N)KTEDTIFLR Hex 3 HexNAc 4

**Mass:**  2879.2976

(5.942e-07)

**Charge States:** 3

**Start Position**: 69

**End Position**: 82

| Profile Score | Tandem Score | TDA q-value | Volume | Spectrum Matches |
| --- | --- | --- | --- | --- |
| -4.9030 | 33.4177 | 0.0105 | 5.4284e+06 | 1 |

**Adduct Signal Fractions**

- **Unmodified**: 5.4284e+06

### QIPLC(C)ANLVPVPITN(N)ATLDQITGK Hex 3 HexNAc 5 Neu5Ac 1

**Mass:**  4368.0515

(-8.597e-07)

**Charge States:** 3, 4

**Start Position**: 19

**End Position**: 43

| Profile Score | Tandem Score | TDA q-value | Volume | Spectrum Matches |
| --- | --- | --- | --- | --- |
| 17.4839 | 32.8010 | 0.0105 | 1.3258e+08 | 6 |

**Adduct Signal Fractions**

- **Unmodified**: 1.3258e+08

### QDQC(C)IYN(N)TTYLNVQR Fuc 3 Hex 6 HexNAc 6

**Mass:**  4543.8635

(1.532e-06)

**Charge States:** 4

**Start Position**: 87

**End Position**: 102

| Profile Score | Tandem Score | TDA q-value | Volume | Spectrum Matches |
| --- | --- | --- | --- | --- |
| -19.8534 | 32.1915 | 0.0117 | 9.3637e+06 | 1 |

**Adduct Signal Fractions**

- **Unmodified**: 9.3637e+06

### LVPVPITN(N)ATLDQITGK Hex 4 HexNAc 6

**Mass:**  3647.7165

(5.526e-04)

**Start Position**: 26

**End Position**: 43

| Profile Score | Tandem Score | TDA q-value | Volume | Spectrum Matches |
| --- | --- | --- | --- | --- |
| 0.0000 | 32.0540 | 0.0117 | 0.0000e+00 | 1 |

Adduct:

No Chromatogram Found

### QDQC(C)IYN(N)TTYLNVQR Fuc 1 Hex 8 HexNAc 6

**Mass:**  4577.8865

(4.459e-04)

**Start Position**: 87

**End Position**: 102

| Profile Score | Tandem Score | TDA q-value | Volume | Spectrum Matches |
| --- | --- | --- | --- | --- |
| 0.0000 | 30.0128 | 0.0208 | 0.0000e+00 | 1 |

Adduct:

No Chromatogram Found

### QDQC(C)IYN(N)TTYLNVQR Fuc 2 Hex 6 HexNAc 8

**Mass:**  4805.9881

(4.228e-04)

**Start Position**: 87

**End Position**: 102

| Profile Score | Tandem Score | TDA q-value | Volume | Spectrum Matches |
| --- | --- | --- | --- | --- |
| 0.0000 | 29.6596 | 0.0208 | 0.0000e+00 | 1 |

Adduct:

No Chromatogram Found

### FTPN(N)KTEDTIFLR Fuc 2 Hex 5 HexNAc 6

**Mass:**  3901.6721

(-1.173e-07)

**Charge States:** 3

**Start Position**: 69

**End Position**: 82

| Profile Score | Tandem Score | TDA q-value | Volume | Spectrum Matches |
| --- | --- | --- | --- | --- |
| -5.4757 | 29.0813 | 0.0231 | 2.4874e+06 | 2 |

**Adduct Signal Fractions**

- **Unmodified**: 2.4874e+06

### LVPVPITN(N)ATLDQITGK Fuc 5 Hex 3 HexNAc 6

**Mass:**  4213.9177

(-2.563e-06)

**Charge States:** 4

**Start Position**: 26

**End Position**: 43

| Profile Score | Tandem Score | TDA q-value | Volume | Spectrum Matches |
| --- | --- | --- | --- | --- |
| -5.4083 | 28.7585 | 0.0241 | 4.2415e+06 | 1 |

**Adduct Signal Fractions**

- **Unmodified**: 4.2415e+06

### YFTPN(N)KTEDTIFLR Hex 4 HexNAc 6

**Mass:**  3610.5756

(1.363e-06)

**Charge States:** 3, 4

**Start Position**: 68

**End Position**: 82

| Profile Score | Tandem Score | TDA q-value | Volume | Spectrum Matches |
| --- | --- | --- | --- | --- |
| -5.8263 | 28.5761 | 0.0287 | 1.5954e+07 | 1 |

**Adduct Signal Fractions**

- **Unmodified**: 1.5954e+07

### FTPN(N)KTEDTIFLR Fuc 1 Hex 7 HexNAc 3

**Mass:**  3471.5249

(2.942e-04)

**Charge States:** 3, 4

**Start Position**: 69

**End Position**: 82

| Profile Score | Tandem Score | TDA q-value | Volume | Spectrum Matches |
| --- | --- | --- | --- | --- |
| -6.3684 | 28.5242 | 0.0321 | 1.8071e+06 | 2 |

**Adduct Signal Fractions**

- **Unmodified**: 1.8071e+06

### SVQEIQATFFYFTPN(N)KTEDTIFLR Fuc 1 Hex 4 HexNAc 5

**Mass:**  4704.1174

(1.392e-06)

**Charge States:** 4

**Start Position**: 58

**End Position**: 82

| Profile Score | Tandem Score | TDA q-value | Volume | Spectrum Matches |
| --- | --- | --- | --- | --- |
| -19.3653 | 28.2192 | 0.0331 | 2.4507e+06 | 1 |

**Adduct Signal Fractions**

- **Unmodified**: 2.4507e+06

### IYN(N)TTYLNVQR Fuc 1 Hex 4 HexNAc 5

**Mass:**  3194.3752

(3.114e-04)

**Start Position**: 91

**End Position**: 102

| Profile Score | Tandem Score | TDA q-value | Volume | Spectrum Matches |
| --- | --- | --- | --- | --- |
| 0.0000 | 27.8524 | 0.0346 | 0.0000e+00 | 1 |

Adduct:

No Chromatogram Found

### QDQC(C)IYN(N)TTYLNVQR Hex 3 HexNAc 5 Neu5Ac 1

**Mass:**  3707.5332

(-1.731e-06)

**Charge States:** 3, 4

**Start Position**: 87

**End Position**: 102

| Profile Score | Tandem Score | TDA q-value | Volume | Spectrum Matches |
| --- | --- | --- | --- | --- |
| 14.2105 | 27.7837 | 0.0346 | 8.4678e+08 | 5 |

**Adduct Signal Fractions**

- **Unmodified**: 8.4678e+08

### IYN(N)TTYLNVQR Fuc 1 Hex 4 HexNAc 6

**Mass:**  3396.4597

(4.971e-07)

**Charge States:** 3

**Start Position**: 91

**End Position**: 102

| Profile Score | Tandem Score | TDA q-value | Volume | Spectrum Matches |
| --- | --- | --- | --- | --- |
| 11.0626 | 27.5925 | 0.0346 | 2.8082e+07 | 2 |

**Adduct Signal Fractions**

- **Unmodified**: 2.8082e+07

### C(C)ANLVPVPITN(N)ATLDQITGK Hex 4 HexNAc 6

**Mass:**  3990.8157

(8.305e-07)

**Charge States:** 3, 4

**Start Position**: 23

**End Position**: 43

| Profile Score | Tandem Score | TDA q-value | Volume | Spectrum Matches |
| --- | --- | --- | --- | --- |
| -4.3900 | 27.4452 | 0.0346 | 1.0495e+07 | 2 |

**Adduct Signal Fractions**

- **Unmodified**: 1.0495e+07

### LVPVPITN(N)ATLDQITGK Hex 3 HexNAc 5

**Mass:**  3282.5764

(6.117e-04)

**Start Position**: 26

**End Position**: 43

| Profile Score | Tandem Score | TDA q-value | Volume | Spectrum Matches |
| --- | --- | --- | --- | --- |
| 0.0000 | 25.3756 | 0.0464 | 0.0000e+00 | 1 |

Adduct:

No Chromatogram Found

### P00738|HPT\_HUMAN

**>P00738|HPT\_HUMAN**  
 `MSALGAVIALLLWGQLFAVDSGNDVTDIADDGCPKPPEIAHGYVEHSVRYQCKNYYKLRTEGDGVYTLNDKKQWINKAVG  
DKLPECEADDGCPKPPEIAHGYVEHSVRYQCKNYYKLRTEGDGVYTLNNEKQWINKAVGDKLPECEAVCGKPKNPANPVQ  
RILGGHLDAKGSFPWQAKMVSHHNLTTGATLINEQWLLTTAKNLFLNHSENATAKDIAPTLTLYVGKKQLVEIEKVVLHP  
NYSQVDIGLIKLKQKVSVNERVMPICLPSKDYAEVGRVGYVSGWGRNANFKFTDHLKYVMLPVADQDQCIRHYEGSTVPE  
KKTPKSPVGVQPILNEHTFCAGMSKYQEDTCYGDAGSAFAVHDLEEDTWYATGILSFDKSCAVAEYGVYVKVTSIQDWVQ  
KTIAEN`

| Site | Type | Count |
| --- | --- | --- |
| 241 | N-linked | 4 |
| 211 | N-linked | 7 |

| Mass | Sequence | MS_2_ Score | `q`-value | MS_1_ Score | Signal | Protein Coordinates | Tandem Scans | Start Time | Apex Time | End Time |
| --- | --- | --- | --- | --- | --- | --- | --- | --- | --- | --- |
| 3092.4828 | VVLHPN(N)YSQVDIGLIK Hex 3 HexNAc 4 | 178.690 | 0.0 | 12.608 | 4.090e+07 | 235 - 251 | 4 | 42.80 | 42.80 | 43.65 |
| 4460.8418 | NLFLNHSEN(N)ATAK Fuc 5 Hex 4 HexNAc 8 | 134.127 | 0.0 | 12.805 | 3.948e+07 | 202 - 215 | 4 | 18.36 | 18.52 | 18.87 |
| 2568.1147 | VVLHPN(N)YSQVD Hex 3 HexNAc 4 | 121.145 | 0.0 | 13.184 | 1.331e+07 | 235 - 246 | 4 | 24.11 | 24.28 | 24.53 |
| 4257.7611 | NLFLNHSEN(N)ATAK Hex 6 HexNAc 9 | 117.433 | 0.0 | -3.130 | 3.352e+07 | 202 - 215 | 3 | 18.52 | 18.69 | 18.87 |
| 4663.9188 | NLFLNHSEN(N)ATAK Fuc 5 Hex 4 HexNAc 9 | 90.425 | 0.0 | -3.727 | 1.637e+07 | 202 - 215 | 2 | 18.26 | 18.36 | 18.52 |
| 3498.6432 | VVLHPN(N)YSQVDIGLIK Hex 3 HexNAc 6 | 65.483 | 0.002 | -19.456 | 7.386e+06 | 235 - 251 | 2 | 42.64 | 42.64 | 42.64 |
| 4567.8681 | NLFLNHSEN(N)ATAK Fuc 1 Hex 7 HexNAc 9 | 42.026 | 0.003 | 0.000 | 0.000e+00 | 202 - 215 | 1 | - | - | - |
| 4054.6832 | NLFLNHSEN(N)ATAK Hex 6 HexNAc 8 | 41.349 | 0.003 | -19.996 | 7.028e+06 | 202 - 215 | 2 | 18.81 | 18.81 | 18.87 |
| 2771.1955 | VVLHPN(N)YSQVD Hex 3 HexNAc 5 | 35.803 | 0.007 | 13.152 | 1.095e+07 | 235 - 246 | 2 | 23.96 | 24.13 | 24.47 |
| 4565.8646 | NLFLNHSEN(N)ATAK Fuc 1 Hex 7 HexNAc 9 | 30.322 | 0.019 | -19.448 | 5.871e+06 | 202 - 215 | 1 | 18.36 | 18.52 | 18.52 |
| 4768.9538 | N(D)LFLNHSEN(N)ATAK Fuc 4 Hex 5 HexNAc 8 Neu5Ac 1 | 27.555 | 0.035 | -5.508 | 1.162e+07 | 202 - 215 | 2 | 18.15 | 18.26 | 18.36 |

### VVLHPN(N)YSQVDIGLIK Hex 3 HexNAc 4

**Mass:**  3092.4831

(9.916e-07)

**Charge States:** 3, 4

**Start Position**: 236

**End Position**: 252

| Profile Score | Tandem Score | TDA q-value | Volume | Spectrum Matches |
| --- | --- | --- | --- | --- |
| 12.6081 | 178.6897 | 0.0000 | 4.0896e+07 | 4 |

**Adduct Signal Fractions**

- **Unmodified**: 4.0896e+07

### NLFLNHSEN(N)ATAK Fuc 5 Hex 4 HexNAc 8

**Mass:**  4460.8410

(-4.417e-06)

**Charge States:** 4

**Start Position**: 203

**End Position**: 216

| Profile Score | Tandem Score | TDA q-value | Volume | Spectrum Matches |
| --- | --- | --- | --- | --- |
| 12.8054 | 134.1274 | 0.0000 | 3.9483e+07 | 4 |

**Adduct Signal Fractions**

- **Unmodified**: 3.9483e+07

### VVLHPN(N)YSQVD Hex 3 HexNAc 4

**Mass:**  2568.1155

(8.042e-07)

**Charge States:** 2, 3

**Start Position**: 236

**End Position**: 247

| Profile Score | Tandem Score | TDA q-value | Volume | Spectrum Matches |
| --- | --- | --- | --- | --- |
| 13.1837 | 121.1450 | 0.0000 | 1.3308e+07 | 4 |

**Adduct Signal Fractions**

- **Unmodified**: 1.3308e+07

### NLFLNHSEN(N)ATAK Hex 6 HexNAc 9

**Mass:**  4257.7639

(9.464e-07)

**Charge States:** 3, 4

**Start Position**: 203

**End Position**: 216

| Profile Score | Tandem Score | TDA q-value | Volume | Spectrum Matches |
| --- | --- | --- | --- | --- |
| -3.1299 | 117.4333 | 0.0000 | 3.3520e+07 | 3 |

**Adduct Signal Fractions**

- **Unmodified**: 3.3520e+07

### NLFLNHSEN(N)ATAK Fuc 5 Hex 4 HexNAc 9

**Mass:**  4663.9186

(-4.900e-06)

**Charge States:** 4

**Start Position**: 203

**End Position**: 216

| Profile Score | Tandem Score | TDA q-value | Volume | Spectrum Matches |
| --- | --- | --- | --- | --- |
| -3.7271 | 90.4254 | 0.0000 | 1.6372e+07 | 2 |

**Adduct Signal Fractions**

- **Unmodified**: 1.6372e+07

### VVLHPN(N)YSQVDIGLIK Hex 3 HexNAc 6

**Mass:**  3498.6410

(6.251e-07)

**Charge States:** 3, 4

**Start Position**: 236

**End Position**: 252

| Profile Score | Tandem Score | TDA q-value | Volume | Spectrum Matches |
| --- | --- | --- | --- | --- |
| -19.4556 | 65.4831 | 0.0020 | 7.3858e+06 | 2 |

**Adduct Signal Fractions**

- **Unmodified**: 7.3858e+06

### NLFLNHSEN(N)ATAK Fuc 1 Hex 7 HexNAc 9

**Mass:**  4567.8681

(4.380e-04)

**Start Position**: 203

**End Position**: 216

| Profile Score | Tandem Score | TDA q-value | Volume | Spectrum Matches |
| --- | --- | --- | --- | --- |
| 0.0000 | 42.0262 | 0.0032 | 0.0000e+00 | 1 |

Adduct:

No Chromatogram Found

### NLFLNHSEN(N)ATAK Hex 6 HexNAc 8

**Mass:**  4054.6813

(2.366e-06)

**Charge States:** 3, 4

**Start Position**: 203

**End Position**: 216

| Profile Score | Tandem Score | TDA q-value | Volume | Spectrum Matches |
| --- | --- | --- | --- | --- |
| -19.9963 | 41.3486 | 0.0032 | 7.0275e+06 | 2 |

**Adduct Signal Fractions**

- **Unmodified**: 7.0275e+06

### VVLHPN(N)YSQVD Hex 3 HexNAc 5

**Mass:**  2771.1954

(2.166e-06)

**Charge States:** 3

**Start Position**: 236

**End Position**: 247

| Profile Score | Tandem Score | TDA q-value | Volume | Spectrum Matches |
| --- | --- | --- | --- | --- |
| 13.1515 | 35.8034 | 0.0071 | 1.0953e+07 | 2 |

**Adduct Signal Fractions**

- **Unmodified**: 1.0953e+07

### NLFLNHSEN(N)ATAK Fuc 1 Hex 7 HexNAc 9

**Mass:**  4565.8679

(-9.689e-08)

**Charge States:** 4

**Start Position**: 203

**End Position**: 216

| Profile Score | Tandem Score | TDA q-value | Volume | Spectrum Matches |
| --- | --- | --- | --- | --- |
| -19.4477 | 30.3216 | 0.0189 | 5.8708e+06 | 1 |

**Adduct Signal Fractions**

- **Unmodified**: 5.8708e+06

### N(D)LFLNHSEN(N)ATAK Fuc 4 Hex 5 HexNAc 8 Neu5Ac 1

**Mass:**  4768.9504

(2.124e-04)

**Charge States:** 4

**Start Position**: 203

**End Position**: 216

| Profile Score | Tandem Score | TDA q-value | Volume | Spectrum Matches |
| --- | --- | --- | --- | --- |
| -5.5076 | 27.5546 | 0.0346 | 1.1624e+07 | 2 |

**Adduct Signal Fractions**

- **Unmodified**: 1.1624e+07

### P05155|IC1\_HUMAN

**>P05155|IC1\_HUMAN**  
 `MASRLTLLTLLLLLLAGDRASSNPNATSSSSQDPESLQDRGEGKVATTVISKMLFVEPILEVSSLPTTNSTTNSATKITA  
NTTDEPTTQPTTEPTTQPTIQPTQPTTQLPTDSPTQPTTGSFCPGPVTLCSDLESHSTEAVLGDALVDFSLKLYHAFSAM  
KKVETNMAFSPFSIASLLTQVLLGAGENTKTNLESILSYPKDFTCVHQALKGFTTKGVTSVSQIFHSPDLAIRDTFVNAS  
RTLYSSSPRVLSNNSDANLELINTWVAKNTNNKISRLLDSLPSDTRLVLLNAIYLSAKWKTTFDPKKTRMEPFHFKNSVI  
KVPMMNSKKYPVAHFIDQTLKAKVGQLQLSHNLSLVILVPQNLKHRLEDMEQALSPSVFKAIMEKLEMSKFQPTLLTLPR  
IKVTTSQDMLSIMEKLEFFDFSYDLNLCGLTEDPDLQVSAMQHQTVLELTETGVEAAAASAISVARTLLVFEVQQPFLFV  
LWDQQHKFPVFMGRVYDPRA`

| Site | Type | Count |
| --- | --- | --- |
| 25 | N-linked | 10 |

| Mass | Sequence | MS_2_ Score | `q`-value | MS_1_ Score | Signal | Protein Coordinates | Tandem Scans | Start Time | Apex Time | End Time |
| --- | --- | --- | --- | --- | --- | --- | --- | --- | --- | --- |
| 3595.6930 | NPN(N)ATSSSSQDPESLQDR Hex 4 HexNAc 5 | 143.912 | 0.0 | -21.844 | 4.453e+07 | 22 - 40 | 6 | 14.14 | 14.14 | 31.38 |
| 3800.5444 | NPN(N)ATSSSSQDPESLQDR Hex 4 HexNAc 6 | 127.706 | 0.0 | 0.000 | 0.000e+00 | 22 - 40 | 1 | - | - | - |
| 3230.3276 | NPN(N)ATSSSSQDPESLQDR Hex 3 HexNAc 4 | 113.394 | 0.0 | 13.086 | 4.168e+07 | 22 - 40 | 1 | 15.36 | 15.46 | 15.68 |
| 3376.3765 | NPN(N)ATSSSSQDPESLQDR Fuc 1 Hex 3 HexNAc 4 | 111.727 | 0.0 | -19.620 | 2.049e+07 | 22 - 40 | 4 | 15.36 | 15.36 | 36.57 |
| 3433.4035 | NPN(N)ATSSSSQDPESLQDR Hex 3 HexNAc 5 | 69.327 | 0.002 | -4.710 | 1.653e+07 | 22 - 40 | 2 | 15.25 | 15.46 | 15.59 |
| 3743.5183 | NPN(N)ATSSSSQDPESLQDR Fuc 1 Hex 4 HexNAc 5 | 63.559 | 0.002 | 0.000 | 0.000e+00 | 22 - 40 | 2 | - | - | - |
| 3798.5353 | NPN(N)ATSSSSQDPESLQDR Hex 4 HexNAc 6 | 53.804 | 0.002 | -21.533 | 7.250e+06 | 22 - 40 | 1 | 14.14 | 14.14 | 14.14 |
| 3231.3316 | NPN(N)ATSSSSQDPESLQDR Hex 3 HexNAc 4 | 45.337 | 0.003 | 0.000 | 0.000e+00 | 22 - 40 | 2 | - | - | - |
| 3613.4939 | NPN(N)ATSSSSQDPESLQDR Fuc 2 Hex 3 HexNAc 3 Neu5Ac 1 | 45.177 | 0.003 | -20.983 | 5.901e+05 | 22 - 40 | 2 | 30.06 | 30.06 | 30.06 |
| 3435.4015 | NPN(N)ATSSSSQDPESLQDR Hex 3 HexNAc 5 | 27.705 | 0.035 | 0.000 | 0.000e+00 | 22 - 40 | 1 | - | - | - |

### NPN(N)ATSSSSQDPESLQDR Hex 4 HexNAc 5

**Mass:**  3595.4566

(7.381e-07)

**Charge States:** 3, 4

**Start Position**: 23

**End Position**: 41

| Profile Score | Tandem Score | TDA q-value | Volume | Spectrum Matches |
| --- | --- | --- | --- | --- |
| -21.8436 | 143.9123 | 0.0000 | 4.4530e+07 | 6 |

**Adduct Signal Fractions**

- **Unmodified**: 4.4530e+07

### NPN(N)ATSSSSQDPESLQDR Hex 4 HexNAc 6

**Mass:**  3800.5444

(5.295e-04)

**Start Position**: 23

**End Position**: 41

| Profile Score | Tandem Score | TDA q-value | Volume | Spectrum Matches |
| --- | --- | --- | --- | --- |
| 0.0000 | 127.7063 | 0.0000 | 0.0000e+00 | 1 |

Adduct:

No Chromatogram Found

### NPN(N)ATSSSSQDPESLQDR Hex 3 HexNAc 4

**Mass:**  3230.3289

(9.298e-07)

**Charge States:** 3

**Start Position**: 23

**End Position**: 41

| Profile Score | Tandem Score | TDA q-value | Volume | Spectrum Matches |
| --- | --- | --- | --- | --- |
| 13.0858 | 113.3937 | 0.0000 | 4.1675e+07 | 1 |

**Adduct Signal Fractions**

- **Unmodified**: 4.1675e+07

### NPN(N)ATSSSSQDPESLQDR Fuc 1 Hex 3 HexNAc 4

**Mass:**  3376.3804

(4.195e-06)

**Charge States:** 3

**Start Position**: 23

**End Position**: 41

| Profile Score | Tandem Score | TDA q-value | Volume | Spectrum Matches |
| --- | --- | --- | --- | --- |
| -19.6201 | 111.7267 | 0.0000 | 2.0490e+07 | 4 |

**Adduct Signal Fractions**

- **Unmodified**: 2.0490e+07

### NPN(N)ATSSSSQDPESLQDR Hex 3 HexNAc 5

**Mass:**  3433.4031

(5.550e-07)

**Charge States:** 3

**Start Position**: 23

**End Position**: 41

| Profile Score | Tandem Score | TDA q-value | Volume | Spectrum Matches |
| --- | --- | --- | --- | --- |
| -4.7098 | 69.3271 | 0.0020 | 1.6526e+07 | 2 |

**Adduct Signal Fractions**

- **Unmodified**: 1.6526e+07

### NPN(N)ATSSSSQDPESLQDR Fuc 1 Hex 4 HexNAc 5

**Mass:**  3743.5183

(2.694e-04)

**Start Position**: 23

**End Position**: 41

| Profile Score | Tandem Score | TDA q-value | Volume | Spectrum Matches |
| --- | --- | --- | --- | --- |
| 0.0000 | 63.5587 | 0.0020 | 0.0000e+00 | 2 |

Adduct:

No Chromatogram Found

### NPN(N)ATSSSSQDPESLQDR Hex 4 HexNAc 6

**Mass:**  3798.5353

(5.343e-07)

**Charge States:** 4

**Start Position**: 23

**End Position**: 41

| Profile Score | Tandem Score | TDA q-value | Volume | Spectrum Matches |
| --- | --- | --- | --- | --- |
| -21.5330 | 53.8038 | 0.0020 | 7.2498e+06 | 1 |

**Adduct Signal Fractions**

- **Unmodified**: 7.2498e+06

### NPN(N)ATSSSSQDPESLQDR Hex 3 HexNAc 4

**Mass:**  3231.3316

(3.126e-04)

**Start Position**: 23

**End Position**: 41

| Profile Score | Tandem Score | TDA q-value | Volume | Spectrum Matches |
| --- | --- | --- | --- | --- |
| 0.0000 | 45.3365 | 0.0032 | 0.0000e+00 | 2 |

Adduct:

No Chromatogram Found

### NPN(N)ATSSSSQDPESLQDR Fuc 2 Hex 3 HexNAc 3 Neu5Ac 1

**Mass:**  3613.4939

(8.413e-04)

**Charge States:** 3

**Start Position**: 23

**End Position**: 41

| Profile Score | Tandem Score | TDA q-value | Volume | Spectrum Matches |
| --- | --- | --- | --- | --- |
| -20.9825 | 45.1770 | 0.0032 | 5.9014e+05 | 2 |

**Adduct Signal Fractions**

- **Unmodified**: 5.9014e+05

### NPN(N)ATSSSSQDPESLQDR Hex 3 HexNAc 5

**Mass:**  3435.4015

(5.826e-04)

**Start Position**: 23

**End Position**: 41

| Profile Score | Tandem Score | TDA q-value | Volume | Spectrum Matches |
| --- | --- | --- | --- | --- |
| 0.0000 | 27.7052 | 0.0346 | 0.0000e+00 | 1 |

Adduct:

No Chromatogram Found

### Q9Y5Y7|LYVE1\_HUMAN

**>Q9Y5Y7|LYVE1\_HUMAN**  
 `MARCFSLVLLLTSIWTTRLLVQGSLRAEELSIQVSCRIMGITLVSKKANQQLNFTEAKEACRLLGLSLAGKDQVETALKA  
SFETCSYGWVGDGFVVISRISPNPKCGKNGVGVLIWKVPVSRQFAAYCYNSSDTWTNSCIPEIITTKDPIFNTQTATQTT  
EFIVSDSTYSVASPYSTIPAPTTTPPAPASTSIPRRKKLICVTEVFMETSTMSTETEPFVENKAAFKNEAAGFGGVPTAL  
LVLALLFFGAAAGLGFCYVKRYVKAFPFTNKNQQKEMIETKVVKEEKANDSNPNEESKKTDKNPEESKSPSKTTVRCLEA  
EV`

| Site | Type | Count |
| --- | --- | --- |
| 53 | N-linked | 9 |

| Mass | Sequence | MS_2_ Score | `q`-value | MS_1_ Score | Signal | Protein Coordinates | Tandem Scans | Start Time | Apex Time | End Time |
| --- | --- | --- | --- | --- | --- | --- | --- | --- | --- | --- |
| 2892.2776 | KANQQLN(N)FTEAK Hex 3 HexNAc 5 | 181.410 | 0.0 | 13.327 | 6.046e+07 | 46 - 58 | 1 | 17.77 | 17.83 | 17.99 |
| 2764.1850 | ANQQLN(N)FTEAK Hex 3 HexNAc 5 | 143.219 | 0.0 | 13.427 | 1.764e+07 | 47 - 58 | 3 | 21.36 | 21.70 | 21.96 |
| 2893.2790 | KANQQLN(N)FTEAK Hex 3 HexNAc 5 | 138.280 | 0.0 | 0.000 | 0.000e+00 | 46 - 58 | 2 | - | - | - |
| 3257.4092 | KANQQLN(N)FTEAK Hex 4 HexNAc 6 | 125.127 | 0.0 | 16.093 | 1.625e+07 | 46 - 58 | 2 | 17.61 | 17.77 | 17.88 |
| 2561.1081 | ANQQLN(N)FTEAK Hex 3 HexNAc 4 | 124.579 | 0.0 | -4.820 | 4.969e+06 | 47 - 58 | 3 | 21.60 | 21.80 | 21.96 |
| 2689.1976 | KANQQLN(N)FTEAK Hex 3 HexNAc 4 | 110.358 | 0.0 | 13.369 | 1.823e+07 | 46 - 58 | 1 | 17.83 | 17.88 | 17.99 |
| 3494.4507 | ANQQLN(N)FTEAK Hex 5 HexNAc 7 | 33.969 | 0.010 | -4.617 | 1.788e+06 | 47 - 58 | 1 | 21.12 | 21.19 | 21.24 |
| 3129.3185 | ANQQLN(N)FTEAK Hex 4 HexNAc 6 | 30.650 | 0.019 | -4.267 | 5.276e+06 | 47 - 58 | 2 | 21.36 | 21.47 | 21.70 |
| 3624.5840 | KANQQLN(N)FTEAK Hex 5 HexNAc 7 | 29.995 | 0.021 | 5.952 | 1.055e+06 | 46 - 58 | 5 | 99.26 | 100.33 | 100.54 |

### KANQQLN(N)FTEAK Hex 3 HexNAc 5

**Mass:**  2892.2768

(8.202e-07)

**Charge States:** 3

**Start Position**: 47

**End Position**: 59

| Profile Score | Tandem Score | TDA q-value | Volume | Spectrum Matches |
| --- | --- | --- | --- | --- |
| 13.3268 | 181.4101 | 0.0000 | 6.0457e+07 | 1 |

**Adduct Signal Fractions**

- **Unmodified**: 6.0457e+07

### ANQQLN(N)FTEAK Hex 3 HexNAc 5

**Mass:**  2764.1837

(1.042e-06)

**Charge States:** 3

**Start Position**: 48

**End Position**: 59

| Profile Score | Tandem Score | TDA q-value | Volume | Spectrum Matches |
| --- | --- | --- | --- | --- |
| 13.4272 | 143.2187 | 0.0000 | 1.7643e+07 | 3 |

**Adduct Signal Fractions**

- **Unmodified**: 1.7643e+07

### KANQQLN(N)FTEAK Hex 3 HexNAc 5

**Mass:**  2893.2790

(6.945e-04)

**Start Position**: 47

**End Position**: 59

| Profile Score | Tandem Score | TDA q-value | Volume | Spectrum Matches |
| --- | --- | --- | --- | --- |
| 0.0000 | 138.2803 | 0.0000 | 0.0000e+00 | 2 |

Adduct:

No Chromatogram Found

### KANQQLN(N)FTEAK Hex 4 HexNAc 6

**Mass:**  3257.4105

(1.064e-06)

**Charge States:** 3

**Start Position**: 47

**End Position**: 59

| Profile Score | Tandem Score | TDA q-value | Volume | Spectrum Matches |
| --- | --- | --- | --- | --- |
| 16.0934 | 125.1272 | 0.0000 | 1.6252e+07 | 2 |

**Adduct Signal Fractions**

- **Unmodified**: 1.6252e+07

### ANQQLN(N)FTEAK Hex 3 HexNAc 4

**Mass:**  2561.1042

(1.074e-06)

**Charge States:** 3

**Start Position**: 48

**End Position**: 59

| Profile Score | Tandem Score | TDA q-value | Volume | Spectrum Matches |
| --- | --- | --- | --- | --- |
| -4.8200 | 124.5789 | 0.0000 | 4.9692e+06 | 3 |

**Adduct Signal Fractions**

- **Unmodified**: 4.9692e+06

### KANQQLN(N)FTEAK Hex 3 HexNAc 4

**Mass:**  2689.1980

(5.220e-07)

**Charge States:** 3

**Start Position**: 47

**End Position**: 59

| Profile Score | Tandem Score | TDA q-value | Volume | Spectrum Matches |
| --- | --- | --- | --- | --- |
| 13.3692 | 110.3584 | 0.0000 | 1.8235e+07 | 1 |

**Adduct Signal Fractions**

- **Unmodified**: 1.8235e+07

### ANQQLN(N)FTEAK Hex 5 HexNAc 7

**Mass:**  3494.4567

(3.032e-07)

**Charge States:** 3

**Start Position**: 48

**End Position**: 59

| Profile Score | Tandem Score | TDA q-value | Volume | Spectrum Matches |
| --- | --- | --- | --- | --- |
| -4.6166 | 33.9692 | 0.0097 | 1.7879e+06 | 1 |

**Adduct Signal Fractions**

- **Unmodified**: 1.7879e+06

### ANQQLN(N)FTEAK Hex 4 HexNAc 6

**Mass:**  3129.3191

(2.607e-07)

**Charge States:** 3

**Start Position**: 48

**End Position**: 59

| Profile Score | Tandem Score | TDA q-value | Volume | Spectrum Matches |
| --- | --- | --- | --- | --- |
| -4.2668 | 30.6496 | 0.0189 | 5.2758e+06 | 2 |

**Adduct Signal Fractions**

- **Unmodified**: 5.2758e+06

### KANQQLN(N)FTEAK Hex 5 HexNAc 7

**Mass:**  3624.5762

(5.552e-04)

**Charge States:** 3, 4

**Start Position**: 47

**End Position**: 59

| Profile Score | Tandem Score | TDA q-value | Volume | Spectrum Matches |
| --- | --- | --- | --- | --- |
| 5.9516 | 29.9955 | 0.0208 | 1.0547e+06 | 5 |

**Adduct Signal Fractions**

- **Unmodified**: 1.0547e+06

### Q12913|PTPRJ\_HUMAN

**>Q12913|PTPRJ\_HUMAN**  
 `MKPAAREARLPPRSPGLRWALPLLLLLLRLGQILCAGGTPSPIPDPSVATVATGENGITQISSTAESFHKQNGTGTPQVE  
TNTSEDGESSGANDSLRTPEQGSNGTDGASQKTPSSTGPSPVFDIKAVSISPTNVILTWKSNDTAASEYKYVVKHKMENE  
KTITVVHQPWCNITGLRPATSYVFSITPGIGNETWGDPRVIKVITEPIPVSDLRVALTGVRKAALSWSNGNGTASCRVLL  
ESIGSHEELTQDSRLQVNISGLKPGVQYNINPYLLQSNKTKGDPLGTEGGLDASNTERSRAGSPTAPVHDESLVGPVDPS  
SGQQSRDTEVLLVGLEPGTRYNATVYSQAANGTEGQPQAIEFRTNAIQVFDVTAVNISATSLTLIWKVSDNESSSNYTYK  
IHVAGETDSSNLNVSEPRAVIPGLRSSTFYNITVCPVLGDIEGTPGFLQVHTPPVPVSDFRVTVVSTTEIGLAWSSHDAE  
SFQMHITQEGAGNSRVEITTNQSIIIGGLFPGTKYCFEIVPKGPNGTEGASRTVCNRTVPSAVFDIHVVYVTTTEMWLDW  
KSPDGASEYVYHLVIESKHGSNHTSTYDKAITLQGLIPGTLYNITISPEVDHVWGDPNSTAQYTRPSNVSNIDVSTNTTA  
ATLSWQNFDDASPTYSYCLLIEKAGNSSNATQVVTDIGITDATVTELIPGSSYTVEIFAQVGDGIKSLEPGRKSFCTDPA  
SMASFDCEVVPKEPALVLKWTCPPGANAGFELEVSSGAWNNATHLESCSSENGTEYRTEVTYLNFSTSYNISITTVSCGK  
MAAPTRNTCTTGITDPPPPDGSPNITSVSHNSVKVKFSGFEASHGPIKAYAVILTTGEAGHPSADVLKYTYEDFKKGASD  
TYVTYLIRTEEKGRSQSLSEVLKYEIDVGNESTTLGYYNGKLEPLGSYRACVAGFTNITFHPQNKGLIDGAESYVSFSRY  
SDAVSLPQDPGVICGAVFGCIFGALVIVTVGGFIFWRKKRKDAKNNEVSFSQIKPKKSKLIRVENFEAYFKKQQADSNCG  
FAEEYEDLKLVGISQPKYAAELAENRGKNRYNNVLPYDISRVKLSVQTHSTDDYINANYMPGYHSKKDFIATQGPLPNTL  
KDFWRMVWEKNVYAIIMLTKCVEQGRTKCEEYWPSKQAQDYGDITVAMTSEIVLPEWTIRDFTVKNIQTSESHPLRQFHF  
TSWPDHGVPDTTDLLINFRYLVRDYMKQSPPESPILVHCSAGVGRTGTFIAIDRLIYQIENENTVDVYGIVYDLRMHRPL  
MVQTEDQYVFLNQCVLDIVRSQKDSKVDLIYQNTTAMTIYENLAPVTTFGKTNGYIA`

| Site | Type | Count |
| --- | --- | --- |
| 525 | N-linked | 1 |
| 413 | N-linked | 3 |

| Mass | Sequence | MS_2_ Score | `q`-value | MS_1_ Score | Signal | Protein Coordinates | Tandem Scans | Start Time | Apex Time | End Time |
| --- | --- | --- | --- | --- | --- | --- | --- | --- | --- | --- |
| 3425.4892 | IHVAGETDSSNLN(N)VSEPR Hex 3 HexNAc 5 | 135.168 | 0.0 | 14.605 | 3.860e+07 | 400 - 418 | 5 | 23.69 | 23.85 | 24.19 |
| 3767.5084 | GPN(N)GTEGASR Fuc 1 Hex 9 HexNAc 6 | 32.874 | 0.011 | -18.938 | 1.377e+07 | 522 - 532 | 1 | 33.58 | 33.75 | 33.75 |
| 3222.4098 | IHVAGETDSSNLN(N)VSEPR Hex 3 HexNAc 4 | 32.439 | 0.011 | 13.409 | 4.585e+06 | 400 - 418 | 3 | 23.85 | 23.96 | 24.16 |
| 3735.5921 | IHVAGETDSSNLN(N)VSEPR Fuc 1 Hex 4 HexNAc 5 | 26.983 | 0.037 | 0.000 | 0.000e+00 | 400 - 418 | 1 | - | - | - |

### IHVAGETDSSNLN(N)VSEPR Hex 3 HexNAc 5

**Mass:**  3425.4874

(1.090e-06)

**Charge States:** 3, 4

**Start Position**: 401

**End Position**: 419

| Profile Score | Tandem Score | TDA q-value | Volume | Spectrum Matches |
| --- | --- | --- | --- | --- |
| 14.6054 | 135.1684 | 0.0000 | 3.8600e+07 | 5 |

**Adduct Signal Fractions**

- **Unmodified**: 3.8600e+07

### GPN(N)GTEGASR Fuc 1 Hex 9 HexNAc 6

**Mass:**  3767.5297

(9.633e-06)

**Charge States:** 4

**Start Position**: 523

**End Position**: 533

| Profile Score | Tandem Score | TDA q-value | Volume | Spectrum Matches |
| --- | --- | --- | --- | --- |
| -18.9378 | 32.8740 | 0.0105 | 1.3771e+07 | 1 |

**Adduct Signal Fractions**

- **Unmodified**: 1.3771e+07

### IHVAGETDSSNLN(N)VSEPR Hex 3 HexNAc 4

**Mass:**  3222.4022

(2.130e-06)

**Charge States:** 3

**Start Position**: 401

**End Position**: 419

| Profile Score | Tandem Score | TDA q-value | Volume | Spectrum Matches |
| --- | --- | --- | --- | --- |
| 13.4089 | 32.4387 | 0.0105 | 4.5852e+06 | 3 |

**Adduct Signal Fractions**

- **Unmodified**: 4.5852e+06

### IHVAGETDSSNLN(N)VSEPR Fuc 1 Hex 4 HexNAc 5

**Mass:**  3735.5921

(5.350e-04)

**Start Position**: 401

**End Position**: 419

| Profile Score | Tandem Score | TDA q-value | Volume | Spectrum Matches |
| --- | --- | --- | --- | --- |
| 0.0000 | 26.9834 | 0.0374 | 0.0000e+00 | 1 |

Adduct:

No Chromatogram Found

### O95497|VNN1\_HUMAN

**>O95497|VNN1\_HUMAN**  
 `MTTQLPAYVAILLFYVSRASCQDTFTAAVYEHAAILPNATLTPVSREEALALMNRNLDILEGAITSAADQGAHIIVTPED  
AIYGWNFNRDSLYPYLEDIPDPEVNWIPCNNRNRFGQTPVQERLSCLAKNNSIYVVANIGDKKPCDTSDPQCPPDGRYQY  
NTDVVFDSQGKLVARYHKQNLFMGENQFNVPKEPEIVTFNTTFGSFGIFTCFDILFHDPAVTLVKDFHVDTIVFPTAWMN  
VLPHLSAVEFHSAWAMGMRVNFLASNIHYPSKKMTGSGIYAPNSSRAFHYDMKTEEGKLLLSQLDSHPSHSAVVNWTSYA  
SSIEALSSGNKEFKGTVFFDEFTFVKLTGVAGNYTVCQKDLCCHLSYKMSENIPNEVYALGAFDGLHTVEGRYYLQICTL  
LKCKTTNLNTCGDSAETASTRFEMFSLSGTFGTQYVFPEVLLSENQLAPGEFQVSTDGRLFSLKPTSGPVLTVTLFGRLY  
EKDWASNASSGLTAQARIIMLIVIAPIVCSLSW`

| Site | Type | Count |
| --- | --- | --- |
| 353 | N-linked | 3 |

| Mass | Sequence | MS_2_ Score | `q`-value | MS_1_ Score | Signal | Protein Coordinates | Tandem Scans | Start Time | Apex Time | End Time |
| --- | --- | --- | --- | --- | --- | --- | --- | --- | --- | --- |
| 2911.2557 | LTGVAGN(N)YTVC(C)QK Hex 3 HexNAc 5 | 104.050 | 0.0 | -4.075 | 5.713e+06 | 346 - 359 | 2 | 22.27 | 22.44 | 22.70 |
| 2708.1771 | LTGVAGN(N)YTVC(C)QK Hex 3 HexNAc 4 | 37.215 | 0.006 | -19.536 | 2.145e+06 | 346 - 359 | 1 | 22.44 | 22.56 | 22.56 |
| 3221.3734 | LTGVAGN(N)YTVC(C)QK Fuc 1 Hex 4 HexNAc 5 | 36.575 | 0.006 | 0.000 | 0.000e+00 | 346 - 359 | 1 | - | - | - |

### LTGVAGN(N)YTVC(C)QK Hex 3 HexNAc 5

**Mass:**  2911.2568

(8.976e-07)

**Charge States:** 3

**Start Position**: 347

**End Position**: 360

| Profile Score | Tandem Score | TDA q-value | Volume | Spectrum Matches |
| --- | --- | --- | --- | --- |
| -4.0755 | 104.0498 | 0.0000 | 5.7128e+06 | 2 |

**Adduct Signal Fractions**

- **Unmodified**: 5.7128e+06

### LTGVAGN(N)YTVC(C)QK Hex 3 HexNAc 4

**Mass:**  2708.1762

(1.816e-06)

**Charge States:** 3

**Start Position**: 347

**End Position**: 360

| Profile Score | Tandem Score | TDA q-value | Volume | Spectrum Matches |
| --- | --- | --- | --- | --- |
| -19.5363 | 37.2151 | 0.0059 | 2.1451e+06 | 1 |

**Adduct Signal Fractions**

- **Unmodified**: 2.1451e+06

### LTGVAGN(N)YTVC(C)QK Fuc 1 Hex 4 HexNAc 5

**Mass:**  3221.3734

(6.243e-04)

**Start Position**: 347

**End Position**: 360

| Profile Score | Tandem Score | TDA q-value | Volume | Spectrum Matches |
| --- | --- | --- | --- | --- |
| 0.0000 | 36.5753 | 0.0059 | 0.0000e+00 | 1 |

Adduct:

No Chromatogram Found

### P11279|LAMP1\_HUMAN

**>P11279|LAMP1\_HUMAN**  
 `MAAPGSARRPLLLLLLLLLLGLMHCASAAMFMVKNGNGTACIMANFSAAFSVNYDTKSGPKNMTFDLPSDATVVLNRSSC  
GKENTSDPSLVIAFGRGHTLTLNFTRNATRYSVQLMSFVYNLSDTHLFPNASSKEIKTVESITDIRADIDKKYRCVSGTQ  
VHMNNVTVTLHDATIQAYLSNSSFSRGETRCEQDRPSPTTAPPAPPSPSPSPVPKSPSVDKYNVSGTNGTCLLASMGLQL  
NLTYERKDNTTVTRLLNINPNKTSASGSCGAHLVTLELHSEGTTVLLFQFGMNASSSRFFLQGIQLNTILPDARDPAFKA  
ANGSLRALQATVGNSYKCNAEEHVRVTKAFSVNIFKVWVQAFKVEGGQFGSVEECLLDENSMLIPIAVGGALAGLVLIVL  
IAYLVGRKRSHAGYQTI`

| Site | Type | Count |
| --- | --- | --- |
| 103 | N-linked | 3 |

| Mass | Sequence | MS_2_ Score | `q`-value | MS_1_ Score | Signal | Protein Coordinates | Tandem Scans | Start Time | Apex Time | End Time |
| --- | --- | --- | --- | --- | --- | --- | --- | --- | --- | --- |
| 2578.1193 | GHTLTLN(N)FTR Hex 5 HexNAc 3 | 134.558 | 0.0 | 12.455 | 3.069e+06 | 96 - 106 | 2 | 30.06 | 30.23 | 30.75 |
| 2416.0683 | GHTLTLN(N)FTR Hex 4 HexNAc 3 | 125.828 | 0.0 | -4.589 | 2.171e+06 | 96 - 106 | 2 | 30.40 | 30.40 | 30.88 |
| 2457.0938 | GHTLTLN(N)FTR Hex 3 HexNAc 4 | 33.093 | 0.011 | -19.542 | 9.772e+05 | 96 - 106 | 1 | 30.57 | 30.57 | 30.75 |

### GHTLTLN(N)FTR Hex 5 HexNAc 3

**Mass:**  2578.1202

(1.324e-06)

**Charge States:** 3

**Start Position**: 97

**End Position**: 107

| Profile Score | Tandem Score | TDA q-value | Volume | Spectrum Matches |
| --- | --- | --- | --- | --- |
| 12.4554 | 134.5584 | 0.0000 | 3.0690e+06 | 2 |

**Adduct Signal Fractions**

- **Unmodified**: 3.0690e+06

### GHTLTLN(N)FTR Hex 4 HexNAc 3

**Mass:**  2416.0698

(2.415e-06)

**Charge States:** 3

**Start Position**: 97

**End Position**: 107

| Profile Score | Tandem Score | TDA q-value | Volume | Spectrum Matches |
| --- | --- | --- | --- | --- |
| -4.5892 | 125.8277 | 0.0000 | 2.1711e+06 | 2 |

**Adduct Signal Fractions**

- **Unmodified**: 2.1711e+06

### GHTLTLN(N)FTR Hex 3 HexNAc 4

**Mass:**  2457.0937

(1.290e-06)

**Charge States:** 3

**Start Position**: 97

**End Position**: 107

| Profile Score | Tandem Score | TDA q-value | Volume | Spectrum Matches |
| --- | --- | --- | --- | --- |
| -19.5424 | 33.0932 | 0.0105 | 9.7725e+05 | 1 |

**Adduct Signal Fractions**

- **Unmodified**: 9.7725e+05

### P21163|PNGF\_ELIMR

**>P21163|PNGF\_ELIMR**  
 `MRKLLIFSISAYLMAGIVSCKGVDSATPVTEDRLALNAVNAPADNTVNIKTFDKVKNAFGDGLSQSAEGTFTFPADVTTV  
KTIKMFIKNECPNKTCDEWDRYANVYVKNKTTGEWYEIGRFITPYWVGTEKLPRGLEIDVTDFKSLLSGNTELKIYTETW  
LAKGREYSVDFDIVYGTPDYKYSAVVPVIQYNKSSIDGVPYGKAHTLGLKKNIQLPTNTEKAYLRTTISGWGHAKPYDAG  
SRGCAEWCFRTHTIAINNANTFQHQLGALGCSANPINNQSPGNWAPDRAGWCPGMAVPTRIDVLNNSLTGSTFSYEYKFQ  
SWTNNGTNGDAFYAISSFVIAKSNTPISAPVVTN`

| Site | Type | Count |
| --- | --- | --- |
| 305 | N-linked | 1 |
| 93 | N-linked | 1 |

| Mass | Sequence | MS_2_ Score | `q`-value | MS_1_ Score | Signal | Protein Coordinates | Tandem Scans | Start Time | Apex Time | End Time |
| --- | --- | --- | --- | --- | --- | --- | --- | --- | --- | --- |
| 3949.6295 | IDVLN(N)NSLTG Fuc 1 Hex 7 HexNAc 8 | 40.761 | 0.003 | 14.097 | 1.007e+08 | 300 - 310 | 2 | 33.25 | 33.75 | 33.91 |
| 3657.4720 | MFIKNEC(C)PN(N)K Fuc 1 Hex 5 HexNAc 7 | 25.698 | 0.046 | 11.112 | 3.158e+08 | 84 - 94 | 4 | 33.91 | 34.07 | 35.05 |

### IDVLN(N)NSLTG Fuc 1 Hex 7 HexNAc 8

**Mass:**  3949.6262

(5.294e-06)

**Charge States:** 3

**Start Position**: 301

**End Position**: 311

| Profile Score | Tandem Score | TDA q-value | Volume | Spectrum Matches |
| --- | --- | --- | --- | --- |
| 14.0972 | 40.7608 | 0.0032 | 1.0068e+08 | 2 |

**Adduct Signal Fractions**

- **Unmodified**: 1.0068e+08

### MFIKNEC(C)PN(N)K Fuc 1 Hex 5 HexNAc 7

**Mass:**  3657.4714

(-2.479e-06)

**Charge States:** 4

**Start Position**: 85

**End Position**: 95

| Profile Score | Tandem Score | TDA q-value | Volume | Spectrum Matches |
| --- | --- | --- | --- | --- |
| 11.1119 | 25.6985 | 0.0464 | 3.1578e+08 | 4 |

**Adduct Signal Fractions**

- **Unmodified**: 3.1578e+08

### Q13201|MMRN1\_HUMAN

**>Q13201|MMRN1\_HUMAN**  
 `MKGARLFVLLSSLWSGGIGLNNSKHSWTIPEDGNSQKTMPSASVPPNKIQSLQILPTTRVMSAEIATTPEARTSEDSLLK  
STLPPSETSAPAEGVRNQTLTSTEKAEGVVKLQNLTLPTNASIKFNPGAESVVLSNSTLKFLQSFARKSNEQATSLNTVG  
GTGGIGGVGGTGGVGNRAPRETYLSRGDSSSSQRTDYQKSNFETTRGKNWCAYVHTRLSPTVILDNQVTYVPGGKGPCGW  
TGGSCPQRSQKISNPVYRMQHKIVTSLDWRCCPGYSGPKCQLRAQEQQSLIHTNQAESHTAVGRGVAEQQQQQGCGDPEV  
MQKMTDQVNYQAMKLTLLQKKIDNISLTVNDVRNTYSSLEGKVSEDKSREFQSLLKGLKSKSINVLIRDIVREQFKIFQN  
DMQETVAQLFKTVSSLSEDLESTRQIIQKVNESVVSIAAQQKFVLVQENRPTLTDIVELRNHIVNVRQEMTLTCEKPIKE  
LEVKQTHLEGALEQEHSRSILYYESLNKTLSKLKEVHEQLLSTEQVSDQKNAPAAESVSNNVTEYMSTLHENIKKQSLMM  
LQMFEDLHIQESKINNLTVSLEMEKESLRGECEDMLSKCRNDFKFQLKDTEENLHVLNQTLAEVLFPMDNKMDKMSEQLN  
DLTYDMEILQPLLEQGASLRQTMTYEQPKEAIVIRKKIENLTSAVNSLNFIIKELTKRHNLLRNEVQGRDDALERRINEY  
ALEMEDGLNKTMTIINNAIDFIQDNYALKETLSTIKDNSEIHHKCTSDMETILTFIPQFHRLNDSIQTLVNDNQRYNFVL  
QVAKTLAGIPRDEKLNQSNFQKMYQMFNETTSQVRKYQQNMSHLEEKLLLTTKISKNFETRLQDIESKVTQTLIPYYISV  
KKGSVVTNERDQALQLQVLNSRFKALEAKSIHLSINFFSLNKTLHEVLTMCHNASTSVSELNATIPKWIKHSLPDIQLLQ  
KGLTEFVEPIIQIKTQAALSNLTCCIDRSLPGSLANVVKSQKQVKSLPKKINALKKPTVNLTTVLIGRTQRNTDNIIYPE  
EYSSCSRHPCQNGGTCINGRTSFTCACRHPFTGDNCTIKLVEENALAPDFSKGSYRYAPMVAFFASHTYGMTIPGPILFN  
NLDVNYGASYTPRTGKFRIPYLGVYVFKYTIESFSAHISGFLVVDGIDKLAFESENINSEIHCDRVLTGDALLELNYGQE  
VWLRLAKGTIPAKFPPVTTFSGYLLYRT`

| Site | Type | Count |
| --- | --- | --- |
| 783 | N-linked | 1 |
| 136 | N-linked | 1 |

| Mass | Sequence | MS_2_ Score | `q`-value | MS_1_ Score | Signal | Protein Coordinates | Tandem Scans | Start Time | Apex Time | End Time |
| --- | --- | --- | --- | --- | --- | --- | --- | --- | --- | --- |
| 3309.4789 | FNPGAESVVLSN(N)STLK Fuc 1 Hex 3 HexNAc 5 | 58.463 | 0.002 | -19.703 | 3.744e+06 | 124 - 140 | 1 | 35.56 | 35.56 | 35.73 |
| 3292.4229 | LN(N)DSIQTLVNDNQR Hex 4 HexNAc 5 | 25.353 | 0.046 | -4.187 | 2.044e+07 | 781 - 795 | 1 | 42.47 | 42.64 | 42.80 |

### FNPGAESVVLSN(N)STLK Fuc 1 Hex 3 HexNAc 5

**Mass:**  3309.4861

(3.174e-06)

**Charge States:** 3

**Start Position**: 125

**End Position**: 141

| Profile Score | Tandem Score | TDA q-value | Volume | Spectrum Matches |
| --- | --- | --- | --- | --- |
| -19.7035 | 58.4628 | 0.0020 | 3.7438e+06 | 1 |

**Adduct Signal Fractions**

- **Unmodified**: 3.7438e+06

### LN(N)DSIQTLVNDNQR Hex 4 HexNAc 5

**Mass:**  3292.4223

(7.220e-07)

**Charge States:** 4

**Start Position**: 782

**End Position**: 796

| Profile Score | Tandem Score | TDA q-value | Volume | Spectrum Matches |
| --- | --- | --- | --- | --- |
| -4.1866 | 25.3533 | 0.0464 | 2.0442e+07 | 1 |

**Adduct Signal Fractions**

- **Unmodified**: 2.0442e+07

### P02790|HEMO\_HUMAN

**>P02790|HEMO\_HUMAN**  
 `MARVLGAPVALGLWSLCWSLAIATPLPPTSAHGNVAEGETKPDPDVTERCSDGWSFDATTLDDNGTMLFFKGEFVWKSHK  
WDRELISERWKNFPSPVDAAFRQGHNSVFLIKGDKVWVYPPEKKEKGYPKLLQDEFPGIPSPLDAAVECHRGECQAEGVL  
FFQGDREWFWDLATGTMKERSWPAVGNCSSALRWLGRYYCFQGNQFLRFDPVRGEVPPRYPRDVRDYFMPCPGRGHGHRN  
GTGHGNSTHHGPEYMRCSPHLVLSALTSDNHGATYAFSGTHYWRLDTSRDGWHSWPIAHQWPQGPSAVDAAFSWEEKLYL  
VQGTQVYVFLTKGGYTLVSGYPKRLEKEVGTPHGIILDSVDAAFICPGSSRLHIMAGRRLWWLDLKSGAQATWTELPWPH  
EKVDGALCMEKSLGPNSCSANGPGLYLIHGPNLYCYSDVEKLNAAKALPQPQNVTSLLGCTH`

| Site | Type | Count |
| --- | --- | --- |
| 187 | N-linked | 1 |
| 453 | N-linked | 1 |

| Mass | Sequence | MS_2_ Score | `q`-value | MS_1_ Score | Signal | Protein Coordinates | Tandem Scans | Start Time | Apex Time | End Time |
| --- | --- | --- | --- | --- | --- | --- | --- | --- | --- | --- |
| 3033.3511 | ALPQPQN(N)VTSLLGC(C)TH Hex 3 HexNAc 4 | 118.946 | 0.0 | 13.361 | 2.173e+07 | 446 - 462 | 3 | 44.44 | 44.78 | 45.27 |
| 2702.1396 | SWPAVGN(N)C(C)SSALR Hex 3 HexNAc 4 | 100.810 | 0.0 | -4.069 | 7.077e+06 | 180 - 193 | 2 | 31.38 | 31.55 | 31.88 |

### ALPQPQN(N)VTSLLGC(C)TH Hex 3 HexNAc 4

**Mass:**  3033.3521

(1.255e-06)

**Charge States:** 3

**Start Position**: 447

**End Position**: 463

| Profile Score | Tandem Score | TDA q-value | Volume | Spectrum Matches |
| --- | --- | --- | --- | --- |
| 13.3606 | 118.9462 | 0.0000 | 2.1726e+07 | 3 |

**Adduct Signal Fractions**

- **Unmodified**: 2.1726e+07

### SWPAVGN(N)C(C)SSALR Hex 3 HexNAc 4

**Mass:**  2702.1390

(1.371e-06)

**Charge States:** 3

**Start Position**: 181

**End Position**: 194

| Profile Score | Tandem Score | TDA q-value | Volume | Spectrum Matches |
| --- | --- | --- | --- | --- |
| -4.0692 | 100.8101 | 0.0000 | 7.0770e+06 | 2 |

**Adduct Signal Fractions**

- **Unmodified**: 7.0770e+06

### Q5R543|HEMO\_PONAB

**>Q5R543|HEMO\_PONAB**  
 `MARALGAPIALGLWSLCWSLAIATPLPPTSAHGNVAEGETKPDPDVTERCSDGWSFDATTLDDNGTMLFFKGEFVWKSHK  
WDRELISERWKNFPSPVDAAFRQGHNSVFLIKGDKVWVYPPEKKEKGYPKLLQDEFPGIPSPLDAAVECHRGECQAEGVL  
FFQGDREWFWDLATGTMKERSWPAVGNCSSALRWLGRYYCFQGNQFLRFDPVRGEVPPRYPRDVRDYFMPCPGRGHGHRN  
GTGHGNGTHHGPEYMRCSPHLVLSALTSDNHGATYAFSGTHYWRLDTSRDGWHSWPIAHQWPQGPSTVDAAFSWEEKLYL  
VQGTQVYVFLTKGGYTLVSGYPKQLEKEVGTPHGIILDSVDAAFICPGSSRLHIMAGRRLWWLDLKSGAQALWTELPWPH  
EKVDGALCVEKSLGPNSCSANGPGLYLIHGPNLYCYSDVEKLNAAKALPQPQNVTSLLGCTH`

| Site | Type | Count |
| --- | --- | --- |
| 187 | N-linked | 1 |
| 453 | N-linked | 1 |

| Mass | Sequence | MS_2_ Score | `q`-value | MS_1_ Score | Signal | Protein Coordinates | Tandem Scans | Start Time | Apex Time | End Time |
| --- | --- | --- | --- | --- | --- | --- | --- | --- | --- | --- |
| 3033.3511 | ALPQPQN(N)VTSLLGC(C)TH Hex 3 HexNAc 4 | 118.946 | 0.0 | 13.361 | 2.173e+07 | 446 - 462 | 3 | 44.44 | 44.78 | 45.27 |
| 2702.1396 | SWPAVGN(N)C(C)SSALR Hex 3 HexNAc 4 | 100.810 | 0.0 | -4.069 | 7.077e+06 | 180 - 193 | 2 | 31.38 | 31.55 | 31.88 |

### ALPQPQN(N)VTSLLGC(C)TH Hex 3 HexNAc 4

**Mass:**  3033.3521

(1.255e-06)

**Charge States:** 3

**Start Position**: 447

**End Position**: 463

| Profile Score | Tandem Score | TDA q-value | Volume | Spectrum Matches |
| --- | --- | --- | --- | --- |
| 13.3606 | 118.9462 | 0.0000 | 2.1726e+07 | 3 |

**Adduct Signal Fractions**

- **Unmodified**: 2.1726e+07

### SWPAVGN(N)C(C)SSALR Hex 3 HexNAc 4

**Mass:**  2702.1390

(1.371e-06)

**Charge States:** 3

**Start Position**: 181

**End Position**: 194

| Profile Score | Tandem Score | TDA q-value | Volume | Spectrum Matches |
| --- | --- | --- | --- | --- |
| -4.0692 | 100.8101 | 0.0000 | 7.0770e+06 | 2 |

**Adduct Signal Fractions**

- **Unmodified**: 7.0770e+06
