# Supplementary material for: Measuring Site-specific Glycosylation Similarity between Influenza a Virus Variants with Statistical Certainty
Source: Mol Cell Proteomics. 2020 Nov 25;19(9):1533–45. doi: 10.1074/mcp.RA120.002031 (PMC8143645; doi:10.1074/mcp.RA120.002031)
Supplement: Supplementary file 1 [file mmc1.zip › mmc1/159250_2_supp_555288_qcjkln.zip!/20190326_aAGPgal_021119_tryp_HILIC_DDA_1ug_02_glycopeptides.html]

## 20190326\_aAGPgal\_021119\_tryp\_HILIC\_DDA\_1ug\_02 @ GlycopeptideHypothesis-805cc742bfc448ae92d143aa41ab1866

**MS^1^ Mass Accuracy:** 10.0 PPM  
**Grouping Tolerance:** 15.0 PPM  
**MS^n^ Mass Accuracy:** 20.0 PPM  
**Minimum `q`-Value For Clustering:** 0.05

**Glycopeptide Hypothesis:**
GlycopeptideHypothesis-805cc742bfc448ae92d143aa41ab1866  
**Sample Searched:**
20190326\_aAGPgal\_021119\_tryp\_HILIC\_DDA\_1ug\_02

| Protein | Matched Glycopeptides |
| --- | --- |
| P19652\|A1AG2\_HUMAN | 157 |
| P02763\|A1AG1\_HUMAN | 152 |
| P00738\|HPT\_HUMAN | 11 |
| P05155\|IC1\_HUMAN | 8 |
| Q9Y5Y7\|LYVE1\_HUMAN | 7 |
| P02790\|HEMO\_HUMAN | 5 |
| Q5R543\|HEMO\_PONAB | 5 |
| P11279\|LAMP1\_HUMAN | 4 |
| Q12913\|PTPRJ\_HUMAN | 3 |
| O95497\|VNN1\_HUMAN | 3 |
| P21163\|PNGF\_ELIMR | 1 |
| Q13201\|MMRN1\_HUMAN | 1 |
| O75144\|ICOSL\_HUMAN | 1 |

### P19652|A1AG2\_HUMAN

**>P19652|A1AG2\_HUMAN**  
 `MALSWVLTVLSLLPLLEAQIPLCANLVPVPITNATLDRITGKWFYIASAFRNEEYNKSVQEIQATFFYFTPNKTEDTIFL  
REYQTRQNQCFYNSSYLNVQRENGTVSRYEGGREHVAHLLFLRDTKTLMFGSYLDDEKNWGLSFYADKPETTKEQLGEFY  
EALDCLCIPRSDVMYTDWKKDKCEPLEKQHEKERKQEEGES`

| Site | Type | Count |
| --- | --- | --- |
| 33 | N-linked | 54 |
| 56 | N-linked | 3 |
| 93 | N-linked | 27 |
| 72 | N-linked | 73 |

| Mass | Sequence | MS_2_ Score | `q`-value | MS_1_ Score | Signal | Protein Coordinates | Tandem Scans | Start Time | Apex Time | End Time |
| --- | --- | --- | --- | --- | --- | --- | --- | --- | --- | --- |
| 2269.9401 | YFTPN(N)K Hex 3 HexNAc 5 | 187.215 | 0.0 | 13.366 | 2.230e+08 | 67 - 73 | 9 | 13.78 | 13.92 | 14.48 |
| 2579.0434 | YFTPN(N)K Fuc 1 Hex 4 HexNAc 5 | 186.195 | 0.0 | 0.000 | 0.000e+00 | 67 - 73 | 1 | - | - | - |
| 2093.8165 | NEEYN(N)K Hex 3 HexNAc 4 | 184.819 | 0.0 | 11.704 | 4.780e+07 | 51 - 57 | 5 | 3.82 | 3.82 | 5.09 |
| 2620.0864 | FYFTPN(N)K Hex 3 HexNAc 6 | 182.864 | 0.0 | 13.086 | 1.141e+08 | 66 - 73 | 12 | 25.06 | 25.40 | 27.88 |
| 2296.8958 | NEEYN(N)K Hex 3 HexNAc 5 | 180.580 | 0.0 | 11.194 | 4.431e+07 | 51 - 57 | 5 | 3.82 | 3.99 | 5.33 |
| 2605.0064 | NEEYN(N)K Fuc 1 Hex 4 HexNAc 5 | 180.189 | 0.0 | 11.957 | 2.209e+07 | 51 - 57 | 4 | 3.82 | 3.82 | 5.06 |
| 3623.5856 | SVQEIQATFFYFTPN(N)K Hex 3 HexNAc 6 | 175.232 | 0.0 | -0.945 | 1.423e+09 | 57 - 73 | 25 | 51.28 | 55.19 | 58.73 |
| 3931.6992 | SVQEIQATFFYFTPN(N)K Fuc 1 Hex 4 HexNAc 6 | 174.868 | 0.0 | 13.709 | 3.813e+08 | 57 - 73 | 14 | 54.51 | 54.68 | 56.87 |
| 2066.8593 | YFTPN(N)K Hex 3 HexNAc 4 | 172.983 | 0.0 | -19.810 | 2.193e+07 | 67 - 73 | 3 | 13.92 | 14.08 | 14.08 |
| 2473.0185 | YFTPN(N)K Hex 3 HexNAc 6 | 172.576 | 0.0 | 14.581 | 3.867e+08 | 67 - 73 | 7 | 13.64 | 13.78 | 14.51 |
| 2838.1510 | YFTPN(N)K Hex 4 HexNAc 7 | 172.169 | 0.0 | -4.355 | 4.768e+07 | 67 - 73 | 3 | 13.35 | 13.52 | 13.78 |
| 2417.0067 | FYFTPN(N)K Hex 3 HexNAc 5 | 169.081 | 0.0 | 12.797 | 5.726e+07 | 66 - 73 | 9 | 25.40 | 25.73 | 27.58 |
| 2985.2208 | FYFTPN(N)K Hex 4 HexNAc 7 | 168.306 | 0.0 | 10.905 | 1.477e+07 | 66 - 73 | 5 | 24.29 | 24.89 | 25.73 |
| 3420.5049 | SVQEIQATFFYFTPN(N)K Hex 3 HexNAc 5 | 167.090 | 0.0 | 14.921 | 7.993e+08 | 57 - 73 | 31 | 51.45 | 55.35 | 59.75 |
| 2909.3664 | LVPVPITN(N)ATLDR Hex 3 HexNAc 5 | 166.413 | 0.0 | 10.589 | 1.558e+10 | 25 - 38 | 539 | 29.93 | 37.10 | 113.56 |
| 4239.8080 | SVQEIQATFFYFTPN(N)K Fuc 2 Hex 5 HexNAc 6 | 165.156 | 0.0 | 13.103 | 5.522e+07 | 57 - 73 | 4 | 54.00 | 54.34 | 55.02 |
| 3217.4801 | LVPVPITN(N)ATLDR Fuc 1 Hex 4 HexNAc 5 | 162.726 | 0.0 | 14.465 | 4.142e+09 | 25 - 38 | 207 | 29.59 | 36.64 | 113.21 |
| 2782.1409 | YFTPN(N)K Fuc 1 Hex 4 HexNAc 6 | 162.605 | 0.042 | -7.397 | 3.062e+06 | 67 - 73 | 5 | 24.72 | 25.90 | 25.90 |
| 3055.4239 | LVPVPITN(N)ATLDR Fuc 1 Hex 3 HexNAc 5 | 162.603 | 0.0 | 11.518 | 7.832e+07 | 25 - 38 | 3 | 36.95 | 37.25 | 37.55 |
| 2706.2922 | LVPVPITN(N)ATLDR Hex 3 HexNAc 4 | 162.434 | 0.0 | 14.529 | 1.224e+09 | 25 - 38 | 16 | 36.64 | 37.41 | 71.29 |
| 3728.6177 | SVQEIQATFFYFTPN(N)K Fuc 1 Hex 4 HexNAc 5 | 161.119 | 0.0 | 12.479 | 2.381e+08 | 57 - 73 | 8 | 54.68 | 54.84 | 56.19 |
| 2939.2408 | ATFFYFTPN(N)K Hex 3 HexNAc 6 | 160.392 | 0.0 | -3.612 | 4.537e+07 | 63 - 73 | 2 | 41.49 | 41.66 | 42.00 |
| 3988.7195 | SVQEIQATFFYFTPN(N)K Hex 4 HexNAc 7 | 160.097 | 0.0 | 10.904 | 2.915e+08 | 57 - 73 | 15 | 54.16 | 54.68 | 57.22 |
| 3071.4164 | LVPVPITN(N)ATLDR Hex 4 HexNAc 5 | 156.944 | 0.0 | -4.543 | 1.226e+08 | 25 - 38 | 2 | 36.49 | 36.80 | 36.95 |
| 3112.4443 | LVPVPITN(N)ATLDR Hex 3 HexNAc 6 | 155.702 | 0.0 | 13.006 | 3.643e+08 | 25 - 38 | 4 | 36.80 | 36.95 | 38.19 |
| 3217.4265 | SVQEIQATFFYFTPN(N)K Hex 3 HexNAc 4 | 155.631 | 0.0 | 13.230 | 9.872e+07 | 57 - 73 | 8 | 55.51 | 55.68 | 57.38 |
| 3420.5581 | LVPVPITN(N)ATLDR Fuc 1 Hex 4 HexNAc 6 | 153.526 | 0.0 | 13.602 | 2.582e+08 | 25 - 38 | 3 | 36.15 | 36.49 | 36.80 |
| 4296.8308 | SVQEIQATFFYFTPN(N)K Fuc 1 Hex 5 HexNAc 7 | 153.517 | 0.0 | 9.888 | 4.212e+07 | 57 - 73 | 5 | 53.66 | 54.16 | 55.02 |
| 3072.4348 | LVPVPITN(N)ATLDR Hex 4 HexNAc 5 | 151.182 | 0.0 | 0.000 | 0.000e+00 | 25 - 38 | 3 | - | - | - |
| 3448.5216 | YFTPN(N)KTEDTIFLR Hex 3 HexNAc 6 | 149.749 | 0.0 | 10.179 | 1.559e+09 | 67 - 81 | 11 | 35.64 | 37.55 | 39.69 |
| 4191.7997 | SVQEIQATFFYFTPN(N)K Hex 4 HexNAc 8 | 149.010 | 0.0 | 13.199 | 3.238e+07 | 57 - 73 | 3 | 54.00 | 54.16 | 54.84 |
| 3274.5010 | LVPVPITN(N)ATLDR Hex 4 HexNAc 6 | 145.048 | 0.0 | 14.263 | 8.407e+08 | 25 - 38 | 9 | 36.32 | 37.55 | 38.52 |
| 3477.5807 | LVPVPITN(N)ATLDR Hex 4 HexNAc 7 | 141.525 | 0.0 | -4.625 | 1.134e+08 | 25 - 38 | 4 | 36.15 | 36.49 | 36.64 |
| 2935.3090 | TPN(N)KTEDTIFLR Hex 3 HexNAc 5 | 140.244 | 0.0 | 12.248 | 4.117e+07 | 69 - 81 | 6 | 25.73 | 26.07 | 27.00 |
| 2796.2828 | VPVPITN(N)ATLDR Hex 3 HexNAc 5 | 138.495 | 0.0 | -2.535 | 7.309e+06 | 26 - 38 | 2 | 30.27 | 30.44 | 30.61 |
| 3756.6307 | YFTPN(N)KTEDTIFLR Fuc 1 Hex 4 HexNAc 6 | 137.450 | 0.0 | -4.168 | 2.883e+08 | 67 - 81 | 6 | 35.31 | 37.10 | 37.55 |
| 3553.5516 | YFTPN(N)KTEDTIFLR Fuc 1 Hex 4 HexNAc 5 | 137.246 | 0.0 | 9.728 | 2.117e+08 | 67 - 81 | 5 | 37.10 | 37.25 | 37.87 |
| 3785.6400 | SVQEIQATFFYFTPN(N)K Hex 4 HexNAc 6 | 137.025 | 0.0 | 10.509 | 4.957e+07 | 57 - 73 | 6 | 54.34 | 54.68 | 56.87 |
| 3254.4765 | C(C)ANLVPVPITN(N)ATLDR Hex 3 HexNAc 5 | 135.969 | 0.0 | 13.035 | 4.876e+08 | 22 - 38 | 9 | 42.34 | 53.83 | 54.51 |
| 3421.4110 | QNQC(C)FYN(N)SSYLNVQR Hex 3 HexNAc 5 | 135.537 | 0.0 | -3.710 | 5.294e+08 | 86 - 101 | 9 | 33.66 | 33.83 | 40.82 |
| 3275.4977 | LVPVPITN(N)ATLDR Hex 4 HexNAc 6 | 135.003 | 0.0 | 0.000 | 0.000e+00 | 25 - 38 | 1 | - | - | - |
| 2736.1603 | ATFFYFTPN(N)K Hex 3 HexNAc 5 | 133.952 | 0.0 | -3.869 | 2.179e+07 | 63 - 73 | 1 | 41.83 | 41.83 | 42.17 |
| 3044.2687 | ATFFYFTPN(N)K Fuc 1 Hex 4 HexNAc 5 | 132.987 | 0.0 | -4.865 | 6.005e+06 | 63 - 73 | 1 | 41.15 | 41.32 | 41.49 |
| 3525.5892 | LVPVPITN(N)ATLDR Fuc 2 Hex 5 HexNAc 5 | 132.929 | 0.0 | -3.629 | 5.457e+07 | 25 - 38 | 2 | 35.98 | 36.15 | 36.32 |
| 3961.6705 | SVQEIQATFFYFTPN(N)K Hex 4 HexNAc 4 Neu5Ac 2 | 131.731 | 0.0 | 12.840 | 5.306e+07 | 57 - 73 | 7 | 55.51 | 55.85 | 56.70 |
| 3151.3773 | PVPITN(N)ATLDR Fuc 2 Hex 4 HexNAc 5 | 131.199 | 0.0 | -5.618 | 1.182e+07 | 27 - 38 | 1 | 40.65 | 40.82 | 41.15 |
| 3138.3890 | TPN(N)KTEDTIFLR Hex 3 HexNAc 6 | 129.398 | 0.0 | 12.528 | 4.322e+07 | 69 - 81 | 5 | 25.57 | 25.90 | 26.25 |
| 3042.3629 | YFTPN(N)KTEDTIFLR Hex 3 HexNAc 4 | 129.080 | 0.0 | -2.987 | 9.010e+07 | 67 - 81 | 2 | 37.87 | 38.03 | 38.19 |
| 3247.3481 | ATFFYFTPN(N)K Fuc 1 Hex 4 HexNAc 6 | 126.835 | 0.0 | -20.785 | 5.852e+06 | 63 - 73 | 1 | 40.99 | 40.99 | 41.15 |
| 3932.6105 | QNQC(C)FYN(N)SSYLNVQR Fuc 1 Hex 4 HexNAc 6 | 126.831 | 0.0 | 11.366 | 4.755e+08 | 86 - 101 | 5 | 33.16 | 33.33 | 39.69 |
| 4066.7533 | YFTPN(N)KTEDTIFLR Fuc 2 Hex 5 HexNAc 6 | 126.562 | 0.0 | 0.000 | 0.000e+00 | 67 - 81 | 1 | - | - | - |
| 3813.6535 | YFTPN(N)KTEDTIFLR Hex 4 HexNAc 7 | 126.424 | 0.0 | 10.380 | 1.748e+08 | 67 - 81 | 6 | 36.32 | 36.95 | 37.87 |
| 3729.5256 | QNQC(C)FYN(N)SSYLNVQR Fuc 1 Hex 4 HexNAc 5 | 126.325 | 0.0 | -5.074 | 1.652e+08 | 86 - 101 | 3 | 33.33 | 33.50 | 40.02 |
| 2966.3885 | NLVPVPITN(N)ATLDR Fuc 1 Hex 3 HexNAc 4 | 125.890 | 0.0 | 15.411 | 1.613e+09 | 24 - 38 | 8 | 37.87 | 38.03 | 39.19 |
| 3781.5288 | ATFFYFTPN(N)K Fuc 3 Hex 8 HexNAc 4 | 124.314 | 0.0 | -21.053 | 9.055e+06 | 63 - 73 | 1 | 54.84 | 54.84 | 55.02 |
| 3624.4980 | QNQC(C)FYN(N)SSYLNVQR Hex 3 HexNAc 6 | 123.492 | 0.0 | -5.060 | 8.152e+08 | 86 - 101 | 2 | 33.50 | 33.66 | 33.98 |
| 3595.5910 | FYFTPN(N)KTEDTIFLR Hex 3 HexNAc 6 | 123.420 | 0.0 | 13.440 | 1.316e+08 | 66 - 81 | 6 | 46.31 | 46.65 | 47.85 |
| 4134.7791 | SVQEIQATFFYFTPN(N)K Fuc 1 Hex 4 HexNAc 7 | 122.553 | 0.0 | -4.524 | 1.261e+07 | 57 - 73 | 3 | 54.16 | 54.51 | 54.68 |
| 3069.3298 | PVPITN(N)ATLDR Fuc 2 Hex 6 HexNAc 3 | 121.032 | 0.0 | 0.000 | 0.000e+00 | 27 - 38 | 1 | - | - | - |
| 3266.4838 | LVPVPITN(N)ATLDR Fuc 1 Hex 5 HexNAc 3 Neu5Ac 1 | 121.014 | 0.0 | 0.000 | 0.000e+00 | 25 - 38 | 1 | - | - | - |
| 4442.8873 | SVQEIQATFFYFTPN(N)K Fuc 2 Hex 5 HexNAc 7 | 118.188 | 0.0 | -19.598 | 3.822e+06 | 57 - 73 | 1 | 54.00 | 54.00 | 54.16 |
| 3392.5107 | FYFTPN(N)KTEDTIFLR Hex 3 HexNAc 5 | 117.888 | 0.0 | 13.545 | 5.658e+07 | 66 - 81 | 5 | 46.48 | 46.82 | 47.50 |
| 3978.6944 | SVQEIQATFFYFTPN(N)K Fuc 1 Hex 5 HexNAc 4 Neu5Ac 1 | 116.217 | 0.0 | -4.937 | 5.887e+06 | 57 - 73 | 1 | 55.68 | 55.85 | 56.19 |
| 3104.3947 | VPVPITN(N)ATLDR Fuc 1 Hex 4 HexNAc 5 | 115.622 | 0.0 | -5.063 | 2.188e+06 | 26 - 38 | 1 | 29.76 | 29.93 | 30.10 |
| 3304.3766 | ATFFYFTPN(N)K Hex 4 HexNAc 7 | 115.403 | 0.0 | -19.822 | 3.428e+06 | 63 - 73 | 1 | 40.82 | 40.82 | 40.99 |
| 3245.4431 | YFTPN(N)KTEDTIFLR Hex 3 HexNAc 5 | 114.001 | 0.0 | 9.439 | 4.430e+07 | 67 - 81 | 5 | 35.81 | 35.98 | 38.85 |
| 3094.4480 | ANLVPVPITN(N)ATLDR Hex 3 HexNAc 5 | 113.956 | 0.0 | -3.582 | 1.636e+07 | 23 - 38 | 2 | 41.83 | 42.00 | 42.34 |
| 3562.5909 | C(C)ANLVPVPITN(N)ATLDR Fuc 1 Hex 4 HexNAc 5 | 112.450 | 0.0 | 10.626 | 6.195e+07 | 22 - 38 | 5 | 42.00 | 42.17 | 42.84 |
| 3381.5407 | LVPVPITN(N)ATLDR Fuc 1 Hex 5 HexNAc 5 | 111.124 | 0.0 | 0.000 | 0.000e+00 | 25 - 38 | 2 | - | - | - |
| 3903.7028 | FYFTPN(N)KTEDTIFLR Fuc 1 Hex 4 HexNAc 6 | 110.841 | 0.0 | 14.340 | 3.015e+07 | 66 - 81 | 4 | 45.97 | 46.14 | 46.65 |
| 3582.6163 | LVPVPITN(N)ATLDR Fuc 1 Hex 5 HexNAc 6 | 109.267 | 0.0 | -5.377 | 4.218e+07 | 25 - 38 | 3 | 35.81 | 36.49 | 36.49 |
| 3786.6070 | TPN(N)KTEDTIFLR Hex 7 HexNAc 6 | 108.999 | 0.0 | 13.601 | 1.059e+08 | 69 - 81 | 4 | 38.19 | 38.35 | 39.02 |
| 3331.5232 | NLVPVPITN(N)ATLDR Fuc 1 Hex 4 HexNAc 5 | 106.338 | 0.0 | -6.088 | 1.739e+07 | 24 - 38 | 2 | 37.55 | 37.71 | 40.99 |
| 3169.4683 | NLVPVPITN(N)ATLDR Fuc 1 Hex 3 HexNAc 5 | 106.206 | 0.0 | -18.374 | 3.764e+07 | 24 - 38 | 1 | 37.71 | 37.87 | 37.87 |
| 4178.7888 | YFTPN(N)KTEDTIFLR Hex 5 HexNAc 8 | 105.817 | 0.0 | -3.427 | 3.477e+07 | 67 - 81 | 3 | 35.98 | 36.15 | 36.32 |
| 3189.4312 | FYFTPN(N)KTEDTIFLR Hex 3 HexNAc 4 | 105.457 | 0.0 | 12.671 | 7.593e+06 | 66 - 81 | 2 | 46.82 | 46.99 | 47.50 |
| 3264.4781 | LVPVPITN(N)ATLDR Fuc 1 Hex 5 HexNAc 3 Neu5Ac 1 | 105.376 | 0.0 | -20.866 | 2.182e+07 | 25 - 38 | 1 | 37.87 | 37.87 | 38.03 |
| 4599.0880 | SVQEIQATFFYFTPN(N)KTEDTIFLR Hex 3 HexNAc 6 | 105.317 | 0.0 | -3.857 | 3.344e+07 | 57 - 81 | 4 | 60.74 | 63.12 | 64.47 |
| 4121.7665 | YFTPN(N)KTEDTIFLR Fuc 1 Hex 5 HexNAc 7 | 104.968 | 0.0 | -4.541 | 3.487e+07 | 67 - 81 | 2 | 35.98 | 36.49 | 36.49 |
| 4556.9328 | SVQEIQATFFYFTPN(N)K Hex 5 HexNAc 9 | 104.722 | 0.0 | -20.312 | 2.348e+06 | 57 - 73 | 1 | 53.49 | 53.49 | 53.49 |
| 4192.7144 | QNQC(C)FYN(N)SSYLNVQR Hex 4 HexNAc 8 | 104.693 | 0.0 | -5.891 | 4.067e+07 | 86 - 101 | 2 | 32.82 | 32.99 | 33.33 |
| 3700.6213 | FYFTPN(N)KTEDTIFLR Fuc 1 Hex 4 HexNAc 5 | 104.244 | 0.0 | -3.525 | 1.624e+07 | 66 - 81 | 4 | 46.14 | 46.31 | 46.65 |
| 3989.6337 | QNQC(C)FYN(N)SSYLNVQR Hex 4 HexNAc 7 | 103.034 | 0.0 | 9.430 | 1.828e+08 | 86 - 101 | 2 | 32.82 | 33.50 | 33.50 |
| 3082.3791 | FTPN(N)KTEDTIFLR Hex 3 HexNAc 5 | 102.515 | 0.0 | 13.867 | 4.658e+07 | 68 - 81 | 3 | 32.14 | 32.31 | 32.82 |
| 3094.3051 | FYN(N)SSYLNVQR Hex 3 HexNAc 6 | 102.113 | 0.0 | 16.412 | 8.541e+06 | 90 - 101 | 3 | 29.45 | 29.76 | 30.10 |
| 3728.6727 | LVPVPITN(N)ATLDR Fuc 2 Hex 5 HexNAc 6 | 101.261 | 0.0 | 10.280 | 2.615e+07 | 25 - 38 | 3 | 35.64 | 36.32 | 36.32 |
| 3285.4579 | FTPN(N)KTEDTIFLR Hex 3 HexNAc 6 | 98.657 | 0.0 | 14.168 | 9.115e+07 | 68 - 81 | 5 | 31.97 | 32.14 | 32.82 |
| 3390.4906 | FTPN(N)KTEDTIFLR Fuc 1 Hex 4 HexNAc 5 | 98.378 | 0.0 | -4.926 | 1.113e+07 | 68 - 81 | 2 | 31.80 | 31.80 | 32.14 |
| 3015.4057 | LVPVPITN(N)ATLDR Fuc 1 Hex 4 HexNAc 4 | 97.929 | 0.0 | 0.000 | 0.000e+00 | 25 - 38 | 1 | - | - | - |
| 2763.3086 | NLVPVPITN(N)ATLDR Fuc 1 Hex 3 HexNAc 3 | 97.742 | 0.0 | 13.977 | 1.231e+08 | 24 - 38 | 4 | 38.19 | 38.35 | 39.02 |
| 3023.4130 | NLVPVPITN(N)ATLDR Hex 3 HexNAc 5 | 97.553 | 0.0 | -4.449 | 1.008e+07 | 24 - 38 | 2 | 41.32 | 41.49 | 41.83 |
| 3785.6921 | LVPVPITN(N)ATLDR Fuc 1 Hex 5 HexNAc 7 | 97.230 | 0.0 | -19.390 | 1.108e+07 | 25 - 38 | 2 | 35.81 | 36.15 | 36.15 |
| 3255.4638 | C(C)AN(D)LVPVPITN(N)ATLDR Hex 3 HexNAc 5 | 88.921 | 0.0 | 7.474 | 6.502e+07 | 22 - 38 | 4 | 43.18 | 44.63 | 44.97 |
| 3960.7256 | FYFTPN(N)KTEDTIFLR Hex 4 HexNAc 7 | 87.605 | 0.002 | 14.095 | 2.316e+07 | 66 - 81 | 4 | 45.63 | 46.14 | 46.48 |
| 3593.5691 | FTPN(N)KTEDTIFLR Fuc 1 Hex 4 HexNAc 6 | 86.433 | 0.002 | -3.715 | 2.030e+07 | 68 - 81 | 4 | 31.46 | 31.63 | 31.97 |
| 2947.2377 | YN(N)SSYLNVQR Hex 3 HexNAc 6 | 85.311 | 0.002 | 12.903 | 1.642e+07 | 91 - 101 | 5 | 22.52 | 22.70 | 22.99 |
| 4381.8676 | YFTPN(N)KTEDTIFLR Hex 5 HexNAc 9 | 83.804 | 0.002 | -5.063 | 9.836e+06 | 67 - 81 | 1 | 35.81 | 35.98 | 36.15 |
| 3244.4133 | TPN(N)KTEDTIFLR Fuc 1 Hex 4 HexNAc 5 | 83.706 | 0.002 | 0.000 | 0.000e+00 | 69 - 81 | 1 | - | - | - |
| 4267.8264 | YFTPN(N)KTEDTIFLR Fuc 2 Hex 5 HexNAc 7 | 81.434 | 0.003 | -20.034 | 2.247e+06 | 67 - 81 | 1 | 36.32 | 36.32 | 36.32 |
| 3446.5007 | TPN(N)KTEDTIFLR Fuc 1 Hex 4 HexNAc 6 | 79.167 | 0.003 | -4.289 | 8.982e+06 | 69 - 81 | 4 | 25.23 | 25.40 | 25.57 |
| 3243.4221 | TPN(N)KTEDTIFLR Fuc 1 Hex 4 HexNAc 5 | 75.903 | 0.003 | -4.939 | 4.206e+06 | 69 - 81 | 3 | 25.40 | 25.57 | 25.73 |
| 4396.0178 | SVQEIQATFFYFTPN(N)KTEDTIFLR Hex 3 HexNAc 5 | 61.755 | 0.003 | -21.526 | 6.211e+06 | 57 - 81 | 1 | 63.29 | 63.46 | 63.46 |
| 3996.8449 | QIPLC(C)ANLVPVPITN(N)ATLDR Hex 3 HexNAc 5 Neu5Ac 1 | 61.142 | 0.003 | 15.738 | 6.015e+08 | 18 - 38 | 11 | 62.09 | 62.77 | 63.96 |
| 4240.7180 | QNQC(C)FYN(N)SSYLNVQR Fuc 2 Hex 5 HexNAc 6 | 55.283 | 0.003 | -18.665 | 2.387e+07 | 86 - 101 | 2 | 32.99 | 32.99 | 33.16 |
| 4922.9745 | QNQC(C)FYN(N)SSYLNVQR Fuc 5 Hex 4 HexNAc 8 | 48.450 | 0.003 | -3.591 | 2.797e+07 | 86 - 101 | 4 | 30.10 | 30.27 | 30.61 |
| 3583.5751 | SVQEIQATFFYFTPN(N)K Hex 4 HexNAc 5 | 47.932 | 0.003 | 0.000 | 0.000e+00 | 57 - 73 | 2 | - | - | - |
| 3051.3985 | C(C)ANLVPVPITN(N)ATLDR Hex 3 HexNAc 4 | 47.858 | 0.003 | -3.063 | 1.468e+07 | 22 - 38 | 2 | 42.67 | 42.84 | 43.18 |
| 4050.7625 | ANLVPVPITN(N)ATLDR Fuc 1 Hex 8 HexNAc 5 | 47.157 | 0.003 | -20.150 | 3.798e+07 | 23 - 38 | 2 | 62.77 | 62.77 | 62.95 |
| 4016.7357 | YFTPN(N)KTEDTIFLR Hex 4 HexNAc 8 | 45.141 | 0.003 | -18.338 | 1.841e+07 | 67 - 81 | 1 | 36.32 | 36.49 | 36.49 |
| 4909.2093 | SVQEIQATFFYFTPN(N)KTEDTIFLR Fuc 1 Hex 4 HexNAc 6 | 44.281 | 0.003 | 0.000 | 0.000e+00 | 57 - 81 | 1 | - | - | - |
| 3625.4817 | QN(D)QC(C)FYN(N)SSYLNVQR Hex 3 HexNAc 6 | 43.374 | 0.003 | -4.205 | 2.291e+08 | 86 - 101 | 3 | 34.63 | 34.80 | 40.34 |
| 4414.8760 | LVPVPITN(N)ATLDR Fuc 1 Hex 6 HexNAc 5 Neu5Ac 3 | 41.805 | 0.003 | -20.605 | 1.709e+06 | 25 - 38 | 1 | 45.47 | 45.47 | 45.63 |
| 3705.7545 | QIPLC(C)ANLVPVPITN(N)ATLDR Hex 3 HexNAc 5 | 41.135 | 0.003 | 13.990 | 5.194e+08 | 18 - 38 | 10 | 62.77 | 63.29 | 64.47 |
| 2879.2996 | FTPN(N)KTEDTIFLR Hex 3 HexNAc 4 | 40.741 | 0.003 | -5.006 | 5.810e+06 | 68 - 81 | 1 | 32.48 | 32.65 | 32.82 |
| 4704.1178 | SVQEIQATFFYFTPN(N)KTEDTIFLR Fuc 1 Hex 4 HexNAc 5 | 40.205 | 0.003 | -5.307 | 3.791e+06 | 57 - 81 | 1 | 62.77 | 62.95 | 63.12 |
| 4557.8462 | QNQC(C)FYN(N)SSYLNVQR Hex 5 HexNAc 9 | 40.160 | 0.003 | -19.008 | 7.797e+06 | 86 - 101 | 1 | 32.48 | 32.48 | 32.48 |
| 4353.8478 | SVQEIQATFFYFTPN(N)K Hex 5 HexNAc 8 | 40.081 | 0.003 | 7.688 | 2.414e+07 | 57 - 73 | 1 | 53.66 | 53.83 | 54.68 |
| 4268.8144 | LVPVPITN(N)ATLDR Hex 6 HexNAc 5 Neu5Ac 3 | 39.030 | 0.004 | 12.185 | 1.105e+07 | 25 - 38 | 1 | 45.47 | 45.63 | 46.14 |
| 4013.8672 | QIPLC(C)ANLVPVPITN(N)ATLDR Fuc 1 Hex 4 HexNAc 5 | 37.473 | 0.006 | 14.150 | 1.541e+08 | 18 - 38 | 5 | 62.26 | 62.77 | 63.29 |
| 3422.4006 | QN(D)QC(C)FYN(N)SSYLNVQR Hex 3 HexNAc 5 | 36.364 | 0.006 | 8.208 | 3.682e+07 | 86 - 101 | 2 | 40.99 | 40.99 | 42.17 |
| 4499.9048 | SVQEIQATFFYFTPN(N)K Fuc 1 Hex 5 HexNAc 8 | 36.043 | 0.006 | -6.327 | 6.475e+06 | 57 - 73 | 2 | 53.49 | 53.66 | 54.00 |
| 4601.0913 | SVQEIQATFFYFTPN(N)KTEDTIFLR Hex 3 HexNAc 6 | 35.675 | 0.008 | 0.000 | 0.000e+00 | 57 - 81 | 1 | - | - | - |
| 4211.8117 | FYFTPN(N)KTEDTIFLR Fuc 2 Hex 5 HexNAc 6 | 35.652 | 0.008 | -4.662 | 5.478e+06 | 66 - 81 | 1 | 45.63 | 45.80 | 45.97 |
| 3545.5640 | C(C)ANLVPVPITN(N)ATLDR Hex 3 HexNAc 5 Neu5Ac 1 | 35.513 | 0.008 | 16.439 | 2.833e+08 | 22 - 38 | 7 | 52.64 | 53.15 | 54.00 |
| 3997.8417 | QIPLC(C)ANLVPVPITN(N)ATLDR Fuc 2 Hex 3 HexNAc 5 | 35.225 | 0.010 | -19.849 | 1.198e+07 | 18 - 38 | 2 | 63.29 | 63.29 | 63.29 |
| 3650.5927 | FTPN(N)KTEDTIFLR Hex 4 HexNAc 7 | 33.870 | 0.013 | -4.590 | 1.137e+07 | 68 - 81 | 3 | 31.29 | 31.63 | 31.80 |
| 2732.2300 | TPN(N)KTEDTIFLR Hex 3 HexNAc 4 | 33.322 | 0.014 | 9.437 | 5.819e+06 | 69 - 81 | 2 | 26.25 | 26.30 | 26.64 |
| 3933.5966 | QN(D)QC(C)FYN(N)SSYLNVQR Fuc 1 Hex 4 HexNAc 6 | 33.081 | 0.014 | -5.467 | 6.022e+07 | 86 - 101 | 7 | 31.80 | 34.46 | 35.14 |
| 3962.5880 | QNQC(C)FYN(N)SSYLNVQR Hex 4 HexNAc 4 Neu5Ac 2 | 33.047 | 0.014 | -4.962 | 6.130e+07 | 86 - 101 | 2 | 34.29 | 34.46 | 34.80 |
| 3271.5039 | ANLVPVPITN(N)ATLDR Fuc 2 Hex 3 HexNAc 3 Neu5Ac 1 | 33.004 | 0.014 | 13.154 | 6.324e+07 | 23 - 38 | 3 | 42.17 | 42.51 | 43.01 |
| 3563.5717 | C(C)AN(D)LVPVPITN(N)ATLDR Fuc 1 Hex 4 HexNAc 5 | 32.579 | 0.014 | 7.938 | 2.722e+07 | 22 - 38 | 4 | 44.03 | 44.17 | 44.63 |
| 3130.4692 | NLVPVPITN(N)ATLDR Fuc 1 Hex 4 HexNAc 4 | 32.566 | 0.014 | 0.000 | 0.000e+00 | 24 - 38 | 1 | - | - | - |
| 3503.5199 | TPN(N)KTEDTIFLR Hex 4 HexNAc 7 | 32.532 | 0.014 | -5.497 | 5.471e+06 | 69 - 81 | 3 | 25.06 | 25.23 | 25.40 |
| 4500.8249 | QNQC(C)FYN(N)SSYLNVQR Fuc 1 Hex 5 HexNAc 8 | 32.229 | 0.018 | -20.804 | 4.442e+06 | 86 - 101 | 1 | 32.48 | 32.65 | 32.65 |
| 3293.4911 | NLVPVPITN(N)ATLDR Fuc 1 Hex 5 HexNAc 4 | 32.224 | 0.018 | -35.652 | 2.007e+06 | 24 - 38 | 1 | 52.64 | 52.64 | 52.81 |
| 3219.4861 | LVPVPITN(N)ATLDR Fuc 1 Hex 4 HexNAc 5 | 31.877 | 0.021 | 0.000 | 0.000e+00 | 25 - 38 | 1 | - | - | - |
| 4208.6726 | QN(D)QC(C)FYN(N)SSYLNVQR Fuc 2 Hex 3 HexNAc 6 Neu5Ac 1 | 31.569 | 0.022 | -21.210 | 1.484e+07 | 86 - 101 | 1 | 39.02 | 39.02 | 39.19 |
| 3235.4990 | LVPVPITN(N)ATLDR Hex 5 HexNAc 5 | 30.771 | 0.024 | -20.737 | 2.443e+05 | 25 - 38 | 25 | 57.73 | 57.73 | 57.73 |
| 7247.1773 | ITGKWFYIASAFRNEEYNKSVQEIQATFFYFTPN(N)K Fuc 2 Hex 8 HexNAc 7 | 30.444 | 0.025 | -19.776 | 1.605e+07 | 38 - 73 | 1 | 55.02 | 55.19 | 55.19 |
| 4006.6451 | QN(D)QC(C)FYN(N)SSYLNVQR Fuc 4 Hex 3 HexNAc 5 | 30.366 | 0.025 | 11.496 | 5.784e+07 | 86 - 101 | 2 | 32.99 | 33.50 | 33.83 |
| 5126.0503 | QNQC(C)FYN(N)SSYLNVQR Fuc 5 Hex 4 HexNAc 9 | 30.330 | 0.026 | 12.351 | 1.915e+07 | 86 - 101 | 4 | 29.76 | 29.93 | 30.61 |
| 2891.2255 | FYN(N)SSYLNVQR Hex 3 HexNAc 5 | 29.885 | 0.032 | -4.446 | 2.958e+06 | 90 - 101 | 1 | 29.76 | 29.93 | 30.10 |
| 3382.3950 | QC(C)FYN(N)SSYLNVQR Hex 3 HexNAc 6 | 28.696 | 0.037 | -20.169 | 5.127e+06 | 88 - 101 | 1 | 33.98 | 33.98 | 33.98 |
| 3901.6834 | FTPN(N)KTEDTIFLR Fuc 2 Hex 5 HexNAc 6 | 28.661 | 0.038 | -19.372 | 2.089e+06 | 68 - 81 | 2 | 31.29 | 31.29 | 31.29 |
| 3608.4564 | QC(C)FYN(N)SSYLNVQR Fuc 1 Hex 6 HexNAc 4 | 28.419 | 0.038 | 14.153 | 6.953e+08 | 88 - 101 | 2 | 40.50 | 40.65 | 42.00 |
| 2744.1591 | YN(N)SSYLNVQR Hex 3 HexNAc 5 | 28.406 | 0.038 | 13.401 | 7.260e+06 | 91 - 101 | 2 | 22.74 | 22.88 | 23.05 |
| 4271.8337 | LVPVPITN(N)ATLDR Fuc 2 Hex 6 HexNAc 5 Neu5Ac 2 | 28.165 | 0.038 | 0.000 | 0.000e+00 | 25 - 38 | 1 | - | - | - |
| 3256.5192 | LVPVPITN(N)ATLDR Fuc 1 Hex 8 HexNAc 2 | 27.715 | 0.039 | -19.454 | 4.857e+06 | 25 - 38 | 3 | 27.56 | 45.30 | 45.47 |
| 3331.4035 | YN(N)SSYLNVQR Fuc 4 Hex 3 HexNAc 5 | 27.696 | 0.039 | 0.000 | 0.000e+00 | 91 - 101 | 1 | - | - | - |
| 3402.4160 | FYN(N)SSYLNVQR Fuc 1 Hex 4 HexNAc 6 | 27.641 | 0.039 | 11.210 | 6.202e+06 | 90 - 101 | 5 | 29.11 | 29.31 | 29.45 |
| 3152.3977 | PVPITN(N)ATLDR Fuc 2 Hex 4 HexNAc 5 | 26.804 | 0.040 | 0.000 | 0.000e+00 | 27 - 38 | 1 | - | - | - |
| 3457.5532 | C(C)ANLVPVPITN(N)ATLDR Hex 3 HexNAc 6 | 26.650 | 0.044 | -19.610 | 1.891e+06 | 22 - 38 | 1 | 42.34 | 42.34 | 42.34 |
| 3948.5642 | QNQC(C)FYN(N)SSYLNVQR Hex 5 HexNAc 6 | 26.096 | 0.045 | -4.908 | 1.006e+08 | 86 - 101 | 4 | 39.53 | 39.69 | 40.02 |
| 3314.4538 | LVPVPITN(N)ATLDR Hex 8 HexNAc 3 | 25.540 | 0.045 | 0.000 | 0.000e+00 | 25 - 38 | 1 | - | - | - |
| 2949.3012 | PVPITN(N)ATLDR Fuc 2 Hex 4 HexNAc 4 | 25.431 | 0.045 | -21.703 | 1.405e+06 | 27 - 38 | 2 | 17.60 | 17.60 | 17.60 |
| 3517.5353 | PVPITN(N)ATLDR Fuc 2 Hex 5 HexNAc 6 | 25.385 | 0.045 | 0.000 | 0.000e+00 | 27 - 38 | 1 | - | - | - |
| 4211.7285 | QN(D)QC(C)FYN(N)SSYLNVQR Fuc 4 Hex 3 HexNAc 6 | 24.890 | 0.050 | 0.000 | 0.000e+00 | 86 - 101 | 1 | - | - | - |

### YFTPN(N)K Hex 3 HexNAc 5

**Mass:**  2269.9422

(1.594e-06)

**Charge States:** 2, 3

**Start Position**: 68

**End Position**: 74

| Profile Score | Tandem Score | TDA q-value | Volume | Spectrum Matches |
| --- | --- | --- | --- | --- |
| 13.3663 | 187.2151 | 0.0000 | 2.2302e+08 | 9 |

**Adduct Signal Fractions**

- **Unmodified**: 2.2302e+08

### YFTPN(N)K Fuc 1 Hex 4 HexNAc 5

**Mass:**  2579.0434

(3.866e-04)

**Start Position**: 68

**End Position**: 74

| Profile Score | Tandem Score | TDA q-value | Volume | Spectrum Matches |
| --- | --- | --- | --- | --- |
| 0.0000 | 186.1954 | 0.0000 | 0.0000e+00 | 1 |

Adduct:

No Chromatogram Found

### NEEYN(N)K Hex 3 HexNAc 4

**Mass:**  2093.8163

(1.414e-07)

**Charge States:** 2, 3

**Start Position**: 52

**End Position**: 58

| Profile Score | Tandem Score | TDA q-value | Volume | Spectrum Matches |
| --- | --- | --- | --- | --- |
| 11.7042 | 184.8191 | 0.0000 | 4.7797e+07 | 5 |

**Adduct Signal Fractions**

- **Unmodified**: 4.7797e+07

### FYFTPN(N)K Hex 3 HexNAc 6

**Mass:**  2620.0863

(1.117e-06)

**Charge States:** 2, 3

**Start Position**: 67

**End Position**: 74

| Profile Score | Tandem Score | TDA q-value | Volume | Spectrum Matches |
| --- | --- | --- | --- | --- |
| 13.0862 | 182.8640 | 0.0000 | 1.1413e+08 | 12 |

**Adduct Signal Fractions**

- **Unmodified**: 1.1413e+08

### NEEYN(N)K Hex 3 HexNAc 5

**Mass:**  2296.8964

(-1.101e-07)

**Charge States:** 2, 3

**Start Position**: 52

**End Position**: 58

| Profile Score | Tandem Score | TDA q-value | Volume | Spectrum Matches |
| --- | --- | --- | --- | --- |
| 11.1939 | 180.5800 | 0.0000 | 4.4311e+07 | 5 |

**Adduct Signal Fractions**

- **Unmodified**: 4.4311e+07

### NEEYN(N)K Fuc 1 Hex 4 HexNAc 5

**Mass:**  2605.0065

(1.851e-07)

**Charge States:** 3

**Start Position**: 52

**End Position**: 58

| Profile Score | Tandem Score | TDA q-value | Volume | Spectrum Matches |
| --- | --- | --- | --- | --- |
| 11.9571 | 180.1890 | 0.0000 | 2.2091e+07 | 4 |

**Adduct Signal Fractions**

- **Unmodified**: 2.2091e+07

### SVQEIQATFFYFTPN(N)K Hex 3 HexNAc 6

**Mass:**  3623.5863

(7.344e-07)

**Charge States:** 3, 4

**Start Position**: 58

**End Position**: 74

| Profile Score | Tandem Score | TDA q-value | Volume | Spectrum Matches |
| --- | --- | --- | --- | --- |
| -0.9451 | 175.2321 | 0.0000 | 1.4232e+09 | 25 |

**Adduct Signal Fractions**

- **Unmodified**: 1.4232e+09

### SVQEIQATFFYFTPN(N)K Fuc 1 Hex 4 HexNAc 6

**Mass:**  3931.7016

(1.984e-06)

**Charge States:** 3, 4

**Start Position**: 58

**End Position**: 74

| Profile Score | Tandem Score | TDA q-value | Volume | Spectrum Matches |
| --- | --- | --- | --- | --- |
| 13.7086 | 174.8684 | 0.0000 | 3.8134e+08 | 14 |

**Adduct Signal Fractions**

- **Unmodified**: 3.8134e+08

### YFTPN(N)K Hex 3 HexNAc 4

**Mass:**  2066.8582

(1.688e-06)

**Charge States:** 2, 3

**Start Position**: 68

**End Position**: 74

| Profile Score | Tandem Score | TDA q-value | Volume | Spectrum Matches |
| --- | --- | --- | --- | --- |
| -19.8102 | 172.9827 | 0.0000 | 2.1926e+07 | 3 |

**Adduct Signal Fractions**

- **Unmodified**: 2.1926e+07

### YFTPN(N)K Hex 3 HexNAc 6

**Mass:**  2473.0175

(2.379e-06)

**Charge States:** 2, 3

**Start Position**: 68

**End Position**: 74

| Profile Score | Tandem Score | TDA q-value | Volume | Spectrum Matches |
| --- | --- | --- | --- | --- |
| 14.5812 | 172.5765 | 0.0000 | 3.8669e+08 | 7 |

**Adduct Signal Fractions**

- **Unmodified**: 3.8669e+08

### YFTPN(N)K Hex 4 HexNAc 7

**Mass:**  2838.1541

(9.481e-07)

**Charge States:** 3

**Start Position**: 68

**End Position**: 74

| Profile Score | Tandem Score | TDA q-value | Volume | Spectrum Matches |
| --- | --- | --- | --- | --- |
| -4.3547 | 172.1686 | 0.0000 | 4.7675e+07 | 3 |

**Adduct Signal Fractions**

- **Unmodified**: 4.7675e+07

### FYFTPN(N)K Hex 3 HexNAc 5

**Mass:**  2417.0075

(1.266e-06)

**Charge States:** 2, 3

**Start Position**: 67

**End Position**: 74

| Profile Score | Tandem Score | TDA q-value | Volume | Spectrum Matches |
| --- | --- | --- | --- | --- |
| 12.7972 | 169.0813 | 0.0000 | 5.7258e+07 | 9 |

**Adduct Signal Fractions**

- **Unmodified**: 5.7258e+07

### FYFTPN(N)K Hex 4 HexNAc 7

**Mass:**  2985.2208

(1.493e-06)

**Charge States:** 3

**Start Position**: 67

**End Position**: 74

| Profile Score | Tandem Score | TDA q-value | Volume | Spectrum Matches |
| --- | --- | --- | --- | --- |
| 10.9049 | 168.3064 | 0.0000 | 1.4771e+07 | 5 |

**Adduct Signal Fractions**

- **Unmodified**: 1.4771e+07

### SVQEIQATFFYFTPN(N)K Hex 3 HexNAc 5

**Mass:**  3420.5048

(8.557e-07)

**Charge States:** 2, 3, 4

**Start Position**: 58

**End Position**: 74

| Profile Score | Tandem Score | TDA q-value | Volume | Spectrum Matches |
| --- | --- | --- | --- | --- |
| 14.9206 | 167.0901 | 0.0000 | 7.9934e+08 | 31 |

**Adduct Signal Fractions**

- **Unmodified**: 7.9934e+08

### LVPVPITN(N)ATLDR Hex 3 HexNAc 5

**Mass:**  2909.3664

(5.107e-07)

**Charge States:** 2, 3, 4

**Start Position**: 26

**End Position**: 39

| Profile Score | Tandem Score | TDA q-value | Volume | Spectrum Matches |
| --- | --- | --- | --- | --- |
| 10.5888 | 166.4130 | 0.0000 | 1.5578e+10 | 539 |

**Adduct Signal Fractions**

- **Unmodified**: 1.5578e+10

### SVQEIQATFFYFTPN(N)K Fuc 2 Hex 5 HexNAc 6

**Mass:**  4239.8088

(1.451e-06)

**Charge States:** 3, 4

**Start Position**: 58

**End Position**: 74

| Profile Score | Tandem Score | TDA q-value | Volume | Spectrum Matches |
| --- | --- | --- | --- | --- |
| 13.1026 | 165.1560 | 0.0000 | 5.5220e+07 | 4 |

**Adduct Signal Fractions**

- **Unmodified**: 5.5220e+07

### LVPVPITN(N)ATLDR Fuc 1 Hex 4 HexNAc 5

**Mass:**  3217.4752

(2.637e-06)

**Charge States:** 2, 3, 4

**Start Position**: 26

**End Position**: 39

| Profile Score | Tandem Score | TDA q-value | Volume | Spectrum Matches |
| --- | --- | --- | --- | --- |
| 14.4652 | 162.7258 | 0.0000 | 4.1418e+09 | 207 |

**Adduct Signal Fractions**

- **Unmodified**: 4.1418e+09

### YFTPN(N)K Fuc 1 Hex 4 HexNAc 6

**Mass:**  2782.1365

(3.621e-04)

**Charge States:** 3

**Start Position**: 68

**End Position**: 74

| Profile Score | Tandem Score | TDA q-value | Volume | Spectrum Matches |
| --- | --- | --- | --- | --- |
| -7.3969 | 162.6053 | 0.0417 | 3.0620e+06 | 5 |

**Adduct Signal Fractions**

- **Unmodified**: 3.0620e+06

### LVPVPITN(N)ATLDR Fuc 1 Hex 3 HexNAc 5

**Mass:**  3055.4221

(1.195e-07)

**Charge States:** 3

**Start Position**: 26

**End Position**: 39

| Profile Score | Tandem Score | TDA q-value | Volume | Spectrum Matches |
| --- | --- | --- | --- | --- |
| 11.5176 | 162.6031 | 0.0000 | 7.8319e+07 | 3 |

**Adduct Signal Fractions**

- **Unmodified**: 7.8319e+07

### LVPVPITN(N)ATLDR Hex 3 HexNAc 4

**Mass:**  2706.2856

(9.250e-07)

**Charge States:** 2, 3, 4

**Start Position**: 26

**End Position**: 39

| Profile Score | Tandem Score | TDA q-value | Volume | Spectrum Matches |
| --- | --- | --- | --- | --- |
| 14.5295 | 162.4339 | 0.0000 | 1.2238e+09 | 16 |

**Adduct Signal Fractions**

- **Unmodified**: 1.2238e+09

### SVQEIQATFFYFTPN(N)K Fuc 1 Hex 4 HexNAc 5

**Mass:**  3728.6188

(9.458e-07)

**Charge States:** 3, 4

**Start Position**: 58

**End Position**: 74

| Profile Score | Tandem Score | TDA q-value | Volume | Spectrum Matches |
| --- | --- | --- | --- | --- |
| 12.4788 | 161.1189 | 0.0000 | 2.3809e+08 | 8 |

**Adduct Signal Fractions**

- **Unmodified**: 2.3809e+08

### ATFFYFTPN(N)K Hex 3 HexNAc 6

**Mass:**  2939.2414

(1.170e-06)

**Charge States:** 3

**Start Position**: 64

**End Position**: 74

| Profile Score | Tandem Score | TDA q-value | Volume | Spectrum Matches |
| --- | --- | --- | --- | --- |
| -3.6124 | 160.3925 | 0.0000 | 4.5366e+07 | 2 |

**Adduct Signal Fractions**

- **Unmodified**: 4.5366e+07

### SVQEIQATFFYFTPN(N)K Hex 4 HexNAc 7

**Mass:**  3988.7202

(1.515e-06)

**Charge States:** 3, 4

**Start Position**: 58

**End Position**: 74

| Profile Score | Tandem Score | TDA q-value | Volume | Spectrum Matches |
| --- | --- | --- | --- | --- |
| 10.9044 | 160.0969 | 0.0000 | 2.9148e+08 | 15 |

**Adduct Signal Fractions**

- **Unmodified**: 2.9148e+08

### LVPVPITN(N)ATLDR Hex 4 HexNAc 5

**Mass:**  3071.4138

(-5.901e-07)

**Charge States:** 3

**Start Position**: 26

**End Position**: 39

| Profile Score | Tandem Score | TDA q-value | Volume | Spectrum Matches |
| --- | --- | --- | --- | --- |
| -4.5431 | 156.9441 | 0.0000 | 1.2263e+08 | 2 |

**Adduct Signal Fractions**

- **Unmodified**: 1.2263e+08

### LVPVPITN(N)ATLDR Hex 3 HexNAc 6

**Mass:**  3112.4441

(1.514e-07)

**Charge States:** 3, 4

**Start Position**: 26

**End Position**: 39

| Profile Score | Tandem Score | TDA q-value | Volume | Spectrum Matches |
| --- | --- | --- | --- | --- |
| 13.0058 | 155.7021 | 0.0000 | 3.6433e+08 | 4 |

**Adduct Signal Fractions**

- **Unmodified**: 3.6433e+08

### SVQEIQATFFYFTPN(N)K Hex 3 HexNAc 4

**Mass:**  3217.4254

(1.290e-06)

**Charge States:** 3, 4

**Start Position**: 58

**End Position**: 74

| Profile Score | Tandem Score | TDA q-value | Volume | Spectrum Matches |
| --- | --- | --- | --- | --- |
| 13.2305 | 155.6313 | 0.0000 | 9.8721e+07 | 8 |

**Adduct Signal Fractions**

- **Unmodified**: 9.8721e+07

### LVPVPITN(N)ATLDR Fuc 1 Hex 4 HexNAc 6

**Mass:**  3420.5564

(1.119e-06)

**Charge States:** 3, 4

**Start Position**: 26

**End Position**: 39

| Profile Score | Tandem Score | TDA q-value | Volume | Spectrum Matches |
| --- | --- | --- | --- | --- |
| 13.6016 | 153.5265 | 0.0000 | 2.5820e+08 | 3 |

**Adduct Signal Fractions**

- **Unmodified**: 2.5820e+08

### SVQEIQATFFYFTPN(N)K Fuc 1 Hex 5 HexNAc 7

**Mass:**  4296.8308

(1.188e-06)

**Charge States:** 4

**Start Position**: 58

**End Position**: 74

| Profile Score | Tandem Score | TDA q-value | Volume | Spectrum Matches |
| --- | --- | --- | --- | --- |
| 9.8877 | 153.5174 | 0.0000 | 4.2117e+07 | 5 |

**Adduct Signal Fractions**

- **Unmodified**: 4.2117e+07

### LVPVPITN(N)ATLDR Hex 4 HexNAc 5

**Mass:**  3072.4348

(3.241e-04)

**Start Position**: 26

**End Position**: 39

| Profile Score | Tandem Score | TDA q-value | Volume | Spectrum Matches |
| --- | --- | --- | --- | --- |
| 0.0000 | 151.1818 | 0.0000 | 0.0000e+00 | 3 |

Adduct:

No Chromatogram Found

### YFTPN(N)KTEDTIFLR Hex 3 HexNAc 6

**Mass:**  3448.5232

(3.755e-07)

**Charge States:** 3, 4

**Start Position**: 68

**End Position**: 82

| Profile Score | Tandem Score | TDA q-value | Volume | Spectrum Matches |
| --- | --- | --- | --- | --- |
| 10.1789 | 149.7491 | 0.0000 | 1.5590e+09 | 11 |

**Adduct Signal Fractions**

- **Unmodified**: 1.5590e+09

### SVQEIQATFFYFTPN(N)K Hex 4 HexNAc 8

**Mass:**  4191.7990

(5.295e-07)

**Charge States:** 3, 4

**Start Position**: 58

**End Position**: 74

| Profile Score | Tandem Score | TDA q-value | Volume | Spectrum Matches |
| --- | --- | --- | --- | --- |
| 13.1992 | 149.0098 | 0.0000 | 3.2383e+07 | 3 |

**Adduct Signal Fractions**

- **Unmodified**: 3.2383e+07

### LVPVPITN(N)ATLDR Hex 4 HexNAc 6

**Mass:**  3274.5020

(1.255e-06)

**Charge States:** 3, 4

**Start Position**: 26

**End Position**: 39

| Profile Score | Tandem Score | TDA q-value | Volume | Spectrum Matches |
| --- | --- | --- | --- | --- |
| 14.2633 | 145.0483 | 0.0000 | 8.4074e+08 | 9 |

**Adduct Signal Fractions**

- **Unmodified**: 8.4074e+08

### LVPVPITN(N)ATLDR Hex 4 HexNAc 7

**Mass:**  3477.5784

(3.092e-06)

**Charge States:** 3, 4

**Start Position**: 26

**End Position**: 39

| Profile Score | Tandem Score | TDA q-value | Volume | Spectrum Matches |
| --- | --- | --- | --- | --- |
| -4.6252 | 141.5250 | 0.0000 | 1.1343e+08 | 4 |

**Adduct Signal Fractions**

- **Unmodified**: 1.1343e+08

### TPN(N)KTEDTIFLR Hex 3 HexNAc 5

**Mass:**  2935.3077

(8.054e-07)

**Charge States:** 3, 4

**Start Position**: 70

**End Position**: 82

| Profile Score | Tandem Score | TDA q-value | Volume | Spectrum Matches |
| --- | --- | --- | --- | --- |
| 12.2476 | 140.2442 | 0.0000 | 4.1170e+07 | 6 |

**Adduct Signal Fractions**

- **Unmodified**: 4.1170e+07

### VPVPITN(N)ATLDR Hex 3 HexNAc 5

**Mass:**  2796.2841

(2.033e-07)

**Charge States:** 3

**Start Position**: 27

**End Position**: 39

| Profile Score | Tandem Score | TDA q-value | Volume | Spectrum Matches |
| --- | --- | --- | --- | --- |
| -2.5355 | 138.4945 | 0.0000 | 7.3087e+06 | 2 |

**Adduct Signal Fractions**

- **Unmodified**: 7.3087e+06

### YFTPN(N)KTEDTIFLR Fuc 1 Hex 4 HexNAc 6

**Mass:**  3756.6325

(8.290e-07)

**Charge States:** 3, 4

**Start Position**: 68

**End Position**: 82

| Profile Score | Tandem Score | TDA q-value | Volume | Spectrum Matches |
| --- | --- | --- | --- | --- |
| -4.1684 | 137.4501 | 0.0000 | 2.8828e+08 | 6 |

**Adduct Signal Fractions**

- **Unmodified**: 2.8828e+08

### YFTPN(N)KTEDTIFLR Fuc 1 Hex 4 HexNAc 5

**Mass:**  3553.5496

(1.196e-06)

**Charge States:** 3, 4

**Start Position**: 68

**End Position**: 82

| Profile Score | Tandem Score | TDA q-value | Volume | Spectrum Matches |
| --- | --- | --- | --- | --- |
| 9.7284 | 137.2462 | 0.0000 | 2.1169e+08 | 5 |

**Adduct Signal Fractions**

- **Unmodified**: 2.1169e+08

### SVQEIQATFFYFTPN(N)K Hex 4 HexNAc 6

**Mass:**  3785.6447

(7.374e-07)

**Charge States:** 3, 4

**Start Position**: 58

**End Position**: 74

| Profile Score | Tandem Score | TDA q-value | Volume | Spectrum Matches |
| --- | --- | --- | --- | --- |
| 10.5093 | 137.0246 | 0.0000 | 4.9567e+07 | 6 |

**Adduct Signal Fractions**

- **Unmodified**: 4.9567e+07

### C(C)ANLVPVPITN(N)ATLDR Hex 3 HexNAc 5

**Mass:**  3254.4761

(1.090e-06)

**Charge States:** 3, 4

**Start Position**: 23

**End Position**: 39

| Profile Score | Tandem Score | TDA q-value | Volume | Spectrum Matches |
| --- | --- | --- | --- | --- |
| 13.0350 | 135.9690 | 0.0000 | 4.8759e+08 | 9 |

**Adduct Signal Fractions**

- **Unmodified**: 4.8759e+08

### QNQC(C)FYN(N)SSYLNVQR Hex 3 HexNAc 5

**Mass:**  3421.4159

(1.224e-06)

**Charge States:** 3, 4

**Start Position**: 87

**End Position**: 102

| Profile Score | Tandem Score | TDA q-value | Volume | Spectrum Matches |
| --- | --- | --- | --- | --- |
| -3.7097 | 135.5374 | 0.0000 | 5.2942e+08 | 9 |

**Adduct Signal Fractions**

- **Unmodified**: 5.2942e+08

### LVPVPITN(N)ATLDR Hex 4 HexNAc 6

**Mass:**  3275.4977

(3.059e-04)

**Start Position**: 26

**End Position**: 39

| Profile Score | Tandem Score | TDA q-value | Volume | Spectrum Matches |
| --- | --- | --- | --- | --- |
| 0.0000 | 135.0029 | 0.0000 | 0.0000e+00 | 1 |

Adduct:

No Chromatogram Found

### ATFFYFTPN(N)K Hex 3 HexNAc 5

**Mass:**  2736.1604

(7.255e-07)

**Charge States:** 3

**Start Position**: 64

**End Position**: 74

| Profile Score | Tandem Score | TDA q-value | Volume | Spectrum Matches |
| --- | --- | --- | --- | --- |
| -3.8688 | 133.9521 | 0.0000 | 2.1791e+07 | 1 |

**Adduct Signal Fractions**

- **Unmodified**: 2.1791e+07

### ATFFYFTPN(N)K Fuc 1 Hex 4 HexNAc 5

**Mass:**  3044.2701

(-1.063e-07)

**Charge States:** 3

**Start Position**: 64

**End Position**: 74

| Profile Score | Tandem Score | TDA q-value | Volume | Spectrum Matches |
| --- | --- | --- | --- | --- |
| -4.8647 | 132.9871 | 0.0000 | 6.0052e+06 | 1 |

**Adduct Signal Fractions**

- **Unmodified**: 6.0052e+06

### LVPVPITN(N)ATLDR Fuc 2 Hex 5 HexNAc 5

**Mass:**  3525.5890

(1.077e-06)

**Charge States:** 4

**Start Position**: 26

**End Position**: 39

| Profile Score | Tandem Score | TDA q-value | Volume | Spectrum Matches |
| --- | --- | --- | --- | --- |
| -3.6291 | 132.9291 | 0.0000 | 5.4569e+07 | 2 |

**Adduct Signal Fractions**

- **Unmodified**: 5.4569e+07

### SVQEIQATFFYFTPN(N)K Hex 4 HexNAc 4 Neu5Ac 2

**Mass:**  3961.6713

(1.363e-06)

**Charge States:** 3, 4

**Start Position**: 58

**End Position**: 74

| Profile Score | Tandem Score | TDA q-value | Volume | Spectrum Matches |
| --- | --- | --- | --- | --- |
| 12.8404 | 131.7311 | 0.0000 | 5.3062e+07 | 7 |

**Adduct Signal Fractions**

- **Unmodified**: 5.3062e+07

### PVPITN(N)ATLDR Fuc 2 Hex 4 HexNAc 5

**Mass:**  3151.3749

(3.153e-07)

**Charge States:** 3

**Start Position**: 28

**End Position**: 39

| Profile Score | Tandem Score | TDA q-value | Volume | Spectrum Matches |
| --- | --- | --- | --- | --- |
| -5.6177 | 131.1994 | 0.0000 | 1.1818e+07 | 1 |

**Adduct Signal Fractions**

- **Unmodified**: 1.1818e+07

### TPN(N)KTEDTIFLR Hex 3 HexNAc 6

**Mass:**  3138.3891

(9.056e-07)

**Charge States:** 3, 4

**Start Position**: 70

**End Position**: 82

| Profile Score | Tandem Score | TDA q-value | Volume | Spectrum Matches |
| --- | --- | --- | --- | --- |
| 12.5277 | 129.3982 | 0.0000 | 4.3220e+07 | 5 |

**Adduct Signal Fractions**

- **Unmodified**: 4.3220e+07

### YFTPN(N)KTEDTIFLR Hex 3 HexNAc 4

**Mass:**  3042.3621

(1.607e-06)

**Charge States:** 3

**Start Position**: 68

**End Position**: 82

| Profile Score | Tandem Score | TDA q-value | Volume | Spectrum Matches |
| --- | --- | --- | --- | --- |
| -2.9874 | 129.0796 | 0.0000 | 9.0099e+07 | 2 |

**Adduct Signal Fractions**

- **Unmodified**: 9.0099e+07

### ATFFYFTPN(N)K Fuc 1 Hex 4 HexNAc 6

**Mass:**  3247.3493

(5.279e-07)

**Charge States:** 3

**Start Position**: 64

**End Position**: 74

| Profile Score | Tandem Score | TDA q-value | Volume | Spectrum Matches |
| --- | --- | --- | --- | --- |
| -20.7849 | 126.8347 | 0.0000 | 5.8521e+06 | 1 |

**Adduct Signal Fractions**

- **Unmodified**: 5.8521e+06

### QNQC(C)FYN(N)SSYLNVQR Fuc 1 Hex 4 HexNAc 6

**Mass:**  3932.6125

(6.616e-07)

**Charge States:** 3, 4

**Start Position**: 87

**End Position**: 102

| Profile Score | Tandem Score | TDA q-value | Volume | Spectrum Matches |
| --- | --- | --- | --- | --- |
| 11.3657 | 126.8314 | 0.0000 | 4.7548e+08 | 5 |

**Adduct Signal Fractions**

- **Unmodified**: 4.7548e+08

### YFTPN(N)KTEDTIFLR Fuc 2 Hex 5 HexNAc 6

**Mass:**  4066.7533

(4.955e-04)

**Start Position**: 68

**End Position**: 82

| Profile Score | Tandem Score | TDA q-value | Volume | Spectrum Matches |
| --- | --- | --- | --- | --- |
| 0.0000 | 126.5616 | 0.0000 | 0.0000e+00 | 1 |

Adduct:

No Chromatogram Found

### YFTPN(N)KTEDTIFLR Hex 4 HexNAc 7

**Mass:**  3813.6511

(1.303e-06)

**Charge States:** 3, 4

**Start Position**: 68

**End Position**: 82

| Profile Score | Tandem Score | TDA q-value | Volume | Spectrum Matches |
| --- | --- | --- | --- | --- |
| 10.3798 | 126.4242 | 0.0000 | 1.7484e+08 | 6 |

**Adduct Signal Fractions**

- **Unmodified**: 1.7484e+08

### QNQC(C)FYN(N)SSYLNVQR Fuc 1 Hex 4 HexNAc 5

**Mass:**  3729.5282

(2.437e-06)

**Charge States:** 3, 4

**Start Position**: 87

**End Position**: 102

| Profile Score | Tandem Score | TDA q-value | Volume | Spectrum Matches |
| --- | --- | --- | --- | --- |
| -5.0739 | 126.3248 | 0.0000 | 1.6516e+08 | 3 |

**Adduct Signal Fractions**

- **Unmodified**: 1.6516e+08

### NLVPVPITN(N)ATLDR Fuc 1 Hex 3 HexNAc 4

**Mass:**  2966.3880

(1.596e-06)

**Charge States:** 2, 3, 4

**Start Position**: 25

**End Position**: 39

| Profile Score | Tandem Score | TDA q-value | Volume | Spectrum Matches |
| --- | --- | --- | --- | --- |
| 15.4109 | 125.8899 | 0.0000 | 1.6131e+09 | 8 |

**Adduct Signal Fractions**

- **Unmodified**: 1.6131e+09

### ATFFYFTPN(N)K Fuc 3 Hex 8 HexNAc 4

**Mass:**  3781.5300

(3.674e-06)

**Charge States:** 4

**Start Position**: 64

**End Position**: 74

| Profile Score | Tandem Score | TDA q-value | Volume | Spectrum Matches |
| --- | --- | --- | --- | --- |
| -21.0529 | 124.3145 | 0.0000 | 9.0554e+06 | 1 |

**Adduct Signal Fractions**

- **Unmodified**: 9.0554e+06

### QNQC(C)FYN(N)SSYLNVQR Hex 3 HexNAc 6

**Mass:**  3624.4975

(7.422e-07)

**Charge States:** 3, 4

**Start Position**: 87

**End Position**: 102

| Profile Score | Tandem Score | TDA q-value | Volume | Spectrum Matches |
| --- | --- | --- | --- | --- |
| -5.0596 | 123.4922 | 0.0000 | 8.1523e+08 | 2 |

**Adduct Signal Fractions**

- **Unmodified**: 8.1523e+08

### FYFTPN(N)KTEDTIFLR Hex 3 HexNAc 6

**Mass:**  3595.5910

(1.519e-06)

**Charge States:** 3, 4

**Start Position**: 67

**End Position**: 82

| Profile Score | Tandem Score | TDA q-value | Volume | Spectrum Matches |
| --- | --- | --- | --- | --- |
| 13.4404 | 123.4202 | 0.0000 | 1.3158e+08 | 6 |

**Adduct Signal Fractions**

- **Unmodified**: 1.3158e+08

### SVQEIQATFFYFTPN(N)K Fuc 1 Hex 4 HexNAc 7

**Mass:**  4134.7790

(2.042e-06)

**Charge States:** 4

**Start Position**: 58

**End Position**: 74

| Profile Score | Tandem Score | TDA q-value | Volume | Spectrum Matches |
| --- | --- | --- | --- | --- |
| -4.5245 | 122.5534 | 0.0000 | 1.2614e+07 | 3 |

**Adduct Signal Fractions**

- **Unmodified**: 1.2614e+07

### PVPITN(N)ATLDR Fuc 2 Hex 6 HexNAc 3

**Mass:**  3069.3298

(9.150e-07)

**Start Position**: 28

**End Position**: 39

| Profile Score | Tandem Score | TDA q-value | Volume | Spectrum Matches |
| --- | --- | --- | --- | --- |
| 0.0000 | 121.0319 | 0.0000 | 0.0000e+00 | 1 |

Adduct:

No Chromatogram Found

### LVPVPITN(N)ATLDR Fuc 1 Hex 5 HexNAc 3 Neu5Ac 1

**Mass:**  3266.4838

(6.187e-04)

**Start Position**: 26

**End Position**: 39

| Profile Score | Tandem Score | TDA q-value | Volume | Spectrum Matches |
| --- | --- | --- | --- | --- |
| 0.0000 | 121.0141 | 0.0000 | 0.0000e+00 | 1 |

Adduct:

No Chromatogram Found

### SVQEIQATFFYFTPN(N)K Fuc 2 Hex 5 HexNAc 7

**Mass:**  4442.8864

(9.557e-07)

**Charge States:** 4

**Start Position**: 58

**End Position**: 74

| Profile Score | Tandem Score | TDA q-value | Volume | Spectrum Matches |
| --- | --- | --- | --- | --- |
| -19.5980 | 118.1883 | 0.0000 | 3.8219e+06 | 1 |

**Adduct Signal Fractions**

- **Unmodified**: 3.8219e+06

### FYFTPN(N)KTEDTIFLR Hex 3 HexNAc 5

**Mass:**  3392.5113

(1.371e-06)

**Charge States:** 3, 4

**Start Position**: 67

**End Position**: 82

| Profile Score | Tandem Score | TDA q-value | Volume | Spectrum Matches |
| --- | --- | --- | --- | --- |
| 13.5454 | 117.8883 | 0.0000 | 5.6579e+07 | 5 |

**Adduct Signal Fractions**

- **Unmodified**: 5.6579e+07

### SVQEIQATFFYFTPN(N)K Fuc 1 Hex 5 HexNAc 4 Neu5Ac 1

**Mass:**  3978.6996

(4.598e-06)

**Charge States:** 3

**Start Position**: 58

**End Position**: 74

| Profile Score | Tandem Score | TDA q-value | Volume | Spectrum Matches |
| --- | --- | --- | --- | --- |
| -4.9373 | 116.2166 | 0.0000 | 5.8867e+06 | 1 |

**Adduct Signal Fractions**

- **Unmodified**: 5.8867e+06

### VPVPITN(N)ATLDR Fuc 1 Hex 4 HexNAc 5

**Mass:**  3104.3933

(9.485e-07)

**Charge States:** 3

**Start Position**: 27

**End Position**: 39

| Profile Score | Tandem Score | TDA q-value | Volume | Spectrum Matches |
| --- | --- | --- | --- | --- |
| -5.0634 | 115.6225 | 0.0000 | 2.1879e+06 | 1 |

**Adduct Signal Fractions**

- **Unmodified**: 2.1879e+06

### ATFFYFTPN(N)K Hex 4 HexNAc 7

**Mass:**  3304.3760

(2.094e-06)

**Charge States:** 3

**Start Position**: 64

**End Position**: 74

| Profile Score | Tandem Score | TDA q-value | Volume | Spectrum Matches |
| --- | --- | --- | --- | --- |
| -19.8216 | 115.4025 | 0.0000 | 3.4277e+06 | 1 |

**Adduct Signal Fractions**

- **Unmodified**: 3.4277e+06

### YFTPN(N)KTEDTIFLR Hex 3 HexNAc 5

**Mass:**  3245.4431

(6.348e-07)

**Charge States:** 3, 4

**Start Position**: 68

**End Position**: 82

| Profile Score | Tandem Score | TDA q-value | Volume | Spectrum Matches |
| --- | --- | --- | --- | --- |
| 9.4386 | 114.0012 | 0.0000 | 4.4304e+07 | 5 |

**Adduct Signal Fractions**

- **Unmodified**: 4.4304e+07

### ANLVPVPITN(N)ATLDR Hex 3 HexNAc 5

**Mass:**  3094.4478

(7.720e-07)

**Charge States:** 3

**Start Position**: 24

**End Position**: 39

| Profile Score | Tandem Score | TDA q-value | Volume | Spectrum Matches |
| --- | --- | --- | --- | --- |
| -3.5825 | 113.9556 | 0.0000 | 1.6360e+07 | 2 |

**Adduct Signal Fractions**

- **Unmodified**: 1.6360e+07

### C(C)ANLVPVPITN(N)ATLDR Fuc 1 Hex 4 HexNAc 5

**Mass:**  3562.5910

(1.941e-06)

**Charge States:** 3, 4

**Start Position**: 23

**End Position**: 39

| Profile Score | Tandem Score | TDA q-value | Volume | Spectrum Matches |
| --- | --- | --- | --- | --- |
| 10.6262 | 112.4496 | 0.0000 | 6.1955e+07 | 5 |

**Adduct Signal Fractions**

- **Unmodified**: 6.1955e+07

### LVPVPITN(N)ATLDR Fuc 1 Hex 5 HexNAc 5

**Mass:**  3381.5407

(5.957e-04)

**Start Position**: 26

**End Position**: 39

| Profile Score | Tandem Score | TDA q-value | Volume | Spectrum Matches |
| --- | --- | --- | --- | --- |
| 0.0000 | 111.1243 | 0.0000 | 0.0000e+00 | 2 |

Adduct:

No Chromatogram Found

### FYFTPN(N)KTEDTIFLR Fuc 1 Hex 4 HexNAc 6

**Mass:**  3903.7038

(2.171e-06)

**Charge States:** 3, 4

**Start Position**: 67

**End Position**: 82

| Profile Score | Tandem Score | TDA q-value | Volume | Spectrum Matches |
| --- | --- | --- | --- | --- |
| 14.3397 | 110.8408 | 0.0000 | 3.0145e+07 | 4 |

**Adduct Signal Fractions**

- **Unmodified**: 3.0145e+07

### LVPVPITN(N)ATLDR Fuc 1 Hex 5 HexNAc 6

**Mass:**  3582.6123

(1.426e-06)

**Charge States:** 3, 4

**Start Position**: 26

**End Position**: 39

| Profile Score | Tandem Score | TDA q-value | Volume | Spectrum Matches |
| --- | --- | --- | --- | --- |
| -5.3771 | 109.2669 | 0.0000 | 4.2184e+07 | 3 |

**Adduct Signal Fractions**

- **Unmodified**: 4.2184e+07

### TPN(N)KTEDTIFLR Hex 7 HexNAc 6

**Mass:**  3786.6061

(2.353e-06)

**Charge States:** 3, 4

**Start Position**: 70

**End Position**: 82

| Profile Score | Tandem Score | TDA q-value | Volume | Spectrum Matches |
| --- | --- | --- | --- | --- |
| 13.6012 | 108.9988 | 0.0000 | 1.0588e+08 | 4 |

**Adduct Signal Fractions**

- **Unmodified**: 1.0588e+08

### NLVPVPITN(N)ATLDR Fuc 1 Hex 4 HexNAc 5

**Mass:**  3331.5217

(3.831e-06)

**Charge States:** 3

**Start Position**: 25

**End Position**: 39

| Profile Score | Tandem Score | TDA q-value | Volume | Spectrum Matches |
| --- | --- | --- | --- | --- |
| -6.0881 | 106.3384 | 0.0000 | 1.7389e+07 | 2 |

**Adduct Signal Fractions**

- **Unmodified**: 1.7389e+07

### NLVPVPITN(N)ATLDR Fuc 1 Hex 3 HexNAc 5

**Mass:**  3169.4676

(1.747e-06)

**Charge States:** 3

**Start Position**: 25

**End Position**: 39

| Profile Score | Tandem Score | TDA q-value | Volume | Spectrum Matches |
| --- | --- | --- | --- | --- |
| -18.3735 | 106.2058 | 0.0000 | 3.7638e+07 | 1 |

**Adduct Signal Fractions**

- **Unmodified**: 3.7638e+07

### YFTPN(N)KTEDTIFLR Hex 5 HexNAc 8

**Mass:**  4178.7883

(1.748e-06)

**Charge States:** 3, 4

**Start Position**: 68

**End Position**: 82

| Profile Score | Tandem Score | TDA q-value | Volume | Spectrum Matches |
| --- | --- | --- | --- | --- |
| -3.4271 | 105.8170 | 0.0000 | 3.4766e+07 | 3 |

**Adduct Signal Fractions**

- **Unmodified**: 3.4766e+07

### FYFTPN(N)KTEDTIFLR Hex 3 HexNAc 4

**Mass:**  3189.4326

(1.566e-06)

**Charge States:** 3

**Start Position**: 67

**End Position**: 82

| Profile Score | Tandem Score | TDA q-value | Volume | Spectrum Matches |
| --- | --- | --- | --- | --- |
| 12.6711 | 105.4573 | 0.0000 | 7.5931e+06 | 2 |

**Adduct Signal Fractions**

- **Unmodified**: 7.5931e+06

### LVPVPITN(N)ATLDR Fuc 1 Hex 5 HexNAc 3 Neu5Ac 1

**Mass:**  3264.4795

(3.867e-06)

**Charge States:** 3

**Start Position**: 26

**End Position**: 39

| Profile Score | Tandem Score | TDA q-value | Volume | Spectrum Matches |
| --- | --- | --- | --- | --- |
| -20.8658 | 105.3756 | 0.0000 | 2.1822e+07 | 1 |

**Adduct Signal Fractions**

- **Unmodified**: 2.1822e+07

### SVQEIQATFFYFTPN(N)KTEDTIFLR Hex 3 HexNAc 6

**Mass:**  4599.0900

(1.332e-06)

**Charge States:** 4

**Start Position**: 58

**End Position**: 82

| Profile Score | Tandem Score | TDA q-value | Volume | Spectrum Matches |
| --- | --- | --- | --- | --- |
| -3.8574 | 105.3167 | 0.0000 | 3.3438e+07 | 4 |

**Adduct Signal Fractions**

- **Unmodified**: 3.3438e+07

### YFTPN(N)KTEDTIFLR Fuc 1 Hex 5 HexNAc 7

**Mass:**  4121.7663

(1.610e-06)

**Charge States:** 3, 4

**Start Position**: 68

**End Position**: 82

| Profile Score | Tandem Score | TDA q-value | Volume | Spectrum Matches |
| --- | --- | --- | --- | --- |
| -4.5412 | 104.9683 | 0.0000 | 3.4872e+07 | 2 |

**Adduct Signal Fractions**

- **Unmodified**: 3.4872e+07

### SVQEIQATFFYFTPN(N)K Hex 5 HexNAc 9

**Mass:**  4556.9328

(1.671e-06)

**Charge States:** 4

**Start Position**: 58

**End Position**: 74

| Profile Score | Tandem Score | TDA q-value | Volume | Spectrum Matches |
| --- | --- | --- | --- | --- |
| -20.3116 | 104.7215 | 0.0000 | 2.3485e+06 | 1 |

**Adduct Signal Fractions**

- **Unmodified**: 2.3485e+06

### QNQC(C)FYN(N)SSYLNVQR Hex 4 HexNAc 8

**Mass:**  4192.7111

(1.566e-06)

**Charge States:** 3, 4

**Start Position**: 87

**End Position**: 102

| Profile Score | Tandem Score | TDA q-value | Volume | Spectrum Matches |
| --- | --- | --- | --- | --- |
| -5.8913 | 104.6933 | 0.0000 | 4.0675e+07 | 2 |

**Adduct Signal Fractions**

- **Unmodified**: 4.0675e+07

### FYFTPN(N)KTEDTIFLR Fuc 1 Hex 4 HexNAc 5

**Mass:**  3700.6210

(1.511e-06)

**Charge States:** 3, 4

**Start Position**: 67

**End Position**: 82

| Profile Score | Tandem Score | TDA q-value | Volume | Spectrum Matches |
| --- | --- | --- | --- | --- |
| -3.5251 | 104.2440 | 0.0000 | 1.6244e+07 | 4 |

**Adduct Signal Fractions**

- **Unmodified**: 1.6244e+07

### QNQC(C)FYN(N)SSYLNVQR Hex 4 HexNAc 7

**Mass:**  3989.6315

(1.389e-06)

**Charge States:** 3, 4

**Start Position**: 87

**End Position**: 102

| Profile Score | Tandem Score | TDA q-value | Volume | Spectrum Matches |
| --- | --- | --- | --- | --- |
| 9.4299 | 103.0339 | 0.0000 | 1.8275e+08 | 2 |

**Adduct Signal Fractions**

- **Unmodified**: 1.8275e+08

### FTPN(N)KTEDTIFLR Hex 3 HexNAc 5

**Mass:**  3082.3786

(1.103e-06)

**Charge States:** 3, 4

**Start Position**: 69

**End Position**: 82

| Profile Score | Tandem Score | TDA q-value | Volume | Spectrum Matches |
| --- | --- | --- | --- | --- |
| 13.8673 | 102.5149 | 0.0000 | 4.6578e+07 | 3 |

**Adduct Signal Fractions**

- **Unmodified**: 4.6578e+07

### FYN(N)SSYLNVQR Hex 3 HexNAc 6

**Mass:**  3094.3050

(1.051e-06)

**Charge States:** 3

**Start Position**: 91

**End Position**: 102

| Profile Score | Tandem Score | TDA q-value | Volume | Spectrum Matches |
| --- | --- | --- | --- | --- |
| 16.4123 | 102.1133 | 0.0000 | 8.5409e+06 | 3 |

**Adduct Signal Fractions**

- **Unmodified**: 8.5409e+06

### LVPVPITN(N)ATLDR Fuc 2 Hex 5 HexNAc 6

**Mass:**  3728.6701

(3.240e-06)

**Charge States:** 3, 4

**Start Position**: 26

**End Position**: 39

| Profile Score | Tandem Score | TDA q-value | Volume | Spectrum Matches |
| --- | --- | --- | --- | --- |
| 10.2801 | 101.2613 | 0.0000 | 2.6149e+07 | 3 |

**Adduct Signal Fractions**

- **Unmodified**: 2.6149e+07

### FTPN(N)KTEDTIFLR Hex 3 HexNAc 6

**Mass:**  3285.4575

(9.724e-07)

**Charge States:** 3, 4

**Start Position**: 69

**End Position**: 82

| Profile Score | Tandem Score | TDA q-value | Volume | Spectrum Matches |
| --- | --- | --- | --- | --- |
| 14.1683 | 98.6572 | 0.0000 | 9.1147e+07 | 5 |

**Adduct Signal Fractions**

- **Unmodified**: 9.1147e+07

### FTPN(N)KTEDTIFLR Fuc 1 Hex 4 HexNAc 5

**Mass:**  3390.4909

(1.465e-06)

**Charge States:** 3, 4

**Start Position**: 69

**End Position**: 82

| Profile Score | Tandem Score | TDA q-value | Volume | Spectrum Matches |
| --- | --- | --- | --- | --- |
| -4.9259 | 98.3779 | 0.0000 | 1.1130e+07 | 2 |

**Adduct Signal Fractions**

- **Unmodified**: 1.1130e+07

### LVPVPITN(N)ATLDR Fuc 1 Hex 4 HexNAc 4

**Mass:**  3015.4057

(3.352e-04)

**Start Position**: 26

**End Position**: 39

| Profile Score | Tandem Score | TDA q-value | Volume | Spectrum Matches |
| --- | --- | --- | --- | --- |
| 0.0000 | 97.9290 | 0.0000 | 0.0000e+00 | 1 |

Adduct:

No Chromatogram Found

### NLVPVPITN(N)ATLDR Fuc 1 Hex 3 HexNAc 3

**Mass:**  2763.3079

(1.199e-06)

**Charge States:** 3

**Start Position**: 25

**End Position**: 39

| Profile Score | Tandem Score | TDA q-value | Volume | Spectrum Matches |
| --- | --- | --- | --- | --- |
| 13.9766 | 97.7424 | 0.0000 | 1.2314e+08 | 4 |

**Adduct Signal Fractions**

- **Unmodified**: 1.2314e+08

### NLVPVPITN(N)ATLDR Hex 3 HexNAc 5

**Mass:**  3023.4126

(1.734e-06)

**Charge States:** 3

**Start Position**: 25

**End Position**: 39

| Profile Score | Tandem Score | TDA q-value | Volume | Spectrum Matches |
| --- | --- | --- | --- | --- |
| -4.4490 | 97.5530 | 0.0000 | 1.0078e+07 | 2 |

**Adduct Signal Fractions**

- **Unmodified**: 1.0078e+07

### LVPVPITN(N)ATLDR Fuc 1 Hex 5 HexNAc 7

**Mass:**  3785.6911

(1.300e-06)

**Charge States:** 4

**Start Position**: 26

**End Position**: 39

| Profile Score | Tandem Score | TDA q-value | Volume | Spectrum Matches |
| --- | --- | --- | --- | --- |
| -19.3900 | 97.2303 | 0.0000 | 1.1076e+07 | 2 |

**Adduct Signal Fractions**

- **Unmodified**: 1.1076e+07

### C(C)AN(D)LVPVPITN(N)ATLDR Hex 3 HexNAc 5

**Mass:**  3255.4608

(3.031e-04)

**Charge States:** 3, 4

**Start Position**: 23

**End Position**: 39

| Profile Score | Tandem Score | TDA q-value | Volume | Spectrum Matches |
| --- | --- | --- | --- | --- |
| 7.4740 | 88.9211 | 0.0000 | 6.5018e+07 | 4 |

**Adduct Signal Fractions**

- **Unmodified**: 6.5018e+07

### FYFTPN(N)KTEDTIFLR Hex 4 HexNAc 7

**Mass:**  3960.7258

(1.635e-06)

**Charge States:** 3, 4

**Start Position**: 67

**End Position**: 82

| Profile Score | Tandem Score | TDA q-value | Volume | Spectrum Matches |
| --- | --- | --- | --- | --- |
| 14.0946 | 87.6053 | 0.0022 | 2.3156e+07 | 4 |

**Adduct Signal Fractions**

- **Unmodified**: 2.3156e+07

### FTPN(N)KTEDTIFLR Fuc 1 Hex 4 HexNAc 6

**Mass:**  3593.5693

(1.109e-06)

**Charge States:** 3, 4

**Start Position**: 69

**End Position**: 82

| Profile Score | Tandem Score | TDA q-value | Volume | Spectrum Matches |
| --- | --- | --- | --- | --- |
| -3.7148 | 86.4331 | 0.0022 | 2.0299e+07 | 4 |

**Adduct Signal Fractions**

- **Unmodified**: 2.0299e+07

### YN(N)SSYLNVQR Hex 3 HexNAc 6

**Mass:**  2947.2381

(1.654e-06)

**Charge States:** 3, 4

**Start Position**: 92

**End Position**: 102

| Profile Score | Tandem Score | TDA q-value | Volume | Spectrum Matches |
| --- | --- | --- | --- | --- |
| 12.9031 | 85.3105 | 0.0022 | 1.6423e+07 | 5 |

**Adduct Signal Fractions**

- **Unmodified**: 1.6423e+07

### YFTPN(N)KTEDTIFLR Hex 5 HexNAc 9

**Mass:**  4381.8688

(1.623e-06)

**Charge States:** 4

**Start Position**: 68

**End Position**: 82

| Profile Score | Tandem Score | TDA q-value | Volume | Spectrum Matches |
| --- | --- | --- | --- | --- |
| -5.0626 | 83.8036 | 0.0022 | 9.8359e+06 | 1 |

**Adduct Signal Fractions**

- **Unmodified**: 9.8359e+06

### TPN(N)KTEDTIFLR Fuc 1 Hex 4 HexNAc 5

**Mass:**  3244.4133

(3.070e-04)

**Start Position**: 70

**End Position**: 82

| Profile Score | Tandem Score | TDA q-value | Volume | Spectrum Matches |
| --- | --- | --- | --- | --- |
| 0.0000 | 83.7063 | 0.0022 | 0.0000e+00 | 1 |

Adduct:

No Chromatogram Found

### YFTPN(N)KTEDTIFLR Fuc 2 Hex 5 HexNAc 7

**Mass:**  4267.8264

(1.749e-06)

**Charge States:** 4

**Start Position**: 68

**End Position**: 82

| Profile Score | Tandem Score | TDA q-value | Volume | Spectrum Matches |
| --- | --- | --- | --- | --- |
| -20.0341 | 81.4343 | 0.0030 | 2.2473e+06 | 1 |

**Adduct Signal Fractions**

- **Unmodified**: 2.2473e+06

### TPN(N)KTEDTIFLR Fuc 1 Hex 4 HexNAc 6

**Mass:**  3446.4999

(1.714e-06)

**Charge States:** 3, 4

**Start Position**: 70

**End Position**: 82

| Profile Score | Tandem Score | TDA q-value | Volume | Spectrum Matches |
| --- | --- | --- | --- | --- |
| -4.2895 | 79.1674 | 0.0030 | 8.9818e+06 | 4 |

**Adduct Signal Fractions**

- **Unmodified**: 8.9818e+06

### TPN(N)KTEDTIFLR Fuc 1 Hex 4 HexNAc 5

**Mass:**  3243.4213

(1.631e-06)

**Charge States:** 3

**Start Position**: 70

**End Position**: 82

| Profile Score | Tandem Score | TDA q-value | Volume | Spectrum Matches |
| --- | --- | --- | --- | --- |
| -4.9391 | 75.9035 | 0.0030 | 4.2060e+06 | 3 |

**Adduct Signal Fractions**

- **Unmodified**: 4.2060e+06

### SVQEIQATFFYFTPN(N)KTEDTIFLR Hex 3 HexNAc 5

**Mass:**  4396.0178

(3.080e-06)

**Charge States:** 4

**Start Position**: 58

**End Position**: 82

| Profile Score | Tandem Score | TDA q-value | Volume | Spectrum Matches |
| --- | --- | --- | --- | --- |
| -21.5260 | 61.7554 | 0.0030 | 6.2106e+06 | 1 |

**Adduct Signal Fractions**

- **Unmodified**: 6.2106e+06

### QIPLC(C)ANLVPVPITN(N)ATLDR Hex 3 HexNAc 5 Neu5Ac 1

**Mass:**  3996.8466

(-6.398e-07)

**Charge States:** 3, 4

**Start Position**: 19

**End Position**: 39

| Profile Score | Tandem Score | TDA q-value | Volume | Spectrum Matches |
| --- | --- | --- | --- | --- |
| 15.7377 | 61.1422 | 0.0030 | 6.0146e+08 | 11 |

**Adduct Signal Fractions**

- **Unmodified**: 6.0146e+08

### QNQC(C)FYN(N)SSYLNVQR Fuc 2 Hex 5 HexNAc 6

**Mass:**  4240.7116

(-6.709e-07)

**Charge States:** 3

**Start Position**: 87

**End Position**: 102

| Profile Score | Tandem Score | TDA q-value | Volume | Spectrum Matches |
| --- | --- | --- | --- | --- |
| -18.6650 | 55.2826 | 0.0030 | 2.3866e+07 | 2 |

**Adduct Signal Fractions**

- **Unmodified**: 2.3866e+07

### QNQC(C)FYN(N)SSYLNVQR Fuc 5 Hex 4 HexNAc 8

**Mass:**  4922.9743

(-4.040e-06)

**Charge States:** 4, 5

**Start Position**: 87

**End Position**: 102

| Profile Score | Tandem Score | TDA q-value | Volume | Spectrum Matches |
| --- | --- | --- | --- | --- |
| -3.5911 | 48.4498 | 0.0030 | 2.7973e+07 | 4 |

**Adduct Signal Fractions**

- **Unmodified**: 2.7973e+07

### SVQEIQATFFYFTPN(N)K Hex 4 HexNAc 5

**Mass:**  3583.5751

(2.848e-04)

**Start Position**: 58

**End Position**: 74

| Profile Score | Tandem Score | TDA q-value | Volume | Spectrum Matches |
| --- | --- | --- | --- | --- |
| 0.0000 | 47.9320 | 0.0030 | 0.0000e+00 | 2 |

Adduct:

No Chromatogram Found

### C(C)ANLVPVPITN(N)ATLDR Hex 3 HexNAc 4

**Mass:**  3051.3971

(1.723e-06)

**Charge States:** 3

**Start Position**: 23

**End Position**: 39

| Profile Score | Tandem Score | TDA q-value | Volume | Spectrum Matches |
| --- | --- | --- | --- | --- |
| -3.0631 | 47.8583 | 0.0030 | 1.4684e+07 | 2 |

**Adduct Signal Fractions**

- **Unmodified**: 1.4684e+07

### ANLVPVPITN(N)ATLDR Fuc 1 Hex 8 HexNAc 5

**Mass:**  4050.7612

(-1.183e-06)

**Charge States:** 3, 4

**Start Position**: 24

**End Position**: 39

| Profile Score | Tandem Score | TDA q-value | Volume | Spectrum Matches |
| --- | --- | --- | --- | --- |
| -20.1502 | 47.1573 | 0.0030 | 3.7976e+07 | 2 |

**Adduct Signal Fractions**

- **Unmodified**: 3.7976e+07

### YFTPN(N)KTEDTIFLR Hex 4 HexNAc 8

**Mass:**  4016.7363

(1.674e-06)

**Charge States:** 4

**Start Position**: 68

**End Position**: 82

| Profile Score | Tandem Score | TDA q-value | Volume | Spectrum Matches |
| --- | --- | --- | --- | --- |
| -18.3376 | 45.1407 | 0.0030 | 1.8415e+07 | 1 |

**Adduct Signal Fractions**

- **Unmodified**: 1.8415e+07

### SVQEIQATFFYFTPN(N)KTEDTIFLR Fuc 1 Hex 4 HexNAc 6

**Mass:**  4909.2093

(4.106e-04)

**Start Position**: 58

**End Position**: 82

| Profile Score | Tandem Score | TDA q-value | Volume | Spectrum Matches |
| --- | --- | --- | --- | --- |
| 0.0000 | 44.2814 | 0.0030 | 0.0000e+00 | 1 |

Adduct:

No Chromatogram Found

### QN(D)QC(C)FYN(N)SSYLNVQR Hex 3 HexNAc 6

**Mass:**  3625.4804

(2.727e-04)

**Charge States:** 3, 4

**Start Position**: 87

**End Position**: 102

| Profile Score | Tandem Score | TDA q-value | Volume | Spectrum Matches |
| --- | --- | --- | --- | --- |
| -4.2049 | 43.3736 | 0.0030 | 2.2912e+08 | 3 |

**Adduct Signal Fractions**

- **Unmodified**: 2.2912e+08

### LVPVPITN(N)ATLDR Fuc 1 Hex 6 HexNAc 5 Neu5Ac 3

**Mass:**  4414.8767

(2.265e-06)

**Charge States:** 4

**Start Position**: 26

**End Position**: 39

| Profile Score | Tandem Score | TDA q-value | Volume | Spectrum Matches |
| --- | --- | --- | --- | --- |
| -20.6054 | 41.8052 | 0.0030 | 1.7092e+06 | 1 |

**Adduct Signal Fractions**

- **Unmodified**: 1.7092e+06

### QIPLC(C)ANLVPVPITN(N)ATLDR Hex 3 HexNAc 5

**Mass:**  3705.7560

(6.987e-07)

**Charge States:** 3, 4

**Start Position**: 19

**End Position**: 39

| Profile Score | Tandem Score | TDA q-value | Volume | Spectrum Matches |
| --- | --- | --- | --- | --- |
| 13.9904 | 41.1353 | 0.0030 | 5.1943e+08 | 10 |

**Adduct Signal Fractions**

- **Unmodified**: 5.1943e+08

### FTPN(N)KTEDTIFLR Hex 3 HexNAc 4

**Mass:**  2879.2999

(1.203e-06)

**Charge States:** 3

**Start Position**: 69

**End Position**: 82

| Profile Score | Tandem Score | TDA q-value | Volume | Spectrum Matches |
| --- | --- | --- | --- | --- |
| -5.0065 | 40.7409 | 0.0030 | 5.8097e+06 | 1 |

**Adduct Signal Fractions**

- **Unmodified**: 5.8097e+06

### SVQEIQATFFYFTPN(N)KTEDTIFLR Fuc 1 Hex 4 HexNAc 5

**Mass:**  4704.1154

(1.087e-07)

**Charge States:** 4

**Start Position**: 58

**End Position**: 82

| Profile Score | Tandem Score | TDA q-value | Volume | Spectrum Matches |
| --- | --- | --- | --- | --- |
| -5.3067 | 40.2046 | 0.0030 | 3.7912e+06 | 1 |

**Adduct Signal Fractions**

- **Unmodified**: 3.7912e+06

### QNQC(C)FYN(N)SSYLNVQR Hex 5 HexNAc 9

**Mass:**  4557.8464

(2.050e-06)

**Charge States:** 3, 4

**Start Position**: 87

**End Position**: 102

| Profile Score | Tandem Score | TDA q-value | Volume | Spectrum Matches |
| --- | --- | --- | --- | --- |
| -19.0080 | 40.1602 | 0.0030 | 7.7969e+06 | 1 |

**Adduct Signal Fractions**

- **Unmodified**: 7.7969e+06

### SVQEIQATFFYFTPN(N)K Hex 5 HexNAc 8

**Mass:**  4353.8459

(2.558e-06)

**Charge States:** 4

**Start Position**: 58

**End Position**: 74

| Profile Score | Tandem Score | TDA q-value | Volume | Spectrum Matches |
| --- | --- | --- | --- | --- |
| 7.6879 | 40.0810 | 0.0030 | 2.4143e+07 | 1 |

**Adduct Signal Fractions**

- **Unmodified**: 2.4143e+07

### LVPVPITN(N)ATLDR Hex 6 HexNAc 5 Neu5Ac 3

**Mass:**  4268.8190

(2.485e-06)

**Charge States:** 3, 4

**Start Position**: 26

**End Position**: 39

| Profile Score | Tandem Score | TDA q-value | Volume | Spectrum Matches |
| --- | --- | --- | --- | --- |
| 12.1854 | 39.0297 | 0.0045 | 1.1045e+07 | 1 |

**Adduct Signal Fractions**

- **Unmodified**: 1.1045e+07

### QIPLC(C)ANLVPVPITN(N)ATLDR Fuc 1 Hex 4 HexNAc 5

**Mass:**  4013.8694

(8.177e-07)

**Charge States:** 3, 4

**Start Position**: 19

**End Position**: 39

| Profile Score | Tandem Score | TDA q-value | Volume | Spectrum Matches |
| --- | --- | --- | --- | --- |
| 14.1505 | 37.4732 | 0.0056 | 1.5406e+08 | 5 |

**Adduct Signal Fractions**

- **Unmodified**: 1.5406e+08

### QN(D)QC(C)FYN(N)SSYLNVQR Hex 3 HexNAc 5

**Mass:**  3422.4054

(2.898e-04)

**Charge States:** 3

**Start Position**: 87

**End Position**: 102

| Profile Score | Tandem Score | TDA q-value | Volume | Spectrum Matches |
| --- | --- | --- | --- | --- |
| 8.2080 | 36.3641 | 0.0056 | 3.6821e+07 | 2 |

**Adduct Signal Fractions**

- **Unmodified**: 3.6821e+07

### SVQEIQATFFYFTPN(N)K Fuc 1 Hex 5 HexNAc 8

**Mass:**  4499.9064

(6.863e-07)

**Charge States:** 4

**Start Position**: 58

**End Position**: 74

| Profile Score | Tandem Score | TDA q-value | Volume | Spectrum Matches |
| --- | --- | --- | --- | --- |
| -6.3274 | 36.0427 | 0.0056 | 6.4748e+06 | 2 |

**Adduct Signal Fractions**

- **Unmodified**: 6.4748e+06

### SVQEIQATFFYFTPN(N)KTEDTIFLR Hex 3 HexNAc 6

**Mass:**  4601.0913

(4.365e-04)

**Start Position**: 58

**End Position**: 82

| Profile Score | Tandem Score | TDA q-value | Volume | Spectrum Matches |
| --- | --- | --- | --- | --- |
| 0.0000 | 35.6746 | 0.0083 | 0.0000e+00 | 1 |

Adduct:

No Chromatogram Found

### FYFTPN(N)KTEDTIFLR Fuc 2 Hex 5 HexNAc 6

**Mass:**  4211.8132

(1.229e-06)

**Charge States:** 4

**Start Position**: 67

**End Position**: 82

| Profile Score | Tandem Score | TDA q-value | Volume | Spectrum Matches |
| --- | --- | --- | --- | --- |
| -4.6621 | 35.6517 | 0.0083 | 5.4781e+06 | 1 |

**Adduct Signal Fractions**

- **Unmodified**: 5.4781e+06

### C(C)ANLVPVPITN(N)ATLDR Hex 3 HexNAc 5 Neu5Ac 1

**Mass:**  3545.5651

(-1.152e-06)

**Charge States:** 3, 4

**Start Position**: 23

**End Position**: 39

| Profile Score | Tandem Score | TDA q-value | Volume | Spectrum Matches |
| --- | --- | --- | --- | --- |
| 16.4389 | 35.5134 | 0.0083 | 2.8329e+08 | 7 |

**Adduct Signal Fractions**

- **Unmodified**: 2.8329e+08

### QIPLC(C)ANLVPVPITN(N)ATLDR Fuc 2 Hex 3 HexNAc 5

**Mass:**  3997.8417

(-7.061e-06)

**Charge States:** 4

**Start Position**: 19

**End Position**: 39

| Profile Score | Tandem Score | TDA q-value | Volume | Spectrum Matches |
| --- | --- | --- | --- | --- |
| -19.8495 | 35.2248 | 0.0096 | 1.1979e+07 | 2 |

**Adduct Signal Fractions**

- **Unmodified**: 1.1979e+07

### FTPN(N)KTEDTIFLR Hex 4 HexNAc 7

**Mass:**  3650.5925

(5.324e-07)

**Charge States:** 3, 4

**Start Position**: 69

**End Position**: 82

| Profile Score | Tandem Score | TDA q-value | Volume | Spectrum Matches |
| --- | --- | --- | --- | --- |
| -4.5901 | 33.8698 | 0.0135 | 1.1374e+07 | 3 |

**Adduct Signal Fractions**

- **Unmodified**: 1.1374e+07

### TPN(N)KTEDTIFLR Hex 3 HexNAc 4

**Mass:**  2732.2307

(3.245e-06)

**Charge States:** 3

**Start Position**: 70

**End Position**: 82

| Profile Score | Tandem Score | TDA q-value | Volume | Spectrum Matches |
| --- | --- | --- | --- | --- |
| 9.4371 | 33.3215 | 0.0142 | 5.8191e+06 | 2 |

**Adduct Signal Fractions**

- **Unmodified**: 5.8191e+06

### QN(D)QC(C)FYN(N)SSYLNVQR Fuc 1 Hex 4 HexNAc 6

**Mass:**  3933.5964

(2.528e-04)

**Charge States:** 3, 4

**Start Position**: 87

**End Position**: 102

| Profile Score | Tandem Score | TDA q-value | Volume | Spectrum Matches |
| --- | --- | --- | --- | --- |
| -5.4673 | 33.0813 | 0.0142 | 6.0221e+07 | 7 |

**Adduct Signal Fractions**

- **Unmodified**: 6.0221e+07

### QNQC(C)FYN(N)SSYLNVQR Hex 4 HexNAc 4 Neu5Ac 2

**Mass:**  3962.5854

(1.685e-06)

**Charge States:** 3, 4

**Start Position**: 87

**End Position**: 102

| Profile Score | Tandem Score | TDA q-value | Volume | Spectrum Matches |
| --- | --- | --- | --- | --- |
| -4.9622 | 33.0471 | 0.0142 | 6.1304e+07 | 2 |

**Adduct Signal Fractions**

- **Unmodified**: 6.1304e+07

### ANLVPVPITN(N)ATLDR Fuc 2 Hex 3 HexNAc 3 Neu5Ac 1

**Mass:**  3271.5038

(2.369e-06)

**Charge States:** 3

**Start Position**: 24

**End Position**: 39

| Profile Score | Tandem Score | TDA q-value | Volume | Spectrum Matches |
| --- | --- | --- | --- | --- |
| 13.1539 | 33.0039 | 0.0142 | 6.3242e+07 | 3 |

**Adduct Signal Fractions**

- **Unmodified**: 6.3242e+07

### C(C)AN(D)LVPVPITN(N)ATLDR Fuc 1 Hex 4 HexNAc 5

**Mass:**  3563.5709

(2.771e-04)

**Charge States:** 3, 4

**Start Position**: 23

**End Position**: 39

| Profile Score | Tandem Score | TDA q-value | Volume | Spectrum Matches |
| --- | --- | --- | --- | --- |
| 7.9378 | 32.5787 | 0.0142 | 2.7221e+07 | 4 |

**Adduct Signal Fractions**

- **Unmodified**: 2.7221e+07

### NLVPVPITN(N)ATLDR Fuc 1 Hex 4 HexNAc 4

**Mass:**  3130.4692

(6.492e-04)

**Start Position**: 25

**End Position**: 39

| Profile Score | Tandem Score | TDA q-value | Volume | Spectrum Matches |
| --- | --- | --- | --- | --- |
| 0.0000 | 32.5660 | 0.0142 | 0.0000e+00 | 1 |

Adduct:

No Chromatogram Found

### TPN(N)KTEDTIFLR Hex 4 HexNAc 7

**Mass:**  3503.5175

(3.213e-06)

**Charge States:** 3, 4

**Start Position**: 70

**End Position**: 82

| Profile Score | Tandem Score | TDA q-value | Volume | Spectrum Matches |
| --- | --- | --- | --- | --- |
| -5.4974 | 32.5322 | 0.0142 | 5.4711e+06 | 3 |

**Adduct Signal Fractions**

- **Unmodified**: 5.4711e+06

### QNQC(C)FYN(N)SSYLNVQR Fuc 1 Hex 5 HexNAc 8

**Mass:**  4500.8269

(1.613e-06)

**Charge States:** 4

**Start Position**: 87

**End Position**: 102

| Profile Score | Tandem Score | TDA q-value | Volume | Spectrum Matches |
| --- | --- | --- | --- | --- |
| -20.8038 | 32.2293 | 0.0178 | 4.4421e+06 | 1 |

**Adduct Signal Fractions**

- **Unmodified**: 4.4421e+06

### NLVPVPITN(N)ATLDR Fuc 1 Hex 5 HexNAc 4

**Mass:**  3293.4889

(9.199e-04)

**Charge States:** 3

**Start Position**: 25

**End Position**: 39

| Profile Score | Tandem Score | TDA q-value | Volume | Spectrum Matches |
| --- | --- | --- | --- | --- |
| -35.6525 | 32.2241 | 0.0178 | 2.0071e+06 | 1 |

**Adduct Signal Fractions**

- **Unmodified**: 2.0071e+06

### LVPVPITN(N)ATLDR Fuc 1 Hex 4 HexNAc 5

**Mass:**  3219.4861

(6.252e-04)

**Start Position**: 26

**End Position**: 39

| Profile Score | Tandem Score | TDA q-value | Volume | Spectrum Matches |
| --- | --- | --- | --- | --- |
| 0.0000 | 31.8767 | 0.0212 | 0.0000e+00 | 1 |

Adduct:

No Chromatogram Found

### QN(D)QC(C)FYN(N)SSYLNVQR Fuc 2 Hex 3 HexNAc 6 Neu5Ac 1

**Mass:**  4208.6726

(2.301e-04)

**Charge States:** 3, 4

**Start Position**: 87

**End Position**: 102

| Profile Score | Tandem Score | TDA q-value | Volume | Spectrum Matches |
| --- | --- | --- | --- | --- |
| -21.2099 | 31.5691 | 0.0224 | 1.4840e+07 | 1 |

**Adduct Signal Fractions**

- **Unmodified**: 1.4840e+07

### LVPVPITN(N)ATLDR Hex 5 HexNAc 5

**Mass:**  3235.4990

(6.295e-04)

**Charge States:** 3

**Start Position**: 26

**End Position**: 39

| Profile Score | Tandem Score | TDA q-value | Volume | Spectrum Matches |
| --- | --- | --- | --- | --- |
| -20.7372 | 30.7715 | 0.0242 | 2.4427e+05 | 25 |

**Adduct Signal Fractions**

- **Unmodified**: 2.4427e+05

### ITGKWFYIASAFRNEEYNKSVQEIQATFFYFTPN(N)K Fuc 2 Hex 8 HexNAc 7

**Mass:**  7247.1768

(-1.708e-06)

**Charge States:** 5

**Start Position**: 39

**End Position**: 74

| Profile Score | Tandem Score | TDA q-value | Volume | Spectrum Matches |
| --- | --- | --- | --- | --- |
| -19.7761 | 30.4438 | 0.0251 | 1.6048e+07 | 1 |

**Adduct Signal Fractions**

- **Unmodified**: 1.6048e+07

### QN(D)QC(C)FYN(N)SSYLNVQR Fuc 4 Hex 3 HexNAc 5

**Mass:**  4006.6428

(2.489e-04)

**Charge States:** 3, 4

**Start Position**: 87

**End Position**: 102

| Profile Score | Tandem Score | TDA q-value | Volume | Spectrum Matches |
| --- | --- | --- | --- | --- |
| 11.4957 | 30.3660 | 0.0251 | 5.7844e+07 | 2 |

**Adduct Signal Fractions**

- **Unmodified**: 5.7844e+07

### QNQC(C)FYN(N)SSYLNVQR Fuc 5 Hex 4 HexNAc 9

**Mass:**  5126.0529

(-5.015e-06)

**Charge States:** 4

**Start Position**: 87

**End Position**: 102

| Profile Score | Tandem Score | TDA q-value | Volume | Spectrum Matches |
| --- | --- | --- | --- | --- |
| 12.3506 | 30.3295 | 0.0262 | 1.9153e+07 | 4 |

**Adduct Signal Fractions**

- **Unmodified**: 1.9153e+07

### FYN(N)SSYLNVQR Hex 3 HexNAc 5

**Mass:**  2891.2269

(1.611e-07)

**Charge States:** 3

**Start Position**: 91

**End Position**: 102

| Profile Score | Tandem Score | TDA q-value | Volume | Spectrum Matches |
| --- | --- | --- | --- | --- |
| -4.4459 | 29.8850 | 0.0319 | 2.9585e+06 | 1 |

**Adduct Signal Fractions**

- **Unmodified**: 2.9585e+06

### QC(C)FYN(N)SSYLNVQR Hex 3 HexNAc 6

**Mass:**  3382.3950

(1.036e-06)

**Charge States:** 3

**Start Position**: 89

**End Position**: 102

| Profile Score | Tandem Score | TDA q-value | Volume | Spectrum Matches |
| --- | --- | --- | --- | --- |
| -20.1691 | 28.6959 | 0.0365 | 5.1269e+06 | 1 |

**Adduct Signal Fractions**

- **Unmodified**: 5.1269e+06

### FTPN(N)KTEDTIFLR Fuc 2 Hex 5 HexNAc 6

**Mass:**  3901.6840

(2.028e-06)

**Charge States:** 3, 4

**Start Position**: 69

**End Position**: 82

| Profile Score | Tandem Score | TDA q-value | Volume | Spectrum Matches |
| --- | --- | --- | --- | --- |
| -19.3716 | 28.6611 | 0.0375 | 2.0889e+06 | 2 |

**Adduct Signal Fractions**

- **Unmodified**: 2.0889e+06

### QC(C)FYN(N)SSYLNVQR Fuc 1 Hex 6 HexNAc 4

**Mass:**  3608.4589

(2.321e-06)

**Charge States:** 3, 4

**Start Position**: 89

**End Position**: 102

| Profile Score | Tandem Score | TDA q-value | Volume | Spectrum Matches |
| --- | --- | --- | --- | --- |
| 14.1534 | 28.4186 | 0.0377 | 6.9532e+08 | 2 |

**Adduct Signal Fractions**

- **Unmodified**: 6.9532e+08

### YN(N)SSYLNVQR Hex 3 HexNAc 5

**Mass:**  2744.1562

(2.564e-06)

**Charge States:** 3

**Start Position**: 92

**End Position**: 102

| Profile Score | Tandem Score | TDA q-value | Volume | Spectrum Matches |
| --- | --- | --- | --- | --- |
| 13.4012 | 28.4060 | 0.0377 | 7.2595e+06 | 2 |

**Adduct Signal Fractions**

- **Unmodified**: 7.2595e+06

### LVPVPITN(N)ATLDR Fuc 2 Hex 6 HexNAc 5 Neu5Ac 2

**Mass:**  4271.8337

(4.695e-04)

**Start Position**: 26

**End Position**: 39

| Profile Score | Tandem Score | TDA q-value | Volume | Spectrum Matches |
| --- | --- | --- | --- | --- |
| 0.0000 | 28.1653 | 0.0377 | 0.0000e+00 | 1 |

Adduct:

No Chromatogram Found

### LVPVPITN(N)ATLDR Fuc 1 Hex 8 HexNAc 2

**Mass:**  3256.4588

(3.373e-06)

**Charge States:** 3, 4

**Start Position**: 26

**End Position**: 39

| Profile Score | Tandem Score | TDA q-value | Volume | Spectrum Matches |
| --- | --- | --- | --- | --- |
| -19.4538 | 27.7147 | 0.0388 | 4.8570e+06 | 3 |

**Adduct Signal Fractions**

- **Unmodified**: 4.8570e+06

### YN(N)SSYLNVQR Fuc 4 Hex 3 HexNAc 5

**Mass:**  3331.4035

(9.065e-04)

**Start Position**: 92

**End Position**: 102

| Profile Score | Tandem Score | TDA q-value | Volume | Spectrum Matches |
| --- | --- | --- | --- | --- |
| 0.0000 | 27.6957 | 0.0388 | 0.0000e+00 | 1 |

Adduct:

No Chromatogram Found

### FYN(N)SSYLNVQR Fuc 1 Hex 4 HexNAc 6

**Mass:**  3402.4165

(1.123e-06)

**Charge States:** 3, 4

**Start Position**: 91

**End Position**: 102

| Profile Score | Tandem Score | TDA q-value | Volume | Spectrum Matches |
| --- | --- | --- | --- | --- |
| 11.2097 | 27.6412 | 0.0388 | 6.2023e+06 | 5 |

**Adduct Signal Fractions**

- **Unmodified**: 6.2023e+06

### PVPITN(N)ATLDR Fuc 2 Hex 4 HexNAc 5

**Mass:**  3152.3977

(3.229e-04)

**Start Position**: 28

**End Position**: 39

| Profile Score | Tandem Score | TDA q-value | Volume | Spectrum Matches |
| --- | --- | --- | --- | --- |
| 0.0000 | 26.8042 | 0.0399 | 0.0000e+00 | 1 |

Adduct:

No Chromatogram Found

### C(C)ANLVPVPITN(N)ATLDR Hex 3 HexNAc 6

**Mass:**  3457.5532

(-2.074e-07)

**Charge States:** 3

**Start Position**: 23

**End Position**: 39

| Profile Score | Tandem Score | TDA q-value | Volume | Spectrum Matches |
| --- | --- | --- | --- | --- |
| -19.6098 | 26.6495 | 0.0441 | 1.8912e+06 | 1 |

**Adduct Signal Fractions**

- **Unmodified**: 1.8912e+06

### QNQC(C)FYN(N)SSYLNVQR Hex 5 HexNAc 6

**Mass:**  3948.5632

(-8.404e-06)

**Charge States:** 3, 4

**Start Position**: 87

**End Position**: 102

| Profile Score | Tandem Score | TDA q-value | Volume | Spectrum Matches |
| --- | --- | --- | --- | --- |
| -4.9084 | 26.0959 | 0.0453 | 1.0055e+08 | 4 |

**Adduct Signal Fractions**

- **Unmodified**: 1.0055e+08

### LVPVPITN(N)ATLDR Hex 8 HexNAc 3

**Mass:**  3314.4538

(2.971e-04)

**Start Position**: 26

**End Position**: 39

| Profile Score | Tandem Score | TDA q-value | Volume | Spectrum Matches |
| --- | --- | --- | --- | --- |
| 0.0000 | 25.5399 | 0.0453 | 0.0000e+00 | 1 |

Adduct:

No Chromatogram Found

### PVPITN(N)ATLDR Fuc 2 Hex 4 HexNAc 4

**Mass:**  2949.3012

(3.440e-04)

**Charge States:** 3

**Start Position**: 28

**End Position**: 39

| Profile Score | Tandem Score | TDA q-value | Volume | Spectrum Matches |
| --- | --- | --- | --- | --- |
| -21.7032 | 25.4313 | 0.0453 | 1.4054e+06 | 2 |

**Adduct Signal Fractions**

- **Unmodified**: 1.4054e+06

### PVPITN(N)ATLDR Fuc 2 Hex 5 HexNAc 6

**Mass:**  3517.5353

(2.909e-04)

**Start Position**: 28

**End Position**: 39

| Profile Score | Tandem Score | TDA q-value | Volume | Spectrum Matches |
| --- | --- | --- | --- | --- |
| 0.0000 | 25.3849 | 0.0453 | 0.0000e+00 | 1 |

Adduct:

No Chromatogram Found

### QN(D)QC(C)FYN(N)SSYLNVQR Fuc 4 Hex 3 HexNAc 6

**Mass:**  4211.7285

(7.137e-04)

**Start Position**: 87

**End Position**: 102

| Profile Score | Tandem Score | TDA q-value | Volume | Spectrum Matches |
| --- | --- | --- | --- | --- |
| 0.0000 | 24.8896 | 0.0497 | 0.0000e+00 | 1 |

Adduct:

No Chromatogram Found

### P02763|A1AG1\_HUMAN

**>P02763|A1AG1\_HUMAN**  
 `MALSWVLTVLSLLPLLEAQIPLCANLVPVPITNATLDQITGKWFYIASAFRNEEYNKSVQEIQATFFYFTPNKTEDTIFL  
REYQTRQDQCIYNTTYLNVQRENGTISRYVGGQEHFAHLLILRDTKTYMLAFDVNDEKNWGLSVYADKPETTKEQLGEFY  
EALDCLRIPKSDVVYTDWKKDKCEPLEKQHEKERKQEEGES`

| Site | Type | Count |
| --- | --- | --- |
| 33 | N-linked | 42 |
| 56 | N-linked | 3 |
| 93 | N-linked | 35 |
| 72 | N-linked | 72 |

| Mass | Sequence | MS_2_ Score | `q`-value | MS_1_ Score | Signal | Protein Coordinates | Tandem Scans | Start Time | Apex Time | End Time |
| --- | --- | --- | --- | --- | --- | --- | --- | --- | --- | --- |
| 2269.9401 | YFTPN(N)K Hex 3 HexNAc 5 | 187.215 | 0.0 | 13.366 | 2.230e+08 | 67 - 73 | 9 | 13.78 | 13.92 | 14.48 |
| 2579.0434 | YFTPN(N)K Fuc 1 Hex 4 HexNAc 5 | 186.195 | 0.0 | 0.000 | 0.000e+00 | 67 - 73 | 1 | - | - | - |
| 2093.8165 | NEEYN(N)K Hex 3 HexNAc 4 | 184.819 | 0.0 | 11.704 | 4.780e+07 | 51 - 57 | 5 | 3.82 | 3.82 | 5.09 |
| 2620.0864 | FYFTPN(N)K Hex 3 HexNAc 6 | 182.864 | 0.0 | 13.086 | 1.141e+08 | 66 - 73 | 12 | 25.06 | 25.40 | 27.88 |
| 2296.8958 | NEEYN(N)K Hex 3 HexNAc 5 | 180.580 | 0.0 | 11.194 | 4.431e+07 | 51 - 57 | 5 | 3.82 | 3.99 | 5.33 |
| 2605.0064 | NEEYN(N)K Fuc 1 Hex 4 HexNAc 5 | 180.189 | 0.0 | 11.957 | 2.209e+07 | 51 - 57 | 4 | 3.82 | 3.82 | 5.06 |
| 3623.5856 | SVQEIQATFFYFTPN(N)K Hex 3 HexNAc 6 | 175.232 | 0.0 | -0.945 | 1.423e+09 | 57 - 73 | 25 | 51.28 | 55.19 | 58.73 |
| 3931.6992 | SVQEIQATFFYFTPN(N)K Fuc 1 Hex 4 HexNAc 6 | 174.868 | 0.0 | 13.709 | 3.813e+08 | 57 - 73 | 14 | 54.51 | 54.68 | 56.87 |
| 2066.8593 | YFTPN(N)K Hex 3 HexNAc 4 | 172.983 | 0.0 | -19.810 | 2.193e+07 | 67 - 73 | 3 | 13.92 | 14.08 | 14.08 |
| 2473.0185 | YFTPN(N)K Hex 3 HexNAc 6 | 172.576 | 0.0 | 14.581 | 3.867e+08 | 67 - 73 | 7 | 13.64 | 13.78 | 14.51 |
| 2838.1510 | YFTPN(N)K Hex 4 HexNAc 7 | 172.169 | 0.0 | -4.355 | 4.768e+07 | 67 - 73 | 3 | 13.35 | 13.52 | 13.78 |
| 2417.0067 | FYFTPN(N)K Hex 3 HexNAc 5 | 169.081 | 0.0 | 12.797 | 5.726e+07 | 66 - 73 | 9 | 25.40 | 25.73 | 27.58 |
| 2985.2208 | FYFTPN(N)K Hex 4 HexNAc 7 | 168.306 | 0.0 | 10.905 | 1.477e+07 | 66 - 73 | 5 | 24.29 | 24.89 | 25.73 |
| 3420.5049 | SVQEIQATFFYFTPN(N)K Hex 3 HexNAc 5 | 167.090 | 0.0 | 14.921 | 7.993e+08 | 57 - 73 | 31 | 51.45 | 55.35 | 59.75 |
| 4239.8080 | SVQEIQATFFYFTPN(N)K Fuc 2 Hex 5 HexNAc 6 | 165.156 | 0.0 | 13.103 | 5.522e+07 | 57 - 73 | 4 | 54.00 | 54.34 | 55.02 |
| 3588.6944 | LVPVPITN(N)ATLDQITGK Fuc 1 Hex 4 HexNAc 5 | 165.095 | 0.0 | 14.917 | 4.428e+09 | 25 - 42 | 107 | 41.83 | 48.69 | 113.03 |
| 2782.1409 | YFTPN(N)K Fuc 1 Hex 4 HexNAc 6 | 162.605 | 0.042 | -7.397 | 3.062e+06 | 67 - 73 | 5 | 24.72 | 25.90 | 25.90 |
| 3728.6177 | SVQEIQATFFYFTPN(N)K Fuc 1 Hex 4 HexNAc 5 | 161.119 | 0.0 | 12.479 | 2.381e+08 | 57 - 73 | 8 | 54.68 | 54.84 | 56.19 |
| 2939.2408 | ATFFYFTPN(N)K Hex 3 HexNAc 6 | 160.392 | 0.0 | -3.612 | 4.537e+07 | 63 - 73 | 2 | 41.49 | 41.66 | 42.00 |
| 3988.7195 | SVQEIQATFFYFTPN(N)K Hex 4 HexNAc 7 | 160.097 | 0.0 | 10.904 | 2.915e+08 | 57 - 73 | 15 | 54.16 | 54.68 | 57.22 |
| 3217.4265 | SVQEIQATFFYFTPN(N)K Hex 3 HexNAc 4 | 155.631 | 0.0 | 13.230 | 9.872e+07 | 57 - 73 | 8 | 55.51 | 55.68 | 57.38 |
| 3280.5746 | LVPVPITN(N)ATLDQITGK Hex 3 HexNAc 5 | 153.674 | 0.0 | 14.247 | 1.500e+10 | 25 - 42 | 526 | 42.17 | 49.00 | 113.40 |
| 4296.8308 | SVQEIQATFFYFTPN(N)K Fuc 1 Hex 5 HexNAc 7 | 153.517 | 0.0 | 9.888 | 4.212e+07 | 57 - 73 | 5 | 53.66 | 54.16 | 55.02 |
| 3077.4918 | LVPVPITN(N)ATLDQITGK Hex 3 HexNAc 4 | 152.781 | 0.0 | 14.602 | 1.452e+09 | 25 - 42 | 26 | 42.34 | 49.46 | 53.49 |
| 3483.6558 | LVPVPITN(N)ATLDQITGK Hex 3 HexNAc 6 | 152.106 | 0.0 | 9.248 | 3.916e+08 | 25 - 42 | 11 | 42.17 | 48.85 | 50.94 |
| 3791.7664 | LVPVPITN(N)ATLDQITGK Fuc 1 Hex 4 HexNAc 6 | 150.220 | 0.0 | 15.108 | 2.059e+08 | 25 - 42 | 5 | 48.18 | 48.52 | 48.85 |
| 3448.5216 | YFTPN(N)KTEDTIFLR Hex 3 HexNAc 6 | 149.749 | 0.0 | 10.179 | 1.559e+09 | 67 - 81 | 11 | 35.64 | 37.55 | 39.69 |
| 4191.7997 | SVQEIQATFFYFTPN(N)K Hex 4 HexNAc 8 | 149.010 | 0.0 | 13.199 | 3.238e+07 | 57 - 73 | 3 | 54.00 | 54.16 | 54.84 |
| 3426.6302 | LVPVPITN(N)ATLDQITGK Fuc 1 Hex 3 HexNAc 5 | 144.664 | 0.0 | -5.095 | 7.435e+07 | 25 - 42 | 3 | 49.31 | 49.31 | 49.61 |
| 3442.6287 | LVPVPITN(N)ATLDQITGK Hex 4 HexNAc 5 | 142.905 | 0.0 | 14.320 | 1.118e+08 | 25 - 42 | 3 | 48.35 | 48.85 | 49.00 |
| 2935.3090 | TPN(N)KTEDTIFLR Hex 3 HexNAc 5 | 140.244 | 0.0 | 12.248 | 4.117e+07 | 69 - 81 | 6 | 25.73 | 26.07 | 27.00 |
| 3984.6623 | QDQC(C)IYN(N)TTYLNVQR Hex 4 HexNAc 7 | 138.559 | 0.0 | 14.381 | 8.304e+08 | 86 - 101 | 8 | 32.82 | 33.50 | 34.46 |
| 4292.7740 | QDQC(C)IYN(N)TTYLNVQR Fuc 1 Hex 5 HexNAc 7 | 138.486 | 0.0 | 14.198 | 1.529e+08 | 86 - 101 | 4 | 32.31 | 32.99 | 33.50 |
| 3927.6393 | QDQC(C)IYN(N)TTYLNVQR Fuc 1 Hex 4 HexNAc 6 | 137.665 | 0.0 | 15.175 | 1.015e+09 | 86 - 101 | 4 | 33.33 | 33.50 | 33.98 |
| 3724.5588 | QDQC(C)IYN(N)TTYLNVQR Fuc 1 Hex 4 HexNAc 5 | 137.604 | 0.0 | 11.020 | 4.845e+08 | 86 - 101 | 4 | 33.50 | 33.66 | 34.63 |
| 3756.6307 | YFTPN(N)KTEDTIFLR Fuc 1 Hex 4 HexNAc 6 | 137.450 | 0.0 | -4.168 | 2.883e+08 | 67 - 81 | 6 | 35.31 | 37.10 | 37.55 |
| 3553.5516 | YFTPN(N)KTEDTIFLR Fuc 1 Hex 4 HexNAc 5 | 137.246 | 0.0 | 9.728 | 2.117e+08 | 67 - 81 | 5 | 37.10 | 37.25 | 37.87 |
| 3785.6400 | SVQEIQATFFYFTPN(N)K Hex 4 HexNAc 6 | 137.025 | 0.0 | 10.509 | 4.957e+07 | 57 - 73 | 6 | 54.34 | 54.68 | 56.87 |
| 3619.5264 | QDQC(C)IYN(N)TTYLNVQR Hex 3 HexNAc 6 | 136.100 | 0.0 | 13.946 | 3.362e+09 | 86 - 101 | 9 | 31.29 | 33.83 | 34.97 |
| 2736.1603 | ATFFYFTPN(N)K Hex 3 HexNAc 5 | 133.952 | 0.0 | -3.869 | 2.179e+07 | 63 - 73 | 1 | 41.83 | 41.83 | 42.17 |
| 4349.7961 | QDQC(C)IYN(N)TTYLNVQR Hex 5 HexNAc 8 | 133.287 | 0.0 | 11.665 | 1.351e+08 | 86 - 101 | 4 | 32.48 | 32.82 | 33.50 |
| 3044.2687 | ATFFYFTPN(N)K Fuc 1 Hex 4 HexNAc 5 | 132.987 | 0.0 | -4.865 | 6.005e+06 | 63 - 73 | 1 | 41.15 | 41.32 | 41.49 |
| 4187.7422 | QDQC(C)IYN(N)TTYLNVQR Hex 4 HexNAc 8 | 132.914 | 0.0 | 13.975 | 1.505e+08 | 86 - 101 | 3 | 32.82 | 32.99 | 33.66 |
| 3848.7903 | LVPVPITN(N)ATLDQITGK Hex 4 HexNAc 7 | 132.728 | 0.0 | 10.972 | 1.150e+08 | 25 - 42 | 6 | 48.02 | 48.52 | 49.00 |
| 3961.6705 | SVQEIQATFFYFTPN(N)K Hex 4 HexNAc 4 Neu5Ac 2 | 131.731 | 0.0 | 12.840 | 5.306e+07 | 57 - 73 | 7 | 55.51 | 55.85 | 56.70 |
| 3416.4469 | QDQC(C)IYN(N)TTYLNVQR Hex 3 HexNAc 5 | 131.078 | 0.0 | 12.958 | 1.719e+09 | 86 - 101 | 7 | 33.83 | 34.14 | 35.14 |
| 3138.3890 | TPN(N)KTEDTIFLR Hex 3 HexNAc 6 | 129.398 | 0.0 | 12.528 | 4.322e+07 | 69 - 81 | 5 | 25.57 | 25.90 | 26.25 |
| 3645.7065 | LVPVPITN(N)ATLDQITGK Hex 4 HexNAc 6 | 129.302 | 0.0 | 13.540 | 8.786e+08 | 25 - 42 | 15 | 42.34 | 49.46 | 51.28 |
| 3042.3629 | YFTPN(N)KTEDTIFLR Hex 3 HexNAc 4 | 129.080 | 0.0 | -2.987 | 9.010e+07 | 67 - 81 | 2 | 37.87 | 38.03 | 38.19 |
| 3896.7974 | LVPVPITN(N)ATLDQITGK Fuc 2 Hex 5 HexNAc 5 | 128.112 | 0.0 | -3.922 | 4.327e+07 | 25 - 42 | 2 | 48.02 | 48.18 | 48.52 |
| 3247.3481 | ATFFYFTPN(N)K Fuc 1 Hex 4 HexNAc 6 | 126.835 | 0.0 | -20.785 | 5.852e+06 | 63 - 73 | 1 | 40.99 | 40.99 | 41.15 |
| 4066.7533 | YFTPN(N)KTEDTIFLR Fuc 2 Hex 5 HexNAc 6 | 126.562 | 0.0 | 0.000 | 0.000e+00 | 67 - 81 | 1 | - | - | - |
| 3813.6535 | YFTPN(N)KTEDTIFLR Hex 4 HexNAc 7 | 126.424 | 0.0 | 10.380 | 1.748e+08 | 67 - 81 | 6 | 36.32 | 36.95 | 37.87 |
| 3781.5288 | ATFFYFTPN(N)K Fuc 3 Hex 8 HexNAc 4 | 124.314 | 0.0 | -21.053 | 9.055e+06 | 63 - 73 | 1 | 54.84 | 54.84 | 55.02 |
| 3595.5910 | FYFTPN(N)KTEDTIFLR Hex 3 HexNAc 6 | 123.420 | 0.0 | 13.440 | 1.316e+08 | 66 - 81 | 6 | 46.31 | 46.65 | 47.85 |
| 3336.5061 | PVPITN(N)ATLDQITGK Fuc 1 Hex 5 HexNAc 4 | 122.637 | 0.0 | 0.000 | 0.000e+00 | 27 - 42 | 1 | - | - | - |
| 4134.7791 | SVQEIQATFFYFTPN(N)K Fuc 1 Hex 4 HexNAc 7 | 122.553 | 0.0 | -4.524 | 1.261e+07 | 57 - 73 | 3 | 54.16 | 54.51 | 54.68 |
| 4235.7505 | QDQC(C)IYN(N)TTYLNVQR Fuc 2 Hex 5 HexNAc 6 | 120.787 | 0.0 | -4.026 | 1.570e+08 | 86 - 101 | 1 | 33.16 | 33.33 | 33.50 |
| 4442.8873 | SVQEIQATFFYFTPN(N)K Fuc 2 Hex 5 HexNAc 7 | 118.188 | 0.0 | -19.598 | 3.822e+06 | 57 - 73 | 1 | 54.00 | 54.00 | 54.16 |
| 3392.5107 | FYFTPN(N)KTEDTIFLR Hex 3 HexNAc 5 | 117.888 | 0.0 | 13.545 | 5.658e+07 | 66 - 81 | 5 | 46.48 | 46.82 | 47.50 |
| 3978.6944 | SVQEIQATFFYFTPN(N)K Fuc 1 Hex 5 HexNAc 4 Neu5Ac 1 | 116.217 | 0.0 | -4.937 | 5.887e+06 | 57 - 73 | 1 | 55.68 | 55.85 | 56.19 |
| 3304.3766 | ATFFYFTPN(N)K Hex 4 HexNAc 7 | 115.403 | 0.0 | -19.822 | 3.428e+06 | 63 - 73 | 1 | 40.82 | 40.82 | 40.99 |
| 3957.6128 | QDQC(C)IYN(N)TTYLNVQR Hex 4 HexNAc 4 Neu5Ac 2 | 114.290 | 0.0 | 14.204 | 2.184e+08 | 86 - 101 | 5 | 34.29 | 34.63 | 35.31 |
| 3245.4431 | YFTPN(N)KTEDTIFLR Hex 3 HexNAc 5 | 114.001 | 0.0 | 9.439 | 4.430e+07 | 67 - 81 | 5 | 35.81 | 35.98 | 38.85 |
| 3213.3679 | QDQC(C)IYN(N)TTYLNVQR Hex 3 HexNAc 4 | 112.866 | 0.0 | 12.565 | 3.391e+07 | 86 - 101 | 3 | 34.14 | 34.29 | 34.80 |
| 3953.8206 | LVPVPITN(N)ATLDQITGK Fuc 1 Hex 5 HexNAc 6 | 111.501 | 0.0 | 8.606 | 3.754e+07 | 25 - 42 | 4 | 47.68 | 48.52 | 48.52 |
| 4099.8755 | LVPVPITN(N)ATLDQITGK Fuc 2 Hex 5 HexNAc 6 | 111.387 | 0.0 | 9.111 | 2.862e+07 | 25 - 42 | 5 | 47.68 | 48.52 | 48.52 |
| 3903.7028 | FYFTPN(N)KTEDTIFLR Fuc 1 Hex 4 HexNAc 6 | 110.841 | 0.0 | 14.340 | 3.015e+07 | 66 - 81 | 4 | 45.97 | 46.14 | 46.65 |
| 3781.5831 | QDQC(C)IYN(N)TTYLNVQR Hex 4 HexNAc 6 | 110.292 | 0.0 | 11.573 | 9.257e+07 | 86 - 101 | 3 | 33.33 | 33.83 | 34.29 |
| 3088.3532 | IYN(N)TTYLNVQR Hex 3 HexNAc 6 | 109.165 | 0.0 | 13.142 | 4.027e+07 | 90 - 101 | 5 | 28.40 | 28.62 | 29.31 |
| 3786.6070 | TPN(N)KTEDTIFLR Hex 7 HexNAc 6 | 108.999 | 0.0 | 13.601 | 1.059e+08 | 69 - 81 | 4 | 38.19 | 38.35 | 39.02 |
| 3648.7313 | LVPVPITN(N)ATLDQITGK Hex 4 HexNAc 6 | 107.538 | 0.0 | 0.000 | 0.000e+00 | 25 - 42 | 1 | - | - | - |
| 3385.6315 | LVPVPITN(N)ATLDQITGK Fuc 1 Hex 4 HexNAc 4 | 106.581 | 0.0 | -19.964 | 1.024e+07 | 25 - 42 | 1 | 48.52 | 48.69 | 48.69 |
| 4178.7888 | YFTPN(N)KTEDTIFLR Hex 5 HexNAc 8 | 105.817 | 0.0 | -3.427 | 3.477e+07 | 67 - 81 | 3 | 35.98 | 36.15 | 36.32 |
| 3189.4312 | FYFTPN(N)KTEDTIFLR Hex 3 HexNAc 4 | 105.457 | 0.0 | 12.671 | 7.593e+06 | 66 - 81 | 2 | 46.82 | 46.99 | 47.50 |
| 4599.0880 | SVQEIQATFFYFTPN(N)KTEDTIFLR Hex 3 HexNAc 6 | 105.317 | 0.0 | -3.857 | 3.344e+07 | 57 - 81 | 4 | 60.74 | 63.12 | 64.47 |
| 4121.7665 | YFTPN(N)KTEDTIFLR Fuc 1 Hex 5 HexNAc 7 | 104.968 | 0.0 | -4.541 | 3.487e+07 | 67 - 81 | 2 | 35.98 | 36.49 | 36.49 |
| 3734.7445 | LVPVPITN(N)ATLDQITGK Fuc 2 Hex 4 HexNAc 5 | 104.884 | 0.0 | 0.000 | 0.000e+00 | 25 - 42 | 1 | - | - | - |
| 4556.9328 | SVQEIQATFFYFTPN(N)K Hex 5 HexNAc 9 | 104.722 | 0.0 | -20.312 | 2.348e+06 | 57 - 73 | 1 | 53.49 | 53.49 | 53.49 |
| 3700.6213 | FYFTPN(N)KTEDTIFLR Fuc 1 Hex 4 HexNAc 5 | 104.244 | 0.0 | -3.525 | 1.624e+07 | 66 - 81 | 4 | 46.14 | 46.31 | 46.65 |
| 3733.7233 | LVPVPITN(N)ATLDQITGK Hex 4 HexNAc 5 Neu5Ac 1 | 103.034 | 0.0 | 12.049 | 1.831e+07 | 25 - 42 | 6 | 51.28 | 51.79 | 52.30 |
| 3845.6778 | PVPITN(N)ATLDQITGK Hex 6 HexNAc 5 Neu5Ac 1 | 103.028 | 0.0 | -4.795 | 7.985e+06 | 27 - 42 | 1 | 52.98 | 53.15 | 53.32 |
| 2885.2736 | IYN(N)TTYLNVQR Hex 3 HexNAc 5 | 102.548 | 0.0 | 13.703 | 3.130e+07 | 90 - 101 | 5 | 28.62 | 28.75 | 29.39 |
| 3082.3791 | FTPN(N)KTEDTIFLR Hex 3 HexNAc 5 | 102.515 | 0.0 | 13.867 | 4.658e+07 | 68 - 81 | 3 | 32.14 | 32.31 | 32.82 |
| 3635.6829 | LVPVPITN(N)ATLDQITGK Fuc 1 Hex 5 HexNAc 3 Neu5Ac 1 | 102.377 | 0.0 | -5.238 | 2.147e+07 | 25 - 42 | 2 | 49.77 | 49.93 | 50.26 |
| 4156.8991 | LVPVPITN(N)ATLDQITGK Fuc 1 Hex 5 HexNAc 7 | 102.183 | 0.0 | 13.289 | 1.914e+07 | 25 - 42 | 3 | 47.50 | 48.18 | 48.52 |
| 4130.7200 | QDQC(C)IYN(N)TTYLNVQR Fuc 1 Hex 4 HexNAc 7 | 101.065 | 0.0 | -4.542 | 5.027e+07 | 86 - 101 | 2 | 32.99 | 33.33 | 33.50 |
| 3285.4579 | FTPN(N)KTEDTIFLR Hex 3 HexNAc 6 | 98.657 | 0.0 | 14.168 | 9.115e+07 | 68 - 81 | 5 | 31.97 | 32.14 | 32.82 |
| 3390.4906 | FTPN(N)KTEDTIFLR Fuc 1 Hex 4 HexNAc 5 | 98.378 | 0.0 | -4.926 | 1.113e+07 | 68 - 81 | 2 | 31.80 | 31.80 | 32.14 |
| 3440.5372 | PVPITN(N)ATLDQITGK Fuc 2 Hex 6 HexNAc 3 | 96.368 | 0.0 | -19.884 | 3.835e+06 | 27 - 42 | 2 | 52.47 | 52.64 | 52.64 |
| 4010.8470 | LVPVPITN(N)ATLDQITGK Hex 5 HexNAc 7 | 95.797 | 0.0 | -5.788 | 8.936e+06 | 25 - 42 | 2 | 48.02 | 48.18 | 48.52 |
| 3750.7430 | LVPVPITN(N)ATLDQITGK Fuc 1 Hex 5 HexNAc 5 | 93.604 | 0.0 | -19.401 | 1.095e+07 | 25 - 42 | 1 | 48.52 | 48.52 | 48.52 |
| 4497.8601 | QDQC(C)IYN(N)TTYLNVQR Fuc 1 Hex 5 HexNAc 8 | 90.735 | 0.0 | 0.000 | 0.000e+00 | 86 - 101 | 1 | - | - | - |
| 4265.7229 | QDQC(C)IYN(N)TTYLNVQR Fuc 1 Hex 5 HexNAc 4 Neu5Ac 2 | 89.781 | 0.0 | -4.977 | 4.272e+07 | 86 - 101 | 2 | 34.14 | 34.29 | 34.63 |
| 3960.7256 | FYFTPN(N)KTEDTIFLR Hex 4 HexNAc 7 | 87.605 | 0.002 | 14.095 | 2.316e+07 | 66 - 81 | 4 | 45.63 | 46.14 | 46.48 |
| 3593.5691 | FTPN(N)KTEDTIFLR Fuc 1 Hex 4 HexNAc 6 | 86.433 | 0.002 | -3.715 | 2.030e+07 | 68 - 81 | 4 | 31.46 | 31.63 | 31.97 |
| 4381.8676 | YFTPN(N)KTEDTIFLR Hex 5 HexNAc 9 | 83.804 | 0.002 | -5.063 | 9.836e+06 | 67 - 81 | 1 | 35.81 | 35.98 | 36.15 |
| 3244.4133 | TPN(N)KTEDTIFLR Fuc 1 Hex 4 HexNAc 5 | 83.706 | 0.002 | 0.000 | 0.000e+00 | 69 - 81 | 1 | - | - | - |
| 3398.3124 | IYN(N)TTYLNVQR Fuc 1 Hex 4 HexNAc 6 | 83.174 | 0.003 | -8.389 | 3.171e+07 | 90 - 101 | 6 | 28.01 | 34.63 | 34.97 |
| 4267.8264 | YFTPN(N)KTEDTIFLR Fuc 2 Hex 5 HexNAc 7 | 81.434 | 0.003 | -20.034 | 2.247e+06 | 67 - 81 | 1 | 36.32 | 36.32 | 36.32 |
| 3446.5007 | TPN(N)KTEDTIFLR Fuc 1 Hex 4 HexNAc 6 | 79.167 | 0.003 | -4.289 | 8.982e+06 | 69 - 81 | 4 | 25.23 | 25.40 | 25.57 |
| 3243.4221 | TPN(N)KTEDTIFLR Fuc 1 Hex 4 HexNAc 5 | 75.903 | 0.003 | -4.939 | 4.206e+06 | 69 - 81 | 3 | 25.40 | 25.57 | 25.73 |
| 3625.6855 | C(C)ANLVPVPITN(N)ATLDQITGK Hex 3 HexNAc 5 | 65.919 | 0.003 | 14.652 | 1.315e+08 | 22 - 42 | 5 | 52.47 | 52.64 | 53.15 |
| 3422.6071 | C(C)ANLVPVPITN(N)ATLDQITGK Hex 3 HexNAc 4 | 63.424 | 0.003 | -3.816 | 1.430e+07 | 22 - 42 | 2 | 52.64 | 52.81 | 53.15 |
| 4396.0178 | SVQEIQATFFYFTPN(N)KTEDTIFLR Hex 3 HexNAc 5 | 61.755 | 0.003 | -21.526 | 6.211e+06 | 57 - 81 | 1 | 63.29 | 63.46 | 63.46 |
| 3933.7978 | C(C)ANLVPVPITN(N)ATLDQITGK Fuc 1 Hex 4 HexNAc 5 | 60.750 | 0.003 | 12.351 | 4.636e+07 | 22 - 42 | 4 | 52.13 | 52.30 | 52.81 |
| 4438.8300 | QDQC(C)IYN(N)TTYLNVQR Fuc 2 Hex 5 HexNAc 7 | 56.135 | 0.003 | -4.665 | 2.724e+07 | 86 - 101 | 2 | 32.82 | 33.16 | 33.33 |
| 3522.5811 | PVPITN(N)ATLDQITGK Fuc 2 Hex 4 HexNAc 5 | 55.999 | 0.003 | -5.405 | 5.693e+06 | 27 - 42 | 2 | 52.64 | 52.81 | 52.98 |
| 4495.8541 | QDQC(C)IYN(N)TTYLNVQR Fuc 1 Hex 5 HexNAc 8 | 48.222 | 0.003 | -4.219 | 3.979e+07 | 86 - 101 | 1 | 32.48 | 32.82 | 32.99 |
| 3583.5751 | SVQEIQATFFYFTPN(N)K Hex 4 HexNAc 5 | 47.932 | 0.003 | 0.000 | 0.000e+00 | 57 - 73 | 2 | - | - | - |
| 3974.6381 | QDQC(C)IYN(N)TTYLNVQR Fuc 1 Hex 5 HexNAc 4 Neu5Ac 1 | 47.061 | 0.003 | -5.558 | 1.198e+07 | 86 - 101 | 1 | 34.46 | 34.46 | 34.80 |
| 3886.7327 | PVPITN(N)ATLDQITGK Hex 5 HexNAc 6 Neu5Ac 1 | 47.000 | 0.003 | -5.143 | 9.611e+06 | 27 - 42 | 1 | 48.18 | 48.52 | 48.52 |
| 3765.5894 | QDQC(C)IYN(N)TTYLNVQR Fuc 1 Hex 3 HexNAc 6 | 46.717 | 0.003 | -18.880 | 1.147e+07 | 86 - 101 | 1 | 33.66 | 33.66 | 33.66 |
| 4552.8753 | QDQC(C)IYN(N)TTYLNVQR Hex 5 HexNAc 9 | 45.704 | 0.003 | -4.919 | 6.780e+07 | 86 - 101 | 2 | 32.48 | 32.65 | 32.99 |
| 4016.7357 | YFTPN(N)KTEDTIFLR Hex 4 HexNAc 8 | 45.141 | 0.003 | -18.338 | 1.841e+07 | 67 - 81 | 1 | 36.32 | 36.49 | 36.49 |
| 4657.9043 | QDQC(C)IYN(N)TTYLNVQR Fuc 1 Hex 6 HexNAc 8 | 44.293 | 0.003 | -4.496 | 1.363e+07 | 86 - 101 | 2 | 32.14 | 32.48 | 32.65 |
| 4909.2093 | SVQEIQATFFYFTPN(N)KTEDTIFLR Fuc 1 Hex 4 HexNAc 6 | 44.281 | 0.003 | 0.000 | 0.000e+00 | 57 - 81 | 1 | - | - | - |
| 2879.2996 | FTPN(N)KTEDTIFLR Hex 3 HexNAc 4 | 40.741 | 0.003 | -5.006 | 5.810e+06 | 68 - 81 | 1 | 32.48 | 32.65 | 32.82 |
| 4704.1178 | SVQEIQATFFYFTPN(N)KTEDTIFLR Fuc 1 Hex 4 HexNAc 5 | 40.205 | 0.003 | -5.307 | 3.791e+06 | 57 - 81 | 1 | 62.77 | 62.95 | 63.12 |
| 4353.8478 | SVQEIQATFFYFTPN(N)K Hex 5 HexNAc 8 | 40.081 | 0.003 | 7.688 | 2.414e+07 | 57 - 73 | 1 | 53.66 | 53.83 | 54.68 |
| 3620.5200 | QDQC(C)IYN(N)TTYLN(D)VQR Hex 3 HexNAc 6 | 39.947 | 0.003 | -20.245 | 9.061e+07 | 86 - 101 | 5 | 32.48 | 35.14 | 35.14 |
| 3792.7733 | LVPVPITN(N)ATLDQITGK Fuc 1 Hex 4 HexNAc 6 | 38.698 | 0.004 | 0.000 | 0.000e+00 | 25 - 42 | 1 | - | - | - |
| 4640.0184 | PVPITN(N)ATLDQITGK Fuc 2 Hex 3 HexNAc 7 Neu5Ac 3 | 38.126 | 0.006 | 11.811 | 5.258e+06 | 27 - 42 | 3 | 57.56 | 57.73 | 58.21 |
| 4213.9204 | LVPVPITN(N)ATLDQITGK Hex 5 HexNAc 8 | 37.177 | 0.006 | 5.828 | 6.635e+06 | 25 - 42 | 3 | 47.50 | 47.68 | 48.35 |
| 4600.8830 | QDQC(C)IYN(N)TTYLNVQR Fuc 2 Hex 6 HexNAc 7 | 36.894 | 0.006 | -5.212 | 1.585e+07 | 86 - 101 | 1 | 32.31 | 32.31 | 32.99 |
| 4860.9845 | QDQC(C)IYN(N)TTYLNVQR Fuc 1 Hex 6 HexNAc 9 | 36.676 | 0.006 | -18.426 | 7.073e+06 | 86 - 101 | 1 | 32.31 | 32.31 | 32.48 |
| 4499.9048 | SVQEIQATFFYFTPN(N)K Fuc 1 Hex 5 HexNAc 8 | 36.043 | 0.006 | -6.327 | 6.475e+06 | 57 - 73 | 2 | 53.49 | 53.66 | 54.00 |
| 4601.0913 | SVQEIQATFFYFTPN(N)KTEDTIFLR Hex 3 HexNAc 6 | 35.675 | 0.008 | 0.000 | 0.000e+00 | 57 - 81 | 1 | - | - | - |
| 4211.8117 | FYFTPN(N)KTEDTIFLR Fuc 2 Hex 5 HexNAc 6 | 35.652 | 0.008 | -4.662 | 5.478e+06 | 66 - 81 | 1 | 45.63 | 45.80 | 45.97 |
| 3650.5927 | FTPN(N)KTEDTIFLR Hex 4 HexNAc 7 | 33.870 | 0.013 | -4.590 | 1.137e+07 | 68 - 81 | 3 | 31.29 | 31.63 | 31.80 |
| 4543.8617 | QDQC(C)IYN(N)TTYLNVQR Fuc 3 Hex 6 HexNAc 6 | 33.692 | 0.014 | -5.508 | 1.491e+07 | 86 - 101 | 1 | 32.82 | 32.99 | 33.16 |
| 3866.8161 | LVPVPITN(N)ATLDQITGK Fuc 4 Hex 3 HexNAc 5 | 33.621 | 0.014 | 0.000 | 0.000e+00 | 25 - 42 | 1 | - | - | - |
| 2732.2300 | TPN(N)KTEDTIFLR Hex 3 HexNAc 4 | 33.322 | 0.014 | 9.437 | 5.819e+06 | 69 - 81 | 2 | 26.25 | 26.30 | 26.64 |
| 4051.8666 | LVPVPITN(N)ATLDQITGK Hex 4 HexNAc 8 | 32.672 | 0.014 | -19.765 | 3.741e+06 | 25 - 42 | 1 | 47.85 | 48.02 | 48.02 |
| 3503.5199 | TPN(N)KTEDTIFLR Hex 4 HexNAc 7 | 32.532 | 0.014 | -5.497 | 5.471e+06 | 69 - 81 | 3 | 25.06 | 25.23 | 25.40 |
| 4368.0496 | QIPLC(C)ANLVPVPITN(N)ATLDQITGK Hex 3 HexNAc 5 Neu5Ac 1 | 32.384 | 0.014 | 13.593 | 2.332e+08 | 18 - 42 | 11 | 63.79 | 68.36 | 69.04 |
| 3236.3038 | QDQC(C)IYN(N)TTYLN Hex 3 HexNAc 6 | 30.310 | 0.026 | -18.928 | 1.218e+07 | 86 - 98 | 1 | 32.65 | 32.82 | 32.82 |
| 2561.1137 | N(N)ATLDQITGK Hex 3 HexNAc 5 | 30.238 | 0.027 | -20.631 | 5.423e+05 | 32 - 42 | 1 | 19.47 | 19.47 | 19.47 |
| 3990.8187 | C(C)ANLVPVPITN(N)ATLDQITGK Hex 4 HexNAc 6 | 29.493 | 0.033 | -4.511 | 1.226e+07 | 22 - 42 | 2 | 53.15 | 53.15 | 53.49 |
| 3928.6284 | QDQC(C)IYN(N)TTYLN(D)VQR Fuc 1 Hex 4 HexNAc 6 | 29.389 | 0.033 | -19.833 | 1.680e+07 | 86 - 101 | 2 | 34.63 | 34.80 | 34.80 |
| 3901.6834 | FTPN(N)KTEDTIFLR Fuc 2 Hex 5 HexNAc 6 | 28.661 | 0.038 | -19.372 | 2.089e+06 | 68 - 81 | 2 | 31.29 | 31.29 | 31.29 |
| 3282.5809 | LVPVPITN(N)ATLDQITGK Hex 3 HexNAc 5 | 28.167 | 0.038 | 0.000 | 0.000e+00 | 25 - 42 | 2 | - | - | - |
| 3193.3824 | IYN(N)TTYLNVQR Fuc 1 Hex 4 HexNAc 5 | 28.127 | 0.038 | 9.508 | 8.064e+06 | 90 - 101 | 1 | 28.27 | 28.33 | 28.62 |
| 3418.4509 | QDQC(C)IYN(N)TTYLNVQR Hex 3 HexNAc 5 | 27.891 | 0.038 | 0.000 | 0.000e+00 | 86 - 101 | 1 | - | - | - |
| 4803.9662 | QDQC(C)IYN(N)TTYLNVQR Fuc 2 Hex 6 HexNAc 8 | 27.502 | 0.039 | -5.641 | 9.196e+06 | 86 - 101 | 2 | 32.14 | 32.48 | 32.65 |
| 4052.7981 | PVPITN(N)ATLDQITGK Fuc 2 Hex 6 HexNAc 6 | 26.950 | 0.040 | -20.677 | 3.378e+06 | 27 - 42 | 5 | 61.75 | 63.12 | 63.12 |
| 4076.9598 | QIPLC(C)ANLVPVPITN(N)ATLDQITGK Hex 3 HexNAc 5 | 26.262 | 0.045 | 13.224 | 1.646e+08 | 18 - 42 | 11 | 68.19 | 68.70 | 71.02 |
| 3707.5331 | QDQC(C)IYN(N)TTYLNVQR Hex 3 HexNAc 5 Neu5Ac 1 | 26.243 | 0.045 | 14.200 | 9.756e+08 | 86 - 101 | 7 | 39.19 | 39.86 | 40.82 |
| 3916.7695 | C(C)ANLVPVPITN(N)ATLDQITGK Hex 3 HexNAc 5 Neu5Ac 1 | 25.804 | 0.045 | 14.335 | 1.643e+08 | 22 - 42 | 8 | 61.07 | 61.41 | 62.09 |
| 3773.6733 | PVPITN(N)ATLDQITGK Fuc 2 Hex 5 HexNAc 4 Neu5Ac 1 | 25.078 | 0.048 | 0.000 | 0.000e+00 | 27 - 42 | 1 | - | - | - |
| 4001.6871 | QDQC(C)IYN(N)TTYLN(D)VQR Fuc 4 Hex 3 HexNAc 5 | 24.723 | 0.050 | -6.037 | 3.855e+07 | 86 - 101 | 3 | 33.16 | 33.16 | 33.66 |
| 4385.0711 | QIPLC(C)ANLVPVPITN(N)ATLDQITGK Fuc 1 Hex 4 HexNAc 5 | 24.617 | 0.050 | 10.594 | 4.688e+07 | 18 - 42 | 4 | 67.85 | 68.36 | 68.70 |

### YFTPN(N)K Hex 3 HexNAc 5

**Mass:**  2269.9422

(1.594e-06)

**Charge States:** 2, 3

**Start Position**: 68

**End Position**: 74

| Profile Score | Tandem Score | TDA q-value | Volume | Spectrum Matches |
| --- | --- | --- | --- | --- |
| 13.3663 | 187.2151 | 0.0000 | 2.2302e+08 | 9 |

**Adduct Signal Fractions**

- **Unmodified**: 2.2302e+08

### YFTPN(N)K Fuc 1 Hex 4 HexNAc 5

**Mass:**  2579.0434

(3.866e-04)

**Start Position**: 68

**End Position**: 74

| Profile Score | Tandem Score | TDA q-value | Volume | Spectrum Matches |
| --- | --- | --- | --- | --- |
| 0.0000 | 186.1954 | 0.0000 | 0.0000e+00 | 1 |

Adduct:

No Chromatogram Found

### NEEYN(N)K Hex 3 HexNAc 4

**Mass:**  2093.8163

(1.414e-07)

**Charge States:** 2, 3

**Start Position**: 52

**End Position**: 58

| Profile Score | Tandem Score | TDA q-value | Volume | Spectrum Matches |
| --- | --- | --- | --- | --- |
| 11.7042 | 184.8191 | 0.0000 | 4.7797e+07 | 5 |

**Adduct Signal Fractions**

- **Unmodified**: 4.7797e+07

### FYFTPN(N)K Hex 3 HexNAc 6

**Mass:**  2620.0863

(1.117e-06)

**Charge States:** 2, 3

**Start Position**: 67

**End Position**: 74

| Profile Score | Tandem Score | TDA q-value | Volume | Spectrum Matches |
| --- | --- | --- | --- | --- |
| 13.0862 | 182.8640 | 0.0000 | 1.1413e+08 | 12 |

**Adduct Signal Fractions**

- **Unmodified**: 1.1413e+08

### NEEYN(N)K Hex 3 HexNAc 5

**Mass:**  2296.8964

(-1.101e-07)

**Charge States:** 2, 3

**Start Position**: 52

**End Position**: 58

| Profile Score | Tandem Score | TDA q-value | Volume | Spectrum Matches |
| --- | --- | --- | --- | --- |
| 11.1939 | 180.5800 | 0.0000 | 4.4311e+07 | 5 |

**Adduct Signal Fractions**

- **Unmodified**: 4.4311e+07

### NEEYN(N)K Fuc 1 Hex 4 HexNAc 5

**Mass:**  2605.0065

(1.851e-07)

**Charge States:** 3

**Start Position**: 52

**End Position**: 58

| Profile Score | Tandem Score | TDA q-value | Volume | Spectrum Matches |
| --- | --- | --- | --- | --- |
| 11.9571 | 180.1890 | 0.0000 | 2.2091e+07 | 4 |

**Adduct Signal Fractions**

- **Unmodified**: 2.2091e+07

### SVQEIQATFFYFTPN(N)K Hex 3 HexNAc 6

**Mass:**  3623.5863

(7.344e-07)

**Charge States:** 3, 4

**Start Position**: 58

**End Position**: 74

| Profile Score | Tandem Score | TDA q-value | Volume | Spectrum Matches |
| --- | --- | --- | --- | --- |
| -0.9451 | 175.2321 | 0.0000 | 1.4232e+09 | 25 |

**Adduct Signal Fractions**

- **Unmodified**: 1.4232e+09

### SVQEIQATFFYFTPN(N)K Fuc 1 Hex 4 HexNAc 6

**Mass:**  3931.7016

(1.984e-06)

**Charge States:** 3, 4

**Start Position**: 58

**End Position**: 74

| Profile Score | Tandem Score | TDA q-value | Volume | Spectrum Matches |
| --- | --- | --- | --- | --- |
| 13.7086 | 174.8684 | 0.0000 | 3.8134e+08 | 14 |

**Adduct Signal Fractions**

- **Unmodified**: 3.8134e+08

### YFTPN(N)K Hex 3 HexNAc 4

**Mass:**  2066.8582

(1.688e-06)

**Charge States:** 2, 3

**Start Position**: 68

**End Position**: 74

| Profile Score | Tandem Score | TDA q-value | Volume | Spectrum Matches |
| --- | --- | --- | --- | --- |
| -19.8102 | 172.9827 | 0.0000 | 2.1926e+07 | 3 |

**Adduct Signal Fractions**

- **Unmodified**: 2.1926e+07

### YFTPN(N)K Hex 3 HexNAc 6

**Mass:**  2473.0175

(2.379e-06)

**Charge States:** 2, 3

**Start Position**: 68

**End Position**: 74

| Profile Score | Tandem Score | TDA q-value | Volume | Spectrum Matches |
| --- | --- | --- | --- | --- |
| 14.5812 | 172.5765 | 0.0000 | 3.8669e+08 | 7 |

**Adduct Signal Fractions**

- **Unmodified**: 3.8669e+08

### YFTPN(N)K Hex 4 HexNAc 7

**Mass:**  2838.1541

(9.481e-07)

**Charge States:** 3

**Start Position**: 68

**End Position**: 74

| Profile Score | Tandem Score | TDA q-value | Volume | Spectrum Matches |
| --- | --- | --- | --- | --- |
| -4.3547 | 172.1686 | 0.0000 | 4.7675e+07 | 3 |

**Adduct Signal Fractions**

- **Unmodified**: 4.7675e+07

### FYFTPN(N)K Hex 3 HexNAc 5

**Mass:**  2417.0075

(1.266e-06)

**Charge States:** 2, 3

**Start Position**: 67

**End Position**: 74

| Profile Score | Tandem Score | TDA q-value | Volume | Spectrum Matches |
| --- | --- | --- | --- | --- |
| 12.7972 | 169.0813 | 0.0000 | 5.7258e+07 | 9 |

**Adduct Signal Fractions**

- **Unmodified**: 5.7258e+07

### FYFTPN(N)K Hex 4 HexNAc 7

**Mass:**  2985.2208

(1.493e-06)

**Charge States:** 3

**Start Position**: 67

**End Position**: 74

| Profile Score | Tandem Score | TDA q-value | Volume | Spectrum Matches |
| --- | --- | --- | --- | --- |
| 10.9049 | 168.3064 | 0.0000 | 1.4771e+07 | 5 |

**Adduct Signal Fractions**

- **Unmodified**: 1.4771e+07

### SVQEIQATFFYFTPN(N)K Hex 3 HexNAc 5

**Mass:**  3420.5048

(8.557e-07)

**Charge States:** 2, 3, 4

**Start Position**: 58

**End Position**: 74

| Profile Score | Tandem Score | TDA q-value | Volume | Spectrum Matches |
| --- | --- | --- | --- | --- |
| 14.9206 | 167.0901 | 0.0000 | 7.9934e+08 | 31 |

**Adduct Signal Fractions**

- **Unmodified**: 7.9934e+08

### SVQEIQATFFYFTPN(N)K Fuc 2 Hex 5 HexNAc 6

**Mass:**  4239.8088

(1.451e-06)

**Charge States:** 3, 4

**Start Position**: 58

**End Position**: 74

| Profile Score | Tandem Score | TDA q-value | Volume | Spectrum Matches |
| --- | --- | --- | --- | --- |
| 13.1026 | 165.1560 | 0.0000 | 5.5220e+07 | 4 |

**Adduct Signal Fractions**

- **Unmodified**: 5.5220e+07

### LVPVPITN(N)ATLDQITGK Fuc 1 Hex 4 HexNAc 5

**Mass:**  3588.6862

(1.852e-06)

**Charge States:** 3, 4

**Start Position**: 26

**End Position**: 43

| Profile Score | Tandem Score | TDA q-value | Volume | Spectrum Matches |
| --- | --- | --- | --- | --- |
| 14.9174 | 165.0946 | 0.0000 | 4.4277e+09 | 107 |

**Adduct Signal Fractions**

- **Unmodified**: 4.4277e+09

### YFTPN(N)K Fuc 1 Hex 4 HexNAc 6

**Mass:**  2782.1365

(3.621e-04)

**Charge States:** 3

**Start Position**: 68

**End Position**: 74

| Profile Score | Tandem Score | TDA q-value | Volume | Spectrum Matches |
| --- | --- | --- | --- | --- |
| -7.3969 | 162.6053 | 0.0417 | 3.0620e+06 | 5 |

**Adduct Signal Fractions**

- **Unmodified**: 3.0620e+06

### SVQEIQATFFYFTPN(N)K Fuc 1 Hex 4 HexNAc 5

**Mass:**  3728.6188

(9.458e-07)

**Charge States:** 3, 4

**Start Position**: 58

**End Position**: 74

| Profile Score | Tandem Score | TDA q-value | Volume | Spectrum Matches |
| --- | --- | --- | --- | --- |
| 12.4788 | 161.1189 | 0.0000 | 2.3809e+08 | 8 |

**Adduct Signal Fractions**

- **Unmodified**: 2.3809e+08

### ATFFYFTPN(N)K Hex 3 HexNAc 6

**Mass:**  2939.2414

(1.170e-06)

**Charge States:** 3

**Start Position**: 64

**End Position**: 74

| Profile Score | Tandem Score | TDA q-value | Volume | Spectrum Matches |
| --- | --- | --- | --- | --- |
| -3.6124 | 160.3925 | 0.0000 | 4.5366e+07 | 2 |

**Adduct Signal Fractions**

- **Unmodified**: 4.5366e+07

### SVQEIQATFFYFTPN(N)K Hex 4 HexNAc 7

**Mass:**  3988.7202

(1.515e-06)

**Charge States:** 3, 4

**Start Position**: 58

**End Position**: 74

| Profile Score | Tandem Score | TDA q-value | Volume | Spectrum Matches |
| --- | --- | --- | --- | --- |
| 10.9044 | 160.0969 | 0.0000 | 2.9148e+08 | 15 |

**Adduct Signal Fractions**

- **Unmodified**: 2.9148e+08

### SVQEIQATFFYFTPN(N)K Hex 3 HexNAc 4

**Mass:**  3217.4254

(1.290e-06)

**Charge States:** 3, 4

**Start Position**: 58

**End Position**: 74

| Profile Score | Tandem Score | TDA q-value | Volume | Spectrum Matches |
| --- | --- | --- | --- | --- |
| 13.2305 | 155.6313 | 0.0000 | 9.8721e+07 | 8 |

**Adduct Signal Fractions**

- **Unmodified**: 9.8721e+07

### LVPVPITN(N)ATLDQITGK Hex 3 HexNAc 5

**Mass:**  3280.5774

(1.556e-06)

**Charge States:** 2, 3, 4

**Start Position**: 26

**End Position**: 43

| Profile Score | Tandem Score | TDA q-value | Volume | Spectrum Matches |
| --- | --- | --- | --- | --- |
| 14.2474 | 153.6743 | 0.0000 | 1.5003e+10 | 526 |

**Adduct Signal Fractions**

- **Unmodified**: 1.5003e+10

### SVQEIQATFFYFTPN(N)K Fuc 1 Hex 5 HexNAc 7

**Mass:**  4296.8308

(1.188e-06)

**Charge States:** 4

**Start Position**: 58

**End Position**: 74

| Profile Score | Tandem Score | TDA q-value | Volume | Spectrum Matches |
| --- | --- | --- | --- | --- |
| 9.8877 | 153.5174 | 0.0000 | 4.2117e+07 | 5 |

**Adduct Signal Fractions**

- **Unmodified**: 4.2117e+07

### LVPVPITN(N)ATLDQITGK Hex 3 HexNAc 4

**Mass:**  3077.4893

(7.772e-07)

**Charge States:** 2, 3, 4

**Start Position**: 26

**End Position**: 43

| Profile Score | Tandem Score | TDA q-value | Volume | Spectrum Matches |
| --- | --- | --- | --- | --- |
| 14.6021 | 152.7806 | 0.0000 | 1.4519e+09 | 26 |

**Adduct Signal Fractions**

- **Unmodified**: 1.4519e+09

### LVPVPITN(N)ATLDQITGK Hex 3 HexNAc 6

**Mass:**  3483.6557

(1.838e-06)

**Charge States:** 3, 4

**Start Position**: 26

**End Position**: 43

| Profile Score | Tandem Score | TDA q-value | Volume | Spectrum Matches |
| --- | --- | --- | --- | --- |
| 9.2475 | 152.1065 | 0.0000 | 3.9157e+08 | 11 |

**Adduct Signal Fractions**

- **Unmodified**: 3.9157e+08

### LVPVPITN(N)ATLDQITGK Fuc 1 Hex 4 HexNAc 6

**Mass:**  3791.7665

(1.986e-06)

**Charge States:** 3, 4

**Start Position**: 26

**End Position**: 43

| Profile Score | Tandem Score | TDA q-value | Volume | Spectrum Matches |
| --- | --- | --- | --- | --- |
| 15.1080 | 150.2199 | 0.0000 | 2.0589e+08 | 5 |

**Adduct Signal Fractions**

- **Unmodified**: 2.0589e+08

### YFTPN(N)KTEDTIFLR Hex 3 HexNAc 6

**Mass:**  3448.5232

(3.755e-07)

**Charge States:** 3, 4

**Start Position**: 68

**End Position**: 82

| Profile Score | Tandem Score | TDA q-value | Volume | Spectrum Matches |
| --- | --- | --- | --- | --- |
| 10.1789 | 149.7491 | 0.0000 | 1.5590e+09 | 11 |

**Adduct Signal Fractions**

- **Unmodified**: 1.5590e+09

### SVQEIQATFFYFTPN(N)K Hex 4 HexNAc 8

**Mass:**  4191.7990

(5.295e-07)

**Charge States:** 3, 4

**Start Position**: 58

**End Position**: 74

| Profile Score | Tandem Score | TDA q-value | Volume | Spectrum Matches |
| --- | --- | --- | --- | --- |
| 13.1992 | 149.0098 | 0.0000 | 3.2383e+07 | 3 |

**Adduct Signal Fractions**

- **Unmodified**: 3.2383e+07

### LVPVPITN(N)ATLDQITGK Fuc 1 Hex 3 HexNAc 5

**Mass:**  3426.6315

(1.186e-06)

**Charge States:** 3, 4

**Start Position**: 26

**End Position**: 43

| Profile Score | Tandem Score | TDA q-value | Volume | Spectrum Matches |
| --- | --- | --- | --- | --- |
| -5.0952 | 144.6642 | 0.0000 | 7.4347e+07 | 3 |

**Adduct Signal Fractions**

- **Unmodified**: 7.4347e+07

### LVPVPITN(N)ATLDQITGK Hex 4 HexNAc 5

**Mass:**  3442.6240

(2.763e-06)

**Charge States:** 3, 4

**Start Position**: 26

**End Position**: 43

| Profile Score | Tandem Score | TDA q-value | Volume | Spectrum Matches |
| --- | --- | --- | --- | --- |
| 14.3204 | 142.9048 | 0.0000 | 1.1182e+08 | 3 |

**Adduct Signal Fractions**

- **Unmodified**: 1.1182e+08

### TPN(N)KTEDTIFLR Hex 3 HexNAc 5

**Mass:**  2935.3077

(8.054e-07)

**Charge States:** 3, 4

**Start Position**: 70

**End Position**: 82

| Profile Score | Tandem Score | TDA q-value | Volume | Spectrum Matches |
| --- | --- | --- | --- | --- |
| 12.2476 | 140.2442 | 0.0000 | 4.1170e+07 | 6 |

**Adduct Signal Fractions**

- **Unmodified**: 4.1170e+07

### QDQC(C)IYN(N)TTYLNVQR Hex 4 HexNAc 7

**Mass:**  3984.6655

(9.240e-07)

**Charge States:** 3, 4

**Start Position**: 87

**End Position**: 102

| Profile Score | Tandem Score | TDA q-value | Volume | Spectrum Matches |
| --- | --- | --- | --- | --- |
| 14.3810 | 138.5594 | 0.0000 | 8.3044e+08 | 8 |

**Adduct Signal Fractions**

- **Unmodified**: 8.3044e+08

### QDQC(C)IYN(N)TTYLNVQR Fuc 1 Hex 5 HexNAc 7

**Mass:**  4292.7756

(2.003e-06)

**Charge States:** 3, 4

**Start Position**: 87

**End Position**: 102

| Profile Score | Tandem Score | TDA q-value | Volume | Spectrum Matches |
| --- | --- | --- | --- | --- |
| 14.1983 | 138.4864 | 0.0000 | 1.5293e+08 | 4 |

**Adduct Signal Fractions**

- **Unmodified**: 1.5293e+08

### QDQC(C)IYN(N)TTYLNVQR Fuc 1 Hex 4 HexNAc 6

**Mass:**  3927.6391

(4.620e-07)

**Charge States:** 3, 4

**Start Position**: 87

**End Position**: 102

| Profile Score | Tandem Score | TDA q-value | Volume | Spectrum Matches |
| --- | --- | --- | --- | --- |
| 15.1750 | 137.6648 | 0.0000 | 1.0153e+09 | 4 |

**Adduct Signal Fractions**

- **Unmodified**: 1.0153e+09

### QDQC(C)IYN(N)TTYLNVQR Fuc 1 Hex 4 HexNAc 5

**Mass:**  3724.5595

(1.804e-06)

**Charge States:** 3, 4

**Start Position**: 87

**End Position**: 102

| Profile Score | Tandem Score | TDA q-value | Volume | Spectrum Matches |
| --- | --- | --- | --- | --- |
| 11.0197 | 137.6037 | 0.0000 | 4.8452e+08 | 4 |

**Adduct Signal Fractions**

- **Unmodified**: 4.8452e+08

### YFTPN(N)KTEDTIFLR Fuc 1 Hex 4 HexNAc 6

**Mass:**  3756.6325

(8.290e-07)

**Charge States:** 3, 4

**Start Position**: 68

**End Position**: 82

| Profile Score | Tandem Score | TDA q-value | Volume | Spectrum Matches |
| --- | --- | --- | --- | --- |
| -4.1684 | 137.4501 | 0.0000 | 2.8828e+08 | 6 |

**Adduct Signal Fractions**

- **Unmodified**: 2.8828e+08

### YFTPN(N)KTEDTIFLR Fuc 1 Hex 4 HexNAc 5

**Mass:**  3553.5496

(1.196e-06)

**Charge States:** 3, 4

**Start Position**: 68

**End Position**: 82

| Profile Score | Tandem Score | TDA q-value | Volume | Spectrum Matches |
| --- | --- | --- | --- | --- |
| 9.7284 | 137.2462 | 0.0000 | 2.1169e+08 | 5 |

**Adduct Signal Fractions**

- **Unmodified**: 2.1169e+08

### SVQEIQATFFYFTPN(N)K Hex 4 HexNAc 6

**Mass:**  3785.6447

(7.374e-07)

**Charge States:** 3, 4

**Start Position**: 58

**End Position**: 74

| Profile Score | Tandem Score | TDA q-value | Volume | Spectrum Matches |
| --- | --- | --- | --- | --- |
| 10.5093 | 137.0246 | 0.0000 | 4.9567e+07 | 6 |

**Adduct Signal Fractions**

- **Unmodified**: 4.9567e+07

### QDQC(C)IYN(N)TTYLNVQR Hex 3 HexNAc 6

**Mass:**  3619.5254

(3.524e-07)

**Charge States:** 3, 4

**Start Position**: 87

**End Position**: 102

| Profile Score | Tandem Score | TDA q-value | Volume | Spectrum Matches |
| --- | --- | --- | --- | --- |
| 13.9461 | 136.1003 | 0.0000 | 3.3622e+09 | 9 |

**Adduct Signal Fractions**

- **Unmodified**: 3.3622e+09

### ATFFYFTPN(N)K Hex 3 HexNAc 5

**Mass:**  2736.1604

(7.255e-07)

**Charge States:** 3

**Start Position**: 64

**End Position**: 74

| Profile Score | Tandem Score | TDA q-value | Volume | Spectrum Matches |
| --- | --- | --- | --- | --- |
| -3.8688 | 133.9521 | 0.0000 | 2.1791e+07 | 1 |

**Adduct Signal Fractions**

- **Unmodified**: 2.1791e+07

### QDQC(C)IYN(N)TTYLNVQR Hex 5 HexNAc 8

**Mass:**  4349.7956

(2.162e-06)

**Charge States:** 3, 4

**Start Position**: 87

**End Position**: 102

| Profile Score | Tandem Score | TDA q-value | Volume | Spectrum Matches |
| --- | --- | --- | --- | --- |
| 11.6649 | 133.2871 | 0.0000 | 1.3513e+08 | 4 |

**Adduct Signal Fractions**

- **Unmodified**: 1.3513e+08

### ATFFYFTPN(N)K Fuc 1 Hex 4 HexNAc 5

**Mass:**  3044.2701

(-1.063e-07)

**Charge States:** 3

**Start Position**: 64

**End Position**: 74

| Profile Score | Tandem Score | TDA q-value | Volume | Spectrum Matches |
| --- | --- | --- | --- | --- |
| -4.8647 | 132.9871 | 0.0000 | 6.0052e+06 | 1 |

**Adduct Signal Fractions**

- **Unmodified**: 6.0052e+06

### QDQC(C)IYN(N)TTYLNVQR Hex 4 HexNAc 8

**Mass:**  4187.7429

(1.305e-06)

**Charge States:** 3, 4

**Start Position**: 87

**End Position**: 102

| Profile Score | Tandem Score | TDA q-value | Volume | Spectrum Matches |
| --- | --- | --- | --- | --- |
| 13.9750 | 132.9143 | 0.0000 | 1.5054e+08 | 3 |

**Adduct Signal Fractions**

- **Unmodified**: 1.5054e+08

### LVPVPITN(N)ATLDQITGK Hex 4 HexNAc 7

**Mass:**  3848.7900

(1.287e-06)

**Charge States:** 3, 4

**Start Position**: 26

**End Position**: 43

| Profile Score | Tandem Score | TDA q-value | Volume | Spectrum Matches |
| --- | --- | --- | --- | --- |
| 10.9718 | 132.7283 | 0.0000 | 1.1500e+08 | 6 |

**Adduct Signal Fractions**

- **Unmodified**: 1.1500e+08

### SVQEIQATFFYFTPN(N)K Hex 4 HexNAc 4 Neu5Ac 2

**Mass:**  3961.6713

(1.363e-06)

**Charge States:** 3, 4

**Start Position**: 58

**End Position**: 74

| Profile Score | Tandem Score | TDA q-value | Volume | Spectrum Matches |
| --- | --- | --- | --- | --- |
| 12.8404 | 131.7311 | 0.0000 | 5.3062e+07 | 7 |

**Adduct Signal Fractions**

- **Unmodified**: 5.3062e+07

### QDQC(C)IYN(N)TTYLNVQR Hex 3 HexNAc 5

**Mass:**  3416.4497

(1.109e-06)

**Charge States:** 3, 4

**Start Position**: 87

**End Position**: 102

| Profile Score | Tandem Score | TDA q-value | Volume | Spectrum Matches |
| --- | --- | --- | --- | --- |
| 12.9583 | 131.0783 | 0.0000 | 1.7191e+09 | 7 |

**Adduct Signal Fractions**

- **Unmodified**: 1.7191e+09

### TPN(N)KTEDTIFLR Hex 3 HexNAc 6

**Mass:**  3138.3891

(9.056e-07)

**Charge States:** 3, 4

**Start Position**: 70

**End Position**: 82

| Profile Score | Tandem Score | TDA q-value | Volume | Spectrum Matches |
| --- | --- | --- | --- | --- |
| 12.5277 | 129.3982 | 0.0000 | 4.3220e+07 | 5 |

**Adduct Signal Fractions**

- **Unmodified**: 4.3220e+07

### LVPVPITN(N)ATLDQITGK Hex 4 HexNAc 6

**Mass:**  3645.7009

(3.715e-06)

**Charge States:** 3, 4

**Start Position**: 26

**End Position**: 43

| Profile Score | Tandem Score | TDA q-value | Volume | Spectrum Matches |
| --- | --- | --- | --- | --- |
| 13.5397 | 129.3024 | 0.0000 | 8.7863e+08 | 15 |

**Adduct Signal Fractions**

- **Unmodified**: 8.7863e+08

### YFTPN(N)KTEDTIFLR Hex 3 HexNAc 4

**Mass:**  3042.3621

(1.607e-06)

**Charge States:** 3

**Start Position**: 68

**End Position**: 82

| Profile Score | Tandem Score | TDA q-value | Volume | Spectrum Matches |
| --- | --- | --- | --- | --- |
| -2.9874 | 129.0796 | 0.0000 | 9.0099e+07 | 2 |

**Adduct Signal Fractions**

- **Unmodified**: 9.0099e+07

### LVPVPITN(N)ATLDQITGK Fuc 2 Hex 5 HexNAc 5

**Mass:**  3896.7985

(1.936e-06)

**Charge States:** 3, 4

**Start Position**: 26

**End Position**: 43

| Profile Score | Tandem Score | TDA q-value | Volume | Spectrum Matches |
| --- | --- | --- | --- | --- |
| -3.9225 | 128.1118 | 0.0000 | 4.3270e+07 | 2 |

**Adduct Signal Fractions**

- **Unmodified**: 4.3270e+07

### ATFFYFTPN(N)K Fuc 1 Hex 4 HexNAc 6

**Mass:**  3247.3493

(5.279e-07)

**Charge States:** 3

**Start Position**: 64

**End Position**: 74

| Profile Score | Tandem Score | TDA q-value | Volume | Spectrum Matches |
| --- | --- | --- | --- | --- |
| -20.7849 | 126.8347 | 0.0000 | 5.8521e+06 | 1 |

**Adduct Signal Fractions**

- **Unmodified**: 5.8521e+06

### YFTPN(N)KTEDTIFLR Fuc 2 Hex 5 HexNAc 6

**Mass:**  4066.7533

(4.955e-04)

**Start Position**: 68

**End Position**: 82

| Profile Score | Tandem Score | TDA q-value | Volume | Spectrum Matches |
| --- | --- | --- | --- | --- |
| 0.0000 | 126.5616 | 0.0000 | 0.0000e+00 | 1 |

Adduct:

No Chromatogram Found

### YFTPN(N)KTEDTIFLR Hex 4 HexNAc 7

**Mass:**  3813.6511

(1.303e-06)

**Charge States:** 3, 4

**Start Position**: 68

**End Position**: 82

| Profile Score | Tandem Score | TDA q-value | Volume | Spectrum Matches |
| --- | --- | --- | --- | --- |
| 10.3798 | 126.4242 | 0.0000 | 1.7484e+08 | 6 |

**Adduct Signal Fractions**

- **Unmodified**: 1.7484e+08

### ATFFYFTPN(N)K Fuc 3 Hex 8 HexNAc 4

**Mass:**  3781.5300

(3.674e-06)

**Charge States:** 4

**Start Position**: 64

**End Position**: 74

| Profile Score | Tandem Score | TDA q-value | Volume | Spectrum Matches |
| --- | --- | --- | --- | --- |
| -21.0529 | 124.3145 | 0.0000 | 9.0554e+06 | 1 |

**Adduct Signal Fractions**

- **Unmodified**: 9.0554e+06

### FYFTPN(N)KTEDTIFLR Hex 3 HexNAc 6

**Mass:**  3595.5910

(1.519e-06)

**Charge States:** 3, 4

**Start Position**: 67

**End Position**: 82

| Profile Score | Tandem Score | TDA q-value | Volume | Spectrum Matches |
| --- | --- | --- | --- | --- |
| 13.4404 | 123.4202 | 0.0000 | 1.3158e+08 | 6 |

**Adduct Signal Fractions**

- **Unmodified**: 1.3158e+08

### PVPITN(N)ATLDQITGK Fuc 1 Hex 5 HexNAc 4

**Mass:**  3336.5061

(3.013e-04)

**Start Position**: 28

**End Position**: 43

| Profile Score | Tandem Score | TDA q-value | Volume | Spectrum Matches |
| --- | --- | --- | --- | --- |
| 0.0000 | 122.6369 | 0.0000 | 0.0000e+00 | 1 |

Adduct:

No Chromatogram Found

### SVQEIQATFFYFTPN(N)K Fuc 1 Hex 4 HexNAc 7

**Mass:**  4134.7790

(2.042e-06)

**Charge States:** 4

**Start Position**: 58

**End Position**: 74

| Profile Score | Tandem Score | TDA q-value | Volume | Spectrum Matches |
| --- | --- | --- | --- | --- |
| -4.5245 | 122.5534 | 0.0000 | 1.2614e+07 | 3 |

**Adduct Signal Fractions**

- **Unmodified**: 1.2614e+07

### QDQC(C)IYN(N)TTYLNVQR Fuc 2 Hex 5 HexNAc 6

**Mass:**  4235.7517

(1.462e-06)

**Charge States:** 3, 4

**Start Position**: 87

**End Position**: 102

| Profile Score | Tandem Score | TDA q-value | Volume | Spectrum Matches |
| --- | --- | --- | --- | --- |
| -4.0261 | 120.7866 | 0.0000 | 1.5695e+08 | 1 |

**Adduct Signal Fractions**

- **Unmodified**: 1.5695e+08

### SVQEIQATFFYFTPN(N)K Fuc 2 Hex 5 HexNAc 7

**Mass:**  4442.8864

(9.557e-07)

**Charge States:** 4

**Start Position**: 58

**End Position**: 74

| Profile Score | Tandem Score | TDA q-value | Volume | Spectrum Matches |
| --- | --- | --- | --- | --- |
| -19.5980 | 118.1883 | 0.0000 | 3.8219e+06 | 1 |

**Adduct Signal Fractions**

- **Unmodified**: 3.8219e+06

### FYFTPN(N)KTEDTIFLR Hex 3 HexNAc 5

**Mass:**  3392.5113

(1.371e-06)

**Charge States:** 3, 4

**Start Position**: 67

**End Position**: 82

| Profile Score | Tandem Score | TDA q-value | Volume | Spectrum Matches |
| --- | --- | --- | --- | --- |
| 13.5454 | 117.8883 | 0.0000 | 5.6579e+07 | 5 |

**Adduct Signal Fractions**

- **Unmodified**: 5.6579e+07

### SVQEIQATFFYFTPN(N)K Fuc 1 Hex 5 HexNAc 4 Neu5Ac 1

**Mass:**  3978.6996

(4.598e-06)

**Charge States:** 3

**Start Position**: 58

**End Position**: 74

| Profile Score | Tandem Score | TDA q-value | Volume | Spectrum Matches |
| --- | --- | --- | --- | --- |
| -4.9373 | 116.2166 | 0.0000 | 5.8867e+06 | 1 |

**Adduct Signal Fractions**

- **Unmodified**: 5.8867e+06

### ATFFYFTPN(N)K Hex 4 HexNAc 7

**Mass:**  3304.3760

(2.094e-06)

**Charge States:** 3

**Start Position**: 64

**End Position**: 74

| Profile Score | Tandem Score | TDA q-value | Volume | Spectrum Matches |
| --- | --- | --- | --- | --- |
| -19.8216 | 115.4025 | 0.0000 | 3.4277e+06 | 1 |

**Adduct Signal Fractions**

- **Unmodified**: 3.4277e+06

### QDQC(C)IYN(N)TTYLNVQR Hex 4 HexNAc 4 Neu5Ac 2

**Mass:**  3957.6137

(1.168e-06)

**Charge States:** 3, 4

**Start Position**: 87

**End Position**: 102

| Profile Score | Tandem Score | TDA q-value | Volume | Spectrum Matches |
| --- | --- | --- | --- | --- |
| 14.2036 | 114.2904 | 0.0000 | 2.1838e+08 | 5 |

**Adduct Signal Fractions**

- **Unmodified**: 2.1838e+08

### YFTPN(N)KTEDTIFLR Hex 3 HexNAc 5

**Mass:**  3245.4431

(6.348e-07)

**Charge States:** 3, 4

**Start Position**: 68

**End Position**: 82

| Profile Score | Tandem Score | TDA q-value | Volume | Spectrum Matches |
| --- | --- | --- | --- | --- |
| 9.4386 | 114.0012 | 0.0000 | 4.4304e+07 | 5 |

**Adduct Signal Fractions**

- **Unmodified**: 4.4304e+07

### QDQC(C)IYN(N)TTYLNVQR Hex 3 HexNAc 4

**Mass:**  3213.3700

(-4.630e-07)

**Charge States:** 3

**Start Position**: 87

**End Position**: 102

| Profile Score | Tandem Score | TDA q-value | Volume | Spectrum Matches |
| --- | --- | --- | --- | --- |
| 12.5655 | 112.8656 | 0.0000 | 3.3911e+07 | 3 |

**Adduct Signal Fractions**

- **Unmodified**: 3.3911e+07

### LVPVPITN(N)ATLDQITGK Fuc 1 Hex 5 HexNAc 6

**Mass:**  3953.8222

(2.477e-06)

**Charge States:** 3, 4

**Start Position**: 26

**End Position**: 43

| Profile Score | Tandem Score | TDA q-value | Volume | Spectrum Matches |
| --- | --- | --- | --- | --- |
| 8.6062 | 111.5009 | 0.0000 | 3.7536e+07 | 4 |

**Adduct Signal Fractions**

- **Unmodified**: 3.7536e+07

### LVPVPITN(N)ATLDQITGK Fuc 2 Hex 5 HexNAc 6

**Mass:**  4099.8752

(1.453e-06)

**Charge States:** 3, 4

**Start Position**: 26

**End Position**: 43

| Profile Score | Tandem Score | TDA q-value | Volume | Spectrum Matches |
| --- | --- | --- | --- | --- |
| 9.1106 | 111.3874 | 0.0000 | 2.8621e+07 | 5 |

**Adduct Signal Fractions**

- **Unmodified**: 2.8621e+07

### FYFTPN(N)KTEDTIFLR Fuc 1 Hex 4 HexNAc 6

**Mass:**  3903.7038

(2.171e-06)

**Charge States:** 3, 4

**Start Position**: 67

**End Position**: 82

| Profile Score | Tandem Score | TDA q-value | Volume | Spectrum Matches |
| --- | --- | --- | --- | --- |
| 14.3397 | 110.8408 | 0.0000 | 3.0145e+07 | 4 |

**Adduct Signal Fractions**

- **Unmodified**: 3.0145e+07

### QDQC(C)IYN(N)TTYLNVQR Hex 4 HexNAc 6

**Mass:**  3781.5811

(1.085e-06)

**Charge States:** 3, 4

**Start Position**: 87

**End Position**: 102

| Profile Score | Tandem Score | TDA q-value | Volume | Spectrum Matches |
| --- | --- | --- | --- | --- |
| 11.5734 | 110.2920 | 0.0000 | 9.2573e+07 | 3 |

**Adduct Signal Fractions**

- **Unmodified**: 9.2573e+07

### IYN(N)TTYLNVQR Hex 3 HexNAc 6

**Mass:**  3088.3529

(1.161e-06)

**Charge States:** 3, 4

**Start Position**: 91

**End Position**: 102

| Profile Score | Tandem Score | TDA q-value | Volume | Spectrum Matches |
| --- | --- | --- | --- | --- |
| 13.1418 | 109.1646 | 0.0000 | 4.0274e+07 | 5 |

**Adduct Signal Fractions**

- **Unmodified**: 4.0274e+07

### TPN(N)KTEDTIFLR Hex 7 HexNAc 6

**Mass:**  3786.6061

(2.353e-06)

**Charge States:** 3, 4

**Start Position**: 70

**End Position**: 82

| Profile Score | Tandem Score | TDA q-value | Volume | Spectrum Matches |
| --- | --- | --- | --- | --- |
| 13.6012 | 108.9988 | 0.0000 | 1.0588e+08 | 4 |

**Adduct Signal Fractions**

- **Unmodified**: 1.0588e+08

### LVPVPITN(N)ATLDQITGK Hex 4 HexNAc 6

**Mass:**  3648.7313

(8.310e-04)

**Start Position**: 26

**End Position**: 43

| Profile Score | Tandem Score | TDA q-value | Volume | Spectrum Matches |
| --- | --- | --- | --- | --- |
| 0.0000 | 107.5379 | 0.0000 | 0.0000e+00 | 1 |

Adduct:

No Chromatogram Found

### LVPVPITN(N)ATLDQITGK Fuc 1 Hex 4 HexNAc 4

**Mass:**  3385.6345

(9.892e-06)

**Charge States:** 3

**Start Position**: 26

**End Position**: 43

| Profile Score | Tandem Score | TDA q-value | Volume | Spectrum Matches |
| --- | --- | --- | --- | --- |
| -19.9639 | 106.5807 | 0.0000 | 1.0239e+07 | 1 |

**Adduct Signal Fractions**

- **Unmodified**: 1.0239e+07

### YFTPN(N)KTEDTIFLR Hex 5 HexNAc 8

**Mass:**  4178.7883

(1.748e-06)

**Charge States:** 3, 4

**Start Position**: 68

**End Position**: 82

| Profile Score | Tandem Score | TDA q-value | Volume | Spectrum Matches |
| --- | --- | --- | --- | --- |
| -3.4271 | 105.8170 | 0.0000 | 3.4766e+07 | 3 |

**Adduct Signal Fractions**

- **Unmodified**: 3.4766e+07

### FYFTPN(N)KTEDTIFLR Hex 3 HexNAc 4

**Mass:**  3189.4326

(1.566e-06)

**Charge States:** 3

**Start Position**: 67

**End Position**: 82

| Profile Score | Tandem Score | TDA q-value | Volume | Spectrum Matches |
| --- | --- | --- | --- | --- |
| 12.6711 | 105.4573 | 0.0000 | 7.5931e+06 | 2 |

**Adduct Signal Fractions**

- **Unmodified**: 7.5931e+06

### SVQEIQATFFYFTPN(N)KTEDTIFLR Hex 3 HexNAc 6

**Mass:**  4599.0900

(1.332e-06)

**Charge States:** 4

**Start Position**: 58

**End Position**: 82

| Profile Score | Tandem Score | TDA q-value | Volume | Spectrum Matches |
| --- | --- | --- | --- | --- |
| -3.8574 | 105.3167 | 0.0000 | 3.3438e+07 | 4 |

**Adduct Signal Fractions**

- **Unmodified**: 3.3438e+07

### YFTPN(N)KTEDTIFLR Fuc 1 Hex 5 HexNAc 7

**Mass:**  4121.7663

(1.610e-06)

**Charge States:** 3, 4

**Start Position**: 68

**End Position**: 82

| Profile Score | Tandem Score | TDA q-value | Volume | Spectrum Matches |
| --- | --- | --- | --- | --- |
| -4.5412 | 104.9683 | 0.0000 | 3.4872e+07 | 2 |

**Adduct Signal Fractions**

- **Unmodified**: 3.4872e+07

### LVPVPITN(N)ATLDQITGK Fuc 2 Hex 4 HexNAc 5

**Mass:**  3734.7445

(1.692e-06)

**Start Position**: 26

**End Position**: 43

| Profile Score | Tandem Score | TDA q-value | Volume | Spectrum Matches |
| --- | --- | --- | --- | --- |
| 0.0000 | 104.8842 | 0.0000 | 0.0000e+00 | 1 |

Adduct:

No Chromatogram Found

### SVQEIQATFFYFTPN(N)K Hex 5 HexNAc 9

**Mass:**  4556.9328

(1.671e-06)

**Charge States:** 4

**Start Position**: 58

**End Position**: 74

| Profile Score | Tandem Score | TDA q-value | Volume | Spectrum Matches |
| --- | --- | --- | --- | --- |
| -20.3116 | 104.7215 | 0.0000 | 2.3485e+06 | 1 |

**Adduct Signal Fractions**

- **Unmodified**: 2.3485e+06

### FYFTPN(N)KTEDTIFLR Fuc 1 Hex 4 HexNAc 5

**Mass:**  3700.6210

(1.511e-06)

**Charge States:** 3, 4

**Start Position**: 67

**End Position**: 82

| Profile Score | Tandem Score | TDA q-value | Volume | Spectrum Matches |
| --- | --- | --- | --- | --- |
| -3.5251 | 104.2440 | 0.0000 | 1.6244e+07 | 4 |

**Adduct Signal Fractions**

- **Unmodified**: 1.6244e+07

### LVPVPITN(N)ATLDQITGK Hex 4 HexNAc 5 Neu5Ac 1

**Mass:**  3733.7228

(1.481e-06)

**Charge States:** 3, 4

**Start Position**: 26

**End Position**: 43

| Profile Score | Tandem Score | TDA q-value | Volume | Spectrum Matches |
| --- | --- | --- | --- | --- |
| 12.0490 | 103.0344 | 0.0000 | 1.8305e+07 | 6 |

**Adduct Signal Fractions**

- **Unmodified**: 1.8305e+07

### PVPITN(N)ATLDQITGK Hex 6 HexNAc 5 Neu5Ac 1

**Mass:**  3845.6764

(1.386e-06)

**Charge States:** 3

**Start Position**: 28

**End Position**: 43

| Profile Score | Tandem Score | TDA q-value | Volume | Spectrum Matches |
| --- | --- | --- | --- | --- |
| -4.7948 | 103.0278 | 0.0000 | 7.9851e+06 | 1 |

**Adduct Signal Fractions**

- **Unmodified**: 7.9851e+06

### IYN(N)TTYLNVQR Hex 3 HexNAc 5

**Mass:**  2885.2734

(1.065e-06)

**Charge States:** 3, 4

**Start Position**: 91

**End Position**: 102

| Profile Score | Tandem Score | TDA q-value | Volume | Spectrum Matches |
| --- | --- | --- | --- | --- |
| 13.7027 | 102.5485 | 0.0000 | 3.1299e+07 | 5 |

**Adduct Signal Fractions**

- **Unmodified**: 3.1299e+07

### FTPN(N)KTEDTIFLR Hex 3 HexNAc 5

**Mass:**  3082.3786

(1.103e-06)

**Charge States:** 3, 4

**Start Position**: 69

**End Position**: 82

| Profile Score | Tandem Score | TDA q-value | Volume | Spectrum Matches |
| --- | --- | --- | --- | --- |
| 13.8673 | 102.5149 | 0.0000 | 4.6578e+07 | 3 |

**Adduct Signal Fractions**

- **Unmodified**: 4.6578e+07

### LVPVPITN(N)ATLDQITGK Fuc 1 Hex 5 HexNAc 3 Neu5Ac 1

**Mass:**  3635.6815

(5.128e-06)

**Charge States:** 3

**Start Position**: 26

**End Position**: 43

| Profile Score | Tandem Score | TDA q-value | Volume | Spectrum Matches |
| --- | --- | --- | --- | --- |
| -5.2383 | 102.3774 | 0.0000 | 2.1473e+07 | 2 |

**Adduct Signal Fractions**

- **Unmodified**: 2.1473e+07

### LVPVPITN(N)ATLDQITGK Fuc 1 Hex 5 HexNAc 7

**Mass:**  4156.8996

(1.819e-06)

**Charge States:** 3, 4

**Start Position**: 26

**End Position**: 43

| Profile Score | Tandem Score | TDA q-value | Volume | Spectrum Matches |
| --- | --- | --- | --- | --- |
| 13.2892 | 102.1830 | 0.0000 | 1.9140e+07 | 3 |

**Adduct Signal Fractions**

- **Unmodified**: 1.9140e+07

### QDQC(C)IYN(N)TTYLNVQR Fuc 1 Hex 4 HexNAc 7

**Mass:**  4130.7219

(2.789e-07)

**Charge States:** 3, 4

**Start Position**: 87

**End Position**: 102

| Profile Score | Tandem Score | TDA q-value | Volume | Spectrum Matches |
| --- | --- | --- | --- | --- |
| -4.5422 | 101.0655 | 0.0000 | 5.0271e+07 | 2 |

**Adduct Signal Fractions**

- **Unmodified**: 5.0271e+07

### FTPN(N)KTEDTIFLR Hex 3 HexNAc 6

**Mass:**  3285.4575

(9.724e-07)

**Charge States:** 3, 4

**Start Position**: 69

**End Position**: 82

| Profile Score | Tandem Score | TDA q-value | Volume | Spectrum Matches |
| --- | --- | --- | --- | --- |
| 14.1683 | 98.6572 | 0.0000 | 9.1147e+07 | 5 |

**Adduct Signal Fractions**

- **Unmodified**: 9.1147e+07

### FTPN(N)KTEDTIFLR Fuc 1 Hex 4 HexNAc 5

**Mass:**  3390.4909

(1.465e-06)

**Charge States:** 3, 4

**Start Position**: 69

**End Position**: 82

| Profile Score | Tandem Score | TDA q-value | Volume | Spectrum Matches |
| --- | --- | --- | --- | --- |
| -4.9259 | 98.3779 | 0.0000 | 1.1130e+07 | 2 |

**Adduct Signal Fractions**

- **Unmodified**: 1.1130e+07

### PVPITN(N)ATLDQITGK Fuc 2 Hex 6 HexNAc 3

**Mass:**  3440.5369

(1.202e-06)

**Charge States:** 3, 4

**Start Position**: 28

**End Position**: 43

| Profile Score | Tandem Score | TDA q-value | Volume | Spectrum Matches |
| --- | --- | --- | --- | --- |
| -19.8836 | 96.3678 | 0.0000 | 3.8347e+06 | 2 |

**Adduct Signal Fractions**

- **Unmodified**: 3.8347e+06

### LVPVPITN(N)ATLDQITGK Hex 5 HexNAc 7

**Mass:**  4010.8449

(3.002e-06)

**Charge States:** 3, 4

**Start Position**: 26

**End Position**: 43

| Profile Score | Tandem Score | TDA q-value | Volume | Spectrum Matches |
| --- | --- | --- | --- | --- |
| -5.7882 | 95.7968 | 0.0000 | 8.9360e+06 | 2 |

**Adduct Signal Fractions**

- **Unmodified**: 8.9360e+06

### LVPVPITN(N)ATLDQITGK Fuc 1 Hex 5 HexNAc 5

**Mass:**  3750.7426

(2.514e-06)

**Charge States:** 3, 4

**Start Position**: 26

**End Position**: 43

| Profile Score | Tandem Score | TDA q-value | Volume | Spectrum Matches |
| --- | --- | --- | --- | --- |
| -19.4007 | 93.6041 | 0.0000 | 1.0947e+07 | 1 |

**Adduct Signal Fractions**

- **Unmodified**: 1.0947e+07

### QDQC(C)IYN(N)TTYLNVQR Fuc 1 Hex 5 HexNAc 8

**Mass:**  4497.8601

(4.479e-04)

**Start Position**: 87

**End Position**: 102

| Profile Score | Tandem Score | TDA q-value | Volume | Spectrum Matches |
| --- | --- | --- | --- | --- |
| 0.0000 | 90.7346 | 0.0000 | 0.0000e+00 | 1 |

Adduct:

No Chromatogram Found

### QDQC(C)IYN(N)TTYLNVQR Fuc 1 Hex 5 HexNAc 4 Neu5Ac 2

**Mass:**  4265.7175

(-5.884e-07)

**Charge States:** 4

**Start Position**: 87

**End Position**: 102

| Profile Score | Tandem Score | TDA q-value | Volume | Spectrum Matches |
| --- | --- | --- | --- | --- |
| -4.9774 | 89.7811 | 0.0000 | 4.2718e+07 | 2 |

**Adduct Signal Fractions**

- **Unmodified**: 4.2718e+07

### FYFTPN(N)KTEDTIFLR Hex 4 HexNAc 7

**Mass:**  3960.7258

(1.635e-06)

**Charge States:** 3, 4

**Start Position**: 67

**End Position**: 82

| Profile Score | Tandem Score | TDA q-value | Volume | Spectrum Matches |
| --- | --- | --- | --- | --- |
| 14.0946 | 87.6053 | 0.0022 | 2.3156e+07 | 4 |

**Adduct Signal Fractions**

- **Unmodified**: 2.3156e+07

### FTPN(N)KTEDTIFLR Fuc 1 Hex 4 HexNAc 6

**Mass:**  3593.5693

(1.109e-06)

**Charge States:** 3, 4

**Start Position**: 69

**End Position**: 82

| Profile Score | Tandem Score | TDA q-value | Volume | Spectrum Matches |
| --- | --- | --- | --- | --- |
| -3.7148 | 86.4331 | 0.0022 | 2.0299e+07 | 4 |

**Adduct Signal Fractions**

- **Unmodified**: 2.0299e+07

### YFTPN(N)KTEDTIFLR Hex 5 HexNAc 9

**Mass:**  4381.8688

(1.623e-06)

**Charge States:** 4

**Start Position**: 68

**End Position**: 82

| Profile Score | Tandem Score | TDA q-value | Volume | Spectrum Matches |
| --- | --- | --- | --- | --- |
| -5.0626 | 83.8036 | 0.0022 | 9.8359e+06 | 1 |

**Adduct Signal Fractions**

- **Unmodified**: 9.8359e+06

### TPN(N)KTEDTIFLR Fuc 1 Hex 4 HexNAc 5

**Mass:**  3244.4133

(3.070e-04)

**Start Position**: 70

**End Position**: 82

| Profile Score | Tandem Score | TDA q-value | Volume | Spectrum Matches |
| --- | --- | --- | --- | --- |
| 0.0000 | 83.7063 | 0.0022 | 0.0000e+00 | 1 |

Adduct:

No Chromatogram Found

### IYN(N)TTYLNVQR Fuc 1 Hex 4 HexNAc 6

**Mass:**  3398.4376

(5.903e-04)

**Charge States:** 3, 4

**Start Position**: 91

**End Position**: 102

| Profile Score | Tandem Score | TDA q-value | Volume | Spectrum Matches |
| --- | --- | --- | --- | --- |
| -8.3892 | 83.1739 | 0.0030 | 3.1713e+07 | 6 |

**Adduct Signal Fractions**

- **Unmodified**: 3.1713e+07

### YFTPN(N)KTEDTIFLR Fuc 2 Hex 5 HexNAc 7

**Mass:**  4267.8264

(1.749e-06)

**Charge States:** 4

**Start Position**: 68

**End Position**: 82

| Profile Score | Tandem Score | TDA q-value | Volume | Spectrum Matches |
| --- | --- | --- | --- | --- |
| -20.0341 | 81.4343 | 0.0030 | 2.2473e+06 | 1 |

**Adduct Signal Fractions**

- **Unmodified**: 2.2473e+06

### TPN(N)KTEDTIFLR Fuc 1 Hex 4 HexNAc 6

**Mass:**  3446.4999

(1.714e-06)

**Charge States:** 3, 4

**Start Position**: 70

**End Position**: 82

| Profile Score | Tandem Score | TDA q-value | Volume | Spectrum Matches |
| --- | --- | --- | --- | --- |
| -4.2895 | 79.1674 | 0.0030 | 8.9818e+06 | 4 |

**Adduct Signal Fractions**

- **Unmodified**: 8.9818e+06

### TPN(N)KTEDTIFLR Fuc 1 Hex 4 HexNAc 5

**Mass:**  3243.4213

(1.631e-06)

**Charge States:** 3

**Start Position**: 70

**End Position**: 82

| Profile Score | Tandem Score | TDA q-value | Volume | Spectrum Matches |
| --- | --- | --- | --- | --- |
| -4.9391 | 75.9035 | 0.0030 | 4.2060e+06 | 3 |

**Adduct Signal Fractions**

- **Unmodified**: 4.2060e+06

### C(C)ANLVPVPITN(N)ATLDQITGK Hex 3 HexNAc 5

**Mass:**  3625.6851

(1.335e-06)

**Charge States:** 3, 4

**Start Position**: 23

**End Position**: 43

| Profile Score | Tandem Score | TDA q-value | Volume | Spectrum Matches |
| --- | --- | --- | --- | --- |
| 14.6516 | 65.9186 | 0.0030 | 1.3154e+08 | 5 |

**Adduct Signal Fractions**

- **Unmodified**: 1.3154e+08

### C(C)ANLVPVPITN(N)ATLDQITGK Hex 3 HexNAc 4

**Mass:**  3422.6054

(1.300e-06)

**Charge States:** 3

**Start Position**: 23

**End Position**: 43

| Profile Score | Tandem Score | TDA q-value | Volume | Spectrum Matches |
| --- | --- | --- | --- | --- |
| -3.8162 | 63.4239 | 0.0030 | 1.4299e+07 | 2 |

**Adduct Signal Fractions**

- **Unmodified**: 1.4299e+07

### SVQEIQATFFYFTPN(N)KTEDTIFLR Hex 3 HexNAc 5

**Mass:**  4396.0178

(3.080e-06)

**Charge States:** 4

**Start Position**: 58

**End Position**: 82

| Profile Score | Tandem Score | TDA q-value | Volume | Spectrum Matches |
| --- | --- | --- | --- | --- |
| -21.5260 | 61.7554 | 0.0030 | 6.2106e+06 | 1 |

**Adduct Signal Fractions**

- **Unmodified**: 6.2106e+06

### C(C)ANLVPVPITN(N)ATLDQITGK Fuc 1 Hex 4 HexNAc 5

**Mass:**  3933.7973

(2.236e-06)

**Charge States:** 3, 4

**Start Position**: 23

**End Position**: 43

| Profile Score | Tandem Score | TDA q-value | Volume | Spectrum Matches |
| --- | --- | --- | --- | --- |
| 12.3514 | 60.7502 | 0.0030 | 4.6356e+07 | 4 |

**Adduct Signal Fractions**

- **Unmodified**: 4.6356e+07

### QDQC(C)IYN(N)TTYLNVQR Fuc 2 Hex 5 HexNAc 7

**Mass:**  4438.8283

(1.664e-06)

**Charge States:** 4

**Start Position**: 87

**End Position**: 102

| Profile Score | Tandem Score | TDA q-value | Volume | Spectrum Matches |
| --- | --- | --- | --- | --- |
| -4.6648 | 56.1352 | 0.0030 | 2.7237e+07 | 2 |

**Adduct Signal Fractions**

- **Unmodified**: 2.7237e+07

### PVPITN(N)ATLDQITGK Fuc 2 Hex 4 HexNAc 5

**Mass:**  3522.5832

(-9.825e-07)

**Charge States:** 3, 4

**Start Position**: 28

**End Position**: 43

| Profile Score | Tandem Score | TDA q-value | Volume | Spectrum Matches |
| --- | --- | --- | --- | --- |
| -5.4045 | 55.9988 | 0.0030 | 5.6928e+06 | 2 |

**Adduct Signal Fractions**

- **Unmodified**: 5.6928e+06

### QDQC(C)IYN(N)TTYLNVQR Fuc 1 Hex 5 HexNAc 8

**Mass:**  4495.8561

(2.127e-06)

**Charge States:** 3, 4

**Start Position**: 87

**End Position**: 102

| Profile Score | Tandem Score | TDA q-value | Volume | Spectrum Matches |
| --- | --- | --- | --- | --- |
| -4.2195 | 48.2222 | 0.0030 | 3.9787e+07 | 1 |

**Adduct Signal Fractions**

- **Unmodified**: 3.9787e+07

### SVQEIQATFFYFTPN(N)K Hex 4 HexNAc 5

**Mass:**  3583.5751

(2.848e-04)

**Start Position**: 58

**End Position**: 74

| Profile Score | Tandem Score | TDA q-value | Volume | Spectrum Matches |
| --- | --- | --- | --- | --- |
| 0.0000 | 47.9320 | 0.0030 | 0.0000e+00 | 2 |

Adduct:

No Chromatogram Found

### QDQC(C)IYN(N)TTYLNVQR Fuc 1 Hex 5 HexNAc 4 Neu5Ac 1

**Mass:**  3974.6402

(3.094e-06)

**Charge States:** 3

**Start Position**: 87

**End Position**: 102

| Profile Score | Tandem Score | TDA q-value | Volume | Spectrum Matches |
| --- | --- | --- | --- | --- |
| -5.5584 | 47.0610 | 0.0030 | 1.1980e+07 | 1 |

**Adduct Signal Fractions**

- **Unmodified**: 1.1980e+07

### PVPITN(N)ATLDQITGK Hex 5 HexNAc 6 Neu5Ac 1

**Mass:**  3886.7314

(8.726e-06)

**Charge States:** 3, 4

**Start Position**: 28

**End Position**: 43

| Profile Score | Tandem Score | TDA q-value | Volume | Spectrum Matches |
| --- | --- | --- | --- | --- |
| -5.1432 | 46.9997 | 0.0030 | 9.6109e+06 | 1 |

**Adduct Signal Fractions**

- **Unmodified**: 9.6109e+06

### QDQC(C)IYN(N)TTYLNVQR Fuc 1 Hex 3 HexNAc 6

**Mass:**  3765.5894

(1.957e-06)

**Charge States:** 3

**Start Position**: 87

**End Position**: 102

| Profile Score | Tandem Score | TDA q-value | Volume | Spectrum Matches |
| --- | --- | --- | --- | --- |
| -18.8797 | 46.7173 | 0.0030 | 1.1472e+07 | 1 |

**Adduct Signal Fractions**

- **Unmodified**: 1.1472e+07

### QDQC(C)IYN(N)TTYLNVQR Hex 5 HexNAc 9

**Mass:**  4552.8767

(1.629e-06)

**Charge States:** 3, 4

**Start Position**: 87

**End Position**: 102

| Profile Score | Tandem Score | TDA q-value | Volume | Spectrum Matches |
| --- | --- | --- | --- | --- |
| -4.9189 | 45.7043 | 0.0030 | 6.7803e+07 | 2 |

**Adduct Signal Fractions**

- **Unmodified**: 6.7803e+07

### YFTPN(N)KTEDTIFLR Hex 4 HexNAc 8

**Mass:**  4016.7363

(1.674e-06)

**Charge States:** 4

**Start Position**: 68

**End Position**: 82

| Profile Score | Tandem Score | TDA q-value | Volume | Spectrum Matches |
| --- | --- | --- | --- | --- |
| -18.3376 | 45.1407 | 0.0030 | 1.8415e+07 | 1 |

**Adduct Signal Fractions**

- **Unmodified**: 1.8415e+07

### QDQC(C)IYN(N)TTYLNVQR Fuc 1 Hex 6 HexNAc 8

**Mass:**  4657.9035

(5.998e-07)

**Charge States:** 4

**Start Position**: 87

**End Position**: 102

| Profile Score | Tandem Score | TDA q-value | Volume | Spectrum Matches |
| --- | --- | --- | --- | --- |
| -4.4957 | 44.2926 | 0.0030 | 1.3631e+07 | 2 |

**Adduct Signal Fractions**

- **Unmodified**: 1.3631e+07

### SVQEIQATFFYFTPN(N)KTEDTIFLR Fuc 1 Hex 4 HexNAc 6

**Mass:**  4909.2093

(4.106e-04)

**Start Position**: 58

**End Position**: 82

| Profile Score | Tandem Score | TDA q-value | Volume | Spectrum Matches |
| --- | --- | --- | --- | --- |
| 0.0000 | 44.2814 | 0.0030 | 0.0000e+00 | 1 |

Adduct:

No Chromatogram Found

### FTPN(N)KTEDTIFLR Hex 3 HexNAc 4

**Mass:**  2879.2999

(1.203e-06)

**Charge States:** 3

**Start Position**: 69

**End Position**: 82

| Profile Score | Tandem Score | TDA q-value | Volume | Spectrum Matches |
| --- | --- | --- | --- | --- |
| -5.0065 | 40.7409 | 0.0030 | 5.8097e+06 | 1 |

**Adduct Signal Fractions**

- **Unmodified**: 5.8097e+06

### SVQEIQATFFYFTPN(N)KTEDTIFLR Fuc 1 Hex 4 HexNAc 5

**Mass:**  4704.1154

(1.087e-07)

**Charge States:** 4

**Start Position**: 58

**End Position**: 82

| Profile Score | Tandem Score | TDA q-value | Volume | Spectrum Matches |
| --- | --- | --- | --- | --- |
| -5.3067 | 40.2046 | 0.0030 | 3.7912e+06 | 1 |

**Adduct Signal Fractions**

- **Unmodified**: 3.7912e+06

### SVQEIQATFFYFTPN(N)K Hex 5 HexNAc 8

**Mass:**  4353.8459

(2.558e-06)

**Charge States:** 4

**Start Position**: 58

**End Position**: 74

| Profile Score | Tandem Score | TDA q-value | Volume | Spectrum Matches |
| --- | --- | --- | --- | --- |
| 7.6879 | 40.0810 | 0.0030 | 2.4143e+07 | 1 |

**Adduct Signal Fractions**

- **Unmodified**: 2.4143e+07

### QDQC(C)IYN(N)TTYLN(D)VQR Hex 3 HexNAc 6

**Mass:**  3620.5197

(2.750e-04)

**Charge States:** 3, 4

**Start Position**: 87

**End Position**: 102

| Profile Score | Tandem Score | TDA q-value | Volume | Spectrum Matches |
| --- | --- | --- | --- | --- |
| -20.2448 | 39.9466 | 0.0030 | 9.0607e+07 | 5 |

**Adduct Signal Fractions**

- **Unmodified**: 9.0607e+07

### LVPVPITN(N)ATLDQITGK Fuc 1 Hex 4 HexNAc 6

**Mass:**  3792.7733

(2.673e-04)

**Start Position**: 26

**End Position**: 43

| Profile Score | Tandem Score | TDA q-value | Volume | Spectrum Matches |
| --- | --- | --- | --- | --- |
| 0.0000 | 38.6978 | 0.0045 | 0.0000e+00 | 1 |

Adduct:

No Chromatogram Found

### PVPITN(N)ATLDQITGK Fuc 2 Hex 3 HexNAc 7 Neu5Ac 3

**Mass:**  4640.0178

(8.975e-06)

**Charge States:** 4

**Start Position**: 28

**End Position**: 43

| Profile Score | Tandem Score | TDA q-value | Volume | Spectrum Matches |
| --- | --- | --- | --- | --- |
| 11.8115 | 38.1257 | 0.0056 | 5.2584e+06 | 3 |

**Adduct Signal Fractions**

- **Unmodified**: 5.2584e+06

### LVPVPITN(N)ATLDQITGK Hex 5 HexNAc 8

**Mass:**  4213.9191

(2.560e-06)

**Charge States:** 4

**Start Position**: 26

**End Position**: 43

| Profile Score | Tandem Score | TDA q-value | Volume | Spectrum Matches |
| --- | --- | --- | --- | --- |
| 5.8282 | 37.1767 | 0.0056 | 6.6351e+06 | 3 |

**Adduct Signal Fractions**

- **Unmodified**: 6.6351e+06

### QDQC(C)IYN(N)TTYLNVQR Fuc 2 Hex 6 HexNAc 7

**Mass:**  4600.8811

(7.477e-07)

**Charge States:** 4

**Start Position**: 87

**End Position**: 102

| Profile Score | Tandem Score | TDA q-value | Volume | Spectrum Matches |
| --- | --- | --- | --- | --- |
| -5.2119 | 36.8937 | 0.0056 | 1.5850e+07 | 1 |

**Adduct Signal Fractions**

- **Unmodified**: 1.5850e+07

### QDQC(C)IYN(N)TTYLNVQR Fuc 1 Hex 6 HexNAc 9

**Mass:**  4860.9831

(9.330e-07)

**Charge States:** 4

**Start Position**: 87

**End Position**: 102

| Profile Score | Tandem Score | TDA q-value | Volume | Spectrum Matches |
| --- | --- | --- | --- | --- |
| -18.4259 | 36.6757 | 0.0056 | 7.0728e+06 | 1 |

**Adduct Signal Fractions**

- **Unmodified**: 7.0728e+06

### SVQEIQATFFYFTPN(N)K Fuc 1 Hex 5 HexNAc 8

**Mass:**  4499.9064

(6.863e-07)

**Charge States:** 4

**Start Position**: 58

**End Position**: 74

| Profile Score | Tandem Score | TDA q-value | Volume | Spectrum Matches |
| --- | --- | --- | --- | --- |
| -6.3274 | 36.0427 | 0.0056 | 6.4748e+06 | 2 |

**Adduct Signal Fractions**

- **Unmodified**: 6.4748e+06

### SVQEIQATFFYFTPN(N)KTEDTIFLR Hex 3 HexNAc 6

**Mass:**  4601.0913

(4.365e-04)

**Start Position**: 58

**End Position**: 82

| Profile Score | Tandem Score | TDA q-value | Volume | Spectrum Matches |
| --- | --- | --- | --- | --- |
| 0.0000 | 35.6746 | 0.0083 | 0.0000e+00 | 1 |

Adduct:

No Chromatogram Found

### FYFTPN(N)KTEDTIFLR Fuc 2 Hex 5 HexNAc 6

**Mass:**  4211.8132

(1.229e-06)

**Charge States:** 4

**Start Position**: 67

**End Position**: 82

| Profile Score | Tandem Score | TDA q-value | Volume | Spectrum Matches |
| --- | --- | --- | --- | --- |
| -4.6621 | 35.6517 | 0.0083 | 5.4781e+06 | 1 |

**Adduct Signal Fractions**

- **Unmodified**: 5.4781e+06

### FTPN(N)KTEDTIFLR Hex 4 HexNAc 7

**Mass:**  3650.5925

(5.324e-07)

**Charge States:** 3, 4

**Start Position**: 69

**End Position**: 82

| Profile Score | Tandem Score | TDA q-value | Volume | Spectrum Matches |
| --- | --- | --- | --- | --- |
| -4.5901 | 33.8698 | 0.0135 | 1.1374e+07 | 3 |

**Adduct Signal Fractions**

- **Unmodified**: 1.1374e+07

### QDQC(C)IYN(N)TTYLNVQR Fuc 3 Hex 6 HexNAc 6

**Mass:**  4543.8630

(1.451e-06)

**Charge States:** 4

**Start Position**: 87

**End Position**: 102

| Profile Score | Tandem Score | TDA q-value | Volume | Spectrum Matches |
| --- | --- | --- | --- | --- |
| -5.5080 | 33.6919 | 0.0142 | 1.4909e+07 | 1 |

**Adduct Signal Fractions**

- **Unmodified**: 1.4909e+07

### LVPVPITN(N)ATLDQITGK Fuc 4 Hex 3 HexNAc 5

**Mass:**  3866.8161

(5.214e-04)

**Start Position**: 26

**End Position**: 43

| Profile Score | Tandem Score | TDA q-value | Volume | Spectrum Matches |
| --- | --- | --- | --- | --- |
| 0.0000 | 33.6206 | 0.0142 | 0.0000e+00 | 1 |

Adduct:

No Chromatogram Found

### TPN(N)KTEDTIFLR Hex 3 HexNAc 4

**Mass:**  2732.2307

(3.245e-06)

**Charge States:** 3

**Start Position**: 70

**End Position**: 82

| Profile Score | Tandem Score | TDA q-value | Volume | Spectrum Matches |
| --- | --- | --- | --- | --- |
| 9.4371 | 33.3215 | 0.0142 | 5.8191e+06 | 2 |

**Adduct Signal Fractions**

- **Unmodified**: 5.8191e+06

### LVPVPITN(N)ATLDQITGK Hex 4 HexNAc 8

**Mass:**  4051.8669

(2.099e-06)

**Charge States:** 4

**Start Position**: 26

**End Position**: 43

| Profile Score | Tandem Score | TDA q-value | Volume | Spectrum Matches |
| --- | --- | --- | --- | --- |
| -19.7654 | 32.6716 | 0.0142 | 3.7414e+06 | 1 |

**Adduct Signal Fractions**

- **Unmodified**: 3.7414e+06

### TPN(N)KTEDTIFLR Hex 4 HexNAc 7

**Mass:**  3503.5175

(3.213e-06)

**Charge States:** 3, 4

**Start Position**: 70

**End Position**: 82

| Profile Score | Tandem Score | TDA q-value | Volume | Spectrum Matches |
| --- | --- | --- | --- | --- |
| -5.4974 | 32.5322 | 0.0142 | 5.4711e+06 | 3 |

**Adduct Signal Fractions**

- **Unmodified**: 5.4711e+06

### QIPLC(C)ANLVPVPITN(N)ATLDQITGK Hex 3 HexNAc 5 Neu5Ac 1

**Mass:**  4368.0515

(-1.190e-06)

**Charge States:** 3, 4

**Start Position**: 19

**End Position**: 43

| Profile Score | Tandem Score | TDA q-value | Volume | Spectrum Matches |
| --- | --- | --- | --- | --- |
| 13.5931 | 32.3836 | 0.0142 | 2.3323e+08 | 11 |

**Adduct Signal Fractions**

- **Unmodified**: 2.3323e+08

### QDQC(C)IYN(N)TTYLN Hex 3 HexNAc 6

**Mass:**  3236.3051

(2.042e-06)

**Charge States:** 3, 4

**Start Position**: 87

**End Position**: 99

| Profile Score | Tandem Score | TDA q-value | Volume | Spectrum Matches |
| --- | --- | --- | --- | --- |
| -18.9279 | 30.3101 | 0.0262 | 1.2178e+07 | 1 |

**Adduct Signal Fractions**

- **Unmodified**: 1.2178e+07

### N(N)ATLDQITGK Hex 3 HexNAc 5

**Mass:**  2561.1137

(8.966e-07)

**Charge States:** 3

**Start Position**: 33

**End Position**: 43

| Profile Score | Tandem Score | TDA q-value | Volume | Spectrum Matches |
| --- | --- | --- | --- | --- |
| -20.6309 | 30.2379 | 0.0274 | 5.4232e+05 | 1 |

**Adduct Signal Fractions**

- **Unmodified**: 5.4232e+05

### C(C)ANLVPVPITN(N)ATLDQITGK Hex 4 HexNAc 6

**Mass:**  3990.8190

(1.677e-06)

**Charge States:** 4

**Start Position**: 23

**End Position**: 43

| Profile Score | Tandem Score | TDA q-value | Volume | Spectrum Matches |
| --- | --- | --- | --- | --- |
| -4.5113 | 29.4927 | 0.0333 | 1.2257e+07 | 2 |

**Adduct Signal Fractions**

- **Unmodified**: 1.2257e+07

### QDQC(C)IYN(N)TTYLN(D)VQR Fuc 1 Hex 4 HexNAc 6

**Mass:**  3928.6279

(2.532e-04)

**Charge States:** 3

**Start Position**: 87

**End Position**: 102

| Profile Score | Tandem Score | TDA q-value | Volume | Spectrum Matches |
| --- | --- | --- | --- | --- |
| -19.8332 | 29.3889 | 0.0333 | 1.6802e+07 | 2 |

**Adduct Signal Fractions**

- **Unmodified**: 1.6802e+07

### FTPN(N)KTEDTIFLR Fuc 2 Hex 5 HexNAc 6

**Mass:**  3901.6840

(2.028e-06)

**Charge States:** 3, 4

**Start Position**: 69

**End Position**: 82

| Profile Score | Tandem Score | TDA q-value | Volume | Spectrum Matches |
| --- | --- | --- | --- | --- |
| -19.3716 | 28.6611 | 0.0375 | 2.0889e+06 | 2 |

**Adduct Signal Fractions**

- **Unmodified**: 2.0889e+06

### LVPVPITN(N)ATLDQITGK Hex 3 HexNAc 5

**Mass:**  3282.5809

(6.119e-04)

**Start Position**: 26

**End Position**: 43

| Profile Score | Tandem Score | TDA q-value | Volume | Spectrum Matches |
| --- | --- | --- | --- | --- |
| 0.0000 | 28.1673 | 0.0377 | 0.0000e+00 | 2 |

Adduct:

No Chromatogram Found

### IYN(N)TTYLNVQR Fuc 1 Hex 4 HexNAc 5

**Mass:**  3193.3830

(2.526e-07)

**Charge States:** 3

**Start Position**: 91

**End Position**: 102

| Profile Score | Tandem Score | TDA q-value | Volume | Spectrum Matches |
| --- | --- | --- | --- | --- |
| 9.5079 | 28.1273 | 0.0377 | 8.0642e+06 | 1 |

**Adduct Signal Fractions**

- **Unmodified**: 8.0642e+06

### QDQC(C)IYN(N)TTYLNVQR Hex 3 HexNAc 5

**Mass:**  3418.4509

(5.872e-04)

**Start Position**: 87

**End Position**: 102

| Profile Score | Tandem Score | TDA q-value | Volume | Spectrum Matches |
| --- | --- | --- | --- | --- |
| 0.0000 | 27.8914 | 0.0385 | 0.0000e+00 | 1 |

Adduct:

No Chromatogram Found

### QDQC(C)IYN(N)TTYLNVQR Fuc 2 Hex 6 HexNAc 8

**Mass:**  4803.9665

(1.899e-06)

**Charge States:** 4

**Start Position**: 87

**End Position**: 102

| Profile Score | Tandem Score | TDA q-value | Volume | Spectrum Matches |
| --- | --- | --- | --- | --- |
| -5.6409 | 27.5019 | 0.0388 | 9.1963e+06 | 2 |

**Adduct Signal Fractions**

- **Unmodified**: 9.1963e+06

### PVPITN(N)ATLDQITGK Fuc 2 Hex 6 HexNAc 6

**Mass:**  4052.7817

(7.495e-04)

**Charge States:** 4

**Start Position**: 28

**End Position**: 43

| Profile Score | Tandem Score | TDA q-value | Volume | Spectrum Matches |
| --- | --- | --- | --- | --- |
| -20.6765 | 26.9499 | 0.0399 | 3.3783e+06 | 5 |

**Adduct Signal Fractions**

- **Unmodified**: 3.3783e+06

### QIPLC(C)ANLVPVPITN(N)ATLDQITGK Hex 3 HexNAc 5

**Mass:**  4076.9650

(5.825e-07)

**Charge States:** 3, 4

**Start Position**: 19

**End Position**: 43

| Profile Score | Tandem Score | TDA q-value | Volume | Spectrum Matches |
| --- | --- | --- | --- | --- |
| 13.2237 | 26.2617 | 0.0453 | 1.6458e+08 | 11 |

**Adduct Signal Fractions**

- **Unmodified**: 1.6458e+08

### QDQC(C)IYN(N)TTYLNVQR Hex 3 HexNAc 5 Neu5Ac 1

**Mass:**  3707.5314

(-9.808e-07)

**Charge States:** 3, 4

**Start Position**: 87

**End Position**: 102

| Profile Score | Tandem Score | TDA q-value | Volume | Spectrum Matches |
| --- | --- | --- | --- | --- |
| 14.2002 | 26.2432 | 0.0453 | 9.7556e+08 | 7 |

**Adduct Signal Fractions**

- **Unmodified**: 9.7556e+08

### C(C)ANLVPVPITN(N)ATLDQITGK Hex 3 HexNAc 5 Neu5Ac 1

**Mass:**  3916.7700

(-1.339e-06)

**Charge States:** 3, 4

**Start Position**: 23

**End Position**: 43

| Profile Score | Tandem Score | TDA q-value | Volume | Spectrum Matches |
| --- | --- | --- | --- | --- |
| 14.3352 | 25.8045 | 0.0453 | 1.6426e+08 | 8 |

**Adduct Signal Fractions**

- **Unmodified**: 1.6426e+08

### PVPITN(N)ATLDQITGK Fuc 2 Hex 5 HexNAc 4 Neu5Ac 1

**Mass:**  3773.6733

(2.700e-04)

**Start Position**: 28

**End Position**: 43

| Profile Score | Tandem Score | TDA q-value | Volume | Spectrum Matches |
| --- | --- | --- | --- | --- |
| 0.0000 | 25.0778 | 0.0480 | 0.0000e+00 | 1 |

Adduct:

No Chromatogram Found

### QDQC(C)IYN(N)TTYLN(D)VQR Fuc 4 Hex 3 HexNAc 5

**Mass:**  4001.6845

(2.520e-04)

**Charge States:** 3

**Start Position**: 87

**End Position**: 102

| Profile Score | Tandem Score | TDA q-value | Volume | Spectrum Matches |
| --- | --- | --- | --- | --- |
| -6.0369 | 24.7227 | 0.0498 | 3.8553e+07 | 3 |

**Adduct Signal Fractions**

- **Unmodified**: 3.8553e+07

### QIPLC(C)ANLVPVPITN(N)ATLDQITGK Fuc 1 Hex 4 HexNAc 5

**Mass:**  4385.0744

(7.838e-07)

**Charge States:** 3, 4

**Start Position**: 19

**End Position**: 43

| Profile Score | Tandem Score | TDA q-value | Volume | Spectrum Matches |
| --- | --- | --- | --- | --- |
| 10.5936 | 24.6167 | 0.0498 | 4.6878e+07 | 4 |

**Adduct Signal Fractions**

- **Unmodified**: 4.6878e+07

### P00738|HPT\_HUMAN

**>P00738|HPT\_HUMAN**  
 `MSALGAVIALLLWGQLFAVDSGNDVTDIADDGCPKPPEIAHGYVEHSVRYQCKNYYKLRTEGDGVYTLNDKKQWINKAVG  
DKLPECEADDGCPKPPEIAHGYVEHSVRYQCKNYYKLRTEGDGVYTLNNEKQWINKAVGDKLPECEAVCGKPKNPANPVQ  
RILGGHLDAKGSFPWQAKMVSHHNLTTGATLINEQWLLTTAKNLFLNHSENATAKDIAPTLTLYVGKKQLVEIEKVVLHP  
NYSQVDIGLIKLKQKVSVNERVMPICLPSKDYAEVGRVGYVSGWGRNANFKFTDHLKYVMLPVADQDQCIRHYEGSTVPE  
KKTPKSPVGVQPILNEHTFCAGMSKYQEDTCYGDAGSAFAVHDLEEDTWYATGILSFDKSCAVAEYGVYVKVTSIQDWVQ  
KTIAEN`

| Site | Type | Count |
| --- | --- | --- |
| 241 | N-linked | 4 |
| 211 | N-linked | 7 |

| Mass | Sequence | MS_2_ Score | `q`-value | MS_1_ Score | Signal | Protein Coordinates | Tandem Scans | Start Time | Apex Time | End Time |
| --- | --- | --- | --- | --- | --- | --- | --- | --- | --- | --- |
| 3092.4826 | VVLHPN(N)YSQVDIGLIK Hex 3 HexNAc 4 | 167.537 | 0.0 | -3.376 | 4.664e+07 | 235 - 251 | 4 | 42.84 | 42.84 | 43.35 |
| 4257.7609 | NLFLNHSEN(N)ATAK Hex 6 HexNAc 9 | 132.893 | 0.0 | 12.267 | 3.630e+07 | 202 - 215 | 5 | 18.33 | 18.50 | 19.15 |
| 4460.8404 | NLFLNHSEN(N)ATAK Fuc 5 Hex 4 HexNAc 8 | 132.450 | 0.0 | 11.924 | 5.278e+07 | 202 - 215 | 5 | 18.13 | 18.33 | 18.91 |
| 2568.1141 | VVLHPN(N)YSQVD Hex 3 HexNAc 4 | 112.091 | 0.0 | 13.444 | 9.525e+06 | 235 - 246 | 2 | 23.91 | 24.08 | 24.29 |
| 4663.9227 | NLFLNHSEN(N)ATAK Fuc 5 Hex 4 HexNAc 9 | 93.854 | 0.0 | -4.022 | 1.719e+07 | 202 - 215 | 2 | 17.99 | 18.13 | 18.33 |
| 3295.5678 | VVLHPN(N)YSQVDIGLIK Hex 3 HexNAc 5 | 60.966 | 0.003 | -19.020 | 4.327e+06 | 235 - 251 | 1 | 42.84 | 42.84 | 42.84 |
| 3498.6447 | VVLHPN(N)YSQVDIGLIK Hex 3 HexNAc 6 | 57.027 | 0.003 | -19.900 | 8.401e+06 | 235 - 251 | 2 | 42.51 | 42.67 | 42.67 |
| 4565.8713 | NLFLNHSEN(N)ATAK Fuc 1 Hex 7 HexNAc 9 | 48.203 | 0.003 | -4.163 | 9.447e+06 | 202 - 215 | 1 | 18.13 | 18.24 | 18.33 |
| 4770.9529 | NLFLNHSEN(N)ATAK Fuc 4 Hex 5 HexNAc 8 Neu5Ac 1 | 29.193 | 0.033 | 0.000 | 0.000e+00 | 202 - 215 | 1 | - | - | - |
| 4054.6834 | NLFLNHSEN(N)ATAK Hex 6 HexNAc 8 | 28.868 | 0.033 | -4.387 | 8.571e+06 | 202 - 215 | 3 | 18.50 | 18.59 | 18.99 |
| 4768.9497 | N(D)LFLNHSEN(N)ATAK Fuc 4 Hex 5 HexNAc 8 Neu5Ac 1 | 28.663 | 0.037 | -5.936 | 1.491e+07 | 202 - 215 | 1 | 17.90 | 17.99 | 18.24 |

### VVLHPN(N)YSQVDIGLIK Hex 3 HexNAc 4

**Mass:**  3092.4823

(8.872e-07)

**Charge States:** 3, 4

**Start Position**: 236

**End Position**: 252

| Profile Score | Tandem Score | TDA q-value | Volume | Spectrum Matches |
| --- | --- | --- | --- | --- |
| -3.3763 | 167.5374 | 0.0000 | 4.6641e+07 | 4 |

**Adduct Signal Fractions**

- **Unmodified**: 4.6641e+07

### NLFLNHSEN(N)ATAK Hex 6 HexNAc 9

**Mass:**  4257.7609

(1.402e-06)

**Charge States:** 4

**Start Position**: 203

**End Position**: 216

| Profile Score | Tandem Score | TDA q-value | Volume | Spectrum Matches |
| --- | --- | --- | --- | --- |
| 12.2665 | 132.8931 | 0.0000 | 3.6304e+07 | 5 |

**Adduct Signal Fractions**

- **Unmodified**: 3.6304e+07

### NLFLNHSEN(N)ATAK Fuc 5 Hex 4 HexNAc 8

**Mass:**  4460.8400

(-4.994e-06)

**Charge States:** 4

**Start Position**: 203

**End Position**: 216

| Profile Score | Tandem Score | TDA q-value | Volume | Spectrum Matches |
| --- | --- | --- | --- | --- |
| 11.9237 | 132.4501 | 0.0000 | 5.2782e+07 | 5 |

**Adduct Signal Fractions**

- **Unmodified**: 5.2782e+07

### VVLHPN(N)YSQVD Hex 3 HexNAc 4

**Mass:**  2568.1144

(7.125e-07)

**Charge States:** 3

**Start Position**: 236

**End Position**: 247

| Profile Score | Tandem Score | TDA q-value | Volume | Spectrum Matches |
| --- | --- | --- | --- | --- |
| 13.4443 | 112.0906 | 0.0000 | 9.5255e+06 | 2 |

**Adduct Signal Fractions**

- **Unmodified**: 9.5255e+06

### NLFLNHSEN(N)ATAK Fuc 5 Hex 4 HexNAc 9

**Mass:**  4663.9221

(-4.056e-06)

**Charge States:** 4

**Start Position**: 203

**End Position**: 216

| Profile Score | Tandem Score | TDA q-value | Volume | Spectrum Matches |
| --- | --- | --- | --- | --- |
| -4.0219 | 93.8540 | 0.0000 | 1.7193e+07 | 2 |

**Adduct Signal Fractions**

- **Unmodified**: 1.7193e+07

### VVLHPN(N)YSQVDIGLIK Hex 3 HexNAc 5

**Mass:**  3295.5678

(2.547e-06)

**Charge States:** 4

**Start Position**: 236

**End Position**: 252

| Profile Score | Tandem Score | TDA q-value | Volume | Spectrum Matches |
| --- | --- | --- | --- | --- |
| -19.0205 | 60.9660 | 0.0030 | 4.3272e+06 | 1 |

**Adduct Signal Fractions**

- **Unmodified**: 4.3272e+06

### VVLHPN(N)YSQVDIGLIK Hex 3 HexNAc 6

**Mass:**  3498.6459

(2.394e-06)

**Charge States:** 3

**Start Position**: 236

**End Position**: 252

| Profile Score | Tandem Score | TDA q-value | Volume | Spectrum Matches |
| --- | --- | --- | --- | --- |
| -19.8998 | 57.0272 | 0.0030 | 8.4013e+06 | 2 |

**Adduct Signal Fractions**

- **Unmodified**: 8.4013e+06

### NLFLNHSEN(N)ATAK Fuc 1 Hex 7 HexNAc 9

**Mass:**  4565.8693

(1.807e-07)

**Charge States:** 4

**Start Position**: 203

**End Position**: 216

| Profile Score | Tandem Score | TDA q-value | Volume | Spectrum Matches |
| --- | --- | --- | --- | --- |
| -4.1627 | 48.2034 | 0.0030 | 9.4466e+06 | 1 |

**Adduct Signal Fractions**

- **Unmodified**: 9.4466e+06

### NLFLNHSEN(N)ATAK Fuc 4 Hex 5 HexNAc 8 Neu5Ac 1

**Mass:**  4770.9529

(6.293e-04)

**Start Position**: 203

**End Position**: 216

| Profile Score | Tandem Score | TDA q-value | Volume | Spectrum Matches |
| --- | --- | --- | --- | --- |
| 0.0000 | 29.1934 | 0.0333 | 0.0000e+00 | 1 |

Adduct:

No Chromatogram Found

### NLFLNHSEN(N)ATAK Hex 6 HexNAc 8

**Mass:**  4054.6828

(3.231e-07)

**Charge States:** 4

**Start Position**: 203

**End Position**: 216

| Profile Score | Tandem Score | TDA q-value | Volume | Spectrum Matches |
| --- | --- | --- | --- | --- |
| -4.3875 | 28.8678 | 0.0333 | 8.5707e+06 | 3 |

**Adduct Signal Fractions**

- **Unmodified**: 8.5707e+06

### N(D)LFLNHSEN(N)ATAK Fuc 4 Hex 5 HexNAc 8 Neu5Ac 1

**Mass:**  4768.9504

(2.088e-04)

**Charge States:** 4

**Start Position**: 203

**End Position**: 216

| Profile Score | Tandem Score | TDA q-value | Volume | Spectrum Matches |
| --- | --- | --- | --- | --- |
| -5.9357 | 28.6633 | 0.0365 | 1.4910e+07 | 1 |

**Adduct Signal Fractions**

- **Unmodified**: 1.4910e+07

### P05155|IC1\_HUMAN

**>P05155|IC1\_HUMAN**  
 `MASRLTLLTLLLLLLAGDRASSNPNATSSSSQDPESLQDRGEGKVATTVISKMLFVEPILEVSSLPTTNSTTNSATKITA  
NTTDEPTTQPTTEPTTQPTIQPTQPTTQLPTDSPTQPTTGSFCPGPVTLCSDLESHSTEAVLGDALVDFSLKLYHAFSAM  
KKVETNMAFSPFSIASLLTQVLLGAGENTKTNLESILSYPKDFTCVHQALKGFTTKGVTSVSQIFHSPDLAIRDTFVNAS  
RTLYSSSPRVLSNNSDANLELINTWVAKNTNNKISRLLDSLPSDTRLVLLNAIYLSAKWKTTFDPKKTRMEPFHFKNSVI  
KVPMMNSKKYPVAHFIDQTLKAKVGQLQLSHNLSLVILVPQNLKHRLEDMEQALSPSVFKAIMEKLEMSKFQPTLLTLPR  
IKVTTSQDMLSIMEKLEFFDFSYDLNLCGLTEDPDLQVSAMQHQTVLELTETGVEAAAASAISVARTLLVFEVQQPFLFV  
LWDQQHKFPVFMGRVYDPRA`

| Site | Type | Count |
| --- | --- | --- |
| 25 | N-linked | 8 |

| Mass | Sequence | MS_2_ Score | `q`-value | MS_1_ Score | Signal | Protein Coordinates | Tandem Scans | Start Time | Apex Time | End Time |
| --- | --- | --- | --- | --- | --- | --- | --- | --- | --- | --- |
| 3595.8415 | NPN(N)ATSSSSQDPESLQDR Hex 4 HexNAc 5 | 141.148 | 0.0 | -6.938 | 5.215e+07 | 22 - 40 | 9 | 13.92 | 13.92 | 31.12 |
| 3433.4015 | NPN(N)ATSSSSQDPESLQDR Hex 3 HexNAc 5 | 121.556 | 0.0 | -5.059 | 1.320e+07 | 22 - 40 | 2 | 15.07 | 15.20 | 15.41 |
| 3376.3839 | NPN(N)ATSSSSQDPESLQDR Fuc 1 Hex 3 HexNAc 4 | 118.004 | 0.0 | -19.869 | 9.976e+06 | 22 - 40 | 1 | 15.07 | 15.07 | 15.20 |
| 3230.3245 | NPN(N)ATSSSSQDPESLQDR Hex 3 HexNAc 4 | 105.396 | 0.0 | 10.879 | 3.558e+07 | 22 - 40 | 3 | 15.07 | 15.20 | 15.56 |
| 3798.5447 | NPN(N)ATSSSSQDPESLQDR Hex 4 HexNAc 6 | 54.813 | 0.003 | -20.772 | 1.776e+07 | 22 - 40 | 3 | 13.78 | 13.92 | 13.92 |
| 3742.5241 | NPN(N)ATSSSSQDPESLQDR Fuc 1 Hex 4 HexNAc 5 | 40.544 | 0.003 | 0.000 | 0.000e+00 | 22 - 40 | 1 | - | - | - |
| 3377.3881 | NPN(N)ATSSSSQDPESLQDR Fuc 1 Hex 3 HexNAc 4 | 28.630 | 0.038 | 0.000 | 0.000e+00 | 22 - 40 | 1 | - | - | - |
| 3580.4596 | NPN(N)ATSSSSQDPESLQDR Fuc 1 Hex 3 HexNAc 5 | 27.384 | 0.040 | 0.000 | 0.000e+00 | 22 - 40 | 1 | - | - | - |

### NPN(N)ATSSSSQDPESLQDR Hex 4 HexNAc 5

**Mass:**  3595.4599

(2.798e-04)

**Charge States:** 3, 4

**Start Position**: 23

**End Position**: 41

| Profile Score | Tandem Score | TDA q-value | Volume | Spectrum Matches |
| --- | --- | --- | --- | --- |
| -6.9384 | 141.1477 | 0.0000 | 5.2145e+07 | 9 |

**Adduct Signal Fractions**

- **Unmodified**: 5.2145e+07

### NPN(N)ATSSSSQDPESLQDR Hex 3 HexNAc 5

**Mass:**  3433.4049

(1.002e-06)

**Charge States:** 3

**Start Position**: 23

**End Position**: 41

| Profile Score | Tandem Score | TDA q-value | Volume | Spectrum Matches |
| --- | --- | --- | --- | --- |
| -5.0592 | 121.5562 | 0.0000 | 1.3196e+07 | 2 |

**Adduct Signal Fractions**

- **Unmodified**: 1.3196e+07

### NPN(N)ATSSSSQDPESLQDR Fuc 1 Hex 3 HexNAc 4

**Mass:**  3376.3833

(1.154e-06)

**Charge States:** 3

**Start Position**: 23

**End Position**: 41

| Profile Score | Tandem Score | TDA q-value | Volume | Spectrum Matches |
| --- | --- | --- | --- | --- |
| -19.8690 | 118.0042 | 0.0000 | 9.9756e+06 | 1 |

**Adduct Signal Fractions**

- **Unmodified**: 9.9756e+06

### NPN(N)ATSSSSQDPESLQDR Hex 3 HexNAc 4

**Mass:**  3230.3263

(1.444e-06)

**Charge States:** 3

**Start Position**: 23

**End Position**: 41

| Profile Score | Tandem Score | TDA q-value | Volume | Spectrum Matches |
| --- | --- | --- | --- | --- |
| 10.8792 | 105.3964 | 0.0000 | 3.5576e+07 | 3 |

**Adduct Signal Fractions**

- **Unmodified**: 3.5576e+07

### NPN(N)ATSSSSQDPESLQDR Hex 4 HexNAc 6

**Mass:**  3798.5495

(1.101e-06)

**Charge States:** 4

**Start Position**: 23

**End Position**: 41

| Profile Score | Tandem Score | TDA q-value | Volume | Spectrum Matches |
| --- | --- | --- | --- | --- |
| -20.7721 | 54.8134 | 0.0030 | 1.7755e+07 | 3 |

**Adduct Signal Fractions**

- **Unmodified**: 1.7755e+07

### NPN(N)ATSSSSQDPESLQDR Fuc 1 Hex 4 HexNAc 5

**Mass:**  3742.5241

(2.706e-04)

**Start Position**: 23

**End Position**: 41

| Profile Score | Tandem Score | TDA q-value | Volume | Spectrum Matches |
| --- | --- | --- | --- | --- |
| 0.0000 | 40.5440 | 0.0030 | 0.0000e+00 | 1 |

Adduct:

No Chromatogram Found

### NPN(N)ATSSSSQDPESLQDR Fuc 1 Hex 3 HexNAc 4

**Mass:**  3377.3881

(2.987e-04)

**Start Position**: 23

**End Position**: 41

| Profile Score | Tandem Score | TDA q-value | Volume | Spectrum Matches |
| --- | --- | --- | --- | --- |
| 0.0000 | 28.6301 | 0.0375 | 0.0000e+00 | 1 |

Adduct:

No Chromatogram Found

### NPN(N)ATSSSSQDPESLQDR Fuc 1 Hex 3 HexNAc 5

**Mass:**  3580.4596

(2.796e-04)

**Start Position**: 23

**End Position**: 41

| Profile Score | Tandem Score | TDA q-value | Volume | Spectrum Matches |
| --- | --- | --- | --- | --- |
| 0.0000 | 27.3843 | 0.0396 | 0.0000e+00 | 1 |

Adduct:

No Chromatogram Found

### Q9Y5Y7|LYVE1\_HUMAN

**>Q9Y5Y7|LYVE1\_HUMAN**  
 `MARCFSLVLLLTSIWTTRLLVQGSLRAEELSIQVSCRIMGITLVSKKANQQLNFTEAKEACRLLGLSLAGKDQVETALKA  
SFETCSYGWVGDGFVVISRISPNPKCGKNGVGVLIWKVPVSRQFAAYCYNSSDTWTNSCIPEIITTKDPIFNTQTATQTT  
EFIVSDSTYSVASPYSTIPAPTTTPPAPASTSIPRRKKLICVTEVFMETSTMSTETEPFVENKAAFKNEAAGFGGVPTAL  
LVLALLFFGAAAGLGFCYVKRYVKAFPFTNKNQQKEMIETKVVKEEKANDSNPNEESKKTDKNPEESKSPSKTTVRCLEA  
EV`

| Site | Type | Count |
| --- | --- | --- |
| 53 | N-linked | 7 |

| Mass | Sequence | MS_2_ Score | `q`-value | MS_1_ Score | Signal | Protein Coordinates | Tandem Scans | Start Time | Apex Time | End Time |
| --- | --- | --- | --- | --- | --- | --- | --- | --- | --- | --- |
| 2892.2779 | KANQQLN(N)FTEAK Hex 3 HexNAc 5 | 171.705 | 0.0 | 12.596 | 3.367e+07 | 46 - 58 | 2 | 17.43 | 17.60 | 17.99 |
| 2689.1987 | KANQQLN(N)FTEAK Hex 3 HexNAc 4 | 135.785 | 0.0 | -4.942 | 8.860e+06 | 46 - 58 | 2 | 17.60 | 17.68 | 17.81 |
| 3259.2357 | KANQQLN(N)FTEAK Hex 4 HexNAc 6 | 133.612 | 0.0 | -24.103 | 7.737e+07 | 46 - 58 | 4 | 17.43 | 53.83 | 54.00 |
| 2764.1843 | ANQQLN(N)FTEAK Hex 3 HexNAc 5 | 130.716 | 0.0 | 11.485 | 1.379e+07 | 47 - 58 | 4 | 21.30 | 21.47 | 21.95 |
| 3129.3183 | ANQQLN(N)FTEAK Hex 4 HexNAc 6 | 113.730 | 0.0 | -4.801 | 4.208e+06 | 47 - 58 | 2 | 21.13 | 21.30 | 21.47 |
| 2561.1113 | ANQQLN(N)FTEAK Hex 3 HexNAc 4 | 39.677 | 0.003 | -4.600 | 5.046e+06 | 47 - 58 | 2 | 21.30 | 21.47 | 21.79 |
| 3624.5168 | KANQQLN(N)FTEAK Hex 5 HexNAc 7 | 37.546 | 0.006 | -7.282 | 2.867e+05 | 46 - 58 | 1 | 26.89 | 26.98 | 27.17 |

### KANQQLN(N)FTEAK Hex 3 HexNAc 5

**Mass:**  2892.2777

(1.010e-06)

**Charge States:** 3

**Start Position**: 47

**End Position**: 59

| Profile Score | Tandem Score | TDA q-value | Volume | Spectrum Matches |
| --- | --- | --- | --- | --- |
| 12.5961 | 171.7052 | 0.0000 | 3.3666e+07 | 2 |

**Adduct Signal Fractions**

- **Unmodified**: 3.3666e+07

### KANQQLN(N)FTEAK Hex 3 HexNAc 4

**Mass:**  2689.2015

(-1.796e-07)

**Charge States:** 3

**Start Position**: 47

**End Position**: 59

| Profile Score | Tandem Score | TDA q-value | Volume | Spectrum Matches |
| --- | --- | --- | --- | --- |
| -4.9419 | 135.7845 | 0.0000 | 8.8601e+06 | 2 |

**Adduct Signal Fractions**

- **Unmodified**: 8.8601e+06

### KANQQLN(N)FTEAK Hex 4 HexNAc 6

**Mass:**  3259.4335

(2.537e-06)

**Charge States:** 3, 4

**Start Position**: 47

**End Position**: 59

| Profile Score | Tandem Score | TDA q-value | Volume | Spectrum Matches |
| --- | --- | --- | --- | --- |
| -24.1031 | 133.6123 | 0.0000 | 7.7368e+07 | 4 |

**Adduct Signal Fractions**

- **Unmodified**: 7.7368e+07

### ANQQLN(N)FTEAK Hex 3 HexNAc 5

**Mass:**  2764.1843

(1.247e-06)

**Charge States:** 3

**Start Position**: 48

**End Position**: 59

| Profile Score | Tandem Score | TDA q-value | Volume | Spectrum Matches |
| --- | --- | --- | --- | --- |
| 11.4848 | 130.7163 | 0.0000 | 1.3794e+07 | 4 |

**Adduct Signal Fractions**

- **Unmodified**: 1.3794e+07

### ANQQLN(N)FTEAK Hex 4 HexNAc 6

**Mass:**  3129.3169

(1.388e-06)

**Charge States:** 3

**Start Position**: 48

**End Position**: 59

| Profile Score | Tandem Score | TDA q-value | Volume | Spectrum Matches |
| --- | --- | --- | --- | --- |
| -4.8011 | 113.7303 | 0.0000 | 4.2075e+06 | 2 |

**Adduct Signal Fractions**

- **Unmodified**: 4.2075e+06

### ANQQLN(N)FTEAK Hex 3 HexNAc 4

**Mass:**  2561.1082

(1.831e-06)

**Charge States:** 3

**Start Position**: 48

**End Position**: 59

| Profile Score | Tandem Score | TDA q-value | Volume | Spectrum Matches |
| --- | --- | --- | --- | --- |
| -4.5996 | 39.6773 | 0.0030 | 5.0457e+06 | 2 |

**Adduct Signal Fractions**

- **Unmodified**: 5.0457e+06

### KANQQLN(N)FTEAK Hex 5 HexNAc 7

**Mass:**  3624.5331

(5.544e-04)

**Charge States:** 4

**Start Position**: 47

**End Position**: 59

| Profile Score | Tandem Score | TDA q-value | Volume | Spectrum Matches |
| --- | --- | --- | --- | --- |
| -7.2824 | 37.5458 | 0.0056 | 2.8671e+05 | 1 |

**Adduct Signal Fractions**

- **Unmodified**: 2.8671e+05

### P02790|HEMO\_HUMAN

**>P02790|HEMO\_HUMAN**  
 `MARVLGAPVALGLWSLCWSLAIATPLPPTSAHGNVAEGETKPDPDVTERCSDGWSFDATTLDDNGTMLFFKGEFVWKSHK  
WDRELISERWKNFPSPVDAAFRQGHNSVFLIKGDKVWVYPPEKKEKGYPKLLQDEFPGIPSPLDAAVECHRGECQAEGVL  
FFQGDREWFWDLATGTMKERSWPAVGNCSSALRWLGRYYCFQGNQFLRFDPVRGEVPPRYPRDVRDYFMPCPGRGHGHRN  
GTGHGNSTHHGPEYMRCSPHLVLSALTSDNHGATYAFSGTHYWRLDTSRDGWHSWPIAHQWPQGPSAVDAAFSWEEKLYL  
VQGTQVYVFLTKGGYTLVSGYPKRLEKEVGTPHGIILDSVDAAFICPGSSRLHIMAGRRLWWLDLKSGAQATWTELPWPH  
EKVDGALCMEKSLGPNSCSANGPGLYLIHGPNLYCYSDVEKLNAAKALPQPQNVTSLLGCTH`

| Site | Type | Count |
| --- | --- | --- |
| 187 | N-linked | 4 |
| 453 | N-linked | 1 |

| Mass | Sequence | MS_2_ Score | `q`-value | MS_1_ Score | Signal | Protein Coordinates | Tandem Scans | Start Time | Apex Time | End Time |
| --- | --- | --- | --- | --- | --- | --- | --- | --- | --- | --- |
| 2702.1398 | SWPAVGN(N)C(C)SSALR Hex 3 HexNAc 4 | 117.475 | 0.0 | 11.558 | 1.030e+07 | 180 - 193 | 2 | 31.12 | 31.29 | 32.14 |
| 3033.3513 | ALPQPQN(N)VTSLLGC(C)TH Hex 3 HexNAc 4 | 107.142 | 0.0 | 13.948 | 2.492e+07 | 446 - 462 | 2 | 44.46 | 44.97 | 45.47 |
| 2905.2189 | SWPAVGN(N)C(C)SSALR Hex 3 HexNAc 5 | 33.278 | 0.014 | -18.236 | 2.513e+06 | 180 - 193 | 1 | 31.12 | 31.12 | 31.29 |
| 2906.2252 | SWPAVGN(N)C(C)SSALR Hex 3 HexNAc 5 | 32.286 | 0.018 | 0.000 | 0.000e+00 | 180 - 193 | 1 | - | - | - |
| 2848.2076 | SWPAVGN(N)C(C)SSALR Fuc 1 Hex 3 HexNAc 4 | 27.218 | 0.040 | 0.000 | 0.000e+00 | 180 - 193 | 1 | - | - | - |

### SWPAVGN(N)C(C)SSALR Hex 3 HexNAc 4

**Mass:**  2702.1399

(1.061e-06)

**Charge States:** 3

**Start Position**: 181

**End Position**: 194

| Profile Score | Tandem Score | TDA q-value | Volume | Spectrum Matches |
| --- | --- | --- | --- | --- |
| 11.5577 | 117.4747 | 0.0000 | 1.0301e+07 | 2 |

**Adduct Signal Fractions**

- **Unmodified**: 1.0301e+07

### ALPQPQN(N)VTSLLGC(C)TH Hex 3 HexNAc 4

**Mass:**  3033.3508

(1.483e-06)

**Charge States:** 3

**Start Position**: 447

**End Position**: 463

| Profile Score | Tandem Score | TDA q-value | Volume | Spectrum Matches |
| --- | --- | --- | --- | --- |
| 13.9482 | 107.1419 | 0.0000 | 2.4922e+07 | 2 |

**Adduct Signal Fractions**

- **Unmodified**: 2.4922e+07

### SWPAVGN(N)C(C)SSALR Hex 3 HexNAc 5

**Mass:**  2905.2174

(1.503e-06)

**Charge States:** 3

**Start Position**: 181

**End Position**: 194

| Profile Score | Tandem Score | TDA q-value | Volume | Spectrum Matches |
| --- | --- | --- | --- | --- |
| -18.2364 | 33.2776 | 0.0142 | 2.5126e+06 | 1 |

**Adduct Signal Fractions**

- **Unmodified**: 2.5126e+06

### SWPAVGN(N)C(C)SSALR Hex 3 HexNAc 5

**Mass:**  2906.2252

(3.470e-04)

**Start Position**: 181

**End Position**: 194

| Profile Score | Tandem Score | TDA q-value | Volume | Spectrum Matches |
| --- | --- | --- | --- | --- |
| 0.0000 | 32.2855 | 0.0178 | 0.0000e+00 | 1 |

Adduct:

No Chromatogram Found

### SWPAVGN(N)C(C)SSALR Fuc 1 Hex 3 HexNAc 4

**Mass:**  2848.2076

(4.255e-06)

**Start Position**: 181

**End Position**: 194

| Profile Score | Tandem Score | TDA q-value | Volume | Spectrum Matches |
| --- | --- | --- | --- | --- |
| 0.0000 | 27.2176 | 0.0396 | 0.0000e+00 | 1 |

Adduct:

No Chromatogram Found

### Q5R543|HEMO\_PONAB

**>Q5R543|HEMO\_PONAB**  
 `MARALGAPIALGLWSLCWSLAIATPLPPTSAHGNVAEGETKPDPDVTERCSDGWSFDATTLDDNGTMLFFKGEFVWKSHK  
WDRELISERWKNFPSPVDAAFRQGHNSVFLIKGDKVWVYPPEKKEKGYPKLLQDEFPGIPSPLDAAVECHRGECQAEGVL  
FFQGDREWFWDLATGTMKERSWPAVGNCSSALRWLGRYYCFQGNQFLRFDPVRGEVPPRYPRDVRDYFMPCPGRGHGHRN  
GTGHGNGTHHGPEYMRCSPHLVLSALTSDNHGATYAFSGTHYWRLDTSRDGWHSWPIAHQWPQGPSTVDAAFSWEEKLYL  
VQGTQVYVFLTKGGYTLVSGYPKQLEKEVGTPHGIILDSVDAAFICPGSSRLHIMAGRRLWWLDLKSGAQALWTELPWPH  
EKVDGALCVEKSLGPNSCSANGPGLYLIHGPNLYCYSDVEKLNAAKALPQPQNVTSLLGCTH`

| Site | Type | Count |
| --- | --- | --- |
| 187 | N-linked | 4 |
| 453 | N-linked | 1 |

| Mass | Sequence | MS_2_ Score | `q`-value | MS_1_ Score | Signal | Protein Coordinates | Tandem Scans | Start Time | Apex Time | End Time |
| --- | --- | --- | --- | --- | --- | --- | --- | --- | --- | --- |
| 2702.1398 | SWPAVGN(N)C(C)SSALR Hex 3 HexNAc 4 | 117.475 | 0.0 | 11.558 | 1.030e+07 | 180 - 193 | 2 | 31.12 | 31.29 | 32.14 |
| 3033.3513 | ALPQPQN(N)VTSLLGC(C)TH Hex 3 HexNAc 4 | 107.142 | 0.0 | 13.948 | 2.492e+07 | 446 - 462 | 2 | 44.46 | 44.97 | 45.47 |
| 2905.2189 | SWPAVGN(N)C(C)SSALR Hex 3 HexNAc 5 | 33.278 | 0.014 | -18.236 | 2.513e+06 | 180 - 193 | 1 | 31.12 | 31.12 | 31.29 |
| 2906.2252 | SWPAVGN(N)C(C)SSALR Hex 3 HexNAc 5 | 32.286 | 0.018 | 0.000 | 0.000e+00 | 180 - 193 | 1 | - | - | - |
| 2848.2076 | SWPAVGN(N)C(C)SSALR Fuc 1 Hex 3 HexNAc 4 | 27.218 | 0.040 | 0.000 | 0.000e+00 | 180 - 193 | 1 | - | - | - |

### SWPAVGN(N)C(C)SSALR Hex 3 HexNAc 4

**Mass:**  2702.1399

(1.061e-06)

**Charge States:** 3

**Start Position**: 181

**End Position**: 194

| Profile Score | Tandem Score | TDA q-value | Volume | Spectrum Matches |
| --- | --- | --- | --- | --- |
| 11.5577 | 117.4747 | 0.0000 | 1.0301e+07 | 2 |

**Adduct Signal Fractions**

- **Unmodified**: 1.0301e+07

### ALPQPQN(N)VTSLLGC(C)TH Hex 3 HexNAc 4

**Mass:**  3033.3508

(1.483e-06)

**Charge States:** 3

**Start Position**: 447

**End Position**: 463

| Profile Score | Tandem Score | TDA q-value | Volume | Spectrum Matches |
| --- | --- | --- | --- | --- |
| 13.9482 | 107.1419 | 0.0000 | 2.4922e+07 | 2 |

**Adduct Signal Fractions**

- **Unmodified**: 2.4922e+07

### SWPAVGN(N)C(C)SSALR Hex 3 HexNAc 5

**Mass:**  2905.2174

(1.503e-06)

**Charge States:** 3

**Start Position**: 181

**End Position**: 194

| Profile Score | Tandem Score | TDA q-value | Volume | Spectrum Matches |
| --- | --- | --- | --- | --- |
| -18.2364 | 33.2776 | 0.0142 | 2.5126e+06 | 1 |

**Adduct Signal Fractions**

- **Unmodified**: 2.5126e+06

### SWPAVGN(N)C(C)SSALR Hex 3 HexNAc 5

**Mass:**  2906.2252

(3.470e-04)

**Start Position**: 181

**End Position**: 194

| Profile Score | Tandem Score | TDA q-value | Volume | Spectrum Matches |
| --- | --- | --- | --- | --- |
| 0.0000 | 32.2855 | 0.0178 | 0.0000e+00 | 1 |

Adduct:

No Chromatogram Found

### SWPAVGN(N)C(C)SSALR Fuc 1 Hex 3 HexNAc 4

**Mass:**  2848.2076

(4.255e-06)

**Start Position**: 181

**End Position**: 194

| Profile Score | Tandem Score | TDA q-value | Volume | Spectrum Matches |
| --- | --- | --- | --- | --- |
| 0.0000 | 27.2176 | 0.0396 | 0.0000e+00 | 1 |

Adduct:

No Chromatogram Found

### P11279|LAMP1\_HUMAN

**>P11279|LAMP1\_HUMAN**  
 `MAAPGSARRPLLLLLLLLLLGLMHCASAAMFMVKNGNGTACIMANFSAAFSVNYDTKSGPKNMTFDLPSDATVVLNRSSC  
GKENTSDPSLVIAFGRGHTLTLNFTRNATRYSVQLMSFVYNLSDTHLFPNASSKEIKTVESITDIRADIDKKYRCVSGTQ  
VHMNNVTVTLHDATIQAYLSNSSFSRGETRCEQDRPSPTTAPPAPPSPSPSPVPKSPSVDKYNVSGTNGTCLLASMGLQL  
NLTYERKDNTTVTRLLNINPNKTSASGSCGAHLVTLELHSEGTTVLLFQFGMNASSSRFFLQGIQLNTILPDARDPAFKA  
ANGSLRALQATVGNSYKCNAEEHVRVTKAFSVNIFKVWVQAFKVEGGQFGSVEECLLDENSMLIPIAVGGALAGLVLIVL  
IAYLVGRKRSHAGYQTI`

| Site | Type | Count |
| --- | --- | --- |
| 103 | N-linked | 4 |

| Mass | Sequence | MS_2_ Score | `q`-value | MS_1_ Score | Signal | Protein Coordinates | Tandem Scans | Start Time | Apex Time | End Time |
| --- | --- | --- | --- | --- | --- | --- | --- | --- | --- | --- |
| 2416.0675 | GHTLTLN(N)FTR Hex 4 HexNAc 3 | 137.262 | 0.0 | -3.739 | 2.728e+06 | 96 - 106 | 2 | 30.27 | 30.27 | 30.61 |
| 2578.1185 | GHTLTLN(N)FTR Hex 5 HexNAc 3 | 126.014 | 0.0 | -3.380 | 3.522e+06 | 96 - 106 | 1 | 29.93 | 30.10 | 30.27 |
| 2457.0943 | GHTLTLN(N)FTR Hex 3 HexNAc 4 | 36.846 | 0.006 | -5.428 | 1.191e+06 | 96 - 106 | 1 | 30.27 | 30.44 | 30.61 |
| 2375.0492 | GHTLTLN(N)FTR Hex 5 HexNAc 2 | 24.934 | 0.050 | -5.611 | 1.808e+06 | 96 - 106 | 1 | 29.93 | 30.10 | 30.27 |

### GHTLTLN(N)FTR Hex 4 HexNAc 3

**Mass:**  2416.0680

(1.667e-06)

**Charge States:** 3

**Start Position**: 97

**End Position**: 107

| Profile Score | Tandem Score | TDA q-value | Volume | Spectrum Matches |
| --- | --- | --- | --- | --- |
| -3.7389 | 137.2618 | 0.0000 | 2.7280e+06 | 2 |

**Adduct Signal Fractions**

- **Unmodified**: 2.7280e+06

### GHTLTLN(N)FTR Hex 5 HexNAc 3

**Mass:**  2578.1195

(1.015e-06)

**Charge States:** 3

**Start Position**: 97

**End Position**: 107

| Profile Score | Tandem Score | TDA q-value | Volume | Spectrum Matches |
| --- | --- | --- | --- | --- |
| -3.3800 | 126.0135 | 0.0000 | 3.5217e+06 | 1 |

**Adduct Signal Fractions**

- **Unmodified**: 3.5217e+06

### GHTLTLN(N)FTR Hex 3 HexNAc 4

**Mass:**  2457.0926

(2.806e-06)

**Charge States:** 3

**Start Position**: 97

**End Position**: 107

| Profile Score | Tandem Score | TDA q-value | Volume | Spectrum Matches |
| --- | --- | --- | --- | --- |
| -5.4283 | 36.8460 | 0.0056 | 1.1908e+06 | 1 |

**Adduct Signal Fractions**

- **Unmodified**: 1.1908e+06

### GHTLTLN(N)FTR Hex 5 HexNAc 2

**Mass:**  2375.0473

(4.133e-06)

**Charge States:** 2, 3

**Start Position**: 97

**End Position**: 107

| Profile Score | Tandem Score | TDA q-value | Volume | Spectrum Matches |
| --- | --- | --- | --- | --- |
| -5.6105 | 24.9336 | 0.0497 | 1.8084e+06 | 1 |

**Adduct Signal Fractions**

- **Unmodified**: 1.8084e+06

### Q12913|PTPRJ\_HUMAN

**>Q12913|PTPRJ\_HUMAN**  
 `MKPAAREARLPPRSPGLRWALPLLLLLLRLGQILCAGGTPSPIPDPSVATVATGENGITQISSTAESFHKQNGTGTPQVE  
TNTSEDGESSGANDSLRTPEQGSNGTDGASQKTPSSTGPSPVFDIKAVSISPTNVILTWKSNDTAASEYKYVVKHKMENE  
KTITVVHQPWCNITGLRPATSYVFSITPGIGNETWGDPRVIKVITEPIPVSDLRVALTGVRKAALSWSNGNGTASCRVLL  
ESIGSHEELTQDSRLQVNISGLKPGVQYNINPYLLQSNKTKGDPLGTEGGLDASNTERSRAGSPTAPVHDESLVGPVDPS  
SGQQSRDTEVLLVGLEPGTRYNATVYSQAANGTEGQPQAIEFRTNAIQVFDVTAVNISATSLTLIWKVSDNESSSNYTYK  
IHVAGETDSSNLNVSEPRAVIPGLRSSTFYNITVCPVLGDIEGTPGFLQVHTPPVPVSDFRVTVVSTTEIGLAWSSHDAE  
SFQMHITQEGAGNSRVEITTNQSIIIGGLFPGTKYCFEIVPKGPNGTEGASRTVCNRTVPSAVFDIHVVYVTTTEMWLDW  
KSPDGASEYVYHLVIESKHGSNHTSTYDKAITLQGLIPGTLYNITISPEVDHVWGDPNSTAQYTRPSNVSNIDVSTNTTA  
ATLSWQNFDDASPTYSYCLLIEKAGNSSNATQVVTDIGITDATVTELIPGSSYTVEIFAQVGDGIKSLEPGRKSFCTDPA  
SMASFDCEVVPKEPALVLKWTCPPGANAGFELEVSSGAWNNATHLESCSSENGTEYRTEVTYLNFSTSYNISITTVSCGK  
MAAPTRNTCTTGITDPPPPDGSPNITSVSHNSVKVKFSGFEASHGPIKAYAVILTTGEAGHPSADVLKYTYEDFKKGASD  
TYVTYLIRTEEKGRSQSLSEVLKYEIDVGNESTTLGYYNGKLEPLGSYRACVAGFTNITFHPQNKGLIDGAESYVSFSRY  
SDAVSLPQDPGVICGAVFGCIFGALVIVTVGGFIFWRKKRKDAKNNEVSFSQIKPKKSKLIRVENFEAYFKKQQADSNCG  
FAEEYEDLKLVGISQPKYAAELAENRGKNRYNNVLPYDISRVKLSVQTHSTDDYINANYMPGYHSKKDFIATQGPLPNTL  
KDFWRMVWEKNVYAIIMLTKCVEQGRTKCEEYWPSKQAQDYGDITVAMTSEIVLPEWTIRDFTVKNIQTSESHPLRQFHF  
TSWPDHGVPDTTDLLINFRYLVRDYMKQSPPESPILVHCSAGVGRTGTFIAIDRLIYQIENENTVDVYGIVYDLRMHRPL  
MVQTEDQYVFLNQCVLDIVRSQKDSKVDLIYQNTTAMTIYENLAPVTTFGKTNGYIA`

| Site | Type | Count |
| --- | --- | --- |
| 525 | N-linked | 1 |
| 413 | N-linked | 2 |

| Mass | Sequence | MS_2_ Score | `q`-value | MS_1_ Score | Signal | Protein Coordinates | Tandem Scans | Start Time | Apex Time | End Time |
| --- | --- | --- | --- | --- | --- | --- | --- | --- | --- | --- |
| 3425.4882 | IHVAGETDSSNLN(N)VSEPR Hex 3 HexNAc 5 | 90.296 | 0.0 | 14.216 | 1.612e+07 | 400 - 418 | 4 | 23.49 | 23.64 | 23.99 |
| 3970.5725 | GPN(N)GTEGASR Fuc 1 Hex 9 HexNAc 7 | 80.080 | 0.003 | -19.277 | 3.902e+07 | 522 - 532 | 1 | 33.33 | 33.50 | 33.50 |
| 3222.4105 | IHVAGETDSSNLN(N)VSEPR Hex 3 HexNAc 4 | 40.036 | 0.003 | 12.202 | 2.898e+06 | 400 - 418 | 2 | 23.64 | 23.77 | 23.91 |

### IHVAGETDSSNLN(N)VSEPR Hex 3 HexNAc 5

**Mass:**  3425.4889

(1.438e-06)

**Charge States:** 3, 4

**Start Position**: 401

**End Position**: 419

| Profile Score | Tandem Score | TDA q-value | Volume | Spectrum Matches |
| --- | --- | --- | --- | --- |
| 14.2158 | 90.2964 | 0.0000 | 1.6119e+07 | 4 |

**Adduct Signal Fractions**

- **Unmodified**: 1.6119e+07

### GPN(N)GTEGASR Fuc 1 Hex 9 HexNAc 7

**Mass:**  3970.5786

(9.608e-06)

**Charge States:** 3, 4

**Start Position**: 523

**End Position**: 533

| Profile Score | Tandem Score | TDA q-value | Volume | Spectrum Matches |
| --- | --- | --- | --- | --- |
| -19.2774 | 80.0798 | 0.0030 | 3.9020e+07 | 1 |

**Adduct Signal Fractions**

- **Unmodified**: 3.9020e+07

### IHVAGETDSSNLN(N)VSEPR Hex 3 HexNAc 4

**Mass:**  3222.4118

(1.784e-06)

**Charge States:** 3

**Start Position**: 401

**End Position**: 419

| Profile Score | Tandem Score | TDA q-value | Volume | Spectrum Matches |
| --- | --- | --- | --- | --- |
| 12.2015 | 40.0362 | 0.0030 | 2.8977e+06 | 2 |

**Adduct Signal Fractions**

- **Unmodified**: 2.8977e+06

### O95497|VNN1\_HUMAN

**>O95497|VNN1\_HUMAN**  
 `MTTQLPAYVAILLFYVSRASCQDTFTAAVYEHAAILPNATLTPVSREEALALMNRNLDILEGAITSAADQGAHIIVTPED  
AIYGWNFNRDSLYPYLEDIPDPEVNWIPCNNRNRFGQTPVQERLSCLAKNNSIYVVANIGDKKPCDTSDPQCPPDGRYQY  
NTDVVFDSQGKLVARYHKQNLFMGENQFNVPKEPEIVTFNTTFGSFGIFTCFDILFHDPAVTLVKDFHVDTIVFPTAWMN  
VLPHLSAVEFHSAWAMGMRVNFLASNIHYPSKKMTGSGIYAPNSSRAFHYDMKTEEGKLLLSQLDSHPSHSAVVNWTSYA  
SSIEALSSGNKEFKGTVFFDEFTFVKLTGVAGNYTVCQKDLCCHLSYKMSENIPNEVYALGAFDGLHTVEGRYYLQICTL  
LKCKTTNLNTCGDSAETASTRFEMFSLSGTFGTQYVFPEVLLSENQLAPGEFQVSTDGRLFSLKPTSGPVLTVTLFGRLY  
EKDWASNASSGLTAQARIIMLIVIAPIVCSLSW`

| Site | Type | Count |
| --- | --- | --- |
| 353 | N-linked | 3 |

| Mass | Sequence | MS_2_ Score | `q`-value | MS_1_ Score | Signal | Protein Coordinates | Tandem Scans | Start Time | Apex Time | End Time |
| --- | --- | --- | --- | --- | --- | --- | --- | --- | --- | --- |
| 2911.2562 | LTGVAGN(N)YTVC(C)QK Hex 3 HexNAc 5 | 130.054 | 0.0 | 9.686 | 6.809e+06 | 346 - 359 | 2 | 22.12 | 22.29 | 22.70 |
| 2708.1752 | LTGVAGN(N)YTVC(C)QK Hex 3 HexNAc 4 | 97.982 | 0.0 | -3.983 | 2.826e+06 | 346 - 359 | 3 | 22.29 | 22.29 | 22.58 |
| 3219.3690 | LTGVAGN(N)YTVC(C)QK Fuc 1 Hex 4 HexNAc 5 | 96.700 | 0.0 | -17.768 | 1.493e+06 | 346 - 359 | 1 | 21.79 | 21.79 | 21.95 |

### LTGVAGN(N)YTVC(C)QK Hex 3 HexNAc 5

**Mass:**  2911.2553

(1.432e-06)

**Charge States:** 3

**Start Position**: 347

**End Position**: 360

| Profile Score | Tandem Score | TDA q-value | Volume | Spectrum Matches |
| --- | --- | --- | --- | --- |
| 9.6862 | 130.0541 | 0.0000 | 6.8091e+06 | 2 |

**Adduct Signal Fractions**

- **Unmodified**: 6.8091e+06

### LTGVAGN(N)YTVC(C)QK Hex 3 HexNAc 4

**Mass:**  2708.1749

(6.316e-07)

**Charge States:** 3

**Start Position**: 347

**End Position**: 360

| Profile Score | Tandem Score | TDA q-value | Volume | Spectrum Matches |
| --- | --- | --- | --- | --- |
| -3.9827 | 97.9823 | 0.0000 | 2.8262e+06 | 3 |

**Adduct Signal Fractions**

- **Unmodified**: 2.8262e+06

### LTGVAGN(N)YTVC(C)QK Fuc 1 Hex 4 HexNAc 5

**Mass:**  3219.3704

(2.228e-06)

**Charge States:** 3

**Start Position**: 347

**End Position**: 360

| Profile Score | Tandem Score | TDA q-value | Volume | Spectrum Matches |
| --- | --- | --- | --- | --- |
| -17.7678 | 96.7000 | 0.0000 | 1.4926e+06 | 1 |

**Adduct Signal Fractions**

- **Unmodified**: 1.4926e+06

### P21163|PNGF\_ELIMR

**>P21163|PNGF\_ELIMR**  
 `MRKLLIFSISAYLMAGIVSCKGVDSATPVTEDRLALNAVNAPADNTVNIKTFDKVKNAFGDGLSQSAEGTFTFPADVTTV  
KTIKMFIKNECPNKTCDEWDRYANVYVKNKTTGEWYEIGRFITPYWVGTEKLPRGLEIDVTDFKSLLSGNTELKIYTETW  
LAKGREYSVDFDIVYGTPDYKYSAVVPVIQYNKSSIDGVPYGKAHTLGLKKNIQLPTNTEKAYLRTTISGWGHAKPYDAG  
SRGCAEWCFRTHTIAINNANTFQHQLGALGCSANPINNQSPGNWAPDRAGWCPGMAVPTRIDVLNNSLTGSTFSYEYKFQ  
SWTNNGTNGDAFYAISSFVIAKSNTPISAPVVTN`

| Site | Type | Count |
| --- | --- | --- |
| 305 | N-linked | 1 |

| Mass | Sequence | MS_2_ Score | `q`-value | MS_1_ Score | Signal | Protein Coordinates | Tandem Scans | Start Time | Apex Time | End Time |
| --- | --- | --- | --- | --- | --- | --- | --- | --- | --- | --- |
| 3949.6263 | IDVLN(N)NSLTG Fuc 1 Hex 7 HexNAc 8 | 44.091 | 0.003 | -4.911 | 7.694e+07 | 300 - 310 | 2 | 33.33 | 33.50 | 33.83 |

### IDVLN(N)NSLTG Fuc 1 Hex 7 HexNAc 8

**Mass:**  3949.6225

(7.268e-06)

**Charge States:** 4

**Start Position**: 301

**End Position**: 311

| Profile Score | Tandem Score | TDA q-value | Volume | Spectrum Matches |
| --- | --- | --- | --- | --- |
| -4.9110 | 44.0906 | 0.0030 | 7.6938e+07 | 2 |

**Adduct Signal Fractions**

- **Unmodified**: 7.6938e+07

### Q13201|MMRN1\_HUMAN

**>Q13201|MMRN1\_HUMAN**  
 `MKGARLFVLLSSLWSGGIGLNNSKHSWTIPEDGNSQKTMPSASVPPNKIQSLQILPTTRVMSAEIATTPEARTSEDSLLK  
STLPPSETSAPAEGVRNQTLTSTEKAEGVVKLQNLTLPTNASIKFNPGAESVVLSNSTLKFLQSFARKSNEQATSLNTVG  
GTGGIGGVGGTGGVGNRAPRETYLSRGDSSSSQRTDYQKSNFETTRGKNWCAYVHTRLSPTVILDNQVTYVPGGKGPCGW  
TGGSCPQRSQKISNPVYRMQHKIVTSLDWRCCPGYSGPKCQLRAQEQQSLIHTNQAESHTAVGRGVAEQQQQQGCGDPEV  
MQKMTDQVNYQAMKLTLLQKKIDNISLTVNDVRNTYSSLEGKVSEDKSREFQSLLKGLKSKSINVLIRDIVREQFKIFQN  
DMQETVAQLFKTVSSLSEDLESTRQIIQKVNESVVSIAAQQKFVLVQENRPTLTDIVELRNHIVNVRQEMTLTCEKPIKE  
LEVKQTHLEGALEQEHSRSILYYESLNKTLSKLKEVHEQLLSTEQVSDQKNAPAAESVSNNVTEYMSTLHENIKKQSLMM  
LQMFEDLHIQESKINNLTVSLEMEKESLRGECEDMLSKCRNDFKFQLKDTEENLHVLNQTLAEVLFPMDNKMDKMSEQLN  
DLTYDMEILQPLLEQGASLRQTMTYEQPKEAIVIRKKIENLTSAVNSLNFIIKELTKRHNLLRNEVQGRDDALERRINEY  
ALEMEDGLNKTMTIINNAIDFIQDNYALKETLSTIKDNSEIHHKCTSDMETILTFIPQFHRLNDSIQTLVNDNQRYNFVL  
QVAKTLAGIPRDEKLNQSNFQKMYQMFNETTSQVRKYQQNMSHLEEKLLLTTKISKNFETRLQDIESKVTQTLIPYYISV  
KKGSVVTNERDQALQLQVLNSRFKALEAKSIHLSINFFSLNKTLHEVLTMCHNASTSVSELNATIPKWIKHSLPDIQLLQ  
KGLTEFVEPIIQIKTQAALSNLTCCIDRSLPGSLANVVKSQKQVKSLPKKINALKKPTVNLTTVLIGRTQRNTDNIIYPE  
EYSSCSRHPCQNGGTCINGRTSFTCACRHPFTGDNCTIKLVEENALAPDFSKGSYRYAPMVAFFASHTYGMTIPGPILFN  
NLDVNYGASYTPRTGKFRIPYLGVYVFKYTIESFSAHISGFLVVDGIDKLAFESENINSEIHCDRVLTGDALLELNYGQE  
VWLRLAKGTIPAKFPPVTTFSGYLLYRT`

| Site | Type | Count |
| --- | --- | --- |
| 136 | N-linked | 1 |

| Mass | Sequence | MS_2_ Score | `q`-value | MS_1_ Score | Signal | Protein Coordinates | Tandem Scans | Start Time | Apex Time | End Time |
| --- | --- | --- | --- | --- | --- | --- | --- | --- | --- | --- |
| 3106.3993 | FNPGAESVVLSN(N)STLK Fuc 1 Hex 3 HexNAc 4 | 58.854 | 0.003 | -18.506 | 3.681e+06 | 124 - 140 | 1 | 35.64 | 35.64 | 35.81 |

### FNPGAESVVLSN(N)STLK Fuc 1 Hex 3 HexNAc 4

**Mass:**  3106.3980

(4.967e-07)

**Charge States:** 3

**Start Position**: 125

**End Position**: 141

| Profile Score | Tandem Score | TDA q-value | Volume | Spectrum Matches |
| --- | --- | --- | --- | --- |
| -18.5063 | 58.8545 | 0.0030 | 3.6809e+06 | 1 |

**Adduct Signal Fractions**

- **Unmodified**: 3.6809e+06

### O75144|ICOSL\_HUMAN

**>O75144|ICOSL\_HUMAN**  
 `MRLGSPGLLFLLFSSLRADTQEKEVRAMVGSDVELSCACPEGSRFDLNDVYVYWQTSESKTVVTYHIPQNSSLENVDSRY  
RNRALMSPAGMLRGDFSLRLFNVTPQDEQKFHCLVLSQSLGFQEVLSVEVTLHVAANFSVPVVSAPHSPSQDELTFTCTS  
INGYPRPNVYWINKTDNSLLDQALQNDTVFLNMRGLYDVVSVLRIARTPSVNIGCCIENVLLQQNLTVGSQTGNDIGERD  
KITENPVSTGEKNAATWSILAVLCLLVVVAVAIGWVCRDRCLQHSYAGAWAVSPETELTGHV`

| Site | Type | Count |
| --- | --- | --- |
| 102 | N-linked | 1 |

| Mass | Sequence | MS_2_ Score | `q`-value | MS_1_ Score | Signal | Protein Coordinates | Tandem Scans | Start Time | Apex Time | End Time |
| --- | --- | --- | --- | --- | --- | --- | --- | --- | --- | --- |
| 2965.2758 | LFN(N)VTPQDEQK Fuc 1 Hex 3 HexNAc 5 | 31.412 | 0.024 | -21.820 | 4.874e+05 | 99 - 110 | 1 | 24.89 | 24.89 | 24.89 |

### LFN(N)VTPQDEQK Fuc 1 Hex 3 HexNAc 5

**Mass:**  2965.2758

(2.033e-06)

**Charge States:** 3

**Start Position**: 100

**End Position**: 111

| Profile Score | Tandem Score | TDA q-value | Volume | Spectrum Matches |
| --- | --- | --- | --- | --- |
| -21.8202 | 31.4120 | 0.0236 | 4.8738e+05 | 1 |

**Adduct Signal Fractions**

- **Unmodified**: 4.8738e+05
