# Supplementary material for: Measuring Site-specific Glycosylation Similarity between Influenza a Virus Variants with Statistical Certainty
Source: Mol Cell Proteomics. 2020 Nov 25;19(9):1533–45. doi: 10.1074/mcp.RA120.002031 (PMC8143645; doi:10.1074/mcp.RA120.002031)
Supplement: Supplementary file 2 [file mmc2.zip › mmc2/159250_2_supp_555289_qcjklv.zip!/20190326_Phil82_021119_chymo_HILIC_DDA_1ug_03_glycopeptides.html]

## 20190326\_Phil82\_021119\_chymo\_HILIC\_DDA\_1ug\_03 @ GlycopeptideHypothesis-7edbe55d76144fb0bac73599522ee8d5

**MS^1^ Mass Accuracy:** 10.0 PPM  
**Grouping Tolerance:** 15.0 PPM  
**MS^n^ Mass Accuracy:** 20.0 PPM  
**Minimum `q`-Value For Clustering:** 0.05

**Glycopeptide Hypothesis:**
GlycopeptideHypothesis-7edbe55d76144fb0bac73599522ee8d5  
**Sample Searched:**
20190326\_Phil82\_021119\_chymo\_HILIC\_DDA\_1ug\_03

| Protein | Matched Glycopeptides |
| --- | --- |
| iav\|AFG99160\|A/Phil/2/1982\_HA | 123 |
| iav\|ADJ41819\|A/Phil/2-MA/1982\_NA | 8 |
| tr\|E1C8K3\|E1C8K3\_CHICK | 2 |
| sp\|Q90734\|ACTN4\_CHICK | 1 |

### iav|AFG99160|A/Phil/2/1982\_HA

**>iav|AFG99160|A/Phil/2/1982\_HA**  
 `MKTIIALSYMFCLVFAQNLPGNDNSTATLCLGHHAVPNGTLVKTITNDQIEVTNATELVQSSSTGRICDSPHRILDGKNC  
TLIDALLGDPHCDGFQNEKWDLFVERSKAFSNCYPYDVPDYASLRSLVASSGTLEFINEGFNWTGVTQSGGSSTCKRGSN  
NSFFSRLNWLYESESKYPVLNVTMPNNGKFDKLYIWGIHHPSTDKEQTNLYIRASGRVTVSTKRSQQTVIPNIGSRPWVR  
GLSSRISIYWTIVKPGDILLINSTGNLIAPRGYFKIRTGKSSIMRSDAPIGTCSSECITPNGSIPNDKPFQNVNKITYGA  
CPRYVKQNTLKLATGMRNVPEKQTRGIFGAIAGFIENGWEGMVDGWYGFRHQNSEGTGQAADLKSTQAAIDQINGKLNRV  
IEKTNEKFHQIEKEFSEVEGRIQDLEKYVEDTKIDLWSYNAELLVALENQHTIDLTDSEMNKLFEKTRKQLRENAEDMGN  
GCFKIYHKCDNACIGSIRNGTYDHDVYRDEALNNRFQIKGVELKSGYKDWILWISFAISCFLLCVVLLGFIMWACQKGNI  
RCNICI`

| Site | Type | Count |
| --- | --- | --- |
| 262 | N-linked | 42 |
| 301 | N-linked | 36 |
| 142 | N-linked | 1 |
| 499 | N-linked | 4 |
| 181 | N-linked | 10 |
| 54 | N-linked | 5 |
| 24 | N-linked | 23 |
| 160 | N-linked | 2 |

| Mass | Sequence | MS_2_ Score | `q`-value | MS_1_ Score | Signal | Protein Coordinates | Tandem Scans | Start Time | Apex Time | End Time |
| --- | --- | --- | --- | --- | --- | --- | --- | --- | --- | --- |
| 3499.5195 | LIN(N)STGNLIAPRGYF Hex 9 HexNAc 2 | 180.448 | 0.0 | -4.113 | 4.119e+07 | 259 - 274 | 1 | 45.54 | 45.54 | 45.88 |
| 2870.1764 | LPGNDN(N)STATL Fuc 1 Hex 5 HexNAc 4 | 174.599 | 0.0 | 13.494 | 6.607e+07 | 18 - 29 | 5 | 22.58 | 23.42 | 24.44 |
| 3132.3656 | LIN(N)STGNLIAPR Hex 9 HexNAc 2 | 171.154 | 0.0 | 11.395 | 2.596e+07 | 259 - 271 | 2 | 28.32 | 28.49 | 28.99 |
| 2695.0887 | LIN(N)STGNL Hex 9 HexNAc 2 | 171.041 | 0.0 | -4.160 | 1.166e+07 | 259 - 267 | 3 | 22.92 | 23.08 | 23.42 |
| 2317.9571 | LPGNDN(N)STATL Hex 5 HexNAc 2 | 167.471 | 0.0 | 11.844 | 1.029e+07 | 18 - 29 | 2 | 23.25 | 23.42 | 23.93 |
| 4068.9295 | TIVKPGDILLIN(N)STGNLIAPR Hex 9 HexNAc 2 | 164.863 | 0.0 | 14.847 | 2.250e+08 | 250 - 271 | 13 | 51.17 | 52.02 | 53.56 |
| 2724.1182 | LPGNDN(N)STATL Hex 5 HexNAc 4 | 160.184 | 0.0 | 12.359 | 1.918e+07 | 18 - 29 | 2 | 22.92 | 23.25 | 23.59 |
| 4289.0182 | TIVKPGDILLIN(N)STGNLIAPRGY Hex 9 HexNAc 2 | 159.704 | 0.0 | 15.612 | 7.505e+08 | 250 - 273 | 31 | 52.71 | 54.59 | 60.97 |
| 3149.3967 | LIN(N)STGNLIAPR Fuc 2 Hex 3 HexNAc 4 Neu5Ac 1 | 159.136 | 0.0 | -19.673 | 5.439e+06 | 259 - 271 | 1 | 28.32 | 28.32 | 28.49 |
| 3159.4276 | TIVKPGDILLIN(N) Hex 9 HexNAc 2 | 158.578 | 0.0 | 13.326 | 1.304e+08 | 250 - 262 | 9 | 43.50 | 45.03 | 46.39 |
| 3352.4507 | LIN(N)STGNLIAPRGY Hex 9 HexNAc 2 | 157.798 | 0.0 | -18.804 | 4.844e+07 | 259 - 273 | 2 | 34.84 | 34.84 | 35.00 |
| 3518.5736 | TIVKPGDILLIN(N)STGN Hex 9 HexNAc 2 | 154.802 | 0.0 | 13.592 | 4.940e+07 | 250 - 266 | 4 | 42.81 | 42.98 | 43.67 |
| 3176.4525 | TIVKPGDILLIN(N) Fuc 2 Hex 3 HexNAc 4 Neu5Ac 1 | 154.206 | 0.0 | 12.467 | 4.670e+07 | 250 - 262 | 5 | 43.67 | 45.03 | 45.54 |
| 3235.3098 | LPGNDN(N)STATL Fuc 1 Hex 6 HexNAc 5 | 153.835 | 0.0 | 12.812 | 1.003e+07 | 18 - 29 | 2 | 22.58 | 23.08 | 23.25 |
| 4085.9551 | TIVKPGDILLIN(N)STGNLIAPR Fuc 2 Hex 3 HexNAc 4 Neu5Ac 1 | 149.400 | 0.0 | 13.471 | 2.800e+07 | 250 - 271 | 3 | 51.68 | 52.02 | 52.37 |
| 4306.0406 | TIVKPGDILLIN(N)STGNLIAPRGY Fuc 2 Hex 3 HexNAc 4 Neu5Ac 1 | 149.079 | 0.0 | 11.616 | 1.103e+08 | 250 - 273 | 3 | 54.42 | 54.59 | 55.28 |
| 3631.6560 | TIVKPGDILLIN(N)STGNL Hex 9 HexNAc 2 | 148.409 | 0.0 | 12.617 | 9.100e+07 | 250 - 267 | 5 | 51.17 | 51.34 | 52.02 |
| 3648.6809 | TIVKPGDILLIN(N)STGNL Fuc 2 Hex 3 HexNAc 4 Neu5Ac 1 | 146.815 | 0.0 | -3.899 | 3.545e+07 | 250 - 267 | 4 | 51.17 | 51.34 | 51.68 |
| 3321.4777 | TIVKPGDILLIN(N) Hex 10 HexNAc 2 | 146.568 | 0.0 | -5.096 | 6.323e+06 | 250 - 262 | 2 | 44.86 | 44.86 | 45.20 |
| 4126.9519 | TIVKPGDILLIN(N)STGNLIAPRGY Hex 8 HexNAc 2 | 144.134 | 0.0 | 12.120 | 7.470e+07 | 250 - 273 | 12 | 54.08 | 54.59 | 56.29 |
| 4451.0671 | TIVKPGDILLIN(N)STGNLIAPRGY Hex 10 HexNAc 2 | 142.612 | 0.0 | 13.381 | 6.894e+07 | 250 - 273 | 5 | 54.25 | 54.59 | 54.93 |
| 3016.2332 | LPGNDN(N)STATL Fuc 2 Hex 5 HexNAc 4 | 140.078 | 0.0 | 11.487 | 1.377e+07 | 18 - 29 | 2 | 22.41 | 23.08 | 23.42 |
| 2683.0906 | LPGNDN(N)STATL Hex 6 HexNAc 3 | 139.912 | 0.0 | 12.013 | 6.712e+06 | 18 - 29 | 1 | 22.92 | 23.08 | 23.59 |
| 4230.9832 | TIVKPGDILLIN(N)STGNLIAPR Hex 10 HexNAc 2 | 135.692 | 0.0 | 12.909 | 1.939e+07 | 250 - 271 | 4 | 51.68 | 51.85 | 52.37 |
| 3906.8773 | TIVKPGDILLIN(N)STGNLIAPR Hex 8 HexNAc 2 | 133.477 | 0.0 | -4.231 | 1.345e+07 | 250 - 271 | 4 | 51.85 | 52.02 | 52.20 |
| 3535.5997 | TIVKPGDILLIN(N)STGN Fuc 2 Hex 3 HexNAc 4 Neu5Ac 1 | 133.072 | 0.0 | 14.370 | 1.849e+07 | 250 - 266 | 2 | 42.81 | 42.98 | 43.50 |
| 3793.7068 | TIVKPGDILLIN(N)STGNL Hex 10 HexNAc 2 | 132.983 | 0.0 | -4.178 | 7.790e+06 | 250 - 267 | 2 | 51.17 | 51.17 | 51.51 |
| 3315.3369 | LPGNDN(N)STATL Fuc 1 Hex 9 HexNAc 3 | 131.899 | 0.0 | -4.838 | 8.525e+07 | 18 - 29 | 1 | 33.70 | 33.86 | 34.02 |
| 3477.4088 | LPGNDN(N)STATL Fuc 2 Hex 3 HexNAc 5 Neu5Ac 2 | 130.345 | 0.0 | 12.627 | 2.121e+08 | 18 - 29 | 3 | 32.55 | 33.06 | 33.39 |
| 3516.9426 | LIN(N)STGNLIAPRGYF Fuc 2 Hex 3 HexNAc 4 Neu5Ac 1 | 129.422 | 0.0 | -6.984 | 1.808e+07 | 259 - 274 | 5 | 28.32 | 45.54 | 45.71 |
| 3386.4420 | IN(N)STGNLIAPRGYF Hex 9 HexNAc 2 | 127.084 | 0.0 | -4.572 | 1.058e+07 | 260 - 274 | 2 | 41.28 | 41.45 | 41.62 |
| 3019.2859 | IN(N)STGNLIAPR Hex 9 HexNAc 2 | 124.040 | 0.0 | -17.130 | 3.268e+06 | 260 - 271 | 1 | 21.74 | 21.74 | 21.74 |
| 2480.0094 | LPGNDN(N)STATL Hex 6 HexNAc 2 | 120.901 | 0.0 | -5.461 | 4.104e+06 | 18 - 29 | 1 | 23.08 | 23.25 | 23.59 |
| 3239.3607 | IN(N)STGNLIAPRGY Hex 9 HexNAc 2 | 120.345 | 0.0 | -18.579 | 5.308e+06 | 260 - 273 | 1 | 29.33 | 29.33 | 29.33 |
| 4683.9293 | RSDAPIGTC(C)SSEC(C)ITPN(N)GSIPNDKPF Hex 9 HexNAc 2 | 116.231 | 0.0 | 10.520 | 4.632e+07 | 284 - 310 | 4 | 35.98 | 36.15 | 36.83 |
| 4521.8809 | RSDAPIGTC(C)SSEC(C)ITPN(N)GSIPNDKPF Hex 8 HexNAc 2 | 113.008 | 0.0 | 10.632 | 5.621e+07 | 284 - 310 | 6 | 35.98 | 36.15 | 36.83 |
| 2950.1763 | LPGNDN(N)STATL Fuc 1 Hex 8 HexNAc 2 | 106.645 | 0.0 | 12.497 | 7.057e+08 | 18 - 29 | 11 | 34.50 | 35.17 | 36.66 |
| 3479.4070 | LPGNDN(N)STATL Fuc 1 Hex 5 HexNAc 7 | 106.203 | 0.0 | 0.000 | 0.000e+00 | 18 - 29 | 1 | - | - | - |
| 3354.3430 | SIRN(N)GTYDHDVY Fuc 2 Hex 5 HexNAc 4 | 104.608 | 0.0 | 0.000 | 0.000e+00 | 495 - 507 | 2 | - | - | - |
| 3769.5293 | LPGNDN(N)STATL Fuc 4 Hex 3 HexNAc 5 Neu5Ac 2 | 103.078 | 0.0 | 11.642 | 1.978e+07 | 18 - 29 | 1 | 32.21 | 32.72 | 32.89 |
| 3129.2990 | LPGNDN(N)STATL Fuc 3 Hex 3 HexNAc 4 Neu5Ac 1 | 102.426 | 0.0 | -20.479 | 4.841e+06 | 18 - 29 | 1 | 33.06 | 33.06 | 33.06 |
| 3469.6003 | TIVKPGDILLIN(N)STGNL Hex 8 HexNAc 2 | 101.834 | 0.0 | -4.966 | 6.232e+06 | 250 - 267 | 2 | 51.34 | 51.34 | 51.68 |
| 3337.4702 | LIN(N)STGNLIAPRGYF Hex 8 HexNAc 2 | 100.024 | 0.0 | -20.133 | 1.718e+06 | 259 - 274 | 1 | 45.54 | 45.71 | 45.71 |
| 3331.3523 | LPGNDN(N)STATL Fuc 1 Hex 3 HexNAc 5 Neu5Ac 2 | 99.767 | 0.0 | -4.512 | 6.121e+07 | 18 - 29 | 1 | 32.72 | 32.89 | 33.23 |
| 3623.4660 | LPGNDN(N)STATL Fuc 3 Hex 3 HexNAc 5 Neu5Ac 2 | 96.376 | 0.0 | 12.048 | 6.988e+07 | 18 - 29 | 2 | 32.21 | 32.89 | 33.23 |
| 4468.0963 | TIVKPGDILLIN(N)STGNLIAPRGY Fuc 2 Hex 4 HexNAc 4 Neu5Ac 1 | 96.078 | 0.0 | -5.541 | 1.160e+07 | 250 - 273 | 1 | 54.42 | 54.59 | 54.76 |
| 4290.0098 | TIVKPGDILLIN(N)STGN(D)LIAPRGY Hex 9 HexNAc 2 | 95.666 | 0.0 | -4.597 | 2.610e+07 | 250 - 273 | 4 | 55.45 | 55.45 | 55.79 |
| 3207.2923 | SIRN(N)GTYDHDVY Fuc 1 Hex 5 HexNAc 4 | 95.173 | 0.0 | -20.558 | 6.825e+06 | 495 - 507 | 1 | 17.22 | 17.22 | 17.35 |
| 3825.5197 | LPGNDN(N)STATL Fuc 1 Hex 3 HexNAc 6 Neu5Ac 3 | 94.460 | 0.0 | 11.615 | 8.599e+07 | 18 - 29 | 4 | 31.87 | 32.72 | 32.89 |
| 4553.9455 | ESESKYPVLN(N)VTM(O)PNN(D)GKFDKLY Hex 9 HexNAc 2 | 91.812 | 0.0 | 11.579 | 5.734e+07 | 171 - 194 | 3 | 40.77 | 41.28 | 41.62 |
| 3201.3597 | TITNDQIEVTN(N)ATEL Hex 7 HexNAc 2 | 91.192 | 0.0 | -17.608 | 7.581e+06 | 43 - 58 | 1 | 38.55 | 38.72 | 38.72 |
| 3753.4828 | LPGNDN(N)STATL Hex 4 HexNAc 7 Neu5Ac 2 | 85.694 | 0.0 | -19.089 | 3.285e+06 | 18 - 29 | 1 | 32.21 | 32.21 | 32.21 |
| 4391.8931 | ESESKYPVLN(N)VTM(O)PNN(D)GKFDKLY Hex 8 HexNAc 2 | 82.687 | 0.0 | 11.490 | 3.963e+07 | 171 - 194 | 2 | 40.94 | 41.28 | 41.62 |
| 3701.5528 | LYESESKYPVLN(N)VTM(O)PN Hex 8 HexNAc 2 | 80.118 | 0.0 | 6.951 | 8.196e+06 | 169 - 186 | 2 | 35.98 | 35.98 | 36.66 |
| 4359.8270 | RSDAPIGTC(C)SSEC(C)ITPN(N)GSIPNDKPF Hex 7 HexNAc 2 | 73.837 | 0.0 | -4.527 | 1.260e+07 | 284 - 310 | 2 | 36.15 | 36.32 | 36.49 |
| 4538.9036 | ITPN(N)GSIPNDKPF Fuc 4 Hex 7 HexNAc 7 | 73.052 | 0.0 | -5.293 | 7.148e+06 | 297 - 310 | 2 | 36.15 | 36.15 | 36.49 |
| 4469.1145 | TIVKPGDILLIN(N)STGNLIAPRGY Fuc 2 Hex 4 HexNAc 4 Neu5Ac 1 | 69.059 | 0.008 | 0.000 | 0.000e+00 | 250 - 273 | 1 | - | - | - |
| 5639.5075 | KIRTGKSSIM(O)RSDAPIGTC(C)SSEC(C)ITPN(N)GSIPNDKPF Hex 8 HexNAc 2 | 68.907 | 0.008 | -5.257 | 5.225e+07 | 274 - 310 | 3 | 31.70 | 31.87 | 32.04 |
| 4446.8053 | ITPN(N)GSIPNDKPF Fuc 1 Hex 5 HexNAc 6 Neu5Ac 3 | 68.686 | 0.008 | -19.389 | 1.271e+07 | 297 - 310 | 2 | 43.67 | 43.84 | 43.84 |
| 5801.5627 | KIRTGKSSIM(O)RSDAPIGTC(C)SSEC(C)ITPN(N)GSIPNDKPF Hex 9 HexNAc 2 | 60.205 | 0.011 | -4.790 | 3.970e+07 | 274 - 310 | 3 | 31.70 | 31.87 | 32.04 |
| 3680.6206 | TIVKPGDILLIN(N)STGN Hex 10 HexNAc 2 | 59.852 | 0.011 | -5.471 | 3.158e+06 | 250 - 266 | 1 | 42.64 | 42.98 | 42.98 |
| 4522.8660 | RSDAPIGTC(C)SSEC(C)ITPN(N)GSIPN(D)DKPF Hex 8 HexNAc 2 | 58.090 | 0.011 | -5.712 | 4.693e+07 | 284 - 310 | 3 | 37.01 | 37.18 | 37.52 |
| 4310.8187 | LYESESKYPVLN(N)VTM(O)PNN(D)GKF Hex 9 HexNAc 2 | 56.292 | 0.011 | -6.308 | 2.872e+07 | 169 - 190 | 4 | 39.91 | 40.25 | 40.42 |
| 3810.7392 | TIVKPGDILLIN(N)STGNL Fuc 2 Hex 4 HexNAc 4 Neu5Ac 1 | 49.097 | 0.011 | -19.358 | 1.859e+06 | 250 - 267 | 1 | 51.17 | 51.17 | 51.17 |
| 3863.6081 | LYESESKYPVLN(N)VTM(O)PN Hex 9 HexNAc 2 | 46.785 | 0.011 | -5.306 | 1.270e+07 | 169 - 186 | 1 | 35.98 | 35.98 | 36.49 |
| 2927.1985 | LPGNDN(N)STATL Hex 5 HexNAc 5 | 46.656 | 0.011 | 10.560 | 1.294e+07 | 18 - 29 | 3 | 22.92 | 23.25 | 23.76 |
| 5477.4550 | KIRTGKSSIM(O)RSDAPIGTC(C)SSEC(C)ITPN(N)GSIPNDKPF Hex 7 HexNAc 2 | 46.520 | 0.011 | -5.980 | 1.021e+07 | 274 - 310 | 2 | 31.70 | 31.87 | 32.04 |
| 4684.9159 | RSDAPIGTC(C)SSEC(C)ITPN(N)GSIPN(D)DKPF Hex 9 HexNAc 2 | 46.395 | 0.011 | 12.448 | 3.459e+07 | 284 - 310 | 3 | 36.83 | 37.01 | 37.52 |
| 4933.2496 | TITNDQIEVTN(N)ATELVQSSSTGRIC(C)DSPHRIL Hex 6 HexNAc 2 | 46.386 | 0.011 | -5.151 | 3.856e+06 | 43 - 75 | 1 | 45.37 | 45.37 | 45.71 |
| 3089.2499 | LPGNDN(N)STATL Hex 6 HexNAc 5 | 46.121 | 0.011 | -20.278 | 1.554e+06 | 18 - 29 | 1 | 22.75 | 22.92 | 22.92 |
| 4700.9530 | RSDAPIGTC(C)SSEC(C)ITPN(N)GSIPNDKPF Fuc 2 Hex 3 HexNAc 4 Neu5Ac 1 | 44.930 | 0.011 | -5.263 | 5.902e+06 | 284 - 310 | 1 | 36.15 | 36.15 | 36.49 |
| 3242.3850 | TITNDQIEVTN(N)ATEL Hex 6 HexNAc 3 | 44.763 | 0.011 | -18.703 | 5.572e+06 | 43 - 58 | 1 | 38.72 | 38.72 | 38.89 |
| 4069.9261 | TIVKPGDILLIN(N)STGN(D)LIAPR Hex 9 HexNAc 2 | 44.601 | 0.011 | -5.181 | 6.289e+06 | 250 - 271 | 3 | 52.88 | 52.88 | 53.22 |
| 3039.3024 | TITNDQIEVTN(N)ATEL Hex 6 HexNAc 2 | 44.062 | 0.011 | -3.672 | 2.054e+07 | 43 - 58 | 3 | 38.72 | 38.89 | 39.06 |
| 4887.1060 | ESESKYPVLN(N)VTM(O)PNN(D)GKFDKLY Fuc 2 Hex 8 HexNAc 3 | 43.164 | 0.011 | 12.914 | 2.201e+08 | 171 - 194 | 7 | 43.33 | 43.84 | 44.35 |
| 5640.4982 | KIRTGKSSIM(O)RSDAPIGTC(C)SSEC(C)ITPN(N)GSIPN(D)DKPF Hex 8 HexNAc 2 | 42.196 | 0.011 | -6.339 | 2.935e+07 | 274 - 310 | 2 | 32.38 | 32.55 | 32.72 |
| 4148.7652 | LYESESKYPVLN(N)VTM(O)PNN(D)GKF Hex 8 HexNAc 2 | 41.685 | 0.011 | -6.270 | 1.860e+07 | 169 - 190 | 4 | 39.91 | 40.25 | 40.42 |
| 3336.3631 | INEGFN(N)WTGVTQ Fuc 1 Hex 5 HexNAc 5 | 41.560 | 0.011 | -20.513 | 2.371e+06 | 136 - 148 | 1 | 42.13 | 42.30 | 42.30 |
| 3606.4428 | LPGNDN(N)STATL Fuc 1 Hex 9 HexNAc 3 Neu5Ac 1 | 40.936 | 0.011 | 13.649 | 2.057e+08 | 18 - 29 | 6 | 32.21 | 32.89 | 33.23 |
| 3727.4686 | SSEC(C)ITPN(N)GSIPN(D)DKPF Hex 9 HexNAc 2 | 40.280 | 0.011 | -20.266 | 1.054e+07 | 293 - 310 | 1 | 34.67 | 34.84 | 34.84 |
| 4840.0040 | SSEC(C)ITPN(N)GSIPNDKPF Fuc 4 Hex 6 HexNAc 7 | 39.776 | 0.011 | -3.536 | 2.190e+07 | 293 - 310 | 3 | 39.40 | 39.57 | 39.74 |
| 3263.3672 | ITPN(N)GSIPNDKPF Hex 9 HexNAc 2 | 39.381 | 0.011 | -4.251 | 1.250e+07 | 297 - 310 | 1 | 29.50 | 29.67 | 29.84 |
| 5802.5510 | KIRTGKSSIM(O)RSDAPIGTC(C)SSEC(C)ITPN(N)GSIPN(D)DKPF Hex 9 HexNAc 2 | 38.850 | 0.011 | -6.099 | 3.908e+07 | 274 - 310 | 2 | 32.21 | 32.38 | 32.72 |
| 3565.4240 | SSEC(C)ITPN(N)GSIPN(D)DKPF Hex 8 HexNAc 2 | 38.668 | 0.011 | -5.950 | 1.808e+07 | 293 - 310 | 1 | 34.50 | 34.84 | 34.84 |
| 4579.7877 | ITPN(N)GSIPNDKPF Fuc 2 Hex 6 HexNAc 8 Neu5Ac 1 | 38.416 | 0.011 | -6.725 | 1.338e+07 | 297 - 310 | 5 | 26.13 | 36.32 | 41.79 |
| 4636.8922 | ITPN(N)GSIPNDKPF Fuc 3 Hex 6 HexNAc 9 | 38.409 | 0.011 | -5.742 | 4.738e+06 | 297 - 310 | 3 | 41.79 | 41.96 | 42.30 |
| 3608.4643 | AC(C)IGSIRN(N)GTYDHDVY Fuc 1 Hex 5 HexNAc 4 | 38.227 | 0.011 | -4.649 | 3.145e+06 | 491 - 507 | 1 | 26.47 | 26.47 | 26.81 |
| 4363.0619 | TIVKPGDILLIN(N)STGNLIAPRGY Fuc 1 Hex 3 HexNAc 5 Neu5Ac 1 | 37.808 | 0.011 | 11.032 | 5.114e+07 | 250 - 273 | 5 | 54.93 | 55.10 | 56.12 |
| 4740.9516 | ITPN(N)GSIPNDKPF Fuc 2 Hex 7 HexNAc 8 Neu5Ac 1 | 37.667 | 0.016 | -4.878 | 6.599e+06 | 297 - 310 | 1 | 36.15 | 36.32 | 36.49 |
| 3523.4518 | IGSIRN(N)GTYDHDVY Fuc 2 Hex 5 HexNAc 4 | 37.236 | 0.016 | -17.034 | 2.561e+06 | 493 - 507 | 1 | 22.07 | 22.07 | 22.07 |
| 3085.2471 | TC(C)KRGSN(N)NSFF Fuc 1 Hex 5 HexNAc 4 | 36.086 | 0.016 | -18.414 | 1.235e+07 | 153 - 164 | 1 | 18.24 | 18.24 | 18.41 |
| 3253.3157 | LPGNDN(N)STATL Hex 7 HexNAc 5 | 35.291 | 0.016 | -23.473 | 3.927e+06 | 18 - 29 | 1 | 27.65 | 27.65 | 27.82 |
| 4360.8112 | RSDAPIGTC(C)SSEC(C)ITPN(N)GSIPN(D)DKPF Hex 7 HexNAc 2 | 34.903 | 0.016 | -6.236 | 1.232e+07 | 284 - 310 | 2 | 37.01 | 37.18 | 37.52 |
| 4579.8846 | ITPN(N)GSIPNDKPF Fuc 4 Hex 6 HexNAc 8 | 34.549 | 0.019 | -5.285 | 8.414e+06 | 297 - 310 | 1 | 37.01 | 37.18 | 37.35 |
| 4840.9840 | SSEC(C)ITPN(N)GSIPN(D)DKPF Fuc 4 Hex 6 HexNAc 7 | 34.301 | 0.019 | -6.542 | 1.442e+07 | 293 - 310 | 3 | 40.25 | 40.42 | 40.77 |
| 4453.1098 | TIVKPGDILLIN(N)STGNLIAPRGY Hex 5 HexNAc 6 | 33.905 | 0.019 | 13.372 | 5.837e+07 | 250 - 273 | 4 | 60.19 | 60.63 | 61.31 |
| 4143.9887 | TIVKPGDILLIN(N)STGNLIAPR Fuc 3 Hex 3 HexNAc 5 | 33.521 | 0.019 | -19.004 | 5.113e+06 | 250 - 271 | 1 | 54.59 | 54.59 | 54.76 |
| 4831.0748 | LYESESKYPVLN(N)VTM(O)PNN(D)GKF Fuc 3 Hex 7 HexNAc 4 | 33.407 | 0.019 | 9.406 | 1.663e+08 | 169 - 190 | 8 | 44.35 | 44.52 | 46.56 |
| 4417.8194 | ITPN(N)GSIPNDKPF Fuc 4 Hex 5 HexNAc 8 | 33.002 | 0.019 | -4.830 | 9.151e+06 | 297 - 310 | 2 | 39.57 | 39.57 | 39.91 |
| 3640.4448 | SEC(C)ITPN(N)GSIPN(D)DKPF Hex 9 HexNAc 2 | 32.715 | 0.019 | -6.250 | 1.408e+07 | 294 - 310 | 1 | 34.67 | 34.84 | 35.00 |
| 3966.9061 | TIVKPGDILLIN(N)STGNLIAPRGY Hex 7 HexNAc 2 | 32.651 | 0.019 | 0.000 | 0.000e+00 | 250 - 273 | 1 | - | - | - |
| 4725.0558 | ESESKYPVLN(N)VTM(O)PNN(D)GKFDKLY Fuc 2 Hex 7 HexNAc 3 | 32.621 | 0.019 | 11.968 | 1.457e+08 | 171 - 194 | 5 | 43.50 | 43.84 | 44.18 |
| 4474.8442 | ITPN(N)GSIPNDKPF Fuc 3 Hex 5 HexNAc 9 | 32.451 | 0.019 | -5.338 | 5.628e+06 | 297 - 310 | 3 | 41.79 | 41.96 | 42.30 |
| 4443.7754 | SSEC(C)ITPN(N)GSIPN(D)DKPF Fuc 1 Hex 5 HexNAc 8 | 31.327 | 0.044 | -6.521 | 2.207e+07 | 293 - 310 | 3 | 38.03 | 38.20 | 38.37 |
| 5002.0565 | SSEC(C)ITPN(N)GSIPNDKPF Fuc 4 Hex 7 HexNAc 7 | 31.256 | 0.027 | -4.267 | 1.519e+07 | 293 - 310 | 2 | 39.40 | 39.40 | 39.74 |
| 4307.0436 | TIVKPGDILLIN(N)STGN(D)LIAPRGY Fuc 2 Hex 3 HexNAc 4 Neu5Ac 1 | 31.256 | 0.027 | -20.648 | 3.158e+06 | 250 - 273 | 1 | 55.45 | 55.45 | 55.62 |
| 4142.9804 | TIVKPGDILLIN(N)STGNLIAPR Fuc 1 Hex 3 HexNAc 5 Neu5Ac 1 | 30.691 | 0.027 | -4.126 | 1.135e+07 | 250 - 271 | 2 | 52.37 | 52.54 | 52.71 |
| 4844.0871 | TIVKPGDILLIN(N)STGNL Fuc 3 Hex 10 HexNAc 5 | 30.310 | 0.027 | -22.814 | 1.537e+05 | 250 - 267 | 12 | 84.17 | 84.17 | 84.17 |
| 2939.2481 | ITPN(N)GSIPNDKPF Hex 7 HexNAc 2 | 30.194 | 0.027 | -18.735 | 1.277e+06 | 297 - 310 | 1 | 29.84 | 29.84 | 29.84 |
| 4521.8653 | ITPN(N)GSIPNDKPF Fuc 3 Hex 6 HexNAc 7 Neu5Ac 1 | 29.902 | 0.027 | -19.930 | 1.942e+06 | 297 - 310 | 1 | 37.69 | 37.69 | 37.69 |
| 3478.3918 | SEC(C)ITPN(N)GSIPN(D)DKPF Hex 8 HexNAc 2 | 29.886 | 0.031 | 10.772 | 1.017e+08 | 294 - 310 | 4 | 34.34 | 34.84 | 35.33 |
| 5788.5781 | FKIRTGKSSIM(O)RSDAPIGTC(C)SSEC(C)ITPN(N)GSIPNDKPF Hex 8 HexNAc 2 | 28.728 | 0.039 | 0.000 | 0.000e+00 | 273 - 310 | 1 | - | - | - |
| 3283.4048 | TITNDQIEVTN(N)ATEL Hex 5 HexNAc 4 | 28.701 | 0.039 | -5.525 | 5.314e+06 | 43 - 58 | 1 | 38.72 | 38.72 | 39.06 |
| 5787.5475 | FKIRTGKSSIM(O)RSDAPIGTC(C)SSEC(C)ITPN(N)GSIPN(D)DKPF Hex 8 HexNAc 2 | 28.259 | 0.039 | 0.000 | 0.000e+00 | 273 - 310 | 1 | - | - | - |
| 3073.2495 | LPGNDN(N)STATL Fuc 1 Hex 5 HexNAc 5 | 28.185 | 0.039 | 4.993 | 6.535e+06 | 18 - 29 | 1 | 23.08 | 23.76 | 23.93 |
| 3086.2465 | TC(C)KRGSN(N)NSFF Fuc 1 Hex 5 HexNAc 4 | 27.617 | 0.041 | 0.000 | 0.000e+00 | 153 - 164 | 1 | - | - | - |
| 4705.9172 | SEC(C)ITPN(N)GSIPN(D)DKPF Fuc 2 Hex 5 HexNAc 9 | 27.478 | 0.041 | -20.613 | 1.156e+06 | 294 - 310 | 1 | 37.01 | 37.01 | 37.01 |
| 4675.8927 | ITPN(N)GSIPNDKPF Fuc 3 Hex 10 HexNAc 6 | 27.433 | 0.041 | -20.390 | 2.549e+06 | 297 - 310 | 1 | 39.06 | 39.06 | 39.06 |
| 3299.3152 | LPGNDN(N)STATL Fuc 2 Hex 8 HexNAc 3 | 27.255 | 0.041 | 12.022 | 1.345e+08 | 18 - 29 | 4 | 34.84 | 35.49 | 36.49 |
| 4365.0793 | TIVKPGDILLIN(N)STGNLIAPRGY Fuc 3 Hex 3 HexNAc 5 | 27.016 | 0.041 | 0.000 | 0.000e+00 | 250 - 273 | 1 | - | - | - |
| 4513.8435 | ITPN(N)GSIPNDKPF Fuc 3 Hex 9 HexNAc 6 | 26.495 | 0.041 | -20.521 | 2.231e+06 | 297 - 310 | 1 | 39.23 | 39.23 | 39.23 |
| 4453.0573 | TIVKPGDILLIN(N)STGN(D)LIAPRGY Hex 10 HexNAc 2 | 25.613 | 0.044 | 0.000 | 0.000e+00 | 250 - 273 | 1 | - | - | - |
| 4944.1290 | ESESKYPVLN(N)VTM(O)PNN(D)GKFDKLY Fuc 1 Hex 8 HexNAc 4 | 24.974 | 0.047 | 11.299 | 5.943e+07 | 171 - 194 | 4 | 43.33 | 43.84 | 44.18 |

### LIN(N)STGNLIAPRGYF Hex 9 HexNAc 2

**Mass:**  3499.5196

(2.051e-06)

**Charge States:** 3

**Start Position**: 260

**End Position**: 275

| Profile Score | Tandem Score | TDA q-value | Volume | Spectrum Matches |
| --- | --- | --- | --- | --- |
| -4.1135 | 180.4477 | 0.0000 | 4.1188e+07 | 1 |

**Adduct Signal Fractions**

- **Unmodified**: 4.1188e+07

### LPGNDN(N)STATL Fuc 1 Hex 5 HexNAc 4

**Mass:**  2870.1778

(1.691e-06)

**Charge States:** 2, 3

**Start Position**: 19

**End Position**: 30

| Profile Score | Tandem Score | TDA q-value | Volume | Spectrum Matches |
| --- | --- | --- | --- | --- |
| 13.4943 | 174.5993 | 0.0000 | 6.6073e+07 | 5 |

**Adduct Signal Fractions**

- **Unmodified**: 6.6073e+07

### LIN(N)STGNLIAPR Hex 9 HexNAc 2

**Mass:**  3132.3649

(2.350e-06)

**Charge States:** 3

**Start Position**: 260

**End Position**: 272

| Profile Score | Tandem Score | TDA q-value | Volume | Spectrum Matches |
| --- | --- | --- | --- | --- |
| 11.3953 | 171.1542 | 0.0000 | 2.5962e+07 | 2 |

**Adduct Signal Fractions**

- **Unmodified**: 2.5962e+07

### LIN(N)STGNL Hex 9 HexNAc 2

**Mass:**  2695.0873

(2.338e-06)

**Charge States:** 2

**Start Position**: 260

**End Position**: 268

| Profile Score | Tandem Score | TDA q-value | Volume | Spectrum Matches |
| --- | --- | --- | --- | --- |
| -4.1601 | 171.0414 | 0.0000 | 1.1660e+07 | 3 |

**Adduct Signal Fractions**

- **Unmodified**: 1.1660e+07

### LPGNDN(N)STATL Hex 5 HexNAc 2

**Mass:**  2317.9574

(2.275e-06)

**Charge States:** 2

**Start Position**: 19

**End Position**: 30

| Profile Score | Tandem Score | TDA q-value | Volume | Spectrum Matches |
| --- | --- | --- | --- | --- |
| 11.8443 | 167.4710 | 0.0000 | 1.0287e+07 | 2 |

**Adduct Signal Fractions**

- **Unmodified**: 1.0287e+07

### TIVKPGDILLIN(N)STGNLIAPR Hex 9 HexNAc 2

**Mass:**  4068.9294

(1.479e-06)

**Charge States:** 3, 4

**Start Position**: 251

**End Position**: 272

| Profile Score | Tandem Score | TDA q-value | Volume | Spectrum Matches |
| --- | --- | --- | --- | --- |
| 14.8472 | 164.8632 | 0.0000 | 2.2503e+08 | 13 |

**Adduct Signal Fractions**

- **Unmodified**: 2.2503e+08

### LPGNDN(N)STATL Hex 5 HexNAc 4

**Mass:**  2724.1208

(3.266e-06)

**Charge States:** 3

**Start Position**: 19

**End Position**: 30

| Profile Score | Tandem Score | TDA q-value | Volume | Spectrum Matches |
| --- | --- | --- | --- | --- |
| 12.3595 | 160.1844 | 0.0000 | 1.9177e+07 | 2 |

**Adduct Signal Fractions**

- **Unmodified**: 1.9177e+07

### TIVKPGDILLIN(N)STGNLIAPRGY Hex 9 HexNAc 2

**Mass:**  4289.0168

(2.009e-06)

**Charge States:** 3, 4, 5

**Start Position**: 251

**End Position**: 274

| Profile Score | Tandem Score | TDA q-value | Volume | Spectrum Matches |
| --- | --- | --- | --- | --- |
| 15.6118 | 159.7043 | 0.0000 | 7.5049e+08 | 31 |

**Adduct Signal Fractions**

- **Unmodified**: 7.5049e+08

### LIN(N)STGNLIAPR Fuc 2 Hex 3 HexNAc 4 Neu5Ac 1

**Mass:**  3149.4054

(-2.057e-06)

**Charge States:** 3

**Start Position**: 260

**End Position**: 272

| Profile Score | Tandem Score | TDA q-value | Volume | Spectrum Matches |
| --- | --- | --- | --- | --- |
| -19.6734 | 159.1364 | 0.0000 | 5.4392e+06 | 1 |

**Adduct Signal Fractions**

- **Unmodified**: 5.4392e+06

### TIVKPGDILLIN(N) Hex 9 HexNAc 2

**Mass:**  3159.4246

(1.549e-06)

**Charge States:** 2, 3

**Start Position**: 251

**End Position**: 263

| Profile Score | Tandem Score | TDA q-value | Volume | Spectrum Matches |
| --- | --- | --- | --- | --- |
| 13.3257 | 158.5779 | 0.0000 | 1.3044e+08 | 9 |

**Adduct Signal Fractions**

- **Unmodified**: 1.3044e+08

### LIN(N)STGNLIAPRGY Hex 9 HexNAc 2

**Mass:**  3352.4522

(2.521e-06)

**Charge States:** 3

**Start Position**: 260

**End Position**: 274

| Profile Score | Tandem Score | TDA q-value | Volume | Spectrum Matches |
| --- | --- | --- | --- | --- |
| -18.8041 | 157.7984 | 0.0000 | 4.8436e+07 | 2 |

**Adduct Signal Fractions**

- **Unmodified**: 4.8436e+07

### TIVKPGDILLIN(N)STGN Hex 9 HexNAc 2

**Mass:**  3518.5731

(2.352e-06)

**Charge States:** 3

**Start Position**: 251

**End Position**: 267

| Profile Score | Tandem Score | TDA q-value | Volume | Spectrum Matches |
| --- | --- | --- | --- | --- |
| 13.5917 | 154.8016 | 0.0000 | 4.9400e+07 | 4 |

**Adduct Signal Fractions**

- **Unmodified**: 4.9400e+07

### TIVKPGDILLIN(N) Fuc 2 Hex 3 HexNAc 4 Neu5Ac 1

**Mass:**  3176.4501

(-6.735e-06)

**Charge States:** 3

**Start Position**: 251

**End Position**: 263

| Profile Score | Tandem Score | TDA q-value | Volume | Spectrum Matches |
| --- | --- | --- | --- | --- |
| 12.4675 | 154.2064 | 0.0000 | 4.6704e+07 | 5 |

**Adduct Signal Fractions**

- **Unmodified**: 4.6704e+07

### LPGNDN(N)STATL Fuc 1 Hex 6 HexNAc 5

**Mass:**  3235.3072

(1.595e-06)

**Charge States:** 3

**Start Position**: 19

**End Position**: 30

| Profile Score | Tandem Score | TDA q-value | Volume | Spectrum Matches |
| --- | --- | --- | --- | --- |
| 12.8118 | 153.8352 | 0.0000 | 1.0034e+07 | 2 |

**Adduct Signal Fractions**

- **Unmodified**: 1.0034e+07

### TIVKPGDILLIN(N)STGNLIAPR Fuc 2 Hex 3 HexNAc 4 Neu5Ac 1

**Mass:**  4085.9555

(-5.124e-06)

**Charge States:** 4

**Start Position**: 251

**End Position**: 272

| Profile Score | Tandem Score | TDA q-value | Volume | Spectrum Matches |
| --- | --- | --- | --- | --- |
| 13.4707 | 149.3996 | 0.0000 | 2.8004e+07 | 3 |

**Adduct Signal Fractions**

- **Unmodified**: 2.8004e+07

### TIVKPGDILLIN(N)STGNLIAPRGY Fuc 2 Hex 3 HexNAc 4 Neu5Ac 1

**Mass:**  4306.0407

(-5.275e-06)

**Charge States:** 4

**Start Position**: 251

**End Position**: 274

| Profile Score | Tandem Score | TDA q-value | Volume | Spectrum Matches |
| --- | --- | --- | --- | --- |
| 11.6162 | 149.0792 | 0.0000 | 1.1031e+08 | 3 |

**Adduct Signal Fractions**

- **Unmodified**: 1.1031e+08

### TIVKPGDILLIN(N)STGNL Hex 9 HexNAc 2

**Mass:**  3631.6572

(1.767e-06)

**Charge States:** 3, 4

**Start Position**: 251

**End Position**: 268

| Profile Score | Tandem Score | TDA q-value | Volume | Spectrum Matches |
| --- | --- | --- | --- | --- |
| 12.6167 | 148.4089 | 0.0000 | 9.1004e+07 | 5 |

**Adduct Signal Fractions**

- **Unmodified**: 9.1004e+07

### TIVKPGDILLIN(N)STGNL Fuc 2 Hex 3 HexNAc 4 Neu5Ac 1

**Mass:**  3648.6809

(-5.511e-06)

**Charge States:** 3

**Start Position**: 251

**End Position**: 268

| Profile Score | Tandem Score | TDA q-value | Volume | Spectrum Matches |
| --- | --- | --- | --- | --- |
| -3.8986 | 146.8150 | 0.0000 | 3.5451e+07 | 4 |

**Adduct Signal Fractions**

- **Unmodified**: 3.5451e+07

### TIVKPGDILLIN(N) Hex 10 HexNAc 2

**Mass:**  3321.4784

(1.647e-06)

**Charge States:** 3

**Start Position**: 251

**End Position**: 263

| Profile Score | Tandem Score | TDA q-value | Volume | Spectrum Matches |
| --- | --- | --- | --- | --- |
| -5.0956 | 146.5682 | 0.0000 | 6.3231e+06 | 2 |

**Adduct Signal Fractions**

- **Unmodified**: 6.3231e+06

### TIVKPGDILLIN(N)STGNLIAPRGY Hex 8 HexNAc 2

**Mass:**  4126.9577

(1.148e-06)

**Charge States:** 3, 4

**Start Position**: 251

**End Position**: 274

| Profile Score | Tandem Score | TDA q-value | Volume | Spectrum Matches |
| --- | --- | --- | --- | --- |
| 12.1198 | 144.1336 | 0.0000 | 7.4705e+07 | 12 |

**Adduct Signal Fractions**

- **Unmodified**: 7.4705e+07

### TIVKPGDILLIN(N)STGNLIAPRGY Hex 10 HexNAc 2

**Mass:**  4451.0661

(1.133e-06)

**Charge States:** 3, 4

**Start Position**: 251

**End Position**: 274

| Profile Score | Tandem Score | TDA q-value | Volume | Spectrum Matches |
| --- | --- | --- | --- | --- |
| 13.3812 | 142.6123 | 0.0000 | 6.8942e+07 | 5 |

**Adduct Signal Fractions**

- **Unmodified**: 6.8942e+07

### LPGNDN(N)STATL Fuc 2 Hex 5 HexNAc 4

**Mass:**  3016.2317

(1.367e-06)

**Charge States:** 3

**Start Position**: 19

**End Position**: 30

| Profile Score | Tandem Score | TDA q-value | Volume | Spectrum Matches |
| --- | --- | --- | --- | --- |
| 11.4867 | 140.0778 | 0.0000 | 1.3774e+07 | 2 |

**Adduct Signal Fractions**

- **Unmodified**: 1.3774e+07

### LPGNDN(N)STATL Hex 6 HexNAc 3

**Mass:**  2683.0876

(8.999e-07)

**Charge States:** 3

**Start Position**: 19

**End Position**: 30

| Profile Score | Tandem Score | TDA q-value | Volume | Spectrum Matches |
| --- | --- | --- | --- | --- |
| 12.0129 | 139.9116 | 0.0000 | 6.7122e+06 | 1 |

**Adduct Signal Fractions**

- **Unmodified**: 6.7122e+06

### TIVKPGDILLIN(N)STGNLIAPR Hex 10 HexNAc 2

**Mass:**  4230.9811

(7.063e-06)

**Charge States:** 3, 4

**Start Position**: 251

**End Position**: 272

| Profile Score | Tandem Score | TDA q-value | Volume | Spectrum Matches |
| --- | --- | --- | --- | --- |
| 12.9093 | 135.6916 | 0.0000 | 1.9387e+07 | 4 |

**Adduct Signal Fractions**

- **Unmodified**: 1.9387e+07

### TIVKPGDILLIN(N)STGNLIAPR Hex 8 HexNAc 2

**Mass:**  3906.8776

(-8.614e-07)

**Charge States:** 3, 4

**Start Position**: 251

**End Position**: 272

| Profile Score | Tandem Score | TDA q-value | Volume | Spectrum Matches |
| --- | --- | --- | --- | --- |
| -4.2310 | 133.4773 | 0.0000 | 1.3447e+07 | 4 |

**Adduct Signal Fractions**

- **Unmodified**: 1.3447e+07

### TIVKPGDILLIN(N)STGN Fuc 2 Hex 3 HexNAc 4 Neu5Ac 1

**Mass:**  3535.5990

(-5.456e-06)

**Charge States:** 3

**Start Position**: 251

**End Position**: 267

| Profile Score | Tandem Score | TDA q-value | Volume | Spectrum Matches |
| --- | --- | --- | --- | --- |
| 14.3700 | 133.0721 | 0.0000 | 1.8491e+07 | 2 |

**Adduct Signal Fractions**

- **Unmodified**: 1.8491e+07

### TIVKPGDILLIN(N)STGNL Hex 10 HexNAc 2

**Mass:**  3793.7074

(1.629e-06)

**Charge States:** 3

**Start Position**: 251

**End Position**: 268

| Profile Score | Tandem Score | TDA q-value | Volume | Spectrum Matches |
| --- | --- | --- | --- | --- |
| -4.1780 | 132.9825 | 0.0000 | 7.7899e+06 | 2 |

**Adduct Signal Fractions**

- **Unmodified**: 7.7899e+06

### LPGNDN(N)STATL Fuc 1 Hex 9 HexNAc 3

**Mass:**  3315.3436

(6.868e-06)

**Charge States:** 3

**Start Position**: 19

**End Position**: 30

| Profile Score | Tandem Score | TDA q-value | Volume | Spectrum Matches |
| --- | --- | --- | --- | --- |
| -4.8380 | 131.8987 | 0.0000 | 8.5249e+07 | 1 |

**Adduct Signal Fractions**

- **Unmodified**: 8.5249e+07

### LPGNDN(N)STATL Fuc 2 Hex 3 HexNAc 5 Neu5Ac 2

**Mass:**  3477.4107

(5.285e-06)

**Charge States:** 3

**Start Position**: 19

**End Position**: 30

| Profile Score | Tandem Score | TDA q-value | Volume | Spectrum Matches |
| --- | --- | --- | --- | --- |
| 12.6270 | 130.3453 | 0.0000 | 2.1206e+08 | 3 |

**Adduct Signal Fractions**

- **Unmodified**: 2.1206e+08

### LIN(N)STGNLIAPRGYF Fuc 2 Hex 3 HexNAc 4 Neu5Ac 1

**Mass:**  3516.5458

(2.845e-04)

**Charge States:** 4

**Start Position**: 260

**End Position**: 275

| Profile Score | Tandem Score | TDA q-value | Volume | Spectrum Matches |
| --- | --- | --- | --- | --- |
| -6.9838 | 129.4221 | 0.0000 | 1.8085e+07 | 5 |

**Adduct Signal Fractions**

- **Unmodified**: 1.8085e+07

### IN(N)STGNLIAPRGYF Hex 9 HexNAc 2

**Mass:**  3386.4417

(3.881e-06)

**Charge States:** 3

**Start Position**: 261

**End Position**: 275

| Profile Score | Tandem Score | TDA q-value | Volume | Spectrum Matches |
| --- | --- | --- | --- | --- |
| -4.5718 | 127.0839 | 0.0000 | 1.0579e+07 | 2 |

**Adduct Signal Fractions**

- **Unmodified**: 1.0579e+07

### IN(N)STGNLIAPR Hex 9 HexNAc 2

**Mass:**  3019.2859

(3.647e-06)

**Charge States:** 3

**Start Position**: 261

**End Position**: 272

| Profile Score | Tandem Score | TDA q-value | Volume | Spectrum Matches |
| --- | --- | --- | --- | --- |
| -17.1303 | 124.0400 | 0.0000 | 3.2682e+06 | 1 |

**Adduct Signal Fractions**

- **Unmodified**: 3.2682e+06

### LPGNDN(N)STATL Hex 6 HexNAc 2

**Mass:**  2480.0079

(8.025e-07)

**Charge States:** 2

**Start Position**: 19

**End Position**: 30

| Profile Score | Tandem Score | TDA q-value | Volume | Spectrum Matches |
| --- | --- | --- | --- | --- |
| -5.4609 | 120.9011 | 0.0000 | 4.1038e+06 | 1 |

**Adduct Signal Fractions**

- **Unmodified**: 4.1038e+06

### IN(N)STGNLIAPRGY Hex 9 HexNAc 2

**Mass:**  3239.3607

(3.557e-07)

**Charge States:** 3

**Start Position**: 261

**End Position**: 274

| Profile Score | Tandem Score | TDA q-value | Volume | Spectrum Matches |
| --- | --- | --- | --- | --- |
| -18.5789 | 120.3446 | 0.0000 | 5.3084e+06 | 1 |

**Adduct Signal Fractions**

- **Unmodified**: 5.3084e+06

### RSDAPIGTC(C)SSEC(C)ITPN(N)GSIPNDKPF Hex 9 HexNAc 2

**Mass:**  4683.9333

(8.235e-07)

**Charge States:** 3, 4

**Start Position**: 285

**End Position**: 311

| Profile Score | Tandem Score | TDA q-value | Volume | Spectrum Matches |
| --- | --- | --- | --- | --- |
| 10.5205 | 116.2313 | 0.0000 | 4.6321e+07 | 4 |

**Adduct Signal Fractions**

- **Unmodified**: 4.6321e+07

### RSDAPIGTC(C)SSEC(C)ITPN(N)GSIPNDKPF Hex 8 HexNAc 2

**Mass:**  4521.8845

(4.845e-06)

**Charge States:** 3, 4

**Start Position**: 285

**End Position**: 311

| Profile Score | Tandem Score | TDA q-value | Volume | Spectrum Matches |
| --- | --- | --- | --- | --- |
| 10.6324 | 113.0082 | 0.0000 | 5.6208e+07 | 6 |

**Adduct Signal Fractions**

- **Unmodified**: 5.6208e+07

### LPGNDN(N)STATL Fuc 1 Hex 8 HexNAc 2

**Mass:**  2950.1742

(4.258e-06)

**Charge States:** 2, 3

**Start Position**: 19

**End Position**: 30

| Profile Score | Tandem Score | TDA q-value | Volume | Spectrum Matches |
| --- | --- | --- | --- | --- |
| 12.4965 | 106.6446 | 0.0000 | 7.0567e+08 | 11 |

**Adduct Signal Fractions**

- **Unmodified**: 7.0567e+08

### LPGNDN(N)STATL Fuc 1 Hex 5 HexNAc 7

**Mass:**  3479.4070

(-2.530e-07)

**Start Position**: 19

**End Position**: 30

| Profile Score | Tandem Score | TDA q-value | Volume | Spectrum Matches |
| --- | --- | --- | --- | --- |
| 0.0000 | 106.2031 | 0.0000 | 0.0000e+00 | 1 |

Adduct:

No Chromatogram Found

### SIRN(N)GTYDHDVY Fuc 2 Hex 5 HexNAc 4

**Mass:**  3354.3430

(2.976e-04)

**Start Position**: 496

**End Position**: 508

| Profile Score | Tandem Score | TDA q-value | Volume | Spectrum Matches |
| --- | --- | --- | --- | --- |
| 0.0000 | 104.6083 | 0.0000 | 0.0000e+00 | 2 |

Adduct:

No Chromatogram Found

### LPGNDN(N)STATL Fuc 4 Hex 3 HexNAc 5 Neu5Ac 2

**Mass:**  3769.5276

(5.555e-06)

**Charge States:** 3

**Start Position**: 19

**End Position**: 30

| Profile Score | Tandem Score | TDA q-value | Volume | Spectrum Matches |
| --- | --- | --- | --- | --- |
| 11.6425 | 103.0776 | 0.0000 | 1.9782e+07 | 1 |

**Adduct Signal Fractions**

- **Unmodified**: 1.9782e+07

### LPGNDN(N)STATL Fuc 3 Hex 3 HexNAc 4 Neu5Ac 1

**Mass:**  3129.2990

(7.586e-06)

**Charge States:** 3

**Start Position**: 19

**End Position**: 30

| Profile Score | Tandem Score | TDA q-value | Volume | Spectrum Matches |
| --- | --- | --- | --- | --- |
| -20.4789 | 102.4263 | 0.0000 | 4.8405e+06 | 1 |

**Adduct Signal Fractions**

- **Unmodified**: 4.8405e+06

### TIVKPGDILLIN(N)STGNL Hex 8 HexNAc 2

**Mass:**  3469.5987

(8.846e-07)

**Charge States:** 3

**Start Position**: 251

**End Position**: 268

| Profile Score | Tandem Score | TDA q-value | Volume | Spectrum Matches |
| --- | --- | --- | --- | --- |
| -4.9664 | 101.8341 | 0.0000 | 6.2316e+06 | 2 |

**Adduct Signal Fractions**

- **Unmodified**: 6.2316e+06

### LIN(N)STGNLIAPRGYF Hex 8 HexNAc 2

**Mass:**  3337.4705

(3.337e-06)

**Charge States:** 3

**Start Position**: 260

**End Position**: 275

| Profile Score | Tandem Score | TDA q-value | Volume | Spectrum Matches |
| --- | --- | --- | --- | --- |
| -20.1329 | 100.0237 | 0.0000 | 1.7182e+06 | 1 |

**Adduct Signal Fractions**

- **Unmodified**: 1.7182e+06

### LPGNDN(N)STATL Fuc 1 Hex 3 HexNAc 5 Neu5Ac 2

**Mass:**  3331.3551

(6.272e-06)

**Charge States:** 3

**Start Position**: 19

**End Position**: 30

| Profile Score | Tandem Score | TDA q-value | Volume | Spectrum Matches |
| --- | --- | --- | --- | --- |
| -4.5117 | 99.7674 | 0.0000 | 6.1209e+07 | 1 |

**Adduct Signal Fractions**

- **Unmodified**: 6.1209e+07

### LPGNDN(N)STATL Fuc 3 Hex 3 HexNAc 5 Neu5Ac 2

**Mass:**  3623.4702

(4.578e-06)

**Charge States:** 3

**Start Position**: 19

**End Position**: 30

| Profile Score | Tandem Score | TDA q-value | Volume | Spectrum Matches |
| --- | --- | --- | --- | --- |
| 12.0477 | 96.3758 | 0.0000 | 6.9881e+07 | 2 |

**Adduct Signal Fractions**

- **Unmodified**: 6.9881e+07

### TIVKPGDILLIN(N)STGNLIAPRGY Fuc 2 Hex 4 HexNAc 4 Neu5Ac 1

**Mass:**  4468.0920

(-4.925e-06)

**Charge States:** 4

**Start Position**: 251

**End Position**: 274

| Profile Score | Tandem Score | TDA q-value | Volume | Spectrum Matches |
| --- | --- | --- | --- | --- |
| -5.5409 | 96.0784 | 0.0000 | 1.1596e+07 | 1 |

**Adduct Signal Fractions**

- **Unmodified**: 1.1596e+07

### TIVKPGDILLIN(N)STGN(D)LIAPRGY Hex 9 HexNAc 2

**Mass:**  4290.0109

(2.328e-04)

**Charge States:** 3

**Start Position**: 251

**End Position**: 274

| Profile Score | Tandem Score | TDA q-value | Volume | Spectrum Matches |
| --- | --- | --- | --- | --- |
| -4.5970 | 95.6663 | 0.0000 | 2.6103e+07 | 4 |

**Adduct Signal Fractions**

- **Unmodified**: 2.6103e+07

### SIRN(N)GTYDHDVY Fuc 1 Hex 5 HexNAc 4

**Mass:**  3207.2964

(2.824e-06)

**Charge States:** 3

**Start Position**: 496

**End Position**: 508

| Profile Score | Tandem Score | TDA q-value | Volume | Spectrum Matches |
| --- | --- | --- | --- | --- |
| -20.5580 | 95.1729 | 0.0000 | 6.8254e+06 | 1 |

**Adduct Signal Fractions**

- **Unmodified**: 6.8254e+06

### LPGNDN(N)STATL Fuc 1 Hex 3 HexNAc 6 Neu5Ac 3

**Mass:**  3825.5172

(2.741e-06)

**Charge States:** 3, 4

**Start Position**: 19

**End Position**: 30

| Profile Score | Tandem Score | TDA q-value | Volume | Spectrum Matches |
| --- | --- | --- | --- | --- |
| 11.6145 | 94.4598 | 0.0000 | 8.5989e+07 | 4 |

**Adduct Signal Fractions**

- **Unmodified**: 8.5989e+07

### ESESKYPVLN(N)VTM(O)PNN(D)GKFDKLY Hex 9 HexNAc 2

**Mass:**  4553.9426

(2.204e-04)

**Charge States:** 4

**Start Position**: 172

**End Position**: 195

| Profile Score | Tandem Score | TDA q-value | Volume | Spectrum Matches |
| --- | --- | --- | --- | --- |
| 11.5786 | 91.8118 | 0.0000 | 5.7342e+07 | 3 |

**Adduct Signal Fractions**

- **Unmodified**: 5.7342e+07

### TITNDQIEVTN(N)ATEL Hex 7 HexNAc 2

**Mass:**  3201.3594

(5.320e-06)

**Charge States:** 3

**Start Position**: 44

**End Position**: 59

| Profile Score | Tandem Score | TDA q-value | Volume | Spectrum Matches |
| --- | --- | --- | --- | --- |
| -17.6082 | 91.1925 | 0.0000 | 7.5814e+06 | 1 |

**Adduct Signal Fractions**

- **Unmodified**: 7.5814e+06

### LPGNDN(N)STATL Hex 4 HexNAc 7 Neu5Ac 2

**Mass:**  3753.4828

(-1.367e-06)

**Charge States:** 3

**Start Position**: 19

**End Position**: 30

| Profile Score | Tandem Score | TDA q-value | Volume | Spectrum Matches |
| --- | --- | --- | --- | --- |
| -19.0892 | 85.6944 | 0.0000 | 3.2848e+06 | 1 |

**Adduct Signal Fractions**

- **Unmodified**: 3.2848e+06

### ESESKYPVLN(N)VTM(O)PNN(D)GKFDKLY Hex 8 HexNAc 2

**Mass:**  4391.8928

(2.276e-04)

**Charge States:** 4

**Start Position**: 172

**End Position**: 195

| Profile Score | Tandem Score | TDA q-value | Volume | Spectrum Matches |
| --- | --- | --- | --- | --- |
| 11.4905 | 82.6874 | 0.0000 | 3.9627e+07 | 2 |

**Adduct Signal Fractions**

- **Unmodified**: 3.9627e+07

### LYESESKYPVLN(N)VTM(O)PN Hex 8 HexNAc 2

**Mass:**  3701.5512

(2.391e-06)

**Charge States:** 3

**Start Position**: 170

**End Position**: 187

| Profile Score | Tandem Score | TDA q-value | Volume | Spectrum Matches |
| --- | --- | --- | --- | --- |
| 6.9512 | 80.1180 | 0.0000 | 8.1964e+06 | 2 |

**Adduct Signal Fractions**

- **Unmodified**: 8.1964e+06

### RSDAPIGTC(C)SSEC(C)ITPN(N)GSIPNDKPF Hex 7 HexNAc 2

**Mass:**  4359.8371

(5.328e-06)

**Charge States:** 3, 4

**Start Position**: 285

**End Position**: 311

| Profile Score | Tandem Score | TDA q-value | Volume | Spectrum Matches |
| --- | --- | --- | --- | --- |
| -4.5270 | 73.8371 | 0.0000 | 1.2598e+07 | 2 |

**Adduct Signal Fractions**

- **Unmodified**: 1.2598e+07

### ITPN(N)GSIPNDKPF Fuc 4 Hex 7 HexNAc 7

**Mass:**  4538.9050

(8.069e-06)

**Charge States:** 3, 4

**Start Position**: 298

**End Position**: 311

| Profile Score | Tandem Score | TDA q-value | Volume | Spectrum Matches |
| --- | --- | --- | --- | --- |
| -5.2927 | 73.0522 | 0.0000 | 7.1484e+06 | 2 |

**Adduct Signal Fractions**

- **Unmodified**: 7.1484e+06

### TIVKPGDILLIN(N)STGNLIAPRGY Fuc 2 Hex 4 HexNAc 4 Neu5Ac 1

**Mass:**  4469.1145

(2.239e-04)

**Start Position**: 251

**End Position**: 274

| Profile Score | Tandem Score | TDA q-value | Volume | Spectrum Matches |
| --- | --- | --- | --- | --- |
| 0.0000 | 69.0589 | 0.0083 | 0.0000e+00 | 1 |

Adduct:

No Chromatogram Found

### KIRTGKSSIM(O)RSDAPIGTC(C)SSEC(C)ITPN(N)GSIPNDKPF Hex 8 HexNAc 2

**Mass:**  5639.5095

(1.583e-06)

**Charge States:** 5

**Start Position**: 275

**End Position**: 311

| Profile Score | Tandem Score | TDA q-value | Volume | Spectrum Matches |
| --- | --- | --- | --- | --- |
| -5.2572 | 68.9070 | 0.0083 | 5.2253e+07 | 3 |

**Adduct Signal Fractions**

- **Unmodified**: 5.2253e+07

### ITPN(N)GSIPNDKPF Fuc 1 Hex 5 HexNAc 6 Neu5Ac 3

**Mass:**  4446.8039

(2.102e-06)

**Charge States:** 3, 4

**Start Position**: 298

**End Position**: 311

| Profile Score | Tandem Score | TDA q-value | Volume | Spectrum Matches |
| --- | --- | --- | --- | --- |
| -19.3887 | 68.6863 | 0.0083 | 1.2713e+07 | 2 |

**Adduct Signal Fractions**

- **Unmodified**: 1.2713e+07

### KIRTGKSSIM(O)RSDAPIGTC(C)SSEC(C)ITPN(N)GSIPNDKPF Hex 9 HexNAc 2

**Mass:**  5801.5609

(2.805e-06)

**Charge States:** 5, 6

**Start Position**: 275

**End Position**: 311

| Profile Score | Tandem Score | TDA q-value | Volume | Spectrum Matches |
| --- | --- | --- | --- | --- |
| -4.7905 | 60.2046 | 0.0112 | 3.9698e+07 | 3 |

**Adduct Signal Fractions**

- **Unmodified**: 3.9698e+07

### TIVKPGDILLIN(N)STGN Hex 10 HexNAc 2

**Mass:**  3680.6218

(6.928e-07)

**Charge States:** 3

**Start Position**: 251

**End Position**: 267

| Profile Score | Tandem Score | TDA q-value | Volume | Spectrum Matches |
| --- | --- | --- | --- | --- |
| -5.4706 | 59.8518 | 0.0112 | 3.1584e+06 | 1 |

**Adduct Signal Fractions**

- **Unmodified**: 3.1584e+06

### RSDAPIGTC(C)SSEC(C)ITPN(N)GSIPN(D)DKPF Hex 8 HexNAc 2

**Mass:**  4522.8708

(2.220e-04)

**Charge States:** 3, 4

**Start Position**: 285

**End Position**: 311

| Profile Score | Tandem Score | TDA q-value | Volume | Spectrum Matches |
| --- | --- | --- | --- | --- |
| -5.7121 | 58.0901 | 0.0112 | 4.6931e+07 | 3 |

**Adduct Signal Fractions**

- **Unmodified**: 4.6931e+07

### LYESESKYPVLN(N)VTM(O)PNN(D)GKF Hex 9 HexNAc 2

**Mass:**  4310.8220

(2.318e-04)

**Charge States:** 3, 4

**Start Position**: 170

**End Position**: 191

| Profile Score | Tandem Score | TDA q-value | Volume | Spectrum Matches |
| --- | --- | --- | --- | --- |
| -6.3082 | 56.2922 | 0.0112 | 2.8717e+07 | 4 |

**Adduct Signal Fractions**

- **Unmodified**: 2.8717e+07

### TIVKPGDILLIN(N)STGNL Fuc 2 Hex 4 HexNAc 4 Neu5Ac 1

**Mass:**  3810.7392

(-3.958e-06)

**Charge States:** 3

**Start Position**: 251

**End Position**: 268

| Profile Score | Tandem Score | TDA q-value | Volume | Spectrum Matches |
| --- | --- | --- | --- | --- |
| -19.3580 | 49.0974 | 0.0112 | 1.8591e+06 | 1 |

**Adduct Signal Fractions**

- **Unmodified**: 1.8591e+06

### LYESESKYPVLN(N)VTM(O)PN Hex 9 HexNAc 2

**Mass:**  3863.6028

(4.629e-06)

**Charge States:** 3

**Start Position**: 170

**End Position**: 187

| Profile Score | Tandem Score | TDA q-value | Volume | Spectrum Matches |
| --- | --- | --- | --- | --- |
| -5.3064 | 46.7852 | 0.0112 | 1.2696e+07 | 1 |

**Adduct Signal Fractions**

- **Unmodified**: 1.2696e+07

### LPGNDN(N)STATL Hex 5 HexNAc 5

**Mass:**  2927.1994

(4.295e-06)

**Charge States:** 3

**Start Position**: 19

**End Position**: 30

| Profile Score | Tandem Score | TDA q-value | Volume | Spectrum Matches |
| --- | --- | --- | --- | --- |
| 10.5599 | 46.6564 | 0.0112 | 1.2939e+07 | 3 |

**Adduct Signal Fractions**

- **Unmodified**: 1.2939e+07

### KIRTGKSSIM(O)RSDAPIGTC(C)SSEC(C)ITPN(N)GSIPNDKPF Hex 7 HexNAc 2

**Mass:**  5477.4587

(3.155e-06)

**Charge States:** 5

**Start Position**: 275

**End Position**: 311

| Profile Score | Tandem Score | TDA q-value | Volume | Spectrum Matches |
| --- | --- | --- | --- | --- |
| -5.9801 | 46.5197 | 0.0112 | 1.0208e+07 | 2 |

**Adduct Signal Fractions**

- **Unmodified**: 1.0208e+07

### RSDAPIGTC(C)SSEC(C)ITPN(N)GSIPN(D)DKPF Hex 9 HexNAc 2

**Mass:**  4684.9191

(2.111e-04)

**Charge States:** 3, 4

**Start Position**: 285

**End Position**: 311

| Profile Score | Tandem Score | TDA q-value | Volume | Spectrum Matches |
| --- | --- | --- | --- | --- |
| 12.4484 | 46.3946 | 0.0112 | 3.4587e+07 | 3 |

**Adduct Signal Fractions**

- **Unmodified**: 3.4587e+07

### TITNDQIEVTN(N)ATELVQSSSTGRIC(C)DSPHRIL Hex 6 HexNAc 2

**Mass:**  4933.2497

(2.116e-06)

**Charge States:** 4

**Start Position**: 44

**End Position**: 76

| Profile Score | Tandem Score | TDA q-value | Volume | Spectrum Matches |
| --- | --- | --- | --- | --- |
| -5.1506 | 46.3862 | 0.0112 | 3.8559e+06 | 1 |

**Adduct Signal Fractions**

- **Unmodified**: 3.8559e+06

### LPGNDN(N)STATL Hex 6 HexNAc 5

**Mass:**  3089.2516

(1.319e-06)

**Charge States:** 3

**Start Position**: 19

**End Position**: 30

| Profile Score | Tandem Score | TDA q-value | Volume | Spectrum Matches |
| --- | --- | --- | --- | --- |
| -20.2785 | 46.1210 | 0.0112 | 1.5544e+06 | 1 |

**Adduct Signal Fractions**

- **Unmodified**: 1.5544e+06

### RSDAPIGTC(C)SSEC(C)ITPN(N)GSIPNDKPF Fuc 2 Hex 3 HexNAc 4 Neu5Ac 1

**Mass:**  4700.9577

(-4.998e-06)

**Charge States:** 3, 4

**Start Position**: 285

**End Position**: 311

| Profile Score | Tandem Score | TDA q-value | Volume | Spectrum Matches |
| --- | --- | --- | --- | --- |
| -5.2634 | 44.9298 | 0.0112 | 5.9020e+06 | 1 |

**Adduct Signal Fractions**

- **Unmodified**: 5.9020e+06

### TITNDQIEVTN(N)ATEL Hex 6 HexNAc 3

**Mass:**  3242.3856

(4.644e-06)

**Charge States:** 3

**Start Position**: 44

**End Position**: 59

| Profile Score | Tandem Score | TDA q-value | Volume | Spectrum Matches |
| --- | --- | --- | --- | --- |
| -18.7035 | 44.7632 | 0.0112 | 5.5722e+06 | 1 |

**Adduct Signal Fractions**

- **Unmodified**: 5.5722e+06

### TIVKPGDILLIN(N)STGN(D)LIAPR Hex 9 HexNAc 2

**Mass:**  4069.9269

(2.466e-04)

**Charge States:** 4

**Start Position**: 251

**End Position**: 272

| Profile Score | Tandem Score | TDA q-value | Volume | Spectrum Matches |
| --- | --- | --- | --- | --- |
| -5.1805 | 44.6012 | 0.0112 | 6.2894e+06 | 3 |

**Adduct Signal Fractions**

- **Unmodified**: 6.2894e+06

### TITNDQIEVTN(N)ATEL Hex 6 HexNAc 2

**Mass:**  3039.3026

(4.311e-06)

**Charge States:** 3

**Start Position**: 44

**End Position**: 59

| Profile Score | Tandem Score | TDA q-value | Volume | Spectrum Matches |
| --- | --- | --- | --- | --- |
| -3.6718 | 44.0620 | 0.0112 | 2.0539e+07 | 3 |

**Adduct Signal Fractions**

- **Unmodified**: 2.0539e+07

### ESESKYPVLN(N)VTM(O)PNN(D)GKFDKLY Fuc 2 Hex 8 HexNAc 3

**Mass:**  4887.1066

(2.087e-04)

**Charge States:** 4, 5

**Start Position**: 172

**End Position**: 195

| Profile Score | Tandem Score | TDA q-value | Volume | Spectrum Matches |
| --- | --- | --- | --- | --- |
| 12.9139 | 43.1644 | 0.0112 | 2.2010e+08 | 7 |

**Adduct Signal Fractions**

- **Unmodified**: 2.2010e+08

### KIRTGKSSIM(O)RSDAPIGTC(C)SSEC(C)ITPN(N)GSIPN(D)DKPF Hex 8 HexNAc 2

**Mass:**  5640.4965

(1.783e-04)

**Charge States:** 5

**Start Position**: 275

**End Position**: 311

| Profile Score | Tandem Score | TDA q-value | Volume | Spectrum Matches |
| --- | --- | --- | --- | --- |
| -6.3394 | 42.1957 | 0.0112 | 2.9354e+07 | 2 |

**Adduct Signal Fractions**

- **Unmodified**: 2.9354e+07

### LYESESKYPVLN(N)VTM(O)PNN(D)GKF Hex 8 HexNAc 2

**Mass:**  4148.7673

(2.397e-04)

**Charge States:** 3, 4

**Start Position**: 170

**End Position**: 191

| Profile Score | Tandem Score | TDA q-value | Volume | Spectrum Matches |
| --- | --- | --- | --- | --- |
| -6.2702 | 41.6846 | 0.0112 | 1.8598e+07 | 4 |

**Adduct Signal Fractions**

- **Unmodified**: 1.8598e+07

### INEGFN(N)WTGVTQ Fuc 1 Hex 5 HexNAc 5

**Mass:**  3336.3620

(3.078e-06)

**Charge States:** 3

**Start Position**: 137

**End Position**: 149

| Profile Score | Tandem Score | TDA q-value | Volume | Spectrum Matches |
| --- | --- | --- | --- | --- |
| -20.5134 | 41.5604 | 0.0112 | 2.3708e+06 | 1 |

**Adduct Signal Fractions**

- **Unmodified**: 2.3708e+06

### LPGNDN(N)STATL Fuc 1 Hex 9 HexNAc 3 Neu5Ac 1

**Mass:**  3606.4455

(9.884e-06)

**Charge States:** 3, 4

**Start Position**: 19

**End Position**: 30

| Profile Score | Tandem Score | TDA q-value | Volume | Spectrum Matches |
| --- | --- | --- | --- | --- |
| 13.6486 | 40.9356 | 0.0112 | 2.0572e+08 | 6 |

**Adduct Signal Fractions**

- **Unmodified**: 2.0572e+08

### SSEC(C)ITPN(N)GSIPN(D)DKPF Hex 9 HexNAc 2

**Mass:**  3727.4683

(2.638e-04)

**Charge States:** 3

**Start Position**: 294

**End Position**: 311

| Profile Score | Tandem Score | TDA q-value | Volume | Spectrum Matches |
| --- | --- | --- | --- | --- |
| -20.2665 | 40.2797 | 0.0112 | 1.0544e+07 | 1 |

**Adduct Signal Fractions**

- **Unmodified**: 1.0544e+07

### SSEC(C)ITPN(N)GSIPNDKPF Fuc 4 Hex 6 HexNAc 7

**Mass:**  4840.0075

(9.899e-06)

**Charge States:** 3, 4

**Start Position**: 294

**End Position**: 311

| Profile Score | Tandem Score | TDA q-value | Volume | Spectrum Matches |
| --- | --- | --- | --- | --- |
| -3.5364 | 39.7765 | 0.0112 | 2.1904e+07 | 3 |

**Adduct Signal Fractions**

- **Unmodified**: 2.1904e+07

### ITPN(N)GSIPNDKPF Hex 9 HexNAc 2

**Mass:**  3263.3651

(7.875e-06)

**Charge States:** 3

**Start Position**: 298

**End Position**: 311

| Profile Score | Tandem Score | TDA q-value | Volume | Spectrum Matches |
| --- | --- | --- | --- | --- |
| -4.2512 | 39.3810 | 0.0112 | 1.2499e+07 | 1 |

**Adduct Signal Fractions**

- **Unmodified**: 1.2499e+07

### KIRTGKSSIM(O)RSDAPIGTC(C)SSEC(C)ITPN(N)GSIPN(D)DKPF Hex 9 HexNAc 2

**Mass:**  5802.5466

(1.729e-04)

**Charge States:** 5, 6

**Start Position**: 275

**End Position**: 311

| Profile Score | Tandem Score | TDA q-value | Volume | Spectrum Matches |
| --- | --- | --- | --- | --- |
| -6.0994 | 38.8505 | 0.0112 | 3.9079e+07 | 2 |

**Adduct Signal Fractions**

- **Unmodified**: 3.9079e+07

### SSEC(C)ITPN(N)GSIPN(D)DKPF Hex 8 HexNAc 2

**Mass:**  3565.4247

(2.783e-04)

**Charge States:** 3

**Start Position**: 294

**End Position**: 311

| Profile Score | Tandem Score | TDA q-value | Volume | Spectrum Matches |
| --- | --- | --- | --- | --- |
| -5.9504 | 38.6679 | 0.0112 | 1.8075e+07 | 1 |

**Adduct Signal Fractions**

- **Unmodified**: 1.8075e+07

### ITPN(N)GSIPNDKPF Fuc 2 Hex 6 HexNAc 8 Neu5Ac 1

**Mass:**  4578.9055

(1.675e-06)

**Charge States:** 3, 4

**Start Position**: 298

**End Position**: 311

| Profile Score | Tandem Score | TDA q-value | Volume | Spectrum Matches |
| --- | --- | --- | --- | --- |
| -6.7245 | 38.4160 | 0.0112 | 1.3380e+07 | 5 |

**Adduct Signal Fractions**

- **Unmodified**: 1.3380e+07

### ITPN(N)GSIPNDKPF Fuc 3 Hex 6 HexNAc 9

**Mass:**  4636.8942

(-5.455e-06)

**Charge States:** 3, 4

**Start Position**: 298

**End Position**: 311

| Profile Score | Tandem Score | TDA q-value | Volume | Spectrum Matches |
| --- | --- | --- | --- | --- |
| -5.7418 | 38.4086 | 0.0112 | 4.7384e+06 | 3 |

**Adduct Signal Fractions**

- **Unmodified**: 4.7384e+06

### AC(C)IGSIRN(N)GTYDHDVY Fuc 1 Hex 5 HexNAc 4

**Mass:**  3608.4625

(5.265e-07)

**Charge States:** 3

**Start Position**: 492

**End Position**: 508

| Profile Score | Tandem Score | TDA q-value | Volume | Spectrum Matches |
| --- | --- | --- | --- | --- |
| -4.6491 | 38.2266 | 0.0112 | 3.1447e+06 | 1 |

**Adduct Signal Fractions**

- **Unmodified**: 3.1447e+06

### TIVKPGDILLIN(N)STGNLIAPRGY Fuc 1 Hex 3 HexNAc 5 Neu5Ac 1

**Mass:**  4363.0632

(-4.447e-06)

**Charge States:** 4

**Start Position**: 251

**End Position**: 274

| Profile Score | Tandem Score | TDA q-value | Volume | Spectrum Matches |
| --- | --- | --- | --- | --- |
| 11.0322 | 37.8081 | 0.0112 | 5.1143e+07 | 5 |

**Adduct Signal Fractions**

- **Unmodified**: 5.1143e+07

### ITPN(N)GSIPNDKPF Fuc 2 Hex 7 HexNAc 8 Neu5Ac 1

**Mass:**  4740.9543

(5.095e-06)

**Charge States:** 3

**Start Position**: 298

**End Position**: 311

| Profile Score | Tandem Score | TDA q-value | Volume | Spectrum Matches |
| --- | --- | --- | --- | --- |
| -4.8775 | 37.6673 | 0.0156 | 6.5990e+06 | 1 |

**Adduct Signal Fractions**

- **Unmodified**: 6.5990e+06

### IGSIRN(N)GTYDHDVY Fuc 2 Hex 5 HexNAc 4

**Mass:**  3523.4518

(3.265e-07)

**Charge States:** 3

**Start Position**: 494

**End Position**: 508

| Profile Score | Tandem Score | TDA q-value | Volume | Spectrum Matches |
| --- | --- | --- | --- | --- |
| -17.0342 | 37.2358 | 0.0156 | 2.5609e+06 | 1 |

**Adduct Signal Fractions**

- **Unmodified**: 2.5609e+06

### TC(C)KRGSN(N)NSFF Fuc 1 Hex 5 HexNAc 4

**Mass:**  3085.2449

(5.458e-06)

**Charge States:** 3

**Start Position**: 154

**End Position**: 165

| Profile Score | Tandem Score | TDA q-value | Volume | Spectrum Matches |
| --- | --- | --- | --- | --- |
| -18.4143 | 36.0856 | 0.0156 | 1.2353e+07 | 1 |

**Adduct Signal Fractions**

- **Unmodified**: 1.2353e+07

### LPGNDN(N)STATL Hex 7 HexNAc 5

**Mass:**  3253.3127

(6.271e-04)

**Charge States:** 4, 5

**Start Position**: 19

**End Position**: 30

| Profile Score | Tandem Score | TDA q-value | Volume | Spectrum Matches |
| --- | --- | --- | --- | --- |
| -23.4733 | 35.2906 | 0.0156 | 3.9271e+06 | 1 |

**Adduct Signal Fractions**

- **Unmodified**: 3.9271e+06

### RSDAPIGTC(C)SSEC(C)ITPN(N)GSIPN(D)DKPF Hex 7 HexNAc 2

**Mass:**  4360.8176

(2.270e-04)

**Charge States:** 3, 4

**Start Position**: 285

**End Position**: 311

| Profile Score | Tandem Score | TDA q-value | Volume | Spectrum Matches |
| --- | --- | --- | --- | --- |
| -6.2356 | 34.9028 | 0.0156 | 1.2324e+07 | 2 |

**Adduct Signal Fractions**

- **Unmodified**: 1.2324e+07

### ITPN(N)GSIPNDKPF Fuc 4 Hex 6 HexNAc 8

**Mass:**  4579.8859

(-1.388e-06)

**Charge States:** 3

**Start Position**: 298

**End Position**: 311

| Profile Score | Tandem Score | TDA q-value | Volume | Spectrum Matches |
| --- | --- | --- | --- | --- |
| -5.2853 | 34.5488 | 0.0191 | 8.4136e+06 | 1 |

**Adduct Signal Fractions**

- **Unmodified**: 8.4136e+06

### SSEC(C)ITPN(N)GSIPN(D)DKPF Fuc 4 Hex 6 HexNAc 7

**Mass:**  4840.9826

(2.087e-04)

**Charge States:** 3, 4

**Start Position**: 294

**End Position**: 311

| Profile Score | Tandem Score | TDA q-value | Volume | Spectrum Matches |
| --- | --- | --- | --- | --- |
| -6.5423 | 34.3005 | 0.0191 | 1.4417e+07 | 3 |

**Adduct Signal Fractions**

- **Unmodified**: 1.4417e+07

### TIVKPGDILLIN(N)STGNLIAPRGY Hex 5 HexNAc 6

**Mass:**  4453.1125

(-7.973e-06)

**Charge States:** 4

**Start Position**: 251

**End Position**: 274

| Profile Score | Tandem Score | TDA q-value | Volume | Spectrum Matches |
| --- | --- | --- | --- | --- |
| 13.3723 | 33.9052 | 0.0191 | 5.8366e+07 | 4 |

**Adduct Signal Fractions**

- **Unmodified**: 5.8366e+07

### TIVKPGDILLIN(N)STGNLIAPR Fuc 3 Hex 3 HexNAc 5

**Mass:**  4143.9909

(-6.624e-06)

**Charge States:** 4

**Start Position**: 251

**End Position**: 272

| Profile Score | Tandem Score | TDA q-value | Volume | Spectrum Matches |
| --- | --- | --- | --- | --- |
| -19.0041 | 33.5207 | 0.0191 | 5.1129e+06 | 1 |

**Adduct Signal Fractions**

- **Unmodified**: 5.1129e+06

### LYESESKYPVLN(N)VTM(O)PNN(D)GKF Fuc 3 Hex 7 HexNAc 4

**Mass:**  4831.0720

(2.117e-04)

**Charge States:** 4

**Start Position**: 170

**End Position**: 191

| Profile Score | Tandem Score | TDA q-value | Volume | Spectrum Matches |
| --- | --- | --- | --- | --- |
| 9.4061 | 33.4072 | 0.0191 | 1.6630e+08 | 8 |

**Adduct Signal Fractions**

- **Unmodified**: 1.6630e+08

### ITPN(N)GSIPNDKPF Fuc 4 Hex 5 HexNAc 8

**Mass:**  4417.8254

(-4.450e-06)

**Charge States:** 3, 4

**Start Position**: 298

**End Position**: 311

| Profile Score | Tandem Score | TDA q-value | Volume | Spectrum Matches |
| --- | --- | --- | --- | --- |
| -4.8297 | 33.0015 | 0.0191 | 9.1513e+06 | 2 |

**Adduct Signal Fractions**

- **Unmodified**: 9.1513e+06

### SEC(C)ITPN(N)GSIPN(D)DKPF Hex 9 HexNAc 2

**Mass:**  3640.4423

(2.724e-04)

**Charge States:** 3

**Start Position**: 295

**End Position**: 311

| Profile Score | Tandem Score | TDA q-value | Volume | Spectrum Matches |
| --- | --- | --- | --- | --- |
| -6.2503 | 32.7151 | 0.0191 | 1.4076e+07 | 1 |

**Adduct Signal Fractions**

- **Unmodified**: 1.4076e+07

### TIVKPGDILLIN(N)STGNLIAPRGY Hex 7 HexNAc 2

**Mass:**  3966.9061

(5.053e-04)

**Start Position**: 251

**End Position**: 274

| Profile Score | Tandem Score | TDA q-value | Volume | Spectrum Matches |
| --- | --- | --- | --- | --- |
| 0.0000 | 32.6513 | 0.0191 | 0.0000e+00 | 1 |

Adduct:

No Chromatogram Found

### ESESKYPVLN(N)VTM(O)PNN(D)GKFDKLY Fuc 2 Hex 7 HexNAc 3

**Mass:**  4725.0573

(2.166e-04)

**Charge States:** 4, 5

**Start Position**: 172

**End Position**: 195

| Profile Score | Tandem Score | TDA q-value | Volume | Spectrum Matches |
| --- | --- | --- | --- | --- |
| 11.9685 | 32.6210 | 0.0191 | 1.4565e+08 | 5 |

**Adduct Signal Fractions**

- **Unmodified**: 1.4565e+08

### ITPN(N)GSIPNDKPF Fuc 3 Hex 5 HexNAc 9

**Mass:**  4474.8493

(-5.018e-06)

**Charge States:** 4

**Start Position**: 298

**End Position**: 311

| Profile Score | Tandem Score | TDA q-value | Volume | Spectrum Matches |
| --- | --- | --- | --- | --- |
| -5.3376 | 32.4510 | 0.0191 | 5.6280e+06 | 3 |

**Adduct Signal Fractions**

- **Unmodified**: 5.6280e+06

### SSEC(C)ITPN(N)GSIPN(D)DKPF Fuc 1 Hex 5 HexNAc 8

**Mass:**  4443.7810

(2.169e-04)

**Charge States:** 5

**Start Position**: 294

**End Position**: 311

| Profile Score | Tandem Score | TDA q-value | Volume | Spectrum Matches |
| --- | --- | --- | --- | --- |
| -6.5210 | 31.3265 | 0.0437 | 2.2067e+07 | 3 |

**Adduct Signal Fractions**

- **Unmodified**: 2.2067e+07

### SSEC(C)ITPN(N)GSIPNDKPF Fuc 4 Hex 7 HexNAc 7

**Mass:**  5002.0583

(9.911e-06)

**Charge States:** 3, 4

**Start Position**: 294

**End Position**: 311

| Profile Score | Tandem Score | TDA q-value | Volume | Spectrum Matches |
| --- | --- | --- | --- | --- |
| -4.2668 | 31.2561 | 0.0270 | 1.5185e+07 | 2 |

**Adduct Signal Fractions**

- **Unmodified**: 1.5185e+07

### TIVKPGDILLIN(N)STGN(D)LIAPRGY Fuc 2 Hex 3 HexNAc 4 Neu5Ac 1

**Mass:**  4307.0412

(2.276e-04)

**Charge States:** 4

**Start Position**: 251

**End Position**: 274

| Profile Score | Tandem Score | TDA q-value | Volume | Spectrum Matches |
| --- | --- | --- | --- | --- |
| -20.6480 | 31.2556 | 0.0270 | 3.1578e+06 | 1 |

**Adduct Signal Fractions**

- **Unmodified**: 3.1578e+06

### TIVKPGDILLIN(N)STGNLIAPR Fuc 1 Hex 3 HexNAc 5 Neu5Ac 1

**Mass:**  4142.9802

(-3.574e-06)

**Charge States:** 4

**Start Position**: 251

**End Position**: 272

| Profile Score | Tandem Score | TDA q-value | Volume | Spectrum Matches |
| --- | --- | --- | --- | --- |
| -4.1260 | 30.6905 | 0.0270 | 1.1353e+07 | 2 |

**Adduct Signal Fractions**

- **Unmodified**: 1.1353e+07

### TIVKPGDILLIN(N)STGNL Fuc 3 Hex 10 HexNAc 5

**Mass:**  4844.0871

(6.136e-04)

**Charge States:** 4

**Start Position**: 251

**End Position**: 268

| Profile Score | Tandem Score | TDA q-value | Volume | Spectrum Matches |
| --- | --- | --- | --- | --- |
| -22.8139 | 30.3100 | 0.0270 | 1.5375e+05 | 12 |

**Adduct Signal Fractions**

- **Unmodified**: 1.5375e+05

### ITPN(N)GSIPNDKPF Hex 7 HexNAc 2

**Mass:**  2939.2481

(1.783e-06)

**Charge States:** 3

**Start Position**: 298

**End Position**: 311

| Profile Score | Tandem Score | TDA q-value | Volume | Spectrum Matches |
| --- | --- | --- | --- | --- |
| -18.7354 | 30.1936 | 0.0270 | 1.2768e+06 | 1 |

**Adduct Signal Fractions**

- **Unmodified**: 1.2768e+06

### ITPN(N)GSIPNDKPF Fuc 3 Hex 6 HexNAc 7 Neu5Ac 1

**Mass:**  4521.8713

(3.331e-06)

**Charge States:** 3, 4

**Start Position**: 298

**End Position**: 311

| Profile Score | Tandem Score | TDA q-value | Volume | Spectrum Matches |
| --- | --- | --- | --- | --- |
| -19.9298 | 29.9022 | 0.0270 | 1.9420e+06 | 1 |

**Adduct Signal Fractions**

- **Unmodified**: 1.9420e+06

### SEC(C)ITPN(N)GSIPN(D)DKPF Hex 8 HexNAc 2

**Mass:**  3478.3917

(2.845e-04)

**Charge States:** 3

**Start Position**: 295

**End Position**: 311

| Profile Score | Tandem Score | TDA q-value | Volume | Spectrum Matches |
| --- | --- | --- | --- | --- |
| 10.7723 | 29.8865 | 0.0311 | 1.0172e+08 | 4 |

**Adduct Signal Fractions**

- **Unmodified**: 1.0172e+08

### FKIRTGKSSIM(O)RSDAPIGTC(C)SSEC(C)ITPN(N)GSIPNDKPF Hex 8 HexNAc 2

**Mass:**  5788.5781

(3.482e-04)

**Start Position**: 274

**End Position**: 311

| Profile Score | Tandem Score | TDA q-value | Volume | Spectrum Matches |
| --- | --- | --- | --- | --- |
| 0.0000 | 28.7281 | 0.0386 | 0.0000e+00 | 1 |

Adduct:

No Chromatogram Found

### TITNDQIEVTN(N)ATEL Hex 5 HexNAc 4

**Mass:**  3283.4093

(3.650e-06)

**Charge States:** 3

**Start Position**: 44

**End Position**: 59

| Profile Score | Tandem Score | TDA q-value | Volume | Spectrum Matches |
| --- | --- | --- | --- | --- |
| -5.5252 | 28.7015 | 0.0386 | 5.3144e+06 | 1 |

**Adduct Signal Fractions**

- **Unmodified**: 5.3144e+06

### FKIRTGKSSIM(O)RSDAPIGTC(C)SSEC(C)ITPN(N)GSIPN(D)DKPF Hex 8 HexNAc 2

**Mass:**  5787.5475

(1.701e-04)

**Start Position**: 274

**End Position**: 311

| Profile Score | Tandem Score | TDA q-value | Volume | Spectrum Matches |
| --- | --- | --- | --- | --- |
| 0.0000 | 28.2591 | 0.0386 | 0.0000e+00 | 1 |

Adduct:

No Chromatogram Found

### LPGNDN(N)STATL Fuc 1 Hex 5 HexNAc 5

**Mass:**  3073.2405

(-2.787e-06)

**Charge States:** 3

**Start Position**: 19

**End Position**: 30

| Profile Score | Tandem Score | TDA q-value | Volume | Spectrum Matches |
| --- | --- | --- | --- | --- |
| 4.9933 | 28.1847 | 0.0386 | 6.5347e+06 | 1 |

**Adduct Signal Fractions**

- **Unmodified**: 6.5347e+06

### TC(C)KRGSN(N)NSFF Fuc 1 Hex 5 HexNAc 4

**Mass:**  3086.2465

(3.286e-04)

**Start Position**: 154

**End Position**: 165

| Profile Score | Tandem Score | TDA q-value | Volume | Spectrum Matches |
| --- | --- | --- | --- | --- |
| 0.0000 | 27.6170 | 0.0412 | 0.0000e+00 | 1 |

Adduct:

No Chromatogram Found

### SEC(C)ITPN(N)GSIPN(D)DKPF Fuc 2 Hex 5 HexNAc 9

**Mass:**  4705.9172

(2.133e-04)

**Charge States:** 4

**Start Position**: 295

**End Position**: 311

| Profile Score | Tandem Score | TDA q-value | Volume | Spectrum Matches |
| --- | --- | --- | --- | --- |
| -20.6133 | 27.4776 | 0.0412 | 1.1560e+06 | 1 |

**Adduct Signal Fractions**

- **Unmodified**: 1.1560e+06

### ITPN(N)GSIPNDKPF Fuc 3 Hex 10 HexNAc 6

**Mass:**  4675.8977

(1.138e-06)

**Charge States:** 3, 4

**Start Position**: 298

**End Position**: 311

| Profile Score | Tandem Score | TDA q-value | Volume | Spectrum Matches |
| --- | --- | --- | --- | --- |
| -20.3904 | 27.4328 | 0.0412 | 2.5491e+06 | 1 |

**Adduct Signal Fractions**

- **Unmodified**: 2.5491e+06

### LPGNDN(N)STATL Fuc 2 Hex 8 HexNAc 3

**Mass:**  3299.3135

(3.136e-06)

**Charge States:** 3

**Start Position**: 19

**End Position**: 30

| Profile Score | Tandem Score | TDA q-value | Volume | Spectrum Matches |
| --- | --- | --- | --- | --- |
| 12.0222 | 27.2551 | 0.0412 | 1.3449e+08 | 4 |

**Adduct Signal Fractions**

- **Unmodified**: 1.3449e+08

### TIVKPGDILLIN(N)STGNLIAPRGY Fuc 3 Hex 3 HexNAc 5

**Mass:**  4365.0793

(2.237e-04)

**Start Position**: 251

**End Position**: 274

| Profile Score | Tandem Score | TDA q-value | Volume | Spectrum Matches |
| --- | --- | --- | --- | --- |
| 0.0000 | 27.0162 | 0.0412 | 0.0000e+00 | 1 |

Adduct:

No Chromatogram Found

### ITPN(N)GSIPNDKPF Fuc 3 Hex 9 HexNAc 6

**Mass:**  4513.8435

(7.827e-07)

**Charge States:** 4

**Start Position**: 298

**End Position**: 311

| Profile Score | Tandem Score | TDA q-value | Volume | Spectrum Matches |
| --- | --- | --- | --- | --- |
| -20.5207 | 26.4948 | 0.0412 | 2.2307e+06 | 1 |

**Adduct Signal Fractions**

- **Unmodified**: 2.2307e+06

### TIVKPGDILLIN(N)STGN(D)LIAPRGY Hex 10 HexNAc 2

**Mass:**  4453.0573

(4.485e-04)

**Start Position**: 251

**End Position**: 274

| Profile Score | Tandem Score | TDA q-value | Volume | Spectrum Matches |
| --- | --- | --- | --- | --- |
| 0.0000 | 25.6129 | 0.0437 | 0.0000e+00 | 1 |

Adduct:

No Chromatogram Found

### ESESKYPVLN(N)VTM(O)PNN(D)GKFDKLY Fuc 1 Hex 8 HexNAc 4

**Mass:**  4944.1281

(2.064e-04)

**Charge States:** 4

**Start Position**: 172

**End Position**: 195

| Profile Score | Tandem Score | TDA q-value | Volume | Spectrum Matches |
| --- | --- | --- | --- | --- |
| 11.2993 | 24.9738 | 0.0472 | 5.9433e+07 | 4 |

**Adduct Signal Fractions**

- **Unmodified**: 5.9433e+07

### iav|ADJ41819|A/Phil/2-MA/1982\_NA

**>iav|ADJ41819|A/Phil/2-MA/1982\_NA**  
 `MNPNQKIITIGSVSLTIATICFLMQIAILVTTVTLHFKQYECSSPPNNQVVPCEPIIIERNITEIVYLTNTTIEKEICPK  
LVEYRNWSKPQCKITGFAPFSKDNSIRLSAGGDIWVTREPYVSCDPGKCYQFALGQGTTLDNKHSNDTIHDRTPYRTLLM  
NELGVPFHLGTRQVCIAWSSSSCHDGKAWLHVCITGYDKNATASFIYDGRLVDSIGSWSKNILRTQESECVCINGTCTVV  
MTDGSASERADTKILFIEEGKIVHISPLSGSAQHVEECSCYPRYPGVRCVCRDNWKGSNRPVVDINVKDYSIVSSYVCSG  
LVGDTPRKNDRSSSSYCRNPNNEKGNHGVKGWAFDDGNDVWMGRTISEESRSGYETFKVIGGWSTPNSKLQINRQVIVDS  
GNRSGYSGIFSVEGKSCINRCFYVELIRGREQETRVWWTSNSIVVFCGTSGTYGTGSWPDGADINLMPI`

| Site | Type | Count |
| --- | --- | --- |
| 402 | N-linked | 4 |
| 146 | N-linked | 4 |

| Mass | Sequence | MS_2_ Score | `q`-value | MS_1_ Score | Signal | Protein Coordinates | Tandem Scans | Start Time | Apex Time | End Time |
| --- | --- | --- | --- | --- | --- | --- | --- | --- | --- | --- |
| 3183.3999 | QINRQVIVDSGN(N)RSGY Hex 6 HexNAc 2 | 150.269 | 0.0 | -6.953 | 3.071e+07 | 390 - 406 | 6 | 21.24 | 29.84 | 30.18 |
| 3345.4477 | QINRQVIVDSGN(N)RSGY Hex 7 HexNAc 2 | 133.436 | 0.0 | -19.924 | 2.648e+06 | 390 - 406 | 1 | 21.24 | 21.24 | 21.24 |
| 3910.7891 | STPNSKLQINRQVIVDSGN(N)RSGY Hex 6 HexNAc 2 | 90.174 | 0.0 | -18.788 | 9.414e+06 | 383 - 406 | 1 | 28.32 | 28.32 | 28.49 |
| 3703.4469 | DN(D)KHSN(N)DTIHDRTPY Fuc 1 Hex 7 HexNAc 3 | 81.413 | 0.0 | 0.000 | 0.000e+00 | 140 - 155 | 1 | - | - | - |
| 4442.8485 | ALGQGTTLDNKHSN(N)DTIHDRTPY Fuc 1 Hex 7 HexNAc 3 | 48.537 | 0.011 | 10.879 | 1.159e+07 | 132 - 155 | 2 | 21.90 | 22.07 | 22.58 |
| 4734.9686 | ALGQGTTLDN(D)KHSN(N)DTIHDRTPY Fuc 1 Hex 7 HexNAc 3 Neu5Ac 1 | 29.812 | 0.031 | -21.445 | 5.836e+06 | 132 - 155 | 1 | 21.74 | 21.74 | 21.90 |
| 3906.7327 | QINRQVIVDSGN(N)RSGY Fuc 3 Hex 4 HexNAc 5 | 27.634 | 0.041 | 9.713 | 1.521e+07 | 390 - 406 | 3 | 30.01 | 30.18 | 30.86 |
| 4588.9111 | ALGQGTTLDNKHSN(N)DTIHDRTPY Fuc 2 Hex 7 HexNAc 3 | 26.178 | 0.044 | -18.972 | 6.352e+06 | 132 - 155 | 1 | 21.90 | 21.90 | 22.07 |

### QINRQVIVDSGN(N)RSGY Hex 6 HexNAc 2

**Mass:**  3183.4015

(2.294e-07)

**Charge States:** 3

**Start Position**: 391

**End Position**: 407

| Profile Score | Tandem Score | TDA q-value | Volume | Spectrum Matches |
| --- | --- | --- | --- | --- |
| -6.9527 | 150.2694 | 0.0000 | 3.0710e+07 | 6 |

**Adduct Signal Fractions**

- **Unmodified**: 3.0710e+07

### QINRQVIVDSGN(N)RSGY Hex 7 HexNAc 2

**Mass:**  3345.4477

(3.828e-07)

**Charge States:** 3

**Start Position**: 391

**End Position**: 407

| Profile Score | Tandem Score | TDA q-value | Volume | Spectrum Matches |
| --- | --- | --- | --- | --- |
| -19.9236 | 133.4364 | 0.0000 | 2.6477e+06 | 1 |

**Adduct Signal Fractions**

- **Unmodified**: 2.6477e+06

### STPNSKLQINRQVIVDSGN(N)RSGY Hex 6 HexNAc 2

**Mass:**  3910.7897

(2.473e-06)

**Charge States:** 4

**Start Position**: 384

**End Position**: 407

| Profile Score | Tandem Score | TDA q-value | Volume | Spectrum Matches |
| --- | --- | --- | --- | --- |
| -18.7884 | 90.1745 | 0.0000 | 9.4143e+06 | 1 |

**Adduct Signal Fractions**

- **Unmodified**: 9.4143e+06

### DN(D)KHSN(N)DTIHDRTPY Fuc 1 Hex 7 HexNAc 3

**Mass:**  3703.4469

(5.302e-04)

**Start Position**: 141

**End Position**: 156

| Profile Score | Tandem Score | TDA q-value | Volume | Spectrum Matches |
| --- | --- | --- | --- | --- |
| 0.0000 | 81.4133 | 0.0000 | 0.0000e+00 | 1 |

Adduct:

No Chromatogram Found

### ALGQGTTLDNKHSN(N)DTIHDRTPY Fuc 1 Hex 7 HexNAc 3

**Mass:**  4442.8474

(-8.806e-06)

**Charge States:** 4

**Start Position**: 133

**End Position**: 156

| Profile Score | Tandem Score | TDA q-value | Volume | Spectrum Matches |
| --- | --- | --- | --- | --- |
| 10.8790 | 48.5373 | 0.0112 | 1.1591e+07 | 2 |

**Adduct Signal Fractions**

- **Unmodified**: 1.1591e+07

### ALGQGTTLDN(D)KHSN(N)DTIHDRTPY Fuc 1 Hex 7 HexNAc 3 Neu5Ac 1

**Mass:**  4734.9645

(2.075e-04)

**Charge States:** 4

**Start Position**: 133

**End Position**: 156

| Profile Score | Tandem Score | TDA q-value | Volume | Spectrum Matches |
| --- | --- | --- | --- | --- |
| -21.4448 | 29.8125 | 0.0311 | 5.8357e+06 | 1 |

**Adduct Signal Fractions**

- **Unmodified**: 5.8357e+06

### QINRQVIVDSGN(N)RSGY Fuc 3 Hex 4 HexNAc 5

**Mass:**  3906.7315

(6.448e-06)

**Charge States:** 5

**Start Position**: 391

**End Position**: 407

| Profile Score | Tandem Score | TDA q-value | Volume | Spectrum Matches |
| --- | --- | --- | --- | --- |
| 9.7133 | 27.6335 | 0.0412 | 1.5208e+07 | 3 |

**Adduct Signal Fractions**

- **Unmodified**: 1.5208e+07

### ALGQGTTLDNKHSN(N)DTIHDRTPY Fuc 2 Hex 7 HexNAc 3

**Mass:**  4588.9118

(-7.142e-06)

**Charge States:** 4

**Start Position**: 133

**End Position**: 156

| Profile Score | Tandem Score | TDA q-value | Volume | Spectrum Matches |
| --- | --- | --- | --- | --- |
| -18.9717 | 26.1775 | 0.0437 | 6.3524e+06 | 1 |

**Adduct Signal Fractions**

- **Unmodified**: 6.3524e+06

### tr|E1C8K3|E1C8K3\_CHICK

**>tr|E1C8K3|E1C8K3\_CHICK**  
 `ALWWRACIEEEGKPVIGVYSNFDPTPDAQTLYKAMKGLGTDEQAIIEVLTKRSNKQRQEIAKSFKAQFGKDLIDSLRSEL  
SGNFERLIVALMYPPYKYEAKELYDAMKGVGTSEDVIIEILASRTKAQIKEIIKAYKEDYGSDLEHDIASETSGYFKQIL  
VCLLQGERDNASLYVDTALARQDAETLFSAGEKIKGTDEIQFITILCKRSATHLLKGMKQSIGRAIDVKAWVVRNNHFNL  
CTRNIRQYFAERLYHALKGAGTDDGTLIRVIVSRNEVDLNLIKDEFRRIAGQPLSSMIVDDTSGDYKTALLNLCGSD`

| Site | Type | Count |
| --- | --- | --- |
| 170 | N-linked | 2 |

| Mass | Sequence | MS_2_ Score | `q`-value | MS_1_ Score | Signal | Protein Coordinates | Tandem Scans | Start Time | Apex Time | End Time |
| --- | --- | --- | --- | --- | --- | --- | --- | --- | --- | --- |
| 2888.2015 | LQGERDN(N)ASL Hex 6 HexNAc 4 | 28.767 | 0.036 | 0.000 | 0.000e+00 | 163 - 173 | 1 | - | - | - |
| 4331.7464 | LQGERDN(N)ASLY Fuc 1 Hex 8 HexNAc 8 | 26.972 | 0.041 | -18.034 | 1.038e+07 | 163 - 174 | 1 | 25.96 | 25.96 | 26.13 |

### LQGERDN(N)ASL Hex 6 HexNAc 4

**Mass:**  2888.2015

(7.018e-04)

**Start Position**: 164

**End Position**: 174

| Profile Score | Tandem Score | TDA q-value | Volume | Spectrum Matches |
| --- | --- | --- | --- | --- |
| 0.0000 | 28.7667 | 0.0356 | 0.0000e+00 | 1 |

Adduct:

No Chromatogram Found

### LQGERDN(N)ASLY Fuc 1 Hex 8 HexNAc 8

**Mass:**  4331.7448

(5.717e-06)

**Charge States:** 4

**Start Position**: 164

**End Position**: 175

| Profile Score | Tandem Score | TDA q-value | Volume | Spectrum Matches |
| --- | --- | --- | --- | --- |
| -18.0337 | 26.9722 | 0.0412 | 1.0384e+07 | 1 |

**Adduct Signal Fractions**

- **Unmodified**: 1.0384e+07

### sp|Q90734|ACTN4\_CHICK

**>sp|Q90734|ACTN4\_CHICK**  
 `MVDYHSAGQPYPYGGNGPGPNGDYMAQEDDWDRDLLLDPAWEKQQRKTFTAWCNSHLRKAGTQIENIDEDFRDGLKLMLL  
LEVISGERLPKPERGKMRVHKINNVNKALDFIASKGVNVVSIGAEEIVDGNAKMTLGMIWTIILRFAIQDISVEETSAKE  
GLLLWCQRKTAPYKNVNVQNFHISWKDGLAFNALIHRHRPELIEYDKLRKDDPVTNLNNAFEVAEKYLDIPKMLDAEDIV  
NTARPDEKAIMTYVSSFYHAFSGAQKAETAANRICKVLAVNQENEHLMEDYEKLASDLLEWIRRTIPWLEDRSPQKTIQE  
MQQKLEDFRDYRRVHKPPKVQEKCQLEINFNTLQTKLRLSNRPAFMPSEGRMVSDINTGWQHLEQAEKGYEEWLLNEIRR  
LEPLDHLAEKFRQKASIHEAWTEGKEAMLKQKDYETATLSDIKALIRKHEAFESDLAAHQDRVEQIAAIAQELNELDYYD  
SPSVNARCQKICDQWDVLGSLTHSRREALEKTEKQLETIDELHLEYAKRAAPFNNWMESAMEDLQDMFIVHTIEEIEGLI  
AAHDQFKATLPDADREREAILGIQREAQRIADLHSIKLSGNNPYTSVTPQVINSKWERVQQLVPTRDRALQDEQSRQQCN  
ERLRRQFAGQANIVGPWMQTKMEEIGRISIEMHGTLEDQLQHLKHYEQSIVDYKPNLELLEHEHQLVEEALIFDNKHTNY  
TMEHIRVGWEQLLTTIARTINEVENQILTRDAKGISQEQMQEFRASFNHFDKDHCGALGPEEFKACLISLGYDVENDRQG  
DAEFNRIMSLVDPNGSGSVTFQAFIDFMSRETTDTDTADQVIASFKVLAGDKNYITAEELRRELPPEQAEYCIARMAPYR  
GPDAAPGALDYKSFSTALYGESDL`

| Site | Type | Count |
| --- | --- | --- |
| 814 | N-linked | 1 |

| Mass | Sequence | MS_2_ Score | `q`-value | MS_1_ Score | Signal | Protein Coordinates | Tandem Scans | Start Time | Apex Time | End Time |
| --- | --- | --- | --- | --- | --- | --- | --- | --- | --- | --- |
| 4606.8090 | VDPN(N)GSGSVTF Fuc 1 Hex 6 HexNAc 9 Neu5Ac 2 | 85.113 | 0.0 | -20.018 | 1.396e+07 | 810 - 821 | 2 | 39.06 | 39.06 | 39.23 |

### VDPN(N)GSGSVTF Fuc 1 Hex 6 HexNAc 9 Neu5Ac 2

**Mass:**  4606.8073

(7.417e-06)

**Charge States:** 4, 5

**Start Position**: 811

**End Position**: 822

| Profile Score | Tandem Score | TDA q-value | Volume | Spectrum Matches |
| --- | --- | --- | --- | --- |
| -20.0182 | 85.1126 | 0.0000 | 1.3956e+07 | 2 |

**Adduct Signal Fractions**

- **Unmodified**: 1.3956e+07
