# Supplementary material for: Measuring Site-specific Glycosylation Similarity between Influenza a Virus Variants with Statistical Certainty
Source: Mol Cell Proteomics. 2020 Nov 25;19(9):1533–45. doi: 10.1074/mcp.RA120.002031 (PMC8143645; doi:10.1074/mcp.RA120.002031)
Supplement: Supplementary file 2 [file mmc2.zip › mmc2/159250_2_supp_555289_qcjklv.zip!/20190326_Phil82gal_021119_chymo_HILIC_DDA_1ug_02_glycopeptides.html]

## 20190326\_Phil82gal\_021119\_chymo\_HILIC\_DDA\_1ug\_02 @ GlycopeptideHypothesis-f7921752f78c47e4989f4648aa55291c

**MS^1^ Mass Accuracy:** 10.0 PPM  
**Grouping Tolerance:** 15.0 PPM  
**MS^n^ Mass Accuracy:** 20.0 PPM  
**Minimum `q`-Value For Clustering:** 0.05

**Glycopeptide Hypothesis:**
GlycopeptideHypothesis-f7921752f78c47e4989f4648aa55291c  
**Sample Searched:**
20190326\_Phil82gal\_021119\_chymo\_HILIC\_DDA\_1ug\_02

| Protein | Matched Glycopeptides |
| --- | --- |
| iav\|AFG99160\|A/Phil/2/1982\_HA | 121 |
| iav\|ADJ41819\|A/Phil/2-MA/1982\_NA | 13 |

### iav|AFG99160|A/Phil/2/1982\_HA

**>iav|AFG99160|A/Phil/2/1982\_HA**  
 `MKTIIALSYMFCLVFAQNLPGNDNSTATLCLGHHAVPNGTLVKTITNDQIEVTNATELVQSSSTGRICDSPHRILDGKNC  
TLIDALLGDPHCDGFQNEKWDLFVERSKAFSNCYPYDVPDYASLRSLVASSGTLEFINEGFNWTGVTQSGGSSTCKRGSN  
NSFFSRLNWLYESESKYPVLNVTMPNNGKFDKLYIWGIHHPSTDKEQTNLYIRASGRVTVSTKRSQQTVIPNIGSRPWVR  
GLSSRISIYWTIVKPGDILLINSTGNLIAPRGYFKIRTGKSSIMRSDAPIGTCSSECITPNGSIPNDKPFQNVNKITYGA  
CPRYVKQNTLKLATGMRNVPEKQTRGIFGAIAGFIENGWEGMVDGWYGFRHQNSEGTGQAADLKSTQAAIDQINGKLNRV  
IEKTNEKFHQIEKEFSEVEGRIQDLEKYVEDTKIDLWSYNAELLVALENQHTIDLTDSEMNKLFEKTRKQLRENAEDMGN  
GCFKIYHKCDNACIGSIRNGTYDHDVYRDEALNNRFQIKGVELKSGYKDWILWISFAISCFLLCVVLLGFIMWACQKGNI  
RCNICI`

| Site | Type | Count |
| --- | --- | --- |
| 262 | N-linked | 42 |
| 301 | N-linked | 34 |
| 499 | N-linked | 6 |
| 181 | N-linked | 10 |
| 54 | N-linked | 7 |
| 24 | N-linked | 13 |
| 160 | N-linked | 9 |

| Mass | Sequence | MS_2_ Score | `q`-value | MS_1_ Score | Signal | Protein Coordinates | Tandem Scans | Start Time | Apex Time | End Time |
| --- | --- | --- | --- | --- | --- | --- | --- | --- | --- | --- |
| 2695.0880 | LIN(N)STGNL Hex 9 HexNAc 2 | 180.270 | 0.0 | -3.331 | 1.204e+07 | 259 - 267 | 2 | 22.88 | 22.88 | 23.22 |
| 3132.3645 | LIN(N)STGNLIAPR Hex 9 HexNAc 2 | 172.477 | 0.0 | 13.008 | 4.049e+07 | 259 - 271 | 4 | 28.11 | 28.11 | 28.78 |
| 4068.9285 | TIVKPGDILLIN(N)STGNLIAPR Hex 9 HexNAc 2 | 171.228 | 0.0 | 14.590 | 2.181e+08 | 250 - 271 | 13 | 49.55 | 51.43 | 52.28 |
| 2546.0673 | LPGNDN(N)STATL Fuc 1 Hex 3 HexNAc 4 | 170.211 | 0.0 | 14.174 | 5.526e+07 | 18 - 29 | 4 | 23.22 | 23.73 | 24.57 |
| 3352.4477 | LIN(N)STGNLIAPRGY Hex 9 HexNAc 2 | 160.686 | 0.0 | -4.542 | 3.451e+07 | 259 - 273 | 1 | 34.62 | 34.62 | 34.95 |
| 3159.4259 | TIVKPGDILLIN(N) Hex 9 HexNAc 2 | 158.418 | 0.0 | 13.008 | 9.636e+07 | 250 - 262 | 8 | 43.26 | 44.79 | 45.63 |
| 4289.0130 | TIVKPGDILLIN(N)STGNLIAPRGY Hex 9 HexNAc 2 | 155.473 | 0.0 | 14.972 | 7.816e+08 | 250 - 273 | 36 | 53.30 | 54.27 | 58.54 |
| 2749.1453 | LPGNDN(N)STATL Fuc 1 Hex 3 HexNAc 5 | 154.322 | 0.0 | 10.738 | 1.365e+07 | 18 - 29 | 3 | 23.22 | 23.73 | 24.24 |
| 2400.0080 | LPGNDN(N)STATL Hex 3 HexNAc 4 | 153.694 | 0.0 | 12.665 | 1.628e+07 | 18 - 29 | 2 | 23.22 | 23.56 | 23.90 |
| 3386.4363 | IN(N)STGNLIAPRGYF Hex 9 HexNAc 2 | 149.450 | 0.0 | -17.268 | 5.987e+06 | 260 - 274 | 1 | 41.05 | 41.22 | 41.22 |
| 4451.0831 | TIVKPGDILLIN(N)STGNLIAPRGY Hex 10 HexNAc 2 | 147.843 | 0.0 | 13.116 | 7.744e+07 | 250 - 273 | 14 | 53.93 | 54.10 | 62.12 |
| 2317.9556 | LPGNDN(N)STATL Hex 5 HexNAc 2 | 147.216 | 0.0 | 11.734 | 9.285e+06 | 18 - 29 | 3 | 22.88 | 23.39 | 23.56 |
| 3101.2996 | ITPN(N)GSIPNDKPF Hex 8 HexNAc 2 | 146.604 | 0.0 | -4.602 | 1.073e+07 | 297 - 310 | 2 | 29.29 | 29.46 | 29.80 |
| 3321.4783 | TIVKPGDILLIN(N) Hex 10 HexNAc 2 | 146.343 | 0.0 | -5.059 | 5.230e+06 | 250 - 262 | 2 | 44.45 | 44.62 | 44.96 |
| 4306.0389 | TIVKPGDILLIN(N)STGNLIAPRGY Fuc 2 Hex 3 HexNAc 4 Neu5Ac 1 | 145.416 | 0.0 | 13.845 | 8.408e+07 | 250 - 273 | 5 | 53.82 | 54.27 | 54.95 |
| 3631.6539 | TIVKPGDILLIN(N)STGNL Hex 9 HexNAc 2 | 145.104 | 0.0 | 13.988 | 8.741e+07 | 250 - 267 | 5 | 50.74 | 50.91 | 51.94 |
| 3518.5709 | TIVKPGDILLIN(N)STGN Hex 9 HexNAc 2 | 144.194 | 0.0 | 12.894 | 3.508e+07 | 250 - 266 | 4 | 42.58 | 42.75 | 43.26 |
| 3176.4521 | TIVKPGDILLIN(N) Fuc 2 Hex 3 HexNAc 4 Neu5Ac 1 | 143.355 | 0.0 | 9.907 | 1.427e+07 | 250 - 262 | 4 | 43.43 | 44.79 | 44.96 |
| 3499.5169 | LIN(N)STGNLIAPRGYF Hex 9 HexNAc 2 | 143.180 | 0.0 | -18.787 | 2.399e+07 | 259 - 274 | 1 | 45.13 | 45.30 | 45.30 |
| 3263.3722 | ITPN(N)GSIPNDKPF Hex 9 HexNAc 2 | 141.080 | 0.0 | -4.694 | 1.183e+07 | 297 - 310 | 2 | 29.29 | 29.46 | 29.63 |
| 2854.1775 | LPGNDN(N)STATL Fuc 2 Hex 4 HexNAc 4 | 139.146 | 0.0 | 11.580 | 1.119e+07 | 18 - 29 | 2 | 22.88 | 23.22 | 23.56 |
| 4230.9814 | TIVKPGDILLIN(N)STGNLIAPR Hex 10 HexNAc 2 | 138.194 | 0.0 | 13.415 | 1.907e+07 | 250 - 271 | 3 | 51.26 | 51.43 | 51.94 |
| 4126.9538 | TIVKPGDILLIN(N)STGNLIAPRGY Hex 8 HexNAc 2 | 136.935 | 0.0 | 12.772 | 6.172e+07 | 250 - 273 | 9 | 53.65 | 54.27 | 55.30 |
| 3906.8755 | TIVKPGDILLIN(N)STGNLIAPR Hex 8 HexNAc 2 | 134.522 | 0.0 | -4.629 | 1.421e+07 | 250 - 271 | 4 | 51.43 | 51.60 | 51.94 |
| 4085.9558 | TIVKPGDILLIN(N)STGNLIAPR Fuc 2 Hex 3 HexNAc 4 Neu5Ac 1 | 133.423 | 0.0 | 12.781 | 1.881e+07 | 250 - 271 | 2 | 51.26 | 51.43 | 51.94 |
| 3239.3633 | IN(N)STGNLIAPRGY Hex 9 HexNAc 2 | 128.918 | 0.0 | -17.274 | 1.005e+07 | 260 - 273 | 1 | 29.12 | 29.29 | 29.29 |
| 3648.6793 | TIVKPGDILLIN(N)STGNL Fuc 2 Hex 3 HexNAc 4 Neu5Ac 1 | 127.178 | 0.0 | -4.296 | 1.405e+07 | 250 - 267 | 2 | 50.74 | 50.91 | 51.26 |
| 3516.5439 | LIN(N)STGNLIAPRGYF Fuc 2 Hex 3 HexNAc 4 Neu5Ac 1 | 124.469 | 0.0 | -19.504 | 3.766e+06 | 259 - 274 | 1 | 45.13 | 45.30 | 45.30 |
| 3537.5941 | TIVKPGDILLIN(N)STGN Fuc 2 Hex 3 HexNAc 4 Neu5Ac 1 | 124.204 | 0.0 | 0.000 | 0.000e+00 | 250 - 266 | 1 | - | - | - |
| 2704.1204 | KRGSN(N)N(D)SFF Fuc 1 Hex 3 HexNAc 5 | 121.405 | 0.0 | -4.722 | 4.347e+07 | 155 - 164 | 8 | 18.20 | 18.53 | 18.53 |
| 2703.1285 | KRGSN(N)NSFF Fuc 1 Hex 3 HexNAc 5 | 119.521 | 0.0 | -2.571 | 6.441e+07 | 155 - 164 | 1 | 18.70 | 18.70 | 19.03 |
| 3564.4342 | SSEC(C)ITPN(N)GSIPNDKPF Hex 8 HexNAc 2 | 118.089 | 0.0 | -5.609 | 1.693e+07 | 293 - 310 | 1 | 33.17 | 33.33 | 33.49 |
| 3191.2945 | SIRN(N)GTYDHDVY Fuc 2 Hex 4 HexNAc 4 | 117.016 | 0.0 | -20.677 | 2.147e+06 | 495 - 507 | 1 | 16.90 | 16.90 | 16.90 |
| 2883.1896 | SIRN(N)GTYDHDVY Fuc 1 Hex 3 HexNAc 4 | 116.747 | 0.0 | -21.408 | 3.517e+06 | 495 - 507 | 1 | 17.23 | 17.35 | 17.35 |
| 4521.8726 | RSDAPIGTC(C)SSEC(C)ITPN(N)GSIPNDKPF Hex 8 HexNAc 2 | 116.318 | 0.0 | 12.245 | 7.419e+07 | 284 - 310 | 4 | 35.78 | 35.94 | 36.62 |
| 2500.0499 | KRGSN(N)NSFF Fuc 1 Hex 3 HexNAc 4 | 115.313 | 0.0 | -19.420 | 1.061e+07 | 155 - 164 | 1 | 18.37 | 18.53 | 18.53 |
| 3011.2395 | KRGSN(N)NSFF Fuc 2 Hex 4 HexNAc 5 | 107.635 | 0.0 | -4.186 | 2.316e+07 | 155 - 164 | 2 | 17.87 | 17.87 | 18.37 |
| 2480.0084 | LPGNDN(N)STATL Hex 6 HexNAc 2 | 107.495 | 0.0 | 0.000 | 0.000e+00 | 18 - 29 | 1 | - | - | - |
| 3361.4071 | IGSIRN(N)GTYDHDVY Fuc 2 Hex 4 HexNAc 4 | 101.108 | 0.0 | -19.638 | 3.547e+06 | 493 - 507 | 1 | 22.04 | 22.21 | 22.21 |
| 2788.1655 | LPGNDN(N)STATL Fuc 1 Hex 7 HexNAc 2 | 99.406 | 0.0 | 11.879 | 3.354e+08 | 18 - 29 | 5 | 33.82 | 34.13 | 34.95 |
| 3162.2910 | LPGNDN(N)STATL Fuc 3 Hex 5 HexNAc 4 | 99.266 | 0.0 | -19.996 | 2.081e+06 | 18 - 29 | 1 | 22.38 | 22.55 | 22.55 |
| 2808.1582 | KRGSN(N)NSFF Fuc 2 Hex 4 HexNAc 4 | 98.875 | 0.0 | -5.255 | 1.069e+07 | 155 - 164 | 2 | 17.59 | 17.59 | 18.03 |
| 3053.2933 | IGSIRN(N)GTYDHDVY Fuc 1 Hex 3 HexNAc 4 | 97.940 | 0.0 | -17.351 | 4.789e+06 | 493 - 507 | 1 | 22.38 | 22.38 | 22.55 |
| 3478.4101 | SEC(C)ITPN(N)GSIPNDKPF Hex 8 HexNAc 2 | 94.415 | 0.0 | 0.000 | 0.000e+00 | 294 - 310 | 1 | - | - | - |
| 4359.8204 | RSDAPIGTC(C)SSEC(C)ITPN(N)GSIPNDKPF Hex 7 HexNAc 2 | 92.160 | 0.0 | -4.966 | 1.812e+07 | 284 - 310 | 3 | 35.94 | 36.10 | 36.27 |
| 3726.4880 | SSEC(C)ITPN(N)GSIPNDKPF Hex 9 HexNAc 2 | 91.478 | 0.0 | -20.165 | 1.096e+07 | 293 - 310 | 1 | 33.17 | 33.33 | 33.33 |
| 3330.3067 | RGSN(N)NSFF Fuc 2 Hex 3 HexNAc 8 | 90.317 | 0.0 | -5.532 | 7.112e+06 | 156 - 164 | 2 | 21.88 | 22.04 | 22.38 |
| 4683.9260 | RSDAPIGTC(C)SSEC(C)ITPN(N)GSIPNDKPF Hex 9 HexNAc 2 | 89.447 | 0.0 | 11.688 | 6.033e+07 | 284 - 310 | 3 | 35.78 | 35.94 | 36.62 |
| 3039.3001 | TITNDQIEVTN(N)ATEL Hex 6 HexNAc 2 | 83.354 | 0.0 | -3.251 | 1.870e+07 | 43 - 58 | 2 | 38.49 | 38.66 | 38.83 |
| 4522.8632 | RSDAPIGTC(C)SSEC(C)ITPN(N)GSIPN(D)DKPF Hex 8 HexNAc 2 | 74.576 | 0.0 | -5.962 | 2.817e+07 | 284 - 310 | 3 | 36.79 | 36.96 | 37.30 |
| 4148.7629 | LYESESKYPVLN(N)VTM(O)PNN(D)GKF Hex 8 HexNAc 2 | 69.455 | 0.0 | 10.819 | 1.762e+07 | 169 - 190 | 3 | 39.68 | 40.02 | 40.37 |
| 4553.9412 | ESESKYPVLN(N)VTM(O)PNN(D)GKFDKLY Hex 9 HexNAc 2 | 67.858 | 0.0 | 11.098 | 3.249e+07 | 171 - 194 | 3 | 40.71 | 41.05 | 41.39 |
| 5801.5595 | KIRTGKSSIM(O)RSDAPIGTC(C)SSEC(C)ITPN(N)GSIPNDKPF Hex 9 HexNAc 2 | 66.796 | 0.0 | -5.118 | 3.355e+07 | 274 - 310 | 3 | 31.48 | 31.65 | 31.99 |
| 4624.8893 | ITPN(N)GSIPNDKPF Hex 7 HexNAc 6 Neu5Ac 3 | 58.831 | 0.0 | 14.112 | 2.668e+07 | 297 - 310 | 4 | 37.47 | 37.98 | 38.32 |
| 5639.5041 | KIRTGKSSIM(O)RSDAPIGTC(C)SSEC(C)ITPN(N)GSIPNDKPF Hex 8 HexNAc 2 | 57.524 | 0.0 | -4.806 | 3.941e+07 | 274 - 310 | 3 | 31.48 | 31.65 | 31.99 |
| 4310.8136 | LYESESKYPVLN(N)VTM(O)PNN(D)GKF Hex 9 HexNAc 2 | 56.502 | 0.0 | -6.276 | 2.432e+07 | 169 - 190 | 3 | 39.68 | 40.02 | 40.20 |
| 3469.6001 | TIVKPGDILLIN(N)STGNL Hex 8 HexNAc 2 | 56.411 | 0.0 | -4.294 | 5.630e+06 | 250 - 267 | 3 | 50.74 | 50.91 | 51.26 |
| 3201.3513 | TITNDQIEVTN(N)ATEL Hex 7 HexNAc 2 | 52.534 | 0.0 | -17.304 | 6.344e+06 | 43 - 58 | 1 | 38.32 | 38.49 | 38.49 |
| 3535.5943 | TIVKPGDILLIN(N)STGN Fuc 2 Hex 3 HexNAc 4 Neu5Ac 1 | 52.209 | 0.0 | -18.606 | 5.117e+06 | 250 - 266 | 1 | 42.75 | 42.75 | 42.92 |
| 4391.8879 | ESESKYPVLN(N)VTM(O)PNN(D)GKFDKLY Hex 8 HexNAc 2 | 51.252 | 0.0 | 9.768 | 2.438e+07 | 171 - 194 | 4 | 40.71 | 41.05 | 41.56 |
| 4684.9162 | RSDAPIGTC(C)SSEC(C)ITPN(N)GSIPN(D)DKPF Hex 9 HexNAc 2 | 51.246 | 0.0 | -6.012 | 2.129e+07 | 284 - 310 | 4 | 36.79 | 36.96 | 37.30 |
| 3863.6006 | LYESESKYPVLN(N)VTM(O)PN Hex 9 HexNAc 2 | 49.873 | 0.0 | -5.283 | 1.290e+07 | 169 - 186 | 1 | 35.62 | 35.78 | 35.94 |
| 3793.7074 | TIVKPGDILLIN(N)STGNL Hex 10 HexNAc 2 | 48.937 | 0.0 | -4.613 | 8.376e+06 | 250 - 267 | 3 | 50.57 | 50.74 | 51.09 |
| 4605.8215 | SSEC(C)ITPN(N)GSIPN(D)DKPF Fuc 1 Hex 6 HexNAc 8 | 47.274 | 0.038 | -6.737 | 1.287e+07 | 293 - 310 | 2 | 37.81 | 37.98 | 38.15 |
| 3964.9040 | TIVKPGDILLIN(N)STGNLIAPRGY Hex 7 HexNAc 2 | 47.015 | 0.0 | -19.897 | 1.912e+06 | 250 - 273 | 2 | 54.44 | 54.44 | 54.44 |
| 4443.7694 | SSEC(C)ITPN(N)GSIPN(D)DKPF Fuc 1 Hex 5 HexNAc 8 | 46.713 | 0.048 | -6.712 | 1.566e+07 | 293 - 310 | 4 | 37.81 | 37.98 | 38.15 |
| 3256.3779 | IGSIRN(N)GTYDHDVY Fuc 1 Hex 3 HexNAc 5 | 45.643 | 0.0 | -20.440 | 2.389e+06 | 493 - 507 | 1 | 22.38 | 22.55 | 22.55 |
| 3701.5506 | LYESESKYPVLN(N)VTM(O)PN Hex 8 HexNAc 2 | 44.851 | 0.0 | 5.995 | 8.177e+06 | 169 - 186 | 1 | 35.78 | 35.78 | 36.45 |
| 3864.6073 | LYESESKYPVLN(N)VTM(O)PN Hex 9 HexNAc 2 | 44.782 | 0.0 | 0.000 | 0.000e+00 | 169 - 186 | 1 | - | - | - |
| 4127.9645 | TIVKPGDILLIN(N)STGN(D)LIAPRGY Hex 8 HexNAc 2 | 44.168 | 0.0 | -19.126 | 7.376e+06 | 250 - 273 | 3 | 54.10 | 54.10 | 54.27 |
| 3680.6218 | TIVKPGDILLIN(N)STGN Hex 10 HexNAc 2 | 42.525 | 0.0 | -18.954 | 2.895e+06 | 250 - 266 | 1 | 42.58 | 42.58 | 42.75 |
| 3388.4342 | TITNDQIEVTN(N)ATEL Fuc 1 Hex 6 HexNAc 3 | 42.360 | 0.0 | -20.031 | 1.967e+06 | 43 - 58 | 2 | 38.32 | 38.32 | 38.49 |
| 3938.6105 | HKC(C)DNAC(C)IGSIRN(N)GTYDHDVY Fuc 1 Hex 3 HexNAc 4 | 42.099 | 0.0 | -20.017 | 7.643e+06 | 486 - 507 | 2 | 23.56 | 23.56 | 23.73 |
| 4602.8541 | ITPN(N)GSIPNDKPF Fuc 3 Hex 9 HexNAc 5 Neu5Ac 1 | 41.001 | 0.0 | 0.000 | 0.000e+00 | 297 - 310 | 1 | - | - | - |
| 2940.2473 | ITPN(N)GSIPNDKPF Hex 7 HexNAc 2 | 39.987 | 0.0 | 0.000 | 0.000e+00 | 297 - 310 | 1 | - | - | - |
| 4467.0945 | TIVKPGDILLIN(N)STGNLIAPRGY Hex 4 HexNAc 4 Neu5Ac 2 | 39.186 | 0.0 | -19.351 | 3.277e+06 | 250 - 273 | 2 | 53.93 | 54.27 | 54.27 |
| 4290.0082 | TIVKPGDILLIN(N)STGN(D)LIAPRGY Hex 9 HexNAc 2 | 39.156 | 0.0 | 7.989 | 1.918e+07 | 250 - 273 | 6 | 55.12 | 55.12 | 55.88 |
| 3565.4200 | SSEC(C)ITPN(N)GSIPN(D)DKPF Hex 8 HexNAc 2 | 38.326 | 0.0 | -6.404 | 9.818e+06 | 293 - 310 | 1 | 34.45 | 34.45 | 34.78 |
| 4831.0756 | ESESKYPVLN(N)VTMPNNGKFDKLY Fuc 2 Hex 4 HexNAc 6 | 37.681 | 0.0 | 11.320 | 1.250e+08 | 171 - 194 | 8 | 44.11 | 44.11 | 45.47 |
| 2877.2491 | TITNDQIEVTN(N)ATEL Hex 5 HexNAc 2 | 35.997 | 0.0 | -3.667 | 9.543e+06 | 43 - 58 | 1 | 38.66 | 38.83 | 39.00 |
| 4250.0221 | TIVKPGDILLIN(N)STGNLIAPR Fuc 2 Hex 4 HexNAc 4 Neu5Ac 1 | 35.363 | 0.0 | 0.000 | 0.000e+00 | 250 - 271 | 1 | - | - | - |
| 5477.4532 | KIRTGKSSIM(O)RSDAPIGTC(C)SSEC(C)ITPN(N)GSIPNDKPF Hex 7 HexNAc 2 | 35.204 | 0.0 | -20.504 | 2.576e+06 | 274 - 310 | 1 | 31.65 | 31.65 | 31.65 |
| 3650.6793 | TIVKPGDILLIN(N)STGNL Fuc 2 Hex 3 HexNAc 4 Neu5Ac 1 | 34.633 | 0.0 | 0.000 | 0.000e+00 | 250 - 267 | 1 | - | - | - |
| 5640.4846 | KIRTGKSSIM(O)RSDAPIGTC(C)SSEC(C)ITPN(N)GSIPN(D)DKPF Hex 8 HexNAc 2 | 34.143 | 0.0 | -20.360 | 1.932e+07 | 274 - 310 | 2 | 32.16 | 32.33 | 32.33 |
| 4538.8889 | ITPN(N)GSIPNDKPF Fuc 4 Hex 7 HexNAc 7 | 33.480 | 0.0 | -20.707 | 2.253e+06 | 297 - 310 | 1 | 35.94 | 35.94 | 35.94 |
| 2626.0697 | LPGNDN(N)STATL Fuc 1 Hex 6 HexNAc 2 | 32.951 | 0.0 | 12.690 | 4.816e+08 | 18 - 29 | 10 | 35.11 | 35.78 | 38.15 |
| 4069.9259 | TIVKPGDILLIN(N)STGN(D)LIAPR Hex 9 HexNAc 2 | 32.716 | 0.0 | -5.681 | 7.001e+06 | 250 - 271 | 1 | 52.28 | 52.45 | 52.79 |
| 4887.1067 | ESESKYPVLN(N)VTM(O)PNN(D)GKFDKLY Fuc 2 Hex 8 HexNAc 3 | 32.654 | 0.0 | -6.015 | 1.212e+08 | 171 - 194 | 4 | 43.26 | 43.60 | 43.77 |
| 5786.5669 | FKIRTGKSSIM(O)RSDAPIGTC(C)SSEC(C)ITPN(N)GSIPNDKPF Hex 8 HexNAc 2 | 32.029 | 0.0 | -20.607 | 1.082e+07 | 273 - 310 | 1 | 35.11 | 35.11 | 35.28 |
| 4839.9999 | SSEC(C)ITPN(N)GSIPNDKPF Fuc 4 Hex 6 HexNAc 7 | 31.782 | 0.0 | -4.304 | 1.919e+07 | 293 - 310 | 2 | 39.17 | 39.34 | 39.68 |
| 4453.1064 | TIVKPGDILLIN(N)STGNLIAPRGY Hex 5 HexNAc 6 | 31.115 | 0.0 | 7.561 | 3.639e+07 | 250 - 273 | 5 | 60.05 | 60.51 | 60.51 |
| 4578.8961 | ITPN(N)GSIPNDKPF Fuc 2 Hex 6 HexNAc 8 Neu5Ac 1 | 31.054 | 0.0 | -20.522 | 4.801e+06 | 297 - 310 | 1 | 36.10 | 36.10 | 36.27 |
| 3339.3463 | SGGSSTC(C)KRGSN(N)NSFF Fuc 1 Hex 3 HexNAc 5 | 31.050 | 0.0 | -19.529 | 9.069e+06 | 148 - 164 | 2 | 18.87 | 18.87 | 19.03 |
| 2603.0899 | LPGNDN(N)STATL Hex 3 HexNAc 5 | 30.787 | 0.0 | 12.639 | 1.182e+07 | 18 - 29 | 3 | 23.22 | 23.56 | 23.90 |
| 3825.5897 | TGVTQSGGSSTC(C)KRGSN(N)NSFF Fuc 1 Hex 3 HexNAc 5 | 30.596 | 0.0 | -4.662 | 1.547e+07 | 143 - 164 | 2 | 21.88 | 22.04 | 22.21 |
| 3337.4573 | LIN(N)STGNLIAPRGYF Hex 8 HexNAc 2 | 30.037 | 0.0 | -21.283 | 1.110e+06 | 259 - 274 | 1 | 45.30 | 45.30 | 45.30 |
| 4933.2520 | TITNDQIEVTN(N)ATELVQSSSTGRIC(C)DSPHRIL Hex 6 HexNAc 2 | 29.826 | 0.0 | -20.386 | 2.958e+06 | 43 - 75 | 1 | 44.96 | 45.13 | 45.13 |
| 5802.5426 | KIRTGKSSIM(O)RSDAPIGTC(C)SSEC(C)ITPN(N)GSIPN(D)DKPF Hex 9 HexNAc 2 | 29.777 | 0.0 | -6.635 | 1.807e+07 | 274 - 310 | 2 | 32.16 | 32.33 | 32.50 |
| 4360.8082 | RSDAPIGTC(C)SSEC(C)ITPN(N)GSIPN(D)DKPF Hex 7 HexNAc 2 | 29.661 | 0.0 | -21.105 | 6.363e+06 | 284 - 310 | 2 | 36.96 | 36.96 | 37.13 |
| 5002.0537 | SSEC(C)ITPN(N)GSIPNDKPF Fuc 4 Hex 7 HexNAc 7 | 29.594 | 0.0 | 10.231 | 1.643e+07 | 293 - 310 | 1 | 39.00 | 39.17 | 39.85 |
| 3160.4261 | TIVKPGDILLIN(N) Hex 9 HexNAc 2 | 27.967 | 0.010 | 0.000 | 0.000e+00 | 250 - 262 | 1 | - | - | - |
| 3019.2742 | IN(N)STGNLIAPR Hex 9 HexNAc 2 | 27.538 | 0.010 | -19.442 | 3.421e+06 | 260 - 271 | 1 | 21.37 | 21.54 | 21.54 |
| 2761.1450 | TC(C)KRGSN(N)NSFF Fuc 1 Hex 3 HexNAc 4 | 27.165 | 0.014 | -18.690 | 9.816e+06 | 153 - 164 | 1 | 18.37 | 18.53 | 18.53 |
| 3356.5109 | TIVKPGDILLIN(N)STGN Hex 8 HexNAc 2 | 26.693 | 0.014 | -19.446 | 7.165e+05 | 250 - 266 | 1 | 42.92 | 42.92 | 42.92 |
| 2959.3032 | TITNDQIEVTN(N)ATEL Hex 3 HexNAc 4 | 26.499 | 0.014 | -4.994 | 5.401e+06 | 43 - 58 | 1 | 38.83 | 39.00 | 39.34 |
| 4365.0737 | TIVKPGDILLIN(N)STGNLIAPRGY Fuc 3 Hex 3 HexNAc 5 | 26.493 | 0.014 | 0.000 | 0.000e+00 | 250 - 273 | 1 | - | - | - |
| 4674.8792 | ITPN(N)GSIPNDKPF Fuc 1 Hex 10 HexNAc 6 Neu5Ac 1 | 26.484 | 0.014 | -5.102 | 3.339e+06 | 297 - 310 | 1 | 37.81 | 37.98 | 38.15 |
| 2975.2057 | LPGNDN(N)STATL Fuc 2 Hex 6 HexNAc 3 | 26.019 | 0.014 | 13.769 | 2.817e+08 | 18 - 29 | 6 | 34.95 | 35.62 | 37.13 |
| 4307.0388 | TIVKPGDILLIN(N)STGN(D)LIAPRGY Fuc 2 Hex 3 HexNAc 4 Neu5Ac 1 | 25.940 | 0.014 | -20.600 | 1.243e+06 | 250 - 273 | 2 | 55.12 | 55.12 | 55.12 |
| 2708.1214 | LPGNDN(N)STATL Fuc 1 Hex 4 HexNAc 4 | 25.909 | 0.014 | -6.128 | 3.085e+06 | 18 - 29 | 1 | 22.88 | 23.05 | 23.22 |
| 4417.8178 | ITPN(N)GSIPNDKPF Fuc 4 Hex 5 HexNAc 8 | 24.892 | 0.026 | -4.899 | 7.364e+06 | 297 - 310 | 2 | 39.34 | 39.34 | 39.85 |
| 4638.9017 | ITPN(N)GSIPNDKPF Fuc 3 Hex 6 HexNAc 9 | 24.640 | 0.026 | 0.000 | 0.000e+00 | 297 - 310 | 1 | - | - | - |
| 4840.9859 | SSEC(C)ITPN(N)GSIPN(D)DKPF Fuc 4 Hex 6 HexNAc 7 | 24.409 | 0.026 | -6.363 | 1.235e+07 | 293 - 310 | 2 | 39.85 | 40.20 | 40.37 |
| 3640.4464 | SEC(C)ITPN(N)GSIPN(D)DKPF Hex 9 HexNAc 2 | 24.181 | 0.030 | -21.050 | 5.374e+06 | 294 - 310 | 1 | 34.62 | 34.62 | 34.78 |
| 4581.8577 | ITPN(N)GSIPNDKPF Fuc 2 Hex 6 HexNAc 8 Neu5Ac 1 | 23.922 | 0.030 | 0.000 | 0.000e+00 | 297 - 310 | 1 | - | - | - |
| 4142.9762 | TIVKPGDILLIN(N)STGNLIAPR Fuc 1 Hex 3 HexNAc 5 Neu5Ac 1 | 23.580 | 0.034 | -20.209 | 1.710e+06 | 250 - 271 | 1 | 51.94 | 51.94 | 52.11 |
| 3080.3302 | TITNDQIEVTN(N)ATEL Hex 5 HexNAc 3 | 23.418 | 0.034 | -4.143 | 5.400e+06 | 43 - 58 | 1 | 38.49 | 38.66 | 38.83 |
| 4363.0622 | TIVKPGDILLIN(N)STGNLIAPRGY Fuc 1 Hex 3 HexNAc 5 Neu5Ac 1 | 23.243 | 0.042 | 8.992 | 1.228e+07 | 250 - 273 | 3 | 54.27 | 54.78 | 55.12 |
| 2830.1677 | LPGNDN(N)STATL Fuc 1 Hex 6 HexNAc 3 | 22.752 | 0.045 | 0.000 | 0.000e+00 | 18 - 29 | 1 | - | - | - |
| 3479.3983 | SEC(C)ITPN(N)GSIPN(D)DKPF Hex 8 HexNAc 2 | 22.672 | 0.048 | 0.000 | 0.000e+00 | 294 - 310 | 1 | - | - | - |
| 4725.0553 | ESESKYPVLN(N)VTM(O)PNN(D)GKFDKLY Fuc 2 Hex 7 HexNAc 3 | 22.478 | 0.048 | 12.594 | 7.658e+07 | 171 - 194 | 5 | 43.09 | 43.60 | 43.77 |

### LIN(N)STGNL Hex 9 HexNAc 2

**Mass:**  2695.0869

(2.683e-06)

**Charge States:** 3

**Start Position**: 260

**End Position**: 268

| Profile Score | Tandem Score | TDA q-value | Volume | Spectrum Matches |
| --- | --- | --- | --- | --- |
| -3.3314 | 180.2701 | 0.0000 | 1.2037e+07 | 2 |

**Adduct Signal Fractions**

- **Unmodified**: 1.2037e+07

### LIN(N)STGNLIAPR Hex 9 HexNAc 2

**Mass:**  3132.3572

(-6.095e-07)

**Charge States:** 3

**Start Position**: 260

**End Position**: 272

| Profile Score | Tandem Score | TDA q-value | Volume | Spectrum Matches |
| --- | --- | --- | --- | --- |
| 13.0079 | 172.4773 | 0.0000 | 4.0491e+07 | 4 |

**Adduct Signal Fractions**

- **Unmodified**: 4.0491e+07

### TIVKPGDILLIN(N)STGNLIAPR Hex 9 HexNAc 2

**Mass:**  4068.9279

(1.334e-06)

**Charge States:** 3, 4, 5

**Start Position**: 251

**End Position**: 272

| Profile Score | Tandem Score | TDA q-value | Volume | Spectrum Matches |
| --- | --- | --- | --- | --- |
| 14.5896 | 171.2281 | 0.0000 | 2.1810e+08 | 13 |

**Adduct Signal Fractions**

- **Unmodified**: 2.1810e+08

### LPGNDN(N)STATL Fuc 1 Hex 3 HexNAc 4

**Mass:**  2546.0672

(1.215e-06)

**Charge States:** 3

**Start Position**: 19

**End Position**: 30

| Profile Score | Tandem Score | TDA q-value | Volume | Spectrum Matches |
| --- | --- | --- | --- | --- |
| 14.1736 | 170.2105 | 0.0000 | 5.5262e+07 | 4 |

**Adduct Signal Fractions**

- **Unmodified**: 5.5262e+07

### LIN(N)STGNLIAPRGY Hex 9 HexNAc 2

**Mass:**  3352.4448

(3.446e-07)

**Charge States:** 3

**Start Position**: 260

**End Position**: 274

| Profile Score | Tandem Score | TDA q-value | Volume | Spectrum Matches |
| --- | --- | --- | --- | --- |
| -4.5418 | 160.6863 | 0.0000 | 3.4507e+07 | 1 |

**Adduct Signal Fractions**

- **Unmodified**: 3.4507e+07

### TIVKPGDILLIN(N) Hex 9 HexNAc 2

**Mass:**  3159.4238

(1.515e-06)

**Charge States:** 3

**Start Position**: 251

**End Position**: 263

| Profile Score | Tandem Score | TDA q-value | Volume | Spectrum Matches |
| --- | --- | --- | --- | --- |
| 13.0084 | 158.4177 | 0.0000 | 9.6359e+07 | 8 |

**Adduct Signal Fractions**

- **Unmodified**: 9.6359e+07

### TIVKPGDILLIN(N)STGNLIAPRGY Hex 9 HexNAc 2

**Mass:**  4289.0158

(1.758e-06)

**Charge States:** 3, 4, 5

**Start Position**: 251

**End Position**: 274

| Profile Score | Tandem Score | TDA q-value | Volume | Spectrum Matches |
| --- | --- | --- | --- | --- |
| 14.9716 | 155.4733 | 0.0000 | 7.8156e+08 | 36 |

**Adduct Signal Fractions**

- **Unmodified**: 7.8156e+08

### LPGNDN(N)STATL Fuc 1 Hex 3 HexNAc 5

**Mass:**  2749.1449

(9.257e-07)

**Charge States:** 3

**Start Position**: 19

**End Position**: 30

| Profile Score | Tandem Score | TDA q-value | Volume | Spectrum Matches |
| --- | --- | --- | --- | --- |
| 10.7379 | 154.3220 | 0.0000 | 1.3650e+07 | 3 |

**Adduct Signal Fractions**

- **Unmodified**: 1.3650e+07

### LPGNDN(N)STATL Hex 3 HexNAc 4

**Mass:**  2400.0077

(6.505e-07)

**Charge States:** 3

**Start Position**: 19

**End Position**: 30

| Profile Score | Tandem Score | TDA q-value | Volume | Spectrum Matches |
| --- | --- | --- | --- | --- |
| 12.6649 | 153.6938 | 0.0000 | 1.6278e+07 | 2 |

**Adduct Signal Fractions**

- **Unmodified**: 1.6278e+07

### IN(N)STGNLIAPRGYF Hex 9 HexNAc 2

**Mass:**  3386.4351

(2.785e-06)

**Charge States:** 3

**Start Position**: 261

**End Position**: 275

| Profile Score | Tandem Score | TDA q-value | Volume | Spectrum Matches |
| --- | --- | --- | --- | --- |
| -17.2680 | 149.4501 | 0.0000 | 5.9873e+06 | 1 |

**Adduct Signal Fractions**

- **Unmodified**: 5.9873e+06

### TIVKPGDILLIN(N)STGNLIAPRGY Hex 10 HexNAc 2

**Mass:**  4451.0661

(1.127e-06)

**Charge States:** 3, 4

**Start Position**: 251

**End Position**: 274

| Profile Score | Tandem Score | TDA q-value | Volume | Spectrum Matches |
| --- | --- | --- | --- | --- |
| 13.1156 | 147.8434 | 0.0000 | 7.7437e+07 | 14 |

**Adduct Signal Fractions**

- **Unmodified**: 7.7437e+07

### LPGNDN(N)STATL Hex 5 HexNAc 2

**Mass:**  2317.9557

(1.115e-07)

**Charge States:** 2, 3

**Start Position**: 19

**End Position**: 30

| Profile Score | Tandem Score | TDA q-value | Volume | Spectrum Matches |
| --- | --- | --- | --- | --- |
| 11.7336 | 147.2159 | 0.0000 | 9.2854e+06 | 3 |

**Adduct Signal Fractions**

- **Unmodified**: 9.2854e+06

### ITPN(N)GSIPNDKPF Hex 8 HexNAc 2

**Mass:**  3101.3032

(8.255e-07)

**Charge States:** 3

**Start Position**: 298

**End Position**: 311

| Profile Score | Tandem Score | TDA q-value | Volume | Spectrum Matches |
| --- | --- | --- | --- | --- |
| -4.6017 | 146.6038 | 0.0000 | 1.0729e+07 | 2 |

**Adduct Signal Fractions**

- **Unmodified**: 1.0729e+07

### TIVKPGDILLIN(N) Hex 10 HexNAc 2

**Mass:**  3321.4799

(2.016e-06)

**Charge States:** 3

**Start Position**: 251

**End Position**: 263

| Profile Score | Tandem Score | TDA q-value | Volume | Spectrum Matches |
| --- | --- | --- | --- | --- |
| -5.0594 | 146.3430 | 0.0000 | 5.2304e+06 | 2 |

**Adduct Signal Fractions**

- **Unmodified**: 5.2304e+06

### TIVKPGDILLIN(N)STGNLIAPRGY Fuc 2 Hex 3 HexNAc 4 Neu5Ac 1

**Mass:**  4306.0378

(-5.478e-06)

**Charge States:** 4, 5

**Start Position**: 251

**End Position**: 274

| Profile Score | Tandem Score | TDA q-value | Volume | Spectrum Matches |
| --- | --- | --- | --- | --- |
| 13.8450 | 145.4158 | 0.0000 | 8.4081e+07 | 5 |

**Adduct Signal Fractions**

- **Unmodified**: 8.4081e+07

### TIVKPGDILLIN(N)STGNL Hex 9 HexNAc 2

**Mass:**  3631.6536

(1.538e-06)

**Charge States:** 3, 4

**Start Position**: 251

**End Position**: 268

| Profile Score | Tandem Score | TDA q-value | Volume | Spectrum Matches |
| --- | --- | --- | --- | --- |
| 13.9883 | 145.1042 | 0.0000 | 8.7414e+07 | 5 |

**Adduct Signal Fractions**

- **Unmodified**: 8.7414e+07

### TIVKPGDILLIN(N)STGN Hex 9 HexNAc 2

**Mass:**  3518.5709

(1.560e-06)

**Charge States:** 3, 4

**Start Position**: 251

**End Position**: 267

| Profile Score | Tandem Score | TDA q-value | Volume | Spectrum Matches |
| --- | --- | --- | --- | --- |
| 12.8941 | 144.1936 | 0.0000 | 3.5077e+07 | 4 |

**Adduct Signal Fractions**

- **Unmodified**: 3.5077e+07

### TIVKPGDILLIN(N) Fuc 2 Hex 3 HexNAc 4 Neu5Ac 1

**Mass:**  3176.4501

(-6.584e-06)

**Charge States:** 3

**Start Position**: 251

**End Position**: 263

| Profile Score | Tandem Score | TDA q-value | Volume | Spectrum Matches |
| --- | --- | --- | --- | --- |
| 9.9070 | 143.3554 | 0.0000 | 1.4272e+07 | 4 |

**Adduct Signal Fractions**

- **Unmodified**: 1.4272e+07

### LIN(N)STGNLIAPRGYF Hex 9 HexNAc 2

**Mass:**  3499.5166

(1.219e-06)

**Charge States:** 3

**Start Position**: 260

**End Position**: 275

| Profile Score | Tandem Score | TDA q-value | Volume | Spectrum Matches |
| --- | --- | --- | --- | --- |
| -18.7873 | 143.1805 | 0.0000 | 2.3989e+07 | 1 |

**Adduct Signal Fractions**

- **Unmodified**: 2.3989e+07

### ITPN(N)GSIPNDKPF Hex 9 HexNAc 2

**Mass:**  3263.3677

(7.652e-06)

**Charge States:** 3

**Start Position**: 298

**End Position**: 311

| Profile Score | Tandem Score | TDA q-value | Volume | Spectrum Matches |
| --- | --- | --- | --- | --- |
| -4.6937 | 141.0801 | 0.0000 | 1.1827e+07 | 2 |

**Adduct Signal Fractions**

- **Unmodified**: 1.1827e+07

### LPGNDN(N)STATL Fuc 2 Hex 4 HexNAc 4

**Mass:**  2854.1784

(1.287e-06)

**Charge States:** 3

**Start Position**: 19

**End Position**: 30

| Profile Score | Tandem Score | TDA q-value | Volume | Spectrum Matches |
| --- | --- | --- | --- | --- |
| 11.5796 | 139.1464 | 0.0000 | 1.1194e+07 | 2 |

**Adduct Signal Fractions**

- **Unmodified**: 1.1194e+07

### TIVKPGDILLIN(N)STGNLIAPR Hex 10 HexNAc 2

**Mass:**  4230.9821

(1.924e-06)

**Charge States:** 3, 4

**Start Position**: 251

**End Position**: 272

| Profile Score | Tandem Score | TDA q-value | Volume | Spectrum Matches |
| --- | --- | --- | --- | --- |
| 13.4152 | 138.1944 | 0.0000 | 1.9071e+07 | 3 |

**Adduct Signal Fractions**

- **Unmodified**: 1.9071e+07

### TIVKPGDILLIN(N)STGNLIAPRGY Hex 8 HexNAc 2

**Mass:**  4126.9582

(3.456e-07)

**Charge States:** 3, 4

**Start Position**: 251

**End Position**: 274

| Profile Score | Tandem Score | TDA q-value | Volume | Spectrum Matches |
| --- | --- | --- | --- | --- |
| 12.7717 | 136.9350 | 0.0000 | 6.1716e+07 | 9 |

**Adduct Signal Fractions**

- **Unmodified**: 6.1716e+07

### TIVKPGDILLIN(N)STGNLIAPR Hex 8 HexNAc 2

**Mass:**  3906.8740

(1.073e-06)

**Charge States:** 3, 4

**Start Position**: 251

**End Position**: 272

| Profile Score | Tandem Score | TDA q-value | Volume | Spectrum Matches |
| --- | --- | --- | --- | --- |
| -4.6288 | 134.5215 | 0.0000 | 1.4207e+07 | 4 |

**Adduct Signal Fractions**

- **Unmodified**: 1.4207e+07

### TIVKPGDILLIN(N)STGNLIAPR Fuc 2 Hex 3 HexNAc 4 Neu5Ac 1

**Mass:**  4085.9560

(-5.245e-06)

**Charge States:** 4

**Start Position**: 251

**End Position**: 272

| Profile Score | Tandem Score | TDA q-value | Volume | Spectrum Matches |
| --- | --- | --- | --- | --- |
| 12.7814 | 133.4228 | 0.0000 | 1.8808e+07 | 2 |

**Adduct Signal Fractions**

- **Unmodified**: 1.8808e+07

### IN(N)STGNLIAPRGY Hex 9 HexNAc 2

**Mass:**  3239.3655

(1.762e-06)

**Charge States:** 3

**Start Position**: 261

**End Position**: 274

| Profile Score | Tandem Score | TDA q-value | Volume | Spectrum Matches |
| --- | --- | --- | --- | --- |
| -17.2737 | 128.9185 | 0.0000 | 1.0048e+07 | 1 |

**Adduct Signal Fractions**

- **Unmodified**: 1.0048e+07

### TIVKPGDILLIN(N)STGNL Fuc 2 Hex 3 HexNAc 4 Neu5Ac 1

**Mass:**  3648.6791

(-6.139e-06)

**Charge States:** 3

**Start Position**: 251

**End Position**: 268

| Profile Score | Tandem Score | TDA q-value | Volume | Spectrum Matches |
| --- | --- | --- | --- | --- |
| -4.2961 | 127.1781 | 0.0000 | 1.4049e+07 | 2 |

**Adduct Signal Fractions**

- **Unmodified**: 1.4049e+07

### LIN(N)STGNLIAPRGYF Fuc 2 Hex 3 HexNAc 4 Neu5Ac 1

**Mass:**  3516.5451

(-6.567e-06)

**Charge States:** 3

**Start Position**: 260

**End Position**: 275

| Profile Score | Tandem Score | TDA q-value | Volume | Spectrum Matches |
| --- | --- | --- | --- | --- |
| -19.5044 | 124.4689 | 0.0000 | 3.7657e+06 | 1 |

**Adduct Signal Fractions**

- **Unmodified**: 3.7657e+06

### TIVKPGDILLIN(N)STGN Fuc 2 Hex 3 HexNAc 4 Neu5Ac 1

**Mass:**  3537.5941

(5.591e-04)

**Start Position**: 251

**End Position**: 267

| Profile Score | Tandem Score | TDA q-value | Volume | Spectrum Matches |
| --- | --- | --- | --- | --- |
| 0.0000 | 124.2042 | 0.0000 | 0.0000e+00 | 1 |

Adduct:

No Chromatogram Found

### KRGSN(N)N(D)SFF Fuc 1 Hex 3 HexNAc 5

**Mass:**  2704.1229

(3.680e-04)

**Charge States:** 3

**Start Position**: 156

**End Position**: 165

| Profile Score | Tandem Score | TDA q-value | Volume | Spectrum Matches |
| --- | --- | --- | --- | --- |
| -4.7215 | 121.4047 | 0.0000 | 4.3473e+07 | 8 |

**Adduct Signal Fractions**

- **Unmodified**: 4.3473e+07

### KRGSN(N)NSFF Fuc 1 Hex 3 HexNAc 5

**Mass:**  2703.1285

(2.374e-07)

**Charge States:** 3

**Start Position**: 156

**End Position**: 165

| Profile Score | Tandem Score | TDA q-value | Volume | Spectrum Matches |
| --- | --- | --- | --- | --- |
| -2.5711 | 119.5210 | 0.0000 | 6.4412e+07 | 1 |

**Adduct Signal Fractions**

- **Unmodified**: 6.4412e+07

### SSEC(C)ITPN(N)GSIPNDKPF Hex 8 HexNAc 2

**Mass:**  3564.4352

(6.294e-07)

**Charge States:** 3

**Start Position**: 294

**End Position**: 311

| Profile Score | Tandem Score | TDA q-value | Volume | Spectrum Matches |
| --- | --- | --- | --- | --- |
| -5.6093 | 118.0890 | 0.0000 | 1.6927e+07 | 1 |

**Adduct Signal Fractions**

- **Unmodified**: 1.6927e+07

### SIRN(N)GTYDHDVY Fuc 2 Hex 4 HexNAc 4

**Mass:**  3191.2945

(6.174e-07)

**Charge States:** 3

**Start Position**: 496

**End Position**: 508

| Profile Score | Tandem Score | TDA q-value | Volume | Spectrum Matches |
| --- | --- | --- | --- | --- |
| -20.6774 | 117.0157 | 0.0000 | 2.1465e+06 | 1 |

**Adduct Signal Fractions**

- **Unmodified**: 2.1465e+06

### SIRN(N)GTYDHDVY Fuc 1 Hex 3 HexNAc 4

**Mass:**  2883.1882

(3.288e-06)

**Charge States:** 3

**Start Position**: 496

**End Position**: 508

| Profile Score | Tandem Score | TDA q-value | Volume | Spectrum Matches |
| --- | --- | --- | --- | --- |
| -21.4084 | 116.7465 | 0.0000 | 3.5171e+06 | 1 |

**Adduct Signal Fractions**

- **Unmodified**: 3.5171e+06

### RSDAPIGTC(C)SSEC(C)ITPN(N)GSIPNDKPF Hex 8 HexNAc 2

**Mass:**  4521.8718

(2.482e-06)

**Charge States:** 3, 4

**Start Position**: 285

**End Position**: 311

| Profile Score | Tandem Score | TDA q-value | Volume | Spectrum Matches |
| --- | --- | --- | --- | --- |
| 12.2448 | 116.3177 | 0.0000 | 7.4186e+07 | 4 |

**Adduct Signal Fractions**

- **Unmodified**: 7.4186e+07

### KRGSN(N)NSFF Fuc 1 Hex 3 HexNAc 4

**Mass:**  2500.0525

(1.520e-06)

**Charge States:** 3

**Start Position**: 156

**End Position**: 165

| Profile Score | Tandem Score | TDA q-value | Volume | Spectrum Matches |
| --- | --- | --- | --- | --- |
| -19.4196 | 115.3130 | 0.0000 | 1.0606e+07 | 1 |

**Adduct Signal Fractions**

- **Unmodified**: 1.0606e+07

### KRGSN(N)NSFF Fuc 2 Hex 4 HexNAc 5

**Mass:**  3011.2401

(1.910e-06)

**Charge States:** 3

**Start Position**: 156

**End Position**: 165

| Profile Score | Tandem Score | TDA q-value | Volume | Spectrum Matches |
| --- | --- | --- | --- | --- |
| -4.1860 | 107.6347 | 0.0000 | 2.3160e+07 | 2 |

**Adduct Signal Fractions**

- **Unmodified**: 2.3160e+07

### LPGNDN(N)STATL Hex 6 HexNAc 2

**Mass:**  2480.0084

(1.026e-06)

**Start Position**: 19

**End Position**: 30

| Profile Score | Tandem Score | TDA q-value | Volume | Spectrum Matches |
| --- | --- | --- | --- | --- |
| 0.0000 | 107.4948 | 0.0000 | 0.0000e+00 | 1 |

Adduct:

No Chromatogram Found

### IGSIRN(N)GTYDHDVY Fuc 2 Hex 4 HexNAc 4

**Mass:**  3361.4082

(2.372e-06)

**Charge States:** 3

**Start Position**: 494

**End Position**: 508

| Profile Score | Tandem Score | TDA q-value | Volume | Spectrum Matches |
| --- | --- | --- | --- | --- |
| -19.6377 | 101.1078 | 0.0000 | 3.5468e+06 | 1 |

**Adduct Signal Fractions**

- **Unmodified**: 3.5468e+06

### LPGNDN(N)STATL Fuc 1 Hex 7 HexNAc 2

**Mass:**  2788.1651

(8.513e-06)

**Charge States:** 2, 3

**Start Position**: 19

**End Position**: 30

| Profile Score | Tandem Score | TDA q-value | Volume | Spectrum Matches |
| --- | --- | --- | --- | --- |
| 11.8787 | 99.4057 | 0.0000 | 3.3537e+08 | 5 |

**Adduct Signal Fractions**

- **Unmodified**: 3.3537e+08

### LPGNDN(N)STATL Fuc 3 Hex 5 HexNAc 4

**Mass:**  3162.2935

(5.784e-07)

**Charge States:** 3

**Start Position**: 19

**End Position**: 30

| Profile Score | Tandem Score | TDA q-value | Volume | Spectrum Matches |
| --- | --- | --- | --- | --- |
| -19.9955 | 99.2663 | 0.0000 | 2.0814e+06 | 1 |

**Adduct Signal Fractions**

- **Unmodified**: 2.0814e+06

### KRGSN(N)NSFF Fuc 2 Hex 4 HexNAc 4

**Mass:**  2808.1527

(1.350e-06)

**Charge States:** 3

**Start Position**: 156

**End Position**: 165

| Profile Score | Tandem Score | TDA q-value | Volume | Spectrum Matches |
| --- | --- | --- | --- | --- |
| -5.2549 | 98.8749 | 0.0000 | 1.0692e+07 | 2 |

**Adduct Signal Fractions**

- **Unmodified**: 1.0692e+07

### IGSIRN(N)GTYDHDVY Fuc 1 Hex 3 HexNAc 4

**Mass:**  3053.2939

(2.231e-06)

**Charge States:** 3

**Start Position**: 494

**End Position**: 508

| Profile Score | Tandem Score | TDA q-value | Volume | Spectrum Matches |
| --- | --- | --- | --- | --- |
| -17.3512 | 97.9402 | 0.0000 | 4.7892e+06 | 1 |

**Adduct Signal Fractions**

- **Unmodified**: 4.7892e+06

### SEC(C)ITPN(N)GSIPNDKPF Hex 8 HexNAc 2

**Mass:**  3478.4101

(2.902e-04)

**Start Position**: 295

**End Position**: 311

| Profile Score | Tandem Score | TDA q-value | Volume | Spectrum Matches |
| --- | --- | --- | --- | --- |
| 0.0000 | 94.4151 | 0.0000 | 0.0000e+00 | 1 |

Adduct:

No Chromatogram Found

### RSDAPIGTC(C)SSEC(C)ITPN(N)GSIPNDKPF Hex 7 HexNAc 2

**Mass:**  4359.8210

(8.455e-07)

**Charge States:** 4

**Start Position**: 285

**End Position**: 311

| Profile Score | Tandem Score | TDA q-value | Volume | Spectrum Matches |
| --- | --- | --- | --- | --- |
| -4.9664 | 92.1596 | 0.0000 | 1.8122e+07 | 3 |

**Adduct Signal Fractions**

- **Unmodified**: 1.8122e+07

### SSEC(C)ITPN(N)GSIPNDKPF Hex 9 HexNAc 2

**Mass:**  3726.4890

(8.794e-07)

**Charge States:** 3

**Start Position**: 294

**End Position**: 311

| Profile Score | Tandem Score | TDA q-value | Volume | Spectrum Matches |
| --- | --- | --- | --- | --- |
| -20.1648 | 91.4784 | 0.0000 | 1.0965e+07 | 1 |

**Adduct Signal Fractions**

- **Unmodified**: 1.0965e+07

### RGSN(N)NSFF Fuc 2 Hex 3 HexNAc 8

**Mass:**  3330.3048

(-8.569e-06)

**Charge States:** 3

**Start Position**: 157

**End Position**: 165

| Profile Score | Tandem Score | TDA q-value | Volume | Spectrum Matches |
| --- | --- | --- | --- | --- |
| -5.5324 | 90.3174 | 0.0000 | 7.1118e+06 | 2 |

**Adduct Signal Fractions**

- **Unmodified**: 7.1118e+06

### RSDAPIGTC(C)SSEC(C)ITPN(N)GSIPNDKPF Hex 9 HexNAc 2

**Mass:**  4683.9255

(1.529e-06)

**Charge States:** 3, 4

**Start Position**: 285

**End Position**: 311

| Profile Score | Tandem Score | TDA q-value | Volume | Spectrum Matches |
| --- | --- | --- | --- | --- |
| 11.6882 | 89.4474 | 0.0000 | 6.0329e+07 | 3 |

**Adduct Signal Fractions**

- **Unmodified**: 6.0329e+07

### TITNDQIEVTN(N)ATEL Hex 6 HexNAc 2

**Mass:**  3039.3014

(2.712e-06)

**Charge States:** 3

**Start Position**: 44

**End Position**: 59

| Profile Score | Tandem Score | TDA q-value | Volume | Spectrum Matches |
| --- | --- | --- | --- | --- |
| -3.2507 | 83.3538 | 0.0000 | 1.8703e+07 | 2 |

**Adduct Signal Fractions**

- **Unmodified**: 1.8703e+07

### RSDAPIGTC(C)SSEC(C)ITPN(N)GSIPN(D)DKPF Hex 8 HexNAc 2

**Mass:**  4522.8630

(2.213e-04)

**Charge States:** 3, 4

**Start Position**: 285

**End Position**: 311

| Profile Score | Tandem Score | TDA q-value | Volume | Spectrum Matches |
| --- | --- | --- | --- | --- |
| -5.9616 | 74.5759 | 0.0000 | 2.8170e+07 | 3 |

**Adduct Signal Fractions**

- **Unmodified**: 2.8170e+07

### LYESESKYPVLN(N)VTM(O)PNN(D)GKF Hex 8 HexNAc 2

**Mass:**  4148.7648

(2.394e-04)

**Charge States:** 3, 4

**Start Position**: 170

**End Position**: 191

| Profile Score | Tandem Score | TDA q-value | Volume | Spectrum Matches |
| --- | --- | --- | --- | --- |
| 10.8188 | 69.4549 | 0.0000 | 1.7624e+07 | 3 |

**Adduct Signal Fractions**

- **Unmodified**: 1.7624e+07

### ESESKYPVLN(N)VTM(O)PNN(D)GKFDKLY Hex 9 HexNAc 2

**Mass:**  4553.9401

(2.188e-04)

**Charge States:** 4

**Start Position**: 172

**End Position**: 195

| Profile Score | Tandem Score | TDA q-value | Volume | Spectrum Matches |
| --- | --- | --- | --- | --- |
| 11.0977 | 67.8582 | 0.0000 | 3.2489e+07 | 3 |

**Adduct Signal Fractions**

- **Unmodified**: 3.2489e+07

### KIRTGKSSIM(O)RSDAPIGTC(C)SSEC(C)ITPN(N)GSIPNDKPF Hex 9 HexNAc 2

**Mass:**  5801.5575

(2.328e-06)

**Charge States:** 5, 6

**Start Position**: 275

**End Position**: 311

| Profile Score | Tandem Score | TDA q-value | Volume | Spectrum Matches |
| --- | --- | --- | --- | --- |
| -5.1175 | 66.7964 | 0.0000 | 3.3552e+07 | 3 |

**Adduct Signal Fractions**

- **Unmodified**: 3.3552e+07

### ITPN(N)GSIPNDKPF Hex 7 HexNAc 6 Neu5Ac 3

**Mass:**  4624.8864

(8.587e-06)

**Charge States:** 3, 4

**Start Position**: 298

**End Position**: 311

| Profile Score | Tandem Score | TDA q-value | Volume | Spectrum Matches |
| --- | --- | --- | --- | --- |
| 14.1123 | 58.8314 | 0.0000 | 2.6683e+07 | 4 |

**Adduct Signal Fractions**

- **Unmodified**: 2.6683e+07

### KIRTGKSSIM(O)RSDAPIGTC(C)SSEC(C)ITPN(N)GSIPNDKPF Hex 8 HexNAc 2

**Mass:**  5639.5022

(1.497e-06)

**Charge States:** 5, 6

**Start Position**: 275

**End Position**: 311

| Profile Score | Tandem Score | TDA q-value | Volume | Spectrum Matches |
| --- | --- | --- | --- | --- |
| -4.8064 | 57.5236 | 0.0000 | 3.9409e+07 | 3 |

**Adduct Signal Fractions**

- **Unmodified**: 3.9409e+07

### LYESESKYPVLN(N)VTM(O)PNN(D)GKF Hex 9 HexNAc 2

**Mass:**  4310.8171

(2.308e-04)

**Charge States:** 3, 4

**Start Position**: 170

**End Position**: 191

| Profile Score | Tandem Score | TDA q-value | Volume | Spectrum Matches |
| --- | --- | --- | --- | --- |
| -6.2763 | 56.5019 | 0.0000 | 2.4324e+07 | 3 |

**Adduct Signal Fractions**

- **Unmodified**: 2.4324e+07

### TIVKPGDILLIN(N)STGNL Hex 8 HexNAc 2

**Mass:**  3469.5983

(2.090e-06)

**Charge States:** 3

**Start Position**: 251

**End Position**: 268

| Profile Score | Tandem Score | TDA q-value | Volume | Spectrum Matches |
| --- | --- | --- | --- | --- |
| -4.2939 | 56.4113 | 0.0000 | 5.6299e+06 | 3 |

**Adduct Signal Fractions**

- **Unmodified**: 5.6299e+06

### TITNDQIEVTN(N)ATEL Hex 7 HexNAc 2

**Mass:**  3201.3502

(2.629e-06)

**Charge States:** 3

**Start Position**: 44

**End Position**: 59

| Profile Score | Tandem Score | TDA q-value | Volume | Spectrum Matches |
| --- | --- | --- | --- | --- |
| -17.3042 | 52.5340 | 0.0000 | 6.3441e+06 | 1 |

**Adduct Signal Fractions**

- **Unmodified**: 6.3441e+06

### TIVKPGDILLIN(N)STGN Fuc 2 Hex 3 HexNAc 4 Neu5Ac 1

**Mass:**  3535.5935

(-5.908e-06)

**Charge States:** 3

**Start Position**: 251

**End Position**: 267

| Profile Score | Tandem Score | TDA q-value | Volume | Spectrum Matches |
| --- | --- | --- | --- | --- |
| -18.6057 | 52.2088 | 0.0000 | 5.1168e+06 | 1 |

**Adduct Signal Fractions**

- **Unmodified**: 5.1168e+06

### ESESKYPVLN(N)VTM(O)PNN(D)GKFDKLY Hex 8 HexNAc 2

**Mass:**  4391.8879

(2.269e-04)

**Charge States:** 4

**Start Position**: 172

**End Position**: 195

| Profile Score | Tandem Score | TDA q-value | Volume | Spectrum Matches |
| --- | --- | --- | --- | --- |
| 9.7678 | 51.2521 | 0.0000 | 2.4375e+07 | 4 |

**Adduct Signal Fractions**

- **Unmodified**: 2.4375e+07

### RSDAPIGTC(C)SSEC(C)ITPN(N)GSIPN(D)DKPF Hex 9 HexNAc 2

**Mass:**  4684.9157

(2.132e-04)

**Charge States:** 3, 4

**Start Position**: 285

**End Position**: 311

| Profile Score | Tandem Score | TDA q-value | Volume | Spectrum Matches |
| --- | --- | --- | --- | --- |
| -6.0123 | 51.2460 | 0.0000 | 2.1291e+07 | 4 |

**Adduct Signal Fractions**

- **Unmodified**: 2.1291e+07

### LYESESKYPVLN(N)VTM(O)PN Hex 9 HexNAc 2

**Mass:**  3863.6035

(2.187e-06)

**Charge States:** 3

**Start Position**: 170

**End Position**: 187

| Profile Score | Tandem Score | TDA q-value | Volume | Spectrum Matches |
| --- | --- | --- | --- | --- |
| -5.2826 | 49.8727 | 0.0000 | 1.2904e+07 | 1 |

**Adduct Signal Fractions**

- **Unmodified**: 1.2904e+07

### TIVKPGDILLIN(N)STGNL Hex 10 HexNAc 2

**Mass:**  3793.7052

(8.508e-07)

**Charge States:** 3

**Start Position**: 251

**End Position**: 268

| Profile Score | Tandem Score | TDA q-value | Volume | Spectrum Matches |
| --- | --- | --- | --- | --- |
| -4.6127 | 48.9374 | 0.0000 | 8.3761e+06 | 3 |

**Adduct Signal Fractions**

- **Unmodified**: 8.3761e+06

### SSEC(C)ITPN(N)GSIPN(D)DKPF Fuc 1 Hex 6 HexNAc 8

**Mass:**  4605.8242

(2.092e-04)

**Charge States:** 5

**Start Position**: 294

**End Position**: 311

| Profile Score | Tandem Score | TDA q-value | Volume | Spectrum Matches |
| --- | --- | --- | --- | --- |
| -6.7373 | 47.2742 | 0.0380 | 1.2872e+07 | 2 |

**Adduct Signal Fractions**

- **Unmodified**: 1.2872e+07

### TIVKPGDILLIN(N)STGNLIAPRGY Hex 7 HexNAc 2

**Mass:**  3964.9084

(1.470e-06)

**Charge States:** 3, 4

**Start Position**: 251

**End Position**: 274

| Profile Score | Tandem Score | TDA q-value | Volume | Spectrum Matches |
| --- | --- | --- | --- | --- |
| -19.8965 | 47.0147 | 0.0000 | 1.9119e+06 | 2 |

**Adduct Signal Fractions**

- **Unmodified**: 1.9119e+06

### SSEC(C)ITPN(N)GSIPN(D)DKPF Fuc 1 Hex 5 HexNAc 8

**Mass:**  4443.7701

(2.175e-04)

**Charge States:** 4, 5

**Start Position**: 294

**End Position**: 311

| Profile Score | Tandem Score | TDA q-value | Volume | Spectrum Matches |
| --- | --- | --- | --- | --- |
| -6.7118 | 46.7133 | 0.0484 | 1.5663e+07 | 4 |

**Adduct Signal Fractions**

- **Unmodified**: 1.5663e+07

### IGSIRN(N)GTYDHDVY Fuc 1 Hex 3 HexNAc 5

**Mass:**  3256.3819

(2.234e-06)

**Charge States:** 3

**Start Position**: 494

**End Position**: 508

| Profile Score | Tandem Score | TDA q-value | Volume | Spectrum Matches |
| --- | --- | --- | --- | --- |
| -20.4399 | 45.6431 | 0.0000 | 2.3888e+06 | 1 |

**Adduct Signal Fractions**

- **Unmodified**: 2.3888e+06

### LYESESKYPVLN(N)VTM(O)PN Hex 8 HexNAc 2

**Mass:**  3701.5504

(2.239e-06)

**Charge States:** 3

**Start Position**: 170

**End Position**: 187

| Profile Score | Tandem Score | TDA q-value | Volume | Spectrum Matches |
| --- | --- | --- | --- | --- |
| 5.9948 | 44.8513 | 0.0000 | 8.1767e+06 | 1 |

**Adduct Signal Fractions**

- **Unmodified**: 8.1767e+06

### LYESESKYPVLN(N)VTM(O)PN Hex 9 HexNAc 2

**Mass:**  3864.6073

(2.620e-04)

**Start Position**: 170

**End Position**: 187

| Profile Score | Tandem Score | TDA q-value | Volume | Spectrum Matches |
| --- | --- | --- | --- | --- |
| 0.0000 | 44.7825 | 0.0000 | 0.0000e+00 | 1 |

Adduct:

No Chromatogram Found

### TIVKPGDILLIN(N)STGN(D)LIAPRGY Hex 8 HexNAc 2

**Mass:**  4127.9650

(2.446e-04)

**Charge States:** 4, 5

**Start Position**: 251

**End Position**: 274

| Profile Score | Tandem Score | TDA q-value | Volume | Spectrum Matches |
| --- | --- | --- | --- | --- |
| -19.1256 | 44.1681 | 0.0000 | 7.3762e+06 | 3 |

**Adduct Signal Fractions**

- **Unmodified**: 7.3762e+06

### TIVKPGDILLIN(N)STGN Hex 10 HexNAc 2

**Mass:**  3680.6237

(1.821e-06)

**Charge States:** 3

**Start Position**: 251

**End Position**: 267

| Profile Score | Tandem Score | TDA q-value | Volume | Spectrum Matches |
| --- | --- | --- | --- | --- |
| -18.9542 | 42.5250 | 0.0000 | 2.8946e+06 | 1 |

**Adduct Signal Fractions**

- **Unmodified**: 2.8946e+06

### TITNDQIEVTN(N)ATEL Fuc 1 Hex 6 HexNAc 3

**Mass:**  3388.4346

(1.795e-06)

**Charge States:** 3

**Start Position**: 44

**End Position**: 59

| Profile Score | Tandem Score | TDA q-value | Volume | Spectrum Matches |
| --- | --- | --- | --- | --- |
| -20.0307 | 42.3602 | 0.0000 | 1.9674e+06 | 2 |

**Adduct Signal Fractions**

- **Unmodified**: 1.9674e+06

### HKC(C)DNAC(C)IGSIRN(N)GTYDHDVY Fuc 1 Hex 3 HexNAc 4

**Mass:**  3938.6047

(2.295e-07)

**Charge States:** 4

**Start Position**: 487

**End Position**: 508

| Profile Score | Tandem Score | TDA q-value | Volume | Spectrum Matches |
| --- | --- | --- | --- | --- |
| -20.0167 | 42.0988 | 0.0000 | 7.6432e+06 | 2 |

**Adduct Signal Fractions**

- **Unmodified**: 7.6432e+06

### ITPN(N)GSIPNDKPF Fuc 3 Hex 9 HexNAc 5 Neu5Ac 1

**Mass:**  4602.8541

(2.169e-04)

**Start Position**: 298

**End Position**: 311

| Profile Score | Tandem Score | TDA q-value | Volume | Spectrum Matches |
| --- | --- | --- | --- | --- |
| 0.0000 | 41.0013 | 0.0000 | 0.0000e+00 | 1 |

Adduct:

No Chromatogram Found

### ITPN(N)GSIPNDKPF Hex 7 HexNAc 2

**Mass:**  2940.2473

(3.417e-04)

**Start Position**: 298

**End Position**: 311

| Profile Score | Tandem Score | TDA q-value | Volume | Spectrum Matches |
| --- | --- | --- | --- | --- |
| 0.0000 | 39.9869 | 0.0000 | 0.0000e+00 | 1 |

Adduct:

No Chromatogram Found

### TIVKPGDILLIN(N)STGNLIAPRGY Hex 4 HexNAc 4 Neu5Ac 2

**Mass:**  4467.0930

(2.147e-06)

**Charge States:** 4

**Start Position**: 251

**End Position**: 274

| Profile Score | Tandem Score | TDA q-value | Volume | Spectrum Matches |
| --- | --- | --- | --- | --- |
| -19.3511 | 39.1856 | 0.0000 | 3.2773e+06 | 2 |

**Adduct Signal Fractions**

- **Unmodified**: 3.2773e+06

### TIVKPGDILLIN(N)STGN(D)LIAPRGY Hex 9 HexNAc 2

**Mass:**  4290.0085

(2.324e-04)

**Charge States:** 3, 4

**Start Position**: 251

**End Position**: 274

| Profile Score | Tandem Score | TDA q-value | Volume | Spectrum Matches |
| --- | --- | --- | --- | --- |
| 7.9886 | 39.1559 | 0.0000 | 1.9182e+07 | 6 |

**Adduct Signal Fractions**

- **Unmodified**: 1.9182e+07

### SSEC(C)ITPN(N)GSIPN(D)DKPF Hex 8 HexNAc 2

**Mass:**  3565.4247

(2.782e-04)

**Charge States:** 3

**Start Position**: 294

**End Position**: 311

| Profile Score | Tandem Score | TDA q-value | Volume | Spectrum Matches |
| --- | --- | --- | --- | --- |
| -6.4044 | 38.3262 | 0.0000 | 9.8179e+06 | 1 |

**Adduct Signal Fractions**

- **Unmodified**: 9.8179e+06

### ESESKYPVLN(N)VTMPNNGKFDKLY Fuc 2 Hex 4 HexNAc 6

**Mass:**  4831.0783

(-8.421e-06)

**Charge States:** 4, 5

**Start Position**: 172

**End Position**: 195

| Profile Score | Tandem Score | TDA q-value | Volume | Spectrum Matches |
| --- | --- | --- | --- | --- |
| 11.3204 | 37.6808 | 0.0000 | 1.2502e+08 | 8 |

**Adduct Signal Fractions**

- **Unmodified**: 1.2502e+08

### TITNDQIEVTN(N)ATEL Hex 5 HexNAc 2

**Mass:**  2877.2495

(3.243e-06)

**Charge States:** 3

**Start Position**: 44

**End Position**: 59

| Profile Score | Tandem Score | TDA q-value | Volume | Spectrum Matches |
| --- | --- | --- | --- | --- |
| -3.6670 | 35.9971 | 0.0000 | 9.5432e+06 | 1 |

**Adduct Signal Fractions**

- **Unmodified**: 9.5432e+06

### TIVKPGDILLIN(N)STGNLIAPR Fuc 2 Hex 4 HexNAc 4 Neu5Ac 1

**Mass:**  4250.0221

(4.691e-04)

**Start Position**: 251

**End Position**: 272

| Profile Score | Tandem Score | TDA q-value | Volume | Spectrum Matches |
| --- | --- | --- | --- | --- |
| 0.0000 | 35.3628 | 0.0000 | 0.0000e+00 | 1 |

Adduct:

No Chromatogram Found

### KIRTGKSSIM(O)RSDAPIGTC(C)SSEC(C)ITPN(N)GSIPNDKPF Hex 7 HexNAc 2

**Mass:**  5477.4532

(2.057e-06)

**Charge States:** 5

**Start Position**: 275

**End Position**: 311

| Profile Score | Tandem Score | TDA q-value | Volume | Spectrum Matches |
| --- | --- | --- | --- | --- |
| -20.5045 | 35.2041 | 0.0000 | 2.5764e+06 | 1 |

**Adduct Signal Fractions**

- **Unmodified**: 2.5764e+06

### TIVKPGDILLIN(N)STGNL Fuc 2 Hex 3 HexNAc 4 Neu5Ac 1

**Mass:**  3650.6793

(5.421e-04)

**Start Position**: 251

**End Position**: 268

| Profile Score | Tandem Score | TDA q-value | Volume | Spectrum Matches |
| --- | --- | --- | --- | --- |
| 0.0000 | 34.6331 | 0.0000 | 0.0000e+00 | 1 |

Adduct:

No Chromatogram Found

### KIRTGKSSIM(O)RSDAPIGTC(C)SSEC(C)ITPN(N)GSIPN(D)DKPF Hex 8 HexNAc 2

**Mass:**  5640.4867

(1.747e-04)

**Charge States:** 5

**Start Position**: 275

**End Position**: 311

| Profile Score | Tandem Score | TDA q-value | Volume | Spectrum Matches |
| --- | --- | --- | --- | --- |
| -20.3602 | 34.1432 | 0.0000 | 1.9320e+07 | 2 |

**Adduct Signal Fractions**

- **Unmodified**: 1.9320e+07

### ITPN(N)GSIPNDKPF Fuc 4 Hex 7 HexNAc 7

**Mass:**  4538.8889

(3.865e-06)

**Charge States:** 4

**Start Position**: 298

**End Position**: 311

| Profile Score | Tandem Score | TDA q-value | Volume | Spectrum Matches |
| --- | --- | --- | --- | --- |
| -20.7073 | 33.4804 | 0.0000 | 2.2528e+06 | 1 |

**Adduct Signal Fractions**

- **Unmodified**: 2.2528e+06

### LPGNDN(N)STATL Fuc 1 Hex 6 HexNAc 2

**Mass:**  2626.0693

(3.811e-06)

**Charge States:** 2, 3

**Start Position**: 19

**End Position**: 30

| Profile Score | Tandem Score | TDA q-value | Volume | Spectrum Matches |
| --- | --- | --- | --- | --- |
| 12.6900 | 32.9506 | 0.0000 | 4.8164e+08 | 10 |

**Adduct Signal Fractions**

- **Unmodified**: 4.8164e+08

### TIVKPGDILLIN(N)STGN(D)LIAPR Hex 9 HexNAc 2

**Mass:**  4069.9250

(2.477e-04)

**Charge States:** 3

**Start Position**: 251

**End Position**: 272

| Profile Score | Tandem Score | TDA q-value | Volume | Spectrum Matches |
| --- | --- | --- | --- | --- |
| -5.6812 | 32.7159 | 0.0000 | 7.0008e+06 | 1 |

**Adduct Signal Fractions**

- **Unmodified**: 7.0008e+06

### ESESKYPVLN(N)VTM(O)PNN(D)GKFDKLY Fuc 2 Hex 8 HexNAc 3

**Mass:**  4887.1047

(2.086e-04)

**Charge States:** 4, 5

**Start Position**: 172

**End Position**: 195

| Profile Score | Tandem Score | TDA q-value | Volume | Spectrum Matches |
| --- | --- | --- | --- | --- |
| -6.0149 | 32.6541 | 0.0000 | 1.2119e+08 | 4 |

**Adduct Signal Fractions**

- **Unmodified**: 1.2119e+08

### FKIRTGKSSIM(O)RSDAPIGTC(C)SSEC(C)ITPN(N)GSIPNDKPF Hex 8 HexNAc 2

**Mass:**  5786.5713

(1.479e-06)

**Charge States:** 5

**Start Position**: 274

**End Position**: 311

| Profile Score | Tandem Score | TDA q-value | Volume | Spectrum Matches |
| --- | --- | --- | --- | --- |
| -20.6066 | 32.0291 | 0.0000 | 1.0820e+07 | 1 |

**Adduct Signal Fractions**

- **Unmodified**: 1.0820e+07

### SSEC(C)ITPN(N)GSIPNDKPF Fuc 4 Hex 6 HexNAc 7

**Mass:**  4840.0012

(9.065e-06)

**Charge States:** 4

**Start Position**: 294

**End Position**: 311

| Profile Score | Tandem Score | TDA q-value | Volume | Spectrum Matches |
| --- | --- | --- | --- | --- |
| -4.3042 | 31.7817 | 0.0000 | 1.9188e+07 | 2 |

**Adduct Signal Fractions**

- **Unmodified**: 1.9188e+07

### TIVKPGDILLIN(N)STGNLIAPRGY Hex 5 HexNAc 6

**Mass:**  4453.1061

(-1.587e-06)

**Charge States:** 4

**Start Position**: 251

**End Position**: 274

| Profile Score | Tandem Score | TDA q-value | Volume | Spectrum Matches |
| --- | --- | --- | --- | --- |
| 7.5606 | 31.1149 | 0.0000 | 3.6393e+07 | 5 |

**Adduct Signal Fractions**

- **Unmodified**: 3.6393e+07

### ITPN(N)GSIPNDKPF Fuc 2 Hex 6 HexNAc 8 Neu5Ac 1

**Mass:**  4578.8937

(3.596e-06)

**Charge States:** 4

**Start Position**: 298

**End Position**: 311

| Profile Score | Tandem Score | TDA q-value | Volume | Spectrum Matches |
| --- | --- | --- | --- | --- |
| -20.5217 | 31.0543 | 0.0000 | 4.8012e+06 | 1 |

**Adduct Signal Fractions**

- **Unmodified**: 4.8012e+06

### SGGSSTC(C)KRGSN(N)NSFF Fuc 1 Hex 3 HexNAc 5

**Mass:**  3339.3484

(9.141e-07)

**Charge States:** 3

**Start Position**: 149

**End Position**: 165

| Profile Score | Tandem Score | TDA q-value | Volume | Spectrum Matches |
| --- | --- | --- | --- | --- |
| -19.5287 | 31.0500 | 0.0000 | 9.0692e+06 | 2 |

**Adduct Signal Fractions**

- **Unmodified**: 9.0692e+06

### LPGNDN(N)STATL Hex 3 HexNAc 5

**Mass:**  2603.0879

(9.375e-07)

**Charge States:** 3

**Start Position**: 19

**End Position**: 30

| Profile Score | Tandem Score | TDA q-value | Volume | Spectrum Matches |
| --- | --- | --- | --- | --- |
| 12.6393 | 30.7874 | 0.0000 | 1.1819e+07 | 3 |

**Adduct Signal Fractions**

- **Unmodified**: 1.1819e+07

### TGVTQSGGSSTC(C)KRGSN(N)NSFF Fuc 1 Hex 3 HexNAc 5

**Mass:**  3825.5917

(2.217e-07)

**Charge States:** 4

**Start Position**: 144

**End Position**: 165

| Profile Score | Tandem Score | TDA q-value | Volume | Spectrum Matches |
| --- | --- | --- | --- | --- |
| -4.6621 | 30.5962 | 0.0000 | 1.5474e+07 | 2 |

**Adduct Signal Fractions**

- **Unmodified**: 1.5474e+07

### LIN(N)STGNLIAPRGYF Hex 8 HexNAc 2

**Mass:**  3337.4573

(-5.969e-07)

**Charge States:** 3

**Start Position**: 260

**End Position**: 275

| Profile Score | Tandem Score | TDA q-value | Volume | Spectrum Matches |
| --- | --- | --- | --- | --- |
| -21.2830 | 30.0370 | 0.0000 | 1.1097e+06 | 1 |

**Adduct Signal Fractions**

- **Unmodified**: 1.1097e+06

### TITNDQIEVTN(N)ATELVQSSSTGRIC(C)DSPHRIL Hex 6 HexNAc 2

**Mass:**  4933.2570

(1.336e-06)

**Charge States:** 4

**Start Position**: 44

**End Position**: 76

| Profile Score | Tandem Score | TDA q-value | Volume | Spectrum Matches |
| --- | --- | --- | --- | --- |
| -20.3865 | 29.8256 | 0.0000 | 2.9583e+06 | 1 |

**Adduct Signal Fractions**

- **Unmodified**: 2.9583e+06

### KIRTGKSSIM(O)RSDAPIGTC(C)SSEC(C)ITPN(N)GSIPN(D)DKPF Hex 9 HexNAc 2

**Mass:**  5802.5362

(1.708e-04)

**Charge States:** 6

**Start Position**: 275

**End Position**: 311

| Profile Score | Tandem Score | TDA q-value | Volume | Spectrum Matches |
| --- | --- | --- | --- | --- |
| -6.6351 | 29.7775 | 0.0000 | 1.8073e+07 | 2 |

**Adduct Signal Fractions**

- **Unmodified**: 1.8073e+07

### RSDAPIGTC(C)SSEC(C)ITPN(N)GSIPN(D)DKPF Hex 7 HexNAc 2

**Mass:**  4360.8044

(2.271e-04)

**Charge States:** 3, 4

**Start Position**: 285

**End Position**: 311

| Profile Score | Tandem Score | TDA q-value | Volume | Spectrum Matches |
| --- | --- | --- | --- | --- |
| -21.1051 | 29.6608 | 0.0000 | 6.3629e+06 | 2 |

**Adduct Signal Fractions**

- **Unmodified**: 6.3629e+06

### SSEC(C)ITPN(N)GSIPNDKPF Fuc 4 Hex 7 HexNAc 7

**Mass:**  5002.0524

(8.720e-06)

**Charge States:** 4

**Start Position**: 294

**End Position**: 311

| Profile Score | Tandem Score | TDA q-value | Volume | Spectrum Matches |
| --- | --- | --- | --- | --- |
| 10.2309 | 29.5943 | 0.0000 | 1.6432e+07 | 1 |

**Adduct Signal Fractions**

- **Unmodified**: 1.6432e+07

### TIVKPGDILLIN(N) Hex 9 HexNAc 2

**Mass:**  3160.4261

(3.184e-04)

**Start Position**: 251

**End Position**: 263

| Profile Score | Tandem Score | TDA q-value | Volume | Spectrum Matches |
| --- | --- | --- | --- | --- |
| 0.0000 | 27.9667 | 0.0099 | 0.0000e+00 | 1 |

Adduct:

No Chromatogram Found

### IN(N)STGNLIAPR Hex 9 HexNAc 2

**Mass:**  3019.2746

(-1.257e-07)

**Charge States:** 3

**Start Position**: 261

**End Position**: 272

| Profile Score | Tandem Score | TDA q-value | Volume | Spectrum Matches |
| --- | --- | --- | --- | --- |
| -19.4419 | 27.5381 | 0.0099 | 3.4214e+06 | 1 |

**Adduct Signal Fractions**

- **Unmodified**: 3.4214e+06

### TC(C)KRGSN(N)NSFF Fuc 1 Hex 3 HexNAc 4

**Mass:**  2761.1475

(7.380e-06)

**Charge States:** 3

**Start Position**: 154

**End Position**: 165

| Profile Score | Tandem Score | TDA q-value | Volume | Spectrum Matches |
| --- | --- | --- | --- | --- |
| -18.6904 | 27.1652 | 0.0137 | 9.8164e+06 | 1 |

**Adduct Signal Fractions**

- **Unmodified**: 9.8164e+06

### TIVKPGDILLIN(N)STGN Hex 8 HexNAc 2

**Mass:**  3356.5109

(-2.053e-07)

**Charge States:** 3

**Start Position**: 251

**End Position**: 267

| Profile Score | Tandem Score | TDA q-value | Volume | Spectrum Matches |
| --- | --- | --- | --- | --- |
| -19.4456 | 26.6932 | 0.0137 | 7.1653e+05 | 1 |

**Adduct Signal Fractions**

- **Unmodified**: 7.1653e+05

### TITNDQIEVTN(N)ATEL Hex 3 HexNAc 4

**Mass:**  2959.3062

(5.581e-06)

**Charge States:** 3

**Start Position**: 44

**End Position**: 59

| Profile Score | Tandem Score | TDA q-value | Volume | Spectrum Matches |
| --- | --- | --- | --- | --- |
| -4.9937 | 26.4992 | 0.0137 | 5.4010e+06 | 1 |

**Adduct Signal Fractions**

- **Unmodified**: 5.4010e+06

### TIVKPGDILLIN(N)STGNLIAPRGY Fuc 3 Hex 3 HexNAc 5

**Mass:**  4365.0737

(2.224e-04)

**Start Position**: 251

**End Position**: 274

| Profile Score | Tandem Score | TDA q-value | Volume | Spectrum Matches |
| --- | --- | --- | --- | --- |
| 0.0000 | 26.4929 | 0.0137 | 0.0000e+00 | 1 |

Adduct:

No Chromatogram Found

### ITPN(N)GSIPNDKPF Fuc 1 Hex 10 HexNAc 6 Neu5Ac 1

**Mass:**  4674.8801

(1.736e-06)

**Charge States:** 4

**Start Position**: 298

**End Position**: 311

| Profile Score | Tandem Score | TDA q-value | Volume | Spectrum Matches |
| --- | --- | --- | --- | --- |
| -5.1019 | 26.4841 | 0.0137 | 3.3388e+06 | 1 |

**Adduct Signal Fractions**

- **Unmodified**: 3.3388e+06

### LPGNDN(N)STATL Fuc 2 Hex 6 HexNAc 3

**Mass:**  2975.2038

(9.734e-07)

**Charge States:** 3

**Start Position**: 19

**End Position**: 30

| Profile Score | Tandem Score | TDA q-value | Volume | Spectrum Matches |
| --- | --- | --- | --- | --- |
| 13.7689 | 26.0188 | 0.0137 | 2.8166e+08 | 6 |

**Adduct Signal Fractions**

- **Unmodified**: 2.8166e+08

### TIVKPGDILLIN(N)STGN(D)LIAPRGY Fuc 2 Hex 3 HexNAc 4 Neu5Ac 1

**Mass:**  4307.0388

(2.270e-04)

**Charge States:** 4

**Start Position**: 251

**End Position**: 274

| Profile Score | Tandem Score | TDA q-value | Volume | Spectrum Matches |
| --- | --- | --- | --- | --- |
| -20.6004 | 25.9403 | 0.0137 | 1.2430e+06 | 2 |

**Adduct Signal Fractions**

- **Unmodified**: 1.2430e+06

### LPGNDN(N)STATL Fuc 1 Hex 4 HexNAc 4

**Mass:**  2708.1218

(1.864e-06)

**Charge States:** 3

**Start Position**: 19

**End Position**: 30

| Profile Score | Tandem Score | TDA q-value | Volume | Spectrum Matches |
| --- | --- | --- | --- | --- |
| -6.1281 | 25.9090 | 0.0137 | 3.0852e+06 | 1 |

**Adduct Signal Fractions**

- **Unmodified**: 3.0852e+06

### ITPN(N)GSIPNDKPF Fuc 4 Hex 5 HexNAc 8

**Mass:**  4417.8161

(-6.607e-06)

**Charge States:** 4

**Start Position**: 298

**End Position**: 311

| Profile Score | Tandem Score | TDA q-value | Volume | Spectrum Matches |
| --- | --- | --- | --- | --- |
| -4.8995 | 24.8919 | 0.0264 | 7.3643e+06 | 2 |

**Adduct Signal Fractions**

- **Unmodified**: 7.3643e+06

### ITPN(N)GSIPNDKPF Fuc 3 Hex 6 HexNAc 9

**Mass:**  4638.9017

(4.275e-04)

**Start Position**: 298

**End Position**: 311

| Profile Score | Tandem Score | TDA q-value | Volume | Spectrum Matches |
| --- | --- | --- | --- | --- |
| 0.0000 | 24.6400 | 0.0264 | 0.0000e+00 | 1 |

Adduct:

No Chromatogram Found

### SSEC(C)ITPN(N)GSIPN(D)DKPF Fuc 4 Hex 6 HexNAc 7

**Mass:**  4840.9899

(2.107e-04)

**Charge States:** 4

**Start Position**: 294

**End Position**: 311

| Profile Score | Tandem Score | TDA q-value | Volume | Spectrum Matches |
| --- | --- | --- | --- | --- |
| -6.3628 | 24.4092 | 0.0264 | 1.2346e+07 | 2 |

**Adduct Signal Fractions**

- **Unmodified**: 1.2346e+07

### SEC(C)ITPN(N)GSIPN(D)DKPF Hex 9 HexNAc 2

**Mass:**  3640.4485

(2.721e-04)

**Charge States:** 3

**Start Position**: 295

**End Position**: 311

| Profile Score | Tandem Score | TDA q-value | Volume | Spectrum Matches |
| --- | --- | --- | --- | --- |
| -21.0501 | 24.1812 | 0.0303 | 5.3736e+06 | 1 |

**Adduct Signal Fractions**

- **Unmodified**: 5.3736e+06

### ITPN(N)GSIPNDKPF Fuc 2 Hex 6 HexNAc 8 Neu5Ac 1

**Mass:**  4581.8577

(6.509e-04)

**Start Position**: 298

**End Position**: 311

| Profile Score | Tandem Score | TDA q-value | Volume | Spectrum Matches |
| --- | --- | --- | --- | --- |
| 0.0000 | 23.9224 | 0.0303 | 0.0000e+00 | 1 |

Adduct:

No Chromatogram Found

### TIVKPGDILLIN(N)STGNLIAPR Fuc 1 Hex 3 HexNAc 5 Neu5Ac 1

**Mass:**  4142.9719

(-6.285e-06)

**Charge States:** 4

**Start Position**: 251

**End Position**: 272

| Profile Score | Tandem Score | TDA q-value | Volume | Spectrum Matches |
| --- | --- | --- | --- | --- |
| -20.2093 | 23.5795 | 0.0339 | 1.7104e+06 | 1 |

**Adduct Signal Fractions**

- **Unmodified**: 1.7104e+06

### TITNDQIEVTN(N)ATEL Hex 5 HexNAc 3

**Mass:**  3080.3292

(3.775e-06)

**Charge States:** 3

**Start Position**: 44

**End Position**: 59

| Profile Score | Tandem Score | TDA q-value | Volume | Spectrum Matches |
| --- | --- | --- | --- | --- |
| -4.1431 | 23.4177 | 0.0339 | 5.3999e+06 | 1 |

**Adduct Signal Fractions**

- **Unmodified**: 5.3999e+06

### TIVKPGDILLIN(N)STGNLIAPRGY Fuc 1 Hex 3 HexNAc 5 Neu5Ac 1

**Mass:**  4363.0622

(-4.658e-06)

**Charge States:** 4

**Start Position**: 251

**End Position**: 274

| Profile Score | Tandem Score | TDA q-value | Volume | Spectrum Matches |
| --- | --- | --- | --- | --- |
| 8.9919 | 23.2430 | 0.0417 | 1.2277e+07 | 3 |

**Adduct Signal Fractions**

- **Unmodified**: 1.2277e+07

### LPGNDN(N)STATL Fuc 1 Hex 6 HexNAc 3

**Mass:**  2830.1677

(3.621e-04)

**Start Position**: 19

**End Position**: 30

| Profile Score | Tandem Score | TDA q-value | Volume | Spectrum Matches |
| --- | --- | --- | --- | --- |
| 0.0000 | 22.7521 | 0.0453 | 0.0000e+00 | 1 |

Adduct:

No Chromatogram Found

### SEC(C)ITPN(N)GSIPN(D)DKPF Hex 8 HexNAc 2

**Mass:**  3479.3983

(5.744e-04)

**Start Position**: 295

**End Position**: 311

| Profile Score | Tandem Score | TDA q-value | Volume | Spectrum Matches |
| --- | --- | --- | --- | --- |
| 0.0000 | 22.6724 | 0.0484 | 0.0000e+00 | 1 |

Adduct:

No Chromatogram Found

### ESESKYPVLN(N)VTM(O)PNN(D)GKFDKLY Fuc 2 Hex 7 HexNAc 3

**Mass:**  4725.0544

(2.155e-04)

**Charge States:** 4, 5

**Start Position**: 172

**End Position**: 195

| Profile Score | Tandem Score | TDA q-value | Volume | Spectrum Matches |
| --- | --- | --- | --- | --- |
| 12.5939 | 22.4783 | 0.0484 | 7.6582e+07 | 5 |

**Adduct Signal Fractions**

- **Unmodified**: 7.6582e+07

### iav|ADJ41819|A/Phil/2-MA/1982\_NA

**>iav|ADJ41819|A/Phil/2-MA/1982\_NA**  
 `MNPNQKIITIGSVSLTIATICFLMQIAILVTTVTLHFKQYECSSPPNNQVVPCEPIIIERNITEIVYLTNTTIEKEICPK  
LVEYRNWSKPQCKITGFAPFSKDNSIRLSAGGDIWVTREPYVSCDPGKCYQFALGQGTTLDNKHSNDTIHDRTPYRTLLM  
NELGVPFHLGTRQVCIAWSSSSCHDGKAWLHVCITGYDKNATASFIYDGRLVDSIGSWSKNILRTQESECVCINGTCTVV  
MTDGSASERADTKILFIEEGKIVHISPLSGSAQHVEECSCYPRYPGVRCVCRDNWKGSNRPVVDINVKDYSIVSSYVCSG  
LVGDTPRKNDRSSSSYCRNPNNEKGNHGVKGWAFDDGNDVWMGRTISEESRSGYETFKVIGGWSTPNSKLQINRQVIVDS  
GNRSGYSGIFSVEGKSCINRCFYVELIRGREQETRVWWTSNSIVVFCGTSGTYGTGSWPDGADINLMPI`

| Site | Type | Count |
| --- | --- | --- |
| 146 | N-linked | 6 |
| 86 | N-linked | 2 |
| 402 | N-linked | 5 |

| Mass | Sequence | MS_2_ Score | `q`-value | MS_1_ Score | Signal | Protein Coordinates | Tandem Scans | Start Time | Apex Time | End Time |
| --- | --- | --- | --- | --- | --- | --- | --- | --- | --- | --- |
| 3183.3964 | QINRQVIVDSGN(N)RSGY Hex 6 HexNAc 2 | 153.629 | 0.0 | -19.006 | 1.881e+07 | 390 - 406 | 4 | 21.04 | 21.20 | 29.80 |
| 3539.4011 | DNKHSN(N)DTIHDRTPY Fuc 1 Hex 6 HexNAc 3 | 125.747 | 0.0 | -20.491 | 2.591e+07 | 140 - 155 | 1 | 12.98 | 12.98 | 13.15 |
| 3345.4509 | QINRQVIVDSGN(N)RSGY Hex 7 HexNAc 2 | 124.106 | 0.0 | -20.164 | 4.887e+06 | 390 - 406 | 2 | 21.04 | 21.04 | 21.20 |
| 3800.6353 | RN(N)WSKPQC(C)KITGFAPF Hex 9 HexNAc 2 | 115.537 | 0.0 | -3.919 | 5.283e+06 | 84 - 100 | 1 | 36.96 | 37.13 | 37.30 |
| 3325.4097 | RN(N)WSKPQC(C)KITGF Hex 8 HexNAc 2 | 104.611 | 0.0 | 0.000 | 0.000e+00 | 84 - 97 | 1 | - | - | - |
| 3910.7848 | STPNSKLQINRQVIVDSGN(N)RSGY Hex 6 HexNAc 2 | 90.296 | 0.0 | -18.589 | 9.510e+06 | 383 - 406 | 1 | 28.11 | 28.11 | 28.28 |
| 4280.7948 | ALGQGTTLDNKHSN(N)DTIHDRTPY Fuc 1 Hex 6 HexNAc 3 | 82.613 | 0.0 | -4.543 | 1.286e+07 | 132 - 155 | 2 | 21.88 | 22.04 | 22.38 |
| 2828.1976 | RQVIVDSGN(N)RSGY Hex 6 HexNAc 2 | 41.472 | 0.0 | 0.000 | 0.000e+00 | 393 - 406 | 1 | - | - | - |
| 3849.5365 | DN(D)KHSN(N)DTIHDRTPY Fuc 2 Hex 7 HexNAc 3 | 41.303 | 0.0 | 0.000 | 0.000e+00 | 140 - 155 | 1 | - | - | - |
| 3540.4021 | DNKHSN(N)DTIHDRTPY Fuc 1 Hex 6 HexNAc 3 | 39.528 | 0.0 | 0.000 | 0.000e+00 | 140 - 155 | 1 | - | - | - |
| 4054.7870 | ALGQGTTLDNKHSN(N)DTIHDRTPY Hex 3 HexNAc 5 | 34.293 | 0.0 | -20.000 | 4.799e+06 | 132 - 155 | 2 | 20.37 | 20.53 | 20.53 |
| 4734.9616 | ALGQGTTLDN(D)KHSN(N)DTIHDRTPY Fuc 1 Hex 7 HexNAc 3 Neu5Ac 1 | 32.312 | 0.0 | -20.599 | 3.370e+06 | 132 - 155 | 1 | 21.54 | 21.54 | 21.54 |
| 3507.5019 | QINRQVIVDSGN(N)RSGY Hex 8 HexNAc 2 | 31.743 | 0.0 | -20.236 | 5.300e+06 | 390 - 406 | 2 | 20.87 | 21.04 | 21.04 |

### QINRQVIVDSGN(N)RSGY Hex 6 HexNAc 2

**Mass:**  3183.3953

(-6.672e-07)

**Charge States:** 3

**Start Position**: 391

**End Position**: 407

| Profile Score | Tandem Score | TDA q-value | Volume | Spectrum Matches |
| --- | --- | --- | --- | --- |
| -19.0057 | 153.6290 | 0.0000 | 1.8812e+07 | 4 |

**Adduct Signal Fractions**

- **Unmodified**: 1.8812e+07

### DNKHSN(N)DTIHDRTPY Fuc 1 Hex 6 HexNAc 3

**Mass:**  3539.4011

(-8.674e-06)

**Charge States:** 4

**Start Position**: 141

**End Position**: 156

| Profile Score | Tandem Score | TDA q-value | Volume | Spectrum Matches |
| --- | --- | --- | --- | --- |
| -20.4911 | 125.7471 | 0.0000 | 2.5911e+07 | 1 |

**Adduct Signal Fractions**

- **Unmodified**: 2.5911e+07

### QINRQVIVDSGN(N)RSGY Hex 7 HexNAc 2

**Mass:**  3345.4499

(9.539e-07)

**Charge States:** 3

**Start Position**: 391

**End Position**: 407

| Profile Score | Tandem Score | TDA q-value | Volume | Spectrum Matches |
| --- | --- | --- | --- | --- |
| -20.1637 | 124.1058 | 0.0000 | 4.8868e+06 | 2 |

**Adduct Signal Fractions**

- **Unmodified**: 4.8868e+06

### RN(N)WSKPQC(C)KITGFAPF Hex 9 HexNAc 2

**Mass:**  3800.6320

(5.304e-06)

**Charge States:** 4

**Start Position**: 85

**End Position**: 101

| Profile Score | Tandem Score | TDA q-value | Volume | Spectrum Matches |
| --- | --- | --- | --- | --- |
| -3.9185 | 115.5374 | 0.0000 | 5.2827e+06 | 1 |

**Adduct Signal Fractions**

- **Unmodified**: 5.2827e+06

### RN(N)WSKPQC(C)KITGF Hex 8 HexNAc 2

**Mass:**  3325.4097

(6.045e-04)

**Start Position**: 85

**End Position**: 98

| Profile Score | Tandem Score | TDA q-value | Volume | Spectrum Matches |
| --- | --- | --- | --- | --- |
| 0.0000 | 104.6110 | 0.0000 | 0.0000e+00 | 1 |

Adduct:

No Chromatogram Found

### STPNSKLQINRQVIVDSGN(N)RSGY Hex 6 HexNAc 2

**Mass:**  3910.7802

(-2.935e-09)

**Charge States:** 4

**Start Position**: 384

**End Position**: 407

| Profile Score | Tandem Score | TDA q-value | Volume | Spectrum Matches |
| --- | --- | --- | --- | --- |
| -18.5892 | 90.2965 | 0.0000 | 9.5102e+06 | 1 |

**Adduct Signal Fractions**

- **Unmodified**: 9.5102e+06

### ALGQGTTLDNKHSN(N)DTIHDRTPY Fuc 1 Hex 6 HexNAc 3

**Mass:**  4280.7932

(-9.481e-06)

**Charge States:** 4

**Start Position**: 133

**End Position**: 156

| Profile Score | Tandem Score | TDA q-value | Volume | Spectrum Matches |
| --- | --- | --- | --- | --- |
| -4.5431 | 82.6134 | 0.0000 | 1.2855e+07 | 2 |

**Adduct Signal Fractions**

- **Unmodified**: 1.2855e+07

### RQVIVDSGN(N)RSGY Hex 6 HexNAc 2

**Mass:**  2828.1976

(-3.732e-06)

**Start Position**: 394

**End Position**: 407

| Profile Score | Tandem Score | TDA q-value | Volume | Spectrum Matches |
| --- | --- | --- | --- | --- |
| 0.0000 | 41.4718 | 0.0000 | 0.0000e+00 | 1 |

Adduct:

No Chromatogram Found

### DN(D)KHSN(N)DTIHDRTPY Fuc 2 Hex 7 HexNAc 3

**Mass:**  3849.5365

(5.183e-04)

**Start Position**: 141

**End Position**: 156

| Profile Score | Tandem Score | TDA q-value | Volume | Spectrum Matches |
| --- | --- | --- | --- | --- |
| 0.0000 | 41.3031 | 0.0000 | 0.0000e+00 | 1 |

Adduct:

No Chromatogram Found

### DNKHSN(N)DTIHDRTPY Fuc 1 Hex 6 HexNAc 3

**Mass:**  3540.4021

(2.742e-04)

**Start Position**: 141

**End Position**: 156

| Profile Score | Tandem Score | TDA q-value | Volume | Spectrum Matches |
| --- | --- | --- | --- | --- |
| 0.0000 | 39.5280 | 0.0000 | 0.0000e+00 | 1 |

Adduct:

No Chromatogram Found

### ALGQGTTLDNKHSN(N)DTIHDRTPY Hex 3 HexNAc 5

**Mass:**  4054.7897

(2.509e-06)

**Charge States:** 5

**Start Position**: 133

**End Position**: 156

| Profile Score | Tandem Score | TDA q-value | Volume | Spectrum Matches |
| --- | --- | --- | --- | --- |
| -19.9999 | 34.2932 | 0.0000 | 4.7994e+06 | 2 |

**Adduct Signal Fractions**

- **Unmodified**: 4.7994e+06

### ALGQGTTLDN(D)KHSN(N)DTIHDRTPY Fuc 1 Hex 7 HexNAc 3 Neu5Ac 1

**Mass:**  4734.9616

(2.069e-04)

**Charge States:** 4

**Start Position**: 133

**End Position**: 156

| Profile Score | Tandem Score | TDA q-value | Volume | Spectrum Matches |
| --- | --- | --- | --- | --- |
| -20.5992 | 32.3117 | 0.0000 | 3.3700e+06 | 1 |

**Adduct Signal Fractions**

- **Unmodified**: 3.3700e+06

### QINRQVIVDSGN(N)RSGY Hex 8 HexNAc 2

**Mass:**  3507.5030

(1.009e-06)

**Charge States:** 3, 4

**Start Position**: 391

**End Position**: 407

| Profile Score | Tandem Score | TDA q-value | Volume | Spectrum Matches |
| --- | --- | --- | --- | --- |
| -20.2356 | 31.7432 | 0.0000 | 5.3001e+06 | 2 |

**Adduct Signal Fractions**

- **Unmodified**: 5.3001e+06
