# Supplementary material for: Measuring Site-specific Glycosylation Similarity between Influenza a Virus Variants with Statistical Certainty
Source: Mol Cell Proteomics. 2020 Nov 25;19(9):1533–45. doi: 10.1074/mcp.RA120.002031 (PMC8143645; doi:10.1074/mcp.RA120.002031)
Supplement: Supplementary file 2 [file mmc2.zip › mmc2/159250_2_supp_555289_qcjklv.zip!/20190326_Phil82gal_021119_chymo_HILIC_DDA_1ug_03_glycopeptides.html]

## 20190326\_Phil82gal\_021119\_chymo\_HILIC\_DDA\_1ug\_03 @ GlycopeptideHypothesis-f9e976c55e814f17a1c399d9fa219201

**MS^1^ Mass Accuracy:** 10.0 PPM  
**Grouping Tolerance:** 15.0 PPM  
**MS^n^ Mass Accuracy:** 20.0 PPM  
**Minimum `q`-Value For Clustering:** 0.05

**Glycopeptide Hypothesis:**
GlycopeptideHypothesis-f9e976c55e814f17a1c399d9fa219201  
**Sample Searched:**
20190326\_Phil82gal\_021119\_chymo\_HILIC\_DDA\_1ug\_03

| Protein | Matched Glycopeptides |
| --- | --- |
| iav\|AFG99160\|A/Phil/2/1982\_HA | 110 |
| iav\|ADJ41819\|A/Phil/2-MA/1982\_NA | 16 |
| sp\|Q90734\|ACTN4\_CHICK | 1 |
| tr\|A0A1D5NZ30\|A0A1D5NZ30\_CHICK | 1 |
| sp\|P15771\|NUCL\_CHICK | 1 |

### iav|AFG99160|A/Phil/2/1982\_HA

**>iav|AFG99160|A/Phil/2/1982\_HA**  
 `MKTIIALSYMFCLVFAQNLPGNDNSTATLCLGHHAVPNGTLVKTITNDQIEVTNATELVQSSSTGRICDSPHRILDGKNC  
TLIDALLGDPHCDGFQNEKWDLFVERSKAFSNCYPYDVPDYASLRSLVASSGTLEFINEGFNWTGVTQSGGSSTCKRGSN  
NSFFSRLNWLYESESKYPVLNVTMPNNGKFDKLYIWGIHHPSTDKEQTNLYIRASGRVTVSTKRSQQTVIPNIGSRPWVR  
GLSSRISIYWTIVKPGDILLINSTGNLIAPRGYFKIRTGKSSIMRSDAPIGTCSSECITPNGSIPNDKPFQNVNKITYGA  
CPRYVKQNTLKLATGMRNVPEKQTRGIFGAIAGFIENGWEGMVDGWYGFRHQNSEGTGQAADLKSTQAAIDQINGKLNRV  
IEKTNEKFHQIEKEFSEVEGRIQDLEKYVEDTKIDLWSYNAELLVALENQHTIDLTDSEMNKLFEKTRKQLRENAEDMGN  
GCFKIYHKCDNACIGSIRNGTYDHDVYRDEALNNRFQIKGVELKSGYKDWILWISFAISCFLLCVVLLGFIMWACQKGNI  
RCNICI`

| Site | Type | Count |
| --- | --- | --- |
| 262 | N-linked | 47 |
| 301 | N-linked | 26 |
| 142 | N-linked | 1 |
| 499 | N-linked | 5 |
| 181 | N-linked | 10 |
| 54 | N-linked | 6 |
| 24 | N-linked | 11 |
| 160 | N-linked | 4 |

| Mass | Sequence | MS_2_ Score | `q`-value | MS_1_ Score | Signal | Protein Coordinates | Tandem Scans | Start Time | Apex Time | End Time |
| --- | --- | --- | --- | --- | --- | --- | --- | --- | --- | --- |
| 2695.0885 | LIN(N)STGNL Hex 9 HexNAc 2 | 180.541 | 0.0 | -2.917 | 1.335e+07 | 259 - 267 | 2 | 22.78 | 22.94 | 23.11 |
| 3132.3664 | LIN(N)STGNLIAPR Hex 9 HexNAc 2 | 171.945 | 0.0 | 14.243 | 3.795e+07 | 259 - 271 | 2 | 27.83 | 28.00 | 28.50 |
| 4068.9292 | TIVKPGDILLIN(N)STGNLIAPR Hex 9 HexNAc 2 | 168.461 | 0.0 | 16.012 | 2.393e+08 | 250 - 271 | 9 | 50.95 | 51.47 | 52.15 |
| 2546.0679 | LPGNDN(N)STATL Fuc 1 Hex 3 HexNAc 4 | 168.450 | 0.0 | 14.189 | 5.640e+07 | 18 - 29 | 4 | 23.11 | 23.79 | 24.63 |
| 4289.0144 | TIVKPGDILLIN(N)STGNLIAPRGY Hex 9 HexNAc 2 | 163.609 | 0.0 | 15.183 | 7.543e+08 | 250 - 273 | 29 | 52.49 | 54.18 | 57.25 |
| 3352.4473 | LIN(N)STGNLIAPRGY Hex 9 HexNAc 2 | 161.396 | 0.0 | -4.369 | 4.191e+07 | 259 - 273 | 1 | 34.34 | 34.34 | 34.84 |
| 3159.4257 | TIVKPGDILLIN(N) Hex 9 HexNAc 2 | 157.152 | 0.0 | 12.979 | 9.188e+07 | 250 - 262 | 9 | 43.15 | 44.68 | 45.35 |
| 2749.1471 | LPGNDN(N)STATL Fuc 1 Hex 3 HexNAc 5 | 155.948 | 0.0 | 13.592 | 1.384e+07 | 18 - 29 | 3 | 23.28 | 23.45 | 24.13 |
| 3518.5700 | TIVKPGDILLIN(N)STGN Hex 9 HexNAc 2 | 154.743 | 0.0 | 13.274 | 3.408e+07 | 250 - 266 | 5 | 42.29 | 42.63 | 43.15 |
| 3176.4535 | TIVKPGDILLIN(N) Fuc 2 Hex 3 HexNAc 4 Neu5Ac 1 | 153.574 | 0.0 | 9.189 | 2.297e+07 | 250 - 262 | 4 | 43.15 | 44.68 | 45.02 |
| 2400.0091 | LPGNDN(N)STATL Hex 3 HexNAc 4 | 152.792 | 0.0 | 11.890 | 1.378e+07 | 18 - 29 | 3 | 23.11 | 23.45 | 23.79 |
| 2317.9561 | LPGNDN(N)STATL Hex 5 HexNAc 2 | 152.730 | 0.0 | -5.126 | 9.183e+06 | 18 - 29 | 4 | 22.94 | 23.11 | 23.45 |
| 4085.9557 | TIVKPGDILLIN(N)STGNLIAPR Fuc 2 Hex 3 HexNAc 4 Neu5Ac 1 | 148.249 | 0.0 | -4.468 | 2.652e+07 | 250 - 271 | 1 | 51.47 | 51.47 | 51.98 |
| 3321.4762 | TIVKPGDILLIN(N) Hex 10 HexNAc 2 | 146.362 | 0.0 | -18.578 | 3.346e+06 | 250 - 262 | 1 | 44.34 | 44.51 | 44.51 |
| 4306.0407 | TIVKPGDILLIN(N)STGNLIAPRGY Fuc 2 Hex 3 HexNAc 4 Neu5Ac 1 | 145.735 | 0.0 | 13.452 | 8.775e+07 | 250 - 273 | 4 | 53.68 | 54.18 | 55.38 |
| 3648.6811 | TIVKPGDILLIN(N)STGNL Fuc 2 Hex 3 HexNAc 4 Neu5Ac 1 | 145.218 | 0.0 | -4.350 | 2.299e+07 | 250 - 267 | 3 | 50.78 | 50.95 | 51.30 |
| 3101.3016 | ITPN(N)GSIPNDKPF Hex 8 HexNAc 2 | 143.035 | 0.0 | -4.139 | 1.131e+07 | 297 - 310 | 2 | 29.18 | 29.34 | 29.51 |
| 4451.0671 | TIVKPGDILLIN(N)STGNLIAPRGY Hex 10 HexNAc 2 | 140.717 | 0.0 | 13.066 | 7.084e+07 | 250 - 273 | 7 | 53.85 | 54.18 | 55.03 |
| 2703.1286 | KRGSN(N)NSFF Fuc 1 Hex 3 HexNAc 5 | 138.588 | 0.0 | -2.648 | 8.489e+07 | 155 - 164 | 2 | 18.78 | 18.78 | 19.11 |
| 2854.1791 | LPGNDN(N)STATL Fuc 2 Hex 4 HexNAc 4 | 138.387 | 0.0 | 11.665 | 1.304e+07 | 18 - 29 | 3 | 22.78 | 23.11 | 23.62 |
| 4126.9560 | TIVKPGDILLIN(N)STGNLIAPRGY Hex 8 HexNAc 2 | 138.366 | 0.0 | 12.052 | 6.347e+07 | 250 - 273 | 10 | 53.85 | 54.35 | 55.96 |
| 4086.9717 | TIVKPGDILLIN(N)STGNLIAPR Fuc 2 Hex 3 HexNAc 4 Neu5Ac 1 | 138.228 | 0.0 | 0.000 | 0.000e+00 | 250 - 271 | 1 | - | - | - |
| 4230.9825 | TIVKPGDILLIN(N)STGNLIAPR Hex 10 HexNAc 2 | 135.704 | 0.0 | -3.918 | 2.035e+07 | 250 - 271 | 3 | 51.30 | 51.47 | 51.81 |
| 3386.4382 | IN(N)STGNLIAPRGYF Hex 9 HexNAc 2 | 134.196 | 0.0 | -3.996 | 7.175e+06 | 260 - 274 | 2 | 40.76 | 40.93 | 41.10 |
| 3906.8745 | TIVKPGDILLIN(N)STGNLIAPR Hex 8 HexNAc 2 | 132.663 | 0.0 | -4.457 | 1.600e+07 | 250 - 271 | 4 | 51.47 | 51.64 | 51.81 |
| 3263.3593 | ITPN(N)GSIPNDKPF Hex 9 HexNAc 2 | 132.521 | 0.0 | -17.981 | 9.235e+06 | 297 - 310 | 1 | 29.18 | 29.18 | 29.34 |
| 3631.6544 | TIVKPGDILLIN(N)STGNL Hex 9 HexNAc 2 | 131.617 | 0.0 | 13.029 | 8.159e+07 | 250 - 267 | 5 | 50.78 | 50.95 | 51.47 |
| 3535.5970 | TIVKPGDILLIN(N)STGN Fuc 2 Hex 3 HexNAc 4 Neu5Ac 1 | 130.783 | 0.0 | -3.788 | 7.808e+06 | 250 - 266 | 2 | 42.29 | 42.63 | 42.80 |
| 3516.5378 | LIN(N)STGNLIAPRGYF Fuc 2 Hex 3 HexNAc 4 Neu5Ac 1 | 130.720 | 0.0 | -19.143 | 5.898e+06 | 259 - 274 | 1 | 45.02 | 45.19 | 45.19 |
| 3019.2766 | IN(N)STGNLIAPR Hex 9 HexNAc 2 | 125.989 | 0.0 | -19.332 | 2.532e+06 | 260 - 271 | 1 | 21.44 | 21.44 | 21.44 |
| 2500.0510 | KRGSN(N)NSFF Fuc 1 Hex 3 HexNAc 4 | 122.098 | 0.0 | -20.244 | 9.336e+06 | 155 - 164 | 1 | 18.61 | 18.61 | 18.78 |
| 3239.3664 | IN(N)STGNLIAPRGY Hex 9 HexNAc 2 | 118.071 | 0.0 | -4.086 | 1.074e+07 | 260 - 273 | 2 | 28.84 | 29.01 | 29.18 |
| 3590.4416 | LPGNDN(N)STATL Fuc 2 Hex 8 HexNAc 3 Neu5Ac 1 | 117.472 | 0.0 | 13.035 | 7.439e+07 | 18 - 29 | 3 | 32.05 | 32.55 | 33.05 |
| 3469.5995 | TIVKPGDILLIN(N)STGNL Hex 8 HexNAc 2 | 116.527 | 0.0 | -4.570 | 5.707e+06 | 250 - 267 | 1 | 50.78 | 50.95 | 51.30 |
| 3502.5244 | LIN(N)STGNLIAPRGYF Hex 9 HexNAc 2 | 108.354 | 0.0 | 0.000 | 0.000e+00 | 259 - 274 | 1 | - | - | - |
| 3282.3319 | LPGNDN(N)STATL Fuc 1 Hex 7 HexNAc 3 Neu5Ac 1 | 107.907 | 0.0 | 13.522 | 1.881e+08 | 18 - 29 | 3 | 32.71 | 33.05 | 33.54 |
| 3053.2953 | IGSIRN(N)GTYDHDVY Fuc 1 Hex 3 HexNAc 4 | 106.466 | 0.0 | -17.872 | 5.345e+06 | 493 - 507 | 1 | 22.28 | 22.28 | 22.44 |
| 3726.4892 | SSEC(C)ITPN(N)GSIPNDKPF Hex 9 HexNAc 2 | 104.791 | 0.0 | -18.289 | 1.220e+07 | 293 - 310 | 1 | 32.88 | 33.05 | 33.05 |
| 2883.1810 | SIRN(N)GTYDHDVY Fuc 1 Hex 3 HexNAc 4 | 104.760 | 0.0 | 0.000 | 0.000e+00 | 495 - 507 | 1 | - | - | - |
| 3369.4701 | LIN(N)STGNLIAPRGY Fuc 2 Hex 3 HexNAc 4 Neu5Ac 1 | 95.458 | 0.0 | -20.828 | 3.905e+06 | 259 - 273 | 1 | 34.34 | 34.51 | 34.51 |
| 4553.9407 | ESESKYPVLN(N)VTM(O)PNN(D)GKFDKLY Hex 9 HexNAc 2 | 93.502 | 0.0 | 11.622 | 3.719e+07 | 171 - 194 | 4 | 40.25 | 40.76 | 41.10 |
| 4391.8888 | ESESKYPVLN(N)VTM(O)PNN(D)GKFDKLY Hex 8 HexNAc 2 | 92.823 | 0.0 | 11.530 | 2.618e+07 | 171 - 194 | 3 | 40.25 | 40.76 | 41.10 |
| 4521.8735 | ITPN(N)GSIPNDKPF Fuc 3 Hex 6 HexNAc 7 Neu5Ac 1 | 91.409 | 0.0 | 12.322 | 7.313e+07 | 297 - 310 | 4 | 35.50 | 35.66 | 36.33 |
| 4683.9240 | ITPN(N)GSIPNDKPF Fuc 3 Hex 7 HexNAc 7 Neu5Ac 1 | 90.043 | 0.0 | 14.068 | 6.244e+07 | 297 - 310 | 3 | 35.50 | 35.66 | 36.16 |
| 4741.9550 | ITPN(N)GSIPNDKPF Fuc 4 Hex 7 HexNAc 8 | 88.929 | 0.0 | -19.960 | 5.087e+06 | 297 - 310 | 1 | 35.82 | 35.82 | 35.99 |
| 4361.8285 | RSDAPIGTC(C)SSEC(C)ITPN(N)GSIPNDKPF Hex 7 HexNAc 2 | 83.112 | 0.0 | 0.000 | 0.000e+00 | 284 - 310 | 1 | - | - | - |
| 3201.3548 | TITNDQIEVTN(N)ATEL Hex 7 HexNAc 2 | 82.990 | 0.0 | -17.213 | 7.283e+06 | 43 - 58 | 1 | 38.04 | 38.04 | 38.21 |
| 4578.8944 | ITPN(N)GSIPNDKPF Fuc 2 Hex 6 HexNAc 8 Neu5Ac 1 | 72.275 | 0.0 | -5.881 | 6.740e+06 | 297 - 310 | 1 | 35.66 | 35.82 | 35.99 |
| 5639.5071 | KIRTGKSSIM(O)RSDAPIGTC(C)SSEC(C)ITPN(N)GSIPNDKPF Hex 8 HexNAc 2 | 71.513 | 0.0 | -4.574 | 4.306e+07 | 274 - 310 | 3 | 31.20 | 31.37 | 31.71 |
| 5801.5598 | KIRTGKSSIM(O)RSDAPIGTC(C)SSEC(C)ITPN(N)GSIPNDKPF Hex 9 HexNAc 2 | 63.853 | 0.0 | -4.621 | 3.342e+07 | 274 - 310 | 3 | 31.20 | 31.37 | 31.54 |
| 4290.0060 | TIVKPGDILLIN(N)STGN(D)LIAPRGY Hex 9 HexNAc 2 | 61.226 | 0.0 | -4.650 | 8.014e+06 | 250 - 273 | 5 | 55.03 | 55.03 | 55.38 |
| 3964.8950 | TIVKPGDILLIN(N)STGNLIAPRGY Hex 7 HexNAc 2 | 57.949 | 0.0 | -6.153 | 2.772e+06 | 250 - 273 | 1 | 54.18 | 54.35 | 54.52 |
| 4468.0977 | TIVKPGDILLIN(N)STGNLIAPRGY Fuc 2 Hex 4 HexNAc 4 Neu5Ac 1 | 57.541 | 0.0 | -5.284 | 8.999e+06 | 250 - 273 | 1 | 54.01 | 54.01 | 54.35 |
| 3863.5960 | LYESESKYPVLN(N)VTM(O)PN Hex 9 HexNAc 2 | 55.377 | 0.0 | -19.736 | 8.464e+06 | 169 - 186 | 1 | 35.50 | 35.50 | 35.66 |
| 4310.8149 | LYESESKYPVLN(N)VTM(O)PNN(D)GKF Hex 9 HexNAc 2 | 54.315 | 0.0 | 10.194 | 2.529e+07 | 169 - 190 | 3 | 39.40 | 39.57 | 40.42 |
| 4470.1077 | TIVKPGDILLIN(N)STGNLIAPRGY Fuc 2 Hex 4 HexNAc 4 Neu5Ac 1 | 53.045 | 0.0 | 0.000 | 0.000e+00 | 250 - 273 | 1 | - | - | - |
| 4624.8894 | ITPN(N)GSIPNDKPF Hex 7 HexNAc 6 Neu5Ac 3 | 51.458 | 0.0 | 12.667 | 3.161e+07 | 297 - 310 | 5 | 37.19 | 37.70 | 38.04 |
| 4454.0569 | TIVKPGDILLIN(N)STGNLIAPRGY Hex 10 HexNAc 2 | 50.325 | 0.0 | 0.000 | 0.000e+00 | 250 - 273 | 1 | - | - | - |
| 4700.9428 | RSDAPIGTC(C)SSEC(C)ITPN(N)GSIPNDKPF Fuc 2 Hex 3 HexNAc 4 Neu5Ac 1 | 49.126 | 0.0 | -19.985 | 7.332e+06 | 284 - 310 | 1 | 35.66 | 35.66 | 35.82 |
| 4248.0144 | TIVKPGDILLIN(N)STGNLIAPR Fuc 2 Hex 4 HexNAc 4 Neu5Ac 1 | 48.809 | 0.0 | -19.311 | 1.917e+06 | 250 - 271 | 1 | 51.47 | 51.47 | 51.47 |
| 3039.3007 | TITNDQIEVTN(N)ATEL Hex 6 HexNAc 2 | 46.670 | 0.0 | -17.187 | 1.639e+07 | 43 - 58 | 1 | 38.21 | 38.38 | 38.38 |
| 4148.7630 | LYESESKYPVLN(N)VTM(O)PNN(D)GKF Hex 8 HexNAc 2 | 46.368 | 0.0 | -6.202 | 1.716e+07 | 169 - 190 | 3 | 39.40 | 39.74 | 39.91 |
| 3793.7098 | TIVKPGDILLIN(N)STGNL Hex 10 HexNAc 2 | 46.309 | 0.0 | -4.508 | 6.508e+06 | 250 - 267 | 3 | 50.63 | 50.78 | 51.12 |
| 3565.4200 | SSEC(C)ITPN(N)GSIPN(D)DKPF Hex 8 HexNAc 2 | 45.706 | 0.0 | -20.579 | 1.113e+07 | 293 - 310 | 1 | 34.18 | 34.34 | 34.34 |
| 4069.9264 | TIVKPGDILLIN(N)STGN(D)LIAPR Hex 9 HexNAc 2 | 45.246 | 0.0 | -4.914 | 7.872e+06 | 250 - 271 | 3 | 52.32 | 52.49 | 52.66 |
| 3256.3751 | IGSIRN(N)GTYDHDVY Fuc 1 Hex 3 HexNAc 5 | 45.002 | 0.0 | -19.448 | 2.954e+06 | 493 - 507 | 1 | 22.28 | 22.44 | 22.44 |
| 3680.6275 | TIVKPGDILLIN(N)STGN Hex 10 HexNAc 2 | 43.615 | 0.0 | -5.060 | 2.931e+06 | 250 - 266 | 1 | 42.29 | 42.46 | 42.63 |
| 4308.0457 | TIVKPGDILLIN(N)STGNLIAPRGY Fuc 2 Hex 3 HexNAc 4 Neu5Ac 1 | 43.512 | 0.0 | 0.000 | 0.000e+00 | 250 - 273 | 2 | - | - | - |
| 4522.8720 | RSDAPIGTC(C)SSEC(C)ITPN(N)GSIPN(D)DKPF Hex 8 HexNAc 2 | 40.960 | 0.0 | -5.796 | 2.389e+07 | 284 - 310 | 3 | 36.50 | 36.67 | 37.02 |
| 4684.9251 | RSDAPIGTC(C)SSEC(C)ITPN(N)GSIPN(D)DKPF Hex 9 HexNAc 2 | 39.699 | 0.0 | -5.792 | 1.840e+07 | 284 - 310 | 3 | 36.33 | 36.50 | 36.85 |
| 3338.4631 | LIN(N)STGNLIAPRGYF Hex 8 HexNAc 2 | 39.589 | 0.0 | 0.000 | 0.000e+00 | 259 - 274 | 1 | - | - | - |
| 2765.1622 | LPGNDN(N)STATL Hex 4 HexNAc 5 | 38.518 | 0.0 | -19.732 | 2.294e+06 | 18 - 29 | 1 | 23.28 | 23.45 | 23.45 |
| 2939.2509 | ITPN(N)GSIPNDKPF Hex 7 HexNAc 2 | 38.441 | 0.0 | -20.489 | 1.374e+06 | 297 - 310 | 1 | 29.34 | 29.34 | 29.34 |
| 3810.7285 | TIVKPGDILLIN(N)STGNL Fuc 2 Hex 4 HexNAc 4 Neu5Ac 1 | 37.866 | 0.0 | -19.794 | 1.104e+06 | 250 - 267 | 1 | 50.78 | 50.78 | 50.78 |
| 3938.6117 | HKC(C)DNAC(C)IGSIRN(N)GTYDHDVY Fuc 1 Hex 3 HexNAc 4 | 37.505 | 0.0 | -19.959 | 8.505e+06 | 486 - 507 | 2 | 23.45 | 23.45 | 23.62 |
| 2603.0909 | LPGNDN(N)STATL Hex 3 HexNAc 5 | 36.130 | 0.0 | 12.905 | 1.449e+07 | 18 - 29 | 2 | 22.94 | 23.62 | 24.13 |
| 3356.5296 | TIVKPGDILLIN(N)STGN Hex 8 HexNAc 2 | 36.068 | 0.0 | -20.470 | 1.117e+06 | 250 - 266 | 1 | 42.63 | 42.63 | 42.80 |
| 3012.2549 | INEGFN(N)WTGVTQ Fuc 1 Hex 3 HexNAc 5 | 35.629 | 0.0 | -19.536 | 3.127e+06 | 136 - 148 | 1 | 42.29 | 42.29 | 42.46 |
| 4831.0723 | ESESKYPVLN(N)VTM(O)PNN(D)GKFDKLY Fuc 1 Hex 3 HexNAc 6 Neu5Ac 1 | 34.839 | 0.0 | 10.151 | 1.137e+08 | 171 - 194 | 8 | 44.00 | 44.00 | 45.35 |
| 4453.1066 | TIVKPGDILLIN(N)STGNLIAPRGY Hex 5 HexNAc 6 | 33.576 | 0.0 | 11.598 | 4.916e+07 | 250 - 273 | 5 | 59.75 | 60.16 | 60.85 |
| 3478.3934 | SEC(C)ITPN(N)GSIPN(D)DKPF Hex 8 HexNAc 2 | 33.183 | 0.0 | -21.445 | 5.652e+06 | 294 - 310 | 1 | 34.34 | 34.34 | 34.51 |
| 4365.0877 | TIVKPGDILLIN(N)STGNLIAPRGY Fuc 3 Hex 3 HexNAc 5 | 32.846 | 0.0 | 0.000 | 0.000e+00 | 250 - 273 | 1 | - | - | - |
| 2877.2470 | TITNDQIEVTN(N)ATEL Hex 5 HexNAc 2 | 32.748 | 0.0 | -3.625 | 9.567e+06 | 43 - 58 | 2 | 38.38 | 38.55 | 38.72 |
| 5802.5457 | KIRTGKSSIM(O)RSDAPIGTC(C)SSEC(C)ITPN(N)GSIPN(D)DKPF Hex 9 HexNAc 2 | 31.754 | 0.005 | -5.930 | 2.864e+07 | 274 - 310 | 3 | 31.88 | 32.05 | 32.22 |
| 4887.1050 | ESESKYPVLN(N)VTM(O)PNN(D)GKFDKLY Fuc 2 Hex 8 HexNAc 3 | 30.322 | 0.009 | 12.158 | 1.167e+08 | 171 - 194 | 4 | 42.98 | 43.49 | 43.66 |
| 4359.8253 | RSDAPIGTC(C)SSEC(C)ITPN(N)GSIPNDKPF Hex 7 HexNAc 2 | 30.120 | 0.009 | -19.460 | 9.603e+06 | 284 - 310 | 1 | 35.82 | 35.82 | 35.99 |
| 4602.8549 | ITPN(N)GSIPNDKPF Fuc 3 Hex 9 HexNAc 5 Neu5Ac 1 | 29.981 | 0.009 | 0.000 | 0.000e+00 | 297 - 310 | 1 | - | - | - |
| 5640.4902 | KIRTGKSSIM(O)RSDAPIGTC(C)SSEC(C)ITPN(N)GSIPN(D)DKPF Hex 8 HexNAc 2 | 29.794 | 0.009 | -6.576 | 1.937e+07 | 274 - 310 | 1 | 31.88 | 32.05 | 32.22 |
| 4610.8541 | ITPN(N)GSIPNDKPF Hex 8 HexNAc 8 Neu5Ac 1 | 29.610 | 0.009 | 0.000 | 0.000e+00 | 297 - 310 | 1 | - | - | - |
| 3080.3308 | TITNDQIEVTN(N)ATEL Hex 5 HexNAc 3 | 29.460 | 0.009 | -19.094 | 5.158e+06 | 43 - 58 | 1 | 38.38 | 38.38 | 38.55 |
| 4675.8973 | ITPN(N)GSIPNDKPF Fuc 3 Hex 10 HexNAc 6 | 29.357 | 0.009 | -20.562 | 3.005e+06 | 297 - 310 | 1 | 38.55 | 38.55 | 38.72 |
| 4360.8199 | RSDAPIGTC(C)SSEC(C)ITPN(N)GSIPN(D)DKPF Hex 7 HexNAc 2 | 28.462 | 0.009 | -6.388 | 5.643e+06 | 284 - 310 | 2 | 36.50 | 36.67 | 36.85 |
| 3388.4374 | TITNDQIEVTN(N)ATEL Fuc 1 Hex 6 HexNAc 3 | 28.437 | 0.009 | -20.311 | 2.404e+06 | 43 - 58 | 1 | 38.04 | 38.04 | 38.21 |
| 4274.0273 | TIVKPGDILLIN(N)STGNLIAPR Hex 4 HexNAc 7 | 27.289 | 0.009 | 14.619 | 2.435e+07 | 250 - 271 | 5 | 59.83 | 60.33 | 60.85 |
| 4538.8934 | ITPN(N)GSIPNDKPF Fuc 4 Hex 7 HexNAc 7 | 27.284 | 0.009 | -20.444 | 7.867e+06 | 297 - 310 | 1 | 35.66 | 35.66 | 35.82 |
| 4307.0407 | TIVKPGDILLIN(N)STGN(D)LIAPRGY Fuc 2 Hex 3 HexNAc 4 Neu5Ac 1 | 26.540 | 0.009 | -19.510 | 1.267e+06 | 250 - 273 | 1 | 55.03 | 55.03 | 55.03 |
| 4436.0817 | TIVKPGDILLIN(N)STGNLIAPR Hex 5 HexNAc 7 | 26.513 | 0.009 | 13.376 | 4.199e+08 | 250 - 271 | 22 | 59.62 | 60.16 | 62.98 |
| 4669.0213 | ESESKYPVLN(N)VTMPNNGKFDKLY Fuc 2 Hex 3 HexNAc 6 | 26.484 | 0.009 | 9.099 | 5.527e+07 | 171 - 194 | 6 | 44.00 | 44.17 | 45.02 |
| 4725.0540 | ESESKYPVLN(N)VTM(O)PNN(D)GKFDKLY Fuc 2 Hex 7 HexNAc 3 | 25.376 | 0.018 | 11.225 | 7.761e+07 | 171 - 194 | 3 | 43.15 | 43.49 | 43.83 |
| 2761.1434 | TC(C)KRGSN(N)NSFF Fuc 1 Hex 3 HexNAc 4 | 24.701 | 0.022 | -20.365 | 7.297e+06 | 153 - 164 | 2 | 18.45 | 18.61 | 18.61 |
| 4246.7257 | HKC(C)DNAC(C)IGSIRN(N)GTYDHDVY Fuc 2 Hex 4 HexNAc 4 | 24.572 | 0.022 | -20.809 | 2.882e+06 | 486 - 507 | 1 | 23.28 | 23.28 | 23.28 |
| 3256.3983 | IN(N)STGNLIAPRGY Fuc 2 Hex 3 HexNAc 4 Neu5Ac 1 | 24.383 | 0.022 | -19.605 | 1.125e+06 | 260 - 273 | 1 | 29.01 | 29.01 | 29.01 |
| 4363.0599 | TIVKPGDILLIN(N)STGNLIAPRGY Fuc 1 Hex 3 HexNAc 5 Neu5Ac 1 | 24.198 | 0.022 | -5.227 | 7.045e+06 | 250 - 273 | 2 | 54.69 | 54.69 | 55.03 |
| 3162.2832 | LPGNDN(N)STATL Fuc 3 Hex 5 HexNAc 4 | 23.962 | 0.022 | -20.213 | 1.044e+06 | 18 - 29 | 1 | 22.44 | 22.44 | 22.44 |
| 5097.3357 | TITNDQIEVTN(N)ATELVQSSSTGRIC(C)DSPHRIL Hex 7 HexNAc 2 | 23.707 | 0.026 | 0.000 | 0.000e+00 | 43 - 75 | 1 | - | - | - |
| 4669.0212 | LIN(N)STGNLIAPRGYF Fuc 1 Hex 6 HexNAc 8 Neu5Ac 1 | 23.661 | 0.030 | -20.485 | 4.457e+06 | 259 - 274 | 1 | 45.35 | 45.35 | 45.35 |
| 3382.3687 | LPGNDN(N)STATL Fuc 2 Hex 6 HexNAc 5 | 23.587 | 0.030 | 0.000 | 0.000e+00 | 18 - 29 | 1 | - | - | - |
| 3727.4837 | SSEC(C)ITPN(N)GSIPN(D)DKPF Hex 9 HexNAc 2 | 23.396 | 0.030 | -20.587 | 4.914e+06 | 293 - 310 | 1 | 34.18 | 34.18 | 34.18 |
| 2965.2094 | TC(C)KRGSN(N)NSFF Fuc 1 Hex 3 HexNAc 5 | 22.961 | 0.038 | 0.000 | 0.000e+00 | 153 - 164 | 1 | - | - | - |
| 4668.0310 | ESESKYPVLN(N)VTMPNNGKFDKLY Hex 3 HexNAc 6 Neu5Ac 1 | 22.409 | 0.038 | 16.708 | 2.582e+08 | 171 - 194 | 6 | 42.98 | 43.49 | 43.83 |

### LIN(N)STGNL Hex 9 HexNAc 2

**Mass:**  2695.0883

(1.558e-06)

**Charge States:** 3

**Start Position**: 260

**End Position**: 268

| Profile Score | Tandem Score | TDA q-value | Volume | Spectrum Matches |
| --- | --- | --- | --- | --- |
| -2.9169 | 180.5410 | 0.0000 | 1.3349e+07 | 2 |

**Adduct Signal Fractions**

- **Unmodified**: 1.3349e+07

### LIN(N)STGNLIAPR Hex 9 HexNAc 2

**Mass:**  3132.3631

(1.272e-06)

**Charge States:** 3

**Start Position**: 260

**End Position**: 272

| Profile Score | Tandem Score | TDA q-value | Volume | Spectrum Matches |
| --- | --- | --- | --- | --- |
| 14.2427 | 171.9446 | 0.0000 | 3.7954e+07 | 2 |

**Adduct Signal Fractions**

- **Unmodified**: 3.7954e+07

### TIVKPGDILLIN(N)STGNLIAPR Hex 9 HexNAc 2

**Mass:**  4068.9289

(1.629e-06)

**Charge States:** 3, 4, 5

**Start Position**: 251

**End Position**: 272

| Profile Score | Tandem Score | TDA q-value | Volume | Spectrum Matches |
| --- | --- | --- | --- | --- |
| 16.0125 | 168.4607 | 0.0000 | 2.3934e+08 | 9 |

**Adduct Signal Fractions**

- **Unmodified**: 2.3934e+08

### LPGNDN(N)STATL Fuc 1 Hex 3 HexNAc 4

**Mass:**  2546.0683

(1.631e-06)

**Charge States:** 2, 3

**Start Position**: 19

**End Position**: 30

| Profile Score | Tandem Score | TDA q-value | Volume | Spectrum Matches |
| --- | --- | --- | --- | --- |
| 14.1892 | 168.4505 | 0.0000 | 5.6400e+07 | 4 |

**Adduct Signal Fractions**

- **Unmodified**: 5.6400e+07

### TIVKPGDILLIN(N)STGNLIAPRGY Hex 9 HexNAc 2

**Mass:**  4289.0158

(1.122e-06)

**Charge States:** 3, 4, 5

**Start Position**: 251

**End Position**: 274

| Profile Score | Tandem Score | TDA q-value | Volume | Spectrum Matches |
| --- | --- | --- | --- | --- |
| 15.1828 | 163.6086 | 0.0000 | 7.5426e+08 | 29 |

**Adduct Signal Fractions**

- **Unmodified**: 7.5426e+08

### LIN(N)STGNLIAPRGY Hex 9 HexNAc 2

**Mass:**  3352.4470

(9.368e-07)

**Charge States:** 3

**Start Position**: 260

**End Position**: 274

| Profile Score | Tandem Score | TDA q-value | Volume | Spectrum Matches |
| --- | --- | --- | --- | --- |
| -4.3693 | 161.3960 | 0.0000 | 4.1913e+07 | 1 |

**Adduct Signal Fractions**

- **Unmodified**: 4.1913e+07

### TIVKPGDILLIN(N) Hex 9 HexNAc 2

**Mass:**  3159.4238

(1.146e-06)

**Charge States:** 2, 3

**Start Position**: 251

**End Position**: 263

| Profile Score | Tandem Score | TDA q-value | Volume | Spectrum Matches |
| --- | --- | --- | --- | --- |
| 12.9794 | 157.1520 | 0.0000 | 9.1881e+07 | 9 |

**Adduct Signal Fractions**

- **Unmodified**: 9.1881e+07

### LPGNDN(N)STATL Fuc 1 Hex 3 HexNAc 5

**Mass:**  2749.1455

(7.490e-07)

**Charge States:** 3

**Start Position**: 19

**End Position**: 30

| Profile Score | Tandem Score | TDA q-value | Volume | Spectrum Matches |
| --- | --- | --- | --- | --- |
| 13.5922 | 155.9475 | 0.0000 | 1.3844e+07 | 3 |

**Adduct Signal Fractions**

- **Unmodified**: 1.3844e+07

### TIVKPGDILLIN(N)STGN Hex 9 HexNAc 2

**Mass:**  3518.5706

(1.742e-06)

**Charge States:** 3, 4

**Start Position**: 251

**End Position**: 267

| Profile Score | Tandem Score | TDA q-value | Volume | Spectrum Matches |
| --- | --- | --- | --- | --- |
| 13.2740 | 154.7434 | 0.0000 | 3.4082e+07 | 5 |

**Adduct Signal Fractions**

- **Unmodified**: 3.4082e+07

### TIVKPGDILLIN(N) Fuc 2 Hex 3 HexNAc 4 Neu5Ac 1

**Mass:**  3176.4508

(-6.646e-06)

**Charge States:** 3

**Start Position**: 251

**End Position**: 263

| Profile Score | Tandem Score | TDA q-value | Volume | Spectrum Matches |
| --- | --- | --- | --- | --- |
| 9.1892 | 153.5739 | 0.0000 | 2.2971e+07 | 4 |

**Adduct Signal Fractions**

- **Unmodified**: 2.2971e+07

### LPGNDN(N)STATL Hex 3 HexNAc 4

**Mass:**  2400.0080

(7.305e-07)

**Charge States:** 3

**Start Position**: 19

**End Position**: 30

| Profile Score | Tandem Score | TDA q-value | Volume | Spectrum Matches |
| --- | --- | --- | --- | --- |
| 11.8901 | 152.7923 | 0.0000 | 1.3776e+07 | 3 |

**Adduct Signal Fractions**

- **Unmodified**: 1.3776e+07

### LPGNDN(N)STATL Hex 5 HexNAc 2

**Mass:**  2317.9581

(6.792e-07)

**Charge States:** 2, 3

**Start Position**: 19

**End Position**: 30

| Profile Score | Tandem Score | TDA q-value | Volume | Spectrum Matches |
| --- | --- | --- | --- | --- |
| -5.1259 | 152.7303 | 0.0000 | 9.1826e+06 | 4 |

**Adduct Signal Fractions**

- **Unmodified**: 9.1826e+06

### TIVKPGDILLIN(N)STGNLIAPR Fuc 2 Hex 3 HexNAc 4 Neu5Ac 1

**Mass:**  4085.9567

(-5.397e-06)

**Charge States:** 4

**Start Position**: 251

**End Position**: 272

| Profile Score | Tandem Score | TDA q-value | Volume | Spectrum Matches |
| --- | --- | --- | --- | --- |
| -4.4677 | 148.2485 | 0.0000 | 2.6516e+07 | 1 |

**Adduct Signal Fractions**

- **Unmodified**: 2.6516e+07

### TIVKPGDILLIN(N) Hex 10 HexNAc 2

**Mass:**  3321.4755

(7.950e-07)

**Charge States:** 3

**Start Position**: 251

**End Position**: 263

| Profile Score | Tandem Score | TDA q-value | Volume | Spectrum Matches |
| --- | --- | --- | --- | --- |
| -18.5784 | 146.3623 | 0.0000 | 3.3457e+06 | 1 |

**Adduct Signal Fractions**

- **Unmodified**: 3.3457e+06

### TIVKPGDILLIN(N)STGNLIAPRGY Fuc 2 Hex 3 HexNAc 4 Neu5Ac 1

**Mass:**  4306.0412

(-4.942e-06)

**Charge States:** 4

**Start Position**: 251

**End Position**: 274

| Profile Score | Tandem Score | TDA q-value | Volume | Spectrum Matches |
| --- | --- | --- | --- | --- |
| 13.4517 | 145.7348 | 0.0000 | 8.7753e+07 | 4 |

**Adduct Signal Fractions**

- **Unmodified**: 8.7753e+07

### TIVKPGDILLIN(N)STGNL Fuc 2 Hex 3 HexNAc 4 Neu5Ac 1

**Mass:**  3648.6806

(-5.849e-06)

**Charge States:** 3

**Start Position**: 251

**End Position**: 268

| Profile Score | Tandem Score | TDA q-value | Volume | Spectrum Matches |
| --- | --- | --- | --- | --- |
| -4.3505 | 145.2179 | 0.0000 | 2.2994e+07 | 3 |

**Adduct Signal Fractions**

- **Unmodified**: 2.2994e+07

### ITPN(N)GSIPNDKPF Hex 8 HexNAc 2

**Mass:**  3101.3021

(2.031e-06)

**Charge States:** 3

**Start Position**: 298

**End Position**: 311

| Profile Score | Tandem Score | TDA q-value | Volume | Spectrum Matches |
| --- | --- | --- | --- | --- |
| -4.1393 | 143.0352 | 0.0000 | 1.1306e+07 | 2 |

**Adduct Signal Fractions**

- **Unmodified**: 1.1306e+07

### TIVKPGDILLIN(N)STGNLIAPRGY Hex 10 HexNAc 2

**Mass:**  4451.0686

(1.665e-06)

**Charge States:** 3, 4, 5

**Start Position**: 251

**End Position**: 274

| Profile Score | Tandem Score | TDA q-value | Volume | Spectrum Matches |
| --- | --- | --- | --- | --- |
| 13.0660 | 140.7168 | 0.0000 | 7.0838e+07 | 7 |

**Adduct Signal Fractions**

- **Unmodified**: 7.0838e+07

### KRGSN(N)NSFF Fuc 1 Hex 3 HexNAc 5

**Mass:**  2703.1280

(-5.274e-08)

**Charge States:** 3

**Start Position**: 156

**End Position**: 165

| Profile Score | Tandem Score | TDA q-value | Volume | Spectrum Matches |
| --- | --- | --- | --- | --- |
| -2.6482 | 138.5885 | 0.0000 | 8.4895e+07 | 2 |

**Adduct Signal Fractions**

- **Unmodified**: 8.4895e+07

### LPGNDN(N)STATL Fuc 2 Hex 4 HexNAc 4

**Mass:**  2854.1790

(1.441e-06)

**Charge States:** 3

**Start Position**: 19

**End Position**: 30

| Profile Score | Tandem Score | TDA q-value | Volume | Spectrum Matches |
| --- | --- | --- | --- | --- |
| 11.6653 | 138.3875 | 0.0000 | 1.3038e+07 | 3 |

**Adduct Signal Fractions**

- **Unmodified**: 1.3038e+07

### TIVKPGDILLIN(N)STGNLIAPRGY Hex 8 HexNAc 2

**Mass:**  4126.9597

(1.032e-06)

**Charge States:** 3, 4

**Start Position**: 251

**End Position**: 274

| Profile Score | Tandem Score | TDA q-value | Volume | Spectrum Matches |
| --- | --- | --- | --- | --- |
| 12.0515 | 138.3662 | 0.0000 | 6.3472e+07 | 10 |

**Adduct Signal Fractions**

- **Unmodified**: 6.3472e+07

### TIVKPGDILLIN(N)STGNLIAPR Fuc 2 Hex 3 HexNAc 4 Neu5Ac 1

**Mass:**  4086.9717

(2.436e-04)

**Start Position**: 251

**End Position**: 272

| Profile Score | Tandem Score | TDA q-value | Volume | Spectrum Matches |
| --- | --- | --- | --- | --- |
| 0.0000 | 138.2277 | 0.0000 | 0.0000e+00 | 1 |

Adduct:

No Chromatogram Found

### TIVKPGDILLIN(N)STGNLIAPR Hex 10 HexNAc 2

**Mass:**  4230.9826

(2.061e-06)

**Charge States:** 4

**Start Position**: 251

**End Position**: 272

| Profile Score | Tandem Score | TDA q-value | Volume | Spectrum Matches |
| --- | --- | --- | --- | --- |
| -3.9181 | 135.7044 | 0.0000 | 2.0352e+07 | 3 |

**Adduct Signal Fractions**

- **Unmodified**: 2.0352e+07

### IN(N)STGNLIAPRGYF Hex 9 HexNAc 2

**Mass:**  3386.4380

(2.841e-06)

**Charge States:** 3

**Start Position**: 261

**End Position**: 275

| Profile Score | Tandem Score | TDA q-value | Volume | Spectrum Matches |
| --- | --- | --- | --- | --- |
| -3.9959 | 134.1957 | 0.0000 | 7.1750e+06 | 2 |

**Adduct Signal Fractions**

- **Unmodified**: 7.1750e+06

### TIVKPGDILLIN(N)STGNLIAPR Hex 8 HexNAc 2

**Mass:**  3906.8737

(-1.104e-06)

**Charge States:** 3, 4

**Start Position**: 251

**End Position**: 272

| Profile Score | Tandem Score | TDA q-value | Volume | Spectrum Matches |
| --- | --- | --- | --- | --- |
| -4.4574 | 132.6634 | 0.0000 | 1.5996e+07 | 4 |

**Adduct Signal Fractions**

- **Unmodified**: 1.5996e+07

### ITPN(N)GSIPNDKPF Hex 9 HexNAc 2

**Mass:**  3263.3637

(1.353e-06)

**Charge States:** 3

**Start Position**: 298

**End Position**: 311

| Profile Score | Tandem Score | TDA q-value | Volume | Spectrum Matches |
| --- | --- | --- | --- | --- |
| -17.9807 | 132.5207 | 0.0000 | 9.2351e+06 | 1 |

**Adduct Signal Fractions**

- **Unmodified**: 9.2351e+06

### TIVKPGDILLIN(N)STGNL Hex 9 HexNAc 2

**Mass:**  3631.6543

(1.634e-06)

**Charge States:** 3, 4

**Start Position**: 251

**End Position**: 268

| Profile Score | Tandem Score | TDA q-value | Volume | Spectrum Matches |
| --- | --- | --- | --- | --- |
| 13.0287 | 131.6169 | 0.0000 | 8.1587e+07 | 5 |

**Adduct Signal Fractions**

- **Unmodified**: 8.1587e+07

### TIVKPGDILLIN(N)STGN Fuc 2 Hex 3 HexNAc 4 Neu5Ac 1

**Mass:**  3535.5994

(-6.414e-06)

**Charge States:** 3

**Start Position**: 251

**End Position**: 267

| Profile Score | Tandem Score | TDA q-value | Volume | Spectrum Matches |
| --- | --- | --- | --- | --- |
| -3.7881 | 130.7829 | 0.0000 | 7.8083e+06 | 2 |

**Adduct Signal Fractions**

- **Unmodified**: 7.8083e+06

### LIN(N)STGNLIAPRGYF Fuc 2 Hex 3 HexNAc 4 Neu5Ac 1

**Mass:**  3516.5359

(-7.098e-06)

**Charge States:** 3

**Start Position**: 260

**End Position**: 275

| Profile Score | Tandem Score | TDA q-value | Volume | Spectrum Matches |
| --- | --- | --- | --- | --- |
| -19.1431 | 130.7199 | 0.0000 | 5.8984e+06 | 1 |

**Adduct Signal Fractions**

- **Unmodified**: 5.8984e+06

### IN(N)STGNLIAPR Hex 9 HexNAc 2

**Mass:**  3019.2766

(5.178e-07)

**Charge States:** 3

**Start Position**: 261

**End Position**: 272

| Profile Score | Tandem Score | TDA q-value | Volume | Spectrum Matches |
| --- | --- | --- | --- | --- |
| -19.3320 | 125.9885 | 0.0000 | 2.5320e+06 | 1 |

**Adduct Signal Fractions**

- **Unmodified**: 2.5320e+06

### KRGSN(N)NSFF Fuc 1 Hex 3 HexNAc 4

**Mass:**  2500.0512

(9.977e-07)

**Charge States:** 3

**Start Position**: 156

**End Position**: 165

| Profile Score | Tandem Score | TDA q-value | Volume | Spectrum Matches |
| --- | --- | --- | --- | --- |
| -20.2437 | 122.0980 | 0.0000 | 9.3357e+06 | 1 |

**Adduct Signal Fractions**

- **Unmodified**: 9.3357e+06

### IN(N)STGNLIAPRGY Hex 9 HexNAc 2

**Mass:**  3239.3673

(2.237e-06)

**Charge States:** 3

**Start Position**: 261

**End Position**: 274

| Profile Score | Tandem Score | TDA q-value | Volume | Spectrum Matches |
| --- | --- | --- | --- | --- |
| -4.0855 | 118.0707 | 0.0000 | 1.0737e+07 | 2 |

**Adduct Signal Fractions**

- **Unmodified**: 1.0737e+07

### LPGNDN(N)STATL Fuc 2 Hex 8 HexNAc 3 Neu5Ac 1

**Mass:**  3590.4369

(9.707e-06)

**Charge States:** 3

**Start Position**: 19

**End Position**: 30

| Profile Score | Tandem Score | TDA q-value | Volume | Spectrum Matches |
| --- | --- | --- | --- | --- |
| 13.0349 | 117.4721 | 0.0000 | 7.4385e+07 | 3 |

**Adduct Signal Fractions**

- **Unmodified**: 7.4385e+07

### TIVKPGDILLIN(N)STGNL Hex 8 HexNAc 2

**Mass:**  3469.6009

(1.520e-06)

**Charge States:** 3

**Start Position**: 251

**End Position**: 268

| Profile Score | Tandem Score | TDA q-value | Volume | Spectrum Matches |
| --- | --- | --- | --- | --- |
| -4.5705 | 116.5268 | 0.0000 | 5.7074e+06 | 1 |

**Adduct Signal Fractions**

- **Unmodified**: 5.7074e+06

### LIN(N)STGNLIAPRGYF Hex 9 HexNAc 2

**Mass:**  3502.5244

(8.607e-04)

**Start Position**: 260

**End Position**: 275

| Profile Score | Tandem Score | TDA q-value | Volume | Spectrum Matches |
| --- | --- | --- | --- | --- |
| 0.0000 | 108.3537 | 0.0000 | 0.0000e+00 | 1 |

Adduct:

No Chromatogram Found

### LPGNDN(N)STATL Fuc 1 Hex 7 HexNAc 3 Neu5Ac 1

**Mass:**  3282.3356

(9.861e-06)

**Charge States:** 3

**Start Position**: 19

**End Position**: 30

| Profile Score | Tandem Score | TDA q-value | Volume | Spectrum Matches |
| --- | --- | --- | --- | --- |
| 13.5217 | 107.9072 | 0.0000 | 1.8815e+08 | 3 |

**Adduct Signal Fractions**

- **Unmodified**: 1.8815e+08

### IGSIRN(N)GTYDHDVY Fuc 1 Hex 3 HexNAc 4

**Mass:**  3053.2954

(2.737e-06)

**Charge States:** 3

**Start Position**: 494

**End Position**: 508

| Profile Score | Tandem Score | TDA q-value | Volume | Spectrum Matches |
| --- | --- | --- | --- | --- |
| -17.8717 | 106.4661 | 0.0000 | 5.3445e+06 | 1 |

**Adduct Signal Fractions**

- **Unmodified**: 5.3445e+06

### SSEC(C)ITPN(N)GSIPNDKPF Hex 9 HexNAc 2

**Mass:**  3726.4916

(1.519e-06)

**Charge States:** 3

**Start Position**: 294

**End Position**: 311

| Profile Score | Tandem Score | TDA q-value | Volume | Spectrum Matches |
| --- | --- | --- | --- | --- |
| -18.2892 | 104.7910 | 0.0000 | 1.2196e+07 | 1 |

**Adduct Signal Fractions**

- **Unmodified**: 1.2196e+07

### SIRN(N)GTYDHDVY Fuc 1 Hex 3 HexNAc 4

**Mass:**  2883.1810

(-2.064e-07)

**Start Position**: 496

**End Position**: 508

| Profile Score | Tandem Score | TDA q-value | Volume | Spectrum Matches |
| --- | --- | --- | --- | --- |
| 0.0000 | 104.7598 | 0.0000 | 0.0000e+00 | 1 |

Adduct:

No Chromatogram Found

### LIN(N)STGNLIAPRGY Fuc 2 Hex 3 HexNAc 4 Neu5Ac 1

**Mass:**  3369.4722

(-7.269e-06)

**Charge States:** 3

**Start Position**: 260

**End Position**: 274

| Profile Score | Tandem Score | TDA q-value | Volume | Spectrum Matches |
| --- | --- | --- | --- | --- |
| -20.8276 | 95.4577 | 0.0000 | 3.9050e+06 | 1 |

**Adduct Signal Fractions**

- **Unmodified**: 3.9050e+06

### ESESKYPVLN(N)VTM(O)PNN(D)GKFDKLY Hex 9 HexNAc 2

**Mass:**  4553.9401

(2.183e-04)

**Charge States:** 4

**Start Position**: 172

**End Position**: 195

| Profile Score | Tandem Score | TDA q-value | Volume | Spectrum Matches |
| --- | --- | --- | --- | --- |
| 11.6217 | 93.5020 | 0.0000 | 3.7190e+07 | 4 |

**Adduct Signal Fractions**

- **Unmodified**: 3.7190e+07

### ESESKYPVLN(N)VTM(O)PNN(D)GKFDKLY Hex 8 HexNAc 2

**Mass:**  4391.8869

(2.266e-04)

**Charge States:** 4

**Start Position**: 172

**End Position**: 195

| Profile Score | Tandem Score | TDA q-value | Volume | Spectrum Matches |
| --- | --- | --- | --- | --- |
| 11.5296 | 92.8227 | 0.0000 | 2.6180e+07 | 3 |

**Adduct Signal Fractions**

- **Unmodified**: 2.6180e+07

### ITPN(N)GSIPNDKPF Fuc 3 Hex 6 HexNAc 7 Neu5Ac 1

**Mass:**  4521.8718

(4.137e-06)

**Charge States:** 3, 4

**Start Position**: 298

**End Position**: 311

| Profile Score | Tandem Score | TDA q-value | Volume | Spectrum Matches |
| --- | --- | --- | --- | --- |
| 12.3219 | 91.4093 | 0.0000 | 7.3126e+07 | 4 |

**Adduct Signal Fractions**

- **Unmodified**: 7.3126e+07

### ITPN(N)GSIPNDKPF Fuc 3 Hex 7 HexNAc 7 Neu5Ac 1

**Mass:**  4683.9240

(3.903e-06)

**Charge States:** 3, 4

**Start Position**: 298

**End Position**: 311

| Profile Score | Tandem Score | TDA q-value | Volume | Spectrum Matches |
| --- | --- | --- | --- | --- |
| 14.0681 | 90.0433 | 0.0000 | 6.2440e+07 | 3 |

**Adduct Signal Fractions**

- **Unmodified**: 6.2440e+07

### ITPN(N)GSIPNDKPF Fuc 4 Hex 7 HexNAc 8

**Mass:**  4741.9528

(4.560e-07)

**Charge States:** 4

**Start Position**: 298

**End Position**: 311

| Profile Score | Tandem Score | TDA q-value | Volume | Spectrum Matches |
| --- | --- | --- | --- | --- |
| -19.9604 | 88.9287 | 0.0000 | 5.0868e+06 | 1 |

**Adduct Signal Fractions**

- **Unmodified**: 5.0868e+06

### RSDAPIGTC(C)SSEC(C)ITPN(N)GSIPNDKPF Hex 7 HexNAc 2

**Mass:**  4361.8285

(4.621e-04)

**Start Position**: 285

**End Position**: 311

| Profile Score | Tandem Score | TDA q-value | Volume | Spectrum Matches |
| --- | --- | --- | --- | --- |
| 0.0000 | 83.1122 | 0.0000 | 0.0000e+00 | 1 |

Adduct:

No Chromatogram Found

### TITNDQIEVTN(N)ATEL Hex 7 HexNAc 2

**Mass:**  3201.3554

(3.476e-06)

**Charge States:** 3

**Start Position**: 44

**End Position**: 59

| Profile Score | Tandem Score | TDA q-value | Volume | Spectrum Matches |
| --- | --- | --- | --- | --- |
| -17.2128 | 82.9899 | 0.0000 | 7.2826e+06 | 1 |

**Adduct Signal Fractions**

- **Unmodified**: 7.2826e+06

### ITPN(N)GSIPNDKPF Fuc 2 Hex 6 HexNAc 8 Neu5Ac 1

**Mass:**  4578.8928

(3.342e-06)

**Charge States:** 4

**Start Position**: 298

**End Position**: 311

| Profile Score | Tandem Score | TDA q-value | Volume | Spectrum Matches |
| --- | --- | --- | --- | --- |
| -5.8811 | 72.2751 | 0.0000 | 6.7400e+06 | 1 |

**Adduct Signal Fractions**

- **Unmodified**: 6.7400e+06

### KIRTGKSSIM(O)RSDAPIGTC(C)SSEC(C)ITPN(N)GSIPNDKPF Hex 8 HexNAc 2

**Mass:**  5639.5071

(2.184e-06)

**Charge States:** 5

**Start Position**: 275

**End Position**: 311

| Profile Score | Tandem Score | TDA q-value | Volume | Spectrum Matches |
| --- | --- | --- | --- | --- |
| -4.5739 | 71.5128 | 0.0000 | 4.3057e+07 | 3 |

**Adduct Signal Fractions**

- **Unmodified**: 4.3057e+07

### KIRTGKSSIM(O)RSDAPIGTC(C)SSEC(C)ITPN(N)GSIPNDKPF Hex 9 HexNAc 2

**Mass:**  5801.5641

(1.542e-06)

**Charge States:** 5, 6

**Start Position**: 275

**End Position**: 311

| Profile Score | Tandem Score | TDA q-value | Volume | Spectrum Matches |
| --- | --- | --- | --- | --- |
| -4.6207 | 63.8528 | 0.0000 | 3.3423e+07 | 3 |

**Adduct Signal Fractions**

- **Unmodified**: 3.3423e+07

### TIVKPGDILLIN(N)STGN(D)LIAPRGY Hex 9 HexNAc 2

**Mass:**  4290.0060

(2.324e-04)

**Charge States:** 3

**Start Position**: 251

**End Position**: 274

| Profile Score | Tandem Score | TDA q-value | Volume | Spectrum Matches |
| --- | --- | --- | --- | --- |
| -4.6497 | 61.2256 | 0.0000 | 8.0142e+06 | 5 |

**Adduct Signal Fractions**

- **Unmodified**: 8.0142e+06

### TIVKPGDILLIN(N)STGNLIAPRGY Hex 7 HexNAc 2

**Mass:**  3964.8959

(-1.671e-06)

**Charge States:** 4

**Start Position**: 251

**End Position**: 274

| Profile Score | Tandem Score | TDA q-value | Volume | Spectrum Matches |
| --- | --- | --- | --- | --- |
| -6.1531 | 57.9485 | 0.0000 | 2.7722e+06 | 1 |

**Adduct Signal Fractions**

- **Unmodified**: 2.7722e+06

### TIVKPGDILLIN(N)STGNLIAPRGY Fuc 2 Hex 4 HexNAc 4 Neu5Ac 1

**Mass:**  4468.0993

(-4.044e-06)

**Charge States:** 4

**Start Position**: 251

**End Position**: 274

| Profile Score | Tandem Score | TDA q-value | Volume | Spectrum Matches |
| --- | --- | --- | --- | --- |
| -5.2836 | 57.5409 | 0.0000 | 8.9995e+06 | 1 |

**Adduct Signal Fractions**

- **Unmodified**: 8.9995e+06

### LYESESKYPVLN(N)VTM(O)PN Hex 9 HexNAc 2

**Mass:**  3863.5940

(-2.560e-07)

**Charge States:** 3

**Start Position**: 170

**End Position**: 187

| Profile Score | Tandem Score | TDA q-value | Volume | Spectrum Matches |
| --- | --- | --- | --- | --- |
| -19.7361 | 55.3772 | 0.0000 | 8.4636e+06 | 1 |

**Adduct Signal Fractions**

- **Unmodified**: 8.4636e+06

### LYESESKYPVLN(N)VTM(O)PNN(D)GKF Hex 9 HexNAc 2

**Mass:**  4310.8186

(2.300e-04)

**Charge States:** 3, 4

**Start Position**: 170

**End Position**: 191

| Profile Score | Tandem Score | TDA q-value | Volume | Spectrum Matches |
| --- | --- | --- | --- | --- |
| 10.1941 | 54.3149 | 0.0000 | 2.5289e+07 | 3 |

**Adduct Signal Fractions**

- **Unmodified**: 2.5289e+07

### TIVKPGDILLIN(N)STGNLIAPRGY Fuc 2 Hex 4 HexNAc 4 Neu5Ac 1

**Mass:**  4470.1077

(4.462e-04)

**Start Position**: 251

**End Position**: 274

| Profile Score | Tandem Score | TDA q-value | Volume | Spectrum Matches |
| --- | --- | --- | --- | --- |
| 0.0000 | 53.0446 | 0.0000 | 0.0000e+00 | 1 |

Adduct:

No Chromatogram Found

### ITPN(N)GSIPNDKPF Hex 7 HexNAc 6 Neu5Ac 3

**Mass:**  4624.8947

(7.399e-06)

**Charge States:** 3, 4

**Start Position**: 298

**End Position**: 311

| Profile Score | Tandem Score | TDA q-value | Volume | Spectrum Matches |
| --- | --- | --- | --- | --- |
| 12.6670 | 51.4578 | 0.0000 | 3.1607e+07 | 5 |

**Adduct Signal Fractions**

- **Unmodified**: 3.1607e+07

### TIVKPGDILLIN(N)STGNLIAPRGY Hex 10 HexNAc 2

**Mass:**  4454.0569

(6.731e-04)

**Start Position**: 251

**End Position**: 274

| Profile Score | Tandem Score | TDA q-value | Volume | Spectrum Matches |
| --- | --- | --- | --- | --- |
| 0.0000 | 50.3252 | 0.0000 | 0.0000e+00 | 1 |

Adduct:

No Chromatogram Found

### RSDAPIGTC(C)SSEC(C)ITPN(N)GSIPNDKPF Fuc 2 Hex 3 HexNAc 4 Neu5Ac 1

**Mass:**  4700.9426

(-6.418e-06)

**Charge States:** 4

**Start Position**: 285

**End Position**: 311

| Profile Score | Tandem Score | TDA q-value | Volume | Spectrum Matches |
| --- | --- | --- | --- | --- |
| -19.9846 | 49.1265 | 0.0000 | 7.3318e+06 | 1 |

**Adduct Signal Fractions**

- **Unmodified**: 7.3318e+06

### TIVKPGDILLIN(N)STGNLIAPR Fuc 2 Hex 4 HexNAc 4 Neu5Ac 1

**Mass:**  4248.0144

(-3.516e-06)

**Charge States:** 4

**Start Position**: 251

**End Position**: 272

| Profile Score | Tandem Score | TDA q-value | Volume | Spectrum Matches |
| --- | --- | --- | --- | --- |
| -19.3106 | 48.8092 | 0.0000 | 1.9173e+06 | 1 |

**Adduct Signal Fractions**

- **Unmodified**: 1.9173e+06

### TITNDQIEVTN(N)ATEL Hex 6 HexNAc 2

**Mass:**  3039.3017

(2.990e-06)

**Charge States:** 2, 3

**Start Position**: 44

**End Position**: 59

| Profile Score | Tandem Score | TDA q-value | Volume | Spectrum Matches |
| --- | --- | --- | --- | --- |
| -17.1873 | 46.6700 | 0.0000 | 1.6386e+07 | 1 |

**Adduct Signal Fractions**

- **Unmodified**: 1.6386e+07

### LYESESKYPVLN(N)VTM(O)PNN(D)GKF Hex 8 HexNAc 2

**Mass:**  4148.7658

(2.400e-04)

**Charge States:** 3, 4

**Start Position**: 170

**End Position**: 191

| Profile Score | Tandem Score | TDA q-value | Volume | Spectrum Matches |
| --- | --- | --- | --- | --- |
| -6.2016 | 46.3677 | 0.0000 | 1.7157e+07 | 3 |

**Adduct Signal Fractions**

- **Unmodified**: 1.7157e+07

### TIVKPGDILLIN(N)STGNL Hex 10 HexNAc 2

**Mass:**  3793.7071

(1.237e-06)

**Charge States:** 3

**Start Position**: 251

**End Position**: 268

| Profile Score | Tandem Score | TDA q-value | Volume | Spectrum Matches |
| --- | --- | --- | --- | --- |
| -4.5082 | 46.3087 | 0.0000 | 6.5081e+06 | 3 |

**Adduct Signal Fractions**

- **Unmodified**: 6.5081e+06

### SSEC(C)ITPN(N)GSIPN(D)DKPF Hex 8 HexNAc 2

**Mass:**  3565.4210

(2.766e-04)

**Charge States:** 3

**Start Position**: 294

**End Position**: 311

| Profile Score | Tandem Score | TDA q-value | Volume | Spectrum Matches |
| --- | --- | --- | --- | --- |
| -20.5793 | 45.7062 | 0.0000 | 1.1132e+07 | 1 |

**Adduct Signal Fractions**

- **Unmodified**: 1.1132e+07

### TIVKPGDILLIN(N)STGN(D)LIAPR Hex 9 HexNAc 2

**Mass:**  4069.9279

(2.461e-04)

**Charge States:** 3

**Start Position**: 251

**End Position**: 272

| Profile Score | Tandem Score | TDA q-value | Volume | Spectrum Matches |
| --- | --- | --- | --- | --- |
| -4.9138 | 45.2458 | 0.0000 | 7.8719e+06 | 3 |

**Adduct Signal Fractions**

- **Unmodified**: 7.8719e+06

### IGSIRN(N)GTYDHDVY Fuc 1 Hex 3 HexNAc 5

**Mass:**  3256.3720

(3.731e-06)

**Charge States:** 3

**Start Position**: 494

**End Position**: 508

| Profile Score | Tandem Score | TDA q-value | Volume | Spectrum Matches |
| --- | --- | --- | --- | --- |
| -19.4483 | 45.0019 | 0.0000 | 2.9537e+06 | 1 |

**Adduct Signal Fractions**

- **Unmodified**: 2.9537e+06

### TIVKPGDILLIN(N)STGN Hex 10 HexNAc 2

**Mass:**  3680.6281

(3.022e-06)

**Charge States:** 3

**Start Position**: 251

**End Position**: 267

| Profile Score | Tandem Score | TDA q-value | Volume | Spectrum Matches |
| --- | --- | --- | --- | --- |
| -5.0603 | 43.6154 | 0.0000 | 2.9306e+06 | 1 |

**Adduct Signal Fractions**

- **Unmodified**: 2.9306e+06

### TIVKPGDILLIN(N)STGNLIAPRGY Fuc 2 Hex 3 HexNAc 4 Neu5Ac 1

**Mass:**  4308.0457

(6.945e-04)

**Start Position**: 251

**End Position**: 274

| Profile Score | Tandem Score | TDA q-value | Volume | Spectrum Matches |
| --- | --- | --- | --- | --- |
| 0.0000 | 43.5118 | 0.0000 | 0.0000e+00 | 2 |

Adduct:

No Chromatogram Found

### RSDAPIGTC(C)SSEC(C)ITPN(N)GSIPN(D)DKPF Hex 8 HexNAc 2

**Mass:**  4522.8767

(2.215e-04)

**Charge States:** 3, 4

**Start Position**: 285

**End Position**: 311

| Profile Score | Tandem Score | TDA q-value | Volume | Spectrum Matches |
| --- | --- | --- | --- | --- |
| -5.7958 | 40.9596 | 0.0000 | 2.3895e+07 | 3 |

**Adduct Signal Fractions**

- **Unmodified**: 2.3895e+07

### RSDAPIGTC(C)SSEC(C)ITPN(N)GSIPN(D)DKPF Hex 9 HexNAc 2

**Mass:**  4684.9274

(2.157e-04)

**Charge States:** 3, 4

**Start Position**: 285

**End Position**: 311

| Profile Score | Tandem Score | TDA q-value | Volume | Spectrum Matches |
| --- | --- | --- | --- | --- |
| -5.7919 | 39.6987 | 0.0000 | 1.8397e+07 | 3 |

**Adduct Signal Fractions**

- **Unmodified**: 1.8397e+07

### LIN(N)STGNLIAPRGYF Hex 8 HexNAc 2

**Mass:**  3338.4631

(3.007e-04)

**Start Position**: 260

**End Position**: 275

| Profile Score | Tandem Score | TDA q-value | Volume | Spectrum Matches |
| --- | --- | --- | --- | --- |
| 0.0000 | 39.5892 | 0.0000 | 0.0000e+00 | 1 |

Adduct:

No Chromatogram Found

### LPGNDN(N)STATL Hex 4 HexNAc 5

**Mass:**  2765.1608

(8.091e-06)

**Charge States:** 3

**Start Position**: 19

**End Position**: 30

| Profile Score | Tandem Score | TDA q-value | Volume | Spectrum Matches |
| --- | --- | --- | --- | --- |
| -19.7315 | 38.5178 | 0.0000 | 2.2938e+06 | 1 |

**Adduct Signal Fractions**

- **Unmodified**: 2.2938e+06

### ITPN(N)GSIPNDKPF Hex 7 HexNAc 2

**Mass:**  2939.2509

(2.711e-06)

**Charge States:** 3

**Start Position**: 298

**End Position**: 311

| Profile Score | Tandem Score | TDA q-value | Volume | Spectrum Matches |
| --- | --- | --- | --- | --- |
| -20.4890 | 38.4409 | 0.0000 | 1.3742e+06 | 1 |

**Adduct Signal Fractions**

- **Unmodified**: 1.3742e+06

### TIVKPGDILLIN(N)STGNL Fuc 2 Hex 4 HexNAc 4 Neu5Ac 1

**Mass:**  3810.7285

(-6.694e-06)

**Charge States:** 3

**Start Position**: 251

**End Position**: 268

| Profile Score | Tandem Score | TDA q-value | Volume | Spectrum Matches |
| --- | --- | --- | --- | --- |
| -19.7938 | 37.8657 | 0.0000 | 1.1038e+06 | 1 |

**Adduct Signal Fractions**

- **Unmodified**: 1.1038e+06

### HKC(C)DNAC(C)IGSIRN(N)GTYDHDVY Fuc 1 Hex 3 HexNAc 4

**Mass:**  3938.6147

(1.360e-06)

**Charge States:** 4

**Start Position**: 487

**End Position**: 508

| Profile Score | Tandem Score | TDA q-value | Volume | Spectrum Matches |
| --- | --- | --- | --- | --- |
| -19.9588 | 37.5047 | 0.0000 | 8.5049e+06 | 2 |

**Adduct Signal Fractions**

- **Unmodified**: 8.5049e+06

### LPGNDN(N)STATL Hex 3 HexNAc 5

**Mass:**  2603.0905

(1.449e-06)

**Charge States:** 3

**Start Position**: 19

**End Position**: 30

| Profile Score | Tandem Score | TDA q-value | Volume | Spectrum Matches |
| --- | --- | --- | --- | --- |
| 12.9054 | 36.1299 | 0.0000 | 1.4486e+07 | 2 |

**Adduct Signal Fractions**

- **Unmodified**: 1.4486e+07

### TIVKPGDILLIN(N)STGN Hex 8 HexNAc 2

**Mass:**  3356.5296

(5.363e-06)

**Charge States:** 3

**Start Position**: 251

**End Position**: 267

| Profile Score | Tandem Score | TDA q-value | Volume | Spectrum Matches |
| --- | --- | --- | --- | --- |
| -20.4696 | 36.0678 | 0.0000 | 1.1173e+06 | 1 |

**Adduct Signal Fractions**

- **Unmodified**: 1.1173e+06

### INEGFN(N)WTGVTQ Fuc 1 Hex 3 HexNAc 5

**Mass:**  3012.2550

(1.897e-06)

**Charge States:** 3

**Start Position**: 137

**End Position**: 149

| Profile Score | Tandem Score | TDA q-value | Volume | Spectrum Matches |
| --- | --- | --- | --- | --- |
| -19.5358 | 35.6293 | 0.0000 | 3.1272e+06 | 1 |

**Adduct Signal Fractions**

- **Unmodified**: 3.1272e+06

### ESESKYPVLN(N)VTM(O)PNN(D)GKFDKLY Fuc 1 Hex 3 HexNAc 6 Neu5Ac 1

**Mass:**  4831.0715

(2.015e-04)

**Charge States:** 4, 5

**Start Position**: 172

**End Position**: 195

| Profile Score | Tandem Score | TDA q-value | Volume | Spectrum Matches |
| --- | --- | --- | --- | --- |
| 10.1512 | 34.8387 | 0.0000 | 1.1368e+08 | 8 |

**Adduct Signal Fractions**

- **Unmodified**: 1.1368e+08

### TIVKPGDILLIN(N)STGNLIAPRGY Hex 5 HexNAc 6

**Mass:**  4453.1066

(-1.749e-06)

**Charge States:** 4

**Start Position**: 251

**End Position**: 274

| Profile Score | Tandem Score | TDA q-value | Volume | Spectrum Matches |
| --- | --- | --- | --- | --- |
| 11.5977 | 33.5760 | 0.0000 | 4.9160e+07 | 5 |

**Adduct Signal Fractions**

- **Unmodified**: 4.9160e+07

### SEC(C)ITPN(N)GSIPN(D)DKPF Hex 8 HexNAc 2

**Mass:**  3478.3980

(2.837e-04)

**Charge States:** 3

**Start Position**: 295

**End Position**: 311

| Profile Score | Tandem Score | TDA q-value | Volume | Spectrum Matches |
| --- | --- | --- | --- | --- |
| -21.4452 | 33.1833 | 0.0000 | 5.6518e+06 | 1 |

**Adduct Signal Fractions**

- **Unmodified**: 5.6518e+06

### TIVKPGDILLIN(N)STGNLIAPRGY Fuc 3 Hex 3 HexNAc 5

**Mass:**  4365.0877

(2.256e-04)

**Start Position**: 251

**End Position**: 274

| Profile Score | Tandem Score | TDA q-value | Volume | Spectrum Matches |
| --- | --- | --- | --- | --- |
| 0.0000 | 32.8465 | 0.0000 | 0.0000e+00 | 1 |

Adduct:

No Chromatogram Found

### TITNDQIEVTN(N)ATEL Hex 5 HexNAc 2

**Mass:**  2877.2470

(3.888e-06)

**Charge States:** 3

**Start Position**: 44

**End Position**: 59

| Profile Score | Tandem Score | TDA q-value | Volume | Spectrum Matches |
| --- | --- | --- | --- | --- |
| -3.6251 | 32.7476 | 0.0000 | 9.5672e+06 | 2 |

**Adduct Signal Fractions**

- **Unmodified**: 9.5672e+06

### KIRTGKSSIM(O)RSDAPIGTC(C)SSEC(C)ITPN(N)GSIPN(D)DKPF Hex 9 HexNAc 2

**Mass:**  5802.5387

(1.723e-04)

**Charge States:** 5, 6

**Start Position**: 275

**End Position**: 311

| Profile Score | Tandem Score | TDA q-value | Volume | Spectrum Matches |
| --- | --- | --- | --- | --- |
| -5.9295 | 31.7537 | 0.0054 | 2.8637e+07 | 3 |

**Adduct Signal Fractions**

- **Unmodified**: 2.8637e+07

### ESESKYPVLN(N)VTM(O)PNN(D)GKFDKLY Fuc 2 Hex 8 HexNAc 3

**Mass:**  4887.1052

(2.084e-04)

**Charge States:** 4, 5

**Start Position**: 172

**End Position**: 195

| Profile Score | Tandem Score | TDA q-value | Volume | Spectrum Matches |
| --- | --- | --- | --- | --- |
| 12.1579 | 30.3221 | 0.0094 | 1.1673e+08 | 4 |

**Adduct Signal Fractions**

- **Unmodified**: 1.1673e+08

### RSDAPIGTC(C)SSEC(C)ITPN(N)GSIPNDKPF Hex 7 HexNAc 2

**Mass:**  4359.8239

(2.106e-06)

**Charge States:** 3, 4

**Start Position**: 285

**End Position**: 311

| Profile Score | Tandem Score | TDA q-value | Volume | Spectrum Matches |
| --- | --- | --- | --- | --- |
| -19.4603 | 30.1198 | 0.0094 | 9.6034e+06 | 1 |

**Adduct Signal Fractions**

- **Unmodified**: 9.6034e+06

### ITPN(N)GSIPNDKPF Fuc 3 Hex 9 HexNAc 5 Neu5Ac 1

**Mass:**  4602.8549

(2.171e-04)

**Start Position**: 298

**End Position**: 311

| Profile Score | Tandem Score | TDA q-value | Volume | Spectrum Matches |
| --- | --- | --- | --- | --- |
| 0.0000 | 29.9809 | 0.0094 | 0.0000e+00 | 1 |

Adduct:

No Chromatogram Found

### KIRTGKSSIM(O)RSDAPIGTC(C)SSEC(C)ITPN(N)GSIPN(D)DKPF Hex 8 HexNAc 2

**Mass:**  5640.4904

(1.766e-04)

**Charge States:** 5

**Start Position**: 275

**End Position**: 311

| Profile Score | Tandem Score | TDA q-value | Volume | Spectrum Matches |
| --- | --- | --- | --- | --- |
| -6.5765 | 29.7942 | 0.0094 | 1.9373e+07 | 1 |

**Adduct Signal Fractions**

- **Unmodified**: 1.9373e+07

### ITPN(N)GSIPNDKPF Hex 8 HexNAc 8 Neu5Ac 1

**Mass:**  4610.8541

(-2.867e-06)

**Start Position**: 298

**End Position**: 311

| Profile Score | Tandem Score | TDA q-value | Volume | Spectrum Matches |
| --- | --- | --- | --- | --- |
| 0.0000 | 29.6101 | 0.0094 | 0.0000e+00 | 1 |

Adduct:

No Chromatogram Found

### TITNDQIEVTN(N)ATEL Hex 5 HexNAc 3

**Mass:**  3080.3292

(5.723e-06)

**Charge States:** 3

**Start Position**: 44

**End Position**: 59

| Profile Score | Tandem Score | TDA q-value | Volume | Spectrum Matches |
| --- | --- | --- | --- | --- |
| -19.0940 | 29.4599 | 0.0094 | 5.1576e+06 | 1 |

**Adduct Signal Fractions**

- **Unmodified**: 5.1576e+06

### ITPN(N)GSIPNDKPF Fuc 3 Hex 10 HexNAc 6

**Mass:**  4675.8898

(-5.849e-07)

**Charge States:** 4

**Start Position**: 298

**End Position**: 311

| Profile Score | Tandem Score | TDA q-value | Volume | Spectrum Matches |
| --- | --- | --- | --- | --- |
| -20.5619 | 29.3567 | 0.0094 | 3.0054e+06 | 1 |

**Adduct Signal Fractions**

- **Unmodified**: 3.0054e+06

### RSDAPIGTC(C)SSEC(C)ITPN(N)GSIPN(D)DKPF Hex 7 HexNAc 2

**Mass:**  4360.8254

(2.320e-04)

**Charge States:** 4

**Start Position**: 285

**End Position**: 311

| Profile Score | Tandem Score | TDA q-value | Volume | Spectrum Matches |
| --- | --- | --- | --- | --- |
| -6.3876 | 28.4622 | 0.0094 | 5.6432e+06 | 2 |

**Adduct Signal Fractions**

- **Unmodified**: 5.6432e+06

### TITNDQIEVTN(N)ATEL Fuc 1 Hex 6 HexNAc 3

**Mass:**  3388.4386

(2.986e-06)

**Charge States:** 3

**Start Position**: 44

**End Position**: 59

| Profile Score | Tandem Score | TDA q-value | Volume | Spectrum Matches |
| --- | --- | --- | --- | --- |
| -20.3112 | 28.4375 | 0.0094 | 2.4043e+06 | 1 |

**Adduct Signal Fractions**

- **Unmodified**: 2.4043e+06

### TIVKPGDILLIN(N)STGNLIAPR Hex 4 HexNAc 7

**Mass:**  4274.0280

(-5.861e-06)

**Charge States:** 3, 4

**Start Position**: 251

**End Position**: 272

| Profile Score | Tandem Score | TDA q-value | Volume | Spectrum Matches |
| --- | --- | --- | --- | --- |
| 14.6192 | 27.2886 | 0.0094 | 2.4350e+07 | 5 |

**Adduct Signal Fractions**

- **Unmodified**: 2.4350e+07

### ITPN(N)GSIPNDKPF Fuc 4 Hex 7 HexNAc 7

**Mass:**  4538.9001

(6.309e-06)

**Charge States:** 4

**Start Position**: 298

**End Position**: 311

| Profile Score | Tandem Score | TDA q-value | Volume | Spectrum Matches |
| --- | --- | --- | --- | --- |
| -20.4439 | 27.2838 | 0.0094 | 7.8671e+06 | 1 |

**Adduct Signal Fractions**

- **Unmodified**: 7.8671e+06

### TIVKPGDILLIN(N)STGN(D)LIAPRGY Fuc 2 Hex 3 HexNAc 4 Neu5Ac 1

**Mass:**  4307.0407

(2.275e-04)

**Charge States:** 4

**Start Position**: 251

**End Position**: 274

| Profile Score | Tandem Score | TDA q-value | Volume | Spectrum Matches |
| --- | --- | --- | --- | --- |
| -19.5103 | 26.5401 | 0.0094 | 1.2674e+06 | 1 |

**Adduct Signal Fractions**

- **Unmodified**: 1.2674e+06

### TIVKPGDILLIN(N)STGNLIAPR Hex 5 HexNAc 7

**Mass:**  4436.0837

(-6.782e-06)

**Charge States:** 3, 4, 5

**Start Position**: 251

**End Position**: 272

| Profile Score | Tandem Score | TDA q-value | Volume | Spectrum Matches |
| --- | --- | --- | --- | --- |
| 13.3764 | 26.5127 | 0.0094 | 4.1994e+08 | 22 |

**Adduct Signal Fractions**

- **Unmodified**: 4.1994e+08

### ESESKYPVLN(N)VTMPNNGKFDKLY Fuc 2 Hex 3 HexNAc 6

**Mass:**  4669.0212

(-9.471e-06)

**Charge States:** 4, 5

**Start Position**: 172

**End Position**: 195

| Profile Score | Tandem Score | TDA q-value | Volume | Spectrum Matches |
| --- | --- | --- | --- | --- |
| 9.0990 | 26.4843 | 0.0094 | 5.5271e+07 | 6 |

**Adduct Signal Fractions**

- **Unmodified**: 5.5271e+07

### ESESKYPVLN(N)VTM(O)PNN(D)GKFDKLY Fuc 2 Hex 7 HexNAc 3

**Mass:**  4725.0529

(2.168e-04)

**Charge States:** 4

**Start Position**: 172

**End Position**: 195

| Profile Score | Tandem Score | TDA q-value | Volume | Spectrum Matches |
| --- | --- | --- | --- | --- |
| 11.2248 | 25.3765 | 0.0184 | 7.7613e+07 | 3 |

**Adduct Signal Fractions**

- **Unmodified**: 7.7613e+07

### TC(C)KRGSN(N)NSFF Fuc 1 Hex 3 HexNAc 4

**Mass:**  2761.1446

(6.321e-06)

**Charge States:** 3

**Start Position**: 154

**End Position**: 165

| Profile Score | Tandem Score | TDA q-value | Volume | Spectrum Matches |
| --- | --- | --- | --- | --- |
| -20.3654 | 24.7009 | 0.0222 | 7.2973e+06 | 2 |

**Adduct Signal Fractions**

- **Unmodified**: 7.2973e+06

### HKC(C)DNAC(C)IGSIRN(N)GTYDHDVY Fuc 2 Hex 4 HexNAc 4

**Mass:**  4246.7268

(1.556e-06)

**Charge States:** 4, 5

**Start Position**: 487

**End Position**: 508

| Profile Score | Tandem Score | TDA q-value | Volume | Spectrum Matches |
| --- | --- | --- | --- | --- |
| -20.8091 | 24.5715 | 0.0222 | 2.8822e+06 | 1 |

**Adduct Signal Fractions**

- **Unmodified**: 2.8822e+06

### IN(N)STGNLIAPRGY Fuc 2 Hex 3 HexNAc 4 Neu5Ac 1

**Mass:**  3256.3983

(-4.410e-06)

**Charge States:** 3

**Start Position**: 261

**End Position**: 274

| Profile Score | Tandem Score | TDA q-value | Volume | Spectrum Matches |
| --- | --- | --- | --- | --- |
| -19.6051 | 24.3833 | 0.0222 | 1.1245e+06 | 1 |

**Adduct Signal Fractions**

- **Unmodified**: 1.1245e+06

### TIVKPGDILLIN(N)STGNLIAPRGY Fuc 1 Hex 3 HexNAc 5 Neu5Ac 1

**Mass:**  4363.0602

(-5.125e-06)

**Charge States:** 4

**Start Position**: 251

**End Position**: 274

| Profile Score | Tandem Score | TDA q-value | Volume | Spectrum Matches |
| --- | --- | --- | --- | --- |
| -5.2268 | 24.1982 | 0.0222 | 7.0452e+06 | 2 |

**Adduct Signal Fractions**

- **Unmodified**: 7.0452e+06

### LPGNDN(N)STATL Fuc 3 Hex 5 HexNAc 4

**Mass:**  3162.2832

(-6.898e-07)

**Charge States:** 3

**Start Position**: 19

**End Position**: 30

| Profile Score | Tandem Score | TDA q-value | Volume | Spectrum Matches |
| --- | --- | --- | --- | --- |
| -20.2127 | 23.9620 | 0.0222 | 1.0444e+06 | 1 |

**Adduct Signal Fractions**

- **Unmodified**: 1.0444e+06

### TITNDQIEVTN(N)ATELVQSSSTGRIC(C)DSPHRIL Hex 7 HexNAc 2

**Mass:**  5097.3357

(4.011e-04)

**Start Position**: 44

**End Position**: 76

| Profile Score | Tandem Score | TDA q-value | Volume | Spectrum Matches |
| --- | --- | --- | --- | --- |
| 0.0000 | 23.7066 | 0.0264 | 0.0000e+00 | 1 |

Adduct:

No Chromatogram Found

### LIN(N)STGNLIAPRGYF Fuc 1 Hex 6 HexNAc 8 Neu5Ac 1

**Mass:**  4669.0212

(8.078e-06)

**Charge States:** 4

**Start Position**: 260

**End Position**: 275

| Profile Score | Tandem Score | TDA q-value | Volume | Spectrum Matches |
| --- | --- | --- | --- | --- |
| -20.4845 | 23.6614 | 0.0302 | 4.4571e+06 | 1 |

**Adduct Signal Fractions**

- **Unmodified**: 4.4571e+06

### LPGNDN(N)STATL Fuc 2 Hex 6 HexNAc 5

**Mass:**  3382.3687

(2.984e-04)

**Start Position**: 19

**End Position**: 30

| Profile Score | Tandem Score | TDA q-value | Volume | Spectrum Matches |
| --- | --- | --- | --- | --- |
| 0.0000 | 23.5870 | 0.0302 | 0.0000e+00 | 1 |

Adduct:

No Chromatogram Found

### SSEC(C)ITPN(N)GSIPN(D)DKPF Hex 9 HexNAc 2

**Mass:**  3727.4837

(2.677e-04)

**Charge States:** 3

**Start Position**: 294

**End Position**: 311

| Profile Score | Tandem Score | TDA q-value | Volume | Spectrum Matches |
| --- | --- | --- | --- | --- |
| -20.5872 | 23.3961 | 0.0302 | 4.9140e+06 | 1 |

**Adduct Signal Fractions**

- **Unmodified**: 4.9140e+06

### TC(C)KRGSN(N)NSFF Fuc 1 Hex 3 HexNAc 5

**Mass:**  2965.2094

(3.384e-04)

**Start Position**: 154

**End Position**: 165

| Profile Score | Tandem Score | TDA q-value | Volume | Spectrum Matches |
| --- | --- | --- | --- | --- |
| 0.0000 | 22.9607 | 0.0381 | 0.0000e+00 | 1 |

Adduct:

No Chromatogram Found

### ESESKYPVLN(N)VTMPNNGKFDKLY Hex 3 HexNAc 6 Neu5Ac 1

**Mass:**  4668.0310

(-3.423e-06)

**Charge States:** 3, 4, 5

**Start Position**: 172

**End Position**: 195

| Profile Score | Tandem Score | TDA q-value | Volume | Spectrum Matches |
| --- | --- | --- | --- | --- |
| 16.7081 | 22.4087 | 0.0381 | 2.5815e+08 | 6 |

**Adduct Signal Fractions**

- **Unmodified**: 2.5815e+08

### iav|ADJ41819|A/Phil/2-MA/1982\_NA

**>iav|ADJ41819|A/Phil/2-MA/1982\_NA**  
 `MNPNQKIITIGSVSLTIATICFLMQIAILVTTVTLHFKQYECSSPPNNQVVPCEPIIIERNITEIVYLTNTTIEKEICPK  
LVEYRNWSKPQCKITGFAPFSKDNSIRLSAGGDIWVTREPYVSCDPGKCYQFALGQGTTLDNKHSNDTIHDRTPYRTLLM  
NELGVPFHLGTRQVCIAWSSSSCHDGKAWLHVCITGYDKNATASFIYDGRLVDSIGSWSKNILRTQESECVCINGTCTVV  
MTDGSASERADTKILFIEEGKIVHISPLSGSAQHVEECSCYPRYPGVRCVCRDNWKGSNRPVVDINVKDYSIVSSYVCSG  
LVGDTPRKNDRSSSSYCRNPNNEKGNHGVKGWAFDDGNDVWMGRTISEESRSGYETFKVIGGWSTPNSKLQINRQVIVDS  
GNRSGYSGIFSVEGKSCINRCFYVELIRGREQETRVWWTSNSIVVFCGTSGTYGTGSWPDGADINLMPI`

| Site | Type | Count |
| --- | --- | --- |
| 146 | N-linked | 8 |
| 200 | N-linked | 1 |
| 86 | N-linked | 2 |
| 402 | N-linked | 5 |

| Mass | Sequence | MS_2_ Score | `q`-value | MS_1_ Score | Signal | Protein Coordinates | Tandem Scans | Start Time | Apex Time | End Time |
| --- | --- | --- | --- | --- | --- | --- | --- | --- | --- | --- |
| 3183.3980 | QINRQVIVDSGN(N)RSGY Hex 6 HexNAc 2 | 171.849 | 0.0 | -9.292 | 2.511e+07 | 390 - 406 | 3 | 21.11 | 21.11 | 29.68 |
| 3323.4077 | RN(N)WSKPQC(C)KITGF Hex 8 HexNAc 2 | 137.984 | 0.0 | -20.371 | 2.646e+06 | 84 - 97 | 1 | 23.96 | 23.96 | 23.96 |
| 3507.5033 | QINRQVIVDSGN(N)RSGY Hex 8 HexNAc 2 | 112.640 | 0.0 | -19.813 | 2.796e+06 | 390 - 406 | 1 | 20.94 | 20.94 | 20.94 |
| 2832.1299 | DKN(N)ATASFIY Hex 8 HexNAc 2 | 107.670 | 0.0 | -19.775 | 8.117e+06 | 197 - 207 | 2 | 18.78 | 18.94 | 18.94 |
| 3847.5063 | DNKHSN(N)DTIHDRTPY Fuc 2 Hex 7 HexNAc 3 | 92.832 | 0.0 | -20.194 | 8.909e+06 | 140 - 155 | 1 | 12.98 | 12.98 | 12.98 |
| 3541.3957 | DN(D)KHSN(N)DTIHDRTPY Fuc 1 Hex 6 HexNAc 3 | 92.768 | 0.0 | 0.000 | 0.000e+00 | 140 - 155 | 1 | - | - | - |
| 3995.5625 | DNKHSN(N)DTIHDRTPY Fuc 1 Hex 7 HexNAc 3 Neu5Ac 1 | 85.896 | 0.0 | 0.000 | 0.000e+00 | 140 - 155 | 1 | - | - | - |
| 3346.5946 | QINRQVIVDSGN(N)RSGY Hex 7 HexNAc 2 | 65.549 | 0.0 | 6.222 | 1.280e+07 | 390 - 406 | 8 | 20.94 | 21.44 | 21.61 |
| 3800.6371 | RN(N)WSKPQC(C)KITGFAPF Hex 9 HexNAc 2 | 62.569 | 0.0 | -4.692 | 5.162e+06 | 84 - 100 | 1 | 36.50 | 36.85 | 37.02 |
| 3910.7884 | STPNSKLQINRQVIVDSGN(N)RSGY Hex 6 HexNAc 2 | 52.270 | 0.0 | -19.785 | 7.278e+06 | 383 - 406 | 1 | 27.83 | 28.00 | 28.00 |
| 4280.7925 | ALGQGTTLDNKHSN(N)DTIHDRTPY Fuc 1 Hex 6 HexNAc 3 | 45.406 | 0.0 | -4.867 | 1.353e+07 | 132 - 155 | 1 | 21.95 | 21.95 | 22.44 |
| 2828.2033 | RQVIVDSGN(N)RSGY Hex 6 HexNAc 2 | 38.872 | 0.0 | -20.241 | 2.060e+06 | 393 - 406 | 1 | 15.18 | 15.18 | 15.18 |
| 4734.9611 | ALGQGTTLDN(D)KHSN(N)DTIHDRTPY Fuc 1 Hex 7 HexNAc 3 Neu5Ac 1 | 31.763 | 0.005 | -20.408 | 5.379e+06 | 132 - 155 | 1 | 21.44 | 21.44 | 21.44 |
| 4588.9099 | ALGQGTTLDNKHSN(N)DTIHDRTPY Fuc 2 Hex 7 HexNAc 3 | 30.350 | 0.009 | -19.931 | 3.080e+06 | 132 - 155 | 1 | 21.61 | 21.61 | 21.61 |
| 4056.8011 | ALGQGTTLDNKHSN(N)DTIHDRTPY Hex 3 HexNAc 5 | 27.728 | 0.009 | 0.000 | 0.000e+00 | 132 - 155 | 1 | - | - | - |
| 4200.8330 | ALGQGTTLDNKHSN(N)DTIHDRTPY Fuc 1 Hex 3 HexNAc 5 | 23.359 | 0.030 | -19.959 | 3.852e+06 | 132 - 155 | 2 | 20.44 | 20.44 | 20.44 |

### QINRQVIVDSGN(N)RSGY Hex 6 HexNAc 2

**Mass:**  3183.3960

(6.826e-07)

**Charge States:** 3

**Start Position**: 391

**End Position**: 407

| Profile Score | Tandem Score | TDA q-value | Volume | Spectrum Matches |
| --- | --- | --- | --- | --- |
| -9.2920 | 171.8487 | 0.0000 | 2.5106e+07 | 3 |

**Adduct Signal Fractions**

- **Unmodified**: 2.5106e+07

### RN(N)WSKPQC(C)KITGF Hex 8 HexNAc 2

**Mass:**  3323.4062

(1.588e-06)

**Charge States:** 3, 4

**Start Position**: 85

**End Position**: 98

| Profile Score | Tandem Score | TDA q-value | Volume | Spectrum Matches |
| --- | --- | --- | --- | --- |
| -20.3707 | 137.9836 | 0.0000 | 2.6463e+06 | 1 |

**Adduct Signal Fractions**

- **Unmodified**: 2.6463e+06

### QINRQVIVDSGN(N)RSGY Hex 8 HexNAc 2

**Mass:**  3507.5033

(1.097e-06)

**Charge States:** 3

**Start Position**: 391

**End Position**: 407

| Profile Score | Tandem Score | TDA q-value | Volume | Spectrum Matches |
| --- | --- | --- | --- | --- |
| -19.8130 | 112.6398 | 0.0000 | 2.7961e+06 | 1 |

**Adduct Signal Fractions**

- **Unmodified**: 2.7961e+06

### DKN(N)ATASFIY Hex 8 HexNAc 2

**Mass:**  2832.1334

(3.556e-04)

**Charge States:** 3

**Start Position**: 198

**End Position**: 208

| Profile Score | Tandem Score | TDA q-value | Volume | Spectrum Matches |
| --- | --- | --- | --- | --- |
| -19.7749 | 107.6702 | 0.0000 | 8.1175e+06 | 2 |

**Adduct Signal Fractions**

- **Unmodified**: 8.1175e+06

### DNKHSN(N)DTIHDRTPY Fuc 2 Hex 7 HexNAc 3

**Mass:**  3847.5063

(-9.374e-06)

**Charge States:** 4

**Start Position**: 141

**End Position**: 156

| Profile Score | Tandem Score | TDA q-value | Volume | Spectrum Matches |
| --- | --- | --- | --- | --- |
| -20.1939 | 92.8315 | 0.0000 | 8.9090e+06 | 1 |

**Adduct Signal Fractions**

- **Unmodified**: 8.9090e+06

### DN(D)KHSN(N)DTIHDRTPY Fuc 1 Hex 6 HexNAc 3

**Mass:**  3541.3957

(5.549e-04)

**Start Position**: 141

**End Position**: 156

| Profile Score | Tandem Score | TDA q-value | Volume | Spectrum Matches |
| --- | --- | --- | --- | --- |
| 0.0000 | 92.7675 | 0.0000 | 0.0000e+00 | 1 |

Adduct:

No Chromatogram Found

### DNKHSN(N)DTIHDRTPY Fuc 1 Hex 7 HexNAc 3 Neu5Ac 1

**Mass:**  3995.5625

(7.470e-04)

**Start Position**: 141

**End Position**: 156

| Profile Score | Tandem Score | TDA q-value | Volume | Spectrum Matches |
| --- | --- | --- | --- | --- |
| 0.0000 | 85.8956 | 0.0000 | 0.0000e+00 | 1 |

Adduct:

No Chromatogram Found

### QINRQVIVDSGN(N)RSGY Hex 7 HexNAc 2

**Mass:**  3345.4469

(1.055e-07)

**Charge States:** 4

**Start Position**: 391

**End Position**: 407

| Profile Score | Tandem Score | TDA q-value | Volume | Spectrum Matches |
| --- | --- | --- | --- | --- |
| 6.2219 | 65.5491 | 0.0000 | 1.2803e+07 | 8 |

**Adduct Signal Fractions**

- **Unmodified**: 1.2803e+07

### RN(N)WSKPQC(C)KITGFAPF Hex 9 HexNAc 2

**Mass:**  3800.6286

(4.406e-06)

**Charge States:** 4

**Start Position**: 85

**End Position**: 101

| Profile Score | Tandem Score | TDA q-value | Volume | Spectrum Matches |
| --- | --- | --- | --- | --- |
| -4.6919 | 62.5695 | 0.0000 | 5.1620e+06 | 1 |

**Adduct Signal Fractions**

- **Unmodified**: 5.1620e+06

### STPNSKLQINRQVIVDSGN(N)RSGY Hex 6 HexNAc 2

**Mass:**  3910.7902

(2.547e-06)

**Charge States:** 4

**Start Position**: 384

**End Position**: 407

| Profile Score | Tandem Score | TDA q-value | Volume | Spectrum Matches |
| --- | --- | --- | --- | --- |
| -19.7845 | 52.2701 | 0.0000 | 7.2784e+06 | 1 |

**Adduct Signal Fractions**

- **Unmodified**: 7.2784e+06

### ALGQGTTLDNKHSN(N)DTIHDRTPY Fuc 1 Hex 6 HexNAc 3

**Mass:**  4280.7922

(-9.679e-06)

**Charge States:** 4

**Start Position**: 133

**End Position**: 156

| Profile Score | Tandem Score | TDA q-value | Volume | Spectrum Matches |
| --- | --- | --- | --- | --- |
| -4.8665 | 45.4064 | 0.0000 | 1.3527e+07 | 1 |

**Adduct Signal Fractions**

- **Unmodified**: 1.3527e+07

### RQVIVDSGN(N)RSGY Hex 6 HexNAc 2

**Mass:**  2828.2033

(-1.706e-06)

**Charge States:** 3

**Start Position**: 394

**End Position**: 407

| Profile Score | Tandem Score | TDA q-value | Volume | Spectrum Matches |
| --- | --- | --- | --- | --- |
| -20.2412 | 38.8719 | 0.0000 | 2.0605e+06 | 1 |

**Adduct Signal Fractions**

- **Unmodified**: 2.0605e+06

### ALGQGTTLDN(D)KHSN(N)DTIHDRTPY Fuc 1 Hex 7 HexNAc 3 Neu5Ac 1

**Mass:**  4734.9611

(2.069e-04)

**Charge States:** 4

**Start Position**: 133

**End Position**: 156

| Profile Score | Tandem Score | TDA q-value | Volume | Spectrum Matches |
| --- | --- | --- | --- | --- |
| -20.4080 | 31.7630 | 0.0054 | 5.3790e+06 | 1 |

**Adduct Signal Fractions**

- **Unmodified**: 5.3790e+06

### ALGQGTTLDNKHSN(N)DTIHDRTPY Fuc 2 Hex 7 HexNAc 3

**Mass:**  4588.9099

(-7.593e-06)

**Charge States:** 4

**Start Position**: 133

**End Position**: 156

| Profile Score | Tandem Score | TDA q-value | Volume | Spectrum Matches |
| --- | --- | --- | --- | --- |
| -19.9308 | 30.3499 | 0.0094 | 3.0797e+06 | 1 |

**Adduct Signal Fractions**

- **Unmodified**: 3.0797e+06

### ALGQGTTLDNKHSN(N)DTIHDRTPY Hex 3 HexNAc 5

**Mass:**  4056.8011

(4.994e-04)

**Start Position**: 133

**End Position**: 156

| Profile Score | Tandem Score | TDA q-value | Volume | Spectrum Matches |
| --- | --- | --- | --- | --- |
| 0.0000 | 27.7284 | 0.0094 | 0.0000e+00 | 1 |

Adduct:

No Chromatogram Found

### ALGQGTTLDNKHSN(N)DTIHDRTPY Fuc 1 Hex 3 HexNAc 5

**Mass:**  4200.8416

(1.779e-06)

**Charge States:** 4, 5

**Start Position**: 133

**End Position**: 156

| Profile Score | Tandem Score | TDA q-value | Volume | Spectrum Matches |
| --- | --- | --- | --- | --- |
| -19.9591 | 23.3595 | 0.0302 | 3.8523e+06 | 2 |

**Adduct Signal Fractions**

- **Unmodified**: 3.8523e+06

### sp|Q90734|ACTN4\_CHICK

**>sp|Q90734|ACTN4\_CHICK**  
 `MVDYHSAGQPYPYGGNGPGPNGDYMAQEDDWDRDLLLDPAWEKQQRKTFTAWCNSHLRKAGTQIENIDEDFRDGLKLMLL  
LEVISGERLPKPERGKMRVHKINNVNKALDFIASKGVNVVSIGAEEIVDGNAKMTLGMIWTIILRFAIQDISVEETSAKE  
GLLLWCQRKTAPYKNVNVQNFHISWKDGLAFNALIHRHRPELIEYDKLRKDDPVTNLNNAFEVAEKYLDIPKMLDAEDIV  
NTARPDEKAIMTYVSSFYHAFSGAQKAETAANRICKVLAVNQENEHLMEDYEKLASDLLEWIRRTIPWLEDRSPQKTIQE  
MQQKLEDFRDYRRVHKPPKVQEKCQLEINFNTLQTKLRLSNRPAFMPSEGRMVSDINTGWQHLEQAEKGYEEWLLNEIRR  
LEPLDHLAEKFRQKASIHEAWTEGKEAMLKQKDYETATLSDIKALIRKHEAFESDLAAHQDRVEQIAAIAQELNELDYYD  
SPSVNARCQKICDQWDVLGSLTHSRREALEKTEKQLETIDELHLEYAKRAAPFNNWMESAMEDLQDMFIVHTIEEIEGLI  
AAHDQFKATLPDADREREAILGIQREAQRIADLHSIKLSGNNPYTSVTPQVINSKWERVQQLVPTRDRALQDEQSRQQCN  
ERLRRQFAGQANIVGPWMQTKMEEIGRISIEMHGTLEDQLQHLKHYEQSIVDYKPNLELLEHEHQLVEEALIFDNKHTNY  
TMEHIRVGWEQLLTTIARTINEVENQILTRDAKGISQEQMQEFRASFNHFDKDHCGALGPEEFKACLISLGYDVENDRQG  
DAEFNRIMSLVDPNGSGSVTFQAFIDFMSRETTDTDTADQVIASFKVLAGDKNYITAEELRRELPPEQAEYCIARMAPYR  
GPDAAPGALDYKSFSTALYGESDL`

| Site | Type | Count |
| --- | --- | --- |
| 814 | N-linked | 1 |

| Mass | Sequence | MS_2_ Score | `q`-value | MS_1_ Score | Signal | Protein Coordinates | Tandem Scans | Start Time | Apex Time | End Time |
| --- | --- | --- | --- | --- | --- | --- | --- | --- | --- | --- |
| 4443.7686 | SLVDPN(N)GSGSVTF Fuc 5 Hex 5 HexNAc 8 | 26.795 | 0.026 | -4.651 | 1.946e+07 | 808 - 821 | 4 | 37.53 | 37.70 | 37.87 |

### SLVDPN(N)GSGSVTF Fuc 5 Hex 5 HexNAc 8

**Mass:**  4443.7676

(-6.663e-06)

**Charge States:** 4, 5

**Start Position**: 809

**End Position**: 822

| Profile Score | Tandem Score | TDA q-value | Volume | Spectrum Matches |
| --- | --- | --- | --- | --- |
| -4.6507 | 26.7954 | 0.0264 | 1.9458e+07 | 4 |

**Adduct Signal Fractions**

- **Unmodified**: 1.9458e+07

### tr|A0A1D5NZ30|A0A1D5NZ30\_CHICK

**>tr|A0A1D5NZ30|A0A1D5NZ30\_CHICK**  
 `MVKLAKTPKNQMKQKKMAPPPKKVEESEEEESSDLEESSGEEVMVPPKKQQKAAVTPAKKAATPAKKAATPAKKAVTPAK  
KAVATPAKKAVAPSPKKAAVVGKGAKNGKNAKKEESEEEDEDDEDDEEDEDEEEESDEEEEPAVPVKPAAKKSAAAVPAK  
KPAVVPAKQESEEEEEEDDEEEDEEDDESEDEAMDTTPAPVKKPTPAKATPAKAKAESEDEEDEEDEDEDEEDEDDEEED  
EEESEDEKPVKEAPGKRKKEMANKSAPEAKKKKTETPASAFSLFVKNLTPTKDYEELRTAIKEFFGKKNLQVSEVRIGSS  
KRFGYVDFLSAEDMDKALQLNGKKLMGLEIKLEKAKSKESLKENKKERDARTLFVKNLPYRVTEDEMKNVFENALEVRLV  
LNKEGSSKGMAYIEFKTEAEAEKALEEKQGTEVDGRAMVIDYTGEKSQQESQKGGGERESKTLIVNNLSYAASEETLQEL  
FKKATSIKMPQNNQGRPKGYAFVEFPTAEDAKEALNSCNNTEIEGRAIRLEFSSPSWQKGNMNARGGFNQQSKTLFVRGL  
SEDTTEETLRESFEGSISARIVTDRDTGSSKGFGFVDFSSPEDAKAAKEAMEDGEIDGNKVTLDFAKPKGEFQRGGGFGG  
GFGGRGGRGGGRGGFGGRGGGRGFGGRGGGFRGSRGGGGDHKPQGKKIKFE`

| Site | Type | Count |
| --- | --- | --- |
| 287 | N-linked | 1 |

| Mass | Sequence | MS_2_ Score | `q`-value | MS_1_ Score | Signal | Protein Coordinates | Tandem Scans | Start Time | Apex Time | End Time |
| --- | --- | --- | --- | --- | --- | --- | --- | --- | --- | --- |
| 3204.3175 | VKN(N)LTPTKDY Hex 10 HexNAc 2 | 25.663 | 0.009 | -18.831 | 4.432e+06 | 284 - 294 | 1 | 29.34 | 29.34 | 29.51 |

### VKN(N)LTPTKDY Hex 10 HexNAc 2

**Mass:**  3204.3149

(-2.013e-06)

**Charge States:** 4

**Start Position**: 285

**End Position**: 295

| Profile Score | Tandem Score | TDA q-value | Volume | Spectrum Matches |
| --- | --- | --- | --- | --- |
| -18.8309 | 25.6633 | 0.0094 | 4.4319e+06 | 1 |

**Adduct Signal Fractions**

- **Unmodified**: 4.4319e+06

### sp|P15771|NUCL\_CHICK

**>sp|P15771|NUCL\_CHICK**  
 `MVKLAKTPKNQMKQKKMAPPPKKVEESEEEESSDLEESSGEEVMVPPKKQQKAAVTPAKKAATPAKKAATPAKKAVTPAK  
KAVATPAKKAVAPSPKKAAVVGKGAKNGKNAKKEESEEEDEDDEDDEEDEDEEEESDEEEEPAVPVKPAAKKSAAAVPAK  
KPAVVPAKQESEEEEEEDDEEEDEEDDESEDEAMDTTPAPVKKPTPAKATPAKAKAESEDEEDEEDEDEDEEDEDDEEED  
EEESEDEKPVKEAPGKRKKEMANKSAPEAKKKKTETPASAFSLFVKNLTPTKDYEELRTAIKEFFGKKNLQVSEVRIGSS  
KRFGYVDFLSAEDMDKALQLNGKKLMGLEIKLEKAKSKESLKENKKERDARTLFVKNLPYRVTEDEMKNVFENALEVRLV  
LNKEGSSKGMAYIEFKTEAEAEKALEEKQGTEVDGRAMVIDYTGEKSQQESQKGGGERESKTLIVNNLSYAASEETLQEL  
FKKATSIKMPQNNQGRPKGYAFVEFPTAEDAKEALNSCNNTEIEGRAIRLEFSSPSWQKGNMNARGGFNQQSKTLFVRGL  
SEDTTEETLRESFEGSISARIVTDRDTGSSKGFGFVDFSSPEDAKAAKEAMEDGEIDGNKVTLDFAKPKGEFQRGGGFGG  
GFGGRGGRGGRGGGRGGFGGRGGGRGFGGRGGGFRGGRGGGGDHKPQGKKIKFE`

| Site | Type | Count |
| --- | --- | --- |
| 287 | N-linked | 1 |

| Mass | Sequence | MS_2_ Score | `q`-value | MS_1_ Score | Signal | Protein Coordinates | Tandem Scans | Start Time | Apex Time | End Time |
| --- | --- | --- | --- | --- | --- | --- | --- | --- | --- | --- |
| 3204.3175 | VKN(N)LTPTKDY Hex 10 HexNAc 2 | 25.663 | 0.009 | -18.831 | 4.432e+06 | 284 - 294 | 1 | 29.34 | 29.34 | 29.51 |

### VKN(N)LTPTKDY Hex 10 HexNAc 2

**Mass:**  3204.3149

(-2.013e-06)

**Charge States:** 4

**Start Position**: 285

**End Position**: 295

| Profile Score | Tandem Score | TDA q-value | Volume | Spectrum Matches |
| --- | --- | --- | --- | --- |
| -18.8309 | 25.6633 | 0.0094 | 4.4319e+06 | 1 |

**Adduct Signal Fractions**

- **Unmodified**: 4.4319e+06
