# Supplementary material for: Measuring Site-specific Glycosylation Similarity between Influenza a Virus Variants with Statistical Certainty
Source: Mol Cell Proteomics. 2020 Nov 25;19(9):1533–45. doi: 10.1074/mcp.RA120.002031 (PMC8143645; doi:10.1074/mcp.RA120.002031)
Supplement: Supplementary file 2 [file mmc2.zip › mmc2/159250_2_supp_555289_qcjklv.zip!/20190326_Phil82gal_021119_chymo_HILIC_DDA_1ug_04_glycopeptides.html]

## 20190326\_Phil82gal\_021119\_chymo\_HILIC\_DDA\_1ug\_04 @ GlycopeptideHypothesis-dd80af9d0799460aa9f8f9518fe2d118

**MS^1^ Mass Accuracy:** 10.0 PPM  
**Grouping Tolerance:** 15.0 PPM  
**MS^n^ Mass Accuracy:** 20.0 PPM  
**Minimum `q`-Value For Clustering:** 0.05

**Glycopeptide Hypothesis:**
GlycopeptideHypothesis-dd80af9d0799460aa9f8f9518fe2d118  
**Sample Searched:**
20190326\_Phil82gal\_021119\_chymo\_HILIC\_DDA\_1ug\_04

| Protein | Matched Glycopeptides |
| --- | --- |
| iav\|AFG99160\|A/Phil/2/1982\_HA | 116 |
| iav\|ADJ41819\|A/Phil/2-MA/1982\_NA | 14 |
| tr\|A0A1D5PUY7\|A0A1D5PUY7\_CHICK | 1 |
| tr\|F1NN63\|F1NN63\_CHICK | 1 |
| tr\|A0A1L1RR19\|A0A1L1RR19\_CHICK | 1 |
| tr\|A0A146F0A0\|A0A146F0A0\_CHICK | 1 |

### iav|AFG99160|A/Phil/2/1982\_HA

**>iav|AFG99160|A/Phil/2/1982\_HA**  
 `MKTIIALSYMFCLVFAQNLPGNDNSTATLCLGHHAVPNGTLVKTITNDQIEVTNATELVQSSSTGRICDSPHRILDGKNC  
TLIDALLGDPHCDGFQNEKWDLFVERSKAFSNCYPYDVPDYASLRSLVASSGTLEFINEGFNWTGVTQSGGSSTCKRGSN  
NSFFSRLNWLYESESKYPVLNVTMPNNGKFDKLYIWGIHHPSTDKEQTNLYIRASGRVTVSTKRSQQTVIPNIGSRPWVR  
GLSSRISIYWTIVKPGDILLINSTGNLIAPRGYFKIRTGKSSIMRSDAPIGTCSSECITPNGSIPNDKPFQNVNKITYGA  
CPRYVKQNTLKLATGMRNVPEKQTRGIFGAIAGFIENGWEGMVDGWYGFRHQNSEGTGQAADLKSTQAAIDQINGKLNRV  
IEKTNEKFHQIEKEFSEVEGRIQDLEKYVEDTKIDLWSYNAELLVALENQHTIDLTDSEMNKLFEKTRKQLRENAEDMGN  
GCFKIYHKCDNACIGSIRNGTYDHDVYRDEALNNRFQIKGVELKSGYKDWILWISFAISCFLLCVVLLGFIMWACQKGNI  
RCNICI`

| Site | Type | Count |
| --- | --- | --- |
| 262 | N-linked | 44 |
| 301 | N-linked | 26 |
| 499 | N-linked | 8 |
| 181 | N-linked | 14 |
| 54 | N-linked | 7 |
| 24 | N-linked | 14 |
| 160 | N-linked | 3 |

| Mass | Sequence | MS_2_ Score | `q`-value | MS_1_ Score | Signal | Protein Coordinates | Tandem Scans | Start Time | Apex Time | End Time |
| --- | --- | --- | --- | --- | --- | --- | --- | --- | --- | --- |
| 3352.4493 | LIN(N)STGNLIAPRGY Hex 9 HexNAc 2 | 171.864 | 0.0 | -4.658 | 4.026e+07 | 259 - 273 | 2 | 34.75 | 34.92 | 35.25 |
| 2546.0673 | LPGNDN(N)STATL Fuc 1 Hex 3 HexNAc 4 | 171.423 | 0.0 | 12.833 | 5.409e+07 | 18 - 29 | 5 | 23.53 | 23.69 | 24.88 |
| 2695.0889 | LIN(N)STGNL Hex 9 HexNAc 2 | 170.928 | 0.0 | -4.464 | 9.463e+06 | 259 - 267 | 2 | 22.86 | 23.02 | 23.36 |
| 2696.0848 | LIN(N)STGNL Hex 9 HexNAc 2 | 166.915 | 0.0 | 0.000 | 0.000e+00 | 259 - 267 | 1 | - | - | - |
| 3132.3637 | LIN(N)STGNLIAPR Hex 9 HexNAc 2 | 164.620 | 0.0 | 12.238 | 3.687e+07 | 259 - 271 | 3 | 28.25 | 28.41 | 29.09 |
| 4842.0892 | TIVKPGDILLIN(N)STGNL Fuc 3 Hex 10 HexNAc 5 | 163.142 | 0.0 | 7.239 | 2.479e+07 | 250 - 267 | 6 | 47.12 | 49.34 | 50.87 |
| 4068.9285 | TIVKPGDILLIN(N)STGNLIAPR Hex 9 HexNAc 2 | 161.680 | 0.0 | 13.219 | 2.362e+08 | 250 - 271 | 12 | 50.87 | 51.55 | 52.41 |
| 3159.4250 | TIVKPGDILLIN(N) Hex 9 HexNAc 2 | 158.483 | 0.0 | 12.905 | 9.550e+07 | 250 - 262 | 7 | 43.56 | 45.09 | 45.77 |
| 4289.0320 | TIVKPGDILLIN(N)STGNLIAPRGY Hex 9 HexNAc 2 | 157.864 | 0.0 | 14.711 | 8.309e+08 | 250 - 273 | 43 | 53.26 | 54.28 | 58.29 |
| 3176.4520 | TIVKPGDILLIN(N) Fuc 2 Hex 3 HexNAc 4 Neu5Ac 1 | 153.908 | 0.0 | 11.843 | 2.678e+07 | 250 - 262 | 4 | 43.56 | 45.09 | 45.43 |
| 2703.1295 | KRGSN(N)NSFF Fuc 1 Hex 3 HexNAc 5 | 153.618 | 0.0 | -3.408 | 8.750e+07 | 155 - 164 | 2 | 18.84 | 18.84 | 19.34 |
| 2749.1463 | LPGNDN(N)STATL Fuc 1 Hex 3 HexNAc 5 | 152.401 | 0.0 | 12.626 | 1.330e+07 | 18 - 29 | 3 | 23.36 | 23.69 | 24.37 |
| 4451.0977 | TIVKPGDILLIN(N)STGNLIAPRGY Hex 10 HexNAc 2 | 152.210 | 0.0 | 11.408 | 6.945e+07 | 250 - 273 | 7 | 53.94 | 54.11 | 61.04 |
| 3386.4380 | IN(N)STGNLIAPRGYF Hex 9 HexNAc 2 | 149.789 | 0.0 | -18.230 | 7.044e+06 | 260 - 274 | 1 | 41.35 | 41.35 | 41.52 |
| 3631.6538 | TIVKPGDILLIN(N)STGNL Hex 9 HexNAc 2 | 149.410 | 0.0 | 12.704 | 8.369e+07 | 250 - 267 | 4 | 50.87 | 51.04 | 51.55 |
| 4126.9551 | TIVKPGDILLIN(N)STGNLIAPRGY Hex 8 HexNAc 2 | 147.038 | 0.0 | 12.265 | 7.499e+07 | 250 - 273 | 11 | 53.77 | 54.28 | 55.48 |
| 3239.3643 | IN(N)STGNLIAPRGY Hex 9 HexNAc 2 | 147.011 | 0.0 | -4.179 | 1.035e+07 | 260 - 273 | 2 | 29.25 | 29.42 | 29.59 |
| 2854.1792 | LPGNDN(N)STATL Fuc 2 Hex 4 HexNAc 4 | 145.021 | 0.0 | 11.413 | 1.011e+07 | 18 - 29 | 2 | 23.02 | 23.36 | 23.69 |
| 3518.5704 | TIVKPGDILLIN(N)STGN Hex 9 HexNAc 2 | 144.987 | 0.0 | 12.293 | 3.339e+07 | 250 - 266 | 4 | 42.88 | 43.05 | 43.56 |
| 4306.0389 | TIVKPGDILLIN(N)STGNLIAPRGY Fuc 2 Hex 3 HexNAc 4 Neu5Ac 1 | 144.799 | 0.0 | 10.802 | 1.044e+08 | 250 - 273 | 3 | 53.60 | 54.28 | 54.80 |
| 2400.0095 | LPGNDN(N)STATL Hex 3 HexNAc 4 | 144.663 | 0.0 | 11.102 | 1.336e+07 | 18 - 29 | 3 | 23.36 | 23.53 | 24.03 |
| 3516.5376 | LIN(N)STGNLIAPRGYF Fuc 2 Hex 3 HexNAc 4 Neu5Ac 1 | 144.058 | 0.0 | -19.646 | 5.584e+06 | 259 - 274 | 1 | 45.43 | 45.60 | 45.60 |
| 3101.2982 | ITPN(N)GSIPNDKPF Hex 8 HexNAc 2 | 142.862 | 0.0 | -3.391 | 1.099e+07 | 297 - 310 | 2 | 29.59 | 29.76 | 29.93 |
| 3499.5149 | LIN(N)STGNLIAPRGYF Hex 9 HexNAc 2 | 142.501 | 0.0 | -19.099 | 2.994e+07 | 259 - 274 | 1 | 45.43 | 45.60 | 45.60 |
| 4085.9567 | TIVKPGDILLIN(N)STGNLIAPR Fuc 2 Hex 3 HexNAc 4 Neu5Ac 1 | 142.377 | 0.0 | 13.189 | 2.633e+07 | 250 - 271 | 3 | 51.38 | 51.55 | 52.07 |
| 2317.9563 | LPGNDN(N)STATL Hex 5 HexNAc 2 | 140.102 | 0.0 | 12.067 | 8.751e+06 | 18 - 29 | 3 | 23.02 | 23.36 | 23.69 |
| 3369.4777 | LIN(N)STGNLIAPRGY Fuc 2 Hex 3 HexNAc 4 Neu5Ac 1 | 136.169 | 0.0 | -20.227 | 4.755e+06 | 259 - 273 | 1 | 34.92 | 34.92 | 34.92 |
| 4230.9825 | TIVKPGDILLIN(N)STGNLIAPR Hex 10 HexNAc 2 | 134.676 | 0.0 | -4.094 | 1.925e+07 | 250 - 271 | 3 | 51.38 | 51.55 | 51.89 |
| 3263.3677 | ITPN(N)GSIPNDKPF Hex 9 HexNAc 2 | 133.607 | 0.0 | -18.225 | 8.961e+06 | 297 - 310 | 1 | 29.59 | 29.59 | 29.76 |
| 3648.6801 | TIVKPGDILLIN(N)STGNL Fuc 2 Hex 3 HexNAc 4 Neu5Ac 1 | 131.701 | 0.0 | -4.219 | 2.245e+07 | 250 - 267 | 2 | 50.87 | 51.04 | 51.38 |
| 3906.8773 | TIVKPGDILLIN(N)STGNLIAPR Hex 8 HexNAc 2 | 129.681 | 0.0 | -4.370 | 1.569e+07 | 250 - 271 | 4 | 51.55 | 51.72 | 51.89 |
| 3793.7080 | TIVKPGDILLIN(N)STGNL Hex 10 HexNAc 2 | 125.979 | 0.0 | -4.370 | 7.150e+06 | 250 - 267 | 3 | 50.87 | 50.87 | 51.21 |
| 3321.4739 | TIVKPGDILLIN(N) Hex 10 HexNAc 2 | 123.876 | 0.0 | -19.927 | 4.654e+06 | 250 - 262 | 1 | 44.92 | 44.92 | 45.09 |
| 4521.8743 | RSDAPIGTC(C)SSEC(C)ITPN(N)GSIPNDKPF Hex 8 HexNAc 2 | 119.624 | 0.0 | 12.091 | 7.504e+07 | 284 - 310 | 4 | 36.08 | 36.24 | 36.92 |
| 3053.2943 | IGSIRN(N)GTYDHDVY Fuc 1 Hex 3 HexNAc 4 | 118.131 | 0.0 | -17.644 | 4.689e+06 | 493 - 507 | 1 | 22.52 | 22.52 | 22.69 |
| 4683.9242 | RSDAPIGTC(C)SSEC(C)ITPN(N)GSIPNDKPF Hex 9 HexNAc 2 | 115.707 | 0.0 | 12.258 | 6.913e+07 | 284 - 310 | 3 | 36.08 | 36.24 | 36.92 |
| 3535.5961 | TIVKPGDILLIN(N)STGN Fuc 2 Hex 3 HexNAc 4 Neu5Ac 1 | 115.681 | 0.0 | -4.853 | 8.593e+06 | 250 - 266 | 2 | 42.88 | 43.05 | 43.22 |
| 3564.4355 | SSEC(C)ITPN(N)GSIPNDKPF Hex 8 HexNAc 2 | 114.320 | 0.0 | -19.509 | 1.398e+07 | 293 - 310 | 1 | 33.47 | 33.47 | 33.63 |
| 3863.5991 | LYESESKYPVLN(N)VTM(O)PN Hex 9 HexNAc 2 | 113.294 | 0.0 | -20.034 | 1.334e+07 | 169 - 186 | 1 | 35.92 | 36.08 | 36.08 |
| 3019.2864 | IN(N)STGNLIAPR Hex 9 HexNAc 2 | 108.514 | 0.0 | -6.188 | 3.363e+06 | 260 - 271 | 1 | 21.51 | 21.68 | 21.85 |
| 2883.1787 | SIRN(N)GTYDHDVY Fuc 1 Hex 3 HexNAc 4 | 105.513 | 0.0 | -20.118 | 2.752e+06 | 495 - 507 | 1 | 17.40 | 17.40 | 17.40 |
| 3726.4902 | SSEC(C)ITPN(N)GSIPNDKPF Hex 9 HexNAc 2 | 105.124 | 0.0 | -19.248 | 9.466e+06 | 293 - 310 | 1 | 33.30 | 33.47 | 33.47 |
| 3469.5996 | TIVKPGDILLIN(N)STGNL Hex 8 HexNAc 2 | 104.537 | 0.0 | -4.881 | 5.471e+06 | 250 - 267 | 1 | 51.04 | 51.04 | 51.38 |
| 3361.4090 | IGSIRN(N)GTYDHDVY Fuc 2 Hex 4 HexNAc 4 | 95.566 | 0.0 | -20.269 | 4.109e+06 | 493 - 507 | 1 | 22.18 | 22.18 | 22.35 |
| 4539.8977 | ITPN(N)GSIPNDKPF Fuc 4 Hex 7 HexNAc 7 | 92.680 | 0.0 | 0.000 | 0.000e+00 | 297 - 310 | 1 | - | - | - |
| 3039.2996 | TITNDQIEVTN(N)ATEL Hex 6 HexNAc 2 | 89.305 | 0.0 | -3.704 | 2.181e+07 | 43 - 58 | 2 | 38.63 | 38.80 | 39.14 |
| 4468.0928 | TIVKPGDILLIN(N)STGNLIAPRGY Fuc 2 Hex 4 HexNAc 4 Neu5Ac 1 | 71.158 | 0.0 | -5.459 | 9.496e+06 | 250 - 273 | 2 | 53.94 | 54.11 | 54.45 |
| 4359.8226 | RSDAPIGTC(C)SSEC(C)ITPN(N)GSIPNDKPF Hex 7 HexNAc 2 | 70.530 | 0.0 | -5.455 | 1.633e+07 | 284 - 310 | 1 | 36.24 | 36.24 | 36.58 |
| 4553.9404 | ESESKYPVLN(N)VTM(O)PNN(D)GKFDKLY Hex 9 HexNAc 2 | 63.872 | 0.0 | 11.181 | 3.792e+07 | 171 - 194 | 4 | 40.84 | 41.35 | 41.69 |
| 5639.5051 | KIRTGKSSIM(O)RSDAPIGTC(C)SSEC(C)ITPN(N)GSIPNDKPF Hex 8 HexNAc 2 | 62.288 | 0.0 | -4.942 | 4.352e+07 | 274 - 310 | 3 | 31.79 | 31.95 | 32.12 |
| 4391.8892 | ESESKYPVLN(N)VTM(O)PNN(D)GKFDKLY Hex 8 HexNAc 2 | 60.470 | 0.0 | 11.366 | 2.595e+07 | 171 - 194 | 3 | 40.84 | 41.35 | 41.69 |
| 5801.5604 | KIRTGKSSIM(O)RSDAPIGTC(C)SSEC(C)ITPN(N)GSIPNDKPF Hex 9 HexNAc 2 | 58.051 | 0.0 | -4.814 | 3.426e+07 | 274 - 310 | 3 | 31.79 | 31.95 | 32.12 |
| 4522.8637 | RSDAPIGTC(C)SSEC(C)ITPN(N)GSIPN(D)DKPF Hex 8 HexNAc 2 | 52.319 | 0.0 | -5.699 | 2.725e+07 | 284 - 310 | 3 | 37.09 | 37.26 | 37.60 |
| 4444.7502 | LPGNDN(N)STATL Fuc 4 Hex 7 HexNAc 8 | 51.353 | 0.019 | -4.319 | 1.266e+07 | 18 - 29 | 2 | 39.14 | 39.31 | 39.48 |
| 4310.8142 | LYESESKYPVLN(N)VTM(O)PNN(D)GKF Hex 9 HexNAc 2 | 51.303 | 0.0 | 9.715 | 2.370e+07 | 169 - 190 | 5 | 39.99 | 40.16 | 41.01 |
| 3938.6161 | HKC(C)DNAC(C)IGSIRN(N)GTYDHDVY Fuc 1 Hex 3 HexNAc 4 | 50.993 | 0.0 | -19.707 | 7.667e+06 | 486 - 507 | 2 | 23.69 | 23.69 | 23.86 |
| 4684.9128 | RSDAPIGTC(C)SSEC(C)ITPN(N)GSIPN(D)DKPF Hex 9 HexNAc 2 | 49.724 | 0.0 | 11.412 | 2.261e+07 | 284 - 310 | 3 | 36.92 | 37.09 | 37.60 |
| 4307.0410 | TIVKPGDILLIN(N)STGN(D)LIAPRGY Fuc 2 Hex 3 HexNAc 4 Neu5Ac 1 | 49.289 | 0.0 | -20.487 | 5.241e+06 | 250 - 273 | 2 | 53.94 | 53.94 | 55.14 |
| 3404.4679 | IN(N)STGNLIAPRGYF Fuc 2 Hex 3 HexNAc 4 Neu5Ac 1 | 48.217 | 0.0 | 0.000 | 0.000e+00 | 260 - 274 | 1 | - | - | - |
| 3680.6268 | TIVKPGDILLIN(N)STGN Hex 10 HexNAc 2 | 46.457 | 0.0 | -5.399 | 3.045e+06 | 250 - 266 | 1 | 42.88 | 42.88 | 43.22 |
| 5786.5658 | FKIRTGKSSIM(O)RSDAPIGTC(C)SSEC(C)ITPN(N)GSIPNDKPF Hex 8 HexNAc 2 | 46.256 | 0.0 | -20.055 | 4.760e+06 | 273 - 310 | 1 | 35.25 | 35.25 | 35.25 |
| 3337.4642 | LIN(N)STGNLIAPRGYF Hex 8 HexNAc 2 | 45.007 | 0.0 | -20.076 | 2.222e+06 | 259 - 274 | 1 | 45.60 | 45.60 | 45.77 |
| 3256.3792 | IGSIRN(N)GTYDHDVY Fuc 1 Hex 3 HexNAc 5 | 44.735 | 0.0 | -20.242 | 2.148e+06 | 493 - 507 | 1 | 22.52 | 22.52 | 22.69 |
| 4933.2463 | TITNDQIEVTN(N)ATELVQSSSTGRIC(C)DSPHRIL Hex 6 HexNAc 2 | 43.491 | 0.0 | -18.631 | 1.554e+06 | 43 - 75 | 1 | 45.26 | 45.26 | 45.26 |
| 4840.0009 | SSEC(C)ITPN(N)GSIPNDKPF Fuc 4 Hex 6 HexNAc 7 | 43.061 | 0.0 | -3.751 | 1.904e+07 | 293 - 310 | 2 | 39.31 | 39.65 | 39.82 |
| 4292.0217 | TIVKPGDILLIN(N)STGNLIAPRGY Hex 9 HexNAc 2 | 43.046 | 0.0 | 0.000 | 0.000e+00 | 250 - 273 | 3 | - | - | - |
| 4354.8537 | VKTITNDQIEVTN(N)ATEL Fuc 3 Hex 5 HexNAc 6 | 42.859 | 0.0 | 0.000 | 0.000e+00 | 41 - 58 | 1 | - | - | - |
| 3201.3518 | TITNDQIEVTN(N)ATEL Hex 7 HexNAc 2 | 41.704 | 0.0 | -4.453 | 1.065e+07 | 43 - 58 | 2 | 38.46 | 38.63 | 38.97 |
| 2877.2466 | TITNDQIEVTN(N)ATEL Hex 5 HexNAc 2 | 41.386 | 0.0 | -4.554 | 9.546e+06 | 43 - 58 | 2 | 38.97 | 39.14 | 39.31 |
| 3565.4181 | SSEC(C)ITPN(N)GSIPN(D)DKPF Hex 8 HexNAc 2 | 40.588 | 0.0 | -21.105 | 1.054e+07 | 293 - 310 | 1 | 34.75 | 34.75 | 34.92 |
| 3284.3611 | AC(C)IGSIRN(N)GTYDHDVY Fuc 1 Hex 3 HexNAc 4 | 40.502 | 0.0 | -19.721 | 1.400e+06 | 491 - 507 | 1 | 26.74 | 26.74 | 26.74 |
| 3486.4210 | LPGNDN(N)STATL Fuc 3 Hex 7 HexNAc 4 | 40.217 | 0.0 | 0.000 | 0.000e+00 | 18 - 29 | 1 | - | - | - |
| 4700.9516 | ITPN(N)GSIPNDKPF Fuc 4 Hex 8 HexNAc 7 | 40.127 | 0.0 | -20.836 | 9.006e+06 | 297 - 310 | 1 | 36.08 | 36.24 | 36.24 |
| 3193.2910 | SIRN(N)GTYDHDVY Fuc 2 Hex 4 HexNAc 4 | 38.776 | 0.0 | 0.000 | 0.000e+00 | 495 - 507 | 1 | - | - | - |
| 4417.8185 | ITPN(N)GSIPNDKPF Fuc 4 Hex 5 HexNAc 8 | 38.003 | 0.0 | -5.724 | 7.311e+06 | 297 - 310 | 2 | 39.48 | 39.65 | 39.99 |
| 4842.0921 | TIVKPGDILLIN(N)STGNL Fuc 1 Hex 5 HexNAc 9 Neu5Ac 1 | 37.460 | 0.0 | -20.977 | 2.438e+06 | 250 - 267 | 1 | 51.72 | 51.72 | 51.89 |
| 3701.5448 | LYESESKYPVLN(N)VTM(O)PN Hex 8 HexNAc 2 | 36.878 | 0.0 | -21.109 | 6.360e+06 | 169 - 186 | 1 | 35.92 | 36.08 | 36.08 |
| 4887.1055 | ESESKYPVLN(N)VTM(O)PNN(D)GKFDKLY Fuc 2 Hex 8 HexNAc 3 | 35.832 | 0.0 | 11.486 | 1.150e+08 | 171 - 194 | 3 | 43.39 | 43.90 | 44.07 |
| 4363.0666 | TIVKPGDILLIN(N)STGNLIAPRGY Fuc 1 Hex 3 HexNAc 5 Neu5Ac 1 | 35.739 | 0.0 | 10.806 | 1.546e+07 | 250 - 273 | 3 | 54.11 | 54.63 | 55.31 |
| 2626.0692 | LPGNDN(N)STATL Fuc 1 Hex 6 HexNAc 2 | 35.678 | 0.0 | 13.200 | 4.638e+08 | 18 - 29 | 10 | 35.25 | 35.92 | 38.12 |
| 5802.5434 | KIRTGKSSIM(O)RSDAPIGTC(C)SSEC(C)ITPN(N)GSIPN(D)DKPF Hex 9 HexNAc 2 | 34.217 | 0.0 | -6.073 | 2.665e+07 | 274 - 310 | 2 | 32.12 | 32.46 | 32.63 |
| 3728.4652 | SSEC(C)ITPN(N)GSIPN(D)DKPF Hex 9 HexNAc 2 | 33.953 | 0.0 | 0.000 | 0.000e+00 | 293 - 310 | 1 | - | - | - |
| 2603.0919 | LPGNDN(N)STATL Hex 3 HexNAc 5 | 33.849 | 0.0 | 10.935 | 1.312e+07 | 18 - 29 | 2 | 23.19 | 23.69 | 24.20 |
| 4148.7643 | LYESESKYPVLN(N)VTM(O)PNN(D)GKF Hex 8 HexNAc 2 | 32.990 | 0.0 | -6.235 | 1.482e+07 | 169 - 190 | 3 | 39.99 | 40.33 | 40.50 |
| 5948.6062 | FKIRTGKSSIM(O)RSDAPIGTC(C)SSEC(C)ITPN(N)GSIPNDKPF Hex 9 HexNAc 2 | 32.802 | 0.0 | -20.419 | 3.588e+06 | 273 - 310 | 1 | 35.25 | 35.25 | 35.25 |
| 3080.3277 | TITNDQIEVTN(N)ATEL Hex 5 HexNAc 3 | 32.164 | 0.006 | -3.961 | 6.140e+06 | 43 - 58 | 1 | 38.80 | 38.97 | 39.14 |
| 4360.8093 | RSDAPIGTC(C)SSEC(C)ITPN(N)GSIPN(D)DKPF Hex 7 HexNAc 2 | 32.161 | 0.006 | -6.251 | 6.773e+06 | 284 - 310 | 1 | 37.09 | 37.26 | 37.60 |
| 4436.0832 | TIVKPGDILLIN(N)STGNLIAPR Hex 5 HexNAc 7 | 32.028 | 0.006 | 15.360 | 4.345e+08 | 250 - 271 | 11 | 59.60 | 60.18 | 60.87 |
| 4452.0669 | TIVKPGDILLIN(N)STGN(D)LIAPRGY Hex 10 HexNAc 2 | 31.963 | 0.006 | -20.794 | 1.157e+06 | 250 - 273 | 4 | 53.77 | 55.31 | 55.31 |
| 2911.2066 | LPGNDN(N)STATL Fuc 1 Hex 4 HexNAc 5 | 31.749 | 0.010 | -18.941 | 1.995e+06 | 18 - 29 | 1 | 23.19 | 23.36 | 23.36 |
| 3160.4467 | TIVKPGDILLIN(N) Hex 9 HexNAc 2 | 31.571 | 0.010 | 0.000 | 0.000e+00 | 250 - 262 | 1 | - | - | - |
| 3356.5145 | TIVKPGDILLIN(N)STGN Hex 8 HexNAc 2 | 30.793 | 0.010 | -20.140 | 8.060e+05 | 250 - 266 | 1 | 43.22 | 43.22 | 43.22 |
| 4274.0272 | TIVKPGDILLIN(N)STGNLIAPR Hex 4 HexNAc 7 | 30.181 | 0.010 | 12.991 | 3.047e+07 | 250 - 271 | 6 | 60.01 | 60.36 | 60.87 |
| 3162.2922 | LPGNDN(N)STATL Fuc 3 Hex 5 HexNAc 4 | 29.841 | 0.010 | -19.635 | 1.839e+06 | 18 - 29 | 1 | 22.52 | 22.69 | 22.69 |
| 5640.4881 | KIRTGKSSIM(O)RSDAPIGTC(C)SSEC(C)ITPN(N)GSIPN(D)DKPF Hex 8 HexNAc 2 | 29.188 | 0.010 | -6.165 | 2.040e+07 | 274 - 310 | 1 | 32.29 | 32.46 | 32.63 |
| 4725.0545 | ESESKYPVLN(N)VTM(O)PNN(D)GKFDKLY Fuc 2 Hex 7 HexNAc 3 | 29.116 | 0.010 | 11.165 | 8.278e+07 | 171 - 194 | 4 | 43.56 | 43.90 | 44.24 |
| 3388.4390 | TITNDQIEVTN(N)ATEL Fuc 1 Hex 6 HexNAc 3 | 27.860 | 0.015 | -5.443 | 3.033e+06 | 43 - 58 | 2 | 38.46 | 38.63 | 38.80 |
| 2761.1415 | TC(C)KRGSN(N)NSFF Fuc 1 Hex 3 HexNAc 4 | 26.542 | 0.019 | -5.334 | 8.434e+06 | 153 - 164 | 2 | 18.34 | 18.67 | 18.67 |
| 4668.0325 | LYESESKYPVLN(N)VTMPNNGKF Fuc 2 Hex 7 HexNAc 4 | 26.413 | 0.019 | 15.543 | 2.666e+08 | 169 - 190 | 7 | 42.71 | 44.07 | 44.24 |
| 3825.5900 | TGVTQSGGSSTC(C)KRGSN(N)NSFF Fuc 1 Hex 3 HexNAc 5 | 26.321 | 0.019 | -19.302 | 1.299e+07 | 143 - 164 | 2 | 22.02 | 22.02 | 22.18 |
| 4669.0223 | ESESKYPVLN(N)VTMPNNGKFDKLY Fuc 2 Hex 3 HexNAc 6 | 26.058 | 0.019 | 8.792 | 6.646e+07 | 171 - 194 | 5 | 44.41 | 44.41 | 45.43 |
| 4832.0916 | ESESKYPVLN(N)VTM(O)PNN(D)GKFDKLY Fuc 3 Hex 3 HexNAc 6 | 25.919 | 0.022 | 0.000 | 0.000e+00 | 171 - 194 | 1 | - | - | - |
| 5477.4559 | KIRTGKSSIM(O)RSDAPIGTC(C)SSEC(C)ITPN(N)GSIPNDKPF Hex 7 HexNAc 2 | 25.743 | 0.022 | -20.289 | 6.413e+06 | 274 - 310 | 2 | 31.79 | 31.95 | 31.95 |
| 4831.0735 | LYESESKYPVLN(N)VTM(O)PNN(D)GKF Fuc 3 Hex 7 HexNAc 4 | 25.543 | 0.022 | 10.503 | 1.281e+08 | 169 - 190 | 9 | 44.41 | 44.41 | 45.77 |
| 2709.1252 | LPGNDN(N)STATL Fuc 1 Hex 4 HexNAc 4 | 25.357 | 0.022 | 0.000 | 0.000e+00 | 18 - 29 | 1 | - | - | - |
| 4453.1083 | TIVKPGDILLIN(N)STGNLIAPRGY Hex 5 HexNAc 6 | 24.980 | 0.022 | 12.848 | 6.610e+07 | 250 - 273 | 5 | 59.85 | 60.18 | 60.87 |
| 4251.0241 | TIVKPGDILLIN(N)STGNLIAPR Fuc 2 Hex 4 HexNAc 4 Neu5Ac 1 | 24.694 | 0.022 | 0.000 | 0.000e+00 | 250 - 271 | 1 | - | - | - |
| 4830.0854 | ESESKYPVLN(N)VTMPNNGKFDKLY Hex 4 HexNAc 6 Neu5Ac 1 | 24.039 | 0.022 | 15.767 | 4.101e+08 | 171 - 194 | 4 | 43.56 | 43.90 | 44.24 |
| 3087.2593 | SIRN(N)GTYDHDVY Fuc 1 Hex 3 HexNAc 5 | 23.777 | 0.025 | 0.000 | 0.000e+00 | 495 - 507 | 1 | - | - | - |
| 4726.9093 | ITPN(N)GSIPNDKPF Fuc 3 Hex 6 HexNAc 8 Neu5Ac 1 | 23.423 | 0.025 | 0.000 | 0.000e+00 | 297 - 310 | 1 | - | - | - |
| 4440.8133 | ITPN(N)GSIPNDKPF Hex 10 HexNAc 7 | 23.365 | 0.025 | 0.000 | 0.000e+00 | 297 - 310 | 1 | - | - | - |
| 3444.3867 | LPGNDN(N)STATL Fuc 1 Hex 8 HexNAc 3 Neu5Ac 1 | 23.277 | 0.025 | 9.435 | 2.301e+07 | 18 - 29 | 3 | 32.46 | 32.80 | 33.13 |
| 3965.9240 | TIVKPGDILLIN(N)STGN(D)LIAPRGY Hex 7 HexNAc 2 | 23.162 | 0.029 | -20.181 | 1.174e+06 | 250 - 273 | 1 | 54.28 | 54.28 | 54.28 |
| 2975.2061 | LPGNDN(N)STATL Fuc 2 Hex 6 HexNAc 3 | 22.913 | 0.029 | 14.409 | 2.416e+08 | 18 - 29 | 7 | 35.25 | 35.75 | 37.26 |
| 4367.8350 | ESESKYPVLN(N)VTM(O)PNN(D)GKF Fuc 2 Hex 8 HexNAc 3 | 22.669 | 0.033 | -6.808 | 5.602e+06 | 171 - 190 | 1 | 39.99 | 40.33 | 40.50 |
| 4474.8456 | ITPN(N)GSIPNDKPF Fuc 3 Hex 5 HexNAc 9 | 22.369 | 0.040 | -20.448 | 2.471e+06 | 297 - 310 | 1 | 42.03 | 42.03 | 42.20 |

### LIN(N)STGNLIAPRGY Hex 9 HexNAc 2

**Mass:**  3352.4500

(1.790e-06)

**Charge States:** 3

**Start Position**: 260

**End Position**: 274

| Profile Score | Tandem Score | TDA q-value | Volume | Spectrum Matches |
| --- | --- | --- | --- | --- |
| -4.6576 | 171.8638 | 0.0000 | 4.0261e+07 | 2 |

**Adduct Signal Fractions**

- **Unmodified**: 4.0261e+07

### LPGNDN(N)STATL Fuc 1 Hex 3 HexNAc 4

**Mass:**  2546.0663

(1.562e-06)

**Charge States:** 2, 3

**Start Position**: 19

**End Position**: 30

| Profile Score | Tandem Score | TDA q-value | Volume | Spectrum Matches |
| --- | --- | --- | --- | --- |
| 12.8326 | 171.4230 | 0.0000 | 5.4086e+07 | 5 |

**Adduct Signal Fractions**

- **Unmodified**: 5.4086e+07

### LIN(N)STGNL Hex 9 HexNAc 2

**Mass:**  2695.0893

(1.838e-06)

**Charge States:** 2, 3

**Start Position**: 260

**End Position**: 268

| Profile Score | Tandem Score | TDA q-value | Volume | Spectrum Matches |
| --- | --- | --- | --- | --- |
| -4.4643 | 170.9279 | 0.0000 | 9.4633e+06 | 2 |

**Adduct Signal Fractions**

- **Unmodified**: 9.4633e+06

### LIN(N)STGNL Hex 9 HexNAc 2

**Mass:**  2696.0848

(3.714e-04)

**Start Position**: 260

**End Position**: 268

| Profile Score | Tandem Score | TDA q-value | Volume | Spectrum Matches |
| --- | --- | --- | --- | --- |
| 0.0000 | 166.9152 | 0.0000 | 0.0000e+00 | 1 |

Adduct:

No Chromatogram Found

### LIN(N)STGNLIAPR Hex 9 HexNAc 2

**Mass:**  3132.3612

(-6.396e-08)

**Charge States:** 3

**Start Position**: 260

**End Position**: 272

| Profile Score | Tandem Score | TDA q-value | Volume | Spectrum Matches |
| --- | --- | --- | --- | --- |
| 12.2380 | 164.6204 | 0.0000 | 3.6867e+07 | 3 |

**Adduct Signal Fractions**

- **Unmodified**: 3.6867e+07

### TIVKPGDILLIN(N)STGNL Fuc 3 Hex 10 HexNAc 5

**Mass:**  4842.0915

(1.996e-04)

**Charge States:** 4

**Start Position**: 251

**End Position**: 268

| Profile Score | Tandem Score | TDA q-value | Volume | Spectrum Matches |
| --- | --- | --- | --- | --- |
| 7.2388 | 163.1421 | 0.0000 | 2.4788e+07 | 6 |

**Adduct Signal Fractions**

- **Unmodified**: 2.4788e+07

### TIVKPGDILLIN(N)STGNLIAPR Hex 9 HexNAc 2

**Mass:**  4068.9287

(1.039e-06)

**Charge States:** 3, 4, 5

**Start Position**: 251

**End Position**: 272

| Profile Score | Tandem Score | TDA q-value | Volume | Spectrum Matches |
| --- | --- | --- | --- | --- |
| 13.2190 | 161.6800 | 0.0000 | 2.3620e+08 | 12 |

**Adduct Signal Fractions**

- **Unmodified**: 2.3620e+08

### TIVKPGDILLIN(N) Hex 9 HexNAc 2

**Mass:**  3159.4242

(1.283e-06)

**Charge States:** 2, 3

**Start Position**: 251

**End Position**: 263

| Profile Score | Tandem Score | TDA q-value | Volume | Spectrum Matches |
| --- | --- | --- | --- | --- |
| 12.9052 | 158.4827 | 0.0000 | 9.5496e+07 | 7 |

**Adduct Signal Fractions**

- **Unmodified**: 9.5496e+07

### TIVKPGDILLIN(N)STGNLIAPRGY Hex 9 HexNAc 2

**Mass:**  4289.0168

(5.076e-07)

**Charge States:** 3, 4, 5

**Start Position**: 251

**End Position**: 274

| Profile Score | Tandem Score | TDA q-value | Volume | Spectrum Matches |
| --- | --- | --- | --- | --- |
| 14.7112 | 157.8636 | 0.0000 | 8.3090e+08 | 43 |

**Adduct Signal Fractions**

- **Unmodified**: 8.3090e+08

### TIVKPGDILLIN(N) Fuc 2 Hex 3 HexNAc 4 Neu5Ac 1

**Mass:**  3176.4508

(-6.950e-06)

**Charge States:** 3

**Start Position**: 251

**End Position**: 263

| Profile Score | Tandem Score | TDA q-value | Volume | Spectrum Matches |
| --- | --- | --- | --- | --- |
| 11.8435 | 153.9085 | 0.0000 | 2.6776e+07 | 4 |

**Adduct Signal Fractions**

- **Unmodified**: 2.6776e+07

### KRGSN(N)NSFF Fuc 1 Hex 3 HexNAc 5

**Mass:**  2703.1296

(5.602e-07)

**Charge States:** 3

**Start Position**: 156

**End Position**: 165

| Profile Score | Tandem Score | TDA q-value | Volume | Spectrum Matches |
| --- | --- | --- | --- | --- |
| -3.4080 | 153.6178 | 0.0000 | 8.7496e+07 | 2 |

**Adduct Signal Fractions**

- **Unmodified**: 8.7496e+07

### LPGNDN(N)STATL Fuc 1 Hex 3 HexNAc 5

**Mass:**  2749.1427

(-2.452e-07)

**Charge States:** 3

**Start Position**: 19

**End Position**: 30

| Profile Score | Tandem Score | TDA q-value | Volume | Spectrum Matches |
| --- | --- | --- | --- | --- |
| 12.6257 | 152.4010 | 0.0000 | 1.3301e+07 | 3 |

**Adduct Signal Fractions**

- **Unmodified**: 1.3301e+07

### TIVKPGDILLIN(N)STGNLIAPRGY Hex 10 HexNAc 2

**Mass:**  4451.0646

(8.300e-07)

**Charge States:** 3, 4

**Start Position**: 251

**End Position**: 274

| Profile Score | Tandem Score | TDA q-value | Volume | Spectrum Matches |
| --- | --- | --- | --- | --- |
| 11.4076 | 152.2100 | 0.0000 | 6.9450e+07 | 7 |

**Adduct Signal Fractions**

- **Unmodified**: 6.9450e+07

### IN(N)STGNLIAPRGYF Hex 9 HexNAc 2

**Mass:**  3386.4380

(2.959e-06)

**Charge States:** 3

**Start Position**: 261

**End Position**: 275

| Profile Score | Tandem Score | TDA q-value | Volume | Spectrum Matches |
| --- | --- | --- | --- | --- |
| -18.2299 | 149.7890 | 0.0000 | 7.0438e+06 | 1 |

**Adduct Signal Fractions**

- **Unmodified**: 7.0438e+06

### TIVKPGDILLIN(N)STGNL Hex 9 HexNAc 2

**Mass:**  3631.6532

(1.277e-06)

**Charge States:** 3, 4

**Start Position**: 251

**End Position**: 268

| Profile Score | Tandem Score | TDA q-value | Volume | Spectrum Matches |
| --- | --- | --- | --- | --- |
| 12.7045 | 149.4105 | 0.0000 | 8.3692e+07 | 4 |

**Adduct Signal Fractions**

- **Unmodified**: 8.3692e+07

### TIVKPGDILLIN(N)STGNLIAPRGY Hex 8 HexNAc 2

**Mass:**  4126.9582

(1.177e-06)

**Charge States:** 3, 4

**Start Position**: 251

**End Position**: 274

| Profile Score | Tandem Score | TDA q-value | Volume | Spectrum Matches |
| --- | --- | --- | --- | --- |
| 12.2654 | 147.0377 | 0.0000 | 7.4992e+07 | 11 |

**Adduct Signal Fractions**

- **Unmodified**: 7.4992e+07

### IN(N)STGNLIAPRGY Hex 9 HexNAc 2

**Mass:**  3239.3662

(-9.027e-07)

**Charge States:** 3

**Start Position**: 261

**End Position**: 274

| Profile Score | Tandem Score | TDA q-value | Volume | Spectrum Matches |
| --- | --- | --- | --- | --- |
| -4.1791 | 147.0113 | 0.0000 | 1.0351e+07 | 2 |

**Adduct Signal Fractions**

- **Unmodified**: 1.0351e+07

### LPGNDN(N)STATL Fuc 2 Hex 4 HexNAc 4

**Mass:**  2854.1810

(2.165e-06)

**Charge States:** 3

**Start Position**: 19

**End Position**: 30

| Profile Score | Tandem Score | TDA q-value | Volume | Spectrum Matches |
| --- | --- | --- | --- | --- |
| 11.4134 | 145.0207 | 0.0000 | 1.0110e+07 | 2 |

**Adduct Signal Fractions**

- **Unmodified**: 1.0110e+07

### TIVKPGDILLIN(N)STGN Hex 9 HexNAc 2

**Mass:**  3518.5706

(1.430e-06)

**Charge States:** 3

**Start Position**: 251

**End Position**: 267

| Profile Score | Tandem Score | TDA q-value | Volume | Spectrum Matches |
| --- | --- | --- | --- | --- |
| 12.2927 | 144.9875 | 0.0000 | 3.3394e+07 | 4 |

**Adduct Signal Fractions**

- **Unmodified**: 3.3394e+07

### TIVKPGDILLIN(N)STGNLIAPRGY Fuc 2 Hex 3 HexNAc 4 Neu5Ac 1

**Mass:**  4306.0383

(-5.354e-06)

**Charge States:** 4

**Start Position**: 251

**End Position**: 274

| Profile Score | Tandem Score | TDA q-value | Volume | Spectrum Matches |
| --- | --- | --- | --- | --- |
| 10.8017 | 144.7986 | 0.0000 | 1.0442e+08 | 3 |

**Adduct Signal Fractions**

- **Unmodified**: 1.0442e+08

### LPGNDN(N)STATL Hex 3 HexNAc 4

**Mass:**  2400.0071

(3.905e-07)

**Charge States:** 3

**Start Position**: 19

**End Position**: 30

| Profile Score | Tandem Score | TDA q-value | Volume | Spectrum Matches |
| --- | --- | --- | --- | --- |
| 11.1023 | 144.6634 | 0.0000 | 1.3358e+07 | 3 |

**Adduct Signal Fractions**

- **Unmodified**: 1.3358e+07

### LIN(N)STGNLIAPRGYF Fuc 2 Hex 3 HexNAc 4 Neu5Ac 1

**Mass:**  3516.5348

(-5.971e-06)

**Charge States:** 3

**Start Position**: 260

**End Position**: 275

| Profile Score | Tandem Score | TDA q-value | Volume | Spectrum Matches |
| --- | --- | --- | --- | --- |
| -19.6456 | 144.0583 | 0.0000 | 5.5845e+06 | 1 |

**Adduct Signal Fractions**

- **Unmodified**: 5.5845e+06

### ITPN(N)GSIPNDKPF Hex 8 HexNAc 2

**Mass:**  3101.2996

(8.873e-07)

**Charge States:** 3

**Start Position**: 298

**End Position**: 311

| Profile Score | Tandem Score | TDA q-value | Volume | Spectrum Matches |
| --- | --- | --- | --- | --- |
| -3.3907 | 142.8621 | 0.0000 | 1.0988e+07 | 2 |

**Adduct Signal Fractions**

- **Unmodified**: 1.0988e+07

### LIN(N)STGNLIAPRGYF Hex 9 HexNAc 2

**Mass:**  3499.5137

(3.788e-07)

**Charge States:** 3

**Start Position**: 260

**End Position**: 275

| Profile Score | Tandem Score | TDA q-value | Volume | Spectrum Matches |
| --- | --- | --- | --- | --- |
| -19.0986 | 142.5013 | 0.0000 | 2.9939e+07 | 1 |

**Adduct Signal Fractions**

- **Unmodified**: 2.9939e+07

### TIVKPGDILLIN(N)STGNLIAPR Fuc 2 Hex 3 HexNAc 4 Neu5Ac 1

**Mass:**  4085.9558

(-4.398e-06)

**Charge States:** 4

**Start Position**: 251

**End Position**: 272

| Profile Score | Tandem Score | TDA q-value | Volume | Spectrum Matches |
| --- | --- | --- | --- | --- |
| 13.1893 | 142.3765 | 0.0000 | 2.6326e+07 | 3 |

**Adduct Signal Fractions**

- **Unmodified**: 2.6326e+07

### LPGNDN(N)STATL Hex 5 HexNAc 2

**Mass:**  2317.9571

(1.253e-06)

**Charge States:** 2

**Start Position**: 19

**End Position**: 30

| Profile Score | Tandem Score | TDA q-value | Volume | Spectrum Matches |
| --- | --- | --- | --- | --- |
| 12.0668 | 140.1020 | 0.0000 | 8.7512e+06 | 3 |

**Adduct Signal Fractions**

- **Unmodified**: 8.7512e+06

### LIN(N)STGNLIAPRGY Fuc 2 Hex 3 HexNAc 4 Neu5Ac 1

**Mass:**  3369.4777

(-5.708e-06)

**Charge States:** 3

**Start Position**: 260

**End Position**: 274

| Profile Score | Tandem Score | TDA q-value | Volume | Spectrum Matches |
| --- | --- | --- | --- | --- |
| -20.2271 | 136.1690 | 0.0000 | 4.7550e+06 | 1 |

**Adduct Signal Fractions**

- **Unmodified**: 4.7550e+06

### TIVKPGDILLIN(N)STGNLIAPR Hex 10 HexNAc 2

**Mass:**  4230.9816

(1.950e-06)

**Charge States:** 3, 4

**Start Position**: 251

**End Position**: 272

| Profile Score | Tandem Score | TDA q-value | Volume | Spectrum Matches |
| --- | --- | --- | --- | --- |
| -4.0944 | 134.6764 | 0.0000 | 1.9254e+07 | 3 |

**Adduct Signal Fractions**

- **Unmodified**: 1.9254e+07

### ITPN(N)GSIPNDKPF Hex 9 HexNAc 2

**Mass:**  3263.3673

(5.738e-06)

**Charge States:** 3

**Start Position**: 298

**End Position**: 311

| Profile Score | Tandem Score | TDA q-value | Volume | Spectrum Matches |
| --- | --- | --- | --- | --- |
| -18.2245 | 133.6069 | 0.0000 | 8.9613e+06 | 1 |

**Adduct Signal Fractions**

- **Unmodified**: 8.9613e+06

### TIVKPGDILLIN(N)STGNL Fuc 2 Hex 3 HexNAc 4 Neu5Ac 1

**Mass:**  3648.6795

(-5.896e-06)

**Charge States:** 3

**Start Position**: 251

**End Position**: 268

| Profile Score | Tandem Score | TDA q-value | Volume | Spectrum Matches |
| --- | --- | --- | --- | --- |
| -4.2187 | 131.7015 | 0.0000 | 2.2452e+07 | 2 |

**Adduct Signal Fractions**

- **Unmodified**: 2.2452e+07

### TIVKPGDILLIN(N)STGNLIAPR Hex 8 HexNAc 2

**Mass:**  3906.8774

(1.352e-06)

**Charge States:** 3, 4

**Start Position**: 251

**End Position**: 272

| Profile Score | Tandem Score | TDA q-value | Volume | Spectrum Matches |
| --- | --- | --- | --- | --- |
| -4.3699 | 129.6805 | 0.0000 | 1.5691e+07 | 4 |

**Adduct Signal Fractions**

- **Unmodified**: 1.5691e+07

### TIVKPGDILLIN(N)STGNL Hex 10 HexNAc 2

**Mass:**  3793.7071

(1.526e-06)

**Charge States:** 3

**Start Position**: 251

**End Position**: 268

| Profile Score | Tandem Score | TDA q-value | Volume | Spectrum Matches |
| --- | --- | --- | --- | --- |
| -4.3701 | 125.9793 | 0.0000 | 7.1498e+06 | 3 |

**Adduct Signal Fractions**

- **Unmodified**: 7.1498e+06

### TIVKPGDILLIN(N) Hex 10 HexNAc 2

**Mass:**  3321.4740

(3.375e-07)

**Charge States:** 3

**Start Position**: 251

**End Position**: 263

| Profile Score | Tandem Score | TDA q-value | Volume | Spectrum Matches |
| --- | --- | --- | --- | --- |
| -19.9270 | 123.8760 | 0.0000 | 4.6543e+06 | 1 |

**Adduct Signal Fractions**

- **Unmodified**: 4.6543e+06

### RSDAPIGTC(C)SSEC(C)ITPN(N)GSIPNDKPF Hex 8 HexNAc 2

**Mass:**  4521.8747

(1.468e-06)

**Charge States:** 3, 4

**Start Position**: 285

**End Position**: 311

| Profile Score | Tandem Score | TDA q-value | Volume | Spectrum Matches |
| --- | --- | --- | --- | --- |
| 12.0910 | 119.6236 | 0.0000 | 7.5042e+07 | 4 |

**Adduct Signal Fractions**

- **Unmodified**: 7.5042e+07

### IGSIRN(N)GTYDHDVY Fuc 1 Hex 3 HexNAc 4

**Mass:**  3053.2948

(2.511e-06)

**Charge States:** 3

**Start Position**: 494

**End Position**: 508

| Profile Score | Tandem Score | TDA q-value | Volume | Spectrum Matches |
| --- | --- | --- | --- | --- |
| -17.6439 | 118.1307 | 0.0000 | 4.6887e+06 | 1 |

**Adduct Signal Fractions**

- **Unmodified**: 4.6887e+06

### RSDAPIGTC(C)SSEC(C)ITPN(N)GSIPNDKPF Hex 9 HexNAc 2

**Mass:**  4683.9260

(3.553e-07)

**Charge States:** 3, 4

**Start Position**: 285

**End Position**: 311

| Profile Score | Tandem Score | TDA q-value | Volume | Spectrum Matches |
| --- | --- | --- | --- | --- |
| 12.2583 | 115.7074 | 0.0000 | 6.9133e+07 | 3 |

**Adduct Signal Fractions**

- **Unmodified**: 6.9133e+07

### TIVKPGDILLIN(N)STGN Fuc 2 Hex 3 HexNAc 4 Neu5Ac 1

**Mass:**  3535.5961

(-6.346e-06)

**Charge States:** 3

**Start Position**: 251

**End Position**: 267

| Profile Score | Tandem Score | TDA q-value | Volume | Spectrum Matches |
| --- | --- | --- | --- | --- |
| -4.8528 | 115.6813 | 0.0000 | 8.5925e+06 | 2 |

**Adduct Signal Fractions**

- **Unmodified**: 8.5925e+06

### SSEC(C)ITPN(N)GSIPNDKPF Hex 8 HexNAc 2

**Mass:**  3564.4381

(1.430e-06)

**Charge States:** 3

**Start Position**: 294

**End Position**: 311

| Profile Score | Tandem Score | TDA q-value | Volume | Spectrum Matches |
| --- | --- | --- | --- | --- |
| -19.5088 | 114.3201 | 0.0000 | 1.3984e+07 | 1 |

**Adduct Signal Fractions**

- **Unmodified**: 1.3984e+07

### LYESESKYPVLN(N)VTM(O)PN Hex 9 HexNAc 2

**Mass:**  3863.5984

(2.124e-06)

**Charge States:** 3, 4

**Start Position**: 170

**End Position**: 187

| Profile Score | Tandem Score | TDA q-value | Volume | Spectrum Matches |
| --- | --- | --- | --- | --- |
| -20.0343 | 113.2935 | 0.0000 | 1.3344e+07 | 1 |

**Adduct Signal Fractions**

- **Unmodified**: 1.3344e+07

### IN(N)STGNLIAPR Hex 9 HexNAc 2

**Mass:**  3019.2780

(1.048e-06)

**Charge States:** 3

**Start Position**: 261

**End Position**: 272

| Profile Score | Tandem Score | TDA q-value | Volume | Spectrum Matches |
| --- | --- | --- | --- | --- |
| -6.1883 | 108.5142 | 0.0000 | 3.3632e+06 | 1 |

**Adduct Signal Fractions**

- **Unmodified**: 3.3632e+06

### SIRN(N)GTYDHDVY Fuc 1 Hex 3 HexNAc 4

**Mass:**  2883.1787

(-1.006e-06)

**Charge States:** 3

**Start Position**: 496

**End Position**: 508

| Profile Score | Tandem Score | TDA q-value | Volume | Spectrum Matches |
| --- | --- | --- | --- | --- |
| -20.1176 | 105.5130 | 0.0000 | 2.7518e+06 | 1 |

**Adduct Signal Fractions**

- **Unmodified**: 2.7518e+06

### SSEC(C)ITPN(N)GSIPNDKPF Hex 9 HexNAc 2

**Mass:**  3726.4901

(1.152e-06)

**Charge States:** 3

**Start Position**: 294

**End Position**: 311

| Profile Score | Tandem Score | TDA q-value | Volume | Spectrum Matches |
| --- | --- | --- | --- | --- |
| -19.2482 | 105.1238 | 0.0000 | 9.4658e+06 | 1 |

**Adduct Signal Fractions**

- **Unmodified**: 9.4658e+06

### TIVKPGDILLIN(N)STGNL Hex 8 HexNAc 2

**Mass:**  3469.5976

(6.193e-07)

**Charge States:** 3

**Start Position**: 251

**End Position**: 268

| Profile Score | Tandem Score | TDA q-value | Volume | Spectrum Matches |
| --- | --- | --- | --- | --- |
| -4.8813 | 104.5366 | 0.0000 | 5.4713e+06 | 1 |

**Adduct Signal Fractions**

- **Unmodified**: 5.4713e+06

### IGSIRN(N)GTYDHDVY Fuc 2 Hex 4 HexNAc 4

**Mass:**  3361.4002

(7.428e-07)

**Charge States:** 3

**Start Position**: 494

**End Position**: 508

| Profile Score | Tandem Score | TDA q-value | Volume | Spectrum Matches |
| --- | --- | --- | --- | --- |
| -20.2693 | 95.5662 | 0.0000 | 4.1085e+06 | 1 |

**Adduct Signal Fractions**

- **Unmodified**: 4.1085e+06

### ITPN(N)GSIPNDKPF Fuc 4 Hex 7 HexNAc 7

**Mass:**  4539.8977

(2.261e-04)

**Start Position**: 298

**End Position**: 311

| Profile Score | Tandem Score | TDA q-value | Volume | Spectrum Matches |
| --- | --- | --- | --- | --- |
| 0.0000 | 92.6805 | 0.0000 | 0.0000e+00 | 1 |

Adduct:

No Chromatogram Found

### TITNDQIEVTN(N)ATEL Hex 6 HexNAc 2

**Mass:**  3039.3004

(3.039e-06)

**Charge States:** 3

**Start Position**: 44

**End Position**: 59

| Profile Score | Tandem Score | TDA q-value | Volume | Spectrum Matches |
| --- | --- | --- | --- | --- |
| -3.7036 | 89.3054 | 0.0000 | 2.1815e+07 | 2 |

**Adduct Signal Fractions**

- **Unmodified**: 2.1815e+07

### TIVKPGDILLIN(N)STGNLIAPRGY Fuc 2 Hex 4 HexNAc 4 Neu5Ac 1

**Mass:**  4468.0939

(-4.131e-06)

**Charge States:** 4

**Start Position**: 251

**End Position**: 274

| Profile Score | Tandem Score | TDA q-value | Volume | Spectrum Matches |
| --- | --- | --- | --- | --- |
| -5.4590 | 71.1580 | 0.0000 | 9.4956e+06 | 2 |

**Adduct Signal Fractions**

- **Unmodified**: 9.4956e+06

### RSDAPIGTC(C)SSEC(C)ITPN(N)GSIPNDKPF Hex 7 HexNAc 2

**Mass:**  4359.8249

(2.493e-06)

**Charge States:** 3, 4

**Start Position**: 285

**End Position**: 311

| Profile Score | Tandem Score | TDA q-value | Volume | Spectrum Matches |
| --- | --- | --- | --- | --- |
| -5.4545 | 70.5301 | 0.0000 | 1.6335e+07 | 1 |

**Adduct Signal Fractions**

- **Unmodified**: 1.6335e+07

### ESESKYPVLN(N)VTM(O)PNN(D)GKFDKLY Hex 9 HexNAc 2

**Mass:**  4553.9406

(2.187e-04)

**Charge States:** 4

**Start Position**: 172

**End Position**: 195

| Profile Score | Tandem Score | TDA q-value | Volume | Spectrum Matches |
| --- | --- | --- | --- | --- |
| 11.1807 | 63.8725 | 0.0000 | 3.7925e+07 | 4 |

**Adduct Signal Fractions**

- **Unmodified**: 3.7925e+07

### KIRTGKSSIM(O)RSDAPIGTC(C)SSEC(C)ITPN(N)GSIPNDKPF Hex 8 HexNAc 2

**Mass:**  5639.5040

(1.449e-06)

**Charge States:** 5

**Start Position**: 275

**End Position**: 311

| Profile Score | Tandem Score | TDA q-value | Volume | Spectrum Matches |
| --- | --- | --- | --- | --- |
| -4.9424 | 62.2878 | 0.0000 | 4.3518e+07 | 3 |

**Adduct Signal Fractions**

- **Unmodified**: 4.3518e+07

### ESESKYPVLN(N)VTM(O)PNN(D)GKFDKLY Hex 8 HexNAc 2

**Mass:**  4391.8889

(2.273e-04)

**Charge States:** 4

**Start Position**: 172

**End Position**: 195

| Profile Score | Tandem Score | TDA q-value | Volume | Spectrum Matches |
| --- | --- | --- | --- | --- |
| 11.3656 | 60.4696 | 0.0000 | 2.5955e+07 | 3 |

**Adduct Signal Fractions**

- **Unmodified**: 2.5955e+07

### KIRTGKSSIM(O)RSDAPIGTC(C)SSEC(C)ITPN(N)GSIPNDKPF Hex 9 HexNAc 2

**Mass:**  5801.5656

(2.204e-06)

**Charge States:** 5

**Start Position**: 275

**End Position**: 311

| Profile Score | Tandem Score | TDA q-value | Volume | Spectrum Matches |
| --- | --- | --- | --- | --- |
| -4.8138 | 58.0510 | 0.0000 | 3.4265e+07 | 3 |

**Adduct Signal Fractions**

- **Unmodified**: 3.4265e+07

### RSDAPIGTC(C)SSEC(C)ITPN(N)GSIPN(D)DKPF Hex 8 HexNAc 2

**Mass:**  4522.8659

(2.215e-04)

**Charge States:** 3, 4

**Start Position**: 285

**End Position**: 311

| Profile Score | Tandem Score | TDA q-value | Volume | Spectrum Matches |
| --- | --- | --- | --- | --- |
| -5.6990 | 52.3191 | 0.0000 | 2.7248e+07 | 3 |

**Adduct Signal Fractions**

- **Unmodified**: 2.7248e+07

### LPGNDN(N)STATL Fuc 4 Hex 7 HexNAc 8

**Mass:**  4444.7551

(-3.379e-06)

**Charge States:** 5

**Start Position**: 19

**End Position**: 30

| Profile Score | Tandem Score | TDA q-value | Volume | Spectrum Matches |
| --- | --- | --- | --- | --- |
| -4.3187 | 51.3527 | 0.0190 | 1.2655e+07 | 2 |

**Adduct Signal Fractions**

- **Unmodified**: 1.2655e+07

### LYESESKYPVLN(N)VTM(O)PNN(D)GKF Hex 9 HexNAc 2

**Mass:**  4310.8161

(2.313e-04)

**Charge States:** 3, 4

**Start Position**: 170

**End Position**: 191

| Profile Score | Tandem Score | TDA q-value | Volume | Spectrum Matches |
| --- | --- | --- | --- | --- |
| 9.7153 | 51.3034 | 0.0000 | 2.3704e+07 | 5 |

**Adduct Signal Fractions**

- **Unmodified**: 2.3704e+07

### HKC(C)DNAC(C)IGSIRN(N)GTYDHDVY Fuc 1 Hex 3 HexNAc 4

**Mass:**  3938.6181

(2.264e-06)

**Charge States:** 4

**Start Position**: 487

**End Position**: 508

| Profile Score | Tandem Score | TDA q-value | Volume | Spectrum Matches |
| --- | --- | --- | --- | --- |
| -19.7069 | 50.9927 | 0.0000 | 7.6672e+06 | 2 |

**Adduct Signal Fractions**

- **Unmodified**: 7.6672e+06

### RSDAPIGTC(C)SSEC(C)ITPN(N)GSIPN(D)DKPF Hex 9 HexNAc 2

**Mass:**  4684.9147

(2.130e-04)

**Charge States:** 3, 4

**Start Position**: 285

**End Position**: 311

| Profile Score | Tandem Score | TDA q-value | Volume | Spectrum Matches |
| --- | --- | --- | --- | --- |
| 11.4116 | 49.7239 | 0.0000 | 2.2605e+07 | 3 |

**Adduct Signal Fractions**

- **Unmodified**: 2.2605e+07

### TIVKPGDILLIN(N)STGN(D)LIAPRGY Fuc 2 Hex 3 HexNAc 4 Neu5Ac 1

**Mass:**  4307.0407

(2.275e-04)

**Charge States:** 4

**Start Position**: 251

**End Position**: 274

| Profile Score | Tandem Score | TDA q-value | Volume | Spectrum Matches |
| --- | --- | --- | --- | --- |
| -20.4867 | 49.2889 | 0.0000 | 5.2409e+06 | 2 |

**Adduct Signal Fractions**

- **Unmodified**: 5.2409e+06

### IN(N)STGNLIAPRGYF Fuc 2 Hex 3 HexNAc 4 Neu5Ac 1

**Mass:**  3404.4679

(2.899e-04)

**Start Position**: 261

**End Position**: 275

| Profile Score | Tandem Score | TDA q-value | Volume | Spectrum Matches |
| --- | --- | --- | --- | --- |
| 0.0000 | 48.2168 | 0.0000 | 0.0000e+00 | 1 |

Adduct:

No Chromatogram Found

### TIVKPGDILLIN(N)STGN Hex 10 HexNAc 2

**Mass:**  3680.6259

(2.354e-06)

**Charge States:** 3

**Start Position**: 251

**End Position**: 267

| Profile Score | Tandem Score | TDA q-value | Volume | Spectrum Matches |
| --- | --- | --- | --- | --- |
| -5.3988 | 46.4573 | 0.0000 | 3.0448e+06 | 1 |

**Adduct Signal Fractions**

- **Unmodified**: 3.0448e+06

### FKIRTGKSSIM(O)RSDAPIGTC(C)SSEC(C)ITPN(N)GSIPNDKPF Hex 8 HexNAc 2

**Mass:**  5786.5658

(4.441e-07)

**Charge States:** 5

**Start Position**: 274

**End Position**: 311

| Profile Score | Tandem Score | TDA q-value | Volume | Spectrum Matches |
| --- | --- | --- | --- | --- |
| -20.0550 | 46.2563 | 0.0000 | 4.7604e+06 | 1 |

**Adduct Signal Fractions**

- **Unmodified**: 4.7604e+06

### LIN(N)STGNLIAPRGYF Hex 8 HexNAc 2

**Mass:**  3337.4562

(-9.314e-07)

**Charge States:** 3

**Start Position**: 260

**End Position**: 275

| Profile Score | Tandem Score | TDA q-value | Volume | Spectrum Matches |
| --- | --- | --- | --- | --- |
| -20.0765 | 45.0074 | 0.0000 | 2.2218e+06 | 1 |

**Adduct Signal Fractions**

- **Unmodified**: 2.2218e+06

### IGSIRN(N)GTYDHDVY Fuc 1 Hex 3 HexNAc 5

**Mass:**  3256.3782

(3.574e-06)

**Charge States:** 3

**Start Position**: 494

**End Position**: 508

| Profile Score | Tandem Score | TDA q-value | Volume | Spectrum Matches |
| --- | --- | --- | --- | --- |
| -20.2424 | 44.7350 | 0.0000 | 2.1484e+06 | 1 |

**Adduct Signal Fractions**

- **Unmodified**: 2.1484e+06

### TITNDQIEVTN(N)ATELVQSSSTGRIC(C)DSPHRIL Hex 6 HexNAc 2

**Mass:**  4933.2463

(1.478e-06)

**Charge States:** 4

**Start Position**: 44

**End Position**: 76

| Profile Score | Tandem Score | TDA q-value | Volume | Spectrum Matches |
| --- | --- | --- | --- | --- |
| -18.6312 | 43.4907 | 0.0000 | 1.5543e+06 | 1 |

**Adduct Signal Fractions**

- **Unmodified**: 1.5543e+06

### SSEC(C)ITPN(N)GSIPNDKPF Fuc 4 Hex 6 HexNAc 7

**Mass:**  4840.0007

(9.240e-06)

**Charge States:** 4

**Start Position**: 294

**End Position**: 311

| Profile Score | Tandem Score | TDA q-value | Volume | Spectrum Matches |
| --- | --- | --- | --- | --- |
| -3.7506 | 43.0610 | 0.0000 | 1.9045e+07 | 2 |

**Adduct Signal Fractions**

- **Unmodified**: 1.9045e+07

### TIVKPGDILLIN(N)STGNLIAPRGY Hex 9 HexNAc 2

**Mass:**  4292.0217

(4.651e-04)

**Start Position**: 251

**End Position**: 274

| Profile Score | Tandem Score | TDA q-value | Volume | Spectrum Matches |
| --- | --- | --- | --- | --- |
| 0.0000 | 43.0455 | 0.0000 | 0.0000e+00 | 3 |

Adduct:

No Chromatogram Found

### VKTITNDQIEVTN(N)ATEL Fuc 3 Hex 5 HexNAc 6

**Mass:**  4354.8537

(-9.029e-06)

**Start Position**: 42

**End Position**: 59

| Profile Score | Tandem Score | TDA q-value | Volume | Spectrum Matches |
| --- | --- | --- | --- | --- |
| 0.0000 | 42.8592 | 0.0000 | 0.0000e+00 | 1 |

Adduct:

No Chromatogram Found

### TITNDQIEVTN(N)ATEL Hex 7 HexNAc 2

**Mass:**  3201.3543

(3.205e-06)

**Charge States:** 3

**Start Position**: 44

**End Position**: 59

| Profile Score | Tandem Score | TDA q-value | Volume | Spectrum Matches |
| --- | --- | --- | --- | --- |
| -4.4531 | 41.7043 | 0.0000 | 1.0652e+07 | 2 |

**Adduct Signal Fractions**

- **Unmodified**: 1.0652e+07

### TITNDQIEVTN(N)ATEL Hex 5 HexNAc 2

**Mass:**  2877.2468

(2.870e-06)

**Charge States:** 3

**Start Position**: 44

**End Position**: 59

| Profile Score | Tandem Score | TDA q-value | Volume | Spectrum Matches |
| --- | --- | --- | --- | --- |
| -4.5541 | 41.3861 | 0.0000 | 9.5463e+06 | 2 |

**Adduct Signal Fractions**

- **Unmodified**: 9.5463e+06

### SSEC(C)ITPN(N)GSIPN(D)DKPF Hex 8 HexNAc 2

**Mass:**  3565.4181

(2.764e-04)

**Charge States:** 3

**Start Position**: 294

**End Position**: 311

| Profile Score | Tandem Score | TDA q-value | Volume | Spectrum Matches |
| --- | --- | --- | --- | --- |
| -21.1048 | 40.5883 | 0.0000 | 1.0537e+07 | 1 |

**Adduct Signal Fractions**

- **Unmodified**: 1.0537e+07

### AC(C)IGSIRN(N)GTYDHDVY Fuc 1 Hex 3 HexNAc 4

**Mass:**  3284.3611

(1.882e-06)

**Charge States:** 3

**Start Position**: 492

**End Position**: 508

| Profile Score | Tandem Score | TDA q-value | Volume | Spectrum Matches |
| --- | --- | --- | --- | --- |
| -19.7205 | 40.5018 | 0.0000 | 1.3996e+06 | 1 |

**Adduct Signal Fractions**

- **Unmodified**: 1.3996e+06

### LPGNDN(N)STATL Fuc 3 Hex 7 HexNAc 4

**Mass:**  3486.4210

(8.540e-06)

**Start Position**: 19

**End Position**: 30

| Profile Score | Tandem Score | TDA q-value | Volume | Spectrum Matches |
| --- | --- | --- | --- | --- |
| 0.0000 | 40.2169 | 0.0000 | 0.0000e+00 | 1 |

Adduct:

No Chromatogram Found

### ITPN(N)GSIPNDKPF Fuc 4 Hex 8 HexNAc 7

**Mass:**  4700.9572

(7.047e-06)

**Charge States:** 4

**Start Position**: 298

**End Position**: 311

| Profile Score | Tandem Score | TDA q-value | Volume | Spectrum Matches |
| --- | --- | --- | --- | --- |
| -20.8362 | 40.1271 | 0.0000 | 9.0061e+06 | 1 |

**Adduct Signal Fractions**

- **Unmodified**: 9.0061e+06

### SIRN(N)GTYDHDVY Fuc 2 Hex 4 HexNAc 4

**Mass:**  3193.2910

(6.263e-04)

**Start Position**: 496

**End Position**: 508

| Profile Score | Tandem Score | TDA q-value | Volume | Spectrum Matches |
| --- | --- | --- | --- | --- |
| 0.0000 | 38.7757 | 0.0000 | 0.0000e+00 | 1 |

Adduct:

No Chromatogram Found

### ITPN(N)GSIPNDKPF Fuc 4 Hex 5 HexNAc 8

**Mass:**  4417.8186

(-5.975e-06)

**Charge States:** 4

**Start Position**: 298

**End Position**: 311

| Profile Score | Tandem Score | TDA q-value | Volume | Spectrum Matches |
| --- | --- | --- | --- | --- |
| -5.7242 | 38.0028 | 0.0000 | 7.3109e+06 | 2 |

**Adduct Signal Fractions**

- **Unmodified**: 7.3109e+06

### TIVKPGDILLIN(N)STGNL Fuc 1 Hex 5 HexNAc 9 Neu5Ac 1

**Mass:**  4842.0871

(-9.744e-06)

**Charge States:** 4

**Start Position**: 251

**End Position**: 268

| Profile Score | Tandem Score | TDA q-value | Volume | Spectrum Matches |
| --- | --- | --- | --- | --- |
| -20.9768 | 37.4598 | 0.0000 | 2.4380e+06 | 1 |

**Adduct Signal Fractions**

- **Unmodified**: 2.4380e+06

### LYESESKYPVLN(N)VTM(O)PN Hex 8 HexNAc 2

**Mass:**  3701.5449

(7.509e-07)

**Charge States:** 3

**Start Position**: 170

**End Position**: 187

| Profile Score | Tandem Score | TDA q-value | Volume | Spectrum Matches |
| --- | --- | --- | --- | --- |
| -21.1093 | 36.8780 | 0.0000 | 6.3598e+06 | 1 |

**Adduct Signal Fractions**

- **Unmodified**: 6.3598e+06

### ESESKYPVLN(N)VTM(O)PNN(D)GKFDKLY Fuc 2 Hex 8 HexNAc 3

**Mass:**  4887.1061

(2.086e-04)

**Charge States:** 4

**Start Position**: 172

**End Position**: 195

| Profile Score | Tandem Score | TDA q-value | Volume | Spectrum Matches |
| --- | --- | --- | --- | --- |
| 11.4860 | 35.8321 | 0.0000 | 1.1502e+08 | 3 |

**Adduct Signal Fractions**

- **Unmodified**: 1.1502e+08

### TIVKPGDILLIN(N)STGNLIAPRGY Fuc 1 Hex 3 HexNAc 5 Neu5Ac 1

**Mass:**  4363.0612

(2.939e-06)

**Charge States:** 4

**Start Position**: 251

**End Position**: 274

| Profile Score | Tandem Score | TDA q-value | Volume | Spectrum Matches |
| --- | --- | --- | --- | --- |
| 10.8056 | 35.7391 | 0.0000 | 1.5462e+07 | 3 |

**Adduct Signal Fractions**

- **Unmodified**: 1.5462e+07

### LPGNDN(N)STATL Fuc 1 Hex 6 HexNAc 2

**Mass:**  2626.0664

(3.594e-06)

**Charge States:** 2, 3

**Start Position**: 19

**End Position**: 30

| Profile Score | Tandem Score | TDA q-value | Volume | Spectrum Matches |
| --- | --- | --- | --- | --- |
| 13.2001 | 35.6777 | 0.0000 | 4.6384e+08 | 10 |

**Adduct Signal Fractions**

- **Unmodified**: 4.6384e+08

### KIRTGKSSIM(O)RSDAPIGTC(C)SSEC(C)ITPN(N)GSIPN(D)DKPF Hex 9 HexNAc 2

**Mass:**  5802.5390

(1.728e-04)

**Charge States:** 5, 6

**Start Position**: 275

**End Position**: 311

| Profile Score | Tandem Score | TDA q-value | Volume | Spectrum Matches |
| --- | --- | --- | --- | --- |
| -6.0728 | 34.2173 | 0.0000 | 2.6646e+07 | 2 |

**Adduct Signal Fractions**

- **Unmodified**: 2.6646e+07

### SSEC(C)ITPN(N)GSIPN(D)DKPF Hex 9 HexNAc 2

**Mass:**  3728.4652

(5.312e-04)

**Start Position**: 294

**End Position**: 311

| Profile Score | Tandem Score | TDA q-value | Volume | Spectrum Matches |
| --- | --- | --- | --- | --- |
| 0.0000 | 33.9527 | 0.0000 | 0.0000e+00 | 1 |

Adduct:

No Chromatogram Found

### LPGNDN(N)STATL Hex 3 HexNAc 5

**Mass:**  2603.0892

(2.705e-06)

**Charge States:** 3

**Start Position**: 19

**End Position**: 30

| Profile Score | Tandem Score | TDA q-value | Volume | Spectrum Matches |
| --- | --- | --- | --- | --- |
| 10.9353 | 33.8495 | 0.0000 | 1.3125e+07 | 2 |

**Adduct Signal Fractions**

- **Unmodified**: 1.3125e+07

### LYESESKYPVLN(N)VTM(O)PNN(D)GKF Hex 8 HexNAc 2

**Mass:**  4148.7648

(2.409e-04)

**Charge States:** 3

**Start Position**: 170

**End Position**: 191

| Profile Score | Tandem Score | TDA q-value | Volume | Spectrum Matches |
| --- | --- | --- | --- | --- |
| -6.2345 | 32.9897 | 0.0000 | 1.4820e+07 | 3 |

**Adduct Signal Fractions**

- **Unmodified**: 1.4820e+07

### FKIRTGKSSIM(O)RSDAPIGTC(C)SSEC(C)ITPN(N)GSIPNDKPF Hex 9 HexNAc 2

**Mass:**  5948.6062

(-1.637e-06)

**Charge States:** 5

**Start Position**: 274

**End Position**: 311

| Profile Score | Tandem Score | TDA q-value | Volume | Spectrum Matches |
| --- | --- | --- | --- | --- |
| -20.4190 | 32.8020 | 0.0000 | 3.5885e+06 | 1 |

**Adduct Signal Fractions**

- **Unmodified**: 3.5885e+06

### TITNDQIEVTN(N)ATEL Hex 5 HexNAc 3

**Mass:**  3080.3289

(1.424e-06)

**Charge States:** 3

**Start Position**: 44

**End Position**: 59

| Profile Score | Tandem Score | TDA q-value | Volume | Spectrum Matches |
| --- | --- | --- | --- | --- |
| -3.9611 | 32.1641 | 0.0058 | 6.1402e+06 | 1 |

**Adduct Signal Fractions**

- **Unmodified**: 6.1402e+06

### RSDAPIGTC(C)SSEC(C)ITPN(N)GSIPN(D)DKPF Hex 7 HexNAc 2

**Mass:**  4360.8122

(2.289e-04)

**Charge States:** 3, 4

**Start Position**: 285

**End Position**: 311

| Profile Score | Tandem Score | TDA q-value | Volume | Spectrum Matches |
| --- | --- | --- | --- | --- |
| -6.2512 | 32.1606 | 0.0058 | 6.7727e+06 | 1 |

**Adduct Signal Fractions**

- **Unmodified**: 6.7727e+06

### TIVKPGDILLIN(N)STGNLIAPR Hex 5 HexNAc 7

**Mass:**  4436.0881

(-5.470e-06)

**Charge States:** 3, 4, 5

**Start Position**: 251

**End Position**: 272

| Profile Score | Tandem Score | TDA q-value | Volume | Spectrum Matches |
| --- | --- | --- | --- | --- |
| 15.3600 | 32.0277 | 0.0058 | 4.3445e+08 | 11 |

**Adduct Signal Fractions**

- **Unmodified**: 4.3445e+08

### TIVKPGDILLIN(N)STGN(D)LIAPRGY Hex 10 HexNAc 2

**Mass:**  4452.0744

(2.263e-04)

**Charge States:** 4

**Start Position**: 251

**End Position**: 274

| Profile Score | Tandem Score | TDA q-value | Volume | Spectrum Matches |
| --- | --- | --- | --- | --- |
| -20.7938 | 31.9633 | 0.0058 | 1.1571e+06 | 4 |

**Adduct Signal Fractions**

- **Unmodified**: 1.1571e+06

### LPGNDN(N)STATL Fuc 1 Hex 4 HexNAc 5

**Mass:**  2911.2066

(3.547e-06)

**Charge States:** 3

**Start Position**: 19

**End Position**: 30

| Profile Score | Tandem Score | TDA q-value | Volume | Spectrum Matches |
| --- | --- | --- | --- | --- |
| -18.9411 | 31.7492 | 0.0103 | 1.9947e+06 | 1 |

**Adduct Signal Fractions**

- **Unmodified**: 1.9947e+06

### TIVKPGDILLIN(N) Hex 9 HexNAc 2

**Mass:**  3160.4467

(3.249e-04)

**Start Position**: 251

**End Position**: 263

| Profile Score | Tandem Score | TDA q-value | Volume | Spectrum Matches |
| --- | --- | --- | --- | --- |
| 0.0000 | 31.5714 | 0.0103 | 0.0000e+00 | 1 |

Adduct:

No Chromatogram Found

### TIVKPGDILLIN(N)STGN Hex 8 HexNAc 2

**Mass:**  3356.5145

(8.916e-07)

**Charge States:** 3

**Start Position**: 251

**End Position**: 267

| Profile Score | Tandem Score | TDA q-value | Volume | Spectrum Matches |
| --- | --- | --- | --- | --- |
| -20.1399 | 30.7934 | 0.0103 | 8.0605e+05 | 1 |

**Adduct Signal Fractions**

- **Unmodified**: 8.0605e+05

### TIVKPGDILLIN(N)STGNLIAPR Hex 4 HexNAc 7

**Mass:**  4274.0280

(-5.893e-06)

**Charge States:** 3, 4

**Start Position**: 251

**End Position**: 272

| Profile Score | Tandem Score | TDA q-value | Volume | Spectrum Matches |
| --- | --- | --- | --- | --- |
| 12.9905 | 30.1806 | 0.0103 | 3.0467e+07 | 6 |

**Adduct Signal Fractions**

- **Unmodified**: 3.0467e+07

### LPGNDN(N)STATL Fuc 3 Hex 5 HexNAc 4

**Mass:**  3162.2986

(4.077e-06)

**Charge States:** 3

**Start Position**: 19

**End Position**: 30

| Profile Score | Tandem Score | TDA q-value | Volume | Spectrum Matches |
| --- | --- | --- | --- | --- |
| -19.6352 | 29.8406 | 0.0103 | 1.8392e+06 | 1 |

**Adduct Signal Fractions**

- **Unmodified**: 1.8392e+06

### KIRTGKSSIM(O)RSDAPIGTC(C)SSEC(C)ITPN(N)GSIPN(D)DKPF Hex 8 HexNAc 2

**Mass:**  5640.4800

(1.747e-04)

**Charge States:** 5

**Start Position**: 275

**End Position**: 311

| Profile Score | Tandem Score | TDA q-value | Volume | Spectrum Matches |
| --- | --- | --- | --- | --- |
| -6.1649 | 29.1883 | 0.0103 | 2.0398e+07 | 1 |

**Adduct Signal Fractions**

- **Unmodified**: 2.0398e+07

### ESESKYPVLN(N)VTM(O)PNN(D)GKFDKLY Fuc 2 Hex 7 HexNAc 3

**Mass:**  4725.0539

(2.161e-04)

**Charge States:** 4, 5

**Start Position**: 172

**End Position**: 195

| Profile Score | Tandem Score | TDA q-value | Volume | Spectrum Matches |
| --- | --- | --- | --- | --- |
| 11.1651 | 29.1162 | 0.0103 | 8.2782e+07 | 4 |

**Adduct Signal Fractions**

- **Unmodified**: 8.2782e+07

### TITNDQIEVTN(N)ATEL Fuc 1 Hex 6 HexNAc 3

**Mass:**  3388.4397

(3.353e-06)

**Charge States:** 3

**Start Position**: 44

**End Position**: 59

| Profile Score | Tandem Score | TDA q-value | Volume | Spectrum Matches |
| --- | --- | --- | --- | --- |
| -5.4431 | 27.8597 | 0.0149 | 3.0332e+06 | 2 |

**Adduct Signal Fractions**

- **Unmodified**: 3.0332e+06

### TC(C)KRGSN(N)NSFF Fuc 1 Hex 3 HexNAc 4

**Mass:**  2761.1413

(5.131e-06)

**Charge States:** 3

**Start Position**: 154

**End Position**: 165

| Profile Score | Tandem Score | TDA q-value | Volume | Spectrum Matches |
| --- | --- | --- | --- | --- |
| -5.3341 | 26.5422 | 0.0190 | 8.4344e+06 | 2 |

**Adduct Signal Fractions**

- **Unmodified**: 8.4344e+06

### LYESESKYPVLN(N)VTMPNNGKF Fuc 2 Hex 7 HexNAc 4

**Mass:**  4668.0329

(7.752e-06)

**Charge States:** 4, 5

**Start Position**: 170

**End Position**: 191

| Profile Score | Tandem Score | TDA q-value | Volume | Spectrum Matches |
| --- | --- | --- | --- | --- |
| 15.5428 | 26.4125 | 0.0190 | 2.6656e+08 | 7 |

**Adduct Signal Fractions**

- **Unmodified**: 2.6656e+08

### TGVTQSGGSSTC(C)KRGSN(N)NSFF Fuc 1 Hex 3 HexNAc 5

**Mass:**  3825.5895

(-9.120e-08)

**Charge States:** 4

**Start Position**: 144

**End Position**: 165

| Profile Score | Tandem Score | TDA q-value | Volume | Spectrum Matches |
| --- | --- | --- | --- | --- |
| -19.3024 | 26.3210 | 0.0190 | 1.2987e+07 | 2 |

**Adduct Signal Fractions**

- **Unmodified**: 1.2987e+07

### ESESKYPVLN(N)VTMPNNGKFDKLY Fuc 2 Hex 3 HexNAc 6

**Mass:**  4669.0241

(-9.383e-06)

**Charge States:** 4, 5

**Start Position**: 172

**End Position**: 195

| Profile Score | Tandem Score | TDA q-value | Volume | Spectrum Matches |
| --- | --- | --- | --- | --- |
| 8.7920 | 26.0585 | 0.0190 | 6.6459e+07 | 5 |

**Adduct Signal Fractions**

- **Unmodified**: 6.6459e+07

### ESESKYPVLN(N)VTM(O)PNN(D)GKFDKLY Fuc 3 Hex 3 HexNAc 6

**Mass:**  4832.0916

(2.013e-04)

**Start Position**: 172

**End Position**: 195

| Profile Score | Tandem Score | TDA q-value | Volume | Spectrum Matches |
| --- | --- | --- | --- | --- |
| 0.0000 | 25.9188 | 0.0218 | 0.0000e+00 | 1 |

Adduct:

No Chromatogram Found

### KIRTGKSSIM(O)RSDAPIGTC(C)SSEC(C)ITPN(N)GSIPNDKPF Hex 7 HexNAc 2

**Mass:**  5477.4581

(3.025e-06)

**Charge States:** 5

**Start Position**: 275

**End Position**: 311

| Profile Score | Tandem Score | TDA q-value | Volume | Spectrum Matches |
| --- | --- | --- | --- | --- |
| -20.2892 | 25.7433 | 0.0218 | 6.4131e+06 | 2 |

**Adduct Signal Fractions**

- **Unmodified**: 6.4131e+06

### LYESESKYPVLN(N)VTM(O)PNN(D)GKF Fuc 3 Hex 7 HexNAc 4

**Mass:**  4831.0754

(2.123e-04)

**Charge States:** 4, 5

**Start Position**: 170

**End Position**: 191

| Profile Score | Tandem Score | TDA q-value | Volume | Spectrum Matches |
| --- | --- | --- | --- | --- |
| 10.5026 | 25.5433 | 0.0218 | 1.2810e+08 | 9 |

**Adduct Signal Fractions**

- **Unmodified**: 1.2810e+08

### LPGNDN(N)STATL Fuc 1 Hex 4 HexNAc 4

**Mass:**  2709.1252

(3.723e-04)

**Start Position**: 19

**End Position**: 30

| Profile Score | Tandem Score | TDA q-value | Volume | Spectrum Matches |
| --- | --- | --- | --- | --- |
| 0.0000 | 25.3572 | 0.0218 | 0.0000e+00 | 1 |

Adduct:

No Chromatogram Found

### TIVKPGDILLIN(N)STGNLIAPRGY Hex 5 HexNAc 6

**Mass:**  4453.1096

(-1.416e-06)

**Charge States:** 4

**Start Position**: 251

**End Position**: 274

| Profile Score | Tandem Score | TDA q-value | Volume | Spectrum Matches |
| --- | --- | --- | --- | --- |
| 12.8480 | 24.9800 | 0.0218 | 6.6096e+07 | 5 |

**Adduct Signal Fractions**

- **Unmodified**: 6.6096e+07

### TIVKPGDILLIN(N)STGNLIAPR Fuc 2 Hex 4 HexNAc 4 Neu5Ac 1

**Mass:**  4251.0241

(7.050e-04)

**Start Position**: 251

**End Position**: 272

| Profile Score | Tandem Score | TDA q-value | Volume | Spectrum Matches |
| --- | --- | --- | --- | --- |
| 0.0000 | 24.6938 | 0.0218 | 0.0000e+00 | 1 |

Adduct:

No Chromatogram Found

### ESESKYPVLN(N)VTMPNNGKFDKLY Hex 4 HexNAc 6 Neu5Ac 1

**Mass:**  4830.0856

(-2.203e-06)

**Charge States:** 3, 4, 5

**Start Position**: 172

**End Position**: 195

| Profile Score | Tandem Score | TDA q-value | Volume | Spectrum Matches |
| --- | --- | --- | --- | --- |
| 15.7674 | 24.0386 | 0.0218 | 4.1009e+08 | 4 |

**Adduct Signal Fractions**

- **Unmodified**: 4.1009e+08

### SIRN(N)GTYDHDVY Fuc 1 Hex 3 HexNAc 5

**Mass:**  3087.2593

(3.235e-04)

**Start Position**: 496

**End Position**: 508

| Profile Score | Tandem Score | TDA q-value | Volume | Spectrum Matches |
| --- | --- | --- | --- | --- |
| 0.0000 | 23.7769 | 0.0253 | 0.0000e+00 | 1 |

Adduct:

No Chromatogram Found

### ITPN(N)GSIPNDKPF Fuc 3 Hex 6 HexNAc 8 Neu5Ac 1

**Mass:**  4726.9093

(4.178e-04)

**Start Position**: 298

**End Position**: 311

| Profile Score | Tandem Score | TDA q-value | Volume | Spectrum Matches |
| --- | --- | --- | --- | --- |
| 0.0000 | 23.4229 | 0.0253 | 0.0000e+00 | 1 |

Adduct:

No Chromatogram Found

### ITPN(N)GSIPNDKPF Hex 10 HexNAc 7

**Mass:**  4440.8133

(3.405e-06)

**Start Position**: 298

**End Position**: 311

| Profile Score | Tandem Score | TDA q-value | Volume | Spectrum Matches |
| --- | --- | --- | --- | --- |
| 0.0000 | 23.3650 | 0.0253 | 0.0000e+00 | 1 |

Adduct:

No Chromatogram Found

### LPGNDN(N)STATL Fuc 1 Hex 8 HexNAc 3 Neu5Ac 1

**Mass:**  3444.3913

(8.920e-06)

**Charge States:** 3

**Start Position**: 19

**End Position**: 30

| Profile Score | Tandem Score | TDA q-value | Volume | Spectrum Matches |
| --- | --- | --- | --- | --- |
| 9.4349 | 23.2775 | 0.0253 | 2.3009e+07 | 3 |

**Adduct Signal Fractions**

- **Unmodified**: 2.3009e+07

### TIVKPGDILLIN(N)STGN(D)LIAPRGY Hex 7 HexNAc 2

**Mass:**  3965.9240

(2.576e-04)

**Charge States:** 4

**Start Position**: 251

**End Position**: 274

| Profile Score | Tandem Score | TDA q-value | Volume | Spectrum Matches |
| --- | --- | --- | --- | --- |
| -20.1808 | 23.1621 | 0.0292 | 1.1743e+06 | 1 |

**Adduct Signal Fractions**

- **Unmodified**: 1.1743e+06

### LPGNDN(N)STATL Fuc 2 Hex 6 HexNAc 3

**Mass:**  2975.2071

(1.204e-06)

**Charge States:** 3

**Start Position**: 19

**End Position**: 30

| Profile Score | Tandem Score | TDA q-value | Volume | Spectrum Matches |
| --- | --- | --- | --- | --- |
| 14.4087 | 22.9132 | 0.0292 | 2.4162e+08 | 7 |

**Adduct Signal Fractions**

- **Unmodified**: 2.4162e+08

### ESESKYPVLN(N)VTM(O)PNN(D)GKF Fuc 2 Hex 8 HexNAc 3

**Mass:**  4367.8371

(2.329e-04)

**Charge States:** 4

**Start Position**: 172

**End Position**: 191

| Profile Score | Tandem Score | TDA q-value | Volume | Spectrum Matches |
| --- | --- | --- | --- | --- |
| -6.8078 | 22.6692 | 0.0329 | 5.6015e+06 | 1 |

**Adduct Signal Fractions**

- **Unmodified**: 5.6015e+06

### ITPN(N)GSIPNDKPF Fuc 3 Hex 5 HexNAc 9

**Mass:**  4474.8474

(-4.287e-06)

**Charge States:** 4

**Start Position**: 298

**End Position**: 311

| Profile Score | Tandem Score | TDA q-value | Volume | Spectrum Matches |
| --- | --- | --- | --- | --- |
| -20.4484 | 22.3692 | 0.0403 | 2.4708e+06 | 1 |

**Adduct Signal Fractions**

- **Unmodified**: 2.4708e+06

### iav|ADJ41819|A/Phil/2-MA/1982\_NA

**>iav|ADJ41819|A/Phil/2-MA/1982\_NA**  
 `MNPNQKIITIGSVSLTIATICFLMQIAILVTTVTLHFKQYECSSPPNNQVVPCEPIIIERNITEIVYLTNTTIEKEICPK  
LVEYRNWSKPQCKITGFAPFSKDNSIRLSAGGDIWVTREPYVSCDPGKCYQFALGQGTTLDNKHSNDTIHDRTPYRTLLM  
NELGVPFHLGTRQVCIAWSSSSCHDGKAWLHVCITGYDKNATASFIYDGRLVDSIGSWSKNILRTQESECVCINGTCTVV  
MTDGSASERADTKILFIEEGKIVHISPLSGSAQHVEECSCYPRYPGVRCVCRDNWKGSNRPVVDINVKDYSIVSSYVCSG  
LVGDTPRKNDRSSSSYCRNPNNEKGNHGVKGWAFDDGNDVWMGRTISEESRSGYETFKVIGGWSTPNSKLQINRQVIVDS  
GNRSGYSGIFSVEGKSCINRCFYVELIRGREQETRVWWTSNSIVVFCGTSGTYGTGSWPDGADINLMPI`

| Site | Type | Count |
| --- | --- | --- |
| 146 | N-linked | 5 |
| 402 | N-linked | 6 |
| 86 | N-linked | 2 |
| 200 | N-linked | 1 |

| Mass | Sequence | MS_2_ Score | `q`-value | MS_1_ Score | Signal | Protein Coordinates | Tandem Scans | Start Time | Apex Time | End Time |
| --- | --- | --- | --- | --- | --- | --- | --- | --- | --- | --- |
| 3183.3968 | QINRQVIVDSGN(N)RSGY Hex 6 HexNAc 2 | 156.881 | 0.0 | -20.550 | 2.187e+07 | 390 - 406 | 3 | 21.18 | 21.34 | 30.10 |
| 3910.7815 | STPNSKLQINRQVIVDSGN(N)RSGY Hex 6 HexNAc 2 | 139.761 | 0.0 | -19.850 | 7.985e+06 | 383 - 406 | 1 | 28.25 | 28.41 | 28.41 |
| 3345.4471 | QINRQVIVDSGN(N)RSGY Hex 7 HexNAc 2 | 123.814 | 0.0 | -20.322 | 4.276e+06 | 390 - 406 | 1 | 21.18 | 21.18 | 21.34 |
| 3323.4067 | RN(N)WSKPQC(C)KITGF Hex 8 HexNAc 2 | 122.136 | 0.0 | -20.499 | 1.789e+06 | 84 - 97 | 1 | 24.20 | 24.20 | 24.20 |
| 2831.1311 | DKN(N)ATASFIY Hex 8 HexNAc 2 | 120.069 | 0.0 | -18.859 | 8.008e+06 | 197 - 207 | 2 | 19.00 | 19.00 | 19.17 |
| 3507.5019 | QINRQVIVDSGN(N)RSGY Hex 8 HexNAc 2 | 107.332 | 0.0 | -19.485 | 1.756e+06 | 390 - 406 | 1 | 21.01 | 21.01 | 21.01 |
| 4280.7939 | ALGQGTTLDNKHSN(N)DTIHDRTPY Fuc 1 Hex 6 HexNAc 3 | 75.221 | 0.0 | -4.577 | 1.210e+07 | 132 - 155 | 2 | 22.02 | 22.18 | 22.52 |
| 3800.6338 | RN(N)WSKPQC(C)KITGFAPF Hex 9 HexNAc 2 | 49.551 | 0.0 | -4.384 | 5.662e+06 | 84 - 100 | 1 | 37.26 | 37.43 | 37.60 |
| 4734.9654 | ALGQGTTLDN(D)KHSN(N)DTIHDRTPY Fuc 1 Hex 7 HexNAc 3 Neu5Ac 1 | 46.812 | 0.0 | -21.126 | 6.065e+06 | 132 - 155 | 1 | 21.51 | 21.68 | 21.68 |
| 2829.2191 | RQVIVDSGN(N)RSGY Hex 6 HexNAc 2 | 39.605 | 0.0 | 0.000 | 0.000e+00 | 393 - 406 | 1 | - | - | - |
| 4054.7863 | ALGQGTTLDNKHSN(N)DTIHDRTPY Hex 3 HexNAc 5 | 31.441 | 0.010 | -19.020 | 3.843e+06 | 132 - 155 | 2 | 20.51 | 20.51 | 20.51 |
| 4200.8413 | ALGQGTTLDNKHSN(N)DTIHDRTPY Fuc 1 Hex 3 HexNAc 5 | 27.637 | 0.015 | -20.673 | 3.346e+06 | 132 - 155 | 2 | 20.51 | 20.51 | 20.67 |
| 4588.9094 | ALGQGTTLDNKHSN(N)DTIHDRTPY Fuc 2 Hex 7 HexNAc 3 | 25.859 | 0.022 | -19.261 | 2.632e+06 | 132 - 155 | 1 | 21.85 | 21.85 | 21.85 |
| 4072.8237 | STPNSKLQINRQVIVDSGN(N)RSGY Hex 7 HexNAc 2 | 25.387 | 0.022 | -20.307 | 2.375e+06 | 383 - 406 | 1 | 28.25 | 28.25 | 28.25 |

### QINRQVIVDSGN(N)RSGY Hex 6 HexNAc 2

**Mass:**  3183.3967

(3.661e-07)

**Charge States:** 3

**Start Position**: 391

**End Position**: 407

| Profile Score | Tandem Score | TDA q-value | Volume | Spectrum Matches |
| --- | --- | --- | --- | --- |
| -20.5505 | 156.8807 | 0.0000 | 2.1868e+07 | 3 |

**Adduct Signal Fractions**

- **Unmodified**: 2.1868e+07

### STPNSKLQINRQVIVDSGN(N)RSGY Hex 6 HexNAc 2

**Mass:**  3910.7853

(-7.117e-07)

**Charge States:** 4

**Start Position**: 384

**End Position**: 407

| Profile Score | Tandem Score | TDA q-value | Volume | Spectrum Matches |
| --- | --- | --- | --- | --- |
| -19.8502 | 139.7611 | 0.0000 | 7.9848e+06 | 1 |

**Adduct Signal Fractions**

- **Unmodified**: 7.9848e+06

### QINRQVIVDSGN(N)RSGY Hex 7 HexNAc 2

**Mass:**  3345.4495

(8.755e-07)

**Charge States:** 3

**Start Position**: 391

**End Position**: 407

| Profile Score | Tandem Score | TDA q-value | Volume | Spectrum Matches |
| --- | --- | --- | --- | --- |
| -20.3218 | 123.8143 | 0.0000 | 4.2759e+06 | 1 |

**Adduct Signal Fractions**

- **Unmodified**: 4.2759e+06

### RN(N)WSKPQC(C)KITGF Hex 8 HexNAc 2

**Mass:**  3323.4067

(1.779e-06)

**Charge States:** 4

**Start Position**: 85

**End Position**: 98

| Profile Score | Tandem Score | TDA q-value | Volume | Spectrum Matches |
| --- | --- | --- | --- | --- |
| -20.4992 | 122.1363 | 0.0000 | 1.7886e+06 | 1 |

**Adduct Signal Fractions**

- **Unmodified**: 1.7886e+06

### DKN(N)ATASFIY Hex 8 HexNAc 2

**Mass:**  2831.1304

(-4.215e-07)

**Charge States:** 3

**Start Position**: 198

**End Position**: 208

| Profile Score | Tandem Score | TDA q-value | Volume | Spectrum Matches |
| --- | --- | --- | --- | --- |
| -18.8587 | 120.0690 | 0.0000 | 8.0078e+06 | 2 |

**Adduct Signal Fractions**

- **Unmodified**: 8.0078e+06

### QINRQVIVDSGN(N)RSGY Hex 8 HexNAc 2

**Mass:**  3507.5019

(7.003e-07)

**Charge States:** 3

**Start Position**: 391

**End Position**: 407

| Profile Score | Tandem Score | TDA q-value | Volume | Spectrum Matches |
| --- | --- | --- | --- | --- |
| -19.4855 | 107.3323 | 0.0000 | 1.7563e+06 | 1 |

**Adduct Signal Fractions**

- **Unmodified**: 1.7563e+06

### ALGQGTTLDNKHSN(N)DTIHDRTPY Fuc 1 Hex 6 HexNAc 3

**Mass:**  4280.7951

(-9.801e-06)

**Charge States:** 4

**Start Position**: 133

**End Position**: 156

| Profile Score | Tandem Score | TDA q-value | Volume | Spectrum Matches |
| --- | --- | --- | --- | --- |
| -4.5770 | 75.2208 | 0.0000 | 1.2105e+07 | 2 |

**Adduct Signal Fractions**

- **Unmodified**: 1.2105e+07

### RN(N)WSKPQC(C)KITGFAPF Hex 9 HexNAc 2

**Mass:**  3800.6311

(5.035e-06)

**Charge States:** 4

**Start Position**: 85

**End Position**: 101

| Profile Score | Tandem Score | TDA q-value | Volume | Spectrum Matches |
| --- | --- | --- | --- | --- |
| -4.3838 | 49.5510 | 0.0000 | 5.6620e+06 | 1 |

**Adduct Signal Fractions**

- **Unmodified**: 5.6620e+06

### ALGQGTTLDN(D)KHSN(N)DTIHDRTPY Fuc 1 Hex 7 HexNAc 3 Neu5Ac 1

**Mass:**  4734.9660

(2.073e-04)

**Charge States:** 4

**Start Position**: 133

**End Position**: 156

| Profile Score | Tandem Score | TDA q-value | Volume | Spectrum Matches |
| --- | --- | --- | --- | --- |
| -21.1264 | 46.8120 | 0.0000 | 6.0650e+06 | 1 |

**Adduct Signal Fractions**

- **Unmodified**: 6.0650e+06

### RQVIVDSGN(N)RSGY Hex 6 HexNAc 2

**Mass:**  2829.2191

(3.575e-04)

**Start Position**: 394

**End Position**: 407

| Profile Score | Tandem Score | TDA q-value | Volume | Spectrum Matches |
| --- | --- | --- | --- | --- |
| 0.0000 | 39.6047 | 0.0000 | 0.0000e+00 | 1 |

Adduct:

No Chromatogram Found

### ALGQGTTLDNKHSN(N)DTIHDRTPY Hex 3 HexNAc 5

**Mass:**  4054.7863

(2.510e-06)

**Charge States:** 4, 5

**Start Position**: 133

**End Position**: 156

| Profile Score | Tandem Score | TDA q-value | Volume | Spectrum Matches |
| --- | --- | --- | --- | --- |
| -19.0198 | 31.4406 | 0.0103 | 3.8432e+06 | 2 |

**Adduct Signal Fractions**

- **Unmodified**: 3.8432e+06

### ALGQGTTLDNKHSN(N)DTIHDRTPY Fuc 1 Hex 3 HexNAc 5

**Mass:**  4200.8440

(2.358e-06)

**Charge States:** 4

**Start Position**: 133

**End Position**: 156

| Profile Score | Tandem Score | TDA q-value | Volume | Spectrum Matches |
| --- | --- | --- | --- | --- |
| -20.6725 | 27.6372 | 0.0149 | 3.3462e+06 | 2 |

**Adduct Signal Fractions**

- **Unmodified**: 3.3462e+06

### ALGQGTTLDNKHSN(N)DTIHDRTPY Fuc 2 Hex 7 HexNAc 3

**Mass:**  4588.9094

(-7.680e-06)

**Charge States:** 4

**Start Position**: 133

**End Position**: 156

| Profile Score | Tandem Score | TDA q-value | Volume | Spectrum Matches |
| --- | --- | --- | --- | --- |
| -19.2611 | 25.8590 | 0.0218 | 2.6320e+06 | 1 |

**Adduct Signal Fractions**

- **Unmodified**: 2.6320e+06

### STPNSKLQINRQVIVDSGN(N)RSGY Hex 7 HexNAc 2

**Mass:**  4072.8237

(-2.289e-06)

**Charge States:** 4

**Start Position**: 384

**End Position**: 407

| Profile Score | Tandem Score | TDA q-value | Volume | Spectrum Matches |
| --- | --- | --- | --- | --- |
| -20.3072 | 25.3865 | 0.0218 | 2.3747e+06 | 1 |

**Adduct Signal Fractions**

- **Unmodified**: 2.3747e+06

### tr|A0A1D5PUY7|A0A1D5PUY7\_CHICK

**>tr|A0A1D5PUY7|A0A1D5PUY7\_CHICK**  
 `MVHIETVKTKAYADQKPGTSGLRKRVTVFQNNAHYAENFIQSILATVPPAERQEATLVVGGDGRFYMKDAIQIIVRIAAA  
NGIGRLVIGQNGILSTPAVSCIIRKIKAIGGIILTASHNPGGPNGDFGIKFNTANGGPAPEGITDKIFQISKKIEEYAIC  
PDLKVDLGTIGKQQFDLENKFKPFTVEIVDSVEAYANMLRNIFDFNALKELLSGKNHLKIRIDAMHGVVGPYVKKILCEE  
LGAPANSAVNCTPLEDFGGHHPDPNLTYAADLVQTMKTGEYDFGAAFDGDGDRNMILGKHGFFVNPSDSVAVIAANIFSI  
PYFQQTGVRGFARSMPTSGALDRVAHATKIALYETPTGWKFFGNLMDANKLSLCGEESFGTGSDHIREKDGLWAVLAWLS  
ILAARKQSVEDIMKDHWQKYGRNFFTRYDYEEVDADAAGKMMKDLETVMFDRSFVGKQLSAGDKVYTVEKADNFEYNDPV  
DGSVSRNQGLRLIFSDGSRIIFRLSGTGSAGATVRLYIDSYEKDAKKIHEDPQHQLKFPLYSSSPCD`

| Site | Type | Count |
| --- | --- | --- |
| 250 | N-linked | 1 |

| Mass | Sequence | MS_2_ Score | `q`-value | MS_1_ Score | Signal | Protein Coordinates | Tandem Scans | Start Time | Apex Time | End Time |
| --- | --- | --- | --- | --- | --- | --- | --- | --- | --- | --- |
| 2715.1302 | GAPANSAVN(N)C(C)TPL Fuc 1 Hex 3 HexNAc 4 | 33.992 | 0.0 | -19.300 | 3.499e+06 | 241 - 254 | 1 | 20.51 | 20.51 | 20.67 |

### GAPANSAVN(N)C(C)TPL Fuc 1 Hex 3 HexNAc 4

**Mass:**  2715.1294

(-7.597e-07)

**Charge States:** 3

**Start Position**: 242

**End Position**: 255

| Profile Score | Tandem Score | TDA q-value | Volume | Spectrum Matches |
| --- | --- | --- | --- | --- |
| -19.2998 | 33.9917 | 0.0000 | 3.4992e+06 | 1 |

**Adduct Signal Fractions**

- **Unmodified**: 3.4992e+06

### tr|F1NN63|F1NN63\_CHICK

**>tr|F1NN63|F1NN63\_CHICK**  
 `MVHIETVKTKAYADQKPGTSGLRKRVTVFQNNAHYAENFIQSILATVPPAERQEATLVVGGDGRFYMKDAIQIIVRIAAA  
NGIGRLVIGQNGILSTPAVSCIIRKIKAIGGIILTASHNPGGPNGDFGIKFNTANGGPAPEGITDKIFQISKKIEEYAIC  
PDLKVDLGTIGKQQFDLENKFKPFTVEIVDSVEAYANMLRNIFDFNALKELLSGKNHLKIRIDAMHGVVGPYVKKILCEE  
LGAPANSAVNCTPLEDFGGHHPDPNLTYAADLVQTMKTGEYDFGAAFDGDGDRNMILGKHGFFVNPSDSVAVIAANIFSI  
PYFQQTGVRGFARSMPTSGALDRVAHATKIALYETPTGWKFFGNLMDANKLSLCGEESFGTGSDHIREKDGLWAVLAWLS  
ILAARKQSVEDIMKDHWQKYGRNFFTRYDYEEVDADAAGKMMKDLETVMFDRSFVGKQLSAGDKVYTVEKADNFEYNDPV  
DGSVSRNQGLRLIFSDGSRIIFRLSGTGSAGATVRLYIDSYEKDAKKIHEDPQVMLAPLISIALKLSQLHERTGRTGPTV  
IT`

| Site | Type | Count |
| --- | --- | --- |
| 250 | N-linked | 1 |

| Mass | Sequence | MS_2_ Score | `q`-value | MS_1_ Score | Signal | Protein Coordinates | Tandem Scans | Start Time | Apex Time | End Time |
| --- | --- | --- | --- | --- | --- | --- | --- | --- | --- | --- |
| 2715.1302 | GAPANSAVN(N)C(C)TPL Fuc 1 Hex 3 HexNAc 4 | 33.992 | 0.0 | -19.300 | 3.499e+06 | 241 - 254 | 1 | 20.51 | 20.51 | 20.67 |

### GAPANSAVN(N)C(C)TPL Fuc 1 Hex 3 HexNAc 4

**Mass:**  2715.1294

(-7.597e-07)

**Charge States:** 3

**Start Position**: 242

**End Position**: 255

| Profile Score | Tandem Score | TDA q-value | Volume | Spectrum Matches |
| --- | --- | --- | --- | --- |
| -19.2998 | 33.9917 | 0.0000 | 3.4992e+06 | 1 |

**Adduct Signal Fractions**

- **Unmodified**: 3.4992e+06

### tr|A0A1L1RR19|A0A1L1RR19\_CHICK

**>tr|A0A1L1RR19|A0A1L1RR19\_CHICK**  
 `MVHIETVKTKAYADQKPGTSGLRKRVTVFQNNAHYAENFIQSILATVPPAERQEATLVVGGDGRFYMKDAIQIIVRIAAA  
NGIGRLVIGQNGILSTPAVSCIIRKIKAIGGIILTASHNPGGPNGDFGIKFNTANGGPAPEGITDKIFQISKKIEEYAIC  
PDLKVDLGTIGKQQFDLENKFKPFTVEIVDSVEAYANMLRNIFDFNALKELLSGKNHLKIRIDAMHGVVGPYVKKILCEE  
LGAPANSAVNCTPLEDFGGHHPDPNLTYAADLVQTMKTGEYDFGAAFDGDGDRNMILGKHGFFVNPSDSVAVIAANIFSI  
PYFQQTGVRGFARSMPTSGALDRVAHATKIALYETPTGWKFFGNLMDANKLSLCGEESFGTGSDHIREKDGLWAVLAWLS  
ILAARKQSVEDIMKDHWQKYGRNFFTRYDYEEVDADAAGKMMKDLETVMFDRSFVGKQLSAGDKVYTVEKADNFEYNDPV  
DGSVSRNQGLRLIFSDGSRIIFRLSGTGSAGATVRLYIDSYEKDAKKIHEDPQVMLAPLISIALKLSQLHERTGRTGPTY  
NIKARPGTKGKKPARRLFYCRVCRYKRNINFICVFKNTARYR`

| Site | Type | Count |
| --- | --- | --- |
| 250 | N-linked | 1 |

| Mass | Sequence | MS_2_ Score | `q`-value | MS_1_ Score | Signal | Protein Coordinates | Tandem Scans | Start Time | Apex Time | End Time |
| --- | --- | --- | --- | --- | --- | --- | --- | --- | --- | --- |
| 2715.1302 | GAPANSAVN(N)C(C)TPL Fuc 1 Hex 3 HexNAc 4 | 33.992 | 0.0 | -19.300 | 3.499e+06 | 241 - 254 | 1 | 20.51 | 20.51 | 20.67 |

### GAPANSAVN(N)C(C)TPL Fuc 1 Hex 3 HexNAc 4

**Mass:**  2715.1294

(-7.597e-07)

**Charge States:** 3

**Start Position**: 242

**End Position**: 255

| Profile Score | Tandem Score | TDA q-value | Volume | Spectrum Matches |
| --- | --- | --- | --- | --- |
| -19.2998 | 33.9917 | 0.0000 | 3.4992e+06 | 1 |

**Adduct Signal Fractions**

- **Unmodified**: 3.4992e+06

### tr|A0A146F0A0|A0A146F0A0\_CHICK

**>tr|A0A146F0A0|A0A146F0A0\_CHICK**  
 `MSRSVSFSSRSVAVPGVSQVRVSSVSSVRGGGSLSKAGGFGSSSLYNLGSANKRISLGGSSSYSVRSGYGYGGMGFGGAI  
APSAGIQEVTVNQSLLTPLNLEIDPNIQRVRKEEKEQIKTLNNRFASFIDKVRFLEQQNKMLETKWSLLQDQKTTRSNIV  
PMFEAYITNLRRQLDGLLNDKGRLEGELRNMQDLVEDFKAKYEDEINKRTTAENEFVVLKKDVDAAYMNKVELEAKVDAL  
TDEINFLRSLYEAELRELQAQISDTSVVLSMDNSRNLDLDSIIAEVKAQYEDIANRSRAEAESWYQSKFEALQVTAGKHG  
DDLRNTKNEITEINRVIQRLQGEIENAKAQRAKMEAAIAEAEERGELAVKDARAKLEELEAALQKAKQDMARQLREYQEL  
MNVKLALDIEIATYRKLLEGEESRLAGDGVGSVNISMVSSSGGGSSGFLGGGVRGGLALGAGMGSGALGFSSGGSTKSYT  
VTTTSSTRSFRK`

| Site | Type | Count |
| --- | --- | --- |
| 92 | N-linked | 1 |

| Mass | Sequence | MS_2_ Score | `q`-value | MS_1_ Score | Signal | Protein Coordinates | Tandem Scans | Start Time | Apex Time | End Time |
| --- | --- | --- | --- | --- | --- | --- | --- | --- | --- | --- |
| 4326.9597 | GGAIAPSAGIQEVTVN(N)QSLLTPLNL Hex 9 HexNAc 2 | 27.749 | 0.015 | 13.606 | 6.943e+07 | 76 - 101 | 7 | 53.94 | 54.28 | 54.97 |

### GGAIAPSAGIQEVTVN(N)QSLLTPLNL Hex 9 HexNAc 2

**Mass:**  4326.9597

(-2.882e-06)

**Charge States:** 4, 5

**Start Position**: 77

**End Position**: 102

| Profile Score | Tandem Score | TDA q-value | Volume | Spectrum Matches |
| --- | --- | --- | --- | --- |
| 13.6057 | 27.7491 | 0.0149 | 6.9430e+07 | 7 |

**Adduct Signal Fractions**

- **Unmodified**: 6.9430e+07
