# Supplementary material for: Measuring Site-specific Glycosylation Similarity between Influenza a Virus Variants with Statistical Certainty
Source: Mol Cell Proteomics. 2020 Nov 25;19(9):1533–45. doi: 10.1074/mcp.RA120.002031 (PMC8143645; doi:10.1074/mcp.RA120.002031)
Supplement: Supplementary file 2 [file mmc2.zip › mmc2/159250_2_supp_555289_qcjklv.zip!/20190301_Phil82_021119_chymo_HILIC_DDA_1ug_01_20190308120227_glycopeptides.html]

## 20190301\_Phil82\_021119\_chymo\_HILIC\_DDA\_1ug\_01\_20190308120227 @ GlycopeptideHypothesis-7233f6403d834841a60b0b72f845f1d9

**MS^1^ Mass Accuracy:** 10.0 PPM  
**Grouping Tolerance:** 15.0 PPM  
**MS^n^ Mass Accuracy:** 20.0 PPM  
**Minimum `q`-Value For Clustering:** 0.05

**Glycopeptide Hypothesis:**
GlycopeptideHypothesis-7233f6403d834841a60b0b72f845f1d9  
**Sample Searched:**
20190301\_Phil82\_021119\_chymo\_HILIC\_DDA\_1ug\_01\_20190308120227

| Protein | Matched Glycopeptides |
| --- | --- |
| iav\|AFG99160\|A/Phil/2/1982\_HA | 117 |
| iav\|ADJ41819\|A/Phil/2-MA/1982\_NA | 15 |
| tr\|F1NB02\|F1NB02\_CHICK | 1 |
| tr\|A0A1D5PT58\|A0A1D5PT58\_CHICK | 1 |
| sp\|P14105\|MYH9\_CHICK | 1 |
| iav\|AAM75156\|A/PR/8/1934\_PB1 | 1 |
| iav\|AGU93028\|A/PR/8/1934\_PB1 | 1 |
| iav\|AAA43581\|A/PR/8/1934\_PB1 | 1 |
| iav\|ACF41842\|A/PR/8/1934\_PB1 | 1 |
| iav\|ADX99655\|A/PR/8-WG/1934\_PB1 | 1 |
| iav\|BAV59609\|A/PR/8/1934\_PB1 | 1 |
| iav\|ABD77683\|A/PR/8/1934\_PB1 | 1 |

### iav|AFG99160|A/Phil/2/1982\_HA

**>iav|AFG99160|A/Phil/2/1982\_HA**  
 `MKTIIALSYMFCLVFAQNLPGNDNSTATLCLGHHAVPNGTLVKTITNDQIEVTNATELVQSSSTGRICDSPHRILDGKNC  
TLIDALLGDPHCDGFQNEKWDLFVERSKAFSNCYPYDVPDYASLRSLVASSGTLEFINEGFNWTGVTQSGGSSTCKRGSN  
NSFFSRLNWLYESESKYPVLNVTMPNNGKFDKLYIWGIHHPSTDKEQTNLYIRASGRVTVSTKRSQQTVIPNIGSRPWVR  
GLSSRISIYWTIVKPGDILLINSTGNLIAPRGYFKIRTGKSSIMRSDAPIGTCSSECITPNGSIPNDKPFQNVNKITYGA  
CPRYVKQNTLKLATGMRNVPEKQTRGIFGAIAGFIENGWEGMVDGWYGFRHQNSEGTGQAADLKSTQAAIDQINGKLNRV  
IEKTNEKFHQIEKEFSEVEGRIQDLEKYVEDTKIDLWSYNAELLVALENQHTIDLTDSEMNKLFEKTRKQLRENAEDMGN  
GCFKIYHKCDNACIGSIRNGTYDHDVYRDEALNNRFQIKGVELKSGYKDWILWISFAISCFLLCVVLLGFIMWACQKGNI  
RCNICI`

| Site | Type | Count |
| --- | --- | --- |
| 262 | N-linked | 43 |
| 301 | N-linked | 33 |
| 142 | N-linked | 3 |
| 499 | N-linked | 3 |
| 181 | N-linked | 9 |
| 54 | N-linked | 6 |
| 24 | N-linked | 14 |
| 160 | N-linked | 6 |

| Mass | Sequence | MS_2_ Score | `q`-value | MS_1_ Score | Signal | Protein Coordinates | Tandem Scans | Start Time | Apex Time | End Time |
| --- | --- | --- | --- | --- | --- | --- | --- | --- | --- | --- |
| 3499.5140 | LIN(N)STGNLIAPRGYF Hex 9 HexNAc 2 | 172.191 | 0.0 | -17.306 | 1.590e+07 | 259 - 274 | 2 | 47.04 | 47.04 | 47.21 |
| 2870.1707 | LPGNDN(N)STATL Fuc 1 Hex 5 HexNAc 4 | 167.453 | 0.0 | 12.645 | 2.720e+07 | 18 - 29 | 3 | 25.95 | 26.46 | 26.96 |
| 3159.4229 | TIVKPGDILLIN(N) Hex 9 HexNAc 2 | 158.358 | 0.0 | 9.834 | 4.297e+07 | 250 - 262 | 5 | 46.06 | 47.55 | 47.89 |
| 2695.0766 | LIN(N)STGNL Hex 9 HexNAc 2 | 158.178 | 0.0 | -20.319 | 5.554e+06 | 259 - 267 | 1 | 26.46 | 26.46 | 26.46 |
| 3352.4445 | LIN(N)STGNLIAPRGY Hex 9 HexNAc 2 | 155.738 | 0.0 | -18.685 | 2.290e+07 | 259 - 273 | 1 | 36.87 | 36.87 | 37.03 |
| 4289.0110 | TIVKPGDILLIN(N)STGNLIAPRGY Hex 9 HexNAc 2 | 155.518 | 0.0 | 17.368 | 5.435e+08 | 250 - 273 | 11 | 53.58 | 55.63 | 56.31 |
| 3176.4484 | TIVKPGDILLIN(N) Fuc 2 Hex 3 HexNAc 4 Neu5Ac 1 | 153.469 | 0.0 | 11.763 | 1.507e+07 | 250 - 262 | 4 | 46.23 | 47.55 | 47.72 |
| 2724.1101 | LPGNDN(N)STATL Hex 5 HexNAc 4 | 151.089 | 0.0 | -5.186 | 8.387e+06 | 18 - 29 | 2 | 26.12 | 26.46 | 26.63 |
| 4085.9523 | TIVKPGDILLIN(N)STGNLIAPR Fuc 2 Hex 3 HexNAc 4 Neu5Ac 1 | 150.832 | 0.0 | -3.234 | 2.299e+07 | 250 - 271 | 2 | 53.07 | 53.24 | 53.41 |
| 4230.9775 | TIVKPGDILLIN(N)STGNLIAPR Hex 10 HexNAc 2 | 150.275 | 0.0 | -4.337 | 1.286e+07 | 250 - 271 | 2 | 53.07 | 53.24 | 53.41 |
| 4068.9256 | TIVKPGDILLIN(N)STGNLIAPR Hex 9 HexNAc 2 | 146.297 | 0.0 | 16.210 | 1.516e+08 | 250 - 271 | 5 | 53.07 | 53.24 | 53.92 |
| 3631.6511 | TIVKPGDILLIN(N)STGNL Hex 9 HexNAc 2 | 145.915 | 0.0 | -3.255 | 7.198e+07 | 250 - 267 | 5 | 52.72 | 52.89 | 53.24 |
| 4306.0370 | TIVKPGDILLIN(N)STGNLIAPRGY Fuc 2 Hex 3 HexNAc 4 Neu5Ac 1 | 142.430 | 0.0 | 15.642 | 6.782e+07 | 250 - 273 | 3 | 55.29 | 55.46 | 55.97 |
| 3132.3631 | LIN(N)STGNLIAPR Hex 9 HexNAc 2 | 141.525 | 0.0 | -16.481 | 7.920e+06 | 259 - 271 | 1 | 31.35 | 31.35 | 31.35 |
| 3386.4321 | IN(N)STGNLIAPRGYF Hex 9 HexNAc 2 | 141.229 | 0.0 | -19.752 | 4.446e+06 | 260 - 274 | 1 | 43.16 | 43.33 | 43.33 |
| 3906.8744 | TIVKPGDILLIN(N)STGNLIAPR Hex 8 HexNAc 2 | 140.718 | 0.0 | -19.110 | 9.371e+06 | 250 - 271 | 2 | 53.24 | 53.41 | 53.41 |
| 4468.0925 | TIVKPGDILLIN(N)STGNLIAPRGY Fuc 2 Hex 4 HexNAc 4 Neu5Ac 1 | 138.574 | 0.0 | -18.622 | 5.953e+06 | 250 - 273 | 1 | 55.29 | 55.46 | 55.46 |
| 4126.9573 | TIVKPGDILLIN(N)STGNLIAPRGY Hex 8 HexNAc 2 | 136.521 | 0.0 | 12.679 | 4.057e+07 | 250 - 273 | 7 | 54.68 | 55.63 | 55.97 |
| 3535.5910 | TIVKPGDILLIN(N)STGN Fuc 2 Hex 3 HexNAc 4 Neu5Ac 1 | 133.703 | 0.0 | -18.859 | 7.505e+06 | 250 - 266 | 3 | 45.21 | 45.38 | 45.38 |
| 3322.4941 | TIVKPGDILLIN(N) Hex 10 HexNAc 2 | 132.761 | 0.0 | 0.000 | 0.000e+00 | 250 - 262 | 1 | - | - | - |
| 3519.5608 | TIVKPGDILLIN(N)STGN Hex 9 HexNAc 2 | 130.193 | 0.0 | 0.000 | 0.000e+00 | 250 - 266 | 1 | - | - | - |
| 3648.6780 | TIVKPGDILLIN(N)STGNL Fuc 2 Hex 3 HexNAc 4 Neu5Ac 1 | 129.370 | 0.0 | -2.794 | 2.729e+07 | 250 - 267 | 3 | 52.72 | 52.89 | 53.07 |
| 3518.5677 | TIVKPGDILLIN(N)STGN Hex 9 HexNAc 2 | 128.041 | 0.0 | -3.099 | 2.370e+07 | 250 - 266 | 3 | 45.21 | 45.38 | 45.55 |
| 3239.3626 | IN(N)STGNLIAPRGY Hex 9 HexNAc 2 | 127.466 | 0.0 | -16.956 | 4.884e+06 | 260 - 273 | 1 | 31.68 | 31.68 | 31.68 |
| 3369.4704 | LIN(N)STGNLIAPRGY Fuc 2 Hex 3 HexNAc 4 Neu5Ac 1 | 126.998 | 0.0 | -20.308 | 4.527e+06 | 259 - 273 | 1 | 36.87 | 36.87 | 36.87 |
| 3016.2289 | LPGNDN(N)STATL Fuc 2 Hex 5 HexNAc 4 | 126.146 | 0.0 | -4.832 | 6.241e+06 | 18 - 29 | 2 | 25.95 | 26.46 | 26.46 |
| 3460.3782 | LPGNDN(N)STATL Hex 9 HexNAc 3 Neu5Ac 1 | 120.907 | 0.0 | 11.936 | 3.554e+08 | 18 - 29 | 10 | 34.05 | 34.73 | 35.38 |
| 3516.5416 | LIN(N)STGNLIAPRGYF Fuc 2 Hex 3 HexNAc 4 Neu5Ac 1 | 120.564 | 0.0 | -19.042 | 4.086e+06 | 259 - 274 | 1 | 47.04 | 47.04 | 47.21 |
| 3469.5958 | TIVKPGDILLIN(N)STGNL Hex 8 HexNAc 2 | 120.328 | 0.0 | -19.624 | 4.870e+06 | 250 - 267 | 2 | 52.89 | 52.89 | 53.07 |
| 3477.4028 | LPGNDN(N)STATL Fuc 2 Hex 3 HexNAc 5 Neu5Ac 2 | 117.653 | 0.0 | 10.868 | 7.208e+07 | 18 - 29 | 2 | 34.22 | 34.73 | 34.89 |
| 3263.3510 | ITPN(N)GSIPNDKPF Hex 9 HexNAc 2 | 116.684 | 0.0 | -19.730 | 3.343e+06 | 297 - 310 | 1 | 32.02 | 32.19 | 32.19 |
| 2317.9560 | LPGNDN(N)STATL Hex 5 HexNAc 2 | 115.798 | 0.0 | -5.684 | 5.204e+06 | 18 - 29 | 2 | 26.63 | 26.63 | 27.13 |
| 3235.3033 | LPGNDN(N)STATL Fuc 1 Hex 6 HexNAc 5 | 113.378 | 0.0 | -5.145 | 4.764e+06 | 18 - 29 | 1 | 25.78 | 26.12 | 26.29 |
| 3019.2792 | IN(N)STGNLIAPR Hex 9 HexNAc 2 | 109.332 | 0.0 | -20.102 | 1.586e+06 | 260 - 271 | 1 | 24.77 | 24.94 | 24.94 |
| 3028.2231 | KRGSN(N)N(D)SFF Fuc 1 Hex 5 HexNAc 5 | 108.828 | 0.0 | -19.764 | 2.210e+07 | 155 - 164 | 3 | 20.92 | 21.09 | 21.09 |
| 2825.1487 | KRGSN(N)N(D)SFF Fuc 1 Hex 5 HexNAc 4 | 106.517 | 0.0 | -19.154 | 7.633e+06 | 155 - 164 | 1 | 20.92 | 20.92 | 20.92 |
| 3129.2960 | LPGNDN(N)STATL Fuc 3 Hex 3 HexNAc 4 Neu5Ac 1 | 105.560 | 0.0 | 10.078 | 4.730e+07 | 18 - 29 | 1 | 34.73 | 35.22 | 35.54 |
| 3623.4616 | LPGNDN(N)STATL Fuc 3 Hex 3 HexNAc 5 Neu5Ac 2 | 102.976 | 0.0 | 11.009 | 2.461e+07 | 18 - 29 | 1 | 33.88 | 34.56 | 34.73 |
| 3101.3002 | ITPN(N)GSIPNDKPF Hex 8 HexNAc 2 | 102.670 | 0.0 | -3.980 | 6.419e+06 | 297 - 310 | 2 | 32.02 | 32.19 | 32.36 |
| 3793.7040 | TIVKPGDILLIN(N)STGNL Hex 10 HexNAc 2 | 99.895 | 0.0 | -19.818 | 5.221e+06 | 250 - 267 | 2 | 52.72 | 52.72 | 52.89 |
| 3331.3465 | LPGNDN(N)STATL Fuc 1 Hex 3 HexNAc 5 Neu5Ac 2 | 99.733 | 0.0 | -19.393 | 1.871e+07 | 18 - 29 | 1 | 34.56 | 34.56 | 34.73 |
| 3207.2887 | SIRN(N)GTYDHDVY Fuc 1 Hex 5 HexNAc 4 | 95.221 | 0.0 | -18.810 | 1.684e+06 | 495 - 507 | 1 | 19.28 | 19.28 | 19.28 |
| 4521.8729 | RSDAPIGTC(C)SSEC(C)ITPN(N)GSIPNDKPF Hex 8 HexNAc 2 | 90.505 | 0.0 | -3.843 | 3.857e+07 | 284 - 310 | 3 | 37.54 | 37.71 | 38.05 |
| 3039.2967 | TITNDQIEVTN(N)ATEL Hex 6 HexNAc 2 | 88.372 | 0.0 | -17.815 | 1.893e+07 | 43 - 58 | 1 | 40.43 | 40.60 | 40.60 |
| 3377.3920 | IGSIRN(N)GTYDHDVY Fuc 1 Hex 5 HexNAc 4 | 84.676 | 0.0 | -18.556 | 3.103e+06 | 493 - 507 | 1 | 24.27 | 24.27 | 24.44 |
| 3353.3489 | SIRN(N)GTYDHDVY Fuc 2 Hex 5 HexNAc 4 | 83.978 | 0.0 | -20.466 | 2.546e+06 | 495 - 507 | 1 | 19.05 | 19.05 | 19.14 |
| 2899.1333 | RGSN(N)NSFF Fuc 1 Hex 5 HexNAc 5 | 78.437 | 0.0 | -19.404 | 5.215e+06 | 156 - 164 | 1 | 26.12 | 26.29 | 26.29 |
| 3091.1955 | KRGSN(N)N(D)SFF Hex 7 HexNAc 3 Neu5Ac 1 | 76.029 | 0.0 | -20.830 | 1.825e+06 | 155 - 164 | 1 | 23.76 | 23.76 | 23.76 |
| 4683.9239 | RSDAPIGTC(C)SSEC(C)ITPN(N)GSIPNDKPF Hex 9 HexNAc 2 | 74.267 | 0.0 | -3.623 | 2.906e+07 | 284 - 310 | 3 | 37.54 | 37.71 | 37.88 |
| 3173.2864 | KRGSN(N)NSFF Fuc 2 Hex 5 HexNAc 5 | 72.451 | 0.0 | -19.485 | 6.611e+06 | 155 - 164 | 2 | 20.76 | 20.76 | 20.76 |
| 4289.9991 | TIVKPGDILLIN(N)STGN(D)LIAPRGY Hex 9 HexNAc 2 | 69.155 | 0.0 | 11.646 | 1.374e+07 | 250 - 273 | 10 | 55.97 | 56.49 | 56.66 |
| 3027.2324 | KRGSN(N)NSFF Fuc 1 Hex 5 HexNAc 5 | 65.008 | 0.0 | -18.303 | 3.974e+07 | 155 - 164 | 1 | 21.26 | 21.26 | 21.43 |
| 4451.0623 | TIVKPGDILLIN(N)STGNLIAPRGY Hex 10 HexNAc 2 | 64.430 | 0.0 | -3.893 | 5.622e+07 | 250 - 273 | 6 | 55.29 | 55.46 | 55.63 |
| 4359.8196 | RSDAPIGTC(C)SSEC(C)ITPN(N)GSIPNDKPF Hex 7 HexNAc 2 | 61.121 | 0.0 | -4.755 | 1.062e+07 | 284 - 310 | 2 | 37.71 | 37.88 | 38.05 |
| 3279.2538 | INEGFN(N)W Fuc 2 Hex 8 HexNAc 4 | 58.935 | 0.0 | -19.910 | 1.753e+07 | 136 - 143 | 1 | 34.89 | 34.89 | 35.06 |
| 3133.1933 | INEGFN(N)W Fuc 1 Hex 8 HexNAc 4 | 58.704 | 0.0 | 11.990 | 7.239e+07 | 136 - 143 | 2 | 34.56 | 35.22 | 35.54 |
| 4148.7588 | LYESESKYPVLN(N)VTM(O)PNN(D)GKF Hex 8 HexNAc 2 | 58.419 | 0.0 | -6.080 | 1.464e+07 | 169 - 190 | 3 | 41.12 | 41.46 | 41.63 |
| 3639.4557 | SEC(C)ITPN(N)GSIPNDKPF Hex 9 HexNAc 2 | 56.442 | 0.0 | -19.699 | 7.547e+06 | 294 - 310 | 2 | 35.70 | 35.70 | 35.87 |
| 3337.4632 | LIN(N)STGNLIAPRGYF Hex 8 HexNAc 2 | 55.041 | 0.0 | -19.785 | 1.058e+06 | 259 - 274 | 1 | 47.21 | 47.21 | 47.21 |
| 4933.2456 | TITNDQIEVTN(N)ATELVQSSSTGRIC(C)DSPHRIL Hex 6 HexNAc 2 | 53.402 | 0.0 | -18.999 | 2.495e+06 | 43 - 75 | 1 | 46.06 | 46.23 | 46.23 |
| 4553.9341 | ESESKYPVLN(N)VTM(O)PNN(D)GKFDKLY Hex 9 HexNAc 2 | 51.906 | 0.0 | -6.067 | 3.112e+07 | 171 - 194 | 3 | 42.14 | 42.48 | 42.65 |
| 3564.4317 | SSEC(C)ITPN(N)GSIPNDKPF Hex 8 HexNAc 2 | 50.522 | 0.0 | -20.285 | 1.006e+07 | 293 - 310 | 1 | 35.54 | 35.70 | 35.70 |
| 3863.5987 | LYESESKYPVLN(N)VTM(O)PN Hex 9 HexNAc 2 | 48.363 | 0.0 | -19.123 | 5.898e+06 | 169 - 186 | 1 | 37.54 | 37.54 | 37.71 |
| 5801.5512 | KIRTGKSSIM(O)RSDAPIGTC(C)SSEC(C)ITPN(N)GSIPNDKPF Hex 9 HexNAc 2 | 47.345 | 0.0 | -19.502 | 2.716e+07 | 274 - 310 | 2 | 32.87 | 32.87 | 33.03 |
| 4522.8613 | RSDAPIGTC(C)SSEC(C)ITPN(N)GSIPN(D)DKPF Hex 8 HexNAc 2 | 47.122 | 0.0 | -5.934 | 3.691e+07 | 284 - 310 | 2 | 38.56 | 38.73 | 38.90 |
| 2997.3701 | TIVKPGDILLIN(N) Hex 8 HexNAc 2 | 44.720 | 0.0 | -6.163 | 1.845e+06 | 250 - 262 | 1 | 47.38 | 47.55 | 47.72 |
| 4391.8813 | ESESKYPVLN(N)VTM(O)PNN(D)GKFDKLY Hex 8 HexNAc 2 | 43.165 | 0.0 | -5.868 | 1.975e+07 | 171 - 194 | 3 | 42.31 | 42.65 | 42.82 |
| 3201.3495 | TITNDQIEVTN(N)ATEL Hex 7 HexNAc 2 | 42.330 | 0.0 | -18.240 | 6.554e+06 | 43 - 58 | 1 | 40.26 | 40.43 | 40.43 |
| 4700.9499 | RSDAPIGTC(C)SSEC(C)ITPN(N)GSIPNDKPF Fuc 2 Hex 3 HexNAc 4 Neu5Ac 1 | 42.327 | 0.0 | -19.180 | 2.501e+06 | 284 - 310 | 1 | 37.71 | 37.71 | 37.71 |
| 4310.8121 | LYESESKYPVLN(N)VTM(O)PNN(D)GKF Hex 9 HexNAc 2 | 42.207 | 0.0 | -6.025 | 1.918e+07 | 169 - 190 | 3 | 41.12 | 41.46 | 41.46 |
| 4579.8674 | ITPN(N)GSIPNDKPF Fuc 4 Hex 6 HexNAc 8 | 42.138 | 0.0 | -2.608 | 1.736e+07 | 297 - 310 | 3 | 38.73 | 41.12 | 41.29 |
| 3701.5491 | LYESESKYPVLN(N)VTM(O)PN Hex 8 HexNAc 2 | 40.994 | 0.0 | -18.311 | 3.871e+06 | 169 - 186 | 3 | 37.54 | 37.71 | 37.71 |
| 3336.3529 | INEGFN(N)WTGVTQ Fuc 1 Hex 5 HexNAc 5 | 40.285 | 0.0 | -19.933 | 2.108e+06 | 136 - 148 | 2 | 43.33 | 43.33 | 43.33 |
| 2877.2427 | TITNDQIEVTN(N)ATEL Hex 5 HexNAc 2 | 40.046 | 0.0 | -17.287 | 7.207e+06 | 43 - 58 | 1 | 40.77 | 40.77 | 40.94 |
| 2939.2501 | ITPN(N)GSIPNDKPF Hex 7 HexNAc 2 | 39.914 | 0.0 | -21.141 | 1.094e+06 | 297 - 310 | 1 | 32.19 | 32.36 | 32.36 |
| 3663.4500 | LPGNDN(N)STATL Hex 9 HexNAc 4 Neu5Ac 1 | 39.387 | 0.0 | -20.724 | 1.877e+07 | 18 - 29 | 2 | 34.73 | 34.89 | 34.89 |
| 4538.8952 | ITPN(N)GSIPNDKPF Fuc 4 Hex 7 HexNAc 7 | 37.603 | 0.0 | -18.837 | 3.409e+06 | 297 - 310 | 1 | 37.71 | 37.71 | 37.71 |
| 4069.9156 | TIVKPGDILLIN(N)STGN(D)LIAPR Hex 9 HexNAc 2 | 36.333 | 0.0 | -20.235 | 3.201e+06 | 250 - 271 | 3 | 54.09 | 54.26 | 54.26 |
| 3299.3110 | LPGNDN(N)STATL Fuc 2 Hex 8 HexNAc 3 | 35.968 | 0.0 | 12.024 | 4.881e+07 | 18 - 29 | 3 | 36.70 | 36.87 | 37.54 |
| 3640.4400 | SEC(C)ITPN(N)GSIPN(D)DKPF Hex 9 HexNAc 2 | 34.942 | 0.0 | -20.838 | 7.317e+06 | 294 - 310 | 1 | 36.87 | 36.87 | 37.03 |
| 5642.5051 | KIRTGKSSIM(O)RSDAPIGTC(C)SSEC(C)ITPN(N)GSIPNDKPF Hex 8 HexNAc 2 | 33.593 | 0.0 | 0.000 | 0.000e+00 | 274 - 310 | 1 | - | - | - |
| 5639.4998 | KIRTGKSSIM(O)RSDAPIGTC(C)SSEC(C)ITPN(N)GSIPNDKPF Hex 8 HexNAc 2 | 33.526 | 0.0 | -19.247 | 3.712e+07 | 274 - 310 | 1 | 32.87 | 33.03 | 33.03 |
| 4363.0558 | TIVKPGDILLIN(N)STGNLIAPRGY Fuc 1 Hex 3 HexNAc 5 Neu5Ac 1 | 32.842 | 0.0 | -4.736 | 2.229e+07 | 250 - 273 | 2 | 55.97 | 56.31 | 56.49 |
| 3727.4670 | SSEC(C)ITPN(N)GSIPN(D)DKPF Hex 9 HexNAc 2 | 32.757 | 0.0 | -21.401 | 5.358e+06 | 293 - 310 | 1 | 36.54 | 36.70 | 36.70 |
| 4684.9089 | RSDAPIGTC(C)SSEC(C)ITPN(N)GSIPN(D)DKPF Hex 9 HexNAc 2 | 32.493 | 0.0 | -6.100 | 2.809e+07 | 284 - 310 | 3 | 38.39 | 38.56 | 38.90 |
| 4142.9715 | TIVKPGDILLIN(N)STGNLIAPR Fuc 1 Hex 3 HexNAc 5 Neu5Ac 1 | 32.100 | 0.0 | -17.789 | 8.938e+06 | 250 - 271 | 2 | 53.92 | 53.92 | 54.09 |
| 3242.3766 | TITNDQIEVTN(N)ATEL Hex 6 HexNAc 3 | 32.068 | 0.0 | -19.877 | 4.727e+06 | 43 - 58 | 1 | 40.43 | 40.60 | 40.60 |
| 4578.8924 | ITPN(N)GSIPNDKPF Fuc 2 Hex 6 HexNAc 8 Neu5Ac 1 | 31.840 | 0.0 | -19.263 | 6.706e+06 | 297 - 310 | 1 | 37.88 | 37.88 | 38.05 |
| 5802.5339 | KIRTGKSSIM(O)RSDAPIGTC(C)SSEC(C)ITPN(N)GSIPN(D)DKPF Hex 9 HexNAc 2 | 31.752 | 0.0 | -20.778 | 1.523e+07 | 274 - 310 | 2 | 33.54 | 33.54 | 33.71 |
| 3825.5132 | LPGNDN(N)STATL Hex 10 HexNAc 4 Neu5Ac 1 | 30.939 | 0.0 | 9.699 | 4.437e+07 | 18 - 29 | 5 | 33.71 | 34.22 | 34.39 |
| 3149.3871 | LIN(N)STGNLIAPR Fuc 2 Hex 3 HexNAc 4 Neu5Ac 1 | 30.844 | 0.0 | -17.291 | 2.039e+06 | 259 - 271 | 1 | 31.35 | 31.35 | 31.35 |
| 4360.8088 | RSDAPIGTC(C)SSEC(C)ITPN(N)GSIPN(D)DKPF Hex 7 HexNAc 2 | 30.834 | 0.0 | -6.211 | 1.087e+07 | 284 - 310 | 2 | 38.56 | 38.73 | 38.90 |
| 4417.8118 | ITPN(N)GSIPNDKPF Fuc 4 Hex 5 HexNAc 8 | 29.639 | 0.006 | -4.098 | 1.709e+07 | 297 - 310 | 2 | 40.94 | 41.12 | 41.29 |
| 2950.1731 | LPGNDN(N)STATL Fuc 1 Hex 8 HexNAc 2 | 29.209 | 0.006 | 12.474 | 2.712e+08 | 18 - 29 | 7 | 36.03 | 36.70 | 37.88 |
| 4668.0328 | ESESKYPVLN(N)VTMPNNGKFDKLY Hex 3 HexNAc 6 Neu5Ac 1 | 28.687 | 0.006 | 15.263 | 1.893e+08 | 171 - 194 | 6 | 44.36 | 44.87 | 45.21 |
| 4887.1019 | ESESKYPVLN(N)VTM(O)PNN(D)GKFDKLY Fuc 2 Hex 8 HexNAc 3 | 28.201 | 0.006 | 11.187 | 1.177e+08 | 171 - 194 | 8 | 43.67 | 44.70 | 45.04 |
| 4248.0075 | TIVKPGDILLIN(N)STGNLIAPR Fuc 2 Hex 4 HexNAc 4 Neu5Ac 1 | 28.189 | 0.013 | -19.839 | 1.259e+06 | 250 - 271 | 1 | 53.24 | 53.24 | 53.24 |
| 3565.4137 | SSEC(C)ITPN(N)GSIPN(D)DKPF Hex 8 HexNAc 2 | 28.072 | 0.013 | -21.464 | 8.305e+06 | 293 - 310 | 1 | 36.70 | 36.70 | 36.87 |
| 3405.4741 | IN(N)STGNLIAPRGYF Fuc 2 Hex 3 HexNAc 4 Neu5Ac 1 | 27.867 | 0.013 | 0.000 | 0.000e+00 | 260 - 274 | 1 | - | - | - |
| 4309.0481 | TIVKPGDILLIN(N)STGNLIAPRGY Fuc 2 Hex 3 HexNAc 4 Neu5Ac 1 | 27.683 | 0.019 | 0.000 | 0.000e+00 | 250 - 273 | 1 | - | - | - |
| 3680.6164 | TIVKPGDILLIN(N)STGN Hex 10 HexNAc 2 | 27.314 | 0.019 | -20.057 | 1.263e+06 | 250 - 266 | 1 | 45.21 | 45.21 | 45.21 |
| 4610.9362 | ITPN(N)GSIPNDKPF Fuc 5 Hex 6 HexNAc 6 Neu5Ac 1 | 27.197 | 0.024 | -21.025 | 5.800e+05 | 297 - 310 | 1 | 38.39 | 38.39 | 38.39 |
| 4701.9378 | RSDAPIGTC(C)SSEC(C)ITPN(N)GSIPN(D)DKPF Fuc 2 Hex 3 HexNAc 4 Neu5Ac 1 | 27.044 | 0.024 | -20.840 | 2.802e+06 | 284 - 310 | 1 | 38.56 | 38.56 | 38.73 |
| 3266.4578 | VKTITNDQIEVTN(N)ATEL Hex 6 HexNAc 2 | 26.658 | 0.024 | -20.335 | 1.399e+06 | 41 - 58 | 1 | 38.05 | 38.05 | 38.05 |
| 3338.4981 | TIVKPGDILLIN(N) Fuc 2 Hex 4 HexNAc 4 Neu5Ac 1 | 26.624 | 0.024 | -19.546 | 3.593e+05 | 250 - 262 | 1 | 47.38 | 47.38 | 47.38 |
| 4676.8696 | ITPN(N)GSIPNDKPF Fuc 3 Hex 10 HexNAc 6 | 26.172 | 0.024 | 12.367 | 9.420e+05 | 297 - 310 | 1 | 77.18 | 77.27 | 77.37 |
| 4474.8324 | ITPN(N)GSIPNDKPF Fuc 3 Hex 5 HexNAc 9 | 25.948 | 0.028 | -20.257 | 3.832e+06 | 297 - 310 | 2 | 42.99 | 42.99 | 43.16 |
| 4453.1049 | TIVKPGDILLIN(N)STGNLIAPRGY Hex 5 HexNAc 6 | 25.888 | 0.028 | 15.329 | 5.608e+07 | 250 - 273 | 3 | 60.89 | 61.31 | 61.48 |
| 3356.5120 | TIVKPGDILLIN(N)STGN Hex 8 HexNAc 2 | 25.853 | 0.028 | -19.947 | 8.615e+05 | 250 - 266 | 1 | 45.38 | 45.38 | 45.38 |
| 4740.9396 | ITPN(N)GSIPNDKPF Fuc 2 Hex 7 HexNAc 8 Neu5Ac 1 | 25.664 | 0.028 | -5.244 | 6.421e+06 | 297 - 310 | 1 | 37.71 | 37.88 | 38.05 |
| 5477.4443 | KIRTGKSSIM(O)RSDAPIGTC(C)SSEC(C)ITPN(N)GSIPNDKPF Hex 7 HexNAc 2 | 25.028 | 0.028 | -19.621 | 5.549e+06 | 274 - 310 | 1 | 32.87 | 33.03 | 33.03 |
| 5640.4843 | KIRTGKSSIM(O)RSDAPIGTC(C)SSEC(C)ITPN(N)GSIPN(D)DKPF Hex 8 HexNAc 2 | 24.578 | 0.028 | -6.507 | 2.376e+07 | 274 - 310 | 3 | 33.54 | 33.71 | 33.88 |
| 3478.3917 | SEC(C)ITPN(N)GSIPN(D)DKPF Hex 8 HexNAc 2 | 24.465 | 0.028 | -21.228 | 1.462e+07 | 294 - 310 | 3 | 35.70 | 35.70 | 37.03 |
| 4436.0789 | TIVKPGDILLIN(N)STGNLIAPR Hex 5 HexNAc 7 | 23.799 | 0.044 | 17.351 | 3.626e+08 | 250 - 271 | 11 | 60.72 | 61.31 | 62.15 |
| 4831.0655 | LYESESKYPVLN(N)VTM(O)PNN(D)GKF Fuc 3 Hex 7 HexNAc 4 | 23.741 | 0.044 | 11.186 | 7.699e+07 | 169 - 190 | 9 | 45.21 | 45.38 | 46.70 |
| 3316.3277 | SEC(C)ITPN(N)GSIPN(D)DKPF Hex 7 HexNAc 2 | 23.520 | 0.044 | -19.913 | 1.007e+07 | 294 - 310 | 1 | 36.87 | 36.87 | 37.03 |
| 4695.9265 | SEC(C)ITPN(N)GSIPNDKPF Fuc 5 Hex 6 HexNAc 6 | 23.282 | 0.049 | -19.233 | 2.384e+06 | 294 - 310 | 1 | 39.07 | 39.07 | 39.07 |

### LIN(N)STGNLIAPRGYF Hex 9 HexNAc 2

**Mass:**  3499.5137

(4.114e-07)

**Charge States:** 3, 4

**Start Position**: 260

**End Position**: 275

| Profile Score | Tandem Score | TDA q-value | Volume | Spectrum Matches |
| --- | --- | --- | --- | --- |
| -17.3059 | 172.1908 | 0.0000 | 1.5905e+07 | 2 |

**Adduct Signal Fractions**

- **Unmodified**: 1.5905e+07

### LPGNDN(N)STATL Fuc 1 Hex 5 HexNAc 4

**Mass:**  2870.1703

(3.827e-07)

**Charge States:** 3

**Start Position**: 19

**End Position**: 30

| Profile Score | Tandem Score | TDA q-value | Volume | Spectrum Matches |
| --- | --- | --- | --- | --- |
| 12.6446 | 167.4525 | 0.0000 | 2.7203e+07 | 3 |

**Adduct Signal Fractions**

- **Unmodified**: 2.7203e+07

### TIVKPGDILLIN(N) Hex 9 HexNAc 2

**Mass:**  3159.4217

(6.262e-07)

**Charge States:** 3

**Start Position**: 251

**End Position**: 263

| Profile Score | Tandem Score | TDA q-value | Volume | Spectrum Matches |
| --- | --- | --- | --- | --- |
| 9.8343 | 158.3580 | 0.0000 | 4.2968e+07 | 5 |

**Adduct Signal Fractions**

- **Unmodified**: 4.2968e+07

### LIN(N)STGNL Hex 9 HexNAc 2

**Mass:**  2695.0739

(-1.216e-06)

**Charge States:** 2, 3

**Start Position**: 260

**End Position**: 268

| Profile Score | Tandem Score | TDA q-value | Volume | Spectrum Matches |
| --- | --- | --- | --- | --- |
| -20.3190 | 158.1784 | 0.0000 | 5.5542e+06 | 1 |

**Adduct Signal Fractions**

- **Unmodified**: 5.5542e+06

### LIN(N)STGNLIAPRGY Hex 9 HexNAc 2

**Mass:**  3352.4441

(1.505e-07)

**Charge States:** 3

**Start Position**: 260

**End Position**: 274

| Profile Score | Tandem Score | TDA q-value | Volume | Spectrum Matches |
| --- | --- | --- | --- | --- |
| -18.6852 | 155.7375 | 0.0000 | 2.2900e+07 | 1 |

**Adduct Signal Fractions**

- **Unmodified**: 2.2900e+07

### TIVKPGDILLIN(N)STGNLIAPRGY Hex 9 HexNAc 2

**Mass:**  4289.0114

(1.468e-06)

**Charge States:** 3, 4, 5

**Start Position**: 251

**End Position**: 274

| Profile Score | Tandem Score | TDA q-value | Volume | Spectrum Matches |
| --- | --- | --- | --- | --- |
| 17.3683 | 155.5184 | 0.0000 | 5.4348e+08 | 11 |

**Adduct Signal Fractions**

- **Unmodified**: 5.4348e+08

### TIVKPGDILLIN(N) Fuc 2 Hex 3 HexNAc 4 Neu5Ac 1

**Mass:**  3176.4472

(-7.328e-06)

**Charge States:** 3

**Start Position**: 251

**End Position**: 263

| Profile Score | Tandem Score | TDA q-value | Volume | Spectrum Matches |
| --- | --- | --- | --- | --- |
| 11.7629 | 153.4685 | 0.0000 | 1.5074e+07 | 4 |

**Adduct Signal Fractions**

- **Unmodified**: 1.5074e+07

### LPGNDN(N)STATL Hex 5 HexNAc 4

**Mass:**  2724.1082

(3.604e-07)

**Charge States:** 3

**Start Position**: 19

**End Position**: 30

| Profile Score | Tandem Score | TDA q-value | Volume | Spectrum Matches |
| --- | --- | --- | --- | --- |
| -5.1864 | 151.0891 | 0.0000 | 8.3866e+06 | 2 |

**Adduct Signal Fractions**

- **Unmodified**: 8.3866e+06

### TIVKPGDILLIN(N)STGNLIAPR Fuc 2 Hex 3 HexNAc 4 Neu5Ac 1

**Mass:**  4085.9526

(-4.920e-06)

**Charge States:** 4

**Start Position**: 251

**End Position**: 272

| Profile Score | Tandem Score | TDA q-value | Volume | Spectrum Matches |
| --- | --- | --- | --- | --- |
| -3.2340 | 150.8316 | 0.0000 | 2.2994e+07 | 2 |

**Adduct Signal Fractions**

- **Unmodified**: 2.2994e+07

### TIVKPGDILLIN(N)STGNLIAPR Hex 10 HexNAc 2

**Mass:**  4230.9782

(6.957e-07)

**Charge States:** 4

**Start Position**: 251

**End Position**: 272

| Profile Score | Tandem Score | TDA q-value | Volume | Spectrum Matches |
| --- | --- | --- | --- | --- |
| -4.3375 | 150.2748 | 0.0000 | 1.2856e+07 | 2 |

**Adduct Signal Fractions**

- **Unmodified**: 1.2856e+07

### TIVKPGDILLIN(N)STGNLIAPR Hex 9 HexNAc 2

**Mass:**  4068.9257

(8.626e-07)

**Charge States:** 3, 4, 5

**Start Position**: 251

**End Position**: 272

| Profile Score | Tandem Score | TDA q-value | Volume | Spectrum Matches |
| --- | --- | --- | --- | --- |
| 16.2105 | 146.2973 | 0.0000 | 1.5158e+08 | 5 |

**Adduct Signal Fractions**

- **Unmodified**: 1.5158e+08

### TIVKPGDILLIN(N)STGNL Hex 9 HexNAc 2

**Mass:**  3631.6518

(3.707e-07)

**Charge States:** 3, 4

**Start Position**: 251

**End Position**: 268

| Profile Score | Tandem Score | TDA q-value | Volume | Spectrum Matches |
| --- | --- | --- | --- | --- |
| -3.2554 | 145.9149 | 0.0000 | 7.1976e+07 | 5 |

**Adduct Signal Fractions**

- **Unmodified**: 7.1976e+07

### TIVKPGDILLIN(N)STGNLIAPRGY Fuc 2 Hex 3 HexNAc 4 Neu5Ac 1

**Mass:**  4306.0363

(-5.524e-06)

**Charge States:** 4

**Start Position**: 251

**End Position**: 274

| Profile Score | Tandem Score | TDA q-value | Volume | Spectrum Matches |
| --- | --- | --- | --- | --- |
| 15.6420 | 142.4304 | 0.0000 | 6.7816e+07 | 3 |

**Adduct Signal Fractions**

- **Unmodified**: 6.7816e+07

### LIN(N)STGNLIAPR Hex 9 HexNAc 2

**Mass:**  3132.3631

(1.252e-06)

**Charge States:** 3

**Start Position**: 260

**End Position**: 272

| Profile Score | Tandem Score | TDA q-value | Volume | Spectrum Matches |
| --- | --- | --- | --- | --- |
| -16.4807 | 141.5253 | 0.0000 | 7.9196e+06 | 1 |

**Adduct Signal Fractions**

- **Unmodified**: 7.9196e+06

### IN(N)STGNLIAPRGYF Hex 9 HexNAc 2

**Mass:**  3386.4318

(1.274e-06)

**Charge States:** 3

**Start Position**: 261

**End Position**: 275

| Profile Score | Tandem Score | TDA q-value | Volume | Spectrum Matches |
| --- | --- | --- | --- | --- |
| -19.7522 | 141.2293 | 0.0000 | 4.4460e+06 | 1 |

**Adduct Signal Fractions**

- **Unmodified**: 4.4460e+06

### TIVKPGDILLIN(N)STGNLIAPR Hex 8 HexNAc 2

**Mass:**  3906.8708

(-5.130e-07)

**Charge States:** 3, 4

**Start Position**: 251

**End Position**: 272

| Profile Score | Tandem Score | TDA q-value | Volume | Spectrum Matches |
| --- | --- | --- | --- | --- |
| -19.1101 | 140.7185 | 0.0000 | 9.3712e+06 | 2 |

**Adduct Signal Fractions**

- **Unmodified**: 9.3712e+06

### TIVKPGDILLIN(N)STGNLIAPRGY Fuc 2 Hex 4 HexNAc 4 Neu5Ac 1

**Mass:**  4468.0915

(-5.090e-06)

**Charge States:** 4

**Start Position**: 251

**End Position**: 274

| Profile Score | Tandem Score | TDA q-value | Volume | Spectrum Matches |
| --- | --- | --- | --- | --- |
| -18.6219 | 138.5736 | 0.0000 | 5.9527e+06 | 1 |

**Adduct Signal Fractions**

- **Unmodified**: 5.9527e+06

### TIVKPGDILLIN(N)STGNLIAPRGY Hex 8 HexNAc 2

**Mass:**  4126.9577

(-8.242e-07)

**Charge States:** 3, 4

**Start Position**: 251

**End Position**: 274

| Profile Score | Tandem Score | TDA q-value | Volume | Spectrum Matches |
| --- | --- | --- | --- | --- |
| 12.6793 | 136.5212 | 0.0000 | 4.0567e+07 | 7 |

**Adduct Signal Fractions**

- **Unmodified**: 4.0567e+07

### TIVKPGDILLIN(N)STGN Fuc 2 Hex 3 HexNAc 4 Neu5Ac 1

**Mass:**  3535.5928

(-7.974e-06)

**Charge States:** 3

**Start Position**: 251

**End Position**: 267

| Profile Score | Tandem Score | TDA q-value | Volume | Spectrum Matches |
| --- | --- | --- | --- | --- |
| -18.8591 | 133.7025 | 0.0000 | 7.5051e+06 | 3 |

**Adduct Signal Fractions**

- **Unmodified**: 7.5051e+06

### TIVKPGDILLIN(N) Hex 10 HexNAc 2

**Mass:**  3322.4941

(3.074e-04)

**Start Position**: 251

**End Position**: 263

| Profile Score | Tandem Score | TDA q-value | Volume | Spectrum Matches |
| --- | --- | --- | --- | --- |
| 0.0000 | 132.7611 | 0.0000 | 0.0000e+00 | 1 |

Adduct:

No Chromatogram Found

### TIVKPGDILLIN(N)STGN Hex 9 HexNAc 2

**Mass:**  3519.5608

(2.832e-04)

**Start Position**: 251

**End Position**: 267

| Profile Score | Tandem Score | TDA q-value | Volume | Spectrum Matches |
| --- | --- | --- | --- | --- |
| 0.0000 | 130.1927 | 0.0000 | 0.0000e+00 | 1 |

Adduct:

No Chromatogram Found

### TIVKPGDILLIN(N)STGNL Fuc 2 Hex 3 HexNAc 4 Neu5Ac 1

**Mass:**  3648.6773

(-6.074e-06)

**Charge States:** 3

**Start Position**: 251

**End Position**: 268

| Profile Score | Tandem Score | TDA q-value | Volume | Spectrum Matches |
| --- | --- | --- | --- | --- |
| -2.7937 | 129.3699 | 0.0000 | 2.7285e+07 | 3 |

**Adduct Signal Fractions**

- **Unmodified**: 2.7285e+07

### TIVKPGDILLIN(N)STGN Hex 9 HexNAc 2

**Mass:**  3518.5673

(8.014e-07)

**Charge States:** 3

**Start Position**: 251

**End Position**: 267

| Profile Score | Tandem Score | TDA q-value | Volume | Spectrum Matches |
| --- | --- | --- | --- | --- |
| -3.0987 | 128.0408 | 0.0000 | 2.3703e+07 | 3 |

**Adduct Signal Fractions**

- **Unmodified**: 2.3703e+07

### IN(N)STGNLIAPRGY Hex 9 HexNAc 2

**Mass:**  3239.3626

(9.246e-07)

**Charge States:** 3

**Start Position**: 261

**End Position**: 274

| Profile Score | Tandem Score | TDA q-value | Volume | Spectrum Matches |
| --- | --- | --- | --- | --- |
| -16.9560 | 127.4658 | 0.0000 | 4.8838e+06 | 1 |

**Adduct Signal Fractions**

- **Unmodified**: 4.8838e+06

### LIN(N)STGNLIAPRGY Fuc 2 Hex 3 HexNAc 4 Neu5Ac 1

**Mass:**  3369.4704

(-7.897e-06)

**Charge States:** 3

**Start Position**: 260

**End Position**: 274

| Profile Score | Tandem Score | TDA q-value | Volume | Spectrum Matches |
| --- | --- | --- | --- | --- |
| -20.3083 | 126.9979 | 0.0000 | 4.5266e+06 | 1 |

**Adduct Signal Fractions**

- **Unmodified**: 4.5266e+06

### LPGNDN(N)STATL Fuc 2 Hex 5 HexNAc 4

**Mass:**  3016.2238

(1.563e-06)

**Charge States:** 3

**Start Position**: 19

**End Position**: 30

| Profile Score | Tandem Score | TDA q-value | Volume | Spectrum Matches |
| --- | --- | --- | --- | --- |
| -4.8316 | 126.1461 | 0.0000 | 6.2409e+06 | 2 |

**Adduct Signal Fractions**

- **Unmodified**: 6.2409e+06

### LPGNDN(N)STATL Hex 9 HexNAc 3 Neu5Ac 1

**Mass:**  3460.3778

(9.410e-06)

**Charge States:** 3, 4

**Start Position**: 19

**End Position**: 30

| Profile Score | Tandem Score | TDA q-value | Volume | Spectrum Matches |
| --- | --- | --- | --- | --- |
| 11.9359 | 120.9069 | 0.0000 | 3.5545e+08 | 10 |

**Adduct Signal Fractions**

- **Unmodified**: 3.5545e+08

### LIN(N)STGNLIAPRGYF Fuc 2 Hex 3 HexNAc 4 Neu5Ac 1

**Mass:**  3516.5396

(-7.314e-06)

**Charge States:** 3

**Start Position**: 260

**End Position**: 275

| Profile Score | Tandem Score | TDA q-value | Volume | Spectrum Matches |
| --- | --- | --- | --- | --- |
| -19.0416 | 120.5645 | 0.0000 | 4.0864e+06 | 1 |

**Adduct Signal Fractions**

- **Unmodified**: 4.0864e+06

### TIVKPGDILLIN(N)STGNL Hex 8 HexNAc 2

**Mass:**  3469.5950

(-1.812e-07)

**Charge States:** 3

**Start Position**: 251

**End Position**: 268

| Profile Score | Tandem Score | TDA q-value | Volume | Spectrum Matches |
| --- | --- | --- | --- | --- |
| -19.6237 | 120.3276 | 0.0000 | 4.8699e+06 | 2 |

**Adduct Signal Fractions**

- **Unmodified**: 4.8699e+06

### LPGNDN(N)STATL Fuc 2 Hex 3 HexNAc 5 Neu5Ac 2

**Mass:**  3477.4033

(3.173e-06)

**Charge States:** 3

**Start Position**: 19

**End Position**: 30

| Profile Score | Tandem Score | TDA q-value | Volume | Spectrum Matches |
| --- | --- | --- | --- | --- |
| 10.8680 | 117.6526 | 0.0000 | 7.2084e+07 | 2 |

**Adduct Signal Fractions**

- **Unmodified**: 7.2084e+07

### ITPN(N)GSIPNDKPF Hex 9 HexNAc 2

**Mass:**  3263.3523

(-3.463e-07)

**Charge States:** 3

**Start Position**: 298

**End Position**: 311

| Profile Score | Tandem Score | TDA q-value | Volume | Spectrum Matches |
| --- | --- | --- | --- | --- |
| -19.7297 | 116.6835 | 0.0000 | 3.3431e+06 | 1 |

**Adduct Signal Fractions**

- **Unmodified**: 3.3431e+06

### LPGNDN(N)STATL Hex 5 HexNAc 2

**Mass:**  2317.9547

(2.498e-07)

**Charge States:** 2, 3

**Start Position**: 19

**End Position**: 30

| Profile Score | Tandem Score | TDA q-value | Volume | Spectrum Matches |
| --- | --- | --- | --- | --- |
| -5.6836 | 115.7985 | 0.0000 | 5.2041e+06 | 2 |

**Adduct Signal Fractions**

- **Unmodified**: 5.2041e+06

### LPGNDN(N)STATL Fuc 1 Hex 6 HexNAc 5

**Mass:**  3235.3028

(2.992e-07)

**Charge States:** 3

**Start Position**: 19

**End Position**: 30

| Profile Score | Tandem Score | TDA q-value | Volume | Spectrum Matches |
| --- | --- | --- | --- | --- |
| -5.1449 | 113.3782 | 0.0000 | 4.7645e+06 | 1 |

**Adduct Signal Fractions**

- **Unmodified**: 4.7645e+06

### IN(N)STGNLIAPR Hex 9 HexNAc 2

**Mass:**  3019.2782

(1.818e-06)

**Charge States:** 3

**Start Position**: 261

**End Position**: 272

| Profile Score | Tandem Score | TDA q-value | Volume | Spectrum Matches |
| --- | --- | --- | --- | --- |
| -20.1021 | 109.3321 | 0.0000 | 1.5857e+06 | 1 |

**Adduct Signal Fractions**

- **Unmodified**: 1.5857e+06

### KRGSN(N)N(D)SFF Fuc 1 Hex 5 HexNAc 5

**Mass:**  3028.2240

(3.271e-04)

**Charge States:** 3

**Start Position**: 156

**End Position**: 165

| Profile Score | Tandem Score | TDA q-value | Volume | Spectrum Matches |
| --- | --- | --- | --- | --- |
| -19.7642 | 108.8278 | 0.0000 | 2.2103e+07 | 3 |

**Adduct Signal Fractions**

- **Unmodified**: 2.2103e+07

### KRGSN(N)N(D)SFF Fuc 1 Hex 5 HexNAc 4

**Mass:**  2825.1487

(3.521e-04)

**Charge States:** 3

**Start Position**: 156

**End Position**: 165

| Profile Score | Tandem Score | TDA q-value | Volume | Spectrum Matches |
| --- | --- | --- | --- | --- |
| -19.1537 | 106.5174 | 0.0000 | 7.6325e+06 | 1 |

**Adduct Signal Fractions**

- **Unmodified**: 7.6325e+06

### LPGNDN(N)STATL Fuc 3 Hex 3 HexNAc 4 Neu5Ac 1

**Mass:**  3129.2971

(6.734e-06)

**Charge States:** 3

**Start Position**: 19

**End Position**: 30

| Profile Score | Tandem Score | TDA q-value | Volume | Spectrum Matches |
| --- | --- | --- | --- | --- |
| 10.0775 | 105.5603 | 0.0000 | 4.7296e+07 | 1 |

**Adduct Signal Fractions**

- **Unmodified**: 4.7296e+07

### LPGNDN(N)STATL Fuc 3 Hex 3 HexNAc 5 Neu5Ac 2

**Mass:**  3623.4618

(3.317e-06)

**Charge States:** 3

**Start Position**: 19

**End Position**: 30

| Profile Score | Tandem Score | TDA q-value | Volume | Spectrum Matches |
| --- | --- | --- | --- | --- |
| 11.0093 | 102.9761 | 0.0000 | 2.4607e+07 | 1 |

**Adduct Signal Fractions**

- **Unmodified**: 2.4607e+07

### ITPN(N)GSIPNDKPF Hex 8 HexNAc 2

**Mass:**  3101.2985

(2.306e-06)

**Charge States:** 3

**Start Position**: 298

**End Position**: 311

| Profile Score | Tandem Score | TDA q-value | Volume | Spectrum Matches |
| --- | --- | --- | --- | --- |
| -3.9796 | 102.6700 | 0.0000 | 6.4187e+06 | 2 |

**Adduct Signal Fractions**

- **Unmodified**: 6.4187e+06

### TIVKPGDILLIN(N)STGNL Hex 10 HexNAc 2

**Mass:**  3793.7034

(5.703e-07)

**Charge States:** 3

**Start Position**: 251

**End Position**: 268

| Profile Score | Tandem Score | TDA q-value | Volume | Spectrum Matches |
| --- | --- | --- | --- | --- |
| -19.8180 | 99.8947 | 0.0000 | 5.2210e+06 | 2 |

**Adduct Signal Fractions**

- **Unmodified**: 5.2210e+06

### LPGNDN(N)STATL Fuc 1 Hex 3 HexNAc 5 Neu5Ac 2

**Mass:**  3331.3460

(3.454e-06)

**Charge States:** 3

**Start Position**: 19

**End Position**: 30

| Profile Score | Tandem Score | TDA q-value | Volume | Spectrum Matches |
| --- | --- | --- | --- | --- |
| -19.3931 | 99.7331 | 0.0000 | 1.8714e+07 | 1 |

**Adduct Signal Fractions**

- **Unmodified**: 1.8714e+07

### SIRN(N)GTYDHDVY Fuc 1 Hex 5 HexNAc 4

**Mass:**  3207.2887

(4.411e-07)

**Charge States:** 3

**Start Position**: 496

**End Position**: 508

| Profile Score | Tandem Score | TDA q-value | Volume | Spectrum Matches |
| --- | --- | --- | --- | --- |
| -18.8099 | 95.2213 | 0.0000 | 1.6835e+06 | 1 |

**Adduct Signal Fractions**

- **Unmodified**: 1.6835e+06

### RSDAPIGTC(C)SSEC(C)ITPN(N)GSIPNDKPF Hex 8 HexNAc 2

**Mass:**  4521.8713

(2.294e-06)

**Charge States:** 3, 4

**Start Position**: 285

**End Position**: 311

| Profile Score | Tandem Score | TDA q-value | Volume | Spectrum Matches |
| --- | --- | --- | --- | --- |
| -3.8426 | 90.5051 | 0.0000 | 3.8570e+07 | 3 |

**Adduct Signal Fractions**

- **Unmodified**: 3.8570e+07

### TITNDQIEVTN(N)ATEL Hex 6 HexNAc 2

**Mass:**  3039.2968

(1.820e-06)

**Charge States:** 3

**Start Position**: 44

**End Position**: 59

| Profile Score | Tandem Score | TDA q-value | Volume | Spectrum Matches |
| --- | --- | --- | --- | --- |
| -17.8147 | 88.3722 | 0.0000 | 1.8934e+07 | 1 |

**Adduct Signal Fractions**

- **Unmodified**: 1.8934e+07

### IGSIRN(N)GTYDHDVY Fuc 1 Hex 5 HexNAc 4

**Mass:**  3377.3900

(-7.920e-07)

**Charge States:** 3

**Start Position**: 494

**End Position**: 508

| Profile Score | Tandem Score | TDA q-value | Volume | Spectrum Matches |
| --- | --- | --- | --- | --- |
| -18.5563 | 84.6764 | 0.0000 | 3.1026e+06 | 1 |

**Adduct Signal Fractions**

- **Unmodified**: 3.1026e+06

### SIRN(N)GTYDHDVY Fuc 2 Hex 5 HexNAc 4

**Mass:**  3353.3446

(-1.052e-07)

**Charge States:** 3

**Start Position**: 496

**End Position**: 508

| Profile Score | Tandem Score | TDA q-value | Volume | Spectrum Matches |
| --- | --- | --- | --- | --- |
| -20.4659 | 83.9782 | 0.0000 | 2.5457e+06 | 1 |

**Adduct Signal Fractions**

- **Unmodified**: 2.5457e+06

### RGSN(N)NSFF Fuc 1 Hex 5 HexNAc 5

**Mass:**  2899.1321

(-2.277e-06)

**Charge States:** 3

**Start Position**: 157

**End Position**: 165

| Profile Score | Tandem Score | TDA q-value | Volume | Spectrum Matches |
| --- | --- | --- | --- | --- |
| -19.4042 | 78.4365 | 0.0000 | 5.2146e+06 | 1 |

**Adduct Signal Fractions**

- **Unmodified**: 5.2146e+06

### KRGSN(N)N(D)SFF Hex 7 HexNAc 3 Neu5Ac 1

**Mass:**  3091.1955

(3.163e-04)

**Charge States:** 3

**Start Position**: 156

**End Position**: 165

| Profile Score | Tandem Score | TDA q-value | Volume | Spectrum Matches |
| --- | --- | --- | --- | --- |
| -20.8304 | 76.0285 | 0.0000 | 1.8246e+06 | 1 |

**Adduct Signal Fractions**

- **Unmodified**: 1.8246e+06

### RSDAPIGTC(C)SSEC(C)ITPN(N)GSIPNDKPF Hex 9 HexNAc 2

**Mass:**  4683.9230

(5.490e-07)

**Charge States:** 3, 4

**Start Position**: 285

**End Position**: 311

| Profile Score | Tandem Score | TDA q-value | Volume | Spectrum Matches |
| --- | --- | --- | --- | --- |
| -3.6233 | 74.2671 | 0.0000 | 2.9056e+07 | 3 |

**Adduct Signal Fractions**

- **Unmodified**: 2.9056e+07

### KRGSN(N)NSFF Fuc 2 Hex 5 HexNAc 5

**Mass:**  3173.2868

(-2.315e-06)

**Charge States:** 3, 4

**Start Position**: 156

**End Position**: 165

| Profile Score | Tandem Score | TDA q-value | Volume | Spectrum Matches |
| --- | --- | --- | --- | --- |
| -19.4852 | 72.4506 | 0.0000 | 6.6109e+06 | 2 |

**Adduct Signal Fractions**

- **Unmodified**: 6.6109e+06

### TIVKPGDILLIN(N)STGN(D)LIAPRGY Hex 9 HexNAc 2

**Mass:**  4289.9973

(2.306e-04)

**Charge States:** 3

**Start Position**: 251

**End Position**: 274

| Profile Score | Tandem Score | TDA q-value | Volume | Spectrum Matches |
| --- | --- | --- | --- | --- |
| 11.6458 | 69.1546 | 0.0000 | 1.3743e+07 | 10 |

**Adduct Signal Fractions**

- **Unmodified**: 1.3743e+07

### KRGSN(N)NSFF Fuc 1 Hex 5 HexNAc 5

**Mass:**  3027.2321

(3.705e-07)

**Charge States:** 3

**Start Position**: 156

**End Position**: 165

| Profile Score | Tandem Score | TDA q-value | Volume | Spectrum Matches |
| --- | --- | --- | --- | --- |
| -18.3033 | 65.0083 | 0.0000 | 3.9735e+07 | 1 |

**Adduct Signal Fractions**

- **Unmodified**: 3.9735e+07

### TIVKPGDILLIN(N)STGNLIAPRGY Hex 10 HexNAc 2

**Mass:**  4451.0632

(-8.524e-07)

**Charge States:** 3, 4

**Start Position**: 251

**End Position**: 274

| Profile Score | Tandem Score | TDA q-value | Volume | Spectrum Matches |
| --- | --- | --- | --- | --- |
| -3.8934 | 64.4302 | 0.0000 | 5.6223e+07 | 6 |

**Adduct Signal Fractions**

- **Unmodified**: 5.6223e+07

### RSDAPIGTC(C)SSEC(C)ITPN(N)GSIPNDKPF Hex 7 HexNAc 2

**Mass:**  4359.8220

(6.878e-07)

**Charge States:** 4

**Start Position**: 285

**End Position**: 311

| Profile Score | Tandem Score | TDA q-value | Volume | Spectrum Matches |
| --- | --- | --- | --- | --- |
| -4.7547 | 61.1208 | 0.0000 | 1.0624e+07 | 2 |

**Adduct Signal Fractions**

- **Unmodified**: 1.0624e+07

### INEGFN(N)W Fuc 2 Hex 8 HexNAc 4

**Mass:**  3279.2499

(4.808e-07)

**Charge States:** 3

**Start Position**: 137

**End Position**: 144

| Profile Score | Tandem Score | TDA q-value | Volume | Spectrum Matches |
| --- | --- | --- | --- | --- |
| -19.9102 | 58.9345 | 0.0000 | 1.7525e+07 | 1 |

**Adduct Signal Fractions**

- **Unmodified**: 1.7525e+07

### INEGFN(N)W Fuc 1 Hex 8 HexNAc 4

**Mass:**  3133.1922

(2.013e-06)

**Charge States:** 3

**Start Position**: 137

**End Position**: 144

| Profile Score | Tandem Score | TDA q-value | Volume | Spectrum Matches |
| --- | --- | --- | --- | --- |
| 11.9896 | 58.7037 | 0.0000 | 7.2392e+07 | 2 |

**Adduct Signal Fractions**

- **Unmodified**: 7.2392e+07

### LYESESKYPVLN(N)VTM(O)PNN(D)GKF Hex 8 HexNAc 2

**Mass:**  4148.7575

(2.390e-04)

**Charge States:** 4

**Start Position**: 170

**End Position**: 191

| Profile Score | Tandem Score | TDA q-value | Volume | Spectrum Matches |
| --- | --- | --- | --- | --- |
| -6.0798 | 58.4193 | 0.0000 | 1.4642e+07 | 3 |

**Adduct Signal Fractions**

- **Unmodified**: 1.4642e+07

### SEC(C)ITPN(N)GSIPNDKPF Hex 9 HexNAc 2

**Mass:**  3639.4539

(7.950e-08)

**Charge States:** 3

**Start Position**: 295

**End Position**: 311

| Profile Score | Tandem Score | TDA q-value | Volume | Spectrum Matches |
| --- | --- | --- | --- | --- |
| -19.6987 | 56.4423 | 0.0000 | 7.5474e+06 | 2 |

**Adduct Signal Fractions**

- **Unmodified**: 7.5474e+06

### LIN(N)STGNLIAPRGYF Hex 8 HexNAc 2

**Mass:**  3337.4632

(1.147e-06)

**Charge States:** 3

**Start Position**: 260

**End Position**: 275

| Profile Score | Tandem Score | TDA q-value | Volume | Spectrum Matches |
| --- | --- | --- | --- | --- |
| -19.7854 | 55.0407 | 0.0000 | 1.0575e+06 | 1 |

**Adduct Signal Fractions**

- **Unmodified**: 1.0575e+06

### TITNDQIEVTN(N)ATELVQSSSTGRIC(C)DSPHRIL Hex 6 HexNAc 2

**Mass:**  4933.2385

(3.773e-06)

**Charge States:** 4

**Start Position**: 44

**End Position**: 76

| Profile Score | Tandem Score | TDA q-value | Volume | Spectrum Matches |
| --- | --- | --- | --- | --- |
| -18.9993 | 53.4019 | 0.0000 | 2.4946e+06 | 1 |

**Adduct Signal Fractions**

- **Unmodified**: 2.4946e+06

### ESESKYPVLN(N)VTM(O)PNN(D)GKFDKLY Hex 9 HexNAc 2

**Mass:**  4553.9328

(2.171e-04)

**Charge States:** 4

**Start Position**: 172

**End Position**: 195

| Profile Score | Tandem Score | TDA q-value | Volume | Spectrum Matches |
| --- | --- | --- | --- | --- |
| -6.0675 | 51.9060 | 0.0000 | 3.1119e+07 | 3 |

**Adduct Signal Fractions**

- **Unmodified**: 3.1119e+07

### SSEC(C)ITPN(N)GSIPNDKPF Hex 8 HexNAc 2

**Mass:**  3564.4275

(-1.545e-06)

**Charge States:** 3

**Start Position**: 294

**End Position**: 311

| Profile Score | Tandem Score | TDA q-value | Volume | Spectrum Matches |
| --- | --- | --- | --- | --- |
| -20.2852 | 50.5217 | 0.0000 | 1.0057e+07 | 1 |

**Adduct Signal Fractions**

- **Unmodified**: 1.0057e+07

### LYESESKYPVLN(N)VTM(O)PN Hex 9 HexNAc 2

**Mass:**  3863.5991

(1.096e-06)

**Charge States:** 3, 4

**Start Position**: 170

**End Position**: 187

| Profile Score | Tandem Score | TDA q-value | Volume | Spectrum Matches |
| --- | --- | --- | --- | --- |
| -19.1227 | 48.3625 | 0.0000 | 5.8976e+06 | 1 |

**Adduct Signal Fractions**

- **Unmodified**: 5.8976e+06

### KIRTGKSSIM(O)RSDAPIGTC(C)SSEC(C)ITPN(N)GSIPNDKPF Hex 9 HexNAc 2

**Mass:**  5801.5499

(4.310e-07)

**Charge States:** 5, 6

**Start Position**: 275

**End Position**: 311

| Profile Score | Tandem Score | TDA q-value | Volume | Spectrum Matches |
| --- | --- | --- | --- | --- |
| -19.5022 | 47.3453 | 0.0000 | 2.7160e+07 | 2 |

**Adduct Signal Fractions**

- **Unmodified**: 2.7160e+07

### RSDAPIGTC(C)SSEC(C)ITPN(N)GSIPN(D)DKPF Hex 8 HexNAc 2

**Mass:**  4522.8630

(2.203e-04)

**Charge States:** 3, 4

**Start Position**: 285

**End Position**: 311

| Profile Score | Tandem Score | TDA q-value | Volume | Spectrum Matches |
| --- | --- | --- | --- | --- |
| -5.9336 | 47.1216 | 0.0000 | 3.6907e+07 | 2 |

**Adduct Signal Fractions**

- **Unmodified**: 3.6907e+07

### TIVKPGDILLIN(N) Hex 8 HexNAc 2

**Mass:**  2997.3675

(2.043e-08)

**Charge States:** 3

**Start Position**: 251

**End Position**: 263

| Profile Score | Tandem Score | TDA q-value | Volume | Spectrum Matches |
| --- | --- | --- | --- | --- |
| -6.1627 | 44.7201 | 0.0000 | 1.8447e+06 | 1 |

**Adduct Signal Fractions**

- **Unmodified**: 1.8447e+06

### ESESKYPVLN(N)VTM(O)PNN(D)GKFDKLY Hex 8 HexNAc 2

**Mass:**  4391.8806

(2.253e-04)

**Charge States:** 4

**Start Position**: 172

**End Position**: 195

| Profile Score | Tandem Score | TDA q-value | Volume | Spectrum Matches |
| --- | --- | --- | --- | --- |
| -5.8678 | 43.1650 | 0.0000 | 1.9746e+07 | 3 |

**Adduct Signal Fractions**

- **Unmodified**: 1.9746e+07

### TITNDQIEVTN(N)ATEL Hex 7 HexNAc 2

**Mass:**  3201.3491

(2.435e-06)

**Charge States:** 3

**Start Position**: 44

**End Position**: 59

| Profile Score | Tandem Score | TDA q-value | Volume | Spectrum Matches |
| --- | --- | --- | --- | --- |
| -18.2399 | 42.3296 | 0.0000 | 6.5538e+06 | 1 |

**Adduct Signal Fractions**

- **Unmodified**: 6.5538e+06

### RSDAPIGTC(C)SSEC(C)ITPN(N)GSIPNDKPF Fuc 2 Hex 3 HexNAc 4 Neu5Ac 1

**Mass:**  4700.9499

(-4.801e-06)

**Charge States:** 4

**Start Position**: 285

**End Position**: 311

| Profile Score | Tandem Score | TDA q-value | Volume | Spectrum Matches |
| --- | --- | --- | --- | --- |
| -19.1795 | 42.3269 | 0.0000 | 2.5005e+06 | 1 |

**Adduct Signal Fractions**

- **Unmodified**: 2.5005e+06

### LYESESKYPVLN(N)VTM(O)PNN(D)GKF Hex 9 HexNAc 2

**Mass:**  4310.8107

(2.292e-04)

**Charge States:** 3, 4

**Start Position**: 170

**End Position**: 191

| Profile Score | Tandem Score | TDA q-value | Volume | Spectrum Matches |
| --- | --- | --- | --- | --- |
| -6.0247 | 42.2071 | 0.0000 | 1.9179e+07 | 3 |

**Adduct Signal Fractions**

- **Unmodified**: 1.9179e+07

### ITPN(N)GSIPNDKPF Fuc 4 Hex 6 HexNAc 8

**Mass:**  4579.8649

(-4.258e-06)

**Charge States:** 3, 4

**Start Position**: 298

**End Position**: 311

| Profile Score | Tandem Score | TDA q-value | Volume | Spectrum Matches |
| --- | --- | --- | --- | --- |
| -2.6076 | 42.1383 | 0.0000 | 1.7360e+07 | 3 |

**Adduct Signal Fractions**

- **Unmodified**: 1.7360e+07

### LYESESKYPVLN(N)VTM(O)PN Hex 8 HexNAc 2

**Mass:**  3701.5479

(2.274e-06)

**Charge States:** 3

**Start Position**: 170

**End Position**: 187

| Profile Score | Tandem Score | TDA q-value | Volume | Spectrum Matches |
| --- | --- | --- | --- | --- |
| -18.3113 | 40.9944 | 0.0000 | 3.8708e+06 | 3 |

**Adduct Signal Fractions**

- **Unmodified**: 3.8708e+06

### INEGFN(N)WTGVTQ Fuc 1 Hex 5 HexNAc 5

**Mass:**  3336.3521

(-8.547e-07)

**Charge States:** 3, 4

**Start Position**: 137

**End Position**: 149

| Profile Score | Tandem Score | TDA q-value | Volume | Spectrum Matches |
| --- | --- | --- | --- | --- |
| -19.9335 | 40.2854 | 0.0000 | 2.1077e+06 | 2 |

**Adduct Signal Fractions**

- **Unmodified**: 2.1077e+06

### TITNDQIEVTN(N)ATEL Hex 5 HexNAc 2

**Mass:**  2877.2435

(1.793e-06)

**Charge States:** 3

**Start Position**: 44

**End Position**: 59

| Profile Score | Tandem Score | TDA q-value | Volume | Spectrum Matches |
| --- | --- | --- | --- | --- |
| -17.2869 | 40.0464 | 0.0000 | 7.2071e+06 | 1 |

**Adduct Signal Fractions**

- **Unmodified**: 7.2071e+06

### ITPN(N)GSIPNDKPF Hex 7 HexNAc 2

**Mass:**  2939.2498

(2.727e-06)

**Charge States:** 3

**Start Position**: 298

**End Position**: 311

| Profile Score | Tandem Score | TDA q-value | Volume | Spectrum Matches |
| --- | --- | --- | --- | --- |
| -21.1410 | 39.9137 | 0.0000 | 1.0942e+06 | 1 |

**Adduct Signal Fractions**

- **Unmodified**: 1.0942e+06

### LPGNDN(N)STATL Hex 9 HexNAc 4 Neu5Ac 1

**Mass:**  3663.4480

(8.051e-06)

**Charge States:** 3

**Start Position**: 19

**End Position**: 30

| Profile Score | Tandem Score | TDA q-value | Volume | Spectrum Matches |
| --- | --- | --- | --- | --- |
| -20.7240 | 39.3869 | 0.0000 | 1.8770e+07 | 2 |

**Adduct Signal Fractions**

- **Unmodified**: 1.8770e+07

### ITPN(N)GSIPNDKPF Fuc 4 Hex 7 HexNAc 7

**Mass:**  4538.8952

(5.266e-06)

**Charge States:** 4

**Start Position**: 298

**End Position**: 311

| Profile Score | Tandem Score | TDA q-value | Volume | Spectrum Matches |
| --- | --- | --- | --- | --- |
| -18.8371 | 37.6034 | 0.0000 | 3.4093e+06 | 1 |

**Adduct Signal Fractions**

- **Unmodified**: 3.4093e+06

### TIVKPGDILLIN(N)STGN(D)LIAPR Hex 9 HexNAc 2

**Mass:**  4069.9150

(2.458e-04)

**Charge States:** 3, 4

**Start Position**: 251

**End Position**: 272

| Profile Score | Tandem Score | TDA q-value | Volume | Spectrum Matches |
| --- | --- | --- | --- | --- |
| -20.2348 | 36.3332 | 0.0000 | 3.2011e+06 | 3 |

**Adduct Signal Fractions**

- **Unmodified**: 3.2011e+06

### LPGNDN(N)STATL Fuc 2 Hex 8 HexNAc 3

**Mass:**  3299.3102

(7.564e-06)

**Charge States:** 3

**Start Position**: 19

**End Position**: 30

| Profile Score | Tandem Score | TDA q-value | Volume | Spectrum Matches |
| --- | --- | --- | --- | --- |
| 12.0245 | 35.9681 | 0.0000 | 4.8809e+07 | 3 |

**Adduct Signal Fractions**

- **Unmodified**: 4.8809e+07

### SEC(C)ITPN(N)GSIPN(D)DKPF Hex 9 HexNAc 2

**Mass:**  3640.4383

(2.705e-04)

**Charge States:** 3

**Start Position**: 295

**End Position**: 311

| Profile Score | Tandem Score | TDA q-value | Volume | Spectrum Matches |
| --- | --- | --- | --- | --- |
| -20.8379 | 34.9424 | 0.0000 | 7.3165e+06 | 1 |

**Adduct Signal Fractions**

- **Unmodified**: 7.3165e+06

### KIRTGKSSIM(O)RSDAPIGTC(C)SSEC(C)ITPN(N)GSIPNDKPF Hex 8 HexNAc 2

**Mass:**  5642.5051

(5.338e-04)

**Start Position**: 275

**End Position**: 311

| Profile Score | Tandem Score | TDA q-value | Volume | Spectrum Matches |
| --- | --- | --- | --- | --- |
| 0.0000 | 33.5929 | 0.0000 | 0.0000e+00 | 1 |

Adduct:

No Chromatogram Found

### KIRTGKSSIM(O)RSDAPIGTC(C)SSEC(C)ITPN(N)GSIPNDKPF Hex 8 HexNAc 2

**Mass:**  5639.4991

(1.894e-06)

**Charge States:** 5, 6

**Start Position**: 275

**End Position**: 311

| Profile Score | Tandem Score | TDA q-value | Volume | Spectrum Matches |
| --- | --- | --- | --- | --- |
| -19.2468 | 33.5257 | 0.0000 | 3.7121e+07 | 1 |

**Adduct Signal Fractions**

- **Unmodified**: 3.7121e+07

### TIVKPGDILLIN(N)STGNLIAPRGY Fuc 1 Hex 3 HexNAc 5 Neu5Ac 1

**Mass:**  4363.0573

(-6.591e-06)

**Charge States:** 4

**Start Position**: 251

**End Position**: 274

| Profile Score | Tandem Score | TDA q-value | Volume | Spectrum Matches |
| --- | --- | --- | --- | --- |
| -4.7362 | 32.8423 | 0.0000 | 2.2287e+07 | 2 |

**Adduct Signal Fractions**

- **Unmodified**: 2.2287e+07

### SSEC(C)ITPN(N)GSIPN(D)DKPF Hex 9 HexNAc 2

**Mass:**  3727.4650

(2.628e-04)

**Charge States:** 3

**Start Position**: 294

**End Position**: 311

| Profile Score | Tandem Score | TDA q-value | Volume | Spectrum Matches |
| --- | --- | --- | --- | --- |
| -21.4008 | 32.7570 | 0.0000 | 5.3579e+06 | 1 |

**Adduct Signal Fractions**

- **Unmodified**: 5.3579e+06

### RSDAPIGTC(C)SSEC(C)ITPN(N)GSIPN(D)DKPF Hex 9 HexNAc 2

**Mass:**  4684.9103

(2.107e-04)

**Charge States:** 3, 4

**Start Position**: 285

**End Position**: 311

| Profile Score | Tandem Score | TDA q-value | Volume | Spectrum Matches |
| --- | --- | --- | --- | --- |
| -6.0995 | 32.4928 | 0.0000 | 2.8094e+07 | 3 |

**Adduct Signal Fractions**

- **Unmodified**: 2.8094e+07

### TIVKPGDILLIN(N)STGNLIAPR Fuc 1 Hex 3 HexNAc 5 Neu5Ac 1

**Mass:**  4142.9714

(-6.383e-06)

**Charge States:** 4

**Start Position**: 251

**End Position**: 272

| Profile Score | Tandem Score | TDA q-value | Volume | Spectrum Matches |
| --- | --- | --- | --- | --- |
| -17.7891 | 32.0997 | 0.0000 | 8.9381e+06 | 2 |

**Adduct Signal Fractions**

- **Unmodified**: 8.9381e+06

### TITNDQIEVTN(N)ATEL Hex 6 HexNAc 3

**Mass:**  3242.3769

(1.718e-06)

**Charge States:** 3

**Start Position**: 44

**End Position**: 59

| Profile Score | Tandem Score | TDA q-value | Volume | Spectrum Matches |
| --- | --- | --- | --- | --- |
| -19.8768 | 32.0685 | 0.0000 | 4.7272e+06 | 1 |

**Adduct Signal Fractions**

- **Unmodified**: 4.7272e+06

### ITPN(N)GSIPNDKPF Fuc 2 Hex 6 HexNAc 8 Neu5Ac 1

**Mass:**  4578.8933

(3.397e-06)

**Charge States:** 4

**Start Position**: 298

**End Position**: 311

| Profile Score | Tandem Score | TDA q-value | Volume | Spectrum Matches |
| --- | --- | --- | --- | --- |
| -19.2633 | 31.8404 | 0.0000 | 6.7065e+06 | 1 |

**Adduct Signal Fractions**

- **Unmodified**: 6.7065e+06

### KIRTGKSSIM(O)RSDAPIGTC(C)SSEC(C)ITPN(N)GSIPN(D)DKPF Hex 9 HexNAc 2

**Mass:**  5802.5350

(1.703e-04)

**Charge States:** 5, 6

**Start Position**: 275

**End Position**: 311

| Profile Score | Tandem Score | TDA q-value | Volume | Spectrum Matches |
| --- | --- | --- | --- | --- |
| -20.7782 | 31.7523 | 0.0000 | 1.5231e+07 | 2 |

**Adduct Signal Fractions**

- **Unmodified**: 1.5231e+07

### LPGNDN(N)STATL Hex 10 HexNAc 4 Neu5Ac 1

**Mass:**  3825.5139

(9.745e-06)

**Charge States:** 3, 4

**Start Position**: 19

**End Position**: 30

| Profile Score | Tandem Score | TDA q-value | Volume | Spectrum Matches |
| --- | --- | --- | --- | --- |
| 9.6990 | 30.9394 | 0.0000 | 4.4372e+07 | 5 |

**Adduct Signal Fractions**

- **Unmodified**: 4.4372e+07

### LIN(N)STGNLIAPR Fuc 2 Hex 3 HexNAc 4 Neu5Ac 1

**Mass:**  3149.3871

(-7.945e-06)

**Charge States:** 3

**Start Position**: 260

**End Position**: 272

| Profile Score | Tandem Score | TDA q-value | Volume | Spectrum Matches |
| --- | --- | --- | --- | --- |
| -17.2910 | 30.8443 | 0.0000 | 2.0388e+06 | 1 |

**Adduct Signal Fractions**

- **Unmodified**: 2.0388e+06

### RSDAPIGTC(C)SSEC(C)ITPN(N)GSIPN(D)DKPF Hex 7 HexNAc 2

**Mass:**  4360.8078

(2.279e-04)

**Charge States:** 3, 4

**Start Position**: 285

**End Position**: 311

| Profile Score | Tandem Score | TDA q-value | Volume | Spectrum Matches |
| --- | --- | --- | --- | --- |
| -6.2109 | 30.8337 | 0.0000 | 1.0869e+07 | 2 |

**Adduct Signal Fractions**

- **Unmodified**: 1.0869e+07

### ITPN(N)GSIPNDKPF Fuc 4 Hex 5 HexNAc 8

**Mass:**  4417.8117

(-6.477e-06)

**Charge States:** 3, 4

**Start Position**: 298

**End Position**: 311

| Profile Score | Tandem Score | TDA q-value | Volume | Spectrum Matches |
| --- | --- | --- | --- | --- |
| -4.0984 | 29.6390 | 0.0065 | 1.7093e+07 | 2 |

**Adduct Signal Fractions**

- **Unmodified**: 1.7093e+07

### LPGNDN(N)STATL Fuc 1 Hex 8 HexNAc 2

**Mass:**  2950.1720

(2.925e-06)

**Charge States:** 3

**Start Position**: 19

**End Position**: 30

| Profile Score | Tandem Score | TDA q-value | Volume | Spectrum Matches |
| --- | --- | --- | --- | --- |
| 12.4738 | 29.2094 | 0.0065 | 2.7120e+08 | 7 |

**Adduct Signal Fractions**

- **Unmodified**: 2.7120e+08

### ESESKYPVLN(N)VTMPNNGKFDKLY Hex 3 HexNAc 6 Neu5Ac 1

**Mass:**  4668.0358

(-2.147e-06)

**Charge States:** 4, 5

**Start Position**: 172

**End Position**: 195

| Profile Score | Tandem Score | TDA q-value | Volume | Spectrum Matches |
| --- | --- | --- | --- | --- |
| 15.2631 | 28.6875 | 0.0065 | 1.8927e+08 | 6 |

**Adduct Signal Fractions**

- **Unmodified**: 1.8927e+08

### ESESKYPVLN(N)VTM(O)PNN(D)GKFDKLY Fuc 2 Hex 8 HexNAc 3

**Mass:**  4887.1037

(2.071e-04)

**Charge States:** 4, 5

**Start Position**: 172

**End Position**: 195

| Profile Score | Tandem Score | TDA q-value | Volume | Spectrum Matches |
| --- | --- | --- | --- | --- |
| 11.1875 | 28.2009 | 0.0065 | 1.1766e+08 | 8 |

**Adduct Signal Fractions**

- **Unmodified**: 1.1766e+08

### TIVKPGDILLIN(N)STGNLIAPR Fuc 2 Hex 4 HexNAc 4 Neu5Ac 1

**Mass:**  4248.0075

(-5.142e-06)

**Charge States:** 4

**Start Position**: 251

**End Position**: 272

| Profile Score | Tandem Score | TDA q-value | Volume | Spectrum Matches |
| --- | --- | --- | --- | --- |
| -19.8392 | 28.1889 | 0.0127 | 1.2587e+06 | 1 |

**Adduct Signal Fractions**

- **Unmodified**: 1.2587e+06

### SSEC(C)ITPN(N)GSIPN(D)DKPF Hex 8 HexNAc 2

**Mass:**  3565.4123

(2.748e-04)

**Charge States:** 3

**Start Position**: 294

**End Position**: 311

| Profile Score | Tandem Score | TDA q-value | Volume | Spectrum Matches |
| --- | --- | --- | --- | --- |
| -21.4642 | 28.0716 | 0.0127 | 8.3046e+06 | 1 |

**Adduct Signal Fractions**

- **Unmodified**: 8.3046e+06

### IN(N)STGNLIAPRGYF Fuc 2 Hex 3 HexNAc 4 Neu5Ac 1

**Mass:**  3405.4741

(5.855e-04)

**Start Position**: 261

**End Position**: 275

| Profile Score | Tandem Score | TDA q-value | Volume | Spectrum Matches |
| --- | --- | --- | --- | --- |
| 0.0000 | 27.8673 | 0.0127 | 0.0000e+00 | 1 |

Adduct:

No Chromatogram Found

### TIVKPGDILLIN(N)STGNLIAPRGY Fuc 2 Hex 3 HexNAc 4 Neu5Ac 1

**Mass:**  4309.0481

(6.936e-04)

**Start Position**: 251

**End Position**: 274

| Profile Score | Tandem Score | TDA q-value | Volume | Spectrum Matches |
| --- | --- | --- | --- | --- |
| 0.0000 | 27.6826 | 0.0186 | 0.0000e+00 | 1 |

Adduct:

No Chromatogram Found

### TIVKPGDILLIN(N)STGN Hex 10 HexNAc 2

**Mass:**  3680.6164

(-2.561e-07)

**Charge States:** 3

**Start Position**: 251

**End Position**: 267

| Profile Score | Tandem Score | TDA q-value | Volume | Spectrum Matches |
| --- | --- | --- | --- | --- |
| -20.0567 | 27.3140 | 0.0186 | 1.2627e+06 | 1 |

**Adduct Signal Fractions**

- **Unmodified**: 1.2627e+06

### ITPN(N)GSIPNDKPF Fuc 5 Hex 6 HexNAc 6 Neu5Ac 1

**Mass:**  4610.9362

(9.510e-06)

**Charge States:** 4

**Start Position**: 298

**End Position**: 311

| Profile Score | Tandem Score | TDA q-value | Volume | Spectrum Matches |
| --- | --- | --- | --- | --- |
| -21.0245 | 27.1965 | 0.0238 | 5.8004e+05 | 1 |

**Adduct Signal Fractions**

- **Unmodified**: 5.8004e+05

### RSDAPIGTC(C)SSEC(C)ITPN(N)GSIPN(D)DKPF Fuc 2 Hex 3 HexNAc 4 Neu5Ac 1

**Mass:**  4701.9411

(2.060e-04)

**Charge States:** 4

**Start Position**: 285

**End Position**: 311

| Profile Score | Tandem Score | TDA q-value | Volume | Spectrum Matches |
| --- | --- | --- | --- | --- |
| -20.8401 | 27.0443 | 0.0238 | 2.8023e+06 | 1 |

**Adduct Signal Fractions**

- **Unmodified**: 2.8023e+06

### VKTITNDQIEVTN(N)ATEL Hex 6 HexNAc 2

**Mass:**  3266.4578

(1.014e-06)

**Charge States:** 3

**Start Position**: 42

**End Position**: 59

| Profile Score | Tandem Score | TDA q-value | Volume | Spectrum Matches |
| --- | --- | --- | --- | --- |
| -20.3350 | 26.6584 | 0.0238 | 1.3995e+06 | 1 |

**Adduct Signal Fractions**

- **Unmodified**: 1.3995e+06

### TIVKPGDILLIN(N) Fuc 2 Hex 4 HexNAc 4 Neu5Ac 1

**Mass:**  3338.4981

(-8.434e-06)

**Charge States:** 3

**Start Position**: 251

**End Position**: 263

| Profile Score | Tandem Score | TDA q-value | Volume | Spectrum Matches |
| --- | --- | --- | --- | --- |
| -19.5458 | 26.6243 | 0.0238 | 3.5930e+05 | 1 |

**Adduct Signal Fractions**

- **Unmodified**: 3.5930e+05

### ITPN(N)GSIPNDKPF Fuc 3 Hex 10 HexNAc 6

**Mass:**  4676.8776

(2.145e-04)

**Charge States:** 4

**Start Position**: 298

**End Position**: 311

| Profile Score | Tandem Score | TDA q-value | Volume | Spectrum Matches |
| --- | --- | --- | --- | --- |
| 12.3674 | 26.1722 | 0.0238 | 9.4200e+05 | 1 |

**Adduct Signal Fractions**

- **Unmodified**: 9.4200e+05

### ITPN(N)GSIPNDKPF Fuc 3 Hex 5 HexNAc 9

**Mass:**  4474.8361

(-6.766e-06)

**Charge States:** 3, 4

**Start Position**: 298

**End Position**: 311

| Profile Score | Tandem Score | TDA q-value | Volume | Spectrum Matches |
| --- | --- | --- | --- | --- |
| -20.2572 | 25.9479 | 0.0279 | 3.8317e+06 | 2 |

**Adduct Signal Fractions**

- **Unmodified**: 3.8317e+06

### TIVKPGDILLIN(N)STGNLIAPRGY Hex 5 HexNAc 6

**Mass:**  4453.1052

(-2.065e-06)

**Charge States:** 4

**Start Position**: 251

**End Position**: 274

| Profile Score | Tandem Score | TDA q-value | Volume | Spectrum Matches |
| --- | --- | --- | --- | --- |
| 15.3288 | 25.8885 | 0.0279 | 5.6078e+07 | 3 |

**Adduct Signal Fractions**

- **Unmodified**: 5.6078e+07

### TIVKPGDILLIN(N)STGN Hex 8 HexNAc 2

**Mass:**  3356.5120

(1.223e-07)

**Charge States:** 3

**Start Position**: 251

**End Position**: 267

| Profile Score | Tandem Score | TDA q-value | Volume | Spectrum Matches |
| --- | --- | --- | --- | --- |
| -19.9471 | 25.8526 | 0.0279 | 8.6152e+05 | 1 |

**Adduct Signal Fractions**

- **Unmodified**: 8.6152e+05

### ITPN(N)GSIPNDKPF Fuc 2 Hex 7 HexNAc 8 Neu5Ac 1

**Mass:**  4740.9401

(1.713e-06)

**Charge States:** 4

**Start Position**: 298

**End Position**: 311

| Profile Score | Tandem Score | TDA q-value | Volume | Spectrum Matches |
| --- | --- | --- | --- | --- |
| -5.2436 | 25.6642 | 0.0279 | 6.4213e+06 | 1 |

**Adduct Signal Fractions**

- **Unmodified**: 6.4213e+06

### KIRTGKSSIM(O)RSDAPIGTC(C)SSEC(C)ITPN(N)GSIPNDKPF Hex 7 HexNAc 2

**Mass:**  5477.4423

(4.403e-08)

**Charge States:** 5

**Start Position**: 275

**End Position**: 311

| Profile Score | Tandem Score | TDA q-value | Volume | Spectrum Matches |
| --- | --- | --- | --- | --- |
| -19.6210 | 25.0283 | 0.0279 | 5.5488e+06 | 1 |

**Adduct Signal Fractions**

- **Unmodified**: 5.5488e+06

### KIRTGKSSIM(O)RSDAPIGTC(C)SSEC(C)ITPN(N)GSIPN(D)DKPF Hex 8 HexNAc 2

**Mass:**  5640.4824

(1.758e-04)

**Charge States:** 5

**Start Position**: 275

**End Position**: 311

| Profile Score | Tandem Score | TDA q-value | Volume | Spectrum Matches |
| --- | --- | --- | --- | --- |
| -6.5073 | 24.5778 | 0.0279 | 2.3759e+07 | 3 |

**Adduct Signal Fractions**

- **Unmodified**: 2.3759e+07

### SEC(C)ITPN(N)GSIPN(D)DKPF Hex 8 HexNAc 2

**Mass:**  3478.3987

(2.895e-04)

**Charge States:** 3

**Start Position**: 295

**End Position**: 311

| Profile Score | Tandem Score | TDA q-value | Volume | Spectrum Matches |
| --- | --- | --- | --- | --- |
| -21.2276 | 24.4646 | 0.0279 | 1.4616e+07 | 3 |

**Adduct Signal Fractions**

- **Unmodified**: 1.4616e+07

### TIVKPGDILLIN(N)STGNLIAPR Hex 5 HexNAc 7

**Mass:**  4436.0822

(-6.086e-06)

**Charge States:** 3, 4, 5

**Start Position**: 251

**End Position**: 272

| Profile Score | Tandem Score | TDA q-value | Volume | Spectrum Matches |
| --- | --- | --- | --- | --- |
| 17.3512 | 23.7990 | 0.0437 | 3.6258e+08 | 11 |

**Adduct Signal Fractions**

- **Unmodified**: 3.6258e+08

### LYESESKYPVLN(N)VTM(O)PNN(D)GKF Fuc 3 Hex 7 HexNAc 4

**Mass:**  4831.0646

(2.101e-04)

**Charge States:** 4, 5

**Start Position**: 170

**End Position**: 191

| Profile Score | Tandem Score | TDA q-value | Volume | Spectrum Matches |
| --- | --- | --- | --- | --- |
| 11.1861 | 23.7407 | 0.0437 | 7.6988e+07 | 9 |

**Adduct Signal Fractions**

- **Unmodified**: 7.6988e+07

### SEC(C)ITPN(N)GSIPN(D)DKPF Hex 7 HexNAc 2

**Mass:**  3316.3299

(2.942e-04)

**Charge States:** 3

**Start Position**: 295

**End Position**: 311

| Profile Score | Tandem Score | TDA q-value | Volume | Spectrum Matches |
| --- | --- | --- | --- | --- |
| -19.9128 | 23.5199 | 0.0437 | 1.0069e+07 | 1 |

**Adduct Signal Fractions**

- **Unmodified**: 1.0069e+07

### SEC(C)ITPN(N)GSIPNDKPF Fuc 5 Hex 6 HexNAc 6

**Mass:**  4695.9265

(5.173e-06)

**Charge States:** 4

**Start Position**: 295

**End Position**: 311

| Profile Score | Tandem Score | TDA q-value | Volume | Spectrum Matches |
| --- | --- | --- | --- | --- |
| -19.2333 | 23.2816 | 0.0489 | 2.3839e+06 | 1 |

**Adduct Signal Fractions**

- **Unmodified**: 2.3839e+06

### iav|ADJ41819|A/Phil/2-MA/1982\_NA

**>iav|ADJ41819|A/Phil/2-MA/1982\_NA**  
 `MNPNQKIITIGSVSLTIATICFLMQIAILVTTVTLHFKQYECSSPPNNQVVPCEPIIIERNITEIVYLTNTTIEKEICPK  
LVEYRNWSKPQCKITGFAPFSKDNSIRLSAGGDIWVTREPYVSCDPGKCYQFALGQGTTLDNKHSNDTIHDRTPYRTLLM  
NELGVPFHLGTRQVCIAWSSSSCHDGKAWLHVCITGYDKNATASFIYDGRLVDSIGSWSKNILRTQESECVCINGTCTVV  
MTDGSASERADTKILFIEEGKIVHISPLSGSAQHVEECSCYPRYPGVRCVCRDNWKGSNRPVVDINVKDYSIVSSYVCSG  
LVGDTPRKNDRSSSSYCRNPNNEKGNHGVKGWAFDDGNDVWMGRTISEESRSGYETFKVIGGWSTPNSKLQINRQVIVDS  
GNRSGYSGIFSVEGKSCINRCFYVELIRGREQETRVWWTSNSIVVFCGTSGTYGTGSWPDGADINLMPI`

| Site | Type | Count |
| --- | --- | --- |
| 402 | N-linked | 6 |
| 200 | N-linked | 1 |
| 86 | N-linked | 2 |
| 146 | N-linked | 6 |

| Mass | Sequence | MS_2_ Score | `q`-value | MS_1_ Score | Signal | Protein Coordinates | Tandem Scans | Start Time | Apex Time | End Time |
| --- | --- | --- | --- | --- | --- | --- | --- | --- | --- | --- |
| 2554.9754 | DKN(N)ATASF Hex 8 HexNAc 2 | 184.643 | 0.0 | 0.000 | 0.000e+00 | 197 - 205 | 2 | - | - | - |
| 3183.4455 | QINRQVIVDSGN(N)RSGY Hex 6 HexNAc 2 | 154.355 | 0.0 | -3.362 | 1.202e+07 | 390 - 406 | 8 | 22.10 | 31.51 | 31.68 |
| 3800.6242 | RN(N)WSKPQC(C)KITGFAPF Hex 9 HexNAc 2 | 152.900 | 0.0 | -18.042 | 2.782e+06 | 84 - 100 | 1 | 38.56 | 38.56 | 38.56 |
| 3638.5693 | RN(N)WSKPQC(C)KITGFAPF Hex 8 HexNAc 2 | 152.458 | 0.0 | -17.585 | 6.323e+06 | 84 - 100 | 1 | 38.56 | 38.73 | 38.73 |
| 3910.7846 | STPNSKLQINRQVIVDSGN(N)RSGY Hex 6 HexNAc 2 | 129.664 | 0.0 | -19.857 | 4.145e+06 | 383 - 406 | 1 | 29.66 | 29.83 | 29.83 |
| 2829.2134 | RQVIVDSGN(N)RSGY Hex 6 HexNAc 2 | 108.549 | 0.0 | 0.000 | 0.000e+00 | 393 - 406 | 1 | - | - | - |
| 3993.5588 | DN(D)KHSN(N)DTIHDRTPY Fuc 1 Hex 7 HexNAc 3 Neu5Ac 1 | 85.987 | 0.0 | -21.176 | 6.039e+06 | 140 - 155 | 1 | 14.51 | 14.51 | 14.51 |
| 3345.4449 | QINRQVIVDSGN(N)RSGY Hex 7 HexNAc 2 | 62.749 | 0.0 | -20.413 | 3.685e+06 | 390 - 406 | 2 | 23.26 | 23.26 | 23.26 |
| 4442.8462 | ALGQGTTLDNKHSN(N)DTIHDRTPY Fuc 1 Hex 7 HexNAc 3 | 61.257 | 0.0 | -20.129 | 6.958e+06 | 132 - 155 | 1 | 24.10 | 24.10 | 24.27 |
| 3507.5006 | QINRQVIVDSGN(N)RSGY Hex 8 HexNAc 2 | 45.796 | 0.0 | -21.200 | 1.744e+06 | 390 - 406 | 1 | 23.09 | 23.09 | 23.09 |
| 3847.4967 | DN(D)KHSN(N)DTIHDRTPY Hex 7 HexNAc 3 Neu5Ac 1 | 37.445 | 0.0 | -20.993 | 8.556e+06 | 140 - 155 | 1 | 14.63 | 14.63 | 14.80 |
| 3702.4489 | DN(D)KHSN(N)DTIHDRTPY Fuc 1 Hex 7 HexNAc 3 | 37.044 | 0.0 | 0.000 | 0.000e+00 | 140 - 155 | 1 | - | - | - |
| 4588.9029 | ALGQGTTLDNKHSN(N)DTIHDRTPY Fuc 2 Hex 7 HexNAc 3 | 34.709 | 0.0 | -20.661 | 3.902e+06 | 132 - 155 | 1 | 23.93 | 24.10 | 24.10 |
| 4072.8310 | STPNSKLQINRQVIVDSGN(N)RSGY Hex 7 HexNAc 2 | 31.327 | 0.0 | -19.523 | 1.656e+06 | 383 - 406 | 1 | 29.66 | 29.66 | 29.66 |
| 4526.9521 | ALGQGTTLDNKHSN(N)DTIHDRTPY Fuc 1 Hex 5 HexNAc 5 | 26.492 | 0.024 | 0.000 | 0.000e+00 | 132 - 155 | 1 | - | - | - |

### DKN(N)ATASF Hex 8 HexNAc 2

**Mass:**  2554.9754

(-1.421e-06)

**Start Position**: 198

**End Position**: 206

| Profile Score | Tandem Score | TDA q-value | Volume | Spectrum Matches |
| --- | --- | --- | --- | --- |
| 0.0000 | 184.6432 | 0.0000 | 0.0000e+00 | 2 |

Adduct:

No Chromatogram Found

### QINRQVIVDSGN(N)RSGY Hex 6 HexNAc 2

**Mass:**  3183.3957

(1.482e-07)

**Charge States:** 3

**Start Position**: 391

**End Position**: 407

| Profile Score | Tandem Score | TDA q-value | Volume | Spectrum Matches |
| --- | --- | --- | --- | --- |
| -3.3616 | 154.3552 | 0.0000 | 1.2017e+07 | 8 |

**Adduct Signal Fractions**

- **Unmodified**: 1.2017e+07

### RN(N)WSKPQC(C)KITGFAPF Hex 9 HexNAc 2

**Mass:**  3800.6242

(3.246e-06)

**Charge States:** 4

**Start Position**: 85

**End Position**: 101

| Profile Score | Tandem Score | TDA q-value | Volume | Spectrum Matches |
| --- | --- | --- | --- | --- |
| -18.0421 | 152.9000 | 0.0000 | 2.7825e+06 | 1 |

**Adduct Signal Fractions**

- **Unmodified**: 2.7825e+06

### RN(N)WSKPQC(C)KITGFAPF Hex 8 HexNAc 2

**Mass:**  3638.5688

(2.638e-06)

**Charge States:** 4

**Start Position**: 85

**End Position**: 101

| Profile Score | Tandem Score | TDA q-value | Volume | Spectrum Matches |
| --- | --- | --- | --- | --- |
| -17.5854 | 152.4581 | 0.0000 | 6.3235e+06 | 1 |

**Adduct Signal Fractions**

- **Unmodified**: 6.3235e+06

### STPNSKLQINRQVIVDSGN(N)RSGY Hex 6 HexNAc 2

**Mass:**  3910.7849

(8.314e-07)

**Charge States:** 4

**Start Position**: 384

**End Position**: 407

| Profile Score | Tandem Score | TDA q-value | Volume | Spectrum Matches |
| --- | --- | --- | --- | --- |
| -19.8573 | 129.6642 | 0.0000 | 4.1448e+06 | 1 |

**Adduct Signal Fractions**

- **Unmodified**: 4.1448e+06

### RQVIVDSGN(N)RSGY Hex 6 HexNAc 2

**Mass:**  2829.2134

(3.554e-04)

**Start Position**: 394

**End Position**: 407

| Profile Score | Tandem Score | TDA q-value | Volume | Spectrum Matches |
| --- | --- | --- | --- | --- |
| 0.0000 | 108.5493 | 0.0000 | 0.0000e+00 | 1 |

Adduct:

No Chromatogram Found

### DN(D)KHSN(N)DTIHDRTPY Fuc 1 Hex 7 HexNAc 3 Neu5Ac 1

**Mass:**  3993.5588

(2.452e-04)

**Charge States:** 4

**Start Position**: 141

**End Position**: 156

| Profile Score | Tandem Score | TDA q-value | Volume | Spectrum Matches |
| --- | --- | --- | --- | --- |
| -21.1760 | 85.9872 | 0.0000 | 6.0386e+06 | 1 |

**Adduct Signal Fractions**

- **Unmodified**: 6.0386e+06

### QINRQVIVDSGN(N)RSGY Hex 7 HexNAc 2

**Mass:**  3345.4440

(-7.211e-07)

**Charge States:** 3, 4

**Start Position**: 391

**End Position**: 407

| Profile Score | Tandem Score | TDA q-value | Volume | Spectrum Matches |
| --- | --- | --- | --- | --- |
| -20.4134 | 62.7489 | 0.0000 | 3.6848e+06 | 2 |

**Adduct Signal Fractions**

- **Unmodified**: 3.6848e+06

### ALGQGTTLDNKHSN(N)DTIHDRTPY Fuc 1 Hex 7 HexNAc 3

**Mass:**  4442.8430

(-9.787e-06)

**Charge States:** 4

**Start Position**: 133

**End Position**: 156

| Profile Score | Tandem Score | TDA q-value | Volume | Spectrum Matches |
| --- | --- | --- | --- | --- |
| -20.1288 | 61.2568 | 0.0000 | 6.9579e+06 | 1 |

**Adduct Signal Fractions**

- **Unmodified**: 6.9579e+06

### QINRQVIVDSGN(N)RSGY Hex 8 HexNAc 2

**Mass:**  3507.5026

(8.762e-07)

**Charge States:** 3, 4

**Start Position**: 391

**End Position**: 407

| Profile Score | Tandem Score | TDA q-value | Volume | Spectrum Matches |
| --- | --- | --- | --- | --- |
| -21.2003 | 45.7957 | 0.0000 | 1.7441e+06 | 1 |

**Adduct Signal Fractions**

- **Unmodified**: 1.7441e+06

### DN(D)KHSN(N)DTIHDRTPY Hex 7 HexNAc 3 Neu5Ac 1

**Mass:**  3847.4960

(2.532e-04)

**Charge States:** 4

**Start Position**: 141

**End Position**: 156

| Profile Score | Tandem Score | TDA q-value | Volume | Spectrum Matches |
| --- | --- | --- | --- | --- |
| -20.9928 | 37.4454 | 0.0000 | 8.5555e+06 | 1 |

**Adduct Signal Fractions**

- **Unmodified**: 8.5555e+06

### DN(D)KHSN(N)DTIHDRTPY Fuc 1 Hex 7 HexNAc 3

**Mass:**  3702.4489

(2.605e-04)

**Start Position**: 141

**End Position**: 156

| Profile Score | Tandem Score | TDA q-value | Volume | Spectrum Matches |
| --- | --- | --- | --- | --- |
| 0.0000 | 37.0442 | 0.0000 | 0.0000e+00 | 1 |

Adduct:

No Chromatogram Found

### ALGQGTTLDNKHSN(N)DTIHDRTPY Fuc 2 Hex 7 HexNAc 3

**Mass:**  4588.9016

(-8.308e-06)

**Charge States:** 4

**Start Position**: 133

**End Position**: 156

| Profile Score | Tandem Score | TDA q-value | Volume | Spectrum Matches |
| --- | --- | --- | --- | --- |
| -20.6611 | 34.7088 | 0.0000 | 3.9019e+06 | 1 |

**Adduct Signal Fractions**

- **Unmodified**: 3.9019e+06

### STPNSKLQINRQVIVDSGN(N)RSGY Hex 7 HexNAc 2

**Mass:**  4072.8310

(-4.957e-07)

**Charge States:** 4

**Start Position**: 384

**End Position**: 407

| Profile Score | Tandem Score | TDA q-value | Volume | Spectrum Matches |
| --- | --- | --- | --- | --- |
| -19.5228 | 31.3267 | 0.0000 | 1.6563e+06 | 1 |

**Adduct Signal Fractions**

- **Unmodified**: 1.6563e+06

### ALGQGTTLDNKHSN(N)DTIHDRTPY Fuc 1 Hex 5 HexNAc 5

**Mass:**  4526.9521

(4.447e-04)

**Start Position**: 133

**End Position**: 156

| Profile Score | Tandem Score | TDA q-value | Volume | Spectrum Matches |
| --- | --- | --- | --- | --- |
| 0.0000 | 26.4920 | 0.0238 | 0.0000e+00 | 1 |

Adduct:

No Chromatogram Found

### tr|F1NB02|F1NB02\_CHICK

**>tr|F1NB02|F1NB02\_CHICK**  
 `GISRDNWHKRRKTGGKRKPYHKKRKYELGRPPANTKIGPRRIHTVRVRGGNKKYRALRLDVGNFSWGSECCTRKTRIIDV  
VYNASNNELVRTKTLVKNCIVLVDSTPYRQWYEAHYALPLGRKKGAKLTPEEEEILNKKRSKKIQKKYDERKKNAKIASI  
LEEQFQQGKLLACIASRPGQCGRADGYVLEGKELEFYLRKIKARKGK`

| Site | Type | Count |
| --- | --- | --- |
| 63 | N-linked | 1 |

| Mass | Sequence | MS_2_ Score | `q`-value | MS_1_ Score | Signal | Protein Coordinates | Tandem Scans | Start Time | Apex Time | End Time |
| --- | --- | --- | --- | --- | --- | --- | --- | --- | --- | --- |
| 4159.6442 | RLDVGN(N)FSW Fuc 1 Hex 8 HexNAc 8 | 67.719 | 0.0 | -18.475 | 9.215e+06 | 57 - 66 | 2 | 26.12 | 26.12 | 26.12 |

### RLDVGN(N)FSW Fuc 1 Hex 8 HexNAc 8

**Mass:**  4159.6452

(-1.371e-06)

**Charge States:** 4, 5

**Start Position**: 58

**End Position**: 67

| Profile Score | Tandem Score | TDA q-value | Volume | Spectrum Matches |
| --- | --- | --- | --- | --- |
| -18.4750 | 67.7187 | 0.0000 | 9.2153e+06 | 2 |

**Adduct Signal Fractions**

- **Unmodified**: 9.2153e+06

### tr|A0A1D5PT58|A0A1D5PT58\_CHICK

**>tr|A0A1D5PT58|A0A1D5PT58\_CHICK**  
 `MGISRDNWHKRRKTGGKRKPYHKKRKYELGRPPANTKIGPRRIHTVRVRGGNKKYRALRLDVGNFSWGSECCTRKTRIID  
VVYNASNNELVRTKTLVKNCIVLVDSTPYRQWYEAHYALPLGRKKGAKLTPEEEEILNKKRSKKIQKKYDERKKNAKIAS  
ILEEQFQQGKLLACIASRPGQCGRADGYVLEGKELEFYLRKIKARKGK`

| Site | Type | Count |
| --- | --- | --- |
| 64 | N-linked | 1 |

| Mass | Sequence | MS_2_ Score | `q`-value | MS_1_ Score | Signal | Protein Coordinates | Tandem Scans | Start Time | Apex Time | End Time |
| --- | --- | --- | --- | --- | --- | --- | --- | --- | --- | --- |
| 4159.6442 | RLDVGN(N)FSW Fuc 1 Hex 8 HexNAc 8 | 67.719 | 0.0 | -18.475 | 9.215e+06 | 58 - 67 | 2 | 26.12 | 26.12 | 26.12 |

### RLDVGN(N)FSW Fuc 1 Hex 8 HexNAc 8

**Mass:**  4159.6452

(-1.371e-06)

**Charge States:** 4, 5

**Start Position**: 59

**End Position**: 68

| Profile Score | Tandem Score | TDA q-value | Volume | Spectrum Matches |
| --- | --- | --- | --- | --- |
| -18.4750 | 67.7187 | 0.0000 | 9.2153e+06 | 2 |

**Adduct Signal Fractions**

- **Unmodified**: 9.2153e+06

### sp|P14105|MYH9\_CHICK

**>sp|P14105|MYH9\_CHICK**  
 `MAQRDADKYLYVDKNIINNPLTQADWAAKKLVWVPSEKSGFEAASLKEEVGDEAIVELAENGKKVKVNKDDIQKMNPPKF  
SKVEDMAELTCLNEASVLHNLKERYYSGLIYTYSGLFCVVINPYKNLPIYSEEIVEMYKGKKRHEMPPHIYAITDTAYRS  
MMQDREDQSILCTGESGAGKTENTKKVIQYLAHVASSHKSKKDQGELERQLLQANPILEAFGNAKTVKNDNSSRFGKFIR  
INFDVNGYIVGANIETYLLEKSRAIRQAKEERTFHIFYYLLSGAGEHLKTDLLLEPYNKYRFLSNGHVTIPGQQDKDMFQ  
ETMEAMRIMGIPDEEQIGLLKVISGVLQLGNIVFKKERNTDQASMPDNTAAQKVSHLLGINVTDFTRGILTPRIKVGRDY  
VQKAQTKEQADFAIEALAKATYEQMFRWLVMRINKALDKTKRQGASFIGILDIAGFEIFELNSFEQLCINYTNEKLQQLF  
NHTMFILEQEEYQNEGIEWNFIDFGLDLQPCIDLIEKPAGPPGILALLDEECWFPKATDKSFVEKVVQEQGTHPKFQKPK  
QLKDKADFCIIHYAGKVDYKADEWLMKNMDPLNDNIATLLHQSSDKFVSELWKDVDRIVGLDQVAGMSETALPGAFKTRK  
GMFRTVGQLYKEQLAKLMATLRNTNPNFVRCIIPNHEKKAGKLDPHLVLDQLRCNGVLEGIRICRQGFPNRVVFQEFRQR  
YEILTPNAIPKGFMDGKQACVLMIKALELDSNLYRIGQSKVFFRAGVLAHLEEERDLKITDVIIGFQACCRGYLARKAFA  
KRQQQLTAMKVLQRNCAAYLKLRNWQWWRLFTKVKPLLQVSRQEEEMMAKEEELIKVKEKQLAAENRLSEMETFQAQLMA  
EKMQLQEQLQAEAELCAEAEEIRARLTAKKQELEEICHDLEARVEEEEERCQHLQAEKKKMQQNIQELEEQLEEEESARQ  
KLQLEKVTTEAKLKKLEEDVIVLEDQNLKLAKEKKLLEDRMSEFTTNLTEEEEKSKSLAKLKNKHEAMITDLEERLRREE  
KQRQELEKTRRKLEGDSSDLHDQIAELQAQIAELKIQLSKKEEELQAALARVEEEAAQKNMALKKIRELESQITELQEDL  
ESERASRNKAEKQKRDLGEELEALKTELEDTLDSTAAQQELRSKREQEVTVLKKTLEDEAKTHEAQIQEMRQKHSQAIEE  
LAEQLEQTKRVKANLEKAKQALESERAELSNEVKVLLQGKGDAEHKRKKVDAQLQELQVKFTEGERVKTELAERVNKLQV  
ELDNVTGLLNQSDSKSIKLAKDFSALESQLQDTQELLQEETRLKLSFSTKLKQTEDEKNALKEQLEEEEEAKRNLEKQIS  
VLQQQAVEARKKMDDGLGCLEIAEEAKKKLQKDLESLTQRYEEKIAAYDKLEKTKTRLQQELDDIAVDLDHQRQTVSNLE  
KKQKKFDQLLAEEKNISAKYAEERDRAEAEAREKETKALSLARALEEAIEQKAELERVNKQFRTEMEDLMSSKDDVGKSV  
HELEKAKRALEQQVEEMKTQLEELEDELQATEDAKLRLEVNQQAMKAQFDRDLLGRDEQNEEKRKQLIRQVREMEVELED  
ERKQRSIAVAARKKLELDLKDLESHIDTANKNRDEAIKHVRKLQAQMKDYMRELEDTRTSREEILAQAKENEKKLKSMEA  
EMIQLQEELAAAERAKRQAQQERDELADEIANSSGKGALAMEEKRRLEARIAQLEEELEEEQGNTEIINDRLKKANLQID  
QMNADLNAERSNAQKNENARQQMERQNKELKLKLQEMESAVKSKYKATITALEAKIVQLEEQLDMETKERQAASKQVRRA  
EKKLKDILLQVDDERRNAEQFKDQADKANMRLKQLKRQLEEAEEEAQRANVRRKLQRELDDATETADAMNREVSSLKSKL  
RRGDLPFVVTRRLVRKGTGECSDEEVDGKAEAGDAKATE`

| Site | Type | Count |
| --- | --- | --- |
| 1284 | N-linked | 1 |

| Mass | Sequence | MS_2_ Score | `q`-value | MS_1_ Score | Signal | Protein Coordinates | Tandem Scans | Start Time | Apex Time | End Time |
| --- | --- | --- | --- | --- | --- | --- | --- | --- | --- | --- |
| 4420.7743 | QVELDN(N)VTGL Fuc 2 Hex 10 HexNAc 7 | 27.544 | 0.019 | -18.459 | 7.214e+06 | 1278 - 1288 | 3 | 25.95 | 25.95 | 25.95 |

### QVELDN(N)VTGL Fuc 2 Hex 10 HexNAc 7

**Mass:**  4420.7745

(4.322e-06)

**Charge States:** 4, 5

**Start Position**: 1279

**End Position**: 1289

| Profile Score | Tandem Score | TDA q-value | Volume | Spectrum Matches |
| --- | --- | --- | --- | --- |
| -18.4586 | 27.5436 | 0.0186 | 7.2144e+06 | 3 |

**Adduct Signal Fractions**

- **Unmodified**: 7.2144e+06

### iav|AAM75156|A/PR/8/1934\_PB1

**>iav|AAM75156|A/PR/8/1934\_PB1**  
 `MDVNPTLLFLKVPAQNAISTTFPYTGDPPYSHGTGTGYTMDTVNRTHQYSEKGRWTTNTETGAPQLNPIDGPLPEDNEPS  
GYAQTDCVLEAMAFLEESHPGIFENSCIETMEVVQQTRVDKLTQGRQTYDWTLNRNQPAATALANTIEVFRSNGLTANES  
GRLIDFLKDVMESMKKEEMGITTHFQRKRRVRDNMTKKMITQRTIGKKKQRLNKRSYLIRALTLNTMTKDAERGKLKRRA  
IATPGMQIRGFVYFVETLARSICEKLEQSGLPVGGNEKKAKLANVVRKMMTNSQDTELSFTITGDNTKWNENQNPRMFLA  
MITYMTRNQPEWFRNVLSIAPIMFSNKMARLGKGYMFESKSMKLRTQIPAEMLASIDLKYFNDSTRKKIEKIRPLLIEGT  
ASLSPGMMMGMFNMLSTVLGVSILNLGQKRYTKTTYWWDGLQSSDDFALIVNAPNHEGIQAGVDRFYRTCKLLGINMSKK  
KSYINRTGTFEFTSFFYRYGFVANFSMELPSFGVSGINESADMSIGVTVIKNNMINNDLGPATAQMALQLFIKDYRYTYR  
CHRGDTQIQTRRSFEIKKLWEQTRSKAGLLVSDGGPNLYNIRNLHIPEVCLKWELMDEDYQGRLCNPLNPFVSHKEIESM  
NNAVMMPAHGPAKNMEYDAVATTHSWIPKRNRSILNTSQRGVLEDEQMYQRCCNLFEKFFPSSSYRRPVGISSMVEAMVS  
RARIDARIDFESGRIKKEEFTEIMKICSTIEELRRQK`

| Site | Type | Count |
| --- | --- | --- |
| 504 | N-linked | 1 |

| Mass | Sequence | MS_2_ Score | `q`-value | MS_1_ Score | Signal | Protein Coordinates | Tandem Scans | Start Time | Apex Time | End Time |
| --- | --- | --- | --- | --- | --- | --- | --- | --- | --- | --- |
| 2867.1319 | VAN(N)FSM Fuc 2 Hex 3 HexNAc 7 | 79.200 | 0.0 | -18.638 | 4.176e+07 | 501 - 507 | 1 | 34.89 | 35.06 | 35.06 |

### VAN(N)FSM Fuc 2 Hex 3 HexNAc 7

**Mass:**  2867.1322

(1.945e-07)

**Charge States:** 3

**Start Position**: 502

**End Position**: 508

| Profile Score | Tandem Score | TDA q-value | Volume | Spectrum Matches |
| --- | --- | --- | --- | --- |
| -18.6384 | 79.2001 | 0.0000 | 4.1760e+07 | 1 |

**Adduct Signal Fractions**

- **Unmodified**: 4.1760e+07

### iav|AGU93028|A/PR/8/1934\_PB1

**>iav|AGU93028|A/PR/8/1934\_PB1**  
 `MDVNPTLLFLKVPAQNAISTTFPYTGDPPYSHGTGTGYTMDTVNRTHQYSEKGRWTTNTETGAPQLNPIDGPLPEDNEPS  
GYAQTDCVLEAMAFLEESHPGIFENSCIETMEVVQQTRVDKLTQGRQTYDWTLNRNQPAATALANTIEVFRSNGLTANES  
GRLIDFLKDVMESMNKEEMGITTHFQRKRRVRDNMTKKMITQRTIGKKKQRLNKRSYLIRALTLNTMTKDAERGKLKRRA  
IATPGMQIRGFVYFVETLARSICEKLEQSGLPVGGNEKKAKLANVVRKMMTNSQDTELSFTITGDNTKWNENQNPRMFLA  
MITYMTRNQPEWFRNVLSIAPIMFSNKMARLGKGYMFESKSMKLRTQIPAEMLASIDLKYFNDSTRKKIEKIRPLLIEGT  
ASLSPGMMMGMFNMLSTVLGVSILNLGQKRYTKTTYWWDGLQSSDDFALIVNAPNHEGIQAGVDRFYRTCKLLGINMSKK  
KSYINRTGTFEFTSFFYRYGFVANFSMELPSFGVSGINESADMSIGVTVIKNNMINNDLGPATAQMALQLFIKDYRYTYR  
CHRGDTQIQTRRSFEIKKLWEQTRSKAGLLVSDGGPNLYNIRNLHIPEVCLKWELMDEDYQGRLCNPLNPFVSHKEIESM  
NNAVMMPAHGPAKNMEYDAVATTHSWIPKRNRSILNTSQRGVLEDEQMYQRCCNLFEKFFPSSSYRRPVGISSMVEAMVS  
RARIDARIDFESGRIKKEEFTEIMKICSTIEELRRQK`

| Site | Type | Count |
| --- | --- | --- |
| 504 | N-linked | 1 |

| Mass | Sequence | MS_2_ Score | `q`-value | MS_1_ Score | Signal | Protein Coordinates | Tandem Scans | Start Time | Apex Time | End Time |
| --- | --- | --- | --- | --- | --- | --- | --- | --- | --- | --- |
| 2867.1319 | VAN(N)FSM Fuc 2 Hex 3 HexNAc 7 | 79.200 | 0.0 | -18.638 | 4.176e+07 | 501 - 507 | 1 | 34.89 | 35.06 | 35.06 |

### VAN(N)FSM Fuc 2 Hex 3 HexNAc 7

**Mass:**  2867.1322

(1.945e-07)

**Charge States:** 3

**Start Position**: 502

**End Position**: 508

| Profile Score | Tandem Score | TDA q-value | Volume | Spectrum Matches |
| --- | --- | --- | --- | --- |
| -18.6384 | 79.2001 | 0.0000 | 4.1760e+07 | 1 |

**Adduct Signal Fractions**

- **Unmodified**: 4.1760e+07

### iav|AAA43581|A/PR/8/1934\_PB1

**>iav|AAA43581|A/PR/8/1934\_PB1**  
 `MDVNPTLLFLKVPAQNAISTTFPYTGDPPYSHGTGTGYTMDTVNRTHQYSEKARWTTNTETGAPQLNPIDGPLPEDNEPS  
GYAQTDCVLEAMAFLEESHPGIFENSCIETMEVVQQTRVDKLTQGRQTYDWTLNRNQPAATALANTIEVFRSNGLTANES  
GRLIDFLKDVMESMKKEEMGITTHFQRKRRVRDNMTKKMITQRTIGKRKQRLNKRSYLIRALTLNTMTKDAERGKLKRRA  
IATPGMQIRGFVYFVETLARSICEKLEQSGLPVGGNEKKAKLANVVRKMMTNSQDTELSLTITGDNTKWNENQNPRMFLA  
MITYMTRNQPEWFRNVLSIAPIMFSNKMARLGKGYMFESKSMKLRTQIPAEMLASIDLKYFNDSTRKKIEKIRPLLIEGT  
ASLSPGMMMGMFNMLSTVLGVSILNLGQKRYTKTTYWWDGLQSSDDFALIVNAPNHEGIQAGVDRFYRTCKLHGINMSKK  
KSYINRTGTFEFTSFFYRYGFVANFSMELPSFGVSGSNESADMSIGVTVIKNNMINNDLGPATAQMALQLFIKDYRYTYR  
CHRGDTQIQTRRSFEIKKLWEQTRSKAGLLVSDGGPNLYNIRNLHIPEVCLKWELMDEDYQGRLCNPLNPFVSHKEIESM  
NNAVMMPAHGPAKNMEYDAVATTHSWIPKRNRSILNTSQRGVLEDEQMYQRCCNLFEKFFPSSSYRRPVGISSMVEAMVS  
RARIDARIDFESGRIKKEEFTEIMKICSTIEELRRQK`

| Site | Type | Count |
| --- | --- | --- |
| 504 | N-linked | 1 |

| Mass | Sequence | MS_2_ Score | `q`-value | MS_1_ Score | Signal | Protein Coordinates | Tandem Scans | Start Time | Apex Time | End Time |
| --- | --- | --- | --- | --- | --- | --- | --- | --- | --- | --- |
| 2867.1319 | VAN(N)FSM Fuc 2 Hex 3 HexNAc 7 | 79.200 | 0.0 | -18.638 | 4.176e+07 | 501 - 507 | 1 | 34.89 | 35.06 | 35.06 |

### VAN(N)FSM Fuc 2 Hex 3 HexNAc 7

**Mass:**  2867.1322

(1.945e-07)

**Charge States:** 3

**Start Position**: 502

**End Position**: 508

| Profile Score | Tandem Score | TDA q-value | Volume | Spectrum Matches |
| --- | --- | --- | --- | --- |
| -18.6384 | 79.2001 | 0.0000 | 4.1760e+07 | 1 |

**Adduct Signal Fractions**

- **Unmodified**: 4.1760e+07

### iav|ACF41842|A/PR/8/1934\_PB1

**>iav|ACF41842|A/PR/8/1934\_PB1**  
 `MDVNPTLLFLKVPAQNAISTTFPYTGDPPYSHGTGTGYTMDTVNRTHQYSEKGRWTTNTETGAPQLNPIDGPLPEDNEPS  
GYAQTDCVLEAMAFLEESHPGIFENSCIETMEVVQQTRVDKLTQGRQTYDWTLNRNQPAATALANTIEVFRSNGLTANES  
GRLIDFLKDVMESMNKEEMGITTHFQRKRRVRDNMTKKMITQRTMGKKKQRLNKRSYLIRALTLNTMTKDAERGKLKRRA  
IATPGMQIRGFVYFVETLARSICEKLEQSGLPVGGNEKKAKLANVVRKMMTNSQDTELSFTITGDNTKWNENQNPRMFLA  
MITYMTRNQPEWFRNVLSIAPIMFSNKMARLGKGYMFESKSMKLRTQIPAEMLASIDLKYFNDSTRKKIEKIRPLLIEGT  
ASLSPGMMMGMFNMLSTVLGVSILNLGQKRYTKTTYWWDGLQSSDDFALIVNAPNHEGIQAGVDRFYRTCKLLGINMSKK  
KSYINRTGTFEFTSFFYRYGFVANFSMELPSFGVSGINESADMSIGVTVIKNNMINNDLGPATAQMALQLFIKDYRYTYR  
CHRGDTQIQTRRSFEIKKLWEQTRSKAGLLVSDGGPNLYNIRNLHIPEVCLKWELMDEDYQGRLCNPLNPFVSHKEIESM  
NNAVMMPAHGPAKNMEYDAVATTHSWIPKRNRSILNTSQRGVLEDEQMYQRCCNLFEKFFPSSSYRRPVGISSMVEAMVS  
RARIDARIDFESGRIKKEEFTEIMKICSTIEELRRQK`

| Site | Type | Count |
| --- | --- | --- |
| 504 | N-linked | 1 |

| Mass | Sequence | MS_2_ Score | `q`-value | MS_1_ Score | Signal | Protein Coordinates | Tandem Scans | Start Time | Apex Time | End Time |
| --- | --- | --- | --- | --- | --- | --- | --- | --- | --- | --- |
| 2867.1319 | VAN(N)FSM Fuc 2 Hex 3 HexNAc 7 | 79.200 | 0.0 | -18.638 | 4.176e+07 | 501 - 507 | 1 | 34.89 | 35.06 | 35.06 |

### VAN(N)FSM Fuc 2 Hex 3 HexNAc 7

**Mass:**  2867.1322

(1.945e-07)

**Charge States:** 3

**Start Position**: 502

**End Position**: 508

| Profile Score | Tandem Score | TDA q-value | Volume | Spectrum Matches |
| --- | --- | --- | --- | --- |
| -18.6384 | 79.2001 | 0.0000 | 4.1760e+07 | 1 |

**Adduct Signal Fractions**

- **Unmodified**: 4.1760e+07

### iav|ADX99655|A/PR/8-WG/1934\_PB1

**>iav|ADX99655|A/PR/8-WG/1934\_PB1**  
 `MDVNPTLLFLKVPAQNAISTTFPYTGDPPYSHGTGTGYTMDTVNRTHQYSEKGRWTTNTETGAPQLNPIDGPLPEDNEPS  
GYAQTDCVLEAMAFLEESHPGIFENSCIETMEVVQQTRVDKLTQGRQTYDWTLNRNQPAATALANTIEVFRSNGLTANES  
GRLIDFLKDVMESMNKEEMGITTHFQRKRRVRDNMTKKMITQRTMGKKKQRLNKRSYLIRALTLNTMTKDAERGKLKRRA  
IATPGMQIRGFVYFVETLARSICEKLEQSGLPVGGNEKKAKLANVVRKMMTNSQDTELSFTITGDNTKWNENQNPRMFLA  
MITYMTRNQPEWFRNVLSIAPIMFSNKMARLGKGYMFESKSMKLRTQIPAEMLASIDLKYFNDSTRKKIEKIRPLLIEGT  
ASLSPGMMMGMFNMLSTVLGVSILNLGQKRYTKTTYWWDGLQSSDDFALIVNAPNHEGIQAGVDRFYRTCKLLGINMSKK  
KSYINRTGTFEFTSFFYRYGFVANFSMELPSFGVSGINESADMSIGVTVIKNNMINNDLGPATAQMALQLFIKDYRYTYR  
CHIGDTQIQTRRSFEIKKLWEQTRSKAGLLVSDGGPNLYNIRNLHIPEVCLKWELMDEDYQGRLCNPLNPFVSHKEIESM  
NNAVMMPAHGPAKNMEYDAVATTHSWIPKRNRSILNTSQRGVLEDEQMYQRCCNLFEKFFPSSSYRRPVGISSMVEAMVS  
RARIDARIDFESGRIKKEEFTEIMKICSTIEELRRQK`

| Site | Type | Count |
| --- | --- | --- |
| 504 | N-linked | 1 |

| Mass | Sequence | MS_2_ Score | `q`-value | MS_1_ Score | Signal | Protein Coordinates | Tandem Scans | Start Time | Apex Time | End Time |
| --- | --- | --- | --- | --- | --- | --- | --- | --- | --- | --- |
| 2867.1319 | VAN(N)FSM Fuc 2 Hex 3 HexNAc 7 | 79.200 | 0.0 | -18.638 | 4.176e+07 | 501 - 507 | 1 | 34.89 | 35.06 | 35.06 |

### VAN(N)FSM Fuc 2 Hex 3 HexNAc 7

**Mass:**  2867.1322

(1.945e-07)

**Charge States:** 3

**Start Position**: 502

**End Position**: 508

| Profile Score | Tandem Score | TDA q-value | Volume | Spectrum Matches |
| --- | --- | --- | --- | --- |
| -18.6384 | 79.2001 | 0.0000 | 4.1760e+07 | 1 |

**Adduct Signal Fractions**

- **Unmodified**: 4.1760e+07

### iav|BAV59609|A/PR/8/1934\_PB1

**>iav|BAV59609|A/PR/8/1934\_PB1**  
 `MDVNPTLLFLKVPAQNAISTTFPYTGDPPYSHGTGTGYTMDTVNRTHQYSEKGRWTTNTETGASQLNPIDGPLPEDNEPS  
GYAQTDCVLEAMAFLEESHPGIFENSCIETIEVVQQTRVDKLTQGRQTYDWTLNRNQPAATALANTIEVFRSNGLTANES  
GRLIDFLKDVMESMNKEEMGITTHFQRKRRVRDNMTKKMITQRTMGKKKQRLNKRSYLIRALTLNTMTKDAERGKLKRRA  
IATPGMQIRGFVYFVETLARSICEKLEQSGLPVGGNEKKAKLANVVRKMMTNSQDTELSFTITGDNTKWNENQNPRMFLA  
MITYMTRNQPEWFRNVLSIAPIMFSNKMARLGKGYMFESKSMKLRTQIPAEMLASIDLKYFNDSTRKKIEKIRPLLIEGT  
ASLSPGMMMGMFNMLSTVLGVSILNLGQKRYTKTTYWWDGLQSSDDFALIVNAPNNEGIQAGVDRFYRTCKLLGINMSKK  
KSYINRTGTFEFTSFFYRYGFVANFSMELPSFGVSGINESADMSIGVTVIKNNMINNDLGPATAQMALQLFIKDYRYTYR  
CHRGDTQIQTRRSFEIKKLWEQTRSKAGLLVSDGGPNLYNIRNLHIPEVCLKWELMDEDYQGRLCNPLNPFVSHKEIESM  
NNAVMMPAHGPAKNMEYDAVATTHSWIPKRNRSILNTSQRGVLEDEQMYQRCCNLFEKFFPSSSYRRPVGISSMVEAMVS  
RARIDARIDFESGRIKKEEFTEIMKICSTIEELRRQK`

| Site | Type | Count |
| --- | --- | --- |
| 504 | N-linked | 1 |

| Mass | Sequence | MS_2_ Score | `q`-value | MS_1_ Score | Signal | Protein Coordinates | Tandem Scans | Start Time | Apex Time | End Time |
| --- | --- | --- | --- | --- | --- | --- | --- | --- | --- | --- |
| 2867.1319 | VAN(N)FSM Fuc 2 Hex 3 HexNAc 7 | 79.200 | 0.0 | -18.638 | 4.176e+07 | 501 - 507 | 1 | 34.89 | 35.06 | 35.06 |

### VAN(N)FSM Fuc 2 Hex 3 HexNAc 7

**Mass:**  2867.1322

(1.945e-07)

**Charge States:** 3

**Start Position**: 502

**End Position**: 508

| Profile Score | Tandem Score | TDA q-value | Volume | Spectrum Matches |
| --- | --- | --- | --- | --- |
| -18.6384 | 79.2001 | 0.0000 | 4.1760e+07 | 1 |

**Adduct Signal Fractions**

- **Unmodified**: 4.1760e+07

### iav|ABD77683|A/PR/8/1934\_PB1

**>iav|ABD77683|A/PR/8/1934\_PB1**  
 `MDVNPTLLFLKVPAQNAISTTFPYTGDPPYSHGTGTGYTMDTVNRTHQYSEKGRWTTNTETGAPQLNPIDGPLPEDNEPS  
GYAQTDCVLEAMAFLEESHPGIFENSCIETMEVVQQTRVDKLTQGRQTYDWTLNRNQPAATALANTIEVFRSNGLTANES  
GRLIDFLKDVMESMNKEEMGITTHFQRKRRVRDNMTKKMITQRTMGKKKQRLNKRSYLIRALTLNTMTKDAERGKLKRRA  
IATPGMQIRGFVYFVETLARSICEKLEQSGLPVGGNEKKAKLANVVRKMMTNSQDTELSFTITGDNTKWNENQNPRMFLA  
MITYMTRNQPEWFRNVLSIAPIMFSNKMARLGKGYMFESKSMKLRTQIPAEMLASIDLKYFNDSTRKKIEKIRSLLIEGT  
ASLSPGMMMGMFNMLSTVLGVSILNLGQKRYTKTTYWWDGLQSSDDFALIVNAPNHEGIQAGVDRFYRTCKLLGINMSKK  
KSYINRTGTFEFTSFFYRYGFVANFSMELPSFGVSGINESADMSIGVTVIKNNMINNDLGPATAQMALQLFIKDYRYTYR  
CHRGDTQIQTRRSFEIKKLWEQTRSKAGLLVSDGGPNLYNIRNLHIPEVCLKWELMDEDYQGRLCNPLNPFVSHKEIESM  
NNAVMMPAHGPAKNMEYDAVATTHSWIPKRNRSILNTSQRGVLEDEQMYQRCCNLFEKFFPSSSYRRPVGISSMVEAMVS  
RARIDARIDFESGRIKKEEFTEIMKICSTIEELRRQK`

| Site | Type | Count |
| --- | --- | --- |
| 504 | N-linked | 1 |

| Mass | Sequence | MS_2_ Score | `q`-value | MS_1_ Score | Signal | Protein Coordinates | Tandem Scans | Start Time | Apex Time | End Time |
| --- | --- | --- | --- | --- | --- | --- | --- | --- | --- | --- |
| 2867.1319 | VAN(N)FSM Fuc 2 Hex 3 HexNAc 7 | 79.200 | 0.0 | -18.638 | 4.176e+07 | 501 - 507 | 1 | 34.89 | 35.06 | 35.06 |

### VAN(N)FSM Fuc 2 Hex 3 HexNAc 7

**Mass:**  2867.1322

(1.945e-07)

**Charge States:** 3

**Start Position**: 502

**End Position**: 508

| Profile Score | Tandem Score | TDA q-value | Volume | Spectrum Matches |
| --- | --- | --- | --- | --- |
| -18.6384 | 79.2001 | 0.0000 | 4.1760e+07 | 1 |

**Adduct Signal Fractions**

- **Unmodified**: 4.1760e+07
