# Supplementary material for: Measuring Site-specific Glycosylation Similarity between Influenza a Virus Variants with Statistical Certainty
Source: Mol Cell Proteomics. 2020 Nov 25;19(9):1533–45. doi: 10.1074/mcp.RA120.002031 (PMC8143645; doi:10.1074/mcp.RA120.002031)
Supplement: Supplementary file 2 [file mmc2.zip › mmc2/159250_2_supp_555289_qcjklv.zip!/20190326_Phil82_021119_chymo_HILIC_DDA_1ug_02_glycopeptides.html]

## 20190326\_Phil82\_021119\_chymo\_HILIC\_DDA\_1ug\_02 @ GlycopeptideHypothesis-ff3e29dc2a30459995ec43521f444fe8

**MS^1^ Mass Accuracy:** 10.0 PPM  
**Grouping Tolerance:** 15.0 PPM  
**MS^n^ Mass Accuracy:** 20.0 PPM  
**Minimum `q`-Value For Clustering:** 0.05

**Glycopeptide Hypothesis:**
GlycopeptideHypothesis-ff3e29dc2a30459995ec43521f444fe8  
**Sample Searched:**
20190326\_Phil82\_021119\_chymo\_HILIC\_DDA\_1ug\_02

| Protein | Matched Glycopeptides |
| --- | --- |
| iav\|AFG99160\|A/Phil/2/1982\_HA | 122 |
| iav\|ADJ41819\|A/Phil/2-MA/1982\_NA | 11 |
| tr\|E1C3D8\|E1C3D8\_CHICK | 2 |
| iav\|BAV59610\|A/PR/8/1934\_PA | 1 |
| iav\|ADX99654\|A/PR/8-WG/1934\_PA | 1 |
| iav\|AGK63052\|A/PR/8/1934\_PA | 1 |
| iav\|CAA24295\|A/PR/8/1934\_PA | 1 |
| iav\|AAM75156\|A/PR/8/1934\_PB1 | 1 |
| iav\|AGU93028\|A/PR/8/1934\_PB1 | 1 |
| iav\|AAA43581\|A/PR/8/1934\_PB1 | 1 |
| iav\|ACF41842\|A/PR/8/1934\_PB1 | 1 |
| iav\|ADX99655\|A/PR/8-WG/1934\_PB1 | 1 |
| iav\|BAV59609\|A/PR/8/1934\_PB1 | 1 |
| iav\|ABD77683\|A/PR/8/1934\_PB1 | 1 |
| tr\|A0A1L1RRT9\|A0A1L1RRT9\_CHICK | 1 |
| sp\|Q5ZKC9\|1433Z\_CHICK | 1 |

### iav|AFG99160|A/Phil/2/1982\_HA

**>iav|AFG99160|A/Phil/2/1982\_HA**  
 `MKTIIALSYMFCLVFAQNLPGNDNSTATLCLGHHAVPNGTLVKTITNDQIEVTNATELVQSSSTGRICDSPHRILDGKNC  
TLIDALLGDPHCDGFQNEKWDLFVERSKAFSNCYPYDVPDYASLRSLVASSGTLEFINEGFNWTGVTQSGGSSTCKRGSN  
NSFFSRLNWLYESESKYPVLNVTMPNNGKFDKLYIWGIHHPSTDKEQTNLYIRASGRVTVSTKRSQQTVIPNIGSRPWVR  
GLSSRISIYWTIVKPGDILLINSTGNLIAPRGYFKIRTGKSSIMRSDAPIGTCSSECITPNGSIPNDKPFQNVNKITYGA  
CPRYVKQNTLKLATGMRNVPEKQTRGIFGAIAGFIENGWEGMVDGWYGFRHQNSEGTGQAADLKSTQAAIDQINGKLNRV  
IEKTNEKFHQIEKEFSEVEGRIQDLEKYVEDTKIDLWSYNAELLVALENQHTIDLTDSEMNKLFEKTRKQLRENAEDMGN  
GCFKIYHKCDNACIGSIRNGTYDHDVYRDEALNNRFQIKGVELKSGYKDWILWISFAISCFLLCVVLLGFIMWACQKGNI  
RCNICI`

| Site | Type | Count |
| --- | --- | --- |
| 262 | N-linked | 41 |
| 301 | N-linked | 32 |
| 142 | N-linked | 2 |
| 499 | N-linked | 5 |
| 181 | N-linked | 9 |
| 54 | N-linked | 6 |
| 24 | N-linked | 19 |
| 160 | N-linked | 8 |

| Mass | Sequence | MS_2_ Score | `q`-value | MS_1_ Score | Signal | Protein Coordinates | Tandem Scans | Start Time | Apex Time | End Time |
| --- | --- | --- | --- | --- | --- | --- | --- | --- | --- | --- |
| 3499.5174 | LIN(N)STGNLIAPRGYF Hex 9 HexNAc 2 | 181.025 | 0.0 | -4.482 | 3.487e+07 | 259 - 274 | 1 | 45.39 | 45.39 | 45.90 |
| 2695.0888 | LIN(N)STGNL Hex 9 HexNAc 2 | 180.221 | 0.0 | -5.166 | 1.093e+07 | 259 - 267 | 2 | 22.95 | 22.95 | 23.29 |
| 2870.4559 | LPGNDN(N)STATL Fuc 1 Hex 5 HexNAc 4 | 175.094 | 0.0 | 11.885 | 1.055e+08 | 18 - 29 | 9 | 22.45 | 23.29 | 25.83 |
| 3132.3628 | LIN(N)STGNLIAPR Hex 9 HexNAc 2 | 172.751 | 0.0 | -2.536 | 2.215e+07 | 259 - 271 | 2 | 28.19 | 28.19 | 28.53 |
| 2724.1163 | LPGNDN(N)STATL Hex 5 HexNAc 4 | 168.508 | 0.0 | 11.937 | 2.189e+07 | 18 - 29 | 3 | 22.79 | 23.12 | 23.63 |
| 4068.9289 | TIVKPGDILLIN(N)STGNLIAPR Hex 9 HexNAc 2 | 168.040 | 0.0 | 14.491 | 2.304e+08 | 250 - 271 | 11 | 50.84 | 51.53 | 52.38 |
| 2317.9547 | LPGNDN(N)STATL Hex 5 HexNAc 2 | 160.129 | 0.0 | 11.140 | 1.268e+07 | 18 - 29 | 3 | 23.12 | 23.46 | 23.80 |
| 3159.4267 | TIVKPGDILLIN(N) Hex 9 HexNAc 2 | 159.215 | 0.0 | 13.555 | 1.224e+08 | 250 - 262 | 8 | 43.52 | 44.88 | 46.07 |
| 3352.4510 | LIN(N)STGNLIAPRGY Hex 9 HexNAc 2 | 158.640 | 0.0 | -5.076 | 4.448e+07 | 259 - 273 | 2 | 34.53 | 34.70 | 35.03 |
| 3160.4377 | TIVKPGDILLIN(N) Hex 9 HexNAc 2 | 156.569 | 0.0 | 0.000 | 0.000e+00 | 250 - 262 | 1 | - | - | - |
| 4289.0131 | TIVKPGDILLIN(N)STGNLIAPRGY Hex 9 HexNAc 2 | 155.716 | 0.0 | 15.040 | 7.236e+08 | 250 - 273 | 26 | 53.58 | 54.27 | 57.22 |
| 3176.4533 | TIVKPGDILLIN(N) Fuc 2 Hex 3 HexNAc 4 Neu5Ac 1 | 154.250 | 0.0 | 8.813 | 3.075e+07 | 250 - 262 | 4 | 43.52 | 44.88 | 45.22 |
| 4306.0384 | TIVKPGDILLIN(N)STGNLIAPRGY Fuc 2 Hex 3 HexNAc 4 Neu5Ac 1 | 150.394 | 0.0 | 13.250 | 7.918e+07 | 250 - 273 | 3 | 53.92 | 54.27 | 54.78 |
| 3631.6534 | TIVKPGDILLIN(N)STGNL Hex 9 HexNAc 2 | 149.773 | 0.0 | 13.303 | 9.472e+07 | 250 - 267 | 6 | 50.84 | 51.02 | 51.70 |
| 3518.5729 | TIVKPGDILLIN(N)STGN Hex 9 HexNAc 2 | 149.737 | 0.0 | 14.044 | 4.394e+07 | 250 - 266 | 6 | 42.66 | 42.83 | 43.35 |
| 3321.4791 | TIVKPGDILLIN(N) Hex 10 HexNAc 2 | 146.544 | 0.0 | -5.212 | 5.245e+06 | 250 - 262 | 2 | 44.71 | 44.88 | 45.05 |
| 2683.0877 | LPGNDN(N)STATL Hex 6 HexNAc 3 | 142.855 | 0.0 | -5.696 | 5.921e+06 | 18 - 29 | 2 | 22.79 | 22.95 | 23.29 |
| 4085.9552 | TIVKPGDILLIN(N)STGNLIAPR Fuc 2 Hex 3 HexNAc 4 Neu5Ac 1 | 140.367 | 0.0 | -3.651 | 2.348e+07 | 250 - 271 | 2 | 51.36 | 51.70 | 51.87 |
| 4451.0659 | TIVKPGDILLIN(N)STGNLIAPRGY Hex 10 HexNAc 2 | 140.290 | 0.0 | 14.264 | 6.585e+07 | 250 - 273 | 7 | 53.92 | 54.27 | 54.78 |
| 3235.3083 | LPGNDN(N)STATL Fuc 1 Hex 6 HexNAc 5 | 139.392 | 0.0 | 12.680 | 1.052e+07 | 18 - 29 | 2 | 22.45 | 22.95 | 23.12 |
| 3016.2325 | LPGNDN(N)STATL Fuc 2 Hex 5 HexNAc 4 | 134.593 | 0.0 | 11.513 | 1.644e+07 | 18 - 29 | 2 | 22.45 | 22.95 | 23.46 |
| 3535.5979 | TIVKPGDILLIN(N)STGN Fuc 2 Hex 3 HexNAc 4 Neu5Ac 1 | 133.548 | 0.0 | -4.051 | 1.089e+07 | 250 - 266 | 2 | 42.83 | 42.83 | 43.18 |
| 3263.3555 | ITPN(N)GSIPNDKPF Hex 9 HexNAc 2 | 133.173 | 0.0 | -4.938 | 1.039e+07 | 297 - 310 | 2 | 29.37 | 29.54 | 29.71 |
| 3906.8799 | TIVKPGDILLIN(N)STGNLIAPR Hex 8 HexNAc 2 | 132.421 | 0.0 | -5.491 | 1.320e+07 | 250 - 271 | 4 | 51.53 | 51.70 | 51.87 |
| 3386.4387 | IN(N)STGNLIAPRGYF Hex 9 HexNAc 2 | 132.294 | 0.0 | -4.168 | 9.839e+06 | 260 - 274 | 2 | 41.13 | 41.30 | 41.47 |
| 4126.9534 | TIVKPGDILLIN(N)STGNLIAPRGY Hex 8 HexNAc 2 | 131.449 | 0.0 | 11.937 | 7.565e+07 | 250 - 273 | 13 | 53.58 | 54.27 | 55.95 |
| 3516.5448 | LIN(N)STGNLIAPRGYF Fuc 2 Hex 3 HexNAc 4 Neu5Ac 1 | 130.818 | 0.0 | -4.939 | 6.027e+06 | 259 - 274 | 2 | 45.22 | 45.39 | 45.56 |
| 3028.2244 | KRGSN(N)N(D)SFF Fuc 1 Hex 5 HexNAc 5 | 130.788 | 0.0 | -19.606 | 4.379e+07 | 155 - 164 | 1 | 18.19 | 18.32 | 18.32 |
| 4230.9811 | TIVKPGDILLIN(N)STGNLIAPR Hex 10 HexNAc 2 | 129.103 | 0.0 | -4.281 | 1.950e+07 | 250 - 271 | 4 | 51.36 | 51.53 | 51.87 |
| 3793.7086 | TIVKPGDILLIN(N)STGNL Hex 10 HexNAc 2 | 127.647 | 0.0 | -4.484 | 6.764e+06 | 250 - 267 | 2 | 50.84 | 51.02 | 51.19 |
| 3648.6791 | TIVKPGDILLIN(N)STGNL Fuc 2 Hex 3 HexNAc 4 Neu5Ac 1 | 127.610 | 0.0 | 11.644 | 2.649e+07 | 250 - 267 | 3 | 50.84 | 51.02 | 51.53 |
| 4521.8871 | RSDAPIGTC(C)SSEC(C)ITPN(N)GSIPNDKPF Hex 8 HexNAc 2 | 123.323 | 0.0 | 10.565 | 5.050e+07 | 284 - 310 | 4 | 35.84 | 36.01 | 37.54 |
| 2899.1364 | RGSN(N)NSFF Fuc 1 Hex 5 HexNAc 5 | 121.317 | 0.0 | 12.841 | 1.722e+07 | 156 - 164 | 2 | 22.45 | 22.79 | 23.12 |
| 3477.4084 | LPGNDN(N)STATL Fuc 2 Hex 3 HexNAc 5 Neu5Ac 2 | 120.855 | 0.0 | 12.910 | 1.608e+08 | 18 - 29 | 2 | 32.25 | 32.93 | 33.25 |
| 3207.2836 | SIRN(N)GTYDHDVY Fuc 1 Hex 5 HexNAc 4 | 116.654 | 0.0 | -20.279 | 2.532e+06 | 495 - 507 | 1 | 17.21 | 17.21 | 17.21 |
| 3377.3982 | IGSIRN(N)GTYDHDVY Fuc 1 Hex 5 HexNAc 4 | 116.176 | 0.0 | -18.644 | 5.489e+06 | 493 - 507 | 1 | 22.11 | 22.11 | 22.28 |
| 3239.3585 | IN(N)STGNLIAPRGY Hex 9 HexNAc 2 | 114.937 | 0.0 | -18.610 | 7.186e+06 | 260 - 273 | 1 | 29.20 | 29.20 | 29.20 |
| 4683.9387 | RSDAPIGTC(C)SSEC(C)ITPN(N)GSIPNDKPF Hex 9 HexNAc 2 | 112.403 | 0.0 | 9.796 | 4.117e+07 | 284 - 310 | 3 | 35.84 | 36.01 | 36.69 |
| 3369.4740 | LIN(N)STGNLIAPRGY Fuc 2 Hex 3 HexNAc 4 Neu5Ac 1 | 103.568 | 0.008 | -20.841 | 3.723e+06 | 259 - 273 | 1 | 34.70 | 34.70 | 34.70 |
| 3019.2809 | IN(N)STGNLIAPR Hex 9 HexNAc 2 | 103.272 | 0.008 | -20.359 | 3.718e+06 | 260 - 271 | 1 | 21.61 | 21.61 | 21.78 |
| 2824.1559 | KRGSN(N)NSFF Fuc 1 Hex 5 HexNAc 4 | 102.543 | 0.0 | -3.736 | 1.550e+07 | 155 - 164 | 2 | 18.19 | 18.19 | 18.64 |
| 3701.5555 | LYESESKYPVLN(N)VTM(O)PN Hex 8 HexNAc 2 | 101.401 | 0.008 | -4.604 | 8.427e+06 | 169 - 186 | 1 | 35.67 | 35.84 | 36.18 |
| 3825.5166 | LPGNDN(N)STATL Fuc 1 Hex 3 HexNAc 6 Neu5Ac 3 | 100.193 | 0.008 | 12.602 | 1.129e+08 | 18 - 29 | 7 | 31.57 | 32.42 | 32.93 |
| 3469.6004 | TIVKPGDILLIN(N)STGNL Hex 8 HexNAc 2 | 99.249 | 0.008 | -5.192 | 6.974e+06 | 250 - 267 | 2 | 51.02 | 51.02 | 51.53 |
| 3623.4634 | LPGNDN(N)STATL Fuc 3 Hex 3 HexNAc 5 Neu5Ac 2 | 98.084 | 0.008 | 11.947 | 4.986e+07 | 18 - 29 | 2 | 32.08 | 32.76 | 33.09 |
| 2941.2685 | ITPN(N)GSIPNDKPF Hex 7 HexNAc 2 | 97.875 | 0.008 | 0.000 | 0.000e+00 | 297 - 310 | 1 | - | - | - |
| 4391.8924 | ESESKYPVLN(N)VTM(O)PNN(D)GKFDKLY Hex 8 HexNAc 2 | 96.133 | 0.008 | 11.953 | 3.375e+07 | 171 - 194 | 5 | 40.62 | 41.13 | 41.47 |
| 3321.3442 | KRGSN(N)N(D)SFF Fuc 3 Hex 5 HexNAc 5 | 94.260 | 0.0 | 0.000 | 0.000e+00 | 155 - 164 | 1 | - | - | - |
| 2696.0632 | RGSN(N)NSFF Fuc 1 Hex 5 HexNAc 4 | 94.219 | 0.0 | -19.940 | 1.856e+06 | 156 - 164 | 1 | 22.45 | 22.62 | 22.62 |
| 3242.3807 | TITNDQIEVTN(N)ATEL Hex 6 HexNAc 3 | 94.030 | 0.008 | -18.785 | 6.622e+06 | 43 - 58 | 1 | 38.57 | 38.57 | 38.74 |
| 3331.3495 | LPGNDN(N)STATL Fuc 1 Hex 3 HexNAc 5 Neu5Ac 2 | 93.324 | 0.008 | -19.248 | 4.333e+07 | 18 - 29 | 1 | 32.76 | 32.76 | 32.93 |
| 3027.2354 | KRGSN(N)NSFF Fuc 1 Hex 5 HexNAc 5 | 93.323 | 0.0 | -3.658 | 6.299e+07 | 155 - 164 | 3 | 18.48 | 18.48 | 18.98 |
| 3174.2897 | KRGSN(N)N(D)SFF Fuc 2 Hex 5 HexNAc 5 | 91.897 | 0.0 | -20.130 | 9.548e+06 | 155 - 164 | 2 | 17.70 | 18.03 | 18.03 |
| 4553.9431 | ESESKYPVLN(N)VTM(O)PNN(D)GKFDKLY Hex 9 HexNAc 2 | 90.001 | 0.008 | 11.155 | 4.987e+07 | 171 - 194 | 3 | 40.79 | 41.13 | 41.47 |
| 3523.4549 | IGSIRN(N)GTYDHDVY Fuc 2 Hex 5 HexNAc 4 | 89.848 | 0.008 | -20.341 | 2.856e+06 | 493 - 507 | 1 | 21.94 | 21.94 | 22.11 |
| 4700.9529 | ITPN(N)GSIPNDKPF Fuc 4 Hex 8 HexNAc 7 | 89.291 | 0.008 | -6.160 | 4.303e+06 | 297 - 310 | 1 | 35.84 | 36.01 | 36.18 |
| 3565.4270 | SSEC(C)ITPN(N)GSIPN(D)DKPF Hex 8 HexNAc 2 | 86.293 | 0.008 | -6.086 | 1.686e+07 | 293 - 310 | 2 | 34.37 | 34.53 | 34.70 |
| 3116.2690 | KRGSN(N)NSFF Fuc 3 Hex 5 HexNAc 4 | 85.859 | 0.0 | -20.225 | 4.658e+06 | 155 - 164 | 1 | 17.37 | 17.37 | 17.53 |
| 4190.6511 | LPGNDN(N)STATL Fuc 1 Hex 4 HexNAc 7 Neu5Ac 3 | 84.544 | 0.008 | 11.077 | 4.221e+07 | 18 - 29 | 3 | 31.23 | 32.08 | 32.42 |
| 3039.3006 | TITNDQIEVTN(N)ATEL Hex 6 HexNAc 2 | 84.071 | 0.008 | -3.468 | 2.613e+07 | 43 - 58 | 2 | 38.57 | 38.74 | 38.91 |
| 4578.9123 | ITPN(N)GSIPNDKPF Fuc 2 Hex 6 HexNAc 8 Neu5Ac 1 | 83.398 | 0.008 | -4.786 | 8.074e+06 | 297 - 310 | 1 | 36.01 | 36.18 | 36.35 |
| 4443.7739 | LPGNDN(N)STATL Fuc 2 Hex 7 HexNAc 8 Neu5Ac 1 | 82.925 | 0.008 | -5.149 | 2.060e+07 | 18 - 29 | 2 | 37.88 | 38.05 | 38.23 |
| 3769.5244 | LPGNDN(N)STATL Fuc 4 Hex 3 HexNAc 5 Neu5Ac 2 | 80.082 | 0.008 | -4.139 | 1.054e+07 | 18 - 29 | 1 | 32.25 | 32.59 | 32.59 |
| 4405.8274 | ITPN(N)GSIPNDKPF Fuc 1 Hex 6 HexNAc 5 Neu5Ac 3 | 77.907 | 0.008 | 14.119 | 2.412e+08 | 297 - 310 | 7 | 37.71 | 38.05 | 38.57 |
| 3971.5766 | LPGNDN(N)STATL Fuc 2 Hex 3 HexNAc 6 Neu5Ac 3 | 76.852 | 0.008 | 12.075 | 3.619e+07 | 18 - 29 | 3 | 31.74 | 32.25 | 32.59 |
| 4359.8378 | RSDAPIGTC(C)SSEC(C)ITPN(N)GSIPNDKPF Hex 7 HexNAc 2 | 70.412 | 0.008 | -4.678 | 1.066e+07 | 284 - 310 | 3 | 36.01 | 36.18 | 36.35 |
| 4522.8670 | RSDAPIGTC(C)SSEC(C)ITPN(N)GSIPN(D)DKPF Hex 8 HexNAc 2 | 68.812 | 0.008 | -5.758 | 4.162e+07 | 284 - 310 | 3 | 36.86 | 37.03 | 37.37 |
| 5639.5108 | KIRTGKSSIM(O)RSDAPIGTC(C)SSEC(C)ITPN(N)GSIPNDKPF Hex 8 HexNAc 2 | 68.510 | 0.008 | -5.170 | 3.932e+07 | 274 - 310 | 2 | 31.57 | 31.74 | 32.08 |
| 4248.0041 | TIVKPGDILLIN(N)STGNLIAPR Fuc 2 Hex 4 HexNAc 4 Neu5Ac 1 | 66.815 | 0.008 | -18.368 | 1.278e+06 | 250 - 271 | 1 | 51.53 | 51.53 | 51.53 |
| 4148.7669 | LYESESKYPVLN(N)VTM(O)PNN(D)GKF Hex 8 HexNAc 2 | 63.779 | 0.008 | 11.657 | 2.155e+07 | 169 - 190 | 4 | 39.76 | 40.10 | 40.44 |
| 4069.9252 | TIVKPGDILLIN(N)STGN(D)LIAPR Hex 9 HexNAc 2 | 61.982 | 0.011 | -19.578 | 7.533e+06 | 250 - 271 | 2 | 52.55 | 52.55 | 52.72 |
| 3680.6257 | TIVKPGDILLIN(N)STGN Hex 10 HexNAc 2 | 59.939 | 0.011 | -5.253 | 3.326e+06 | 250 - 266 | 2 | 42.66 | 42.83 | 43.00 |
| 4470.1189 | TIVKPGDILLIN(N)STGNLIAPRGY Fuc 2 Hex 4 HexNAc 4 Neu5Ac 1 | 59.761 | 0.011 | 0.000 | 0.000e+00 | 250 - 273 | 2 | - | - | - |
| 4310.8182 | LYESESKYPVLN(N)VTM(O)PNN(D)GKF Hex 9 HexNAc 2 | 59.371 | 0.011 | -6.275 | 3.136e+07 | 169 - 190 | 4 | 39.76 | 40.10 | 40.27 |
| 5801.5635 | KIRTGKSSIM(O)RSDAPIGTC(C)SSEC(C)ITPN(N)GSIPNDKPF Hex 9 HexNAc 2 | 56.675 | 0.011 | -4.943 | 3.347e+07 | 274 - 310 | 2 | 31.57 | 31.57 | 31.91 |
| 4290.0073 | TIVKPGDILLIN(N)STGN(D)LIAPRGY Hex 9 HexNAc 2 | 53.528 | 0.011 | -4.468 | 1.705e+07 | 250 - 273 | 2 | 55.12 | 55.12 | 55.46 |
| 3863.6127 | LYESESKYPVLN(N)VTM(O)PN Hex 9 HexNAc 2 | 52.406 | 0.011 | -5.346 | 1.328e+07 | 169 - 186 | 1 | 35.84 | 35.84 | 36.35 |
| 3727.4782 | SSEC(C)ITPN(N)GSIPN(D)DKPF Hex 9 HexNAc 2 | 48.536 | 0.011 | -5.898 | 1.362e+07 | 293 - 310 | 1 | 34.37 | 34.53 | 34.70 |
| 3810.7262 | TIVKPGDILLIN(N)STGNL Fuc 2 Hex 4 HexNAc 4 Neu5Ac 1 | 47.586 | 0.011 | -19.546 | 1.871e+06 | 250 - 267 | 2 | 50.84 | 51.02 | 51.02 |
| 3478.3950 | SEC(C)ITPN(N)GSIPN(D)DKPF Hex 8 HexNAc 2 | 45.986 | 0.011 | 10.414 | 7.229e+07 | 294 - 310 | 3 | 34.20 | 34.70 | 35.19 |
| 4933.2443 | TITNDQIEVTN(N)ATELVQSSSTGRIC(C)DSPHRIL Hex 6 HexNAc 2 | 45.947 | 0.011 | -19.576 | 2.081e+06 | 43 - 75 | 1 | 45.22 | 45.22 | 45.22 |
| 3336.3632 | INEGFN(N)WTGVTQ Fuc 1 Hex 5 HexNAc 5 | 45.509 | 0.011 | -19.991 | 2.715e+06 | 136 - 148 | 1 | 41.98 | 42.15 | 42.15 |
| 3201.3571 | TITNDQIEVTN(N)ATEL Hex 7 HexNAc 2 | 45.226 | 0.011 | -17.004 | 9.571e+06 | 43 - 58 | 1 | 38.40 | 38.40 | 38.57 |
| 3162.2859 | LPGNDN(N)STATL Fuc 3 Hex 5 HexNAc 4 | 43.980 | 0.011 | -19.870 | 2.271e+06 | 18 - 29 | 1 | 22.62 | 22.79 | 22.79 |
| 4684.9146 | RSDAPIGTC(C)SSEC(C)ITPN(N)GSIPN(D)DKPF Hex 9 HexNAc 2 | 43.754 | 0.011 | 12.732 | 3.046e+07 | 284 - 310 | 2 | 36.69 | 37.03 | 37.37 |
| 3477.4110 | SEC(C)ITPN(N)GSIPNDKPF Hex 8 HexNAc 2 | 43.217 | 0.011 | -19.662 | 2.017e+07 | 294 - 310 | 1 | 33.57 | 33.72 | 33.72 |
| 3353.3482 | SIRN(N)GTYDHDVY Fuc 2 Hex 5 HexNAc 4 | 43.079 | 0.011 | -20.870 | 3.770e+06 | 495 - 507 | 1 | 16.97 | 16.97 | 17.04 |
| 3640.4526 | SEC(C)ITPN(N)GSIPN(D)DKPF Hex 9 HexNAc 2 | 42.736 | 0.011 | -6.558 | 9.939e+06 | 294 - 310 | 1 | 34.37 | 34.70 | 34.70 |
| 4740.9602 | ITPN(N)GSIPNDKPF Fuc 2 Hex 7 HexNAc 8 Neu5Ac 1 | 42.585 | 0.011 | -4.746 | 5.772e+06 | 297 - 310 | 1 | 36.01 | 36.18 | 36.35 |
| 5642.5231 | KIRTGKSSIM(O)RSDAPIGTC(C)SSEC(C)ITPN(N)GSIPNDKPF Hex 8 HexNAc 2 | 41.566 | 0.011 | 0.000 | 0.000e+00 | 274 - 310 | 1 | - | - | - |
| 4070.9301 | TIVKPGDILLIN(N)STGNLIAPR Hex 9 HexNAc 2 | 41.246 | 0.011 | 0.000 | 0.000e+00 | 250 - 271 | 1 | - | - | - |
| 5640.4984 | KIRTGKSSIM(O)RSDAPIGTC(C)SSEC(C)ITPN(N)GSIPN(D)DKPF Hex 8 HexNAc 2 | 40.464 | 0.011 | -21.048 | 2.811e+07 | 274 - 310 | 1 | 32.25 | 32.25 | 32.42 |
| 4686.9249 | RSDAPIGTC(C)SSEC(C)ITPN(N)GSIPN(D)DKPF Hex 9 HexNAc 2 | 39.272 | 0.011 | 0.000 | 0.000e+00 | 284 - 310 | 1 | - | - | - |
| 4676.9041 | ITPN(N)GSIPNDKPF Fuc 3 Hex 10 HexNAc 6 | 37.860 | 0.011 | 0.000 | 0.000e+00 | 297 - 310 | 1 | - | - | - |
| 4887.1042 | ESESKYPVLN(N)VTM(O)PNN(D)GKFDKLY Fuc 2 Hex 8 HexNAc 3 | 37.462 | 0.011 | 12.946 | 2.008e+08 | 171 - 194 | 14 | 42.66 | 43.69 | 44.20 |
| 3965.8965 | TIVKPGDILLIN(N)STGNLIAPRGY Hex 7 HexNAc 2 | 37.311 | 0.015 | 0.000 | 0.000e+00 | 250 - 273 | 1 | - | - | - |
| 4452.0584 | TIVKPGDILLIN(N)STGN(D)LIAPRGY Hex 10 HexNAc 2 | 37.109 | 0.015 | -20.452 | 2.535e+05 | 250 - 273 | 2 | 53.92 | 53.92 | 53.92 |
| 3608.4604 | AC(C)IGSIRN(N)GTYDHDVY Fuc 1 Hex 5 HexNAc 4 | 36.474 | 0.015 | -6.040 | 3.594e+06 | 491 - 507 | 2 | 26.17 | 26.34 | 26.51 |
| 5002.0554 | SSEC(C)ITPN(N)GSIPNDKPF Fuc 4 Hex 7 HexNAc 7 | 35.792 | 0.015 | -4.087 | 1.754e+07 | 293 - 310 | 2 | 39.25 | 39.25 | 39.76 |
| 4360.8140 | RSDAPIGTC(C)SSEC(C)ITPN(N)GSIPN(D)DKPF Hex 7 HexNAc 2 | 35.529 | 0.015 | -5.998 | 1.008e+07 | 284 - 310 | 4 | 36.86 | 37.03 | 37.37 |
| 5802.5468 | KIRTGKSSIM(O)RSDAPIGTC(C)SSEC(C)ITPN(N)GSIPN(D)DKPF Hex 9 HexNAc 2 | 34.791 | 0.015 | -6.120 | 3.221e+07 | 274 - 310 | 2 | 32.08 | 32.42 | 32.59 |
| 2950.1768 | LPGNDN(N)STATL Fuc 1 Hex 8 HexNAc 2 | 34.491 | 0.015 | 12.675 | 7.001e+08 | 18 - 29 | 7 | 34.37 | 35.03 | 37.03 |
| 4538.8989 | ITPN(N)GSIPNDKPF Fuc 4 Hex 7 HexNAc 7 | 33.978 | 0.015 | -8.160 | 6.193e+06 | 297 - 310 | 2 | 36.01 | 36.01 | 37.03 |
| 3606.4395 | LPGNDN(N)STATL Fuc 1 Hex 9 HexNAc 3 Neu5Ac 1 | 33.872 | 0.015 | 13.998 | 2.580e+08 | 18 - 29 | 6 | 32.08 | 32.76 | 33.25 |
| 4512.8401 | ITPN(N)GSIPNDKPF Fuc 1 Hex 9 HexNAc 6 Neu5Ac 1 | 33.503 | 0.015 | -5.378 | 9.837e+06 | 297 - 310 | 2 | 38.91 | 39.08 | 39.25 |
| 2927.1989 | LPGNDN(N)STATL Hex 5 HexNAc 5 | 33.458 | 0.015 | -4.785 | 1.190e+07 | 18 - 29 | 2 | 22.95 | 23.12 | 23.46 |
| 4840.0021 | SSEC(C)ITPN(N)GSIPNDKPF Fuc 4 Hex 6 HexNAc 7 | 31.813 | 0.024 | -3.938 | 2.193e+07 | 293 - 310 | 2 | 39.25 | 39.25 | 39.59 |
| 5477.4636 | KIRTGKSSIM(O)RSDAPIGTC(C)SSEC(C)ITPN(N)GSIPNDKPF Hex 7 HexNAc 2 | 31.521 | 0.027 | -19.845 | 6.691e+06 | 274 - 310 | 1 | 31.57 | 31.74 | 31.74 |
| 4683.9151 | ITPN(N)GSIPNDKPF Fuc 3 Hex 7 HexNAc 7 Neu5Ac 1 | 31.454 | 0.027 | -20.899 | 1.579e+06 | 297 - 310 | 1 | 37.37 | 37.37 | 37.54 |
| 4725.0533 | ESESKYPVLN(N)VTM(O)PNN(D)GKFDKLY Fuc 2 Hex 7 HexNAc 3 | 31.268 | 0.027 | 13.566 | 1.266e+08 | 171 - 194 | 5 | 43.18 | 43.86 | 44.03 |
| 2877.2505 | TITNDQIEVTN(N)ATEL Hex 5 HexNAc 2 | 31.191 | 0.027 | -2.979 | 1.094e+07 | 43 - 58 | 2 | 38.74 | 38.91 | 39.08 |
| 4142.9791 | TIVKPGDILLIN(N)STGNLIAPR Fuc 1 Hex 3 HexNAc 5 Neu5Ac 1 | 30.722 | 0.027 | -18.761 | 6.662e+06 | 250 - 271 | 1 | 52.04 | 52.21 | 52.21 |
| 4652.9345 | ITPN(N)GSIPNDKPF Fuc 2 Hex 7 HexNAc 9 | 30.698 | 0.027 | 0.000 | 0.000e+00 | 297 - 310 | 1 | - | - | - |
| 3089.2521 | LPGNDN(N)STATL Hex 6 HexNAc 5 | 29.629 | 0.027 | -5.451 | 2.510e+06 | 18 - 29 | 1 | 22.45 | 22.79 | 22.79 |
| 4143.9887 | TIVKPGDILLIN(N)STGNLIAPR Fuc 3 Hex 3 HexNAc 5 | 29.219 | 0.027 | -19.641 | 4.375e+06 | 250 - 271 | 2 | 54.27 | 54.27 | 54.43 |
| 4363.0624 | TIVKPGDILLIN(N)STGNLIAPRGY Fuc 1 Hex 3 HexNAc 5 Neu5Ac 1 | 28.743 | 0.027 | 10.046 | 3.197e+07 | 250 - 273 | 5 | 54.27 | 54.78 | 55.63 |
| 4831.0725 | ESESKYPVLN(N)VTM(O)PNN(D)GKFDKLY Fuc 1 Hex 3 HexNAc 6 Neu5Ac 1 | 27.293 | 0.035 | 10.207 | 1.210e+08 | 171 - 194 | 7 | 44.37 | 44.37 | 45.90 |
| 3388.4401 | TITNDQIEVTN(N)ATEL Fuc 1 Hex 6 HexNAc 3 | 26.667 | 0.039 | -19.886 | 1.650e+06 | 43 - 58 | 1 | 38.40 | 38.40 | 38.40 |
| 4742.9821 | ITPN(N)GSIPNDKPF Fuc 4 Hex 7 HexNAc 8 | 26.431 | 0.042 | 0.000 | 0.000e+00 | 297 - 310 | 1 | - | - | - |
| 4307.0399 | TIVKPGDILLIN(N)STGN(D)LIAPRGY Fuc 2 Hex 3 HexNAc 4 Neu5Ac 1 | 26.255 | 0.042 | -5.735 | 2.802e+06 | 250 - 273 | 2 | 55.12 | 55.12 | 55.46 |
| 4453.1076 | TIVKPGDILLIN(N)STGNLIAPRGY Hex 5 HexNAc 6 | 26.178 | 0.042 | 11.516 | 4.950e+07 | 250 - 273 | 3 | 60.07 | 60.42 | 61.10 |
| 2905.1722 | INEGFN(N)WTGVTQ Hex 7 HexNAc 2 | 25.765 | 0.046 | -20.778 | 6.148e+05 | 136 - 148 | 1 | 42.83 | 42.83 | 42.83 |

### LIN(N)STGNLIAPRGYF Hex 9 HexNAc 2

**Mass:**  3499.5174

(1.459e-06)

**Charge States:** 3

**Start Position**: 260

**End Position**: 275

| Profile Score | Tandem Score | TDA q-value | Volume | Spectrum Matches |
| --- | --- | --- | --- | --- |
| -4.4822 | 181.0248 | 0.0000 | 3.4871e+07 | 1 |

**Adduct Signal Fractions**

- **Unmodified**: 3.4871e+07

### LIN(N)STGNL Hex 9 HexNAc 2

**Mass:**  2695.0889

(1.845e-06)

**Charge States:** 2

**Start Position**: 260

**End Position**: 268

| Profile Score | Tandem Score | TDA q-value | Volume | Spectrum Matches |
| --- | --- | --- | --- | --- |
| -5.1657 | 180.2209 | 0.0000 | 1.0929e+07 | 2 |

**Adduct Signal Fractions**

- **Unmodified**: 1.0929e+07

### LPGNDN(N)STATL Fuc 1 Hex 5 HexNAc 4

**Mass:**  2870.1771

(1.415e-06)

**Charge States:** 3

**Start Position**: 19

**End Position**: 30

| Profile Score | Tandem Score | TDA q-value | Volume | Spectrum Matches |
| --- | --- | --- | --- | --- |
| 11.8846 | 175.0944 | 0.0000 | 1.0548e+08 | 9 |

**Adduct Signal Fractions**

- **Unmodified**: 1.0548e+08

### LIN(N)STGNLIAPR Hex 9 HexNAc 2

**Mass:**  3132.3634

(1.442e-06)

**Charge States:** 3

**Start Position**: 260

**End Position**: 272

| Profile Score | Tandem Score | TDA q-value | Volume | Spectrum Matches |
| --- | --- | --- | --- | --- |
| -2.5356 | 172.7513 | 0.0000 | 2.2149e+07 | 2 |

**Adduct Signal Fractions**

- **Unmodified**: 2.2149e+07

### LPGNDN(N)STATL Hex 5 HexNAc 4

**Mass:**  2724.1171

(1.927e-06)

**Charge States:** 3

**Start Position**: 19

**End Position**: 30

| Profile Score | Tandem Score | TDA q-value | Volume | Spectrum Matches |
| --- | --- | --- | --- | --- |
| 11.9372 | 168.5080 | 0.0000 | 2.1888e+07 | 3 |

**Adduct Signal Fractions**

- **Unmodified**: 2.1888e+07

### TIVKPGDILLIN(N)STGNLIAPR Hex 9 HexNAc 2

**Mass:**  4068.9304

(1.703e-06)

**Charge States:** 3, 4, 5

**Start Position**: 251

**End Position**: 272

| Profile Score | Tandem Score | TDA q-value | Volume | Spectrum Matches |
| --- | --- | --- | --- | --- |
| 14.4912 | 168.0398 | 0.0000 | 2.3038e+08 | 11 |

**Adduct Signal Fractions**

- **Unmodified**: 2.3038e+08

### LPGNDN(N)STATL Hex 5 HexNAc 2

**Mass:**  2317.9557

(-3.548e-07)

**Charge States:** 2, 3

**Start Position**: 19

**End Position**: 30

| Profile Score | Tandem Score | TDA q-value | Volume | Spectrum Matches |
| --- | --- | --- | --- | --- |
| 11.1404 | 160.1294 | 0.0000 | 1.2680e+07 | 3 |

**Adduct Signal Fractions**

- **Unmodified**: 1.2680e+07

### TIVKPGDILLIN(N) Hex 9 HexNAc 2

**Mass:**  3159.4246

(2.290e-06)

**Charge States:** 2, 3

**Start Position**: 251

**End Position**: 263

| Profile Score | Tandem Score | TDA q-value | Volume | Spectrum Matches |
| --- | --- | --- | --- | --- |
| 13.5549 | 159.2149 | 0.0000 | 1.2243e+08 | 8 |

**Adduct Signal Fractions**

- **Unmodified**: 1.2243e+08

### LIN(N)STGNLIAPRGY Hex 9 HexNAc 2

**Mass:**  3352.4518

(2.400e-06)

**Charge States:** 3

**Start Position**: 260

**End Position**: 274

| Profile Score | Tandem Score | TDA q-value | Volume | Spectrum Matches |
| --- | --- | --- | --- | --- |
| -5.0760 | 158.6404 | 0.0000 | 4.4482e+07 | 2 |

**Adduct Signal Fractions**

- **Unmodified**: 4.4482e+07

### TIVKPGDILLIN(N) Hex 9 HexNAc 2

**Mass:**  3160.4377

(3.220e-04)

**Start Position**: 251

**End Position**: 263

| Profile Score | Tandem Score | TDA q-value | Volume | Spectrum Matches |
| --- | --- | --- | --- | --- |
| 0.0000 | 156.5694 | 0.0000 | 0.0000e+00 | 1 |

Adduct:

No Chromatogram Found

### TIVKPGDILLIN(N)STGNLIAPRGY Hex 9 HexNAc 2

**Mass:**  4289.0158

(2.012e-06)

**Charge States:** 3, 4, 5

**Start Position**: 251

**End Position**: 274

| Profile Score | Tandem Score | TDA q-value | Volume | Spectrum Matches |
| --- | --- | --- | --- | --- |
| 15.0400 | 155.7164 | 0.0000 | 7.2359e+08 | 26 |

**Adduct Signal Fractions**

- **Unmodified**: 7.2359e+08

### TIVKPGDILLIN(N) Fuc 2 Hex 3 HexNAc 4 Neu5Ac 1

**Mass:**  3176.4527

(-6.130e-06)

**Charge States:** 3

**Start Position**: 251

**End Position**: 263

| Profile Score | Tandem Score | TDA q-value | Volume | Spectrum Matches |
| --- | --- | --- | --- | --- |
| 8.8129 | 154.2498 | 0.0000 | 3.0749e+07 | 4 |

**Adduct Signal Fractions**

- **Unmodified**: 3.0749e+07

### TIVKPGDILLIN(N)STGNLIAPRGY Fuc 2 Hex 3 HexNAc 4 Neu5Ac 1

**Mass:**  4306.0388

(-5.388e-06)

**Charge States:** 4

**Start Position**: 251

**End Position**: 274

| Profile Score | Tandem Score | TDA q-value | Volume | Spectrum Matches |
| --- | --- | --- | --- | --- |
| 13.2501 | 150.3944 | 0.0000 | 7.9177e+07 | 3 |

**Adduct Signal Fractions**

- **Unmodified**: 7.9177e+07

### TIVKPGDILLIN(N)STGNL Hex 9 HexNAc 2

**Mass:**  3631.6518

(2.171e-06)

**Charge States:** 3, 4

**Start Position**: 251

**End Position**: 268

| Profile Score | Tandem Score | TDA q-value | Volume | Spectrum Matches |
| --- | --- | --- | --- | --- |
| 13.3026 | 149.7732 | 0.0000 | 9.4716e+07 | 6 |

**Adduct Signal Fractions**

- **Unmodified**: 9.4716e+07

### TIVKPGDILLIN(N)STGN Hex 9 HexNAc 2

**Mass:**  3518.5728

(2.498e-06)

**Charge States:** 3, 4

**Start Position**: 251

**End Position**: 267

| Profile Score | Tandem Score | TDA q-value | Volume | Spectrum Matches |
| --- | --- | --- | --- | --- |
| 14.0439 | 149.7374 | 0.0000 | 4.3945e+07 | 6 |

**Adduct Signal Fractions**

- **Unmodified**: 4.3945e+07

### TIVKPGDILLIN(N) Hex 10 HexNAc 2

**Mass:**  3321.4780

(2.598e-06)

**Charge States:** 3

**Start Position**: 251

**End Position**: 263

| Profile Score | Tandem Score | TDA q-value | Volume | Spectrum Matches |
| --- | --- | --- | --- | --- |
| -5.2117 | 146.5437 | 0.0000 | 5.2452e+06 | 2 |

**Adduct Signal Fractions**

- **Unmodified**: 5.2452e+06

### LPGNDN(N)STATL Hex 6 HexNAc 3

**Mass:**  2683.0861

(-2.521e-06)

**Charge States:** 3

**Start Position**: 19

**End Position**: 30

| Profile Score | Tandem Score | TDA q-value | Volume | Spectrum Matches |
| --- | --- | --- | --- | --- |
| -5.6958 | 142.8547 | 0.0000 | 5.9209e+06 | 2 |

**Adduct Signal Fractions**

- **Unmodified**: 5.9209e+06

### TIVKPGDILLIN(N)STGNLIAPR Fuc 2 Hex 3 HexNAc 4 Neu5Ac 1

**Mass:**  4085.9528

(-4.763e-06)

**Charge States:** 4

**Start Position**: 251

**End Position**: 272

| Profile Score | Tandem Score | TDA q-value | Volume | Spectrum Matches |
| --- | --- | --- | --- | --- |
| -3.6508 | 140.3668 | 0.0000 | 2.3479e+07 | 2 |

**Adduct Signal Fractions**

- **Unmodified**: 2.3479e+07

### TIVKPGDILLIN(N)STGNLIAPRGY Hex 10 HexNAc 2

**Mass:**  4451.0656

(1.004e-06)

**Charge States:** 3, 4

**Start Position**: 251

**End Position**: 274

| Profile Score | Tandem Score | TDA q-value | Volume | Spectrum Matches |
| --- | --- | --- | --- | --- |
| 14.2639 | 140.2896 | 0.0000 | 6.5855e+07 | 7 |

**Adduct Signal Fractions**

- **Unmodified**: 6.5855e+07

### LPGNDN(N)STATL Fuc 1 Hex 6 HexNAc 5

**Mass:**  3235.3082

(2.898e-06)

**Charge States:** 3

**Start Position**: 19

**End Position**: 30

| Profile Score | Tandem Score | TDA q-value | Volume | Spectrum Matches |
| --- | --- | --- | --- | --- |
| 12.6803 | 139.3924 | 0.0000 | 1.0518e+07 | 2 |

**Adduct Signal Fractions**

- **Unmodified**: 1.0518e+07

### LPGNDN(N)STATL Fuc 2 Hex 5 HexNAc 4

**Mass:**  3016.2328

(3.713e-07)

**Charge States:** 3

**Start Position**: 19

**End Position**: 30

| Profile Score | Tandem Score | TDA q-value | Volume | Spectrum Matches |
| --- | --- | --- | --- | --- |
| 11.5131 | 134.5931 | 0.0000 | 1.6436e+07 | 2 |

**Adduct Signal Fractions**

- **Unmodified**: 1.6436e+07

### TIVKPGDILLIN(N)STGN Fuc 2 Hex 3 HexNAc 4 Neu5Ac 1

**Mass:**  3535.5990

(-5.144e-06)

**Charge States:** 3

**Start Position**: 251

**End Position**: 267

| Profile Score | Tandem Score | TDA q-value | Volume | Spectrum Matches |
| --- | --- | --- | --- | --- |
| -4.0515 | 133.5477 | 0.0000 | 1.0888e+07 | 2 |

**Adduct Signal Fractions**

- **Unmodified**: 1.0888e+07

### ITPN(N)GSIPNDKPF Hex 9 HexNAc 2

**Mass:**  3263.3545

(1.118e-06)

**Charge States:** 3

**Start Position**: 298

**End Position**: 311

| Profile Score | Tandem Score | TDA q-value | Volume | Spectrum Matches |
| --- | --- | --- | --- | --- |
| -4.9384 | 133.1730 | 0.0000 | 1.0390e+07 | 2 |

**Adduct Signal Fractions**

- **Unmodified**: 1.0390e+07

### TIVKPGDILLIN(N)STGNLIAPR Hex 8 HexNAc 2

**Mass:**  3906.8796

(2.157e-06)

**Charge States:** 3, 4

**Start Position**: 251

**End Position**: 272

| Profile Score | Tandem Score | TDA q-value | Volume | Spectrum Matches |
| --- | --- | --- | --- | --- |
| -5.4905 | 132.4210 | 0.0000 | 1.3195e+07 | 4 |

**Adduct Signal Fractions**

- **Unmodified**: 1.3195e+07

### IN(N)STGNLIAPRGYF Hex 9 HexNAc 2

**Mass:**  3386.4376

(3.698e-06)

**Charge States:** 3

**Start Position**: 261

**End Position**: 275

| Profile Score | Tandem Score | TDA q-value | Volume | Spectrum Matches |
| --- | --- | --- | --- | --- |
| -4.1678 | 132.2941 | 0.0000 | 9.8394e+06 | 2 |

**Adduct Signal Fractions**

- **Unmodified**: 9.8394e+06

### TIVKPGDILLIN(N)STGNLIAPRGY Hex 8 HexNAc 2

**Mass:**  4126.9611

(1.133e-06)

**Charge States:** 3, 4

**Start Position**: 251

**End Position**: 274

| Profile Score | Tandem Score | TDA q-value | Volume | Spectrum Matches |
| --- | --- | --- | --- | --- |
| 11.9367 | 131.4488 | 0.0000 | 7.5652e+07 | 13 |

**Adduct Signal Fractions**

- **Unmodified**: 7.5652e+07

### LIN(N)STGNLIAPRGYF Fuc 2 Hex 3 HexNAc 4 Neu5Ac 1

**Mass:**  3516.5443

(-3.868e-06)

**Charge States:** 3

**Start Position**: 260

**End Position**: 275

| Profile Score | Tandem Score | TDA q-value | Volume | Spectrum Matches |
| --- | --- | --- | --- | --- |
| -4.9391 | 130.8183 | 0.0000 | 6.0265e+06 | 2 |

**Adduct Signal Fractions**

- **Unmodified**: 6.0265e+06

### KRGSN(N)N(D)SFF Fuc 1 Hex 5 HexNAc 5

**Mass:**  3028.2269

(3.281e-04)

**Charge States:** 3

**Start Position**: 156

**End Position**: 165

| Profile Score | Tandem Score | TDA q-value | Volume | Spectrum Matches |
| --- | --- | --- | --- | --- |
| -19.6061 | 130.7876 | 0.0000 | 4.3787e+07 | 1 |

**Adduct Signal Fractions**

- **Unmodified**: 4.3787e+07

### TIVKPGDILLIN(N)STGNLIAPR Hex 10 HexNAc 2

**Mass:**  4230.9826

(1.519e-06)

**Charge States:** 3, 4

**Start Position**: 251

**End Position**: 272

| Profile Score | Tandem Score | TDA q-value | Volume | Spectrum Matches |
| --- | --- | --- | --- | --- |
| -4.2808 | 129.1026 | 0.0000 | 1.9498e+07 | 4 |

**Adduct Signal Fractions**

- **Unmodified**: 1.9498e+07

### TIVKPGDILLIN(N)STGNL Hex 10 HexNAc 2

**Mass:**  3793.7067

(1.908e-06)

**Charge States:** 3

**Start Position**: 251

**End Position**: 268

| Profile Score | Tandem Score | TDA q-value | Volume | Spectrum Matches |
| --- | --- | --- | --- | --- |
| -4.4836 | 127.6473 | 0.0000 | 6.7641e+06 | 2 |

**Adduct Signal Fractions**

- **Unmodified**: 6.7641e+06

### TIVKPGDILLIN(N)STGNL Fuc 2 Hex 3 HexNAc 4 Neu5Ac 1

**Mass:**  3648.6787

(-4.353e-06)

**Charge States:** 3

**Start Position**: 251

**End Position**: 268

| Profile Score | Tandem Score | TDA q-value | Volume | Spectrum Matches |
| --- | --- | --- | --- | --- |
| 11.6435 | 127.6096 | 0.0000 | 2.6493e+07 | 3 |

**Adduct Signal Fractions**

- **Unmodified**: 2.6493e+07

### RSDAPIGTC(C)SSEC(C)ITPN(N)GSIPNDKPF Hex 8 HexNAc 2

**Mass:**  4521.8928

(5.796e-06)

**Charge States:** 3, 4

**Start Position**: 285

**End Position**: 311

| Profile Score | Tandem Score | TDA q-value | Volume | Spectrum Matches |
| --- | --- | --- | --- | --- |
| 10.5652 | 123.3230 | 0.0000 | 5.0503e+07 | 4 |

**Adduct Signal Fractions**

- **Unmodified**: 5.0503e+07

### RGSN(N)NSFF Fuc 1 Hex 5 HexNAc 5

**Mass:**  2899.1365

(-1.592e-06)

**Charge States:** 3

**Start Position**: 157

**End Position**: 165

| Profile Score | Tandem Score | TDA q-value | Volume | Spectrum Matches |
| --- | --- | --- | --- | --- |
| 12.8406 | 121.3169 | 0.0000 | 1.7222e+07 | 2 |

**Adduct Signal Fractions**

- **Unmodified**: 1.7222e+07

### LPGNDN(N)STATL Fuc 2 Hex 3 HexNAc 5 Neu5Ac 2

**Mass:**  3477.4085

(4.674e-06)

**Charge States:** 3

**Start Position**: 19

**End Position**: 30

| Profile Score | Tandem Score | TDA q-value | Volume | Spectrum Matches |
| --- | --- | --- | --- | --- |
| 12.9096 | 120.8555 | 0.0000 | 1.6076e+08 | 2 |

**Adduct Signal Fractions**

- **Unmodified**: 1.6076e+08

### SIRN(N)GTYDHDVY Fuc 1 Hex 5 HexNAc 4

**Mass:**  3207.2836

(-1.172e-06)

**Charge States:** 3

**Start Position**: 496

**End Position**: 508

| Profile Score | Tandem Score | TDA q-value | Volume | Spectrum Matches |
| --- | --- | --- | --- | --- |
| -20.2786 | 116.6541 | 0.0000 | 2.5325e+06 | 1 |

**Adduct Signal Fractions**

- **Unmodified**: 2.5325e+06

### IGSIRN(N)GTYDHDVY Fuc 1 Hex 5 HexNAc 4

**Mass:**  3377.3985

(1.684e-06)

**Charge States:** 3

**Start Position**: 494

**End Position**: 508

| Profile Score | Tandem Score | TDA q-value | Volume | Spectrum Matches |
| --- | --- | --- | --- | --- |
| -18.6441 | 116.1762 | 0.0000 | 5.4891e+06 | 1 |

**Adduct Signal Fractions**

- **Unmodified**: 5.4891e+06

### IN(N)STGNLIAPRGY Hex 9 HexNAc 2

**Mass:**  3239.3585

(-3.600e-07)

**Charge States:** 3

**Start Position**: 261

**End Position**: 274

| Profile Score | Tandem Score | TDA q-value | Volume | Spectrum Matches |
| --- | --- | --- | --- | --- |
| -18.6104 | 114.9374 | 0.0000 | 7.1864e+06 | 1 |

**Adduct Signal Fractions**

- **Unmodified**: 7.1864e+06

### RSDAPIGTC(C)SSEC(C)ITPN(N)GSIPNDKPF Hex 9 HexNAc 2

**Mass:**  4683.9445

(3.579e-06)

**Charge States:** 3, 4

**Start Position**: 285

**End Position**: 311

| Profile Score | Tandem Score | TDA q-value | Volume | Spectrum Matches |
| --- | --- | --- | --- | --- |
| 9.7958 | 112.4033 | 0.0000 | 4.1172e+07 | 3 |

**Adduct Signal Fractions**

- **Unmodified**: 4.1172e+07

### LIN(N)STGNLIAPRGY Fuc 2 Hex 3 HexNAc 4 Neu5Ac 1

**Mass:**  3369.4740

(-6.727e-06)

**Charge States:** 3

**Start Position**: 260

**End Position**: 274

| Profile Score | Tandem Score | TDA q-value | Volume | Spectrum Matches |
| --- | --- | --- | --- | --- |
| -20.8413 | 103.5678 | 0.0079 | 3.7235e+06 | 1 |

**Adduct Signal Fractions**

- **Unmodified**: 3.7235e+06

### IN(N)STGNLIAPR Hex 9 HexNAc 2

**Mass:**  3019.2802

(1.749e-06)

**Charge States:** 3

**Start Position**: 261

**End Position**: 272

| Profile Score | Tandem Score | TDA q-value | Volume | Spectrum Matches |
| --- | --- | --- | --- | --- |
| -20.3589 | 103.2717 | 0.0079 | 3.7179e+06 | 1 |

**Adduct Signal Fractions**

- **Unmodified**: 3.7179e+06

### KRGSN(N)NSFF Fuc 1 Hex 5 HexNAc 4

**Mass:**  2824.1555

(3.874e-07)

**Charge States:** 3

**Start Position**: 156

**End Position**: 165

| Profile Score | Tandem Score | TDA q-value | Volume | Spectrum Matches |
| --- | --- | --- | --- | --- |
| -3.7362 | 102.5430 | 0.0000 | 1.5501e+07 | 2 |

**Adduct Signal Fractions**

- **Unmodified**: 1.5501e+07

### LYESESKYPVLN(N)VTM(O)PN Hex 8 HexNAc 2

**Mass:**  3701.5523

(2.730e-06)

**Charge States:** 3

**Start Position**: 170

**End Position**: 187

| Profile Score | Tandem Score | TDA q-value | Volume | Spectrum Matches |
| --- | --- | --- | --- | --- |
| -4.6044 | 101.4009 | 0.0079 | 8.4269e+06 | 1 |

**Adduct Signal Fractions**

- **Unmodified**: 8.4269e+06

### LPGNDN(N)STATL Fuc 1 Hex 3 HexNAc 6 Neu5Ac 3

**Mass:**  3825.5147

(1.896e-06)

**Charge States:** 3, 4

**Start Position**: 19

**End Position**: 30

| Profile Score | Tandem Score | TDA q-value | Volume | Spectrum Matches |
| --- | --- | --- | --- | --- |
| 12.6024 | 100.1934 | 0.0079 | 1.1293e+08 | 7 |

**Adduct Signal Fractions**

- **Unmodified**: 1.1293e+08

### TIVKPGDILLIN(N)STGNL Hex 8 HexNAc 2

**Mass:**  3469.6027

(1.411e-06)

**Charge States:** 3

**Start Position**: 251

**End Position**: 268

| Profile Score | Tandem Score | TDA q-value | Volume | Spectrum Matches |
| --- | --- | --- | --- | --- |
| -5.1918 | 99.2492 | 0.0079 | 6.9745e+06 | 2 |

**Adduct Signal Fractions**

- **Unmodified**: 6.9745e+06

### LPGNDN(N)STATL Fuc 3 Hex 3 HexNAc 5 Neu5Ac 2

**Mass:**  3623.4629

(3.640e-06)

**Charge States:** 3

**Start Position**: 19

**End Position**: 30

| Profile Score | Tandem Score | TDA q-value | Volume | Spectrum Matches |
| --- | --- | --- | --- | --- |
| 11.9474 | 98.0838 | 0.0079 | 4.9863e+07 | 2 |

**Adduct Signal Fractions**

- **Unmodified**: 4.9863e+07

### ITPN(N)GSIPNDKPF Hex 7 HexNAc 2

**Mass:**  2941.2685

(6.892e-04)

**Start Position**: 298

**End Position**: 311

| Profile Score | Tandem Score | TDA q-value | Volume | Spectrum Matches |
| --- | --- | --- | --- | --- |
| 0.0000 | 97.8750 | 0.0079 | 0.0000e+00 | 1 |

Adduct:

No Chromatogram Found

### ESESKYPVLN(N)VTM(O)PNN(D)GKFDKLY Hex 8 HexNAc 2

**Mass:**  4391.8933

(2.281e-04)

**Charge States:** 4

**Start Position**: 172

**End Position**: 195

| Profile Score | Tandem Score | TDA q-value | Volume | Spectrum Matches |
| --- | --- | --- | --- | --- |
| 11.9531 | 96.1334 | 0.0079 | 3.3746e+07 | 5 |

**Adduct Signal Fractions**

- **Unmodified**: 3.3746e+07

### KRGSN(N)N(D)SFF Fuc 3 Hex 5 HexNAc 5

**Mass:**  3321.3442

(6.009e-04)

**Start Position**: 156

**End Position**: 165

| Profile Score | Tandem Score | TDA q-value | Volume | Spectrum Matches |
| --- | --- | --- | --- | --- |
| 0.0000 | 94.2602 | 0.0000 | 0.0000e+00 | 1 |

Adduct:

No Chromatogram Found

### RGSN(N)NSFF Fuc 1 Hex 5 HexNAc 4

**Mass:**  2696.0630

(1.346e-06)

**Charge States:** 3

**Start Position**: 157

**End Position**: 165

| Profile Score | Tandem Score | TDA q-value | Volume | Spectrum Matches |
| --- | --- | --- | --- | --- |
| -19.9399 | 94.2187 | 0.0000 | 1.8557e+06 | 1 |

**Adduct Signal Fractions**

- **Unmodified**: 1.8557e+06

### TITNDQIEVTN(N)ATEL Hex 6 HexNAc 3

**Mass:**  3242.3845

(4.253e-06)

**Charge States:** 3

**Start Position**: 44

**End Position**: 59

| Profile Score | Tandem Score | TDA q-value | Volume | Spectrum Matches |
| --- | --- | --- | --- | --- |
| -18.7852 | 94.0299 | 0.0079 | 6.6220e+06 | 1 |

**Adduct Signal Fractions**

- **Unmodified**: 6.6220e+06

### LPGNDN(N)STATL Fuc 1 Hex 3 HexNAc 5 Neu5Ac 2

**Mass:**  3331.3489

(4.430e-06)

**Charge States:** 3

**Start Position**: 19

**End Position**: 30

| Profile Score | Tandem Score | TDA q-value | Volume | Spectrum Matches |
| --- | --- | --- | --- | --- |
| -19.2479 | 93.3235 | 0.0079 | 4.3332e+07 | 1 |

**Adduct Signal Fractions**

- **Unmodified**: 4.3332e+07

### KRGSN(N)NSFF Fuc 1 Hex 5 HexNAc 5

**Mass:**  3027.2354

(2.399e-07)

**Charge States:** 3

**Start Position**: 156

**End Position**: 165

| Profile Score | Tandem Score | TDA q-value | Volume | Spectrum Matches |
| --- | --- | --- | --- | --- |
| -3.6578 | 93.3232 | 0.0000 | 6.2993e+07 | 3 |

**Adduct Signal Fractions**

- **Unmodified**: 6.2993e+07

### KRGSN(N)N(D)SFF Fuc 2 Hex 5 HexNAc 5

**Mass:**  3174.2909

(3.138e-04)

**Charge States:** 3

**Start Position**: 156

**End Position**: 165

| Profile Score | Tandem Score | TDA q-value | Volume | Spectrum Matches |
| --- | --- | --- | --- | --- |
| -20.1301 | 91.8975 | 0.0000 | 9.5480e+06 | 2 |

**Adduct Signal Fractions**

- **Unmodified**: 9.5480e+06

### ESESKYPVLN(N)VTM(O)PNN(D)GKFDKLY Hex 9 HexNAc 2

**Mass:**  4553.9426

(2.194e-04)

**Charge States:** 4

**Start Position**: 172

**End Position**: 195

| Profile Score | Tandem Score | TDA q-value | Volume | Spectrum Matches |
| --- | --- | --- | --- | --- |
| 11.1549 | 90.0014 | 0.0079 | 4.9867e+07 | 3 |

**Adduct Signal Fractions**

- **Unmodified**: 4.9867e+07

### IGSIRN(N)GTYDHDVY Fuc 2 Hex 5 HexNAc 4

**Mass:**  3523.4547

(1.125e-06)

**Charge States:** 3

**Start Position**: 494

**End Position**: 508

| Profile Score | Tandem Score | TDA q-value | Volume | Spectrum Matches |
| --- | --- | --- | --- | --- |
| -20.3413 | 89.8478 | 0.0079 | 2.8557e+06 | 1 |

**Adduct Signal Fractions**

- **Unmodified**: 2.8557e+06

### ITPN(N)GSIPNDKPF Fuc 4 Hex 8 HexNAc 7

**Mass:**  4700.9645

(8.601e-06)

**Charge States:** 4

**Start Position**: 298

**End Position**: 311

| Profile Score | Tandem Score | TDA q-value | Volume | Spectrum Matches |
| --- | --- | --- | --- | --- |
| -6.1603 | 89.2912 | 0.0079 | 4.3032e+06 | 1 |

**Adduct Signal Fractions**

- **Unmodified**: 4.3032e+06

### SSEC(C)ITPN(N)GSIPN(D)DKPF Hex 8 HexNAc 2

**Mass:**  3565.4291

(2.795e-04)

**Charge States:** 3

**Start Position**: 294

**End Position**: 311

| Profile Score | Tandem Score | TDA q-value | Volume | Spectrum Matches |
| --- | --- | --- | --- | --- |
| -6.0857 | 86.2931 | 0.0079 | 1.6863e+07 | 2 |

**Adduct Signal Fractions**

- **Unmodified**: 1.6863e+07

### KRGSN(N)NSFF Fuc 3 Hex 5 HexNAc 4

**Mass:**  3116.2717

(5.400e-07)

**Charge States:** 3

**Start Position**: 156

**End Position**: 165

| Profile Score | Tandem Score | TDA q-value | Volume | Spectrum Matches |
| --- | --- | --- | --- | --- |
| -20.2254 | 85.8587 | 0.0000 | 4.6581e+06 | 1 |

**Adduct Signal Fractions**

- **Unmodified**: 4.6581e+06

### LPGNDN(N)STATL Fuc 1 Hex 4 HexNAc 7 Neu5Ac 3

**Mass:**  4190.6525

(2.312e-06)

**Charge States:** 3, 4

**Start Position**: 19

**End Position**: 30

| Profile Score | Tandem Score | TDA q-value | Volume | Spectrum Matches |
| --- | --- | --- | --- | --- |
| 11.0773 | 84.5441 | 0.0079 | 4.2210e+07 | 3 |

**Adduct Signal Fractions**

- **Unmodified**: 4.2210e+07

### TITNDQIEVTN(N)ATEL Hex 6 HexNAc 2

**Mass:**  3039.3008

(3.583e-06)

**Charge States:** 3

**Start Position**: 44

**End Position**: 59

| Profile Score | Tandem Score | TDA q-value | Volume | Spectrum Matches |
| --- | --- | --- | --- | --- |
| -3.4684 | 84.0708 | 0.0079 | 2.6129e+07 | 2 |

**Adduct Signal Fractions**

- **Unmodified**: 2.6129e+07

### ITPN(N)GSIPNDKPF Fuc 2 Hex 6 HexNAc 8 Neu5Ac 1

**Mass:**  4578.9157

(6.545e-06)

**Charge States:** 4

**Start Position**: 298

**End Position**: 311

| Profile Score | Tandem Score | TDA q-value | Volume | Spectrum Matches |
| --- | --- | --- | --- | --- |
| -4.7864 | 83.3976 | 0.0079 | 8.0745e+06 | 1 |

**Adduct Signal Fractions**

- **Unmodified**: 8.0745e+06

### LPGNDN(N)STATL Fuc 2 Hex 7 HexNAc 8 Neu5Ac 1

**Mass:**  4443.7759

(6.028e-06)

**Charge States:** 5

**Start Position**: 19

**End Position**: 30

| Profile Score | Tandem Score | TDA q-value | Volume | Spectrum Matches |
| --- | --- | --- | --- | --- |
| -5.1490 | 82.9249 | 0.0079 | 2.0597e+07 | 2 |

**Adduct Signal Fractions**

- **Unmodified**: 2.0597e+07

### LPGNDN(N)STATL Fuc 4 Hex 3 HexNAc 5 Neu5Ac 2

**Mass:**  3769.5239

(3.914e-06)

**Charge States:** 3

**Start Position**: 19

**End Position**: 30

| Profile Score | Tandem Score | TDA q-value | Volume | Spectrum Matches |
| --- | --- | --- | --- | --- |
| -4.1389 | 80.0823 | 0.0079 | 1.0541e+07 | 1 |

**Adduct Signal Fractions**

- **Unmodified**: 1.0541e+07

### ITPN(N)GSIPNDKPF Fuc 1 Hex 6 HexNAc 5 Neu5Ac 3

**Mass:**  4405.8356

(9.899e-06)

**Charge States:** 3, 4

**Start Position**: 298

**End Position**: 311

| Profile Score | Tandem Score | TDA q-value | Volume | Spectrum Matches |
| --- | --- | --- | --- | --- |
| 14.1190 | 77.9067 | 0.0079 | 2.4116e+08 | 7 |

**Adduct Signal Fractions**

- **Unmodified**: 2.4116e+08

### LPGNDN(N)STATL Fuc 2 Hex 3 HexNAc 6 Neu5Ac 3

**Mass:**  3971.5793

(1.795e-06)

**Charge States:** 3, 4

**Start Position**: 19

**End Position**: 30

| Profile Score | Tandem Score | TDA q-value | Volume | Spectrum Matches |
| --- | --- | --- | --- | --- |
| 12.0746 | 76.8516 | 0.0079 | 3.6192e+07 | 3 |

**Adduct Signal Fractions**

- **Unmodified**: 3.6192e+07

### RSDAPIGTC(C)SSEC(C)ITPN(N)GSIPNDKPF Hex 7 HexNAc 2

**Mass:**  4359.8395

(6.849e-06)

**Charge States:** 3, 4

**Start Position**: 285

**End Position**: 311

| Profile Score | Tandem Score | TDA q-value | Volume | Spectrum Matches |
| --- | --- | --- | --- | --- |
| -4.6780 | 70.4124 | 0.0079 | 1.0659e+07 | 3 |

**Adduct Signal Fractions**

- **Unmodified**: 1.0659e+07

### RSDAPIGTC(C)SSEC(C)ITPN(N)GSIPN(D)DKPF Hex 8 HexNAc 2

**Mass:**  4522.8703

(2.219e-04)

**Charge States:** 3, 4

**Start Position**: 285

**End Position**: 311

| Profile Score | Tandem Score | TDA q-value | Volume | Spectrum Matches |
| --- | --- | --- | --- | --- |
| -5.7584 | 68.8116 | 0.0079 | 4.1622e+07 | 3 |

**Adduct Signal Fractions**

- **Unmodified**: 4.1622e+07

### KIRTGKSSIM(O)RSDAPIGTC(C)SSEC(C)ITPN(N)GSIPNDKPF Hex 8 HexNAc 2

**Mass:**  5639.5156

(3.746e-06)

**Charge States:** 5

**Start Position**: 275

**End Position**: 311

| Profile Score | Tandem Score | TDA q-value | Volume | Spectrum Matches |
| --- | --- | --- | --- | --- |
| -5.1698 | 68.5102 | 0.0079 | 3.9322e+07 | 2 |

**Adduct Signal Fractions**

- **Unmodified**: 3.9322e+07

### TIVKPGDILLIN(N)STGNLIAPR Fuc 2 Hex 4 HexNAc 4 Neu5Ac 1

**Mass:**  4248.0041

(-5.881e-06)

**Charge States:** 4

**Start Position**: 251

**End Position**: 272

| Profile Score | Tandem Score | TDA q-value | Volume | Spectrum Matches |
| --- | --- | --- | --- | --- |
| -18.3676 | 66.8148 | 0.0079 | 1.2777e+06 | 1 |

**Adduct Signal Fractions**

- **Unmodified**: 1.2777e+06

### LYESESKYPVLN(N)VTM(O)PNN(D)GKF Hex 8 HexNAc 2

**Mass:**  4148.7707

(2.404e-04)

**Charge States:** 3, 4

**Start Position**: 170

**End Position**: 191

| Profile Score | Tandem Score | TDA q-value | Volume | Spectrum Matches |
| --- | --- | --- | --- | --- |
| 11.6574 | 63.7793 | 0.0079 | 2.1545e+07 | 4 |

**Adduct Signal Fractions**

- **Unmodified**: 2.1545e+07

### TIVKPGDILLIN(N)STGN(D)LIAPR Hex 9 HexNAc 2

**Mass:**  4069.9218

(2.454e-04)

**Charge States:** 3, 4

**Start Position**: 251

**End Position**: 272

| Profile Score | Tandem Score | TDA q-value | Volume | Spectrum Matches |
| --- | --- | --- | --- | --- |
| -19.5783 | 61.9825 | 0.0112 | 7.5328e+06 | 2 |

**Adduct Signal Fractions**

- **Unmodified**: 7.5328e+06

### TIVKPGDILLIN(N)STGN Hex 10 HexNAc 2

**Mass:**  3680.6251

(2.799e-06)

**Charge States:** 3

**Start Position**: 251

**End Position**: 267

| Profile Score | Tandem Score | TDA q-value | Volume | Spectrum Matches |
| --- | --- | --- | --- | --- |
| -5.2527 | 59.9394 | 0.0112 | 3.3260e+06 | 2 |

**Adduct Signal Fractions**

- **Unmodified**: 3.3260e+06

### TIVKPGDILLIN(N)STGNLIAPRGY Fuc 2 Hex 4 HexNAc 4 Neu5Ac 1

**Mass:**  4470.1189

(6.698e-04)

**Start Position**: 251

**End Position**: 274

| Profile Score | Tandem Score | TDA q-value | Volume | Spectrum Matches |
| --- | --- | --- | --- | --- |
| 0.0000 | 59.7605 | 0.0112 | 0.0000e+00 | 2 |

Adduct:

No Chromatogram Found

### LYESESKYPVLN(N)VTM(O)PNN(D)GKF Hex 9 HexNAc 2

**Mass:**  4310.8239

(2.320e-04)

**Charge States:** 3, 4

**Start Position**: 170

**End Position**: 191

| Profile Score | Tandem Score | TDA q-value | Volume | Spectrum Matches |
| --- | --- | --- | --- | --- |
| -6.2747 | 59.3709 | 0.0112 | 3.1360e+07 | 4 |

**Adduct Signal Fractions**

- **Unmodified**: 3.1360e+07

### KIRTGKSSIM(O)RSDAPIGTC(C)SSEC(C)ITPN(N)GSIPNDKPF Hex 9 HexNAc 2

**Mass:**  5801.5652

(3.096e-06)

**Charge States:** 5

**Start Position**: 275

**End Position**: 311

| Profile Score | Tandem Score | TDA q-value | Volume | Spectrum Matches |
| --- | --- | --- | --- | --- |
| -4.9434 | 56.6747 | 0.0112 | 3.3471e+07 | 2 |

**Adduct Signal Fractions**

- **Unmodified**: 3.3471e+07

### TIVKPGDILLIN(N)STGN(D)LIAPRGY Hex 9 HexNAc 2

**Mass:**  4290.0080

(2.325e-04)

**Charge States:** 3

**Start Position**: 251

**End Position**: 274

| Profile Score | Tandem Score | TDA q-value | Volume | Spectrum Matches |
| --- | --- | --- | --- | --- |
| -4.4680 | 53.5277 | 0.0112 | 1.7053e+07 | 2 |

**Adduct Signal Fractions**

- **Unmodified**: 1.7053e+07

### LYESESKYPVLN(N)VTM(O)PN Hex 9 HexNAc 2

**Mass:**  3863.6076

(3.292e-06)

**Charge States:** 3

**Start Position**: 170

**End Position**: 187

| Profile Score | Tandem Score | TDA q-value | Volume | Spectrum Matches |
| --- | --- | --- | --- | --- |
| -5.3457 | 52.4059 | 0.0112 | 1.3281e+07 | 1 |

**Adduct Signal Fractions**

- **Unmodified**: 1.3281e+07

### SSEC(C)ITPN(N)GSIPN(D)DKPF Hex 9 HexNAc 2

**Mass:**  3727.4793

(2.655e-04)

**Charge States:** 3

**Start Position**: 294

**End Position**: 311

| Profile Score | Tandem Score | TDA q-value | Volume | Spectrum Matches |
| --- | --- | --- | --- | --- |
| -5.8984 | 48.5363 | 0.0112 | 1.3624e+07 | 1 |

**Adduct Signal Fractions**

- **Unmodified**: 1.3624e+07

### TIVKPGDILLIN(N)STGNL Fuc 2 Hex 4 HexNAc 4 Neu5Ac 1

**Mass:**  3810.7168

(-3.446e-06)

**Charge States:** 3

**Start Position**: 251

**End Position**: 268

| Profile Score | Tandem Score | TDA q-value | Volume | Spectrum Matches |
| --- | --- | --- | --- | --- |
| -19.5457 | 47.5859 | 0.0112 | 1.8706e+06 | 2 |

**Adduct Signal Fractions**

- **Unmodified**: 1.8706e+06

### SEC(C)ITPN(N)GSIPN(D)DKPF Hex 8 HexNAc 2

**Mass:**  3478.3939

(2.855e-04)

**Charge States:** 3

**Start Position**: 295

**End Position**: 311

| Profile Score | Tandem Score | TDA q-value | Volume | Spectrum Matches |
| --- | --- | --- | --- | --- |
| 10.4142 | 45.9858 | 0.0112 | 7.2286e+07 | 3 |

**Adduct Signal Fractions**

- **Unmodified**: 7.2286e+07

### TITNDQIEVTN(N)ATELVQSSSTGRIC(C)DSPHRIL Hex 6 HexNAc 2

**Mass:**  4933.2443

(1.028e-06)

**Charge States:** 4

**Start Position**: 44

**End Position**: 76

| Profile Score | Tandem Score | TDA q-value | Volume | Spectrum Matches |
| --- | --- | --- | --- | --- |
| -19.5763 | 45.9470 | 0.0112 | 2.0809e+06 | 1 |

**Adduct Signal Fractions**

- **Unmodified**: 2.0809e+06

### INEGFN(N)WTGVTQ Fuc 1 Hex 5 HexNAc 5

**Mass:**  3336.3609

(4.128e-06)

**Charge States:** 3

**Start Position**: 137

**End Position**: 149

| Profile Score | Tandem Score | TDA q-value | Volume | Spectrum Matches |
| --- | --- | --- | --- | --- |
| -19.9910 | 45.5094 | 0.0112 | 2.7150e+06 | 1 |

**Adduct Signal Fractions**

- **Unmodified**: 2.7150e+06

### TITNDQIEVTN(N)ATEL Hex 7 HexNAc 2

**Mass:**  3201.3576

(4.208e-06)

**Charge States:** 3

**Start Position**: 44

**End Position**: 59

| Profile Score | Tandem Score | TDA q-value | Volume | Spectrum Matches |
| --- | --- | --- | --- | --- |
| -17.0041 | 45.2256 | 0.0112 | 9.5707e+06 | 1 |

**Adduct Signal Fractions**

- **Unmodified**: 9.5707e+06

### LPGNDN(N)STATL Fuc 3 Hex 5 HexNAc 4

**Mass:**  3162.2818

(1.865e-06)

**Charge States:** 3

**Start Position**: 19

**End Position**: 30

| Profile Score | Tandem Score | TDA q-value | Volume | Spectrum Matches |
| --- | --- | --- | --- | --- |
| -19.8705 | 43.9797 | 0.0112 | 2.2705e+06 | 1 |

**Adduct Signal Fractions**

- **Unmodified**: 2.2705e+06

### RSDAPIGTC(C)SSEC(C)ITPN(N)GSIPN(D)DKPF Hex 9 HexNAc 2

**Mass:**  4684.9147

(2.114e-04)

**Charge States:** 3, 4

**Start Position**: 285

**End Position**: 311

| Profile Score | Tandem Score | TDA q-value | Volume | Spectrum Matches |
| --- | --- | --- | --- | --- |
| 12.7319 | 43.7542 | 0.0112 | 3.0462e+07 | 2 |

**Adduct Signal Fractions**

- **Unmodified**: 3.0462e+07

### SEC(C)ITPN(N)GSIPNDKPF Hex 8 HexNAc 2

**Mass:**  3477.4118

(2.745e-06)

**Charge States:** 3

**Start Position**: 295

**End Position**: 311

| Profile Score | Tandem Score | TDA q-value | Volume | Spectrum Matches |
| --- | --- | --- | --- | --- |
| -19.6616 | 43.2169 | 0.0112 | 2.0171e+07 | 1 |

**Adduct Signal Fractions**

- **Unmodified**: 2.0171e+07

### SIRN(N)GTYDHDVY Fuc 2 Hex 5 HexNAc 4

**Mass:**  3353.3468

(4.565e-07)

**Charge States:** 3

**Start Position**: 496

**End Position**: 508

| Profile Score | Tandem Score | TDA q-value | Volume | Spectrum Matches |
| --- | --- | --- | --- | --- |
| -20.8698 | 43.0792 | 0.0112 | 3.7699e+06 | 1 |

**Adduct Signal Fractions**

- **Unmodified**: 3.7699e+06

### SEC(C)ITPN(N)GSIPN(D)DKPF Hex 9 HexNAc 2

**Mass:**  3640.4485

(2.733e-04)

**Charge States:** 3

**Start Position**: 295

**End Position**: 311

| Profile Score | Tandem Score | TDA q-value | Volume | Spectrum Matches |
| --- | --- | --- | --- | --- |
| -6.5583 | 42.7363 | 0.0112 | 9.9392e+06 | 1 |

**Adduct Signal Fractions**

- **Unmodified**: 9.9392e+06

### ITPN(N)GSIPNDKPF Fuc 2 Hex 7 HexNAc 8 Neu5Ac 1

**Mass:**  4740.9641

(5.792e-06)

**Charge States:** 3

**Start Position**: 298

**End Position**: 311

| Profile Score | Tandem Score | TDA q-value | Volume | Spectrum Matches |
| --- | --- | --- | --- | --- |
| -4.7456 | 42.5853 | 0.0112 | 5.7721e+06 | 1 |

**Adduct Signal Fractions**

- **Unmodified**: 5.7721e+06

### KIRTGKSSIM(O)RSDAPIGTC(C)SSEC(C)ITPN(N)GSIPNDKPF Hex 8 HexNAc 2

**Mass:**  5642.5231

(5.370e-04)

**Start Position**: 275

**End Position**: 311

| Profile Score | Tandem Score | TDA q-value | Volume | Spectrum Matches |
| --- | --- | --- | --- | --- |
| 0.0000 | 41.5657 | 0.0112 | 0.0000e+00 | 1 |

Adduct:

No Chromatogram Found

### TIVKPGDILLIN(N)STGNLIAPR Hex 9 HexNAc 2

**Mass:**  4070.9301

(4.932e-04)

**Start Position**: 251

**End Position**: 272

| Profile Score | Tandem Score | TDA q-value | Volume | Spectrum Matches |
| --- | --- | --- | --- | --- |
| 0.0000 | 41.2457 | 0.0112 | 0.0000e+00 | 1 |

Adduct:

No Chromatogram Found

### KIRTGKSSIM(O)RSDAPIGTC(C)SSEC(C)ITPN(N)GSIPN(D)DKPF Hex 8 HexNAc 2

**Mass:**  5640.4989

(1.774e-04)

**Charge States:** 5, 7

**Start Position**: 275

**End Position**: 311

| Profile Score | Tandem Score | TDA q-value | Volume | Spectrum Matches |
| --- | --- | --- | --- | --- |
| -21.0476 | 40.4642 | 0.0112 | 2.8109e+07 | 1 |

**Adduct Signal Fractions**

- **Unmodified**: 2.8109e+07

### RSDAPIGTC(C)SSEC(C)ITPN(N)GSIPN(D)DKPF Hex 9 HexNAc 2

**Mass:**  4686.9249

(6.416e-04)

**Start Position**: 285

**End Position**: 311

| Profile Score | Tandem Score | TDA q-value | Volume | Spectrum Matches |
| --- | --- | --- | --- | --- |
| 0.0000 | 39.2720 | 0.0112 | 0.0000e+00 | 1 |

Adduct:

No Chromatogram Found

### ITPN(N)GSIPNDKPF Fuc 3 Hex 10 HexNAc 6

**Mass:**  4676.9041

(2.163e-04)

**Start Position**: 298

**End Position**: 311

| Profile Score | Tandem Score | TDA q-value | Volume | Spectrum Matches |
| --- | --- | --- | --- | --- |
| 0.0000 | 37.8595 | 0.0112 | 0.0000e+00 | 1 |

Adduct:

No Chromatogram Found

### ESESKYPVLN(N)VTM(O)PNN(D)GKFDKLY Fuc 2 Hex 8 HexNAc 3

**Mass:**  4887.1052

(2.081e-04)

**Charge States:** 4, 5

**Start Position**: 172

**End Position**: 195

| Profile Score | Tandem Score | TDA q-value | Volume | Spectrum Matches |
| --- | --- | --- | --- | --- |
| 12.9461 | 37.4619 | 0.0112 | 2.0085e+08 | 14 |

**Adduct Signal Fractions**

- **Unmodified**: 2.0085e+08

### TIVKPGDILLIN(N)STGNLIAPRGY Hex 7 HexNAc 2

**Mass:**  3965.8965

(2.507e-04)

**Start Position**: 251

**End Position**: 274

| Profile Score | Tandem Score | TDA q-value | Volume | Spectrum Matches |
| --- | --- | --- | --- | --- |
| 0.0000 | 37.3109 | 0.0151 | 0.0000e+00 | 1 |

Adduct:

No Chromatogram Found

### TIVKPGDILLIN(N)STGN(D)LIAPRGY Hex 10 HexNAc 2

**Mass:**  4452.0584

(2.240e-04)

**Charge States:** 3

**Start Position**: 251

**End Position**: 274

| Profile Score | Tandem Score | TDA q-value | Volume | Spectrum Matches |
| --- | --- | --- | --- | --- |
| -20.4522 | 37.1090 | 0.0151 | 2.5355e+05 | 2 |

**Adduct Signal Fractions**

- **Unmodified**: 2.5355e+05

### AC(C)IGSIRN(N)GTYDHDVY Fuc 1 Hex 5 HexNAc 4

**Mass:**  3608.4618

(3.744e-07)

**Charge States:** 3

**Start Position**: 492

**End Position**: 508

| Profile Score | Tandem Score | TDA q-value | Volume | Spectrum Matches |
| --- | --- | --- | --- | --- |
| -6.0403 | 36.4739 | 0.0151 | 3.5945e+06 | 2 |

**Adduct Signal Fractions**

- **Unmodified**: 3.5945e+06

### SSEC(C)ITPN(N)GSIPNDKPF Fuc 4 Hex 7 HexNAc 7

**Mass:**  5002.0529

(8.879e-06)

**Charge States:** 4

**Start Position**: 294

**End Position**: 311

| Profile Score | Tandem Score | TDA q-value | Volume | Spectrum Matches |
| --- | --- | --- | --- | --- |
| -4.0868 | 35.7915 | 0.0151 | 1.7540e+07 | 2 |

**Adduct Signal Fractions**

- **Unmodified**: 1.7540e+07

### RSDAPIGTC(C)SSEC(C)ITPN(N)GSIPN(D)DKPF Hex 7 HexNAc 2

**Mass:**  4360.8171

(2.301e-04)

**Charge States:** 3, 4

**Start Position**: 285

**End Position**: 311

| Profile Score | Tandem Score | TDA q-value | Volume | Spectrum Matches |
| --- | --- | --- | --- | --- |
| -5.9979 | 35.5293 | 0.0151 | 1.0083e+07 | 4 |

**Adduct Signal Fractions**

- **Unmodified**: 1.0083e+07

### KIRTGKSSIM(O)RSDAPIGTC(C)SSEC(C)ITPN(N)GSIPN(D)DKPF Hex 9 HexNAc 2

**Mass:**  5802.5430

(1.721e-04)

**Charge States:** 5, 6

**Start Position**: 275

**End Position**: 311

| Profile Score | Tandem Score | TDA q-value | Volume | Spectrum Matches |
| --- | --- | --- | --- | --- |
| -6.1195 | 34.7907 | 0.0151 | 3.2206e+07 | 2 |

**Adduct Signal Fractions**

- **Unmodified**: 3.2206e+07

### LPGNDN(N)STATL Fuc 1 Hex 8 HexNAc 2

**Mass:**  2950.1748

(4.875e-06)

**Charge States:** 2, 3

**Start Position**: 19

**End Position**: 30

| Profile Score | Tandem Score | TDA q-value | Volume | Spectrum Matches |
| --- | --- | --- | --- | --- |
| 12.6753 | 34.4905 | 0.0151 | 7.0008e+08 | 7 |

**Adduct Signal Fractions**

- **Unmodified**: 7.0008e+08

### ITPN(N)GSIPNDKPF Fuc 4 Hex 7 HexNAc 7

**Mass:**  4538.9069

(7.885e-06)

**Charge States:** 4

**Start Position**: 298

**End Position**: 311

| Profile Score | Tandem Score | TDA q-value | Volume | Spectrum Matches |
| --- | --- | --- | --- | --- |
| -8.1595 | 33.9778 | 0.0151 | 6.1926e+06 | 2 |

**Adduct Signal Fractions**

- **Unmodified**: 6.1926e+06

### LPGNDN(N)STATL Fuc 1 Hex 9 HexNAc 3 Neu5Ac 1

**Mass:**  3606.4385

(9.163e-06)

**Charge States:** 3, 4

**Start Position**: 19

**End Position**: 30

| Profile Score | Tandem Score | TDA q-value | Volume | Spectrum Matches |
| --- | --- | --- | --- | --- |
| 13.9983 | 33.8721 | 0.0151 | 2.5800e+08 | 6 |

**Adduct Signal Fractions**

- **Unmodified**: 2.5800e+08

### ITPN(N)GSIPNDKPF Fuc 1 Hex 9 HexNAc 6 Neu5Ac 1

**Mass:**  4512.8464

(5.608e-06)

**Charge States:** 4

**Start Position**: 298

**End Position**: 311

| Profile Score | Tandem Score | TDA q-value | Volume | Spectrum Matches |
| --- | --- | --- | --- | --- |
| -5.3778 | 33.5033 | 0.0151 | 9.8373e+06 | 2 |

**Adduct Signal Fractions**

- **Unmodified**: 9.8373e+06

### LPGNDN(N)STATL Hex 5 HexNAc 5

**Mass:**  2927.1991

(3.225e-06)

**Charge States:** 3

**Start Position**: 19

**End Position**: 30

| Profile Score | Tandem Score | TDA q-value | Volume | Spectrum Matches |
| --- | --- | --- | --- | --- |
| -4.7854 | 33.4579 | 0.0151 | 1.1905e+07 | 2 |

**Adduct Signal Fractions**

- **Unmodified**: 1.1905e+07

### SSEC(C)ITPN(N)GSIPNDKPF Fuc 4 Hex 6 HexNAc 7

**Mass:**  4840.0021

(9.622e-06)

**Charge States:** 4

**Start Position**: 294

**End Position**: 311

| Profile Score | Tandem Score | TDA q-value | Volume | Spectrum Matches |
| --- | --- | --- | --- | --- |
| -3.9376 | 31.8129 | 0.0244 | 2.1933e+07 | 2 |

**Adduct Signal Fractions**

- **Unmodified**: 2.1933e+07

### KIRTGKSSIM(O)RSDAPIGTC(C)SSEC(C)ITPN(N)GSIPNDKPF Hex 7 HexNAc 2

**Mass:**  5477.4661

(4.425e-06)

**Charge States:** 5

**Start Position**: 275

**End Position**: 311

| Profile Score | Tandem Score | TDA q-value | Volume | Spectrum Matches |
| --- | --- | --- | --- | --- |
| -19.8455 | 31.5207 | 0.0268 | 6.6914e+06 | 1 |

**Adduct Signal Fractions**

- **Unmodified**: 6.6914e+06

### ITPN(N)GSIPNDKPF Fuc 3 Hex 7 HexNAc 7 Neu5Ac 1

**Mass:**  4683.9167

(1.650e-06)

**Charge States:** 3, 4

**Start Position**: 298

**End Position**: 311

| Profile Score | Tandem Score | TDA q-value | Volume | Spectrum Matches |
| --- | --- | --- | --- | --- |
| -20.8994 | 31.4537 | 0.0268 | 1.5787e+06 | 1 |

**Adduct Signal Fractions**

- **Unmodified**: 1.5787e+06

### ESESKYPVLN(N)VTM(O)PNN(D)GKFDKLY Fuc 2 Hex 7 HexNAc 3

**Mass:**  4725.0539

(2.159e-04)

**Charge States:** 4, 5

**Start Position**: 172

**End Position**: 195

| Profile Score | Tandem Score | TDA q-value | Volume | Spectrum Matches |
| --- | --- | --- | --- | --- |
| 13.5656 | 31.2677 | 0.0268 | 1.2659e+08 | 5 |

**Adduct Signal Fractions**

- **Unmodified**: 1.2659e+08

### TITNDQIEVTN(N)ATEL Hex 5 HexNAc 2

**Mass:**  2877.2508

(4.376e-06)

**Charge States:** 3

**Start Position**: 44

**End Position**: 59

| Profile Score | Tandem Score | TDA q-value | Volume | Spectrum Matches |
| --- | --- | --- | --- | --- |
| -2.9786 | 31.1906 | 0.0268 | 1.0941e+07 | 2 |

**Adduct Signal Fractions**

- **Unmodified**: 1.0941e+07

### TIVKPGDILLIN(N)STGNLIAPR Fuc 1 Hex 3 HexNAc 5 Neu5Ac 1

**Mass:**  4142.9797

(-4.705e-06)

**Charge States:** 4

**Start Position**: 251

**End Position**: 272

| Profile Score | Tandem Score | TDA q-value | Volume | Spectrum Matches |
| --- | --- | --- | --- | --- |
| -18.7613 | 30.7218 | 0.0268 | 6.6618e+06 | 1 |

**Adduct Signal Fractions**

- **Unmodified**: 6.6618e+06

### ITPN(N)GSIPNDKPF Fuc 2 Hex 7 HexNAc 9

**Mass:**  4652.9345

(4.348e-06)

**Start Position**: 298

**End Position**: 311

| Profile Score | Tandem Score | TDA q-value | Volume | Spectrum Matches |
| --- | --- | --- | --- | --- |
| 0.0000 | 30.6983 | 0.0268 | 0.0000e+00 | 1 |

Adduct:

No Chromatogram Found

### LPGNDN(N)STATL Hex 6 HexNAc 5

**Mass:**  3089.2542

(3.324e-06)

**Charge States:** 3

**Start Position**: 19

**End Position**: 30

| Profile Score | Tandem Score | TDA q-value | Volume | Spectrum Matches |
| --- | --- | --- | --- | --- |
| -5.4514 | 29.6289 | 0.0268 | 2.5097e+06 | 1 |

**Adduct Signal Fractions**

- **Unmodified**: 2.5097e+06

### TIVKPGDILLIN(N)STGNLIAPR Fuc 3 Hex 3 HexNAc 5

**Mass:**  4143.9894

(-5.651e-06)

**Charge States:** 4

**Start Position**: 251

**End Position**: 272

| Profile Score | Tandem Score | TDA q-value | Volume | Spectrum Matches |
| --- | --- | --- | --- | --- |
| -19.6408 | 29.2187 | 0.0268 | 4.3746e+06 | 2 |

**Adduct Signal Fractions**

- **Unmodified**: 4.3746e+06

### TIVKPGDILLIN(N)STGNLIAPRGY Fuc 1 Hex 3 HexNAc 5 Neu5Ac 1

**Mass:**  4363.0637

(-4.710e-06)

**Charge States:** 4

**Start Position**: 251

**End Position**: 274

| Profile Score | Tandem Score | TDA q-value | Volume | Spectrum Matches |
| --- | --- | --- | --- | --- |
| 10.0459 | 28.7435 | 0.0268 | 3.1975e+07 | 5 |

**Adduct Signal Fractions**

- **Unmodified**: 3.1975e+07

### ESESKYPVLN(N)VTM(O)PNN(D)GKFDKLY Fuc 1 Hex 3 HexNAc 6 Neu5Ac 1

**Mass:**  4831.0734

(2.018e-04)

**Charge States:** 4, 5

**Start Position**: 172

**End Position**: 195

| Profile Score | Tandem Score | TDA q-value | Volume | Spectrum Matches |
| --- | --- | --- | --- | --- |
| 10.2074 | 27.2928 | 0.0351 | 1.2099e+08 | 7 |

**Adduct Signal Fractions**

- **Unmodified**: 1.2099e+08

### TITNDQIEVTN(N)ATEL Fuc 1 Hex 6 HexNAc 3

**Mass:**  3388.4401

(3.445e-06)

**Charge States:** 3

**Start Position**: 44

**End Position**: 59

| Profile Score | Tandem Score | TDA q-value | Volume | Spectrum Matches |
| --- | --- | --- | --- | --- |
| -19.8864 | 26.6670 | 0.0391 | 1.6495e+06 | 1 |

**Adduct Signal Fractions**

- **Unmodified**: 1.6495e+06

### ITPN(N)GSIPNDKPF Fuc 4 Hex 7 HexNAc 8

**Mass:**  4742.9821

(2.175e-04)

**Start Position**: 298

**End Position**: 311

| Profile Score | Tandem Score | TDA q-value | Volume | Spectrum Matches |
| --- | --- | --- | --- | --- |
| 0.0000 | 26.4307 | 0.0424 | 0.0000e+00 | 1 |

Adduct:

No Chromatogram Found

### TIVKPGDILLIN(N)STGN(D)LIAPRGY Fuc 2 Hex 3 HexNAc 4 Neu5Ac 1

**Mass:**  4307.0373

(2.266e-04)

**Charge States:** 4

**Start Position**: 251

**End Position**: 274

| Profile Score | Tandem Score | TDA q-value | Volume | Spectrum Matches |
| --- | --- | --- | --- | --- |
| -5.7350 | 26.2550 | 0.0424 | 2.8025e+06 | 2 |

**Adduct Signal Fractions**

- **Unmodified**: 2.8025e+06

### TIVKPGDILLIN(N)STGNLIAPRGY Hex 5 HexNAc 6

**Mass:**  4453.1081

(-1.566e-06)

**Charge States:** 4

**Start Position**: 251

**End Position**: 274

| Profile Score | Tandem Score | TDA q-value | Volume | Spectrum Matches |
| --- | --- | --- | --- | --- |
| 11.5157 | 26.1777 | 0.0424 | 4.9497e+07 | 3 |

**Adduct Signal Fractions**

- **Unmodified**: 4.9497e+07

### INEGFN(N)WTGVTQ Hex 7 HexNAc 2

**Mass:**  2905.1722

(2.639e-06)

**Charge States:** 3

**Start Position**: 137

**End Position**: 149

| Profile Score | Tandem Score | TDA q-value | Volume | Spectrum Matches |
| --- | --- | --- | --- | --- |
| -20.7785 | 25.7650 | 0.0462 | 6.1481e+05 | 1 |

**Adduct Signal Fractions**

- **Unmodified**: 6.1481e+05

### iav|ADJ41819|A/Phil/2-MA/1982\_NA

**>iav|ADJ41819|A/Phil/2-MA/1982\_NA**  
 `MNPNQKIITIGSVSLTIATICFLMQIAILVTTVTLHFKQYECSSPPNNQVVPCEPIIIERNITEIVYLTNTTIEKEICPK  
LVEYRNWSKPQCKITGFAPFSKDNSIRLSAGGDIWVTREPYVSCDPGKCYQFALGQGTTLDNKHSNDTIHDRTPYRTLLM  
NELGVPFHLGTRQVCIAWSSSSCHDGKAWLHVCITGYDKNATASFIYDGRLVDSIGSWSKNILRTQESECVCINGTCTVV  
MTDGSASERADTKILFIEEGKIVHISPLSGSAQHVEECSCYPRYPGVRCVCRDNWKGSNRPVVDINVKDYSIVSSYVCSG  
LVGDTPRKNDRSSSSYCRNPNNEKGNHGVKGWAFDDGNDVWMGRTISEESRSGYETFKVIGGWSTPNSKLQINRQVIVDS  
GNRSGYSGIFSVEGKSCINRCFYVELIRGREQETRVWWTSNSIVVFCGTSGTYGTGSWPDGADINLMPI`

| Site | Type | Count |
| --- | --- | --- |
| 402 | N-linked | 5 |
| 86 | N-linked | 3 |
| 146 | N-linked | 3 |

| Mass | Sequence | MS_2_ Score | `q`-value | MS_1_ Score | Signal | Protein Coordinates | Tandem Scans | Start Time | Apex Time | End Time |
| --- | --- | --- | --- | --- | --- | --- | --- | --- | --- | --- |
| 3183.3990 | QINRQVIVDSGN(N)RSGY Hex 6 HexNAc 2 | 172.299 | 0.0 | -18.729 | 2.358e+07 | 390 - 406 | 3 | 21.27 | 21.27 | 29.88 |
| 3638.5786 | RN(N)WSKPQC(C)KITGFAPF Hex 8 HexNAc 2 | 152.003 | 0.0 | -3.653 | 7.586e+06 | 84 - 100 | 1 | 37.20 | 37.20 | 37.54 |
| 3800.6353 | RN(N)WSKPQC(C)KITGFAPF Hex 9 HexNAc 2 | 144.982 | 0.0 | -3.942 | 6.933e+06 | 84 - 100 | 1 | 37.03 | 37.20 | 37.54 |
| 4442.8516 | ALGQGTTLDNKHSN(N)DTIHDRTPY Fuc 1 Hex 7 HexNAc 3 | 75.745 | 0.008 | -17.048 | 9.497e+06 | 132 - 155 | 2 | 21.94 | 21.94 | 22.11 |
| 3910.7855 | STPNSKLQINRQVIVDSGN(N)RSGY Hex 6 HexNAc 2 | 57.992 | 0.011 | -4.286 | 1.007e+07 | 383 - 406 | 1 | 28.19 | 28.19 | 28.53 |
| 3476.5214 | RN(N)WSKPQC(C)KITGFAPF Hex 7 HexNAc 2 | 48.852 | 0.011 | -20.534 | 8.113e+05 | 84 - 100 | 1 | 37.37 | 37.37 | 37.54 |
| 4588.9054 | ALGQGTTLDNKHSN(N)DTIHDRTPY Fuc 2 Hex 7 HexNAc 3 | 46.539 | 0.011 | -19.039 | 6.056e+06 | 132 - 155 | 1 | 21.78 | 21.78 | 21.94 |
| 3345.4507 | QINRQVIVDSGN(N)RSGY Hex 7 HexNAc 2 | 43.841 | 0.011 | -19.760 | 4.137e+06 | 390 - 406 | 1 | 21.11 | 21.27 | 21.27 |
| 4734.9636 | ALGQGTTLDN(D)KHSN(N)DTIHDRTPY Fuc 1 Hex 7 HexNAc 3 Neu5Ac 1 | 38.561 | 0.011 | -21.303 | 3.361e+06 | 132 - 155 | 1 | 21.61 | 21.61 | 21.61 |
| 3507.5075 | QINRQVIVDSGN(N)RSGY Hex 8 HexNAc 2 | 32.528 | 0.024 | -20.434 | 2.965e+06 | 390 - 406 | 1 | 21.11 | 21.11 | 21.11 |
| 3348.4601 | QINRQVIVDSGN(N)RSGY Hex 7 HexNAc 2 | 28.222 | 0.027 | 0.000 | 0.000e+00 | 390 - 406 | 1 | - | - | - |

### QINRQVIVDSGN(N)RSGY Hex 6 HexNAc 2

**Mass:**  3183.4011

(2.297e-06)

**Charge States:** 3

**Start Position**: 391

**End Position**: 407

| Profile Score | Tandem Score | TDA q-value | Volume | Spectrum Matches |
| --- | --- | --- | --- | --- |
| -18.7286 | 172.2995 | 0.0000 | 2.3585e+07 | 3 |

**Adduct Signal Fractions**

- **Unmodified**: 2.3585e+07

### RN(N)WSKPQC(C)KITGFAPF Hex 8 HexNAc 2

**Mass:**  3638.5795

(5.587e-06)

**Charge States:** 4

**Start Position**: 85

**End Position**: 101

| Profile Score | Tandem Score | TDA q-value | Volume | Spectrum Matches |
| --- | --- | --- | --- | --- |
| -3.6533 | 152.0027 | 0.0000 | 7.5865e+06 | 1 |

**Adduct Signal Fractions**

- **Unmodified**: 7.5865e+06

### RN(N)WSKPQC(C)KITGFAPF Hex 9 HexNAc 2

**Mass:**  3800.6311

(8.692e-06)

**Charge States:** 4

**Start Position**: 85

**End Position**: 101

| Profile Score | Tandem Score | TDA q-value | Volume | Spectrum Matches |
| --- | --- | --- | --- | --- |
| -3.9421 | 144.9821 | 0.0000 | 6.9327e+06 | 1 |

**Adduct Signal Fractions**

- **Unmodified**: 6.9327e+06

### ALGQGTTLDNKHSN(N)DTIHDRTPY Fuc 1 Hex 7 HexNAc 3

**Mass:**  4442.8513

(-7.976e-06)

**Charge States:** 4

**Start Position**: 133

**End Position**: 156

| Profile Score | Tandem Score | TDA q-value | Volume | Spectrum Matches |
| --- | --- | --- | --- | --- |
| -17.0483 | 75.7447 | 0.0079 | 9.4971e+06 | 2 |

**Adduct Signal Fractions**

- **Unmodified**: 9.4971e+06

### STPNSKLQINRQVIVDSGN(N)RSGY Hex 6 HexNAc 2

**Mass:**  3910.7866

(1.615e-06)

**Charge States:** 4

**Start Position**: 384

**End Position**: 407

| Profile Score | Tandem Score | TDA q-value | Volume | Spectrum Matches |
| --- | --- | --- | --- | --- |
| -4.2856 | 57.9919 | 0.0112 | 1.0068e+07 | 1 |

**Adduct Signal Fractions**

- **Unmodified**: 1.0068e+07

### RN(N)WSKPQC(C)KITGFAPF Hex 7 HexNAc 2

**Mass:**  3476.5222

(4.595e-06)

**Charge States:** 4

**Start Position**: 85

**End Position**: 101

| Profile Score | Tandem Score | TDA q-value | Volume | Spectrum Matches |
| --- | --- | --- | --- | --- |
| -20.5340 | 48.8517 | 0.0112 | 8.1131e+05 | 1 |

**Adduct Signal Fractions**

- **Unmodified**: 8.1131e+05

### ALGQGTTLDNKHSN(N)DTIHDRTPY Fuc 2 Hex 7 HexNAc 3

**Mass:**  4588.9069

(-8.181e-06)

**Charge States:** 4

**Start Position**: 133

**End Position**: 156

| Profile Score | Tandem Score | TDA q-value | Volume | Spectrum Matches |
| --- | --- | --- | --- | --- |
| -19.0386 | 46.5388 | 0.0112 | 6.0557e+06 | 1 |

**Adduct Signal Fractions**

- **Unmodified**: 6.0557e+06

### QINRQVIVDSGN(N)RSGY Hex 7 HexNAc 2

**Mass:**  3345.4502

(1.113e-06)

**Charge States:** 3

**Start Position**: 391

**End Position**: 407

| Profile Score | Tandem Score | TDA q-value | Volume | Spectrum Matches |
| --- | --- | --- | --- | --- |
| -19.7601 | 43.8410 | 0.0112 | 4.1366e+06 | 1 |

**Adduct Signal Fractions**

- **Unmodified**: 4.1366e+06

### ALGQGTTLDN(D)KHSN(N)DTIHDRTPY Fuc 1 Hex 7 HexNAc 3 Neu5Ac 1

**Mass:**  4734.9636

(2.073e-04)

**Charge States:** 4

**Start Position**: 133

**End Position**: 156

| Profile Score | Tandem Score | TDA q-value | Volume | Spectrum Matches |
| --- | --- | --- | --- | --- |
| -21.3033 | 38.5613 | 0.0112 | 3.3606e+06 | 1 |

**Adduct Signal Fractions**

- **Unmodified**: 3.3606e+06

### QINRQVIVDSGN(N)RSGY Hex 8 HexNAc 2

**Mass:**  3507.5044

(1.421e-06)

**Charge States:** 3, 4

**Start Position**: 391

**End Position**: 407

| Profile Score | Tandem Score | TDA q-value | Volume | Spectrum Matches |
| --- | --- | --- | --- | --- |
| -20.4338 | 32.5284 | 0.0244 | 2.9649e+06 | 1 |

**Adduct Signal Fractions**

- **Unmodified**: 2.9649e+06

### QINRQVIVDSGN(N)RSGY Hex 7 HexNAc 2

**Mass:**  3348.4601

(9.008e-04)

**Start Position**: 391

**End Position**: 407

| Profile Score | Tandem Score | TDA q-value | Volume | Spectrum Matches |
| --- | --- | --- | --- | --- |
| 0.0000 | 28.2223 | 0.0268 | 0.0000e+00 | 1 |

Adduct:

No Chromatogram Found

### tr|E1C3D8|E1C3D8\_CHICK

**>tr|E1C3D8|E1C3D8\_CHICK**  
 `MESNGTFLGCNSLKDDKDLKFLLKGSQLLKVKSSSWRKERFYKLQEDCKTIWQESKKMLRSPDSQIFSIEDIRDVRSGHK  
TEGMEKYAKDVPEYRCFSIIFKDQRKNLDLIASSEDDANHWVCGLGKIIAHSNSMNQKQKLQHWIHTCLRKADKNKDNKM  
SFKELKNFLKEVNIEVDDYHAKEIFQYCDKSKTEALEDDEIEEFYRILTERKEIDTIFQKYSDAEGLMSCQNLVRFLYET  
QQEEDAVVAAPPLIQRYEPSERAKKRNAMTKDGFLMYLLSDDGNIFNTSHRKVYQDMTQPLSHYFVSSSHNTYLMDDQLT  
GPSSTEAYIRALTKGCRCVELDCWDGPNSEPIIYHGYTLTSKILFSDVIKAIKNYAFKTSPYPVIISLENHCSLEQQKVM  
AQHMTTILQDMLLVAPIDGSKSQFPSPEQLKGKILVKGKKLSRQEDPTGTNGNNNLEAEDVSDEDEAAEMEDESVKTEVQ  
QKGKSDTLKLAKELSDTVVYCKSVHFNGFEDPSHPRAFYEMSSFTESKALKLAQESGNSFILHNIRHLSRIYPAGWRTDS  
SNYNPVDLWNVGCQIVALNFQTGGTEMDVYQGRFQDNGFSGYVLKPEFLRDEQTKFNPKSITEGTWGTKKKLLLKIISGQ  
QLPKVNKSKNSIVDPRVTIEIHGVQQDNNKKQTKVIENNGFNPNWDEEFTFDIEIPVLALVRFVVEDFDMSTKNDFIGQY  
TVPFTSLKQGYRHIHLLTKNGDPYSSSTLFVYVNIQDCD`

| Site | Type | Count |
| --- | --- | --- |
| 4 | N-linked | 2 |

| Mass | Sequence | MS_2_ Score | `q`-value | MS_1_ Score | Signal | Protein Coordinates | Tandem Scans | Start Time | Apex Time | End Time |
| --- | --- | --- | --- | --- | --- | --- | --- | --- | --- | --- |
| 3752.4974 | ESN(N)GTFL Fuc 4 Hex 3 HexNAc 8 Neu5Ac 1 | 106.334 | 0.0 | 11.621 | 7.407e+07 | 1 - 8 | 2 | 31.91 | 32.59 | 33.09 |
| 2909.1498 | ESN(N)GTFL Fuc 3 Hex 3 HexNAc 6 | 97.932 | 0.0 | 12.716 | 2.912e+08 | 1 - 8 | 8 | 34.53 | 35.19 | 36.52 |

### ESN(N)GTFL Fuc 4 Hex 3 HexNAc 8 Neu5Ac 1

**Mass:**  3752.4995

(7.328e-06)

**Charge States:** 3

**Start Position**: 2

**End Position**: 9

| Profile Score | Tandem Score | TDA q-value | Volume | Spectrum Matches |
| --- | --- | --- | --- | --- |
| 11.6205 | 106.3341 | 0.0000 | 7.4073e+07 | 2 |

**Adduct Signal Fractions**

- **Unmodified**: 7.4073e+07

### ESN(N)GTFL Fuc 3 Hex 3 HexNAc 6

**Mass:**  2909.1472

(1.084e-07)

**Charge States:** 2, 3

**Start Position**: 2

**End Position**: 9

| Profile Score | Tandem Score | TDA q-value | Volume | Spectrum Matches |
| --- | --- | --- | --- | --- |
| 12.7162 | 97.9318 | 0.0000 | 2.9122e+08 | 8 |

**Adduct Signal Fractions**

- **Unmodified**: 2.9122e+08

### iav|BAV59610|A/PR/8/1934\_PA

**>iav|BAV59610|A/PR/8/1934\_PA**  
 `MEDFVRQCFNPMIVELAEKTMKEYGEDLKIETNKFAAICTHLEICFMYSDFHFINEQGESIIVELDDPNALLKHRFEIIE  
GRDRTMAWTVVNSICNTTGAEKPKFLPDLYDYKENRFIEIGVTRREVHIYYLEKANKIKSEKTHIHIFSFTGEEMATKAD  
YTLDEESRARIKTRLFTIRQEMASRGLWDSFRQSERGEETIEERFEITGTMRKLADQSLPPNFSSLENFRAYVDGFEPNG  
YIEGKLSQMSKEVNARIEPFLKTTPRPLRLPNGPPCSQRSKFLLMDALKLSIEDPSHEGEGIPLYDAIKCMRTFFGWKEP  
NVVKPHEKGINPNYLLSWKQVLAELQDIENEEKIPKTKNMKKTSQLKWALGENMAPEKVDFDDCKDVGDLKQYDSDEPEL  
RSLASWIQNEFNKACELTDSSWIELDEIGEDVAPIEHIASMRRNYFTSEVSHCRATEYIMKGVYINTALLNASCAAMDDF  
QLIPMISKCRTKEGRRKTNLYGFIIKGRSHLRNDTDVVNFVSMEFSLTDPRLEPHKWEKYCVLEIGDMLIRSAIGQVSRP  
MFLYVRTNGTSKIKMKWGMEMRRCLLQSLQQIESMIEAESSVKEKDMTKEFFENKSETWPIGESPKGVEESSIGKVCRTL  
LAKSVFNSLYASPQLEGFSAESRKLLLIVQALRDNLEPGTFDLGGLYDAIEECLINDPWVLLNASWFNSFLTHALS`

| Site | Type | Count |
| --- | --- | --- |
| 703 | N-linked | 1 |

| Mass | Sequence | MS_2_ Score | `q`-value | MS_1_ Score | Signal | Protein Coordinates | Tandem Scans | Start Time | Apex Time | End Time |
| --- | --- | --- | --- | --- | --- | --- | --- | --- | --- | --- |
| 4444.7519 | LN(N)ASWF Fuc 4 Hex 8 HexNAc 9 | 97.885 | 0.0 | -4.292 | 1.737e+07 | 701 - 707 | 2 | 38.91 | 39.08 | 39.25 |

### LN(N)ASWF Fuc 4 Hex 8 HexNAc 9

**Mass:**  4444.7507

(7.173e-06)

**Charge States:** 5

**Start Position**: 702

**End Position**: 708

| Profile Score | Tandem Score | TDA q-value | Volume | Spectrum Matches |
| --- | --- | --- | --- | --- |
| -4.2917 | 97.8847 | 0.0000 | 1.7374e+07 | 2 |

**Adduct Signal Fractions**

- **Unmodified**: 1.7374e+07

### iav|ADX99654|A/PR/8-WG/1934\_PA

**>iav|ADX99654|A/PR/8-WG/1934\_PA**  
 `MEDFVRQCFNPMIVELAEKTMKEYGEDLKIETNKFAAICTHLEVCFMYSDFHFINEQGESIIVELGDPNALLKHRFEIIE  
GRDRTMAWTVVNSICNTTGAEKPKFLPDLYDYKENRFIEIGVTRREVHIYYLEKANKIKSEKTHIHIFSFTGEEMATKAD  
YTLDEESRARIKTRLFTIRQEMASRGLWDSFRQSERGEETIEERFEITGTMRKLADQSPPPNFSSLENFRAYVDGFEPNG  
YIEGKLSQMSKEVNARIEPFLKTTPRPLRLPNGPPCSQRSKFLLMDALKLSIEDPSHEGEGIPLYDAIKCMRTFFGWKEP  
NVVKPHEKGINPNYLLSWKQVLAELQDIENEEKIPKTKNMKKTSQLKWALGENMAPEKVDFDDCKDVGDLKQYDSDEPEL  
RSLASWIQNEFNKACELTDSSWIELDEIGEDVAPIEHIASMRRNYFTSEVSHCRATEYIMKGVYINTALLNASCAAMDDF  
QLIPMISKCRTKEGRRKTNLYGFIIKGRSHLRNDTDVVNFVSMEFSLTDPRLEPHKWEKYCVLEIGDMLIRSAIGQVSRP  
MFLYVRTNGTSKIKMKWGMEMRRCLLQSLQQIESMIEAESSVKEKDMTKEFFENKSETWPIGESPKGVEESSIGKVCRTL  
LAKSVFNSLYASPQLEGFSAESRKLLLIVQALRDNLEPGTFDLGGLYEAIEECLINDPWVLLNASWFNSFLTHALS`

| Site | Type | Count |
| --- | --- | --- |
| 703 | N-linked | 1 |

| Mass | Sequence | MS_2_ Score | `q`-value | MS_1_ Score | Signal | Protein Coordinates | Tandem Scans | Start Time | Apex Time | End Time |
| --- | --- | --- | --- | --- | --- | --- | --- | --- | --- | --- |
| 4444.7519 | LN(N)ASWF Fuc 4 Hex 8 HexNAc 9 | 97.885 | 0.0 | -4.292 | 1.737e+07 | 701 - 707 | 2 | 38.91 | 39.08 | 39.25 |

### LN(N)ASWF Fuc 4 Hex 8 HexNAc 9

**Mass:**  4444.7507

(7.173e-06)

**Charge States:** 5

**Start Position**: 702

**End Position**: 708

| Profile Score | Tandem Score | TDA q-value | Volume | Spectrum Matches |
| --- | --- | --- | --- | --- |
| -4.2917 | 97.8847 | 0.0000 | 1.7374e+07 | 2 |

**Adduct Signal Fractions**

- **Unmodified**: 1.7374e+07

### iav|AGK63052|A/PR/8/1934\_PA

**>iav|AGK63052|A/PR/8/1934\_PA**  
 `MEDFVRQCFNPMIVELAEKTMKEYGEDLKIETNKFAAICTHLEVCFMYSDFHFINEQGESIIVELGDPNALLKHRFEIIE  
GRDRTMAWTVVNSICNTTGAEKPKFLPDLYDYKENRFIEIGVTRREVHIYYLEKANKIKSEKTHIHIFSFTGEEMATKAD  
YTLDEESRARIKTRLFTIRQEMASRGLWDSFRQSERGEETIEERFEITGTMRKLADQSLPPNFSSLENFRAYVDGFEPNG  
YIEGKLSQMSKEVNARIEPFLKTTPRPLRLPNGPPCSQRSKFLLMDALKLSIEDPSHEGEGIPLYDAIKCMRTFFGWKEP  
NVVKPHEKGINPNYLLSWKQVLAELQDIENEEKIPKTKNMKKTSQLKWALGENMAPEKVDFDDCKDVGDLKQYDSDEPEL  
RSLASWIQNEFNKACELTDSSWIELDEIGEDVAPIEHIASMRRNYFTSEVSHCRATEYIMKGVYINTALLNASCAAMDDF  
QLIPMISKCRTKEGRRKTNLYGFIIKGRSHLRNDTDVVNFVSMEFSLTDPRLEPHKWEKYCVLEIGDMLIRSAIGQVSRP  
MFLYVRTNGTSKIKMKWGMEMRRCLLQSLQQIESMIEAESSVKEKDMTKEFFENKSETWPIGESPKGVEESSIGKVCRTL  
LAKSVFNSLYASPQLEGFSAESRKLLLIVQALRDNLEPGTFDLGGLYEAIEECLINDPWVLLNASWFNSFLTHALS`

| Site | Type | Count |
| --- | --- | --- |
| 703 | N-linked | 1 |

| Mass | Sequence | MS_2_ Score | `q`-value | MS_1_ Score | Signal | Protein Coordinates | Tandem Scans | Start Time | Apex Time | End Time |
| --- | --- | --- | --- | --- | --- | --- | --- | --- | --- | --- |
| 4444.7519 | LN(N)ASWF Fuc 4 Hex 8 HexNAc 9 | 97.885 | 0.0 | -4.292 | 1.737e+07 | 701 - 707 | 2 | 38.91 | 39.08 | 39.25 |

### LN(N)ASWF Fuc 4 Hex 8 HexNAc 9

**Mass:**  4444.7507

(7.173e-06)

**Charge States:** 5

**Start Position**: 702

**End Position**: 708

| Profile Score | Tandem Score | TDA q-value | Volume | Spectrum Matches |
| --- | --- | --- | --- | --- |
| -4.2917 | 97.8847 | 0.0000 | 1.7374e+07 | 2 |

**Adduct Signal Fractions**

- **Unmodified**: 1.7374e+07

### iav|CAA24295|A/PR/8/1934\_PA

**>iav|CAA24295|A/PR/8/1934\_PA**  
 `MEDFVRQCFNPMIVELAEKTMKEYGEDLKIETNKFAAICTHLEVCFMYSDFHFINEQGESIIVELGDPNALLKHRFEIIE  
GRDRTMAWTVVNSICNTTGAEKPKFLPDLYDYKENRFIEIGVTRREVHIYYLEKANKIKSEKTHIHIFSFTGEEMATKAD  
YTLDEESRARIKTRLFTIRQEMASRGLWDSFRQSERGEETIEERFEITGTMRKLADQSLPPNFSSLENFRAYVDGFEPNG  
YIEGKLSQMSKEVNARIEPFLKTTPRPLRLPNGPPCSQRSKFLLMDALKLSIEDPSHEGEGIPLYDAIKCMRTFFGWKEP  
NVVKPHEKGINPNYLLSWKQVLAELQDIENEEKIPKTKNMKKTSQLKWALGENMAPEKVDFDDCKDVGDLKQYDSDEPEL  
RSLASWIQNEFNKACELTDSSWIELDEIGEDVAPIEHIASMRRNYFTSEVSHCRATEYIMKGVYINTALLNASCAAMDDF  
QLIPMISKCRTKEGRRKTNLYGFIIKGRSHLRNDTDVVNFVSMEFSLTDPRLEPHKWEKYCVLEIGDMLLRSAIGQVSRP  
MFLYVRTNGTSKIKMKWGMEMRRCLLQSLQQIESMIEAESSVKEKDMTKEFFENKSETWPIGESPKGVEESSIGKVCRTL  
LAKSVFNSLYASPQLEGFSAESRKLLLIVQALRDNLEPGTFDLGGLYEAIEECLINDPWVLLNASWFNSFLTHALS`

| Site | Type | Count |
| --- | --- | --- |
| 703 | N-linked | 1 |

| Mass | Sequence | MS_2_ Score | `q`-value | MS_1_ Score | Signal | Protein Coordinates | Tandem Scans | Start Time | Apex Time | End Time |
| --- | --- | --- | --- | --- | --- | --- | --- | --- | --- | --- |
| 4444.7519 | LN(N)ASWF Fuc 4 Hex 8 HexNAc 9 | 97.885 | 0.0 | -4.292 | 1.737e+07 | 701 - 707 | 2 | 38.91 | 39.08 | 39.25 |

### LN(N)ASWF Fuc 4 Hex 8 HexNAc 9

**Mass:**  4444.7507

(7.173e-06)

**Charge States:** 5

**Start Position**: 702

**End Position**: 708

| Profile Score | Tandem Score | TDA q-value | Volume | Spectrum Matches |
| --- | --- | --- | --- | --- |
| -4.2917 | 97.8847 | 0.0000 | 1.7374e+07 | 2 |

**Adduct Signal Fractions**

- **Unmodified**: 1.7374e+07

### iav|AAM75156|A/PR/8/1934\_PB1

**>iav|AAM75156|A/PR/8/1934\_PB1**  
 `MDVNPTLLFLKVPAQNAISTTFPYTGDPPYSHGTGTGYTMDTVNRTHQYSEKGRWTTNTETGAPQLNPIDGPLPEDNEPS  
GYAQTDCVLEAMAFLEESHPGIFENSCIETMEVVQQTRVDKLTQGRQTYDWTLNRNQPAATALANTIEVFRSNGLTANES  
GRLIDFLKDVMESMKKEEMGITTHFQRKRRVRDNMTKKMITQRTIGKKKQRLNKRSYLIRALTLNTMTKDAERGKLKRRA  
IATPGMQIRGFVYFVETLARSICEKLEQSGLPVGGNEKKAKLANVVRKMMTNSQDTELSFTITGDNTKWNENQNPRMFLA  
MITYMTRNQPEWFRNVLSIAPIMFSNKMARLGKGYMFESKSMKLRTQIPAEMLASIDLKYFNDSTRKKIEKIRPLLIEGT  
ASLSPGMMMGMFNMLSTVLGVSILNLGQKRYTKTTYWWDGLQSSDDFALIVNAPNHEGIQAGVDRFYRTCKLLGINMSKK  
KSYINRTGTFEFTSFFYRYGFVANFSMELPSFGVSGINESADMSIGVTVIKNNMINNDLGPATAQMALQLFIKDYRYTYR  
CHRGDTQIQTRRSFEIKKLWEQTRSKAGLLVSDGGPNLYNIRNLHIPEVCLKWELMDEDYQGRLCNPLNPFVSHKEIESM  
NNAVMMPAHGPAKNMEYDAVATTHSWIPKRNRSILNTSQRGVLEDEQMYQRCCNLFEKFFPSSSYRRPVGISSMVEAMVS  
RARIDARIDFESGRIKKEEFTEIMKICSTIEELRRQK`

| Site | Type | Count |
| --- | --- | --- |
| 504 | N-linked | 1 |

| Mass | Sequence | MS_2_ Score | `q`-value | MS_1_ Score | Signal | Protein Coordinates | Tandem Scans | Start Time | Apex Time | End Time |
| --- | --- | --- | --- | --- | --- | --- | --- | --- | --- | --- |
| 4362.7335 | RYGFVAN(N)FSM Fuc 2 Hex 9 HexNAc 7 | 30.817 | 0.027 | -5.228 | 7.473e+06 | 497 - 507 | 2 | 26.00 | 26.17 | 26.34 |

### RYGFVAN(N)FSM Fuc 2 Hex 9 HexNAc 7

**Mass:**  4362.7336

(6.729e-06)

**Charge States:** 4

**Start Position**: 498

**End Position**: 508

| Profile Score | Tandem Score | TDA q-value | Volume | Spectrum Matches |
| --- | --- | --- | --- | --- |
| -5.2284 | 30.8168 | 0.0268 | 7.4731e+06 | 2 |

**Adduct Signal Fractions**

- **Unmodified**: 7.4731e+06

### iav|AGU93028|A/PR/8/1934\_PB1

**>iav|AGU93028|A/PR/8/1934\_PB1**  
 `MDVNPTLLFLKVPAQNAISTTFPYTGDPPYSHGTGTGYTMDTVNRTHQYSEKGRWTTNTETGAPQLNPIDGPLPEDNEPS  
GYAQTDCVLEAMAFLEESHPGIFENSCIETMEVVQQTRVDKLTQGRQTYDWTLNRNQPAATALANTIEVFRSNGLTANES  
GRLIDFLKDVMESMNKEEMGITTHFQRKRRVRDNMTKKMITQRTIGKKKQRLNKRSYLIRALTLNTMTKDAERGKLKRRA  
IATPGMQIRGFVYFVETLARSICEKLEQSGLPVGGNEKKAKLANVVRKMMTNSQDTELSFTITGDNTKWNENQNPRMFLA  
MITYMTRNQPEWFRNVLSIAPIMFSNKMARLGKGYMFESKSMKLRTQIPAEMLASIDLKYFNDSTRKKIEKIRPLLIEGT  
ASLSPGMMMGMFNMLSTVLGVSILNLGQKRYTKTTYWWDGLQSSDDFALIVNAPNHEGIQAGVDRFYRTCKLLGINMSKK  
KSYINRTGTFEFTSFFYRYGFVANFSMELPSFGVSGINESADMSIGVTVIKNNMINNDLGPATAQMALQLFIKDYRYTYR  
CHRGDTQIQTRRSFEIKKLWEQTRSKAGLLVSDGGPNLYNIRNLHIPEVCLKWELMDEDYQGRLCNPLNPFVSHKEIESM  
NNAVMMPAHGPAKNMEYDAVATTHSWIPKRNRSILNTSQRGVLEDEQMYQRCCNLFEKFFPSSSYRRPVGISSMVEAMVS  
RARIDARIDFESGRIKKEEFTEIMKICSTIEELRRQK`

| Site | Type | Count |
| --- | --- | --- |
| 504 | N-linked | 1 |

| Mass | Sequence | MS_2_ Score | `q`-value | MS_1_ Score | Signal | Protein Coordinates | Tandem Scans | Start Time | Apex Time | End Time |
| --- | --- | --- | --- | --- | --- | --- | --- | --- | --- | --- |
| 4362.7335 | RYGFVAN(N)FSM Fuc 2 Hex 9 HexNAc 7 | 30.817 | 0.027 | -5.228 | 7.473e+06 | 497 - 507 | 2 | 26.00 | 26.17 | 26.34 |

### RYGFVAN(N)FSM Fuc 2 Hex 9 HexNAc 7

**Mass:**  4362.7336

(6.729e-06)

**Charge States:** 4

**Start Position**: 498

**End Position**: 508

| Profile Score | Tandem Score | TDA q-value | Volume | Spectrum Matches |
| --- | --- | --- | --- | --- |
| -5.2284 | 30.8168 | 0.0268 | 7.4731e+06 | 2 |

**Adduct Signal Fractions**

- **Unmodified**: 7.4731e+06

### iav|AAA43581|A/PR/8/1934\_PB1

**>iav|AAA43581|A/PR/8/1934\_PB1**  
 `MDVNPTLLFLKVPAQNAISTTFPYTGDPPYSHGTGTGYTMDTVNRTHQYSEKARWTTNTETGAPQLNPIDGPLPEDNEPS  
GYAQTDCVLEAMAFLEESHPGIFENSCIETMEVVQQTRVDKLTQGRQTYDWTLNRNQPAATALANTIEVFRSNGLTANES  
GRLIDFLKDVMESMKKEEMGITTHFQRKRRVRDNMTKKMITQRTIGKRKQRLNKRSYLIRALTLNTMTKDAERGKLKRRA  
IATPGMQIRGFVYFVETLARSICEKLEQSGLPVGGNEKKAKLANVVRKMMTNSQDTELSLTITGDNTKWNENQNPRMFLA  
MITYMTRNQPEWFRNVLSIAPIMFSNKMARLGKGYMFESKSMKLRTQIPAEMLASIDLKYFNDSTRKKIEKIRPLLIEGT  
ASLSPGMMMGMFNMLSTVLGVSILNLGQKRYTKTTYWWDGLQSSDDFALIVNAPNHEGIQAGVDRFYRTCKLHGINMSKK  
KSYINRTGTFEFTSFFYRYGFVANFSMELPSFGVSGSNESADMSIGVTVIKNNMINNDLGPATAQMALQLFIKDYRYTYR  
CHRGDTQIQTRRSFEIKKLWEQTRSKAGLLVSDGGPNLYNIRNLHIPEVCLKWELMDEDYQGRLCNPLNPFVSHKEIESM  
NNAVMMPAHGPAKNMEYDAVATTHSWIPKRNRSILNTSQRGVLEDEQMYQRCCNLFEKFFPSSSYRRPVGISSMVEAMVS  
RARIDARIDFESGRIKKEEFTEIMKICSTIEELRRQK`

| Site | Type | Count |
| --- | --- | --- |
| 504 | N-linked | 1 |

| Mass | Sequence | MS_2_ Score | `q`-value | MS_1_ Score | Signal | Protein Coordinates | Tandem Scans | Start Time | Apex Time | End Time |
| --- | --- | --- | --- | --- | --- | --- | --- | --- | --- | --- |
| 4362.7335 | RYGFVAN(N)FSM Fuc 2 Hex 9 HexNAc 7 | 30.817 | 0.027 | -5.228 | 7.473e+06 | 497 - 507 | 2 | 26.00 | 26.17 | 26.34 |

### RYGFVAN(N)FSM Fuc 2 Hex 9 HexNAc 7

**Mass:**  4362.7336

(6.729e-06)

**Charge States:** 4

**Start Position**: 498

**End Position**: 508

| Profile Score | Tandem Score | TDA q-value | Volume | Spectrum Matches |
| --- | --- | --- | --- | --- |
| -5.2284 | 30.8168 | 0.0268 | 7.4731e+06 | 2 |

**Adduct Signal Fractions**

- **Unmodified**: 7.4731e+06

### iav|ACF41842|A/PR/8/1934\_PB1

**>iav|ACF41842|A/PR/8/1934\_PB1**  
 `MDVNPTLLFLKVPAQNAISTTFPYTGDPPYSHGTGTGYTMDTVNRTHQYSEKGRWTTNTETGAPQLNPIDGPLPEDNEPS  
GYAQTDCVLEAMAFLEESHPGIFENSCIETMEVVQQTRVDKLTQGRQTYDWTLNRNQPAATALANTIEVFRSNGLTANES  
GRLIDFLKDVMESMNKEEMGITTHFQRKRRVRDNMTKKMITQRTMGKKKQRLNKRSYLIRALTLNTMTKDAERGKLKRRA  
IATPGMQIRGFVYFVETLARSICEKLEQSGLPVGGNEKKAKLANVVRKMMTNSQDTELSFTITGDNTKWNENQNPRMFLA  
MITYMTRNQPEWFRNVLSIAPIMFSNKMARLGKGYMFESKSMKLRTQIPAEMLASIDLKYFNDSTRKKIEKIRPLLIEGT  
ASLSPGMMMGMFNMLSTVLGVSILNLGQKRYTKTTYWWDGLQSSDDFALIVNAPNHEGIQAGVDRFYRTCKLLGINMSKK  
KSYINRTGTFEFTSFFYRYGFVANFSMELPSFGVSGINESADMSIGVTVIKNNMINNDLGPATAQMALQLFIKDYRYTYR  
CHRGDTQIQTRRSFEIKKLWEQTRSKAGLLVSDGGPNLYNIRNLHIPEVCLKWELMDEDYQGRLCNPLNPFVSHKEIESM  
NNAVMMPAHGPAKNMEYDAVATTHSWIPKRNRSILNTSQRGVLEDEQMYQRCCNLFEKFFPSSSYRRPVGISSMVEAMVS  
RARIDARIDFESGRIKKEEFTEIMKICSTIEELRRQK`

| Site | Type | Count |
| --- | --- | --- |
| 504 | N-linked | 1 |

| Mass | Sequence | MS_2_ Score | `q`-value | MS_1_ Score | Signal | Protein Coordinates | Tandem Scans | Start Time | Apex Time | End Time |
| --- | --- | --- | --- | --- | --- | --- | --- | --- | --- | --- |
| 4362.7335 | RYGFVAN(N)FSM Fuc 2 Hex 9 HexNAc 7 | 30.817 | 0.027 | -5.228 | 7.473e+06 | 497 - 507 | 2 | 26.00 | 26.17 | 26.34 |

### RYGFVAN(N)FSM Fuc 2 Hex 9 HexNAc 7

**Mass:**  4362.7336

(6.729e-06)

**Charge States:** 4

**Start Position**: 498

**End Position**: 508

| Profile Score | Tandem Score | TDA q-value | Volume | Spectrum Matches |
| --- | --- | --- | --- | --- |
| -5.2284 | 30.8168 | 0.0268 | 7.4731e+06 | 2 |

**Adduct Signal Fractions**

- **Unmodified**: 7.4731e+06

### iav|ADX99655|A/PR/8-WG/1934\_PB1

**>iav|ADX99655|A/PR/8-WG/1934\_PB1**  
 `MDVNPTLLFLKVPAQNAISTTFPYTGDPPYSHGTGTGYTMDTVNRTHQYSEKGRWTTNTETGAPQLNPIDGPLPEDNEPS  
GYAQTDCVLEAMAFLEESHPGIFENSCIETMEVVQQTRVDKLTQGRQTYDWTLNRNQPAATALANTIEVFRSNGLTANES  
GRLIDFLKDVMESMNKEEMGITTHFQRKRRVRDNMTKKMITQRTMGKKKQRLNKRSYLIRALTLNTMTKDAERGKLKRRA  
IATPGMQIRGFVYFVETLARSICEKLEQSGLPVGGNEKKAKLANVVRKMMTNSQDTELSFTITGDNTKWNENQNPRMFLA  
MITYMTRNQPEWFRNVLSIAPIMFSNKMARLGKGYMFESKSMKLRTQIPAEMLASIDLKYFNDSTRKKIEKIRPLLIEGT  
ASLSPGMMMGMFNMLSTVLGVSILNLGQKRYTKTTYWWDGLQSSDDFALIVNAPNHEGIQAGVDRFYRTCKLLGINMSKK  
KSYINRTGTFEFTSFFYRYGFVANFSMELPSFGVSGINESADMSIGVTVIKNNMINNDLGPATAQMALQLFIKDYRYTYR  
CHIGDTQIQTRRSFEIKKLWEQTRSKAGLLVSDGGPNLYNIRNLHIPEVCLKWELMDEDYQGRLCNPLNPFVSHKEIESM  
NNAVMMPAHGPAKNMEYDAVATTHSWIPKRNRSILNTSQRGVLEDEQMYQRCCNLFEKFFPSSSYRRPVGISSMVEAMVS  
RARIDARIDFESGRIKKEEFTEIMKICSTIEELRRQK`

| Site | Type | Count |
| --- | --- | --- |
| 504 | N-linked | 1 |

| Mass | Sequence | MS_2_ Score | `q`-value | MS_1_ Score | Signal | Protein Coordinates | Tandem Scans | Start Time | Apex Time | End Time |
| --- | --- | --- | --- | --- | --- | --- | --- | --- | --- | --- |
| 4362.7335 | RYGFVAN(N)FSM Fuc 2 Hex 9 HexNAc 7 | 30.817 | 0.027 | -5.228 | 7.473e+06 | 497 - 507 | 2 | 26.00 | 26.17 | 26.34 |

### RYGFVAN(N)FSM Fuc 2 Hex 9 HexNAc 7

**Mass:**  4362.7336

(6.729e-06)

**Charge States:** 4

**Start Position**: 498

**End Position**: 508

| Profile Score | Tandem Score | TDA q-value | Volume | Spectrum Matches |
| --- | --- | --- | --- | --- |
| -5.2284 | 30.8168 | 0.0268 | 7.4731e+06 | 2 |

**Adduct Signal Fractions**

- **Unmodified**: 7.4731e+06

### iav|BAV59609|A/PR/8/1934\_PB1

**>iav|BAV59609|A/PR/8/1934\_PB1**  
 `MDVNPTLLFLKVPAQNAISTTFPYTGDPPYSHGTGTGYTMDTVNRTHQYSEKGRWTTNTETGASQLNPIDGPLPEDNEPS  
GYAQTDCVLEAMAFLEESHPGIFENSCIETIEVVQQTRVDKLTQGRQTYDWTLNRNQPAATALANTIEVFRSNGLTANES  
GRLIDFLKDVMESMNKEEMGITTHFQRKRRVRDNMTKKMITQRTMGKKKQRLNKRSYLIRALTLNTMTKDAERGKLKRRA  
IATPGMQIRGFVYFVETLARSICEKLEQSGLPVGGNEKKAKLANVVRKMMTNSQDTELSFTITGDNTKWNENQNPRMFLA  
MITYMTRNQPEWFRNVLSIAPIMFSNKMARLGKGYMFESKSMKLRTQIPAEMLASIDLKYFNDSTRKKIEKIRPLLIEGT  
ASLSPGMMMGMFNMLSTVLGVSILNLGQKRYTKTTYWWDGLQSSDDFALIVNAPNNEGIQAGVDRFYRTCKLLGINMSKK  
KSYINRTGTFEFTSFFYRYGFVANFSMELPSFGVSGINESADMSIGVTVIKNNMINNDLGPATAQMALQLFIKDYRYTYR  
CHRGDTQIQTRRSFEIKKLWEQTRSKAGLLVSDGGPNLYNIRNLHIPEVCLKWELMDEDYQGRLCNPLNPFVSHKEIESM  
NNAVMMPAHGPAKNMEYDAVATTHSWIPKRNRSILNTSQRGVLEDEQMYQRCCNLFEKFFPSSSYRRPVGISSMVEAMVS  
RARIDARIDFESGRIKKEEFTEIMKICSTIEELRRQK`

| Site | Type | Count |
| --- | --- | --- |
| 504 | N-linked | 1 |

| Mass | Sequence | MS_2_ Score | `q`-value | MS_1_ Score | Signal | Protein Coordinates | Tandem Scans | Start Time | Apex Time | End Time |
| --- | --- | --- | --- | --- | --- | --- | --- | --- | --- | --- |
| 4362.7335 | RYGFVAN(N)FSM Fuc 2 Hex 9 HexNAc 7 | 30.817 | 0.027 | -5.228 | 7.473e+06 | 497 - 507 | 2 | 26.00 | 26.17 | 26.34 |

### RYGFVAN(N)FSM Fuc 2 Hex 9 HexNAc 7

**Mass:**  4362.7336

(6.729e-06)

**Charge States:** 4

**Start Position**: 498

**End Position**: 508

| Profile Score | Tandem Score | TDA q-value | Volume | Spectrum Matches |
| --- | --- | --- | --- | --- |
| -5.2284 | 30.8168 | 0.0268 | 7.4731e+06 | 2 |

**Adduct Signal Fractions**

- **Unmodified**: 7.4731e+06

### iav|ABD77683|A/PR/8/1934\_PB1

**>iav|ABD77683|A/PR/8/1934\_PB1**  
 `MDVNPTLLFLKVPAQNAISTTFPYTGDPPYSHGTGTGYTMDTVNRTHQYSEKGRWTTNTETGAPQLNPIDGPLPEDNEPS  
GYAQTDCVLEAMAFLEESHPGIFENSCIETMEVVQQTRVDKLTQGRQTYDWTLNRNQPAATALANTIEVFRSNGLTANES  
GRLIDFLKDVMESMNKEEMGITTHFQRKRRVRDNMTKKMITQRTMGKKKQRLNKRSYLIRALTLNTMTKDAERGKLKRRA  
IATPGMQIRGFVYFVETLARSICEKLEQSGLPVGGNEKKAKLANVVRKMMTNSQDTELSFTITGDNTKWNENQNPRMFLA  
MITYMTRNQPEWFRNVLSIAPIMFSNKMARLGKGYMFESKSMKLRTQIPAEMLASIDLKYFNDSTRKKIEKIRSLLIEGT  
ASLSPGMMMGMFNMLSTVLGVSILNLGQKRYTKTTYWWDGLQSSDDFALIVNAPNHEGIQAGVDRFYRTCKLLGINMSKK  
KSYINRTGTFEFTSFFYRYGFVANFSMELPSFGVSGINESADMSIGVTVIKNNMINNDLGPATAQMALQLFIKDYRYTYR  
CHRGDTQIQTRRSFEIKKLWEQTRSKAGLLVSDGGPNLYNIRNLHIPEVCLKWELMDEDYQGRLCNPLNPFVSHKEIESM  
NNAVMMPAHGPAKNMEYDAVATTHSWIPKRNRSILNTSQRGVLEDEQMYQRCCNLFEKFFPSSSYRRPVGISSMVEAMVS  
RARIDARIDFESGRIKKEEFTEIMKICSTIEELRRQK`

| Site | Type | Count |
| --- | --- | --- |
| 504 | N-linked | 1 |

| Mass | Sequence | MS_2_ Score | `q`-value | MS_1_ Score | Signal | Protein Coordinates | Tandem Scans | Start Time | Apex Time | End Time |
| --- | --- | --- | --- | --- | --- | --- | --- | --- | --- | --- |
| 4362.7335 | RYGFVAN(N)FSM Fuc 2 Hex 9 HexNAc 7 | 30.817 | 0.027 | -5.228 | 7.473e+06 | 497 - 507 | 2 | 26.00 | 26.17 | 26.34 |

### RYGFVAN(N)FSM Fuc 2 Hex 9 HexNAc 7

**Mass:**  4362.7336

(6.729e-06)

**Charge States:** 4

**Start Position**: 498

**End Position**: 508

| Profile Score | Tandem Score | TDA q-value | Volume | Spectrum Matches |
| --- | --- | --- | --- | --- |
| -5.2284 | 30.8168 | 0.0268 | 7.4731e+06 | 2 |

**Adduct Signal Fractions**

- **Unmodified**: 7.4731e+06

### tr|A0A1L1RRT9|A0A1L1RRT9\_CHICK

**>tr|A0A1L1RRT9|A0A1L1RRT9\_CHICK**  
 `MASCMKSVTEQGAELSNEERNLLSVAYKNVVGARRSSWRVVSSIEQKTEGAEKKQQMAREYREKIETELRDICNDVLSLL  
EKFLIPNASQAESKVFYLKMKGDYYRYLAEVAAGDDKKGIVEQSQQAYQEAFEISKKEMQPTHPIRLGLALNFSVFYYEI  
LNSPEKACSLAKTAFDEAIAELDTLSEESYKDSTLIMQLLRDNLTLWTSDTQGDEAEAGEGGEN`

| Site | Type | Count |
| --- | --- | --- |
| 87 | N-linked | 1 |

| Mass | Sequence | MS_2_ Score | `q`-value | MS_1_ Score | Signal | Protein Coordinates | Tandem Scans | Start Time | Apex Time | End Time |
| --- | --- | --- | --- | --- | --- | --- | --- | --- | --- | --- |
| 3346.4538 | IPN(N)ASQAESKVF Fuc 1 Hex 3 HexNAc 7 | 26.082 | 0.042 | -9.592 | 6.876e+07 | 84 - 96 | 12 | 17.37 | 17.37 | 26.00 |

### IPN(N)ASQAESKVF Fuc 1 Hex 3 HexNAc 7

**Mass:**  3346.4553

(9.033e-04)

**Charge States:** 4, 5

**Start Position**: 85

**End Position**: 97

| Profile Score | Tandem Score | TDA q-value | Volume | Spectrum Matches |
| --- | --- | --- | --- | --- |
| -9.5915 | 26.0820 | 0.0424 | 6.8760e+07 | 12 |

**Adduct Signal Fractions**

- **Unmodified**: 6.8760e+07

### sp|Q5ZKC9|1433Z\_CHICK

**>sp|Q5ZKC9|1433Z\_CHICK**  
 `MDKNELVQKAKLAEQAERYDDMASCMKSVTEQGAELSNEERNLLSVAYKNVVGARRSSWRVVSSIEQKTEGAEKKQQMAR  
EYREKIETELRDICNDVLSLLEKFLIPNASQAESKVFYLKMKGDYYRYLAEVAAGDDKKGIVEQSQQAYQEAFEISKKEM  
QPTHPIRLGLALNFSVFYYEILNSPEKACSLAKTAFDEAIAELDTLSEESYKDSTLIMQLLRDNLTLWTSDTQGDEAEAG  
EGGEN`

| Site | Type | Count |
| --- | --- | --- |
| 108 | N-linked | 1 |

| Mass | Sequence | MS_2_ Score | `q`-value | MS_1_ Score | Signal | Protein Coordinates | Tandem Scans | Start Time | Apex Time | End Time |
| --- | --- | --- | --- | --- | --- | --- | --- | --- | --- | --- |
| 3346.4538 | IPN(N)ASQAESKVF Fuc 1 Hex 3 HexNAc 7 | 26.082 | 0.042 | -9.592 | 6.876e+07 | 105 - 117 | 12 | 17.37 | 17.37 | 26.00 |

### IPN(N)ASQAESKVF Fuc 1 Hex 3 HexNAc 7

**Mass:**  3346.4553

(9.033e-04)

**Charge States:** 4, 5

**Start Position**: 106

**End Position**: 118

| Profile Score | Tandem Score | TDA q-value | Volume | Spectrum Matches |
| --- | --- | --- | --- | --- |
| -9.5915 | 26.0820 | 0.0424 | 6.8760e+07 | 12 |

**Adduct Signal Fractions**

- **Unmodified**: 6.8760e+07
