# Supplementary material for: Measuring Site-specific Glycosylation Similarity between Influenza a Virus Variants with Statistical Certainty
Source: Mol Cell Proteomics. 2020 Nov 25;19(9):1533–45. doi: 10.1074/mcp.RA120.002031 (PMC8143645; doi:10.1074/mcp.RA120.002031)
Supplement: Supplementary file 2 [file mmc2.zip › mmc2/159250_2_supp_555290_qcjklq.zip!/20190326_Phil82_032219_tryp_HILIC_DDA_1ug_04_glycopeptides.html]

## 20190326\_Phil82\_032219\_tryp\_HILIC\_DDA\_1ug\_04 @ GlycopeptideHypothesis-416d3d8c35094398b28a0360d60928ae

**MS^1^ Mass Accuracy:** 10.0 PPM  
**Grouping Tolerance:** 15.0 PPM  
**MS^n^ Mass Accuracy:** 20.0 PPM  
**Minimum `q`-Value For Clustering:** 0.05

**Glycopeptide Hypothesis:**
GlycopeptideHypothesis-416d3d8c35094398b28a0360d60928ae  
**Sample Searched:**
20190326\_Phil82\_032219\_tryp\_HILIC\_DDA\_1ug\_04

| Protein | Matched Glycopeptides |
| --- | --- |
| iav\|AFG99160\|A/Phil/2/1982\_HA | 76 |
| iav\|ADJ41819\|A/Phil/2-MA/1982\_NA | 5 |
| tr\|Q5F491\|Q5F491\_CHICK | 1 |

### iav|AFG99160|A/Phil/2/1982\_HA

**>iav|AFG99160|A/Phil/2/1982\_HA**  
 `MKTIIALSYMFCLVFAQNLPGNDNSTATLCLGHHAVPNGTLVKTITNDQIEVTNATELVQSSSTGRICDSPHRILDGKNC  
TLIDALLGDPHCDGFQNEKWDLFVERSKAFSNCYPYDVPDYASLRSLVASSGTLEFINEGFNWTGVTQSGGSSTCKRGSN  
NSFFSRLNWLYESESKYPVLNVTMPNNGKFDKLYIWGIHHPSTDKEQTNLYIRASGRVTVSTKRSQQTVIPNIGSRPWVR  
GLSSRISIYWTIVKPGDILLINSTGNLIAPRGYFKIRTGKSSIMRSDAPIGTCSSECITPNGSIPNDKPFQNVNKITYGA  
CPRYVKQNTLKLATGMRNVPEKQTRGIFGAIAGFIENGWEGMVDGWYGFRHQNSEGTGQAADLKSTQAAIDQINGKLNRV  
IEKTNEKFHQIEKEFSEVEGRIQDLEKYVEDTKIDLWSYNAELLVALENQHTIDLTDSEMNKLFEKTRKQLRENAEDMGN  
GCFKIYHKCDNACIGSIRNGTYDHDVYRDEALNNRFQIKGVELKSGYKDWILWISFAISCFLLCVVLLGFIMWACQKGNI  
RCNICI`

| Site | Type | Count |
| --- | --- | --- |
| 262 | N-linked | 2 |
| 301 | N-linked | 2 |
| 499 | N-linked | 36 |
| 181 | N-linked | 9 |
| 54 | N-linked | 7 |
| 160 | N-linked | 20 |

| Mass | Sequence | MS_2_ Score | `q`-value | MS_1_ Score | Signal | Protein Coordinates | Tandem Scans | Start Time | Apex Time | End Time |
| --- | --- | --- | --- | --- | --- | --- | --- | --- | --- | --- |
| 3007.1729 | N(N)GTYDHDVYR Fuc 1 Hex 5 HexNAc 4 | 169.257 | 0.0 | 13.910 | 2.601e+08 | 498 - 508 | 5 | 12.94 | 13.27 | 14.10 |
| 3142.2749 | RGSN(N)NSFFSR Fuc 1 Hex 5 HexNAc 5 | 163.075 | 0.0 | 13.314 | 1.699e+08 | 156 - 166 | 7 | 16.06 | 17.08 | 17.42 |
| 3299.2908 | N(N)GTYDHDVYR Fuc 3 Hex 5 HexNAc 4 | 159.518 | 0.0 | -4.359 | 8.913e+07 | 498 - 508 | 3 | 12.94 | 12.94 | 13.43 |
| 3153.2287 | N(N)GTYDHDVYR Fuc 2 Hex 5 HexNAc 4 | 153.537 | 0.0 | 14.367 | 2.154e+08 | 498 - 508 | 5 | 12.77 | 13.11 | 13.60 |
| 3372.3046 | N(N)GTYDHDVYR Fuc 1 Hex 6 HexNAc 5 | 149.724 | 0.0 | 13.043 | 1.229e+08 | 498 - 508 | 4 | 12.94 | 13.11 | 13.60 |
| 2986.1728 | GSN(N)NSFFSR Fuc 1 Hex 5 HexNAc 5 | 144.270 | 0.0 | 13.118 | 2.557e+08 | 157 - 166 | 7 | 19.12 | 20.47 | 20.64 |
| 3199.2961 | RGSN(N)NSFFSR Hex 5 HexNAc 6 | 140.389 | 0.0 | 12.477 | 6.317e+07 | 156 - 166 | 4 | 16.57 | 17.25 | 17.42 |
| 2939.1961 | RGSN(N)NSFFSR Fuc 1 Hex 5 HexNAc 4 | 139.350 | 0.0 | -2.311 | 2.663e+07 | 156 - 166 | 2 | 16.74 | 16.91 | 17.08 |
| 4068.9269 | TIVKPGDILLIN(N)STGNLIAPR Hex 9 HexNAc 2 | 137.737 | 0.0 | 15.182 | 2.829e+08 | 250 - 271 | 13 | 51.17 | 51.94 | 53.32 |
| 3288.3331 | RGSN(N)NSFFSR Fuc 2 Hex 5 HexNAc 5 | 137.683 | 0.0 | 12.601 | 5.103e+07 | 156 - 166 | 3 | 16.23 | 16.74 | 16.91 |
| 3210.2523 | N(N)GTYDHDVYR Fuc 1 Hex 5 HexNAc 5 | 137.368 | 0.0 | 13.415 | 1.799e+08 | 498 - 508 | 9 | 12.63 | 13.27 | 14.43 |
| 3231.3114 | RGSN(N)NSFFSR Fuc 3 Hex 5 HexNAc 4 | 135.768 | 0.0 | -3.421 | 8.931e+06 | 156 - 166 | 3 | 16.23 | 16.40 | 16.57 |
| 3965.6113 | N(N)GTYDHDVYRDEALNNR Fuc 2 Hex 5 HexNAc 4 | 133.965 | 0.0 | 16.322 | 2.577e+08 | 498 - 515 | 3 | 17.76 | 18.27 | 18.44 |
| 3587.3681 | RGSN(N)NSFFSR Fuc 1 Hex 9 HexNAc 4 | 132.893 | 0.0 | 11.859 | 2.035e+07 | 156 - 166 | 3 | 18.44 | 19.12 | 19.46 |
| 3165.2962 | YPVLN(N)VTM(O)PNN(D)GK Hex 8 HexNAc 2 | 131.846 | 0.0 | 11.964 | 4.605e+08 | 176 - 189 | 6 | 28.43 | 28.93 | 29.61 |
| 3327.3489 | YPVLN(N)VTM(O)PNN(D)GK Hex 9 HexNAc 2 | 129.970 | 0.0 | 12.223 | 8.065e+08 | 176 - 189 | 7 | 28.43 | 28.93 | 29.61 |
| 3061.1731 | RGSN(N)N(D)SFFSR Fuc 1 Hex 7 HexNAc 3 | 128.144 | 0.0 | -19.342 | 5.776e+06 | 156 - 166 | 1 | 19.97 | 19.97 | 19.97 |
| 3664.4267 | N(N)GTYDHDVYR Fuc 3 Hex 6 HexNAc 5 | 127.518 | 0.0 | -19.523 | 2.261e+07 | 498 - 508 | 1 | 12.94 | 12.94 | 13.11 |
| 5111.1361 | SDAPIGTC(C)SSEC(C)ITPN(N)GSIPNDKPFQNVNK Hex 9 HexNAc 2 | 126.886 | 0.0 | 12.676 | 1.034e+08 | 285 - 315 | 5 | 34.90 | 35.07 | 35.75 |
| 3344.3766 | YPVLN(N)VTM(O)PNN(D)GK Fuc 2 Hex 3 HexNAc 4 Neu5Ac 1 | 125.484 | 0.0 | 12.142 | 2.286e+08 | 176 - 189 | 4 | 28.27 | 28.93 | 29.44 |
| 3003.2420 | YPVLN(N)VTM(O)PNN(D)GK Hex 7 HexNAc 2 | 124.675 | 0.0 | 10.888 | 4.894e+07 | 176 - 189 | 3 | 28.60 | 28.93 | 29.61 |
| 2783.0940 | GSN(N)NSFFSR Fuc 1 Hex 5 HexNAc 4 | 123.728 | 0.0 | -3.011 | 4.509e+07 | 157 - 166 | 2 | 19.97 | 20.14 | 20.31 |
| 4949.0848 | SDAPIGTC(C)SSEC(C)ITPN(N)GSIPNDKPFQNVNK Hex 8 HexNAc 2 | 122.606 | 0.0 | 12.784 | 1.231e+08 | 285 - 315 | 5 | 34.90 | 35.07 | 35.92 |
| 3819.5529 | N(N)GTYDHDVYRDEALNNR Fuc 1 Hex 5 HexNAc 4 | 122.579 | 0.0 | -2.540 | 2.930e+08 | 498 - 515 | 3 | 18.10 | 18.44 | 18.61 |
| 4190.8298 | TITNDQIEVTN(N)ATELVQSSSTGR Fuc 1 Hex 6 HexNAc 3 | 122.425 | 0.0 | 13.435 | 2.813e+08 | 43 - 66 | 6 | 39.34 | 39.51 | 40.36 |
| 3401.3982 | YPVLN(N)VTM(O)PNN(D)GK Fuc 1 Hex 3 HexNAc 5 Neu5Ac 1 | 122.375 | 0.0 | 10.448 | 2.559e+08 | 176 - 189 | 7 | 27.08 | 28.43 | 29.27 |
| 3434.3906 | RGSN(N)NSFFSR Fuc 3 Hex 5 HexNAc 5 | 119.504 | 0.0 | -4.970 | 1.159e+07 | 156 - 166 | 3 | 16.23 | 16.40 | 16.74 |
| 3076.2079 | RGSN(N)NSFFSR Hex 8 HexNAc 3 | 118.014 | 0.0 | -18.528 | 3.584e+06 | 156 - 166 | 1 | 19.29 | 19.29 | 19.29 |
| 3555.4883 | YPVLN(N)VTM(O)PNN(D)GKFDK Hex 8 HexNAc 2 | 117.664 | 0.0 | 11.900 | 1.190e+08 | 176 - 192 | 6 | 31.65 | 32.16 | 32.50 |
| 3519.3785 | N(N)GTYDHDVYR Fuc 2 Hex 6 HexNAc 5 | 117.302 | 0.0 | 0.000 | 0.000e+00 | 498 - 508 | 1 | - | - | - |
| 2861.1156 | N(N)GTYDHDVYR Hex 5 HexNAc 4 | 117.100 | 0.0 | -20.633 | 7.749e+06 | 498 - 508 | 2 | 13.11 | 13.27 | 13.27 |
| 4022.6335 | N(N)GTYDHDVYRDEALNNR Fuc 1 Hex 5 HexNAc 5 | 116.621 | 0.0 | 11.636 | 2.110e+08 | 498 - 515 | 5 | 17.42 | 18.61 | 18.78 |
| 3489.4066 | YPVLN(N)VTM(O)PNN(D)GK Hex 10 HexNAc 2 | 116.618 | 0.0 | -6.137 | 2.310e+07 | 176 - 189 | 2 | 28.43 | 28.60 | 28.93 |
| 4044.7701 | TITNDQIEVTN(N)ATELVQSSSTGR Hex 6 HexNAc 3 | 115.379 | 0.0 | 13.661 | 3.511e+08 | 43 - 66 | 8 | 39.51 | 39.68 | 41.05 |
| 4111.6707 | N(N)GTYDHDVYRDEALNNR Fuc 3 Hex 5 HexNAc 4 | 115.309 | 0.0 | -2.208 | 1.292e+08 | 498 - 515 | 2 | 17.93 | 18.10 | 18.27 |
| 3358.3093 | N(N)GTYDHDVYR Fuc 2 Hex 5 HexNAc 5 | 114.702 | 0.0 | 0.000 | 0.000e+00 | 498 - 508 | 1 | - | - | - |
| 3345.3537 | RGSN(N)NSFFSR Fuc 1 Hex 5 HexNAc 6 | 114.534 | 0.0 | 10.605 | 1.753e+07 | 156 - 166 | 2 | 16.23 | 16.74 | 16.91 |
| 3412.4688 | DQIEVTN(N)ATELVQSSSTGR Hex 6 HexNAc 2 | 113.445 | 0.0 | -19.859 | 4.421e+06 | 47 - 66 | 1 | 36.44 | 36.44 | 36.61 |
| 4476.8021 | N(N)GTYDHDVYRDEALNNR Fuc 3 Hex 6 HexNAc 5 | 112.306 | 0.0 | -3.654 | 5.299e+07 | 498 - 515 | 2 | 17.76 | 18.10 | 18.27 |
| 3841.6899 | TITNDQIEVTN(N)ATELVQSSSTGR Hex 6 HexNAc 2 | 111.866 | 0.0 | 14.058 | 9.386e+08 | 43 - 66 | 12 | 38.83 | 39.68 | 40.88 |
| 4410.7011 | N(N)GTYDHDVYRDEALNNR Fuc 2 Hex 9 HexNAc 3 | 111.165 | 0.0 | -5.040 | 1.562e+07 | 498 - 515 | 1 | 19.80 | 19.80 | 20.31 |
| 4184.6866 | N(N)GTYDHDVYRDEALNNR Fuc 1 Hex 6 HexNAc 5 | 110.339 | 0.0 | -2.695 | 1.465e+08 | 498 - 515 | 2 | 17.93 | 18.27 | 18.44 |
| 4622.8601 | N(N)GTYDHDVYRDEALNNR Fuc 4 Hex 6 HexNAc 5 | 109.947 | 0.0 | -3.869 | 2.214e+07 | 498 - 515 | 2 | 17.76 | 17.93 | 18.10 |
| 3627.6310 | PGDILLIN(N)STGNLIAPR Hex 9 HexNAc 2 | 108.444 | 0.0 | 14.161 | 2.702e+07 | 254 - 271 | 4 | 49.69 | 49.97 | 50.34 |
| 3289.3214 | RGSN(N)N(D)SFFSR Fuc 2 Hex 5 HexNAc 5 | 108.433 | 0.0 | -5.652 | 1.669e+07 | 156 - 166 | 3 | 17.08 | 17.25 | 17.42 |
| 4085.7959 | TITNDQIEVTN(N)ATELVQSSSTGR Hex 5 HexNAc 4 | 108.322 | 0.0 | 13.829 | 4.050e+08 | 43 - 66 | 10 | 38.66 | 39.51 | 40.88 |
| 4168.6909 | N(N)GTYDHDVYRDEALNNR Fuc 2 Hex 5 HexNAc 5 | 107.583 | 0.0 | 12.155 | 8.880e+07 | 498 - 515 | 2 | 17.42 | 18.27 | 18.61 |
| 3356.3100 | N(N)GTYDHDVYR Fuc 2 Hex 5 HexNAc 5 | 106.991 | 0.0 | 11.306 | 6.633e+07 | 498 - 508 | 4 | 12.63 | 13.11 | 13.60 |
| 3206.2476 | RGSN(N)NSFFSR Hex 7 HexNAc 3 Neu5Ac 1 | 106.686 | 0.0 | 8.929 | 2.356e+07 | 156 - 166 | 6 | 19.29 | 19.63 | 20.47 |
| 3746.3968 | N(N)GTYDHDVYR Fuc 1 Hex 9 HexNAc 3 Neu5Ac 1 | 105.753 | 0.0 | 0.000 | 0.000e+00 | 498 - 508 | 1 | - | - | - |
| 4264.6424 | N(N)GTYDHDVYRDEALNNR Fuc 1 Hex 9 HexNAc 3 | 104.858 | 0.012 | 10.971 | 2.839e+07 | 498 - 515 | 2 | 19.80 | 19.97 | 20.47 |
| 4387.7659 | N(N)GTYDHDVYRDEALNNR Fuc 1 Hex 6 HexNAc 6 | 104.492 | 0.012 | 10.802 | 4.851e+07 | 498 - 515 | 3 | 17.59 | 18.44 | 18.61 |
| 3518.3621 | N(N)GTYDHDVYR Fuc 2 Hex 6 HexNAc 5 | 103.518 | 0.018 | -4.880 | 4.737e+07 | 498 - 508 | 2 | 12.94 | 13.11 | 13.27 |
| 4003.7447 | TITNDQIEVTN(N)ATELVQSSSTGR Hex 7 HexNAc 2 | 103.512 | 0.018 | 13.955 | 5.702e+08 | 43 - 66 | 10 | 38.66 | 39.51 | 40.71 |
| 4330.7457 | N(N)GTYDHDVYRDEALNNR Fuc 2 Hex 6 HexNAc 5 | 103.174 | 0.018 | -1.194 | 6.702e+07 | 498 - 515 | 2 | 17.93 | 18.10 | 18.27 |
| 3600.3460 | N(N)GTYDHDVYR Fuc 2 Hex 9 HexNAc 3 | 103.063 | 0.018 | 0.000 | 0.000e+00 | 498 - 508 | 1 | - | - | - |
| 3141.2520 | YPVLN(N)VTM(O)PNN Hex 9 HexNAc 2 | 102.876 | 0.018 | 10.695 | 6.316e+06 | 176 - 187 | 3 | 32.33 | 32.50 | 33.19 |
| 4549.8212 | N(N)GTYDHDVYRDEALNNR Fuc 1 Hex 7 HexNAc 6 | 102.481 | 0.018 | -4.379 | 2.081e+07 | 498 - 515 | 1 | 17.76 | 18.10 | 18.10 |
| 3575.3831 | N(N)GTYDHDVYR Fuc 1 Hex 6 HexNAc 6 | 102.311 | 0.018 | -5.120 | 3.059e+07 | 498 - 508 | 4 | 13.27 | 13.27 | 13.76 |
| 2980.2225 | RGSN(N)NSFFSR Fuc 1 Hex 4 HexNAc 5 | 101.793 | 0.018 | -4.875 | 5.749e+06 | 156 - 166 | 1 | 16.91 | 16.91 | 17.25 |
| 3665.4357 | N(N)GTYDHDVYR Fuc 3 Hex 6 HexNAc 5 | 101.282 | 0.018 | 0.000 | 0.000e+00 | 498 - 508 | 1 | - | - | - |
| 3154.2283 | N(N)GTYDHDVYR Fuc 2 Hex 5 HexNAc 4 | 100.831 | 0.018 | 0.000 | 0.000e+00 | 498 - 508 | 1 | - | - | - |
| 3346.3453 | RGSN(N)N(D)SFFSR Fuc 1 Hex 5 HexNAc 6 | 100.330 | 0.018 | -20.311 | 4.551e+06 | 156 - 166 | 3 | 17.25 | 17.25 | 17.42 |
| 2979.1981 | YPVLN(N)VTM(O)PNN Hex 8 HexNAc 2 | 99.197 | 0.018 | -4.627 | 2.434e+06 | 176 - 187 | 2 | 32.50 | 32.68 | 32.85 |
| 3304.3267 | RGSN(N)NSFFSR Fuc 1 Hex 6 HexNAc 5 | 97.866 | 0.018 | 14.998 | 1.317e+07 | 156 - 166 | 3 | 16.40 | 17.08 | 17.25 |
| 3733.4265 | RGSN(N)NSFFSR Fuc 2 Hex 9 HexNAc 4 | 97.196 | 0.026 | 9.377 | 1.030e+07 | 156 - 166 | 1 | 18.27 | 18.61 | 19.12 |
| 3885.4966 | N(N)GTYDHDVYR Fuc 2 Hex 7 HexNAc 6 | 95.330 | 0.032 | 0.000 | 0.000e+00 | 498 - 508 | 1 | - | - | - |
| 3810.4852 | N(N)GTYDHDVYR Fuc 4 Hex 6 HexNAc 5 | 95.239 | 0.032 | -20.185 | 9.931e+06 | 498 - 508 | 1 | 12.77 | 12.77 | 12.94 |
| 3737.4484 | N(N)GTYDHDVYR Fuc 1 Hex 7 HexNAc 6 | 94.649 | 0.032 | -20.893 | 1.041e+07 | 498 - 508 | 2 | 12.94 | 12.94 | 12.94 |
| 3368.2872 | RGSN(N)N(D)SFFSR Hex 8 HexNAc 3 Neu5Ac 1 | 94.106 | 0.032 | -7.302 | 6.122e+06 | 156 - 166 | 1 | 19.46 | 19.46 | 19.80 |
| 3298.2739 | N(N)GTYDHDVYR Fuc 1 Hex 5 HexNAc 4 Neu5Ac 1 | 93.107 | 0.032 | -20.529 | 3.463e+06 | 498 - 508 | 1 | 14.10 | 14.27 | 14.27 |
| 4695.8774 | N(N)GTYDHDVYRDEALNNR Fuc 2 Hex 7 HexNAc 6 | 90.841 | 0.044 | -3.763 | 1.303e+07 | 498 - 515 | 1 | 17.76 | 17.93 | 18.10 |
| 4533.8230 | N(N)GTYDHDVYRDEALNNR Fuc 2 Hex 6 HexNAc 6 | 90.003 | 0.044 | 8.958 | 5.319e+07 | 498 - 515 | 6 | 17.25 | 18.27 | 18.95 |
| 3491.4132 | RGSN(N)NSFFSR Fuc 2 Hex 5 HexNAc 6 | 89.409 | 0.044 | -19.455 | 3.928e+06 | 156 - 166 | 1 | 16.40 | 16.40 | 16.57 |
| 4288.8742 | TITNDQIEVTN(N)ATELVQSSSTGR Hex 5 HexNAc 5 | 89.237 | 0.044 | 13.827 | 1.970e+08 | 43 - 66 | 9 | 38.83 | 39.51 | 40.88 |
| 4113.6781 | N(N)GTYDHDVYRDEALNNR Fuc 3 Hex 5 HexNAc 4 | 89.050 | 0.044 | 0.000 | 0.000e+00 | 498 - 515 | 1 | - | - | - |

### N(N)GTYDHDVYR Fuc 1 Hex 5 HexNAc 4

**Mass:**  3007.1738

(9.110e-07)

**Charge States:** 3

**Start Position**: 499

**End Position**: 509

| Profile Score | Tandem Score | TDA q-value | Volume | Spectrum Matches |
| --- | --- | --- | --- | --- |
| 13.9100 | 169.2574 | 0.0000 | 2.6010e+08 | 5 |

**Adduct Signal Fractions**

- **Unmodified**: 2.6010e+08

### RGSN(N)NSFFSR Fuc 1 Hex 5 HexNAc 5

**Mass:**  3142.2746

(8.199e-07)

**Charge States:** 3, 4

**Start Position**: 157

**End Position**: 167

| Profile Score | Tandem Score | TDA q-value | Volume | Spectrum Matches |
| --- | --- | --- | --- | --- |
| 13.3137 | 163.0745 | 0.0000 | 1.6994e+08 | 7 |

**Adduct Signal Fractions**

- **Unmodified**: 1.6994e+08

### N(N)GTYDHDVYR Fuc 3 Hex 5 HexNAc 4

**Mass:**  3299.2937

(2.031e-06)

**Charge States:** 3

**Start Position**: 499

**End Position**: 509

| Profile Score | Tandem Score | TDA q-value | Volume | Spectrum Matches |
| --- | --- | --- | --- | --- |
| -4.3592 | 159.5182 | 0.0000 | 8.9127e+07 | 3 |

**Adduct Signal Fractions**

- **Unmodified**: 8.9127e+07

### N(N)GTYDHDVYR Fuc 2 Hex 5 HexNAc 4

**Mass:**  3153.2272

(-5.472e-07)

**Charge States:** 3

**Start Position**: 499

**End Position**: 509

| Profile Score | Tandem Score | TDA q-value | Volume | Spectrum Matches |
| --- | --- | --- | --- | --- |
| 14.3669 | 153.5370 | 0.0000 | 2.1537e+08 | 5 |

**Adduct Signal Fractions**

- **Unmodified**: 2.1537e+08

### N(N)GTYDHDVYR Fuc 1 Hex 6 HexNAc 5

**Mass:**  3372.3026

(-1.947e-07)

**Charge States:** 3

**Start Position**: 499

**End Position**: 509

| Profile Score | Tandem Score | TDA q-value | Volume | Spectrum Matches |
| --- | --- | --- | --- | --- |
| 13.0427 | 149.7235 | 0.0000 | 1.2291e+08 | 4 |

**Adduct Signal Fractions**

- **Unmodified**: 1.2291e+08

### GSN(N)NSFFSR Fuc 1 Hex 5 HexNAc 5

**Mass:**  2986.1726

(4.144e-07)

**Charge States:** 3, 4

**Start Position**: 158

**End Position**: 167

| Profile Score | Tandem Score | TDA q-value | Volume | Spectrum Matches |
| --- | --- | --- | --- | --- |
| 13.1178 | 144.2697 | 0.0000 | 2.5573e+08 | 7 |

**Adduct Signal Fractions**

- **Unmodified**: 2.5573e+08

### RGSN(N)NSFFSR Hex 5 HexNAc 6

**Mass:**  3199.2951

(5.569e-07)

**Charge States:** 3

**Start Position**: 157

**End Position**: 167

| Profile Score | Tandem Score | TDA q-value | Volume | Spectrum Matches |
| --- | --- | --- | --- | --- |
| 12.4772 | 140.3887 | 0.0000 | 6.3174e+07 | 4 |

**Adduct Signal Fractions**

- **Unmodified**: 6.3174e+07

### RGSN(N)NSFFSR Fuc 1 Hex 5 HexNAc 4

**Mass:**  2939.1963

(1.014e-06)

**Charge States:** 3

**Start Position**: 157

**End Position**: 167

| Profile Score | Tandem Score | TDA q-value | Volume | Spectrum Matches |
| --- | --- | --- | --- | --- |
| -2.3106 | 139.3499 | 0.0000 | 2.6631e+07 | 2 |

**Adduct Signal Fractions**

- **Unmodified**: 2.6631e+07

### TIVKPGDILLIN(N)STGNLIAPR Hex 9 HexNAc 2

**Mass:**  4068.9267

(8.701e-07)

**Charge States:** 3, 4, 5

**Start Position**: 251

**End Position**: 272

| Profile Score | Tandem Score | TDA q-value | Volume | Spectrum Matches |
| --- | --- | --- | --- | --- |
| 15.1816 | 137.7368 | 0.0000 | 2.8287e+08 | 13 |

**Adduct Signal Fractions**

- **Unmodified**: 2.8287e+08

### RGSN(N)NSFFSR Fuc 2 Hex 5 HexNAc 5

**Mass:**  3288.3334

(1.131e-06)

**Charge States:** 3

**Start Position**: 157

**End Position**: 167

| Profile Score | Tandem Score | TDA q-value | Volume | Spectrum Matches |
| --- | --- | --- | --- | --- |
| 12.6007 | 137.6831 | 0.0000 | 5.1028e+07 | 3 |

**Adduct Signal Fractions**

- **Unmodified**: 5.1028e+07

### N(N)GTYDHDVYR Fuc 1 Hex 5 HexNAc 5

**Mass:**  3210.2532

(7.982e-07)

**Charge States:** 3, 4

**Start Position**: 499

**End Position**: 509

| Profile Score | Tandem Score | TDA q-value | Volume | Spectrum Matches |
| --- | --- | --- | --- | --- |
| 13.4151 | 137.3679 | 0.0000 | 1.7991e+08 | 9 |

**Adduct Signal Fractions**

- **Unmodified**: 1.7991e+08

### RGSN(N)NSFFSR Fuc 3 Hex 5 HexNAc 4

**Mass:**  3231.3111

(1.680e-06)

**Charge States:** 3

**Start Position**: 157

**End Position**: 167

| Profile Score | Tandem Score | TDA q-value | Volume | Spectrum Matches |
| --- | --- | --- | --- | --- |
| -3.4209 | 135.7679 | 0.0000 | 8.9309e+06 | 3 |

**Adduct Signal Fractions**

- **Unmodified**: 8.9309e+06

### N(N)GTYDHDVYRDEALNNR Fuc 2 Hex 5 HexNAc 4

**Mass:**  3965.6105

(9.763e-07)

**Charge States:** 3, 4

**Start Position**: 499

**End Position**: 516

| Profile Score | Tandem Score | TDA q-value | Volume | Spectrum Matches |
| --- | --- | --- | --- | --- |
| 16.3217 | 133.9654 | 0.0000 | 2.5770e+08 | 3 |

**Adduct Signal Fractions**

- **Unmodified**: 2.5770e+08

### RGSN(N)NSFFSR Fuc 1 Hex 9 HexNAc 4

**Mass:**  3587.3648

(-8.586e-06)

**Charge States:** 3

**Start Position**: 157

**End Position**: 167

| Profile Score | Tandem Score | TDA q-value | Volume | Spectrum Matches |
| --- | --- | --- | --- | --- |
| 11.8588 | 132.8933 | 0.0000 | 2.0347e+07 | 3 |

**Adduct Signal Fractions**

- **Unmodified**: 2.0347e+07

### YPVLN(N)VTM(O)PNN(D)GK Hex 8 HexNAc 2

**Mass:**  3165.2960

(3.115e-04)

**Charge States:** 2, 3

**Start Position**: 177

**End Position**: 190

| Profile Score | Tandem Score | TDA q-value | Volume | Spectrum Matches |
| --- | --- | --- | --- | --- |
| 11.9642 | 131.8464 | 0.0000 | 4.6051e+08 | 6 |

**Adduct Signal Fractions**

- **Unmodified**: 4.6051e+08

### YPVLN(N)VTM(O)PNN(D)GK Hex 9 HexNAc 2

**Mass:**  3327.3484

(2.964e-04)

**Charge States:** 2, 3, 4

**Start Position**: 177

**End Position**: 190

| Profile Score | Tandem Score | TDA q-value | Volume | Spectrum Matches |
| --- | --- | --- | --- | --- |
| 12.2229 | 129.9703 | 0.0000 | 8.0651e+08 | 7 |

**Adduct Signal Fractions**

- **Unmodified**: 8.0651e+08

### RGSN(N)N(D)SFFSR Fuc 1 Hex 7 HexNAc 3

**Mass:**  3061.1731

(3.119e-04)

**Charge States:** 3

**Start Position**: 157

**End Position**: 167

| Profile Score | Tandem Score | TDA q-value | Volume | Spectrum Matches |
| --- | --- | --- | --- | --- |
| -19.3415 | 128.1436 | 0.0000 | 5.7759e+06 | 1 |

**Adduct Signal Fractions**

- **Unmodified**: 5.7759e+06

### N(N)GTYDHDVYR Fuc 3 Hex 6 HexNAc 5

**Mass:**  3664.4298

(2.928e-06)

**Charge States:** 3

**Start Position**: 499

**End Position**: 509

| Profile Score | Tandem Score | TDA q-value | Volume | Spectrum Matches |
| --- | --- | --- | --- | --- |
| -19.5231 | 127.5180 | 0.0000 | 2.2606e+07 | 1 |

**Adduct Signal Fractions**

- **Unmodified**: 2.2606e+07

### SDAPIGTC(C)SSEC(C)ITPN(N)GSIPNDKPFQNVNK Hex 9 HexNAc 2

**Mass:**  5111.1369

(1.864e-06)

**Charge States:** 4

**Start Position**: 286

**End Position**: 316

| Profile Score | Tandem Score | TDA q-value | Volume | Spectrum Matches |
| --- | --- | --- | --- | --- |
| 12.6760 | 126.8856 | 0.0000 | 1.0338e+08 | 5 |

**Adduct Signal Fractions**

- **Unmodified**: 1.0338e+08

### YPVLN(N)VTM(O)PNN(D)GK Fuc 2 Hex 3 HexNAc 4 Neu5Ac 1

**Mass:**  3344.3747

(2.869e-04)

**Charge States:** 3

**Start Position**: 177

**End Position**: 190

| Profile Score | Tandem Score | TDA q-value | Volume | Spectrum Matches |
| --- | --- | --- | --- | --- |
| 12.1415 | 125.4842 | 0.0000 | 2.2860e+08 | 4 |

**Adduct Signal Fractions**

- **Unmodified**: 2.2860e+08

### YPVLN(N)VTM(O)PNN(D)GK Hex 7 HexNAc 2

**Mass:**  3003.2422

(3.280e-04)

**Charge States:** 3

**Start Position**: 177

**End Position**: 190

| Profile Score | Tandem Score | TDA q-value | Volume | Spectrum Matches |
| --- | --- | --- | --- | --- |
| 10.8877 | 124.6746 | 0.0000 | 4.8939e+07 | 3 |

**Adduct Signal Fractions**

- **Unmodified**: 4.8939e+07

### GSN(N)NSFFSR Fuc 1 Hex 5 HexNAc 4

**Mass:**  2783.0949

(9.636e-07)

**Charge States:** 3

**Start Position**: 158

**End Position**: 167

| Profile Score | Tandem Score | TDA q-value | Volume | Spectrum Matches |
| --- | --- | --- | --- | --- |
| -3.0111 | 123.7284 | 0.0000 | 4.5085e+07 | 2 |

**Adduct Signal Fractions**

- **Unmodified**: 4.5085e+07

### SDAPIGTC(C)SSEC(C)ITPN(N)GSIPNDKPFQNVNK Hex 8 HexNAc 2

**Mass:**  4949.0856

(2.437e-06)

**Charge States:** 3, 4

**Start Position**: 286

**End Position**: 316

| Profile Score | Tandem Score | TDA q-value | Volume | Spectrum Matches |
| --- | --- | --- | --- | --- |
| 12.7843 | 122.6058 | 0.0000 | 1.2311e+08 | 5 |

**Adduct Signal Fractions**

- **Unmodified**: 1.2311e+08

### N(N)GTYDHDVYRDEALNNR Fuc 1 Hex 5 HexNAc 4

**Mass:**  3819.5524

(9.482e-07)

**Charge States:** 3, 4

**Start Position**: 499

**End Position**: 516

| Profile Score | Tandem Score | TDA q-value | Volume | Spectrum Matches |
| --- | --- | --- | --- | --- |
| -2.5401 | 122.5791 | 0.0000 | 2.9304e+08 | 3 |

**Adduct Signal Fractions**

- **Unmodified**: 2.9304e+08

### TITNDQIEVTN(N)ATELVQSSSTGR Fuc 1 Hex 6 HexNAc 3

**Mass:**  4190.8317

(1.452e-06)

**Charge States:** 3, 4

**Start Position**: 44

**End Position**: 67

| Profile Score | Tandem Score | TDA q-value | Volume | Spectrum Matches |
| --- | --- | --- | --- | --- |
| 13.4353 | 122.4246 | 0.0000 | 2.8125e+08 | 6 |

**Adduct Signal Fractions**

- **Unmodified**: 2.8125e+08

### YPVLN(N)VTM(O)PNN(D)GK Fuc 1 Hex 3 HexNAc 5 Neu5Ac 1

**Mass:**  3401.3970

(2.826e-04)

**Charge States:** 3

**Start Position**: 177

**End Position**: 190

| Profile Score | Tandem Score | TDA q-value | Volume | Spectrum Matches |
| --- | --- | --- | --- | --- |
| 10.4482 | 122.3751 | 0.0000 | 2.5593e+08 | 7 |

**Adduct Signal Fractions**

- **Unmodified**: 2.5593e+08

### RGSN(N)NSFFSR Fuc 3 Hex 5 HexNAc 5

**Mass:**  3434.3911

(2.010e-06)

**Charge States:** 3

**Start Position**: 157

**End Position**: 167

| Profile Score | Tandem Score | TDA q-value | Volume | Spectrum Matches |
| --- | --- | --- | --- | --- |
| -4.9699 | 119.5039 | 0.0000 | 1.1593e+07 | 3 |

**Adduct Signal Fractions**

- **Unmodified**: 1.1593e+07

### RGSN(N)NSFFSR Hex 8 HexNAc 3

**Mass:**  3076.2079

(-1.842e-06)

**Charge States:** 3

**Start Position**: 157

**End Position**: 167

| Profile Score | Tandem Score | TDA q-value | Volume | Spectrum Matches |
| --- | --- | --- | --- | --- |
| -18.5276 | 118.0141 | 0.0000 | 3.5843e+06 | 1 |

**Adduct Signal Fractions**

- **Unmodified**: 3.5843e+06

### YPVLN(N)VTM(O)PNN(D)GKFDK Hex 8 HexNAc 2

**Mass:**  3555.4876

(2.778e-04)

**Charge States:** 3, 4

**Start Position**: 177

**End Position**: 193

| Profile Score | Tandem Score | TDA q-value | Volume | Spectrum Matches |
| --- | --- | --- | --- | --- |
| 11.8995 | 117.6645 | 0.0000 | 1.1898e+08 | 6 |

**Adduct Signal Fractions**

- **Unmodified**: 1.1898e+08

### N(N)GTYDHDVYR Fuc 2 Hex 6 HexNAc 5

**Mass:**  3519.3785

(2.891e-04)

**Start Position**: 499

**End Position**: 509

| Profile Score | Tandem Score | TDA q-value | Volume | Spectrum Matches |
| --- | --- | --- | --- | --- |
| 0.0000 | 117.3020 | 0.0000 | 0.0000e+00 | 1 |

Adduct:

No Chromatogram Found

### N(N)GTYDHDVYR Hex 5 HexNAc 4

**Mass:**  2861.1159

(5.879e-07)

**Charge States:** 3

**Start Position**: 499

**End Position**: 509

| Profile Score | Tandem Score | TDA q-value | Volume | Spectrum Matches |
| --- | --- | --- | --- | --- |
| -20.6334 | 117.1002 | 0.0000 | 7.7489e+06 | 2 |

**Adduct Signal Fractions**

- **Unmodified**: 7.7489e+06

### N(N)GTYDHDVYRDEALNNR Fuc 1 Hex 5 HexNAc 5

**Mass:**  4022.6330

(1.092e-06)

**Charge States:** 3, 4

**Start Position**: 499

**End Position**: 516

| Profile Score | Tandem Score | TDA q-value | Volume | Spectrum Matches |
| --- | --- | --- | --- | --- |
| 11.6355 | 116.6209 | 0.0000 | 2.1098e+08 | 5 |

**Adduct Signal Fractions**

- **Unmodified**: 2.1098e+08

### YPVLN(N)VTM(O)PNN(D)GK Hex 10 HexNAc 2

**Mass:**  3489.4077

(2.839e-04)

**Charge States:** 3

**Start Position**: 177

**End Position**: 190

| Profile Score | Tandem Score | TDA q-value | Volume | Spectrum Matches |
| --- | --- | --- | --- | --- |
| -6.1375 | 116.6177 | 0.0000 | 2.3096e+07 | 2 |

**Adduct Signal Fractions**

- **Unmodified**: 2.3096e+07

### TITNDQIEVTN(N)ATELVQSSSTGR Hex 6 HexNAc 3

**Mass:**  4044.7700

(2.354e-06)

**Charge States:** 3, 4

**Start Position**: 44

**End Position**: 67

| Profile Score | Tandem Score | TDA q-value | Volume | Spectrum Matches |
| --- | --- | --- | --- | --- |
| 13.6605 | 115.3792 | 0.0000 | 3.5105e+08 | 8 |

**Adduct Signal Fractions**

- **Unmodified**: 3.5105e+08

### N(N)GTYDHDVYRDEALNNR Fuc 3 Hex 5 HexNAc 4

**Mass:**  4111.6711

(1.524e-06)

**Charge States:** 4

**Start Position**: 499

**End Position**: 516

| Profile Score | Tandem Score | TDA q-value | Volume | Spectrum Matches |
| --- | --- | --- | --- | --- |
| -2.2080 | 115.3092 | 0.0000 | 1.2922e+08 | 2 |

**Adduct Signal Fractions**

- **Unmodified**: 1.2922e+08

### N(N)GTYDHDVYR Fuc 2 Hex 5 HexNAc 5

**Mass:**  3358.3093

(5.962e-04)

**Start Position**: 499

**End Position**: 509

| Profile Score | Tandem Score | TDA q-value | Volume | Spectrum Matches |
| --- | --- | --- | --- | --- |
| 0.0000 | 114.7019 | 0.0000 | 0.0000e+00 | 1 |

Adduct:

No Chromatogram Found

### RGSN(N)NSFFSR Fuc 1 Hex 5 HexNAc 6

**Mass:**  3345.3532

(5.820e-07)

**Charge States:** 3

**Start Position**: 157

**End Position**: 167

| Profile Score | Tandem Score | TDA q-value | Volume | Spectrum Matches |
| --- | --- | --- | --- | --- |
| 10.6049 | 114.5338 | 0.0000 | 1.7528e+07 | 2 |

**Adduct Signal Fractions**

- **Unmodified**: 1.7528e+07

### DQIEVTN(N)ATELVQSSSTGR Hex 6 HexNAc 2

**Mass:**  3412.4690

(2.031e-06)

**Charge States:** 3

**Start Position**: 48

**End Position**: 67

| Profile Score | Tandem Score | TDA q-value | Volume | Spectrum Matches |
| --- | --- | --- | --- | --- |
| -19.8586 | 113.4452 | 0.0000 | 4.4207e+06 | 1 |

**Adduct Signal Fractions**

- **Unmodified**: 4.4207e+06

### N(N)GTYDHDVYRDEALNNR Fuc 3 Hex 6 HexNAc 5

**Mass:**  4476.8029

(1.317e-06)

**Charge States:** 4

**Start Position**: 499

**End Position**: 516

| Profile Score | Tandem Score | TDA q-value | Volume | Spectrum Matches |
| --- | --- | --- | --- | --- |
| -3.6541 | 112.3064 | 0.0000 | 5.2993e+07 | 2 |

**Adduct Signal Fractions**

- **Unmodified**: 5.2993e+07

### TITNDQIEVTN(N)ATELVQSSSTGR Hex 6 HexNAc 2

**Mass:**  3841.6909

(-1.657e-07)

**Charge States:** 3, 4

**Start Position**: 44

**End Position**: 67

| Profile Score | Tandem Score | TDA q-value | Volume | Spectrum Matches |
| --- | --- | --- | --- | --- |
| 14.0577 | 111.8664 | 0.0000 | 9.3856e+08 | 12 |

**Adduct Signal Fractions**

- **Unmodified**: 9.3856e+08

### N(N)GTYDHDVYRDEALNNR Fuc 2 Hex 9 HexNAc 3

**Mass:**  4410.7033

(-7.966e-06)

**Charge States:** 4

**Start Position**: 499

**End Position**: 516

| Profile Score | Tandem Score | TDA q-value | Volume | Spectrum Matches |
| --- | --- | --- | --- | --- |
| -5.0396 | 111.1650 | 0.0000 | 1.5616e+07 | 1 |

**Adduct Signal Fractions**

- **Unmodified**: 1.5616e+07

### N(N)GTYDHDVYRDEALNNR Fuc 1 Hex 6 HexNAc 5

**Mass:**  4184.6862

(1.864e-06)

**Charge States:** 4

**Start Position**: 499

**End Position**: 516

| Profile Score | Tandem Score | TDA q-value | Volume | Spectrum Matches |
| --- | --- | --- | --- | --- |
| -2.6951 | 110.3388 | 0.0000 | 1.4648e+08 | 2 |

**Adduct Signal Fractions**

- **Unmodified**: 1.4648e+08

### N(N)GTYDHDVYRDEALNNR Fuc 4 Hex 6 HexNAc 5

**Mass:**  4622.8610

(1.588e-06)

**Charge States:** 4

**Start Position**: 499

**End Position**: 516

| Profile Score | Tandem Score | TDA q-value | Volume | Spectrum Matches |
| --- | --- | --- | --- | --- |
| -3.8692 | 109.9474 | 0.0000 | 2.2137e+07 | 2 |

**Adduct Signal Fractions**

- **Unmodified**: 2.2137e+07

### PGDILLIN(N)STGNLIAPR Hex 9 HexNAc 2

**Mass:**  3627.6315

(6.076e-07)

**Charge States:** 3, 4

**Start Position**: 255

**End Position**: 272

| Profile Score | Tandem Score | TDA q-value | Volume | Spectrum Matches |
| --- | --- | --- | --- | --- |
| 14.1615 | 108.4444 | 0.0000 | 2.7024e+07 | 4 |

**Adduct Signal Fractions**

- **Unmodified**: 2.7024e+07

### RGSN(N)N(D)SFFSR Fuc 2 Hex 5 HexNAc 5

**Mass:**  3289.3200

(3.025e-04)

**Charge States:** 3

**Start Position**: 157

**End Position**: 167

| Profile Score | Tandem Score | TDA q-value | Volume | Spectrum Matches |
| --- | --- | --- | --- | --- |
| -5.6516 | 108.4327 | 0.0000 | 1.6693e+07 | 3 |

**Adduct Signal Fractions**

- **Unmodified**: 1.6693e+07

### TITNDQIEVTN(N)ATELVQSSSTGR Hex 5 HexNAc 4

**Mass:**  4085.7998

(2.159e-06)

**Charge States:** 3, 4

**Start Position**: 44

**End Position**: 67

| Profile Score | Tandem Score | TDA q-value | Volume | Spectrum Matches |
| --- | --- | --- | --- | --- |
| 13.8293 | 108.3219 | 0.0000 | 4.0496e+08 | 10 |

**Adduct Signal Fractions**

- **Unmodified**: 4.0496e+08

### N(N)GTYDHDVYRDEALNNR Fuc 2 Hex 5 HexNAc 5

**Mass:**  4168.6921

(1.439e-06)

**Charge States:** 4

**Start Position**: 499

**End Position**: 516

| Profile Score | Tandem Score | TDA q-value | Volume | Spectrum Matches |
| --- | --- | --- | --- | --- |
| 12.1551 | 107.5831 | 0.0000 | 8.8801e+07 | 2 |

**Adduct Signal Fractions**

- **Unmodified**: 8.8801e+07

### N(N)GTYDHDVYR Fuc 2 Hex 5 HexNAc 5

**Mass:**  3356.3065

(1.562e-06)

**Charge States:** 3

**Start Position**: 499

**End Position**: 509

| Profile Score | Tandem Score | TDA q-value | Volume | Spectrum Matches |
| --- | --- | --- | --- | --- |
| 11.3059 | 106.9915 | 0.0000 | 6.6333e+07 | 4 |

**Adduct Signal Fractions**

- **Unmodified**: 6.6333e+07

### RGSN(N)NSFFSR Hex 7 HexNAc 3 Neu5Ac 1

**Mass:**  3206.2370

(3.070e-04)

**Charge States:** 3

**Start Position**: 157

**End Position**: 167

| Profile Score | Tandem Score | TDA q-value | Volume | Spectrum Matches |
| --- | --- | --- | --- | --- |
| 8.9287 | 106.6863 | 0.0000 | 2.3561e+07 | 6 |

**Adduct Signal Fractions**

- **Unmodified**: 2.3561e+07

### N(N)GTYDHDVYR Fuc 1 Hex 9 HexNAc 3 Neu5Ac 1

**Mass:**  3746.3968

(8.010e-04)

**Start Position**: 499

**End Position**: 509

| Profile Score | Tandem Score | TDA q-value | Volume | Spectrum Matches |
| --- | --- | --- | --- | --- |
| 0.0000 | 105.7534 | 0.0000 | 0.0000e+00 | 1 |

Adduct:

No Chromatogram Found

### N(N)GTYDHDVYRDEALNNR Fuc 1 Hex 9 HexNAc 3

**Mass:**  4264.6442

(-8.508e-06)

**Charge States:** 3, 4

**Start Position**: 499

**End Position**: 516

| Profile Score | Tandem Score | TDA q-value | Volume | Spectrum Matches |
| --- | --- | --- | --- | --- |
| 10.9711 | 104.8576 | 0.0119 | 2.8388e+07 | 2 |

**Adduct Signal Fractions**

- **Unmodified**: 2.8388e+07

### N(N)GTYDHDVYRDEALNNR Fuc 1 Hex 6 HexNAc 6

**Mass:**  4387.7653

(9.497e-07)

**Charge States:** 4

**Start Position**: 499

**End Position**: 516

| Profile Score | Tandem Score | TDA q-value | Volume | Spectrum Matches |
| --- | --- | --- | --- | --- |
| 10.8024 | 104.4924 | 0.0119 | 4.8507e+07 | 3 |

**Adduct Signal Fractions**

- **Unmodified**: 4.8507e+07

### N(N)GTYDHDVYR Fuc 2 Hex 6 HexNAc 5

**Mass:**  3518.3593

(1.207e-06)

**Charge States:** 3

**Start Position**: 499

**End Position**: 509

| Profile Score | Tandem Score | TDA q-value | Volume | Spectrum Matches |
| --- | --- | --- | --- | --- |
| -4.8799 | 103.5177 | 0.0179 | 4.7374e+07 | 2 |

**Adduct Signal Fractions**

- **Unmodified**: 4.7374e+07

### TITNDQIEVTN(N)ATELVQSSSTGR Hex 7 HexNAc 2

**Mass:**  4003.7470

(1.411e-06)

**Charge States:** 3, 4

**Start Position**: 44

**End Position**: 67

| Profile Score | Tandem Score | TDA q-value | Volume | Spectrum Matches |
| --- | --- | --- | --- | --- |
| 13.9548 | 103.5117 | 0.0179 | 5.7017e+08 | 10 |

**Adduct Signal Fractions**

- **Unmodified**: 5.7017e+08

### N(N)GTYDHDVYRDEALNNR Fuc 2 Hex 6 HexNAc 5

**Mass:**  4330.7468

(1.831e-06)

**Charge States:** 4

**Start Position**: 499

**End Position**: 516

| Profile Score | Tandem Score | TDA q-value | Volume | Spectrum Matches |
| --- | --- | --- | --- | --- |
| -1.1939 | 103.1742 | 0.0179 | 6.7023e+07 | 2 |

**Adduct Signal Fractions**

- **Unmodified**: 6.7023e+07

### N(N)GTYDHDVYR Fuc 2 Hex 9 HexNAc 3

**Mass:**  3600.3460

(5.516e-04)

**Start Position**: 499

**End Position**: 509

| Profile Score | Tandem Score | TDA q-value | Volume | Spectrum Matches |
| --- | --- | --- | --- | --- |
| 0.0000 | 103.0631 | 0.0179 | 0.0000e+00 | 1 |

Adduct:

No Chromatogram Found

### YPVLN(N)VTM(O)PNN Hex 9 HexNAc 2

**Mass:**  3141.2528

(2.023e-06)

**Charge States:** 3

**Start Position**: 177

**End Position**: 188

| Profile Score | Tandem Score | TDA q-value | Volume | Spectrum Matches |
| --- | --- | --- | --- | --- |
| 10.6951 | 102.8756 | 0.0179 | 6.3155e+06 | 3 |

**Adduct Signal Fractions**

- **Unmodified**: 6.3155e+06

### N(N)GTYDHDVYRDEALNNR Fuc 1 Hex 7 HexNAc 6

**Mass:**  4549.8205

(1.950e-06)

**Charge States:** 4

**Start Position**: 499

**End Position**: 516

| Profile Score | Tandem Score | TDA q-value | Volume | Spectrum Matches |
| --- | --- | --- | --- | --- |
| -4.3786 | 102.4806 | 0.0179 | 2.0812e+07 | 1 |

**Adduct Signal Fractions**

- **Unmodified**: 2.0812e+07

### N(N)GTYDHDVYR Fuc 1 Hex 6 HexNAc 6

**Mass:**  3575.3842

(4.557e-07)

**Charge States:** 3

**Start Position**: 499

**End Position**: 509

| Profile Score | Tandem Score | TDA q-value | Volume | Spectrum Matches |
| --- | --- | --- | --- | --- |
| -5.1200 | 102.3106 | 0.0179 | 3.0586e+07 | 4 |

**Adduct Signal Fractions**

- **Unmodified**: 3.0586e+07

### RGSN(N)NSFFSR Fuc 1 Hex 4 HexNAc 5

**Mass:**  2980.2216

(8.552e-07)

**Charge States:** 3

**Start Position**: 157

**End Position**: 167

| Profile Score | Tandem Score | TDA q-value | Volume | Spectrum Matches |
| --- | --- | --- | --- | --- |
| -4.8746 | 101.7932 | 0.0179 | 5.7486e+06 | 1 |

**Adduct Signal Fractions**

- **Unmodified**: 5.7486e+06

### N(N)GTYDHDVYR Fuc 3 Hex 6 HexNAc 5

**Mass:**  3665.4357

(2.774e-04)

**Start Position**: 499

**End Position**: 509

| Profile Score | Tandem Score | TDA q-value | Volume | Spectrum Matches |
| --- | --- | --- | --- | --- |
| 0.0000 | 101.2823 | 0.0179 | 0.0000e+00 | 1 |

Adduct:

No Chromatogram Found

### N(N)GTYDHDVYR Fuc 2 Hex 5 HexNAc 4

**Mass:**  3154.2283

(3.169e-04)

**Start Position**: 499

**End Position**: 509

| Profile Score | Tandem Score | TDA q-value | Volume | Spectrum Matches |
| --- | --- | --- | --- | --- |
| 0.0000 | 100.8306 | 0.0179 | 0.0000e+00 | 1 |

Adduct:

No Chromatogram Found

### RGSN(N)N(D)SFFSR Fuc 1 Hex 5 HexNAc 6

**Mass:**  3346.3474

(3.024e-04)

**Charge States:** 3

**Start Position**: 157

**End Position**: 167

| Profile Score | Tandem Score | TDA q-value | Volume | Spectrum Matches |
| --- | --- | --- | --- | --- |
| -20.3113 | 100.3295 | 0.0179 | 4.5505e+06 | 3 |

**Adduct Signal Fractions**

- **Unmodified**: 4.5505e+06

### YPVLN(N)VTM(O)PNN Hex 8 HexNAc 2

**Mass:**  2979.1953

(1.109e-06)

**Charge States:** 3

**Start Position**: 177

**End Position**: 188

| Profile Score | Tandem Score | TDA q-value | Volume | Spectrum Matches |
| --- | --- | --- | --- | --- |
| -4.6265 | 99.1971 | 0.0179 | 2.4339e+06 | 2 |

**Adduct Signal Fractions**

- **Unmodified**: 2.4339e+06

### RGSN(N)NSFFSR Fuc 1 Hex 6 HexNAc 5

**Mass:**  3304.3247

(-2.845e-06)

**Charge States:** 3

**Start Position**: 157

**End Position**: 167

| Profile Score | Tandem Score | TDA q-value | Volume | Spectrum Matches |
| --- | --- | --- | --- | --- |
| 14.9977 | 97.8662 | 0.0179 | 1.3170e+07 | 3 |

**Adduct Signal Fractions**

- **Unmodified**: 1.3170e+07

### RGSN(N)NSFFSR Fuc 2 Hex 9 HexNAc 4

**Mass:**  3733.4262

(-9.485e-06)

**Charge States:** 3

**Start Position**: 157

**End Position**: 167

| Profile Score | Tandem Score | TDA q-value | Volume | Spectrum Matches |
| --- | --- | --- | --- | --- |
| 9.3770 | 97.1965 | 0.0259 | 1.0301e+07 | 1 |

**Adduct Signal Fractions**

- **Unmodified**: 1.0301e+07

### N(N)GTYDHDVYR Fuc 2 Hex 7 HexNAc 6

**Mass:**  3885.4966

(5.158e-04)

**Start Position**: 499

**End Position**: 509

| Profile Score | Tandem Score | TDA q-value | Volume | Spectrum Matches |
| --- | --- | --- | --- | --- |
| 0.0000 | 95.3303 | 0.0320 | 0.0000e+00 | 1 |

Adduct:

No Chromatogram Found

### N(N)GTYDHDVYR Fuc 4 Hex 6 HexNAc 5

**Mass:**  3810.4795

(6.322e-07)

**Charge States:** 3

**Start Position**: 499

**End Position**: 509

| Profile Score | Tandem Score | TDA q-value | Volume | Spectrum Matches |
| --- | --- | --- | --- | --- |
| -20.1845 | 95.2393 | 0.0320 | 9.9310e+06 | 1 |

**Adduct Signal Fractions**

- **Unmodified**: 9.9310e+06

### N(N)GTYDHDVYR Fuc 1 Hex 7 HexNAc 6

**Mass:**  3737.4516

(4.273e-06)

**Charge States:** 3, 4

**Start Position**: 499

**End Position**: 509

| Profile Score | Tandem Score | TDA q-value | Volume | Spectrum Matches |
| --- | --- | --- | --- | --- |
| -20.8931 | 94.6490 | 0.0320 | 1.0411e+07 | 2 |

**Adduct Signal Fractions**

- **Unmodified**: 1.0411e+07

### RGSN(N)N(D)SFFSR Hex 8 HexNAc 3 Neu5Ac 1

**Mass:**  3368.2886

(2.909e-04)

**Charge States:** 3

**Start Position**: 157

**End Position**: 167

| Profile Score | Tandem Score | TDA q-value | Volume | Spectrum Matches |
| --- | --- | --- | --- | --- |
| -7.3019 | 94.1055 | 0.0320 | 6.1224e+06 | 1 |

**Adduct Signal Fractions**

- **Unmodified**: 6.1224e+06

### N(N)GTYDHDVYR Fuc 1 Hex 5 HexNAc 4 Neu5Ac 1

**Mass:**  3298.2687

(5.553e-06)

**Charge States:** 3

**Start Position**: 499

**End Position**: 509

| Profile Score | Tandem Score | TDA q-value | Volume | Spectrum Matches |
| --- | --- | --- | --- | --- |
| -20.5289 | 93.1070 | 0.0320 | 3.4627e+06 | 1 |

**Adduct Signal Fractions**

- **Unmodified**: 3.4627e+06

### N(N)GTYDHDVYRDEALNNR Fuc 2 Hex 7 HexNAc 6

**Mass:**  4695.8786

(1.181e-06)

**Charge States:** 4

**Start Position**: 499

**End Position**: 516

| Profile Score | Tandem Score | TDA q-value | Volume | Spectrum Matches |
| --- | --- | --- | --- | --- |
| -3.7633 | 90.8408 | 0.0441 | 1.3030e+07 | 1 |

**Adduct Signal Fractions**

- **Unmodified**: 1.3030e+07

### N(N)GTYDHDVYRDEALNNR Fuc 2 Hex 6 HexNAc 6

**Mass:**  4533.8234

(1.137e-06)

**Charge States:** 4

**Start Position**: 499

**End Position**: 516

| Profile Score | Tandem Score | TDA q-value | Volume | Spectrum Matches |
| --- | --- | --- | --- | --- |
| 8.9583 | 90.0025 | 0.0441 | 5.3186e+07 | 6 |

**Adduct Signal Fractions**

- **Unmodified**: 5.3186e+07

### RGSN(N)NSFFSR Fuc 2 Hex 5 HexNAc 6

**Mass:**  3491.4127

(1.066e-06)

**Charge States:** 3

**Start Position**: 157

**End Position**: 167

| Profile Score | Tandem Score | TDA q-value | Volume | Spectrum Matches |
| --- | --- | --- | --- | --- |
| -19.4546 | 89.4089 | 0.0441 | 3.9275e+06 | 1 |

**Adduct Signal Fractions**

- **Unmodified**: 3.9275e+06

### TITNDQIEVTN(N)ATELVQSSSTGR Hex 5 HexNAc 5

**Mass:**  4288.8786

(2.055e-06)

**Charge States:** 3, 4

**Start Position**: 44

**End Position**: 67

| Profile Score | Tandem Score | TDA q-value | Volume | Spectrum Matches |
| --- | --- | --- | --- | --- |
| 13.8268 | 89.2367 | 0.0441 | 1.9703e+08 | 9 |

**Adduct Signal Fractions**

- **Unmodified**: 1.9703e+08

### N(N)GTYDHDVYRDEALNNR Fuc 3 Hex 5 HexNAc 4

**Mass:**  4113.6781

(4.897e-04)

**Start Position**: 499

**End Position**: 516

| Profile Score | Tandem Score | TDA q-value | Volume | Spectrum Matches |
| --- | --- | --- | --- | --- |
| 0.0000 | 89.0501 | 0.0441 | 0.0000e+00 | 1 |

Adduct:

No Chromatogram Found

### iav|ADJ41819|A/Phil/2-MA/1982\_NA

**>iav|ADJ41819|A/Phil/2-MA/1982\_NA**  
 `MNPNQKIITIGSVSLTIATICFLMQIAILVTTVTLHFKQYECSSPPNNQVVPCEPIIIERNITEIVYLTNTTIEKEICPK  
LVEYRNWSKPQCKITGFAPFSKDNSIRLSAGGDIWVTREPYVSCDPGKCYQFALGQGTTLDNKHSNDTIHDRTPYRTLLM  
NELGVPFHLGTRQVCIAWSSSSCHDGKAWLHVCITGYDKNATASFIYDGRLVDSIGSWSKNILRTQESECVCINGTCTVV  
MTDGSASERADTKILFIEEGKIVHISPLSGSAQHVEECSCYPRYPGVRCVCRDNWKGSNRPVVDINVKDYSIVSSYVCSG  
LVGDTPRKNDRSSSSYCRNPNNEKGNHGVKGWAFDDGNDVWMGRTISEESRSGYETFKVIGGWSTPNSKLQINRQVIVDS  
GNRSGYSGIFSVEGKSCINRCFYVELIRGREQETRVWWTSNSIVVFCGTSGTYGTGSWPDGADINLMPI`

| Site | Type | Count |
| --- | --- | --- |
| 402 | N-linked | 2 |
| 200 | N-linked | 3 |

| Mass | Sequence | MS_2_ Score | `q`-value | MS_1_ Score | Signal | Protein Coordinates | Tandem Scans | Start Time | Apex Time | End Time |
| --- | --- | --- | --- | --- | --- | --- | --- | --- | --- | --- |
| 2364.9938 | QVIVDSGN(N)R Hex 6 HexNAc 2 | 176.283 | 0.0 | -21.301 | 8.317e+06 | 394 - 403 | 1 | 12.94 | 12.94 | 13.11 |
| 2689.0955 | QVIVDSGN(N)R Hex 8 HexNAc 2 | 170.735 | 0.0 | -21.456 | 5.608e+06 | 394 - 403 | 1 | 12.77 | 12.94 | 12.94 |
| 2916.1570 | N(N)ATASFIYDGR Hex 8 HexNAc 2 | 134.311 | 0.0 | 12.740 | 3.722e+07 | 199 - 210 | 4 | 25.72 | 25.72 | 26.40 |
| 3078.2107 | N(N)ATASFIYDGR Hex 9 HexNAc 2 | 105.142 | 0.012 | -4.417 | 1.069e+07 | 199 - 210 | 2 | 25.55 | 25.72 | 25.89 |
| 2754.1046 | N(N)ATASFIYDGR Hex 7 HexNAc 2 | 104.482 | 0.018 | -3.179 | 1.416e+07 | 199 - 210 | 3 | 25.89 | 26.06 | 26.40 |

### QVIVDSGN(N)R Hex 6 HexNAc 2

**Mass:**  2364.9903

(1.500e-06)

**Charge States:** 2

**Start Position**: 395

**End Position**: 404

| Profile Score | Tandem Score | TDA q-value | Volume | Spectrum Matches |
| --- | --- | --- | --- | --- |
| -21.3011 | 176.2827 | 0.0000 | 8.3169e+06 | 1 |

**Adduct Signal Fractions**

- **Unmodified**: 8.3169e+06

### QVIVDSGN(N)R Hex 8 HexNAc 2

**Mass:**  2689.0962

(-5.360e-07)

**Charge States:** 3

**Start Position**: 395

**End Position**: 404

| Profile Score | Tandem Score | TDA q-value | Volume | Spectrum Matches |
| --- | --- | --- | --- | --- |
| -21.4559 | 170.7346 | 0.0000 | 5.6085e+06 | 1 |

**Adduct Signal Fractions**

- **Unmodified**: 5.6085e+06

### N(N)ATASFIYDGR Hex 8 HexNAc 2

**Mass:**  2916.1571

(1.015e-06)

**Charge States:** 2, 3

**Start Position**: 200

**End Position**: 211

| Profile Score | Tandem Score | TDA q-value | Volume | Spectrum Matches |
| --- | --- | --- | --- | --- |
| 12.7401 | 134.3114 | 0.0000 | 3.7219e+07 | 4 |

**Adduct Signal Fractions**

- **Unmodified**: 3.7219e+07

### N(N)ATASFIYDGR Hex 9 HexNAc 2

**Mass:**  3078.2103

(1.464e-06)

**Charge States:** 3

**Start Position**: 200

**End Position**: 211

| Profile Score | Tandem Score | TDA q-value | Volume | Spectrum Matches |
| --- | --- | --- | --- | --- |
| -4.4172 | 105.1422 | 0.0119 | 1.0695e+07 | 2 |

**Adduct Signal Fractions**

- **Unmodified**: 1.0695e+07

### N(N)ATASFIYDGR Hex 7 HexNAc 2

**Mass:**  2754.1045

(1.007e-06)

**Charge States:** 3

**Start Position**: 200

**End Position**: 211

| Profile Score | Tandem Score | TDA q-value | Volume | Spectrum Matches |
| --- | --- | --- | --- | --- |
| -3.1785 | 104.4816 | 0.0179 | 1.4164e+07 | 3 |

**Adduct Signal Fractions**

- **Unmodified**: 1.4164e+07

### tr|Q5F491|Q5F491\_CHICK

**>tr|Q5F491|Q5F491\_CHICK**  
 `MSHVAVENALSLDQQFSGLDLNSSDSQSEGSATSKGRYIPPHLRNREASKQGFDSGGWSTSRDKDAYSSFGARSDRGAKS  
SFFDRGNGSRGGRYEERGRGSDYDRSGFGRFDRGGNSRWCDKSDEDDWSKPLPPSERLEQELFSGSNTGINFEKYDDIPV  
EATGSNCPPHIESFSDVDMGEIIMGNIELTRYTRPTPVQKHAIPIIKEKRDLMACAQTGSGKTAAFLLPILSQIYADGPG  
DALRAMKENGRYGRRKQYPISLVLAPTRELAVQIYEEARKFAYRSRVRPCVVYGGADIGQQIRDLERGCHLLVATPGRLV  
DMMERGKIGLDFCKYLVLDEADRMLDMGFEPQIRRIVEQDTMPPKGVRHTMMFSATFPKEIQMLARDFLDEYIFLAVGRV  
GSTSENITQKVVWVEELDKRSFLLDLLNATGKDSLTLVFVETKKGADALEDFLYHEGYACTSIHGDRSQRDREEALHQFR  
SGKSPILVATAVAARGLDISNVKHVINFDLPSDIEEYVHRIGRTGRVGNLGLATSFFNERNINITKDLLDLLVEAKQEVP  
SWLENMAYEQHHKGGGSRGRSKSRFTGGFGARDYRTSSGAGSSSFSSSRPASGRTGGSGSRGFGGGGYGGFYNSDGYGGN  
YNSQGVDWWGN`

| Site | Type | Count |
| --- | --- | --- |
| 87 | N-linked | 1 |

| Mass | Sequence | MS_2_ Score | `q`-value | MS_1_ Score | Signal | Protein Coordinates | Tandem Scans | Start Time | Apex Time | End Time |
| --- | --- | --- | --- | --- | --- | --- | --- | --- | --- | --- |
| 3200.2867 | SSFFDRGN(N)GSR Fuc 1 Hex 5 HexNAc 5 | 104.996 | 0.012 | -4.449 | 1.609e+07 | 79 - 90 | 1 | 17.59 | 17.59 | 17.93 |

### SSFFDRGN(N)GSR Fuc 1 Hex 5 HexNAc 5

**Mass:**  3200.2864

(2.847e-06)

**Charge States:** 3

**Start Position**: 80

**End Position**: 91

| Profile Score | Tandem Score | TDA q-value | Volume | Spectrum Matches |
| --- | --- | --- | --- | --- |
| -4.4488 | 104.9960 | 0.0119 | 1.6090e+07 | 1 |

**Adduct Signal Fractions**

- **Unmodified**: 1.6090e+07
