# Supplementary material for: Measuring Site-specific Glycosylation Similarity between Influenza a Virus Variants with Statistical Certainty
Source: Mol Cell Proteomics. 2020 Nov 25;19(9):1533–45. doi: 10.1074/mcp.RA120.002031 (PMC8143645; doi:10.1074/mcp.RA120.002031)
Supplement: Supplementary file 2 [file mmc2.zip › mmc2/159250_2_supp_555290_qcjklq.zip!/20190326_Phil82_032219_tryp_HILIC_DDA_02_glycopeptides.html]

## 20190326\_Phil82\_032219\_tryp\_HILIC\_DDA\_02 @ GlycopeptideHypothesis-2fbad353d4e94676830b64e47b25b3ed

**MS^1^ Mass Accuracy:** 10.0 PPM  
**Grouping Tolerance:** 15.0 PPM  
**MS^n^ Mass Accuracy:** 20.0 PPM  
**Minimum `q`-Value For Clustering:** 0.05

**Glycopeptide Hypothesis:**
GlycopeptideHypothesis-2fbad353d4e94676830b64e47b25b3ed  
**Sample Searched:**
20190326\_Phil82\_032219\_tryp\_HILIC\_DDA\_02

| Protein | Matched Glycopeptides |
| --- | --- |
| iav\|AFG99160\|A/Phil/2/1982\_HA | 73 |
| iav\|ADJ41819\|A/Phil/2-MA/1982\_NA | 5 |

### iav|AFG99160|A/Phil/2/1982\_HA

**>iav|AFG99160|A/Phil/2/1982\_HA**  
 `MKTIIALSYMFCLVFAQNLPGNDNSTATLCLGHHAVPNGTLVKTITNDQIEVTNATELVQSSSTGRICDSPHRILDGKNC  
TLIDALLGDPHCDGFQNEKWDLFVERSKAFSNCYPYDVPDYASLRSLVASSGTLEFINEGFNWTGVTQSGGSSTCKRGSN  
NSFFSRLNWLYESESKYPVLNVTMPNNGKFDKLYIWGIHHPSTDKEQTNLYIRASGRVTVSTKRSQQTVIPNIGSRPWVR  
GLSSRISIYWTIVKPGDILLINSTGNLIAPRGYFKIRTGKSSIMRSDAPIGTCSSECITPNGSIPNDKPFQNVNKITYGA  
CPRYVKQNTLKLATGMRNVPEKQTRGIFGAIAGFIENGWEGMVDGWYGFRHQNSEGTGQAADLKSTQAAIDQINGKLNRV  
IEKTNEKFHQIEKEFSEVEGRIQDLEKYVEDTKIDLWSYNAELLVALENQHTIDLTDSEMNKLFEKTRKQLRENAEDMGN  
GCFKIYHKCDNACIGSIRNGTYDHDVYRDEALNNRFQIKGVELKSGYKDWILWISFAISCFLLCVVLLGFIMWACQKGNI  
RCNICI`

| Site | Type | Count |
| --- | --- | --- |
| 262 | N-linked | 1 |
| 301 | N-linked | 3 |
| 499 | N-linked | 31 |
| 181 | N-linked | 11 |
| 54 | N-linked | 8 |
| 160 | N-linked | 19 |

| Mass | Sequence | MS_2_ Score | `q`-value | MS_1_ Score | Signal | Protein Coordinates | Tandem Scans | Start Time | Apex Time | End Time |
| --- | --- | --- | --- | --- | --- | --- | --- | --- | --- | --- |
| 3153.2303 | N(N)GTYDHDVYR Fuc 2 Hex 5 HexNAc 4 | 160.367 | 0.0 | 13.075 | 1.455e+08 | 498 - 508 | 4 | 12.57 | 12.57 | 13.40 |
| 3142.2754 | RGSN(N)NSFFSR Fuc 1 Hex 5 HexNAc 5 | 150.740 | 0.0 | 13.616 | 1.384e+08 | 156 - 166 | 7 | 15.73 | 16.58 | 16.92 |
| 4068.9274 | TIVKPGDILLIN(N)STGNLIAPR Hex 9 HexNAc 2 | 150.226 | 0.0 | 15.565 | 4.080e+08 | 250 - 271 | 17 | 50.85 | 51.53 | 53.39 |
| 2986.1721 | GSN(N)NSFFSR Fuc 1 Hex 5 HexNAc 5 | 148.836 | 0.0 | 12.173 | 2.794e+08 | 157 - 166 | 8 | 18.62 | 19.98 | 20.32 |
| 3007.1728 | N(N)GTYDHDVYR Fuc 1 Hex 5 HexNAc 4 | 148.058 | 0.0 | 13.892 | 2.374e+08 | 498 - 508 | 7 | 12.57 | 12.74 | 13.57 |
| 3085.2526 | RGSN(N)NSFFSR Fuc 2 Hex 5 HexNAc 4 | 137.619 | 0.0 | -4.325 | 1.211e+07 | 156 - 166 | 2 | 16.07 | 16.24 | 16.58 |
| 2939.1956 | RGSN(N)NSFFSR Fuc 1 Hex 5 HexNAc 4 | 134.314 | 0.0 | -3.659 | 2.663e+07 | 156 - 166 | 3 | 16.24 | 16.41 | 16.75 |
| 3434.6552 | RGSN(N)NSFFSR Fuc 3 Hex 5 HexNAc 5 | 132.656 | 0.0 | -4.926 | 1.376e+07 | 156 - 166 | 4 | 15.90 | 15.90 | 16.58 |
| 3327.3502 | YPVLN(N)VTM(O)PNN(D)GK Hex 9 HexNAc 2 | 130.796 | 0.0 | 13.484 | 8.832e+08 | 176 - 189 | 8 | 28.10 | 28.43 | 29.26 |
| 3061.1760 | RGSN(N)N(D)SFFSR Fuc 1 Hex 7 HexNAc 3 | 129.950 | 0.0 | -19.001 | 5.827e+06 | 156 - 166 | 1 | 19.64 | 19.64 | 19.64 |
| 3165.2971 | YPVLN(N)VTM(O)PNN(D)GK Hex 8 HexNAc 2 | 129.789 | 0.0 | 11.537 | 5.138e+08 | 176 - 189 | 7 | 28.10 | 28.59 | 29.09 |
| 3299.2885 | N(N)GTYDHDVYR Fuc 3 Hex 5 HexNAc 4 | 129.749 | 0.0 | -4.749 | 6.388e+07 | 498 - 508 | 2 | 12.41 | 12.57 | 12.90 |
| 3141.2527 | YPVLN(N)VTM(O)PNN Hex 9 HexNAc 2 | 129.482 | 0.0 | 12.984 | 8.178e+06 | 176 - 187 | 3 | 31.99 | 32.33 | 33.02 |
| 2979.1995 | YPVLN(N)VTM(O)PNN Hex 8 HexNAc 2 | 129.140 | 0.0 | -4.661 | 3.022e+06 | 176 - 187 | 1 | 32.16 | 32.50 | 32.50 |
| 3210.2525 | N(N)GTYDHDVYR Fuc 1 Hex 5 HexNAc 5 | 128.979 | 0.0 | 12.230 | 1.607e+08 | 498 - 508 | 9 | 12.24 | 12.90 | 13.57 |
| 3372.3049 | N(N)GTYDHDVYR Fuc 1 Hex 6 HexNAc 5 | 128.312 | 0.0 | -4.580 | 1.021e+08 | 498 - 508 | 4 | 12.57 | 12.74 | 13.07 |
| 4949.0861 | SDAPIGTC(C)SSEC(C)ITPN(N)GSIPNDKPFQNVNK Hex 8 HexNAc 2 | 128.070 | 0.0 | 12.909 | 1.428e+08 | 285 - 315 | 7 | 34.72 | 34.89 | 35.41 |
| 3003.2445 | YPVLN(N)VTM(O)PNN(D)GK Hex 7 HexNAc 2 | 127.653 | 0.0 | 11.133 | 6.191e+07 | 176 - 189 | 4 | 28.26 | 28.76 | 29.26 |
| 3199.2974 | RGSN(N)NSFFSR Hex 5 HexNAc 6 | 127.634 | 0.0 | 12.534 | 5.672e+07 | 156 - 166 | 6 | 15.90 | 16.75 | 17.09 |
| 3344.3771 | YPVLN(N)VTM(O)PNN(D)GK Fuc 2 Hex 3 HexNAc 4 Neu5Ac 1 | 126.452 | 0.0 | 11.885 | 1.228e+08 | 176 - 189 | 2 | 28.10 | 28.59 | 29.09 |
| 3717.5433 | YPVLN(N)VTM(O)PNN(D)GKFDK Hex 9 HexNAc 2 | 125.564 | 0.0 | 12.921 | 1.961e+08 | 176 - 192 | 6 | 31.31 | 31.82 | 32.33 |
| 3555.4891 | YPVLN(N)VTM(O)PNN(D)GKFDK Hex 8 HexNAc 2 | 124.834 | 0.0 | 11.415 | 1.121e+08 | 176 - 192 | 6 | 31.48 | 31.99 | 32.33 |
| 4190.8272 | TITNDQIEVTN(N)ATELVQSSSTGR Fuc 1 Hex 6 HexNAc 3 | 124.253 | 0.0 | 13.502 | 3.251e+08 | 43 - 66 | 9 | 38.48 | 39.33 | 40.52 |
| 3288.3333 | RGSN(N)NSFFSR Fuc 2 Hex 5 HexNAc 5 | 123.911 | 0.0 | 12.384 | 4.483e+07 | 156 - 166 | 5 | 15.73 | 16.24 | 16.58 |
| 3965.6100 | N(N)GTYDHDVYRDEALNNR Fuc 2 Hex 5 HexNAc 4 | 122.051 | 0.0 | 15.036 | 2.528e+08 | 498 - 515 | 6 | 17.43 | 17.77 | 18.11 |
| 3518.3626 | N(N)GTYDHDVYR Fuc 2 Hex 6 HexNAc 5 | 121.985 | 0.0 | -4.518 | 3.992e+07 | 498 - 508 | 2 | 12.41 | 12.57 | 12.74 |
| 3664.4209 | N(N)GTYDHDVYR Fuc 3 Hex 6 HexNAc 5 | 121.091 | 0.0 | -5.970 | 2.740e+07 | 498 - 508 | 3 | 12.41 | 12.57 | 12.74 |
| 3356.3104 | N(N)GTYDHDVYR Fuc 2 Hex 5 HexNAc 5 | 120.532 | 0.0 | 10.818 | 5.016e+07 | 498 - 508 | 5 | 12.41 | 12.74 | 13.24 |
| 5111.1375 | SDAPIGTC(C)SSEC(C)ITPN(N)GSIPNDKPFQNVNK Hex 9 HexNAc 2 | 119.273 | 0.0 | 11.532 | 1.240e+08 | 285 - 315 | 6 | 34.72 | 34.89 | 35.58 |
| 2841.1925 | YPVLN(N)VTM(O)PNN(D)GK Hex 6 HexNAc 2 | 118.586 | 0.0 | -6.046 | 6.884e+06 | 176 - 189 | 2 | 28.76 | 28.76 | 29.09 |
| 4003.7428 | TITNDQIEVTN(N)ATELVQSSSTGR Hex 7 HexNAc 2 | 118.544 | 0.0 | 13.886 | 5.917e+08 | 43 - 66 | 16 | 37.97 | 39.33 | 40.52 |
| 3819.5515 | N(N)GTYDHDVYRDEALNNR Fuc 1 Hex 5 HexNAc 4 | 117.996 | 0.0 | 16.315 | 3.366e+08 | 498 - 515 | 5 | 17.26 | 17.94 | 18.28 |
| 2980.2252 | RGSN(N)NSFFSR Fuc 1 Hex 4 HexNAc 5 | 117.676 | 0.0 | -4.539 | 5.140e+06 | 156 - 166 | 2 | 16.41 | 16.58 | 16.75 |
| 3520.3645 | N(N)GTYDHDVYR Fuc 2 Hex 6 HexNAc 5 | 117.491 | 0.0 | 0.000 | 0.000e+00 | 498 - 508 | 1 | - | - | - |
| 3679.6364 | TITNDQIEVTN(N)ATELVQSSSTGR Hex 5 HexNAc 2 | 117.000 | 0.0 | 14.485 | 3.088e+08 | 43 - 66 | 8 | 39.33 | 39.67 | 40.87 |
| 4476.8020 | N(N)GTYDHDVYRDEALNNR Fuc 3 Hex 6 HexNAc 5 | 115.918 | 0.0 | 12.561 | 5.207e+07 | 498 - 515 | 3 | 17.26 | 17.60 | 17.94 |
| 4022.6333 | N(N)GTYDHDVYRDEALNNR Fuc 1 Hex 5 HexNAc 5 | 114.252 | 0.0 | 8.762 | 2.077e+08 | 498 - 515 | 5 | 16.92 | 18.11 | 18.28 |
| 4111.6688 | N(N)GTYDHDVYRDEALNNR Fuc 3 Hex 5 HexNAc 4 | 113.721 | 0.0 | -3.616 | 1.136e+08 | 498 - 515 | 2 | 17.43 | 17.77 | 17.94 |
| 3401.3987 | YPVLN(N)VTM(O)PNN(D)GK Fuc 1 Hex 3 HexNAc 5 Neu5Ac 1 | 113.092 | 0.0 | 10.283 | 1.404e+08 | 176 - 189 | 8 | 26.91 | 28.10 | 28.92 |
| 3345.3562 | RGSN(N)NSFFSR Fuc 1 Hex 5 HexNAc 6 | 112.597 | 0.0 | -4.531 | 1.572e+07 | 156 - 166 | 2 | 16.24 | 16.41 | 16.58 |
| 4044.7688 | TITNDQIEVTN(N)ATELVQSSSTGR Hex 6 HexNAc 3 | 111.471 | 0.0 | 13.518 | 3.697e+08 | 43 - 66 | 12 | 38.48 | 39.33 | 40.70 |
| 3206.2387 | RGSN(N)N(D)SFFSR Hex 7 HexNAc 3 Neu5Ac 1 | 110.937 | 0.0 | 17.801 | 2.071e+07 | 156 - 166 | 3 | 18.28 | 18.45 | 19.47 |
| 3489.4092 | YPVLN(N)VTM(O)PNN(D)GK Hex 10 HexNAc 2 | 109.934 | 0.0 | -6.202 | 2.416e+07 | 176 - 189 | 2 | 28.26 | 28.43 | 28.76 |
| 3576.3910 | N(N)GTYDHDVYR Fuc 1 Hex 6 HexNAc 6 | 109.792 | 0.0 | 0.000 | 0.000e+00 | 498 - 508 | 2 | - | - | - |
| 4085.7946 | TITNDQIEVTN(N)ATELVQSSSTGR Hex 5 HexNAc 4 | 109.702 | 0.0 | 13.534 | 4.215e+08 | 43 - 66 | 12 | 38.48 | 39.33 | 40.52 |
| 4231.8522 | TITNDQIEVTN(N)ATELVQSSSTGR Fuc 1 Hex 5 HexNAc 4 | 109.540 | 0.0 | 13.519 | 1.810e+08 | 43 - 66 | 7 | 38.99 | 39.16 | 40.70 |
| 3300.2961 | N(N)GTYDHDVYR Fuc 3 Hex 5 HexNAc 4 | 109.173 | 0.0 | 0.000 | 0.000e+00 | 498 - 508 | 1 | - | - | - |
| 3304.3275 | RGSN(N)NSFFSR Fuc 1 Hex 6 HexNAc 5 | 109.015 | 0.0 | 7.106 | 9.451e+06 | 156 - 166 | 3 | 15.90 | 16.75 | 16.75 |
| 2861.1145 | N(N)GTYDHDVYR Hex 5 HexNAc 4 | 107.511 | 0.0 | -19.656 | 7.474e+06 | 498 - 508 | 1 | 12.74 | 12.74 | 12.74 |
| 3841.6892 | TITNDQIEVTN(N)ATELVQSSSTGR Hex 6 HexNAc 2 | 106.697 | 0.0 | 13.724 | 9.062e+08 | 43 - 66 | 16 | 38.14 | 39.50 | 40.70 |
| 4330.7424 | N(N)GTYDHDVYRDEALNNR Fuc 2 Hex 6 HexNAc 5 | 106.429 | 0.0 | -3.785 | 6.305e+07 | 498 - 515 | 2 | 17.43 | 17.60 | 17.94 |
| 4184.6839 | N(N)GTYDHDVYRDEALNNR Fuc 1 Hex 6 HexNAc 5 | 106.189 | 0.0 | 14.594 | 1.507e+08 | 498 - 515 | 2 | 17.43 | 17.77 | 18.11 |
| 4311.8109 | TITNDQIEVTN(N)ATELVQSSSTGR Fuc 1 Hex 8 HexNAc 2 | 105.890 | 0.009 | 13.675 | 5.181e+07 | 43 - 66 | 7 | 41.89 | 42.06 | 42.92 |
| 4264.6421 | N(N)GTYDHDVYRDEALNNR Fuc 1 Hex 9 HexNAc 3 | 105.695 | 0.009 | -4.867 | 2.873e+07 | 498 - 515 | 3 | 19.47 | 19.64 | 19.98 |
| 4387.7641 | N(N)GTYDHDVYRDEALNNR Fuc 1 Hex 6 HexNAc 6 | 105.546 | 0.009 | 6.985 | 4.659e+07 | 498 - 515 | 4 | 17.09 | 17.94 | 18.11 |
| 3143.2647 | RGSN(N)N(D)SFFSR Fuc 1 Hex 5 HexNAc 5 | 105.200 | 0.009 | -5.371 | 4.978e+07 | 156 - 166 | 3 | 17.09 | 17.09 | 17.43 |
| 2940.1835 | RGSN(N)N(D)SFFSR Fuc 1 Hex 5 HexNAc 4 | 103.833 | 0.009 | -5.628 | 7.030e+06 | 156 - 166 | 2 | 16.92 | 16.92 | 17.26 |
| 3231.3124 | RGSN(N)NSFFSR Fuc 3 Hex 5 HexNAc 4 | 103.681 | 0.009 | -4.462 | 9.957e+06 | 156 - 166 | 3 | 15.73 | 15.90 | 16.24 |
| 4622.8623 | N(N)GTYDHDVYRDEALNNR Fuc 4 Hex 6 HexNAc 5 | 103.348 | 0.015 | -3.380 | 1.892e+07 | 498 - 515 | 2 | 17.26 | 17.43 | 17.60 |
| 3899.5090 | N(N)GTYDHDVYRDEALNNR Fuc 1 Hex 8 HexNAc 2 | 103.151 | 0.015 | 15.001 | 3.399e+07 | 498 - 515 | 3 | 19.64 | 19.98 | 20.32 |
| 4396.6813 | N(N)GTYDHDVYR Fuc 3 Hex 8 HexNAc 7 | 102.047 | 0.015 | 0.000 | 0.000e+00 | 498 - 508 | 1 | - | - | - |
| 4533.8214 | N(N)GTYDHDVYRDEALNNR Fuc 2 Hex 6 HexNAc 6 | 100.787 | 0.015 | 9.419 | 4.461e+07 | 498 - 515 | 5 | 16.92 | 17.94 | 18.62 |
| 4410.7024 | N(N)GTYDHDVYRDEALNNR Fuc 2 Hex 9 HexNAc 3 | 100.587 | 0.015 | -4.251 | 2.031e+07 | 498 - 515 | 2 | 19.47 | 19.64 | 19.81 |
| 4549.8179 | N(N)GTYDHDVYRDEALNNR Fuc 1 Hex 7 HexNAc 6 | 98.811 | 0.015 | -4.089 | 1.820e+07 | 498 - 515 | 1 | 17.43 | 17.60 | 17.77 |
| 3721.4425 | N(N)GTYDHDVYR Fuc 2 Hex 6 HexNAc 6 | 97.019 | 0.015 | -5.910 | 1.682e+07 | 498 - 508 | 3 | 12.74 | 12.74 | 13.07 |
| 3128.1570 | N(N)GTYDHDVYR Fuc 1 Hex 7 HexNAc 3 | 96.813 | 0.015 | -4.998 | 4.715e+06 | 498 - 508 | 2 | 14.07 | 14.07 | 14.41 |
| 4168.6906 | N(N)GTYDHDVYRDEALNNR Fuc 2 Hex 5 HexNAc 5 | 95.087 | 0.021 | 11.685 | 8.207e+07 | 498 - 515 | 3 | 16.92 | 17.94 | 18.11 |
| 2752.1120 | RGSN(N)NSFFSR Hex 6 HexNAc 3 | 94.666 | 0.021 | -20.224 | 1.120e+06 | 156 - 166 | 1 | 16.41 | 16.41 | 16.41 |
| 2996.2161 | RGSN(N)NSFFSR Hex 5 HexNAc 5 | 93.988 | 0.021 | -4.455 | 1.383e+07 | 156 - 166 | 2 | 16.41 | 16.58 | 16.92 |
| 3810.4764 | N(N)GTYDHDVYR Fuc 4 Hex 6 HexNAc 5 | 93.425 | 0.021 | -18.838 | 1.282e+07 | 498 - 508 | 2 | 12.41 | 12.41 | 12.41 |
| 3368.2892 | RGSN(N)N(D)SFFSR Hex 8 HexNAc 3 Neu5Ac 1 | 93.348 | 0.021 | -6.527 | 1.057e+07 | 156 - 166 | 2 | 18.45 | 18.45 | 19.13 |
| 4787.0321 | SDAPIGTC(C)SSEC(C)ITPN(N)GSIPNDKPFQNVNK Hex 7 HexNAc 2 | 91.548 | 0.032 | 12.880 | 3.208e+07 | 285 - 315 | 3 | 34.72 | 34.89 | 35.41 |
| 3530.3501 | RGSN(N)NSFFSR Fuc 2 Hex 9 HexNAc 3 | 91.449 | 0.032 | -20.189 | 3.821e+06 | 156 - 166 | 1 | 17.94 | 17.94 | 18.11 |

### N(N)GTYDHDVYR Fuc 2 Hex 5 HexNAc 4

**Mass:**  3153.2301

(3.854e-07)

**Charge States:** 3

**Start Position**: 499

**End Position**: 509

| Profile Score | Tandem Score | TDA q-value | Volume | Spectrum Matches |
| --- | --- | --- | --- | --- |
| 13.0748 | 160.3668 | 0.0000 | 1.4553e+08 | 4 |

**Adduct Signal Fractions**

- **Unmodified**: 1.4553e+08

### RGSN(N)NSFFSR Fuc 1 Hex 5 HexNAc 5

**Mass:**  3142.2746

(8.925e-07)

**Charge States:** 3, 4

**Start Position**: 157

**End Position**: 167

| Profile Score | Tandem Score | TDA q-value | Volume | Spectrum Matches |
| --- | --- | --- | --- | --- |
| 13.6160 | 150.7402 | 0.0000 | 1.3836e+08 | 7 |

**Adduct Signal Fractions**

- **Unmodified**: 1.3836e+08

### TIVKPGDILLIN(N)STGNLIAPR Hex 9 HexNAc 2

**Mass:**  4068.9282

(6.514e-07)

**Charge States:** 3, 4, 5

**Start Position**: 251

**End Position**: 272

| Profile Score | Tandem Score | TDA q-value | Volume | Spectrum Matches |
| --- | --- | --- | --- | --- |
| 15.5652 | 150.2261 | 0.0000 | 4.0802e+08 | 17 |

**Adduct Signal Fractions**

- **Unmodified**: 4.0802e+08

### GSN(N)NSFFSR Fuc 1 Hex 5 HexNAc 5

**Mass:**  2986.1711

(8.329e-07)

**Charge States:** 3, 4

**Start Position**: 158

**End Position**: 167

| Profile Score | Tandem Score | TDA q-value | Volume | Spectrum Matches |
| --- | --- | --- | --- | --- |
| 12.1730 | 148.8360 | 0.0000 | 2.7940e+08 | 8 |

**Adduct Signal Fractions**

- **Unmodified**: 2.7940e+08

### N(N)GTYDHDVYR Fuc 1 Hex 5 HexNAc 4

**Mass:**  3007.1726

(5.583e-07)

**Charge States:** 3, 4

**Start Position**: 499

**End Position**: 509

| Profile Score | Tandem Score | TDA q-value | Volume | Spectrum Matches |
| --- | --- | --- | --- | --- |
| 13.8915 | 148.0579 | 0.0000 | 2.3744e+08 | 7 |

**Adduct Signal Fractions**

- **Unmodified**: 2.3744e+08

### RGSN(N)NSFFSR Fuc 2 Hex 5 HexNAc 4

**Mass:**  3085.2522

(2.035e-06)

**Charge States:** 3

**Start Position**: 157

**End Position**: 167

| Profile Score | Tandem Score | TDA q-value | Volume | Spectrum Matches |
| --- | --- | --- | --- | --- |
| -4.3254 | 137.6188 | 0.0000 | 1.2110e+07 | 2 |

**Adduct Signal Fractions**

- **Unmodified**: 1.2110e+07

### RGSN(N)NSFFSR Fuc 1 Hex 5 HexNAc 4

**Mass:**  2939.1959

(1.273e-06)

**Charge States:** 3

**Start Position**: 157

**End Position**: 167

| Profile Score | Tandem Score | TDA q-value | Volume | Spectrum Matches |
| --- | --- | --- | --- | --- |
| -3.6594 | 134.3142 | 0.0000 | 2.6628e+07 | 3 |

**Adduct Signal Fractions**

- **Unmodified**: 2.6628e+07

### RGSN(N)NSFFSR Fuc 3 Hex 5 HexNAc 5

**Mass:**  3434.3937

(2.927e-04)

**Charge States:** 3

**Start Position**: 157

**End Position**: 167

| Profile Score | Tandem Score | TDA q-value | Volume | Spectrum Matches |
| --- | --- | --- | --- | --- |
| -4.9264 | 132.6558 | 0.0000 | 1.3757e+07 | 4 |

**Adduct Signal Fractions**

- **Unmodified**: 1.3757e+07

### YPVLN(N)VTM(O)PNN(D)GK Hex 9 HexNAc 2

**Mass:**  3327.3510

(2.971e-04)

**Charge States:** 2, 3, 4

**Start Position**: 177

**End Position**: 190

| Profile Score | Tandem Score | TDA q-value | Volume | Spectrum Matches |
| --- | --- | --- | --- | --- |
| 13.4844 | 130.7959 | 0.0000 | 8.8317e+08 | 8 |

**Adduct Signal Fractions**

- **Unmodified**: 8.8317e+08

### RGSN(N)N(D)SFFSR Fuc 1 Hex 7 HexNAc 3

**Mass:**  3061.1760

(3.128e-04)

**Charge States:** 3

**Start Position**: 157

**End Position**: 167

| Profile Score | Tandem Score | TDA q-value | Volume | Spectrum Matches |
| --- | --- | --- | --- | --- |
| -19.0013 | 129.9500 | 0.0000 | 5.8271e+06 | 1 |

**Adduct Signal Fractions**

- **Unmodified**: 5.8271e+06

### YPVLN(N)VTM(O)PNN(D)GK Hex 8 HexNAc 2

**Mass:**  3165.2964

(3.121e-04)

**Charge States:** 2, 3, 4

**Start Position**: 177

**End Position**: 190

| Profile Score | Tandem Score | TDA q-value | Volume | Spectrum Matches |
| --- | --- | --- | --- | --- |
| 11.5370 | 129.7887 | 0.0000 | 5.1379e+08 | 7 |

**Adduct Signal Fractions**

- **Unmodified**: 5.1379e+08

### N(N)GTYDHDVYR Fuc 3 Hex 5 HexNAc 4

**Mass:**  3299.2893

(5.423e-07)

**Charge States:** 3

**Start Position**: 499

**End Position**: 509

| Profile Score | Tandem Score | TDA q-value | Volume | Spectrum Matches |
| --- | --- | --- | --- | --- |
| -4.7492 | 129.7490 | 0.0000 | 6.3885e+07 | 2 |

**Adduct Signal Fractions**

- **Unmodified**: 6.3885e+07

### YPVLN(N)VTM(O)PNN Hex 9 HexNAc 2

**Mass:**  3141.2521

(8.058e-07)

**Charge States:** 3

**Start Position**: 177

**End Position**: 188

| Profile Score | Tandem Score | TDA q-value | Volume | Spectrum Matches |
| --- | --- | --- | --- | --- |
| 12.9843 | 129.4818 | 0.0000 | 8.1777e+06 | 3 |

**Adduct Signal Fractions**

- **Unmodified**: 8.1777e+06

### YPVLN(N)VTM(O)PNN Hex 8 HexNAc 2

**Mass:**  2979.1970

(2.817e-06)

**Charge States:** 3

**Start Position**: 177

**End Position**: 188

| Profile Score | Tandem Score | TDA q-value | Volume | Spectrum Matches |
| --- | --- | --- | --- | --- |
| -4.6610 | 129.1399 | 0.0000 | 3.0219e+06 | 1 |

**Adduct Signal Fractions**

- **Unmodified**: 3.0219e+06

### N(N)GTYDHDVYR Fuc 1 Hex 5 HexNAc 5

**Mass:**  3210.2525

(5.959e-07)

**Charge States:** 3, 4

**Start Position**: 499

**End Position**: 509

| Profile Score | Tandem Score | TDA q-value | Volume | Spectrum Matches |
| --- | --- | --- | --- | --- |
| 12.2296 | 128.9785 | 0.0000 | 1.6069e+08 | 9 |

**Adduct Signal Fractions**

- **Unmodified**: 1.6069e+08

### N(N)GTYDHDVYR Fuc 1 Hex 6 HexNAc 5

**Mass:**  3372.3037

(3.087e-07)

**Charge States:** 3, 4

**Start Position**: 499

**End Position**: 509

| Profile Score | Tandem Score | TDA q-value | Volume | Spectrum Matches |
| --- | --- | --- | --- | --- |
| -4.5799 | 128.3116 | 0.0000 | 1.0207e+08 | 4 |

**Adduct Signal Fractions**

- **Unmodified**: 1.0207e+08

### SDAPIGTC(C)SSEC(C)ITPN(N)GSIPNDKPFQNVNK Hex 8 HexNAc 2

**Mass:**  4949.0856

(2.966e-06)

**Charge States:** 4, 5

**Start Position**: 286

**End Position**: 316

| Profile Score | Tandem Score | TDA q-value | Volume | Spectrum Matches |
| --- | --- | --- | --- | --- |
| 12.9094 | 128.0698 | 0.0000 | 1.4280e+08 | 7 |

**Adduct Signal Fractions**

- **Unmodified**: 1.4280e+08

### YPVLN(N)VTM(O)PNN(D)GK Hex 7 HexNAc 2

**Mass:**  3003.2437

(3.286e-04)

**Charge States:** 3

**Start Position**: 177

**End Position**: 190

| Profile Score | Tandem Score | TDA q-value | Volume | Spectrum Matches |
| --- | --- | --- | --- | --- |
| 11.1327 | 127.6527 | 0.0000 | 6.1909e+07 | 4 |

**Adduct Signal Fractions**

- **Unmodified**: 6.1909e+07

### RGSN(N)NSFFSR Hex 5 HexNAc 6

**Mass:**  3199.2973

(7.993e-07)

**Charge States:** 3, 4

**Start Position**: 157

**End Position**: 167

| Profile Score | Tandem Score | TDA q-value | Volume | Spectrum Matches |
| --- | --- | --- | --- | --- |
| 12.5342 | 127.6340 | 0.0000 | 5.6725e+07 | 6 |

**Adduct Signal Fractions**

- **Unmodified**: 5.6725e+07

### YPVLN(N)VTM(O)PNN(D)GK Fuc 2 Hex 3 HexNAc 4 Neu5Ac 1

**Mass:**  3344.3765

(2.881e-04)

**Charge States:** 3

**Start Position**: 177

**End Position**: 190

| Profile Score | Tandem Score | TDA q-value | Volume | Spectrum Matches |
| --- | --- | --- | --- | --- |
| 11.8853 | 126.4520 | 0.0000 | 1.2284e+08 | 2 |

**Adduct Signal Fractions**

- **Unmodified**: 1.2284e+08

### YPVLN(N)VTM(O)PNN(D)GKFDK Hex 9 HexNAc 2

**Mass:**  3717.5440

(2.666e-04)

**Charge States:** 3, 4

**Start Position**: 177

**End Position**: 193

| Profile Score | Tandem Score | TDA q-value | Volume | Spectrum Matches |
| --- | --- | --- | --- | --- |
| 12.9205 | 125.5635 | 0.0000 | 1.9613e+08 | 6 |

**Adduct Signal Fractions**

- **Unmodified**: 1.9613e+08

### YPVLN(N)VTM(O)PNN(D)GKFDK Hex 8 HexNAc 2

**Mass:**  3555.4883

(2.780e-04)

**Charge States:** 3, 4

**Start Position**: 177

**End Position**: 193

| Profile Score | Tandem Score | TDA q-value | Volume | Spectrum Matches |
| --- | --- | --- | --- | --- |
| 11.4153 | 124.8345 | 0.0000 | 1.1210e+08 | 6 |

**Adduct Signal Fractions**

- **Unmodified**: 1.1210e+08

### TITNDQIEVTN(N)ATELVQSSSTGR Fuc 1 Hex 6 HexNAc 3

**Mass:**  4190.8264

(2.110e-06)

**Charge States:** 3, 4

**Start Position**: 44

**End Position**: 67

| Profile Score | Tandem Score | TDA q-value | Volume | Spectrum Matches |
| --- | --- | --- | --- | --- |
| 13.5020 | 124.2532 | 0.0000 | 3.2511e+08 | 9 |

**Adduct Signal Fractions**

- **Unmodified**: 3.2511e+08

### RGSN(N)NSFFSR Fuc 2 Hex 5 HexNAc 5

**Mass:**  3288.3323

(8.979e-07)

**Charge States:** 3, 4

**Start Position**: 157

**End Position**: 167

| Profile Score | Tandem Score | TDA q-value | Volume | Spectrum Matches |
| --- | --- | --- | --- | --- |
| 12.3841 | 123.9106 | 0.0000 | 4.4830e+07 | 5 |

**Adduct Signal Fractions**

- **Unmodified**: 4.4830e+07

### N(N)GTYDHDVYRDEALNNR Fuc 2 Hex 5 HexNAc 4

**Mass:**  3965.6108

(1.002e-06)

**Charge States:** 4

**Start Position**: 499

**End Position**: 516

| Profile Score | Tandem Score | TDA q-value | Volume | Spectrum Matches |
| --- | --- | --- | --- | --- |
| 15.0362 | 122.0507 | 0.0000 | 2.5280e+08 | 6 |

**Adduct Signal Fractions**

- **Unmodified**: 2.5280e+08

### N(N)GTYDHDVYR Fuc 2 Hex 6 HexNAc 5

**Mass:**  3518.3637

(-1.740e-07)

**Charge States:** 4

**Start Position**: 499

**End Position**: 509

| Profile Score | Tandem Score | TDA q-value | Volume | Spectrum Matches |
| --- | --- | --- | --- | --- |
| -4.5183 | 121.9854 | 0.0000 | 3.9918e+07 | 2 |

**Adduct Signal Fractions**

- **Unmodified**: 3.9918e+07

### N(N)GTYDHDVYR Fuc 3 Hex 6 HexNAc 5

**Mass:**  3664.4225

(-5.517e-07)

**Charge States:** 4

**Start Position**: 499

**End Position**: 509

| Profile Score | Tandem Score | TDA q-value | Volume | Spectrum Matches |
| --- | --- | --- | --- | --- |
| -5.9701 | 121.0912 | 0.0000 | 2.7400e+07 | 3 |

**Adduct Signal Fractions**

- **Unmodified**: 2.7400e+07

### N(N)GTYDHDVYR Fuc 2 Hex 5 HexNAc 5

**Mass:**  3356.3120

(2.010e-06)

**Charge States:** 3

**Start Position**: 499

**End Position**: 509

| Profile Score | Tandem Score | TDA q-value | Volume | Spectrum Matches |
| --- | --- | --- | --- | --- |
| 10.8178 | 120.5322 | 0.0000 | 5.0163e+07 | 5 |

**Adduct Signal Fractions**

- **Unmodified**: 5.0163e+07

### SDAPIGTC(C)SSEC(C)ITPN(N)GSIPNDKPFQNVNK Hex 9 HexNAc 2

**Mass:**  5111.1359

(1.977e-06)

**Charge States:** 4, 5

**Start Position**: 286

**End Position**: 316

| Profile Score | Tandem Score | TDA q-value | Volume | Spectrum Matches |
| --- | --- | --- | --- | --- |
| 11.5318 | 119.2731 | 0.0000 | 1.2404e+08 | 6 |

**Adduct Signal Fractions**

- **Unmodified**: 1.2404e+08

### YPVLN(N)VTM(O)PNN(D)GK Hex 6 HexNAc 2

**Mass:**  2841.1898

(3.470e-04)

**Charge States:** 3

**Start Position**: 177

**End Position**: 190

| Profile Score | Tandem Score | TDA q-value | Volume | Spectrum Matches |
| --- | --- | --- | --- | --- |
| -6.0457 | 118.5856 | 0.0000 | 6.8837e+06 | 2 |

**Adduct Signal Fractions**

- **Unmodified**: 6.8837e+06

### TITNDQIEVTN(N)ATELVQSSSTGR Hex 7 HexNAc 2

**Mass:**  4003.7436

(1.488e-06)

**Charge States:** 3, 4

**Start Position**: 44

**End Position**: 67

| Profile Score | Tandem Score | TDA q-value | Volume | Spectrum Matches |
| --- | --- | --- | --- | --- |
| 13.8862 | 118.5435 | 0.0000 | 5.9169e+08 | 16 |

**Adduct Signal Fractions**

- **Unmodified**: 5.9169e+08

### N(N)GTYDHDVYRDEALNNR Fuc 1 Hex 5 HexNAc 4

**Mass:**  3819.5510

(5.871e-07)

**Charge States:** 4, 5

**Start Position**: 499

**End Position**: 516

| Profile Score | Tandem Score | TDA q-value | Volume | Spectrum Matches |
| --- | --- | --- | --- | --- |
| 16.3150 | 117.9955 | 0.0000 | 3.3659e+08 | 5 |

**Adduct Signal Fractions**

- **Unmodified**: 3.3659e+08

### RGSN(N)NSFFSR Fuc 1 Hex 4 HexNAc 5

**Mass:**  2980.2253

(1.611e-06)

**Charge States:** 3

**Start Position**: 157

**End Position**: 167

| Profile Score | Tandem Score | TDA q-value | Volume | Spectrum Matches |
| --- | --- | --- | --- | --- |
| -4.5389 | 117.6757 | 0.0000 | 5.1396e+06 | 2 |

**Adduct Signal Fractions**

- **Unmodified**: 5.1396e+06

### N(N)GTYDHDVYR Fuc 2 Hex 6 HexNAc 5

**Mass:**  3520.3645

(5.694e-04)

**Start Position**: 499

**End Position**: 509

| Profile Score | Tandem Score | TDA q-value | Volume | Spectrum Matches |
| --- | --- | --- | --- | --- |
| 0.0000 | 117.4906 | 0.0000 | 0.0000e+00 | 1 |

Adduct:

No Chromatogram Found

### TITNDQIEVTN(N)ATELVQSSSTGR Hex 5 HexNAc 2

**Mass:**  3679.6382

(1.126e-06)

**Charge States:** 3, 4

**Start Position**: 44

**End Position**: 67

| Profile Score | Tandem Score | TDA q-value | Volume | Spectrum Matches |
| --- | --- | --- | --- | --- |
| 14.4850 | 116.9997 | 0.0000 | 3.0878e+08 | 8 |

**Adduct Signal Fractions**

- **Unmodified**: 3.0878e+08

### N(N)GTYDHDVYRDEALNNR Fuc 3 Hex 6 HexNAc 5

**Mass:**  4476.8044

(1.707e-06)

**Charge States:** 4

**Start Position**: 499

**End Position**: 516

| Profile Score | Tandem Score | TDA q-value | Volume | Spectrum Matches |
| --- | --- | --- | --- | --- |
| 12.5609 | 115.9182 | 0.0000 | 5.2065e+07 | 3 |

**Adduct Signal Fractions**

- **Unmodified**: 5.2065e+07

### N(N)GTYDHDVYRDEALNNR Fuc 1 Hex 5 HexNAc 5

**Mass:**  4022.6340

(4.694e-08)

**Charge States:** 4

**Start Position**: 499

**End Position**: 516

| Profile Score | Tandem Score | TDA q-value | Volume | Spectrum Matches |
| --- | --- | --- | --- | --- |
| 8.7616 | 114.2523 | 0.0000 | 2.0765e+08 | 5 |

**Adduct Signal Fractions**

- **Unmodified**: 2.0765e+08

### N(N)GTYDHDVYRDEALNNR Fuc 3 Hex 5 HexNAc 4

**Mass:**  4111.6682

(2.035e-06)

**Charge States:** 4

**Start Position**: 499

**End Position**: 516

| Profile Score | Tandem Score | TDA q-value | Volume | Spectrum Matches |
| --- | --- | --- | --- | --- |
| -3.6161 | 113.7207 | 0.0000 | 1.1359e+08 | 2 |

**Adduct Signal Fractions**

- **Unmodified**: 1.1359e+08

### YPVLN(N)VTM(O)PNN(D)GK Fuc 1 Hex 3 HexNAc 5 Neu5Ac 1

**Mass:**  3401.3959

(2.824e-04)

**Charge States:** 3

**Start Position**: 177

**End Position**: 190

| Profile Score | Tandem Score | TDA q-value | Volume | Spectrum Matches |
| --- | --- | --- | --- | --- |
| 10.2825 | 113.0922 | 0.0000 | 1.4038e+08 | 8 |

**Adduct Signal Fractions**

- **Unmodified**: 1.4038e+08

### RGSN(N)NSFFSR Fuc 1 Hex 5 HexNAc 6

**Mass:**  3345.3557

(3.436e-07)

**Charge States:** 3

**Start Position**: 157

**End Position**: 167

| Profile Score | Tandem Score | TDA q-value | Volume | Spectrum Matches |
| --- | --- | --- | --- | --- |
| -4.5306 | 112.5972 | 0.0000 | 1.5718e+07 | 2 |

**Adduct Signal Fractions**

- **Unmodified**: 1.5718e+07

### TITNDQIEVTN(N)ATELVQSSSTGR Hex 6 HexNAc 3

**Mass:**  4044.7685

(1.153e-06)

**Charge States:** 3, 4

**Start Position**: 44

**End Position**: 67

| Profile Score | Tandem Score | TDA q-value | Volume | Spectrum Matches |
| --- | --- | --- | --- | --- |
| 13.5176 | 111.4708 | 0.0000 | 3.6973e+08 | 12 |

**Adduct Signal Fractions**

- **Unmodified**: 3.6973e+08

### RGSN(N)N(D)SFFSR Hex 7 HexNAc 3 Neu5Ac 1

**Mass:**  3206.2381

(3.049e-04)

**Charge States:** 3

**Start Position**: 157

**End Position**: 167

| Profile Score | Tandem Score | TDA q-value | Volume | Spectrum Matches |
| --- | --- | --- | --- | --- |
| 17.8014 | 110.9369 | 0.0000 | 2.0712e+07 | 3 |

**Adduct Signal Fractions**

- **Unmodified**: 2.0712e+07

### YPVLN(N)VTM(O)PNN(D)GK Hex 10 HexNAc 2

**Mass:**  3489.4063

(2.840e-04)

**Charge States:** 3

**Start Position**: 177

**End Position**: 190

| Profile Score | Tandem Score | TDA q-value | Volume | Spectrum Matches |
| --- | --- | --- | --- | --- |
| -6.2021 | 109.9344 | 0.0000 | 2.4162e+07 | 2 |

**Adduct Signal Fractions**

- **Unmodified**: 2.4162e+07

### N(N)GTYDHDVYR Fuc 1 Hex 6 HexNAc 6

**Mass:**  3576.3910

(2.820e-04)

**Start Position**: 499

**End Position**: 509

| Profile Score | Tandem Score | TDA q-value | Volume | Spectrum Matches |
| --- | --- | --- | --- | --- |
| 0.0000 | 109.7920 | 0.0000 | 0.0000e+00 | 2 |

Adduct:

No Chromatogram Found

### TITNDQIEVTN(N)ATELVQSSSTGR Hex 5 HexNAc 4

**Mass:**  4085.7949

(1.579e-06)

**Charge States:** 3, 4

**Start Position**: 44

**End Position**: 67

| Profile Score | Tandem Score | TDA q-value | Volume | Spectrum Matches |
| --- | --- | --- | --- | --- |
| 13.5341 | 109.7020 | 0.0000 | 4.2150e+08 | 12 |

**Adduct Signal Fractions**

- **Unmodified**: 4.2150e+08

### TITNDQIEVTN(N)ATELVQSSSTGR Fuc 1 Hex 5 HexNAc 4

**Mass:**  4231.8552

(1.536e-06)

**Charge States:** 3, 4

**Start Position**: 44

**End Position**: 67

| Profile Score | Tandem Score | TDA q-value | Volume | Spectrum Matches |
| --- | --- | --- | --- | --- |
| 13.5189 | 109.5401 | 0.0000 | 1.8100e+08 | 7 |

**Adduct Signal Fractions**

- **Unmodified**: 1.8100e+08

### N(N)GTYDHDVYR Fuc 3 Hex 5 HexNAc 4

**Mass:**  3300.2961

(3.059e-04)

**Start Position**: 499

**End Position**: 509

| Profile Score | Tandem Score | TDA q-value | Volume | Spectrum Matches |
| --- | --- | --- | --- | --- |
| 0.0000 | 109.1726 | 0.0000 | 0.0000e+00 | 1 |

Adduct:

No Chromatogram Found

### RGSN(N)NSFFSR Fuc 1 Hex 6 HexNAc 5

**Mass:**  3304.3273

(3.621e-07)

**Charge States:** 3

**Start Position**: 157

**End Position**: 167

| Profile Score | Tandem Score | TDA q-value | Volume | Spectrum Matches |
| --- | --- | --- | --- | --- |
| 7.1064 | 109.0149 | 0.0000 | 9.4510e+06 | 3 |

**Adduct Signal Fractions**

- **Unmodified**: 9.4510e+06

### N(N)GTYDHDVYR Hex 5 HexNAc 4

**Mass:**  2861.1145

(4.315e-07)

**Charge States:** 3

**Start Position**: 499

**End Position**: 509

| Profile Score | Tandem Score | TDA q-value | Volume | Spectrum Matches |
| --- | --- | --- | --- | --- |
| -19.6558 | 107.5113 | 0.0000 | 7.4739e+06 | 1 |

**Adduct Signal Fractions**

- **Unmodified**: 7.4739e+06

### TITNDQIEVTN(N)ATELVQSSSTGR Hex 6 HexNAc 2

**Mass:**  3841.6898

(1.332e-06)

**Charge States:** 3, 4

**Start Position**: 44

**End Position**: 67

| Profile Score | Tandem Score | TDA q-value | Volume | Spectrum Matches |
| --- | --- | --- | --- | --- |
| 13.7238 | 106.6972 | 0.0000 | 9.0616e+08 | 16 |

**Adduct Signal Fractions**

- **Unmodified**: 9.0616e+08

### N(N)GTYDHDVYRDEALNNR Fuc 2 Hex 6 HexNAc 5

**Mass:**  4330.7463

(1.816e-06)

**Charge States:** 4

**Start Position**: 499

**End Position**: 516

| Profile Score | Tandem Score | TDA q-value | Volume | Spectrum Matches |
| --- | --- | --- | --- | --- |
| -3.7854 | 106.4289 | 0.0000 | 6.3048e+07 | 2 |

**Adduct Signal Fractions**

- **Unmodified**: 6.3048e+07

### N(N)GTYDHDVYRDEALNNR Fuc 1 Hex 6 HexNAc 5

**Mass:**  4184.6848

(1.724e-06)

**Charge States:** 4

**Start Position**: 499

**End Position**: 516

| Profile Score | Tandem Score | TDA q-value | Volume | Spectrum Matches |
| --- | --- | --- | --- | --- |
| 14.5941 | 106.1889 | 0.0000 | 1.5070e+08 | 2 |

**Adduct Signal Fractions**

- **Unmodified**: 1.5070e+08

### TITNDQIEVTN(N)ATELVQSSSTGR Fuc 1 Hex 8 HexNAc 2

**Mass:**  4311.8142

(-8.193e-06)

**Charge States:** 3, 4

**Start Position**: 44

**End Position**: 67

| Profile Score | Tandem Score | TDA q-value | Volume | Spectrum Matches |
| --- | --- | --- | --- | --- |
| 13.6749 | 105.8902 | 0.0086 | 5.1805e+07 | 7 |

**Adduct Signal Fractions**

- **Unmodified**: 5.1805e+07

### N(N)GTYDHDVYRDEALNNR Fuc 1 Hex 9 HexNAc 3

**Mass:**  4264.6442

(-8.555e-06)

**Charge States:** 4

**Start Position**: 499

**End Position**: 516

| Profile Score | Tandem Score | TDA q-value | Volume | Spectrum Matches |
| --- | --- | --- | --- | --- |
| -4.8666 | 105.6955 | 0.0086 | 2.8726e+07 | 3 |

**Adduct Signal Fractions**

- **Unmodified**: 2.8726e+07

### N(N)GTYDHDVYRDEALNNR Fuc 1 Hex 6 HexNAc 6

**Mass:**  4387.7624

(1.410e-08)

**Charge States:** 4

**Start Position**: 499

**End Position**: 516

| Profile Score | Tandem Score | TDA q-value | Volume | Spectrum Matches |
| --- | --- | --- | --- | --- |
| 6.9851 | 105.5461 | 0.0086 | 4.6591e+07 | 4 |

**Adduct Signal Fractions**

- **Unmodified**: 4.6591e+07

### RGSN(N)N(D)SFFSR Fuc 1 Hex 5 HexNAc 5

**Mass:**  3143.2648

(3.158e-04)

**Charge States:** 3, 4

**Start Position**: 157

**End Position**: 167

| Profile Score | Tandem Score | TDA q-value | Volume | Spectrum Matches |
| --- | --- | --- | --- | --- |
| -5.3707 | 105.2004 | 0.0086 | 4.9775e+07 | 3 |

**Adduct Signal Fractions**

- **Unmodified**: 4.9775e+07

### RGSN(N)N(D)SFFSR Fuc 1 Hex 5 HexNAc 4

**Mass:**  2940.1842

(3.374e-04)

**Charge States:** 3

**Start Position**: 157

**End Position**: 167

| Profile Score | Tandem Score | TDA q-value | Volume | Spectrum Matches |
| --- | --- | --- | --- | --- |
| -5.6278 | 103.8328 | 0.0086 | 7.0301e+06 | 2 |

**Adduct Signal Fractions**

- **Unmodified**: 7.0301e+06

### RGSN(N)NSFFSR Fuc 3 Hex 5 HexNAc 4

**Mass:**  3231.3136

(2.005e-07)

**Charge States:** 3

**Start Position**: 157

**End Position**: 167

| Profile Score | Tandem Score | TDA q-value | Volume | Spectrum Matches |
| --- | --- | --- | --- | --- |
| -4.4621 | 103.6806 | 0.0086 | 9.9569e+06 | 3 |

**Adduct Signal Fractions**

- **Unmodified**: 9.9569e+06

### N(N)GTYDHDVYRDEALNNR Fuc 4 Hex 6 HexNAc 5

**Mass:**  4622.8625

(2.594e-06)

**Charge States:** 4

**Start Position**: 499

**End Position**: 516

| Profile Score | Tandem Score | TDA q-value | Volume | Spectrum Matches |
| --- | --- | --- | --- | --- |
| -3.3802 | 103.3481 | 0.0147 | 1.8916e+07 | 2 |

**Adduct Signal Fractions**

- **Unmodified**: 1.8916e+07

### N(N)GTYDHDVYRDEALNNR Fuc 1 Hex 8 HexNAc 2

**Mass:**  3899.5085

(-9.885e-06)

**Charge States:** 3, 4

**Start Position**: 499

**End Position**: 516

| Profile Score | Tandem Score | TDA q-value | Volume | Spectrum Matches |
| --- | --- | --- | --- | --- |
| 15.0013 | 103.1507 | 0.0147 | 3.3985e+07 | 3 |

**Adduct Signal Fractions**

- **Unmodified**: 3.3985e+07

### N(N)GTYDHDVYR Fuc 3 Hex 8 HexNAc 7

**Mass:**  4396.6813

(4.546e-04)

**Start Position**: 499

**End Position**: 509

| Profile Score | Tandem Score | TDA q-value | Volume | Spectrum Matches |
| --- | --- | --- | --- | --- |
| 0.0000 | 102.0469 | 0.0147 | 0.0000e+00 | 1 |

Adduct:

No Chromatogram Found

### N(N)GTYDHDVYRDEALNNR Fuc 2 Hex 6 HexNAc 6

**Mass:**  4533.8195

(3.535e-07)

**Charge States:** 4

**Start Position**: 499

**End Position**: 516

| Profile Score | Tandem Score | TDA q-value | Volume | Spectrum Matches |
| --- | --- | --- | --- | --- |
| 9.4194 | 100.7871 | 0.0147 | 4.4614e+07 | 5 |

**Adduct Signal Fractions**

- **Unmodified**: 4.4614e+07

### N(N)GTYDHDVYRDEALNNR Fuc 2 Hex 9 HexNAc 3

**Mass:**  4410.7028

(-8.484e-06)

**Charge States:** 4

**Start Position**: 499

**End Position**: 516

| Profile Score | Tandem Score | TDA q-value | Volume | Spectrum Matches |
| --- | --- | --- | --- | --- |
| -4.2506 | 100.5866 | 0.0147 | 2.0313e+07 | 2 |

**Adduct Signal Fractions**

- **Unmodified**: 2.0313e+07

### N(N)GTYDHDVYRDEALNNR Fuc 1 Hex 7 HexNAc 6

**Mass:**  4549.8195

(1.377e-06)

**Charge States:** 4

**Start Position**: 499

**End Position**: 516

| Profile Score | Tandem Score | TDA q-value | Volume | Spectrum Matches |
| --- | --- | --- | --- | --- |
| -4.0889 | 98.8111 | 0.0147 | 1.8201e+07 | 1 |

**Adduct Signal Fractions**

- **Unmodified**: 1.8201e+07

### N(N)GTYDHDVYR Fuc 2 Hex 6 HexNAc 6

**Mass:**  3721.4445

(1.063e-06)

**Charge States:** 3, 4

**Start Position**: 499

**End Position**: 509

| Profile Score | Tandem Score | TDA q-value | Volume | Spectrum Matches |
| --- | --- | --- | --- | --- |
| -5.9104 | 97.0192 | 0.0147 | 1.6817e+07 | 3 |

**Adduct Signal Fractions**

- **Unmodified**: 1.6817e+07

### N(N)GTYDHDVYR Fuc 1 Hex 7 HexNAc 3

**Mass:**  3128.1590

(-9.729e-06)

**Charge States:** 3

**Start Position**: 499

**End Position**: 509

| Profile Score | Tandem Score | TDA q-value | Volume | Spectrum Matches |
| --- | --- | --- | --- | --- |
| -4.9979 | 96.8125 | 0.0147 | 4.7154e+06 | 2 |

**Adduct Signal Fractions**

- **Unmodified**: 4.7154e+06

### N(N)GTYDHDVYRDEALNNR Fuc 2 Hex 5 HexNAc 5

**Mass:**  4168.6892

(9.346e-07)

**Charge States:** 4

**Start Position**: 499

**End Position**: 516

| Profile Score | Tandem Score | TDA q-value | Volume | Spectrum Matches |
| --- | --- | --- | --- | --- |
| 11.6845 | 95.0871 | 0.0207 | 8.2068e+07 | 3 |

**Adduct Signal Fractions**

- **Unmodified**: 8.2068e+07

### RGSN(N)NSFFSR Hex 6 HexNAc 3

**Mass:**  2752.1120

(1.380e-06)

**Charge States:** 3

**Start Position**: 157

**End Position**: 167

| Profile Score | Tandem Score | TDA q-value | Volume | Spectrum Matches |
| --- | --- | --- | --- | --- |
| -20.2236 | 94.6662 | 0.0207 | 1.1200e+06 | 1 |

**Adduct Signal Fractions**

- **Unmodified**: 1.1200e+06

### RGSN(N)NSFFSR Hex 5 HexNAc 5

**Mass:**  2996.2177

(9.974e-07)

**Charge States:** 3

**Start Position**: 157

**End Position**: 167

| Profile Score | Tandem Score | TDA q-value | Volume | Spectrum Matches |
| --- | --- | --- | --- | --- |
| -4.4552 | 93.9877 | 0.0207 | 1.3827e+07 | 2 |

**Adduct Signal Fractions**

- **Unmodified**: 1.3827e+07

### N(N)GTYDHDVYR Fuc 4 Hex 6 HexNAc 5

**Mass:**  3810.4794

(6.216e-07)

**Charge States:** 3, 4

**Start Position**: 499

**End Position**: 509

| Profile Score | Tandem Score | TDA q-value | Volume | Spectrum Matches |
| --- | --- | --- | --- | --- |
| -18.8381 | 93.4252 | 0.0207 | 1.2819e+07 | 2 |

**Adduct Signal Fractions**

- **Unmodified**: 1.2819e+07

### RGSN(N)N(D)SFFSR Hex 8 HexNAc 3 Neu5Ac 1

**Mass:**  3368.2915

(2.917e-04)

**Charge States:** 3

**Start Position**: 157

**End Position**: 167

| Profile Score | Tandem Score | TDA q-value | Volume | Spectrum Matches |
| --- | --- | --- | --- | --- |
| -6.5266 | 93.3475 | 0.0207 | 1.0565e+07 | 2 |

**Adduct Signal Fractions**

- **Unmodified**: 1.0565e+07

### SDAPIGTC(C)SSEC(C)ITPN(N)GSIPNDKPFQNVNK Hex 7 HexNAc 2

**Mass:**  4787.0314

(4.348e-06)

**Charge States:** 4

**Start Position**: 286

**End Position**: 316

| Profile Score | Tandem Score | TDA q-value | Volume | Spectrum Matches |
| --- | --- | --- | --- | --- |
| 12.8798 | 91.5482 | 0.0318 | 3.2084e+07 | 3 |

**Adduct Signal Fractions**

- **Unmodified**: 3.2084e+07

### RGSN(N)NSFFSR Fuc 2 Hex 9 HexNAc 3

**Mass:**  3530.3498

(-9.282e-06)

**Charge States:** 3

**Start Position**: 157

**End Position**: 167

| Profile Score | Tandem Score | TDA q-value | Volume | Spectrum Matches |
| --- | --- | --- | --- | --- |
| -20.1888 | 91.4486 | 0.0318 | 3.8208e+06 | 1 |

**Adduct Signal Fractions**

- **Unmodified**: 3.8208e+06

### iav|ADJ41819|A/Phil/2-MA/1982\_NA

**>iav|ADJ41819|A/Phil/2-MA/1982\_NA**  
 `MNPNQKIITIGSVSLTIATICFLMQIAILVTTVTLHFKQYECSSPPNNQVVPCEPIIIERNITEIVYLTNTTIEKEICPK  
LVEYRNWSKPQCKITGFAPFSKDNSIRLSAGGDIWVTREPYVSCDPGKCYQFALGQGTTLDNKHSNDTIHDRTPYRTLLM  
NELGVPFHLGTRQVCIAWSSSSCHDGKAWLHVCITGYDKNATASFIYDGRLVDSIGSWSKNILRTQESECVCINGTCTVV  
MTDGSASERADTKILFIEEGKIVHISPLSGSAQHVEECSCYPRYPGVRCVCRDNWKGSNRPVVDINVKDYSIVSSYVCSG  
LVGDTPRKNDRSSSSYCRNPNNEKGNHGVKGWAFDDGNDVWMGRTISEESRSGYETFKVIGGWSTPNSKLQINRQVIVDS  
GNRSGYSGIFSVEGKSCINRCFYVELIRGREQETRVWWTSNSIVVFCGTSGTYGTGSWPDGADINLMPI`

| Site | Type | Count |
| --- | --- | --- |
| 402 | N-linked | 2 |
| 200 | N-linked | 3 |

| Mass | Sequence | MS_2_ Score | `q`-value | MS_1_ Score | Signal | Protein Coordinates | Tandem Scans | Start Time | Apex Time | End Time |
| --- | --- | --- | --- | --- | --- | --- | --- | --- | --- | --- |
| 2690.1007 | QVIVDSGN(N)R Hex 8 HexNAc 2 | 172.030 | 0.0 | 0.000 | 0.000e+00 | 394 - 403 | 1 | - | - | - |
| 2364.9984 | QVIVDSGN(N)R Hex 6 HexNAc 2 | 152.975 | 0.0 | -21.321 | 3.549e+06 | 394 - 403 | 1 | 12.57 | 12.57 | 12.57 |
| 2916.1580 | N(N)ATASFIYDGR Hex 8 HexNAc 2 | 136.864 | 0.0 | 11.456 | 5.562e+07 | 199 - 210 | 4 | 23.87 | 25.56 | 26.06 |
| 3078.2106 | N(N)ATASFIYDGR Hex 9 HexNAc 2 | 125.516 | 0.0 | -3.532 | 1.449e+07 | 199 - 210 | 2 | 25.22 | 25.39 | 25.56 |
| 2754.1044 | N(N)ATASFIYDGR Hex 7 HexNAc 2 | 118.478 | 0.0 | 13.746 | 2.024e+07 | 199 - 210 | 3 | 25.22 | 25.73 | 26.23 |

### QVIVDSGN(N)R Hex 8 HexNAc 2

**Mass:**  2690.1007

(3.737e-04)

**Start Position**: 395

**End Position**: 404

| Profile Score | Tandem Score | TDA q-value | Volume | Spectrum Matches |
| --- | --- | --- | --- | --- |
| 0.0000 | 172.0296 | 0.0000 | 0.0000e+00 | 1 |

Adduct:

No Chromatogram Found

### QVIVDSGN(N)R Hex 6 HexNAc 2

**Mass:**  2364.9984

(3.465e-06)

**Charge States:** 2

**Start Position**: 395

**End Position**: 404

| Profile Score | Tandem Score | TDA q-value | Volume | Spectrum Matches |
| --- | --- | --- | --- | --- |
| -21.3206 | 152.9751 | 0.0000 | 3.5492e+06 | 1 |

**Adduct Signal Fractions**

- **Unmodified**: 3.5492e+06

### N(N)ATASFIYDGR Hex 8 HexNAc 2

**Mass:**  2916.1583

(1.571e-06)

**Charge States:** 3

**Start Position**: 200

**End Position**: 211

| Profile Score | Tandem Score | TDA q-value | Volume | Spectrum Matches |
| --- | --- | --- | --- | --- |
| 11.4560 | 136.8641 | 0.0000 | 5.5624e+07 | 4 |

**Adduct Signal Fractions**

- **Unmodified**: 5.5624e+07

### N(N)ATASFIYDGR Hex 9 HexNAc 2

**Mass:**  3078.2100

(1.644e-06)

**Charge States:** 3

**Start Position**: 200

**End Position**: 211

| Profile Score | Tandem Score | TDA q-value | Volume | Spectrum Matches |
| --- | --- | --- | --- | --- |
| -3.5323 | 125.5155 | 0.0000 | 1.4493e+07 | 2 |

**Adduct Signal Fractions**

- **Unmodified**: 1.4493e+07

### N(N)ATASFIYDGR Hex 7 HexNAc 2

**Mass:**  2754.1045

(1.370e-06)

**Charge States:** 3

**Start Position**: 200

**End Position**: 211

| Profile Score | Tandem Score | TDA q-value | Volume | Spectrum Matches |
| --- | --- | --- | --- | --- |
| 13.7459 | 118.4785 | 0.0000 | 2.0244e+07 | 3 |

**Adduct Signal Fractions**

- **Unmodified**: 2.0244e+07
