# Supplementary material for: Measuring Site-specific Glycosylation Similarity between Influenza a Virus Variants with Statistical Certainty
Source: Mol Cell Proteomics. 2020 Nov 25;19(9):1533–45. doi: 10.1074/mcp.RA120.002031 (PMC8143645; doi:10.1074/mcp.RA120.002031)
Supplement: Supplementary file 2 [file mmc2.zip › mmc2/159250_2_supp_555290_qcjklq.zip!/20190326_Phil82gal_032219_tryp_HILIC_DDA_1ug_02_glycopeptides.html]

## 20190326\_Phil82gal\_032219\_tryp\_HILIC\_DDA\_1ug\_02 @ GlycopeptideHypothesis-3e3201fa0d7e4481a0dbc3996163f1e4

**MS^1^ Mass Accuracy:** 10.0 PPM  
**Grouping Tolerance:** 15.0 PPM  
**MS^n^ Mass Accuracy:** 20.0 PPM  
**Minimum `q`-Value For Clustering:** 0.05

**Glycopeptide Hypothesis:**
GlycopeptideHypothesis-3e3201fa0d7e4481a0dbc3996163f1e4  
**Sample Searched:**
20190326\_Phil82gal\_032219\_tryp\_HILIC\_DDA\_1ug\_02

| Protein | Matched Glycopeptides |
| --- | --- |
| iav\|AFG99160\|A/Phil/2/1982\_HA | 75 |
| iav\|ADJ41819\|A/Phil/2-MA/1982\_NA | 4 |

### iav|AFG99160|A/Phil/2/1982\_HA

**>iav|AFG99160|A/Phil/2/1982\_HA**  
 `MKTIIALSYMFCLVFAQNLPGNDNSTATLCLGHHAVPNGTLVKTITNDQIEVTNATELVQSSSTGRICDSPHRILDGKNC  
TLIDALLGDPHCDGFQNEKWDLFVERSKAFSNCYPYDVPDYASLRSLVASSGTLEFINEGFNWTGVTQSGGSSTCKRGSN  
NSFFSRLNWLYESESKYPVLNVTMPNNGKFDKLYIWGIHHPSTDKEQTNLYIRASGRVTVSTKRSQQTVIPNIGSRPWVR  
GLSSRISIYWTIVKPGDILLINSTGNLIAPRGYFKIRTGKSSIMRSDAPIGTCSSECITPNGSIPNDKPFQNVNKITYGA  
CPRYVKQNTLKLATGMRNVPEKQTRGIFGAIAGFIENGWEGMVDGWYGFRHQNSEGTGQAADLKSTQAAIDQINGKLNRV  
IEKTNEKFHQIEKEFSEVEGRIQDLEKYVEDTKIDLWSYNAELLVALENQHTIDLTDSEMNKLFEKTRKQLRENAEDMGN  
GCFKIYHKCDNACIGSIRNGTYDHDVYRDEALNNRFQIKGVELKSGYKDWILWISFAISCFLLCVVLLGFIMWACQKGNI  
RCNICI`

| Site | Type | Count |
| --- | --- | --- |
| 262 | N-linked | 5 |
| 301 | N-linked | 4 |
| 499 | N-linked | 27 |
| 181 | N-linked | 12 |
| 54 | N-linked | 8 |
| 160 | N-linked | 19 |

| Mass | Sequence | MS_2_ Score | `q`-value | MS_1_ Score | Signal | Protein Coordinates | Tandem Scans | Start Time | Apex Time | End Time |
| --- | --- | --- | --- | --- | --- | --- | --- | --- | --- | --- |
| 4068.9283 | TIVKPGDILLIN(N)STGNLIAPR Hex 9 HexNAc 2 | 162.912 | 0.0 | 13.319 | 1.818e+08 | 250 - 271 | 19 | 50.72 | 51.56 | 52.95 |
| 3126.2836 | RGSN(N)NSFFSR Fuc 2 Hex 4 HexNAc 5 | 161.340 | 0.0 | 14.059 | 3.179e+07 | 156 - 166 | 4 | 16.08 | 16.42 | 16.75 |
| 2615.0934 | RGSN(N)NSFFSR Fuc 1 Hex 3 HexNAc 4 | 160.250 | 0.0 | -4.618 | 1.154e+07 | 156 - 166 | 1 | 16.75 | 16.75 | 17.09 |
| 2662.0687 | GSN(N)NSFFSR Fuc 1 Hex 3 HexNAc 5 | 155.111 | 0.0 | 13.494 | 2.256e+08 | 157 - 166 | 5 | 19.65 | 20.33 | 20.67 |
| 2818.1738 | RGSN(N)NSFFSR Fuc 1 Hex 3 HexNAc 5 | 144.883 | 0.0 | 14.447 | 1.011e+08 | 156 - 166 | 5 | 16.08 | 16.92 | 17.26 |
| 3327.4205 | YPVLN(N)VTM(O)PNN(D)GK Hex 9 HexNAc 2 | 143.224 | 0.0 | 10.013 | 1.103e+09 | 176 - 189 | 9 | 27.98 | 28.32 | 30.00 |
| 2819.1620 | RGSN(N)N(D)SFFSR Fuc 1 Hex 3 HexNAc 5 | 142.662 | 0.0 | 8.046 | 3.576e+07 | 156 - 166 | 1 | 17.43 | 17.43 | 18.46 |
| 3141.2531 | YPVLN(N)VTM(O)PNN Hex 9 HexNAc 2 | 142.246 | 0.0 | -4.122 | 5.065e+06 | 176 - 187 | 2 | 31.89 | 32.06 | 32.23 |
| 3344.3768 | YPVLN(N)VTM(O)PNN(D)GK Fuc 2 Hex 3 HexNAc 4 Neu5Ac 1 | 140.362 | 0.0 | 11.242 | 2.745e+08 | 176 - 189 | 3 | 27.98 | 28.32 | 28.98 |
| 2899.1220 | RGSN(N)N(D)SFFSR Fuc 1 Hex 6 HexNAc 3 | 139.820 | 0.0 | -20.039 | 4.733e+06 | 156 - 166 | 1 | 19.82 | 19.82 | 19.99 |
| 3627.6323 | PGDILLIN(N)STGNLIAPR Hex 9 HexNAc 2 | 139.633 | 0.0 | -3.570 | 1.570e+07 | 254 - 271 | 4 | 49.26 | 49.43 | 49.77 |
| 4085.9534 | TIVKPGDILLIN(N)STGNLIAPR Fuc 2 Hex 3 HexNAc 4 Neu5Ac 1 | 136.269 | 0.0 | 12.130 | 2.503e+07 | 250 - 271 | 3 | 51.27 | 51.56 | 52.66 |
| 2970.1826 | GSN(N)NSFFSR Fuc 2 Hex 4 HexNAc 5 | 133.851 | 0.0 | 13.799 | 8.472e+07 | 157 - 166 | 3 | 19.14 | 19.65 | 19.99 |
| 4230.9797 | TIVKPGDILLIN(N)STGNLIAPR Hex 10 HexNAc 2 | 132.903 | 0.0 | 13.837 | 2.370e+07 | 250 - 271 | 5 | 51.23 | 51.45 | 51.73 |
| 3644.6615 | PGDILLIN(N)STGNLIAPR Fuc 2 Hex 3 HexNAc 4 Neu5Ac 1 | 130.550 | 0.0 | -4.449 | 6.384e+06 | 254 - 271 | 3 | 49.26 | 49.43 | 49.77 |
| 3165.2980 | YPVLN(N)VTM(O)PNN(D)GK Hex 8 HexNAc 2 | 130.416 | 0.0 | 12.037 | 5.957e+08 | 176 - 189 | 7 | 27.98 | 28.32 | 29.15 |
| 2991.1942 | N(N)GTYDHDVYR Fuc 2 Hex 4 HexNAc 4 | 129.705 | 0.0 | -3.901 | 1.361e+07 | 498 - 508 | 2 | 12.69 | 12.86 | 13.20 |
| 3412.4713 | DQIEVTN(N)ATELVQSSSTGR Hex 6 HexNAc 2 | 128.813 | 0.0 | -4.393 | 8.582e+06 | 47 - 66 | 1 | 35.82 | 35.99 | 36.17 |
| 4949.0887 | SDAPIGTC(C)SSEC(C)ITPN(N)GSIPNDKPFQNVNK Hex 8 HexNAc 2 | 127.723 | 0.0 | 13.189 | 2.857e+08 | 285 - 315 | 8 | 34.45 | 34.62 | 35.31 |
| 3717.5432 | YPVLN(N)VTM(O)PNN(D)GKFDK Hex 9 HexNAc 2 | 127.520 | 0.0 | 12.019 | 3.173e+08 | 176 - 192 | 6 | 31.03 | 31.71 | 32.06 |
| 3502.3796 | N(N)GTYDHDVYR Fuc 3 Hex 5 HexNAc 5 | 126.723 | 0.0 | -19.833 | 2.667e+06 | 498 - 508 | 1 | 12.52 | 12.69 | 12.69 |
| 3003.2446 | YPVLN(N)VTM(O)PNN(D)GK Hex 7 HexNAc 2 | 124.951 | 0.0 | 10.681 | 7.121e+07 | 176 - 189 | 4 | 27.98 | 28.48 | 29.15 |
| 3425.3209 | RGSN(N)NSFFSR Fuc 1 Hex 8 HexNAc 4 | 122.827 | 0.0 | 12.835 | 9.863e+06 | 156 - 166 | 2 | 18.46 | 18.97 | 19.14 |
| 3698.5312 | N(N)GTYDHDVYRDEALNNR Fuc 1 Hex 3 HexNAc 5 | 122.433 | 0.0 | 15.238 | 1.443e+08 | 498 - 515 | 4 | 17.77 | 18.29 | 18.46 |
| 3841.6950 | TITNDQIEVTN(N)ATELVQSSSTGR Hex 6 HexNAc 2 | 122.148 | 0.0 | 13.832 | 1.282e+09 | 43 - 66 | 14 | 38.39 | 39.24 | 40.43 |
| 3183.3036 | RGSN(N)NSFFSR Fuc 1 Hex 4 HexNAc 6 | 121.920 | 0.0 | 13.567 | 1.415e+07 | 156 - 166 | 5 | 16.25 | 16.42 | 17.09 |
| 3555.4896 | YPVLN(N)VTM(O)PNN(D)GKFDK Hex 8 HexNAc 2 | 120.634 | 0.0 | 12.797 | 1.771e+08 | 176 - 192 | 7 | 31.20 | 31.71 | 32.40 |
| 2886.1648 | N(N)GTYDHDVYR Fuc 1 Hex 3 HexNAc 5 | 119.589 | 0.0 | -4.548 | 7.338e+06 | 498 - 508 | 1 | 12.86 | 13.03 | 13.20 |
| 3495.4505 | N(N)GTYDHDVYRDEALNNR Fuc 1 Hex 3 HexNAc 4 | 117.686 | 0.0 | 16.973 | 1.735e+08 | 498 - 515 | 5 | 17.77 | 18.29 | 18.80 |
| 3803.5630 | N(N)GTYDHDVYRDEALNNR Fuc 2 Hex 4 HexNAc 4 | 116.890 | 0.0 | -2.005 | 1.845e+08 | 498 - 515 | 5 | 17.60 | 17.95 | 18.12 |
| 3489.4079 | YPVLN(N)VTM(O)PNN(D)GK Hex 10 HexNAc 2 | 116.771 | 0.0 | 10.879 | 3.406e+07 | 176 - 189 | 3 | 27.98 | 28.32 | 28.64 |
| 2875.1948 | RGSN(N)NSFFSR Hex 3 HexNAc 6 | 115.155 | 0.0 | -3.059 | 3.564e+07 | 156 - 166 | 3 | 16.75 | 17.09 | 17.26 |
| 3761.6953 | TITNDQIEVTN(N)ATELVQSSSTGR Hex 3 HexNAc 4 | 114.448 | 0.0 | 14.095 | 2.999e+08 | 43 - 66 | 8 | 39.24 | 39.41 | 40.60 |
| 3299.3012 | N(N)GTYDHDVYR Fuc 3 Hex 5 HexNAc 4 | 113.685 | 0.0 | -4.539 | 7.294e+06 | 498 - 508 | 2 | 12.52 | 12.52 | 12.86 |
| 2979.2025 | YPVLN(N)VTM(O)PNN Hex 8 HexNAc 2 | 112.914 | 0.0 | -4.361 | 3.793e+06 | 176 - 187 | 2 | 31.89 | 32.23 | 32.40 |
| 4003.7473 | TITNDQIEVTN(N)ATELVQSSSTGR Hex 7 HexNAc 2 | 112.765 | 0.0 | 14.049 | 8.523e+08 | 43 - 66 | 14 | 38.22 | 39.07 | 40.26 |
| 3882.7192 | TITNDQIEVTN(N)ATELVQSSSTGR Hex 5 HexNAc 3 | 112.727 | 0.0 | 13.180 | 4.189e+08 | 43 - 66 | 11 | 38.39 | 39.24 | 41.12 |
| 4190.8326 | TITNDQIEVTN(N)ATELVQSSSTGR Fuc 1 Hex 6 HexNAc 3 | 112.554 | 0.0 | 14.262 | 4.620e+08 | 43 - 66 | 11 | 38.22 | 38.90 | 40.26 |
| 4111.6754 | N(N)GTYDHDVYRDEALNNR Fuc 3 Hex 5 HexNAc 4 | 111.581 | 0.0 | -2.340 | 9.444e+07 | 498 - 515 | 3 | 17.26 | 17.60 | 17.77 |
| 2683.0848 | N(N)GTYDHDVYR Fuc 1 Hex 3 HexNAc 4 | 111.493 | 0.0 | -5.441 | 4.311e+06 | 498 - 508 | 2 | 12.86 | 13.03 | 13.20 |
| 3231.3176 | RGSN(N)NSFFSR Fuc 3 Hex 5 HexNAc 4 | 110.563 | 0.0 | -16.754 | 5.077e+06 | 156 - 166 | 2 | 15.74 | 15.91 | 15.91 |
| 4377.9179 | TITNDQIEVTN(N)ATELVQSSSTGR Fuc 2 Hex 5 HexNAc 4 | 110.111 | 0.0 | -4.703 | 6.980e+07 | 43 - 66 | 2 | 38.73 | 38.73 | 39.07 |
| 2923.2067 | RGSN(N)NSFFSR Fuc 2 Hex 4 HexNAc 4 | 110.045 | 0.0 | -4.286 | 8.518e+06 | 156 - 166 | 3 | 16.08 | 16.42 | 16.59 |
| 5111.1390 | SDAPIGTC(C)SSEC(C)ITPN(N)GSIPNDKPFQNVNK Hex 9 HexNAc 2 | 109.389 | 0.0 | 11.973 | 2.447e+08 | 285 - 315 | 5 | 34.45 | 34.62 | 35.31 |
| 3194.2707 | N(N)GTYDHDVYR Fuc 2 Hex 4 HexNAc 5 | 108.766 | 0.0 | -4.301 | 5.381e+06 | 498 - 508 | 2 | 12.69 | 12.86 | 13.03 |
| 4576.9509 | LNWLYESESKYPVLN(N)VTM(O)PNN(D)GK Hex 9 HexNAc 2 | 108.208 | 0.0 | 8.709 | 3.276e+07 | 166 - 189 | 6 | 48.63 | 48.80 | 49.60 |
| 5128.1597 | SDAPIGTC(C)SSEC(C)ITPN(N)GSIPNDKPFQNVNK Fuc 2 Hex 3 HexNAc 4 Neu5Ac 1 | 107.839 | 0.0 | -4.060 | 7.583e+07 | 285 - 315 | 2 | 34.45 | 34.62 | 34.97 |
| 3810.4895 | N(N)GTYDHDVYR Fuc 4 Hex 6 HexNAc 5 | 107.786 | 0.0 | -20.273 | 7.127e+05 | 498 - 508 | 1 | 12.36 | 12.36 | 12.52 |
| 3401.4000 | YPVLN(N)VTM(O)PNN(D)GK Fuc 1 Hex 3 HexNAc 5 Neu5Ac 1 | 107.506 | 0.0 | 12.850 | 2.665e+08 | 176 - 189 | 7 | 26.63 | 27.98 | 28.81 |
| 3206.2371 | RGSN(N)N(D)SFFSR Hex 7 HexNAc 3 Neu5Ac 1 | 104.433 | 0.0 | 9.965 | 1.738e+07 | 156 - 166 | 3 | 18.29 | 18.46 | 19.31 |
| 2925.0936 | N(N)GTYDHDVYR Fuc 1 Hex 7 HexNAc 2 | 103.698 | 0.0 | -3.732 | 4.169e+06 | 498 - 508 | 2 | 14.05 | 14.22 | 14.39 |
| 4787.0342 | SDAPIGTC(C)SSEC(C)ITPN(N)GSIPNDKPFQNVNK Hex 7 HexNAc 2 | 103.648 | 0.0 | 14.045 | 6.432e+07 | 285 - 315 | 2 | 34.45 | 34.62 | 35.14 |
| 4006.6432 | N(N)GTYDHDVYRDEALNNR Fuc 2 Hex 4 HexNAc 5 | 102.689 | 0.009 | -3.210 | 7.317e+07 | 498 - 515 | 3 | 17.60 | 17.95 | 18.12 |
| 3290.2227 | N(N)GTYDHDVYR Fuc 1 Hex 8 HexNAc 3 | 102.632 | 0.009 | -20.720 | 9.796e+05 | 498 - 508 | 1 | 13.88 | 13.88 | 13.88 |
| 2841.1908 | YPVLN(N)VTM(O)PNN(D)GK Hex 6 HexNAc 2 | 102.594 | 0.009 | -7.033 | 6.092e+06 | 176 - 189 | 1 | 28.48 | 28.64 | 28.98 |
| 3493.4254 | RGSN(N)NSFFSR Fuc 2 Hex 5 HexNAc 6 | 101.857 | 0.009 | 0.000 | 0.000e+00 | 156 - 166 | 1 | - | - | - |
| 4622.8638 | N(N)GTYDHDVYRDEALNNR Fuc 4 Hex 6 HexNAc 5 | 100.834 | 0.009 | -3.825 | 1.391e+07 | 498 - 515 | 2 | 17.26 | 17.43 | 17.60 |
| 4314.7531 | N(N)GTYDHDVYRDEALNNR Fuc 3 Hex 5 HexNAc 5 | 99.772 | 0.009 | -3.422 | 4.522e+07 | 498 - 515 | 2 | 17.43 | 17.60 | 17.95 |
| 3436.2801 | N(N)GTYDHDVYR Fuc 2 Hex 8 HexNAc 3 | 99.685 | 0.009 | -20.903 | 1.378e+06 | 498 - 508 | 1 | 13.71 | 13.71 | 13.88 |
| 3494.2766 | N(N)GTYDHDVYR Fuc 1 Hex 8 HexNAc 4 | 99.076 | 0.009 | 9.255 | 3.033e+07 | 498 - 508 | 5 | 30.35 | 30.69 | 31.37 |
| 3352.2960 | RGSN(N)N(D)SFFSR Fuc 1 Hex 7 HexNAc 3 Neu5Ac 1 | 96.523 | 0.009 | -6.609 | 8.323e+06 | 156 - 166 | 2 | 17.95 | 17.95 | 18.46 |
| 3436.4065 | RGSN(N)N(D)SFFSR Fuc 3 Hex 5 HexNAc 5 | 94.895 | 0.009 | 0.000 | 0.000e+00 | 156 - 166 | 1 | - | - | - |
| 3128.1681 | N(N)GTYDHDVYR Fuc 1 Hex 7 HexNAc 3 | 94.525 | 0.016 | -19.997 | 2.038e+06 | 498 - 508 | 1 | 14.05 | 14.22 | 14.22 |
| 3274.2282 | N(N)GTYDHDVYR Fuc 2 Hex 7 HexNAc 3 | 94.428 | 0.016 | -20.833 | 1.097e+06 | 498 - 508 | 1 | 13.88 | 13.88 | 14.05 |
| 3901.6101 | N(N)GTYDHDVYRDEALNNR Fuc 1 Hex 3 HexNAc 6 | 94.129 | 0.016 | 11.022 | 2.656e+07 | 498 - 515 | 3 | 17.95 | 18.29 | 18.80 |
| 2672.1162 | RGSN(N)NSFFSR Hex 3 HexNAc 5 | 93.721 | 0.016 | -5.178 | 6.820e+06 | 156 - 166 | 2 | 16.75 | 16.92 | 17.26 |
| 3288.3404 | RGSN(N)NSFFSR Fuc 2 Hex 5 HexNAc 5 | 93.061 | 0.016 | -6.065 | 2.640e+06 | 156 - 166 | 2 | 15.91 | 15.91 | 16.42 |
| 2980.2264 | RGSN(N)NSFFSR Fuc 1 Hex 4 HexNAc 5 | 92.974 | 0.022 | 10.768 | 8.836e+06 | 156 - 166 | 3 | 16.25 | 16.92 | 17.09 |
| 3860.5833 | N(N)GTYDHDVYRDEALNNR Fuc 1 Hex 4 HexNAc 5 | 92.845 | 0.022 | 12.921 | 1.543e+08 | 498 - 515 | 6 | 17.26 | 17.60 | 19.31 |
| 3737.4586 | N(N)GTYDHDVYRDEALNNR Fuc 1 Hex 7 HexNAc 2 | 91.231 | 0.022 | -3.960 | 1.874e+07 | 498 - 515 | 4 | 19.82 | 19.99 | 20.33 |
| 4209.7203 | N(N)GTYDHDVYRDEALNNR Fuc 2 Hex 4 HexNAc 6 | 89.102 | 0.022 | 12.115 | 2.243e+07 | 498 - 515 | 3 | 17.60 | 17.95 | 18.63 |
| 4102.5927 | N(N)GTYDHDVYRDEALNNR Fuc 1 Hex 8 HexNAc 3 | 87.423 | 0.036 | -5.273 | 1.054e+07 | 498 - 515 | 1 | 19.48 | 19.65 | 19.82 |
| 3940.5346 | N(N)GTYDHDVYRDEALNNR Fuc 1 Hex 7 HexNAc 3 | 86.995 | 0.036 | 8.867 | 2.667e+07 | 498 - 515 | 5 | 19.31 | 19.82 | 20.67 |
| 3679.6408 | TITNDQIEVTN(N)ATELVQSSSTGR Hex 5 HexNAc 2 | 85.894 | 0.048 | 14.014 | 4.660e+08 | 43 - 66 | 9 | 38.56 | 39.41 | 40.60 |
| 3048.2161 | N(N)GTYDHDVYR Fuc 1 Hex 4 HexNAc 5 | 84.999 | 0.048 | 5.043 | 9.468e+06 | 498 - 508 | 3 | 12.52 | 13.37 | 13.54 |

### TIVKPGDILLIN(N)STGNLIAPR Hex 9 HexNAc 2

**Mass:**  4068.9274

(1.011e-06)

**Charge States:** 3, 4, 5

**Start Position**: 251

**End Position**: 272

| Profile Score | Tandem Score | TDA q-value | Volume | Spectrum Matches |
| --- | --- | --- | --- | --- |
| 13.3190 | 162.9125 | 0.0000 | 1.8179e+08 | 19 |

**Adduct Signal Fractions**

- **Unmodified**: 1.8179e+08

### RGSN(N)NSFFSR Fuc 2 Hex 4 HexNAc 5

**Mass:**  3126.2836

(3.439e-06)

**Charge States:** 3, 4

**Start Position**: 157

**End Position**: 167

| Profile Score | Tandem Score | TDA q-value | Volume | Spectrum Matches |
| --- | --- | --- | --- | --- |
| 14.0592 | 161.3398 | 0.0000 | 3.1788e+07 | 4 |

**Adduct Signal Fractions**

- **Unmodified**: 3.1788e+07

### RGSN(N)NSFFSR Fuc 1 Hex 3 HexNAc 4

**Mass:**  2615.0940

(2.735e-06)

**Charge States:** 3

**Start Position**: 157

**End Position**: 167

| Profile Score | Tandem Score | TDA q-value | Volume | Spectrum Matches |
| --- | --- | --- | --- | --- |
| -4.6176 | 160.2497 | 0.0000 | 1.1540e+07 | 1 |

**Adduct Signal Fractions**

- **Unmodified**: 1.1540e+07

### GSN(N)NSFFSR Fuc 1 Hex 3 HexNAc 5

**Mass:**  2662.0689

(1.052e-06)

**Charge States:** 2, 3

**Start Position**: 158

**End Position**: 167

| Profile Score | Tandem Score | TDA q-value | Volume | Spectrum Matches |
| --- | --- | --- | --- | --- |
| 13.4945 | 155.1115 | 0.0000 | 2.2562e+08 | 5 |

**Adduct Signal Fractions**

- **Unmodified**: 2.2562e+08

### RGSN(N)NSFFSR Fuc 1 Hex 3 HexNAc 5

**Mass:**  2818.1738

(2.632e-06)

**Charge States:** 3

**Start Position**: 157

**End Position**: 167

| Profile Score | Tandem Score | TDA q-value | Volume | Spectrum Matches |
| --- | --- | --- | --- | --- |
| 14.4470 | 144.8831 | 0.0000 | 1.0113e+08 | 5 |

**Adduct Signal Fractions**

- **Unmodified**: 1.0113e+08

### YPVLN(N)VTM(O)PNN(D)GK Hex 9 HexNAc 2

**Mass:**  3327.3513

(2.966e-04)

**Charge States:** 2, 3

**Start Position**: 177

**End Position**: 190

| Profile Score | Tandem Score | TDA q-value | Volume | Spectrum Matches |
| --- | --- | --- | --- | --- |
| 10.0133 | 143.2236 | 0.0000 | 1.1031e+09 | 9 |

**Adduct Signal Fractions**

- **Unmodified**: 1.1031e+09

### RGSN(N)N(D)SFFSR Fuc 1 Hex 3 HexNAc 5

**Mass:**  2819.1619

(3.533e-04)

**Charge States:** 3

**Start Position**: 157

**End Position**: 167

| Profile Score | Tandem Score | TDA q-value | Volume | Spectrum Matches |
| --- | --- | --- | --- | --- |
| 8.0460 | 142.6620 | 0.0000 | 3.5759e+07 | 1 |

**Adduct Signal Fractions**

- **Unmodified**: 3.5759e+07

### YPVLN(N)VTM(O)PNN Hex 9 HexNAc 2

**Mass:**  3141.2514

(1.381e-06)

**Charge States:** 3

**Start Position**: 177

**End Position**: 188

| Profile Score | Tandem Score | TDA q-value | Volume | Spectrum Matches |
| --- | --- | --- | --- | --- |
| -4.1220 | 142.2457 | 0.0000 | 5.0645e+06 | 2 |

**Adduct Signal Fractions**

- **Unmodified**: 5.0645e+06

### YPVLN(N)VTM(O)PNN(D)GK Fuc 2 Hex 3 HexNAc 4 Neu5Ac 1

**Mass:**  3344.3761

(2.873e-04)

**Charge States:** 3

**Start Position**: 177

**End Position**: 190

| Profile Score | Tandem Score | TDA q-value | Volume | Spectrum Matches |
| --- | --- | --- | --- | --- |
| 11.2421 | 140.3624 | 0.0000 | 2.7446e+08 | 3 |

**Adduct Signal Fractions**

- **Unmodified**: 2.7446e+08

### RGSN(N)N(D)SFFSR Fuc 1 Hex 6 HexNAc 3

**Mass:**  2899.1215

(3.297e-04)

**Charge States:** 3

**Start Position**: 157

**End Position**: 167

| Profile Score | Tandem Score | TDA q-value | Volume | Spectrum Matches |
| --- | --- | --- | --- | --- |
| -20.0392 | 139.8195 | 0.0000 | 4.7330e+06 | 1 |

**Adduct Signal Fractions**

- **Unmodified**: 4.7330e+06

### PGDILLIN(N)STGNLIAPR Hex 9 HexNAc 2

**Mass:**  3627.6326

(1.027e-06)

**Charge States:** 3, 4

**Start Position**: 255

**End Position**: 272

| Profile Score | Tandem Score | TDA q-value | Volume | Spectrum Matches |
| --- | --- | --- | --- | --- |
| -3.5700 | 139.6328 | 0.0000 | 1.5703e+07 | 4 |

**Adduct Signal Fractions**

- **Unmodified**: 1.5703e+07

### TIVKPGDILLIN(N)STGNLIAPR Fuc 2 Hex 3 HexNAc 4 Neu5Ac 1

**Mass:**  4085.9528

(-5.735e-06)

**Charge States:** 4

**Start Position**: 251

**End Position**: 272

| Profile Score | Tandem Score | TDA q-value | Volume | Spectrum Matches |
| --- | --- | --- | --- | --- |
| 12.1297 | 136.2694 | 0.0000 | 2.5034e+07 | 3 |

**Adduct Signal Fractions**

- **Unmodified**: 2.5034e+07

### GSN(N)NSFFSR Fuc 2 Hex 4 HexNAc 5

**Mass:**  2970.1831

(2.424e-06)

**Charge States:** 3

**Start Position**: 158

**End Position**: 167

| Profile Score | Tandem Score | TDA q-value | Volume | Spectrum Matches |
| --- | --- | --- | --- | --- |
| 13.7989 | 133.8508 | 0.0000 | 8.4719e+07 | 3 |

**Adduct Signal Fractions**

- **Unmodified**: 8.4719e+07

### TIVKPGDILLIN(N)STGNLIAPR Hex 10 HexNAc 2

**Mass:**  4230.9787

(6.339e-07)

**Charge States:** 3, 4

**Start Position**: 251

**End Position**: 272

| Profile Score | Tandem Score | TDA q-value | Volume | Spectrum Matches |
| --- | --- | --- | --- | --- |
| 13.8367 | 132.9031 | 0.0000 | 2.3695e+07 | 5 |

**Adduct Signal Fractions**

- **Unmodified**: 2.3695e+07

### PGDILLIN(N)STGNLIAPR Fuc 2 Hex 3 HexNAc 4 Neu5Ac 1

**Mass:**  3644.6614

(-5.784e-06)

**Charge States:** 3

**Start Position**: 255

**End Position**: 272

| Profile Score | Tandem Score | TDA q-value | Volume | Spectrum Matches |
| --- | --- | --- | --- | --- |
| -4.4488 | 130.5499 | 0.0000 | 6.3843e+06 | 3 |

**Adduct Signal Fractions**

- **Unmodified**: 6.3843e+06

### YPVLN(N)VTM(O)PNN(D)GK Hex 8 HexNAc 2

**Mass:**  3165.2975

(3.121e-04)

**Charge States:** 2, 3

**Start Position**: 177

**End Position**: 190

| Profile Score | Tandem Score | TDA q-value | Volume | Spectrum Matches |
| --- | --- | --- | --- | --- |
| 12.0367 | 130.4157 | 0.0000 | 5.9574e+08 | 7 |

**Adduct Signal Fractions**

- **Unmodified**: 5.9574e+08

### N(N)GTYDHDVYR Fuc 2 Hex 4 HexNAc 4

**Mass:**  2991.1931

(6.395e-06)

**Charge States:** 3

**Start Position**: 499

**End Position**: 509

| Profile Score | Tandem Score | TDA q-value | Volume | Spectrum Matches |
| --- | --- | --- | --- | --- |
| -3.9007 | 129.7048 | 0.0000 | 1.3614e+07 | 2 |

**Adduct Signal Fractions**

- **Unmodified**: 1.3614e+07

### DQIEVTN(N)ATELVQSSSTGR Hex 6 HexNAc 2

**Mass:**  3412.4727

(2.128e-06)

**Charge States:** 3

**Start Position**: 48

**End Position**: 67

| Profile Score | Tandem Score | TDA q-value | Volume | Spectrum Matches |
| --- | --- | --- | --- | --- |
| -4.3928 | 128.8133 | 0.0000 | 8.5820e+06 | 1 |

**Adduct Signal Fractions**

- **Unmodified**: 8.5820e+06

### SDAPIGTC(C)SSEC(C)ITPN(N)GSIPNDKPFQNVNK Hex 8 HexNAc 2

**Mass:**  4949.0891

(3.333e-06)

**Charge States:** 3, 4

**Start Position**: 286

**End Position**: 316

| Profile Score | Tandem Score | TDA q-value | Volume | Spectrum Matches |
| --- | --- | --- | --- | --- |
| 13.1887 | 127.7233 | 0.0000 | 2.8570e+08 | 8 |

**Adduct Signal Fractions**

- **Unmodified**: 2.8570e+08

### YPVLN(N)VTM(O)PNN(D)GKFDK Hex 9 HexNAc 2

**Mass:**  3717.5410

(2.668e-04)

**Charge States:** 3, 4

**Start Position**: 177

**End Position**: 193

| Profile Score | Tandem Score | TDA q-value | Volume | Spectrum Matches |
| --- | --- | --- | --- | --- |
| 12.0188 | 127.5202 | 0.0000 | 3.1726e+08 | 6 |

**Adduct Signal Fractions**

- **Unmodified**: 3.1726e+08

### N(N)GTYDHDVYR Fuc 3 Hex 5 HexNAc 5

**Mass:**  3502.3800

(3.540e-06)

**Charge States:** 3

**Start Position**: 499

**End Position**: 509

| Profile Score | Tandem Score | TDA q-value | Volume | Spectrum Matches |
| --- | --- | --- | --- | --- |
| -19.8331 | 126.7228 | 0.0000 | 2.6674e+06 | 1 |

**Adduct Signal Fractions**

- **Unmodified**: 2.6674e+06

### YPVLN(N)VTM(O)PNN(D)GK Hex 7 HexNAc 2

**Mass:**  3003.2422

(3.291e-04)

**Charge States:** 3

**Start Position**: 177

**End Position**: 190

| Profile Score | Tandem Score | TDA q-value | Volume | Spectrum Matches |
| --- | --- | --- | --- | --- |
| 10.6805 | 124.9508 | 0.0000 | 7.1207e+07 | 4 |

**Adduct Signal Fractions**

- **Unmodified**: 7.1207e+07

### RGSN(N)NSFFSR Fuc 1 Hex 8 HexNAc 4

**Mass:**  3425.3190

(-9.753e-06)

**Charge States:** 3

**Start Position**: 157

**End Position**: 167

| Profile Score | Tandem Score | TDA q-value | Volume | Spectrum Matches |
| --- | --- | --- | --- | --- |
| 12.8351 | 122.8266 | 0.0000 | 9.8625e+06 | 2 |

**Adduct Signal Fractions**

- **Unmodified**: 9.8625e+06

### N(N)GTYDHDVYRDEALNNR Fuc 1 Hex 3 HexNAc 5

**Mass:**  3698.5324

(2.337e-06)

**Charge States:** 3, 4

**Start Position**: 499

**End Position**: 516

| Profile Score | Tandem Score | TDA q-value | Volume | Spectrum Matches |
| --- | --- | --- | --- | --- |
| 15.2380 | 122.4330 | 0.0000 | 1.4430e+08 | 4 |

**Adduct Signal Fractions**

- **Unmodified**: 1.4430e+08

### TITNDQIEVTN(N)ATELVQSSSTGR Hex 6 HexNAc 2

**Mass:**  3841.6946

(3.206e-06)

**Charge States:** 3, 4

**Start Position**: 44

**End Position**: 67

| Profile Score | Tandem Score | TDA q-value | Volume | Spectrum Matches |
| --- | --- | --- | --- | --- |
| 13.8317 | 122.1480 | 0.0000 | 1.2819e+09 | 14 |

**Adduct Signal Fractions**

- **Unmodified**: 1.2819e+09

### RGSN(N)NSFFSR Fuc 1 Hex 4 HexNAc 6

**Mass:**  3183.3052

(2.800e-06)

**Charge States:** 3

**Start Position**: 157

**End Position**: 167

| Profile Score | Tandem Score | TDA q-value | Volume | Spectrum Matches |
| --- | --- | --- | --- | --- |
| 13.5674 | 121.9199 | 0.0000 | 1.4147e+07 | 5 |

**Adduct Signal Fractions**

- **Unmodified**: 1.4147e+07

### YPVLN(N)VTM(O)PNN(D)GKFDK Hex 8 HexNAc 2

**Mass:**  3555.4887

(2.784e-04)

**Charge States:** 3, 4

**Start Position**: 177

**End Position**: 193

| Profile Score | Tandem Score | TDA q-value | Volume | Spectrum Matches |
| --- | --- | --- | --- | --- |
| 12.7972 | 120.6336 | 0.0000 | 1.7714e+08 | 7 |

**Adduct Signal Fractions**

- **Unmodified**: 1.7714e+08

### N(N)GTYDHDVYR Fuc 1 Hex 3 HexNAc 5

**Mass:**  2886.1660

(7.346e-06)

**Charge States:** 3

**Start Position**: 499

**End Position**: 509

| Profile Score | Tandem Score | TDA q-value | Volume | Spectrum Matches |
| --- | --- | --- | --- | --- |
| -4.5478 | 119.5887 | 0.0000 | 7.3376e+06 | 1 |

**Adduct Signal Fractions**

- **Unmodified**: 7.3376e+06

### N(N)GTYDHDVYRDEALNNR Fuc 1 Hex 3 HexNAc 4

**Mass:**  3495.4519

(2.480e-06)

**Charge States:** 3, 4

**Start Position**: 499

**End Position**: 516

| Profile Score | Tandem Score | TDA q-value | Volume | Spectrum Matches |
| --- | --- | --- | --- | --- |
| 16.9727 | 117.6863 | 0.0000 | 1.7347e+08 | 5 |

**Adduct Signal Fractions**

- **Unmodified**: 1.7347e+08

### N(N)GTYDHDVYRDEALNNR Fuc 2 Hex 4 HexNAc 4

**Mass:**  3803.5632

(2.438e-06)

**Charge States:** 3, 4

**Start Position**: 499

**End Position**: 516

| Profile Score | Tandem Score | TDA q-value | Volume | Spectrum Matches |
| --- | --- | --- | --- | --- |
| -2.0048 | 116.8899 | 0.0000 | 1.8453e+08 | 5 |

**Adduct Signal Fractions**

- **Unmodified**: 1.8453e+08

### YPVLN(N)VTM(O)PNN(D)GK Hex 10 HexNAc 2

**Mass:**  3489.4099

(2.842e-04)

**Charge States:** 3

**Start Position**: 177

**End Position**: 190

| Profile Score | Tandem Score | TDA q-value | Volume | Spectrum Matches |
| --- | --- | --- | --- | --- |
| 10.8788 | 116.7710 | 0.0000 | 3.4059e+07 | 3 |

**Adduct Signal Fractions**

- **Unmodified**: 3.4059e+07

### RGSN(N)NSFFSR Hex 3 HexNAc 6

**Mass:**  2875.1951

(2.045e-06)

**Charge States:** 3

**Start Position**: 157

**End Position**: 167

| Profile Score | Tandem Score | TDA q-value | Volume | Spectrum Matches |
| --- | --- | --- | --- | --- |
| -3.0588 | 115.1551 | 0.0000 | 3.5644e+07 | 3 |

**Adduct Signal Fractions**

- **Unmodified**: 3.5644e+07

### TITNDQIEVTN(N)ATELVQSSSTGR Hex 3 HexNAc 4

**Mass:**  3761.6962

(2.967e-06)

**Charge States:** 3, 4

**Start Position**: 44

**End Position**: 67

| Profile Score | Tandem Score | TDA q-value | Volume | Spectrum Matches |
| --- | --- | --- | --- | --- |
| 14.0950 | 114.4476 | 0.0000 | 2.9986e+08 | 8 |

**Adduct Signal Fractions**

- **Unmodified**: 2.9986e+08

### N(N)GTYDHDVYR Fuc 3 Hex 5 HexNAc 4

**Mass:**  3299.3007

(4.206e-06)

**Charge States:** 3

**Start Position**: 499

**End Position**: 509

| Profile Score | Tandem Score | TDA q-value | Volume | Spectrum Matches |
| --- | --- | --- | --- | --- |
| -4.5390 | 113.6848 | 0.0000 | 7.2937e+06 | 2 |

**Adduct Signal Fractions**

- **Unmodified**: 7.2937e+06

### YPVLN(N)VTM(O)PNN Hex 8 HexNAc 2

**Mass:**  2979.2016

(2.968e-06)

**Charge States:** 3

**Start Position**: 177

**End Position**: 188

| Profile Score | Tandem Score | TDA q-value | Volume | Spectrum Matches |
| --- | --- | --- | --- | --- |
| -4.3607 | 112.9139 | 0.0000 | 3.7934e+06 | 2 |

**Adduct Signal Fractions**

- **Unmodified**: 3.7934e+06

### TITNDQIEVTN(N)ATELVQSSSTGR Hex 7 HexNAc 2

**Mass:**  4003.7477

(3.623e-06)

**Charge States:** 3, 4

**Start Position**: 44

**End Position**: 67

| Profile Score | Tandem Score | TDA q-value | Volume | Spectrum Matches |
| --- | --- | --- | --- | --- |
| 14.0488 | 112.7650 | 0.0000 | 8.5226e+08 | 14 |

**Adduct Signal Fractions**

- **Unmodified**: 8.5226e+08

### TITNDQIEVTN(N)ATELVQSSSTGR Hex 5 HexNAc 3

**Mass:**  3882.7194

(2.909e-06)

**Charge States:** 3, 4

**Start Position**: 44

**End Position**: 67

| Profile Score | Tandem Score | TDA q-value | Volume | Spectrum Matches |
| --- | --- | --- | --- | --- |
| 13.1800 | 112.7269 | 0.0000 | 4.1893e+08 | 11 |

**Adduct Signal Fractions**

- **Unmodified**: 4.1893e+08

### TITNDQIEVTN(N)ATELVQSSSTGR Fuc 1 Hex 6 HexNAc 3

**Mass:**  4190.8352

(3.213e-06)

**Charge States:** 3, 4

**Start Position**: 44

**End Position**: 67

| Profile Score | Tandem Score | TDA q-value | Volume | Spectrum Matches |
| --- | --- | --- | --- | --- |
| 14.2623 | 112.5539 | 0.0000 | 4.6196e+08 | 11 |

**Adduct Signal Fractions**

- **Unmodified**: 4.6196e+08

### N(N)GTYDHDVYRDEALNNR Fuc 3 Hex 5 HexNAc 4

**Mass:**  4111.6769

(3.025e-06)

**Charge States:** 4

**Start Position**: 499

**End Position**: 516

| Profile Score | Tandem Score | TDA q-value | Volume | Spectrum Matches |
| --- | --- | --- | --- | --- |
| -2.3405 | 111.5813 | 0.0000 | 9.4439e+07 | 3 |

**Adduct Signal Fractions**

- **Unmodified**: 9.4439e+07

### N(N)GTYDHDVYR Fuc 1 Hex 3 HexNAc 4

**Mass:**  2683.0856

(7.319e-06)

**Charge States:** 3

**Start Position**: 499

**End Position**: 509

| Profile Score | Tandem Score | TDA q-value | Volume | Spectrum Matches |
| --- | --- | --- | --- | --- |
| -5.4408 | 111.4926 | 0.0000 | 4.3108e+06 | 2 |

**Adduct Signal Fractions**

- **Unmodified**: 4.3108e+06

### RGSN(N)NSFFSR Fuc 3 Hex 5 HexNAc 4

**Mass:**  3231.3187

(1.622e-06)

**Charge States:** 3

**Start Position**: 157

**End Position**: 167

| Profile Score | Tandem Score | TDA q-value | Volume | Spectrum Matches |
| --- | --- | --- | --- | --- |
| -16.7539 | 110.5630 | 0.0000 | 5.0772e+06 | 2 |

**Adduct Signal Fractions**

- **Unmodified**: 5.0772e+06

### TITNDQIEVTN(N)ATELVQSSSTGR Fuc 2 Hex 5 HexNAc 4

**Mass:**  4377.9206

(3.243e-06)

**Charge States:** 4

**Start Position**: 44

**End Position**: 67

| Profile Score | Tandem Score | TDA q-value | Volume | Spectrum Matches |
| --- | --- | --- | --- | --- |
| -4.7028 | 110.1107 | 0.0000 | 6.9803e+07 | 2 |

**Adduct Signal Fractions**

- **Unmodified**: 6.9803e+07

### RGSN(N)NSFFSR Fuc 2 Hex 4 HexNAc 4

**Mass:**  2923.2042

(6.396e-06)

**Charge States:** 3

**Start Position**: 157

**End Position**: 167

| Profile Score | Tandem Score | TDA q-value | Volume | Spectrum Matches |
| --- | --- | --- | --- | --- |
| -4.2862 | 110.0449 | 0.0000 | 8.5183e+06 | 3 |

**Adduct Signal Fractions**

- **Unmodified**: 8.5183e+06

### SDAPIGTC(C)SSEC(C)ITPN(N)GSIPNDKPFQNVNK Hex 9 HexNAc 2

**Mass:**  5111.1394

(2.586e-06)

**Charge States:** 3, 4, 5

**Start Position**: 286

**End Position**: 316

| Profile Score | Tandem Score | TDA q-value | Volume | Spectrum Matches |
| --- | --- | --- | --- | --- |
| 11.9729 | 109.3893 | 0.0000 | 2.4469e+08 | 5 |

**Adduct Signal Fractions**

- **Unmodified**: 2.4469e+08

### N(N)GTYDHDVYR Fuc 2 Hex 4 HexNAc 5

**Mass:**  3194.2714

(4.514e-06)

**Charge States:** 3

**Start Position**: 499

**End Position**: 509

| Profile Score | Tandem Score | TDA q-value | Volume | Spectrum Matches |
| --- | --- | --- | --- | --- |
| -4.3008 | 108.7660 | 0.0000 | 5.3815e+06 | 2 |

**Adduct Signal Fractions**

- **Unmodified**: 5.3815e+06

### LNWLYESESKYPVLN(N)VTM(O)PNN(D)GK Hex 9 HexNAc 2

**Mass:**  4576.9504

(2.169e-04)

**Charge States:** 3, 4

**Start Position**: 167

**End Position**: 190

| Profile Score | Tandem Score | TDA q-value | Volume | Spectrum Matches |
| --- | --- | --- | --- | --- |
| 8.7089 | 108.2078 | 0.0000 | 3.2755e+07 | 6 |

**Adduct Signal Fractions**

- **Unmodified**: 3.2755e+07

### SDAPIGTC(C)SSEC(C)ITPN(N)GSIPNDKPFQNVNK Fuc 2 Hex 3 HexNAc 4 Neu5Ac 1

**Mass:**  5128.1618

(-3.376e-06)

**Charge States:** 4

**Start Position**: 286

**End Position**: 316

| Profile Score | Tandem Score | TDA q-value | Volume | Spectrum Matches |
| --- | --- | --- | --- | --- |
| -4.0599 | 107.8392 | 0.0000 | 7.5833e+07 | 2 |

**Adduct Signal Fractions**

- **Unmodified**: 7.5833e+07

### N(N)GTYDHDVYR Fuc 4 Hex 6 HexNAc 5

**Mass:**  3810.4909

(3.596e-06)

**Charge States:** 3

**Start Position**: 499

**End Position**: 509

| Profile Score | Tandem Score | TDA q-value | Volume | Spectrum Matches |
| --- | --- | --- | --- | --- |
| -20.2726 | 107.7864 | 0.0000 | 7.1265e+05 | 1 |

**Adduct Signal Fractions**

- **Unmodified**: 7.1265e+05

### YPVLN(N)VTM(O)PNN(D)GK Fuc 1 Hex 3 HexNAc 5 Neu5Ac 1

**Mass:**  3401.3981

(2.835e-04)

**Charge States:** 3

**Start Position**: 177

**End Position**: 190

| Profile Score | Tandem Score | TDA q-value | Volume | Spectrum Matches |
| --- | --- | --- | --- | --- |
| 12.8497 | 107.5062 | 0.0000 | 2.6652e+08 | 7 |

**Adduct Signal Fractions**

- **Unmodified**: 2.6652e+08

### RGSN(N)N(D)SFFSR Hex 7 HexNAc 3 Neu5Ac 1

**Mass:**  3206.2403

(3.055e-04)

**Charge States:** 3

**Start Position**: 157

**End Position**: 167

| Profile Score | Tandem Score | TDA q-value | Volume | Spectrum Matches |
| --- | --- | --- | --- | --- |
| 9.9648 | 104.4332 | 0.0000 | 1.7382e+07 | 3 |

**Adduct Signal Fractions**

- **Unmodified**: 1.7382e+07

### N(N)GTYDHDVYR Fuc 1 Hex 7 HexNAc 2

**Mass:**  2925.0943

(-7.789e-06)

**Charge States:** 3

**Start Position**: 499

**End Position**: 509

| Profile Score | Tandem Score | TDA q-value | Volume | Spectrum Matches |
| --- | --- | --- | --- | --- |
| -3.7323 | 103.6975 | 0.0000 | 4.1686e+06 | 2 |

**Adduct Signal Fractions**

- **Unmodified**: 4.1686e+06

### SDAPIGTC(C)SSEC(C)ITPN(N)GSIPNDKPFQNVNK Hex 7 HexNAc 2

**Mass:**  4787.0353

(2.665e-06)

**Charge States:** 4

**Start Position**: 286

**End Position**: 316

| Profile Score | Tandem Score | TDA q-value | Volume | Spectrum Matches |
| --- | --- | --- | --- | --- |
| 14.0454 | 103.6476 | 0.0000 | 6.4323e+07 | 2 |

**Adduct Signal Fractions**

- **Unmodified**: 6.4323e+07

### N(N)GTYDHDVYRDEALNNR Fuc 2 Hex 4 HexNAc 5

**Mass:**  4006.6447

(2.643e-06)

**Charge States:** 4

**Start Position**: 499

**End Position**: 516

| Profile Score | Tandem Score | TDA q-value | Volume | Spectrum Matches |
| --- | --- | --- | --- | --- |
| -3.2104 | 102.6887 | 0.0085 | 7.3172e+07 | 3 |

**Adduct Signal Fractions**

- **Unmodified**: 7.3172e+07

### N(N)GTYDHDVYR Fuc 1 Hex 8 HexNAc 3

**Mass:**  3290.2227

(-8.415e-06)

**Charge States:** 3

**Start Position**: 499

**End Position**: 509

| Profile Score | Tandem Score | TDA q-value | Volume | Spectrum Matches |
| --- | --- | --- | --- | --- |
| -20.7197 | 102.6321 | 0.0085 | 9.7964e+05 | 1 |

**Adduct Signal Fractions**

- **Unmodified**: 9.7964e+05

### YPVLN(N)VTM(O)PNN(D)GK Hex 6 HexNAc 2

**Mass:**  2841.1887

(3.478e-04)

**Charge States:** 3

**Start Position**: 177

**End Position**: 190

| Profile Score | Tandem Score | TDA q-value | Volume | Spectrum Matches |
| --- | --- | --- | --- | --- |
| -7.0327 | 102.5937 | 0.0085 | 6.0924e+06 | 1 |

**Adduct Signal Fractions**

- **Unmodified**: 6.0924e+06

### RGSN(N)NSFFSR Fuc 2 Hex 5 HexNAc 6

**Mass:**  3493.4254

(5.775e-04)

**Start Position**: 157

**End Position**: 167

| Profile Score | Tandem Score | TDA q-value | Volume | Spectrum Matches |
| --- | --- | --- | --- | --- |
| 0.0000 | 101.8573 | 0.0085 | 0.0000e+00 | 1 |

Adduct:

No Chromatogram Found

### N(N)GTYDHDVYRDEALNNR Fuc 4 Hex 6 HexNAc 5

**Mass:**  4622.8630

(2.079e-06)

**Charge States:** 4

**Start Position**: 499

**End Position**: 516

| Profile Score | Tandem Score | TDA q-value | Volume | Spectrum Matches |
| --- | --- | --- | --- | --- |
| -3.8252 | 100.8337 | 0.0085 | 1.3912e+07 | 2 |

**Adduct Signal Fractions**

- **Unmodified**: 1.3912e+07

### N(N)GTYDHDVYRDEALNNR Fuc 3 Hex 5 HexNAc 5

**Mass:**  4314.7565

(1.180e-06)

**Charge States:** 4

**Start Position**: 499

**End Position**: 516

| Profile Score | Tandem Score | TDA q-value | Volume | Spectrum Matches |
| --- | --- | --- | --- | --- |
| -3.4215 | 99.7723 | 0.0085 | 4.5218e+07 | 2 |

**Adduct Signal Fractions**

- **Unmodified**: 4.5218e+07

### N(N)GTYDHDVYR Fuc 2 Hex 8 HexNAc 3

**Mass:**  3436.2852

(-6.699e-06)

**Charge States:** 3

**Start Position**: 499

**End Position**: 509

| Profile Score | Tandem Score | TDA q-value | Volume | Spectrum Matches |
| --- | --- | --- | --- | --- |
| -20.9033 | 99.6855 | 0.0085 | 1.3780e+06 | 1 |

**Adduct Signal Fractions**

- **Unmodified**: 1.3780e+06

### N(N)GTYDHDVYR Fuc 1 Hex 8 HexNAc 4

**Mass:**  3494.2780

(2.804e-04)

**Charge States:** 3

**Start Position**: 499

**End Position**: 509

| Profile Score | Tandem Score | TDA q-value | Volume | Spectrum Matches |
| --- | --- | --- | --- | --- |
| 9.2552 | 99.0757 | 0.0085 | 3.0329e+07 | 5 |

**Adduct Signal Fractions**

- **Unmodified**: 3.0329e+07

### RGSN(N)N(D)SFFSR Fuc 1 Hex 7 HexNAc 3 Neu5Ac 1

**Mass:**  3352.2965

(2.931e-04)

**Charge States:** 3

**Start Position**: 157

**End Position**: 167

| Profile Score | Tandem Score | TDA q-value | Volume | Spectrum Matches |
| --- | --- | --- | --- | --- |
| -6.6086 | 96.5231 | 0.0085 | 8.3234e+06 | 2 |

**Adduct Signal Fractions**

- **Unmodified**: 8.3234e+06

### RGSN(N)N(D)SFFSR Fuc 3 Hex 5 HexNAc 5

**Mass:**  3436.4065

(5.878e-04)

**Start Position**: 157

**End Position**: 167

| Profile Score | Tandem Score | TDA q-value | Volume | Spectrum Matches |
| --- | --- | --- | --- | --- |
| 0.0000 | 94.8950 | 0.0085 | 0.0000e+00 | 1 |

Adduct:

No Chromatogram Found

### N(N)GTYDHDVYR Fuc 1 Hex 7 HexNAc 3

**Mass:**  3128.1703

(-8.626e-06)

**Charge States:** 3

**Start Position**: 499

**End Position**: 509

| Profile Score | Tandem Score | TDA q-value | Volume | Spectrum Matches |
| --- | --- | --- | --- | --- |
| -19.9970 | 94.5254 | 0.0160 | 2.0381e+06 | 1 |

**Adduct Signal Fractions**

- **Unmodified**: 2.0381e+06

### N(N)GTYDHDVYR Fuc 2 Hex 7 HexNAc 3

**Mass:**  3274.2306

(-7.498e-06)

**Charge States:** 3

**Start Position**: 499

**End Position**: 509

| Profile Score | Tandem Score | TDA q-value | Volume | Spectrum Matches |
| --- | --- | --- | --- | --- |
| -20.8330 | 94.4277 | 0.0160 | 1.0972e+06 | 1 |

**Adduct Signal Fractions**

- **Unmodified**: 1.0972e+06

### N(N)GTYDHDVYRDEALNNR Fuc 1 Hex 3 HexNAc 6

**Mass:**  3901.6093

(3.139e-06)

**Charge States:** 4

**Start Position**: 499

**End Position**: 516

| Profile Score | Tandem Score | TDA q-value | Volume | Spectrum Matches |
| --- | --- | --- | --- | --- |
| 11.0217 | 94.1286 | 0.0160 | 2.6564e+07 | 3 |

**Adduct Signal Fractions**

- **Unmodified**: 2.6564e+07

### RGSN(N)NSFFSR Hex 3 HexNAc 5

**Mass:**  2672.1168

(2.982e-06)

**Charge States:** 3

**Start Position**: 157

**End Position**: 167

| Profile Score | Tandem Score | TDA q-value | Volume | Spectrum Matches |
| --- | --- | --- | --- | --- |
| -5.1783 | 93.7212 | 0.0160 | 6.8197e+06 | 2 |

**Adduct Signal Fractions**

- **Unmodified**: 6.8197e+06

### RGSN(N)NSFFSR Fuc 2 Hex 5 HexNAc 5

**Mass:**  3288.3429

(4.024e-06)

**Charge States:** 3

**Start Position**: 157

**End Position**: 167

| Profile Score | Tandem Score | TDA q-value | Volume | Spectrum Matches |
| --- | --- | --- | --- | --- |
| -6.0653 | 93.0608 | 0.0160 | 2.6403e+06 | 2 |

**Adduct Signal Fractions**

- **Unmodified**: 2.6403e+06

### RGSN(N)NSFFSR Fuc 1 Hex 4 HexNAc 5

**Mass:**  2980.2268

(2.957e-06)

**Charge States:** 3

**Start Position**: 157

**End Position**: 167

| Profile Score | Tandem Score | TDA q-value | Volume | Spectrum Matches |
| --- | --- | --- | --- | --- |
| 10.7681 | 92.9745 | 0.0219 | 8.8359e+06 | 3 |

**Adduct Signal Fractions**

- **Unmodified**: 8.8359e+06

### N(N)GTYDHDVYRDEALNNR Fuc 1 Hex 4 HexNAc 5

**Mass:**  3860.5888

(1.519e-06)

**Charge States:** 3, 4

**Start Position**: 499

**End Position**: 516

| Profile Score | Tandem Score | TDA q-value | Volume | Spectrum Matches |
| --- | --- | --- | --- | --- |
| 12.9209 | 92.8453 | 0.0219 | 1.5430e+08 | 6 |

**Adduct Signal Fractions**

- **Unmodified**: 1.5430e+08

### N(N)GTYDHDVYRDEALNNR Fuc 1 Hex 7 HexNAc 2

**Mass:**  3737.4623

(-9.348e-06)

**Charge States:** 3, 4

**Start Position**: 499

**End Position**: 516

| Profile Score | Tandem Score | TDA q-value | Volume | Spectrum Matches |
| --- | --- | --- | --- | --- |
| -3.9602 | 91.2313 | 0.0219 | 1.8742e+07 | 4 |

**Adduct Signal Fractions**

- **Unmodified**: 1.8742e+07

### N(N)GTYDHDVYRDEALNNR Fuc 2 Hex 4 HexNAc 6

**Mass:**  4209.7228

(2.617e-06)

**Charge States:** 4

**Start Position**: 499

**End Position**: 516

| Profile Score | Tandem Score | TDA q-value | Volume | Spectrum Matches |
| --- | --- | --- | --- | --- |
| 12.1154 | 89.1023 | 0.0219 | 2.2427e+07 | 3 |

**Adduct Signal Fractions**

- **Unmodified**: 2.2427e+07

### N(N)GTYDHDVYRDEALNNR Fuc 1 Hex 8 HexNAc 3

**Mass:**  4102.5944

(-8.160e-06)

**Charge States:** 3

**Start Position**: 499

**End Position**: 516

| Profile Score | Tandem Score | TDA q-value | Volume | Spectrum Matches |
| --- | --- | --- | --- | --- |
| -5.2729 | 87.4232 | 0.0357 | 1.0542e+07 | 1 |

**Adduct Signal Fractions**

- **Unmodified**: 1.0542e+07

### N(N)GTYDHDVYRDEALNNR Fuc 1 Hex 7 HexNAc 3

**Mass:**  3940.5395

(-9.045e-06)

**Charge States:** 3, 4

**Start Position**: 499

**End Position**: 516

| Profile Score | Tandem Score | TDA q-value | Volume | Spectrum Matches |
| --- | --- | --- | --- | --- |
| 8.8670 | 86.9952 | 0.0357 | 2.6667e+07 | 5 |

**Adduct Signal Fractions**

- **Unmodified**: 2.6667e+07

### TITNDQIEVTN(N)ATELVQSSSTGR Hex 5 HexNAc 2

**Mass:**  3679.6422

(2.072e-06)

**Charge States:** 3, 4

**Start Position**: 44

**End Position**: 67

| Profile Score | Tandem Score | TDA q-value | Volume | Spectrum Matches |
| --- | --- | --- | --- | --- |
| 14.0142 | 85.8937 | 0.0476 | 4.6603e+08 | 9 |

**Adduct Signal Fractions**

- **Unmodified**: 4.6603e+08

### N(N)GTYDHDVYR Fuc 1 Hex 4 HexNAc 5

**Mass:**  3048.2157

(7.850e-06)

**Charge States:** 3

**Start Position**: 499

**End Position**: 509

| Profile Score | Tandem Score | TDA q-value | Volume | Spectrum Matches |
| --- | --- | --- | --- | --- |
| 5.0428 | 84.9989 | 0.0476 | 9.4679e+06 | 3 |

**Adduct Signal Fractions**

- **Unmodified**: 9.4679e+06

### iav|ADJ41819|A/Phil/2-MA/1982\_NA

**>iav|ADJ41819|A/Phil/2-MA/1982\_NA**  
 `MNPNQKIITIGSVSLTIATICFLMQIAILVTTVTLHFKQYECSSPPNNQVVPCEPIIIERNITEIVYLTNTTIEKEICPK  
LVEYRNWSKPQCKITGFAPFSKDNSIRLSAGGDIWVTREPYVSCDPGKCYQFALGQGTTLDNKHSNDTIHDRTPYRTLLM  
NELGVPFHLGTRQVCIAWSSSSCHDGKAWLHVCITGYDKNATASFIYDGRLVDSIGSWSKNILRTQESECVCINGTCTVV  
MTDGSASERADTKILFIEEGKIVHISPLSGSAQHVEECSCYPRYPGVRCVCRDNWKGSNRPVVDINVKDYSIVSSYVCSG  
LVGDTPRKNDRSSSSYCRNPNNEKGNHGVKGWAFDDGNDVWMGRTISEESRSGYETFKVIGGWSTPNSKLQINRQVIVDS  
GNRSGYSGIFSVEGKSCINRCFYVELIRGREQETRVWWTSNSIVVFCGTSGTYGTGSWPDGADINLMPI`

| Site | Type | Count |
| --- | --- | --- |
| 402 | N-linked | 1 |
| 200 | N-linked | 3 |

| Mass | Sequence | MS_2_ Score | `q`-value | MS_1_ Score | Signal | Protein Coordinates | Tandem Scans | Start Time | Apex Time | End Time |
| --- | --- | --- | --- | --- | --- | --- | --- | --- | --- | --- |
| 2364.9980 | QVIVDSGN(N)R Hex 6 HexNAc 2 | 133.483 | 0.0 | -20.264 | 3.568e+06 | 394 - 403 | 3 | 19.48 | 19.48 | 19.65 |
| 2916.1590 | N(N)ATASFIYDGR Hex 8 HexNAc 2 | 122.568 | 0.0 | 14.850 | 3.718e+07 | 199 - 210 | 5 | 25.10 | 25.44 | 25.95 |
| 2754.1062 | N(N)ATASFIYDGR Hex 7 HexNAc 2 | 118.107 | 0.0 | 11.794 | 1.365e+07 | 199 - 210 | 3 | 25.44 | 25.61 | 26.12 |
| 3095.2404 | N(N)ATASFIYDGR Fuc 2 Hex 3 HexNAc 4 Neu5Ac 1 | 109.124 | 0.0 | -19.336 | 2.453e+06 | 199 - 210 | 1 | 25.10 | 25.27 | 25.27 |

### QVIVDSGN(N)R Hex 6 HexNAc 2

**Mass:**  2364.9994

(7.126e-06)

**Charge States:** 3

**Start Position**: 395

**End Position**: 404

| Profile Score | Tandem Score | TDA q-value | Volume | Spectrum Matches |
| --- | --- | --- | --- | --- |
| -20.2643 | 133.4827 | 0.0000 | 3.5681e+06 | 3 |

**Adduct Signal Fractions**

- **Unmodified**: 3.5681e+06

### N(N)ATASFIYDGR Hex 8 HexNAc 2

**Mass:**  2916.1591

(1.007e-06)

**Charge States:** 3

**Start Position**: 200

**End Position**: 211

| Profile Score | Tandem Score | TDA q-value | Volume | Spectrum Matches |
| --- | --- | --- | --- | --- |
| 14.8501 | 122.5677 | 0.0000 | 3.7185e+07 | 5 |

**Adduct Signal Fractions**

- **Unmodified**: 3.7185e+07

### N(N)ATASFIYDGR Hex 7 HexNAc 2

**Mass:**  2754.1054

(1.942e-06)

**Charge States:** 3

**Start Position**: 200

**End Position**: 211

| Profile Score | Tandem Score | TDA q-value | Volume | Spectrum Matches |
| --- | --- | --- | --- | --- |
| 11.7940 | 118.1071 | 0.0000 | 1.3653e+07 | 3 |

**Adduct Signal Fractions**

- **Unmodified**: 1.3653e+07

### N(N)ATASFIYDGR Fuc 2 Hex 3 HexNAc 4 Neu5Ac 1

**Mass:**  3095.2395

(-6.642e-06)

**Charge States:** 3

**Start Position**: 200

**End Position**: 211

| Profile Score | Tandem Score | TDA q-value | Volume | Spectrum Matches |
| --- | --- | --- | --- | --- |
| -19.3362 | 109.1243 | 0.0000 | 2.4531e+06 | 1 |

**Adduct Signal Fractions**

- **Unmodified**: 2.4531e+06
