# Supplementary material for: Measuring Site-specific Glycosylation Similarity between Influenza a Virus Variants with Statistical Certainty
Source: Mol Cell Proteomics. 2020 Nov 25;19(9):1533–45. doi: 10.1074/mcp.RA120.002031 (PMC8143645; doi:10.1074/mcp.RA120.002031)
Supplement: Supplementary file 2 [file mmc2.zip › mmc2/159250_2_supp_555290_qcjklq.zip!/20190326_Phil82gal_032219_tryp_HILIC_DDA_1ug_03_glycopeptides.html]

## 20190326\_Phil82gal\_032219\_tryp\_HILIC\_DDA\_1ug\_03 @ GlycopeptideHypothesis-d37267e4f4fa4a6fa9f5536c5d50c490

**MS^1^ Mass Accuracy:** 10.0 PPM  
**Grouping Tolerance:** 15.0 PPM  
**MS^n^ Mass Accuracy:** 20.0 PPM  
**Minimum `q`-Value For Clustering:** 0.05

**Glycopeptide Hypothesis:**
GlycopeptideHypothesis-d37267e4f4fa4a6fa9f5536c5d50c490  
**Sample Searched:**
20190326\_Phil82gal\_032219\_tryp\_HILIC\_DDA\_1ug\_03

| Protein | Matched Glycopeptides |
| --- | --- |
| iav\|AFG99160\|A/Phil/2/1982\_HA | 82 |
| iav\|ADJ41819\|A/Phil/2-MA/1982\_NA | 3 |

### iav|AFG99160|A/Phil/2/1982\_HA

**>iav|AFG99160|A/Phil/2/1982\_HA**  
 `MKTIIALSYMFCLVFAQNLPGNDNSTATLCLGHHAVPNGTLVKTITNDQIEVTNATELVQSSSTGRICDSPHRILDGKNC  
TLIDALLGDPHCDGFQNEKWDLFVERSKAFSNCYPYDVPDYASLRSLVASSGTLEFINEGFNWTGVTQSGGSSTCKRGSN  
NSFFSRLNWLYESESKYPVLNVTMPNNGKFDKLYIWGIHHPSTDKEQTNLYIRASGRVTVSTKRSQQTVIPNIGSRPWVR  
GLSSRISIYWTIVKPGDILLINSTGNLIAPRGYFKIRTGKSSIMRSDAPIGTCSSECITPNGSIPNDKPFQNVNKITYGA  
CPRYVKQNTLKLATGMRNVPEKQTRGIFGAIAGFIENGWEGMVDGWYGFRHQNSEGTGQAADLKSTQAAIDQINGKLNRV  
IEKTNEKFHQIEKEFSEVEGRIQDLEKYVEDTKIDLWSYNAELLVALENQHTIDLTDSEMNKLFEKTRKQLRENAEDMGN  
GCFKIYHKCDNACIGSIRNGTYDHDVYRDEALNNRFQIKGVELKSGYKDWILWISFAISCFLLCVVLLGFIMWACQKGNI  
RCNICI`

| Site | Type | Count |
| --- | --- | --- |
| 262 | N-linked | 5 |
| 301 | N-linked | 3 |
| 499 | N-linked | 27 |
| 181 | N-linked | 13 |
| 54 | N-linked | 12 |
| 160 | N-linked | 22 |

| Mass | Sequence | MS_2_ Score | `q`-value | MS_1_ Score | Signal | Protein Coordinates | Tandem Scans | Start Time | Apex Time | End Time |
| --- | --- | --- | --- | --- | --- | --- | --- | --- | --- | --- |
| 4068.9280 | TIVKPGDILLIN(N)STGNLIAPR Hex 9 HexNAc 2 | 156.913 | 0.0 | 14.791 | 3.080e+08 | 250 - 271 | 18 | 51.09 | 51.82 | 53.99 |
| 2818.1737 | RGSN(N)NSFFSR Fuc 1 Hex 3 HexNAc 5 | 147.264 | 0.0 | 14.582 | 1.024e+08 | 156 - 166 | 4 | 16.89 | 17.40 | 17.74 |
| 3327.3510 | YPVLN(N)VTM(O)PNN(D)GK Hex 9 HexNAc 2 | 142.720 | 0.0 | 10.886 | 9.987e+08 | 176 - 189 | 6 | 28.46 | 28.79 | 29.46 |
| 3627.6325 | PGDILLIN(N)STGNLIAPR Hex 9 HexNAc 2 | 141.856 | 0.0 | 10.615 | 1.479e+07 | 254 - 271 | 5 | 49.71 | 49.88 | 50.48 |
| 2819.1630 | RGSN(N)N(D)SFFSR Fuc 1 Hex 3 HexNAc 5 | 139.484 | 0.0 | 7.918 | 3.493e+07 | 156 - 166 | 1 | 17.91 | 17.91 | 18.93 |
| 3344.3770 | YPVLN(N)VTM(O)PNN(D)GK Fuc 2 Hex 3 HexNAc 4 Neu5Ac 1 | 139.362 | 0.0 | 11.639 | 2.643e+08 | 176 - 189 | 3 | 28.46 | 28.96 | 29.46 |
| 2662.0691 | GSN(N)NSFFSR Fuc 1 Hex 3 HexNAc 5 | 139.015 | 0.0 | 13.250 | 2.101e+08 | 157 - 166 | 5 | 20.13 | 20.81 | 21.15 |
| 4230.9787 | TIVKPGDILLIN(N)STGNLIAPR Hex 10 HexNAc 2 | 137.399 | 0.0 | 13.774 | 3.222e+07 | 250 - 271 | 5 | 51.46 | 51.78 | 52.15 |
| 4085.9542 | TIVKPGDILLIN(N)STGNLIAPR Fuc 2 Hex 3 HexNAc 4 Neu5Ac 1 | 132.429 | 0.0 | 12.915 | 4.004e+07 | 250 - 271 | 5 | 51.54 | 51.78 | 52.34 |
| 2991.2024 | N(N)GTYDHDVYR Fuc 2 Hex 4 HexNAc 4 | 132.068 | 0.0 | 13.739 | 3.131e+07 | 498 - 508 | 2 | 13.00 | 13.17 | 13.68 |
| 3165.2972 | YPVLN(N)VTM(O)PNN(D)GK Hex 8 HexNAc 2 | 130.788 | 0.0 | 11.375 | 5.643e+08 | 176 - 189 | 5 | 28.63 | 28.96 | 29.79 |
| 3126.2858 | RGSN(N)NSFFSR Fuc 2 Hex 4 HexNAc 5 | 127.574 | 0.0 | 13.559 | 3.137e+07 | 156 - 166 | 3 | 16.55 | 16.89 | 17.23 |
| 3803.5636 | N(N)GTYDHDVYRDEALNNR Fuc 2 Hex 4 HexNAc 4 | 127.113 | 0.0 | -2.013 | 2.012e+08 | 498 - 515 | 4 | 18.08 | 18.42 | 18.59 |
| 3698.5298 | N(N)GTYDHDVYRDEALNNR Fuc 1 Hex 3 HexNAc 5 | 126.500 | 0.0 | 20.584 | 1.441e+08 | 498 - 515 | 4 | 18.25 | 18.59 | 18.93 |
| 3003.2434 | YPVLN(N)VTM(O)PNN(D)GK Hex 7 HexNAc 2 | 125.600 | 0.0 | 11.021 | 6.905e+07 | 176 - 189 | 4 | 28.63 | 29.12 | 29.79 |
| 2970.1822 | GSN(N)NSFFSR Fuc 2 Hex 4 HexNAc 5 | 124.098 | 0.0 | 13.336 | 7.144e+07 | 157 - 166 | 3 | 19.62 | 20.13 | 20.30 |
| 3646.6651 | PGDILLIN(N)STGNLIAPR Fuc 2 Hex 3 HexNAc 4 Neu5Ac 1 | 124.020 | 0.0 | 0.000 | 0.000e+00 | 254 - 271 | 1 | - | - | - |
| 3717.5431 | YPVLN(N)VTM(O)PNN(D)GKFDK Hex 9 HexNAc 2 | 123.684 | 0.0 | 13.300 | 3.414e+08 | 176 - 192 | 8 | 31.68 | 32.19 | 32.87 |
| 3425.3210 | RGSN(N)NSFFSR Fuc 1 Hex 8 HexNAc 4 | 123.669 | 0.0 | 12.063 | 1.146e+07 | 156 - 166 | 2 | 18.76 | 19.10 | 19.44 |
| 3194.2792 | N(N)GTYDHDVYR Fuc 2 Hex 4 HexNAc 5 | 122.847 | 0.0 | -18.304 | 9.080e+06 | 498 - 508 | 2 | 13.17 | 13.17 | 13.34 |
| 4949.0869 | SDAPIGTC(C)SSEC(C)ITPN(N)GSIPNDKPFQNVNK Hex 8 HexNAc 2 | 122.190 | 0.0 | 13.238 | 2.776e+08 | 285 - 315 | 7 | 34.93 | 35.10 | 35.78 |
| 5111.1382 | SDAPIGTC(C)SSEC(C)ITPN(N)GSIPNDKPFQNVNK Hex 9 HexNAc 2 | 121.577 | 0.0 | 13.193 | 2.400e+08 | 285 - 315 | 5 | 34.93 | 35.10 | 35.78 |
| 3555.4901 | YPVLN(N)VTM(O)PNN(D)GKFDK Hex 8 HexNAc 2 | 120.928 | 0.0 | 11.537 | 1.854e+08 | 176 - 192 | 6 | 31.85 | 32.19 | 32.70 |
| 5128.1587 | SDAPIGTC(C)SSEC(C)ITPN(N)GSIPNDKPFQNVNK Fuc 2 Hex 3 HexNAc 4 Neu5Ac 1 | 117.814 | 0.0 | 11.549 | 8.171e+07 | 285 - 315 | 3 | 34.93 | 35.10 | 35.78 |
| 3141.2537 | YPVLN(N)VTM(O)PNN Hex 9 HexNAc 2 | 117.570 | 0.0 | 8.951 | 5.188e+06 | 176 - 187 | 3 | 32.53 | 32.53 | 33.22 |
| 4190.8330 | TITNDQIEVTN(N)ATELVQSSSTGR Fuc 1 Hex 6 HexNAc 3 | 117.348 | 0.0 | 13.544 | 4.325e+08 | 43 - 66 | 13 | 38.70 | 39.55 | 40.74 |
| 3495.4491 | N(N)GTYDHDVYRDEALNNR Fuc 1 Hex 3 HexNAc 4 | 117.299 | 0.0 | 16.678 | 1.706e+08 | 498 - 515 | 4 | 18.25 | 18.59 | 18.93 |
| 4622.8646 | N(N)GTYDHDVYRDEALNNR Fuc 4 Hex 6 HexNAc 5 | 115.876 | 0.0 | -4.820 | 1.428e+07 | 498 - 515 | 2 | 17.74 | 17.91 | 18.25 |
| 3489.4047 | YPVLN(N)VTM(O)PNN(D)GK Hex 10 HexNAc 2 | 114.772 | 0.0 | 9.878 | 3.617e+07 | 176 - 189 | 3 | 28.46 | 28.79 | 29.46 |
| 3231.3171 | RGSN(N)NSFFSR Fuc 3 Hex 5 HexNAc 4 | 114.335 | 0.0 | -3.709 | 5.652e+06 | 156 - 166 | 2 | 16.21 | 16.38 | 16.55 |
| 3412.4678 | DQIEVTN(N)ATELVQSSSTGR Hex 6 HexNAc 2 | 113.447 | 0.0 | -5.162 | 7.051e+06 | 47 - 66 | 1 | 36.30 | 36.47 | 36.64 |
| 2683.0894 | N(N)GTYDHDVYR Fuc 1 Hex 3 HexNAc 4 | 113.239 | 0.0 | -4.672 | 1.011e+07 | 498 - 508 | 1 | 13.34 | 13.34 | 13.68 |
| 2615.2543 | RGSN(N)NSFFSR Fuc 1 Hex 3 HexNAc 4 | 113.200 | 0.0 | 8.102 | 1.400e+07 | 156 - 166 | 4 | 17.23 | 17.23 | 17.91 |
| 4111.6757 | N(N)GTYDHDVYRDEALNNR Fuc 3 Hex 5 HexNAc 4 | 112.808 | 0.0 | -2.456 | 1.053e+08 | 498 - 515 | 4 | 17.74 | 18.08 | 18.25 |
| 2886.1721 | N(N)GTYDHDVYR Fuc 1 Hex 3 HexNAc 5 | 110.695 | 0.0 | -4.103 | 1.194e+07 | 498 - 508 | 1 | 13.34 | 13.51 | 13.68 |
| 2980.2261 | RGSN(N)NSFFSR Fuc 1 Hex 4 HexNAc 5 | 110.449 | 0.0 | 9.917 | 9.019e+06 | 156 - 166 | 3 | 16.72 | 17.40 | 17.57 |
| 4377.9174 | TITNDQIEVTN(N)ATELVQSSSTGR Fuc 2 Hex 5 HexNAc 4 | 110.165 | 0.0 | -5.012 | 6.716e+07 | 43 - 66 | 2 | 39.21 | 39.38 | 39.72 |
| 3679.6404 | TITNDQIEVTN(N)ATELVQSSSTGR Hex 5 HexNAc 2 | 109.352 | 0.0 | 15.451 | 4.652e+08 | 43 - 66 | 12 | 39.72 | 39.89 | 41.09 |
| 4006.6451 | N(N)GTYDHDVYRDEALNNR Fuc 2 Hex 4 HexNAc 5 | 109.202 | 0.0 | -3.069 | 7.759e+07 | 498 - 515 | 2 | 18.08 | 18.42 | 18.59 |
| 2841.1918 | YPVLN(N)VTM(O)PNN(D)GK Hex 6 HexNAc 2 | 109.200 | 0.0 | -6.482 | 3.035e+06 | 176 - 189 | 1 | 29.29 | 29.29 | 29.63 |
| 3299.3118 | N(N)GTYDHDVYR Fuc 3 Hex 5 HexNAc 4 | 109.010 | 0.0 | -4.776 | 1.391e+07 | 498 - 508 | 3 | 12.83 | 13.00 | 13.17 |
| 3158.2826 | YPVLN(N)VTM(O)PNN Fuc 2 Hex 3 HexNAc 4 Neu5Ac 1 | 108.723 | 0.0 | -6.573 | 1.699e+06 | 176 - 187 | 1 | 32.53 | 32.53 | 33.22 |
| 2875.1947 | RGSN(N)NSFFSR Hex 3 HexNAc 6 | 108.508 | 0.0 | -2.718 | 3.456e+07 | 156 - 166 | 2 | 17.23 | 17.57 | 17.74 |
| 2979.1992 | YPVLN(N)VTM(O)PNN Hex 8 HexNAc 2 | 106.612 | 0.0 | -4.398 | 2.608e+06 | 176 - 187 | 1 | 32.53 | 32.53 | 32.87 |
| 3274.2351 | N(N)GTYDHDVYR Fuc 2 Hex 7 HexNAc 3 | 105.001 | 0.0 | -20.998 | 9.890e+05 | 498 - 508 | 1 | 14.35 | 14.35 | 14.52 |
| 2925.1007 | N(N)GTYDHDVYR Fuc 1 Hex 7 HexNAc 2 | 104.913 | 0.008 | -4.913 | 5.380e+06 | 498 - 508 | 2 | 14.52 | 14.52 | 14.86 |
| 4003.7476 | TITNDQIEVTN(N)ATELVQSSSTGR Hex 7 HexNAc 2 | 104.520 | 0.008 | 13.822 | 8.212e+08 | 43 - 66 | 13 | 38.70 | 39.55 | 40.74 |
| 3514.3506 | RGSN(N)N(D)SFFSR Fuc 1 Hex 8 HexNAc 3 Neu5Ac 1 | 104.425 | 0.008 | -6.586 | 5.930e+06 | 156 - 166 | 1 | 18.25 | 18.42 | 18.59 |
| 3183.3071 | RGSN(N)NSFFSR Fuc 1 Hex 4 HexNAc 6 | 104.137 | 0.008 | -3.766 | 1.189e+07 | 156 - 166 | 2 | 16.72 | 16.89 | 17.23 |
| 3491.4173 | RGSN(N)NSFFSR Fuc 2 Hex 5 HexNAc 6 | 104.006 | 0.008 | -20.858 | 1.454e+06 | 156 - 166 | 1 | 16.38 | 16.55 | 16.55 |
| 3401.3996 | YPVLN(N)VTM(O)PNN(D)GK Fuc 1 Hex 3 HexNAc 5 Neu5Ac 1 | 103.698 | 0.008 | 11.588 | 2.611e+08 | 176 - 189 | 8 | 27.27 | 28.63 | 29.79 |
| 3502.3863 | N(N)GTYDHDVYR Fuc 3 Hex 5 HexNAc 5 | 103.622 | 0.008 | -19.220 | 4.679e+06 | 498 - 508 | 1 | 13.00 | 13.00 | 13.17 |
| 3841.6940 | TITNDQIEVTN(N)ATELVQSSSTGR Hex 6 HexNAc 2 | 103.160 | 0.008 | 14.011 | 1.268e+09 | 43 - 66 | 14 | 38.87 | 39.72 | 40.92 |
| 3571.3678 | RGSN(N)N(D)SFFSR Hex 8 HexNAc 4 Neu5Ac 1 | 102.879 | 0.008 | 8.094 | 9.673e+06 | 156 - 166 | 2 | 18.42 | 18.93 | 19.27 |
| 3128.1799 | N(N)GTYDHDVYR Fuc 1 Hex 7 HexNAc 3 | 101.398 | 0.008 | -5.640 | 3.084e+06 | 498 - 508 | 2 | 14.52 | 14.69 | 14.86 |
| 3435.3864 | RGSN(N)N(D)SFFSR Fuc 3 Hex 5 HexNAc 5 | 100.270 | 0.008 | -20.879 | 1.615e+06 | 156 - 166 | 2 | 16.72 | 16.89 | 16.89 |
| 2923.2090 | RGSN(N)NSFFSR Fuc 2 Hex 4 HexNAc 4 | 99.238 | 0.008 | -4.252 | 7.798e+06 | 156 - 166 | 3 | 16.55 | 16.89 | 17.06 |
| 3206.2371 | RGSN(N)N(D)SFFSR Hex 7 HexNAc 3 Neu5Ac 1 | 98.801 | 0.008 | 2.799 | 1.611e+07 | 156 - 166 | 4 | 18.76 | 18.93 | 19.79 |
| 3810.4923 | N(N)GTYDHDVYR Fuc 4 Hex 6 HexNAc 5 | 97.889 | 0.008 | -16.259 | 1.332e+06 | 498 - 508 | 1 | 12.83 | 12.83 | 12.83 |
| 3436.2880 | N(N)GTYDHDVYR Fuc 2 Hex 8 HexNAc 3 | 96.852 | 0.008 | -21.001 | 8.939e+05 | 498 - 508 | 1 | 14.18 | 14.18 | 14.35 |
| 4314.7553 | N(N)GTYDHDVYRDEALNNR Fuc 3 Hex 5 HexNAc 5 | 96.694 | 0.008 | -2.060 | 4.242e+07 | 498 - 515 | 3 | 17.91 | 18.08 | 18.25 |
| 3434.3964 | RGSN(N)NSFFSR Fuc 3 Hex 5 HexNAc 5 | 96.687 | 0.008 | -19.270 | 6.261e+06 | 156 - 166 | 2 | 16.38 | 16.38 | 16.55 |
| 3393.4364 | YPVLN(N)VTM(O)PNN(D)GKFDK Hex 7 HexNAc 2 | 96.042 | 0.008 | 11.495 | 1.636e+07 | 176 - 192 | 2 | 32.02 | 32.36 | 32.70 |
| 3737.4588 | N(N)GTYDHDVYRDEALNNR Fuc 1 Hex 7 HexNAc 2 | 95.232 | 0.008 | -4.332 | 2.253e+07 | 498 - 515 | 4 | 20.30 | 20.46 | 20.81 |
| 3397.3515 | N(N)GTYDHDVYR Fuc 2 Hex 4 HexNAc 6 | 95.092 | 0.008 | -21.126 | 5.578e+05 | 498 - 508 | 1 | 13.34 | 13.34 | 13.34 |
| 3290.2340 | N(N)GTYDHDVYR Fuc 1 Hex 8 HexNAc 3 | 93.860 | 0.008 | 0.000 | 0.000e+00 | 498 - 508 | 2 | - | - | - |
| 3940.5383 | N(N)GTYDHDVYRDEALNNR Fuc 1 Hex 7 HexNAc 3 | 93.137 | 0.008 | 10.583 | 1.945e+07 | 498 - 515 | 3 | 19.79 | 20.30 | 20.64 |
| 3860.5839 | N(N)GTYDHDVYRDEALNNR Fuc 1 Hex 4 HexNAc 5 | 91.321 | 0.015 | 12.828 | 1.079e+08 | 498 - 515 | 5 | 17.74 | 18.08 | 18.93 |
| 3882.7193 | TITNDQIEVTN(N)ATELVQSSSTGR Hex 5 HexNAc 3 | 91.271 | 0.015 | 12.816 | 4.155e+08 | 43 - 66 | 11 | 38.87 | 39.72 | 41.09 |
| 3089.2516 | N(N)GTYDHDVYR Fuc 1 Hex 3 HexNAc 6 | 91.077 | 0.015 | -19.753 | 1.099e+06 | 498 - 508 | 1 | 13.51 | 13.51 | 13.51 |
| 3761.6933 | TITNDQIEVTN(N)ATELVQSSSTGR Hex 3 HexNAc 4 | 90.426 | 0.015 | 14.034 | 3.169e+08 | 43 - 66 | 7 | 39.72 | 39.89 | 41.09 |
| 4250.6621 | N(N)GTYDHDVYRDEALNNR Fuc 2 Hex 8 HexNAc 3 | 87.749 | 0.021 | 0.000 | 0.000e+00 | 498 - 515 | 1 | - | - | - |
| 4086.5943 | N(N)GTYDHDVYRDEALNNR Fuc 2 Hex 7 HexNAc 3 | 87.252 | 0.021 | -5.203 | 9.815e+06 | 498 - 515 | 1 | 20.13 | 20.13 | 20.46 |
| 3048.2230 | N(N)GTYDHDVYR Fuc 1 Hex 4 HexNAc 5 | 86.272 | 0.021 | 14.450 | 1.441e+07 | 498 - 508 | 4 | 13.00 | 13.00 | 14.01 |
| 4473.8815 | TITNDQIEVTN(N)ATELVQSSSTGR Fuc 1 Hex 9 HexNAc 2 | 83.954 | 0.021 | -20.113 | 2.782e+06 | 43 - 66 | 1 | 42.12 | 42.12 | 42.12 |
| 3352.2948 | RGSN(N)N(D)SFFSR Fuc 1 Hex 7 HexNAc 3 Neu5Ac 1 | 83.625 | 0.021 | -6.080 | 8.821e+06 | 156 - 166 | 1 | 18.42 | 18.42 | 18.93 |
| 3720.6650 | TITNDQIEVTN(N)ATELVQSSSTGR Hex 4 HexNAc 3 | 78.645 | 0.033 | -3.926 | 5.373e+07 | 43 - 66 | 1 | 39.72 | 39.89 | 40.06 |
| 2898.1783 | RGSN(N)NSFFSR Fuc 1 Hex 6 HexNAc 3 | 77.767 | 0.038 | -20.289 | 2.750e+05 | 156 - 166 | 1 | 16.55 | 16.55 | 16.55 |
| 3964.7731 | TITNDQIEVTN(N)ATELVQSSSTGR Hex 3 HexNAc 5 | 75.798 | 0.038 | 14.385 | 1.474e+08 | 43 - 66 | 5 | 39.72 | 39.89 | 41.09 |
| 4149.7605 | TITNDQIEVTN(N)ATELVQSSSTGR Fuc 1 Hex 7 HexNAc 2 | 75.455 | 0.038 | 14.754 | 5.445e+07 | 43 - 66 | 6 | 42.46 | 42.63 | 43.31 |
| 3288.3427 | RGSN(N)NSFFSR Fuc 2 Hex 5 HexNAc 5 | 75.241 | 0.038 | -20.078 | 2.009e+06 | 156 - 166 | 1 | 16.38 | 16.38 | 16.55 |
| 3021.2598 | RGSN(N)NSFFSR Fuc 1 Hex 3 HexNAc 6 | 74.944 | 0.044 | -21.104 | 1.481e+06 | 156 - 166 | 1 | 16.89 | 17.06 | 17.06 |

### TIVKPGDILLIN(N)STGNLIAPR Hex 9 HexNAc 2

**Mass:**  4068.9289

(8.814e-07)

**Charge States:** 3, 4, 5

**Start Position**: 251

**End Position**: 272

| Profile Score | Tandem Score | TDA q-value | Volume | Spectrum Matches |
| --- | --- | --- | --- | --- |
| 14.7906 | 156.9127 | 0.0000 | 3.0802e+08 | 18 |

**Adduct Signal Fractions**

- **Unmodified**: 3.0802e+08

### RGSN(N)NSFFSR Fuc 1 Hex 3 HexNAc 5

**Mass:**  2818.1738

(3.465e-06)

**Charge States:** 3

**Start Position**: 157

**End Position**: 167

| Profile Score | Tandem Score | TDA q-value | Volume | Spectrum Matches |
| --- | --- | --- | --- | --- |
| 14.5818 | 147.2643 | 0.0000 | 1.0241e+08 | 4 |

**Adduct Signal Fractions**

- **Unmodified**: 1.0241e+08

### YPVLN(N)VTM(O)PNN(D)GK Hex 9 HexNAc 2

**Mass:**  3327.3506

(2.966e-04)

**Charge States:** 2, 3

**Start Position**: 177

**End Position**: 190

| Profile Score | Tandem Score | TDA q-value | Volume | Spectrum Matches |
| --- | --- | --- | --- | --- |
| 10.8862 | 142.7200 | 0.0000 | 9.9865e+08 | 6 |

**Adduct Signal Fractions**

- **Unmodified**: 9.9865e+08

### PGDILLIN(N)STGNLIAPR Hex 9 HexNAc 2

**Mass:**  3627.6311

(1.879e-06)

**Charge States:** 3, 4

**Start Position**: 255

**End Position**: 272

| Profile Score | Tandem Score | TDA q-value | Volume | Spectrum Matches |
| --- | --- | --- | --- | --- |
| 10.6150 | 141.8555 | 0.0000 | 1.4790e+07 | 5 |

**Adduct Signal Fractions**

- **Unmodified**: 1.4790e+07

### RGSN(N)N(D)SFFSR Fuc 1 Hex 3 HexNAc 5

**Mass:**  2819.1626

(3.536e-04)

**Charge States:** 3

**Start Position**: 157

**End Position**: 167

| Profile Score | Tandem Score | TDA q-value | Volume | Spectrum Matches |
| --- | --- | --- | --- | --- |
| 7.9175 | 139.4841 | 0.0000 | 3.4935e+07 | 1 |

**Adduct Signal Fractions**

- **Unmodified**: 3.4935e+07

### YPVLN(N)VTM(O)PNN(D)GK Fuc 2 Hex 3 HexNAc 4 Neu5Ac 1

**Mass:**  3344.3750

(2.879e-04)

**Charge States:** 3

**Start Position**: 177

**End Position**: 190

| Profile Score | Tandem Score | TDA q-value | Volume | Spectrum Matches |
| --- | --- | --- | --- | --- |
| 11.6388 | 139.3620 | 0.0000 | 2.6434e+08 | 3 |

**Adduct Signal Fractions**

- **Unmodified**: 2.6434e+08

### GSN(N)NSFFSR Fuc 1 Hex 3 HexNAc 5

**Mass:**  2662.0702

(9.929e-07)

**Charge States:** 2, 3

**Start Position**: 158

**End Position**: 167

| Profile Score | Tandem Score | TDA q-value | Volume | Spectrum Matches |
| --- | --- | --- | --- | --- |
| 13.2496 | 139.0150 | 0.0000 | 2.1007e+08 | 5 |

**Adduct Signal Fractions**

- **Unmodified**: 2.1007e+08

### TIVKPGDILLIN(N)STGNLIAPR Hex 10 HexNAc 2

**Mass:**  4230.9802

(8.159e-07)

**Charge States:** 3, 4

**Start Position**: 251

**End Position**: 272

| Profile Score | Tandem Score | TDA q-value | Volume | Spectrum Matches |
| --- | --- | --- | --- | --- |
| 13.7739 | 137.3989 | 0.0000 | 3.2219e+07 | 5 |

**Adduct Signal Fractions**

- **Unmodified**: 3.2219e+07

### TIVKPGDILLIN(N)STGNLIAPR Fuc 2 Hex 3 HexNAc 4 Neu5Ac 1

**Mass:**  4085.9514

(-5.301e-06)

**Charge States:** 3, 4

**Start Position**: 251

**End Position**: 272

| Profile Score | Tandem Score | TDA q-value | Volume | Spectrum Matches |
| --- | --- | --- | --- | --- |
| 12.9147 | 132.4292 | 0.0000 | 4.0038e+07 | 5 |

**Adduct Signal Fractions**

- **Unmodified**: 4.0038e+07

### N(N)GTYDHDVYR Fuc 2 Hex 4 HexNAc 4

**Mass:**  2991.2023

(8.728e-06)

**Charge States:** 3

**Start Position**: 499

**End Position**: 509

| Profile Score | Tandem Score | TDA q-value | Volume | Spectrum Matches |
| --- | --- | --- | --- | --- |
| 13.7392 | 132.0681 | 0.0000 | 3.1307e+07 | 2 |

**Adduct Signal Fractions**

- **Unmodified**: 3.1307e+07

### YPVLN(N)VTM(O)PNN(D)GK Hex 8 HexNAc 2

**Mass:**  3165.2960

(3.123e-04)

**Charge States:** 2, 3

**Start Position**: 177

**End Position**: 190

| Profile Score | Tandem Score | TDA q-value | Volume | Spectrum Matches |
| --- | --- | --- | --- | --- |
| 11.3745 | 130.7881 | 0.0000 | 5.6432e+08 | 5 |

**Adduct Signal Fractions**

- **Unmodified**: 5.6432e+08

### RGSN(N)NSFFSR Fuc 2 Hex 4 HexNAc 5

**Mass:**  3126.2884

(3.391e-06)

**Charge States:** 3, 4

**Start Position**: 157

**End Position**: 167

| Profile Score | Tandem Score | TDA q-value | Volume | Spectrum Matches |
| --- | --- | --- | --- | --- |
| 13.5595 | 127.5740 | 0.0000 | 3.1370e+07 | 3 |

**Adduct Signal Fractions**

- **Unmodified**: 3.1370e+07

### N(N)GTYDHDVYRDEALNNR Fuc 2 Hex 4 HexNAc 4

**Mass:**  3803.5629

(2.371e-06)

**Charge States:** 3, 4

**Start Position**: 499

**End Position**: 516

| Profile Score | Tandem Score | TDA q-value | Volume | Spectrum Matches |
| --- | --- | --- | --- | --- |
| -2.0132 | 127.1132 | 0.0000 | 2.0124e+08 | 4 |

**Adduct Signal Fractions**

- **Unmodified**: 2.0124e+08

### N(N)GTYDHDVYRDEALNNR Fuc 1 Hex 3 HexNAc 5

**Mass:**  3698.5292

(1.807e-06)

**Charge States:** 3, 4

**Start Position**: 499

**End Position**: 516

| Profile Score | Tandem Score | TDA q-value | Volume | Spectrum Matches |
| --- | --- | --- | --- | --- |
| 20.5837 | 126.4997 | 0.0000 | 1.4408e+08 | 4 |

**Adduct Signal Fractions**

- **Unmodified**: 1.4408e+08

### YPVLN(N)VTM(O)PNN(D)GK Hex 7 HexNAc 2

**Mass:**  3003.2409

(3.277e-04)

**Charge States:** 3

**Start Position**: 177

**End Position**: 190

| Profile Score | Tandem Score | TDA q-value | Volume | Spectrum Matches |
| --- | --- | --- | --- | --- |
| 11.0210 | 125.6002 | 0.0000 | 6.9047e+07 | 4 |

**Adduct Signal Fractions**

- **Unmodified**: 6.9047e+07

### GSN(N)NSFFSR Fuc 2 Hex 4 HexNAc 5

**Mass:**  2970.1820

(2.448e-06)

**Charge States:** 2, 3

**Start Position**: 158

**End Position**: 167

| Profile Score | Tandem Score | TDA q-value | Volume | Spectrum Matches |
| --- | --- | --- | --- | --- |
| 13.3364 | 124.0981 | 0.0000 | 7.1436e+07 | 3 |

**Adduct Signal Fractions**

- **Unmodified**: 7.1436e+07

### PGDILLIN(N)STGNLIAPR Fuc 2 Hex 3 HexNAc 4 Neu5Ac 1

**Mass:**  3646.6651

(5.443e-04)

**Start Position**: 255

**End Position**: 272

| Profile Score | Tandem Score | TDA q-value | Volume | Spectrum Matches |
| --- | --- | --- | --- | --- |
| 0.0000 | 124.0202 | 0.0000 | 0.0000e+00 | 1 |

Adduct:

No Chromatogram Found

### YPVLN(N)VTM(O)PNN(D)GKFDK Hex 9 HexNAc 2

**Mass:**  3717.5407

(2.662e-04)

**Charge States:** 3, 4

**Start Position**: 177

**End Position**: 193

| Profile Score | Tandem Score | TDA q-value | Volume | Spectrum Matches |
| --- | --- | --- | --- | --- |
| 13.2997 | 123.6836 | 0.0000 | 3.4139e+08 | 8 |

**Adduct Signal Fractions**

- **Unmodified**: 3.4139e+08

### RGSN(N)NSFFSR Fuc 1 Hex 8 HexNAc 4

**Mass:**  3425.3183

(-9.053e-06)

**Charge States:** 3

**Start Position**: 157

**End Position**: 167

| Profile Score | Tandem Score | TDA q-value | Volume | Spectrum Matches |
| --- | --- | --- | --- | --- |
| 12.0635 | 123.6690 | 0.0000 | 1.1464e+07 | 2 |

**Adduct Signal Fractions**

- **Unmodified**: 1.1464e+07

### N(N)GTYDHDVYR Fuc 2 Hex 4 HexNAc 5

**Mass:**  3194.2791

(7.320e-06)

**Charge States:** 3

**Start Position**: 499

**End Position**: 509

| Profile Score | Tandem Score | TDA q-value | Volume | Spectrum Matches |
| --- | --- | --- | --- | --- |
| -18.3041 | 122.8466 | 0.0000 | 9.0796e+06 | 2 |

**Adduct Signal Fractions**

- **Unmodified**: 9.0796e+06

### SDAPIGTC(C)SSEC(C)ITPN(N)GSIPNDKPFQNVNK Hex 8 HexNAc 2

**Mass:**  4949.0881

(2.858e-06)

**Charge States:** 3, 4

**Start Position**: 286

**End Position**: 316

| Profile Score | Tandem Score | TDA q-value | Volume | Spectrum Matches |
| --- | --- | --- | --- | --- |
| 13.2376 | 122.1895 | 0.0000 | 2.7755e+08 | 7 |

**Adduct Signal Fractions**

- **Unmodified**: 2.7755e+08

### SDAPIGTC(C)SSEC(C)ITPN(N)GSIPNDKPFQNVNK Hex 9 HexNAc 2

**Mass:**  5111.1384

(2.343e-06)

**Charge States:** 3, 4

**Start Position**: 286

**End Position**: 316

| Profile Score | Tandem Score | TDA q-value | Volume | Spectrum Matches |
| --- | --- | --- | --- | --- |
| 13.1934 | 121.5766 | 0.0000 | 2.3996e+08 | 5 |

**Adduct Signal Fractions**

- **Unmodified**: 2.3996e+08

### YPVLN(N)VTM(O)PNN(D)GKFDK Hex 8 HexNAc 2

**Mass:**  3555.4898

(2.784e-04)

**Charge States:** 3, 4

**Start Position**: 177

**End Position**: 193

| Profile Score | Tandem Score | TDA q-value | Volume | Spectrum Matches |
| --- | --- | --- | --- | --- |
| 11.5371 | 120.9283 | 0.0000 | 1.8537e+08 | 6 |

**Adduct Signal Fractions**

- **Unmodified**: 1.8537e+08

### SDAPIGTC(C)SSEC(C)ITPN(N)GSIPNDKPFQNVNK Fuc 2 Hex 3 HexNAc 4 Neu5Ac 1

**Mass:**  5128.1608

(-5.432e-06)

**Charge States:** 4

**Start Position**: 286

**End Position**: 316

| Profile Score | Tandem Score | TDA q-value | Volume | Spectrum Matches |
| --- | --- | --- | --- | --- |
| 11.5492 | 117.8142 | 0.0000 | 8.1708e+07 | 3 |

**Adduct Signal Fractions**

- **Unmodified**: 8.1708e+07

### YPVLN(N)VTM(O)PNN Hex 9 HexNAc 2

**Mass:**  3141.2536

(2.287e-06)

**Charge States:** 3

**Start Position**: 177

**End Position**: 188

| Profile Score | Tandem Score | TDA q-value | Volume | Spectrum Matches |
| --- | --- | --- | --- | --- |
| 8.9507 | 117.5705 | 0.0000 | 5.1884e+06 | 3 |

**Adduct Signal Fractions**

- **Unmodified**: 5.1884e+06

### TITNDQIEVTN(N)ATELVQSSSTGR Fuc 1 Hex 6 HexNAc 3

**Mass:**  4190.8352

(2.955e-06)

**Charge States:** 3, 4

**Start Position**: 44

**End Position**: 67

| Profile Score | Tandem Score | TDA q-value | Volume | Spectrum Matches |
| --- | --- | --- | --- | --- |
| 13.5436 | 117.3479 | 0.0000 | 4.3254e+08 | 13 |

**Adduct Signal Fractions**

- **Unmodified**: 4.3254e+08

### N(N)GTYDHDVYRDEALNNR Fuc 1 Hex 3 HexNAc 4

**Mass:**  3495.4489

(2.493e-06)

**Charge States:** 3, 4

**Start Position**: 499

**End Position**: 516

| Profile Score | Tandem Score | TDA q-value | Volume | Spectrum Matches |
| --- | --- | --- | --- | --- |
| 16.6783 | 117.2992 | 0.0000 | 1.7058e+08 | 4 |

**Adduct Signal Fractions**

- **Unmodified**: 1.7058e+08

### N(N)GTYDHDVYRDEALNNR Fuc 4 Hex 6 HexNAc 5

**Mass:**  4622.8659

(2.307e-06)

**Charge States:** 4

**Start Position**: 499

**End Position**: 516

| Profile Score | Tandem Score | TDA q-value | Volume | Spectrum Matches |
| --- | --- | --- | --- | --- |
| -4.8204 | 115.8755 | 0.0000 | 1.4285e+07 | 2 |

**Adduct Signal Fractions**

- **Unmodified**: 1.4285e+07

### YPVLN(N)VTM(O)PNN(D)GK Hex 10 HexNAc 2

**Mass:**  3489.4026

(2.829e-04)

**Charge States:** 3

**Start Position**: 177

**End Position**: 190

| Profile Score | Tandem Score | TDA q-value | Volume | Spectrum Matches |
| --- | --- | --- | --- | --- |
| 9.8780 | 114.7716 | 0.0000 | 3.6174e+07 | 3 |

**Adduct Signal Fractions**

- **Unmodified**: 3.6174e+07

### RGSN(N)NSFFSR Fuc 3 Hex 5 HexNAc 4

**Mass:**  3231.3184

(1.421e-06)

**Charge States:** 3

**Start Position**: 157

**End Position**: 167

| Profile Score | Tandem Score | TDA q-value | Volume | Spectrum Matches |
| --- | --- | --- | --- | --- |
| -3.7087 | 114.3347 | 0.0000 | 5.6523e+06 | 2 |

**Adduct Signal Fractions**

- **Unmodified**: 5.6523e+06

### DQIEVTN(N)ATELVQSSSTGR Hex 6 HexNAc 2

**Mass:**  3412.4690

(1.975e-06)

**Charge States:** 3

**Start Position**: 48

**End Position**: 67

| Profile Score | Tandem Score | TDA q-value | Volume | Spectrum Matches |
| --- | --- | --- | --- | --- |
| -5.1619 | 113.4467 | 0.0000 | 7.0512e+06 | 1 |

**Adduct Signal Fractions**

- **Unmodified**: 7.0512e+06

### N(N)GTYDHDVYR Fuc 1 Hex 3 HexNAc 4

**Mass:**  2683.0885

(9.727e-06)

**Charge States:** 3

**Start Position**: 499

**End Position**: 509

| Profile Score | Tandem Score | TDA q-value | Volume | Spectrum Matches |
| --- | --- | --- | --- | --- |
| -4.6716 | 113.2387 | 0.0000 | 1.0111e+07 | 1 |

**Adduct Signal Fractions**

- **Unmodified**: 1.0111e+07

### RGSN(N)NSFFSR Fuc 1 Hex 3 HexNAc 4

**Mass:**  2615.0963

(2.964e-06)

**Charge States:** 3

**Start Position**: 157

**End Position**: 167

| Profile Score | Tandem Score | TDA q-value | Volume | Spectrum Matches |
| --- | --- | --- | --- | --- |
| 8.1024 | 113.2000 | 0.0000 | 1.3998e+07 | 4 |

**Adduct Signal Fractions**

- **Unmodified**: 1.3998e+07

### N(N)GTYDHDVYRDEALNNR Fuc 3 Hex 5 HexNAc 4

**Mass:**  4111.6765

(2.927e-06)

**Charge States:** 3, 4

**Start Position**: 499

**End Position**: 516

| Profile Score | Tandem Score | TDA q-value | Volume | Spectrum Matches |
| --- | --- | --- | --- | --- |
| -2.4558 | 112.8081 | 0.0000 | 1.0532e+08 | 4 |

**Adduct Signal Fractions**

- **Unmodified**: 1.0532e+08

### N(N)GTYDHDVYR Fuc 1 Hex 3 HexNAc 5

**Mass:**  2886.1712

(9.143e-06)

**Charge States:** 3

**Start Position**: 499

**End Position**: 509

| Profile Score | Tandem Score | TDA q-value | Volume | Spectrum Matches |
| --- | --- | --- | --- | --- |
| -4.1031 | 110.6952 | 0.0000 | 1.1939e+07 | 1 |

**Adduct Signal Fractions**

- **Unmodified**: 1.1939e+07

### RGSN(N)NSFFSR Fuc 1 Hex 4 HexNAc 5

**Mass:**  2980.2231

(1.835e-06)

**Charge States:** 3

**Start Position**: 157

**End Position**: 167

| Profile Score | Tandem Score | TDA q-value | Volume | Spectrum Matches |
| --- | --- | --- | --- | --- |
| 9.9168 | 110.4494 | 0.0000 | 9.0194e+06 | 3 |

**Adduct Signal Fractions**

- **Unmodified**: 9.0194e+06

### TITNDQIEVTN(N)ATELVQSSSTGR Fuc 2 Hex 5 HexNAc 4

**Mass:**  4377.9186

(3.035e-06)

**Charge States:** 3, 4

**Start Position**: 44

**End Position**: 67

| Profile Score | Tandem Score | TDA q-value | Volume | Spectrum Matches |
| --- | --- | --- | --- | --- |
| -5.0121 | 110.1653 | 0.0000 | 6.7161e+07 | 2 |

**Adduct Signal Fractions**

- **Unmodified**: 6.7161e+07

### TITNDQIEVTN(N)ATELVQSSSTGR Hex 5 HexNAc 2

**Mass:**  3679.6411

(2.781e-06)

**Charge States:** 3, 4

**Start Position**: 44

**End Position**: 67

| Profile Score | Tandem Score | TDA q-value | Volume | Spectrum Matches |
| --- | --- | --- | --- | --- |
| 15.4506 | 109.3515 | 0.0000 | 4.6519e+08 | 12 |

**Adduct Signal Fractions**

- **Unmodified**: 4.6519e+08

### N(N)GTYDHDVYRDEALNNR Fuc 2 Hex 4 HexNAc 5

**Mass:**  4006.6459

(3.357e-06)

**Charge States:** 4

**Start Position**: 499

**End Position**: 516

| Profile Score | Tandem Score | TDA q-value | Volume | Spectrum Matches |
| --- | --- | --- | --- | --- |
| -3.0694 | 109.2021 | 0.0000 | 7.7593e+07 | 2 |

**Adduct Signal Fractions**

- **Unmodified**: 7.7593e+07

### YPVLN(N)VTM(O)PNN(D)GK Hex 6 HexNAc 2

**Mass:**  2841.1908

(3.473e-04)

**Charge States:** 3

**Start Position**: 177

**End Position**: 190

| Profile Score | Tandem Score | TDA q-value | Volume | Spectrum Matches |
| --- | --- | --- | --- | --- |
| -6.4816 | 109.2003 | 0.0000 | 3.0353e+06 | 1 |

**Adduct Signal Fractions**

- **Unmodified**: 3.0353e+06

### N(N)GTYDHDVYR Fuc 3 Hex 5 HexNAc 4

**Mass:**  3299.3124

(5.212e-06)

**Charge States:** 3

**Start Position**: 499

**End Position**: 509

| Profile Score | Tandem Score | TDA q-value | Volume | Spectrum Matches |
| --- | --- | --- | --- | --- |
| -4.7756 | 109.0103 | 0.0000 | 1.3914e+07 | 3 |

**Adduct Signal Fractions**

- **Unmodified**: 1.3914e+07

### YPVLN(N)VTM(O)PNN Fuc 2 Hex 3 HexNAc 4 Neu5Ac 1

**Mass:**  3158.2772

(-7.056e-06)

**Charge States:** 3

**Start Position**: 177

**End Position**: 188

| Profile Score | Tandem Score | TDA q-value | Volume | Spectrum Matches |
| --- | --- | --- | --- | --- |
| -6.5732 | 108.7235 | 0.0000 | 1.6992e+06 | 1 |

**Adduct Signal Fractions**

- **Unmodified**: 1.6992e+06

### RGSN(N)NSFFSR Hex 3 HexNAc 6

**Mass:**  2875.1947

(2.504e-06)

**Charge States:** 3

**Start Position**: 157

**End Position**: 167

| Profile Score | Tandem Score | TDA q-value | Volume | Spectrum Matches |
| --- | --- | --- | --- | --- |
| -2.7178 | 108.5079 | 0.0000 | 3.4558e+07 | 2 |

**Adduct Signal Fractions**

- **Unmodified**: 3.4558e+07

### YPVLN(N)VTM(O)PNN Hex 8 HexNAc 2

**Mass:**  2979.1948

(1.691e-06)

**Charge States:** 3

**Start Position**: 177

**End Position**: 188

| Profile Score | Tandem Score | TDA q-value | Volume | Spectrum Matches |
| --- | --- | --- | --- | --- |
| -4.3976 | 106.6120 | 0.0000 | 2.6082e+06 | 1 |

**Adduct Signal Fractions**

- **Unmodified**: 2.6082e+06

### N(N)GTYDHDVYR Fuc 2 Hex 7 HexNAc 3

**Mass:**  3274.2328

(-6.874e-06)

**Charge States:** 3

**Start Position**: 499

**End Position**: 509

| Profile Score | Tandem Score | TDA q-value | Volume | Spectrum Matches |
| --- | --- | --- | --- | --- |
| -20.9980 | 105.0009 | 0.0000 | 9.8901e+05 | 1 |

**Adduct Signal Fractions**

- **Unmodified**: 9.8901e+05

### N(N)GTYDHDVYR Fuc 1 Hex 7 HexNAc 2

**Mass:**  2925.1030

(-5.151e-06)

**Charge States:** 3

**Start Position**: 499

**End Position**: 509

| Profile Score | Tandem Score | TDA q-value | Volume | Spectrum Matches |
| --- | --- | --- | --- | --- |
| -4.9131 | 104.9129 | 0.0078 | 5.3804e+06 | 2 |

**Adduct Signal Fractions**

- **Unmodified**: 5.3804e+06

### TITNDQIEVTN(N)ATELVQSSSTGR Hex 7 HexNAc 2

**Mass:**  4003.7503

(2.352e-06)

**Charge States:** 3, 4

**Start Position**: 44

**End Position**: 67

| Profile Score | Tandem Score | TDA q-value | Volume | Spectrum Matches |
| --- | --- | --- | --- | --- |
| 13.8222 | 104.5199 | 0.0078 | 8.2121e+08 | 13 |

**Adduct Signal Fractions**

- **Unmodified**: 8.2121e+08

### RGSN(N)N(D)SFFSR Fuc 1 Hex 8 HexNAc 3 Neu5Ac 1

**Mass:**  3514.3478

(2.791e-04)

**Charge States:** 3

**Start Position**: 157

**End Position**: 167

| Profile Score | Tandem Score | TDA q-value | Volume | Spectrum Matches |
| --- | --- | --- | --- | --- |
| -6.5861 | 104.4252 | 0.0078 | 5.9300e+06 | 1 |

**Adduct Signal Fractions**

- **Unmodified**: 5.9300e+06

### RGSN(N)NSFFSR Fuc 1 Hex 4 HexNAc 6

**Mass:**  3183.3096

(1.896e-06)

**Charge States:** 3

**Start Position**: 157

**End Position**: 167

| Profile Score | Tandem Score | TDA q-value | Volume | Spectrum Matches |
| --- | --- | --- | --- | --- |
| -3.7656 | 104.1371 | 0.0078 | 1.1893e+07 | 2 |

**Adduct Signal Fractions**

- **Unmodified**: 1.1893e+07

### RGSN(N)NSFFSR Fuc 2 Hex 5 HexNAc 6

**Mass:**  3491.4197

(1.428e-06)

**Charge States:** 3

**Start Position**: 157

**End Position**: 167

| Profile Score | Tandem Score | TDA q-value | Volume | Spectrum Matches |
| --- | --- | --- | --- | --- |
| -20.8580 | 104.0062 | 0.0078 | 1.4544e+06 | 1 |

**Adduct Signal Fractions**

- **Unmodified**: 1.4544e+06

### YPVLN(N)VTM(O)PNN(D)GK Fuc 1 Hex 3 HexNAc 5 Neu5Ac 1

**Mass:**  3401.4018

(2.827e-04)

**Charge States:** 3

**Start Position**: 177

**End Position**: 190

| Profile Score | Tandem Score | TDA q-value | Volume | Spectrum Matches |
| --- | --- | --- | --- | --- |
| 11.5879 | 103.6975 | 0.0078 | 2.6109e+08 | 8 |

**Adduct Signal Fractions**

- **Unmodified**: 2.6109e+08

### N(N)GTYDHDVYR Fuc 3 Hex 5 HexNAc 5

**Mass:**  3502.3892

(6.564e-06)

**Charge States:** 3

**Start Position**: 499

**End Position**: 509

| Profile Score | Tandem Score | TDA q-value | Volume | Spectrum Matches |
| --- | --- | --- | --- | --- |
| -19.2197 | 103.6218 | 0.0078 | 4.6793e+06 | 1 |

**Adduct Signal Fractions**

- **Unmodified**: 4.6793e+06

### TITNDQIEVTN(N)ATELVQSSSTGR Hex 6 HexNAc 2

**Mass:**  3841.6950

(2.032e-06)

**Charge States:** 3, 4

**Start Position**: 44

**End Position**: 67

| Profile Score | Tandem Score | TDA q-value | Volume | Spectrum Matches |
| --- | --- | --- | --- | --- |
| 14.0112 | 103.1596 | 0.0078 | 1.2677e+09 | 14 |

**Adduct Signal Fractions**

- **Unmodified**: 1.2677e+09

### RGSN(N)N(D)SFFSR Hex 8 HexNAc 4 Neu5Ac 1

**Mass:**  3571.3723

(2.724e-04)

**Charge States:** 3

**Start Position**: 157

**End Position**: 167

| Profile Score | Tandem Score | TDA q-value | Volume | Spectrum Matches |
| --- | --- | --- | --- | --- |
| 8.0939 | 102.8791 | 0.0078 | 9.6725e+06 | 2 |

**Adduct Signal Fractions**

- **Unmodified**: 9.6725e+06

### N(N)GTYDHDVYR Fuc 1 Hex 7 HexNAc 3

**Mass:**  3128.1791

(-4.699e-06)

**Charge States:** 3

**Start Position**: 499

**End Position**: 509

| Profile Score | Tandem Score | TDA q-value | Volume | Spectrum Matches |
| --- | --- | --- | --- | --- |
| -5.6402 | 101.3980 | 0.0078 | 3.0841e+06 | 2 |

**Adduct Signal Fractions**

- **Unmodified**: 3.0841e+06

### RGSN(N)N(D)SFFSR Fuc 3 Hex 5 HexNAc 5

**Mass:**  3435.3843

(2.926e-04)

**Charge States:** 3

**Start Position**: 157

**End Position**: 167

| Profile Score | Tandem Score | TDA q-value | Volume | Spectrum Matches |
| --- | --- | --- | --- | --- |
| -20.8790 | 100.2704 | 0.0078 | 1.6150e+06 | 2 |

**Adduct Signal Fractions**

- **Unmodified**: 1.6150e+06

### RGSN(N)NSFFSR Fuc 2 Hex 4 HexNAc 4

**Mass:**  2923.2077

(5.414e-06)

**Charge States:** 3

**Start Position**: 157

**End Position**: 167

| Profile Score | Tandem Score | TDA q-value | Volume | Spectrum Matches |
| --- | --- | --- | --- | --- |
| -4.2519 | 99.2384 | 0.0078 | 7.7978e+06 | 3 |

**Adduct Signal Fractions**

- **Unmodified**: 7.7978e+06

### RGSN(N)N(D)SFFSR Hex 7 HexNAc 3 Neu5Ac 1

**Mass:**  3206.2410

(3.042e-04)

**Charge States:** 3

**Start Position**: 157

**End Position**: 167

| Profile Score | Tandem Score | TDA q-value | Volume | Spectrum Matches |
| --- | --- | --- | --- | --- |
| 2.7987 | 98.8010 | 0.0078 | 1.6106e+07 | 4 |

**Adduct Signal Fractions**

- **Unmodified**: 1.6106e+07

### N(N)GTYDHDVYR Fuc 4 Hex 6 HexNAc 5

**Mass:**  3810.4923

(4.038e-06)

**Charge States:** 3

**Start Position**: 499

**End Position**: 509

| Profile Score | Tandem Score | TDA q-value | Volume | Spectrum Matches |
| --- | --- | --- | --- | --- |
| -16.2585 | 97.8894 | 0.0078 | 1.3323e+06 | 1 |

**Adduct Signal Fractions**

- **Unmodified**: 1.3323e+06

### N(N)GTYDHDVYR Fuc 2 Hex 8 HexNAc 3

**Mass:**  3436.2896

(-5.449e-06)

**Charge States:** 3

**Start Position**: 499

**End Position**: 509

| Profile Score | Tandem Score | TDA q-value | Volume | Spectrum Matches |
| --- | --- | --- | --- | --- |
| -21.0011 | 96.8524 | 0.0078 | 8.9391e+05 | 1 |

**Adduct Signal Fractions**

- **Unmodified**: 8.9391e+05

### N(N)GTYDHDVYRDEALNNR Fuc 3 Hex 5 HexNAc 5

**Mass:**  4314.7556

(2.679e-06)

**Charge States:** 4

**Start Position**: 499

**End Position**: 516

| Profile Score | Tandem Score | TDA q-value | Volume | Spectrum Matches |
| --- | --- | --- | --- | --- |
| -2.0597 | 96.6942 | 0.0078 | 4.2423e+07 | 3 |

**Adduct Signal Fractions**

- **Unmodified**: 4.2423e+07

### RGSN(N)NSFFSR Fuc 3 Hex 5 HexNAc 5

**Mass:**  3434.3955

(2.575e-06)

**Charge States:** 3

**Start Position**: 157

**End Position**: 167

| Profile Score | Tandem Score | TDA q-value | Volume | Spectrum Matches |
| --- | --- | --- | --- | --- |
| -19.2699 | 96.6866 | 0.0078 | 6.2611e+06 | 2 |

**Adduct Signal Fractions**

- **Unmodified**: 6.2611e+06

### YPVLN(N)VTM(O)PNN(D)GKFDK Hex 7 HexNAc 2

**Mass:**  3393.4359

(2.923e-04)

**Charge States:** 3

**Start Position**: 177

**End Position**: 193

| Profile Score | Tandem Score | TDA q-value | Volume | Spectrum Matches |
| --- | --- | --- | --- | --- |
| 11.4954 | 96.0417 | 0.0078 | 1.6358e+07 | 2 |

**Adduct Signal Fractions**

- **Unmodified**: 1.6358e+07

### N(N)GTYDHDVYRDEALNNR Fuc 1 Hex 7 HexNAc 2

**Mass:**  3737.4578

(-9.223e-06)

**Charge States:** 3, 4

**Start Position**: 499

**End Position**: 516

| Profile Score | Tandem Score | TDA q-value | Volume | Spectrum Matches |
| --- | --- | --- | --- | --- |
| -4.3324 | 95.2320 | 0.0078 | 2.2535e+07 | 4 |

**Adduct Signal Fractions**

- **Unmodified**: 2.2535e+07

### N(N)GTYDHDVYR Fuc 2 Hex 4 HexNAc 6

**Mass:**  3397.3515

(4.848e-06)

**Charge States:** 3

**Start Position**: 499

**End Position**: 509

| Profile Score | Tandem Score | TDA q-value | Volume | Spectrum Matches |
| --- | --- | --- | --- | --- |
| -21.1264 | 95.0916 | 0.0078 | 5.5775e+05 | 1 |

**Adduct Signal Fractions**

- **Unmodified**: 5.5775e+05

### N(N)GTYDHDVYR Fuc 1 Hex 8 HexNAc 3

**Mass:**  3290.2340

(6.065e-04)

**Start Position**: 499

**End Position**: 509

| Profile Score | Tandem Score | TDA q-value | Volume | Spectrum Matches |
| --- | --- | --- | --- | --- |
| 0.0000 | 93.8600 | 0.0078 | 0.0000e+00 | 2 |

Adduct:

No Chromatogram Found

### N(N)GTYDHDVYRDEALNNR Fuc 1 Hex 7 HexNAc 3

**Mass:**  3940.5402

(-8.833e-06)

**Charge States:** 3, 4

**Start Position**: 499

**End Position**: 516

| Profile Score | Tandem Score | TDA q-value | Volume | Spectrum Matches |
| --- | --- | --- | --- | --- |
| 10.5826 | 93.1373 | 0.0078 | 1.9450e+07 | 3 |

**Adduct Signal Fractions**

- **Unmodified**: 1.9450e+07

### N(N)GTYDHDVYRDEALNNR Fuc 1 Hex 4 HexNAc 5

**Mass:**  3860.5869

(2.244e-06)

**Charge States:** 3, 4

**Start Position**: 499

**End Position**: 516

| Profile Score | Tandem Score | TDA q-value | Volume | Spectrum Matches |
| --- | --- | --- | --- | --- |
| 12.8281 | 91.3215 | 0.0149 | 1.0788e+08 | 5 |

**Adduct Signal Fractions**

- **Unmodified**: 1.0788e+08

### TITNDQIEVTN(N)ATELVQSSSTGR Hex 5 HexNAc 3

**Mass:**  3882.7212

(2.244e-06)

**Charge States:** 3, 4

**Start Position**: 44

**End Position**: 67

| Profile Score | Tandem Score | TDA q-value | Volume | Spectrum Matches |
| --- | --- | --- | --- | --- |
| 12.8164 | 91.2710 | 0.0149 | 4.1553e+08 | 11 |

**Adduct Signal Fractions**

- **Unmodified**: 4.1553e+08

### N(N)GTYDHDVYR Fuc 1 Hex 3 HexNAc 6

**Mass:**  3089.2516

(8.821e-06)

**Charge States:** 3

**Start Position**: 499

**End Position**: 509

| Profile Score | Tandem Score | TDA q-value | Volume | Spectrum Matches |
| --- | --- | --- | --- | --- |
| -19.7533 | 91.0766 | 0.0149 | 1.0993e+06 | 1 |

**Adduct Signal Fractions**

- **Unmodified**: 1.0993e+06

### TITNDQIEVTN(N)ATELVQSSSTGR Hex 3 HexNAc 4

**Mass:**  3761.6933

(2.457e-06)

**Charge States:** 3, 4

**Start Position**: 44

**End Position**: 67

| Profile Score | Tandem Score | TDA q-value | Volume | Spectrum Matches |
| --- | --- | --- | --- | --- |
| 14.0343 | 90.4256 | 0.0149 | 3.1691e+08 | 7 |

**Adduct Signal Fractions**

- **Unmodified**: 3.1691e+08

### N(N)GTYDHDVYRDEALNNR Fuc 2 Hex 8 HexNAc 3

**Mass:**  4250.6621

(4.652e-04)

**Start Position**: 499

**End Position**: 516

| Profile Score | Tandem Score | TDA q-value | Volume | Spectrum Matches |
| --- | --- | --- | --- | --- |
| 0.0000 | 87.7494 | 0.0208 | 0.0000e+00 | 1 |

Adduct:

No Chromatogram Found

### N(N)GTYDHDVYRDEALNNR Fuc 2 Hex 7 HexNAc 3

**Mass:**  4086.5956

(-9.418e-06)

**Charge States:** 3, 4

**Start Position**: 499

**End Position**: 516

| Profile Score | Tandem Score | TDA q-value | Volume | Spectrum Matches |
| --- | --- | --- | --- | --- |
| -5.2033 | 87.2522 | 0.0208 | 9.8151e+06 | 1 |

**Adduct Signal Fractions**

- **Unmodified**: 9.8151e+06

### N(N)GTYDHDVYR Fuc 1 Hex 4 HexNAc 5

**Mass:**  3048.2221

(8.326e-06)

**Charge States:** 3

**Start Position**: 499

**End Position**: 509

| Profile Score | Tandem Score | TDA q-value | Volume | Spectrum Matches |
| --- | --- | --- | --- | --- |
| 14.4502 | 86.2715 | 0.0208 | 1.4410e+07 | 4 |

**Adduct Signal Fractions**

- **Unmodified**: 1.4410e+07

### TITNDQIEVTN(N)ATELVQSSSTGR Fuc 1 Hex 9 HexNAc 2

**Mass:**  4473.8815

(-4.394e-06)

**Charge States:** 4

**Start Position**: 44

**End Position**: 67

| Profile Score | Tandem Score | TDA q-value | Volume | Spectrum Matches |
| --- | --- | --- | --- | --- |
| -20.1128 | 83.9541 | 0.0208 | 2.7820e+06 | 1 |

**Adduct Signal Fractions**

- **Unmodified**: 2.7820e+06

### RGSN(N)N(D)SFFSR Fuc 1 Hex 7 HexNAc 3 Neu5Ac 1

**Mass:**  3352.2969

(2.933e-04)

**Charge States:** 3

**Start Position**: 157

**End Position**: 167

| Profile Score | Tandem Score | TDA q-value | Volume | Spectrum Matches |
| --- | --- | --- | --- | --- |
| -6.0798 | 83.6245 | 0.0208 | 8.8206e+06 | 1 |

**Adduct Signal Fractions**

- **Unmodified**: 8.8206e+06

### TITNDQIEVTN(N)ATELVQSSSTGR Hex 4 HexNAc 3

**Mass:**  3720.6652

(1.823e-06)

**Charge States:** 3

**Start Position**: 44

**End Position**: 67

| Profile Score | Tandem Score | TDA q-value | Volume | Spectrum Matches |
| --- | --- | --- | --- | --- |
| -3.9261 | 78.6451 | 0.0331 | 5.3725e+07 | 1 |

**Adduct Signal Fractions**

- **Unmodified**: 5.3725e+07

### RGSN(N)NSFFSR Fuc 1 Hex 6 HexNAc 3

**Mass:**  2898.1783

(4.276e-06)

**Charge States:** 3

**Start Position**: 157

**End Position**: 167

| Profile Score | Tandem Score | TDA q-value | Volume | Spectrum Matches |
| --- | --- | --- | --- | --- |
| -20.2885 | 77.7669 | 0.0377 | 2.7505e+05 | 1 |

**Adduct Signal Fractions**

- **Unmodified**: 2.7505e+05

### TITNDQIEVTN(N)ATELVQSSSTGR Hex 3 HexNAc 5

**Mass:**  3964.7739

(2.419e-06)

**Charge States:** 3, 4

**Start Position**: 44

**End Position**: 67

| Profile Score | Tandem Score | TDA q-value | Volume | Spectrum Matches |
| --- | --- | --- | --- | --- |
| 14.3848 | 75.7984 | 0.0377 | 1.4738e+08 | 5 |

**Adduct Signal Fractions**

- **Unmodified**: 1.4738e+08

### TITNDQIEVTN(N)ATELVQSSSTGR Fuc 1 Hex 7 HexNAc 2

**Mass:**  4149.7615

(-8.049e-06)

**Charge States:** 3, 4

**Start Position**: 44

**End Position**: 67

| Profile Score | Tandem Score | TDA q-value | Volume | Spectrum Matches |
| --- | --- | --- | --- | --- |
| 14.7536 | 75.4551 | 0.0377 | 5.4447e+07 | 6 |

**Adduct Signal Fractions**

- **Unmodified**: 5.4447e+07

### RGSN(N)NSFFSR Fuc 2 Hex 5 HexNAc 5

**Mass:**  3288.3444

(4.376e-06)

**Charge States:** 3

**Start Position**: 157

**End Position**: 167

| Profile Score | Tandem Score | TDA q-value | Volume | Spectrum Matches |
| --- | --- | --- | --- | --- |
| -20.0780 | 75.2410 | 0.0377 | 2.0095e+06 | 1 |

**Adduct Signal Fractions**

- **Unmodified**: 2.0095e+06

### RGSN(N)NSFFSR Fuc 1 Hex 3 HexNAc 6

**Mass:**  3021.2539

(7.149e-06)

**Charge States:** 3

**Start Position**: 157

**End Position**: 167

| Profile Score | Tandem Score | TDA q-value | Volume | Spectrum Matches |
| --- | --- | --- | --- | --- |
| -21.1035 | 74.9439 | 0.0437 | 1.4808e+06 | 1 |

**Adduct Signal Fractions**

- **Unmodified**: 1.4808e+06

### iav|ADJ41819|A/Phil/2-MA/1982\_NA

**>iav|ADJ41819|A/Phil/2-MA/1982\_NA**  
 `MNPNQKIITIGSVSLTIATICFLMQIAILVTTVTLHFKQYECSSPPNNQVVPCEPIIIERNITEIVYLTNTTIEKEICPK  
LVEYRNWSKPQCKITGFAPFSKDNSIRLSAGGDIWVTREPYVSCDPGKCYQFALGQGTTLDNKHSNDTIHDRTPYRTLLM  
NELGVPFHLGTRQVCIAWSSSSCHDGKAWLHVCITGYDKNATASFIYDGRLVDSIGSWSKNILRTQESECVCINGTCTVV  
MTDGSASERADTKILFIEEGKIVHISPLSGSAQHVEECSCYPRYPGVRCVCRDNWKGSNRPVVDINVKDYSIVSSYVCSG  
LVGDTPRKNDRSSSSYCRNPNNEKGNHGVKGWAFDDGNDVWMGRTISEESRSGYETFKVIGGWSTPNSKLQINRQVIVDS  
GNRSGYSGIFSVEGKSCINRCFYVELIRGREQETRVWWTSNSIVVFCGTSGTYGTGSWPDGADINLMPI`

| Site | Type | Count |
| --- | --- | --- |
| 200 | N-linked | 3 |

| Mass | Sequence | MS_2_ Score | `q`-value | MS_1_ Score | Signal | Protein Coordinates | Tandem Scans | Start Time | Apex Time | End Time |
| --- | --- | --- | --- | --- | --- | --- | --- | --- | --- | --- |
| 2916.1595 | N(N)ATASFIYDGR Hex 8 HexNAc 2 | 119.213 | 0.0 | 14.917 | 3.273e+07 | 199 - 210 | 3 | 25.57 | 25.91 | 26.25 |
| 2754.1065 | N(N)ATASFIYDGR Hex 7 HexNAc 2 | 117.397 | 0.0 | -4.306 | 1.223e+07 | 199 - 210 | 2 | 25.91 | 26.08 | 26.25 |
| 3095.2382 | N(N)ATASFIYDGR Fuc 2 Hex 3 HexNAc 4 Neu5Ac 1 | 91.942 | 0.008 | -19.959 | 2.539e+06 | 199 - 210 | 1 | 25.74 | 25.74 | 25.91 |

### N(N)ATASFIYDGR Hex 8 HexNAc 2

**Mass:**  2916.1598

(1.394e-06)

**Charge States:** 2, 3

**Start Position**: 200

**End Position**: 211

| Profile Score | Tandem Score | TDA q-value | Volume | Spectrum Matches |
| --- | --- | --- | --- | --- |
| 14.9171 | 119.2127 | 0.0000 | 3.2734e+07 | 3 |

**Adduct Signal Fractions**

- **Unmodified**: 3.2734e+07

### N(N)ATASFIYDGR Hex 7 HexNAc 2

**Mass:**  2754.1063

(1.707e-06)

**Charge States:** 2, 3

**Start Position**: 200

**End Position**: 211

| Profile Score | Tandem Score | TDA q-value | Volume | Spectrum Matches |
| --- | --- | --- | --- | --- |
| -4.3055 | 117.3974 | 0.0000 | 1.2229e+07 | 2 |

**Adduct Signal Fractions**

- **Unmodified**: 1.2229e+07

### N(N)ATASFIYDGR Fuc 2 Hex 3 HexNAc 4 Neu5Ac 1

**Mass:**  3095.2384

(-6.976e-06)

**Charge States:** 3

**Start Position**: 200

**End Position**: 211

| Profile Score | Tandem Score | TDA q-value | Volume | Spectrum Matches |
| --- | --- | --- | --- | --- |
| -19.9591 | 91.9419 | 0.0078 | 2.5387e+06 | 1 |

**Adduct Signal Fractions**

- **Unmodified**: 2.5387e+06
