# Supplementary material for: Measuring Site-specific Glycosylation Similarity between Influenza a Virus Variants with Statistical Certainty
Source: Mol Cell Proteomics. 2020 Nov 25;19(9):1533–45. doi: 10.1074/mcp.RA120.002031 (PMC8143645; doi:10.1074/mcp.RA120.002031)
Supplement: Supplementary file 2 [file mmc2.zip › mmc2/159250_2_supp_555290_qcjklq.zip!/20190326_Phil82gal_032219_tryp_HILIC_DDA_1ug_04_glycopeptides.html]

## 20190326\_Phil82gal\_032219\_tryp\_HILIC\_DDA\_1ug\_04 @ GlycopeptideHypothesis-99149fae792e45319eafc7c7abf6e287

**MS^1^ Mass Accuracy:** 10.0 PPM  
**Grouping Tolerance:** 15.0 PPM  
**MS^n^ Mass Accuracy:** 20.0 PPM  
**Minimum `q`-Value For Clustering:** 0.05

**Glycopeptide Hypothesis:**
GlycopeptideHypothesis-99149fae792e45319eafc7c7abf6e287  
**Sample Searched:**
20190326\_Phil82gal\_032219\_tryp\_HILIC\_DDA\_1ug\_04

| Protein | Matched Glycopeptides |
| --- | --- |
| iav\|AFG99160\|A/Phil/2/1982\_HA | 67 |
| iav\|ADJ41819\|A/Phil/2-MA/1982\_NA | 2 |

### iav|AFG99160|A/Phil/2/1982\_HA

**>iav|AFG99160|A/Phil/2/1982\_HA**  
 `MKTIIALSYMFCLVFAQNLPGNDNSTATLCLGHHAVPNGTLVKTITNDQIEVTNATELVQSSSTGRICDSPHRILDGKNC  
TLIDALLGDPHCDGFQNEKWDLFVERSKAFSNCYPYDVPDYASLRSLVASSGTLEFINEGFNWTGVTQSGGSSTCKRGSN  
NSFFSRLNWLYESESKYPVLNVTMPNNGKFDKLYIWGIHHPSTDKEQTNLYIRASGRVTVSTKRSQQTVIPNIGSRPWVR  
GLSSRISIYWTIVKPGDILLINSTGNLIAPRGYFKIRTGKSSIMRSDAPIGTCSSECITPNGSIPNDKPFQNVNKITYGA  
CPRYVKQNTLKLATGMRNVPEKQTRGIFGAIAGFIENGWEGMVDGWYGFRHQNSEGTGQAADLKSTQAAIDQINGKLNRV  
IEKTNEKFHQIEKEFSEVEGRIQDLEKYVEDTKIDLWSYNAELLVALENQHTIDLTDSEMNKLFEKTRKQLRENAEDMGN  
GCFKIYHKCDNACIGSIRNGTYDHDVYRDEALNNRFQIKGVELKSGYKDWILWISFAISCFLLCVVLLGFIMWACQKGNI  
RCNICI`

| Site | Type | Count |
| --- | --- | --- |
| 262 | N-linked | 6 |
| 301 | N-linked | 4 |
| 499 | N-linked | 24 |
| 181 | N-linked | 11 |
| 54 | N-linked | 8 |
| 160 | N-linked | 14 |

| Mass | Sequence | MS_2_ Score | `q`-value | MS_1_ Score | Signal | Protein Coordinates | Tandem Scans | Start Time | Apex Time | End Time |
| --- | --- | --- | --- | --- | --- | --- | --- | --- | --- | --- |
| 2818.1735 | RGSN(N)NSFFSR Fuc 1 Hex 3 HexNAc 5 | 174.090 | 0.0 | 14.462 | 9.965e+07 | 156 - 166 | 4 | 16.89 | 17.40 | 17.74 |
| 3627.6324 | PGDILLIN(N)STGNLIAPR Hex 9 HexNAc 2 | 152.593 | 0.0 | 13.530 | 1.128e+07 | 254 - 271 | 4 | 49.48 | 49.76 | 50.04 |
| 4068.9284 | TIVKPGDILLIN(N)STGNLIAPR Hex 9 HexNAc 2 | 152.042 | 0.0 | 14.709 | 2.037e+08 | 250 - 271 | 19 | 50.98 | 51.74 | 53.63 |
| 3126.2859 | RGSN(N)NSFFSR Fuc 2 Hex 4 HexNAc 5 | 151.004 | 0.0 | 13.728 | 3.280e+07 | 156 - 166 | 3 | 16.55 | 16.89 | 17.23 |
| 2923.2078 | RGSN(N)NSFFSR Fuc 2 Hex 4 HexNAc 4 | 149.568 | 0.0 | -4.520 | 8.655e+06 | 156 - 166 | 2 | 16.55 | 16.89 | 17.06 |
| 3644.6595 | PGDILLIN(N)STGNLIAPR Fuc 2 Hex 3 HexNAc 4 Neu5Ac 1 | 142.549 | 0.0 | -16.892 | 5.972e+06 | 254 - 271 | 1 | 49.59 | 49.76 | 49.76 |
| 3327.3509 | YPVLN(N)VTM(O)PNN(D)GK Hex 9 HexNAc 2 | 140.884 | 0.0 | 12.224 | 9.096e+08 | 176 - 189 | 8 | 28.29 | 28.96 | 29.63 |
| 2683.0822 | N(N)GTYDHDVYR Fuc 1 Hex 3 HexNAc 4 | 140.804 | 0.0 | -18.749 | 1.082e+07 | 498 - 508 | 1 | 13.34 | 13.34 | 13.51 |
| 2662.0693 | GSN(N)NSFFSR Fuc 1 Hex 3 HexNAc 5 | 139.676 | 0.0 | 13.545 | 1.932e+08 | 157 - 166 | 5 | 19.62 | 20.98 | 21.15 |
| 3344.3763 | YPVLN(N)VTM(O)PNN(D)GK Fuc 2 Hex 3 HexNAc 4 Neu5Ac 1 | 138.992 | 0.0 | 11.475 | 3.455e+08 | 176 - 189 | 4 | 28.63 | 28.96 | 29.63 |
| 4085.9542 | TIVKPGDILLIN(N)STGNLIAPR Fuc 2 Hex 3 HexNAc 4 Neu5Ac 1 | 137.961 | 0.0 | 13.785 | 3.003e+07 | 250 - 271 | 4 | 51.46 | 51.74 | 52.54 |
| 2615.0940 | RGSN(N)NSFFSR Fuc 1 Hex 3 HexNAc 4 | 136.782 | 0.0 | -3.942 | 1.125e+07 | 156 - 166 | 2 | 17.23 | 17.23 | 17.57 |
| 5128.1592 | SDAPIGTC(C)SSEC(C)ITPN(N)GSIPNDKPFQNVNK Fuc 2 Hex 3 HexNAc 4 Neu5Ac 1 | 131.643 | 0.0 | 11.673 | 9.377e+07 | 285 - 315 | 3 | 34.93 | 35.10 | 35.62 |
| 3165.2974 | YPVLN(N)VTM(O)PNN(D)GK Hex 8 HexNAc 2 | 131.287 | 0.0 | 12.316 | 5.141e+08 | 176 - 189 | 8 | 28.63 | 28.96 | 29.80 |
| 2819.1624 | RGSN(N)N(D)SFFSR Fuc 1 Hex 3 HexNAc 5 | 131.242 | 0.0 | -19.830 | 2.336e+07 | 156 - 166 | 1 | 17.91 | 17.91 | 18.08 |
| 3495.4488 | N(N)GTYDHDVYRDEALNNR Fuc 1 Hex 3 HexNAc 4 | 130.951 | 0.0 | -2.876 | 1.567e+08 | 498 - 515 | 4 | 18.42 | 18.76 | 18.93 |
| 3717.5432 | YPVLN(N)VTM(O)PNN(D)GKFDK Hex 9 HexNAc 2 | 129.313 | 0.0 | 12.768 | 3.351e+08 | 176 - 192 | 7 | 31.68 | 32.19 | 32.88 |
| 2991.1940 | N(N)GTYDHDVYR Fuc 2 Hex 4 HexNAc 4 | 128.740 | 0.0 | -3.559 | 2.513e+07 | 498 - 508 | 2 | 13.00 | 13.17 | 13.51 |
| 4230.9786 | TIVKPGDILLIN(N)STGNLIAPR Hex 10 HexNAc 2 | 127.618 | 0.0 | 13.814 | 2.127e+07 | 250 - 271 | 5 | 51.42 | 51.66 | 52.11 |
| 4003.7458 | TITNDQIEVTN(N)ATELVQSSSTGR Hex 7 HexNAc 2 | 126.114 | 0.0 | 13.655 | 6.152e+08 | 43 - 66 | 13 | 38.53 | 39.38 | 40.58 |
| 3555.4902 | YPVLN(N)VTM(O)PNN(D)GKFDK Hex 8 HexNAc 2 | 125.084 | 0.0 | 12.274 | 1.888e+08 | 176 - 192 | 6 | 31.85 | 32.19 | 32.88 |
| 3003.2454 | YPVLN(N)VTM(O)PNN(D)GK Hex 7 HexNAc 2 | 124.956 | 0.0 | 11.940 | 5.719e+07 | 176 - 189 | 4 | 28.63 | 29.29 | 29.80 |
| 3401.3992 | YPVLN(N)VTM(O)PNN(D)GK Fuc 1 Hex 3 HexNAc 5 Neu5Ac 1 | 122.970 | 0.0 | 9.882 | 3.410e+08 | 176 - 189 | 7 | 27.44 | 28.46 | 29.46 |
| 3222.2386 | RGSN(N)NSFFSR Fuc 1 Hex 8 HexNAc 3 | 122.726 | 0.0 | -4.939 | 5.336e+06 | 156 - 166 | 2 | 18.93 | 18.93 | 19.27 |
| 3299.3022 | N(N)GTYDHDVYR Fuc 3 Hex 5 HexNAc 4 | 122.622 | 0.0 | -4.225 | 1.210e+07 | 498 - 508 | 2 | 12.83 | 13.00 | 13.17 |
| 2899.1223 | RGSN(N)N(D)SFFSR Fuc 1 Hex 6 HexNAc 3 | 121.980 | 0.0 | -5.533 | 6.104e+06 | 156 - 166 | 1 | 20.30 | 20.30 | 20.81 |
| 3141.2530 | YPVLN(N)VTM(O)PNN Hex 9 HexNAc 2 | 121.891 | 0.0 | 9.454 | 4.129e+06 | 176 - 187 | 2 | 32.53 | 32.70 | 33.22 |
| 4190.8312 | TITNDQIEVTN(N)ATELVQSSSTGR Fuc 1 Hex 6 HexNAc 3 | 121.300 | 0.0 | 13.374 | 2.974e+08 | 43 - 66 | 10 | 38.53 | 39.38 | 40.41 |
| 5111.1380 | SDAPIGTC(C)SSEC(C)ITPN(N)GSIPNDKPFQNVNK Hex 9 HexNAc 2 | 117.977 | 0.0 | 12.154 | 1.749e+08 | 285 - 315 | 6 | 34.93 | 35.10 | 35.62 |
| 3882.7189 | TITNDQIEVTN(N)ATELVQSSSTGR Hex 5 HexNAc 3 | 117.211 | 0.0 | 11.094 | 2.144e+08 | 43 - 66 | 4 | 38.87 | 39.55 | 39.89 |
| 3412.4692 | DQIEVTN(N)ATELVQSSSTGR Hex 6 HexNAc 2 | 116.375 | 0.0 | -19.021 | 7.026e+06 | 47 - 66 | 1 | 36.30 | 36.30 | 36.47 |
| 3629.6470 | PGDILLIN(N)STGNLIAPR Hex 9 HexNAc 2 | 116.286 | 0.0 | 0.000 | 0.000e+00 | 254 - 271 | 1 | - | - | - |
| 3698.5310 | N(N)GTYDHDVYRDEALNNR Fuc 1 Hex 3 HexNAc 5 | 112.892 | 0.0 | -2.894 | 1.255e+08 | 498 - 515 | 5 | 18.42 | 18.76 | 18.93 |
| 3803.5642 | N(N)GTYDHDVYRDEALNNR Fuc 2 Hex 4 HexNAc 4 | 112.890 | 0.0 | 16.233 | 2.147e+08 | 498 - 515 | 5 | 18.08 | 18.59 | 18.76 |
| 4787.0287 | SDAPIGTC(C)SSEC(C)ITPN(N)GSIPNDKPFQNVNK Hex 7 HexNAc 2 | 112.118 | 0.0 | -4.437 | 4.466e+07 | 285 - 315 | 3 | 34.93 | 35.10 | 35.44 |
| 3206.2376 | RGSN(N)N(D)SFFSR Hex 7 HexNAc 3 Neu5Ac 1 | 108.703 | 0.0 | 5.537 | 1.432e+07 | 156 - 166 | 3 | 18.76 | 18.93 | 19.79 |
| 4069.8034 | TITNDQIEVTN(N)ATELVQSSSTGR Fuc 1 Hex 4 HexNAc 4 | 108.004 | 0.0 | 13.245 | 1.161e+08 | 43 - 66 | 3 | 39.21 | 39.55 | 40.24 |
| 3558.6143 | TITNDQIEVTN(N)ATELVQSSSTGR Hex 3 HexNAc 3 | 107.965 | 0.0 | 12.444 | 7.354e+07 | 43 - 66 | 4 | 39.89 | 39.89 | 40.58 |
| 4006.6440 | N(N)GTYDHDVYRDEALNNR Fuc 2 Hex 4 HexNAc 5 | 107.904 | 0.0 | 15.239 | 8.382e+07 | 498 - 515 | 4 | 18.08 | 18.42 | 18.76 |
| 4949.0850 | SDAPIGTC(C)SSEC(C)ITPN(N)GSIPNDKPFQNVNK Hex 8 HexNAc 2 | 107.079 | 0.0 | 11.914 | 2.036e+08 | 285 - 315 | 6 | 34.93 | 35.10 | 35.79 |
| 4111.6755 | N(N)GTYDHDVYRDEALNNR Fuc 3 Hex 5 HexNAc 4 | 107.071 | 0.0 | -2.592 | 1.053e+08 | 498 - 515 | 3 | 17.91 | 18.25 | 18.42 |
| 4314.7544 | N(N)GTYDHDVYRDEALNNR Fuc 3 Hex 5 HexNAc 5 | 106.253 | 0.0 | -3.401 | 4.390e+07 | 498 - 515 | 3 | 17.91 | 18.25 | 18.42 |
| 2673.1201 | RGSN(N)NSFFSR Hex 3 HexNAc 5 | 106.116 | 0.0 | 0.000 | 0.000e+00 | 156 - 166 | 1 | - | - | - |
| 3194.2702 | N(N)GTYDHDVYR Fuc 2 Hex 4 HexNAc 5 | 105.928 | 0.0 | -3.722 | 8.672e+06 | 498 - 508 | 2 | 13.00 | 13.17 | 13.34 |
| 4622.8624 | N(N)GTYDHDVYRDEALNNR Fuc 4 Hex 6 HexNAc 5 | 104.395 | 0.0 | -3.773 | 1.508e+07 | 498 - 515 | 1 | 17.74 | 17.91 | 18.08 |
| 3503.3767 | N(N)GTYDHDVYR Fuc 3 Hex 5 HexNAc 5 | 104.169 | 0.0 | 0.000 | 0.000e+00 | 498 - 508 | 1 | - | - | - |
| 4576.9490 | LNWLYESESKYPVLN(N)VTM(O)PNN(D)GK Hex 9 HexNAc 2 | 104.132 | 0.0 | 13.228 | 2.607e+07 | 166 - 189 | 5 | 48.81 | 48.98 | 49.48 |
| 3489.4059 | YPVLN(N)VTM(O)PNN(D)GK Hex 10 HexNAc 2 | 103.485 | 0.0 | 11.309 | 2.700e+07 | 176 - 189 | 2 | 28.46 | 28.96 | 29.13 |
| 3393.4367 | YPVLN(N)VTM(O)PNN(D)GKFDK Hex 7 HexNAc 2 | 103.061 | 0.0 | 9.496 | 1.720e+07 | 176 - 192 | 3 | 32.02 | 32.36 | 32.88 |
| 2886.1640 | N(N)GTYDHDVYR Fuc 1 Hex 3 HexNAc 5 | 102.750 | 0.0 | -4.491 | 1.342e+07 | 498 - 508 | 1 | 13.17 | 13.34 | 13.68 |
| 3436.2855 | N(N)GTYDHDVYR Fuc 2 Hex 8 HexNAc 3 | 100.409 | 0.0 | -20.905 | 1.063e+06 | 498 - 508 | 1 | 14.18 | 14.18 | 14.35 |
| 3860.5840 | N(N)GTYDHDVYRDEALNNR Fuc 1 Hex 4 HexNAc 5 | 99.532 | 0.0 | 11.414 | 1.649e+08 | 498 - 515 | 8 | 17.91 | 18.25 | 19.79 |
| 3810.4887 | N(N)GTYDHDVYR Fuc 4 Hex 6 HexNAc 5 | 99.385 | 0.0 | -19.801 | 5.495e+05 | 498 - 508 | 1 | 12.66 | 12.66 | 12.66 |
| 3128.1699 | N(N)GTYDHDVYR Fuc 1 Hex 7 HexNAc 3 | 98.498 | 0.0 | -20.397 | 2.224e+06 | 498 - 508 | 1 | 14.52 | 14.69 | 14.69 |
| 2925.0915 | N(N)GTYDHDVYR Fuc 1 Hex 7 HexNAc 2 | 98.335 | 0.0 | -4.974 | 3.614e+06 | 498 - 508 | 1 | 14.52 | 14.52 | 14.86 |
| 2980.2287 | RGSN(N)NSFFSR Fuc 1 Hex 4 HexNAc 5 | 98.042 | 0.0 | 9.738 | 8.624e+06 | 156 - 166 | 3 | 16.89 | 17.40 | 17.57 |
| 3274.2282 | N(N)GTYDHDVYR Fuc 2 Hex 7 HexNAc 3 | 97.652 | 0.0 | -20.438 | 1.113e+06 | 498 - 508 | 1 | 14.35 | 14.35 | 14.52 |
| 3496.4436 | N(N)GTYDHDVYRDEALN(D)NR Fuc 1 Hex 3 HexNAc 4 | 97.626 | 0.0 | 8.685 | 4.911e+07 | 498 - 515 | 4 | 19.10 | 19.10 | 20.47 |
| 3291.2356 | N(N)GTYDHDVYR Fuc 1 Hex 8 HexNAc 3 | 97.434 | 0.0 | 0.000 | 0.000e+00 | 498 - 508 | 1 | - | - | - |
| 3425.3235 | RGSN(N)NSFFSR Fuc 1 Hex 8 HexNAc 4 | 96.964 | 0.009 | -4.865 | 8.575e+06 | 156 - 166 | 3 | 18.93 | 19.10 | 19.44 |
| 3737.4612 | N(N)GTYDHDVYRDEALNNR Fuc 1 Hex 7 HexNAc 2 | 95.136 | 0.025 | -3.953 | 1.831e+07 | 498 - 515 | 4 | 20.30 | 20.47 | 20.81 |
| 3352.3005 | RGSN(N)N(D)SFFSR Fuc 1 Hex 7 HexNAc 3 Neu5Ac 1 | 94.552 | 0.025 | -20.945 | 6.053e+06 | 156 - 166 | 1 | 18.42 | 18.59 | 18.59 |
| 3183.3061 | RGSN(N)NSFFSR Fuc 1 Hex 4 HexNAc 6 | 94.473 | 0.025 | 11.988 | 1.407e+07 | 156 - 166 | 3 | 16.72 | 16.89 | 17.40 |
| 3679.6402 | TITNDQIEVTN(N)ATELVQSSSTGR Hex 5 HexNAc 2 | 94.087 | 0.031 | 14.816 | 3.365e+08 | 43 - 66 | 10 | 39.55 | 39.72 | 40.75 |
| 3841.6932 | TITNDQIEVTN(N)ATELVQSSSTGR Hex 6 HexNAc 2 | 94.086 | 0.031 | 13.336 | 9.527e+08 | 43 - 66 | 14 | 38.70 | 39.55 | 40.75 |
| 3892.4710 | N(N)GTYDHDVYR Fuc 2 Hex 9 HexNAc 3 Neu5Ac 1 | 90.095 | 0.044 | 0.000 | 0.000e+00 | 498 - 508 | 1 | - | - | - |
| 3901.6091 | N(N)GTYDHDVYRDEALNNR Fuc 1 Hex 3 HexNAc 6 | 89.832 | 0.044 | 11.208 | 2.720e+07 | 498 - 515 | 3 | 18.42 | 18.76 | 19.44 |

### RGSN(N)NSFFSR Fuc 1 Hex 3 HexNAc 5

**Mass:**  2818.1744

(2.123e-06)

**Charge States:** 3

**Start Position**: 157

**End Position**: 167

| Profile Score | Tandem Score | TDA q-value | Volume | Spectrum Matches |
| --- | --- | --- | --- | --- |
| 14.4618 | 174.0897 | 0.0000 | 9.9650e+07 | 4 |

**Adduct Signal Fractions**

- **Unmodified**: 9.9650e+07

### PGDILLIN(N)STGNLIAPR Hex 9 HexNAc 2

**Mass:**  3627.6330

(1.277e-06)

**Charge States:** 3, 4

**Start Position**: 255

**End Position**: 272

| Profile Score | Tandem Score | TDA q-value | Volume | Spectrum Matches |
| --- | --- | --- | --- | --- |
| 13.5295 | 152.5927 | 0.0000 | 1.1279e+07 | 4 |

**Adduct Signal Fractions**

- **Unmodified**: 1.1279e+07

### TIVKPGDILLIN(N)STGNLIAPR Hex 9 HexNAc 2

**Mass:**  4068.9292

(1.355e-06)

**Charge States:** 3, 4, 5

**Start Position**: 251

**End Position**: 272

| Profile Score | Tandem Score | TDA q-value | Volume | Spectrum Matches |
| --- | --- | --- | --- | --- |
| 14.7093 | 152.0418 | 0.0000 | 2.0365e+08 | 19 |

**Adduct Signal Fractions**

- **Unmodified**: 2.0365e+08

### RGSN(N)NSFFSR Fuc 2 Hex 4 HexNAc 5

**Mass:**  3126.2873

(2.928e-06)

**Charge States:** 3

**Start Position**: 157

**End Position**: 167

| Profile Score | Tandem Score | TDA q-value | Volume | Spectrum Matches |
| --- | --- | --- | --- | --- |
| 13.7278 | 151.0043 | 0.0000 | 3.2798e+07 | 3 |

**Adduct Signal Fractions**

- **Unmodified**: 3.2798e+07

### RGSN(N)NSFFSR Fuc 2 Hex 4 HexNAc 4

**Mass:**  2923.2083

(3.669e-06)

**Charge States:** 3

**Start Position**: 157

**End Position**: 167

| Profile Score | Tandem Score | TDA q-value | Volume | Spectrum Matches |
| --- | --- | --- | --- | --- |
| -4.5195 | 149.5677 | 0.0000 | 8.6549e+06 | 2 |

**Adduct Signal Fractions**

- **Unmodified**: 8.6549e+06

### PGDILLIN(N)STGNLIAPR Fuc 2 Hex 3 HexNAc 4 Neu5Ac 1

**Mass:**  3644.6599

(-6.154e-06)

**Charge States:** 3

**Start Position**: 255

**End Position**: 272

| Profile Score | Tandem Score | TDA q-value | Volume | Spectrum Matches |
| --- | --- | --- | --- | --- |
| -16.8920 | 142.5493 | 0.0000 | 5.9718e+06 | 1 |

**Adduct Signal Fractions**

- **Unmodified**: 5.9718e+06

### YPVLN(N)VTM(O)PNN(D)GK Hex 9 HexNAc 2

**Mass:**  3327.3517

(2.982e-04)

**Charge States:** 2, 3

**Start Position**: 177

**End Position**: 190

| Profile Score | Tandem Score | TDA q-value | Volume | Spectrum Matches |
| --- | --- | --- | --- | --- |
| 12.2238 | 140.8841 | 0.0000 | 9.0964e+08 | 8 |

**Adduct Signal Fractions**

- **Unmodified**: 9.0964e+08

### N(N)GTYDHDVYR Fuc 1 Hex 3 HexNAc 4

**Mass:**  2683.0821

(6.191e-06)

**Charge States:** 3

**Start Position**: 499

**End Position**: 509

| Profile Score | Tandem Score | TDA q-value | Volume | Spectrum Matches |
| --- | --- | --- | --- | --- |
| -18.7489 | 140.8038 | 0.0000 | 1.0817e+07 | 1 |

**Adduct Signal Fractions**

- **Unmodified**: 1.0817e+07

### GSN(N)NSFFSR Fuc 1 Hex 3 HexNAc 5

**Mass:**  2662.0691

(1.500e-06)

**Charge States:** 2, 3

**Start Position**: 158

**End Position**: 167

| Profile Score | Tandem Score | TDA q-value | Volume | Spectrum Matches |
| --- | --- | --- | --- | --- |
| 13.5452 | 139.6756 | 0.0000 | 1.9318e+08 | 5 |

**Adduct Signal Fractions**

- **Unmodified**: 1.9318e+08

### YPVLN(N)VTM(O)PNN(D)GK Fuc 2 Hex 3 HexNAc 4 Neu5Ac 1

**Mass:**  3344.3769

(2.875e-04)

**Charge States:** 3

**Start Position**: 177

**End Position**: 190

| Profile Score | Tandem Score | TDA q-value | Volume | Spectrum Matches |
| --- | --- | --- | --- | --- |
| 11.4752 | 138.9924 | 0.0000 | 3.4554e+08 | 4 |

**Adduct Signal Fractions**

- **Unmodified**: 3.4554e+08

### TIVKPGDILLIN(N)STGNLIAPR Fuc 2 Hex 3 HexNAc 4 Neu5Ac 1

**Mass:**  4085.9548

(-5.819e-06)

**Charge States:** 4

**Start Position**: 251

**End Position**: 272

| Profile Score | Tandem Score | TDA q-value | Volume | Spectrum Matches |
| --- | --- | --- | --- | --- |
| 13.7849 | 137.9613 | 0.0000 | 3.0028e+07 | 4 |

**Adduct Signal Fractions**

- **Unmodified**: 3.0028e+07

### RGSN(N)NSFFSR Fuc 1 Hex 3 HexNAc 4

**Mass:**  2615.0947

(2.957e-06)

**Charge States:** 3

**Start Position**: 157

**End Position**: 167

| Profile Score | Tandem Score | TDA q-value | Volume | Spectrum Matches |
| --- | --- | --- | --- | --- |
| -3.9420 | 136.7822 | 0.0000 | 1.1250e+07 | 2 |

**Adduct Signal Fractions**

- **Unmodified**: 1.1250e+07

### SDAPIGTC(C)SSEC(C)ITPN(N)GSIPNDKPFQNVNK Fuc 2 Hex 3 HexNAc 4 Neu5Ac 1

**Mass:**  5128.1603

(-3.737e-06)

**Charge States:** 4

**Start Position**: 286

**End Position**: 316

| Profile Score | Tandem Score | TDA q-value | Volume | Spectrum Matches |
| --- | --- | --- | --- | --- |
| 11.6726 | 131.6433 | 0.0000 | 9.3766e+07 | 3 |

**Adduct Signal Fractions**

- **Unmodified**: 9.3766e+07

### YPVLN(N)VTM(O)PNN(D)GK Hex 8 HexNAc 2

**Mass:**  3165.2979

(3.122e-04)

**Charge States:** 2, 3

**Start Position**: 177

**End Position**: 190

| Profile Score | Tandem Score | TDA q-value | Volume | Spectrum Matches |
| --- | --- | --- | --- | --- |
| 12.3155 | 131.2867 | 0.0000 | 5.1408e+08 | 8 |

**Adduct Signal Fractions**

- **Unmodified**: 5.1408e+08

### RGSN(N)N(D)SFFSR Fuc 1 Hex 3 HexNAc 5

**Mass:**  2819.1619

(3.533e-04)

**Charge States:** 3

**Start Position**: 157

**End Position**: 167

| Profile Score | Tandem Score | TDA q-value | Volume | Spectrum Matches |
| --- | --- | --- | --- | --- |
| -19.8301 | 131.2417 | 0.0000 | 2.3362e+07 | 1 |

**Adduct Signal Fractions**

- **Unmodified**: 2.3362e+07

### N(N)GTYDHDVYRDEALNNR Fuc 1 Hex 3 HexNAc 4

**Mass:**  3495.4472

(1.180e-06)

**Charge States:** 3, 4

**Start Position**: 499

**End Position**: 516

| Profile Score | Tandem Score | TDA q-value | Volume | Spectrum Matches |
| --- | --- | --- | --- | --- |
| -2.8761 | 130.9512 | 0.0000 | 1.5666e+08 | 4 |

**Adduct Signal Fractions**

- **Unmodified**: 1.5666e+08

### YPVLN(N)VTM(O)PNN(D)GKFDK Hex 9 HexNAc 2

**Mass:**  3717.5440

(2.666e-04)

**Charge States:** 3, 4

**Start Position**: 177

**End Position**: 193

| Profile Score | Tandem Score | TDA q-value | Volume | Spectrum Matches |
| --- | --- | --- | --- | --- |
| 12.7682 | 129.3135 | 0.0000 | 3.3515e+08 | 7 |

**Adduct Signal Fractions**

- **Unmodified**: 3.3515e+08

### N(N)GTYDHDVYR Fuc 2 Hex 4 HexNAc 4

**Mass:**  2991.1939

(6.105e-06)

**Charge States:** 3

**Start Position**: 499

**End Position**: 509

| Profile Score | Tandem Score | TDA q-value | Volume | Spectrum Matches |
| --- | --- | --- | --- | --- |
| -3.5590 | 128.7399 | 0.0000 | 2.5133e+07 | 2 |

**Adduct Signal Fractions**

- **Unmodified**: 2.5133e+07

### TIVKPGDILLIN(N)STGNLIAPR Hex 10 HexNAc 2

**Mass:**  4230.9763

(1.044e-08)

**Charge States:** 3, 4

**Start Position**: 251

**End Position**: 272

| Profile Score | Tandem Score | TDA q-value | Volume | Spectrum Matches |
| --- | --- | --- | --- | --- |
| 13.8144 | 127.6179 | 0.0000 | 2.1270e+07 | 5 |

**Adduct Signal Fractions**

- **Unmodified**: 2.1270e+07

### TITNDQIEVTN(N)ATELVQSSSTGR Hex 7 HexNAc 2

**Mass:**  4003.7473

(3.003e-06)

**Charge States:** 3, 4

**Start Position**: 44

**End Position**: 67

| Profile Score | Tandem Score | TDA q-value | Volume | Spectrum Matches |
| --- | --- | --- | --- | --- |
| 13.6550 | 126.1137 | 0.0000 | 6.1524e+08 | 13 |

**Adduct Signal Fractions**

- **Unmodified**: 6.1524e+08

### YPVLN(N)VTM(O)PNN(D)GKFDK Hex 8 HexNAc 2

**Mass:**  3555.4898

(2.784e-04)

**Charge States:** 3, 4

**Start Position**: 177

**End Position**: 193

| Profile Score | Tandem Score | TDA q-value | Volume | Spectrum Matches |
| --- | --- | --- | --- | --- |
| 12.2737 | 125.0840 | 0.0000 | 1.8878e+08 | 6 |

**Adduct Signal Fractions**

- **Unmodified**: 1.8878e+08

### YPVLN(N)VTM(O)PNN(D)GK Hex 7 HexNAc 2

**Mass:**  3003.2453

(3.280e-04)

**Charge States:** 3

**Start Position**: 177

**End Position**: 190

| Profile Score | Tandem Score | TDA q-value | Volume | Spectrum Matches |
| --- | --- | --- | --- | --- |
| 11.9403 | 124.9557 | 0.0000 | 5.7190e+07 | 4 |

**Adduct Signal Fractions**

- **Unmodified**: 5.7190e+07

### YPVLN(N)VTM(O)PNN(D)GK Fuc 1 Hex 3 HexNAc 5 Neu5Ac 1

**Mass:**  3401.3985

(2.818e-04)

**Charge States:** 3

**Start Position**: 177

**End Position**: 190

| Profile Score | Tandem Score | TDA q-value | Volume | Spectrum Matches |
| --- | --- | --- | --- | --- |
| 9.8824 | 122.9696 | 0.0000 | 3.4103e+08 | 7 |

**Adduct Signal Fractions**

- **Unmodified**: 3.4103e+08

### RGSN(N)NSFFSR Fuc 1 Hex 8 HexNAc 3

**Mass:**  3222.2415

(-9.337e-06)

**Charge States:** 3

**Start Position**: 157

**End Position**: 167

| Profile Score | Tandem Score | TDA q-value | Volume | Spectrum Matches |
| --- | --- | --- | --- | --- |
| -4.9391 | 122.7256 | 0.0000 | 5.3360e+06 | 2 |

**Adduct Signal Fractions**

- **Unmodified**: 5.3360e+06

### N(N)GTYDHDVYR Fuc 3 Hex 5 HexNAc 4

**Mass:**  3299.3021

(4.993e-06)

**Charge States:** 3

**Start Position**: 499

**End Position**: 509

| Profile Score | Tandem Score | TDA q-value | Volume | Spectrum Matches |
| --- | --- | --- | --- | --- |
| -4.2251 | 122.6216 | 0.0000 | 1.2095e+07 | 2 |

**Adduct Signal Fractions**

- **Unmodified**: 1.2095e+07

### RGSN(N)N(D)SFFSR Fuc 1 Hex 6 HexNAc 3

**Mass:**  2899.1218

(3.298e-04)

**Charge States:** 3

**Start Position**: 157

**End Position**: 167

| Profile Score | Tandem Score | TDA q-value | Volume | Spectrum Matches |
| --- | --- | --- | --- | --- |
| -5.5333 | 121.9799 | 0.0000 | 6.1036e+06 | 1 |

**Adduct Signal Fractions**

- **Unmodified**: 6.1036e+06

### YPVLN(N)VTM(O)PNN Hex 9 HexNAc 2

**Mass:**  3141.2521

(1.519e-06)

**Charge States:** 3

**Start Position**: 177

**End Position**: 188

| Profile Score | Tandem Score | TDA q-value | Volume | Spectrum Matches |
| --- | --- | --- | --- | --- |
| 9.4537 | 121.8912 | 0.0000 | 4.1294e+06 | 2 |

**Adduct Signal Fractions**

- **Unmodified**: 4.1294e+06

### TITNDQIEVTN(N)ATELVQSSSTGR Fuc 1 Hex 6 HexNAc 3

**Mass:**  4190.8313

(3.361e-06)

**Charge States:** 3, 4

**Start Position**: 44

**End Position**: 67

| Profile Score | Tandem Score | TDA q-value | Volume | Spectrum Matches |
| --- | --- | --- | --- | --- |
| 13.3739 | 121.3004 | 0.0000 | 2.9740e+08 | 10 |

**Adduct Signal Fractions**

- **Unmodified**: 2.9740e+08

### SDAPIGTC(C)SSEC(C)ITPN(N)GSIPNDKPFQNVNK Hex 9 HexNAc 2

**Mass:**  5111.1359

(3.528e-06)

**Charge States:** 3, 4

**Start Position**: 286

**End Position**: 316

| Profile Score | Tandem Score | TDA q-value | Volume | Spectrum Matches |
| --- | --- | --- | --- | --- |
| 12.1538 | 117.9770 | 0.0000 | 1.7486e+08 | 6 |

**Adduct Signal Fractions**

- **Unmodified**: 1.7486e+08

### TITNDQIEVTN(N)ATELVQSSSTGR Hex 5 HexNAc 3

**Mass:**  3882.7183

(2.149e-06)

**Charge States:** 3, 4

**Start Position**: 44

**End Position**: 67

| Profile Score | Tandem Score | TDA q-value | Volume | Spectrum Matches |
| --- | --- | --- | --- | --- |
| 11.0943 | 117.2106 | 0.0000 | 2.1436e+08 | 4 |

**Adduct Signal Fractions**

- **Unmodified**: 2.1436e+08

### DQIEVTN(N)ATELVQSSSTGR Hex 6 HexNAc 2

**Mass:**  3412.4690

(1.957e-06)

**Charge States:** 3

**Start Position**: 48

**End Position**: 67

| Profile Score | Tandem Score | TDA q-value | Volume | Spectrum Matches |
| --- | --- | --- | --- | --- |
| -19.0212 | 116.3752 | 0.0000 | 7.0259e+06 | 1 |

**Adduct Signal Fractions**

- **Unmodified**: 7.0259e+06

### PGDILLIN(N)STGNLIAPR Hex 9 HexNAc 2

**Mass:**  3629.6470

(5.565e-04)

**Start Position**: 255

**End Position**: 272

| Profile Score | Tandem Score | TDA q-value | Volume | Spectrum Matches |
| --- | --- | --- | --- | --- |
| 0.0000 | 116.2864 | 0.0000 | 0.0000e+00 | 1 |

Adduct:

No Chromatogram Found

### N(N)GTYDHDVYRDEALNNR Fuc 1 Hex 3 HexNAc 5

**Mass:**  3698.5300

(3.020e-06)

**Charge States:** 3, 4

**Start Position**: 499

**End Position**: 516

| Profile Score | Tandem Score | TDA q-value | Volume | Spectrum Matches |
| --- | --- | --- | --- | --- |
| -2.8936 | 112.8920 | 0.0000 | 1.2547e+08 | 5 |

**Adduct Signal Fractions**

- **Unmodified**: 1.2547e+08

### N(N)GTYDHDVYRDEALNNR Fuc 2 Hex 4 HexNAc 4

**Mass:**  3803.5642

(3.682e-06)

**Charge States:** 3, 4

**Start Position**: 499

**End Position**: 516

| Profile Score | Tandem Score | TDA q-value | Volume | Spectrum Matches |
| --- | --- | --- | --- | --- |
| 16.2331 | 112.8902 | 0.0000 | 2.1468e+08 | 5 |

**Adduct Signal Fractions**

- **Unmodified**: 2.1468e+08

### SDAPIGTC(C)SSEC(C)ITPN(N)GSIPNDKPFQNVNK Hex 7 HexNAc 2

**Mass:**  4787.0266

(3.540e-06)

**Charge States:** 3, 4

**Start Position**: 286

**End Position**: 316

| Profile Score | Tandem Score | TDA q-value | Volume | Spectrum Matches |
| --- | --- | --- | --- | --- |
| -4.4372 | 112.1176 | 0.0000 | 4.4660e+07 | 3 |

**Adduct Signal Fractions**

- **Unmodified**: 4.4660e+07

### RGSN(N)N(D)SFFSR Hex 7 HexNAc 3 Neu5Ac 1

**Mass:**  3206.2406

(3.051e-04)

**Charge States:** 3

**Start Position**: 157

**End Position**: 167

| Profile Score | Tandem Score | TDA q-value | Volume | Spectrum Matches |
| --- | --- | --- | --- | --- |
| 5.5372 | 108.7025 | 0.0000 | 1.4324e+07 | 3 |

**Adduct Signal Fractions**

- **Unmodified**: 1.4324e+07

### TITNDQIEVTN(N)ATELVQSSSTGR Fuc 1 Hex 4 HexNAc 4

**Mass:**  4069.8026

(2.191e-06)

**Charge States:** 3, 4

**Start Position**: 44

**End Position**: 67

| Profile Score | Tandem Score | TDA q-value | Volume | Spectrum Matches |
| --- | --- | --- | --- | --- |
| 13.2454 | 108.0039 | 0.0000 | 1.1615e+08 | 3 |

**Adduct Signal Fractions**

- **Unmodified**: 1.1615e+08

### TITNDQIEVTN(N)ATELVQSSSTGR Hex 3 HexNAc 3

**Mass:**  3558.6150

(2.682e-06)

**Charge States:** 3, 4

**Start Position**: 44

**End Position**: 67

| Profile Score | Tandem Score | TDA q-value | Volume | Spectrum Matches |
| --- | --- | --- | --- | --- |
| 12.4438 | 107.9649 | 0.0000 | 7.3545e+07 | 4 |

**Adduct Signal Fractions**

- **Unmodified**: 7.3545e+07

### N(N)GTYDHDVYRDEALNNR Fuc 2 Hex 4 HexNAc 5

**Mass:**  4006.6450

(2.912e-06)

**Charge States:** 4

**Start Position**: 499

**End Position**: 516

| Profile Score | Tandem Score | TDA q-value | Volume | Spectrum Matches |
| --- | --- | --- | --- | --- |
| 15.2391 | 107.9040 | 0.0000 | 8.3818e+07 | 4 |

**Adduct Signal Fractions**

- **Unmodified**: 8.3818e+07

### SDAPIGTC(C)SSEC(C)ITPN(N)GSIPNDKPFQNVNK Hex 8 HexNAc 2

**Mass:**  4949.0847

(3.038e-06)

**Charge States:** 3, 4

**Start Position**: 286

**End Position**: 316

| Profile Score | Tandem Score | TDA q-value | Volume | Spectrum Matches |
| --- | --- | --- | --- | --- |
| 11.9143 | 107.0787 | 0.0000 | 2.0359e+08 | 6 |

**Adduct Signal Fractions**

- **Unmodified**: 2.0359e+08

### N(N)GTYDHDVYRDEALNNR Fuc 3 Hex 5 HexNAc 4

**Mass:**  4111.6755

(2.689e-06)

**Charge States:** 3, 4

**Start Position**: 499

**End Position**: 516

| Profile Score | Tandem Score | TDA q-value | Volume | Spectrum Matches |
| --- | --- | --- | --- | --- |
| -2.5919 | 107.0714 | 0.0000 | 1.0531e+08 | 3 |

**Adduct Signal Fractions**

- **Unmodified**: 1.0531e+08

### N(N)GTYDHDVYRDEALNNR Fuc 3 Hex 5 HexNAc 5

**Mass:**  4314.7551

(2.595e-06)

**Charge States:** 4

**Start Position**: 499

**End Position**: 516

| Profile Score | Tandem Score | TDA q-value | Volume | Spectrum Matches |
| --- | --- | --- | --- | --- |
| -3.4010 | 106.2531 | 0.0000 | 4.3895e+07 | 3 |

**Adduct Signal Fractions**

- **Unmodified**: 4.3895e+07

### RGSN(N)NSFFSR Hex 3 HexNAc 5

**Mass:**  2673.1201

(3.786e-04)

**Start Position**: 157

**End Position**: 167

| Profile Score | Tandem Score | TDA q-value | Volume | Spectrum Matches |
| --- | --- | --- | --- | --- |
| 0.0000 | 106.1158 | 0.0000 | 0.0000e+00 | 1 |

Adduct:

No Chromatogram Found

### N(N)GTYDHDVYR Fuc 2 Hex 4 HexNAc 5

**Mass:**  3194.2703

(4.965e-06)

**Charge States:** 3

**Start Position**: 499

**End Position**: 509

| Profile Score | Tandem Score | TDA q-value | Volume | Spectrum Matches |
| --- | --- | --- | --- | --- |
| -3.7219 | 105.9280 | 0.0000 | 8.6718e+06 | 2 |

**Adduct Signal Fractions**

- **Unmodified**: 8.6718e+06

### N(N)GTYDHDVYRDEALNNR Fuc 4 Hex 6 HexNAc 5

**Mass:**  4622.8615

(1.456e-06)

**Charge States:** 4

**Start Position**: 499

**End Position**: 516

| Profile Score | Tandem Score | TDA q-value | Volume | Spectrum Matches |
| --- | --- | --- | --- | --- |
| -3.7733 | 104.3953 | 0.0000 | 1.5081e+07 | 1 |

**Adduct Signal Fractions**

- **Unmodified**: 1.5081e+07

### N(N)GTYDHDVYR Fuc 3 Hex 5 HexNAc 5

**Mass:**  3503.3767

(2.885e-04)

**Start Position**: 499

**End Position**: 509

| Profile Score | Tandem Score | TDA q-value | Volume | Spectrum Matches |
| --- | --- | --- | --- | --- |
| 0.0000 | 104.1687 | 0.0000 | 0.0000e+00 | 1 |

Adduct:

No Chromatogram Found

### LNWLYESESKYPVLN(N)VTM(O)PNN(D)GK Hex 9 HexNAc 2

**Mass:**  4576.9470

(2.155e-04)

**Charge States:** 3, 4

**Start Position**: 167

**End Position**: 190

| Profile Score | Tandem Score | TDA q-value | Volume | Spectrum Matches |
| --- | --- | --- | --- | --- |
| 13.2283 | 104.1318 | 0.0000 | 2.6065e+07 | 5 |

**Adduct Signal Fractions**

- **Unmodified**: 2.6065e+07

### YPVLN(N)VTM(O)PNN(D)GK Hex 10 HexNAc 2

**Mass:**  3489.4066

(2.844e-04)

**Charge States:** 3

**Start Position**: 177

**End Position**: 190

| Profile Score | Tandem Score | TDA q-value | Volume | Spectrum Matches |
| --- | --- | --- | --- | --- |
| 11.3093 | 103.4854 | 0.0000 | 2.7000e+07 | 2 |

**Adduct Signal Fractions**

- **Unmodified**: 2.7000e+07

### YPVLN(N)VTM(O)PNN(D)GKFDK Hex 7 HexNAc 2

**Mass:**  3393.4363

(2.915e-04)

**Charge States:** 3

**Start Position**: 177

**End Position**: 193

| Profile Score | Tandem Score | TDA q-value | Volume | Spectrum Matches |
| --- | --- | --- | --- | --- |
| 9.4964 | 103.0611 | 0.0000 | 1.7204e+07 | 3 |

**Adduct Signal Fractions**

- **Unmodified**: 1.7204e+07

### N(N)GTYDHDVYR Fuc 1 Hex 3 HexNAc 5

**Mass:**  2886.1637

(6.435e-06)

**Charge States:** 3

**Start Position**: 499

**End Position**: 509

| Profile Score | Tandem Score | TDA q-value | Volume | Spectrum Matches |
| --- | --- | --- | --- | --- |
| -4.4905 | 102.7497 | 0.0000 | 1.3422e+07 | 1 |

**Adduct Signal Fractions**

- **Unmodified**: 1.3422e+07

### N(N)GTYDHDVYR Fuc 2 Hex 8 HexNAc 3

**Mass:**  3436.2852

(-6.676e-06)

**Charge States:** 3

**Start Position**: 499

**End Position**: 509

| Profile Score | Tandem Score | TDA q-value | Volume | Spectrum Matches |
| --- | --- | --- | --- | --- |
| -20.9051 | 100.4093 | 0.0000 | 1.0626e+06 | 1 |

**Adduct Signal Fractions**

- **Unmodified**: 1.0626e+06

### N(N)GTYDHDVYRDEALNNR Fuc 1 Hex 4 HexNAc 5

**Mass:**  3860.5859

(1.885e-06)

**Charge States:** 3, 4

**Start Position**: 499

**End Position**: 516

| Profile Score | Tandem Score | TDA q-value | Volume | Spectrum Matches |
| --- | --- | --- | --- | --- |
| 11.4140 | 99.5324 | 0.0000 | 1.6486e+08 | 8 |

**Adduct Signal Fractions**

- **Unmodified**: 1.6486e+08

### N(N)GTYDHDVYR Fuc 4 Hex 6 HexNAc 5

**Mass:**  3810.4887

(3.063e-06)

**Charge States:** 3

**Start Position**: 499

**End Position**: 509

| Profile Score | Tandem Score | TDA q-value | Volume | Spectrum Matches |
| --- | --- | --- | --- | --- |
| -19.8013 | 99.3850 | 0.0000 | 5.4953e+05 | 1 |

**Adduct Signal Fractions**

- **Unmodified**: 5.4953e+05

### N(N)GTYDHDVYR Fuc 1 Hex 7 HexNAc 3

**Mass:**  3128.1725

(-9.689e-06)

**Charge States:** 3

**Start Position**: 499

**End Position**: 509

| Profile Score | Tandem Score | TDA q-value | Volume | Spectrum Matches |
| --- | --- | --- | --- | --- |
| -20.3973 | 98.4978 | 0.0000 | 2.2238e+06 | 1 |

**Adduct Signal Fractions**

- **Unmodified**: 2.2238e+06

### N(N)GTYDHDVYR Fuc 1 Hex 7 HexNAc 2

**Mass:**  2925.0893

(-9.797e-06)

**Charge States:** 3

**Start Position**: 499

**End Position**: 509

| Profile Score | Tandem Score | TDA q-value | Volume | Spectrum Matches |
| --- | --- | --- | --- | --- |
| -4.9740 | 98.3346 | 0.0000 | 3.6139e+06 | 1 |

**Adduct Signal Fractions**

- **Unmodified**: 3.6139e+06

### RGSN(N)NSFFSR Fuc 1 Hex 4 HexNAc 5

**Mass:**  2980.2268

(4.531e-06)

**Charge States:** 3

**Start Position**: 157

**End Position**: 167

| Profile Score | Tandem Score | TDA q-value | Volume | Spectrum Matches |
| --- | --- | --- | --- | --- |
| 9.7380 | 98.0419 | 0.0000 | 8.6241e+06 | 3 |

**Adduct Signal Fractions**

- **Unmodified**: 8.6241e+06

### N(N)GTYDHDVYR Fuc 2 Hex 7 HexNAc 3

**Mass:**  3274.2302

(-7.618e-06)

**Charge States:** 3

**Start Position**: 499

**End Position**: 509

| Profile Score | Tandem Score | TDA q-value | Volume | Spectrum Matches |
| --- | --- | --- | --- | --- |
| -20.4379 | 97.6521 | 0.0000 | 1.1129e+06 | 1 |

**Adduct Signal Fractions**

- **Unmodified**: 1.1129e+06

### N(N)GTYDHDVYRDEALN(D)NR Fuc 1 Hex 3 HexNAc 4

**Mass:**  3496.4433

(2.861e-04)

**Charge States:** 4

**Start Position**: 499

**End Position**: 516

| Profile Score | Tandem Score | TDA q-value | Volume | Spectrum Matches |
| --- | --- | --- | --- | --- |
| 8.6853 | 97.6258 | 0.0000 | 4.9110e+07 | 4 |

**Adduct Signal Fractions**

- **Unmodified**: 4.9110e+07

### N(N)GTYDHDVYR Fuc 1 Hex 8 HexNAc 3

**Mass:**  3291.2356

(2.995e-04)

**Start Position**: 499

**End Position**: 509

| Profile Score | Tandem Score | TDA q-value | Volume | Spectrum Matches |
| --- | --- | --- | --- | --- |
| 0.0000 | 97.4342 | 0.0000 | 0.0000e+00 | 1 |

Adduct:

No Chromatogram Found

### RGSN(N)NSFFSR Fuc 1 Hex 8 HexNAc 4

**Mass:**  3425.3241

(-8.469e-06)

**Charge States:** 3

**Start Position**: 157

**End Position**: 167

| Profile Score | Tandem Score | TDA q-value | Volume | Spectrum Matches |
| --- | --- | --- | --- | --- |
| -4.8651 | 96.9639 | 0.0089 | 8.5755e+06 | 3 |

**Adduct Signal Fractions**

- **Unmodified**: 8.5755e+06

### N(N)GTYDHDVYRDEALNNR Fuc 1 Hex 7 HexNAc 2

**Mass:**  3737.4596

(-9.627e-06)

**Charge States:** 3, 4

**Start Position**: 499

**End Position**: 516

| Profile Score | Tandem Score | TDA q-value | Volume | Spectrum Matches |
| --- | --- | --- | --- | --- |
| -3.9535 | 95.1363 | 0.0250 | 1.8308e+07 | 4 |

**Adduct Signal Fractions**

- **Unmodified**: 1.8308e+07

### RGSN(N)N(D)SFFSR Fuc 1 Hex 7 HexNAc 3 Neu5Ac 1

**Mass:**  3352.2995

(2.940e-04)

**Charge States:** 3

**Start Position**: 157

**End Position**: 167

| Profile Score | Tandem Score | TDA q-value | Volume | Spectrum Matches |
| --- | --- | --- | --- | --- |
| -20.9453 | 94.5522 | 0.0250 | 6.0526e+06 | 1 |

**Adduct Signal Fractions**

- **Unmodified**: 6.0526e+06

### RGSN(N)NSFFSR Fuc 1 Hex 4 HexNAc 6

**Mass:**  3183.3067

(3.115e-06)

**Charge States:** 3

**Start Position**: 157

**End Position**: 167

| Profile Score | Tandem Score | TDA q-value | Volume | Spectrum Matches |
| --- | --- | --- | --- | --- |
| 11.9881 | 94.4731 | 0.0250 | 1.4073e+07 | 3 |

**Adduct Signal Fractions**

- **Unmodified**: 1.4073e+07

### TITNDQIEVTN(N)ATELVQSSSTGR Hex 5 HexNAc 2

**Mass:**  3679.6408

(2.154e-06)

**Charge States:** 3, 4

**Start Position**: 44

**End Position**: 67

| Profile Score | Tandem Score | TDA q-value | Volume | Spectrum Matches |
| --- | --- | --- | --- | --- |
| 14.8156 | 94.0872 | 0.0315 | 3.3647e+08 | 10 |

**Adduct Signal Fractions**

- **Unmodified**: 3.3647e+08

### TITNDQIEVTN(N)ATELVQSSSTGR Hex 6 HexNAc 2

**Mass:**  3841.6939

(2.777e-06)

**Charge States:** 3, 4

**Start Position**: 44

**End Position**: 67

| Profile Score | Tandem Score | TDA q-value | Volume | Spectrum Matches |
| --- | --- | --- | --- | --- |
| 13.3362 | 94.0861 | 0.0315 | 9.5266e+08 | 14 |

**Adduct Signal Fractions**

- **Unmodified**: 9.5266e+08

### N(N)GTYDHDVYR Fuc 2 Hex 9 HexNAc 3 Neu5Ac 1

**Mass:**  3892.4710

(7.751e-04)

**Start Position**: 499

**End Position**: 509

| Profile Score | Tandem Score | TDA q-value | Volume | Spectrum Matches |
| --- | --- | --- | --- | --- |
| 0.0000 | 90.0952 | 0.0444 | 0.0000e+00 | 1 |

Adduct:

No Chromatogram Found

### N(N)GTYDHDVYRDEALNNR Fuc 1 Hex 3 HexNAc 6

**Mass:**  3901.6081

(2.888e-06)

**Charge States:** 4

**Start Position**: 499

**End Position**: 516

| Profile Score | Tandem Score | TDA q-value | Volume | Spectrum Matches |
| --- | --- | --- | --- | --- |
| 11.2079 | 89.8316 | 0.0444 | 2.7203e+07 | 3 |

**Adduct Signal Fractions**

- **Unmodified**: 2.7203e+07

### iav|ADJ41819|A/Phil/2-MA/1982\_NA

**>iav|ADJ41819|A/Phil/2-MA/1982\_NA**  
 `MNPNQKIITIGSVSLTIATICFLMQIAILVTTVTLHFKQYECSSPPNNQVVPCEPIIIERNITEIVYLTNTTIEKEICPK  
LVEYRNWSKPQCKITGFAPFSKDNSIRLSAGGDIWVTREPYVSCDPGKCYQFALGQGTTLDNKHSNDTIHDRTPYRTLLM  
NELGVPFHLGTRQVCIAWSSSSCHDGKAWLHVCITGYDKNATASFIYDGRLVDSIGSWSKNILRTQESECVCINGTCTVV  
MTDGSASERADTKILFIEEGKIVHISPLSGSAQHVEECSCYPRYPGVRCVCRDNWKGSNRPVVDINVKDYSIVSSYVCSG  
LVGDTPRKNDRSSSSYCRNPNNEKGNHGVKGWAFDDGNDVWMGRTISEESRSGYETFKVIGGWSTPNSKLQINRQVIVDS  
GNRSGYSGIFSVEGKSCINRCFYVELIRGREQETRVWWTSNSIVVFCGTSGTYGTGSWPDGADINLMPI`

| Site | Type | Count |
| --- | --- | --- |
| 200 | N-linked | 2 |

| Mass | Sequence | MS_2_ Score | `q`-value | MS_1_ Score | Signal | Protein Coordinates | Tandem Scans | Start Time | Apex Time | End Time |
| --- | --- | --- | --- | --- | --- | --- | --- | --- | --- | --- |
| 2916.1596 | N(N)ATASFIYDGR Hex 8 HexNAc 2 | 115.563 | 0.0 | 14.517 | 3.131e+07 | 199 - 210 | 4 | 25.74 | 25.91 | 26.43 |
| 2754.1049 | N(N)ATASFIYDGR Hex 7 HexNAc 2 | 103.652 | 0.0 | -4.145 | 1.210e+07 | 199 - 210 | 3 | 25.91 | 26.09 | 26.43 |

### N(N)ATASFIYDGR Hex 8 HexNAc 2

**Mass:**  2916.1582

(2.006e-06)

**Charge States:** 2, 3

**Start Position**: 200

**End Position**: 211

| Profile Score | Tandem Score | TDA q-value | Volume | Spectrum Matches |
| --- | --- | --- | --- | --- |
| 14.5171 | 115.5632 | 0.0000 | 3.1310e+07 | 4 |

**Adduct Signal Fractions**

- **Unmodified**: 3.1310e+07

### N(N)ATASFIYDGR Hex 7 HexNAc 2

**Mass:**  2754.1056

(1.935e-06)

**Charge States:** 3

**Start Position**: 200

**End Position**: 211

| Profile Score | Tandem Score | TDA q-value | Volume | Spectrum Matches |
| --- | --- | --- | --- | --- |
| -4.1447 | 103.6522 | 0.0000 | 1.2099e+07 | 3 |

**Adduct Signal Fractions**

- **Unmodified**: 1.2099e+07
