# Supplementary material for: Measuring Site-specific Glycosylation Similarity between Influenza a Virus Variants with Statistical Certainty
Source: Mol Cell Proteomics. 2020 Nov 25;19(9):1533–45. doi: 10.1074/mcp.RA120.002031 (PMC8143645; doi:10.1074/mcp.RA120.002031)
Supplement: Supplementary file 2 [file mmc2.zip › mmc2/159250_2_supp_555290_qcjklq.zip!/20190326_Phil82_032219_tryp_HILIC_DDA_1ug_03_glycopeptides.html]

## 20190326\_Phil82\_032219\_tryp\_HILIC\_DDA\_1ug\_03 @ GlycopeptideHypothesis-42ca2cbdf5454f99abaff476fb08356b

**MS^1^ Mass Accuracy:** 10.0 PPM  
**Grouping Tolerance:** 15.0 PPM  
**MS^n^ Mass Accuracy:** 20.0 PPM  
**Minimum `q`-Value For Clustering:** 0.05

**Glycopeptide Hypothesis:**
GlycopeptideHypothesis-42ca2cbdf5454f99abaff476fb08356b  
**Sample Searched:**
20190326\_Phil82\_032219\_tryp\_HILIC\_DDA\_1ug\_03

| Protein | Matched Glycopeptides |
| --- | --- |
| iav\|AFG99160\|A/Phil/2/1982\_HA | 72 |
| iav\|ADJ41819\|A/Phil/2-MA/1982\_NA | 6 |
| tr\|Q5F491\|Q5F491\_CHICK | 1 |

### iav|AFG99160|A/Phil/2/1982\_HA

**>iav|AFG99160|A/Phil/2/1982\_HA**  
 `MKTIIALSYMFCLVFAQNLPGNDNSTATLCLGHHAVPNGTLVKTITNDQIEVTNATELVQSSSTGRICDSPHRILDGKNC  
TLIDALLGDPHCDGFQNEKWDLFVERSKAFSNCYPYDVPDYASLRSLVASSGTLEFINEGFNWTGVTQSGGSSTCKRGSN  
NSFFSRLNWLYESESKYPVLNVTMPNNGKFDKLYIWGIHHPSTDKEQTNLYIRASGRVTVSTKRSQQTVIPNIGSRPWVR  
GLSSRISIYWTIVKPGDILLINSTGNLIAPRGYFKIRTGKSSIMRSDAPIGTCSSECITPNGSIPNDKPFQNVNKITYGA  
CPRYVKQNTLKLATGMRNVPEKQTRGIFGAIAGFIENGWEGMVDGWYGFRHQNSEGTGQAADLKSTQAAIDQINGKLNRV  
IEKTNEKFHQIEKEFSEVEGRIQDLEKYVEDTKIDLWSYNAELLVALENQHTIDLTDSEMNKLFEKTRKQLRENAEDMGN  
GCFKIYHKCDNACIGSIRNGTYDHDVYRDEALNNRFQIKGVELKSGYKDWILWISFAISCFLLCVVLLGFIMWACQKGNI  
RCNICI`

| Site | Type | Count |
| --- | --- | --- |
| 262 | N-linked | 1 |
| 301 | N-linked | 3 |
| 499 | N-linked | 33 |
| 181 | N-linked | 10 |
| 54 | N-linked | 5 |
| 38 | N-linked | 1 |
| 160 | N-linked | 19 |

| Mass | Sequence | MS_2_ Score | `q`-value | MS_1_ Score | Signal | Protein Coordinates | Tandem Scans | Start Time | Apex Time | End Time |
| --- | --- | --- | --- | --- | --- | --- | --- | --- | --- | --- |
| 3007.1742 | N(N)GTYDHDVYR Fuc 1 Hex 5 HexNAc 4 | 172.859 | 0.0 | 13.816 | 2.281e+08 | 498 - 508 | 5 | 12.64 | 12.81 | 13.97 |
| 3142.2756 | RGSN(N)NSFFSR Fuc 1 Hex 5 HexNAc 5 | 170.525 | 0.0 | 13.899 | 1.809e+08 | 156 - 166 | 5 | 15.82 | 16.67 | 16.83 |
| 3153.2315 | N(N)GTYDHDVYR Fuc 2 Hex 5 HexNAc 4 | 159.035 | 0.0 | 11.763 | 1.602e+08 | 498 - 508 | 3 | 12.64 | 12.81 | 13.30 |
| 3143.2659 | RGSN(N)N(D)SFFSR Fuc 1 Hex 5 HexNAc 5 | 155.046 | 0.0 | -5.430 | 5.873e+07 | 156 - 166 | 5 | 17.00 | 17.17 | 17.34 |
| 3061.1759 | RGSN(N)N(D)SFFSR Fuc 1 Hex 7 HexNAc 3 | 145.045 | 0.0 | -19.602 | 3.942e+06 | 156 - 166 | 1 | 19.39 | 19.39 | 19.39 |
| 3518.3624 | N(N)GTYDHDVYR Fuc 2 Hex 6 HexNAc 5 | 141.228 | 0.0 | -5.701 | 3.203e+07 | 498 - 508 | 3 | 12.64 | 12.64 | 12.97 |
| 3210.2535 | N(N)GTYDHDVYR Fuc 1 Hex 5 HexNAc 5 | 140.623 | 0.0 | 12.118 | 1.730e+08 | 498 - 508 | 8 | 12.47 | 12.97 | 13.97 |
| 3199.2980 | RGSN(N)NSFFSR Hex 5 HexNAc 6 | 139.526 | 0.0 | -3.602 | 6.772e+07 | 156 - 166 | 3 | 16.50 | 16.83 | 17.00 |
| 3344.3765 | YPVLN(N)VTM(O)PNN(D)GK Fuc 2 Hex 3 HexNAc 4 Neu5Ac 1 | 138.771 | 0.0 | 12.043 | 1.626e+08 | 176 - 189 | 2 | 27.67 | 28.01 | 28.67 |
| 2986.1730 | GSN(N)NSFFSR Fuc 1 Hex 5 HexNAc 5 | 138.325 | 0.0 | 13.657 | 2.783e+08 | 157 - 166 | 8 | 18.53 | 19.89 | 20.06 |
| 3372.3057 | N(N)GTYDHDVYR Fuc 1 Hex 6 HexNAc 5 | 137.378 | 0.0 | 12.689 | 1.105e+08 | 498 - 508 | 5 | 12.64 | 12.81 | 13.30 |
| 3288.3333 | RGSN(N)NSFFSR Fuc 2 Hex 5 HexNAc 5 | 137.027 | 0.0 | 12.734 | 5.003e+07 | 156 - 166 | 5 | 15.82 | 16.33 | 16.50 |
| 4068.9271 | TIVKPGDILLIN(N)STGNLIAPR Hex 9 HexNAc 2 | 135.027 | 0.0 | 15.273 | 2.974e+08 | 250 - 271 | 15 | 50.65 | 51.41 | 52.69 |
| 2939.1962 | RGSN(N)NSFFSR Fuc 1 Hex 5 HexNAc 4 | 134.801 | 0.0 | -3.095 | 2.752e+07 | 156 - 166 | 3 | 16.33 | 16.50 | 16.67 |
| 3434.3920 | RGSN(N)NSFFSR Fuc 3 Hex 5 HexNAc 5 | 132.671 | 0.0 | -4.936 | 1.223e+07 | 156 - 166 | 3 | 15.82 | 15.99 | 16.33 |
| 3165.2967 | YPVLN(N)VTM(O)PNN(D)GK Hex 8 HexNAc 2 | 130.840 | 0.0 | 11.685 | 4.507e+08 | 176 - 189 | 7 | 27.51 | 28.17 | 28.67 |
| 3327.3495 | YPVLN(N)VTM(O)PNN(D)GK Hex 9 HexNAc 2 | 130.204 | 0.0 | 12.784 | 7.854e+08 | 176 - 189 | 9 | 27.67 | 28.01 | 28.84 |
| 3299.2878 | N(N)GTYDHDVYR Fuc 3 Hex 5 HexNAc 4 | 129.975 | 0.0 | -4.752 | 7.400e+07 | 498 - 508 | 3 | 12.47 | 12.64 | 12.97 |
| 2783.0935 | GSN(N)NSFFSR Fuc 1 Hex 5 HexNAc 4 | 129.947 | 0.0 | -3.698 | 4.932e+07 | 157 - 166 | 2 | 19.39 | 19.72 | 19.89 |
| 3003.2429 | YPVLN(N)VTM(O)PNN(D)GK Hex 7 HexNAc 2 | 128.270 | 0.0 | 10.536 | 5.255e+07 | 176 - 189 | 3 | 27.84 | 28.34 | 28.84 |
| 3555.4891 | YPVLN(N)VTM(O)PNN(D)GKFDK Hex 8 HexNAc 2 | 127.146 | 0.0 | 12.360 | 1.048e+08 | 176 - 192 | 6 | 31.06 | 31.40 | 31.91 |
| 3819.5532 | N(N)GTYDHDVYRDEALNNR Fuc 1 Hex 5 HexNAc 4 | 126.604 | 0.0 | 15.714 | 3.687e+08 | 498 - 515 | 7 | 16.83 | 17.85 | 18.19 |
| 3345.3562 | RGSN(N)NSFFSR Fuc 1 Hex 5 HexNAc 6 | 126.155 | 0.0 | -4.640 | 1.775e+07 | 156 - 166 | 3 | 16.33 | 16.50 | 16.67 |
| 4022.6331 | N(N)GTYDHDVYRDEALNNR Fuc 1 Hex 5 HexNAc 5 | 124.643 | 0.0 | 10.704 | 2.237e+08 | 498 - 515 | 4 | 17.00 | 18.02 | 18.19 |
| 3717.5433 | YPVLN(N)VTM(O)PNN(D)GKFDK Hex 9 HexNAc 2 | 123.270 | 0.0 | 12.604 | 1.941e+08 | 176 - 192 | 6 | 30.89 | 31.40 | 31.91 |
| 3085.2532 | RGSN(N)NSFFSR Fuc 2 Hex 5 HexNAc 4 | 122.868 | 0.0 | -3.393 | 1.451e+07 | 156 - 166 | 1 | 16.16 | 16.16 | 16.50 |
| 3231.3126 | RGSN(N)NSFFSR Fuc 3 Hex 5 HexNAc 4 | 122.317 | 0.0 | -3.065 | 1.022e+07 | 156 - 166 | 2 | 15.82 | 15.99 | 16.16 |
| 3141.2520 | YPVLN(N)VTM(O)PNN Hex 9 HexNAc 2 | 121.469 | 0.0 | 10.110 | 6.524e+06 | 176 - 187 | 3 | 31.57 | 31.91 | 32.59 |
| 4184.6859 | N(N)GTYDHDVYRDEALNNR Fuc 1 Hex 6 HexNAc 5 | 118.475 | 0.0 | -3.444 | 1.716e+08 | 498 - 515 | 2 | 17.51 | 17.69 | 18.02 |
| 2980.2236 | RGSN(N)NSFFSR Fuc 1 Hex 4 HexNAc 5 | 118.279 | 0.0 | -19.321 | 4.813e+06 | 156 - 166 | 1 | 16.50 | 16.50 | 16.67 |
| 3666.4301 | N(N)GTYDHDVYR Fuc 3 Hex 6 HexNAc 5 | 116.852 | 0.0 | 0.000 | 0.000e+00 | 498 - 508 | 1 | - | - | - |
| 2841.1925 | YPVLN(N)VTM(O)PNN(D)GK Hex 6 HexNAc 2 | 116.431 | 0.0 | -5.952 | 5.684e+06 | 176 - 189 | 1 | 28.34 | 28.50 | 28.84 |
| 5111.1364 | SDAPIGTC(C)SSEC(C)ITPN(N)GSIPNDKPFQNVNK Hex 9 HexNAc 2 | 115.281 | 0.0 | 12.338 | 1.280e+08 | 285 - 315 | 6 | 34.13 | 34.30 | 35.16 |
| 3575.3857 | N(N)GTYDHDVYR Fuc 1 Hex 6 HexNAc 6 | 114.731 | 0.0 | -4.901 | 3.614e+07 | 498 - 508 | 4 | 12.81 | 12.97 | 13.30 |
| 3965.6110 | N(N)GTYDHDVYRDEALNNR Fuc 2 Hex 5 HexNAc 4 | 114.414 | 0.0 | 16.761 | 2.949e+08 | 498 - 515 | 5 | 17.17 | 17.85 | 18.02 |
| 4190.8279 | TITNDQIEVTN(N)ATELVQSSSTGR Fuc 1 Hex 6 HexNAc 3 | 113.804 | 0.0 | 13.268 | 2.846e+08 | 43 - 66 | 9 | 38.75 | 38.92 | 40.11 |
| 4476.8048 | N(N)GTYDHDVYRDEALNNR Fuc 3 Hex 6 HexNAc 5 | 112.338 | 0.0 | -4.045 | 4.936e+07 | 498 - 515 | 2 | 17.17 | 17.51 | 17.69 |
| 3249.3321 | HAVPN(N)GTLVK Fuc 1 Hex 9 HexNAc 3 | 112.171 | 0.0 | 8.577 | 3.342e+06 | 33 - 43 | 2 | 29.18 | 29.86 | 30.20 |
| 3412.4649 | DQIEVTN(N)ATELVQSSSTGR Hex 6 HexNAc 2 | 111.438 | 0.0 | -5.237 | 5.081e+06 | 47 - 66 | 1 | 35.67 | 35.67 | 36.01 |
| 4330.7452 | N(N)GTYDHDVYRDEALNNR Fuc 2 Hex 6 HexNAc 5 | 111.284 | 0.0 | -3.108 | 7.091e+07 | 498 - 515 | 2 | 17.34 | 17.69 | 17.85 |
| 4085.7947 | TITNDQIEVTN(N)ATELVQSSSTGR Hex 5 HexNAc 4 | 110.217 | 0.0 | 13.453 | 4.157e+08 | 43 - 66 | 11 | 38.07 | 38.92 | 40.11 |
| 4111.6714 | N(N)GTYDHDVYRDEALNNR Fuc 3 Hex 5 HexNAc 4 | 108.914 | 0.0 | -3.110 | 1.376e+08 | 498 - 515 | 2 | 17.34 | 17.69 | 17.85 |
| 4949.0838 | SDAPIGTC(C)SSEC(C)ITPN(N)GSIPNDKPFQNVNK Hex 8 HexNAc 2 | 107.938 | 0.0 | 11.223 | 1.395e+08 | 285 - 315 | 5 | 34.30 | 34.47 | 34.99 |
| 3489.4067 | YPVLN(N)VTM(O)PNN(D)GK Hex 10 HexNAc 2 | 107.569 | 0.0 | 10.986 | 2.785e+07 | 176 - 189 | 2 | 27.67 | 27.84 | 28.34 |
| 4003.7437 | TITNDQIEVTN(N)ATELVQSSSTGR Hex 7 HexNAc 2 | 107.283 | 0.0 | 13.702 | 6.090e+08 | 43 - 66 | 14 | 38.07 | 38.92 | 40.11 |
| 4622.8623 | N(N)GTYDHDVYRDEALNNR Fuc 4 Hex 6 HexNAc 5 | 107.011 | 0.0 | -3.703 | 2.344e+07 | 498 - 515 | 1 | 17.17 | 17.34 | 17.51 |
| 4387.7646 | N(N)GTYDHDVYRDEALNNR Fuc 1 Hex 6 HexNAc 6 | 105.867 | 0.0 | 6.653 | 5.767e+07 | 498 - 515 | 3 | 17.00 | 17.85 | 18.02 |
| 2996.2171 | RGSN(N)NSFFSR Hex 5 HexNAc 5 | 104.827 | 0.0 | -3.819 | 1.481e+07 | 156 - 166 | 2 | 16.50 | 16.67 | 16.83 |
| 3899.5085 | N(N)GTYDHDVYRDEALNNR Fuc 1 Hex 8 HexNAc 2 | 104.562 | 0.0 | -4.468 | 3.161e+07 | 498 - 515 | 2 | 19.55 | 19.72 | 20.06 |
| 3356.3130 | N(N)GTYDHDVYR Fuc 2 Hex 5 HexNAc 5 | 104.247 | 0.0 | -5.084 | 4.551e+07 | 498 - 508 | 4 | 12.81 | 12.81 | 13.30 |
| 4264.6418 | N(N)GTYDHDVYRDEALNNR Fuc 1 Hex 9 HexNAc 3 | 103.744 | 0.0 | -4.487 | 3.077e+07 | 498 - 515 | 2 | 19.22 | 19.39 | 19.72 |
| 3357.3082 | N(N)GTYDHDVYR Fuc 2 Hex 5 HexNAc 5 | 103.486 | 0.0 | 0.000 | 0.000e+00 | 498 - 508 | 1 | - | - | - |
| 5128.1576 | SDAPIGTC(C)SSEC(C)ITPN(N)GSIPNDKPFQNVNK Fuc 2 Hex 3 HexNAc 4 Neu5Ac 1 | 102.884 | 0.0 | -4.462 | 2.563e+07 | 285 - 315 | 3 | 34.30 | 34.30 | 34.81 |
| 3966.6018 | N(N)GTYDHDVYRDEALN(D)NR Fuc 2 Hex 5 HexNAc 4 | 102.582 | 0.0 | 6.632 | 8.518e+07 | 498 - 515 | 3 | 18.19 | 18.19 | 19.22 |
| 3009.1816 | N(N)GTYDHDVYR Fuc 1 Hex 5 HexNAc 4 | 101.404 | 0.0 | 0.000 | 0.000e+00 | 498 - 508 | 1 | - | - | - |
| 3304.3276 | RGSN(N)NSFFSR Fuc 1 Hex 6 HexNAc 5 | 101.247 | 0.0 | 6.982 | 1.143e+07 | 156 - 166 | 3 | 15.99 | 16.67 | 16.83 |
| 3401.3984 | YPVLN(N)VTM(O)PNN(D)GK Fuc 1 Hex 3 HexNAc 5 Neu5Ac 1 | 101.205 | 0.0 | 12.129 | 2.043e+08 | 176 - 189 | 7 | 26.49 | 27.67 | 28.67 |
| 3841.6906 | TITNDQIEVTN(N)ATELVQSSSTGR Hex 6 HexNAc 2 | 101.029 | 0.0 | 14.218 | 1.045e+09 | 43 - 66 | 15 | 37.73 | 39.09 | 40.28 |
| 4410.7049 | N(N)GTYDHDVYRDEALNNR Fuc 2 Hex 9 HexNAc 3 | 100.746 | 0.0 | -4.852 | 1.849e+07 | 498 - 515 | 2 | 19.22 | 19.22 | 19.55 |
| 3810.4794 | N(N)GTYDHDVYR Fuc 4 Hex 6 HexNAc 5 | 100.695 | 0.0 | -20.098 | 1.310e+07 | 498 - 508 | 2 | 12.47 | 12.47 | 12.47 |
| 3664.4207 | N(N)GTYDHDVYR Fuc 3 Hex 6 HexNAc 5 | 98.855 | 0.0 | -5.261 | 3.110e+07 | 498 - 508 | 2 | 12.47 | 12.64 | 12.81 |
| 3352.3023 | RGSN(N)N(D)SFFSR Fuc 1 Hex 7 HexNAc 3 Neu5Ac 1 | 98.765 | 0.0 | -6.523 | 9.502e+06 | 156 - 166 | 1 | 17.85 | 18.02 | 18.36 |
| 3723.4717 | N(N)GTYDHDVYR Fuc 2 Hex 6 HexNAc 6 | 98.753 | 0.0 | 0.000 | 0.000e+00 | 498 - 508 | 1 | - | - | - |
| 4168.6917 | N(N)GTYDHDVYRDEALNNR Fuc 2 Hex 5 HexNAc 5 | 97.984 | 0.0 | 12.289 | 9.073e+07 | 498 - 515 | 4 | 16.83 | 17.85 | 18.02 |
| 4104.5809 | N(N)GTYDHDVYR Fuc 1 Hex 8 HexNAc 7 | 95.956 | 0.008 | 0.000 | 0.000e+00 | 498 - 508 | 1 | - | - | - |
| 3206.2401 | RGSN(N)N(D)SFFSR Hex 7 HexNAc 3 Neu5Ac 1 | 95.280 | 0.015 | 3.529 | 1.939e+07 | 156 - 166 | 3 | 18.36 | 18.36 | 19.22 |
| 3737.4369 | N(N)GTYDHDVYR Fuc 1 Hex 7 HexNAc 6 | 93.774 | 0.015 | -20.711 | 1.193e+07 | 498 - 508 | 2 | 12.47 | 12.64 | 12.64 |
| 4549.8204 | N(N)GTYDHDVYRDEALNNR Fuc 1 Hex 7 HexNAc 6 | 93.129 | 0.022 | -4.506 | 2.471e+07 | 498 - 515 | 1 | 17.34 | 17.51 | 17.69 |
| 2861.1146 | N(N)GTYDHDVYR Hex 5 HexNAc 4 | 92.845 | 0.022 | -20.009 | 7.100e+06 | 498 - 508 | 1 | 12.81 | 12.81 | 12.97 |
| 3491.4135 | RGSN(N)NSFFSR Fuc 2 Hex 5 HexNAc 6 | 89.658 | 0.048 | -19.762 | 4.230e+06 | 156 - 166 | 1 | 15.99 | 16.16 | 16.16 |
| 3721.4455 | N(N)GTYDHDVYR Fuc 2 Hex 6 HexNAc 6 | 89.352 | 0.048 | -5.705 | 1.329e+07 | 498 - 508 | 2 | 12.81 | 12.81 | 13.14 |
| 3289.3226 | RGSN(N)N(D)SFFSR Fuc 2 Hex 5 HexNAc 5 | 88.813 | 0.048 | -5.914 | 1.805e+07 | 156 - 166 | 2 | 16.67 | 16.83 | 17.00 |

### N(N)GTYDHDVYR Fuc 1 Hex 5 HexNAc 4

**Mass:**  3007.1748

(1.227e-06)

**Charge States:** 3

**Start Position**: 499

**End Position**: 509

| Profile Score | Tandem Score | TDA q-value | Volume | Spectrum Matches |
| --- | --- | --- | --- | --- |
| 13.8159 | 172.8587 | 0.0000 | 2.2812e+08 | 5 |

**Adduct Signal Fractions**

- **Unmodified**: 2.2812e+08

### RGSN(N)NSFFSR Fuc 1 Hex 5 HexNAc 5

**Mass:**  3142.2753

(1.096e-06)

**Charge States:** 3, 4

**Start Position**: 157

**End Position**: 167

| Profile Score | Tandem Score | TDA q-value | Volume | Spectrum Matches |
| --- | --- | --- | --- | --- |
| 13.8995 | 170.5253 | 0.0000 | 1.8093e+08 | 5 |

**Adduct Signal Fractions**

- **Unmodified**: 1.8093e+08

### N(N)GTYDHDVYR Fuc 2 Hex 5 HexNAc 4

**Mass:**  3153.2331

(1.311e-06)

**Charge States:** 3

**Start Position**: 499

**End Position**: 509

| Profile Score | Tandem Score | TDA q-value | Volume | Spectrum Matches |
| --- | --- | --- | --- | --- |
| 11.7630 | 159.0350 | 0.0000 | 1.6018e+08 | 3 |

**Adduct Signal Fractions**

- **Unmodified**: 1.6018e+08

### RGSN(N)N(D)SFFSR Fuc 1 Hex 5 HexNAc 5

**Mass:**  3143.2637

(3.179e-04)

**Charge States:** 3, 4

**Start Position**: 157

**End Position**: 167

| Profile Score | Tandem Score | TDA q-value | Volume | Spectrum Matches |
| --- | --- | --- | --- | --- |
| -5.4304 | 155.0462 | 0.0000 | 5.8726e+07 | 5 |

**Adduct Signal Fractions**

- **Unmodified**: 5.8726e+07

### RGSN(N)N(D)SFFSR Fuc 1 Hex 7 HexNAc 3

**Mass:**  3061.1759

(3.127e-04)

**Charge States:** 3

**Start Position**: 157

**End Position**: 167

| Profile Score | Tandem Score | TDA q-value | Volume | Spectrum Matches |
| --- | --- | --- | --- | --- |
| -19.6019 | 145.0453 | 0.0000 | 3.9420e+06 | 1 |

**Adduct Signal Fractions**

- **Unmodified**: 3.9420e+06

### N(N)GTYDHDVYR Fuc 2 Hex 6 HexNAc 5

**Mass:**  3518.3604

(-2.757e-07)

**Charge States:** 3

**Start Position**: 499

**End Position**: 509

| Profile Score | Tandem Score | TDA q-value | Volume | Spectrum Matches |
| --- | --- | --- | --- | --- |
| -5.7010 | 141.2277 | 0.0000 | 3.2027e+07 | 3 |

**Adduct Signal Fractions**

- **Unmodified**: 3.2027e+07

### N(N)GTYDHDVYR Fuc 1 Hex 5 HexNAc 5

**Mass:**  3210.2536

(9.241e-07)

**Charge States:** 3, 4

**Start Position**: 499

**End Position**: 509

| Profile Score | Tandem Score | TDA q-value | Volume | Spectrum Matches |
| --- | --- | --- | --- | --- |
| 12.1182 | 140.6231 | 0.0000 | 1.7300e+08 | 8 |

**Adduct Signal Fractions**

- **Unmodified**: 1.7300e+08

### RGSN(N)NSFFSR Hex 5 HexNAc 6

**Mass:**  3199.2980

(1.423e-06)

**Charge States:** 3, 4

**Start Position**: 157

**End Position**: 167

| Profile Score | Tandem Score | TDA q-value | Volume | Spectrum Matches |
| --- | --- | --- | --- | --- |
| -3.6016 | 139.5261 | 0.0000 | 6.7715e+07 | 3 |

**Adduct Signal Fractions**

- **Unmodified**: 6.7715e+07

### YPVLN(N)VTM(O)PNN(D)GK Fuc 2 Hex 3 HexNAc 4 Neu5Ac 1

**Mass:**  3344.3750

(2.870e-04)

**Charge States:** 3

**Start Position**: 177

**End Position**: 190

| Profile Score | Tandem Score | TDA q-value | Volume | Spectrum Matches |
| --- | --- | --- | --- | --- |
| 12.0429 | 138.7714 | 0.0000 | 1.6261e+08 | 2 |

**Adduct Signal Fractions**

- **Unmodified**: 1.6261e+08

### GSN(N)NSFFSR Fuc 1 Hex 5 HexNAc 5

**Mass:**  2986.1731

(7.674e-07)

**Charge States:** 3, 4

**Start Position**: 158

**End Position**: 167

| Profile Score | Tandem Score | TDA q-value | Volume | Spectrum Matches |
| --- | --- | --- | --- | --- |
| 13.6574 | 138.3248 | 0.0000 | 2.7830e+08 | 8 |

**Adduct Signal Fractions**

- **Unmodified**: 2.7830e+08

### N(N)GTYDHDVYR Fuc 1 Hex 6 HexNAc 5

**Mass:**  3372.3078

(9.682e-07)

**Charge States:** 3, 4

**Start Position**: 499

**End Position**: 509

| Profile Score | Tandem Score | TDA q-value | Volume | Spectrum Matches |
| --- | --- | --- | --- | --- |
| 12.6890 | 137.3782 | 0.0000 | 1.1048e+08 | 5 |

**Adduct Signal Fractions**

- **Unmodified**: 1.1048e+08

### RGSN(N)NSFFSR Fuc 2 Hex 5 HexNAc 5

**Mass:**  3288.3327

(1.191e-06)

**Charge States:** 3, 4

**Start Position**: 157

**End Position**: 167

| Profile Score | Tandem Score | TDA q-value | Volume | Spectrum Matches |
| --- | --- | --- | --- | --- |
| 12.7345 | 137.0267 | 0.0000 | 5.0026e+07 | 5 |

**Adduct Signal Fractions**

- **Unmodified**: 5.0026e+07

### TIVKPGDILLIN(N)STGNLIAPR Hex 9 HexNAc 2

**Mass:**  4068.9257

(8.969e-07)

**Charge States:** 3, 4, 5

**Start Position**: 251

**End Position**: 272

| Profile Score | Tandem Score | TDA q-value | Volume | Spectrum Matches |
| --- | --- | --- | --- | --- |
| 15.2731 | 135.0267 | 0.0000 | 2.9740e+08 | 15 |

**Adduct Signal Fractions**

- **Unmodified**: 2.9740e+08

### RGSN(N)NSFFSR Fuc 1 Hex 5 HexNAc 4

**Mass:**  2939.1963

(1.042e-06)

**Charge States:** 3

**Start Position**: 157

**End Position**: 167

| Profile Score | Tandem Score | TDA q-value | Volume | Spectrum Matches |
| --- | --- | --- | --- | --- |
| -3.0950 | 134.8014 | 0.0000 | 2.7523e+07 | 3 |

**Adduct Signal Fractions**

- **Unmodified**: 2.7523e+07

### RGSN(N)NSFFSR Fuc 3 Hex 5 HexNAc 5

**Mass:**  3434.3922

(2.507e-06)

**Charge States:** 3

**Start Position**: 157

**End Position**: 167

| Profile Score | Tandem Score | TDA q-value | Volume | Spectrum Matches |
| --- | --- | --- | --- | --- |
| -4.9359 | 132.6714 | 0.0000 | 1.2227e+07 | 3 |

**Adduct Signal Fractions**

- **Unmodified**: 1.2227e+07

### YPVLN(N)VTM(O)PNN(D)GK Hex 8 HexNAc 2

**Mass:**  3165.2949

(3.114e-04)

**Charge States:** 2, 3, 4

**Start Position**: 177

**End Position**: 190

| Profile Score | Tandem Score | TDA q-value | Volume | Spectrum Matches |
| --- | --- | --- | --- | --- |
| 11.6853 | 130.8404 | 0.0000 | 4.5070e+08 | 7 |

**Adduct Signal Fractions**

- **Unmodified**: 4.5070e+08

### YPVLN(N)VTM(O)PNN(D)GK Hex 9 HexNAc 2

**Mass:**  3327.3491

(2.965e-04)

**Charge States:** 2, 3, 4

**Start Position**: 177

**End Position**: 190

| Profile Score | Tandem Score | TDA q-value | Volume | Spectrum Matches |
| --- | --- | --- | --- | --- |
| 12.7840 | 130.2041 | 0.0000 | 7.8537e+08 | 9 |

**Adduct Signal Fractions**

- **Unmodified**: 7.8537e+08

### N(N)GTYDHDVYR Fuc 3 Hex 5 HexNAc 4

**Mass:**  3299.2886

(-4.393e-07)

**Charge States:** 3

**Start Position**: 499

**End Position**: 509

| Profile Score | Tandem Score | TDA q-value | Volume | Spectrum Matches |
| --- | --- | --- | --- | --- |
| -4.7521 | 129.9745 | 0.0000 | 7.3995e+07 | 3 |

**Adduct Signal Fractions**

- **Unmodified**: 7.3995e+07

### GSN(N)NSFFSR Fuc 1 Hex 5 HexNAc 4

**Mass:**  2783.0936

(1.020e-06)

**Charge States:** 3

**Start Position**: 158

**End Position**: 167

| Profile Score | Tandem Score | TDA q-value | Volume | Spectrum Matches |
| --- | --- | --- | --- | --- |
| -3.6977 | 129.9468 | 0.0000 | 4.9323e+07 | 2 |

**Adduct Signal Fractions**

- **Unmodified**: 4.9323e+07

### YPVLN(N)VTM(O)PNN(D)GK Hex 7 HexNAc 2

**Mass:**  3003.2428

(3.283e-04)

**Charge States:** 3

**Start Position**: 177

**End Position**: 190

| Profile Score | Tandem Score | TDA q-value | Volume | Spectrum Matches |
| --- | --- | --- | --- | --- |
| 10.5362 | 128.2700 | 0.0000 | 5.2549e+07 | 3 |

**Adduct Signal Fractions**

- **Unmodified**: 5.2549e+07

### YPVLN(N)VTM(O)PNN(D)GKFDK Hex 8 HexNAc 2

**Mass:**  3555.4890

(2.782e-04)

**Charge States:** 3, 4

**Start Position**: 177

**End Position**: 193

| Profile Score | Tandem Score | TDA q-value | Volume | Spectrum Matches |
| --- | --- | --- | --- | --- |
| 12.3603 | 127.1458 | 0.0000 | 1.0481e+08 | 6 |

**Adduct Signal Fractions**

- **Unmodified**: 1.0481e+08

### N(N)GTYDHDVYRDEALNNR Fuc 1 Hex 5 HexNAc 4

**Mass:**  3819.5534

(1.236e-06)

**Charge States:** 3, 4

**Start Position**: 499

**End Position**: 516

| Profile Score | Tandem Score | TDA q-value | Volume | Spectrum Matches |
| --- | --- | --- | --- | --- |
| 15.7137 | 126.6036 | 0.0000 | 3.6874e+08 | 7 |

**Adduct Signal Fractions**

- **Unmodified**: 3.6874e+08

### RGSN(N)NSFFSR Fuc 1 Hex 5 HexNAc 6

**Mass:**  3345.3557

(7.669e-07)

**Charge States:** 3

**Start Position**: 157

**End Position**: 167

| Profile Score | Tandem Score | TDA q-value | Volume | Spectrum Matches |
| --- | --- | --- | --- | --- |
| -4.6400 | 126.1554 | 0.0000 | 1.7755e+07 | 3 |

**Adduct Signal Fractions**

- **Unmodified**: 1.7755e+07

### N(N)GTYDHDVYRDEALNNR Fuc 1 Hex 5 HexNAc 5

**Mass:**  4022.6328

(1.122e-06)

**Charge States:** 3, 4

**Start Position**: 499

**End Position**: 516

| Profile Score | Tandem Score | TDA q-value | Volume | Spectrum Matches |
| --- | --- | --- | --- | --- |
| 10.7038 | 124.6432 | 0.0000 | 2.2367e+08 | 4 |

**Adduct Signal Fractions**

- **Unmodified**: 2.2367e+08

### YPVLN(N)VTM(O)PNN(D)GKFDK Hex 9 HexNAc 2

**Mass:**  3717.5436

(2.665e-04)

**Charge States:** 3, 4

**Start Position**: 177

**End Position**: 193

| Profile Score | Tandem Score | TDA q-value | Volume | Spectrum Matches |
| --- | --- | --- | --- | --- |
| 12.6037 | 123.2701 | 0.0000 | 1.9409e+08 | 6 |

**Adduct Signal Fractions**

- **Unmodified**: 1.9409e+08

### RGSN(N)NSFFSR Fuc 2 Hex 5 HexNAc 4

**Mass:**  3085.2530

(8.175e-07)

**Charge States:** 3

**Start Position**: 157

**End Position**: 167

| Profile Score | Tandem Score | TDA q-value | Volume | Spectrum Matches |
| --- | --- | --- | --- | --- |
| -3.3930 | 122.8680 | 0.0000 | 1.4506e+07 | 1 |

**Adduct Signal Fractions**

- **Unmodified**: 1.4506e+07

### RGSN(N)NSFFSR Fuc 3 Hex 5 HexNAc 4

**Mass:**  3231.3118

(1.840e-06)

**Charge States:** 3

**Start Position**: 157

**End Position**: 167

| Profile Score | Tandem Score | TDA q-value | Volume | Spectrum Matches |
| --- | --- | --- | --- | --- |
| -3.0654 | 122.3170 | 0.0000 | 1.0218e+07 | 2 |

**Adduct Signal Fractions**

- **Unmodified**: 1.0218e+07

### YPVLN(N)VTM(O)PNN Hex 9 HexNAc 2

**Mass:**  3141.2528

(2.153e-06)

**Charge States:** 3

**Start Position**: 177

**End Position**: 188

| Profile Score | Tandem Score | TDA q-value | Volume | Spectrum Matches |
| --- | --- | --- | --- | --- |
| 10.1096 | 121.4685 | 0.0000 | 6.5237e+06 | 3 |

**Adduct Signal Fractions**

- **Unmodified**: 6.5237e+06

### N(N)GTYDHDVYRDEALNNR Fuc 1 Hex 6 HexNAc 5

**Mass:**  4184.6872

(1.847e-06)

**Charge States:** 4

**Start Position**: 499

**End Position**: 516

| Profile Score | Tandem Score | TDA q-value | Volume | Spectrum Matches |
| --- | --- | --- | --- | --- |
| -3.4440 | 118.4751 | 0.0000 | 1.7163e+08 | 2 |

**Adduct Signal Fractions**

- **Unmodified**: 1.7163e+08

### RGSN(N)NSFFSR Fuc 1 Hex 4 HexNAc 5

**Mass:**  2980.2246

(1.871e-06)

**Charge States:** 3

**Start Position**: 157

**End Position**: 167

| Profile Score | Tandem Score | TDA q-value | Volume | Spectrum Matches |
| --- | --- | --- | --- | --- |
| -19.3208 | 118.2792 | 0.0000 | 4.8133e+06 | 1 |

**Adduct Signal Fractions**

- **Unmodified**: 4.8133e+06

### N(N)GTYDHDVYR Fuc 3 Hex 6 HexNAc 5

**Mass:**  3666.4301

(5.488e-04)

**Start Position**: 499

**End Position**: 509

| Profile Score | Tandem Score | TDA q-value | Volume | Spectrum Matches |
| --- | --- | --- | --- | --- |
| 0.0000 | 116.8524 | 0.0000 | 0.0000e+00 | 1 |

Adduct:

No Chromatogram Found

### YPVLN(N)VTM(O)PNN(D)GK Hex 6 HexNAc 2

**Mass:**  2841.1887

(3.466e-04)

**Charge States:** 3

**Start Position**: 177

**End Position**: 190

| Profile Score | Tandem Score | TDA q-value | Volume | Spectrum Matches |
| --- | --- | --- | --- | --- |
| -5.9524 | 116.4312 | 0.0000 | 5.6838e+06 | 1 |

**Adduct Signal Fractions**

- **Unmodified**: 5.6838e+06

### SDAPIGTC(C)SSEC(C)ITPN(N)GSIPNDKPFQNVNK Hex 9 HexNAc 2

**Mass:**  5111.1369

(1.871e-06)

**Charge States:** 4, 5

**Start Position**: 286

**End Position**: 316

| Profile Score | Tandem Score | TDA q-value | Volume | Spectrum Matches |
| --- | --- | --- | --- | --- |
| 12.3382 | 115.2813 | 0.0000 | 1.2803e+08 | 6 |

**Adduct Signal Fractions**

- **Unmodified**: 1.2803e+08

### N(N)GTYDHDVYR Fuc 1 Hex 6 HexNAc 6

**Mass:**  3575.3840

(2.458e-06)

**Charge States:** 3, 4

**Start Position**: 499

**End Position**: 509

| Profile Score | Tandem Score | TDA q-value | Volume | Spectrum Matches |
| --- | --- | --- | --- | --- |
| -4.9008 | 114.7306 | 0.0000 | 3.6139e+07 | 4 |

**Adduct Signal Fractions**

- **Unmodified**: 3.6139e+07

### N(N)GTYDHDVYRDEALNNR Fuc 2 Hex 5 HexNAc 4

**Mass:**  3965.6127

(9.870e-07)

**Charge States:** 4

**Start Position**: 499

**End Position**: 516

| Profile Score | Tandem Score | TDA q-value | Volume | Spectrum Matches |
| --- | --- | --- | --- | --- |
| 16.7608 | 114.4140 | 0.0000 | 2.9490e+08 | 5 |

**Adduct Signal Fractions**

- **Unmodified**: 2.9490e+08

### TITNDQIEVTN(N)ATELVQSSSTGR Fuc 1 Hex 6 HexNAc 3

**Mass:**  4190.8273

(2.044e-06)

**Charge States:** 3, 4

**Start Position**: 44

**End Position**: 67

| Profile Score | Tandem Score | TDA q-value | Volume | Spectrum Matches |
| --- | --- | --- | --- | --- |
| 13.2684 | 113.8039 | 0.0000 | 2.8463e+08 | 9 |

**Adduct Signal Fractions**

- **Unmodified**: 2.8463e+08

### N(N)GTYDHDVYRDEALNNR Fuc 3 Hex 6 HexNAc 5

**Mass:**  4476.8054

(1.696e-06)

**Charge States:** 4

**Start Position**: 499

**End Position**: 516

| Profile Score | Tandem Score | TDA q-value | Volume | Spectrum Matches |
| --- | --- | --- | --- | --- |
| -4.0446 | 112.3383 | 0.0000 | 4.9363e+07 | 2 |

**Adduct Signal Fractions**

- **Unmodified**: 4.9363e+07

### HAVPN(N)GTLVK Fuc 1 Hex 9 HexNAc 3

**Mass:**  3249.3374

(3.031e-04)

**Charge States:** 3

**Start Position**: 34

**End Position**: 44

| Profile Score | Tandem Score | TDA q-value | Volume | Spectrum Matches |
| --- | --- | --- | --- | --- |
| 8.5768 | 112.1710 | 0.0000 | 3.3418e+06 | 2 |

**Adduct Signal Fractions**

- **Unmodified**: 3.3418e+06

### DQIEVTN(N)ATELVQSSSTGR Hex 6 HexNAc 2

**Mass:**  3412.4679

(1.642e-06)

**Charge States:** 3

**Start Position**: 48

**End Position**: 67

| Profile Score | Tandem Score | TDA q-value | Volume | Spectrum Matches |
| --- | --- | --- | --- | --- |
| -5.2365 | 111.4377 | 0.0000 | 5.0813e+06 | 1 |

**Adduct Signal Fractions**

- **Unmodified**: 5.0813e+06

### N(N)GTYDHDVYRDEALNNR Fuc 2 Hex 6 HexNAc 5

**Mass:**  4330.7463

(1.662e-06)

**Charge States:** 4

**Start Position**: 499

**End Position**: 516

| Profile Score | Tandem Score | TDA q-value | Volume | Spectrum Matches |
| --- | --- | --- | --- | --- |
| -3.1084 | 111.2843 | 0.0000 | 7.0910e+07 | 2 |

**Adduct Signal Fractions**

- **Unmodified**: 7.0910e+07

### TITNDQIEVTN(N)ATELVQSSSTGR Hex 5 HexNAc 4

**Mass:**  4085.7958

(1.673e-06)

**Charge States:** 3, 4

**Start Position**: 44

**End Position**: 67

| Profile Score | Tandem Score | TDA q-value | Volume | Spectrum Matches |
| --- | --- | --- | --- | --- |
| 13.4529 | 110.2166 | 0.0000 | 4.1566e+08 | 11 |

**Adduct Signal Fractions**

- **Unmodified**: 4.1566e+08

### N(N)GTYDHDVYRDEALNNR Fuc 3 Hex 5 HexNAc 4

**Mass:**  4111.6730

(2.035e-06)

**Charge States:** 4

**Start Position**: 499

**End Position**: 516

| Profile Score | Tandem Score | TDA q-value | Volume | Spectrum Matches |
| --- | --- | --- | --- | --- |
| -3.1104 | 108.9144 | 0.0000 | 1.3756e+08 | 2 |

**Adduct Signal Fractions**

- **Unmodified**: 1.3756e+08

### SDAPIGTC(C)SSEC(C)ITPN(N)GSIPNDKPFQNVNK Hex 8 HexNAc 2

**Mass:**  4949.0837

(2.477e-06)

**Charge States:** 3, 4, 5

**Start Position**: 286

**End Position**: 316

| Profile Score | Tandem Score | TDA q-value | Volume | Spectrum Matches |
| --- | --- | --- | --- | --- |
| 11.2227 | 107.9379 | 0.0000 | 1.3954e+08 | 5 |

**Adduct Signal Fractions**

- **Unmodified**: 1.3954e+08

### YPVLN(N)VTM(O)PNN(D)GK Hex 10 HexNAc 2

**Mass:**  3489.3997

(2.842e-04)

**Charge States:** 3

**Start Position**: 177

**End Position**: 190

| Profile Score | Tandem Score | TDA q-value | Volume | Spectrum Matches |
| --- | --- | --- | --- | --- |
| 10.9856 | 107.5689 | 0.0000 | 2.7854e+07 | 2 |

**Adduct Signal Fractions**

- **Unmodified**: 2.7854e+07

### TITNDQIEVTN(N)ATELVQSSSTGR Hex 7 HexNAc 2

**Mass:**  4003.7440

(1.503e-06)

**Charge States:** 3, 4

**Start Position**: 44

**End Position**: 67

| Profile Score | Tandem Score | TDA q-value | Volume | Spectrum Matches |
| --- | --- | --- | --- | --- |
| 13.7016 | 107.2829 | 0.0000 | 6.0897e+08 | 14 |

**Adduct Signal Fractions**

- **Unmodified**: 6.0897e+08

### N(N)GTYDHDVYRDEALNNR Fuc 4 Hex 6 HexNAc 5

**Mass:**  4622.8625

(1.701e-06)

**Charge States:** 4

**Start Position**: 499

**End Position**: 516

| Profile Score | Tandem Score | TDA q-value | Volume | Spectrum Matches |
| --- | --- | --- | --- | --- |
| -3.7028 | 107.0110 | 0.0000 | 2.3435e+07 | 1 |

**Adduct Signal Fractions**

- **Unmodified**: 2.3435e+07

### N(N)GTYDHDVYRDEALNNR Fuc 1 Hex 6 HexNAc 6

**Mass:**  4387.7634

(6.488e-07)

**Charge States:** 4

**Start Position**: 499

**End Position**: 516

| Profile Score | Tandem Score | TDA q-value | Volume | Spectrum Matches |
| --- | --- | --- | --- | --- |
| 6.6532 | 105.8668 | 0.0000 | 5.7669e+07 | 3 |

**Adduct Signal Fractions**

- **Unmodified**: 5.7669e+07

### RGSN(N)NSFFSR Hex 5 HexNAc 5

**Mass:**  2996.2163

(1.870e-06)

**Charge States:** 3

**Start Position**: 157

**End Position**: 167

| Profile Score | Tandem Score | TDA q-value | Volume | Spectrum Matches |
| --- | --- | --- | --- | --- |
| -3.8185 | 104.8273 | 0.0000 | 1.4810e+07 | 2 |

**Adduct Signal Fractions**

- **Unmodified**: 1.4810e+07

### N(N)GTYDHDVYRDEALNNR Fuc 1 Hex 8 HexNAc 2

**Mass:**  3899.5090

(-9.595e-06)

**Charge States:** 3, 4

**Start Position**: 499

**End Position**: 516

| Profile Score | Tandem Score | TDA q-value | Volume | Spectrum Matches |
| --- | --- | --- | --- | --- |
| -4.4685 | 104.5623 | 0.0000 | 3.1609e+07 | 2 |

**Adduct Signal Fractions**

- **Unmodified**: 3.1609e+07

### N(N)GTYDHDVYR Fuc 2 Hex 5 HexNAc 5

**Mass:**  3356.3153

(-1.452e-07)

**Charge States:** 3

**Start Position**: 499

**End Position**: 509

| Profile Score | Tandem Score | TDA q-value | Volume | Spectrum Matches |
| --- | --- | --- | --- | --- |
| -5.0840 | 104.2466 | 0.0000 | 4.5508e+07 | 4 |

**Adduct Signal Fractions**

- **Unmodified**: 4.5508e+07

### N(N)GTYDHDVYRDEALNNR Fuc 1 Hex 9 HexNAc 3

**Mass:**  4264.6437

(-7.966e-06)

**Charge States:** 4

**Start Position**: 499

**End Position**: 516

| Profile Score | Tandem Score | TDA q-value | Volume | Spectrum Matches |
| --- | --- | --- | --- | --- |
| -4.4874 | 103.7436 | 0.0000 | 3.0768e+07 | 2 |

**Adduct Signal Fractions**

- **Unmodified**: 3.0768e+07

### N(N)GTYDHDVYR Fuc 2 Hex 5 HexNAc 5

**Mass:**  3357.3082

(2.979e-04)

**Start Position**: 499

**End Position**: 509

| Profile Score | Tandem Score | TDA q-value | Volume | Spectrum Matches |
| --- | --- | --- | --- | --- |
| 0.0000 | 103.4864 | 0.0000 | 0.0000e+00 | 1 |

Adduct:

No Chromatogram Found

### SDAPIGTC(C)SSEC(C)ITPN(N)GSIPNDKPFQNVNK Fuc 2 Hex 3 HexNAc 4 Neu5Ac 1

**Mass:**  5128.1574

(-4.288e-06)

**Charge States:** 4

**Start Position**: 286

**End Position**: 316

| Profile Score | Tandem Score | TDA q-value | Volume | Spectrum Matches |
| --- | --- | --- | --- | --- |
| -4.4620 | 102.8835 | 0.0000 | 2.5627e+07 | 3 |

**Adduct Signal Fractions**

- **Unmodified**: 2.5627e+07

### N(N)GTYDHDVYRDEALN(D)NR Fuc 2 Hex 5 HexNAc 4

**Mass:**  3966.5996

(2.504e-04)

**Charge States:** 4

**Start Position**: 499

**End Position**: 516

| Profile Score | Tandem Score | TDA q-value | Volume | Spectrum Matches |
| --- | --- | --- | --- | --- |
| 6.6324 | 102.5825 | 0.0000 | 8.5180e+07 | 3 |

**Adduct Signal Fractions**

- **Unmodified**: 8.5180e+07

### N(N)GTYDHDVYR Fuc 1 Hex 5 HexNAc 4

**Mass:**  3009.1816

(6.685e-04)

**Start Position**: 499

**End Position**: 509

| Profile Score | Tandem Score | TDA q-value | Volume | Spectrum Matches |
| --- | --- | --- | --- | --- |
| 0.0000 | 101.4037 | 0.0000 | 0.0000e+00 | 1 |

Adduct:

No Chromatogram Found

### RGSN(N)NSFFSR Fuc 1 Hex 6 HexNAc 5

**Mass:**  3304.3269

(6.882e-07)

**Charge States:** 3

**Start Position**: 157

**End Position**: 167

| Profile Score | Tandem Score | TDA q-value | Volume | Spectrum Matches |
| --- | --- | --- | --- | --- |
| 6.9819 | 101.2470 | 0.0000 | 1.1429e+07 | 3 |

**Adduct Signal Fractions**

- **Unmodified**: 1.1429e+07

### YPVLN(N)VTM(O)PNN(D)GK Fuc 1 Hex 3 HexNAc 5 Neu5Ac 1

**Mass:**  3401.3963

(2.827e-04)

**Charge States:** 3

**Start Position**: 177

**End Position**: 190

| Profile Score | Tandem Score | TDA q-value | Volume | Spectrum Matches |
| --- | --- | --- | --- | --- |
| 12.1295 | 101.2049 | 0.0000 | 2.0427e+08 | 7 |

**Adduct Signal Fractions**

- **Unmodified**: 2.0427e+08

### TITNDQIEVTN(N)ATELVQSSSTGR Hex 6 HexNAc 2

**Mass:**  3841.6924

(1.683e-06)

**Charge States:** 3, 4

**Start Position**: 44

**End Position**: 67

| Profile Score | Tandem Score | TDA q-value | Volume | Spectrum Matches |
| --- | --- | --- | --- | --- |
| 14.2183 | 101.0289 | 0.0000 | 1.0451e+09 | 15 |

**Adduct Signal Fractions**

- **Unmodified**: 1.0451e+09

### N(N)GTYDHDVYRDEALNNR Fuc 2 Hex 9 HexNAc 3

**Mass:**  4410.7028

(-7.599e-06)

**Charge States:** 4

**Start Position**: 499

**End Position**: 516

| Profile Score | Tandem Score | TDA q-value | Volume | Spectrum Matches |
| --- | --- | --- | --- | --- |
| -4.8515 | 100.7460 | 0.0000 | 1.8491e+07 | 2 |

**Adduct Signal Fractions**

- **Unmodified**: 1.8491e+07

### N(N)GTYDHDVYR Fuc 4 Hex 6 HexNAc 5

**Mass:**  3810.4797

(6.797e-07)

**Charge States:** 3, 4

**Start Position**: 499

**End Position**: 509

| Profile Score | Tandem Score | TDA q-value | Volume | Spectrum Matches |
| --- | --- | --- | --- | --- |
| -20.0982 | 100.6946 | 0.0000 | 1.3103e+07 | 2 |

**Adduct Signal Fractions**

- **Unmodified**: 1.3103e+07

### N(N)GTYDHDVYR Fuc 3 Hex 6 HexNAc 5

**Mass:**  3664.4174

(8.107e-07)

**Charge States:** 3

**Start Position**: 499

**End Position**: 509

| Profile Score | Tandem Score | TDA q-value | Volume | Spectrum Matches |
| --- | --- | --- | --- | --- |
| -5.2609 | 98.8553 | 0.0000 | 3.1099e+07 | 2 |

**Adduct Signal Fractions**

- **Unmodified**: 3.1099e+07

### RGSN(N)N(D)SFFSR Fuc 1 Hex 7 HexNAc 3 Neu5Ac 1

**Mass:**  3352.2998

(2.941e-04)

**Charge States:** 3

**Start Position**: 157

**End Position**: 167

| Profile Score | Tandem Score | TDA q-value | Volume | Spectrum Matches |
| --- | --- | --- | --- | --- |
| -6.5235 | 98.7646 | 0.0000 | 9.5016e+06 | 1 |

**Adduct Signal Fractions**

- **Unmodified**: 9.5016e+06

### N(N)GTYDHDVYR Fuc 2 Hex 6 HexNAc 6

**Mass:**  3723.4717

(5.458e-04)

**Start Position**: 499

**End Position**: 509

| Profile Score | Tandem Score | TDA q-value | Volume | Spectrum Matches |
| --- | --- | --- | --- | --- |
| 0.0000 | 98.7525 | 0.0000 | 0.0000e+00 | 1 |

Adduct:

No Chromatogram Found

### N(N)GTYDHDVYRDEALNNR Fuc 2 Hex 5 HexNAc 5

**Mass:**  4168.6896

(9.046e-07)

**Charge States:** 4

**Start Position**: 499

**End Position**: 516

| Profile Score | Tandem Score | TDA q-value | Volume | Spectrum Matches |
| --- | --- | --- | --- | --- |
| 12.2889 | 97.9840 | 0.0000 | 9.0732e+07 | 4 |

**Adduct Signal Fractions**

- **Unmodified**: 9.0732e+07

### N(N)GTYDHDVYR Fuc 1 Hex 8 HexNAc 7

**Mass:**  4104.5809

(4.907e-04)

**Start Position**: 499

**End Position**: 509

| Profile Score | Tandem Score | TDA q-value | Volume | Spectrum Matches |
| --- | --- | --- | --- | --- |
| 0.0000 | 95.9556 | 0.0079 | 0.0000e+00 | 1 |

Adduct:

No Chromatogram Found

### RGSN(N)N(D)SFFSR Hex 7 HexNAc 3 Neu5Ac 1

**Mass:**  3206.2395

(3.068e-04)

**Charge States:** 3

**Start Position**: 157

**End Position**: 167

| Profile Score | Tandem Score | TDA q-value | Volume | Spectrum Matches |
| --- | --- | --- | --- | --- |
| 3.5294 | 95.2796 | 0.0150 | 1.9386e+07 | 3 |

**Adduct Signal Fractions**

- **Unmodified**: 1.9386e+07

### N(N)GTYDHDVYR Fuc 1 Hex 7 HexNAc 6

**Mass:**  3737.4370

(2.193e-06)

**Charge States:** 4

**Start Position**: 499

**End Position**: 509

| Profile Score | Tandem Score | TDA q-value | Volume | Spectrum Matches |
| --- | --- | --- | --- | --- |
| -20.7110 | 93.7744 | 0.0150 | 1.1927e+07 | 2 |

**Adduct Signal Fractions**

- **Unmodified**: 1.1927e+07

### N(N)GTYDHDVYRDEALNNR Fuc 1 Hex 7 HexNAc 6

**Mass:**  4549.8215

(1.789e-06)

**Charge States:** 4

**Start Position**: 499

**End Position**: 516

| Profile Score | Tandem Score | TDA q-value | Volume | Spectrum Matches |
| --- | --- | --- | --- | --- |
| -4.5057 | 93.1289 | 0.0216 | 2.4712e+07 | 1 |

**Adduct Signal Fractions**

- **Unmodified**: 2.4712e+07

### N(N)GTYDHDVYR Hex 5 HexNAc 4

**Mass:**  2861.1134

(6.152e-08)

**Charge States:** 3

**Start Position**: 499

**End Position**: 509

| Profile Score | Tandem Score | TDA q-value | Volume | Spectrum Matches |
| --- | --- | --- | --- | --- |
| -20.0090 | 92.8446 | 0.0216 | 7.1004e+06 | 1 |

**Adduct Signal Fractions**

- **Unmodified**: 7.1004e+06

### RGSN(N)NSFFSR Fuc 2 Hex 5 HexNAc 6

**Mass:**  3491.4116

(2.146e-06)

**Charge States:** 3

**Start Position**: 157

**End Position**: 167

| Profile Score | Tandem Score | TDA q-value | Volume | Spectrum Matches |
| --- | --- | --- | --- | --- |
| -19.7616 | 89.6579 | 0.0483 | 4.2300e+06 | 1 |

**Adduct Signal Fractions**

- **Unmodified**: 4.2300e+06

### N(N)GTYDHDVYR Fuc 2 Hex 6 HexNAc 6

**Mass:**  3721.4462

(1.545e-06)

**Charge States:** 3, 4

**Start Position**: 499

**End Position**: 509

| Profile Score | Tandem Score | TDA q-value | Volume | Spectrum Matches |
| --- | --- | --- | --- | --- |
| -5.7048 | 89.3520 | 0.0483 | 1.3293e+07 | 2 |

**Adduct Signal Fractions**

- **Unmodified**: 1.3293e+07

### RGSN(N)N(D)SFFSR Fuc 2 Hex 5 HexNAc 5

**Mass:**  3289.3214

(3.016e-04)

**Charge States:** 4

**Start Position**: 157

**End Position**: 167

| Profile Score | Tandem Score | TDA q-value | Volume | Spectrum Matches |
| --- | --- | --- | --- | --- |
| -5.9140 | 88.8132 | 0.0483 | 1.8054e+07 | 2 |

**Adduct Signal Fractions**

- **Unmodified**: 1.8054e+07

### iav|ADJ41819|A/Phil/2-MA/1982\_NA

**>iav|ADJ41819|A/Phil/2-MA/1982\_NA**  
 `MNPNQKIITIGSVSLTIATICFLMQIAILVTTVTLHFKQYECSSPPNNQVVPCEPIIIERNITEIVYLTNTTIEKEICPK  
LVEYRNWSKPQCKITGFAPFSKDNSIRLSAGGDIWVTREPYVSCDPGKCYQFALGQGTTLDNKHSNDTIHDRTPYRTLLM  
NELGVPFHLGTRQVCIAWSSSSCHDGKAWLHVCITGYDKNATASFIYDGRLVDSIGSWSKNILRTQESECVCINGTCTVV  
MTDGSASERADTKILFIEEGKIVHISPLSGSAQHVEECSCYPRYPGVRCVCRDNWKGSNRPVVDINVKDYSIVSSYVCSG  
LVGDTPRKNDRSSSSYCRNPNNEKGNHGVKGWAFDDGNDVWMGRTISEESRSGYETFKVIGGWSTPNSKLQINRQVIVDS  
GNRSGYSGIFSVEGKSCINRCFYVELIRGREQETRVWWTSNSIVVFCGTSGTYGTGSWPDGADINLMPI`

| Site | Type | Count |
| --- | --- | --- |
| 402 | N-linked | 2 |
| 200 | N-linked | 4 |

| Mass | Sequence | MS_2_ Score | `q`-value | MS_1_ Score | Signal | Protein Coordinates | Tandem Scans | Start Time | Apex Time | End Time |
| --- | --- | --- | --- | --- | --- | --- | --- | --- | --- | --- |
| 2364.9919 | QVIVDSGN(N)R Hex 6 HexNAc 2 | 172.255 | 0.0 | -20.906 | 9.299e+06 | 394 - 403 | 1 | 12.64 | 12.64 | 12.64 |
| 2689.0999 | QVIVDSGN(N)R Hex 8 HexNAc 2 | 168.497 | 0.0 | -21.433 | 2.750e+06 | 394 - 403 | 1 | 12.47 | 12.47 | 12.47 |
| 2916.1578 | N(N)ATASFIYDGR Hex 8 HexNAc 2 | 129.068 | 0.0 | 14.080 | 5.671e+07 | 199 - 210 | 4 | 24.96 | 25.13 | 25.98 |
| 3078.2109 | N(N)ATASFIYDGR Hex 9 HexNAc 2 | 114.596 | 0.0 | -4.339 | 1.569e+07 | 199 - 210 | 1 | 24.96 | 24.96 | 25.47 |
| 2755.1023 | N(N)ATASFIYDGR Hex 7 HexNAc 2 | 107.696 | 0.0 | 0.000 | 0.000e+00 | 199 - 210 | 1 | - | - | - |
| 2754.1052 | N(N)ATASFIYDGR Hex 7 HexNAc 2 | 105.486 | 0.0 | 13.361 | 1.944e+07 | 199 - 210 | 2 | 25.30 | 25.30 | 26.15 |

### QVIVDSGN(N)R Hex 6 HexNAc 2

**Mass:**  2364.9906

(1.427e-06)

**Charge States:** 2, 3

**Start Position**: 395

**End Position**: 404

| Profile Score | Tandem Score | TDA q-value | Volume | Spectrum Matches |
| --- | --- | --- | --- | --- |
| -20.9056 | 172.2549 | 0.0000 | 9.2987e+06 | 1 |

**Adduct Signal Fractions**

- **Unmodified**: 9.2987e+06

### QVIVDSGN(N)R Hex 8 HexNAc 2

**Mass:**  2689.0999

(1.525e-06)

**Charge States:** 3

**Start Position**: 395

**End Position**: 404

| Profile Score | Tandem Score | TDA q-value | Volume | Spectrum Matches |
| --- | --- | --- | --- | --- |
| -21.4329 | 168.4966 | 0.0000 | 2.7501e+06 | 1 |

**Adduct Signal Fractions**

- **Unmodified**: 2.7501e+06

### N(N)ATASFIYDGR Hex 8 HexNAc 2

**Mass:**  2916.1574

(1.124e-06)

**Charge States:** 3

**Start Position**: 200

**End Position**: 211

| Profile Score | Tandem Score | TDA q-value | Volume | Spectrum Matches |
| --- | --- | --- | --- | --- |
| 14.0798 | 129.0680 | 0.0000 | 5.6707e+07 | 4 |

**Adduct Signal Fractions**

- **Unmodified**: 5.6707e+07

### N(N)ATASFIYDGR Hex 9 HexNAc 2

**Mass:**  3078.2111

(1.323e-06)

**Charge States:** 3

**Start Position**: 200

**End Position**: 211

| Profile Score | Tandem Score | TDA q-value | Volume | Spectrum Matches |
| --- | --- | --- | --- | --- |
| -4.3385 | 114.5956 | 0.0000 | 1.5691e+07 | 1 |

**Adduct Signal Fractions**

- **Unmodified**: 1.5691e+07

### N(N)ATASFIYDGR Hex 7 HexNAc 2

**Mass:**  2755.1023

(3.635e-04)

**Start Position**: 200

**End Position**: 211

| Profile Score | Tandem Score | TDA q-value | Volume | Spectrum Matches |
| --- | --- | --- | --- | --- |
| 0.0000 | 107.6958 | 0.0000 | 0.0000e+00 | 1 |

Adduct:

No Chromatogram Found

### N(N)ATASFIYDGR Hex 7 HexNAc 2

**Mass:**  2754.1054

(1.134e-06)

**Charge States:** 3

**Start Position**: 200

**End Position**: 211

| Profile Score | Tandem Score | TDA q-value | Volume | Spectrum Matches |
| --- | --- | --- | --- | --- |
| 13.3606 | 105.4856 | 0.0000 | 1.9445e+07 | 2 |

**Adduct Signal Fractions**

- **Unmodified**: 1.9445e+07

### tr|Q5F491|Q5F491\_CHICK

**>tr|Q5F491|Q5F491\_CHICK**  
 `MSHVAVENALSLDQQFSGLDLNSSDSQSEGSATSKGRYIPPHLRNREASKQGFDSGGWSTSRDKDAYSSFGARSDRGAKS  
SFFDRGNGSRGGRYEERGRGSDYDRSGFGRFDRGGNSRWCDKSDEDDWSKPLPPSERLEQELFSGSNTGINFEKYDDIPV  
EATGSNCPPHIESFSDVDMGEIIMGNIELTRYTRPTPVQKHAIPIIKEKRDLMACAQTGSGKTAAFLLPILSQIYADGPG  
DALRAMKENGRYGRRKQYPISLVLAPTRELAVQIYEEARKFAYRSRVRPCVVYGGADIGQQIRDLERGCHLLVATPGRLV  
DMMERGKIGLDFCKYLVLDEADRMLDMGFEPQIRRIVEQDTMPPKGVRHTMMFSATFPKEIQMLARDFLDEYIFLAVGRV  
GSTSENITQKVVWVEELDKRSFLLDLLNATGKDSLTLVFVETKKGADALEDFLYHEGYACTSIHGDRSQRDREEALHQFR  
SGKSPILVATAVAARGLDISNVKHVINFDLPSDIEEYVHRIGRTGRVGNLGLATSFFNERNINITKDLLDLLVEAKQEVP  
SWLENMAYEQHHKGGGSRGRSKSRFTGGFGARDYRTSSGAGSSSFSSSRPASGRTGGSGSRGFGGGGYGGFYNSDGYGGN  
YNSQGVDWWGN`

| Site | Type | Count |
| --- | --- | --- |
| 87 | N-linked | 1 |

| Mass | Sequence | MS_2_ Score | `q`-value | MS_1_ Score | Signal | Protein Coordinates | Tandem Scans | Start Time | Apex Time | End Time |
| --- | --- | --- | --- | --- | --- | --- | --- | --- | --- | --- |
| 3200.2870 | SSFFDRGN(N)GSR Fuc 1 Hex 5 HexNAc 5 | 97.525 | 0.0 | -18.315 | 1.925e+07 | 79 - 90 | 2 | 17.17 | 17.17 | 17.34 |

### SSFFDRGN(N)GSR Fuc 1 Hex 5 HexNAc 5

**Mass:**  3200.2868

(3.457e-06)

**Charge States:** 3, 4

**Start Position**: 80

**End Position**: 91

| Profile Score | Tandem Score | TDA q-value | Volume | Spectrum Matches |
| --- | --- | --- | --- | --- |
| -18.3155 | 97.5251 | 0.0000 | 1.9255e+07 | 2 |

**Adduct Signal Fractions**

- **Unmodified**: 1.9255e+07
