# Supplementary material for: Measuring Site-specific Glycosylation Similarity between Influenza a Virus Variants with Statistical Certainty
Source: Mol Cell Proteomics. 2020 Nov 25;19(9):1533–45. doi: 10.1074/mcp.RA120.002031 (PMC8143645; doi:10.1074/mcp.RA120.002031)
Supplement: Supplementary file 2 [file mmc2.zip › mmc2/159250_2_supp_555291_qcjklr.zip!/20190301_5B8eggL2_021119_chymo_HILIC_DDA_1ug_02_glycopeptides.html]

## 20190301\_5B8eggL2\_021119\_chymo\_HILIC\_DDA\_1ug\_02 @ GlycopeptideHypothesis-0b4bd4e337ec455597a62a031fa6bf44

**MS^1^ Mass Accuracy:** 10.0 PPM  
**Grouping Tolerance:** 15.0 PPM  
**MS^n^ Mass Accuracy:** 20.0 PPM  
**Minimum `q`-Value For Clustering:** 0.05

**Glycopeptide Hypothesis:**
GlycopeptideHypothesis-0b4bd4e337ec455597a62a031fa6bf44  
**Sample Searched:**
20190301\_5B8eggL2\_021119\_chymo\_HILIC\_DDA\_1ug\_02

| Protein | Matched Glycopeptides |
| --- | --- |
| cus\|SWZHA\_5B8\|A/Switzerland/9715293/2013 | 102 |
| cus\|SWZNA\|A/Switzerland/9715293/2013 | 36 |
| iav\|ACF41842\|A/Puerto | 1 |
| iav\|AGU93028\|A/Puerto | 1 |
| iav\|AFM71854\|A/Puerto | 1 |
| iav\|AAM75156\|A/Puerto | 1 |
| iav\|ADX99655\|A/Puerto | 1 |
| iav\|ACV49553\|A/Puerto | 1 |
| iav\|BAV59609\|A/Puerto | 1 |

### cus|SWZHA\_5B8|A/Switzerland/9715293/2013

**>cus|SWZHA\_5B8|A/Switzerland/9715293/2013**  
 `QKLPGNDNSTATLCLGHHAVPNGTIVKTITNDRIEVTNATELVQNSSIGEICDSPHQILDGENCTLIDALLGDPQCDGFQ  
NKKWDLFVERSKAYSNCYPYDVPDYASLRSLVASSGTLEFNNESFNWAGVTHNGTSSSCRRGSNSSFFSRLNWLTHLNSK  
YPALNVTMPNNEQFDKLYIWGVHHPVTDKDQIFLYAQSSGRITVSTKRSQQAVIPNIGSRPRIRNIPSRISIYWTIVKPG  
DILLINSTGNLIAPRGYFKIRSGKSSIMRSDAPIGKCKSECITPNGSIPNDKPFQNVNRITYGACPRYVKQSTLKLATGM  
RNVPERQTRGIFGAIAGFIENGWEGMVDGWYGFRHQNSEGRGQAADLKSTQAAIDQINGKLNRLIGKTNEKFHQIEKEFS  
EVEGRIQDLEKYVEDTKIDLWSYNAELLVALENQHTIDLTDSEMNKLFEKTKKQLRENAEDMGNGCFKIYHKCDNACIGS  
IRNGTYDHDVYRDEALNNRFQIKGVELKSGYKDWILWISFAISCFLLCVALLGFIMWACQKGNIRCNICI`

| Site | Type | Count |
| --- | --- | --- |
| 483 | N-linked | 1 |
| 165 | N-linked | 5 |
| 38 | N-linked | 3 |
| 8 | N-linked | 1 |
| 144 | N-linked | 7 |
| 246 | N-linked | 49 |
| 133 | N-linked | 16 |
| 285 | N-linked | 19 |
| 22 | N-linked | 1 |

| Mass | Sequence | MS_2_ Score | `q`-value | MS_1_ Score | Signal | Protein Coordinates | Tandem Scans | Start Time | Apex Time | End Time |
| --- | --- | --- | --- | --- | --- | --- | --- | --- | --- | --- |
| 3499.5192 | LIN(N)STGNLIAPRGYF Hex 9 HexNAc 2 | 176.057 | 0.0 | -3.027 | 9.165e+07 | 243 - 258 | 3 | 47.90 | 47.90 | 48.23 |
| 3352.4503 | LIN(N)STGNLIAPRGY Hex 9 HexNAc 2 | 175.496 | 0.0 | 14.904 | 8.029e+07 | 243 - 257 | 3 | 37.72 | 37.89 | 38.40 |
| 2695.0873 | LIN(N)STGNL Hex 9 HexNAc 2 | 169.909 | 0.0 | -19.440 | 1.200e+07 | 243 - 251 | 1 | 27.48 | 27.65 | 27.65 |
| 4068.9248 | TIVKPGDILLIN(N)STGNLIAPR Hex 9 HexNAc 2 | 167.888 | 0.0 | 17.085 | 2.009e+09 | 234 - 255 | 17 | 52.15 | 54.03 | 112.89 |
| 2533.0340 | LIN(N)STGNL Hex 8 HexNAc 2 | 165.945 | 0.0 | -20.336 | 7.976e+05 | 243 - 251 | 2 | 27.65 | 27.65 | 27.65 |
| 3906.8727 | TIVKPGDILLIN(N)STGNLIAPR Hex 8 HexNAc 2 | 163.329 | 0.0 | 15.569 | 2.309e+08 | 234 - 255 | 5 | 53.86 | 54.03 | 54.87 |
| 3017.1378 | NWAGVTHN(N)GTSSSC(C) Hex 7 HexNAc 2 | 161.423 | 0.0 | -18.375 | 8.262e+06 | 125 - 139 | 2 | 18.97 | 18.97 | 19.13 |
| 4126.9607 | TIVKPGDILLIN(N)STGNLIAPRGY Hex 8 HexNAc 2 | 161.344 | 0.0 | 15.015 | 5.744e+08 | 234 - 257 | 16 | 55.55 | 56.40 | 58.79 |
| 4230.9782 | TIVKPGDILLIN(N)STGNLIAPR Hex 10 HexNAc 2 | 161.311 | 0.0 | -3.544 | 9.106e+07 | 234 - 255 | 2 | 53.86 | 53.86 | 54.36 |
| 3159.4266 | TIVKPGDILLIN(N) Hex 9 HexNAc 2 | 158.305 | 0.0 | 11.251 | 1.559e+08 | 234 - 246 | 5 | 47.07 | 48.41 | 48.92 |
| 4289.0173 | TIVKPGDILLIN(N)STGNLIAPRGY Hex 9 HexNAc 2 | 157.639 | 0.0 | 17.926 | 4.106e+09 | 234 - 257 | 34 | 54.36 | 56.23 | 112.89 |
| 4306.0363 | TIVKPGDILLIN(N)STGNLIAPRGY Fuc 2 Hex 3 HexNAc 4 Neu5Ac 1 | 157.308 | 0.0 | 14.161 | 2.983e+08 | 234 - 257 | 4 | 55.89 | 56.23 | 56.73 |
| 2770.0728 | NWAGVTHN(N)GTSS Hex 7 HexNAc 2 | 156.892 | 0.0 | -18.150 | 4.332e+06 | 125 - 137 | 1 | 18.47 | 18.47 | 18.47 |
| 3337.4639 | LIN(N)STGNLIAPRGYF Hex 8 HexNAc 2 | 154.594 | 0.0 | -17.940 | 6.384e+06 | 243 - 258 | 1 | 48.06 | 48.06 | 48.06 |
| 3190.3978 | LIN(N)STGNLIAPRGY Hex 8 HexNAc 2 | 154.290 | 0.0 | -16.805 | 8.840e+06 | 243 - 257 | 1 | 37.89 | 37.89 | 38.06 |
| 4085.9503 | TIVKPGDILLIN(N)STGNLIAPR Fuc 2 Hex 3 HexNAc 4 Neu5Ac 1 | 154.221 | 0.0 | 13.219 | 1.424e+08 | 234 - 255 | 3 | 53.86 | 54.03 | 54.70 |
| 3176.4526 | TIVKPGDILLIN(N) Fuc 2 Hex 3 HexNAc 4 Neu5Ac 1 | 154.120 | 0.0 | 13.562 | 3.319e+07 | 234 - 246 | 4 | 47.07 | 48.41 | 48.58 |
| 3648.6793 | TIVKPGDILLIN(N)STGNL Fuc 2 Hex 3 HexNAc 4 Neu5Ac 1 | 153.258 | 0.0 | -4.001 | 1.014e+08 | 234 - 251 | 2 | 53.52 | 53.69 | 53.86 |
| 4451.0660 | TIVKPGDILLIN(N)STGNLIAPRGY Hex 10 HexNAc 2 | 152.386 | 0.0 | 16.048 | 1.866e+08 | 234 - 257 | 10 | 55.89 | 56.06 | 60.95 |
| 3431.4081 | AGVTHN(N)GTSSSC(C)RRGSNSSFF Hex 5 HexNAc 2 | 152.351 | 0.0 | -3.425 | 1.021e+08 | 127 - 148 | 2 | 21.98 | 22.14 | 22.31 |
| 3518.5728 | TIVKPGDILLIN(N)STGN Hex 9 HexNAc 2 | 149.168 | 0.0 | 15.985 | 1.774e+08 | 234 - 250 | 5 | 45.88 | 46.22 | 46.56 |
| 4427.9430 | RSDAPIGKC(C)KSEC(C)ITPN(N)GSIPNDKPF Hex 7 HexNAc 2 | 148.978 | 0.0 | 16.263 | 3.570e+08 | 268 - 294 | 3 | 30.84 | 31.00 | 31.51 |
| 4203.7806 | NSKYPALN(N)VTM(O)PNNEQFDKLY Hex 8 HexNAc 2 | 147.917 | 0.0 | -2.183 | 1.140e+08 | 157 - 178 | 4 | 43.50 | 43.84 | 44.01 |
| 3631.6534 | TIVKPGDILLIN(N)STGNL Hex 9 HexNAc 2 | 147.605 | 0.0 | 17.033 | 4.877e+08 | 234 - 251 | 9 | 53.00 | 53.69 | 55.21 |
| 2997.3748 | TIVKPGDILLIN(N) Hex 8 HexNAc 2 | 147.211 | 0.0 | 12.256 | 1.358e+07 | 234 - 246 | 2 | 48.06 | 48.41 | 48.75 |
| 3535.5970 | TIVKPGDILLIN(N)STGN Fuc 2 Hex 3 HexNAc 4 Neu5Ac 1 | 147.121 | 0.0 | -3.951 | 4.121e+07 | 234 - 250 | 2 | 46.05 | 46.22 | 46.56 |
| 3516.5447 | LIN(N)STGNLIAPRGYF Fuc 2 Hex 3 HexNAc 4 Neu5Ac 1 | 144.719 | 0.0 | -18.837 | 1.368e+07 | 243 - 258 | 1 | 47.90 | 47.90 | 48.06 |
| 4752.0494 | RSDAPIGKC(C)KSEC(C)ITPN(N)GSIPNDKPF Hex 9 HexNAc 2 | 142.988 | 0.0 | 14.725 | 3.563e+08 | 268 - 294 | 3 | 30.84 | 31.00 | 31.51 |
| 3793.7058 | TIVKPGDILLIN(N)STGNL Hex 10 HexNAc 2 | 142.539 | 0.0 | -19.013 | 1.665e+07 | 234 - 251 | 2 | 53.52 | 53.52 | 53.69 |
| 3731.5475 | NWAGVTHN(N)GTSSSC(C)RRGSNSSFF Hex 5 HexNAc 2 | 140.540 | 0.0 | -17.157 | 2.961e+07 | 125 - 148 | 3 | 31.51 | 31.51 | 31.68 |
| 4055.6459 | NWAGVTHNGTSSSC(C)RRGSN(N)SSFF Hex 7 HexNAc 2 | 140.313 | 0.0 | -2.769 | 7.254e+07 | 125 - 148 | 2 | 31.17 | 31.17 | 31.68 |
| 3443.4418 | KSEC(C)ITPN(N)GSIPNDKPF Hex 7 HexNAc 2 | 139.502 | 0.0 | -5.014 | 3.698e+07 | 277 - 294 | 1 | 32.67 | 32.67 | 32.99 |
| 3964.9083 | TIVKPGDILLIN(N)STGNLIAPRGY Hex 7 HexNAc 2 | 139.217 | 0.0 | -4.027 | 4.239e+07 | 234 - 257 | 4 | 56.23 | 56.56 | 56.73 |
| 3469.5986 | TIVKPGDILLIN(N)STGNL Hex 8 HexNAc 2 | 139.202 | 0.0 | -4.076 | 5.607e+07 | 234 - 251 | 2 | 53.52 | 53.69 | 54.03 |
| 3132.3667 | LIN(N)STGNLIAPR Hex 9 HexNAc 2 | 138.709 | 0.0 | -16.510 | 3.055e+07 | 243 - 255 | 1 | 32.19 | 32.19 | 32.19 |
| 2857.1077 | NWAGVTHN(N)GTSSS Hex 7 HexNAc 2 | 137.448 | 0.0 | -19.536 | 2.817e+06 | 125 - 138 | 2 | 18.30 | 18.30 | 18.30 |
| 3755.5132 | AGVTHN(N)GTSSSC(C)RRGSNSSFF Hex 7 HexNAc 2 | 135.174 | 0.0 | 15.909 | 2.585e+08 | 127 - 148 | 3 | 21.14 | 21.64 | 22.14 |
| 4589.9939 | RSDAPIGKC(C)KSEC(C)ITPN(N)GSIPNDKPF Hex 8 HexNAc 2 | 129.853 | 0.008 | -2.715 | 2.161e+08 | 268 - 294 | 3 | 30.84 | 31.00 | 31.34 |
| 3640.8059 | TIVKPGDILLIN(N)STGNLIAPRGY Hex 5 HexNAc 2 | 129.832 | 0.008 | -19.090 | 1.517e+07 | 234 - 257 | 2 | 56.90 | 56.90 | 57.07 |
| 3019.2815 | IN(N)STGNLIAPR Hex 9 HexNAc 2 | 129.309 | 0.008 | -18.480 | 4.802e+06 | 244 - 255 | 1 | 25.98 | 25.98 | 25.98 |
| 4089.7383 | SKYPALN(N)VTM(O)PNNEQFDKLY Hex 8 HexNAc 2 | 129.058 | 0.008 | -2.141 | 4.472e+07 | 158 - 178 | 4 | 43.67 | 43.84 | 44.01 |
| 3356.5172 | TIVKPGDILLIN(N)STGN Hex 8 HexNAc 2 | 128.468 | 0.008 | -3.950 | 1.956e+07 | 234 - 250 | 3 | 46.05 | 46.22 | 46.39 |
| 3605.5006 | KSEC(C)ITPN(N)GSIPNDKPF Hex 8 HexNAc 2 | 128.050 | 0.008 | -18.566 | 1.125e+07 | 277 - 294 | 1 | 32.67 | 32.67 | 32.67 |
| 3593.4618 | AGVTHNGTSSSC(C)RRGSN(N)SSFF Hex 6 HexNAc 2 | 127.132 | 0.008 | -3.897 | 6.658e+07 | 127 - 148 | 1 | 21.64 | 21.81 | 22.14 |
| 4265.8846 | RSDAPIGKC(C)KSEC(C)ITPN(N)GSIPNDKPF Hex 6 HexNAc 2 | 124.730 | 0.008 | -3.551 | 3.723e+07 | 268 - 294 | 1 | 31.00 | 31.17 | 31.34 |
| 3386.4325 | IN(N)STGNLIAPRGYF Hex 9 HexNAc 2 | 124.660 | 0.008 | -17.104 | 1.003e+07 | 244 - 258 | 1 | 44.18 | 44.18 | 44.18 |
| 3229.3933 | TITNDRIEVTN(N)ATEL Hex 7 HexNAc 2 | 123.285 | 0.008 | -18.003 | 3.550e+06 | 27 - 42 | 1 | 37.55 | 37.55 | 37.72 |
| 3369.4759 | LIN(N)STGNLIAPRGY Fuc 2 Hex 3 HexNAc 4 Neu5Ac 1 | 121.760 | 0.008 | -18.486 | 1.141e+07 | 243 - 257 | 1 | 37.72 | 37.89 | 37.89 |
| 2905.2866 | TITNDRIEVTN(N)ATEL Hex 5 HexNAc 2 | 121.040 | 0.008 | -18.448 | 4.076e+06 | 27 - 42 | 1 | 37.89 | 37.89 | 38.06 |
| 4429.9457 | TIVKPGDILLIN(N) Fuc 5 Hex 3 HexNAc 8 Neu5Ac 1 | 119.885 | 0.008 | 0.000 | 0.000e+00 | 234 - 246 | 1 | - | - | - |
| 3680.6255 | TIVKPGDILLIN(N)STGN Hex 10 HexNAc 2 | 118.142 | 0.008 | -4.725 | 6.813e+06 | 234 - 250 | 1 | 45.88 | 46.05 | 46.22 |
| 2872.1295 | RGSN(N)SSFF Fuc 1 Hex 5 HexNAc 5 | 111.275 | 0.0 | -19.962 | 3.586e+06 | 140 - 148 | 1 | 26.81 | 26.81 | 26.81 |
| 3067.3412 | TITNDRIEVTN(N)ATEL Hex 6 HexNAc 2 | 109.449 | 0.008 | -19.506 | 3.848e+06 | 27 - 42 | 1 | 37.72 | 37.72 | 37.89 |
| 3917.5655 | AGVTHN(N)GTSSSC(C)RRGSNSSFF Hex 8 HexNAc 2 | 108.899 | 0.012 | -17.688 | 2.777e+07 | 127 - 148 | 1 | 21.48 | 21.64 | 21.64 |
| 3639.4579 | SEC(C)ITPN(N)GSIPNDKPF Hex 9 HexNAc 2 | 105.294 | 0.012 | -18.930 | 3.486e+06 | 278 - 294 | 1 | 36.71 | 36.71 | 36.88 |
| 4069.9215 | TIVKPGDILLIN(N)STGN(D)LIAPR Hex 9 HexNAc 2 | 104.468 | 0.012 | -19.791 | 1.234e+07 | 234 - 255 | 3 | 55.04 | 55.04 | 55.04 |
| 3810.7318 | TIVKPGDILLIN(N)STGNL Fuc 2 Hex 4 HexNAc 4 Neu5Ac 1 | 103.203 | 0.012 | -16.927 | 2.159e+06 | 234 - 251 | 1 | 53.52 | 53.52 | 53.52 |
| 3473.5034 | C(C)LGHHAVPN(N)GTIVK Fuc 1 Hex 5 HexNAc 5 | 99.574 | 0.012 | -20.079 | 3.869e+06 | 13 - 27 | 1 | 19.80 | 19.80 | 19.97 |
| 3477.4055 | SEC(C)ITPN(N)GSIPNDKPF Hex 8 HexNAc 2 | 92.855 | 0.012 | -20.157 | 8.280e+05 | 278 - 294 | 1 | 36.71 | 36.71 | 36.71 |
| 2272.9352 | RRGSN(N)SSFF Hex 5 HexNAc 2 | 88.291 | 0.0 | -20.284 | 2.578e+06 | 139 - 148 | 1 | 22.14 | 22.14 | 22.14 |
| 3420.7136 | TIVKPGDILLIN(N)STGNLIAPR Hex 5 HexNAc 2 | 81.943 | 0.012 | -4.706 | 6.950e+06 | 234 - 255 | 2 | 54.53 | 54.70 | 54.87 |
| 4753.0347 | RSDAPIGKC(C)KSEC(C)ITPN(N)GSIPN(D)DKPF Hex 9 HexNAc 2 | 80.373 | 0.012 | -5.624 | 6.577e+07 | 268 - 294 | 1 | 31.68 | 31.68 | 32.02 |
| 4428.9274 | RSDAPIGKC(C)KSEC(C)ITPN(N)GSIPN(D)DKPF Hex 7 HexNAc 2 | 79.808 | 0.012 | -5.568 | 7.066e+07 | 268 - 294 | 2 | 31.68 | 31.85 | 32.02 |
| 4125.9508 | TIVKPGDILLIN(N)STGNLIAPRG Hex 9 HexNAc 2 | 74.381 | 0.015 | 16.081 | 5.317e+08 | 234 - 256 | 11 | 53.69 | 54.87 | 55.72 |
| 5531.5551 | KIRSGKSSIM(O)RSDAPIGKC(C)KSEC(C)ITPN(N)GSIPNDKPF Hex 7 HexNAc 2 | 73.023 | 0.015 | -3.806 | 5.333e+07 | 258 - 294 | 1 | 27.82 | 27.98 | 28.15 |
| 3802.8551 | TIVKPGDILLIN(N)STGNLIAPRGY Hex 6 HexNAc 2 | 71.866 | 0.015 | -5.229 | 6.410e+06 | 234 - 257 | 2 | 56.56 | 56.73 | 56.90 |
| 4590.9853 | RSDAPIGKC(C)KSEC(C)ITPN(N)GSIPN(D)DKPF Hex 8 HexNAc 2 | 66.113 | 0.015 | -5.690 | 4.169e+07 | 268 - 294 | 2 | 31.68 | 31.85 | 32.02 |
| 3472.4323 | AGVTHN(N)GTSSSC(C)RRGSNSSFF Hex 4 HexNAc 3 | 65.520 | 0.015 | -18.158 | 1.499e+07 | 127 - 148 | 1 | 22.14 | 22.14 | 22.14 |
| 3958.5940 | AGVTHN(N)GTSSSC(C)RRGSNSSFF Hex 7 HexNAc 3 | 64.410 | 0.015 | -18.143 | 2.555e+07 | 127 - 148 | 1 | 21.64 | 21.64 | 21.81 |
| 5134.2638 | SGKSSIM(O)RSDAPIGKC(C)KSEC(C)ITPN(N)GSIPNDKPF Hex 7 HexNAc 2 | 62.164 | 0.015 | -4.679 | 1.947e+07 | 261 - 294 | 1 | 30.33 | 30.50 | 30.67 |
| 3837.5676 | AGVTHN(N)GTSSSC(C)RRGSNSSFF Hex 5 HexNAc 4 | 60.453 | 0.015 | -4.489 | 4.423e+07 | 127 - 148 | 2 | 21.81 | 22.14 | 22.31 |
| 4591.9986 | RSDAPIGKC(C)KSEC(C)ITPN(N)GSIPN(D)DKPF Hex 8 HexNAc 2 | 58.041 | 0.015 | 0.000 | 0.000e+00 | 268 - 294 | 1 | - | - | - |
| 4161.6720 | AGVTHN(N)GTSSSC(C)RRGSNSSFF Hex 7 HexNAc 4 | 55.094 | 0.015 | -4.242 | 3.049e+07 | 127 - 148 | 1 | 21.48 | 21.64 | 21.81 |
| 4204.7636 | N(D)SKYPALN(N)VTM(O)PNNEQFDKLY Hex 8 HexNAc 2 | 54.362 | 0.015 | -6.107 | 2.424e+07 | 157 - 178 | 2 | 46.39 | 46.56 | 46.73 |
| 4187.7868 | NSKYPALN(N)VTMPNNEQFDKLY Hex 8 HexNAc 2 | 52.383 | 0.015 | -18.477 | 3.920e+06 | 157 - 178 | 2 | 49.60 | 49.60 | 49.77 |
| 5416.4395 | RSDAPIGKC(C)KSEC(C)ITPN(N)GSIPNDKPFQNVNRITY Hex 7 HexNAc 2 | 52.299 | 0.015 | -20.205 | 8.393e+06 | 268 - 302 | 2 | 36.54 | 36.54 | 36.71 |
| 3918.6298 | NWAGVTHN(N)GTSSSC(C)RRGSNSSFF Fuc 1 Hex 4 HexNAc 3 | 50.340 | 0.015 | -18.320 | 3.999e+06 | 125 - 148 | 1 | 31.51 | 31.51 | 31.51 |
| 4040.6477 | AGVTHN(N)GTSSSC(C)RRGSNSSFF Hex 5 HexNAc 5 | 49.050 | 0.015 | -3.542 | 4.834e+07 | 127 - 148 | 1 | 21.81 | 21.98 | 22.31 |
| 3315.3546 | SEC(C)ITPN(N)GSIPNDKPF Hex 7 HexNAc 2 | 48.869 | 0.015 | -18.658 | 4.016e+06 | 278 - 294 | 1 | 36.71 | 36.88 | 36.88 |
| 4103.8299 | RSDAPIGKC(C)KSEC(C)ITPN(N)GSIPNDKPF Hex 5 HexNAc 2 | 48.510 | 0.015 | -19.144 | 1.171e+07 | 268 - 294 | 1 | 31.17 | 31.17 | 31.34 |
| 3618.4897 | AGVTHN(N)GTSSSC(C)RRGSNSSFF Fuc 1 Hex 4 HexNAc 3 | 46.920 | 0.019 | -4.800 | 1.613e+07 | 127 - 148 | 1 | 21.81 | 21.98 | 22.14 |
| 4468.0946 | TIVKPGDILLIN(N)STGNLIAPRGY Fuc 2 Hex 4 HexNAc 4 Neu5Ac 1 | 45.732 | 0.019 | -19.707 | 1.237e+07 | 234 - 257 | 1 | 56.06 | 56.06 | 56.23 |
| 2693.0326 | NWAGVTHN(N)GTSSSC(C) Hex 5 HexNAc 2 | 45.503 | 0.024 | -18.215 | 2.053e+06 | 125 - 139 | 2 | 19.47 | 19.47 | 19.47 |
| 3353.4385 | LIN(N)STGN(D)LIAPRGY Hex 9 HexNAc 2 | 43.376 | 0.026 | -5.689 | 3.034e+06 | 243 - 257 | 1 | 38.58 | 38.92 | 38.92 |
| 4345.9029 | LIN(N)STGNLIAPRGY Fuc 4 Hex 4 HexNAc 8 | 43.373 | 0.026 | -21.403 | 1.102e+06 | 243 - 257 | 2 | 49.43 | 49.43 | 49.43 |
| 3756.4979 | AGVTHN(D)GTSSSC(C)RRGSN(N)SSFF Hex 7 HexNAc 2 | 43.119 | 0.026 | 9.122 | 6.606e+07 | 127 - 148 | 2 | 22.81 | 22.97 | 23.64 |
| 4290.0059 | TIVKPGDILLIN(N)STGN(D)LIAPRGY Hex 9 HexNAc 2 | 42.832 | 0.026 | -19.545 | 4.969e+07 | 234 - 257 | 67 | 57.25 | 57.25 | 57.42 |
| 3744.8266 | TIVKPGDILLIN(N)STGNLIAPR Hex 7 HexNAc 2 | 42.652 | 0.026 | -19.400 | 6.622e+06 | 234 - 255 | 1 | 54.19 | 54.19 | 54.36 |
| 4041.7290 | NSKYPALN(N)VTM(O)PNNEQFDKLY Hex 7 HexNAc 2 | 42.213 | 0.026 | -18.129 | 1.889e+07 | 157 - 178 | 2 | 43.84 | 43.84 | 44.01 |
| 3582.7764 | TIVKPGDILLIN(N)STGNLIAPR Hex 6 HexNAc 2 | 42.092 | 0.026 | -19.621 | 1.828e+06 | 234 - 255 | 2 | 54.53 | 54.53 | 54.53 |
| 4288.0084 | TIVKPGDILLIN(N)STGNLIAPR Hex 3 HexNAc 5 Neu5Ac 2 | 40.350 | 0.026 | -18.141 | 1.762e+07 | 234 - 255 | 1 | 54.70 | 54.70 | 54.87 |
| 4408.7790 | HKC(C)DNAC(C)IGSIRN(N)GTYDHDVY Fuc 2 Hex 5 HexNAc 4 | 40.158 | 0.031 | -17.822 | 1.321e+07 | 470 - 491 | 2 | 26.15 | 26.15 | 26.15 |
| 4266.8869 | RSDAPIGKC(C)KSEC(C)ITPN(N)GSIPN(D)DKPF Hex 6 HexNAc 2 | 39.525 | 0.033 | -18.542 | 2.371e+06 | 268 - 294 | 1 | 31.00 | 31.00 | 31.00 |
| 4142.9763 | TIVKPGDILLIN(N)STGNLIAPR Fuc 1 Hex 3 HexNAc 5 Neu5Ac 1 | 39.059 | 0.033 | -3.985 | 4.304e+07 | 234 - 255 | 3 | 54.53 | 54.70 | 55.04 |
| 4137.7035 | NWAGVTHN(N)GTSSSC(C)RRGSNSSFF Hex 5 HexNAc 4 | 38.925 | 0.033 | -4.265 | 1.458e+07 | 125 - 148 | 1 | 31.17 | 31.34 | 31.68 |
| 3942.5982 | AGVTHNGTSSSC(C)RRGSN(N)SSFF Fuc 1 Hex 6 HexNAc 3 | 38.112 | 0.033 | -17.461 | 1.893e+07 | 127 - 148 | 1 | 21.64 | 21.64 | 21.81 |
| 4669.9736 | KSEC(C)ITPN(N)GSIPNDKPF Hex 4 HexNAc 9 Neu5Ac 1 | 37.299 | 0.033 | 0.000 | 0.000e+00 | 277 - 294 | 1 | - | - | - |
| 3309.5551 | TIVKPGDILLIN(N)STGNL Hex 7 HexNAc 2 | 36.126 | 0.033 | 0.000 | 0.000e+00 | 234 - 251 | 1 | - | - | - |
| 2744.1655 | QKLPGNDN(N)STATL Hex 3 HexNAc 3 Neu5Ac 1 | 35.839 | 0.041 | 13.696 | 1.601e+08 | 0 - 13 | 4 | 32.51 | 32.82 | 33.33 |
| 3404.5237 | TIVKPGDILLIN(N)STG Hex 9 HexNAc 2 | 35.765 | 0.045 | -20.112 | 5.302e+06 | 234 - 249 | 1 | 47.23 | 47.23 | 47.23 |
| 3432.3920 | AGVTHN(D)GTSSSC(C)RRGSN(N)SSFF Hex 5 HexNAc 2 | 35.552 | 0.048 | -6.315 | 2.322e+07 | 127 - 148 | 2 | 23.31 | 23.31 | 23.64 |
| 4274.0307 | TIVKPGDILLIN(N)STGNLIAPRG Fuc 1 Hex 4 HexNAc 6 | 35.349 | 0.048 | 16.342 | 4.050e+08 | 234 - 256 | 12 | 61.42 | 62.10 | 62.61 |

### LIN(N)STGNLIAPRGYF Hex 9 HexNAc 2

**Mass:**  3499.5196

(2.149e-06)

**Charge States:** 3

**Start Position**: 244

**End Position**: 259

| Profile Score | Tandem Score | TDA q-value | Volume | Spectrum Matches |
| --- | --- | --- | --- | --- |
| -3.0267 | 176.0573 | 0.0000 | 9.1649e+07 | 3 |

**Adduct Signal Fractions**

- **Unmodified**: 9.1649e+07

### LIN(N)STGNLIAPRGY Hex 9 HexNAc 2

**Mass:**  3352.4507

(2.214e-06)

**Charge States:** 3

**Start Position**: 244

**End Position**: 258

| Profile Score | Tandem Score | TDA q-value | Volume | Spectrum Matches |
| --- | --- | --- | --- | --- |
| 14.9037 | 175.4960 | 0.0000 | 8.0291e+07 | 3 |

**Adduct Signal Fractions**

- **Unmodified**: 8.0291e+07

### LIN(N)STGNL Hex 9 HexNAc 2

**Mass:**  2695.0882

(1.568e-06)

**Charge States:** 2, 3

**Start Position**: 244

**End Position**: 252

| Profile Score | Tandem Score | TDA q-value | Volume | Spectrum Matches |
| --- | --- | --- | --- | --- |
| -19.4401 | 169.9095 | 0.0000 | 1.2000e+07 | 1 |

**Adduct Signal Fractions**

- **Unmodified**: 1.2000e+07

### TIVKPGDILLIN(N)STGNLIAPR Hex 9 HexNAc 2

**Mass:**  4068.9226

(-1.984e-07)

**Charge States:** 3, 4, 5

**Start Position**: 235

**End Position**: 256

| Profile Score | Tandem Score | TDA q-value | Volume | Spectrum Matches |
| --- | --- | --- | --- | --- |
| 17.0851 | 167.8881 | 0.0000 | 2.0086e+09 | 17 |

**Adduct Signal Fractions**

- **Unmodified**: 2.0086e+09

### LIN(N)STGNL Hex 8 HexNAc 2

**Mass:**  2533.0340

(1.177e-06)

**Charge States:** 2

**Start Position**: 244

**End Position**: 252

| Profile Score | Tandem Score | TDA q-value | Volume | Spectrum Matches |
| --- | --- | --- | --- | --- |
| -20.3360 | 165.9450 | 0.0000 | 7.9764e+05 | 2 |

**Adduct Signal Fractions**

- **Unmodified**: 7.9764e+05

### TIVKPGDILLIN(N)STGNLIAPR Hex 8 HexNAc 2

**Mass:**  3906.8696

(1.713e-06)

**Charge States:** 3, 4

**Start Position**: 235

**End Position**: 256

| Profile Score | Tandem Score | TDA q-value | Volume | Spectrum Matches |
| --- | --- | --- | --- | --- |
| 15.5686 | 163.3289 | 0.0000 | 2.3095e+08 | 5 |

**Adduct Signal Fractions**

- **Unmodified**: 2.3095e+08

### NWAGVTHN(N)GTSSSC(C) Hex 7 HexNAc 2

**Mass:**  3017.1384

(1.564e-06)

**Charge States:** 3

**Start Position**: 126

**End Position**: 140

| Profile Score | Tandem Score | TDA q-value | Volume | Spectrum Matches |
| --- | --- | --- | --- | --- |
| -18.3746 | 161.4227 | 0.0000 | 8.2619e+06 | 2 |

**Adduct Signal Fractions**

- **Unmodified**: 8.2619e+06

### TIVKPGDILLIN(N)STGNLIAPRGY Hex 8 HexNAc 2

**Mass:**  4126.9641

(2.105e-06)

**Charge States:** 3, 4, 5

**Start Position**: 235

**End Position**: 258

| Profile Score | Tandem Score | TDA q-value | Volume | Spectrum Matches |
| --- | --- | --- | --- | --- |
| 15.0147 | 161.3445 | 0.0000 | 5.7442e+08 | 16 |

**Adduct Signal Fractions**

- **Unmodified**: 5.7442e+08

### TIVKPGDILLIN(N)STGNLIAPR Hex 10 HexNAc 2

**Mass:**  4230.9802

(9.032e-07)

**Charge States:** 4

**Start Position**: 235

**End Position**: 256

| Profile Score | Tandem Score | TDA q-value | Volume | Spectrum Matches |
| --- | --- | --- | --- | --- |
| -3.5442 | 161.3112 | 0.0000 | 9.1065e+07 | 2 |

**Adduct Signal Fractions**

- **Unmodified**: 9.1065e+07

### TIVKPGDILLIN(N) Hex 9 HexNAc 2

**Mass:**  3159.4260

(2.053e-06)

**Charge States:** 2, 3

**Start Position**: 235

**End Position**: 247

| Profile Score | Tandem Score | TDA q-value | Volume | Spectrum Matches |
| --- | --- | --- | --- | --- |
| 11.2508 | 158.3054 | 0.0000 | 1.5589e+08 | 5 |

**Adduct Signal Fractions**

- **Unmodified**: 1.5589e+08

### TIVKPGDILLIN(N)STGNLIAPRGY Hex 9 HexNAc 2

**Mass:**  4289.0212

(2.891e-06)

**Charge States:** 3, 4, 5

**Start Position**: 235

**End Position**: 258

| Profile Score | Tandem Score | TDA q-value | Volume | Spectrum Matches |
| --- | --- | --- | --- | --- |
| 17.9263 | 157.6391 | 0.0000 | 4.1060e+09 | 34 |

**Adduct Signal Fractions**

- **Unmodified**: 4.1060e+09

### TIVKPGDILLIN(N)STGNLIAPRGY Fuc 2 Hex 3 HexNAc 4 Neu5Ac 1

**Mass:**  4306.0324

(-6.743e-06)

**Charge States:** 4, 5

**Start Position**: 235

**End Position**: 258

| Profile Score | Tandem Score | TDA q-value | Volume | Spectrum Matches |
| --- | --- | --- | --- | --- |
| 14.1612 | 157.3081 | 0.0000 | 2.9831e+08 | 4 |

**Adduct Signal Fractions**

- **Unmodified**: 2.9831e+08

### NWAGVTHN(N)GTSS Hex 7 HexNAc 2

**Mass:**  2770.0728

(6.498e-07)

**Charge States:** 3

**Start Position**: 126

**End Position**: 138

| Profile Score | Tandem Score | TDA q-value | Volume | Spectrum Matches |
| --- | --- | --- | --- | --- |
| -18.1502 | 156.8917 | 0.0000 | 4.3317e+06 | 1 |

**Adduct Signal Fractions**

- **Unmodified**: 4.3317e+06

### LIN(N)STGNLIAPRGYF Hex 8 HexNAc 2

**Mass:**  3337.4639

(1.352e-06)

**Charge States:** 3

**Start Position**: 244

**End Position**: 259

| Profile Score | Tandem Score | TDA q-value | Volume | Spectrum Matches |
| --- | --- | --- | --- | --- |
| -17.9396 | 154.5936 | 0.0000 | 6.3843e+06 | 1 |

**Adduct Signal Fractions**

- **Unmodified**: 6.3843e+06

### LIN(N)STGNLIAPRGY Hex 8 HexNAc 2

**Mass:**  3190.3972

(1.943e-06)

**Charge States:** 3

**Start Position**: 244

**End Position**: 258

| Profile Score | Tandem Score | TDA q-value | Volume | Spectrum Matches |
| --- | --- | --- | --- | --- |
| -16.8053 | 154.2898 | 0.0000 | 8.8400e+06 | 1 |

**Adduct Signal Fractions**

- **Unmodified**: 8.8400e+06

### TIVKPGDILLIN(N)STGNLIAPR Fuc 2 Hex 3 HexNAc 4 Neu5Ac 1

**Mass:**  4085.9475

(-5.879e-06)

**Charge States:** 4

**Start Position**: 235

**End Position**: 256

| Profile Score | Tandem Score | TDA q-value | Volume | Spectrum Matches |
| --- | --- | --- | --- | --- |
| 13.2191 | 154.2211 | 0.0000 | 1.4241e+08 | 3 |

**Adduct Signal Fractions**

- **Unmodified**: 1.4241e+08

### TIVKPGDILLIN(N) Fuc 2 Hex 3 HexNAc 4 Neu5Ac 1

**Mass:**  3176.4530

(-6.898e-06)

**Charge States:** 3

**Start Position**: 235

**End Position**: 247

| Profile Score | Tandem Score | TDA q-value | Volume | Spectrum Matches |
| --- | --- | --- | --- | --- |
| 13.5625 | 154.1205 | 0.0000 | 3.3190e+07 | 4 |

**Adduct Signal Fractions**

- **Unmodified**: 3.3190e+07

### TIVKPGDILLIN(N)STGNL Fuc 2 Hex 3 HexNAc 4 Neu5Ac 1

**Mass:**  3648.6795

(-5.819e-06)

**Charge States:** 3

**Start Position**: 235

**End Position**: 252

| Profile Score | Tandem Score | TDA q-value | Volume | Spectrum Matches |
| --- | --- | --- | --- | --- |
| -4.0008 | 153.2580 | 0.0000 | 1.0139e+08 | 2 |

**Adduct Signal Fractions**

- **Unmodified**: 1.0139e+08

### TIVKPGDILLIN(N)STGNLIAPRGY Hex 10 HexNAc 2

**Mass:**  4451.0656

(1.040e-06)

**Charge States:** 3, 4

**Start Position**: 235

**End Position**: 258

| Profile Score | Tandem Score | TDA q-value | Volume | Spectrum Matches |
| --- | --- | --- | --- | --- |
| 16.0481 | 152.3863 | 0.0000 | 1.8657e+08 | 10 |

**Adduct Signal Fractions**

- **Unmodified**: 1.8657e+08

### AGVTHN(N)GTSSSC(C)RRGSNSSFF Hex 5 HexNAc 2

**Mass:**  3431.4072

(1.033e-06)

**Charge States:** 4

**Start Position**: 128

**End Position**: 149

| Profile Score | Tandem Score | TDA q-value | Volume | Spectrum Matches |
| --- | --- | --- | --- | --- |
| -3.4246 | 152.3513 | 0.0000 | 1.0214e+08 | 2 |

**Adduct Signal Fractions**

- **Unmodified**: 1.0214e+08

### TIVKPGDILLIN(N)STGN Hex 9 HexNAc 2

**Mass:**  3518.5724

(2.301e-06)

**Charge States:** 3, 4

**Start Position**: 235

**End Position**: 251

| Profile Score | Tandem Score | TDA q-value | Volume | Spectrum Matches |
| --- | --- | --- | --- | --- |
| 15.9850 | 149.1680 | 0.0000 | 1.7736e+08 | 5 |

**Adduct Signal Fractions**

- **Unmodified**: 1.7736e+08

### RSDAPIGKC(C)KSEC(C)ITPN(N)GSIPNDKPF Hex 7 HexNAc 2

**Mass:**  4427.9473

(5.258e-06)

**Charge States:** 4, 5

**Start Position**: 269

**End Position**: 295

| Profile Score | Tandem Score | TDA q-value | Volume | Spectrum Matches |
| --- | --- | --- | --- | --- |
| 16.2630 | 148.9782 | 0.0000 | 3.5697e+08 | 3 |

**Adduct Signal Fractions**

- **Unmodified**: 3.5697e+08

### NSKYPALN(N)VTM(O)PNNEQFDKLY Hex 8 HexNAc 2

**Mass:**  4203.7814

(3.248e-06)

**Charge States:** 3, 4

**Start Position**: 158

**End Position**: 179

| Profile Score | Tandem Score | TDA q-value | Volume | Spectrum Matches |
| --- | --- | --- | --- | --- |
| -2.1825 | 147.9175 | 0.0000 | 1.1403e+08 | 4 |

**Adduct Signal Fractions**

- **Unmodified**: 1.1403e+08

### TIVKPGDILLIN(N)STGNL Hex 9 HexNAc 2

**Mass:**  3631.6540

(1.027e-06)

**Charge States:** 3, 4

**Start Position**: 235

**End Position**: 252

| Profile Score | Tandem Score | TDA q-value | Volume | Spectrum Matches |
| --- | --- | --- | --- | --- |
| 17.0327 | 147.6047 | 0.0000 | 4.8772e+08 | 9 |

**Adduct Signal Fractions**

- **Unmodified**: 4.8772e+08

### TIVKPGDILLIN(N) Hex 8 HexNAc 2

**Mass:**  2997.3737

(2.094e-06)

**Charge States:** 3

**Start Position**: 235

**End Position**: 247

| Profile Score | Tandem Score | TDA q-value | Volume | Spectrum Matches |
| --- | --- | --- | --- | --- |
| 12.2557 | 147.2108 | 0.0000 | 1.3578e+07 | 2 |

**Adduct Signal Fractions**

- **Unmodified**: 1.3578e+07

### TIVKPGDILLIN(N)STGN Fuc 2 Hex 3 HexNAc 4 Neu5Ac 1

**Mass:**  3535.5972

(-6.494e-06)

**Charge States:** 3

**Start Position**: 235

**End Position**: 251

| Profile Score | Tandem Score | TDA q-value | Volume | Spectrum Matches |
| --- | --- | --- | --- | --- |
| -3.9512 | 147.1212 | 0.0000 | 4.1205e+07 | 2 |

**Adduct Signal Fractions**

- **Unmodified**: 4.1205e+07

### LIN(N)STGNLIAPRGYF Fuc 2 Hex 3 HexNAc 4 Neu5Ac 1

**Mass:**  3516.5451

(-5.771e-06)

**Charge States:** 3

**Start Position**: 244

**End Position**: 259

| Profile Score | Tandem Score | TDA q-value | Volume | Spectrum Matches |
| --- | --- | --- | --- | --- |
| -18.8375 | 144.7187 | 0.0000 | 1.3680e+07 | 1 |

**Adduct Signal Fractions**

- **Unmodified**: 1.3680e+07

### RSDAPIGKC(C)KSEC(C)ITPN(N)GSIPNDKPF Hex 9 HexNAc 2

**Mass:**  4752.0504

(5.700e-06)

**Charge States:** 4, 5

**Start Position**: 269

**End Position**: 295

| Profile Score | Tandem Score | TDA q-value | Volume | Spectrum Matches |
| --- | --- | --- | --- | --- |
| 14.7252 | 142.9879 | 0.0000 | 3.5626e+08 | 3 |

**Adduct Signal Fractions**

- **Unmodified**: 3.5626e+08

### TIVKPGDILLIN(N)STGNL Hex 10 HexNAc 2

**Mass:**  3793.7067

(1.407e-06)

**Charge States:** 3

**Start Position**: 235

**End Position**: 252

| Profile Score | Tandem Score | TDA q-value | Volume | Spectrum Matches |
| --- | --- | --- | --- | --- |
| -19.0133 | 142.5391 | 0.0000 | 1.6655e+07 | 2 |

**Adduct Signal Fractions**

- **Unmodified**: 1.6655e+07

### NWAGVTHN(N)GTSSSC(C)RRGSNSSFF Hex 5 HexNAc 2

**Mass:**  3731.5495

(5.853e-06)

**Charge States:** 3, 4

**Start Position**: 126

**End Position**: 149

| Profile Score | Tandem Score | TDA q-value | Volume | Spectrum Matches |
| --- | --- | --- | --- | --- |
| -17.1572 | 140.5396 | 0.0000 | 2.9609e+07 | 3 |

**Adduct Signal Fractions**

- **Unmodified**: 2.9609e+07

### NWAGVTHNGTSSSC(C)RRGSN(N)SSFF Hex 7 HexNAc 2

**Mass:**  4055.6455

(3.036e-06)

**Charge States:** 4

**Start Position**: 126

**End Position**: 149

| Profile Score | Tandem Score | TDA q-value | Volume | Spectrum Matches |
| --- | --- | --- | --- | --- |
| -2.7686 | 140.3130 | 0.0000 | 7.2538e+07 | 2 |

**Adduct Signal Fractions**

- **Unmodified**: 7.2538e+07

### KSEC(C)ITPN(N)GSIPNDKPF Hex 7 HexNAc 2

**Mass:**  3443.4453

(6.938e-07)

**Charge States:** 3

**Start Position**: 278

**End Position**: 295

| Profile Score | Tandem Score | TDA q-value | Volume | Spectrum Matches |
| --- | --- | --- | --- | --- |
| -5.0140 | 139.5020 | 0.0000 | 3.6984e+07 | 1 |

**Adduct Signal Fractions**

- **Unmodified**: 3.6984e+07

### TIVKPGDILLIN(N)STGNLIAPRGY Hex 7 HexNAc 2

**Mass:**  3964.9096

(1.756e-06)

**Charge States:** 3, 4

**Start Position**: 235

**End Position**: 258

| Profile Score | Tandem Score | TDA q-value | Volume | Spectrum Matches |
| --- | --- | --- | --- | --- |
| -4.0268 | 139.2173 | 0.0000 | 4.2387e+07 | 4 |

**Adduct Signal Fractions**

- **Unmodified**: 4.2387e+07

### TIVKPGDILLIN(N)STGNL Hex 8 HexNAc 2

**Mass:**  3469.6005

(1.432e-06)

**Charge States:** 3

**Start Position**: 235

**End Position**: 252

| Profile Score | Tandem Score | TDA q-value | Volume | Spectrum Matches |
| --- | --- | --- | --- | --- |
| -4.0762 | 139.2023 | 0.0000 | 5.6073e+07 | 2 |

**Adduct Signal Fractions**

- **Unmodified**: 5.6073e+07

### LIN(N)STGNLIAPR Hex 9 HexNAc 2

**Mass:**  3132.3667

(2.489e-06)

**Charge States:** 3

**Start Position**: 244

**End Position**: 256

| Profile Score | Tandem Score | TDA q-value | Volume | Spectrum Matches |
| --- | --- | --- | --- | --- |
| -16.5096 | 138.7093 | 0.0000 | 3.0550e+07 | 1 |

**Adduct Signal Fractions**

- **Unmodified**: 3.0550e+07

### NWAGVTHN(N)GTSSS Hex 7 HexNAc 2

**Mass:**  2857.1088

(2.037e-06)

**Charge States:** 2, 3

**Start Position**: 126

**End Position**: 139

| Profile Score | Tandem Score | TDA q-value | Volume | Spectrum Matches |
| --- | --- | --- | --- | --- |
| -19.5357 | 137.4483 | 0.0000 | 2.8173e+06 | 2 |

**Adduct Signal Fractions**

- **Unmodified**: 2.8173e+06

### AGVTHN(N)GTSSSC(C)RRGSNSSFF Hex 7 HexNAc 2

**Mass:**  3755.5126

(1.208e-06)

**Charge States:** 4

**Start Position**: 128

**End Position**: 149

| Profile Score | Tandem Score | TDA q-value | Volume | Spectrum Matches |
| --- | --- | --- | --- | --- |
| 15.9088 | 135.1742 | 0.0000 | 2.5847e+08 | 3 |

**Adduct Signal Fractions**

- **Unmodified**: 2.5847e+08

### RSDAPIGKC(C)KSEC(C)ITPN(N)GSIPNDKPF Hex 8 HexNAc 2

**Mass:**  4589.9963

(4.540e-06)

**Charge States:** 4, 5

**Start Position**: 269

**End Position**: 295

| Profile Score | Tandem Score | TDA q-value | Volume | Spectrum Matches |
| --- | --- | --- | --- | --- |
| -2.7153 | 129.8527 | 0.0077 | 2.1606e+08 | 3 |

**Adduct Signal Fractions**

- **Unmodified**: 2.1606e+08

### TIVKPGDILLIN(N)STGNLIAPRGY Hex 5 HexNAc 2

**Mass:**  3640.8039

(1.389e-06)

**Charge States:** 3, 4

**Start Position**: 235

**End Position**: 258

| Profile Score | Tandem Score | TDA q-value | Volume | Spectrum Matches |
| --- | --- | --- | --- | --- |
| -19.0898 | 129.8320 | 0.0077 | 1.5168e+07 | 2 |

**Adduct Signal Fractions**

- **Unmodified**: 1.5168e+07

### IN(N)STGNLIAPR Hex 9 HexNAc 2

**Mass:**  3019.2815

(2.158e-06)

**Charge States:** 3

**Start Position**: 245

**End Position**: 256

| Profile Score | Tandem Score | TDA q-value | Volume | Spectrum Matches |
| --- | --- | --- | --- | --- |
| -18.4804 | 129.3095 | 0.0077 | 4.8024e+06 | 1 |

**Adduct Signal Fractions**

- **Unmodified**: 4.8024e+06

### SKYPALN(N)VTM(O)PNNEQFDKLY Hex 8 HexNAc 2

**Mass:**  4089.7397

(2.968e-06)

**Charge States:** 3, 4

**Start Position**: 159

**End Position**: 179

| Profile Score | Tandem Score | TDA q-value | Volume | Spectrum Matches |
| --- | --- | --- | --- | --- |
| -2.1406 | 129.0577 | 0.0077 | 4.4723e+07 | 4 |

**Adduct Signal Fractions**

- **Unmodified**: 4.4723e+07

### TIVKPGDILLIN(N)STGN Hex 8 HexNAc 2

**Mass:**  3356.5167

(7.941e-07)

**Charge States:** 3

**Start Position**: 235

**End Position**: 251

| Profile Score | Tandem Score | TDA q-value | Volume | Spectrum Matches |
| --- | --- | --- | --- | --- |
| -3.9501 | 128.4684 | 0.0077 | 1.9556e+07 | 3 |

**Adduct Signal Fractions**

- **Unmodified**: 1.9556e+07

### KSEC(C)ITPN(N)GSIPNDKPF Hex 8 HexNAc 2

**Mass:**  3605.5006

(1.321e-06)

**Charge States:** 3

**Start Position**: 278

**End Position**: 295

| Profile Score | Tandem Score | TDA q-value | Volume | Spectrum Matches |
| --- | --- | --- | --- | --- |
| -18.5661 | 128.0502 | 0.0077 | 1.1251e+07 | 1 |

**Adduct Signal Fractions**

- **Unmodified**: 1.1251e+07

### AGVTHNGTSSSC(C)RRGSN(N)SSFF Hex 6 HexNAc 2

**Mass:**  3593.4616

(9.913e-07)

**Charge States:** 4

**Start Position**: 128

**End Position**: 149

| Profile Score | Tandem Score | TDA q-value | Volume | Spectrum Matches |
| --- | --- | --- | --- | --- |
| -3.8973 | 127.1321 | 0.0077 | 6.6581e+07 | 1 |

**Adduct Signal Fractions**

- **Unmodified**: 6.6581e+07

### RSDAPIGKC(C)KSEC(C)ITPN(N)GSIPNDKPF Hex 6 HexNAc 2

**Mass:**  4265.8852

(2.637e-06)

**Charge States:** 5

**Start Position**: 269

**End Position**: 295

| Profile Score | Tandem Score | TDA q-value | Volume | Spectrum Matches |
| --- | --- | --- | --- | --- |
| -3.5514 | 124.7303 | 0.0077 | 3.7232e+07 | 1 |

**Adduct Signal Fractions**

- **Unmodified**: 3.7232e+07

### IN(N)STGNLIAPRGYF Hex 9 HexNAc 2

**Mass:**  3386.4325

(1.262e-06)

**Charge States:** 3

**Start Position**: 245

**End Position**: 259

| Profile Score | Tandem Score | TDA q-value | Volume | Spectrum Matches |
| --- | --- | --- | --- | --- |
| -17.1037 | 124.6605 | 0.0077 | 1.0029e+07 | 1 |

**Adduct Signal Fractions**

- **Unmodified**: 1.0029e+07

### TITNDRIEVTN(N)ATEL Hex 7 HexNAc 2

**Mass:**  3229.3925

(1.839e-06)

**Charge States:** 3

**Start Position**: 28

**End Position**: 43

| Profile Score | Tandem Score | TDA q-value | Volume | Spectrum Matches |
| --- | --- | --- | --- | --- |
| -18.0026 | 123.2849 | 0.0077 | 3.5499e+06 | 1 |

**Adduct Signal Fractions**

- **Unmodified**: 3.5499e+06

### LIN(N)STGNLIAPRGY Fuc 2 Hex 3 HexNAc 4 Neu5Ac 1

**Mass:**  3369.4748

(-6.601e-06)

**Charge States:** 3

**Start Position**: 244

**End Position**: 258

| Profile Score | Tandem Score | TDA q-value | Volume | Spectrum Matches |
| --- | --- | --- | --- | --- |
| -18.4864 | 121.7598 | 0.0077 | 1.1413e+07 | 1 |

**Adduct Signal Fractions**

- **Unmodified**: 1.1413e+07

### TITNDRIEVTN(N)ATEL Hex 5 HexNAc 2

**Mass:**  2905.2877

(2.305e-06)

**Charge States:** 3

**Start Position**: 28

**End Position**: 43

| Profile Score | Tandem Score | TDA q-value | Volume | Spectrum Matches |
| --- | --- | --- | --- | --- |
| -18.4476 | 121.0398 | 0.0077 | 4.0758e+06 | 1 |

**Adduct Signal Fractions**

- **Unmodified**: 4.0758e+06

### TIVKPGDILLIN(N) Fuc 5 Hex 3 HexNAc 8 Neu5Ac 1

**Mass:**  4429.9457

(6.734e-04)

**Start Position**: 235

**End Position**: 247

| Profile Score | Tandem Score | TDA q-value | Volume | Spectrum Matches |
| --- | --- | --- | --- | --- |
| 0.0000 | 119.8849 | 0.0077 | 0.0000e+00 | 1 |

Adduct:

No Chromatogram Found

### TIVKPGDILLIN(N)STGN Hex 10 HexNAc 2

**Mass:**  3680.6255

(2.294e-06)

**Charge States:** 3

**Start Position**: 235

**End Position**: 251

| Profile Score | Tandem Score | TDA q-value | Volume | Spectrum Matches |
| --- | --- | --- | --- | --- |
| -4.7255 | 118.1416 | 0.0077 | 6.8134e+06 | 1 |

**Adduct Signal Fractions**

- **Unmodified**: 6.8134e+06

### RGSN(N)SSFF Fuc 1 Hex 5 HexNAc 5

**Mass:**  2872.1295

(5.865e-07)

**Charge States:** 3

**Start Position**: 141

**End Position**: 149

| Profile Score | Tandem Score | TDA q-value | Volume | Spectrum Matches |
| --- | --- | --- | --- | --- |
| -19.9621 | 111.2747 | 0.0000 | 3.5865e+06 | 1 |

**Adduct Signal Fractions**

- **Unmodified**: 3.5865e+06

### TITNDRIEVTN(N)ATEL Hex 6 HexNAc 2

**Mass:**  3067.3425

(2.821e-06)

**Charge States:** 3

**Start Position**: 28

**End Position**: 43

| Profile Score | Tandem Score | TDA q-value | Volume | Spectrum Matches |
| --- | --- | --- | --- | --- |
| -19.5061 | 109.4488 | 0.0077 | 3.8482e+06 | 1 |

**Adduct Signal Fractions**

- **Unmodified**: 3.8482e+06

### AGVTHN(N)GTSSSC(C)RRGSNSSFF Hex 8 HexNAc 2

**Mass:**  3917.5661

(3.049e-08)

**Charge States:** 4

**Start Position**: 128

**End Position**: 149

| Profile Score | Tandem Score | TDA q-value | Volume | Spectrum Matches |
| --- | --- | --- | --- | --- |
| -17.6883 | 108.8991 | 0.0124 | 2.7771e+07 | 1 |

**Adduct Signal Fractions**

- **Unmodified**: 2.7771e+07

### SEC(C)ITPN(N)GSIPNDKPF Hex 9 HexNAc 2

**Mass:**  3639.4561

(6.860e-07)

**Charge States:** 3

**Start Position**: 279

**End Position**: 295

| Profile Score | Tandem Score | TDA q-value | Volume | Spectrum Matches |
| --- | --- | --- | --- | --- |
| -18.9299 | 105.2939 | 0.0124 | 3.4863e+06 | 1 |

**Adduct Signal Fractions**

- **Unmodified**: 3.4863e+06

### TIVKPGDILLIN(N)STGN(D)LIAPR Hex 9 HexNAc 2

**Mass:**  4069.9230

(2.457e-04)

**Charge States:** 3, 4

**Start Position**: 235

**End Position**: 256

| Profile Score | Tandem Score | TDA q-value | Volume | Spectrum Matches |
| --- | --- | --- | --- | --- |
| -19.7908 | 104.4677 | 0.0124 | 1.2343e+07 | 3 |

**Adduct Signal Fractions**

- **Unmodified**: 1.2343e+07

### TIVKPGDILLIN(N)STGNL Fuc 2 Hex 4 HexNAc 4 Neu5Ac 1

**Mass:**  3810.7318

(-5.893e-06)

**Charge States:** 3

**Start Position**: 235

**End Position**: 252

| Profile Score | Tandem Score | TDA q-value | Volume | Spectrum Matches |
| --- | --- | --- | --- | --- |
| -16.9269 | 103.2026 | 0.0124 | 2.1594e+06 | 1 |

**Adduct Signal Fractions**

- **Unmodified**: 2.1594e+06

### C(C)LGHHAVPN(N)GTIVK Fuc 1 Hex 5 HexNAc 5

**Mass:**  3473.5053

(1.138e-06)

**Charge States:** 4

**Start Position**: 14

**End Position**: 28

| Profile Score | Tandem Score | TDA q-value | Volume | Spectrum Matches |
| --- | --- | --- | --- | --- |
| -20.0794 | 99.5737 | 0.0124 | 3.8693e+06 | 1 |

**Adduct Signal Fractions**

- **Unmodified**: 3.8693e+06

### SEC(C)ITPN(N)GSIPNDKPF Hex 8 HexNAc 2

**Mass:**  3477.4055

(1.296e-06)

**Charge States:** 3

**Start Position**: 279

**End Position**: 295

| Profile Score | Tandem Score | TDA q-value | Volume | Spectrum Matches |
| --- | --- | --- | --- | --- |
| -20.1565 | 92.8546 | 0.0124 | 8.2800e+05 | 1 |

**Adduct Signal Fractions**

- **Unmodified**: 8.2800e+05

### RRGSN(N)SSFF Hex 5 HexNAc 2

**Mass:**  2272.9352

(1.006e-06)

**Charge States:** 3

**Start Position**: 140

**End Position**: 149

| Profile Score | Tandem Score | TDA q-value | Volume | Spectrum Matches |
| --- | --- | --- | --- | --- |
| -20.2836 | 88.2912 | 0.0000 | 2.5775e+06 | 1 |

**Adduct Signal Fractions**

- **Unmodified**: 2.5775e+06

### TIVKPGDILLIN(N)STGNLIAPR Hex 5 HexNAc 2

**Mass:**  3420.7143

(6.515e-07)

**Charge States:** 4

**Start Position**: 235

**End Position**: 256

| Profile Score | Tandem Score | TDA q-value | Volume | Spectrum Matches |
| --- | --- | --- | --- | --- |
| -4.7062 | 81.9429 | 0.0124 | 6.9497e+06 | 2 |

**Adduct Signal Fractions**

- **Unmodified**: 6.9497e+06

### RSDAPIGKC(C)KSEC(C)ITPN(N)GSIPN(D)DKPF Hex 9 HexNAc 2

**Mass:**  4753.0386

(2.123e-04)

**Charge States:** 4

**Start Position**: 269

**End Position**: 295

| Profile Score | Tandem Score | TDA q-value | Volume | Spectrum Matches |
| --- | --- | --- | --- | --- |
| -5.6240 | 80.3731 | 0.0124 | 6.5768e+07 | 1 |

**Adduct Signal Fractions**

- **Unmodified**: 6.5768e+07

### RSDAPIGKC(C)KSEC(C)ITPN(N)GSIPN(D)DKPF Hex 7 HexNAc 2

**Mass:**  4428.9282

(2.287e-04)

**Charge States:** 4, 5

**Start Position**: 269

**End Position**: 295

| Profile Score | Tandem Score | TDA q-value | Volume | Spectrum Matches |
| --- | --- | --- | --- | --- |
| -5.5679 | 79.8083 | 0.0124 | 7.0662e+07 | 2 |

**Adduct Signal Fractions**

- **Unmodified**: 7.0662e+07

### TIVKPGDILLIN(N)STGNLIAPRG Hex 9 HexNAc 2

**Mass:**  4125.9504

(1.984e-06)

**Charge States:** 3, 4

**Start Position**: 235

**End Position**: 257

| Profile Score | Tandem Score | TDA q-value | Volume | Spectrum Matches |
| --- | --- | --- | --- | --- |
| 16.0806 | 74.3814 | 0.0146 | 5.3167e+08 | 11 |

**Adduct Signal Fractions**

- **Unmodified**: 5.3167e+08

### KIRSGKSSIM(O)RSDAPIGKC(C)KSEC(C)ITPN(N)GSIPNDKPF Hex 7 HexNAc 2

**Mass:**  5531.5608

(4.444e-06)

**Charge States:** 6

**Start Position**: 259

**End Position**: 295

| Profile Score | Tandem Score | TDA q-value | Volume | Spectrum Matches |
| --- | --- | --- | --- | --- |
| -3.8061 | 73.0235 | 0.0146 | 5.3334e+07 | 1 |

**Adduct Signal Fractions**

- **Unmodified**: 5.3334e+07

### TIVKPGDILLIN(N)STGNLIAPRGY Hex 6 HexNAc 2

**Mass:**  3802.8569

(1.895e-06)

**Charge States:** 4

**Start Position**: 235

**End Position**: 258

| Profile Score | Tandem Score | TDA q-value | Volume | Spectrum Matches |
| --- | --- | --- | --- | --- |
| -5.2287 | 71.8655 | 0.0146 | 6.4100e+06 | 2 |

**Adduct Signal Fractions**

- **Unmodified**: 6.4100e+06

### RSDAPIGKC(C)KSEC(C)ITPN(N)GSIPN(D)DKPF Hex 8 HexNAc 2

**Mass:**  4590.9875

(2.201e-04)

**Charge States:** 5

**Start Position**: 269

**End Position**: 295

| Profile Score | Tandem Score | TDA q-value | Volume | Spectrum Matches |
| --- | --- | --- | --- | --- |
| -5.6899 | 66.1128 | 0.0146 | 4.1691e+07 | 2 |

**Adduct Signal Fractions**

- **Unmodified**: 4.1691e+07

### AGVTHN(N)GTSSSC(C)RRGSNSSFF Hex 4 HexNAc 3

**Mass:**  3472.4323

(1.146e-07)

**Charge States:** 4

**Start Position**: 128

**End Position**: 149

| Profile Score | Tandem Score | TDA q-value | Volume | Spectrum Matches |
| --- | --- | --- | --- | --- |
| -18.1576 | 65.5205 | 0.0146 | 1.4986e+07 | 1 |

**Adduct Signal Fractions**

- **Unmodified**: 1.4986e+07

### AGVTHN(N)GTSSSC(C)RRGSNSSFF Hex 7 HexNAc 3

**Mass:**  3958.5932

(7.258e-07)

**Charge States:** 4

**Start Position**: 128

**End Position**: 149

| Profile Score | Tandem Score | TDA q-value | Volume | Spectrum Matches |
| --- | --- | --- | --- | --- |
| -18.1434 | 64.4098 | 0.0146 | 2.5547e+07 | 1 |

**Adduct Signal Fractions**

- **Unmodified**: 2.5547e+07

### SGKSSIM(O)RSDAPIGKC(C)KSEC(C)ITPN(N)GSIPNDKPF Hex 7 HexNAc 2

**Mass:**  5134.2599

(7.192e-07)

**Charge States:** 5

**Start Position**: 262

**End Position**: 295

| Profile Score | Tandem Score | TDA q-value | Volume | Spectrum Matches |
| --- | --- | --- | --- | --- |
| -4.6786 | 62.1639 | 0.0146 | 1.9465e+07 | 1 |

**Adduct Signal Fractions**

- **Unmodified**: 1.9465e+07

### AGVTHN(N)GTSSSC(C)RRGSNSSFF Hex 5 HexNAc 4

**Mass:**  3837.5673

(1.334e-06)

**Charge States:** 4

**Start Position**: 128

**End Position**: 149

| Profile Score | Tandem Score | TDA q-value | Volume | Spectrum Matches |
| --- | --- | --- | --- | --- |
| -4.4891 | 60.4530 | 0.0146 | 4.4230e+07 | 2 |

**Adduct Signal Fractions**

- **Unmodified**: 4.4230e+07

### RSDAPIGKC(C)KSEC(C)ITPN(N)GSIPN(D)DKPF Hex 8 HexNAc 2

**Mass:**  4591.9986

(4.404e-04)

**Start Position**: 269

**End Position**: 295

| Profile Score | Tandem Score | TDA q-value | Volume | Spectrum Matches |
| --- | --- | --- | --- | --- |
| 0.0000 | 58.0407 | 0.0146 | 0.0000e+00 | 1 |

Adduct:

No Chromatogram Found

### AGVTHN(N)GTSSSC(C)RRGSNSSFF Hex 7 HexNAc 4

**Mass:**  4161.6716

(4.791e-07)

**Charge States:** 4

**Start Position**: 128

**End Position**: 149

| Profile Score | Tandem Score | TDA q-value | Volume | Spectrum Matches |
| --- | --- | --- | --- | --- |
| -4.2415 | 55.0944 | 0.0146 | 3.0491e+07 | 1 |

**Adduct Signal Fractions**

- **Unmodified**: 3.0491e+07

### N(D)SKYPALN(N)VTM(O)PNNEQFDKLY Hex 8 HexNAc 2

**Mass:**  4204.7658

(2.364e-04)

**Charge States:** 3, 4

**Start Position**: 158

**End Position**: 179

| Profile Score | Tandem Score | TDA q-value | Volume | Spectrum Matches |
| --- | --- | --- | --- | --- |
| -6.1070 | 54.3617 | 0.0146 | 2.4239e+07 | 2 |

**Adduct Signal Fractions**

- **Unmodified**: 2.4239e+07

### NSKYPALN(N)VTMPNNEQFDKLY Hex 8 HexNAc 2

**Mass:**  4187.7837

(2.361e-06)

**Charge States:** 3, 4

**Start Position**: 158

**End Position**: 179

| Profile Score | Tandem Score | TDA q-value | Volume | Spectrum Matches |
| --- | --- | --- | --- | --- |
| -18.4774 | 52.3832 | 0.0146 | 3.9195e+06 | 2 |

**Adduct Signal Fractions**

- **Unmodified**: 3.9195e+06

### RSDAPIGKC(C)KSEC(C)ITPN(N)GSIPNDKPFQNVNRITY Hex 7 HexNAc 2

**Mass:**  5416.4364

(6.016e-07)

**Charge States:** 5

**Start Position**: 269

**End Position**: 303

| Profile Score | Tandem Score | TDA q-value | Volume | Spectrum Matches |
| --- | --- | --- | --- | --- |
| -20.2046 | 52.2995 | 0.0146 | 8.3928e+06 | 2 |

**Adduct Signal Fractions**

- **Unmodified**: 8.3928e+06

### NWAGVTHN(N)GTSSSC(C)RRGSNSSFF Fuc 1 Hex 4 HexNAc 3

**Mass:**  3918.6298

(4.536e-06)

**Charge States:** 4

**Start Position**: 126

**End Position**: 149

| Profile Score | Tandem Score | TDA q-value | Volume | Spectrum Matches |
| --- | --- | --- | --- | --- |
| -18.3196 | 50.3403 | 0.0146 | 3.9994e+06 | 1 |

**Adduct Signal Fractions**

- **Unmodified**: 3.9994e+06

### AGVTHN(N)GTSSSC(C)RRGSNSSFF Hex 5 HexNAc 5

**Mass:**  4040.6472

(1.400e-06)

**Charge States:** 4

**Start Position**: 128

**End Position**: 149

| Profile Score | Tandem Score | TDA q-value | Volume | Spectrum Matches |
| --- | --- | --- | --- | --- |
| -3.5416 | 49.0497 | 0.0146 | 4.8344e+07 | 1 |

**Adduct Signal Fractions**

- **Unmodified**: 4.8344e+07

### SEC(C)ITPN(N)GSIPNDKPF Hex 7 HexNAc 2

**Mass:**  3315.3554

(2.194e-06)

**Charge States:** 3

**Start Position**: 279

**End Position**: 295

| Profile Score | Tandem Score | TDA q-value | Volume | Spectrum Matches |
| --- | --- | --- | --- | --- |
| -18.6580 | 48.8693 | 0.0146 | 4.0159e+06 | 1 |

**Adduct Signal Fractions**

- **Unmodified**: 4.0159e+06

### RSDAPIGKC(C)KSEC(C)ITPN(N)GSIPNDKPF Hex 5 HexNAc 2

**Mass:**  4103.8296

(2.430e-06)

**Charge States:** 4, 5

**Start Position**: 269

**End Position**: 295

| Profile Score | Tandem Score | TDA q-value | Volume | Spectrum Matches |
| --- | --- | --- | --- | --- |
| -19.1444 | 48.5105 | 0.0146 | 1.1711e+07 | 1 |

**Adduct Signal Fractions**

- **Unmodified**: 1.1711e+07

### AGVTHN(N)GTSSSC(C)RRGSNSSFF Fuc 1 Hex 4 HexNAc 3

**Mass:**  3618.4907

(2.224e-07)

**Charge States:** 4

**Start Position**: 128

**End Position**: 149

| Profile Score | Tandem Score | TDA q-value | Volume | Spectrum Matches |
| --- | --- | --- | --- | --- |
| -4.7995 | 46.9198 | 0.0189 | 1.6133e+07 | 1 |

**Adduct Signal Fractions**

- **Unmodified**: 1.6133e+07

### TIVKPGDILLIN(N)STGNLIAPRGY Fuc 2 Hex 4 HexNAc 4 Neu5Ac 1

**Mass:**  4468.0978

(-5.877e-06)

**Charge States:** 4

**Start Position**: 235

**End Position**: 258

| Profile Score | Tandem Score | TDA q-value | Volume | Spectrum Matches |
| --- | --- | --- | --- | --- |
| -19.7065 | 45.7316 | 0.0189 | 1.2366e+07 | 1 |

**Adduct Signal Fractions**

- **Unmodified**: 1.2366e+07

### NWAGVTHN(N)GTSSSC(C) Hex 5 HexNAc 2

**Mass:**  2693.0326

(1.687e-06)

**Charge States:** 3

**Start Position**: 126

**End Position**: 140

| Profile Score | Tandem Score | TDA q-value | Volume | Spectrum Matches |
| --- | --- | --- | --- | --- |
| -18.2155 | 45.5026 | 0.0236 | 2.0526e+06 | 2 |

**Adduct Signal Fractions**

- **Unmodified**: 2.0526e+06

### LIN(N)STGN(D)LIAPRGY Hex 9 HexNAc 2

**Mass:**  3353.4453

(2.947e-04)

**Charge States:** 3

**Start Position**: 244

**End Position**: 258

| Profile Score | Tandem Score | TDA q-value | Volume | Spectrum Matches |
| --- | --- | --- | --- | --- |
| -5.6892 | 43.3760 | 0.0263 | 3.0344e+06 | 1 |

**Adduct Signal Fractions**

- **Unmodified**: 3.0344e+06

### LIN(N)STGNLIAPRGY Fuc 4 Hex 4 HexNAc 8

**Mass:**  4345.9036

(2.336e-04)

**Charge States:** 4, 5

**Start Position**: 244

**End Position**: 258

| Profile Score | Tandem Score | TDA q-value | Volume | Spectrum Matches |
| --- | --- | --- | --- | --- |
| -21.4032 | 43.3729 | 0.0263 | 1.1015e+06 | 2 |

**Adduct Signal Fractions**

- **Unmodified**: 1.1015e+06

### AGVTHN(D)GTSSSC(C)RRGSN(N)SSFF Hex 7 HexNAc 2

**Mass:**  3756.5007

(2.635e-04)

**Charge States:** 4

**Start Position**: 128

**End Position**: 149

| Profile Score | Tandem Score | TDA q-value | Volume | Spectrum Matches |
| --- | --- | --- | --- | --- |
| 9.1219 | 43.1190 | 0.0263 | 6.6059e+07 | 2 |

**Adduct Signal Fractions**

- **Unmodified**: 6.6059e+07

### TIVKPGDILLIN(N)STGN(D)LIAPRGY Hex 9 HexNAc 2

**Mass:**  4290.0061

(2.322e-04)

**Charge States:** 3, 4, 5

**Start Position**: 235

**End Position**: 258

| Profile Score | Tandem Score | TDA q-value | Volume | Spectrum Matches |
| --- | --- | --- | --- | --- |
| -19.5451 | 42.8319 | 0.0263 | 4.9690e+07 | 67 |

**Adduct Signal Fractions**

- **Unmodified**: 4.9690e+07

### TIVKPGDILLIN(N)STGNLIAPR Hex 7 HexNAc 2

**Mass:**  3744.8276

(2.029e-06)

**Charge States:** 4

**Start Position**: 235

**End Position**: 256

| Profile Score | Tandem Score | TDA q-value | Volume | Spectrum Matches |
| --- | --- | --- | --- | --- |
| -19.3996 | 42.6519 | 0.0263 | 6.6220e+06 | 1 |

**Adduct Signal Fractions**

- **Unmodified**: 6.6220e+06

### NSKYPALN(N)VTM(O)PNNEQFDKLY Hex 7 HexNAc 2

**Mass:**  4041.7290

(2.662e-06)

**Charge States:** 3, 4

**Start Position**: 158

**End Position**: 179

| Profile Score | Tandem Score | TDA q-value | Volume | Spectrum Matches |
| --- | --- | --- | --- | --- |
| -18.1291 | 42.2129 | 0.0263 | 1.8890e+07 | 2 |

**Adduct Signal Fractions**

- **Unmodified**: 1.8890e+07

### TIVKPGDILLIN(N)STGNLIAPR Hex 6 HexNAc 2

**Mass:**  3582.7719

(1.949e-06)

**Charge States:** 3, 4

**Start Position**: 235

**End Position**: 256

| Profile Score | Tandem Score | TDA q-value | Volume | Spectrum Matches |
| --- | --- | --- | --- | --- |
| -19.6208 | 42.0925 | 0.0263 | 1.8279e+06 | 2 |

**Adduct Signal Fractions**

- **Unmodified**: 1.8279e+06

### TIVKPGDILLIN(N)STGNLIAPR Hex 3 HexNAc 5 Neu5Ac 2

**Mass:**  4288.0080

(-6.339e-06)

**Charge States:** 4

**Start Position**: 235

**End Position**: 256

| Profile Score | Tandem Score | TDA q-value | Volume | Spectrum Matches |
| --- | --- | --- | --- | --- |
| -18.1413 | 40.3497 | 0.0263 | 1.7617e+07 | 1 |

**Adduct Signal Fractions**

- **Unmodified**: 1.7617e+07

### HKC(C)DNAC(C)IGSIRN(N)GTYDHDVY Fuc 2 Hex 5 HexNAc 4

**Mass:**  4408.7774

(2.053e-06)

**Charge States:** 4, 5

**Start Position**: 471

**End Position**: 492

| Profile Score | Tandem Score | TDA q-value | Volume | Spectrum Matches |
| --- | --- | --- | --- | --- |
| -17.8221 | 40.1582 | 0.0306 | 1.3213e+07 | 2 |

**Adduct Signal Fractions**

- **Unmodified**: 1.3213e+07

### RSDAPIGKC(C)KSEC(C)ITPN(N)GSIPN(D)DKPF Hex 6 HexNAc 2

**Mass:**  4266.8869

(2.381e-04)

**Charge States:** 4

**Start Position**: 269

**End Position**: 295

| Profile Score | Tandem Score | TDA q-value | Volume | Spectrum Matches |
| --- | --- | --- | --- | --- |
| -18.5422 | 39.5249 | 0.0327 | 2.3711e+06 | 1 |

**Adduct Signal Fractions**

- **Unmodified**: 2.3711e+06

### TIVKPGDILLIN(N)STGNLIAPR Fuc 1 Hex 3 HexNAc 5 Neu5Ac 1

**Mass:**  4142.9763

(-4.947e-06)

**Charge States:** 4

**Start Position**: 235

**End Position**: 256

| Profile Score | Tandem Score | TDA q-value | Volume | Spectrum Matches |
| --- | --- | --- | --- | --- |
| -3.9849 | 39.0595 | 0.0327 | 4.3044e+07 | 3 |

**Adduct Signal Fractions**

- **Unmodified**: 4.3044e+07

### NWAGVTHN(N)GTSSSC(C)RRGSNSSFF Hex 5 HexNAc 4

**Mass:**  4137.7019

(4.762e-06)

**Charge States:** 4

**Start Position**: 126

**End Position**: 149

| Profile Score | Tandem Score | TDA q-value | Volume | Spectrum Matches |
| --- | --- | --- | --- | --- |
| -4.2652 | 38.9253 | 0.0327 | 1.4583e+07 | 1 |

**Adduct Signal Fractions**

- **Unmodified**: 1.4583e+07

### AGVTHNGTSSSC(C)RRGSN(N)SSFF Fuc 1 Hex 6 HexNAc 3

**Mass:**  3942.5983

(7.072e-07)

**Charge States:** 4

**Start Position**: 128

**End Position**: 149

| Profile Score | Tandem Score | TDA q-value | Volume | Spectrum Matches |
| --- | --- | --- | --- | --- |
| -17.4613 | 38.1119 | 0.0327 | 1.8925e+07 | 1 |

**Adduct Signal Fractions**

- **Unmodified**: 1.8925e+07

### KSEC(C)ITPN(N)GSIPNDKPF Hex 4 HexNAc 9 Neu5Ac 1

**Mass:**  4669.9736

(8.135e-06)

**Start Position**: 278

**End Position**: 295

| Profile Score | Tandem Score | TDA q-value | Volume | Spectrum Matches |
| --- | --- | --- | --- | --- |
| 0.0000 | 37.2987 | 0.0327 | 0.0000e+00 | 1 |

Adduct:

No Chromatogram Found

### TIVKPGDILLIN(N)STGNL Hex 7 HexNAc 2

**Mass:**  3309.5551

(6.084e-04)

**Start Position**: 235

**End Position**: 252

| Profile Score | Tandem Score | TDA q-value | Volume | Spectrum Matches |
| --- | --- | --- | --- | --- |
| 0.0000 | 36.1259 | 0.0327 | 0.0000e+00 | 1 |

Adduct:

No Chromatogram Found

### QKLPGNDN(N)STATL Hex 3 HexNAc 3 Neu5Ac 1

**Mass:**  2744.1626

(-4.802e-06)

**Charge States:** 3

**Start Position**: 1

**End Position**: 14

| Profile Score | Tandem Score | TDA q-value | Volume | Spectrum Matches |
| --- | --- | --- | --- | --- |
| 13.6964 | 35.8393 | 0.0408 | 1.6014e+08 | 4 |

**Adduct Signal Fractions**

- **Unmodified**: 1.6014e+08

### TIVKPGDILLIN(N)STG Hex 9 HexNAc 2

**Mass:**  3404.5237

(7.223e-07)

**Charge States:** 3

**Start Position**: 235

**End Position**: 250

| Profile Score | Tandem Score | TDA q-value | Volume | Spectrum Matches |
| --- | --- | --- | --- | --- |
| -20.1123 | 35.7647 | 0.0447 | 5.3018e+06 | 1 |

**Adduct Signal Fractions**

- **Unmodified**: 5.3018e+06

### AGVTHN(D)GTSSSC(C)RRGSN(N)SSFF Hex 5 HexNAc 2

**Mass:**  3432.3915

(2.874e-04)

**Charge States:** 4

**Start Position**: 128

**End Position**: 149

| Profile Score | Tandem Score | TDA q-value | Volume | Spectrum Matches |
| --- | --- | --- | --- | --- |
| -6.3149 | 35.5523 | 0.0482 | 2.3219e+07 | 2 |

**Adduct Signal Fractions**

- **Unmodified**: 2.3219e+07

### TIVKPGDILLIN(N)STGNLIAPRG Fuc 1 Hex 4 HexNAc 6

**Mass:**  4274.0324

(-5.435e-06)

**Charge States:** 3, 4, 5

**Start Position**: 235

**End Position**: 257

| Profile Score | Tandem Score | TDA q-value | Volume | Spectrum Matches |
| --- | --- | --- | --- | --- |
| 16.3425 | 35.3488 | 0.0482 | 4.0500e+08 | 12 |

**Adduct Signal Fractions**

- **Unmodified**: 4.0500e+08

### cus|SWZNA|A/Switzerland/9715293/2013

**>cus|SWZNA|A/Switzerland/9715293/2013**  
 `MNPNQKIITIGSVSLTISTICFFMQIAILITTVTLHFKQYEFNSPPNNQVMLCEPTIIERNITEIVYLTNTTIEKEICPK  
PAEYRNWSKPQCGITGFAPFSKDNSIRLSAGGDIWVTREPYVSCDPDKCYQFALGQGTTLNNVHSNNTVRDRTPYRTLLM  
NELGVPFHLGTKQVCIAWSSSSCHDGKAWLHVCITGDDKNATASFIYNGRLVDSVVSWSKDILRTQESECVCINGTCTVV  
MTDGSASGKADTKILFIEEGKIVHTSTLSGSAQHVEECSCYPRYPGVRCVCRDNWKGSNRPIVDINIKDHSIVSSYVCSG  
LVGDTPRKNDSSSSSHCLDPNNEEGGHGVKGWAFDDGNDVWMGRTINETSRLGYETFKVIEGWSNPKSKLQTNRQVIVDR  
GDRSGYSGIFSVEGKSCINRCFYVELIRGRKEETEVLWTSNSIVVFCGTSGTYGTGSWPDGADLNLMPI`

| Site | Type | Count |
| --- | --- | --- |
| 146 | N-linked | 16 |
| 86 | N-linked | 3 |
| 367 | N-linked | 14 |
| 200 | N-linked | 3 |

| Mass | Sequence | MS_2_ Score | `q`-value | MS_1_ Score | Signal | Protein Coordinates | Tandem Scans | Start Time | Apex Time | End Time |
| --- | --- | --- | --- | --- | --- | --- | --- | --- | --- | --- |
| 3232.3235 | TIN(N)ETSRLGYETF Hex 8 HexNAc 2 | 180.444 | 0.0 | -2.513 | 4.711e+07 | 364 - 377 | 2 | 37.05 | 37.22 | 37.39 |
| 3567.4899 | RN(N)WSKPQC(C)GITGFAPF Hex 8 HexNAc 2 | 175.433 | 0.0 | -4.206 | 3.327e+07 | 84 - 100 | 2 | 45.88 | 46.05 | 46.22 |
| 3612.4656 | LHVC(C)ITGDDKN(N)ATASF Hex 9 HexNAc 2 | 170.524 | 0.0 | -18.820 | 2.312e+07 | 189 - 205 | 2 | 34.85 | 34.85 | 35.01 |
| 3414.3924 | RN(N)WSKPQC(C)GITGF Hex 9 HexNAc 2 | 170.511 | 0.0 | -17.817 | 1.092e+07 | 84 - 97 | 1 | 31.68 | 31.68 | 31.68 |
| 3729.5450 | RN(N)WSKPQC(C)GITGFAPF Hex 9 HexNAc 2 | 166.088 | 0.0 | -3.550 | 3.342e+07 | 84 - 100 | 3 | 45.88 | 46.05 | 46.22 |
| 3430.4232 | M(O)GRTIN(N)ETSRLGYETF Hex 7 HexNAc 2 | 164.985 | 0.0 | 17.317 | 3.566e+08 | 361 - 377 | 3 | 32.67 | 32.82 | 33.50 |
| 3450.4196 | LHVC(C)ITGDDKN(N)ATASF Hex 8 HexNAc 2 | 164.862 | 0.0 | -17.615 | 1.458e+07 | 189 - 205 | 2 | 35.01 | 35.01 | 35.18 |
| 3592.4755 | M(O)GRTIN(N)ETSRLGYETF Hex 8 HexNAc 2 | 163.276 | 0.0 | 16.831 | 5.798e+08 | 361 - 377 | 3 | 32.67 | 32.67 | 33.33 |
| 3268.3760 | M(O)GRTIN(N)ETSRLGYETF Hex 6 HexNAc 2 | 163.122 | 0.0 | -2.280 | 1.746e+08 | 361 - 377 | 3 | 32.99 | 33.16 | 33.50 |
| 3106.3234 | M(O)GRTIN(N)ETSRLGYETF Hex 5 HexNAc 2 | 153.706 | 0.0 | -17.277 | 2.258e+07 | 361 - 377 | 1 | 33.33 | 33.33 | 33.33 |
| 3288.3663 | LHVC(C)ITGDDKN(N)ATASF Hex 7 HexNAc 2 | 146.655 | 0.0 | -19.684 | 2.335e+06 | 189 - 205 | 1 | 35.01 | 35.01 | 35.18 |
| 3215.3182 | M(O)GRTIN(N)ETSRLGY Hex 8 HexNAc 2 | 143.798 | 0.0 | -2.735 | 4.984e+07 | 361 - 374 | 1 | 22.64 | 22.64 | 22.97 |
| 2908.2190 | TIN(N)ETSRLGYETF Hex 6 HexNAc 2 | 143.116 | 0.0 | -18.436 | 7.083e+06 | 364 - 377 | 1 | 37.39 | 37.55 | 37.55 |
| 2995.2383 | M(O)GRTIN(N)ETSRL Hex 8 HexNAc 2 | 141.105 | 0.0 | -17.833 | 8.660e+06 | 361 - 372 | 1 | 18.30 | 18.30 | 18.47 |
| 3070.2718 | TIN(N)ETSRLGYETF Hex 7 HexNAc 2 | 138.876 | 0.0 | -2.811 | 2.094e+07 | 364 - 377 | 2 | 37.22 | 37.22 | 37.55 |
| 3283.3786 | GRTIN(N)ETSRLGYETF Hex 7 HexNAc 2 | 136.141 | 0.0 | -19.557 | 6.152e+06 | 362 - 377 | 1 | 32.67 | 32.67 | 32.67 |
| 3576.4995 | MGRTIN(N)ETSRLGYETF Hex 8 HexNAc 2 | 136.089 | 0.0 | -18.742 | 7.165e+06 | 361 - 377 | 1 | 35.18 | 35.35 | 35.35 |
| 4141.7141 | NNVHSN(N)NTVRDRTPY Hex 7 HexNAc 6 | 134.276 | 0.0 | 0.000 | 0.000e+00 | 140 - 155 | 1 | - | - | - |
| 3414.4456 | MGRTIN(N)ETSRLGYETF Hex 7 HexNAc 2 | 130.273 | 0.008 | -18.938 | 4.113e+06 | 361 - 377 | 1 | 35.35 | 35.35 | 35.52 |
| 3053.2678 | M(O)GRTIN(N)ETSRLGY Hex 7 HexNAc 2 | 120.967 | 0.008 | -3.317 | 3.600e+07 | 361 - 374 | 2 | 22.64 | 22.81 | 22.97 |
| 3920.0660 | NNVHSN(N)NTVRDRTPY Fuc 1 Hex 6 HexNAc 5 | 109.029 | 0.012 | 7.110 | 9.528e+07 | 140 - 155 | 7 | 13.71 | 13.71 | 17.12 |
| 3252.3967 | MGRTIN(N)ETSRLGYETF Hex 6 HexNAc 2 | 107.406 | 0.012 | -19.034 | 2.560e+06 | 361 - 377 | 1 | 35.52 | 35.52 | 35.52 |
| 4284.7524 | NNVHSN(N)NTVRDRTPY Fuc 1 Hex 7 HexNAc 6 | 100.959 | 0.012 | -18.664 | 1.897e+07 | 140 - 155 | 1 | 13.55 | 13.71 | 13.71 |
| 3999.5827 | NNVHSN(N)NTVRDRTPY Fuc 1 Hex 9 HexNAc 3 | 97.333 | 0.012 | -4.668 | 3.156e+07 | 140 - 155 | 2 | 14.84 | 15.01 | 15.17 |
| 4065.6806 | NNVHSN(N)NTVRDRTPY Fuc 2 Hex 6 HexNAc 5 | 95.302 | 0.012 | -19.058 | 8.229e+06 | 140 - 155 | 1 | 13.71 | 13.71 | 13.71 |
| 4365.0374 | NNVHSN(N)NTVRDRTPY Fuc 1 Hex 10 HexNAc 4 | 85.256 | 0.012 | -20.683 | 1.285e+07 | 140 - 155 | 3 | 14.68 | 14.84 | 16.67 |
| 4510.7712 | NNVHSN(N)NTVRDRTPY Fuc 2 Hex 10 HexNAc 4 | 83.718 | 0.012 | -21.347 | 5.214e+06 | 140 - 155 | 1 | 14.51 | 14.51 | 14.51 |
| 4503.8366 | NNVHSN(N)N(D)TVRDRTPY Fuc 3 Hex 6 HexNAc 5 Neu5Ac 1 | 78.325 | 0.012 | -22.253 | 3.564e+06 | 140 - 155 | 1 | 13.55 | 13.55 | 13.55 |
| 4430.8073 | NNVHSN(N)NTVRDRTPY Fuc 2 Hex 7 HexNAc 6 | 74.021 | 0.015 | -19.855 | 3.487e+06 | 140 - 155 | 1 | 13.55 | 13.55 | 13.55 |
| 4218.6532 | NNVHSN(N)NTVRDRTPY Hex 10 HexNAc 4 | 54.709 | 0.015 | -5.043 | 1.642e+07 | 140 - 155 | 2 | 14.68 | 14.84 | 15.17 |
| 3773.5666 | NNVHSN(N)NTVRDRTPY Hex 6 HexNAc 5 | 54.419 | 0.015 | -4.196 | 6.151e+07 | 140 - 155 | 2 | 13.71 | 13.87 | 14.04 |
| 3554.4936 | NNVHSN(N)NTVRDRTPY Fuc 1 Hex 5 HexNAc 4 | 43.359 | 0.026 | -20.268 | 1.654e+07 | 140 - 155 | 1 | 13.87 | 13.87 | 14.04 |
| 4146.6263 | NNVHSN(N)NTVRDRTPY Hex 9 HexNAc 3 Neu5Ac 1 | 40.368 | 0.026 | -35.652 | 3.972e+06 | 140 - 155 | 5 | 16.95 | 16.95 | 17.12 |
| 3855.5461 | NNVHSN(N)NTVRDRTPY Hex 9 HexNAc 3 | 40.356 | 0.026 | 0.000 | 0.000e+00 | 140 - 155 | 1 | - | - | - |
| 3702.5505 | NNVHSN(N)NTVRDRTPY Fuc 2 Hex 5 HexNAc 4 | 39.408 | 0.033 | 0.000 | 0.000e+00 | 140 - 155 | 1 | - | - | - |
| 4650.8898 | NNVHSN(N)N(D)TVRDRTPY Fuc 1 Hex 8 HexNAc 7 | 35.667 | 0.048 | -20.619 | 2.746e+06 | 140 - 155 | 1 | 13.55 | 13.55 | 13.55 |

### TIN(N)ETSRLGYETF Hex 8 HexNAc 2

**Mass:**  3232.3233

(2.207e-06)

**Charge States:** 3

**Start Position**: 365

**End Position**: 378

| Profile Score | Tandem Score | TDA q-value | Volume | Spectrum Matches |
| --- | --- | --- | --- | --- |
| -2.5135 | 180.4437 | 0.0000 | 4.7110e+07 | 2 |

**Adduct Signal Fractions**

- **Unmodified**: 4.7110e+07

### RN(N)WSKPQC(C)GITGFAPF Hex 8 HexNAc 2

**Mass:**  3567.4901

(-4.757e-08)

**Charge States:** 3

**Start Position**: 85

**End Position**: 101

| Profile Score | Tandem Score | TDA q-value | Volume | Spectrum Matches |
| --- | --- | --- | --- | --- |
| -4.2061 | 175.4330 | 0.0000 | 3.3267e+07 | 2 |

**Adduct Signal Fractions**

- **Unmodified**: 3.3267e+07

### LHVC(C)ITGDDKN(N)ATASF Hex 9 HexNAc 2

**Mass:**  3612.4590

(1.356e-06)

**Charge States:** 3, 4

**Start Position**: 190

**End Position**: 206

| Profile Score | Tandem Score | TDA q-value | Volume | Spectrum Matches |
| --- | --- | --- | --- | --- |
| -18.8202 | 170.5239 | 0.0000 | 2.3118e+07 | 2 |

**Adduct Signal Fractions**

- **Unmodified**: 2.3118e+07

### RN(N)WSKPQC(C)GITGF Hex 9 HexNAc 2

**Mass:**  3414.3924

(3.567e-06)

**Charge States:** 3

**Start Position**: 85

**End Position**: 98

| Profile Score | Tandem Score | TDA q-value | Volume | Spectrum Matches |
| --- | --- | --- | --- | --- |
| -17.8172 | 170.5107 | 0.0000 | 1.0923e+07 | 1 |

**Adduct Signal Fractions**

- **Unmodified**: 1.0923e+07

### RN(N)WSKPQC(C)GITGFAPF Hex 9 HexNAc 2

**Mass:**  3729.5454

(1.873e-06)

**Charge States:** 3

**Start Position**: 85

**End Position**: 101

| Profile Score | Tandem Score | TDA q-value | Volume | Spectrum Matches |
| --- | --- | --- | --- | --- |
| -3.5504 | 166.0881 | 0.0000 | 3.3424e+07 | 3 |

**Adduct Signal Fractions**

- **Unmodified**: 3.3424e+07

### M(O)GRTIN(N)ETSRLGYETF Hex 7 HexNAc 2

**Mass:**  3430.4203

(-6.759e-07)

**Charge States:** 3

**Start Position**: 362

**End Position**: 378

| Profile Score | Tandem Score | TDA q-value | Volume | Spectrum Matches |
| --- | --- | --- | --- | --- |
| 17.3171 | 164.9846 | 0.0000 | 3.5660e+08 | 3 |

**Adduct Signal Fractions**

- **Unmodified**: 3.5660e+08

### LHVC(C)ITGDDKN(N)ATASF Hex 8 HexNAc 2

**Mass:**  3450.4187

(5.108e-06)

**Charge States:** 3

**Start Position**: 190

**End Position**: 206

| Profile Score | Tandem Score | TDA q-value | Volume | Spectrum Matches |
| --- | --- | --- | --- | --- |
| -17.6149 | 164.8620 | 0.0000 | 1.4577e+07 | 2 |

**Adduct Signal Fractions**

- **Unmodified**: 1.4577e+07

### M(O)GRTIN(N)ETSRLGYETF Hex 8 HexNAc 2

**Mass:**  3592.4778

(5.953e-07)

**Charge States:** 3

**Start Position**: 362

**End Position**: 378

| Profile Score | Tandem Score | TDA q-value | Volume | Spectrum Matches |
| --- | --- | --- | --- | --- |
| 16.8308 | 163.2762 | 0.0000 | 5.7976e+08 | 3 |

**Adduct Signal Fractions**

- **Unmodified**: 5.7976e+08

### M(O)GRTIN(N)ETSRLGYETF Hex 6 HexNAc 2

**Mass:**  3268.3764

(1.034e-06)

**Charge States:** 3

**Start Position**: 362

**End Position**: 378

| Profile Score | Tandem Score | TDA q-value | Volume | Spectrum Matches |
| --- | --- | --- | --- | --- |
| -2.2797 | 163.1218 | 0.0000 | 1.7457e+08 | 3 |

**Adduct Signal Fractions**

- **Unmodified**: 1.7457e+08

### M(O)GRTIN(N)ETSRLGYETF Hex 5 HexNAc 2

**Mass:**  3106.3236

(2.173e-06)

**Charge States:** 3, 4

**Start Position**: 362

**End Position**: 378

| Profile Score | Tandem Score | TDA q-value | Volume | Spectrum Matches |
| --- | --- | --- | --- | --- |
| -17.2768 | 153.7056 | 0.0000 | 2.2585e+07 | 1 |

**Adduct Signal Fractions**

- **Unmodified**: 2.2585e+07

### LHVC(C)ITGDDKN(N)ATASF Hex 7 HexNAc 2

**Mass:**  3288.3642

(4.760e-06)

**Charge States:** 3

**Start Position**: 190

**End Position**: 206

| Profile Score | Tandem Score | TDA q-value | Volume | Spectrum Matches |
| --- | --- | --- | --- | --- |
| -19.6841 | 146.6553 | 0.0000 | 2.3351e+06 | 1 |

**Adduct Signal Fractions**

- **Unmodified**: 2.3351e+06

### M(O)GRTIN(N)ETSRLGY Hex 8 HexNAc 2

**Mass:**  3215.3172

(1.101e-07)

**Charge States:** 3

**Start Position**: 362

**End Position**: 375

| Profile Score | Tandem Score | TDA q-value | Volume | Spectrum Matches |
| --- | --- | --- | --- | --- |
| -2.7352 | 143.7978 | 0.0000 | 4.9841e+07 | 1 |

**Adduct Signal Fractions**

- **Unmodified**: 4.9841e+07

### TIN(N)ETSRLGYETF Hex 6 HexNAc 2

**Mass:**  2908.2198

(2.733e-06)

**Charge States:** 3

**Start Position**: 365

**End Position**: 378

| Profile Score | Tandem Score | TDA q-value | Volume | Spectrum Matches |
| --- | --- | --- | --- | --- |
| -18.4362 | 143.1159 | 0.0000 | 7.0834e+06 | 1 |

**Adduct Signal Fractions**

- **Unmodified**: 7.0834e+06

### M(O)GRTIN(N)ETSRL Hex 8 HexNAc 2

**Mass:**  2995.2363

(1.425e-06)

**Charge States:** 3

**Start Position**: 362

**End Position**: 373

| Profile Score | Tandem Score | TDA q-value | Volume | Spectrum Matches |
| --- | --- | --- | --- | --- |
| -17.8327 | 141.1053 | 0.0000 | 8.6601e+06 | 1 |

**Adduct Signal Fractions**

- **Unmodified**: 8.6601e+06

### TIN(N)ETSRLGYETF Hex 7 HexNAc 2

**Mass:**  3070.2731

(1.915e-06)

**Charge States:** 3

**Start Position**: 365

**End Position**: 378

| Profile Score | Tandem Score | TDA q-value | Volume | Spectrum Matches |
| --- | --- | --- | --- | --- |
| -2.8107 | 138.8757 | 0.0000 | 2.0942e+07 | 2 |

**Adduct Signal Fractions**

- **Unmodified**: 2.0942e+07

### GRTIN(N)ETSRLGYETF Hex 7 HexNAc 2

**Mass:**  3283.3786

(-2.636e-06)

**Charge States:** 3

**Start Position**: 363

**End Position**: 378

| Profile Score | Tandem Score | TDA q-value | Volume | Spectrum Matches |
| --- | --- | --- | --- | --- |
| -19.5574 | 136.1415 | 0.0000 | 6.1521e+06 | 1 |

**Adduct Signal Fractions**

- **Unmodified**: 6.1521e+06

### MGRTIN(N)ETSRLGYETF Hex 8 HexNAc 2

**Mass:**  3576.4993

(5.272e-06)

**Charge States:** 3

**Start Position**: 362

**End Position**: 378

| Profile Score | Tandem Score | TDA q-value | Volume | Spectrum Matches |
| --- | --- | --- | --- | --- |
| -18.7420 | 136.0888 | 0.0000 | 7.1655e+06 | 1 |

**Adduct Signal Fractions**

- **Unmodified**: 7.1655e+06

### NNVHSN(N)NTVRDRTPY Hex 7 HexNAc 6

**Mass:**  4141.7141

(7.291e-04)

**Start Position**: 141

**End Position**: 156

| Profile Score | Tandem Score | TDA q-value | Volume | Spectrum Matches |
| --- | --- | --- | --- | --- |
| 0.0000 | 134.2758 | 0.0000 | 0.0000e+00 | 1 |

Adduct:

No Chromatogram Found

### MGRTIN(N)ETSRLGYETF Hex 7 HexNAc 2

**Mass:**  3414.4455

(5.212e-06)

**Charge States:** 3

**Start Position**: 362

**End Position**: 378

| Profile Score | Tandem Score | TDA q-value | Volume | Spectrum Matches |
| --- | --- | --- | --- | --- |
| -18.9377 | 130.2733 | 0.0077 | 4.1132e+06 | 1 |

**Adduct Signal Fractions**

- **Unmodified**: 4.1132e+06

### M(O)GRTIN(N)ETSRLGY Hex 7 HexNAc 2

**Mass:**  3053.2672

(2.689e-06)

**Charge States:** 3

**Start Position**: 362

**End Position**: 375

| Profile Score | Tandem Score | TDA q-value | Volume | Spectrum Matches |
| --- | --- | --- | --- | --- |
| -3.3170 | 120.9669 | 0.0077 | 3.6003e+07 | 2 |

**Adduct Signal Fractions**

- **Unmodified**: 3.6003e+07

### NNVHSN(N)NTVRDRTPY Fuc 1 Hex 6 HexNAc 5

**Mass:**  3919.6218

(2.556e-04)

**Charge States:** 4

**Start Position**: 141

**End Position**: 156

| Profile Score | Tandem Score | TDA q-value | Volume | Spectrum Matches |
| --- | --- | --- | --- | --- |
| 7.1100 | 109.0288 | 0.0124 | 9.5280e+07 | 7 |

**Adduct Signal Fractions**

- **Unmodified**: 9.5280e+07

### MGRTIN(N)ETSRLGYETF Hex 6 HexNAc 2

**Mass:**  3252.3967

(6.702e-06)

**Charge States:** 3

**Start Position**: 362

**End Position**: 378

| Profile Score | Tandem Score | TDA q-value | Volume | Spectrum Matches |
| --- | --- | --- | --- | --- |
| -19.0337 | 107.4060 | 0.0124 | 2.5599e+06 | 1 |

**Adduct Signal Fractions**

- **Unmodified**: 2.5599e+06

### NNVHSN(N)NTVRDRTPY Fuc 1 Hex 7 HexNAc 6

**Mass:**  4284.7482

(5.810e-07)

**Charge States:** 4

**Start Position**: 141

**End Position**: 156

| Profile Score | Tandem Score | TDA q-value | Volume | Spectrum Matches |
| --- | --- | --- | --- | --- |
| -18.6639 | 100.9585 | 0.0124 | 1.8973e+07 | 1 |

**Adduct Signal Fractions**

- **Unmodified**: 1.8973e+07

### NNVHSN(N)NTVRDRTPY Fuc 1 Hex 9 HexNAc 3

**Mass:**  3999.5817

(-9.567e-06)

**Charge States:** 4

**Start Position**: 141

**End Position**: 156

| Profile Score | Tandem Score | TDA q-value | Volume | Spectrum Matches |
| --- | --- | --- | --- | --- |
| -4.6679 | 97.3327 | 0.0124 | 3.1560e+07 | 2 |

**Adduct Signal Fractions**

- **Unmodified**: 3.1560e+07

### NNVHSN(N)NTVRDRTPY Fuc 2 Hex 6 HexNAc 5

**Mass:**  4065.6806

(6.149e-08)

**Charge States:** 4

**Start Position**: 141

**End Position**: 156

| Profile Score | Tandem Score | TDA q-value | Volume | Spectrum Matches |
| --- | --- | --- | --- | --- |
| -19.0580 | 95.3022 | 0.0124 | 8.2290e+06 | 1 |

**Adduct Signal Fractions**

- **Unmodified**: 8.2290e+06

### NNVHSN(N)NTVRDRTPY Fuc 1 Hex 10 HexNAc 4

**Mass:**  4364.7165

(-9.692e-06)

**Charge States:** 4

**Start Position**: 141

**End Position**: 156

| Profile Score | Tandem Score | TDA q-value | Volume | Spectrum Matches |
| --- | --- | --- | --- | --- |
| -20.6830 | 85.2557 | 0.0124 | 1.2848e+07 | 3 |

**Adduct Signal Fractions**

- **Unmodified**: 1.2848e+07

### NNVHSN(N)NTVRDRTPY Fuc 2 Hex 10 HexNAc 4

**Mass:**  4510.7712

(-9.052e-06)

**Charge States:** 4

**Start Position**: 141

**End Position**: 156

| Profile Score | Tandem Score | TDA q-value | Volume | Spectrum Matches |
| --- | --- | --- | --- | --- |
| -21.3466 | 83.7182 | 0.0124 | 5.2137e+06 | 1 |

**Adduct Signal Fractions**

- **Unmodified**: 5.2137e+06

### NNVHSN(N)N(D)TVRDRTPY Fuc 3 Hex 6 HexNAc 5 Neu5Ac 1

**Mass:**  4503.8366

(2.227e-04)

**Charge States:** 4

**Start Position**: 141

**End Position**: 156

| Profile Score | Tandem Score | TDA q-value | Volume | Spectrum Matches |
| --- | --- | --- | --- | --- |
| -22.2531 | 78.3251 | 0.0124 | 3.5643e+06 | 1 |

**Adduct Signal Fractions**

- **Unmodified**: 3.5643e+06

### NNVHSN(N)NTVRDRTPY Fuc 2 Hex 7 HexNAc 6

**Mass:**  4430.8073

(-1.189e-06)

**Charge States:** 4

**Start Position**: 141

**End Position**: 156

| Profile Score | Tandem Score | TDA q-value | Volume | Spectrum Matches |
| --- | --- | --- | --- | --- |
| -19.8546 | 74.0211 | 0.0146 | 3.4872e+06 | 1 |

**Adduct Signal Fractions**

- **Unmodified**: 3.4872e+06

### NNVHSN(N)NTVRDRTPY Hex 10 HexNAc 4

**Mass:**  4218.6530

(-8.919e-06)

**Charge States:** 4

**Start Position**: 141

**End Position**: 156

| Profile Score | Tandem Score | TDA q-value | Volume | Spectrum Matches |
| --- | --- | --- | --- | --- |
| -5.0430 | 54.7092 | 0.0146 | 1.6421e+07 | 2 |

**Adduct Signal Fractions**

- **Unmodified**: 1.6421e+07

### NNVHSN(N)NTVRDRTPY Hex 6 HexNAc 5

**Mass:**  3773.5668

(3.817e-08)

**Charge States:** 4

**Start Position**: 141

**End Position**: 156

| Profile Score | Tandem Score | TDA q-value | Volume | Spectrum Matches |
| --- | --- | --- | --- | --- |
| -4.1965 | 54.4191 | 0.0146 | 6.1507e+07 | 2 |

**Adduct Signal Fractions**

- **Unmodified**: 6.1507e+07

### NNVHSN(N)NTVRDRTPY Fuc 1 Hex 5 HexNAc 4

**Mass:**  3554.4912

(2.497e-07)

**Charge States:** 4

**Start Position**: 141

**End Position**: 156

| Profile Score | Tandem Score | TDA q-value | Volume | Spectrum Matches |
| --- | --- | --- | --- | --- |
| -20.2678 | 43.3588 | 0.0263 | 1.6536e+07 | 1 |

**Adduct Signal Fractions**

- **Unmodified**: 1.6536e+07

### NNVHSN(N)NTVRDRTPY Hex 9 HexNAc 3 Neu5Ac 1

**Mass:**  4146.6213

(4.774e-04)

**Charge States:** 4

**Start Position**: 141

**End Position**: 156

| Profile Score | Tandem Score | TDA q-value | Volume | Spectrum Matches |
| --- | --- | --- | --- | --- |
| -35.6525 | 40.3682 | 0.0263 | 3.9715e+06 | 5 |

**Adduct Signal Fractions**

- **Unmodified**: 3.9715e+06

### NNVHSN(N)NTVRDRTPY Hex 9 HexNAc 3

**Mass:**  3855.5461

(5.143e-04)

**Start Position**: 141

**End Position**: 156

| Profile Score | Tandem Score | TDA q-value | Volume | Spectrum Matches |
| --- | --- | --- | --- | --- |
| 0.0000 | 40.3556 | 0.0263 | 0.0000e+00 | 1 |

Adduct:

No Chromatogram Found

### NNVHSN(N)NTVRDRTPY Fuc 2 Hex 5 HexNAc 4

**Mass:**  3702.5505

(5.411e-04)

**Start Position**: 141

**End Position**: 156

| Profile Score | Tandem Score | TDA q-value | Volume | Spectrum Matches |
| --- | --- | --- | --- | --- |
| 0.0000 | 39.4082 | 0.0327 | 0.0000e+00 | 1 |

Adduct:

No Chromatogram Found

### NNVHSN(N)N(D)TVRDRTPY Fuc 1 Hex 8 HexNAc 7

**Mass:**  4650.8898

(2.157e-04)

**Charge States:** 4

**Start Position**: 141

**End Position**: 156

| Profile Score | Tandem Score | TDA q-value | Volume | Spectrum Matches |
| --- | --- | --- | --- | --- |
| -20.6186 | 35.6671 | 0.0482 | 2.7458e+06 | 1 |

**Adduct Signal Fractions**

- **Unmodified**: 2.7458e+06

### iav|ACF41842|A/Puerto

**>iav|ACF41842|A/Puerto**  
 `MDVNPTLLFLKVPAQNAISTTFPYTGDPPYSHGTGTGYTMDTVNRTHQYSEKGRWTTNTETGAPQLNPIDGPLPEDNEPS  
GYAQTDCVLEAMAFLEESHPGIFENSCIETMEVVQQTRVDKLTQGRQTYDWTLNRNQPAATALANTIEVFRSNGLTANES  
GRLIDFLKDVMESMNKEEMGITTHFQRKRRVRDNMTKKMITQRTMGKKKQRLNKRSYLIRALTLNTMTKDAERGKLKRRA  
IATPGMQIRGFVYFVETLARSICEKLEQSGLPVGGNEKKAKLANVVRKMMTNSQDTELSFTITGDNTKWNENQNPRMFLA  
MITYMTRNQPEWFRNVLSIAPIMFSNKMARLGKGYMFESKSMKLRTQIPAEMLASIDLKYFNDSTRKKIEKIRPLLIEGT  
ASLSPGMMMGMFNMLSTVLGVSILNLGQKRYTKTTYWWDGLQSSDDFALIVNAPNHEGIQAGVDRFYRTCKLLGINMSKK  
KSYINRTGTFEFTSFFYRYGFVANFSMELPSFGVSGINESADMSIGVTVIKNNMINNDLGPATAQMALQLFIKDYRYTYR  
CHRGDTQIQTRRSFEIKKLWEQTRSKAGLLVSDGGPNLYNIRNLHIPEVCLKWELMDEDYQGRLCNPLNPFVSHKEIESM  
NNAVMMPAHGPAKNMEYDAVATTHSWIPKRNRSILNTSQRGVLEDEQMYQRCCNLFEKFFPSSSYRRPVGISSMVEAMVS  
RARIDARIDFESGRIKKEEFTEIMKICSTIEELRRQK`

| Site | Type | Count |
| --- | --- | --- |
| 476 | N-linked | 1 |

| Mass | Sequence | MS_2_ Score | `q`-value | MS_1_ Score | Signal | Protein Coordinates | Tandem Scans | Start Time | Apex Time | End Time |
| --- | --- | --- | --- | --- | --- | --- | --- | --- | --- | --- |
| 3977.6300 | GIN(N)MSKKKSY Fuc 1 Hex 9 HexNAc 6 | 46.256 | 0.019 | -5.930 | 1.361e+07 | 473 - 483 | 2 | 15.17 | 15.34 | 15.67 |

### GIN(N)MSKKKSY Fuc 1 Hex 9 HexNAc 6

**Mass:**  3977.6306

(2.410e-06)

**Charge States:** 4

**Start Position**: 474

**End Position**: 484

| Profile Score | Tandem Score | TDA q-value | Volume | Spectrum Matches |
| --- | --- | --- | --- | --- |
| -5.9303 | 46.2564 | 0.0189 | 1.3606e+07 | 2 |

**Adduct Signal Fractions**

- **Unmodified**: 1.3606e+07

### iav|AGU93028|A/Puerto

**>iav|AGU93028|A/Puerto**  
 `MDVNPTLLFLKVPAQNAISTTFPYTGDPPYSHGTGTGYTMDTVNRTHQYSEKGRWTTNTETGAPQLNPIDGPLPEDNEPS  
GYAQTDCVLEAMAFLEESHPGIFENSCIETMEVVQQTRVDKLTQGRQTYDWTLNRNQPAATALANTIEVFRSNGLTANES  
GRLIDFLKDVMESMNKEEMGITTHFQRKRRVRDNMTKKMITQRTIGKKKQRLNKRSYLIRALTLNTMTKDAERGKLKRRA  
IATPGMQIRGFVYFVETLARSICEKLEQSGLPVGGNEKKAKLANVVRKMMTNSQDTELSFTITGDNTKWNENQNPRMFLA  
MITYMTRNQPEWFRNVLSIAPIMFSNKMARLGKGYMFESKSMKLRTQIPAEMLASIDLKYFNDSTRKKIEKIRPLLIEGT  
ASLSPGMMMGMFNMLSTVLGVSILNLGQKRYTKTTYWWDGLQSSDDFALIVNAPNHEGIQAGVDRFYRTCKLLGINMSKK  
KSYINRTGTFEFTSFFYRYGFVANFSMELPSFGVSGINESADMSIGVTVIKNNMINNDLGPATAQMALQLFIKDYRYTYR  
CHRGDTQIQTRRSFEIKKLWEQTRSKAGLLVSDGGPNLYNIRNLHIPEVCLKWELMDEDYQGRLCNPLNPFVSHKEIESM  
NNAVMMPAHGPAKNMEYDAVATTHSWIPKRNRSILNTSQRGVLEDEQMYQRCCNLFEKFFPSSSYRRPVGISSMVEAMVS  
RARIDARIDFESGRIKKEEFTEIMKICSTIEELRRQK`

| Site | Type | Count |
| --- | --- | --- |
| 476 | N-linked | 1 |

| Mass | Sequence | MS_2_ Score | `q`-value | MS_1_ Score | Signal | Protein Coordinates | Tandem Scans | Start Time | Apex Time | End Time |
| --- | --- | --- | --- | --- | --- | --- | --- | --- | --- | --- |
| 3977.6300 | GIN(N)MSKKKSY Fuc 1 Hex 9 HexNAc 6 | 46.256 | 0.019 | -5.930 | 1.361e+07 | 473 - 483 | 2 | 15.17 | 15.34 | 15.67 |

### GIN(N)MSKKKSY Fuc 1 Hex 9 HexNAc 6

**Mass:**  3977.6306

(2.410e-06)

**Charge States:** 4

**Start Position**: 474

**End Position**: 484

| Profile Score | Tandem Score | TDA q-value | Volume | Spectrum Matches |
| --- | --- | --- | --- | --- |
| -5.9303 | 46.2564 | 0.0189 | 1.3606e+07 | 2 |

**Adduct Signal Fractions**

- **Unmodified**: 1.3606e+07

### iav|AFM71854|A/Puerto

**>iav|AFM71854|A/Puerto**  
 `MDVNPTLLFLKVPAQNAISTTFPYTGDPPYSHGTGTGYTMDTVNRTHQYSEKGRWTTNTETGAPQLNPIDGPLPEDNEPS  
GYAQTDCVLEAMAFLEESHPGIFENSCIETMEVVQQTRVDKLTQGRQTYDWTLNRNQPAATALANTIEVFRSNGLTANES  
GRLIDFLKDVMESMNKEEMGITTHFQRKRRVRDNMTKKMITQRTMGKKKQRLNKRSYLIRALTLNTMTKDAERGKLKRRA  
IATPGMQIRGFVYFVETLARSICEKLEQSGLPVGGNEKKAKLANVVRKMMTNSQDTELSFTITGDNTKWNENQNPRMFLA  
MITYMTRNQPEWFRNVLSIAPIMFSNKMARLGKGYMFESKSMKLRTQIPAEMLASIDLKYFNDSTRKKIEKIRPLLIEGT  
ASLSPGMMMGMFNMLSTVLGVSILNLGQKRYTKTTYWWDGLQSSDDFALIVNAPNHEGIQAGVDRFYRTCKLLGINMSKK  
KSYINRTGTFEFTSFFYRYGFVANFSMELPSFGVSGINESADMSIGVTVIKNNMINNDLGPATAQMALQLFIKDYRYTYR  
CHRGDTQIQTRRSFEIKKLWEQTRSKAGLLVSDGGPNLYNIRNLHIPEVCLKWELMDEDYQGRLCNPLNPFVSHKEIESM  
NNAVMMPAHGPAKNMEYDAVATTHSWIPKRNRSILNTSQRGVLEDEQMYQRCCNLFEKFFPSSSYRRPVGISSMVEAMVS  
RARIDARIDFESGRIKKEEFTEIMKICSTIEELRRQK`

| Site | Type | Count |
| --- | --- | --- |
| 476 | N-linked | 1 |

| Mass | Sequence | MS_2_ Score | `q`-value | MS_1_ Score | Signal | Protein Coordinates | Tandem Scans | Start Time | Apex Time | End Time |
| --- | --- | --- | --- | --- | --- | --- | --- | --- | --- | --- |
| 3977.6300 | GIN(N)MSKKKSY Fuc 1 Hex 9 HexNAc 6 | 46.256 | 0.019 | -5.930 | 1.361e+07 | 473 - 483 | 2 | 15.17 | 15.34 | 15.67 |

### GIN(N)MSKKKSY Fuc 1 Hex 9 HexNAc 6

**Mass:**  3977.6306

(2.410e-06)

**Charge States:** 4

**Start Position**: 474

**End Position**: 484

| Profile Score | Tandem Score | TDA q-value | Volume | Spectrum Matches |
| --- | --- | --- | --- | --- |
| -5.9303 | 46.2564 | 0.0189 | 1.3606e+07 | 2 |

**Adduct Signal Fractions**

- **Unmodified**: 1.3606e+07

### iav|AAM75156|A/Puerto

**>iav|AAM75156|A/Puerto**  
 `MDVNPTLLFLKVPAQNAISTTFPYTGDPPYSHGTGTGYTMDTVNRTHQYSEKGRWTTNTETGAPQLNPIDGPLPEDNEPS  
GYAQTDCVLEAMAFLEESHPGIFENSCIETMEVVQQTRVDKLTQGRQTYDWTLNRNQPAATALANTIEVFRSNGLTANES  
GRLIDFLKDVMESMKKEEMGITTHFQRKRRVRDNMTKKMITQRTIGKKKQRLNKRSYLIRALTLNTMTKDAERGKLKRRA  
IATPGMQIRGFVYFVETLARSICEKLEQSGLPVGGNEKKAKLANVVRKMMTNSQDTELSFTITGDNTKWNENQNPRMFLA  
MITYMTRNQPEWFRNVLSIAPIMFSNKMARLGKGYMFESKSMKLRTQIPAEMLASIDLKYFNDSTRKKIEKIRPLLIEGT  
ASLSPGMMMGMFNMLSTVLGVSILNLGQKRYTKTTYWWDGLQSSDDFALIVNAPNHEGIQAGVDRFYRTCKLLGINMSKK  
KSYINRTGTFEFTSFFYRYGFVANFSMELPSFGVSGINESADMSIGVTVIKNNMINNDLGPATAQMALQLFIKDYRYTYR  
CHRGDTQIQTRRSFEIKKLWEQTRSKAGLLVSDGGPNLYNIRNLHIPEVCLKWELMDEDYQGRLCNPLNPFVSHKEIESM  
NNAVMMPAHGPAKNMEYDAVATTHSWIPKRNRSILNTSQRGVLEDEQMYQRCCNLFEKFFPSSSYRRPVGISSMVEAMVS  
RARIDARIDFESGRIKKEEFTEIMKICSTIEELRRQK`

| Site | Type | Count |
| --- | --- | --- |
| 476 | N-linked | 1 |

| Mass | Sequence | MS_2_ Score | `q`-value | MS_1_ Score | Signal | Protein Coordinates | Tandem Scans | Start Time | Apex Time | End Time |
| --- | --- | --- | --- | --- | --- | --- | --- | --- | --- | --- |
| 3977.6300 | GIN(N)MSKKKSY Fuc 1 Hex 9 HexNAc 6 | 46.256 | 0.019 | -5.930 | 1.361e+07 | 473 - 483 | 2 | 15.17 | 15.34 | 15.67 |

### GIN(N)MSKKKSY Fuc 1 Hex 9 HexNAc 6

**Mass:**  3977.6306

(2.410e-06)

**Charge States:** 4

**Start Position**: 474

**End Position**: 484

| Profile Score | Tandem Score | TDA q-value | Volume | Spectrum Matches |
| --- | --- | --- | --- | --- |
| -5.9303 | 46.2564 | 0.0189 | 1.3606e+07 | 2 |

**Adduct Signal Fractions**

- **Unmodified**: 1.3606e+07

### iav|ADX99655|A/Puerto

**>iav|ADX99655|A/Puerto**  
 `MDVNPTLLFLKVPAQNAISTTFPYTGDPPYSHGTGTGYTMDTVNRTHQYSEKGRWTTNTETGAPQLNPIDGPLPEDNEPS  
GYAQTDCVLEAMAFLEESHPGIFENSCIETMEVVQQTRVDKLTQGRQTYDWTLNRNQPAATALANTIEVFRSNGLTANES  
GRLIDFLKDVMESMNKEEMGITTHFQRKRRVRDNMTKKMITQRTMGKKKQRLNKRSYLIRALTLNTMTKDAERGKLKRRA  
IATPGMQIRGFVYFVETLARSICEKLEQSGLPVGGNEKKAKLANVVRKMMTNSQDTELSFTITGDNTKWNENQNPRMFLA  
MITYMTRNQPEWFRNVLSIAPIMFSNKMARLGKGYMFESKSMKLRTQIPAEMLASIDLKYFNDSTRKKIEKIRPLLIEGT  
ASLSPGMMMGMFNMLSTVLGVSILNLGQKRYTKTTYWWDGLQSSDDFALIVNAPNHEGIQAGVDRFYRTCKLLGINMSKK  
KSYINRTGTFEFTSFFYRYGFVANFSMELPSFGVSGINESADMSIGVTVIKNNMINNDLGPATAQMALQLFIKDYRYTYR  
CHIGDTQIQTRRSFEIKKLWEQTRSKAGLLVSDGGPNLYNIRNLHIPEVCLKWELMDEDYQGRLCNPLNPFVSHKEIESM  
NNAVMMPAHGPAKNMEYDAVATTHSWIPKRNRSILNTSQRGVLEDEQMYQRCCNLFEKFFPSSSYRRPVGISSMVEAMVS  
RARIDARIDFESGRIKKEEFTEIMKICSTIEELRRQK`

| Site | Type | Count |
| --- | --- | --- |
| 476 | N-linked | 1 |

| Mass | Sequence | MS_2_ Score | `q`-value | MS_1_ Score | Signal | Protein Coordinates | Tandem Scans | Start Time | Apex Time | End Time |
| --- | --- | --- | --- | --- | --- | --- | --- | --- | --- | --- |
| 3977.6300 | GIN(N)MSKKKSY Fuc 1 Hex 9 HexNAc 6 | 46.256 | 0.019 | -5.930 | 1.361e+07 | 473 - 483 | 2 | 15.17 | 15.34 | 15.67 |

### GIN(N)MSKKKSY Fuc 1 Hex 9 HexNAc 6

**Mass:**  3977.6306

(2.410e-06)

**Charge States:** 4

**Start Position**: 474

**End Position**: 484

| Profile Score | Tandem Score | TDA q-value | Volume | Spectrum Matches |
| --- | --- | --- | --- | --- |
| -5.9303 | 46.2564 | 0.0189 | 1.3606e+07 | 2 |

**Adduct Signal Fractions**

- **Unmodified**: 1.3606e+07

### iav|ACV49553|A/Puerto

**>iav|ACV49553|A/Puerto**  
 `MDVNPTLLFLKVPAQNAISTTFPYTGDPPYSHGTGTGYTMDTVNRTHQYSEKGRWTTNTETGAPQLNPIDGPLPEDNEPS  
GYAQTDCVLEAMAFLEESHPGIFENSCIETMEVVQQTRVDKLTQGRQTYDWTLNRNQPAATALANTIEVFRSNGLTANES  
GRLIDFLKDVMESMNKEEMGITTHFQRKRRVRDNMTKKMITQRTMGKKKQRLNKRSYLIRALTLNTMTKDAERGKLKRRA  
IATPGMQIRGFVYFVETLARSICEKLEQSGLPVGGNEKKAKLANVVRKMMTNSQDTELSFTITGDNTKWNENQNPRMFLA  
MITYMTRNQPEWFRNVLSIAPIMFSNKMARLGKGYMFESKSMKLRTQIPAEMLASIDLKYFNDSTRKKIEKIRPLLIEGT  
ASLSPGMMMGMFNMLSTVLGVSILNLGQKRYTKTTYWWDGLQSSDDFALIVNAPNHEGIQAGVDRFYRTCKLLGINMSKK  
KSYINRTGTFEFTSFFYRYGFVANFSMELPSFGVSGINESADMSIGVTVIKNNMINNDLGPATAQMALQLFIKDYRYTYR  
CHRGDTQIQTRRSFEIKKLWEQTRSKAGLLVSDGGPNLYNIRNLHIPEVCLKWELMDEDYQGRLCNPLNPFVSHKEIESM  
NNAVMMPAHGPAKNMEYDAVATTHSWIPKRNRSILNTSQRGVLEDEQMYQRCCNLFEKFFPSSSYRRPVGISSMVEAMVS  
RARIDARIDFESGRIKKEEFTEIMKICSTIEELRRQK`

| Site | Type | Count |
| --- | --- | --- |
| 476 | N-linked | 1 |

| Mass | Sequence | MS_2_ Score | `q`-value | MS_1_ Score | Signal | Protein Coordinates | Tandem Scans | Start Time | Apex Time | End Time |
| --- | --- | --- | --- | --- | --- | --- | --- | --- | --- | --- |
| 3977.6300 | GIN(N)MSKKKSY Fuc 1 Hex 9 HexNAc 6 | 46.256 | 0.019 | -5.930 | 1.361e+07 | 473 - 483 | 2 | 15.17 | 15.34 | 15.67 |

### GIN(N)MSKKKSY Fuc 1 Hex 9 HexNAc 6

**Mass:**  3977.6306

(2.410e-06)

**Charge States:** 4

**Start Position**: 474

**End Position**: 484

| Profile Score | Tandem Score | TDA q-value | Volume | Spectrum Matches |
| --- | --- | --- | --- | --- |
| -5.9303 | 46.2564 | 0.0189 | 1.3606e+07 | 2 |

**Adduct Signal Fractions**

- **Unmodified**: 1.3606e+07

### iav|BAV59609|A/Puerto

**>iav|BAV59609|A/Puerto**  
 `MDVNPTLLFLKVPAQNAISTTFPYTGDPPYSHGTGTGYTMDTVNRTHQYSEKGRWTTNTETGASQLNPIDGPLPEDNEPS  
GYAQTDCVLEAMAFLEESHPGIFENSCIETIEVVQQTRVDKLTQGRQTYDWTLNRNQPAATALANTIEVFRSNGLTANES  
GRLIDFLKDVMESMNKEEMGITTHFQRKRRVRDNMTKKMITQRTMGKKKQRLNKRSYLIRALTLNTMTKDAERGKLKRRA  
IATPGMQIRGFVYFVETLARSICEKLEQSGLPVGGNEKKAKLANVVRKMMTNSQDTELSFTITGDNTKWNENQNPRMFLA  
MITYMTRNQPEWFRNVLSIAPIMFSNKMARLGKGYMFESKSMKLRTQIPAEMLASIDLKYFNDSTRKKIEKIRPLLIEGT  
ASLSPGMMMGMFNMLSTVLGVSILNLGQKRYTKTTYWWDGLQSSDDFALIVNAPNNEGIQAGVDRFYRTCKLLGINMSKK  
KSYINRTGTFEFTSFFYRYGFVANFSMELPSFGVSGINESADMSIGVTVIKNNMINNDLGPATAQMALQLFIKDYRYTYR  
CHRGDTQIQTRRSFEIKKLWEQTRSKAGLLVSDGGPNLYNIRNLHIPEVCLKWELMDEDYQGRLCNPLNPFVSHKEIESM  
NNAVMMPAHGPAKNMEYDAVATTHSWIPKRNRSILNTSQRGVLEDEQMYQRCCNLFEKFFPSSSYRRPVGISSMVEAMVS  
RARIDARIDFESGRIKKEEFTEIMKICSTIEELRRQK`

| Site | Type | Count |
| --- | --- | --- |
| 476 | N-linked | 1 |

| Mass | Sequence | MS_2_ Score | `q`-value | MS_1_ Score | Signal | Protein Coordinates | Tandem Scans | Start Time | Apex Time | End Time |
| --- | --- | --- | --- | --- | --- | --- | --- | --- | --- | --- |
| 3977.6300 | GIN(N)MSKKKSY Fuc 1 Hex 9 HexNAc 6 | 46.256 | 0.019 | -5.930 | 1.361e+07 | 473 - 483 | 2 | 15.17 | 15.34 | 15.67 |

### GIN(N)MSKKKSY Fuc 1 Hex 9 HexNAc 6

**Mass:**  3977.6306

(2.410e-06)

**Charge States:** 4

**Start Position**: 474

**End Position**: 484

| Profile Score | Tandem Score | TDA q-value | Volume | Spectrum Matches |
| --- | --- | --- | --- | --- |
| -5.9303 | 46.2564 | 0.0189 | 1.3606e+07 | 2 |

**Adduct Signal Fractions**

- **Unmodified**: 1.3606e+07
