# Supplementary material for: Measuring Site-specific Glycosylation Similarity between Influenza a Virus Variants with Statistical Certainty
Source: Mol Cell Proteomics. 2020 Nov 25;19(9):1533–45. doi: 10.1074/mcp.RA120.002031 (PMC8143645; doi:10.1074/mcp.RA120.002031)
Supplement: Supplementary file 2 [file mmc2.zip › mmc2/159250_2_supp_555291_qcjklr.zip!/20190301_5B8eggL2_021119_chymo_HILIC_DDA_1ug_03_glycopeptides.html]

## 20190301\_5B8eggL2\_021119\_chymo\_HILIC\_DDA\_1ug\_03 @ GlycopeptideHypothesis-12d760f891904e879d518259c983d567

**MS^1^ Mass Accuracy:** 10.0 PPM  
**Grouping Tolerance:** 15.0 PPM  
**MS^n^ Mass Accuracy:** 20.0 PPM  
**Minimum `q`-Value For Clustering:** 0.05

**Glycopeptide Hypothesis:**
GlycopeptideHypothesis-12d760f891904e879d518259c983d567  
**Sample Searched:**
20190301\_5B8eggL2\_021119\_chymo\_HILIC\_DDA\_1ug\_03

| Protein | Matched Glycopeptides |
| --- | --- |
| cus\|SWZHA\_5B8\|A/Switzerland/9715293/2013 | 102 |
| cus\|SWZNA\|A/Switzerland/9715293/2013 | 32 |
| iav\|ACF41842\|A/Puerto | 2 |
| iav\|AGU93028\|A/Puerto | 2 |
| iav\|AFM71854\|A/Puerto | 2 |
| iav\|AAM75156\|A/Puerto | 2 |
| iav\|ADX99655\|A/Puerto | 2 |
| iav\|ACV49553\|A/Puerto | 2 |
| iav\|BAV59609\|A/Puerto | 2 |

### cus|SWZHA\_5B8|A/Switzerland/9715293/2013

**>cus|SWZHA\_5B8|A/Switzerland/9715293/2013**  
 `QKLPGNDNSTATLCLGHHAVPNGTIVKTITNDRIEVTNATELVQNSSIGEICDSPHQILDGENCTLIDALLGDPQCDGFQ  
NKKWDLFVERSKAYSNCYPYDVPDYASLRSLVASSGTLEFNNESFNWAGVTHNGTSSSCRRGSNSSFFSRLNWLTHLNSK  
YPALNVTMPNNEQFDKLYIWGVHHPVTDKDQIFLYAQSSGRITVSTKRSQQAVIPNIGSRPRIRNIPSRISIYWTIVKPG  
DILLINSTGNLIAPRGYFKIRSGKSSIMRSDAPIGKCKSECITPNGSIPNDKPFQNVNRITYGACPRYVKQSTLKLATGM  
RNVPERQTRGIFGAIAGFIENGWEGMVDGWYGFRHQNSEGRGQAADLKSTQAAIDQINGKLNRLIGKTNEKFHQIEKEFS  
EVEGRIQDLEKYVEDTKIDLWSYNAELLVALENQHTIDLTDSEMNKLFEKTKKQLRENAEDMGNGCFKIYHKCDNACIGS  
IRNGTYDHDVYRDEALNNRFQIKGVELKSGYKDWILWISFAISCFLLCVALLGFIMWACQKGNIRCNICI`

| Site | Type | Count |
| --- | --- | --- |
| 483 | N-linked | 4 |
| 165 | N-linked | 6 |
| 8 | N-linked | 1 |
| 144 | N-linked | 6 |
| 246 | N-linked | 41 |
| 133 | N-linked | 24 |
| 285 | N-linked | 20 |

| Mass | Sequence | MS_2_ Score | `q`-value | MS_1_ Score | Signal | Protein Coordinates | Tandem Scans | Start Time | Apex Time | End Time |
| --- | --- | --- | --- | --- | --- | --- | --- | --- | --- | --- |
| 3516.5435 | LIN(N)STGNLIAPRGYF Fuc 2 Hex 3 HexNAc 4 Neu5Ac 1 | 189.821 | 0.0 | -17.944 | 5.036e+07 | 243 - 258 | 1 | 47.08 | 47.08 | 47.25 |
| 2695.0858 | LIN(N)STGNL Hex 9 HexNAc 2 | 184.530 | 0.0 | -19.160 | 2.408e+07 | 243 - 251 | 1 | 26.55 | 26.55 | 26.72 |
| 3499.5170 | LIN(N)STGNLIAPRGYF Hex 9 HexNAc 2 | 178.412 | 0.0 | -3.207 | 1.807e+08 | 243 - 258 | 2 | 47.08 | 47.08 | 47.41 |
| 3352.4524 | LIN(N)STGNLIAPRGY Hex 9 HexNAc 2 | 172.231 | 0.0 | -2.627 | 1.063e+08 | 243 - 257 | 3 | 36.80 | 36.97 | 37.14 |
| 3906.8775 | TIVKPGDILLIN(N)STGNLIAPR Hex 8 HexNAc 2 | 167.770 | 0.0 | 16.419 | 3.548e+08 | 234 - 255 | 5 | 53.02 | 53.19 | 53.87 |
| 3369.4788 | LIN(N)STGNLIAPRGY Fuc 2 Hex 3 HexNAc 4 Neu5Ac 1 | 164.209 | 0.0 | -18.011 | 2.495e+07 | 243 - 257 | 1 | 36.80 | 36.97 | 36.97 |
| 3661.5676 | LIN(N)STGNLIAPRGYF Hex 10 HexNAc 2 | 164.016 | 0.0 | -20.166 | 8.641e+06 | 243 - 258 | 1 | 46.92 | 47.08 | 47.08 |
| 4451.0687 | TIVKPGDILLIN(N)STGNLIAPRGY Hex 10 HexNAc 2 | 163.226 | 0.0 | 15.272 | 2.537e+08 | 234 - 257 | 7 | 55.06 | 55.23 | 55.73 |
| 4055.6511 | NWAGVTHN(N)GTSSSC(C)RRGSNSSFF Hex 7 HexNAc 2 | 162.389 | 0.0 | -15.460 | 5.517e+07 | 125 - 148 | 1 | 30.38 | 30.55 | 30.55 |
| 4126.9630 | TIVKPGDILLIN(N)STGNLIAPRGY Hex 8 HexNAc 2 | 160.091 | 0.0 | 13.885 | 7.757e+08 | 234 - 257 | 11 | 55.06 | 55.40 | 56.41 |
| 4230.9815 | TIVKPGDILLIN(N)STGNLIAPR Hex 10 HexNAc 2 | 160.049 | 0.0 | -3.482 | 1.282e+08 | 234 - 255 | 2 | 52.85 | 53.02 | 53.36 |
| 4085.9519 | TIVKPGDILLIN(N)STGNLIAPR Fuc 2 Hex 3 HexNAc 4 Neu5Ac 1 | 159.961 | 0.0 | 14.890 | 3.063e+08 | 234 - 255 | 3 | 52.85 | 53.02 | 53.87 |
| 4752.0486 | RSDAPIGKC(C)KSEC(C)ITPN(N)GSIPNDKPF Hex 9 HexNAc 2 | 159.480 | 0.0 | -2.786 | 2.890e+08 | 268 - 294 | 3 | 29.70 | 29.87 | 30.21 |
| 4068.9397 | TIVKPGDILLIN(N)STGNLIAPR Hex 9 HexNAc 2 | 159.199 | 0.0 | 19.187 | 2.875e+09 | 234 - 255 | 15 | 51.16 | 53.02 | 54.89 |
| 4289.0247 | TIVKPGDILLIN(N)STGNLIAPRGY Hex 9 HexNAc 2 | 159.128 | 0.0 | 18.407 | 5.610e+09 | 234 - 257 | 25 | 53.53 | 55.40 | 58.28 |
| 3159.4285 | TIVKPGDILLIN(N) Hex 9 HexNAc 2 | 159.086 | 0.0 | 11.382 | 2.160e+08 | 234 - 246 | 6 | 45.93 | 47.41 | 47.92 |
| 3190.3987 | LIN(N)STGNLIAPRGY Hex 8 HexNAc 2 | 157.344 | 0.0 | -17.699 | 1.261e+07 | 243 - 257 | 1 | 36.97 | 36.97 | 37.14 |
| 3767.5559 | KSEC(C)ITPN(N)GSIPNDKPF Hex 9 HexNAc 2 | 156.127 | 0.0 | -18.810 | 1.492e+07 | 277 - 294 | 1 | 31.56 | 31.56 | 31.73 |
| 4306.0380 | TIVKPGDILLIN(N)STGNLIAPRGY Fuc 2 Hex 3 HexNAc 4 Neu5Ac 1 | 155.359 | 0.0 | 16.718 | 5.839e+08 | 234 - 257 | 4 | 54.89 | 55.40 | 56.24 |
| 3755.5155 | AGVTHN(N)GTSSSC(C)RRGSNSSFF Hex 7 HexNAc 2 | 154.345 | 0.0 | 16.438 | 5.288e+08 | 127 - 148 | 3 | 20.97 | 21.29 | 21.79 |
| 3337.4666 | LIN(N)STGNLIAPRGYF Hex 8 HexNAc 2 | 154.006 | 0.0 | -19.369 | 1.647e+07 | 243 - 258 | 1 | 47.08 | 47.25 | 47.25 |
| 3386.4395 | IN(N)STGNLIAPRGYF Hex 9 HexNAc 2 | 153.519 | 0.0 | -18.025 | 6.287e+06 | 244 - 258 | 1 | 43.23 | 43.23 | 43.40 |
| 3648.6822 | TIVKPGDILLIN(N)STGNL Fuc 2 Hex 3 HexNAc 4 Neu5Ac 1 | 151.831 | 0.0 | -3.564 | 1.876e+08 | 234 - 251 | 2 | 52.52 | 52.69 | 53.02 |
| 3356.5191 | TIVKPGDILLIN(N)STGN Hex 8 HexNAc 2 | 149.806 | 0.0 | -3.447 | 2.322e+07 | 234 - 250 | 1 | 45.08 | 45.08 | 45.42 |
| 3535.5973 | TIVKPGDILLIN(N)STGN Fuc 2 Hex 3 HexNAc 4 Neu5Ac 1 | 149.579 | 0.0 | -3.621 | 8.477e+07 | 234 - 250 | 2 | 44.92 | 45.08 | 45.25 |
| 4427.9425 | RSDAPIGKC(C)KSEC(C)ITPN(N)GSIPNDKPF Hex 7 HexNAc 2 | 149.417 | 0.0 | -2.313 | 2.869e+08 | 268 - 294 | 6 | 29.87 | 30.04 | 30.21 |
| 3431.4091 | AGVTHN(N)GTSSSC(C)RRGSNSSFF Hex 5 HexNAc 2 | 148.698 | 0.0 | -2.267 | 2.287e+08 | 127 - 148 | 3 | 21.45 | 21.62 | 21.95 |
| 3176.4538 | TIVKPGDILLIN(N) Fuc 2 Hex 3 HexNAc 4 Neu5Ac 1 | 147.890 | 0.0 | 10.074 | 6.053e+07 | 234 - 246 | 4 | 45.93 | 47.41 | 47.58 |
| 2997.3762 | TIVKPGDILLIN(N) Hex 8 HexNAc 2 | 147.795 | 0.0 | -18.619 | 1.249e+07 | 234 - 246 | 1 | 47.41 | 47.41 | 47.58 |
| 4589.9919 | RSDAPIGKC(C)KSEC(C)ITPN(N)GSIPNDKPF Hex 8 HexNAc 2 | 147.626 | 0.0 | -2.388 | 1.768e+08 | 268 - 294 | 3 | 29.87 | 30.04 | 30.21 |
| 3593.4631 | AGVTHN(N)GTSSSC(C)RRGSNSSFF Hex 6 HexNAc 2 | 147.480 | 0.0 | -3.934 | 1.488e+08 | 127 - 148 | 1 | 21.29 | 21.45 | 21.79 |
| 3518.5719 | TIVKPGDILLIN(N)STGN Hex 9 HexNAc 2 | 146.858 | 0.0 | 16.408 | 2.552e+08 | 234 - 250 | 4 | 44.92 | 45.08 | 45.59 |
| 3631.6560 | TIVKPGDILLIN(N)STGNL Hex 9 HexNAc 2 | 146.069 | 0.0 | 18.207 | 7.178e+08 | 234 - 251 | 8 | 52.18 | 52.69 | 54.55 |
| 3893.6024 | NWAGVTHNGTSSSC(C)RRGSN(N)SSFF Hex 6 HexNAc 2 | 143.603 | 0.0 | -19.054 | 1.366e+07 | 125 - 148 | 1 | 30.55 | 30.55 | 30.72 |
| 4265.8851 | RSDAPIGKC(C)KSEC(C)ITPN(N)GSIPNDKPF Hex 6 HexNAc 2 | 143.216 | 0.0 | -3.493 | 3.008e+07 | 268 - 294 | 2 | 30.04 | 30.21 | 30.38 |
| 3731.5385 | NWAGVTHN(N)GTSSSC(C)RRGSNSSFF Hex 5 HexNAc 2 | 142.631 | 0.0 | -16.791 | 1.782e+07 | 125 - 148 | 3 | 30.72 | 30.72 | 30.72 |
| 3605.5001 | KSEC(C)ITPN(N)GSIPNDKPF Hex 8 HexNAc 2 | 141.678 | 0.0 | -18.546 | 8.544e+06 | 277 - 294 | 1 | 31.56 | 31.56 | 31.73 |
| 3917.5684 | AGVTHN(N)GTSSSC(C)RRGSNSSFF Hex 8 HexNAc 2 | 141.201 | 0.0 | -17.731 | 5.074e+07 | 127 - 148 | 1 | 21.12 | 21.12 | 21.29 |
| 2857.1056 | NWAGVTHN(N)GTSSS Hex 7 HexNAc 2 | 140.623 | 0.0 | -20.131 | 8.060e+06 | 125 - 138 | 1 | 17.67 | 17.84 | 17.84 |
| 3174.2449 | NWAGVTHN(N)GTSSSC(C)R Hex 7 HexNAc 2 | 140.139 | 0.0 | 0.000 | 0.000e+00 | 125 - 140 | 1 | - | - | - |
| 2825.1514 | RRGSN(N)SSFF Fuc 1 Hex 5 HexNAc 4 | 136.725 | 0.0 | -20.779 | 7.325e+06 | 139 - 148 | 1 | 21.29 | 21.29 | 21.45 |
| 3469.6039 | TIVKPGDILLIN(N)STGNL Hex 8 HexNAc 2 | 136.546 | 0.008 | -3.077 | 7.739e+07 | 234 - 251 | 3 | 52.69 | 52.85 | 53.02 |
| 3964.9106 | TIVKPGDILLIN(N)STGNLIAPRGY Hex 7 HexNAc 2 | 136.454 | 0.008 | -3.304 | 4.933e+07 | 234 - 257 | 2 | 55.57 | 55.57 | 55.90 |
| 4203.7867 | NSKYPALN(N)VTM(O)PNNEQFDKLY Hex 8 HexNAc 2 | 135.909 | 0.008 | 17.779 | 1.215e+08 | 157 - 178 | 4 | 42.72 | 42.89 | 43.40 |
| 4096.6760 | NWAGVTHN(N)GTSSSC(C)RRGSNSSFF Hex 6 HexNAc 3 | 134.403 | 0.008 | -3.849 | 1.488e+07 | 125 - 148 | 1 | 30.38 | 30.55 | 30.72 |
| 4087.6439 | SC(C)RRGSN(N)SSFF Fuc 1 Hex 5 HexNAc 9 | 133.126 | 0.008 | -17.259 | 1.293e+07 | 137 - 148 | 1 | 30.38 | 30.55 | 30.55 |
| 4262.7220 | HKC(C)DNAC(C)IGSIRN(N)GTYDHDVY Fuc 1 Hex 5 HexNAc 4 | 132.392 | 0.008 | -19.294 | 2.909e+07 | 470 - 491 | 2 | 25.24 | 25.24 | 25.40 |
| 4248.0144 | TIVKPGDILLIN(N)STGNLIAPR Fuc 2 Hex 4 HexNAc 4 Neu5Ac 1 | 131.369 | 0.008 | -17.507 | 1.080e+07 | 234 - 255 | 1 | 53.02 | 53.02 | 53.02 |
| 3793.7094 | TIVKPGDILLIN(N)STGNL Hex 10 HexNAc 2 | 129.366 | 0.008 | -4.415 | 2.972e+07 | 234 - 251 | 3 | 52.52 | 52.69 | 52.85 |
| 3640.8040 | TIVKPGDILLIN(N)STGNLIAPRGY Hex 5 HexNAc 2 | 119.854 | 0.015 | -4.796 | 2.448e+07 | 234 - 257 | 3 | 55.90 | 56.07 | 56.41 |
| 4242.7400 | NWAGVTHN(N)GTSSSC(C)RRGSNSSFF Fuc 1 Hex 6 HexNAc 3 | 119.409 | 0.015 | -18.614 | 5.373e+06 | 125 - 148 | 1 | 30.38 | 30.38 | 30.38 |
| 3680.6241 | TIVKPGDILLIN(N)STGN Hex 10 HexNAc 2 | 118.594 | 0.015 | -18.602 | 6.779e+06 | 234 - 250 | 1 | 44.92 | 45.08 | 45.08 |
| 3472.4349 | AGVTHN(N)GTSSSC(C)RRGSNSSFF Hex 4 HexNAc 3 | 117.744 | 0.015 | -18.383 | 3.448e+07 | 127 - 148 | 1 | 21.62 | 21.79 | 21.79 |
| 3942.5965 | AGVTHN(N)GTSSSC(C)RRGSNSSFF Fuc 1 Hex 6 HexNAc 3 | 117.415 | 0.015 | -19.899 | 2.437e+07 | 127 - 148 | 1 | 21.12 | 21.29 | 21.29 |
| 3639.4669 | SEC(C)ITPN(N)GSIPNDKPF Hex 9 HexNAc 2 | 117.244 | 0.015 | -4.710 | 4.564e+06 | 278 - 294 | 1 | 35.46 | 35.63 | 35.80 |
| 3999.6217 | AGVTHN(N)GTSSSC(C)RRGSNSSFF Hex 6 HexNAc 4 | 115.078 | 0.015 | -5.071 | 4.432e+07 | 127 - 148 | 1 | 21.29 | 21.45 | 21.62 |
| 3017.1364 | NWAGVTHN(N)GTSSSC(C) Hex 7 HexNAc 2 | 109.656 | 0.029 | -20.285 | 7.942e+06 | 125 - 139 | 1 | 18.33 | 18.33 | 18.33 |
| 4069.9252 | TIVKPGDILLIN(N)STGN(D)LIAPR Hex 9 HexNAc 2 | 102.210 | 0.035 | -19.148 | 2.055e+07 | 234 - 255 | 2 | 54.04 | 54.04 | 54.21 |
| 4161.6753 | AGVTHN(N)GTSSSC(C)RRGSNSSFF Hex 7 HexNAc 4 | 99.644 | 0.038 | -3.389 | 6.017e+07 | 127 - 148 | 1 | 21.12 | 21.29 | 21.45 |
| 3377.3919 | IGSIRN(N)GTYDHDVY Fuc 1 Hex 5 HexNAc 4 | 97.624 | 0.038 | -19.712 | 5.165e+06 | 477 - 491 | 1 | 24.41 | 24.41 | 24.41 |
| 3444.4476 | KSEC(C)ITPN(N)GSIPN(D)DKPF Hex 7 HexNAc 2 | 96.195 | 0.038 | -20.417 | 1.703e+07 | 277 - 294 | 2 | 31.73 | 31.73 | 32.91 |
| 3697.6554 | TIVKPGDILLIN(N)STGN Fuc 2 Hex 4 HexNAc 4 Neu5Ac 1 | 95.832 | 0.038 | -21.125 | 6.857e+05 | 234 - 250 | 1 | 44.92 | 44.92 | 44.92 |
| 3744.8292 | TIVKPGDILLIN(N)STGNLIAPR Hex 7 HexNAc 2 | 91.818 | 0.039 | -19.318 | 1.371e+07 | 234 - 255 | 2 | 53.36 | 53.36 | 53.53 |
| 3523.4558 | IGSIRN(N)GTYDHDVY Fuc 2 Hex 5 HexNAc 4 | 91.385 | 0.039 | -19.737 | 3.232e+06 | 477 - 491 | 1 | 24.25 | 24.25 | 24.25 |
| 5134.2839 | SGKSSIM(O)RSDAPIGKC(C)KSEC(C)ITPN(N)GSIPNDKPF Hex 7 HexNAc 2 | 90.510 | 0.039 | -18.530 | 1.185e+07 | 261 - 294 | 1 | 29.53 | 29.53 | 29.70 |
| 4465.7977 | HKC(C)DNAC(C)IGSIRN(N)GTYDHDVY Fuc 1 Hex 5 HexNAc 5 | 89.751 | 0.039 | -20.678 | 8.450e+06 | 470 - 491 | 2 | 25.73 | 25.73 | 25.73 |
| 4753.0297 | RSDAPIGKC(C)KSEC(C)ITPN(N)GSIPN(D)DKPF Hex 9 HexNAc 2 | 88.119 | 0.039 | -5.542 | 4.206e+07 | 268 - 294 | 2 | 30.55 | 30.72 | 30.89 |
| 3420.7203 | TIVKPGDILLIN(N)STGNLIAPR Hex 5 HexNAc 2 | 84.961 | 0.039 | -5.029 | 9.419e+06 | 234 - 255 | 2 | 53.70 | 53.87 | 54.04 |
| 4428.9195 | RSDAPIGKC(C)KSEC(C)ITPN(N)GSIPN(D)DKPF Hex 7 HexNAc 2 | 84.657 | 0.039 | -5.629 | 3.727e+07 | 268 - 294 | 2 | 30.72 | 30.89 | 31.06 |
| 4125.9598 | TIVKPGDILLIN(N)STGNLIAPRG Hex 9 HexNAc 2 | 84.417 | 0.039 | 14.029 | 6.495e+08 | 234 - 256 | 10 | 52.85 | 53.70 | 54.89 |
| 2898.2181 | QKLPGNDN(N)STATL Hex 7 HexNAc 2 | 84.136 | 0.039 | -18.409 | 2.723e+07 | 0 - 13 | 1 | 30.89 | 30.89 | 31.06 |
| 3802.8572 | TIVKPGDILLIN(N)STGNLIAPRGY Hex 6 HexNAc 2 | 82.217 | 0.039 | -19.462 | 1.034e+07 | 234 - 257 | 2 | 55.73 | 55.90 | 55.90 |
| 3837.5670 | AGVTHNGTSSSC(C)RRGSN(N)SSFF Hex 5 HexNAc 4 | 79.654 | 0.039 | -3.480 | 8.764e+07 | 127 - 148 | 2 | 21.45 | 21.62 | 21.79 |
| 4742.9938 | KSEC(C)ITPN(N)GSIPNDKPF Fuc 3 Hex 3 HexNAc 8 Neu5Ac 1 | 79.397 | 0.040 | -4.927 | 2.117e+07 | 277 - 294 | 1 | 31.90 | 31.90 | 32.41 |
| 5855.6734 | KIRSGKSSIM(O)RSDAPIGKC(C)KSEC(C)ITPN(N)GSIPNDKPF Hex 9 HexNAc 2 | 79.284 | 0.040 | -17.900 | 4.586e+07 | 258 - 294 | 3 | 27.06 | 27.23 | 27.23 |
| 4089.7417 | SKYPALN(N)VTM(O)PNNEQFDKLY Hex 8 HexNAc 2 | 77.546 | 0.040 | -2.465 | 4.735e+07 | 158 - 178 | 4 | 42.72 | 42.89 | 43.05 |
| 4461.8042 | NWAGVTHN(N)GTSSSC(C)RRGSNSSFF Hex 7 HexNAc 4 | 77.250 | 0.040 | -19.194 | 8.302e+06 | 125 - 148 | 1 | 30.38 | 30.38 | 30.55 |
| 5296.3333 | SGKSSIM(O)RSDAPIGKC(C)KSEC(C)ITPN(N)GSIPNDKPF Hex 8 HexNAc 2 | 76.203 | 0.040 | -18.920 | 3.503e+06 | 261 - 294 | 1 | 29.53 | 29.53 | 29.70 |
| 4040.6469 | AGVTHN(N)GTSSSC(C)RRGSNSSFF Hex 5 HexNAc 5 | 74.578 | 0.040 | -4.571 | 8.410e+07 | 127 - 148 | 1 | 21.29 | 21.45 | 21.62 |
| 5458.3887 | SGKSSIM(O)RSDAPIGKC(C)KSEC(C)ITPN(N)GSIPNDKPF Hex 9 HexNAc 2 | 73.153 | 0.040 | -4.427 | 1.191e+07 | 261 - 294 | 2 | 29.36 | 29.53 | 29.70 |
| 4041.7408 | NSKYPALN(N)VTM(O)PNNEQFDKLY Hex 7 HexNAc 2 | 73.024 | 0.040 | -4.137 | 1.818e+07 | 157 - 178 | 2 | 42.72 | 42.89 | 43.23 |
| 4590.9778 | RSDAPIGKC(C)KSEC(C)ITPN(N)GSIPN(D)DKPF Hex 8 HexNAc 2 | 72.415 | 0.040 | -20.182 | 1.955e+07 | 268 - 294 | 1 | 30.72 | 30.72 | 30.89 |
| 5693.6182 | KIRSGKSSIM(O)RSDAPIGKC(C)KSEC(C)ITPN(N)GSIPNDKPF Hex 8 HexNAc 2 | 71.807 | 0.040 | -4.388 | 2.840e+07 | 258 - 294 | 2 | 27.06 | 27.23 | 27.40 |
| 3618.4952 | AGVTHN(N)GTSSSC(C)RRGSNSSFF Fuc 1 Hex 4 HexNAc 3 | 69.443 | 0.040 | -5.053 | 3.079e+07 | 127 - 148 | 1 | 21.45 | 21.45 | 21.79 |
| 5531.5659 | KIRSGKSSIM(O)RSDAPIGKC(C)KSEC(C)ITPN(N)GSIPNDKPF Hex 7 HexNAc 2 | 67.696 | 0.040 | -4.488 | 5.349e+07 | 258 - 294 | 3 | 27.06 | 27.23 | 27.40 |
| 4307.0374 | TIVKPGDILLIN(N)STGN(D)LIAPRGY Fuc 2 Hex 3 HexNAc 4 Neu5Ac 1 | 65.409 | 0.040 | -20.028 | 5.193e+06 | 234 - 257 | 1 | 56.41 | 56.41 | 56.58 |
| 4290.0048 | TIVKPGDILLIN(N)STGN(D)LIAPRGY Hex 9 HexNAc 2 | 63.750 | 0.040 | -19.697 | 5.936e+07 | 234 - 257 | 12 | 56.41 | 56.41 | 56.58 |
| 4187.7885 | NSKYPALN(N)VTMPNNEQFDKLY Hex 8 HexNAc 2 | 63.734 | 0.040 | -19.084 | 3.386e+06 | 157 - 178 | 2 | 48.78 | 48.78 | 48.95 |
| 3582.7722 | TIVKPGDILLIN(N)STGNLIAPR Hex 6 HexNAc 2 | 63.227 | 0.040 | -19.343 | 2.312e+06 | 234 - 255 | 1 | 53.53 | 53.53 | 53.53 |
| 4317.8499 | SKYPALN(N)VTM(O)PNNEQFDKLY Fuc 1 Hex 6 HexNAc 4 | 60.207 | 0.040 | 12.479 | 4.542e+07 | 158 - 178 | 5 | 44.58 | 45.08 | 46.10 |
| 4204.7680 | N(D)SKYPALN(N)VTM(O)PNNEQFDKLY Hex 8 HexNAc 2 | 59.375 | 0.040 | -6.121 | 2.123e+07 | 157 - 178 | 3 | 45.42 | 45.59 | 45.76 |
| 4299.7524 | NWAGVTHN(N)GTSSSC(C)RRGSNSSFF Hex 6 HexNAc 4 | 58.501 | 0.040 | -19.091 | 4.467e+06 | 125 - 148 | 1 | 30.55 | 30.55 | 30.72 |
| 4112.6637 | AGVTHN(N)GTSSSC(C)RRGSNSSFF Fuc 1 Hex 4 HexNAc 4 Neu5Ac 1 | 57.923 | 0.040 | -19.452 | 4.033e+06 | 127 - 148 | 1 | 31.39 | 31.56 | 31.56 |
| 3983.6242 | AGVTHN(N)GTSSSC(C)RRGSNSSFF Fuc 1 Hex 5 HexNAc 4 | 56.589 | 0.040 | -5.750 | 2.035e+07 | 127 - 148 | 1 | 21.29 | 21.29 | 21.62 |
| 3315.3646 | SEC(C)ITPN(N)GSIPNDKPF Hex 7 HexNAc 2 | 55.803 | 0.040 | -19.629 | 3.971e+06 | 278 - 294 | 1 | 35.80 | 35.80 | 35.97 |
| 3432.3921 | AGVTHN(D)GTSSSC(C)RRGSN(N)SSFF Hex 5 HexNAc 2 | 55.233 | 0.040 | -6.394 | 4.904e+07 | 127 - 148 | 1 | 22.61 | 22.77 | 23.10 |
| 3958.5979 | AGVTHN(N)GTSSSC(C)RRGSNSSFF Hex 7 HexNAc 3 | 54.561 | 0.044 | -19.239 | 3.717e+07 | 127 - 148 | 1 | 21.29 | 21.29 | 21.45 |
| 3514.5049 | LIN(N)STGNLIAPRGY Hex 10 HexNAc 2 | 54.521 | 0.047 | -19.493 | 2.704e+06 | 243 - 257 | 1 | 36.80 | 36.80 | 36.80 |
| 4646.8727 | NNESFNWAGVTHN(N)GTSSSC(C)RRGSNSSFF Hex 7 HexNAc 2 | 53.297 | 0.047 | -18.981 | 2.116e+06 | 120 - 148 | 1 | 40.35 | 40.35 | 40.35 |
| 4646.8727 | NNESFNWAGVTHNGTSSSC(C)RRGSN(N)SSFF Hex 7 HexNAc 2 | 53.297 | 0.047 | -18.981 | 2.116e+06 | 120 - 148 | 1 | 40.35 | 40.35 | 40.35 |
| 4137.7016 | NWAGVTHN(N)GTSSSC(C)RRGSNSSFF Hex 5 HexNAc 4 | 52.355 | 0.047 | -17.865 | 1.131e+07 | 125 - 148 | 1 | 30.55 | 30.72 | 30.72 |
| 5416.4694 | RSDAPIGKC(C)KSEC(C)ITPN(N)GSIPNDKPFQNVNRITY Hex 7 HexNAc 2 | 49.726 | 0.049 | -19.066 | 5.069e+06 | 268 - 302 | 2 | 35.63 | 35.63 | 35.63 |

### LIN(N)STGNLIAPRGYF Fuc 2 Hex 3 HexNAc 4 Neu5Ac 1

**Mass:**  3516.5432

(-6.240e-06)

**Charge States:** 3

**Start Position**: 244

**End Position**: 259

| Profile Score | Tandem Score | TDA q-value | Volume | Spectrum Matches |
| --- | --- | --- | --- | --- |
| -17.9441 | 189.8213 | 0.0000 | 5.0356e+07 | 1 |

**Adduct Signal Fractions**

- **Unmodified**: 5.0356e+07

### LIN(N)STGNL Hex 9 HexNAc 2

**Mass:**  2695.0885

(1.678e-06)

**Charge States:** 2, 3

**Start Position**: 244

**End Position**: 252

| Profile Score | Tandem Score | TDA q-value | Volume | Spectrum Matches |
| --- | --- | --- | --- | --- |
| -19.1601 | 184.5297 | 0.0000 | 2.4081e+07 | 1 |

**Adduct Signal Fractions**

- **Unmodified**: 2.4081e+07

### LIN(N)STGNLIAPRGYF Hex 9 HexNAc 2

**Mass:**  3499.5170

(1.415e-06)

**Charge States:** 3

**Start Position**: 244

**End Position**: 259

| Profile Score | Tandem Score | TDA q-value | Volume | Spectrum Matches |
| --- | --- | --- | --- | --- |
| -3.2074 | 178.4116 | 0.0000 | 1.8066e+08 | 2 |

**Adduct Signal Fractions**

- **Unmodified**: 1.8066e+08

### LIN(N)STGNLIAPRGY Hex 9 HexNAc 2

**Mass:**  3352.4507

(4.023e-06)

**Charge States:** 3

**Start Position**: 244

**End Position**: 258

| Profile Score | Tandem Score | TDA q-value | Volume | Spectrum Matches |
| --- | --- | --- | --- | --- |
| -2.6271 | 172.2306 | 0.0000 | 1.0625e+08 | 3 |

**Adduct Signal Fractions**

- **Unmodified**: 1.0625e+08

### TIVKPGDILLIN(N)STGNLIAPR Hex 8 HexNAc 2

**Mass:**  3906.8767

(2.355e-06)

**Charge States:** 3, 4, 5

**Start Position**: 235

**End Position**: 256

| Profile Score | Tandem Score | TDA q-value | Volume | Spectrum Matches |
| --- | --- | --- | --- | --- |
| 16.4195 | 167.7702 | 0.0000 | 3.5482e+08 | 5 |

**Adduct Signal Fractions**

- **Unmodified**: 3.5482e+08

### LIN(N)STGNLIAPRGY Fuc 2 Hex 3 HexNAc 4 Neu5Ac 1

**Mass:**  3369.4777

(-4.405e-06)

**Charge States:** 3

**Start Position**: 244

**End Position**: 258

| Profile Score | Tandem Score | TDA q-value | Volume | Spectrum Matches |
| --- | --- | --- | --- | --- |
| -18.0113 | 164.2089 | 0.0000 | 2.4950e+07 | 1 |

**Adduct Signal Fractions**

- **Unmodified**: 2.4950e+07

### LIN(N)STGNLIAPRGYF Hex 10 HexNAc 2

**Mass:**  3661.5683

(3.659e-07)

**Charge States:** 3

**Start Position**: 244

**End Position**: 259

| Profile Score | Tandem Score | TDA q-value | Volume | Spectrum Matches |
| --- | --- | --- | --- | --- |
| -20.1656 | 164.0161 | 0.0000 | 8.6409e+06 | 1 |

**Adduct Signal Fractions**

- **Unmodified**: 8.6409e+06

### TIVKPGDILLIN(N)STGNLIAPRGY Hex 10 HexNAc 2

**Mass:**  4451.0710

(2.200e-06)

**Charge States:** 3, 4

**Start Position**: 235

**End Position**: 258

| Profile Score | Tandem Score | TDA q-value | Volume | Spectrum Matches |
| --- | --- | --- | --- | --- |
| 15.2725 | 163.2261 | 0.0000 | 2.5371e+08 | 7 |

**Adduct Signal Fractions**

- **Unmodified**: 2.5371e+08

### NWAGVTHN(N)GTSSSC(C)RRGSNSSFF Hex 7 HexNAc 2

**Mass:**  4055.6525

(4.356e-06)

**Charge States:** 3, 4

**Start Position**: 126

**End Position**: 149

| Profile Score | Tandem Score | TDA q-value | Volume | Spectrum Matches |
| --- | --- | --- | --- | --- |
| -15.4600 | 162.3892 | 0.0000 | 5.5170e+07 | 1 |

**Adduct Signal Fractions**

- **Unmodified**: 5.5170e+07

### TIVKPGDILLIN(N)STGNLIAPRGY Hex 8 HexNAc 2

**Mass:**  4126.9685

(1.449e-06)

**Charge States:** 3, 4, 5

**Start Position**: 235

**End Position**: 258

| Profile Score | Tandem Score | TDA q-value | Volume | Spectrum Matches |
| --- | --- | --- | --- | --- |
| 13.8849 | 160.0910 | 0.0000 | 7.7565e+08 | 11 |

**Adduct Signal Fractions**

- **Unmodified**: 7.7565e+08

### TIVKPGDILLIN(N)STGNLIAPR Hex 10 HexNAc 2

**Mass:**  4230.9841

(2.674e-06)

**Charge States:** 3, 4

**Start Position**: 235

**End Position**: 256

| Profile Score | Tandem Score | TDA q-value | Volume | Spectrum Matches |
| --- | --- | --- | --- | --- |
| -3.4823 | 160.0494 | 0.0000 | 1.2822e+08 | 2 |

**Adduct Signal Fractions**

- **Unmodified**: 1.2822e+08

### TIVKPGDILLIN(N)STGNLIAPR Fuc 2 Hex 3 HexNAc 4 Neu5Ac 1

**Mass:**  4085.9479

(-3.243e-06)

**Charge States:** 4

**Start Position**: 235

**End Position**: 256

| Profile Score | Tandem Score | TDA q-value | Volume | Spectrum Matches |
| --- | --- | --- | --- | --- |
| 14.8904 | 159.9605 | 0.0000 | 3.0630e+08 | 3 |

**Adduct Signal Fractions**

- **Unmodified**: 3.0630e+08

### RSDAPIGKC(C)KSEC(C)ITPN(N)GSIPNDKPF Hex 9 HexNAc 2

**Mass:**  4752.0550

(1.402e-06)

**Charge States:** 4, 5

**Start Position**: 269

**End Position**: 295

| Profile Score | Tandem Score | TDA q-value | Volume | Spectrum Matches |
| --- | --- | --- | --- | --- |
| -2.7855 | 159.4804 | 0.0000 | 2.8898e+08 | 3 |

**Adduct Signal Fractions**

- **Unmodified**: 2.8898e+08

### TIVKPGDILLIN(N)STGNLIAPR Hex 9 HexNAc 2

**Mass:**  4068.9589

(1.382e-06)

**Charge States:** 3, 4, 5

**Start Position**: 235

**End Position**: 256

| Profile Score | Tandem Score | TDA q-value | Volume | Spectrum Matches |
| --- | --- | --- | --- | --- |
| 19.1870 | 159.1987 | 0.0000 | 2.8748e+09 | 15 |

**Adduct Signal Fractions**

- **Unmodified**: 2.8748e+09

### TIVKPGDILLIN(N)STGNLIAPRGY Hex 9 HexNAc 2

**Mass:**  4289.0192

(4.282e-06)

**Charge States:** 3, 4, 5

**Start Position**: 235

**End Position**: 258

| Profile Score | Tandem Score | TDA q-value | Volume | Spectrum Matches |
| --- | --- | --- | --- | --- |
| 18.4073 | 159.1278 | 0.0000 | 5.6098e+09 | 25 |

**Adduct Signal Fractions**

- **Unmodified**: 5.6098e+09

### TIVKPGDILLIN(N) Hex 9 HexNAc 2

**Mass:**  3159.4315

(1.977e-06)

**Charge States:** 2, 3

**Start Position**: 235

**End Position**: 247

| Profile Score | Tandem Score | TDA q-value | Volume | Spectrum Matches |
| --- | --- | --- | --- | --- |
| 11.3823 | 159.0863 | 0.0000 | 2.1599e+08 | 6 |

**Adduct Signal Fractions**

- **Unmodified**: 2.1599e+08

### LIN(N)STGNLIAPRGY Hex 8 HexNAc 2

**Mass:**  3190.3983

(2.270e-06)

**Charge States:** 3

**Start Position**: 244

**End Position**: 258

| Profile Score | Tandem Score | TDA q-value | Volume | Spectrum Matches |
| --- | --- | --- | --- | --- |
| -17.6986 | 157.3442 | 0.0000 | 1.2612e+07 | 1 |

**Adduct Signal Fractions**

- **Unmodified**: 1.2612e+07

### KSEC(C)ITPN(N)GSIPNDKPF Hex 9 HexNAc 2

**Mass:**  3767.5548

(1.631e-06)

**Charge States:** 3

**Start Position**: 278

**End Position**: 295

| Profile Score | Tandem Score | TDA q-value | Volume | Spectrum Matches |
| --- | --- | --- | --- | --- |
| -18.8098 | 156.1273 | 0.0000 | 1.4916e+07 | 1 |

**Adduct Signal Fractions**

- **Unmodified**: 1.4916e+07

### TIVKPGDILLIN(N)STGNLIAPRGY Fuc 2 Hex 3 HexNAc 4 Neu5Ac 1

**Mass:**  4306.0383

(-4.318e-06)

**Charge States:** 4, 5

**Start Position**: 235

**End Position**: 258

| Profile Score | Tandem Score | TDA q-value | Volume | Spectrum Matches |
| --- | --- | --- | --- | --- |
| 16.7184 | 155.3593 | 0.0000 | 5.8389e+08 | 4 |

**Adduct Signal Fractions**

- **Unmodified**: 5.8389e+08

### AGVTHN(N)GTSSSC(C)RRGSNSSFF Hex 7 HexNAc 2

**Mass:**  3755.5156

(9.518e-07)

**Charge States:** 4

**Start Position**: 128

**End Position**: 149

| Profile Score | Tandem Score | TDA q-value | Volume | Spectrum Matches |
| --- | --- | --- | --- | --- |
| 16.4377 | 154.3448 | 0.0000 | 5.2880e+08 | 3 |

**Adduct Signal Fractions**

- **Unmodified**: 5.2880e+08

### LIN(N)STGNLIAPRGYF Hex 8 HexNAc 2

**Mass:**  3337.4661

(2.013e-06)

**Charge States:** 3

**Start Position**: 244

**End Position**: 259

| Profile Score | Tandem Score | TDA q-value | Volume | Spectrum Matches |
| --- | --- | --- | --- | --- |
| -19.3685 | 154.0057 | 0.0000 | 1.6469e+07 | 1 |

**Adduct Signal Fractions**

- **Unmodified**: 1.6469e+07

### IN(N)STGNLIAPRGYF Hex 9 HexNAc 2

**Mass:**  3386.4417

(3.998e-06)

**Charge States:** 3

**Start Position**: 245

**End Position**: 259

| Profile Score | Tandem Score | TDA q-value | Volume | Spectrum Matches |
| --- | --- | --- | --- | --- |
| -18.0252 | 153.5192 | 0.0000 | 6.2867e+06 | 1 |

**Adduct Signal Fractions**

- **Unmodified**: 6.2867e+06

### TIVKPGDILLIN(N)STGNL Fuc 2 Hex 3 HexNAc 4 Neu5Ac 1

**Mass:**  3648.6813

(-5.085e-06)

**Charge States:** 3

**Start Position**: 235

**End Position**: 252

| Profile Score | Tandem Score | TDA q-value | Volume | Spectrum Matches |
| --- | --- | --- | --- | --- |
| -3.5639 | 151.8309 | 0.0000 | 1.8764e+08 | 2 |

**Adduct Signal Fractions**

- **Unmodified**: 1.8764e+08

### TIVKPGDILLIN(N)STGN Hex 8 HexNAc 2

**Mass:**  3356.5171

(1.715e-06)

**Charge States:** 3

**Start Position**: 235

**End Position**: 251

| Profile Score | Tandem Score | TDA q-value | Volume | Spectrum Matches |
| --- | --- | --- | --- | --- |
| -3.4469 | 149.8059 | 0.0000 | 2.3220e+07 | 1 |

**Adduct Signal Fractions**

- **Unmodified**: 2.3220e+07

### TIVKPGDILLIN(N)STGN Fuc 2 Hex 3 HexNAc 4 Neu5Ac 1

**Mass:**  3535.5968

(-5.505e-06)

**Charge States:** 3

**Start Position**: 235

**End Position**: 251

| Profile Score | Tandem Score | TDA q-value | Volume | Spectrum Matches |
| --- | --- | --- | --- | --- |
| -3.6213 | 149.5788 | 0.0000 | 8.4774e+07 | 2 |

**Adduct Signal Fractions**

- **Unmodified**: 8.4774e+07

### RSDAPIGKC(C)KSEC(C)ITPN(N)GSIPNDKPF Hex 7 HexNAc 2

**Mass:**  4427.9461

(2.232e-06)

**Charge States:** 4, 5

**Start Position**: 269

**End Position**: 295

| Profile Score | Tandem Score | TDA q-value | Volume | Spectrum Matches |
| --- | --- | --- | --- | --- |
| -2.3134 | 149.4169 | 0.0000 | 2.8690e+08 | 6 |

**Adduct Signal Fractions**

- **Unmodified**: 2.8690e+08

### AGVTHN(N)GTSSSC(C)RRGSNSSFF Hex 5 HexNAc 2

**Mass:**  3431.4094

(1.208e-06)

**Charge States:** 4

**Start Position**: 128

**End Position**: 149

| Profile Score | Tandem Score | TDA q-value | Volume | Spectrum Matches |
| --- | --- | --- | --- | --- |
| -2.2669 | 148.6982 | 0.0000 | 2.2870e+08 | 3 |

**Adduct Signal Fractions**

- **Unmodified**: 2.2870e+08

### TIVKPGDILLIN(N) Fuc 2 Hex 3 HexNAc 4 Neu5Ac 1

**Mass:**  3176.4563

(-6.622e-06)

**Charge States:** 3

**Start Position**: 235

**End Position**: 247

| Profile Score | Tandem Score | TDA q-value | Volume | Spectrum Matches |
| --- | --- | --- | --- | --- |
| 10.0740 | 147.8903 | 0.0000 | 6.0532e+07 | 4 |

**Adduct Signal Fractions**

- **Unmodified**: 6.0532e+07

### TIVKPGDILLIN(N) Hex 8 HexNAc 2

**Mass:**  2997.3755

(2.742e-06)

**Charge States:** 3

**Start Position**: 235

**End Position**: 247

| Profile Score | Tandem Score | TDA q-value | Volume | Spectrum Matches |
| --- | --- | --- | --- | --- |
| -18.6192 | 147.7950 | 0.0000 | 1.2487e+07 | 1 |

**Adduct Signal Fractions**

- **Unmodified**: 1.2487e+07

### RSDAPIGKC(C)KSEC(C)ITPN(N)GSIPNDKPF Hex 8 HexNAc 2

**Mass:**  4589.9941

(7.807e-07)

**Charge States:** 4, 5

**Start Position**: 269

**End Position**: 295

| Profile Score | Tandem Score | TDA q-value | Volume | Spectrum Matches |
| --- | --- | --- | --- | --- |
| -2.3875 | 147.6255 | 0.0000 | 1.7679e+08 | 3 |

**Adduct Signal Fractions**

- **Unmodified**: 1.7679e+08

### AGVTHN(N)GTSSSC(C)RRGSNSSFF Hex 6 HexNAc 2

**Mass:**  3593.4623

(1.135e-06)

**Charge States:** 4

**Start Position**: 128

**End Position**: 149

| Profile Score | Tandem Score | TDA q-value | Volume | Spectrum Matches |
| --- | --- | --- | --- | --- |
| -3.9338 | 147.4801 | 0.0000 | 1.4877e+08 | 1 |

**Adduct Signal Fractions**

- **Unmodified**: 1.4877e+08

### TIVKPGDILLIN(N)STGN Hex 9 HexNAc 2

**Mass:**  3518.5713

(2.379e-06)

**Charge States:** 2, 3, 4

**Start Position**: 235

**End Position**: 251

| Profile Score | Tandem Score | TDA q-value | Volume | Spectrum Matches |
| --- | --- | --- | --- | --- |
| 16.4077 | 146.8584 | 0.0000 | 2.5519e+08 | 4 |

**Adduct Signal Fractions**

- **Unmodified**: 2.5519e+08

### TIVKPGDILLIN(N)STGNL Hex 9 HexNAc 2

**Mass:**  3631.6558

(2.435e-06)

**Charge States:** 3

**Start Position**: 235

**End Position**: 252

| Profile Score | Tandem Score | TDA q-value | Volume | Spectrum Matches |
| --- | --- | --- | --- | --- |
| 18.2074 | 146.0689 | 0.0000 | 7.1783e+08 | 8 |

**Adduct Signal Fractions**

- **Unmodified**: 7.1783e+08

### NWAGVTHNGTSSSC(C)RRGSN(N)SSFF Hex 6 HexNAc 2

**Mass:**  3893.6049

(6.312e-06)

**Charge States:** 3, 4

**Start Position**: 126

**End Position**: 149

| Profile Score | Tandem Score | TDA q-value | Volume | Spectrum Matches |
| --- | --- | --- | --- | --- |
| -19.0542 | 143.6028 | 0.0000 | 1.3658e+07 | 1 |

**Adduct Signal Fractions**

- **Unmodified**: 1.3658e+07

### RSDAPIGKC(C)KSEC(C)ITPN(N)GSIPNDKPF Hex 6 HexNAc 2

**Mass:**  4265.8886

(2.341e-06)

**Charge States:** 5

**Start Position**: 269

**End Position**: 295

| Profile Score | Tandem Score | TDA q-value | Volume | Spectrum Matches |
| --- | --- | --- | --- | --- |
| -3.4929 | 143.2156 | 0.0000 | 3.0083e+07 | 2 |

**Adduct Signal Fractions**

- **Unmodified**: 3.0083e+07

### NWAGVTHN(N)GTSSSC(C)RRGSNSSFF Hex 5 HexNAc 2

**Mass:**  3731.5390

(3.059e-06)

**Charge States:** 3, 4

**Start Position**: 126

**End Position**: 149

| Profile Score | Tandem Score | TDA q-value | Volume | Spectrum Matches |
| --- | --- | --- | --- | --- |
| -16.7911 | 142.6314 | 0.0000 | 1.7824e+07 | 3 |

**Adduct Signal Fractions**

- **Unmodified**: 1.7824e+07

### KSEC(C)ITPN(N)GSIPNDKPF Hex 8 HexNAc 2

**Mass:**  3605.4973

(1.889e-06)

**Charge States:** 3

**Start Position**: 278

**End Position**: 295

| Profile Score | Tandem Score | TDA q-value | Volume | Spectrum Matches |
| --- | --- | --- | --- | --- |
| -18.5456 | 141.6778 | 0.0000 | 8.5436e+06 | 1 |

**Adduct Signal Fractions**

- **Unmodified**: 8.5436e+06

### AGVTHN(N)GTSSSC(C)RRGSNSSFF Hex 8 HexNAc 2

**Mass:**  3917.5698

(1.501e-06)

**Charge States:** 4

**Start Position**: 128

**End Position**: 149

| Profile Score | Tandem Score | TDA q-value | Volume | Spectrum Matches |
| --- | --- | --- | --- | --- |
| -17.7308 | 141.2009 | 0.0000 | 5.0741e+07 | 1 |

**Adduct Signal Fractions**

- **Unmodified**: 5.0741e+07

### NWAGVTHN(N)GTSSS Hex 7 HexNAc 2

**Mass:**  2857.1081

(-9.089e-07)

**Charge States:** 3

**Start Position**: 126

**End Position**: 139

| Profile Score | Tandem Score | TDA q-value | Volume | Spectrum Matches |
| --- | --- | --- | --- | --- |
| -20.1314 | 140.6225 | 0.0000 | 8.0597e+06 | 1 |

**Adduct Signal Fractions**

- **Unmodified**: 8.0597e+06

### NWAGVTHN(N)GTSSSC(C)R Hex 7 HexNAc 2

**Mass:**  3174.2449

(3.183e-04)

**Start Position**: 126

**End Position**: 141

| Profile Score | Tandem Score | TDA q-value | Volume | Spectrum Matches |
| --- | --- | --- | --- | --- |
| 0.0000 | 140.1389 | 0.0000 | 0.0000e+00 | 1 |

Adduct:

No Chromatogram Found

### RRGSN(N)SSFF Fuc 1 Hex 5 HexNAc 4

**Mass:**  2825.1504

(2.906e-07)

**Charge States:** 3

**Start Position**: 140

**End Position**: 149

| Profile Score | Tandem Score | TDA q-value | Volume | Spectrum Matches |
| --- | --- | --- | --- | --- |
| -20.7795 | 136.7247 | 0.0000 | 7.3254e+06 | 1 |

**Adduct Signal Fractions**

- **Unmodified**: 7.3254e+06

### TIVKPGDILLIN(N)STGNL Hex 8 HexNAc 2

**Mass:**  3469.6045

(1.862e-06)

**Charge States:** 3

**Start Position**: 235

**End Position**: 252

| Profile Score | Tandem Score | TDA q-value | Volume | Spectrum Matches |
| --- | --- | --- | --- | --- |
| -3.0770 | 136.5462 | 0.0079 | 7.7395e+07 | 3 |

**Adduct Signal Fractions**

- **Unmodified**: 7.7395e+07

### TIVKPGDILLIN(N)STGNLIAPRGY Hex 7 HexNAc 2

**Mass:**  3964.9113

(2.326e-06)

**Charge States:** 3, 4

**Start Position**: 235

**End Position**: 258

| Profile Score | Tandem Score | TDA q-value | Volume | Spectrum Matches |
| --- | --- | --- | --- | --- |
| -3.3038 | 136.4541 | 0.0079 | 4.9330e+07 | 2 |

**Adduct Signal Fractions**

- **Unmodified**: 4.9330e+07

### NSKYPALN(N)VTM(O)PNNEQFDKLY Hex 8 HexNAc 2

**Mass:**  4203.7932

(3.399e-06)

**Charge States:** 3, 4

**Start Position**: 158

**End Position**: 179

| Profile Score | Tandem Score | TDA q-value | Volume | Spectrum Matches |
| --- | --- | --- | --- | --- |
| 17.7791 | 135.9092 | 0.0079 | 1.2145e+08 | 4 |

**Adduct Signal Fractions**

- **Unmodified**: 1.2145e+08

### NWAGVTHN(N)GTSSSC(C)RRGSNSSFF Hex 6 HexNAc 3

**Mass:**  4096.6769

(4.171e-06)

**Charge States:** 4

**Start Position**: 126

**End Position**: 149

| Profile Score | Tandem Score | TDA q-value | Volume | Spectrum Matches |
| --- | --- | --- | --- | --- |
| -3.8489 | 134.4033 | 0.0079 | 1.4884e+07 | 1 |

**Adduct Signal Fractions**

- **Unmodified**: 1.4884e+07

### SC(C)RRGSN(N)SSFF Fuc 1 Hex 5 HexNAc 9

**Mass:**  4087.6447

(8.040e-06)

**Charge States:** 4

**Start Position**: 138

**End Position**: 149

| Profile Score | Tandem Score | TDA q-value | Volume | Spectrum Matches |
| --- | --- | --- | --- | --- |
| -17.2590 | 133.1262 | 0.0079 | 1.2929e+07 | 1 |

**Adduct Signal Fractions**

- **Unmodified**: 1.2929e+07

### HKC(C)DNAC(C)IGSIRN(N)GTYDHDVY Fuc 1 Hex 5 HexNAc 4

**Mass:**  4262.7238

(1.408e-06)

**Charge States:** 5

**Start Position**: 471

**End Position**: 492

| Profile Score | Tandem Score | TDA q-value | Volume | Spectrum Matches |
| --- | --- | --- | --- | --- |
| -19.2944 | 132.3924 | 0.0079 | 2.9093e+07 | 2 |

**Adduct Signal Fractions**

- **Unmodified**: 2.9093e+07

### TIVKPGDILLIN(N)STGNLIAPR Fuc 2 Hex 4 HexNAc 4 Neu5Ac 1

**Mass:**  4248.0144

(-3.532e-06)

**Charge States:** 4

**Start Position**: 235

**End Position**: 256

| Profile Score | Tandem Score | TDA q-value | Volume | Spectrum Matches |
| --- | --- | --- | --- | --- |
| -17.5071 | 131.3695 | 0.0079 | 1.0798e+07 | 1 |

**Adduct Signal Fractions**

- **Unmodified**: 1.0798e+07

### TIVKPGDILLIN(N)STGNL Hex 10 HexNAc 2

**Mass:**  3793.7096

(2.068e-06)

**Charge States:** 3

**Start Position**: 235

**End Position**: 252

| Profile Score | Tandem Score | TDA q-value | Volume | Spectrum Matches |
| --- | --- | --- | --- | --- |
| -4.4154 | 129.3661 | 0.0079 | 2.9722e+07 | 3 |

**Adduct Signal Fractions**

- **Unmodified**: 2.9722e+07

### TIVKPGDILLIN(N)STGNLIAPRGY Hex 5 HexNAc 2

**Mass:**  3640.8030

(2.549e-06)

**Charge States:** 3, 4

**Start Position**: 235

**End Position**: 258

| Profile Score | Tandem Score | TDA q-value | Volume | Spectrum Matches |
| --- | --- | --- | --- | --- |
| -4.7963 | 119.8544 | 0.0146 | 2.4482e+07 | 3 |

**Adduct Signal Fractions**

- **Unmodified**: 2.4482e+07

### NWAGVTHN(N)GTSSSC(C)RRGSNSSFF Fuc 1 Hex 6 HexNAc 3

**Mass:**  4242.7409

(5.449e-06)

**Charge States:** 3, 4

**Start Position**: 126

**End Position**: 149

| Profile Score | Tandem Score | TDA q-value | Volume | Spectrum Matches |
| --- | --- | --- | --- | --- |
| -18.6139 | 119.4094 | 0.0146 | 5.3730e+06 | 1 |

**Adduct Signal Fractions**

- **Unmodified**: 5.3730e+06

### TIVKPGDILLIN(N)STGN Hex 10 HexNAc 2

**Mass:**  3680.6244

(1.833e-06)

**Charge States:** 3

**Start Position**: 235

**End Position**: 251

| Profile Score | Tandem Score | TDA q-value | Volume | Spectrum Matches |
| --- | --- | --- | --- | --- |
| -18.6022 | 118.5944 | 0.0146 | 6.7789e+06 | 1 |

**Adduct Signal Fractions**

- **Unmodified**: 6.7789e+06

### AGVTHN(N)GTSSSC(C)RRGSNSSFF Hex 4 HexNAc 3

**Mass:**  3472.4333

(1.714e-06)

**Charge States:** 4

**Start Position**: 128

**End Position**: 149

| Profile Score | Tandem Score | TDA q-value | Volume | Spectrum Matches |
| --- | --- | --- | --- | --- |
| -18.3826 | 117.7437 | 0.0146 | 3.4483e+07 | 1 |

**Adduct Signal Fractions**

- **Unmodified**: 3.4483e+07

### AGVTHN(N)GTSSSC(C)RRGSNSSFF Fuc 1 Hex 6 HexNAc 3

**Mass:**  3942.5952

(9.511e-07)

**Charge States:** 4

**Start Position**: 128

**End Position**: 149

| Profile Score | Tandem Score | TDA q-value | Volume | Spectrum Matches |
| --- | --- | --- | --- | --- |
| -19.8990 | 117.4155 | 0.0146 | 2.4369e+07 | 1 |

**Adduct Signal Fractions**

- **Unmodified**: 2.4369e+07

### SEC(C)ITPN(N)GSIPNDKPF Hex 9 HexNAc 2

**Mass:**  3639.4667

(3.602e-06)

**Charge States:** 3

**Start Position**: 279

**End Position**: 295

| Profile Score | Tandem Score | TDA q-value | Volume | Spectrum Matches |
| --- | --- | --- | --- | --- |
| -4.7095 | 117.2441 | 0.0146 | 4.5641e+06 | 1 |

**Adduct Signal Fractions**

- **Unmodified**: 4.5641e+06

### AGVTHN(N)GTSSSC(C)RRGSNSSFF Hex 6 HexNAc 4

**Mass:**  3999.6230

(8.924e-07)

**Charge States:** 4

**Start Position**: 128

**End Position**: 149

| Profile Score | Tandem Score | TDA q-value | Volume | Spectrum Matches |
| --- | --- | --- | --- | --- |
| -5.0710 | 115.0777 | 0.0146 | 4.4320e+07 | 1 |

**Adduct Signal Fractions**

- **Unmodified**: 4.4320e+07

### NWAGVTHN(N)GTSSSC(C) Hex 7 HexNAc 2

**Mass:**  3017.1364

(9.325e-07)

**Charge States:** 3

**Start Position**: 126

**End Position**: 140

| Profile Score | Tandem Score | TDA q-value | Volume | Spectrum Matches |
| --- | --- | --- | --- | --- |
| -20.2848 | 109.6564 | 0.0286 | 7.9425e+06 | 1 |

**Adduct Signal Fractions**

- **Unmodified**: 7.9425e+06

### TIVKPGDILLIN(N)STGN(D)LIAPR Hex 9 HexNAc 2

**Mass:**  4069.9284

(2.448e-04)

**Charge States:** 3, 4

**Start Position**: 235

**End Position**: 256

| Profile Score | Tandem Score | TDA q-value | Volume | Spectrum Matches |
| --- | --- | --- | --- | --- |
| -19.1481 | 102.2099 | 0.0347 | 2.0552e+07 | 2 |

**Adduct Signal Fractions**

- **Unmodified**: 2.0552e+07

### AGVTHN(N)GTSSSC(C)RRGSNSSFF Hex 7 HexNAc 4

**Mass:**  4161.6750

(1.794e-06)

**Charge States:** 4

**Start Position**: 128

**End Position**: 149

| Profile Score | Tandem Score | TDA q-value | Volume | Spectrum Matches |
| --- | --- | --- | --- | --- |
| -3.3889 | 99.6437 | 0.0380 | 6.0166e+07 | 1 |

**Adduct Signal Fractions**

- **Unmodified**: 6.0166e+07

### IGSIRN(N)GTYDHDVY Fuc 1 Hex 5 HexNAc 4

**Mass:**  3377.3919

(-2.589e-07)

**Charge States:** 3

**Start Position**: 478

**End Position**: 492

| Profile Score | Tandem Score | TDA q-value | Volume | Spectrum Matches |
| --- | --- | --- | --- | --- |
| -19.7115 | 97.6240 | 0.0380 | 5.1651e+06 | 1 |

**Adduct Signal Fractions**

- **Unmodified**: 5.1651e+06

### KSEC(C)ITPN(N)GSIPN(D)DKPF Hex 7 HexNAc 2

**Mass:**  3444.4484

(2.904e-04)

**Charge States:** 3

**Start Position**: 278

**End Position**: 295

| Profile Score | Tandem Score | TDA q-value | Volume | Spectrum Matches |
| --- | --- | --- | --- | --- |
| -20.4173 | 96.1955 | 0.0380 | 1.7029e+07 | 2 |

**Adduct Signal Fractions**

- **Unmodified**: 1.7029e+07

### TIVKPGDILLIN(N)STGN Fuc 2 Hex 4 HexNAc 4 Neu5Ac 1

**Mass:**  3697.6554

(-3.995e-06)

**Charge States:** 3

**Start Position**: 235

**End Position**: 251

| Profile Score | Tandem Score | TDA q-value | Volume | Spectrum Matches |
| --- | --- | --- | --- | --- |
| -21.1247 | 95.8324 | 0.0380 | 6.8573e+05 | 1 |

**Adduct Signal Fractions**

- **Unmodified**: 6.8573e+05

### TIVKPGDILLIN(N)STGNLIAPR Hex 7 HexNAc 2

**Mass:**  3744.8295

(3.173e-06)

**Charge States:** 3, 4

**Start Position**: 235

**End Position**: 256

| Profile Score | Tandem Score | TDA q-value | Volume | Spectrum Matches |
| --- | --- | --- | --- | --- |
| -19.3183 | 91.8183 | 0.0393 | 1.3709e+07 | 2 |

**Adduct Signal Fractions**

- **Unmodified**: 1.3709e+07

### IGSIRN(N)GTYDHDVY Fuc 2 Hex 5 HexNAc 4

**Mass:**  3523.4558

(1.449e-06)

**Charge States:** 3

**Start Position**: 478

**End Position**: 492

| Profile Score | Tandem Score | TDA q-value | Volume | Spectrum Matches |
| --- | --- | --- | --- | --- |
| -19.7373 | 91.3846 | 0.0393 | 3.2316e+06 | 1 |

**Adduct Signal Fractions**

- **Unmodified**: 3.2316e+06

### SGKSSIM(O)RSDAPIGKC(C)KSEC(C)ITPN(N)GSIPNDKPF Hex 7 HexNAc 2

**Mass:**  5134.2855

(5.727e-06)

**Charge States:** 5

**Start Position**: 262

**End Position**: 295

| Profile Score | Tandem Score | TDA q-value | Volume | Spectrum Matches |
| --- | --- | --- | --- | --- |
| -18.5302 | 90.5100 | 0.0393 | 1.1848e+07 | 1 |

**Adduct Signal Fractions**

- **Unmodified**: 1.1848e+07

### HKC(C)DNAC(C)IGSIRN(N)GTYDHDVY Fuc 1 Hex 5 HexNAc 5

**Mass:**  4465.7971

(3.992e-06)

**Charge States:** 4, 5

**Start Position**: 471

**End Position**: 492

| Profile Score | Tandem Score | TDA q-value | Volume | Spectrum Matches |
| --- | --- | --- | --- | --- |
| -20.6778 | 89.7509 | 0.0393 | 8.4504e+06 | 2 |

**Adduct Signal Fractions**

- **Unmodified**: 8.4504e+06

### RSDAPIGKC(C)KSEC(C)ITPN(N)GSIPN(D)DKPF Hex 9 HexNAc 2

**Mass:**  4753.0285

(2.126e-04)

**Charge States:** 5

**Start Position**: 269

**End Position**: 295

| Profile Score | Tandem Score | TDA q-value | Volume | Spectrum Matches |
| --- | --- | --- | --- | --- |
| -5.5415 | 88.1191 | 0.0393 | 4.2056e+07 | 2 |

**Adduct Signal Fractions**

- **Unmodified**: 4.2056e+07

### TIVKPGDILLIN(N)STGNLIAPR Hex 5 HexNAc 2

**Mass:**  3420.7165

(1.299e-06)

**Charge States:** 4

**Start Position**: 235

**End Position**: 256

| Profile Score | Tandem Score | TDA q-value | Volume | Spectrum Matches |
| --- | --- | --- | --- | --- |
| -5.0287 | 84.9612 | 0.0393 | 9.4193e+06 | 2 |

**Adduct Signal Fractions**

- **Unmodified**: 9.4193e+06

### RSDAPIGKC(C)KSEC(C)ITPN(N)GSIPN(D)DKPF Hex 7 HexNAc 2

**Mass:**  4428.9196

(2.257e-04)

**Charge States:** 5

**Start Position**: 269

**End Position**: 295

| Profile Score | Tandem Score | TDA q-value | Volume | Spectrum Matches |
| --- | --- | --- | --- | --- |
| -5.6294 | 84.6567 | 0.0393 | 3.7274e+07 | 2 |

**Adduct Signal Fractions**

- **Unmodified**: 3.7274e+07

### TIVKPGDILLIN(N)STGNLIAPRG Hex 9 HexNAc 2

**Mass:**  4125.9694

(4.877e-06)

**Charge States:** 3, 4

**Start Position**: 235

**End Position**: 257

| Profile Score | Tandem Score | TDA q-value | Volume | Spectrum Matches |
| --- | --- | --- | --- | --- |
| 14.0293 | 84.4170 | 0.0393 | 6.4954e+08 | 10 |

**Adduct Signal Fractions**

- **Unmodified**: 6.4954e+08

### QKLPGNDN(N)STATL Hex 7 HexNAc 2

**Mass:**  2898.2190

(2.278e-06)

**Charge States:** 3

**Start Position**: 1

**End Position**: 14

| Profile Score | Tandem Score | TDA q-value | Volume | Spectrum Matches |
| --- | --- | --- | --- | --- |
| -18.4092 | 84.1364 | 0.0393 | 2.7227e+07 | 1 |

**Adduct Signal Fractions**

- **Unmodified**: 2.7227e+07

### TIVKPGDILLIN(N)STGNLIAPRGY Hex 6 HexNAc 2

**Mass:**  3802.8603

(-6.384e-07)

**Charge States:** 3, 4

**Start Position**: 235

**End Position**: 258

| Profile Score | Tandem Score | TDA q-value | Volume | Spectrum Matches |
| --- | --- | --- | --- | --- |
| -19.4616 | 82.2168 | 0.0393 | 1.0337e+07 | 2 |

**Adduct Signal Fractions**

- **Unmodified**: 1.0337e+07

### AGVTHNGTSSSC(C)RRGSN(N)SSFF Hex 5 HexNAc 4

**Mass:**  3837.5668

(1.236e-06)

**Charge States:** 4

**Start Position**: 128

**End Position**: 149

| Profile Score | Tandem Score | TDA q-value | Volume | Spectrum Matches |
| --- | --- | --- | --- | --- |
| -3.4804 | 79.6544 | 0.0393 | 8.7642e+07 | 2 |

**Adduct Signal Fractions**

- **Unmodified**: 8.7642e+07

### KSEC(C)ITPN(N)GSIPNDKPF Fuc 3 Hex 3 HexNAc 8 Neu5Ac 1

**Mass:**  4743.0002

(4.826e-06)

**Charge States:** 4, 5

**Start Position**: 278

**End Position**: 295

| Profile Score | Tandem Score | TDA q-value | Volume | Spectrum Matches |
| --- | --- | --- | --- | --- |
| -4.9271 | 79.3967 | 0.0396 | 2.1165e+07 | 1 |

**Adduct Signal Fractions**

- **Unmodified**: 2.1165e+07

### KIRSGKSSIM(O)RSDAPIGKC(C)KSEC(C)ITPN(N)GSIPNDKPF Hex 9 HexNAc 2

**Mass:**  5855.6733

(6.301e-06)

**Charge States:** 5, 6, 7

**Start Position**: 259

**End Position**: 295

| Profile Score | Tandem Score | TDA q-value | Volume | Spectrum Matches |
| --- | --- | --- | --- | --- |
| -17.9000 | 79.2840 | 0.0396 | 4.5864e+07 | 3 |

**Adduct Signal Fractions**

- **Unmodified**: 4.5864e+07

### SKYPALN(N)VTM(O)PNNEQFDKLY Hex 8 HexNAc 2

**Mass:**  4089.7497

(4.416e-06)

**Charge States:** 3, 4

**Start Position**: 159

**End Position**: 179

| Profile Score | Tandem Score | TDA q-value | Volume | Spectrum Matches |
| --- | --- | --- | --- | --- |
| -2.4648 | 77.5459 | 0.0396 | 4.7350e+07 | 4 |

**Adduct Signal Fractions**

- **Unmodified**: 4.7350e+07

### NWAGVTHN(N)GTSSSC(C)RRGSNSSFF Hex 7 HexNAc 4

**Mass:**  4461.8049

(2.879e-06)

**Charge States:** 4

**Start Position**: 126

**End Position**: 149

| Profile Score | Tandem Score | TDA q-value | Volume | Spectrum Matches |
| --- | --- | --- | --- | --- |
| -19.1939 | 77.2502 | 0.0396 | 8.3022e+06 | 1 |

**Adduct Signal Fractions**

- **Unmodified**: 8.3022e+06

### SGKSSIM(O)RSDAPIGKC(C)KSEC(C)ITPN(N)GSIPNDKPF Hex 8 HexNAc 2

**Mass:**  5296.3308

(4.152e-06)

**Charge States:** 5

**Start Position**: 262

**End Position**: 295

| Profile Score | Tandem Score | TDA q-value | Volume | Spectrum Matches |
| --- | --- | --- | --- | --- |
| -18.9203 | 76.2033 | 0.0396 | 3.5033e+06 | 1 |

**Adduct Signal Fractions**

- **Unmodified**: 3.5033e+06

### AGVTHN(N)GTSSSC(C)RRGSNSSFF Hex 5 HexNAc 5

**Mass:**  4040.6467

(7.972e-07)

**Charge States:** 4

**Start Position**: 128

**End Position**: 149

| Profile Score | Tandem Score | TDA q-value | Volume | Spectrum Matches |
| --- | --- | --- | --- | --- |
| -4.5708 | 74.5776 | 0.0396 | 8.4095e+07 | 1 |

**Adduct Signal Fractions**

- **Unmodified**: 8.4095e+07

### SGKSSIM(O)RSDAPIGKC(C)KSEC(C)ITPN(N)GSIPNDKPF Hex 9 HexNAc 2

**Mass:**  5458.3852

(4.347e-06)

**Charge States:** 5

**Start Position**: 262

**End Position**: 295

| Profile Score | Tandem Score | TDA q-value | Volume | Spectrum Matches |
| --- | --- | --- | --- | --- |
| -4.4272 | 73.1525 | 0.0396 | 1.1907e+07 | 2 |

**Adduct Signal Fractions**

- **Unmodified**: 1.1907e+07

### NSKYPALN(N)VTM(O)PNNEQFDKLY Hex 7 HexNAc 2

**Mass:**  4041.7463

(6.924e-06)

**Charge States:** 4

**Start Position**: 158

**End Position**: 179

| Profile Score | Tandem Score | TDA q-value | Volume | Spectrum Matches |
| --- | --- | --- | --- | --- |
| -4.1366 | 73.0238 | 0.0396 | 1.8183e+07 | 2 |

**Adduct Signal Fractions**

- **Unmodified**: 1.8183e+07

### RSDAPIGKC(C)KSEC(C)ITPN(N)GSIPN(D)DKPF Hex 8 HexNAc 2

**Mass:**  4590.9853

(2.197e-04)

**Charge States:** 4, 5

**Start Position**: 269

**End Position**: 295

| Profile Score | Tandem Score | TDA q-value | Volume | Spectrum Matches |
| --- | --- | --- | --- | --- |
| -20.1821 | 72.4150 | 0.0396 | 1.9549e+07 | 1 |

**Adduct Signal Fractions**

- **Unmodified**: 1.9549e+07

### KIRSGKSSIM(O)RSDAPIGKC(C)KSEC(C)ITPN(N)GSIPNDKPF Hex 8 HexNAc 2

**Mass:**  5693.6194

(6.993e-06)

**Charge States:** 6

**Start Position**: 259

**End Position**: 295

| Profile Score | Tandem Score | TDA q-value | Volume | Spectrum Matches |
| --- | --- | --- | --- | --- |
| -4.3884 | 71.8069 | 0.0396 | 2.8403e+07 | 2 |

**Adduct Signal Fractions**

- **Unmodified**: 2.8403e+07

### AGVTHN(N)GTSSSC(C)RRGSNSSFF Fuc 1 Hex 4 HexNAc 3

**Mass:**  3618.4941

(1.539e-06)

**Charge States:** 4

**Start Position**: 128

**End Position**: 149

| Profile Score | Tandem Score | TDA q-value | Volume | Spectrum Matches |
| --- | --- | --- | --- | --- |
| -5.0526 | 69.4427 | 0.0396 | 3.0785e+07 | 1 |

**Adduct Signal Fractions**

- **Unmodified**: 3.0785e+07

### KIRSGKSSIM(O)RSDAPIGKC(C)KSEC(C)ITPN(N)GSIPNDKPF Hex 7 HexNAc 2

**Mass:**  5531.5685

(7.667e-06)

**Charge States:** 6

**Start Position**: 259

**End Position**: 295

| Profile Score | Tandem Score | TDA q-value | Volume | Spectrum Matches |
| --- | --- | --- | --- | --- |
| -4.4877 | 67.6957 | 0.0396 | 5.3492e+07 | 3 |

**Adduct Signal Fractions**

- **Unmodified**: 5.3492e+07

### TIVKPGDILLIN(N)STGN(D)LIAPRGY Fuc 2 Hex 3 HexNAc 4 Neu5Ac 1

**Mass:**  4307.0344

(2.259e-04)

**Charge States:** 4

**Start Position**: 235

**End Position**: 258

| Profile Score | Tandem Score | TDA q-value | Volume | Spectrum Matches |
| --- | --- | --- | --- | --- |
| -20.0276 | 65.4094 | 0.0396 | 5.1929e+06 | 1 |

**Adduct Signal Fractions**

- **Unmodified**: 5.1929e+06

### TIVKPGDILLIN(N)STGN(D)LIAPRGY Hex 9 HexNAc 2

**Mass:**  4290.0056

(2.317e-04)

**Charge States:** 3, 4, 5

**Start Position**: 235

**End Position**: 258

| Profile Score | Tandem Score | TDA q-value | Volume | Spectrum Matches |
| --- | --- | --- | --- | --- |
| -19.6972 | 63.7503 | 0.0396 | 5.9365e+07 | 12 |

**Adduct Signal Fractions**

- **Unmodified**: 5.9365e+07

### NSKYPALN(N)VTMPNNEQFDKLY Hex 8 HexNAc 2

**Mass:**  4187.7866

(2.502e-06)

**Charge States:** 3, 4

**Start Position**: 158

**End Position**: 179

| Profile Score | Tandem Score | TDA q-value | Volume | Spectrum Matches |
| --- | --- | --- | --- | --- |
| -19.0837 | 63.7335 | 0.0396 | 3.3856e+06 | 2 |

**Adduct Signal Fractions**

- **Unmodified**: 3.3856e+06

### TIVKPGDILLIN(N)STGNLIAPR Hex 6 HexNAc 2

**Mass:**  3582.7739

(2.496e-06)

**Charge States:** 3, 4

**Start Position**: 235

**End Position**: 256

| Profile Score | Tandem Score | TDA q-value | Volume | Spectrum Matches |
| --- | --- | --- | --- | --- |
| -19.3426 | 63.2270 | 0.0396 | 2.3117e+06 | 1 |

**Adduct Signal Fractions**

- **Unmodified**: 2.3117e+06

### SKYPALN(N)VTM(O)PNNEQFDKLY Fuc 1 Hex 6 HexNAc 4

**Mass:**  4317.8498

(5.861e-06)

**Charge States:** 3, 4

**Start Position**: 159

**End Position**: 179

| Profile Score | Tandem Score | TDA q-value | Volume | Spectrum Matches |
| --- | --- | --- | --- | --- |
| 12.4794 | 60.2066 | 0.0396 | 4.5417e+07 | 5 |

**Adduct Signal Fractions**

- **Unmodified**: 4.5417e+07

### N(D)SKYPALN(N)VTM(O)PNNEQFDKLY Hex 8 HexNAc 2

**Mass:**  4204.7717

(2.374e-04)

**Charge States:** 3

**Start Position**: 158

**End Position**: 179

| Profile Score | Tandem Score | TDA q-value | Volume | Spectrum Matches |
| --- | --- | --- | --- | --- |
| -6.1209 | 59.3751 | 0.0396 | 2.1231e+07 | 3 |

**Adduct Signal Fractions**

- **Unmodified**: 2.1231e+07

### NWAGVTHN(N)GTSSSC(C)RRGSNSSFF Hex 6 HexNAc 4

**Mass:**  4299.7570

(4.172e-06)

**Charge States:** 4

**Start Position**: 126

**End Position**: 149

| Profile Score | Tandem Score | TDA q-value | Volume | Spectrum Matches |
| --- | --- | --- | --- | --- |
| -19.0910 | 58.5007 | 0.0396 | 4.4674e+06 | 1 |

**Adduct Signal Fractions**

- **Unmodified**: 4.4674e+06

### AGVTHN(N)GTSSSC(C)RRGSNSSFF Fuc 1 Hex 4 HexNAc 4 Neu5Ac 1

**Mass:**  4112.6623

(1.020e-07)

**Charge States:** 4

**Start Position**: 128

**End Position**: 149

| Profile Score | Tandem Score | TDA q-value | Volume | Spectrum Matches |
| --- | --- | --- | --- | --- |
| -19.4518 | 57.9227 | 0.0396 | 4.0326e+06 | 1 |

**Adduct Signal Fractions**

- **Unmodified**: 4.0326e+06

### AGVTHN(N)GTSSSC(C)RRGSNSSFF Fuc 1 Hex 5 HexNAc 4

**Mass:**  3983.6274

(1.327e-06)

**Charge States:** 4

**Start Position**: 128

**End Position**: 149

| Profile Score | Tandem Score | TDA q-value | Volume | Spectrum Matches |
| --- | --- | --- | --- | --- |
| -5.7503 | 56.5892 | 0.0396 | 2.0345e+07 | 1 |

**Adduct Signal Fractions**

- **Unmodified**: 2.0345e+07

### SEC(C)ITPN(N)GSIPNDKPF Hex 7 HexNAc 2

**Mass:**  3315.3653

(5.127e-06)

**Charge States:** 3

**Start Position**: 279

**End Position**: 295

| Profile Score | Tandem Score | TDA q-value | Volume | Spectrum Matches |
| --- | --- | --- | --- | --- |
| -19.6289 | 55.8030 | 0.0396 | 3.9711e+06 | 1 |

**Adduct Signal Fractions**

- **Unmodified**: 3.9711e+06

### AGVTHN(D)GTSSSC(C)RRGSN(N)SSFF Hex 5 HexNAc 2

**Mass:**  3432.3908

(2.866e-04)

**Charge States:** 4

**Start Position**: 128

**End Position**: 149

| Profile Score | Tandem Score | TDA q-value | Volume | Spectrum Matches |
| --- | --- | --- | --- | --- |
| -6.3938 | 55.2334 | 0.0396 | 4.9038e+07 | 1 |

**Adduct Signal Fractions**

- **Unmodified**: 4.9038e+07

### AGVTHN(N)GTSSSC(C)RRGSNSSFF Hex 7 HexNAc 3

**Mass:**  3958.5981

(1.933e-06)

**Charge States:** 4

**Start Position**: 128

**End Position**: 149

| Profile Score | Tandem Score | TDA q-value | Volume | Spectrum Matches |
| --- | --- | --- | --- | --- |
| -19.2390 | 54.5606 | 0.0441 | 3.7167e+07 | 1 |

**Adduct Signal Fractions**

- **Unmodified**: 3.7167e+07

### LIN(N)STGNLIAPRGY Hex 10 HexNAc 2

**Mass:**  3514.5049

(2.371e-06)

**Charge States:** 3

**Start Position**: 244

**End Position**: 258

| Profile Score | Tandem Score | TDA q-value | Volume | Spectrum Matches |
| --- | --- | --- | --- | --- |
| -19.4927 | 54.5212 | 0.0470 | 2.7041e+06 | 1 |

**Adduct Signal Fractions**

- **Unmodified**: 2.7041e+06

### NNESFNWAGVTHN(N)GTSSSC(C)RRGSNSSFF Hex 7 HexNAc 2

**Mass:**  4646.8727

(2.285e-06)

**Charge States:** 4

**Start Position**: 121

**End Position**: 149

| Profile Score | Tandem Score | TDA q-value | Volume | Spectrum Matches |
| --- | --- | --- | --- | --- |
| -18.9812 | 53.2969 | 0.0470 | 2.1159e+06 | 1 |

**Adduct Signal Fractions**

- **Unmodified**: 2.1159e+06

### NNESFNWAGVTHNGTSSSC(C)RRGSN(N)SSFF Hex 7 HexNAc 2

**Mass:**  4646.8727

(2.285e-06)

**Charge States:** 4

**Start Position**: 121

**End Position**: 149

| Profile Score | Tandem Score | TDA q-value | Volume | Spectrum Matches |
| --- | --- | --- | --- | --- |
| -18.9812 | 53.2969 | 0.0470 | 2.1159e+06 | 1 |

**Adduct Signal Fractions**

- **Unmodified**: 2.1159e+06

### NWAGVTHN(N)GTSSSC(C)RRGSNSSFF Hex 5 HexNAc 4

**Mass:**  4137.7009

(3.822e-06)

**Charge States:** 4

**Start Position**: 126

**End Position**: 149

| Profile Score | Tandem Score | TDA q-value | Volume | Spectrum Matches |
| --- | --- | --- | --- | --- |
| -17.8652 | 52.3546 | 0.0470 | 1.1305e+07 | 1 |

**Adduct Signal Fractions**

- **Unmodified**: 1.1305e+07

### RSDAPIGKC(C)KSEC(C)ITPN(N)GSIPNDKPFQNVNRITY Hex 7 HexNAc 2

**Mass:**  5416.4675

(6.304e-06)

**Charge States:** 5, 6

**Start Position**: 269

**End Position**: 303

| Profile Score | Tandem Score | TDA q-value | Volume | Spectrum Matches |
| --- | --- | --- | --- | --- |
| -19.0657 | 49.7263 | 0.0494 | 5.0685e+06 | 2 |

**Adduct Signal Fractions**

- **Unmodified**: 5.0685e+06

### cus|SWZNA|A/Switzerland/9715293/2013

**>cus|SWZNA|A/Switzerland/9715293/2013**  
 `MNPNQKIITIGSVSLTISTICFFMQIAILITTVTLHFKQYEFNSPPNNQVMLCEPTIIERNITEIVYLTNTTIEKEICPK  
PAEYRNWSKPQCGITGFAPFSKDNSIRLSAGGDIWVTREPYVSCDPDKCYQFALGQGTTLNNVHSNNTVRDRTPYRTLLM  
NELGVPFHLGTKQVCIAWSSSSCHDGKAWLHVCITGDDKNATASFIYNGRLVDSVVSWSKDILRTQESECVCINGTCTVV  
MTDGSASGKADTKILFIEEGKIVHTSTLSGSAQHVEECSCYPRYPGVRCVCRDNWKGSNRPIVDINIKDHSIVSSYVCSG  
LVGDTPRKNDSSSSSHCLDPNNEEGGHGVKGWAFDDGNDVWMGRTINETSRLGYETFKVIEGWSNPKSKLQTNRQVIVDR  
GDRSGYSGIFSVEGKSCINRCFYVELIRGRKEETEVLWTSNSIVVFCGTSGTYGTGSWPDGADLNLMPI`

| Site | Type | Count |
| --- | --- | --- |
| 146 | N-linked | 6 |
| 86 | N-linked | 4 |
| 367 | N-linked | 19 |
| 200 | N-linked | 3 |

| Mass | Sequence | MS_2_ Score | `q`-value | MS_1_ Score | Signal | Protein Coordinates | Tandem Scans | Start Time | Apex Time | End Time |
| --- | --- | --- | --- | --- | --- | --- | --- | --- | --- | --- |
| 3450.4220 | LHVC(C)ITGDDKN(N)ATASF Hex 8 HexNAc 2 | 188.438 | 0.0 | -18.031 | 1.303e+07 | 189 - 205 | 2 | 33.93 | 33.93 | 34.10 |
| 3612.4746 | LHVC(C)ITGDDKN(N)ATASF Hex 9 HexNAc 2 | 187.252 | 0.0 | -17.483 | 1.688e+07 | 189 - 205 | 2 | 33.76 | 33.93 | 33.93 |
| 3232.3282 | TIN(N)ETSRLGYETF Hex 8 HexNAc 2 | 178.777 | 0.0 | -2.702 | 5.289e+07 | 364 - 377 | 2 | 36.14 | 36.30 | 36.47 |
| 3729.5473 | RN(N)WSKPQC(C)GITGFAPF Hex 9 HexNAc 2 | 178.701 | 0.0 | -3.835 | 4.123e+07 | 84 - 100 | 1 | 45.08 | 45.25 | 45.59 |
| 3567.4950 | RN(N)WSKPQC(C)GITGFAPF Hex 8 HexNAc 2 | 175.860 | 0.0 | -2.997 | 3.269e+07 | 84 - 100 | 2 | 45.25 | 45.42 | 45.59 |
| 2908.2227 | TIN(N)ETSRLGYETF Hex 6 HexNAc 2 | 169.308 | 0.0 | -18.993 | 9.995e+06 | 364 - 377 | 1 | 36.47 | 36.47 | 36.63 |
| 3430.4308 | M(O)GRTIN(N)ETSRLGYETF Hex 7 HexNAc 2 | 163.974 | 0.0 | 16.936 | 2.486e+08 | 361 - 377 | 3 | 31.90 | 32.07 | 32.58 |
| 3592.4796 | M(O)GRTIN(N)ETSRLGYETF Hex 8 HexNAc 2 | 163.962 | 0.0 | 17.742 | 4.440e+08 | 361 - 377 | 4 | 31.73 | 31.90 | 32.41 |
| 3070.2803 | TIN(N)ETSRLGYETF Hex 7 HexNAc 2 | 158.333 | 0.0 | -16.143 | 2.440e+07 | 364 - 377 | 3 | 36.30 | 36.47 | 36.47 |
| 3268.3789 | M(O)GRTIN(N)ETSRLGYETF Hex 6 HexNAc 2 | 156.623 | 0.0 | 16.750 | 1.449e+08 | 361 - 377 | 3 | 32.07 | 32.24 | 32.75 |
| 3288.3667 | LHVC(C)ITGDDKN(N)ATASF Hex 7 HexNAc 2 | 156.140 | 0.0 | -18.369 | 1.921e+06 | 189 - 205 | 1 | 34.10 | 34.10 | 34.10 |
| 3053.2665 | M(O)GRTIN(N)ETSRLGY Hex 7 HexNAc 2 | 151.864 | 0.0 | -2.184 | 8.128e+07 | 361 - 374 | 2 | 21.79 | 21.95 | 22.11 |
| 3414.4462 | MGRTIN(N)ETSRLGYETF Hex 7 HexNAc 2 | 144.551 | 0.0 | -18.782 | 5.246e+06 | 361 - 377 | 1 | 34.44 | 34.44 | 34.61 |
| 3215.3201 | M(O)GRTIN(N)ETSRLGY Hex 8 HexNAc 2 | 141.147 | 0.0 | -15.855 | 1.433e+08 | 361 - 374 | 1 | 21.79 | 21.79 | 21.95 |
| 3576.4978 | MGRTIN(N)ETSRLGYETF Hex 8 HexNAc 2 | 138.836 | 0.008 | -18.153 | 1.335e+07 | 361 - 377 | 1 | 34.27 | 34.44 | 34.44 |
| 2891.2137 | M(O)GRTIN(N)ETSRLGY Hex 6 HexNAc 2 | 134.093 | 0.008 | -17.867 | 4.623e+07 | 361 - 374 | 2 | 21.95 | 22.11 | 22.11 |
| 2671.1274 | M(O)GRTIN(N)ETSRL Hex 6 HexNAc 2 | 129.542 | 0.008 | -20.708 | 7.174e+06 | 361 - 372 | 1 | 18.01 | 18.17 | 18.17 |
| 2995.2364 | M(O)GRTIN(N)ETSRL Hex 8 HexNAc 2 | 125.713 | 0.008 | -18.671 | 2.720e+07 | 361 - 372 | 1 | 17.84 | 17.84 | 18.01 |
| 3106.3325 | M(O)GRTIN(N)ETSRLGYETF Hex 5 HexNAc 2 | 124.571 | 0.008 | -19.061 | 1.390e+07 | 361 - 377 | 1 | 32.41 | 32.41 | 32.58 |
| 3773.5707 | NNVHSN(N)NTVRDRTPY Hex 6 HexNAc 5 | 121.759 | 0.008 | -19.068 | 7.387e+07 | 140 - 155 | 2 | 14.22 | 14.22 | 14.38 |
| 3252.3860 | MGRTIN(N)ETSRLGYETF Hex 6 HexNAc 2 | 121.277 | 0.015 | -19.193 | 2.377e+06 | 361 - 377 | 1 | 34.61 | 34.61 | 34.78 |
| 4286.7565 | NNVHSN(N)NTVRDRTPY Fuc 1 Hex 7 HexNAc 6 | 115.923 | 0.015 | 0.000 | 0.000e+00 | 140 - 155 | 1 | - | - | - |
| 2833.1807 | M(O)GRTIN(N)ETSRL Hex 7 HexNAc 2 | 109.131 | 0.035 | -18.782 | 1.317e+07 | 361 - 372 | 1 | 17.84 | 18.01 | 18.01 |
| 4366.7189 | NNVHSN(N)NTVRDRTPY Fuc 1 Hex 10 HexNAc 4 | 106.941 | 0.035 | 0.000 | 0.000e+00 | 140 - 155 | 1 | - | - | - |
| 3919.6266 | NNVHSN(N)NTVRDRTPY Fuc 1 Hex 6 HexNAc 5 | 105.666 | 0.035 | -19.130 | 6.633e+07 | 140 - 155 | 1 | 14.22 | 14.22 | 14.38 |
| 2729.1612 | M(O)GRTIN(N)ETSRLGY Hex 5 HexNAc 2 | 101.751 | 0.038 | -17.441 | 8.099e+06 | 361 - 374 | 1 | 22.28 | 22.28 | 22.28 |
| 4138.7014 | NNVHSN(N)NTVRDRTPY Hex 7 HexNAc 6 | 98.105 | 0.038 | -16.555 | 3.586e+07 | 140 - 155 | 1 | 14.22 | 14.22 | 14.22 |
| 3658.5366 | M(O)GRTIN(N)ETSRLGYETF Fuc 1 Hex 5 HexNAc 4 | 95.980 | 0.038 | -18.743 | 1.044e+07 | 361 - 377 | 1 | 31.73 | 31.90 | 31.90 |
| 4432.8149 | NNVHSN(N)NTVRDRTPY Fuc 2 Hex 7 HexNAc 6 | 92.571 | 0.039 | 0.000 | 0.000e+00 | 140 - 155 | 1 | - | - | - |
| 3487.4579 | RN(N)WSKPQC(C)GITGFAPF Hex 5 HexNAc 4 | 60.909 | 0.040 | -3.969 | 7.024e+07 | 84 - 100 | 3 | 32.91 | 33.08 | 33.25 |
| 4721.8823 | GRTIN(N)ETSRL Fuc 3 Hex 7 HexNAc 7 Neu5Ac 2 | 60.005 | 0.040 | -18.577 | 2.526e+07 | 362 - 372 | 2 | 42.04 | 42.04 | 42.21 |
| 3649.5128 | RN(N)WSKPQC(C)GITGFAPF Hex 6 HexNAc 4 | 57.051 | 0.040 | -2.295 | 1.266e+08 | 84 - 100 | 3 | 32.75 | 32.91 | 33.25 |

### LHVC(C)ITGDDKN(N)ATASF Hex 8 HexNAc 2

**Mass:**  3450.4220

(6.007e-06)

**Charge States:** 3

**Start Position**: 190

**End Position**: 206

| Profile Score | Tandem Score | TDA q-value | Volume | Spectrum Matches |
| --- | --- | --- | --- | --- |
| -18.0313 | 188.4378 | 0.0000 | 1.3026e+07 | 2 |

**Adduct Signal Fractions**

- **Unmodified**: 1.3026e+07

### LHVC(C)ITGDDKN(N)ATASF Hex 9 HexNAc 2

**Mass:**  3612.4737

(6.823e-06)

**Charge States:** 3

**Start Position**: 190

**End Position**: 206

| Profile Score | Tandem Score | TDA q-value | Volume | Spectrum Matches |
| --- | --- | --- | --- | --- |
| -17.4828 | 187.2524 | 0.0000 | 1.6884e+07 | 2 |

**Adduct Signal Fractions**

- **Unmodified**: 1.6884e+07

### TIN(N)ETSRLGYETF Hex 8 HexNAc 2

**Mass:**  3232.3361

(5.759e-06)

**Charge States:** 3

**Start Position**: 365

**End Position**: 378

| Profile Score | Tandem Score | TDA q-value | Volume | Spectrum Matches |
| --- | --- | --- | --- | --- |
| -2.7022 | 178.7768 | 0.0000 | 5.2888e+07 | 2 |

**Adduct Signal Fractions**

- **Unmodified**: 5.2888e+07

### RN(N)WSKPQC(C)GITGFAPF Hex 9 HexNAc 2

**Mass:**  3729.5469

(2.259e-06)

**Charge States:** 3

**Start Position**: 85

**End Position**: 101

| Profile Score | Tandem Score | TDA q-value | Volume | Spectrum Matches |
| --- | --- | --- | --- | --- |
| -3.8354 | 178.7011 | 0.0000 | 4.1234e+07 | 1 |

**Adduct Signal Fractions**

- **Unmodified**: 4.1234e+07

### RN(N)WSKPQC(C)GITGFAPF Hex 8 HexNAc 2

**Mass:**  3567.4953

(1.230e-06)

**Charge States:** 3

**Start Position**: 85

**End Position**: 101

| Profile Score | Tandem Score | TDA q-value | Volume | Spectrum Matches |
| --- | --- | --- | --- | --- |
| -2.9971 | 175.8602 | 0.0000 | 3.2686e+07 | 2 |

**Adduct Signal Fractions**

- **Unmodified**: 3.2686e+07

### TIN(N)ETSRLGYETF Hex 6 HexNAc 2

**Mass:**  2908.2255

(4.659e-06)

**Charge States:** 2, 3

**Start Position**: 365

**End Position**: 378

| Profile Score | Tandem Score | TDA q-value | Volume | Spectrum Matches |
| --- | --- | --- | --- | --- |
| -18.9931 | 169.3083 | 0.0000 | 9.9953e+06 | 1 |

**Adduct Signal Fractions**

- **Unmodified**: 9.9953e+06

### M(O)GRTIN(N)ETSRLGYETF Hex 7 HexNAc 2

**Mass:**  3430.4309

(2.098e-06)

**Charge States:** 3, 4

**Start Position**: 362

**End Position**: 378

| Profile Score | Tandem Score | TDA q-value | Volume | Spectrum Matches |
| --- | --- | --- | --- | --- |
| 16.9359 | 163.9737 | 0.0000 | 2.4860e+08 | 3 |

**Adduct Signal Fractions**

- **Unmodified**: 2.4860e+08

### M(O)GRTIN(N)ETSRLGYETF Hex 8 HexNAc 2

**Mass:**  3592.4778

(2.467e-06)

**Charge States:** 3, 4

**Start Position**: 362

**End Position**: 378

| Profile Score | Tandem Score | TDA q-value | Volume | Spectrum Matches |
| --- | --- | --- | --- | --- |
| 17.7423 | 163.9624 | 0.0000 | 4.4397e+08 | 4 |

**Adduct Signal Fractions**

- **Unmodified**: 4.4397e+08

### TIN(N)ETSRLGYETF Hex 7 HexNAc 2

**Mass:**  3070.2811

(5.434e-06)

**Charge States:** 2, 3

**Start Position**: 365

**End Position**: 378

| Profile Score | Tandem Score | TDA q-value | Volume | Spectrum Matches |
| --- | --- | --- | --- | --- |
| -16.1431 | 158.3333 | 0.0000 | 2.4402e+07 | 3 |

**Adduct Signal Fractions**

- **Unmodified**: 2.4402e+07

### M(O)GRTIN(N)ETSRLGYETF Hex 6 HexNAc 2

**Mass:**  3268.3782

(3.736e-06)

**Charge States:** 3

**Start Position**: 362

**End Position**: 378

| Profile Score | Tandem Score | TDA q-value | Volume | Spectrum Matches |
| --- | --- | --- | --- | --- |
| 16.7500 | 156.6227 | 0.0000 | 1.4490e+08 | 3 |

**Adduct Signal Fractions**

- **Unmodified**: 1.4490e+08

### LHVC(C)ITGDDKN(N)ATASF Hex 7 HexNAc 2

**Mass:**  3288.3667

(5.522e-06)

**Charge States:** 3

**Start Position**: 190

**End Position**: 206

| Profile Score | Tandem Score | TDA q-value | Volume | Spectrum Matches |
| --- | --- | --- | --- | --- |
| -18.3692 | 156.1400 | 0.0000 | 1.9213e+06 | 1 |

**Adduct Signal Fractions**

- **Unmodified**: 1.9213e+06

### M(O)GRTIN(N)ETSRLGY Hex 7 HexNAc 2

**Mass:**  3053.2664

(6.069e-07)

**Charge States:** 3

**Start Position**: 362

**End Position**: 375

| Profile Score | Tandem Score | TDA q-value | Volume | Spectrum Matches |
| --- | --- | --- | --- | --- |
| -2.1845 | 151.8642 | 0.0000 | 8.1281e+07 | 2 |

**Adduct Signal Fractions**

- **Unmodified**: 8.1281e+07

### MGRTIN(N)ETSRLGYETF Hex 7 HexNAc 2

**Mass:**  3414.4447

(4.922e-06)

**Charge States:** 3

**Start Position**: 362

**End Position**: 378

| Profile Score | Tandem Score | TDA q-value | Volume | Spectrum Matches |
| --- | --- | --- | --- | --- |
| -18.7820 | 144.5513 | 0.0000 | 5.2460e+06 | 1 |

**Adduct Signal Fractions**

- **Unmodified**: 5.2460e+06

### M(O)GRTIN(N)ETSRLGY Hex 8 HexNAc 2

**Mass:**  3215.3201

(1.074e-06)

**Charge States:** 3

**Start Position**: 362

**End Position**: 375

| Profile Score | Tandem Score | TDA q-value | Volume | Spectrum Matches |
| --- | --- | --- | --- | --- |
| -15.8553 | 141.1473 | 0.0000 | 1.4330e+08 | 1 |

**Adduct Signal Fractions**

- **Unmodified**: 1.4330e+08

### MGRTIN(N)ETSRLGYETF Hex 8 HexNAc 2

**Mass:**  3576.4975

(2.862e-06)

**Charge States:** 3

**Start Position**: 362

**End Position**: 378

| Profile Score | Tandem Score | TDA q-value | Volume | Spectrum Matches |
| --- | --- | --- | --- | --- |
| -18.1528 | 138.8364 | 0.0079 | 1.3349e+07 | 1 |

**Adduct Signal Fractions**

- **Unmodified**: 1.3349e+07

### M(O)GRTIN(N)ETSRLGY Hex 6 HexNAc 2

**Mass:**  2891.2137

(1.034e-06)

**Charge States:** 3

**Start Position**: 362

**End Position**: 375

| Profile Score | Tandem Score | TDA q-value | Volume | Spectrum Matches |
| --- | --- | --- | --- | --- |
| -17.8673 | 134.0927 | 0.0079 | 4.6233e+07 | 2 |

**Adduct Signal Fractions**

- **Unmodified**: 4.6233e+07

### M(O)GRTIN(N)ETSRL Hex 6 HexNAc 2

**Mass:**  2671.1284

(-1.176e-07)

**Charge States:** 3

**Start Position**: 362

**End Position**: 373

| Profile Score | Tandem Score | TDA q-value | Volume | Spectrum Matches |
| --- | --- | --- | --- | --- |
| -20.7077 | 129.5423 | 0.0079 | 7.1735e+06 | 1 |

**Adduct Signal Fractions**

- **Unmodified**: 7.1735e+06

### M(O)GRTIN(N)ETSRL Hex 8 HexNAc 2

**Mass:**  2995.2361

(1.375e-06)

**Charge States:** 3

**Start Position**: 362

**End Position**: 373

| Profile Score | Tandem Score | TDA q-value | Volume | Spectrum Matches |
| --- | --- | --- | --- | --- |
| -18.6709 | 125.7133 | 0.0079 | 2.7202e+07 | 1 |

**Adduct Signal Fractions**

- **Unmodified**: 2.7202e+07

### M(O)GRTIN(N)ETSRLGYETF Hex 5 HexNAc 2

**Mass:**  3106.3313

(4.547e-06)

**Charge States:** 3

**Start Position**: 362

**End Position**: 378

| Profile Score | Tandem Score | TDA q-value | Volume | Spectrum Matches |
| --- | --- | --- | --- | --- |
| -19.0606 | 124.5714 | 0.0079 | 1.3903e+07 | 1 |

**Adduct Signal Fractions**

- **Unmodified**: 1.3903e+07

### NNVHSN(N)NTVRDRTPY Hex 6 HexNAc 5

**Mass:**  3773.5717

(1.939e-06)

**Charge States:** 4

**Start Position**: 141

**End Position**: 156

| Profile Score | Tandem Score | TDA q-value | Volume | Spectrum Matches |
| --- | --- | --- | --- | --- |
| -19.0684 | 121.7589 | 0.0079 | 7.3870e+07 | 2 |

**Adduct Signal Fractions**

- **Unmodified**: 7.3870e+07

### MGRTIN(N)ETSRLGYETF Hex 6 HexNAc 2

**Mass:**  3252.3909

(4.934e-06)

**Charge States:** 3

**Start Position**: 362

**End Position**: 378

| Profile Score | Tandem Score | TDA q-value | Volume | Spectrum Matches |
| --- | --- | --- | --- | --- |
| -19.1929 | 121.2770 | 0.0146 | 2.3774e+06 | 1 |

**Adduct Signal Fractions**

- **Unmodified**: 2.3774e+06

### NNVHSN(N)NTVRDRTPY Fuc 1 Hex 7 HexNAc 6

**Mass:**  4286.7565

(4.672e-04)

**Start Position**: 141

**End Position**: 156

| Profile Score | Tandem Score | TDA q-value | Volume | Spectrum Matches |
| --- | --- | --- | --- | --- |
| 0.0000 | 115.9234 | 0.0146 | 0.0000e+00 | 1 |

Adduct:

No Chromatogram Found

### M(O)GRTIN(N)ETSRL Hex 7 HexNAc 2

**Mass:**  2833.1814

(7.610e-07)

**Charge States:** 3

**Start Position**: 362

**End Position**: 373

| Profile Score | Tandem Score | TDA q-value | Volume | Spectrum Matches |
| --- | --- | --- | --- | --- |
| -18.7820 | 109.1315 | 0.0347 | 1.3171e+07 | 1 |

**Adduct Signal Fractions**

- **Unmodified**: 1.3171e+07

### NNVHSN(N)NTVRDRTPY Fuc 1 Hex 10 HexNAc 4

**Mass:**  4366.7189

(4.501e-04)

**Start Position**: 141

**End Position**: 156

| Profile Score | Tandem Score | TDA q-value | Volume | Spectrum Matches |
| --- | --- | --- | --- | --- |
| 0.0000 | 106.9413 | 0.0347 | 0.0000e+00 | 1 |

Adduct:

No Chromatogram Found

### NNVHSN(N)NTVRDRTPY Fuc 1 Hex 6 HexNAc 5

**Mass:**  3919.6274

(1.280e-06)

**Charge States:** 4

**Start Position**: 141

**End Position**: 156

| Profile Score | Tandem Score | TDA q-value | Volume | Spectrum Matches |
| --- | --- | --- | --- | --- |
| -19.1304 | 105.6663 | 0.0347 | 6.6334e+07 | 1 |

**Adduct Signal Fractions**

- **Unmodified**: 6.6334e+07

### M(O)GRTIN(N)ETSRLGY Hex 5 HexNAc 2

**Mass:**  2729.1612

(1.026e-06)

**Charge States:** 3

**Start Position**: 362

**End Position**: 375

| Profile Score | Tandem Score | TDA q-value | Volume | Spectrum Matches |
| --- | --- | --- | --- | --- |
| -17.4410 | 101.7508 | 0.0380 | 8.0993e+06 | 1 |

**Adduct Signal Fractions**

- **Unmodified**: 8.0993e+06

### NNVHSN(N)NTVRDRTPY Hex 7 HexNAc 6

**Mass:**  4138.7014

(1.169e-06)

**Charge States:** 4

**Start Position**: 141

**End Position**: 156

| Profile Score | Tandem Score | TDA q-value | Volume | Spectrum Matches |
| --- | --- | --- | --- | --- |
| -16.5548 | 98.1046 | 0.0380 | 3.5865e+07 | 1 |

**Adduct Signal Fractions**

- **Unmodified**: 3.5865e+07

### M(O)GRTIN(N)ETSRLGYETF Fuc 1 Hex 5 HexNAc 4

**Mass:**  3658.5364

(7.175e-07)

**Charge States:** 3

**Start Position**: 362

**End Position**: 378

| Profile Score | Tandem Score | TDA q-value | Volume | Spectrum Matches |
| --- | --- | --- | --- | --- |
| -18.7427 | 95.9797 | 0.0380 | 1.0443e+07 | 1 |

**Adduct Signal Fractions**

- **Unmodified**: 1.0443e+07

### NNVHSN(N)NTVRDRTPY Fuc 2 Hex 7 HexNAc 6

**Mass:**  4432.8149

(4.519e-04)

**Start Position**: 141

**End Position**: 156

| Profile Score | Tandem Score | TDA q-value | Volume | Spectrum Matches |
| --- | --- | --- | --- | --- |
| 0.0000 | 92.5709 | 0.0393 | 0.0000e+00 | 1 |

Adduct:

No Chromatogram Found

### RN(N)WSKPQC(C)GITGFAPF Hex 5 HexNAc 4

**Mass:**  3487.4588

(-8.973e-06)

**Charge States:** 3

**Start Position**: 85

**End Position**: 101

| Profile Score | Tandem Score | TDA q-value | Volume | Spectrum Matches |
| --- | --- | --- | --- | --- |
| -3.9689 | 60.9086 | 0.0396 | 7.0243e+07 | 3 |

**Adduct Signal Fractions**

- **Unmodified**: 7.0243e+07

### GRTIN(N)ETSRL Fuc 3 Hex 7 HexNAc 7 Neu5Ac 2

**Mass:**  4721.8862

(-4.012e-06)

**Charge States:** 4, 5

**Start Position**: 363

**End Position**: 373

| Profile Score | Tandem Score | TDA q-value | Volume | Spectrum Matches |
| --- | --- | --- | --- | --- |
| -18.5771 | 60.0053 | 0.0396 | 2.5261e+07 | 2 |

**Adduct Signal Fractions**

- **Unmodified**: 2.5261e+07

### RN(N)WSKPQC(C)GITGFAPF Hex 6 HexNAc 4

**Mass:**  3649.5133

(-6.995e-06)

**Charge States:** 3

**Start Position**: 85

**End Position**: 101

| Profile Score | Tandem Score | TDA q-value | Volume | Spectrum Matches |
| --- | --- | --- | --- | --- |
| -2.2946 | 57.0508 | 0.0396 | 1.2658e+08 | 3 |

**Adduct Signal Fractions**

- **Unmodified**: 1.2658e+08

### iav|ACF41842|A/Puerto

**>iav|ACF41842|A/Puerto**  
 `MDVNPTLLFLKVPAQNAISTTFPYTGDPPYSHGTGTGYTMDTVNRTHQYSEKGRWTTNTETGAPQLNPIDGPLPEDNEPS  
GYAQTDCVLEAMAFLEESHPGIFENSCIETMEVVQQTRVDKLTQGRQTYDWTLNRNQPAATALANTIEVFRSNGLTANES  
GRLIDFLKDVMESMNKEEMGITTHFQRKRRVRDNMTKKMITQRTMGKKKQRLNKRSYLIRALTLNTMTKDAERGKLKRRA  
IATPGMQIRGFVYFVETLARSICEKLEQSGLPVGGNEKKAKLANVVRKMMTNSQDTELSFTITGDNTKWNENQNPRMFLA  
MITYMTRNQPEWFRNVLSIAPIMFSNKMARLGKGYMFESKSMKLRTQIPAEMLASIDLKYFNDSTRKKIEKIRPLLIEGT  
ASLSPGMMMGMFNMLSTVLGVSILNLGQKRYTKTTYWWDGLQSSDDFALIVNAPNHEGIQAGVDRFYRTCKLLGINMSKK  
KSYINRTGTFEFTSFFYRYGFVANFSMELPSFGVSGINESADMSIGVTVIKNNMINNDLGPATAQMALQLFIKDYRYTYR  
CHRGDTQIQTRRSFEIKKLWEQTRSKAGLLVSDGGPNLYNIRNLHIPEVCLKWELMDEDYQGRLCNPLNPFVSHKEIESM  
NNAVMMPAHGPAKNMEYDAVATTHSWIPKRNRSILNTSQRGVLEDEQMYQRCCNLFEKFFPSSSYRRPVGISSMVEAMVS  
RARIDARIDFESGRIKKEEFTEIMKICSTIEELRRQK`

| Site | Type | Count |
| --- | --- | --- |
| 476 | N-linked | 2 |

| Mass | Sequence | MS_2_ Score | `q`-value | MS_1_ Score | Signal | Protein Coordinates | Tandem Scans | Start Time | Apex Time | End Time |
| --- | --- | --- | --- | --- | --- | --- | --- | --- | --- | --- |
| 3977.6313 | GIN(N)MSKKKSY Fuc 1 Hex 9 HexNAc 6 | 69.707 | 0.040 | -21.612 | 3.549e+06 | 473 - 483 | 2 | 15.05 | 15.05 | 15.05 |
| 3978.6313 | GIN(N)MSKKKSY Fuc 1 Hex 9 HexNAc 6 | 50.566 | 0.049 | 0.000 | 0.000e+00 | 473 - 483 | 1 | - | - | - |

### GIN(N)MSKKKSY Fuc 1 Hex 9 HexNAc 6

**Mass:**  3977.6313

(2.528e-06)

**Charge States:** 4

**Start Position**: 474

**End Position**: 484

| Profile Score | Tandem Score | TDA q-value | Volume | Spectrum Matches |
| --- | --- | --- | --- | --- |
| -21.6119 | 69.7072 | 0.0396 | 3.5493e+06 | 2 |

**Adduct Signal Fractions**

- **Unmodified**: 3.5493e+06

### GIN(N)MSKKKSY Fuc 1 Hex 9 HexNAc 6

**Mass:**  3978.6313

(2.539e-04)

**Start Position**: 474

**End Position**: 484

| Profile Score | Tandem Score | TDA q-value | Volume | Spectrum Matches |
| --- | --- | --- | --- | --- |
| 0.0000 | 50.5663 | 0.0494 | 0.0000e+00 | 1 |

Adduct:

No Chromatogram Found

### iav|AGU93028|A/Puerto

**>iav|AGU93028|A/Puerto**  
 `MDVNPTLLFLKVPAQNAISTTFPYTGDPPYSHGTGTGYTMDTVNRTHQYSEKGRWTTNTETGAPQLNPIDGPLPEDNEPS  
GYAQTDCVLEAMAFLEESHPGIFENSCIETMEVVQQTRVDKLTQGRQTYDWTLNRNQPAATALANTIEVFRSNGLTANES  
GRLIDFLKDVMESMNKEEMGITTHFQRKRRVRDNMTKKMITQRTIGKKKQRLNKRSYLIRALTLNTMTKDAERGKLKRRA  
IATPGMQIRGFVYFVETLARSICEKLEQSGLPVGGNEKKAKLANVVRKMMTNSQDTELSFTITGDNTKWNENQNPRMFLA  
MITYMTRNQPEWFRNVLSIAPIMFSNKMARLGKGYMFESKSMKLRTQIPAEMLASIDLKYFNDSTRKKIEKIRPLLIEGT  
ASLSPGMMMGMFNMLSTVLGVSILNLGQKRYTKTTYWWDGLQSSDDFALIVNAPNHEGIQAGVDRFYRTCKLLGINMSKK  
KSYINRTGTFEFTSFFYRYGFVANFSMELPSFGVSGINESADMSIGVTVIKNNMINNDLGPATAQMALQLFIKDYRYTYR  
CHRGDTQIQTRRSFEIKKLWEQTRSKAGLLVSDGGPNLYNIRNLHIPEVCLKWELMDEDYQGRLCNPLNPFVSHKEIESM  
NNAVMMPAHGPAKNMEYDAVATTHSWIPKRNRSILNTSQRGVLEDEQMYQRCCNLFEKFFPSSSYRRPVGISSMVEAMVS  
RARIDARIDFESGRIKKEEFTEIMKICSTIEELRRQK`

| Site | Type | Count |
| --- | --- | --- |
| 476 | N-linked | 2 |

| Mass | Sequence | MS_2_ Score | `q`-value | MS_1_ Score | Signal | Protein Coordinates | Tandem Scans | Start Time | Apex Time | End Time |
| --- | --- | --- | --- | --- | --- | --- | --- | --- | --- | --- |
| 3977.6313 | GIN(N)MSKKKSY Fuc 1 Hex 9 HexNAc 6 | 69.707 | 0.040 | -21.612 | 3.549e+06 | 473 - 483 | 2 | 15.05 | 15.05 | 15.05 |
| 3978.6313 | GIN(N)MSKKKSY Fuc 1 Hex 9 HexNAc 6 | 50.566 | 0.049 | 0.000 | 0.000e+00 | 473 - 483 | 1 | - | - | - |

### GIN(N)MSKKKSY Fuc 1 Hex 9 HexNAc 6

**Mass:**  3977.6313

(2.528e-06)

**Charge States:** 4

**Start Position**: 474

**End Position**: 484

| Profile Score | Tandem Score | TDA q-value | Volume | Spectrum Matches |
| --- | --- | --- | --- | --- |
| -21.6119 | 69.7072 | 0.0396 | 3.5493e+06 | 2 |

**Adduct Signal Fractions**

- **Unmodified**: 3.5493e+06

### GIN(N)MSKKKSY Fuc 1 Hex 9 HexNAc 6

**Mass:**  3978.6313

(2.539e-04)

**Start Position**: 474

**End Position**: 484

| Profile Score | Tandem Score | TDA q-value | Volume | Spectrum Matches |
| --- | --- | --- | --- | --- |
| 0.0000 | 50.5663 | 0.0494 | 0.0000e+00 | 1 |

Adduct:

No Chromatogram Found

### iav|AFM71854|A/Puerto

**>iav|AFM71854|A/Puerto**  
 `MDVNPTLLFLKVPAQNAISTTFPYTGDPPYSHGTGTGYTMDTVNRTHQYSEKGRWTTNTETGAPQLNPIDGPLPEDNEPS  
GYAQTDCVLEAMAFLEESHPGIFENSCIETMEVVQQTRVDKLTQGRQTYDWTLNRNQPAATALANTIEVFRSNGLTANES  
GRLIDFLKDVMESMNKEEMGITTHFQRKRRVRDNMTKKMITQRTMGKKKQRLNKRSYLIRALTLNTMTKDAERGKLKRRA  
IATPGMQIRGFVYFVETLARSICEKLEQSGLPVGGNEKKAKLANVVRKMMTNSQDTELSFTITGDNTKWNENQNPRMFLA  
MITYMTRNQPEWFRNVLSIAPIMFSNKMARLGKGYMFESKSMKLRTQIPAEMLASIDLKYFNDSTRKKIEKIRPLLIEGT  
ASLSPGMMMGMFNMLSTVLGVSILNLGQKRYTKTTYWWDGLQSSDDFALIVNAPNHEGIQAGVDRFYRTCKLLGINMSKK  
KSYINRTGTFEFTSFFYRYGFVANFSMELPSFGVSGINESADMSIGVTVIKNNMINNDLGPATAQMALQLFIKDYRYTYR  
CHRGDTQIQTRRSFEIKKLWEQTRSKAGLLVSDGGPNLYNIRNLHIPEVCLKWELMDEDYQGRLCNPLNPFVSHKEIESM  
NNAVMMPAHGPAKNMEYDAVATTHSWIPKRNRSILNTSQRGVLEDEQMYQRCCNLFEKFFPSSSYRRPVGISSMVEAMVS  
RARIDARIDFESGRIKKEEFTEIMKICSTIEELRRQK`

| Site | Type | Count |
| --- | --- | --- |
| 476 | N-linked | 2 |

| Mass | Sequence | MS_2_ Score | `q`-value | MS_1_ Score | Signal | Protein Coordinates | Tandem Scans | Start Time | Apex Time | End Time |
| --- | --- | --- | --- | --- | --- | --- | --- | --- | --- | --- |
| 3977.6313 | GIN(N)MSKKKSY Fuc 1 Hex 9 HexNAc 6 | 69.707 | 0.040 | -21.612 | 3.549e+06 | 473 - 483 | 2 | 15.05 | 15.05 | 15.05 |
| 3978.6313 | GIN(N)MSKKKSY Fuc 1 Hex 9 HexNAc 6 | 50.566 | 0.049 | 0.000 | 0.000e+00 | 473 - 483 | 1 | - | - | - |

### GIN(N)MSKKKSY Fuc 1 Hex 9 HexNAc 6

**Mass:**  3977.6313

(2.528e-06)

**Charge States:** 4

**Start Position**: 474

**End Position**: 484

| Profile Score | Tandem Score | TDA q-value | Volume | Spectrum Matches |
| --- | --- | --- | --- | --- |
| -21.6119 | 69.7072 | 0.0396 | 3.5493e+06 | 2 |

**Adduct Signal Fractions**

- **Unmodified**: 3.5493e+06

### GIN(N)MSKKKSY Fuc 1 Hex 9 HexNAc 6

**Mass:**  3978.6313

(2.539e-04)

**Start Position**: 474

**End Position**: 484

| Profile Score | Tandem Score | TDA q-value | Volume | Spectrum Matches |
| --- | --- | --- | --- | --- |
| 0.0000 | 50.5663 | 0.0494 | 0.0000e+00 | 1 |

Adduct:

No Chromatogram Found

### iav|AAM75156|A/Puerto

**>iav|AAM75156|A/Puerto**  
 `MDVNPTLLFLKVPAQNAISTTFPYTGDPPYSHGTGTGYTMDTVNRTHQYSEKGRWTTNTETGAPQLNPIDGPLPEDNEPS  
GYAQTDCVLEAMAFLEESHPGIFENSCIETMEVVQQTRVDKLTQGRQTYDWTLNRNQPAATALANTIEVFRSNGLTANES  
GRLIDFLKDVMESMKKEEMGITTHFQRKRRVRDNMTKKMITQRTIGKKKQRLNKRSYLIRALTLNTMTKDAERGKLKRRA  
IATPGMQIRGFVYFVETLARSICEKLEQSGLPVGGNEKKAKLANVVRKMMTNSQDTELSFTITGDNTKWNENQNPRMFLA  
MITYMTRNQPEWFRNVLSIAPIMFSNKMARLGKGYMFESKSMKLRTQIPAEMLASIDLKYFNDSTRKKIEKIRPLLIEGT  
ASLSPGMMMGMFNMLSTVLGVSILNLGQKRYTKTTYWWDGLQSSDDFALIVNAPNHEGIQAGVDRFYRTCKLLGINMSKK  
KSYINRTGTFEFTSFFYRYGFVANFSMELPSFGVSGINESADMSIGVTVIKNNMINNDLGPATAQMALQLFIKDYRYTYR  
CHRGDTQIQTRRSFEIKKLWEQTRSKAGLLVSDGGPNLYNIRNLHIPEVCLKWELMDEDYQGRLCNPLNPFVSHKEIESM  
NNAVMMPAHGPAKNMEYDAVATTHSWIPKRNRSILNTSQRGVLEDEQMYQRCCNLFEKFFPSSSYRRPVGISSMVEAMVS  
RARIDARIDFESGRIKKEEFTEIMKICSTIEELRRQK`

| Site | Type | Count |
| --- | --- | --- |
| 476 | N-linked | 2 |

| Mass | Sequence | MS_2_ Score | `q`-value | MS_1_ Score | Signal | Protein Coordinates | Tandem Scans | Start Time | Apex Time | End Time |
| --- | --- | --- | --- | --- | --- | --- | --- | --- | --- | --- |
| 3977.6313 | GIN(N)MSKKKSY Fuc 1 Hex 9 HexNAc 6 | 69.707 | 0.040 | -21.612 | 3.549e+06 | 473 - 483 | 2 | 15.05 | 15.05 | 15.05 |
| 3978.6313 | GIN(N)MSKKKSY Fuc 1 Hex 9 HexNAc 6 | 50.566 | 0.049 | 0.000 | 0.000e+00 | 473 - 483 | 1 | - | - | - |

### GIN(N)MSKKKSY Fuc 1 Hex 9 HexNAc 6

**Mass:**  3977.6313

(2.528e-06)

**Charge States:** 4

**Start Position**: 474

**End Position**: 484

| Profile Score | Tandem Score | TDA q-value | Volume | Spectrum Matches |
| --- | --- | --- | --- | --- |
| -21.6119 | 69.7072 | 0.0396 | 3.5493e+06 | 2 |

**Adduct Signal Fractions**

- **Unmodified**: 3.5493e+06

### GIN(N)MSKKKSY Fuc 1 Hex 9 HexNAc 6

**Mass:**  3978.6313

(2.539e-04)

**Start Position**: 474

**End Position**: 484

| Profile Score | Tandem Score | TDA q-value | Volume | Spectrum Matches |
| --- | --- | --- | --- | --- |
| 0.0000 | 50.5663 | 0.0494 | 0.0000e+00 | 1 |

Adduct:

No Chromatogram Found

### iav|ADX99655|A/Puerto

**>iav|ADX99655|A/Puerto**  
 `MDVNPTLLFLKVPAQNAISTTFPYTGDPPYSHGTGTGYTMDTVNRTHQYSEKGRWTTNTETGAPQLNPIDGPLPEDNEPS  
GYAQTDCVLEAMAFLEESHPGIFENSCIETMEVVQQTRVDKLTQGRQTYDWTLNRNQPAATALANTIEVFRSNGLTANES  
GRLIDFLKDVMESMNKEEMGITTHFQRKRRVRDNMTKKMITQRTMGKKKQRLNKRSYLIRALTLNTMTKDAERGKLKRRA  
IATPGMQIRGFVYFVETLARSICEKLEQSGLPVGGNEKKAKLANVVRKMMTNSQDTELSFTITGDNTKWNENQNPRMFLA  
MITYMTRNQPEWFRNVLSIAPIMFSNKMARLGKGYMFESKSMKLRTQIPAEMLASIDLKYFNDSTRKKIEKIRPLLIEGT  
ASLSPGMMMGMFNMLSTVLGVSILNLGQKRYTKTTYWWDGLQSSDDFALIVNAPNHEGIQAGVDRFYRTCKLLGINMSKK  
KSYINRTGTFEFTSFFYRYGFVANFSMELPSFGVSGINESADMSIGVTVIKNNMINNDLGPATAQMALQLFIKDYRYTYR  
CHIGDTQIQTRRSFEIKKLWEQTRSKAGLLVSDGGPNLYNIRNLHIPEVCLKWELMDEDYQGRLCNPLNPFVSHKEIESM  
NNAVMMPAHGPAKNMEYDAVATTHSWIPKRNRSILNTSQRGVLEDEQMYQRCCNLFEKFFPSSSYRRPVGISSMVEAMVS  
RARIDARIDFESGRIKKEEFTEIMKICSTIEELRRQK`

| Site | Type | Count |
| --- | --- | --- |
| 476 | N-linked | 2 |

| Mass | Sequence | MS_2_ Score | `q`-value | MS_1_ Score | Signal | Protein Coordinates | Tandem Scans | Start Time | Apex Time | End Time |
| --- | --- | --- | --- | --- | --- | --- | --- | --- | --- | --- |
| 3977.6313 | GIN(N)MSKKKSY Fuc 1 Hex 9 HexNAc 6 | 69.707 | 0.040 | -21.612 | 3.549e+06 | 473 - 483 | 2 | 15.05 | 15.05 | 15.05 |
| 3978.6313 | GIN(N)MSKKKSY Fuc 1 Hex 9 HexNAc 6 | 50.566 | 0.049 | 0.000 | 0.000e+00 | 473 - 483 | 1 | - | - | - |

### GIN(N)MSKKKSY Fuc 1 Hex 9 HexNAc 6

**Mass:**  3977.6313

(2.528e-06)

**Charge States:** 4

**Start Position**: 474

**End Position**: 484

| Profile Score | Tandem Score | TDA q-value | Volume | Spectrum Matches |
| --- | --- | --- | --- | --- |
| -21.6119 | 69.7072 | 0.0396 | 3.5493e+06 | 2 |

**Adduct Signal Fractions**

- **Unmodified**: 3.5493e+06

### GIN(N)MSKKKSY Fuc 1 Hex 9 HexNAc 6

**Mass:**  3978.6313

(2.539e-04)

**Start Position**: 474

**End Position**: 484

| Profile Score | Tandem Score | TDA q-value | Volume | Spectrum Matches |
| --- | --- | --- | --- | --- |
| 0.0000 | 50.5663 | 0.0494 | 0.0000e+00 | 1 |

Adduct:

No Chromatogram Found

### iav|ACV49553|A/Puerto

**>iav|ACV49553|A/Puerto**  
 `MDVNPTLLFLKVPAQNAISTTFPYTGDPPYSHGTGTGYTMDTVNRTHQYSEKGRWTTNTETGAPQLNPIDGPLPEDNEPS  
GYAQTDCVLEAMAFLEESHPGIFENSCIETMEVVQQTRVDKLTQGRQTYDWTLNRNQPAATALANTIEVFRSNGLTANES  
GRLIDFLKDVMESMNKEEMGITTHFQRKRRVRDNMTKKMITQRTMGKKKQRLNKRSYLIRALTLNTMTKDAERGKLKRRA  
IATPGMQIRGFVYFVETLARSICEKLEQSGLPVGGNEKKAKLANVVRKMMTNSQDTELSFTITGDNTKWNENQNPRMFLA  
MITYMTRNQPEWFRNVLSIAPIMFSNKMARLGKGYMFESKSMKLRTQIPAEMLASIDLKYFNDSTRKKIEKIRPLLIEGT  
ASLSPGMMMGMFNMLSTVLGVSILNLGQKRYTKTTYWWDGLQSSDDFALIVNAPNHEGIQAGVDRFYRTCKLLGINMSKK  
KSYINRTGTFEFTSFFYRYGFVANFSMELPSFGVSGINESADMSIGVTVIKNNMINNDLGPATAQMALQLFIKDYRYTYR  
CHRGDTQIQTRRSFEIKKLWEQTRSKAGLLVSDGGPNLYNIRNLHIPEVCLKWELMDEDYQGRLCNPLNPFVSHKEIESM  
NNAVMMPAHGPAKNMEYDAVATTHSWIPKRNRSILNTSQRGVLEDEQMYQRCCNLFEKFFPSSSYRRPVGISSMVEAMVS  
RARIDARIDFESGRIKKEEFTEIMKICSTIEELRRQK`

| Site | Type | Count |
| --- | --- | --- |
| 476 | N-linked | 2 |

| Mass | Sequence | MS_2_ Score | `q`-value | MS_1_ Score | Signal | Protein Coordinates | Tandem Scans | Start Time | Apex Time | End Time |
| --- | --- | --- | --- | --- | --- | --- | --- | --- | --- | --- |
| 3977.6313 | GIN(N)MSKKKSY Fuc 1 Hex 9 HexNAc 6 | 69.707 | 0.040 | -21.612 | 3.549e+06 | 473 - 483 | 2 | 15.05 | 15.05 | 15.05 |
| 3978.6313 | GIN(N)MSKKKSY Fuc 1 Hex 9 HexNAc 6 | 50.566 | 0.049 | 0.000 | 0.000e+00 | 473 - 483 | 1 | - | - | - |

### GIN(N)MSKKKSY Fuc 1 Hex 9 HexNAc 6

**Mass:**  3977.6313

(2.528e-06)

**Charge States:** 4

**Start Position**: 474

**End Position**: 484

| Profile Score | Tandem Score | TDA q-value | Volume | Spectrum Matches |
| --- | --- | --- | --- | --- |
| -21.6119 | 69.7072 | 0.0396 | 3.5493e+06 | 2 |

**Adduct Signal Fractions**

- **Unmodified**: 3.5493e+06

### GIN(N)MSKKKSY Fuc 1 Hex 9 HexNAc 6

**Mass:**  3978.6313

(2.539e-04)

**Start Position**: 474

**End Position**: 484

| Profile Score | Tandem Score | TDA q-value | Volume | Spectrum Matches |
| --- | --- | --- | --- | --- |
| 0.0000 | 50.5663 | 0.0494 | 0.0000e+00 | 1 |

Adduct:

No Chromatogram Found

### iav|BAV59609|A/Puerto

**>iav|BAV59609|A/Puerto**  
 `MDVNPTLLFLKVPAQNAISTTFPYTGDPPYSHGTGTGYTMDTVNRTHQYSEKGRWTTNTETGASQLNPIDGPLPEDNEPS  
GYAQTDCVLEAMAFLEESHPGIFENSCIETIEVVQQTRVDKLTQGRQTYDWTLNRNQPAATALANTIEVFRSNGLTANES  
GRLIDFLKDVMESMNKEEMGITTHFQRKRRVRDNMTKKMITQRTMGKKKQRLNKRSYLIRALTLNTMTKDAERGKLKRRA  
IATPGMQIRGFVYFVETLARSICEKLEQSGLPVGGNEKKAKLANVVRKMMTNSQDTELSFTITGDNTKWNENQNPRMFLA  
MITYMTRNQPEWFRNVLSIAPIMFSNKMARLGKGYMFESKSMKLRTQIPAEMLASIDLKYFNDSTRKKIEKIRPLLIEGT  
ASLSPGMMMGMFNMLSTVLGVSILNLGQKRYTKTTYWWDGLQSSDDFALIVNAPNNEGIQAGVDRFYRTCKLLGINMSKK  
KSYINRTGTFEFTSFFYRYGFVANFSMELPSFGVSGINESADMSIGVTVIKNNMINNDLGPATAQMALQLFIKDYRYTYR  
CHRGDTQIQTRRSFEIKKLWEQTRSKAGLLVSDGGPNLYNIRNLHIPEVCLKWELMDEDYQGRLCNPLNPFVSHKEIESM  
NNAVMMPAHGPAKNMEYDAVATTHSWIPKRNRSILNTSQRGVLEDEQMYQRCCNLFEKFFPSSSYRRPVGISSMVEAMVS  
RARIDARIDFESGRIKKEEFTEIMKICSTIEELRRQK`

| Site | Type | Count |
| --- | --- | --- |
| 476 | N-linked | 2 |

| Mass | Sequence | MS_2_ Score | `q`-value | MS_1_ Score | Signal | Protein Coordinates | Tandem Scans | Start Time | Apex Time | End Time |
| --- | --- | --- | --- | --- | --- | --- | --- | --- | --- | --- |
| 3977.6313 | GIN(N)MSKKKSY Fuc 1 Hex 9 HexNAc 6 | 69.707 | 0.040 | -21.612 | 3.549e+06 | 473 - 483 | 2 | 15.05 | 15.05 | 15.05 |
| 3978.6313 | GIN(N)MSKKKSY Fuc 1 Hex 9 HexNAc 6 | 50.566 | 0.049 | 0.000 | 0.000e+00 | 473 - 483 | 1 | - | - | - |

### GIN(N)MSKKKSY Fuc 1 Hex 9 HexNAc 6

**Mass:**  3977.6313

(2.528e-06)

**Charge States:** 4

**Start Position**: 474

**End Position**: 484

| Profile Score | Tandem Score | TDA q-value | Volume | Spectrum Matches |
| --- | --- | --- | --- | --- |
| -21.6119 | 69.7072 | 0.0396 | 3.5493e+06 | 2 |

**Adduct Signal Fractions**

- **Unmodified**: 3.5493e+06

### GIN(N)MSKKKSY Fuc 1 Hex 9 HexNAc 6

**Mass:**  3978.6313

(2.539e-04)

**Start Position**: 474

**End Position**: 484

| Profile Score | Tandem Score | TDA q-value | Volume | Spectrum Matches |
| --- | --- | --- | --- | --- |
| 0.0000 | 50.5663 | 0.0494 | 0.0000e+00 | 1 |

Adduct:

No Chromatogram Found
