# Supplementary material for: Measuring Site-specific Glycosylation Similarity between Influenza a Virus Variants with Statistical Certainty
Source: Mol Cell Proteomics. 2020 Nov 25;19(9):1533–45. doi: 10.1074/mcp.RA120.002031 (PMC8143645; doi:10.1074/mcp.RA120.002031)
Supplement: Supplementary file 2 [file mmc2.zip › mmc2/159250_2_supp_555291_qcjklr.zip!/20190301_SWZeggL2_021119_chymo_HILIC_DDA_1ug_01_glycopeptides.html]

## 20190301\_SWZeggL2\_021119\_chymo\_HILIC\_DDA\_1ug\_01 @ GlycopeptideHypothesis-92f1f4322b084b56a04083a111b8ab77

**MS^1^ Mass Accuracy:** 10.0 PPM  
**Grouping Tolerance:** 15.0 PPM  
**MS^n^ Mass Accuracy:** 20.0 PPM  
**Minimum `q`-Value For Clustering:** 0.05

**Glycopeptide Hypothesis:**
GlycopeptideHypothesis-92f1f4322b084b56a04083a111b8ab77  
**Sample Searched:**
20190301\_SWZeggL2\_021119\_chymo\_HILIC\_DDA\_1ug\_01

| Protein | Matched Glycopeptides |
| --- | --- |
| cus\|SWZHA\|A/Switzerland/9715293/2013 | 103 |
| cus\|SWZHA\_5B8\|A/Switzerland/9715293/2013 | 83 |
| cus\|SWZNA\|A/Switzerland/9715293/2013 | 38 |
| tr\|A0A1D5PV37\|A0A1D5PV37\_CHICK | 2 |
| tr\|A0A1D5P780\|A0A1D5P780\_CHICK | 2 |
| sp\|P02845\|VIT2\_CHICK | 2 |
| tr\|F1NFL6\|F1NFL6\_CHICK | 2 |
| sp\|P09244\|TBB7\_CHICK | 1 |
| tr\|E1C8K3\|E1C8K3\_CHICK | 1 |
| tr\|F1NUU6\|F1NUU6\_CHICK | 1 |
| tr\|A0A1D5P474\|A0A1D5P474\_CHICK | 1 |
| tr\|E1BXM5\|E1BXM5\_CHICK | 1 |
| sp\|O42273\|TENP\_CHICK | 1 |
| tr\|A0A1L1RMA4\|A0A1L1RMA4\_CHICK | 1 |

### cus|SWZHA|A/Switzerland/9715293/2013

**>cus|SWZHA|A/Switzerland/9715293/2013**  
 `QKLPGNDNSTATLCLGHHAVPNGTIVKTITNDRIEVTNATELVQNSSIGEICDSPHQILDGENCTLIDALLGDPQCDGFQ  
NKKWDLFVERSKAYSNCYPYDVPDYASLRSLVASSGTLEFNNESFNWAGVTQNGTSSSCRRGSNSSFFSRLNWLTHLNSK  
YPALNVTMPNNEQFDKLYIWGVHHPVTDKDQIFLYAQSSGRITVSTKRSQQAVIPNIGYRPRIRDIPSRISIYWTIVKPG  
DILLINSTGNLIAPRGYFKIRSGKSSIMRSDAPIGKCKSECITPNGSIPNDKPFQNVNRITYGACPRYVKQSTLKLATGM  
RNVPERQTRGIFGAIAGFIENGWEGMVDGWYGFRHQNSEGRGQAADLKSTQAAIDQINGKLNRLIGKTNEKFHQIEKEFS  
EVEGRIQDLEKYVEDTKIDLWSYNAELLVALENQHTIDLTDSEMNKLFEKTKKQLRENAEDMGNGCFKIYHKCDNACIGS  
IRNGTYDHDVYRDEALNNRFQIKGVELKSGYKDWILWISFAISCFLLCVALLGFIMWACQKGNIRCNICI`

| Site | Type | Count |
| --- | --- | --- |
| 483 | N-linked | 5 |
| 133 | N-linked | 19 |
| 38 | N-linked | 5 |
| 8 | N-linked | 3 |
| 144 | N-linked | 8 |
| 246 | N-linked | 45 |
| 165 | N-linked | 13 |
| 285 | N-linked | 4 |
| 22 | N-linked | 1 |

| Mass | Sequence | MS_2_ Score | `q`-value | MS_1_ Score | Signal | Protein Coordinates | Tandem Scans | Start Time | Apex Time | End Time |
| --- | --- | --- | --- | --- | --- | --- | --- | --- | --- | --- |
| 2695.0875 | LIN(N)STGNL Hex 9 HexNAc 2 | 188.203 | 0.0 | -4.225 | 9.956e+07 | 243 - 251 | 2 | 26.20 | 26.36 | 26.53 |
| 3516.5422 | LIN(N)STGNLIAPRGYF Fuc 2 Hex 3 HexNAc 4 Neu5Ac 1 | 185.714 | 0.0 | -17.407 | 5.841e+07 | 243 - 258 | 1 | 46.76 | 46.76 | 46.92 |
| 3499.5148 | LIN(N)STGNLIAPRGYF Hex 9 HexNAc 2 | 181.072 | 0.0 | -1.872 | 3.809e+08 | 243 - 258 | 3 | 46.76 | 46.76 | 47.26 |
| 3352.4469 | LIN(N)STGNLIAPRGY Hex 9 HexNAc 2 | 173.965 | 0.0 | -17.289 | 3.163e+08 | 243 - 257 | 2 | 36.57 | 36.57 | 36.73 |
| 4068.9241 | TIVKPGDILLIN(N)STGNLIAPR Hex 9 HexNAc 2 | 173.328 | 0.0 | 17.938 | 1.904e+09 | 234 - 255 | 13 | 52.36 | 53.03 | 55.07 |
| 3028.2314 | RRGSN(N)SSFF Fuc 1 Hex 5 HexNAc 5 | 170.390 | 0.0 | -18.773 | 2.824e+07 | 139 - 148 | 1 | 20.79 | 20.95 | 20.95 |
| 2712.1148 | LIN(N)STGNL Fuc 2 Hex 3 HexNAc 4 Neu5Ac 1 | 168.174 | 0.0 | -19.546 | 1.336e+07 | 243 - 251 | 2 | 26.36 | 26.36 | 26.36 |
| 3008.1347 | NWAGVTQN(N)GTSSSC(C) Hex 7 HexNAc 2 | 167.542 | 0.0 | -3.240 | 2.898e+07 | 125 - 139 | 1 | 22.91 | 22.91 | 23.23 |
| 3337.4655 | LIN(N)STGNLIAPRGYF Hex 8 HexNAc 2 | 166.761 | 0.0 | -4.432 | 2.153e+07 | 243 - 258 | 1 | 46.76 | 46.92 | 47.09 |
| 2317.9572 | LPGNDN(N)STATL Hex 5 HexNAc 2 | 164.872 | 0.0 | -5.715 | 1.752e+07 | 2 - 13 | 2 | 26.36 | 26.53 | 26.86 |
| 3906.8734 | TIVKPGDILLIN(N)STGNLIAPR Hex 8 HexNAc 2 | 163.121 | 0.0 | 14.116 | 1.129e+08 | 234 - 255 | 5 | 53.03 | 53.19 | 53.71 |
| 3369.4723 | LIN(N)STGNLIAPRGY Fuc 2 Hex 3 HexNAc 4 Neu5Ac 1 | 163.101 | 0.0 | -17.049 | 5.398e+07 | 243 - 257 | 1 | 36.57 | 36.57 | 36.73 |
| 4289.0130 | TIVKPGDILLIN(N)STGNLIAPRGY Hex 9 HexNAc 2 | 162.338 | 0.0 | 18.525 | 3.507e+09 | 234 - 257 | 25 | 53.53 | 55.41 | 58.53 |
| 3422.4070 | AGVTQN(N)GTSSSC(C)RRGSNSSFF Hex 5 HexNAc 2 | 162.329 | 0.0 | 15.564 | 4.221e+08 | 127 - 148 | 5 | 24.88 | 25.54 | 25.71 |
| 4451.0798 | TIVKPGDILLIN(N)STGNLIAPRGY Hex 10 HexNAc 2 | 161.776 | 0.0 | 15.376 | 3.318e+08 | 234 - 257 | 13 | 55.07 | 55.24 | 62.13 |
| 4230.9752 | TIVKPGDILLIN(N)STGNLIAPR Hex 10 HexNAc 2 | 160.882 | 0.0 | -3.696 | 1.727e+08 | 234 - 255 | 5 | 52.86 | 53.03 | 53.36 |
| 3386.4296 | IN(N)STGNLIAPRGYF Hex 9 HexNAc 2 | 160.140 | 0.0 | -19.202 | 1.872e+07 | 244 - 258 | 2 | 42.94 | 43.11 | 43.11 |
| 4365.8334 | NSKYPALN(N)VTM(O)PNNEQFDKLY Hex 9 HexNAc 2 | 159.848 | 0.0 | -2.615 | 2.962e+08 | 157 - 178 | 3 | 42.27 | 42.61 | 42.61 |
| 4126.9563 | TIVKPGDILLIN(N)STGNLIAPRGY Hex 8 HexNAc 2 | 159.608 | 0.0 | 14.918 | 3.372e+08 | 234 - 257 | 12 | 54.73 | 55.41 | 56.60 |
| 4089.7377 | SKYPALN(N)VTM(O)PNNEQFDKLY Hex 8 HexNAc 2 | 159.230 | 0.0 | -2.186 | 8.227e+07 | 158 - 178 | 4 | 42.44 | 42.61 | 42.78 |
| 3159.4237 | TIVKPGDILLIN(N) Hex 9 HexNAc 2 | 158.298 | 0.0 | 10.967 | 2.657e+08 | 234 - 246 | 6 | 45.80 | 47.26 | 47.76 |
| 4085.9497 | TIVKPGDILLIN(N)STGNLIAPR Fuc 2 Hex 3 HexNAc 4 Neu5Ac 1 | 156.807 | 0.0 | 14.075 | 1.298e+08 | 234 - 255 | 2 | 52.86 | 53.03 | 53.71 |
| 3584.4612 | AGVTQN(N)GTSSSC(C)RRGSNSSFF Hex 6 HexNAc 2 | 156.181 | 0.0 | 15.501 | 2.511e+08 | 127 - 148 | 2 | 25.04 | 25.21 | 25.71 |
| 3164.2351 | NWAGVTQN(N)GTSSSC(C)R Hex 7 HexNAc 2 | 155.744 | 0.0 | -17.526 | 1.843e+07 | 125 - 140 | 2 | 19.82 | 19.82 | 19.82 |
| 3514.5047 | LIN(N)STGNLIAPRGY Hex 10 HexNAc 2 | 155.700 | 0.0 | -3.637 | 3.657e+07 | 243 - 257 | 2 | 36.40 | 36.57 | 36.73 |
| 3746.5129 | AGVTQN(N)GTSSSC(C)RRGSNSSFF Hex 7 HexNAc 2 | 154.926 | 0.0 | 17.457 | 7.553e+08 | 127 - 148 | 4 | 24.55 | 25.04 | 25.54 |
| 4203.7809 | NSKYPALN(N)VTM(O)PNNEQFDKLY Hex 8 HexNAc 2 | 153.972 | 0.0 | 17.557 | 2.875e+08 | 157 - 178 | 4 | 42.10 | 42.61 | 42.78 |
| 3176.4461 | TIVKPGDILLIN(N) Fuc 2 Hex 3 HexNAc 4 Neu5Ac 1 | 153.335 | 0.0 | 11.290 | 4.865e+07 | 234 - 246 | 1 | 46.76 | 47.26 | 47.60 |
| 3270.4171 | TITNDRIEVTN(N)ATEL Hex 6 HexNAc 3 | 152.826 | 0.0 | -19.450 | 2.563e+07 | 27 - 42 | 1 | 36.40 | 36.57 | 36.57 |
| 3631.6501 | TIVKPGDILLIN(N)STGNL Hex 9 HexNAc 2 | 151.647 | 0.0 | 17.601 | 1.079e+09 | 234 - 251 | 10 | 52.01 | 52.70 | 54.73 |
| 3321.4759 | TIVKPGDILLIN(N) Hex 10 HexNAc 2 | 150.837 | 0.0 | -5.115 | 1.713e+07 | 234 - 246 | 1 | 46.76 | 47.09 | 47.26 |
| 4251.7897 | SKYPALN(N)VTM(O)PNNEQFDKLY Hex 9 HexNAc 2 | 150.524 | 0.0 | -2.386 | 8.857e+07 | 158 - 178 | 3 | 42.44 | 42.61 | 42.78 |
| 2848.1046 | NWAGVTQN(N)GTSSS Hex 7 HexNAc 2 | 150.361 | 0.0 | -19.325 | 9.657e+06 | 125 - 138 | 1 | 22.26 | 22.26 | 22.26 |
| 3518.5723 | TIVKPGDILLIN(N)STGN Hex 9 HexNAc 2 | 149.931 | 0.0 | 16.511 | 3.389e+08 | 234 - 250 | 5 | 44.78 | 45.12 | 45.63 |
| 4306.0360 | TIVKPGDILLIN(N)STGNLIAPRGY Fuc 2 Hex 3 HexNAc 4 Neu5Ac 1 | 149.913 | 0.0 | 14.572 | 2.523e+08 | 234 - 257 | 5 | 55.07 | 55.41 | 56.08 |
| 3680.6248 | TIVKPGDILLIN(N)STGN Hex 10 HexNAc 2 | 149.110 | 0.0 | -4.351 | 2.298e+07 | 234 - 250 | 1 | 44.78 | 44.95 | 45.12 |
| 3535.5982 | TIVKPGDILLIN(N)STGN Fuc 2 Hex 3 HexNAc 4 Neu5Ac 1 | 148.221 | 0.0 | -4.110 | 6.518e+07 | 234 - 250 | 1 | 44.95 | 45.12 | 45.29 |
| 3793.7038 | TIVKPGDILLIN(N)STGNL Hex 10 HexNAc 2 | 147.600 | 0.0 | -3.362 | 9.465e+07 | 234 - 251 | 3 | 52.36 | 52.53 | 52.86 |
| 2272.9358 | RRGSN(N)SSFF Hex 5 HexNAc 2 | 147.597 | 0.0 | -19.560 | 1.395e+07 | 139 - 148 | 1 | 20.95 | 20.95 | 21.10 |
| 2825.1525 | RRGSN(N)SSFF Fuc 1 Hex 5 HexNAc 4 | 144.840 | 0.0 | -19.079 | 1.603e+07 | 139 - 148 | 1 | 20.63 | 20.63 | 20.79 |
| 3633.4727 | HKC(C)DNAC(C)IGSIRN(N)GTY Fuc 1 Hex 5 HexNAc 4 | 144.456 | 0.0 | -21.056 | 8.830e+06 | 470 - 486 | 1 | 20.31 | 20.31 | 20.47 |
| 4046.6358 | NWAGVTQN(N)GTSSSC(C)RRGSNSSFF Hex 7 HexNAc 2 | 144.043 | 0.0 | -2.667 | 3.970e+08 | 125 - 148 | 4 | 35.10 | 35.27 | 35.59 |
| 4752.0333 | RSDAPIGKC(C)KSEC(C)ITPN(N)GSIPNDKPF Hex 9 HexNAc 2 | 144.021 | 0.0 | -2.829 | 2.985e+08 | 268 - 294 | 2 | 29.67 | 29.67 | 30.00 |
| 3469.5969 | TIVKPGDILLIN(N)STGNL Hex 8 HexNAc 2 | 142.831 | 0.0 | -3.816 | 8.483e+07 | 234 - 251 | 2 | 52.53 | 52.70 | 53.03 |
| 3648.6761 | TIVKPGDILLIN(N)STGNL Fuc 2 Hex 3 HexNAc 4 Neu5Ac 1 | 142.762 | 0.0 | -3.973 | 1.893e+08 | 234 - 251 | 2 | 52.53 | 52.70 | 53.03 |
| 4589.9783 | RSDAPIGKC(C)KSEC(C)ITPN(N)GSIPNDKPF Hex 8 HexNAc 2 | 142.363 | 0.0 | -3.998 | 1.317e+08 | 268 - 294 | 2 | 29.67 | 29.83 | 30.00 |
| 2905.2883 | TITNDRIEVTN(N)ATEL Hex 5 HexNAc 2 | 142.038 | 0.0 | -17.514 | 3.671e+07 | 27 - 42 | 1 | 36.73 | 36.73 | 36.90 |
| 3640.7994 | TIVKPGDILLIN(N)STGNLIAPRGY Hex 5 HexNAc 2 | 138.884 | 0.007 | 16.119 | 4.182e+07 | 234 - 257 | 4 | 55.91 | 56.08 | 56.60 |
| 3420.7150 | TIVKPGDILLIN(N)STGNLIAPR Hex 5 HexNAc 2 | 138.747 | 0.007 | -3.531 | 2.283e+07 | 234 - 255 | 3 | 53.71 | 53.88 | 54.05 |
| 3207.2892 | SIRN(N)GTYDHDVY Fuc 1 Hex 5 HexNAc 4 | 136.180 | 0.007 | -19.038 | 1.472e+07 | 479 - 491 | 2 | 19.16 | 19.16 | 19.33 |
| 3473.5031 | C(C)LGHHAVPN(N)GTIVK Fuc 1 Hex 5 HexNAc 5 | 135.025 | 0.007 | -18.683 | 3.839e+07 | 13 - 27 | 1 | 18.51 | 18.67 | 18.67 |
| 2999.3800 | TIVKPGDILLIN(N) Hex 8 HexNAc 2 | 134.763 | 0.007 | 0.000 | 0.000e+00 | 234 - 246 | 1 | - | - | - |
| 3108.3679 | TITNDRIEVTN(N)ATEL Hex 5 HexNAc 3 | 134.744 | 0.007 | -17.172 | 1.281e+07 | 27 - 42 | 1 | 36.73 | 36.73 | 36.90 |
| 4427.9258 | RSDAPIGKC(C)KSEC(C)ITPN(N)GSIPNDKPF Hex 7 HexNAc 2 | 134.730 | 0.007 | -3.910 | 1.628e+08 | 268 - 294 | 3 | 29.67 | 29.83 | 30.00 |
| 3229.3892 | TITNDRIEVTN(N)ATEL Hex 7 HexNAc 2 | 134.644 | 0.007 | -17.441 | 4.228e+07 | 27 - 42 | 1 | 36.40 | 36.40 | 36.57 |
| 4202.7651 | NSKYPALN(N)VTM(O)PNNEQFDKL Hex 9 HexNAc 2 | 134.232 | 0.007 | -17.247 | 2.976e+07 | 157 - 177 | 2 | 40.09 | 40.25 | 40.25 |
| 3964.9076 | TIVKPGDILLIN(N)STGNLIAPRGY Hex 7 HexNAc 2 | 131.655 | 0.007 | -3.842 | 1.440e+07 | 234 - 257 | 3 | 55.57 | 55.57 | 55.91 |
| 3722.5275 | NWAGVTQN(N)GTSSSC(C)RRGSNSSFF Hex 5 HexNAc 2 | 131.520 | 0.007 | -4.011 | 2.438e+08 | 125 - 148 | 4 | 35.59 | 35.75 | 36.07 |
| 3190.3990 | LIN(N)STGNLIAPRGY Hex 8 HexNAc 2 | 130.926 | 0.007 | -18.313 | 1.482e+07 | 243 - 257 | 1 | 36.73 | 36.73 | 36.90 |
| 2481.9654 | RGSN(N)SSFF Hex 6 HexNAc 3 | 130.596 | 0.0 | -20.107 | 6.142e+06 | 140 - 148 | 1 | 25.54 | 25.54 | 25.54 |
| 3884.5829 | NWAGVTQN(N)GTSSSC(C)RRGSNSSFF Hex 6 HexNAc 2 | 130.083 | 0.007 | -17.729 | 1.160e+08 | 125 - 148 | 2 | 35.43 | 35.59 | 35.59 |
| 2983.4399 | TIVKPGDILLIN(N)STGNL Hex 5 HexNAc 2 | 129.599 | 0.007 | -3.562 | 1.183e+07 | 234 - 251 | 1 | 53.36 | 53.53 | 53.71 |
| 3810.7244 | TIVKPGDILLIN(N)STGNL Fuc 2 Hex 4 HexNAc 4 Neu5Ac 1 | 126.829 | 0.011 | -18.901 | 1.669e+07 | 234 - 251 | 1 | 52.53 | 52.53 | 52.70 |
| 4187.7824 | NSKYPALN(N)VTMPNNEQFDKLY Hex 8 HexNAc 2 | 124.568 | 0.011 | -18.353 | 1.035e+07 | 157 - 178 | 4 | 48.61 | 48.61 | 48.78 |
| 3787.5415 | AGVTQN(N)GTSSSC(C)RRGSNSSFF Hex 6 HexNAc 3 | 124.025 | 0.011 | -4.238 | 1.417e+08 | 127 - 148 | 3 | 25.04 | 25.21 | 25.54 |
| 3747.4976 | AGVTQN(N)GTSSSC(C)RRGSN(D)SSFF Hex 7 HexNAc 2 | 121.850 | 0.011 | 10.045 | 3.135e+08 | 127 - 148 | 4 | 26.04 | 26.20 | 27.20 |
| 4069.9188 | TIVKPGDILLIN(N)STGN(D)LIAPR Hex 9 HexNAc 2 | 120.835 | 0.011 | -5.379 | 1.745e+07 | 234 - 255 | 3 | 54.05 | 54.05 | 54.39 |
| 3949.5941 | AGVTQN(N)GTSSSC(C)RRGSNSSFF Hex 7 HexNAc 3 | 119.422 | 0.011 | -3.771 | 7.654e+07 | 127 - 148 | 2 | 24.88 | 24.88 | 25.21 |
| 2638.0658 | RRGSN(N)SSFF Hex 6 HexNAc 3 | 118.895 | 0.0 | -16.069 | 9.254e+06 | 139 - 148 | 1 | 20.63 | 20.63 | 20.63 |
| 4290.0001 | TIVKPGDILLIN(N)STGN(D)LIAPRGY Hex 9 HexNAc 2 | 113.951 | 0.011 | -19.754 | 3.288e+07 | 234 - 257 | 21 | 56.26 | 56.43 | 56.43 |
| 2608.1092 | KLPGNDN(N)STATL Hex 6 HexNAc 2 | 112.936 | 0.011 | -19.876 | 3.109e+06 | 1 - 13 | 1 | 22.42 | 22.42 | 22.42 |
| 3308.5315 | TIVKPGDILLIN(N)STGNL Hex 7 HexNAc 2 | 112.032 | 0.011 | 0.000 | 0.000e+00 | 234 - 251 | 1 | - | - | - |
| 4248.0021 | TIVKPGDILLIN(N)STGNLIAPR Fuc 2 Hex 4 HexNAc 4 Neu5Ac 1 | 111.075 | 0.011 | -17.749 | 1.104e+07 | 234 - 255 | 1 | 53.03 | 53.03 | 53.03 |
| 3347.4979 | TIVKPGDILLIN(N)ST Hex 9 HexNAc 2 | 110.709 | 0.011 | -19.647 | 1.147e+07 | 234 - 248 | 1 | 46.44 | 46.60 | 46.60 |
| 3423.3938 | AGVTQN(N)GTSSSC(C)RRGSN(D)SSFF Hex 5 HexNAc 2 | 106.287 | 0.011 | -5.998 | 1.038e+08 | 127 - 148 | 3 | 26.53 | 26.70 | 27.03 |
| 4627.8565 | HKC(C)DNAC(C)IGSIRN(N)GTYDHDVY Fuc 1 Hex 6 HexNAc 5 | 106.227 | 0.011 | -21.686 | 5.908e+06 | 470 - 491 | 1 | 24.71 | 24.88 | 24.88 |
| 3661.5679 | LIN(N)STGNLIAPRGYF Fuc 1 Hex 3 HexNAc 4 Neu5Ac 2 | 103.593 | 0.011 | -18.372 | 3.660e+07 | 243 - 258 | 1 | 46.76 | 46.76 | 46.92 |
| 4204.7609 | N(D)SKYPALN(N)VTM(O)PNNEQFDKLY Hex 8 HexNAc 2 | 102.446 | 0.011 | -6.185 | 1.070e+08 | 157 - 178 | 3 | 45.29 | 45.46 | 45.63 |
| 2744.1658 | QKLPGNDN(N)STATL Hex 3 HexNAc 3 Neu5Ac 1 | 97.550 | 0.017 | 14.850 | 3.818e+08 | 0 - 13 | 4 | 31.31 | 31.63 | 32.46 |
| 4366.8141 | N(D)SKYPALN(N)VTM(O)PNNEQFDKLY Hex 9 HexNAc 2 | 93.995 | 0.027 | -6.107 | 1.224e+08 | 157 - 178 | 2 | 45.12 | 45.29 | 45.46 |
| 3967.9337 | TIVKPGDILLIN(N)STGNLIAPRGY Hex 7 HexNAc 2 | 93.247 | 0.027 | 0.000 | 0.000e+00 | 234 - 257 | 1 | - | - | - |
| 3763.5549 | NWAGVTQN(N)GTSSSC(C)RRGSNSSFF Hex 4 HexNAc 3 | 92.346 | 0.027 | -19.756 | 5.098e+07 | 125 - 148 | 2 | 35.75 | 35.75 | 35.92 |
| 2682.0463 | SC(C)RRGSN(N)SSFF Hex 6 HexNAc 2 | 91.422 | 0.032 | -21.216 | 3.361e+06 | 137 - 148 | 1 | 20.47 | 20.63 | 20.63 |
| 3500.5018 | LIN(N)STGN(D)LIAPRGYF Hex 9 HexNAc 2 | 89.061 | 0.032 | -5.874 | 9.255e+06 | 243 - 258 | 1 | 48.10 | 48.44 | 48.44 |
| 3377.3926 | IGSIRN(N)GTYDHDVY Fuc 1 Hex 5 HexNAc 4 | 88.272 | 0.032 | -18.318 | 7.015e+06 | 477 - 491 | 1 | 24.22 | 24.22 | 24.22 |
| 4753.0327 | RSDAPIGKC(C)KSEC(C)ITPN(N)GSIPN(D)DKPF Hex 9 HexNAc 2 | 83.602 | 0.036 | -5.934 | 3.409e+07 | 268 - 294 | 1 | 30.33 | 30.50 | 30.67 |
| 4342.6783 | HKC(C)DNAC(C)IGSIRN(N)GTYDHDVY Fuc 1 Hex 8 HexNAc 2 | 79.826 | 0.039 | -19.756 | 6.636e+06 | 470 - 491 | 1 | 26.86 | 27.03 | 27.03 |
| 4040.7128 | NSKYPALN(N)VTM(O)PNNEQFDKL Hex 8 HexNAc 2 | 79.133 | 0.039 | -18.788 | 2.460e+07 | 157 - 177 | 2 | 40.25 | 40.25 | 40.42 |
| 4187.9667 | IVKPGDILLIN(N)STGNLIAPRGY Hex 9 HexNAc 2 | 78.911 | 0.039 | -18.919 | 4.835e+06 | 235 - 257 | 1 | 54.73 | 54.90 | 54.90 |
| 3625.4884 | AGVTQN(N)GTSSSC(C)RRGSNSSFF Hex 5 HexNAc 3 | 74.957 | 0.039 | -19.478 | 2.167e+07 | 127 - 148 | 1 | 25.21 | 25.38 | 25.38 |
| 4153.8027 | LNSKYPALN(N)VTM(O)PNNEQFDKL Hex 8 HexNAc 2 | 74.549 | 0.039 | -17.099 | 1.494e+07 | 156 - 177 | 2 | 41.76 | 41.76 | 41.93 |
| 3582.7713 | TIVKPGDILLIN(N)STGNLIAPR Hex 6 HexNAc 2 | 73.026 | 0.039 | -19.396 | 3.749e+06 | 234 - 255 | 1 | 53.53 | 53.53 | 53.71 |
| 3802.8541 | TIVKPGDILLIN(N)STGNLIAPRGY Hex 6 HexNAc 2 | 72.091 | 0.042 | -18.276 | 1.080e+07 | 234 - 257 | 4 | 55.74 | 55.91 | 55.91 |
| 4315.8514 | LNSKYPALN(N)VTM(O)PNNEQFDKL Hex 9 HexNAc 2 | 70.444 | 0.042 | -15.958 | 1.544e+07 | 156 - 177 | 1 | 41.76 | 41.76 | 41.76 |
| 3098.3008 | AGVTQN(N)GTSSSC(C)RRGSNSSFF Hex 3 HexNAc 2 | 69.678 | 0.042 | -18.750 | 1.840e+07 | 127 - 148 | 2 | 25.88 | 26.04 | 26.04 |
| 3828.5677 | AGVTQN(N)GTSSSC(C)RRGSNSSFF Hex 5 HexNAc 4 | 69.677 | 0.045 | 16.377 | 1.617e+08 | 127 - 148 | 4 | 25.04 | 25.54 | 25.71 |
| 4047.6222 | NWAGVTQN(N)GTSSSC(C)RRGSN(D)SSFF Hex 7 HexNAc 2 | 65.892 | 0.042 | 10.649 | 1.418e+08 | 125 - 148 | 3 | 36.40 | 36.57 | 37.41 |
| 4349.8368 | NSKYPALN(N)VTMPNNEQFDKLY Hex 9 HexNAc 2 | 63.435 | 0.045 | -4.694 | 1.099e+07 | 157 - 178 | 3 | 48.44 | 48.61 | 48.78 |
| 4041.7307 | NSKYPALN(N)VTM(O)PNNEQFDKLY Hex 7 HexNAc 2 | 62.532 | 0.045 | -4.208 | 1.896e+07 | 157 - 178 | 1 | 42.44 | 42.78 | 42.78 |
| 3275.2982 | SC(C)RRGSN(N)SSFF Fuc 1 Hex 5 HexNAc 5 | 56.542 | 0.045 | -20.178 | 1.452e+07 | 137 - 148 | 1 | 20.79 | 20.79 | 20.79 |
| 3311.4438 | TITNDRIEVTN(N)ATEL Hex 5 HexNAc 4 | 56.325 | 0.045 | -20.043 | 1.594e+07 | 27 - 42 | 1 | 36.40 | 36.40 | 36.40 |
| 4128.6899 | NWAGVTQN(N)GTSSSC(C)RRGSNSSFF Hex 5 HexNAc 4 | 56.093 | 0.045 | -4.936 | 8.837e+07 | 125 - 148 | 2 | 35.10 | 35.75 | 35.75 |
| 4290.7458 | NWAGVTQNGTSSSC(C)RRGSN(N)SSFF Hex 6 HexNAc 4 | 54.153 | 0.045 | -4.809 | 4.101e+07 | 125 - 148 | 2 | 35.27 | 35.43 | 35.59 |

### LIN(N)STGNL Hex 9 HexNAc 2

**Mass:**  2695.0874

(1.548e-06)

**Charge States:** 2, 3

**Start Position**: 244

**End Position**: 252

| Profile Score | Tandem Score | TDA q-value | Volume | Spectrum Matches |
| --- | --- | --- | --- | --- |
| -4.2249 | 188.2026 | 0.0000 | 9.9560e+07 | 2 |

**Adduct Signal Fractions**

- **Unmodified**: 9.9560e+07

### LIN(N)STGNLIAPRGYF Fuc 2 Hex 3 HexNAc 4 Neu5Ac 1

**Mass:**  3516.5429

(-6.365e-06)

**Charge States:** 3

**Start Position**: 244

**End Position**: 259

| Profile Score | Tandem Score | TDA q-value | Volume | Spectrum Matches |
| --- | --- | --- | --- | --- |
| -17.4071 | 185.7137 | 0.0000 | 5.8415e+07 | 1 |

**Adduct Signal Fractions**

- **Unmodified**: 5.8415e+07

### LIN(N)STGNLIAPRGYF Hex 9 HexNAc 2

**Mass:**  3499.5152

(7.985e-07)

**Charge States:** 3, 4

**Start Position**: 244

**End Position**: 259

| Profile Score | Tandem Score | TDA q-value | Volume | Spectrum Matches |
| --- | --- | --- | --- | --- |
| -1.8723 | 181.0717 | 0.0000 | 3.8090e+08 | 3 |

**Adduct Signal Fractions**

- **Unmodified**: 3.8090e+08

### LIN(N)STGNLIAPRGY Hex 9 HexNAc 2

**Mass:**  3352.4459

(6.359e-07)

**Charge States:** 2, 3, 4

**Start Position**: 244

**End Position**: 258

| Profile Score | Tandem Score | TDA q-value | Volume | Spectrum Matches |
| --- | --- | --- | --- | --- |
| -17.2892 | 173.9650 | 0.0000 | 3.1634e+08 | 2 |

**Adduct Signal Fractions**

- **Unmodified**: 3.1634e+08

### TIVKPGDILLIN(N)STGNLIAPR Hex 9 HexNAc 2

**Mass:**  4068.9238

(1.048e-07)

**Charge States:** 3, 4, 5

**Start Position**: 235

**End Position**: 256

| Profile Score | Tandem Score | TDA q-value | Volume | Spectrum Matches |
| --- | --- | --- | --- | --- |
| 17.9383 | 173.3284 | 0.0000 | 1.9044e+09 | 13 |

**Adduct Signal Fractions**

- **Unmodified**: 1.9044e+09

### RRGSN(N)SSFF Fuc 1 Hex 5 HexNAc 5

**Mass:**  3028.2330

(2.062e-07)

**Charge States:** 3

**Start Position**: 140

**End Position**: 149

| Profile Score | Tandem Score | TDA q-value | Volume | Spectrum Matches |
| --- | --- | --- | --- | --- |
| -18.7725 | 170.3896 | 0.0000 | 2.8238e+07 | 1 |

**Adduct Signal Fractions**

- **Unmodified**: 2.8238e+07

### LIN(N)STGNL Fuc 2 Hex 3 HexNAc 4 Neu5Ac 1

**Mass:**  2712.1157

(-7.830e-06)

**Charge States:** 2, 3

**Start Position**: 244

**End Position**: 252

| Profile Score | Tandem Score | TDA q-value | Volume | Spectrum Matches |
| --- | --- | --- | --- | --- |
| -19.5459 | 168.1737 | 0.0000 | 1.3363e+07 | 2 |

**Adduct Signal Fractions**

- **Unmodified**: 1.3363e+07

### NWAGVTQN(N)GTSSSC(C) Hex 7 HexNAc 2

**Mass:**  3008.1326

(-2.342e-07)

**Charge States:** 3

**Start Position**: 126

**End Position**: 140

| Profile Score | Tandem Score | TDA q-value | Volume | Spectrum Matches |
| --- | --- | --- | --- | --- |
| -3.2397 | 167.5423 | 0.0000 | 2.8976e+07 | 1 |

**Adduct Signal Fractions**

- **Unmodified**: 2.8976e+07

### LIN(N)STGNLIAPRGYF Hex 8 HexNAc 2

**Mass:**  3337.4624

(9.049e-07)

**Charge States:** 3

**Start Position**: 244

**End Position**: 259

| Profile Score | Tandem Score | TDA q-value | Volume | Spectrum Matches |
| --- | --- | --- | --- | --- |
| -4.4315 | 166.7611 | 0.0000 | 2.1533e+07 | 1 |

**Adduct Signal Fractions**

- **Unmodified**: 2.1533e+07

### LPGNDN(N)STATL Hex 5 HexNAc 2

**Mass:**  2317.9596

(9.050e-07)

**Charge States:** 3

**Start Position**: 3

**End Position**: 14

| Profile Score | Tandem Score | TDA q-value | Volume | Spectrum Matches |
| --- | --- | --- | --- | --- |
| -5.7155 | 164.8724 | 0.0000 | 1.7519e+07 | 2 |

**Adduct Signal Fractions**

- **Unmodified**: 1.7519e+07

### TIVKPGDILLIN(N)STGNLIAPR Hex 8 HexNAc 2

**Mass:**  3906.8737

(1.534e-07)

**Charge States:** 3, 4

**Start Position**: 235

**End Position**: 256

| Profile Score | Tandem Score | TDA q-value | Volume | Spectrum Matches |
| --- | --- | --- | --- | --- |
| 14.1164 | 163.1211 | 0.0000 | 1.1291e+08 | 5 |

**Adduct Signal Fractions**

- **Unmodified**: 1.1291e+08

### LIN(N)STGNLIAPRGY Fuc 2 Hex 3 HexNAc 4 Neu5Ac 1

**Mass:**  3369.4722

(-7.269e-06)

**Charge States:** 3

**Start Position**: 244

**End Position**: 258

| Profile Score | Tandem Score | TDA q-value | Volume | Spectrum Matches |
| --- | --- | --- | --- | --- |
| -17.0488 | 163.1008 | 0.0000 | 5.3977e+07 | 1 |

**Adduct Signal Fractions**

- **Unmodified**: 5.3977e+07

### TIVKPGDILLIN(N)STGNLIAPRGY Hex 9 HexNAc 2

**Mass:**  4289.0124

(9.225e-07)

**Charge States:** 3, 4, 5

**Start Position**: 235

**End Position**: 258

| Profile Score | Tandem Score | TDA q-value | Volume | Spectrum Matches |
| --- | --- | --- | --- | --- |
| 18.5248 | 162.3376 | 0.0000 | 3.5071e+09 | 25 |

**Adduct Signal Fractions**

- **Unmodified**: 3.5071e+09

### AGVTQN(N)GTSSSC(C)RRGSNSSFF Hex 5 HexNAc 2

**Mass:**  3422.4061

(7.889e-07)

**Charge States:** 3, 4

**Start Position**: 128

**End Position**: 149

| Profile Score | Tandem Score | TDA q-value | Volume | Spectrum Matches |
| --- | --- | --- | --- | --- |
| 15.5642 | 162.3289 | 0.0000 | 4.2212e+08 | 5 |

**Adduct Signal Fractions**

- **Unmodified**: 4.2212e+08

### TIVKPGDILLIN(N)STGNLIAPRGY Hex 10 HexNAc 2

**Mass:**  4451.0646

(7.937e-07)

**Charge States:** 3, 4, 5

**Start Position**: 235

**End Position**: 258

| Profile Score | Tandem Score | TDA q-value | Volume | Spectrum Matches |
| --- | --- | --- | --- | --- |
| 15.3757 | 161.7763 | 0.0000 | 3.3181e+08 | 13 |

**Adduct Signal Fractions**

- **Unmodified**: 3.3181e+08

### TIVKPGDILLIN(N)STGNLIAPR Hex 10 HexNAc 2

**Mass:**  4230.9748

(-1.026e-06)

**Charge States:** 3, 4

**Start Position**: 235

**End Position**: 256

| Profile Score | Tandem Score | TDA q-value | Volume | Spectrum Matches |
| --- | --- | --- | --- | --- |
| -3.6962 | 160.8820 | 0.0000 | 1.7268e+08 | 5 |

**Adduct Signal Fractions**

- **Unmodified**: 1.7268e+08

### IN(N)STGNLIAPRGYF Hex 9 HexNAc 2

**Mass:**  3386.4296

(4.245e-07)

**Charge States:** 3

**Start Position**: 245

**End Position**: 259

| Profile Score | Tandem Score | TDA q-value | Volume | Spectrum Matches |
| --- | --- | --- | --- | --- |
| -19.2020 | 160.1403 | 0.0000 | 1.8716e+07 | 2 |

**Adduct Signal Fractions**

- **Unmodified**: 1.8716e+07

### NSKYPALN(N)VTM(O)PNNEQFDKLY Hex 9 HexNAc 2

**Mass:**  4365.8361

(2.860e-06)

**Charge States:** 3, 4

**Start Position**: 158

**End Position**: 179

| Profile Score | Tandem Score | TDA q-value | Volume | Spectrum Matches |
| --- | --- | --- | --- | --- |
| -2.6146 | 159.8481 | 0.0000 | 2.9615e+08 | 3 |

**Adduct Signal Fractions**

- **Unmodified**: 2.9615e+08

### TIVKPGDILLIN(N)STGNLIAPRGY Hex 8 HexNAc 2

**Mass:**  4126.9582

(5.694e-07)

**Charge States:** 3, 4, 5

**Start Position**: 235

**End Position**: 258

| Profile Score | Tandem Score | TDA q-value | Volume | Spectrum Matches |
| --- | --- | --- | --- | --- |
| 14.9176 | 159.6083 | 0.0000 | 3.3721e+08 | 12 |

**Adduct Signal Fractions**

- **Unmodified**: 3.3721e+08

### SKYPALN(N)VTM(O)PNNEQFDKLY Hex 8 HexNAc 2

**Mass:**  4089.7385

(3.542e-06)

**Charge States:** 3, 4

**Start Position**: 159

**End Position**: 179

| Profile Score | Tandem Score | TDA q-value | Volume | Spectrum Matches |
| --- | --- | --- | --- | --- |
| -2.1856 | 159.2304 | 0.0000 | 8.2272e+07 | 4 |

**Adduct Signal Fractions**

- **Unmodified**: 8.2272e+07

### TIVKPGDILLIN(N) Hex 9 HexNAc 2

**Mass:**  3159.4242

(2.290e-06)

**Charge States:** 2, 3

**Start Position**: 235

**End Position**: 247

| Profile Score | Tandem Score | TDA q-value | Volume | Spectrum Matches |
| --- | --- | --- | --- | --- |
| 10.9665 | 158.2985 | 0.0000 | 2.6569e+08 | 6 |

**Adduct Signal Fractions**

- **Unmodified**: 2.6569e+08

### TIVKPGDILLIN(N)STGNLIAPR Fuc 2 Hex 3 HexNAc 4 Neu5Ac 1

**Mass:**  4085.9492

(-6.755e-06)

**Charge States:** 4

**Start Position**: 235

**End Position**: 256

| Profile Score | Tandem Score | TDA q-value | Volume | Spectrum Matches |
| --- | --- | --- | --- | --- |
| 14.0750 | 156.8065 | 0.0000 | 1.2985e+08 | 2 |

**Adduct Signal Fractions**

- **Unmodified**: 1.2985e+08

### AGVTQN(N)GTSSSC(C)RRGSNSSFF Hex 6 HexNAc 2

**Mass:**  3584.4607

(1.548e-06)

**Charge States:** 3, 4

**Start Position**: 128

**End Position**: 149

| Profile Score | Tandem Score | TDA q-value | Volume | Spectrum Matches |
| --- | --- | --- | --- | --- |
| 15.5013 | 156.1805 | 0.0000 | 2.5110e+08 | 2 |

**Adduct Signal Fractions**

- **Unmodified**: 2.5110e+08

### NWAGVTQN(N)GTSSSC(C)R Hex 7 HexNAc 2

**Mass:**  3164.2351

(2.439e-07)

**Charge States:** 3

**Start Position**: 126

**End Position**: 141

| Profile Score | Tandem Score | TDA q-value | Volume | Spectrum Matches |
| --- | --- | --- | --- | --- |
| -17.5258 | 155.7436 | 0.0000 | 1.8432e+07 | 2 |

**Adduct Signal Fractions**

- **Unmodified**: 1.8432e+07

### LIN(N)STGNLIAPRGY Hex 10 HexNAc 2

**Mass:**  3514.4979

(4.289e-06)

**Charge States:** 3

**Start Position**: 244

**End Position**: 258

| Profile Score | Tandem Score | TDA q-value | Volume | Spectrum Matches |
| --- | --- | --- | --- | --- |
| -3.6368 | 155.7001 | 0.0000 | 3.6575e+07 | 2 |

**Adduct Signal Fractions**

- **Unmodified**: 3.6575e+07

### AGVTQN(N)GTSSSC(C)RRGSNSSFF Hex 7 HexNAc 2

**Mass:**  3746.5131

(1.019e-06)

**Charge States:** 3, 4

**Start Position**: 128

**End Position**: 149

| Profile Score | Tandem Score | TDA q-value | Volume | Spectrum Matches |
| --- | --- | --- | --- | --- |
| 17.4566 | 154.9257 | 0.0000 | 7.5527e+08 | 4 |

**Adduct Signal Fractions**

- **Unmodified**: 7.5527e+08

### NSKYPALN(N)VTM(O)PNNEQFDKLY Hex 8 HexNAc 2

**Mass:**  4203.7824

(2.681e-06)

**Charge States:** 3, 4

**Start Position**: 158

**End Position**: 179

| Profile Score | Tandem Score | TDA q-value | Volume | Spectrum Matches |
| --- | --- | --- | --- | --- |
| 17.5565 | 153.9716 | 0.0000 | 2.8746e+08 | 4 |

**Adduct Signal Fractions**

- **Unmodified**: 2.8746e+08

### TIVKPGDILLIN(N) Fuc 2 Hex 3 HexNAc 4 Neu5Ac 1

**Mass:**  3176.4497

(-7.345e-06)

**Charge States:** 3

**Start Position**: 235

**End Position**: 247

| Profile Score | Tandem Score | TDA q-value | Volume | Spectrum Matches |
| --- | --- | --- | --- | --- |
| 11.2896 | 153.3354 | 0.0000 | 4.8651e+07 | 1 |

**Adduct Signal Fractions**

- **Unmodified**: 4.8651e+07

### TITNDRIEVTN(N)ATEL Hex 6 HexNAc 3

**Mass:**  3270.4169

(1.422e-06)

**Charge States:** 3

**Start Position**: 28

**End Position**: 43

| Profile Score | Tandem Score | TDA q-value | Volume | Spectrum Matches |
| --- | --- | --- | --- | --- |
| -19.4503 | 152.8261 | 0.0000 | 2.5631e+07 | 1 |

**Adduct Signal Fractions**

- **Unmodified**: 2.5631e+07

### TIVKPGDILLIN(N)STGNL Hex 9 HexNAc 2

**Mass:**  3631.6499

(1.209e-06)

**Charge States:** 3, 4

**Start Position**: 235

**End Position**: 252

| Profile Score | Tandem Score | TDA q-value | Volume | Spectrum Matches |
| --- | --- | --- | --- | --- |
| 17.6014 | 151.6469 | 0.0000 | 1.0793e+09 | 10 |

**Adduct Signal Fractions**

- **Unmodified**: 1.0793e+09

### TIVKPGDILLIN(N) Hex 10 HexNAc 2

**Mass:**  3321.4784

(1.652e-06)

**Charge States:** 3

**Start Position**: 235

**End Position**: 247

| Profile Score | Tandem Score | TDA q-value | Volume | Spectrum Matches |
| --- | --- | --- | --- | --- |
| -5.1151 | 150.8368 | 0.0000 | 1.7126e+07 | 1 |

**Adduct Signal Fractions**

- **Unmodified**: 1.7126e+07

### SKYPALN(N)VTM(O)PNNEQFDKLY Hex 9 HexNAc 2

**Mass:**  4251.7907

(3.752e-06)

**Charge States:** 3, 4

**Start Position**: 159

**End Position**: 179

| Profile Score | Tandem Score | TDA q-value | Volume | Spectrum Matches |
| --- | --- | --- | --- | --- |
| -2.3857 | 150.5245 | 0.0000 | 8.8572e+07 | 3 |

**Adduct Signal Fractions**

- **Unmodified**: 8.8572e+07

### NWAGVTQN(N)GTSSS Hex 7 HexNAc 2

**Mass:**  2848.1046

(6.936e-07)

**Charge States:** 3

**Start Position**: 126

**End Position**: 139

| Profile Score | Tandem Score | TDA q-value | Volume | Spectrum Matches |
| --- | --- | --- | --- | --- |
| -19.3254 | 150.3611 | 0.0000 | 9.6566e+06 | 1 |

**Adduct Signal Fractions**

- **Unmodified**: 9.6566e+06

### TIVKPGDILLIN(N)STGN Hex 9 HexNAc 2

**Mass:**  3518.5717

(2.686e-06)

**Charge States:** 3, 4

**Start Position**: 235

**End Position**: 251

| Profile Score | Tandem Score | TDA q-value | Volume | Spectrum Matches |
| --- | --- | --- | --- | --- |
| 16.5113 | 149.9312 | 0.0000 | 3.3890e+08 | 5 |

**Adduct Signal Fractions**

- **Unmodified**: 3.3890e+08

### TIVKPGDILLIN(N)STGNLIAPRGY Fuc 2 Hex 3 HexNAc 4 Neu5Ac 1

**Mass:**  4306.0363

(-6.233e-06)

**Charge States:** 4, 5

**Start Position**: 235

**End Position**: 258

| Profile Score | Tandem Score | TDA q-value | Volume | Spectrum Matches |
| --- | --- | --- | --- | --- |
| 14.5723 | 149.9134 | 0.0000 | 2.5234e+08 | 5 |

**Adduct Signal Fractions**

- **Unmodified**: 2.5234e+08

### TIVKPGDILLIN(N)STGN Hex 10 HexNAc 2

**Mass:**  3680.6237

(1.768e-06)

**Charge States:** 3

**Start Position**: 235

**End Position**: 251

| Profile Score | Tandem Score | TDA q-value | Volume | Spectrum Matches |
| --- | --- | --- | --- | --- |
| -4.3510 | 149.1098 | 0.0000 | 2.2982e+07 | 1 |

**Adduct Signal Fractions**

- **Unmodified**: 2.2982e+07

### TIVKPGDILLIN(N)STGN Fuc 2 Hex 3 HexNAc 4 Neu5Ac 1

**Mass:**  3535.5979

(-5.003e-06)

**Charge States:** 3

**Start Position**: 235

**End Position**: 251

| Profile Score | Tandem Score | TDA q-value | Volume | Spectrum Matches |
| --- | --- | --- | --- | --- |
| -4.1100 | 148.2210 | 0.0000 | 6.5185e+07 | 1 |

**Adduct Signal Fractions**

- **Unmodified**: 6.5185e+07

### TIVKPGDILLIN(N)STGNL Hex 10 HexNAc 2

**Mass:**  3793.7038

(8.386e-07)

**Charge States:** 3, 4

**Start Position**: 235

**End Position**: 252

| Profile Score | Tandem Score | TDA q-value | Volume | Spectrum Matches |
| --- | --- | --- | --- | --- |
| -3.3620 | 147.6003 | 0.0000 | 9.4655e+07 | 3 |

**Adduct Signal Fractions**

- **Unmodified**: 9.4655e+07

### RRGSN(N)SSFF Hex 5 HexNAc 2

**Mass:**  2272.9365

(1.591e-06)

**Charge States:** 3

**Start Position**: 140

**End Position**: 149

| Profile Score | Tandem Score | TDA q-value | Volume | Spectrum Matches |
| --- | --- | --- | --- | --- |
| -19.5601 | 147.5974 | 0.0000 | 1.3954e+07 | 1 |

**Adduct Signal Fractions**

- **Unmodified**: 1.3954e+07

### RRGSN(N)SSFF Fuc 1 Hex 5 HexNAc 4

**Mass:**  2825.1516

(7.038e-07)

**Charge States:** 3

**Start Position**: 140

**End Position**: 149

| Profile Score | Tandem Score | TDA q-value | Volume | Spectrum Matches |
| --- | --- | --- | --- | --- |
| -19.0789 | 144.8401 | 0.0000 | 1.6027e+07 | 1 |

**Adduct Signal Fractions**

- **Unmodified**: 1.6027e+07

### HKC(C)DNAC(C)IGSIRN(N)GTY Fuc 1 Hex 5 HexNAc 4

**Mass:**  3633.4760

(1.583e-06)

**Charge States:** 4

**Start Position**: 471

**End Position**: 487

| Profile Score | Tandem Score | TDA q-value | Volume | Spectrum Matches |
| --- | --- | --- | --- | --- |
| -21.0563 | 144.4564 | 0.0000 | 8.8297e+06 | 1 |

**Adduct Signal Fractions**

- **Unmodified**: 8.8297e+06

### NWAGVTQN(N)GTSSSC(C)RRGSNSSFF Hex 7 HexNAc 2

**Mass:**  4046.6350

(3.472e-06)

**Charge States:** 3, 4

**Start Position**: 126

**End Position**: 149

| Profile Score | Tandem Score | TDA q-value | Volume | Spectrum Matches |
| --- | --- | --- | --- | --- |
| -2.6674 | 144.0430 | 0.0000 | 3.9695e+08 | 4 |

**Adduct Signal Fractions**

- **Unmodified**: 3.9695e+08

### RSDAPIGKC(C)KSEC(C)ITPN(N)GSIPNDKPF Hex 9 HexNAc 2

**Mass:**  4752.0370

(1.485e-06)

**Charge States:** 5

**Start Position**: 269

**End Position**: 295

| Profile Score | Tandem Score | TDA q-value | Volume | Spectrum Matches |
| --- | --- | --- | --- | --- |
| -2.8287 | 144.0210 | 0.0000 | 2.9848e+08 | 2 |

**Adduct Signal Fractions**

- **Unmodified**: 2.9848e+08

### TIVKPGDILLIN(N)STGNL Hex 8 HexNAc 2

**Mass:**  3469.5954

(-3.476e-08)

**Charge States:** 3, 4

**Start Position**: 235

**End Position**: 252

| Profile Score | Tandem Score | TDA q-value | Volume | Spectrum Matches |
| --- | --- | --- | --- | --- |
| -3.8156 | 142.8314 | 0.0000 | 8.4829e+07 | 2 |

**Adduct Signal Fractions**

- **Unmodified**: 8.4829e+07

### TIVKPGDILLIN(N)STGNL Fuc 2 Hex 3 HexNAc 4 Neu5Ac 1

**Mass:**  3648.6758

(-6.366e-06)

**Charge States:** 3

**Start Position**: 235

**End Position**: 252

| Profile Score | Tandem Score | TDA q-value | Volume | Spectrum Matches |
| --- | --- | --- | --- | --- |
| -3.9731 | 142.7624 | 0.0000 | 1.8930e+08 | 2 |

**Adduct Signal Fractions**

- **Unmodified**: 1.8930e+08

### RSDAPIGKC(C)KSEC(C)ITPN(N)GSIPNDKPF Hex 8 HexNAc 2

**Mass:**  4589.9755

(1.037e-06)

**Charge States:** 5

**Start Position**: 269

**End Position**: 295

| Profile Score | Tandem Score | TDA q-value | Volume | Spectrum Matches |
| --- | --- | --- | --- | --- |
| -3.9975 | 142.3632 | 0.0000 | 1.3173e+08 | 2 |

**Adduct Signal Fractions**

- **Unmodified**: 1.3173e+08

### TITNDRIEVTN(N)ATEL Hex 5 HexNAc 2

**Mass:**  2905.2877

(2.343e-06)

**Charge States:** 2, 3

**Start Position**: 28

**End Position**: 43

| Profile Score | Tandem Score | TDA q-value | Volume | Spectrum Matches |
| --- | --- | --- | --- | --- |
| -17.5138 | 142.0385 | 0.0000 | 3.6712e+07 | 1 |

**Adduct Signal Fractions**

- **Unmodified**: 3.6712e+07

### TIVKPGDILLIN(N)STGNLIAPRGY Hex 5 HexNAc 2

**Mass:**  3640.7980

(1.501e-06)

**Charge States:** 3, 4

**Start Position**: 235

**End Position**: 258

| Profile Score | Tandem Score | TDA q-value | Volume | Spectrum Matches |
| --- | --- | --- | --- | --- |
| 16.1191 | 138.8840 | 0.0075 | 4.1822e+07 | 4 |

**Adduct Signal Fractions**

- **Unmodified**: 4.1822e+07

### TIVKPGDILLIN(N)STGNLIAPR Hex 5 HexNAc 2

**Mass:**  3420.7150

(1.127e-06)

**Charge States:** 4

**Start Position**: 235

**End Position**: 256

| Profile Score | Tandem Score | TDA q-value | Volume | Spectrum Matches |
| --- | --- | --- | --- | --- |
| -3.5311 | 138.7473 | 0.0075 | 2.2827e+07 | 3 |

**Adduct Signal Fractions**

- **Unmodified**: 2.2827e+07

### SIRN(N)GTYDHDVY Fuc 1 Hex 5 HexNAc 4

**Mass:**  3207.2884

(3.493e-07)

**Charge States:** 3, 4

**Start Position**: 480

**End Position**: 492

| Profile Score | Tandem Score | TDA q-value | Volume | Spectrum Matches |
| --- | --- | --- | --- | --- |
| -19.0382 | 136.1804 | 0.0075 | 1.4723e+07 | 2 |

**Adduct Signal Fractions**

- **Unmodified**: 1.4723e+07

### C(C)LGHHAVPN(N)GTIVK Fuc 1 Hex 5 HexNAc 5

**Mass:**  3473.5029

(5.008e-07)

**Charge States:** 4

**Start Position**: 14

**End Position**: 28

| Profile Score | Tandem Score | TDA q-value | Volume | Spectrum Matches |
| --- | --- | --- | --- | --- |
| -18.6830 | 135.0253 | 0.0075 | 3.8385e+07 | 1 |

**Adduct Signal Fractions**

- **Unmodified**: 3.8385e+07

### TIVKPGDILLIN(N) Hex 8 HexNAc 2

**Mass:**  2999.3800

(6.715e-04)

**Start Position**: 235

**End Position**: 247

| Profile Score | Tandem Score | TDA q-value | Volume | Spectrum Matches |
| --- | --- | --- | --- | --- |
| 0.0000 | 134.7626 | 0.0075 | 0.0000e+00 | 1 |

Adduct:

No Chromatogram Found

### TITNDRIEVTN(N)ATEL Hex 5 HexNAc 3

**Mass:**  3108.3671

(2.153e-06)

**Charge States:** 3

**Start Position**: 28

**End Position**: 43

| Profile Score | Tandem Score | TDA q-value | Volume | Spectrum Matches |
| --- | --- | --- | --- | --- |
| -17.1724 | 134.7439 | 0.0075 | 1.2814e+07 | 1 |

**Adduct Signal Fractions**

- **Unmodified**: 1.2814e+07

### RSDAPIGKC(C)KSEC(C)ITPN(N)GSIPNDKPF Hex 7 HexNAc 2

**Mass:**  4427.9223

(1.680e-06)

**Charge States:** 4, 5

**Start Position**: 269

**End Position**: 295

| Profile Score | Tandem Score | TDA q-value | Volume | Spectrum Matches |
| --- | --- | --- | --- | --- |
| -3.9097 | 134.7297 | 0.0075 | 1.6279e+08 | 3 |

**Adduct Signal Fractions**

- **Unmodified**: 1.6279e+08

### TITNDRIEVTN(N)ATEL Hex 7 HexNAc 2

**Mass:**  3229.3906

(1.208e-06)

**Charge States:** 3

**Start Position**: 28

**End Position**: 43

| Profile Score | Tandem Score | TDA q-value | Volume | Spectrum Matches |
| --- | --- | --- | --- | --- |
| -17.4406 | 134.6436 | 0.0075 | 4.2279e+07 | 1 |

**Adduct Signal Fractions**

- **Unmodified**: 4.2279e+07

### NSKYPALN(N)VTM(O)PNNEQFDKL Hex 9 HexNAc 2

**Mass:**  4202.7639

(8.390e-07)

**Charge States:** 3, 4

**Start Position**: 158

**End Position**: 178

| Profile Score | Tandem Score | TDA q-value | Volume | Spectrum Matches |
| --- | --- | --- | --- | --- |
| -17.2467 | 134.2319 | 0.0075 | 2.9764e+07 | 2 |

**Adduct Signal Fractions**

- **Unmodified**: 2.9764e+07

### TIVKPGDILLIN(N)STGNLIAPRGY Hex 7 HexNAc 2

**Mass:**  3964.9084

(1.452e-06)

**Charge States:** 3, 4

**Start Position**: 235

**End Position**: 258

| Profile Score | Tandem Score | TDA q-value | Volume | Spectrum Matches |
| --- | --- | --- | --- | --- |
| -3.8415 | 131.6551 | 0.0075 | 1.4400e+07 | 3 |

**Adduct Signal Fractions**

- **Unmodified**: 1.4400e+07

### NWAGVTQN(N)GTSSSC(C)RRGSNSSFF Hex 5 HexNAc 2

**Mass:**  3722.5275

(5.114e-08)

**Charge States:** 3, 4

**Start Position**: 126

**End Position**: 149

| Profile Score | Tandem Score | TDA q-value | Volume | Spectrum Matches |
| --- | --- | --- | --- | --- |
| -4.0111 | 131.5197 | 0.0075 | 2.4376e+08 | 4 |

**Adduct Signal Fractions**

- **Unmodified**: 2.4376e+08

### LIN(N)STGNLIAPRGY Hex 8 HexNAc 2

**Mass:**  3190.3983

(2.286e-06)

**Charge States:** 3

**Start Position**: 244

**End Position**: 258

| Profile Score | Tandem Score | TDA q-value | Volume | Spectrum Matches |
| --- | --- | --- | --- | --- |
| -18.3127 | 130.9262 | 0.0075 | 1.4824e+07 | 1 |

**Adduct Signal Fractions**

- **Unmodified**: 1.4824e+07

### RGSN(N)SSFF Hex 6 HexNAc 3

**Mass:**  2481.9688

(1.922e-06)

**Charge States:** 2, 3

**Start Position**: 141

**End Position**: 149

| Profile Score | Tandem Score | TDA q-value | Volume | Spectrum Matches |
| --- | --- | --- | --- | --- |
| -20.1072 | 130.5957 | 0.0000 | 6.1421e+06 | 1 |

**Adduct Signal Fractions**

- **Unmodified**: 6.1421e+06

### NWAGVTQN(N)GTSSSC(C)RRGSNSSFF Hex 6 HexNAc 2

**Mass:**  3884.5837

(1.280e-06)

**Charge States:** 3, 4

**Start Position**: 126

**End Position**: 149

| Profile Score | Tandem Score | TDA q-value | Volume | Spectrum Matches |
| --- | --- | --- | --- | --- |
| -17.7293 | 130.0826 | 0.0075 | 1.1601e+08 | 2 |

**Adduct Signal Fractions**

- **Unmodified**: 1.1601e+08

### TIVKPGDILLIN(N)STGNL Hex 5 HexNAc 2

**Mass:**  2983.4388

(5.871e-07)

**Charge States:** 3

**Start Position**: 235

**End Position**: 252

| Profile Score | Tandem Score | TDA q-value | Volume | Spectrum Matches |
| --- | --- | --- | --- | --- |
| -3.5620 | 129.5990 | 0.0075 | 1.1826e+07 | 1 |

**Adduct Signal Fractions**

- **Unmodified**: 1.1826e+07

### TIVKPGDILLIN(N)STGNL Fuc 2 Hex 4 HexNAc 4 Neu5Ac 1

**Mass:**  3810.7190

(-9.205e-06)

**Charge States:** 3

**Start Position**: 235

**End Position**: 252

| Profile Score | Tandem Score | TDA q-value | Volume | Spectrum Matches |
| --- | --- | --- | --- | --- |
| -18.9008 | 126.8289 | 0.0112 | 1.6687e+07 | 1 |

**Adduct Signal Fractions**

- **Unmodified**: 1.6687e+07

### NSKYPALN(N)VTMPNNEQFDKLY Hex 8 HexNAc 2

**Mass:**  4187.7824

(1.492e-06)

**Charge States:** 3, 4

**Start Position**: 158

**End Position**: 179

| Profile Score | Tandem Score | TDA q-value | Volume | Spectrum Matches |
| --- | --- | --- | --- | --- |
| -18.3529 | 124.5683 | 0.0112 | 1.0348e+07 | 4 |

**Adduct Signal Fractions**

- **Unmodified**: 1.0348e+07

### AGVTQN(N)GTSSSC(C)RRGSNSSFF Hex 6 HexNAc 3

**Mass:**  3787.5424

(9.998e-07)

**Charge States:** 3

**Start Position**: 128

**End Position**: 149

| Profile Score | Tandem Score | TDA q-value | Volume | Spectrum Matches |
| --- | --- | --- | --- | --- |
| -4.2380 | 124.0255 | 0.0112 | 1.4168e+08 | 3 |

**Adduct Signal Fractions**

- **Unmodified**: 1.4168e+08

### AGVTQN(N)GTSSSC(C)RRGSN(D)SSFF Hex 7 HexNAc 2

**Mass:**  3747.4978

(2.628e-04)

**Charge States:** 3, 4

**Start Position**: 128

**End Position**: 149

| Profile Score | Tandem Score | TDA q-value | Volume | Spectrum Matches |
| --- | --- | --- | --- | --- |
| 10.0450 | 121.8503 | 0.0112 | 3.1354e+08 | 4 |

**Adduct Signal Fractions**

- **Unmodified**: 3.1354e+08

### TIVKPGDILLIN(N)STGN(D)LIAPR Hex 9 HexNAc 2

**Mass:**  4069.9182

(2.445e-04)

**Charge States:** 3

**Start Position**: 235

**End Position**: 256

| Profile Score | Tandem Score | TDA q-value | Volume | Spectrum Matches |
| --- | --- | --- | --- | --- |
| -5.3785 | 120.8353 | 0.0112 | 1.7449e+07 | 3 |

**Adduct Signal Fractions**

- **Unmodified**: 1.7449e+07

### AGVTQN(N)GTSSSC(C)RRGSNSSFF Hex 7 HexNAc 3

**Mass:**  3949.5952

(1.311e-06)

**Charge States:** 3

**Start Position**: 128

**End Position**: 149

| Profile Score | Tandem Score | TDA q-value | Volume | Spectrum Matches |
| --- | --- | --- | --- | --- |
| -3.7711 | 119.4218 | 0.0112 | 7.6543e+07 | 2 |

**Adduct Signal Fractions**

- **Unmodified**: 7.6543e+07

### RRGSN(N)SSFF Hex 6 HexNAc 3

**Mass:**  2638.0658

(2.731e-07)

**Charge States:** 3

**Start Position**: 140

**End Position**: 149

| Profile Score | Tandem Score | TDA q-value | Volume | Spectrum Matches |
| --- | --- | --- | --- | --- |
| -16.0687 | 118.8952 | 0.0000 | 9.2541e+06 | 1 |

**Adduct Signal Fractions**

- **Unmodified**: 9.2541e+06

### TIVKPGDILLIN(N)STGN(D)LIAPRGY Hex 9 HexNAc 2

**Mass:**  4290.0002

(2.312e-04)

**Charge States:** 3, 5

**Start Position**: 235

**End Position**: 258

| Profile Score | Tandem Score | TDA q-value | Volume | Spectrum Matches |
| --- | --- | --- | --- | --- |
| -19.7541 | 113.9510 | 0.0112 | 3.2877e+07 | 21 |

**Adduct Signal Fractions**

- **Unmodified**: 3.2877e+07

### KLPGNDN(N)STATL Hex 6 HexNAc 2

**Mass:**  2608.1092

(3.225e-06)

**Charge States:** 3

**Start Position**: 2

**End Position**: 14

| Profile Score | Tandem Score | TDA q-value | Volume | Spectrum Matches |
| --- | --- | --- | --- | --- |
| -19.8763 | 112.9363 | 0.0112 | 3.1090e+06 | 1 |

**Adduct Signal Fractions**

- **Unmodified**: 3.1090e+06

### TIVKPGDILLIN(N)STGNL Hex 7 HexNAc 2

**Mass:**  3308.5315

(2.989e-04)

**Start Position**: 235

**End Position**: 252

| Profile Score | Tandem Score | TDA q-value | Volume | Spectrum Matches |
| --- | --- | --- | --- | --- |
| 0.0000 | 112.0319 | 0.0112 | 0.0000e+00 | 1 |

Adduct:

No Chromatogram Found

### TIVKPGDILLIN(N)STGNLIAPR Fuc 2 Hex 4 HexNAc 4 Neu5Ac 1

**Mass:**  4248.0021

(-6.351e-06)

**Charge States:** 4

**Start Position**: 235

**End Position**: 256

| Profile Score | Tandem Score | TDA q-value | Volume | Spectrum Matches |
| --- | --- | --- | --- | --- |
| -17.7493 | 111.0749 | 0.0112 | 1.1042e+07 | 1 |

**Adduct Signal Fractions**

- **Unmodified**: 1.1042e+07

### TIVKPGDILLIN(N)ST Hex 9 HexNAc 2

**Mass:**  3347.4995

(-1.357e-07)

**Charge States:** 3

**Start Position**: 235

**End Position**: 249

| Profile Score | Tandem Score | TDA q-value | Volume | Spectrum Matches |
| --- | --- | --- | --- | --- |
| -19.6473 | 110.7085 | 0.0112 | 1.1465e+07 | 1 |

**Adduct Signal Fractions**

- **Unmodified**: 1.1465e+07

### AGVTQN(N)GTSSSC(C)RRGSN(D)SSFF Hex 5 HexNAc 2

**Mass:**  3423.3931

(2.880e-04)

**Charge States:** 3, 4

**Start Position**: 128

**End Position**: 149

| Profile Score | Tandem Score | TDA q-value | Volume | Spectrum Matches |
| --- | --- | --- | --- | --- |
| -5.9982 | 106.2870 | 0.0112 | 1.0377e+08 | 3 |

**Adduct Signal Fractions**

- **Unmodified**: 1.0377e+08

### HKC(C)DNAC(C)IGSIRN(N)GTYDHDVY Fuc 1 Hex 6 HexNAc 5

**Mass:**  4627.8512

(3.589e-06)

**Charge States:** 5

**Start Position**: 471

**End Position**: 492

| Profile Score | Tandem Score | TDA q-value | Volume | Spectrum Matches |
| --- | --- | --- | --- | --- |
| -21.6861 | 106.2269 | 0.0112 | 5.9075e+06 | 1 |

**Adduct Signal Fractions**

- **Unmodified**: 5.9075e+06

### LIN(N)STGNLIAPRGYF Fuc 1 Hex 3 HexNAc 4 Neu5Ac 2

**Mass:**  3661.5683

(-9.387e-06)

**Charge States:** 3

**Start Position**: 244

**End Position**: 259

| Profile Score | Tandem Score | TDA q-value | Volume | Spectrum Matches |
| --- | --- | --- | --- | --- |
| -18.3722 | 103.5931 | 0.0112 | 3.6600e+07 | 1 |

**Adduct Signal Fractions**

- **Unmodified**: 3.6600e+07

### N(D)SKYPALN(N)VTM(O)PNNEQFDKLY Hex 8 HexNAc 2

**Mass:**  4204.7595

(2.361e-04)

**Charge States:** 3, 4

**Start Position**: 158

**End Position**: 179

| Profile Score | Tandem Score | TDA q-value | Volume | Spectrum Matches |
| --- | --- | --- | --- | --- |
| -6.1850 | 102.4456 | 0.0112 | 1.0698e+08 | 3 |

**Adduct Signal Fractions**

- **Unmodified**: 1.0698e+08

### QKLPGNDN(N)STATL Hex 3 HexNAc 3 Neu5Ac 1

**Mass:**  2744.1655

(-4.633e-06)

**Charge States:** 3

**Start Position**: 1

**End Position**: 14

| Profile Score | Tandem Score | TDA q-value | Volume | Spectrum Matches |
| --- | --- | --- | --- | --- |
| 14.8499 | 97.5496 | 0.0168 | 3.8183e+08 | 4 |

**Adduct Signal Fractions**

- **Unmodified**: 3.8183e+08

### N(D)SKYPALN(N)VTM(O)PNNEQFDKLY Hex 9 HexNAc 2

**Mass:**  4366.8176

(2.269e-04)

**Charge States:** 4

**Start Position**: 158

**End Position**: 179

| Profile Score | Tandem Score | TDA q-value | Volume | Spectrum Matches |
| --- | --- | --- | --- | --- |
| -6.1070 | 93.9955 | 0.0270 | 1.2238e+08 | 2 |

**Adduct Signal Fractions**

- **Unmodified**: 1.2238e+08

### TIVKPGDILLIN(N)STGNLIAPRGY Hex 7 HexNAc 2

**Mass:**  3967.9337

(7.645e-04)

**Start Position**: 235

**End Position**: 258

| Profile Score | Tandem Score | TDA q-value | Volume | Spectrum Matches |
| --- | --- | --- | --- | --- |
| 0.0000 | 93.2467 | 0.0270 | 0.0000e+00 | 1 |

Adduct:

No Chromatogram Found

### NWAGVTQN(N)GTSSSC(C)RRGSNSSFF Hex 4 HexNAc 3

**Mass:**  3763.5556

(4.815e-07)

**Charge States:** 4

**Start Position**: 126

**End Position**: 149

| Profile Score | Tandem Score | TDA q-value | Volume | Spectrum Matches |
| --- | --- | --- | --- | --- |
| -19.7558 | 92.3462 | 0.0270 | 5.0979e+07 | 2 |

**Adduct Signal Fractions**

- **Unmodified**: 5.0979e+07

### SC(C)RRGSN(N)SSFF Hex 6 HexNAc 2

**Mass:**  2682.0498

(-2.349e-06)

**Charge States:** 3

**Start Position**: 138

**End Position**: 149

| Profile Score | Tandem Score | TDA q-value | Volume | Spectrum Matches |
| --- | --- | --- | --- | --- |
| -21.2161 | 91.4224 | 0.0316 | 3.3607e+06 | 1 |

**Adduct Signal Fractions**

- **Unmodified**: 3.3607e+06

### LIN(N)STGN(D)LIAPRGYF Hex 9 HexNAc 2

**Mass:**  3500.5010

(2.828e-04)

**Charge States:** 3

**Start Position**: 244

**End Position**: 259

| Profile Score | Tandem Score | TDA q-value | Volume | Spectrum Matches |
| --- | --- | --- | --- | --- |
| -5.8739 | 89.0610 | 0.0316 | 9.2554e+06 | 1 |

**Adduct Signal Fractions**

- **Unmodified**: 9.2554e+06

### IGSIRN(N)GTYDHDVY Fuc 1 Hex 5 HexNAc 4

**Mass:**  3377.3926

(-4.511e-08)

**Charge States:** 3

**Start Position**: 478

**End Position**: 492

| Profile Score | Tandem Score | TDA q-value | Volume | Spectrum Matches |
| --- | --- | --- | --- | --- |
| -18.3183 | 88.2724 | 0.0316 | 7.0150e+06 | 1 |

**Adduct Signal Fractions**

- **Unmodified**: 7.0150e+06

### RSDAPIGKC(C)KSEC(C)ITPN(N)GSIPN(D)DKPF Hex 9 HexNAc 2

**Mass:**  4753.0331

(2.126e-04)

**Charge States:** 5

**Start Position**: 269

**End Position**: 295

| Profile Score | Tandem Score | TDA q-value | Volume | Spectrum Matches |
| --- | --- | --- | --- | --- |
| -5.9340 | 83.6022 | 0.0359 | 3.4088e+07 | 1 |

**Adduct Signal Fractions**

- **Unmodified**: 3.4088e+07

### HKC(C)DNAC(C)IGSIRN(N)GTYDHDVY Fuc 1 Hex 8 HexNAc 2

**Mass:**  4342.6755

(-5.819e-06)

**Charge States:** 4

**Start Position**: 471

**End Position**: 492

| Profile Score | Tandem Score | TDA q-value | Volume | Spectrum Matches |
| --- | --- | --- | --- | --- |
| -19.7563 | 79.8255 | 0.0386 | 6.6360e+06 | 1 |

**Adduct Signal Fractions**

- **Unmodified**: 6.6360e+06

### NSKYPALN(N)VTM(O)PNNEQFDKL Hex 8 HexNAc 2

**Mass:**  4040.7121

(1.102e-06)

**Charge States:** 3, 4

**Start Position**: 158

**End Position**: 178

| Profile Score | Tandem Score | TDA q-value | Volume | Spectrum Matches |
| --- | --- | --- | --- | --- |
| -18.7884 | 79.1332 | 0.0386 | 2.4605e+07 | 2 |

**Adduct Signal Fractions**

- **Unmodified**: 2.4605e+07

### IVKPGDILLIN(N)STGNLIAPRGY Hex 9 HexNAc 2

**Mass:**  4187.9650

(2.119e-06)

**Charge States:** 3, 4

**Start Position**: 236

**End Position**: 258

| Profile Score | Tandem Score | TDA q-value | Volume | Spectrum Matches |
| --- | --- | --- | --- | --- |
| -18.9192 | 78.9106 | 0.0386 | 4.8348e+06 | 1 |

**Adduct Signal Fractions**

- **Unmodified**: 4.8348e+06

### AGVTQN(N)GTSSSC(C)RRGSNSSFF Hex 5 HexNAc 3

**Mass:**  3625.4862

(1.583e-06)

**Charge States:** 3

**Start Position**: 128

**End Position**: 149

| Profile Score | Tandem Score | TDA q-value | Volume | Spectrum Matches |
| --- | --- | --- | --- | --- |
| -19.4780 | 74.9572 | 0.0386 | 2.1673e+07 | 1 |

**Adduct Signal Fractions**

- **Unmodified**: 2.1673e+07

### LNSKYPALN(N)VTM(O)PNNEQFDKL Hex 8 HexNAc 2

**Mass:**  4153.8024

(2.535e-06)

**Charge States:** 3, 4

**Start Position**: 157

**End Position**: 178

| Profile Score | Tandem Score | TDA q-value | Volume | Spectrum Matches |
| --- | --- | --- | --- | --- |
| -17.0987 | 74.5489 | 0.0386 | 1.4941e+07 | 2 |

**Adduct Signal Fractions**

- **Unmodified**: 1.4941e+07

### TIVKPGDILLIN(N)STGNLIAPR Hex 6 HexNAc 2

**Mass:**  3582.7695

(1.238e-06)

**Charge States:** 4

**Start Position**: 235

**End Position**: 256

| Profile Score | Tandem Score | TDA q-value | Volume | Spectrum Matches |
| --- | --- | --- | --- | --- |
| -19.3961 | 73.0264 | 0.0386 | 3.7490e+06 | 1 |

**Adduct Signal Fractions**

- **Unmodified**: 3.7490e+06

### TIVKPGDILLIN(N)STGNLIAPRGY Hex 6 HexNAc 2

**Mass:**  3802.8549

(6.084e-07)

**Charge States:** 3, 4

**Start Position**: 235

**End Position**: 258

| Profile Score | Tandem Score | TDA q-value | Volume | Spectrum Matches |
| --- | --- | --- | --- | --- |
| -18.2763 | 72.0905 | 0.0419 | 1.0796e+07 | 4 |

**Adduct Signal Fractions**

- **Unmodified**: 1.0796e+07

### LNSKYPALN(N)VTM(O)PNNEQFDKL Hex 9 HexNAc 2

**Mass:**  4315.8542

(-2.242e-07)

**Charge States:** 3, 4

**Start Position**: 157

**End Position**: 178

| Profile Score | Tandem Score | TDA q-value | Volume | Spectrum Matches |
| --- | --- | --- | --- | --- |
| -15.9577 | 70.4444 | 0.0419 | 1.5442e+07 | 1 |

**Adduct Signal Fractions**

- **Unmodified**: 1.5442e+07

### AGVTQN(N)GTSSSC(C)RRGSNSSFF Hex 3 HexNAc 2

**Mass:**  3098.3010

(1.311e-06)

**Charge States:** 3

**Start Position**: 128

**End Position**: 149

| Profile Score | Tandem Score | TDA q-value | Volume | Spectrum Matches |
| --- | --- | --- | --- | --- |
| -18.7496 | 69.6783 | 0.0419 | 1.8401e+07 | 2 |

**Adduct Signal Fractions**

- **Unmodified**: 1.8401e+07

### AGVTQN(N)GTSSSC(C)RRGSNSSFF Hex 5 HexNAc 4

**Mass:**  3828.5683

(9.953e-07)

**Charge States:** 3, 4

**Start Position**: 128

**End Position**: 149

| Profile Score | Tandem Score | TDA q-value | Volume | Spectrum Matches |
| --- | --- | --- | --- | --- |
| 16.3769 | 69.6774 | 0.0453 | 1.6175e+08 | 4 |

**Adduct Signal Fractions**

- **Unmodified**: 1.6175e+08

### NWAGVTQN(N)GTSSSC(C)RRGSN(D)SSFF Hex 7 HexNAc 2

**Mass:**  4047.6215

(2.445e-04)

**Charge States:** 3, 4

**Start Position**: 126

**End Position**: 149

| Profile Score | Tandem Score | TDA q-value | Volume | Spectrum Matches |
| --- | --- | --- | --- | --- |
| 10.6493 | 65.8923 | 0.0419 | 1.4182e+08 | 3 |

**Adduct Signal Fractions**

- **Unmodified**: 1.4182e+08

### NSKYPALN(N)VTMPNNEQFDKLY Hex 9 HexNAc 2

**Mass:**  4349.8376

(2.544e-06)

**Charge States:** 3

**Start Position**: 158

**End Position**: 179

| Profile Score | Tandem Score | TDA q-value | Volume | Spectrum Matches |
| --- | --- | --- | --- | --- |
| -4.6940 | 63.4350 | 0.0453 | 1.0993e+07 | 3 |

**Adduct Signal Fractions**

- **Unmodified**: 1.0993e+07

### NSKYPALN(N)VTM(O)PNNEQFDKLY Hex 7 HexNAc 2

**Mass:**  4041.7316

(3.326e-06)

**Charge States:** 3, 4

**Start Position**: 158

**End Position**: 179

| Profile Score | Tandem Score | TDA q-value | Volume | Spectrum Matches |
| --- | --- | --- | --- | --- |
| -4.2084 | 62.5315 | 0.0453 | 1.8962e+07 | 1 |

**Adduct Signal Fractions**

- **Unmodified**: 1.8962e+07

### SC(C)RRGSN(N)SSFF Fuc 1 Hex 5 HexNAc 5

**Mass:**  3275.2992

(2.255e-06)

**Charge States:** 3, 4

**Start Position**: 138

**End Position**: 149

| Profile Score | Tandem Score | TDA q-value | Volume | Spectrum Matches |
| --- | --- | --- | --- | --- |
| -20.1779 | 56.5424 | 0.0453 | 1.4515e+07 | 1 |

**Adduct Signal Fractions**

- **Unmodified**: 1.4515e+07

### TITNDRIEVTN(N)ATEL Hex 5 HexNAc 4

**Mass:**  3311.4442

(1.412e-06)

**Charge States:** 3, 4

**Start Position**: 28

**End Position**: 43

| Profile Score | Tandem Score | TDA q-value | Volume | Spectrum Matches |
| --- | --- | --- | --- | --- |
| -20.0432 | 56.3254 | 0.0453 | 1.5944e+07 | 1 |

**Adduct Signal Fractions**

- **Unmodified**: 1.5944e+07

### NWAGVTQN(N)GTSSSC(C)RRGSNSSFF Hex 5 HexNAc 4

**Mass:**  4128.6857

(-4.173e-08)

**Charge States:** 3, 4

**Start Position**: 126

**End Position**: 149

| Profile Score | Tandem Score | TDA q-value | Volume | Spectrum Matches |
| --- | --- | --- | --- | --- |
| -4.9355 | 56.0928 | 0.0453 | 8.8370e+07 | 2 |

**Adduct Signal Fractions**

- **Unmodified**: 8.8370e+07

### NWAGVTQNGTSSSC(C)RRGSN(N)SSFF Hex 6 HexNAc 4

**Mass:**  4290.7458

(1.414e-06)

**Charge States:** 4

**Start Position**: 126

**End Position**: 149

| Profile Score | Tandem Score | TDA q-value | Volume | Spectrum Matches |
| --- | --- | --- | --- | --- |
| -4.8087 | 54.1530 | 0.0453 | 4.1014e+07 | 2 |

**Adduct Signal Fractions**

- **Unmodified**: 4.1014e+07

### cus|SWZHA\_5B8|A/Switzerland/9715293/2013

**>cus|SWZHA\_5B8|A/Switzerland/9715293/2013**  
 `QKLPGNDNSTATLCLGHHAVPNGTIVKTITNDRIEVTNATELVQNSSIGEICDSPHQILDGENCTLIDALLGDPQCDGFQ  
NKKWDLFVERSKAYSNCYPYDVPDYASLRSLVASSGTLEFNNESFNWAGVTHNGTSSSCRRGSNSSFFSRLNWLTHLNSK  
YPALNVTMPNNEQFDKLYIWGVHHPVTDKDQIFLYAQSSGRITVSTKRSQQAVIPNIGSRPRIRNIPSRISIYWTIVKPG  
DILLINSTGNLIAPRGYFKIRSGKSSIMRSDAPIGKCKSECITPNGSIPNDKPFQNVNRITYGACPRYVKQSTLKLATGM  
RNVPERQTRGIFGAIAGFIENGWEGMVDGWYGFRHQNSEGRGQAADLKSTQAAIDQINGKLNRLIGKTNEKFHQIEKEFS  
EVEGRIQDLEKYVEDTKIDLWSYNAELLVALENQHTIDLTDSEMNKLFEKTKKQLRENAEDMGNGCFKIYHKCDNACIGS  
IRNGTYDHDVYRDEALNNRFQIKGVELKSGYKDWILWISFAISCFLLCVALLGFIMWACQKGNIRCNICI`

| Site | Type | Count |
| --- | --- | --- |
| 483 | N-linked | 5 |
| 165 | N-linked | 13 |
| 38 | N-linked | 5 |
| 8 | N-linked | 3 |
| 144 | N-linked | 7 |
| 246 | N-linked | 45 |
| 285 | N-linked | 4 |
| 22 | N-linked | 1 |

| Mass | Sequence | MS_2_ Score | `q`-value | MS_1_ Score | Signal | Protein Coordinates | Tandem Scans | Start Time | Apex Time | End Time |
| --- | --- | --- | --- | --- | --- | --- | --- | --- | --- | --- |
| 2695.0875 | LIN(N)STGNL Hex 9 HexNAc 2 | 188.203 | 0.0 | -4.225 | 9.956e+07 | 243 - 251 | 2 | 26.20 | 26.36 | 26.53 |
| 3516.5422 | LIN(N)STGNLIAPRGYF Fuc 2 Hex 3 HexNAc 4 Neu5Ac 1 | 185.714 | 0.0 | -17.407 | 5.841e+07 | 243 - 258 | 1 | 46.76 | 46.76 | 46.92 |
| 3499.5148 | LIN(N)STGNLIAPRGYF Hex 9 HexNAc 2 | 181.072 | 0.0 | -1.872 | 3.809e+08 | 243 - 258 | 3 | 46.76 | 46.76 | 47.26 |
| 3352.4469 | LIN(N)STGNLIAPRGY Hex 9 HexNAc 2 | 173.965 | 0.0 | -17.289 | 3.163e+08 | 243 - 257 | 2 | 36.57 | 36.57 | 36.73 |
| 4068.9241 | TIVKPGDILLIN(N)STGNLIAPR Hex 9 HexNAc 2 | 173.328 | 0.0 | 17.938 | 1.904e+09 | 234 - 255 | 13 | 52.36 | 53.03 | 55.07 |
| 3028.2314 | RRGSN(N)SSFF Fuc 1 Hex 5 HexNAc 5 | 170.390 | 0.0 | -18.773 | 2.824e+07 | 139 - 148 | 1 | 20.79 | 20.95 | 20.95 |
| 2712.1148 | LIN(N)STGNL Fuc 2 Hex 3 HexNAc 4 Neu5Ac 1 | 168.174 | 0.0 | -19.546 | 1.336e+07 | 243 - 251 | 2 | 26.36 | 26.36 | 26.36 |
| 3337.4655 | LIN(N)STGNLIAPRGYF Hex 8 HexNAc 2 | 166.761 | 0.0 | -4.432 | 2.153e+07 | 243 - 258 | 1 | 46.76 | 46.92 | 47.09 |
| 2317.9572 | LPGNDN(N)STATL Hex 5 HexNAc 2 | 164.872 | 0.0 | -5.715 | 1.752e+07 | 2 - 13 | 2 | 26.36 | 26.53 | 26.86 |
| 3906.8734 | TIVKPGDILLIN(N)STGNLIAPR Hex 8 HexNAc 2 | 163.121 | 0.0 | 14.116 | 1.129e+08 | 234 - 255 | 5 | 53.03 | 53.19 | 53.71 |
| 3369.4723 | LIN(N)STGNLIAPRGY Fuc 2 Hex 3 HexNAc 4 Neu5Ac 1 | 163.101 | 0.0 | -17.049 | 5.398e+07 | 243 - 257 | 1 | 36.57 | 36.57 | 36.73 |
| 4289.0130 | TIVKPGDILLIN(N)STGNLIAPRGY Hex 9 HexNAc 2 | 162.338 | 0.0 | 18.525 | 3.507e+09 | 234 - 257 | 25 | 53.53 | 55.41 | 58.53 |
| 4451.0798 | TIVKPGDILLIN(N)STGNLIAPRGY Hex 10 HexNAc 2 | 161.776 | 0.0 | 15.376 | 3.318e+08 | 234 - 257 | 13 | 55.07 | 55.24 | 62.13 |
| 4230.9752 | TIVKPGDILLIN(N)STGNLIAPR Hex 10 HexNAc 2 | 160.882 | 0.0 | -3.696 | 1.727e+08 | 234 - 255 | 5 | 52.86 | 53.03 | 53.36 |
| 3386.4296 | IN(N)STGNLIAPRGYF Hex 9 HexNAc 2 | 160.140 | 0.0 | -19.202 | 1.872e+07 | 244 - 258 | 2 | 42.94 | 43.11 | 43.11 |
| 4365.8334 | NSKYPALN(N)VTM(O)PNNEQFDKLY Hex 9 HexNAc 2 | 159.848 | 0.0 | -2.615 | 2.962e+08 | 157 - 178 | 3 | 42.27 | 42.61 | 42.61 |
| 4126.9563 | TIVKPGDILLIN(N)STGNLIAPRGY Hex 8 HexNAc 2 | 159.608 | 0.0 | 14.918 | 3.372e+08 | 234 - 257 | 12 | 54.73 | 55.41 | 56.60 |
| 4089.7377 | SKYPALN(N)VTM(O)PNNEQFDKLY Hex 8 HexNAc 2 | 159.230 | 0.0 | -2.186 | 8.227e+07 | 158 - 178 | 4 | 42.44 | 42.61 | 42.78 |
| 3159.4237 | TIVKPGDILLIN(N) Hex 9 HexNAc 2 | 158.298 | 0.0 | 10.967 | 2.657e+08 | 234 - 246 | 6 | 45.80 | 47.26 | 47.76 |
| 4085.9497 | TIVKPGDILLIN(N)STGNLIAPR Fuc 2 Hex 3 HexNAc 4 Neu5Ac 1 | 156.807 | 0.0 | 14.075 | 1.298e+08 | 234 - 255 | 2 | 52.86 | 53.03 | 53.71 |
| 3514.5047 | LIN(N)STGNLIAPRGY Hex 10 HexNAc 2 | 155.700 | 0.0 | -3.637 | 3.657e+07 | 243 - 257 | 2 | 36.40 | 36.57 | 36.73 |
| 4203.7809 | NSKYPALN(N)VTM(O)PNNEQFDKLY Hex 8 HexNAc 2 | 153.972 | 0.0 | 17.557 | 2.875e+08 | 157 - 178 | 4 | 42.10 | 42.61 | 42.78 |
| 3176.4461 | TIVKPGDILLIN(N) Fuc 2 Hex 3 HexNAc 4 Neu5Ac 1 | 153.335 | 0.0 | 11.290 | 4.865e+07 | 234 - 246 | 1 | 46.76 | 47.26 | 47.60 |
| 3270.4171 | TITNDRIEVTN(N)ATEL Hex 6 HexNAc 3 | 152.826 | 0.0 | -19.450 | 2.563e+07 | 27 - 42 | 1 | 36.40 | 36.57 | 36.57 |
| 3631.6501 | TIVKPGDILLIN(N)STGNL Hex 9 HexNAc 2 | 151.647 | 0.0 | 17.601 | 1.079e+09 | 234 - 251 | 10 | 52.01 | 52.70 | 54.73 |
| 3321.4759 | TIVKPGDILLIN(N) Hex 10 HexNAc 2 | 150.837 | 0.0 | -5.115 | 1.713e+07 | 234 - 246 | 1 | 46.76 | 47.09 | 47.26 |
| 4251.7897 | SKYPALN(N)VTM(O)PNNEQFDKLY Hex 9 HexNAc 2 | 150.524 | 0.0 | -2.386 | 8.857e+07 | 158 - 178 | 3 | 42.44 | 42.61 | 42.78 |
| 3518.5723 | TIVKPGDILLIN(N)STGN Hex 9 HexNAc 2 | 149.931 | 0.0 | 16.511 | 3.389e+08 | 234 - 250 | 5 | 44.78 | 45.12 | 45.63 |
| 4306.0360 | TIVKPGDILLIN(N)STGNLIAPRGY Fuc 2 Hex 3 HexNAc 4 Neu5Ac 1 | 149.913 | 0.0 | 14.572 | 2.523e+08 | 234 - 257 | 5 | 55.07 | 55.41 | 56.08 |
| 3680.6248 | TIVKPGDILLIN(N)STGN Hex 10 HexNAc 2 | 149.110 | 0.0 | -4.351 | 2.298e+07 | 234 - 250 | 1 | 44.78 | 44.95 | 45.12 |
| 3535.5982 | TIVKPGDILLIN(N)STGN Fuc 2 Hex 3 HexNAc 4 Neu5Ac 1 | 148.221 | 0.0 | -4.110 | 6.518e+07 | 234 - 250 | 1 | 44.95 | 45.12 | 45.29 |
| 3793.7038 | TIVKPGDILLIN(N)STGNL Hex 10 HexNAc 2 | 147.600 | 0.0 | -3.362 | 9.465e+07 | 234 - 251 | 3 | 52.36 | 52.53 | 52.86 |
| 2272.9358 | RRGSN(N)SSFF Hex 5 HexNAc 2 | 147.597 | 0.0 | -19.560 | 1.395e+07 | 139 - 148 | 1 | 20.95 | 20.95 | 21.10 |
| 2825.1525 | RRGSN(N)SSFF Fuc 1 Hex 5 HexNAc 4 | 144.840 | 0.0 | -19.079 | 1.603e+07 | 139 - 148 | 1 | 20.63 | 20.63 | 20.79 |
| 3633.4727 | HKC(C)DNAC(C)IGSIRN(N)GTY Fuc 1 Hex 5 HexNAc 4 | 144.456 | 0.0 | -21.056 | 8.830e+06 | 470 - 486 | 1 | 20.31 | 20.31 | 20.47 |
| 4752.0333 | RSDAPIGKC(C)KSEC(C)ITPN(N)GSIPNDKPF Hex 9 HexNAc 2 | 144.021 | 0.0 | -2.829 | 2.985e+08 | 268 - 294 | 2 | 29.67 | 29.67 | 30.00 |
| 3469.5969 | TIVKPGDILLIN(N)STGNL Hex 8 HexNAc 2 | 142.831 | 0.0 | -3.816 | 8.483e+07 | 234 - 251 | 2 | 52.53 | 52.70 | 53.03 |
| 3648.6761 | TIVKPGDILLIN(N)STGNL Fuc 2 Hex 3 HexNAc 4 Neu5Ac 1 | 142.762 | 0.0 | -3.973 | 1.893e+08 | 234 - 251 | 2 | 52.53 | 52.70 | 53.03 |
| 4589.9783 | RSDAPIGKC(C)KSEC(C)ITPN(N)GSIPNDKPF Hex 8 HexNAc 2 | 142.363 | 0.0 | -3.998 | 1.317e+08 | 268 - 294 | 2 | 29.67 | 29.83 | 30.00 |
| 2905.2883 | TITNDRIEVTN(N)ATEL Hex 5 HexNAc 2 | 142.038 | 0.0 | -17.514 | 3.671e+07 | 27 - 42 | 1 | 36.73 | 36.73 | 36.90 |
| 3640.7994 | TIVKPGDILLIN(N)STGNLIAPRGY Hex 5 HexNAc 2 | 138.884 | 0.007 | 16.119 | 4.182e+07 | 234 - 257 | 4 | 55.91 | 56.08 | 56.60 |
| 3420.7150 | TIVKPGDILLIN(N)STGNLIAPR Hex 5 HexNAc 2 | 138.747 | 0.007 | -3.531 | 2.283e+07 | 234 - 255 | 3 | 53.71 | 53.88 | 54.05 |
| 3207.2892 | SIRN(N)GTYDHDVY Fuc 1 Hex 5 HexNAc 4 | 136.180 | 0.007 | -19.038 | 1.472e+07 | 479 - 491 | 2 | 19.16 | 19.16 | 19.33 |
| 3473.5031 | C(C)LGHHAVPN(N)GTIVK Fuc 1 Hex 5 HexNAc 5 | 135.025 | 0.007 | -18.683 | 3.839e+07 | 13 - 27 | 1 | 18.51 | 18.67 | 18.67 |
| 2999.3800 | TIVKPGDILLIN(N) Hex 8 HexNAc 2 | 134.763 | 0.007 | 0.000 | 0.000e+00 | 234 - 246 | 1 | - | - | - |
| 3108.3679 | TITNDRIEVTN(N)ATEL Hex 5 HexNAc 3 | 134.744 | 0.007 | -17.172 | 1.281e+07 | 27 - 42 | 1 | 36.73 | 36.73 | 36.90 |
| 4427.9258 | RSDAPIGKC(C)KSEC(C)ITPN(N)GSIPNDKPF Hex 7 HexNAc 2 | 134.730 | 0.007 | -3.910 | 1.628e+08 | 268 - 294 | 3 | 29.67 | 29.83 | 30.00 |
| 3229.3892 | TITNDRIEVTN(N)ATEL Hex 7 HexNAc 2 | 134.644 | 0.007 | -17.441 | 4.228e+07 | 27 - 42 | 1 | 36.40 | 36.40 | 36.57 |
| 4202.7651 | NSKYPALN(N)VTM(O)PNNEQFDKL Hex 9 HexNAc 2 | 134.232 | 0.007 | -17.247 | 2.976e+07 | 157 - 177 | 2 | 40.09 | 40.25 | 40.25 |
| 3964.9076 | TIVKPGDILLIN(N)STGNLIAPRGY Hex 7 HexNAc 2 | 131.655 | 0.007 | -3.842 | 1.440e+07 | 234 - 257 | 3 | 55.57 | 55.57 | 55.91 |
| 3190.3990 | LIN(N)STGNLIAPRGY Hex 8 HexNAc 2 | 130.926 | 0.007 | -18.313 | 1.482e+07 | 243 - 257 | 1 | 36.73 | 36.73 | 36.90 |
| 2481.9654 | RGSN(N)SSFF Hex 6 HexNAc 3 | 130.596 | 0.0 | -20.107 | 6.142e+06 | 140 - 148 | 1 | 25.54 | 25.54 | 25.54 |
| 2983.4399 | TIVKPGDILLIN(N)STGNL Hex 5 HexNAc 2 | 129.599 | 0.007 | -3.562 | 1.183e+07 | 234 - 251 | 1 | 53.36 | 53.53 | 53.71 |
| 3810.7244 | TIVKPGDILLIN(N)STGNL Fuc 2 Hex 4 HexNAc 4 Neu5Ac 1 | 126.829 | 0.011 | -18.901 | 1.669e+07 | 234 - 251 | 1 | 52.53 | 52.53 | 52.70 |
| 4187.7824 | NSKYPALN(N)VTMPNNEQFDKLY Hex 8 HexNAc 2 | 124.568 | 0.011 | -18.353 | 1.035e+07 | 157 - 178 | 4 | 48.61 | 48.61 | 48.78 |
| 4069.9188 | TIVKPGDILLIN(N)STGN(D)LIAPR Hex 9 HexNAc 2 | 120.835 | 0.011 | -5.379 | 1.745e+07 | 234 - 255 | 3 | 54.05 | 54.05 | 54.39 |
| 2638.0658 | RRGSN(N)SSFF Hex 6 HexNAc 3 | 118.895 | 0.0 | -16.069 | 9.254e+06 | 139 - 148 | 1 | 20.63 | 20.63 | 20.63 |
| 4290.0001 | TIVKPGDILLIN(N)STGN(D)LIAPRGY Hex 9 HexNAc 2 | 113.951 | 0.011 | -19.754 | 3.288e+07 | 234 - 257 | 21 | 56.26 | 56.43 | 56.43 |
| 2608.1092 | KLPGNDN(N)STATL Hex 6 HexNAc 2 | 112.936 | 0.011 | -19.876 | 3.109e+06 | 1 - 13 | 1 | 22.42 | 22.42 | 22.42 |
| 3308.5315 | TIVKPGDILLIN(N)STGNL Hex 7 HexNAc 2 | 112.032 | 0.011 | 0.000 | 0.000e+00 | 234 - 251 | 1 | - | - | - |
| 4248.0021 | TIVKPGDILLIN(N)STGNLIAPR Fuc 2 Hex 4 HexNAc 4 Neu5Ac 1 | 111.075 | 0.011 | -17.749 | 1.104e+07 | 234 - 255 | 1 | 53.03 | 53.03 | 53.03 |
| 3347.4979 | TIVKPGDILLIN(N)ST Hex 9 HexNAc 2 | 110.709 | 0.011 | -19.647 | 1.147e+07 | 234 - 248 | 1 | 46.44 | 46.60 | 46.60 |
| 4627.8565 | HKC(C)DNAC(C)IGSIRN(N)GTYDHDVY Fuc 1 Hex 6 HexNAc 5 | 106.227 | 0.011 | -21.686 | 5.908e+06 | 470 - 491 | 1 | 24.71 | 24.88 | 24.88 |
| 3661.5679 | LIN(N)STGNLIAPRGYF Fuc 1 Hex 3 HexNAc 4 Neu5Ac 2 | 103.593 | 0.011 | -18.372 | 3.660e+07 | 243 - 258 | 1 | 46.76 | 46.76 | 46.92 |
| 4204.7609 | N(D)SKYPALN(N)VTM(O)PNNEQFDKLY Hex 8 HexNAc 2 | 102.446 | 0.011 | -6.185 | 1.070e+08 | 157 - 178 | 3 | 45.29 | 45.46 | 45.63 |
| 2744.1658 | QKLPGNDN(N)STATL Hex 3 HexNAc 3 Neu5Ac 1 | 97.550 | 0.017 | 14.850 | 3.818e+08 | 0 - 13 | 4 | 31.31 | 31.63 | 32.46 |
| 4366.8141 | N(D)SKYPALN(N)VTM(O)PNNEQFDKLY Hex 9 HexNAc 2 | 93.995 | 0.027 | -6.107 | 1.224e+08 | 157 - 178 | 2 | 45.12 | 45.29 | 45.46 |
| 3967.9337 | TIVKPGDILLIN(N)STGNLIAPRGY Hex 7 HexNAc 2 | 93.247 | 0.027 | 0.000 | 0.000e+00 | 234 - 257 | 1 | - | - | - |
| 2682.0463 | SC(C)RRGSN(N)SSFF Hex 6 HexNAc 2 | 91.422 | 0.032 | -21.216 | 3.361e+06 | 137 - 148 | 1 | 20.47 | 20.63 | 20.63 |
| 3500.5018 | LIN(N)STGN(D)LIAPRGYF Hex 9 HexNAc 2 | 89.061 | 0.032 | -5.874 | 9.255e+06 | 243 - 258 | 1 | 48.10 | 48.44 | 48.44 |
| 3377.3926 | IGSIRN(N)GTYDHDVY Fuc 1 Hex 5 HexNAc 4 | 88.272 | 0.032 | -18.318 | 7.015e+06 | 477 - 491 | 1 | 24.22 | 24.22 | 24.22 |
| 4753.0327 | RSDAPIGKC(C)KSEC(C)ITPN(N)GSIPN(D)DKPF Hex 9 HexNAc 2 | 83.602 | 0.036 | -5.934 | 3.409e+07 | 268 - 294 | 1 | 30.33 | 30.50 | 30.67 |
| 4342.6783 | HKC(C)DNAC(C)IGSIRN(N)GTYDHDVY Fuc 1 Hex 8 HexNAc 2 | 79.826 | 0.039 | -19.756 | 6.636e+06 | 470 - 491 | 1 | 26.86 | 27.03 | 27.03 |
| 4040.7128 | NSKYPALN(N)VTM(O)PNNEQFDKL Hex 8 HexNAc 2 | 79.133 | 0.039 | -18.788 | 2.460e+07 | 157 - 177 | 2 | 40.25 | 40.25 | 40.42 |
| 4187.9667 | IVKPGDILLIN(N)STGNLIAPRGY Hex 9 HexNAc 2 | 78.911 | 0.039 | -18.919 | 4.835e+06 | 235 - 257 | 1 | 54.73 | 54.90 | 54.90 |
| 4153.8027 | LNSKYPALN(N)VTM(O)PNNEQFDKL Hex 8 HexNAc 2 | 74.549 | 0.039 | -17.099 | 1.494e+07 | 156 - 177 | 2 | 41.76 | 41.76 | 41.93 |
| 3582.7713 | TIVKPGDILLIN(N)STGNLIAPR Hex 6 HexNAc 2 | 73.026 | 0.039 | -19.396 | 3.749e+06 | 234 - 255 | 1 | 53.53 | 53.53 | 53.71 |
| 3802.8541 | TIVKPGDILLIN(N)STGNLIAPRGY Hex 6 HexNAc 2 | 72.091 | 0.042 | -18.276 | 1.080e+07 | 234 - 257 | 4 | 55.74 | 55.91 | 55.91 |
| 4315.8514 | LNSKYPALN(N)VTM(O)PNNEQFDKL Hex 9 HexNAc 2 | 70.444 | 0.042 | -15.958 | 1.544e+07 | 156 - 177 | 1 | 41.76 | 41.76 | 41.76 |
| 4349.8368 | NSKYPALN(N)VTMPNNEQFDKLY Hex 9 HexNAc 2 | 63.435 | 0.045 | -4.694 | 1.099e+07 | 157 - 178 | 3 | 48.44 | 48.61 | 48.78 |
| 4041.7307 | NSKYPALN(N)VTM(O)PNNEQFDKLY Hex 7 HexNAc 2 | 62.532 | 0.045 | -4.208 | 1.896e+07 | 157 - 178 | 1 | 42.44 | 42.78 | 42.78 |
| 3275.2982 | SC(C)RRGSN(N)SSFF Fuc 1 Hex 5 HexNAc 5 | 56.542 | 0.045 | -20.178 | 1.452e+07 | 137 - 148 | 1 | 20.79 | 20.79 | 20.79 |
| 3311.4438 | TITNDRIEVTN(N)ATEL Hex 5 HexNAc 4 | 56.325 | 0.045 | -20.043 | 1.594e+07 | 27 - 42 | 1 | 36.40 | 36.40 | 36.40 |

### LIN(N)STGNL Hex 9 HexNAc 2

**Mass:**  2695.0874

(1.548e-06)

**Charge States:** 2, 3

**Start Position**: 244

**End Position**: 252

| Profile Score | Tandem Score | TDA q-value | Volume | Spectrum Matches |
| --- | --- | --- | --- | --- |
| -4.2249 | 188.2026 | 0.0000 | 9.9560e+07 | 2 |

**Adduct Signal Fractions**

- **Unmodified**: 9.9560e+07

### LIN(N)STGNLIAPRGYF Fuc 2 Hex 3 HexNAc 4 Neu5Ac 1

**Mass:**  3516.5429

(-6.365e-06)

**Charge States:** 3

**Start Position**: 244

**End Position**: 259

| Profile Score | Tandem Score | TDA q-value | Volume | Spectrum Matches |
| --- | --- | --- | --- | --- |
| -17.4071 | 185.7137 | 0.0000 | 5.8415e+07 | 1 |

**Adduct Signal Fractions**

- **Unmodified**: 5.8415e+07

### LIN(N)STGNLIAPRGYF Hex 9 HexNAc 2

**Mass:**  3499.5152

(7.985e-07)

**Charge States:** 3, 4

**Start Position**: 244

**End Position**: 259

| Profile Score | Tandem Score | TDA q-value | Volume | Spectrum Matches |
| --- | --- | --- | --- | --- |
| -1.8723 | 181.0717 | 0.0000 | 3.8090e+08 | 3 |

**Adduct Signal Fractions**

- **Unmodified**: 3.8090e+08

### LIN(N)STGNLIAPRGY Hex 9 HexNAc 2

**Mass:**  3352.4459

(6.359e-07)

**Charge States:** 2, 3, 4

**Start Position**: 244

**End Position**: 258

| Profile Score | Tandem Score | TDA q-value | Volume | Spectrum Matches |
| --- | --- | --- | --- | --- |
| -17.2892 | 173.9650 | 0.0000 | 3.1634e+08 | 2 |

**Adduct Signal Fractions**

- **Unmodified**: 3.1634e+08

### TIVKPGDILLIN(N)STGNLIAPR Hex 9 HexNAc 2

**Mass:**  4068.9238

(1.048e-07)

**Charge States:** 3, 4, 5

**Start Position**: 235

**End Position**: 256

| Profile Score | Tandem Score | TDA q-value | Volume | Spectrum Matches |
| --- | --- | --- | --- | --- |
| 17.9383 | 173.3284 | 0.0000 | 1.9044e+09 | 13 |

**Adduct Signal Fractions**

- **Unmodified**: 1.9044e+09

### RRGSN(N)SSFF Fuc 1 Hex 5 HexNAc 5

**Mass:**  3028.2330

(2.062e-07)

**Charge States:** 3

**Start Position**: 140

**End Position**: 149

| Profile Score | Tandem Score | TDA q-value | Volume | Spectrum Matches |
| --- | --- | --- | --- | --- |
| -18.7725 | 170.3896 | 0.0000 | 2.8238e+07 | 1 |

**Adduct Signal Fractions**

- **Unmodified**: 2.8238e+07

### LIN(N)STGNL Fuc 2 Hex 3 HexNAc 4 Neu5Ac 1

**Mass:**  2712.1157

(-7.830e-06)

**Charge States:** 2, 3

**Start Position**: 244

**End Position**: 252

| Profile Score | Tandem Score | TDA q-value | Volume | Spectrum Matches |
| --- | --- | --- | --- | --- |
| -19.5459 | 168.1737 | 0.0000 | 1.3363e+07 | 2 |

**Adduct Signal Fractions**

- **Unmodified**: 1.3363e+07

### LIN(N)STGNLIAPRGYF Hex 8 HexNAc 2

**Mass:**  3337.4624

(9.049e-07)

**Charge States:** 3

**Start Position**: 244

**End Position**: 259

| Profile Score | Tandem Score | TDA q-value | Volume | Spectrum Matches |
| --- | --- | --- | --- | --- |
| -4.4315 | 166.7611 | 0.0000 | 2.1533e+07 | 1 |

**Adduct Signal Fractions**

- **Unmodified**: 2.1533e+07

### LPGNDN(N)STATL Hex 5 HexNAc 2

**Mass:**  2317.9596

(9.050e-07)

**Charge States:** 3

**Start Position**: 3

**End Position**: 14

| Profile Score | Tandem Score | TDA q-value | Volume | Spectrum Matches |
| --- | --- | --- | --- | --- |
| -5.7155 | 164.8724 | 0.0000 | 1.7519e+07 | 2 |

**Adduct Signal Fractions**

- **Unmodified**: 1.7519e+07

### TIVKPGDILLIN(N)STGNLIAPR Hex 8 HexNAc 2

**Mass:**  3906.8737

(1.534e-07)

**Charge States:** 3, 4

**Start Position**: 235

**End Position**: 256

| Profile Score | Tandem Score | TDA q-value | Volume | Spectrum Matches |
| --- | --- | --- | --- | --- |
| 14.1164 | 163.1211 | 0.0000 | 1.1291e+08 | 5 |

**Adduct Signal Fractions**

- **Unmodified**: 1.1291e+08

### LIN(N)STGNLIAPRGY Fuc 2 Hex 3 HexNAc 4 Neu5Ac 1

**Mass:**  3369.4722

(-7.269e-06)

**Charge States:** 3

**Start Position**: 244

**End Position**: 258

| Profile Score | Tandem Score | TDA q-value | Volume | Spectrum Matches |
| --- | --- | --- | --- | --- |
| -17.0488 | 163.1008 | 0.0000 | 5.3977e+07 | 1 |

**Adduct Signal Fractions**

- **Unmodified**: 5.3977e+07

### TIVKPGDILLIN(N)STGNLIAPRGY Hex 9 HexNAc 2

**Mass:**  4289.0124

(9.225e-07)

**Charge States:** 3, 4, 5

**Start Position**: 235

**End Position**: 258

| Profile Score | Tandem Score | TDA q-value | Volume | Spectrum Matches |
| --- | --- | --- | --- | --- |
| 18.5248 | 162.3376 | 0.0000 | 3.5071e+09 | 25 |

**Adduct Signal Fractions**

- **Unmodified**: 3.5071e+09

### TIVKPGDILLIN(N)STGNLIAPRGY Hex 10 HexNAc 2

**Mass:**  4451.0646

(7.937e-07)

**Charge States:** 3, 4, 5

**Start Position**: 235

**End Position**: 258

| Profile Score | Tandem Score | TDA q-value | Volume | Spectrum Matches |
| --- | --- | --- | --- | --- |
| 15.3757 | 161.7763 | 0.0000 | 3.3181e+08 | 13 |

**Adduct Signal Fractions**

- **Unmodified**: 3.3181e+08

### TIVKPGDILLIN(N)STGNLIAPR Hex 10 HexNAc 2

**Mass:**  4230.9748

(-1.026e-06)

**Charge States:** 3, 4

**Start Position**: 235

**End Position**: 256

| Profile Score | Tandem Score | TDA q-value | Volume | Spectrum Matches |
| --- | --- | --- | --- | --- |
| -3.6962 | 160.8820 | 0.0000 | 1.7268e+08 | 5 |

**Adduct Signal Fractions**

- **Unmodified**: 1.7268e+08

### IN(N)STGNLIAPRGYF Hex 9 HexNAc 2

**Mass:**  3386.4296

(4.245e-07)

**Charge States:** 3

**Start Position**: 245

**End Position**: 259

| Profile Score | Tandem Score | TDA q-value | Volume | Spectrum Matches |
| --- | --- | --- | --- | --- |
| -19.2020 | 160.1403 | 0.0000 | 1.8716e+07 | 2 |

**Adduct Signal Fractions**

- **Unmodified**: 1.8716e+07

### NSKYPALN(N)VTM(O)PNNEQFDKLY Hex 9 HexNAc 2

**Mass:**  4365.8361

(2.860e-06)

**Charge States:** 3, 4

**Start Position**: 158

**End Position**: 179

| Profile Score | Tandem Score | TDA q-value | Volume | Spectrum Matches |
| --- | --- | --- | --- | --- |
| -2.6146 | 159.8481 | 0.0000 | 2.9615e+08 | 3 |

**Adduct Signal Fractions**

- **Unmodified**: 2.9615e+08

### TIVKPGDILLIN(N)STGNLIAPRGY Hex 8 HexNAc 2

**Mass:**  4126.9582

(5.694e-07)

**Charge States:** 3, 4, 5

**Start Position**: 235

**End Position**: 258

| Profile Score | Tandem Score | TDA q-value | Volume | Spectrum Matches |
| --- | --- | --- | --- | --- |
| 14.9176 | 159.6083 | 0.0000 | 3.3721e+08 | 12 |

**Adduct Signal Fractions**

- **Unmodified**: 3.3721e+08

### SKYPALN(N)VTM(O)PNNEQFDKLY Hex 8 HexNAc 2

**Mass:**  4089.7385

(3.542e-06)

**Charge States:** 3, 4

**Start Position**: 159

**End Position**: 179

| Profile Score | Tandem Score | TDA q-value | Volume | Spectrum Matches |
| --- | --- | --- | --- | --- |
| -2.1856 | 159.2304 | 0.0000 | 8.2272e+07 | 4 |

**Adduct Signal Fractions**

- **Unmodified**: 8.2272e+07

### TIVKPGDILLIN(N) Hex 9 HexNAc 2

**Mass:**  3159.4242

(2.290e-06)

**Charge States:** 2, 3

**Start Position**: 235

**End Position**: 247

| Profile Score | Tandem Score | TDA q-value | Volume | Spectrum Matches |
| --- | --- | --- | --- | --- |
| 10.9665 | 158.2985 | 0.0000 | 2.6569e+08 | 6 |

**Adduct Signal Fractions**

- **Unmodified**: 2.6569e+08

### TIVKPGDILLIN(N)STGNLIAPR Fuc 2 Hex 3 HexNAc 4 Neu5Ac 1

**Mass:**  4085.9492

(-6.755e-06)

**Charge States:** 4

**Start Position**: 235

**End Position**: 256

| Profile Score | Tandem Score | TDA q-value | Volume | Spectrum Matches |
| --- | --- | --- | --- | --- |
| 14.0750 | 156.8065 | 0.0000 | 1.2985e+08 | 2 |

**Adduct Signal Fractions**

- **Unmodified**: 1.2985e+08

### LIN(N)STGNLIAPRGY Hex 10 HexNAc 2

**Mass:**  3514.4979

(4.289e-06)

**Charge States:** 3

**Start Position**: 244

**End Position**: 258

| Profile Score | Tandem Score | TDA q-value | Volume | Spectrum Matches |
| --- | --- | --- | --- | --- |
| -3.6368 | 155.7001 | 0.0000 | 3.6575e+07 | 2 |

**Adduct Signal Fractions**

- **Unmodified**: 3.6575e+07

### NSKYPALN(N)VTM(O)PNNEQFDKLY Hex 8 HexNAc 2

**Mass:**  4203.7824

(2.681e-06)

**Charge States:** 3, 4

**Start Position**: 158

**End Position**: 179

| Profile Score | Tandem Score | TDA q-value | Volume | Spectrum Matches |
| --- | --- | --- | --- | --- |
| 17.5565 | 153.9716 | 0.0000 | 2.8746e+08 | 4 |

**Adduct Signal Fractions**

- **Unmodified**: 2.8746e+08

### TIVKPGDILLIN(N) Fuc 2 Hex 3 HexNAc 4 Neu5Ac 1

**Mass:**  3176.4497

(-7.345e-06)

**Charge States:** 3

**Start Position**: 235

**End Position**: 247

| Profile Score | Tandem Score | TDA q-value | Volume | Spectrum Matches |
| --- | --- | --- | --- | --- |
| 11.2896 | 153.3354 | 0.0000 | 4.8651e+07 | 1 |

**Adduct Signal Fractions**

- **Unmodified**: 4.8651e+07

### TITNDRIEVTN(N)ATEL Hex 6 HexNAc 3

**Mass:**  3270.4169

(1.422e-06)

**Charge States:** 3

**Start Position**: 28

**End Position**: 43

| Profile Score | Tandem Score | TDA q-value | Volume | Spectrum Matches |
| --- | --- | --- | --- | --- |
| -19.4503 | 152.8261 | 0.0000 | 2.5631e+07 | 1 |

**Adduct Signal Fractions**

- **Unmodified**: 2.5631e+07

### TIVKPGDILLIN(N)STGNL Hex 9 HexNAc 2

**Mass:**  3631.6499

(1.209e-06)

**Charge States:** 3, 4

**Start Position**: 235

**End Position**: 252

| Profile Score | Tandem Score | TDA q-value | Volume | Spectrum Matches |
| --- | --- | --- | --- | --- |
| 17.6014 | 151.6469 | 0.0000 | 1.0793e+09 | 10 |

**Adduct Signal Fractions**

- **Unmodified**: 1.0793e+09

### TIVKPGDILLIN(N) Hex 10 HexNAc 2

**Mass:**  3321.4784

(1.652e-06)

**Charge States:** 3

**Start Position**: 235

**End Position**: 247

| Profile Score | Tandem Score | TDA q-value | Volume | Spectrum Matches |
| --- | --- | --- | --- | --- |
| -5.1151 | 150.8368 | 0.0000 | 1.7126e+07 | 1 |

**Adduct Signal Fractions**

- **Unmodified**: 1.7126e+07

### SKYPALN(N)VTM(O)PNNEQFDKLY Hex 9 HexNAc 2

**Mass:**  4251.7907

(3.752e-06)

**Charge States:** 3, 4

**Start Position**: 159

**End Position**: 179

| Profile Score | Tandem Score | TDA q-value | Volume | Spectrum Matches |
| --- | --- | --- | --- | --- |
| -2.3857 | 150.5245 | 0.0000 | 8.8572e+07 | 3 |

**Adduct Signal Fractions**

- **Unmodified**: 8.8572e+07

### TIVKPGDILLIN(N)STGN Hex 9 HexNAc 2

**Mass:**  3518.5717

(2.686e-06)

**Charge States:** 3, 4

**Start Position**: 235

**End Position**: 251

| Profile Score | Tandem Score | TDA q-value | Volume | Spectrum Matches |
| --- | --- | --- | --- | --- |
| 16.5113 | 149.9312 | 0.0000 | 3.3890e+08 | 5 |

**Adduct Signal Fractions**

- **Unmodified**: 3.3890e+08

### TIVKPGDILLIN(N)STGNLIAPRGY Fuc 2 Hex 3 HexNAc 4 Neu5Ac 1

**Mass:**  4306.0363

(-6.233e-06)

**Charge States:** 4, 5

**Start Position**: 235

**End Position**: 258

| Profile Score | Tandem Score | TDA q-value | Volume | Spectrum Matches |
| --- | --- | --- | --- | --- |
| 14.5723 | 149.9134 | 0.0000 | 2.5234e+08 | 5 |

**Adduct Signal Fractions**

- **Unmodified**: 2.5234e+08

### TIVKPGDILLIN(N)STGN Hex 10 HexNAc 2

**Mass:**  3680.6237

(1.768e-06)

**Charge States:** 3

**Start Position**: 235

**End Position**: 251

| Profile Score | Tandem Score | TDA q-value | Volume | Spectrum Matches |
| --- | --- | --- | --- | --- |
| -4.3510 | 149.1098 | 0.0000 | 2.2982e+07 | 1 |

**Adduct Signal Fractions**

- **Unmodified**: 2.2982e+07

### TIVKPGDILLIN(N)STGN Fuc 2 Hex 3 HexNAc 4 Neu5Ac 1

**Mass:**  3535.5979

(-5.003e-06)

**Charge States:** 3

**Start Position**: 235

**End Position**: 251

| Profile Score | Tandem Score | TDA q-value | Volume | Spectrum Matches |
| --- | --- | --- | --- | --- |
| -4.1100 | 148.2210 | 0.0000 | 6.5185e+07 | 1 |

**Adduct Signal Fractions**

- **Unmodified**: 6.5185e+07

### TIVKPGDILLIN(N)STGNL Hex 10 HexNAc 2

**Mass:**  3793.7038

(8.386e-07)

**Charge States:** 3, 4

**Start Position**: 235

**End Position**: 252

| Profile Score | Tandem Score | TDA q-value | Volume | Spectrum Matches |
| --- | --- | --- | --- | --- |
| -3.3620 | 147.6003 | 0.0000 | 9.4655e+07 | 3 |

**Adduct Signal Fractions**

- **Unmodified**: 9.4655e+07

### RRGSN(N)SSFF Hex 5 HexNAc 2

**Mass:**  2272.9365

(1.591e-06)

**Charge States:** 3

**Start Position**: 140

**End Position**: 149

| Profile Score | Tandem Score | TDA q-value | Volume | Spectrum Matches |
| --- | --- | --- | --- | --- |
| -19.5601 | 147.5974 | 0.0000 | 1.3954e+07 | 1 |

**Adduct Signal Fractions**

- **Unmodified**: 1.3954e+07

### RRGSN(N)SSFF Fuc 1 Hex 5 HexNAc 4

**Mass:**  2825.1516

(7.038e-07)

**Charge States:** 3

**Start Position**: 140

**End Position**: 149

| Profile Score | Tandem Score | TDA q-value | Volume | Spectrum Matches |
| --- | --- | --- | --- | --- |
| -19.0789 | 144.8401 | 0.0000 | 1.6027e+07 | 1 |

**Adduct Signal Fractions**

- **Unmodified**: 1.6027e+07

### HKC(C)DNAC(C)IGSIRN(N)GTY Fuc 1 Hex 5 HexNAc 4

**Mass:**  3633.4760

(1.583e-06)

**Charge States:** 4

**Start Position**: 471

**End Position**: 487

| Profile Score | Tandem Score | TDA q-value | Volume | Spectrum Matches |
| --- | --- | --- | --- | --- |
| -21.0563 | 144.4564 | 0.0000 | 8.8297e+06 | 1 |

**Adduct Signal Fractions**

- **Unmodified**: 8.8297e+06

### RSDAPIGKC(C)KSEC(C)ITPN(N)GSIPNDKPF Hex 9 HexNAc 2

**Mass:**  4752.0370

(1.485e-06)

**Charge States:** 5

**Start Position**: 269

**End Position**: 295

| Profile Score | Tandem Score | TDA q-value | Volume | Spectrum Matches |
| --- | --- | --- | --- | --- |
| -2.8287 | 144.0210 | 0.0000 | 2.9848e+08 | 2 |

**Adduct Signal Fractions**

- **Unmodified**: 2.9848e+08

### TIVKPGDILLIN(N)STGNL Hex 8 HexNAc 2

**Mass:**  3469.5954

(-3.476e-08)

**Charge States:** 3, 4

**Start Position**: 235

**End Position**: 252

| Profile Score | Tandem Score | TDA q-value | Volume | Spectrum Matches |
| --- | --- | --- | --- | --- |
| -3.8156 | 142.8314 | 0.0000 | 8.4829e+07 | 2 |

**Adduct Signal Fractions**

- **Unmodified**: 8.4829e+07

### TIVKPGDILLIN(N)STGNL Fuc 2 Hex 3 HexNAc 4 Neu5Ac 1

**Mass:**  3648.6758

(-6.366e-06)

**Charge States:** 3

**Start Position**: 235

**End Position**: 252

| Profile Score | Tandem Score | TDA q-value | Volume | Spectrum Matches |
| --- | --- | --- | --- | --- |
| -3.9731 | 142.7624 | 0.0000 | 1.8930e+08 | 2 |

**Adduct Signal Fractions**

- **Unmodified**: 1.8930e+08

### RSDAPIGKC(C)KSEC(C)ITPN(N)GSIPNDKPF Hex 8 HexNAc 2

**Mass:**  4589.9755

(1.037e-06)

**Charge States:** 5

**Start Position**: 269

**End Position**: 295

| Profile Score | Tandem Score | TDA q-value | Volume | Spectrum Matches |
| --- | --- | --- | --- | --- |
| -3.9975 | 142.3632 | 0.0000 | 1.3173e+08 | 2 |

**Adduct Signal Fractions**

- **Unmodified**: 1.3173e+08

### TITNDRIEVTN(N)ATEL Hex 5 HexNAc 2

**Mass:**  2905.2877

(2.343e-06)

**Charge States:** 2, 3

**Start Position**: 28

**End Position**: 43

| Profile Score | Tandem Score | TDA q-value | Volume | Spectrum Matches |
| --- | --- | --- | --- | --- |
| -17.5138 | 142.0385 | 0.0000 | 3.6712e+07 | 1 |

**Adduct Signal Fractions**

- **Unmodified**: 3.6712e+07

### TIVKPGDILLIN(N)STGNLIAPRGY Hex 5 HexNAc 2

**Mass:**  3640.7980

(1.501e-06)

**Charge States:** 3, 4

**Start Position**: 235

**End Position**: 258

| Profile Score | Tandem Score | TDA q-value | Volume | Spectrum Matches |
| --- | --- | --- | --- | --- |
| 16.1191 | 138.8840 | 0.0075 | 4.1822e+07 | 4 |

**Adduct Signal Fractions**

- **Unmodified**: 4.1822e+07

### TIVKPGDILLIN(N)STGNLIAPR Hex 5 HexNAc 2

**Mass:**  3420.7150

(1.127e-06)

**Charge States:** 4

**Start Position**: 235

**End Position**: 256

| Profile Score | Tandem Score | TDA q-value | Volume | Spectrum Matches |
| --- | --- | --- | --- | --- |
| -3.5311 | 138.7473 | 0.0075 | 2.2827e+07 | 3 |

**Adduct Signal Fractions**

- **Unmodified**: 2.2827e+07

### SIRN(N)GTYDHDVY Fuc 1 Hex 5 HexNAc 4

**Mass:**  3207.2884

(3.493e-07)

**Charge States:** 3, 4

**Start Position**: 480

**End Position**: 492

| Profile Score | Tandem Score | TDA q-value | Volume | Spectrum Matches |
| --- | --- | --- | --- | --- |
| -19.0382 | 136.1804 | 0.0075 | 1.4723e+07 | 2 |

**Adduct Signal Fractions**

- **Unmodified**: 1.4723e+07

### C(C)LGHHAVPN(N)GTIVK Fuc 1 Hex 5 HexNAc 5

**Mass:**  3473.5029

(5.008e-07)

**Charge States:** 4

**Start Position**: 14

**End Position**: 28

| Profile Score | Tandem Score | TDA q-value | Volume | Spectrum Matches |
| --- | --- | --- | --- | --- |
| -18.6830 | 135.0253 | 0.0075 | 3.8385e+07 | 1 |

**Adduct Signal Fractions**

- **Unmodified**: 3.8385e+07

### TIVKPGDILLIN(N) Hex 8 HexNAc 2

**Mass:**  2999.3800

(6.715e-04)

**Start Position**: 235

**End Position**: 247

| Profile Score | Tandem Score | TDA q-value | Volume | Spectrum Matches |
| --- | --- | --- | --- | --- |
| 0.0000 | 134.7626 | 0.0075 | 0.0000e+00 | 1 |

Adduct:

No Chromatogram Found

### TITNDRIEVTN(N)ATEL Hex 5 HexNAc 3

**Mass:**  3108.3671

(2.153e-06)

**Charge States:** 3

**Start Position**: 28

**End Position**: 43

| Profile Score | Tandem Score | TDA q-value | Volume | Spectrum Matches |
| --- | --- | --- | --- | --- |
| -17.1724 | 134.7439 | 0.0075 | 1.2814e+07 | 1 |

**Adduct Signal Fractions**

- **Unmodified**: 1.2814e+07

### RSDAPIGKC(C)KSEC(C)ITPN(N)GSIPNDKPF Hex 7 HexNAc 2

**Mass:**  4427.9223

(1.680e-06)

**Charge States:** 4, 5

**Start Position**: 269

**End Position**: 295

| Profile Score | Tandem Score | TDA q-value | Volume | Spectrum Matches |
| --- | --- | --- | --- | --- |
| -3.9097 | 134.7297 | 0.0075 | 1.6279e+08 | 3 |

**Adduct Signal Fractions**

- **Unmodified**: 1.6279e+08

### TITNDRIEVTN(N)ATEL Hex 7 HexNAc 2

**Mass:**  3229.3906

(1.208e-06)

**Charge States:** 3

**Start Position**: 28

**End Position**: 43

| Profile Score | Tandem Score | TDA q-value | Volume | Spectrum Matches |
| --- | --- | --- | --- | --- |
| -17.4406 | 134.6436 | 0.0075 | 4.2279e+07 | 1 |

**Adduct Signal Fractions**

- **Unmodified**: 4.2279e+07

### NSKYPALN(N)VTM(O)PNNEQFDKL Hex 9 HexNAc 2

**Mass:**  4202.7639

(8.390e-07)

**Charge States:** 3, 4

**Start Position**: 158

**End Position**: 178

| Profile Score | Tandem Score | TDA q-value | Volume | Spectrum Matches |
| --- | --- | --- | --- | --- |
| -17.2467 | 134.2319 | 0.0075 | 2.9764e+07 | 2 |

**Adduct Signal Fractions**

- **Unmodified**: 2.9764e+07

### TIVKPGDILLIN(N)STGNLIAPRGY Hex 7 HexNAc 2

**Mass:**  3964.9084

(1.452e-06)

**Charge States:** 3, 4

**Start Position**: 235

**End Position**: 258

| Profile Score | Tandem Score | TDA q-value | Volume | Spectrum Matches |
| --- | --- | --- | --- | --- |
| -3.8415 | 131.6551 | 0.0075 | 1.4400e+07 | 3 |

**Adduct Signal Fractions**

- **Unmodified**: 1.4400e+07

### LIN(N)STGNLIAPRGY Hex 8 HexNAc 2

**Mass:**  3190.3983

(2.286e-06)

**Charge States:** 3

**Start Position**: 244

**End Position**: 258

| Profile Score | Tandem Score | TDA q-value | Volume | Spectrum Matches |
| --- | --- | --- | --- | --- |
| -18.3127 | 130.9262 | 0.0075 | 1.4824e+07 | 1 |

**Adduct Signal Fractions**

- **Unmodified**: 1.4824e+07

### RGSN(N)SSFF Hex 6 HexNAc 3

**Mass:**  2481.9688

(1.922e-06)

**Charge States:** 2, 3

**Start Position**: 141

**End Position**: 149

| Profile Score | Tandem Score | TDA q-value | Volume | Spectrum Matches |
| --- | --- | --- | --- | --- |
| -20.1072 | 130.5957 | 0.0000 | 6.1421e+06 | 1 |

**Adduct Signal Fractions**

- **Unmodified**: 6.1421e+06

### TIVKPGDILLIN(N)STGNL Hex 5 HexNAc 2

**Mass:**  2983.4388

(5.871e-07)

**Charge States:** 3

**Start Position**: 235

**End Position**: 252

| Profile Score | Tandem Score | TDA q-value | Volume | Spectrum Matches |
| --- | --- | --- | --- | --- |
| -3.5620 | 129.5990 | 0.0075 | 1.1826e+07 | 1 |

**Adduct Signal Fractions**

- **Unmodified**: 1.1826e+07

### TIVKPGDILLIN(N)STGNL Fuc 2 Hex 4 HexNAc 4 Neu5Ac 1

**Mass:**  3810.7190

(-9.205e-06)

**Charge States:** 3

**Start Position**: 235

**End Position**: 252

| Profile Score | Tandem Score | TDA q-value | Volume | Spectrum Matches |
| --- | --- | --- | --- | --- |
| -18.9008 | 126.8289 | 0.0112 | 1.6687e+07 | 1 |

**Adduct Signal Fractions**

- **Unmodified**: 1.6687e+07

### NSKYPALN(N)VTMPNNEQFDKLY Hex 8 HexNAc 2

**Mass:**  4187.7824

(1.492e-06)

**Charge States:** 3, 4

**Start Position**: 158

**End Position**: 179

| Profile Score | Tandem Score | TDA q-value | Volume | Spectrum Matches |
| --- | --- | --- | --- | --- |
| -18.3529 | 124.5683 | 0.0112 | 1.0348e+07 | 4 |

**Adduct Signal Fractions**

- **Unmodified**: 1.0348e+07

### TIVKPGDILLIN(N)STGN(D)LIAPR Hex 9 HexNAc 2

**Mass:**  4069.9182

(2.445e-04)

**Charge States:** 3

**Start Position**: 235

**End Position**: 256

| Profile Score | Tandem Score | TDA q-value | Volume | Spectrum Matches |
| --- | --- | --- | --- | --- |
| -5.3785 | 120.8353 | 0.0112 | 1.7449e+07 | 3 |

**Adduct Signal Fractions**

- **Unmodified**: 1.7449e+07

### RRGSN(N)SSFF Hex 6 HexNAc 3

**Mass:**  2638.0658

(2.731e-07)

**Charge States:** 3

**Start Position**: 140

**End Position**: 149

| Profile Score | Tandem Score | TDA q-value | Volume | Spectrum Matches |
| --- | --- | --- | --- | --- |
| -16.0687 | 118.8952 | 0.0000 | 9.2541e+06 | 1 |

**Adduct Signal Fractions**

- **Unmodified**: 9.2541e+06

### TIVKPGDILLIN(N)STGN(D)LIAPRGY Hex 9 HexNAc 2

**Mass:**  4290.0002

(2.312e-04)

**Charge States:** 3, 5

**Start Position**: 235

**End Position**: 258

| Profile Score | Tandem Score | TDA q-value | Volume | Spectrum Matches |
| --- | --- | --- | --- | --- |
| -19.7541 | 113.9510 | 0.0112 | 3.2877e+07 | 21 |

**Adduct Signal Fractions**

- **Unmodified**: 3.2877e+07

### KLPGNDN(N)STATL Hex 6 HexNAc 2

**Mass:**  2608.1092

(3.225e-06)

**Charge States:** 3

**Start Position**: 2

**End Position**: 14

| Profile Score | Tandem Score | TDA q-value | Volume | Spectrum Matches |
| --- | --- | --- | --- | --- |
| -19.8763 | 112.9363 | 0.0112 | 3.1090e+06 | 1 |

**Adduct Signal Fractions**

- **Unmodified**: 3.1090e+06

### TIVKPGDILLIN(N)STGNL Hex 7 HexNAc 2

**Mass:**  3308.5315

(2.989e-04)

**Start Position**: 235

**End Position**: 252

| Profile Score | Tandem Score | TDA q-value | Volume | Spectrum Matches |
| --- | --- | --- | --- | --- |
| 0.0000 | 112.0319 | 0.0112 | 0.0000e+00 | 1 |

Adduct:

No Chromatogram Found

### TIVKPGDILLIN(N)STGNLIAPR Fuc 2 Hex 4 HexNAc 4 Neu5Ac 1

**Mass:**  4248.0021

(-6.351e-06)

**Charge States:** 4

**Start Position**: 235

**End Position**: 256

| Profile Score | Tandem Score | TDA q-value | Volume | Spectrum Matches |
| --- | --- | --- | --- | --- |
| -17.7493 | 111.0749 | 0.0112 | 1.1042e+07 | 1 |

**Adduct Signal Fractions**

- **Unmodified**: 1.1042e+07

### TIVKPGDILLIN(N)ST Hex 9 HexNAc 2

**Mass:**  3347.4995

(-1.357e-07)

**Charge States:** 3

**Start Position**: 235

**End Position**: 249

| Profile Score | Tandem Score | TDA q-value | Volume | Spectrum Matches |
| --- | --- | --- | --- | --- |
| -19.6473 | 110.7085 | 0.0112 | 1.1465e+07 | 1 |

**Adduct Signal Fractions**

- **Unmodified**: 1.1465e+07

### HKC(C)DNAC(C)IGSIRN(N)GTYDHDVY Fuc 1 Hex 6 HexNAc 5

**Mass:**  4627.8512

(3.589e-06)

**Charge States:** 5

**Start Position**: 471

**End Position**: 492

| Profile Score | Tandem Score | TDA q-value | Volume | Spectrum Matches |
| --- | --- | --- | --- | --- |
| -21.6861 | 106.2269 | 0.0112 | 5.9075e+06 | 1 |

**Adduct Signal Fractions**

- **Unmodified**: 5.9075e+06

### LIN(N)STGNLIAPRGYF Fuc 1 Hex 3 HexNAc 4 Neu5Ac 2

**Mass:**  3661.5683

(-9.387e-06)

**Charge States:** 3

**Start Position**: 244

**End Position**: 259

| Profile Score | Tandem Score | TDA q-value | Volume | Spectrum Matches |
| --- | --- | --- | --- | --- |
| -18.3722 | 103.5931 | 0.0112 | 3.6600e+07 | 1 |

**Adduct Signal Fractions**

- **Unmodified**: 3.6600e+07

### N(D)SKYPALN(N)VTM(O)PNNEQFDKLY Hex 8 HexNAc 2

**Mass:**  4204.7595

(2.361e-04)

**Charge States:** 3, 4

**Start Position**: 158

**End Position**: 179

| Profile Score | Tandem Score | TDA q-value | Volume | Spectrum Matches |
| --- | --- | --- | --- | --- |
| -6.1850 | 102.4456 | 0.0112 | 1.0698e+08 | 3 |

**Adduct Signal Fractions**

- **Unmodified**: 1.0698e+08

### QKLPGNDN(N)STATL Hex 3 HexNAc 3 Neu5Ac 1

**Mass:**  2744.1655

(-4.633e-06)

**Charge States:** 3

**Start Position**: 1

**End Position**: 14

| Profile Score | Tandem Score | TDA q-value | Volume | Spectrum Matches |
| --- | --- | --- | --- | --- |
| 14.8499 | 97.5496 | 0.0168 | 3.8183e+08 | 4 |

**Adduct Signal Fractions**

- **Unmodified**: 3.8183e+08

### N(D)SKYPALN(N)VTM(O)PNNEQFDKLY Hex 9 HexNAc 2

**Mass:**  4366.8176

(2.269e-04)

**Charge States:** 4

**Start Position**: 158

**End Position**: 179

| Profile Score | Tandem Score | TDA q-value | Volume | Spectrum Matches |
| --- | --- | --- | --- | --- |
| -6.1070 | 93.9955 | 0.0270 | 1.2238e+08 | 2 |

**Adduct Signal Fractions**

- **Unmodified**: 1.2238e+08

### TIVKPGDILLIN(N)STGNLIAPRGY Hex 7 HexNAc 2

**Mass:**  3967.9337

(7.645e-04)

**Start Position**: 235

**End Position**: 258

| Profile Score | Tandem Score | TDA q-value | Volume | Spectrum Matches |
| --- | --- | --- | --- | --- |
| 0.0000 | 93.2467 | 0.0270 | 0.0000e+00 | 1 |

Adduct:

No Chromatogram Found

### SC(C)RRGSN(N)SSFF Hex 6 HexNAc 2

**Mass:**  2682.0498

(-2.349e-06)

**Charge States:** 3

**Start Position**: 138

**End Position**: 149

| Profile Score | Tandem Score | TDA q-value | Volume | Spectrum Matches |
| --- | --- | --- | --- | --- |
| -21.2161 | 91.4224 | 0.0316 | 3.3607e+06 | 1 |

**Adduct Signal Fractions**

- **Unmodified**: 3.3607e+06

### LIN(N)STGN(D)LIAPRGYF Hex 9 HexNAc 2

**Mass:**  3500.5010

(2.828e-04)

**Charge States:** 3

**Start Position**: 244

**End Position**: 259

| Profile Score | Tandem Score | TDA q-value | Volume | Spectrum Matches |
| --- | --- | --- | --- | --- |
| -5.8739 | 89.0610 | 0.0316 | 9.2554e+06 | 1 |

**Adduct Signal Fractions**

- **Unmodified**: 9.2554e+06

### IGSIRN(N)GTYDHDVY Fuc 1 Hex 5 HexNAc 4

**Mass:**  3377.3926

(-4.511e-08)

**Charge States:** 3

**Start Position**: 478

**End Position**: 492

| Profile Score | Tandem Score | TDA q-value | Volume | Spectrum Matches |
| --- | --- | --- | --- | --- |
| -18.3183 | 88.2724 | 0.0316 | 7.0150e+06 | 1 |

**Adduct Signal Fractions**

- **Unmodified**: 7.0150e+06

### RSDAPIGKC(C)KSEC(C)ITPN(N)GSIPN(D)DKPF Hex 9 HexNAc 2

**Mass:**  4753.0331

(2.126e-04)

**Charge States:** 5

**Start Position**: 269

**End Position**: 295

| Profile Score | Tandem Score | TDA q-value | Volume | Spectrum Matches |
| --- | --- | --- | --- | --- |
| -5.9340 | 83.6022 | 0.0359 | 3.4088e+07 | 1 |

**Adduct Signal Fractions**

- **Unmodified**: 3.4088e+07

### HKC(C)DNAC(C)IGSIRN(N)GTYDHDVY Fuc 1 Hex 8 HexNAc 2

**Mass:**  4342.6755

(-5.819e-06)

**Charge States:** 4

**Start Position**: 471

**End Position**: 492

| Profile Score | Tandem Score | TDA q-value | Volume | Spectrum Matches |
| --- | --- | --- | --- | --- |
| -19.7563 | 79.8255 | 0.0386 | 6.6360e+06 | 1 |

**Adduct Signal Fractions**

- **Unmodified**: 6.6360e+06

### NSKYPALN(N)VTM(O)PNNEQFDKL Hex 8 HexNAc 2

**Mass:**  4040.7121

(1.102e-06)

**Charge States:** 3, 4

**Start Position**: 158

**End Position**: 178

| Profile Score | Tandem Score | TDA q-value | Volume | Spectrum Matches |
| --- | --- | --- | --- | --- |
| -18.7884 | 79.1332 | 0.0386 | 2.4605e+07 | 2 |

**Adduct Signal Fractions**

- **Unmodified**: 2.4605e+07

### IVKPGDILLIN(N)STGNLIAPRGY Hex 9 HexNAc 2

**Mass:**  4187.9650

(2.119e-06)

**Charge States:** 3, 4

**Start Position**: 236

**End Position**: 258

| Profile Score | Tandem Score | TDA q-value | Volume | Spectrum Matches |
| --- | --- | --- | --- | --- |
| -18.9192 | 78.9106 | 0.0386 | 4.8348e+06 | 1 |

**Adduct Signal Fractions**

- **Unmodified**: 4.8348e+06

### LNSKYPALN(N)VTM(O)PNNEQFDKL Hex 8 HexNAc 2

**Mass:**  4153.8024

(2.535e-06)

**Charge States:** 3, 4

**Start Position**: 157

**End Position**: 178

| Profile Score | Tandem Score | TDA q-value | Volume | Spectrum Matches |
| --- | --- | --- | --- | --- |
| -17.0987 | 74.5489 | 0.0386 | 1.4941e+07 | 2 |

**Adduct Signal Fractions**

- **Unmodified**: 1.4941e+07

### TIVKPGDILLIN(N)STGNLIAPR Hex 6 HexNAc 2

**Mass:**  3582.7695

(1.238e-06)

**Charge States:** 4

**Start Position**: 235

**End Position**: 256

| Profile Score | Tandem Score | TDA q-value | Volume | Spectrum Matches |
| --- | --- | --- | --- | --- |
| -19.3961 | 73.0264 | 0.0386 | 3.7490e+06 | 1 |

**Adduct Signal Fractions**

- **Unmodified**: 3.7490e+06

### TIVKPGDILLIN(N)STGNLIAPRGY Hex 6 HexNAc 2

**Mass:**  3802.8549

(6.084e-07)

**Charge States:** 3, 4

**Start Position**: 235

**End Position**: 258

| Profile Score | Tandem Score | TDA q-value | Volume | Spectrum Matches |
| --- | --- | --- | --- | --- |
| -18.2763 | 72.0905 | 0.0419 | 1.0796e+07 | 4 |

**Adduct Signal Fractions**

- **Unmodified**: 1.0796e+07

### LNSKYPALN(N)VTM(O)PNNEQFDKL Hex 9 HexNAc 2

**Mass:**  4315.8542

(-2.242e-07)

**Charge States:** 3, 4

**Start Position**: 157

**End Position**: 178

| Profile Score | Tandem Score | TDA q-value | Volume | Spectrum Matches |
| --- | --- | --- | --- | --- |
| -15.9577 | 70.4444 | 0.0419 | 1.5442e+07 | 1 |

**Adduct Signal Fractions**

- **Unmodified**: 1.5442e+07

### NSKYPALN(N)VTMPNNEQFDKLY Hex 9 HexNAc 2

**Mass:**  4349.8376

(2.544e-06)

**Charge States:** 3

**Start Position**: 158

**End Position**: 179

| Profile Score | Tandem Score | TDA q-value | Volume | Spectrum Matches |
| --- | --- | --- | --- | --- |
| -4.6940 | 63.4350 | 0.0453 | 1.0993e+07 | 3 |

**Adduct Signal Fractions**

- **Unmodified**: 1.0993e+07

### NSKYPALN(N)VTM(O)PNNEQFDKLY Hex 7 HexNAc 2

**Mass:**  4041.7316

(3.326e-06)

**Charge States:** 3, 4

**Start Position**: 158

**End Position**: 179

| Profile Score | Tandem Score | TDA q-value | Volume | Spectrum Matches |
| --- | --- | --- | --- | --- |
| -4.2084 | 62.5315 | 0.0453 | 1.8962e+07 | 1 |

**Adduct Signal Fractions**

- **Unmodified**: 1.8962e+07

### SC(C)RRGSN(N)SSFF Fuc 1 Hex 5 HexNAc 5

**Mass:**  3275.2992

(2.255e-06)

**Charge States:** 3, 4

**Start Position**: 138

**End Position**: 149

| Profile Score | Tandem Score | TDA q-value | Volume | Spectrum Matches |
| --- | --- | --- | --- | --- |
| -20.1779 | 56.5424 | 0.0453 | 1.4515e+07 | 1 |

**Adduct Signal Fractions**

- **Unmodified**: 1.4515e+07

### TITNDRIEVTN(N)ATEL Hex 5 HexNAc 4

**Mass:**  3311.4442

(1.412e-06)

**Charge States:** 3, 4

**Start Position**: 28

**End Position**: 43

| Profile Score | Tandem Score | TDA q-value | Volume | Spectrum Matches |
| --- | --- | --- | --- | --- |
| -20.0432 | 56.3254 | 0.0453 | 1.5944e+07 | 1 |

**Adduct Signal Fractions**

- **Unmodified**: 1.5944e+07

### cus|SWZNA|A/Switzerland/9715293/2013

**>cus|SWZNA|A/Switzerland/9715293/2013**  
 `MNPNQKIITIGSVSLTISTICFFMQIAILITTVTLHFKQYEFNSPPNNQVMLCEPTIIERNITEIVYLTNTTIEKEICPK  
PAEYRNWSKPQCGITGFAPFSKDNSIRLSAGGDIWVTREPYVSCDPDKCYQFALGQGTTLNNVHSNNTVRDRTPYRTLLM  
NELGVPFHLGTKQVCIAWSSSSCHDGKAWLHVCITGDDKNATASFIYNGRLVDSVVSWSKDILRTQESECVCINGTCTVV  
MTDGSASGKADTKILFIEEGKIVHTSTLSGSAQHVEECSCYPRYPGVRCVCRDNWKGSNRPIVDINIKDHSIVSSYVCSG  
LVGDTPRKNDSSSSSHCLDPNNEEGGHGVKGWAFDDGNDVWMGRTINETSRLGYETFKVIEGWSNPKSKLQTNRQVIVDR  
GDRSGYSGIFSVEGKSCINRCFYVELIRGRKEETEVLWTSNSIVVFCGTSGTYGTGSWPDGADLNLMPI`

| Site | Type | Count |
| --- | --- | --- |
| 329 | N-linked | 3 |
| 146 | N-linked | 11 |
| 86 | N-linked | 4 |
| 367 | N-linked | 17 |
| 200 | N-linked | 3 |

| Mass | Sequence | MS_2_ Score | `q`-value | MS_1_ Score | Signal | Protein Coordinates | Tandem Scans | Start Time | Apex Time | End Time |
| --- | --- | --- | --- | --- | --- | --- | --- | --- | --- | --- |
| 3252.3352 | RN(N)WSKPQC(C)GITGF Hex 8 HexNAc 2 | 197.800 | 0.0 | -16.752 | 1.658e+07 | 84 - 97 | 1 | 30.50 | 30.50 | 30.50 |
| 3450.4062 | LHVC(C)ITGDDKN(N)ATASF Hex 8 HexNAc 2 | 195.877 | 0.0 | -3.991 | 8.485e+07 | 189 - 205 | 2 | 33.60 | 33.76 | 33.93 |
| 3612.4589 | LHVC(C)ITGDDKN(N)ATASF Hex 9 HexNAc 2 | 195.274 | 0.0 | -17.363 | 1.098e+08 | 189 - 205 | 3 | 33.60 | 33.76 | 33.76 |
| 3232.3170 | TIN(N)ETSRLGYETF Hex 8 HexNAc 2 | 189.466 | 0.0 | -18.046 | 9.217e+07 | 364 - 377 | 1 | 35.92 | 35.92 | 36.07 |
| 3414.3891 | RN(N)WSKPQC(C)GITGF Hex 9 HexNAc 2 | 186.580 | 0.0 | -17.912 | 2.702e+07 | 84 - 97 | 1 | 30.33 | 30.50 | 30.50 |
| 3729.5457 | RN(N)WSKPQC(C)GITGFAPF Hex 9 HexNAc 2 | 185.709 | 0.0 | -2.038 | 7.827e+07 | 84 - 100 | 2 | 44.61 | 44.78 | 44.95 |
| 3567.4922 | RN(N)WSKPQC(C)GITGFAPF Hex 8 HexNAc 2 | 171.832 | 0.0 | -4.006 | 7.334e+07 | 84 - 100 | 3 | 44.78 | 44.95 | 45.12 |
| 3068.2823 | GRTIN(N)ETSRLGY Hex 8 HexNAc 2 | 170.893 | 0.0 | -17.652 | 1.229e+07 | 362 - 374 | 1 | 20.95 | 20.95 | 21.10 |
| 3268.3722 | M(O)GRTIN(N)ETSRLGYETF Hex 6 HexNAc 2 | 170.071 | 0.0 | -2.869 | 2.809e+08 | 361 - 377 | 3 | 31.63 | 31.79 | 32.12 |
| 3070.2665 | TIN(N)ETSRLGYETF Hex 7 HexNAc 2 | 168.432 | 0.0 | -4.102 | 3.931e+07 | 364 - 377 | 1 | 35.92 | 36.07 | 36.23 |
| 3215.3181 | M(O)GRTIN(N)ETSRLGY Hex 8 HexNAc 2 | 167.961 | 0.0 | -1.967 | 3.585e+08 | 361 - 374 | 4 | 21.27 | 21.44 | 21.77 |
| 2995.2333 | M(O)GRTIN(N)ETSRL Hex 8 HexNAc 2 | 164.049 | 0.0 | -3.252 | 5.598e+07 | 361 - 372 | 1 | 17.05 | 17.21 | 17.38 |
| 3592.4763 | M(O)GRTIN(N)ETSRLGYETF Hex 8 HexNAc 2 | 161.492 | 0.0 | -3.174 | 7.836e+08 | 361 - 377 | 3 | 31.16 | 31.31 | 31.63 |
| 3106.3234 | M(O)GRTIN(N)ETSRLGYETF Hex 5 HexNAc 2 | 160.923 | 0.0 | -18.581 | 6.519e+07 | 361 - 377 | 1 | 31.95 | 32.12 | 32.12 |
| 3430.4229 | M(O)GRTIN(N)ETSRLGYETF Hex 7 HexNAc 2 | 156.501 | 0.0 | 16.022 | 5.008e+08 | 361 - 377 | 3 | 31.31 | 31.63 | 32.12 |
| 2891.2114 | M(O)GRTIN(N)ETSRLGY Hex 6 HexNAc 2 | 152.718 | 0.0 | -17.183 | 7.569e+07 | 361 - 374 | 2 | 21.60 | 21.60 | 21.77 |
| 3305.3759 | VC(C)SGLVGDTPRKN(N)DSSSSSH Hex 5 HexNAc 2 | 147.648 | 0.0 | -19.268 | 2.021e+07 | 316 - 336 | 1 | 14.58 | 14.74 | 14.74 |
| 3053.2646 | M(O)GRTIN(N)ETSRLGY Hex 7 HexNAc 2 | 144.505 | 0.0 | -2.672 | 1.952e+08 | 361 - 374 | 1 | 21.44 | 21.60 | 21.77 |
| 2729.1605 | M(O)GRTIN(N)ETSRLGY Hex 5 HexNAc 2 | 144.373 | 0.0 | -16.943 | 1.637e+07 | 361 - 374 | 1 | 21.77 | 21.93 | 21.93 |
| 3576.4845 | MGRTIN(N)ETSRLGYETF Hex 8 HexNAc 2 | 143.069 | 0.0 | -18.663 | 4.149e+07 | 361 - 377 | 1 | 33.93 | 34.09 | 34.09 |
| 2833.1807 | M(O)GRTIN(N)ETSRL Hex 7 HexNAc 2 | 135.532 | 0.007 | -16.973 | 2.901e+07 | 361 - 372 | 1 | 17.21 | 17.38 | 17.38 |
| 2671.1277 | M(O)GRTIN(N)ETSRL Hex 6 HexNAc 2 | 135.204 | 0.007 | -17.328 | 1.219e+07 | 361 - 372 | 1 | 17.38 | 17.38 | 17.38 |
| 3773.5669 | NNVHSN(N)NTVRDRTPY Hex 6 HexNAc 5 | 134.433 | 0.007 | -3.633 | 1.396e+08 | 140 - 155 | 2 | 12.77 | 12.93 | 13.09 |
| 3199.3233 | MGRTIN(N)ETSRLGY Hex 8 HexNAc 2 | 130.464 | 0.007 | -17.070 | 1.079e+07 | 361 - 374 | 1 | 24.22 | 24.22 | 24.22 |
| 3414.4328 | MGRTIN(N)ETSRLGYETF Hex 7 HexNAc 2 | 127.083 | 0.011 | -18.546 | 1.909e+07 | 361 - 377 | 2 | 34.09 | 34.09 | 34.26 |
| 4138.6977 | NNVHSN(N)NTVRDRTPY Hex 7 HexNAc 6 | 120.608 | 0.011 | -3.538 | 8.333e+07 | 140 - 155 | 2 | 12.62 | 12.77 | 12.93 |
| 4511.7624 | NNVHSN(N)NTVRDRTPY Fuc 2 Hex 10 HexNAc 4 | 117.070 | 0.011 | -21.172 | 3.127e+06 | 140 - 155 | 3 | 15.41 | 15.41 | 15.41 |
| 4284.7580 | NNVHSN(N)NTVRDRTPY Fuc 1 Hex 7 HexNAc 6 | 116.988 | 0.011 | -20.426 | 1.301e+07 | 140 - 155 | 1 | 12.62 | 12.62 | 12.62 |
| 3919.6248 | NNVHSN(N)NTVRDRTPY Fuc 1 Hex 6 HexNAc 5 | 107.358 | 0.011 | -3.900 | 7.562e+07 | 140 - 155 | 2 | 12.77 | 12.77 | 13.09 |
| 4218.6543 | NNVHSN(N)NTVRDRTPY Hex 10 HexNAc 4 | 94.149 | 0.027 | 11.829 | 4.651e+07 | 140 - 155 | 3 | 13.58 | 13.75 | 14.24 |
| 3999.5820 | NNVHSN(N)NTVRDRTPY Fuc 1 Hex 9 HexNAc 3 | 85.107 | 0.036 | -4.383 | 5.672e+07 | 140 - 155 | 3 | 13.75 | 13.91 | 14.24 |
| 4065.6787 | NNVHSN(N)NTVRDRTPY Fuc 2 Hex 6 HexNAc 5 | 83.360 | 0.036 | -21.622 | 7.071e+06 | 140 - 155 | 1 | 12.77 | 12.77 | 12.77 |
| 3554.4926 | NNVHSN(N)NTVRDRTPY Fuc 1 Hex 5 HexNAc 4 | 80.325 | 0.036 | -19.108 | 3.449e+07 | 140 - 155 | 1 | 12.93 | 13.09 | 13.09 |
| 4364.7113 | NNVHSN(N)NTVRDRTPY Fuc 1 Hex 10 HexNAc 4 | 77.204 | 0.039 | -5.836 | 2.278e+07 | 140 - 155 | 2 | 13.58 | 13.75 | 13.91 |
| 3914.6157 | VC(C)SGLVGDTPRKN(N)DSSSSSH Hex 5 HexNAc 5 | 65.327 | 0.045 | -17.690 | 2.663e+07 | 316 - 336 | 1 | 14.74 | 14.74 | 14.74 |
| 3408.4334 | NNVHSN(N)NTVRDRTPY Hex 5 HexNAc 4 | 56.472 | 0.045 | -18.755 | 5.669e+07 | 140 - 155 | 2 | 12.93 | 13.09 | 13.09 |
| 3288.3523 | LHVC(C)ITGDDKN(N)ATASF Hex 7 HexNAc 2 | 54.004 | 0.045 | -19.979 | 1.514e+07 | 189 - 205 | 1 | 33.76 | 33.93 | 33.93 |
| 3711.5372 | VC(C)SGLVGDTPRKN(N)DSSSSSH Hex 5 HexNAc 4 | 51.957 | 0.045 | -19.566 | 1.121e+07 | 316 - 336 | 1 | 14.58 | 14.58 | 14.74 |

### RN(N)WSKPQC(C)GITGF Hex 8 HexNAc 2

**Mass:**  3252.3352

(2.468e-06)

**Charge States:** 3

**Start Position**: 85

**End Position**: 98

| Profile Score | Tandem Score | TDA q-value | Volume | Spectrum Matches |
| --- | --- | --- | --- | --- |
| -16.7519 | 197.8004 | 0.0000 | 1.6583e+07 | 1 |

**Adduct Signal Fractions**

- **Unmodified**: 1.6583e+07

### LHVC(C)ITGDDKN(N)ATASF Hex 8 HexNAc 2

**Mass:**  3450.4055

(1.189e-06)

**Charge States:** 3

**Start Position**: 190

**End Position**: 206

| Profile Score | Tandem Score | TDA q-value | Volume | Spectrum Matches |
| --- | --- | --- | --- | --- |
| -3.9905 | 195.8774 | 0.0000 | 8.4852e+07 | 2 |

**Adduct Signal Fractions**

- **Unmodified**: 8.4852e+07

### LHVC(C)ITGDDKN(N)ATASF Hex 9 HexNAc 2

**Mass:**  3612.4579

(1.007e-06)

**Charge States:** 3, 4

**Start Position**: 190

**End Position**: 206

| Profile Score | Tandem Score | TDA q-value | Volume | Spectrum Matches |
| --- | --- | --- | --- | --- |
| -17.3632 | 195.2741 | 0.0000 | 1.0985e+08 | 3 |

**Adduct Signal Fractions**

- **Unmodified**: 1.0985e+08

### TIN(N)ETSRLGYETF Hex 8 HexNAc 2

**Mass:**  3232.3167

(-2.266e-07)

**Charge States:** 3

**Start Position**: 365

**End Position**: 378

| Profile Score | Tandem Score | TDA q-value | Volume | Spectrum Matches |
| --- | --- | --- | --- | --- |
| -18.0458 | 189.4665 | 0.0000 | 9.2173e+07 | 1 |

**Adduct Signal Fractions**

- **Unmodified**: 9.2173e+07

### RN(N)WSKPQC(C)GITGF Hex 9 HexNAc 2

**Mass:**  3414.3891

(2.560e-06)

**Charge States:** 3

**Start Position**: 85

**End Position**: 98

| Profile Score | Tandem Score | TDA q-value | Volume | Spectrum Matches |
| --- | --- | --- | --- | --- |
| -17.9123 | 186.5801 | 0.0000 | 2.7024e+07 | 1 |

**Adduct Signal Fractions**

- **Unmodified**: 2.7024e+07

### RN(N)WSKPQC(C)GITGFAPF Hex 9 HexNAc 2

**Mass:**  3729.5451

(1.779e-06)

**Charge States:** 3

**Start Position**: 85

**End Position**: 101

| Profile Score | Tandem Score | TDA q-value | Volume | Spectrum Matches |
| --- | --- | --- | --- | --- |
| -2.0380 | 185.7087 | 0.0000 | 7.8272e+07 | 2 |

**Adduct Signal Fractions**

- **Unmodified**: 7.8272e+07

### RN(N)WSKPQC(C)GITGFAPF Hex 8 HexNAc 2

**Mass:**  3567.4927

(1.781e-06)

**Charge States:** 3

**Start Position**: 85

**End Position**: 101

| Profile Score | Tandem Score | TDA q-value | Volume | Spectrum Matches |
| --- | --- | --- | --- | --- |
| -4.0064 | 171.8320 | 0.0000 | 7.3336e+07 | 3 |

**Adduct Signal Fractions**

- **Unmodified**: 7.3336e+07

### GRTIN(N)ETSRLGY Hex 8 HexNAc 2

**Mass:**  3068.2820

(2.024e-07)

**Charge States:** 3

**Start Position**: 363

**End Position**: 375

| Profile Score | Tandem Score | TDA q-value | Volume | Spectrum Matches |
| --- | --- | --- | --- | --- |
| -17.6521 | 170.8925 | 0.0000 | 1.2290e+07 | 1 |

**Adduct Signal Fractions**

- **Unmodified**: 1.2290e+07

### M(O)GRTIN(N)ETSRLGYETF Hex 6 HexNAc 2

**Mass:**  3268.3712

(-2.765e-07)

**Charge States:** 3, 4

**Start Position**: 362

**End Position**: 378

| Profile Score | Tandem Score | TDA q-value | Volume | Spectrum Matches |
| --- | --- | --- | --- | --- |
| -2.8692 | 170.0712 | 0.0000 | 2.8095e+08 | 3 |

**Adduct Signal Fractions**

- **Unmodified**: 2.8095e+08

### TIN(N)ETSRLGYETF Hex 7 HexNAc 2

**Mass:**  3070.2661

(4.956e-07)

**Charge States:** 3

**Start Position**: 365

**End Position**: 378

| Profile Score | Tandem Score | TDA q-value | Volume | Spectrum Matches |
| --- | --- | --- | --- | --- |
| -4.1020 | 168.4318 | 0.0000 | 3.9305e+07 | 1 |

**Adduct Signal Fractions**

- **Unmodified**: 3.9305e+07

### M(O)GRTIN(N)ETSRLGY Hex 8 HexNAc 2

**Mass:**  3215.3183

(3.072e-07)

**Charge States:** 3

**Start Position**: 362

**End Position**: 375

| Profile Score | Tandem Score | TDA q-value | Volume | Spectrum Matches |
| --- | --- | --- | --- | --- |
| -1.9669 | 167.9612 | 0.0000 | 3.5848e+08 | 4 |

**Adduct Signal Fractions**

- **Unmodified**: 3.5848e+08

### M(O)GRTIN(N)ETSRL Hex 8 HexNAc 2

**Mass:**  2995.2334

(4.308e-07)

**Charge States:** 3

**Start Position**: 362

**End Position**: 373

| Profile Score | Tandem Score | TDA q-value | Volume | Spectrum Matches |
| --- | --- | --- | --- | --- |
| -3.2518 | 164.0493 | 0.0000 | 5.5982e+07 | 1 |

**Adduct Signal Fractions**

- **Unmodified**: 5.5982e+07

### M(O)GRTIN(N)ETSRLGYETF Hex 8 HexNAc 2

**Mass:**  3592.4771

(6.215e-07)

**Charge States:** 3, 4

**Start Position**: 362

**End Position**: 378

| Profile Score | Tandem Score | TDA q-value | Volume | Spectrum Matches |
| --- | --- | --- | --- | --- |
| -3.1742 | 161.4915 | 0.0000 | 7.8360e+08 | 3 |

**Adduct Signal Fractions**

- **Unmodified**: 7.8360e+08

### M(O)GRTIN(N)ETSRLGYETF Hex 5 HexNAc 2

**Mass:**  3106.3225

(2.461e-06)

**Charge States:** 3, 4

**Start Position**: 362

**End Position**: 378

| Profile Score | Tandem Score | TDA q-value | Volume | Spectrum Matches |
| --- | --- | --- | --- | --- |
| -18.5806 | 160.9233 | 0.0000 | 6.5190e+07 | 1 |

**Adduct Signal Fractions**

- **Unmodified**: 6.5190e+07

### M(O)GRTIN(N)ETSRLGYETF Hex 7 HexNAc 2

**Mass:**  3430.4225

(-1.760e-07)

**Charge States:** 3

**Start Position**: 362

**End Position**: 378

| Profile Score | Tandem Score | TDA q-value | Volume | Spectrum Matches |
| --- | --- | --- | --- | --- |
| 16.0218 | 156.5006 | 0.0000 | 5.0083e+08 | 3 |

**Adduct Signal Fractions**

- **Unmodified**: 5.0083e+08

### M(O)GRTIN(N)ETSRLGY Hex 6 HexNAc 2

**Mass:**  2891.2110

(-5.075e-08)

**Charge States:** 3

**Start Position**: 362

**End Position**: 375

| Profile Score | Tandem Score | TDA q-value | Volume | Spectrum Matches |
| --- | --- | --- | --- | --- |
| -17.1828 | 152.7176 | 0.0000 | 7.5687e+07 | 2 |

**Adduct Signal Fractions**

- **Unmodified**: 7.5687e+07

### VC(C)SGLVGDTPRKN(N)DSSSSSH Hex 5 HexNAc 2

**Mass:**  3305.3762

(9.022e-07)

**Charge States:** 4

**Start Position**: 317

**End Position**: 337

| Profile Score | Tandem Score | TDA q-value | Volume | Spectrum Matches |
| --- | --- | --- | --- | --- |
| -19.2685 | 147.6484 | 0.0000 | 2.0209e+07 | 1 |

**Adduct Signal Fractions**

- **Unmodified**: 2.0209e+07

### M(O)GRTIN(N)ETSRLGY Hex 7 HexNAc 2

**Mass:**  3053.2642

(4.054e-07)

**Charge States:** 3

**Start Position**: 362

**End Position**: 375

| Profile Score | Tandem Score | TDA q-value | Volume | Spectrum Matches |
| --- | --- | --- | --- | --- |
| -2.6724 | 144.5053 | 0.0000 | 1.9518e+08 | 1 |

**Adduct Signal Fractions**

- **Unmodified**: 1.9518e+08

### M(O)GRTIN(N)ETSRLGY Hex 5 HexNAc 2

**Mass:**  2729.1593

(1.599e-06)

**Charge States:** 3

**Start Position**: 362

**End Position**: 375

| Profile Score | Tandem Score | TDA q-value | Volume | Spectrum Matches |
| --- | --- | --- | --- | --- |
| -16.9431 | 144.3727 | 0.0000 | 1.6373e+07 | 1 |

**Adduct Signal Fractions**

- **Unmodified**: 1.6373e+07

### MGRTIN(N)ETSRLGYETF Hex 8 HexNAc 2

**Mass:**  3576.4839

(9.812e-07)

**Charge States:** 3

**Start Position**: 362

**End Position**: 378

| Profile Score | Tandem Score | TDA q-value | Volume | Spectrum Matches |
| --- | --- | --- | --- | --- |
| -18.6633 | 143.0693 | 0.0000 | 4.1493e+07 | 1 |

**Adduct Signal Fractions**

- **Unmodified**: 4.1493e+07

### M(O)GRTIN(N)ETSRL Hex 7 HexNAc 2

**Mass:**  2833.1808

(4.606e-07)

**Charge States:** 3

**Start Position**: 362

**End Position**: 373

| Profile Score | Tandem Score | TDA q-value | Volume | Spectrum Matches |
| --- | --- | --- | --- | --- |
| -16.9730 | 135.5322 | 0.0075 | 2.9010e+07 | 1 |

**Adduct Signal Fractions**

- **Unmodified**: 2.9010e+07

### M(O)GRTIN(N)ETSRL Hex 6 HexNAc 2

**Mass:**  2671.1277

(5.167e-07)

**Charge States:** 3

**Start Position**: 362

**End Position**: 373

| Profile Score | Tandem Score | TDA q-value | Volume | Spectrum Matches |
| --- | --- | --- | --- | --- |
| -17.3277 | 135.2037 | 0.0075 | 1.2189e+07 | 1 |

**Adduct Signal Fractions**

- **Unmodified**: 1.2189e+07

### NNVHSN(N)NTVRDRTPY Hex 6 HexNAc 5

**Mass:**  3773.5698

(-3.346e-07)

**Charge States:** 4

**Start Position**: 141

**End Position**: 156

| Profile Score | Tandem Score | TDA q-value | Volume | Spectrum Matches |
| --- | --- | --- | --- | --- |
| -3.6326 | 134.4331 | 0.0075 | 1.3958e+08 | 2 |

**Adduct Signal Fractions**

- **Unmodified**: 1.3958e+08

### MGRTIN(N)ETSRLGY Hex 8 HexNAc 2

**Mass:**  3199.3233

(4.515e-07)

**Charge States:** 3

**Start Position**: 362

**End Position**: 375

| Profile Score | Tandem Score | TDA q-value | Volume | Spectrum Matches |
| --- | --- | --- | --- | --- |
| -17.0703 | 130.4644 | 0.0075 | 1.0788e+07 | 1 |

**Adduct Signal Fractions**

- **Unmodified**: 1.0788e+07

### MGRTIN(N)ETSRLGYETF Hex 7 HexNAc 2

**Mass:**  3414.4319

(1.173e-06)

**Charge States:** 3

**Start Position**: 362

**End Position**: 378

| Profile Score | Tandem Score | TDA q-value | Volume | Spectrum Matches |
| --- | --- | --- | --- | --- |
| -18.5462 | 127.0835 | 0.0112 | 1.9093e+07 | 2 |

**Adduct Signal Fractions**

- **Unmodified**: 1.9093e+07

### NNVHSN(N)NTVRDRTPY Hex 7 HexNAc 6

**Mass:**  4138.6970

(5.096e-07)

**Charge States:** 4

**Start Position**: 141

**End Position**: 156

| Profile Score | Tandem Score | TDA q-value | Volume | Spectrum Matches |
| --- | --- | --- | --- | --- |
| -3.5385 | 120.6078 | 0.0112 | 8.3326e+07 | 2 |

**Adduct Signal Fractions**

- **Unmodified**: 8.3326e+07

### NNVHSN(N)NTVRDRTPY Fuc 2 Hex 10 HexNAc 4

**Mass:**  4511.7624

(2.132e-04)

**Charge States:** 4

**Start Position**: 141

**End Position**: 156

| Profile Score | Tandem Score | TDA q-value | Volume | Spectrum Matches |
| --- | --- | --- | --- | --- |
| -21.1718 | 117.0701 | 0.0112 | 3.1274e+06 | 3 |

**Adduct Signal Fractions**

- **Unmodified**: 3.1274e+06

### NNVHSN(N)NTVRDRTPY Fuc 1 Hex 7 HexNAc 6

**Mass:**  4284.7580

(7.958e-07)

**Charge States:** 4

**Start Position**: 141

**End Position**: 156

| Profile Score | Tandem Score | TDA q-value | Volume | Spectrum Matches |
| --- | --- | --- | --- | --- |
| -20.4255 | 116.9876 | 0.0112 | 1.3013e+07 | 1 |

**Adduct Signal Fractions**

- **Unmodified**: 1.3013e+07

### NNVHSN(N)NTVRDRTPY Fuc 1 Hex 6 HexNAc 5

**Mass:**  3919.6240

(3.974e-07)

**Charge States:** 4

**Start Position**: 141

**End Position**: 156

| Profile Score | Tandem Score | TDA q-value | Volume | Spectrum Matches |
| --- | --- | --- | --- | --- |
| -3.9003 | 107.3582 | 0.0112 | 7.5622e+07 | 2 |

**Adduct Signal Fractions**

- **Unmodified**: 7.5622e+07

### NNVHSN(N)NTVRDRTPY Hex 10 HexNAc 4

**Mass:**  4218.6530

(-9.609e-06)

**Charge States:** 4

**Start Position**: 141

**End Position**: 156

| Profile Score | Tandem Score | TDA q-value | Volume | Spectrum Matches |
| --- | --- | --- | --- | --- |
| 11.8292 | 94.1492 | 0.0270 | 4.6514e+07 | 3 |

**Adduct Signal Fractions**

- **Unmodified**: 4.6514e+07

### NNVHSN(N)NTVRDRTPY Fuc 1 Hex 9 HexNAc 3

**Mass:**  3999.5830

(-9.939e-06)

**Charge States:** 4

**Start Position**: 141

**End Position**: 156

| Profile Score | Tandem Score | TDA q-value | Volume | Spectrum Matches |
| --- | --- | --- | --- | --- |
| -4.3831 | 85.1075 | 0.0359 | 5.6717e+07 | 3 |

**Adduct Signal Fractions**

- **Unmodified**: 5.6717e+07

### NNVHSN(N)NTVRDRTPY Fuc 2 Hex 6 HexNAc 5

**Mass:**  4065.6787

(-4.124e-07)

**Charge States:** 4

**Start Position**: 141

**End Position**: 156

| Profile Score | Tandem Score | TDA q-value | Volume | Spectrum Matches |
| --- | --- | --- | --- | --- |
| -21.6218 | 83.3603 | 0.0359 | 7.0708e+06 | 1 |

**Adduct Signal Fractions**

- **Unmodified**: 7.0708e+06

### NNVHSN(N)NTVRDRTPY Fuc 1 Hex 5 HexNAc 4

**Mass:**  3554.4899

(1.460e-06)

**Charge States:** 4

**Start Position**: 141

**End Position**: 156

| Profile Score | Tandem Score | TDA q-value | Volume | Spectrum Matches |
| --- | --- | --- | --- | --- |
| -19.1083 | 80.3249 | 0.0359 | 3.4488e+07 | 1 |

**Adduct Signal Fractions**

- **Unmodified**: 3.4488e+07

### NNVHSN(N)NTVRDRTPY Fuc 1 Hex 10 HexNAc 4

**Mass:**  4364.7097

(-8.548e-06)

**Charge States:** 4

**Start Position**: 141

**End Position**: 156

| Profile Score | Tandem Score | TDA q-value | Volume | Spectrum Matches |
| --- | --- | --- | --- | --- |
| -5.8361 | 77.2038 | 0.0386 | 2.2780e+07 | 2 |

**Adduct Signal Fractions**

- **Unmodified**: 2.2780e+07

### VC(C)SGLVGDTPRKN(N)DSSSSSH Hex 5 HexNAc 5

**Mass:**  3914.6157

(1.326e-06)

**Charge States:** 4

**Start Position**: 317

**End Position**: 337

| Profile Score | Tandem Score | TDA q-value | Volume | Spectrum Matches |
| --- | --- | --- | --- | --- |
| -17.6895 | 65.3270 | 0.0453 | 2.6632e+07 | 1 |

**Adduct Signal Fractions**

- **Unmodified**: 2.6632e+07

### NNVHSN(N)NTVRDRTPY Hex 5 HexNAc 4

**Mass:**  3408.4328

(1.121e-06)

**Charge States:** 4

**Start Position**: 141

**End Position**: 156

| Profile Score | Tandem Score | TDA q-value | Volume | Spectrum Matches |
| --- | --- | --- | --- | --- |
| -18.7551 | 56.4718 | 0.0453 | 5.6695e+07 | 2 |

**Adduct Signal Fractions**

- **Unmodified**: 5.6695e+07

### LHVC(C)ITGDDKN(N)ATASF Hex 7 HexNAc 2

**Mass:**  3288.3524

(1.244e-06)

**Charge States:** 3

**Start Position**: 190

**End Position**: 206

| Profile Score | Tandem Score | TDA q-value | Volume | Spectrum Matches |
| --- | --- | --- | --- | --- |
| -19.9791 | 54.0037 | 0.0453 | 1.5142e+07 | 1 |

**Adduct Signal Fractions**

- **Unmodified**: 1.5142e+07

### VC(C)SGLVGDTPRKN(N)DSSSSSH Hex 5 HexNAc 4

**Mass:**  3711.5393

(2.181e-06)

**Charge States:** 4

**Start Position**: 317

**End Position**: 337

| Profile Score | Tandem Score | TDA q-value | Volume | Spectrum Matches |
| --- | --- | --- | --- | --- |
| -19.5661 | 51.9572 | 0.0453 | 1.1208e+07 | 1 |

**Adduct Signal Fractions**

- **Unmodified**: 1.1208e+07

### tr|A0A1D5PV37|A0A1D5PV37\_CHICK

**>tr|A0A1D5PV37|A0A1D5PV37\_CHICK**  
 `MPGRALFTFAMRGIILALVLTLVGSQKFDIDPGFNSRRSYLYNYEGSMLNGLQDRSLGKAGVRLSSKLEISGLPENAYLL  
KVRSPQVEEYNGVWPRDPFTRSSKITQVISSCFTRLFKFEYSSGRIGNIYAPEDCPDLCVNIVRGILNMFQMTIKKSQNV  
YELQEAGIGGICHARYVIQEDRKNSRIYVTRTVDLNNCQEKVQKSIGMAYIYPCPVDVMKERLTKGTTAFSYKLKQSDSG  
TLITDVSSRQVYQISPFNEPTGVAVMEARQQLTLVEVRSERGSAPDVPMQNYGSLRYRFPAVLPQMPLQLIKTKNPEQRI  
IETLQHIVLNNQQDFHDDVSYRFLEVVQLCRIANADNLESIWRQVSDKPRYRRWLLSAVSASGTTETLKFLKNRIRNDDL  
NYIQTLLTVSLTLHLLQADEHTLPIAADLMTSSRIQKNPVLQQVACLGYSSVVNRYCSQTSACPKEALQPIHDLADEAIS  
RGREDKMKLALKCIGNMGEPASLKRILKFLPISSSSAADIPVHIQIDAITALKKIAWKDPKTVQGYLIQILADQSLPPEV  
RMMACAVIFETRPALALITTIANVAMKESNMQVASFVYSHMKSLSKSRLPFMYNISSACNIALKLLSPKLDSMSYRYSKV  
IRADTYFDNYRVGATGEIFVVNSPRTMFPSAIISKLMANSAGSVADLVEVGIRVEGLADVIMKRNIPFAEYPTYKQIKEL  
GKALQGWKELPTETPLVSAYLKILGQEVAFININKELLQQVMKTVVEPADRNAAIKRIANQIRNSIAGQWTQPVWMGELR  
YVVPSCLGLPLEYGSYTTALARAAVSVEGKMTPPLTGDFRLSQLLESTMQIRSDLKPSLYVHTVATMGVNTEYFQHAVEI  
QGEVQTRMPMKFDAKIDVKLKNLKIETNPCREETEIVVGRHKAFAVSRNIGELGVEKRTSILPEDAPLDVTEEPFQTSER  
ASREHFAMQGPDSMPRKQSHSSREDLRRSTGKRAHKRDICLKMHHIGCQLCFSRRSRDASFIQNTYLHKLIGEHEAKIVL  
MPVHTDADIEKIQLEIQAGAKAASKIIDATSSGPKEEKKPSLYENIKAKLKKILGIENVFKVGDFEYGR`

| Site | Type | Count |
| --- | --- | --- |
| 614 | N-linked | 2 |

| Mass | Sequence | MS_2_ Score | `q`-value | MS_1_ Score | Signal | Protein Coordinates | Tandem Scans | Start Time | Apex Time | End Time |
| --- | --- | --- | --- | --- | --- | --- | --- | --- | --- | --- |
| 4532.7463 | N(N)ISSAC(C)NIAL Fuc 2 Hex 6 HexNAc 8 Neu5Ac 2 | 88.854 | 0.032 | -4.664 | 4.192e+07 | 613 - 623 | 2 | 42.94 | 43.11 | 43.27 |
| 4370.6985 | N(N)ISSAC(C)NIAL Fuc 2 Hex 5 HexNAc 8 Neu5Ac 2 | 56.245 | 0.045 | -18.902 | 1.339e+07 | 613 - 623 | 1 | 41.93 | 41.93 | 42.10 |

### N(N)ISSAC(C)NIAL Fuc 2 Hex 6 HexNAc 8 Neu5Ac 2

**Mass:**  4532.7512

(-6.829e-06)

**Charge States:** 4

**Start Position**: 614

**End Position**: 624

| Profile Score | Tandem Score | TDA q-value | Volume | Spectrum Matches |
| --- | --- | --- | --- | --- |
| -4.6639 | 88.8537 | 0.0316 | 4.1922e+07 | 2 |

**Adduct Signal Fractions**

- **Unmodified**: 4.1922e+07

### N(N)ISSAC(C)NIAL Fuc 2 Hex 5 HexNAc 8 Neu5Ac 2

**Mass:**  4370.7009

(-5.134e-06)

**Charge States:** 4

**Start Position**: 614

**End Position**: 624

| Profile Score | Tandem Score | TDA q-value | Volume | Spectrum Matches |
| --- | --- | --- | --- | --- |
| -18.9016 | 56.2455 | 0.0453 | 1.3388e+07 | 1 |

**Adduct Signal Fractions**

- **Unmodified**: 1.3388e+07

### tr|A0A1D5P780|A0A1D5P780\_CHICK

**>tr|A0A1D5P780|A0A1D5P780\_CHICK**  
 `MRGIILALVLTLVDPGFNSRRSYLYNYEGSMLNGLQDRSLGKAGVRLSSKLEISGLPENAYLLKVRSPQVEEYNGVWPRD  
PFTRSSKITQVISSCFTRLFKFEYSSGRIGNIYAPEDCPDLCVNIVRGILNMFQMTIKKSQNVYELQEAGIGGICHARYV  
IQEDRKNSRIYVTRTVDLNNCQEKVQKSIGMAYIYPCPVDVMKERLTKGTTAFSYKLKQSDSGTLITDVSSRQVYQISPF  
NEPTGVAVMEARQQLTLVEVRSERGSAPDVPMQNYGSLRYRFPAVLPQMPLQLIKTKNPEQRIIETLQHIVLNNQQDFHD  
DVSYRFLEVVQLCRIANADNLESIWRQVSDKPRYRRWLLSAVSASGTTETLKFLKNRIRNDDLNYIQTLLTVSLTLHLLQ  
ADEHTLPIAADLMTSSRIQKNPVLQQVACLGYSSVVNRYCSQTSACPKEALQPIHDLADEAISRGREDKMKLALKCIGNM  
GEPASLKRILKFLPISSSSAADIPVHIQIDAITALKKIAWKDPKTVQGYLIQILADQSLPPEVRMMACAVIFETRPALAL  
ITTIANVAMKESNMQVASFVYSHMKSLSKSRLPFMYNISSACNIALKLLSPKLDSMSYRYSKVIRADTYFDNYRVGATGE  
IFVVNSPRTMFPSAIISKLMANSAGSVADLVEVGIRVEGLADVIMKRNIPFAEYPTYKQIKELGKALQGWKELPTETPLV  
SAYLKILGQEVAFININKELLQQVMKTVVEPADRNAAIKRIANQIRNSIAGQWTQPVWMGELRYVVPSCLGLPLEYGSYT  
TALARAAVSVEGKMTPPLTGDFRLSQLLESTMQIRSDLKPSLYVHTVATMGVNTEYFQHAVEIQGEVQTRMPMKFDAKID  
VKLKNLKIETNPCREETEIVVGRHKAFAVSRNIGELGVEKRTSILPEDAPLDVTEEPFQTSERASREHFAMQGPDSMPRK  
QSHSSREDLRRSTGKRAHKRDICLKMHHIGCQLCFSRRSRDASFIQNTYLHKLIGEHEAKIVLMPVHTDADIDKIQLEIQ  
AGSRAAARIITEVNPESEEEDESSPYEDIQAKLKRILGIDSMFKVANKTRHPKNRPSKKGNTVLAEFGTEPDAKTSSSSS  
SASSTATSSSSSSASSPNRKKPMDEEENDQVKQARNKDASSSSRSSKSSNSSKRSSSKSSNSSKRSSSSSSSSSSSSSSS  
SRSSSSSSSSSSNSKSSSSSSKSSSSSSRSRSSSKSSSSSSSSSSSSSSKSSSSRSSSSSSKSSSHHSHSHHSGHLNGSS  
SSSSSSRSVSHHSHEHHSGHLEDDSSSSSSSSVLSKIWGRHEIYQYRFRSAHRQEFPKRKLPGDRATSRYSSTRSSHDTS  
RAASWPKFLGDIKTPVLAAFLHGISNNKKTGGLQLVVYADTDSVRPRVQVFVTNLTDSSKWKLCADASVRNAHKAVAYVK  
WGWDCRDYKVSTELVTGRFAGHPAAQVKLEWPKVPSNVRSVVEWFYEFVPGAAFMLGFSERMDKNPSRQARMVVALTSPR  
TCDVVVKLPDMILYQKAVRLPLSLPVGPRIPASELQSPIWNVFAEAPSAVLENLKARCSVSYNKIKTFNEVKFNYSMPAN  
CYHILVQDCSSELKFLVMMKSAGEATNLKAINIKIGSHEIDMHPVNGQVKLLVDGAESPTANISLISAGASLWIHNENQG  
FALAAPGHGIDKLYFDGKTITIQVPLWMAGKTCGICGKYDAECEQEYRMPNGYLAKNAVSFGHSWILEEAPCRGACKLHR  
SFVKLEKTVQLAGVDSKCYSTEPVLRCAKGCSATKTTPVTVGFHCLPADSANSLTDKQMKYDQKSEDMQDTVDAHTACSC  
ENEECST`

| Site | Type | Count |
| --- | --- | --- |
| 597 | N-linked | 2 |

| Mass | Sequence | MS_2_ Score | `q`-value | MS_1_ Score | Signal | Protein Coordinates | Tandem Scans | Start Time | Apex Time | End Time |
| --- | --- | --- | --- | --- | --- | --- | --- | --- | --- | --- |
| 4532.7463 | N(N)ISSAC(C)NIAL Fuc 2 Hex 6 HexNAc 8 Neu5Ac 2 | 88.854 | 0.032 | -4.664 | 4.192e+07 | 596 - 606 | 2 | 42.94 | 43.11 | 43.27 |
| 4370.6985 | N(N)ISSAC(C)NIAL Fuc 2 Hex 5 HexNAc 8 Neu5Ac 2 | 56.245 | 0.045 | -18.902 | 1.339e+07 | 596 - 606 | 1 | 41.93 | 41.93 | 42.10 |

### N(N)ISSAC(C)NIAL Fuc 2 Hex 6 HexNAc 8 Neu5Ac 2

**Mass:**  4532.7512

(-6.829e-06)

**Charge States:** 4

**Start Position**: 597

**End Position**: 607

| Profile Score | Tandem Score | TDA q-value | Volume | Spectrum Matches |
| --- | --- | --- | --- | --- |
| -4.6639 | 88.8537 | 0.0316 | 4.1922e+07 | 2 |

**Adduct Signal Fractions**

- **Unmodified**: 4.1922e+07

### N(N)ISSAC(C)NIAL Fuc 2 Hex 5 HexNAc 8 Neu5Ac 2

**Mass:**  4370.7009

(-5.134e-06)

**Charge States:** 4

**Start Position**: 597

**End Position**: 607

| Profile Score | Tandem Score | TDA q-value | Volume | Spectrum Matches |
| --- | --- | --- | --- | --- |
| -18.9016 | 56.2455 | 0.0453 | 1.3388e+07 | 1 |

**Adduct Signal Fractions**

- **Unmodified**: 1.3388e+07

### sp|P02845|VIT2\_CHICK

**>sp|P02845|VIT2\_CHICK**  
 `MRGIILALVLTLVGSQKFDIDPGFNSRRSYLYNYEGSMLNGLQDRSLGKAGVRLSSKLEISGLPENAYLLKVRSPQVEEY  
NGVWPRDPFTRSSKITQVISSCFTRLFKFEYSSGRIGNIYAPEDCPDLCVNIVRGILNMFQMTIKKSQNVYELQEAGIGG  
ICHARYVIQEDRKNSRIYVTRTVDLNNCQEKVQKSIGMAYIYPCPVDVMKERLTKGTTAFSYKLKQSDSGTLITDVSSRQ  
VYQISPFNEPTGVAVMEARQQLTLVEVRSERGSAPDVPMQNYGSLRYRFPAVLPQMPLQLIKTKNPEQRIVETLQHIVLN  
NQQDFHDDVSYRFLEVVQLCRIANADNLESIWRQVSDKPRYRRWLLSAVSASGTTETLKFLKNRIRNDDLNYIQTLLTVS  
LTLHLLQADEHTLPIAADLMTSSRIQKNPVLQQVACLGYSSVVNRYCSQTSACPKEALQPIHDLADEAISRGREDKMKLA  
LKCIGNMGEPASLKRILKFLPISSSSAADIPVHIQIDAITALKKIAWKDPKTVQGYLIQILADQSLPPEVRMMACAVIFE  
TRPALALITTIANVAMKESNMQVASFVYSHMKSLSKSRLPFMYNISSACNIALKLLSPKLDSMSYRYSKVIRADTYFDNY  
RVGATGEIFVVNSPRTMFPSAIISKLMANSAGSVADLVEVGIRVEGLADVIMKRNIPFAEYPTYKQIKELGKALQGWKEL  
PTETPLVSAYLKILGQEVAFININKELLQQVMKTVVEPADRNAAIKRIANQIRNSIAGQWTQPVWMGELRYVVPSCLGLP  
LEYGSYTTALARAAVSVEGKMTPPLTGDFRLSQLLESTMQIRSDLKPSLYVHTVATMGVNTEYFQHAVEIQGEVQTRMPM  
KFDAKIDVKLKNLKIETNPCREETEIVVGRHKAFAVSRNIGELGVEKRTSILPEDAPLDVTEEPFQTSERASREHFAMQG  
PDSMPRKQSHSSREDLRRSTGKRAHKRDICLKMHHIGCQLCFSRRSRDASFIQNTYLHKLIGEHEAKIVLMPVHTDADID  
KIQLEIQAGSRAAARIITEVNPESEEEDESSPYEDIQAKLKRILGIDSMFKVANKTRHPKNRPSKKGNTVLAEFGTEPDA  
KTSSSSSSASSTATSSSSSSASSPNRKKPMDEEENDQVKQARNKDASSSSRSSKSSNSSKRSSSKSSNSSKRSSSSSSSS  
SSSSRSSSSSSSSSSNSKSSSSSSKSSSSSSRSRSSSKSSSSSSSSSSSSSSKSSSSRSSSSSSKSSSHHSHSHHSGHLN  
GSSSSSSSSRSVSHHSHEHHSGHLEDDSSSSSSSSVLSKIWGRHEIYQYRFRSAHRQEFPKRKLPGDRATSRYSSTRSSH  
DTSRAASWPKFLGDIKTPVLAAFLHGISNNKKTGGLQLVVYADTDSVRPRVQVFVTNLTDSSKWKLCADASVRNAHKAVA  
YVKWGWDCRDYKVSTELVTGRFAGHPAAQVKLEWPKVPSNVRSVVEWFYEFVPGAAFMLGFSERMDKNPSRQARMVVALT  
SPRTCDVVVKLPDIILYQKAVRLPLSLPVGPRIPASELQPPIWNVFAEAPSAVLENLKARCSVSYNKIKTFNEVKFNYSM  
PANCYHILVQDCSSELKFLVMMKSAGEATNLKAINIKIGSHEIDMHPVNGQVKLLVDGAESPTANISLISAGASLWIHNE  
NQGFALAAPGHGIDKLYFDGKTITIQVPLWMAGKTCGICGKYDAECEQEYRMPNGYLAKNAVSFGHSWILEEAPCRGACK  
LHRSFVKLEKTVQLAGVDSKCYSTEPVLRCAKGCSATKTTPVTVGFHCLPADSANSLTDKQMKYDQKSEDMQDTVDAHTT  
CSCENEECST`

| Site | Type | Count |
| --- | --- | --- |
| 604 | N-linked | 2 |

| Mass | Sequence | MS_2_ Score | `q`-value | MS_1_ Score | Signal | Protein Coordinates | Tandem Scans | Start Time | Apex Time | End Time |
| --- | --- | --- | --- | --- | --- | --- | --- | --- | --- | --- |
| 4532.7463 | N(N)ISSAC(C)NIAL Fuc 2 Hex 6 HexNAc 8 Neu5Ac 2 | 88.854 | 0.032 | -4.664 | 4.192e+07 | 603 - 613 | 2 | 42.94 | 43.11 | 43.27 |
| 4370.6985 | N(N)ISSAC(C)NIAL Fuc 2 Hex 5 HexNAc 8 Neu5Ac 2 | 56.245 | 0.045 | -18.902 | 1.339e+07 | 603 - 613 | 1 | 41.93 | 41.93 | 42.10 |

### N(N)ISSAC(C)NIAL Fuc 2 Hex 6 HexNAc 8 Neu5Ac 2

**Mass:**  4532.7512

(-6.829e-06)

**Charge States:** 4

**Start Position**: 604

**End Position**: 614

| Profile Score | Tandem Score | TDA q-value | Volume | Spectrum Matches |
| --- | --- | --- | --- | --- |
| -4.6639 | 88.8537 | 0.0316 | 4.1922e+07 | 2 |

**Adduct Signal Fractions**

- **Unmodified**: 4.1922e+07

### N(N)ISSAC(C)NIAL Fuc 2 Hex 5 HexNAc 8 Neu5Ac 2

**Mass:**  4370.7009

(-5.134e-06)

**Charge States:** 4

**Start Position**: 604

**End Position**: 614

| Profile Score | Tandem Score | TDA q-value | Volume | Spectrum Matches |
| --- | --- | --- | --- | --- |
| -18.9016 | 56.2455 | 0.0453 | 1.3388e+07 | 1 |

**Adduct Signal Fractions**

- **Unmodified**: 1.3388e+07

### tr|F1NFL6|F1NFL6\_CHICK

**>tr|F1NFL6|F1NFL6\_CHICK**  
 `MRGIILALVLTLVGSQKFDIDPGFNSRRSYLYNYEGSMLNGLQDRSLGKAGVRLSSKLEISGLPENAYLLKVRSPQVEEY  
NGVWPRDPFTRSSKITQVISSCFTRLFKFEYSSGRIGNIYAPEDCPDLCVNIVRGILNMFQMTIKKSQNVYELQEAGIGG  
ICHARYVIQEDRKNSRIYVTRTVDLNNCQEKVQKSIGMAYIYPCPVDVMKERLTKGTTAFSYKLKQSDSGTLITDVSSRQ  
VYQISPFNEPTGVAVMEARQQLTLVEVRSERGSAPDVPMQNYGSLRYRFPAVLPQMPLQLIKTKNPEQRIIETLQHIVLN  
NQQDFHDDVSYRFLEVVQLCRIANADNLESIWRQVSDKPRYRRWLLSAVSASGTTETLKFLKNRIRNDDLNYIQTLLTVS  
LTLHLLQADEHTLPIAADLMTSSRIQKNPVLQQVACLGYSSVVNRYCSQTSACPKEALQPIHDLADEAISRGREDKMKLA  
LKCIGNMGEPASLKRILKFLPISSSSAADIPVHIQIDAITALKKIAWKDPKTVQGYLIQILADQSLPPEVRMMACAVIFE  
TRPALALITTIANVAMKESNMQVASFVYSHMKSLSKSRLPFMYNISSACNIALKLLSPKLDSMSYRYSKVIRADTYFDNY  
RVGATGEIFVVNSPRTMFPSAIISKLMANSAGSVADLVEVGIRVEGLADVIMKRNIPFAEYPTYKQIKELGKALQGWKEL  
PTETPLVSAYLKILGQEVAFININKELLQQVMKTVVEPADRNAAIKRIANQIRNSIAGQWTQPVWMGELRYVVPSCLGLP  
LEYGSYTTALARAAVSVEGKMTPPLTGDFRLSQLLESTMQIRSDLKPSLYVHTVATMGVNTEYFQHAVEIQGEVQTRMPM  
KFDAKIDVKLKNLKIETNPCREETEIVVGRHKAFAVSRNIGELGVEKRTSILPEDAPLDVTEEPFQTSERASREHFAMQG  
PDSMPRKQSHSSREDLRRSTGKRAHKRDICLKMHHIGCQLCFSRRSRDASFIQNTYLHKLIGEHEAKIVLMPVHTDADID  
KIQLEIQAGSRAAARIITEVNPESEEEDESSPYEDIQAKLKRILGIDSMFKVANKTRHPKNRPSKKGNTVLAEFGTEPDA  
KTSSSSSSASSTATSSSSSSASSPNRKKPMDEEENDQVKQARNKDASSSSRSSKSSNSSKRSSSKSSNSSKRSSSSSSSS  
SSSSSSSSRSSSSSSSSSSNSKSSSSSSKSSSSSSRSRSSSKSSSSSSSSSSSSSSKSSSSRSSSSSSKSSSHHSHSHHS  
GHLNGSSSSSSSSRSVSHHSHEHHSGHLEDDSSSSSSSSVLSKIWGRHEIYQYRFRSAHRQEFPKRKLPGDRATSRYSST  
RSSHDTSRAASWPKFLGDIKTPVLAAFLHGISNNKKTGGLQLVVYADTDSVRPRVQVFVTNLTDSSKWKLCADASVRNAH  
KAVAYVKWGWDCRDYKVSTELVTGRFAGHPAAQVKLEWPKVPSNVRSVVEWFYEFVPGAAFMLGFSERMDKNPSRQARMV  
VALTSPRTCDVVVKLPDMILYQKAVRLPLSLPVGPRIPASELQSPIWNVFAEAPSAVLENLKARCSVSYNKIKTFNEVKF  
NYSMPANCYHILVQDCSSELKFLVMMKSAGEATNLKAINIKIGSHEIDMHPVNGQVKLLVDGAESPTANISLISAGASLW  
IHNENQGFALAAPGHGIDKLYFDGKTITIQVPLWMAGKTCGICGKYDAECEQEYRMPNGYLAKNAVSFGHSWILEEAPCR  
GACKLHRSFVKLEKTVQLAGVDSKCYSTEPVLRCAKGCSATKTTPVTVGFHCLPADSANSLTDKQMKYDQKSEDMQDTVD  
AHTACSCENEECST`

| Site | Type | Count |
| --- | --- | --- |
| 604 | N-linked | 2 |

| Mass | Sequence | MS_2_ Score | `q`-value | MS_1_ Score | Signal | Protein Coordinates | Tandem Scans | Start Time | Apex Time | End Time |
| --- | --- | --- | --- | --- | --- | --- | --- | --- | --- | --- |
| 4532.7463 | N(N)ISSAC(C)NIAL Fuc 2 Hex 6 HexNAc 8 Neu5Ac 2 | 88.854 | 0.032 | -4.664 | 4.192e+07 | 603 - 613 | 2 | 42.94 | 43.11 | 43.27 |
| 4370.6985 | N(N)ISSAC(C)NIAL Fuc 2 Hex 5 HexNAc 8 Neu5Ac 2 | 56.245 | 0.045 | -18.902 | 1.339e+07 | 603 - 613 | 1 | 41.93 | 41.93 | 42.10 |

### N(N)ISSAC(C)NIAL Fuc 2 Hex 6 HexNAc 8 Neu5Ac 2

**Mass:**  4532.7512

(-6.829e-06)

**Charge States:** 4

**Start Position**: 604

**End Position**: 614

| Profile Score | Tandem Score | TDA q-value | Volume | Spectrum Matches |
| --- | --- | --- | --- | --- |
| -4.6639 | 88.8537 | 0.0316 | 4.1922e+07 | 2 |

**Adduct Signal Fractions**

- **Unmodified**: 4.1922e+07

### N(N)ISSAC(C)NIAL Fuc 2 Hex 5 HexNAc 8 Neu5Ac 2

**Mass:**  4370.7009

(-5.134e-06)

**Charge States:** 4

**Start Position**: 604

**End Position**: 614

| Profile Score | Tandem Score | TDA q-value | Volume | Spectrum Matches |
| --- | --- | --- | --- | --- |
| -18.9016 | 56.2455 | 0.0453 | 1.3388e+07 | 1 |

**Adduct Signal Fractions**

- **Unmodified**: 1.3388e+07

### sp|P09244|TBB7\_CHICK

**>sp|P09244|TBB7\_CHICK**  
 `MREIVHIQAGQCGNQIGAKFWEVISDEHGIDPTGTYHGDSDLQLDRISVYYNEATGGKYVPRAILVDLEPGTMDSVRSGP  
FGQIFRPDNFVFGQSGAGNNWAKGHYTEGAELVDSVLDVVRKEAESCDCLQGFQLTHSLGGGTGSGMGTLLISKIREEYP  
DRIMNTFSVVPSPKVSDTVVEPYNATLSVHQLVENTDETYCIDNEALYDICFRTLKLTTPTYGDLNHLVSATMSGVTTCL  
RFPGQLNADLRKLAVNMVPFPRLHFFMPGFAPLTSRGSQQYRALTVPELTQQVFDAKNMMAACDPRHGRYLTVAAVFRGR  
MSMKEVDEQMLNVQNKNSSYFVEWIPNNVKTAVCDIPPRGLKMAVTFIGNSTAIQELFKRISEQFTAMFRRKAFLHWYTG  
EGMDEMEFTEAESNMNDLVSEYQQYQDATAEEEEDFGEEAEEEA`

| Site | Type | Count |
| --- | --- | --- |
| 337 | N-linked | 1 |

| Mass | Sequence | MS_2_ Score | `q`-value | MS_1_ Score | Signal | Protein Coordinates | Tandem Scans | Start Time | Apex Time | End Time |
| --- | --- | --- | --- | --- | --- | --- | --- | --- | --- | --- |
| 4078.6251 | NVQNKN(N)SSYF Fuc 3 Hex 7 HexNAc 5 Neu5Ac 1 | 95.149 | 0.027 | -19.428 | 7.258e+07 | 331 - 341 | 2 | 35.27 | 35.27 | 35.43 |

### NVQNKN(N)SSYF Fuc 3 Hex 7 HexNAc 5 Neu5Ac 1

**Mass:**  4078.6215

(7.039e-06)

**Charge States:** 3, 4

**Start Position**: 332

**End Position**: 342

| Profile Score | Tandem Score | TDA q-value | Volume | Spectrum Matches |
| --- | --- | --- | --- | --- |
| -19.4279 | 95.1488 | 0.0270 | 7.2580e+07 | 2 |

**Adduct Signal Fractions**

- **Unmodified**: 7.2580e+07

### tr|E1C8K3|E1C8K3\_CHICK

**>tr|E1C8K3|E1C8K3\_CHICK**  
 `ALWWRACIEEEGKPVIGVYSNFDPTPDAQTLYKAMKGLGTDEQAIIEVLTKRSNKQRQEIAKSFKAQFGKDLIDSLRSEL  
SGNFERLIVALMYPPYKYEAKELYDAMKGVGTSEDVIIEILASRTKAQIKEIIKAYKEDYGSDLEHDIASETSGYFKQIL  
VCLLQGERDNASLYVDTALARQDAETLFSAGEKIKGTDEIQFITILCKRSATHLLKGMKQSIGRAIDVKAWVVRNNHFNL  
CTRNIRQYFAERLYHALKGAGTDDGTLIRVIVSRNEVDLNLIKDEFRRIAGQPLSSMIVDDTSGDYKTALLNLCGSD`

| Site | Type | Count |
| --- | --- | --- |
| 170 | N-linked | 1 |

| Mass | Sequence | MS_2_ Score | `q`-value | MS_1_ Score | Signal | Protein Coordinates | Tandem Scans | Start Time | Apex Time | End Time |
| --- | --- | --- | --- | --- | --- | --- | --- | --- | --- | --- |
| 4370.6888 | LQGERDN(N)ASLY Fuc 2 Hex 5 HexNAc 7 Neu5Ac 2 | 99.308 | 0.011 | -17.705 | 3.616e+07 | 163 - 174 | 1 | 43.11 | 43.27 | 43.27 |

### LQGERDN(N)ASLY Fuc 2 Hex 5 HexNAc 7 Neu5Ac 2

**Mass:**  4370.6862

(-8.901e-06)

**Charge States:** 4

**Start Position**: 164

**End Position**: 175

| Profile Score | Tandem Score | TDA q-value | Volume | Spectrum Matches |
| --- | --- | --- | --- | --- |
| -17.7054 | 99.3076 | 0.0112 | 3.6158e+07 | 1 |

**Adduct Signal Fractions**

- **Unmodified**: 3.6158e+07

### tr|F1NUU6|F1NUU6\_CHICK

**>tr|F1NUU6|F1NUU6\_CHICK**  
 `MGCSTILVFLTLLSFASAHRKLLLFLLDGFRFDYIDDAELESLPGFRDIIGMGVKVDYMTPDFPSLSYPNYYTLMTGRHC  
EVHQMIGNYMWDPKTNVSFDIGVNKESLLPLWWNGSEPLWVTMTKEKKNVFMYYWPGCEVEILGVRPSYCREYFSVPTDQ  
NFADAISDALEALRNGSAEMAAVYYERIDVEGHHYGPWSEQRKSALKELDKAMSNMITQIKNKGLQHDVNVLLFSDHGMT  
DISWTDKVIELKNYINMSDTIQMKDRGPVVSLWPVPEKHTEIYQKLKAVEHMNVYNIQDIPERFFYKKGKFVSPLTLVAE  
EGWFIVESRDKLPFWENGTGKREAWQNGWHGYDNELMEMRGIFLAYGPDFRSNYRAPPIRSVDVYNVMCSLAGVQPQPNN  
GSWSRVECMLKNTAVRVPLCTRSGCVLALTLLLLFWLL`

| Site | Type | Count |
| --- | --- | --- |
| 175 | N-linked | 1 |

| Mass | Sequence | MS_2_ Score | `q`-value | MS_1_ Score | Signal | Protein Coordinates | Tandem Scans | Start Time | Apex Time | End Time |
| --- | --- | --- | --- | --- | --- | --- | --- | --- | --- | --- |
| 4962.9337 | RN(N)GSAEMAAVY Fuc 2 Hex 8 HexNAc 8 Neu5Ac 2 | 58.704 | 0.045 | -19.043 | 5.012e+07 | 173 - 184 | 1 | 21.93 | 21.93 | 22.10 |

### RN(N)GSAEMAAVY Fuc 2 Hex 8 HexNAc 8 Neu5Ac 2

**Mass:**  4962.9352

(7.422e-06)

**Charge States:** 3, 4

**Start Position**: 174

**End Position**: 185

| Profile Score | Tandem Score | TDA q-value | Volume | Spectrum Matches |
| --- | --- | --- | --- | --- |
| -19.0433 | 58.7041 | 0.0453 | 5.0120e+07 | 1 |

**Adduct Signal Fractions**

- **Unmodified**: 5.0120e+07

### tr|A0A1D5P474|A0A1D5P474\_CHICK

**>tr|A0A1D5P474|A0A1D5P474\_CHICK**  
 `MGALLALLDPVQPTRAPDCGGILTPLGLSYLAEVSKPHAEVVLRQDLMAQRASDLFLGSMEPSRNRITSVKVADLWLSVV  
PEAGLRLGIEVELRVAPLHAVPMPVRISIRADLHVDMGPDGNLQLLTSACRPTVQAQSTREAESKSSRSILDKVVDVDKL  
CLDVSKLLLFPNEQLMSLTALFPVTPNCQLQYLPLAAPVFSKQGIALSLQTTFQVAGAVVPVPVSPVPFSMPELASTSTS  
HLILALSEHFYTSLYFTLERAGAFNMTIPSMLTTATLAQKITQVGSLYHEDLPITLSAALRSSPRVVLEEGRAALKLFLT  
VHIGVGSPDFQSFLSVSAVSEPQDCSSASLTHG`

| Site | Type | Count |
| --- | --- | --- |
| 265 | N-linked | 1 |

| Mass | Sequence | MS_2_ Score | `q`-value | MS_1_ Score | Signal | Protein Coordinates | Tandem Scans | Start Time | Apex Time | End Time |
| --- | --- | --- | --- | --- | --- | --- | --- | --- | --- | --- |
| 3575.4193 | N(N)MTIPSM Fuc 4 Hex 3 HexNAc 7 Neu5Ac 1 | 128.923 | 0.0 | -19.627 | 1.663e+07 | 264 - 271 | 1 | 29.02 | 29.18 | 29.18 |

### N(N)MTIPSM Fuc 4 Hex 3 HexNAc 7 Neu5Ac 1

**Mass:**  3575.4204

(7.958e-06)

**Charge States:** 3

**Start Position**: 265

**End Position**: 272

| Profile Score | Tandem Score | TDA q-value | Volume | Spectrum Matches |
| --- | --- | --- | --- | --- |
| -19.6269 | 128.9233 | 0.0000 | 1.6633e+07 | 1 |

**Adduct Signal Fractions**

- **Unmodified**: 1.6633e+07

### tr|E1BXM5|E1BXM5\_CHICK

**>tr|E1BXM5|E1BXM5\_CHICK**  
 `MGALLALLDPVQPTRAPDCGGILTPLGLSYLAEVSKPHAEVVLRQDLMPKEPQTCSLAPWSPAGNRITSVKVADLWLSVV  
PEAGLRLGIEVELRVAPLHAVPMPVRISIRADLHVDMGPDGNLQLLTSACRPTVQAQSTREAESKSSRSILDKVVDVDKL  
CLDVSKLLLFPNEQLMSLTALFPVTPNCQLQYLPLAAPVFSKQGIALSLQTTFQVAGAVVPVPVSPVPFSMPELASTSTS  
HLILALSEHFYTSLYFTLERAGAFNMTIPSMLTTATLAQKITQVGSLYHEDLPITLSAALRSSPRVVLEEGRAALKLFLT  
VHIGVGSPDFQSFLSVSADVTAGLQLSVSDTRMMISTAVIEDAELSLAASNVGLVRAALLEELFLAPVCQQVPAWMDDVL  
REGVHLPHLSHFTYTDVNVVVHKDYVLVPCKLKLRSTMA`

| Site | Type | Count |
| --- | --- | --- |
| 265 | N-linked | 1 |

| Mass | Sequence | MS_2_ Score | `q`-value | MS_1_ Score | Signal | Protein Coordinates | Tandem Scans | Start Time | Apex Time | End Time |
| --- | --- | --- | --- | --- | --- | --- | --- | --- | --- | --- |
| 3575.4193 | N(N)MTIPSM Fuc 4 Hex 3 HexNAc 7 Neu5Ac 1 | 128.923 | 0.0 | -19.627 | 1.663e+07 | 264 - 271 | 1 | 29.02 | 29.18 | 29.18 |

### N(N)MTIPSM Fuc 4 Hex 3 HexNAc 7 Neu5Ac 1

**Mass:**  3575.4204

(7.958e-06)

**Charge States:** 3

**Start Position**: 265

**End Position**: 272

| Profile Score | Tandem Score | TDA q-value | Volume | Spectrum Matches |
| --- | --- | --- | --- | --- |
| -19.6269 | 128.9233 | 0.0000 | 1.6633e+07 | 1 |

**Adduct Signal Fractions**

- **Unmodified**: 1.6633e+07

### sp|O42273|TENP\_CHICK

**>sp|O42273|TENP\_CHICK**  
 `MGALLALLDPVQPTRAPDCGGILTPLGLSYLAEVSKPHAEVVLRQDLMPKEPQTCSLAPWSPAGTELPAVKVADLWLSVI  
PEAGLRLGIEVELRIAPLHTVPMPVRISIRADLHVDMGPDGNLQLLTSACRPTVQAQSTREAESKSSRSILDKVVDVDKL  
CLDVSKLLLFPNEQLMSLTALFPVTPNCQLQYLALAAPVFSKQGIALSLQTTFQVAGAVVPVPVSPVPFSMPELASTSTS  
HLILALSEHFYTSLYFTLERAGAFNMTIPSMLTTATLAQKITQVGSLYHEDLPITLSAALRSSPRVVLEEGRAALKLFLT  
VHIGAGSPDFQSFLSVSADVTRAGLQLSVSDTRMMISTAVIEDAELSLAASNVGLVRAALLEELFLAPVCQQVPAWMDDV  
LREGVHLPHMSHFTYTDVNVVVHKDYVLVPCKLKLRSTMA`

| Site | Type | Count |
| --- | --- | --- |
| 265 | N-linked | 1 |

| Mass | Sequence | MS_2_ Score | `q`-value | MS_1_ Score | Signal | Protein Coordinates | Tandem Scans | Start Time | Apex Time | End Time |
| --- | --- | --- | --- | --- | --- | --- | --- | --- | --- | --- |
| 3575.4193 | N(N)MTIPSM Fuc 4 Hex 3 HexNAc 7 Neu5Ac 1 | 128.923 | 0.0 | -19.627 | 1.663e+07 | 264 - 271 | 1 | 29.02 | 29.18 | 29.18 |

### N(N)MTIPSM Fuc 4 Hex 3 HexNAc 7 Neu5Ac 1

**Mass:**  3575.4204

(7.958e-06)

**Charge States:** 3

**Start Position**: 265

**End Position**: 272

| Profile Score | Tandem Score | TDA q-value | Volume | Spectrum Matches |
| --- | --- | --- | --- | --- |
| -19.6269 | 128.9233 | 0.0000 | 1.6633e+07 | 1 |

**Adduct Signal Fractions**

- **Unmodified**: 1.6633e+07

### tr|A0A1L1RMA4|A0A1L1RMA4\_CHICK

**>tr|A0A1L1RMA4|A0A1L1RMA4\_CHICK**  
 `MGTANRKGSAVPLLCTMGALLALLDPVQPTRAPDCGGILTPLGLSYLAEVSKPHAEVVLRQDLMAQRASDLFLGSMEPSR  
NRITSVKVADLWLSVVPEAGLRLGIEVELRVAPLHAVPMPVRISIRADLHVDMGPDGNLQLLTSACRPTVQAQSTREAES  
KSSRSILDKVVDVDKLCLDVSKLLLFPNEQLMSLTALFPVTPNCQLQYLPLAAPVFSKQGIALSLQVAGAVVPVPVSPVP  
FSMPELASTSTSHLILALSEHFYTSLYFTLERAGAFNMTIPSMLTTATLAQKITQVGSLYHEDLPITLSAALRSSPRVVL  
EEGRAALKLFLTVHIGVGSPDFQSFLSVSADVTAGLQLSVSDTRMMISTAVIEDAELSLAASNVGLVRAALLEELFLAPV  
CQQVPAWMDDVLREGVHLPHLSHFTYTDVNVVVHKDYVLVPCKLKLRSTMA`

| Site | Type | Count |
| --- | --- | --- |
| 277 | N-linked | 1 |

| Mass | Sequence | MS_2_ Score | `q`-value | MS_1_ Score | Signal | Protein Coordinates | Tandem Scans | Start Time | Apex Time | End Time |
| --- | --- | --- | --- | --- | --- | --- | --- | --- | --- | --- |
| 3575.4193 | N(N)MTIPSM Fuc 4 Hex 3 HexNAc 7 Neu5Ac 1 | 128.923 | 0.0 | -19.627 | 1.663e+07 | 276 - 283 | 1 | 29.02 | 29.18 | 29.18 |

### N(N)MTIPSM Fuc 4 Hex 3 HexNAc 7 Neu5Ac 1

**Mass:**  3575.4204

(7.958e-06)

**Charge States:** 3

**Start Position**: 277

**End Position**: 284

| Profile Score | Tandem Score | TDA q-value | Volume | Spectrum Matches |
| --- | --- | --- | --- | --- |
| -19.6269 | 128.9233 | 0.0000 | 1.6633e+07 | 1 |

**Adduct Signal Fractions**

- **Unmodified**: 1.6633e+07
