# Supplementary material for: Measuring Site-specific Glycosylation Similarity between Influenza a Virus Variants with Statistical Certainty
Source: Mol Cell Proteomics. 2020 Nov 25;19(9):1533–45. doi: 10.1074/mcp.RA120.002031 (PMC8143645; doi:10.1074/mcp.RA120.002031)
Supplement: Supplementary file 2 [file mmc2.zip › mmc2/159250_2_supp_555291_qcjklr.zip!/20190301_SWZeggL2_021119_chymo_HILIC_DDA_1ug_02_glycopeptides.html]

## 20190301\_SWZeggL2\_021119\_chymo\_HILIC\_DDA\_1ug\_02 @ GlycopeptideHypothesis-713e689f83b544a5b6e892601cc2179b

**MS^1^ Mass Accuracy:** 10.0 PPM  
**Grouping Tolerance:** 15.0 PPM  
**MS^n^ Mass Accuracy:** 20.0 PPM  
**Minimum `q`-Value For Clustering:** 0.05

**Glycopeptide Hypothesis:**
GlycopeptideHypothesis-713e689f83b544a5b6e892601cc2179b  
**Sample Searched:**
20190301\_SWZeggL2\_021119\_chymo\_HILIC\_DDA\_1ug\_02

| Protein | Matched Glycopeptides |
| --- | --- |
| cus\|SWZHA\|A/Switzerland/9715293/2013 | 106 |
| cus\|SWZHA\_5B8\|A/Switzerland/9715293/2013 | 83 |
| cus\|SWZNA\|A/Switzerland/9715293/2013 | 37 |

### cus|SWZHA|A/Switzerland/9715293/2013

**>cus|SWZHA|A/Switzerland/9715293/2013**  
 `QKLPGNDNSTATLCLGHHAVPNGTIVKTITNDRIEVTNATELVQNSSIGEICDSPHQILDGENCTLIDALLGDPQCDGFQ  
NKKWDLFVERSKAYSNCYPYDVPDYASLRSLVASSGTLEFNNESFNWAGVTQNGTSSSCRRGSNSSFFSRLNWLTHLNSK  
YPALNVTMPNNEQFDKLYIWGVHHPVTDKDQIFLYAQSSGRITVSTKRSQQAVIPNIGYRPRIRDIPSRISIYWTIVKPG  
DILLINSTGNLIAPRGYFKIRSGKSSIMRSDAPIGKCKSECITPNGSIPNDKPFQNVNRITYGACPRYVKQSTLKLATGM  
RNVPERQTRGIFGAIAGFIENGWEGMVDGWYGFRHQNSEGRGQAADLKSTQAAIDQINGKLNRLIGKTNEKFHQIEKEFS  
EVEGRIQDLEKYVEDTKIDLWSYNAELLVALENQHTIDLTDSEMNKLFEKTKKQLRENAEDMGNGCFKIYHKCDNACIGS  
IRNGTYDHDVYRDEALNNRFQIKGVELKSGYKDWILWISFAISCFLLCVALLGFIMWACQKGNIRCNICI`

| Site | Type | Count |
| --- | --- | --- |
| 483 | N-linked | 3 |
| 133 | N-linked | 22 |
| 38 | N-linked | 6 |
| 8 | N-linked | 1 |
| 144 | N-linked | 6 |
| 246 | N-linked | 43 |
| 122 | N-linked | 2 |
| 165 | N-linked | 15 |
| 285 | N-linked | 6 |
| 22 | N-linked | 2 |

| Mass | Sequence | MS_2_ Score | `q`-value | MS_1_ Score | Signal | Protein Coordinates | Tandem Scans | Start Time | Apex Time | End Time |
| --- | --- | --- | --- | --- | --- | --- | --- | --- | --- | --- |
| 2695.0875 | LIN(N)STGNL Hex 9 HexNAc 2 | 188.192 | 0.0 | -18.441 | 5.771e+07 | 243 - 251 | 2 | 27.40 | 27.40 | 27.56 |
| 3661.5649 | LIN(N)STGNLIAPRGYF Hex 10 HexNAc 2 | 184.743 | 0.0 | -17.499 | 3.256e+07 | 243 - 258 | 1 | 47.55 | 47.72 | 47.72 |
| 3499.5179 | LIN(N)STGNLIAPRGYF Hex 9 HexNAc 2 | 181.976 | 0.0 | 17.745 | 3.775e+08 | 243 - 258 | 4 | 47.55 | 47.72 | 48.22 |
| 3516.5440 | LIN(N)STGNLIAPRGYF Fuc 2 Hex 3 HexNAc 4 Neu5Ac 1 | 178.455 | 0.0 | -17.662 | 4.867e+07 | 243 - 258 | 1 | 47.72 | 47.72 | 47.88 |
| 3352.4496 | LIN(N)STGNLIAPRGY Hex 9 HexNAc 2 | 173.211 | 0.0 | 15.835 | 2.397e+08 | 243 - 257 | 3 | 37.49 | 37.66 | 38.16 |
| 3132.3710 | LIN(N)STGNLIAPR Hex 9 HexNAc 2 | 171.070 | 0.0 | -17.874 | 7.887e+07 | 243 - 255 | 1 | 31.89 | 31.89 | 31.89 |
| 3746.5117 | AGVTQN(N)GTSSSC(C)RRGSNSSFF Hex 7 HexNAc 2 | 169.768 | 0.0 | 16.343 | 6.188e+08 | 127 - 148 | 5 | 25.57 | 26.07 | 26.57 |
| 4068.9311 | TIVKPGDILLIN(N)STGNLIAPR Hex 9 HexNAc 2 | 168.727 | 0.0 | 18.493 | 2.013e+09 | 234 - 255 | 14 | 53.14 | 53.82 | 55.86 |
| 3386.4333 | IN(N)STGNLIAPRGYF Hex 9 HexNAc 2 | 168.264 | 0.0 | -18.346 | 2.082e+07 | 244 - 258 | 1 | 43.89 | 44.06 | 44.06 |
| 4203.7828 | NSKYPALN(N)VTM(O)PNNEQFDKLY Hex 8 HexNAc 2 | 166.793 | 0.0 | 18.530 | 2.976e+08 | 157 - 178 | 4 | 43.05 | 43.55 | 43.72 |
| 2317.9565 | LPGNDN(N)STATL Hex 5 HexNAc 2 | 165.509 | 0.0 | -20.107 | 7.194e+06 | 2 - 13 | 2 | 27.56 | 27.56 | 27.73 |
| 3337.4693 | LIN(N)STGNLIAPRGYF Hex 8 HexNAc 2 | 162.369 | 0.0 | -4.495 | 2.751e+07 | 243 - 258 | 1 | 47.72 | 47.88 | 48.05 |
| 4230.9805 | TIVKPGDILLIN(N)STGNLIAPR Hex 10 HexNAc 2 | 161.401 | 0.0 | 14.440 | 1.928e+08 | 234 - 255 | 5 | 53.65 | 53.82 | 54.32 |
| 3369.4745 | LIN(N)STGNLIAPRGY Fuc 2 Hex 3 HexNAc 4 Neu5Ac 1 | 161.040 | 0.0 | -18.836 | 2.634e+07 | 243 - 257 | 1 | 37.49 | 37.66 | 37.66 |
| 3422.4099 | AGVTQN(N)GTSSSC(C)RRGSNSSFF Hex 5 HexNAc 2 | 160.646 | 0.0 | 15.467 | 4.482e+08 | 127 - 148 | 4 | 25.91 | 26.57 | 26.90 |
| 4365.8348 | NSKYPALN(N)VTM(O)PNNEQFDKLY Hex 9 HexNAc 2 | 160.263 | 0.0 | 16.608 | 3.689e+08 | 157 - 178 | 4 | 43.05 | 43.38 | 43.72 |
| 3584.4617 | AGVTQN(N)GTSSSC(C)RRGSNSSFF Hex 6 HexNAc 2 | 159.110 | 0.0 | 14.745 | 1.989e+08 | 127 - 148 | 3 | 25.91 | 26.23 | 26.57 |
| 4289.0184 | TIVKPGDILLIN(N)STGNLIAPRGY Hex 9 HexNAc 2 | 158.376 | 0.0 | 18.705 | 3.716e+09 | 234 - 257 | 22 | 54.32 | 56.20 | 58.72 |
| 3159.4268 | TIVKPGDILLIN(N) Hex 9 HexNAc 2 | 158.134 | 0.0 | 11.067 | 2.505e+08 | 234 - 246 | 3 | 46.76 | 48.22 | 48.72 |
| 4126.9607 | TIVKPGDILLIN(N)STGNLIAPRGY Hex 8 HexNAc 2 | 157.458 | 0.0 | 14.599 | 3.558e+08 | 234 - 257 | 13 | 55.52 | 56.20 | 57.56 |
| 4306.0372 | TIVKPGDILLIN(N)STGNLIAPRGY Fuc 2 Hex 3 HexNAc 4 Neu5Ac 1 | 157.419 | 0.0 | 12.272 | 2.600e+08 | 234 - 257 | 5 | 55.69 | 56.20 | 57.56 |
| 2905.2900 | TITNDRIEVTN(N)ATEL Hex 5 HexNAc 2 | 157.278 | 0.0 | -3.185 | 3.287e+07 | 27 - 42 | 2 | 37.66 | 37.82 | 37.99 |
| 2997.3727 | TIVKPGDILLIN(N) Hex 8 HexNAc 2 | 156.906 | 0.0 | 10.911 | 1.324e+07 | 234 - 246 | 2 | 47.88 | 48.22 | 48.55 |
| 2848.1060 | NWAGVTQN(N)GTSSS Hex 7 HexNAc 2 | 156.857 | 0.0 | -18.483 | 1.109e+07 | 125 - 138 | 2 | 23.29 | 23.46 | 23.46 |
| 4451.0683 | TIVKPGDILLIN(N)STGNLIAPRGY Hex 10 HexNAc 2 | 155.298 | 0.0 | 15.841 | 3.546e+08 | 234 - 257 | 7 | 55.69 | 56.03 | 56.70 |
| 3906.8767 | TIVKPGDILLIN(N)STGNLIAPR Hex 8 HexNAc 2 | 155.096 | 0.0 | -3.232 | 1.446e+08 | 234 - 255 | 4 | 53.82 | 53.99 | 54.32 |
| 2116.8346 | RGSN(N)SSFF Hex 5 HexNAc 2 | 154.880 | 0.0 | -19.250 | 6.277e+06 | 140 - 148 | 1 | 26.90 | 27.06 | 27.06 |
| 3648.6796 | TIVKPGDILLIN(N)STGNL Fuc 2 Hex 3 HexNAc 4 Neu5Ac 1 | 152.556 | 0.0 | -3.955 | 2.080e+08 | 234 - 251 | 2 | 53.31 | 53.48 | 53.82 |
| 3229.3941 | TITNDRIEVTN(N)ATEL Hex 7 HexNAc 2 | 152.251 | 0.0 | -17.082 | 3.644e+07 | 27 - 42 | 1 | 37.32 | 37.49 | 37.49 |
| 4085.9511 | TIVKPGDILLIN(N)STGNLIAPR Fuc 2 Hex 3 HexNAc 4 Neu5Ac 1 | 151.739 | 0.0 | 14.730 | 1.398e+08 | 234 - 255 | 2 | 53.65 | 53.82 | 54.49 |
| 3631.6538 | TIVKPGDILLIN(N)STGNL Hex 9 HexNAc 2 | 151.047 | 0.0 | 18.109 | 1.200e+09 | 234 - 251 | 10 | 52.80 | 53.48 | 55.69 |
| 4089.7401 | SKYPALN(N)VTM(O)PNNEQFDKLY Hex 8 HexNAc 2 | 150.689 | 0.0 | -2.647 | 8.393e+07 | 158 - 178 | 2 | 43.38 | 43.55 | 43.72 |
| 3321.4777 | TIVKPGDILLIN(N) Hex 10 HexNAc 2 | 150.354 | 0.0 | -17.294 | 8.864e+06 | 234 - 246 | 1 | 48.05 | 48.05 | 48.05 |
| 3518.5741 | TIVKPGDILLIN(N)STGN Hex 9 HexNAc 2 | 149.942 | 0.0 | 16.194 | 3.167e+08 | 234 - 250 | 3 | 45.74 | 46.08 | 46.42 |
| 3270.4260 | C(C)LGHHAVPN(N)GTIVK Fuc 1 Hex 5 HexNAc 4 | 149.528 | 0.0 | -19.150 | 7.485e+06 | 13 - 27 | 1 | 19.67 | 19.67 | 19.84 |
| 4046.6411 | NWAGVTQN(N)GTSSSC(C)RRGSNSSFF Hex 7 HexNAc 2 | 148.052 | 0.0 | 17.371 | 2.618e+08 | 125 - 148 | 4 | 35.85 | 36.35 | 36.51 |
| 3347.5022 | TIVKPGDILLIN(N)ST Hex 9 HexNAc 2 | 147.964 | 0.0 | -19.896 | 1.420e+07 | 234 - 248 | 1 | 47.39 | 47.39 | 47.55 |
| 3793.7073 | TIVKPGDILLIN(N)STGNL Hex 10 HexNAc 2 | 145.463 | 0.0 | -4.215 | 8.874e+07 | 234 - 251 | 4 | 53.31 | 53.48 | 53.82 |
| 3535.5982 | TIVKPGDILLIN(N)STGN Fuc 2 Hex 3 HexNAc 4 Neu5Ac 1 | 145.407 | 0.0 | -4.413 | 5.586e+07 | 234 - 250 | 1 | 45.91 | 46.08 | 46.25 |
| 2272.9352 | RRGSN(N)SSFF Hex 5 HexNAc 2 | 144.891 | 0.0 | -19.412 | 1.057e+07 | 139 - 148 | 1 | 21.97 | 22.13 | 22.13 |
| 3164.2360 | NWAGVTQN(N)GTSSSC(C)R Hex 7 HexNAc 2 | 144.339 | 0.0 | -19.311 | 1.520e+07 | 125 - 140 | 1 | 20.83 | 20.99 | 20.99 |
| 3176.4496 | TIVKPGDILLIN(N) Fuc 2 Hex 3 HexNAc 4 Neu5Ac 1 | 143.586 | 0.0 | 10.480 | 4.262e+07 | 234 - 246 | 2 | 46.92 | 48.22 | 48.55 |
| 3469.5990 | TIVKPGDILLIN(N)STGNL Hex 8 HexNAc 2 | 142.156 | 0.0 | -3.271 | 9.776e+07 | 234 - 251 | 3 | 53.48 | 53.65 | 53.82 |
| 3473.5012 | C(C)LGHHAVPN(N)GTIVK Fuc 1 Hex 5 HexNAc 5 | 141.399 | 0.0 | -19.089 | 2.252e+07 | 13 - 27 | 1 | 19.67 | 19.84 | 19.84 |
| 3514.5100 | LIN(N)STGNLIAPRGY Hex 10 HexNAc 2 | 140.941 | 0.0 | -18.359 | 2.984e+07 | 243 - 257 | 1 | 37.49 | 37.49 | 37.66 |
| 3008.1351 | NWAGVTQN(N)GTSSSC(C) Hex 7 HexNAc 2 | 139.477 | 0.0 | -4.379 | 1.762e+07 | 125 - 139 | 1 | 23.94 | 24.10 | 24.26 |
| 3908.5700 | AGVTQN(N)GTSSSC(C)RRGSNSSFF Hex 8 HexNAc 2 | 139.163 | 0.0 | -3.178 | 7.893e+07 | 127 - 148 | 2 | 25.74 | 25.91 | 26.07 |
| 4752.0367 | RSDAPIGKC(C)KSEC(C)ITPN(N)GSIPNDKPF Hex 9 HexNAc 2 | 137.850 | 0.0 | -2.742 | 2.923e+08 | 268 - 294 | 3 | 30.55 | 30.72 | 30.88 |
| 3028.2300 | RRGSN(N)SSFF Fuc 1 Hex 5 HexNAc 5 | 136.949 | 0.0 | -19.292 | 2.631e+07 | 139 - 148 | 1 | 21.97 | 21.97 | 22.13 |
| 2563.9568 | EFNN(N)ESFNW Hex 6 HexNAc 2 | 135.728 | 0.0 | -18.130 | 8.949e+06 | 118 - 127 | 2 | 44.90 | 44.90 | 45.07 |
| 3270.4203 | TITNDRIEVTN(N)ATEL Hex 6 HexNAc 3 | 135.604 | 0.0 | -4.694 | 2.882e+07 | 27 - 42 | 1 | 37.49 | 37.49 | 37.82 |
| 3420.7168 | TIVKPGDILLIN(N)STGNLIAPR Hex 5 HexNAc 2 | 135.584 | 0.0 | -4.409 | 2.204e+07 | 234 - 255 | 3 | 54.49 | 54.66 | 54.84 |
| 4427.9309 | RSDAPIGKC(C)KSEC(C)ITPN(N)GSIPNDKPF Hex 7 HexNAc 2 | 132.819 | 0.007 | -3.601 | 1.722e+08 | 268 - 294 | 2 | 30.72 | 30.88 | 31.05 |
| 2669.0553 | RGSN(N)SSFF Fuc 1 Hex 5 HexNAc 4 | 130.542 | 0.0 | -19.988 | 4.892e+06 | 140 - 148 | 1 | 26.40 | 26.40 | 26.40 |
| 3884.5851 | NWAGVTQN(N)GTSSSC(C)RRGSNSSFF Hex 6 HexNAc 2 | 129.705 | 0.007 | -4.161 | 1.139e+08 | 125 - 148 | 3 | 36.35 | 36.51 | 36.68 |
| 3787.5420 | AGVTQN(N)GTSSSC(C)RRGSNSSFF Hex 6 HexNAc 3 | 129.690 | 0.007 | -4.421 | 1.074e+08 | 127 - 148 | 2 | 26.07 | 26.23 | 26.40 |
| 4589.9824 | RSDAPIGKC(C)KSEC(C)ITPN(N)GSIPNDKPF Hex 8 HexNAc 2 | 129.479 | 0.007 | -3.432 | 1.232e+08 | 268 - 294 | 1 | 30.72 | 30.72 | 31.05 |
| 2983.4422 | TIVKPGDILLIN(N)STGNL Hex 5 HexNAc 2 | 127.164 | 0.007 | -3.304 | 1.243e+07 | 234 - 251 | 1 | 54.15 | 54.32 | 54.49 |
| 2825.1478 | RRGSN(N)SSFF Fuc 1 Hex 5 HexNAc 4 | 126.060 | 0.0 | -17.814 | 9.929e+06 | 139 - 148 | 1 | 21.81 | 21.81 | 21.81 |
| 4202.7675 | NSKYPALN(N)VTM(O)PNNEQFDKL Hex 9 HexNAc 2 | 124.369 | 0.007 | -3.547 | 3.601e+07 | 157 - 177 | 2 | 41.02 | 41.19 | 41.36 |
| 4349.8375 | NSKYPALN(N)VTMPNNEQFDKLY Hex 9 HexNAc 2 | 124.146 | 0.007 | -18.839 | 7.203e+06 | 157 - 178 | 2 | 49.40 | 49.40 | 49.57 |
| 3722.5301 | NWAGVTQN(N)GTSSSC(C)RRGSNSSFF Hex 5 HexNAc 2 | 123.019 | 0.007 | -2.402 | 2.184e+08 | 125 - 148 | 3 | 36.68 | 36.68 | 36.99 |
| 4262.7191 | HKC(C)DNAC(C)IGSIRN(N)GTYDHDVY Fuc 1 Hex 5 HexNAc 4 | 119.160 | 0.007 | -3.312 | 8.106e+07 | 470 - 491 | 1 | 25.91 | 26.07 | 26.23 |
| 4204.7626 | N(D)SKYPALN(N)VTM(O)PNNEQFDKLY Hex 8 HexNAc 2 | 117.656 | 0.007 | -6.102 | 1.033e+08 | 157 - 178 | 4 | 46.08 | 46.42 | 46.59 |
| 3949.5958 | AGVTQN(N)GTSSSC(C)RRGSNSSFF Hex 7 HexNAc 3 | 117.259 | 0.007 | -3.615 | 6.152e+07 | 127 - 148 | 2 | 25.91 | 25.91 | 26.23 |
| 4040.7231 | NSKYPALN(N)VTM(O)PNNEQFDKL Hex 8 HexNAc 2 | 115.997 | 0.007 | -4.992 | 2.487e+07 | 157 - 177 | 2 | 41.19 | 41.36 | 41.52 |
| 3500.5083 | LIN(N)STGN(D)LIAPRGYF Hex 9 HexNAc 2 | 113.671 | 0.012 | -20.577 | 5.020e+06 | 243 - 258 | 2 | 49.23 | 49.23 | 49.23 |
| 3207.2899 | SIRN(N)GTYDHDVY Fuc 1 Hex 5 HexNAc 4 | 109.405 | 0.012 | -16.988 | 9.002e+06 | 479 - 491 | 1 | 20.33 | 20.33 | 20.49 |
| 3640.8032 | TIVKPGDILLIN(N)STGNLIAPRGY Hex 5 HexNAc 2 | 106.634 | 0.012 | -3.118 | 6.938e+07 | 234 - 257 | 5 | 56.70 | 56.88 | 57.22 |
| 4290.0073 | TIVKPGDILLIN(N)STGN(D)LIAPRGY Hex 9 HexNAc 2 | 105.820 | 0.012 | -19.426 | 6.377e+07 | 234 - 257 | 16 | 57.05 | 57.22 | 57.22 |
| 3404.5210 | TIVKPGDILLIN(N)STG Hex 9 HexNAc 2 | 103.978 | 0.012 | -20.716 | 1.109e+07 | 234 - 249 | 1 | 46.92 | 46.92 | 47.08 |
| 3747.4983 | AGVTQN(N)GTSSSC(C)RRGSN(D)SSFF Hex 7 HexNAc 2 | 101.856 | 0.012 | 10.547 | 2.696e+08 | 127 - 148 | 4 | 27.06 | 27.23 | 28.24 |
| 3966.9321 | TIVKPGDILLIN(N)STGNLIAPRGY Hex 7 HexNAc 2 | 95.138 | 0.027 | 0.000 | 0.000e+00 | 234 - 257 | 1 | - | - | - |
| 4366.8152 | N(D)SKYPALN(N)VTM(O)PNNEQFDKLY Hex 9 HexNAc 2 | 92.584 | 0.027 | -6.194 | 1.149e+08 | 157 - 178 | 2 | 46.08 | 46.25 | 46.42 |
| 3353.4307 | LIN(N)STGN(D)LIAPRGY Hex 9 HexNAc 2 | 91.873 | 0.027 | -20.295 | 9.829e+06 | 243 - 257 | 1 | 38.50 | 38.67 | 38.67 |
| 4249.7266 | NWAGVTQN(N)GTSSSC(C)RRGSNSSFF Hex 7 HexNAc 3 | 88.777 | 0.027 | -17.564 | 2.608e+07 | 125 - 148 | 2 | 36.18 | 36.18 | 36.35 |
| 3235.3702 | RSLVASSGTLEFNN(N)ESF Hex 6 HexNAc 2 | 87.496 | 0.027 | -20.353 | 9.791e+06 | 108 - 125 | 1 | 44.56 | 44.56 | 44.73 |
| 4248.0081 | TIVKPGDILLIN(N)STGNLIAPR Fuc 2 Hex 4 HexNAc 4 Neu5Ac 1 | 86.953 | 0.027 | -19.072 | 1.193e+07 | 234 - 255 | 1 | 53.65 | 53.82 | 53.82 |
| 4153.8048 | LNSKYPALN(N)VTM(O)PNNEQFDKL Hex 8 HexNAc 2 | 85.006 | 0.032 | -17.796 | 1.186e+07 | 156 - 177 | 2 | 42.71 | 42.88 | 42.88 |
| 4753.0353 | RSDAPIGKC(C)KSEC(C)ITPN(N)GSIPN(D)DKPF Hex 9 HexNAc 2 | 84.748 | 0.032 | -5.845 | 3.570e+07 | 268 - 294 | 1 | 31.21 | 31.38 | 31.55 |
| 4087.6634 | NWAGVTQN(N)GTSSSC(C)RRGSNSSFF Hex 6 HexNAc 3 | 83.358 | 0.040 | -3.108 | 8.704e+07 | 125 - 148 | 1 | 36.18 | 36.35 | 36.51 |
| 3802.8562 | TIVKPGDILLIN(N)STGNLIAPRGY Hex 6 HexNAc 2 | 82.403 | 0.040 | -4.403 | 1.356e+07 | 234 - 257 | 3 | 56.53 | 56.70 | 56.88 |
| 3964.9072 | TIVKPGDILLIN(N)STGNLIAPRGY Hex 7 HexNAc 2 | 78.693 | 0.040 | -4.592 | 1.594e+07 | 234 - 257 | 2 | 56.36 | 56.36 | 56.70 |
| 3609.4947 | AGVTQN(N)GTSSSC(C)RRGSNSSFF Fuc 1 Hex 4 HexNAc 3 | 78.071 | 0.040 | -4.804 | 4.963e+07 | 127 - 148 | 1 | 26.23 | 26.57 | 26.73 |
| 3585.4463 | AGVTQN(N)GTSSSC(C)RRGSN(D)SSFF Hex 6 HexNAc 2 | 77.881 | 0.040 | 11.838 | 5.950e+07 | 127 - 148 | 2 | 27.23 | 27.40 | 27.90 |
| 3398.4263 | NWAGVTQN(N)GTSSSC(C)RRGSNSSFF Hex 3 HexNAc 2 | 75.159 | 0.040 | -19.159 | 9.888e+06 | 125 - 148 | 1 | 37.15 | 37.15 | 37.15 |
| 4465.7951 | HKC(C)DNAC(C)IGSIRN(N)GTYDHDVY Fuc 1 Hex 5 HexNAc 5 | 67.576 | 0.043 | -19.461 | 2.464e+07 | 470 - 491 | 1 | 26.07 | 26.07 | 26.23 |
| 3763.5592 | NWAGVTQN(N)GTSSSC(C)RRGSNSSFF Hex 4 HexNAc 3 | 67.409 | 0.043 | -19.519 | 5.255e+07 | 125 - 148 | 2 | 36.68 | 36.84 | 36.84 |
| 3423.3958 | AGVTQN(N)GTSSSC(C)RRGSN(D)SSFF Hex 5 HexNAc 2 | 65.950 | 0.043 | 9.647 | 9.482e+07 | 127 - 148 | 4 | 27.40 | 27.73 | 28.40 |
| 3717.6181 | NSKYPALN(N)VTM(O)PNNEQFDKLY Hex 5 HexNAc 2 | 65.269 | 0.046 | -18.168 | 8.725e+06 | 157 - 178 | 2 | 43.89 | 44.06 | 44.06 |
| 4527.8889 | NSKYPALN(N)VTM(O)PNNEQFDKLY Hex 10 HexNAc 2 | 64.101 | 0.046 | -19.021 | 2.579e+06 | 157 - 178 | 1 | 43.38 | 43.38 | 43.38 |
| 4103.6640 | AGVTQN(N)GTSSSC(C)RRGSNSSFF Fuc 1 Hex 4 HexNAc 4 Neu5Ac 1 | 63.353 | 0.046 | -3.508 | 8.029e+07 | 127 - 148 | 2 | 37.15 | 37.32 | 37.49 |
| 4428.9297 | RSDAPIGKC(C)KSEC(C)ITPN(N)GSIPN(D)DKPF Hex 7 HexNAc 2 | 62.433 | 0.046 | -6.104 | 2.193e+07 | 268 - 294 | 1 | 31.38 | 31.55 | 31.72 |
| 4041.7310 | NSKYPALN(N)VTM(O)PNNEQFDKLY Hex 7 HexNAc 2 | 60.927 | 0.046 | -4.213 | 2.004e+07 | 157 - 178 | 1 | 43.38 | 43.55 | 43.72 |
| 4290.7454 | NWAGVTQNGTSSSC(C)RRGSN(N)SSFF Hex 6 HexNAc 4 | 60.429 | 0.046 | -5.439 | 3.431e+07 | 125 - 148 | 1 | 36.18 | 36.35 | 36.68 |
| 3311.4494 | TITNDRIEVTN(N)ATEL Hex 5 HexNAc 4 | 60.374 | 0.046 | -20.171 | 1.085e+07 | 27 - 42 | 1 | 37.32 | 37.49 | 37.49 |
| 3605.5116 | KSEC(C)ITPN(N)GSIPNDKPF Hex 8 HexNAc 2 | 60.224 | 0.046 | -20.483 | 8.684e+06 | 277 - 294 | 1 | 32.37 | 32.37 | 32.37 |
| 4315.8493 | LNSKYPALN(N)VTM(O)PNNEQFDKL Hex 9 HexNAc 2 | 60.120 | 0.046 | -18.016 | 1.210e+07 | 156 - 177 | 1 | 42.54 | 42.71 | 42.71 |
| 4253.7025 | IN(N)STGNLIAPRGY Fuc 1 Hex 7 HexNAc 5 Neu5Ac 2 | 59.868 | 0.046 | 0.000 | 0.000e+00 | 244 - 257 | 1 | - | - | - |
| 3108.3709 | TITNDRIEVTN(N)ATEL Hex 5 HexNAc 3 | 58.488 | 0.046 | -18.678 | 9.717e+06 | 27 - 42 | 1 | 37.66 | 37.82 | 37.82 |
| 4125.9521 | TIVKPGDILLIN(N)STGNLIAPRG Hex 9 HexNAc 2 | 57.558 | 0.046 | 16.347 | 4.153e+08 | 234 - 256 | 10 | 53.65 | 54.66 | 55.52 |
| 4718.0121 | THLN(D)SKYPALN(N)VTM(O)PNNEQFDKL Fuc 3 Hex 3 HexNAc 4 Neu5Ac 1 | 57.540 | 0.046 | 7.187 | 1.607e+08 | 154 - 177 | 5 | 42.37 | 43.55 | 43.89 |
| 4069.9234 | TIVKPGDILLIN(N)STGN(D)LIAPR Hex 9 HexNAc 2 | 56.918 | 0.046 | -19.599 | 2.419e+07 | 234 - 255 | 4 | 54.84 | 54.84 | 55.01 |
| 3463.4364 | AGVTQN(N)GTSSSC(C)RRGSNSSFF Hex 4 HexNAc 3 | 56.103 | 0.046 | -3.964 | 1.071e+08 | 127 - 148 | 2 | 26.40 | 26.57 | 26.90 |
| 4317.8459 | SKYPALN(N)VTM(O)PNNEQFDKLY Fuc 1 Hex 6 HexNAc 4 | 55.369 | 0.046 | -4.090 | 4.438e+07 | 158 - 178 | 2 | 46.59 | 46.59 | 46.92 |
| 3416.4721 | TITNDRIEVTN(N)ATEL Fuc 1 Hex 6 HexNAc 3 | 54.534 | 0.049 | -21.308 | 4.636e+06 | 27 - 42 | 1 | 37.32 | 37.32 | 37.32 |

### LIN(N)STGNL Hex 9 HexNAc 2

**Mass:**  2695.0892

(1.967e-06)

**Charge States:** 2, 3

**Start Position**: 244

**End Position**: 252

| Profile Score | Tandem Score | TDA q-value | Volume | Spectrum Matches |
| --- | --- | --- | --- | --- |
| -18.4405 | 188.1922 | 0.0000 | 5.7714e+07 | 2 |

**Adduct Signal Fractions**

- **Unmodified**: 5.7714e+07

### LIN(N)STGNLIAPRGYF Hex 10 HexNAc 2

**Mass:**  3661.5683

(-1.268e-06)

**Charge States:** 3

**Start Position**: 244

**End Position**: 259

| Profile Score | Tandem Score | TDA q-value | Volume | Spectrum Matches |
| --- | --- | --- | --- | --- |
| -17.4989 | 184.7426 | 0.0000 | 3.2558e+07 | 1 |

**Adduct Signal Fractions**

- **Unmodified**: 3.2558e+07

### LIN(N)STGNLIAPRGYF Hex 9 HexNAc 2

**Mass:**  3499.5174

(2.288e-06)

**Charge States:** 3

**Start Position**: 244

**End Position**: 259

| Profile Score | Tandem Score | TDA q-value | Volume | Spectrum Matches |
| --- | --- | --- | --- | --- |
| 17.7449 | 181.9755 | 0.0000 | 3.7754e+08 | 4 |

**Adduct Signal Fractions**

- **Unmodified**: 3.7754e+08

### LIN(N)STGNLIAPRGYF Fuc 2 Hex 3 HexNAc 4 Neu5Ac 1

**Mass:**  3516.5436

(-6.203e-06)

**Charge States:** 3

**Start Position**: 244

**End Position**: 259

| Profile Score | Tandem Score | TDA q-value | Volume | Spectrum Matches |
| --- | --- | --- | --- | --- |
| -17.6621 | 178.4552 | 0.0000 | 4.8673e+07 | 1 |

**Adduct Signal Fractions**

- **Unmodified**: 4.8673e+07

### LIN(N)STGNLIAPRGY Hex 9 HexNAc 2

**Mass:**  3352.4489

(2.160e-06)

**Charge States:** 2, 3

**Start Position**: 244

**End Position**: 258

| Profile Score | Tandem Score | TDA q-value | Volume | Spectrum Matches |
| --- | --- | --- | --- | --- |
| 15.8345 | 173.2108 | 0.0000 | 2.3966e+08 | 3 |

**Adduct Signal Fractions**

- **Unmodified**: 2.3966e+08

### LIN(N)STGNLIAPR Hex 9 HexNAc 2

**Mass:**  3132.3707

(3.732e-06)

**Charge States:** 2, 3

**Start Position**: 244

**End Position**: 256

| Profile Score | Tandem Score | TDA q-value | Volume | Spectrum Matches |
| --- | --- | --- | --- | --- |
| -17.8742 | 171.0698 | 0.0000 | 7.8869e+07 | 1 |

**Adduct Signal Fractions**

- **Unmodified**: 7.8869e+07

### AGVTQN(N)GTSSSC(C)RRGSNSSFF Hex 7 HexNAc 2

**Mass:**  3746.5102

(-1.351e-07)

**Charge States:** 3, 4

**Start Position**: 128

**End Position**: 149

| Profile Score | Tandem Score | TDA q-value | Volume | Spectrum Matches |
| --- | --- | --- | --- | --- |
| 16.3431 | 169.7675 | 0.0000 | 6.1879e+08 | 5 |

**Adduct Signal Fractions**

- **Unmodified**: 6.1879e+08

### TIVKPGDILLIN(N)STGNLIAPR Hex 9 HexNAc 2

**Mass:**  4068.9326

(2.457e-06)

**Charge States:** 3, 4, 5

**Start Position**: 235

**End Position**: 256

| Profile Score | Tandem Score | TDA q-value | Volume | Spectrum Matches |
| --- | --- | --- | --- | --- |
| 18.4931 | 168.7265 | 0.0000 | 2.0126e+09 | 14 |

**Adduct Signal Fractions**

- **Unmodified**: 2.0126e+09

### IN(N)STGNLIAPRGYF Hex 9 HexNAc 2

**Mass:**  3386.4395

(-6.607e-07)

**Charge States:** 3

**Start Position**: 245

**End Position**: 259

| Profile Score | Tandem Score | TDA q-value | Volume | Spectrum Matches |
| --- | --- | --- | --- | --- |
| -18.3461 | 168.2640 | 0.0000 | 2.0821e+07 | 1 |

**Adduct Signal Fractions**

- **Unmodified**: 2.0821e+07

### NSKYPALN(N)VTM(O)PNNEQFDKLY Hex 8 HexNAc 2

**Mass:**  4203.7878

(3.327e-06)

**Charge States:** 3, 4

**Start Position**: 158

**End Position**: 179

| Profile Score | Tandem Score | TDA q-value | Volume | Spectrum Matches |
| --- | --- | --- | --- | --- |
| 18.5304 | 166.7929 | 0.0000 | 2.9760e+08 | 4 |

**Adduct Signal Fractions**

- **Unmodified**: 2.9760e+08

### LPGNDN(N)STATL Hex 5 HexNAc 2

**Mass:**  2317.9562

(1.352e-06)

**Charge States:** 3

**Start Position**: 3

**End Position**: 14

| Profile Score | Tandem Score | TDA q-value | Volume | Spectrum Matches |
| --- | --- | --- | --- | --- |
| -20.1072 | 165.5091 | 0.0000 | 7.1944e+06 | 2 |

**Adduct Signal Fractions**

- **Unmodified**: 7.1944e+06

### LIN(N)STGNLIAPRGYF Hex 8 HexNAc 2

**Mass:**  3337.4676

(2.461e-06)

**Charge States:** 3

**Start Position**: 244

**End Position**: 259

| Profile Score | Tandem Score | TDA q-value | Volume | Spectrum Matches |
| --- | --- | --- | --- | --- |
| -4.4950 | 162.3694 | 0.0000 | 2.7508e+07 | 1 |

**Adduct Signal Fractions**

- **Unmodified**: 2.7508e+07

### TIVKPGDILLIN(N)STGNLIAPR Hex 10 HexNAc 2

**Mass:**  4230.9816

(1.061e-06)

**Charge States:** 3, 4

**Start Position**: 235

**End Position**: 256

| Profile Score | Tandem Score | TDA q-value | Volume | Spectrum Matches |
| --- | --- | --- | --- | --- |
| 14.4403 | 161.4006 | 0.0000 | 1.9280e+08 | 5 |

**Adduct Signal Fractions**

- **Unmodified**: 1.9280e+08

### LIN(N)STGNLIAPRGY Fuc 2 Hex 3 HexNAc 4 Neu5Ac 1

**Mass:**  3369.4744

(-6.506e-06)

**Charge States:** 3

**Start Position**: 244

**End Position**: 258

| Profile Score | Tandem Score | TDA q-value | Volume | Spectrum Matches |
| --- | --- | --- | --- | --- |
| -18.8356 | 161.0401 | 0.0000 | 2.6336e+07 | 1 |

**Adduct Signal Fractions**

- **Unmodified**: 2.6336e+07

### AGVTQN(N)GTSSSC(C)RRGSNSSFF Hex 5 HexNAc 2

**Mass:**  3422.4109

(1.744e-06)

**Charge States:** 3, 4

**Start Position**: 128

**End Position**: 149

| Profile Score | Tandem Score | TDA q-value | Volume | Spectrum Matches |
| --- | --- | --- | --- | --- |
| 15.4670 | 160.6456 | 0.0000 | 4.4819e+08 | 4 |

**Adduct Signal Fractions**

- **Unmodified**: 4.4819e+08

### NSKYPALN(N)VTM(O)PNNEQFDKLY Hex 9 HexNAc 2

**Mass:**  4365.8361

(1.910e-06)

**Charge States:** 3, 4

**Start Position**: 158

**End Position**: 179

| Profile Score | Tandem Score | TDA q-value | Volume | Spectrum Matches |
| --- | --- | --- | --- | --- |
| 16.6077 | 160.2627 | 0.0000 | 3.6891e+08 | 4 |

**Adduct Signal Fractions**

- **Unmodified**: 3.6891e+08

### AGVTQN(N)GTSSSC(C)RRGSNSSFF Hex 6 HexNAc 2

**Mass:**  3584.4629

(1.445e-06)

**Charge States:** 3

**Start Position**: 128

**End Position**: 149

| Profile Score | Tandem Score | TDA q-value | Volume | Spectrum Matches |
| --- | --- | --- | --- | --- |
| 14.7446 | 159.1098 | 0.0000 | 1.9886e+08 | 3 |

**Adduct Signal Fractions**

- **Unmodified**: 1.9886e+08

### TIVKPGDILLIN(N)STGNLIAPRGY Hex 9 HexNAc 2

**Mass:**  4289.0168

(1.998e-06)

**Charge States:** 3, 4, 5

**Start Position**: 235

**End Position**: 258

| Profile Score | Tandem Score | TDA q-value | Volume | Spectrum Matches |
| --- | --- | --- | --- | --- |
| 18.7055 | 158.3761 | 0.0000 | 3.7161e+09 | 22 |

**Adduct Signal Fractions**

- **Unmodified**: 3.7161e+09

### TIVKPGDILLIN(N) Hex 9 HexNAc 2

**Mass:**  3159.4253

(1.653e-06)

**Charge States:** 2, 3

**Start Position**: 235

**End Position**: 247

| Profile Score | Tandem Score | TDA q-value | Volume | Spectrum Matches |
| --- | --- | --- | --- | --- |
| 11.0666 | 158.1340 | 0.0000 | 2.5052e+08 | 3 |

**Adduct Signal Fractions**

- **Unmodified**: 2.5052e+08

### TIVKPGDILLIN(N)STGNLIAPRGY Hex 8 HexNAc 2

**Mass:**  4126.9611

(2.095e-06)

**Charge States:** 3, 4, 5

**Start Position**: 235

**End Position**: 258

| Profile Score | Tandem Score | TDA q-value | Volume | Spectrum Matches |
| --- | --- | --- | --- | --- |
| 14.5993 | 157.4580 | 0.0000 | 3.5578e+08 | 13 |

**Adduct Signal Fractions**

- **Unmodified**: 3.5578e+08

### TIVKPGDILLIN(N)STGNLIAPRGY Fuc 2 Hex 3 HexNAc 4 Neu5Ac 1

**Mass:**  4306.0349

(-5.518e-06)

**Charge States:** 4

**Start Position**: 235

**End Position**: 258

| Profile Score | Tandem Score | TDA q-value | Volume | Spectrum Matches |
| --- | --- | --- | --- | --- |
| 12.2723 | 157.4188 | 0.0000 | 2.6004e+08 | 5 |

**Adduct Signal Fractions**

- **Unmodified**: 2.6004e+08

### TITNDRIEVTN(N)ATEL Hex 5 HexNAc 2

**Mass:**  2905.2910

(1.295e-06)

**Charge States:** 3

**Start Position**: 28

**End Position**: 43

| Profile Score | Tandem Score | TDA q-value | Volume | Spectrum Matches |
| --- | --- | --- | --- | --- |
| -3.1850 | 157.2783 | 0.0000 | 3.2867e+07 | 2 |

**Adduct Signal Fractions**

- **Unmodified**: 3.2867e+07

### TIVKPGDILLIN(N) Hex 8 HexNAc 2

**Mass:**  2997.3717

(1.406e-06)

**Charge States:** 3

**Start Position**: 235

**End Position**: 247

| Profile Score | Tandem Score | TDA q-value | Volume | Spectrum Matches |
| --- | --- | --- | --- | --- |
| 10.9113 | 156.9062 | 0.0000 | 1.3241e+07 | 2 |

**Adduct Signal Fractions**

- **Unmodified**: 1.3241e+07

### NWAGVTQN(N)GTSSS Hex 7 HexNAc 2

**Mass:**  2848.1065

(9.097e-07)

**Charge States:** 3

**Start Position**: 126

**End Position**: 139

| Profile Score | Tandem Score | TDA q-value | Volume | Spectrum Matches |
| --- | --- | --- | --- | --- |
| -18.4830 | 156.8567 | 0.0000 | 1.1090e+07 | 2 |

**Adduct Signal Fractions**

- **Unmodified**: 1.1090e+07

### TIVKPGDILLIN(N)STGNLIAPRGY Hex 10 HexNAc 2

**Mass:**  4451.0690

(1.239e-06)

**Charge States:** 3, 4

**Start Position**: 235

**End Position**: 258

| Profile Score | Tandem Score | TDA q-value | Volume | Spectrum Matches |
| --- | --- | --- | --- | --- |
| 15.8409 | 155.2980 | 0.0000 | 3.5463e+08 | 7 |

**Adduct Signal Fractions**

- **Unmodified**: 3.5463e+08

### TIVKPGDILLIN(N)STGNLIAPR Hex 8 HexNAc 2

**Mass:**  3906.8737

(2.334e-06)

**Charge States:** 3, 4

**Start Position**: 235

**End Position**: 256

| Profile Score | Tandem Score | TDA q-value | Volume | Spectrum Matches |
| --- | --- | --- | --- | --- |
| -3.2321 | 155.0964 | 0.0000 | 1.4459e+08 | 4 |

**Adduct Signal Fractions**

- **Unmodified**: 1.4459e+08

### RGSN(N)SSFF Hex 5 HexNAc 2

**Mass:**  2116.8360

(2.016e-06)

**Charge States:** 2

**Start Position**: 141

**End Position**: 149

| Profile Score | Tandem Score | TDA q-value | Volume | Spectrum Matches |
| --- | --- | --- | --- | --- |
| -19.2504 | 154.8805 | 0.0000 | 6.2767e+06 | 1 |

**Adduct Signal Fractions**

- **Unmodified**: 6.2767e+06

### TIVKPGDILLIN(N)STGNL Fuc 2 Hex 3 HexNAc 4 Neu5Ac 1

**Mass:**  3648.6798

(-4.683e-06)

**Charge States:** 3

**Start Position**: 235

**End Position**: 252

| Profile Score | Tandem Score | TDA q-value | Volume | Spectrum Matches |
| --- | --- | --- | --- | --- |
| -3.9547 | 152.5555 | 0.0000 | 2.0797e+08 | 2 |

**Adduct Signal Fractions**

- **Unmodified**: 2.0797e+08

### TITNDRIEVTN(N)ATEL Hex 7 HexNAc 2

**Mass:**  3229.3950

(1.941e-06)

**Charge States:** 3

**Start Position**: 28

**End Position**: 43

| Profile Score | Tandem Score | TDA q-value | Volume | Spectrum Matches |
| --- | --- | --- | --- | --- |
| -17.0823 | 152.2509 | 0.0000 | 3.6435e+07 | 1 |

**Adduct Signal Fractions**

- **Unmodified**: 3.6435e+07

### TIVKPGDILLIN(N)STGNLIAPR Fuc 2 Hex 3 HexNAc 4 Neu5Ac 1

**Mass:**  4085.9511

(-6.359e-06)

**Charge States:** 4

**Start Position**: 235

**End Position**: 256

| Profile Score | Tandem Score | TDA q-value | Volume | Spectrum Matches |
| --- | --- | --- | --- | --- |
| 14.7296 | 151.7394 | 0.0000 | 1.3976e+08 | 2 |

**Adduct Signal Fractions**

- **Unmodified**: 1.3976e+08

### TIVKPGDILLIN(N)STGNL Hex 9 HexNAc 2

**Mass:**  3631.6543

(1.942e-06)

**Charge States:** 3, 4

**Start Position**: 235

**End Position**: 252

| Profile Score | Tandem Score | TDA q-value | Volume | Spectrum Matches |
| --- | --- | --- | --- | --- |
| 18.1091 | 151.0471 | 0.0000 | 1.1998e+09 | 10 |

**Adduct Signal Fractions**

- **Unmodified**: 1.1998e+09

### SKYPALN(N)VTM(O)PNNEQFDKLY Hex 8 HexNAc 2

**Mass:**  4089.7458

(3.015e-06)

**Charge States:** 3, 4

**Start Position**: 159

**End Position**: 179

| Profile Score | Tandem Score | TDA q-value | Volume | Spectrum Matches |
| --- | --- | --- | --- | --- |
| -2.6470 | 150.6886 | 0.0000 | 8.3932e+07 | 2 |

**Adduct Signal Fractions**

- **Unmodified**: 8.3932e+07

### TIVKPGDILLIN(N) Hex 10 HexNAc 2

**Mass:**  3321.4777

(1.441e-06)

**Charge States:** 3

**Start Position**: 235

**End Position**: 247

| Profile Score | Tandem Score | TDA q-value | Volume | Spectrum Matches |
| --- | --- | --- | --- | --- |
| -17.2941 | 150.3545 | 0.0000 | 8.8639e+06 | 1 |

**Adduct Signal Fractions**

- **Unmodified**: 8.8639e+06

### TIVKPGDILLIN(N)STGN Hex 9 HexNAc 2

**Mass:**  3518.5739

(3.177e-06)

**Charge States:** 3, 4

**Start Position**: 235

**End Position**: 251

| Profile Score | Tandem Score | TDA q-value | Volume | Spectrum Matches |
| --- | --- | --- | --- | --- |
| 16.1938 | 149.9422 | 0.0000 | 3.1673e+08 | 3 |

**Adduct Signal Fractions**

- **Unmodified**: 3.1673e+08

### C(C)LGHHAVPN(N)GTIVK Fuc 1 Hex 5 HexNAc 4

**Mass:**  3270.4252

(1.012e-06)

**Charge States:** 4

**Start Position**: 14

**End Position**: 28

| Profile Score | Tandem Score | TDA q-value | Volume | Spectrum Matches |
| --- | --- | --- | --- | --- |
| -19.1497 | 149.5285 | 0.0000 | 7.4847e+06 | 1 |

**Adduct Signal Fractions**

- **Unmodified**: 7.4847e+06

### NWAGVTQN(N)GTSSSC(C)RRGSNSSFF Hex 7 HexNAc 2

**Mass:**  4046.6389

(2.880e-06)

**Charge States:** 3, 4

**Start Position**: 126

**End Position**: 149

| Profile Score | Tandem Score | TDA q-value | Volume | Spectrum Matches |
| --- | --- | --- | --- | --- |
| 17.3708 | 148.0516 | 0.0000 | 2.6180e+08 | 4 |

**Adduct Signal Fractions**

- **Unmodified**: 2.6180e+08

### TIVKPGDILLIN(N)ST Hex 9 HexNAc 2

**Mass:**  3347.5076

(2.297e-06)

**Charge States:** 3

**Start Position**: 235

**End Position**: 249

| Profile Score | Tandem Score | TDA q-value | Volume | Spectrum Matches |
| --- | --- | --- | --- | --- |
| -19.8957 | 147.9638 | 0.0000 | 1.4199e+07 | 1 |

**Adduct Signal Fractions**

- **Unmodified**: 1.4199e+07

### TIVKPGDILLIN(N)STGNL Hex 10 HexNAc 2

**Mass:**  3793.7071

(1.973e-06)

**Charge States:** 3

**Start Position**: 235

**End Position**: 252

| Profile Score | Tandem Score | TDA q-value | Volume | Spectrum Matches |
| --- | --- | --- | --- | --- |
| -4.2154 | 145.4631 | 0.0000 | 8.8743e+07 | 4 |

**Adduct Signal Fractions**

- **Unmodified**: 8.8743e+07

### TIVKPGDILLIN(N)STGN Fuc 2 Hex 3 HexNAc 4 Neu5Ac 1

**Mass:**  3535.5983

(-5.401e-06)

**Charge States:** 3

**Start Position**: 235

**End Position**: 251

| Profile Score | Tandem Score | TDA q-value | Volume | Spectrum Matches |
| --- | --- | --- | --- | --- |
| -4.4129 | 145.4066 | 0.0000 | 5.5864e+07 | 1 |

**Adduct Signal Fractions**

- **Unmodified**: 5.5864e+07

### RRGSN(N)SSFF Hex 5 HexNAc 2

**Mass:**  2272.9317

(-5.144e-07)

**Charge States:** 3

**Start Position**: 140

**End Position**: 149

| Profile Score | Tandem Score | TDA q-value | Volume | Spectrum Matches |
| --- | --- | --- | --- | --- |
| -19.4121 | 144.8915 | 0.0000 | 1.0570e+07 | 1 |

**Adduct Signal Fractions**

- **Unmodified**: 1.0570e+07

### NWAGVTQN(N)GTSSSC(C)R Hex 7 HexNAc 2

**Mass:**  3164.2366

(1.224e-07)

**Charge States:** 3

**Start Position**: 126

**End Position**: 141

| Profile Score | Tandem Score | TDA q-value | Volume | Spectrum Matches |
| --- | --- | --- | --- | --- |
| -19.3111 | 144.3387 | 0.0000 | 1.5201e+07 | 1 |

**Adduct Signal Fractions**

- **Unmodified**: 1.5201e+07

### TIVKPGDILLIN(N) Fuc 2 Hex 3 HexNAc 4 Neu5Ac 1

**Mass:**  3176.4516

(-6.820e-06)

**Charge States:** 3

**Start Position**: 235

**End Position**: 247

| Profile Score | Tandem Score | TDA q-value | Volume | Spectrum Matches |
| --- | --- | --- | --- | --- |
| 10.4797 | 143.5855 | 0.0000 | 4.2616e+07 | 2 |

**Adduct Signal Fractions**

- **Unmodified**: 4.2616e+07

### TIVKPGDILLIN(N)STGNL Hex 8 HexNAc 2

**Mass:**  3469.5987

(1.230e-06)

**Charge States:** 3, 4

**Start Position**: 235

**End Position**: 252

| Profile Score | Tandem Score | TDA q-value | Volume | Spectrum Matches |
| --- | --- | --- | --- | --- |
| -3.2705 | 142.1557 | 0.0000 | 9.7758e+07 | 3 |

**Adduct Signal Fractions**

- **Unmodified**: 9.7758e+07

### C(C)LGHHAVPN(N)GTIVK Fuc 1 Hex 5 HexNAc 5

**Mass:**  3473.5014

(-8.166e-07)

**Charge States:** 4

**Start Position**: 14

**End Position**: 28

| Profile Score | Tandem Score | TDA q-value | Volume | Spectrum Matches |
| --- | --- | --- | --- | --- |
| -19.0893 | 141.3988 | 0.0000 | 2.2516e+07 | 1 |

**Adduct Signal Fractions**

- **Unmodified**: 2.2516e+07

### LIN(N)STGNLIAPRGY Hex 10 HexNAc 2

**Mass:**  3514.5089

(3.533e-06)

**Charge States:** 3

**Start Position**: 244

**End Position**: 258

| Profile Score | Tandem Score | TDA q-value | Volume | Spectrum Matches |
| --- | --- | --- | --- | --- |
| -18.3594 | 140.9414 | 0.0000 | 2.9843e+07 | 1 |

**Adduct Signal Fractions**

- **Unmodified**: 2.9843e+07

### NWAGVTQN(N)GTSSSC(C) Hex 7 HexNAc 2

**Mass:**  3008.1344

(3.522e-07)

**Charge States:** 3

**Start Position**: 126

**End Position**: 140

| Profile Score | Tandem Score | TDA q-value | Volume | Spectrum Matches |
| --- | --- | --- | --- | --- |
| -4.3792 | 139.4772 | 0.0000 | 1.7623e+07 | 1 |

**Adduct Signal Fractions**

- **Unmodified**: 1.7623e+07

### AGVTQN(N)GTSSSC(C)RRGSNSSFF Hex 8 HexNAc 2

**Mass:**  3908.5698

(1.907e-06)

**Charge States:** 3

**Start Position**: 128

**End Position**: 149

| Profile Score | Tandem Score | TDA q-value | Volume | Spectrum Matches |
| --- | --- | --- | --- | --- |
| -3.1784 | 139.1627 | 0.0000 | 7.8927e+07 | 2 |

**Adduct Signal Fractions**

- **Unmodified**: 7.8927e+07

### RSDAPIGKC(C)KSEC(C)ITPN(N)GSIPNDKPF Hex 9 HexNAc 2

**Mass:**  4752.0364

(9.928e-07)

**Charge States:** 5

**Start Position**: 269

**End Position**: 295

| Profile Score | Tandem Score | TDA q-value | Volume | Spectrum Matches |
| --- | --- | --- | --- | --- |
| -2.7418 | 137.8502 | 0.0000 | 2.9232e+08 | 3 |

**Adduct Signal Fractions**

- **Unmodified**: 2.9232e+08

### RRGSN(N)SSFF Fuc 1 Hex 5 HexNAc 5

**Mass:**  3028.2301

(3.758e-07)

**Charge States:** 3, 4

**Start Position**: 140

**End Position**: 149

| Profile Score | Tandem Score | TDA q-value | Volume | Spectrum Matches |
| --- | --- | --- | --- | --- |
| -19.2917 | 136.9488 | 0.0000 | 2.6314e+07 | 1 |

**Adduct Signal Fractions**

- **Unmodified**: 2.6314e+07

### EFNN(N)ESFNW Hex 6 HexNAc 2

**Mass:**  2563.9594

(2.529e-06)

**Charge States:** 2, 3

**Start Position**: 119

**End Position**: 128

| Profile Score | Tandem Score | TDA q-value | Volume | Spectrum Matches |
| --- | --- | --- | --- | --- |
| -18.1299 | 135.7280 | 0.0000 | 8.9495e+06 | 2 |

**Adduct Signal Fractions**

- **Unmodified**: 8.9495e+06

### TITNDRIEVTN(N)ATEL Hex 6 HexNAc 3

**Mass:**  3270.4206

(2.247e-06)

**Charge States:** 3

**Start Position**: 28

**End Position**: 43

| Profile Score | Tandem Score | TDA q-value | Volume | Spectrum Matches |
| --- | --- | --- | --- | --- |
| -4.6936 | 135.6038 | 0.0000 | 2.8819e+07 | 1 |

**Adduct Signal Fractions**

- **Unmodified**: 2.8819e+07

### TIVKPGDILLIN(N)STGNLIAPR Hex 5 HexNAc 2

**Mass:**  3420.7148

(2.607e-06)

**Charge States:** 3, 4

**Start Position**: 235

**End Position**: 256

| Profile Score | Tandem Score | TDA q-value | Volume | Spectrum Matches |
| --- | --- | --- | --- | --- |
| -4.4093 | 135.5844 | 0.0000 | 2.2042e+07 | 3 |

**Adduct Signal Fractions**

- **Unmodified**: 2.2042e+07

### RSDAPIGKC(C)KSEC(C)ITPN(N)GSIPNDKPF Hex 7 HexNAc 2

**Mass:**  4427.9308

(1.463e-06)

**Charge States:** 5

**Start Position**: 269

**End Position**: 295

| Profile Score | Tandem Score | TDA q-value | Volume | Spectrum Matches |
| --- | --- | --- | --- | --- |
| -3.6013 | 132.8194 | 0.0067 | 1.7221e+08 | 2 |

**Adduct Signal Fractions**

- **Unmodified**: 1.7221e+08

### RGSN(N)SSFF Fuc 1 Hex 5 HexNAc 4

**Mass:**  2669.0553

(2.556e-06)

**Charge States:** 3

**Start Position**: 141

**End Position**: 149

| Profile Score | Tandem Score | TDA q-value | Volume | Spectrum Matches |
| --- | --- | --- | --- | --- |
| -19.9879 | 130.5416 | 0.0000 | 4.8924e+06 | 1 |

**Adduct Signal Fractions**

- **Unmodified**: 4.8924e+06

### NWAGVTQN(N)GTSSSC(C)RRGSNSSFF Hex 6 HexNAc 2

**Mass:**  3884.5863

(1.648e-06)

**Charge States:** 3, 4

**Start Position**: 126

**End Position**: 149

| Profile Score | Tandem Score | TDA q-value | Volume | Spectrum Matches |
| --- | --- | --- | --- | --- |
| -4.1613 | 129.7054 | 0.0067 | 1.1387e+08 | 3 |

**Adduct Signal Fractions**

- **Unmodified**: 1.1387e+08

### AGVTQN(N)GTSSSC(C)RRGSNSSFF Hex 6 HexNAc 3

**Mass:**  3787.5446

(2.871e-07)

**Charge States:** 3

**Start Position**: 128

**End Position**: 149

| Profile Score | Tandem Score | TDA q-value | Volume | Spectrum Matches |
| --- | --- | --- | --- | --- |
| -4.4211 | 129.6897 | 0.0067 | 1.0745e+08 | 2 |

**Adduct Signal Fractions**

- **Unmodified**: 1.0745e+08

### RSDAPIGKC(C)KSEC(C)ITPN(N)GSIPNDKPF Hex 8 HexNAc 2

**Mass:**  4589.9819

(1.042e-06)

**Charge States:** 5

**Start Position**: 269

**End Position**: 295

| Profile Score | Tandem Score | TDA q-value | Volume | Spectrum Matches |
| --- | --- | --- | --- | --- |
| -3.4321 | 129.4788 | 0.0067 | 1.2324e+08 | 1 |

**Adduct Signal Fractions**

- **Unmodified**: 1.2324e+08

### TIVKPGDILLIN(N)STGNL Hex 5 HexNAc 2

**Mass:**  2983.4428

(1.906e-06)

**Charge States:** 3

**Start Position**: 235

**End Position**: 252

| Profile Score | Tandem Score | TDA q-value | Volume | Spectrum Matches |
| --- | --- | --- | --- | --- |
| -3.3044 | 127.1643 | 0.0067 | 1.2434e+07 | 1 |

**Adduct Signal Fractions**

- **Unmodified**: 1.2434e+07

### RRGSN(N)SSFF Fuc 1 Hex 5 HexNAc 4

**Mass:**  2825.1478

(-6.326e-07)

**Charge States:** 3

**Start Position**: 140

**End Position**: 149

| Profile Score | Tandem Score | TDA q-value | Volume | Spectrum Matches |
| --- | --- | --- | --- | --- |
| -17.8140 | 126.0600 | 0.0000 | 9.9294e+06 | 1 |

**Adduct Signal Fractions**

- **Unmodified**: 9.9294e+06

### NSKYPALN(N)VTM(O)PNNEQFDKL Hex 9 HexNAc 2

**Mass:**  4202.7697

(1.412e-07)

**Charge States:** 3, 4

**Start Position**: 158

**End Position**: 178

| Profile Score | Tandem Score | TDA q-value | Volume | Spectrum Matches |
| --- | --- | --- | --- | --- |
| -3.5467 | 124.3692 | 0.0067 | 3.6011e+07 | 2 |

**Adduct Signal Fractions**

- **Unmodified**: 3.6011e+07

### NSKYPALN(N)VTMPNNEQFDKLY Hex 9 HexNAc 2

**Mass:**  4349.8371

(3.757e-06)

**Charge States:** 3, 4

**Start Position**: 158

**End Position**: 179

| Profile Score | Tandem Score | TDA q-value | Volume | Spectrum Matches |
| --- | --- | --- | --- | --- |
| -18.8389 | 124.1460 | 0.0067 | 7.2034e+06 | 2 |

**Adduct Signal Fractions**

- **Unmodified**: 7.2034e+06

### NWAGVTQN(N)GTSSSC(C)RRGSNSSFF Hex 5 HexNAc 2

**Mass:**  3722.5318

(1.215e-06)

**Charge States:** 3

**Start Position**: 126

**End Position**: 149

| Profile Score | Tandem Score | TDA q-value | Volume | Spectrum Matches |
| --- | --- | --- | --- | --- |
| -2.4015 | 123.0191 | 0.0067 | 2.1835e+08 | 3 |

**Adduct Signal Fractions**

- **Unmodified**: 2.1835e+08

### HKC(C)DNAC(C)IGSIRN(N)GTYDHDVY Fuc 1 Hex 5 HexNAc 4

**Mass:**  4262.7175

(9.706e-07)

**Charge States:** 4

**Start Position**: 471

**End Position**: 492

| Profile Score | Tandem Score | TDA q-value | Volume | Spectrum Matches |
| --- | --- | --- | --- | --- |
| -3.3122 | 119.1598 | 0.0067 | 8.1062e+07 | 1 |

**Adduct Signal Fractions**

- **Unmodified**: 8.1062e+07

### N(D)SKYPALN(N)VTM(O)PNNEQFDKLY Hex 8 HexNAc 2

**Mass:**  4204.7644

(2.355e-04)

**Charge States:** 3, 4

**Start Position**: 158

**End Position**: 179

| Profile Score | Tandem Score | TDA q-value | Volume | Spectrum Matches |
| --- | --- | --- | --- | --- |
| -6.1018 | 117.6557 | 0.0067 | 1.0326e+08 | 4 |

**Adduct Signal Fractions**

- **Unmodified**: 1.0326e+08

### AGVTQN(N)GTSSSC(C)RRGSNSSFF Hex 7 HexNAc 3

**Mass:**  3949.5981

(2.043e-06)

**Charge States:** 3

**Start Position**: 128

**End Position**: 149

| Profile Score | Tandem Score | TDA q-value | Volume | Spectrum Matches |
| --- | --- | --- | --- | --- |
| -3.6154 | 117.2589 | 0.0067 | 6.1516e+07 | 2 |

**Adduct Signal Fractions**

- **Unmodified**: 6.1516e+07

### NSKYPALN(N)VTM(O)PNNEQFDKL Hex 8 HexNAc 2

**Mass:**  4040.7228

(1.710e-06)

**Charge States:** 4

**Start Position**: 158

**End Position**: 178

| Profile Score | Tandem Score | TDA q-value | Volume | Spectrum Matches |
| --- | --- | --- | --- | --- |
| -4.9924 | 115.9972 | 0.0067 | 2.4872e+07 | 2 |

**Adduct Signal Fractions**

- **Unmodified**: 2.4872e+07

### LIN(N)STGN(D)LIAPRGYF Hex 9 HexNAc 2

**Mass:**  3500.5083

(2.846e-04)

**Charge States:** 3

**Start Position**: 244

**End Position**: 259

| Profile Score | Tandem Score | TDA q-value | Volume | Spectrum Matches |
| --- | --- | --- | --- | --- |
| -20.5767 | 113.6706 | 0.0120 | 5.0202e+06 | 2 |

**Adduct Signal Fractions**

- **Unmodified**: 5.0202e+06

### SIRN(N)GTYDHDVY Fuc 1 Hex 5 HexNAc 4

**Mass:**  3207.2905

(1.037e-06)

**Charge States:** 3

**Start Position**: 480

**End Position**: 492

| Profile Score | Tandem Score | TDA q-value | Volume | Spectrum Matches |
| --- | --- | --- | --- | --- |
| -16.9875 | 109.4049 | 0.0120 | 9.0020e+06 | 1 |

**Adduct Signal Fractions**

- **Unmodified**: 9.0020e+06

### TIVKPGDILLIN(N)STGNLIAPRGY Hex 5 HexNAc 2

**Mass:**  3640.8044

(2.007e-06)

**Charge States:** 3, 4

**Start Position**: 235

**End Position**: 258

| Profile Score | Tandem Score | TDA q-value | Volume | Spectrum Matches |
| --- | --- | --- | --- | --- |
| -3.1183 | 106.6336 | 0.0120 | 6.9383e+07 | 5 |

**Adduct Signal Fractions**

- **Unmodified**: 6.9383e+07

### TIVKPGDILLIN(N)STGN(D)LIAPRGY Hex 9 HexNAc 2

**Mass:**  4290.0046

(2.323e-04)

**Charge States:** 3, 4

**Start Position**: 235

**End Position**: 258

| Profile Score | Tandem Score | TDA q-value | Volume | Spectrum Matches |
| --- | --- | --- | --- | --- |
| -19.4261 | 105.8199 | 0.0120 | 6.3774e+07 | 16 |

**Adduct Signal Fractions**

- **Unmodified**: 6.3774e+07

### TIVKPGDILLIN(N)STG Hex 9 HexNAc 2

**Mass:**  3404.5200

(-3.932e-07)

**Charge States:** 3

**Start Position**: 235

**End Position**: 250

| Profile Score | Tandem Score | TDA q-value | Volume | Spectrum Matches |
| --- | --- | --- | --- | --- |
| -20.7156 | 103.9783 | 0.0120 | 1.1094e+07 | 1 |

**Adduct Signal Fractions**

- **Unmodified**: 1.1094e+07

### AGVTQN(N)GTSSSC(C)RRGSN(D)SSFF Hex 7 HexNAc 2

**Mass:**  3747.4975

(2.634e-04)

**Charge States:** 3, 4

**Start Position**: 128

**End Position**: 149

| Profile Score | Tandem Score | TDA q-value | Volume | Spectrum Matches |
| --- | --- | --- | --- | --- |
| 10.5473 | 101.8559 | 0.0120 | 2.6963e+08 | 4 |

**Adduct Signal Fractions**

- **Unmodified**: 2.6963e+08

### TIVKPGDILLIN(N)STGNLIAPRGY Hex 7 HexNAc 2

**Mass:**  3966.9321

(5.119e-04)

**Start Position**: 235

**End Position**: 258

| Profile Score | Tandem Score | TDA q-value | Volume | Spectrum Matches |
| --- | --- | --- | --- | --- |
| 0.0000 | 95.1384 | 0.0272 | 0.0000e+00 | 1 |

Adduct:

No Chromatogram Found

### N(D)SKYPALN(N)VTM(O)PNNEQFDKLY Hex 9 HexNAc 2

**Mass:**  4366.8166

(2.274e-04)

**Charge States:** 3, 4

**Start Position**: 158

**End Position**: 179

| Profile Score | Tandem Score | TDA q-value | Volume | Spectrum Matches |
| --- | --- | --- | --- | --- |
| -6.1942 | 92.5840 | 0.0272 | 1.1493e+08 | 2 |

**Adduct Signal Fractions**

- **Unmodified**: 1.1493e+08

### LIN(N)STGN(D)LIAPRGY Hex 9 HexNAc 2

**Mass:**  3353.4300

(2.942e-04)

**Charge States:** 3

**Start Position**: 244

**End Position**: 258

| Profile Score | Tandem Score | TDA q-value | Volume | Spectrum Matches |
| --- | --- | --- | --- | --- |
| -20.2951 | 91.8731 | 0.0272 | 9.8288e+06 | 1 |

**Adduct Signal Fractions**

- **Unmodified**: 9.8288e+06

### NWAGVTQN(N)GTSSSC(C)RRGSNSSFF Hex 7 HexNAc 3

**Mass:**  4249.7321

(4.731e-06)

**Charge States:** 4

**Start Position**: 126

**End Position**: 149

| Profile Score | Tandem Score | TDA q-value | Volume | Spectrum Matches |
| --- | --- | --- | --- | --- |
| -17.5644 | 88.7774 | 0.0272 | 2.6084e+07 | 2 |

**Adduct Signal Fractions**

- **Unmodified**: 2.6084e+07

### RSLVASSGTLEFNN(N)ESF Hex 6 HexNAc 2

**Mass:**  3235.3698

(1.152e-06)

**Charge States:** 3

**Start Position**: 109

**End Position**: 126

| Profile Score | Tandem Score | TDA q-value | Volume | Spectrum Matches |
| --- | --- | --- | --- | --- |
| -20.3526 | 87.4959 | 0.0272 | 9.7910e+06 | 1 |

**Adduct Signal Fractions**

- **Unmodified**: 9.7910e+06

### TIVKPGDILLIN(N)STGNLIAPR Fuc 2 Hex 4 HexNAc 4 Neu5Ac 1

**Mass:**  4248.0114

(-4.196e-06)

**Charge States:** 4

**Start Position**: 235

**End Position**: 256

| Profile Score | Tandem Score | TDA q-value | Volume | Spectrum Matches |
| --- | --- | --- | --- | --- |
| -19.0718 | 86.9534 | 0.0272 | 1.1931e+07 | 1 |

**Adduct Signal Fractions**

- **Unmodified**: 1.1931e+07

### LNSKYPALN(N)VTM(O)PNNEQFDKL Hex 8 HexNAc 2

**Mass:**  4153.8127

(1.166e-06)

**Charge States:** 3, 4

**Start Position**: 157

**End Position**: 178

| Profile Score | Tandem Score | TDA q-value | Volume | Spectrum Matches |
| --- | --- | --- | --- | --- |
| -17.7955 | 85.0062 | 0.0321 | 1.1859e+07 | 2 |

**Adduct Signal Fractions**

- **Unmodified**: 1.1859e+07

### RSDAPIGKC(C)KSEC(C)ITPN(N)GSIPN(D)DKPF Hex 9 HexNAc 2

**Mass:**  4753.0386

(2.130e-04)

**Charge States:** 5

**Start Position**: 269

**End Position**: 295

| Profile Score | Tandem Score | TDA q-value | Volume | Spectrum Matches |
| --- | --- | --- | --- | --- |
| -5.8450 | 84.7479 | 0.0321 | 3.5703e+07 | 1 |

**Adduct Signal Fractions**

- **Unmodified**: 3.5703e+07

### NWAGVTQN(N)GTSSSC(C)RRGSNSSFF Hex 6 HexNAc 3

**Mass:**  4087.6630

(8.808e-07)

**Charge States:** 3

**Start Position**: 126

**End Position**: 149

| Profile Score | Tandem Score | TDA q-value | Volume | Spectrum Matches |
| --- | --- | --- | --- | --- |
| -3.1082 | 83.3584 | 0.0398 | 8.7041e+07 | 1 |

**Adduct Signal Fractions**

- **Unmodified**: 8.7041e+07

### TIVKPGDILLIN(N)STGNLIAPRGY Hex 6 HexNAc 2

**Mass:**  3802.8579

(1.681e-06)

**Charge States:** 4

**Start Position**: 235

**End Position**: 258

| Profile Score | Tandem Score | TDA q-value | Volume | Spectrum Matches |
| --- | --- | --- | --- | --- |
| -4.4028 | 82.4034 | 0.0398 | 1.3555e+07 | 3 |

**Adduct Signal Fractions**

- **Unmodified**: 1.3555e+07

### TIVKPGDILLIN(N)STGNLIAPRGY Hex 7 HexNAc 2

**Mass:**  3964.9025

(2.350e-06)

**Charge States:** 4

**Start Position**: 235

**End Position**: 258

| Profile Score | Tandem Score | TDA q-value | Volume | Spectrum Matches |
| --- | --- | --- | --- | --- |
| -4.5917 | 78.6932 | 0.0398 | 1.5936e+07 | 2 |

**Adduct Signal Fractions**

- **Unmodified**: 1.5936e+07

### AGVTQN(N)GTSSSC(C)RRGSNSSFF Fuc 1 Hex 4 HexNAc 3

**Mass:**  3609.4982

(2.445e-06)

**Charge States:** 3, 4

**Start Position**: 128

**End Position**: 149

| Profile Score | Tandem Score | TDA q-value | Volume | Spectrum Matches |
| --- | --- | --- | --- | --- |
| -4.8039 | 78.0712 | 0.0398 | 4.9631e+07 | 1 |

**Adduct Signal Fractions**

- **Unmodified**: 4.9631e+07

### AGVTQN(N)GTSSSC(C)RRGSN(D)SSFF Hex 6 HexNAc 2

**Mass:**  3585.4488

(2.764e-04)

**Charge States:** 3

**Start Position**: 128

**End Position**: 149

| Profile Score | Tandem Score | TDA q-value | Volume | Spectrum Matches |
| --- | --- | --- | --- | --- |
| 11.8382 | 77.8814 | 0.0398 | 5.9498e+07 | 2 |

**Adduct Signal Fractions**

- **Unmodified**: 5.9498e+07

### NWAGVTQN(N)GTSSSC(C)RRGSNSSFF Hex 3 HexNAc 2

**Mass:**  3398.4256

(1.120e-06)

**Charge States:** 3, 4

**Start Position**: 126

**End Position**: 149

| Profile Score | Tandem Score | TDA q-value | Volume | Spectrum Matches |
| --- | --- | --- | --- | --- |
| -19.1588 | 75.1588 | 0.0398 | 9.8876e+06 | 1 |

**Adduct Signal Fractions**

- **Unmodified**: 9.8876e+06

### HKC(C)DNAC(C)IGSIRN(N)GTYDHDVY Fuc 1 Hex 5 HexNAc 5

**Mass:**  4465.7983

(-1.675e-06)

**Charge States:** 5

**Start Position**: 471

**End Position**: 492

| Profile Score | Tandem Score | TDA q-value | Volume | Spectrum Matches |
| --- | --- | --- | --- | --- |
| -19.4613 | 67.5757 | 0.0429 | 2.4642e+07 | 1 |

**Adduct Signal Fractions**

- **Unmodified**: 2.4642e+07

### NWAGVTQN(N)GTSSSC(C)RRGSNSSFF Hex 4 HexNAc 3

**Mass:**  3763.5580

(2.346e-06)

**Charge States:** 3, 4

**Start Position**: 126

**End Position**: 149

| Profile Score | Tandem Score | TDA q-value | Volume | Spectrum Matches |
| --- | --- | --- | --- | --- |
| -19.5186 | 67.4088 | 0.0429 | 5.2553e+07 | 2 |

**Adduct Signal Fractions**

- **Unmodified**: 5.2553e+07

### AGVTQN(N)GTSSSC(C)RRGSN(D)SSFF Hex 5 HexNAc 2

**Mass:**  3423.3957

(2.886e-04)

**Charge States:** 3, 4

**Start Position**: 128

**End Position**: 149

| Profile Score | Tandem Score | TDA q-value | Volume | Spectrum Matches |
| --- | --- | --- | --- | --- |
| 9.6467 | 65.9496 | 0.0429 | 9.4825e+07 | 4 |

**Adduct Signal Fractions**

- **Unmodified**: 9.4825e+07

### NSKYPALN(N)VTM(O)PNNEQFDKLY Hex 5 HexNAc 2

**Mass:**  3717.6260

(-6.321e-07)

**Charge States:** 3

**Start Position**: 158

**End Position**: 179

| Profile Score | Tandem Score | TDA q-value | Volume | Spectrum Matches |
| --- | --- | --- | --- | --- |
| -18.1684 | 65.2689 | 0.0458 | 8.7252e+06 | 2 |

**Adduct Signal Fractions**

- **Unmodified**: 8.7252e+06

### NSKYPALN(N)VTM(O)PNNEQFDKLY Hex 10 HexNAc 2

**Mass:**  4527.8889

(2.710e-06)

**Charge States:** 3

**Start Position**: 158

**End Position**: 179

| Profile Score | Tandem Score | TDA q-value | Volume | Spectrum Matches |
| --- | --- | --- | --- | --- |
| -19.0213 | 64.1012 | 0.0458 | 2.5795e+06 | 1 |

**Adduct Signal Fractions**

- **Unmodified**: 2.5795e+06

### AGVTQN(N)GTSSSC(C)RRGSNSSFF Fuc 1 Hex 4 HexNAc 4 Neu5Ac 1

**Mass:**  4103.6613

(-7.344e-07)

**Charge States:** 3, 4

**Start Position**: 128

**End Position**: 149

| Profile Score | Tandem Score | TDA q-value | Volume | Spectrum Matches |
| --- | --- | --- | --- | --- |
| -3.5076 | 63.3534 | 0.0458 | 8.0289e+07 | 2 |

**Adduct Signal Fractions**

- **Unmodified**: 8.0289e+07

### RSDAPIGKC(C)KSEC(C)ITPN(N)GSIPN(D)DKPF Hex 7 HexNAc 2

**Mass:**  4428.9248

(2.288e-04)

**Charge States:** 5

**Start Position**: 269

**End Position**: 295

| Profile Score | Tandem Score | TDA q-value | Volume | Spectrum Matches |
| --- | --- | --- | --- | --- |
| -6.1042 | 62.4333 | 0.0458 | 2.1929e+07 | 1 |

**Adduct Signal Fractions**

- **Unmodified**: 2.1929e+07

### NSKYPALN(N)VTM(O)PNNEQFDKLY Hex 7 HexNAc 2

**Mass:**  4041.7287

(3.028e-06)

**Charge States:** 3, 4

**Start Position**: 158

**End Position**: 179

| Profile Score | Tandem Score | TDA q-value | Volume | Spectrum Matches |
| --- | --- | --- | --- | --- |
| -4.2131 | 60.9272 | 0.0458 | 2.0042e+07 | 1 |

**Adduct Signal Fractions**

- **Unmodified**: 2.0042e+07

### NWAGVTQNGTSSSC(C)RRGSN(N)SSFF Hex 6 HexNAc 4

**Mass:**  4290.7434

(1.061e-06)

**Charge States:** 4

**Start Position**: 126

**End Position**: 149

| Profile Score | Tandem Score | TDA q-value | Volume | Spectrum Matches |
| --- | --- | --- | --- | --- |
| -5.4388 | 60.4287 | 0.0458 | 3.4307e+07 | 1 |

**Adduct Signal Fractions**

- **Unmodified**: 3.4307e+07

### TITNDRIEVTN(N)ATEL Hex 5 HexNAc 4

**Mass:**  3311.4505

(2.509e-06)

**Charge States:** 3

**Start Position**: 28

**End Position**: 43

| Profile Score | Tandem Score | TDA q-value | Volume | Spectrum Matches |
| --- | --- | --- | --- | --- |
| -20.1707 | 60.3736 | 0.0458 | 1.0847e+07 | 1 |

**Adduct Signal Fractions**

- **Unmodified**: 1.0847e+07

### KSEC(C)ITPN(N)GSIPNDKPF Hex 8 HexNAc 2

**Mass:**  3605.5116

(4.333e-06)

**Charge States:** 3

**Start Position**: 278

**End Position**: 295

| Profile Score | Tandem Score | TDA q-value | Volume | Spectrum Matches |
| --- | --- | --- | --- | --- |
| -20.4827 | 60.2242 | 0.0458 | 8.6841e+06 | 1 |

**Adduct Signal Fractions**

- **Unmodified**: 8.6841e+06

### LNSKYPALN(N)VTM(O)PNNEQFDKL Hex 9 HexNAc 2

**Mass:**  4315.8513

(1.549e-06)

**Charge States:** 4

**Start Position**: 157

**End Position**: 178

| Profile Score | Tandem Score | TDA q-value | Volume | Spectrum Matches |
| --- | --- | --- | --- | --- |
| -18.0162 | 60.1197 | 0.0458 | 1.2103e+07 | 1 |

**Adduct Signal Fractions**

- **Unmodified**: 1.2103e+07

### IN(N)STGNLIAPRGY Fuc 1 Hex 7 HexNAc 5 Neu5Ac 2

**Mass:**  4253.7025

(2.261e-04)

**Start Position**: 245

**End Position**: 258

| Profile Score | Tandem Score | TDA q-value | Volume | Spectrum Matches |
| --- | --- | --- | --- | --- |
| 0.0000 | 59.8680 | 0.0458 | 0.0000e+00 | 1 |

Adduct:

No Chromatogram Found

### TITNDRIEVTN(N)ATEL Hex 5 HexNAc 3

**Mass:**  3108.3726

(3.948e-06)

**Charge States:** 3

**Start Position**: 28

**End Position**: 43

| Profile Score | Tandem Score | TDA q-value | Volume | Spectrum Matches |
| --- | --- | --- | --- | --- |
| -18.6781 | 58.4877 | 0.0458 | 9.7170e+06 | 1 |

**Adduct Signal Fractions**

- **Unmodified**: 9.7170e+06

### TIVKPGDILLIN(N)STGNLIAPRG Hex 9 HexNAc 2

**Mass:**  4125.9553

(1.557e-06)

**Charge States:** 3, 4

**Start Position**: 235

**End Position**: 257

| Profile Score | Tandem Score | TDA q-value | Volume | Spectrum Matches |
| --- | --- | --- | --- | --- |
| 16.3466 | 57.5578 | 0.0458 | 4.1533e+08 | 10 |

**Adduct Signal Fractions**

- **Unmodified**: 4.1533e+08

### THLN(D)SKYPALN(N)VTM(O)PNNEQFDKL Fuc 3 Hex 3 HexNAc 4 Neu5Ac 1

**Mass:**  4718.0173

(2.025e-04)

**Charge States:** 4, 5

**Start Position**: 155

**End Position**: 178

| Profile Score | Tandem Score | TDA q-value | Volume | Spectrum Matches |
| --- | --- | --- | --- | --- |
| 7.1867 | 57.5401 | 0.0458 | 1.6069e+08 | 5 |

**Adduct Signal Fractions**

- **Unmodified**: 1.6069e+08

### TIVKPGDILLIN(N)STGN(D)LIAPR Hex 9 HexNAc 2

**Mass:**  4069.9250

(2.455e-04)

**Charge States:** 3, 4

**Start Position**: 235

**End Position**: 256

| Profile Score | Tandem Score | TDA q-value | Volume | Spectrum Matches |
| --- | --- | --- | --- | --- |
| -19.5990 | 56.9179 | 0.0458 | 2.4194e+07 | 4 |

**Adduct Signal Fractions**

- **Unmodified**: 2.4194e+07

### AGVTQN(N)GTSSSC(C)RRGSNSSFF Hex 4 HexNAc 3

**Mass:**  3463.4375

(2.413e-06)

**Charge States:** 3

**Start Position**: 128

**End Position**: 149

| Profile Score | Tandem Score | TDA q-value | Volume | Spectrum Matches |
| --- | --- | --- | --- | --- |
| -3.9639 | 56.1033 | 0.0458 | 1.0710e+08 | 2 |

**Adduct Signal Fractions**

- **Unmodified**: 1.0710e+08

### SKYPALN(N)VTM(O)PNNEQFDKLY Fuc 1 Hex 6 HexNAc 4

**Mass:**  4317.8518

(1.485e-06)

**Charge States:** 4

**Start Position**: 159

**End Position**: 179

| Profile Score | Tandem Score | TDA q-value | Volume | Spectrum Matches |
| --- | --- | --- | --- | --- |
| -4.0904 | 55.3686 | 0.0458 | 4.4383e+07 | 2 |

**Adduct Signal Fractions**

- **Unmodified**: 4.4383e+07

### TITNDRIEVTN(N)ATEL Fuc 1 Hex 6 HexNAc 3

**Mass:**  3416.4721

(3.047e-07)

**Charge States:** 3

**Start Position**: 28

**End Position**: 43

| Profile Score | Tandem Score | TDA q-value | Volume | Spectrum Matches |
| --- | --- | --- | --- | --- |
| -21.3078 | 54.5340 | 0.0494 | 4.6356e+06 | 1 |

**Adduct Signal Fractions**

- **Unmodified**: 4.6356e+06

### cus|SWZHA\_5B8|A/Switzerland/9715293/2013

**>cus|SWZHA\_5B8|A/Switzerland/9715293/2013**  
 `QKLPGNDNSTATLCLGHHAVPNGTIVKTITNDRIEVTNATELVQNSSIGEICDSPHQILDGENCTLIDALLGDPQCDGFQ  
NKKWDLFVERSKAYSNCYPYDVPDYASLRSLVASSGTLEFNNESFNWAGVTHNGTSSSCRRGSNSSFFSRLNWLTHLNSK  
YPALNVTMPNNEQFDKLYIWGVHHPVTDKDQIFLYAQSSGRITVSTKRSQQAVIPNIGSRPRIRNIPSRISIYWTIVKPG  
DILLINSTGNLIAPRGYFKIRSGKSSIMRSDAPIGKCKSECITPNGSIPNDKPFQNVNRITYGACPRYVKQSTLKLATGM  
RNVPERQTRGIFGAIAGFIENGWEGMVDGWYGFRHQNSEGRGQAADLKSTQAAIDQINGKLNRLIGKTNEKFHQIEKEFS  
EVEGRIQDLEKYVEDTKIDLWSYNAELLVALENQHTIDLTDSEMNKLFEKTKKQLRENAEDMGNGCFKIYHKCDNACIGS  
IRNGTYDHDVYRDEALNNRFQIKGVELKSGYKDWILWISFAISCFLLCVALLGFIMWACQKGNIRCNICI`

| Site | Type | Count |
| --- | --- | --- |
| 483 | N-linked | 3 |
| 165 | N-linked | 15 |
| 38 | N-linked | 6 |
| 8 | N-linked | 1 |
| 144 | N-linked | 5 |
| 246 | N-linked | 43 |
| 122 | N-linked | 2 |
| 285 | N-linked | 6 |
| 22 | N-linked | 2 |

| Mass | Sequence | MS_2_ Score | `q`-value | MS_1_ Score | Signal | Protein Coordinates | Tandem Scans | Start Time | Apex Time | End Time |
| --- | --- | --- | --- | --- | --- | --- | --- | --- | --- | --- |
| 2695.0875 | LIN(N)STGNL Hex 9 HexNAc 2 | 188.192 | 0.0 | -18.441 | 5.771e+07 | 243 - 251 | 2 | 27.40 | 27.40 | 27.56 |
| 3661.5649 | LIN(N)STGNLIAPRGYF Hex 10 HexNAc 2 | 184.743 | 0.0 | -17.499 | 3.256e+07 | 243 - 258 | 1 | 47.55 | 47.72 | 47.72 |
| 3499.5179 | LIN(N)STGNLIAPRGYF Hex 9 HexNAc 2 | 181.976 | 0.0 | 17.745 | 3.775e+08 | 243 - 258 | 4 | 47.55 | 47.72 | 48.22 |
| 3516.5440 | LIN(N)STGNLIAPRGYF Fuc 2 Hex 3 HexNAc 4 Neu5Ac 1 | 178.455 | 0.0 | -17.662 | 4.867e+07 | 243 - 258 | 1 | 47.72 | 47.72 | 47.88 |
| 3352.4496 | LIN(N)STGNLIAPRGY Hex 9 HexNAc 2 | 173.211 | 0.0 | 15.835 | 2.397e+08 | 243 - 257 | 3 | 37.49 | 37.66 | 38.16 |
| 3132.3710 | LIN(N)STGNLIAPR Hex 9 HexNAc 2 | 171.070 | 0.0 | -17.874 | 7.887e+07 | 243 - 255 | 1 | 31.89 | 31.89 | 31.89 |
| 4068.9311 | TIVKPGDILLIN(N)STGNLIAPR Hex 9 HexNAc 2 | 168.727 | 0.0 | 18.493 | 2.013e+09 | 234 - 255 | 14 | 53.14 | 53.82 | 55.86 |
| 3386.4333 | IN(N)STGNLIAPRGYF Hex 9 HexNAc 2 | 168.264 | 0.0 | -18.346 | 2.082e+07 | 244 - 258 | 1 | 43.89 | 44.06 | 44.06 |
| 4203.7828 | NSKYPALN(N)VTM(O)PNNEQFDKLY Hex 8 HexNAc 2 | 166.793 | 0.0 | 18.530 | 2.976e+08 | 157 - 178 | 4 | 43.05 | 43.55 | 43.72 |
| 2317.9565 | LPGNDN(N)STATL Hex 5 HexNAc 2 | 165.509 | 0.0 | -20.107 | 7.194e+06 | 2 - 13 | 2 | 27.56 | 27.56 | 27.73 |
| 3337.4693 | LIN(N)STGNLIAPRGYF Hex 8 HexNAc 2 | 162.369 | 0.0 | -4.495 | 2.751e+07 | 243 - 258 | 1 | 47.72 | 47.88 | 48.05 |
| 4230.9805 | TIVKPGDILLIN(N)STGNLIAPR Hex 10 HexNAc 2 | 161.401 | 0.0 | 14.440 | 1.928e+08 | 234 - 255 | 5 | 53.65 | 53.82 | 54.32 |
| 3369.4745 | LIN(N)STGNLIAPRGY Fuc 2 Hex 3 HexNAc 4 Neu5Ac 1 | 161.040 | 0.0 | -18.836 | 2.634e+07 | 243 - 257 | 1 | 37.49 | 37.66 | 37.66 |
| 4365.8348 | NSKYPALN(N)VTM(O)PNNEQFDKLY Hex 9 HexNAc 2 | 160.263 | 0.0 | 16.608 | 3.689e+08 | 157 - 178 | 4 | 43.05 | 43.38 | 43.72 |
| 4289.0184 | TIVKPGDILLIN(N)STGNLIAPRGY Hex 9 HexNAc 2 | 158.376 | 0.0 | 18.705 | 3.716e+09 | 234 - 257 | 22 | 54.32 | 56.20 | 58.72 |
| 3159.4268 | TIVKPGDILLIN(N) Hex 9 HexNAc 2 | 158.134 | 0.0 | 11.067 | 2.505e+08 | 234 - 246 | 3 | 46.76 | 48.22 | 48.72 |
| 4126.9607 | TIVKPGDILLIN(N)STGNLIAPRGY Hex 8 HexNAc 2 | 157.458 | 0.0 | 14.599 | 3.558e+08 | 234 - 257 | 13 | 55.52 | 56.20 | 57.56 |
| 4306.0372 | TIVKPGDILLIN(N)STGNLIAPRGY Fuc 2 Hex 3 HexNAc 4 Neu5Ac 1 | 157.419 | 0.0 | 12.272 | 2.600e+08 | 234 - 257 | 5 | 55.69 | 56.20 | 57.56 |
| 2905.2900 | TITNDRIEVTN(N)ATEL Hex 5 HexNAc 2 | 157.278 | 0.0 | -3.185 | 3.287e+07 | 27 - 42 | 2 | 37.66 | 37.82 | 37.99 |
| 2997.3727 | TIVKPGDILLIN(N) Hex 8 HexNAc 2 | 156.906 | 0.0 | 10.911 | 1.324e+07 | 234 - 246 | 2 | 47.88 | 48.22 | 48.55 |
| 4451.0683 | TIVKPGDILLIN(N)STGNLIAPRGY Hex 10 HexNAc 2 | 155.298 | 0.0 | 15.841 | 3.546e+08 | 234 - 257 | 7 | 55.69 | 56.03 | 56.70 |
| 3906.8767 | TIVKPGDILLIN(N)STGNLIAPR Hex 8 HexNAc 2 | 155.096 | 0.0 | -3.232 | 1.446e+08 | 234 - 255 | 4 | 53.82 | 53.99 | 54.32 |
| 2116.8346 | RGSN(N)SSFF Hex 5 HexNAc 2 | 154.880 | 0.0 | -19.250 | 6.277e+06 | 140 - 148 | 1 | 26.90 | 27.06 | 27.06 |
| 3648.6796 | TIVKPGDILLIN(N)STGNL Fuc 2 Hex 3 HexNAc 4 Neu5Ac 1 | 152.556 | 0.0 | -3.955 | 2.080e+08 | 234 - 251 | 2 | 53.31 | 53.48 | 53.82 |
| 3229.3941 | TITNDRIEVTN(N)ATEL Hex 7 HexNAc 2 | 152.251 | 0.0 | -17.082 | 3.644e+07 | 27 - 42 | 1 | 37.32 | 37.49 | 37.49 |
| 4085.9511 | TIVKPGDILLIN(N)STGNLIAPR Fuc 2 Hex 3 HexNAc 4 Neu5Ac 1 | 151.739 | 0.0 | 14.730 | 1.398e+08 | 234 - 255 | 2 | 53.65 | 53.82 | 54.49 |
| 3631.6538 | TIVKPGDILLIN(N)STGNL Hex 9 HexNAc 2 | 151.047 | 0.0 | 18.109 | 1.200e+09 | 234 - 251 | 10 | 52.80 | 53.48 | 55.69 |
| 4089.7401 | SKYPALN(N)VTM(O)PNNEQFDKLY Hex 8 HexNAc 2 | 150.689 | 0.0 | -2.647 | 8.393e+07 | 158 - 178 | 2 | 43.38 | 43.55 | 43.72 |
| 3321.4777 | TIVKPGDILLIN(N) Hex 10 HexNAc 2 | 150.354 | 0.0 | -17.294 | 8.864e+06 | 234 - 246 | 1 | 48.05 | 48.05 | 48.05 |
| 3518.5741 | TIVKPGDILLIN(N)STGN Hex 9 HexNAc 2 | 149.942 | 0.0 | 16.194 | 3.167e+08 | 234 - 250 | 3 | 45.74 | 46.08 | 46.42 |
| 3270.4260 | C(C)LGHHAVPN(N)GTIVK Fuc 1 Hex 5 HexNAc 4 | 149.528 | 0.0 | -19.150 | 7.485e+06 | 13 - 27 | 1 | 19.67 | 19.67 | 19.84 |
| 3347.5022 | TIVKPGDILLIN(N)ST Hex 9 HexNAc 2 | 147.964 | 0.0 | -19.896 | 1.420e+07 | 234 - 248 | 1 | 47.39 | 47.39 | 47.55 |
| 3793.7073 | TIVKPGDILLIN(N)STGNL Hex 10 HexNAc 2 | 145.463 | 0.0 | -4.215 | 8.874e+07 | 234 - 251 | 4 | 53.31 | 53.48 | 53.82 |
| 3535.5982 | TIVKPGDILLIN(N)STGN Fuc 2 Hex 3 HexNAc 4 Neu5Ac 1 | 145.407 | 0.0 | -4.413 | 5.586e+07 | 234 - 250 | 1 | 45.91 | 46.08 | 46.25 |
| 2272.9352 | RRGSN(N)SSFF Hex 5 HexNAc 2 | 144.891 | 0.0 | -19.412 | 1.057e+07 | 139 - 148 | 1 | 21.97 | 22.13 | 22.13 |
| 3176.4496 | TIVKPGDILLIN(N) Fuc 2 Hex 3 HexNAc 4 Neu5Ac 1 | 143.586 | 0.0 | 10.480 | 4.262e+07 | 234 - 246 | 2 | 46.92 | 48.22 | 48.55 |
| 3469.5990 | TIVKPGDILLIN(N)STGNL Hex 8 HexNAc 2 | 142.156 | 0.0 | -3.271 | 9.776e+07 | 234 - 251 | 3 | 53.48 | 53.65 | 53.82 |
| 3473.5012 | C(C)LGHHAVPN(N)GTIVK Fuc 1 Hex 5 HexNAc 5 | 141.399 | 0.0 | -19.089 | 2.252e+07 | 13 - 27 | 1 | 19.67 | 19.84 | 19.84 |
| 3514.5100 | LIN(N)STGNLIAPRGY Hex 10 HexNAc 2 | 140.941 | 0.0 | -18.359 | 2.984e+07 | 243 - 257 | 1 | 37.49 | 37.49 | 37.66 |
| 4752.0367 | RSDAPIGKC(C)KSEC(C)ITPN(N)GSIPNDKPF Hex 9 HexNAc 2 | 137.850 | 0.0 | -2.742 | 2.923e+08 | 268 - 294 | 3 | 30.55 | 30.72 | 30.88 |
| 3028.2300 | RRGSN(N)SSFF Fuc 1 Hex 5 HexNAc 5 | 136.949 | 0.0 | -19.292 | 2.631e+07 | 139 - 148 | 1 | 21.97 | 21.97 | 22.13 |
| 2563.9568 | EFNN(N)ESFNW Hex 6 HexNAc 2 | 135.728 | 0.0 | -18.130 | 8.949e+06 | 118 - 127 | 2 | 44.90 | 44.90 | 45.07 |
| 3270.4203 | TITNDRIEVTN(N)ATEL Hex 6 HexNAc 3 | 135.604 | 0.0 | -4.694 | 2.882e+07 | 27 - 42 | 1 | 37.49 | 37.49 | 37.82 |
| 3420.7168 | TIVKPGDILLIN(N)STGNLIAPR Hex 5 HexNAc 2 | 135.584 | 0.0 | -4.409 | 2.204e+07 | 234 - 255 | 3 | 54.49 | 54.66 | 54.84 |
| 4427.9309 | RSDAPIGKC(C)KSEC(C)ITPN(N)GSIPNDKPF Hex 7 HexNAc 2 | 132.819 | 0.007 | -3.601 | 1.722e+08 | 268 - 294 | 2 | 30.72 | 30.88 | 31.05 |
| 2669.0553 | RGSN(N)SSFF Fuc 1 Hex 5 HexNAc 4 | 130.542 | 0.0 | -19.988 | 4.892e+06 | 140 - 148 | 1 | 26.40 | 26.40 | 26.40 |
| 4589.9824 | RSDAPIGKC(C)KSEC(C)ITPN(N)GSIPNDKPF Hex 8 HexNAc 2 | 129.479 | 0.007 | -3.432 | 1.232e+08 | 268 - 294 | 1 | 30.72 | 30.72 | 31.05 |
| 2983.4422 | TIVKPGDILLIN(N)STGNL Hex 5 HexNAc 2 | 127.164 | 0.007 | -3.304 | 1.243e+07 | 234 - 251 | 1 | 54.15 | 54.32 | 54.49 |
| 2825.1478 | RRGSN(N)SSFF Fuc 1 Hex 5 HexNAc 4 | 126.060 | 0.0 | -17.814 | 9.929e+06 | 139 - 148 | 1 | 21.81 | 21.81 | 21.81 |
| 4202.7675 | NSKYPALN(N)VTM(O)PNNEQFDKL Hex 9 HexNAc 2 | 124.369 | 0.007 | -3.547 | 3.601e+07 | 157 - 177 | 2 | 41.02 | 41.19 | 41.36 |
| 4349.8375 | NSKYPALN(N)VTMPNNEQFDKLY Hex 9 HexNAc 2 | 124.146 | 0.007 | -18.839 | 7.203e+06 | 157 - 178 | 2 | 49.40 | 49.40 | 49.57 |
| 4262.7191 | HKC(C)DNAC(C)IGSIRN(N)GTYDHDVY Fuc 1 Hex 5 HexNAc 4 | 119.160 | 0.007 | -3.312 | 8.106e+07 | 470 - 491 | 1 | 25.91 | 26.07 | 26.23 |
| 4204.7626 | N(D)SKYPALN(N)VTM(O)PNNEQFDKLY Hex 8 HexNAc 2 | 117.656 | 0.007 | -6.102 | 1.033e+08 | 157 - 178 | 4 | 46.08 | 46.42 | 46.59 |
| 4040.7231 | NSKYPALN(N)VTM(O)PNNEQFDKL Hex 8 HexNAc 2 | 115.997 | 0.007 | -4.992 | 2.487e+07 | 157 - 177 | 2 | 41.19 | 41.36 | 41.52 |
| 3500.5083 | LIN(N)STGN(D)LIAPRGYF Hex 9 HexNAc 2 | 113.671 | 0.012 | -20.577 | 5.020e+06 | 243 - 258 | 2 | 49.23 | 49.23 | 49.23 |
| 3207.2899 | SIRN(N)GTYDHDVY Fuc 1 Hex 5 HexNAc 4 | 109.405 | 0.012 | -16.988 | 9.002e+06 | 479 - 491 | 1 | 20.33 | 20.33 | 20.49 |
| 3640.8032 | TIVKPGDILLIN(N)STGNLIAPRGY Hex 5 HexNAc 2 | 106.634 | 0.012 | -3.118 | 6.938e+07 | 234 - 257 | 5 | 56.70 | 56.88 | 57.22 |
| 4290.0073 | TIVKPGDILLIN(N)STGN(D)LIAPRGY Hex 9 HexNAc 2 | 105.820 | 0.012 | -19.426 | 6.377e+07 | 234 - 257 | 16 | 57.05 | 57.22 | 57.22 |
| 3404.5210 | TIVKPGDILLIN(N)STG Hex 9 HexNAc 2 | 103.978 | 0.012 | -20.716 | 1.109e+07 | 234 - 249 | 1 | 46.92 | 46.92 | 47.08 |
| 3966.9321 | TIVKPGDILLIN(N)STGNLIAPRGY Hex 7 HexNAc 2 | 95.138 | 0.027 | 0.000 | 0.000e+00 | 234 - 257 | 1 | - | - | - |
| 4366.8152 | N(D)SKYPALN(N)VTM(O)PNNEQFDKLY Hex 9 HexNAc 2 | 92.584 | 0.027 | -6.194 | 1.149e+08 | 157 - 178 | 2 | 46.08 | 46.25 | 46.42 |
| 3353.4307 | LIN(N)STGN(D)LIAPRGY Hex 9 HexNAc 2 | 91.873 | 0.027 | -20.295 | 9.829e+06 | 243 - 257 | 1 | 38.50 | 38.67 | 38.67 |
| 3235.3702 | RSLVASSGTLEFNN(N)ESF Hex 6 HexNAc 2 | 87.496 | 0.027 | -20.353 | 9.791e+06 | 108 - 125 | 1 | 44.56 | 44.56 | 44.73 |
| 4248.0081 | TIVKPGDILLIN(N)STGNLIAPR Fuc 2 Hex 4 HexNAc 4 Neu5Ac 1 | 86.953 | 0.027 | -19.072 | 1.193e+07 | 234 - 255 | 1 | 53.65 | 53.82 | 53.82 |
| 4153.8048 | LNSKYPALN(N)VTM(O)PNNEQFDKL Hex 8 HexNAc 2 | 85.006 | 0.032 | -17.796 | 1.186e+07 | 156 - 177 | 2 | 42.71 | 42.88 | 42.88 |
| 4753.0353 | RSDAPIGKC(C)KSEC(C)ITPN(N)GSIPN(D)DKPF Hex 9 HexNAc 2 | 84.748 | 0.032 | -5.845 | 3.570e+07 | 268 - 294 | 1 | 31.21 | 31.38 | 31.55 |
| 3802.8562 | TIVKPGDILLIN(N)STGNLIAPRGY Hex 6 HexNAc 2 | 82.403 | 0.040 | -4.403 | 1.356e+07 | 234 - 257 | 3 | 56.53 | 56.70 | 56.88 |
| 3964.9072 | TIVKPGDILLIN(N)STGNLIAPRGY Hex 7 HexNAc 2 | 78.693 | 0.040 | -4.592 | 1.594e+07 | 234 - 257 | 2 | 56.36 | 56.36 | 56.70 |
| 4465.7951 | HKC(C)DNAC(C)IGSIRN(N)GTYDHDVY Fuc 1 Hex 5 HexNAc 5 | 67.576 | 0.043 | -19.461 | 2.464e+07 | 470 - 491 | 1 | 26.07 | 26.07 | 26.23 |
| 3717.6181 | NSKYPALN(N)VTM(O)PNNEQFDKLY Hex 5 HexNAc 2 | 65.269 | 0.046 | -18.168 | 8.725e+06 | 157 - 178 | 2 | 43.89 | 44.06 | 44.06 |
| 4527.8889 | NSKYPALN(N)VTM(O)PNNEQFDKLY Hex 10 HexNAc 2 | 64.101 | 0.046 | -19.021 | 2.579e+06 | 157 - 178 | 1 | 43.38 | 43.38 | 43.38 |
| 4428.9297 | RSDAPIGKC(C)KSEC(C)ITPN(N)GSIPN(D)DKPF Hex 7 HexNAc 2 | 62.433 | 0.046 | -6.104 | 2.193e+07 | 268 - 294 | 1 | 31.38 | 31.55 | 31.72 |
| 4041.7310 | NSKYPALN(N)VTM(O)PNNEQFDKLY Hex 7 HexNAc 2 | 60.927 | 0.046 | -4.213 | 2.004e+07 | 157 - 178 | 1 | 43.38 | 43.55 | 43.72 |
| 3311.4494 | TITNDRIEVTN(N)ATEL Hex 5 HexNAc 4 | 60.374 | 0.046 | -20.171 | 1.085e+07 | 27 - 42 | 1 | 37.32 | 37.49 | 37.49 |
| 3605.5116 | KSEC(C)ITPN(N)GSIPNDKPF Hex 8 HexNAc 2 | 60.224 | 0.046 | -20.483 | 8.684e+06 | 277 - 294 | 1 | 32.37 | 32.37 | 32.37 |
| 4315.8493 | LNSKYPALN(N)VTM(O)PNNEQFDKL Hex 9 HexNAc 2 | 60.120 | 0.046 | -18.016 | 1.210e+07 | 156 - 177 | 1 | 42.54 | 42.71 | 42.71 |
| 4253.7025 | IN(N)STGNLIAPRGY Fuc 1 Hex 7 HexNAc 5 Neu5Ac 2 | 59.868 | 0.046 | 0.000 | 0.000e+00 | 244 - 257 | 1 | - | - | - |
| 3108.3709 | TITNDRIEVTN(N)ATEL Hex 5 HexNAc 3 | 58.488 | 0.046 | -18.678 | 9.717e+06 | 27 - 42 | 1 | 37.66 | 37.82 | 37.82 |
| 4125.9521 | TIVKPGDILLIN(N)STGNLIAPRG Hex 9 HexNAc 2 | 57.558 | 0.046 | 16.347 | 4.153e+08 | 234 - 256 | 10 | 53.65 | 54.66 | 55.52 |
| 4718.0121 | THLN(D)SKYPALN(N)VTM(O)PNNEQFDKL Fuc 3 Hex 3 HexNAc 4 Neu5Ac 1 | 57.540 | 0.046 | 7.187 | 1.607e+08 | 154 - 177 | 5 | 42.37 | 43.55 | 43.89 |
| 4069.9234 | TIVKPGDILLIN(N)STGN(D)LIAPR Hex 9 HexNAc 2 | 56.918 | 0.046 | -19.599 | 2.419e+07 | 234 - 255 | 4 | 54.84 | 54.84 | 55.01 |
| 4317.8459 | SKYPALN(N)VTM(O)PNNEQFDKLY Fuc 1 Hex 6 HexNAc 4 | 55.369 | 0.046 | -4.090 | 4.438e+07 | 158 - 178 | 2 | 46.59 | 46.59 | 46.92 |
| 3416.4721 | TITNDRIEVTN(N)ATEL Fuc 1 Hex 6 HexNAc 3 | 54.534 | 0.049 | -21.308 | 4.636e+06 | 27 - 42 | 1 | 37.32 | 37.32 | 37.32 |

### LIN(N)STGNL Hex 9 HexNAc 2

**Mass:**  2695.0892

(1.967e-06)

**Charge States:** 2, 3

**Start Position**: 244

**End Position**: 252

| Profile Score | Tandem Score | TDA q-value | Volume | Spectrum Matches |
| --- | --- | --- | --- | --- |
| -18.4405 | 188.1922 | 0.0000 | 5.7714e+07 | 2 |

**Adduct Signal Fractions**

- **Unmodified**: 5.7714e+07

### LIN(N)STGNLIAPRGYF Hex 10 HexNAc 2

**Mass:**  3661.5683

(-1.268e-06)

**Charge States:** 3

**Start Position**: 244

**End Position**: 259

| Profile Score | Tandem Score | TDA q-value | Volume | Spectrum Matches |
| --- | --- | --- | --- | --- |
| -17.4989 | 184.7426 | 0.0000 | 3.2558e+07 | 1 |

**Adduct Signal Fractions**

- **Unmodified**: 3.2558e+07

### LIN(N)STGNLIAPRGYF Hex 9 HexNAc 2

**Mass:**  3499.5174

(2.288e-06)

**Charge States:** 3

**Start Position**: 244

**End Position**: 259

| Profile Score | Tandem Score | TDA q-value | Volume | Spectrum Matches |
| --- | --- | --- | --- | --- |
| 17.7449 | 181.9755 | 0.0000 | 3.7754e+08 | 4 |

**Adduct Signal Fractions**

- **Unmodified**: 3.7754e+08

### LIN(N)STGNLIAPRGYF Fuc 2 Hex 3 HexNAc 4 Neu5Ac 1

**Mass:**  3516.5436

(-6.203e-06)

**Charge States:** 3

**Start Position**: 244

**End Position**: 259

| Profile Score | Tandem Score | TDA q-value | Volume | Spectrum Matches |
| --- | --- | --- | --- | --- |
| -17.6621 | 178.4552 | 0.0000 | 4.8673e+07 | 1 |

**Adduct Signal Fractions**

- **Unmodified**: 4.8673e+07

### LIN(N)STGNLIAPRGY Hex 9 HexNAc 2

**Mass:**  3352.4489

(2.160e-06)

**Charge States:** 2, 3

**Start Position**: 244

**End Position**: 258

| Profile Score | Tandem Score | TDA q-value | Volume | Spectrum Matches |
| --- | --- | --- | --- | --- |
| 15.8345 | 173.2108 | 0.0000 | 2.3966e+08 | 3 |

**Adduct Signal Fractions**

- **Unmodified**: 2.3966e+08

### LIN(N)STGNLIAPR Hex 9 HexNAc 2

**Mass:**  3132.3707

(3.732e-06)

**Charge States:** 2, 3

**Start Position**: 244

**End Position**: 256

| Profile Score | Tandem Score | TDA q-value | Volume | Spectrum Matches |
| --- | --- | --- | --- | --- |
| -17.8742 | 171.0698 | 0.0000 | 7.8869e+07 | 1 |

**Adduct Signal Fractions**

- **Unmodified**: 7.8869e+07

### TIVKPGDILLIN(N)STGNLIAPR Hex 9 HexNAc 2

**Mass:**  4068.9326

(2.457e-06)

**Charge States:** 3, 4, 5

**Start Position**: 235

**End Position**: 256

| Profile Score | Tandem Score | TDA q-value | Volume | Spectrum Matches |
| --- | --- | --- | --- | --- |
| 18.4931 | 168.7265 | 0.0000 | 2.0126e+09 | 14 |

**Adduct Signal Fractions**

- **Unmodified**: 2.0126e+09

### IN(N)STGNLIAPRGYF Hex 9 HexNAc 2

**Mass:**  3386.4395

(-6.607e-07)

**Charge States:** 3

**Start Position**: 245

**End Position**: 259

| Profile Score | Tandem Score | TDA q-value | Volume | Spectrum Matches |
| --- | --- | --- | --- | --- |
| -18.3461 | 168.2640 | 0.0000 | 2.0821e+07 | 1 |

**Adduct Signal Fractions**

- **Unmodified**: 2.0821e+07

### NSKYPALN(N)VTM(O)PNNEQFDKLY Hex 8 HexNAc 2

**Mass:**  4203.7878

(3.327e-06)

**Charge States:** 3, 4

**Start Position**: 158

**End Position**: 179

| Profile Score | Tandem Score | TDA q-value | Volume | Spectrum Matches |
| --- | --- | --- | --- | --- |
| 18.5304 | 166.7929 | 0.0000 | 2.9760e+08 | 4 |

**Adduct Signal Fractions**

- **Unmodified**: 2.9760e+08

### LPGNDN(N)STATL Hex 5 HexNAc 2

**Mass:**  2317.9562

(1.352e-06)

**Charge States:** 3

**Start Position**: 3

**End Position**: 14

| Profile Score | Tandem Score | TDA q-value | Volume | Spectrum Matches |
| --- | --- | --- | --- | --- |
| -20.1072 | 165.5091 | 0.0000 | 7.1944e+06 | 2 |

**Adduct Signal Fractions**

- **Unmodified**: 7.1944e+06

### LIN(N)STGNLIAPRGYF Hex 8 HexNAc 2

**Mass:**  3337.4676

(2.461e-06)

**Charge States:** 3

**Start Position**: 244

**End Position**: 259

| Profile Score | Tandem Score | TDA q-value | Volume | Spectrum Matches |
| --- | --- | --- | --- | --- |
| -4.4950 | 162.3694 | 0.0000 | 2.7508e+07 | 1 |

**Adduct Signal Fractions**

- **Unmodified**: 2.7508e+07

### TIVKPGDILLIN(N)STGNLIAPR Hex 10 HexNAc 2

**Mass:**  4230.9816

(1.061e-06)

**Charge States:** 3, 4

**Start Position**: 235

**End Position**: 256

| Profile Score | Tandem Score | TDA q-value | Volume | Spectrum Matches |
| --- | --- | --- | --- | --- |
| 14.4403 | 161.4006 | 0.0000 | 1.9280e+08 | 5 |

**Adduct Signal Fractions**

- **Unmodified**: 1.9280e+08

### LIN(N)STGNLIAPRGY Fuc 2 Hex 3 HexNAc 4 Neu5Ac 1

**Mass:**  3369.4744

(-6.506e-06)

**Charge States:** 3

**Start Position**: 244

**End Position**: 258

| Profile Score | Tandem Score | TDA q-value | Volume | Spectrum Matches |
| --- | --- | --- | --- | --- |
| -18.8356 | 161.0401 | 0.0000 | 2.6336e+07 | 1 |

**Adduct Signal Fractions**

- **Unmodified**: 2.6336e+07

### NSKYPALN(N)VTM(O)PNNEQFDKLY Hex 9 HexNAc 2

**Mass:**  4365.8361

(1.910e-06)

**Charge States:** 3, 4

**Start Position**: 158

**End Position**: 179

| Profile Score | Tandem Score | TDA q-value | Volume | Spectrum Matches |
| --- | --- | --- | --- | --- |
| 16.6077 | 160.2627 | 0.0000 | 3.6891e+08 | 4 |

**Adduct Signal Fractions**

- **Unmodified**: 3.6891e+08

### TIVKPGDILLIN(N)STGNLIAPRGY Hex 9 HexNAc 2

**Mass:**  4289.0168

(1.998e-06)

**Charge States:** 3, 4, 5

**Start Position**: 235

**End Position**: 258

| Profile Score | Tandem Score | TDA q-value | Volume | Spectrum Matches |
| --- | --- | --- | --- | --- |
| 18.7055 | 158.3761 | 0.0000 | 3.7161e+09 | 22 |

**Adduct Signal Fractions**

- **Unmodified**: 3.7161e+09

### TIVKPGDILLIN(N) Hex 9 HexNAc 2

**Mass:**  3159.4253

(1.653e-06)

**Charge States:** 2, 3

**Start Position**: 235

**End Position**: 247

| Profile Score | Tandem Score | TDA q-value | Volume | Spectrum Matches |
| --- | --- | --- | --- | --- |
| 11.0666 | 158.1340 | 0.0000 | 2.5052e+08 | 3 |

**Adduct Signal Fractions**

- **Unmodified**: 2.5052e+08

### TIVKPGDILLIN(N)STGNLIAPRGY Hex 8 HexNAc 2

**Mass:**  4126.9611

(2.095e-06)

**Charge States:** 3, 4, 5

**Start Position**: 235

**End Position**: 258

| Profile Score | Tandem Score | TDA q-value | Volume | Spectrum Matches |
| --- | --- | --- | --- | --- |
| 14.5993 | 157.4580 | 0.0000 | 3.5578e+08 | 13 |

**Adduct Signal Fractions**

- **Unmodified**: 3.5578e+08

### TIVKPGDILLIN(N)STGNLIAPRGY Fuc 2 Hex 3 HexNAc 4 Neu5Ac 1

**Mass:**  4306.0349

(-5.518e-06)

**Charge States:** 4

**Start Position**: 235

**End Position**: 258

| Profile Score | Tandem Score | TDA q-value | Volume | Spectrum Matches |
| --- | --- | --- | --- | --- |
| 12.2723 | 157.4188 | 0.0000 | 2.6004e+08 | 5 |

**Adduct Signal Fractions**

- **Unmodified**: 2.6004e+08

### TITNDRIEVTN(N)ATEL Hex 5 HexNAc 2

**Mass:**  2905.2910

(1.295e-06)

**Charge States:** 3

**Start Position**: 28

**End Position**: 43

| Profile Score | Tandem Score | TDA q-value | Volume | Spectrum Matches |
| --- | --- | --- | --- | --- |
| -3.1850 | 157.2783 | 0.0000 | 3.2867e+07 | 2 |

**Adduct Signal Fractions**

- **Unmodified**: 3.2867e+07

### TIVKPGDILLIN(N) Hex 8 HexNAc 2

**Mass:**  2997.3717

(1.406e-06)

**Charge States:** 3

**Start Position**: 235

**End Position**: 247

| Profile Score | Tandem Score | TDA q-value | Volume | Spectrum Matches |
| --- | --- | --- | --- | --- |
| 10.9113 | 156.9062 | 0.0000 | 1.3241e+07 | 2 |

**Adduct Signal Fractions**

- **Unmodified**: 1.3241e+07

### TIVKPGDILLIN(N)STGNLIAPRGY Hex 10 HexNAc 2

**Mass:**  4451.0690

(1.239e-06)

**Charge States:** 3, 4

**Start Position**: 235

**End Position**: 258

| Profile Score | Tandem Score | TDA q-value | Volume | Spectrum Matches |
| --- | --- | --- | --- | --- |
| 15.8409 | 155.2980 | 0.0000 | 3.5463e+08 | 7 |

**Adduct Signal Fractions**

- **Unmodified**: 3.5463e+08

### TIVKPGDILLIN(N)STGNLIAPR Hex 8 HexNAc 2

**Mass:**  3906.8737

(2.334e-06)

**Charge States:** 3, 4

**Start Position**: 235

**End Position**: 256

| Profile Score | Tandem Score | TDA q-value | Volume | Spectrum Matches |
| --- | --- | --- | --- | --- |
| -3.2321 | 155.0964 | 0.0000 | 1.4459e+08 | 4 |

**Adduct Signal Fractions**

- **Unmodified**: 1.4459e+08

### RGSN(N)SSFF Hex 5 HexNAc 2

**Mass:**  2116.8360

(2.016e-06)

**Charge States:** 2

**Start Position**: 141

**End Position**: 149

| Profile Score | Tandem Score | TDA q-value | Volume | Spectrum Matches |
| --- | --- | --- | --- | --- |
| -19.2504 | 154.8805 | 0.0000 | 6.2767e+06 | 1 |

**Adduct Signal Fractions**

- **Unmodified**: 6.2767e+06

### TIVKPGDILLIN(N)STGNL Fuc 2 Hex 3 HexNAc 4 Neu5Ac 1

**Mass:**  3648.6798

(-4.683e-06)

**Charge States:** 3

**Start Position**: 235

**End Position**: 252

| Profile Score | Tandem Score | TDA q-value | Volume | Spectrum Matches |
| --- | --- | --- | --- | --- |
| -3.9547 | 152.5555 | 0.0000 | 2.0797e+08 | 2 |

**Adduct Signal Fractions**

- **Unmodified**: 2.0797e+08

### TITNDRIEVTN(N)ATEL Hex 7 HexNAc 2

**Mass:**  3229.3950

(1.941e-06)

**Charge States:** 3

**Start Position**: 28

**End Position**: 43

| Profile Score | Tandem Score | TDA q-value | Volume | Spectrum Matches |
| --- | --- | --- | --- | --- |
| -17.0823 | 152.2509 | 0.0000 | 3.6435e+07 | 1 |

**Adduct Signal Fractions**

- **Unmodified**: 3.6435e+07

### TIVKPGDILLIN(N)STGNLIAPR Fuc 2 Hex 3 HexNAc 4 Neu5Ac 1

**Mass:**  4085.9511

(-6.359e-06)

**Charge States:** 4

**Start Position**: 235

**End Position**: 256

| Profile Score | Tandem Score | TDA q-value | Volume | Spectrum Matches |
| --- | --- | --- | --- | --- |
| 14.7296 | 151.7394 | 0.0000 | 1.3976e+08 | 2 |

**Adduct Signal Fractions**

- **Unmodified**: 1.3976e+08

### TIVKPGDILLIN(N)STGNL Hex 9 HexNAc 2

**Mass:**  3631.6543

(1.942e-06)

**Charge States:** 3, 4

**Start Position**: 235

**End Position**: 252

| Profile Score | Tandem Score | TDA q-value | Volume | Spectrum Matches |
| --- | --- | --- | --- | --- |
| 18.1091 | 151.0471 | 0.0000 | 1.1998e+09 | 10 |

**Adduct Signal Fractions**

- **Unmodified**: 1.1998e+09

### SKYPALN(N)VTM(O)PNNEQFDKLY Hex 8 HexNAc 2

**Mass:**  4089.7458

(3.015e-06)

**Charge States:** 3, 4

**Start Position**: 159

**End Position**: 179

| Profile Score | Tandem Score | TDA q-value | Volume | Spectrum Matches |
| --- | --- | --- | --- | --- |
| -2.6470 | 150.6886 | 0.0000 | 8.3932e+07 | 2 |

**Adduct Signal Fractions**

- **Unmodified**: 8.3932e+07

### TIVKPGDILLIN(N) Hex 10 HexNAc 2

**Mass:**  3321.4777

(1.441e-06)

**Charge States:** 3

**Start Position**: 235

**End Position**: 247

| Profile Score | Tandem Score | TDA q-value | Volume | Spectrum Matches |
| --- | --- | --- | --- | --- |
| -17.2941 | 150.3545 | 0.0000 | 8.8639e+06 | 1 |

**Adduct Signal Fractions**

- **Unmodified**: 8.8639e+06

### TIVKPGDILLIN(N)STGN Hex 9 HexNAc 2

**Mass:**  3518.5739

(3.177e-06)

**Charge States:** 3, 4

**Start Position**: 235

**End Position**: 251

| Profile Score | Tandem Score | TDA q-value | Volume | Spectrum Matches |
| --- | --- | --- | --- | --- |
| 16.1938 | 149.9422 | 0.0000 | 3.1673e+08 | 3 |

**Adduct Signal Fractions**

- **Unmodified**: 3.1673e+08

### C(C)LGHHAVPN(N)GTIVK Fuc 1 Hex 5 HexNAc 4

**Mass:**  3270.4252

(1.012e-06)

**Charge States:** 4

**Start Position**: 14

**End Position**: 28

| Profile Score | Tandem Score | TDA q-value | Volume | Spectrum Matches |
| --- | --- | --- | --- | --- |
| -19.1497 | 149.5285 | 0.0000 | 7.4847e+06 | 1 |

**Adduct Signal Fractions**

- **Unmodified**: 7.4847e+06

### TIVKPGDILLIN(N)ST Hex 9 HexNAc 2

**Mass:**  3347.5076

(2.297e-06)

**Charge States:** 3

**Start Position**: 235

**End Position**: 249

| Profile Score | Tandem Score | TDA q-value | Volume | Spectrum Matches |
| --- | --- | --- | --- | --- |
| -19.8957 | 147.9638 | 0.0000 | 1.4199e+07 | 1 |

**Adduct Signal Fractions**

- **Unmodified**: 1.4199e+07

### TIVKPGDILLIN(N)STGNL Hex 10 HexNAc 2

**Mass:**  3793.7071

(1.973e-06)

**Charge States:** 3

**Start Position**: 235

**End Position**: 252

| Profile Score | Tandem Score | TDA q-value | Volume | Spectrum Matches |
| --- | --- | --- | --- | --- |
| -4.2154 | 145.4631 | 0.0000 | 8.8743e+07 | 4 |

**Adduct Signal Fractions**

- **Unmodified**: 8.8743e+07

### TIVKPGDILLIN(N)STGN Fuc 2 Hex 3 HexNAc 4 Neu5Ac 1

**Mass:**  3535.5983

(-5.401e-06)

**Charge States:** 3

**Start Position**: 235

**End Position**: 251

| Profile Score | Tandem Score | TDA q-value | Volume | Spectrum Matches |
| --- | --- | --- | --- | --- |
| -4.4129 | 145.4066 | 0.0000 | 5.5864e+07 | 1 |

**Adduct Signal Fractions**

- **Unmodified**: 5.5864e+07

### RRGSN(N)SSFF Hex 5 HexNAc 2

**Mass:**  2272.9317

(-5.144e-07)

**Charge States:** 3

**Start Position**: 140

**End Position**: 149

| Profile Score | Tandem Score | TDA q-value | Volume | Spectrum Matches |
| --- | --- | --- | --- | --- |
| -19.4121 | 144.8915 | 0.0000 | 1.0570e+07 | 1 |

**Adduct Signal Fractions**

- **Unmodified**: 1.0570e+07

### TIVKPGDILLIN(N) Fuc 2 Hex 3 HexNAc 4 Neu5Ac 1

**Mass:**  3176.4516

(-6.820e-06)

**Charge States:** 3

**Start Position**: 235

**End Position**: 247

| Profile Score | Tandem Score | TDA q-value | Volume | Spectrum Matches |
| --- | --- | --- | --- | --- |
| 10.4797 | 143.5855 | 0.0000 | 4.2616e+07 | 2 |

**Adduct Signal Fractions**

- **Unmodified**: 4.2616e+07

### TIVKPGDILLIN(N)STGNL Hex 8 HexNAc 2

**Mass:**  3469.5987

(1.230e-06)

**Charge States:** 3, 4

**Start Position**: 235

**End Position**: 252

| Profile Score | Tandem Score | TDA q-value | Volume | Spectrum Matches |
| --- | --- | --- | --- | --- |
| -3.2705 | 142.1557 | 0.0000 | 9.7758e+07 | 3 |

**Adduct Signal Fractions**

- **Unmodified**: 9.7758e+07

### C(C)LGHHAVPN(N)GTIVK Fuc 1 Hex 5 HexNAc 5

**Mass:**  3473.5014

(-8.166e-07)

**Charge States:** 4

**Start Position**: 14

**End Position**: 28

| Profile Score | Tandem Score | TDA q-value | Volume | Spectrum Matches |
| --- | --- | --- | --- | --- |
| -19.0893 | 141.3988 | 0.0000 | 2.2516e+07 | 1 |

**Adduct Signal Fractions**

- **Unmodified**: 2.2516e+07

### LIN(N)STGNLIAPRGY Hex 10 HexNAc 2

**Mass:**  3514.5089

(3.533e-06)

**Charge States:** 3

**Start Position**: 244

**End Position**: 258

| Profile Score | Tandem Score | TDA q-value | Volume | Spectrum Matches |
| --- | --- | --- | --- | --- |
| -18.3594 | 140.9414 | 0.0000 | 2.9843e+07 | 1 |

**Adduct Signal Fractions**

- **Unmodified**: 2.9843e+07

### RSDAPIGKC(C)KSEC(C)ITPN(N)GSIPNDKPF Hex 9 HexNAc 2

**Mass:**  4752.0364

(9.928e-07)

**Charge States:** 5

**Start Position**: 269

**End Position**: 295

| Profile Score | Tandem Score | TDA q-value | Volume | Spectrum Matches |
| --- | --- | --- | --- | --- |
| -2.7418 | 137.8502 | 0.0000 | 2.9232e+08 | 3 |

**Adduct Signal Fractions**

- **Unmodified**: 2.9232e+08

### RRGSN(N)SSFF Fuc 1 Hex 5 HexNAc 5

**Mass:**  3028.2301

(3.758e-07)

**Charge States:** 3, 4

**Start Position**: 140

**End Position**: 149

| Profile Score | Tandem Score | TDA q-value | Volume | Spectrum Matches |
| --- | --- | --- | --- | --- |
| -19.2917 | 136.9488 | 0.0000 | 2.6314e+07 | 1 |

**Adduct Signal Fractions**

- **Unmodified**: 2.6314e+07

### EFNN(N)ESFNW Hex 6 HexNAc 2

**Mass:**  2563.9594

(2.529e-06)

**Charge States:** 2, 3

**Start Position**: 119

**End Position**: 128

| Profile Score | Tandem Score | TDA q-value | Volume | Spectrum Matches |
| --- | --- | --- | --- | --- |
| -18.1299 | 135.7280 | 0.0000 | 8.9495e+06 | 2 |

**Adduct Signal Fractions**

- **Unmodified**: 8.9495e+06

### TITNDRIEVTN(N)ATEL Hex 6 HexNAc 3

**Mass:**  3270.4206

(2.247e-06)

**Charge States:** 3

**Start Position**: 28

**End Position**: 43

| Profile Score | Tandem Score | TDA q-value | Volume | Spectrum Matches |
| --- | --- | --- | --- | --- |
| -4.6936 | 135.6038 | 0.0000 | 2.8819e+07 | 1 |

**Adduct Signal Fractions**

- **Unmodified**: 2.8819e+07

### TIVKPGDILLIN(N)STGNLIAPR Hex 5 HexNAc 2

**Mass:**  3420.7148

(2.607e-06)

**Charge States:** 3, 4

**Start Position**: 235

**End Position**: 256

| Profile Score | Tandem Score | TDA q-value | Volume | Spectrum Matches |
| --- | --- | --- | --- | --- |
| -4.4093 | 135.5844 | 0.0000 | 2.2042e+07 | 3 |

**Adduct Signal Fractions**

- **Unmodified**: 2.2042e+07

### RSDAPIGKC(C)KSEC(C)ITPN(N)GSIPNDKPF Hex 7 HexNAc 2

**Mass:**  4427.9308

(1.463e-06)

**Charge States:** 5

**Start Position**: 269

**End Position**: 295

| Profile Score | Tandem Score | TDA q-value | Volume | Spectrum Matches |
| --- | --- | --- | --- | --- |
| -3.6013 | 132.8194 | 0.0067 | 1.7221e+08 | 2 |

**Adduct Signal Fractions**

- **Unmodified**: 1.7221e+08

### RGSN(N)SSFF Fuc 1 Hex 5 HexNAc 4

**Mass:**  2669.0553

(2.556e-06)

**Charge States:** 3

**Start Position**: 141

**End Position**: 149

| Profile Score | Tandem Score | TDA q-value | Volume | Spectrum Matches |
| --- | --- | --- | --- | --- |
| -19.9879 | 130.5416 | 0.0000 | 4.8924e+06 | 1 |

**Adduct Signal Fractions**

- **Unmodified**: 4.8924e+06

### RSDAPIGKC(C)KSEC(C)ITPN(N)GSIPNDKPF Hex 8 HexNAc 2

**Mass:**  4589.9819

(1.042e-06)

**Charge States:** 5

**Start Position**: 269

**End Position**: 295

| Profile Score | Tandem Score | TDA q-value | Volume | Spectrum Matches |
| --- | --- | --- | --- | --- |
| -3.4321 | 129.4788 | 0.0067 | 1.2324e+08 | 1 |

**Adduct Signal Fractions**

- **Unmodified**: 1.2324e+08

### TIVKPGDILLIN(N)STGNL Hex 5 HexNAc 2

**Mass:**  2983.4428

(1.906e-06)

**Charge States:** 3

**Start Position**: 235

**End Position**: 252

| Profile Score | Tandem Score | TDA q-value | Volume | Spectrum Matches |
| --- | --- | --- | --- | --- |
| -3.3044 | 127.1643 | 0.0067 | 1.2434e+07 | 1 |

**Adduct Signal Fractions**

- **Unmodified**: 1.2434e+07

### RRGSN(N)SSFF Fuc 1 Hex 5 HexNAc 4

**Mass:**  2825.1478

(-6.326e-07)

**Charge States:** 3

**Start Position**: 140

**End Position**: 149

| Profile Score | Tandem Score | TDA q-value | Volume | Spectrum Matches |
| --- | --- | --- | --- | --- |
| -17.8140 | 126.0600 | 0.0000 | 9.9294e+06 | 1 |

**Adduct Signal Fractions**

- **Unmodified**: 9.9294e+06

### NSKYPALN(N)VTM(O)PNNEQFDKL Hex 9 HexNAc 2

**Mass:**  4202.7697

(1.412e-07)

**Charge States:** 3, 4

**Start Position**: 158

**End Position**: 178

| Profile Score | Tandem Score | TDA q-value | Volume | Spectrum Matches |
| --- | --- | --- | --- | --- |
| -3.5467 | 124.3692 | 0.0067 | 3.6011e+07 | 2 |

**Adduct Signal Fractions**

- **Unmodified**: 3.6011e+07

### NSKYPALN(N)VTMPNNEQFDKLY Hex 9 HexNAc 2

**Mass:**  4349.8371

(3.757e-06)

**Charge States:** 3, 4

**Start Position**: 158

**End Position**: 179

| Profile Score | Tandem Score | TDA q-value | Volume | Spectrum Matches |
| --- | --- | --- | --- | --- |
| -18.8389 | 124.1460 | 0.0067 | 7.2034e+06 | 2 |

**Adduct Signal Fractions**

- **Unmodified**: 7.2034e+06

### HKC(C)DNAC(C)IGSIRN(N)GTYDHDVY Fuc 1 Hex 5 HexNAc 4

**Mass:**  4262.7175

(9.706e-07)

**Charge States:** 4

**Start Position**: 471

**End Position**: 492

| Profile Score | Tandem Score | TDA q-value | Volume | Spectrum Matches |
| --- | --- | --- | --- | --- |
| -3.3122 | 119.1598 | 0.0067 | 8.1062e+07 | 1 |

**Adduct Signal Fractions**

- **Unmodified**: 8.1062e+07

### N(D)SKYPALN(N)VTM(O)PNNEQFDKLY Hex 8 HexNAc 2

**Mass:**  4204.7644

(2.355e-04)

**Charge States:** 3, 4

**Start Position**: 158

**End Position**: 179

| Profile Score | Tandem Score | TDA q-value | Volume | Spectrum Matches |
| --- | --- | --- | --- | --- |
| -6.1018 | 117.6557 | 0.0067 | 1.0326e+08 | 4 |

**Adduct Signal Fractions**

- **Unmodified**: 1.0326e+08

### NSKYPALN(N)VTM(O)PNNEQFDKL Hex 8 HexNAc 2

**Mass:**  4040.7228

(1.710e-06)

**Charge States:** 4

**Start Position**: 158

**End Position**: 178

| Profile Score | Tandem Score | TDA q-value | Volume | Spectrum Matches |
| --- | --- | --- | --- | --- |
| -4.9924 | 115.9972 | 0.0067 | 2.4872e+07 | 2 |

**Adduct Signal Fractions**

- **Unmodified**: 2.4872e+07

### LIN(N)STGN(D)LIAPRGYF Hex 9 HexNAc 2

**Mass:**  3500.5083

(2.846e-04)

**Charge States:** 3

**Start Position**: 244

**End Position**: 259

| Profile Score | Tandem Score | TDA q-value | Volume | Spectrum Matches |
| --- | --- | --- | --- | --- |
| -20.5767 | 113.6706 | 0.0120 | 5.0202e+06 | 2 |

**Adduct Signal Fractions**

- **Unmodified**: 5.0202e+06

### SIRN(N)GTYDHDVY Fuc 1 Hex 5 HexNAc 4

**Mass:**  3207.2905

(1.037e-06)

**Charge States:** 3

**Start Position**: 480

**End Position**: 492

| Profile Score | Tandem Score | TDA q-value | Volume | Spectrum Matches |
| --- | --- | --- | --- | --- |
| -16.9875 | 109.4049 | 0.0120 | 9.0020e+06 | 1 |

**Adduct Signal Fractions**

- **Unmodified**: 9.0020e+06

### TIVKPGDILLIN(N)STGNLIAPRGY Hex 5 HexNAc 2

**Mass:**  3640.8044

(2.007e-06)

**Charge States:** 3, 4

**Start Position**: 235

**End Position**: 258

| Profile Score | Tandem Score | TDA q-value | Volume | Spectrum Matches |
| --- | --- | --- | --- | --- |
| -3.1183 | 106.6336 | 0.0120 | 6.9383e+07 | 5 |

**Adduct Signal Fractions**

- **Unmodified**: 6.9383e+07

### TIVKPGDILLIN(N)STGN(D)LIAPRGY Hex 9 HexNAc 2

**Mass:**  4290.0046

(2.323e-04)

**Charge States:** 3, 4

**Start Position**: 235

**End Position**: 258

| Profile Score | Tandem Score | TDA q-value | Volume | Spectrum Matches |
| --- | --- | --- | --- | --- |
| -19.4261 | 105.8199 | 0.0120 | 6.3774e+07 | 16 |

**Adduct Signal Fractions**

- **Unmodified**: 6.3774e+07

### TIVKPGDILLIN(N)STG Hex 9 HexNAc 2

**Mass:**  3404.5200

(-3.932e-07)

**Charge States:** 3

**Start Position**: 235

**End Position**: 250

| Profile Score | Tandem Score | TDA q-value | Volume | Spectrum Matches |
| --- | --- | --- | --- | --- |
| -20.7156 | 103.9783 | 0.0120 | 1.1094e+07 | 1 |

**Adduct Signal Fractions**

- **Unmodified**: 1.1094e+07

### TIVKPGDILLIN(N)STGNLIAPRGY Hex 7 HexNAc 2

**Mass:**  3966.9321

(5.119e-04)

**Start Position**: 235

**End Position**: 258

| Profile Score | Tandem Score | TDA q-value | Volume | Spectrum Matches |
| --- | --- | --- | --- | --- |
| 0.0000 | 95.1384 | 0.0272 | 0.0000e+00 | 1 |

Adduct:

No Chromatogram Found

### N(D)SKYPALN(N)VTM(O)PNNEQFDKLY Hex 9 HexNAc 2

**Mass:**  4366.8166

(2.274e-04)

**Charge States:** 3, 4

**Start Position**: 158

**End Position**: 179

| Profile Score | Tandem Score | TDA q-value | Volume | Spectrum Matches |
| --- | --- | --- | --- | --- |
| -6.1942 | 92.5840 | 0.0272 | 1.1493e+08 | 2 |

**Adduct Signal Fractions**

- **Unmodified**: 1.1493e+08

### LIN(N)STGN(D)LIAPRGY Hex 9 HexNAc 2

**Mass:**  3353.4300

(2.942e-04)

**Charge States:** 3

**Start Position**: 244

**End Position**: 258

| Profile Score | Tandem Score | TDA q-value | Volume | Spectrum Matches |
| --- | --- | --- | --- | --- |
| -20.2951 | 91.8731 | 0.0272 | 9.8288e+06 | 1 |

**Adduct Signal Fractions**

- **Unmodified**: 9.8288e+06

### RSLVASSGTLEFNN(N)ESF Hex 6 HexNAc 2

**Mass:**  3235.3698

(1.152e-06)

**Charge States:** 3

**Start Position**: 109

**End Position**: 126

| Profile Score | Tandem Score | TDA q-value | Volume | Spectrum Matches |
| --- | --- | --- | --- | --- |
| -20.3526 | 87.4959 | 0.0272 | 9.7910e+06 | 1 |

**Adduct Signal Fractions**

- **Unmodified**: 9.7910e+06

### TIVKPGDILLIN(N)STGNLIAPR Fuc 2 Hex 4 HexNAc 4 Neu5Ac 1

**Mass:**  4248.0114

(-4.196e-06)

**Charge States:** 4

**Start Position**: 235

**End Position**: 256

| Profile Score | Tandem Score | TDA q-value | Volume | Spectrum Matches |
| --- | --- | --- | --- | --- |
| -19.0718 | 86.9534 | 0.0272 | 1.1931e+07 | 1 |

**Adduct Signal Fractions**

- **Unmodified**: 1.1931e+07

### LNSKYPALN(N)VTM(O)PNNEQFDKL Hex 8 HexNAc 2

**Mass:**  4153.8127

(1.166e-06)

**Charge States:** 3, 4

**Start Position**: 157

**End Position**: 178

| Profile Score | Tandem Score | TDA q-value | Volume | Spectrum Matches |
| --- | --- | --- | --- | --- |
| -17.7955 | 85.0062 | 0.0321 | 1.1859e+07 | 2 |

**Adduct Signal Fractions**

- **Unmodified**: 1.1859e+07

### RSDAPIGKC(C)KSEC(C)ITPN(N)GSIPN(D)DKPF Hex 9 HexNAc 2

**Mass:**  4753.0386

(2.130e-04)

**Charge States:** 5

**Start Position**: 269

**End Position**: 295

| Profile Score | Tandem Score | TDA q-value | Volume | Spectrum Matches |
| --- | --- | --- | --- | --- |
| -5.8450 | 84.7479 | 0.0321 | 3.5703e+07 | 1 |

**Adduct Signal Fractions**

- **Unmodified**: 3.5703e+07

### TIVKPGDILLIN(N)STGNLIAPRGY Hex 6 HexNAc 2

**Mass:**  3802.8579

(1.681e-06)

**Charge States:** 4

**Start Position**: 235

**End Position**: 258

| Profile Score | Tandem Score | TDA q-value | Volume | Spectrum Matches |
| --- | --- | --- | --- | --- |
| -4.4028 | 82.4034 | 0.0398 | 1.3555e+07 | 3 |

**Adduct Signal Fractions**

- **Unmodified**: 1.3555e+07

### TIVKPGDILLIN(N)STGNLIAPRGY Hex 7 HexNAc 2

**Mass:**  3964.9025

(2.350e-06)

**Charge States:** 4

**Start Position**: 235

**End Position**: 258

| Profile Score | Tandem Score | TDA q-value | Volume | Spectrum Matches |
| --- | --- | --- | --- | --- |
| -4.5917 | 78.6932 | 0.0398 | 1.5936e+07 | 2 |

**Adduct Signal Fractions**

- **Unmodified**: 1.5936e+07

### HKC(C)DNAC(C)IGSIRN(N)GTYDHDVY Fuc 1 Hex 5 HexNAc 5

**Mass:**  4465.7983

(-1.675e-06)

**Charge States:** 5

**Start Position**: 471

**End Position**: 492

| Profile Score | Tandem Score | TDA q-value | Volume | Spectrum Matches |
| --- | --- | --- | --- | --- |
| -19.4613 | 67.5757 | 0.0429 | 2.4642e+07 | 1 |

**Adduct Signal Fractions**

- **Unmodified**: 2.4642e+07

### NSKYPALN(N)VTM(O)PNNEQFDKLY Hex 5 HexNAc 2

**Mass:**  3717.6260

(-6.321e-07)

**Charge States:** 3

**Start Position**: 158

**End Position**: 179

| Profile Score | Tandem Score | TDA q-value | Volume | Spectrum Matches |
| --- | --- | --- | --- | --- |
| -18.1684 | 65.2689 | 0.0458 | 8.7252e+06 | 2 |

**Adduct Signal Fractions**

- **Unmodified**: 8.7252e+06

### NSKYPALN(N)VTM(O)PNNEQFDKLY Hex 10 HexNAc 2

**Mass:**  4527.8889

(2.710e-06)

**Charge States:** 3

**Start Position**: 158

**End Position**: 179

| Profile Score | Tandem Score | TDA q-value | Volume | Spectrum Matches |
| --- | --- | --- | --- | --- |
| -19.0213 | 64.1012 | 0.0458 | 2.5795e+06 | 1 |

**Adduct Signal Fractions**

- **Unmodified**: 2.5795e+06

### RSDAPIGKC(C)KSEC(C)ITPN(N)GSIPN(D)DKPF Hex 7 HexNAc 2

**Mass:**  4428.9248

(2.288e-04)

**Charge States:** 5

**Start Position**: 269

**End Position**: 295

| Profile Score | Tandem Score | TDA q-value | Volume | Spectrum Matches |
| --- | --- | --- | --- | --- |
| -6.1042 | 62.4333 | 0.0458 | 2.1929e+07 | 1 |

**Adduct Signal Fractions**

- **Unmodified**: 2.1929e+07

### NSKYPALN(N)VTM(O)PNNEQFDKLY Hex 7 HexNAc 2

**Mass:**  4041.7287

(3.028e-06)

**Charge States:** 3, 4

**Start Position**: 158

**End Position**: 179

| Profile Score | Tandem Score | TDA q-value | Volume | Spectrum Matches |
| --- | --- | --- | --- | --- |
| -4.2131 | 60.9272 | 0.0458 | 2.0042e+07 | 1 |

**Adduct Signal Fractions**

- **Unmodified**: 2.0042e+07

### TITNDRIEVTN(N)ATEL Hex 5 HexNAc 4

**Mass:**  3311.4505

(2.509e-06)

**Charge States:** 3

**Start Position**: 28

**End Position**: 43

| Profile Score | Tandem Score | TDA q-value | Volume | Spectrum Matches |
| --- | --- | --- | --- | --- |
| -20.1707 | 60.3736 | 0.0458 | 1.0847e+07 | 1 |

**Adduct Signal Fractions**

- **Unmodified**: 1.0847e+07

### KSEC(C)ITPN(N)GSIPNDKPF Hex 8 HexNAc 2

**Mass:**  3605.5116

(4.333e-06)

**Charge States:** 3

**Start Position**: 278

**End Position**: 295

| Profile Score | Tandem Score | TDA q-value | Volume | Spectrum Matches |
| --- | --- | --- | --- | --- |
| -20.4827 | 60.2242 | 0.0458 | 8.6841e+06 | 1 |

**Adduct Signal Fractions**

- **Unmodified**: 8.6841e+06

### LNSKYPALN(N)VTM(O)PNNEQFDKL Hex 9 HexNAc 2

**Mass:**  4315.8513

(1.549e-06)

**Charge States:** 4

**Start Position**: 157

**End Position**: 178

| Profile Score | Tandem Score | TDA q-value | Volume | Spectrum Matches |
| --- | --- | --- | --- | --- |
| -18.0162 | 60.1197 | 0.0458 | 1.2103e+07 | 1 |

**Adduct Signal Fractions**

- **Unmodified**: 1.2103e+07

### IN(N)STGNLIAPRGY Fuc 1 Hex 7 HexNAc 5 Neu5Ac 2

**Mass:**  4253.7025

(2.261e-04)

**Start Position**: 245

**End Position**: 258

| Profile Score | Tandem Score | TDA q-value | Volume | Spectrum Matches |
| --- | --- | --- | --- | --- |
| 0.0000 | 59.8680 | 0.0458 | 0.0000e+00 | 1 |

Adduct:

No Chromatogram Found

### TITNDRIEVTN(N)ATEL Hex 5 HexNAc 3

**Mass:**  3108.3726

(3.948e-06)

**Charge States:** 3

**Start Position**: 28

**End Position**: 43

| Profile Score | Tandem Score | TDA q-value | Volume | Spectrum Matches |
| --- | --- | --- | --- | --- |
| -18.6781 | 58.4877 | 0.0458 | 9.7170e+06 | 1 |

**Adduct Signal Fractions**

- **Unmodified**: 9.7170e+06

### TIVKPGDILLIN(N)STGNLIAPRG Hex 9 HexNAc 2

**Mass:**  4125.9553

(1.557e-06)

**Charge States:** 3, 4

**Start Position**: 235

**End Position**: 257

| Profile Score | Tandem Score | TDA q-value | Volume | Spectrum Matches |
| --- | --- | --- | --- | --- |
| 16.3466 | 57.5578 | 0.0458 | 4.1533e+08 | 10 |

**Adduct Signal Fractions**

- **Unmodified**: 4.1533e+08

### THLN(D)SKYPALN(N)VTM(O)PNNEQFDKL Fuc 3 Hex 3 HexNAc 4 Neu5Ac 1

**Mass:**  4718.0173

(2.025e-04)

**Charge States:** 4, 5

**Start Position**: 155

**End Position**: 178

| Profile Score | Tandem Score | TDA q-value | Volume | Spectrum Matches |
| --- | --- | --- | --- | --- |
| 7.1867 | 57.5401 | 0.0458 | 1.6069e+08 | 5 |

**Adduct Signal Fractions**

- **Unmodified**: 1.6069e+08

### TIVKPGDILLIN(N)STGN(D)LIAPR Hex 9 HexNAc 2

**Mass:**  4069.9250

(2.455e-04)

**Charge States:** 3, 4

**Start Position**: 235

**End Position**: 256

| Profile Score | Tandem Score | TDA q-value | Volume | Spectrum Matches |
| --- | --- | --- | --- | --- |
| -19.5990 | 56.9179 | 0.0458 | 2.4194e+07 | 4 |

**Adduct Signal Fractions**

- **Unmodified**: 2.4194e+07

### SKYPALN(N)VTM(O)PNNEQFDKLY Fuc 1 Hex 6 HexNAc 4

**Mass:**  4317.8518

(1.485e-06)

**Charge States:** 4

**Start Position**: 159

**End Position**: 179

| Profile Score | Tandem Score | TDA q-value | Volume | Spectrum Matches |
| --- | --- | --- | --- | --- |
| -4.0904 | 55.3686 | 0.0458 | 4.4383e+07 | 2 |

**Adduct Signal Fractions**

- **Unmodified**: 4.4383e+07

### TITNDRIEVTN(N)ATEL Fuc 1 Hex 6 HexNAc 3

**Mass:**  3416.4721

(3.047e-07)

**Charge States:** 3

**Start Position**: 28

**End Position**: 43

| Profile Score | Tandem Score | TDA q-value | Volume | Spectrum Matches |
| --- | --- | --- | --- | --- |
| -21.3078 | 54.5340 | 0.0494 | 4.6356e+06 | 1 |

**Adduct Signal Fractions**

- **Unmodified**: 4.6356e+06

### cus|SWZNA|A/Switzerland/9715293/2013

**>cus|SWZNA|A/Switzerland/9715293/2013**  
 `MNPNQKIITIGSVSLTISTICFFMQIAILITTVTLHFKQYEFNSPPNNQVMLCEPTIIERNITEIVYLTNTTIEKEICPK  
PAEYRNWSKPQCGITGFAPFSKDNSIRLSAGGDIWVTREPYVSCDPDKCYQFALGQGTTLNNVHSNNTVRDRTPYRTLLM  
NELGVPFHLGTKQVCIAWSSSSCHDGKAWLHVCITGDDKNATASFIYNGRLVDSVVSWSKDILRTQESECVCINGTCTVV  
MTDGSASGKADTKILFIEEGKIVHTSTLSGSAQHVEECSCYPRYPGVRCVCRDNWKGSNRPIVDINIKDHSIVSSYVCSG  
LVGDTPRKNDSSSSSHCLDPNNEEGGHGVKGWAFDDGNDVWMGRTINETSRLGYETFKVIEGWSNPKSKLQTNRQVIVDR  
GDRSGYSGIFSVEGKSCINRCFYVELIRGRKEETEVLWTSNSIVVFCGTSGTYGTGSWPDGADLNLMPI`

| Site | Type | Count |
| --- | --- | --- |
| 329 | N-linked | 2 |
| 146 | N-linked | 9 |
| 86 | N-linked | 4 |
| 367 | N-linked | 19 |
| 200 | N-linked | 3 |

| Mass | Sequence | MS_2_ Score | `q`-value | MS_1_ Score | Signal | Protein Coordinates | Tandem Scans | Start Time | Apex Time | End Time |
| --- | --- | --- | --- | --- | --- | --- | --- | --- | --- | --- |
| 3612.4595 | LHVC(C)ITGDDKN(N)ATASF Hex 9 HexNAc 2 | 196.223 | 0.0 | -2.876 | 1.262e+08 | 189 - 205 | 2 | 34.67 | 34.67 | 35.17 |
| 3450.4073 | LHVC(C)ITGDDKN(N)ATASF Hex 8 HexNAc 2 | 195.980 | 0.0 | -3.818 | 8.562e+07 | 189 - 205 | 2 | 34.67 | 34.84 | 35.00 |
| 3252.3339 | RN(N)WSKPQC(C)GITGF Hex 8 HexNAc 2 | 188.231 | 0.0 | -17.816 | 3.328e+07 | 84 - 97 | 1 | 31.38 | 31.55 | 31.55 |
| 3414.3909 | RN(N)WSKPQC(C)GITGF Hex 9 HexNAc 2 | 186.621 | 0.0 | -18.113 | 2.741e+07 | 84 - 97 | 1 | 31.38 | 31.55 | 31.55 |
| 3567.4944 | RN(N)WSKPQC(C)GITGFAPF Hex 8 HexNAc 2 | 180.803 | 0.0 | -17.393 | 6.269e+07 | 84 - 100 | 2 | 45.74 | 45.74 | 45.91 |
| 3729.5463 | RN(N)WSKPQC(C)GITGFAPF Hex 9 HexNAc 2 | 180.794 | 0.0 | -2.709 | 6.725e+07 | 84 - 100 | 3 | 45.57 | 45.74 | 46.08 |
| 3232.3214 | TIN(N)ETSRLGYETF Hex 8 HexNAc 2 | 178.039 | 0.0 | -3.406 | 7.533e+07 | 364 - 377 | 2 | 36.84 | 36.99 | 37.15 |
| 3288.3536 | LHVC(C)ITGDDKN(N)ATASF Hex 7 HexNAc 2 | 175.691 | 0.0 | -19.088 | 1.773e+07 | 189 - 205 | 1 | 34.84 | 34.84 | 35.00 |
| 3268.3783 | M(O)GRTIN(N)ETSRLGYETF Hex 6 HexNAc 2 | 168.534 | 0.0 | -3.262 | 2.213e+08 | 361 - 377 | 3 | 32.53 | 32.70 | 33.03 |
| 3430.4315 | M(O)GRTIN(N)ETSRLGYETF Hex 7 HexNAc 2 | 167.413 | 0.0 | 15.300 | 3.510e+08 | 361 - 377 | 2 | 32.21 | 32.37 | 33.03 |
| 3576.4887 | MGRTIN(N)ETSRLGYETF Hex 8 HexNAc 2 | 158.258 | 0.0 | -17.298 | 3.606e+07 | 361 - 377 | 1 | 35.00 | 35.00 | 35.17 |
| 3592.4847 | M(O)GRTIN(N)ETSRLGYETF Hex 8 HexNAc 2 | 156.619 | 0.0 | -2.942 | 5.972e+08 | 361 - 377 | 3 | 32.04 | 32.21 | 32.53 |
| 3070.2703 | TIN(N)ETSRLGYETF Hex 7 HexNAc 2 | 156.264 | 0.0 | -4.208 | 3.360e+07 | 364 - 377 | 2 | 36.99 | 37.15 | 37.32 |
| 2729.1611 | M(O)GRTIN(N)ETSRLGY Hex 5 HexNAc 2 | 150.989 | 0.0 | -17.751 | 1.876e+07 | 361 - 374 | 1 | 22.96 | 22.96 | 23.13 |
| 2908.2168 | TIN(N)ETSRLGYETF Hex 6 HexNAc 2 | 150.699 | 0.0 | -19.499 | 1.134e+07 | 364 - 377 | 1 | 37.15 | 37.32 | 37.32 |
| 2891.2138 | M(O)GRTIN(N)ETSRLGY Hex 6 HexNAc 2 | 142.963 | 0.0 | -2.990 | 6.300e+07 | 361 - 374 | 1 | 22.63 | 22.79 | 22.96 |
| 3305.3727 | VC(C)SGLVGDTPRKN(N)DSSSSSH Hex 5 HexNAc 2 | 141.082 | 0.0 | -21.019 | 1.713e+06 | 316 - 336 | 1 | 15.70 | 15.70 | 15.70 |
| 3215.3150 | M(O)GRTIN(N)ETSRLGY Hex 8 HexNAc 2 | 138.741 | 0.0 | -1.755 | 2.524e+08 | 361 - 374 | 3 | 22.46 | 22.63 | 22.96 |
| 3414.4323 | MGRTIN(N)ETSRLGYETF Hex 7 HexNAc 2 | 137.811 | 0.0 | -18.074 | 1.184e+07 | 361 - 377 | 1 | 35.17 | 35.17 | 35.17 |
| 3053.2629 | M(O)GRTIN(N)ETSRLGY Hex 7 HexNAc 2 | 137.168 | 0.0 | -1.947 | 1.563e+08 | 361 - 374 | 2 | 22.46 | 22.63 | 22.96 |
| 2833.1820 | M(O)GRTIN(N)ETSRL Hex 7 HexNAc 2 | 127.879 | 0.007 | -17.441 | 2.187e+07 | 361 - 372 | 1 | 18.36 | 18.36 | 18.52 |
| 2995.2342 | M(O)GRTIN(N)ETSRL Hex 8 HexNAc 2 | 125.654 | 0.007 | -2.932 | 4.381e+07 | 361 - 372 | 1 | 18.19 | 18.36 | 18.52 |
| 3068.2828 | GRTIN(N)ETSRLGY Hex 8 HexNAc 2 | 125.475 | 0.007 | -18.547 | 1.018e+07 | 362 - 374 | 1 | 22.13 | 22.13 | 22.29 |
| 3408.4335 | NNVHSN(N)NTVRDRTPY Hex 5 HexNAc 4 | 124.434 | 0.007 | -18.116 | 4.278e+07 | 140 - 155 | 2 | 14.20 | 14.20 | 14.37 |
| 2944.2747 | M(O)GRTIN(N)ETSRLGYETF Hex 4 HexNAc 2 | 122.726 | 0.007 | -16.727 | 1.613e+07 | 361 - 377 | 1 | 33.19 | 33.36 | 33.36 |
| 4284.7564 | NNVHSN(N)NTVRDRTPY Fuc 1 Hex 7 HexNAc 6 | 117.799 | 0.007 | -4.943 | 2.417e+07 | 140 - 155 | 2 | 13.79 | 13.91 | 14.05 |
| 3445.4544 | GRTIN(N)ETSRLGYETF Hex 8 HexNAc 2 | 109.200 | 0.012 | -19.096 | 1.042e+07 | 362 - 377 | 1 | 32.04 | 32.04 | 32.04 |
| 3999.5828 | NNVHSN(N)NTVRDRTPY Fuc 1 Hex 9 HexNAc 3 | 106.758 | 0.012 | -4.434 | 3.020e+07 | 140 - 155 | 3 | 14.87 | 15.20 | 15.37 |
| 3773.5679 | NNVHSN(N)NTVRDRTPY Hex 6 HexNAc 5 | 105.776 | 0.012 | -3.499 | 1.111e+08 | 140 - 155 | 3 | 13.91 | 14.05 | 14.37 |
| 4138.7019 | NNVHSN(N)NTVRDRTPY Hex 7 HexNAc 6 | 101.774 | 0.012 | -19.144 | 4.302e+07 | 140 - 155 | 1 | 13.91 | 13.91 | 14.05 |
| 4364.7170 | NNVHSN(N)NTVRDRTPY Fuc 1 Hex 10 HexNAc 4 | 101.351 | 0.012 | -5.401 | 1.456e+07 | 140 - 155 | 2 | 14.70 | 14.87 | 15.03 |
| 4218.6572 | NNVHSN(N)NTVRDRTPY Hex 10 HexNAc 4 | 100.034 | 0.012 | 11.932 | 3.345e+07 | 140 - 155 | 4 | 14.70 | 14.87 | 15.37 |
| 2671.1279 | M(O)GRTIN(N)ETSRL Hex 6 HexNAc 2 | 98.223 | 0.018 | -19.273 | 8.027e+06 | 361 - 372 | 1 | 18.52 | 18.52 | 18.52 |
| 3919.6264 | NNVHSN(N)NTVRDRTPY Fuc 1 Hex 6 HexNAc 5 | 96.970 | 0.024 | -4.444 | 5.581e+07 | 140 - 155 | 2 | 13.91 | 13.91 | 14.37 |
| 2906.2315 | GRTIN(N)ETSRLGY Hex 7 HexNAc 2 | 91.325 | 0.027 | -15.477 | 2.905e+06 | 362 - 374 | 1 | 22.29 | 22.29 | 22.29 |
| 3914.6166 | VC(C)SGLVGDTPRKN(N)DSSSSSH Hex 5 HexNAc 5 | 79.663 | 0.040 | -17.340 | 1.756e+07 | 316 - 336 | 1 | 15.87 | 15.87 | 15.87 |
| 4065.6848 | NNVHSN(N)NTVRDRTPY Fuc 2 Hex 6 HexNAc 5 | 68.378 | 0.043 | -21.786 | 5.892e+06 | 140 - 155 | 1 | 13.91 | 13.91 | 13.91 |

### LHVC(C)ITGDDKN(N)ATASF Hex 9 HexNAc 2

**Mass:**  3612.4594

(1.477e-06)

**Charge States:** 3

**Start Position**: 190

**End Position**: 206

| Profile Score | Tandem Score | TDA q-value | Volume | Spectrum Matches |
| --- | --- | --- | --- | --- |
| -2.8758 | 196.2234 | 0.0000 | 1.2616e+08 | 2 |

**Adduct Signal Fractions**

- **Unmodified**: 1.2616e+08

### LHVC(C)ITGDDKN(N)ATASF Hex 8 HexNAc 2

**Mass:**  3450.4070

(1.586e-06)

**Charge States:** 3

**Start Position**: 190

**End Position**: 206

| Profile Score | Tandem Score | TDA q-value | Volume | Spectrum Matches |
| --- | --- | --- | --- | --- |
| -3.8180 | 195.9801 | 0.0000 | 8.5624e+07 | 2 |

**Adduct Signal Fractions**

- **Unmodified**: 8.5624e+07

### RN(N)WSKPQC(C)GITGF Hex 8 HexNAc 2

**Mass:**  3252.3345

(2.215e-06)

**Charge States:** 3

**Start Position**: 85

**End Position**: 98

| Profile Score | Tandem Score | TDA q-value | Volume | Spectrum Matches |
| --- | --- | --- | --- | --- |
| -17.8156 | 188.2312 | 0.0000 | 3.3283e+07 | 1 |

**Adduct Signal Fractions**

- **Unmodified**: 3.3283e+07

### RN(N)WSKPQC(C)GITGF Hex 9 HexNAc 2

**Mass:**  3414.3880

(4.289e-06)

**Charge States:** 3

**Start Position**: 85

**End Position**: 98

| Profile Score | Tandem Score | TDA q-value | Volume | Spectrum Matches |
| --- | --- | --- | --- | --- |
| -18.1133 | 186.6210 | 0.0000 | 2.7407e+07 | 1 |

**Adduct Signal Fractions**

- **Unmodified**: 2.7407e+07

### RN(N)WSKPQC(C)GITGFAPF Hex 8 HexNAc 2

**Mass:**  3567.4945

(2.484e-06)

**Charge States:** 3, 4

**Start Position**: 85

**End Position**: 101

| Profile Score | Tandem Score | TDA q-value | Volume | Spectrum Matches |
| --- | --- | --- | --- | --- |
| -17.3926 | 180.8026 | 0.0000 | 6.2695e+07 | 2 |

**Adduct Signal Fractions**

- **Unmodified**: 6.2695e+07

### RN(N)WSKPQC(C)GITGFAPF Hex 9 HexNAc 2

**Mass:**  3729.5462

(1.527e-06)

**Charge States:** 3

**Start Position**: 85

**End Position**: 101

| Profile Score | Tandem Score | TDA q-value | Volume | Spectrum Matches |
| --- | --- | --- | --- | --- |
| -2.7088 | 180.7944 | 0.0000 | 6.7252e+07 | 3 |

**Adduct Signal Fractions**

- **Unmodified**: 6.7252e+07

### TIN(N)ETSRLGYETF Hex 8 HexNAc 2

**Mass:**  3232.3233

(5.633e-07)

**Charge States:** 3

**Start Position**: 365

**End Position**: 378

| Profile Score | Tandem Score | TDA q-value | Volume | Spectrum Matches |
| --- | --- | --- | --- | --- |
| -3.4057 | 178.0392 | 0.0000 | 7.5334e+07 | 2 |

**Adduct Signal Fractions**

- **Unmodified**: 7.5334e+07

### LHVC(C)ITGDDKN(N)ATASF Hex 7 HexNAc 2

**Mass:**  3288.3524

(1.174e-06)

**Charge States:** 3

**Start Position**: 190

**End Position**: 206

| Profile Score | Tandem Score | TDA q-value | Volume | Spectrum Matches |
| --- | --- | --- | --- | --- |
| -19.0876 | 175.6911 | 0.0000 | 1.7734e+07 | 1 |

**Adduct Signal Fractions**

- **Unmodified**: 1.7734e+07

### M(O)GRTIN(N)ETSRLGYETF Hex 6 HexNAc 2

**Mass:**  3268.3793

(1.827e-06)

**Charge States:** 3

**Start Position**: 362

**End Position**: 378

| Profile Score | Tandem Score | TDA q-value | Volume | Spectrum Matches |
| --- | --- | --- | --- | --- |
| -3.2621 | 168.5339 | 0.0000 | 2.2132e+08 | 3 |

**Adduct Signal Fractions**

- **Unmodified**: 2.2132e+08

### M(O)GRTIN(N)ETSRLGYETF Hex 7 HexNAc 2

**Mass:**  3430.4309

(2.601e-06)

**Charge States:** 3

**Start Position**: 362

**End Position**: 378

| Profile Score | Tandem Score | TDA q-value | Volume | Spectrum Matches |
| --- | --- | --- | --- | --- |
| 15.3001 | 167.4129 | 0.0000 | 3.5102e+08 | 2 |

**Adduct Signal Fractions**

- **Unmodified**: 3.5102e+08

### MGRTIN(N)ETSRLGYETF Hex 8 HexNAc 2

**Mass:**  3576.4890

(2.359e-06)

**Charge States:** 3

**Start Position**: 362

**End Position**: 378

| Profile Score | Tandem Score | TDA q-value | Volume | Spectrum Matches |
| --- | --- | --- | --- | --- |
| -17.2984 | 158.2579 | 0.0000 | 3.6062e+07 | 1 |

**Adduct Signal Fractions**

- **Unmodified**: 3.6062e+07

### M(O)GRTIN(N)ETSRLGYETF Hex 8 HexNAc 2

**Mass:**  3592.4840

(3.178e-06)

**Charge States:** 3

**Start Position**: 362

**End Position**: 378

| Profile Score | Tandem Score | TDA q-value | Volume | Spectrum Matches |
| --- | --- | --- | --- | --- |
| -2.9417 | 156.6185 | 0.0000 | 5.9715e+08 | 3 |

**Adduct Signal Fractions**

- **Unmodified**: 5.9715e+08

### TIN(N)ETSRLGYETF Hex 7 HexNAc 2

**Mass:**  3070.2698

(1.684e-06)

**Charge States:** 3

**Start Position**: 365

**End Position**: 378

| Profile Score | Tandem Score | TDA q-value | Volume | Spectrum Matches |
| --- | --- | --- | --- | --- |
| -4.2084 | 156.2638 | 0.0000 | 3.3602e+07 | 2 |

**Adduct Signal Fractions**

- **Unmodified**: 3.3602e+07

### M(O)GRTIN(N)ETSRLGY Hex 5 HexNAc 2

**Mass:**  2729.1612

(1.008e-06)

**Charge States:** 3

**Start Position**: 362

**End Position**: 375

| Profile Score | Tandem Score | TDA q-value | Volume | Spectrum Matches |
| --- | --- | --- | --- | --- |
| -17.7513 | 150.9889 | 0.0000 | 1.8762e+07 | 1 |

**Adduct Signal Fractions**

- **Unmodified**: 1.8762e+07

### TIN(N)ETSRLGYETF Hex 6 HexNAc 2

**Mass:**  2908.2160

(2.123e-06)

**Charge States:** 3

**Start Position**: 365

**End Position**: 378

| Profile Score | Tandem Score | TDA q-value | Volume | Spectrum Matches |
| --- | --- | --- | --- | --- |
| -19.4992 | 150.6991 | 0.0000 | 1.1343e+07 | 1 |

**Adduct Signal Fractions**

- **Unmodified**: 1.1343e+07

### M(O)GRTIN(N)ETSRLGY Hex 6 HexNAc 2

**Mass:**  2891.2135

(7.956e-07)

**Charge States:** 3

**Start Position**: 362

**End Position**: 375

| Profile Score | Tandem Score | TDA q-value | Volume | Spectrum Matches |
| --- | --- | --- | --- | --- |
| -2.9896 | 142.9634 | 0.0000 | 6.3002e+07 | 1 |

**Adduct Signal Fractions**

- **Unmodified**: 6.3002e+07

### VC(C)SGLVGDTPRKN(N)DSSSSSH Hex 5 HexNAc 2

**Mass:**  3305.3727

(1.019e-07)

**Charge States:** 4

**Start Position**: 317

**End Position**: 337

| Profile Score | Tandem Score | TDA q-value | Volume | Spectrum Matches |
| --- | --- | --- | --- | --- |
| -21.0188 | 141.0816 | 0.0000 | 1.7129e+06 | 1 |

**Adduct Signal Fractions**

- **Unmodified**: 1.7129e+06

### M(O)GRTIN(N)ETSRLGY Hex 8 HexNAc 2

**Mass:**  3215.3135

(3.871e-07)

**Charge States:** 3

**Start Position**: 362

**End Position**: 375

| Profile Score | Tandem Score | TDA q-value | Volume | Spectrum Matches |
| --- | --- | --- | --- | --- |
| -1.7550 | 138.7406 | 0.0000 | 2.5243e+08 | 3 |

**Adduct Signal Fractions**

- **Unmodified**: 2.5243e+08

### MGRTIN(N)ETSRLGYETF Hex 7 HexNAc 2

**Mass:**  3414.4326

(1.476e-06)

**Charge States:** 3, 4

**Start Position**: 362

**End Position**: 378

| Profile Score | Tandem Score | TDA q-value | Volume | Spectrum Matches |
| --- | --- | --- | --- | --- |
| -18.0740 | 137.8108 | 0.0000 | 1.1844e+07 | 1 |

**Adduct Signal Fractions**

- **Unmodified**: 1.1844e+07

### M(O)GRTIN(N)ETSRLGY Hex 7 HexNAc 2

**Mass:**  3053.2619

(1.512e-07)

**Charge States:** 3

**Start Position**: 362

**End Position**: 375

| Profile Score | Tandem Score | TDA q-value | Volume | Spectrum Matches |
| --- | --- | --- | --- | --- |
| -1.9470 | 137.1681 | 0.0000 | 1.5633e+08 | 2 |

**Adduct Signal Fractions**

- **Unmodified**: 1.5633e+08

### M(O)GRTIN(N)ETSRL Hex 7 HexNAc 2

**Mass:**  2833.1817

(8.958e-07)

**Charge States:** 3

**Start Position**: 362

**End Position**: 373

| Profile Score | Tandem Score | TDA q-value | Volume | Spectrum Matches |
| --- | --- | --- | --- | --- |
| -17.4412 | 127.8792 | 0.0067 | 2.1866e+07 | 1 |

**Adduct Signal Fractions**

- **Unmodified**: 2.1866e+07

### M(O)GRTIN(N)ETSRL Hex 8 HexNAc 2

**Mass:**  2995.2341

(7.686e-07)

**Charge States:** 3

**Start Position**: 362

**End Position**: 373

| Profile Score | Tandem Score | TDA q-value | Volume | Spectrum Matches |
| --- | --- | --- | --- | --- |
| -2.9318 | 125.6540 | 0.0067 | 4.3806e+07 | 1 |

**Adduct Signal Fractions**

- **Unmodified**: 4.3806e+07

### GRTIN(N)ETSRLGY Hex 8 HexNAc 2

**Mass:**  3068.2829

(4.628e-07)

**Charge States:** 3

**Start Position**: 363

**End Position**: 375

| Profile Score | Tandem Score | TDA q-value | Volume | Spectrum Matches |
| --- | --- | --- | --- | --- |
| -18.5469 | 125.4748 | 0.0067 | 1.0181e+07 | 1 |

**Adduct Signal Fractions**

- **Unmodified**: 1.0181e+07

### NNVHSN(N)NTVRDRTPY Hex 5 HexNAc 4

**Mass:**  3408.4333

(2.768e-07)

**Charge States:** 4

**Start Position**: 141

**End Position**: 156

| Profile Score | Tandem Score | TDA q-value | Volume | Spectrum Matches |
| --- | --- | --- | --- | --- |
| -18.1160 | 124.4344 | 0.0067 | 4.2776e+07 | 2 |

**Adduct Signal Fractions**

- **Unmodified**: 4.2776e+07

### M(O)GRTIN(N)ETSRLGYETF Hex 4 HexNAc 2

**Mass:**  2944.2740

(3.343e-06)

**Charge States:** 3

**Start Position**: 362

**End Position**: 378

| Profile Score | Tandem Score | TDA q-value | Volume | Spectrum Matches |
| --- | --- | --- | --- | --- |
| -16.7267 | 122.7263 | 0.0067 | 1.6133e+07 | 1 |

**Adduct Signal Fractions**

- **Unmodified**: 1.6133e+07

### NNVHSN(N)NTVRDRTPY Fuc 1 Hex 7 HexNAc 6

**Mass:**  4284.7561

(-5.497e-07)

**Charge States:** 4

**Start Position**: 141

**End Position**: 156

| Profile Score | Tandem Score | TDA q-value | Volume | Spectrum Matches |
| --- | --- | --- | --- | --- |
| -4.9434 | 117.7994 | 0.0067 | 2.4171e+07 | 2 |

**Adduct Signal Fractions**

- **Unmodified**: 2.4171e+07

### GRTIN(N)ETSRLGYETF Hex 8 HexNAc 2

**Mass:**  3445.4544

(4.142e-06)

**Charge States:** 3

**Start Position**: 363

**End Position**: 378

| Profile Score | Tandem Score | TDA q-value | Volume | Spectrum Matches |
| --- | --- | --- | --- | --- |
| -19.0959 | 109.1996 | 0.0120 | 1.0424e+07 | 1 |

**Adduct Signal Fractions**

- **Unmodified**: 1.0424e+07

### NNVHSN(N)NTVRDRTPY Fuc 1 Hex 9 HexNAc 3

**Mass:**  3999.5864

(-8.943e-06)

**Charge States:** 4

**Start Position**: 141

**End Position**: 156

| Profile Score | Tandem Score | TDA q-value | Volume | Spectrum Matches |
| --- | --- | --- | --- | --- |
| -4.4341 | 106.7580 | 0.0120 | 3.0196e+07 | 3 |

**Adduct Signal Fractions**

- **Unmodified**: 3.0196e+07

### NNVHSN(N)NTVRDRTPY Hex 6 HexNAc 5

**Mass:**  3773.5678

(8.578e-07)

**Charge States:** 4

**Start Position**: 141

**End Position**: 156

| Profile Score | Tandem Score | TDA q-value | Volume | Spectrum Matches |
| --- | --- | --- | --- | --- |
| -3.4987 | 105.7758 | 0.0120 | 1.1114e+08 | 3 |

**Adduct Signal Fractions**

- **Unmodified**: 1.1114e+08

### NNVHSN(N)NTVRDRTPY Hex 7 HexNAc 6

**Mass:**  4138.7028

(1.495e-06)

**Charge States:** 4

**Start Position**: 141

**End Position**: 156

| Profile Score | Tandem Score | TDA q-value | Volume | Spectrum Matches |
| --- | --- | --- | --- | --- |
| -19.1436 | 101.7739 | 0.0120 | 4.3019e+07 | 1 |

**Adduct Signal Fractions**

- **Unmodified**: 4.3019e+07

### NNVHSN(N)NTVRDRTPY Fuc 1 Hex 10 HexNAc 4

**Mass:**  4364.7165

(-8.736e-06)

**Charge States:** 4

**Start Position**: 141

**End Position**: 156

| Profile Score | Tandem Score | TDA q-value | Volume | Spectrum Matches |
| --- | --- | --- | --- | --- |
| -5.4006 | 101.3515 | 0.0120 | 1.4562e+07 | 2 |

**Adduct Signal Fractions**

- **Unmodified**: 1.4562e+07

### NNVHSN(N)NTVRDRTPY Hex 10 HexNAc 4

**Mass:**  4218.6569

(-9.061e-06)

**Charge States:** 4

**Start Position**: 141

**End Position**: 156

| Profile Score | Tandem Score | TDA q-value | Volume | Spectrum Matches |
| --- | --- | --- | --- | --- |
| 11.9317 | 100.0338 | 0.0120 | 3.3450e+07 | 4 |

**Adduct Signal Fractions**

- **Unmodified**: 3.3450e+07

### M(O)GRTIN(N)ETSRL Hex 6 HexNAc 2

**Mass:**  2671.1279

(5.433e-07)

**Charge States:** 3

**Start Position**: 362

**End Position**: 373

| Profile Score | Tandem Score | TDA q-value | Volume | Spectrum Matches |
| --- | --- | --- | --- | --- |
| -19.2726 | 98.2234 | 0.0179 | 8.0268e+06 | 1 |

**Adduct Signal Fractions**

- **Unmodified**: 8.0268e+06

### NNVHSN(N)NTVRDRTPY Fuc 1 Hex 6 HexNAc 5

**Mass:**  3919.6264

(1.039e-06)

**Charge States:** 4

**Start Position**: 141

**End Position**: 156

| Profile Score | Tandem Score | TDA q-value | Volume | Spectrum Matches |
| --- | --- | --- | --- | --- |
| -4.4439 | 96.9698 | 0.0235 | 5.5812e+07 | 2 |

**Adduct Signal Fractions**

- **Unmodified**: 5.5812e+07

### GRTIN(N)ETSRLGY Hex 7 HexNAc 2

**Mass:**  2906.2315

(9.797e-07)

**Charge States:** 3

**Start Position**: 363

**End Position**: 375

| Profile Score | Tandem Score | TDA q-value | Volume | Spectrum Matches |
| --- | --- | --- | --- | --- |
| -15.4766 | 91.3253 | 0.0272 | 2.9048e+06 | 1 |

**Adduct Signal Fractions**

- **Unmodified**: 2.9048e+06

### VC(C)SGLVGDTPRKN(N)DSSSSSH Hex 5 HexNAc 5

**Mass:**  3914.6166

(1.584e-06)

**Charge States:** 4

**Start Position**: 317

**End Position**: 337

| Profile Score | Tandem Score | TDA q-value | Volume | Spectrum Matches |
| --- | --- | --- | --- | --- |
| -17.3404 | 79.6630 | 0.0398 | 1.7565e+07 | 1 |

**Adduct Signal Fractions**

- **Unmodified**: 1.7565e+07

### NNVHSN(N)NTVRDRTPY Fuc 2 Hex 6 HexNAc 5

**Mass:**  4065.6848

(1.105e-06)

**Charge States:** 4

**Start Position**: 141

**End Position**: 156

| Profile Score | Tandem Score | TDA q-value | Volume | Spectrum Matches |
| --- | --- | --- | --- | --- |
| -21.7863 | 68.3779 | 0.0429 | 5.8919e+06 | 1 |

**Adduct Signal Fractions**

- **Unmodified**: 5.8919e+06
