# Supplementary material for: Measuring Site-specific Glycosylation Similarity between Influenza a Virus Variants with Statistical Certainty
Source: Mol Cell Proteomics. 2020 Nov 25;19(9):1533–45. doi: 10.1074/mcp.RA120.002031 (PMC8143645; doi:10.1074/mcp.RA120.002031)
Supplement: Supplementary file 2 [file mmc2.zip › mmc2/159250_2_supp_555291_qcjklr.zip!/20190326_SWZeggL2_021119_chymo_HILIC_DDA_1ug_01_glycopeptides.html]

## 20190326\_SWZeggL2\_021119\_chymo\_HILIC\_DDA\_1ug\_01 @ GlycopeptideHypothesis-ec2041d509454643beafecd5053d4028

**MS^1^ Mass Accuracy:** 10.0 PPM  
**Grouping Tolerance:** 15.0 PPM  
**MS^n^ Mass Accuracy:** 20.0 PPM  
**Minimum `q`-Value For Clustering:** 0.05

**Glycopeptide Hypothesis:**
GlycopeptideHypothesis-ec2041d509454643beafecd5053d4028  
**Sample Searched:**
20190326\_SWZeggL2\_021119\_chymo\_HILIC\_DDA\_1ug\_01

| Protein | Matched Glycopeptides |
| --- | --- |
| cus\|SWZHA\|A/Switzerland/9715293/2013 | 100 |
| cus\|SWZHA\_5B8\|A/Switzerland/9715293/2013 | 76 |
| cus\|SWZNA\|A/Switzerland/9715293/2013 | 32 |
| iav\|AGU93026\|A/Puerto | 3 |
| iav\|AFM71853\|A/Puerto | 3 |
| iav\|ACV49552\|A/Puerto | 3 |
| iav\|AGK63052\|A/Puerto | 3 |
| iav\|ABD77682\|A/Puerto | 3 |
| iav\|AAM75157\|A/Puerto | 3 |
| iav\|AEX92880\|A/Puerto | 3 |
| iav\|CAA24295\|A/Puerto | 3 |
| iav\|ADX99654\|A/Puerto | 3 |
| iav\|ABO21708\|A/Puerto | 3 |
| iav\|BAV59610\|A/Puerto | 3 |
| iav\|ACF41841\|A/Puerto | 3 |
| tr\|A0A1D5PV37\|A0A1D5PV37\_CHICK | 1 |
| tr\|A0A1D5P780\|A0A1D5P780\_CHICK | 1 |
| sp\|P02845\|VIT2\_CHICK | 1 |
| tr\|F1NFL6\|F1NFL6\_CHICK | 1 |
| tr\|A0A1D5P6L2\|A0A1D5P6L2\_CHICK | 1 |
| tr\|A0A1L1RK76\|A0A1L1RK76\_CHICK | 1 |
| tr\|A0A1D5P198\|A0A1D5P198\_CHICK | 1 |
| tr\|A0A1D5NW27\|A0A1D5NW27\_CHICK | 1 |
| iav\|ACF41842\|A/Puerto | 1 |
| iav\|AGU93028\|A/Puerto | 1 |
| iav\|AFM71854\|A/Puerto | 1 |
| iav\|ABO21706\|A/Puerto | 1 |
| iav\|ABD77683\|A/Puerto | 1 |
| iav\|AAM75156\|A/Puerto | 1 |
| iav\|ADX99655\|A/Puerto | 1 |
| iav\|ACV49553\|A/Puerto | 1 |
| iav\|BAV59609\|A/Puerto | 1 |
| iav\|AGK63053\|A/Puerto | 1 |

### cus|SWZHA|A/Switzerland/9715293/2013

**>cus|SWZHA|A/Switzerland/9715293/2013**  
 `QKLPGNDNSTATLCLGHHAVPNGTIVKTITNDRIEVTNATELVQNSSIGEICDSPHQILDGENCTLIDALLGDPQCDGFQ  
NKKWDLFVERSKAYSNCYPYDVPDYASLRSLVASSGTLEFNNESFNWAGVTQNGTSSSCRRGSNSSFFSRLNWLTHLNSK  
YPALNVTMPNNEQFDKLYIWGVHHPVTDKDQIFLYAQSSGRITVSTKRSQQAVIPNIGYRPRIRDIPSRISIYWTIVKPG  
DILLINSTGNLIAPRGYFKIRSGKSSIMRSDAPIGKCKSECITPNGSIPNDKPFQNVNRITYGACPRYVKQSTLKLATGM  
RNVPERQTRGIFGAIAGFIENGWEGMVDGWYGFRHQNSEGRGQAADLKSTQAAIDQINGKLNRLIGKTNEKFHQIEKEFS  
EVEGRIQDLEKYVEDTKIDLWSYNAELLVALENQHTIDLTDSEMNKLFEKTKKQLRENAEDMGNGCFKIYHKCDNACIGS  
IRNGTYDHDVYRDEALNNRFQIKGVELKSGYKDWILWISFAISCFLLCVALLGFIMWACQKGNIRCNICI`

| Site | Type | Count |
| --- | --- | --- |
| 483 | N-linked | 3 |
| 133 | N-linked | 19 |
| 38 | N-linked | 3 |
| 8 | N-linked | 2 |
| 144 | N-linked | 6 |
| 246 | N-linked | 46 |
| 122 | N-linked | 1 |
| 165 | N-linked | 16 |
| 285 | N-linked | 3 |
| 22 | N-linked | 1 |

| Mass | Sequence | MS_2_ Score | `q`-value | MS_1_ Score | Signal | Protein Coordinates | Tandem Scans | Start Time | Apex Time | End Time |
| --- | --- | --- | --- | --- | --- | --- | --- | --- | --- | --- |
| 2695.0883 | LIN(N)STGNL Hex 9 HexNAc 2 | 187.010 | 0.0 | -4.475 | 5.551e+07 | 243 - 251 | 3 | 23.07 | 23.23 | 23.56 |
| 3519.5614 | LIN(N)STGNLIAPRGYF Fuc 2 Hex 3 HexNAc 4 Neu5Ac 1 | 184.281 | 0.0 | 0.000 | 0.000e+00 | 243 - 258 | 1 | - | - | - |
| 3132.3710 | LIN(N)STGNLIAPR Hex 9 HexNAc 2 | 181.606 | 0.0 | -2.753 | 5.135e+07 | 243 - 255 | 2 | 28.21 | 28.38 | 28.54 |
| 3499.5171 | LIN(N)STGNLIAPRGYF Hex 9 HexNAc 2 | 181.268 | 0.0 | 12.618 | 3.216e+08 | 243 - 258 | 5 | 45.25 | 45.41 | 46.54 |
| 2712.1149 | LIN(N)STGNL Fuc 2 Hex 3 HexNAc 4 Neu5Ac 1 | 180.426 | 0.0 | -4.569 | 1.841e+07 | 243 - 251 | 2 | 23.07 | 23.23 | 23.39 |
| 3516.5451 | LIN(N)STGNLIAPRGYF Fuc 2 Hex 3 HexNAc 4 Neu5Ac 1 | 180.399 | 0.0 | 13.740 | 1.572e+08 | 243 - 258 | 2 | 45.25 | 45.41 | 45.89 |
| 3352.4401 | LIN(N)STGNLIAPRGY Hex 9 HexNAc 2 | 178.612 | 0.0 | 14.899 | 2.580e+08 | 243 - 257 | 3 | 34.68 | 34.83 | 35.66 |
| 3369.4669 | LIN(N)STGNLIAPRGY Fuc 2 Hex 3 HexNAc 4 Neu5Ac 1 | 174.482 | 0.0 | 15.576 | 1.276e+08 | 243 - 257 | 4 | 34.68 | 34.83 | 35.50 |
| 4068.9333 | TIVKPGDILLIN(N)STGNLIAPR Hex 9 HexNAc 2 | 172.886 | 0.0 | 15.855 | 1.522e+09 | 234 - 255 | 23 | 49.94 | 51.64 | 54.20 |
| 3149.3994 | LIN(N)STGNLIAPR Fuc 2 Hex 3 HexNAc 4 Neu5Ac 1 | 171.673 | 0.0 | -3.934 | 1.971e+07 | 243 - 255 | 1 | 28.21 | 28.38 | 28.54 |
| 3337.4660 | LIN(N)STGNLIAPRGYF Hex 8 HexNAc 2 | 171.084 | 0.0 | -19.552 | 1.894e+07 | 243 - 258 | 1 | 45.57 | 45.57 | 45.73 |
| 3908.5706 | AGVTQN(N)GTSSSC(C)RRGSNSSFF Hex 8 HexNAc 2 | 170.147 | 0.0 | -3.622 | 9.784e+07 | 127 - 148 | 1 | 22.23 | 22.40 | 22.73 |
| 3661.5737 | LIN(N)STGNLIAPRGYF Hex 10 HexNAc 2 | 168.083 | 0.0 | -19.966 | 2.415e+07 | 243 - 258 | 1 | 45.25 | 45.25 | 45.41 |
| 3746.5140 | AGVTQN(N)GTSSSC(C)RRGSNSSFF Hex 7 HexNAc 2 | 168.023 | 0.0 | 15.827 | 1.003e+09 | 127 - 148 | 5 | 22.07 | 22.40 | 23.07 |
| 3386.4380 | IN(N)STGNLIAPRGYF Hex 9 HexNAc 2 | 165.849 | 0.0 | -19.942 | 1.333e+07 | 244 - 258 | 1 | 41.23 | 41.40 | 41.40 |
| 4126.9600 | TIVKPGDILLIN(N)STGNLIAPRGY Hex 8 HexNAc 2 | 164.669 | 0.0 | 12.952 | 2.842e+08 | 234 - 257 | 15 | 53.86 | 54.53 | 56.41 |
| 3422.4036 | AGVTQN(N)GTSSSC(C)RRGSNSSFF Hex 5 HexNAc 2 | 164.155 | 0.0 | 14.890 | 5.143e+08 | 127 - 148 | 4 | 22.40 | 22.90 | 23.56 |
| 2761.0694 | NWAGVTQN(N)GTSS Hex 7 HexNAc 2 | 161.636 | 0.0 | -4.999 | 2.103e+07 | 125 - 137 | 2 | 20.28 | 20.44 | 20.61 |
| 3159.4262 | TIVKPGDILLIN(N) Hex 9 HexNAc 2 | 159.555 | 0.0 | 12.779 | 1.815e+08 | 234 - 246 | 4 | 44.42 | 44.92 | 45.57 |
| 4230.9825 | TIVKPGDILLIN(N)STGNLIAPR Hex 10 HexNAc 2 | 158.479 | 0.0 | 13.526 | 1.274e+08 | 234 - 255 | 5 | 51.47 | 51.64 | 52.84 |
| 4289.0156 | TIVKPGDILLIN(N)STGNLIAPRGY Hex 9 HexNAc 2 | 158.253 | 0.0 | 16.223 | 2.989e+09 | 234 - 257 | 37 | 52.49 | 54.37 | 58.92 |
| 4365.8319 | NSKYPALN(N)VTM(O)PNNEQFDKLY Hex 9 HexNAc 2 | 157.954 | 0.0 | 14.510 | 3.789e+08 | 157 - 178 | 4 | 41.40 | 41.90 | 42.23 |
| 3514.4942 | LIN(N)STGNLIAPRGY Hex 10 HexNAc 2 | 157.833 | 0.0 | -5.190 | 2.090e+07 | 243 - 257 | 1 | 34.51 | 34.68 | 34.83 |
| 4085.9572 | TIVKPGDILLIN(N)STGNLIAPR Fuc 2 Hex 3 HexNAc 4 Neu5Ac 1 | 157.461 | 0.0 | 12.944 | 2.283e+08 | 234 - 255 | 4 | 51.47 | 51.64 | 53.01 |
| 3176.4528 | TIVKPGDILLIN(N) Fuc 2 Hex 3 HexNAc 4 Neu5Ac 1 | 155.486 | 0.0 | 11.868 | 8.871e+07 | 234 - 246 | 3 | 44.42 | 44.92 | 45.25 |
| 3906.8790 | TIVKPGDILLIN(N)STGNLIAPR Hex 8 HexNAc 2 | 155.260 | 0.0 | 12.886 | 1.116e+08 | 234 - 255 | 4 | 51.64 | 51.81 | 52.32 |
| 3648.6840 | TIVKPGDILLIN(N)STGNL Fuc 2 Hex 3 HexNAc 4 Neu5Ac 1 | 152.486 | 0.0 | 13.249 | 4.821e+08 | 234 - 251 | 6 | 50.97 | 51.14 | 52.84 |
| 2857.1356 | LIN(N)STGNL Hex 10 HexNAc 2 | 152.460 | 0.0 | -20.028 | 1.625e+06 | 243 - 251 | 1 | 23.23 | 23.23 | 23.23 |
| 4306.0397 | TIVKPGDILLIN(N)STGNLIAPRGY Fuc 2 Hex 3 HexNAc 4 Neu5Ac 1 | 152.238 | 0.0 | 13.918 | 5.074e+08 | 234 - 257 | 9 | 53.86 | 54.37 | 56.75 |
| 3584.4580 | AGVTQN(N)GTSSSC(C)RRGSNSSFF Hex 6 HexNAc 2 | 151.761 | 0.0 | 14.279 | 3.045e+08 | 127 - 148 | 3 | 22.23 | 22.73 | 23.23 |
| 3631.6600 | TIVKPGDILLIN(N)STGNL Hex 9 HexNAc 2 | 150.825 | 0.0 | 15.892 | 9.462e+08 | 234 - 251 | 13 | 50.45 | 51.14 | 54.03 |
| 2905.2769 | TITNDRIEVTN(N)ATEL Hex 5 HexNAc 2 | 150.611 | 0.0 | -17.728 | 2.704e+07 | 27 - 42 | 1 | 34.68 | 34.83 | 34.83 |
| 3473.5030 | C(C)LGHHAVPN(N)GTIVK Fuc 1 Hex 5 HexNAc 5 | 149.874 | 0.0 | -5.115 | 1.708e+07 | 13 - 27 | 1 | 16.70 | 16.87 | 17.03 |
| 4251.7888 | SKYPALN(N)VTM(O)PNNEQFDKLY Hex 9 HexNAc 2 | 148.973 | 0.0 | 13.067 | 1.220e+08 | 158 - 178 | 3 | 41.56 | 41.90 | 42.40 |
| 4752.0445 | RSDAPIGKC(C)KSEC(C)ITPN(N)GSIPNDKPF Hex 9 HexNAc 2 | 147.773 | 0.0 | 14.968 | 2.400e+08 | 268 - 294 | 4 | 27.88 | 28.04 | 28.54 |
| 4451.0650 | TIVKPGDILLIN(N)STGNLIAPRGY Hex 10 HexNAc 2 | 146.989 | 0.0 | 13.390 | 2.745e+08 | 234 - 257 | 11 | 54.03 | 54.20 | 55.73 |
| 3793.7118 | TIVKPGDILLIN(N)STGNL Hex 10 HexNAc 2 | 145.778 | 0.0 | 14.558 | 7.586e+07 | 234 - 251 | 4 | 50.79 | 50.97 | 51.47 |
| 4089.7369 | SKYPALN(N)VTM(O)PNNEQFDKLY Hex 8 HexNAc 2 | 145.533 | 0.0 | 13.294 | 1.235e+08 | 158 - 178 | 3 | 41.56 | 41.90 | 42.23 |
| 4248.0116 | TIVKPGDILLIN(N)STGNLIAPR Fuc 2 Hex 4 HexNAc 4 Neu5Ac 1 | 145.347 | 0.0 | -4.285 | 1.918e+07 | 234 - 255 | 1 | 51.47 | 51.64 | 51.98 |
| 4203.7793 | NSKYPALN(N)VTM(O)PNNEQFDKLY Hex 8 HexNAc 2 | 144.052 | 0.0 | 16.125 | 4.258e+08 | 157 - 178 | 5 | 41.06 | 41.90 | 42.40 |
| 3164.2349 | NWAGVTQN(N)GTSSSC(C)R Hex 7 HexNAc 2 | 143.737 | 0.0 | -18.278 | 1.357e+07 | 125 - 140 | 1 | 17.84 | 17.84 | 18.01 |
| 4470.1257 | TIVKPGDILLIN(N)STGNLIAPRGY Fuc 2 Hex 4 HexNAc 4 Neu5Ac 1 | 142.806 | 0.0 | 0.000 | 0.000e+00 | 234 - 257 | 1 | - | - | - |
| 3533.5490 | LIN(N)STGNLIAPRGY Fuc 2 Hex 4 HexNAc 4 Neu5Ac 1 | 142.200 | 0.0 | -7.410 | 1.524e+07 | 243 - 257 | 3 | 31.17 | 31.50 | 31.50 |
| 2116.8313 | RGSN(N)SSFF Hex 5 HexNAc 2 | 139.749 | 0.0 | -3.917 | 1.322e+07 | 140 - 148 | 1 | 22.40 | 22.57 | 22.73 |
| 3190.3947 | LIN(N)STGNLIAPRGY Hex 8 HexNAc 2 | 139.196 | 0.0 | -19.739 | 1.362e+07 | 243 - 257 | 1 | 34.83 | 35.00 | 35.00 |
| 3420.7174 | TIVKPGDILLIN(N)STGNLIAPR Hex 5 HexNAc 2 | 139.096 | 0.0 | -3.894 | 2.096e+07 | 234 - 255 | 3 | 52.32 | 52.32 | 52.84 |
| 4468.0960 | TIVKPGDILLIN(N)STGNLIAPRGY Fuc 2 Hex 4 HexNAc 4 Neu5Ac 1 | 138.571 | 0.0 | -4.528 | 4.884e+07 | 234 - 257 | 2 | 54.20 | 54.20 | 54.70 |
| 3067.3366 | TITNDRIEVTN(N)ATEL Hex 6 HexNAc 2 | 138.168 | 0.0 | -19.696 | 1.522e+07 | 27 - 42 | 1 | 34.51 | 34.68 | 34.68 |
| 3469.6067 | TIVKPGDILLIN(N)STGNL Hex 8 HexNAc 2 | 137.352 | 0.0 | 14.081 | 7.256e+07 | 234 - 251 | 4 | 50.97 | 51.14 | 51.64 |
| 3008.1325 | NWAGVTQN(N)GTSSSC(C) Hex 7 HexNAc 2 | 135.854 | 0.0 | -4.425 | 1.782e+07 | 125 - 139 | 1 | 20.94 | 21.26 | 21.26 |
| 3640.8046 | TIVKPGDILLIN(N)STGNLIAPRGY Hex 5 HexNAc 2 | 134.719 | 0.0 | 12.704 | 4.130e+07 | 234 - 257 | 5 | 54.87 | 55.04 | 55.90 |
| 2436.9673 | NWAGVTQN(N)GTSS Hex 5 HexNAc 2 | 134.328 | 0.0 | -20.279 | 8.008e+06 | 125 - 137 | 1 | 20.94 | 20.94 | 21.10 |
| 2849.1019 | NWAGVTQN(N)GTSSS Hex 7 HexNAc 2 | 134.018 | 0.0 | 0.000 | 0.000e+00 | 125 - 138 | 1 | - | - | - |
| 2563.9536 | EFNN(N)ESFNW Hex 6 HexNAc 2 | 133.371 | 0.0 | -4.396 | 5.327e+06 | 118 - 127 | 2 | 42.57 | 42.74 | 42.91 |
| 4589.9912 | RSDAPIGKC(C)KSEC(C)ITPN(N)GSIPNDKPF Hex 8 HexNAc 2 | 133.196 | 0.0 | -3.988 | 9.923e+07 | 268 - 294 | 2 | 28.04 | 28.04 | 28.38 |
| 4427.9353 | RSDAPIGKC(C)KSEC(C)ITPN(N)GSIPNDKPF Hex 7 HexNAc 2 | 131.175 | 0.0 | -4.114 | 1.104e+08 | 268 - 294 | 3 | 28.04 | 28.21 | 28.54 |
| 3207.2925 | SIRN(N)GTYDHDVY Fuc 1 Hex 5 HexNAc 4 | 129.786 | 0.0 | -18.155 | 1.282e+07 | 479 - 491 | 1 | 17.36 | 17.36 | 17.52 |
| 3810.7344 | TIVKPGDILLIN(N)STGNL Fuc 2 Hex 4 HexNAc 4 Neu5Ac 1 | 129.135 | 0.0 | -18.515 | 1.872e+07 | 234 - 251 | 1 | 50.97 | 50.97 | 50.97 |
| 3518.5768 | TIVKPGDILLIN(N)STGN Hex 9 HexNAc 2 | 128.227 | 0.0 | 14.363 | 1.823e+08 | 234 - 250 | 3 | 42.74 | 42.91 | 43.76 |
| 3884.5868 | NWAGVTQNGTSSSC(C)RRGSN(N)SSFF Hex 6 HexNAc 2 | 126.598 | 0.0 | -3.807 | 4.887e+07 | 125 - 148 | 3 | 34.00 | 34.17 | 34.51 |
| 4262.7208 | HKC(C)DNAC(C)IGSIRN(N)GTYDHDVY Fuc 1 Hex 5 HexNAc 4 | 126.010 | 0.0 | -3.889 | 1.100e+08 | 470 - 491 | 2 | 23.39 | 23.56 | 23.73 |
| 3423.3949 | AGVTQN(N)GTSSSC(C)RRGSN(D)SSFF Hex 5 HexNAc 2 | 121.072 | 0.0 | 10.708 | 2.022e+08 | 127 - 148 | 3 | 23.73 | 24.06 | 24.89 |
| 3609.4875 | AGVTQNGTSSSC(C)RRGSN(N)SSFF Fuc 1 Hex 4 HexNAc 3 | 120.726 | 0.0 | 12.485 | 5.611e+07 | 127 - 148 | 1 | 22.73 | 23.07 | 23.39 |
| 4040.7202 | NSKYPALN(N)VTM(O)PNNEQFDKL Hex 8 HexNAc 2 | 119.901 | 0.0 | -3.959 | 1.465e+07 | 157 - 177 | 1 | 39.20 | 39.20 | 39.53 |
| 3270.4107 | TITNDRIEVTN(N)ATEL Hex 6 HexNAc 3 | 115.285 | 0.0 | -5.609 | 1.648e+07 | 27 - 42 | 1 | 34.51 | 34.68 | 34.83 |
| 2997.3821 | TIVKPGDILLIN(N) Hex 8 HexNAc 2 | 109.791 | 0.005 | -18.581 | 6.984e+06 | 234 - 246 | 1 | 45.09 | 45.09 | 45.09 |
| 4290.0084 | TIVKPGDILLIN(N)STGN(D)LIAPRGY Hex 9 HexNAc 2 | 109.760 | 0.005 | -18.882 | 1.386e+07 | 234 - 257 | 4 | 55.38 | 55.38 | 55.38 |
| 3747.5001 | AGVTQN(N)GTSSSC(C)RRGSN(D)SSFF Hex 7 HexNAc 2 | 108.769 | 0.005 | 10.894 | 3.757e+08 | 127 - 148 | 6 | 23.23 | 23.56 | 24.72 |
| 2733.0339 | N(N)VTMPNNEQF Hex 7 HexNAc 2 | 99.788 | 0.005 | -20.648 | 6.312e+06 | 164 - 174 | 1 | 23.23 | 23.23 | 23.39 |
| 3098.3120 | AGVTQN(N)GTSSSC(C)RRGSNSSFF Hex 3 HexNAc 2 | 99.453 | 0.005 | -18.547 | 2.646e+07 | 127 - 148 | 4 | 23.39 | 23.56 | 23.56 |
| 3500.5073 | LIN(N)STGN(D)LIAPRGYF Hex 9 HexNAc 2 | 98.067 | 0.005 | -5.932 | 8.569e+06 | 243 - 258 | 1 | 46.71 | 46.88 | 47.05 |
| 4366.8147 | N(D)SKYPALN(N)VTM(O)PNNEQFDKLY Hex 9 HexNAc 2 | 96.938 | 0.005 | 9.102 | 2.235e+08 | 157 - 178 | 5 | 42.40 | 44.76 | 45.09 |
| 2744.1717 | QKLPGNDN(N)STATL Hex 3 HexNAc 3 Neu5Ac 1 | 95.934 | 0.005 | 13.195 | 3.220e+08 | 0 - 13 | 4 | 29.35 | 29.51 | 30.33 |
| 4046.6422 | NWAGVTQN(N)GTSSSC(C)RRGSNSSFF Hex 7 HexNAc 2 | 94.006 | 0.005 | 13.258 | 1.621e+08 | 125 - 148 | 3 | 33.67 | 33.83 | 34.51 |
| 3802.8571 | TIVKPGDILLIN(N)STGNLIAPRGY Hex 6 HexNAc 2 | 92.463 | 0.009 | -4.589 | 1.387e+07 | 234 - 257 | 2 | 54.70 | 54.87 | 55.21 |
| 4153.8074 | LNSKYPALN(N)VTM(O)PNNEQFDKL Hex 8 HexNAc 2 | 89.413 | 0.009 | -3.822 | 1.159e+07 | 156 - 177 | 3 | 40.72 | 40.89 | 41.23 |
| 3722.5291 | NWAGVTQNGTSSSC(C)RRGSN(N)SSFF Hex 5 HexNAc 2 | 88.688 | 0.009 | 12.295 | 1.496e+08 | 125 - 148 | 4 | 34.17 | 34.34 | 34.83 |
| 3060.2758 | QKLPGNDN(N)STATL Hex 8 HexNAc 2 | 78.601 | 0.018 | -2.488 | 2.991e+07 | 0 - 13 | 1 | 28.38 | 28.54 | 28.71 |
| 4249.7212 | NWAGVTQNGTSSSC(C)RRGSN(N)SSFF Hex 7 HexNAc 3 | 76.478 | 0.022 | -4.528 | 2.346e+07 | 125 - 148 | 1 | 33.67 | 33.83 | 34.17 |
| 4249.7212 | NWAGVTQN(N)GTSSSC(C)RRGSNSSFF Hex 7 HexNAc 3 | 76.478 | 0.022 | -4.528 | 2.346e+07 | 125 - 148 | 1 | 33.67 | 33.83 | 34.17 |
| 3964.9087 | TIVKPGDILLIN(N)STGNLIAPRGY Hex 7 HexNAc 2 | 76.024 | 0.022 | 8.186 | 1.498e+07 | 234 - 257 | 2 | 54.53 | 54.53 | 55.21 |
| 4202.7671 | NSKYPALN(N)VTM(O)PNNEQFDKL Hex 9 HexNAc 2 | 69.972 | 0.026 | -3.440 | 1.591e+07 | 157 - 177 | 1 | 39.03 | 39.20 | 39.36 |
| 4718.0102 | THLN(D)SKYPALN(N)VTM(O)PNNEQFDKL Fuc 3 Hex 3 HexNAc 4 Neu5Ac 1 | 68.448 | 0.026 | 8.228 | 2.184e+08 | 154 - 177 | 3 | 41.06 | 41.90 | 42.57 |
| 4204.7651 | N(D)SKYPALN(N)VTM(O)PNNEQFDKLY Hex 8 HexNAc 2 | 66.845 | 0.026 | 8.701 | 2.341e+08 | 157 - 178 | 6 | 42.23 | 44.76 | 45.09 |
| 2983.4456 | TIVKPGDILLIN(N)STGNL Hex 5 HexNAc 2 | 66.138 | 0.026 | -4.297 | 1.244e+07 | 234 - 251 | 1 | 51.98 | 51.98 | 52.32 |
| 3463.4307 | AGVTQN(N)GTSSSC(C)RRGSNSSFF Hex 4 HexNAc 3 | 64.845 | 0.026 | 12.501 | 1.165e+08 | 127 - 148 | 2 | 22.57 | 23.07 | 23.56 |
| 4143.9888 | TIVKPGDILLIN(N)STGNLIAPR Fuc 3 Hex 3 HexNAc 5 | 63.860 | 0.026 | 10.797 | 3.963e+07 | 234 - 255 | 3 | 54.20 | 54.53 | 55.04 |
| 4369.8016 | N(D)SKYPALN(N)VTM(O)PNN(D)EQFDKLY Hex 4 HexNAc 6 | 62.130 | 0.026 | 0.000 | 0.000e+00 | 157 - 178 | 1 | - | - | - |
| 3723.5244 | NWAGVTQN(D)GTSSSC(C)RRGSN(N)SSFF Hex 5 HexNAc 2 | 60.645 | 0.026 | 10.331 | 3.781e+07 | 125 - 148 | 4 | 35.33 | 35.50 | 36.17 |
| 4087.6668 | NWAGVTQN(N)GTSSSC(C)RRGSNSSFF Hex 6 HexNAc 3 | 60.241 | 0.026 | 14.633 | 2.908e+07 | 125 - 148 | 2 | 33.83 | 34.00 | 34.51 |
| 4103.6484 | AGVTQN(N)GTSSSC(C)RRGSNSSFF Fuc 1 Hex 4 HexNAc 4 Neu5Ac 1 | 58.823 | 0.026 | 13.156 | 6.360e+07 | 127 - 148 | 2 | 34.51 | 34.68 | 35.16 |
| 4317.8466 | SKYPALN(N)VTM(O)PNNEQFDKLY Fuc 1 Hex 6 HexNAc 4 | 56.947 | 0.030 | 12.454 | 1.720e+08 | 158 - 178 | 7 | 43.93 | 45.09 | 45.25 |
| 3828.5630 | AGVTQN(N)GTSSSC(C)RRGSNSSFF Hex 5 HexNAc 4 | 56.844 | 0.030 | 14.210 | 1.617e+08 | 127 - 148 | 4 | 22.57 | 22.90 | 23.23 |
| 4315.8586 | LNSKYPALN(N)VTM(O)PNNEQFDKL Hex 9 HexNAc 2 | 56.170 | 0.033 | -3.050 | 1.431e+07 | 156 - 177 | 2 | 40.72 | 40.89 | 41.06 |
| 3717.6227 | NSKYPALN(N)VTM(O)PNNEQFDKLY Hex 5 HexNAc 2 | 54.928 | 0.035 | -4.837 | 1.835e+07 | 157 - 178 | 1 | 42.07 | 42.23 | 42.57 |
| 4125.9528 | TIVKPGDILLIN(N)STGNLIAPRG Hex 9 HexNAc 2 | 53.786 | 0.035 | 12.861 | 3.556e+08 | 234 - 256 | 14 | 51.30 | 52.32 | 53.86 |
| 4187.9648 | IVKPGDILLIN(N)STGNLIAPRGY Hex 9 HexNAc 2 | 51.429 | 0.041 | -19.226 | 3.066e+06 | 235 - 257 | 2 | 53.52 | 53.69 | 53.69 |
| 2840.1348 | NWAGVTQN(N)GTSSSC(C)R Hex 5 HexNAc 2 | 50.605 | 0.041 | -20.437 | 6.681e+06 | 125 - 140 | 1 | 18.34 | 18.34 | 18.50 |
| 4465.8022 | HKC(C)DNAC(C)IGSIRN(N)GTYDHDVY Fuc 1 Hex 5 HexNAc 5 | 49.349 | 0.043 | 12.328 | 5.822e+07 | 470 - 491 | 1 | 23.39 | 23.56 | 24.06 |
| 4956.0035 | N(N)VTMPNNEQFDKLY Fuc 3 Hex 8 HexNAc 6 Neu5Ac 1 | 46.646 | 0.049 | -3.189 | 1.104e+08 | 164 - 178 | 2 | 46.37 | 46.54 | 46.71 |

### LIN(N)STGNL Hex 9 HexNAc 2

**Mass:**  2695.0880

(-5.247e-07)

**Charge States:** 2

**Start Position**: 244

**End Position**: 252

| Profile Score | Tandem Score | TDA q-value | Volume | Spectrum Matches |
| --- | --- | --- | --- | --- |
| -4.4755 | 187.0098 | 0.0000 | 5.5511e+07 | 3 |

**Adduct Signal Fractions**

- **Unmodified**: 5.5511e+07

### LIN(N)STGNLIAPRGYF Fuc 2 Hex 3 HexNAc 4 Neu5Ac 1

**Mass:**  3519.5614

(8.520e-04)

**Start Position**: 244

**End Position**: 259

| Profile Score | Tandem Score | TDA q-value | Volume | Spectrum Matches |
| --- | --- | --- | --- | --- |
| 0.0000 | 184.2809 | 0.0000 | 0.0000e+00 | 1 |

Adduct:

No Chromatogram Found

### LIN(N)STGNLIAPR Hex 9 HexNAc 2

**Mass:**  3132.3726

(4.283e-06)

**Charge States:** 3

**Start Position**: 244

**End Position**: 256

| Profile Score | Tandem Score | TDA q-value | Volume | Spectrum Matches |
| --- | --- | --- | --- | --- |
| -2.7526 | 181.6060 | 0.0000 | 5.1350e+07 | 2 |

**Adduct Signal Fractions**

- **Unmodified**: 5.1350e+07

### LIN(N)STGNLIAPRGYF Hex 9 HexNAc 2

**Mass:**  3499.5177

(1.473e-06)

**Charge States:** 3

**Start Position**: 244

**End Position**: 259

| Profile Score | Tandem Score | TDA q-value | Volume | Spectrum Matches |
| --- | --- | --- | --- | --- |
| 12.6181 | 181.2677 | 0.0000 | 3.2159e+08 | 5 |

**Adduct Signal Fractions**

- **Unmodified**: 3.2159e+08

### LIN(N)STGNL Fuc 2 Hex 3 HexNAc 4 Neu5Ac 1

**Mass:**  2712.1148

(-8.127e-06)

**Charge States:** 2, 3

**Start Position**: 244

**End Position**: 252

| Profile Score | Tandem Score | TDA q-value | Volume | Spectrum Matches |
| --- | --- | --- | --- | --- |
| -4.5693 | 180.4256 | 0.0000 | 1.8406e+07 | 2 |

**Adduct Signal Fractions**

- **Unmodified**: 1.8406e+07

### LIN(N)STGNLIAPRGYF Fuc 2 Hex 3 HexNAc 4 Neu5Ac 1

**Mass:**  3516.5447

(-5.759e-06)

**Charge States:** 3

**Start Position**: 244

**End Position**: 259

| Profile Score | Tandem Score | TDA q-value | Volume | Spectrum Matches |
| --- | --- | --- | --- | --- |
| 13.7399 | 180.3994 | 0.0000 | 1.5715e+08 | 2 |

**Adduct Signal Fractions**

- **Unmodified**: 1.5715e+08

### LIN(N)STGNLIAPRGY Hex 9 HexNAc 2

**Mass:**  3352.4394

(-1.793e-06)

**Charge States:** 3

**Start Position**: 244

**End Position**: 258

| Profile Score | Tandem Score | TDA q-value | Volume | Spectrum Matches |
| --- | --- | --- | --- | --- |
| 14.8987 | 178.6124 | 0.0000 | 2.5796e+08 | 3 |

**Adduct Signal Fractions**

- **Unmodified**: 2.5796e+08

### LIN(N)STGNLIAPRGY Fuc 2 Hex 3 HexNAc 4 Neu5Ac 1

**Mass:**  3369.4656

(-9.097e-06)

**Charge States:** 3

**Start Position**: 244

**End Position**: 258

| Profile Score | Tandem Score | TDA q-value | Volume | Spectrum Matches |
| --- | --- | --- | --- | --- |
| 15.5756 | 174.4817 | 0.0000 | 1.2755e+08 | 4 |

**Adduct Signal Fractions**

- **Unmodified**: 1.2755e+08

### TIVKPGDILLIN(N)STGNLIAPR Hex 9 HexNAc 2

**Mass:**  4068.9389

(3.100e-06)

**Charge States:** 3, 4, 5

**Start Position**: 235

**End Position**: 256

| Profile Score | Tandem Score | TDA q-value | Volume | Spectrum Matches |
| --- | --- | --- | --- | --- |
| 15.8551 | 172.8858 | 0.0000 | 1.5225e+09 | 23 |

**Adduct Signal Fractions**

- **Unmodified**: 1.5225e+09

### LIN(N)STGNLIAPR Fuc 2 Hex 3 HexNAc 4 Neu5Ac 1

**Mass:**  3149.3981

(-4.474e-06)

**Charge States:** 3

**Start Position**: 244

**End Position**: 256

| Profile Score | Tandem Score | TDA q-value | Volume | Spectrum Matches |
| --- | --- | --- | --- | --- |
| -3.9336 | 171.6733 | 0.0000 | 1.9709e+07 | 1 |

**Adduct Signal Fractions**

- **Unmodified**: 1.9709e+07

### LIN(N)STGNLIAPRGYF Hex 8 HexNAc 2

**Mass:**  3337.4661

(2.002e-06)

**Charge States:** 3

**Start Position**: 244

**End Position**: 259

| Profile Score | Tandem Score | TDA q-value | Volume | Spectrum Matches |
| --- | --- | --- | --- | --- |
| -19.5515 | 171.0841 | 0.0000 | 1.8940e+07 | 1 |

**Adduct Signal Fractions**

- **Unmodified**: 1.8940e+07

### AGVTQN(N)GTSSSC(C)RRGSNSSFF Hex 8 HexNAc 2

**Mass:**  3908.5728

(4.332e-07)

**Charge States:** 3

**Start Position**: 128

**End Position**: 149

| Profile Score | Tandem Score | TDA q-value | Volume | Spectrum Matches |
| --- | --- | --- | --- | --- |
| -3.6221 | 170.1469 | 0.0000 | 9.7835e+07 | 1 |

**Adduct Signal Fractions**

- **Unmodified**: 9.7835e+07

### LIN(N)STGNLIAPRGYF Hex 10 HexNAc 2

**Mass:**  3661.5727

(2.071e-06)

**Charge States:** 3

**Start Position**: 244

**End Position**: 259

| Profile Score | Tandem Score | TDA q-value | Volume | Spectrum Matches |
| --- | --- | --- | --- | --- |
| -19.9664 | 168.0829 | 0.0000 | 2.4151e+07 | 1 |

**Adduct Signal Fractions**

- **Unmodified**: 2.4151e+07

### AGVTQN(N)GTSSSC(C)RRGSNSSFF Hex 7 HexNAc 2

**Mass:**  3746.5182

(-3.845e-08)

**Charge States:** 3, 4

**Start Position**: 128

**End Position**: 149

| Profile Score | Tandem Score | TDA q-value | Volume | Spectrum Matches |
| --- | --- | --- | --- | --- |
| 15.8275 | 168.0228 | 0.0000 | 1.0031e+09 | 5 |

**Adduct Signal Fractions**

- **Unmodified**: 1.0031e+09

### IN(N)STGNLIAPRGYF Hex 9 HexNAc 2

**Mass:**  3386.4358

(4.344e-06)

**Charge States:** 3

**Start Position**: 245

**End Position**: 259

| Profile Score | Tandem Score | TDA q-value | Volume | Spectrum Matches |
| --- | --- | --- | --- | --- |
| -19.9417 | 165.8495 | 0.0000 | 1.3326e+07 | 1 |

**Adduct Signal Fractions**

- **Unmodified**: 1.3326e+07

### TIVKPGDILLIN(N)STGNLIAPRGY Hex 8 HexNAc 2

**Mass:**  4126.9660

(6.861e-07)

**Charge States:** 3, 4

**Start Position**: 235

**End Position**: 258

| Profile Score | Tandem Score | TDA q-value | Volume | Spectrum Matches |
| --- | --- | --- | --- | --- |
| 12.9524 | 164.6691 | 0.0000 | 2.8420e+08 | 15 |

**Adduct Signal Fractions**

- **Unmodified**: 2.8420e+08

### AGVTQN(N)GTSSSC(C)RRGSNSSFF Hex 5 HexNAc 2

**Mass:**  3422.4021

(1.194e-06)

**Charge States:** 3

**Start Position**: 128

**End Position**: 149

| Profile Score | Tandem Score | TDA q-value | Volume | Spectrum Matches |
| --- | --- | --- | --- | --- |
| 14.8897 | 164.1548 | 0.0000 | 5.1430e+08 | 4 |

**Adduct Signal Fractions**

- **Unmodified**: 5.1430e+08

### NWAGVTQN(N)GTSS Hex 7 HexNAc 2

**Mass:**  2761.0651

(6.255e-07)

**Charge States:** 2

**Start Position**: 126

**End Position**: 138

| Profile Score | Tandem Score | TDA q-value | Volume | Spectrum Matches |
| --- | --- | --- | --- | --- |
| -4.9989 | 161.6361 | 0.0000 | 2.1031e+07 | 2 |

**Adduct Signal Fractions**

- **Unmodified**: 2.1031e+07

### TIVKPGDILLIN(N) Hex 9 HexNAc 2

**Mass:**  3159.4260

(1.905e-06)

**Charge States:** 3

**Start Position**: 235

**End Position**: 247

| Profile Score | Tandem Score | TDA q-value | Volume | Spectrum Matches |
| --- | --- | --- | --- | --- |
| 12.7786 | 159.5551 | 0.0000 | 1.8147e+08 | 4 |

**Adduct Signal Fractions**

- **Unmodified**: 1.8147e+08

### TIVKPGDILLIN(N)STGNLIAPR Hex 10 HexNAc 2

**Mass:**  4230.9846

(1.201e-06)

**Charge States:** 3, 4

**Start Position**: 235

**End Position**: 256

| Profile Score | Tandem Score | TDA q-value | Volume | Spectrum Matches |
| --- | --- | --- | --- | --- |
| 13.5264 | 158.4794 | 0.0000 | 1.2736e+08 | 5 |

**Adduct Signal Fractions**

- **Unmodified**: 1.2736e+08

### TIVKPGDILLIN(N)STGNLIAPRGY Hex 9 HexNAc 2

**Mass:**  4289.0178

(1.029e-06)

**Charge States:** 3, 4, 5

**Start Position**: 235

**End Position**: 258

| Profile Score | Tandem Score | TDA q-value | Volume | Spectrum Matches |
| --- | --- | --- | --- | --- |
| 16.2234 | 158.2527 | 0.0000 | 2.9890e+09 | 37 |

**Adduct Signal Fractions**

- **Unmodified**: 2.9890e+09

### NSKYPALN(N)VTM(O)PNNEQFDKLY Hex 9 HexNAc 2

**Mass:**  4365.8292

(4.122e-06)

**Charge States:** 3, 4

**Start Position**: 158

**End Position**: 179

| Profile Score | Tandem Score | TDA q-value | Volume | Spectrum Matches |
| --- | --- | --- | --- | --- |
| 14.5099 | 157.9540 | 0.0000 | 3.7886e+08 | 4 |

**Adduct Signal Fractions**

- **Unmodified**: 3.7886e+08

### LIN(N)STGNLIAPRGY Hex 10 HexNAc 2

**Mass:**  3514.4932

(-1.017e-06)

**Charge States:** 3

**Start Position**: 244

**End Position**: 258

| Profile Score | Tandem Score | TDA q-value | Volume | Spectrum Matches |
| --- | --- | --- | --- | --- |
| -5.1898 | 157.8333 | 0.0000 | 2.0903e+07 | 1 |

**Adduct Signal Fractions**

- **Unmodified**: 2.0903e+07

### TIVKPGDILLIN(N)STGNLIAPR Fuc 2 Hex 3 HexNAc 4 Neu5Ac 1

**Mass:**  4085.9543

(-4.642e-06)

**Charge States:** 4

**Start Position**: 235

**End Position**: 256

| Profile Score | Tandem Score | TDA q-value | Volume | Spectrum Matches |
| --- | --- | --- | --- | --- |
| 12.9436 | 157.4613 | 0.0000 | 2.2826e+08 | 4 |

**Adduct Signal Fractions**

- **Unmodified**: 2.2826e+08

### TIVKPGDILLIN(N) Fuc 2 Hex 3 HexNAc 4 Neu5Ac 1

**Mass:**  3176.4538

(-6.272e-06)

**Charge States:** 3

**Start Position**: 235

**End Position**: 247

| Profile Score | Tandem Score | TDA q-value | Volume | Spectrum Matches |
| --- | --- | --- | --- | --- |
| 11.8681 | 155.4858 | 0.0000 | 8.8711e+07 | 3 |

**Adduct Signal Fractions**

- **Unmodified**: 8.8711e+07

### TIVKPGDILLIN(N)STGNLIAPR Hex 8 HexNAc 2

**Mass:**  3906.8780

(1.988e-06)

**Charge States:** 3, 4

**Start Position**: 235

**End Position**: 256

| Profile Score | Tandem Score | TDA q-value | Volume | Spectrum Matches |
| --- | --- | --- | --- | --- |
| 12.8856 | 155.2597 | 0.0000 | 1.1159e+08 | 4 |

**Adduct Signal Fractions**

- **Unmodified**: 1.1159e+08

### TIVKPGDILLIN(N)STGNL Fuc 2 Hex 3 HexNAc 4 Neu5Ac 1

**Mass:**  3648.6839

(-4.438e-06)

**Charge States:** 3

**Start Position**: 235

**End Position**: 252

| Profile Score | Tandem Score | TDA q-value | Volume | Spectrum Matches |
| --- | --- | --- | --- | --- |
| 13.2488 | 152.4863 | 0.0000 | 4.8207e+08 | 6 |

**Adduct Signal Fractions**

- **Unmodified**: 4.8207e+08

### LIN(N)STGNL Hex 10 HexNAc 2

**Mass:**  2857.1356

(-4.044e-07)

**Charge States:** 2

**Start Position**: 244

**End Position**: 252

| Profile Score | Tandem Score | TDA q-value | Volume | Spectrum Matches |
| --- | --- | --- | --- | --- |
| -20.0280 | 152.4600 | 0.0000 | 1.6254e+06 | 1 |

**Adduct Signal Fractions**

- **Unmodified**: 1.6254e+06

### TIVKPGDILLIN(N)STGNLIAPRGY Fuc 2 Hex 3 HexNAc 4 Neu5Ac 1

**Mass:**  4306.0358

(-5.264e-06)

**Charge States:** 3, 4

**Start Position**: 235

**End Position**: 258

| Profile Score | Tandem Score | TDA q-value | Volume | Spectrum Matches |
| --- | --- | --- | --- | --- |
| 13.9184 | 152.2383 | 0.0000 | 5.0744e+08 | 9 |

**Adduct Signal Fractions**

- **Unmodified**: 5.0744e+08

### AGVTQN(N)GTSSSC(C)RRGSNSSFF Hex 6 HexNAc 2

**Mass:**  3584.4596

(6.912e-07)

**Charge States:** 3, 4

**Start Position**: 128

**End Position**: 149

| Profile Score | Tandem Score | TDA q-value | Volume | Spectrum Matches |
| --- | --- | --- | --- | --- |
| 14.2794 | 151.7607 | 0.0000 | 3.0449e+08 | 3 |

**Adduct Signal Fractions**

- **Unmodified**: 3.0449e+08

### TIVKPGDILLIN(N)STGNL Hex 9 HexNAc 2

**Mass:**  3631.6620

(3.072e-06)

**Charge States:** 2, 3, 4

**Start Position**: 235

**End Position**: 252

| Profile Score | Tandem Score | TDA q-value | Volume | Spectrum Matches |
| --- | --- | --- | --- | --- |
| 15.8919 | 150.8255 | 0.0000 | 9.4617e+08 | 13 |

**Adduct Signal Fractions**

- **Unmodified**: 9.4617e+08

### TITNDRIEVTN(N)ATEL Hex 5 HexNAc 2

**Mass:**  2905.2775

(-1.635e-06)

**Charge States:** 3

**Start Position**: 28

**End Position**: 43

| Profile Score | Tandem Score | TDA q-value | Volume | Spectrum Matches |
| --- | --- | --- | --- | --- |
| -17.7280 | 150.6112 | 0.0000 | 2.7045e+07 | 1 |

**Adduct Signal Fractions**

- **Unmodified**: 2.7045e+07

### C(C)LGHHAVPN(N)GTIVK Fuc 1 Hex 5 HexNAc 5

**Mass:**  3473.5036

(1.555e-06)

**Charge States:** 4

**Start Position**: 14

**End Position**: 28

| Profile Score | Tandem Score | TDA q-value | Volume | Spectrum Matches |
| --- | --- | --- | --- | --- |
| -5.1148 | 149.8736 | 0.0000 | 1.7076e+07 | 1 |

**Adduct Signal Fractions**

- **Unmodified**: 1.7076e+07

### SKYPALN(N)VTM(O)PNNEQFDKLY Hex 9 HexNAc 2

**Mass:**  4251.7864

(1.558e-06)

**Charge States:** 3, 4

**Start Position**: 159

**End Position**: 179

| Profile Score | Tandem Score | TDA q-value | Volume | Spectrum Matches |
| --- | --- | --- | --- | --- |
| 13.0670 | 148.9725 | 0.0000 | 1.2201e+08 | 3 |

**Adduct Signal Fractions**

- **Unmodified**: 1.2201e+08

### RSDAPIGKC(C)KSEC(C)ITPN(N)GSIPNDKPF Hex 9 HexNAc 2

**Mass:**  4752.0428

(2.746e-06)

**Charge States:** 4, 5

**Start Position**: 269

**End Position**: 295

| Profile Score | Tandem Score | TDA q-value | Volume | Spectrum Matches |
| --- | --- | --- | --- | --- |
| 14.9680 | 147.7730 | 0.0000 | 2.4001e+08 | 4 |

**Adduct Signal Fractions**

- **Unmodified**: 2.4001e+08

### TIVKPGDILLIN(N)STGNLIAPRGY Hex 10 HexNAc 2

**Mass:**  4451.0666

(9.641e-07)

**Charge States:** 3, 4

**Start Position**: 235

**End Position**: 258

| Profile Score | Tandem Score | TDA q-value | Volume | Spectrum Matches |
| --- | --- | --- | --- | --- |
| 13.3896 | 146.9894 | 0.0000 | 2.7446e+08 | 11 |

**Adduct Signal Fractions**

- **Unmodified**: 2.7446e+08

### TIVKPGDILLIN(N)STGNL Hex 10 HexNAc 2

**Mass:**  3793.7103

(4.576e-06)

**Charge States:** 3

**Start Position**: 235

**End Position**: 252

| Profile Score | Tandem Score | TDA q-value | Volume | Spectrum Matches |
| --- | --- | --- | --- | --- |
| 14.5582 | 145.7781 | 0.0000 | 7.5860e+07 | 4 |

**Adduct Signal Fractions**

- **Unmodified**: 7.5860e+07

### SKYPALN(N)VTM(O)PNNEQFDKLY Hex 8 HexNAc 2

**Mass:**  4089.7362

(1.963e-06)

**Charge States:** 3, 4

**Start Position**: 159

**End Position**: 179

| Profile Score | Tandem Score | TDA q-value | Volume | Spectrum Matches |
| --- | --- | --- | --- | --- |
| 13.2936 | 145.5325 | 0.0000 | 1.2351e+08 | 3 |

**Adduct Signal Fractions**

- **Unmodified**: 1.2351e+08

### TIVKPGDILLIN(N)STGNLIAPR Fuc 2 Hex 4 HexNAc 4 Neu5Ac 1

**Mass:**  4248.0104

(-3.409e-06)

**Charge States:** 4

**Start Position**: 235

**End Position**: 256

| Profile Score | Tandem Score | TDA q-value | Volume | Spectrum Matches |
| --- | --- | --- | --- | --- |
| -4.2854 | 145.3469 | 0.0000 | 1.9184e+07 | 1 |

**Adduct Signal Fractions**

- **Unmodified**: 1.9184e+07

### NSKYPALN(N)VTM(O)PNNEQFDKLY Hex 8 HexNAc 2

**Mass:**  4203.7783

(2.653e-06)

**Charge States:** 3, 4

**Start Position**: 158

**End Position**: 179

| Profile Score | Tandem Score | TDA q-value | Volume | Spectrum Matches |
| --- | --- | --- | --- | --- |
| 16.1248 | 144.0520 | 0.0000 | 4.2577e+08 | 5 |

**Adduct Signal Fractions**

- **Unmodified**: 4.2577e+08

### NWAGVTQN(N)GTSSSC(C)R Hex 7 HexNAc 2

**Mass:**  3164.2366

(6.918e-07)

**Charge States:** 3

**Start Position**: 126

**End Position**: 141

| Profile Score | Tandem Score | TDA q-value | Volume | Spectrum Matches |
| --- | --- | --- | --- | --- |
| -18.2778 | 143.7366 | 0.0000 | 1.3570e+07 | 1 |

**Adduct Signal Fractions**

- **Unmodified**: 1.3570e+07

### TIVKPGDILLIN(N)STGNLIAPRGY Fuc 2 Hex 4 HexNAc 4 Neu5Ac 1

**Mass:**  4470.1257

(4.502e-04)

**Start Position**: 235

**End Position**: 258

| Profile Score | Tandem Score | TDA q-value | Volume | Spectrum Matches |
| --- | --- | --- | --- | --- |
| 0.0000 | 142.8058 | 0.0000 | 0.0000e+00 | 1 |

Adduct:

No Chromatogram Found

### LIN(N)STGNLIAPRGY Fuc 2 Hex 4 HexNAc 4 Neu5Ac 1

**Mass:**  3533.5495

(5.602e-04)

**Charge States:** 4

**Start Position**: 244

**End Position**: 258

| Profile Score | Tandem Score | TDA q-value | Volume | Spectrum Matches |
| --- | --- | --- | --- | --- |
| -7.4101 | 142.2004 | 0.0000 | 1.5235e+07 | 3 |

**Adduct Signal Fractions**

- **Unmodified**: 1.5235e+07

### RGSN(N)SSFF Hex 5 HexNAc 2

**Mass:**  2116.8292

(-1.270e-06)

**Charge States:** 2

**Start Position**: 141

**End Position**: 149

| Profile Score | Tandem Score | TDA q-value | Volume | Spectrum Matches |
| --- | --- | --- | --- | --- |
| -3.9173 | 139.7489 | 0.0000 | 1.3215e+07 | 1 |

**Adduct Signal Fractions**

- **Unmodified**: 1.3215e+07

### LIN(N)STGNLIAPRGY Hex 8 HexNAc 2

**Mass:**  3190.3969

(-4.509e-07)

**Charge States:** 3

**Start Position**: 244

**End Position**: 258

| Profile Score | Tandem Score | TDA q-value | Volume | Spectrum Matches |
| --- | --- | --- | --- | --- |
| -19.7388 | 139.1957 | 0.0000 | 1.3623e+07 | 1 |

**Adduct Signal Fractions**

- **Unmodified**: 1.3623e+07

### TIVKPGDILLIN(N)STGNLIAPR Hex 5 HexNAc 2

**Mass:**  3420.7157

(2.332e-06)

**Charge States:** 3, 4

**Start Position**: 235

**End Position**: 256

| Profile Score | Tandem Score | TDA q-value | Volume | Spectrum Matches |
| --- | --- | --- | --- | --- |
| -3.8936 | 139.0961 | 0.0000 | 2.0959e+07 | 3 |

**Adduct Signal Fractions**

- **Unmodified**: 2.0959e+07

### TIVKPGDILLIN(N)STGNLIAPRGY Fuc 2 Hex 4 HexNAc 4 Neu5Ac 1

**Mass:**  4468.0949

(-4.507e-06)

**Charge States:** 4

**Start Position**: 235

**End Position**: 258

| Profile Score | Tandem Score | TDA q-value | Volume | Spectrum Matches |
| --- | --- | --- | --- | --- |
| -4.5279 | 138.5714 | 0.0000 | 4.8839e+07 | 2 |

**Adduct Signal Fractions**

- **Unmodified**: 4.8839e+07

### TITNDRIEVTN(N)ATEL Hex 6 HexNAc 2

**Mass:**  3067.3339

(2.711e-06)

**Charge States:** 3

**Start Position**: 28

**End Position**: 43

| Profile Score | Tandem Score | TDA q-value | Volume | Spectrum Matches |
| --- | --- | --- | --- | --- |
| -19.6955 | 138.1675 | 0.0000 | 1.5220e+07 | 1 |

**Adduct Signal Fractions**

- **Unmodified**: 1.5220e+07

### TIVKPGDILLIN(N)STGNL Hex 8 HexNAc 2

**Mass:**  3469.6100

(2.627e-06)

**Charge States:** 3, 4

**Start Position**: 235

**End Position**: 252

| Profile Score | Tandem Score | TDA q-value | Volume | Spectrum Matches |
| --- | --- | --- | --- | --- |
| 14.0813 | 137.3519 | 0.0000 | 7.2556e+07 | 4 |

**Adduct Signal Fractions**

- **Unmodified**: 7.2556e+07

### NWAGVTQN(N)GTSSSC(C) Hex 7 HexNAc 2

**Mass:**  3008.1326

(1.333e-06)

**Charge States:** 3

**Start Position**: 126

**End Position**: 140

| Profile Score | Tandem Score | TDA q-value | Volume | Spectrum Matches |
| --- | --- | --- | --- | --- |
| -4.4253 | 135.8544 | 0.0000 | 1.7815e+07 | 1 |

**Adduct Signal Fractions**

- **Unmodified**: 1.7815e+07

### TIVKPGDILLIN(N)STGNLIAPRGY Hex 5 HexNAc 2

**Mass:**  3640.8026

(1.587e-06)

**Charge States:** 3, 4

**Start Position**: 235

**End Position**: 258

| Profile Score | Tandem Score | TDA q-value | Volume | Spectrum Matches |
| --- | --- | --- | --- | --- |
| 12.7044 | 134.7194 | 0.0000 | 4.1295e+07 | 5 |

**Adduct Signal Fractions**

- **Unmodified**: 4.1295e+07

### NWAGVTQN(N)GTSS Hex 5 HexNAc 2

**Mass:**  2436.9681

(1.228e-06)

**Charge States:** 2

**Start Position**: 126

**End Position**: 138

| Profile Score | Tandem Score | TDA q-value | Volume | Spectrum Matches |
| --- | --- | --- | --- | --- |
| -20.2789 | 134.3278 | 0.0000 | 8.0076e+06 | 1 |

**Adduct Signal Fractions**

- **Unmodified**: 8.0076e+06

### NWAGVTQN(N)GTSSS Hex 7 HexNAc 2

**Mass:**  2849.1019

(3.508e-04)

**Start Position**: 126

**End Position**: 139

| Profile Score | Tandem Score | TDA q-value | Volume | Spectrum Matches |
| --- | --- | --- | --- | --- |
| 0.0000 | 134.0177 | 0.0000 | 0.0000e+00 | 1 |

Adduct:

No Chromatogram Found

### EFNN(N)ESFNW Hex 6 HexNAc 2

**Mass:**  2563.9510

(1.055e-06)

**Charge States:** 2

**Start Position**: 119

**End Position**: 128

| Profile Score | Tandem Score | TDA q-value | Volume | Spectrum Matches |
| --- | --- | --- | --- | --- |
| -4.3964 | 133.3713 | 0.0000 | 5.3272e+06 | 2 |

**Adduct Signal Fractions**

- **Unmodified**: 5.3272e+06

### RSDAPIGKC(C)KSEC(C)ITPN(N)GSIPNDKPF Hex 8 HexNAc 2

**Mass:**  4589.9868

(2.128e-06)

**Charge States:** 4, 5

**Start Position**: 269

**End Position**: 295

| Profile Score | Tandem Score | TDA q-value | Volume | Spectrum Matches |
| --- | --- | --- | --- | --- |
| -3.9876 | 133.1961 | 0.0000 | 9.9225e+07 | 2 |

**Adduct Signal Fractions**

- **Unmodified**: 9.9225e+07

### RSDAPIGKC(C)KSEC(C)ITPN(N)GSIPNDKPF Hex 7 HexNAc 2

**Mass:**  4427.9354

(2.685e-06)

**Charge States:** 4, 5

**Start Position**: 269

**End Position**: 295

| Profile Score | Tandem Score | TDA q-value | Volume | Spectrum Matches |
| --- | --- | --- | --- | --- |
| -4.1135 | 131.1753 | 0.0000 | 1.1037e+08 | 3 |

**Adduct Signal Fractions**

- **Unmodified**: 1.1037e+08

### SIRN(N)GTYDHDVY Fuc 1 Hex 5 HexNAc 4

**Mass:**  3207.2938

(2.016e-06)

**Charge States:** 3

**Start Position**: 480

**End Position**: 492

| Profile Score | Tandem Score | TDA q-value | Volume | Spectrum Matches |
| --- | --- | --- | --- | --- |
| -18.1549 | 129.7864 | 0.0000 | 1.2825e+07 | 1 |

**Adduct Signal Fractions**

- **Unmodified**: 1.2825e+07

### TIVKPGDILLIN(N)STGNL Fuc 2 Hex 4 HexNAc 4 Neu5Ac 1

**Mass:**  3810.7344

(-5.221e-06)

**Charge States:** 3

**Start Position**: 235

**End Position**: 252

| Profile Score | Tandem Score | TDA q-value | Volume | Spectrum Matches |
| --- | --- | --- | --- | --- |
| -18.5149 | 129.1345 | 0.0000 | 1.8720e+07 | 1 |

**Adduct Signal Fractions**

- **Unmodified**: 1.8720e+07

### TIVKPGDILLIN(N)STGN Hex 9 HexNAc 2

**Mass:**  3518.5761

(3.705e-06)

**Charge States:** 3

**Start Position**: 235

**End Position**: 251

| Profile Score | Tandem Score | TDA q-value | Volume | Spectrum Matches |
| --- | --- | --- | --- | --- |
| 14.3635 | 128.2265 | 0.0000 | 1.8229e+08 | 3 |

**Adduct Signal Fractions**

- **Unmodified**: 1.8229e+08

### NWAGVTQNGTSSSC(C)RRGSN(N)SSFF Hex 6 HexNAc 2

**Mass:**  3884.5871

(2.599e-06)

**Charge States:** 3, 4

**Start Position**: 126

**End Position**: 149

| Profile Score | Tandem Score | TDA q-value | Volume | Spectrum Matches |
| --- | --- | --- | --- | --- |
| -3.8072 | 126.5980 | 0.0000 | 4.8869e+07 | 3 |

**Adduct Signal Fractions**

- **Unmodified**: 4.8869e+07

### HKC(C)DNAC(C)IGSIRN(N)GTYDHDVY Fuc 1 Hex 5 HexNAc 4

**Mass:**  4262.7204

(1.320e-06)

**Charge States:** 4, 5

**Start Position**: 471

**End Position**: 492

| Profile Score | Tandem Score | TDA q-value | Volume | Spectrum Matches |
| --- | --- | --- | --- | --- |
| -3.8887 | 126.0101 | 0.0000 | 1.0995e+08 | 2 |

**Adduct Signal Fractions**

- **Unmodified**: 1.0995e+08

### AGVTQN(N)GTSSSC(C)RRGSN(D)SSFF Hex 5 HexNAc 2

**Mass:**  3423.3942

(2.918e-04)

**Charge States:** 3

**Start Position**: 128

**End Position**: 149

| Profile Score | Tandem Score | TDA q-value | Volume | Spectrum Matches |
| --- | --- | --- | --- | --- |
| 10.7085 | 121.0723 | 0.0000 | 2.0221e+08 | 3 |

**Adduct Signal Fractions**

- **Unmodified**: 2.0221e+08

### AGVTQNGTSSSC(C)RRGSN(N)SSFF Fuc 1 Hex 4 HexNAc 3

**Mass:**  3609.4832

(-1.707e-06)

**Charge States:** 3

**Start Position**: 128

**End Position**: 149

| Profile Score | Tandem Score | TDA q-value | Volume | Spectrum Matches |
| --- | --- | --- | --- | --- |
| 12.4851 | 120.7262 | 0.0000 | 5.6108e+07 | 1 |

**Adduct Signal Fractions**

- **Unmodified**: 5.6108e+07

### NSKYPALN(N)VTM(O)PNNEQFDKL Hex 8 HexNAc 2

**Mass:**  4040.7156

(1.921e-06)

**Charge States:** 3, 4

**Start Position**: 158

**End Position**: 178

| Profile Score | Tandem Score | TDA q-value | Volume | Spectrum Matches |
| --- | --- | --- | --- | --- |
| -3.9594 | 119.9012 | 0.0000 | 1.4651e+07 | 1 |

**Adduct Signal Fractions**

- **Unmodified**: 1.4651e+07

### TITNDRIEVTN(N)ATEL Hex 6 HexNAc 3

**Mass:**  3270.4037

(2.505e-06)

**Charge States:** 3

**Start Position**: 28

**End Position**: 43

| Profile Score | Tandem Score | TDA q-value | Volume | Spectrum Matches |
| --- | --- | --- | --- | --- |
| -5.6094 | 115.2853 | 0.0000 | 1.6480e+07 | 1 |

**Adduct Signal Fractions**

- **Unmodified**: 1.6480e+07

### TIVKPGDILLIN(N) Hex 8 HexNAc 2

**Mass:**  2997.3821

(4.885e-06)

**Charge States:** 3

**Start Position**: 235

**End Position**: 247

| Profile Score | Tandem Score | TDA q-value | Volume | Spectrum Matches |
| --- | --- | --- | --- | --- |
| -18.5813 | 109.7905 | 0.0051 | 6.9837e+06 | 1 |

**Adduct Signal Fractions**

- **Unmodified**: 6.9837e+06

### TIVKPGDILLIN(N)STGN(D)LIAPRGY Hex 9 HexNAc 2

**Mass:**  4290.0082

(2.332e-04)

**Charge States:** 3, 5

**Start Position**: 235

**End Position**: 258

| Profile Score | Tandem Score | TDA q-value | Volume | Spectrum Matches |
| --- | --- | --- | --- | --- |
| -18.8818 | 109.7598 | 0.0051 | 1.3863e+07 | 4 |

**Adduct Signal Fractions**

- **Unmodified**: 1.3863e+07

### AGVTQN(N)GTSSSC(C)RRGSN(D)SSFF Hex 7 HexNAc 2

**Mass:**  3747.4986

(2.636e-04)

**Charge States:** 3, 4

**Start Position**: 128

**End Position**: 149

| Profile Score | Tandem Score | TDA q-value | Volume | Spectrum Matches |
| --- | --- | --- | --- | --- |
| 10.8941 | 108.7688 | 0.0051 | 3.7569e+08 | 6 |

**Adduct Signal Fractions**

- **Unmodified**: 3.7569e+08

### N(N)VTMPNNEQF Hex 7 HexNAc 2

**Mass:**  2733.0353

(-4.229e-06)

**Charge States:** 3

**Start Position**: 165

**End Position**: 175

| Profile Score | Tandem Score | TDA q-value | Volume | Spectrum Matches |
| --- | --- | --- | --- | --- |
| -20.6485 | 99.7879 | 0.0051 | 6.3122e+06 | 1 |

**Adduct Signal Fractions**

- **Unmodified**: 6.3122e+06

### AGVTQN(N)GTSSSC(C)RRGSNSSFF Hex 3 HexNAc 2

**Mass:**  3098.3131

(3.476e-06)

**Charge States:** 3

**Start Position**: 128

**End Position**: 149

| Profile Score | Tandem Score | TDA q-value | Volume | Spectrum Matches |
| --- | --- | --- | --- | --- |
| -18.5473 | 99.4528 | 0.0051 | 2.6461e+07 | 4 |

**Adduct Signal Fractions**

- **Unmodified**: 2.6461e+07

### LIN(N)STGN(D)LIAPRGYF Hex 9 HexNAc 2

**Mass:**  3500.5054

(2.838e-04)

**Charge States:** 3

**Start Position**: 244

**End Position**: 259

| Profile Score | Tandem Score | TDA q-value | Volume | Spectrum Matches |
| --- | --- | --- | --- | --- |
| -5.9321 | 98.0669 | 0.0051 | 8.5694e+06 | 1 |

**Adduct Signal Fractions**

- **Unmodified**: 8.5694e+06

### N(D)SKYPALN(N)VTM(O)PNNEQFDKLY Hex 9 HexNAc 2

**Mass:**  4366.8107

(2.267e-04)

**Charge States:** 3, 4

**Start Position**: 158

**End Position**: 179

| Profile Score | Tandem Score | TDA q-value | Volume | Spectrum Matches |
| --- | --- | --- | --- | --- |
| 9.1020 | 96.9383 | 0.0051 | 2.2349e+08 | 5 |

**Adduct Signal Fractions**

- **Unmodified**: 2.2349e+08

### QKLPGNDN(N)STATL Hex 3 HexNAc 3 Neu5Ac 1

**Mass:**  2744.1718

(-1.203e-06)

**Charge States:** 2, 3

**Start Position**: 1

**End Position**: 14

| Profile Score | Tandem Score | TDA q-value | Volume | Spectrum Matches |
| --- | --- | --- | --- | --- |
| 13.1955 | 95.9338 | 0.0051 | 3.2201e+08 | 4 |

**Adduct Signal Fractions**

- **Unmodified**: 3.2201e+08

### NWAGVTQN(N)GTSSSC(C)RRGSNSSFF Hex 7 HexNAc 2

**Mass:**  4046.6391

(2.586e-06)

**Charge States:** 3, 4

**Start Position**: 126

**End Position**: 149

| Profile Score | Tandem Score | TDA q-value | Volume | Spectrum Matches |
| --- | --- | --- | --- | --- |
| 13.2583 | 94.0063 | 0.0051 | 1.6214e+08 | 3 |

**Adduct Signal Fractions**

- **Unmodified**: 1.6214e+08

### TIVKPGDILLIN(N)STGNLIAPRGY Hex 6 HexNAc 2

**Mass:**  3802.8568

(9.530e-07)

**Charge States:** 3, 4

**Start Position**: 235

**End Position**: 258

| Profile Score | Tandem Score | TDA q-value | Volume | Spectrum Matches |
| --- | --- | --- | --- | --- |
| -4.5895 | 92.4626 | 0.0094 | 1.3872e+07 | 2 |

**Adduct Signal Fractions**

- **Unmodified**: 1.3872e+07

### LNSKYPALN(N)VTM(O)PNNEQFDKL Hex 8 HexNAc 2

**Mass:**  4153.8048

(5.236e-06)

**Charge States:** 3, 4

**Start Position**: 157

**End Position**: 178

| Profile Score | Tandem Score | TDA q-value | Volume | Spectrum Matches |
| --- | --- | --- | --- | --- |
| -3.8222 | 89.4125 | 0.0094 | 1.1591e+07 | 3 |

**Adduct Signal Fractions**

- **Unmodified**: 1.1591e+07

### NWAGVTQNGTSSSC(C)RRGSN(N)SSFF Hex 5 HexNAc 2

**Mass:**  3722.5303

(5.669e-07)

**Charge States:** 3

**Start Position**: 126

**End Position**: 149

| Profile Score | Tandem Score | TDA q-value | Volume | Spectrum Matches |
| --- | --- | --- | --- | --- |
| 12.2952 | 88.6882 | 0.0094 | 1.4961e+08 | 4 |

**Adduct Signal Fractions**

- **Unmodified**: 1.4961e+08

### QKLPGNDN(N)STATL Hex 8 HexNAc 2

**Mass:**  3060.2774

(4.020e-06)

**Charge States:** 3

**Start Position**: 1

**End Position**: 14

| Profile Score | Tandem Score | TDA q-value | Volume | Spectrum Matches |
| --- | --- | --- | --- | --- |
| -2.4877 | 78.6013 | 0.0179 | 2.9906e+07 | 1 |

**Adduct Signal Fractions**

- **Unmodified**: 2.9906e+07

### NWAGVTQNGTSSSC(C)RRGSN(N)SSFF Hex 7 HexNAc 3

**Mass:**  4249.7169

(3.615e-06)

**Charge States:** 3, 4

**Start Position**: 126

**End Position**: 149

| Profile Score | Tandem Score | TDA q-value | Volume | Spectrum Matches |
| --- | --- | --- | --- | --- |
| -4.5279 | 76.4781 | 0.0218 | 2.3456e+07 | 1 |

**Adduct Signal Fractions**

- **Unmodified**: 2.3456e+07

### NWAGVTQN(N)GTSSSC(C)RRGSNSSFF Hex 7 HexNAc 3

**Mass:**  4249.7169

(3.615e-06)

**Charge States:** 3, 4

**Start Position**: 126

**End Position**: 149

| Profile Score | Tandem Score | TDA q-value | Volume | Spectrum Matches |
| --- | --- | --- | --- | --- |
| -4.5279 | 76.4781 | 0.0218 | 2.3456e+07 | 1 |

**Adduct Signal Fractions**

- **Unmodified**: 2.3456e+07

### TIVKPGDILLIN(N)STGNLIAPRGY Hex 7 HexNAc 2

**Mass:**  3964.9092

(1.236e-06)

**Charge States:** 3, 4

**Start Position**: 235

**End Position**: 258

| Profile Score | Tandem Score | TDA q-value | Volume | Spectrum Matches |
| --- | --- | --- | --- | --- |
| 8.1856 | 76.0239 | 0.0218 | 1.4982e+07 | 2 |

**Adduct Signal Fractions**

- **Unmodified**: 1.4982e+07

### NSKYPALN(N)VTM(O)PNNEQFDKL Hex 9 HexNAc 2

**Mass:**  4202.7636

(2.765e-06)

**Charge States:** 4

**Start Position**: 158

**End Position**: 178

| Profile Score | Tandem Score | TDA q-value | Volume | Spectrum Matches |
| --- | --- | --- | --- | --- |
| -3.4404 | 69.9724 | 0.0262 | 1.5912e+07 | 1 |

**Adduct Signal Fractions**

- **Unmodified**: 1.5912e+07

### THLN(D)SKYPALN(N)VTM(O)PNNEQFDKL Fuc 3 Hex 3 HexNAc 4 Neu5Ac 1

**Mass:**  4718.0085

(2.014e-04)

**Charge States:** 3, 4

**Start Position**: 155

**End Position**: 178

| Profile Score | Tandem Score | TDA q-value | Volume | Spectrum Matches |
| --- | --- | --- | --- | --- |
| 8.2281 | 68.4481 | 0.0262 | 2.1844e+08 | 3 |

**Adduct Signal Fractions**

- **Unmodified**: 2.1844e+08

### N(D)SKYPALN(N)VTM(O)PNNEQFDKLY Hex 8 HexNAc 2

**Mass:**  4204.7616

(2.363e-04)

**Charge States:** 3, 4

**Start Position**: 158

**End Position**: 179

| Profile Score | Tandem Score | TDA q-value | Volume | Spectrum Matches |
| --- | --- | --- | --- | --- |
| 8.7011 | 66.8448 | 0.0262 | 2.3405e+08 | 6 |

**Adduct Signal Fractions**

- **Unmodified**: 2.3405e+08

### TIVKPGDILLIN(N)STGNL Hex 5 HexNAc 2

**Mass:**  2983.4452

(2.719e-06)

**Charge States:** 3

**Start Position**: 235

**End Position**: 252

| Profile Score | Tandem Score | TDA q-value | Volume | Spectrum Matches |
| --- | --- | --- | --- | --- |
| -4.2973 | 66.1376 | 0.0262 | 1.2443e+07 | 1 |

**Adduct Signal Fractions**

- **Unmodified**: 1.2443e+07

### AGVTQN(N)GTSSSC(C)RRGSNSSFF Hex 4 HexNAc 3

**Mass:**  3463.4276

(1.105e-06)

**Charge States:** 3

**Start Position**: 128

**End Position**: 149

| Profile Score | Tandem Score | TDA q-value | Volume | Spectrum Matches |
| --- | --- | --- | --- | --- |
| 12.5007 | 64.8448 | 0.0262 | 1.1654e+08 | 2 |

**Adduct Signal Fractions**

- **Unmodified**: 1.1654e+08

### TIVKPGDILLIN(N)STGNLIAPR Fuc 3 Hex 3 HexNAc 5

**Mass:**  4143.9929

(-7.483e-06)

**Charge States:** 4

**Start Position**: 235

**End Position**: 256

| Profile Score | Tandem Score | TDA q-value | Volume | Spectrum Matches |
| --- | --- | --- | --- | --- |
| 10.7970 | 63.8604 | 0.0262 | 3.9630e+07 | 3 |

**Adduct Signal Fractions**

- **Unmodified**: 3.9630e+07

### N(D)SKYPALN(N)VTM(O)PNN(D)EQFDKLY Hex 4 HexNAc 6

**Mass:**  4369.8016

(4.406e-04)

**Start Position**: 158

**End Position**: 179

| Profile Score | Tandem Score | TDA q-value | Volume | Spectrum Matches |
| --- | --- | --- | --- | --- |
| 0.0000 | 62.1298 | 0.0262 | 0.0000e+00 | 1 |

Adduct:

No Chromatogram Found

### NWAGVTQN(D)GTSSSC(C)RRGSN(N)SSFF Hex 5 HexNAc 2

**Mass:**  3723.5136

(2.704e-04)

**Charge States:** 3

**Start Position**: 126

**End Position**: 149

| Profile Score | Tandem Score | TDA q-value | Volume | Spectrum Matches |
| --- | --- | --- | --- | --- |
| 10.3308 | 60.6452 | 0.0262 | 3.7814e+07 | 4 |

**Adduct Signal Fractions**

- **Unmodified**: 3.7814e+07

### NWAGVTQN(N)GTSSSC(C)RRGSNSSFF Hex 6 HexNAc 3

**Mass:**  4087.6693

(3.497e-06)

**Charge States:** 3

**Start Position**: 126

**End Position**: 149

| Profile Score | Tandem Score | TDA q-value | Volume | Spectrum Matches |
| --- | --- | --- | --- | --- |
| 14.6332 | 60.2411 | 0.0262 | 2.9082e+07 | 2 |

**Adduct Signal Fractions**

- **Unmodified**: 2.9082e+07

### AGVTQN(N)GTSSSC(C)RRGSNSSFF Fuc 1 Hex 4 HexNAc 4 Neu5Ac 1

**Mass:**  4103.6486

(-3.929e-06)

**Charge States:** 3

**Start Position**: 128

**End Position**: 149

| Profile Score | Tandem Score | TDA q-value | Volume | Spectrum Matches |
| --- | --- | --- | --- | --- |
| 13.1558 | 58.8226 | 0.0262 | 6.3597e+07 | 2 |

**Adduct Signal Fractions**

- **Unmodified**: 6.3597e+07

### SKYPALN(N)VTM(O)PNNEQFDKLY Fuc 1 Hex 6 HexNAc 4

**Mass:**  4317.8446

(2.053e-06)

**Charge States:** 3, 4

**Start Position**: 159

**End Position**: 179

| Profile Score | Tandem Score | TDA q-value | Volume | Spectrum Matches |
| --- | --- | --- | --- | --- |
| 12.4540 | 56.9466 | 0.0295 | 1.7197e+08 | 7 |

**Adduct Signal Fractions**

- **Unmodified**: 1.7197e+08

### AGVTQN(N)GTSSSC(C)RRGSNSSFF Hex 5 HexNAc 4

**Mass:**  3828.5619

(-7.351e-07)

**Charge States:** 3, 4

**Start Position**: 128

**End Position**: 149

| Profile Score | Tandem Score | TDA q-value | Volume | Spectrum Matches |
| --- | --- | --- | --- | --- |
| 14.2100 | 56.8436 | 0.0295 | 1.6167e+08 | 4 |

**Adduct Signal Fractions**

- **Unmodified**: 1.6167e+08

### LNSKYPALN(N)VTM(O)PNNEQFDKL Hex 9 HexNAc 2

**Mass:**  4315.8654

(2.086e-06)

**Charge States:** 3, 4

**Start Position**: 157

**End Position**: 178

| Profile Score | Tandem Score | TDA q-value | Volume | Spectrum Matches |
| --- | --- | --- | --- | --- |
| -3.0499 | 56.1702 | 0.0331 | 1.4313e+07 | 2 |

**Adduct Signal Fractions**

- **Unmodified**: 1.4313e+07

### NSKYPALN(N)VTM(O)PNNEQFDKLY Hex 5 HexNAc 2

**Mass:**  3717.6256

(3.558e-06)

**Charge States:** 3

**Start Position**: 158

**End Position**: 179

| Profile Score | Tandem Score | TDA q-value | Volume | Spectrum Matches |
| --- | --- | --- | --- | --- |
| -4.8372 | 54.9283 | 0.0350 | 1.8354e+07 | 1 |

**Adduct Signal Fractions**

- **Unmodified**: 1.8354e+07

### TIVKPGDILLIN(N)STGNLIAPRG Hex 9 HexNAc 2

**Mass:**  4125.9543

(6.100e-07)

**Charge States:** 3, 4

**Start Position**: 235

**End Position**: 257

| Profile Score | Tandem Score | TDA q-value | Volume | Spectrum Matches |
| --- | --- | --- | --- | --- |
| 12.8614 | 53.7856 | 0.0350 | 3.5559e+08 | 14 |

**Adduct Signal Fractions**

- **Unmodified**: 3.5559e+08

### IVKPGDILLIN(N)STGNLIAPRGY Hex 9 HexNAc 2

**Mass:**  4187.9672

(1.573e-06)

**Charge States:** 3, 4

**Start Position**: 236

**End Position**: 258

| Profile Score | Tandem Score | TDA q-value | Volume | Spectrum Matches |
| --- | --- | --- | --- | --- |
| -19.2259 | 51.4294 | 0.0411 | 3.0662e+06 | 2 |

**Adduct Signal Fractions**

- **Unmodified**: 3.0662e+06

### NWAGVTQN(N)GTSSSC(C)R Hex 5 HexNAc 2

**Mass:**  2840.1328

(1.401e-06)

**Charge States:** 3

**Start Position**: 126

**End Position**: 141

| Profile Score | Tandem Score | TDA q-value | Volume | Spectrum Matches |
| --- | --- | --- | --- | --- |
| -20.4372 | 50.6049 | 0.0411 | 6.6813e+06 | 1 |

**Adduct Signal Fractions**

- **Unmodified**: 6.6813e+06

### HKC(C)DNAC(C)IGSIRN(N)GTYDHDVY Fuc 1 Hex 5 HexNAc 5

**Mass:**  4465.8010

(1.633e-06)

**Charge States:** 4, 5

**Start Position**: 471

**End Position**: 492

| Profile Score | Tandem Score | TDA q-value | Volume | Spectrum Matches |
| --- | --- | --- | --- | --- |
| 12.3284 | 49.3495 | 0.0433 | 5.8219e+07 | 1 |

**Adduct Signal Fractions**

- **Unmodified**: 5.8219e+07

### N(N)VTMPNNEQFDKLY Fuc 3 Hex 8 HexNAc 6 Neu5Ac 1

**Mass:**  4956.0041

(8.088e-06)

**Charge States:** 4

**Start Position**: 165

**End Position**: 179

| Profile Score | Tandem Score | TDA q-value | Volume | Spectrum Matches |
| --- | --- | --- | --- | --- |
| -3.1891 | 46.6459 | 0.0489 | 1.1041e+08 | 2 |

**Adduct Signal Fractions**

- **Unmodified**: 1.1041e+08

### cus|SWZHA\_5B8|A/Switzerland/9715293/2013

**>cus|SWZHA\_5B8|A/Switzerland/9715293/2013**  
 `QKLPGNDNSTATLCLGHHAVPNGTIVKTITNDRIEVTNATELVQNSSIGEICDSPHQILDGENCTLIDALLGDPQCDGFQ  
NKKWDLFVERSKAYSNCYPYDVPDYASLRSLVASSGTLEFNNESFNWAGVTHNGTSSSCRRGSNSSFFSRLNWLTHLNSK  
YPALNVTMPNNEQFDKLYIWGVHHPVTDKDQIFLYAQSSGRITVSTKRSQQAVIPNIGSRPRIRNIPSRISIYWTIVKPG  
DILLINSTGNLIAPRGYFKIRSGKSSIMRSDAPIGKCKSECITPNGSIPNDKPFQNVNRITYGACPRYVKQSTLKLATGM  
RNVPERQTRGIFGAIAGFIENGWEGMVDGWYGFRHQNSEGRGQAADLKSTQAAIDQINGKLNRLIGKTNEKFHQIEKEFS  
EVEGRIQDLEKYVEDTKIDLWSYNAELLVALENQHTIDLTDSEMNKLFEKTKKQLRENAEDMGNGCFKIYHKCDNACIGS  
IRNGTYDHDVYRDEALNNRFQIKGVELKSGYKDWILWISFAISCFLLCVALLGFIMWACQKGNIRCNICI`

| Site | Type | Count |
| --- | --- | --- |
| 483 | N-linked | 3 |
| 165 | N-linked | 16 |
| 38 | N-linked | 3 |
| 8 | N-linked | 2 |
| 144 | N-linked | 1 |
| 22 | N-linked | 1 |
| 122 | N-linked | 1 |
| 285 | N-linked | 3 |
| 246 | N-linked | 46 |

| Mass | Sequence | MS_2_ Score | `q`-value | MS_1_ Score | Signal | Protein Coordinates | Tandem Scans | Start Time | Apex Time | End Time |
| --- | --- | --- | --- | --- | --- | --- | --- | --- | --- | --- |
| 2695.0883 | LIN(N)STGNL Hex 9 HexNAc 2 | 187.010 | 0.0 | -4.475 | 5.551e+07 | 243 - 251 | 3 | 23.07 | 23.23 | 23.56 |
| 3519.5614 | LIN(N)STGNLIAPRGYF Fuc 2 Hex 3 HexNAc 4 Neu5Ac 1 | 184.281 | 0.0 | 0.000 | 0.000e+00 | 243 - 258 | 1 | - | - | - |
| 3132.3710 | LIN(N)STGNLIAPR Hex 9 HexNAc 2 | 181.606 | 0.0 | -2.753 | 5.135e+07 | 243 - 255 | 2 | 28.21 | 28.38 | 28.54 |
| 3499.5171 | LIN(N)STGNLIAPRGYF Hex 9 HexNAc 2 | 181.268 | 0.0 | 12.618 | 3.216e+08 | 243 - 258 | 5 | 45.25 | 45.41 | 46.54 |
| 2712.1149 | LIN(N)STGNL Fuc 2 Hex 3 HexNAc 4 Neu5Ac 1 | 180.426 | 0.0 | -4.569 | 1.841e+07 | 243 - 251 | 2 | 23.07 | 23.23 | 23.39 |
| 3516.5451 | LIN(N)STGNLIAPRGYF Fuc 2 Hex 3 HexNAc 4 Neu5Ac 1 | 180.399 | 0.0 | 13.740 | 1.572e+08 | 243 - 258 | 2 | 45.25 | 45.41 | 45.89 |
| 3352.4401 | LIN(N)STGNLIAPRGY Hex 9 HexNAc 2 | 178.612 | 0.0 | 14.899 | 2.580e+08 | 243 - 257 | 3 | 34.68 | 34.83 | 35.66 |
| 3369.4669 | LIN(N)STGNLIAPRGY Fuc 2 Hex 3 HexNAc 4 Neu5Ac 1 | 174.482 | 0.0 | 15.576 | 1.276e+08 | 243 - 257 | 4 | 34.68 | 34.83 | 35.50 |
| 4068.9333 | TIVKPGDILLIN(N)STGNLIAPR Hex 9 HexNAc 2 | 172.886 | 0.0 | 15.855 | 1.522e+09 | 234 - 255 | 23 | 49.94 | 51.64 | 54.20 |
| 3149.3994 | LIN(N)STGNLIAPR Fuc 2 Hex 3 HexNAc 4 Neu5Ac 1 | 171.673 | 0.0 | -3.934 | 1.971e+07 | 243 - 255 | 1 | 28.21 | 28.38 | 28.54 |
| 3337.4660 | LIN(N)STGNLIAPRGYF Hex 8 HexNAc 2 | 171.084 | 0.0 | -19.552 | 1.894e+07 | 243 - 258 | 1 | 45.57 | 45.57 | 45.73 |
| 3661.5737 | LIN(N)STGNLIAPRGYF Hex 10 HexNAc 2 | 168.083 | 0.0 | -19.966 | 2.415e+07 | 243 - 258 | 1 | 45.25 | 45.25 | 45.41 |
| 3386.4380 | IN(N)STGNLIAPRGYF Hex 9 HexNAc 2 | 165.849 | 0.0 | -19.942 | 1.333e+07 | 244 - 258 | 1 | 41.23 | 41.40 | 41.40 |
| 4126.9600 | TIVKPGDILLIN(N)STGNLIAPRGY Hex 8 HexNAc 2 | 164.669 | 0.0 | 12.952 | 2.842e+08 | 234 - 257 | 15 | 53.86 | 54.53 | 56.41 |
| 3159.4262 | TIVKPGDILLIN(N) Hex 9 HexNAc 2 | 159.555 | 0.0 | 12.779 | 1.815e+08 | 234 - 246 | 4 | 44.42 | 44.92 | 45.57 |
| 4230.9825 | TIVKPGDILLIN(N)STGNLIAPR Hex 10 HexNAc 2 | 158.479 | 0.0 | 13.526 | 1.274e+08 | 234 - 255 | 5 | 51.47 | 51.64 | 52.84 |
| 4289.0156 | TIVKPGDILLIN(N)STGNLIAPRGY Hex 9 HexNAc 2 | 158.253 | 0.0 | 16.223 | 2.989e+09 | 234 - 257 | 37 | 52.49 | 54.37 | 58.92 |
| 4365.8319 | NSKYPALN(N)VTM(O)PNNEQFDKLY Hex 9 HexNAc 2 | 157.954 | 0.0 | 14.510 | 3.789e+08 | 157 - 178 | 4 | 41.40 | 41.90 | 42.23 |
| 3514.4942 | LIN(N)STGNLIAPRGY Hex 10 HexNAc 2 | 157.833 | 0.0 | -5.190 | 2.090e+07 | 243 - 257 | 1 | 34.51 | 34.68 | 34.83 |
| 4085.9572 | TIVKPGDILLIN(N)STGNLIAPR Fuc 2 Hex 3 HexNAc 4 Neu5Ac 1 | 157.461 | 0.0 | 12.944 | 2.283e+08 | 234 - 255 | 4 | 51.47 | 51.64 | 53.01 |
| 3176.4528 | TIVKPGDILLIN(N) Fuc 2 Hex 3 HexNAc 4 Neu5Ac 1 | 155.486 | 0.0 | 11.868 | 8.871e+07 | 234 - 246 | 3 | 44.42 | 44.92 | 45.25 |
| 3906.8790 | TIVKPGDILLIN(N)STGNLIAPR Hex 8 HexNAc 2 | 155.260 | 0.0 | 12.886 | 1.116e+08 | 234 - 255 | 4 | 51.64 | 51.81 | 52.32 |
| 3648.6840 | TIVKPGDILLIN(N)STGNL Fuc 2 Hex 3 HexNAc 4 Neu5Ac 1 | 152.486 | 0.0 | 13.249 | 4.821e+08 | 234 - 251 | 6 | 50.97 | 51.14 | 52.84 |
| 2857.1356 | LIN(N)STGNL Hex 10 HexNAc 2 | 152.460 | 0.0 | -20.028 | 1.625e+06 | 243 - 251 | 1 | 23.23 | 23.23 | 23.23 |
| 4306.0397 | TIVKPGDILLIN(N)STGNLIAPRGY Fuc 2 Hex 3 HexNAc 4 Neu5Ac 1 | 152.238 | 0.0 | 13.918 | 5.074e+08 | 234 - 257 | 9 | 53.86 | 54.37 | 56.75 |
| 3631.6600 | TIVKPGDILLIN(N)STGNL Hex 9 HexNAc 2 | 150.825 | 0.0 | 15.892 | 9.462e+08 | 234 - 251 | 13 | 50.45 | 51.14 | 54.03 |
| 2905.2769 | TITNDRIEVTN(N)ATEL Hex 5 HexNAc 2 | 150.611 | 0.0 | -17.728 | 2.704e+07 | 27 - 42 | 1 | 34.68 | 34.83 | 34.83 |
| 3473.5030 | C(C)LGHHAVPN(N)GTIVK Fuc 1 Hex 5 HexNAc 5 | 149.874 | 0.0 | -5.115 | 1.708e+07 | 13 - 27 | 1 | 16.70 | 16.87 | 17.03 |
| 4251.7888 | SKYPALN(N)VTM(O)PNNEQFDKLY Hex 9 HexNAc 2 | 148.973 | 0.0 | 13.067 | 1.220e+08 | 158 - 178 | 3 | 41.56 | 41.90 | 42.40 |
| 4752.0445 | RSDAPIGKC(C)KSEC(C)ITPN(N)GSIPNDKPF Hex 9 HexNAc 2 | 147.773 | 0.0 | 14.968 | 2.400e+08 | 268 - 294 | 4 | 27.88 | 28.04 | 28.54 |
| 4451.0650 | TIVKPGDILLIN(N)STGNLIAPRGY Hex 10 HexNAc 2 | 146.989 | 0.0 | 13.390 | 2.745e+08 | 234 - 257 | 11 | 54.03 | 54.20 | 55.73 |
| 3793.7118 | TIVKPGDILLIN(N)STGNL Hex 10 HexNAc 2 | 145.778 | 0.0 | 14.558 | 7.586e+07 | 234 - 251 | 4 | 50.79 | 50.97 | 51.47 |
| 4089.7369 | SKYPALN(N)VTM(O)PNNEQFDKLY Hex 8 HexNAc 2 | 145.533 | 0.0 | 13.294 | 1.235e+08 | 158 - 178 | 3 | 41.56 | 41.90 | 42.23 |
| 4248.0116 | TIVKPGDILLIN(N)STGNLIAPR Fuc 2 Hex 4 HexNAc 4 Neu5Ac 1 | 145.347 | 0.0 | -4.285 | 1.918e+07 | 234 - 255 | 1 | 51.47 | 51.64 | 51.98 |
| 4203.7793 | NSKYPALN(N)VTM(O)PNNEQFDKLY Hex 8 HexNAc 2 | 144.052 | 0.0 | 16.125 | 4.258e+08 | 157 - 178 | 5 | 41.06 | 41.90 | 42.40 |
| 4470.1257 | TIVKPGDILLIN(N)STGNLIAPRGY Fuc 2 Hex 4 HexNAc 4 Neu5Ac 1 | 142.806 | 0.0 | 0.000 | 0.000e+00 | 234 - 257 | 1 | - | - | - |
| 3533.5490 | LIN(N)STGNLIAPRGY Fuc 2 Hex 4 HexNAc 4 Neu5Ac 1 | 142.200 | 0.0 | -7.410 | 1.524e+07 | 243 - 257 | 3 | 31.17 | 31.50 | 31.50 |
| 2116.8313 | RGSN(N)SSFF Hex 5 HexNAc 2 | 139.749 | 0.0 | -3.917 | 1.322e+07 | 140 - 148 | 1 | 22.40 | 22.57 | 22.73 |
| 3190.3947 | LIN(N)STGNLIAPRGY Hex 8 HexNAc 2 | 139.196 | 0.0 | -19.739 | 1.362e+07 | 243 - 257 | 1 | 34.83 | 35.00 | 35.00 |
| 3420.7174 | TIVKPGDILLIN(N)STGNLIAPR Hex 5 HexNAc 2 | 139.096 | 0.0 | -3.894 | 2.096e+07 | 234 - 255 | 3 | 52.32 | 52.32 | 52.84 |
| 4468.0960 | TIVKPGDILLIN(N)STGNLIAPRGY Fuc 2 Hex 4 HexNAc 4 Neu5Ac 1 | 138.571 | 0.0 | -4.528 | 4.884e+07 | 234 - 257 | 2 | 54.20 | 54.20 | 54.70 |
| 3067.3366 | TITNDRIEVTN(N)ATEL Hex 6 HexNAc 2 | 138.168 | 0.0 | -19.696 | 1.522e+07 | 27 - 42 | 1 | 34.51 | 34.68 | 34.68 |
| 3469.6067 | TIVKPGDILLIN(N)STGNL Hex 8 HexNAc 2 | 137.352 | 0.0 | 14.081 | 7.256e+07 | 234 - 251 | 4 | 50.97 | 51.14 | 51.64 |
| 3640.8046 | TIVKPGDILLIN(N)STGNLIAPRGY Hex 5 HexNAc 2 | 134.719 | 0.0 | 12.704 | 4.130e+07 | 234 - 257 | 5 | 54.87 | 55.04 | 55.90 |
| 2563.9536 | EFNN(N)ESFNW Hex 6 HexNAc 2 | 133.371 | 0.0 | -4.396 | 5.327e+06 | 118 - 127 | 2 | 42.57 | 42.74 | 42.91 |
| 4589.9912 | RSDAPIGKC(C)KSEC(C)ITPN(N)GSIPNDKPF Hex 8 HexNAc 2 | 133.196 | 0.0 | -3.988 | 9.923e+07 | 268 - 294 | 2 | 28.04 | 28.04 | 28.38 |
| 4427.9353 | RSDAPIGKC(C)KSEC(C)ITPN(N)GSIPNDKPF Hex 7 HexNAc 2 | 131.175 | 0.0 | -4.114 | 1.104e+08 | 268 - 294 | 3 | 28.04 | 28.21 | 28.54 |
| 3207.2925 | SIRN(N)GTYDHDVY Fuc 1 Hex 5 HexNAc 4 | 129.786 | 0.0 | -18.155 | 1.282e+07 | 479 - 491 | 1 | 17.36 | 17.36 | 17.52 |
| 3810.7344 | TIVKPGDILLIN(N)STGNL Fuc 2 Hex 4 HexNAc 4 Neu5Ac 1 | 129.135 | 0.0 | -18.515 | 1.872e+07 | 234 - 251 | 1 | 50.97 | 50.97 | 50.97 |
| 3518.5768 | TIVKPGDILLIN(N)STGN Hex 9 HexNAc 2 | 128.227 | 0.0 | 14.363 | 1.823e+08 | 234 - 250 | 3 | 42.74 | 42.91 | 43.76 |
| 4262.7208 | HKC(C)DNAC(C)IGSIRN(N)GTYDHDVY Fuc 1 Hex 5 HexNAc 4 | 126.010 | 0.0 | -3.889 | 1.100e+08 | 470 - 491 | 2 | 23.39 | 23.56 | 23.73 |
| 4040.7202 | NSKYPALN(N)VTM(O)PNNEQFDKL Hex 8 HexNAc 2 | 119.901 | 0.0 | -3.959 | 1.465e+07 | 157 - 177 | 1 | 39.20 | 39.20 | 39.53 |
| 3270.4107 | TITNDRIEVTN(N)ATEL Hex 6 HexNAc 3 | 115.285 | 0.0 | -5.609 | 1.648e+07 | 27 - 42 | 1 | 34.51 | 34.68 | 34.83 |
| 2997.3821 | TIVKPGDILLIN(N) Hex 8 HexNAc 2 | 109.791 | 0.005 | -18.581 | 6.984e+06 | 234 - 246 | 1 | 45.09 | 45.09 | 45.09 |
| 4290.0084 | TIVKPGDILLIN(N)STGN(D)LIAPRGY Hex 9 HexNAc 2 | 109.760 | 0.005 | -18.882 | 1.386e+07 | 234 - 257 | 4 | 55.38 | 55.38 | 55.38 |
| 2733.0339 | N(N)VTMPNNEQF Hex 7 HexNAc 2 | 99.788 | 0.005 | -20.648 | 6.312e+06 | 164 - 174 | 1 | 23.23 | 23.23 | 23.39 |
| 3500.5073 | LIN(N)STGN(D)LIAPRGYF Hex 9 HexNAc 2 | 98.067 | 0.005 | -5.932 | 8.569e+06 | 243 - 258 | 1 | 46.71 | 46.88 | 47.05 |
| 4366.8147 | N(D)SKYPALN(N)VTM(O)PNNEQFDKLY Hex 9 HexNAc 2 | 96.938 | 0.005 | 9.102 | 2.235e+08 | 157 - 178 | 5 | 42.40 | 44.76 | 45.09 |
| 2744.1717 | QKLPGNDN(N)STATL Hex 3 HexNAc 3 Neu5Ac 1 | 95.934 | 0.005 | 13.195 | 3.220e+08 | 0 - 13 | 4 | 29.35 | 29.51 | 30.33 |
| 3802.8571 | TIVKPGDILLIN(N)STGNLIAPRGY Hex 6 HexNAc 2 | 92.463 | 0.009 | -4.589 | 1.387e+07 | 234 - 257 | 2 | 54.70 | 54.87 | 55.21 |
| 4153.8074 | LNSKYPALN(N)VTM(O)PNNEQFDKL Hex 8 HexNAc 2 | 89.413 | 0.009 | -3.822 | 1.159e+07 | 156 - 177 | 3 | 40.72 | 40.89 | 41.23 |
| 3060.2758 | QKLPGNDN(N)STATL Hex 8 HexNAc 2 | 78.601 | 0.018 | -2.488 | 2.991e+07 | 0 - 13 | 1 | 28.38 | 28.54 | 28.71 |
| 3964.9087 | TIVKPGDILLIN(N)STGNLIAPRGY Hex 7 HexNAc 2 | 76.024 | 0.022 | 8.186 | 1.498e+07 | 234 - 257 | 2 | 54.53 | 54.53 | 55.21 |
| 4202.7671 | NSKYPALN(N)VTM(O)PNNEQFDKL Hex 9 HexNAc 2 | 69.972 | 0.026 | -3.440 | 1.591e+07 | 157 - 177 | 1 | 39.03 | 39.20 | 39.36 |
| 4718.0102 | THLN(D)SKYPALN(N)VTM(O)PNNEQFDKL Fuc 3 Hex 3 HexNAc 4 Neu5Ac 1 | 68.448 | 0.026 | 8.228 | 2.184e+08 | 154 - 177 | 3 | 41.06 | 41.90 | 42.57 |
| 4204.7651 | N(D)SKYPALN(N)VTM(O)PNNEQFDKLY Hex 8 HexNAc 2 | 66.845 | 0.026 | 8.701 | 2.341e+08 | 157 - 178 | 6 | 42.23 | 44.76 | 45.09 |
| 2983.4456 | TIVKPGDILLIN(N)STGNL Hex 5 HexNAc 2 | 66.138 | 0.026 | -4.297 | 1.244e+07 | 234 - 251 | 1 | 51.98 | 51.98 | 52.32 |
| 4143.9888 | TIVKPGDILLIN(N)STGNLIAPR Fuc 3 Hex 3 HexNAc 5 | 63.860 | 0.026 | 10.797 | 3.963e+07 | 234 - 255 | 3 | 54.20 | 54.53 | 55.04 |
| 4369.8016 | N(D)SKYPALN(N)VTM(O)PNN(D)EQFDKLY Hex 4 HexNAc 6 | 62.130 | 0.026 | 0.000 | 0.000e+00 | 157 - 178 | 1 | - | - | - |
| 4317.8466 | SKYPALN(N)VTM(O)PNNEQFDKLY Fuc 1 Hex 6 HexNAc 4 | 56.947 | 0.030 | 12.454 | 1.720e+08 | 158 - 178 | 7 | 43.93 | 45.09 | 45.25 |
| 4315.8586 | LNSKYPALN(N)VTM(O)PNNEQFDKL Hex 9 HexNAc 2 | 56.170 | 0.033 | -3.050 | 1.431e+07 | 156 - 177 | 2 | 40.72 | 40.89 | 41.06 |
| 3717.6227 | NSKYPALN(N)VTM(O)PNNEQFDKLY Hex 5 HexNAc 2 | 54.928 | 0.035 | -4.837 | 1.835e+07 | 157 - 178 | 1 | 42.07 | 42.23 | 42.57 |
| 4125.9528 | TIVKPGDILLIN(N)STGNLIAPRG Hex 9 HexNAc 2 | 53.786 | 0.035 | 12.861 | 3.556e+08 | 234 - 256 | 14 | 51.30 | 52.32 | 53.86 |
| 4187.9648 | IVKPGDILLIN(N)STGNLIAPRGY Hex 9 HexNAc 2 | 51.429 | 0.041 | -19.226 | 3.066e+06 | 235 - 257 | 2 | 53.52 | 53.69 | 53.69 |
| 4465.8022 | HKC(C)DNAC(C)IGSIRN(N)GTYDHDVY Fuc 1 Hex 5 HexNAc 5 | 49.349 | 0.043 | 12.328 | 5.822e+07 | 470 - 491 | 1 | 23.39 | 23.56 | 24.06 |
| 4956.0035 | N(N)VTMPNNEQFDKLY Fuc 3 Hex 8 HexNAc 6 Neu5Ac 1 | 46.646 | 0.049 | -3.189 | 1.104e+08 | 164 - 178 | 2 | 46.37 | 46.54 | 46.71 |

### LIN(N)STGNL Hex 9 HexNAc 2

**Mass:**  2695.0880

(-5.247e-07)

**Charge States:** 2

**Start Position**: 244

**End Position**: 252

| Profile Score | Tandem Score | TDA q-value | Volume | Spectrum Matches |
| --- | --- | --- | --- | --- |
| -4.4755 | 187.0098 | 0.0000 | 5.5511e+07 | 3 |

**Adduct Signal Fractions**

- **Unmodified**: 5.5511e+07

### LIN(N)STGNLIAPRGYF Fuc 2 Hex 3 HexNAc 4 Neu5Ac 1

**Mass:**  3519.5614

(8.520e-04)

**Start Position**: 244

**End Position**: 259

| Profile Score | Tandem Score | TDA q-value | Volume | Spectrum Matches |
| --- | --- | --- | --- | --- |
| 0.0000 | 184.2809 | 0.0000 | 0.0000e+00 | 1 |

Adduct:

No Chromatogram Found

### LIN(N)STGNLIAPR Hex 9 HexNAc 2

**Mass:**  3132.3726

(4.283e-06)

**Charge States:** 3

**Start Position**: 244

**End Position**: 256

| Profile Score | Tandem Score | TDA q-value | Volume | Spectrum Matches |
| --- | --- | --- | --- | --- |
| -2.7526 | 181.6060 | 0.0000 | 5.1350e+07 | 2 |

**Adduct Signal Fractions**

- **Unmodified**: 5.1350e+07

### LIN(N)STGNLIAPRGYF Hex 9 HexNAc 2

**Mass:**  3499.5177

(1.473e-06)

**Charge States:** 3

**Start Position**: 244

**End Position**: 259

| Profile Score | Tandem Score | TDA q-value | Volume | Spectrum Matches |
| --- | --- | --- | --- | --- |
| 12.6181 | 181.2677 | 0.0000 | 3.2159e+08 | 5 |

**Adduct Signal Fractions**

- **Unmodified**: 3.2159e+08

### LIN(N)STGNL Fuc 2 Hex 3 HexNAc 4 Neu5Ac 1

**Mass:**  2712.1148

(-8.127e-06)

**Charge States:** 2, 3

**Start Position**: 244

**End Position**: 252

| Profile Score | Tandem Score | TDA q-value | Volume | Spectrum Matches |
| --- | --- | --- | --- | --- |
| -4.5693 | 180.4256 | 0.0000 | 1.8406e+07 | 2 |

**Adduct Signal Fractions**

- **Unmodified**: 1.8406e+07

### LIN(N)STGNLIAPRGYF Fuc 2 Hex 3 HexNAc 4 Neu5Ac 1

**Mass:**  3516.5447

(-5.759e-06)

**Charge States:** 3

**Start Position**: 244

**End Position**: 259

| Profile Score | Tandem Score | TDA q-value | Volume | Spectrum Matches |
| --- | --- | --- | --- | --- |
| 13.7399 | 180.3994 | 0.0000 | 1.5715e+08 | 2 |

**Adduct Signal Fractions**

- **Unmodified**: 1.5715e+08

### LIN(N)STGNLIAPRGY Hex 9 HexNAc 2

**Mass:**  3352.4394

(-1.793e-06)

**Charge States:** 3

**Start Position**: 244

**End Position**: 258

| Profile Score | Tandem Score | TDA q-value | Volume | Spectrum Matches |
| --- | --- | --- | --- | --- |
| 14.8987 | 178.6124 | 0.0000 | 2.5796e+08 | 3 |

**Adduct Signal Fractions**

- **Unmodified**: 2.5796e+08

### LIN(N)STGNLIAPRGY Fuc 2 Hex 3 HexNAc 4 Neu5Ac 1

**Mass:**  3369.4656

(-9.097e-06)

**Charge States:** 3

**Start Position**: 244

**End Position**: 258

| Profile Score | Tandem Score | TDA q-value | Volume | Spectrum Matches |
| --- | --- | --- | --- | --- |
| 15.5756 | 174.4817 | 0.0000 | 1.2755e+08 | 4 |

**Adduct Signal Fractions**

- **Unmodified**: 1.2755e+08

### TIVKPGDILLIN(N)STGNLIAPR Hex 9 HexNAc 2

**Mass:**  4068.9389

(3.100e-06)

**Charge States:** 3, 4, 5

**Start Position**: 235

**End Position**: 256

| Profile Score | Tandem Score | TDA q-value | Volume | Spectrum Matches |
| --- | --- | --- | --- | --- |
| 15.8551 | 172.8858 | 0.0000 | 1.5225e+09 | 23 |

**Adduct Signal Fractions**

- **Unmodified**: 1.5225e+09

### LIN(N)STGNLIAPR Fuc 2 Hex 3 HexNAc 4 Neu5Ac 1

**Mass:**  3149.3981

(-4.474e-06)

**Charge States:** 3

**Start Position**: 244

**End Position**: 256

| Profile Score | Tandem Score | TDA q-value | Volume | Spectrum Matches |
| --- | --- | --- | --- | --- |
| -3.9336 | 171.6733 | 0.0000 | 1.9709e+07 | 1 |

**Adduct Signal Fractions**

- **Unmodified**: 1.9709e+07

### LIN(N)STGNLIAPRGYF Hex 8 HexNAc 2

**Mass:**  3337.4661

(2.002e-06)

**Charge States:** 3

**Start Position**: 244

**End Position**: 259

| Profile Score | Tandem Score | TDA q-value | Volume | Spectrum Matches |
| --- | --- | --- | --- | --- |
| -19.5515 | 171.0841 | 0.0000 | 1.8940e+07 | 1 |

**Adduct Signal Fractions**

- **Unmodified**: 1.8940e+07

### LIN(N)STGNLIAPRGYF Hex 10 HexNAc 2

**Mass:**  3661.5727

(2.071e-06)

**Charge States:** 3

**Start Position**: 244

**End Position**: 259

| Profile Score | Tandem Score | TDA q-value | Volume | Spectrum Matches |
| --- | --- | --- | --- | --- |
| -19.9664 | 168.0829 | 0.0000 | 2.4151e+07 | 1 |

**Adduct Signal Fractions**

- **Unmodified**: 2.4151e+07

### IN(N)STGNLIAPRGYF Hex 9 HexNAc 2

**Mass:**  3386.4358

(4.344e-06)

**Charge States:** 3

**Start Position**: 245

**End Position**: 259

| Profile Score | Tandem Score | TDA q-value | Volume | Spectrum Matches |
| --- | --- | --- | --- | --- |
| -19.9417 | 165.8495 | 0.0000 | 1.3326e+07 | 1 |

**Adduct Signal Fractions**

- **Unmodified**: 1.3326e+07

### TIVKPGDILLIN(N)STGNLIAPRGY Hex 8 HexNAc 2

**Mass:**  4126.9660

(6.861e-07)

**Charge States:** 3, 4

**Start Position**: 235

**End Position**: 258

| Profile Score | Tandem Score | TDA q-value | Volume | Spectrum Matches |
| --- | --- | --- | --- | --- |
| 12.9524 | 164.6691 | 0.0000 | 2.8420e+08 | 15 |

**Adduct Signal Fractions**

- **Unmodified**: 2.8420e+08

### TIVKPGDILLIN(N) Hex 9 HexNAc 2

**Mass:**  3159.4260

(1.905e-06)

**Charge States:** 3

**Start Position**: 235

**End Position**: 247

| Profile Score | Tandem Score | TDA q-value | Volume | Spectrum Matches |
| --- | --- | --- | --- | --- |
| 12.7786 | 159.5551 | 0.0000 | 1.8147e+08 | 4 |

**Adduct Signal Fractions**

- **Unmodified**: 1.8147e+08

### TIVKPGDILLIN(N)STGNLIAPR Hex 10 HexNAc 2

**Mass:**  4230.9846

(1.201e-06)

**Charge States:** 3, 4

**Start Position**: 235

**End Position**: 256

| Profile Score | Tandem Score | TDA q-value | Volume | Spectrum Matches |
| --- | --- | --- | --- | --- |
| 13.5264 | 158.4794 | 0.0000 | 1.2736e+08 | 5 |

**Adduct Signal Fractions**

- **Unmodified**: 1.2736e+08

### TIVKPGDILLIN(N)STGNLIAPRGY Hex 9 HexNAc 2

**Mass:**  4289.0178

(1.029e-06)

**Charge States:** 3, 4, 5

**Start Position**: 235

**End Position**: 258

| Profile Score | Tandem Score | TDA q-value | Volume | Spectrum Matches |
| --- | --- | --- | --- | --- |
| 16.2234 | 158.2527 | 0.0000 | 2.9890e+09 | 37 |

**Adduct Signal Fractions**

- **Unmodified**: 2.9890e+09

### NSKYPALN(N)VTM(O)PNNEQFDKLY Hex 9 HexNAc 2

**Mass:**  4365.8292

(4.122e-06)

**Charge States:** 3, 4

**Start Position**: 158

**End Position**: 179

| Profile Score | Tandem Score | TDA q-value | Volume | Spectrum Matches |
| --- | --- | --- | --- | --- |
| 14.5099 | 157.9540 | 0.0000 | 3.7886e+08 | 4 |

**Adduct Signal Fractions**

- **Unmodified**: 3.7886e+08

### LIN(N)STGNLIAPRGY Hex 10 HexNAc 2

**Mass:**  3514.4932

(-1.017e-06)

**Charge States:** 3

**Start Position**: 244

**End Position**: 258

| Profile Score | Tandem Score | TDA q-value | Volume | Spectrum Matches |
| --- | --- | --- | --- | --- |
| -5.1898 | 157.8333 | 0.0000 | 2.0903e+07 | 1 |

**Adduct Signal Fractions**

- **Unmodified**: 2.0903e+07

### TIVKPGDILLIN(N)STGNLIAPR Fuc 2 Hex 3 HexNAc 4 Neu5Ac 1

**Mass:**  4085.9543

(-4.642e-06)

**Charge States:** 4

**Start Position**: 235

**End Position**: 256

| Profile Score | Tandem Score | TDA q-value | Volume | Spectrum Matches |
| --- | --- | --- | --- | --- |
| 12.9436 | 157.4613 | 0.0000 | 2.2826e+08 | 4 |

**Adduct Signal Fractions**

- **Unmodified**: 2.2826e+08

### TIVKPGDILLIN(N) Fuc 2 Hex 3 HexNAc 4 Neu5Ac 1

**Mass:**  3176.4538

(-6.272e-06)

**Charge States:** 3

**Start Position**: 235

**End Position**: 247

| Profile Score | Tandem Score | TDA q-value | Volume | Spectrum Matches |
| --- | --- | --- | --- | --- |
| 11.8681 | 155.4858 | 0.0000 | 8.8711e+07 | 3 |

**Adduct Signal Fractions**

- **Unmodified**: 8.8711e+07

### TIVKPGDILLIN(N)STGNLIAPR Hex 8 HexNAc 2

**Mass:**  3906.8780

(1.988e-06)

**Charge States:** 3, 4

**Start Position**: 235

**End Position**: 256

| Profile Score | Tandem Score | TDA q-value | Volume | Spectrum Matches |
| --- | --- | --- | --- | --- |
| 12.8856 | 155.2597 | 0.0000 | 1.1159e+08 | 4 |

**Adduct Signal Fractions**

- **Unmodified**: 1.1159e+08

### TIVKPGDILLIN(N)STGNL Fuc 2 Hex 3 HexNAc 4 Neu5Ac 1

**Mass:**  3648.6839

(-4.438e-06)

**Charge States:** 3

**Start Position**: 235

**End Position**: 252

| Profile Score | Tandem Score | TDA q-value | Volume | Spectrum Matches |
| --- | --- | --- | --- | --- |
| 13.2488 | 152.4863 | 0.0000 | 4.8207e+08 | 6 |

**Adduct Signal Fractions**

- **Unmodified**: 4.8207e+08

### LIN(N)STGNL Hex 10 HexNAc 2

**Mass:**  2857.1356

(-4.044e-07)

**Charge States:** 2

**Start Position**: 244

**End Position**: 252

| Profile Score | Tandem Score | TDA q-value | Volume | Spectrum Matches |
| --- | --- | --- | --- | --- |
| -20.0280 | 152.4600 | 0.0000 | 1.6254e+06 | 1 |

**Adduct Signal Fractions**

- **Unmodified**: 1.6254e+06

### TIVKPGDILLIN(N)STGNLIAPRGY Fuc 2 Hex 3 HexNAc 4 Neu5Ac 1

**Mass:**  4306.0358

(-5.264e-06)

**Charge States:** 3, 4

**Start Position**: 235

**End Position**: 258

| Profile Score | Tandem Score | TDA q-value | Volume | Spectrum Matches |
| --- | --- | --- | --- | --- |
| 13.9184 | 152.2383 | 0.0000 | 5.0744e+08 | 9 |

**Adduct Signal Fractions**

- **Unmodified**: 5.0744e+08

### TIVKPGDILLIN(N)STGNL Hex 9 HexNAc 2

**Mass:**  3631.6620

(3.072e-06)

**Charge States:** 2, 3, 4

**Start Position**: 235

**End Position**: 252

| Profile Score | Tandem Score | TDA q-value | Volume | Spectrum Matches |
| --- | --- | --- | --- | --- |
| 15.8919 | 150.8255 | 0.0000 | 9.4617e+08 | 13 |

**Adduct Signal Fractions**

- **Unmodified**: 9.4617e+08

### TITNDRIEVTN(N)ATEL Hex 5 HexNAc 2

**Mass:**  2905.2775

(-1.635e-06)

**Charge States:** 3

**Start Position**: 28

**End Position**: 43

| Profile Score | Tandem Score | TDA q-value | Volume | Spectrum Matches |
| --- | --- | --- | --- | --- |
| -17.7280 | 150.6112 | 0.0000 | 2.7045e+07 | 1 |

**Adduct Signal Fractions**

- **Unmodified**: 2.7045e+07

### C(C)LGHHAVPN(N)GTIVK Fuc 1 Hex 5 HexNAc 5

**Mass:**  3473.5036

(1.555e-06)

**Charge States:** 4

**Start Position**: 14

**End Position**: 28

| Profile Score | Tandem Score | TDA q-value | Volume | Spectrum Matches |
| --- | --- | --- | --- | --- |
| -5.1148 | 149.8736 | 0.0000 | 1.7076e+07 | 1 |

**Adduct Signal Fractions**

- **Unmodified**: 1.7076e+07

### SKYPALN(N)VTM(O)PNNEQFDKLY Hex 9 HexNAc 2

**Mass:**  4251.7864

(1.558e-06)

**Charge States:** 3, 4

**Start Position**: 159

**End Position**: 179

| Profile Score | Tandem Score | TDA q-value | Volume | Spectrum Matches |
| --- | --- | --- | --- | --- |
| 13.0670 | 148.9725 | 0.0000 | 1.2201e+08 | 3 |

**Adduct Signal Fractions**

- **Unmodified**: 1.2201e+08

### RSDAPIGKC(C)KSEC(C)ITPN(N)GSIPNDKPF Hex 9 HexNAc 2

**Mass:**  4752.0428

(2.746e-06)

**Charge States:** 4, 5

**Start Position**: 269

**End Position**: 295

| Profile Score | Tandem Score | TDA q-value | Volume | Spectrum Matches |
| --- | --- | --- | --- | --- |
| 14.9680 | 147.7730 | 0.0000 | 2.4001e+08 | 4 |

**Adduct Signal Fractions**

- **Unmodified**: 2.4001e+08

### TIVKPGDILLIN(N)STGNLIAPRGY Hex 10 HexNAc 2

**Mass:**  4451.0666

(9.641e-07)

**Charge States:** 3, 4

**Start Position**: 235

**End Position**: 258

| Profile Score | Tandem Score | TDA q-value | Volume | Spectrum Matches |
| --- | --- | --- | --- | --- |
| 13.3896 | 146.9894 | 0.0000 | 2.7446e+08 | 11 |

**Adduct Signal Fractions**

- **Unmodified**: 2.7446e+08

### TIVKPGDILLIN(N)STGNL Hex 10 HexNAc 2

**Mass:**  3793.7103

(4.576e-06)

**Charge States:** 3

**Start Position**: 235

**End Position**: 252

| Profile Score | Tandem Score | TDA q-value | Volume | Spectrum Matches |
| --- | --- | --- | --- | --- |
| 14.5582 | 145.7781 | 0.0000 | 7.5860e+07 | 4 |

**Adduct Signal Fractions**

- **Unmodified**: 7.5860e+07

### SKYPALN(N)VTM(O)PNNEQFDKLY Hex 8 HexNAc 2

**Mass:**  4089.7362

(1.963e-06)

**Charge States:** 3, 4

**Start Position**: 159

**End Position**: 179

| Profile Score | Tandem Score | TDA q-value | Volume | Spectrum Matches |
| --- | --- | --- | --- | --- |
| 13.2936 | 145.5325 | 0.0000 | 1.2351e+08 | 3 |

**Adduct Signal Fractions**

- **Unmodified**: 1.2351e+08

### TIVKPGDILLIN(N)STGNLIAPR Fuc 2 Hex 4 HexNAc 4 Neu5Ac 1

**Mass:**  4248.0104

(-3.409e-06)

**Charge States:** 4

**Start Position**: 235

**End Position**: 256

| Profile Score | Tandem Score | TDA q-value | Volume | Spectrum Matches |
| --- | --- | --- | --- | --- |
| -4.2854 | 145.3469 | 0.0000 | 1.9184e+07 | 1 |

**Adduct Signal Fractions**

- **Unmodified**: 1.9184e+07

### NSKYPALN(N)VTM(O)PNNEQFDKLY Hex 8 HexNAc 2

**Mass:**  4203.7783

(2.653e-06)

**Charge States:** 3, 4

**Start Position**: 158

**End Position**: 179

| Profile Score | Tandem Score | TDA q-value | Volume | Spectrum Matches |
| --- | --- | --- | --- | --- |
| 16.1248 | 144.0520 | 0.0000 | 4.2577e+08 | 5 |

**Adduct Signal Fractions**

- **Unmodified**: 4.2577e+08

### TIVKPGDILLIN(N)STGNLIAPRGY Fuc 2 Hex 4 HexNAc 4 Neu5Ac 1

**Mass:**  4470.1257

(4.502e-04)

**Start Position**: 235

**End Position**: 258

| Profile Score | Tandem Score | TDA q-value | Volume | Spectrum Matches |
| --- | --- | --- | --- | --- |
| 0.0000 | 142.8058 | 0.0000 | 0.0000e+00 | 1 |

Adduct:

No Chromatogram Found

### LIN(N)STGNLIAPRGY Fuc 2 Hex 4 HexNAc 4 Neu5Ac 1

**Mass:**  3533.5495

(5.602e-04)

**Charge States:** 4

**Start Position**: 244

**End Position**: 258

| Profile Score | Tandem Score | TDA q-value | Volume | Spectrum Matches |
| --- | --- | --- | --- | --- |
| -7.4101 | 142.2004 | 0.0000 | 1.5235e+07 | 3 |

**Adduct Signal Fractions**

- **Unmodified**: 1.5235e+07

### RGSN(N)SSFF Hex 5 HexNAc 2

**Mass:**  2116.8292

(-1.270e-06)

**Charge States:** 2

**Start Position**: 141

**End Position**: 149

| Profile Score | Tandem Score | TDA q-value | Volume | Spectrum Matches |
| --- | --- | --- | --- | --- |
| -3.9173 | 139.7489 | 0.0000 | 1.3215e+07 | 1 |

**Adduct Signal Fractions**

- **Unmodified**: 1.3215e+07

### LIN(N)STGNLIAPRGY Hex 8 HexNAc 2

**Mass:**  3190.3969

(-4.509e-07)

**Charge States:** 3

**Start Position**: 244

**End Position**: 258

| Profile Score | Tandem Score | TDA q-value | Volume | Spectrum Matches |
| --- | --- | --- | --- | --- |
| -19.7388 | 139.1957 | 0.0000 | 1.3623e+07 | 1 |

**Adduct Signal Fractions**

- **Unmodified**: 1.3623e+07

### TIVKPGDILLIN(N)STGNLIAPR Hex 5 HexNAc 2

**Mass:**  3420.7157

(2.332e-06)

**Charge States:** 3, 4

**Start Position**: 235

**End Position**: 256

| Profile Score | Tandem Score | TDA q-value | Volume | Spectrum Matches |
| --- | --- | --- | --- | --- |
| -3.8936 | 139.0961 | 0.0000 | 2.0959e+07 | 3 |

**Adduct Signal Fractions**

- **Unmodified**: 2.0959e+07

### TIVKPGDILLIN(N)STGNLIAPRGY Fuc 2 Hex 4 HexNAc 4 Neu5Ac 1

**Mass:**  4468.0949

(-4.507e-06)

**Charge States:** 4

**Start Position**: 235

**End Position**: 258

| Profile Score | Tandem Score | TDA q-value | Volume | Spectrum Matches |
| --- | --- | --- | --- | --- |
| -4.5279 | 138.5714 | 0.0000 | 4.8839e+07 | 2 |

**Adduct Signal Fractions**

- **Unmodified**: 4.8839e+07

### TITNDRIEVTN(N)ATEL Hex 6 HexNAc 2

**Mass:**  3067.3339

(2.711e-06)

**Charge States:** 3

**Start Position**: 28

**End Position**: 43

| Profile Score | Tandem Score | TDA q-value | Volume | Spectrum Matches |
| --- | --- | --- | --- | --- |
| -19.6955 | 138.1675 | 0.0000 | 1.5220e+07 | 1 |

**Adduct Signal Fractions**

- **Unmodified**: 1.5220e+07

### TIVKPGDILLIN(N)STGNL Hex 8 HexNAc 2

**Mass:**  3469.6100

(2.627e-06)

**Charge States:** 3, 4

**Start Position**: 235

**End Position**: 252

| Profile Score | Tandem Score | TDA q-value | Volume | Spectrum Matches |
| --- | --- | --- | --- | --- |
| 14.0813 | 137.3519 | 0.0000 | 7.2556e+07 | 4 |

**Adduct Signal Fractions**

- **Unmodified**: 7.2556e+07

### TIVKPGDILLIN(N)STGNLIAPRGY Hex 5 HexNAc 2

**Mass:**  3640.8026

(1.587e-06)

**Charge States:** 3, 4

**Start Position**: 235

**End Position**: 258

| Profile Score | Tandem Score | TDA q-value | Volume | Spectrum Matches |
| --- | --- | --- | --- | --- |
| 12.7044 | 134.7194 | 0.0000 | 4.1295e+07 | 5 |

**Adduct Signal Fractions**

- **Unmodified**: 4.1295e+07

### EFNN(N)ESFNW Hex 6 HexNAc 2

**Mass:**  2563.9510

(1.055e-06)

**Charge States:** 2

**Start Position**: 119

**End Position**: 128

| Profile Score | Tandem Score | TDA q-value | Volume | Spectrum Matches |
| --- | --- | --- | --- | --- |
| -4.3964 | 133.3713 | 0.0000 | 5.3272e+06 | 2 |

**Adduct Signal Fractions**

- **Unmodified**: 5.3272e+06

### RSDAPIGKC(C)KSEC(C)ITPN(N)GSIPNDKPF Hex 8 HexNAc 2

**Mass:**  4589.9868

(2.128e-06)

**Charge States:** 4, 5

**Start Position**: 269

**End Position**: 295

| Profile Score | Tandem Score | TDA q-value | Volume | Spectrum Matches |
| --- | --- | --- | --- | --- |
| -3.9876 | 133.1961 | 0.0000 | 9.9225e+07 | 2 |

**Adduct Signal Fractions**

- **Unmodified**: 9.9225e+07

### RSDAPIGKC(C)KSEC(C)ITPN(N)GSIPNDKPF Hex 7 HexNAc 2

**Mass:**  4427.9354

(2.685e-06)

**Charge States:** 4, 5

**Start Position**: 269

**End Position**: 295

| Profile Score | Tandem Score | TDA q-value | Volume | Spectrum Matches |
| --- | --- | --- | --- | --- |
| -4.1135 | 131.1753 | 0.0000 | 1.1037e+08 | 3 |

**Adduct Signal Fractions**

- **Unmodified**: 1.1037e+08

### SIRN(N)GTYDHDVY Fuc 1 Hex 5 HexNAc 4

**Mass:**  3207.2938

(2.016e-06)

**Charge States:** 3

**Start Position**: 480

**End Position**: 492

| Profile Score | Tandem Score | TDA q-value | Volume | Spectrum Matches |
| --- | --- | --- | --- | --- |
| -18.1549 | 129.7864 | 0.0000 | 1.2825e+07 | 1 |

**Adduct Signal Fractions**

- **Unmodified**: 1.2825e+07

### TIVKPGDILLIN(N)STGNL Fuc 2 Hex 4 HexNAc 4 Neu5Ac 1

**Mass:**  3810.7344

(-5.221e-06)

**Charge States:** 3

**Start Position**: 235

**End Position**: 252

| Profile Score | Tandem Score | TDA q-value | Volume | Spectrum Matches |
| --- | --- | --- | --- | --- |
| -18.5149 | 129.1345 | 0.0000 | 1.8720e+07 | 1 |

**Adduct Signal Fractions**

- **Unmodified**: 1.8720e+07

### TIVKPGDILLIN(N)STGN Hex 9 HexNAc 2

**Mass:**  3518.5761

(3.705e-06)

**Charge States:** 3

**Start Position**: 235

**End Position**: 251

| Profile Score | Tandem Score | TDA q-value | Volume | Spectrum Matches |
| --- | --- | --- | --- | --- |
| 14.3635 | 128.2265 | 0.0000 | 1.8229e+08 | 3 |

**Adduct Signal Fractions**

- **Unmodified**: 1.8229e+08

### HKC(C)DNAC(C)IGSIRN(N)GTYDHDVY Fuc 1 Hex 5 HexNAc 4

**Mass:**  4262.7204

(1.320e-06)

**Charge States:** 4, 5

**Start Position**: 471

**End Position**: 492

| Profile Score | Tandem Score | TDA q-value | Volume | Spectrum Matches |
| --- | --- | --- | --- | --- |
| -3.8887 | 126.0101 | 0.0000 | 1.0995e+08 | 2 |

**Adduct Signal Fractions**

- **Unmodified**: 1.0995e+08

### NSKYPALN(N)VTM(O)PNNEQFDKL Hex 8 HexNAc 2

**Mass:**  4040.7156

(1.921e-06)

**Charge States:** 3, 4

**Start Position**: 158

**End Position**: 178

| Profile Score | Tandem Score | TDA q-value | Volume | Spectrum Matches |
| --- | --- | --- | --- | --- |
| -3.9594 | 119.9012 | 0.0000 | 1.4651e+07 | 1 |

**Adduct Signal Fractions**

- **Unmodified**: 1.4651e+07

### TITNDRIEVTN(N)ATEL Hex 6 HexNAc 3

**Mass:**  3270.4037

(2.505e-06)

**Charge States:** 3

**Start Position**: 28

**End Position**: 43

| Profile Score | Tandem Score | TDA q-value | Volume | Spectrum Matches |
| --- | --- | --- | --- | --- |
| -5.6094 | 115.2853 | 0.0000 | 1.6480e+07 | 1 |

**Adduct Signal Fractions**

- **Unmodified**: 1.6480e+07

### TIVKPGDILLIN(N) Hex 8 HexNAc 2

**Mass:**  2997.3821

(4.885e-06)

**Charge States:** 3

**Start Position**: 235

**End Position**: 247

| Profile Score | Tandem Score | TDA q-value | Volume | Spectrum Matches |
| --- | --- | --- | --- | --- |
| -18.5813 | 109.7905 | 0.0051 | 6.9837e+06 | 1 |

**Adduct Signal Fractions**

- **Unmodified**: 6.9837e+06

### TIVKPGDILLIN(N)STGN(D)LIAPRGY Hex 9 HexNAc 2

**Mass:**  4290.0082

(2.332e-04)

**Charge States:** 3, 5

**Start Position**: 235

**End Position**: 258

| Profile Score | Tandem Score | TDA q-value | Volume | Spectrum Matches |
| --- | --- | --- | --- | --- |
| -18.8818 | 109.7598 | 0.0051 | 1.3863e+07 | 4 |

**Adduct Signal Fractions**

- **Unmodified**: 1.3863e+07

### N(N)VTMPNNEQF Hex 7 HexNAc 2

**Mass:**  2733.0353

(-4.229e-06)

**Charge States:** 3

**Start Position**: 165

**End Position**: 175

| Profile Score | Tandem Score | TDA q-value | Volume | Spectrum Matches |
| --- | --- | --- | --- | --- |
| -20.6485 | 99.7879 | 0.0051 | 6.3122e+06 | 1 |

**Adduct Signal Fractions**

- **Unmodified**: 6.3122e+06

### LIN(N)STGN(D)LIAPRGYF Hex 9 HexNAc 2

**Mass:**  3500.5054

(2.838e-04)

**Charge States:** 3

**Start Position**: 244

**End Position**: 259

| Profile Score | Tandem Score | TDA q-value | Volume | Spectrum Matches |
| --- | --- | --- | --- | --- |
| -5.9321 | 98.0669 | 0.0051 | 8.5694e+06 | 1 |

**Adduct Signal Fractions**

- **Unmodified**: 8.5694e+06

### N(D)SKYPALN(N)VTM(O)PNNEQFDKLY Hex 9 HexNAc 2

**Mass:**  4366.8107

(2.267e-04)

**Charge States:** 3, 4

**Start Position**: 158

**End Position**: 179

| Profile Score | Tandem Score | TDA q-value | Volume | Spectrum Matches |
| --- | --- | --- | --- | --- |
| 9.1020 | 96.9383 | 0.0051 | 2.2349e+08 | 5 |

**Adduct Signal Fractions**

- **Unmodified**: 2.2349e+08

### QKLPGNDN(N)STATL Hex 3 HexNAc 3 Neu5Ac 1

**Mass:**  2744.1718

(-1.203e-06)

**Charge States:** 2, 3

**Start Position**: 1

**End Position**: 14

| Profile Score | Tandem Score | TDA q-value | Volume | Spectrum Matches |
| --- | --- | --- | --- | --- |
| 13.1955 | 95.9338 | 0.0051 | 3.2201e+08 | 4 |

**Adduct Signal Fractions**

- **Unmodified**: 3.2201e+08

### TIVKPGDILLIN(N)STGNLIAPRGY Hex 6 HexNAc 2

**Mass:**  3802.8568

(9.530e-07)

**Charge States:** 3, 4

**Start Position**: 235

**End Position**: 258

| Profile Score | Tandem Score | TDA q-value | Volume | Spectrum Matches |
| --- | --- | --- | --- | --- |
| -4.5895 | 92.4626 | 0.0094 | 1.3872e+07 | 2 |

**Adduct Signal Fractions**

- **Unmodified**: 1.3872e+07

### LNSKYPALN(N)VTM(O)PNNEQFDKL Hex 8 HexNAc 2

**Mass:**  4153.8048

(5.236e-06)

**Charge States:** 3, 4

**Start Position**: 157

**End Position**: 178

| Profile Score | Tandem Score | TDA q-value | Volume | Spectrum Matches |
| --- | --- | --- | --- | --- |
| -3.8222 | 89.4125 | 0.0094 | 1.1591e+07 | 3 |

**Adduct Signal Fractions**

- **Unmodified**: 1.1591e+07

### QKLPGNDN(N)STATL Hex 8 HexNAc 2

**Mass:**  3060.2774

(4.020e-06)

**Charge States:** 3

**Start Position**: 1

**End Position**: 14

| Profile Score | Tandem Score | TDA q-value | Volume | Spectrum Matches |
| --- | --- | --- | --- | --- |
| -2.4877 | 78.6013 | 0.0179 | 2.9906e+07 | 1 |

**Adduct Signal Fractions**

- **Unmodified**: 2.9906e+07

### TIVKPGDILLIN(N)STGNLIAPRGY Hex 7 HexNAc 2

**Mass:**  3964.9092

(1.236e-06)

**Charge States:** 3, 4

**Start Position**: 235

**End Position**: 258

| Profile Score | Tandem Score | TDA q-value | Volume | Spectrum Matches |
| --- | --- | --- | --- | --- |
| 8.1856 | 76.0239 | 0.0218 | 1.4982e+07 | 2 |

**Adduct Signal Fractions**

- **Unmodified**: 1.4982e+07

### NSKYPALN(N)VTM(O)PNNEQFDKL Hex 9 HexNAc 2

**Mass:**  4202.7636

(2.765e-06)

**Charge States:** 4

**Start Position**: 158

**End Position**: 178

| Profile Score | Tandem Score | TDA q-value | Volume | Spectrum Matches |
| --- | --- | --- | --- | --- |
| -3.4404 | 69.9724 | 0.0262 | 1.5912e+07 | 1 |

**Adduct Signal Fractions**

- **Unmodified**: 1.5912e+07

### THLN(D)SKYPALN(N)VTM(O)PNNEQFDKL Fuc 3 Hex 3 HexNAc 4 Neu5Ac 1

**Mass:**  4718.0085

(2.014e-04)

**Charge States:** 3, 4

**Start Position**: 155

**End Position**: 178

| Profile Score | Tandem Score | TDA q-value | Volume | Spectrum Matches |
| --- | --- | --- | --- | --- |
| 8.2281 | 68.4481 | 0.0262 | 2.1844e+08 | 3 |

**Adduct Signal Fractions**

- **Unmodified**: 2.1844e+08

### N(D)SKYPALN(N)VTM(O)PNNEQFDKLY Hex 8 HexNAc 2

**Mass:**  4204.7616

(2.363e-04)

**Charge States:** 3, 4

**Start Position**: 158

**End Position**: 179

| Profile Score | Tandem Score | TDA q-value | Volume | Spectrum Matches |
| --- | --- | --- | --- | --- |
| 8.7011 | 66.8448 | 0.0262 | 2.3405e+08 | 6 |

**Adduct Signal Fractions**

- **Unmodified**: 2.3405e+08

### TIVKPGDILLIN(N)STGNL Hex 5 HexNAc 2

**Mass:**  2983.4452

(2.719e-06)

**Charge States:** 3

**Start Position**: 235

**End Position**: 252

| Profile Score | Tandem Score | TDA q-value | Volume | Spectrum Matches |
| --- | --- | --- | --- | --- |
| -4.2973 | 66.1376 | 0.0262 | 1.2443e+07 | 1 |

**Adduct Signal Fractions**

- **Unmodified**: 1.2443e+07

### TIVKPGDILLIN(N)STGNLIAPR Fuc 3 Hex 3 HexNAc 5

**Mass:**  4143.9929

(-7.483e-06)

**Charge States:** 4

**Start Position**: 235

**End Position**: 256

| Profile Score | Tandem Score | TDA q-value | Volume | Spectrum Matches |
| --- | --- | --- | --- | --- |
| 10.7970 | 63.8604 | 0.0262 | 3.9630e+07 | 3 |

**Adduct Signal Fractions**

- **Unmodified**: 3.9630e+07

### N(D)SKYPALN(N)VTM(O)PNN(D)EQFDKLY Hex 4 HexNAc 6

**Mass:**  4369.8016

(4.406e-04)

**Start Position**: 158

**End Position**: 179

| Profile Score | Tandem Score | TDA q-value | Volume | Spectrum Matches |
| --- | --- | --- | --- | --- |
| 0.0000 | 62.1298 | 0.0262 | 0.0000e+00 | 1 |

Adduct:

No Chromatogram Found

### SKYPALN(N)VTM(O)PNNEQFDKLY Fuc 1 Hex 6 HexNAc 4

**Mass:**  4317.8446

(2.053e-06)

**Charge States:** 3, 4

**Start Position**: 159

**End Position**: 179

| Profile Score | Tandem Score | TDA q-value | Volume | Spectrum Matches |
| --- | --- | --- | --- | --- |
| 12.4540 | 56.9466 | 0.0295 | 1.7197e+08 | 7 |

**Adduct Signal Fractions**

- **Unmodified**: 1.7197e+08

### LNSKYPALN(N)VTM(O)PNNEQFDKL Hex 9 HexNAc 2

**Mass:**  4315.8654

(2.086e-06)

**Charge States:** 3, 4

**Start Position**: 157

**End Position**: 178

| Profile Score | Tandem Score | TDA q-value | Volume | Spectrum Matches |
| --- | --- | --- | --- | --- |
| -3.0499 | 56.1702 | 0.0331 | 1.4313e+07 | 2 |

**Adduct Signal Fractions**

- **Unmodified**: 1.4313e+07

### NSKYPALN(N)VTM(O)PNNEQFDKLY Hex 5 HexNAc 2

**Mass:**  3717.6256

(3.558e-06)

**Charge States:** 3

**Start Position**: 158

**End Position**: 179

| Profile Score | Tandem Score | TDA q-value | Volume | Spectrum Matches |
| --- | --- | --- | --- | --- |
| -4.8372 | 54.9283 | 0.0350 | 1.8354e+07 | 1 |

**Adduct Signal Fractions**

- **Unmodified**: 1.8354e+07

### TIVKPGDILLIN(N)STGNLIAPRG Hex 9 HexNAc 2

**Mass:**  4125.9543

(6.100e-07)

**Charge States:** 3, 4

**Start Position**: 235

**End Position**: 257

| Profile Score | Tandem Score | TDA q-value | Volume | Spectrum Matches |
| --- | --- | --- | --- | --- |
| 12.8614 | 53.7856 | 0.0350 | 3.5559e+08 | 14 |

**Adduct Signal Fractions**

- **Unmodified**: 3.5559e+08

### IVKPGDILLIN(N)STGNLIAPRGY Hex 9 HexNAc 2

**Mass:**  4187.9672

(1.573e-06)

**Charge States:** 3, 4

**Start Position**: 236

**End Position**: 258

| Profile Score | Tandem Score | TDA q-value | Volume | Spectrum Matches |
| --- | --- | --- | --- | --- |
| -19.2259 | 51.4294 | 0.0411 | 3.0662e+06 | 2 |

**Adduct Signal Fractions**

- **Unmodified**: 3.0662e+06

### HKC(C)DNAC(C)IGSIRN(N)GTYDHDVY Fuc 1 Hex 5 HexNAc 5

**Mass:**  4465.8010

(1.633e-06)

**Charge States:** 4, 5

**Start Position**: 471

**End Position**: 492

| Profile Score | Tandem Score | TDA q-value | Volume | Spectrum Matches |
| --- | --- | --- | --- | --- |
| 12.3284 | 49.3495 | 0.0433 | 5.8219e+07 | 1 |

**Adduct Signal Fractions**

- **Unmodified**: 5.8219e+07

### N(N)VTMPNNEQFDKLY Fuc 3 Hex 8 HexNAc 6 Neu5Ac 1

**Mass:**  4956.0041

(8.088e-06)

**Charge States:** 4

**Start Position**: 165

**End Position**: 179

| Profile Score | Tandem Score | TDA q-value | Volume | Spectrum Matches |
| --- | --- | --- | --- | --- |
| -3.1891 | 46.6459 | 0.0489 | 1.1041e+08 | 2 |

**Adduct Signal Fractions**

- **Unmodified**: 1.1041e+08

### cus|SWZNA|A/Switzerland/9715293/2013

**>cus|SWZNA|A/Switzerland/9715293/2013**  
 `MNPNQKIITIGSVSLTISTICFFMQIAILITTVTLHFKQYEFNSPPNNQVMLCEPTIIERNITEIVYLTNTTIEKEICPK  
PAEYRNWSKPQCGITGFAPFSKDNSIRLSAGGDIWVTREPYVSCDPDKCYQFALGQGTTLNNVHSNNTVRDRTPYRTLLM  
NELGVPFHLGTKQVCIAWSSSSCHDGKAWLHVCITGDDKNATASFIYNGRLVDSVVSWSKDILRTQESECVCINGTCTVV  
MTDGSASGKADTKILFIEEGKIVHTSTLSGSAQHVEECSCYPRYPGVRCVCRDNWKGSNRPIVDINIKDHSIVSSYVCSG  
LVGDTPRKNDSSSSSHCLDPNNEEGGHGVKGWAFDDGNDVWMGRTINETSRLGYETFKVIEGWSNPKSKLQTNRQVIVDR  
GDRSGYSGIFSVEGKSCINRCFYVELIRGRKEETEVLWTSNSIVVFCGTSGTYGTGSWPDGADLNLMPI`

| Site | Type | Count |
| --- | --- | --- |
| 329 | N-linked | 1 |
| 146 | N-linked | 9 |
| 86 | N-linked | 3 |
| 367 | N-linked | 16 |
| 200 | N-linked | 3 |

| Mass | Sequence | MS_2_ Score | `q`-value | MS_1_ Score | Signal | Protein Coordinates | Tandem Scans | Start Time | Apex Time | End Time |
| --- | --- | --- | --- | --- | --- | --- | --- | --- | --- | --- |
| 3450.4025 | LHVC(C)ITGDDKN(N)ATASF Hex 8 HexNAc 2 | 192.977 | 0.0 | -5.073 | 2.267e+07 | 189 - 205 | 1 | 31.83 | 32.00 | 32.17 |
| 3612.4553 | LHVC(C)ITGDDKN(N)ATASF Hex 9 HexNAc 2 | 192.277 | 0.0 | -4.749 | 3.291e+07 | 189 - 205 | 1 | 31.66 | 31.83 | 32.17 |
| 3567.4977 | RN(N)WSKPQC(C)GITGFAPF Hex 8 HexNAc 2 | 182.210 | 0.0 | 14.728 | 6.935e+07 | 84 - 100 | 2 | 43.25 | 43.42 | 43.93 |
| 3729.5491 | RN(N)WSKPQC(C)GITGFAPF Hex 9 HexNAc 2 | 180.480 | 0.0 | -2.104 | 7.679e+07 | 84 - 100 | 2 | 43.25 | 43.42 | 43.76 |
| 3414.3836 | RN(N)WSKPQC(C)GITGF Hex 9 HexNAc 2 | 178.060 | 0.0 | -17.137 | 1.892e+07 | 84 - 97 | 1 | 28.71 | 28.88 | 28.88 |
| 3268.3802 | M(O)GRTIN(N)ETSRLGYETF Hex 6 HexNAc 2 | 170.782 | 0.0 | 12.466 | 1.425e+08 | 361 - 377 | 3 | 30.00 | 30.33 | 30.84 |
| 3106.3305 | M(O)GRTIN(N)ETSRLGYETF Hex 5 HexNAc 2 | 166.353 | 0.0 | -5.478 | 2.751e+07 | 361 - 377 | 1 | 30.50 | 30.50 | 31.01 |
| 3232.3281 | TIN(N)ETSRLGYETF Hex 8 HexNAc 2 | 166.009 | 0.0 | -17.353 | 1.541e+07 | 364 - 377 | 1 | 34.00 | 34.17 | 34.17 |
| 3430.4269 | M(O)GRTIN(N)ETSRLGYETF Hex 7 HexNAc 2 | 164.774 | 0.0 | 13.655 | 5.420e+08 | 361 - 377 | 3 | 29.83 | 30.00 | 31.50 |
| 3592.4791 | M(O)GRTIN(N)ETSRLGYETF Hex 8 HexNAc 2 | 152.258 | 0.0 | 15.555 | 1.187e+09 | 361 - 377 | 7 | 28.21 | 29.83 | 31.50 |
| 2833.1812 | M(O)GRTIN(N)ETSRL Hex 7 HexNAc 2 | 146.473 | 0.0 | -4.251 | 2.456e+07 | 361 - 372 | 2 | 15.40 | 15.40 | 15.73 |
| 3215.3191 | M(O)GRTIN(N)ETSRLGY Hex 8 HexNAc 2 | 146.041 | 0.0 | 13.397 | 3.587e+08 | 361 - 374 | 4 | 19.15 | 19.48 | 20.44 |
| 3288.3528 | LHVC(C)ITGDDKN(N)ATASF Hex 7 HexNAc 2 | 144.994 | 0.0 | -19.540 | 3.125e+06 | 189 - 205 | 1 | 32.00 | 32.00 | 32.00 |
| 3068.2851 | GRTIN(N)ETSRLGY Hex 8 HexNAc 2 | 144.603 | 0.0 | -19.186 | 1.042e+07 | 362 - 374 | 1 | 18.99 | 18.99 | 19.15 |
| 2995.2348 | M(O)GRTIN(N)ETSRL Hex 8 HexNAc 2 | 144.275 | 0.0 | -1.949 | 5.448e+07 | 361 - 372 | 2 | 15.24 | 15.40 | 15.73 |
| 2729.1592 | M(O)GRTIN(N)ETSRLGY Hex 5 HexNAc 2 | 140.441 | 0.0 | -18.077 | 1.870e+07 | 361 - 374 | 1 | 19.79 | 19.96 | 19.96 |
| 3053.2661 | M(O)GRTIN(N)ETSRLGY Hex 7 HexNAc 2 | 136.954 | 0.0 | 14.448 | 2.009e+08 | 361 - 374 | 3 | 19.32 | 19.48 | 19.96 |
| 3576.4972 | MGRTIN(N)ETSRLGYETF Hex 8 HexNAc 2 | 133.000 | 0.0 | -19.189 | 4.887e+06 | 361 - 377 | 2 | 32.50 | 32.50 | 32.67 |
| 2891.2138 | M(O)GRTIN(N)ETSRLGY Hex 6 HexNAc 2 | 129.365 | 0.0 | -3.378 | 9.124e+07 | 361 - 374 | 2 | 19.64 | 19.64 | 20.12 |
| 2671.1278 | M(O)GRTIN(N)ETSRL Hex 6 HexNAc 2 | 127.518 | 0.0 | -19.760 | 1.115e+07 | 361 - 372 | 1 | 15.57 | 15.57 | 15.73 |
| 4139.6884 | NNVHSN(N)NTVRDRTPY Hex 7 HexNAc 6 | 124.557 | 0.0 | -6.599 | 4.680e+07 | 140 - 155 | 4 | 12.94 | 13.27 | 13.44 |
| 3999.9482 | NNVHSN(N)NTVRDRTPY Fuc 1 Hex 9 HexNAc 3 | 118.188 | 0.0 | -20.012 | 2.501e+07 | 140 - 155 | 4 | 13.11 | 13.11 | 14.43 |
| 3773.5700 | NNVHSN(N)NTVRDRTPY Hex 6 HexNAc 5 | 114.912 | 0.0 | -4.420 | 1.248e+08 | 140 - 155 | 3 | 12.21 | 12.29 | 12.62 |
| 4218.6622 | NNVHSN(N)NTVRDRTPY Hex 10 HexNAc 4 | 106.761 | 0.005 | -5.360 | 2.959e+07 | 140 - 155 | 3 | 12.78 | 12.94 | 13.11 |
| 4331.7811 | MGRTIN(N)ETSRLGYETF Fuc 1 Hex 8 HexNAc 5 | 105.315 | 0.005 | -4.742 | 1.429e+07 | 361 - 377 | 1 | 33.83 | 34.00 | 34.17 |
| 3919.9816 | NNVHSN(N)NTVRDRTPY Fuc 1 Hex 6 HexNAc 5 | 100.320 | 0.005 | 2.455 | 1.184e+08 | 140 - 155 | 5 | 12.19 | 12.29 | 13.44 |
| 3658.5385 | M(O)GRTIN(N)ETSRLGYETF Fuc 1 Hex 5 HexNAc 4 | 90.980 | 0.009 | -3.690 | 2.978e+07 | 361 - 377 | 2 | 29.83 | 30.00 | 30.17 |
| 4286.7653 | NNVHSN(N)N(D)TVRDRTPY Fuc 1 Hex 7 HexNAc 6 | 81.912 | 0.014 | 0.000 | 0.000e+00 | 140 - 155 | 1 | - | - | - |
| 4364.7233 | NNVHSN(N)NTVRDRTPY Fuc 1 Hex 10 HexNAc 4 | 79.186 | 0.018 | -20.137 | 7.696e+06 | 140 - 155 | 1 | 12.94 | 12.94 | 12.94 |
| 3854.4980 | NNVHSN(N)NTVRDRTPY Hex 9 HexNAc 3 | 58.886 | 0.026 | -6.232 | 2.672e+07 | 140 - 155 | 3 | 14.26 | 14.59 | 14.75 |
| 3408.4378 | NNVHSN(N)NTVRDRTPY Hex 5 HexNAc 4 | 49.431 | 0.043 | -19.504 | 3.707e+07 | 140 - 155 | 2 | 12.46 | 12.46 | 12.46 |
| 3305.3727 | VC(C)SGLVGDTPRKN(N)DSSSSSH Hex 5 HexNAc 2 | 48.019 | 0.046 | -19.646 | 7.708e+06 | 316 - 336 | 1 | 13.27 | 13.27 | 13.27 |

### LHVC(C)ITGDDKN(N)ATASF Hex 8 HexNAc 2

**Mass:**  3450.4070

(1.190e-06)

**Charge States:** 3

**Start Position**: 190

**End Position**: 206

| Profile Score | Tandem Score | TDA q-value | Volume | Spectrum Matches |
| --- | --- | --- | --- | --- |
| -5.0733 | 192.9772 | 0.0000 | 2.2671e+07 | 1 |

**Adduct Signal Fractions**

- **Unmodified**: 2.2671e+07

### LHVC(C)ITGDDKN(N)ATASF Hex 9 HexNAc 2

**Mass:**  3612.4575

(9.984e-07)

**Charge States:** 3

**Start Position**: 190

**End Position**: 206

| Profile Score | Tandem Score | TDA q-value | Volume | Spectrum Matches |
| --- | --- | --- | --- | --- |
| -4.7487 | 192.2768 | 0.0000 | 3.2906e+07 | 1 |

**Adduct Signal Fractions**

- **Unmodified**: 3.2906e+07

### RN(N)WSKPQC(C)GITGFAPF Hex 8 HexNAc 2

**Mass:**  3567.4993

(3.804e-06)

**Charge States:** 3

**Start Position**: 85

**End Position**: 101

| Profile Score | Tandem Score | TDA q-value | Volume | Spectrum Matches |
| --- | --- | --- | --- | --- |
| 14.7278 | 182.2096 | 0.0000 | 6.9348e+07 | 2 |

**Adduct Signal Fractions**

- **Unmodified**: 6.9348e+07

### RN(N)WSKPQC(C)GITGFAPF Hex 9 HexNAc 2

**Mass:**  3729.5495

(3.097e-06)

**Charge States:** 3

**Start Position**: 85

**End Position**: 101

| Profile Score | Tandem Score | TDA q-value | Volume | Spectrum Matches |
| --- | --- | --- | --- | --- |
| -2.1040 | 180.4799 | 0.0000 | 7.6785e+07 | 2 |

**Adduct Signal Fractions**

- **Unmodified**: 7.6785e+07

### RN(N)WSKPQC(C)GITGF Hex 9 HexNAc 2

**Mass:**  3414.3854

(3.725e-07)

**Charge States:** 3

**Start Position**: 85

**End Position**: 98

| Profile Score | Tandem Score | TDA q-value | Volume | Spectrum Matches |
| --- | --- | --- | --- | --- |
| -17.1372 | 178.0601 | 0.0000 | 1.8916e+07 | 1 |

**Adduct Signal Fractions**

- **Unmodified**: 1.8916e+07

### M(O)GRTIN(N)ETSRLGYETF Hex 6 HexNAc 2

**Mass:**  3268.3804

(3.531e-06)

**Charge States:** 3

**Start Position**: 362

**End Position**: 378

| Profile Score | Tandem Score | TDA q-value | Volume | Spectrum Matches |
| --- | --- | --- | --- | --- |
| 12.4663 | 170.7817 | 0.0000 | 1.4246e+08 | 3 |

**Adduct Signal Fractions**

- **Unmodified**: 1.4246e+08

### M(O)GRTIN(N)ETSRLGYETF Hex 5 HexNAc 2

**Mass:**  3106.3295

(4.042e-06)

**Charge States:** 3

**Start Position**: 362

**End Position**: 378

| Profile Score | Tandem Score | TDA q-value | Volume | Spectrum Matches |
| --- | --- | --- | --- | --- |
| -5.4777 | 166.3528 | 0.0000 | 2.7513e+07 | 1 |

**Adduct Signal Fractions**

- **Unmodified**: 2.7513e+07

### TIN(N)ETSRLGYETF Hex 8 HexNAc 2

**Mass:**  3232.3291

(3.620e-06)

**Charge States:** 2, 3

**Start Position**: 365

**End Position**: 378

| Profile Score | Tandem Score | TDA q-value | Volume | Spectrum Matches |
| --- | --- | --- | --- | --- |
| -17.3527 | 166.0088 | 0.0000 | 1.5406e+07 | 1 |

**Adduct Signal Fractions**

- **Unmodified**: 1.5406e+07

### M(O)GRTIN(N)ETSRLGYETF Hex 7 HexNAc 2

**Mass:**  3430.4240

(3.271e-06)

**Charge States:** 3

**Start Position**: 362

**End Position**: 378

| Profile Score | Tandem Score | TDA q-value | Volume | Spectrum Matches |
| --- | --- | --- | --- | --- |
| 13.6549 | 164.7739 | 0.0000 | 5.4198e+08 | 3 |

**Adduct Signal Fractions**

- **Unmodified**: 5.4198e+08

### M(O)GRTIN(N)ETSRLGYETF Hex 8 HexNAc 2

**Mass:**  3592.4793

(5.709e-07)

**Charge States:** 3, 4

**Start Position**: 362

**End Position**: 378

| Profile Score | Tandem Score | TDA q-value | Volume | Spectrum Matches |
| --- | --- | --- | --- | --- |
| 15.5550 | 152.2584 | 0.0000 | 1.1867e+09 | 7 |

**Adduct Signal Fractions**

- **Unmodified**: 1.1867e+09

### M(O)GRTIN(N)ETSRL Hex 7 HexNAc 2

**Mass:**  2833.1815

(8.281e-07)

**Charge States:** 3

**Start Position**: 362

**End Position**: 373

| Profile Score | Tandem Score | TDA q-value | Volume | Spectrum Matches |
| --- | --- | --- | --- | --- |
| -4.2510 | 146.4731 | 0.0000 | 2.4565e+07 | 2 |

**Adduct Signal Fractions**

- **Unmodified**: 2.4565e+07

### M(O)GRTIN(N)ETSRLGY Hex 8 HexNAc 2

**Mass:**  3215.3183

(5.775e-07)

**Charge States:** 3

**Start Position**: 362

**End Position**: 375

| Profile Score | Tandem Score | TDA q-value | Volume | Spectrum Matches |
| --- | --- | --- | --- | --- |
| 13.3974 | 146.0405 | 0.0000 | 3.5869e+08 | 4 |

**Adduct Signal Fractions**

- **Unmodified**: 3.5869e+08

### LHVC(C)ITGDDKN(N)ATASF Hex 7 HexNAc 2

**Mass:**  3288.3528

(1.312e-06)

**Charge States:** 3

**Start Position**: 190

**End Position**: 206

| Profile Score | Tandem Score | TDA q-value | Volume | Spectrum Matches |
| --- | --- | --- | --- | --- |
| -19.5395 | 144.9937 | 0.0000 | 3.1251e+06 | 1 |

**Adduct Signal Fractions**

- **Unmodified**: 3.1251e+06

### GRTIN(N)ETSRLGY Hex 8 HexNAc 2

**Mass:**  3068.2848

(1.099e-06)

**Charge States:** 3

**Start Position**: 363

**End Position**: 375

| Profile Score | Tandem Score | TDA q-value | Volume | Spectrum Matches |
| --- | --- | --- | --- | --- |
| -19.1863 | 144.6026 | 0.0000 | 1.0416e+07 | 1 |

**Adduct Signal Fractions**

- **Unmodified**: 1.0416e+07

### M(O)GRTIN(N)ETSRL Hex 8 HexNAc 2

**Mass:**  2995.2343

(2.882e-06)

**Charge States:** 3

**Start Position**: 362

**End Position**: 373

| Profile Score | Tandem Score | TDA q-value | Volume | Spectrum Matches |
| --- | --- | --- | --- | --- |
| -1.9492 | 144.2749 | 0.0000 | 5.4477e+07 | 2 |

**Adduct Signal Fractions**

- **Unmodified**: 5.4477e+07

### M(O)GRTIN(N)ETSRLGY Hex 5 HexNAc 2

**Mass:**  2729.1606

(-2.516e-07)

**Charge States:** 3

**Start Position**: 362

**End Position**: 375

| Profile Score | Tandem Score | TDA q-value | Volume | Spectrum Matches |
| --- | --- | --- | --- | --- |
| -18.0768 | 140.4407 | 0.0000 | 1.8697e+07 | 1 |

**Adduct Signal Fractions**

- **Unmodified**: 1.8697e+07

### M(O)GRTIN(N)ETSRLGY Hex 7 HexNAc 2

**Mass:**  3053.2655

(1.290e-06)

**Charge States:** 3

**Start Position**: 362

**End Position**: 375

| Profile Score | Tandem Score | TDA q-value | Volume | Spectrum Matches |
| --- | --- | --- | --- | --- |
| 14.4483 | 136.9544 | 0.0000 | 2.0091e+08 | 3 |

**Adduct Signal Fractions**

- **Unmodified**: 2.0091e+08

### MGRTIN(N)ETSRLGYETF Hex 8 HexNAc 2

**Mass:**  3576.4975

(4.722e-06)

**Charge States:** 3

**Start Position**: 362

**End Position**: 378

| Profile Score | Tandem Score | TDA q-value | Volume | Spectrum Matches |
| --- | --- | --- | --- | --- |
| -19.1893 | 133.0001 | 0.0000 | 4.8865e+06 | 2 |

**Adduct Signal Fractions**

- **Unmodified**: 4.8865e+06

### M(O)GRTIN(N)ETSRLGY Hex 6 HexNAc 2

**Mass:**  2891.2137

(8.897e-07)

**Charge States:** 3

**Start Position**: 362

**End Position**: 375

| Profile Score | Tandem Score | TDA q-value | Volume | Spectrum Matches |
| --- | --- | --- | --- | --- |
| -3.3776 | 129.3654 | 0.0000 | 9.1238e+07 | 2 |

**Adduct Signal Fractions**

- **Unmodified**: 9.1238e+07

### M(O)GRTIN(N)ETSRL Hex 6 HexNAc 2

**Mass:**  2671.1266

(5.889e-08)

**Charge States:** 3

**Start Position**: 362

**End Position**: 373

| Profile Score | Tandem Score | TDA q-value | Volume | Spectrum Matches |
| --- | --- | --- | --- | --- |
| -19.7604 | 127.5182 | 0.0000 | 1.1148e+07 | 1 |

**Adduct Signal Fractions**

- **Unmodified**: 1.1148e+07

### NNVHSN(N)NTVRDRTPY Hex 7 HexNAc 6

**Mass:**  4139.6848

(2.425e-04)

**Charge States:** 4

**Start Position**: 141

**End Position**: 156

| Profile Score | Tandem Score | TDA q-value | Volume | Spectrum Matches |
| --- | --- | --- | --- | --- |
| -6.5991 | 124.5571 | 0.0000 | 4.6803e+07 | 4 |

**Adduct Signal Fractions**

- **Unmodified**: 4.6803e+07

### NNVHSN(N)NTVRDRTPY Fuc 1 Hex 9 HexNAc 3

**Mass:**  3999.5791

(4.930e-04)

**Charge States:** 4

**Start Position**: 141

**End Position**: 156

| Profile Score | Tandem Score | TDA q-value | Volume | Spectrum Matches |
| --- | --- | --- | --- | --- |
| -20.0117 | 118.1883 | 0.0000 | 2.5008e+07 | 4 |

**Adduct Signal Fractions**

- **Unmodified**: 2.5008e+07

### NNVHSN(N)NTVRDRTPY Hex 6 HexNAc 5

**Mass:**  3773.5688

(3.052e-06)

**Charge States:** 4

**Start Position**: 141

**End Position**: 156

| Profile Score | Tandem Score | TDA q-value | Volume | Spectrum Matches |
| --- | --- | --- | --- | --- |
| -4.4201 | 114.9119 | 0.0000 | 1.2480e+08 | 3 |

**Adduct Signal Fractions**

- **Unmodified**: 1.2480e+08

### NNVHSN(N)NTVRDRTPY Hex 10 HexNAc 4

**Mass:**  4218.6682

(-8.805e-06)

**Charge States:** 4

**Start Position**: 141

**End Position**: 156

| Profile Score | Tandem Score | TDA q-value | Volume | Spectrum Matches |
| --- | --- | --- | --- | --- |
| -5.3604 | 106.7607 | 0.0051 | 2.9593e+07 | 3 |

**Adduct Signal Fractions**

- **Unmodified**: 2.9593e+07

### MGRTIN(N)ETSRLGYETF Fuc 1 Hex 8 HexNAc 5

**Mass:**  4331.7849

(1.907e-06)

**Charge States:** 3

**Start Position**: 362

**End Position**: 378

| Profile Score | Tandem Score | TDA q-value | Volume | Spectrum Matches |
| --- | --- | --- | --- | --- |
| -4.7419 | 105.3154 | 0.0051 | 1.4292e+07 | 1 |

**Adduct Signal Fractions**

- **Unmodified**: 1.4292e+07

### NNVHSN(N)NTVRDRTPY Fuc 1 Hex 6 HexNAc 5

**Mass:**  3919.6289

(4.256e-07)

**Charge States:** 4

**Start Position**: 141

**End Position**: 156

| Profile Score | Tandem Score | TDA q-value | Volume | Spectrum Matches |
| --- | --- | --- | --- | --- |
| 2.4551 | 100.3202 | 0.0051 | 1.1844e+08 | 5 |

**Adduct Signal Fractions**

- **Unmodified**: 1.1844e+08

### M(O)GRTIN(N)ETSRLGYETF Fuc 1 Hex 5 HexNAc 4

**Mass:**  3658.5360

(2.903e-06)

**Charge States:** 3

**Start Position**: 362

**End Position**: 378

| Profile Score | Tandem Score | TDA q-value | Volume | Spectrum Matches |
| --- | --- | --- | --- | --- |
| -3.6904 | 90.9796 | 0.0094 | 2.9782e+07 | 2 |

**Adduct Signal Fractions**

- **Unmodified**: 2.9782e+07

### NNVHSN(N)N(D)TVRDRTPY Fuc 1 Hex 7 HexNAc 6

**Mass:**  4286.7653

(4.693e-04)

**Start Position**: 141

**End Position**: 156

| Profile Score | Tandem Score | TDA q-value | Volume | Spectrum Matches |
| --- | --- | --- | --- | --- |
| 0.0000 | 81.9123 | 0.0137 | 0.0000e+00 | 1 |

Adduct:

No Chromatogram Found

### NNVHSN(N)NTVRDRTPY Fuc 1 Hex 10 HexNAc 4

**Mass:**  4364.7233

(-7.077e-06)

**Charge States:** 4

**Start Position**: 141

**End Position**: 156

| Profile Score | Tandem Score | TDA q-value | Volume | Spectrum Matches |
| --- | --- | --- | --- | --- |
| -20.1367 | 79.1862 | 0.0179 | 7.6958e+06 | 1 |

**Adduct Signal Fractions**

- **Unmodified**: 7.6958e+06

### NNVHSN(N)NTVRDRTPY Hex 9 HexNAc 3

**Mass:**  3854.4955

(2.511e-04)

**Charge States:** 3, 4

**Start Position**: 141

**End Position**: 156

| Profile Score | Tandem Score | TDA q-value | Volume | Spectrum Matches |
| --- | --- | --- | --- | --- |
| -6.2317 | 58.8864 | 0.0262 | 2.6720e+07 | 3 |

**Adduct Signal Fractions**

- **Unmodified**: 2.6720e+07

### NNVHSN(N)NTVRDRTPY Hex 5 HexNAc 4

**Mass:**  3408.4372

(2.293e-06)

**Charge States:** 3, 4

**Start Position**: 141

**End Position**: 156

| Profile Score | Tandem Score | TDA q-value | Volume | Spectrum Matches |
| --- | --- | --- | --- | --- |
| -19.5041 | 49.4308 | 0.0433 | 3.7073e+07 | 2 |

**Adduct Signal Fractions**

- **Unmodified**: 3.7073e+07

### VC(C)SGLVGDTPRKN(N)DSSSSSH Hex 5 HexNAc 2

**Mass:**  3305.3727

(1.565e-07)

**Charge States:** 4

**Start Position**: 317

**End Position**: 337

| Profile Score | Tandem Score | TDA q-value | Volume | Spectrum Matches |
| --- | --- | --- | --- | --- |
| -19.6457 | 48.0193 | 0.0456 | 7.7085e+06 | 1 |

**Adduct Signal Fractions**

- **Unmodified**: 7.7085e+06

### iav|AGU93026|A/Puerto

**>iav|AGU93026|A/Puerto**  
 `MEDFVRQCFNPMIVELAEKTMKEYGEDLKIETNKFAAICTHLEVCFMYSDFHFINEQGESIIVELGDPNALLKHRFEIIE  
GRDRTMAWTVVNSICNTTGAEKPKFLPDLYDYKENRFIEIGVTRREVHIYYLEKANKIKSEKTHIHIFSFTGEEMATKAD  
YTLDEESRARIKTRLFTIRQEMASRGLWDSFRQSERGEETIEERFEITGTMRKLADQSLPPNFSSLENFRAYVDGFEPNG  
YIEGKLSQMSKEVNARIEPFLKTTPRPLRLPNGPPCSQRSKFLLMDALKLSIEDPSHEGEGIPLYDAIKCMRTFFGWKEP  
NVVKPHEKGINPNYLLSWKQVLAELQDIENEEKIPKTKNMKKTSQLKWALGENMAPEKVDFDDCKDVGDLKQYDSDEPEL  
RSLASWIQNEFNKACELTDSSWIELDEIGEDVAPIEHIASMRRNYFTSEVSHCRATEYIMKGVYINTALLNASCAAMDDF  
QLIPMISKCRTKEGRRKTNLYGFIIKGRSHLRNDTDVVNFVSMEFSLTDPRLEPHKWEKYCVLEIGDMLIRSAIGQVSRP  
MFLYVRTNGTSKIKMKWGMEMRRCLLQSLQQIESMIEAESSVKEKDMTKEFFENKSETWPIGESPKGVEESSIGKVCRTL  
LAKSVFNSLYASPQLEGFSAESRKLLLIVQALRDNLEPGTFDLGGLYEAIEECLINDPWVLLNASWFNSFLTHALS`

| Site | Type | Count |
| --- | --- | --- |
| 513 | N-linked | 1 |
| 703 | N-linked | 2 |

| Mass | Sequence | MS_2_ Score | `q`-value | MS_1_ Score | Signal | Protein Coordinates | Tandem Scans | Start Time | Apex Time | End Time |
| --- | --- | --- | --- | --- | --- | --- | --- | --- | --- | --- |
| 4184.6209 | LN(N)ASWF Fuc 5 Hex 8 HexNAc 7 | 130.343 | 0.0 | 0.000 | 0.000e+00 | 701 - 707 | 1 | - | - | - |
| 3575.3813 | LN(N)ASWF Hex 10 HexNAc 6 | 128.990 | 0.0 | -19.087 | 9.991e+06 | 701 - 707 | 1 | 25.71 | 25.88 | 25.88 |
| 4047.6195 | RN(N)DTDVVNFVSMEF Fuc 1 Hex 10 HexNAc 3 | 82.356 | 0.014 | 11.489 | 1.325e+08 | 511 - 525 | 4 | 34.68 | 35.00 | 36.68 |

### LN(N)ASWF Fuc 5 Hex 8 HexNAc 7

**Mass:**  4184.6209

(-2.930e-07)

**Start Position**: 702

**End Position**: 708

| Profile Score | Tandem Score | TDA q-value | Volume | Spectrum Matches |
| --- | --- | --- | --- | --- |
| 0.0000 | 130.3434 | 0.0000 | 0.0000e+00 | 1 |

Adduct:

No Chromatogram Found

### LN(N)ASWF Hex 10 HexNAc 6

**Mass:**  3575.3864

(7.661e-06)

**Charge States:** 3

**Start Position**: 702

**End Position**: 708

| Profile Score | Tandem Score | TDA q-value | Volume | Spectrum Matches |
| --- | --- | --- | --- | --- |
| -19.0874 | 128.9895 | 0.0000 | 9.9906e+06 | 1 |

**Adduct Signal Fractions**

- **Unmodified**: 9.9906e+06

### RN(N)DTDVVNFVSMEF Fuc 1 Hex 10 HexNAc 3

**Mass:**  4047.6128

(8.003e-06)

**Charge States:** 3, 4

**Start Position**: 512

**End Position**: 526

| Profile Score | Tandem Score | TDA q-value | Volume | Spectrum Matches |
| --- | --- | --- | --- | --- |
| 11.4887 | 82.3564 | 0.0137 | 1.3254e+08 | 4 |

**Adduct Signal Fractions**

- **Unmodified**: 1.3254e+08

### iav|AFM71853|A/Puerto

**>iav|AFM71853|A/Puerto**  
 `MEDFVRQCFNPMIVELAEKTMKEYGEDLKIETNKFAAICTHLEVCFMYSDFHFINEQGESIIVELGDPNALLKHRFEIIE  
GRDRTMAWTVVNSICNTTGAEKPKFLPDLYDYKENRFIEIGVTRREVHIYYLEKANKIKSEKTHIHIFSFTGEEMATKAD  
YTLDEESRARIKTRLFTIRQEMASRGLWDSFRQSERGEETIEERFEITGTMRKLADQSLPPNFSSLENFRAYVDGFEPNG  
YIEGKLSQMSKEVNARIEPFLKTTPRPLRLPNGPPCSQRSKFLLMDALKLSIEDPSHEGEGIPLYDAIKCMRTFFGWKEP  
NVVKPHEKGINPNYLLSWKQVLAELQDIENEEKIPKTKNMKKTSQLKWALGENMAPEKVDFDDCKDVGDLKQYDSDEPEL  
RSLASWIQNEFNKACELTDSSWIELDEIGEDVAPIEHIASMRRNYFTSEVSHCRATEYIMKGVYINTALLNASCAAMDDF  
QLIPMISKCRTKEGRRKTNLYGFIIKGRSHLRNDTDVVNFVSMEFSLTDPRLEPHKWEKYCVLEIGDMLIRSAIGQVSRP  
MFLYVRTNGTSKIKMKWGMEMRRCLLQSLQQIESMIEAESSVKEKDMTKEFFENKSETWPIGESPKGVEESSIGKVCRTL  
LAKSVFNSLYASPQLEGFSAESRKLLLIVQALRDNLEPGTFDLGGLYEAIEECLINDPWVLLNASWFNSFLTHALS`

| Site | Type | Count |
| --- | --- | --- |
| 513 | N-linked | 1 |
| 703 | N-linked | 2 |

| Mass | Sequence | MS_2_ Score | `q`-value | MS_1_ Score | Signal | Protein Coordinates | Tandem Scans | Start Time | Apex Time | End Time |
| --- | --- | --- | --- | --- | --- | --- | --- | --- | --- | --- |
| 4184.6209 | LN(N)ASWF Fuc 5 Hex 8 HexNAc 7 | 130.343 | 0.0 | 0.000 | 0.000e+00 | 701 - 707 | 1 | - | - | - |
| 3575.3813 | LN(N)ASWF Hex 10 HexNAc 6 | 128.990 | 0.0 | -19.087 | 9.991e+06 | 701 - 707 | 1 | 25.71 | 25.88 | 25.88 |
| 4047.6195 | RN(N)DTDVVNFVSMEF Fuc 1 Hex 10 HexNAc 3 | 82.356 | 0.014 | 11.489 | 1.325e+08 | 511 - 525 | 4 | 34.68 | 35.00 | 36.68 |

### LN(N)ASWF Fuc 5 Hex 8 HexNAc 7

**Mass:**  4184.6209

(-2.930e-07)

**Start Position**: 702

**End Position**: 708

| Profile Score | Tandem Score | TDA q-value | Volume | Spectrum Matches |
| --- | --- | --- | --- | --- |
| 0.0000 | 130.3434 | 0.0000 | 0.0000e+00 | 1 |

Adduct:

No Chromatogram Found

### LN(N)ASWF Hex 10 HexNAc 6

**Mass:**  3575.3864

(7.661e-06)

**Charge States:** 3

**Start Position**: 702

**End Position**: 708

| Profile Score | Tandem Score | TDA q-value | Volume | Spectrum Matches |
| --- | --- | --- | --- | --- |
| -19.0874 | 128.9895 | 0.0000 | 9.9906e+06 | 1 |

**Adduct Signal Fractions**

- **Unmodified**: 9.9906e+06

### RN(N)DTDVVNFVSMEF Fuc 1 Hex 10 HexNAc 3

**Mass:**  4047.6128

(8.003e-06)

**Charge States:** 3, 4

**Start Position**: 512

**End Position**: 526

| Profile Score | Tandem Score | TDA q-value | Volume | Spectrum Matches |
| --- | --- | --- | --- | --- |
| 11.4887 | 82.3564 | 0.0137 | 1.3254e+08 | 4 |

**Adduct Signal Fractions**

- **Unmodified**: 1.3254e+08

### iav|ACV49552|A/Puerto

**>iav|ACV49552|A/Puerto**  
 `MEDFVRQCFNPMIVELAEKTMKEYGEDLKIETNKFAAICTHLEVCFMYSDFHFINEQGESIIVELGDPNALLKHRFEIIE  
GRDRTMAWTVVNSICNTTGAEKPKFLPDLYDYKENRFIEIGVTRREVHIYYLEKANKIKSEKTHIHIFSFTGEEMATKAD  
YTLDEESRARIKTRLFTIRQEMASRGLWDSFRQSERGEETIEERFEITGTMRKLADQSLPPNFSSLENFRAYVDGFEPNG  
YIEGKLSQMSKEVNARIEPFLKTTPRPLRLPNGPPCSQRSKFLLMDALKLSIEDPSHEGEGIPLYDAIKCMRTFFGWKEP  
NVVKPHEKGINPNYLLSWKQVLAELQDIENEEKIPKTKNMKKTSQLKWALGENMAPEKVDFDDCKDVGDLKQYDSDEPEL  
RSLASWIQNEFNKACELTDSSWIELDEIGEDVAPIEHIASMRRNYFTSEVSHCRATEYIMKGVYINTALLNASCAAMDDF  
QLIPMISKCRTKEGRRKTNLYGFIIKGRSHLRNDTDVVNFVSMEFSLTDPRLEPHKWEKYCVLEIGDMLIRSAIGQVSRP  
MFLYVRTNGTSKIKMKWGMEMRRCLLQSLQQIESMIEAESSVKEKDMTKEFFENKSETWPIGESPKGVEESSIGKVCRTL  
LAKSVFNSLYASPQLEGFSAESRKLLLIVQALRDNLEPGTFDLGGLYEAIEECLINDPWVLLNASWFNSFLTHALS`

| Site | Type | Count |
| --- | --- | --- |
| 513 | N-linked | 1 |
| 703 | N-linked | 2 |

| Mass | Sequence | MS_2_ Score | `q`-value | MS_1_ Score | Signal | Protein Coordinates | Tandem Scans | Start Time | Apex Time | End Time |
| --- | --- | --- | --- | --- | --- | --- | --- | --- | --- | --- |
| 4184.6209 | LN(N)ASWF Fuc 5 Hex 8 HexNAc 7 | 130.343 | 0.0 | 0.000 | 0.000e+00 | 701 - 707 | 1 | - | - | - |
| 3575.3813 | LN(N)ASWF Hex 10 HexNAc 6 | 128.990 | 0.0 | -19.087 | 9.991e+06 | 701 - 707 | 1 | 25.71 | 25.88 | 25.88 |
| 4047.6195 | RN(N)DTDVVNFVSMEF Fuc 1 Hex 10 HexNAc 3 | 82.356 | 0.014 | 11.489 | 1.325e+08 | 511 - 525 | 4 | 34.68 | 35.00 | 36.68 |

### LN(N)ASWF Fuc 5 Hex 8 HexNAc 7

**Mass:**  4184.6209

(-2.930e-07)

**Start Position**: 702

**End Position**: 708

| Profile Score | Tandem Score | TDA q-value | Volume | Spectrum Matches |
| --- | --- | --- | --- | --- |
| 0.0000 | 130.3434 | 0.0000 | 0.0000e+00 | 1 |

Adduct:

No Chromatogram Found

### LN(N)ASWF Hex 10 HexNAc 6

**Mass:**  3575.3864

(7.661e-06)

**Charge States:** 3

**Start Position**: 702

**End Position**: 708

| Profile Score | Tandem Score | TDA q-value | Volume | Spectrum Matches |
| --- | --- | --- | --- | --- |
| -19.0874 | 128.9895 | 0.0000 | 9.9906e+06 | 1 |

**Adduct Signal Fractions**

- **Unmodified**: 9.9906e+06

### RN(N)DTDVVNFVSMEF Fuc 1 Hex 10 HexNAc 3

**Mass:**  4047.6128

(8.003e-06)

**Charge States:** 3, 4

**Start Position**: 512

**End Position**: 526

| Profile Score | Tandem Score | TDA q-value | Volume | Spectrum Matches |
| --- | --- | --- | --- | --- |
| 11.4887 | 82.3564 | 0.0137 | 1.3254e+08 | 4 |

**Adduct Signal Fractions**

- **Unmodified**: 1.3254e+08

### iav|AGK63052|A/Puerto

**>iav|AGK63052|A/Puerto**  
 `MEDFVRQCFNPMIVELAEKTMKEYGEDLKIETNKFAAICTHLEVCFMYSDFHFINEQGESIIVELGDPNALLKHRFEIIE  
GRDRTMAWTVVNSICNTTGAEKPKFLPDLYDYKENRFIEIGVTRREVHIYYLEKANKIKSEKTHIHIFSFTGEEMATKAD  
YTLDEESRARIKTRLFTIRQEMASRGLWDSFRQSERGEETIEERFEITGTMRKLADQSLPPNFSSLENFRAYVDGFEPNG  
YIEGKLSQMSKEVNARIEPFLKTTPRPLRLPNGPPCSQRSKFLLMDALKLSIEDPSHEGEGIPLYDAIKCMRTFFGWKEP  
NVVKPHEKGINPNYLLSWKQVLAELQDIENEEKIPKTKNMKKTSQLKWALGENMAPEKVDFDDCKDVGDLKQYDSDEPEL  
RSLASWIQNEFNKACELTDSSWIELDEIGEDVAPIEHIASMRRNYFTSEVSHCRATEYIMKGVYINTALLNASCAAMDDF  
QLIPMISKCRTKEGRRKTNLYGFIIKGRSHLRNDTDVVNFVSMEFSLTDPRLEPHKWEKYCVLEIGDMLIRSAIGQVSRP  
MFLYVRTNGTSKIKMKWGMEMRRCLLQSLQQIESMIEAESSVKEKDMTKEFFENKSETWPIGESPKGVEESSIGKVCRTL  
LAKSVFNSLYASPQLEGFSAESRKLLLIVQALRDNLEPGTFDLGGLYEAIEECLINDPWVLLNASWFNSFLTHALS`

| Site | Type | Count |
| --- | --- | --- |
| 513 | N-linked | 1 |
| 703 | N-linked | 2 |

| Mass | Sequence | MS_2_ Score | `q`-value | MS_1_ Score | Signal | Protein Coordinates | Tandem Scans | Start Time | Apex Time | End Time |
| --- | --- | --- | --- | --- | --- | --- | --- | --- | --- | --- |
| 4184.6209 | LN(N)ASWF Fuc 5 Hex 8 HexNAc 7 | 130.343 | 0.0 | 0.000 | 0.000e+00 | 701 - 707 | 1 | - | - | - |
| 3575.3813 | LN(N)ASWF Hex 10 HexNAc 6 | 128.990 | 0.0 | -19.087 | 9.991e+06 | 701 - 707 | 1 | 25.71 | 25.88 | 25.88 |
| 4047.6195 | RN(N)DTDVVNFVSMEF Fuc 1 Hex 10 HexNAc 3 | 82.356 | 0.014 | 11.489 | 1.325e+08 | 511 - 525 | 4 | 34.68 | 35.00 | 36.68 |

### LN(N)ASWF Fuc 5 Hex 8 HexNAc 7

**Mass:**  4184.6209

(-2.930e-07)

**Start Position**: 702

**End Position**: 708

| Profile Score | Tandem Score | TDA q-value | Volume | Spectrum Matches |
| --- | --- | --- | --- | --- |
| 0.0000 | 130.3434 | 0.0000 | 0.0000e+00 | 1 |

Adduct:

No Chromatogram Found

### LN(N)ASWF Hex 10 HexNAc 6

**Mass:**  3575.3864

(7.661e-06)

**Charge States:** 3

**Start Position**: 702

**End Position**: 708

| Profile Score | Tandem Score | TDA q-value | Volume | Spectrum Matches |
| --- | --- | --- | --- | --- |
| -19.0874 | 128.9895 | 0.0000 | 9.9906e+06 | 1 |

**Adduct Signal Fractions**

- **Unmodified**: 9.9906e+06

### RN(N)DTDVVNFVSMEF Fuc 1 Hex 10 HexNAc 3

**Mass:**  4047.6128

(8.003e-06)

**Charge States:** 3, 4

**Start Position**: 512

**End Position**: 526

| Profile Score | Tandem Score | TDA q-value | Volume | Spectrum Matches |
| --- | --- | --- | --- | --- |
| 11.4887 | 82.3564 | 0.0137 | 1.3254e+08 | 4 |

**Adduct Signal Fractions**

- **Unmodified**: 1.3254e+08

### iav|ABD77682|A/Puerto

**>iav|ABD77682|A/Puerto**  
 `MEDFVRQCFNPMIVELAEKTMKEYGEDLKIETNKFAAICTHLEVCFMYSDFHFINEQGESIIVELGDPNALLKHRFEIIE  
GRDRTMAWTVVNSICNTTGAEKPKFLPDLYDYKENRFIEIGVTRREVHIYYLEKANKIKSEKTHIHIFSFTGEEMATKAD  
YTLDEESRARIKTRLFTIRQEMASRGLWDSFRQSERGEETIEERFEITGTMRKLADQSLPPNFSSLENFRAYVDGFEPNG  
YIEGKLSQMSKEVNARIEPFLKTTPRPLRLPNGPPCSQRSKFLLMDALKLSIEDPSHEGEGIPLYDAIKCMRTFFGWKEP  
NVVKPHEKGINPNYLLSWKQVLAELQDIENEEKIPKTKNMKKTSQLKWALGENMAPEKVDFDDCKDVGDLKQYDSDEPEL  
RSLASWIQNEFNKACELTDSSWIELDEIGEDVAPIEHIASMRRNYFTSEVSHCRATEYIMKGVYINTALLNASCAAMDDF  
QLIPMISKCRTKEGRRKTNLYGFIIKGRSHLRNDTDVVNFVSMEFSLTDPRLEPHKWEKYCVLEIGDMLIRSAIGQVSRP  
MFLYVRTNGTSKIKMKWGMEMRRCLLQSLQQIESMIEAESSVKEKDMTKEFFENKSETWPIGESPKGVEESSIGKVCRTL  
LAKSVFNSLYASPQLEGFSAESRKLLLIVQALRDNLEPGTFDLGGLYEAIEECLINDPWVLLNASWFNSFLTHALS`

| Site | Type | Count |
| --- | --- | --- |
| 513 | N-linked | 1 |
| 703 | N-linked | 2 |

| Mass | Sequence | MS_2_ Score | `q`-value | MS_1_ Score | Signal | Protein Coordinates | Tandem Scans | Start Time | Apex Time | End Time |
| --- | --- | --- | --- | --- | --- | --- | --- | --- | --- | --- |
| 4184.6209 | LN(N)ASWF Fuc 5 Hex 8 HexNAc 7 | 130.343 | 0.0 | 0.000 | 0.000e+00 | 701 - 707 | 1 | - | - | - |
| 3575.3813 | LN(N)ASWF Hex 10 HexNAc 6 | 128.990 | 0.0 | -19.087 | 9.991e+06 | 701 - 707 | 1 | 25.71 | 25.88 | 25.88 |
| 4047.6195 | RN(N)DTDVVNFVSMEF Fuc 1 Hex 10 HexNAc 3 | 82.356 | 0.014 | 11.489 | 1.325e+08 | 511 - 525 | 4 | 34.68 | 35.00 | 36.68 |

### LN(N)ASWF Fuc 5 Hex 8 HexNAc 7

**Mass:**  4184.6209

(-2.930e-07)

**Start Position**: 702

**End Position**: 708

| Profile Score | Tandem Score | TDA q-value | Volume | Spectrum Matches |
| --- | --- | --- | --- | --- |
| 0.0000 | 130.3434 | 0.0000 | 0.0000e+00 | 1 |

Adduct:

No Chromatogram Found

### LN(N)ASWF Hex 10 HexNAc 6

**Mass:**  3575.3864

(7.661e-06)

**Charge States:** 3

**Start Position**: 702

**End Position**: 708

| Profile Score | Tandem Score | TDA q-value | Volume | Spectrum Matches |
| --- | --- | --- | --- | --- |
| -19.0874 | 128.9895 | 0.0000 | 9.9906e+06 | 1 |

**Adduct Signal Fractions**

- **Unmodified**: 9.9906e+06

### RN(N)DTDVVNFVSMEF Fuc 1 Hex 10 HexNAc 3

**Mass:**  4047.6128

(8.003e-06)

**Charge States:** 3, 4

**Start Position**: 512

**End Position**: 526

| Profile Score | Tandem Score | TDA q-value | Volume | Spectrum Matches |
| --- | --- | --- | --- | --- |
| 11.4887 | 82.3564 | 0.0137 | 1.3254e+08 | 4 |

**Adduct Signal Fractions**

- **Unmodified**: 1.3254e+08

### iav|AAM75157|A/Puerto

**>iav|AAM75157|A/Puerto**  
 `MEDFVRQCFNPMIVELAEKTMKEYGEDLKIETNKFAAICTHLEVCFMYSDFHFINEQGESIIVELGDPNALLKHRFEIIE  
GRDRTMAWTVVNSICNTTGAEKPKFLPDLYDYKENRFIEIGVTRREVHIYYLEKANKIKSEKTHIHIFSFTGEEMATKAD  
YTLDEESRARIKTRLFTIRQEMASRGLWDSFRQSERGEETIEERFEITGTMRKLADQSLPPNFSSLENFRAYVDGFEPNG  
YIEGKLSQMSKEVNARIEPFLKTTPRPLRLPNGPPCSQRSKFLLMDALKLSIEDPSHEGEGIPLYDAIKCMRTFFGWKEP  
NVVKPHEKGINPNYLLSWKQVLAELQDIENEEKIPKTKNMKKTSQLKWALGENMAPEKVDFDDCKDVGDLKQYDSDEPEL  
RSLASWIQNEFNKACELTDSSWIELDEIGEDVAPIEHIASMRRNYFTSEVSHCRATEYIMKGVYINTALLNASCAAMDDF  
QLIPMISKCRTKEGRRKTNLYGFIIKGRSHLRNDTDVVNFVSMEFSLTDPRLEPHKWEKYCVLEIGDMLIRSAIGQVSRP  
MFLYVRTNGTSKIKMKWGMEMRRCLLQSLQQIESMIEAESSVKEKDMTKEFFENKSETWPIGESPKGVEESSIGKVCRTL  
LAKSVFNSLYASPQLEGFSAESRKLLLIVQALRDNLEPGTFDLGGLYEAIEECLINDPWVLLNASWFNSFLTHALS`

| Site | Type | Count |
| --- | --- | --- |
| 513 | N-linked | 1 |
| 703 | N-linked | 2 |

| Mass | Sequence | MS_2_ Score | `q`-value | MS_1_ Score | Signal | Protein Coordinates | Tandem Scans | Start Time | Apex Time | End Time |
| --- | --- | --- | --- | --- | --- | --- | --- | --- | --- | --- |
| 4184.6209 | LN(N)ASWF Fuc 5 Hex 8 HexNAc 7 | 130.343 | 0.0 | 0.000 | 0.000e+00 | 701 - 707 | 1 | - | - | - |
| 3575.3813 | LN(N)ASWF Hex 10 HexNAc 6 | 128.990 | 0.0 | -19.087 | 9.991e+06 | 701 - 707 | 1 | 25.71 | 25.88 | 25.88 |
| 4047.6195 | RN(N)DTDVVNFVSMEF Fuc 1 Hex 10 HexNAc 3 | 82.356 | 0.014 | 11.489 | 1.325e+08 | 511 - 525 | 4 | 34.68 | 35.00 | 36.68 |

### LN(N)ASWF Fuc 5 Hex 8 HexNAc 7

**Mass:**  4184.6209

(-2.930e-07)

**Start Position**: 702

**End Position**: 708

| Profile Score | Tandem Score | TDA q-value | Volume | Spectrum Matches |
| --- | --- | --- | --- | --- |
| 0.0000 | 130.3434 | 0.0000 | 0.0000e+00 | 1 |

Adduct:

No Chromatogram Found

### LN(N)ASWF Hex 10 HexNAc 6

**Mass:**  3575.3864

(7.661e-06)

**Charge States:** 3

**Start Position**: 702

**End Position**: 708

| Profile Score | Tandem Score | TDA q-value | Volume | Spectrum Matches |
| --- | --- | --- | --- | --- |
| -19.0874 | 128.9895 | 0.0000 | 9.9906e+06 | 1 |

**Adduct Signal Fractions**

- **Unmodified**: 9.9906e+06

### RN(N)DTDVVNFVSMEF Fuc 1 Hex 10 HexNAc 3

**Mass:**  4047.6128

(8.003e-06)

**Charge States:** 3, 4

**Start Position**: 512

**End Position**: 526

| Profile Score | Tandem Score | TDA q-value | Volume | Spectrum Matches |
| --- | --- | --- | --- | --- |
| 11.4887 | 82.3564 | 0.0137 | 1.3254e+08 | 4 |

**Adduct Signal Fractions**

- **Unmodified**: 1.3254e+08

### iav|AEX92880|A/Puerto

**>iav|AEX92880|A/Puerto**  
 `MEDFVRQCFNPMIVELAEKTMKEYGEDLKIETNKFAAICTHLEVCFMYSDFHFINEQGESIIVELGDPNALLKHRFEIIE  
GRDRTMAWTVVNSICNTTGAEKPKFLPDLYDYKENRFIEIGVTRREVHIYYLEKANKIKSEKTHIHIFSFTGEEMATKAD  
YTLDEESRARIKTRLFTIRQEMASRGLWDSFRQSERGEETIEERFEITGTMRKLADQSLPPNFSSLENFRAYVDGFEPNG  
YIEGKLSQMSKEVNARIEPFLKTTPRPLRLPNGPPCSQRSKFLLMDALKLSIEDPSHEGEGIPLYDAIKCMRTFFGWKEP  
NVVKPHEKGINPNYLLSWKQVLAELQDIENEEKIPKTKNMKKTSQLKWALGENMAPEKVDFDDCKDVGDLKQYDSDEPEL  
RSLASWIQNEFNKACELTDSSWIELDEIGEDVAPIEHIASMRRNYFTSEVSHCRATEYIMKGVYINTALLNASCAAMDDF  
QLIPMISKCRTKEGRRKTNLYGFIIKGRSHLRNDTDVVNFVSMEFSLTDPRLEPHKWEKYCVLEIGDMLIRSAIGQVSRP  
MFLYVRTNGTSKIKMKWGMEMRRCLLQSLQQIESMIEAESSVKEKDMTKEFFENKSETWPIGESPKGVEESSIGKVCRTL  
LAKSVFNSLYASPQLEGFSAESRKLLLIVQALRDNLEPGTFDLGGLYEAIEECLINDPWVLLNASWFNSFLTHALS`

| Site | Type | Count |
| --- | --- | --- |
| 513 | N-linked | 1 |
| 703 | N-linked | 2 |

| Mass | Sequence | MS_2_ Score | `q`-value | MS_1_ Score | Signal | Protein Coordinates | Tandem Scans | Start Time | Apex Time | End Time |
| --- | --- | --- | --- | --- | --- | --- | --- | --- | --- | --- |
| 4184.6209 | LN(N)ASWF Fuc 5 Hex 8 HexNAc 7 | 130.343 | 0.0 | 0.000 | 0.000e+00 | 701 - 707 | 1 | - | - | - |
| 3575.3813 | LN(N)ASWF Hex 10 HexNAc 6 | 128.990 | 0.0 | -19.087 | 9.991e+06 | 701 - 707 | 1 | 25.71 | 25.88 | 25.88 |
| 4047.6195 | RN(N)DTDVVNFVSMEF Fuc 1 Hex 10 HexNAc 3 | 82.356 | 0.014 | 11.489 | 1.325e+08 | 511 - 525 | 4 | 34.68 | 35.00 | 36.68 |

### LN(N)ASWF Fuc 5 Hex 8 HexNAc 7

**Mass:**  4184.6209

(-2.930e-07)

**Start Position**: 702

**End Position**: 708

| Profile Score | Tandem Score | TDA q-value | Volume | Spectrum Matches |
| --- | --- | --- | --- | --- |
| 0.0000 | 130.3434 | 0.0000 | 0.0000e+00 | 1 |

Adduct:

No Chromatogram Found

### LN(N)ASWF Hex 10 HexNAc 6

**Mass:**  3575.3864

(7.661e-06)

**Charge States:** 3

**Start Position**: 702

**End Position**: 708

| Profile Score | Tandem Score | TDA q-value | Volume | Spectrum Matches |
| --- | --- | --- | --- | --- |
| -19.0874 | 128.9895 | 0.0000 | 9.9906e+06 | 1 |

**Adduct Signal Fractions**

- **Unmodified**: 9.9906e+06

### RN(N)DTDVVNFVSMEF Fuc 1 Hex 10 HexNAc 3

**Mass:**  4047.6128

(8.003e-06)

**Charge States:** 3, 4

**Start Position**: 512

**End Position**: 526

| Profile Score | Tandem Score | TDA q-value | Volume | Spectrum Matches |
| --- | --- | --- | --- | --- |
| 11.4887 | 82.3564 | 0.0137 | 1.3254e+08 | 4 |

**Adduct Signal Fractions**

- **Unmodified**: 1.3254e+08

### iav|CAA24295|A/Puerto

**>iav|CAA24295|A/Puerto**  
 `MEDFVRQCFNPMIVELAEKTMKEYGEDLKIETNKFAAICTHLEVCFMYSDFHFINEQGESIIVELGDPNALLKHRFEIIE  
GRDRTMAWTVVNSICNTTGAEKPKFLPDLYDYKENRFIEIGVTRREVHIYYLEKANKIKSEKTHIHIFSFTGEEMATKAD  
YTLDEESRARIKTRLFTIRQEMASRGLWDSFRQSERGEETIEERFEITGTMRKLADQSLPPNFSSLENFRAYVDGFEPNG  
YIEGKLSQMSKEVNARIEPFLKTTPRPLRLPNGPPCSQRSKFLLMDALKLSIEDPSHEGEGIPLYDAIKCMRTFFGWKEP  
NVVKPHEKGINPNYLLSWKQVLAELQDIENEEKIPKTKNMKKTSQLKWALGENMAPEKVDFDDCKDVGDLKQYDSDEPEL  
RSLASWIQNEFNKACELTDSSWIELDEIGEDVAPIEHIASMRRNYFTSEVSHCRATEYIMKGVYINTALLNASCAAMDDF  
QLIPMISKCRTKEGRRKTNLYGFIIKGRSHLRNDTDVVNFVSMEFSLTDPRLEPHKWEKYCVLEIGDMLLRSAIGQVSRP  
MFLYVRTNGTSKIKMKWGMEMRRCLLQSLQQIESMIEAESSVKEKDMTKEFFENKSETWPIGESPKGVEESSIGKVCRTL  
LAKSVFNSLYASPQLEGFSAESRKLLLIVQALRDNLEPGTFDLGGLYEAIEECLINDPWVLLNASWFNSFLTHALS`

| Site | Type | Count |
| --- | --- | --- |
| 513 | N-linked | 1 |
| 703 | N-linked | 2 |

| Mass | Sequence | MS_2_ Score | `q`-value | MS_1_ Score | Signal | Protein Coordinates | Tandem Scans | Start Time | Apex Time | End Time |
| --- | --- | --- | --- | --- | --- | --- | --- | --- | --- | --- |
| 4184.6209 | LN(N)ASWF Fuc 5 Hex 8 HexNAc 7 | 130.343 | 0.0 | 0.000 | 0.000e+00 | 701 - 707 | 1 | - | - | - |
| 3575.3813 | LN(N)ASWF Hex 10 HexNAc 6 | 128.990 | 0.0 | -19.087 | 9.991e+06 | 701 - 707 | 1 | 25.71 | 25.88 | 25.88 |
| 4047.6195 | RN(N)DTDVVNFVSMEF Fuc 1 Hex 10 HexNAc 3 | 82.356 | 0.014 | 11.489 | 1.325e+08 | 511 - 525 | 4 | 34.68 | 35.00 | 36.68 |

### LN(N)ASWF Fuc 5 Hex 8 HexNAc 7

**Mass:**  4184.6209

(-2.930e-07)

**Start Position**: 702

**End Position**: 708

| Profile Score | Tandem Score | TDA q-value | Volume | Spectrum Matches |
| --- | --- | --- | --- | --- |
| 0.0000 | 130.3434 | 0.0000 | 0.0000e+00 | 1 |

Adduct:

No Chromatogram Found

### LN(N)ASWF Hex 10 HexNAc 6

**Mass:**  3575.3864

(7.661e-06)

**Charge States:** 3

**Start Position**: 702

**End Position**: 708

| Profile Score | Tandem Score | TDA q-value | Volume | Spectrum Matches |
| --- | --- | --- | --- | --- |
| -19.0874 | 128.9895 | 0.0000 | 9.9906e+06 | 1 |

**Adduct Signal Fractions**

- **Unmodified**: 9.9906e+06

### RN(N)DTDVVNFVSMEF Fuc 1 Hex 10 HexNAc 3

**Mass:**  4047.6128

(8.003e-06)

**Charge States:** 3, 4

**Start Position**: 512

**End Position**: 526

| Profile Score | Tandem Score | TDA q-value | Volume | Spectrum Matches |
| --- | --- | --- | --- | --- |
| 11.4887 | 82.3564 | 0.0137 | 1.3254e+08 | 4 |

**Adduct Signal Fractions**

- **Unmodified**: 1.3254e+08

### iav|ADX99654|A/Puerto

**>iav|ADX99654|A/Puerto**  
 `MEDFVRQCFNPMIVELAEKTMKEYGEDLKIETNKFAAICTHLEVCFMYSDFHFINEQGESIIVELGDPNALLKHRFEIIE  
GRDRTMAWTVVNSICNTTGAEKPKFLPDLYDYKENRFIEIGVTRREVHIYYLEKANKIKSEKTHIHIFSFTGEEMATKAD  
YTLDEESRARIKTRLFTIRQEMASRGLWDSFRQSERGEETIEERFEITGTMRKLADQSPPPNFSSLENFRAYVDGFEPNG  
YIEGKLSQMSKEVNARIEPFLKTTPRPLRLPNGPPCSQRSKFLLMDALKLSIEDPSHEGEGIPLYDAIKCMRTFFGWKEP  
NVVKPHEKGINPNYLLSWKQVLAELQDIENEEKIPKTKNMKKTSQLKWALGENMAPEKVDFDDCKDVGDLKQYDSDEPEL  
RSLASWIQNEFNKACELTDSSWIELDEIGEDVAPIEHIASMRRNYFTSEVSHCRATEYIMKGVYINTALLNASCAAMDDF  
QLIPMISKCRTKEGRRKTNLYGFIIKGRSHLRNDTDVVNFVSMEFSLTDPRLEPHKWEKYCVLEIGDMLIRSAIGQVSRP  
MFLYVRTNGTSKIKMKWGMEMRRCLLQSLQQIESMIEAESSVKEKDMTKEFFENKSETWPIGESPKGVEESSIGKVCRTL  
LAKSVFNSLYASPQLEGFSAESRKLLLIVQALRDNLEPGTFDLGGLYEAIEECLINDPWVLLNASWFNSFLTHALS`

| Site | Type | Count |
| --- | --- | --- |
| 513 | N-linked | 1 |
| 703 | N-linked | 2 |

| Mass | Sequence | MS_2_ Score | `q`-value | MS_1_ Score | Signal | Protein Coordinates | Tandem Scans | Start Time | Apex Time | End Time |
| --- | --- | --- | --- | --- | --- | --- | --- | --- | --- | --- |
| 4184.6209 | LN(N)ASWF Fuc 5 Hex 8 HexNAc 7 | 130.343 | 0.0 | 0.000 | 0.000e+00 | 701 - 707 | 1 | - | - | - |
| 3575.3813 | LN(N)ASWF Hex 10 HexNAc 6 | 128.990 | 0.0 | -19.087 | 9.991e+06 | 701 - 707 | 1 | 25.71 | 25.88 | 25.88 |
| 4047.6195 | RN(N)DTDVVNFVSMEF Fuc 1 Hex 10 HexNAc 3 | 82.356 | 0.014 | 11.489 | 1.325e+08 | 511 - 525 | 4 | 34.68 | 35.00 | 36.68 |

### LN(N)ASWF Fuc 5 Hex 8 HexNAc 7

**Mass:**  4184.6209

(-2.930e-07)

**Start Position**: 702

**End Position**: 708

| Profile Score | Tandem Score | TDA q-value | Volume | Spectrum Matches |
| --- | --- | --- | --- | --- |
| 0.0000 | 130.3434 | 0.0000 | 0.0000e+00 | 1 |

Adduct:

No Chromatogram Found

### LN(N)ASWF Hex 10 HexNAc 6

**Mass:**  3575.3864

(7.661e-06)

**Charge States:** 3

**Start Position**: 702

**End Position**: 708

| Profile Score | Tandem Score | TDA q-value | Volume | Spectrum Matches |
| --- | --- | --- | --- | --- |
| -19.0874 | 128.9895 | 0.0000 | 9.9906e+06 | 1 |

**Adduct Signal Fractions**

- **Unmodified**: 9.9906e+06

### RN(N)DTDVVNFVSMEF Fuc 1 Hex 10 HexNAc 3

**Mass:**  4047.6128

(8.003e-06)

**Charge States:** 3, 4

**Start Position**: 512

**End Position**: 526

| Profile Score | Tandem Score | TDA q-value | Volume | Spectrum Matches |
| --- | --- | --- | --- | --- |
| 11.4887 | 82.3564 | 0.0137 | 1.3254e+08 | 4 |

**Adduct Signal Fractions**

- **Unmodified**: 1.3254e+08

### iav|ABO21708|A/Puerto

**>iav|ABO21708|A/Puerto**  
 `MEDFVRQCFNPMIVELAEKTMKEYGEDLKIETNKFAAICTHLEVCFMYSDFHFINEQGESIIVELGDPNALLKHRFEIIE  
GRDRTMAWTVVNSICNTTGAEKPKFLPDLYDYKENRFIEIGVTRREVHIYYLEKANKIKSEKTHIHIFSFTGEEMATKAD  
YTLDEESRARIKTRLFTIRQEMASRGLWDSFRQSERGEETIEERFEITGTMRKLADQSLPPNFSSLENFRAYVDGFEPNG  
YIEGKLSQMSKEVNARIEPFLKTTPRPLRLPNGPPCSQRSKFLLMDALKLSIEDPSHEGEGIPLYDAIKCMRTFFGWKEP  
NVVKPHEKGINPNYLLSWKQVLAELQDIENEEKIPKTKNMKKTSQLKWALGENMAPEKVDFDDCKDVGDLKQYDSDEPEL  
RSLASWIQNEFNKACELTDSSWIELDEIGEDVAPIEHIASMRRNYFTSEVSHCRATEYIMKGVYINTALLNASCAAMDDF  
QLIPMISKCRTKEGRRKTNLYGFIIKGRSHLRNDTDVVNFVSMEFSLTDPRLEPHKWEKYCVLEIGDMLIRSAIGQVSRP  
MFLYVRTNGTSKIKMKWGMEMRRCLLQSLQQIESMIEAESSVKEKDMTKEFFENKSETWPIGESPKGVEESSIGKVCRTL  
LAKSVFNSLYASPQLEGFSAESRKLLLIVQALRDNLEPGTFDLGGLYEAIEECLINDPWVLLNASWFNSFLTHALS`

| Site | Type | Count |
| --- | --- | --- |
| 513 | N-linked | 1 |
| 703 | N-linked | 2 |

| Mass | Sequence | MS_2_ Score | `q`-value | MS_1_ Score | Signal | Protein Coordinates | Tandem Scans | Start Time | Apex Time | End Time |
| --- | --- | --- | --- | --- | --- | --- | --- | --- | --- | --- |
| 4184.6209 | LN(N)ASWF Fuc 5 Hex 8 HexNAc 7 | 130.343 | 0.0 | 0.000 | 0.000e+00 | 701 - 707 | 1 | - | - | - |
| 3575.3813 | LN(N)ASWF Hex 10 HexNAc 6 | 128.990 | 0.0 | -19.087 | 9.991e+06 | 701 - 707 | 1 | 25.71 | 25.88 | 25.88 |
| 4047.6195 | RN(N)DTDVVNFVSMEF Fuc 1 Hex 10 HexNAc 3 | 82.356 | 0.014 | 11.489 | 1.325e+08 | 511 - 525 | 4 | 34.68 | 35.00 | 36.68 |

### LN(N)ASWF Fuc 5 Hex 8 HexNAc 7

**Mass:**  4184.6209

(-2.930e-07)

**Start Position**: 702

**End Position**: 708

| Profile Score | Tandem Score | TDA q-value | Volume | Spectrum Matches |
| --- | --- | --- | --- | --- |
| 0.0000 | 130.3434 | 0.0000 | 0.0000e+00 | 1 |

Adduct:

No Chromatogram Found

### LN(N)ASWF Hex 10 HexNAc 6

**Mass:**  3575.3864

(7.661e-06)

**Charge States:** 3

**Start Position**: 702

**End Position**: 708

| Profile Score | Tandem Score | TDA q-value | Volume | Spectrum Matches |
| --- | --- | --- | --- | --- |
| -19.0874 | 128.9895 | 0.0000 | 9.9906e+06 | 1 |

**Adduct Signal Fractions**

- **Unmodified**: 9.9906e+06

### RN(N)DTDVVNFVSMEF Fuc 1 Hex 10 HexNAc 3

**Mass:**  4047.6128

(8.003e-06)

**Charge States:** 3, 4

**Start Position**: 512

**End Position**: 526

| Profile Score | Tandem Score | TDA q-value | Volume | Spectrum Matches |
| --- | --- | --- | --- | --- |
| 11.4887 | 82.3564 | 0.0137 | 1.3254e+08 | 4 |

**Adduct Signal Fractions**

- **Unmodified**: 1.3254e+08

### iav|BAV59610|A/Puerto

**>iav|BAV59610|A/Puerto**  
 `MEDFVRQCFNPMIVELAEKTMKEYGEDLKIETNKFAAICTHLEICFMYSDFHFINEQGESIIVELDDPNALLKHRFEIIE  
GRDRTMAWTVVNSICNTTGAEKPKFLPDLYDYKENRFIEIGVTRREVHIYYLEKANKIKSEKTHIHIFSFTGEEMATKAD  
YTLDEESRARIKTRLFTIRQEMASRGLWDSFRQSERGEETIEERFEITGTMRKLADQSLPPNFSSLENFRAYVDGFEPNG  
YIEGKLSQMSKEVNARIEPFLKTTPRPLRLPNGPPCSQRSKFLLMDALKLSIEDPSHEGEGIPLYDAIKCMRTFFGWKEP  
NVVKPHEKGINPNYLLSWKQVLAELQDIENEEKIPKTKNMKKTSQLKWALGENMAPEKVDFDDCKDVGDLKQYDSDEPEL  
RSLASWIQNEFNKACELTDSSWIELDEIGEDVAPIEHIASMRRNYFTSEVSHCRATEYIMKGVYINTALLNASCAAMDDF  
QLIPMISKCRTKEGRRKTNLYGFIIKGRSHLRNDTDVVNFVSMEFSLTDPRLEPHKWEKYCVLEIGDMLIRSAIGQVSRP  
MFLYVRTNGTSKIKMKWGMEMRRCLLQSLQQIESMIEAESSVKEKDMTKEFFENKSETWPIGESPKGVEESSIGKVCRTL  
LAKSVFNSLYASPQLEGFSAESRKLLLIVQALRDNLEPGTFDLGGLYDAIEECLINDPWVLLNASWFNSFLTHALS`

| Site | Type | Count |
| --- | --- | --- |
| 513 | N-linked | 1 |
| 703 | N-linked | 2 |

| Mass | Sequence | MS_2_ Score | `q`-value | MS_1_ Score | Signal | Protein Coordinates | Tandem Scans | Start Time | Apex Time | End Time |
| --- | --- | --- | --- | --- | --- | --- | --- | --- | --- | --- |
| 4184.6209 | LN(N)ASWF Fuc 5 Hex 8 HexNAc 7 | 130.343 | 0.0 | 0.000 | 0.000e+00 | 701 - 707 | 1 | - | - | - |
| 3575.3813 | LN(N)ASWF Hex 10 HexNAc 6 | 128.990 | 0.0 | -19.087 | 9.991e+06 | 701 - 707 | 1 | 25.71 | 25.88 | 25.88 |
| 4047.6195 | RN(N)DTDVVNFVSMEF Fuc 1 Hex 10 HexNAc 3 | 82.356 | 0.014 | 11.489 | 1.325e+08 | 511 - 525 | 4 | 34.68 | 35.00 | 36.68 |

### LN(N)ASWF Fuc 5 Hex 8 HexNAc 7

**Mass:**  4184.6209

(-2.930e-07)

**Start Position**: 702

**End Position**: 708

| Profile Score | Tandem Score | TDA q-value | Volume | Spectrum Matches |
| --- | --- | --- | --- | --- |
| 0.0000 | 130.3434 | 0.0000 | 0.0000e+00 | 1 |

Adduct:

No Chromatogram Found

### LN(N)ASWF Hex 10 HexNAc 6

**Mass:**  3575.3864

(7.661e-06)

**Charge States:** 3

**Start Position**: 702

**End Position**: 708

| Profile Score | Tandem Score | TDA q-value | Volume | Spectrum Matches |
| --- | --- | --- | --- | --- |
| -19.0874 | 128.9895 | 0.0000 | 9.9906e+06 | 1 |

**Adduct Signal Fractions**

- **Unmodified**: 9.9906e+06

### RN(N)DTDVVNFVSMEF Fuc 1 Hex 10 HexNAc 3

**Mass:**  4047.6128

(8.003e-06)

**Charge States:** 3, 4

**Start Position**: 512

**End Position**: 526

| Profile Score | Tandem Score | TDA q-value | Volume | Spectrum Matches |
| --- | --- | --- | --- | --- |
| 11.4887 | 82.3564 | 0.0137 | 1.3254e+08 | 4 |

**Adduct Signal Fractions**

- **Unmodified**: 1.3254e+08

### iav|ACF41841|A/Puerto

**>iav|ACF41841|A/Puerto**  
 `MEDFVRQCFNPMIVELAEKTMKEYGEDLKIETNKFAAICTHLEVCFMYSDFHFINEQGESIIVELGDPNALLKHRFEIIE  
GRDRTMAWTVVNSICNTTGAEKPKFLPDLYDYKENRFIEIGVTRREVHIYYLEKANKIKSEKTHIHIFSFTGEEMATKAD  
YTLDEESRARIKTRLFTIRQEMASRGLWDSFRQSERGEETIEERFEITGTMRKLADQSLPPNFSSLENFRAYVDGFEPNG  
YIEGKLSQMSKEVNARIEPFLKTTPRPLRLPNGPPCSQRSKFLLMDALKLSIEDPSHEGEGIPLYDAIKCMRTFFGWKEP  
NVVKPHEKGINPNYLLSWKQVLAELQDIENEEKIPKTKNMKKTSQLKWALGENMAPEKVDFDDCKDVGDLKQYDSDEPEL  
RSLASWIQNEFNKACELTDSSWIELDEIGEDVAPIEHIASMRRNYFTSEVSHCRATEYIMKGVYINTALLNASCAAMDDF  
QLIPMISKCRTKEGRRKTNLYGFIIKGRSHLRNDTDVVNFVSMEFSLTDPRLEPHKWEKYCVLEIGDMLIRSAIGQVSRP  
MFLYVRTNGTSKIKMKWGMEMRRCLLQSLQQIESMIEAESSVKEKDMTKEFFENKSETWPIGESPKGVEESSIGKVCRTL  
LAKSVFNSLYASPQLEGFSAESRKLLLIVQALRDNLEPGTFDLGGLYEAIEECLINDPWVLLNASWFNSFLTHALS`

| Site | Type | Count |
| --- | --- | --- |
| 513 | N-linked | 1 |
| 703 | N-linked | 2 |

| Mass | Sequence | MS_2_ Score | `q`-value | MS_1_ Score | Signal | Protein Coordinates | Tandem Scans | Start Time | Apex Time | End Time |
| --- | --- | --- | --- | --- | --- | --- | --- | --- | --- | --- |
| 4184.6209 | LN(N)ASWF Fuc 5 Hex 8 HexNAc 7 | 130.343 | 0.0 | 0.000 | 0.000e+00 | 701 - 707 | 1 | - | - | - |
| 3575.3813 | LN(N)ASWF Hex 10 HexNAc 6 | 128.990 | 0.0 | -19.087 | 9.991e+06 | 701 - 707 | 1 | 25.71 | 25.88 | 25.88 |
| 4047.6195 | RN(N)DTDVVNFVSMEF Fuc 1 Hex 10 HexNAc 3 | 82.356 | 0.014 | 11.489 | 1.325e+08 | 511 - 525 | 4 | 34.68 | 35.00 | 36.68 |

### LN(N)ASWF Fuc 5 Hex 8 HexNAc 7

**Mass:**  4184.6209

(-2.930e-07)

**Start Position**: 702

**End Position**: 708

| Profile Score | Tandem Score | TDA q-value | Volume | Spectrum Matches |
| --- | --- | --- | --- | --- |
| 0.0000 | 130.3434 | 0.0000 | 0.0000e+00 | 1 |

Adduct:

No Chromatogram Found

### LN(N)ASWF Hex 10 HexNAc 6

**Mass:**  3575.3864

(7.661e-06)

**Charge States:** 3

**Start Position**: 702

**End Position**: 708

| Profile Score | Tandem Score | TDA q-value | Volume | Spectrum Matches |
| --- | --- | --- | --- | --- |
| -19.0874 | 128.9895 | 0.0000 | 9.9906e+06 | 1 |

**Adduct Signal Fractions**

- **Unmodified**: 9.9906e+06

### RN(N)DTDVVNFVSMEF Fuc 1 Hex 10 HexNAc 3

**Mass:**  4047.6128

(8.003e-06)

**Charge States:** 3, 4

**Start Position**: 512

**End Position**: 526

| Profile Score | Tandem Score | TDA q-value | Volume | Spectrum Matches |
| --- | --- | --- | --- | --- |
| 11.4887 | 82.3564 | 0.0137 | 1.3254e+08 | 4 |

**Adduct Signal Fractions**

- **Unmodified**: 1.3254e+08

### tr|A0A1D5PV37|A0A1D5PV37\_CHICK

**>tr|A0A1D5PV37|A0A1D5PV37\_CHICK**  
 `MPGRALFTFAMRGIILALVLTLVGSQKFDIDPGFNSRRSYLYNYEGSMLNGLQDRSLGKAGVRLSSKLEISGLPENAYLL  
KVRSPQVEEYNGVWPRDPFTRSSKITQVISSCFTRLFKFEYSSGRIGNIYAPEDCPDLCVNIVRGILNMFQMTIKKSQNV  
YELQEAGIGGICHARYVIQEDRKNSRIYVTRTVDLNNCQEKVQKSIGMAYIYPCPVDVMKERLTKGTTAFSYKLKQSDSG  
TLITDVSSRQVYQISPFNEPTGVAVMEARQQLTLVEVRSERGSAPDVPMQNYGSLRYRFPAVLPQMPLQLIKTKNPEQRI  
IETLQHIVLNNQQDFHDDVSYRFLEVVQLCRIANADNLESIWRQVSDKPRYRRWLLSAVSASGTTETLKFLKNRIRNDDL  
NYIQTLLTVSLTLHLLQADEHTLPIAADLMTSSRIQKNPVLQQVACLGYSSVVNRYCSQTSACPKEALQPIHDLADEAIS  
RGREDKMKLALKCIGNMGEPASLKRILKFLPISSSSAADIPVHIQIDAITALKKIAWKDPKTVQGYLIQILADQSLPPEV  
RMMACAVIFETRPALALITTIANVAMKESNMQVASFVYSHMKSLSKSRLPFMYNISSACNIALKLLSPKLDSMSYRYSKV  
IRADTYFDNYRVGATGEIFVVNSPRTMFPSAIISKLMANSAGSVADLVEVGIRVEGLADVIMKRNIPFAEYPTYKQIKEL  
GKALQGWKELPTETPLVSAYLKILGQEVAFININKELLQQVMKTVVEPADRNAAIKRIANQIRNSIAGQWTQPVWMGELR  
YVVPSCLGLPLEYGSYTTALARAAVSVEGKMTPPLTGDFRLSQLLESTMQIRSDLKPSLYVHTVATMGVNTEYFQHAVEI  
QGEVQTRMPMKFDAKIDVKLKNLKIETNPCREETEIVVGRHKAFAVSRNIGELGVEKRTSILPEDAPLDVTEEPFQTSER  
ASREHFAMQGPDSMPRKQSHSSREDLRRSTGKRAHKRDICLKMHHIGCQLCFSRRSRDASFIQNTYLHKLIGEHEAKIVL  
MPVHTDADIEKIQLEIQAGAKAASKIIDATSSGPKEEKKPSLYENIKAKLKKILGIENVFKVGDFEYGR`

| Site | Type | Count |
| --- | --- | --- |
| 614 | N-linked | 1 |

| Mass | Sequence | MS_2_ Score | `q`-value | MS_1_ Score | Signal | Protein Coordinates | Tandem Scans | Start Time | Apex Time | End Time |
| --- | --- | --- | --- | --- | --- | --- | --- | --- | --- | --- |
| 4532.7497 | N(N)ISSAC(C)NIAL Fuc 2 Hex 6 HexNAc 8 Neu5Ac 2 | 77.353 | 0.018 | 9.848 | 5.756e+07 | 613 - 623 | 3 | 41.23 | 42.57 | 42.91 |

### N(N)ISSAC(C)NIAL Fuc 2 Hex 6 HexNAc 8 Neu5Ac 2

**Mass:**  4532.7561

(-4.183e-06)

**Charge States:** 4

**Start Position**: 614

**End Position**: 624

| Profile Score | Tandem Score | TDA q-value | Volume | Spectrum Matches |
| --- | --- | --- | --- | --- |
| 9.8481 | 77.3525 | 0.0179 | 5.7558e+07 | 3 |

**Adduct Signal Fractions**

- **Unmodified**: 5.7558e+07

### tr|A0A1D5P780|A0A1D5P780\_CHICK

**>tr|A0A1D5P780|A0A1D5P780\_CHICK**  
 `MRGIILALVLTLVDPGFNSRRSYLYNYEGSMLNGLQDRSLGKAGVRLSSKLEISGLPENAYLLKVRSPQVEEYNGVWPRD  
PFTRSSKITQVISSCFTRLFKFEYSSGRIGNIYAPEDCPDLCVNIVRGILNMFQMTIKKSQNVYELQEAGIGGICHARYV  
IQEDRKNSRIYVTRTVDLNNCQEKVQKSIGMAYIYPCPVDVMKERLTKGTTAFSYKLKQSDSGTLITDVSSRQVYQISPF  
NEPTGVAVMEARQQLTLVEVRSERGSAPDVPMQNYGSLRYRFPAVLPQMPLQLIKTKNPEQRIIETLQHIVLNNQQDFHD  
DVSYRFLEVVQLCRIANADNLESIWRQVSDKPRYRRWLLSAVSASGTTETLKFLKNRIRNDDLNYIQTLLTVSLTLHLLQ  
ADEHTLPIAADLMTSSRIQKNPVLQQVACLGYSSVVNRYCSQTSACPKEALQPIHDLADEAISRGREDKMKLALKCIGNM  
GEPASLKRILKFLPISSSSAADIPVHIQIDAITALKKIAWKDPKTVQGYLIQILADQSLPPEVRMMACAVIFETRPALAL  
ITTIANVAMKESNMQVASFVYSHMKSLSKSRLPFMYNISSACNIALKLLSPKLDSMSYRYSKVIRADTYFDNYRVGATGE  
IFVVNSPRTMFPSAIISKLMANSAGSVADLVEVGIRVEGLADVIMKRNIPFAEYPTYKQIKELGKALQGWKELPTETPLV  
SAYLKILGQEVAFININKELLQQVMKTVVEPADRNAAIKRIANQIRNSIAGQWTQPVWMGELRYVVPSCLGLPLEYGSYT  
TALARAAVSVEGKMTPPLTGDFRLSQLLESTMQIRSDLKPSLYVHTVATMGVNTEYFQHAVEIQGEVQTRMPMKFDAKID  
VKLKNLKIETNPCREETEIVVGRHKAFAVSRNIGELGVEKRTSILPEDAPLDVTEEPFQTSERASREHFAMQGPDSMPRK  
QSHSSREDLRRSTGKRAHKRDICLKMHHIGCQLCFSRRSRDASFIQNTYLHKLIGEHEAKIVLMPVHTDADIDKIQLEIQ  
AGSRAAARIITEVNPESEEEDESSPYEDIQAKLKRILGIDSMFKVANKTRHPKNRPSKKGNTVLAEFGTEPDAKTSSSSS  
SASSTATSSSSSSASSPNRKKPMDEEENDQVKQARNKDASSSSRSSKSSNSSKRSSSKSSNSSKRSSSSSSSSSSSSSSS  
SRSSSSSSSSSSNSKSSSSSSKSSSSSSRSRSSSKSSSSSSSSSSSSSSKSSSSRSSSSSSKSSSHHSHSHHSGHLNGSS  
SSSSSSRSVSHHSHEHHSGHLEDDSSSSSSSSVLSKIWGRHEIYQYRFRSAHRQEFPKRKLPGDRATSRYSSTRSSHDTS  
RAASWPKFLGDIKTPVLAAFLHGISNNKKTGGLQLVVYADTDSVRPRVQVFVTNLTDSSKWKLCADASVRNAHKAVAYVK  
WGWDCRDYKVSTELVTGRFAGHPAAQVKLEWPKVPSNVRSVVEWFYEFVPGAAFMLGFSERMDKNPSRQARMVVALTSPR  
TCDVVVKLPDMILYQKAVRLPLSLPVGPRIPASELQSPIWNVFAEAPSAVLENLKARCSVSYNKIKTFNEVKFNYSMPAN  
CYHILVQDCSSELKFLVMMKSAGEATNLKAINIKIGSHEIDMHPVNGQVKLLVDGAESPTANISLISAGASLWIHNENQG  
FALAAPGHGIDKLYFDGKTITIQVPLWMAGKTCGICGKYDAECEQEYRMPNGYLAKNAVSFGHSWILEEAPCRGACKLHR  
SFVKLEKTVQLAGVDSKCYSTEPVLRCAKGCSATKTTPVTVGFHCLPADSANSLTDKQMKYDQKSEDMQDTVDAHTACSC  
ENEECST`

| Site | Type | Count |
| --- | --- | --- |
| 597 | N-linked | 1 |

| Mass | Sequence | MS_2_ Score | `q`-value | MS_1_ Score | Signal | Protein Coordinates | Tandem Scans | Start Time | Apex Time | End Time |
| --- | --- | --- | --- | --- | --- | --- | --- | --- | --- | --- |
| 4532.7497 | N(N)ISSAC(C)NIAL Fuc 2 Hex 6 HexNAc 8 Neu5Ac 2 | 77.353 | 0.018 | 9.848 | 5.756e+07 | 596 - 606 | 3 | 41.23 | 42.57 | 42.91 |

### N(N)ISSAC(C)NIAL Fuc 2 Hex 6 HexNAc 8 Neu5Ac 2

**Mass:**  4532.7561

(-4.183e-06)

**Charge States:** 4

**Start Position**: 597

**End Position**: 607

| Profile Score | Tandem Score | TDA q-value | Volume | Spectrum Matches |
| --- | --- | --- | --- | --- |
| 9.8481 | 77.3525 | 0.0179 | 5.7558e+07 | 3 |

**Adduct Signal Fractions**

- **Unmodified**: 5.7558e+07

### sp|P02845|VIT2\_CHICK

**>sp|P02845|VIT2\_CHICK**  
 `MRGIILALVLTLVGSQKFDIDPGFNSRRSYLYNYEGSMLNGLQDRSLGKAGVRLSSKLEISGLPENAYLLKVRSPQVEEY  
NGVWPRDPFTRSSKITQVISSCFTRLFKFEYSSGRIGNIYAPEDCPDLCVNIVRGILNMFQMTIKKSQNVYELQEAGIGG  
ICHARYVIQEDRKNSRIYVTRTVDLNNCQEKVQKSIGMAYIYPCPVDVMKERLTKGTTAFSYKLKQSDSGTLITDVSSRQ  
VYQISPFNEPTGVAVMEARQQLTLVEVRSERGSAPDVPMQNYGSLRYRFPAVLPQMPLQLIKTKNPEQRIVETLQHIVLN  
NQQDFHDDVSYRFLEVVQLCRIANADNLESIWRQVSDKPRYRRWLLSAVSASGTTETLKFLKNRIRNDDLNYIQTLLTVS  
LTLHLLQADEHTLPIAADLMTSSRIQKNPVLQQVACLGYSSVVNRYCSQTSACPKEALQPIHDLADEAISRGREDKMKLA  
LKCIGNMGEPASLKRILKFLPISSSSAADIPVHIQIDAITALKKIAWKDPKTVQGYLIQILADQSLPPEVRMMACAVIFE  
TRPALALITTIANVAMKESNMQVASFVYSHMKSLSKSRLPFMYNISSACNIALKLLSPKLDSMSYRYSKVIRADTYFDNY  
RVGATGEIFVVNSPRTMFPSAIISKLMANSAGSVADLVEVGIRVEGLADVIMKRNIPFAEYPTYKQIKELGKALQGWKEL  
PTETPLVSAYLKILGQEVAFININKELLQQVMKTVVEPADRNAAIKRIANQIRNSIAGQWTQPVWMGELRYVVPSCLGLP  
LEYGSYTTALARAAVSVEGKMTPPLTGDFRLSQLLESTMQIRSDLKPSLYVHTVATMGVNTEYFQHAVEIQGEVQTRMPM  
KFDAKIDVKLKNLKIETNPCREETEIVVGRHKAFAVSRNIGELGVEKRTSILPEDAPLDVTEEPFQTSERASREHFAMQG  
PDSMPRKQSHSSREDLRRSTGKRAHKRDICLKMHHIGCQLCFSRRSRDASFIQNTYLHKLIGEHEAKIVLMPVHTDADID  
KIQLEIQAGSRAAARIITEVNPESEEEDESSPYEDIQAKLKRILGIDSMFKVANKTRHPKNRPSKKGNTVLAEFGTEPDA  
KTSSSSSSASSTATSSSSSSASSPNRKKPMDEEENDQVKQARNKDASSSSRSSKSSNSSKRSSSKSSNSSKRSSSSSSSS  
SSSSRSSSSSSSSSSNSKSSSSSSKSSSSSSRSRSSSKSSSSSSSSSSSSSSKSSSSRSSSSSSKSSSHHSHSHHSGHLN  
GSSSSSSSSRSVSHHSHEHHSGHLEDDSSSSSSSSVLSKIWGRHEIYQYRFRSAHRQEFPKRKLPGDRATSRYSSTRSSH  
DTSRAASWPKFLGDIKTPVLAAFLHGISNNKKTGGLQLVVYADTDSVRPRVQVFVTNLTDSSKWKLCADASVRNAHKAVA  
YVKWGWDCRDYKVSTELVTGRFAGHPAAQVKLEWPKVPSNVRSVVEWFYEFVPGAAFMLGFSERMDKNPSRQARMVVALT  
SPRTCDVVVKLPDIILYQKAVRLPLSLPVGPRIPASELQPPIWNVFAEAPSAVLENLKARCSVSYNKIKTFNEVKFNYSM  
PANCYHILVQDCSSELKFLVMMKSAGEATNLKAINIKIGSHEIDMHPVNGQVKLLVDGAESPTANISLISAGASLWIHNE  
NQGFALAAPGHGIDKLYFDGKTITIQVPLWMAGKTCGICGKYDAECEQEYRMPNGYLAKNAVSFGHSWILEEAPCRGACK  
LHRSFVKLEKTVQLAGVDSKCYSTEPVLRCAKGCSATKTTPVTVGFHCLPADSANSLTDKQMKYDQKSEDMQDTVDAHTT  
CSCENEECST`

| Site | Type | Count |
| --- | --- | --- |
| 604 | N-linked | 1 |

| Mass | Sequence | MS_2_ Score | `q`-value | MS_1_ Score | Signal | Protein Coordinates | Tandem Scans | Start Time | Apex Time | End Time |
| --- | --- | --- | --- | --- | --- | --- | --- | --- | --- | --- |
| 4532.7497 | N(N)ISSAC(C)NIAL Fuc 2 Hex 6 HexNAc 8 Neu5Ac 2 | 77.353 | 0.018 | 9.848 | 5.756e+07 | 603 - 613 | 3 | 41.23 | 42.57 | 42.91 |

### N(N)ISSAC(C)NIAL Fuc 2 Hex 6 HexNAc 8 Neu5Ac 2

**Mass:**  4532.7561

(-4.183e-06)

**Charge States:** 4

**Start Position**: 604

**End Position**: 614

| Profile Score | Tandem Score | TDA q-value | Volume | Spectrum Matches |
| --- | --- | --- | --- | --- |
| 9.8481 | 77.3525 | 0.0179 | 5.7558e+07 | 3 |

**Adduct Signal Fractions**

- **Unmodified**: 5.7558e+07

### tr|F1NFL6|F1NFL6\_CHICK

**>tr|F1NFL6|F1NFL6\_CHICK**  
 `MRGIILALVLTLVGSQKFDIDPGFNSRRSYLYNYEGSMLNGLQDRSLGKAGVRLSSKLEISGLPENAYLLKVRSPQVEEY  
NGVWPRDPFTRSSKITQVISSCFTRLFKFEYSSGRIGNIYAPEDCPDLCVNIVRGILNMFQMTIKKSQNVYELQEAGIGG  
ICHARYVIQEDRKNSRIYVTRTVDLNNCQEKVQKSIGMAYIYPCPVDVMKERLTKGTTAFSYKLKQSDSGTLITDVSSRQ  
VYQISPFNEPTGVAVMEARQQLTLVEVRSERGSAPDVPMQNYGSLRYRFPAVLPQMPLQLIKTKNPEQRIIETLQHIVLN  
NQQDFHDDVSYRFLEVVQLCRIANADNLESIWRQVSDKPRYRRWLLSAVSASGTTETLKFLKNRIRNDDLNYIQTLLTVS  
LTLHLLQADEHTLPIAADLMTSSRIQKNPVLQQVACLGYSSVVNRYCSQTSACPKEALQPIHDLADEAISRGREDKMKLA  
LKCIGNMGEPASLKRILKFLPISSSSAADIPVHIQIDAITALKKIAWKDPKTVQGYLIQILADQSLPPEVRMMACAVIFE  
TRPALALITTIANVAMKESNMQVASFVYSHMKSLSKSRLPFMYNISSACNIALKLLSPKLDSMSYRYSKVIRADTYFDNY  
RVGATGEIFVVNSPRTMFPSAIISKLMANSAGSVADLVEVGIRVEGLADVIMKRNIPFAEYPTYKQIKELGKALQGWKEL  
PTETPLVSAYLKILGQEVAFININKELLQQVMKTVVEPADRNAAIKRIANQIRNSIAGQWTQPVWMGELRYVVPSCLGLP  
LEYGSYTTALARAAVSVEGKMTPPLTGDFRLSQLLESTMQIRSDLKPSLYVHTVATMGVNTEYFQHAVEIQGEVQTRMPM  
KFDAKIDVKLKNLKIETNPCREETEIVVGRHKAFAVSRNIGELGVEKRTSILPEDAPLDVTEEPFQTSERASREHFAMQG  
PDSMPRKQSHSSREDLRRSTGKRAHKRDICLKMHHIGCQLCFSRRSRDASFIQNTYLHKLIGEHEAKIVLMPVHTDADID  
KIQLEIQAGSRAAARIITEVNPESEEEDESSPYEDIQAKLKRILGIDSMFKVANKTRHPKNRPSKKGNTVLAEFGTEPDA  
KTSSSSSSASSTATSSSSSSASSPNRKKPMDEEENDQVKQARNKDASSSSRSSKSSNSSKRSSSKSSNSSKRSSSSSSSS  
SSSSSSSSRSSSSSSSSSSNSKSSSSSSKSSSSSSRSRSSSKSSSSSSSSSSSSSSKSSSSRSSSSSSKSSSHHSHSHHS  
GHLNGSSSSSSSSRSVSHHSHEHHSGHLEDDSSSSSSSSVLSKIWGRHEIYQYRFRSAHRQEFPKRKLPGDRATSRYSST  
RSSHDTSRAASWPKFLGDIKTPVLAAFLHGISNNKKTGGLQLVVYADTDSVRPRVQVFVTNLTDSSKWKLCADASVRNAH  
KAVAYVKWGWDCRDYKVSTELVTGRFAGHPAAQVKLEWPKVPSNVRSVVEWFYEFVPGAAFMLGFSERMDKNPSRQARMV  
VALTSPRTCDVVVKLPDMILYQKAVRLPLSLPVGPRIPASELQSPIWNVFAEAPSAVLENLKARCSVSYNKIKTFNEVKF  
NYSMPANCYHILVQDCSSELKFLVMMKSAGEATNLKAINIKIGSHEIDMHPVNGQVKLLVDGAESPTANISLISAGASLW  
IHNENQGFALAAPGHGIDKLYFDGKTITIQVPLWMAGKTCGICGKYDAECEQEYRMPNGYLAKNAVSFGHSWILEEAPCR  
GACKLHRSFVKLEKTVQLAGVDSKCYSTEPVLRCAKGCSATKTTPVTVGFHCLPADSANSLTDKQMKYDQKSEDMQDTVD  
AHTACSCENEECST`

| Site | Type | Count |
| --- | --- | --- |
| 604 | N-linked | 1 |

| Mass | Sequence | MS_2_ Score | `q`-value | MS_1_ Score | Signal | Protein Coordinates | Tandem Scans | Start Time | Apex Time | End Time |
| --- | --- | --- | --- | --- | --- | --- | --- | --- | --- | --- |
| 4532.7497 | N(N)ISSAC(C)NIAL Fuc 2 Hex 6 HexNAc 8 Neu5Ac 2 | 77.353 | 0.018 | 9.848 | 5.756e+07 | 603 - 613 | 3 | 41.23 | 42.57 | 42.91 |

### N(N)ISSAC(C)NIAL Fuc 2 Hex 6 HexNAc 8 Neu5Ac 2

**Mass:**  4532.7561

(-4.183e-06)

**Charge States:** 4

**Start Position**: 604

**End Position**: 614

| Profile Score | Tandem Score | TDA q-value | Volume | Spectrum Matches |
| --- | --- | --- | --- | --- |
| 9.8481 | 77.3525 | 0.0179 | 5.7558e+07 | 3 |

**Adduct Signal Fractions**

- **Unmodified**: 5.7558e+07

### tr|A0A1D5P6L2|A0A1D5P6L2\_CHICK

**>tr|A0A1D5P6L2|A0A1D5P6L2\_CHICK**  
 `MPSDKTIGGGDDSFNTFFSETGAGKHVPRAVFVDLEPTVIDEVRTGTYRQLFHPEQLITGKEDAANNYARGHYTIGKEII  
DLVLDRIRKLGFLVFHSFGGGTGSGFTSLLMERLSVDYGKKSKLEFSIYPAPQVSTAVVEPYNSILTTHTTLEHSDCAFM  
VDNEAIYDICRRNLDIERPTYTNLNRLIGQIVSSITASLRFDGALNVDLTEFQTNLVPYPRIHFPLATYAPVISAEKAYH  
EQLSVAEITNACFEPANQMVKCDPRHGKYMACCLLYRGDVVPKDVNAAIATIKTKRTIQFVDWCPTGFKVGINYQPPTVV  
PGGDLAKVQRAVCMLSNTTAIAEAWARLDHKFDLMYAKRAFVHWYVGEGMEEGEFSEAREDMAALEKDYEEVGVDSVEGE  
GEEEERSTEGSQRCYSCSYRQQS`

| Site | Type | Count |
| --- | --- | --- |
| 337 | N-linked | 1 |

| Mass | Sequence | MS_2_ Score | `q`-value | MS_1_ Score | Signal | Protein Coordinates | Tandem Scans | Start Time | Apex Time | End Time |
| --- | --- | --- | --- | --- | --- | --- | --- | --- | --- | --- |
| 4646.8116 | LSN(N)TTAIAEAW Fuc 2 Hex 6 HexNAc 8 Neu5Ac 2 | 74.848 | 0.026 | -6.001 | 1.984e+07 | 334 - 345 | 1 | 45.89 | 45.89 | 46.21 |

### LSN(N)TTAIAEAW Fuc 2 Hex 6 HexNAc 8 Neu5Ac 2

**Mass:**  4646.8093

(-6.824e-06)

**Charge States:** 4

**Start Position**: 335

**End Position**: 346

| Profile Score | Tandem Score | TDA q-value | Volume | Spectrum Matches |
| --- | --- | --- | --- | --- |
| -6.0013 | 74.8480 | 0.0255 | 1.9837e+07 | 1 |

**Adduct Signal Fractions**

- **Unmodified**: 1.9837e+07

### tr|A0A1L1RK76|A0A1L1RK76\_CHICK

**>tr|A0A1L1RK76|A0A1L1RK76\_CHICK**  
 `MRECISIHVGQAGVQIGNACWELYCLEHGIQPDGQMPSDKTIGGGDDSFNTFFSETGAGKHVPRAVFVDLEPTVIDEVRT  
GTYRQLFHPEQLITGKEDAANNYARGHYTIGKEIIDLVLDRIRKLADQCTGLQGFLVFHSFGGGTGSGFTSLLMERLSVD  
YGKKSKLEFSIYPAPQVSTAVVEPYNSILTTHTTLEHSDCAFMVDNEAIYDICRRNLDIERPTYTNLNRLISQIVSSITA  
SLRFDGALNVDLTEFQTNLVPYPRIHFPLATYAPVISAEKAYHEQLSVAEITNACFEPANQMVKCDPRHGKYMACCLLYR  
GDVVPKDVNAAIATIKTKRSIQFVDWCPTGFKVGINYQPPTVVPGGDLAKVQRAVCMLSNTTAIAEAWARLDHKFDLMYA  
KRAFVHWYVGEGMEEGEFSEAREDMATLLLLCVSMNKKHSL`

| Site | Type | Count |
| --- | --- | --- |
| 380 | N-linked | 1 |

| Mass | Sequence | MS_2_ Score | `q`-value | MS_1_ Score | Signal | Protein Coordinates | Tandem Scans | Start Time | Apex Time | End Time |
| --- | --- | --- | --- | --- | --- | --- | --- | --- | --- | --- |
| 4646.8116 | LSN(N)TTAIAEAW Fuc 2 Hex 6 HexNAc 8 Neu5Ac 2 | 74.848 | 0.026 | -6.001 | 1.984e+07 | 377 - 388 | 1 | 45.89 | 45.89 | 46.21 |

### LSN(N)TTAIAEAW Fuc 2 Hex 6 HexNAc 8 Neu5Ac 2

**Mass:**  4646.8093

(-6.824e-06)

**Charge States:** 4

**Start Position**: 378

**End Position**: 389

| Profile Score | Tandem Score | TDA q-value | Volume | Spectrum Matches |
| --- | --- | --- | --- | --- |
| -6.0013 | 74.8480 | 0.0255 | 1.9837e+07 | 1 |

**Adduct Signal Fractions**

- **Unmodified**: 1.9837e+07

### tr|A0A1D5P198|A0A1D5P198\_CHICK

**>tr|A0A1D5P198|A0A1D5P198\_CHICK**  
 `MRECISIHVGQAGVQIGNACWELYCLEHGIQPDGQMPSDKTIGGGDDSFNTFFSETGAGKHVPRAVFVDLEPTVIDEVRT  
GTYRQLFHPEQLITGKEDAANNYARGHYTIGKEIIDLVLDRIRKLADQCTGLQGFLVFHSFGGGTGSGFTSLLMERLSVD  
YGKKSKLEFSIYPAPQVSTAVVEPYNSILTTHTTLEHSDCAFMVDNEAIYDICRRNLDIERPTYTNLNRLISQIVSSITA  
SLRFDGALNVDLTEFQTNLVPYPRIHFPLATYAPVISAEKAYHEQLSVAEITNACFEPANQMVKCDPRHGKYMACCLLYR  
GDVVPKDVNAAIATIKTKRSIQFVDWCPTGFKVGINYQPPTVVPGGDLAKVQRAVCMLSNTTAIAEAWARLDHKFDLMYA  
KRAFVHWYVGEGMEEGEFSEAREDMAALEKDYEEVGVDSVEGEGEEEGEEY`

| Site | Type | Count |
| --- | --- | --- |
| 380 | N-linked | 1 |

| Mass | Sequence | MS_2_ Score | `q`-value | MS_1_ Score | Signal | Protein Coordinates | Tandem Scans | Start Time | Apex Time | End Time |
| --- | --- | --- | --- | --- | --- | --- | --- | --- | --- | --- |
| 4646.8116 | LSN(N)TTAIAEAW Fuc 2 Hex 6 HexNAc 8 Neu5Ac 2 | 74.848 | 0.026 | -6.001 | 1.984e+07 | 377 - 388 | 1 | 45.89 | 45.89 | 46.21 |

### LSN(N)TTAIAEAW Fuc 2 Hex 6 HexNAc 8 Neu5Ac 2

**Mass:**  4646.8093

(-6.824e-06)

**Charge States:** 4

**Start Position**: 378

**End Position**: 389

| Profile Score | Tandem Score | TDA q-value | Volume | Spectrum Matches |
| --- | --- | --- | --- | --- |
| -6.0013 | 74.8480 | 0.0255 | 1.9837e+07 | 1 |

**Adduct Signal Fractions**

- **Unmodified**: 1.9837e+07

### tr|A0A1D5NW27|A0A1D5NW27\_CHICK

**>tr|A0A1D5NW27|A0A1D5NW27\_CHICK**  
 `MRECISIHVGQAGVQIGNACWELYCLEHGIQPDGQMPSDKTIGGGDDSFNTFFSETGAGKHVPRAVFVDLEPTVIDEVRT  
GTYRQLFHPEQLITGKEDAANNYARGHYTIGKEIIDLVLDRIRKLADQCTGLQGFLVFHSFGGGTGSGFTSLLMERLSVD  
YGKKSKLEFSIYPAPQVSTAVVEPYNSILTTHTTLEHSDCAFMVDNEAIYDICRRNLDIERPTYTNLNRLIGQIVSSITA  
SLRFDGALNVDLTEFQTNLVPYPRIHFPLATYAPVISAEKAYHEQLSVAEITNACFEPANQMVKCDPRHGKYMACCLLYR  
GDVVPKDVNAAIATIKTKRTIQFVDWCPTGFKVGINYQPPTVVPGGDLAKVQRAVCMLSNTTAIAEAWARLDHKFDLMYA  
KRAFVHWYVGEGMEEGEFSEAREDMAALEKDYEEVGVDSVEGEGEEEGEEY`

| Site | Type | Count |
| --- | --- | --- |
| 380 | N-linked | 1 |

| Mass | Sequence | MS_2_ Score | `q`-value | MS_1_ Score | Signal | Protein Coordinates | Tandem Scans | Start Time | Apex Time | End Time |
| --- | --- | --- | --- | --- | --- | --- | --- | --- | --- | --- |
| 4646.8116 | LSN(N)TTAIAEAW Fuc 2 Hex 6 HexNAc 8 Neu5Ac 2 | 74.848 | 0.026 | -6.001 | 1.984e+07 | 377 - 388 | 1 | 45.89 | 45.89 | 46.21 |

### LSN(N)TTAIAEAW Fuc 2 Hex 6 HexNAc 8 Neu5Ac 2

**Mass:**  4646.8093

(-6.824e-06)

**Charge States:** 4

**Start Position**: 378

**End Position**: 389

| Profile Score | Tandem Score | TDA q-value | Volume | Spectrum Matches |
| --- | --- | --- | --- | --- |
| -6.0013 | 74.8480 | 0.0255 | 1.9837e+07 | 1 |

**Adduct Signal Fractions**

- **Unmodified**: 1.9837e+07

### iav|ACF41842|A/Puerto

**>iav|ACF41842|A/Puerto**  
 `MDVNPTLLFLKVPAQNAISTTFPYTGDPPYSHGTGTGYTMDTVNRTHQYSEKGRWTTNTETGAPQLNPIDGPLPEDNEPS  
GYAQTDCVLEAMAFLEESHPGIFENSCIETMEVVQQTRVDKLTQGRQTYDWTLNRNQPAATALANTIEVFRSNGLTANES  
GRLIDFLKDVMESMNKEEMGITTHFQRKRRVRDNMTKKMITQRTMGKKKQRLNKRSYLIRALTLNTMTKDAERGKLKRRA  
IATPGMQIRGFVYFVETLARSICEKLEQSGLPVGGNEKKAKLANVVRKMMTNSQDTELSFTITGDNTKWNENQNPRMFLA  
MITYMTRNQPEWFRNVLSIAPIMFSNKMARLGKGYMFESKSMKLRTQIPAEMLASIDLKYFNDSTRKKIEKIRPLLIEGT  
ASLSPGMMMGMFNMLSTVLGVSILNLGQKRYTKTTYWWDGLQSSDDFALIVNAPNHEGIQAGVDRFYRTCKLLGINMSKK  
KSYINRTGTFEFTSFFYRYGFVANFSMELPSFGVSGINESADMSIGVTVIKNNMINNDLGPATAQMALQLFIKDYRYTYR  
CHRGDTQIQTRRSFEIKKLWEQTRSKAGLLVSDGGPNLYNIRNLHIPEVCLKWELMDEDYQGRLCNPLNPFVSHKEIESM  
NNAVMMPAHGPAKNMEYDAVATTHSWIPKRNRSILNTSQRGVLEDEQMYQRCCNLFEKFFPSSSYRRPVGISSMVEAMVS  
RARIDARIDFESGRIKKEEFTEIMKICSTIEELRRQK`

| Site | Type | Count |
| --- | --- | --- |
| 476 | N-linked | 1 |

| Mass | Sequence | MS_2_ Score | `q`-value | MS_1_ Score | Signal | Protein Coordinates | Tandem Scans | Start Time | Apex Time | End Time |
| --- | --- | --- | --- | --- | --- | --- | --- | --- | --- | --- |
| 3977.6335 | GIN(N)MSKKKSY Fuc 1 Hex 9 HexNAc 6 | 65.888 | 0.026 | -19.982 | 3.918e+06 | 473 - 483 | 1 | 13.11 | 13.11 | 13.11 |

### GIN(N)MSKKKSY Fuc 1 Hex 9 HexNAc 6

**Mass:**  3977.6335

(3.077e-06)

**Charge States:** 4

**Start Position**: 474

**End Position**: 484

| Profile Score | Tandem Score | TDA q-value | Volume | Spectrum Matches |
| --- | --- | --- | --- | --- |
| -19.9822 | 65.8883 | 0.0262 | 3.9179e+06 | 1 |

**Adduct Signal Fractions**

- **Unmodified**: 3.9179e+06

### iav|AGU93028|A/Puerto

**>iav|AGU93028|A/Puerto**  
 `MDVNPTLLFLKVPAQNAISTTFPYTGDPPYSHGTGTGYTMDTVNRTHQYSEKGRWTTNTETGAPQLNPIDGPLPEDNEPS  
GYAQTDCVLEAMAFLEESHPGIFENSCIETMEVVQQTRVDKLTQGRQTYDWTLNRNQPAATALANTIEVFRSNGLTANES  
GRLIDFLKDVMESMNKEEMGITTHFQRKRRVRDNMTKKMITQRTIGKKKQRLNKRSYLIRALTLNTMTKDAERGKLKRRA  
IATPGMQIRGFVYFVETLARSICEKLEQSGLPVGGNEKKAKLANVVRKMMTNSQDTELSFTITGDNTKWNENQNPRMFLA  
MITYMTRNQPEWFRNVLSIAPIMFSNKMARLGKGYMFESKSMKLRTQIPAEMLASIDLKYFNDSTRKKIEKIRPLLIEGT  
ASLSPGMMMGMFNMLSTVLGVSILNLGQKRYTKTTYWWDGLQSSDDFALIVNAPNHEGIQAGVDRFYRTCKLLGINMSKK  
KSYINRTGTFEFTSFFYRYGFVANFSMELPSFGVSGINESADMSIGVTVIKNNMINNDLGPATAQMALQLFIKDYRYTYR  
CHRGDTQIQTRRSFEIKKLWEQTRSKAGLLVSDGGPNLYNIRNLHIPEVCLKWELMDEDYQGRLCNPLNPFVSHKEIESM  
NNAVMMPAHGPAKNMEYDAVATTHSWIPKRNRSILNTSQRGVLEDEQMYQRCCNLFEKFFPSSSYRRPVGISSMVEAMVS  
RARIDARIDFESGRIKKEEFTEIMKICSTIEELRRQK`

| Site | Type | Count |
| --- | --- | --- |
| 476 | N-linked | 1 |

| Mass | Sequence | MS_2_ Score | `q`-value | MS_1_ Score | Signal | Protein Coordinates | Tandem Scans | Start Time | Apex Time | End Time |
| --- | --- | --- | --- | --- | --- | --- | --- | --- | --- | --- |
| 3977.6335 | GIN(N)MSKKKSY Fuc 1 Hex 9 HexNAc 6 | 65.888 | 0.026 | -19.982 | 3.918e+06 | 473 - 483 | 1 | 13.11 | 13.11 | 13.11 |

### GIN(N)MSKKKSY Fuc 1 Hex 9 HexNAc 6

**Mass:**  3977.6335

(3.077e-06)

**Charge States:** 4

**Start Position**: 474

**End Position**: 484

| Profile Score | Tandem Score | TDA q-value | Volume | Spectrum Matches |
| --- | --- | --- | --- | --- |
| -19.9822 | 65.8883 | 0.0262 | 3.9179e+06 | 1 |

**Adduct Signal Fractions**

- **Unmodified**: 3.9179e+06

### iav|AFM71854|A/Puerto

**>iav|AFM71854|A/Puerto**  
 `MDVNPTLLFLKVPAQNAISTTFPYTGDPPYSHGTGTGYTMDTVNRTHQYSEKGRWTTNTETGAPQLNPIDGPLPEDNEPS  
GYAQTDCVLEAMAFLEESHPGIFENSCIETMEVVQQTRVDKLTQGRQTYDWTLNRNQPAATALANTIEVFRSNGLTANES  
GRLIDFLKDVMESMNKEEMGITTHFQRKRRVRDNMTKKMITQRTMGKKKQRLNKRSYLIRALTLNTMTKDAERGKLKRRA  
IATPGMQIRGFVYFVETLARSICEKLEQSGLPVGGNEKKAKLANVVRKMMTNSQDTELSFTITGDNTKWNENQNPRMFLA  
MITYMTRNQPEWFRNVLSIAPIMFSNKMARLGKGYMFESKSMKLRTQIPAEMLASIDLKYFNDSTRKKIEKIRPLLIEGT  
ASLSPGMMMGMFNMLSTVLGVSILNLGQKRYTKTTYWWDGLQSSDDFALIVNAPNHEGIQAGVDRFYRTCKLLGINMSKK  
KSYINRTGTFEFTSFFYRYGFVANFSMELPSFGVSGINESADMSIGVTVIKNNMINNDLGPATAQMALQLFIKDYRYTYR  
CHRGDTQIQTRRSFEIKKLWEQTRSKAGLLVSDGGPNLYNIRNLHIPEVCLKWELMDEDYQGRLCNPLNPFVSHKEIESM  
NNAVMMPAHGPAKNMEYDAVATTHSWIPKRNRSILNTSQRGVLEDEQMYQRCCNLFEKFFPSSSYRRPVGISSMVEAMVS  
RARIDARIDFESGRIKKEEFTEIMKICSTIEELRRQK`

| Site | Type | Count |
| --- | --- | --- |
| 476 | N-linked | 1 |

| Mass | Sequence | MS_2_ Score | `q`-value | MS_1_ Score | Signal | Protein Coordinates | Tandem Scans | Start Time | Apex Time | End Time |
| --- | --- | --- | --- | --- | --- | --- | --- | --- | --- | --- |
| 3977.6335 | GIN(N)MSKKKSY Fuc 1 Hex 9 HexNAc 6 | 65.888 | 0.026 | -19.982 | 3.918e+06 | 473 - 483 | 1 | 13.11 | 13.11 | 13.11 |

### GIN(N)MSKKKSY Fuc 1 Hex 9 HexNAc 6

**Mass:**  3977.6335

(3.077e-06)

**Charge States:** 4

**Start Position**: 474

**End Position**: 484

| Profile Score | Tandem Score | TDA q-value | Volume | Spectrum Matches |
| --- | --- | --- | --- | --- |
| -19.9822 | 65.8883 | 0.0262 | 3.9179e+06 | 1 |

**Adduct Signal Fractions**

- **Unmodified**: 3.9179e+06

### iav|ABO21706|A/Puerto

**>iav|ABO21706|A/Puerto**  
 `MDVNPTLLFLKVPAQNAISTTFPYTGDPPYSHGTGTGYTMDTVNRTHQYSEKGRWTTNTETGAPQLNPIDGPLPEDNEPS  
GYAQTDCVLEAMAFLEESHPGIFENSCIETMEVVQQTRVDKLTQGRQTYDWTLNRNQPAATALANTIEVFRSNGLTANES  
GRLIDFLKDVMESMNKEEMGITTHFQRKRRVRDNMTKKMITQRTMGKKKQRLNKRSYLIRALTLNTMTKDAERGKLKRRA  
IATPGMQIRGFVYFVETLARSICEKLEQSGLPVGGNEKKAKLANVVRKMMTNSQDTELSFTITGDNTKWNENQNPRMFLA  
MITYMTRNQPEWFRNVLSIAPIMFSNKMARLGKGYMFESKSMKLRTQIPAEMLASIDLKYFNDSTRKKIEKIRSLLIEGT  
ASLSPGMMMGMFNMLSTVLGVSILNLGQKRYTKTTYWWDGLQSSDDFALIVNAPNHEGIQAGVDRFYRTCKLLGINMSKK  
KSYINRTGTFEFTSFFYRYGFVANFSMELPSFGVSGINESADMSIGVTVIKNNMINNDLGPATAQMALQLFIKDYRYTYR  
CHRGDTQIQTRRSFEIKKLWEQTRSKAGLLVSDGGPNLYNIRNLHIPEVCLKWELMDEDYQGRLCNPLNPFVSHKEIESM  
NNAVMMPAHGPAKNMEYDAVATTHSWIPKRNRSILNTSQRGVLEDEQMYQRCCNLFEKFFPSSSYRRPVGISSMVEAMVS  
RARIDARIDFESGRIKKEEFTEIMKICSTIEELRRQK`

| Site | Type | Count |
| --- | --- | --- |
| 476 | N-linked | 1 |

| Mass | Sequence | MS_2_ Score | `q`-value | MS_1_ Score | Signal | Protein Coordinates | Tandem Scans | Start Time | Apex Time | End Time |
| --- | --- | --- | --- | --- | --- | --- | --- | --- | --- | --- |
| 3977.6335 | GIN(N)MSKKKSY Fuc 1 Hex 9 HexNAc 6 | 65.888 | 0.026 | -19.982 | 3.918e+06 | 473 - 483 | 1 | 13.11 | 13.11 | 13.11 |

### GIN(N)MSKKKSY Fuc 1 Hex 9 HexNAc 6

**Mass:**  3977.6335

(3.077e-06)

**Charge States:** 4

**Start Position**: 474

**End Position**: 484

| Profile Score | Tandem Score | TDA q-value | Volume | Spectrum Matches |
| --- | --- | --- | --- | --- |
| -19.9822 | 65.8883 | 0.0262 | 3.9179e+06 | 1 |

**Adduct Signal Fractions**

- **Unmodified**: 3.9179e+06

### iav|ABD77683|A/Puerto

**>iav|ABD77683|A/Puerto**  
 `MDVNPTLLFLKVPAQNAISTTFPYTGDPPYSHGTGTGYTMDTVNRTHQYSEKGRWTTNTETGAPQLNPIDGPLPEDNEPS  
GYAQTDCVLEAMAFLEESHPGIFENSCIETMEVVQQTRVDKLTQGRQTYDWTLNRNQPAATALANTIEVFRSNGLTANES  
GRLIDFLKDVMESMNKEEMGITTHFQRKRRVRDNMTKKMITQRTMGKKKQRLNKRSYLIRALTLNTMTKDAERGKLKRRA  
IATPGMQIRGFVYFVETLARSICEKLEQSGLPVGGNEKKAKLANVVRKMMTNSQDTELSFTITGDNTKWNENQNPRMFLA  
MITYMTRNQPEWFRNVLSIAPIMFSNKMARLGKGYMFESKSMKLRTQIPAEMLASIDLKYFNDSTRKKIEKIRSLLIEGT  
ASLSPGMMMGMFNMLSTVLGVSILNLGQKRYTKTTYWWDGLQSSDDFALIVNAPNHEGIQAGVDRFYRTCKLLGINMSKK  
KSYINRTGTFEFTSFFYRYGFVANFSMELPSFGVSGINESADMSIGVTVIKNNMINNDLGPATAQMALQLFIKDYRYTYR  
CHRGDTQIQTRRSFEIKKLWEQTRSKAGLLVSDGGPNLYNIRNLHIPEVCLKWELMDEDYQGRLCNPLNPFVSHKEIESM  
NNAVMMPAHGPAKNMEYDAVATTHSWIPKRNRSILNTSQRGVLEDEQMYQRCCNLFEKFFPSSSYRRPVGISSMVEAMVS  
RARIDARIDFESGRIKKEEFTEIMKICSTIEELRRQK`

| Site | Type | Count |
| --- | --- | --- |
| 476 | N-linked | 1 |

| Mass | Sequence | MS_2_ Score | `q`-value | MS_1_ Score | Signal | Protein Coordinates | Tandem Scans | Start Time | Apex Time | End Time |
| --- | --- | --- | --- | --- | --- | --- | --- | --- | --- | --- |
| 3977.6335 | GIN(N)MSKKKSY Fuc 1 Hex 9 HexNAc 6 | 65.888 | 0.026 | -19.982 | 3.918e+06 | 473 - 483 | 1 | 13.11 | 13.11 | 13.11 |

### GIN(N)MSKKKSY Fuc 1 Hex 9 HexNAc 6

**Mass:**  3977.6335

(3.077e-06)

**Charge States:** 4

**Start Position**: 474

**End Position**: 484

| Profile Score | Tandem Score | TDA q-value | Volume | Spectrum Matches |
| --- | --- | --- | --- | --- |
| -19.9822 | 65.8883 | 0.0262 | 3.9179e+06 | 1 |

**Adduct Signal Fractions**

- **Unmodified**: 3.9179e+06

### iav|AAM75156|A/Puerto

**>iav|AAM75156|A/Puerto**  
 `MDVNPTLLFLKVPAQNAISTTFPYTGDPPYSHGTGTGYTMDTVNRTHQYSEKGRWTTNTETGAPQLNPIDGPLPEDNEPS  
GYAQTDCVLEAMAFLEESHPGIFENSCIETMEVVQQTRVDKLTQGRQTYDWTLNRNQPAATALANTIEVFRSNGLTANES  
GRLIDFLKDVMESMKKEEMGITTHFQRKRRVRDNMTKKMITQRTIGKKKQRLNKRSYLIRALTLNTMTKDAERGKLKRRA  
IATPGMQIRGFVYFVETLARSICEKLEQSGLPVGGNEKKAKLANVVRKMMTNSQDTELSFTITGDNTKWNENQNPRMFLA  
MITYMTRNQPEWFRNVLSIAPIMFSNKMARLGKGYMFESKSMKLRTQIPAEMLASIDLKYFNDSTRKKIEKIRPLLIEGT  
ASLSPGMMMGMFNMLSTVLGVSILNLGQKRYTKTTYWWDGLQSSDDFALIVNAPNHEGIQAGVDRFYRTCKLLGINMSKK  
KSYINRTGTFEFTSFFYRYGFVANFSMELPSFGVSGINESADMSIGVTVIKNNMINNDLGPATAQMALQLFIKDYRYTYR  
CHRGDTQIQTRRSFEIKKLWEQTRSKAGLLVSDGGPNLYNIRNLHIPEVCLKWELMDEDYQGRLCNPLNPFVSHKEIESM  
NNAVMMPAHGPAKNMEYDAVATTHSWIPKRNRSILNTSQRGVLEDEQMYQRCCNLFEKFFPSSSYRRPVGISSMVEAMVS  
RARIDARIDFESGRIKKEEFTEIMKICSTIEELRRQK`

| Site | Type | Count |
| --- | --- | --- |
| 476 | N-linked | 1 |

| Mass | Sequence | MS_2_ Score | `q`-value | MS_1_ Score | Signal | Protein Coordinates | Tandem Scans | Start Time | Apex Time | End Time |
| --- | --- | --- | --- | --- | --- | --- | --- | --- | --- | --- |
| 3977.6335 | GIN(N)MSKKKSY Fuc 1 Hex 9 HexNAc 6 | 65.888 | 0.026 | -19.982 | 3.918e+06 | 473 - 483 | 1 | 13.11 | 13.11 | 13.11 |

### GIN(N)MSKKKSY Fuc 1 Hex 9 HexNAc 6

**Mass:**  3977.6335

(3.077e-06)

**Charge States:** 4

**Start Position**: 474

**End Position**: 484

| Profile Score | Tandem Score | TDA q-value | Volume | Spectrum Matches |
| --- | --- | --- | --- | --- |
| -19.9822 | 65.8883 | 0.0262 | 3.9179e+06 | 1 |

**Adduct Signal Fractions**

- **Unmodified**: 3.9179e+06

### iav|ADX99655|A/Puerto

**>iav|ADX99655|A/Puerto**  
 `MDVNPTLLFLKVPAQNAISTTFPYTGDPPYSHGTGTGYTMDTVNRTHQYSEKGRWTTNTETGAPQLNPIDGPLPEDNEPS  
GYAQTDCVLEAMAFLEESHPGIFENSCIETMEVVQQTRVDKLTQGRQTYDWTLNRNQPAATALANTIEVFRSNGLTANES  
GRLIDFLKDVMESMNKEEMGITTHFQRKRRVRDNMTKKMITQRTMGKKKQRLNKRSYLIRALTLNTMTKDAERGKLKRRA  
IATPGMQIRGFVYFVETLARSICEKLEQSGLPVGGNEKKAKLANVVRKMMTNSQDTELSFTITGDNTKWNENQNPRMFLA  
MITYMTRNQPEWFRNVLSIAPIMFSNKMARLGKGYMFESKSMKLRTQIPAEMLASIDLKYFNDSTRKKIEKIRPLLIEGT  
ASLSPGMMMGMFNMLSTVLGVSILNLGQKRYTKTTYWWDGLQSSDDFALIVNAPNHEGIQAGVDRFYRTCKLLGINMSKK  
KSYINRTGTFEFTSFFYRYGFVANFSMELPSFGVSGINESADMSIGVTVIKNNMINNDLGPATAQMALQLFIKDYRYTYR  
CHIGDTQIQTRRSFEIKKLWEQTRSKAGLLVSDGGPNLYNIRNLHIPEVCLKWELMDEDYQGRLCNPLNPFVSHKEIESM  
NNAVMMPAHGPAKNMEYDAVATTHSWIPKRNRSILNTSQRGVLEDEQMYQRCCNLFEKFFPSSSYRRPVGISSMVEAMVS  
RARIDARIDFESGRIKKEEFTEIMKICSTIEELRRQK`

| Site | Type | Count |
| --- | --- | --- |
| 476 | N-linked | 1 |

| Mass | Sequence | MS_2_ Score | `q`-value | MS_1_ Score | Signal | Protein Coordinates | Tandem Scans | Start Time | Apex Time | End Time |
| --- | --- | --- | --- | --- | --- | --- | --- | --- | --- | --- |
| 3977.6335 | GIN(N)MSKKKSY Fuc 1 Hex 9 HexNAc 6 | 65.888 | 0.026 | -19.982 | 3.918e+06 | 473 - 483 | 1 | 13.11 | 13.11 | 13.11 |

### GIN(N)MSKKKSY Fuc 1 Hex 9 HexNAc 6

**Mass:**  3977.6335

(3.077e-06)

**Charge States:** 4

**Start Position**: 474

**End Position**: 484

| Profile Score | Tandem Score | TDA q-value | Volume | Spectrum Matches |
| --- | --- | --- | --- | --- |
| -19.9822 | 65.8883 | 0.0262 | 3.9179e+06 | 1 |

**Adduct Signal Fractions**

- **Unmodified**: 3.9179e+06

### iav|ACV49553|A/Puerto

**>iav|ACV49553|A/Puerto**  
 `MDVNPTLLFLKVPAQNAISTTFPYTGDPPYSHGTGTGYTMDTVNRTHQYSEKGRWTTNTETGAPQLNPIDGPLPEDNEPS  
GYAQTDCVLEAMAFLEESHPGIFENSCIETMEVVQQTRVDKLTQGRQTYDWTLNRNQPAATALANTIEVFRSNGLTANES  
GRLIDFLKDVMESMNKEEMGITTHFQRKRRVRDNMTKKMITQRTMGKKKQRLNKRSYLIRALTLNTMTKDAERGKLKRRA  
IATPGMQIRGFVYFVETLARSICEKLEQSGLPVGGNEKKAKLANVVRKMMTNSQDTELSFTITGDNTKWNENQNPRMFLA  
MITYMTRNQPEWFRNVLSIAPIMFSNKMARLGKGYMFESKSMKLRTQIPAEMLASIDLKYFNDSTRKKIEKIRPLLIEGT  
ASLSPGMMMGMFNMLSTVLGVSILNLGQKRYTKTTYWWDGLQSSDDFALIVNAPNHEGIQAGVDRFYRTCKLLGINMSKK  
KSYINRTGTFEFTSFFYRYGFVANFSMELPSFGVSGINESADMSIGVTVIKNNMINNDLGPATAQMALQLFIKDYRYTYR  
CHRGDTQIQTRRSFEIKKLWEQTRSKAGLLVSDGGPNLYNIRNLHIPEVCLKWELMDEDYQGRLCNPLNPFVSHKEIESM  
NNAVMMPAHGPAKNMEYDAVATTHSWIPKRNRSILNTSQRGVLEDEQMYQRCCNLFEKFFPSSSYRRPVGISSMVEAMVS  
RARIDARIDFESGRIKKEEFTEIMKICSTIEELRRQK`

| Site | Type | Count |
| --- | --- | --- |
| 476 | N-linked | 1 |

| Mass | Sequence | MS_2_ Score | `q`-value | MS_1_ Score | Signal | Protein Coordinates | Tandem Scans | Start Time | Apex Time | End Time |
| --- | --- | --- | --- | --- | --- | --- | --- | --- | --- | --- |
| 3977.6335 | GIN(N)MSKKKSY Fuc 1 Hex 9 HexNAc 6 | 65.888 | 0.026 | -19.982 | 3.918e+06 | 473 - 483 | 1 | 13.11 | 13.11 | 13.11 |

### GIN(N)MSKKKSY Fuc 1 Hex 9 HexNAc 6

**Mass:**  3977.6335

(3.077e-06)

**Charge States:** 4

**Start Position**: 474

**End Position**: 484

| Profile Score | Tandem Score | TDA q-value | Volume | Spectrum Matches |
| --- | --- | --- | --- | --- |
| -19.9822 | 65.8883 | 0.0262 | 3.9179e+06 | 1 |

**Adduct Signal Fractions**

- **Unmodified**: 3.9179e+06

### iav|BAV59609|A/Puerto

**>iav|BAV59609|A/Puerto**  
 `MDVNPTLLFLKVPAQNAISTTFPYTGDPPYSHGTGTGYTMDTVNRTHQYSEKGRWTTNTETGASQLNPIDGPLPEDNEPS  
GYAQTDCVLEAMAFLEESHPGIFENSCIETIEVVQQTRVDKLTQGRQTYDWTLNRNQPAATALANTIEVFRSNGLTANES  
GRLIDFLKDVMESMNKEEMGITTHFQRKRRVRDNMTKKMITQRTMGKKKQRLNKRSYLIRALTLNTMTKDAERGKLKRRA  
IATPGMQIRGFVYFVETLARSICEKLEQSGLPVGGNEKKAKLANVVRKMMTNSQDTELSFTITGDNTKWNENQNPRMFLA  
MITYMTRNQPEWFRNVLSIAPIMFSNKMARLGKGYMFESKSMKLRTQIPAEMLASIDLKYFNDSTRKKIEKIRPLLIEGT  
ASLSPGMMMGMFNMLSTVLGVSILNLGQKRYTKTTYWWDGLQSSDDFALIVNAPNNEGIQAGVDRFYRTCKLLGINMSKK  
KSYINRTGTFEFTSFFYRYGFVANFSMELPSFGVSGINESADMSIGVTVIKNNMINNDLGPATAQMALQLFIKDYRYTYR  
CHRGDTQIQTRRSFEIKKLWEQTRSKAGLLVSDGGPNLYNIRNLHIPEVCLKWELMDEDYQGRLCNPLNPFVSHKEIESM  
NNAVMMPAHGPAKNMEYDAVATTHSWIPKRNRSILNTSQRGVLEDEQMYQRCCNLFEKFFPSSSYRRPVGISSMVEAMVS  
RARIDARIDFESGRIKKEEFTEIMKICSTIEELRRQK`

| Site | Type | Count |
| --- | --- | --- |
| 476 | N-linked | 1 |

| Mass | Sequence | MS_2_ Score | `q`-value | MS_1_ Score | Signal | Protein Coordinates | Tandem Scans | Start Time | Apex Time | End Time |
| --- | --- | --- | --- | --- | --- | --- | --- | --- | --- | --- |
| 3977.6335 | GIN(N)MSKKKSY Fuc 1 Hex 9 HexNAc 6 | 65.888 | 0.026 | -19.982 | 3.918e+06 | 473 - 483 | 1 | 13.11 | 13.11 | 13.11 |

### GIN(N)MSKKKSY Fuc 1 Hex 9 HexNAc 6

**Mass:**  3977.6335

(3.077e-06)

**Charge States:** 4

**Start Position**: 474

**End Position**: 484

| Profile Score | Tandem Score | TDA q-value | Volume | Spectrum Matches |
| --- | --- | --- | --- | --- |
| -19.9822 | 65.8883 | 0.0262 | 3.9179e+06 | 1 |

**Adduct Signal Fractions**

- **Unmodified**: 3.9179e+06

### iav|AGK63053|A/Puerto

**>iav|AGK63053|A/Puerto**  
 `MDVNPTLLFLKVPTQNAISTTFPYTGDPPYSHGTGTGYTMDTVNRTHQYSEKGRWTTNTETGAPQLNPIDGPLPEDNEPS  
GYAQTDCVLEAMAFLEESHPGIFENSCIETMEVVQQTRVDKLTQGRQTYDWTLNRNQPAATALANTIEVFRSNGLTANES  
GRLIDFLKDVMESMNKEEMGITTHFQRKRRVRDNMTKKMITQRTMGKKKQRLNKRSYLIRALTLNTMTKDAERGKLKRRA  
IATPGMQIRGFVYFVETLARSICEKLEQSGLPVGGNEKKAKLANVVRKMMTNSQDTELSFTITGDNTKWNENQNPRMFLA  
MITYMTRNQPEWFRNVLSIAPIMFSNKMARLGKGYMFESKSMKLRTQIPAEMLASIDLKYFNDSTRKKIEKIRPLLIEGT  
ASLSPGMMMGMFNMLSTVLGVSILNLGQKRYTKTTYWWDGLQSSDDFALIVNAPNHEGIQAGVDRFYRTCKLLGINMSKK  
KSYINRTGTFEFTSFFYRYGFVANFSMELPSFGVSGINESADMSIGVTVIKNNMINNDLGPATAQMALQLFIKDYRYTYR  
CHRGDTQIQTRRSFEIKKLWEQTRSKAGLLVSDGGPNLYNIRNLHIPEVCLKWELMDEDYQGRLCNPLNPFVSHKEIESM  
NNAVMMPAHGPAKNMEYDAVATTHSWIPKRNRSILNTSQRGVLEDEQMYQRCCNLFEKFFPSSSYRRPVGISSMVEAMVS  
RARIDARIDFESGRIKKEEFTEIMKICSTIEELRRQK`

| Site | Type | Count |
| --- | --- | --- |
| 476 | N-linked | 1 |

| Mass | Sequence | MS_2_ Score | `q`-value | MS_1_ Score | Signal | Protein Coordinates | Tandem Scans | Start Time | Apex Time | End Time |
| --- | --- | --- | --- | --- | --- | --- | --- | --- | --- | --- |
| 3977.6335 | GIN(N)MSKKKSY Fuc 1 Hex 9 HexNAc 6 | 65.888 | 0.026 | -19.982 | 3.918e+06 | 473 - 483 | 1 | 13.11 | 13.11 | 13.11 |

### GIN(N)MSKKKSY Fuc 1 Hex 9 HexNAc 6

**Mass:**  3977.6335

(3.077e-06)

**Charge States:** 4

**Start Position**: 474

**End Position**: 484

| Profile Score | Tandem Score | TDA q-value | Volume | Spectrum Matches |
| --- | --- | --- | --- | --- |
| -19.9822 | 65.8883 | 0.0262 | 3.9179e+06 | 1 |

**Adduct Signal Fractions**

- **Unmodified**: 3.9179e+06
