# Supplementary material for: Measuring Site-specific Glycosylation Similarity between Influenza a Virus Variants with Statistical Certainty
Source: Mol Cell Proteomics. 2020 Nov 25;19(9):1533–45. doi: 10.1074/mcp.RA120.002031 (PMC8143645; doi:10.1074/mcp.RA120.002031)
Supplement: Supplementary file 2 [file mmc2.zip › mmc2/159250_2_supp_555291_qcjklr.zip!/20190301_5B8eggL2_021119_chymo_HILIC_DDA_1ug_01_20190312055232_glycopeptides.html]

## 20190301\_5B8eggL2\_021119\_chymo\_HILIC\_DDA\_1ug\_01\_20190312055232 @ GlycopeptideHypothesis-d68556561be34579bbe32f339c3ea9d2

**MS^1^ Mass Accuracy:** 10.0 PPM  
**Grouping Tolerance:** 15.0 PPM  
**MS^n^ Mass Accuracy:** 20.0 PPM  
**Minimum `q`-Value For Clustering:** 0.05

**Glycopeptide Hypothesis:**
GlycopeptideHypothesis-d68556561be34579bbe32f339c3ea9d2  
**Sample Searched:**
20190301\_5B8eggL2\_021119\_chymo\_HILIC\_DDA\_1ug\_01\_20190312055232

| Protein | Matched Glycopeptides |
| --- | --- |
| cus\|SWZHA\_5B8\|A/Switzerland/9715293/2013 | 62 |
| cus\|SWZNA\|A/Switzerland/9715293/2013 | 29 |
| tr\|F1NUU6\|F1NUU6\_CHICK | 1 |

### cus|SWZHA\_5B8|A/Switzerland/9715293/2013

**>cus|SWZHA\_5B8|A/Switzerland/9715293/2013**  
 `QKLPGNDNSTATLCLGHHAVPNGTIVKTITNDRIEVTNATELVQNSSIGEICDSPHQILDGENCTLIDALLGDPQCDGFQ  
NKKWDLFVERSKAYSNCYPYDVPDYASLRSLVASSGTLEFNNESFNWAGVTHNGTSSSCRRGSNSSFFSRLNWLTHLNSK  
YPALNVTMPNNEQFDKLYIWGVHHPVTDKDQIFLYAQSSGRITVSTKRSQQAVIPNIGSRPRIRNIPSRISIYWTIVKPG  
DILLINSTGNLIAPRGYFKIRSGKSSIMRSDAPIGKCKSECITPNGSIPNDKPFQNVNRITYGACPRYVKQSTLKLATGM  
RNVPERQTRGIFGAIAGFIENGWEGMVDGWYGFRHQNSEGRGQAADLKSTQAAIDQINGKLNRLIGKTNEKFHQIEKEFS  
EVEGRIQDLEKYVEDTKIDLWSYNAELLVALENQHTIDLTDSEMNKLFEKTKKQLRENAEDMGNGCFKIYHKCDNACIGS  
IRNGTYDHDVYRDEALNNRFQIKGVELKSGYKDWILWISFAISCFLLCVALLGFIMWACQKGNIRCNICI`

| Site | Type | Count |
| --- | --- | --- |
| 483 | N-linked | 2 |
| 133 | N-linked | 14 |
| 38 | N-linked | 3 |
| 8 | N-linked | 1 |
| 144 | N-linked | 3 |
| 246 | N-linked | 18 |
| 165 | N-linked | 4 |
| 285 | N-linked | 16 |
| 22 | N-linked | 1 |

| Mass | Sequence | MS_2_ Score | `q`-value | MS_1_ Score | Signal | Protein Coordinates | Tandem Scans | Start Time | Apex Time | End Time |
| --- | --- | --- | --- | --- | --- | --- | --- | --- | --- | --- |
| 3499.5165 | LIN(N)STGNLIAPRGYF Hex 9 HexNAc 2 | 181.523 | 0.0 | -2.716 | 9.686e+07 | 243 - 258 | 4 | 47.30 | 47.47 | 47.81 |
| 2695.0864 | LIN(N)STGNL Hex 9 HexNAc 2 | 181.418 | 0.0 | -19.145 | 1.947e+07 | 243 - 251 | 3 | 27.26 | 27.26 | 27.43 |
| 3352.4519 | LIN(N)STGNLIAPRGY Hex 9 HexNAc 2 | 175.865 | 0.0 | -2.477 | 8.566e+07 | 243 - 257 | 2 | 37.30 | 37.47 | 37.64 |
| 3516.5438 | LIN(N)STGNLIAPRGYF Fuc 2 Hex 3 HexNAc 4 Neu5Ac 1 | 175.673 | 0.0 | -18.070 | 1.989e+07 | 243 - 258 | 2 | 47.30 | 47.47 | 47.47 |
| 3369.4776 | LIN(N)STGNLIAPRGY Fuc 2 Hex 3 HexNAc 4 Neu5Ac 1 | 167.269 | 0.0 | -17.547 | 1.642e+07 | 243 - 257 | 4 | 37.30 | 37.47 | 37.47 |
| 3017.1346 | NWAGVTHN(N)GTSSSC(C) Hex 7 HexNAc 2 | 163.477 | 0.0 | -18.753 | 7.601e+06 | 125 - 139 | 1 | 18.81 | 18.81 | 18.81 |
| 3159.4254 | TIVKPGDILLIN(N) Hex 9 HexNAc 2 | 158.424 | 0.0 | 13.016 | 1.604e+08 | 234 - 246 | 6 | 46.47 | 47.81 | 48.32 |
| 3431.4080 | AGVTHNGTSSSC(C)RRGSN(N)SSFF Hex 5 HexNAc 2 | 156.058 | 0.0 | -3.749 | 1.443e+08 | 127 - 148 | 3 | 21.63 | 21.80 | 22.12 |
| 3176.4506 | TIVKPGDILLIN(N) Fuc 2 Hex 3 HexNAc 4 Neu5Ac 1 | 154.752 | 0.0 | 12.865 | 3.796e+07 | 234 - 246 | 4 | 46.63 | 47.81 | 48.15 |
| 3386.4324 | IN(N)STGNLIAPRGYF Hex 9 HexNAc 2 | 152.465 | 0.0 | -19.027 | 7.089e+06 | 244 - 258 | 2 | 43.57 | 43.74 | 43.74 |
| 3755.5139 | AGVTHNGTSSSC(C)RRGSN(N)SSFF Hex 7 HexNAc 2 | 151.592 | 0.0 | 15.570 | 3.808e+08 | 127 - 148 | 4 | 20.97 | 21.46 | 21.80 |
| 3593.4610 | AGVTHN(N)GTSSSC(C)RRGSNSSFF Hex 6 HexNAc 2 | 148.775 | 0.0 | -3.377 | 9.926e+07 | 127 - 148 | 1 | 21.46 | 21.63 | 21.80 |
| 3518.5713 | TIVKPGDILLIN(N)STGN Hex 9 HexNAc 2 | 146.592 | 0.0 | 16.393 | 1.691e+08 | 234 - 250 | 4 | 45.44 | 45.79 | 46.30 |
| 3535.5952 | TIVKPGDILLIN(N)STGN Fuc 2 Hex 3 HexNAc 4 Neu5Ac 1 | 146.163 | 0.0 | -3.252 | 4.382e+07 | 234 - 250 | 2 | 45.44 | 45.79 | 45.96 |
| 3337.4650 | LIN(N)STGNLIAPRGYF Hex 8 HexNAc 2 | 145.867 | 0.0 | -19.428 | 7.367e+06 | 243 - 258 | 2 | 47.47 | 47.47 | 47.64 |
| 3190.4005 | LIN(N)STGNLIAPRGY Hex 8 HexNAc 2 | 145.705 | 0.0 | -17.332 | 1.026e+07 | 243 - 257 | 1 | 37.47 | 37.47 | 37.64 |
| 4752.0478 | RSDAPIGKC(C)KSEC(C)ITPN(N)GSIPNDKPF Hex 9 HexNAc 2 | 144.452 | 0.0 | -2.082 | 3.872e+08 | 268 - 294 | 2 | 30.43 | 30.60 | 30.77 |
| 3767.5544 | KSEC(C)ITPN(N)GSIPNDKPF Hex 9 HexNAc 2 | 144.083 | 0.0 | -4.701 | 3.539e+07 | 277 - 294 | 1 | 32.10 | 32.26 | 32.42 |
| 4427.9434 | RSDAPIGKC(C)KSEC(C)ITPN(N)GSIPNDKPF Hex 7 HexNAc 2 | 143.845 | 0.0 | -2.490 | 4.143e+08 | 268 - 294 | 2 | 30.60 | 30.77 | 30.94 |
| 2997.3737 | TIVKPGDILLIN(N) Hex 8 HexNAc 2 | 143.627 | 0.0 | -4.577 | 1.020e+07 | 234 - 246 | 1 | 47.64 | 47.98 | 48.15 |
| 3605.4918 | KSEC(C)ITPN(N)GSIPNDKPF Hex 8 HexNAc 2 | 141.554 | 0.0 | -20.068 | 1.069e+07 | 277 - 294 | 1 | 32.26 | 32.26 | 32.26 |
| 3443.4456 | KSEC(C)ITPN(N)GSIPNDKPF Hex 7 HexNAc 2 | 140.883 | 0.0 | -19.402 | 3.305e+07 | 277 - 294 | 1 | 32.42 | 32.42 | 32.58 |
| 4203.7798 | NSKYPALN(N)VTM(O)PNNEQFDKLY Hex 8 HexNAc 2 | 140.444 | 0.0 | -1.975 | 1.156e+08 | 157 - 178 | 4 | 43.07 | 43.23 | 43.57 |
| 4089.7362 | SKYPALN(N)VTM(O)PNNEQFDKLY Hex 8 HexNAc 2 | 140.441 | 0.0 | -3.022 | 4.577e+07 | 158 - 178 | 2 | 43.07 | 43.23 | 43.57 |
| 3356.5181 | TIVKPGDILLIN(N)STGN Hex 8 HexNAc 2 | 137.627 | 0.0 | -3.618 | 1.991e+07 | 234 - 250 | 2 | 45.62 | 45.79 | 45.96 |
| 3229.3959 | TITNDRIEVTN(N)ATEL Hex 7 HexNAc 2 | 136.627 | 0.0 | -17.232 | 3.546e+06 | 27 - 42 | 1 | 37.13 | 37.13 | 37.30 |
| 4265.8870 | RSDAPIGKC(C)KSEC(C)ITPN(N)GSIPNDKPF Hex 6 HexNAc 2 | 136.327 | 0.0 | -18.053 | 4.459e+07 | 268 - 294 | 2 | 30.77 | 30.77 | 30.94 |
| 4055.6473 | NWAGVTHN(N)GTSSSC(C)RRGSNSSFF Hex 7 HexNAc 2 | 132.971 | 0.0 | -2.226 | 9.722e+07 | 125 - 148 | 2 | 30.77 | 30.94 | 31.28 |
| 2770.0755 | NWAGVTHN(N)GTSS Hex 7 HexNAc 2 | 127.628 | 0.011 | -19.906 | 7.145e+06 | 125 - 137 | 2 | 18.32 | 18.32 | 18.32 |
| 2905.2894 | TITNDRIEVTN(N)ATEL Hex 5 HexNAc 2 | 124.944 | 0.011 | -16.962 | 4.133e+06 | 27 - 42 | 1 | 37.47 | 37.47 | 37.64 |
| 3731.5471 | NWAGVTHN(N)GTSSSC(C)RRGSNSSFF Hex 5 HexNAc 2 | 124.916 | 0.011 | -16.204 | 2.893e+07 | 125 - 148 | 1 | 31.28 | 31.28 | 31.28 |
| 4589.9934 | RSDAPIGKC(C)KSEC(C)ITPN(N)GSIPNDKPF Hex 8 HexNAc 2 | 124.374 | 0.011 | -2.924 | 2.413e+08 | 268 - 294 | 2 | 30.60 | 30.77 | 30.94 |
| 3917.5664 | AGVTHN(N)GTSSSC(C)RRGSNSSFF Hex 8 HexNAc 2 | 123.856 | 0.011 | -18.588 | 3.502e+07 | 127 - 148 | 1 | 21.30 | 21.46 | 21.46 |
| 3661.5686 | LIN(N)STGNLIAPRGYF Hex 10 HexNAc 2 | 123.207 | 0.011 | -18.114 | 4.132e+06 | 243 - 258 | 1 | 47.30 | 47.30 | 47.30 |
| 3173.2388 | NWAGVTHN(N)GTSSSC(C)R Hex 7 HexNAc 2 | 121.623 | 0.011 | -17.773 | 3.995e+06 | 125 - 140 | 1 | 16.33 | 16.33 | 16.33 |
| 3680.6226 | TIVKPGDILLIN(N)STGN Hex 10 HexNAc 2 | 119.734 | 0.011 | -4.930 | 6.850e+06 | 234 - 250 | 1 | 45.44 | 45.62 | 45.79 |
| 3472.4376 | AGVTHN(N)GTSSSC(C)RRGSNSSFF Hex 4 HexNAc 3 | 116.086 | 0.011 | -18.336 | 2.530e+07 | 127 - 148 | 1 | 21.80 | 21.96 | 21.96 |
| 3473.4985 | C(C)LGHHAVPN(N)GTIVK Fuc 1 Hex 5 HexNAc 5 | 116.015 | 0.011 | -19.627 | 5.955e+06 | 13 - 27 | 1 | 19.63 | 19.63 | 19.80 |
| 3639.4560 | SEC(C)ITPN(N)GSIPNDKPF Hex 9 HexNAc 2 | 109.675 | 0.011 | -5.844 | 5.993e+06 | 278 - 294 | 1 | 36.13 | 36.29 | 36.45 |
| 3067.3426 | TITNDRIEVTN(N)ATEL Hex 6 HexNAc 2 | 109.408 | 0.011 | -18.611 | 4.228e+06 | 27 - 42 | 1 | 37.30 | 37.30 | 37.47 |
| 5134.2710 | SGKSSIM(O)RSDAPIGKC(C)KSEC(C)ITPN(N)GSIPNDKPF Hex 7 HexNAc 2 | 107.133 | 0.011 | -19.139 | 1.780e+07 | 261 - 294 | 1 | 30.09 | 30.26 | 30.26 |
| 4429.9369 | TIVKPGDILLIN(N) Fuc 5 Hex 3 HexNAc 8 Neu5Ac 1 | 106.615 | 0.011 | 0.000 | 0.000e+00 | 234 - 246 | 1 | - | - | - |
| 3404.5255 | TIVKPGDILLIN(N)STG Hex 9 HexNAc 2 | 98.957 | 0.011 | -20.380 | 4.798e+06 | 234 - 249 | 1 | 46.63 | 46.63 | 46.63 |
| 3315.3540 | SEC(C)ITPN(N)GSIPNDKPF Hex 7 HexNAc 2 | 94.317 | 0.011 | -4.642 | 5.464e+06 | 278 - 294 | 1 | 36.29 | 36.45 | 36.62 |
| 2736.1613 | QKLPGNDN(N)STATL Hex 6 HexNAc 2 | 91.689 | 0.011 | -19.370 | 7.971e+07 | 0 - 13 | 1 | 31.94 | 31.94 | 32.10 |
| 4753.0320 | RSDAPIGKC(C)KSEC(C)ITPN(N)GSIPN(D)DKPF Hex 9 HexNAc 2 | 83.639 | 0.019 | -5.498 | 7.116e+07 | 268 - 294 | 1 | 31.28 | 31.45 | 31.61 |
| 4428.9266 | RSDAPIGKC(C)KSEC(C)ITPN(N)GSIPN(D)DKPF Hex 7 HexNAc 2 | 81.900 | 0.019 | -20.250 | 6.105e+07 | 268 - 294 | 1 | 31.45 | 31.45 | 31.61 |
| 4461.7994 | NWAGVTHN(N)GTSSSC(C)RRGSNSSFF Hex 7 HexNAc 4 | 81.770 | 0.019 | -4.909 | 9.634e+06 | 125 - 148 | 1 | 30.77 | 30.94 | 31.11 |
| 5855.6560 | KIRSGKSSIM(O)RSDAPIGKC(C)KSEC(C)ITPN(N)GSIPNDKPF Hex 9 HexNAc 2 | 78.220 | 0.019 | -18.109 | 6.867e+07 | 258 - 294 | 1 | 27.60 | 27.77 | 27.77 |
| 4591.9911 | RSDAPIGKC(C)KSEC(C)ITPN(N)GSIPN(D)DKPF Hex 8 HexNAc 2 | 75.886 | 0.019 | 0.000 | 0.000e+00 | 268 - 294 | 1 | - | - | - |
| 3796.5419 | AGVTHN(N)GTSSSC(C)RRGSNSSFF Hex 6 HexNAc 3 | 72.053 | 0.019 | -4.924 | 4.985e+07 | 127 - 148 | 1 | 21.46 | 21.63 | 21.80 |
| 4262.7207 | HKC(C)DNAC(C)IGSIRN(N)GTYDHDVY Fuc 1 Hex 5 HexNAc 4 | 68.160 | 0.019 | -18.680 | 2.758e+07 | 470 - 491 | 2 | 25.94 | 25.94 | 26.10 |
| 3893.6007 | NWAGVTHN(N)GTSSSC(C)RRGSNSSFF Hex 6 HexNAc 2 | 65.461 | 0.026 | -16.855 | 2.006e+07 | 125 - 148 | 1 | 31.11 | 31.11 | 31.11 |
| 4041.7269 | NSKYPALN(N)VTM(O)PNNEQFDKLY Hex 7 HexNAc 2 | 63.520 | 0.026 | -4.336 | 1.653e+07 | 157 - 178 | 3 | 43.23 | 43.23 | 43.57 |
| 3958.5968 | AGVTHN(N)GTSSSC(C)RRGSNSSFF Hex 7 HexNAc 3 | 62.810 | 0.026 | -18.818 | 3.461e+07 | 127 - 148 | 1 | 21.46 | 21.46 | 21.63 |
| 4408.7761 | HKC(C)DNAC(C)IGSIRN(N)GTYDHDVY Fuc 2 Hex 5 HexNAc 4 | 59.654 | 0.026 | -19.136 | 1.854e+07 | 470 - 491 | 3 | 25.94 | 25.94 | 25.94 |
| 4404.7829 | NWAGVTHN(N)GTSSSC(C)RRGSNSSFF Fuc 1 Hex 7 HexNAc 3 | 57.565 | 0.026 | -18.939 | 4.370e+06 | 125 - 148 | 1 | 30.77 | 30.77 | 30.77 |
| 4161.6725 | AGVTHN(N)GTSSSC(C)RRGSNSSFF Hex 7 HexNAc 4 | 57.169 | 0.026 | -4.080 | 4.159e+07 | 127 - 148 | 1 | 21.30 | 21.46 | 21.63 |
| 3756.4983 | AGVTHN(D)GTSSSC(C)RRGSN(N)SSFF Hex 7 HexNAc 2 | 55.153 | 0.026 | 9.596 | 1.093e+08 | 127 - 148 | 3 | 22.62 | 22.79 | 23.45 |
| 4204.7630 | N(D)SKYPALN(N)VTM(O)PNNEQFDKLY Hex 8 HexNAc 2 | 53.644 | 0.026 | -20.573 | 2.018e+07 | 157 - 178 | 3 | 45.96 | 45.96 | 46.13 |
| 3477.4022 | SEC(C)ITPN(N)GSIPNDKPF Hex 8 HexNAc 2 | 51.100 | 0.048 | -19.725 | 2.680e+06 | 278 - 294 | 1 | 36.29 | 36.29 | 36.45 |
| 5740.5518 | RSDAPIGKC(C)KSEC(C)ITPN(N)GSIPNDKPFQNVNRITY Hex 9 HexNAc 2 | 51.011 | 0.048 | -21.512 | 3.919e+06 | 268 - 302 | 2 | 35.96 | 36.13 | 36.13 |

### LIN(N)STGNLIAPRGYF Hex 9 HexNAc 2

**Mass:**  3499.5163

(1.130e-06)

**Charge States:** 3

**Start Position**: 244

**End Position**: 259

| Profile Score | Tandem Score | TDA q-value | Volume | Spectrum Matches |
| --- | --- | --- | --- | --- |
| -2.7164 | 181.5233 | 0.0000 | 9.6863e+07 | 4 |

**Adduct Signal Fractions**

- **Unmodified**: 9.6863e+07

### LIN(N)STGNL Hex 9 HexNAc 2

**Mass:**  2695.0821

(2.137e-06)

**Charge States:** 2, 3

**Start Position**: 244

**End Position**: 252

| Profile Score | Tandem Score | TDA q-value | Volume | Spectrum Matches |
| --- | --- | --- | --- | --- |
| -19.1448 | 181.4179 | 0.0000 | 1.9473e+07 | 3 |

**Adduct Signal Fractions**

- **Unmodified**: 1.9473e+07

### LIN(N)STGNLIAPRGY Hex 9 HexNAc 2

**Mass:**  3352.4525

(2.197e-06)

**Charge States:** 3

**Start Position**: 244

**End Position**: 258

| Profile Score | Tandem Score | TDA q-value | Volume | Spectrum Matches |
| --- | --- | --- | --- | --- |
| -2.4769 | 175.8647 | 0.0000 | 8.5658e+07 | 2 |

**Adduct Signal Fractions**

- **Unmodified**: 8.5658e+07

### LIN(N)STGNLIAPRGYF Fuc 2 Hex 3 HexNAc 4 Neu5Ac 1

**Mass:**  3516.5443

(-5.899e-06)

**Charge States:** 3

**Start Position**: 244

**End Position**: 259

| Profile Score | Tandem Score | TDA q-value | Volume | Spectrum Matches |
| --- | --- | --- | --- | --- |
| -18.0699 | 175.6731 | 0.0000 | 1.9890e+07 | 2 |

**Adduct Signal Fractions**

- **Unmodified**: 1.9890e+07

### LIN(N)STGNLIAPRGY Fuc 2 Hex 3 HexNAc 4 Neu5Ac 1

**Mass:**  3369.4777

(-5.839e-06)

**Charge States:** 3

**Start Position**: 244

**End Position**: 258

| Profile Score | Tandem Score | TDA q-value | Volume | Spectrum Matches |
| --- | --- | --- | --- | --- |
| -17.5469 | 167.2690 | 0.0000 | 1.6422e+07 | 4 |

**Adduct Signal Fractions**

- **Unmodified**: 1.6422e+07

### NWAGVTHN(N)GTSSSC(C) Hex 7 HexNAc 2

**Mass:**  3017.1346

(2.716e-07)

**Charge States:** 3

**Start Position**: 126

**End Position**: 140

| Profile Score | Tandem Score | TDA q-value | Volume | Spectrum Matches |
| --- | --- | --- | --- | --- |
| -18.7529 | 163.4766 | 0.0000 | 7.6014e+06 | 1 |

**Adduct Signal Fractions**

- **Unmodified**: 7.6014e+06

### TIVKPGDILLIN(N) Hex 9 HexNAc 2

**Mass:**  3159.4249

(1.469e-06)

**Charge States:** 2, 3

**Start Position**: 235

**End Position**: 247

| Profile Score | Tandem Score | TDA q-value | Volume | Spectrum Matches |
| --- | --- | --- | --- | --- |
| 13.0161 | 158.4241 | 0.0000 | 1.6035e+08 | 6 |

**Adduct Signal Fractions**

- **Unmodified**: 1.6035e+08

### AGVTHNGTSSSC(C)RRGSN(N)SSFF Hex 5 HexNAc 2

**Mass:**  3431.4091

(1.104e-06)

**Charge States:** 4

**Start Position**: 128

**End Position**: 149

| Profile Score | Tandem Score | TDA q-value | Volume | Spectrum Matches |
| --- | --- | --- | --- | --- |
| -3.7489 | 156.0582 | 0.0000 | 1.4427e+08 | 3 |

**Adduct Signal Fractions**

- **Unmodified**: 1.4427e+08

### TIVKPGDILLIN(N) Fuc 2 Hex 3 HexNAc 4 Neu5Ac 1

**Mass:**  3176.4508

(-7.003e-06)

**Charge States:** 3

**Start Position**: 235

**End Position**: 247

| Profile Score | Tandem Score | TDA q-value | Volume | Spectrum Matches |
| --- | --- | --- | --- | --- |
| 12.8655 | 154.7518 | 0.0000 | 3.7963e+07 | 4 |

**Adduct Signal Fractions**

- **Unmodified**: 3.7963e+07

### IN(N)STGNLIAPRGYF Hex 9 HexNAc 2

**Mass:**  3386.4314

(1.875e-06)

**Charge States:** 3

**Start Position**: 245

**End Position**: 259

| Profile Score | Tandem Score | TDA q-value | Volume | Spectrum Matches |
| --- | --- | --- | --- | --- |
| -19.0267 | 152.4653 | 0.0000 | 7.0895e+06 | 2 |

**Adduct Signal Fractions**

- **Unmodified**: 7.0895e+06

### AGVTHNGTSSSC(C)RRGSN(N)SSFF Hex 7 HexNAc 2

**Mass:**  3755.5139

(7.178e-07)

**Charge States:** 4

**Start Position**: 128

**End Position**: 149

| Profile Score | Tandem Score | TDA q-value | Volume | Spectrum Matches |
| --- | --- | --- | --- | --- |
| 15.5697 | 151.5919 | 0.0000 | 3.8079e+08 | 4 |

**Adduct Signal Fractions**

- **Unmodified**: 3.8079e+08

### AGVTHN(N)GTSSSC(C)RRGSNSSFF Hex 6 HexNAc 2

**Mass:**  3593.4611

(8.581e-07)

**Charge States:** 4

**Start Position**: 128

**End Position**: 149

| Profile Score | Tandem Score | TDA q-value | Volume | Spectrum Matches |
| --- | --- | --- | --- | --- |
| -3.3767 | 148.7749 | 0.0000 | 9.9264e+07 | 1 |

**Adduct Signal Fractions**

- **Unmodified**: 9.9264e+07

### TIVKPGDILLIN(N)STGN Hex 9 HexNAc 2

**Mass:**  3518.5706

(1.801e-06)

**Charge States:** 3

**Start Position**: 235

**End Position**: 251

| Profile Score | Tandem Score | TDA q-value | Volume | Spectrum Matches |
| --- | --- | --- | --- | --- |
| 16.3933 | 146.5918 | 0.0000 | 1.6910e+08 | 4 |

**Adduct Signal Fractions**

- **Unmodified**: 1.6910e+08

### TIVKPGDILLIN(N)STGN Fuc 2 Hex 3 HexNAc 4 Neu5Ac 1

**Mass:**  3535.5968

(-5.644e-06)

**Charge States:** 3

**Start Position**: 235

**End Position**: 251

| Profile Score | Tandem Score | TDA q-value | Volume | Spectrum Matches |
| --- | --- | --- | --- | --- |
| -3.2524 | 146.1629 | 0.0000 | 4.3816e+07 | 2 |

**Adduct Signal Fractions**

- **Unmodified**: 4.3816e+07

### LIN(N)STGNLIAPRGYF Hex 8 HexNAc 2

**Mass:**  3337.4632

(1.101e-06)

**Charge States:** 3

**Start Position**: 244

**End Position**: 259

| Profile Score | Tandem Score | TDA q-value | Volume | Spectrum Matches |
| --- | --- | --- | --- | --- |
| -19.4277 | 145.8673 | 0.0000 | 7.3672e+06 | 2 |

**Adduct Signal Fractions**

- **Unmodified**: 7.3672e+06

### LIN(N)STGNLIAPRGY Hex 8 HexNAc 2

**Mass:**  3190.4005

(3.039e-06)

**Charge States:** 3

**Start Position**: 244

**End Position**: 258

| Profile Score | Tandem Score | TDA q-value | Volume | Spectrum Matches |
| --- | --- | --- | --- | --- |
| -17.3324 | 145.7055 | 0.0000 | 1.0261e+07 | 1 |

**Adduct Signal Fractions**

- **Unmodified**: 1.0261e+07

### RSDAPIGKC(C)KSEC(C)ITPN(N)GSIPNDKPF Hex 9 HexNAc 2

**Mass:**  4752.0504

(4.302e-06)

**Charge States:** 4, 5

**Start Position**: 269

**End Position**: 295

| Profile Score | Tandem Score | TDA q-value | Volume | Spectrum Matches |
| --- | --- | --- | --- | --- |
| -2.0821 | 144.4516 | 0.0000 | 3.8719e+08 | 2 |

**Adduct Signal Fractions**

- **Unmodified**: 3.8719e+08

### KSEC(C)ITPN(N)GSIPNDKPF Hex 9 HexNAc 2

**Mass:**  3767.5515

(7.069e-07)

**Charge States:** 3

**Start Position**: 278

**End Position**: 295

| Profile Score | Tandem Score | TDA q-value | Volume | Spectrum Matches |
| --- | --- | --- | --- | --- |
| -4.7006 | 144.0831 | 0.0000 | 3.5393e+07 | 1 |

**Adduct Signal Fractions**

- **Unmodified**: 3.5393e+07

### RSDAPIGKC(C)KSEC(C)ITPN(N)GSIPNDKPF Hex 7 HexNAc 2

**Mass:**  4427.9485

(1.326e-06)

**Charge States:** 4, 5

**Start Position**: 269

**End Position**: 295

| Profile Score | Tandem Score | TDA q-value | Volume | Spectrum Matches |
| --- | --- | --- | --- | --- |
| -2.4896 | 143.8453 | 0.0000 | 4.1428e+08 | 2 |

**Adduct Signal Fractions**

- **Unmodified**: 4.1428e+08

### TIVKPGDILLIN(N) Hex 8 HexNAc 2

**Mass:**  2997.3724

(2.602e-06)

**Charge States:** 3

**Start Position**: 235

**End Position**: 247

| Profile Score | Tandem Score | TDA q-value | Volume | Spectrum Matches |
| --- | --- | --- | --- | --- |
| -4.5769 | 143.6269 | 0.0000 | 1.0203e+07 | 1 |

**Adduct Signal Fractions**

- **Unmodified**: 1.0203e+07

### KSEC(C)ITPN(N)GSIPNDKPF Hex 8 HexNAc 2

**Mass:**  3605.4918

(-1.099e-06)

**Charge States:** 3

**Start Position**: 278

**End Position**: 295

| Profile Score | Tandem Score | TDA q-value | Volume | Spectrum Matches |
| --- | --- | --- | --- | --- |
| -20.0685 | 141.5538 | 0.0000 | 1.0690e+07 | 1 |

**Adduct Signal Fractions**

- **Unmodified**: 1.0690e+07

### KSEC(C)ITPN(N)GSIPNDKPF Hex 7 HexNAc 2

**Mass:**  3443.4453

(6.898e-07)

**Charge States:** 3

**Start Position**: 278

**End Position**: 295

| Profile Score | Tandem Score | TDA q-value | Volume | Spectrum Matches |
| --- | --- | --- | --- | --- |
| -19.4023 | 140.8833 | 0.0000 | 3.3050e+07 | 1 |

**Adduct Signal Fractions**

- **Unmodified**: 3.3050e+07

### NSKYPALN(N)VTM(O)PNNEQFDKLY Hex 8 HexNAc 2

**Mass:**  4203.7814

(2.269e-06)

**Charge States:** 3, 4

**Start Position**: 158

**End Position**: 179

| Profile Score | Tandem Score | TDA q-value | Volume | Spectrum Matches |
| --- | --- | --- | --- | --- |
| -1.9751 | 140.4436 | 0.0000 | 1.1557e+08 | 4 |

**Adduct Signal Fractions**

- **Unmodified**: 1.1557e+08

### SKYPALN(N)VTM(O)PNNEQFDKLY Hex 8 HexNAc 2

**Mass:**  4089.7348

(2.566e-06)

**Charge States:** 3, 4

**Start Position**: 159

**End Position**: 179

| Profile Score | Tandem Score | TDA q-value | Volume | Spectrum Matches |
| --- | --- | --- | --- | --- |
| -3.0216 | 140.4410 | 0.0000 | 4.5768e+07 | 2 |

**Adduct Signal Fractions**

- **Unmodified**: 4.5768e+07

### TIVKPGDILLIN(N)STGN Hex 8 HexNAc 2

**Mass:**  3356.5171

(2.293e-06)

**Charge States:** 3

**Start Position**: 235

**End Position**: 251

| Profile Score | Tandem Score | TDA q-value | Volume | Spectrum Matches |
| --- | --- | --- | --- | --- |
| -3.6184 | 137.6267 | 0.0000 | 1.9909e+07 | 2 |

**Adduct Signal Fractions**

- **Unmodified**: 1.9909e+07

### TITNDRIEVTN(N)ATEL Hex 7 HexNAc 2

**Mass:**  3229.3961

(2.990e-06)

**Charge States:** 3

**Start Position**: 28

**End Position**: 43

| Profile Score | Tandem Score | TDA q-value | Volume | Spectrum Matches |
| --- | --- | --- | --- | --- |
| -17.2321 | 136.6265 | 0.0000 | 3.5459e+06 | 1 |

**Adduct Signal Fractions**

- **Unmodified**: 3.5459e+06

### RSDAPIGKC(C)KSEC(C)ITPN(N)GSIPNDKPF Hex 6 HexNAc 2

**Mass:**  4265.8865

(2.677e-06)

**Charge States:** 4, 5

**Start Position**: 269

**End Position**: 295

| Profile Score | Tandem Score | TDA q-value | Volume | Spectrum Matches |
| --- | --- | --- | --- | --- |
| -18.0531 | 136.3270 | 0.0000 | 4.4594e+07 | 2 |

**Adduct Signal Fractions**

- **Unmodified**: 4.4594e+07

### NWAGVTHN(N)GTSSSC(C)RRGSNSSFF Hex 7 HexNAc 2

**Mass:**  4055.6474

(3.468e-06)

**Charge States:** 4

**Start Position**: 126

**End Position**: 149

| Profile Score | Tandem Score | TDA q-value | Volume | Spectrum Matches |
| --- | --- | --- | --- | --- |
| -2.2255 | 132.9711 | 0.0000 | 9.7225e+07 | 2 |

**Adduct Signal Fractions**

- **Unmodified**: 9.7225e+07

### NWAGVTHN(N)GTSS Hex 7 HexNAc 2

**Mass:**  2770.0757

(1.719e-06)

**Charge States:** 2, 3

**Start Position**: 126

**End Position**: 138

| Profile Score | Tandem Score | TDA q-value | Volume | Spectrum Matches |
| --- | --- | --- | --- | --- |
| -19.9055 | 127.6279 | 0.0105 | 7.1447e+06 | 2 |

**Adduct Signal Fractions**

- **Unmodified**: 7.1447e+06

### TITNDRIEVTN(N)ATEL Hex 5 HexNAc 2

**Mass:**  2905.2896

(2.945e-06)

**Charge States:** 3

**Start Position**: 28

**End Position**: 43

| Profile Score | Tandem Score | TDA q-value | Volume | Spectrum Matches |
| --- | --- | --- | --- | --- |
| -16.9620 | 124.9437 | 0.0105 | 4.1334e+06 | 1 |

**Adduct Signal Fractions**

- **Unmodified**: 4.1334e+06

### NWAGVTHN(N)GTSSSC(C)RRGSNSSFF Hex 5 HexNAc 2

**Mass:**  3731.5476

(5.369e-06)

**Charge States:** 3, 4

**Start Position**: 126

**End Position**: 149

| Profile Score | Tandem Score | TDA q-value | Volume | Spectrum Matches |
| --- | --- | --- | --- | --- |
| -16.2037 | 124.9157 | 0.0105 | 2.8932e+07 | 1 |

**Adduct Signal Fractions**

- **Unmodified**: 2.8932e+07

### RSDAPIGKC(C)KSEC(C)ITPN(N)GSIPNDKPF Hex 8 HexNAc 2

**Mass:**  4589.9978

(4.512e-06)

**Charge States:** 4, 5

**Start Position**: 269

**End Position**: 295

| Profile Score | Tandem Score | TDA q-value | Volume | Spectrum Matches |
| --- | --- | --- | --- | --- |
| -2.9244 | 124.3740 | 0.0105 | 2.4127e+08 | 2 |

**Adduct Signal Fractions**

- **Unmodified**: 2.4127e+08

### AGVTHN(N)GTSSSC(C)RRGSNSSFF Hex 8 HexNAc 2

**Mass:**  3917.5671

(8.500e-07)

**Charge States:** 4

**Start Position**: 128

**End Position**: 149

| Profile Score | Tandem Score | TDA q-value | Volume | Spectrum Matches |
| --- | --- | --- | --- | --- |
| -18.5883 | 123.8556 | 0.0105 | 3.5022e+07 | 1 |

**Adduct Signal Fractions**

- **Unmodified**: 3.5022e+07

### LIN(N)STGNLIAPRGYF Hex 10 HexNAc 2

**Mass:**  3661.5686

(9.778e-07)

**Charge States:** 3

**Start Position**: 244

**End Position**: 259

| Profile Score | Tandem Score | TDA q-value | Volume | Spectrum Matches |
| --- | --- | --- | --- | --- |
| -18.1141 | 123.2069 | 0.0105 | 4.1320e+06 | 1 |

**Adduct Signal Fractions**

- **Unmodified**: 4.1320e+06

### NWAGVTHN(N)GTSSSC(C)R Hex 7 HexNAc 2

**Mass:**  3173.2388

(1.299e-06)

**Charge States:** 3

**Start Position**: 126

**End Position**: 141

| Profile Score | Tandem Score | TDA q-value | Volume | Spectrum Matches |
| --- | --- | --- | --- | --- |
| -17.7733 | 121.6234 | 0.0105 | 3.9953e+06 | 1 |

**Adduct Signal Fractions**

- **Unmodified**: 3.9953e+06

### TIVKPGDILLIN(N)STGN Hex 10 HexNAc 2

**Mass:**  3680.6211

(2.154e-06)

**Charge States:** 3

**Start Position**: 235

**End Position**: 251

| Profile Score | Tandem Score | TDA q-value | Volume | Spectrum Matches |
| --- | --- | --- | --- | --- |
| -4.9296 | 119.7336 | 0.0105 | 6.8500e+06 | 1 |

**Adduct Signal Fractions**

- **Unmodified**: 6.8500e+06

### AGVTHN(N)GTSSSC(C)RRGSNSSFF Hex 4 HexNAc 3

**Mass:**  3472.4382

(1.298e-06)

**Charge States:** 4

**Start Position**: 128

**End Position**: 149

| Profile Score | Tandem Score | TDA q-value | Volume | Spectrum Matches |
| --- | --- | --- | --- | --- |
| -18.3359 | 116.0864 | 0.0105 | 2.5305e+07 | 1 |

**Adduct Signal Fractions**

- **Unmodified**: 2.5305e+07

### C(C)LGHHAVPN(N)GTIVK Fuc 1 Hex 5 HexNAc 5

**Mass:**  3473.5014

(7.707e-08)

**Charge States:** 4

**Start Position**: 14

**End Position**: 28

| Profile Score | Tandem Score | TDA q-value | Volume | Spectrum Matches |
| --- | --- | --- | --- | --- |
| -19.6270 | 116.0152 | 0.0105 | 5.9547e+06 | 1 |

**Adduct Signal Fractions**

- **Unmodified**: 5.9547e+06

### SEC(C)ITPN(N)GSIPNDKPF Hex 9 HexNAc 2

**Mass:**  3639.4579

(-2.338e-07)

**Charge States:** 3

**Start Position**: 279

**End Position**: 295

| Profile Score | Tandem Score | TDA q-value | Volume | Spectrum Matches |
| --- | --- | --- | --- | --- |
| -5.8443 | 109.6747 | 0.0105 | 5.9930e+06 | 1 |

**Adduct Signal Fractions**

- **Unmodified**: 5.9930e+06

### TITNDRIEVTN(N)ATEL Hex 6 HexNAc 2

**Mass:**  3067.3427

(2.921e-06)

**Charge States:** 3

**Start Position**: 28

**End Position**: 43

| Profile Score | Tandem Score | TDA q-value | Volume | Spectrum Matches |
| --- | --- | --- | --- | --- |
| -18.6112 | 109.4081 | 0.0105 | 4.2280e+06 | 1 |

**Adduct Signal Fractions**

- **Unmodified**: 4.2280e+06

### SGKSSIM(O)RSDAPIGKC(C)KSEC(C)ITPN(N)GSIPNDKPF Hex 7 HexNAc 2

**Mass:**  5134.2703

(2.717e-06)

**Charge States:** 5, 6

**Start Position**: 262

**End Position**: 295

| Profile Score | Tandem Score | TDA q-value | Volume | Spectrum Matches |
| --- | --- | --- | --- | --- |
| -19.1386 | 107.1332 | 0.0105 | 1.7796e+07 | 1 |

**Adduct Signal Fractions**

- **Unmodified**: 1.7796e+07

### TIVKPGDILLIN(N) Fuc 5 Hex 3 HexNAc 8 Neu5Ac 1

**Mass:**  4429.9369

(6.714e-04)

**Start Position**: 235

**End Position**: 247

| Profile Score | Tandem Score | TDA q-value | Volume | Spectrum Matches |
| --- | --- | --- | --- | --- |
| 0.0000 | 106.6150 | 0.0105 | 0.0000e+00 | 1 |

Adduct:

No Chromatogram Found

### TIVKPGDILLIN(N)STG Hex 9 HexNAc 2

**Mass:**  3404.5255

(1.274e-06)

**Charge States:** 3

**Start Position**: 235

**End Position**: 250

| Profile Score | Tandem Score | TDA q-value | Volume | Spectrum Matches |
| --- | --- | --- | --- | --- |
| -20.3798 | 98.9575 | 0.0105 | 4.7984e+06 | 1 |

**Adduct Signal Fractions**

- **Unmodified**: 4.7984e+06

### SEC(C)ITPN(N)GSIPNDKPF Hex 7 HexNAc 2

**Mass:**  3315.3543

(1.850e-06)

**Charge States:** 3

**Start Position**: 279

**End Position**: 295

| Profile Score | Tandem Score | TDA q-value | Volume | Spectrum Matches |
| --- | --- | --- | --- | --- |
| -4.6418 | 94.3170 | 0.0105 | 5.4635e+06 | 1 |

**Adduct Signal Fractions**

- **Unmodified**: 5.4635e+06

### QKLPGNDN(N)STATL Hex 6 HexNAc 2

**Mass:**  2736.1618

(6.348e-07)

**Charge States:** 3

**Start Position**: 1

**End Position**: 14

| Profile Score | Tandem Score | TDA q-value | Volume | Spectrum Matches |
| --- | --- | --- | --- | --- |
| -19.3696 | 91.6891 | 0.0105 | 7.9714e+07 | 1 |

**Adduct Signal Fractions**

- **Unmodified**: 7.9714e+07

### RSDAPIGKC(C)KSEC(C)ITPN(N)GSIPN(D)DKPF Hex 9 HexNAc 2

**Mass:**  4753.0328

(2.122e-04)

**Charge States:** 4, 5

**Start Position**: 269

**End Position**: 295

| Profile Score | Tandem Score | TDA q-value | Volume | Spectrum Matches |
| --- | --- | --- | --- | --- |
| -5.4982 | 83.6386 | 0.0187 | 7.1162e+07 | 1 |

**Adduct Signal Fractions**

- **Unmodified**: 7.1162e+07

### RSDAPIGKC(C)KSEC(C)ITPN(N)GSIPN(D)DKPF Hex 7 HexNAc 2

**Mass:**  4428.9291

(2.269e-04)

**Charge States:** 4, 5

**Start Position**: 269

**End Position**: 295

| Profile Score | Tandem Score | TDA q-value | Volume | Spectrum Matches |
| --- | --- | --- | --- | --- |
| -20.2498 | 81.9004 | 0.0187 | 6.1046e+07 | 1 |

**Adduct Signal Fractions**

- **Unmodified**: 6.1046e+07

### NWAGVTHN(N)GTSSSC(C)RRGSNSSFF Hex 7 HexNAc 4

**Mass:**  4461.8000

(8.372e-07)

**Charge States:** 4

**Start Position**: 126

**End Position**: 149

| Profile Score | Tandem Score | TDA q-value | Volume | Spectrum Matches |
| --- | --- | --- | --- | --- |
| -4.9091 | 81.7699 | 0.0187 | 9.6342e+06 | 1 |

**Adduct Signal Fractions**

- **Unmodified**: 9.6342e+06

### KIRSGKSSIM(O)RSDAPIGKC(C)KSEC(C)ITPN(N)GSIPNDKPF Hex 9 HexNAc 2

**Mass:**  5855.6597

(3.012e-06)

**Charge States:** 5, 6, 7

**Start Position**: 259

**End Position**: 295

| Profile Score | Tandem Score | TDA q-value | Volume | Spectrum Matches |
| --- | --- | --- | --- | --- |
| -18.1093 | 78.2195 | 0.0187 | 6.8672e+07 | 1 |

**Adduct Signal Fractions**

- **Unmodified**: 6.8672e+07

### RSDAPIGKC(C)KSEC(C)ITPN(N)GSIPN(D)DKPF Hex 8 HexNAc 2

**Mass:**  4591.9911

(4.388e-04)

**Start Position**: 269

**End Position**: 295

| Profile Score | Tandem Score | TDA q-value | Volume | Spectrum Matches |
| --- | --- | --- | --- | --- |
| 0.0000 | 75.8858 | 0.0187 | 0.0000e+00 | 1 |

Adduct:

No Chromatogram Found

### AGVTHN(N)GTSSSC(C)RRGSNSSFF Hex 6 HexNAc 3

**Mass:**  3796.5415

(1.046e-06)

**Charge States:** 4

**Start Position**: 128

**End Position**: 149

| Profile Score | Tandem Score | TDA q-value | Volume | Spectrum Matches |
| --- | --- | --- | --- | --- |
| -4.9237 | 72.0531 | 0.0187 | 4.9854e+07 | 1 |

**Adduct Signal Fractions**

- **Unmodified**: 4.9854e+07

### HKC(C)DNAC(C)IGSIRN(N)GTYDHDVY Fuc 1 Hex 5 HexNAc 4

**Mass:**  4262.7175

(5.943e-07)

**Charge States:** 4, 5

**Start Position**: 471

**End Position**: 492

| Profile Score | Tandem Score | TDA q-value | Volume | Spectrum Matches |
| --- | --- | --- | --- | --- |
| -18.6799 | 68.1598 | 0.0187 | 2.7577e+07 | 2 |

**Adduct Signal Fractions**

- **Unmodified**: 2.7577e+07

### NWAGVTHN(N)GTSSSC(C)RRGSNSSFF Hex 6 HexNAc 2

**Mass:**  3893.6015

(5.397e-06)

**Charge States:** 3, 4

**Start Position**: 126

**End Position**: 149

| Profile Score | Tandem Score | TDA q-value | Volume | Spectrum Matches |
| --- | --- | --- | --- | --- |
| -16.8551 | 65.4615 | 0.0256 | 2.0057e+07 | 1 |

**Adduct Signal Fractions**

- **Unmodified**: 2.0057e+07

### NSKYPALN(N)VTM(O)PNNEQFDKLY Hex 7 HexNAc 2

**Mass:**  4041.7260

(2.242e-06)

**Charge States:** 3, 4

**Start Position**: 158

**End Position**: 179

| Profile Score | Tandem Score | TDA q-value | Volume | Spectrum Matches |
| --- | --- | --- | --- | --- |
| -4.3365 | 63.5202 | 0.0256 | 1.6534e+07 | 3 |

**Adduct Signal Fractions**

- **Unmodified**: 1.6534e+07

### AGVTHN(N)GTSSSC(C)RRGSNSSFF Hex 7 HexNAc 3

**Mass:**  3958.5956

(1.346e-06)

**Charge States:** 4

**Start Position**: 128

**End Position**: 149

| Profile Score | Tandem Score | TDA q-value | Volume | Spectrum Matches |
| --- | --- | --- | --- | --- |
| -18.8176 | 62.8103 | 0.0256 | 3.4608e+07 | 1 |

**Adduct Signal Fractions**

- **Unmodified**: 3.4608e+07

### HKC(C)DNAC(C)IGSIRN(N)GTYDHDVY Fuc 2 Hex 5 HexNAc 4

**Mass:**  4408.7765

(8.079e-07)

**Charge States:** 4, 5

**Start Position**: 471

**End Position**: 492

| Profile Score | Tandem Score | TDA q-value | Volume | Spectrum Matches |
| --- | --- | --- | --- | --- |
| -19.1357 | 59.6542 | 0.0256 | 1.8538e+07 | 3 |

**Adduct Signal Fractions**

- **Unmodified**: 1.8538e+07

### NWAGVTHN(N)GTSSSC(C)RRGSNSSFF Fuc 1 Hex 7 HexNAc 3

**Mass:**  4404.7829

(2.838e-06)

**Charge States:** 4

**Start Position**: 126

**End Position**: 149

| Profile Score | Tandem Score | TDA q-value | Volume | Spectrum Matches |
| --- | --- | --- | --- | --- |
| -18.9390 | 57.5653 | 0.0256 | 4.3699e+06 | 1 |

**Adduct Signal Fractions**

- **Unmodified**: 4.3699e+06

### AGVTHN(N)GTSSSC(C)RRGSNSSFF Hex 7 HexNAc 4

**Mass:**  4161.6730

(8.295e-07)

**Charge States:** 4

**Start Position**: 128

**End Position**: 149

| Profile Score | Tandem Score | TDA q-value | Volume | Spectrum Matches |
| --- | --- | --- | --- | --- |
| -4.0802 | 57.1687 | 0.0256 | 4.1587e+07 | 1 |

**Adduct Signal Fractions**

- **Unmodified**: 4.1587e+07

### AGVTHN(D)GTSSSC(C)RRGSN(N)SSFF Hex 7 HexNAc 2

**Mass:**  3756.4987

(2.634e-04)

**Charge States:** 4

**Start Position**: 128

**End Position**: 149

| Profile Score | Tandem Score | TDA q-value | Volume | Spectrum Matches |
| --- | --- | --- | --- | --- |
| 9.5957 | 55.1535 | 0.0256 | 1.0931e+08 | 3 |

**Adduct Signal Fractions**

- **Unmodified**: 1.0931e+08

### N(D)SKYPALN(N)VTM(O)PNNEQFDKLY Hex 8 HexNAc 2

**Mass:**  4204.7644

(2.360e-04)

**Charge States:** 3, 4

**Start Position**: 158

**End Position**: 179

| Profile Score | Tandem Score | TDA q-value | Volume | Spectrum Matches |
| --- | --- | --- | --- | --- |
| -20.5726 | 53.6443 | 0.0256 | 2.0178e+07 | 3 |

**Adduct Signal Fractions**

- **Unmodified**: 2.0178e+07

### SEC(C)ITPN(N)GSIPNDKPF Hex 8 HexNAc 2

**Mass:**  3477.4019

(2.421e-07)

**Charge States:** 3

**Start Position**: 279

**End Position**: 295

| Profile Score | Tandem Score | TDA q-value | Volume | Spectrum Matches |
| --- | --- | --- | --- | --- |
| -19.7252 | 51.1005 | 0.0480 | 2.6805e+06 | 1 |

**Adduct Signal Fractions**

- **Unmodified**: 2.6805e+06

### RSDAPIGKC(C)KSEC(C)ITPN(N)GSIPNDKPFQNVNRITY Hex 9 HexNAc 2

**Mass:**  5740.5447

(5.064e-06)

**Charge States:** 5

**Start Position**: 269

**End Position**: 303

| Profile Score | Tandem Score | TDA q-value | Volume | Spectrum Matches |
| --- | --- | --- | --- | --- |
| -21.5116 | 51.0114 | 0.0480 | 3.9191e+06 | 2 |

**Adduct Signal Fractions**

- **Unmodified**: 3.9191e+06

### cus|SWZNA|A/Switzerland/9715293/2013

**>cus|SWZNA|A/Switzerland/9715293/2013**  
 `MNPNQKIITIGSVSLTISTICFFMQIAILITTVTLHFKQYEFNSPPNNQVMLCEPTIIERNITEIVYLTNTTIEKEICPK  
PAEYRNWSKPQCGITGFAPFSKDNSIRLSAGGDIWVTREPYVSCDPDKCYQFALGQGTTLNNVHSNNTVRDRTPYRTLLM  
NELGVPFHLGTKQVCIAWSSSSCHDGKAWLHVCITGDDKNATASFIYNGRLVDSVVSWSKDILRTQESECVCINGTCTVV  
MTDGSASGKADTKILFIEEGKIVHTSTLSGSAQHVEECSCYPRYPGVRCVCRDNWKGSNRPIVDINIKDHSIVSSYVCSG  
LVGDTPRKNDSSSSSHCLDPNNEEGGHGVKGWAFDDGNDVWMGRTINETSRLGYETFKVIEGWSNPKSKLQTNRQVIVDR  
GDRSGYSGIFSVEGKSCINRCFYVELIRGRKEETEVLWTSNSIVVFCGTSGTYGTGSWPDGADLNLMPI`

| Site | Type | Count |
| --- | --- | --- |
| 146 | N-linked | 11 |
| 86 | N-linked | 3 |
| 367 | N-linked | 13 |
| 200 | N-linked | 2 |

| Mass | Sequence | MS_2_ Score | `q`-value | MS_1_ Score | Signal | Protein Coordinates | Tandem Scans | Start Time | Apex Time | End Time |
| --- | --- | --- | --- | --- | --- | --- | --- | --- | --- | --- |
| 3612.4599 | LHVC(C)ITGDDKN(N)ATASF Hex 9 HexNAc 2 | 193.627 | 0.0 | -17.114 | 2.986e+07 | 189 - 205 | 2 | 34.43 | 34.60 | 34.60 |
| 3232.3204 | TIN(N)ETSRLGYETF Hex 8 HexNAc 2 | 181.420 | 0.0 | -3.400 | 5.377e+07 | 364 - 377 | 3 | 36.62 | 36.79 | 36.96 |
| 3414.3913 | RN(N)WSKPQC(C)GITGF Hex 9 HexNAc 2 | 179.748 | 0.0 | -19.013 | 1.389e+07 | 84 - 97 | 2 | 31.28 | 31.45 | 31.45 |
| 3450.4080 | LHVC(C)ITGDDKN(N)ATASF Hex 8 HexNAc 2 | 179.672 | 0.0 | -18.667 | 2.465e+07 | 189 - 205 | 2 | 34.60 | 34.60 | 34.77 |
| 3268.3773 | M(O)GRTIN(N)ETSRLGYETF Hex 6 HexNAc 2 | 171.647 | 0.0 | -1.867 | 2.784e+08 | 361 - 377 | 2 | 32.58 | 32.75 | 32.91 |
| 3430.4275 | M(O)GRTIN(N)ETSRLGYETF Hex 7 HexNAc 2 | 164.226 | 0.0 | -2.083 | 4.870e+08 | 361 - 377 | 3 | 32.42 | 32.58 | 32.91 |
| 3567.4900 | RN(N)WSKPQC(C)GITGFAPF Hex 8 HexNAc 2 | 161.410 | 0.0 | -17.890 | 3.440e+07 | 84 - 100 | 2 | 45.44 | 45.62 | 45.62 |
| 3729.5440 | RN(N)WSKPQC(C)GITGFAPF Hex 9 HexNAc 2 | 161.131 | 0.0 | -2.528 | 3.813e+07 | 84 - 100 | 3 | 45.28 | 45.44 | 45.62 |
| 2908.2211 | TIN(N)ETSRLGYETF Hex 6 HexNAc 2 | 150.613 | 0.0 | -4.585 | 7.358e+06 | 364 - 377 | 1 | 36.96 | 37.13 | 37.30 |
| 3592.4778 | M(O)GRTIN(N)ETSRLGYETF Hex 8 HexNAc 2 | 149.322 | 0.0 | 18.448 | 7.829e+08 | 361 - 377 | 3 | 32.26 | 32.42 | 33.08 |
| 3215.3176 | M(O)GRTIN(N)ETSRLGY Hex 8 HexNAc 2 | 147.251 | 0.0 | -2.203 | 9.511e+07 | 361 - 374 | 1 | 22.29 | 22.46 | 22.62 |
| 2995.2355 | M(O)GRTIN(N)ETSRL Hex 8 HexNAc 2 | 146.238 | 0.0 | -17.215 | 1.470e+07 | 361 - 372 | 1 | 18.15 | 18.15 | 18.32 |
| 3414.4378 | MGRTIN(N)ETSRLGYETF Hex 7 HexNAc 2 | 142.185 | 0.0 | -17.653 | 6.231e+06 | 361 - 377 | 1 | 34.94 | 34.94 | 35.11 |
| 3070.2700 | TIN(N)ETSRLGYETF Hex 7 HexNAc 2 | 140.828 | 0.0 | -3.619 | 2.105e+07 | 364 - 377 | 1 | 36.79 | 36.79 | 37.13 |
| 3053.2661 | M(O)GRTIN(N)ETSRLGY Hex 7 HexNAc 2 | 135.601 | 0.0 | -16.546 | 5.655e+07 | 361 - 374 | 1 | 22.46 | 22.62 | 22.62 |
| 3576.4906 | MGRTIN(N)ETSRLGYETF Hex 8 HexNAc 2 | 134.257 | 0.0 | -18.402 | 1.215e+07 | 361 - 377 | 1 | 34.77 | 34.94 | 34.94 |
| 2891.2142 | M(O)GRTIN(N)ETSRLGY Hex 6 HexNAc 2 | 125.887 | 0.011 | -18.522 | 2.657e+07 | 361 - 374 | 1 | 22.62 | 22.62 | 22.79 |
| 3919.6257 | NNVHSN(N)NTVRDRTPY Fuc 1 Hex 6 HexNAc 5 | 119.168 | 0.011 | -3.792 | 9.361e+07 | 140 - 155 | 3 | 13.52 | 13.52 | 13.84 |
| 4284.7596 | NNVHSN(N)NTVRDRTPY Fuc 1 Hex 7 HexNAc 6 | 112.578 | 0.011 | -18.684 | 3.106e+07 | 140 - 155 | 1 | 13.36 | 13.52 | 13.52 |
| 4138.7000 | NNVHSN(N)NTVRDRTPY Hex 7 HexNAc 6 | 107.391 | 0.011 | -17.653 | 5.079e+07 | 140 - 155 | 1 | 13.52 | 13.52 | 13.68 |
| 4510.7701 | NNVHSN(N)NTVRDRTPY Fuc 2 Hex 10 HexNAc 4 | 104.735 | 0.011 | -20.948 | 8.331e+06 | 140 - 155 | 1 | 14.34 | 14.34 | 14.50 |
| 3773.5653 | NNVHSN(N)NTVRDRTPY Hex 6 HexNAc 5 | 103.712 | 0.011 | -4.115 | 9.651e+07 | 140 - 155 | 3 | 13.52 | 13.68 | 13.84 |
| 4506.8844 | NNVHSN(N)NTVRDRTPY Hex 8 HexNAc 7 | 98.033 | 0.011 | -22.621 | 3.307e+06 | 140 - 155 | 2 | 36.29 | 36.29 | 36.29 |
| 4364.7194 | NNVHSN(N)NTVRDRTPY Fuc 1 Hex 10 HexNAc 4 | 97.782 | 0.011 | -6.014 | 1.715e+07 | 140 - 155 | 2 | 14.50 | 14.84 | 14.84 |
| 4065.6845 | NNVHSN(N)NTVRDRTPY Fuc 2 Hex 6 HexNAc 5 | 97.660 | 0.011 | -19.099 | 1.591e+07 | 140 - 155 | 1 | 13.52 | 13.52 | 13.52 |
| 4431.8209 | NNVHSN(N)NTVRDRTPY Fuc 2 Hex 7 HexNAc 6 | 88.951 | 0.011 | 0.000 | 0.000e+00 | 140 - 155 | 1 | - | - | - |
| 4000.5676 | NNVHSN(N)NTVRDRTPY Fuc 1 Hex 9 HexNAc 3 | 86.794 | 0.019 | -6.334 | 2.847e+07 | 140 - 155 | 4 | 16.82 | 16.98 | 17.15 |
| 4649.8908 | NNVHSN(N)NTVRDRTPY Fuc 1 Hex 8 HexNAc 7 | 72.845 | 0.019 | -21.795 | 4.075e+06 | 140 - 155 | 1 | 13.36 | 13.36 | 13.36 |
| 3252.3865 | MGRTIN(N)ETSRLGYETF Hex 6 HexNAc 2 | 51.606 | 0.042 | -17.943 | 3.693e+06 | 361 - 377 | 1 | 35.11 | 35.11 | 35.11 |

### LHVC(C)ITGDDKN(N)ATASF Hex 9 HexNAc 2

**Mass:**  3612.4594

(1.549e-06)

**Charge States:** 3

**Start Position**: 190

**End Position**: 206

| Profile Score | Tandem Score | TDA q-value | Volume | Spectrum Matches |
| --- | --- | --- | --- | --- |
| -17.1142 | 193.6269 | 0.0000 | 2.9862e+07 | 2 |

**Adduct Signal Fractions**

- **Unmodified**: 2.9862e+07

### TIN(N)ETSRLGYETF Hex 8 HexNAc 2

**Mass:**  3232.3192

(2.442e-06)

**Charge States:** 3

**Start Position**: 365

**End Position**: 378

| Profile Score | Tandem Score | TDA q-value | Volume | Spectrum Matches |
| --- | --- | --- | --- | --- |
| -3.4005 | 181.4199 | 0.0000 | 5.3773e+07 | 3 |

**Adduct Signal Fractions**

- **Unmodified**: 5.3773e+07

### RN(N)WSKPQC(C)GITGF Hex 9 HexNAc 2

**Mass:**  3414.3909

(3.910e-06)

**Charge States:** 3

**Start Position**: 85

**End Position**: 98

| Profile Score | Tandem Score | TDA q-value | Volume | Spectrum Matches |
| --- | --- | --- | --- | --- |
| -19.0132 | 179.7483 | 0.0000 | 1.3889e+07 | 2 |

**Adduct Signal Fractions**

- **Unmodified**: 1.3889e+07

### LHVC(C)ITGDDKN(N)ATASF Hex 8 HexNAc 2

**Mass:**  3450.4070

(1.712e-06)

**Charge States:** 3

**Start Position**: 190

**End Position**: 206

| Profile Score | Tandem Score | TDA q-value | Volume | Spectrum Matches |
| --- | --- | --- | --- | --- |
| -18.6666 | 179.6719 | 0.0000 | 2.4653e+07 | 2 |

**Adduct Signal Fractions**

- **Unmodified**: 2.4653e+07

### M(O)GRTIN(N)ETSRLGYETF Hex 6 HexNAc 2

**Mass:**  3268.3771

(2.237e-06)

**Charge States:** 3

**Start Position**: 362

**End Position**: 378

| Profile Score | Tandem Score | TDA q-value | Volume | Spectrum Matches |
| --- | --- | --- | --- | --- |
| -1.8671 | 171.6474 | 0.0000 | 2.7842e+08 | 2 |

**Adduct Signal Fractions**

- **Unmodified**: 2.7842e+08

### M(O)GRTIN(N)ETSRLGYETF Hex 7 HexNAc 2

**Mass:**  3430.4276

(7.534e-07)

**Charge States:** 3

**Start Position**: 362

**End Position**: 378

| Profile Score | Tandem Score | TDA q-value | Volume | Spectrum Matches |
| --- | --- | --- | --- | --- |
| -2.0831 | 164.2262 | 0.0000 | 4.8703e+08 | 3 |

**Adduct Signal Fractions**

- **Unmodified**: 4.8703e+08

### RN(N)WSKPQC(C)GITGFAPF Hex 8 HexNAc 2

**Mass:**  3567.4890

(1.790e-06)

**Charge States:** 3

**Start Position**: 85

**End Position**: 101

| Profile Score | Tandem Score | TDA q-value | Volume | Spectrum Matches |
| --- | --- | --- | --- | --- |
| -17.8902 | 161.4096 | 0.0000 | 3.4401e+07 | 2 |

**Adduct Signal Fractions**

- **Unmodified**: 3.4401e+07

### RN(N)WSKPQC(C)GITGFAPF Hex 9 HexNAc 2

**Mass:**  3729.5432

(2.667e-06)

**Charge States:** 3

**Start Position**: 85

**End Position**: 101

| Profile Score | Tandem Score | TDA q-value | Volume | Spectrum Matches |
| --- | --- | --- | --- | --- |
| -2.5277 | 161.1315 | 0.0000 | 3.8128e+07 | 3 |

**Adduct Signal Fractions**

- **Unmodified**: 3.8128e+07

### TIN(N)ETSRLGYETF Hex 6 HexNAc 2

**Mass:**  2908.2215

(2.959e-06)

**Charge States:** 3

**Start Position**: 365

**End Position**: 378

| Profile Score | Tandem Score | TDA q-value | Volume | Spectrum Matches |
| --- | --- | --- | --- | --- |
| -4.5850 | 150.6133 | 0.0000 | 7.3579e+06 | 1 |

**Adduct Signal Fractions**

- **Unmodified**: 7.3579e+06

### M(O)GRTIN(N)ETSRLGYETF Hex 8 HexNAc 2

**Mass:**  3592.4767

(1.680e-06)

**Charge States:** 3

**Start Position**: 362

**End Position**: 378

| Profile Score | Tandem Score | TDA q-value | Volume | Spectrum Matches |
| --- | --- | --- | --- | --- |
| 18.4482 | 149.3223 | 0.0000 | 7.8288e+08 | 3 |

**Adduct Signal Fractions**

- **Unmodified**: 7.8288e+08

### M(O)GRTIN(N)ETSRLGY Hex 8 HexNAc 2

**Mass:**  3215.3172

(1.389e-07)

**Charge States:** 3

**Start Position**: 362

**End Position**: 375

| Profile Score | Tandem Score | TDA q-value | Volume | Spectrum Matches |
| --- | --- | --- | --- | --- |
| -2.2031 | 147.2508 | 0.0000 | 9.5112e+07 | 1 |

**Adduct Signal Fractions**

- **Unmodified**: 9.5112e+07

### M(O)GRTIN(N)ETSRL Hex 8 HexNAc 2

**Mass:**  2995.2357

(1.270e-06)

**Charge States:** 3

**Start Position**: 362

**End Position**: 373

| Profile Score | Tandem Score | TDA q-value | Volume | Spectrum Matches |
| --- | --- | --- | --- | --- |
| -17.2150 | 146.2376 | 0.0000 | 1.4698e+07 | 1 |

**Adduct Signal Fractions**

- **Unmodified**: 1.4698e+07

### MGRTIN(N)ETSRLGYETF Hex 7 HexNAc 2

**Mass:**  3414.4374

(2.830e-06)

**Charge States:** 3

**Start Position**: 362

**End Position**: 378

| Profile Score | Tandem Score | TDA q-value | Volume | Spectrum Matches |
| --- | --- | --- | --- | --- |
| -17.6529 | 142.1847 | 0.0000 | 6.2306e+06 | 1 |

**Adduct Signal Fractions**

- **Unmodified**: 6.2306e+06

### TIN(N)ETSRLGYETF Hex 7 HexNAc 2

**Mass:**  3070.2672

(2.713e-06)

**Charge States:** 3

**Start Position**: 365

**End Position**: 378

| Profile Score | Tandem Score | TDA q-value | Volume | Spectrum Matches |
| --- | --- | --- | --- | --- |
| -3.6185 | 140.8284 | 0.0000 | 2.1055e+07 | 1 |

**Adduct Signal Fractions**

- **Unmodified**: 2.1055e+07

### M(O)GRTIN(N)ETSRLGY Hex 7 HexNAc 2

**Mass:**  3053.2668

(-2.233e-07)

**Charge States:** 3

**Start Position**: 362

**End Position**: 375

| Profile Score | Tandem Score | TDA q-value | Volume | Spectrum Matches |
| --- | --- | --- | --- | --- |
| -16.5457 | 135.6009 | 0.0000 | 5.6554e+07 | 1 |

**Adduct Signal Fractions**

- **Unmodified**: 5.6554e+07

### MGRTIN(N)ETSRLGYETF Hex 8 HexNAc 2

**Mass:**  3576.4890

(3.910e-06)

**Charge States:** 3

**Start Position**: 362

**End Position**: 378

| Profile Score | Tandem Score | TDA q-value | Volume | Spectrum Matches |
| --- | --- | --- | --- | --- |
| -18.4018 | 134.2565 | 0.0000 | 1.2153e+07 | 1 |

**Adduct Signal Fractions**

- **Unmodified**: 1.2153e+07

### M(O)GRTIN(N)ETSRLGY Hex 6 HexNAc 2

**Mass:**  2891.2135

(8.182e-07)

**Charge States:** 3

**Start Position**: 362

**End Position**: 375

| Profile Score | Tandem Score | TDA q-value | Volume | Spectrum Matches |
| --- | --- | --- | --- | --- |
| -18.5215 | 125.8870 | 0.0105 | 2.6568e+07 | 1 |

**Adduct Signal Fractions**

- **Unmodified**: 2.6568e+07

### NNVHSN(N)NTVRDRTPY Fuc 1 Hex 6 HexNAc 5

**Mass:**  3919.6257

(8.743e-07)

**Charge States:** 4

**Start Position**: 141

**End Position**: 156

| Profile Score | Tandem Score | TDA q-value | Volume | Spectrum Matches |
| --- | --- | --- | --- | --- |
| -3.7924 | 119.1675 | 0.0105 | 9.3614e+07 | 3 |

**Adduct Signal Fractions**

- **Unmodified**: 9.3614e+07

### NNVHSN(N)NTVRDRTPY Fuc 1 Hex 7 HexNAc 6

**Mass:**  4284.7570

(2.009e-06)

**Charge States:** 4

**Start Position**: 141

**End Position**: 156

| Profile Score | Tandem Score | TDA q-value | Volume | Spectrum Matches |
| --- | --- | --- | --- | --- |
| -18.6844 | 112.5777 | 0.0105 | 3.1055e+07 | 1 |

**Adduct Signal Fractions**

- **Unmodified**: 3.1055e+07

### NNVHSN(N)NTVRDRTPY Hex 7 HexNAc 6

**Mass:**  4138.7004

(9.519e-07)

**Charge States:** 4

**Start Position**: 141

**End Position**: 156

| Profile Score | Tandem Score | TDA q-value | Volume | Spectrum Matches |
| --- | --- | --- | --- | --- |
| -17.6527 | 107.3907 | 0.0105 | 5.0792e+07 | 1 |

**Adduct Signal Fractions**

- **Unmodified**: 5.0792e+07

### NNVHSN(N)NTVRDRTPY Fuc 2 Hex 10 HexNAc 4

**Mass:**  4510.7678

(-9.873e-06)

**Charge States:** 4

**Start Position**: 141

**End Position**: 156

| Profile Score | Tandem Score | TDA q-value | Volume | Spectrum Matches |
| --- | --- | --- | --- | --- |
| -20.9482 | 104.7346 | 0.0105 | 8.3314e+06 | 1 |

**Adduct Signal Fractions**

- **Unmodified**: 8.3314e+06

### NNVHSN(N)NTVRDRTPY Hex 6 HexNAc 5

**Mass:**  3773.5651

(4.641e-07)

**Charge States:** 4

**Start Position**: 141

**End Position**: 156

| Profile Score | Tandem Score | TDA q-value | Volume | Spectrum Matches |
| --- | --- | --- | --- | --- |
| -4.1146 | 103.7122 | 0.0105 | 9.6513e+07 | 3 |

**Adduct Signal Fractions**

- **Unmodified**: 9.6513e+07

### NNVHSN(N)NTVRDRTPY Hex 8 HexNAc 7

**Mass:**  4506.8844

(6.712e-04)

**Charge States:** 5

**Start Position**: 141

**End Position**: 156

| Profile Score | Tandem Score | TDA q-value | Volume | Spectrum Matches |
| --- | --- | --- | --- | --- |
| -22.6209 | 98.0327 | 0.0105 | 3.3074e+06 | 2 |

**Adduct Signal Fractions**

- **Unmodified**: 3.3074e+06

### NNVHSN(N)NTVRDRTPY Fuc 1 Hex 10 HexNAc 4

**Mass:**  4364.7194

(-7.145e-06)

**Charge States:** 4

**Start Position**: 141

**End Position**: 156

| Profile Score | Tandem Score | TDA q-value | Volume | Spectrum Matches |
| --- | --- | --- | --- | --- |
| -6.0137 | 97.7815 | 0.0105 | 1.7149e+07 | 2 |

**Adduct Signal Fractions**

- **Unmodified**: 1.7149e+07

### NNVHSN(N)NTVRDRTPY Fuc 2 Hex 6 HexNAc 5

**Mass:**  4065.6845

(1.056e-06)

**Charge States:** 4

**Start Position**: 141

**End Position**: 156

| Profile Score | Tandem Score | TDA q-value | Volume | Spectrum Matches |
| --- | --- | --- | --- | --- |
| -19.0988 | 97.6597 | 0.0105 | 1.5911e+07 | 1 |

**Adduct Signal Fractions**

- **Unmodified**: 1.5911e+07

### NNVHSN(N)NTVRDRTPY Fuc 2 Hex 7 HexNAc 6

**Mass:**  4431.8209

(2.276e-04)

**Start Position**: 141

**End Position**: 156

| Profile Score | Tandem Score | TDA q-value | Volume | Spectrum Matches |
| --- | --- | --- | --- | --- |
| 0.0000 | 88.9505 | 0.0105 | 0.0000e+00 | 1 |

Adduct:

No Chromatogram Found

### NNVHSN(N)NTVRDRTPY Fuc 1 Hex 9 HexNAc 3

**Mass:**  4000.5676

(2.410e-04)

**Charge States:** 4

**Start Position**: 141

**End Position**: 156

| Profile Score | Tandem Score | TDA q-value | Volume | Spectrum Matches |
| --- | --- | --- | --- | --- |
| -6.3338 | 86.7936 | 0.0187 | 2.8472e+07 | 4 |

**Adduct Signal Fractions**

- **Unmodified**: 2.8472e+07

### NNVHSN(N)NTVRDRTPY Fuc 1 Hex 8 HexNAc 7

**Mass:**  4649.8908

(8.815e-07)

**Charge States:** 4

**Start Position**: 141

**End Position**: 156

| Profile Score | Tandem Score | TDA q-value | Volume | Spectrum Matches |
| --- | --- | --- | --- | --- |
| -21.7948 | 72.8449 | 0.0187 | 4.0750e+06 | 1 |

**Adduct Signal Fractions**

- **Unmodified**: 4.0750e+06

### MGRTIN(N)ETSRLGYETF Hex 6 HexNAc 2

**Mass:**  3252.3865

(3.561e-06)

**Charge States:** 3

**Start Position**: 362

**End Position**: 378

| Profile Score | Tandem Score | TDA q-value | Volume | Spectrum Matches |
| --- | --- | --- | --- | --- |
| -17.9435 | 51.6056 | 0.0417 | 3.6926e+06 | 1 |

**Adduct Signal Fractions**

- **Unmodified**: 3.6926e+06

### tr|F1NUU6|F1NUU6\_CHICK

**>tr|F1NUU6|F1NUU6\_CHICK**  
 `MGCSTILVFLTLLSFASAHRKLLLFLLDGFRFDYIDDAELESLPGFRDIIGMGVKVDYMTPDFPSLSYPNYYTLMTGRHC  
EVHQMIGNYMWDPKTNVSFDIGVNKESLLPLWWNGSEPLWVTMTKEKKNVFMYYWPGCEVEILGVRPSYCREYFSVPTDQ  
NFADAISDALEALRNGSAEMAAVYYERIDVEGHHYGPWSEQRKSALKELDKAMSNMITQIKNKGLQHDVNVLLFSDHGMT  
DISWTDKVIELKNYINMSDTIQMKDRGPVVSLWPVPEKHTEIYQKLKAVEHMNVYNIQDIPERFFYKKGKFVSPLTLVAE  
EGWFIVESRDKLPFWENGTGKREAWQNGWHGYDNELMEMRGIFLAYGPDFRSNYRAPPIRSVDVYNVMCSLAGVQPQPNN  
GSWSRVECMLKNTAVRVPLCTRSGCVLALTLLLLFWLL`

| Site | Type | Count |
| --- | --- | --- |
| 175 | N-linked | 1 |

| Mass | Sequence | MS_2_ Score | `q`-value | MS_1_ Score | Signal | Protein Coordinates | Tandem Scans | Start Time | Apex Time | End Time |
| --- | --- | --- | --- | --- | --- | --- | --- | --- | --- | --- |
| 3584.4239 | RN(N)GSAEMAAVY Fuc 1 Hex 9 HexNAc 4 | 88.277 | 0.019 | -4.836 | 1.532e+07 | 173 - 184 | 2 | 25.11 | 25.28 | 25.44 |

### RN(N)GSAEMAAVY Fuc 1 Hex 9 HexNAc 4

**Mass:**  3584.4256

(9.848e-06)

**Charge States:** 3

**Start Position**: 174

**End Position**: 185

| Profile Score | Tandem Score | TDA q-value | Volume | Spectrum Matches |
| --- | --- | --- | --- | --- |
| -4.8362 | 88.2766 | 0.0187 | 1.5321e+07 | 2 |

**Adduct Signal Fractions**

- **Unmodified**: 1.5321e+07
