# Supplementary material for: Measuring Site-specific Glycosylation Similarity between Influenza a Virus Variants with Statistical Certainty
Source: Mol Cell Proteomics. 2020 Nov 25;19(9):1533–45. doi: 10.1074/mcp.RA120.002031 (PMC8143645; doi:10.1074/mcp.RA120.002031)
Supplement: Supplementary file 2 [file mmc2.zip › mmc2/159250_2_supp_555292_qcjkl5.zip!/20190301_5B8eggL2_021119_tryp_HILIC_DDA_1ug_02_glycopeptides.html]

## 20190301\_5B8eggL2\_021119\_tryp\_HILIC\_DDA\_1ug\_02 @ GlycopeptideHypothesis-112f0f6ccc3b47668dc9e5badafef90e

**MS^1^ Mass Accuracy:** 10.0 PPM  
**Grouping Tolerance:** 15.0 PPM  
**MS^n^ Mass Accuracy:** 20.0 PPM  
**Minimum `q`-Value For Clustering:** 0.05

**Glycopeptide Hypothesis:**
GlycopeptideHypothesis-112f0f6ccc3b47668dc9e5badafef90e  
**Sample Searched:**
20190301\_5B8eggL2\_021119\_tryp\_HILIC\_DDA\_1ug\_02

| Protein | Matched Glycopeptides |
| --- | --- |
| cus\|SWZHA\_5B8\|A/Switzerland/9715293/2013 | 157 |
| cus\|SWZHA\|A/Switzerland/9715293/2013 | 136 |
| cus\|SWZNA\|A/Switzerland/9715293/2013 | 11 |
| tr\|E1C041\|E1C041\_CHICK | 5 |
| tr\|F1NV02\|F1NV02\_CHICK | 1 |
| tr\|A0A1D5P780\|A0A1D5P780\_CHICK | 1 |
| sp\|P02845\|VIT2\_CHICK | 1 |
| tr\|F1NFL6\|F1NFL6\_CHICK | 1 |
| tr\|A0A1D5PNI3\|A0A1D5PNI3\_CHICK | 1 |
| tr\|A0A1D5PUR1\|A0A1D5PUR1\_CHICK | 1 |

### cus|SWZHA\_5B8|A/Switzerland/9715293/2013

**>cus|SWZHA\_5B8|A/Switzerland/9715293/2013**  
 `QKLPGNDNSTATLCLGHHAVPNGTIVKTITNDRIEVTNATELVQNSSIGEICDSPHQILDGENCTLIDALLGDPQCDGFQ  
NKKWDLFVERSKAYSNCYPYDVPDYASLRSLVASSGTLEFNNESFNWAGVTHNGTSSSCRRGSNSSFFSRLNWLTHLNSK  
YPALNVTMPNNEQFDKLYIWGVHHPVTDKDQIFLYAQSSGRITVSTKRSQQAVIPNIGSRPRIRNIPSRISIYWTIVKPG  
DILLINSTGNLIAPRGYFKIRSGKSSIMRSDAPIGKCKSECITPNGSIPNDKPFQNVNRITYGACPRYVKQSTLKLATGM  
RNVPERQTRGIFGAIAGFIENGWEGMVDGWYGFRHQNSEGRGQAADLKSTQAAIDQINGKLNRLIGKTNEKFHQIEKEFS  
EVEGRIQDLEKYVEDTKIDLWSYNAELLVALENQHTIDLTDSEMNKLFEKTKKQLRENAEDMGNGCFKIYHKCDNACIGS  
IRNGTYDHDVYRDEALNNRFQIKGVELKSGYKDWILWISFAISCFLLCVALLGFIMWACQKGNIRCNICI`

| Site | Type | Count |
| --- | --- | --- |
| 483 | N-linked | 50 |
| 165 | N-linked | 14 |
| 144 | N-linked | 48 |
| 246 | N-linked | 13 |
| 133 | N-linked | 18 |
| 285 | N-linked | 13 |
| 22 | N-linked | 1 |

| Mass | Sequence | MS_2_ Score | `q`-value | MS_1_ Score | Signal | Protein Coordinates | Tandem Scans | Start Time | Apex Time | End Time |
| --- | --- | --- | --- | --- | --- | --- | --- | --- | --- | --- |
| 3172.2867 | RGSN(N)SSFFSR Hex 5 HexNAc 6 | 189.046 | 0.0 | -18.689 | 1.666e+08 | 140 - 150 | 2 | 19.20 | 19.82 | 19.98 |
| 2522.0197 | RGSN(N)SSFFSR Hex 6 HexNAc 2 | 188.071 | 0.0 | -2.983 | 4.586e+08 | 140 - 150 | 7 | 19.03 | 19.67 | 19.67 |
| 2684.0728 | RGSN(N)SSFFSR Hex 7 HexNAc 2 | 187.594 | 0.0 | 13.172 | 2.725e+08 | 140 - 150 | 2 | 18.70 | 19.52 | 19.67 |
| 2359.9664 | RGSN(N)SSFFSR Hex 5 HexNAc 2 | 187.502 | 0.0 | 11.791 | 3.942e+08 | 140 - 150 | 2 | 19.20 | 19.82 | 19.98 |
| 2725.0996 | RGSN(N)SSFFSR Hex 6 HexNAc 3 | 185.104 | 0.0 | 13.687 | 5.833e+08 | 140 - 150 | 5 | 19.03 | 19.67 | 19.98 |
| 3058.2420 | RGSN(N)SSFFSR Fuc 2 Hex 5 HexNAc 4 | 180.960 | 0.0 | 13.653 | 3.153e+08 | 140 - 150 | 2 | 18.70 | 19.37 | 19.37 |
| 3153.2329 | N(N)GTYDHDVYR Fuc 2 Hex 5 HexNAc 4 | 180.501 | 0.0 | -2.093 | 3.150e+08 | 482 - 492 | 3 | 15.38 | 15.38 | 15.86 |
| 3261.3211 | RGSN(N)SSFFSR Fuc 2 Hex 5 HexNAc 5 | 180.090 | 0.0 | 13.603 | 4.050e+08 | 140 - 150 | 4 | 18.70 | 19.37 | 19.67 |
| 2846.1264 | RGSN(N)SSFFSR Hex 8 HexNAc 2 | 180.005 | 0.0 | -17.423 | 9.750e+07 | 140 - 150 | 1 | 19.37 | 19.37 | 19.37 |
| 2912.1840 | RGSN(N)SSFFSR Fuc 1 Hex 5 HexNAc 4 | 178.604 | 0.0 | 13.938 | 8.835e+08 | 140 - 150 | 4 | 18.87 | 19.52 | 20.26 |
| 3480.3969 | RGSN(N)SSFFSR Fuc 1 Hex 6 HexNAc 6 | 177.909 | 0.0 | -4.398 | 7.037e+07 | 140 - 150 | 2 | 19.03 | 19.37 | 19.37 |
| 2969.2063 | RGSN(N)SSFFSR Hex 5 HexNAc 5 | 176.833 | 0.0 | 11.081 | 2.917e+08 | 140 - 150 | 2 | 19.03 | 19.82 | 19.98 |
| 2871.1569 | RGSN(N)SSFFSR Fuc 1 Hex 6 HexNAc 3 | 174.716 | 0.0 | 15.209 | 3.382e+08 | 140 - 150 | 3 | 18.70 | 19.37 | 19.67 |
| 2709.1058 | RGSN(N)SSFFSR Fuc 1 Hex 5 HexNAc 3 | 172.419 | 0.0 | -18.104 | 6.777e+07 | 140 - 150 | 1 | 19.67 | 19.67 | 19.82 |
| 2766.1259 | RGSN(N)SSFFSR Hex 5 HexNAc 4 | 172.012 | 0.0 | -18.111 | 8.979e+07 | 140 - 150 | 1 | 19.67 | 19.67 | 19.82 |
| 3582.4658 | YPALN(N)VTMPNNEQFDK Hex 8 HexNAc 2 | 171.966 | 0.0 | -1.736 | 3.745e+08 | 160 - 176 | 8 | 41.99 | 42.32 | 42.49 |
| 2547.0510 | RGSN(N)SSFFSR Fuc 1 Hex 4 HexNAc 3 | 171.901 | 0.0 | -20.010 | 2.363e+07 | 140 - 150 | 1 | 20.12 | 20.12 | 20.12 |
| 3760.5099 | YPALN(N)VTM(O)PNNEQFDK Hex 9 HexNAc 2 | 171.505 | 0.0 | 13.528 | 3.085e+08 | 160 - 176 | 4 | 34.34 | 34.51 | 35.00 |
| 3115.2636 | RGSN(N)SSFFSR Fuc 1 Hex 5 HexNAc 5 | 171.144 | 0.0 | 14.271 | 1.582e+09 | 140 - 150 | 6 | 19.03 | 19.82 | 20.72 |
| 3204.3001 | RGSN(N)SSFFSR Fuc 3 Hex 5 HexNAc 4 | 171.008 | 0.0 | -17.809 | 1.319e+08 | 140 - 150 | 1 | 19.03 | 19.20 | 19.20 |
| 3598.4617 | YPALN(N)VTM(O)PNNEQFDK Hex 8 HexNAc 2 | 170.509 | 0.0 | -0.697 | 3.429e+09 | 160 - 176 | 15 | 34.18 | 34.67 | 112.87 |
| 3436.4033 | YPALN(N)VTM(O)PNNEQFDK Hex 7 HexNAc 2 | 168.029 | 0.0 | 14.355 | 6.222e+08 | 160 - 176 | 4 | 34.34 | 34.83 | 35.16 |
| 4068.9337 | TIVKPGDILLIN(N)STGNLIAPR Hex 9 HexNAc 2 | 165.046 | 0.0 | 17.964 | 6.197e+08 | 234 - 255 | 7 | 52.63 | 53.14 | 53.83 |
| 3277.3165 | RGSN(N)SSFFSR Fuc 1 Hex 6 HexNAc 5 | 160.990 | 0.0 | -3.978 | 1.121e+08 | 140 - 150 | 4 | 19.03 | 19.20 | 19.37 |
| 3007.1750 | N(N)GTYDHDVYR Fuc 1 Hex 5 HexNAc 4 | 160.824 | 0.0 | 16.137 | 5.201e+08 | 482 - 492 | 5 | 15.38 | 15.54 | 16.03 |
| 3178.2321 | RGSN(N)SSFFSR Hex 7 HexNAc 3 Neu5Ac 1 | 160.665 | 0.0 | -20.426 | 7.455e+06 | 140 - 150 | 1 | 22.35 | 22.35 | 22.35 |
| 5018.0827 | SLVASSGTLEFNNESFNWAGVTHN(N)GTSSSC(C)R Hex 8 HexNAc 2 | 160.222 | 0.0 | -3.649 | 1.298e+08 | 109 - 140 | 2 | 49.23 | 49.23 | 49.57 |
| 2928.1800 | RGSN(N)SSFFSR Hex 6 HexNAc 4 | 160.171 | 0.0 | 11.739 | 1.304e+08 | 140 - 150 | 1 | 19.03 | 19.52 | 19.98 |
| 3210.2548 | N(N)GTYDHDVYR Fuc 1 Hex 5 HexNAc 5 | 159.254 | 0.0 | -3.365 | 2.080e+08 | 482 - 492 | 5 | 15.54 | 15.70 | 16.03 |
| 4063.5808 | RGSN(N)SSFFSR Fuc 3 Hex 6 HexNAc 6 Neu5Ac 1 | 158.992 | 0.0 | -20.819 | 1.467e+07 | 140 - 150 | 1 | 21.85 | 21.85 | 21.85 |
| 3420.4120 | YPALN(N)VTMPNNEQFDK Hex 7 HexNAc 2 | 158.963 | 0.0 | -3.217 | 6.271e+07 | 160 - 176 | 3 | 42.16 | 42.32 | 42.49 |
| 2568.9979 | GSN(N)SSFFSR Hex 6 HexNAc 3 | 158.274 | 0.0 | -3.316 | 2.674e+08 | 141 - 150 | 3 | 23.00 | 23.15 | 23.31 |
| 3906.8786 | TIVKPGDILLIN(N)STGNLIAPR Hex 8 HexNAc 2 | 157.814 | 0.0 | -18.434 | 5.777e+07 | 234 - 255 | 2 | 53.14 | 53.14 | 53.31 |
| 2693.1059 | RGSN(N)SSFFSR Fuc 2 Hex 4 HexNAc 3 | 157.566 | 0.0 | -17.296 | 2.351e+07 | 140 - 150 | 1 | 19.82 | 19.82 | 19.82 |
| 2756.0832 | GSN(N)SSFFSR Fuc 1 Hex 5 HexNAc 4 | 155.980 | 0.0 | 12.287 | 3.116e+08 | 141 - 150 | 4 | 22.18 | 23.00 | 23.77 |
| 3777.5388 | YPALN(N)VTM(O)PNNEQFDK Fuc 2 Hex 3 HexNAc 4 Neu5Ac 1 | 155.518 | 0.0 | -5.223 | 3.780e+07 | 160 - 176 | 1 | 34.51 | 34.51 | 34.83 |
| 2690.0237 | GSN(N)SSFFSR Hex 8 HexNAc 2 | 155.434 | 0.0 | -18.804 | 2.923e+07 | 141 - 150 | 1 | 22.68 | 22.68 | 22.68 |
| 3274.3497 | YPALN(N)VTM(O)PNNEQFDK Hex 6 HexNAc 2 | 155.283 | 0.0 | -5.624 | 4.116e+07 | 160 - 176 | 1 | 34.83 | 35.00 | 35.16 |
| 4085.9574 | TIVKPGDILLIN(N)STGNLIAPR Fuc 2 Hex 3 HexNAc 4 Neu5Ac 1 | 155.157 | 0.0 | -3.530 | 5.876e+07 | 234 - 255 | 2 | 52.97 | 53.14 | 53.31 |
| 4376.8440 | C(C)KSEC(C)ITPN(N)GSIPNDKPFQNVNR Hex 8 HexNAc 2 | 155.028 | 0.0 | -3.533 | 1.399e+08 | 276 - 299 | 1 | 29.14 | 29.31 | 29.47 |
| 3112.2989 | YPALN(N)VTM(O)PNNEQFDK Hex 5 HexNAc 2 | 154.715 | 0.0 | -3.521 | 4.223e+07 | 160 - 176 | 1 | 35.16 | 35.33 | 35.49 |
| 3048.1970 | GSN(N)SSFFSR Fuc 3 Hex 5 HexNAc 4 | 154.487 | 0.0 | -18.467 | 5.005e+07 | 141 - 150 | 1 | 22.18 | 22.18 | 22.35 |
| 2959.1621 | GSN(N)SSFFSR Fuc 1 Hex 5 HexNAc 5 | 154.188 | 0.0 | 13.621 | 9.092e+08 | 141 - 150 | 5 | 22.35 | 23.15 | 23.77 |
| 2553.0027 | GSN(N)SSFFSR Fuc 1 Hex 5 HexNAc 3 | 152.964 | 0.0 | -19.571 | 3.018e+07 | 141 - 150 | 1 | 23.15 | 23.15 | 23.31 |
| 3744.5195 | YPALN(N)VTMPNNEQFDK Hex 9 HexNAc 2 | 152.849 | 0.0 | -4.939 | 2.832e+07 | 160 - 176 | 3 | 41.99 | 42.32 | 42.32 |
| 3007.3189 | HAVPN(N)GTIVK Fuc 1 Hex 5 HexNAc 5 | 151.370 | 0.0 | 0.000 | 0.000e+00 | 17 - 27 | 1 | - | - | - |
| 2715.0549 | GSN(N)SSFFSR Fuc 1 Hex 6 HexNAc 3 | 149.446 | 0.0 | -4.339 | 1.127e+08 | 141 - 150 | 2 | 22.68 | 22.84 | 23.00 |
| 2365.9178 | GSN(N)SSFFSR Hex 6 HexNAc 2 | 148.842 | 0.0 | -4.019 | 2.181e+08 | 141 - 150 | 3 | 23.00 | 23.15 | 23.46 |
| 4748.2959 | ISIYWTIVKPGDILLIN(N)STGNLIAPR Fuc 2 Hex 3 HexNAc 4 Neu5Ac 1 | 148.667 | 0.0 | 13.381 | 1.923e+08 | 229 - 255 | 4 | 68.40 | 68.91 | 69.08 |
| 4052.7388 | C(C)KSEC(C)ITPN(N)GSIPNDKPFQNVNR Hex 6 HexNAc 2 | 148.655 | 0.0 | -18.276 | 3.278e+07 | 276 - 299 | 2 | 29.31 | 29.31 | 29.47 |
| 2203.8657 | GSN(N)SSFFSR Hex 5 HexNAc 2 | 148.592 | 0.0 | -2.772 | 2.887e+08 | 141 - 150 | 3 | 23.31 | 23.46 | 23.77 |
| 4531.9241 | SLVASSGTLEFNNESFNWAGVTHN(N)GTSSSC(C)R Hex 5 HexNAc 2 | 148.349 | 0.0 | 15.373 | 1.740e+08 | 109 - 140 | 4 | 49.57 | 49.74 | 50.25 |
| 3779.5122 | N(N)GTYDHDVYRDEALN(D)NR Fuc 1 Hex 6 HexNAc 3 | 148.254 | 0.0 | -20.785 | 3.711e+07 | 482 - 499 | 3 | 20.72 | 20.72 | 20.72 |
| 4731.2724 | ISIYWTIVKPGDILLIN(N)STGNLIAPR Hex 9 HexNAc 2 | 148.106 | 0.0 | 17.085 | 8.550e+08 | 229 - 255 | 8 | 68.40 | 68.91 | 69.42 |
| 4538.8961 | C(C)KSEC(C)ITPN(N)GSIPNDKPFQNVNR Hex 9 HexNAc 2 | 148.097 | 0.0 | -3.908 | 1.970e+08 | 276 - 299 | 2 | 29.14 | 29.14 | 29.47 |
| 4230.9874 | TIVKPGDILLIN(N)STGNLIAPR Hex 10 HexNAc 2 | 148.060 | 0.0 | -17.675 | 1.675e+07 | 234 - 255 | 1 | 52.97 | 53.14 | 53.14 |
| 4214.7907 | C(C)KSEC(C)ITPN(N)GSIPNDKPFQNVNR Hex 7 HexNAc 2 | 147.670 | 0.0 | -3.567 | 2.358e+08 | 276 - 299 | 4 | 29.14 | 29.31 | 29.47 |
| 3113.3095 | YPALN(N)VTM(O)PNNEQFDK Hex 5 HexNAc 2 | 147.640 | 0.0 | 0.000 | 0.000e+00 | 160 - 176 | 1 | - | - | - |
| 4693.9769 | SLVASSGTLEFNNESFNWAGVTHN(N)GTSSSC(C)R Hex 6 HexNAc 2 | 147.418 | 0.0 | -3.169 | 1.503e+08 | 109 - 140 | 2 | 49.40 | 49.57 | 49.91 |
| 4048.6379 | RGSN(N)SSFFSR Fuc 1 Hex 7 HexNAc 8 | 145.192 | 0.0 | -19.342 | 2.841e+07 | 140 - 150 | 1 | 22.84 | 22.84 | 22.84 |
| 3423.3732 | RGSN(N)SSFFSR Fuc 2 Hex 6 HexNAc 5 | 144.097 | 0.0 | -5.563 | 2.878e+07 | 140 - 150 | 1 | 18.87 | 19.03 | 19.37 |
| 4394.7033 | RGSN(N)SSFFSR Fuc 1 Hex 5 HexNAc 7 Neu5Ac 3 | 143.964 | 0.0 | -18.547 | 2.714e+07 | 140 - 150 | 1 | 21.69 | 21.69 | 21.69 |
| 2813.1041 | GSN(N)SSFFSR Hex 5 HexNAc 5 | 143.782 | 0.0 | 8.503 | 2.094e+08 | 141 - 150 | 3 | 22.84 | 23.31 | 23.46 |
| 3299.2900 | N(N)GTYDHDVYR Fuc 3 Hex 5 HexNAc 4 | 143.580 | 0.0 | -18.789 | 8.697e+07 | 482 - 492 | 2 | 15.23 | 15.38 | 15.38 |
| 4569.2161 | ISIYWTIVKPGDILLIN(N)STGNLIAPR Hex 8 HexNAc 2 | 143.367 | 0.0 | -4.057 | 8.710e+07 | 229 - 255 | 5 | 68.74 | 68.91 | 69.25 |
| 3820.5385 | N(N)GTYDHDVYRDEALN(D)NR Fuc 1 Hex 5 HexNAc 4 | 142.361 | 0.0 | 4.427 | 1.330e+09 | 482 - 499 | 13 | 20.72 | 20.87 | 21.52 |
| 3778.5278 | N(N)GTYDHDVYRDEALNNR Fuc 1 Hex 6 HexNAc 3 | 141.966 | 0.0 | 12.968 | 3.345e+08 | 482 - 499 | 1 | 19.98 | 20.12 | 20.57 |
| 3318.3428 | RGSN(N)SSFFSR Fuc 1 Hex 5 HexNAc 6 | 140.815 | 0.0 | -19.721 | 5.816e+07 | 140 - 150 | 1 | 19.52 | 19.52 | 19.67 |
| 5262.1840 | SLVASSGTLEFNNESFNWAGVTHN(N)GTSSSC(C)R Hex 7 HexNAc 4 | 140.304 | 0.0 | -4.892 | 4.073e+07 | 109 - 140 | 1 | 49.23 | 49.23 | 49.57 |
| 3967.8858 | IVKPGDILLIN(N)STGNLIAPR Hex 9 HexNAc 2 | 139.947 | 0.0 | -18.113 | 1.125e+07 | 235 - 255 | 1 | 52.29 | 52.46 | 52.46 |
| 3454.4199 | N(N)GTYDHDVYRDEALNNR Fuc 1 Hex 4 HexNAc 3 | 139.477 | 0.0 | -4.238 | 1.321e+08 | 482 - 499 | 1 | 20.41 | 20.57 | 20.72 |
| 3334.3328 | RGSN(N)SSFFSR Hex 6 HexNAc 6 | 139.454 | 0.0 | -18.481 | 1.403e+07 | 140 - 150 | 1 | 19.20 | 19.20 | 19.20 |
| 2902.1406 | GSN(N)SSFFSR Fuc 2 Hex 5 HexNAc 4 | 139.095 | 0.0 | -18.627 | 1.040e+08 | 141 - 150 | 3 | 22.51 | 22.68 | 22.68 |
| 3105.2217 | GSN(N)SSFFSR Fuc 2 Hex 5 HexNAc 5 | 138.644 | 0.0 | -18.737 | 9.978e+07 | 141 - 150 | 1 | 22.68 | 22.68 | 22.84 |
| 3965.6111 | N(N)GTYDHDVYRDEALNNR Fuc 2 Hex 5 HexNAc 4 | 138.287 | 0.0 | 0.136 | 2.178e+09 | 482 - 499 | 5 | 19.82 | 20.12 | 112.87 |
| 2772.0762 | GSN(N)SSFFSR Hex 6 HexNAc 4 | 137.455 | 0.0 | -5.119 | 2.949e+07 | 141 - 150 | 1 | 23.15 | 23.46 | 23.46 |
| 2406.9428 | GSN(N)SSFFSR Hex 5 HexNAc 3 | 137.306 | 0.0 | -5.382 | 3.447e+07 | 141 - 150 | 1 | 23.31 | 23.46 | 23.62 |
| 3437.3917 | YPALN(N)VTM(O)PN(D)NEQFDK Hex 7 HexNAc 2 | 136.546 | 0.0 | 9.263 | 1.011e+08 | 160 - 176 | 4 | 35.16 | 35.49 | 36.33 |
| 3632.4680 | N(N)GTYDHDVYRDEALNNR Hex 6 HexNAc 3 | 136.320 | 0.0 | -3.315 | 3.415e+08 | 482 - 499 | 3 | 20.26 | 20.41 | 20.72 |
| 3017.2157 | RGSN(N)SSFFSR Fuc 2 Hex 6 HexNAc 3 | 136.165 | 0.0 | -17.878 | 2.919e+07 | 140 - 150 | 2 | 19.37 | 19.37 | 19.37 |
| 3599.4451 | YPALN(N)VTM(O)PN(D)NEQFDK Hex 8 HexNAc 2 | 135.824 | 0.0 | 9.814 | 5.734e+08 | 160 - 176 | 7 | 35.16 | 35.49 | 36.84 |
| 3267.3352 | N(N)GTYDHDVYRDEALNNR Hex 5 HexNAc 2 | 135.720 | 0.0 | -5.182 | 1.130e+08 | 482 - 499 | 2 | 20.41 | 20.57 | 20.72 |
| 3926.6664 | SEC(C)ITPN(N)GSIPNDKPFQNVNR Hex 7 HexNAc 2 | 135.520 | 0.0 | 15.714 | 1.866e+09 | 278 - 299 | 5 | 31.87 | 32.03 | 32.51 |
| 3627.6373 | PGDILLIN(N)STGNLIAPR Hex 9 HexNAc 2 | 135.342 | 0.0 | -20.070 | 2.572e+07 | 238 - 255 | 1 | 51.27 | 51.44 | 51.44 |
| 5567.7740 | IRNIPSRISIYWTIVKPGDILLIN(N)STGNLIAPR Hex 9 HexNAc 2 | 135.007 | 0.0 | 13.631 | 1.485e+08 | 222 - 255 | 5 | 63.72 | 63.84 | 64.19 |
| 5298.5889 | NIPSRISIYWTIVKPGDILLIN(N)STGNLIAPR Hex 9 HexNAc 2 | 134.676 | 0.0 | -17.281 | 4.063e+07 | 224 - 255 | 4 | 66.78 | 66.95 | 66.95 |
| 3600.4790 | N(N)GTYDHDVYRDEALNNR Fuc 2 Hex 4 HexNAc 3 | 133.590 | 0.0 | -4.071 | 9.557e+07 | 482 - 499 | 1 | 20.41 | 20.41 | 20.72 |
| 4022.6311 | N(N)GTYDHDVYRDEALNNR Fuc 1 Hex 5 HexNAc 5 | 133.119 | 0.0 | 20.913 | 2.407e+09 | 482 - 499 | 7 | 19.52 | 20.41 | 20.57 |
| 5059.1063 | SLVASSGTLEFNNESFNWAGVTHN(N)GTSSSC(C)R Hex 7 HexNAc 3 | 133.089 | 0.0 | -18.464 | 3.865e+07 | 109 - 140 | 1 | 49.23 | 49.23 | 49.40 |
| 4897.0466 | SLVASSGTLEFNNESFNWAGVTHN(N)GTSSSC(C)R Hex 6 HexNAc 3 | 133.056 | 0.0 | -18.797 | 6.642e+07 | 109 - 140 | 1 | 49.40 | 49.40 | 49.57 |
| 4248.6471 | N(N)GTYDHDVYRDEALNNR Fuc 2 Hex 8 HexNAc 3 | 132.549 | 0.0 | -3.875 | 1.847e+08 | 482 - 499 | 3 | 21.36 | 21.85 | 21.85 |
| 3819.5521 | N(N)GTYDHDVYRDEALNNR Fuc 1 Hex 5 HexNAc 4 | 131.954 | 0.0 | 2.077 | 4.273e+09 | 482 - 499 | 6 | 19.98 | 20.26 | 112.87 |
| 4856.0367 | SLVASSGTLEFNNESFNWAGVTHN(N)GTSSSC(C)R Hex 7 HexNAc 2 | 130.882 | 0.0 | 16.398 | 6.213e+08 | 109 - 140 | 4 | 49.23 | 49.40 | 49.91 |
| 3626.4611 | RGSN(N)SSFFSR Fuc 2 Hex 6 HexNAc 6 | 130.716 | 0.0 | -20.072 | 3.751e+06 | 140 - 150 | 2 | 19.20 | 19.20 | 19.20 |
| 3616.4734 | N(N)GTYDHDVYRDEALNNR Fuc 1 Hex 5 HexNAc 3 | 130.288 | 0.0 | -4.257 | 1.465e+08 | 482 - 499 | 1 | 20.26 | 20.41 | 20.72 |
| 2820.0899 | N(N)GTYDHDVYR Hex 6 HexNAc 3 | 130.134 | 0.0 | -17.289 | 2.415e+07 | 482 - 492 | 2 | 15.54 | 15.54 | 15.54 |
| 3764.6127 | SEC(C)ITPN(N)GSIPNDKPFQNVNR Hex 6 HexNAc 2 | 130.007 | 0.0 | -2.777 | 2.539e+08 | 278 - 299 | 2 | 32.19 | 32.19 | 32.51 |
| 6236.5181 | SLVASSGTLEFNNESFNWAGVTHN(N)GTSSSC(C)R Hex 8 HexNAc 8 | 129.989 | 0.0 | 0.000 | 0.000e+00 | 109 - 140 | 1 | - | - | - |
| 4387.7637 | N(N)GTYDHDVYRDEALNNR Fuc 1 Hex 6 HexNAc 6 | 129.953 | 0.0 | -4.569 | 1.243e+08 | 482 - 499 | 1 | 20.12 | 20.26 | 20.41 |
| 4088.7187 | SEC(C)ITPN(N)GSIPNDKPFQNVNR Hex 8 HexNAc 2 | 129.733 | 0.0 | 13.860 | 1.088e+09 | 278 - 299 | 5 | 31.87 | 32.03 | 32.51 |
| 2527.9635 | GSN(N)SSFFSR Hex 7 HexNAc 2 | 129.553 | 0.038 | -4.817 | 5.100e+07 | 141 - 150 | 1 | 22.68 | 22.84 | 23.00 |
| 3899.5080 | N(N)GTYDHDVYRDEALNNR Fuc 1 Hex 8 HexNAc 2 | 128.256 | 0.0 | 13.561 | 7.389e+08 | 482 - 499 | 5 | 21.69 | 22.02 | 22.68 |
| 4330.7416 | N(N)GTYDHDVYRDEALNNR Fuc 2 Hex 6 HexNAc 5 | 128.060 | 0.0 | -4.558 | 1.662e+08 | 482 - 499 | 1 | 19.82 | 19.98 | 20.12 |
| 3673.4954 | N(N)GTYDHDVYRDEALNNR Hex 5 HexNAc 4 | 128.006 | 0.0 | -4.006 | 1.979e+08 | 482 - 499 | 2 | 20.26 | 20.41 | 20.57 |
| 4184.6856 | N(N)GTYDHDVYRDEALNNR Fuc 1 Hex 6 HexNAc 5 | 127.629 | 0.0 | 11.963 | 4.486e+08 | 482 - 499 | 2 | 19.82 | 19.98 | 20.41 |
| 5205.1640 | SLVASSGTLEFNNESFNWAGVTHN(N)GTSSSC(C)R Fuc 1 Hex 7 HexNAc 3 | 127.326 | 0.0 | -18.922 | 1.207e+07 | 109 - 140 | 1 | 49.06 | 49.23 | 49.23 |
| 3966.5972 | N(N)GTYDHDVYRDEALN(D)NR Fuc 2 Hex 5 HexNAc 4 | 126.603 | 0.0 | 4.869 | 8.642e+08 | 482 - 499 | 4 | 20.57 | 20.72 | 21.36 |
| 4250.7716 | SEC(C)ITPN(N)GSIPNDKPFQNVNR Hex 9 HexNAc 2 | 126.439 | 0.0 | -2.448 | 1.670e+09 | 278 - 299 | 4 | 31.87 | 31.87 | 32.35 |
| 3407.3803 | RGSN(N)SSFFSR Fuc 3 Hex 5 HexNAc 5 | 125.430 | 0.0 | -18.173 | 5.573e+07 | 140 - 150 | 2 | 19.03 | 19.20 | 19.20 |
| 4112.6563 | N(N)GTYDHDVYRDEALN(D)NR Fuc 3 Hex 5 HexNAc 4 | 123.796 | 0.0 | 8.827 | 3.320e+08 | 482 - 499 | 3 | 20.41 | 20.57 | 21.36 |
| 3762.5304 | N(N)GTYDHDVYRDEALNNR Fuc 2 Hex 5 HexNAc 3 | 122.412 | 0.0 | -4.465 | 7.200e+07 | 482 - 499 | 1 | 20.12 | 20.26 | 20.41 |
| 4572.9445 | SLVASSGTLEFNNESFNWAGVTHN(N)GTSSSC(C)R Hex 4 HexNAc 3 | 122.362 | 0.0 | -4.666 | 3.102e+07 | 109 - 140 | 1 | 49.57 | 49.74 | 50.08 |
| 4111.6690 | N(N)GTYDHDVYRDEALNNR Fuc 3 Hex 5 HexNAc 4 | 122.196 | 0.0 | -2.675 | 6.350e+08 | 482 - 499 | 2 | 19.82 | 19.98 | 20.26 |
| 4264.6394 | N(N)GTYDHDVYRDEALNNR Fuc 1 Hex 9 HexNAc 3 | 122.141 | 0.0 | 7.113 | 1.552e+08 | 482 - 499 | 3 | 21.52 | 21.69 | 22.18 |
| 3356.3128 | N(N)GTYDHDVYR Fuc 2 Hex 5 HexNAc 5 | 121.256 | 0.0 | -19.097 | 4.659e+07 | 482 - 492 | 3 | 15.38 | 15.54 | 15.54 |
| 4168.6900 | N(N)GTYDHDVYRDEALNNR Fuc 2 Hex 5 HexNAc 5 | 119.896 | 0.0 | 11.554 | 6.072e+08 | 482 - 499 | 4 | 19.37 | 20.12 | 20.57 |
| 5100.1322 | SLVASSGTLEFNNESFNWAGVTHN(N)GTSSSC(C)R Hex 6 HexNAc 4 | 119.246 | 0.0 | -4.593 | 3.164e+07 | 109 - 140 | 1 | 49.23 | 49.40 | 49.74 |
| 3602.5604 | SEC(C)ITPN(N)GSIPNDKPFQNVNR Hex 5 HexNAc 2 | 119.152 | 0.0 | -18.792 | 9.990e+07 | 278 - 299 | 2 | 32.35 | 32.35 | 32.51 |
| 4267.7974 | SEC(C)ITPN(N)GSIPNDKPFQNVNR Fuc 2 Hex 3 HexNAc 4 Neu5Ac 1 | 118.809 | 0.0 | -18.952 | 9.893e+07 | 278 - 299 | 1 | 31.87 | 31.87 | 32.03 |
| 3090.2313 | RGSN(N)SSFFSR Hex 7 HexNAc 4 | 118.490 | 0.0 | -18.640 | 2.004e+07 | 140 - 150 | 1 | 19.52 | 19.52 | 19.67 |
| 4410.7011 | N(N)GTYDHDVYRDEALNNR Fuc 2 Hex 9 HexNAc 3 | 118.140 | 0.0 | -4.477 | 1.177e+08 | 482 - 499 | 1 | 21.52 | 21.69 | 21.85 |
| 3794.5210 | N(N)GTYDHDVYRDEALNNR Hex 7 HexNAc 3 | 118.057 | 0.0 | -19.888 | 4.381e+07 | 482 - 499 | 1 | 20.12 | 20.26 | 20.26 |
| 4314.7482 | N(N)GTYDHDVYRDEALNNR Fuc 3 Hex 5 HexNAc 5 | 115.880 | 0.0 | -17.763 | 3.555e+07 | 482 - 499 | 1 | 19.98 | 19.98 | 19.98 |
| 3470.4151 | N(N)GTYDHDVYRDEALNNR Hex 5 HexNAc 3 | 115.285 | 0.0 | -4.938 | 5.447e+07 | 482 - 499 | 1 | 20.41 | 20.57 | 20.72 |
| 4102.5882 | N(N)GTYDHDVYRDEALNNR Fuc 1 Hex 8 HexNAc 3 | 114.315 | 0.0 | 7.392 | 4.621e+08 | 482 - 499 | 7 | 21.20 | 22.02 | 22.68 |
| 3591.4420 | N(N)GTYDHDVYRDEALNNR Hex 7 HexNAc 2 | 113.805 | 0.004 | -17.733 | 4.651e+07 | 482 - 499 | 1 | 20.12 | 20.12 | 20.26 |
| 4169.6779 | N(N)GTYDHDVYRDEALN(D)NR Fuc 2 Hex 5 HexNAc 5 | 113.559 | 0.004 | -20.080 | 1.188e+08 | 482 - 499 | 1 | 20.72 | 20.72 | 20.87 |
| 2966.1470 | N(N)GTYDHDVYR Fuc 1 Hex 6 HexNAc 3 | 113.481 | 0.004 | -17.635 | 4.309e+07 | 482 - 492 | 1 | 15.38 | 15.38 | 15.54 |
| 4481.9033 | N(N)GTYDHDVYRDEALNNRFQIK Fuc 2 Hex 5 HexNAc 4 | 112.831 | 0.004 | -4.868 | 6.808e+07 | 482 - 503 | 2 | 31.71 | 31.87 | 32.19 |
| 2642.0465 | N(N)GTYDHDVYR Fuc 1 Hex 4 HexNAc 3 | 112.155 | 0.008 | -21.261 | 4.927e+06 | 482 - 492 | 1 | 15.70 | 15.70 | 15.70 |
| 4734.9895 | SLVASSGTLEFNNESFNWAGVTHN(N)GTSSSC(C)R Hex 5 HexNAc 3 | 112.011 | 0.012 | -18.708 | 1.417e+07 | 109 - 140 | 1 | 49.57 | 49.57 | 49.74 |
| 3372.3076 | N(N)GTYDHDVYR Fuc 1 Hex 6 HexNAc 5 | 110.459 | 0.012 | -4.942 | 6.897e+07 | 482 - 492 | 3 | 15.38 | 15.38 | 15.70 |
| 2861.1163 | N(N)GTYDHDVYR Hex 5 HexNAc 4 | 110.291 | 0.012 | -18.413 | 1.466e+07 | 482 - 492 | 1 | 15.54 | 15.54 | 15.54 |
| 3583.4483 | YPALN(N)VTMPN(D)NEQFDK Hex 8 HexNAc 2 | 109.507 | 0.012 | -6.227 | 3.546e+07 | 160 - 176 | 2 | 43.17 | 43.33 | 43.50 |
| 4467.7209 | N(N)GTYDHDVYRDEALNNR Fuc 1 Hex 9 HexNAc 4 | 109.322 | 0.012 | -17.133 | 2.217e+07 | 482 - 499 | 1 | 21.85 | 21.85 | 21.85 |
| 3876.5728 | N(N)GTYDHDVYRDEALNNR Hex 5 HexNAc 5 | 108.927 | 0.012 | -18.498 | 8.457e+07 | 482 - 499 | 1 | 20.41 | 20.41 | 20.41 |
| 3059.2459 | RGSN(N)SSFFSR Fuc 2 Hex 5 HexNAc 4 | 108.339 | 0.012 | 0.000 | 0.000e+00 | 140 - 150 | 1 | - | - | - |
| 4110.6491 | N(N)GTYDHDVYRDEALNNR Fuc 1 Hex 5 HexNAc 4 Neu5Ac 1 | 105.310 | 0.028 | -19.100 | 1.145e+07 | 482 - 499 | 1 | 21.69 | 21.69 | 21.69 |
| 6234.4982 | SLVASSGTLEFNNESFNWAGVTHN(N)GTSSSC(C)R Fuc 1 Hex 6 HexNAc 6 Neu5Ac 2 | 103.741 | 0.032 | -4.722 | 3.709e+07 | 109 - 140 | 1 | 46.01 | 46.18 | 46.34 |
| 5141.1594 | SLVASSGTLEFNNESFNWAGVTHN(N)GTSSSC(C)R Hex 5 HexNAc 5 | 103.688 | 0.032 | 12.752 | 6.536e+07 | 109 - 140 | 2 | 49.06 | 49.40 | 49.74 |
| 4251.7565 | SEC(C)ITPN(N)GSIPN(D)DKPFQNVNR Hex 9 HexNAc 2 | 103.682 | 0.032 | 8.947 | 5.284e+08 | 278 - 299 | 7 | 32.51 | 33.34 | 33.51 |
| 6396.5506 | SLVASSGTLEFNNESFNWAGVTHN(N)GTSSSC(C)R Fuc 1 Hex 7 HexNAc 6 Neu5Ac 2 | 103.520 | 0.032 | -4.144 | 6.847e+07 | 109 - 140 | 2 | 45.84 | 46.01 | 46.18 |
| 4613.7764 | N(N)GTYDHDVYRDEALNNR Fuc 2 Hex 9 HexNAc 4 | 103.104 | 0.032 | -4.995 | 3.495e+07 | 482 - 499 | 1 | 21.52 | 21.69 | 21.85 |
| 4629.7743 | N(N)GTYDHDVYRDEALNNR Fuc 1 Hex 10 HexNAc 4 | 102.382 | 0.032 | -5.059 | 3.115e+07 | 482 - 499 | 1 | 21.36 | 21.52 | 21.69 |
| 5043.1060 | SLVASSGTLEFNNESFNWAGVTHN(N)GTSSSC(C)R Fuc 1 Hex 6 HexNAc 3 | 101.760 | 0.032 | -5.284 | 2.339e+07 | 109 - 140 | 1 | 49.06 | 49.23 | 49.40 |
| 3633.4539 | N(N)GTYDHDVYRDEALN(D)NR Hex 6 HexNAc 3 | 101.344 | 0.032 | -6.207 | 7.365e+07 | 482 - 499 | 2 | 20.87 | 20.87 | 21.52 |
| 4445.7915 | RGSN(N)SSFFSR Fuc 4 Hex 8 HexNAc 7 | 100.511 | 0.032 | -20.022 | 1.731e+07 | 140 - 150 | 1 | 21.04 | 21.20 | 21.20 |
| 4893.3257 | ISIYWTIVKPGDILLIN(N)STGNLIAPR Hex 10 HexNAc 2 | 99.708 | 0.032 | -4.142 | 3.933e+07 | 229 - 255 | 4 | 68.57 | 68.74 | 68.91 |
| 4805.1207 | LNWLTHLNSKYPALN(N)VTM(O)PNNEQFDK Hex 8 HexNAc 2 | 99.448 | 0.032 | -17.623 | 4.132e+07 | 150 - 176 | 2 | 47.87 | 47.87 | 48.04 |
| 4556.7577 | N(N)GTYDHDVYR Fuc 3 Hex 9 HexNAc 7 | 98.253 | 0.032 | -3.860 | 1.019e+08 | 482 - 492 | 1 | 21.36 | 21.52 | 21.85 |
| 6558.6108 | SLVASSGTLEFNNESFNWAGVTHN(N)GTSSSC(C)R Fuc 1 Hex 8 HexNAc 6 Neu5Ac 2 | 97.713 | 0.032 | -19.265 | 2.948e+07 | 109 - 140 | 2 | 45.84 | 46.01 | 46.01 |
| 3835.5496 | N(N)GTYDHDVYRDEALNNR Hex 6 HexNAc 4 | 96.960 | 0.032 | -6.565 | 2.372e+07 | 482 - 499 | 1 | 20.26 | 20.26 | 20.57 |
| 4857.0158 | SLVASSGTLEFN(D)NESFNWAGVTHN(N)GTSSSC(C)R Hex 7 HexNAc 2 | 93.563 | 0.041 | 8.570 | 6.120e+07 | 109 - 140 | 4 | 49.06 | 50.76 | 51.10 |
| 5405.7203 | IRNIPSRISIYWTIVKPGDILLIN(N)STGNLIAPR Hex 8 HexNAc 2 | 93.264 | 0.041 | -4.044 | 1.453e+07 | 222 - 255 | 3 | 63.84 | 63.97 | 64.19 |
| 4089.7040 | SEC(C)ITPN(N)GSIPN(D)DKPFQNVNR Hex 8 HexNAc 2 | 92.652 | 0.041 | 9.976 | 3.091e+08 | 278 - 299 | 5 | 32.68 | 33.34 | 33.51 |
| 3927.6518 | SEC(C)ITPN(N)GSIPN(D)DKPFQNVNR Hex 7 HexNAc 2 | 92.601 | 0.041 | 11.066 | 5.645e+08 | 278 - 299 | 7 | 32.68 | 33.51 | 33.67 |
| 3518.3645 | N(N)GTYDHDVYR Fuc 2 Hex 6 HexNAc 5 | 91.686 | 0.044 | -19.779 | 1.978e+07 | 482 - 492 | 1 | 15.38 | 15.38 | 15.38 |

### RGSN(N)SSFFSR Hex 5 HexNAc 6

**Mass:**  3172.2899

(-4.736e-07)

**Charge States:** 3

**Start Position**: 141

**End Position**: 151

| Profile Score | Tandem Score | TDA q-value | Volume | Spectrum Matches |
| --- | --- | --- | --- | --- |
| -18.6890 | 189.0458 | 0.0000 | 1.6657e+08 | 2 |

**Adduct Signal Fractions**

- **Unmodified**: 1.6657e+08

### RGSN(N)SSFFSR Hex 6 HexNAc 2

**Mass:**  2522.0200

(6.925e-07)

**Charge States:** 3

**Start Position**: 141

**End Position**: 151

| Profile Score | Tandem Score | TDA q-value | Volume | Spectrum Matches |
| --- | --- | --- | --- | --- |
| -2.9829 | 188.0709 | 0.0000 | 4.5858e+08 | 7 |

**Adduct Signal Fractions**

- **Unmodified**: 4.5858e+08

### RGSN(N)SSFFSR Hex 7 HexNAc 2

**Mass:**  2684.0727

(6.305e-07)

**Charge States:** 3

**Start Position**: 141

**End Position**: 151

| Profile Score | Tandem Score | TDA q-value | Volume | Spectrum Matches |
| --- | --- | --- | --- | --- |
| 13.1722 | 187.5943 | 0.0000 | 2.7247e+08 | 2 |

**Adduct Signal Fractions**

- **Unmodified**: 2.7247e+08

### RGSN(N)SSFFSR Hex 5 HexNAc 2

**Mass:**  2359.9663

(5.791e-07)

**Charge States:** 3

**Start Position**: 141

**End Position**: 151

| Profile Score | Tandem Score | TDA q-value | Volume | Spectrum Matches |
| --- | --- | --- | --- | --- |
| 11.7911 | 187.5019 | 0.0000 | 3.9425e+08 | 2 |

**Adduct Signal Fractions**

- **Unmodified**: 3.9425e+08

### RGSN(N)SSFFSR Hex 6 HexNAc 3

**Mass:**  2725.0995

(1.024e-06)

**Charge States:** 3

**Start Position**: 141

**End Position**: 151

| Profile Score | Tandem Score | TDA q-value | Volume | Spectrum Matches |
| --- | --- | --- | --- | --- |
| 13.6872 | 185.1040 | 0.0000 | 5.8326e+08 | 5 |

**Adduct Signal Fractions**

- **Unmodified**: 5.8326e+08

### RGSN(N)SSFFSR Fuc 2 Hex 5 HexNAc 4

**Mass:**  3058.2420

(5.766e-07)

**Charge States:** 3

**Start Position**: 141

**End Position**: 151

| Profile Score | Tandem Score | TDA q-value | Volume | Spectrum Matches |
| --- | --- | --- | --- | --- |
| 13.6525 | 180.9597 | 0.0000 | 3.1527e+08 | 2 |

**Adduct Signal Fractions**

- **Unmodified**: 3.1527e+08

### N(N)GTYDHDVYR Fuc 2 Hex 5 HexNAc 4

**Mass:**  3153.2327

(1.164e-06)

**Charge States:** 3

**Start Position**: 483

**End Position**: 493

| Profile Score | Tandem Score | TDA q-value | Volume | Spectrum Matches |
| --- | --- | --- | --- | --- |
| -2.0928 | 180.5015 | 0.0000 | 3.1500e+08 | 3 |

**Adduct Signal Fractions**

- **Unmodified**: 3.1500e+08

### RGSN(N)SSFFSR Fuc 2 Hex 5 HexNAc 5

**Mass:**  3261.3213

(6.752e-07)

**Charge States:** 3

**Start Position**: 141

**End Position**: 151

| Profile Score | Tandem Score | TDA q-value | Volume | Spectrum Matches |
| --- | --- | --- | --- | --- |
| 13.6028 | 180.0905 | 0.0000 | 4.0498e+08 | 4 |

**Adduct Signal Fractions**

- **Unmodified**: 4.0498e+08

### RGSN(N)SSFFSR Hex 8 HexNAc 2

**Mass:**  2846.1264

(1.023e-06)

**Charge States:** 3

**Start Position**: 141

**End Position**: 151

| Profile Score | Tandem Score | TDA q-value | Volume | Spectrum Matches |
| --- | --- | --- | --- | --- |
| -17.4235 | 180.0047 | 0.0000 | 9.7495e+07 | 1 |

**Adduct Signal Fractions**

- **Unmodified**: 9.7495e+07

### RGSN(N)SSFFSR Fuc 1 Hex 5 HexNAc 4

**Mass:**  2912.1835

(2.078e-06)

**Charge States:** 3

**Start Position**: 141

**End Position**: 151

| Profile Score | Tandem Score | TDA q-value | Volume | Spectrum Matches |
| --- | --- | --- | --- | --- |
| 13.9382 | 178.6039 | 0.0000 | 8.8351e+08 | 4 |

**Adduct Signal Fractions**

- **Unmodified**: 8.8351e+08

### RGSN(N)SSFFSR Fuc 1 Hex 6 HexNAc 6

**Mass:**  3480.3960

(8.568e-07)

**Charge States:** 3, 4

**Start Position**: 141

**End Position**: 151

| Profile Score | Tandem Score | TDA q-value | Volume | Spectrum Matches |
| --- | --- | --- | --- | --- |
| -4.3977 | 177.9086 | 0.0000 | 7.0371e+07 | 2 |

**Adduct Signal Fractions**

- **Unmodified**: 7.0371e+07

### RGSN(N)SSFFSR Hex 5 HexNAc 5

**Mass:**  2969.2062

(1.112e-06)

**Charge States:** 3

**Start Position**: 141

**End Position**: 151

| Profile Score | Tandem Score | TDA q-value | Volume | Spectrum Matches |
| --- | --- | --- | --- | --- |
| 11.0806 | 176.8326 | 0.0000 | 2.9170e+08 | 2 |

**Adduct Signal Fractions**

- **Unmodified**: 2.9170e+08

### RGSN(N)SSFFSR Fuc 1 Hex 6 HexNAc 3

**Mass:**  2871.1571

(6.923e-07)

**Charge States:** 3

**Start Position**: 141

**End Position**: 151

| Profile Score | Tandem Score | TDA q-value | Volume | Spectrum Matches |
| --- | --- | --- | --- | --- |
| 15.2088 | 174.7164 | 0.0000 | 3.3825e+08 | 3 |

**Adduct Signal Fractions**

- **Unmodified**: 3.3825e+08

### RGSN(N)SSFFSR Fuc 1 Hex 5 HexNAc 3

**Mass:**  2709.1056

(1.253e-06)

**Charge States:** 3

**Start Position**: 141

**End Position**: 151

| Profile Score | Tandem Score | TDA q-value | Volume | Spectrum Matches |
| --- | --- | --- | --- | --- |
| -18.1038 | 172.4187 | 0.0000 | 6.7771e+07 | 1 |

**Adduct Signal Fractions**

- **Unmodified**: 6.7771e+07

### RGSN(N)SSFFSR Hex 5 HexNAc 4

**Mass:**  2766.1265

(1.001e-06)

**Charge States:** 3

**Start Position**: 141

**End Position**: 151

| Profile Score | Tandem Score | TDA q-value | Volume | Spectrum Matches |
| --- | --- | --- | --- | --- |
| -18.1112 | 172.0124 | 0.0000 | 8.9786e+07 | 1 |

**Adduct Signal Fractions**

- **Unmodified**: 8.9786e+07

### YPALN(N)VTMPNNEQFDK Hex 8 HexNAc 2

**Mass:**  3582.4649

(1.710e-06)

**Charge States:** 3, 4

**Start Position**: 161

**End Position**: 177

| Profile Score | Tandem Score | TDA q-value | Volume | Spectrum Matches |
| --- | --- | --- | --- | --- |
| -1.7363 | 171.9664 | 0.0000 | 3.7454e+08 | 8 |

**Adduct Signal Fractions**

- **Unmodified**: 3.7454e+08

### RGSN(N)SSFFSR Fuc 1 Hex 4 HexNAc 3

**Mass:**  2547.0510

(6.153e-07)

**Charge States:** 3

**Start Position**: 141

**End Position**: 151

| Profile Score | Tandem Score | TDA q-value | Volume | Spectrum Matches |
| --- | --- | --- | --- | --- |
| -20.0098 | 171.9011 | 0.0000 | 2.3635e+07 | 1 |

**Adduct Signal Fractions**

- **Unmodified**: 2.3635e+07

### YPALN(N)VTM(O)PNNEQFDK Hex 9 HexNAc 2

**Mass:**  3760.5111

(9.867e-07)

**Charge States:** 3, 4

**Start Position**: 161

**End Position**: 177

| Profile Score | Tandem Score | TDA q-value | Volume | Spectrum Matches |
| --- | --- | --- | --- | --- |
| 13.5277 | 171.5050 | 0.0000 | 3.0846e+08 | 4 |

**Adduct Signal Fractions**

- **Unmodified**: 3.0846e+08

### RGSN(N)SSFFSR Fuc 1 Hex 5 HexNAc 5

**Mass:**  3115.2636

(8.332e-07)

**Charge States:** 3, 4

**Start Position**: 141

**End Position**: 151

| Profile Score | Tandem Score | TDA q-value | Volume | Spectrum Matches |
| --- | --- | --- | --- | --- |
| 14.2714 | 171.1441 | 0.0000 | 1.5817e+09 | 6 |

**Adduct Signal Fractions**

- **Unmodified**: 1.5817e+09

### RGSN(N)SSFFSR Fuc 3 Hex 5 HexNAc 4

**Mass:**  3204.2997

(8.809e-07)

**Charge States:** 3, 4

**Start Position**: 141

**End Position**: 151

| Profile Score | Tandem Score | TDA q-value | Volume | Spectrum Matches |
| --- | --- | --- | --- | --- |
| -17.8087 | 171.0084 | 0.0000 | 1.3186e+08 | 1 |

**Adduct Signal Fractions**

- **Unmodified**: 1.3186e+08

### YPALN(N)VTM(O)PNNEQFDK Hex 8 HexNAc 2

**Mass:**  3598.4573

(1.008e-06)

**Charge States:** 2, 3, 4

**Start Position**: 161

**End Position**: 177

| Profile Score | Tandem Score | TDA q-value | Volume | Spectrum Matches |
| --- | --- | --- | --- | --- |
| -0.6967 | 170.5088 | 0.0000 | 3.4290e+09 | 15 |

**Adduct Signal Fractions**

- **Unmodified**: 3.4290e+09

### YPALN(N)VTM(O)PNNEQFDK Hex 7 HexNAc 2

**Mass:**  3436.4038

(1.909e-07)

**Charge States:** 3, 4

**Start Position**: 161

**End Position**: 177

| Profile Score | Tandem Score | TDA q-value | Volume | Spectrum Matches |
| --- | --- | --- | --- | --- |
| 14.3549 | 168.0286 | 0.0000 | 6.2224e+08 | 4 |

**Adduct Signal Fractions**

- **Unmodified**: 6.2224e+08

### TIVKPGDILLIN(N)STGNLIAPR Hex 9 HexNAc 2

**Mass:**  4068.9355

(2.881e-06)

**Charge States:** 3, 4, 5

**Start Position**: 235

**End Position**: 256

| Profile Score | Tandem Score | TDA q-value | Volume | Spectrum Matches |
| --- | --- | --- | --- | --- |
| 17.9643 | 165.0457 | 0.0000 | 6.1967e+08 | 7 |

**Adduct Signal Fractions**

- **Unmodified**: 6.1967e+08

### RGSN(N)SSFFSR Fuc 1 Hex 6 HexNAc 5

**Mass:**  3277.3159

(3.681e-07)

**Charge States:** 3

**Start Position**: 141

**End Position**: 151

| Profile Score | Tandem Score | TDA q-value | Volume | Spectrum Matches |
| --- | --- | --- | --- | --- |
| -3.9776 | 160.9900 | 0.0000 | 1.1213e+08 | 4 |

**Adduct Signal Fractions**

- **Unmodified**: 1.1213e+08

### N(N)GTYDHDVYR Fuc 1 Hex 5 HexNAc 4

**Mass:**  3007.1749

(1.787e-06)

**Charge States:** 3

**Start Position**: 483

**End Position**: 493

| Profile Score | Tandem Score | TDA q-value | Volume | Spectrum Matches |
| --- | --- | --- | --- | --- |
| 16.1369 | 160.8236 | 0.0000 | 5.2007e+08 | 5 |

**Adduct Signal Fractions**

- **Unmodified**: 5.2007e+08

### RGSN(N)SSFFSR Hex 7 HexNAc 3 Neu5Ac 1

**Mass:**  3178.2321

(-4.183e-06)

**Charge States:** 3

**Start Position**: 141

**End Position**: 151

| Profile Score | Tandem Score | TDA q-value | Volume | Spectrum Matches |
| --- | --- | --- | --- | --- |
| -20.4264 | 160.6654 | 0.0000 | 7.4546e+06 | 1 |

**Adduct Signal Fractions**

- **Unmodified**: 7.4546e+06

### SLVASSGTLEFNNESFNWAGVTHN(N)GTSSSC(C)R Hex 8 HexNAc 2

**Mass:**  5018.0827

(3.221e-06)

**Charge States:** 4

**Start Position**: 110

**End Position**: 141

| Profile Score | Tandem Score | TDA q-value | Volume | Spectrum Matches |
| --- | --- | --- | --- | --- |
| -3.6490 | 160.2221 | 0.0000 | 1.2975e+08 | 2 |

**Adduct Signal Fractions**

- **Unmodified**: 1.2975e+08

### RGSN(N)SSFFSR Hex 6 HexNAc 4

**Mass:**  2928.1798

(1.093e-06)

**Charge States:** 3

**Start Position**: 141

**End Position**: 151

| Profile Score | Tandem Score | TDA q-value | Volume | Spectrum Matches |
| --- | --- | --- | --- | --- |
| 11.7390 | 160.1707 | 0.0000 | 1.3041e+08 | 1 |

**Adduct Signal Fractions**

- **Unmodified**: 1.3041e+08

### N(N)GTYDHDVYR Fuc 1 Hex 5 HexNAc 5

**Mass:**  3210.2536

(1.507e-06)

**Charge States:** 3, 4

**Start Position**: 483

**End Position**: 493

| Profile Score | Tandem Score | TDA q-value | Volume | Spectrum Matches |
| --- | --- | --- | --- | --- |
| -3.3654 | 159.2536 | 0.0000 | 2.0797e+08 | 5 |

**Adduct Signal Fractions**

- **Unmodified**: 2.0797e+08

### RGSN(N)SSFFSR Fuc 3 Hex 6 HexNAc 6 Neu5Ac 1

**Mass:**  4063.5808

(-5.808e-06)

**Charge States:** 4

**Start Position**: 141

**End Position**: 151

| Profile Score | Tandem Score | TDA q-value | Volume | Spectrum Matches |
| --- | --- | --- | --- | --- |
| -20.8185 | 158.9921 | 0.0000 | 1.4667e+07 | 1 |

**Adduct Signal Fractions**

- **Unmodified**: 1.4667e+07

### YPALN(N)VTMPNNEQFDK Hex 7 HexNAc 2

**Mass:**  3420.4107

(2.030e-06)

**Charge States:** 3, 4

**Start Position**: 161

**End Position**: 177

| Profile Score | Tandem Score | TDA q-value | Volume | Spectrum Matches |
| --- | --- | --- | --- | --- |
| -3.2167 | 158.9633 | 0.0000 | 6.2713e+07 | 3 |

**Adduct Signal Fractions**

- **Unmodified**: 6.2713e+07

### GSN(N)SSFFSR Hex 6 HexNAc 3

**Mass:**  2568.9975

(1.731e-07)

**Charge States:** 3

**Start Position**: 142

**End Position**: 151

| Profile Score | Tandem Score | TDA q-value | Volume | Spectrum Matches |
| --- | --- | --- | --- | --- |
| -3.3165 | 158.2737 | 0.0000 | 2.6741e+08 | 3 |

**Adduct Signal Fractions**

- **Unmodified**: 2.6741e+08

### TIVKPGDILLIN(N)STGNLIAPR Hex 8 HexNAc 2

**Mass:**  3906.8796

(2.286e-06)

**Charge States:** 3, 4

**Start Position**: 235

**End Position**: 256

| Profile Score | Tandem Score | TDA q-value | Volume | Spectrum Matches |
| --- | --- | --- | --- | --- |
| -18.4338 | 157.8137 | 0.0000 | 5.7765e+07 | 2 |

**Adduct Signal Fractions**

- **Unmodified**: 5.7765e+07

### RGSN(N)SSFFSR Fuc 2 Hex 4 HexNAc 3

**Mass:**  2693.1059

(-5.343e-07)

**Charge States:** 3

**Start Position**: 141

**End Position**: 151

| Profile Score | Tandem Score | TDA q-value | Volume | Spectrum Matches |
| --- | --- | --- | --- | --- |
| -17.2965 | 157.5661 | 0.0000 | 2.3508e+07 | 1 |

**Adduct Signal Fractions**

- **Unmodified**: 2.3508e+07

### GSN(N)SSFFSR Fuc 1 Hex 5 HexNAc 4

**Mass:**  2756.0828

(2.960e-07)

**Charge States:** 3

**Start Position**: 142

**End Position**: 151

| Profile Score | Tandem Score | TDA q-value | Volume | Spectrum Matches |
| --- | --- | --- | --- | --- |
| 12.2867 | 155.9804 | 0.0000 | 3.1163e+08 | 4 |

**Adduct Signal Fractions**

- **Unmodified**: 3.1163e+08

### YPALN(N)VTM(O)PNNEQFDK Fuc 2 Hex 3 HexNAc 4 Neu5Ac 1

**Mass:**  3777.5374

(-5.898e-06)

**Charge States:** 3

**Start Position**: 161

**End Position**: 177

| Profile Score | Tandem Score | TDA q-value | Volume | Spectrum Matches |
| --- | --- | --- | --- | --- |
| -5.2226 | 155.5181 | 0.0000 | 3.7804e+07 | 1 |

**Adduct Signal Fractions**

- **Unmodified**: 3.7804e+07

### GSN(N)SSFFSR Hex 8 HexNAc 2

**Mass:**  2690.0240

(5.946e-07)

**Charge States:** 2, 3

**Start Position**: 142

**End Position**: 151

| Profile Score | Tandem Score | TDA q-value | Volume | Spectrum Matches |
| --- | --- | --- | --- | --- |
| -18.8040 | 155.4343 | 0.0000 | 2.9231e+07 | 1 |

**Adduct Signal Fractions**

- **Unmodified**: 2.9231e+07

### YPALN(N)VTM(O)PNNEQFDK Hex 6 HexNAc 2

**Mass:**  3274.3485

(2.882e-06)

**Charge States:** 3

**Start Position**: 161

**End Position**: 177

| Profile Score | Tandem Score | TDA q-value | Volume | Spectrum Matches |
| --- | --- | --- | --- | --- |
| -5.6241 | 155.2832 | 0.0000 | 4.1160e+07 | 1 |

**Adduct Signal Fractions**

- **Unmodified**: 4.1160e+07

### TIVKPGDILLIN(N)STGNLIAPR Fuc 2 Hex 3 HexNAc 4 Neu5Ac 1

**Mass:**  4085.9567

(-3.934e-06)

**Charge States:** 4

**Start Position**: 235

**End Position**: 256

| Profile Score | Tandem Score | TDA q-value | Volume | Spectrum Matches |
| --- | --- | --- | --- | --- |
| -3.5295 | 155.1565 | 0.0000 | 5.8763e+07 | 2 |

**Adduct Signal Fractions**

- **Unmodified**: 5.8763e+07

### C(C)KSEC(C)ITPN(N)GSIPNDKPFQNVNR Hex 8 HexNAc 2

**Mass:**  4376.8449

(5.487e-07)

**Charge States:** 4

**Start Position**: 277

**End Position**: 300

| Profile Score | Tandem Score | TDA q-value | Volume | Spectrum Matches |
| --- | --- | --- | --- | --- |
| -3.5326 | 155.0275 | 0.0000 | 1.3987e+08 | 1 |

**Adduct Signal Fractions**

- **Unmodified**: 1.3987e+08

### YPALN(N)VTM(O)PNNEQFDK Hex 5 HexNAc 2

**Mass:**  3112.2991

(1.265e-06)

**Charge States:** 3

**Start Position**: 161

**End Position**: 177

| Profile Score | Tandem Score | TDA q-value | Volume | Spectrum Matches |
| --- | --- | --- | --- | --- |
| -3.5209 | 154.7150 | 0.0000 | 4.2231e+07 | 1 |

**Adduct Signal Fractions**

- **Unmodified**: 4.2231e+07

### GSN(N)SSFFSR Fuc 3 Hex 5 HexNAc 4

**Mass:**  3048.1972

(2.698e-07)

**Charge States:** 3

**Start Position**: 142

**End Position**: 151

| Profile Score | Tandem Score | TDA q-value | Volume | Spectrum Matches |
| --- | --- | --- | --- | --- |
| -18.4669 | 154.4868 | 0.0000 | 5.0047e+07 | 1 |

**Adduct Signal Fractions**

- **Unmodified**: 5.0047e+07

### GSN(N)SSFFSR Fuc 1 Hex 5 HexNAc 5

**Mass:**  2959.1621

(7.345e-07)

**Charge States:** 2, 3, 4

**Start Position**: 142

**End Position**: 151

| Profile Score | Tandem Score | TDA q-value | Volume | Spectrum Matches |
| --- | --- | --- | --- | --- |
| 13.6214 | 154.1880 | 0.0000 | 9.0917e+08 | 5 |

**Adduct Signal Fractions**

- **Unmodified**: 9.0917e+08

### GSN(N)SSFFSR Fuc 1 Hex 5 HexNAc 3

**Mass:**  2553.0062

(2.517e-07)

**Charge States:** 3

**Start Position**: 142

**End Position**: 151

| Profile Score | Tandem Score | TDA q-value | Volume | Spectrum Matches |
| --- | --- | --- | --- | --- |
| -19.5714 | 152.9639 | 0.0000 | 3.0179e+07 | 1 |

**Adduct Signal Fractions**

- **Unmodified**: 3.0179e+07

### YPALN(N)VTMPNNEQFDK Hex 9 HexNAc 2

**Mass:**  3744.5176

(1.671e-06)

**Charge States:** 3

**Start Position**: 161

**End Position**: 177

| Profile Score | Tandem Score | TDA q-value | Volume | Spectrum Matches |
| --- | --- | --- | --- | --- |
| -4.9393 | 152.8493 | 0.0000 | 2.8321e+07 | 3 |

**Adduct Signal Fractions**

- **Unmodified**: 2.8321e+07

### HAVPN(N)GTIVK Fuc 1 Hex 5 HexNAc 5

**Mass:**  3007.3189

(3.369e-04)

**Start Position**: 18

**End Position**: 28

| Profile Score | Tandem Score | TDA q-value | Volume | Spectrum Matches |
| --- | --- | --- | --- | --- |
| 0.0000 | 151.3700 | 0.0000 | 0.0000e+00 | 1 |

Adduct:

No Chromatogram Found

### GSN(N)SSFFSR Fuc 1 Hex 6 HexNAc 3

**Mass:**  2715.0545

(-7.119e-07)

**Charge States:** 3

**Start Position**: 142

**End Position**: 151

| Profile Score | Tandem Score | TDA q-value | Volume | Spectrum Matches |
| --- | --- | --- | --- | --- |
| -4.3389 | 149.4462 | 0.0000 | 1.1268e+08 | 2 |

**Adduct Signal Fractions**

- **Unmodified**: 1.1268e+08

### GSN(N)SSFFSR Hex 6 HexNAc 2

**Mass:**  2365.9183

(2.334e-08)

**Charge States:** 2

**Start Position**: 142

**End Position**: 151

| Profile Score | Tandem Score | TDA q-value | Volume | Spectrum Matches |
| --- | --- | --- | --- | --- |
| -4.0191 | 148.8422 | 0.0000 | 2.1812e+08 | 3 |

**Adduct Signal Fractions**

- **Unmodified**: 2.1812e+08

### ISIYWTIVKPGDILLIN(N)STGNLIAPR Fuc 2 Hex 3 HexNAc 4 Neu5Ac 1

**Mass:**  4748.2971

(-4.564e-06)

**Charge States:** 4

**Start Position**: 230

**End Position**: 256

| Profile Score | Tandem Score | TDA q-value | Volume | Spectrum Matches |
| --- | --- | --- | --- | --- |
| 13.3809 | 148.6666 | 0.0000 | 1.9232e+08 | 4 |

**Adduct Signal Fractions**

- **Unmodified**: 1.9232e+08

### C(C)KSEC(C)ITPN(N)GSIPNDKPFQNVNR Hex 6 HexNAc 2

**Mass:**  4052.7404

(1.383e-06)

**Charge States:** 4

**Start Position**: 277

**End Position**: 300

| Profile Score | Tandem Score | TDA q-value | Volume | Spectrum Matches |
| --- | --- | --- | --- | --- |
| -18.2756 | 148.6549 | 0.0000 | 3.2778e+07 | 2 |

**Adduct Signal Fractions**

- **Unmodified**: 3.2778e+07

### GSN(N)SSFFSR Hex 5 HexNAc 2

**Mass:**  2203.8656

(8.486e-07)

**Charge States:** 2, 3

**Start Position**: 142

**End Position**: 151

| Profile Score | Tandem Score | TDA q-value | Volume | Spectrum Matches |
| --- | --- | --- | --- | --- |
| -2.7715 | 148.5922 | 0.0000 | 2.8871e+08 | 3 |

**Adduct Signal Fractions**

- **Unmodified**: 2.8871e+08

### SLVASSGTLEFNNESFNWAGVTHN(N)GTSSSC(C)R Hex 5 HexNAc 2

**Mass:**  4531.9260

(2.000e-06)

**Charge States:** 3, 4

**Start Position**: 110

**End Position**: 141

| Profile Score | Tandem Score | TDA q-value | Volume | Spectrum Matches |
| --- | --- | --- | --- | --- |
| 15.3735 | 148.3487 | 0.0000 | 1.7397e+08 | 4 |

**Adduct Signal Fractions**

- **Unmodified**: 1.7397e+08

### N(N)GTYDHDVYRDEALN(D)NR Fuc 1 Hex 6 HexNAc 3

**Mass:**  3779.5122

(2.619e-04)

**Charge States:** 4

**Start Position**: 483

**End Position**: 500

| Profile Score | Tandem Score | TDA q-value | Volume | Spectrum Matches |
| --- | --- | --- | --- | --- |
| -20.7853 | 148.2539 | 0.0000 | 3.7111e+07 | 3 |

**Adduct Signal Fractions**

- **Unmodified**: 3.7111e+07

### ISIYWTIVKPGDILLIN(N)STGNLIAPR Hex 9 HexNAc 2

**Mass:**  4731.2746

(8.459e-07)

**Charge States:** 3, 4, 5

**Start Position**: 230

**End Position**: 256

| Profile Score | Tandem Score | TDA q-value | Volume | Spectrum Matches |
| --- | --- | --- | --- | --- |
| 17.0854 | 148.1059 | 0.0000 | 8.5500e+08 | 8 |

**Adduct Signal Fractions**

- **Unmodified**: 8.5500e+08

### C(C)KSEC(C)ITPN(N)GSIPNDKPFQNVNR Hex 9 HexNAc 2

**Mass:**  4538.8952

(4.338e-07)

**Charge States:** 4

**Start Position**: 277

**End Position**: 300

| Profile Score | Tandem Score | TDA q-value | Volume | Spectrum Matches |
| --- | --- | --- | --- | --- |
| -3.9084 | 148.0975 | 0.0000 | 1.9703e+08 | 2 |

**Adduct Signal Fractions**

- **Unmodified**: 1.9703e+08

### TIVKPGDILLIN(N)STGNLIAPR Hex 10 HexNAc 2

**Mass:**  4230.9875

(2.490e-06)

**Charge States:** 4

**Start Position**: 235

**End Position**: 256

| Profile Score | Tandem Score | TDA q-value | Volume | Spectrum Matches |
| --- | --- | --- | --- | --- |
| -17.6754 | 148.0604 | 0.0000 | 1.6753e+07 | 1 |

**Adduct Signal Fractions**

- **Unmodified**: 1.6753e+07

### C(C)KSEC(C)ITPN(N)GSIPNDKPFQNVNR Hex 7 HexNAc 2

**Mass:**  4214.7907

(2.002e-07)

**Charge States:** 4

**Start Position**: 277

**End Position**: 300

| Profile Score | Tandem Score | TDA q-value | Volume | Spectrum Matches |
| --- | --- | --- | --- | --- |
| -3.5666 | 147.6701 | 0.0000 | 2.3584e+08 | 4 |

**Adduct Signal Fractions**

- **Unmodified**: 2.3584e+08

### YPALN(N)VTM(O)PNNEQFDK Hex 5 HexNAc 2

**Mass:**  3113.3095

(3.259e-04)

**Start Position**: 161

**End Position**: 177

| Profile Score | Tandem Score | TDA q-value | Volume | Spectrum Matches |
| --- | --- | --- | --- | --- |
| 0.0000 | 147.6402 | 0.0000 | 0.0000e+00 | 1 |

Adduct:

No Chromatogram Found

### SLVASSGTLEFNNESFNWAGVTHN(N)GTSSSC(C)R Hex 6 HexNAc 2

**Mass:**  4693.9782

(3.883e-06)

**Charge States:** 3, 4

**Start Position**: 110

**End Position**: 141

| Profile Score | Tandem Score | TDA q-value | Volume | Spectrum Matches |
| --- | --- | --- | --- | --- |
| -3.1685 | 147.4184 | 0.0000 | 1.5031e+08 | 2 |

**Adduct Signal Fractions**

- **Unmodified**: 1.5031e+08

### RGSN(N)SSFFSR Fuc 1 Hex 7 HexNAc 8

**Mass:**  4048.6379

(8.177e-06)

**Charge States:** 4

**Start Position**: 141

**End Position**: 151

| Profile Score | Tandem Score | TDA q-value | Volume | Spectrum Matches |
| --- | --- | --- | --- | --- |
| -19.3423 | 145.1918 | 0.0000 | 2.8408e+07 | 1 |

**Adduct Signal Fractions**

- **Unmodified**: 2.8408e+07

### RGSN(N)SSFFSR Fuc 2 Hex 6 HexNAc 5

**Mass:**  3423.3737

(7.031e-07)

**Charge States:** 3

**Start Position**: 141

**End Position**: 151

| Profile Score | Tandem Score | TDA q-value | Volume | Spectrum Matches |
| --- | --- | --- | --- | --- |
| -5.5630 | 144.0966 | 0.0000 | 2.8779e+07 | 1 |

**Adduct Signal Fractions**

- **Unmodified**: 2.8779e+07

### RGSN(N)SSFFSR Fuc 1 Hex 5 HexNAc 7 Neu5Ac 3

**Mass:**  4394.7033

(-6.646e-07)

**Charge States:** 4

**Start Position**: 141

**End Position**: 151

| Profile Score | Tandem Score | TDA q-value | Volume | Spectrum Matches |
| --- | --- | --- | --- | --- |
| -18.5469 | 143.9638 | 0.0000 | 2.7137e+07 | 1 |

**Adduct Signal Fractions**

- **Unmodified**: 2.7137e+07

### GSN(N)SSFFSR Hex 5 HexNAc 5

**Mass:**  2813.1040

(1.459e-06)

**Charge States:** 2, 3

**Start Position**: 142

**End Position**: 151

| Profile Score | Tandem Score | TDA q-value | Volume | Spectrum Matches |
| --- | --- | --- | --- | --- |
| 8.5031 | 143.7821 | 0.0000 | 2.0935e+08 | 3 |

**Adduct Signal Fractions**

- **Unmodified**: 2.0935e+08

### N(N)GTYDHDVYR Fuc 3 Hex 5 HexNAc 4

**Mass:**  3299.2890

(1.200e-06)

**Charge States:** 3, 4

**Start Position**: 483

**End Position**: 493

| Profile Score | Tandem Score | TDA q-value | Volume | Spectrum Matches |
| --- | --- | --- | --- | --- |
| -18.7889 | 143.5797 | 0.0000 | 8.6970e+07 | 2 |

**Adduct Signal Fractions**

- **Unmodified**: 8.6970e+07

### ISIYWTIVKPGDILLIN(N)STGNLIAPR Hex 8 HexNAc 2

**Mass:**  4569.2180

(1.055e-06)

**Charge States:** 3, 4

**Start Position**: 230

**End Position**: 256

| Profile Score | Tandem Score | TDA q-value | Volume | Spectrum Matches |
| --- | --- | --- | --- | --- |
| -4.0572 | 143.3672 | 0.0000 | 8.7101e+07 | 5 |

**Adduct Signal Fractions**

- **Unmodified**: 8.7101e+07

### N(N)GTYDHDVYRDEALN(D)NR Fuc 1 Hex 5 HexNAc 4

**Mass:**  3820.5363

(2.595e-04)

**Charge States:** 3, 4

**Start Position**: 483

**End Position**: 500

| Profile Score | Tandem Score | TDA q-value | Volume | Spectrum Matches |
| --- | --- | --- | --- | --- |
| 4.4274 | 142.3615 | 0.0000 | 1.3296e+09 | 13 |

**Adduct Signal Fractions**

- **Unmodified**: 1.3296e+09

### N(N)GTYDHDVYRDEALNNR Fuc 1 Hex 6 HexNAc 3

**Mass:**  3778.5275

(1.371e-06)

**Charge States:** 4

**Start Position**: 483

**End Position**: 500

| Profile Score | Tandem Score | TDA q-value | Volume | Spectrum Matches |
| --- | --- | --- | --- | --- |
| 12.9683 | 141.9662 | 0.0000 | 3.3451e+08 | 1 |

**Adduct Signal Fractions**

- **Unmodified**: 3.3451e+08

### RGSN(N)SSFFSR Fuc 1 Hex 5 HexNAc 6

**Mass:**  3318.3433

(8.523e-07)

**Charge States:** 3

**Start Position**: 141

**End Position**: 151

| Profile Score | Tandem Score | TDA q-value | Volume | Spectrum Matches |
| --- | --- | --- | --- | --- |
| -19.7210 | 140.8152 | 0.0000 | 5.8157e+07 | 1 |

**Adduct Signal Fractions**

- **Unmodified**: 5.8157e+07

### SLVASSGTLEFNNESFNWAGVTHN(N)GTSSSC(C)R Hex 7 HexNAc 4

**Mass:**  5262.1838

(2.183e-06)

**Charge States:** 4

**Start Position**: 110

**End Position**: 141

| Profile Score | Tandem Score | TDA q-value | Volume | Spectrum Matches |
| --- | --- | --- | --- | --- |
| -4.8923 | 140.3036 | 0.0000 | 4.0729e+07 | 1 |

**Adduct Signal Fractions**

- **Unmodified**: 4.0729e+07

### IVKPGDILLIN(N)STGNLIAPR Hex 9 HexNAc 2

**Mass:**  3967.8867

(2.210e-06)

**Charge States:** 4

**Start Position**: 236

**End Position**: 256

| Profile Score | Tandem Score | TDA q-value | Volume | Spectrum Matches |
| --- | --- | --- | --- | --- |
| -18.1128 | 139.9472 | 0.0000 | 1.1250e+07 | 1 |

**Adduct Signal Fractions**

- **Unmodified**: 1.1250e+07

### N(N)GTYDHDVYRDEALNNR Fuc 1 Hex 4 HexNAc 3

**Mass:**  3454.4196

(9.004e-07)

**Charge States:** 4

**Start Position**: 483

**End Position**: 500

| Profile Score | Tandem Score | TDA q-value | Volume | Spectrum Matches |
| --- | --- | --- | --- | --- |
| -4.2377 | 139.4767 | 0.0000 | 1.3208e+08 | 1 |

**Adduct Signal Fractions**

- **Unmodified**: 1.3208e+08

### RGSN(N)SSFFSR Hex 6 HexNAc 6

**Mass:**  3334.3328

(-7.907e-07)

**Charge States:** 3

**Start Position**: 141

**End Position**: 151

| Profile Score | Tandem Score | TDA q-value | Volume | Spectrum Matches |
| --- | --- | --- | --- | --- |
| -18.4811 | 139.4540 | 0.0000 | 1.4029e+07 | 1 |

**Adduct Signal Fractions**

- **Unmodified**: 1.4029e+07

### GSN(N)SSFFSR Fuc 2 Hex 5 HexNAc 4

**Mass:**  2902.1409

(6.444e-07)

**Charge States:** 2, 3

**Start Position**: 142

**End Position**: 151

| Profile Score | Tandem Score | TDA q-value | Volume | Spectrum Matches |
| --- | --- | --- | --- | --- |
| -18.6274 | 139.0945 | 0.0000 | 1.0402e+08 | 3 |

**Adduct Signal Fractions**

- **Unmodified**: 1.0402e+08

### GSN(N)SSFFSR Fuc 2 Hex 5 HexNAc 5

**Mass:**  3105.2210

(9.756e-07)

**Charge States:** 3

**Start Position**: 142

**End Position**: 151

| Profile Score | Tandem Score | TDA q-value | Volume | Spectrum Matches |
| --- | --- | --- | --- | --- |
| -18.7371 | 138.6443 | 0.0000 | 9.9780e+07 | 1 |

**Adduct Signal Fractions**

- **Unmodified**: 9.9780e+07

### N(N)GTYDHDVYRDEALNNR Fuc 2 Hex 5 HexNAc 4

**Mass:**  3965.6108

(1.196e-06)

**Charge States:** 3, 4, 5

**Start Position**: 483

**End Position**: 500

| Profile Score | Tandem Score | TDA q-value | Volume | Spectrum Matches |
| --- | --- | --- | --- | --- |
| 0.1363 | 138.2868 | 0.0000 | 2.1784e+09 | 5 |

**Adduct Signal Fractions**

- **Unmodified**: 2.1784e+09

### GSN(N)SSFFSR Hex 6 HexNAc 4

**Mass:**  2772.0769

(4.880e-07)

**Charge States:** 3

**Start Position**: 142

**End Position**: 151

| Profile Score | Tandem Score | TDA q-value | Volume | Spectrum Matches |
| --- | --- | --- | --- | --- |
| -5.1189 | 137.4554 | 0.0000 | 2.9490e+07 | 1 |

**Adduct Signal Fractions**

- **Unmodified**: 2.9490e+07

### GSN(N)SSFFSR Hex 5 HexNAc 3

**Mass:**  2406.9415

(-6.962e-07)

**Charge States:** 3

**Start Position**: 142

**End Position**: 151

| Profile Score | Tandem Score | TDA q-value | Volume | Spectrum Matches |
| --- | --- | --- | --- | --- |
| -5.3822 | 137.3061 | 0.0000 | 3.4473e+07 | 1 |

**Adduct Signal Fractions**

- **Unmodified**: 3.4473e+07

### YPALN(N)VTM(O)PN(D)NEQFDK Hex 7 HexNAc 2

**Mass:**  3437.3911

(2.882e-04)

**Charge States:** 3, 4

**Start Position**: 161

**End Position**: 177

| Profile Score | Tandem Score | TDA q-value | Volume | Spectrum Matches |
| --- | --- | --- | --- | --- |
| 9.2634 | 136.5455 | 0.0000 | 1.0105e+08 | 4 |

**Adduct Signal Fractions**

- **Unmodified**: 1.0105e+08

### N(N)GTYDHDVYRDEALNNR Hex 6 HexNAc 3

**Mass:**  3632.4680

(1.169e-06)

**Charge States:** 3, 4

**Start Position**: 483

**End Position**: 500

| Profile Score | Tandem Score | TDA q-value | Volume | Spectrum Matches |
| --- | --- | --- | --- | --- |
| -3.3146 | 136.3196 | 0.0000 | 3.4151e+08 | 3 |

**Adduct Signal Fractions**

- **Unmodified**: 3.4151e+08

### RGSN(N)SSFFSR Fuc 2 Hex 6 HexNAc 3

**Mass:**  3017.2157

(8.801e-07)

**Charge States:** 3

**Start Position**: 141

**End Position**: 151

| Profile Score | Tandem Score | TDA q-value | Volume | Spectrum Matches |
| --- | --- | --- | --- | --- |
| -17.8782 | 136.1648 | 0.0000 | 2.9193e+07 | 2 |

**Adduct Signal Fractions**

- **Unmodified**: 2.9193e+07

### YPALN(N)VTM(O)PN(D)NEQFDK Hex 8 HexNAc 2

**Mass:**  3599.4442

(2.755e-04)

**Charge States:** 3, 4

**Start Position**: 161

**End Position**: 177

| Profile Score | Tandem Score | TDA q-value | Volume | Spectrum Matches |
| --- | --- | --- | --- | --- |
| 9.8145 | 135.8236 | 0.0000 | 5.7342e+08 | 7 |

**Adduct Signal Fractions**

- **Unmodified**: 5.7342e+08

### N(N)GTYDHDVYRDEALNNR Hex 5 HexNAc 2

**Mass:**  3267.3352

(9.304e-07)

**Charge States:** 4

**Start Position**: 483

**End Position**: 500

| Profile Score | Tandem Score | TDA q-value | Volume | Spectrum Matches |
| --- | --- | --- | --- | --- |
| -5.1823 | 135.7199 | 0.0000 | 1.1300e+08 | 2 |

**Adduct Signal Fractions**

- **Unmodified**: 1.1300e+08

### SEC(C)ITPN(N)GSIPNDKPFQNVNR Hex 7 HexNAc 2

**Mass:**  3926.6672

(9.771e-07)

**Charge States:** 3, 4

**Start Position**: 279

**End Position**: 300

| Profile Score | Tandem Score | TDA q-value | Volume | Spectrum Matches |
| --- | --- | --- | --- | --- |
| 15.7143 | 135.5203 | 0.0000 | 1.8657e+09 | 5 |

**Adduct Signal Fractions**

- **Unmodified**: 1.8657e+09

### PGDILLIN(N)STGNLIAPR Hex 9 HexNAc 2

**Mass:**  3627.6384

(2.754e-06)

**Charge States:** 3, 4

**Start Position**: 239

**End Position**: 256

| Profile Score | Tandem Score | TDA q-value | Volume | Spectrum Matches |
| --- | --- | --- | --- | --- |
| -20.0697 | 135.3423 | 0.0000 | 2.5723e+07 | 1 |

**Adduct Signal Fractions**

- **Unmodified**: 2.5723e+07

### IRNIPSRISIYWTIVKPGDILLIN(N)STGNLIAPR Hex 9 HexNAc 2

**Mass:**  5567.7755

(1.991e-06)

**Charge States:** 5, 6, 7

**Start Position**: 223

**End Position**: 256

| Profile Score | Tandem Score | TDA q-value | Volume | Spectrum Matches |
| --- | --- | --- | --- | --- |
| 13.6312 | 135.0065 | 0.0000 | 1.4854e+08 | 5 |

**Adduct Signal Fractions**

- **Unmodified**: 1.4854e+08

### NIPSRISIYWTIVKPGDILLIN(N)STGNLIAPR Hex 9 HexNAc 2

**Mass:**  5298.5895

(1.812e-06)

**Charge States:** 4, 5, 6

**Start Position**: 225

**End Position**: 256

| Profile Score | Tandem Score | TDA q-value | Volume | Spectrum Matches |
| --- | --- | --- | --- | --- |
| -17.2807 | 134.6756 | 0.0000 | 4.0626e+07 | 4 |

**Adduct Signal Fractions**

- **Unmodified**: 4.0626e+07

### N(N)GTYDHDVYRDEALNNR Fuc 2 Hex 4 HexNAc 3

**Mass:**  3600.4794

(1.334e-06)

**Charge States:** 4

**Start Position**: 483

**End Position**: 500

| Profile Score | Tandem Score | TDA q-value | Volume | Spectrum Matches |
| --- | --- | --- | --- | --- |
| -4.0707 | 133.5902 | 0.0000 | 9.5569e+07 | 1 |

**Adduct Signal Fractions**

- **Unmodified**: 9.5569e+07

### N(N)GTYDHDVYRDEALNNR Fuc 1 Hex 5 HexNAc 5

**Mass:**  4022.6308

(5.812e-07)

**Charge States:** 3, 4, 5

**Start Position**: 483

**End Position**: 500

| Profile Score | Tandem Score | TDA q-value | Volume | Spectrum Matches |
| --- | --- | --- | --- | --- |
| 20.9128 | 133.1189 | 0.0000 | 2.4066e+09 | 7 |

**Adduct Signal Fractions**

- **Unmodified**: 2.4066e+09

### SLVASSGTLEFNNESFNWAGVTHN(N)GTSSSC(C)R Hex 7 HexNAc 3

**Mass:**  5059.1047

(2.278e-06)

**Charge States:** 4

**Start Position**: 110

**End Position**: 141

| Profile Score | Tandem Score | TDA q-value | Volume | Spectrum Matches |
| --- | --- | --- | --- | --- |
| -18.4639 | 133.0891 | 0.0000 | 3.8646e+07 | 1 |

**Adduct Signal Fractions**

- **Unmodified**: 3.8646e+07

### SLVASSGTLEFNNESFNWAGVTHN(N)GTSSSC(C)R Hex 6 HexNAc 3

**Mass:**  4897.0451

(1.034e-06)

**Charge States:** 4

**Start Position**: 110

**End Position**: 141

| Profile Score | Tandem Score | TDA q-value | Volume | Spectrum Matches |
| --- | --- | --- | --- | --- |
| -18.7970 | 133.0560 | 0.0000 | 6.6423e+07 | 1 |

**Adduct Signal Fractions**

- **Unmodified**: 6.6423e+07

### N(N)GTYDHDVYRDEALNNR Fuc 2 Hex 8 HexNAc 3

**Mass:**  4248.6472

(-8.590e-06)

**Charge States:** 3, 4

**Start Position**: 483

**End Position**: 500

| Profile Score | Tandem Score | TDA q-value | Volume | Spectrum Matches |
| --- | --- | --- | --- | --- |
| -3.8754 | 132.5494 | 0.0000 | 1.8474e+08 | 3 |

**Adduct Signal Fractions**

- **Unmodified**: 1.8474e+08

### N(N)GTYDHDVYRDEALNNR Fuc 1 Hex 5 HexNAc 4

**Mass:**  3819.5517

(2.818e-06)

**Charge States:** 3, 4, 5

**Start Position**: 483

**End Position**: 500

| Profile Score | Tandem Score | TDA q-value | Volume | Spectrum Matches |
| --- | --- | --- | --- | --- |
| 2.0769 | 131.9541 | 0.0000 | 4.2729e+09 | 6 |

**Adduct Signal Fractions**

- **Unmodified**: 4.2729e+09

### SLVASSGTLEFNNESFNWAGVTHN(N)GTSSSC(C)R Hex 7 HexNAc 2

**Mass:**  4856.0417

(4.988e-06)

**Charge States:** 3, 4, 5

**Start Position**: 110

**End Position**: 141

| Profile Score | Tandem Score | TDA q-value | Volume | Spectrum Matches |
| --- | --- | --- | --- | --- |
| 16.3976 | 130.8819 | 0.0000 | 6.2128e+08 | 4 |

**Adduct Signal Fractions**

- **Unmodified**: 6.2128e+08

### RGSN(N)SSFFSR Fuc 2 Hex 6 HexNAc 6

**Mass:**  3626.4611

(2.768e-06)

**Charge States:** 3

**Start Position**: 141

**End Position**: 151

| Profile Score | Tandem Score | TDA q-value | Volume | Spectrum Matches |
| --- | --- | --- | --- | --- |
| -20.0719 | 130.7161 | 0.0000 | 3.7509e+06 | 2 |

**Adduct Signal Fractions**

- **Unmodified**: 3.7509e+06

### N(N)GTYDHDVYRDEALNNR Fuc 1 Hex 5 HexNAc 3

**Mass:**  3616.4748

(5.468e-07)

**Charge States:** 4

**Start Position**: 483

**End Position**: 500

| Profile Score | Tandem Score | TDA q-value | Volume | Spectrum Matches |
| --- | --- | --- | --- | --- |
| -4.2568 | 130.2881 | 0.0000 | 1.4651e+08 | 1 |

**Adduct Signal Fractions**

- **Unmodified**: 1.4651e+08

### N(N)GTYDHDVYR Hex 6 HexNAc 3

**Mass:**  2820.0899

(1.135e-06)

**Charge States:** 3

**Start Position**: 483

**End Position**: 493

| Profile Score | Tandem Score | TDA q-value | Volume | Spectrum Matches |
| --- | --- | --- | --- | --- |
| -17.2892 | 130.1340 | 0.0000 | 2.4153e+07 | 2 |

**Adduct Signal Fractions**

- **Unmodified**: 2.4153e+07

### SEC(C)ITPN(N)GSIPNDKPFQNVNR Hex 6 HexNAc 2

**Mass:**  3764.6138

(1.239e-06)

**Charge States:** 3, 4

**Start Position**: 279

**End Position**: 300

| Profile Score | Tandem Score | TDA q-value | Volume | Spectrum Matches |
| --- | --- | --- | --- | --- |
| -2.7773 | 130.0072 | 0.0000 | 2.5393e+08 | 2 |

**Adduct Signal Fractions**

- **Unmodified**: 2.5393e+08

### SLVASSGTLEFNNESFNWAGVTHN(N)GTSSSC(C)R Hex 8 HexNAc 8

**Mass:**  6236.5181

(-3.933e-06)

**Start Position**: 110

**End Position**: 141

| Profile Score | Tandem Score | TDA q-value | Volume | Spectrum Matches |
| --- | --- | --- | --- | --- |
| 0.0000 | 129.9885 | 0.0000 | 0.0000e+00 | 1 |

Adduct:

No Chromatogram Found

### N(N)GTYDHDVYRDEALNNR Fuc 1 Hex 6 HexNAc 6

**Mass:**  4387.7639

(1.239e-08)

**Charge States:** 4

**Start Position**: 483

**End Position**: 500

| Profile Score | Tandem Score | TDA q-value | Volume | Spectrum Matches |
| --- | --- | --- | --- | --- |
| -4.5687 | 129.9534 | 0.0000 | 1.2430e+08 | 1 |

**Adduct Signal Fractions**

- **Unmodified**: 1.2430e+08

### SEC(C)ITPN(N)GSIPNDKPFQNVNR Hex 8 HexNAc 2

**Mass:**  4088.7167

(9.629e-07)

**Charge States:** 3, 4

**Start Position**: 279

**End Position**: 300

| Profile Score | Tandem Score | TDA q-value | Volume | Spectrum Matches |
| --- | --- | --- | --- | --- |
| 13.8596 | 129.7329 | 0.0000 | 1.0883e+09 | 5 |

**Adduct Signal Fractions**

- **Unmodified**: 1.0883e+09

### GSN(N)SSFFSR Hex 7 HexNAc 2

**Mass:**  2527.9618

(-3.107e-06)

**Charge States:** 2

**Start Position**: 142

**End Position**: 151

| Profile Score | Tandem Score | TDA q-value | Volume | Spectrum Matches |
| --- | --- | --- | --- | --- |
| -4.8170 | 129.5528 | 0.0385 | 5.1001e+07 | 1 |

**Adduct Signal Fractions**

- **Unmodified**: 5.1001e+07

### N(N)GTYDHDVYRDEALNNR Fuc 1 Hex 8 HexNAc 2

**Mass:**  3899.5082

(-9.971e-06)

**Charge States:** 3, 4

**Start Position**: 483

**End Position**: 500

| Profile Score | Tandem Score | TDA q-value | Volume | Spectrum Matches |
| --- | --- | --- | --- | --- |
| 13.5606 | 128.2563 | 0.0000 | 7.3893e+08 | 5 |

**Adduct Signal Fractions**

- **Unmodified**: 7.3893e+08

### N(N)GTYDHDVYRDEALNNR Fuc 2 Hex 6 HexNAc 5

**Mass:**  4330.7404

(3.351e-07)

**Charge States:** 4

**Start Position**: 483

**End Position**: 500

| Profile Score | Tandem Score | TDA q-value | Volume | Spectrum Matches |
| --- | --- | --- | --- | --- |
| -4.5583 | 128.0601 | 0.0000 | 1.6621e+08 | 1 |

**Adduct Signal Fractions**

- **Unmodified**: 1.6621e+08

### N(N)GTYDHDVYRDEALNNR Hex 5 HexNAc 4

**Mass:**  3673.4960

(1.106e-06)

**Charge States:** 4

**Start Position**: 483

**End Position**: 500

| Profile Score | Tandem Score | TDA q-value | Volume | Spectrum Matches |
| --- | --- | --- | --- | --- |
| -4.0062 | 128.0065 | 0.0000 | 1.9786e+08 | 2 |

**Adduct Signal Fractions**

- **Unmodified**: 1.9786e+08

### N(N)GTYDHDVYRDEALNNR Fuc 1 Hex 6 HexNAc 5

**Mass:**  4184.6862

(1.346e-06)

**Charge States:** 4

**Start Position**: 483

**End Position**: 500

| Profile Score | Tandem Score | TDA q-value | Volume | Spectrum Matches |
| --- | --- | --- | --- | --- |
| 11.9632 | 127.6285 | 0.0000 | 4.4863e+08 | 2 |

**Adduct Signal Fractions**

- **Unmodified**: 4.4863e+08

### SLVASSGTLEFNNESFNWAGVTHN(N)GTSSSC(C)R Fuc 1 Hex 7 HexNAc 3

**Mass:**  5205.1652

(1.132e-06)

**Charge States:** 4

**Start Position**: 110

**End Position**: 141

| Profile Score | Tandem Score | TDA q-value | Volume | Spectrum Matches |
| --- | --- | --- | --- | --- |
| -18.9219 | 127.3261 | 0.0000 | 1.2075e+07 | 1 |

**Adduct Signal Fractions**

- **Unmodified**: 1.2075e+07

### N(N)GTYDHDVYRDEALN(D)NR Fuc 2 Hex 5 HexNAc 4

**Mass:**  3966.5952

(2.497e-04)

**Charge States:** 3, 4

**Start Position**: 483

**End Position**: 500

| Profile Score | Tandem Score | TDA q-value | Volume | Spectrum Matches |
| --- | --- | --- | --- | --- |
| 4.8691 | 126.6033 | 0.0000 | 8.6420e+08 | 4 |

**Adduct Signal Fractions**

- **Unmodified**: 8.6420e+08

### SEC(C)ITPN(N)GSIPNDKPFQNVNR Hex 9 HexNAc 2

**Mass:**  4250.7727

(6.830e-07)

**Charge States:** 3, 4

**Start Position**: 279

**End Position**: 300

| Profile Score | Tandem Score | TDA q-value | Volume | Spectrum Matches |
| --- | --- | --- | --- | --- |
| -2.4475 | 126.4385 | 0.0000 | 1.6697e+09 | 4 |

**Adduct Signal Fractions**

- **Unmodified**: 1.6697e+09

### RGSN(N)SSFFSR Fuc 3 Hex 5 HexNAc 5

**Mass:**  3407.3790

(7.007e-07)

**Charge States:** 3

**Start Position**: 141

**End Position**: 151

| Profile Score | Tandem Score | TDA q-value | Volume | Spectrum Matches |
| --- | --- | --- | --- | --- |
| -18.1734 | 125.4305 | 0.0000 | 5.5735e+07 | 2 |

**Adduct Signal Fractions**

- **Unmodified**: 5.5735e+07

### N(N)GTYDHDVYRDEALN(D)NR Fuc 3 Hex 5 HexNAc 4

**Mass:**  4112.6545

(2.420e-04)

**Charge States:** 4

**Start Position**: 483

**End Position**: 500

| Profile Score | Tandem Score | TDA q-value | Volume | Spectrum Matches |
| --- | --- | --- | --- | --- |
| 8.8275 | 123.7963 | 0.0000 | 3.3197e+08 | 3 |

**Adduct Signal Fractions**

- **Unmodified**: 3.3197e+08

### N(N)GTYDHDVYRDEALNNR Fuc 2 Hex 5 HexNAc 3

**Mass:**  3762.5312

(1.047e-06)

**Charge States:** 4

**Start Position**: 483

**End Position**: 500

| Profile Score | Tandem Score | TDA q-value | Volume | Spectrum Matches |
| --- | --- | --- | --- | --- |
| -4.4650 | 122.4123 | 0.0000 | 7.2002e+07 | 1 |

**Adduct Signal Fractions**

- **Unmodified**: 7.2002e+07

### SLVASSGTLEFNNESFNWAGVTHN(N)GTSSSC(C)R Hex 4 HexNAc 3

**Mass:**  4572.9460

(2.534e-06)

**Charge States:** 4

**Start Position**: 110

**End Position**: 141

| Profile Score | Tandem Score | TDA q-value | Volume | Spectrum Matches |
| --- | --- | --- | --- | --- |
| -4.6661 | 122.3616 | 0.0000 | 3.1017e+07 | 1 |

**Adduct Signal Fractions**

- **Unmodified**: 3.1017e+07

### N(N)GTYDHDVYRDEALNNR Fuc 3 Hex 5 HexNAc 4

**Mass:**  4111.6686

(9.565e-07)

**Charge States:** 4

**Start Position**: 483

**End Position**: 500

| Profile Score | Tandem Score | TDA q-value | Volume | Spectrum Matches |
| --- | --- | --- | --- | --- |
| -2.6747 | 122.1958 | 0.0000 | 6.3501e+08 | 2 |

**Adduct Signal Fractions**

- **Unmodified**: 6.3501e+08

### N(N)GTYDHDVYRDEALNNR Fuc 1 Hex 9 HexNAc 3

**Mass:**  4264.6413

(-9.290e-06)

**Charge States:** 3, 4

**Start Position**: 483

**End Position**: 500

| Profile Score | Tandem Score | TDA q-value | Volume | Spectrum Matches |
| --- | --- | --- | --- | --- |
| 7.1128 | 122.1409 | 0.0000 | 1.5515e+08 | 3 |

**Adduct Signal Fractions**

- **Unmodified**: 1.5515e+08

### N(N)GTYDHDVYR Fuc 2 Hex 5 HexNAc 5

**Mass:**  3356.3113

(2.029e-06)

**Charge States:** 3

**Start Position**: 483

**End Position**: 493

| Profile Score | Tandem Score | TDA q-value | Volume | Spectrum Matches |
| --- | --- | --- | --- | --- |
| -19.0975 | 121.2565 | 0.0000 | 4.6592e+07 | 3 |

**Adduct Signal Fractions**

- **Unmodified**: 4.6592e+07

### N(N)GTYDHDVYRDEALNNR Fuc 2 Hex 5 HexNAc 5

**Mass:**  4168.6911

(1.159e-06)

**Charge States:** 3, 4

**Start Position**: 483

**End Position**: 500

| Profile Score | Tandem Score | TDA q-value | Volume | Spectrum Matches |
| --- | --- | --- | --- | --- |
| 11.5541 | 119.8958 | 0.0000 | 6.0720e+08 | 4 |

**Adduct Signal Fractions**

- **Unmodified**: 6.0720e+08

### SLVASSGTLEFNNESFNWAGVTHN(N)GTSSSC(C)R Hex 6 HexNAc 4

**Mass:**  5100.1320

(2.497e-06)

**Charge States:** 4

**Start Position**: 110

**End Position**: 141

| Profile Score | Tandem Score | TDA q-value | Volume | Spectrum Matches |
| --- | --- | --- | --- | --- |
| -4.5930 | 119.2458 | 0.0000 | 3.1637e+07 | 1 |

**Adduct Signal Fractions**

- **Unmodified**: 3.1637e+07

### SEC(C)ITPN(N)GSIPNDKPFQNVNR Hex 5 HexNAc 2

**Mass:**  3602.5607

(1.831e-07)

**Charge States:** 3, 4

**Start Position**: 279

**End Position**: 300

| Profile Score | Tandem Score | TDA q-value | Volume | Spectrum Matches |
| --- | --- | --- | --- | --- |
| -18.7915 | 119.1517 | 0.0000 | 9.9902e+07 | 2 |

**Adduct Signal Fractions**

- **Unmodified**: 9.9902e+07

### SEC(C)ITPN(N)GSIPNDKPFQNVNR Fuc 2 Hex 3 HexNAc 4 Neu5Ac 1

**Mass:**  4267.7985

(-5.816e-06)

**Charge States:** 4

**Start Position**: 279

**End Position**: 300

| Profile Score | Tandem Score | TDA q-value | Volume | Spectrum Matches |
| --- | --- | --- | --- | --- |
| -18.9524 | 118.8085 | 0.0000 | 9.8927e+07 | 1 |

**Adduct Signal Fractions**

- **Unmodified**: 9.8927e+07

### RGSN(N)SSFFSR Hex 7 HexNAc 4

**Mass:**  3090.2290

(1.894e-06)

**Charge States:** 3

**Start Position**: 141

**End Position**: 151

| Profile Score | Tandem Score | TDA q-value | Volume | Spectrum Matches |
| --- | --- | --- | --- | --- |
| -18.6401 | 118.4901 | 0.0000 | 2.0037e+07 | 1 |

**Adduct Signal Fractions**

- **Unmodified**: 2.0037e+07

### N(N)GTYDHDVYRDEALNNR Fuc 2 Hex 9 HexNAc 3

**Mass:**  4410.7009

(-8.467e-06)

**Charge States:** 4

**Start Position**: 483

**End Position**: 500

| Profile Score | Tandem Score | TDA q-value | Volume | Spectrum Matches |
| --- | --- | --- | --- | --- |
| -4.4766 | 118.1404 | 0.0000 | 1.1767e+08 | 1 |

**Adduct Signal Fractions**

- **Unmodified**: 1.1767e+08

### N(N)GTYDHDVYRDEALNNR Hex 7 HexNAc 3

**Mass:**  3794.5217

(1.199e-06)

**Charge States:** 4

**Start Position**: 483

**End Position**: 500

| Profile Score | Tandem Score | TDA q-value | Volume | Spectrum Matches |
| --- | --- | --- | --- | --- |
| -19.8884 | 118.0571 | 0.0000 | 4.3807e+07 | 1 |

**Adduct Signal Fractions**

- **Unmodified**: 4.3807e+07

### N(N)GTYDHDVYRDEALNNR Fuc 3 Hex 5 HexNAc 5

**Mass:**  4314.7482

(1.007e-06)

**Charge States:** 4

**Start Position**: 483

**End Position**: 500

| Profile Score | Tandem Score | TDA q-value | Volume | Spectrum Matches |
| --- | --- | --- | --- | --- |
| -17.7629 | 115.8801 | 0.0000 | 3.5554e+07 | 1 |

**Adduct Signal Fractions**

- **Unmodified**: 3.5554e+07

### N(N)GTYDHDVYRDEALNNR Hex 5 HexNAc 3

**Mass:**  3470.4147

(8.966e-07)

**Charge States:** 4

**Start Position**: 483

**End Position**: 500

| Profile Score | Tandem Score | TDA q-value | Volume | Spectrum Matches |
| --- | --- | --- | --- | --- |
| -4.9382 | 115.2852 | 0.0000 | 5.4472e+07 | 1 |

**Adduct Signal Fractions**

- **Unmodified**: 5.4472e+07

### N(N)GTYDHDVYRDEALNNR Fuc 1 Hex 8 HexNAc 3

**Mass:**  4102.5876

(-9.904e-06)

**Charge States:** 3, 4

**Start Position**: 483

**End Position**: 500

| Profile Score | Tandem Score | TDA q-value | Volume | Spectrum Matches |
| --- | --- | --- | --- | --- |
| 7.3916 | 114.3151 | 0.0000 | 4.6207e+08 | 7 |

**Adduct Signal Fractions**

- **Unmodified**: 4.6207e+08

### N(N)GTYDHDVYRDEALNNR Hex 7 HexNAc 2

**Mass:**  3591.4433

(7.123e-07)

**Charge States:** 4

**Start Position**: 483

**End Position**: 500

| Profile Score | Tandem Score | TDA q-value | Volume | Spectrum Matches |
| --- | --- | --- | --- | --- |
| -17.7326 | 113.8051 | 0.0042 | 4.6506e+07 | 1 |

**Adduct Signal Fractions**

- **Unmodified**: 4.6506e+07

### N(N)GTYDHDVYRDEALN(D)NR Fuc 2 Hex 5 HexNAc 5

**Mass:**  4169.6755

(2.373e-04)

**Charge States:** 4

**Start Position**: 483

**End Position**: 500

| Profile Score | Tandem Score | TDA q-value | Volume | Spectrum Matches |
| --- | --- | --- | --- | --- |
| -20.0800 | 113.5585 | 0.0042 | 1.1880e+08 | 1 |

**Adduct Signal Fractions**

- **Unmodified**: 1.1880e+08

### N(N)GTYDHDVYR Fuc 1 Hex 6 HexNAc 3

**Mass:**  2966.1469

(7.723e-07)

**Charge States:** 3

**Start Position**: 483

**End Position**: 493

| Profile Score | Tandem Score | TDA q-value | Volume | Spectrum Matches |
| --- | --- | --- | --- | --- |
| -17.6352 | 113.4811 | 0.0042 | 4.3092e+07 | 1 |

**Adduct Signal Fractions**

- **Unmodified**: 4.3092e+07

### N(N)GTYDHDVYRDEALNNRFQIK Fuc 2 Hex 5 HexNAc 4

**Mass:**  4481.9189

(-5.696e-07)

**Charge States:** 5

**Start Position**: 483

**End Position**: 504

| Profile Score | Tandem Score | TDA q-value | Volume | Spectrum Matches |
| --- | --- | --- | --- | --- |
| -4.8685 | 112.8307 | 0.0042 | 6.8078e+07 | 2 |

**Adduct Signal Fractions**

- **Unmodified**: 6.8078e+07

### N(N)GTYDHDVYR Fuc 1 Hex 4 HexNAc 3

**Mass:**  2642.0465

(2.845e-06)

**Charge States:** 3

**Start Position**: 483

**End Position**: 493

| Profile Score | Tandem Score | TDA q-value | Volume | Spectrum Matches |
| --- | --- | --- | --- | --- |
| -21.2606 | 112.1546 | 0.0084 | 4.9272e+06 | 1 |

**Adduct Signal Fractions**

- **Unmodified**: 4.9272e+06

### SLVASSGTLEFNNESFNWAGVTHN(N)GTSSSC(C)R Hex 5 HexNAc 3

**Mass:**  4734.9865

(-1.707e-07)

**Charge States:** 4

**Start Position**: 110

**End Position**: 141

| Profile Score | Tandem Score | TDA q-value | Volume | Spectrum Matches |
| --- | --- | --- | --- | --- |
| -18.7085 | 112.0110 | 0.0119 | 1.4168e+07 | 1 |

**Adduct Signal Fractions**

- **Unmodified**: 1.4168e+07

### N(N)GTYDHDVYR Fuc 1 Hex 6 HexNAc 5

**Mass:**  3372.3074

(2.311e-06)

**Charge States:** 3

**Start Position**: 483

**End Position**: 493

| Profile Score | Tandem Score | TDA q-value | Volume | Spectrum Matches |
| --- | --- | --- | --- | --- |
| -4.9424 | 110.4591 | 0.0119 | 6.8966e+07 | 3 |

**Adduct Signal Fractions**

- **Unmodified**: 6.8966e+07

### N(N)GTYDHDVYR Hex 5 HexNAc 4

**Mass:**  2861.1163

(1.042e-06)

**Charge States:** 3

**Start Position**: 483

**End Position**: 493

| Profile Score | Tandem Score | TDA q-value | Volume | Spectrum Matches |
| --- | --- | --- | --- | --- |
| -18.4135 | 110.2913 | 0.0119 | 1.4655e+07 | 1 |

**Adduct Signal Fractions**

- **Unmodified**: 1.4655e+07

### YPALN(N)VTMPN(D)NEQFDK Hex 8 HexNAc 2

**Mass:**  3583.4467

(2.765e-04)

**Charge States:** 3

**Start Position**: 161

**End Position**: 177

| Profile Score | Tandem Score | TDA q-value | Volume | Spectrum Matches |
| --- | --- | --- | --- | --- |
| -6.2267 | 109.5071 | 0.0119 | 3.5464e+07 | 2 |

**Adduct Signal Fractions**

- **Unmodified**: 3.5464e+07

### N(N)GTYDHDVYRDEALNNR Fuc 1 Hex 9 HexNAc 4

**Mass:**  4467.7209

(-8.813e-06)

**Charge States:** 4

**Start Position**: 483

**End Position**: 500

| Profile Score | Tandem Score | TDA q-value | Volume | Spectrum Matches |
| --- | --- | --- | --- | --- |
| -17.1330 | 109.3223 | 0.0119 | 2.2170e+07 | 1 |

**Adduct Signal Fractions**

- **Unmodified**: 2.2170e+07

### N(N)GTYDHDVYRDEALNNR Hex 5 HexNAc 5

**Mass:**  3876.5727

(6.013e-07)

**Charge States:** 3, 4

**Start Position**: 483

**End Position**: 500

| Profile Score | Tandem Score | TDA q-value | Volume | Spectrum Matches |
| --- | --- | --- | --- | --- |
| -18.4984 | 108.9273 | 0.0119 | 8.4569e+07 | 1 |

**Adduct Signal Fractions**

- **Unmodified**: 8.4569e+07

### RGSN(N)SSFFSR Fuc 2 Hex 5 HexNAc 4

**Mass:**  3059.2459

(3.291e-04)

**Start Position**: 141

**End Position**: 151

| Profile Score | Tandem Score | TDA q-value | Volume | Spectrum Matches |
| --- | --- | --- | --- | --- |
| 0.0000 | 108.3394 | 0.0119 | 0.0000e+00 | 1 |

Adduct:

No Chromatogram Found

### N(N)GTYDHDVYRDEALNNR Fuc 1 Hex 5 HexNAc 4 Neu5Ac 1

**Mass:**  4110.6491

(1.184e-06)

**Charge States:** 4

**Start Position**: 483

**End Position**: 500

| Profile Score | Tandem Score | TDA q-value | Volume | Spectrum Matches |
| --- | --- | --- | --- | --- |
| -19.1001 | 105.3097 | 0.0276 | 1.1447e+07 | 1 |

**Adduct Signal Fractions**

- **Unmodified**: 1.1447e+07

### SLVASSGTLEFNNESFNWAGVTHN(N)GTSSSC(C)R Fuc 1 Hex 6 HexNAc 6 Neu5Ac 2

**Mass:**  6234.5026

(-6.178e-06)

**Charge States:** 4

**Start Position**: 110

**End Position**: 141

| Profile Score | Tandem Score | TDA q-value | Volume | Spectrum Matches |
| --- | --- | --- | --- | --- |
| -4.7223 | 103.7411 | 0.0319 | 3.7087e+07 | 1 |

**Adduct Signal Fractions**

- **Unmodified**: 3.7087e+07

### SLVASSGTLEFNNESFNWAGVTHN(N)GTSSSC(C)R Hex 5 HexNAc 5

**Mass:**  5141.1603

(3.209e-06)

**Charge States:** 4

**Start Position**: 110

**End Position**: 141

| Profile Score | Tandem Score | TDA q-value | Volume | Spectrum Matches |
| --- | --- | --- | --- | --- |
| 12.7519 | 103.6876 | 0.0319 | 6.5360e+07 | 2 |

**Adduct Signal Fractions**

- **Unmodified**: 6.5360e+07

### SEC(C)ITPN(N)GSIPN(D)DKPFQNVNR Hex 9 HexNAc 2

**Mass:**  4251.7561

(2.324e-04)

**Charge States:** 3, 4

**Start Position**: 279

**End Position**: 300

| Profile Score | Tandem Score | TDA q-value | Volume | Spectrum Matches |
| --- | --- | --- | --- | --- |
| 8.9474 | 103.6825 | 0.0319 | 5.2840e+08 | 7 |

**Adduct Signal Fractions**

- **Unmodified**: 5.2840e+08

### SLVASSGTLEFNNESFNWAGVTHN(N)GTSSSC(C)R Fuc 1 Hex 7 HexNAc 6 Neu5Ac 2

**Mass:**  6396.5495

(-4.751e-06)

**Charge States:** 4

**Start Position**: 110

**End Position**: 141

| Profile Score | Tandem Score | TDA q-value | Volume | Spectrum Matches |
| --- | --- | --- | --- | --- |
| -4.1438 | 103.5200 | 0.0319 | 6.8467e+07 | 2 |

**Adduct Signal Fractions**

- **Unmodified**: 6.8467e+07

### N(N)GTYDHDVYRDEALNNR Fuc 2 Hex 9 HexNAc 4

**Mass:**  4613.7731

(-9.747e-06)

**Charge States:** 4

**Start Position**: 483

**End Position**: 500

| Profile Score | Tandem Score | TDA q-value | Volume | Spectrum Matches |
| --- | --- | --- | --- | --- |
| -4.9951 | 103.1038 | 0.0319 | 3.4952e+07 | 1 |

**Adduct Signal Fractions**

- **Unmodified**: 3.4952e+07

### N(N)GTYDHDVYRDEALNNR Fuc 1 Hex 10 HexNAc 4

**Mass:**  4629.7736

(-8.460e-06)

**Charge States:** 4

**Start Position**: 483

**End Position**: 500

| Profile Score | Tandem Score | TDA q-value | Volume | Spectrum Matches |
| --- | --- | --- | --- | --- |
| -5.0589 | 102.3822 | 0.0319 | 3.1154e+07 | 1 |

**Adduct Signal Fractions**

- **Unmodified**: 3.1154e+07

### SLVASSGTLEFNNESFNWAGVTHN(N)GTSSSC(C)R Fuc 1 Hex 6 HexNAc 3

**Mass:**  5043.1086

(2.102e-06)

**Charge States:** 4

**Start Position**: 110

**End Position**: 141

| Profile Score | Tandem Score | TDA q-value | Volume | Spectrum Matches |
| --- | --- | --- | --- | --- |
| -5.2840 | 101.7604 | 0.0319 | 2.3394e+07 | 1 |

**Adduct Signal Fractions**

- **Unmodified**: 2.3394e+07

### N(N)GTYDHDVYRDEALN(D)NR Hex 6 HexNAc 3

**Mass:**  3633.4523

(2.720e-04)

**Charge States:** 4

**Start Position**: 483

**End Position**: 500

| Profile Score | Tandem Score | TDA q-value | Volume | Spectrum Matches |
| --- | --- | --- | --- | --- |
| -6.2071 | 101.3437 | 0.0319 | 7.3654e+07 | 2 |

**Adduct Signal Fractions**

- **Unmodified**: 7.3654e+07

### RGSN(N)SSFFSR Fuc 4 Hex 8 HexNAc 7

**Mass:**  4445.7927

(9.212e-06)

**Charge States:** 4

**Start Position**: 141

**End Position**: 151

| Profile Score | Tandem Score | TDA q-value | Volume | Spectrum Matches |
| --- | --- | --- | --- | --- |
| -20.0224 | 100.5108 | 0.0319 | 1.7307e+07 | 1 |

**Adduct Signal Fractions**

- **Unmodified**: 1.7307e+07

### ISIYWTIVKPGDILLIN(N)STGNLIAPR Hex 10 HexNAc 2

**Mass:**  4893.3239

(1.913e-07)

**Charge States:** 4

**Start Position**: 230

**End Position**: 256

| Profile Score | Tandem Score | TDA q-value | Volume | Spectrum Matches |
| --- | --- | --- | --- | --- |
| -4.1416 | 99.7082 | 0.0319 | 3.9331e+07 | 4 |

**Adduct Signal Fractions**

- **Unmodified**: 3.9331e+07

### LNWLTHLNSKYPALN(N)VTM(O)PNNEQFDK Hex 8 HexNAc 2

**Mass:**  4805.1218

(3.604e-06)

**Charge States:** 4, 5

**Start Position**: 151

**End Position**: 177

| Profile Score | Tandem Score | TDA q-value | Volume | Spectrum Matches |
| --- | --- | --- | --- | --- |
| -17.6225 | 99.4480 | 0.0319 | 4.1322e+07 | 2 |

**Adduct Signal Fractions**

- **Unmodified**: 4.1322e+07

### N(N)GTYDHDVYR Fuc 3 Hex 9 HexNAc 7

**Mass:**  4556.7575

(4.611e-06)

**Charge States:** 4

**Start Position**: 483

**End Position**: 493

| Profile Score | Tandem Score | TDA q-value | Volume | Spectrum Matches |
| --- | --- | --- | --- | --- |
| -3.8603 | 98.2528 | 0.0319 | 1.0191e+08 | 1 |

**Adduct Signal Fractions**

- **Unmodified**: 1.0191e+08

### SLVASSGTLEFNNESFNWAGVTHN(N)GTSSSC(C)R Fuc 1 Hex 8 HexNAc 6 Neu5Ac 2

**Mass:**  6558.6130

(-4.120e-06)

**Charge States:** 4, 5

**Start Position**: 110

**End Position**: 141

| Profile Score | Tandem Score | TDA q-value | Volume | Spectrum Matches |
| --- | --- | --- | --- | --- |
| -19.2655 | 97.7135 | 0.0319 | 2.9482e+07 | 2 |

**Adduct Signal Fractions**

- **Unmodified**: 2.9482e+07

### N(N)GTYDHDVYRDEALNNR Hex 6 HexNAc 4

**Mass:**  3835.5539

(1.502e-06)

**Charge States:** 4

**Start Position**: 483

**End Position**: 500

| Profile Score | Tandem Score | TDA q-value | Volume | Spectrum Matches |
| --- | --- | --- | --- | --- |
| -6.5645 | 96.9599 | 0.0319 | 2.3722e+07 | 1 |

**Adduct Signal Fractions**

- **Unmodified**: 2.3722e+07

### SLVASSGTLEFN(D)NESFNWAGVTHN(N)GTSSSC(C)R Hex 7 HexNAc 2

**Mass:**  4857.0134

(2.070e-04)

**Charge States:** 3, 4

**Start Position**: 110

**End Position**: 141

| Profile Score | Tandem Score | TDA q-value | Volume | Spectrum Matches |
| --- | --- | --- | --- | --- |
| 8.5697 | 93.5625 | 0.0412 | 6.1204e+07 | 4 |

**Adduct Signal Fractions**

- **Unmodified**: 6.1204e+07

### IRNIPSRISIYWTIVKPGDILLIN(N)STGNLIAPR Hex 8 HexNAc 2

**Mass:**  5405.7211

(1.732e-06)

**Charge States:** 5

**Start Position**: 223

**End Position**: 256

| Profile Score | Tandem Score | TDA q-value | Volume | Spectrum Matches |
| --- | --- | --- | --- | --- |
| -4.0444 | 93.2645 | 0.0412 | 1.4533e+07 | 3 |

**Adduct Signal Fractions**

- **Unmodified**: 1.4533e+07

### SEC(C)ITPN(N)GSIPN(D)DKPFQNVNR Hex 8 HexNAc 2

**Mass:**  4089.7050

(2.419e-04)

**Charge States:** 3, 4

**Start Position**: 279

**End Position**: 300

| Profile Score | Tandem Score | TDA q-value | Volume | Spectrum Matches |
| --- | --- | --- | --- | --- |
| 9.9760 | 92.6519 | 0.0412 | 3.0909e+08 | 5 |

**Adduct Signal Fractions**

- **Unmodified**: 3.0909e+08

### SEC(C)ITPN(N)GSIPN(D)DKPFQNVNR Hex 7 HexNAc 2

**Mass:**  3927.6523

(2.520e-04)

**Charge States:** 3, 4, 5

**Start Position**: 279

**End Position**: 300

| Profile Score | Tandem Score | TDA q-value | Volume | Spectrum Matches |
| --- | --- | --- | --- | --- |
| 11.0663 | 92.6014 | 0.0412 | 5.6447e+08 | 7 |

**Adduct Signal Fractions**

- **Unmodified**: 5.6447e+08

### N(N)GTYDHDVYR Fuc 2 Hex 6 HexNAc 5

**Mass:**  3518.3655

(1.186e-06)

**Charge States:** 3, 4

**Start Position**: 483

**End Position**: 493

| Profile Score | Tandem Score | TDA q-value | Volume | Spectrum Matches |
| --- | --- | --- | --- | --- |
| -19.7795 | 91.6859 | 0.0442 | 1.9780e+07 | 1 |

**Adduct Signal Fractions**

- **Unmodified**: 1.9780e+07

### cus|SWZHA|A/Switzerland/9715293/2013

**>cus|SWZHA|A/Switzerland/9715293/2013**  
 `QKLPGNDNSTATLCLGHHAVPNGTIVKTITNDRIEVTNATELVQNSSIGEICDSPHQILDGENCTLIDALLGDPQCDGFQ  
NKKWDLFVERSKAYSNCYPYDVPDYASLRSLVASSGTLEFNNESFNWAGVTQNGTSSSCRRGSNSSFFSRLNWLTHLNSK  
YPALNVTMPNNEQFDKLYIWGVHHPVTDKDQIFLYAQSSGRITVSTKRSQQAVIPNIGYRPRIRDIPSRISIYWTIVKPG  
DILLINSTGNLIAPRGYFKIRSGKSSIMRSDAPIGKCKSECITPNGSIPNDKPFQNVNRITYGACPRYVKQSTLKLATGM  
RNVPERQTRGIFGAIAGFIENGWEGMVDGWYGFRHQNSEGRGQAADLKSTQAAIDQINGKLNRLIGKTNEKFHQIEKEFS  
EVEGRIQDLEKYVEDTKIDLWSYNAELLVALENQHTIDLTDSEMNKLFEKTKKQLRENAEDMGNGCFKIYHKCDNACIGS  
IRNGTYDHDVYRDEALNNRFQIKGVELKSGYKDWILWISFAISCFLLCVALLGFIMWACQKGNIRCNICI`

| Site | Type | Count |
| --- | --- | --- |
| 483 | N-linked | 50 |
| 165 | N-linked | 14 |
| 144 | N-linked | 48 |
| 22 | N-linked | 1 |
| 285 | N-linked | 13 |
| 246 | N-linked | 10 |

| Mass | Sequence | MS_2_ Score | `q`-value | MS_1_ Score | Signal | Protein Coordinates | Tandem Scans | Start Time | Apex Time | End Time |
| --- | --- | --- | --- | --- | --- | --- | --- | --- | --- | --- |
| 3172.2867 | RGSN(N)SSFFSR Hex 5 HexNAc 6 | 189.046 | 0.0 | -18.689 | 1.666e+08 | 140 - 150 | 2 | 19.20 | 19.82 | 19.98 |
| 2522.0197 | RGSN(N)SSFFSR Hex 6 HexNAc 2 | 188.071 | 0.0 | -2.983 | 4.586e+08 | 140 - 150 | 7 | 19.03 | 19.67 | 19.67 |
| 2684.0728 | RGSN(N)SSFFSR Hex 7 HexNAc 2 | 187.594 | 0.0 | 13.172 | 2.725e+08 | 140 - 150 | 2 | 18.70 | 19.52 | 19.67 |
| 2359.9664 | RGSN(N)SSFFSR Hex 5 HexNAc 2 | 187.502 | 0.0 | 11.791 | 3.942e+08 | 140 - 150 | 2 | 19.20 | 19.82 | 19.98 |
| 2725.0996 | RGSN(N)SSFFSR Hex 6 HexNAc 3 | 185.104 | 0.0 | 13.687 | 5.833e+08 | 140 - 150 | 5 | 19.03 | 19.67 | 19.98 |
| 3058.2420 | RGSN(N)SSFFSR Fuc 2 Hex 5 HexNAc 4 | 180.960 | 0.0 | 13.653 | 3.153e+08 | 140 - 150 | 2 | 18.70 | 19.37 | 19.37 |
| 3153.2329 | N(N)GTYDHDVYR Fuc 2 Hex 5 HexNAc 4 | 180.501 | 0.0 | -2.093 | 3.150e+08 | 482 - 492 | 3 | 15.38 | 15.38 | 15.86 |
| 3261.3211 | RGSN(N)SSFFSR Fuc 2 Hex 5 HexNAc 5 | 180.090 | 0.0 | 13.603 | 4.050e+08 | 140 - 150 | 4 | 18.70 | 19.37 | 19.67 |
| 2846.1264 | RGSN(N)SSFFSR Hex 8 HexNAc 2 | 180.005 | 0.0 | -17.423 | 9.750e+07 | 140 - 150 | 1 | 19.37 | 19.37 | 19.37 |
| 2912.1840 | RGSN(N)SSFFSR Fuc 1 Hex 5 HexNAc 4 | 178.604 | 0.0 | 13.938 | 8.835e+08 | 140 - 150 | 4 | 18.87 | 19.52 | 20.26 |
| 3480.3969 | RGSN(N)SSFFSR Fuc 1 Hex 6 HexNAc 6 | 177.909 | 0.0 | -4.398 | 7.037e+07 | 140 - 150 | 2 | 19.03 | 19.37 | 19.37 |
| 2969.2063 | RGSN(N)SSFFSR Hex 5 HexNAc 5 | 176.833 | 0.0 | 11.081 | 2.917e+08 | 140 - 150 | 2 | 19.03 | 19.82 | 19.98 |
| 2871.1569 | RGSN(N)SSFFSR Fuc 1 Hex 6 HexNAc 3 | 174.716 | 0.0 | 15.209 | 3.382e+08 | 140 - 150 | 3 | 18.70 | 19.37 | 19.67 |
| 2709.1058 | RGSN(N)SSFFSR Fuc 1 Hex 5 HexNAc 3 | 172.419 | 0.0 | -18.104 | 6.777e+07 | 140 - 150 | 1 | 19.67 | 19.67 | 19.82 |
| 2766.1259 | RGSN(N)SSFFSR Hex 5 HexNAc 4 | 172.012 | 0.0 | -18.111 | 8.979e+07 | 140 - 150 | 1 | 19.67 | 19.67 | 19.82 |
| 3582.4658 | YPALN(N)VTMPNNEQFDK Hex 8 HexNAc 2 | 171.966 | 0.0 | -1.736 | 3.745e+08 | 160 - 176 | 8 | 41.99 | 42.32 | 42.49 |
| 2547.0510 | RGSN(N)SSFFSR Fuc 1 Hex 4 HexNAc 3 | 171.901 | 0.0 | -20.010 | 2.363e+07 | 140 - 150 | 1 | 20.12 | 20.12 | 20.12 |
| 3760.5099 | YPALN(N)VTM(O)PNNEQFDK Hex 9 HexNAc 2 | 171.505 | 0.0 | 13.528 | 3.085e+08 | 160 - 176 | 4 | 34.34 | 34.51 | 35.00 |
| 3115.2636 | RGSN(N)SSFFSR Fuc 1 Hex 5 HexNAc 5 | 171.144 | 0.0 | 14.271 | 1.582e+09 | 140 - 150 | 6 | 19.03 | 19.82 | 20.72 |
| 3204.3001 | RGSN(N)SSFFSR Fuc 3 Hex 5 HexNAc 4 | 171.008 | 0.0 | -17.809 | 1.319e+08 | 140 - 150 | 1 | 19.03 | 19.20 | 19.20 |
| 3598.4617 | YPALN(N)VTM(O)PNNEQFDK Hex 8 HexNAc 2 | 170.509 | 0.0 | -0.697 | 3.429e+09 | 160 - 176 | 15 | 34.18 | 34.67 | 112.87 |
| 3436.4033 | YPALN(N)VTM(O)PNNEQFDK Hex 7 HexNAc 2 | 168.029 | 0.0 | 14.355 | 6.222e+08 | 160 - 176 | 4 | 34.34 | 34.83 | 35.16 |
| 4068.9337 | TIVKPGDILLIN(N)STGNLIAPR Hex 9 HexNAc 2 | 165.046 | 0.0 | 17.964 | 6.197e+08 | 234 - 255 | 7 | 52.63 | 53.14 | 53.83 |
| 3277.3165 | RGSN(N)SSFFSR Fuc 1 Hex 6 HexNAc 5 | 160.990 | 0.0 | -3.978 | 1.121e+08 | 140 - 150 | 4 | 19.03 | 19.20 | 19.37 |
| 3007.1750 | N(N)GTYDHDVYR Fuc 1 Hex 5 HexNAc 4 | 160.824 | 0.0 | 16.137 | 5.201e+08 | 482 - 492 | 5 | 15.38 | 15.54 | 16.03 |
| 3178.2321 | RGSN(N)SSFFSR Hex 7 HexNAc 3 Neu5Ac 1 | 160.665 | 0.0 | -20.426 | 7.455e+06 | 140 - 150 | 1 | 22.35 | 22.35 | 22.35 |
| 2928.1800 | RGSN(N)SSFFSR Hex 6 HexNAc 4 | 160.171 | 0.0 | 11.739 | 1.304e+08 | 140 - 150 | 1 | 19.03 | 19.52 | 19.98 |
| 3210.2548 | N(N)GTYDHDVYR Fuc 1 Hex 5 HexNAc 5 | 159.254 | 0.0 | -3.365 | 2.080e+08 | 482 - 492 | 5 | 15.54 | 15.70 | 16.03 |
| 4063.5808 | RGSN(N)SSFFSR Fuc 3 Hex 6 HexNAc 6 Neu5Ac 1 | 158.992 | 0.0 | -20.819 | 1.467e+07 | 140 - 150 | 1 | 21.85 | 21.85 | 21.85 |
| 3420.4120 | YPALN(N)VTMPNNEQFDK Hex 7 HexNAc 2 | 158.963 | 0.0 | -3.217 | 6.271e+07 | 160 - 176 | 3 | 42.16 | 42.32 | 42.49 |
| 2568.9979 | GSN(N)SSFFSR Hex 6 HexNAc 3 | 158.274 | 0.0 | -3.316 | 2.674e+08 | 141 - 150 | 3 | 23.00 | 23.15 | 23.31 |
| 3906.8786 | TIVKPGDILLIN(N)STGNLIAPR Hex 8 HexNAc 2 | 157.814 | 0.0 | -18.434 | 5.777e+07 | 234 - 255 | 2 | 53.14 | 53.14 | 53.31 |
| 2693.1059 | RGSN(N)SSFFSR Fuc 2 Hex 4 HexNAc 3 | 157.566 | 0.0 | -17.296 | 2.351e+07 | 140 - 150 | 1 | 19.82 | 19.82 | 19.82 |
| 2756.0832 | GSN(N)SSFFSR Fuc 1 Hex 5 HexNAc 4 | 155.980 | 0.0 | 12.287 | 3.116e+08 | 141 - 150 | 4 | 22.18 | 23.00 | 23.77 |
| 3777.5388 | YPALN(N)VTM(O)PNNEQFDK Fuc 2 Hex 3 HexNAc 4 Neu5Ac 1 | 155.518 | 0.0 | -5.223 | 3.780e+07 | 160 - 176 | 1 | 34.51 | 34.51 | 34.83 |
| 2690.0237 | GSN(N)SSFFSR Hex 8 HexNAc 2 | 155.434 | 0.0 | -18.804 | 2.923e+07 | 141 - 150 | 1 | 22.68 | 22.68 | 22.68 |
| 3274.3497 | YPALN(N)VTM(O)PNNEQFDK Hex 6 HexNAc 2 | 155.283 | 0.0 | -5.624 | 4.116e+07 | 160 - 176 | 1 | 34.83 | 35.00 | 35.16 |
| 4085.9574 | TIVKPGDILLIN(N)STGNLIAPR Fuc 2 Hex 3 HexNAc 4 Neu5Ac 1 | 155.157 | 0.0 | -3.530 | 5.876e+07 | 234 - 255 | 2 | 52.97 | 53.14 | 53.31 |
| 4376.8440 | C(C)KSEC(C)ITPN(N)GSIPNDKPFQNVNR Hex 8 HexNAc 2 | 155.028 | 0.0 | -3.533 | 1.399e+08 | 276 - 299 | 1 | 29.14 | 29.31 | 29.47 |
| 3112.2989 | YPALN(N)VTM(O)PNNEQFDK Hex 5 HexNAc 2 | 154.715 | 0.0 | -3.521 | 4.223e+07 | 160 - 176 | 1 | 35.16 | 35.33 | 35.49 |
| 3048.1970 | GSN(N)SSFFSR Fuc 3 Hex 5 HexNAc 4 | 154.487 | 0.0 | -18.467 | 5.005e+07 | 141 - 150 | 1 | 22.18 | 22.18 | 22.35 |
| 2959.1621 | GSN(N)SSFFSR Fuc 1 Hex 5 HexNAc 5 | 154.188 | 0.0 | 13.621 | 9.092e+08 | 141 - 150 | 5 | 22.35 | 23.15 | 23.77 |
| 2553.0027 | GSN(N)SSFFSR Fuc 1 Hex 5 HexNAc 3 | 152.964 | 0.0 | -19.571 | 3.018e+07 | 141 - 150 | 1 | 23.15 | 23.15 | 23.31 |
| 3744.5195 | YPALN(N)VTMPNNEQFDK Hex 9 HexNAc 2 | 152.849 | 0.0 | -4.939 | 2.832e+07 | 160 - 176 | 3 | 41.99 | 42.32 | 42.32 |
| 3007.3189 | HAVPN(N)GTIVK Fuc 1 Hex 5 HexNAc 5 | 151.370 | 0.0 | 0.000 | 0.000e+00 | 17 - 27 | 1 | - | - | - |
| 2715.0549 | GSN(N)SSFFSR Fuc 1 Hex 6 HexNAc 3 | 149.446 | 0.0 | -4.339 | 1.127e+08 | 141 - 150 | 2 | 22.68 | 22.84 | 23.00 |
| 2365.9178 | GSN(N)SSFFSR Hex 6 HexNAc 2 | 148.842 | 0.0 | -4.019 | 2.181e+08 | 141 - 150 | 3 | 23.00 | 23.15 | 23.46 |
| 4748.2959 | ISIYWTIVKPGDILLIN(N)STGNLIAPR Fuc 2 Hex 3 HexNAc 4 Neu5Ac 1 | 148.667 | 0.0 | 13.381 | 1.923e+08 | 229 - 255 | 4 | 68.40 | 68.91 | 69.08 |
| 4052.7388 | C(C)KSEC(C)ITPN(N)GSIPNDKPFQNVNR Hex 6 HexNAc 2 | 148.655 | 0.0 | -18.276 | 3.278e+07 | 276 - 299 | 2 | 29.31 | 29.31 | 29.47 |
| 2203.8657 | GSN(N)SSFFSR Hex 5 HexNAc 2 | 148.592 | 0.0 | -2.772 | 2.887e+08 | 141 - 150 | 3 | 23.31 | 23.46 | 23.77 |
| 3779.5122 | N(N)GTYDHDVYRDEALN(D)NR Fuc 1 Hex 6 HexNAc 3 | 148.254 | 0.0 | -20.785 | 3.711e+07 | 482 - 499 | 3 | 20.72 | 20.72 | 20.72 |
| 4731.2724 | ISIYWTIVKPGDILLIN(N)STGNLIAPR Hex 9 HexNAc 2 | 148.106 | 0.0 | 17.085 | 8.550e+08 | 229 - 255 | 8 | 68.40 | 68.91 | 69.42 |
| 4538.8961 | C(C)KSEC(C)ITPN(N)GSIPNDKPFQNVNR Hex 9 HexNAc 2 | 148.097 | 0.0 | -3.908 | 1.970e+08 | 276 - 299 | 2 | 29.14 | 29.14 | 29.47 |
| 4230.9874 | TIVKPGDILLIN(N)STGNLIAPR Hex 10 HexNAc 2 | 148.060 | 0.0 | -17.675 | 1.675e+07 | 234 - 255 | 1 | 52.97 | 53.14 | 53.14 |
| 4214.7907 | C(C)KSEC(C)ITPN(N)GSIPNDKPFQNVNR Hex 7 HexNAc 2 | 147.670 | 0.0 | -3.567 | 2.358e+08 | 276 - 299 | 4 | 29.14 | 29.31 | 29.47 |
| 3113.3095 | YPALN(N)VTM(O)PNNEQFDK Hex 5 HexNAc 2 | 147.640 | 0.0 | 0.000 | 0.000e+00 | 160 - 176 | 1 | - | - | - |
| 4048.6379 | RGSN(N)SSFFSR Fuc 1 Hex 7 HexNAc 8 | 145.192 | 0.0 | -19.342 | 2.841e+07 | 140 - 150 | 1 | 22.84 | 22.84 | 22.84 |
| 3423.3732 | RGSN(N)SSFFSR Fuc 2 Hex 6 HexNAc 5 | 144.097 | 0.0 | -5.563 | 2.878e+07 | 140 - 150 | 1 | 18.87 | 19.03 | 19.37 |
| 4394.7033 | RGSN(N)SSFFSR Fuc 1 Hex 5 HexNAc 7 Neu5Ac 3 | 143.964 | 0.0 | -18.547 | 2.714e+07 | 140 - 150 | 1 | 21.69 | 21.69 | 21.69 |
| 2813.1041 | GSN(N)SSFFSR Hex 5 HexNAc 5 | 143.782 | 0.0 | 8.503 | 2.094e+08 | 141 - 150 | 3 | 22.84 | 23.31 | 23.46 |
| 3299.2900 | N(N)GTYDHDVYR Fuc 3 Hex 5 HexNAc 4 | 143.580 | 0.0 | -18.789 | 8.697e+07 | 482 - 492 | 2 | 15.23 | 15.38 | 15.38 |
| 4569.2161 | ISIYWTIVKPGDILLIN(N)STGNLIAPR Hex 8 HexNAc 2 | 143.367 | 0.0 | -4.057 | 8.710e+07 | 229 - 255 | 5 | 68.74 | 68.91 | 69.25 |
| 3820.5385 | N(N)GTYDHDVYRDEALN(D)NR Fuc 1 Hex 5 HexNAc 4 | 142.361 | 0.0 | 4.427 | 1.330e+09 | 482 - 499 | 13 | 20.72 | 20.87 | 21.52 |
| 3778.5278 | N(N)GTYDHDVYRDEALNNR Fuc 1 Hex 6 HexNAc 3 | 141.966 | 0.0 | 12.968 | 3.345e+08 | 482 - 499 | 1 | 19.98 | 20.12 | 20.57 |
| 3318.3428 | RGSN(N)SSFFSR Fuc 1 Hex 5 HexNAc 6 | 140.815 | 0.0 | -19.721 | 5.816e+07 | 140 - 150 | 1 | 19.52 | 19.52 | 19.67 |
| 3967.8858 | IVKPGDILLIN(N)STGNLIAPR Hex 9 HexNAc 2 | 139.947 | 0.0 | -18.113 | 1.125e+07 | 235 - 255 | 1 | 52.29 | 52.46 | 52.46 |
| 3454.4199 | N(N)GTYDHDVYRDEALNNR Fuc 1 Hex 4 HexNAc 3 | 139.477 | 0.0 | -4.238 | 1.321e+08 | 482 - 499 | 1 | 20.41 | 20.57 | 20.72 |
| 3334.3328 | RGSN(N)SSFFSR Hex 6 HexNAc 6 | 139.454 | 0.0 | -18.481 | 1.403e+07 | 140 - 150 | 1 | 19.20 | 19.20 | 19.20 |
| 2902.1406 | GSN(N)SSFFSR Fuc 2 Hex 5 HexNAc 4 | 139.095 | 0.0 | -18.627 | 1.040e+08 | 141 - 150 | 3 | 22.51 | 22.68 | 22.68 |
| 3105.2217 | GSN(N)SSFFSR Fuc 2 Hex 5 HexNAc 5 | 138.644 | 0.0 | -18.737 | 9.978e+07 | 141 - 150 | 1 | 22.68 | 22.68 | 22.84 |
| 3965.6111 | N(N)GTYDHDVYRDEALNNR Fuc 2 Hex 5 HexNAc 4 | 138.287 | 0.0 | 0.136 | 2.178e+09 | 482 - 499 | 5 | 19.82 | 20.12 | 112.87 |
| 2772.0762 | GSN(N)SSFFSR Hex 6 HexNAc 4 | 137.455 | 0.0 | -5.119 | 2.949e+07 | 141 - 150 | 1 | 23.15 | 23.46 | 23.46 |
| 2406.9428 | GSN(N)SSFFSR Hex 5 HexNAc 3 | 137.306 | 0.0 | -5.382 | 3.447e+07 | 141 - 150 | 1 | 23.31 | 23.46 | 23.62 |
| 3437.3917 | YPALN(N)VTM(O)PN(D)NEQFDK Hex 7 HexNAc 2 | 136.546 | 0.0 | 9.263 | 1.011e+08 | 160 - 176 | 4 | 35.16 | 35.49 | 36.33 |
| 3632.4680 | N(N)GTYDHDVYRDEALNNR Hex 6 HexNAc 3 | 136.320 | 0.0 | -3.315 | 3.415e+08 | 482 - 499 | 3 | 20.26 | 20.41 | 20.72 |
| 3017.2157 | RGSN(N)SSFFSR Fuc 2 Hex 6 HexNAc 3 | 136.165 | 0.0 | -17.878 | 2.919e+07 | 140 - 150 | 2 | 19.37 | 19.37 | 19.37 |
| 3599.4451 | YPALN(N)VTM(O)PN(D)NEQFDK Hex 8 HexNAc 2 | 135.824 | 0.0 | 9.814 | 5.734e+08 | 160 - 176 | 7 | 35.16 | 35.49 | 36.84 |
| 3267.3352 | N(N)GTYDHDVYRDEALNNR Hex 5 HexNAc 2 | 135.720 | 0.0 | -5.182 | 1.130e+08 | 482 - 499 | 2 | 20.41 | 20.57 | 20.72 |
| 3926.6664 | SEC(C)ITPN(N)GSIPNDKPFQNVNR Hex 7 HexNAc 2 | 135.520 | 0.0 | 15.714 | 1.866e+09 | 278 - 299 | 5 | 31.87 | 32.03 | 32.51 |
| 3627.6373 | PGDILLIN(N)STGNLIAPR Hex 9 HexNAc 2 | 135.342 | 0.0 | -20.070 | 2.572e+07 | 238 - 255 | 1 | 51.27 | 51.44 | 51.44 |
| 3600.4790 | N(N)GTYDHDVYRDEALNNR Fuc 2 Hex 4 HexNAc 3 | 133.590 | 0.0 | -4.071 | 9.557e+07 | 482 - 499 | 1 | 20.41 | 20.41 | 20.72 |
| 4022.6311 | N(N)GTYDHDVYRDEALNNR Fuc 1 Hex 5 HexNAc 5 | 133.119 | 0.0 | 20.913 | 2.407e+09 | 482 - 499 | 7 | 19.52 | 20.41 | 20.57 |
| 4248.6471 | N(N)GTYDHDVYRDEALNNR Fuc 2 Hex 8 HexNAc 3 | 132.549 | 0.0 | -3.875 | 1.847e+08 | 482 - 499 | 3 | 21.36 | 21.85 | 21.85 |
| 3819.5521 | N(N)GTYDHDVYRDEALNNR Fuc 1 Hex 5 HexNAc 4 | 131.954 | 0.0 | 2.077 | 4.273e+09 | 482 - 499 | 6 | 19.98 | 20.26 | 112.87 |
| 3626.4611 | RGSN(N)SSFFSR Fuc 2 Hex 6 HexNAc 6 | 130.716 | 0.0 | -20.072 | 3.751e+06 | 140 - 150 | 2 | 19.20 | 19.20 | 19.20 |
| 3616.4734 | N(N)GTYDHDVYRDEALNNR Fuc 1 Hex 5 HexNAc 3 | 130.288 | 0.0 | -4.257 | 1.465e+08 | 482 - 499 | 1 | 20.26 | 20.41 | 20.72 |
| 2820.0899 | N(N)GTYDHDVYR Hex 6 HexNAc 3 | 130.134 | 0.0 | -17.289 | 2.415e+07 | 482 - 492 | 2 | 15.54 | 15.54 | 15.54 |
| 3764.6127 | SEC(C)ITPN(N)GSIPNDKPFQNVNR Hex 6 HexNAc 2 | 130.007 | 0.0 | -2.777 | 2.539e+08 | 278 - 299 | 2 | 32.19 | 32.19 | 32.51 |
| 4387.7637 | N(N)GTYDHDVYRDEALNNR Fuc 1 Hex 6 HexNAc 6 | 129.953 | 0.0 | -4.569 | 1.243e+08 | 482 - 499 | 1 | 20.12 | 20.26 | 20.41 |
| 4088.7187 | SEC(C)ITPN(N)GSIPNDKPFQNVNR Hex 8 HexNAc 2 | 129.733 | 0.0 | 13.860 | 1.088e+09 | 278 - 299 | 5 | 31.87 | 32.03 | 32.51 |
| 2527.9635 | GSN(N)SSFFSR Hex 7 HexNAc 2 | 129.553 | 0.038 | -4.817 | 5.100e+07 | 141 - 150 | 1 | 22.68 | 22.84 | 23.00 |
| 3899.5080 | N(N)GTYDHDVYRDEALNNR Fuc 1 Hex 8 HexNAc 2 | 128.256 | 0.0 | 13.561 | 7.389e+08 | 482 - 499 | 5 | 21.69 | 22.02 | 22.68 |
| 4330.7416 | N(N)GTYDHDVYRDEALNNR Fuc 2 Hex 6 HexNAc 5 | 128.060 | 0.0 | -4.558 | 1.662e+08 | 482 - 499 | 1 | 19.82 | 19.98 | 20.12 |
| 3673.4954 | N(N)GTYDHDVYRDEALNNR Hex 5 HexNAc 4 | 128.006 | 0.0 | -4.006 | 1.979e+08 | 482 - 499 | 2 | 20.26 | 20.41 | 20.57 |
| 4184.6856 | N(N)GTYDHDVYRDEALNNR Fuc 1 Hex 6 HexNAc 5 | 127.629 | 0.0 | 11.963 | 4.486e+08 | 482 - 499 | 2 | 19.82 | 19.98 | 20.41 |
| 3966.5972 | N(N)GTYDHDVYRDEALN(D)NR Fuc 2 Hex 5 HexNAc 4 | 126.603 | 0.0 | 4.869 | 8.642e+08 | 482 - 499 | 4 | 20.57 | 20.72 | 21.36 |
| 4250.7716 | SEC(C)ITPN(N)GSIPNDKPFQNVNR Hex 9 HexNAc 2 | 126.439 | 0.0 | -2.448 | 1.670e+09 | 278 - 299 | 4 | 31.87 | 31.87 | 32.35 |
| 3407.3803 | RGSN(N)SSFFSR Fuc 3 Hex 5 HexNAc 5 | 125.430 | 0.0 | -18.173 | 5.573e+07 | 140 - 150 | 2 | 19.03 | 19.20 | 19.20 |
| 4112.6563 | N(N)GTYDHDVYRDEALN(D)NR Fuc 3 Hex 5 HexNAc 4 | 123.796 | 0.0 | 8.827 | 3.320e+08 | 482 - 499 | 3 | 20.41 | 20.57 | 21.36 |
| 3762.5304 | N(N)GTYDHDVYRDEALNNR Fuc 2 Hex 5 HexNAc 3 | 122.412 | 0.0 | -4.465 | 7.200e+07 | 482 - 499 | 1 | 20.12 | 20.26 | 20.41 |
| 4111.6690 | N(N)GTYDHDVYRDEALNNR Fuc 3 Hex 5 HexNAc 4 | 122.196 | 0.0 | -2.675 | 6.350e+08 | 482 - 499 | 2 | 19.82 | 19.98 | 20.26 |
| 4264.6394 | N(N)GTYDHDVYRDEALNNR Fuc 1 Hex 9 HexNAc 3 | 122.141 | 0.0 | 7.113 | 1.552e+08 | 482 - 499 | 3 | 21.52 | 21.69 | 22.18 |
| 3356.3128 | N(N)GTYDHDVYR Fuc 2 Hex 5 HexNAc 5 | 121.256 | 0.0 | -19.097 | 4.659e+07 | 482 - 492 | 3 | 15.38 | 15.54 | 15.54 |
| 4168.6900 | N(N)GTYDHDVYRDEALNNR Fuc 2 Hex 5 HexNAc 5 | 119.896 | 0.0 | 11.554 | 6.072e+08 | 482 - 499 | 4 | 19.37 | 20.12 | 20.57 |
| 3602.5604 | SEC(C)ITPN(N)GSIPNDKPFQNVNR Hex 5 HexNAc 2 | 119.152 | 0.0 | -18.792 | 9.990e+07 | 278 - 299 | 2 | 32.35 | 32.35 | 32.51 |
| 4267.7974 | SEC(C)ITPN(N)GSIPNDKPFQNVNR Fuc 2 Hex 3 HexNAc 4 Neu5Ac 1 | 118.809 | 0.0 | -18.952 | 9.893e+07 | 278 - 299 | 1 | 31.87 | 31.87 | 32.03 |
| 3090.2313 | RGSN(N)SSFFSR Hex 7 HexNAc 4 | 118.490 | 0.0 | -18.640 | 2.004e+07 | 140 - 150 | 1 | 19.52 | 19.52 | 19.67 |
| 4410.7011 | N(N)GTYDHDVYRDEALNNR Fuc 2 Hex 9 HexNAc 3 | 118.140 | 0.0 | -4.477 | 1.177e+08 | 482 - 499 | 1 | 21.52 | 21.69 | 21.85 |
| 3794.5210 | N(N)GTYDHDVYRDEALNNR Hex 7 HexNAc 3 | 118.057 | 0.0 | -19.888 | 4.381e+07 | 482 - 499 | 1 | 20.12 | 20.26 | 20.26 |
| 4314.7482 | N(N)GTYDHDVYRDEALNNR Fuc 3 Hex 5 HexNAc 5 | 115.880 | 0.0 | -17.763 | 3.555e+07 | 482 - 499 | 1 | 19.98 | 19.98 | 19.98 |
| 3470.4151 | N(N)GTYDHDVYRDEALNNR Hex 5 HexNAc 3 | 115.285 | 0.0 | -4.938 | 5.447e+07 | 482 - 499 | 1 | 20.41 | 20.57 | 20.72 |
| 4102.5882 | N(N)GTYDHDVYRDEALNNR Fuc 1 Hex 8 HexNAc 3 | 114.315 | 0.0 | 7.392 | 4.621e+08 | 482 - 499 | 7 | 21.20 | 22.02 | 22.68 |
| 3591.4420 | N(N)GTYDHDVYRDEALNNR Hex 7 HexNAc 2 | 113.805 | 0.004 | -17.733 | 4.651e+07 | 482 - 499 | 1 | 20.12 | 20.12 | 20.26 |
| 4169.6779 | N(N)GTYDHDVYRDEALN(D)NR Fuc 2 Hex 5 HexNAc 5 | 113.559 | 0.004 | -20.080 | 1.188e+08 | 482 - 499 | 1 | 20.72 | 20.72 | 20.87 |
| 2966.1470 | N(N)GTYDHDVYR Fuc 1 Hex 6 HexNAc 3 | 113.481 | 0.004 | -17.635 | 4.309e+07 | 482 - 492 | 1 | 15.38 | 15.38 | 15.54 |
| 4481.9033 | N(N)GTYDHDVYRDEALNNRFQIK Fuc 2 Hex 5 HexNAc 4 | 112.831 | 0.004 | -4.868 | 6.808e+07 | 482 - 503 | 2 | 31.71 | 31.87 | 32.19 |
| 2642.0465 | N(N)GTYDHDVYR Fuc 1 Hex 4 HexNAc 3 | 112.155 | 0.008 | -21.261 | 4.927e+06 | 482 - 492 | 1 | 15.70 | 15.70 | 15.70 |
| 3372.3076 | N(N)GTYDHDVYR Fuc 1 Hex 6 HexNAc 5 | 110.459 | 0.012 | -4.942 | 6.897e+07 | 482 - 492 | 3 | 15.38 | 15.38 | 15.70 |
| 2861.1163 | N(N)GTYDHDVYR Hex 5 HexNAc 4 | 110.291 | 0.012 | -18.413 | 1.466e+07 | 482 - 492 | 1 | 15.54 | 15.54 | 15.54 |
| 3583.4483 | YPALN(N)VTMPN(D)NEQFDK Hex 8 HexNAc 2 | 109.507 | 0.012 | -6.227 | 3.546e+07 | 160 - 176 | 2 | 43.17 | 43.33 | 43.50 |
| 4467.7209 | N(N)GTYDHDVYRDEALNNR Fuc 1 Hex 9 HexNAc 4 | 109.322 | 0.012 | -17.133 | 2.217e+07 | 482 - 499 | 1 | 21.85 | 21.85 | 21.85 |
| 3876.5728 | N(N)GTYDHDVYRDEALNNR Hex 5 HexNAc 5 | 108.927 | 0.012 | -18.498 | 8.457e+07 | 482 - 499 | 1 | 20.41 | 20.41 | 20.41 |
| 3059.2459 | RGSN(N)SSFFSR Fuc 2 Hex 5 HexNAc 4 | 108.339 | 0.012 | 0.000 | 0.000e+00 | 140 - 150 | 1 | - | - | - |
| 4110.6491 | N(N)GTYDHDVYRDEALNNR Fuc 1 Hex 5 HexNAc 4 Neu5Ac 1 | 105.310 | 0.028 | -19.100 | 1.145e+07 | 482 - 499 | 1 | 21.69 | 21.69 | 21.69 |
| 4251.7565 | SEC(C)ITPN(N)GSIPN(D)DKPFQNVNR Hex 9 HexNAc 2 | 103.682 | 0.032 | 8.947 | 5.284e+08 | 278 - 299 | 7 | 32.51 | 33.34 | 33.51 |
| 4613.7764 | N(N)GTYDHDVYRDEALNNR Fuc 2 Hex 9 HexNAc 4 | 103.104 | 0.032 | -4.995 | 3.495e+07 | 482 - 499 | 1 | 21.52 | 21.69 | 21.85 |
| 4629.7743 | N(N)GTYDHDVYRDEALNNR Fuc 1 Hex 10 HexNAc 4 | 102.382 | 0.032 | -5.059 | 3.115e+07 | 482 - 499 | 1 | 21.36 | 21.52 | 21.69 |
| 3633.4539 | N(N)GTYDHDVYRDEALN(D)NR Hex 6 HexNAc 3 | 101.344 | 0.032 | -6.207 | 7.365e+07 | 482 - 499 | 2 | 20.87 | 20.87 | 21.52 |
| 4445.7915 | RGSN(N)SSFFSR Fuc 4 Hex 8 HexNAc 7 | 100.511 | 0.032 | -20.022 | 1.731e+07 | 140 - 150 | 1 | 21.04 | 21.20 | 21.20 |
| 4893.3257 | ISIYWTIVKPGDILLIN(N)STGNLIAPR Hex 10 HexNAc 2 | 99.708 | 0.032 | -4.142 | 3.933e+07 | 229 - 255 | 4 | 68.57 | 68.74 | 68.91 |
| 4805.1207 | LNWLTHLNSKYPALN(N)VTM(O)PNNEQFDK Hex 8 HexNAc 2 | 99.448 | 0.032 | -17.623 | 4.132e+07 | 150 - 176 | 2 | 47.87 | 47.87 | 48.04 |
| 4556.7577 | N(N)GTYDHDVYR Fuc 3 Hex 9 HexNAc 7 | 98.253 | 0.032 | -3.860 | 1.019e+08 | 482 - 492 | 1 | 21.36 | 21.52 | 21.85 |
| 3835.5496 | N(N)GTYDHDVYRDEALNNR Hex 6 HexNAc 4 | 96.960 | 0.032 | -6.565 | 2.372e+07 | 482 - 499 | 1 | 20.26 | 20.26 | 20.57 |
| 4089.7040 | SEC(C)ITPN(N)GSIPN(D)DKPFQNVNR Hex 8 HexNAc 2 | 92.652 | 0.041 | 9.976 | 3.091e+08 | 278 - 299 | 5 | 32.68 | 33.34 | 33.51 |
| 3927.6518 | SEC(C)ITPN(N)GSIPN(D)DKPFQNVNR Hex 7 HexNAc 2 | 92.601 | 0.041 | 11.066 | 5.645e+08 | 278 - 299 | 7 | 32.68 | 33.51 | 33.67 |
| 3518.3645 | N(N)GTYDHDVYR Fuc 2 Hex 6 HexNAc 5 | 91.686 | 0.044 | -19.779 | 1.978e+07 | 482 - 492 | 1 | 15.38 | 15.38 | 15.38 |

### RGSN(N)SSFFSR Hex 5 HexNAc 6

**Mass:**  3172.2899

(-4.736e-07)

**Charge States:** 3

**Start Position**: 141

**End Position**: 151

| Profile Score | Tandem Score | TDA q-value | Volume | Spectrum Matches |
| --- | --- | --- | --- | --- |
| -18.6890 | 189.0458 | 0.0000 | 1.6657e+08 | 2 |

**Adduct Signal Fractions**

- **Unmodified**: 1.6657e+08

### RGSN(N)SSFFSR Hex 6 HexNAc 2

**Mass:**  2522.0200

(6.925e-07)

**Charge States:** 3

**Start Position**: 141

**End Position**: 151

| Profile Score | Tandem Score | TDA q-value | Volume | Spectrum Matches |
| --- | --- | --- | --- | --- |
| -2.9829 | 188.0709 | 0.0000 | 4.5858e+08 | 7 |

**Adduct Signal Fractions**

- **Unmodified**: 4.5858e+08

### RGSN(N)SSFFSR Hex 7 HexNAc 2

**Mass:**  2684.0727

(6.305e-07)

**Charge States:** 3

**Start Position**: 141

**End Position**: 151

| Profile Score | Tandem Score | TDA q-value | Volume | Spectrum Matches |
| --- | --- | --- | --- | --- |
| 13.1722 | 187.5943 | 0.0000 | 2.7247e+08 | 2 |

**Adduct Signal Fractions**

- **Unmodified**: 2.7247e+08

### RGSN(N)SSFFSR Hex 5 HexNAc 2

**Mass:**  2359.9663

(5.791e-07)

**Charge States:** 3

**Start Position**: 141

**End Position**: 151

| Profile Score | Tandem Score | TDA q-value | Volume | Spectrum Matches |
| --- | --- | --- | --- | --- |
| 11.7911 | 187.5019 | 0.0000 | 3.9425e+08 | 2 |

**Adduct Signal Fractions**

- **Unmodified**: 3.9425e+08

### RGSN(N)SSFFSR Hex 6 HexNAc 3

**Mass:**  2725.0995

(1.024e-06)

**Charge States:** 3

**Start Position**: 141

**End Position**: 151

| Profile Score | Tandem Score | TDA q-value | Volume | Spectrum Matches |
| --- | --- | --- | --- | --- |
| 13.6872 | 185.1040 | 0.0000 | 5.8326e+08 | 5 |

**Adduct Signal Fractions**

- **Unmodified**: 5.8326e+08

### RGSN(N)SSFFSR Fuc 2 Hex 5 HexNAc 4

**Mass:**  3058.2420

(5.766e-07)

**Charge States:** 3

**Start Position**: 141

**End Position**: 151

| Profile Score | Tandem Score | TDA q-value | Volume | Spectrum Matches |
| --- | --- | --- | --- | --- |
| 13.6525 | 180.9597 | 0.0000 | 3.1527e+08 | 2 |

**Adduct Signal Fractions**

- **Unmodified**: 3.1527e+08

### N(N)GTYDHDVYR Fuc 2 Hex 5 HexNAc 4

**Mass:**  3153.2327

(1.164e-06)

**Charge States:** 3

**Start Position**: 483

**End Position**: 493

| Profile Score | Tandem Score | TDA q-value | Volume | Spectrum Matches |
| --- | --- | --- | --- | --- |
| -2.0928 | 180.5015 | 0.0000 | 3.1500e+08 | 3 |

**Adduct Signal Fractions**

- **Unmodified**: 3.1500e+08

### RGSN(N)SSFFSR Fuc 2 Hex 5 HexNAc 5

**Mass:**  3261.3213

(6.752e-07)

**Charge States:** 3

**Start Position**: 141

**End Position**: 151

| Profile Score | Tandem Score | TDA q-value | Volume | Spectrum Matches |
| --- | --- | --- | --- | --- |
| 13.6028 | 180.0905 | 0.0000 | 4.0498e+08 | 4 |

**Adduct Signal Fractions**

- **Unmodified**: 4.0498e+08

### RGSN(N)SSFFSR Hex 8 HexNAc 2

**Mass:**  2846.1264

(1.023e-06)

**Charge States:** 3

**Start Position**: 141

**End Position**: 151

| Profile Score | Tandem Score | TDA q-value | Volume | Spectrum Matches |
| --- | --- | --- | --- | --- |
| -17.4235 | 180.0047 | 0.0000 | 9.7495e+07 | 1 |

**Adduct Signal Fractions**

- **Unmodified**: 9.7495e+07

### RGSN(N)SSFFSR Fuc 1 Hex 5 HexNAc 4

**Mass:**  2912.1835

(2.078e-06)

**Charge States:** 3

**Start Position**: 141

**End Position**: 151

| Profile Score | Tandem Score | TDA q-value | Volume | Spectrum Matches |
| --- | --- | --- | --- | --- |
| 13.9382 | 178.6039 | 0.0000 | 8.8351e+08 | 4 |

**Adduct Signal Fractions**

- **Unmodified**: 8.8351e+08

### RGSN(N)SSFFSR Fuc 1 Hex 6 HexNAc 6

**Mass:**  3480.3960

(8.568e-07)

**Charge States:** 3, 4

**Start Position**: 141

**End Position**: 151

| Profile Score | Tandem Score | TDA q-value | Volume | Spectrum Matches |
| --- | --- | --- | --- | --- |
| -4.3977 | 177.9086 | 0.0000 | 7.0371e+07 | 2 |

**Adduct Signal Fractions**

- **Unmodified**: 7.0371e+07

### RGSN(N)SSFFSR Hex 5 HexNAc 5

**Mass:**  2969.2062

(1.112e-06)

**Charge States:** 3

**Start Position**: 141

**End Position**: 151

| Profile Score | Tandem Score | TDA q-value | Volume | Spectrum Matches |
| --- | --- | --- | --- | --- |
| 11.0806 | 176.8326 | 0.0000 | 2.9170e+08 | 2 |

**Adduct Signal Fractions**

- **Unmodified**: 2.9170e+08

### RGSN(N)SSFFSR Fuc 1 Hex 6 HexNAc 3

**Mass:**  2871.1571

(6.923e-07)

**Charge States:** 3

**Start Position**: 141

**End Position**: 151

| Profile Score | Tandem Score | TDA q-value | Volume | Spectrum Matches |
| --- | --- | --- | --- | --- |
| 15.2088 | 174.7164 | 0.0000 | 3.3825e+08 | 3 |

**Adduct Signal Fractions**

- **Unmodified**: 3.3825e+08

### RGSN(N)SSFFSR Fuc 1 Hex 5 HexNAc 3

**Mass:**  2709.1056

(1.253e-06)

**Charge States:** 3

**Start Position**: 141

**End Position**: 151

| Profile Score | Tandem Score | TDA q-value | Volume | Spectrum Matches |
| --- | --- | --- | --- | --- |
| -18.1038 | 172.4187 | 0.0000 | 6.7771e+07 | 1 |

**Adduct Signal Fractions**

- **Unmodified**: 6.7771e+07

### RGSN(N)SSFFSR Hex 5 HexNAc 4

**Mass:**  2766.1265

(1.001e-06)

**Charge States:** 3

**Start Position**: 141

**End Position**: 151

| Profile Score | Tandem Score | TDA q-value | Volume | Spectrum Matches |
| --- | --- | --- | --- | --- |
| -18.1112 | 172.0124 | 0.0000 | 8.9786e+07 | 1 |

**Adduct Signal Fractions**

- **Unmodified**: 8.9786e+07

### YPALN(N)VTMPNNEQFDK Hex 8 HexNAc 2

**Mass:**  3582.4649

(1.710e-06)

**Charge States:** 3, 4

**Start Position**: 161

**End Position**: 177

| Profile Score | Tandem Score | TDA q-value | Volume | Spectrum Matches |
| --- | --- | --- | --- | --- |
| -1.7363 | 171.9664 | 0.0000 | 3.7454e+08 | 8 |

**Adduct Signal Fractions**

- **Unmodified**: 3.7454e+08

### RGSN(N)SSFFSR Fuc 1 Hex 4 HexNAc 3

**Mass:**  2547.0510

(6.153e-07)

**Charge States:** 3

**Start Position**: 141

**End Position**: 151

| Profile Score | Tandem Score | TDA q-value | Volume | Spectrum Matches |
| --- | --- | --- | --- | --- |
| -20.0098 | 171.9011 | 0.0000 | 2.3635e+07 | 1 |

**Adduct Signal Fractions**

- **Unmodified**: 2.3635e+07

### YPALN(N)VTM(O)PNNEQFDK Hex 9 HexNAc 2

**Mass:**  3760.5111

(9.867e-07)

**Charge States:** 3, 4

**Start Position**: 161

**End Position**: 177

| Profile Score | Tandem Score | TDA q-value | Volume | Spectrum Matches |
| --- | --- | --- | --- | --- |
| 13.5277 | 171.5050 | 0.0000 | 3.0846e+08 | 4 |

**Adduct Signal Fractions**

- **Unmodified**: 3.0846e+08

### RGSN(N)SSFFSR Fuc 1 Hex 5 HexNAc 5

**Mass:**  3115.2636

(8.332e-07)

**Charge States:** 3, 4

**Start Position**: 141

**End Position**: 151

| Profile Score | Tandem Score | TDA q-value | Volume | Spectrum Matches |
| --- | --- | --- | --- | --- |
| 14.2714 | 171.1441 | 0.0000 | 1.5817e+09 | 6 |

**Adduct Signal Fractions**

- **Unmodified**: 1.5817e+09

### RGSN(N)SSFFSR Fuc 3 Hex 5 HexNAc 4

**Mass:**  3204.2997

(8.809e-07)

**Charge States:** 3, 4

**Start Position**: 141

**End Position**: 151

| Profile Score | Tandem Score | TDA q-value | Volume | Spectrum Matches |
| --- | --- | --- | --- | --- |
| -17.8087 | 171.0084 | 0.0000 | 1.3186e+08 | 1 |

**Adduct Signal Fractions**

- **Unmodified**: 1.3186e+08

### YPALN(N)VTM(O)PNNEQFDK Hex 8 HexNAc 2

**Mass:**  3598.4573

(1.008e-06)

**Charge States:** 2, 3, 4

**Start Position**: 161

**End Position**: 177

| Profile Score | Tandem Score | TDA q-value | Volume | Spectrum Matches |
| --- | --- | --- | --- | --- |
| -0.6967 | 170.5088 | 0.0000 | 3.4290e+09 | 15 |

**Adduct Signal Fractions**

- **Unmodified**: 3.4290e+09

### YPALN(N)VTM(O)PNNEQFDK Hex 7 HexNAc 2

**Mass:**  3436.4038

(1.909e-07)

**Charge States:** 3, 4

**Start Position**: 161

**End Position**: 177

| Profile Score | Tandem Score | TDA q-value | Volume | Spectrum Matches |
| --- | --- | --- | --- | --- |
| 14.3549 | 168.0286 | 0.0000 | 6.2224e+08 | 4 |

**Adduct Signal Fractions**

- **Unmodified**: 6.2224e+08

### TIVKPGDILLIN(N)STGNLIAPR Hex 9 HexNAc 2

**Mass:**  4068.9355

(2.881e-06)

**Charge States:** 3, 4, 5

**Start Position**: 235

**End Position**: 256

| Profile Score | Tandem Score | TDA q-value | Volume | Spectrum Matches |
| --- | --- | --- | --- | --- |
| 17.9643 | 165.0457 | 0.0000 | 6.1967e+08 | 7 |

**Adduct Signal Fractions**

- **Unmodified**: 6.1967e+08

### RGSN(N)SSFFSR Fuc 1 Hex 6 HexNAc 5

**Mass:**  3277.3159

(3.681e-07)

**Charge States:** 3

**Start Position**: 141

**End Position**: 151

| Profile Score | Tandem Score | TDA q-value | Volume | Spectrum Matches |
| --- | --- | --- | --- | --- |
| -3.9776 | 160.9900 | 0.0000 | 1.1213e+08 | 4 |

**Adduct Signal Fractions**

- **Unmodified**: 1.1213e+08

### N(N)GTYDHDVYR Fuc 1 Hex 5 HexNAc 4

**Mass:**  3007.1749

(1.787e-06)

**Charge States:** 3

**Start Position**: 483

**End Position**: 493

| Profile Score | Tandem Score | TDA q-value | Volume | Spectrum Matches |
| --- | --- | --- | --- | --- |
| 16.1369 | 160.8236 | 0.0000 | 5.2007e+08 | 5 |

**Adduct Signal Fractions**

- **Unmodified**: 5.2007e+08

### RGSN(N)SSFFSR Hex 7 HexNAc 3 Neu5Ac 1

**Mass:**  3178.2321

(-4.183e-06)

**Charge States:** 3

**Start Position**: 141

**End Position**: 151

| Profile Score | Tandem Score | TDA q-value | Volume | Spectrum Matches |
| --- | --- | --- | --- | --- |
| -20.4264 | 160.6654 | 0.0000 | 7.4546e+06 | 1 |

**Adduct Signal Fractions**

- **Unmodified**: 7.4546e+06

### RGSN(N)SSFFSR Hex 6 HexNAc 4

**Mass:**  2928.1798

(1.093e-06)

**Charge States:** 3

**Start Position**: 141

**End Position**: 151

| Profile Score | Tandem Score | TDA q-value | Volume | Spectrum Matches |
| --- | --- | --- | --- | --- |
| 11.7390 | 160.1707 | 0.0000 | 1.3041e+08 | 1 |

**Adduct Signal Fractions**

- **Unmodified**: 1.3041e+08

### N(N)GTYDHDVYR Fuc 1 Hex 5 HexNAc 5

**Mass:**  3210.2536

(1.507e-06)

**Charge States:** 3, 4

**Start Position**: 483

**End Position**: 493

| Profile Score | Tandem Score | TDA q-value | Volume | Spectrum Matches |
| --- | --- | --- | --- | --- |
| -3.3654 | 159.2536 | 0.0000 | 2.0797e+08 | 5 |

**Adduct Signal Fractions**

- **Unmodified**: 2.0797e+08

### RGSN(N)SSFFSR Fuc 3 Hex 6 HexNAc 6 Neu5Ac 1

**Mass:**  4063.5808

(-5.808e-06)

**Charge States:** 4

**Start Position**: 141

**End Position**: 151

| Profile Score | Tandem Score | TDA q-value | Volume | Spectrum Matches |
| --- | --- | --- | --- | --- |
| -20.8185 | 158.9921 | 0.0000 | 1.4667e+07 | 1 |

**Adduct Signal Fractions**

- **Unmodified**: 1.4667e+07

### YPALN(N)VTMPNNEQFDK Hex 7 HexNAc 2

**Mass:**  3420.4107

(2.030e-06)

**Charge States:** 3, 4

**Start Position**: 161

**End Position**: 177

| Profile Score | Tandem Score | TDA q-value | Volume | Spectrum Matches |
| --- | --- | --- | --- | --- |
| -3.2167 | 158.9633 | 0.0000 | 6.2713e+07 | 3 |

**Adduct Signal Fractions**

- **Unmodified**: 6.2713e+07

### GSN(N)SSFFSR Hex 6 HexNAc 3

**Mass:**  2568.9975

(1.731e-07)

**Charge States:** 3

**Start Position**: 142

**End Position**: 151

| Profile Score | Tandem Score | TDA q-value | Volume | Spectrum Matches |
| --- | --- | --- | --- | --- |
| -3.3165 | 158.2737 | 0.0000 | 2.6741e+08 | 3 |

**Adduct Signal Fractions**

- **Unmodified**: 2.6741e+08

### TIVKPGDILLIN(N)STGNLIAPR Hex 8 HexNAc 2

**Mass:**  3906.8796

(2.286e-06)

**Charge States:** 3, 4

**Start Position**: 235

**End Position**: 256

| Profile Score | Tandem Score | TDA q-value | Volume | Spectrum Matches |
| --- | --- | --- | --- | --- |
| -18.4338 | 157.8137 | 0.0000 | 5.7765e+07 | 2 |

**Adduct Signal Fractions**

- **Unmodified**: 5.7765e+07

### RGSN(N)SSFFSR Fuc 2 Hex 4 HexNAc 3

**Mass:**  2693.1059

(-5.343e-07)

**Charge States:** 3

**Start Position**: 141

**End Position**: 151

| Profile Score | Tandem Score | TDA q-value | Volume | Spectrum Matches |
| --- | --- | --- | --- | --- |
| -17.2965 | 157.5661 | 0.0000 | 2.3508e+07 | 1 |

**Adduct Signal Fractions**

- **Unmodified**: 2.3508e+07

### GSN(N)SSFFSR Fuc 1 Hex 5 HexNAc 4

**Mass:**  2756.0828

(2.960e-07)

**Charge States:** 3

**Start Position**: 142

**End Position**: 151

| Profile Score | Tandem Score | TDA q-value | Volume | Spectrum Matches |
| --- | --- | --- | --- | --- |
| 12.2867 | 155.9804 | 0.0000 | 3.1163e+08 | 4 |

**Adduct Signal Fractions**

- **Unmodified**: 3.1163e+08

### YPALN(N)VTM(O)PNNEQFDK Fuc 2 Hex 3 HexNAc 4 Neu5Ac 1

**Mass:**  3777.5374

(-5.898e-06)

**Charge States:** 3

**Start Position**: 161

**End Position**: 177

| Profile Score | Tandem Score | TDA q-value | Volume | Spectrum Matches |
| --- | --- | --- | --- | --- |
| -5.2226 | 155.5181 | 0.0000 | 3.7804e+07 | 1 |

**Adduct Signal Fractions**

- **Unmodified**: 3.7804e+07

### GSN(N)SSFFSR Hex 8 HexNAc 2

**Mass:**  2690.0240

(5.946e-07)

**Charge States:** 2, 3

**Start Position**: 142

**End Position**: 151

| Profile Score | Tandem Score | TDA q-value | Volume | Spectrum Matches |
| --- | --- | --- | --- | --- |
| -18.8040 | 155.4343 | 0.0000 | 2.9231e+07 | 1 |

**Adduct Signal Fractions**

- **Unmodified**: 2.9231e+07

### YPALN(N)VTM(O)PNNEQFDK Hex 6 HexNAc 2

**Mass:**  3274.3485

(2.882e-06)

**Charge States:** 3

**Start Position**: 161

**End Position**: 177

| Profile Score | Tandem Score | TDA q-value | Volume | Spectrum Matches |
| --- | --- | --- | --- | --- |
| -5.6241 | 155.2832 | 0.0000 | 4.1160e+07 | 1 |

**Adduct Signal Fractions**

- **Unmodified**: 4.1160e+07

### TIVKPGDILLIN(N)STGNLIAPR Fuc 2 Hex 3 HexNAc 4 Neu5Ac 1

**Mass:**  4085.9567

(-3.934e-06)

**Charge States:** 4

**Start Position**: 235

**End Position**: 256

| Profile Score | Tandem Score | TDA q-value | Volume | Spectrum Matches |
| --- | --- | --- | --- | --- |
| -3.5295 | 155.1565 | 0.0000 | 5.8763e+07 | 2 |

**Adduct Signal Fractions**

- **Unmodified**: 5.8763e+07

### C(C)KSEC(C)ITPN(N)GSIPNDKPFQNVNR Hex 8 HexNAc 2

**Mass:**  4376.8449

(5.487e-07)

**Charge States:** 4

**Start Position**: 277

**End Position**: 300

| Profile Score | Tandem Score | TDA q-value | Volume | Spectrum Matches |
| --- | --- | --- | --- | --- |
| -3.5326 | 155.0275 | 0.0000 | 1.3987e+08 | 1 |

**Adduct Signal Fractions**

- **Unmodified**: 1.3987e+08

### YPALN(N)VTM(O)PNNEQFDK Hex 5 HexNAc 2

**Mass:**  3112.2991

(1.265e-06)

**Charge States:** 3

**Start Position**: 161

**End Position**: 177

| Profile Score | Tandem Score | TDA q-value | Volume | Spectrum Matches |
| --- | --- | --- | --- | --- |
| -3.5209 | 154.7150 | 0.0000 | 4.2231e+07 | 1 |

**Adduct Signal Fractions**

- **Unmodified**: 4.2231e+07

### GSN(N)SSFFSR Fuc 3 Hex 5 HexNAc 4

**Mass:**  3048.1972

(2.698e-07)

**Charge States:** 3

**Start Position**: 142

**End Position**: 151

| Profile Score | Tandem Score | TDA q-value | Volume | Spectrum Matches |
| --- | --- | --- | --- | --- |
| -18.4669 | 154.4868 | 0.0000 | 5.0047e+07 | 1 |

**Adduct Signal Fractions**

- **Unmodified**: 5.0047e+07

### GSN(N)SSFFSR Fuc 1 Hex 5 HexNAc 5

**Mass:**  2959.1621

(7.345e-07)

**Charge States:** 2, 3, 4

**Start Position**: 142

**End Position**: 151

| Profile Score | Tandem Score | TDA q-value | Volume | Spectrum Matches |
| --- | --- | --- | --- | --- |
| 13.6214 | 154.1880 | 0.0000 | 9.0917e+08 | 5 |

**Adduct Signal Fractions**

- **Unmodified**: 9.0917e+08

### GSN(N)SSFFSR Fuc 1 Hex 5 HexNAc 3

**Mass:**  2553.0062

(2.517e-07)

**Charge States:** 3

**Start Position**: 142

**End Position**: 151

| Profile Score | Tandem Score | TDA q-value | Volume | Spectrum Matches |
| --- | --- | --- | --- | --- |
| -19.5714 | 152.9639 | 0.0000 | 3.0179e+07 | 1 |

**Adduct Signal Fractions**

- **Unmodified**: 3.0179e+07

### YPALN(N)VTMPNNEQFDK Hex 9 HexNAc 2

**Mass:**  3744.5176

(1.671e-06)

**Charge States:** 3

**Start Position**: 161

**End Position**: 177

| Profile Score | Tandem Score | TDA q-value | Volume | Spectrum Matches |
| --- | --- | --- | --- | --- |
| -4.9393 | 152.8493 | 0.0000 | 2.8321e+07 | 3 |

**Adduct Signal Fractions**

- **Unmodified**: 2.8321e+07

### HAVPN(N)GTIVK Fuc 1 Hex 5 HexNAc 5

**Mass:**  3007.3189

(3.369e-04)

**Start Position**: 18

**End Position**: 28

| Profile Score | Tandem Score | TDA q-value | Volume | Spectrum Matches |
| --- | --- | --- | --- | --- |
| 0.0000 | 151.3700 | 0.0000 | 0.0000e+00 | 1 |

Adduct:

No Chromatogram Found

### GSN(N)SSFFSR Fuc 1 Hex 6 HexNAc 3

**Mass:**  2715.0545

(-7.119e-07)

**Charge States:** 3

**Start Position**: 142

**End Position**: 151

| Profile Score | Tandem Score | TDA q-value | Volume | Spectrum Matches |
| --- | --- | --- | --- | --- |
| -4.3389 | 149.4462 | 0.0000 | 1.1268e+08 | 2 |

**Adduct Signal Fractions**

- **Unmodified**: 1.1268e+08

### GSN(N)SSFFSR Hex 6 HexNAc 2

**Mass:**  2365.9183

(2.334e-08)

**Charge States:** 2

**Start Position**: 142

**End Position**: 151

| Profile Score | Tandem Score | TDA q-value | Volume | Spectrum Matches |
| --- | --- | --- | --- | --- |
| -4.0191 | 148.8422 | 0.0000 | 2.1812e+08 | 3 |

**Adduct Signal Fractions**

- **Unmodified**: 2.1812e+08

### ISIYWTIVKPGDILLIN(N)STGNLIAPR Fuc 2 Hex 3 HexNAc 4 Neu5Ac 1

**Mass:**  4748.2971

(-4.564e-06)

**Charge States:** 4

**Start Position**: 230

**End Position**: 256

| Profile Score | Tandem Score | TDA q-value | Volume | Spectrum Matches |
| --- | --- | --- | --- | --- |
| 13.3809 | 148.6666 | 0.0000 | 1.9232e+08 | 4 |

**Adduct Signal Fractions**

- **Unmodified**: 1.9232e+08

### C(C)KSEC(C)ITPN(N)GSIPNDKPFQNVNR Hex 6 HexNAc 2

**Mass:**  4052.7404

(1.383e-06)

**Charge States:** 4

**Start Position**: 277

**End Position**: 300

| Profile Score | Tandem Score | TDA q-value | Volume | Spectrum Matches |
| --- | --- | --- | --- | --- |
| -18.2756 | 148.6549 | 0.0000 | 3.2778e+07 | 2 |

**Adduct Signal Fractions**

- **Unmodified**: 3.2778e+07

### GSN(N)SSFFSR Hex 5 HexNAc 2

**Mass:**  2203.8656

(8.486e-07)

**Charge States:** 2, 3

**Start Position**: 142

**End Position**: 151

| Profile Score | Tandem Score | TDA q-value | Volume | Spectrum Matches |
| --- | --- | --- | --- | --- |
| -2.7715 | 148.5922 | 0.0000 | 2.8871e+08 | 3 |

**Adduct Signal Fractions**

- **Unmodified**: 2.8871e+08

### N(N)GTYDHDVYRDEALN(D)NR Fuc 1 Hex 6 HexNAc 3

**Mass:**  3779.5122

(2.619e-04)

**Charge States:** 4

**Start Position**: 483

**End Position**: 500

| Profile Score | Tandem Score | TDA q-value | Volume | Spectrum Matches |
| --- | --- | --- | --- | --- |
| -20.7853 | 148.2539 | 0.0000 | 3.7111e+07 | 3 |

**Adduct Signal Fractions**

- **Unmodified**: 3.7111e+07

### ISIYWTIVKPGDILLIN(N)STGNLIAPR Hex 9 HexNAc 2

**Mass:**  4731.2746

(8.459e-07)

**Charge States:** 3, 4, 5

**Start Position**: 230

**End Position**: 256

| Profile Score | Tandem Score | TDA q-value | Volume | Spectrum Matches |
| --- | --- | --- | --- | --- |
| 17.0854 | 148.1059 | 0.0000 | 8.5500e+08 | 8 |

**Adduct Signal Fractions**

- **Unmodified**: 8.5500e+08

### C(C)KSEC(C)ITPN(N)GSIPNDKPFQNVNR Hex 9 HexNAc 2

**Mass:**  4538.8952

(4.338e-07)

**Charge States:** 4

**Start Position**: 277

**End Position**: 300

| Profile Score | Tandem Score | TDA q-value | Volume | Spectrum Matches |
| --- | --- | --- | --- | --- |
| -3.9084 | 148.0975 | 0.0000 | 1.9703e+08 | 2 |

**Adduct Signal Fractions**

- **Unmodified**: 1.9703e+08

### TIVKPGDILLIN(N)STGNLIAPR Hex 10 HexNAc 2

**Mass:**  4230.9875

(2.490e-06)

**Charge States:** 4

**Start Position**: 235

**End Position**: 256

| Profile Score | Tandem Score | TDA q-value | Volume | Spectrum Matches |
| --- | --- | --- | --- | --- |
| -17.6754 | 148.0604 | 0.0000 | 1.6753e+07 | 1 |

**Adduct Signal Fractions**

- **Unmodified**: 1.6753e+07

### C(C)KSEC(C)ITPN(N)GSIPNDKPFQNVNR Hex 7 HexNAc 2

**Mass:**  4214.7907

(2.002e-07)

**Charge States:** 4

**Start Position**: 277

**End Position**: 300

| Profile Score | Tandem Score | TDA q-value | Volume | Spectrum Matches |
| --- | --- | --- | --- | --- |
| -3.5666 | 147.6701 | 0.0000 | 2.3584e+08 | 4 |

**Adduct Signal Fractions**

- **Unmodified**: 2.3584e+08

### YPALN(N)VTM(O)PNNEQFDK Hex 5 HexNAc 2

**Mass:**  3113.3095

(3.259e-04)

**Start Position**: 161

**End Position**: 177

| Profile Score | Tandem Score | TDA q-value | Volume | Spectrum Matches |
| --- | --- | --- | --- | --- |
| 0.0000 | 147.6402 | 0.0000 | 0.0000e+00 | 1 |

Adduct:

No Chromatogram Found

### RGSN(N)SSFFSR Fuc 1 Hex 7 HexNAc 8

**Mass:**  4048.6379

(8.177e-06)

**Charge States:** 4

**Start Position**: 141

**End Position**: 151

| Profile Score | Tandem Score | TDA q-value | Volume | Spectrum Matches |
| --- | --- | --- | --- | --- |
| -19.3423 | 145.1918 | 0.0000 | 2.8408e+07 | 1 |

**Adduct Signal Fractions**

- **Unmodified**: 2.8408e+07

### RGSN(N)SSFFSR Fuc 2 Hex 6 HexNAc 5

**Mass:**  3423.3737

(7.031e-07)

**Charge States:** 3

**Start Position**: 141

**End Position**: 151

| Profile Score | Tandem Score | TDA q-value | Volume | Spectrum Matches |
| --- | --- | --- | --- | --- |
| -5.5630 | 144.0966 | 0.0000 | 2.8779e+07 | 1 |

**Adduct Signal Fractions**

- **Unmodified**: 2.8779e+07

### RGSN(N)SSFFSR Fuc 1 Hex 5 HexNAc 7 Neu5Ac 3

**Mass:**  4394.7033

(-6.646e-07)

**Charge States:** 4

**Start Position**: 141

**End Position**: 151

| Profile Score | Tandem Score | TDA q-value | Volume | Spectrum Matches |
| --- | --- | --- | --- | --- |
| -18.5469 | 143.9638 | 0.0000 | 2.7137e+07 | 1 |

**Adduct Signal Fractions**

- **Unmodified**: 2.7137e+07

### GSN(N)SSFFSR Hex 5 HexNAc 5

**Mass:**  2813.1040

(1.459e-06)

**Charge States:** 2, 3

**Start Position**: 142

**End Position**: 151

| Profile Score | Tandem Score | TDA q-value | Volume | Spectrum Matches |
| --- | --- | --- | --- | --- |
| 8.5031 | 143.7821 | 0.0000 | 2.0935e+08 | 3 |

**Adduct Signal Fractions**

- **Unmodified**: 2.0935e+08

### N(N)GTYDHDVYR Fuc 3 Hex 5 HexNAc 4

**Mass:**  3299.2890

(1.200e-06)

**Charge States:** 3, 4

**Start Position**: 483

**End Position**: 493

| Profile Score | Tandem Score | TDA q-value | Volume | Spectrum Matches |
| --- | --- | --- | --- | --- |
| -18.7889 | 143.5797 | 0.0000 | 8.6970e+07 | 2 |

**Adduct Signal Fractions**

- **Unmodified**: 8.6970e+07

### ISIYWTIVKPGDILLIN(N)STGNLIAPR Hex 8 HexNAc 2

**Mass:**  4569.2180

(1.055e-06)

**Charge States:** 3, 4

**Start Position**: 230

**End Position**: 256

| Profile Score | Tandem Score | TDA q-value | Volume | Spectrum Matches |
| --- | --- | --- | --- | --- |
| -4.0572 | 143.3672 | 0.0000 | 8.7101e+07 | 5 |

**Adduct Signal Fractions**

- **Unmodified**: 8.7101e+07

### N(N)GTYDHDVYRDEALN(D)NR Fuc 1 Hex 5 HexNAc 4

**Mass:**  3820.5363

(2.595e-04)

**Charge States:** 3, 4

**Start Position**: 483

**End Position**: 500

| Profile Score | Tandem Score | TDA q-value | Volume | Spectrum Matches |
| --- | --- | --- | --- | --- |
| 4.4274 | 142.3615 | 0.0000 | 1.3296e+09 | 13 |

**Adduct Signal Fractions**

- **Unmodified**: 1.3296e+09

### N(N)GTYDHDVYRDEALNNR Fuc 1 Hex 6 HexNAc 3

**Mass:**  3778.5275

(1.371e-06)

**Charge States:** 4

**Start Position**: 483

**End Position**: 500

| Profile Score | Tandem Score | TDA q-value | Volume | Spectrum Matches |
| --- | --- | --- | --- | --- |
| 12.9683 | 141.9662 | 0.0000 | 3.3451e+08 | 1 |

**Adduct Signal Fractions**

- **Unmodified**: 3.3451e+08

### RGSN(N)SSFFSR Fuc 1 Hex 5 HexNAc 6

**Mass:**  3318.3433

(8.523e-07)

**Charge States:** 3

**Start Position**: 141

**End Position**: 151

| Profile Score | Tandem Score | TDA q-value | Volume | Spectrum Matches |
| --- | --- | --- | --- | --- |
| -19.7210 | 140.8152 | 0.0000 | 5.8157e+07 | 1 |

**Adduct Signal Fractions**

- **Unmodified**: 5.8157e+07

### IVKPGDILLIN(N)STGNLIAPR Hex 9 HexNAc 2

**Mass:**  3967.8867

(2.210e-06)

**Charge States:** 4

**Start Position**: 236

**End Position**: 256

| Profile Score | Tandem Score | TDA q-value | Volume | Spectrum Matches |
| --- | --- | --- | --- | --- |
| -18.1128 | 139.9472 | 0.0000 | 1.1250e+07 | 1 |

**Adduct Signal Fractions**

- **Unmodified**: 1.1250e+07

### N(N)GTYDHDVYRDEALNNR Fuc 1 Hex 4 HexNAc 3

**Mass:**  3454.4196

(9.004e-07)

**Charge States:** 4

**Start Position**: 483

**End Position**: 500

| Profile Score | Tandem Score | TDA q-value | Volume | Spectrum Matches |
| --- | --- | --- | --- | --- |
| -4.2377 | 139.4767 | 0.0000 | 1.3208e+08 | 1 |

**Adduct Signal Fractions**

- **Unmodified**: 1.3208e+08

### RGSN(N)SSFFSR Hex 6 HexNAc 6

**Mass:**  3334.3328

(-7.907e-07)

**Charge States:** 3

**Start Position**: 141

**End Position**: 151

| Profile Score | Tandem Score | TDA q-value | Volume | Spectrum Matches |
| --- | --- | --- | --- | --- |
| -18.4811 | 139.4540 | 0.0000 | 1.4029e+07 | 1 |

**Adduct Signal Fractions**

- **Unmodified**: 1.4029e+07

### GSN(N)SSFFSR Fuc 2 Hex 5 HexNAc 4

**Mass:**  2902.1409

(6.444e-07)

**Charge States:** 2, 3

**Start Position**: 142

**End Position**: 151

| Profile Score | Tandem Score | TDA q-value | Volume | Spectrum Matches |
| --- | --- | --- | --- | --- |
| -18.6274 | 139.0945 | 0.0000 | 1.0402e+08 | 3 |

**Adduct Signal Fractions**

- **Unmodified**: 1.0402e+08

### GSN(N)SSFFSR Fuc 2 Hex 5 HexNAc 5

**Mass:**  3105.2210

(9.756e-07)

**Charge States:** 3

**Start Position**: 142

**End Position**: 151

| Profile Score | Tandem Score | TDA q-value | Volume | Spectrum Matches |
| --- | --- | --- | --- | --- |
| -18.7371 | 138.6443 | 0.0000 | 9.9780e+07 | 1 |

**Adduct Signal Fractions**

- **Unmodified**: 9.9780e+07

### N(N)GTYDHDVYRDEALNNR Fuc 2 Hex 5 HexNAc 4

**Mass:**  3965.6108

(1.196e-06)

**Charge States:** 3, 4, 5

**Start Position**: 483

**End Position**: 500

| Profile Score | Tandem Score | TDA q-value | Volume | Spectrum Matches |
| --- | --- | --- | --- | --- |
| 0.1363 | 138.2868 | 0.0000 | 2.1784e+09 | 5 |

**Adduct Signal Fractions**

- **Unmodified**: 2.1784e+09

### GSN(N)SSFFSR Hex 6 HexNAc 4

**Mass:**  2772.0769

(4.880e-07)

**Charge States:** 3

**Start Position**: 142

**End Position**: 151

| Profile Score | Tandem Score | TDA q-value | Volume | Spectrum Matches |
| --- | --- | --- | --- | --- |
| -5.1189 | 137.4554 | 0.0000 | 2.9490e+07 | 1 |

**Adduct Signal Fractions**

- **Unmodified**: 2.9490e+07

### GSN(N)SSFFSR Hex 5 HexNAc 3

**Mass:**  2406.9415

(-6.962e-07)

**Charge States:** 3

**Start Position**: 142

**End Position**: 151

| Profile Score | Tandem Score | TDA q-value | Volume | Spectrum Matches |
| --- | --- | --- | --- | --- |
| -5.3822 | 137.3061 | 0.0000 | 3.4473e+07 | 1 |

**Adduct Signal Fractions**

- **Unmodified**: 3.4473e+07

### YPALN(N)VTM(O)PN(D)NEQFDK Hex 7 HexNAc 2

**Mass:**  3437.3911

(2.882e-04)

**Charge States:** 3, 4

**Start Position**: 161

**End Position**: 177

| Profile Score | Tandem Score | TDA q-value | Volume | Spectrum Matches |
| --- | --- | --- | --- | --- |
| 9.2634 | 136.5455 | 0.0000 | 1.0105e+08 | 4 |

**Adduct Signal Fractions**

- **Unmodified**: 1.0105e+08

### N(N)GTYDHDVYRDEALNNR Hex 6 HexNAc 3

**Mass:**  3632.4680

(1.169e-06)

**Charge States:** 3, 4

**Start Position**: 483

**End Position**: 500

| Profile Score | Tandem Score | TDA q-value | Volume | Spectrum Matches |
| --- | --- | --- | --- | --- |
| -3.3146 | 136.3196 | 0.0000 | 3.4151e+08 | 3 |

**Adduct Signal Fractions**

- **Unmodified**: 3.4151e+08

### RGSN(N)SSFFSR Fuc 2 Hex 6 HexNAc 3

**Mass:**  3017.2157

(8.801e-07)

**Charge States:** 3

**Start Position**: 141

**End Position**: 151

| Profile Score | Tandem Score | TDA q-value | Volume | Spectrum Matches |
| --- | --- | --- | --- | --- |
| -17.8782 | 136.1648 | 0.0000 | 2.9193e+07 | 2 |

**Adduct Signal Fractions**

- **Unmodified**: 2.9193e+07

### YPALN(N)VTM(O)PN(D)NEQFDK Hex 8 HexNAc 2

**Mass:**  3599.4442

(2.755e-04)

**Charge States:** 3, 4

**Start Position**: 161

**End Position**: 177

| Profile Score | Tandem Score | TDA q-value | Volume | Spectrum Matches |
| --- | --- | --- | --- | --- |
| 9.8145 | 135.8236 | 0.0000 | 5.7342e+08 | 7 |

**Adduct Signal Fractions**

- **Unmodified**: 5.7342e+08

### N(N)GTYDHDVYRDEALNNR Hex 5 HexNAc 2

**Mass:**  3267.3352

(9.304e-07)

**Charge States:** 4

**Start Position**: 483

**End Position**: 500

| Profile Score | Tandem Score | TDA q-value | Volume | Spectrum Matches |
| --- | --- | --- | --- | --- |
| -5.1823 | 135.7199 | 0.0000 | 1.1300e+08 | 2 |

**Adduct Signal Fractions**

- **Unmodified**: 1.1300e+08

### SEC(C)ITPN(N)GSIPNDKPFQNVNR Hex 7 HexNAc 2

**Mass:**  3926.6672

(9.771e-07)

**Charge States:** 3, 4

**Start Position**: 279

**End Position**: 300

| Profile Score | Tandem Score | TDA q-value | Volume | Spectrum Matches |
| --- | --- | --- | --- | --- |
| 15.7143 | 135.5203 | 0.0000 | 1.8657e+09 | 5 |

**Adduct Signal Fractions**

- **Unmodified**: 1.8657e+09

### PGDILLIN(N)STGNLIAPR Hex 9 HexNAc 2

**Mass:**  3627.6384

(2.754e-06)

**Charge States:** 3, 4

**Start Position**: 239

**End Position**: 256

| Profile Score | Tandem Score | TDA q-value | Volume | Spectrum Matches |
| --- | --- | --- | --- | --- |
| -20.0697 | 135.3423 | 0.0000 | 2.5723e+07 | 1 |

**Adduct Signal Fractions**

- **Unmodified**: 2.5723e+07

### N(N)GTYDHDVYRDEALNNR Fuc 2 Hex 4 HexNAc 3

**Mass:**  3600.4794

(1.334e-06)

**Charge States:** 4

**Start Position**: 483

**End Position**: 500

| Profile Score | Tandem Score | TDA q-value | Volume | Spectrum Matches |
| --- | --- | --- | --- | --- |
| -4.0707 | 133.5902 | 0.0000 | 9.5569e+07 | 1 |

**Adduct Signal Fractions**

- **Unmodified**: 9.5569e+07

### N(N)GTYDHDVYRDEALNNR Fuc 1 Hex 5 HexNAc 5

**Mass:**  4022.6308

(5.812e-07)

**Charge States:** 3, 4, 5

**Start Position**: 483

**End Position**: 500

| Profile Score | Tandem Score | TDA q-value | Volume | Spectrum Matches |
| --- | --- | --- | --- | --- |
| 20.9128 | 133.1189 | 0.0000 | 2.4066e+09 | 7 |

**Adduct Signal Fractions**

- **Unmodified**: 2.4066e+09

### N(N)GTYDHDVYRDEALNNR Fuc 2 Hex 8 HexNAc 3

**Mass:**  4248.6472

(-8.590e-06)

**Charge States:** 3, 4

**Start Position**: 483

**End Position**: 500

| Profile Score | Tandem Score | TDA q-value | Volume | Spectrum Matches |
| --- | --- | --- | --- | --- |
| -3.8754 | 132.5494 | 0.0000 | 1.8474e+08 | 3 |

**Adduct Signal Fractions**

- **Unmodified**: 1.8474e+08

### N(N)GTYDHDVYRDEALNNR Fuc 1 Hex 5 HexNAc 4

**Mass:**  3819.5517

(2.818e-06)

**Charge States:** 3, 4, 5

**Start Position**: 483

**End Position**: 500

| Profile Score | Tandem Score | TDA q-value | Volume | Spectrum Matches |
| --- | --- | --- | --- | --- |
| 2.0769 | 131.9541 | 0.0000 | 4.2729e+09 | 6 |

**Adduct Signal Fractions**

- **Unmodified**: 4.2729e+09

### RGSN(N)SSFFSR Fuc 2 Hex 6 HexNAc 6

**Mass:**  3626.4611

(2.768e-06)

**Charge States:** 3

**Start Position**: 141

**End Position**: 151

| Profile Score | Tandem Score | TDA q-value | Volume | Spectrum Matches |
| --- | --- | --- | --- | --- |
| -20.0719 | 130.7161 | 0.0000 | 3.7509e+06 | 2 |

**Adduct Signal Fractions**

- **Unmodified**: 3.7509e+06

### N(N)GTYDHDVYRDEALNNR Fuc 1 Hex 5 HexNAc 3

**Mass:**  3616.4748

(5.468e-07)

**Charge States:** 4

**Start Position**: 483

**End Position**: 500

| Profile Score | Tandem Score | TDA q-value | Volume | Spectrum Matches |
| --- | --- | --- | --- | --- |
| -4.2568 | 130.2881 | 0.0000 | 1.4651e+08 | 1 |

**Adduct Signal Fractions**

- **Unmodified**: 1.4651e+08

### N(N)GTYDHDVYR Hex 6 HexNAc 3

**Mass:**  2820.0899

(1.135e-06)

**Charge States:** 3

**Start Position**: 483

**End Position**: 493

| Profile Score | Tandem Score | TDA q-value | Volume | Spectrum Matches |
| --- | --- | --- | --- | --- |
| -17.2892 | 130.1340 | 0.0000 | 2.4153e+07 | 2 |

**Adduct Signal Fractions**

- **Unmodified**: 2.4153e+07

### SEC(C)ITPN(N)GSIPNDKPFQNVNR Hex 6 HexNAc 2

**Mass:**  3764.6138

(1.239e-06)

**Charge States:** 3, 4

**Start Position**: 279

**End Position**: 300

| Profile Score | Tandem Score | TDA q-value | Volume | Spectrum Matches |
| --- | --- | --- | --- | --- |
| -2.7773 | 130.0072 | 0.0000 | 2.5393e+08 | 2 |

**Adduct Signal Fractions**

- **Unmodified**: 2.5393e+08

### N(N)GTYDHDVYRDEALNNR Fuc 1 Hex 6 HexNAc 6

**Mass:**  4387.7639

(1.239e-08)

**Charge States:** 4

**Start Position**: 483

**End Position**: 500

| Profile Score | Tandem Score | TDA q-value | Volume | Spectrum Matches |
| --- | --- | --- | --- | --- |
| -4.5687 | 129.9534 | 0.0000 | 1.2430e+08 | 1 |

**Adduct Signal Fractions**

- **Unmodified**: 1.2430e+08

### SEC(C)ITPN(N)GSIPNDKPFQNVNR Hex 8 HexNAc 2

**Mass:**  4088.7167

(9.629e-07)

**Charge States:** 3, 4

**Start Position**: 279

**End Position**: 300

| Profile Score | Tandem Score | TDA q-value | Volume | Spectrum Matches |
| --- | --- | --- | --- | --- |
| 13.8596 | 129.7329 | 0.0000 | 1.0883e+09 | 5 |

**Adduct Signal Fractions**

- **Unmodified**: 1.0883e+09

### GSN(N)SSFFSR Hex 7 HexNAc 2

**Mass:**  2527.9618

(-3.107e-06)

**Charge States:** 2

**Start Position**: 142

**End Position**: 151

| Profile Score | Tandem Score | TDA q-value | Volume | Spectrum Matches |
| --- | --- | --- | --- | --- |
| -4.8170 | 129.5528 | 0.0385 | 5.1001e+07 | 1 |

**Adduct Signal Fractions**

- **Unmodified**: 5.1001e+07

### N(N)GTYDHDVYRDEALNNR Fuc 1 Hex 8 HexNAc 2

**Mass:**  3899.5082

(-9.971e-06)

**Charge States:** 3, 4

**Start Position**: 483

**End Position**: 500

| Profile Score | Tandem Score | TDA q-value | Volume | Spectrum Matches |
| --- | --- | --- | --- | --- |
| 13.5606 | 128.2563 | 0.0000 | 7.3893e+08 | 5 |

**Adduct Signal Fractions**

- **Unmodified**: 7.3893e+08

### N(N)GTYDHDVYRDEALNNR Fuc 2 Hex 6 HexNAc 5

**Mass:**  4330.7404

(3.351e-07)

**Charge States:** 4

**Start Position**: 483

**End Position**: 500

| Profile Score | Tandem Score | TDA q-value | Volume | Spectrum Matches |
| --- | --- | --- | --- | --- |
| -4.5583 | 128.0601 | 0.0000 | 1.6621e+08 | 1 |

**Adduct Signal Fractions**

- **Unmodified**: 1.6621e+08

### N(N)GTYDHDVYRDEALNNR Hex 5 HexNAc 4

**Mass:**  3673.4960

(1.106e-06)

**Charge States:** 4

**Start Position**: 483

**End Position**: 500

| Profile Score | Tandem Score | TDA q-value | Volume | Spectrum Matches |
| --- | --- | --- | --- | --- |
| -4.0062 | 128.0065 | 0.0000 | 1.9786e+08 | 2 |

**Adduct Signal Fractions**

- **Unmodified**: 1.9786e+08

### N(N)GTYDHDVYRDEALNNR Fuc 1 Hex 6 HexNAc 5

**Mass:**  4184.6862

(1.346e-06)

**Charge States:** 4

**Start Position**: 483

**End Position**: 500

| Profile Score | Tandem Score | TDA q-value | Volume | Spectrum Matches |
| --- | --- | --- | --- | --- |
| 11.9632 | 127.6285 | 0.0000 | 4.4863e+08 | 2 |

**Adduct Signal Fractions**

- **Unmodified**: 4.4863e+08

### N(N)GTYDHDVYRDEALN(D)NR Fuc 2 Hex 5 HexNAc 4

**Mass:**  3966.5952

(2.497e-04)

**Charge States:** 3, 4

**Start Position**: 483

**End Position**: 500

| Profile Score | Tandem Score | TDA q-value | Volume | Spectrum Matches |
| --- | --- | --- | --- | --- |
| 4.8691 | 126.6033 | 0.0000 | 8.6420e+08 | 4 |

**Adduct Signal Fractions**

- **Unmodified**: 8.6420e+08

### SEC(C)ITPN(N)GSIPNDKPFQNVNR Hex 9 HexNAc 2

**Mass:**  4250.7727

(6.830e-07)

**Charge States:** 3, 4

**Start Position**: 279

**End Position**: 300

| Profile Score | Tandem Score | TDA q-value | Volume | Spectrum Matches |
| --- | --- | --- | --- | --- |
| -2.4475 | 126.4385 | 0.0000 | 1.6697e+09 | 4 |

**Adduct Signal Fractions**

- **Unmodified**: 1.6697e+09

### RGSN(N)SSFFSR Fuc 3 Hex 5 HexNAc 5

**Mass:**  3407.3790

(7.007e-07)

**Charge States:** 3

**Start Position**: 141

**End Position**: 151

| Profile Score | Tandem Score | TDA q-value | Volume | Spectrum Matches |
| --- | --- | --- | --- | --- |
| -18.1734 | 125.4305 | 0.0000 | 5.5735e+07 | 2 |

**Adduct Signal Fractions**

- **Unmodified**: 5.5735e+07

### N(N)GTYDHDVYRDEALN(D)NR Fuc 3 Hex 5 HexNAc 4

**Mass:**  4112.6545

(2.420e-04)

**Charge States:** 4

**Start Position**: 483

**End Position**: 500

| Profile Score | Tandem Score | TDA q-value | Volume | Spectrum Matches |
| --- | --- | --- | --- | --- |
| 8.8275 | 123.7963 | 0.0000 | 3.3197e+08 | 3 |

**Adduct Signal Fractions**

- **Unmodified**: 3.3197e+08

### N(N)GTYDHDVYRDEALNNR Fuc 2 Hex 5 HexNAc 3

**Mass:**  3762.5312

(1.047e-06)

**Charge States:** 4

**Start Position**: 483

**End Position**: 500

| Profile Score | Tandem Score | TDA q-value | Volume | Spectrum Matches |
| --- | --- | --- | --- | --- |
| -4.4650 | 122.4123 | 0.0000 | 7.2002e+07 | 1 |

**Adduct Signal Fractions**

- **Unmodified**: 7.2002e+07

### N(N)GTYDHDVYRDEALNNR Fuc 3 Hex 5 HexNAc 4

**Mass:**  4111.6686

(9.565e-07)

**Charge States:** 4

**Start Position**: 483

**End Position**: 500

| Profile Score | Tandem Score | TDA q-value | Volume | Spectrum Matches |
| --- | --- | --- | --- | --- |
| -2.6747 | 122.1958 | 0.0000 | 6.3501e+08 | 2 |

**Adduct Signal Fractions**

- **Unmodified**: 6.3501e+08

### N(N)GTYDHDVYRDEALNNR Fuc 1 Hex 9 HexNAc 3

**Mass:**  4264.6413

(-9.290e-06)

**Charge States:** 3, 4

**Start Position**: 483

**End Position**: 500

| Profile Score | Tandem Score | TDA q-value | Volume | Spectrum Matches |
| --- | --- | --- | --- | --- |
| 7.1128 | 122.1409 | 0.0000 | 1.5515e+08 | 3 |

**Adduct Signal Fractions**

- **Unmodified**: 1.5515e+08

### N(N)GTYDHDVYR Fuc 2 Hex 5 HexNAc 5

**Mass:**  3356.3113

(2.029e-06)

**Charge States:** 3

**Start Position**: 483

**End Position**: 493

| Profile Score | Tandem Score | TDA q-value | Volume | Spectrum Matches |
| --- | --- | --- | --- | --- |
| -19.0975 | 121.2565 | 0.0000 | 4.6592e+07 | 3 |

**Adduct Signal Fractions**

- **Unmodified**: 4.6592e+07

### N(N)GTYDHDVYRDEALNNR Fuc 2 Hex 5 HexNAc 5

**Mass:**  4168.6911

(1.159e-06)

**Charge States:** 3, 4

**Start Position**: 483

**End Position**: 500

| Profile Score | Tandem Score | TDA q-value | Volume | Spectrum Matches |
| --- | --- | --- | --- | --- |
| 11.5541 | 119.8958 | 0.0000 | 6.0720e+08 | 4 |

**Adduct Signal Fractions**

- **Unmodified**: 6.0720e+08

### SEC(C)ITPN(N)GSIPNDKPFQNVNR Hex 5 HexNAc 2

**Mass:**  3602.5607

(1.831e-07)

**Charge States:** 3, 4

**Start Position**: 279

**End Position**: 300

| Profile Score | Tandem Score | TDA q-value | Volume | Spectrum Matches |
| --- | --- | --- | --- | --- |
| -18.7915 | 119.1517 | 0.0000 | 9.9902e+07 | 2 |

**Adduct Signal Fractions**

- **Unmodified**: 9.9902e+07

### SEC(C)ITPN(N)GSIPNDKPFQNVNR Fuc 2 Hex 3 HexNAc 4 Neu5Ac 1

**Mass:**  4267.7985

(-5.816e-06)

**Charge States:** 4

**Start Position**: 279

**End Position**: 300

| Profile Score | Tandem Score | TDA q-value | Volume | Spectrum Matches |
| --- | --- | --- | --- | --- |
| -18.9524 | 118.8085 | 0.0000 | 9.8927e+07 | 1 |

**Adduct Signal Fractions**

- **Unmodified**: 9.8927e+07

### RGSN(N)SSFFSR Hex 7 HexNAc 4

**Mass:**  3090.2290

(1.894e-06)

**Charge States:** 3

**Start Position**: 141

**End Position**: 151

| Profile Score | Tandem Score | TDA q-value | Volume | Spectrum Matches |
| --- | --- | --- | --- | --- |
| -18.6401 | 118.4901 | 0.0000 | 2.0037e+07 | 1 |

**Adduct Signal Fractions**

- **Unmodified**: 2.0037e+07

### N(N)GTYDHDVYRDEALNNR Fuc 2 Hex 9 HexNAc 3

**Mass:**  4410.7009

(-8.467e-06)

**Charge States:** 4

**Start Position**: 483

**End Position**: 500

| Profile Score | Tandem Score | TDA q-value | Volume | Spectrum Matches |
| --- | --- | --- | --- | --- |
| -4.4766 | 118.1404 | 0.0000 | 1.1767e+08 | 1 |

**Adduct Signal Fractions**

- **Unmodified**: 1.1767e+08

### N(N)GTYDHDVYRDEALNNR Hex 7 HexNAc 3

**Mass:**  3794.5217

(1.199e-06)

**Charge States:** 4

**Start Position**: 483

**End Position**: 500

| Profile Score | Tandem Score | TDA q-value | Volume | Spectrum Matches |
| --- | --- | --- | --- | --- |
| -19.8884 | 118.0571 | 0.0000 | 4.3807e+07 | 1 |

**Adduct Signal Fractions**

- **Unmodified**: 4.3807e+07

### N(N)GTYDHDVYRDEALNNR Fuc 3 Hex 5 HexNAc 5

**Mass:**  4314.7482

(1.007e-06)

**Charge States:** 4

**Start Position**: 483

**End Position**: 500

| Profile Score | Tandem Score | TDA q-value | Volume | Spectrum Matches |
| --- | --- | --- | --- | --- |
| -17.7629 | 115.8801 | 0.0000 | 3.5554e+07 | 1 |

**Adduct Signal Fractions**

- **Unmodified**: 3.5554e+07

### N(N)GTYDHDVYRDEALNNR Hex 5 HexNAc 3

**Mass:**  3470.4147

(8.966e-07)

**Charge States:** 4

**Start Position**: 483

**End Position**: 500

| Profile Score | Tandem Score | TDA q-value | Volume | Spectrum Matches |
| --- | --- | --- | --- | --- |
| -4.9382 | 115.2852 | 0.0000 | 5.4472e+07 | 1 |

**Adduct Signal Fractions**

- **Unmodified**: 5.4472e+07

### N(N)GTYDHDVYRDEALNNR Fuc 1 Hex 8 HexNAc 3

**Mass:**  4102.5876

(-9.904e-06)

**Charge States:** 3, 4

**Start Position**: 483

**End Position**: 500

| Profile Score | Tandem Score | TDA q-value | Volume | Spectrum Matches |
| --- | --- | --- | --- | --- |
| 7.3916 | 114.3151 | 0.0000 | 4.6207e+08 | 7 |

**Adduct Signal Fractions**

- **Unmodified**: 4.6207e+08

### N(N)GTYDHDVYRDEALNNR Hex 7 HexNAc 2

**Mass:**  3591.4433

(7.123e-07)

**Charge States:** 4

**Start Position**: 483

**End Position**: 500

| Profile Score | Tandem Score | TDA q-value | Volume | Spectrum Matches |
| --- | --- | --- | --- | --- |
| -17.7326 | 113.8051 | 0.0042 | 4.6506e+07 | 1 |

**Adduct Signal Fractions**

- **Unmodified**: 4.6506e+07

### N(N)GTYDHDVYRDEALN(D)NR Fuc 2 Hex 5 HexNAc 5

**Mass:**  4169.6755

(2.373e-04)

**Charge States:** 4

**Start Position**: 483

**End Position**: 500

| Profile Score | Tandem Score | TDA q-value | Volume | Spectrum Matches |
| --- | --- | --- | --- | --- |
| -20.0800 | 113.5585 | 0.0042 | 1.1880e+08 | 1 |

**Adduct Signal Fractions**

- **Unmodified**: 1.1880e+08

### N(N)GTYDHDVYR Fuc 1 Hex 6 HexNAc 3

**Mass:**  2966.1469

(7.723e-07)

**Charge States:** 3

**Start Position**: 483

**End Position**: 493

| Profile Score | Tandem Score | TDA q-value | Volume | Spectrum Matches |
| --- | --- | --- | --- | --- |
| -17.6352 | 113.4811 | 0.0042 | 4.3092e+07 | 1 |

**Adduct Signal Fractions**

- **Unmodified**: 4.3092e+07

### N(N)GTYDHDVYRDEALNNRFQIK Fuc 2 Hex 5 HexNAc 4

**Mass:**  4481.9189

(-5.696e-07)

**Charge States:** 5

**Start Position**: 483

**End Position**: 504

| Profile Score | Tandem Score | TDA q-value | Volume | Spectrum Matches |
| --- | --- | --- | --- | --- |
| -4.8685 | 112.8307 | 0.0042 | 6.8078e+07 | 2 |

**Adduct Signal Fractions**

- **Unmodified**: 6.8078e+07

### N(N)GTYDHDVYR Fuc 1 Hex 4 HexNAc 3

**Mass:**  2642.0465

(2.845e-06)

**Charge States:** 3

**Start Position**: 483

**End Position**: 493

| Profile Score | Tandem Score | TDA q-value | Volume | Spectrum Matches |
| --- | --- | --- | --- | --- |
| -21.2606 | 112.1546 | 0.0084 | 4.9272e+06 | 1 |

**Adduct Signal Fractions**

- **Unmodified**: 4.9272e+06

### N(N)GTYDHDVYR Fuc 1 Hex 6 HexNAc 5

**Mass:**  3372.3074

(2.311e-06)

**Charge States:** 3

**Start Position**: 483

**End Position**: 493

| Profile Score | Tandem Score | TDA q-value | Volume | Spectrum Matches |
| --- | --- | --- | --- | --- |
| -4.9424 | 110.4591 | 0.0119 | 6.8966e+07 | 3 |

**Adduct Signal Fractions**

- **Unmodified**: 6.8966e+07

### N(N)GTYDHDVYR Hex 5 HexNAc 4

**Mass:**  2861.1163

(1.042e-06)

**Charge States:** 3

**Start Position**: 483

**End Position**: 493

| Profile Score | Tandem Score | TDA q-value | Volume | Spectrum Matches |
| --- | --- | --- | --- | --- |
| -18.4135 | 110.2913 | 0.0119 | 1.4655e+07 | 1 |

**Adduct Signal Fractions**

- **Unmodified**: 1.4655e+07

### YPALN(N)VTMPN(D)NEQFDK Hex 8 HexNAc 2

**Mass:**  3583.4467

(2.765e-04)

**Charge States:** 3

**Start Position**: 161

**End Position**: 177

| Profile Score | Tandem Score | TDA q-value | Volume | Spectrum Matches |
| --- | --- | --- | --- | --- |
| -6.2267 | 109.5071 | 0.0119 | 3.5464e+07 | 2 |

**Adduct Signal Fractions**

- **Unmodified**: 3.5464e+07

### N(N)GTYDHDVYRDEALNNR Fuc 1 Hex 9 HexNAc 4

**Mass:**  4467.7209

(-8.813e-06)

**Charge States:** 4

**Start Position**: 483

**End Position**: 500

| Profile Score | Tandem Score | TDA q-value | Volume | Spectrum Matches |
| --- | --- | --- | --- | --- |
| -17.1330 | 109.3223 | 0.0119 | 2.2170e+07 | 1 |

**Adduct Signal Fractions**

- **Unmodified**: 2.2170e+07

### N(N)GTYDHDVYRDEALNNR Hex 5 HexNAc 5

**Mass:**  3876.5727

(6.013e-07)

**Charge States:** 3, 4

**Start Position**: 483

**End Position**: 500

| Profile Score | Tandem Score | TDA q-value | Volume | Spectrum Matches |
| --- | --- | --- | --- | --- |
| -18.4984 | 108.9273 | 0.0119 | 8.4569e+07 | 1 |

**Adduct Signal Fractions**

- **Unmodified**: 8.4569e+07

### RGSN(N)SSFFSR Fuc 2 Hex 5 HexNAc 4

**Mass:**  3059.2459

(3.291e-04)

**Start Position**: 141

**End Position**: 151

| Profile Score | Tandem Score | TDA q-value | Volume | Spectrum Matches |
| --- | --- | --- | --- | --- |
| 0.0000 | 108.3394 | 0.0119 | 0.0000e+00 | 1 |

Adduct:

No Chromatogram Found

### N(N)GTYDHDVYRDEALNNR Fuc 1 Hex 5 HexNAc 4 Neu5Ac 1

**Mass:**  4110.6491

(1.184e-06)

**Charge States:** 4

**Start Position**: 483

**End Position**: 500

| Profile Score | Tandem Score | TDA q-value | Volume | Spectrum Matches |
| --- | --- | --- | --- | --- |
| -19.1001 | 105.3097 | 0.0276 | 1.1447e+07 | 1 |

**Adduct Signal Fractions**

- **Unmodified**: 1.1447e+07

### SEC(C)ITPN(N)GSIPN(D)DKPFQNVNR Hex 9 HexNAc 2

**Mass:**  4251.7561

(2.324e-04)

**Charge States:** 3, 4

**Start Position**: 279

**End Position**: 300

| Profile Score | Tandem Score | TDA q-value | Volume | Spectrum Matches |
| --- | --- | --- | --- | --- |
| 8.9474 | 103.6825 | 0.0319 | 5.2840e+08 | 7 |

**Adduct Signal Fractions**

- **Unmodified**: 5.2840e+08

### N(N)GTYDHDVYRDEALNNR Fuc 2 Hex 9 HexNAc 4

**Mass:**  4613.7731

(-9.747e-06)

**Charge States:** 4

**Start Position**: 483

**End Position**: 500

| Profile Score | Tandem Score | TDA q-value | Volume | Spectrum Matches |
| --- | --- | --- | --- | --- |
| -4.9951 | 103.1038 | 0.0319 | 3.4952e+07 | 1 |

**Adduct Signal Fractions**

- **Unmodified**: 3.4952e+07

### N(N)GTYDHDVYRDEALNNR Fuc 1 Hex 10 HexNAc 4

**Mass:**  4629.7736

(-8.460e-06)

**Charge States:** 4

**Start Position**: 483

**End Position**: 500

| Profile Score | Tandem Score | TDA q-value | Volume | Spectrum Matches |
| --- | --- | --- | --- | --- |
| -5.0589 | 102.3822 | 0.0319 | 3.1154e+07 | 1 |

**Adduct Signal Fractions**

- **Unmodified**: 3.1154e+07

### N(N)GTYDHDVYRDEALN(D)NR Hex 6 HexNAc 3

**Mass:**  3633.4523

(2.720e-04)

**Charge States:** 4

**Start Position**: 483

**End Position**: 500

| Profile Score | Tandem Score | TDA q-value | Volume | Spectrum Matches |
| --- | --- | --- | --- | --- |
| -6.2071 | 101.3437 | 0.0319 | 7.3654e+07 | 2 |

**Adduct Signal Fractions**

- **Unmodified**: 7.3654e+07

### RGSN(N)SSFFSR Fuc 4 Hex 8 HexNAc 7

**Mass:**  4445.7927

(9.212e-06)

**Charge States:** 4

**Start Position**: 141

**End Position**: 151

| Profile Score | Tandem Score | TDA q-value | Volume | Spectrum Matches |
| --- | --- | --- | --- | --- |
| -20.0224 | 100.5108 | 0.0319 | 1.7307e+07 | 1 |

**Adduct Signal Fractions**

- **Unmodified**: 1.7307e+07

### ISIYWTIVKPGDILLIN(N)STGNLIAPR Hex 10 HexNAc 2

**Mass:**  4893.3239

(1.913e-07)

**Charge States:** 4

**Start Position**: 230

**End Position**: 256

| Profile Score | Tandem Score | TDA q-value | Volume | Spectrum Matches |
| --- | --- | --- | --- | --- |
| -4.1416 | 99.7082 | 0.0319 | 3.9331e+07 | 4 |

**Adduct Signal Fractions**

- **Unmodified**: 3.9331e+07

### LNWLTHLNSKYPALN(N)VTM(O)PNNEQFDK Hex 8 HexNAc 2

**Mass:**  4805.1218

(3.604e-06)

**Charge States:** 4, 5

**Start Position**: 151

**End Position**: 177

| Profile Score | Tandem Score | TDA q-value | Volume | Spectrum Matches |
| --- | --- | --- | --- | --- |
| -17.6225 | 99.4480 | 0.0319 | 4.1322e+07 | 2 |

**Adduct Signal Fractions**

- **Unmodified**: 4.1322e+07

### N(N)GTYDHDVYR Fuc 3 Hex 9 HexNAc 7

**Mass:**  4556.7575

(4.611e-06)

**Charge States:** 4

**Start Position**: 483

**End Position**: 493

| Profile Score | Tandem Score | TDA q-value | Volume | Spectrum Matches |
| --- | --- | --- | --- | --- |
| -3.8603 | 98.2528 | 0.0319 | 1.0191e+08 | 1 |

**Adduct Signal Fractions**

- **Unmodified**: 1.0191e+08

### N(N)GTYDHDVYRDEALNNR Hex 6 HexNAc 4

**Mass:**  3835.5539

(1.502e-06)

**Charge States:** 4

**Start Position**: 483

**End Position**: 500

| Profile Score | Tandem Score | TDA q-value | Volume | Spectrum Matches |
| --- | --- | --- | --- | --- |
| -6.5645 | 96.9599 | 0.0319 | 2.3722e+07 | 1 |

**Adduct Signal Fractions**

- **Unmodified**: 2.3722e+07

### SEC(C)ITPN(N)GSIPN(D)DKPFQNVNR Hex 8 HexNAc 2

**Mass:**  4089.7050

(2.419e-04)

**Charge States:** 3, 4

**Start Position**: 279

**End Position**: 300

| Profile Score | Tandem Score | TDA q-value | Volume | Spectrum Matches |
| --- | --- | --- | --- | --- |
| 9.9760 | 92.6519 | 0.0412 | 3.0909e+08 | 5 |

**Adduct Signal Fractions**

- **Unmodified**: 3.0909e+08

### SEC(C)ITPN(N)GSIPN(D)DKPFQNVNR Hex 7 HexNAc 2

**Mass:**  3927.6523

(2.520e-04)

**Charge States:** 3, 4, 5

**Start Position**: 279

**End Position**: 300

| Profile Score | Tandem Score | TDA q-value | Volume | Spectrum Matches |
| --- | --- | --- | --- | --- |
| 11.0663 | 92.6014 | 0.0412 | 5.6447e+08 | 7 |

**Adduct Signal Fractions**

- **Unmodified**: 5.6447e+08

### N(N)GTYDHDVYR Fuc 2 Hex 6 HexNAc 5

**Mass:**  3518.3655

(1.186e-06)

**Charge States:** 3, 4

**Start Position**: 483

**End Position**: 493

| Profile Score | Tandem Score | TDA q-value | Volume | Spectrum Matches |
| --- | --- | --- | --- | --- |
| -19.7795 | 91.6859 | 0.0442 | 1.9780e+07 | 1 |

**Adduct Signal Fractions**

- **Unmodified**: 1.9780e+07

### cus|SWZNA|A/Switzerland/9715293/2013

**>cus|SWZNA|A/Switzerland/9715293/2013**  
 `MNPNQKIITIGSVSLTISTICFFMQIAILITTVTLHFKQYEFNSPPNNQVMLCEPTIIERNITEIVYLTNTTIEKEICPK  
PAEYRNWSKPQCGITGFAPFSKDNSIRLSAGGDIWVTREPYVSCDPDKCYQFALGQGTTLNNVHSNNTVRDRTPYRTLLM  
NELGVPFHLGTKQVCIAWSSSSCHDGKAWLHVCITGDDKNATASFIYNGRLVDSVVSWSKDILRTQESECVCINGTCTVV  
MTDGSASGKADTKILFIEEGKIVHTSTLSGSAQHVEECSCYPRYPGVRCVCRDNWKGSNRPIVDINIKDHSIVSSYVCSG  
LVGDTPRKNDSSSSSHCLDPNNEEGGHGVKGWAFDDGNDVWMGRTINETSRLGYETFKVIEGWSNPKSKLQTNRQVIVDR  
GDRSGYSGIFSVEGKSCINRCFYVELIRGRKEETEVLWTSNSIVVFCGTSGTYGTGSWPDGADLNLMPI`

| Site | Type | Count |
| --- | --- | --- |
| 86 | N-linked | 9 |
| 367 | N-linked | 1 |
| 200 | N-linked | 1 |

| Mass | Sequence | MS_2_ Score | `q`-value | MS_1_ Score | Signal | Protein Coordinates | Tandem Scans | Start Time | Apex Time | End Time |
| --- | --- | --- | --- | --- | --- | --- | --- | --- | --- | --- |
| 2521.9925 | TIN(N)ETSR Hex 8 HexNAc 2 | 173.478 | 0.0 | -17.072 | 7.320e+06 | 364 - 371 | 4 | 13.76 | 13.76 | 13.76 |
| 3411.3935 | N(N)WSKPQC(C)GITGFAPF Hex 8 HexNAc 2 | 163.886 | 0.0 | -3.087 | 1.554e+08 | 85 - 100 | 3 | 51.61 | 51.78 | 51.95 |
| 3788.5727 | N(N)WSKPQC(C)GITGFAPFSK Hex 9 HexNAc 2 | 156.832 | 0.0 | -1.262 | 6.717e+08 | 85 - 102 | 4 | 42.83 | 43.00 | 43.33 |
| 3626.5166 | N(N)WSKPQC(C)GITGFAPFSK Hex 8 HexNAc 2 | 150.939 | 0.0 | -2.121 | 7.875e+08 | 85 - 102 | 4 | 43.00 | 43.17 | 43.50 |
| 3590.4754 | N(N)WSKPQC(C)GITGFAPF Fuc 2 Hex 3 HexNAc 4 Neu5Ac 1 | 140.851 | 0.0 | -18.957 | 2.585e+07 | 85 - 100 | 1 | 51.44 | 51.61 | 51.61 |
| 3464.4619 | N(N)WSKPQC(C)GITGFAPFSK Hex 7 HexNAc 2 | 135.843 | 0.0 | -4.576 | 5.397e+07 | 85 - 102 | 2 | 43.17 | 43.33 | 43.50 |
| 3805.5992 | N(N)WSKPQC(C)GITGFAPFSK Fuc 2 Hex 3 HexNAc 4 Neu5Ac 1 | 128.526 | 0.0 | -18.879 | 3.748e+07 | 85 - 102 | 3 | 42.83 | 43.00 | 43.00 |
| 3573.4460 | N(N)WSKPQC(C)GITGFAPF Hex 9 HexNAc 2 | 124.935 | 0.0 | -2.905 | 1.762e+08 | 85 - 100 | 3 | 51.44 | 51.61 | 51.78 |
| 4472.8956 | AWLHVC(C)ITGDDKN(N)ATASFIYNGR Hex 9 HexNAc 2 | 122.106 | 0.0 | -20.142 | 4.228e+06 | 187 - 210 | 1 | 46.18 | 46.34 | 46.34 |
| 3249.3393 | N(N)WSKPQC(C)GITGFAPF Hex 7 HexNAc 2 | 112.290 | 0.004 | -19.605 | 4.344e+06 | 85 - 100 | 1 | 51.95 | 51.95 | 51.95 |
| 3302.4130 | N(N)WSKPQC(C)GITGFAPFSK Hex 6 HexNAc 2 | 94.250 | 0.039 | -20.026 | 3.343e+06 | 85 - 102 | 1 | 43.33 | 43.50 | 43.50 |

### TIN(N)ETSR Hex 8 HexNAc 2

**Mass:**  2521.9925

(1.038e-06)

**Charge States:** 2

**Start Position**: 365

**End Position**: 372

| Profile Score | Tandem Score | TDA q-value | Volume | Spectrum Matches |
| --- | --- | --- | --- | --- |
| -17.0715 | 173.4776 | 0.0000 | 7.3200e+06 | 4 |

**Adduct Signal Fractions**

- **Unmodified**: 7.3200e+06

### N(N)WSKPQC(C)GITGFAPF Hex 8 HexNAc 2

**Mass:**  3411.3938

(2.513e-06)

**Charge States:** 3

**Start Position**: 86

**End Position**: 101

| Profile Score | Tandem Score | TDA q-value | Volume | Spectrum Matches |
| --- | --- | --- | --- | --- |
| -3.0874 | 163.8857 | 0.0000 | 1.5536e+08 | 3 |

**Adduct Signal Fractions**

- **Unmodified**: 1.5536e+08

### N(N)WSKPQC(C)GITGFAPFSK Hex 9 HexNAc 2

**Mass:**  3788.5709

(3.359e-06)

**Charge States:** 3, 4

**Start Position**: 86

**End Position**: 103

| Profile Score | Tandem Score | TDA q-value | Volume | Spectrum Matches |
| --- | --- | --- | --- | --- |
| -1.2624 | 156.8323 | 0.0000 | 6.7169e+08 | 4 |

**Adduct Signal Fractions**

- **Unmodified**: 6.7169e+08

### N(N)WSKPQC(C)GITGFAPFSK Hex 8 HexNAc 2

**Mass:**  3626.5149

(2.752e-06)

**Charge States:** 3, 4

**Start Position**: 86

**End Position**: 103

| Profile Score | Tandem Score | TDA q-value | Volume | Spectrum Matches |
| --- | --- | --- | --- | --- |
| -2.1210 | 150.9395 | 0.0000 | 7.8750e+08 | 4 |

**Adduct Signal Fractions**

- **Unmodified**: 7.8750e+08

### N(N)WSKPQC(C)GITGFAPF Fuc 2 Hex 3 HexNAc 4 Neu5Ac 1

**Mass:**  3590.4750

(-3.165e-06)

**Charge States:** 3

**Start Position**: 86

**End Position**: 101

| Profile Score | Tandem Score | TDA q-value | Volume | Spectrum Matches |
| --- | --- | --- | --- | --- |
| -18.9567 | 140.8511 | 0.0000 | 2.5850e+07 | 1 |

**Adduct Signal Fractions**

- **Unmodified**: 2.5850e+07

### N(N)WSKPQC(C)GITGFAPFSK Hex 7 HexNAc 2

**Mass:**  3464.4618

(1.270e-06)

**Charge States:** 3

**Start Position**: 86

**End Position**: 103

| Profile Score | Tandem Score | TDA q-value | Volume | Spectrum Matches |
| --- | --- | --- | --- | --- |
| -4.5755 | 135.8431 | 0.0000 | 5.3972e+07 | 2 |

**Adduct Signal Fractions**

- **Unmodified**: 5.3972e+07

### N(N)WSKPQC(C)GITGFAPFSK Fuc 2 Hex 3 HexNAc 4 Neu5Ac 1

**Mass:**  3805.5998

(-4.627e-06)

**Charge States:** 4

**Start Position**: 86

**End Position**: 103

| Profile Score | Tandem Score | TDA q-value | Volume | Spectrum Matches |
| --- | --- | --- | --- | --- |
| -18.8790 | 128.5260 | 0.0000 | 3.7479e+07 | 3 |

**Adduct Signal Fractions**

- **Unmodified**: 3.7479e+07

### N(N)WSKPQC(C)GITGFAPF Hex 9 HexNAc 2

**Mass:**  3573.4462

(2.053e-06)

**Charge States:** 3

**Start Position**: 86

**End Position**: 101

| Profile Score | Tandem Score | TDA q-value | Volume | Spectrum Matches |
| --- | --- | --- | --- | --- |
| -2.9054 | 124.9346 | 0.0000 | 1.7620e+08 | 3 |

**Adduct Signal Fractions**

- **Unmodified**: 1.7620e+08

### AWLHVC(C)ITGDDKN(N)ATASFIYNGR Hex 9 HexNAc 2

**Mass:**  4472.9006

(1.067e-06)

**Charge States:** 4

**Start Position**: 188

**End Position**: 211

| Profile Score | Tandem Score | TDA q-value | Volume | Spectrum Matches |
| --- | --- | --- | --- | --- |
| -20.1421 | 122.1060 | 0.0000 | 4.2282e+06 | 1 |

**Adduct Signal Fractions**

- **Unmodified**: 4.2282e+06

### N(N)WSKPQC(C)GITGFAPF Hex 7 HexNAc 2

**Mass:**  3249.3393

(2.331e-06)

**Charge States:** 3

**Start Position**: 86

**End Position**: 101

| Profile Score | Tandem Score | TDA q-value | Volume | Spectrum Matches |
| --- | --- | --- | --- | --- |
| -19.6047 | 112.2902 | 0.0042 | 4.3436e+06 | 1 |

**Adduct Signal Fractions**

- **Unmodified**: 4.3436e+06

### N(N)WSKPQC(C)GITGFAPFSK Hex 6 HexNAc 2

**Mass:**  3302.4146

(2.617e-06)

**Charge States:** 3

**Start Position**: 86

**End Position**: 103

| Profile Score | Tandem Score | TDA q-value | Volume | Spectrum Matches |
| --- | --- | --- | --- | --- |
| -20.0258 | 94.2504 | 0.0389 | 3.3432e+06 | 1 |

**Adduct Signal Fractions**

- **Unmodified**: 3.3432e+06

### tr|E1C041|E1C041\_CHICK

**>tr|E1C041|E1C041\_CHICK**  
 `MRTGAGEAVGVLCLVSLVAALDYKYHHSEELEAYLKEVHAAYPALTHLHSIGRSVEGRDLWVLVLGRFPTQHKIGIPEFK  
YVANMHGDETVGREILLHLIDHLVTNYGRDPVITRLLNNTRIHIMPTMNPDGFEATVVPDCYYSRGRYNKNGEDLNRNFP  
DAFENNNNLIQPETQAVINWIKNETFVLSANLHGGALVASYTFDNGNSVTGTSNGYSRSPDDDVFIHLAKTYSFNHASMY  
KGTGCDSKQTFPDGITNGYSWYQLEGGMQDYNYVWGQCFEITLELSCCKYPPAEQLEKFWRDNKVALVEYIKQVHLGVKG  
QVTDKNGNPIPNAIVEAKGRPHICPYRTNEHGEYFLLLLPGKYVINATVPGFKSMLKTVDIPDNTANFSALKQDFSFPEV  
SVKPRPASCPRTPLYQELERSSAAVKPTLHILVLVIVTISILK`

| Site | Type | Count |
| --- | --- | --- |
| 387 | N-linked | 1 |
| 366 | N-linked | 4 |

| Mass | Sequence | MS_2_ Score | `q`-value | MS_1_ Score | Signal | Protein Coordinates | Tandem Scans | Start Time | Apex Time | End Time |
| --- | --- | --- | --- | --- | --- | --- | --- | --- | --- | --- |
| 3072.2988 | YVIN(N)ATVPGFK Hex 9 HexNAc 2 | 166.865 | 0.0 | -2.240 | 7.652e+07 | 362 - 373 | 3 | 40.66 | 40.83 | 41.00 |
| 3089.3259 | YVIN(N)ATVPGFK Fuc 2 Hex 3 HexNAc 4 Neu5Ac 1 | 155.556 | 0.0 | -19.075 | 1.232e+07 | 362 - 373 | 1 | 40.83 | 40.83 | 41.00 |
| 3234.3515 | YVIN(N)ATVPGFK Hex 10 HexNAc 2 | 151.783 | 0.0 | -18.373 | 8.206e+06 | 362 - 373 | 1 | 40.66 | 40.83 | 40.83 |
| 2910.2457 | YVIN(N)ATVPGFK Hex 8 HexNAc 2 | 145.253 | 0.0 | -17.796 | 2.270e+07 | 362 - 373 | 2 | 40.83 | 41.00 | 41.00 |
| 3576.5303 | TVDIPDNTAN(N)FSALK Fuc 1 Hex 5 HexNAc 5 | 135.011 | 0.0 | -17.551 | 9.054e+06 | 377 - 392 | 2 | 40.16 | 40.16 | 40.16 |

### YVIN(N)ATVPGFK Hex 9 HexNAc 2

**Mass:**  3072.2986

(1.792e-06)

**Charge States:** 3

**Start Position**: 363

**End Position**: 374

| Profile Score | Tandem Score | TDA q-value | Volume | Spectrum Matches |
| --- | --- | --- | --- | --- |
| -2.2398 | 166.8651 | 0.0000 | 7.6522e+07 | 3 |

**Adduct Signal Fractions**

- **Unmodified**: 7.6522e+07

### YVIN(N)ATVPGFK Fuc 2 Hex 3 HexNAc 4 Neu5Ac 1

**Mass:**  3089.3259

(-6.893e-06)

**Charge States:** 3

**Start Position**: 363

**End Position**: 374

| Profile Score | Tandem Score | TDA q-value | Volume | Spectrum Matches |
| --- | --- | --- | --- | --- |
| -19.0753 | 155.5562 | 0.0000 | 1.2317e+07 | 1 |

**Adduct Signal Fractions**

- **Unmodified**: 1.2317e+07

### YVIN(N)ATVPGFK Hex 10 HexNAc 2

**Mass:**  3234.3513

(1.524e-06)

**Charge States:** 3

**Start Position**: 363

**End Position**: 374

| Profile Score | Tandem Score | TDA q-value | Volume | Spectrum Matches |
| --- | --- | --- | --- | --- |
| -18.3728 | 151.7827 | 0.0000 | 8.2061e+06 | 1 |

**Adduct Signal Fractions**

- **Unmodified**: 8.2061e+06

### YVIN(N)ATVPGFK Hex 8 HexNAc 2

**Mass:**  2910.2455

(2.065e-06)

**Charge States:** 3

**Start Position**: 363

**End Position**: 374

| Profile Score | Tandem Score | TDA q-value | Volume | Spectrum Matches |
| --- | --- | --- | --- | --- |
| -17.7958 | 145.2531 | 0.0000 | 2.2702e+07 | 2 |

**Adduct Signal Fractions**

- **Unmodified**: 2.2702e+07

### TVDIPDNTAN(N)FSALK Fuc 1 Hex 5 HexNAc 5

**Mass:**  3576.5297

(2.144e-06)

**Charge States:** 3, 4

**Start Position**: 378

**End Position**: 393

| Profile Score | Tandem Score | TDA q-value | Volume | Spectrum Matches |
| --- | --- | --- | --- | --- |
| -17.5512 | 135.0112 | 0.0000 | 9.0539e+06 | 2 |

**Adduct Signal Fractions**

- **Unmodified**: 9.0539e+06

### tr|F1NV02|F1NV02\_CHICK

**>tr|F1NV02|F1NV02\_CHICK**  
 `MGPVQLLLLLLLLSSGVLTQEGTPENGNPGCSKDAARFKSLRKYVYLYEAETSSGITGTADSHSGSKITCKVELEVPQLC  
QFILRTMHCSLRETFGVDSERRAMLRKSKNSDDFANAMSKHELRFSTQDGTKVKLYPEKDEPLNVLNLKRGIISALLAPT  
ETEENIKTISMDTVYGKCDSEVEFKSRRGSVAEDISINRNLKACDNFSPIRDYVSPVAIVKGLNIPLSTLLSSTQSCHYS  
IDAKKKHIRDVVCSEKHLFLPSSYKNQYGMMTEVNQTLKLEDNQRMNNRNPDGDELEEKGLALESTDAKFSRQGDAVLKI  
LQELQKLTASQQNQQRAKLFYKFVSGLRSLHNSTLGSLVPKMMETSSSITIQALIQCGTPECYSAVLQILRTGNVNPLVV  
DLVTYTLGLLPSPTPKRIREILNMAQYQPSRASFYGLSHAVTKFYSEKMIVTEEITDVADFMVSLLGTDCSGDAELTYLT  
LRAIGNMGAVMEKAKPSLKASLKTCIRNQAASLSVQKAAIQAFRKMTITEEDRSALLKEFQEGDAPTDKRLATYLILMKN  
PSPADLAKIMRILTREKNEQVKSFVASHIANILDSDEVGIEDLKSHVEEALKGNEVPTAKDFRKFSQNYQVSKRVSVPGL  
NPISAKVEGNVIFDPSSYVPKETMLKTTLNLYGFGPSDIFELGLDGKGFEPTLEALFGEKGFFPDTASKALYWVDGKVPE  
QVSKALFDYFGYSHDGKQDQEITKAVILNLEKLIKELSKKEAPEGRAFLRILGEELGYMKLSDFKLLGSVALECIKTLQR  
IPEIIAQAISKGVDKDLFVHYMFMDNEFELPTGAGLQLKFALSGIVTPGAKVAVKLHQKSMQAELIAKPSVAVEFVTHLG  
INMPEFARSGVEMNSNIFHESGIEAHVSVKAGQLKFSIPAPKTPTKLLSISNTLHLVSPAKTEEIPPLIENREFSTSCKP  
FISGLNFCTKLLYSNASSMEAAPYYPLTGETRFEVEIVSTGEVKEYSASANYDLQREGTDLVDTLKFAVQAEGVKQHEAT  
LTFKYNRDRKILTSDVSIPDVDVDFGTNFRITDESVSGKKAYTFVIDFNNKKISEVTLTGQIRYAGIEEAMLRGTVSIPR  
LQTELKTEALVNYSPTKGYLQMSSSVGTHGNTVSKRVLLRYDSEKAELEWNSGATAAVGRMSSAFQVDFSDYSKTLEKYA  
NELLDRKVALTDMTMRHIVSQFIVATNTWLQKASKDVPYAQTLQAKLSGLQELNIQKIKLPVITIPEELFLKSEGRIKYS  
FNKNSFLINIPLPFGGRSSHDIRVPQTVKTPRLVIESMGINIPSQEYRMPTFTVPESYPLLVPLFGALEASASVHSNYYN  
WTAAYTLTNSSTEKTARIGTTYAVNADSVFELLSYNMKGSGEASSSRNGFTCAYENHLKHRLLTSDFKMSRTKSYEPTSV  
SNCTIFLMASSALGPQLSFSSDVVSEKTNNMNINNVRIEGQLEVASVFARSVYTMSSSYNEKRRVLEGKSNLRLDSSYLQ  
ATNHLSGRYTDGVFSITSASDVQNGLLKNTASLKYENSQLKITSETNGRYLHLAAVNKLEFLLSKKMAALRSEYQATYKQ  
TQCYALFTGSLNSQDLVFNTDFSLTDQRNRAAHKSSLNVNQYGLASSATTNVQFSPLTMQSEMNAKLDTSGGSVSLSSSG  
RYGKNNAKFNVGGRVSLTEITLGSEYQSTILGMDNKHVLNFRINKEGLKFSNNLQGSLKEIKLEYTNDLNIPGLSLTFVS  
KLDNSFSFDKFHKHVFDLQLQPRSLTAKLNNNIKYTKTEVSNKAELLLEPLKLNLGGNVRAAYGTDEVRHTYAITYADLT  
ANFKTDTVANVQGAAVSHRVNLNVAGLASSITMNTNCDSKSLRFSNALRSTMAPFTITADVHTNGNGKLIALGEHTGDLY  
SKILFKAEPLAFTFSHDYRGSTSHSFKSMRRYSTQLDNKFHMLFTPSEQSSAWKLKSQLNNNIYSQDINAYNDAEKIGVE  
LSGRALADLSVVDTAIRLPFTSEEVNVIDVLGLRDSVSEPQEFSISGSVKYDKNKDMHVINLPFLEHFPVYFEQIRGAIL  
STLQAVQNYLKNIDIDQYMKKYKATLDEFPQHLNDYMDKLDLKGRASTIKTNLIAFTKDYRITSDDLEIILEKALDNLQE  
ILLQLQVYLVQIEQYIKENYDQFDINALIAQLLDKIVEKMTALDEKYKIRVTVVDTIQKLQFFLNQYYPSNIGSSTMTWI  
KNIDDEYRITGRIKENLEQLKIQIQNIDIRSFAENLKRKIKITDVKQLLEKLKRSLPIKKMKEVLEQIKDFILSWMEEYE  
VSEKISAFRGHMHKLIVKYEIDKHVYFLLDRMIELLNQYRIRETVRKMTTYLRKIDLKTCFDKIVSLIDDAVKKVQTFDY  
EMMIKKLNKFLDMIIKKLKSFDYNQFVDDTNNKIQEIIQKINEELRNLELPQKAEALKQYMRDFNAVVSKYVEQLRDTKL  
VAIINWLKELIDSTTFTNLKAKVNEHLEDLRERISDMDISKEFEWYLQKISQFYNSVVIYISEQWNIAFKKIVTLAEKYD  
LKNWAENLNQFIETGFKVPEIRTVIVTIPAFEFSLRSLREATFRTPDFIVPLTDLKIPSYEINIRRLKDMKIPAKFTTPE  
FTVLNSFKVPSYTIDLNEIKFQIVRMIDQLISGEFQLPAIDLYFKDLKMRDMPFSEISFPELQMPQLEIPELLIPKLNLN  
EFQIPDLKIPEFQLPRIPHTVTAPTFGKLSGAFRVASPFFTLSTQAEVHNTTASANSPEFVTSLSAQATSKLDFLVFSVI  
ADSHILAPEMKQLKLKNSMKVSHKFLKIDHTNEVVFLGTSVSGEAETRAKFSAIKNSIELQNNLMVNLQRKIQMQSGTAY  
SHRLNIPEADFSSQADLVNNMTTEVEAGRISFTSNGKGNWKWTSPNFSDEGTHNSHATFRVDGPILIFFADYRINDRYLK  
VSQSMRYECGFLSYATLQVQSEIESQRVGRSILNVKGTGQLGGMKVELTGSHNARLNGRITGTVNNDFFFLVQPFEIRLL  
TNNDGNVKISFPMKLTGKIDFLNNYGLSLSSSVQQVSWQATGRFNQYRYSHNMSAGNNDDRIEAHVEMSGDANLDFLNIP  
LTIPQLHIPYTGIQTPQLKDYSLWEQAGLKNLLKTTRQSFDLNLNAQYEKNKDMHVIPLPLATVHEALNKYIIFFNKYFE  
RGRNTALDFLTKSYNEAKTKFDKYKIQTSLNKLPRTFRIPGYTIPIVNIEVSPFTAEMPAFGYVLPKEISTTGFTVPFIG  
FSVPSYTLVLPSLELPVLHVPQDLRTLKLPRFRINSPSNQILIPAMGNITYDFSFKSSVITLTANAGLFNQSDIAGHLSI  
SSSSVIDALQFKLDGSTSLTRKRGLKLATALSLNNNKFLGGSHDNSISLTKKNLEASMMTNAKINTPVFKMNFSQELSGN  
TKSKPTISSGLKVTYDFTTPKHGISAKGGVAHKLALETLTSYLSVETSTKGNIDGAIYTGNSFSGALDHEANTYLHANGV  
RSSLKLEANSKVDGLWNSEMKEILAVEASTSRVYAVWEHNGKNFARYTPLFTTTGSQKCKATFELAPWTVSADLQIQVTQ  
PNSFLDTASVNQVVLMKVSPTDQKVGWKGEGQIQSLSLRHDMQLSNEKSNAKFDISGSLEGYMDFLKRINCAISKKSLWD  
ILKLDVTTVADRKHYLNASASFIYRKSDDGYFFPMPVIRLSDGFTFSIPELHLKAPSPVLSTPEFRVPFSTLQVPAYTID  
LRNIKIPQTLNIMPFDVNLPTLPKLRFPRVDVGANYITLEEYKIPYFEVTVPEYQITVSQFTLPKSISLGSFHVDLDEVA  
NKIADFDLPTITIPEQKIEIPPLKVSLPAGIYIPSFGALTGSLKVASPLYNVTWRTDLTNKKESFEVSIDSTCSSTLQFL  
EYDLNVVSNYKYEEGKFVKKTVGSFAHRDLSANYIEDLALQGFGTVENTASLDVISPTFADVHVRYQMTDNMISSTVSSP  
SAGILGFLVQIETDTLKEKFYYRTLSAPQKDIDILKSEISFRNSDIIQIKLNWKEDAAKDLLLGLKEKVPKMTSAVYKCV  
NRYYKEHMGLDISDATVIMKNILQNNADKAYMFAARQVDQMDVQLRTAANEASEKYQEMKVKARQLYKRAAEQAEQIDYQ  
RIKARLLDATVDLMEEYHSKIRHLIDSVIEFLKTTKFQIPGLSEKYTGEELYLMATEKAAKTADICLSKLQEYFDALIAA  
INELEVRVPASETILRGRNVLDQIKEMLKHLQEKIRQTFVTLQEADFAGKLNQLKQVVQKTFQKAGNMVRSLQSKNFEDI  
KVQMQQLYKDAMASDYAHKLRSLAENVKKYISQMKNFSQKTLQKVSENLQQLVLYIKALREEYFDPTTLGWSVKYYEVED  
KVLGLLKNLMDTLVIWYNEYAKDLSDLVTRLTDQVRELVENYRQEYYDLITDVEGKGRQKVMELSSAAQEKIRYWSAVAK  
RKINEHNRQVKAKLQEIYGQLSDSQEKLINVAKMLIDLTVEKYSTFMKYIFELLRWFEQATADSIKPYIAVREGELRIDV  
PFDWEYINQMPQKSREALRNKVELTRALIQQGVEQGTRKWEEMQAFIDEQLATEQLSFQQIVENIQKRMKT`

| Site | Type | Count |
| --- | --- | --- |
| 3891 | N-linked | 1 |

| Mass | Sequence | MS_2_ Score | `q`-value | MS_1_ Score | Signal | Protein Coordinates | Tandem Scans | Start Time | Apex Time | End Time |
| --- | --- | --- | --- | --- | --- | --- | --- | --- | --- | --- |
| 2521.1155 | VASPLYN(N)VTWR Hex 5 HexNAc 2 | 138.966 | 0.0 | 0.000 | 0.000e+00 | 3884 - 3895 | 1 | - | - | - |

### VASPLYN(N)VTWR Hex 5 HexNAc 2

**Mass:**  2521.1155

(1.939e-06)

**Start Position**: 3885

**End Position**: 3896

| Profile Score | Tandem Score | TDA q-value | Volume | Spectrum Matches |
| --- | --- | --- | --- | --- |
| 0.0000 | 138.9661 | 0.0000 | 0.0000e+00 | 1 |

Adduct:

No Chromatogram Found

### tr|A0A1D5P780|A0A1D5P780\_CHICK

**>tr|A0A1D5P780|A0A1D5P780\_CHICK**  
 `MRGIILALVLTLVDPGFNSRRSYLYNYEGSMLNGLQDRSLGKAGVRLSSKLEISGLPENAYLLKVRSPQVEEYNGVWPRD  
PFTRSSKITQVISSCFTRLFKFEYSSGRIGNIYAPEDCPDLCVNIVRGILNMFQMTIKKSQNVYELQEAGIGGICHARYV  
IQEDRKNSRIYVTRTVDLNNCQEKVQKSIGMAYIYPCPVDVMKERLTKGTTAFSYKLKQSDSGTLITDVSSRQVYQISPF  
NEPTGVAVMEARQQLTLVEVRSERGSAPDVPMQNYGSLRYRFPAVLPQMPLQLIKTKNPEQRIIETLQHIVLNNQQDFHD  
DVSYRFLEVVQLCRIANADNLESIWRQVSDKPRYRRWLLSAVSASGTTETLKFLKNRIRNDDLNYIQTLLTVSLTLHLLQ  
ADEHTLPIAADLMTSSRIQKNPVLQQVACLGYSSVVNRYCSQTSACPKEALQPIHDLADEAISRGREDKMKLALKCIGNM  
GEPASLKRILKFLPISSSSAADIPVHIQIDAITALKKIAWKDPKTVQGYLIQILADQSLPPEVRMMACAVIFETRPALAL  
ITTIANVAMKESNMQVASFVYSHMKSLSKSRLPFMYNISSACNIALKLLSPKLDSMSYRYSKVIRADTYFDNYRVGATGE  
IFVVNSPRTMFPSAIISKLMANSAGSVADLVEVGIRVEGLADVIMKRNIPFAEYPTYKQIKELGKALQGWKELPTETPLV  
SAYLKILGQEVAFININKELLQQVMKTVVEPADRNAAIKRIANQIRNSIAGQWTQPVWMGELRYVVPSCLGLPLEYGSYT  
TALARAAVSVEGKMTPPLTGDFRLSQLLESTMQIRSDLKPSLYVHTVATMGVNTEYFQHAVEIQGEVQTRMPMKFDAKID  
VKLKNLKIETNPCREETEIVVGRHKAFAVSRNIGELGVEKRTSILPEDAPLDVTEEPFQTSERASREHFAMQGPDSMPRK  
QSHSSREDLRRSTGKRAHKRDICLKMHHIGCQLCFSRRSRDASFIQNTYLHKLIGEHEAKIVLMPVHTDADIDKIQLEIQ  
AGSRAAARIITEVNPESEEEDESSPYEDIQAKLKRILGIDSMFKVANKTRHPKNRPSKKGNTVLAEFGTEPDAKTSSSSS  
SASSTATSSSSSSASSPNRKKPMDEEENDQVKQARNKDASSSSRSSKSSNSSKRSSSKSSNSSKRSSSSSSSSSSSSSSS  
SRSSSSSSSSSSNSKSSSSSSKSSSSSSRSRSSSKSSSSSSSSSSSSSSKSSSSRSSSSSSKSSSHHSHSHHSGHLNGSS  
SSSSSSRSVSHHSHEHHSGHLEDDSSSSSSSSVLSKIWGRHEIYQYRFRSAHRQEFPKRKLPGDRATSRYSSTRSSHDTS  
RAASWPKFLGDIKTPVLAAFLHGISNNKKTGGLQLVVYADTDSVRPRVQVFVTNLTDSSKWKLCADASVRNAHKAVAYVK  
WGWDCRDYKVSTELVTGRFAGHPAAQVKLEWPKVPSNVRSVVEWFYEFVPGAAFMLGFSERMDKNPSRQARMVVALTSPR  
TCDVVVKLPDMILYQKAVRLPLSLPVGPRIPASELQSPIWNVFAEAPSAVLENLKARCSVSYNKIKTFNEVKFNYSMPAN  
CYHILVQDCSSELKFLVMMKSAGEATNLKAINIKIGSHEIDMHPVNGQVKLLVDGAESPTANISLISAGASLWIHNENQG  
FALAAPGHGIDKLYFDGKTITIQVPLWMAGKTCGICGKYDAECEQEYRMPNGYLAKNAVSFGHSWILEEAPCRGACKLHR  
SFVKLEKTVQLAGVDSKCYSTEPVLRCAKGCSATKTTPVTVGFHCLPADSANSLTDKQMKYDQKSEDMQDTVDAHTACSC  
ENEECST`

| Site | Type | Count |
| --- | --- | --- |
| 1414 | N-linked | 1 |

| Mass | Sequence | MS_2_ Score | `q`-value | MS_1_ Score | Signal | Protein Coordinates | Tandem Scans | Start Time | Apex Time | End Time |
| --- | --- | --- | --- | --- | --- | --- | --- | --- | --- | --- |
| 2815.2323 | VQVFVTN(N)LTDSSK Hex 6 HexNAc 2 | 124.791 | 0.0 | -19.390 | 3.818e+06 | 1407 - 1420 | 1 | 36.84 | 36.84 | 36.84 |

### VQVFVTN(N)LTDSSK Hex 6 HexNAc 2

**Mass:**  2815.2323

(1.923e-06)

**Charge States:** 3

**Start Position**: 1408

**End Position**: 1421

| Profile Score | Tandem Score | TDA q-value | Volume | Spectrum Matches |
| --- | --- | --- | --- | --- |
| -19.3901 | 124.7910 | 0.0000 | 3.8183e+06 | 1 |

**Adduct Signal Fractions**

- **Unmodified**: 3.8183e+06

### sp|P02845|VIT2\_CHICK

**>sp|P02845|VIT2\_CHICK**  
 `MRGIILALVLTLVGSQKFDIDPGFNSRRSYLYNYEGSMLNGLQDRSLGKAGVRLSSKLEISGLPENAYLLKVRSPQVEEY  
NGVWPRDPFTRSSKITQVISSCFTRLFKFEYSSGRIGNIYAPEDCPDLCVNIVRGILNMFQMTIKKSQNVYELQEAGIGG  
ICHARYVIQEDRKNSRIYVTRTVDLNNCQEKVQKSIGMAYIYPCPVDVMKERLTKGTTAFSYKLKQSDSGTLITDVSSRQ  
VYQISPFNEPTGVAVMEARQQLTLVEVRSERGSAPDVPMQNYGSLRYRFPAVLPQMPLQLIKTKNPEQRIVETLQHIVLN  
NQQDFHDDVSYRFLEVVQLCRIANADNLESIWRQVSDKPRYRRWLLSAVSASGTTETLKFLKNRIRNDDLNYIQTLLTVS  
LTLHLLQADEHTLPIAADLMTSSRIQKNPVLQQVACLGYSSVVNRYCSQTSACPKEALQPIHDLADEAISRGREDKMKLA  
LKCIGNMGEPASLKRILKFLPISSSSAADIPVHIQIDAITALKKIAWKDPKTVQGYLIQILADQSLPPEVRMMACAVIFE  
TRPALALITTIANVAMKESNMQVASFVYSHMKSLSKSRLPFMYNISSACNIALKLLSPKLDSMSYRYSKVIRADTYFDNY  
RVGATGEIFVVNSPRTMFPSAIISKLMANSAGSVADLVEVGIRVEGLADVIMKRNIPFAEYPTYKQIKELGKALQGWKEL  
PTETPLVSAYLKILGQEVAFININKELLQQVMKTVVEPADRNAAIKRIANQIRNSIAGQWTQPVWMGELRYVVPSCLGLP  
LEYGSYTTALARAAVSVEGKMTPPLTGDFRLSQLLESTMQIRSDLKPSLYVHTVATMGVNTEYFQHAVEIQGEVQTRMPM  
KFDAKIDVKLKNLKIETNPCREETEIVVGRHKAFAVSRNIGELGVEKRTSILPEDAPLDVTEEPFQTSERASREHFAMQG  
PDSMPRKQSHSSREDLRRSTGKRAHKRDICLKMHHIGCQLCFSRRSRDASFIQNTYLHKLIGEHEAKIVLMPVHTDADID  
KIQLEIQAGSRAAARIITEVNPESEEEDESSPYEDIQAKLKRILGIDSMFKVANKTRHPKNRPSKKGNTVLAEFGTEPDA  
KTSSSSSSASSTATSSSSSSASSPNRKKPMDEEENDQVKQARNKDASSSSRSSKSSNSSKRSSSKSSNSSKRSSSSSSSS  
SSSSRSSSSSSSSSSNSKSSSSSSKSSSSSSRSRSSSKSSSSSSSSSSSSSSKSSSSRSSSSSSKSSSHHSHSHHSGHLN  
GSSSSSSSSRSVSHHSHEHHSGHLEDDSSSSSSSSVLSKIWGRHEIYQYRFRSAHRQEFPKRKLPGDRATSRYSSTRSSH  
DTSRAASWPKFLGDIKTPVLAAFLHGISNNKKTGGLQLVVYADTDSVRPRVQVFVTNLTDSSKWKLCADASVRNAHKAVA  
YVKWGWDCRDYKVSTELVTGRFAGHPAAQVKLEWPKVPSNVRSVVEWFYEFVPGAAFMLGFSERMDKNPSRQARMVVALT  
SPRTCDVVVKLPDIILYQKAVRLPLSLPVGPRIPASELQPPIWNVFAEAPSAVLENLKARCSVSYNKIKTFNEVKFNYSM  
PANCYHILVQDCSSELKFLVMMKSAGEATNLKAINIKIGSHEIDMHPVNGQVKLLVDGAESPTANISLISAGASLWIHNE  
NQGFALAAPGHGIDKLYFDGKTITIQVPLWMAGKTCGICGKYDAECEQEYRMPNGYLAKNAVSFGHSWILEEAPCRGACK  
LHRSFVKLEKTVQLAGVDSKCYSTEPVLRCAKGCSATKTTPVTVGFHCLPADSANSLTDKQMKYDQKSEDMQDTVDAHTT  
CSCENEECST`

| Site | Type | Count |
| --- | --- | --- |
| 1417 | N-linked | 1 |

| Mass | Sequence | MS_2_ Score | `q`-value | MS_1_ Score | Signal | Protein Coordinates | Tandem Scans | Start Time | Apex Time | End Time |
| --- | --- | --- | --- | --- | --- | --- | --- | --- | --- | --- |
| 2815.2323 | VQVFVTN(N)LTDSSK Hex 6 HexNAc 2 | 124.791 | 0.0 | -19.390 | 3.818e+06 | 1410 - 1423 | 1 | 36.84 | 36.84 | 36.84 |

### VQVFVTN(N)LTDSSK Hex 6 HexNAc 2

**Mass:**  2815.2323

(1.923e-06)

**Charge States:** 3

**Start Position**: 1411

**End Position**: 1424

| Profile Score | Tandem Score | TDA q-value | Volume | Spectrum Matches |
| --- | --- | --- | --- | --- |
| -19.3901 | 124.7910 | 0.0000 | 3.8183e+06 | 1 |

**Adduct Signal Fractions**

- **Unmodified**: 3.8183e+06

### tr|F1NFL6|F1NFL6\_CHICK

**>tr|F1NFL6|F1NFL6\_CHICK**  
 `MRGIILALVLTLVGSQKFDIDPGFNSRRSYLYNYEGSMLNGLQDRSLGKAGVRLSSKLEISGLPENAYLLKVRSPQVEEY  
NGVWPRDPFTRSSKITQVISSCFTRLFKFEYSSGRIGNIYAPEDCPDLCVNIVRGILNMFQMTIKKSQNVYELQEAGIGG  
ICHARYVIQEDRKNSRIYVTRTVDLNNCQEKVQKSIGMAYIYPCPVDVMKERLTKGTTAFSYKLKQSDSGTLITDVSSRQ  
VYQISPFNEPTGVAVMEARQQLTLVEVRSERGSAPDVPMQNYGSLRYRFPAVLPQMPLQLIKTKNPEQRIIETLQHIVLN  
NQQDFHDDVSYRFLEVVQLCRIANADNLESIWRQVSDKPRYRRWLLSAVSASGTTETLKFLKNRIRNDDLNYIQTLLTVS  
LTLHLLQADEHTLPIAADLMTSSRIQKNPVLQQVACLGYSSVVNRYCSQTSACPKEALQPIHDLADEAISRGREDKMKLA  
LKCIGNMGEPASLKRILKFLPISSSSAADIPVHIQIDAITALKKIAWKDPKTVQGYLIQILADQSLPPEVRMMACAVIFE  
TRPALALITTIANVAMKESNMQVASFVYSHMKSLSKSRLPFMYNISSACNIALKLLSPKLDSMSYRYSKVIRADTYFDNY  
RVGATGEIFVVNSPRTMFPSAIISKLMANSAGSVADLVEVGIRVEGLADVIMKRNIPFAEYPTYKQIKELGKALQGWKEL  
PTETPLVSAYLKILGQEVAFININKELLQQVMKTVVEPADRNAAIKRIANQIRNSIAGQWTQPVWMGELRYVVPSCLGLP  
LEYGSYTTALARAAVSVEGKMTPPLTGDFRLSQLLESTMQIRSDLKPSLYVHTVATMGVNTEYFQHAVEIQGEVQTRMPM  
KFDAKIDVKLKNLKIETNPCREETEIVVGRHKAFAVSRNIGELGVEKRTSILPEDAPLDVTEEPFQTSERASREHFAMQG  
PDSMPRKQSHSSREDLRRSTGKRAHKRDICLKMHHIGCQLCFSRRSRDASFIQNTYLHKLIGEHEAKIVLMPVHTDADID  
KIQLEIQAGSRAAARIITEVNPESEEEDESSPYEDIQAKLKRILGIDSMFKVANKTRHPKNRPSKKGNTVLAEFGTEPDA  
KTSSSSSSASSTATSSSSSSASSPNRKKPMDEEENDQVKQARNKDASSSSRSSKSSNSSKRSSSKSSNSSKRSSSSSSSS  
SSSSSSSSRSSSSSSSSSSNSKSSSSSSKSSSSSSRSRSSSKSSSSSSSSSSSSSSKSSSSRSSSSSSKSSSHHSHSHHS  
GHLNGSSSSSSSSRSVSHHSHEHHSGHLEDDSSSSSSSSVLSKIWGRHEIYQYRFRSAHRQEFPKRKLPGDRATSRYSST  
RSSHDTSRAASWPKFLGDIKTPVLAAFLHGISNNKKTGGLQLVVYADTDSVRPRVQVFVTNLTDSSKWKLCADASVRNAH  
KAVAYVKWGWDCRDYKVSTELVTGRFAGHPAAQVKLEWPKVPSNVRSVVEWFYEFVPGAAFMLGFSERMDKNPSRQARMV  
VALTSPRTCDVVVKLPDMILYQKAVRLPLSLPVGPRIPASELQSPIWNVFAEAPSAVLENLKARCSVSYNKIKTFNEVKF  
NYSMPANCYHILVQDCSSELKFLVMMKSAGEATNLKAINIKIGSHEIDMHPVNGQVKLLVDGAESPTANISLISAGASLW  
IHNENQGFALAAPGHGIDKLYFDGKTITIQVPLWMAGKTCGICGKYDAECEQEYRMPNGYLAKNAVSFGHSWILEEAPCR  
GACKLHRSFVKLEKTVQLAGVDSKCYSTEPVLRCAKGCSATKTTPVTVGFHCLPADSANSLTDKQMKYDQKSEDMQDTVD  
AHTACSCENEECST`

| Site | Type | Count |
| --- | --- | --- |
| 1421 | N-linked | 1 |

| Mass | Sequence | MS_2_ Score | `q`-value | MS_1_ Score | Signal | Protein Coordinates | Tandem Scans | Start Time | Apex Time | End Time |
| --- | --- | --- | --- | --- | --- | --- | --- | --- | --- | --- |
| 2815.2323 | VQVFVTN(N)LTDSSK Hex 6 HexNAc 2 | 124.791 | 0.0 | -19.390 | 3.818e+06 | 1414 - 1427 | 1 | 36.84 | 36.84 | 36.84 |

### VQVFVTN(N)LTDSSK Hex 6 HexNAc 2

**Mass:**  2815.2323

(1.923e-06)

**Charge States:** 3

**Start Position**: 1415

**End Position**: 1428

| Profile Score | Tandem Score | TDA q-value | Volume | Spectrum Matches |
| --- | --- | --- | --- | --- |
| -19.3901 | 124.7910 | 0.0000 | 3.8183e+06 | 1 |

**Adduct Signal Fractions**

- **Unmodified**: 3.8183e+06

### tr|A0A1D5PNI3|A0A1D5PNI3\_CHICK

**>tr|A0A1D5PNI3|A0A1D5PNI3\_CHICK**  
 `MTTSTLAQTTSLPGTSTPTPASSTLMTTSTPAQTSLMPGHFKAQLLHLLHFTTTSTPAQTTSVPASSSPTPASSALPTAT  
TPAQTPGTSSPTPASSALPTTTTPAETTTFPATSSTTPASSAPPTTSTPAQTTSLPRTSTPTPASSALTTTTTPEQTTTV  
PGTSTPTPASSTVLTTSTPVQTTSMPGISSPTPASSALLTKSTPAQTTTVPATSSPTPASSALPTTTTPEQTTTESGTST  
PTPASSGPPTTSTPAETTSFPATSSPTPASSTLMTTSTPAQTTTVPGTSSPTPVSSALLTTRTLAQTKASLQLNSSPTSV  
SSALSTTSTPAETTTVPGTSIPTAASSTLLTTSTLAQTTSFPATSTPTPSSSTLMTTSTRAETKTSLQLNSSPTPASLAL  
PTTSTPAQTTSVPTSSSPTPASSALSTTSTPALTTALRSTSQIMSPSSPATSPSPTATSAGTVTTAQETTSPAGTTNALT  
STIRTTSTQTLSSSSPPYTTAALSYTAIPTSTPATPTTSAPQTTIASSSTPTESTALLTTTVPSTTQDISSQTAQTSNAA  
TSSTTNSATSARLTRTHAETTSSTTTFTSHSRPSASQTSTPGTTISPLPATTAQQTTAPAGTTPPTTASTTLFSTRETSS  
KEEETSTVVTSSQTPASSVLPSTSTPAQTTSMPSTSSPTPASSTLMTTSTLAQTTSLPGTSVPTPASSAPRTTTTPAQTT  
SVPGNSSPIPPYSTLLTTSTLAQTTSLPGTSTPTPASSTLMTTSTPAETKTSLLLNSSPNICIFCSSDYNHSCTDNISAR  
HFKPNSCTAIKHSCRKQTFITTFTSRARTSASWTTTPGTTTSALTATTARETTTPAGTTHPHNSNIETTSTSTIKAPTRP  
PTTPAGHERFTVNFTITNLPYDSDLQIPHSAKLNATQRVMTTLLNRLLKDTSIGPSFLGCATTAFR`

| Site | Type | Count |
| --- | --- | --- |
| 893 | N-linked | 1 |

| Mass | Sequence | MS_2_ Score | `q`-value | MS_1_ Score | Signal | Protein Coordinates | Tandem Scans | Start Time | Apex Time | End Time |
| --- | --- | --- | --- | --- | --- | --- | --- | --- | --- | --- |
| 4161.8681 | FTVN(N)FTITNLPYDSDLQIPHSAK Hex 7 HexNAc 2 | 104.166 | 0.031 | 0.000 | 0.000e+00 | 889 - 912 | 1 | - | - | - |

### FTVN(N)FTITNLPYDSDLQIPHSAK Hex 7 HexNAc 2

**Mass:**  4161.8681

(2.457e-04)

**Start Position**: 890

**End Position**: 913

| Profile Score | Tandem Score | TDA q-value | Volume | Spectrum Matches |
| --- | --- | --- | --- | --- |
| 0.0000 | 104.1659 | 0.0312 | 0.0000e+00 | 1 |

Adduct:

No Chromatogram Found

### tr|A0A1D5PUR1|A0A1D5PUR1\_CHICK

**>tr|A0A1D5PUR1|A0A1D5PUR1\_CHICK**  
 `MTTSTLAQTTSLPGTSTPTPASSTLMTTSTPAQTSLMPGHFKAQLLHLLHFTTTSTPAQTTSVPASSSPTPASSALPTAT  
TPAQTPGTSSPTPASSALPTTTTPAETTTFPATSSTTPASSAPPTTSTPAQTTSLPRTSTPTPASSALTTTTTPEQTTTV  
PGTSTPTPASSTVLTTSTPVQTTSMPGISSPTPASSALLTKSTPAQTTTVPATSSPTPASSALPTTTTPEQTTTESGTST  
PTPASSGPPTTSTPAETTSVPGTSYPNPASSSLMTTSTLAQTTSLPATSTPSLASSTLMTKSTFAPTTALPGTSTPTPAS  
SAPPTTSTPAQTTSVPGTSTATSASSTLLTTSTPAQTTSFPATSSPTPASSTLMTTSTPAQTTTVPGTSSPTPVSSALLT  
TRTLAQTKASLQLNSSPTSVSSALSTTSTPAETTTVPGTSIPTAASSTLLTTSTLAQTTSFPATSTPTPSSSTLMTTSTR  
AETKTSLQLNSSPTPASLALPTTSTPAQTTSVPTSSSPTPASSALSTTSTPALTTALRSTSQIMSPSSPATSPSPTATSA  
GTVTTAQETTSPAGTTNALTSTIRTTSTQTLSSSSPPYTTAALSYTAIPTSTPATPTTSAPQTTIASSSTPTESTALLTT  
TVPSTTQDISSQTAQTSNAATSSTTNSATSARLTRTHAETTSSTTTFTSHSRPSASQTSTPGTTISPLPATTAQQTTAPA  
GTTPPTTASTTLFSTRETSSKEEETSTVVTSSQTPASSVLPSTSTPAQTTSMPSTSSPTPASSTLMTTSTLAQTTSLPGT  
SVPTPASSAPRTTTTPAQTTSVPGNSSPIPPYSTLLTTSTLAQTTSLPGTSTPTPASSTLMTTSTPAETKTSLLLNSSPN  
ICIFCSSDYNHSCTDNISARHFKPNSCTAIKHSCRKQTFITTFTSRARTSASWTTTPGTTTSALTATTARETTTPAGTTH  
PHNSNIETTSTSTIKAPTRPPTTPAGHERFTVNFTITNLPYDSDLQIPHSAKLNATQRVMTTLLNRLLKDTSIGPSFLGC  
ATTAFR`

| Site | Type | Count |
| --- | --- | --- |
| 993 | N-linked | 1 |

| Mass | Sequence | MS_2_ Score | `q`-value | MS_1_ Score | Signal | Protein Coordinates | Tandem Scans | Start Time | Apex Time | End Time |
| --- | --- | --- | --- | --- | --- | --- | --- | --- | --- | --- |
| 4161.8681 | FTVN(N)FTITNLPYDSDLQIPHSAK Hex 7 HexNAc 2 | 104.166 | 0.031 | 0.000 | 0.000e+00 | 989 - 1012 | 1 | - | - | - |

### FTVN(N)FTITNLPYDSDLQIPHSAK Hex 7 HexNAc 2

**Mass:**  4161.8681

(2.457e-04)

**Start Position**: 990

**End Position**: 1013

| Profile Score | Tandem Score | TDA q-value | Volume | Spectrum Matches |
| --- | --- | --- | --- | --- |
| 0.0000 | 104.1659 | 0.0312 | 0.0000e+00 | 1 |

Adduct:

No Chromatogram Found
