# Supplementary material for: Measuring Site-specific Glycosylation Similarity between Influenza a Virus Variants with Statistical Certainty
Source: Mol Cell Proteomics. 2020 Nov 25;19(9):1533–45. doi: 10.1074/mcp.RA120.002031 (PMC8143645; doi:10.1074/mcp.RA120.002031)
Supplement: Supplementary file 2 [file mmc2.zip › mmc2/159250_2_supp_555292_qcjkl5.zip!/20190301_5B8eggL2_021119_tryp_HILIC_DDA_1ug_03_glycopeptides.html]

## 20190301\_5B8eggL2\_021119\_tryp\_HILIC\_DDA\_1ug\_03 @ GlycopeptideHypothesis-a44591715a5e44e5a30d1d9c429c6882

**MS^1^ Mass Accuracy:** 10.0 PPM  
**Grouping Tolerance:** 15.0 PPM  
**MS^n^ Mass Accuracy:** 20.0 PPM  
**Minimum `q`-Value For Clustering:** 0.05

**Glycopeptide Hypothesis:**
GlycopeptideHypothesis-a44591715a5e44e5a30d1d9c429c6882  
**Sample Searched:**
20190301\_5B8eggL2\_021119\_tryp\_HILIC\_DDA\_1ug\_03

| Protein | Matched Glycopeptides |
| --- | --- |
| cus\|SWZHA\_5B8\|A/Switzerland/9715293/2013 | 146 |
| cus\|SWZHA\|A/Switzerland/9715293/2013 | 129 |
| cus\|SWZNA\|A/Switzerland/9715293/2013 | 12 |
| tr\|E1C041\|E1C041\_CHICK | 6 |
| tr\|A0A1D5PGT3\|A0A1D5PGT3\_CHICK | 2 |
| tr\|A0A1D5P0L1\|A0A1D5P0L1\_CHICK | 2 |
| tr\|A0A1D5PBT7\|A0A1D5PBT7\_CHICK | 2 |
| tr\|A0A1D5PRN6\|A0A1D5PRN6\_CHICK | 2 |
| tr\|A0A1D5P780\|A0A1D5P780\_CHICK | 2 |
| sp\|P02845\|VIT2\_CHICK | 2 |
| tr\|F1NFL6\|F1NFL6\_CHICK | 2 |
| tr\|F1NV02\|F1NV02\_CHICK | 1 |
| tr\|A0A1D5PNI3\|A0A1D5PNI3\_CHICK | 1 |
| tr\|A0A1D5PUR1\|A0A1D5PUR1\_CHICK | 1 |

### cus|SWZHA\_5B8|A/Switzerland/9715293/2013

**>cus|SWZHA\_5B8|A/Switzerland/9715293/2013**  
 `QKLPGNDNSTATLCLGHHAVPNGTIVKTITNDRIEVTNATELVQNSSIGEICDSPHQILDGENCTLIDALLGDPQCDGFQ  
NKKWDLFVERSKAYSNCYPYDVPDYASLRSLVASSGTLEFNNESFNWAGVTHNGTSSSCRRGSNSSFFSRLNWLTHLNSK  
YPALNVTMPNNEQFDKLYIWGVHHPVTDKDQIFLYAQSSGRITVSTKRSQQAVIPNIGSRPRIRNIPSRISIYWTIVKPG  
DILLINSTGNLIAPRGYFKIRSGKSSIMRSDAPIGKCKSECITPNGSIPNDKPFQNVNRITYGACPRYVKQSTLKLATGM  
RNVPERQTRGIFGAIAGFIENGWEGMVDGWYGFRHQNSEGRGQAADLKSTQAAIDQINGKLNRLIGKTNEKFHQIEKEFS  
EVEGRIQDLEKYVEDTKIDLWSYNAELLVALENQHTIDLTDSEMNKLFEKTKKQLRENAEDMGNGCFKIYHKCDNACIGS  
IRNGTYDHDVYRDEALNNRFQIKGVELKSGYKDWILWISFAISCFLLCVALLGFIMWACQKGNIRCNICI`

| Site | Type | Count |
| --- | --- | --- |
| 483 | N-linked | 48 |
| 165 | N-linked | 12 |
| 144 | N-linked | 44 |
| 246 | N-linked | 11 |
| 133 | N-linked | 15 |
| 285 | N-linked | 13 |
| 22 | N-linked | 3 |

| Mass | Sequence | MS_2_ Score | `q`-value | MS_1_ Score | Signal | Protein Coordinates | Tandem Scans | Start Time | Apex Time | End Time |
| --- | --- | --- | --- | --- | --- | --- | --- | --- | --- | --- |
| 2522.0187 | RGSN(N)SSFFSR Hex 6 HexNAc 2 | 193.768 | 0.0 | -3.260 | 5.115e+08 | 140 - 150 | 4 | 19.22 | 19.86 | 20.01 |
| 2359.9656 | RGSN(N)SSFFSR Hex 5 HexNAc 2 | 193.228 | 0.0 | 11.947 | 4.709e+08 | 140 - 150 | 3 | 19.38 | 20.16 | 20.31 |
| 2766.1238 | RGSN(N)SSFFSR Hex 5 HexNAc 4 | 189.579 | 0.0 | -3.834 | 1.114e+08 | 140 - 150 | 1 | 20.01 | 20.01 | 20.31 |
| 3172.2842 | RGSN(N)SSFFSR Hex 5 HexNAc 6 | 188.247 | 0.0 | -7.298 | 2.262e+08 | 140 - 150 | 2 | 19.54 | 20.16 | 20.31 |
| 2846.1184 | RGSN(N)SSFFSR Hex 8 HexNAc 2 | 188.138 | 0.0 | -17.985 | 1.005e+08 | 140 - 150 | 2 | 19.54 | 19.71 | 22.37 |
| 2725.0985 | RGSN(N)SSFFSR Hex 6 HexNAc 3 | 183.251 | 0.0 | -3.713 | 6.190e+08 | 140 - 150 | 4 | 19.22 | 20.01 | 20.01 |
| 3261.3203 | RGSN(N)SSFFSR Fuc 2 Hex 5 HexNAc 5 | 182.202 | 0.0 | 14.008 | 4.348e+08 | 140 - 150 | 4 | 19.05 | 19.71 | 20.01 |
| 3582.4698 | YPALN(N)VTMPNNEQFDK Hex 8 HexNAc 2 | 178.356 | 0.0 | 18.224 | 3.368e+08 | 160 - 176 | 5 | 42.21 | 42.72 | 42.89 |
| 2684.0718 | RGSN(N)SSFFSR Hex 7 HexNAc 2 | 178.260 | 0.0 | 12.046 | 2.924e+08 | 140 - 150 | 2 | 19.05 | 19.71 | 20.01 |
| 3115.2626 | RGSN(N)SSFFSR Fuc 1 Hex 5 HexNAc 5 | 174.660 | 0.0 | 13.157 | 1.885e+09 | 140 - 150 | 6 | 19.22 | 20.01 | 20.31 |
| 3407.3814 | RGSN(N)SSFFSR Fuc 3 Hex 5 HexNAc 5 | 173.807 | 0.0 | -17.593 | 4.596e+07 | 140 - 150 | 1 | 19.38 | 19.38 | 19.54 |
| 3058.2417 | RGSN(N)SSFFSR Fuc 2 Hex 5 HexNAc 4 | 173.104 | 0.0 | -17.706 | 2.857e+08 | 140 - 150 | 3 | 19.54 | 19.71 | 19.71 |
| 3277.3168 | RGSN(N)SSFFSR Fuc 1 Hex 6 HexNAc 5 | 173.075 | 0.0 | -3.744 | 1.040e+08 | 140 - 150 | 2 | 19.38 | 19.54 | 19.71 |
| 3598.4732 | YPALN(N)VTM(O)PNNEQFDK Hex 8 HexNAc 2 | 171.687 | 0.0 | 16.803 | 1.825e+09 | 160 - 176 | 22 | 34.44 | 34.95 | 113.40 |
| 3760.5151 | YPALN(N)VTM(O)PNNEQFDK Hex 9 HexNAc 2 | 171.319 | 0.0 | 15.489 | 1.576e+08 | 160 - 176 | 5 | 34.61 | 34.95 | 35.28 |
| 3436.4156 | YPALN(N)VTM(O)PNNEQFDK Hex 7 HexNAc 2 | 171.200 | 0.0 | 15.915 | 3.479e+08 | 160 - 176 | 4 | 34.78 | 35.11 | 35.45 |
| 3204.3012 | RGSN(N)SSFFSR Fuc 3 Hex 5 HexNAc 4 | 169.938 | 0.0 | -4.286 | 1.638e+08 | 140 - 150 | 2 | 18.72 | 19.38 | 19.54 |
| 2969.2055 | RGSN(N)SSFFSR Hex 5 HexNAc 5 | 167.777 | 0.0 | 12.042 | 3.110e+08 | 140 - 150 | 2 | 19.38 | 20.01 | 20.31 |
| 4068.9355 | TIVKPGDILLIN(N)STGNLIAPR Hex 9 HexNAc 2 | 167.512 | 0.0 | 19.243 | 6.085e+08 | 234 - 255 | 5 | 52.81 | 53.49 | 54.18 |
| 2855.1612 | RGSN(N)SSFFSR Fuc 2 Hex 5 HexNAc 3 | 165.538 | 0.0 | -18.653 | 2.692e+07 | 140 - 150 | 1 | 19.86 | 19.86 | 19.86 |
| 2709.1046 | RGSN(N)SSFFSR Fuc 1 Hex 5 HexNAc 3 | 164.072 | 0.0 | -4.237 | 8.869e+07 | 140 - 150 | 1 | 19.86 | 20.01 | 20.16 |
| 2871.1562 | RGSN(N)SSFFSR Fuc 1 Hex 6 HexNAc 3 | 163.919 | 0.0 | 10.515 | 3.969e+08 | 140 - 150 | 2 | 19.05 | 19.71 | 19.86 |
| 2756.0830 | GSN(N)SSFFSR Fuc 1 Hex 5 HexNAc 4 | 163.050 | 0.0 | 12.047 | 3.908e+08 | 141 - 150 | 3 | 23.04 | 23.19 | 24.10 |
| 2912.1825 | RGSN(N)SSFFSR Fuc 1 Hex 5 HexNAc 4 | 162.197 | 0.0 | 13.977 | 9.188e+08 | 140 - 150 | 4 | 19.22 | 19.86 | 20.16 |
| 2547.0560 | RGSN(N)SSFFSR Fuc 1 Hex 4 HexNAc 3 | 161.266 | 0.0 | -18.934 | 2.375e+07 | 140 - 150 | 1 | 20.46 | 20.46 | 20.46 |
| 3420.4165 | YPALN(N)VTMPNNEQFDK Hex 7 HexNAc 2 | 159.418 | 0.0 | -3.277 | 5.481e+07 | 160 - 176 | 2 | 42.38 | 42.72 | 42.89 |
| 3560.3593 | RGSN(N)SSFFSR Fuc 1 Hex 9 HexNAc 4 | 158.605 | 0.0 | 10.829 | 8.519e+07 | 140 - 150 | 1 | 21.38 | 21.54 | 22.37 |
| 4085.9617 | TIVKPGDILLIN(N)STGNLIAPR Fuc 2 Hex 3 HexNAc 4 Neu5Ac 1 | 157.203 | 0.0 | -3.075 | 7.946e+07 | 234 - 255 | 2 | 53.32 | 53.49 | 53.66 |
| 4856.0342 | SLVASSGTLEFNNESFNWAGVTHN(N)GTSSSC(C)R Hex 7 HexNAc 2 | 155.936 | 0.0 | -2.248 | 5.253e+08 | 109 - 140 | 4 | 49.58 | 49.58 | 50.09 |
| 3744.5213 | YPALN(N)VTMPNNEQFDK Hex 9 HexNAc 2 | 155.413 | 0.0 | -4.073 | 3.045e+07 | 160 - 176 | 2 | 42.38 | 42.55 | 42.72 |
| 4376.8407 | C(C)KSEC(C)ITPN(N)GSIPNDKPFQNVNR Hex 8 HexNAc 2 | 155.304 | 0.0 | -17.162 | 1.511e+08 | 276 - 299 | 1 | 29.47 | 29.63 | 29.63 |
| 3503.3339 | RGSN(N)SSFFSR Fuc 2 Hex 9 HexNAc 3 | 155.200 | 0.0 | -3.892 | 6.640e+07 | 140 - 150 | 2 | 21.22 | 21.38 | 21.54 |
| 3007.1758 | N(N)GTYDHDVYR Fuc 1 Hex 5 HexNAc 4 | 154.693 | 0.0 | 14.788 | 4.754e+08 | 482 - 492 | 4 | 15.72 | 15.72 | 16.36 |
| 3274.3635 | YPALN(N)VTM(O)PNNEQFDK Hex 6 HexNAc 2 | 154.094 | 0.0 | 11.964 | 2.388e+07 | 160 - 176 | 2 | 34.95 | 35.28 | 35.62 |
| 2203.8642 | GSN(N)SSFFSR Hex 5 HexNAc 2 | 153.887 | 0.0 | 13.079 | 3.438e+08 | 141 - 150 | 3 | 23.64 | 23.79 | 24.41 |
| 3008.1553 | RGSN(N)SSFFSR Hex 9 HexNAc 2 | 153.138 | 0.0 | -20.654 | 7.745e+06 | 140 - 150 | 1 | 21.86 | 21.86 | 21.86 |
| 3006.3096 | HAVPN(N)GTIVK Fuc 1 Hex 5 HexNAc 5 | 152.094 | 0.0 | -20.144 | 9.221e+06 | 17 - 27 | 1 | 16.70 | 16.86 | 16.86 |
| 3372.7047 | N(N)GTYDHDVYR Fuc 1 Hex 6 HexNAc 5 | 152.044 | 0.0 | -21.786 | 7.488e+07 | 482 - 492 | 4 | 15.58 | 15.72 | 21.70 |
| 2966.1438 | N(N)GTYDHDVYR Fuc 1 Hex 6 HexNAc 3 | 152.015 | 0.0 | -20.136 | 1.735e+07 | 482 - 492 | 1 | 15.58 | 15.58 | 15.58 |
| 5059.1019 | SLVASSGTLEFNNESFNWAGVTHN(N)GTSSSC(C)R Hex 7 HexNAc 3 | 151.738 | 0.0 | -18.470 | 4.250e+07 | 109 - 140 | 1 | 49.58 | 49.58 | 49.75 |
| 4230.9889 | TIVKPGDILLIN(N)STGNLIAPR Hex 10 HexNAc 2 | 150.699 | 0.0 | -19.398 | 2.129e+07 | 234 - 255 | 2 | 53.32 | 53.32 | 53.49 |
| 3334.3350 | RGSN(N)SSFFSR Hex 6 HexNAc 6 | 150.507 | 0.0 | -19.465 | 1.801e+07 | 140 - 150 | 1 | 19.54 | 19.54 | 19.54 |
| 2715.0561 | GSN(N)SSFFSR Fuc 1 Hex 6 HexNAc 3 | 149.917 | 0.0 | -3.812 | 1.567e+08 | 141 - 150 | 2 | 23.04 | 23.04 | 23.33 |
| 3210.2552 | N(N)GTYDHDVYR Fuc 1 Hex 5 HexNAc 5 | 149.868 | 0.0 | -2.265 | 2.449e+08 | 482 - 492 | 3 | 15.87 | 15.87 | 16.36 |
| 4052.7348 | C(C)KSEC(C)ITPN(N)GSIPNDKPFQNVNR Hex 6 HexNAc 2 | 149.846 | 0.0 | -19.053 | 3.089e+07 | 276 - 299 | 1 | 29.63 | 29.63 | 29.80 |
| 3632.4667 | N(N)GTYDHDVYRDEALNNR Hex 6 HexNAc 3 | 149.660 | 0.0 | -3.443 | 3.288e+08 | 482 - 499 | 3 | 20.61 | 20.76 | 21.06 |
| 4214.7889 | C(C)KSEC(C)ITPN(N)GSIPNDKPFQNVNR Hex 7 HexNAc 2 | 149.629 | 0.0 | -2.923 | 3.049e+08 | 276 - 299 | 3 | 29.47 | 29.63 | 29.80 |
| 3906.8814 | TIVKPGDILLIN(N)STGNLIAPR Hex 8 HexNAc 2 | 149.127 | 0.0 | -3.606 | 6.072e+07 | 234 - 255 | 3 | 53.32 | 53.49 | 53.66 |
| 2365.9189 | GSN(N)SSFFSR Hex 6 HexNAc 2 | 148.899 | 0.0 | -3.275 | 2.380e+08 | 141 - 150 | 2 | 23.33 | 23.48 | 23.79 |
| 2813.1041 | GSN(N)SSFFSR Hex 5 HexNAc 5 | 148.700 | 0.0 | 11.787 | 2.406e+08 | 141 - 150 | 2 | 23.04 | 23.64 | 24.10 |
| 3761.5519 | YPALN(N)VTMPNNEQFDK Fuc 2 Hex 3 HexNAc 4 Neu5Ac 1 | 148.563 | 0.0 | -18.750 | 7.459e+06 | 160 - 176 | 1 | 42.55 | 42.55 | 42.72 |
| 4693.9802 | SLVASSGTLEFNNESFNWAGVTHN(N)GTSSSC(C)R Hex 6 HexNAc 2 | 148.556 | 0.0 | -3.156 | 1.284e+08 | 109 - 140 | 3 | 49.75 | 49.92 | 50.26 |
| 4185.6745 | RGSN(N)SSFFSR Fuc 1 Hex 3 HexNAc 9 Neu5Ac 2 | 148.355 | 0.0 | -4.088 | 1.823e+08 | 140 - 150 | 3 | 21.06 | 21.06 | 21.54 |
| 2568.9979 | GSN(N)SSFFSR Hex 6 HexNAc 3 | 147.908 | 0.0 | 12.673 | 3.330e+08 | 141 - 150 | 3 | 23.33 | 23.48 | 23.95 |
| 4731.2829 | ISIYWTIVKPGDILLIN(N)STGNLIAPR Hex 9 HexNAc 2 | 147.546 | 0.0 | 18.663 | 8.639e+08 | 229 - 255 | 9 | 68.48 | 69.17 | 69.68 |
| 3423.3739 | RGSN(N)SSFFSR Fuc 2 Hex 6 HexNAc 5 | 147.496 | 0.0 | -5.134 | 2.611e+07 | 140 - 150 | 1 | 19.22 | 19.38 | 19.54 |
| 4538.8925 | C(C)KSEC(C)ITPN(N)GSIPNDKPFQNVNR Hex 9 HexNAc 2 | 146.948 | 0.0 | -18.274 | 2.061e+08 | 276 - 299 | 1 | 29.47 | 29.63 | 29.63 |
| 3017.2132 | RGSN(N)SSFFSR Fuc 2 Hex 6 HexNAc 3 | 145.214 | 0.0 | -19.268 | 2.854e+07 | 140 - 150 | 1 | 19.54 | 19.71 | 19.71 |
| 3105.2201 | GSN(N)SSFFSR Fuc 2 Hex 5 HexNAc 5 | 145.207 | 0.0 | -3.417 | 1.250e+08 | 141 - 150 | 1 | 22.87 | 23.04 | 23.19 |
| 2928.1781 | RGSN(N)SSFFSR Hex 6 HexNAc 4 | 144.715 | 0.0 | 12.814 | 1.306e+08 | 140 - 150 | 2 | 19.38 | 19.86 | 20.16 |
| 4748.3020 | ISIYWTIVKPGDILLIN(N)STGNLIAPR Fuc 2 Hex 3 HexNAc 4 Neu5Ac 1 | 143.893 | 0.0 | 15.036 | 2.274e+08 | 229 - 255 | 8 | 68.48 | 69.17 | 69.51 |
| 4111.6680 | N(N)GTYDHDVYRDEALNNR Fuc 3 Hex 5 HexNAc 4 | 143.236 | 0.0 | 14.911 | 6.881e+08 | 482 - 499 | 3 | 20.01 | 20.31 | 20.61 |
| 2872.1308 | RGSN(N)SSFF Fuc 1 Hex 5 HexNAc 5 | 142.825 | 0.0 | -18.570 | 1.513e+07 | 140 - 148 | 1 | 26.52 | 26.52 | 26.68 |
| 3267.3338 | N(N)GTYDHDVYRDEALNNR Hex 5 HexNAc 2 | 141.073 | 0.0 | -20.055 | 1.104e+08 | 482 - 499 | 1 | 20.91 | 20.91 | 21.06 |
| 5567.7750 | IRNIPSRISIYWTIVKPGDILLIN(N)STGNLIAPR Hex 9 HexNAc 2 | 140.549 | 0.0 | -2.433 | 1.234e+08 | 222 - 255 | 6 | 63.93 | 64.22 | 64.35 |
| 4569.2241 | ISIYWTIVKPGDILLIN(N)STGNLIAPR Hex 8 HexNAc 2 | 140.047 | 0.0 | -2.569 | 9.463e+07 | 229 - 255 | 5 | 68.99 | 69.17 | 69.51 |
| 3112.3180 | YPALN(N)VTM(O)PNNEQFDK Hex 5 HexNAc 2 | 139.855 | 0.0 | -4.672 | 1.313e+07 | 160 - 176 | 2 | 35.28 | 35.62 | 35.79 |
| 3778.5268 | N(N)GTYDHDVYRDEALNNR Fuc 1 Hex 6 HexNAc 3 | 139.248 | 0.0 | 13.153 | 3.214e+08 | 482 - 499 | 2 | 20.31 | 20.46 | 20.91 |
| 3935.5993 | RGSN(N)SSFFSR Fuc 3 Hex 7 HexNAc 6 | 139.021 | 0.0 | 0.000 | 0.000e+00 | 140 - 150 | 1 | - | - | - |
| 2610.0249 | GSN(N)SSFFSR Hex 5 HexNAc 4 | 137.950 | 0.045 | -19.555 | 4.296e+07 | 141 - 150 | 2 | 23.33 | 23.48 | 23.48 |
| 3153.2316 | N(N)GTYDHDVYR Fuc 2 Hex 5 HexNAc 4 | 136.976 | 0.0 | -3.760 | 3.200e+08 | 482 - 492 | 3 | 15.58 | 15.72 | 16.04 |
| 3899.5158 | N(N)GTYDHDVYRDEALNNR Fuc 1 Hex 8 HexNAc 2 | 136.679 | 0.0 | 13.604 | 7.330e+08 | 482 - 499 | 2 | 22.03 | 22.37 | 22.70 |
| 2959.1627 | GSN(N)SSFFSR Fuc 1 Hex 5 HexNAc 5 | 136.337 | 0.045 | 13.202 | 9.913e+08 | 141 - 150 | 3 | 22.70 | 23.48 | 23.79 |
| 3300.2893 | N(N)GTYDHDVYR Fuc 3 Hex 5 HexNAc 4 | 135.437 | 0.0 | 0.000 | 0.000e+00 | 482 - 492 | 2 | - | - | - |
| 3437.4127 | YPALN(N)VTM(O)PN(D)NEQFDK Hex 7 HexNAc 2 | 134.846 | 0.0 | 6.457 | 2.512e+07 | 160 - 176 | 3 | 35.62 | 35.79 | 36.64 |
| 3965.6102 | N(N)GTYDHDVYRDEALNNR Fuc 2 Hex 5 HexNAc 4 | 133.596 | 0.0 | 15.283 | 2.157e+09 | 482 - 499 | 4 | 20.16 | 20.46 | 20.76 |
| 2406.9435 | GSN(N)SSFFSR Hex 5 HexNAc 3 | 131.858 | 0.045 | -20.051 | 3.837e+07 | 141 - 150 | 2 | 23.79 | 23.79 | 23.95 |
| 2820.0913 | N(N)GTYDHDVYR Hex 6 HexNAc 3 | 131.808 | 0.0 | -18.067 | 3.580e+07 | 482 - 492 | 2 | 15.72 | 15.72 | 15.87 |
| 4102.5961 | N(N)GTYDHDVYRDEALNNR Fuc 1 Hex 8 HexNAc 3 | 131.777 | 0.0 | 11.260 | 3.462e+08 | 482 - 499 | 4 | 21.54 | 22.37 | 22.70 |
| 3819.5506 | N(N)GTYDHDVYRDEALNNR Fuc 1 Hex 5 HexNAc 4 | 131.428 | 0.0 | 16.671 | 4.737e+09 | 482 - 499 | 7 | 20.16 | 20.61 | 21.06 |
| 3616.4712 | N(N)GTYDHDVYRDEALNNR Fuc 1 Hex 5 HexNAc 3 | 131.320 | 0.0 | -3.329 | 1.475e+08 | 482 - 499 | 2 | 20.61 | 20.76 | 21.06 |
| 3764.6165 | SEC(C)ITPN(N)GSIPNDKPFQNVNR Hex 6 HexNAc 2 | 130.789 | 0.0 | -3.168 | 2.504e+08 | 278 - 299 | 2 | 32.41 | 32.58 | 32.74 |
| 3599.4670 | YPALN(N)VTM(O)PN(D)NEQFDK Hex 8 HexNAc 2 | 130.636 | 0.0 | 7.908 | 2.044e+08 | 160 - 176 | 9 | 35.45 | 35.79 | 37.15 |
| 3356.3108 | N(N)GTYDHDVYR Fuc 2 Hex 5 HexNAc 5 | 130.257 | 0.0 | -19.114 | 6.421e+07 | 482 - 492 | 2 | 15.72 | 15.72 | 15.87 |
| 4531.9287 | SLVASSGTLEFNNESFNWAGVTHN(N)GTSSSC(C)R Hex 5 HexNAc 2 | 130.181 | 0.0 | -2.869 | 1.603e+08 | 109 - 140 | 3 | 49.92 | 50.09 | 50.43 |
| 3454.4198 | N(N)GTYDHDVYRDEALNNR Fuc 1 Hex 4 HexNAc 3 | 130.063 | 0.0 | -18.909 | 1.236e+08 | 482 - 499 | 1 | 20.91 | 21.06 | 21.06 |
| 2772.0812 | GSN(N)SSFFSR Hex 6 HexNAc 4 | 129.530 | 0.045 | 6.628 | 7.385e+07 | 141 - 150 | 2 | 23.04 | 23.33 | 23.79 |
| 5018.0823 | SLVASSGTLEFNNESFNWAGVTHN(N)GTSSSC(C)R Hex 8 HexNAc 2 | 129.414 | 0.0 | -18.484 | 1.134e+08 | 109 - 140 | 1 | 49.58 | 49.58 | 49.75 |
| 5298.5888 | NIPSRISIYWTIVKPGDILLIN(N)STGNLIAPR Hex 9 HexNAc 2 | 128.755 | 0.0 | -3.222 | 4.117e+07 | 224 - 255 | 5 | 66.94 | 67.11 | 67.28 |
| 4022.6296 | N(N)GTYDHDVYRDEALNNR Fuc 1 Hex 5 HexNAc 5 | 128.429 | 0.0 | 14.988 | 2.637e+09 | 482 - 499 | 7 | 19.86 | 20.76 | 21.06 |
| 3926.6691 | SEC(C)ITPN(N)GSIPNDKPFQNVNR Hex 7 HexNAc 2 | 127.979 | 0.0 | 17.748 | 1.940e+09 | 278 - 299 | 4 | 32.25 | 32.41 | 32.91 |
| 3470.4133 | N(N)GTYDHDVYRDEALNNR Hex 5 HexNAc 3 | 127.920 | 0.0 | -19.201 | 5.359e+07 | 482 - 499 | 1 | 20.91 | 20.91 | 21.06 |
| 3967.8852 | IVKPGDILLIN(N)STGNLIAPR Hex 9 HexNAc 2 | 126.693 | 0.0 | -18.773 | 1.170e+07 | 235 - 255 | 1 | 52.64 | 52.64 | 52.81 |
| 4088.7218 | SEC(C)ITPN(N)GSIPNDKPFQNVNR Hex 8 HexNAc 2 | 126.577 | 0.0 | 17.591 | 8.791e+08 | 278 - 299 | 3 | 32.25 | 32.41 | 32.91 |
| 3673.4941 | N(N)GTYDHDVYRDEALNNR Hex 5 HexNAc 4 | 126.112 | 0.0 | -4.310 | 2.189e+08 | 482 - 499 | 2 | 20.61 | 20.76 | 21.06 |
| 5141.1598 | SLVASSGTLEFNNESFNWAGVTHN(N)GTSSSC(C)R Hex 5 HexNAc 5 | 125.867 | 0.0 | -4.429 | 5.195e+07 | 109 - 140 | 1 | 49.58 | 49.58 | 49.92 |
| 3245.3311 | RGSN(N)SSFFSR Fuc 3 Hex 4 HexNAc 5 | 125.652 | 0.0 | -20.755 | 3.285e+06 | 140 - 150 | 1 | 19.38 | 19.38 | 19.38 |
| 4410.7055 | N(N)GTYDHDVYRDEALNNR Fuc 2 Hex 9 HexNAc 3 | 125.348 | 0.0 | -4.189 | 1.043e+08 | 482 - 499 | 1 | 21.86 | 22.03 | 22.37 |
| 4184.6828 | N(N)GTYDHDVYRDEALNNR Fuc 1 Hex 6 HexNAc 5 | 125.346 | 0.0 | 10.988 | 5.569e+08 | 482 - 499 | 3 | 20.16 | 20.31 | 20.91 |
| 4250.7764 | SEC(C)ITPN(N)GSIPNDKPFQNVNR Hex 9 HexNAc 2 | 124.695 | 0.0 | 17.002 | 1.001e+09 | 278 - 299 | 5 | 32.08 | 32.41 | 32.74 |
| 6237.5216 | SLVASSGTLEFNNESFNWAGVTHN(N)GTSSSC(C)R Hex 8 HexNAc 8 | 124.592 | 0.0 | 0.000 | 0.000e+00 | 109 - 140 | 1 | - | - | - |
| 3779.5153 | N(N)GTYDHDVYRDEALN(D)NR Fuc 1 Hex 6 HexNAc 3 | 124.336 | 0.0 | -6.006 | 1.058e+08 | 482 - 499 | 2 | 21.06 | 21.06 | 21.70 |
| 3820.5416 | N(N)GTYDHDVYRDEALN(D)NR Fuc 1 Hex 5 HexNAc 4 | 124.186 | 0.0 | 7.197 | 1.495e+09 | 482 - 499 | 9 | 21.22 | 21.22 | 22.03 |
| 4264.6481 | N(N)GTYDHDVYRDEALNNR Fuc 1 Hex 9 HexNAc 3 | 123.333 | 0.0 | 11.999 | 2.159e+08 | 482 - 499 | 4 | 21.86 | 22.20 | 22.53 |
| 5262.1840 | SLVASSGTLEFNNESFNWAGVTHN(N)GTSSSC(C)R Hex 7 HexNAc 4 | 123.206 | 0.0 | -4.863 | 3.575e+07 | 109 - 140 | 2 | 49.41 | 49.58 | 49.75 |
| 4476.8013 | N(N)GTYDHDVYRDEALNNR Fuc 3 Hex 6 HexNAc 5 | 121.926 | 0.0 | -18.348 | 9.765e+07 | 482 - 499 | 1 | 20.16 | 20.16 | 20.31 |
| 5100.1379 | SLVASSGTLEFNNESFNWAGVTHN(N)GTSSSC(C)R Hex 6 HexNAc 4 | 121.447 | 0.0 | -5.029 | 2.746e+07 | 109 - 140 | 1 | 49.58 | 49.75 | 49.92 |
| 3600.4767 | N(N)GTYDHDVYRDEALNNR Fuc 2 Hex 4 HexNAc 3 | 120.644 | 0.0 | -2.927 | 9.291e+07 | 482 - 499 | 2 | 20.76 | 20.76 | 21.06 |
| 3436.2746 | N(N)GTYDHDVYR Fuc 2 Hex 8 HexNAc 3 | 120.628 | 0.0 | -16.879 | 1.390e+07 | 482 - 492 | 1 | 16.70 | 16.70 | 16.70 |
| 4267.8019 | SEC(C)ITPN(N)GSIPNDKPFQNVNR Fuc 2 Hex 3 HexNAc 4 Neu5Ac 1 | 120.502 | 0.0 | -18.691 | 5.224e+07 | 278 - 299 | 1 | 32.25 | 32.41 | 32.41 |
| 3762.5284 | N(N)GTYDHDVYRDEALNNR Fuc 2 Hex 5 HexNAc 3 | 119.840 | 0.0 | -5.383 | 7.494e+07 | 482 - 499 | 1 | 20.61 | 20.61 | 20.91 |
| 2950.1497 | N(N)GTYDHDVYR Fuc 2 Hex 5 HexNAc 3 | 118.812 | 0.0 | -17.813 | 8.840e+06 | 482 - 492 | 1 | 15.72 | 15.72 | 15.72 |
| 4387.7634 | N(N)GTYDHDVYRDEALNNR Fuc 1 Hex 6 HexNAc 6 | 118.235 | 0.0 | -3.402 | 1.225e+08 | 482 - 499 | 1 | 20.46 | 20.61 | 20.76 |
| 3602.5693 | SEC(C)ITPN(N)GSIPNDKPFQNVNR Hex 5 HexNAc 2 | 118.018 | 0.0 | -18.680 | 4.527e+07 | 278 - 299 | 1 | 32.74 | 32.74 | 32.91 |
| 4168.6880 | N(N)GTYDHDVYRDEALNNR Fuc 2 Hex 5 HexNAc 5 | 115.581 | 0.008 | 11.391 | 6.399e+08 | 482 - 499 | 4 | 19.86 | 20.46 | 20.91 |
| 3583.4539 | YPALN(N)VTMPN(D)NEQFDK Hex 8 HexNAc 2 | 115.230 | 0.008 | -5.933 | 3.073e+07 | 160 - 176 | 1 | 43.40 | 43.57 | 43.72 |
| 5205.1601 | SLVASSGTLEFNNESFNWAGVTHN(N)GTSSSC(C)R Fuc 1 Hex 7 HexNAc 3 | 114.780 | 0.008 | -20.438 | 8.929e+06 | 109 - 140 | 1 | 49.41 | 49.41 | 49.58 |
| 3966.5999 | N(N)GTYDHDVYRDEALN(D)NR Fuc 2 Hex 5 HexNAc 4 | 114.704 | 0.008 | 3.925 | 1.092e+09 | 482 - 499 | 5 | 20.91 | 21.06 | 21.86 |
| 3452.2701 | N(N)GTYDHDVYR Fuc 1 Hex 9 HexNAc 3 | 113.908 | 0.008 | -19.802 | 1.487e+07 | 482 - 492 | 1 | 16.53 | 16.70 | 16.70 |
| 4330.7413 | N(N)GTYDHDVYRDEALNNR Fuc 2 Hex 6 HexNAc 5 | 113.097 | 0.008 | -4.515 | 1.543e+08 | 482 - 499 | 1 | 20.16 | 20.31 | 20.46 |
| 4467.7307 | N(N)GTYDHDVYRDEALNNR Fuc 1 Hex 9 HexNAc 4 | 111.992 | 0.008 | 5.147 | 5.865e+07 | 482 - 499 | 1 | 21.70 | 22.20 | 22.37 |
| 6396.5528 | SLVASSGTLEFNNESFNWAGVTHN(N)GTSSSC(C)R Fuc 1 Hex 7 HexNAc 6 Neu5Ac 2 | 110.820 | 0.008 | -4.727 | 5.220e+07 | 109 - 140 | 2 | 46.19 | 46.36 | 46.52 |
| 6251.5249 | SLVASSGTLEFNNESFNWAGVTHN(N)GTSSSC(C)R Fuc 2 Hex 7 HexNAc 6 Neu5Ac 1 | 109.463 | 0.008 | -19.666 | 1.376e+07 | 109 - 140 | 1 | 46.36 | 46.36 | 46.52 |
| 3633.4551 | N(N)GTYDHDVYRDEALN(D)NR Hex 6 HexNAc 3 | 107.988 | 0.008 | -5.862 | 6.543e+07 | 482 - 499 | 1 | 21.22 | 21.22 | 21.54 |
| 2548.0397 | RGSN(N)SSFFSR Fuc 1 Hex 4 HexNAc 3 | 107.838 | 0.008 | 0.000 | 0.000e+00 | 140 - 150 | 1 | - | - | - |
| 2643.0553 | N(N)GTYDHDVYR Fuc 1 Hex 4 HexNAc 3 | 106.013 | 0.008 | 0.000 | 0.000e+00 | 482 - 492 | 1 | - | - | - |
| 4335.8680 | N(N)GTYDHDVYRDEALNNRFQIK Fuc 1 Hex 5 HexNAc 4 | 105.420 | 0.008 | -17.610 | 2.490e+07 | 482 - 503 | 1 | 32.25 | 32.25 | 32.25 |
| 4938.0805 | SLVASSGTLEFNNESFNWAGVTHN(N)GTSSSC(C)R Hex 5 HexNAc 4 | 105.322 | 0.008 | -5.412 | 4.646e+07 | 109 - 140 | 2 | 49.58 | 49.92 | 49.92 |
| 4023.6241 | N(N)GTYDHDVYRDEALN(D)NR Fuc 1 Hex 5 HexNAc 5 | 105.298 | 0.008 | 8.605 | 3.555e+08 | 482 - 499 | 1 | 21.86 | 21.86 | 23.04 |
| 4248.6510 | N(N)GTYDHDVYRDEALNNR Fuc 2 Hex 8 HexNAc 3 | 104.712 | 0.008 | -3.822 | 2.234e+08 | 482 - 499 | 1 | 21.86 | 22.20 | 22.37 |
| 2861.1149 | N(N)GTYDHDVYR Hex 5 HexNAc 4 | 104.226 | 0.012 | -20.320 | 1.636e+07 | 482 - 492 | 1 | 15.72 | 15.87 | 15.87 |
| 3518.3608 | N(N)GTYDHDVYR Fuc 2 Hex 6 HexNAc 5 | 102.524 | 0.012 | -20.104 | 2.296e+07 | 482 - 492 | 2 | 15.58 | 15.58 | 15.58 |
| 3233.3290 | HAVPN(N)GTIVK Hex 8 HexNAc 3 Neu5Ac 1 | 101.682 | 0.012 | 0.000 | 0.000e+00 | 17 - 27 | 1 | - | - | - |
| 5012.1340 | SLVASSGTLEFNNESFNWAGVTHN(N)GTSSSC(C)RR Hex 7 HexNAc 2 | 98.880 | 0.012 | -18.393 | 1.686e+07 | 109 - 141 | 1 | 47.20 | 47.20 | 47.37 |
| 2454.9570 | N(N)GTYDHDVYR Hex 5 HexNAc 2 | 98.162 | 0.012 | 0.000 | 0.000e+00 | 482 - 492 | 1 | - | - | - |
| 3480.3968 | RGSN(N)SSFFSR Fuc 1 Hex 6 HexNAc 6 | 98.037 | 0.015 | -5.124 | 8.047e+07 | 140 - 150 | 2 | 19.38 | 19.38 | 19.86 |
| 4394.7109 | RGSN(N)SSFFSR Fuc 1 Hex 5 HexNAc 7 Neu5Ac 3 | 96.378 | 0.015 | -17.591 | 3.190e+07 | 140 - 150 | 1 | 22.03 | 22.03 | 22.03 |
| 4910.3560 | ISIYWTIVKPGDILLIN(N)STGNLIAPR Fuc 2 Hex 4 HexNAc 4 Neu5Ac 1 | 95.441 | 0.019 | -4.676 | 1.090e+07 | 229 - 255 | 2 | 68.65 | 68.99 | 69.17 |
| 4112.6564 | N(N)GTYDHDVYRDEALN(D)NR Fuc 3 Hex 5 HexNAc 4 | 94.879 | 0.019 | 10.462 | 3.733e+08 | 482 - 499 | 3 | 20.76 | 20.91 | 21.70 |
| 3314.4785 | LGHHAVPN(N)GTIVK Fuc 1 Hex 5 HexNAc 5 | 94.064 | 0.030 | 0.000 | 0.000e+00 | 14 - 27 | 1 | - | - | - |
| 3251.2210 | N(N)GTYDHDVYR Fuc 1 Hex 9 HexNAc 2 | 93.460 | 0.033 | 0.000 | 0.000e+00 | 482 - 492 | 1 | - | - | - |
| 4251.7647 | SEC(C)ITPN(N)GSIPN(D)DKPFQNVNR Hex 9 HexNAc 2 | 92.197 | 0.036 | 6.849 | 1.853e+08 | 278 - 299 | 5 | 32.91 | 33.59 | 33.76 |
| 4857.0192 | SLVASSGTLEFN(D)NESFNWAGVTHN(N)GTSSSC(C)R Hex 7 HexNAc 2 | 92.049 | 0.036 | 9.426 | 4.871e+07 | 109 - 140 | 4 | 50.09 | 50.94 | 51.11 |
| 3927.6625 | SEC(C)ITPN(N)GSIPN(D)DKPFQNVNR Hex 7 HexNAc 2 | 91.602 | 0.036 | 11.002 | 2.599e+08 | 278 - 299 | 5 | 32.91 | 33.76 | 34.10 |
| 3267.2763 | N(N)GTYDHDVYR Hex 5 HexNAc 6 | 89.864 | 0.043 | -18.808 | 3.716e+07 | 482 - 492 | 2 | 16.53 | 16.53 | 16.70 |
| 4089.7162 | SEC(C)ITPN(N)GSIPN(D)DKPFQNVNR Hex 8 HexNAc 2 | 89.740 | 0.046 | 10.808 | 1.473e+08 | 278 - 299 | 4 | 32.91 | 33.76 | 33.93 |

### RGSN(N)SSFFSR Hex 6 HexNAc 2

**Mass:**  2522.0185

(-2.011e-07)

**Charge States:** 3

**Start Position**: 141

**End Position**: 151

| Profile Score | Tandem Score | TDA q-value | Volume | Spectrum Matches |
| --- | --- | --- | --- | --- |
| -3.2600 | 193.7682 | 0.0000 | 5.1154e+08 | 4 |

**Adduct Signal Fractions**

- **Unmodified**: 5.1154e+08

### RGSN(N)SSFFSR Hex 5 HexNAc 2

**Mass:**  2359.9654

(8.481e-07)

**Charge States:** 3

**Start Position**: 141

**End Position**: 151

| Profile Score | Tandem Score | TDA q-value | Volume | Spectrum Matches |
| --- | --- | --- | --- | --- |
| 11.9473 | 193.2276 | 0.0000 | 4.7089e+08 | 3 |

**Adduct Signal Fractions**

- **Unmodified**: 4.7089e+08

### RGSN(N)SSFFSR Hex 5 HexNAc 4

**Mass:**  2766.1248

(3.952e-07)

**Charge States:** 3

**Start Position**: 141

**End Position**: 151

| Profile Score | Tandem Score | TDA q-value | Volume | Spectrum Matches |
| --- | --- | --- | --- | --- |
| -3.8344 | 189.5788 | 0.0000 | 1.1137e+08 | 1 |

**Adduct Signal Fractions**

- **Unmodified**: 1.1137e+08

### RGSN(N)SSFFSR Hex 5 HexNAc 6

**Mass:**  3172.2848

(-1.013e-06)

**Charge States:** 3

**Start Position**: 141

**End Position**: 151

| Profile Score | Tandem Score | TDA q-value | Volume | Spectrum Matches |
| --- | --- | --- | --- | --- |
| -7.2981 | 188.2475 | 0.0000 | 2.2622e+08 | 2 |

**Adduct Signal Fractions**

- **Unmodified**: 2.2622e+08

### RGSN(N)SSFFSR Hex 8 HexNAc 2

**Mass:**  2846.1247

(1.022e-06)

**Charge States:** 3

**Start Position**: 141

**End Position**: 151

| Profile Score | Tandem Score | TDA q-value | Volume | Spectrum Matches |
| --- | --- | --- | --- | --- |
| -17.9854 | 188.1376 | 0.0000 | 1.0055e+08 | 2 |

**Adduct Signal Fractions**

- **Unmodified**: 1.0055e+08

### RGSN(N)SSFFSR Hex 6 HexNAc 3

**Mass:**  2725.0988

(3.336e-07)

**Charge States:** 3

**Start Position**: 141

**End Position**: 151

| Profile Score | Tandem Score | TDA q-value | Volume | Spectrum Matches |
| --- | --- | --- | --- | --- |
| -3.7128 | 183.2509 | 0.0000 | 6.1903e+08 | 4 |

**Adduct Signal Fractions**

- **Unmodified**: 6.1903e+08

### RGSN(N)SSFFSR Fuc 2 Hex 5 HexNAc 5

**Mass:**  3261.3198

(3.402e-08)

**Charge States:** 3

**Start Position**: 141

**End Position**: 151

| Profile Score | Tandem Score | TDA q-value | Volume | Spectrum Matches |
| --- | --- | --- | --- | --- |
| 14.0082 | 182.2022 | 0.0000 | 4.3483e+08 | 4 |

**Adduct Signal Fractions**

- **Unmodified**: 4.3483e+08

### YPALN(N)VTMPNNEQFDK Hex 8 HexNAc 2

**Mass:**  3582.4722

(3.746e-06)

**Charge States:** 3, 4

**Start Position**: 161

**End Position**: 177

| Profile Score | Tandem Score | TDA q-value | Volume | Spectrum Matches |
| --- | --- | --- | --- | --- |
| 18.2239 | 178.3558 | 0.0000 | 3.3676e+08 | 5 |

**Adduct Signal Fractions**

- **Unmodified**: 3.3676e+08

### RGSN(N)SSFFSR Hex 7 HexNAc 2

**Mass:**  2684.0718

(4.232e-07)

**Charge States:** 3

**Start Position**: 141

**End Position**: 151

| Profile Score | Tandem Score | TDA q-value | Volume | Spectrum Matches |
| --- | --- | --- | --- | --- |
| 12.0456 | 178.2603 | 0.0000 | 2.9245e+08 | 2 |

**Adduct Signal Fractions**

- **Unmodified**: 2.9245e+08

### RGSN(N)SSFFSR Fuc 1 Hex 5 HexNAc 5

**Mass:**  3115.2621

(1.844e-06)

**Charge States:** 2, 3, 4

**Start Position**: 141

**End Position**: 151

| Profile Score | Tandem Score | TDA q-value | Volume | Spectrum Matches |
| --- | --- | --- | --- | --- |
| 13.1566 | 174.6602 | 0.0000 | 1.8854e+09 | 6 |

**Adduct Signal Fractions**

- **Unmodified**: 1.8854e+09

### RGSN(N)SSFFSR Fuc 3 Hex 5 HexNAc 5

**Mass:**  3407.3820

(1.474e-06)

**Charge States:** 3

**Start Position**: 141

**End Position**: 151

| Profile Score | Tandem Score | TDA q-value | Volume | Spectrum Matches |
| --- | --- | --- | --- | --- |
| -17.5930 | 173.8069 | 0.0000 | 4.5960e+07 | 1 |

**Adduct Signal Fractions**

- **Unmodified**: 4.5960e+07

### RGSN(N)SSFFSR Fuc 2 Hex 5 HexNAc 4

**Mass:**  3058.2410

(1.141e-06)

**Charge States:** 3, 4

**Start Position**: 141

**End Position**: 151

| Profile Score | Tandem Score | TDA q-value | Volume | Spectrum Matches |
| --- | --- | --- | --- | --- |
| -17.7063 | 173.1043 | 0.0000 | 2.8572e+08 | 3 |

**Adduct Signal Fractions**

- **Unmodified**: 2.8572e+08

### RGSN(N)SSFFSR Fuc 1 Hex 6 HexNAc 5

**Mass:**  3277.3178

(9.987e-07)

**Charge States:** 3

**Start Position**: 141

**End Position**: 151

| Profile Score | Tandem Score | TDA q-value | Volume | Spectrum Matches |
| --- | --- | --- | --- | --- |
| -3.7442 | 173.0750 | 0.0000 | 1.0397e+08 | 2 |

**Adduct Signal Fractions**

- **Unmodified**: 1.0397e+08

### YPALN(N)VTM(O)PNNEQFDK Hex 8 HexNAc 2

**Mass:**  3598.4785

(3.990e-06)

**Charge States:** 2, 3, 4

**Start Position**: 161

**End Position**: 177

| Profile Score | Tandem Score | TDA q-value | Volume | Spectrum Matches |
| --- | --- | --- | --- | --- |
| 16.8034 | 171.6873 | 0.0000 | 1.8245e+09 | 22 |

**Adduct Signal Fractions**

- **Unmodified**: 1.8245e+09

### YPALN(N)VTM(O)PNNEQFDK Hex 9 HexNAc 2

**Mass:**  3760.5108

(1.176e-06)

**Charge States:** 3, 4

**Start Position**: 161

**End Position**: 177

| Profile Score | Tandem Score | TDA q-value | Volume | Spectrum Matches |
| --- | --- | --- | --- | --- |
| 15.4894 | 171.3189 | 0.0000 | 1.5763e+08 | 5 |

**Adduct Signal Fractions**

- **Unmodified**: 1.5763e+08

### YPALN(N)VTM(O)PNNEQFDK Hex 7 HexNAc 2

**Mass:**  3436.4166

(4.633e-06)

**Charge States:** 2, 3, 4

**Start Position**: 161

**End Position**: 177

| Profile Score | Tandem Score | TDA q-value | Volume | Spectrum Matches |
| --- | --- | --- | --- | --- |
| 15.9153 | 171.1999 | 0.0000 | 3.4790e+08 | 4 |

**Adduct Signal Fractions**

- **Unmodified**: 3.4790e+08

### RGSN(N)SSFFSR Fuc 3 Hex 5 HexNAc 4

**Mass:**  3204.3012

(1.216e-06)

**Charge States:** 3

**Start Position**: 141

**End Position**: 151

| Profile Score | Tandem Score | TDA q-value | Volume | Spectrum Matches |
| --- | --- | --- | --- | --- |
| -4.2860 | 169.9383 | 0.0000 | 1.6385e+08 | 2 |

**Adduct Signal Fractions**

- **Unmodified**: 1.6385e+08

### RGSN(N)SSFFSR Hex 5 HexNAc 5

**Mass:**  2969.2051

(6.676e-07)

**Charge States:** 3

**Start Position**: 141

**End Position**: 151

| Profile Score | Tandem Score | TDA q-value | Volume | Spectrum Matches |
| --- | --- | --- | --- | --- |
| 12.0423 | 167.7772 | 0.0000 | 3.1101e+08 | 2 |

**Adduct Signal Fractions**

- **Unmodified**: 3.1101e+08

### TIVKPGDILLIN(N)STGNLIAPR Hex 9 HexNAc 2

**Mass:**  4068.9360

(3.094e-06)

**Charge States:** 3, 4, 5

**Start Position**: 235

**End Position**: 256

| Profile Score | Tandem Score | TDA q-value | Volume | Spectrum Matches |
| --- | --- | --- | --- | --- |
| 19.2426 | 167.5123 | 0.0000 | 6.0855e+08 | 5 |

**Adduct Signal Fractions**

- **Unmodified**: 6.0855e+08

### RGSN(N)SSFFSR Fuc 2 Hex 5 HexNAc 3

**Mass:**  2855.1612

(3.713e-07)

**Charge States:** 3

**Start Position**: 141

**End Position**: 151

| Profile Score | Tandem Score | TDA q-value | Volume | Spectrum Matches |
| --- | --- | --- | --- | --- |
| -18.6532 | 165.5378 | 0.0000 | 2.6921e+07 | 1 |

**Adduct Signal Fractions**

- **Unmodified**: 2.6921e+07

### RGSN(N)SSFFSR Fuc 1 Hex 5 HexNAc 3

**Mass:**  2709.1049

(9.841e-07)

**Charge States:** 3

**Start Position**: 141

**End Position**: 151

| Profile Score | Tandem Score | TDA q-value | Volume | Spectrum Matches |
| --- | --- | --- | --- | --- |
| -4.2373 | 164.0725 | 0.0000 | 8.8690e+07 | 1 |

**Adduct Signal Fractions**

- **Unmodified**: 8.8690e+07

### RGSN(N)SSFFSR Fuc 1 Hex 6 HexNAc 3

**Mass:**  2871.1565

(5.167e-07)

**Charge States:** 3

**Start Position**: 141

**End Position**: 151

| Profile Score | Tandem Score | TDA q-value | Volume | Spectrum Matches |
| --- | --- | --- | --- | --- |
| 10.5155 | 163.9185 | 0.0000 | 3.9687e+08 | 2 |

**Adduct Signal Fractions**

- **Unmodified**: 3.9687e+08

### GSN(N)SSFFSR Fuc 1 Hex 5 HexNAc 4

**Mass:**  2756.0837

(1.314e-06)

**Charge States:** 3

**Start Position**: 142

**End Position**: 151

| Profile Score | Tandem Score | TDA q-value | Volume | Spectrum Matches |
| --- | --- | --- | --- | --- |
| 12.0475 | 163.0499 | 0.0000 | 3.9084e+08 | 3 |

**Adduct Signal Fractions**

- **Unmodified**: 3.9084e+08

### RGSN(N)SSFFSR Fuc 1 Hex 5 HexNAc 4

**Mass:**  2912.1820

(5.270e-07)

**Charge States:** 3

**Start Position**: 141

**End Position**: 151

| Profile Score | Tandem Score | TDA q-value | Volume | Spectrum Matches |
| --- | --- | --- | --- | --- |
| 13.9766 | 162.1966 | 0.0000 | 9.1883e+08 | 4 |

**Adduct Signal Fractions**

- **Unmodified**: 9.1883e+08

### RGSN(N)SSFFSR Fuc 1 Hex 4 HexNAc 3

**Mass:**  2547.0560

(2.538e-06)

**Charge States:** 3

**Start Position**: 141

**End Position**: 151

| Profile Score | Tandem Score | TDA q-value | Volume | Spectrum Matches |
| --- | --- | --- | --- | --- |
| -18.9342 | 161.2664 | 0.0000 | 2.3751e+07 | 1 |

**Adduct Signal Fractions**

- **Unmodified**: 2.3751e+07

### YPALN(N)VTMPNNEQFDK Hex 7 HexNAc 2

**Mass:**  3420.4173

(3.730e-06)

**Charge States:** 3

**Start Position**: 161

**End Position**: 177

| Profile Score | Tandem Score | TDA q-value | Volume | Spectrum Matches |
| --- | --- | --- | --- | --- |
| -3.2775 | 159.4179 | 0.0000 | 5.4809e+07 | 2 |

**Adduct Signal Fractions**

- **Unmodified**: 5.4809e+07

### RGSN(N)SSFFSR Fuc 1 Hex 9 HexNAc 4

**Mass:**  3560.3582

(-9.720e-06)

**Charge States:** 3

**Start Position**: 141

**End Position**: 151

| Profile Score | Tandem Score | TDA q-value | Volume | Spectrum Matches |
| --- | --- | --- | --- | --- |
| 10.8286 | 158.6045 | 0.0000 | 8.5186e+07 | 1 |

**Adduct Signal Fractions**

- **Unmodified**: 8.5186e+07

### TIVKPGDILLIN(N)STGNLIAPR Fuc 2 Hex 3 HexNAc 4 Neu5Ac 1

**Mass:**  4085.9599

(-3.163e-06)

**Charge States:** 4

**Start Position**: 235

**End Position**: 256

| Profile Score | Tandem Score | TDA q-value | Volume | Spectrum Matches |
| --- | --- | --- | --- | --- |
| -3.0745 | 157.2027 | 0.0000 | 7.9458e+07 | 2 |

**Adduct Signal Fractions**

- **Unmodified**: 7.9458e+07

### SLVASSGTLEFNNESFNWAGVTHN(N)GTSSSC(C)R Hex 7 HexNAc 2

**Mass:**  4856.0383

(7.272e-07)

**Charge States:** 3, 4

**Start Position**: 110

**End Position**: 141

| Profile Score | Tandem Score | TDA q-value | Volume | Spectrum Matches |
| --- | --- | --- | --- | --- |
| -2.2483 | 155.9357 | 0.0000 | 5.2527e+08 | 4 |

**Adduct Signal Fractions**

- **Unmodified**: 5.2527e+08

### YPALN(N)VTMPNNEQFDK Hex 9 HexNAc 2

**Mass:**  3744.5205

(3.402e-06)

**Charge States:** 3

**Start Position**: 161

**End Position**: 177

| Profile Score | Tandem Score | TDA q-value | Volume | Spectrum Matches |
| --- | --- | --- | --- | --- |
| -4.0731 | 155.4127 | 0.0000 | 3.0454e+07 | 2 |

**Adduct Signal Fractions**

- **Unmodified**: 3.0454e+07

### C(C)KSEC(C)ITPN(N)GSIPNDKPFQNVNR Hex 8 HexNAc 2

**Mass:**  4376.8420

(-4.431e-07)

**Charge States:** 4

**Start Position**: 277

**End Position**: 300

| Profile Score | Tandem Score | TDA q-value | Volume | Spectrum Matches |
| --- | --- | --- | --- | --- |
| -17.1617 | 155.3036 | 0.0000 | 1.5110e+08 | 1 |

**Adduct Signal Fractions**

- **Unmodified**: 1.5110e+08

### RGSN(N)SSFFSR Fuc 2 Hex 9 HexNAc 3

**Mass:**  3503.3325

(-9.675e-06)

**Charge States:** 3

**Start Position**: 141

**End Position**: 151

| Profile Score | Tandem Score | TDA q-value | Volume | Spectrum Matches |
| --- | --- | --- | --- | --- |
| -3.8918 | 155.2002 | 0.0000 | 6.6398e+07 | 2 |

**Adduct Signal Fractions**

- **Unmodified**: 6.6398e+07

### N(N)GTYDHDVYR Fuc 1 Hex 5 HexNAc 4

**Mass:**  3007.1755

(1.460e-06)

**Charge States:** 3

**Start Position**: 483

**End Position**: 493

| Profile Score | Tandem Score | TDA q-value | Volume | Spectrum Matches |
| --- | --- | --- | --- | --- |
| 14.7884 | 154.6934 | 0.0000 | 4.7540e+08 | 4 |

**Adduct Signal Fractions**

- **Unmodified**: 4.7540e+08

### YPALN(N)VTM(O)PNNEQFDK Hex 6 HexNAc 2

**Mass:**  3274.3646

(5.041e-06)

**Charge States:** 3

**Start Position**: 161

**End Position**: 177

| Profile Score | Tandem Score | TDA q-value | Volume | Spectrum Matches |
| --- | --- | --- | --- | --- |
| 11.9640 | 154.0942 | 0.0000 | 2.3878e+07 | 2 |

**Adduct Signal Fractions**

- **Unmodified**: 2.3878e+07

### GSN(N)SSFFSR Hex 5 HexNAc 2

**Mass:**  2203.8641

(1.125e-07)

**Charge States:** 2, 3

**Start Position**: 142

**End Position**: 151

| Profile Score | Tandem Score | TDA q-value | Volume | Spectrum Matches |
| --- | --- | --- | --- | --- |
| 13.0786 | 153.8869 | 0.0000 | 3.4381e+08 | 3 |

**Adduct Signal Fractions**

- **Unmodified**: 3.4381e+08

### RGSN(N)SSFFSR Hex 9 HexNAc 2

**Mass:**  3008.1553

(-6.976e-06)

**Charge States:** 3

**Start Position**: 141

**End Position**: 151

| Profile Score | Tandem Score | TDA q-value | Volume | Spectrum Matches |
| --- | --- | --- | --- | --- |
| -20.6542 | 153.1382 | 0.0000 | 7.7449e+06 | 1 |

**Adduct Signal Fractions**

- **Unmodified**: 7.7449e+06

### HAVPN(N)GTIVK Fuc 1 Hex 5 HexNAc 5

**Mass:**  3006.3067

(2.836e-06)

**Charge States:** 3

**Start Position**: 18

**End Position**: 28

| Profile Score | Tandem Score | TDA q-value | Volume | Spectrum Matches |
| --- | --- | --- | --- | --- |
| -20.1436 | 152.0945 | 0.0000 | 9.2211e+06 | 1 |

**Adduct Signal Fractions**

- **Unmodified**: 9.2211e+06

### N(N)GTYDHDVYR Fuc 1 Hex 6 HexNAc 5

**Mass:**  3372.3085

(-3.536e-06)

**Charge States:** 3

**Start Position**: 483

**End Position**: 493

| Profile Score | Tandem Score | TDA q-value | Volume | Spectrum Matches |
| --- | --- | --- | --- | --- |
| -21.7860 | 152.0439 | 0.0000 | 7.4881e+07 | 4 |

**Adduct Signal Fractions**

- **Unmodified**: 7.4881e+07

### N(N)GTYDHDVYR Fuc 1 Hex 6 HexNAc 3

**Mass:**  2966.1438

(-2.794e-07)

**Charge States:** 3

**Start Position**: 483

**End Position**: 493

| Profile Score | Tandem Score | TDA q-value | Volume | Spectrum Matches |
| --- | --- | --- | --- | --- |
| -20.1362 | 152.0151 | 0.0000 | 1.7351e+07 | 1 |

**Adduct Signal Fractions**

- **Unmodified**: 1.7351e+07

### SLVASSGTLEFNNESFNWAGVTHN(N)GTSSSC(C)R Hex 7 HexNAc 3

**Mass:**  5059.1022

(1.846e-06)

**Charge States:** 4

**Start Position**: 110

**End Position**: 141

| Profile Score | Tandem Score | TDA q-value | Volume | Spectrum Matches |
| --- | --- | --- | --- | --- |
| -18.4699 | 151.7381 | 0.0000 | 4.2496e+07 | 1 |

**Adduct Signal Fractions**

- **Unmodified**: 4.2496e+07

### TIVKPGDILLIN(N)STGNLIAPR Hex 10 HexNAc 2

**Mass:**  4230.9919

(3.652e-06)

**Charge States:** 4

**Start Position**: 235

**End Position**: 256

| Profile Score | Tandem Score | TDA q-value | Volume | Spectrum Matches |
| --- | --- | --- | --- | --- |
| -19.3976 | 150.6985 | 0.0000 | 2.1293e+07 | 2 |

**Adduct Signal Fractions**

- **Unmodified**: 2.1293e+07

### RGSN(N)SSFFSR Hex 6 HexNAc 6

**Mass:**  3334.3350

(-5.027e-08)

**Charge States:** 3

**Start Position**: 141

**End Position**: 151

| Profile Score | Tandem Score | TDA q-value | Volume | Spectrum Matches |
| --- | --- | --- | --- | --- |
| -19.4655 | 150.5067 | 0.0000 | 1.8006e+07 | 1 |

**Adduct Signal Fractions**

- **Unmodified**: 1.8006e+07

### GSN(N)SSFFSR Fuc 1 Hex 6 HexNAc 3

**Mass:**  2715.0556

(1.072e-06)

**Charge States:** 2, 3

**Start Position**: 142

**End Position**: 151

| Profile Score | Tandem Score | TDA q-value | Volume | Spectrum Matches |
| --- | --- | --- | --- | --- |
| -3.8120 | 149.9170 | 0.0000 | 1.5670e+08 | 2 |

**Adduct Signal Fractions**

- **Unmodified**: 1.5670e+08

### N(N)GTYDHDVYR Fuc 1 Hex 5 HexNAc 5

**Mass:**  3210.2558

(1.611e-06)

**Charge States:** 3

**Start Position**: 483

**End Position**: 493

| Profile Score | Tandem Score | TDA q-value | Volume | Spectrum Matches |
| --- | --- | --- | --- | --- |
| -2.2647 | 149.8677 | 0.0000 | 2.4486e+08 | 3 |

**Adduct Signal Fractions**

- **Unmodified**: 2.4486e+08

### C(C)KSEC(C)ITPN(N)GSIPNDKPFQNVNR Hex 6 HexNAc 2

**Mass:**  4052.7343

(-1.393e-07)

**Charge States:** 4

**Start Position**: 277

**End Position**: 300

| Profile Score | Tandem Score | TDA q-value | Volume | Spectrum Matches |
| --- | --- | --- | --- | --- |
| -19.0530 | 149.8456 | 0.0000 | 3.0887e+07 | 1 |

**Adduct Signal Fractions**

- **Unmodified**: 3.0887e+07

### N(N)GTYDHDVYRDEALNNR Hex 6 HexNAc 3

**Mass:**  3632.4677

(7.051e-07)

**Charge States:** 4

**Start Position**: 483

**End Position**: 500

| Profile Score | Tandem Score | TDA q-value | Volume | Spectrum Matches |
| --- | --- | --- | --- | --- |
| -3.4434 | 149.6602 | 0.0000 | 3.2883e+08 | 3 |

**Adduct Signal Fractions**

- **Unmodified**: 3.2883e+08

### C(C)KSEC(C)ITPN(N)GSIPNDKPFQNVNR Hex 7 HexNAc 2

**Mass:**  4214.7893

(-7.962e-07)

**Charge States:** 4

**Start Position**: 277

**End Position**: 300

| Profile Score | Tandem Score | TDA q-value | Volume | Spectrum Matches |
| --- | --- | --- | --- | --- |
| -2.9225 | 149.6288 | 0.0000 | 3.0487e+08 | 3 |

**Adduct Signal Fractions**

- **Unmodified**: 3.0487e+08

### TIVKPGDILLIN(N)STGNLIAPR Hex 8 HexNAc 2

**Mass:**  3906.8813

(3.807e-06)

**Charge States:** 4

**Start Position**: 235

**End Position**: 256

| Profile Score | Tandem Score | TDA q-value | Volume | Spectrum Matches |
| --- | --- | --- | --- | --- |
| -3.6064 | 149.1272 | 0.0000 | 6.0719e+07 | 3 |

**Adduct Signal Fractions**

- **Unmodified**: 6.0719e+07

### GSN(N)SSFFSR Hex 6 HexNAc 2

**Mass:**  2365.9195

(-4.855e-07)

**Charge States:** 2, 3

**Start Position**: 142

**End Position**: 151

| Profile Score | Tandem Score | TDA q-value | Volume | Spectrum Matches |
| --- | --- | --- | --- | --- |
| -3.2752 | 148.8987 | 0.0000 | 2.3802e+08 | 2 |

**Adduct Signal Fractions**

- **Unmodified**: 2.3802e+08

### GSN(N)SSFFSR Hex 5 HexNAc 5

**Mass:**  2813.1042

(7.878e-07)

**Charge States:** 2, 3

**Start Position**: 142

**End Position**: 151

| Profile Score | Tandem Score | TDA q-value | Volume | Spectrum Matches |
| --- | --- | --- | --- | --- |
| 11.7871 | 148.6997 | 0.0000 | 2.4058e+08 | 2 |

**Adduct Signal Fractions**

- **Unmodified**: 2.4058e+08

### YPALN(N)VTMPNNEQFDK Fuc 2 Hex 3 HexNAc 4 Neu5Ac 1

**Mass:**  3761.5486

(-4.225e-06)

**Charge States:** 3

**Start Position**: 161

**End Position**: 177

| Profile Score | Tandem Score | TDA q-value | Volume | Spectrum Matches |
| --- | --- | --- | --- | --- |
| -18.7500 | 148.5634 | 0.0000 | 7.4587e+06 | 1 |

**Adduct Signal Fractions**

- **Unmodified**: 7.4587e+06

### SLVASSGTLEFNNESFNWAGVTHN(N)GTSSSC(C)R Hex 6 HexNAc 2

**Mass:**  4693.9836

(4.623e-06)

**Charge States:** 4

**Start Position**: 110

**End Position**: 141

| Profile Score | Tandem Score | TDA q-value | Volume | Spectrum Matches |
| --- | --- | --- | --- | --- |
| -3.1561 | 148.5562 | 0.0000 | 1.2839e+08 | 3 |

**Adduct Signal Fractions**

- **Unmodified**: 1.2839e+08

### RGSN(N)SSFFSR Fuc 1 Hex 3 HexNAc 9 Neu5Ac 2

**Mass:**  4185.6750

(2.262e-06)

**Charge States:** 4

**Start Position**: 141

**End Position**: 151

| Profile Score | Tandem Score | TDA q-value | Volume | Spectrum Matches |
| --- | --- | --- | --- | --- |
| -4.0883 | 148.3546 | 0.0000 | 1.8228e+08 | 3 |

**Adduct Signal Fractions**

- **Unmodified**: 1.8228e+08

### GSN(N)SSFFSR Hex 6 HexNAc 3

**Mass:**  2568.9995

(-4.381e-07)

**Charge States:** 2, 3

**Start Position**: 142

**End Position**: 151

| Profile Score | Tandem Score | TDA q-value | Volume | Spectrum Matches |
| --- | --- | --- | --- | --- |
| 12.6727 | 147.9083 | 0.0000 | 3.3299e+08 | 3 |

**Adduct Signal Fractions**

- **Unmodified**: 3.3299e+08

### ISIYWTIVKPGDILLIN(N)STGNLIAPR Hex 9 HexNAc 2

**Mass:**  4731.2810

(2.063e-06)

**Charge States:** 3, 4, 5

**Start Position**: 230

**End Position**: 256

| Profile Score | Tandem Score | TDA q-value | Volume | Spectrum Matches |
| --- | --- | --- | --- | --- |
| 18.6635 | 147.5462 | 0.0000 | 8.6389e+08 | 9 |

**Adduct Signal Fractions**

- **Unmodified**: 8.6389e+08

### RGSN(N)SSFFSR Fuc 2 Hex 6 HexNAc 5

**Mass:**  3423.3740

(6.731e-07)

**Charge States:** 3

**Start Position**: 141

**End Position**: 151

| Profile Score | Tandem Score | TDA q-value | Volume | Spectrum Matches |
| --- | --- | --- | --- | --- |
| -5.1344 | 147.4956 | 0.0000 | 2.6108e+07 | 1 |

**Adduct Signal Fractions**

- **Unmodified**: 2.6108e+07

### C(C)KSEC(C)ITPN(N)GSIPNDKPFQNVNR Hex 9 HexNAc 2

**Mass:**  4538.8937

(-5.011e-07)

**Charge States:** 4

**Start Position**: 277

**End Position**: 300

| Profile Score | Tandem Score | TDA q-value | Volume | Spectrum Matches |
| --- | --- | --- | --- | --- |
| -18.2743 | 146.9481 | 0.0000 | 2.0613e+08 | 1 |

**Adduct Signal Fractions**

- **Unmodified**: 2.0613e+08

### RGSN(N)SSFFSR Fuc 2 Hex 6 HexNAc 3

**Mass:**  3017.2117

(8.069e-07)

**Charge States:** 3

**Start Position**: 141

**End Position**: 151

| Profile Score | Tandem Score | TDA q-value | Volume | Spectrum Matches |
| --- | --- | --- | --- | --- |
| -19.2684 | 145.2142 | 0.0000 | 2.8539e+07 | 1 |

**Adduct Signal Fractions**

- **Unmodified**: 2.8539e+07

### GSN(N)SSFFSR Fuc 2 Hex 5 HexNAc 5

**Mass:**  3105.2191

(3.884e-07)

**Charge States:** 3

**Start Position**: 142

**End Position**: 151

| Profile Score | Tandem Score | TDA q-value | Volume | Spectrum Matches |
| --- | --- | --- | --- | --- |
| -3.4173 | 145.2074 | 0.0000 | 1.2504e+08 | 1 |

**Adduct Signal Fractions**

- **Unmodified**: 1.2504e+08

### RGSN(N)SSFFSR Hex 6 HexNAc 4

**Mass:**  2928.1765

(9.626e-07)

**Charge States:** 3

**Start Position**: 141

**End Position**: 151

| Profile Score | Tandem Score | TDA q-value | Volume | Spectrum Matches |
| --- | --- | --- | --- | --- |
| 12.8141 | 144.7148 | 0.0000 | 1.3064e+08 | 2 |

**Adduct Signal Fractions**

- **Unmodified**: 1.3064e+08

### ISIYWTIVKPGDILLIN(N)STGNLIAPR Fuc 2 Hex 3 HexNAc 4 Neu5Ac 1

**Mass:**  4748.3019

(-3.247e-06)

**Charge States:** 4

**Start Position**: 230

**End Position**: 256

| Profile Score | Tandem Score | TDA q-value | Volume | Spectrum Matches |
| --- | --- | --- | --- | --- |
| 15.0358 | 143.8934 | 0.0000 | 2.2744e+08 | 8 |

**Adduct Signal Fractions**

- **Unmodified**: 2.2744e+08

### N(N)GTYDHDVYRDEALNNR Fuc 3 Hex 5 HexNAc 4

**Mass:**  4111.6677

(8.441e-07)

**Charge States:** 4

**Start Position**: 483

**End Position**: 500

| Profile Score | Tandem Score | TDA q-value | Volume | Spectrum Matches |
| --- | --- | --- | --- | --- |
| 14.9110 | 143.2357 | 0.0000 | 6.8811e+08 | 3 |

**Adduct Signal Fractions**

- **Unmodified**: 6.8811e+08

### RGSN(N)SSFF Fuc 1 Hex 5 HexNAc 5

**Mass:**  2872.1295

(5.562e-07)

**Charge States:** 3

**Start Position**: 141

**End Position**: 149

| Profile Score | Tandem Score | TDA q-value | Volume | Spectrum Matches |
| --- | --- | --- | --- | --- |
| -18.5699 | 142.8248 | 0.0000 | 1.5133e+07 | 1 |

**Adduct Signal Fractions**

- **Unmodified**: 1.5133e+07

### N(N)GTYDHDVYRDEALNNR Hex 5 HexNAc 2

**Mass:**  3267.3320

(-6.432e-08)

**Charge States:** 3, 4

**Start Position**: 483

**End Position**: 500

| Profile Score | Tandem Score | TDA q-value | Volume | Spectrum Matches |
| --- | --- | --- | --- | --- |
| -20.0547 | 141.0733 | 0.0000 | 1.1036e+08 | 1 |

**Adduct Signal Fractions**

- **Unmodified**: 1.1036e+08

### IRNIPSRISIYWTIVKPGDILLIN(N)STGNLIAPR Hex 9 HexNAc 2

**Mass:**  5567.7749

(2.097e-06)

**Charge States:** 4, 5, 6

**Start Position**: 223

**End Position**: 256

| Profile Score | Tandem Score | TDA q-value | Volume | Spectrum Matches |
| --- | --- | --- | --- | --- |
| -2.4333 | 140.5490 | 0.0000 | 1.2342e+08 | 6 |

**Adduct Signal Fractions**

- **Unmodified**: 1.2342e+08

### ISIYWTIVKPGDILLIN(N)STGNLIAPR Hex 8 HexNAc 2

**Mass:**  4569.2253

(3.221e-06)

**Charge States:** 3, 4, 5

**Start Position**: 230

**End Position**: 256

| Profile Score | Tandem Score | TDA q-value | Volume | Spectrum Matches |
| --- | --- | --- | --- | --- |
| -2.5693 | 140.0469 | 0.0000 | 9.4631e+07 | 5 |

**Adduct Signal Fractions**

- **Unmodified**: 9.4631e+07

### YPALN(N)VTM(O)PNNEQFDK Hex 5 HexNAc 2

**Mass:**  3112.3189

(6.775e-06)

**Charge States:** 3

**Start Position**: 161

**End Position**: 177

| Profile Score | Tandem Score | TDA q-value | Volume | Spectrum Matches |
| --- | --- | --- | --- | --- |
| -4.6719 | 139.8547 | 0.0000 | 1.3130e+07 | 2 |

**Adduct Signal Fractions**

- **Unmodified**: 1.3130e+07

### N(N)GTYDHDVYRDEALNNR Fuc 1 Hex 6 HexNAc 3

**Mass:**  3778.5275

(1.382e-06)

**Charge States:** 4

**Start Position**: 483

**End Position**: 500

| Profile Score | Tandem Score | TDA q-value | Volume | Spectrum Matches |
| --- | --- | --- | --- | --- |
| 13.1532 | 139.2485 | 0.0000 | 3.2135e+08 | 2 |

**Adduct Signal Fractions**

- **Unmodified**: 3.2135e+08

### RGSN(N)SSFFSR Fuc 3 Hex 7 HexNAc 6

**Mass:**  3935.5993

(2.637e-04)

**Start Position**: 141

**End Position**: 151

| Profile Score | Tandem Score | TDA q-value | Volume | Spectrum Matches |
| --- | --- | --- | --- | --- |
| 0.0000 | 139.0206 | 0.0000 | 0.0000e+00 | 1 |

Adduct:

No Chromatogram Found

### GSN(N)SSFFSR Hex 5 HexNAc 4

**Mass:**  2610.0261

(7.732e-07)

**Charge States:** 3

**Start Position**: 142

**End Position**: 151

| Profile Score | Tandem Score | TDA q-value | Volume | Spectrum Matches |
| --- | --- | --- | --- | --- |
| -19.5553 | 137.9504 | 0.0455 | 4.2961e+07 | 2 |

**Adduct Signal Fractions**

- **Unmodified**: 4.2961e+07

### N(N)GTYDHDVYR Fuc 2 Hex 5 HexNAc 4

**Mass:**  3153.2334

(-6.439e-07)

**Charge States:** 3

**Start Position**: 483

**End Position**: 493

| Profile Score | Tandem Score | TDA q-value | Volume | Spectrum Matches |
| --- | --- | --- | --- | --- |
| -3.7601 | 136.9756 | 0.0000 | 3.1998e+08 | 3 |

**Adduct Signal Fractions**

- **Unmodified**: 3.1998e+08

### N(N)GTYDHDVYRDEALNNR Fuc 1 Hex 8 HexNAc 2

**Mass:**  3899.5190

(-8.110e-06)

**Charge States:** 3, 4

**Start Position**: 483

**End Position**: 500

| Profile Score | Tandem Score | TDA q-value | Volume | Spectrum Matches |
| --- | --- | --- | --- | --- |
| 13.6036 | 136.6785 | 0.0000 | 7.3297e+08 | 2 |

**Adduct Signal Fractions**

- **Unmodified**: 7.3297e+08

### GSN(N)SSFFSR Fuc 1 Hex 5 HexNAc 5

**Mass:**  2959.1629

(-6.451e-07)

**Charge States:** 2, 3

**Start Position**: 142

**End Position**: 151

| Profile Score | Tandem Score | TDA q-value | Volume | Spectrum Matches |
| --- | --- | --- | --- | --- |
| 13.2017 | 136.3372 | 0.0455 | 9.9126e+08 | 3 |

**Adduct Signal Fractions**

- **Unmodified**: 9.9126e+08

### N(N)GTYDHDVYR Fuc 3 Hex 5 HexNAc 4

**Mass:**  3300.2893

(6.047e-04)

**Start Position**: 483

**End Position**: 493

| Profile Score | Tandem Score | TDA q-value | Volume | Spectrum Matches |
| --- | --- | --- | --- | --- |
| 0.0000 | 135.4368 | 0.0000 | 0.0000e+00 | 2 |

Adduct:

No Chromatogram Found

### YPALN(N)VTM(O)PN(D)NEQFDK Hex 7 HexNAc 2

**Mass:**  3437.4113

(2.940e-04)

**Charge States:** 3

**Start Position**: 161

**End Position**: 177

| Profile Score | Tandem Score | TDA q-value | Volume | Spectrum Matches |
| --- | --- | --- | --- | --- |
| 6.4573 | 134.8458 | 0.0000 | 2.5124e+07 | 3 |

**Adduct Signal Fractions**

- **Unmodified**: 2.5124e+07

### N(N)GTYDHDVYRDEALNNR Fuc 2 Hex 5 HexNAc 4

**Mass:**  3965.6103

(8.563e-07)

**Charge States:** 3, 4

**Start Position**: 483

**End Position**: 500

| Profile Score | Tandem Score | TDA q-value | Volume | Spectrum Matches |
| --- | --- | --- | --- | --- |
| 15.2830 | 133.5964 | 0.0000 | 2.1566e+09 | 4 |

**Adduct Signal Fractions**

- **Unmodified**: 2.1566e+09

### GSN(N)SSFFSR Hex 5 HexNAc 3

**Mass:**  2406.9424

(-3.168e-07)

**Charge States:** 2, 3

**Start Position**: 142

**End Position**: 151

| Profile Score | Tandem Score | TDA q-value | Volume | Spectrum Matches |
| --- | --- | --- | --- | --- |
| -20.0509 | 131.8583 | 0.0455 | 3.8373e+07 | 2 |

**Adduct Signal Fractions**

- **Unmodified**: 3.8373e+07

### N(N)GTYDHDVYR Hex 6 HexNAc 3

**Mass:**  2820.0913

(1.650e-06)

**Charge States:** 3

**Start Position**: 483

**End Position**: 493

| Profile Score | Tandem Score | TDA q-value | Volume | Spectrum Matches |
| --- | --- | --- | --- | --- |
| -18.0667 | 131.8080 | 0.0000 | 3.5796e+07 | 2 |

**Adduct Signal Fractions**

- **Unmodified**: 3.5796e+07

### N(N)GTYDHDVYRDEALNNR Fuc 1 Hex 8 HexNAc 3

**Mass:**  4102.6003

(-6.729e-06)

**Charge States:** 3, 4

**Start Position**: 483

**End Position**: 500

| Profile Score | Tandem Score | TDA q-value | Volume | Spectrum Matches |
| --- | --- | --- | --- | --- |
| 11.2602 | 131.7769 | 0.0000 | 3.4625e+08 | 4 |

**Adduct Signal Fractions**

- **Unmodified**: 3.4625e+08

### N(N)GTYDHDVYRDEALNNR Fuc 1 Hex 5 HexNAc 4

**Mass:**  3819.5500

(1.208e-06)

**Charge States:** 3, 4, 5

**Start Position**: 483

**End Position**: 500

| Profile Score | Tandem Score | TDA q-value | Volume | Spectrum Matches |
| --- | --- | --- | --- | --- |
| 16.6707 | 131.4278 | 0.0000 | 4.7370e+09 | 7 |

**Adduct Signal Fractions**

- **Unmodified**: 4.7370e+09

### N(N)GTYDHDVYRDEALNNR Fuc 1 Hex 5 HexNAc 3

**Mass:**  3616.4721

(5.915e-07)

**Charge States:** 4

**Start Position**: 483

**End Position**: 500

| Profile Score | Tandem Score | TDA q-value | Volume | Spectrum Matches |
| --- | --- | --- | --- | --- |
| -3.3291 | 131.3200 | 0.0000 | 1.4748e+08 | 2 |

**Adduct Signal Fractions**

- **Unmodified**: 1.4748e+08

### SEC(C)ITPN(N)GSIPNDKPFQNVNR Hex 6 HexNAc 2

**Mass:**  3764.6145

(2.205e-06)

**Charge States:** 3, 4

**Start Position**: 279

**End Position**: 300

| Profile Score | Tandem Score | TDA q-value | Volume | Spectrum Matches |
| --- | --- | --- | --- | --- |
| -3.1678 | 130.7891 | 0.0000 | 2.5042e+08 | 2 |

**Adduct Signal Fractions**

- **Unmodified**: 2.5042e+08

### YPALN(N)VTM(O)PN(D)NEQFDK Hex 8 HexNAc 2

**Mass:**  3599.4633

(2.824e-04)

**Charge States:** 2, 3, 4

**Start Position**: 161

**End Position**: 177

| Profile Score | Tandem Score | TDA q-value | Volume | Spectrum Matches |
| --- | --- | --- | --- | --- |
| 7.9083 | 130.6362 | 0.0000 | 2.0440e+08 | 9 |

**Adduct Signal Fractions**

- **Unmodified**: 2.0440e+08

### N(N)GTYDHDVYR Fuc 2 Hex 5 HexNAc 5

**Mass:**  3356.3106

(6.610e-07)

**Charge States:** 3

**Start Position**: 483

**End Position**: 493

| Profile Score | Tandem Score | TDA q-value | Volume | Spectrum Matches |
| --- | --- | --- | --- | --- |
| -19.1138 | 130.2570 | 0.0000 | 6.4210e+07 | 2 |

**Adduct Signal Fractions**

- **Unmodified**: 6.4210e+07

### SLVASSGTLEFNNESFNWAGVTHN(N)GTSSSC(C)R Hex 5 HexNAc 2

**Mass:**  4531.9313

(5.295e-06)

**Charge States:** 3, 4

**Start Position**: 110

**End Position**: 141

| Profile Score | Tandem Score | TDA q-value | Volume | Spectrum Matches |
| --- | --- | --- | --- | --- |
| -2.8688 | 130.1807 | 0.0000 | 1.6028e+08 | 3 |

**Adduct Signal Fractions**

- **Unmodified**: 1.6028e+08

### N(N)GTYDHDVYRDEALNNR Fuc 1 Hex 4 HexNAc 3

**Mass:**  3454.4216

(-2.333e-07)

**Charge States:** 3, 4

**Start Position**: 483

**End Position**: 500

| Profile Score | Tandem Score | TDA q-value | Volume | Spectrum Matches |
| --- | --- | --- | --- | --- |
| -18.9088 | 130.0632 | 0.0000 | 1.2360e+08 | 1 |

**Adduct Signal Fractions**

- **Unmodified**: 1.2360e+08

### GSN(N)SSFFSR Hex 6 HexNAc 4

**Mass:**  2772.0868

(4.052e-06)

**Charge States:** 3

**Start Position**: 142

**End Position**: 151

| Profile Score | Tandem Score | TDA q-value | Volume | Spectrum Matches |
| --- | --- | --- | --- | --- |
| 6.6283 | 129.5301 | 0.0455 | 7.3848e+07 | 2 |

**Adduct Signal Fractions**

- **Unmodified**: 7.3848e+07

### SLVASSGTLEFNNESFNWAGVTHN(N)GTSSSC(C)R Hex 8 HexNAc 2

**Mass:**  5018.0827

(3.207e-06)

**Charge States:** 3, 4

**Start Position**: 110

**End Position**: 141

| Profile Score | Tandem Score | TDA q-value | Volume | Spectrum Matches |
| --- | --- | --- | --- | --- |
| -18.4840 | 129.4140 | 0.0000 | 1.1340e+08 | 1 |

**Adduct Signal Fractions**

- **Unmodified**: 1.1340e+08

### NIPSRISIYWTIVKPGDILLIN(N)STGNLIAPR Hex 9 HexNAc 2

**Mass:**  5298.5895

(5.735e-07)

**Charge States:** 4, 5

**Start Position**: 225

**End Position**: 256

| Profile Score | Tandem Score | TDA q-value | Volume | Spectrum Matches |
| --- | --- | --- | --- | --- |
| -3.2218 | 128.7550 | 0.0000 | 4.1174e+07 | 5 |

**Adduct Signal Fractions**

- **Unmodified**: 4.1174e+07

### N(N)GTYDHDVYRDEALNNR Fuc 1 Hex 5 HexNAc 5

**Mass:**  4022.6296

(-7.936e-08)

**Charge States:** 3, 4

**Start Position**: 483

**End Position**: 500

| Profile Score | Tandem Score | TDA q-value | Volume | Spectrum Matches |
| --- | --- | --- | --- | --- |
| 14.9877 | 128.4289 | 0.0000 | 2.6374e+09 | 7 |

**Adduct Signal Fractions**

- **Unmodified**: 2.6374e+09

### SEC(C)ITPN(N)GSIPNDKPFQNVNR Hex 7 HexNAc 2

**Mass:**  3926.6687

(1.618e-06)

**Charge States:** 3, 4

**Start Position**: 279

**End Position**: 300

| Profile Score | Tandem Score | TDA q-value | Volume | Spectrum Matches |
| --- | --- | --- | --- | --- |
| 17.7475 | 127.9791 | 0.0000 | 1.9403e+09 | 4 |

**Adduct Signal Fractions**

- **Unmodified**: 1.9403e+09

### N(N)GTYDHDVYRDEALNNR Hex 5 HexNAc 3

**Mass:**  3470.4108

(-2.047e-07)

**Charge States:** 4

**Start Position**: 483

**End Position**: 500

| Profile Score | Tandem Score | TDA q-value | Volume | Spectrum Matches |
| --- | --- | --- | --- | --- |
| -19.2015 | 127.9202 | 0.0000 | 5.3593e+07 | 1 |

**Adduct Signal Fractions**

- **Unmodified**: 5.3593e+07

### IVKPGDILLIN(N)STGNLIAPR Hex 9 HexNAc 2

**Mass:**  3967.8879

(3.056e-06)

**Charge States:** 4

**Start Position**: 236

**End Position**: 256

| Profile Score | Tandem Score | TDA q-value | Volume | Spectrum Matches |
| --- | --- | --- | --- | --- |
| -18.7730 | 126.6935 | 0.0000 | 1.1698e+07 | 1 |

**Adduct Signal Fractions**

- **Unmodified**: 1.1698e+07

### SEC(C)ITPN(N)GSIPNDKPFQNVNR Hex 8 HexNAc 2

**Mass:**  4088.7218

(2.157e-06)

**Charge States:** 3, 4

**Start Position**: 279

**End Position**: 300

| Profile Score | Tandem Score | TDA q-value | Volume | Spectrum Matches |
| --- | --- | --- | --- | --- |
| 17.5908 | 126.5775 | 0.0000 | 8.7905e+08 | 3 |

**Adduct Signal Fractions**

- **Unmodified**: 8.7905e+08

### N(N)GTYDHDVYRDEALNNR Hex 5 HexNAc 4

**Mass:**  3673.4953

(3.063e-07)

**Charge States:** 3, 4

**Start Position**: 483

**End Position**: 500

| Profile Score | Tandem Score | TDA q-value | Volume | Spectrum Matches |
| --- | --- | --- | --- | --- |
| -4.3096 | 126.1120 | 0.0000 | 2.1893e+08 | 2 |

**Adduct Signal Fractions**

- **Unmodified**: 2.1893e+08

### SLVASSGTLEFNNESFNWAGVTHN(N)GTSSSC(C)R Hex 5 HexNAc 5

**Mass:**  5141.1574

(2.253e-06)

**Charge States:** 4

**Start Position**: 110

**End Position**: 141

| Profile Score | Tandem Score | TDA q-value | Volume | Spectrum Matches |
| --- | --- | --- | --- | --- |
| -4.4288 | 125.8667 | 0.0000 | 5.1946e+07 | 1 |

**Adduct Signal Fractions**

- **Unmodified**: 5.1946e+07

### RGSN(N)SSFFSR Fuc 3 Hex 4 HexNAc 5

**Mass:**  3245.3311

(2.221e-06)

**Charge States:** 3

**Start Position**: 141

**End Position**: 151

| Profile Score | Tandem Score | TDA q-value | Volume | Spectrum Matches |
| --- | --- | --- | --- | --- |
| -20.7547 | 125.6519 | 0.0000 | 3.2845e+06 | 1 |

**Adduct Signal Fractions**

- **Unmodified**: 3.2845e+06

### N(N)GTYDHDVYRDEALNNR Fuc 2 Hex 9 HexNAc 3

**Mass:**  4410.7092

(-8.031e-06)

**Charge States:** 3, 4

**Start Position**: 483

**End Position**: 500

| Profile Score | Tandem Score | TDA q-value | Volume | Spectrum Matches |
| --- | --- | --- | --- | --- |
| -4.1888 | 125.3483 | 0.0000 | 1.0432e+08 | 1 |

**Adduct Signal Fractions**

- **Unmodified**: 1.0432e+08

### N(N)GTYDHDVYRDEALNNR Fuc 1 Hex 6 HexNAc 5

**Mass:**  4184.6848

(7.546e-07)

**Charge States:** 4

**Start Position**: 483

**End Position**: 500

| Profile Score | Tandem Score | TDA q-value | Volume | Spectrum Matches |
| --- | --- | --- | --- | --- |
| 10.9881 | 125.3463 | 0.0000 | 5.5685e+08 | 3 |

**Adduct Signal Fractions**

- **Unmodified**: 5.5685e+08

### SEC(C)ITPN(N)GSIPNDKPFQNVNR Hex 9 HexNAc 2

**Mass:**  4250.7746

(1.587e-06)

**Charge States:** 3, 4

**Start Position**: 279

**End Position**: 300

| Profile Score | Tandem Score | TDA q-value | Volume | Spectrum Matches |
| --- | --- | --- | --- | --- |
| 17.0015 | 124.6955 | 0.0000 | 1.0006e+09 | 5 |

**Adduct Signal Fractions**

- **Unmodified**: 1.0006e+09

### SLVASSGTLEFNNESFNWAGVTHN(N)GTSSSC(C)R Hex 8 HexNAc 8

**Mass:**  6237.5216

(1.570e-04)

**Start Position**: 110

**End Position**: 141

| Profile Score | Tandem Score | TDA q-value | Volume | Spectrum Matches |
| --- | --- | --- | --- | --- |
| 0.0000 | 124.5923 | 0.0000 | 0.0000e+00 | 1 |

Adduct:

No Chromatogram Found

### N(N)GTYDHDVYRDEALN(D)NR Fuc 1 Hex 6 HexNAc 3

**Mass:**  3779.5153

(2.628e-04)

**Charge States:** 4

**Start Position**: 483

**End Position**: 500

| Profile Score | Tandem Score | TDA q-value | Volume | Spectrum Matches |
| --- | --- | --- | --- | --- |
| -6.0058 | 124.3364 | 0.0000 | 1.0579e+08 | 2 |

**Adduct Signal Fractions**

- **Unmodified**: 1.0579e+08

### N(N)GTYDHDVYRDEALN(D)NR Fuc 1 Hex 5 HexNAc 4

**Mass:**  3820.5400

(2.589e-04)

**Charge States:** 3, 4

**Start Position**: 483

**End Position**: 500

| Profile Score | Tandem Score | TDA q-value | Volume | Spectrum Matches |
| --- | --- | --- | --- | --- |
| 7.1967 | 124.1859 | 0.0000 | 1.4946e+09 | 9 |

**Adduct Signal Fractions**

- **Unmodified**: 1.4946e+09

### N(N)GTYDHDVYRDEALNNR Fuc 1 Hex 9 HexNAc 3

**Mass:**  4264.6520

(-7.547e-06)

**Charge States:** 3, 4

**Start Position**: 483

**End Position**: 500

| Profile Score | Tandem Score | TDA q-value | Volume | Spectrum Matches |
| --- | --- | --- | --- | --- |
| 11.9994 | 123.3330 | 0.0000 | 2.1595e+08 | 4 |

**Adduct Signal Fractions**

- **Unmodified**: 2.1595e+08

### SLVASSGTLEFNNESFNWAGVTHN(N)GTSSSC(C)R Hex 7 HexNAc 4

**Mass:**  5262.1843

(2.300e-06)

**Charge States:** 4

**Start Position**: 110

**End Position**: 141

| Profile Score | Tandem Score | TDA q-value | Volume | Spectrum Matches |
| --- | --- | --- | --- | --- |
| -4.8635 | 123.2062 | 0.0000 | 3.5748e+07 | 2 |

**Adduct Signal Fractions**

- **Unmodified**: 3.5748e+07

### N(N)GTYDHDVYRDEALNNR Fuc 3 Hex 6 HexNAc 5

**Mass:**  4476.8005

(8.580e-07)

**Charge States:** 4

**Start Position**: 483

**End Position**: 500

| Profile Score | Tandem Score | TDA q-value | Volume | Spectrum Matches |
| --- | --- | --- | --- | --- |
| -18.3475 | 121.9255 | 0.0000 | 9.7653e+07 | 1 |

**Adduct Signal Fractions**

- **Unmodified**: 9.7653e+07

### SLVASSGTLEFNNESFNWAGVTHN(N)GTSSSC(C)R Hex 6 HexNAc 4

**Mass:**  5100.1408

(4.196e-06)

**Charge States:** 4

**Start Position**: 110

**End Position**: 141

| Profile Score | Tandem Score | TDA q-value | Volume | Spectrum Matches |
| --- | --- | --- | --- | --- |
| -5.0291 | 121.4472 | 0.0000 | 2.7458e+07 | 1 |

**Adduct Signal Fractions**

- **Unmodified**: 2.7458e+07

### N(N)GTYDHDVYRDEALNNR Fuc 2 Hex 4 HexNAc 3

**Mass:**  3600.4777

(8.938e-07)

**Charge States:** 4

**Start Position**: 483

**End Position**: 500

| Profile Score | Tandem Score | TDA q-value | Volume | Spectrum Matches |
| --- | --- | --- | --- | --- |
| -2.9265 | 120.6436 | 0.0000 | 9.2910e+07 | 2 |

**Adduct Signal Fractions**

- **Unmodified**: 9.2910e+07

### N(N)GTYDHDVYR Fuc 2 Hex 8 HexNAc 3

**Mass:**  3436.2746

(-9.799e-06)

**Charge States:** 3

**Start Position**: 483

**End Position**: 493

| Profile Score | Tandem Score | TDA q-value | Volume | Spectrum Matches |
| --- | --- | --- | --- | --- |
| -16.8790 | 120.6279 | 0.0000 | 1.3900e+07 | 1 |

**Adduct Signal Fractions**

- **Unmodified**: 1.3900e+07

### SEC(C)ITPN(N)GSIPNDKPFQNVNR Fuc 2 Hex 3 HexNAc 4 Neu5Ac 1

**Mass:**  4267.8015

(-4.248e-06)

**Charge States:** 4

**Start Position**: 279

**End Position**: 300

| Profile Score | Tandem Score | TDA q-value | Volume | Spectrum Matches |
| --- | --- | --- | --- | --- |
| -18.6910 | 120.5018 | 0.0000 | 5.2242e+07 | 1 |

**Adduct Signal Fractions**

- **Unmodified**: 5.2242e+07

### N(N)GTYDHDVYRDEALNNR Fuc 2 Hex 5 HexNAc 3

**Mass:**  3762.5300

(6.902e-07)

**Charge States:** 4

**Start Position**: 483

**End Position**: 500

| Profile Score | Tandem Score | TDA q-value | Volume | Spectrum Matches |
| --- | --- | --- | --- | --- |
| -5.3834 | 119.8404 | 0.0000 | 7.4942e+07 | 1 |

**Adduct Signal Fractions**

- **Unmodified**: 7.4942e+07

### N(N)GTYDHDVYR Fuc 2 Hex 5 HexNAc 3

**Mass:**  2950.1497

(-8.101e-10)

**Charge States:** 3

**Start Position**: 483

**End Position**: 493

| Profile Score | Tandem Score | TDA q-value | Volume | Spectrum Matches |
| --- | --- | --- | --- | --- |
| -17.8135 | 118.8116 | 0.0000 | 8.8399e+06 | 1 |

**Adduct Signal Fractions**

- **Unmodified**: 8.8399e+06

### N(N)GTYDHDVYRDEALNNR Fuc 1 Hex 6 HexNAc 6

**Mass:**  4387.7634

(6.972e-07)

**Charge States:** 4

**Start Position**: 483

**End Position**: 500

| Profile Score | Tandem Score | TDA q-value | Volume | Spectrum Matches |
| --- | --- | --- | --- | --- |
| -3.4023 | 118.2348 | 0.0000 | 1.2251e+08 | 1 |

**Adduct Signal Fractions**

- **Unmodified**: 1.2251e+08

### SEC(C)ITPN(N)GSIPNDKPFQNVNR Hex 5 HexNAc 2

**Mass:**  3602.5658

(2.565e-06)

**Charge States:** 3, 4

**Start Position**: 279

**End Position**: 300

| Profile Score | Tandem Score | TDA q-value | Volume | Spectrum Matches |
| --- | --- | --- | --- | --- |
| -18.6800 | 118.0176 | 0.0000 | 4.5275e+07 | 1 |

**Adduct Signal Fractions**

- **Unmodified**: 4.5275e+07

### N(N)GTYDHDVYRDEALNNR Fuc 2 Hex 5 HexNAc 5

**Mass:**  4168.6892

(1.171e-06)

**Charge States:** 4

**Start Position**: 483

**End Position**: 500

| Profile Score | Tandem Score | TDA q-value | Volume | Spectrum Matches |
| --- | --- | --- | --- | --- |
| 11.3912 | 115.5813 | 0.0081 | 6.3992e+08 | 4 |

**Adduct Signal Fractions**

- **Unmodified**: 6.3992e+08

### YPALN(N)VTMPN(D)NEQFDK Hex 8 HexNAc 2

**Mass:**  3583.4544

(2.779e-04)

**Charge States:** 3

**Start Position**: 161

**End Position**: 177

| Profile Score | Tandem Score | TDA q-value | Volume | Spectrum Matches |
| --- | --- | --- | --- | --- |
| -5.9334 | 115.2300 | 0.0081 | 3.0732e+07 | 1 |

**Adduct Signal Fractions**

- **Unmodified**: 3.0732e+07

### SLVASSGTLEFNNESFNWAGVTHN(N)GTSSSC(C)R Fuc 1 Hex 7 HexNAc 3

**Mass:**  5205.1608

(1.894e-06)

**Charge States:** 4

**Start Position**: 110

**End Position**: 141

| Profile Score | Tandem Score | TDA q-value | Volume | Spectrum Matches |
| --- | --- | --- | --- | --- |
| -20.4380 | 114.7801 | 0.0081 | 8.9292e+06 | 1 |

**Adduct Signal Fractions**

- **Unmodified**: 8.9292e+06

### N(N)GTYDHDVYRDEALN(D)NR Fuc 2 Hex 5 HexNAc 4

**Mass:**  3966.5991

(2.506e-04)

**Charge States:** 3, 4

**Start Position**: 483

**End Position**: 500

| Profile Score | Tandem Score | TDA q-value | Volume | Spectrum Matches |
| --- | --- | --- | --- | --- |
| 3.9248 | 114.7043 | 0.0081 | 1.0916e+09 | 5 |

**Adduct Signal Fractions**

- **Unmodified**: 1.0916e+09

### N(N)GTYDHDVYR Fuc 1 Hex 9 HexNAc 3

**Mass:**  3452.2699

(-9.467e-06)

**Charge States:** 3

**Start Position**: 483

**End Position**: 493

| Profile Score | Tandem Score | TDA q-value | Volume | Spectrum Matches |
| --- | --- | --- | --- | --- |
| -19.8023 | 113.9081 | 0.0081 | 1.4867e+07 | 1 |

**Adduct Signal Fractions**

- **Unmodified**: 1.4867e+07

### N(N)GTYDHDVYRDEALNNR Fuc 2 Hex 6 HexNAc 5

**Mass:**  4330.7424

(8.107e-07)

**Charge States:** 4

**Start Position**: 483

**End Position**: 500

| Profile Score | Tandem Score | TDA q-value | Volume | Spectrum Matches |
| --- | --- | --- | --- | --- |
| -4.5148 | 113.0971 | 0.0081 | 1.5430e+08 | 1 |

**Adduct Signal Fractions**

- **Unmodified**: 1.5430e+08

### N(N)GTYDHDVYRDEALNNR Fuc 1 Hex 9 HexNAc 4

**Mass:**  4467.7311

(-6.526e-06)

**Charge States:** 4

**Start Position**: 483

**End Position**: 500

| Profile Score | Tandem Score | TDA q-value | Volume | Spectrum Matches |
| --- | --- | --- | --- | --- |
| 5.1468 | 111.9923 | 0.0081 | 5.8650e+07 | 1 |

**Adduct Signal Fractions**

- **Unmodified**: 5.8650e+07

### SLVASSGTLEFNNESFNWAGVTHN(N)GTSSSC(C)R Fuc 1 Hex 7 HexNAc 6 Neu5Ac 2

**Mass:**  6396.5485

(-4.882e-06)

**Charge States:** 4

**Start Position**: 110

**End Position**: 141

| Profile Score | Tandem Score | TDA q-value | Volume | Spectrum Matches |
| --- | --- | --- | --- | --- |
| -4.7268 | 110.8203 | 0.0081 | 5.2195e+07 | 2 |

**Adduct Signal Fractions**

- **Unmodified**: 5.2195e+07

### SLVASSGTLEFNNESFNWAGVTHN(N)GTSSSC(C)R Fuc 2 Hex 7 HexNAc 6 Neu5Ac 1

**Mass:**  6251.5231

(-3.029e-06)

**Charge States:** 4

**Start Position**: 110

**End Position**: 141

| Profile Score | Tandem Score | TDA q-value | Volume | Spectrum Matches |
| --- | --- | --- | --- | --- |
| -19.6660 | 109.4634 | 0.0081 | 1.3762e+07 | 1 |

**Adduct Signal Fractions**

- **Unmodified**: 1.3762e+07

### N(N)GTYDHDVYRDEALN(D)NR Hex 6 HexNAc 3

**Mass:**  3633.4582

(2.736e-04)

**Charge States:** 4

**Start Position**: 483

**End Position**: 500

| Profile Score | Tandem Score | TDA q-value | Volume | Spectrum Matches |
| --- | --- | --- | --- | --- |
| -5.8625 | 107.9879 | 0.0081 | 6.5434e+07 | 1 |

**Adduct Signal Fractions**

- **Unmodified**: 6.5434e+07

### RGSN(N)SSFFSR Fuc 1 Hex 4 HexNAc 3

**Mass:**  2548.0397

(3.888e-04)

**Start Position**: 141

**End Position**: 151

| Profile Score | Tandem Score | TDA q-value | Volume | Spectrum Matches |
| --- | --- | --- | --- | --- |
| 0.0000 | 107.8376 | 0.0081 | 0.0000e+00 | 1 |

Adduct:

No Chromatogram Found

### N(N)GTYDHDVYR Fuc 1 Hex 4 HexNAc 3

**Mass:**  2643.0553

(3.847e-04)

**Start Position**: 483

**End Position**: 493

| Profile Score | Tandem Score | TDA q-value | Volume | Spectrum Matches |
| --- | --- | --- | --- | --- |
| 0.0000 | 106.0125 | 0.0081 | 0.0000e+00 | 1 |

Adduct:

No Chromatogram Found

### N(N)GTYDHDVYRDEALNNRFQIK Fuc 1 Hex 5 HexNAc 4

**Mass:**  4335.8682

(3.077e-06)

**Charge States:** 5, 6

**Start Position**: 483

**End Position**: 504

| Profile Score | Tandem Score | TDA q-value | Volume | Spectrum Matches |
| --- | --- | --- | --- | --- |
| -17.6101 | 105.4205 | 0.0081 | 2.4904e+07 | 1 |

**Adduct Signal Fractions**

- **Unmodified**: 2.4904e+07

### SLVASSGTLEFNNESFNWAGVTHN(N)GTSSSC(C)R Hex 5 HexNAc 4

**Mass:**  4938.0881

(4.370e-06)

**Charge States:** 4

**Start Position**: 110

**End Position**: 141

| Profile Score | Tandem Score | TDA q-value | Volume | Spectrum Matches |
| --- | --- | --- | --- | --- |
| -5.4116 | 105.3223 | 0.0081 | 4.6459e+07 | 2 |

**Adduct Signal Fractions**

- **Unmodified**: 4.6459e+07

### N(N)GTYDHDVYRDEALN(D)NR Fuc 1 Hex 5 HexNAc 5

**Mass:**  4023.6227

(2.473e-04)

**Charge States:** 3, 4

**Start Position**: 483

**End Position**: 500

| Profile Score | Tandem Score | TDA q-value | Volume | Spectrum Matches |
| --- | --- | --- | --- | --- |
| 8.6047 | 105.2978 | 0.0081 | 3.5550e+08 | 1 |

**Adduct Signal Fractions**

- **Unmodified**: 3.5550e+08

### N(N)GTYDHDVYRDEALNNR Fuc 2 Hex 8 HexNAc 3

**Mass:**  4248.6511

(-8.231e-06)

**Charge States:** 3, 4

**Start Position**: 483

**End Position**: 500

| Profile Score | Tandem Score | TDA q-value | Volume | Spectrum Matches |
| --- | --- | --- | --- | --- |
| -3.8219 | 104.7122 | 0.0081 | 2.2344e+08 | 1 |

**Adduct Signal Fractions**

- **Unmodified**: 2.2344e+08

### N(N)GTYDHDVYR Hex 5 HexNAc 4

**Mass:**  2861.1121

(1.838e-06)

**Charge States:** 3

**Start Position**: 483

**End Position**: 493

| Profile Score | Tandem Score | TDA q-value | Volume | Spectrum Matches |
| --- | --- | --- | --- | --- |
| -20.3196 | 104.2262 | 0.0116 | 1.6355e+07 | 1 |

**Adduct Signal Fractions**

- **Unmodified**: 1.6355e+07

### N(N)GTYDHDVYR Fuc 2 Hex 6 HexNAc 5

**Mass:**  3518.3589

(-6.462e-07)

**Charge States:** 3, 4

**Start Position**: 483

**End Position**: 493

| Profile Score | Tandem Score | TDA q-value | Volume | Spectrum Matches |
| --- | --- | --- | --- | --- |
| -20.1040 | 102.5243 | 0.0116 | 2.2964e+07 | 2 |

**Adduct Signal Fractions**

- **Unmodified**: 2.2964e+07

### HAVPN(N)GTIVK Hex 8 HexNAc 3 Neu5Ac 1

**Mass:**  3233.3290

(6.145e-04)

**Start Position**: 18

**End Position**: 28

| Profile Score | Tandem Score | TDA q-value | Volume | Spectrum Matches |
| --- | --- | --- | --- | --- |
| 0.0000 | 101.6817 | 0.0116 | 0.0000e+00 | 1 |

Adduct:

No Chromatogram Found

### SLVASSGTLEFNNESFNWAGVTHN(N)GTSSSC(C)RR Hex 7 HexNAc 2

**Mass:**  5012.1320

(3.459e-06)

**Charge States:** 4, 5

**Start Position**: 110

**End Position**: 142

| Profile Score | Tandem Score | TDA q-value | Volume | Spectrum Matches |
| --- | --- | --- | --- | --- |
| -18.3933 | 98.8796 | 0.0116 | 1.6864e+07 | 1 |

**Adduct Signal Fractions**

- **Unmodified**: 1.6864e+07

### N(N)GTYDHDVYR Hex 5 HexNAc 2

**Mass:**  2454.9570

(1.020e-06)

**Start Position**: 483

**End Position**: 493

| Profile Score | Tandem Score | TDA q-value | Volume | Spectrum Matches |
| --- | --- | --- | --- | --- |
| 0.0000 | 98.1618 | 0.0116 | 0.0000e+00 | 1 |

Adduct:

No Chromatogram Found

### RGSN(N)SSFFSR Fuc 1 Hex 6 HexNAc 6

**Mass:**  3480.3967

(1.729e-06)

**Charge States:** 3, 4

**Start Position**: 141

**End Position**: 151

| Profile Score | Tandem Score | TDA q-value | Volume | Spectrum Matches |
| --- | --- | --- | --- | --- |
| -5.1244 | 98.0365 | 0.0149 | 8.0469e+07 | 2 |

**Adduct Signal Fractions**

- **Unmodified**: 8.0469e+07

### RGSN(N)SSFFSR Fuc 1 Hex 5 HexNAc 7 Neu5Ac 3

**Mass:**  4394.7121

(1.423e-06)

**Charge States:** 3, 4

**Start Position**: 141

**End Position**: 151

| Profile Score | Tandem Score | TDA q-value | Volume | Spectrum Matches |
| --- | --- | --- | --- | --- |
| -17.5913 | 96.3782 | 0.0149 | 3.1895e+07 | 1 |

**Adduct Signal Fractions**

- **Unmodified**: 3.1895e+07

### ISIYWTIVKPGDILLIN(N)STGNLIAPR Fuc 2 Hex 4 HexNAc 4 Neu5Ac 1

**Mass:**  4910.3581

(-2.223e-06)

**Charge States:** 4

**Start Position**: 230

**End Position**: 256

| Profile Score | Tandem Score | TDA q-value | Volume | Spectrum Matches |
| --- | --- | --- | --- | --- |
| -4.6759 | 95.4413 | 0.0185 | 1.0895e+07 | 2 |

**Adduct Signal Fractions**

- **Unmodified**: 1.0895e+07

### N(N)GTYDHDVYRDEALN(D)NR Fuc 3 Hex 5 HexNAc 4

**Mass:**  4112.6520

(2.398e-04)

**Charge States:** 3, 4

**Start Position**: 483

**End Position**: 500

| Profile Score | Tandem Score | TDA q-value | Volume | Spectrum Matches |
| --- | --- | --- | --- | --- |
| 10.4625 | 94.8792 | 0.0185 | 3.7326e+08 | 3 |

**Adduct Signal Fractions**

- **Unmodified**: 3.7326e+08

### LGHHAVPN(N)GTIVK Fuc 1 Hex 5 HexNAc 5

**Mass:**  3314.4785

(3.042e-04)

**Start Position**: 15

**End Position**: 28

| Profile Score | Tandem Score | TDA q-value | Volume | Spectrum Matches |
| --- | --- | --- | --- | --- |
| 0.0000 | 94.0643 | 0.0295 | 0.0000e+00 | 1 |

Adduct:

No Chromatogram Found

### N(N)GTYDHDVYR Fuc 1 Hex 9 HexNAc 2

**Mass:**  3251.2210

(6.147e-04)

**Start Position**: 483

**End Position**: 493

| Profile Score | Tandem Score | TDA q-value | Volume | Spectrum Matches |
| --- | --- | --- | --- | --- |
| 0.0000 | 93.4596 | 0.0331 | 0.0000e+00 | 1 |

Adduct:

No Chromatogram Found

### SEC(C)ITPN(N)GSIPN(D)DKPFQNVNR Hex 9 HexNAc 2

**Mass:**  4251.7634

(2.342e-04)

**Charge States:** 3, 4

**Start Position**: 279

**End Position**: 300

| Profile Score | Tandem Score | TDA q-value | Volume | Spectrum Matches |
| --- | --- | --- | --- | --- |
| 6.8494 | 92.1967 | 0.0361 | 1.8534e+08 | 5 |

**Adduct Signal Fractions**

- **Unmodified**: 1.8534e+08

### SLVASSGTLEFN(D)NESFNWAGVTHN(N)GTSSSC(C)R Hex 7 HexNAc 2

**Mass:**  4857.0178

(2.070e-04)

**Charge States:** 3, 4

**Start Position**: 110

**End Position**: 141

| Profile Score | Tandem Score | TDA q-value | Volume | Spectrum Matches |
| --- | --- | --- | --- | --- |
| 9.4263 | 92.0494 | 0.0361 | 4.8714e+07 | 4 |

**Adduct Signal Fractions**

- **Unmodified**: 4.8714e+07

### SEC(C)ITPN(N)GSIPN(D)DKPFQNVNR Hex 7 HexNAc 2

**Mass:**  3927.6586

(2.561e-04)

**Charge States:** 3, 4

**Start Position**: 279

**End Position**: 300

| Profile Score | Tandem Score | TDA q-value | Volume | Spectrum Matches |
| --- | --- | --- | --- | --- |
| 11.0023 | 91.6018 | 0.0361 | 2.5987e+08 | 5 |

**Adduct Signal Fractions**

- **Unmodified**: 2.5987e+08

### N(N)GTYDHDVYR Hex 5 HexNAc 6

**Mass:**  3267.2752

(9.439e-07)

**Charge States:** 3

**Start Position**: 483

**End Position**: 493

| Profile Score | Tandem Score | TDA q-value | Volume | Spectrum Matches |
| --- | --- | --- | --- | --- |
| -18.8076 | 89.8644 | 0.0432 | 3.7165e+07 | 2 |

**Adduct Signal Fractions**

- **Unmodified**: 3.7165e+07

### SEC(C)ITPN(N)GSIPN(D)DKPFQNVNR Hex 8 HexNAc 2

**Mass:**  4089.7209

(2.452e-04)

**Charge States:** 3, 4

**Start Position**: 279

**End Position**: 300

| Profile Score | Tandem Score | TDA q-value | Volume | Spectrum Matches |
| --- | --- | --- | --- | --- |
| 10.8082 | 89.7396 | 0.0463 | 1.4732e+08 | 4 |

**Adduct Signal Fractions**

- **Unmodified**: 1.4732e+08

### cus|SWZHA|A/Switzerland/9715293/2013

**>cus|SWZHA|A/Switzerland/9715293/2013**  
 `QKLPGNDNSTATLCLGHHAVPNGTIVKTITNDRIEVTNATELVQNSSIGEICDSPHQILDGENCTLIDALLGDPQCDGFQ  
NKKWDLFVERSKAYSNCYPYDVPDYASLRSLVASSGTLEFNNESFNWAGVTQNGTSSSCRRGSNSSFFSRLNWLTHLNSK  
YPALNVTMPNNEQFDKLYIWGVHHPVTDKDQIFLYAQSSGRITVSTKRSQQAVIPNIGYRPRIRDIPSRISIYWTIVKPG  
DILLINSTGNLIAPRGYFKIRSGKSSIMRSDAPIGKCKSECITPNGSIPNDKPFQNVNRITYGACPRYVKQSTLKLATGM  
RNVPERQTRGIFGAIAGFIENGWEGMVDGWYGFRHQNSEGRGQAADLKSTQAAIDQINGKLNRLIGKTNEKFHQIEKEFS  
EVEGRIQDLEKYVEDTKIDLWSYNAELLVALENQHTIDLTDSEMNKLFEKTKKQLRENAEDMGNGCFKIYHKCDNACIGS  
IRNGTYDHDVYRDEALNNRFQIKGVELKSGYKDWILWISFAISCFLLCVALLGFIMWACQKGNIRCNICI`

| Site | Type | Count |
| --- | --- | --- |
| 483 | N-linked | 48 |
| 165 | N-linked | 12 |
| 144 | N-linked | 44 |
| 22 | N-linked | 3 |
| 285 | N-linked | 13 |
| 246 | N-linked | 9 |

| Mass | Sequence | MS_2_ Score | `q`-value | MS_1_ Score | Signal | Protein Coordinates | Tandem Scans | Start Time | Apex Time | End Time |
| --- | --- | --- | --- | --- | --- | --- | --- | --- | --- | --- |
| 2522.0187 | RGSN(N)SSFFSR Hex 6 HexNAc 2 | 193.768 | 0.0 | -3.260 | 5.115e+08 | 140 - 150 | 4 | 19.22 | 19.86 | 20.01 |
| 2359.9656 | RGSN(N)SSFFSR Hex 5 HexNAc 2 | 193.228 | 0.0 | 11.947 | 4.709e+08 | 140 - 150 | 3 | 19.38 | 20.16 | 20.31 |
| 2766.1238 | RGSN(N)SSFFSR Hex 5 HexNAc 4 | 189.579 | 0.0 | -3.834 | 1.114e+08 | 140 - 150 | 1 | 20.01 | 20.01 | 20.31 |
| 3172.2842 | RGSN(N)SSFFSR Hex 5 HexNAc 6 | 188.247 | 0.0 | -7.298 | 2.262e+08 | 140 - 150 | 2 | 19.54 | 20.16 | 20.31 |
| 2846.1184 | RGSN(N)SSFFSR Hex 8 HexNAc 2 | 188.138 | 0.0 | -17.985 | 1.005e+08 | 140 - 150 | 2 | 19.54 | 19.71 | 22.37 |
| 2725.0985 | RGSN(N)SSFFSR Hex 6 HexNAc 3 | 183.251 | 0.0 | -3.713 | 6.190e+08 | 140 - 150 | 4 | 19.22 | 20.01 | 20.01 |
| 3261.3203 | RGSN(N)SSFFSR Fuc 2 Hex 5 HexNAc 5 | 182.202 | 0.0 | 14.008 | 4.348e+08 | 140 - 150 | 4 | 19.05 | 19.71 | 20.01 |
| 3582.4698 | YPALN(N)VTMPNNEQFDK Hex 8 HexNAc 2 | 178.356 | 0.0 | 18.224 | 3.368e+08 | 160 - 176 | 5 | 42.21 | 42.72 | 42.89 |
| 2684.0718 | RGSN(N)SSFFSR Hex 7 HexNAc 2 | 178.260 | 0.0 | 12.046 | 2.924e+08 | 140 - 150 | 2 | 19.05 | 19.71 | 20.01 |
| 3115.2626 | RGSN(N)SSFFSR Fuc 1 Hex 5 HexNAc 5 | 174.660 | 0.0 | 13.157 | 1.885e+09 | 140 - 150 | 6 | 19.22 | 20.01 | 20.31 |
| 3407.3814 | RGSN(N)SSFFSR Fuc 3 Hex 5 HexNAc 5 | 173.807 | 0.0 | -17.593 | 4.596e+07 | 140 - 150 | 1 | 19.38 | 19.38 | 19.54 |
| 3058.2417 | RGSN(N)SSFFSR Fuc 2 Hex 5 HexNAc 4 | 173.104 | 0.0 | -17.706 | 2.857e+08 | 140 - 150 | 3 | 19.54 | 19.71 | 19.71 |
| 3277.3168 | RGSN(N)SSFFSR Fuc 1 Hex 6 HexNAc 5 | 173.075 | 0.0 | -3.744 | 1.040e+08 | 140 - 150 | 2 | 19.38 | 19.54 | 19.71 |
| 3598.4732 | YPALN(N)VTM(O)PNNEQFDK Hex 8 HexNAc 2 | 171.687 | 0.0 | 16.803 | 1.825e+09 | 160 - 176 | 22 | 34.44 | 34.95 | 113.40 |
| 3760.5151 | YPALN(N)VTM(O)PNNEQFDK Hex 9 HexNAc 2 | 171.319 | 0.0 | 15.489 | 1.576e+08 | 160 - 176 | 5 | 34.61 | 34.95 | 35.28 |
| 3436.4156 | YPALN(N)VTM(O)PNNEQFDK Hex 7 HexNAc 2 | 171.200 | 0.0 | 15.915 | 3.479e+08 | 160 - 176 | 4 | 34.78 | 35.11 | 35.45 |
| 3204.3012 | RGSN(N)SSFFSR Fuc 3 Hex 5 HexNAc 4 | 169.938 | 0.0 | -4.286 | 1.638e+08 | 140 - 150 | 2 | 18.72 | 19.38 | 19.54 |
| 2969.2055 | RGSN(N)SSFFSR Hex 5 HexNAc 5 | 167.777 | 0.0 | 12.042 | 3.110e+08 | 140 - 150 | 2 | 19.38 | 20.01 | 20.31 |
| 4068.9355 | TIVKPGDILLIN(N)STGNLIAPR Hex 9 HexNAc 2 | 167.512 | 0.0 | 19.243 | 6.085e+08 | 234 - 255 | 5 | 52.81 | 53.49 | 54.18 |
| 2855.1612 | RGSN(N)SSFFSR Fuc 2 Hex 5 HexNAc 3 | 165.538 | 0.0 | -18.653 | 2.692e+07 | 140 - 150 | 1 | 19.86 | 19.86 | 19.86 |
| 2709.1046 | RGSN(N)SSFFSR Fuc 1 Hex 5 HexNAc 3 | 164.072 | 0.0 | -4.237 | 8.869e+07 | 140 - 150 | 1 | 19.86 | 20.01 | 20.16 |
| 2871.1562 | RGSN(N)SSFFSR Fuc 1 Hex 6 HexNAc 3 | 163.919 | 0.0 | 10.515 | 3.969e+08 | 140 - 150 | 2 | 19.05 | 19.71 | 19.86 |
| 2756.0830 | GSN(N)SSFFSR Fuc 1 Hex 5 HexNAc 4 | 163.050 | 0.0 | 12.047 | 3.908e+08 | 141 - 150 | 3 | 23.04 | 23.19 | 24.10 |
| 2912.1825 | RGSN(N)SSFFSR Fuc 1 Hex 5 HexNAc 4 | 162.197 | 0.0 | 13.977 | 9.188e+08 | 140 - 150 | 4 | 19.22 | 19.86 | 20.16 |
| 2547.0560 | RGSN(N)SSFFSR Fuc 1 Hex 4 HexNAc 3 | 161.266 | 0.0 | -18.934 | 2.375e+07 | 140 - 150 | 1 | 20.46 | 20.46 | 20.46 |
| 3420.4165 | YPALN(N)VTMPNNEQFDK Hex 7 HexNAc 2 | 159.418 | 0.0 | -3.277 | 5.481e+07 | 160 - 176 | 2 | 42.38 | 42.72 | 42.89 |
| 3560.3593 | RGSN(N)SSFFSR Fuc 1 Hex 9 HexNAc 4 | 158.605 | 0.0 | 10.829 | 8.519e+07 | 140 - 150 | 1 | 21.38 | 21.54 | 22.37 |
| 4085.9617 | TIVKPGDILLIN(N)STGNLIAPR Fuc 2 Hex 3 HexNAc 4 Neu5Ac 1 | 157.203 | 0.0 | -3.075 | 7.946e+07 | 234 - 255 | 2 | 53.32 | 53.49 | 53.66 |
| 3744.5213 | YPALN(N)VTMPNNEQFDK Hex 9 HexNAc 2 | 155.413 | 0.0 | -4.073 | 3.045e+07 | 160 - 176 | 2 | 42.38 | 42.55 | 42.72 |
| 4376.8407 | C(C)KSEC(C)ITPN(N)GSIPNDKPFQNVNR Hex 8 HexNAc 2 | 155.304 | 0.0 | -17.162 | 1.511e+08 | 276 - 299 | 1 | 29.47 | 29.63 | 29.63 |
| 3503.3339 | RGSN(N)SSFFSR Fuc 2 Hex 9 HexNAc 3 | 155.200 | 0.0 | -3.892 | 6.640e+07 | 140 - 150 | 2 | 21.22 | 21.38 | 21.54 |
| 3007.1758 | N(N)GTYDHDVYR Fuc 1 Hex 5 HexNAc 4 | 154.693 | 0.0 | 14.788 | 4.754e+08 | 482 - 492 | 4 | 15.72 | 15.72 | 16.36 |
| 3274.3635 | YPALN(N)VTM(O)PNNEQFDK Hex 6 HexNAc 2 | 154.094 | 0.0 | 11.964 | 2.388e+07 | 160 - 176 | 2 | 34.95 | 35.28 | 35.62 |
| 2203.8642 | GSN(N)SSFFSR Hex 5 HexNAc 2 | 153.887 | 0.0 | 13.079 | 3.438e+08 | 141 - 150 | 3 | 23.64 | 23.79 | 24.41 |
| 3008.1553 | RGSN(N)SSFFSR Hex 9 HexNAc 2 | 153.138 | 0.0 | -20.654 | 7.745e+06 | 140 - 150 | 1 | 21.86 | 21.86 | 21.86 |
| 3006.3096 | HAVPN(N)GTIVK Fuc 1 Hex 5 HexNAc 5 | 152.094 | 0.0 | -20.144 | 9.221e+06 | 17 - 27 | 1 | 16.70 | 16.86 | 16.86 |
| 3372.7047 | N(N)GTYDHDVYR Fuc 1 Hex 6 HexNAc 5 | 152.044 | 0.0 | -21.786 | 7.488e+07 | 482 - 492 | 4 | 15.58 | 15.72 | 21.70 |
| 2966.1438 | N(N)GTYDHDVYR Fuc 1 Hex 6 HexNAc 3 | 152.015 | 0.0 | -20.136 | 1.735e+07 | 482 - 492 | 1 | 15.58 | 15.58 | 15.58 |
| 4230.9889 | TIVKPGDILLIN(N)STGNLIAPR Hex 10 HexNAc 2 | 150.699 | 0.0 | -19.398 | 2.129e+07 | 234 - 255 | 2 | 53.32 | 53.32 | 53.49 |
| 3334.3350 | RGSN(N)SSFFSR Hex 6 HexNAc 6 | 150.507 | 0.0 | -19.465 | 1.801e+07 | 140 - 150 | 1 | 19.54 | 19.54 | 19.54 |
| 2715.0561 | GSN(N)SSFFSR Fuc 1 Hex 6 HexNAc 3 | 149.917 | 0.0 | -3.812 | 1.567e+08 | 141 - 150 | 2 | 23.04 | 23.04 | 23.33 |
| 3210.2552 | N(N)GTYDHDVYR Fuc 1 Hex 5 HexNAc 5 | 149.868 | 0.0 | -2.265 | 2.449e+08 | 482 - 492 | 3 | 15.87 | 15.87 | 16.36 |
| 4052.7348 | C(C)KSEC(C)ITPN(N)GSIPNDKPFQNVNR Hex 6 HexNAc 2 | 149.846 | 0.0 | -19.053 | 3.089e+07 | 276 - 299 | 1 | 29.63 | 29.63 | 29.80 |
| 3632.4667 | N(N)GTYDHDVYRDEALNNR Hex 6 HexNAc 3 | 149.660 | 0.0 | -3.443 | 3.288e+08 | 482 - 499 | 3 | 20.61 | 20.76 | 21.06 |
| 4214.7889 | C(C)KSEC(C)ITPN(N)GSIPNDKPFQNVNR Hex 7 HexNAc 2 | 149.629 | 0.0 | -2.923 | 3.049e+08 | 276 - 299 | 3 | 29.47 | 29.63 | 29.80 |
| 3906.8814 | TIVKPGDILLIN(N)STGNLIAPR Hex 8 HexNAc 2 | 149.127 | 0.0 | -3.606 | 6.072e+07 | 234 - 255 | 3 | 53.32 | 53.49 | 53.66 |
| 2365.9189 | GSN(N)SSFFSR Hex 6 HexNAc 2 | 148.899 | 0.0 | -3.275 | 2.380e+08 | 141 - 150 | 2 | 23.33 | 23.48 | 23.79 |
| 2813.1041 | GSN(N)SSFFSR Hex 5 HexNAc 5 | 148.700 | 0.0 | 11.787 | 2.406e+08 | 141 - 150 | 2 | 23.04 | 23.64 | 24.10 |
| 3761.5519 | YPALN(N)VTMPNNEQFDK Fuc 2 Hex 3 HexNAc 4 Neu5Ac 1 | 148.563 | 0.0 | -18.750 | 7.459e+06 | 160 - 176 | 1 | 42.55 | 42.55 | 42.72 |
| 4185.6745 | RGSN(N)SSFFSR Fuc 1 Hex 3 HexNAc 9 Neu5Ac 2 | 148.355 | 0.0 | -4.088 | 1.823e+08 | 140 - 150 | 3 | 21.06 | 21.06 | 21.54 |
| 2568.9979 | GSN(N)SSFFSR Hex 6 HexNAc 3 | 147.908 | 0.0 | 12.673 | 3.330e+08 | 141 - 150 | 3 | 23.33 | 23.48 | 23.95 |
| 4731.2829 | ISIYWTIVKPGDILLIN(N)STGNLIAPR Hex 9 HexNAc 2 | 147.546 | 0.0 | 18.663 | 8.639e+08 | 229 - 255 | 9 | 68.48 | 69.17 | 69.68 |
| 3423.3739 | RGSN(N)SSFFSR Fuc 2 Hex 6 HexNAc 5 | 147.496 | 0.0 | -5.134 | 2.611e+07 | 140 - 150 | 1 | 19.22 | 19.38 | 19.54 |
| 4538.8925 | C(C)KSEC(C)ITPN(N)GSIPNDKPFQNVNR Hex 9 HexNAc 2 | 146.948 | 0.0 | -18.274 | 2.061e+08 | 276 - 299 | 1 | 29.47 | 29.63 | 29.63 |
| 3017.2132 | RGSN(N)SSFFSR Fuc 2 Hex 6 HexNAc 3 | 145.214 | 0.0 | -19.268 | 2.854e+07 | 140 - 150 | 1 | 19.54 | 19.71 | 19.71 |
| 3105.2201 | GSN(N)SSFFSR Fuc 2 Hex 5 HexNAc 5 | 145.207 | 0.0 | -3.417 | 1.250e+08 | 141 - 150 | 1 | 22.87 | 23.04 | 23.19 |
| 2928.1781 | RGSN(N)SSFFSR Hex 6 HexNAc 4 | 144.715 | 0.0 | 12.814 | 1.306e+08 | 140 - 150 | 2 | 19.38 | 19.86 | 20.16 |
| 4748.3020 | ISIYWTIVKPGDILLIN(N)STGNLIAPR Fuc 2 Hex 3 HexNAc 4 Neu5Ac 1 | 143.893 | 0.0 | 15.036 | 2.274e+08 | 229 - 255 | 8 | 68.48 | 69.17 | 69.51 |
| 4111.6680 | N(N)GTYDHDVYRDEALNNR Fuc 3 Hex 5 HexNAc 4 | 143.236 | 0.0 | 14.911 | 6.881e+08 | 482 - 499 | 3 | 20.01 | 20.31 | 20.61 |
| 2872.1308 | RGSN(N)SSFF Fuc 1 Hex 5 HexNAc 5 | 142.825 | 0.0 | -18.570 | 1.513e+07 | 140 - 148 | 1 | 26.52 | 26.52 | 26.68 |
| 3267.3338 | N(N)GTYDHDVYRDEALNNR Hex 5 HexNAc 2 | 141.073 | 0.0 | -20.055 | 1.104e+08 | 482 - 499 | 1 | 20.91 | 20.91 | 21.06 |
| 4569.2241 | ISIYWTIVKPGDILLIN(N)STGNLIAPR Hex 8 HexNAc 2 | 140.047 | 0.0 | -2.569 | 9.463e+07 | 229 - 255 | 5 | 68.99 | 69.17 | 69.51 |
| 3112.3180 | YPALN(N)VTM(O)PNNEQFDK Hex 5 HexNAc 2 | 139.855 | 0.0 | -4.672 | 1.313e+07 | 160 - 176 | 2 | 35.28 | 35.62 | 35.79 |
| 3778.5268 | N(N)GTYDHDVYRDEALNNR Fuc 1 Hex 6 HexNAc 3 | 139.248 | 0.0 | 13.153 | 3.214e+08 | 482 - 499 | 2 | 20.31 | 20.46 | 20.91 |
| 3935.5993 | RGSN(N)SSFFSR Fuc 3 Hex 7 HexNAc 6 | 139.021 | 0.0 | 0.000 | 0.000e+00 | 140 - 150 | 1 | - | - | - |
| 2610.0249 | GSN(N)SSFFSR Hex 5 HexNAc 4 | 137.950 | 0.045 | -19.555 | 4.296e+07 | 141 - 150 | 2 | 23.33 | 23.48 | 23.48 |
| 3153.2316 | N(N)GTYDHDVYR Fuc 2 Hex 5 HexNAc 4 | 136.976 | 0.0 | -3.760 | 3.200e+08 | 482 - 492 | 3 | 15.58 | 15.72 | 16.04 |
| 3899.5158 | N(N)GTYDHDVYRDEALNNR Fuc 1 Hex 8 HexNAc 2 | 136.679 | 0.0 | 13.604 | 7.330e+08 | 482 - 499 | 2 | 22.03 | 22.37 | 22.70 |
| 2959.1627 | GSN(N)SSFFSR Fuc 1 Hex 5 HexNAc 5 | 136.337 | 0.045 | 13.202 | 9.913e+08 | 141 - 150 | 3 | 22.70 | 23.48 | 23.79 |
| 3300.2893 | N(N)GTYDHDVYR Fuc 3 Hex 5 HexNAc 4 | 135.437 | 0.0 | 0.000 | 0.000e+00 | 482 - 492 | 2 | - | - | - |
| 3437.4127 | YPALN(N)VTM(O)PN(D)NEQFDK Hex 7 HexNAc 2 | 134.846 | 0.0 | 6.457 | 2.512e+07 | 160 - 176 | 3 | 35.62 | 35.79 | 36.64 |
| 3965.6102 | N(N)GTYDHDVYRDEALNNR Fuc 2 Hex 5 HexNAc 4 | 133.596 | 0.0 | 15.283 | 2.157e+09 | 482 - 499 | 4 | 20.16 | 20.46 | 20.76 |
| 2406.9435 | GSN(N)SSFFSR Hex 5 HexNAc 3 | 131.858 | 0.045 | -20.051 | 3.837e+07 | 141 - 150 | 2 | 23.79 | 23.79 | 23.95 |
| 2820.0913 | N(N)GTYDHDVYR Hex 6 HexNAc 3 | 131.808 | 0.0 | -18.067 | 3.580e+07 | 482 - 492 | 2 | 15.72 | 15.72 | 15.87 |
| 4102.5961 | N(N)GTYDHDVYRDEALNNR Fuc 1 Hex 8 HexNAc 3 | 131.777 | 0.0 | 11.260 | 3.462e+08 | 482 - 499 | 4 | 21.54 | 22.37 | 22.70 |
| 3819.5506 | N(N)GTYDHDVYRDEALNNR Fuc 1 Hex 5 HexNAc 4 | 131.428 | 0.0 | 16.671 | 4.737e+09 | 482 - 499 | 7 | 20.16 | 20.61 | 21.06 |
| 3616.4712 | N(N)GTYDHDVYRDEALNNR Fuc 1 Hex 5 HexNAc 3 | 131.320 | 0.0 | -3.329 | 1.475e+08 | 482 - 499 | 2 | 20.61 | 20.76 | 21.06 |
| 3764.6165 | SEC(C)ITPN(N)GSIPNDKPFQNVNR Hex 6 HexNAc 2 | 130.789 | 0.0 | -3.168 | 2.504e+08 | 278 - 299 | 2 | 32.41 | 32.58 | 32.74 |
| 3599.4670 | YPALN(N)VTM(O)PN(D)NEQFDK Hex 8 HexNAc 2 | 130.636 | 0.0 | 7.908 | 2.044e+08 | 160 - 176 | 9 | 35.45 | 35.79 | 37.15 |
| 3356.3108 | N(N)GTYDHDVYR Fuc 2 Hex 5 HexNAc 5 | 130.257 | 0.0 | -19.114 | 6.421e+07 | 482 - 492 | 2 | 15.72 | 15.72 | 15.87 |
| 3454.4198 | N(N)GTYDHDVYRDEALNNR Fuc 1 Hex 4 HexNAc 3 | 130.063 | 0.0 | -18.909 | 1.236e+08 | 482 - 499 | 1 | 20.91 | 21.06 | 21.06 |
| 2772.0812 | GSN(N)SSFFSR Hex 6 HexNAc 4 | 129.530 | 0.045 | 6.628 | 7.385e+07 | 141 - 150 | 2 | 23.04 | 23.33 | 23.79 |
| 4022.6296 | N(N)GTYDHDVYRDEALNNR Fuc 1 Hex 5 HexNAc 5 | 128.429 | 0.0 | 14.988 | 2.637e+09 | 482 - 499 | 7 | 19.86 | 20.76 | 21.06 |
| 3926.6691 | SEC(C)ITPN(N)GSIPNDKPFQNVNR Hex 7 HexNAc 2 | 127.979 | 0.0 | 17.748 | 1.940e+09 | 278 - 299 | 4 | 32.25 | 32.41 | 32.91 |
| 3470.4133 | N(N)GTYDHDVYRDEALNNR Hex 5 HexNAc 3 | 127.920 | 0.0 | -19.201 | 5.359e+07 | 482 - 499 | 1 | 20.91 | 20.91 | 21.06 |
| 3967.8852 | IVKPGDILLIN(N)STGNLIAPR Hex 9 HexNAc 2 | 126.693 | 0.0 | -18.773 | 1.170e+07 | 235 - 255 | 1 | 52.64 | 52.64 | 52.81 |
| 4088.7218 | SEC(C)ITPN(N)GSIPNDKPFQNVNR Hex 8 HexNAc 2 | 126.577 | 0.0 | 17.591 | 8.791e+08 | 278 - 299 | 3 | 32.25 | 32.41 | 32.91 |
| 3673.4941 | N(N)GTYDHDVYRDEALNNR Hex 5 HexNAc 4 | 126.112 | 0.0 | -4.310 | 2.189e+08 | 482 - 499 | 2 | 20.61 | 20.76 | 21.06 |
| 3245.3311 | RGSN(N)SSFFSR Fuc 3 Hex 4 HexNAc 5 | 125.652 | 0.0 | -20.755 | 3.285e+06 | 140 - 150 | 1 | 19.38 | 19.38 | 19.38 |
| 4410.7055 | N(N)GTYDHDVYRDEALNNR Fuc 2 Hex 9 HexNAc 3 | 125.348 | 0.0 | -4.189 | 1.043e+08 | 482 - 499 | 1 | 21.86 | 22.03 | 22.37 |
| 4184.6828 | N(N)GTYDHDVYRDEALNNR Fuc 1 Hex 6 HexNAc 5 | 125.346 | 0.0 | 10.988 | 5.569e+08 | 482 - 499 | 3 | 20.16 | 20.31 | 20.91 |
| 4250.7764 | SEC(C)ITPN(N)GSIPNDKPFQNVNR Hex 9 HexNAc 2 | 124.695 | 0.0 | 17.002 | 1.001e+09 | 278 - 299 | 5 | 32.08 | 32.41 | 32.74 |
| 3779.5153 | N(N)GTYDHDVYRDEALN(D)NR Fuc 1 Hex 6 HexNAc 3 | 124.336 | 0.0 | -6.006 | 1.058e+08 | 482 - 499 | 2 | 21.06 | 21.06 | 21.70 |
| 3820.5416 | N(N)GTYDHDVYRDEALN(D)NR Fuc 1 Hex 5 HexNAc 4 | 124.186 | 0.0 | 7.197 | 1.495e+09 | 482 - 499 | 9 | 21.22 | 21.22 | 22.03 |
| 4264.6481 | N(N)GTYDHDVYRDEALNNR Fuc 1 Hex 9 HexNAc 3 | 123.333 | 0.0 | 11.999 | 2.159e+08 | 482 - 499 | 4 | 21.86 | 22.20 | 22.53 |
| 4476.8013 | N(N)GTYDHDVYRDEALNNR Fuc 3 Hex 6 HexNAc 5 | 121.926 | 0.0 | -18.348 | 9.765e+07 | 482 - 499 | 1 | 20.16 | 20.16 | 20.31 |
| 3600.4767 | N(N)GTYDHDVYRDEALNNR Fuc 2 Hex 4 HexNAc 3 | 120.644 | 0.0 | -2.927 | 9.291e+07 | 482 - 499 | 2 | 20.76 | 20.76 | 21.06 |
| 3436.2746 | N(N)GTYDHDVYR Fuc 2 Hex 8 HexNAc 3 | 120.628 | 0.0 | -16.879 | 1.390e+07 | 482 - 492 | 1 | 16.70 | 16.70 | 16.70 |
| 4267.8019 | SEC(C)ITPN(N)GSIPNDKPFQNVNR Fuc 2 Hex 3 HexNAc 4 Neu5Ac 1 | 120.502 | 0.0 | -18.691 | 5.224e+07 | 278 - 299 | 1 | 32.25 | 32.41 | 32.41 |
| 3762.5284 | N(N)GTYDHDVYRDEALNNR Fuc 2 Hex 5 HexNAc 3 | 119.840 | 0.0 | -5.383 | 7.494e+07 | 482 - 499 | 1 | 20.61 | 20.61 | 20.91 |
| 2950.1497 | N(N)GTYDHDVYR Fuc 2 Hex 5 HexNAc 3 | 118.812 | 0.0 | -17.813 | 8.840e+06 | 482 - 492 | 1 | 15.72 | 15.72 | 15.72 |
| 4387.7634 | N(N)GTYDHDVYRDEALNNR Fuc 1 Hex 6 HexNAc 6 | 118.235 | 0.0 | -3.402 | 1.225e+08 | 482 - 499 | 1 | 20.46 | 20.61 | 20.76 |
| 3602.5693 | SEC(C)ITPN(N)GSIPNDKPFQNVNR Hex 5 HexNAc 2 | 118.018 | 0.0 | -18.680 | 4.527e+07 | 278 - 299 | 1 | 32.74 | 32.74 | 32.91 |
| 4168.6880 | N(N)GTYDHDVYRDEALNNR Fuc 2 Hex 5 HexNAc 5 | 115.581 | 0.008 | 11.391 | 6.399e+08 | 482 - 499 | 4 | 19.86 | 20.46 | 20.91 |
| 3583.4539 | YPALN(N)VTMPN(D)NEQFDK Hex 8 HexNAc 2 | 115.230 | 0.008 | -5.933 | 3.073e+07 | 160 - 176 | 1 | 43.40 | 43.57 | 43.72 |
| 3966.5999 | N(N)GTYDHDVYRDEALN(D)NR Fuc 2 Hex 5 HexNAc 4 | 114.704 | 0.008 | 3.925 | 1.092e+09 | 482 - 499 | 5 | 20.91 | 21.06 | 21.86 |
| 3452.2701 | N(N)GTYDHDVYR Fuc 1 Hex 9 HexNAc 3 | 113.908 | 0.008 | -19.802 | 1.487e+07 | 482 - 492 | 1 | 16.53 | 16.70 | 16.70 |
| 4330.7413 | N(N)GTYDHDVYRDEALNNR Fuc 2 Hex 6 HexNAc 5 | 113.097 | 0.008 | -4.515 | 1.543e+08 | 482 - 499 | 1 | 20.16 | 20.31 | 20.46 |
| 4467.7307 | N(N)GTYDHDVYRDEALNNR Fuc 1 Hex 9 HexNAc 4 | 111.992 | 0.008 | 5.147 | 5.865e+07 | 482 - 499 | 1 | 21.70 | 22.20 | 22.37 |
| 3633.4551 | N(N)GTYDHDVYRDEALN(D)NR Hex 6 HexNAc 3 | 107.988 | 0.008 | -5.862 | 6.543e+07 | 482 - 499 | 1 | 21.22 | 21.22 | 21.54 |
| 2548.0397 | RGSN(N)SSFFSR Fuc 1 Hex 4 HexNAc 3 | 107.838 | 0.008 | 0.000 | 0.000e+00 | 140 - 150 | 1 | - | - | - |
| 2643.0553 | N(N)GTYDHDVYR Fuc 1 Hex 4 HexNAc 3 | 106.013 | 0.008 | 0.000 | 0.000e+00 | 482 - 492 | 1 | - | - | - |
| 4335.8680 | N(N)GTYDHDVYRDEALNNRFQIK Fuc 1 Hex 5 HexNAc 4 | 105.420 | 0.008 | -17.610 | 2.490e+07 | 482 - 503 | 1 | 32.25 | 32.25 | 32.25 |
| 4023.6241 | N(N)GTYDHDVYRDEALN(D)NR Fuc 1 Hex 5 HexNAc 5 | 105.298 | 0.008 | 8.605 | 3.555e+08 | 482 - 499 | 1 | 21.86 | 21.86 | 23.04 |
| 4248.6510 | N(N)GTYDHDVYRDEALNNR Fuc 2 Hex 8 HexNAc 3 | 104.712 | 0.008 | -3.822 | 2.234e+08 | 482 - 499 | 1 | 21.86 | 22.20 | 22.37 |
| 2861.1149 | N(N)GTYDHDVYR Hex 5 HexNAc 4 | 104.226 | 0.012 | -20.320 | 1.636e+07 | 482 - 492 | 1 | 15.72 | 15.87 | 15.87 |
| 3518.3608 | N(N)GTYDHDVYR Fuc 2 Hex 6 HexNAc 5 | 102.524 | 0.012 | -20.104 | 2.296e+07 | 482 - 492 | 2 | 15.58 | 15.58 | 15.58 |
| 3233.3290 | HAVPN(N)GTIVK Hex 8 HexNAc 3 Neu5Ac 1 | 101.682 | 0.012 | 0.000 | 0.000e+00 | 17 - 27 | 1 | - | - | - |
| 2454.9570 | N(N)GTYDHDVYR Hex 5 HexNAc 2 | 98.162 | 0.012 | 0.000 | 0.000e+00 | 482 - 492 | 1 | - | - | - |
| 3480.3968 | RGSN(N)SSFFSR Fuc 1 Hex 6 HexNAc 6 | 98.037 | 0.015 | -5.124 | 8.047e+07 | 140 - 150 | 2 | 19.38 | 19.38 | 19.86 |
| 4394.7109 | RGSN(N)SSFFSR Fuc 1 Hex 5 HexNAc 7 Neu5Ac 3 | 96.378 | 0.015 | -17.591 | 3.190e+07 | 140 - 150 | 1 | 22.03 | 22.03 | 22.03 |
| 4910.3560 | ISIYWTIVKPGDILLIN(N)STGNLIAPR Fuc 2 Hex 4 HexNAc 4 Neu5Ac 1 | 95.441 | 0.019 | -4.676 | 1.090e+07 | 229 - 255 | 2 | 68.65 | 68.99 | 69.17 |
| 4112.6564 | N(N)GTYDHDVYRDEALN(D)NR Fuc 3 Hex 5 HexNAc 4 | 94.879 | 0.019 | 10.462 | 3.733e+08 | 482 - 499 | 3 | 20.76 | 20.91 | 21.70 |
| 3314.4785 | LGHHAVPN(N)GTIVK Fuc 1 Hex 5 HexNAc 5 | 94.064 | 0.030 | 0.000 | 0.000e+00 | 14 - 27 | 1 | - | - | - |
| 3251.2210 | N(N)GTYDHDVYR Fuc 1 Hex 9 HexNAc 2 | 93.460 | 0.033 | 0.000 | 0.000e+00 | 482 - 492 | 1 | - | - | - |
| 4251.7647 | SEC(C)ITPN(N)GSIPN(D)DKPFQNVNR Hex 9 HexNAc 2 | 92.197 | 0.036 | 6.849 | 1.853e+08 | 278 - 299 | 5 | 32.91 | 33.59 | 33.76 |
| 3927.6625 | SEC(C)ITPN(N)GSIPN(D)DKPFQNVNR Hex 7 HexNAc 2 | 91.602 | 0.036 | 11.002 | 2.599e+08 | 278 - 299 | 5 | 32.91 | 33.76 | 34.10 |
| 3267.2763 | N(N)GTYDHDVYR Hex 5 HexNAc 6 | 89.864 | 0.043 | -18.808 | 3.716e+07 | 482 - 492 | 2 | 16.53 | 16.53 | 16.70 |
| 4089.7162 | SEC(C)ITPN(N)GSIPN(D)DKPFQNVNR Hex 8 HexNAc 2 | 89.740 | 0.046 | 10.808 | 1.473e+08 | 278 - 299 | 4 | 32.91 | 33.76 | 33.93 |

### RGSN(N)SSFFSR Hex 6 HexNAc 2

**Mass:**  2522.0185

(-2.011e-07)

**Charge States:** 3

**Start Position**: 141

**End Position**: 151

| Profile Score | Tandem Score | TDA q-value | Volume | Spectrum Matches |
| --- | --- | --- | --- | --- |
| -3.2600 | 193.7682 | 0.0000 | 5.1154e+08 | 4 |

**Adduct Signal Fractions**

- **Unmodified**: 5.1154e+08

### RGSN(N)SSFFSR Hex 5 HexNAc 2

**Mass:**  2359.9654

(8.481e-07)

**Charge States:** 3

**Start Position**: 141

**End Position**: 151

| Profile Score | Tandem Score | TDA q-value | Volume | Spectrum Matches |
| --- | --- | --- | --- | --- |
| 11.9473 | 193.2276 | 0.0000 | 4.7089e+08 | 3 |

**Adduct Signal Fractions**

- **Unmodified**: 4.7089e+08

### RGSN(N)SSFFSR Hex 5 HexNAc 4

**Mass:**  2766.1248

(3.952e-07)

**Charge States:** 3

**Start Position**: 141

**End Position**: 151

| Profile Score | Tandem Score | TDA q-value | Volume | Spectrum Matches |
| --- | --- | --- | --- | --- |
| -3.8344 | 189.5788 | 0.0000 | 1.1137e+08 | 1 |

**Adduct Signal Fractions**

- **Unmodified**: 1.1137e+08

### RGSN(N)SSFFSR Hex 5 HexNAc 6

**Mass:**  3172.2848

(-1.013e-06)

**Charge States:** 3

**Start Position**: 141

**End Position**: 151

| Profile Score | Tandem Score | TDA q-value | Volume | Spectrum Matches |
| --- | --- | --- | --- | --- |
| -7.2981 | 188.2475 | 0.0000 | 2.2622e+08 | 2 |

**Adduct Signal Fractions**

- **Unmodified**: 2.2622e+08

### RGSN(N)SSFFSR Hex 8 HexNAc 2

**Mass:**  2846.1247

(1.022e-06)

**Charge States:** 3

**Start Position**: 141

**End Position**: 151

| Profile Score | Tandem Score | TDA q-value | Volume | Spectrum Matches |
| --- | --- | --- | --- | --- |
| -17.9854 | 188.1376 | 0.0000 | 1.0055e+08 | 2 |

**Adduct Signal Fractions**

- **Unmodified**: 1.0055e+08

### RGSN(N)SSFFSR Hex 6 HexNAc 3

**Mass:**  2725.0988

(3.336e-07)

**Charge States:** 3

**Start Position**: 141

**End Position**: 151

| Profile Score | Tandem Score | TDA q-value | Volume | Spectrum Matches |
| --- | --- | --- | --- | --- |
| -3.7128 | 183.2509 | 0.0000 | 6.1903e+08 | 4 |

**Adduct Signal Fractions**

- **Unmodified**: 6.1903e+08

### RGSN(N)SSFFSR Fuc 2 Hex 5 HexNAc 5

**Mass:**  3261.3198

(3.402e-08)

**Charge States:** 3

**Start Position**: 141

**End Position**: 151

| Profile Score | Tandem Score | TDA q-value | Volume | Spectrum Matches |
| --- | --- | --- | --- | --- |
| 14.0082 | 182.2022 | 0.0000 | 4.3483e+08 | 4 |

**Adduct Signal Fractions**

- **Unmodified**: 4.3483e+08

### YPALN(N)VTMPNNEQFDK Hex 8 HexNAc 2

**Mass:**  3582.4722

(3.746e-06)

**Charge States:** 3, 4

**Start Position**: 161

**End Position**: 177

| Profile Score | Tandem Score | TDA q-value | Volume | Spectrum Matches |
| --- | --- | --- | --- | --- |
| 18.2239 | 178.3558 | 0.0000 | 3.3676e+08 | 5 |

**Adduct Signal Fractions**

- **Unmodified**: 3.3676e+08

### RGSN(N)SSFFSR Hex 7 HexNAc 2

**Mass:**  2684.0718

(4.232e-07)

**Charge States:** 3

**Start Position**: 141

**End Position**: 151

| Profile Score | Tandem Score | TDA q-value | Volume | Spectrum Matches |
| --- | --- | --- | --- | --- |
| 12.0456 | 178.2603 | 0.0000 | 2.9245e+08 | 2 |

**Adduct Signal Fractions**

- **Unmodified**: 2.9245e+08

### RGSN(N)SSFFSR Fuc 1 Hex 5 HexNAc 5

**Mass:**  3115.2621

(1.844e-06)

**Charge States:** 2, 3, 4

**Start Position**: 141

**End Position**: 151

| Profile Score | Tandem Score | TDA q-value | Volume | Spectrum Matches |
| --- | --- | --- | --- | --- |
| 13.1566 | 174.6602 | 0.0000 | 1.8854e+09 | 6 |

**Adduct Signal Fractions**

- **Unmodified**: 1.8854e+09

### RGSN(N)SSFFSR Fuc 3 Hex 5 HexNAc 5

**Mass:**  3407.3820

(1.474e-06)

**Charge States:** 3

**Start Position**: 141

**End Position**: 151

| Profile Score | Tandem Score | TDA q-value | Volume | Spectrum Matches |
| --- | --- | --- | --- | --- |
| -17.5930 | 173.8069 | 0.0000 | 4.5960e+07 | 1 |

**Adduct Signal Fractions**

- **Unmodified**: 4.5960e+07

### RGSN(N)SSFFSR Fuc 2 Hex 5 HexNAc 4

**Mass:**  3058.2410

(1.141e-06)

**Charge States:** 3, 4

**Start Position**: 141

**End Position**: 151

| Profile Score | Tandem Score | TDA q-value | Volume | Spectrum Matches |
| --- | --- | --- | --- | --- |
| -17.7063 | 173.1043 | 0.0000 | 2.8572e+08 | 3 |

**Adduct Signal Fractions**

- **Unmodified**: 2.8572e+08

### RGSN(N)SSFFSR Fuc 1 Hex 6 HexNAc 5

**Mass:**  3277.3178

(9.987e-07)

**Charge States:** 3

**Start Position**: 141

**End Position**: 151

| Profile Score | Tandem Score | TDA q-value | Volume | Spectrum Matches |
| --- | --- | --- | --- | --- |
| -3.7442 | 173.0750 | 0.0000 | 1.0397e+08 | 2 |

**Adduct Signal Fractions**

- **Unmodified**: 1.0397e+08

### YPALN(N)VTM(O)PNNEQFDK Hex 8 HexNAc 2

**Mass:**  3598.4785

(3.990e-06)

**Charge States:** 2, 3, 4

**Start Position**: 161

**End Position**: 177

| Profile Score | Tandem Score | TDA q-value | Volume | Spectrum Matches |
| --- | --- | --- | --- | --- |
| 16.8034 | 171.6873 | 0.0000 | 1.8245e+09 | 22 |

**Adduct Signal Fractions**

- **Unmodified**: 1.8245e+09

### YPALN(N)VTM(O)PNNEQFDK Hex 9 HexNAc 2

**Mass:**  3760.5108

(1.176e-06)

**Charge States:** 3, 4

**Start Position**: 161

**End Position**: 177

| Profile Score | Tandem Score | TDA q-value | Volume | Spectrum Matches |
| --- | --- | --- | --- | --- |
| 15.4894 | 171.3189 | 0.0000 | 1.5763e+08 | 5 |

**Adduct Signal Fractions**

- **Unmodified**: 1.5763e+08

### YPALN(N)VTM(O)PNNEQFDK Hex 7 HexNAc 2

**Mass:**  3436.4166

(4.633e-06)

**Charge States:** 2, 3, 4

**Start Position**: 161

**End Position**: 177

| Profile Score | Tandem Score | TDA q-value | Volume | Spectrum Matches |
| --- | --- | --- | --- | --- |
| 15.9153 | 171.1999 | 0.0000 | 3.4790e+08 | 4 |

**Adduct Signal Fractions**

- **Unmodified**: 3.4790e+08

### RGSN(N)SSFFSR Fuc 3 Hex 5 HexNAc 4

**Mass:**  3204.3012

(1.216e-06)

**Charge States:** 3

**Start Position**: 141

**End Position**: 151

| Profile Score | Tandem Score | TDA q-value | Volume | Spectrum Matches |
| --- | --- | --- | --- | --- |
| -4.2860 | 169.9383 | 0.0000 | 1.6385e+08 | 2 |

**Adduct Signal Fractions**

- **Unmodified**: 1.6385e+08

### RGSN(N)SSFFSR Hex 5 HexNAc 5

**Mass:**  2969.2051

(6.676e-07)

**Charge States:** 3

**Start Position**: 141

**End Position**: 151

| Profile Score | Tandem Score | TDA q-value | Volume | Spectrum Matches |
| --- | --- | --- | --- | --- |
| 12.0423 | 167.7772 | 0.0000 | 3.1101e+08 | 2 |

**Adduct Signal Fractions**

- **Unmodified**: 3.1101e+08

### TIVKPGDILLIN(N)STGNLIAPR Hex 9 HexNAc 2

**Mass:**  4068.9360

(3.094e-06)

**Charge States:** 3, 4, 5

**Start Position**: 235

**End Position**: 256

| Profile Score | Tandem Score | TDA q-value | Volume | Spectrum Matches |
| --- | --- | --- | --- | --- |
| 19.2426 | 167.5123 | 0.0000 | 6.0855e+08 | 5 |

**Adduct Signal Fractions**

- **Unmodified**: 6.0855e+08

### RGSN(N)SSFFSR Fuc 2 Hex 5 HexNAc 3

**Mass:**  2855.1612

(3.713e-07)

**Charge States:** 3

**Start Position**: 141

**End Position**: 151

| Profile Score | Tandem Score | TDA q-value | Volume | Spectrum Matches |
| --- | --- | --- | --- | --- |
| -18.6532 | 165.5378 | 0.0000 | 2.6921e+07 | 1 |

**Adduct Signal Fractions**

- **Unmodified**: 2.6921e+07

### RGSN(N)SSFFSR Fuc 1 Hex 5 HexNAc 3

**Mass:**  2709.1049

(9.841e-07)

**Charge States:** 3

**Start Position**: 141

**End Position**: 151

| Profile Score | Tandem Score | TDA q-value | Volume | Spectrum Matches |
| --- | --- | --- | --- | --- |
| -4.2373 | 164.0725 | 0.0000 | 8.8690e+07 | 1 |

**Adduct Signal Fractions**

- **Unmodified**: 8.8690e+07

### RGSN(N)SSFFSR Fuc 1 Hex 6 HexNAc 3

**Mass:**  2871.1565

(5.167e-07)

**Charge States:** 3

**Start Position**: 141

**End Position**: 151

| Profile Score | Tandem Score | TDA q-value | Volume | Spectrum Matches |
| --- | --- | --- | --- | --- |
| 10.5155 | 163.9185 | 0.0000 | 3.9687e+08 | 2 |

**Adduct Signal Fractions**

- **Unmodified**: 3.9687e+08

### GSN(N)SSFFSR Fuc 1 Hex 5 HexNAc 4

**Mass:**  2756.0837

(1.314e-06)

**Charge States:** 3

**Start Position**: 142

**End Position**: 151

| Profile Score | Tandem Score | TDA q-value | Volume | Spectrum Matches |
| --- | --- | --- | --- | --- |
| 12.0475 | 163.0499 | 0.0000 | 3.9084e+08 | 3 |

**Adduct Signal Fractions**

- **Unmodified**: 3.9084e+08

### RGSN(N)SSFFSR Fuc 1 Hex 5 HexNAc 4

**Mass:**  2912.1820

(5.270e-07)

**Charge States:** 3

**Start Position**: 141

**End Position**: 151

| Profile Score | Tandem Score | TDA q-value | Volume | Spectrum Matches |
| --- | --- | --- | --- | --- |
| 13.9766 | 162.1966 | 0.0000 | 9.1883e+08 | 4 |

**Adduct Signal Fractions**

- **Unmodified**: 9.1883e+08

### RGSN(N)SSFFSR Fuc 1 Hex 4 HexNAc 3

**Mass:**  2547.0560

(2.538e-06)

**Charge States:** 3

**Start Position**: 141

**End Position**: 151

| Profile Score | Tandem Score | TDA q-value | Volume | Spectrum Matches |
| --- | --- | --- | --- | --- |
| -18.9342 | 161.2664 | 0.0000 | 2.3751e+07 | 1 |

**Adduct Signal Fractions**

- **Unmodified**: 2.3751e+07

### YPALN(N)VTMPNNEQFDK Hex 7 HexNAc 2

**Mass:**  3420.4173

(3.730e-06)

**Charge States:** 3

**Start Position**: 161

**End Position**: 177

| Profile Score | Tandem Score | TDA q-value | Volume | Spectrum Matches |
| --- | --- | --- | --- | --- |
| -3.2775 | 159.4179 | 0.0000 | 5.4809e+07 | 2 |

**Adduct Signal Fractions**

- **Unmodified**: 5.4809e+07

### RGSN(N)SSFFSR Fuc 1 Hex 9 HexNAc 4

**Mass:**  3560.3582

(-9.720e-06)

**Charge States:** 3

**Start Position**: 141

**End Position**: 151

| Profile Score | Tandem Score | TDA q-value | Volume | Spectrum Matches |
| --- | --- | --- | --- | --- |
| 10.8286 | 158.6045 | 0.0000 | 8.5186e+07 | 1 |

**Adduct Signal Fractions**

- **Unmodified**: 8.5186e+07

### TIVKPGDILLIN(N)STGNLIAPR Fuc 2 Hex 3 HexNAc 4 Neu5Ac 1

**Mass:**  4085.9599

(-3.163e-06)

**Charge States:** 4

**Start Position**: 235

**End Position**: 256

| Profile Score | Tandem Score | TDA q-value | Volume | Spectrum Matches |
| --- | --- | --- | --- | --- |
| -3.0745 | 157.2027 | 0.0000 | 7.9458e+07 | 2 |

**Adduct Signal Fractions**

- **Unmodified**: 7.9458e+07

### YPALN(N)VTMPNNEQFDK Hex 9 HexNAc 2

**Mass:**  3744.5205

(3.402e-06)

**Charge States:** 3

**Start Position**: 161

**End Position**: 177

| Profile Score | Tandem Score | TDA q-value | Volume | Spectrum Matches |
| --- | --- | --- | --- | --- |
| -4.0731 | 155.4127 | 0.0000 | 3.0454e+07 | 2 |

**Adduct Signal Fractions**

- **Unmodified**: 3.0454e+07

### C(C)KSEC(C)ITPN(N)GSIPNDKPFQNVNR Hex 8 HexNAc 2

**Mass:**  4376.8420

(-4.431e-07)

**Charge States:** 4

**Start Position**: 277

**End Position**: 300

| Profile Score | Tandem Score | TDA q-value | Volume | Spectrum Matches |
| --- | --- | --- | --- | --- |
| -17.1617 | 155.3036 | 0.0000 | 1.5110e+08 | 1 |

**Adduct Signal Fractions**

- **Unmodified**: 1.5110e+08

### RGSN(N)SSFFSR Fuc 2 Hex 9 HexNAc 3

**Mass:**  3503.3325

(-9.675e-06)

**Charge States:** 3

**Start Position**: 141

**End Position**: 151

| Profile Score | Tandem Score | TDA q-value | Volume | Spectrum Matches |
| --- | --- | --- | --- | --- |
| -3.8918 | 155.2002 | 0.0000 | 6.6398e+07 | 2 |

**Adduct Signal Fractions**

- **Unmodified**: 6.6398e+07

### N(N)GTYDHDVYR Fuc 1 Hex 5 HexNAc 4

**Mass:**  3007.1755

(1.460e-06)

**Charge States:** 3

**Start Position**: 483

**End Position**: 493

| Profile Score | Tandem Score | TDA q-value | Volume | Spectrum Matches |
| --- | --- | --- | --- | --- |
| 14.7884 | 154.6934 | 0.0000 | 4.7540e+08 | 4 |

**Adduct Signal Fractions**

- **Unmodified**: 4.7540e+08

### YPALN(N)VTM(O)PNNEQFDK Hex 6 HexNAc 2

**Mass:**  3274.3646

(5.041e-06)

**Charge States:** 3

**Start Position**: 161

**End Position**: 177

| Profile Score | Tandem Score | TDA q-value | Volume | Spectrum Matches |
| --- | --- | --- | --- | --- |
| 11.9640 | 154.0942 | 0.0000 | 2.3878e+07 | 2 |

**Adduct Signal Fractions**

- **Unmodified**: 2.3878e+07

### GSN(N)SSFFSR Hex 5 HexNAc 2

**Mass:**  2203.8641

(1.125e-07)

**Charge States:** 2, 3

**Start Position**: 142

**End Position**: 151

| Profile Score | Tandem Score | TDA q-value | Volume | Spectrum Matches |
| --- | --- | --- | --- | --- |
| 13.0786 | 153.8869 | 0.0000 | 3.4381e+08 | 3 |

**Adduct Signal Fractions**

- **Unmodified**: 3.4381e+08

### RGSN(N)SSFFSR Hex 9 HexNAc 2

**Mass:**  3008.1553

(-6.976e-06)

**Charge States:** 3

**Start Position**: 141

**End Position**: 151

| Profile Score | Tandem Score | TDA q-value | Volume | Spectrum Matches |
| --- | --- | --- | --- | --- |
| -20.6542 | 153.1382 | 0.0000 | 7.7449e+06 | 1 |

**Adduct Signal Fractions**

- **Unmodified**: 7.7449e+06

### HAVPN(N)GTIVK Fuc 1 Hex 5 HexNAc 5

**Mass:**  3006.3067

(2.836e-06)

**Charge States:** 3

**Start Position**: 18

**End Position**: 28

| Profile Score | Tandem Score | TDA q-value | Volume | Spectrum Matches |
| --- | --- | --- | --- | --- |
| -20.1436 | 152.0945 | 0.0000 | 9.2211e+06 | 1 |

**Adduct Signal Fractions**

- **Unmodified**: 9.2211e+06

### N(N)GTYDHDVYR Fuc 1 Hex 6 HexNAc 5

**Mass:**  3372.3085

(-3.536e-06)

**Charge States:** 3

**Start Position**: 483

**End Position**: 493

| Profile Score | Tandem Score | TDA q-value | Volume | Spectrum Matches |
| --- | --- | --- | --- | --- |
| -21.7860 | 152.0439 | 0.0000 | 7.4881e+07 | 4 |

**Adduct Signal Fractions**

- **Unmodified**: 7.4881e+07

### N(N)GTYDHDVYR Fuc 1 Hex 6 HexNAc 3

**Mass:**  2966.1438

(-2.794e-07)

**Charge States:** 3

**Start Position**: 483

**End Position**: 493

| Profile Score | Tandem Score | TDA q-value | Volume | Spectrum Matches |
| --- | --- | --- | --- | --- |
| -20.1362 | 152.0151 | 0.0000 | 1.7351e+07 | 1 |

**Adduct Signal Fractions**

- **Unmodified**: 1.7351e+07

### TIVKPGDILLIN(N)STGNLIAPR Hex 10 HexNAc 2

**Mass:**  4230.9919

(3.652e-06)

**Charge States:** 4

**Start Position**: 235

**End Position**: 256

| Profile Score | Tandem Score | TDA q-value | Volume | Spectrum Matches |
| --- | --- | --- | --- | --- |
| -19.3976 | 150.6985 | 0.0000 | 2.1293e+07 | 2 |

**Adduct Signal Fractions**

- **Unmodified**: 2.1293e+07

### RGSN(N)SSFFSR Hex 6 HexNAc 6

**Mass:**  3334.3350

(-5.027e-08)

**Charge States:** 3

**Start Position**: 141

**End Position**: 151

| Profile Score | Tandem Score | TDA q-value | Volume | Spectrum Matches |
| --- | --- | --- | --- | --- |
| -19.4655 | 150.5067 | 0.0000 | 1.8006e+07 | 1 |

**Adduct Signal Fractions**

- **Unmodified**: 1.8006e+07

### GSN(N)SSFFSR Fuc 1 Hex 6 HexNAc 3

**Mass:**  2715.0556

(1.072e-06)

**Charge States:** 2, 3

**Start Position**: 142

**End Position**: 151

| Profile Score | Tandem Score | TDA q-value | Volume | Spectrum Matches |
| --- | --- | --- | --- | --- |
| -3.8120 | 149.9170 | 0.0000 | 1.5670e+08 | 2 |

**Adduct Signal Fractions**

- **Unmodified**: 1.5670e+08

### N(N)GTYDHDVYR Fuc 1 Hex 5 HexNAc 5

**Mass:**  3210.2558

(1.611e-06)

**Charge States:** 3

**Start Position**: 483

**End Position**: 493

| Profile Score | Tandem Score | TDA q-value | Volume | Spectrum Matches |
| --- | --- | --- | --- | --- |
| -2.2647 | 149.8677 | 0.0000 | 2.4486e+08 | 3 |

**Adduct Signal Fractions**

- **Unmodified**: 2.4486e+08

### C(C)KSEC(C)ITPN(N)GSIPNDKPFQNVNR Hex 6 HexNAc 2

**Mass:**  4052.7343

(-1.393e-07)

**Charge States:** 4

**Start Position**: 277

**End Position**: 300

| Profile Score | Tandem Score | TDA q-value | Volume | Spectrum Matches |
| --- | --- | --- | --- | --- |
| -19.0530 | 149.8456 | 0.0000 | 3.0887e+07 | 1 |

**Adduct Signal Fractions**

- **Unmodified**: 3.0887e+07

### N(N)GTYDHDVYRDEALNNR Hex 6 HexNAc 3

**Mass:**  3632.4677

(7.051e-07)

**Charge States:** 4

**Start Position**: 483

**End Position**: 500

| Profile Score | Tandem Score | TDA q-value | Volume | Spectrum Matches |
| --- | --- | --- | --- | --- |
| -3.4434 | 149.6602 | 0.0000 | 3.2883e+08 | 3 |

**Adduct Signal Fractions**

- **Unmodified**: 3.2883e+08

### C(C)KSEC(C)ITPN(N)GSIPNDKPFQNVNR Hex 7 HexNAc 2

**Mass:**  4214.7893

(-7.962e-07)

**Charge States:** 4

**Start Position**: 277

**End Position**: 300

| Profile Score | Tandem Score | TDA q-value | Volume | Spectrum Matches |
| --- | --- | --- | --- | --- |
| -2.9225 | 149.6288 | 0.0000 | 3.0487e+08 | 3 |

**Adduct Signal Fractions**

- **Unmodified**: 3.0487e+08

### TIVKPGDILLIN(N)STGNLIAPR Hex 8 HexNAc 2

**Mass:**  3906.8813

(3.807e-06)

**Charge States:** 4

**Start Position**: 235

**End Position**: 256

| Profile Score | Tandem Score | TDA q-value | Volume | Spectrum Matches |
| --- | --- | --- | --- | --- |
| -3.6064 | 149.1272 | 0.0000 | 6.0719e+07 | 3 |

**Adduct Signal Fractions**

- **Unmodified**: 6.0719e+07

### GSN(N)SSFFSR Hex 6 HexNAc 2

**Mass:**  2365.9195

(-4.855e-07)

**Charge States:** 2, 3

**Start Position**: 142

**End Position**: 151

| Profile Score | Tandem Score | TDA q-value | Volume | Spectrum Matches |
| --- | --- | --- | --- | --- |
| -3.2752 | 148.8987 | 0.0000 | 2.3802e+08 | 2 |

**Adduct Signal Fractions**

- **Unmodified**: 2.3802e+08

### GSN(N)SSFFSR Hex 5 HexNAc 5

**Mass:**  2813.1042

(7.878e-07)

**Charge States:** 2, 3

**Start Position**: 142

**End Position**: 151

| Profile Score | Tandem Score | TDA q-value | Volume | Spectrum Matches |
| --- | --- | --- | --- | --- |
| 11.7871 | 148.6997 | 0.0000 | 2.4058e+08 | 2 |

**Adduct Signal Fractions**

- **Unmodified**: 2.4058e+08

### YPALN(N)VTMPNNEQFDK Fuc 2 Hex 3 HexNAc 4 Neu5Ac 1

**Mass:**  3761.5486

(-4.225e-06)

**Charge States:** 3

**Start Position**: 161

**End Position**: 177

| Profile Score | Tandem Score | TDA q-value | Volume | Spectrum Matches |
| --- | --- | --- | --- | --- |
| -18.7500 | 148.5634 | 0.0000 | 7.4587e+06 | 1 |

**Adduct Signal Fractions**

- **Unmodified**: 7.4587e+06

### RGSN(N)SSFFSR Fuc 1 Hex 3 HexNAc 9 Neu5Ac 2

**Mass:**  4185.6750

(2.262e-06)

**Charge States:** 4

**Start Position**: 141

**End Position**: 151

| Profile Score | Tandem Score | TDA q-value | Volume | Spectrum Matches |
| --- | --- | --- | --- | --- |
| -4.0883 | 148.3546 | 0.0000 | 1.8228e+08 | 3 |

**Adduct Signal Fractions**

- **Unmodified**: 1.8228e+08

### GSN(N)SSFFSR Hex 6 HexNAc 3

**Mass:**  2568.9995

(-4.381e-07)

**Charge States:** 2, 3

**Start Position**: 142

**End Position**: 151

| Profile Score | Tandem Score | TDA q-value | Volume | Spectrum Matches |
| --- | --- | --- | --- | --- |
| 12.6727 | 147.9083 | 0.0000 | 3.3299e+08 | 3 |

**Adduct Signal Fractions**

- **Unmodified**: 3.3299e+08

### ISIYWTIVKPGDILLIN(N)STGNLIAPR Hex 9 HexNAc 2

**Mass:**  4731.2810

(2.063e-06)

**Charge States:** 3, 4, 5

**Start Position**: 230

**End Position**: 256

| Profile Score | Tandem Score | TDA q-value | Volume | Spectrum Matches |
| --- | --- | --- | --- | --- |
| 18.6635 | 147.5462 | 0.0000 | 8.6389e+08 | 9 |

**Adduct Signal Fractions**

- **Unmodified**: 8.6389e+08

### RGSN(N)SSFFSR Fuc 2 Hex 6 HexNAc 5

**Mass:**  3423.3740

(6.731e-07)

**Charge States:** 3

**Start Position**: 141

**End Position**: 151

| Profile Score | Tandem Score | TDA q-value | Volume | Spectrum Matches |
| --- | --- | --- | --- | --- |
| -5.1344 | 147.4956 | 0.0000 | 2.6108e+07 | 1 |

**Adduct Signal Fractions**

- **Unmodified**: 2.6108e+07

### C(C)KSEC(C)ITPN(N)GSIPNDKPFQNVNR Hex 9 HexNAc 2

**Mass:**  4538.8937

(-5.011e-07)

**Charge States:** 4

**Start Position**: 277

**End Position**: 300

| Profile Score | Tandem Score | TDA q-value | Volume | Spectrum Matches |
| --- | --- | --- | --- | --- |
| -18.2743 | 146.9481 | 0.0000 | 2.0613e+08 | 1 |

**Adduct Signal Fractions**

- **Unmodified**: 2.0613e+08

### RGSN(N)SSFFSR Fuc 2 Hex 6 HexNAc 3

**Mass:**  3017.2117

(8.069e-07)

**Charge States:** 3

**Start Position**: 141

**End Position**: 151

| Profile Score | Tandem Score | TDA q-value | Volume | Spectrum Matches |
| --- | --- | --- | --- | --- |
| -19.2684 | 145.2142 | 0.0000 | 2.8539e+07 | 1 |

**Adduct Signal Fractions**

- **Unmodified**: 2.8539e+07

### GSN(N)SSFFSR Fuc 2 Hex 5 HexNAc 5

**Mass:**  3105.2191

(3.884e-07)

**Charge States:** 3

**Start Position**: 142

**End Position**: 151

| Profile Score | Tandem Score | TDA q-value | Volume | Spectrum Matches |
| --- | --- | --- | --- | --- |
| -3.4173 | 145.2074 | 0.0000 | 1.2504e+08 | 1 |

**Adduct Signal Fractions**

- **Unmodified**: 1.2504e+08

### RGSN(N)SSFFSR Hex 6 HexNAc 4

**Mass:**  2928.1765

(9.626e-07)

**Charge States:** 3

**Start Position**: 141

**End Position**: 151

| Profile Score | Tandem Score | TDA q-value | Volume | Spectrum Matches |
| --- | --- | --- | --- | --- |
| 12.8141 | 144.7148 | 0.0000 | 1.3064e+08 | 2 |

**Adduct Signal Fractions**

- **Unmodified**: 1.3064e+08

### ISIYWTIVKPGDILLIN(N)STGNLIAPR Fuc 2 Hex 3 HexNAc 4 Neu5Ac 1

**Mass:**  4748.3019

(-3.247e-06)

**Charge States:** 4

**Start Position**: 230

**End Position**: 256

| Profile Score | Tandem Score | TDA q-value | Volume | Spectrum Matches |
| --- | --- | --- | --- | --- |
| 15.0358 | 143.8934 | 0.0000 | 2.2744e+08 | 8 |

**Adduct Signal Fractions**

- **Unmodified**: 2.2744e+08

### N(N)GTYDHDVYRDEALNNR Fuc 3 Hex 5 HexNAc 4

**Mass:**  4111.6677

(8.441e-07)

**Charge States:** 4

**Start Position**: 483

**End Position**: 500

| Profile Score | Tandem Score | TDA q-value | Volume | Spectrum Matches |
| --- | --- | --- | --- | --- |
| 14.9110 | 143.2357 | 0.0000 | 6.8811e+08 | 3 |

**Adduct Signal Fractions**

- **Unmodified**: 6.8811e+08

### RGSN(N)SSFF Fuc 1 Hex 5 HexNAc 5

**Mass:**  2872.1295

(5.562e-07)

**Charge States:** 3

**Start Position**: 141

**End Position**: 149

| Profile Score | Tandem Score | TDA q-value | Volume | Spectrum Matches |
| --- | --- | --- | --- | --- |
| -18.5699 | 142.8248 | 0.0000 | 1.5133e+07 | 1 |

**Adduct Signal Fractions**

- **Unmodified**: 1.5133e+07

### N(N)GTYDHDVYRDEALNNR Hex 5 HexNAc 2

**Mass:**  3267.3320

(-6.432e-08)

**Charge States:** 3, 4

**Start Position**: 483

**End Position**: 500

| Profile Score | Tandem Score | TDA q-value | Volume | Spectrum Matches |
| --- | --- | --- | --- | --- |
| -20.0547 | 141.0733 | 0.0000 | 1.1036e+08 | 1 |

**Adduct Signal Fractions**

- **Unmodified**: 1.1036e+08

### ISIYWTIVKPGDILLIN(N)STGNLIAPR Hex 8 HexNAc 2

**Mass:**  4569.2253

(3.221e-06)

**Charge States:** 3, 4, 5

**Start Position**: 230

**End Position**: 256

| Profile Score | Tandem Score | TDA q-value | Volume | Spectrum Matches |
| --- | --- | --- | --- | --- |
| -2.5693 | 140.0469 | 0.0000 | 9.4631e+07 | 5 |

**Adduct Signal Fractions**

- **Unmodified**: 9.4631e+07

### YPALN(N)VTM(O)PNNEQFDK Hex 5 HexNAc 2

**Mass:**  3112.3189

(6.775e-06)

**Charge States:** 3

**Start Position**: 161

**End Position**: 177

| Profile Score | Tandem Score | TDA q-value | Volume | Spectrum Matches |
| --- | --- | --- | --- | --- |
| -4.6719 | 139.8547 | 0.0000 | 1.3130e+07 | 2 |

**Adduct Signal Fractions**

- **Unmodified**: 1.3130e+07

### N(N)GTYDHDVYRDEALNNR Fuc 1 Hex 6 HexNAc 3

**Mass:**  3778.5275

(1.382e-06)

**Charge States:** 4

**Start Position**: 483

**End Position**: 500

| Profile Score | Tandem Score | TDA q-value | Volume | Spectrum Matches |
| --- | --- | --- | --- | --- |
| 13.1532 | 139.2485 | 0.0000 | 3.2135e+08 | 2 |

**Adduct Signal Fractions**

- **Unmodified**: 3.2135e+08

### RGSN(N)SSFFSR Fuc 3 Hex 7 HexNAc 6

**Mass:**  3935.5993

(2.637e-04)

**Start Position**: 141

**End Position**: 151

| Profile Score | Tandem Score | TDA q-value | Volume | Spectrum Matches |
| --- | --- | --- | --- | --- |
| 0.0000 | 139.0206 | 0.0000 | 0.0000e+00 | 1 |

Adduct:

No Chromatogram Found

### GSN(N)SSFFSR Hex 5 HexNAc 4

**Mass:**  2610.0261

(7.732e-07)

**Charge States:** 3

**Start Position**: 142

**End Position**: 151

| Profile Score | Tandem Score | TDA q-value | Volume | Spectrum Matches |
| --- | --- | --- | --- | --- |
| -19.5553 | 137.9504 | 0.0455 | 4.2961e+07 | 2 |

**Adduct Signal Fractions**

- **Unmodified**: 4.2961e+07

### N(N)GTYDHDVYR Fuc 2 Hex 5 HexNAc 4

**Mass:**  3153.2334

(-6.439e-07)

**Charge States:** 3

**Start Position**: 483

**End Position**: 493

| Profile Score | Tandem Score | TDA q-value | Volume | Spectrum Matches |
| --- | --- | --- | --- | --- |
| -3.7601 | 136.9756 | 0.0000 | 3.1998e+08 | 3 |

**Adduct Signal Fractions**

- **Unmodified**: 3.1998e+08

### N(N)GTYDHDVYRDEALNNR Fuc 1 Hex 8 HexNAc 2

**Mass:**  3899.5190

(-8.110e-06)

**Charge States:** 3, 4

**Start Position**: 483

**End Position**: 500

| Profile Score | Tandem Score | TDA q-value | Volume | Spectrum Matches |
| --- | --- | --- | --- | --- |
| 13.6036 | 136.6785 | 0.0000 | 7.3297e+08 | 2 |

**Adduct Signal Fractions**

- **Unmodified**: 7.3297e+08

### GSN(N)SSFFSR Fuc 1 Hex 5 HexNAc 5

**Mass:**  2959.1629

(-6.451e-07)

**Charge States:** 2, 3

**Start Position**: 142

**End Position**: 151

| Profile Score | Tandem Score | TDA q-value | Volume | Spectrum Matches |
| --- | --- | --- | --- | --- |
| 13.2017 | 136.3372 | 0.0455 | 9.9126e+08 | 3 |

**Adduct Signal Fractions**

- **Unmodified**: 9.9126e+08

### N(N)GTYDHDVYR Fuc 3 Hex 5 HexNAc 4

**Mass:**  3300.2893

(6.047e-04)

**Start Position**: 483

**End Position**: 493

| Profile Score | Tandem Score | TDA q-value | Volume | Spectrum Matches |
| --- | --- | --- | --- | --- |
| 0.0000 | 135.4368 | 0.0000 | 0.0000e+00 | 2 |

Adduct:

No Chromatogram Found

### YPALN(N)VTM(O)PN(D)NEQFDK Hex 7 HexNAc 2

**Mass:**  3437.4113

(2.940e-04)

**Charge States:** 3

**Start Position**: 161

**End Position**: 177

| Profile Score | Tandem Score | TDA q-value | Volume | Spectrum Matches |
| --- | --- | --- | --- | --- |
| 6.4573 | 134.8458 | 0.0000 | 2.5124e+07 | 3 |

**Adduct Signal Fractions**

- **Unmodified**: 2.5124e+07

### N(N)GTYDHDVYRDEALNNR Fuc 2 Hex 5 HexNAc 4

**Mass:**  3965.6103

(8.563e-07)

**Charge States:** 3, 4

**Start Position**: 483

**End Position**: 500

| Profile Score | Tandem Score | TDA q-value | Volume | Spectrum Matches |
| --- | --- | --- | --- | --- |
| 15.2830 | 133.5964 | 0.0000 | 2.1566e+09 | 4 |

**Adduct Signal Fractions**

- **Unmodified**: 2.1566e+09

### GSN(N)SSFFSR Hex 5 HexNAc 3

**Mass:**  2406.9424

(-3.168e-07)

**Charge States:** 2, 3

**Start Position**: 142

**End Position**: 151

| Profile Score | Tandem Score | TDA q-value | Volume | Spectrum Matches |
| --- | --- | --- | --- | --- |
| -20.0509 | 131.8583 | 0.0455 | 3.8373e+07 | 2 |

**Adduct Signal Fractions**

- **Unmodified**: 3.8373e+07

### N(N)GTYDHDVYR Hex 6 HexNAc 3

**Mass:**  2820.0913

(1.650e-06)

**Charge States:** 3

**Start Position**: 483

**End Position**: 493

| Profile Score | Tandem Score | TDA q-value | Volume | Spectrum Matches |
| --- | --- | --- | --- | --- |
| -18.0667 | 131.8080 | 0.0000 | 3.5796e+07 | 2 |

**Adduct Signal Fractions**

- **Unmodified**: 3.5796e+07

### N(N)GTYDHDVYRDEALNNR Fuc 1 Hex 8 HexNAc 3

**Mass:**  4102.6003

(-6.729e-06)

**Charge States:** 3, 4

**Start Position**: 483

**End Position**: 500

| Profile Score | Tandem Score | TDA q-value | Volume | Spectrum Matches |
| --- | --- | --- | --- | --- |
| 11.2602 | 131.7769 | 0.0000 | 3.4625e+08 | 4 |

**Adduct Signal Fractions**

- **Unmodified**: 3.4625e+08

### N(N)GTYDHDVYRDEALNNR Fuc 1 Hex 5 HexNAc 4

**Mass:**  3819.5500

(1.208e-06)

**Charge States:** 3, 4, 5

**Start Position**: 483

**End Position**: 500

| Profile Score | Tandem Score | TDA q-value | Volume | Spectrum Matches |
| --- | --- | --- | --- | --- |
| 16.6707 | 131.4278 | 0.0000 | 4.7370e+09 | 7 |

**Adduct Signal Fractions**

- **Unmodified**: 4.7370e+09

### N(N)GTYDHDVYRDEALNNR Fuc 1 Hex 5 HexNAc 3

**Mass:**  3616.4721

(5.915e-07)

**Charge States:** 4

**Start Position**: 483

**End Position**: 500

| Profile Score | Tandem Score | TDA q-value | Volume | Spectrum Matches |
| --- | --- | --- | --- | --- |
| -3.3291 | 131.3200 | 0.0000 | 1.4748e+08 | 2 |

**Adduct Signal Fractions**

- **Unmodified**: 1.4748e+08

### SEC(C)ITPN(N)GSIPNDKPFQNVNR Hex 6 HexNAc 2

**Mass:**  3764.6145

(2.205e-06)

**Charge States:** 3, 4

**Start Position**: 279

**End Position**: 300

| Profile Score | Tandem Score | TDA q-value | Volume | Spectrum Matches |
| --- | --- | --- | --- | --- |
| -3.1678 | 130.7891 | 0.0000 | 2.5042e+08 | 2 |

**Adduct Signal Fractions**

- **Unmodified**: 2.5042e+08

### YPALN(N)VTM(O)PN(D)NEQFDK Hex 8 HexNAc 2

**Mass:**  3599.4633

(2.824e-04)

**Charge States:** 2, 3, 4

**Start Position**: 161

**End Position**: 177

| Profile Score | Tandem Score | TDA q-value | Volume | Spectrum Matches |
| --- | --- | --- | --- | --- |
| 7.9083 | 130.6362 | 0.0000 | 2.0440e+08 | 9 |

**Adduct Signal Fractions**

- **Unmodified**: 2.0440e+08

### N(N)GTYDHDVYR Fuc 2 Hex 5 HexNAc 5

**Mass:**  3356.3106

(6.610e-07)

**Charge States:** 3

**Start Position**: 483

**End Position**: 493

| Profile Score | Tandem Score | TDA q-value | Volume | Spectrum Matches |
| --- | --- | --- | --- | --- |
| -19.1138 | 130.2570 | 0.0000 | 6.4210e+07 | 2 |

**Adduct Signal Fractions**

- **Unmodified**: 6.4210e+07

### N(N)GTYDHDVYRDEALNNR Fuc 1 Hex 4 HexNAc 3

**Mass:**  3454.4216

(-2.333e-07)

**Charge States:** 3, 4

**Start Position**: 483

**End Position**: 500

| Profile Score | Tandem Score | TDA q-value | Volume | Spectrum Matches |
| --- | --- | --- | --- | --- |
| -18.9088 | 130.0632 | 0.0000 | 1.2360e+08 | 1 |

**Adduct Signal Fractions**

- **Unmodified**: 1.2360e+08

### GSN(N)SSFFSR Hex 6 HexNAc 4

**Mass:**  2772.0868

(4.052e-06)

**Charge States:** 3

**Start Position**: 142

**End Position**: 151

| Profile Score | Tandem Score | TDA q-value | Volume | Spectrum Matches |
| --- | --- | --- | --- | --- |
| 6.6283 | 129.5301 | 0.0455 | 7.3848e+07 | 2 |

**Adduct Signal Fractions**

- **Unmodified**: 7.3848e+07

### N(N)GTYDHDVYRDEALNNR Fuc 1 Hex 5 HexNAc 5

**Mass:**  4022.6296

(-7.936e-08)

**Charge States:** 3, 4

**Start Position**: 483

**End Position**: 500

| Profile Score | Tandem Score | TDA q-value | Volume | Spectrum Matches |
| --- | --- | --- | --- | --- |
| 14.9877 | 128.4289 | 0.0000 | 2.6374e+09 | 7 |

**Adduct Signal Fractions**

- **Unmodified**: 2.6374e+09

### SEC(C)ITPN(N)GSIPNDKPFQNVNR Hex 7 HexNAc 2

**Mass:**  3926.6687

(1.618e-06)

**Charge States:** 3, 4

**Start Position**: 279

**End Position**: 300

| Profile Score | Tandem Score | TDA q-value | Volume | Spectrum Matches |
| --- | --- | --- | --- | --- |
| 17.7475 | 127.9791 | 0.0000 | 1.9403e+09 | 4 |

**Adduct Signal Fractions**

- **Unmodified**: 1.9403e+09

### N(N)GTYDHDVYRDEALNNR Hex 5 HexNAc 3

**Mass:**  3470.4108

(-2.047e-07)

**Charge States:** 4

**Start Position**: 483

**End Position**: 500

| Profile Score | Tandem Score | TDA q-value | Volume | Spectrum Matches |
| --- | --- | --- | --- | --- |
| -19.2015 | 127.9202 | 0.0000 | 5.3593e+07 | 1 |

**Adduct Signal Fractions**

- **Unmodified**: 5.3593e+07

### IVKPGDILLIN(N)STGNLIAPR Hex 9 HexNAc 2

**Mass:**  3967.8879

(3.056e-06)

**Charge States:** 4

**Start Position**: 236

**End Position**: 256

| Profile Score | Tandem Score | TDA q-value | Volume | Spectrum Matches |
| --- | --- | --- | --- | --- |
| -18.7730 | 126.6935 | 0.0000 | 1.1698e+07 | 1 |

**Adduct Signal Fractions**

- **Unmodified**: 1.1698e+07

### SEC(C)ITPN(N)GSIPNDKPFQNVNR Hex 8 HexNAc 2

**Mass:**  4088.7218

(2.157e-06)

**Charge States:** 3, 4

**Start Position**: 279

**End Position**: 300

| Profile Score | Tandem Score | TDA q-value | Volume | Spectrum Matches |
| --- | --- | --- | --- | --- |
| 17.5908 | 126.5775 | 0.0000 | 8.7905e+08 | 3 |

**Adduct Signal Fractions**

- **Unmodified**: 8.7905e+08

### N(N)GTYDHDVYRDEALNNR Hex 5 HexNAc 4

**Mass:**  3673.4953

(3.063e-07)

**Charge States:** 3, 4

**Start Position**: 483

**End Position**: 500

| Profile Score | Tandem Score | TDA q-value | Volume | Spectrum Matches |
| --- | --- | --- | --- | --- |
| -4.3096 | 126.1120 | 0.0000 | 2.1893e+08 | 2 |

**Adduct Signal Fractions**

- **Unmodified**: 2.1893e+08

### RGSN(N)SSFFSR Fuc 3 Hex 4 HexNAc 5

**Mass:**  3245.3311

(2.221e-06)

**Charge States:** 3

**Start Position**: 141

**End Position**: 151

| Profile Score | Tandem Score | TDA q-value | Volume | Spectrum Matches |
| --- | --- | --- | --- | --- |
| -20.7547 | 125.6519 | 0.0000 | 3.2845e+06 | 1 |

**Adduct Signal Fractions**

- **Unmodified**: 3.2845e+06

### N(N)GTYDHDVYRDEALNNR Fuc 2 Hex 9 HexNAc 3

**Mass:**  4410.7092

(-8.031e-06)

**Charge States:** 3, 4

**Start Position**: 483

**End Position**: 500

| Profile Score | Tandem Score | TDA q-value | Volume | Spectrum Matches |
| --- | --- | --- | --- | --- |
| -4.1888 | 125.3483 | 0.0000 | 1.0432e+08 | 1 |

**Adduct Signal Fractions**

- **Unmodified**: 1.0432e+08

### N(N)GTYDHDVYRDEALNNR Fuc 1 Hex 6 HexNAc 5

**Mass:**  4184.6848

(7.546e-07)

**Charge States:** 4

**Start Position**: 483

**End Position**: 500

| Profile Score | Tandem Score | TDA q-value | Volume | Spectrum Matches |
| --- | --- | --- | --- | --- |
| 10.9881 | 125.3463 | 0.0000 | 5.5685e+08 | 3 |

**Adduct Signal Fractions**

- **Unmodified**: 5.5685e+08

### SEC(C)ITPN(N)GSIPNDKPFQNVNR Hex 9 HexNAc 2

**Mass:**  4250.7746

(1.587e-06)

**Charge States:** 3, 4

**Start Position**: 279

**End Position**: 300

| Profile Score | Tandem Score | TDA q-value | Volume | Spectrum Matches |
| --- | --- | --- | --- | --- |
| 17.0015 | 124.6955 | 0.0000 | 1.0006e+09 | 5 |

**Adduct Signal Fractions**

- **Unmodified**: 1.0006e+09

### N(N)GTYDHDVYRDEALN(D)NR Fuc 1 Hex 6 HexNAc 3

**Mass:**  3779.5153

(2.628e-04)

**Charge States:** 4

**Start Position**: 483

**End Position**: 500

| Profile Score | Tandem Score | TDA q-value | Volume | Spectrum Matches |
| --- | --- | --- | --- | --- |
| -6.0058 | 124.3364 | 0.0000 | 1.0579e+08 | 2 |

**Adduct Signal Fractions**

- **Unmodified**: 1.0579e+08

### N(N)GTYDHDVYRDEALN(D)NR Fuc 1 Hex 5 HexNAc 4

**Mass:**  3820.5400

(2.589e-04)

**Charge States:** 3, 4

**Start Position**: 483

**End Position**: 500

| Profile Score | Tandem Score | TDA q-value | Volume | Spectrum Matches |
| --- | --- | --- | --- | --- |
| 7.1967 | 124.1859 | 0.0000 | 1.4946e+09 | 9 |

**Adduct Signal Fractions**

- **Unmodified**: 1.4946e+09

### N(N)GTYDHDVYRDEALNNR Fuc 1 Hex 9 HexNAc 3

**Mass:**  4264.6520

(-7.547e-06)

**Charge States:** 3, 4

**Start Position**: 483

**End Position**: 500

| Profile Score | Tandem Score | TDA q-value | Volume | Spectrum Matches |
| --- | --- | --- | --- | --- |
| 11.9994 | 123.3330 | 0.0000 | 2.1595e+08 | 4 |

**Adduct Signal Fractions**

- **Unmodified**: 2.1595e+08

### N(N)GTYDHDVYRDEALNNR Fuc 3 Hex 6 HexNAc 5

**Mass:**  4476.8005

(8.580e-07)

**Charge States:** 4

**Start Position**: 483

**End Position**: 500

| Profile Score | Tandem Score | TDA q-value | Volume | Spectrum Matches |
| --- | --- | --- | --- | --- |
| -18.3475 | 121.9255 | 0.0000 | 9.7653e+07 | 1 |

**Adduct Signal Fractions**

- **Unmodified**: 9.7653e+07

### N(N)GTYDHDVYRDEALNNR Fuc 2 Hex 4 HexNAc 3

**Mass:**  3600.4777

(8.938e-07)

**Charge States:** 4

**Start Position**: 483

**End Position**: 500

| Profile Score | Tandem Score | TDA q-value | Volume | Spectrum Matches |
| --- | --- | --- | --- | --- |
| -2.9265 | 120.6436 | 0.0000 | 9.2910e+07 | 2 |

**Adduct Signal Fractions**

- **Unmodified**: 9.2910e+07

### N(N)GTYDHDVYR Fuc 2 Hex 8 HexNAc 3

**Mass:**  3436.2746

(-9.799e-06)

**Charge States:** 3

**Start Position**: 483

**End Position**: 493

| Profile Score | Tandem Score | TDA q-value | Volume | Spectrum Matches |
| --- | --- | --- | --- | --- |
| -16.8790 | 120.6279 | 0.0000 | 1.3900e+07 | 1 |

**Adduct Signal Fractions**

- **Unmodified**: 1.3900e+07

### SEC(C)ITPN(N)GSIPNDKPFQNVNR Fuc 2 Hex 3 HexNAc 4 Neu5Ac 1

**Mass:**  4267.8015

(-4.248e-06)

**Charge States:** 4

**Start Position**: 279

**End Position**: 300

| Profile Score | Tandem Score | TDA q-value | Volume | Spectrum Matches |
| --- | --- | --- | --- | --- |
| -18.6910 | 120.5018 | 0.0000 | 5.2242e+07 | 1 |

**Adduct Signal Fractions**

- **Unmodified**: 5.2242e+07

### N(N)GTYDHDVYRDEALNNR Fuc 2 Hex 5 HexNAc 3

**Mass:**  3762.5300

(6.902e-07)

**Charge States:** 4

**Start Position**: 483

**End Position**: 500

| Profile Score | Tandem Score | TDA q-value | Volume | Spectrum Matches |
| --- | --- | --- | --- | --- |
| -5.3834 | 119.8404 | 0.0000 | 7.4942e+07 | 1 |

**Adduct Signal Fractions**

- **Unmodified**: 7.4942e+07

### N(N)GTYDHDVYR Fuc 2 Hex 5 HexNAc 3

**Mass:**  2950.1497

(-8.101e-10)

**Charge States:** 3

**Start Position**: 483

**End Position**: 493

| Profile Score | Tandem Score | TDA q-value | Volume | Spectrum Matches |
| --- | --- | --- | --- | --- |
| -17.8135 | 118.8116 | 0.0000 | 8.8399e+06 | 1 |

**Adduct Signal Fractions**

- **Unmodified**: 8.8399e+06

### N(N)GTYDHDVYRDEALNNR Fuc 1 Hex 6 HexNAc 6

**Mass:**  4387.7634

(6.972e-07)

**Charge States:** 4

**Start Position**: 483

**End Position**: 500

| Profile Score | Tandem Score | TDA q-value | Volume | Spectrum Matches |
| --- | --- | --- | --- | --- |
| -3.4023 | 118.2348 | 0.0000 | 1.2251e+08 | 1 |

**Adduct Signal Fractions**

- **Unmodified**: 1.2251e+08

### SEC(C)ITPN(N)GSIPNDKPFQNVNR Hex 5 HexNAc 2

**Mass:**  3602.5658

(2.565e-06)

**Charge States:** 3, 4

**Start Position**: 279

**End Position**: 300

| Profile Score | Tandem Score | TDA q-value | Volume | Spectrum Matches |
| --- | --- | --- | --- | --- |
| -18.6800 | 118.0176 | 0.0000 | 4.5275e+07 | 1 |

**Adduct Signal Fractions**

- **Unmodified**: 4.5275e+07

### N(N)GTYDHDVYRDEALNNR Fuc 2 Hex 5 HexNAc 5

**Mass:**  4168.6892

(1.171e-06)

**Charge States:** 4

**Start Position**: 483

**End Position**: 500

| Profile Score | Tandem Score | TDA q-value | Volume | Spectrum Matches |
| --- | --- | --- | --- | --- |
| 11.3912 | 115.5813 | 0.0081 | 6.3992e+08 | 4 |

**Adduct Signal Fractions**

- **Unmodified**: 6.3992e+08

### YPALN(N)VTMPN(D)NEQFDK Hex 8 HexNAc 2

**Mass:**  3583.4544

(2.779e-04)

**Charge States:** 3

**Start Position**: 161

**End Position**: 177

| Profile Score | Tandem Score | TDA q-value | Volume | Spectrum Matches |
| --- | --- | --- | --- | --- |
| -5.9334 | 115.2300 | 0.0081 | 3.0732e+07 | 1 |

**Adduct Signal Fractions**

- **Unmodified**: 3.0732e+07

### N(N)GTYDHDVYRDEALN(D)NR Fuc 2 Hex 5 HexNAc 4

**Mass:**  3966.5991

(2.506e-04)

**Charge States:** 3, 4

**Start Position**: 483

**End Position**: 500

| Profile Score | Tandem Score | TDA q-value | Volume | Spectrum Matches |
| --- | --- | --- | --- | --- |
| 3.9248 | 114.7043 | 0.0081 | 1.0916e+09 | 5 |

**Adduct Signal Fractions**

- **Unmodified**: 1.0916e+09

### N(N)GTYDHDVYR Fuc 1 Hex 9 HexNAc 3

**Mass:**  3452.2699

(-9.467e-06)

**Charge States:** 3

**Start Position**: 483

**End Position**: 493

| Profile Score | Tandem Score | TDA q-value | Volume | Spectrum Matches |
| --- | --- | --- | --- | --- |
| -19.8023 | 113.9081 | 0.0081 | 1.4867e+07 | 1 |

**Adduct Signal Fractions**

- **Unmodified**: 1.4867e+07

### N(N)GTYDHDVYRDEALNNR Fuc 2 Hex 6 HexNAc 5

**Mass:**  4330.7424

(8.107e-07)

**Charge States:** 4

**Start Position**: 483

**End Position**: 500

| Profile Score | Tandem Score | TDA q-value | Volume | Spectrum Matches |
| --- | --- | --- | --- | --- |
| -4.5148 | 113.0971 | 0.0081 | 1.5430e+08 | 1 |

**Adduct Signal Fractions**

- **Unmodified**: 1.5430e+08

### N(N)GTYDHDVYRDEALNNR Fuc 1 Hex 9 HexNAc 4

**Mass:**  4467.7311

(-6.526e-06)

**Charge States:** 4

**Start Position**: 483

**End Position**: 500

| Profile Score | Tandem Score | TDA q-value | Volume | Spectrum Matches |
| --- | --- | --- | --- | --- |
| 5.1468 | 111.9923 | 0.0081 | 5.8650e+07 | 1 |

**Adduct Signal Fractions**

- **Unmodified**: 5.8650e+07

### N(N)GTYDHDVYRDEALN(D)NR Hex 6 HexNAc 3

**Mass:**  3633.4582

(2.736e-04)

**Charge States:** 4

**Start Position**: 483

**End Position**: 500

| Profile Score | Tandem Score | TDA q-value | Volume | Spectrum Matches |
| --- | --- | --- | --- | --- |
| -5.8625 | 107.9879 | 0.0081 | 6.5434e+07 | 1 |

**Adduct Signal Fractions**

- **Unmodified**: 6.5434e+07

### RGSN(N)SSFFSR Fuc 1 Hex 4 HexNAc 3

**Mass:**  2548.0397

(3.888e-04)

**Start Position**: 141

**End Position**: 151

| Profile Score | Tandem Score | TDA q-value | Volume | Spectrum Matches |
| --- | --- | --- | --- | --- |
| 0.0000 | 107.8376 | 0.0081 | 0.0000e+00 | 1 |

Adduct:

No Chromatogram Found

### N(N)GTYDHDVYR Fuc 1 Hex 4 HexNAc 3

**Mass:**  2643.0553

(3.847e-04)

**Start Position**: 483

**End Position**: 493

| Profile Score | Tandem Score | TDA q-value | Volume | Spectrum Matches |
| --- | --- | --- | --- | --- |
| 0.0000 | 106.0125 | 0.0081 | 0.0000e+00 | 1 |

Adduct:

No Chromatogram Found

### N(N)GTYDHDVYRDEALNNRFQIK Fuc 1 Hex 5 HexNAc 4

**Mass:**  4335.8682

(3.077e-06)

**Charge States:** 5, 6

**Start Position**: 483

**End Position**: 504

| Profile Score | Tandem Score | TDA q-value | Volume | Spectrum Matches |
| --- | --- | --- | --- | --- |
| -17.6101 | 105.4205 | 0.0081 | 2.4904e+07 | 1 |

**Adduct Signal Fractions**

- **Unmodified**: 2.4904e+07

### N(N)GTYDHDVYRDEALN(D)NR Fuc 1 Hex 5 HexNAc 5

**Mass:**  4023.6227

(2.473e-04)

**Charge States:** 3, 4

**Start Position**: 483

**End Position**: 500

| Profile Score | Tandem Score | TDA q-value | Volume | Spectrum Matches |
| --- | --- | --- | --- | --- |
| 8.6047 | 105.2978 | 0.0081 | 3.5550e+08 | 1 |

**Adduct Signal Fractions**

- **Unmodified**: 3.5550e+08

### N(N)GTYDHDVYRDEALNNR Fuc 2 Hex 8 HexNAc 3

**Mass:**  4248.6511

(-8.231e-06)

**Charge States:** 3, 4

**Start Position**: 483

**End Position**: 500

| Profile Score | Tandem Score | TDA q-value | Volume | Spectrum Matches |
| --- | --- | --- | --- | --- |
| -3.8219 | 104.7122 | 0.0081 | 2.2344e+08 | 1 |

**Adduct Signal Fractions**

- **Unmodified**: 2.2344e+08

### N(N)GTYDHDVYR Hex 5 HexNAc 4

**Mass:**  2861.1121

(1.838e-06)

**Charge States:** 3

**Start Position**: 483

**End Position**: 493

| Profile Score | Tandem Score | TDA q-value | Volume | Spectrum Matches |
| --- | --- | --- | --- | --- |
| -20.3196 | 104.2262 | 0.0116 | 1.6355e+07 | 1 |

**Adduct Signal Fractions**

- **Unmodified**: 1.6355e+07

### N(N)GTYDHDVYR Fuc 2 Hex 6 HexNAc 5

**Mass:**  3518.3589

(-6.462e-07)

**Charge States:** 3, 4

**Start Position**: 483

**End Position**: 493

| Profile Score | Tandem Score | TDA q-value | Volume | Spectrum Matches |
| --- | --- | --- | --- | --- |
| -20.1040 | 102.5243 | 0.0116 | 2.2964e+07 | 2 |

**Adduct Signal Fractions**

- **Unmodified**: 2.2964e+07

### HAVPN(N)GTIVK Hex 8 HexNAc 3 Neu5Ac 1

**Mass:**  3233.3290

(6.145e-04)

**Start Position**: 18

**End Position**: 28

| Profile Score | Tandem Score | TDA q-value | Volume | Spectrum Matches |
| --- | --- | --- | --- | --- |
| 0.0000 | 101.6817 | 0.0116 | 0.0000e+00 | 1 |

Adduct:

No Chromatogram Found

### N(N)GTYDHDVYR Hex 5 HexNAc 2

**Mass:**  2454.9570

(1.020e-06)

**Start Position**: 483

**End Position**: 493

| Profile Score | Tandem Score | TDA q-value | Volume | Spectrum Matches |
| --- | --- | --- | --- | --- |
| 0.0000 | 98.1618 | 0.0116 | 0.0000e+00 | 1 |

Adduct:

No Chromatogram Found

### RGSN(N)SSFFSR Fuc 1 Hex 6 HexNAc 6

**Mass:**  3480.3967

(1.729e-06)

**Charge States:** 3, 4

**Start Position**: 141

**End Position**: 151

| Profile Score | Tandem Score | TDA q-value | Volume | Spectrum Matches |
| --- | --- | --- | --- | --- |
| -5.1244 | 98.0365 | 0.0149 | 8.0469e+07 | 2 |

**Adduct Signal Fractions**

- **Unmodified**: 8.0469e+07

### RGSN(N)SSFFSR Fuc 1 Hex 5 HexNAc 7 Neu5Ac 3

**Mass:**  4394.7121

(1.423e-06)

**Charge States:** 3, 4

**Start Position**: 141

**End Position**: 151

| Profile Score | Tandem Score | TDA q-value | Volume | Spectrum Matches |
| --- | --- | --- | --- | --- |
| -17.5913 | 96.3782 | 0.0149 | 3.1895e+07 | 1 |

**Adduct Signal Fractions**

- **Unmodified**: 3.1895e+07

### ISIYWTIVKPGDILLIN(N)STGNLIAPR Fuc 2 Hex 4 HexNAc 4 Neu5Ac 1

**Mass:**  4910.3581

(-2.223e-06)

**Charge States:** 4

**Start Position**: 230

**End Position**: 256

| Profile Score | Tandem Score | TDA q-value | Volume | Spectrum Matches |
| --- | --- | --- | --- | --- |
| -4.6759 | 95.4413 | 0.0185 | 1.0895e+07 | 2 |

**Adduct Signal Fractions**

- **Unmodified**: 1.0895e+07

### N(N)GTYDHDVYRDEALN(D)NR Fuc 3 Hex 5 HexNAc 4

**Mass:**  4112.6520

(2.398e-04)

**Charge States:** 3, 4

**Start Position**: 483

**End Position**: 500

| Profile Score | Tandem Score | TDA q-value | Volume | Spectrum Matches |
| --- | --- | --- | --- | --- |
| 10.4625 | 94.8792 | 0.0185 | 3.7326e+08 | 3 |

**Adduct Signal Fractions**

- **Unmodified**: 3.7326e+08

### LGHHAVPN(N)GTIVK Fuc 1 Hex 5 HexNAc 5

**Mass:**  3314.4785

(3.042e-04)

**Start Position**: 15

**End Position**: 28

| Profile Score | Tandem Score | TDA q-value | Volume | Spectrum Matches |
| --- | --- | --- | --- | --- |
| 0.0000 | 94.0643 | 0.0295 | 0.0000e+00 | 1 |

Adduct:

No Chromatogram Found

### N(N)GTYDHDVYR Fuc 1 Hex 9 HexNAc 2

**Mass:**  3251.2210

(6.147e-04)

**Start Position**: 483

**End Position**: 493

| Profile Score | Tandem Score | TDA q-value | Volume | Spectrum Matches |
| --- | --- | --- | --- | --- |
| 0.0000 | 93.4596 | 0.0331 | 0.0000e+00 | 1 |

Adduct:

No Chromatogram Found

### SEC(C)ITPN(N)GSIPN(D)DKPFQNVNR Hex 9 HexNAc 2

**Mass:**  4251.7634

(2.342e-04)

**Charge States:** 3, 4

**Start Position**: 279

**End Position**: 300

| Profile Score | Tandem Score | TDA q-value | Volume | Spectrum Matches |
| --- | --- | --- | --- | --- |
| 6.8494 | 92.1967 | 0.0361 | 1.8534e+08 | 5 |

**Adduct Signal Fractions**

- **Unmodified**: 1.8534e+08

### SEC(C)ITPN(N)GSIPN(D)DKPFQNVNR Hex 7 HexNAc 2

**Mass:**  3927.6586

(2.561e-04)

**Charge States:** 3, 4

**Start Position**: 279

**End Position**: 300

| Profile Score | Tandem Score | TDA q-value | Volume | Spectrum Matches |
| --- | --- | --- | --- | --- |
| 11.0023 | 91.6018 | 0.0361 | 2.5987e+08 | 5 |

**Adduct Signal Fractions**

- **Unmodified**: 2.5987e+08

### N(N)GTYDHDVYR Hex 5 HexNAc 6

**Mass:**  3267.2752

(9.439e-07)

**Charge States:** 3

**Start Position**: 483

**End Position**: 493

| Profile Score | Tandem Score | TDA q-value | Volume | Spectrum Matches |
| --- | --- | --- | --- | --- |
| -18.8076 | 89.8644 | 0.0432 | 3.7165e+07 | 2 |

**Adduct Signal Fractions**

- **Unmodified**: 3.7165e+07

### SEC(C)ITPN(N)GSIPN(D)DKPFQNVNR Hex 8 HexNAc 2

**Mass:**  4089.7209

(2.452e-04)

**Charge States:** 3, 4

**Start Position**: 279

**End Position**: 300

| Profile Score | Tandem Score | TDA q-value | Volume | Spectrum Matches |
| --- | --- | --- | --- | --- |
| 10.8082 | 89.7396 | 0.0463 | 1.4732e+08 | 4 |

**Adduct Signal Fractions**

- **Unmodified**: 1.4732e+08

### cus|SWZNA|A/Switzerland/9715293/2013

**>cus|SWZNA|A/Switzerland/9715293/2013**  
 `MNPNQKIITIGSVSLTISTICFFMQIAILITTVTLHFKQYEFNSPPNNQVMLCEPTIIERNITEIVYLTNTTIEKEICPK  
PAEYRNWSKPQCGITGFAPFSKDNSIRLSAGGDIWVTREPYVSCDPDKCYQFALGQGTTLNNVHSNNTVRDRTPYRTLLM  
NELGVPFHLGTKQVCIAWSSSSCHDGKAWLHVCITGDDKNATASFIYNGRLVDSVVSWSKDILRTQESECVCINGTCTVV  
MTDGSASGKADTKILFIEEGKIVHTSTLSGSAQHVEECSCYPRYPGVRCVCRDNWKGSNRPIVDINIKDHSIVSSYVCSG  
LVGDTPRKNDSSSSSHCLDPNNEEGGHGVKGWAFDDGNDVWMGRTINETSRLGYETFKVIEGWSNPKSKLQTNRQVIVDR  
GDRSGYSGIFSVEGKSCINRCFYVELIRGRKEETEVLWTSNSIVVFCGTSGTYGTGSWPDGADLNLMPI`

| Site | Type | Count |
| --- | --- | --- |
| 329 | N-linked | 1 |
| 86 | N-linked | 8 |
| 367 | N-linked | 2 |
| 200 | N-linked | 1 |

| Mass | Sequence | MS_2_ Score | `q`-value | MS_1_ Score | Signal | Protein Coordinates | Tandem Scans | Start Time | Apex Time | End Time |
| --- | --- | --- | --- | --- | --- | --- | --- | --- | --- | --- |
| 2521.9923 | TIN(N)ETSR Hex 8 HexNAc 2 | 175.465 | 0.0 | -18.543 | 1.098e+07 | 364 - 371 | 5 | 13.97 | 13.97 | 13.97 |
| 3626.5218 | N(N)WSKPQC(C)GITGFAPFSK Hex 8 HexNAc 2 | 166.591 | 0.0 | 18.297 | 8.054e+08 | 85 - 102 | 5 | 43.23 | 43.40 | 44.06 |
| 3411.3956 | N(N)WSKPQC(C)GITGFAPF Hex 8 HexNAc 2 | 162.590 | 0.0 | -17.225 | 1.400e+08 | 85 - 100 | 3 | 51.96 | 51.96 | 52.13 |
| 3788.5744 | N(N)WSKPQC(C)GITGFAPFSK Hex 9 HexNAc 2 | 158.568 | 0.0 | -1.454 | 7.721e+08 | 85 - 102 | 4 | 43.23 | 43.40 | 43.57 |
| 3573.4443 | N(N)WSKPQC(C)GITGFAPF Hex 9 HexNAc 2 | 151.026 | 0.0 | -3.262 | 1.616e+08 | 85 - 100 | 3 | 51.79 | 51.96 | 52.13 |
| 3805.6002 | N(N)WSKPQC(C)GITGFAPFSK Fuc 2 Hex 3 HexNAc 4 Neu5Ac 1 | 148.614 | 0.0 | -18.193 | 4.802e+07 | 85 - 102 | 2 | 43.23 | 43.40 | 43.40 |
| 3464.4673 | N(N)WSKPQC(C)GITGFAPFSK Hex 7 HexNAc 2 | 143.838 | 0.0 | -17.309 | 5.252e+07 | 85 - 102 | 1 | 43.57 | 43.57 | 43.72 |
| 3590.4721 | N(N)WSKPQC(C)GITGFAPF Fuc 2 Hex 3 HexNAc 4 Neu5Ac 1 | 143.072 | 0.0 | -17.436 | 4.515e+07 | 85 - 100 | 1 | 51.79 | 51.96 | 51.96 |
| 3670.4731 | KN(N)DSSSSSHC(C)LDPNNEEGGHGVK Hex 5 HexNAc 2 | 139.171 | 0.0 | -19.855 | 6.333e+07 | 327 - 350 | 2 | 14.80 | 14.80 | 14.80 |
| 3318.3424 | TIN(N)ETSR Fuc 1 Hex 7 HexNAc 6 | 132.342 | 0.045 | -4.983 | 5.256e+07 | 364 - 371 | 1 | 19.54 | 19.86 | 19.86 |
| 4472.9025 | AWLHVC(C)ITGDDKN(N)ATASFIYNGR Hex 9 HexNAc 2 | 123.089 | 0.0 | -19.725 | 5.076e+06 | 187 - 210 | 1 | 46.52 | 46.69 | 46.69 |
| 3249.3389 | N(N)WSKPQC(C)GITGFAPF Hex 7 HexNAc 2 | 110.728 | 0.008 | -20.239 | 5.373e+06 | 85 - 100 | 2 | 52.13 | 52.13 | 52.30 |

### TIN(N)ETSR Hex 8 HexNAc 2

**Mass:**  2521.9923

(9.415e-07)

**Charge States:** 2

**Start Position**: 365

**End Position**: 372

| Profile Score | Tandem Score | TDA q-value | Volume | Spectrum Matches |
| --- | --- | --- | --- | --- |
| -18.5427 | 175.4654 | 0.0000 | 1.0980e+07 | 5 |

**Adduct Signal Fractions**

- **Unmodified**: 1.0980e+07

### N(N)WSKPQC(C)GITGFAPFSK Hex 8 HexNAc 2

**Mass:**  3626.5244

(3.540e-06)

**Charge States:** 3, 4

**Start Position**: 86

**End Position**: 103

| Profile Score | Tandem Score | TDA q-value | Volume | Spectrum Matches |
| --- | --- | --- | --- | --- |
| 18.2970 | 166.5911 | 0.0000 | 8.0542e+08 | 5 |

**Adduct Signal Fractions**

- **Unmodified**: 8.0542e+08

### N(N)WSKPQC(C)GITGFAPF Hex 8 HexNAc 2

**Mass:**  3411.3964

(3.514e-06)

**Charge States:** 2, 3, 4

**Start Position**: 86

**End Position**: 101

| Profile Score | Tandem Score | TDA q-value | Volume | Spectrum Matches |
| --- | --- | --- | --- | --- |
| -17.2251 | 162.5897 | 0.0000 | 1.4003e+08 | 3 |

**Adduct Signal Fractions**

- **Unmodified**: 1.4003e+08

### N(N)WSKPQC(C)GITGFAPFSK Hex 9 HexNAc 2

**Mass:**  3788.5731

(2.949e-06)

**Charge States:** 3, 4

**Start Position**: 86

**End Position**: 103

| Profile Score | Tandem Score | TDA q-value | Volume | Spectrum Matches |
| --- | --- | --- | --- | --- |
| -1.4535 | 158.5681 | 0.0000 | 7.7211e+08 | 4 |

**Adduct Signal Fractions**

- **Unmodified**: 7.7211e+08

### N(N)WSKPQC(C)GITGFAPF Hex 9 HexNAc 2

**Mass:**  3573.4477

(6.123e-07)

**Charge States:** 3

**Start Position**: 86

**End Position**: 101

| Profile Score | Tandem Score | TDA q-value | Volume | Spectrum Matches |
| --- | --- | --- | --- | --- |
| -3.2625 | 151.0264 | 0.0000 | 1.6155e+08 | 3 |

**Adduct Signal Fractions**

- **Unmodified**: 1.6155e+08

### N(N)WSKPQC(C)GITGFAPFSK Fuc 2 Hex 3 HexNAc 4 Neu5Ac 1

**Mass:**  3805.6015

(-3.946e-06)

**Charge States:** 4

**Start Position**: 86

**End Position**: 103

| Profile Score | Tandem Score | TDA q-value | Volume | Spectrum Matches |
| --- | --- | --- | --- | --- |
| -18.1926 | 148.6140 | 0.0000 | 4.8019e+07 | 2 |

**Adduct Signal Fractions**

- **Unmodified**: 4.8019e+07

### N(N)WSKPQC(C)GITGFAPFSK Hex 7 HexNAc 2

**Mass:**  3464.4680

(2.694e-06)

**Charge States:** 3

**Start Position**: 86

**End Position**: 103

| Profile Score | Tandem Score | TDA q-value | Volume | Spectrum Matches |
| --- | --- | --- | --- | --- |
| -17.3095 | 143.8381 | 0.0000 | 5.2519e+07 | 1 |

**Adduct Signal Fractions**

- **Unmodified**: 5.2519e+07

### N(N)WSKPQC(C)GITGFAPF Fuc 2 Hex 3 HexNAc 4 Neu5Ac 1

**Mass:**  3590.4754

(-6.338e-06)

**Charge States:** 3

**Start Position**: 86

**End Position**: 101

| Profile Score | Tandem Score | TDA q-value | Volume | Spectrum Matches |
| --- | --- | --- | --- | --- |
| -17.4363 | 143.0720 | 0.0000 | 4.5147e+07 | 1 |

**Adduct Signal Fractions**

- **Unmodified**: 4.5147e+07

### KN(N)DSSSSSHC(C)LDPNNEEGGHGVK Hex 5 HexNAc 2

**Mass:**  3670.4730

(9.228e-07)

**Charge States:** 4, 5

**Start Position**: 328

**End Position**: 351

| Profile Score | Tandem Score | TDA q-value | Volume | Spectrum Matches |
| --- | --- | --- | --- | --- |
| -19.8547 | 139.1712 | 0.0000 | 6.3334e+07 | 2 |

**Adduct Signal Fractions**

- **Unmodified**: 6.3334e+07

### TIN(N)ETSR Fuc 1 Hex 7 HexNAc 6

**Mass:**  3318.3411

(8.651e-06)

**Charge States:** 3

**Start Position**: 365

**End Position**: 372

| Profile Score | Tandem Score | TDA q-value | Volume | Spectrum Matches |
| --- | --- | --- | --- | --- |
| -4.9826 | 132.3420 | 0.0455 | 5.2558e+07 | 1 |

**Adduct Signal Fractions**

- **Unmodified**: 5.2558e+07

### AWLHVC(C)ITGDDKN(N)ATASFIYNGR Hex 9 HexNAc 2

**Mass:**  4472.9016

(4.535e-06)

**Charge States:** 4

**Start Position**: 188

**End Position**: 211

| Profile Score | Tandem Score | TDA q-value | Volume | Spectrum Matches |
| --- | --- | --- | --- | --- |
| -19.7251 | 123.0889 | 0.0000 | 5.0758e+06 | 1 |

**Adduct Signal Fractions**

- **Unmodified**: 5.0758e+06

### N(N)WSKPQC(C)GITGFAPF Hex 7 HexNAc 2

**Mass:**  3249.3374

(1.818e-06)

**Charge States:** 3

**Start Position**: 86

**End Position**: 101

| Profile Score | Tandem Score | TDA q-value | Volume | Spectrum Matches |
| --- | --- | --- | --- | --- |
| -20.2392 | 110.7284 | 0.0081 | 5.3731e+06 | 2 |

**Adduct Signal Fractions**

- **Unmodified**: 5.3731e+06

### tr|E1C041|E1C041\_CHICK

**>tr|E1C041|E1C041\_CHICK**  
 `MRTGAGEAVGVLCLVSLVAALDYKYHHSEELEAYLKEVHAAYPALTHLHSIGRSVEGRDLWVLVLGRFPTQHKIGIPEFK  
YVANMHGDETVGREILLHLIDHLVTNYGRDPVITRLLNNTRIHIMPTMNPDGFEATVVPDCYYSRGRYNKNGEDLNRNFP  
DAFENNNNLIQPETQAVINWIKNETFVLSANLHGGALVASYTFDNGNSVTGTSNGYSRSPDDDVFIHLAKTYSFNHASMY  
KGTGCDSKQTFPDGITNGYSWYQLEGGMQDYNYVWGQCFEITLELSCCKYPPAEQLEKFWRDNKVALVEYIKQVHLGVKG  
QVTDKNGNPIPNAIVEAKGRPHICPYRTNEHGEYFLLLLPGKYVINATVPGFKSMLKTVDIPDNTANFSALKQDFSFPEV  
SVKPRPASCPRTPLYQELERSSAAVKPTLHILVLVIVTISILK`

| Site | Type | Count |
| --- | --- | --- |
| 387 | N-linked | 1 |
| 366 | N-linked | 5 |

| Mass | Sequence | MS_2_ Score | `q`-value | MS_1_ Score | Signal | Protein Coordinates | Tandem Scans | Start Time | Apex Time | End Time |
| --- | --- | --- | --- | --- | --- | --- | --- | --- | --- | --- |
| 3234.3609 | YVIN(N)ATVPGFK Hex 10 HexNAc 2 | 158.646 | 0.0 | -18.193 | 5.301e+06 | 362 - 373 | 2 | 41.05 | 41.05 | 41.22 |
| 2910.2499 | YVIN(N)ATVPGFK Hex 8 HexNAc 2 | 150.734 | 0.0 | -18.841 | 1.684e+07 | 362 - 373 | 2 | 41.22 | 41.22 | 41.38 |
| 3072.3046 | YVIN(N)ATVPGFK Hex 9 HexNAc 2 | 149.211 | 0.0 | -2.359 | 5.673e+07 | 362 - 373 | 3 | 41.05 | 41.22 | 41.38 |
| 3089.3320 | YVIN(N)ATVPGFK Fuc 2 Hex 3 HexNAc 4 Neu5Ac 1 | 147.530 | 0.0 | -16.699 | 1.310e+07 | 362 - 373 | 1 | 41.05 | 41.22 | 41.22 |
| 3576.5348 | TVDIPDNTAN(N)FSALK Fuc 1 Hex 5 HexNAc 5 | 132.638 | 0.0 | -19.131 | 3.325e+06 | 377 - 392 | 2 | 40.37 | 40.54 | 40.54 |
| 3251.3893 | YVIN(N)ATVPGFK Fuc 2 Hex 4 HexNAc 4 Neu5Ac 1 | 111.954 | 0.008 | -19.248 | 7.740e+05 | 362 - 373 | 1 | 41.05 | 41.05 | 41.05 |

### YVIN(N)ATVPGFK Hex 10 HexNAc 2

**Mass:**  3234.3616

(4.466e-06)

**Charge States:** 3

**Start Position**: 363

**End Position**: 374

| Profile Score | Tandem Score | TDA q-value | Volume | Spectrum Matches |
| --- | --- | --- | --- | --- |
| -18.1935 | 158.6459 | 0.0000 | 5.3011e+06 | 2 |

**Adduct Signal Fractions**

- **Unmodified**: 5.3011e+06

### YVIN(N)ATVPGFK Hex 8 HexNAc 2

**Mass:**  2910.2519

(3.577e-06)

**Charge States:** 3

**Start Position**: 363

**End Position**: 374

| Profile Score | Tandem Score | TDA q-value | Volume | Spectrum Matches |
| --- | --- | --- | --- | --- |
| -18.8415 | 150.7337 | 0.0000 | 1.6838e+07 | 2 |

**Adduct Signal Fractions**

- **Unmodified**: 1.6838e+07

### YVIN(N)ATVPGFK Hex 9 HexNAc 2

**Mass:**  3072.3056

(1.724e-06)

**Charge States:** 3

**Start Position**: 363

**End Position**: 374

| Profile Score | Tandem Score | TDA q-value | Volume | Spectrum Matches |
| --- | --- | --- | --- | --- |
| -2.3592 | 149.2111 | 0.0000 | 5.6725e+07 | 3 |

**Adduct Signal Fractions**

- **Unmodified**: 5.6725e+07

### YVIN(N)ATVPGFK Fuc 2 Hex 3 HexNAc 4 Neu5Ac 1

**Mass:**  3089.3325

(-5.820e-06)

**Charge States:** 3

**Start Position**: 363

**End Position**: 374

| Profile Score | Tandem Score | TDA q-value | Volume | Spectrum Matches |
| --- | --- | --- | --- | --- |
| -16.6991 | 147.5296 | 0.0000 | 1.3104e+07 | 1 |

**Adduct Signal Fractions**

- **Unmodified**: 1.3104e+07

### TVDIPDNTAN(N)FSALK Fuc 1 Hex 5 HexNAc 5

**Mass:**  3576.5348

(5.204e-06)

**Charge States:** 3

**Start Position**: 378

**End Position**: 393

| Profile Score | Tandem Score | TDA q-value | Volume | Spectrum Matches |
| --- | --- | --- | --- | --- |
| -19.1306 | 132.6382 | 0.0000 | 3.3250e+06 | 2 |

**Adduct Signal Fractions**

- **Unmodified**: 3.3250e+06

### YVIN(N)ATVPGFK Fuc 2 Hex 4 HexNAc 4 Neu5Ac 1

**Mass:**  3251.3893

(-3.341e-06)

**Charge States:** 3

**Start Position**: 363

**End Position**: 374

| Profile Score | Tandem Score | TDA q-value | Volume | Spectrum Matches |
| --- | --- | --- | --- | --- |
| -19.2477 | 111.9536 | 0.0081 | 7.7396e+05 | 1 |

**Adduct Signal Fractions**

- **Unmodified**: 7.7396e+05

### tr|A0A1D5PGT3|A0A1D5PGT3\_CHICK

**>tr|A0A1D5PGT3|A0A1D5PGT3\_CHICK**  
 `MVLPGPPGMARSEEVHRLTENVYKTIMEQFNPSLRNFISMGKTYEKALASMTYAAKGYFDALVKMGELASESQGSKELGD  
VLFQMAEVHRQIQNQLEEMLKSFHNELLTQLEQKVELDSRYLSAALKKYQTEQRNKGDSLDKCQAELKKLRKKSQGSKNP  
QKYSDKELQYIEAISNKQGELENYVSDGYKTALTEERRRFCFLVEKQCAVAKSAITYHAKGKEMLTQKLPLWQQSCSDPN  
KIPDRAIQLMQQMAASSNGTIMPSPLSTSKSNLIISDPIPGAKPLPVPPELAPFVGRMQEAMPAVNGVSGPDSDDYNHWS  
EVKPAQPKSLSPPQPQKQLSDSYSNTLPVRKNVTPKNSYASAENKTLPRSSSMAAGLERNGRTRVQAIFSHAAGDNSTLL  
SFKEGDLITLLVPEARDGWHYGESEKTKLRGWFPFSYTRVLDNDGSERVHASLQQGKSSSTGNLLDKDDIAIPPPDYGMS  
SQAFPSQNVNTFKQRPYSVAVPAFSQDYLMPYGCAIKDYSSGLCQPPPAHYKPYARSTAGLDDYGTRSVSSPDVEVARF`

| Site | Type | Count |
| --- | --- | --- |
| 352 | N-linked | 2 |

| Mass | Sequence | MS_2_ Score | `q`-value | MS_1_ Score | Signal | Protein Coordinates | Tandem Scans | Start Time | Apex Time | End Time |
| --- | --- | --- | --- | --- | --- | --- | --- | --- | --- | --- |
| 4425.7682 | KN(N)VTPK Fuc 2 Hex 10 HexNAc 9 | 152.468 | 0.0 | -19.936 | 4.067e+07 | 350 - 356 | 2 | 14.80 | 14.80 | 14.80 |
| 4279.7120 | KN(N)VTPK Fuc 1 Hex 10 HexNAc 9 | 147.014 | 0.0 | -17.864 | 8.390e+07 | 350 - 356 | 2 | 14.80 | 14.80 | 14.80 |

### KN(N)VTPK Fuc 2 Hex 10 HexNAc 9

**Mass:**  4425.7684

(-6.865e-07)

**Charge States:** 4, 5

**Start Position**: 351

**End Position**: 357

| Profile Score | Tandem Score | TDA q-value | Volume | Spectrum Matches |
| --- | --- | --- | --- | --- |
| -19.9357 | 152.4677 | 0.0000 | 4.0668e+07 | 2 |

**Adduct Signal Fractions**

- **Unmodified**: 4.0668e+07

### KN(N)VTPK Fuc 1 Hex 10 HexNAc 9

**Mass:**  4279.7112

(2.435e-07)

**Charge States:** 4, 5

**Start Position**: 351

**End Position**: 357

| Profile Score | Tandem Score | TDA q-value | Volume | Spectrum Matches |
| --- | --- | --- | --- | --- |
| -17.8644 | 147.0140 | 0.0000 | 8.3903e+07 | 2 |

**Adduct Signal Fractions**

- **Unmodified**: 8.3903e+07

### tr|A0A1D5P0L1|A0A1D5P0L1\_CHICK

**>tr|A0A1D5P0L1|A0A1D5P0L1\_CHICK**  
 `MVLPGPPGMARSEEVHRLTENVYKTIMEQFNPSLRNFISMGKTYEKALASMTYAAKGYFDALVKMGELASESQGSKELGD  
VLFQMAEVHRQIQNQLEEMLKSFHNELLTQLEQKVELDSRYLSAALKKYQTEQRNKGDSLDKCQAELKKLRKKSQGSKNP  
QKYSDKELQYIEAISNKQGELENYVSDGYKTALTEERRRFCFLVEKQCAVAKSAITYHAKGKEMLTQKLPLWQQSCSDPN  
KIPDRAIQLMQQMAASSNGTIMPSPLSTSKSNLIISDPIPGAKPLPVPPELAPFVGRMQEAMPAVNGVSGPDSDDYNHWS  
EVKPAQPKSLSPPQPQKQLSDSYSNTLPVRKNVTPKNSYASAENKTLPRSSSMAAGLERNGRTRVQAIFSHAAGDNSTLL  
SFKEGDLITLLVPEARDGWHYGESEKTKLRGWFPFSYTRVLDNDGSERVHASLQQGKSSSTGNLLDKDDIAIPPPDYGMS  
SQAFPSQNVNTFKQRPYSVAVPAFSQDYLMPYGCAIKDYSSGLCQPPPAHYKPYARSTAGLDDYGTRSVSRNPFANVQLK  
PTVTNDRSAPLIS`

| Site | Type | Count |
| --- | --- | --- |
| 352 | N-linked | 2 |

| Mass | Sequence | MS_2_ Score | `q`-value | MS_1_ Score | Signal | Protein Coordinates | Tandem Scans | Start Time | Apex Time | End Time |
| --- | --- | --- | --- | --- | --- | --- | --- | --- | --- | --- |
| 4425.7682 | KN(N)VTPK Fuc 2 Hex 10 HexNAc 9 | 152.468 | 0.0 | -19.936 | 4.067e+07 | 350 - 356 | 2 | 14.80 | 14.80 | 14.80 |
| 4279.7120 | KN(N)VTPK Fuc 1 Hex 10 HexNAc 9 | 147.014 | 0.0 | -17.864 | 8.390e+07 | 350 - 356 | 2 | 14.80 | 14.80 | 14.80 |

### KN(N)VTPK Fuc 2 Hex 10 HexNAc 9

**Mass:**  4425.7684

(-6.865e-07)

**Charge States:** 4, 5

**Start Position**: 351

**End Position**: 357

| Profile Score | Tandem Score | TDA q-value | Volume | Spectrum Matches |
| --- | --- | --- | --- | --- |
| -19.9357 | 152.4677 | 0.0000 | 4.0668e+07 | 2 |

**Adduct Signal Fractions**

- **Unmodified**: 4.0668e+07

### KN(N)VTPK Fuc 1 Hex 10 HexNAc 9

**Mass:**  4279.7112

(2.435e-07)

**Charge States:** 4, 5

**Start Position**: 351

**End Position**: 357

| Profile Score | Tandem Score | TDA q-value | Volume | Spectrum Matches |
| --- | --- | --- | --- | --- |
| -17.8644 | 147.0140 | 0.0000 | 8.3903e+07 | 2 |

**Adduct Signal Fractions**

- **Unmodified**: 8.3903e+07

### tr|A0A1D5PBT7|A0A1D5PBT7\_CHICK

**>tr|A0A1D5PBT7|A0A1D5PBT7\_CHICK**  
 `MVLPGPPGMARSEEVHRLTENVYKTIMEQFNPSLRNFISMGKTYEKALASMTYAAKGYFDALVKMGELASESQGSKELGD  
VLFQMAEVHRQIQNQLEEMLKSFHNELLTQLEQKVELDSRYLSAALKKYQTEQRNKGDSLDKCQAELKKLRKKSQGSKNP  
QKYSDKELQYIEAISNKQGELENYVSDGYKTALTEERRRFCFLVEKQCAVAKSAITYHAKGKEMLTQKLPLWQQSCSDPN  
KIPDRAIQLMQQMAASSNGTIMPSPLSTSKSNLIISDPIPGAKPLPVPPELAPFVGRMQEAMPAVNGVSGPDSDDYNHWS  
EVKPAQPKSLSPPQPQKQLSDSYSNTLPVRKNVTPKNSYASAENKTLPRSSSMAAGLERNGRTRVQAIFSHAAGDNSTLL  
SFKEGDLITLLVPEARDGWHYGESEKTKLRGWFPFSYTRVLDNDGSERVHASLQQGKSSSTGNLLDKDDIAIPPPDYGMS  
SQAFPSQNVNTFKQRPYSVAVPAFSQGLDDYGTRSVSSGSSSLVSTV`

| Site | Type | Count |
| --- | --- | --- |
| 352 | N-linked | 2 |

| Mass | Sequence | MS_2_ Score | `q`-value | MS_1_ Score | Signal | Protein Coordinates | Tandem Scans | Start Time | Apex Time | End Time |
| --- | --- | --- | --- | --- | --- | --- | --- | --- | --- | --- |
| 4425.7682 | KN(N)VTPK Fuc 2 Hex 10 HexNAc 9 | 152.468 | 0.0 | -19.936 | 4.067e+07 | 350 - 356 | 2 | 14.80 | 14.80 | 14.80 |
| 4279.7120 | KN(N)VTPK Fuc 1 Hex 10 HexNAc 9 | 147.014 | 0.0 | -17.864 | 8.390e+07 | 350 - 356 | 2 | 14.80 | 14.80 | 14.80 |

### KN(N)VTPK Fuc 2 Hex 10 HexNAc 9

**Mass:**  4425.7684

(-6.865e-07)

**Charge States:** 4, 5

**Start Position**: 351

**End Position**: 357

| Profile Score | Tandem Score | TDA q-value | Volume | Spectrum Matches |
| --- | --- | --- | --- | --- |
| -19.9357 | 152.4677 | 0.0000 | 4.0668e+07 | 2 |

**Adduct Signal Fractions**

- **Unmodified**: 4.0668e+07

### KN(N)VTPK Fuc 1 Hex 10 HexNAc 9

**Mass:**  4279.7112

(2.435e-07)

**Charge States:** 4, 5

**Start Position**: 351

**End Position**: 357

| Profile Score | Tandem Score | TDA q-value | Volume | Spectrum Matches |
| --- | --- | --- | --- | --- |
| -17.8644 | 147.0140 | 0.0000 | 8.3903e+07 | 2 |

**Adduct Signal Fractions**

- **Unmodified**: 8.3903e+07

### tr|A0A1D5PRN6|A0A1D5PRN6\_CHICK

**>tr|A0A1D5PRN6|A0A1D5PRN6\_CHICK**  
 `MVLPGPPGMARSEEVHRLTENVYKTIMEQFNPSLRNFISMGKTYEKALASMTYAAKGYFDALVKMGELASESQGSKELGD  
VLFQMAEVHRQIQNQLEEMLKSFHNELLTQLEQKVELDSRYLSAALKKYQTEQRNKGDSLDKCQAELKKLRKKSQGSKNP  
QKYSDKELQYIEAISNKQGELENYVSDGYKTALTEERRRFCFLVEKQCAVAKSAITYHAKGKEMLTQKLPLWQQSCSDPN  
KIPDRAIQLMQQMAASSNGTIMPSPLSTSKSNLIISDPIPGAKPLPVPPELAPFVGRMQEAMPAVNGVSGPDSDDYNHWS  
EVKPAQPKSLSPPQPQKQLSDSYSNTLPVRKNVTPKNSYASAENKTLPRSSSMAAGLERNGRTRVQAIFSHAAGDNSTLL  
SFKEGDLITLLVPEARDGWHYGESEKTKLRGWFPFSYTRVLDNDGSERVHASLQQGKSSSTGNLLDKDDIAIPPPDYGMS  
SQAFPSQNVNTFKQRPYSVAVPAFSQDYLMPYGCAIKDYSSGLCQPPPAHYKPYARSTAGLDDYGTRSVSSGSSSLVSTV`

| Site | Type | Count |
| --- | --- | --- |
| 352 | N-linked | 2 |

| Mass | Sequence | MS_2_ Score | `q`-value | MS_1_ Score | Signal | Protein Coordinates | Tandem Scans | Start Time | Apex Time | End Time |
| --- | --- | --- | --- | --- | --- | --- | --- | --- | --- | --- |
| 4425.7682 | KN(N)VTPK Fuc 2 Hex 10 HexNAc 9 | 152.468 | 0.0 | -19.936 | 4.067e+07 | 350 - 356 | 2 | 14.80 | 14.80 | 14.80 |
| 4279.7120 | KN(N)VTPK Fuc 1 Hex 10 HexNAc 9 | 147.014 | 0.0 | -17.864 | 8.390e+07 | 350 - 356 | 2 | 14.80 | 14.80 | 14.80 |

### KN(N)VTPK Fuc 2 Hex 10 HexNAc 9

**Mass:**  4425.7684

(-6.865e-07)

**Charge States:** 4, 5

**Start Position**: 351

**End Position**: 357

| Profile Score | Tandem Score | TDA q-value | Volume | Spectrum Matches |
| --- | --- | --- | --- | --- |
| -19.9357 | 152.4677 | 0.0000 | 4.0668e+07 | 2 |

**Adduct Signal Fractions**

- **Unmodified**: 4.0668e+07

### KN(N)VTPK Fuc 1 Hex 10 HexNAc 9

**Mass:**  4279.7112

(2.435e-07)

**Charge States:** 4, 5

**Start Position**: 351

**End Position**: 357

| Profile Score | Tandem Score | TDA q-value | Volume | Spectrum Matches |
| --- | --- | --- | --- | --- |
| -17.8644 | 147.0140 | 0.0000 | 8.3903e+07 | 2 |

**Adduct Signal Fractions**

- **Unmodified**: 8.3903e+07

### tr|A0A1D5P780|A0A1D5P780\_CHICK

**>tr|A0A1D5P780|A0A1D5P780\_CHICK**  
 `MRGIILALVLTLVDPGFNSRRSYLYNYEGSMLNGLQDRSLGKAGVRLSSKLEISGLPENAYLLKVRSPQVEEYNGVWPRD  
PFTRSSKITQVISSCFTRLFKFEYSSGRIGNIYAPEDCPDLCVNIVRGILNMFQMTIKKSQNVYELQEAGIGGICHARYV  
IQEDRKNSRIYVTRTVDLNNCQEKVQKSIGMAYIYPCPVDVMKERLTKGTTAFSYKLKQSDSGTLITDVSSRQVYQISPF  
NEPTGVAVMEARQQLTLVEVRSERGSAPDVPMQNYGSLRYRFPAVLPQMPLQLIKTKNPEQRIIETLQHIVLNNQQDFHD  
DVSYRFLEVVQLCRIANADNLESIWRQVSDKPRYRRWLLSAVSASGTTETLKFLKNRIRNDDLNYIQTLLTVSLTLHLLQ  
ADEHTLPIAADLMTSSRIQKNPVLQQVACLGYSSVVNRYCSQTSACPKEALQPIHDLADEAISRGREDKMKLALKCIGNM  
GEPASLKRILKFLPISSSSAADIPVHIQIDAITALKKIAWKDPKTVQGYLIQILADQSLPPEVRMMACAVIFETRPALAL  
ITTIANVAMKESNMQVASFVYSHMKSLSKSRLPFMYNISSACNIALKLLSPKLDSMSYRYSKVIRADTYFDNYRVGATGE  
IFVVNSPRTMFPSAIISKLMANSAGSVADLVEVGIRVEGLADVIMKRNIPFAEYPTYKQIKELGKALQGWKELPTETPLV  
SAYLKILGQEVAFININKELLQQVMKTVVEPADRNAAIKRIANQIRNSIAGQWTQPVWMGELRYVVPSCLGLPLEYGSYT  
TALARAAVSVEGKMTPPLTGDFRLSQLLESTMQIRSDLKPSLYVHTVATMGVNTEYFQHAVEIQGEVQTRMPMKFDAKID  
VKLKNLKIETNPCREETEIVVGRHKAFAVSRNIGELGVEKRTSILPEDAPLDVTEEPFQTSERASREHFAMQGPDSMPRK  
QSHSSREDLRRSTGKRAHKRDICLKMHHIGCQLCFSRRSRDASFIQNTYLHKLIGEHEAKIVLMPVHTDADIDKIQLEIQ  
AGSRAAARIITEVNPESEEEDESSPYEDIQAKLKRILGIDSMFKVANKTRHPKNRPSKKGNTVLAEFGTEPDAKTSSSSS  
SASSTATSSSSSSASSPNRKKPMDEEENDQVKQARNKDASSSSRSSKSSNSSKRSSSKSSNSSKRSSSSSSSSSSSSSSS  
SRSSSSSSSSSSNSKSSSSSSKSSSSSSRSRSSSKSSSSSSSSSSSSSSKSSSSRSSSSSSKSSSHHSHSHHSGHLNGSS  
SSSSSSRSVSHHSHEHHSGHLEDDSSSSSSSSVLSKIWGRHEIYQYRFRSAHRQEFPKRKLPGDRATSRYSSTRSSHDTS  
RAASWPKFLGDIKTPVLAAFLHGISNNKKTGGLQLVVYADTDSVRPRVQVFVTNLTDSSKWKLCADASVRNAHKAVAYVK  
WGWDCRDYKVSTELVTGRFAGHPAAQVKLEWPKVPSNVRSVVEWFYEFVPGAAFMLGFSERMDKNPSRQARMVVALTSPR  
TCDVVVKLPDMILYQKAVRLPLSLPVGPRIPASELQSPIWNVFAEAPSAVLENLKARCSVSYNKIKTFNEVKFNYSMPAN  
CYHILVQDCSSELKFLVMMKSAGEATNLKAINIKIGSHEIDMHPVNGQVKLLVDGAESPTANISLISAGASLWIHNENQG  
FALAAPGHGIDKLYFDGKTITIQVPLWMAGKTCGICGKYDAECEQEYRMPNGYLAKNAVSFGHSWILEEAPCRGACKLHR  
SFVKLEKTVQLAGVDSKCYSTEPVLRCAKGCSATKTTPVTVGFHCLPADSANSLTDKQMKYDQKSEDMQDTVDAHTACSC  
ENEECST`

| Site | Type | Count |
| --- | --- | --- |
| 1414 | N-linked | 2 |

| Mass | Sequence | MS_2_ Score | `q`-value | MS_1_ Score | Signal | Protein Coordinates | Tandem Scans | Start Time | Apex Time | End Time |
| --- | --- | --- | --- | --- | --- | --- | --- | --- | --- | --- |
| 2815.2520 | VQVFVTN(N)LTDSSK Hex 6 HexNAc 2 | 137.454 | 0.0 | -19.470 | 1.023e+06 | 1407 - 1420 | 1 | 37.15 | 37.15 | 37.15 |
| 2653.1969 | VQVFVTN(N)LTDSSK Hex 5 HexNAc 2 | 129.750 | 0.0 | -20.505 | 4.939e+05 | 1407 - 1420 | 1 | 37.32 | 37.32 | 37.32 |

### VQVFVTN(N)LTDSSK Hex 6 HexNAc 2

**Mass:**  2815.2520

(8.982e-06)

**Charge States:** 3

**Start Position**: 1408

**End Position**: 1421

| Profile Score | Tandem Score | TDA q-value | Volume | Spectrum Matches |
| --- | --- | --- | --- | --- |
| -19.4697 | 137.4540 | 0.0000 | 1.0234e+06 | 1 |

**Adduct Signal Fractions**

- **Unmodified**: 1.0234e+06

### VQVFVTN(N)LTDSSK Hex 5 HexNAc 2

**Mass:**  2653.1969

(8.622e-06)

**Charge States:** 3

**Start Position**: 1408

**End Position**: 1421

| Profile Score | Tandem Score | TDA q-value | Volume | Spectrum Matches |
| --- | --- | --- | --- | --- |
| -20.5046 | 129.7500 | 0.0000 | 4.9394e+05 | 1 |

**Adduct Signal Fractions**

- **Unmodified**: 4.9394e+05

### sp|P02845|VIT2\_CHICK

**>sp|P02845|VIT2\_CHICK**  
 `MRGIILALVLTLVGSQKFDIDPGFNSRRSYLYNYEGSMLNGLQDRSLGKAGVRLSSKLEISGLPENAYLLKVRSPQVEEY  
NGVWPRDPFTRSSKITQVISSCFTRLFKFEYSSGRIGNIYAPEDCPDLCVNIVRGILNMFQMTIKKSQNVYELQEAGIGG  
ICHARYVIQEDRKNSRIYVTRTVDLNNCQEKVQKSIGMAYIYPCPVDVMKERLTKGTTAFSYKLKQSDSGTLITDVSSRQ  
VYQISPFNEPTGVAVMEARQQLTLVEVRSERGSAPDVPMQNYGSLRYRFPAVLPQMPLQLIKTKNPEQRIVETLQHIVLN  
NQQDFHDDVSYRFLEVVQLCRIANADNLESIWRQVSDKPRYRRWLLSAVSASGTTETLKFLKNRIRNDDLNYIQTLLTVS  
LTLHLLQADEHTLPIAADLMTSSRIQKNPVLQQVACLGYSSVVNRYCSQTSACPKEALQPIHDLADEAISRGREDKMKLA  
LKCIGNMGEPASLKRILKFLPISSSSAADIPVHIQIDAITALKKIAWKDPKTVQGYLIQILADQSLPPEVRMMACAVIFE  
TRPALALITTIANVAMKESNMQVASFVYSHMKSLSKSRLPFMYNISSACNIALKLLSPKLDSMSYRYSKVIRADTYFDNY  
RVGATGEIFVVNSPRTMFPSAIISKLMANSAGSVADLVEVGIRVEGLADVIMKRNIPFAEYPTYKQIKELGKALQGWKEL  
PTETPLVSAYLKILGQEVAFININKELLQQVMKTVVEPADRNAAIKRIANQIRNSIAGQWTQPVWMGELRYVVPSCLGLP  
LEYGSYTTALARAAVSVEGKMTPPLTGDFRLSQLLESTMQIRSDLKPSLYVHTVATMGVNTEYFQHAVEIQGEVQTRMPM  
KFDAKIDVKLKNLKIETNPCREETEIVVGRHKAFAVSRNIGELGVEKRTSILPEDAPLDVTEEPFQTSERASREHFAMQG  
PDSMPRKQSHSSREDLRRSTGKRAHKRDICLKMHHIGCQLCFSRRSRDASFIQNTYLHKLIGEHEAKIVLMPVHTDADID  
KIQLEIQAGSRAAARIITEVNPESEEEDESSPYEDIQAKLKRILGIDSMFKVANKTRHPKNRPSKKGNTVLAEFGTEPDA  
KTSSSSSSASSTATSSSSSSASSPNRKKPMDEEENDQVKQARNKDASSSSRSSKSSNSSKRSSSKSSNSSKRSSSSSSSS  
SSSSRSSSSSSSSSSNSKSSSSSSKSSSSSSRSRSSSKSSSSSSSSSSSSSSKSSSSRSSSSSSKSSSHHSHSHHSGHLN  
GSSSSSSSSRSVSHHSHEHHSGHLEDDSSSSSSSSVLSKIWGRHEIYQYRFRSAHRQEFPKRKLPGDRATSRYSSTRSSH  
DTSRAASWPKFLGDIKTPVLAAFLHGISNNKKTGGLQLVVYADTDSVRPRVQVFVTNLTDSSKWKLCADASVRNAHKAVA  
YVKWGWDCRDYKVSTELVTGRFAGHPAAQVKLEWPKVPSNVRSVVEWFYEFVPGAAFMLGFSERMDKNPSRQARMVVALT  
SPRTCDVVVKLPDIILYQKAVRLPLSLPVGPRIPASELQPPIWNVFAEAPSAVLENLKARCSVSYNKIKTFNEVKFNYSM  
PANCYHILVQDCSSELKFLVMMKSAGEATNLKAINIKIGSHEIDMHPVNGQVKLLVDGAESPTANISLISAGASLWIHNE  
NQGFALAAPGHGIDKLYFDGKTITIQVPLWMAGKTCGICGKYDAECEQEYRMPNGYLAKNAVSFGHSWILEEAPCRGACK  
LHRSFVKLEKTVQLAGVDSKCYSTEPVLRCAKGCSATKTTPVTVGFHCLPADSANSLTDKQMKYDQKSEDMQDTVDAHTT  
CSCENEECST`

| Site | Type | Count |
| --- | --- | --- |
| 1417 | N-linked | 2 |

| Mass | Sequence | MS_2_ Score | `q`-value | MS_1_ Score | Signal | Protein Coordinates | Tandem Scans | Start Time | Apex Time | End Time |
| --- | --- | --- | --- | --- | --- | --- | --- | --- | --- | --- |
| 2815.2520 | VQVFVTN(N)LTDSSK Hex 6 HexNAc 2 | 137.454 | 0.0 | -19.470 | 1.023e+06 | 1410 - 1423 | 1 | 37.15 | 37.15 | 37.15 |
| 2653.1969 | VQVFVTN(N)LTDSSK Hex 5 HexNAc 2 | 129.750 | 0.0 | -20.505 | 4.939e+05 | 1410 - 1423 | 1 | 37.32 | 37.32 | 37.32 |

### VQVFVTN(N)LTDSSK Hex 6 HexNAc 2

**Mass:**  2815.2520

(8.982e-06)

**Charge States:** 3

**Start Position**: 1411

**End Position**: 1424

| Profile Score | Tandem Score | TDA q-value | Volume | Spectrum Matches |
| --- | --- | --- | --- | --- |
| -19.4697 | 137.4540 | 0.0000 | 1.0234e+06 | 1 |

**Adduct Signal Fractions**

- **Unmodified**: 1.0234e+06

### VQVFVTN(N)LTDSSK Hex 5 HexNAc 2

**Mass:**  2653.1969

(8.622e-06)

**Charge States:** 3

**Start Position**: 1411

**End Position**: 1424

| Profile Score | Tandem Score | TDA q-value | Volume | Spectrum Matches |
| --- | --- | --- | --- | --- |
| -20.5046 | 129.7500 | 0.0000 | 4.9394e+05 | 1 |

**Adduct Signal Fractions**

- **Unmodified**: 4.9394e+05

### tr|F1NFL6|F1NFL6\_CHICK

**>tr|F1NFL6|F1NFL6\_CHICK**  
 `MRGIILALVLTLVGSQKFDIDPGFNSRRSYLYNYEGSMLNGLQDRSLGKAGVRLSSKLEISGLPENAYLLKVRSPQVEEY  
NGVWPRDPFTRSSKITQVISSCFTRLFKFEYSSGRIGNIYAPEDCPDLCVNIVRGILNMFQMTIKKSQNVYELQEAGIGG  
ICHARYVIQEDRKNSRIYVTRTVDLNNCQEKVQKSIGMAYIYPCPVDVMKERLTKGTTAFSYKLKQSDSGTLITDVSSRQ  
VYQISPFNEPTGVAVMEARQQLTLVEVRSERGSAPDVPMQNYGSLRYRFPAVLPQMPLQLIKTKNPEQRIIETLQHIVLN  
NQQDFHDDVSYRFLEVVQLCRIANADNLESIWRQVSDKPRYRRWLLSAVSASGTTETLKFLKNRIRNDDLNYIQTLLTVS  
LTLHLLQADEHTLPIAADLMTSSRIQKNPVLQQVACLGYSSVVNRYCSQTSACPKEALQPIHDLADEAISRGREDKMKLA  
LKCIGNMGEPASLKRILKFLPISSSSAADIPVHIQIDAITALKKIAWKDPKTVQGYLIQILADQSLPPEVRMMACAVIFE  
TRPALALITTIANVAMKESNMQVASFVYSHMKSLSKSRLPFMYNISSACNIALKLLSPKLDSMSYRYSKVIRADTYFDNY  
RVGATGEIFVVNSPRTMFPSAIISKLMANSAGSVADLVEVGIRVEGLADVIMKRNIPFAEYPTYKQIKELGKALQGWKEL  
PTETPLVSAYLKILGQEVAFININKELLQQVMKTVVEPADRNAAIKRIANQIRNSIAGQWTQPVWMGELRYVVPSCLGLP  
LEYGSYTTALARAAVSVEGKMTPPLTGDFRLSQLLESTMQIRSDLKPSLYVHTVATMGVNTEYFQHAVEIQGEVQTRMPM  
KFDAKIDVKLKNLKIETNPCREETEIVVGRHKAFAVSRNIGELGVEKRTSILPEDAPLDVTEEPFQTSERASREHFAMQG  
PDSMPRKQSHSSREDLRRSTGKRAHKRDICLKMHHIGCQLCFSRRSRDASFIQNTYLHKLIGEHEAKIVLMPVHTDADID  
KIQLEIQAGSRAAARIITEVNPESEEEDESSPYEDIQAKLKRILGIDSMFKVANKTRHPKNRPSKKGNTVLAEFGTEPDA  
KTSSSSSSASSTATSSSSSSASSPNRKKPMDEEENDQVKQARNKDASSSSRSSKSSNSSKRSSSKSSNSSKRSSSSSSSS  
SSSSSSSSRSSSSSSSSSSNSKSSSSSSKSSSSSSRSRSSSKSSSSSSSSSSSSSSKSSSSRSSSSSSKSSSHHSHSHHS  
GHLNGSSSSSSSSRSVSHHSHEHHSGHLEDDSSSSSSSSVLSKIWGRHEIYQYRFRSAHRQEFPKRKLPGDRATSRYSST  
RSSHDTSRAASWPKFLGDIKTPVLAAFLHGISNNKKTGGLQLVVYADTDSVRPRVQVFVTNLTDSSKWKLCADASVRNAH  
KAVAYVKWGWDCRDYKVSTELVTGRFAGHPAAQVKLEWPKVPSNVRSVVEWFYEFVPGAAFMLGFSERMDKNPSRQARMV  
VALTSPRTCDVVVKLPDMILYQKAVRLPLSLPVGPRIPASELQSPIWNVFAEAPSAVLENLKARCSVSYNKIKTFNEVKF  
NYSMPANCYHILVQDCSSELKFLVMMKSAGEATNLKAINIKIGSHEIDMHPVNGQVKLLVDGAESPTANISLISAGASLW  
IHNENQGFALAAPGHGIDKLYFDGKTITIQVPLWMAGKTCGICGKYDAECEQEYRMPNGYLAKNAVSFGHSWILEEAPCR  
GACKLHRSFVKLEKTVQLAGVDSKCYSTEPVLRCAKGCSATKTTPVTVGFHCLPADSANSLTDKQMKYDQKSEDMQDTVD  
AHTACSCENEECST`

| Site | Type | Count |
| --- | --- | --- |
| 1421 | N-linked | 2 |

| Mass | Sequence | MS_2_ Score | `q`-value | MS_1_ Score | Signal | Protein Coordinates | Tandem Scans | Start Time | Apex Time | End Time |
| --- | --- | --- | --- | --- | --- | --- | --- | --- | --- | --- |
| 2815.2520 | VQVFVTN(N)LTDSSK Hex 6 HexNAc 2 | 137.454 | 0.0 | -19.470 | 1.023e+06 | 1414 - 1427 | 1 | 37.15 | 37.15 | 37.15 |
| 2653.1969 | VQVFVTN(N)LTDSSK Hex 5 HexNAc 2 | 129.750 | 0.0 | -20.505 | 4.939e+05 | 1414 - 1427 | 1 | 37.32 | 37.32 | 37.32 |

### VQVFVTN(N)LTDSSK Hex 6 HexNAc 2

**Mass:**  2815.2520

(8.982e-06)

**Charge States:** 3

**Start Position**: 1415

**End Position**: 1428

| Profile Score | Tandem Score | TDA q-value | Volume | Spectrum Matches |
| --- | --- | --- | --- | --- |
| -19.4697 | 137.4540 | 0.0000 | 1.0234e+06 | 1 |

**Adduct Signal Fractions**

- **Unmodified**: 1.0234e+06

### VQVFVTN(N)LTDSSK Hex 5 HexNAc 2

**Mass:**  2653.1969

(8.622e-06)

**Charge States:** 3

**Start Position**: 1415

**End Position**: 1428

| Profile Score | Tandem Score | TDA q-value | Volume | Spectrum Matches |
| --- | --- | --- | --- | --- |
| -20.5046 | 129.7500 | 0.0000 | 4.9394e+05 | 1 |

**Adduct Signal Fractions**

- **Unmodified**: 4.9394e+05

### tr|F1NV02|F1NV02\_CHICK

**>tr|F1NV02|F1NV02\_CHICK**  
 `MGPVQLLLLLLLLSSGVLTQEGTPENGNPGCSKDAARFKSLRKYVYLYEAETSSGITGTADSHSGSKITCKVELEVPQLC  
QFILRTMHCSLRETFGVDSERRAMLRKSKNSDDFANAMSKHELRFSTQDGTKVKLYPEKDEPLNVLNLKRGIISALLAPT  
ETEENIKTISMDTVYGKCDSEVEFKSRRGSVAEDISINRNLKACDNFSPIRDYVSPVAIVKGLNIPLSTLLSSTQSCHYS  
IDAKKKHIRDVVCSEKHLFLPSSYKNQYGMMTEVNQTLKLEDNQRMNNRNPDGDELEEKGLALESTDAKFSRQGDAVLKI  
LQELQKLTASQQNQQRAKLFYKFVSGLRSLHNSTLGSLVPKMMETSSSITIQALIQCGTPECYSAVLQILRTGNVNPLVV  
DLVTYTLGLLPSPTPKRIREILNMAQYQPSRASFYGLSHAVTKFYSEKMIVTEEITDVADFMVSLLGTDCSGDAELTYLT  
LRAIGNMGAVMEKAKPSLKASLKTCIRNQAASLSVQKAAIQAFRKMTITEEDRSALLKEFQEGDAPTDKRLATYLILMKN  
PSPADLAKIMRILTREKNEQVKSFVASHIANILDSDEVGIEDLKSHVEEALKGNEVPTAKDFRKFSQNYQVSKRVSVPGL  
NPISAKVEGNVIFDPSSYVPKETMLKTTLNLYGFGPSDIFELGLDGKGFEPTLEALFGEKGFFPDTASKALYWVDGKVPE  
QVSKALFDYFGYSHDGKQDQEITKAVILNLEKLIKELSKKEAPEGRAFLRILGEELGYMKLSDFKLLGSVALECIKTLQR  
IPEIIAQAISKGVDKDLFVHYMFMDNEFELPTGAGLQLKFALSGIVTPGAKVAVKLHQKSMQAELIAKPSVAVEFVTHLG  
INMPEFARSGVEMNSNIFHESGIEAHVSVKAGQLKFSIPAPKTPTKLLSISNTLHLVSPAKTEEIPPLIENREFSTSCKP  
FISGLNFCTKLLYSNASSMEAAPYYPLTGETRFEVEIVSTGEVKEYSASANYDLQREGTDLVDTLKFAVQAEGVKQHEAT  
LTFKYNRDRKILTSDVSIPDVDVDFGTNFRITDESVSGKKAYTFVIDFNNKKISEVTLTGQIRYAGIEEAMLRGTVSIPR  
LQTELKTEALVNYSPTKGYLQMSSSVGTHGNTVSKRVLLRYDSEKAELEWNSGATAAVGRMSSAFQVDFSDYSKTLEKYA  
NELLDRKVALTDMTMRHIVSQFIVATNTWLQKASKDVPYAQTLQAKLSGLQELNIQKIKLPVITIPEELFLKSEGRIKYS  
FNKNSFLINIPLPFGGRSSHDIRVPQTVKTPRLVIESMGINIPSQEYRMPTFTVPESYPLLVPLFGALEASASVHSNYYN  
WTAAYTLTNSSTEKTARIGTTYAVNADSVFELLSYNMKGSGEASSSRNGFTCAYENHLKHRLLTSDFKMSRTKSYEPTSV  
SNCTIFLMASSALGPQLSFSSDVVSEKTNNMNINNVRIEGQLEVASVFARSVYTMSSSYNEKRRVLEGKSNLRLDSSYLQ  
ATNHLSGRYTDGVFSITSASDVQNGLLKNTASLKYENSQLKITSETNGRYLHLAAVNKLEFLLSKKMAALRSEYQATYKQ  
TQCYALFTGSLNSQDLVFNTDFSLTDQRNRAAHKSSLNVNQYGLASSATTNVQFSPLTMQSEMNAKLDTSGGSVSLSSSG  
RYGKNNAKFNVGGRVSLTEITLGSEYQSTILGMDNKHVLNFRINKEGLKFSNNLQGSLKEIKLEYTNDLNIPGLSLTFVS  
KLDNSFSFDKFHKHVFDLQLQPRSLTAKLNNNIKYTKTEVSNKAELLLEPLKLNLGGNVRAAYGTDEVRHTYAITYADLT  
ANFKTDTVANVQGAAVSHRVNLNVAGLASSITMNTNCDSKSLRFSNALRSTMAPFTITADVHTNGNGKLIALGEHTGDLY  
SKILFKAEPLAFTFSHDYRGSTSHSFKSMRRYSTQLDNKFHMLFTPSEQSSAWKLKSQLNNNIYSQDINAYNDAEKIGVE  
LSGRALADLSVVDTAIRLPFTSEEVNVIDVLGLRDSVSEPQEFSISGSVKYDKNKDMHVINLPFLEHFPVYFEQIRGAIL  
STLQAVQNYLKNIDIDQYMKKYKATLDEFPQHLNDYMDKLDLKGRASTIKTNLIAFTKDYRITSDDLEIILEKALDNLQE  
ILLQLQVYLVQIEQYIKENYDQFDINALIAQLLDKIVEKMTALDEKYKIRVTVVDTIQKLQFFLNQYYPSNIGSSTMTWI  
KNIDDEYRITGRIKENLEQLKIQIQNIDIRSFAENLKRKIKITDVKQLLEKLKRSLPIKKMKEVLEQIKDFILSWMEEYE  
VSEKISAFRGHMHKLIVKYEIDKHVYFLLDRMIELLNQYRIRETVRKMTTYLRKIDLKTCFDKIVSLIDDAVKKVQTFDY  
EMMIKKLNKFLDMIIKKLKSFDYNQFVDDTNNKIQEIIQKINEELRNLELPQKAEALKQYMRDFNAVVSKYVEQLRDTKL  
VAIINWLKELIDSTTFTNLKAKVNEHLEDLRERISDMDISKEFEWYLQKISQFYNSVVIYISEQWNIAFKKIVTLAEKYD  
LKNWAENLNQFIETGFKVPEIRTVIVTIPAFEFSLRSLREATFRTPDFIVPLTDLKIPSYEINIRRLKDMKIPAKFTTPE  
FTVLNSFKVPSYTIDLNEIKFQIVRMIDQLISGEFQLPAIDLYFKDLKMRDMPFSEISFPELQMPQLEIPELLIPKLNLN  
EFQIPDLKIPEFQLPRIPHTVTAPTFGKLSGAFRVASPFFTLSTQAEVHNTTASANSPEFVTSLSAQATSKLDFLVFSVI  
ADSHILAPEMKQLKLKNSMKVSHKFLKIDHTNEVVFLGTSVSGEAETRAKFSAIKNSIELQNNLMVNLQRKIQMQSGTAY  
SHRLNIPEADFSSQADLVNNMTTEVEAGRISFTSNGKGNWKWTSPNFSDEGTHNSHATFRVDGPILIFFADYRINDRYLK  
VSQSMRYECGFLSYATLQVQSEIESQRVGRSILNVKGTGQLGGMKVELTGSHNARLNGRITGTVNNDFFFLVQPFEIRLL  
TNNDGNVKISFPMKLTGKIDFLNNYGLSLSSSVQQVSWQATGRFNQYRYSHNMSAGNNDDRIEAHVEMSGDANLDFLNIP  
LTIPQLHIPYTGIQTPQLKDYSLWEQAGLKNLLKTTRQSFDLNLNAQYEKNKDMHVIPLPLATVHEALNKYIIFFNKYFE  
RGRNTALDFLTKSYNEAKTKFDKYKIQTSLNKLPRTFRIPGYTIPIVNIEVSPFTAEMPAFGYVLPKEISTTGFTVPFIG  
FSVPSYTLVLPSLELPVLHVPQDLRTLKLPRFRINSPSNQILIPAMGNITYDFSFKSSVITLTANAGLFNQSDIAGHLSI  
SSSSVIDALQFKLDGSTSLTRKRGLKLATALSLNNNKFLGGSHDNSISLTKKNLEASMMTNAKINTPVFKMNFSQELSGN  
TKSKPTISSGLKVTYDFTTPKHGISAKGGVAHKLALETLTSYLSVETSTKGNIDGAIYTGNSFSGALDHEANTYLHANGV  
RSSLKLEANSKVDGLWNSEMKEILAVEASTSRVYAVWEHNGKNFARYTPLFTTTGSQKCKATFELAPWTVSADLQIQVTQ  
PNSFLDTASVNQVVLMKVSPTDQKVGWKGEGQIQSLSLRHDMQLSNEKSNAKFDISGSLEGYMDFLKRINCAISKKSLWD  
ILKLDVTTVADRKHYLNASASFIYRKSDDGYFFPMPVIRLSDGFTFSIPELHLKAPSPVLSTPEFRVPFSTLQVPAYTID  
LRNIKIPQTLNIMPFDVNLPTLPKLRFPRVDVGANYITLEEYKIPYFEVTVPEYQITVSQFTLPKSISLGSFHVDLDEVA  
NKIADFDLPTITIPEQKIEIPPLKVSLPAGIYIPSFGALTGSLKVASPLYNVTWRTDLTNKKESFEVSIDSTCSSTLQFL  
EYDLNVVSNYKYEEGKFVKKTVGSFAHRDLSANYIEDLALQGFGTVENTASLDVISPTFADVHVRYQMTDNMISSTVSSP  
SAGILGFLVQIETDTLKEKFYYRTLSAPQKDIDILKSEISFRNSDIIQIKLNWKEDAAKDLLLGLKEKVPKMTSAVYKCV  
NRYYKEHMGLDISDATVIMKNILQNNADKAYMFAARQVDQMDVQLRTAANEASEKYQEMKVKARQLYKRAAEQAEQIDYQ  
RIKARLLDATVDLMEEYHSKIRHLIDSVIEFLKTTKFQIPGLSEKYTGEELYLMATEKAAKTADICLSKLQEYFDALIAA  
INELEVRVPASETILRGRNVLDQIKEMLKHLQEKIRQTFVTLQEADFAGKLNQLKQVVQKTFQKAGNMVRSLQSKNFEDI  
KVQMQQLYKDAMASDYAHKLRSLAENVKKYISQMKNFSQKTLQKVSENLQQLVLYIKALREEYFDPTTLGWSVKYYEVED  
KVLGLLKNLMDTLVIWYNEYAKDLSDLVTRLTDQVRELVENYRQEYYDLITDVEGKGRQKVMELSSAAQEKIRYWSAVAK  
RKINEHNRQVKAKLQEIYGQLSDSQEKLINVAKMLIDLTVEKYSTFMKYIFELLRWFEQATADSIKPYIAVREGELRIDV  
PFDWEYINQMPQKSREALRNKVELTRALIQQGVEQGTRKWEEMQAFIDEQLATEQLSFQQIVENIQKRMKT`

| Site | Type | Count |
| --- | --- | --- |
| 3891 | N-linked | 1 |

| Mass | Sequence | MS_2_ Score | `q`-value | MS_1_ Score | Signal | Protein Coordinates | Tandem Scans | Start Time | Apex Time | End Time |
| --- | --- | --- | --- | --- | --- | --- | --- | --- | --- | --- |
| 2521.1143 | VASPLYN(N)VTWR Hex 5 HexNAc 2 | 119.753 | 0.0 | -20.150 | 1.317e+06 | 3884 - 3895 | 1 | 42.04 | 42.04 | 42.04 |

### VASPLYN(N)VTWR Hex 5 HexNAc 2

**Mass:**  2521.1143

(1.501e-06)

**Charge States:** 3

**Start Position**: 3885

**End Position**: 3896

| Profile Score | Tandem Score | TDA q-value | Volume | Spectrum Matches |
| --- | --- | --- | --- | --- |
| -20.1504 | 119.7525 | 0.0000 | 1.3168e+06 | 1 |

**Adduct Signal Fractions**

- **Unmodified**: 1.3168e+06

### tr|A0A1D5PNI3|A0A1D5PNI3\_CHICK

**>tr|A0A1D5PNI3|A0A1D5PNI3\_CHICK**  
 `MTTSTLAQTTSLPGTSTPTPASSTLMTTSTPAQTSLMPGHFKAQLLHLLHFTTTSTPAQTTSVPASSSPTPASSALPTAT  
TPAQTPGTSSPTPASSALPTTTTPAETTTFPATSSTTPASSAPPTTSTPAQTTSLPRTSTPTPASSALTTTTTPEQTTTV  
PGTSTPTPASSTVLTTSTPVQTTSMPGISSPTPASSALLTKSTPAQTTTVPATSSPTPASSALPTTTTPEQTTTESGTST  
PTPASSGPPTTSTPAETTSFPATSSPTPASSTLMTTSTPAQTTTVPGTSSPTPVSSALLTTRTLAQTKASLQLNSSPTSV  
SSALSTTSTPAETTTVPGTSIPTAASSTLLTTSTLAQTTSFPATSTPTPSSSTLMTTSTRAETKTSLQLNSSPTPASLAL  
PTTSTPAQTTSVPTSSSPTPASSALSTTSTPALTTALRSTSQIMSPSSPATSPSPTATSAGTVTTAQETTSPAGTTNALT  
STIRTTSTQTLSSSSPPYTTAALSYTAIPTSTPATPTTSAPQTTIASSSTPTESTALLTTTVPSTTQDISSQTAQTSNAA  
TSSTTNSATSARLTRTHAETTSSTTTFTSHSRPSASQTSTPGTTISPLPATTAQQTTAPAGTTPPTTASTTLFSTRETSS  
KEEETSTVVTSSQTPASSVLPSTSTPAQTTSMPSTSSPTPASSTLMTTSTLAQTTSLPGTSVPTPASSAPRTTTTPAQTT  
SVPGNSSPIPPYSTLLTTSTLAQTTSLPGTSTPTPASSTLMTTSTPAETKTSLLLNSSPNICIFCSSDYNHSCTDNISAR  
HFKPNSCTAIKHSCRKQTFITTFTSRARTSASWTTTPGTTTSALTATTARETTTPAGTTHPHNSNIETTSTSTIKAPTRP  
PTTPAGHERFTVNFTITNLPYDSDLQIPHSAKLNATQRVMTTLLNRLLKDTSIGPSFLGCATTAFR`

| Site | Type | Count |
| --- | --- | --- |
| 893 | N-linked | 1 |

| Mass | Sequence | MS_2_ Score | `q`-value | MS_1_ Score | Signal | Protein Coordinates | Tandem Scans | Start Time | Apex Time | End Time |
| --- | --- | --- | --- | --- | --- | --- | --- | --- | --- | --- |
| 4160.8531 | FTVN(N)FTITNLPYDSDLQIPHSAK Hex 7 HexNAc 2 | 115.652 | 0.008 | -18.022 | 1.310e+07 | 889 - 912 | 2 | 58.26 | 58.43 | 58.43 |

### FTVN(N)FTITNLPYDSDLQIPHSAK Hex 7 HexNAc 2

**Mass:**  4160.8552

(1.510e-06)

**Charge States:** 4

**Start Position**: 890

**End Position**: 913

| Profile Score | Tandem Score | TDA q-value | Volume | Spectrum Matches |
| --- | --- | --- | --- | --- |
| -18.0222 | 115.6520 | 0.0081 | 1.3103e+07 | 2 |

**Adduct Signal Fractions**

- **Unmodified**: 1.3103e+07

### tr|A0A1D5PUR1|A0A1D5PUR1\_CHICK

**>tr|A0A1D5PUR1|A0A1D5PUR1\_CHICK**  
 `MTTSTLAQTTSLPGTSTPTPASSTLMTTSTPAQTSLMPGHFKAQLLHLLHFTTTSTPAQTTSVPASSSPTPASSALPTAT  
TPAQTPGTSSPTPASSALPTTTTPAETTTFPATSSTTPASSAPPTTSTPAQTTSLPRTSTPTPASSALTTTTTPEQTTTV  
PGTSTPTPASSTVLTTSTPVQTTSMPGISSPTPASSALLTKSTPAQTTTVPATSSPTPASSALPTTTTPEQTTTESGTST  
PTPASSGPPTTSTPAETTSVPGTSYPNPASSSLMTTSTLAQTTSLPATSTPSLASSTLMTKSTFAPTTALPGTSTPTPAS  
SAPPTTSTPAQTTSVPGTSTATSASSTLLTTSTPAQTTSFPATSSPTPASSTLMTTSTPAQTTTVPGTSSPTPVSSALLT  
TRTLAQTKASLQLNSSPTSVSSALSTTSTPAETTTVPGTSIPTAASSTLLTTSTLAQTTSFPATSTPTPSSSTLMTTSTR  
AETKTSLQLNSSPTPASLALPTTSTPAQTTSVPTSSSPTPASSALSTTSTPALTTALRSTSQIMSPSSPATSPSPTATSA  
GTVTTAQETTSPAGTTNALTSTIRTTSTQTLSSSSPPYTTAALSYTAIPTSTPATPTTSAPQTTIASSSTPTESTALLTT  
TVPSTTQDISSQTAQTSNAATSSTTNSATSARLTRTHAETTSSTTTFTSHSRPSASQTSTPGTTISPLPATTAQQTTAPA  
GTTPPTTASTTLFSTRETSSKEEETSTVVTSSQTPASSVLPSTSTPAQTTSMPSTSSPTPASSTLMTTSTLAQTTSLPGT  
SVPTPASSAPRTTTTPAQTTSVPGNSSPIPPYSTLLTTSTLAQTTSLPGTSTPTPASSTLMTTSTPAETKTSLLLNSSPN  
ICIFCSSDYNHSCTDNISARHFKPNSCTAIKHSCRKQTFITTFTSRARTSASWTTTPGTTTSALTATTARETTTPAGTTH  
PHNSNIETTSTSTIKAPTRPPTTPAGHERFTVNFTITNLPYDSDLQIPHSAKLNATQRVMTTLLNRLLKDTSIGPSFLGC  
ATTAFR`

| Site | Type | Count |
| --- | --- | --- |
| 993 | N-linked | 1 |

| Mass | Sequence | MS_2_ Score | `q`-value | MS_1_ Score | Signal | Protein Coordinates | Tandem Scans | Start Time | Apex Time | End Time |
| --- | --- | --- | --- | --- | --- | --- | --- | --- | --- | --- |
| 4160.8531 | FTVN(N)FTITNLPYDSDLQIPHSAK Hex 7 HexNAc 2 | 115.652 | 0.008 | -18.022 | 1.310e+07 | 989 - 1012 | 2 | 58.26 | 58.43 | 58.43 |

### FTVN(N)FTITNLPYDSDLQIPHSAK Hex 7 HexNAc 2

**Mass:**  4160.8552

(1.510e-06)

**Charge States:** 4

**Start Position**: 990

**End Position**: 1013

| Profile Score | Tandem Score | TDA q-value | Volume | Spectrum Matches |
| --- | --- | --- | --- | --- |
| -18.0222 | 115.6520 | 0.0081 | 1.3103e+07 | 2 |

**Adduct Signal Fractions**

- **Unmodified**: 1.3103e+07
