# Supplementary material for: Measuring Site-specific Glycosylation Similarity between Influenza a Virus Variants with Statistical Certainty
Source: Mol Cell Proteomics. 2020 Nov 25;19(9):1533–45. doi: 10.1074/mcp.RA120.002031 (PMC8143645; doi:10.1074/mcp.RA120.002031)
Supplement: Supplementary file 2 [file mmc2.zip › mmc2/159250_2_supp_555292_qcjkl5.zip!/20190301_5B8eggL2_021119_tryp_HILIC_DDA_1ug_01_glycopeptides.html]

## 20190301\_5B8eggL2\_021119\_tryp\_HILIC\_DDA\_1ug\_01 @ GlycopeptideHypothesis-b78003cc67624f92867ef06e780b6aab

**MS^1^ Mass Accuracy:** 10.0 PPM  
**Grouping Tolerance:** 15.0 PPM  
**MS^n^ Mass Accuracy:** 20.0 PPM  
**Minimum `q`-Value For Clustering:** 0.05

**Glycopeptide Hypothesis:**
GlycopeptideHypothesis-b78003cc67624f92867ef06e780b6aab  
**Sample Searched:**
20190301\_5B8eggL2\_021119\_tryp\_HILIC\_DDA\_1ug\_01

| Protein | Matched Glycopeptides |
| --- | --- |
| cus\|SWZHA\_5B8\|A/Switzerland/9715293/2013 | 164 |
| cus\|SWZHA\|A/Switzerland/9715293/2013 | 146 |
| cus\|SWZNA\|A/Switzerland/9715293/2013 | 19 |
| tr\|E1C041\|E1C041\_CHICK | 4 |
| tr\|A0A1D5PGT3\|A0A1D5PGT3\_CHICK | 1 |
| tr\|A0A1D5P0L1\|A0A1D5P0L1\_CHICK | 1 |
| tr\|A0A1D5PBT7\|A0A1D5PBT7\_CHICK | 1 |
| tr\|A0A1D5PRN6\|A0A1D5PRN6\_CHICK | 1 |
| tr\|A0A1D5P780\|A0A1D5P780\_CHICK | 1 |
| sp\|P02845\|VIT2\_CHICK | 1 |
| tr\|F1NFL6\|F1NFL6\_CHICK | 1 |

### cus|SWZHA\_5B8|A/Switzerland/9715293/2013

**>cus|SWZHA\_5B8|A/Switzerland/9715293/2013**  
 `QKLPGNDNSTATLCLGHHAVPNGTIVKTITNDRIEVTNATELVQNSSIGEICDSPHQILDGENCTLIDALLGDPQCDGFQ  
NKKWDLFVERSKAYSNCYPYDVPDYASLRSLVASSGTLEFNNESFNWAGVTHNGTSSSCRRGSNSSFFSRLNWLTHLNSK  
YPALNVTMPNNEQFDKLYIWGVHHPVTDKDQIFLYAQSSGRITVSTKRSQQAVIPNIGSRPRIRNIPSRISIYWTIVKPG  
DILLINSTGNLIAPRGYFKIRSGKSSIMRSDAPIGKCKSECITPNGSIPNDKPFQNVNRITYGACPRYVKQSTLKLATGM  
RNVPERQTRGIFGAIAGFIENGWEGMVDGWYGFRHQNSEGRGQAADLKSTQAAIDQINGKLNRLIGKTNEKFHQIEKEFS  
EVEGRIQDLEKYVEDTKIDLWSYNAELLVALENQHTIDLTDSEMNKLFEKTKKQLRENAEDMGNGCFKIYHKCDNACIGS  
IRNGTYDHDVYRDEALNNRFQIKGVELKSGYKDWILWISFAISCFLLCVALLGFIMWACQKGNIRCNICI`

| Site | Type | Count |
| --- | --- | --- |
| 483 | N-linked | 55 |
| 165 | N-linked | 14 |
| 38 | N-linked | 1 |
| 144 | N-linked | 48 |
| 246 | N-linked | 15 |
| 133 | N-linked | 16 |
| 285 | N-linked | 14 |
| 22 | N-linked | 1 |

| Mass | Sequence | MS_2_ Score | `q`-value | MS_1_ Score | Signal | Protein Coordinates | Tandem Scans | Start Time | Apex Time | End Time |
| --- | --- | --- | --- | --- | --- | --- | --- | --- | --- | --- |
| 2547.0504 | RGSN(N)SSFFSR Fuc 1 Hex 4 HexNAc 3 | 192.551 | 0.0 | -4.996 | 5.388e+07 | 140 - 150 | 2 | 19.55 | 20.03 | 20.03 |
| 2522.0186 | RGSN(N)SSFFSR Hex 6 HexNAc 2 | 187.745 | 0.0 | -2.516 | 3.694e+08 | 140 - 150 | 3 | 19.06 | 19.39 | 19.55 |
| 2684.0732 | RGSN(N)SSFFSR Hex 7 HexNAc 2 | 187.187 | 0.0 | 12.351 | 1.951e+08 | 140 - 150 | 4 | 18.39 | 19.23 | 19.39 |
| 2871.1576 | RGSN(N)SSFFSR Fuc 1 Hex 6 HexNAc 3 | 182.895 | 0.0 | 14.803 | 2.509e+08 | 140 - 150 | 4 | 18.22 | 19.06 | 19.39 |
| 2912.1825 | RGSN(N)SSFFSR Fuc 1 Hex 5 HexNAc 4 | 181.650 | 0.0 | 14.114 | 6.666e+08 | 140 - 150 | 7 | 18.56 | 19.39 | 20.20 |
| 3115.2626 | RGSN(N)SSFFSR Fuc 1 Hex 5 HexNAc 5 | 180.569 | 0.0 | 14.508 | 1.563e+09 | 140 - 150 | 8 | 18.56 | 19.70 | 20.20 |
| 2359.9658 | RGSN(N)SSFFSR Hex 5 HexNAc 2 | 178.787 | 0.0 | 13.212 | 4.030e+08 | 140 - 150 | 4 | 18.90 | 19.70 | 20.03 |
| 2725.0980 | RGSN(N)SSFFSR Hex 6 HexNAc 3 | 178.109 | 0.0 | 14.634 | 4.578e+08 | 140 - 150 | 4 | 18.56 | 19.39 | 19.55 |
| 3436.4109 | YPALN(N)VTM(O)PNNEQFDK Hex 7 HexNAc 2 | 177.098 | 0.0 | 15.501 | 5.389e+08 | 160 - 176 | 5 | 35.14 | 35.47 | 35.81 |
| 3598.4682 | YPALN(N)VTM(O)PNNEQFDK Hex 8 HexNAc 2 | 176.212 | 0.0 | 3.140 | 2.675e+09 | 160 - 176 | 7 | 34.80 | 35.31 | 112.83 |
| 2563.0462 | RGSN(N)SSFFSR Hex 5 HexNAc 3 | 173.704 | 0.0 | -3.801 | 5.759e+07 | 140 - 150 | 1 | 19.55 | 19.70 | 19.86 |
| 3760.5158 | YPALN(N)VTM(O)PNNEQFDK Hex 9 HexNAc 2 | 172.800 | 0.0 | 14.596 | 2.315e+08 | 160 - 176 | 4 | 34.97 | 35.31 | 35.64 |
| 2709.1028 | RGSN(N)SSFFSR Fuc 1 Hex 5 HexNAc 3 | 170.682 | 0.0 | -3.753 | 7.876e+07 | 140 - 150 | 2 | 19.39 | 19.39 | 19.86 |
| 3058.2427 | RGSN(N)SSFFSR Fuc 2 Hex 5 HexNAc 4 | 170.114 | 0.0 | 14.584 | 2.337e+08 | 140 - 150 | 4 | 18.39 | 19.06 | 19.23 |
| 2766.1252 | RGSN(N)SSFFSR Hex 5 HexNAc 4 | 167.709 | 0.0 | -3.932 | 9.122e+07 | 140 - 150 | 2 | 19.39 | 19.55 | 20.03 |
| 2400.9928 | RGSN(N)SSFFSR Hex 4 HexNAc 3 | 166.588 | 0.0 | -19.581 | 1.368e+07 | 140 - 150 | 1 | 20.03 | 20.03 | 20.03 |
| 3582.5528 | YPALN(N)VTMPNNEQFDK Hex 8 HexNAc 2 | 166.026 | 0.0 | 12.696 | 3.046e+08 | 160 - 176 | 8 | 42.69 | 43.03 | 44.21 |
| 4085.9555 | TIVKPGDILLIN(N)STGNLIAPR Fuc 2 Hex 3 HexNAc 4 Neu5Ac 1 | 164.824 | 0.0 | -17.883 | 4.489e+07 | 234 - 255 | 1 | 53.73 | 53.73 | 53.90 |
| 4068.9306 | TIVKPGDILLIN(N)STGNLIAPR Hex 9 HexNAc 2 | 164.431 | 0.0 | 19.101 | 4.872e+08 | 234 - 255 | 5 | 53.21 | 53.73 | 54.41 |
| 3204.3002 | RGSN(N)SSFFSR Fuc 3 Hex 5 HexNAc 4 | 162.992 | 0.0 | -2.872 | 8.324e+07 | 140 - 150 | 2 | 18.73 | 18.90 | 19.06 |
| 3153.2315 | N(N)GTYDHDVYR Fuc 2 Hex 5 HexNAc 4 | 160.895 | 0.0 | 15.565 | 2.631e+08 | 482 - 492 | 5 | 14.50 | 14.50 | 15.14 |
| 3423.3728 | RGSN(N)SSFFSR Fuc 2 Hex 6 HexNAc 5 | 159.764 | 0.0 | -20.097 | 1.162e+07 | 140 - 150 | 1 | 18.56 | 18.73 | 18.73 |
| 3278.3221 | RGSN(N)SSFFSR Fuc 1 Hex 6 HexNAc 5 | 159.653 | 0.0 | 0.000 | 0.000e+00 | 140 - 150 | 1 | - | - | - |
| 4856.0269 | SLVASSGTLEFNNESFNWAGVTHN(N)GTSSSC(C)R Hex 7 HexNAc 2 | 159.440 | 0.0 | -1.662 | 5.457e+08 | 109 - 140 | 3 | 49.98 | 49.98 | 50.49 |
| 3906.8782 | TIVKPGDILLIN(N)STGNLIAPR Hex 8 HexNAc 2 | 159.423 | 0.0 | -3.316 | 5.211e+07 | 234 - 255 | 3 | 53.73 | 53.90 | 54.07 |
| 2953.2100 | RGSN(N)SSFFSR Fuc 1 Hex 4 HexNAc 5 | 158.502 | 0.0 | -4.283 | 5.371e+07 | 140 - 150 | 2 | 19.39 | 19.55 | 19.86 |
| 3261.3219 | RGSN(N)SSFFSR Fuc 2 Hex 5 HexNAc 5 | 158.360 | 0.0 | 13.968 | 3.148e+08 | 140 - 150 | 5 | 18.39 | 19.23 | 19.39 |
| 2527.9656 | GSN(N)SSFFSR Hex 7 HexNAc 2 | 158.330 | 0.0 | -5.120 | 3.999e+07 | 141 - 150 | 1 | 22.78 | 22.94 | 23.11 |
| 3420.4105 | YPALN(N)VTMPNNEQFDK Hex 7 HexNAc 2 | 155.533 | 0.0 | -3.237 | 4.252e+07 | 160 - 176 | 3 | 42.69 | 43.03 | 43.20 |
| 4376.8446 | C(C)KSEC(C)ITPN(N)GSIPNDKPFQNVNR Hex 8 HexNAc 2 | 155.184 | 0.0 | -3.881 | 1.376e+08 | 276 - 299 | 1 | 29.66 | 29.66 | 29.99 |
| 2756.0817 | GSN(N)SSFFSR Fuc 1 Hex 5 HexNAc 4 | 155.043 | 0.0 | 11.847 | 2.522e+08 | 141 - 150 | 2 | 22.94 | 22.94 | 23.90 |
| 4538.8973 | C(C)KSEC(C)ITPN(N)GSIPNDKPFQNVNR Hex 9 HexNAc 2 | 154.476 | 0.0 | -3.717 | 2.292e+08 | 276 - 299 | 3 | 29.50 | 29.66 | 29.83 |
| 3407.3813 | RGSN(N)SSFFSR Fuc 3 Hex 5 HexNAc 5 | 154.260 | 0.0 | -18.509 | 3.239e+07 | 140 - 150 | 3 | 18.73 | 18.90 | 18.90 |
| 2969.2045 | RGSN(N)SSFFSR Hex 5 HexNAc 5 | 154.224 | 0.0 | 12.946 | 2.980e+08 | 140 - 150 | 3 | 18.73 | 19.70 | 19.86 |
| 3778.5267 | N(N)GTYDHDVYRDEALNNR Fuc 1 Hex 6 HexNAc 3 | 153.311 | 0.0 | -3.624 | 2.971e+08 | 482 - 499 | 2 | 20.65 | 20.65 | 21.11 |
| 2928.1777 | RGSN(N)SSFFSR Hex 6 HexNAc 4 | 152.623 | 0.0 | 9.607 | 1.161e+08 | 140 - 150 | 2 | 19.23 | 19.39 | 20.03 |
| 3112.3065 | YPALN(N)VTM(O)PNNEQFDK Hex 5 HexNAc 2 | 151.800 | 0.0 | 12.942 | 3.132e+07 | 160 - 176 | 2 | 35.47 | 35.98 | 36.15 |
| 3007.1745 | N(N)GTYDHDVYR Fuc 1 Hex 5 HexNAc 4 | 151.798 | 0.0 | 16.144 | 4.201e+08 | 482 - 492 | 6 | 14.50 | 14.82 | 15.31 |
| 4214.7922 | C(C)KSEC(C)ITPN(N)GSIPNDKPFQNVNR Hex 7 HexNAc 2 | 151.758 | 0.0 | -17.484 | 2.394e+08 | 276 - 299 | 1 | 29.66 | 29.83 | 29.83 |
| 4731.2739 | ISIYWTIVKPGDILLIN(N)STGNLIAPR Hex 9 HexNAc 2 | 151.247 | 0.0 | 19.938 | 8.766e+08 | 229 - 255 | 10 | 68.58 | 69.36 | 69.85 |
| 3274.3575 | YPALN(N)VTM(O)PNNEQFDK Hex 6 HexNAc 2 | 150.623 | 0.0 | 12.604 | 4.201e+07 | 160 - 176 | 3 | 35.31 | 35.64 | 35.98 |
| 2365.9177 | GSN(N)SSFFSR Hex 6 HexNAc 2 | 150.460 | 0.0 | -3.698 | 1.255e+08 | 141 - 150 | 2 | 22.94 | 23.26 | 23.26 |
| 2846.1269 | RGSN(N)SSFFSR Hex 8 HexNAc 2 | 149.909 | 0.0 | -3.088 | 7.197e+07 | 140 - 150 | 1 | 18.90 | 19.06 | 19.23 |
| 5043.1077 | SLVASSGTLEFNNESFNWAGVTHN(N)GTSSSC(C)R Fuc 1 Hex 6 HexNAc 3 | 148.908 | 0.0 | -18.685 | 2.779e+07 | 109 - 140 | 1 | 49.81 | 49.98 | 49.98 |
| 4531.9187 | SLVASSGTLEFNNESFNWAGVTHN(N)GTSSSC(C)R Hex 5 HexNAc 2 | 148.811 | 0.0 | 15.727 | 1.735e+08 | 109 - 140 | 4 | 50.15 | 50.32 | 50.83 |
| 2203.8645 | GSN(N)SSFFSR Hex 5 HexNAc 2 | 148.743 | 0.0 | -2.280 | 2.013e+08 | 141 - 150 | 3 | 23.26 | 23.59 | 23.75 |
| 3017.2163 | RGSN(N)SSFFSR Fuc 2 Hex 6 HexNAc 3 | 147.861 | 0.0 | -17.894 | 1.807e+07 | 140 - 150 | 1 | 19.06 | 19.06 | 19.06 |
| 3074.2355 | RGSN(N)SSFFSR Fuc 1 Hex 6 HexNAc 4 | 147.736 | 0.0 | -5.259 | 3.861e+07 | 140 - 150 | 2 | 19.06 | 19.70 | 19.86 |
| 4893.3257 | ISIYWTIVKPGDILLIN(N)STGNLIAPR Hex 10 HexNAc 2 | 147.623 | 0.0 | -4.106 | 4.252e+07 | 229 - 255 | 4 | 69.02 | 69.19 | 69.53 |
| 3172.2854 | RGSN(N)SSFFSR Hex 5 HexNAc 6 | 147.236 | 0.0 | -2.092 | 2.472e+08 | 140 - 150 | 2 | 19.70 | 19.86 | 20.03 |
| 4569.2192 | ISIYWTIVKPGDILLIN(N)STGNLIAPR Hex 8 HexNAc 2 | 146.263 | 0.0 | 15.516 | 9.151e+07 | 229 - 255 | 5 | 69.02 | 69.36 | 69.70 |
| 2959.1623 | GSN(N)SSFFSR Fuc 1 Hex 5 HexNAc 5 | 144.682 | 0.0 | 13.094 | 6.069e+08 | 141 - 150 | 5 | 22.61 | 23.26 | 23.75 |
| 2855.1602 | RGSN(N)SSFFSR Fuc 2 Hex 5 HexNAc 3 | 143.885 | 0.0 | -17.295 | 2.300e+07 | 140 - 150 | 1 | 19.39 | 19.39 | 19.39 |
| 4748.2969 | ISIYWTIVKPGDILLIN(N)STGNLIAPR Fuc 2 Hex 3 HexNAc 4 Neu5Ac 1 | 143.225 | 0.0 | 19.502 | 1.505e+08 | 229 - 255 | 3 | 69.19 | 69.36 | 69.85 |
| 3899.5114 | N(N)GTYDHDVYRDEALNNR Fuc 1 Hex 8 HexNAc 2 | 142.507 | 0.0 | 13.853 | 6.512e+08 | 482 - 499 | 3 | 22.27 | 22.61 | 22.94 |
| 3965.6101 | N(N)GTYDHDVYRDEALNNR Fuc 2 Hex 5 HexNAc 4 | 141.997 | 0.0 | 17.083 | 2.403e+09 | 482 - 499 | 4 | 20.35 | 20.65 | 20.96 |
| 4230.9816 | TIVKPGDILLIN(N)STGNLIAPR Hex 10 HexNAc 2 | 141.435 | 0.0 | -19.163 | 1.652e+07 | 234 - 255 | 2 | 53.56 | 53.73 | 53.73 |
| 3627.6336 | PGDILLIN(N)STGNLIAPR Hex 9 HexNAc 2 | 141.327 | 0.0 | -18.503 | 2.537e+07 | 238 - 255 | 3 | 52.02 | 52.02 | 52.19 |
| 3267.3355 | N(N)GTYDHDVYRDEALNNR Hex 5 HexNAc 2 | 141.244 | 0.0 | -4.851 | 9.602e+07 | 482 - 499 | 1 | 20.96 | 21.11 | 21.28 |
| 4693.9707 | SLVASSGTLEFNNESFNWAGVTHN(N)GTSSSC(C)R Hex 6 HexNAc 2 | 140.999 | 0.0 | -3.454 | 1.326e+08 | 109 - 140 | 3 | 50.15 | 50.32 | 50.66 |
| 3356.3131 | N(N)GTYDHDVYR Fuc 2 Hex 5 HexNAc 5 | 140.452 | 0.0 | -19.137 | 5.374e+07 | 482 - 492 | 4 | 14.66 | 14.66 | 14.82 |
| 5141.1561 | SLVASSGTLEFNNESFNWAGVTHN(N)GTSSSC(C)R Hex 5 HexNAc 5 | 140.275 | 0.0 | 11.809 | 6.188e+07 | 109 - 140 | 3 | 49.98 | 49.98 | 50.66 |
| 3277.3155 | RGSN(N)SSFFSR Fuc 1 Hex 6 HexNAc 5 | 140.002 | 0.0 | -3.774 | 8.049e+07 | 140 - 150 | 3 | 18.73 | 18.90 | 19.23 |
| 3967.8799 | IVKPGDILLIN(N)STGNLIAPR Hex 9 HexNAc 2 | 138.589 | 0.0 | -18.347 | 9.414e+06 | 235 - 255 | 2 | 53.04 | 53.04 | 53.21 |
| 3008.3236 | HAVPN(N)GTIVK Fuc 1 Hex 5 HexNAc 5 | 138.124 | 0.0 | 0.000 | 0.000e+00 | 17 - 27 | 1 | - | - | - |
| 3454.4201 | N(N)GTYDHDVYRDEALNNR Fuc 1 Hex 4 HexNAc 3 | 137.699 | 0.0 | -3.000 | 8.758e+07 | 482 - 499 | 1 | 20.96 | 21.28 | 21.28 |
| 3777.5420 | YPALN(N)VTM(O)PNNEQFDK Fuc 2 Hex 3 HexNAc 4 Neu5Ac 1 | 137.455 | 0.0 | -18.197 | 1.653e+07 | 160 - 176 | 1 | 35.14 | 35.31 | 35.31 |
| 3299.2877 | N(N)GTYDHDVYR Fuc 3 Hex 5 HexNAc 4 | 136.394 | 0.0 | -19.073 | 8.878e+07 | 482 - 492 | 2 | 14.35 | 14.35 | 14.50 |
| 5262.1828 | SLVASSGTLEFNNESFNWAGVTHN(N)GTSSSC(C)R Hex 7 HexNAc 4 | 135.858 | 0.0 | 13.073 | 4.360e+07 | 109 - 140 | 3 | 49.63 | 49.98 | 50.32 |
| 3599.4518 | YPALN(N)VTM(O)PN(D)NEQFDK Hex 8 HexNAc 2 | 135.779 | 0.0 | 9.640 | 3.209e+08 | 160 - 176 | 27 | 35.81 | 36.15 | 37.34 |
| 4248.6497 | N(N)GTYDHDVYRDEALNNR Fuc 2 Hex 8 HexNAc 3 | 135.368 | 0.0 | 11.575 | 1.855e+08 | 482 - 499 | 2 | 21.77 | 22.44 | 22.78 |
| 3632.4656 | N(N)GTYDHDVYRDEALNNR Hex 6 HexNAc 3 | 135.262 | 0.0 | -3.901 | 2.274e+08 | 482 - 499 | 3 | 20.65 | 20.96 | 21.11 |
| 4572.9390 | SLVASSGTLEFNNESFNWAGVTHN(N)GTSSSC(C)R Hex 4 HexNAc 3 | 133.841 | 0.0 | -4.018 | 2.775e+07 | 109 - 140 | 1 | 50.32 | 50.49 | 50.66 |
| 3819.5501 | N(N)GTYDHDVYRDEALNNR Fuc 1 Hex 5 HexNAc 4 | 133.678 | 0.0 | 16.117 | 3.378e+09 | 482 - 499 | 6 | 20.50 | 20.80 | 21.28 |
| 3706.4150 | RGSN(N)SSFFSR Fuc 2 Hex 9 HexNAc 4 | 132.856 | 0.0 | 9.312 | 3.226e+07 | 140 - 150 | 2 | 21.11 | 21.28 | 21.77 |
| 3673.4923 | N(N)GTYDHDVYRDEALNNR Hex 5 HexNAc 4 | 132.477 | 0.0 | 13.013 | 1.752e+08 | 482 - 499 | 3 | 20.65 | 20.96 | 21.28 |
| 4088.7191 | SEC(C)ITPN(N)GSIPNDKPFQNVNR Hex 8 HexNAc 2 | 132.310 | 0.0 | 16.325 | 9.453e+08 | 278 - 299 | 4 | 32.45 | 32.61 | 33.11 |
| 3090.2327 | RGSN(N)SSFFSR Hex 7 HexNAc 4 | 132.124 | 0.0 | -19.496 | 1.752e+07 | 140 - 150 | 1 | 19.23 | 19.39 | 19.39 |
| 3518.3639 | N(N)GTYDHDVYR Fuc 2 Hex 6 HexNAc 5 | 131.894 | 0.0 | -19.755 | 2.006e+07 | 482 - 492 | 2 | 14.50 | 14.50 | 14.50 |
| 3926.6684 | SEC(C)ITPN(N)GSIPNDKPFQNVNR Hex 7 HexNAc 2 | 131.470 | 0.0 | 16.149 | 1.580e+09 | 278 - 299 | 6 | 32.45 | 32.61 | 33.11 |
| 2966.1475 | N(N)GTYDHDVYR Fuc 1 Hex 6 HexNAc 3 | 131.091 | 0.0 | -18.003 | 3.579e+07 | 482 - 492 | 1 | 14.50 | 14.50 | 14.66 |
| 4185.6732 | RGSN(N)SSFFSR Fuc 1 Hex 3 HexNAc 9 Neu5Ac 2 | 130.506 | 0.0 | -17.685 | 4.991e+07 | 140 - 150 | 1 | 21.77 | 21.77 | 21.94 |
| 4264.6437 | N(N)GTYDHDVYRDEALNNR Fuc 1 Hex 9 HexNAc 3 | 130.103 | 0.0 | -3.379 | 1.513e+08 | 482 - 499 | 2 | 22.27 | 22.27 | 22.78 |
| 2820.0893 | N(N)GTYDHDVYR Hex 6 HexNAc 3 | 129.951 | 0.0 | -18.914 | 1.997e+07 | 482 - 492 | 1 | 14.66 | 14.66 | 14.66 |
| 4022.6301 | N(N)GTYDHDVYRDEALNNR Fuc 1 Hex 5 HexNAc 5 | 129.623 | 0.0 | 10.621 | 2.208e+09 | 482 - 499 | 7 | 19.86 | 20.96 | 21.28 |
| 4250.7727 | SEC(C)ITPN(N)GSIPNDKPFQNVNR Hex 9 HexNAc 2 | 129.278 | 0.0 | -1.638 | 1.438e+09 | 278 - 299 | 3 | 32.45 | 32.45 | 32.94 |
| 3764.6174 | SEC(C)ITPN(N)GSIPNDKPFQNVNR Hex 6 HexNAc 2 | 129.272 | 0.0 | -2.891 | 2.213e+08 | 278 - 299 | 3 | 32.61 | 32.77 | 33.11 |
| 5018.0793 | SLVASSGTLEFNNESFNWAGVTHN(N)GTSSSC(C)R Hex 8 HexNAc 2 | 128.618 | 0.0 | -3.367 | 1.246e+08 | 109 - 140 | 3 | 49.81 | 49.98 | 50.32 |
| 4330.7424 | N(N)GTYDHDVYRDEALNNR Fuc 2 Hex 6 HexNAc 5 | 128.209 | 0.0 | 13.543 | 2.475e+08 | 482 - 499 | 1 | 20.35 | 20.50 | 20.96 |
| 3600.4777 | N(N)GTYDHDVYRDEALNNR Fuc 2 Hex 4 HexNAc 3 | 128.165 | 0.0 | -16.840 | 7.534e+07 | 482 - 499 | 2 | 20.96 | 20.96 | 21.11 |
| 2693.1077 | RGSN(N)SSFFSR Fuc 2 Hex 4 HexNAc 3 | 127.462 | 0.0 | -18.359 | 2.218e+07 | 140 - 150 | 1 | 19.70 | 19.70 | 19.70 |
| 3480.3978 | RGSN(N)SSFFSR Fuc 1 Hex 6 HexNAc 6 | 126.299 | 0.0 | -4.716 | 4.814e+07 | 140 - 150 | 3 | 18.73 | 19.23 | 19.23 |
| 4476.8006 | N(N)GTYDHDVYRDEALNNR Fuc 3 Hex 6 HexNAc 5 | 126.139 | 0.0 | -4.353 | 1.245e+08 | 482 - 499 | 1 | 20.35 | 20.50 | 20.65 |
| 5059.1046 | SLVASSGTLEFNNESFNWAGVTHN(N)GTSSSC(C)R Hex 7 HexNAc 3 | 125.576 | 0.0 | -4.270 | 4.729e+07 | 109 - 140 | 1 | 49.81 | 49.98 | 50.15 |
| 4048.6397 | RGSN(N)SSFFSR Fuc 1 Hex 7 HexNAc 8 | 125.486 | 0.0 | -18.144 | 5.510e+07 | 140 - 150 | 1 | 23.42 | 23.42 | 23.59 |
| 5298.5852 | NIPSRISIYWTIVKPGDILLIN(N)STGNLIAPR Hex 9 HexNAc 2 | 124.971 | 0.0 | -2.908 | 3.401e+07 | 224 - 255 | 4 | 67.08 | 67.39 | 67.56 |
| 4184.6844 | N(N)GTYDHDVYRDEALNNR Fuc 1 Hex 6 HexNAc 5 | 124.693 | 0.0 | 11.420 | 6.097e+08 | 482 - 499 | 3 | 20.50 | 20.65 | 21.11 |
| 3616.4721 | N(N)GTYDHDVYRDEALNNR Fuc 1 Hex 5 HexNAc 3 | 124.205 | 0.0 | 12.382 | 1.122e+08 | 482 - 499 | 2 | 20.80 | 21.11 | 21.44 |
| 6234.4947 | SLVASSGTLEFNNESFNWAGVTHN(N)GTSSSC(C)R Fuc 1 Hex 6 HexNAc 6 Neu5Ac 2 | 123.414 | 0.0 | -18.875 | 3.786e+07 | 109 - 140 | 2 | 46.74 | 46.74 | 46.91 |
| 3211.2617 | N(N)GTYDHDVYR Fuc 1 Hex 5 HexNAc 5 | 123.157 | 0.0 | 0.000 | 0.000e+00 | 482 - 492 | 1 | - | - | - |
| 3602.5666 | SEC(C)ITPN(N)GSIPNDKPFQNVNR Hex 5 HexNAc 2 | 122.741 | 0.0 | -4.235 | 6.902e+07 | 278 - 299 | 2 | 32.77 | 32.94 | 33.11 |
| 5567.7697 | IRNIPSRISIYWTIVKPGDILLIN(N)STGNLIAPR Hex 9 HexNAc 2 | 122.056 | 0.0 | 13.234 | 1.229e+08 | 222 - 255 | 5 | 64.00 | 64.20 | 64.61 |
| 4102.5902 | N(N)GTYDHDVYRDEALNNR Fuc 1 Hex 8 HexNAc 3 | 121.156 | 0.0 | 11.022 | 3.417e+08 | 482 - 499 | 5 | 21.60 | 22.61 | 23.11 |
| 3762.5291 | N(N)GTYDHDVYRDEALNNR Fuc 2 Hex 5 HexNAc 3 | 120.998 | 0.0 | -4.264 | 5.741e+07 | 482 - 499 | 1 | 20.80 | 20.80 | 21.11 |
| 4111.6681 | N(N)GTYDHDVYRDEALNNR Fuc 3 Hex 5 HexNAc 4 | 120.915 | 0.0 | 15.727 | 9.365e+08 | 482 - 499 | 3 | 20.35 | 20.50 | 20.96 |
| 3744.5159 | YPALN(N)VTMPNNEQFDK Hex 9 HexNAc 2 | 120.572 | 0.0 | -4.523 | 2.403e+07 | 160 - 176 | 3 | 42.69 | 42.86 | 43.03 |
| 3414.3042 | RGSN(N)SSFFSR Hex 9 HexNAc 4 | 120.212 | 0.0 | -4.653 | 1.582e+07 | 140 - 150 | 1 | 21.44 | 21.60 | 21.77 |
| 4897.0431 | SLVASSGTLEFNNESFNWAGVTHN(N)GTSSSC(C)R Hex 6 HexNAc 3 | 120.077 | 0.0 | -4.598 | 6.158e+07 | 109 - 140 | 1 | 49.98 | 50.15 | 50.32 |
| 3210.2535 | N(N)GTYDHDVYR Fuc 1 Hex 5 HexNAc 5 | 119.999 | 0.0 | 10.250 | 1.991e+08 | 482 - 492 | 6 | 14.82 | 14.98 | 15.81 |
| 4912.0910 | SEC(C)ITPN(N)GSIPNDKPFQNVNR Fuc 2 Hex 5 HexNAc 7 | 119.740 | 0.0 | -6.382 | 1.334e+06 | 278 - 299 | 1 | 72.45 | 72.66 | 72.72 |
| 4387.7631 | N(N)GTYDHDVYRDEALNNR Fuc 1 Hex 6 HexNAc 6 | 119.599 | 0.0 | 7.062 | 1.596e+08 | 482 - 499 | 2 | 20.03 | 20.80 | 20.96 |
| 4267.7988 | SEC(C)ITPN(N)GSIPNDKPFQNVNR Fuc 2 Hex 3 HexNAc 4 Neu5Ac 1 | 118.874 | 0.0 | -21.113 | 4.557e+07 | 278 - 299 | 1 | 32.45 | 32.45 | 32.61 |
| 3099.2658 | RGSN(N)SSFFSR Fuc 2 Hex 4 HexNAc 5 | 118.395 | 0.0 | -18.371 | 1.592e+07 | 140 - 150 | 1 | 19.06 | 19.23 | 19.23 |
| 3267.2739 | N(N)GTYDHDVYR Hex 5 HexNAc 6 | 117.749 | 0.0 | -5.679 | 2.985e+07 | 482 - 492 | 3 | 14.35 | 14.66 | 14.82 |
| 4168.6873 | N(N)GTYDHDVYRDEALNNR Fuc 2 Hex 5 HexNAc 5 | 117.118 | 0.0 | -3.208 | 4.334e+08 | 482 - 499 | 2 | 20.65 | 20.80 | 21.11 |
| 5205.1581 | SLVASSGTLEFNNESFNWAGVTHN(N)GTSSSC(C)R Fuc 1 Hex 7 HexNAc 3 | 115.507 | 0.0 | -18.875 | 1.155e+07 | 109 - 140 | 1 | 49.81 | 49.81 | 49.98 |
| 4743.2696 | TIVKPGDILLIN(N)STGNLIAPR Fuc 4 Hex 4 HexNAc 5 Neu5Ac 1 | 114.465 | 0.0 | 13.979 | 5.224e+07 | 234 - 255 | 6 | 72.66 | 73.30 | 73.75 |
| 3966.5985 | N(N)GTYDHDVYRDEALN(D)NR Fuc 2 Hex 5 HexNAc 4 | 114.372 | 0.0 | 6.385 | 6.913e+08 | 482 - 499 | 4 | 21.11 | 21.28 | 21.94 |
| 3779.5152 | N(N)GTYDHDVYRDEALN(D)NR Fuc 1 Hex 6 HexNAc 3 | 114.197 | 0.0 | -5.772 | 7.151e+07 | 482 - 499 | 2 | 21.28 | 21.28 | 21.94 |
| 4410.7024 | N(N)GTYDHDVYRDEALNNR Fuc 2 Hex 9 HexNAc 3 | 113.309 | 0.0 | -4.357 | 1.058e+08 | 482 - 499 | 2 | 22.11 | 22.27 | 22.61 |
| 2861.1179 | N(N)GTYDHDVYR Hex 5 HexNAc 4 | 112.854 | 0.0 | -19.169 | 1.546e+07 | 482 - 492 | 1 | 14.66 | 14.82 | 14.82 |
| 4857.0118 | SLVASSGTLEFN(D)NESFNWAGVTHN(N)GTSSSC(C)R Hex 7 HexNAc 2 | 112.727 | 0.0 | 11.318 | 5.288e+07 | 109 - 140 | 4 | 49.81 | 51.51 | 51.68 |
| 3437.3979 | YPALN(N)VTM(O)PN(D)NEQFDK Hex 7 HexNAc 2 | 112.643 | 0.0 | 10.605 | 5.022e+07 | 160 - 176 | 3 | 35.81 | 36.15 | 37.00 |
| 3128.2003 | N(N)GTYDHDVYR Fuc 1 Hex 7 HexNAc 3 | 112.578 | 0.0 | -19.624 | 5.275e+06 | 482 - 492 | 1 | 14.35 | 14.35 | 14.35 |
| 3940.5839 | N(N)GTYDHDVYRDEALNNR Fuc 1 Hex 7 HexNAc 3 | 112.202 | 0.0 | -19.016 | 7.130e+07 | 482 - 499 | 1 | 20.50 | 20.65 | 20.65 |
| 3633.4552 | N(N)GTYDHDVYRDEALN(D)NR Hex 6 HexNAc 3 | 112.192 | 0.0 | -5.940 | 5.836e+07 | 482 - 499 | 1 | 21.44 | 21.44 | 22.11 |
| 3372.3056 | N(N)GTYDHDVYR Fuc 1 Hex 6 HexNAc 5 | 112.191 | 0.0 | -4.325 | 6.090e+07 | 482 - 492 | 4 | 14.50 | 14.66 | 14.98 |
| 2788.0980 | N(N)GTYDHDVYR Fuc 2 Hex 4 HexNAc 3 | 111.725 | 0.0 | -18.684 | 5.185e+06 | 482 - 492 | 1 | 14.82 | 14.82 | 14.82 |
| 4467.7224 | N(N)GTYDHDVYRDEALNNR Fuc 1 Hex 9 HexNAc 4 | 111.523 | 0.0 | 8.819 | 5.121e+07 | 482 - 499 | 1 | 21.94 | 22.44 | 22.61 |
| 3318.3409 | RGSN(N)SSFFSR Fuc 1 Hex 5 HexNAc 6 | 110.892 | 0.0 | -5.076 | 4.413e+07 | 140 - 150 | 1 | 19.23 | 19.39 | 19.55 |
| 3794.5221 | N(N)GTYDHDVYRDEALNNR Hex 7 HexNAc 3 | 109.697 | 0.0 | -19.662 | 4.700e+07 | 482 - 499 | 2 | 20.65 | 20.80 | 20.80 |
| 4314.7491 | N(N)GTYDHDVYRDEALNNR Fuc 3 Hex 5 HexNAc 5 | 108.365 | 0.0 | -18.494 | 7.415e+07 | 482 - 499 | 2 | 20.50 | 20.65 | 20.65 |
| 4137.6801 | RGSN(N)SSFFSR Fuc 3 Hex 7 HexNAc 7 | 107.776 | 0.0 | -20.476 | 3.167e+07 | 140 - 150 | 1 | 22.94 | 23.11 | 23.11 |
| 3258.3573 | YPALN(N)VTMPNNEQFDK Hex 6 HexNAc 2 | 107.258 | 0.004 | -20.347 | 2.144e+06 | 160 - 176 | 1 | 43.20 | 43.20 | 43.37 |
| 3575.3869 | N(N)GTYDHDVYR Fuc 1 Hex 6 HexNAc 6 | 106.451 | 0.007 | -19.577 | 1.388e+07 | 482 - 492 | 2 | 14.82 | 14.98 | 14.98 |
| 4533.8258 | N(N)GTYDHDVYRDEALNNR Fuc 2 Hex 6 HexNAc 6 | 106.025 | 0.007 | -19.646 | 4.795e+07 | 482 - 499 | 1 | 20.65 | 20.65 | 20.80 |
| 3761.5413 | YPALN(N)VTMPNNEQFDK Fuc 2 Hex 3 HexNAc 4 Neu5Ac 1 | 103.970 | 0.007 | -19.774 | 2.661e+06 | 160 - 176 | 1 | 42.86 | 42.86 | 42.86 |
| 4046.5557 | RGSN(N)SSFFSR Fuc 2 Hex 5 HexNAc 6 Neu5Ac 2 | 103.533 | 0.007 | 10.381 | 1.062e+08 | 140 - 150 | 1 | 22.94 | 22.94 | 23.75 |
| 3820.5389 | N(N)GTYDHDVYRDEALN(D)NR Fuc 1 Hex 5 HexNAc 4 | 102.764 | 0.007 | -5.663 | 1.046e+09 | 482 - 499 | 19 | 21.44 | 21.44 | 22.11 |
| 4622.8581 | N(N)GTYDHDVYRDEALNNR Fuc 4 Hex 6 HexNAc 5 | 102.501 | 0.007 | -18.767 | 3.892e+07 | 482 - 499 | 1 | 20.35 | 20.35 | 20.35 |
| 4910.3514 | ISIYWTIVKPGDILLIN(N)STGNLIAPR Fuc 2 Hex 4 HexNAc 4 Neu5Ac 1 | 102.212 | 0.007 | -19.634 | 8.561e+06 | 229 - 255 | 1 | 69.19 | 69.19 | 69.36 |
| 4549.8187 | N(N)GTYDHDVYRDEALNNR Fuc 1 Hex 7 HexNAc 6 | 101.785 | 0.007 | -20.935 | 3.493e+07 | 482 - 499 | 1 | 20.35 | 20.50 | 20.50 |
| 3991.6214 | RGSN(N)SSFFSR Fuc 2 Hex 7 HexNAc 7 | 100.584 | 0.010 | -19.641 | 7.578e+07 | 140 - 150 | 1 | 23.11 | 23.26 | 23.26 |
| 5100.1310 | SLVASSGTLEFNNESFNWAGVTHN(N)GTSSSC(C)R Hex 6 HexNAc 4 | 100.328 | 0.010 | -18.879 | 2.551e+07 | 109 - 140 | 2 | 49.98 | 50.15 | 50.15 |
| 3324.2985 | RGSN(N)SSFFSR Fuc 1 Hex 7 HexNAc 3 Neu5Ac 1 | 98.407 | 0.010 | -5.460 | 1.599e+07 | 140 - 150 | 2 | 22.27 | 22.61 | 22.61 |
| 4805.1179 | LNWLTHLNSKYPALN(N)VTM(O)PNNEQFDK Hex 8 HexNAc 2 | 98.054 | 0.010 | -17.763 | 3.871e+07 | 150 - 176 | 2 | 48.44 | 48.44 | 48.61 |
| 4045.5690 | RGSN(N)SSFFSR Hex 5 HexNAc 6 Neu5Ac 3 | 97.517 | 0.010 | 14.388 | 5.843e+08 | 140 - 150 | 3 | 22.11 | 22.44 | 22.78 |
| 4539.8795 | C(C)KSEC(C)ITPN(N)GSIPN(D)DKPFQNVNR Hex 9 HexNAc 2 | 96.720 | 0.010 | -6.384 | 4.437e+07 | 276 - 299 | 1 | 30.96 | 31.12 | 31.29 |
| 4733.2711 | ISIYWTIVKPGDILLIN(N)STGNLIAPR Hex 9 HexNAc 2 | 96.321 | 0.010 | 0.000 | 0.000e+00 | 229 - 255 | 1 | - | - | - |
| 3664.4196 | N(N)GTYDHDVYR Fuc 3 Hex 6 HexNAc 5 | 95.972 | 0.010 | -21.365 | 6.264e+06 | 482 - 492 | 1 | 14.35 | 14.35 | 14.35 |
| 2982.1401 | N(N)GTYDHDVYR Hex 7 HexNAc 3 | 95.710 | 0.010 | -20.322 | 6.969e+06 | 482 - 492 | 1 | 14.50 | 14.50 | 14.66 |
| 4089.7124 | SEC(C)ITPN(N)GSIPN(D)DKPFQNVNR Hex 8 HexNAc 2 | 95.656 | 0.010 | 9.532 | 1.815e+08 | 278 - 299 | 3 | 33.11 | 34.13 | 34.13 |
| 2642.0390 | N(N)GTYDHDVYR Fuc 1 Hex 4 HexNAc 3 | 92.993 | 0.016 | -19.506 | 3.824e+06 | 482 - 492 | 1 | 14.98 | 14.98 | 14.98 |
| 3927.6598 | SEC(C)ITPN(N)GSIPN(D)DKPFQNVNR Hex 7 HexNAc 2 | 92.958 | 0.016 | 11.693 | 3.346e+08 | 278 - 299 | 4 | 33.28 | 34.13 | 34.30 |
| 2454.9580 | N(N)GTYDHDVYR Hex 5 HexNAc 2 | 91.518 | 0.021 | -20.435 | 4.752e+06 | 482 - 492 | 1 | 14.82 | 14.82 | 14.82 |
| 4251.7638 | SEC(C)ITPN(N)GSIPN(D)DKPFQNVNR Hex 9 HexNAc 2 | 91.406 | 0.021 | 6.461 | 2.437e+08 | 278 - 299 | 4 | 33.11 | 33.96 | 34.13 |
| 2658.0419 | N(N)GTYDHDVYR Hex 5 HexNAc 3 | 90.960 | 0.021 | -20.001 | 3.641e+06 | 482 - 492 | 1 | 14.82 | 14.82 | 14.82 |
| 2546.1057 | IEVTN(N)ATELVQN Hex 5 HexNAc 2 | 89.036 | 0.021 | -19.921 | 1.456e+06 | 33 - 45 | 1 | 36.15 | 36.15 | 36.15 |
| 6072.4525 | SLVASSGTLEFNNESFNWAGVTHN(N)GTSSSC(C)R Fuc 1 Hex 5 HexNAc 6 Neu5Ac 2 | 88.099 | 0.024 | -18.833 | 1.706e+07 | 109 - 140 | 2 | 46.91 | 46.91 | 47.08 |
| 2887.1522 | RGSN(N)SSFFSR Hex 7 HexNAc 3 | 86.000 | 0.027 | -18.669 | 1.089e+07 | 140 - 150 | 1 | 19.23 | 19.23 | 19.23 |
| 6396.5508 | SLVASSGTLEFNNESFNWAGVTHN(N)GTSSSC(C)R Fuc 1 Hex 7 HexNAc 6 Neu5Ac 2 | 84.418 | 0.035 | -3.781 | 4.683e+07 | 109 - 140 | 2 | 46.58 | 46.74 | 46.91 |
| 4008.5869 | N(N)GTYDHDVYR Fuc 2 Hex 4 HexNAc 9 | 83.428 | 0.041 | 0.000 | 0.000e+00 | 482 - 492 | 1 | - | - | - |
| 4112.6562 | N(N)GTYDHDVYRDEALN(D)NR Fuc 3 Hex 5 HexNAc 4 | 83.246 | 0.041 | -5.567 | 2.346e+08 | 482 - 499 | 2 | 21.11 | 21.11 | 21.77 |
| 4274.6618 | RGSN(N)SSFFSR Fuc 2 Hex 10 HexNAc 6 | 81.804 | 0.043 | -19.296 | 2.973e+06 | 140 - 150 | 1 | 24.87 | 24.87 | 24.87 |

### RGSN(N)SSFFSR Fuc 1 Hex 4 HexNAc 3

**Mass:**  2547.0510

(6.508e-07)

**Charge States:** 3

**Start Position**: 141

**End Position**: 151

| Profile Score | Tandem Score | TDA q-value | Volume | Spectrum Matches |
| --- | --- | --- | --- | --- |
| -4.9956 | 192.5506 | 0.0000 | 5.3877e+07 | 2 |

**Adduct Signal Fractions**

- **Unmodified**: 5.3877e+07

### RGSN(N)SSFFSR Hex 6 HexNAc 2

**Mass:**  2522.0185

(1.115e-06)

**Charge States:** 3

**Start Position**: 141

**End Position**: 151

| Profile Score | Tandem Score | TDA q-value | Volume | Spectrum Matches |
| --- | --- | --- | --- | --- |
| -2.5158 | 187.7451 | 0.0000 | 3.6936e+08 | 3 |

**Adduct Signal Fractions**

- **Unmodified**: 3.6936e+08

### RGSN(N)SSFFSR Hex 7 HexNAc 2

**Mass:**  2684.0733

(9.619e-07)

**Charge States:** 3

**Start Position**: 141

**End Position**: 151

| Profile Score | Tandem Score | TDA q-value | Volume | Spectrum Matches |
| --- | --- | --- | --- | --- |
| 12.3506 | 187.1874 | 0.0000 | 1.9512e+08 | 4 |

**Adduct Signal Fractions**

- **Unmodified**: 1.9512e+08

### RGSN(N)SSFFSR Fuc 1 Hex 6 HexNAc 3

**Mass:**  2871.1582

(1.072e-06)

**Charge States:** 3

**Start Position**: 141

**End Position**: 151

| Profile Score | Tandem Score | TDA q-value | Volume | Spectrum Matches |
| --- | --- | --- | --- | --- |
| 14.8032 | 182.8952 | 0.0000 | 2.5088e+08 | 4 |

**Adduct Signal Fractions**

- **Unmodified**: 2.5088e+08

### RGSN(N)SSFFSR Fuc 1 Hex 5 HexNAc 4

**Mass:**  2912.1817

(9.044e-07)

**Charge States:** 3

**Start Position**: 141

**End Position**: 151

| Profile Score | Tandem Score | TDA q-value | Volume | Spectrum Matches |
| --- | --- | --- | --- | --- |
| 14.1140 | 181.6497 | 0.0000 | 6.6662e+08 | 7 |

**Adduct Signal Fractions**

- **Unmodified**: 6.6662e+08

### RGSN(N)SSFFSR Fuc 1 Hex 5 HexNAc 5

**Mass:**  3115.2621

(5.407e-07)

**Charge States:** 3, 4

**Start Position**: 141

**End Position**: 151

| Profile Score | Tandem Score | TDA q-value | Volume | Spectrum Matches |
| --- | --- | --- | --- | --- |
| 14.5079 | 180.5694 | 0.0000 | 1.5630e+09 | 8 |

**Adduct Signal Fractions**

- **Unmodified**: 1.5630e+09

### RGSN(N)SSFFSR Hex 5 HexNAc 2

**Mass:**  2359.9654

(5.595e-07)

**Charge States:** 3

**Start Position**: 141

**End Position**: 151

| Profile Score | Tandem Score | TDA q-value | Volume | Spectrum Matches |
| --- | --- | --- | --- | --- |
| 13.2119 | 178.7873 | 0.0000 | 4.0297e+08 | 4 |

**Adduct Signal Fractions**

- **Unmodified**: 4.0297e+08

### RGSN(N)SSFFSR Hex 6 HexNAc 3

**Mass:**  2725.0977

(-1.898e-07)

**Charge States:** 3

**Start Position**: 141

**End Position**: 151

| Profile Score | Tandem Score | TDA q-value | Volume | Spectrum Matches |
| --- | --- | --- | --- | --- |
| 14.6345 | 178.1088 | 0.0000 | 4.5779e+08 | 4 |

**Adduct Signal Fractions**

- **Unmodified**: 4.5779e+08

### YPALN(N)VTM(O)PNNEQFDK Hex 7 HexNAc 2

**Mass:**  3436.4126

(3.463e-06)

**Charge States:** 2, 3, 4

**Start Position**: 161

**End Position**: 177

| Profile Score | Tandem Score | TDA q-value | Volume | Spectrum Matches |
| --- | --- | --- | --- | --- |
| 15.5006 | 177.0981 | 0.0000 | 5.3888e+08 | 5 |

**Adduct Signal Fractions**

- **Unmodified**: 5.3888e+08

### YPALN(N)VTM(O)PNNEQFDK Hex 8 HexNAc 2

**Mass:**  3598.4752

(2.875e-06)

**Charge States:** 2, 3, 4

**Start Position**: 161

**End Position**: 177

| Profile Score | Tandem Score | TDA q-value | Volume | Spectrum Matches |
| --- | --- | --- | --- | --- |
| 3.1404 | 176.2119 | 0.0000 | 2.6753e+09 | 7 |

**Adduct Signal Fractions**

- **Unmodified**: 2.6753e+09

### RGSN(N)SSFFSR Hex 5 HexNAc 3

**Mass:**  2563.0460

(6.692e-07)

**Charge States:** 3

**Start Position**: 141

**End Position**: 151

| Profile Score | Tandem Score | TDA q-value | Volume | Spectrum Matches |
| --- | --- | --- | --- | --- |
| -3.8011 | 173.7038 | 0.0000 | 5.7586e+07 | 1 |

**Adduct Signal Fractions**

- **Unmodified**: 5.7586e+07

### YPALN(N)VTM(O)PNNEQFDK Hex 9 HexNAc 2

**Mass:**  3760.5159

(2.292e-06)

**Charge States:** 3, 4

**Start Position**: 161

**End Position**: 177

| Profile Score | Tandem Score | TDA q-value | Volume | Spectrum Matches |
| --- | --- | --- | --- | --- |
| 14.5962 | 172.8004 | 0.0000 | 2.3152e+08 | 4 |

**Adduct Signal Fractions**

- **Unmodified**: 2.3152e+08

### RGSN(N)SSFFSR Fuc 1 Hex 5 HexNAc 3

**Mass:**  2709.1034

(3.935e-07)

**Charge States:** 3

**Start Position**: 141

**End Position**: 151

| Profile Score | Tandem Score | TDA q-value | Volume | Spectrum Matches |
| --- | --- | --- | --- | --- |
| -3.7535 | 170.6815 | 0.0000 | 7.8760e+07 | 2 |

**Adduct Signal Fractions**

- **Unmodified**: 7.8760e+07

### RGSN(N)SSFFSR Fuc 2 Hex 5 HexNAc 4

**Mass:**  3058.2427

(1.256e-06)

**Charge States:** 3

**Start Position**: 141

**End Position**: 151

| Profile Score | Tandem Score | TDA q-value | Volume | Spectrum Matches |
| --- | --- | --- | --- | --- |
| 14.5839 | 170.1136 | 0.0000 | 2.3374e+08 | 4 |

**Adduct Signal Fractions**

- **Unmodified**: 2.3374e+08

### RGSN(N)SSFFSR Hex 5 HexNAc 4

**Mass:**  2766.1254

(3.885e-07)

**Charge States:** 3

**Start Position**: 141

**End Position**: 151

| Profile Score | Tandem Score | TDA q-value | Volume | Spectrum Matches |
| --- | --- | --- | --- | --- |
| -3.9319 | 167.7085 | 0.0000 | 9.1216e+07 | 2 |

**Adduct Signal Fractions**

- **Unmodified**: 9.1216e+07

### RGSN(N)SSFFSR Hex 4 HexNAc 3

**Mass:**  2400.9928

(4.905e-07)

**Charge States:** 3

**Start Position**: 141

**End Position**: 151

| Profile Score | Tandem Score | TDA q-value | Volume | Spectrum Matches |
| --- | --- | --- | --- | --- |
| -19.5807 | 166.5880 | 0.0000 | 1.3682e+07 | 1 |

**Adduct Signal Fractions**

- **Unmodified**: 1.3682e+07

### YPALN(N)VTMPNNEQFDK Hex 8 HexNAc 2

**Mass:**  3582.4641

(1.475e-06)

**Charge States:** 3, 4

**Start Position**: 161

**End Position**: 177

| Profile Score | Tandem Score | TDA q-value | Volume | Spectrum Matches |
| --- | --- | --- | --- | --- |
| 12.6961 | 166.0260 | 0.0000 | 3.0460e+08 | 8 |

**Adduct Signal Fractions**

- **Unmodified**: 3.0460e+08

### TIVKPGDILLIN(N)STGNLIAPR Fuc 2 Hex 3 HexNAc 4 Neu5Ac 1

**Mass:**  4085.9555

(-5.101e-06)

**Charge States:** 4

**Start Position**: 235

**End Position**: 256

| Profile Score | Tandem Score | TDA q-value | Volume | Spectrum Matches |
| --- | --- | --- | --- | --- |
| -17.8830 | 164.8242 | 0.0000 | 4.4893e+07 | 1 |

**Adduct Signal Fractions**

- **Unmodified**: 4.4893e+07

### TIVKPGDILLIN(N)STGNLIAPR Hex 9 HexNAc 2

**Mass:**  4068.9309

(1.612e-06)

**Charge States:** 3, 4

**Start Position**: 235

**End Position**: 256

| Profile Score | Tandem Score | TDA q-value | Volume | Spectrum Matches |
| --- | --- | --- | --- | --- |
| 19.1007 | 164.4311 | 0.0000 | 4.8723e+08 | 5 |

**Adduct Signal Fractions**

- **Unmodified**: 4.8723e+08

### RGSN(N)SSFFSR Fuc 3 Hex 5 HexNAc 4

**Mass:**  3204.3001

(1.079e-06)

**Charge States:** 3

**Start Position**: 141

**End Position**: 151

| Profile Score | Tandem Score | TDA q-value | Volume | Spectrum Matches |
| --- | --- | --- | --- | --- |
| -2.8720 | 162.9923 | 0.0000 | 8.3240e+07 | 2 |

**Adduct Signal Fractions**

- **Unmodified**: 8.3240e+07

### N(N)GTYDHDVYR Fuc 2 Hex 5 HexNAc 4

**Mass:**  3153.2320

(4.713e-07)

**Charge States:** 3

**Start Position**: 483

**End Position**: 493

| Profile Score | Tandem Score | TDA q-value | Volume | Spectrum Matches |
| --- | --- | --- | --- | --- |
| 15.5653 | 160.8949 | 0.0000 | 2.6313e+08 | 5 |

**Adduct Signal Fractions**

- **Unmodified**: 2.6313e+08

### RGSN(N)SSFFSR Fuc 2 Hex 6 HexNAc 5

**Mass:**  3423.3748

(1.828e-07)

**Charge States:** 3, 4

**Start Position**: 141

**End Position**: 151

| Profile Score | Tandem Score | TDA q-value | Volume | Spectrum Matches |
| --- | --- | --- | --- | --- |
| -20.0967 | 159.7642 | 0.0000 | 1.1616e+07 | 1 |

**Adduct Signal Fractions**

- **Unmodified**: 1.1616e+07

### RGSN(N)SSFFSR Fuc 1 Hex 6 HexNAc 5

**Mass:**  3278.3221

(3.076e-04)

**Start Position**: 141

**End Position**: 151

| Profile Score | Tandem Score | TDA q-value | Volume | Spectrum Matches |
| --- | --- | --- | --- | --- |
| 0.0000 | 159.6527 | 0.0000 | 0.0000e+00 | 1 |

Adduct:

No Chromatogram Found

### SLVASSGTLEFNNESFNWAGVTHN(N)GTSSSC(C)R Hex 7 HexNAc 2

**Mass:**  4856.0300

(1.882e-06)

**Charge States:** 3, 4

**Start Position**: 110

**End Position**: 141

| Profile Score | Tandem Score | TDA q-value | Volume | Spectrum Matches |
| --- | --- | --- | --- | --- |
| -1.6621 | 159.4401 | 0.0000 | 5.4571e+08 | 3 |

**Adduct Signal Fractions**

- **Unmodified**: 5.4571e+08

### TIVKPGDILLIN(N)STGNLIAPR Hex 8 HexNAc 2

**Mass:**  3906.8791

(1.733e-06)

**Charge States:** 4

**Start Position**: 235

**End Position**: 256

| Profile Score | Tandem Score | TDA q-value | Volume | Spectrum Matches |
| --- | --- | --- | --- | --- |
| -3.3159 | 159.4225 | 0.0000 | 5.2110e+07 | 3 |

**Adduct Signal Fractions**

- **Unmodified**: 5.2110e+07

### RGSN(N)SSFFSR Fuc 1 Hex 4 HexNAc 5

**Mass:**  2953.2092

(1.195e-06)

**Charge States:** 3

**Start Position**: 141

**End Position**: 151

| Profile Score | Tandem Score | TDA q-value | Volume | Spectrum Matches |
| --- | --- | --- | --- | --- |
| -4.2827 | 158.5016 | 0.0000 | 5.3706e+07 | 2 |

**Adduct Signal Fractions**

- **Unmodified**: 5.3706e+07

### RGSN(N)SSFFSR Fuc 2 Hex 5 HexNAc 5

**Mass:**  3261.3217

(8.735e-07)

**Charge States:** 3, 4

**Start Position**: 141

**End Position**: 151

| Profile Score | Tandem Score | TDA q-value | Volume | Spectrum Matches |
| --- | --- | --- | --- | --- |
| 13.9685 | 158.3598 | 0.0000 | 3.1477e+08 | 5 |

**Adduct Signal Fractions**

- **Unmodified**: 3.1477e+08

### GSN(N)SSFFSR Hex 7 HexNAc 2

**Mass:**  2527.9703

(2.971e-07)

**Charge States:** 2

**Start Position**: 142

**End Position**: 151

| Profile Score | Tandem Score | TDA q-value | Volume | Spectrum Matches |
| --- | --- | --- | --- | --- |
| -5.1203 | 158.3305 | 0.0000 | 3.9991e+07 | 1 |

**Adduct Signal Fractions**

- **Unmodified**: 3.9991e+07

### YPALN(N)VTMPNNEQFDK Hex 7 HexNAc 2

**Mass:**  3420.4099

(1.609e-06)

**Charge States:** 3

**Start Position**: 161

**End Position**: 177

| Profile Score | Tandem Score | TDA q-value | Volume | Spectrum Matches |
| --- | --- | --- | --- | --- |
| -3.2375 | 155.5333 | 0.0000 | 4.2517e+07 | 3 |

**Adduct Signal Fractions**

- **Unmodified**: 4.2517e+07

### C(C)KSEC(C)ITPN(N)GSIPNDKPFQNVNR Hex 8 HexNAc 2

**Mass:**  4376.8444

(8.521e-07)

**Charge States:** 4

**Start Position**: 277

**End Position**: 300

| Profile Score | Tandem Score | TDA q-value | Volume | Spectrum Matches |
| --- | --- | --- | --- | --- |
| -3.8808 | 155.1839 | 0.0000 | 1.3761e+08 | 1 |

**Adduct Signal Fractions**

- **Unmodified**: 1.3761e+08

### GSN(N)SSFFSR Fuc 1 Hex 5 HexNAc 4

**Mass:**  2756.0824

(7.186e-07)

**Charge States:** 3

**Start Position**: 142

**End Position**: 151

| Profile Score | Tandem Score | TDA q-value | Volume | Spectrum Matches |
| --- | --- | --- | --- | --- |
| 11.8469 | 155.0426 | 0.0000 | 2.5216e+08 | 2 |

**Adduct Signal Fractions**

- **Unmodified**: 2.5216e+08

### C(C)KSEC(C)ITPN(N)GSIPNDKPFQNVNR Hex 9 HexNAc 2

**Mass:**  4538.8977

(-8.052e-07)

**Charge States:** 4

**Start Position**: 277

**End Position**: 300

| Profile Score | Tandem Score | TDA q-value | Volume | Spectrum Matches |
| --- | --- | --- | --- | --- |
| -3.7168 | 154.4760 | 0.0000 | 2.2917e+08 | 3 |

**Adduct Signal Fractions**

- **Unmodified**: 2.2917e+08

### RGSN(N)SSFFSR Fuc 3 Hex 5 HexNAc 5

**Mass:**  3407.3798

(3.486e-06)

**Charge States:** 3, 4

**Start Position**: 141

**End Position**: 151

| Profile Score | Tandem Score | TDA q-value | Volume | Spectrum Matches |
| --- | --- | --- | --- | --- |
| -18.5087 | 154.2605 | 0.0000 | 3.2394e+07 | 3 |

**Adduct Signal Fractions**

- **Unmodified**: 3.2394e+07

### RGSN(N)SSFFSR Hex 5 HexNAc 5

**Mass:**  2969.2036

(5.628e-07)

**Charge States:** 3, 4

**Start Position**: 141

**End Position**: 151

| Profile Score | Tandem Score | TDA q-value | Volume | Spectrum Matches |
| --- | --- | --- | --- | --- |
| 12.9465 | 154.2240 | 0.0000 | 2.9795e+08 | 3 |

**Adduct Signal Fractions**

- **Unmodified**: 2.9795e+08

### N(N)GTYDHDVYRDEALNNR Fuc 1 Hex 6 HexNAc 3

**Mass:**  3778.5278

(1.442e-06)

**Charge States:** 4

**Start Position**: 483

**End Position**: 500

| Profile Score | Tandem Score | TDA q-value | Volume | Spectrum Matches |
| --- | --- | --- | --- | --- |
| -3.6244 | 153.3110 | 0.0000 | 2.9715e+08 | 2 |

**Adduct Signal Fractions**

- **Unmodified**: 2.9715e+08

### RGSN(N)SSFFSR Hex 6 HexNAc 4

**Mass:**  2928.1756

(-6.265e-07)

**Charge States:** 3

**Start Position**: 141

**End Position**: 151

| Profile Score | Tandem Score | TDA q-value | Volume | Spectrum Matches |
| --- | --- | --- | --- | --- |
| 9.6071 | 152.6227 | 0.0000 | 1.1608e+08 | 2 |

**Adduct Signal Fractions**

- **Unmodified**: 1.1608e+08

### YPALN(N)VTM(O)PNNEQFDK Hex 5 HexNAc 2

**Mass:**  3112.3075

(3.926e-06)

**Charge States:** 3

**Start Position**: 161

**End Position**: 177

| Profile Score | Tandem Score | TDA q-value | Volume | Spectrum Matches |
| --- | --- | --- | --- | --- |
| 12.9425 | 151.7996 | 0.0000 | 3.1322e+07 | 2 |

**Adduct Signal Fractions**

- **Unmodified**: 3.1322e+07

### N(N)GTYDHDVYR Fuc 1 Hex 5 HexNAc 4

**Mass:**  3007.1744

(-1.024e-06)

**Charge States:** 3, 4

**Start Position**: 483

**End Position**: 493

| Profile Score | Tandem Score | TDA q-value | Volume | Spectrum Matches |
| --- | --- | --- | --- | --- |
| 16.1445 | 151.7980 | 0.0000 | 4.2008e+08 | 6 |

**Adduct Signal Fractions**

- **Unmodified**: 4.2008e+08

### C(C)KSEC(C)ITPN(N)GSIPNDKPFQNVNR Hex 7 HexNAc 2

**Mass:**  4214.7932

(5.565e-07)

**Charge States:** 3, 4, 5

**Start Position**: 277

**End Position**: 300

| Profile Score | Tandem Score | TDA q-value | Volume | Spectrum Matches |
| --- | --- | --- | --- | --- |
| -17.4842 | 151.7581 | 0.0000 | 2.3944e+08 | 1 |

**Adduct Signal Fractions**

- **Unmodified**: 2.3944e+08

### ISIYWTIVKPGDILLIN(N)STGNLIAPR Hex 9 HexNAc 2

**Mass:**  4731.2751

(7.743e-07)

**Charge States:** 3, 4, 5

**Start Position**: 230

**End Position**: 256

| Profile Score | Tandem Score | TDA q-value | Volume | Spectrum Matches |
| --- | --- | --- | --- | --- |
| 19.9380 | 151.2474 | 0.0000 | 8.7664e+08 | 10 |

**Adduct Signal Fractions**

- **Unmodified**: 8.7664e+08

### YPALN(N)VTM(O)PNNEQFDK Hex 6 HexNAc 2

**Mass:**  3274.3581

(3.017e-06)

**Charge States:** 3

**Start Position**: 161

**End Position**: 177

| Profile Score | Tandem Score | TDA q-value | Volume | Spectrum Matches |
| --- | --- | --- | --- | --- |
| 12.6040 | 150.6227 | 0.0000 | 4.2009e+07 | 3 |

**Adduct Signal Fractions**

- **Unmodified**: 4.2009e+07

### GSN(N)SSFFSR Hex 6 HexNAc 2

**Mass:**  2365.9186

(-6.407e-07)

**Charge States:** 2

**Start Position**: 142

**End Position**: 151

| Profile Score | Tandem Score | TDA q-value | Volume | Spectrum Matches |
| --- | --- | --- | --- | --- |
| -3.6984 | 150.4600 | 0.0000 | 1.2551e+08 | 2 |

**Adduct Signal Fractions**

- **Unmodified**: 1.2551e+08

### RGSN(N)SSFFSR Hex 8 HexNAc 2

**Mass:**  2846.1271

(1.304e-06)

**Charge States:** 3

**Start Position**: 141

**End Position**: 151

| Profile Score | Tandem Score | TDA q-value | Volume | Spectrum Matches |
| --- | --- | --- | --- | --- |
| -3.0880 | 149.9087 | 0.0000 | 7.1970e+07 | 1 |

**Adduct Signal Fractions**

- **Unmodified**: 7.1970e+07

### SLVASSGTLEFNNESFNWAGVTHN(N)GTSSSC(C)R Fuc 1 Hex 6 HexNAc 3

**Mass:**  5043.1081

(1.337e-06)

**Charge States:** 4

**Start Position**: 110

**End Position**: 141

| Profile Score | Tandem Score | TDA q-value | Volume | Spectrum Matches |
| --- | --- | --- | --- | --- |
| -18.6851 | 148.9077 | 0.0000 | 2.7793e+07 | 1 |

**Adduct Signal Fractions**

- **Unmodified**: 2.7793e+07

### SLVASSGTLEFNNESFNWAGVTHN(N)GTSSSC(C)R Hex 5 HexNAc 2

**Mass:**  4531.9172

(3.200e-06)

**Charge States:** 3, 4

**Start Position**: 110

**End Position**: 141

| Profile Score | Tandem Score | TDA q-value | Volume | Spectrum Matches |
| --- | --- | --- | --- | --- |
| 15.7268 | 148.8108 | 0.0000 | 1.7353e+08 | 4 |

**Adduct Signal Fractions**

- **Unmodified**: 1.7353e+08

### GSN(N)SSFFSR Hex 5 HexNAc 2

**Mass:**  2203.8646

(1.877e-07)

**Charge States:** 2, 3

**Start Position**: 142

**End Position**: 151

| Profile Score | Tandem Score | TDA q-value | Volume | Spectrum Matches |
| --- | --- | --- | --- | --- |
| -2.2803 | 148.7433 | 0.0000 | 2.0125e+08 | 3 |

**Adduct Signal Fractions**

- **Unmodified**: 2.0125e+08

### RGSN(N)SSFFSR Fuc 2 Hex 6 HexNAc 3

**Mass:**  3017.2163

(1.087e-06)

**Charge States:** 3

**Start Position**: 141

**End Position**: 151

| Profile Score | Tandem Score | TDA q-value | Volume | Spectrum Matches |
| --- | --- | --- | --- | --- |
| -17.8940 | 147.8611 | 0.0000 | 1.8067e+07 | 1 |

**Adduct Signal Fractions**

- **Unmodified**: 1.8067e+07

### RGSN(N)SSFFSR Fuc 1 Hex 6 HexNAc 4

**Mass:**  3074.2326

(-6.253e-07)

**Charge States:** 3

**Start Position**: 141

**End Position**: 151

| Profile Score | Tandem Score | TDA q-value | Volume | Spectrum Matches |
| --- | --- | --- | --- | --- |
| -5.2591 | 147.7356 | 0.0000 | 3.8606e+07 | 2 |

**Adduct Signal Fractions**

- **Unmodified**: 3.8606e+07

### ISIYWTIVKPGDILLIN(N)STGNLIAPR Hex 10 HexNAc 2

**Mass:**  4893.3254

(1.306e-06)

**Charge States:** 4

**Start Position**: 230

**End Position**: 256

| Profile Score | Tandem Score | TDA q-value | Volume | Spectrum Matches |
| --- | --- | --- | --- | --- |
| -4.1061 | 147.6235 | 0.0000 | 4.2517e+07 | 4 |

**Adduct Signal Fractions**

- **Unmodified**: 4.2517e+07

### RGSN(N)SSFFSR Hex 5 HexNAc 6

**Mass:**  3172.2852

(8.048e-07)

**Charge States:** 3

**Start Position**: 141

**End Position**: 151

| Profile Score | Tandem Score | TDA q-value | Volume | Spectrum Matches |
| --- | --- | --- | --- | --- |
| -2.0918 | 147.2361 | 0.0000 | 2.4724e+08 | 2 |

**Adduct Signal Fractions**

- **Unmodified**: 2.4724e+08

### ISIYWTIVKPGDILLIN(N)STGNLIAPR Hex 8 HexNAc 2

**Mass:**  4569.2194

(1.374e-06)

**Charge States:** 4, 5

**Start Position**: 230

**End Position**: 256

| Profile Score | Tandem Score | TDA q-value | Volume | Spectrum Matches |
| --- | --- | --- | --- | --- |
| 15.5162 | 146.2627 | 0.0000 | 9.1510e+07 | 5 |

**Adduct Signal Fractions**

- **Unmodified**: 9.1510e+07

### GSN(N)SSFFSR Fuc 1 Hex 5 HexNAc 5

**Mass:**  2959.1629

(6.772e-07)

**Charge States:** 3

**Start Position**: 142

**End Position**: 151

| Profile Score | Tandem Score | TDA q-value | Volume | Spectrum Matches |
| --- | --- | --- | --- | --- |
| 13.0938 | 144.6823 | 0.0000 | 6.0685e+08 | 5 |

**Adduct Signal Fractions**

- **Unmodified**: 6.0685e+08

### RGSN(N)SSFFSR Fuc 2 Hex 5 HexNAc 3

**Mass:**  2855.1602

(2.574e-08)

**Charge States:** 3

**Start Position**: 141

**End Position**: 151

| Profile Score | Tandem Score | TDA q-value | Volume | Spectrum Matches |
| --- | --- | --- | --- | --- |
| -17.2945 | 143.8853 | 0.0000 | 2.3003e+07 | 1 |

**Adduct Signal Fractions**

- **Unmodified**: 2.3003e+07

### ISIYWTIVKPGDILLIN(N)STGNLIAPR Fuc 2 Hex 3 HexNAc 4 Neu5Ac 1

**Mass:**  4748.2956

(-4.944e-06)

**Charge States:** 4

**Start Position**: 230

**End Position**: 256

| Profile Score | Tandem Score | TDA q-value | Volume | Spectrum Matches |
| --- | --- | --- | --- | --- |
| 19.5023 | 143.2254 | 0.0000 | 1.5050e+08 | 3 |

**Adduct Signal Fractions**

- **Unmodified**: 1.5050e+08

### N(N)GTYDHDVYRDEALNNR Fuc 1 Hex 8 HexNAc 2

**Mass:**  3899.5141

(-9.234e-06)

**Charge States:** 3, 4

**Start Position**: 483

**End Position**: 500

| Profile Score | Tandem Score | TDA q-value | Volume | Spectrum Matches |
| --- | --- | --- | --- | --- |
| 13.8532 | 142.5070 | 0.0000 | 6.5117e+08 | 3 |

**Adduct Signal Fractions**

- **Unmodified**: 6.5117e+08

### N(N)GTYDHDVYRDEALNNR Fuc 2 Hex 5 HexNAc 4

**Mass:**  3965.6105

(5.995e-07)

**Charge States:** 4

**Start Position**: 483

**End Position**: 500

| Profile Score | Tandem Score | TDA q-value | Volume | Spectrum Matches |
| --- | --- | --- | --- | --- |
| 17.0830 | 141.9970 | 0.0000 | 2.4031e+09 | 4 |

**Adduct Signal Fractions**

- **Unmodified**: 2.4031e+09

### TIVKPGDILLIN(N)STGNLIAPR Hex 10 HexNAc 2

**Mass:**  4230.9831

(4.531e-06)

**Charge States:** 4

**Start Position**: 235

**End Position**: 256

| Profile Score | Tandem Score | TDA q-value | Volume | Spectrum Matches |
| --- | --- | --- | --- | --- |
| -19.1627 | 141.4347 | 0.0000 | 1.6522e+07 | 2 |

**Adduct Signal Fractions**

- **Unmodified**: 1.6522e+07

### PGDILLIN(N)STGNLIAPR Hex 9 HexNAc 2

**Mass:**  3627.6337

(1.507e-06)

**Charge States:** 3

**Start Position**: 239

**End Position**: 256

| Profile Score | Tandem Score | TDA q-value | Volume | Spectrum Matches |
| --- | --- | --- | --- | --- |
| -18.5031 | 141.3272 | 0.0000 | 2.5371e+07 | 3 |

**Adduct Signal Fractions**

- **Unmodified**: 2.5371e+07

### N(N)GTYDHDVYRDEALNNR Hex 5 HexNAc 2

**Mass:**  3267.3359

(1.108e-06)

**Charge States:** 4

**Start Position**: 483

**End Position**: 500

| Profile Score | Tandem Score | TDA q-value | Volume | Spectrum Matches |
| --- | --- | --- | --- | --- |
| -4.8506 | 141.2443 | 0.0000 | 9.6018e+07 | 1 |

**Adduct Signal Fractions**

- **Unmodified**: 9.6018e+07

### SLVASSGTLEFNNESFNWAGVTHN(N)GTSSSC(C)R Hex 6 HexNAc 2

**Mass:**  4693.9728

(1.506e-06)

**Charge States:** 3, 4

**Start Position**: 110

**End Position**: 141

| Profile Score | Tandem Score | TDA q-value | Volume | Spectrum Matches |
| --- | --- | --- | --- | --- |
| -3.4545 | 140.9992 | 0.0000 | 1.3264e+08 | 3 |

**Adduct Signal Fractions**

- **Unmodified**: 1.3264e+08

### N(N)GTYDHDVYR Fuc 2 Hex 5 HexNAc 5

**Mass:**  3356.3128

(1.349e-06)

**Charge States:** 3, 4

**Start Position**: 483

**End Position**: 493

| Profile Score | Tandem Score | TDA q-value | Volume | Spectrum Matches |
| --- | --- | --- | --- | --- |
| -19.1373 | 140.4520 | 0.0000 | 5.3740e+07 | 4 |

**Adduct Signal Fractions**

- **Unmodified**: 5.3740e+07

### SLVASSGTLEFNNESFNWAGVTHN(N)GTSSSC(C)R Hex 5 HexNAc 5

**Mass:**  5141.1579

(2.285e-06)

**Charge States:** 4

**Start Position**: 110

**End Position**: 141

| Profile Score | Tandem Score | TDA q-value | Volume | Spectrum Matches |
| --- | --- | --- | --- | --- |
| 11.8091 | 140.2750 | 0.0000 | 6.1876e+07 | 3 |

**Adduct Signal Fractions**

- **Unmodified**: 6.1876e+07

### RGSN(N)SSFFSR Fuc 1 Hex 6 HexNAc 5

**Mass:**  3277.3163

(6.881e-07)

**Charge States:** 3

**Start Position**: 141

**End Position**: 151

| Profile Score | Tandem Score | TDA q-value | Volume | Spectrum Matches |
| --- | --- | --- | --- | --- |
| -3.7741 | 140.0018 | 0.0000 | 8.0495e+07 | 3 |

**Adduct Signal Fractions**

- **Unmodified**: 8.0495e+07

### IVKPGDILLIN(N)STGNLIAPR Hex 9 HexNAc 2

**Mass:**  3967.8808

(1.256e-06)

**Charge States:** 4

**Start Position**: 236

**End Position**: 256

| Profile Score | Tandem Score | TDA q-value | Volume | Spectrum Matches |
| --- | --- | --- | --- | --- |
| -18.3474 | 138.5892 | 0.0000 | 9.4137e+06 | 2 |

**Adduct Signal Fractions**

- **Unmodified**: 9.4137e+06

### HAVPN(N)GTIVK Fuc 1 Hex 5 HexNAc 5

**Mass:**  3008.3236

(6.711e-04)

**Start Position**: 18

**End Position**: 28

| Profile Score | Tandem Score | TDA q-value | Volume | Spectrum Matches |
| --- | --- | --- | --- | --- |
| 0.0000 | 138.1240 | 0.0000 | 0.0000e+00 | 1 |

Adduct:

No Chromatogram Found

### N(N)GTYDHDVYRDEALNNR Fuc 1 Hex 4 HexNAc 3

**Mass:**  3454.4199

(1.262e-06)

**Charge States:** 4

**Start Position**: 483

**End Position**: 500

| Profile Score | Tandem Score | TDA q-value | Volume | Spectrum Matches |
| --- | --- | --- | --- | --- |
| -3.0003 | 137.6993 | 0.0000 | 8.7585e+07 | 1 |

**Adduct Signal Fractions**

- **Unmodified**: 8.7585e+07

### YPALN(N)VTM(O)PNNEQFDK Fuc 2 Hex 3 HexNAc 4 Neu5Ac 1

**Mass:**  3777.5403

(-5.067e-06)

**Charge States:** 3

**Start Position**: 161

**End Position**: 177

| Profile Score | Tandem Score | TDA q-value | Volume | Spectrum Matches |
| --- | --- | --- | --- | --- |
| -18.1974 | 137.4550 | 0.0000 | 1.6530e+07 | 1 |

**Adduct Signal Fractions**

- **Unmodified**: 1.6530e+07

### N(N)GTYDHDVYR Fuc 3 Hex 5 HexNAc 4

**Mass:**  3299.2868

(-8.528e-08)

**Charge States:** 3, 4

**Start Position**: 483

**End Position**: 493

| Profile Score | Tandem Score | TDA q-value | Volume | Spectrum Matches |
| --- | --- | --- | --- | --- |
| -19.0732 | 136.3943 | 0.0000 | 8.8780e+07 | 2 |

**Adduct Signal Fractions**

- **Unmodified**: 8.8780e+07

### SLVASSGTLEFNNESFNWAGVTHN(N)GTSSSC(C)R Hex 7 HexNAc 4

**Mass:**  5262.1857

(2.540e-06)

**Charge States:** 4

**Start Position**: 110

**End Position**: 141

| Profile Score | Tandem Score | TDA q-value | Volume | Spectrum Matches |
| --- | --- | --- | --- | --- |
| 13.0728 | 135.8580 | 0.0000 | 4.3605e+07 | 3 |

**Adduct Signal Fractions**

- **Unmodified**: 4.3605e+07

### YPALN(N)VTM(O)PN(D)NEQFDK Hex 8 HexNAc 2

**Mass:**  3599.4523

(2.770e-04)

**Charge States:** 2, 3, 4

**Start Position**: 161

**End Position**: 177

| Profile Score | Tandem Score | TDA q-value | Volume | Spectrum Matches |
| --- | --- | --- | --- | --- |
| 9.6400 | 135.7791 | 0.0000 | 3.2087e+08 | 27 |

**Adduct Signal Fractions**

- **Unmodified**: 3.2087e+08

### N(N)GTYDHDVYRDEALNNR Fuc 2 Hex 8 HexNAc 3

**Mass:**  4248.6501

(-8.395e-06)

**Charge States:** 3, 4

**Start Position**: 483

**End Position**: 500

| Profile Score | Tandem Score | TDA q-value | Volume | Spectrum Matches |
| --- | --- | --- | --- | --- |
| 11.5747 | 135.3678 | 0.0000 | 1.8553e+08 | 2 |

**Adduct Signal Fractions**

- **Unmodified**: 1.8553e+08

### N(N)GTYDHDVYRDEALNNR Hex 6 HexNAc 3

**Mass:**  3632.4658

(3.700e-07)

**Charge States:** 4

**Start Position**: 483

**End Position**: 500

| Profile Score | Tandem Score | TDA q-value | Volume | Spectrum Matches |
| --- | --- | --- | --- | --- |
| -3.9010 | 135.2617 | 0.0000 | 2.2741e+08 | 3 |

**Adduct Signal Fractions**

- **Unmodified**: 2.2741e+08

### SLVASSGTLEFNNESFNWAGVTHN(N)GTSSSC(C)R Hex 4 HexNAc 3

**Mass:**  4572.9406

(-5.256e-07)

**Charge States:** 4

**Start Position**: 110

**End Position**: 141

| Profile Score | Tandem Score | TDA q-value | Volume | Spectrum Matches |
| --- | --- | --- | --- | --- |
| -4.0178 | 133.8413 | 0.0000 | 2.7753e+07 | 1 |

**Adduct Signal Fractions**

- **Unmodified**: 2.7753e+07

### N(N)GTYDHDVYRDEALNNR Fuc 1 Hex 5 HexNAc 4

**Mass:**  3819.5498

(5.985e-07)

**Charge States:** 3, 4, 5

**Start Position**: 483

**End Position**: 500

| Profile Score | Tandem Score | TDA q-value | Volume | Spectrum Matches |
| --- | --- | --- | --- | --- |
| 16.1167 | 133.6776 | 0.0000 | 3.3785e+09 | 6 |

**Adduct Signal Fractions**

- **Unmodified**: 3.3785e+09

### RGSN(N)SSFFSR Fuc 2 Hex 9 HexNAc 4

**Mass:**  3706.4090

(-8.421e-06)

**Charge States:** 3

**Start Position**: 141

**End Position**: 151

| Profile Score | Tandem Score | TDA q-value | Volume | Spectrum Matches |
| --- | --- | --- | --- | --- |
| 9.3122 | 132.8555 | 0.0000 | 3.2256e+07 | 2 |

**Adduct Signal Fractions**

- **Unmodified**: 3.2256e+07

### N(N)GTYDHDVYRDEALNNR Hex 5 HexNAc 4

**Mass:**  3673.4924

(-1.171e-07)

**Charge States:** 4

**Start Position**: 483

**End Position**: 500

| Profile Score | Tandem Score | TDA q-value | Volume | Spectrum Matches |
| --- | --- | --- | --- | --- |
| 13.0127 | 132.4771 | 0.0000 | 1.7520e+08 | 3 |

**Adduct Signal Fractions**

- **Unmodified**: 1.7520e+08

### SEC(C)ITPN(N)GSIPNDKPFQNVNR Hex 8 HexNAc 2

**Mass:**  4088.7170

(1.306e-06)

**Charge States:** 3, 4

**Start Position**: 279

**End Position**: 300

| Profile Score | Tandem Score | TDA q-value | Volume | Spectrum Matches |
| --- | --- | --- | --- | --- |
| 16.3255 | 132.3095 | 0.0000 | 9.4531e+08 | 4 |

**Adduct Signal Fractions**

- **Unmodified**: 9.4531e+08

### RGSN(N)SSFFSR Hex 7 HexNAc 4

**Mass:**  3090.2323

(9.053e-07)

**Charge States:** 3

**Start Position**: 141

**End Position**: 151

| Profile Score | Tandem Score | TDA q-value | Volume | Spectrum Matches |
| --- | --- | --- | --- | --- |
| -19.4958 | 132.1240 | 0.0000 | 1.7522e+07 | 1 |

**Adduct Signal Fractions**

- **Unmodified**: 1.7522e+07

### N(N)GTYDHDVYR Fuc 2 Hex 6 HexNAc 5

**Mass:**  3518.3637

(6.556e-07)

**Charge States:** 3, 4

**Start Position**: 483

**End Position**: 493

| Profile Score | Tandem Score | TDA q-value | Volume | Spectrum Matches |
| --- | --- | --- | --- | --- |
| -19.7549 | 131.8935 | 0.0000 | 2.0062e+07 | 2 |

**Adduct Signal Fractions**

- **Unmodified**: 2.0062e+07

### SEC(C)ITPN(N)GSIPNDKPFQNVNR Hex 7 HexNAc 2

**Mass:**  3926.6682

(1.794e-06)

**Charge States:** 3, 4

**Start Position**: 279

**End Position**: 300

| Profile Score | Tandem Score | TDA q-value | Volume | Spectrum Matches |
| --- | --- | --- | --- | --- |
| 16.1490 | 131.4705 | 0.0000 | 1.5801e+09 | 6 |

**Adduct Signal Fractions**

- **Unmodified**: 1.5801e+09

### N(N)GTYDHDVYR Fuc 1 Hex 6 HexNAc 3

**Mass:**  2966.1476

(1.028e-06)

**Charge States:** 3

**Start Position**: 483

**End Position**: 493

| Profile Score | Tandem Score | TDA q-value | Volume | Spectrum Matches |
| --- | --- | --- | --- | --- |
| -18.0030 | 131.0909 | 0.0000 | 3.5788e+07 | 1 |

**Adduct Signal Fractions**

- **Unmodified**: 3.5788e+07

### RGSN(N)SSFFSR Fuc 1 Hex 3 HexNAc 9 Neu5Ac 2

**Mass:**  4185.6726

(2.154e-06)

**Charge States:** 4

**Start Position**: 141

**End Position**: 151

| Profile Score | Tandem Score | TDA q-value | Volume | Spectrum Matches |
| --- | --- | --- | --- | --- |
| -17.6853 | 130.5065 | 0.0000 | 4.9908e+07 | 1 |

**Adduct Signal Fractions**

- **Unmodified**: 4.9908e+07

### N(N)GTYDHDVYRDEALNNR Fuc 1 Hex 9 HexNAc 3

**Mass:**  4264.6452

(-8.339e-06)

**Charge States:** 4

**Start Position**: 483

**End Position**: 500

| Profile Score | Tandem Score | TDA q-value | Volume | Spectrum Matches |
| --- | --- | --- | --- | --- |
| -3.3790 | 130.1030 | 0.0000 | 1.5132e+08 | 2 |

**Adduct Signal Fractions**

- **Unmodified**: 1.5132e+08

### N(N)GTYDHDVYR Hex 6 HexNAc 3

**Mass:**  2820.0893

(9.621e-07)

**Charge States:** 3

**Start Position**: 483

**End Position**: 493

| Profile Score | Tandem Score | TDA q-value | Volume | Spectrum Matches |
| --- | --- | --- | --- | --- |
| -18.9141 | 129.9509 | 0.0000 | 1.9966e+07 | 1 |

**Adduct Signal Fractions**

- **Unmodified**: 1.9966e+07

### N(N)GTYDHDVYRDEALNNR Fuc 1 Hex 5 HexNAc 5

**Mass:**  4022.6284

(2.793e-08)

**Charge States:** 3, 4, 5

**Start Position**: 483

**End Position**: 500

| Profile Score | Tandem Score | TDA q-value | Volume | Spectrum Matches |
| --- | --- | --- | --- | --- |
| 10.6215 | 129.6230 | 0.0000 | 2.2084e+09 | 7 |

**Adduct Signal Fractions**

- **Unmodified**: 2.2084e+09

### SEC(C)ITPN(N)GSIPNDKPFQNVNR Hex 9 HexNAc 2

**Mass:**  4250.7761

(1.961e-06)

**Charge States:** 3, 4

**Start Position**: 279

**End Position**: 300

| Profile Score | Tandem Score | TDA q-value | Volume | Spectrum Matches |
| --- | --- | --- | --- | --- |
| -1.6379 | 129.2779 | 0.0000 | 1.4383e+09 | 3 |

**Adduct Signal Fractions**

- **Unmodified**: 1.4383e+09

### SEC(C)ITPN(N)GSIPNDKPFQNVNR Hex 6 HexNAc 2

**Mass:**  3764.6201

(1.825e-06)

**Charge States:** 3, 4

**Start Position**: 279

**End Position**: 300

| Profile Score | Tandem Score | TDA q-value | Volume | Spectrum Matches |
| --- | --- | --- | --- | --- |
| -2.8912 | 129.2717 | 0.0000 | 2.2129e+08 | 3 |

**Adduct Signal Fractions**

- **Unmodified**: 2.2129e+08

### SLVASSGTLEFNNESFNWAGVTHN(N)GTSSSC(C)R Hex 8 HexNAc 2

**Mass:**  5018.0798

(2.641e-06)

**Charge States:** 4

**Start Position**: 110

**End Position**: 141

| Profile Score | Tandem Score | TDA q-value | Volume | Spectrum Matches |
| --- | --- | --- | --- | --- |
| -3.3668 | 128.6184 | 0.0000 | 1.2460e+08 | 3 |

**Adduct Signal Fractions**

- **Unmodified**: 1.2460e+08

### N(N)GTYDHDVYRDEALNNR Fuc 2 Hex 6 HexNAc 5

**Mass:**  4330.7419

(7.426e-07)

**Charge States:** 4

**Start Position**: 483

**End Position**: 500

| Profile Score | Tandem Score | TDA q-value | Volume | Spectrum Matches |
| --- | --- | --- | --- | --- |
| 13.5430 | 128.2094 | 0.0000 | 2.4748e+08 | 1 |

**Adduct Signal Fractions**

- **Unmodified**: 2.4748e+08

### N(N)GTYDHDVYRDEALNNR Fuc 2 Hex 4 HexNAc 3

**Mass:**  3600.4768

(5.918e-07)

**Charge States:** 4

**Start Position**: 483

**End Position**: 500

| Profile Score | Tandem Score | TDA q-value | Volume | Spectrum Matches |
| --- | --- | --- | --- | --- |
| -16.8400 | 128.1653 | 0.0000 | 7.5336e+07 | 2 |

**Adduct Signal Fractions**

- **Unmodified**: 7.5336e+07

### RGSN(N)SSFFSR Fuc 2 Hex 4 HexNAc 3

**Mass:**  2693.1077

(1.058e-07)

**Charge States:** 3

**Start Position**: 141

**End Position**: 151

| Profile Score | Tandem Score | TDA q-value | Volume | Spectrum Matches |
| --- | --- | --- | --- | --- |
| -18.3592 | 127.4619 | 0.0000 | 2.2176e+07 | 1 |

**Adduct Signal Fractions**

- **Unmodified**: 2.2176e+07

### RGSN(N)SSFFSR Fuc 1 Hex 6 HexNAc 6

**Mass:**  3480.3945

(9.978e-07)

**Charge States:** 3, 4

**Start Position**: 141

**End Position**: 151

| Profile Score | Tandem Score | TDA q-value | Volume | Spectrum Matches |
| --- | --- | --- | --- | --- |
| -4.7156 | 126.2987 | 0.0000 | 4.8137e+07 | 3 |

**Adduct Signal Fractions**

- **Unmodified**: 4.8137e+07

### N(N)GTYDHDVYRDEALNNR Fuc 3 Hex 6 HexNAc 5

**Mass:**  4476.7995

(1.694e-06)

**Charge States:** 4

**Start Position**: 483

**End Position**: 500

| Profile Score | Tandem Score | TDA q-value | Volume | Spectrum Matches |
| --- | --- | --- | --- | --- |
| -4.3527 | 126.1391 | 0.0000 | 1.2450e+08 | 1 |

**Adduct Signal Fractions**

- **Unmodified**: 1.2450e+08

### SLVASSGTLEFNNESFNWAGVTHN(N)GTSSSC(C)R Hex 7 HexNAc 3

**Mass:**  5059.1057

(1.346e-06)

**Charge States:** 4

**Start Position**: 110

**End Position**: 141

| Profile Score | Tandem Score | TDA q-value | Volume | Spectrum Matches |
| --- | --- | --- | --- | --- |
| -4.2703 | 125.5759 | 0.0000 | 4.7288e+07 | 1 |

**Adduct Signal Fractions**

- **Unmodified**: 4.7288e+07

### RGSN(N)SSFFSR Fuc 1 Hex 7 HexNAc 8

**Mass:**  4048.6357

(7.609e-06)

**Charge States:** 4

**Start Position**: 141

**End Position**: 151

| Profile Score | Tandem Score | TDA q-value | Volume | Spectrum Matches |
| --- | --- | --- | --- | --- |
| -18.1438 | 125.4858 | 0.0000 | 5.5097e+07 | 1 |

**Adduct Signal Fractions**

- **Unmodified**: 5.5097e+07

### NIPSRISIYWTIVKPGDILLIN(N)STGNLIAPR Hex 9 HexNAc 2

**Mass:**  5298.5856

(1.083e-06)

**Charge States:** 4, 5

**Start Position**: 225

**End Position**: 256

| Profile Score | Tandem Score | TDA q-value | Volume | Spectrum Matches |
| --- | --- | --- | --- | --- |
| -2.9080 | 124.9714 | 0.0000 | 3.4012e+07 | 4 |

**Adduct Signal Fractions**

- **Unmodified**: 3.4012e+07

### N(N)GTYDHDVYRDEALNNR Fuc 1 Hex 6 HexNAc 5

**Mass:**  4184.6852

(6.421e-07)

**Charge States:** 4

**Start Position**: 483

**End Position**: 500

| Profile Score | Tandem Score | TDA q-value | Volume | Spectrum Matches |
| --- | --- | --- | --- | --- |
| 11.4200 | 124.6928 | 0.0000 | 6.0972e+08 | 3 |

**Adduct Signal Fractions**

- **Unmodified**: 6.0972e+08

### N(N)GTYDHDVYRDEALNNR Fuc 1 Hex 5 HexNAc 3

**Mass:**  3616.4746

(1.387e-06)

**Charge States:** 4

**Start Position**: 483

**End Position**: 500

| Profile Score | Tandem Score | TDA q-value | Volume | Spectrum Matches |
| --- | --- | --- | --- | --- |
| 12.3823 | 124.2055 | 0.0000 | 1.1224e+08 | 2 |

**Adduct Signal Fractions**

- **Unmodified**: 1.1224e+08

### SLVASSGTLEFNNESFNWAGVTHN(N)GTSSSC(C)R Fuc 1 Hex 6 HexNAc 6 Neu5Ac 2

**Mass:**  6234.4909

(-5.816e-06)

**Charge States:** 4, 5

**Start Position**: 110

**End Position**: 141

| Profile Score | Tandem Score | TDA q-value | Volume | Spectrum Matches |
| --- | --- | --- | --- | --- |
| -18.8751 | 123.4142 | 0.0000 | 3.7856e+07 | 2 |

**Adduct Signal Fractions**

- **Unmodified**: 3.7856e+07

### N(N)GTYDHDVYR Fuc 1 Hex 5 HexNAc 5

**Mass:**  3211.2617

(3.150e-04)

**Start Position**: 483

**End Position**: 493

| Profile Score | Tandem Score | TDA q-value | Volume | Spectrum Matches |
| --- | --- | --- | --- | --- |
| 0.0000 | 123.1572 | 0.0000 | 0.0000e+00 | 1 |

Adduct:

No Chromatogram Found

### SEC(C)ITPN(N)GSIPNDKPFQNVNR Hex 5 HexNAc 2

**Mass:**  3602.5651

(3.057e-06)

**Charge States:** 4

**Start Position**: 279

**End Position**: 300

| Profile Score | Tandem Score | TDA q-value | Volume | Spectrum Matches |
| --- | --- | --- | --- | --- |
| -4.2348 | 122.7406 | 0.0000 | 6.9018e+07 | 2 |

**Adduct Signal Fractions**

- **Unmodified**: 6.9018e+07

### IRNIPSRISIYWTIVKPGDILLIN(N)STGNLIAPR Hex 9 HexNAc 2

**Mass:**  5567.7694

(8.049e-07)

**Charge States:** 4, 5, 6, 7

**Start Position**: 223

**End Position**: 256

| Profile Score | Tandem Score | TDA q-value | Volume | Spectrum Matches |
| --- | --- | --- | --- | --- |
| 13.2338 | 122.0558 | 0.0000 | 1.2295e+08 | 5 |

**Adduct Signal Fractions**

- **Unmodified**: 1.2295e+08

### N(N)GTYDHDVYRDEALNNR Fuc 1 Hex 8 HexNAc 3

**Mass:**  4102.5915

(-9.120e-06)

**Charge States:** 3, 4

**Start Position**: 483

**End Position**: 500

| Profile Score | Tandem Score | TDA q-value | Volume | Spectrum Matches |
| --- | --- | --- | --- | --- |
| 11.0217 | 121.1555 | 0.0000 | 3.4167e+08 | 5 |

**Adduct Signal Fractions**

- **Unmodified**: 3.4167e+08

### N(N)GTYDHDVYRDEALNNR Fuc 2 Hex 5 HexNAc 3

**Mass:**  3762.5300

(7.107e-07)

**Charge States:** 4

**Start Position**: 483

**End Position**: 500

| Profile Score | Tandem Score | TDA q-value | Volume | Spectrum Matches |
| --- | --- | --- | --- | --- |
| -4.2644 | 120.9983 | 0.0000 | 5.7408e+07 | 1 |

**Adduct Signal Fractions**

- **Unmodified**: 5.7408e+07

### N(N)GTYDHDVYRDEALNNR Fuc 3 Hex 5 HexNAc 4

**Mass:**  4111.6677

(7.547e-07)

**Charge States:** 4

**Start Position**: 483

**End Position**: 500

| Profile Score | Tandem Score | TDA q-value | Volume | Spectrum Matches |
| --- | --- | --- | --- | --- |
| 15.7272 | 120.9145 | 0.0000 | 9.3651e+08 | 3 |

**Adduct Signal Fractions**

- **Unmodified**: 9.3651e+08

### YPALN(N)VTMPNNEQFDK Hex 9 HexNAc 2

**Mass:**  3744.5165

(1.593e-06)

**Charge States:** 3

**Start Position**: 161

**End Position**: 177

| Profile Score | Tandem Score | TDA q-value | Volume | Spectrum Matches |
| --- | --- | --- | --- | --- |
| -4.5228 | 120.5715 | 0.0000 | 2.4029e+07 | 3 |

**Adduct Signal Fractions**

- **Unmodified**: 2.4029e+07

### RGSN(N)SSFFSR Hex 9 HexNAc 4

**Mass:**  3414.3092

(-7.509e-06)

**Charge States:** 3

**Start Position**: 141

**End Position**: 151

| Profile Score | Tandem Score | TDA q-value | Volume | Spectrum Matches |
| --- | --- | --- | --- | --- |
| -4.6533 | 120.2115 | 0.0000 | 1.5816e+07 | 1 |

**Adduct Signal Fractions**

- **Unmodified**: 1.5816e+07

### SLVASSGTLEFNNESFNWAGVTHN(N)GTSSSC(C)R Hex 6 HexNAc 3

**Mass:**  4897.0456

(-7.680e-07)

**Charge States:** 4

**Start Position**: 110

**End Position**: 141

| Profile Score | Tandem Score | TDA q-value | Volume | Spectrum Matches |
| --- | --- | --- | --- | --- |
| -4.5979 | 120.0767 | 0.0000 | 6.1577e+07 | 1 |

**Adduct Signal Fractions**

- **Unmodified**: 6.1577e+07

### N(N)GTYDHDVYR Fuc 1 Hex 5 HexNAc 5

**Mass:**  3210.2528

(2.071e-06)

**Charge States:** 3, 4

**Start Position**: 483

**End Position**: 493

| Profile Score | Tandem Score | TDA q-value | Volume | Spectrum Matches |
| --- | --- | --- | --- | --- |
| 10.2504 | 119.9988 | 0.0000 | 1.9905e+08 | 6 |

**Adduct Signal Fractions**

- **Unmodified**: 1.9905e+08

### SEC(C)ITPN(N)GSIPNDKPFQNVNR Fuc 2 Hex 5 HexNAc 7

**Mass:**  4912.0931

(4.157e-04)

**Charge States:** 4

**Start Position**: 279

**End Position**: 300

| Profile Score | Tandem Score | TDA q-value | Volume | Spectrum Matches |
| --- | --- | --- | --- | --- |
| -6.3819 | 119.7401 | 0.0000 | 1.3342e+06 | 1 |

**Adduct Signal Fractions**

- **Unmodified**: 1.3342e+06

### N(N)GTYDHDVYRDEALNNR Fuc 1 Hex 6 HexNAc 6

**Mass:**  4387.7653

(1.176e-06)

**Charge States:** 4

**Start Position**: 483

**End Position**: 500

| Profile Score | Tandem Score | TDA q-value | Volume | Spectrum Matches |
| --- | --- | --- | --- | --- |
| 7.0621 | 119.5992 | 0.0000 | 1.5964e+08 | 2 |

**Adduct Signal Fractions**

- **Unmodified**: 1.5964e+08

### SEC(C)ITPN(N)GSIPNDKPFQNVNR Fuc 2 Hex 3 HexNAc 4 Neu5Ac 1

**Mass:**  4267.7985

(-5.084e-06)

**Charge States:** 4

**Start Position**: 279

**End Position**: 300

| Profile Score | Tandem Score | TDA q-value | Volume | Spectrum Matches |
| --- | --- | --- | --- | --- |
| -21.1132 | 118.8737 | 0.0000 | 4.5567e+07 | 1 |

**Adduct Signal Fractions**

- **Unmodified**: 4.5567e+07

### RGSN(N)SSFFSR Fuc 2 Hex 4 HexNAc 5

**Mass:**  3099.2671

(2.865e-07)

**Charge States:** 3

**Start Position**: 141

**End Position**: 151

| Profile Score | Tandem Score | TDA q-value | Volume | Spectrum Matches |
| --- | --- | --- | --- | --- |
| -18.3711 | 118.3946 | 0.0000 | 1.5922e+07 | 1 |

**Adduct Signal Fractions**

- **Unmodified**: 1.5922e+07

### N(N)GTYDHDVYR Hex 5 HexNAc 6

**Mass:**  3267.2744

(4.575e-07)

**Charge States:** 3

**Start Position**: 483

**End Position**: 493

| Profile Score | Tandem Score | TDA q-value | Volume | Spectrum Matches |
| --- | --- | --- | --- | --- |
| -5.6793 | 117.7487 | 0.0000 | 2.9854e+07 | 3 |

**Adduct Signal Fractions**

- **Unmodified**: 2.9854e+07

### N(N)GTYDHDVYRDEALNNR Fuc 2 Hex 5 HexNAc 5

**Mass:**  4168.6857

(1.035e-06)

**Charge States:** 4

**Start Position**: 483

**End Position**: 500

| Profile Score | Tandem Score | TDA q-value | Volume | Spectrum Matches |
| --- | --- | --- | --- | --- |
| -3.2080 | 117.1181 | 0.0000 | 4.3341e+08 | 2 |

**Adduct Signal Fractions**

- **Unmodified**: 4.3341e+08

### SLVASSGTLEFNNESFNWAGVTHN(N)GTSSSC(C)R Fuc 1 Hex 7 HexNAc 3

**Mass:**  5205.1564

(1.068e-06)

**Charge States:** 4

**Start Position**: 110

**End Position**: 141

| Profile Score | Tandem Score | TDA q-value | Volume | Spectrum Matches |
| --- | --- | --- | --- | --- |
| -18.8753 | 115.5065 | 0.0000 | 1.1545e+07 | 1 |

**Adduct Signal Fractions**

- **Unmodified**: 1.1545e+07

### TIVKPGDILLIN(N)STGNLIAPR Fuc 4 Hex 4 HexNAc 5 Neu5Ac 1

**Mass:**  4743.2702

(9.607e-06)

**Charge States:** 3, 4

**Start Position**: 235

**End Position**: 256

| Profile Score | Tandem Score | TDA q-value | Volume | Spectrum Matches |
| --- | --- | --- | --- | --- |
| 13.9786 | 114.4653 | 0.0000 | 5.2235e+07 | 6 |

**Adduct Signal Fractions**

- **Unmodified**: 5.2235e+07

### N(N)GTYDHDVYRDEALN(D)NR Fuc 2 Hex 5 HexNAc 4

**Mass:**  3966.5974

(2.503e-04)

**Charge States:** 3, 4

**Start Position**: 483

**End Position**: 500

| Profile Score | Tandem Score | TDA q-value | Volume | Spectrum Matches |
| --- | --- | --- | --- | --- |
| 6.3855 | 114.3721 | 0.0000 | 6.9127e+08 | 4 |

**Adduct Signal Fractions**

- **Unmodified**: 6.9127e+08

### N(N)GTYDHDVYRDEALN(D)NR Fuc 1 Hex 6 HexNAc 3

**Mass:**  3779.5144

(2.626e-04)

**Charge States:** 4

**Start Position**: 483

**End Position**: 500

| Profile Score | Tandem Score | TDA q-value | Volume | Spectrum Matches |
| --- | --- | --- | --- | --- |
| -5.7720 | 114.1971 | 0.0000 | 7.1511e+07 | 2 |

**Adduct Signal Fractions**

- **Unmodified**: 7.1511e+07

### N(N)GTYDHDVYRDEALNNR Fuc 2 Hex 9 HexNAc 3

**Mass:**  4410.7038

(-8.407e-06)

**Charge States:** 3, 4

**Start Position**: 483

**End Position**: 500

| Profile Score | Tandem Score | TDA q-value | Volume | Spectrum Matches |
| --- | --- | --- | --- | --- |
| -4.3575 | 113.3093 | 0.0000 | 1.0581e+08 | 2 |

**Adduct Signal Fractions**

- **Unmodified**: 1.0581e+08

### N(N)GTYDHDVYR Hex 5 HexNAc 4

**Mass:**  2861.1189

(8.125e-07)

**Charge States:** 3

**Start Position**: 483

**End Position**: 493

| Profile Score | Tandem Score | TDA q-value | Volume | Spectrum Matches |
| --- | --- | --- | --- | --- |
| -19.1687 | 112.8543 | 0.0000 | 1.5462e+07 | 1 |

**Adduct Signal Fractions**

- **Unmodified**: 1.5462e+07

### SLVASSGTLEFN(D)NESFNWAGVTHN(N)GTSSSC(C)R Hex 7 HexNAc 2

**Mass:**  4857.0065

(2.069e-04)

**Charge States:** 3, 4

**Start Position**: 110

**End Position**: 141

| Profile Score | Tandem Score | TDA q-value | Volume | Spectrum Matches |
| --- | --- | --- | --- | --- |
| 11.3179 | 112.7271 | 0.0000 | 5.2879e+07 | 4 |

**Adduct Signal Fractions**

- **Unmodified**: 5.2879e+07

### YPALN(N)VTM(O)PN(D)NEQFDK Hex 7 HexNAc 2

**Mass:**  3437.3966

(2.897e-04)

**Charge States:** 3, 4

**Start Position**: 161

**End Position**: 177

| Profile Score | Tandem Score | TDA q-value | Volume | Spectrum Matches |
| --- | --- | --- | --- | --- |
| 10.6049 | 112.6425 | 0.0000 | 5.0218e+07 | 3 |

**Adduct Signal Fractions**

- **Unmodified**: 5.0218e+07

### N(N)GTYDHDVYR Fuc 1 Hex 7 HexNAc 3

**Mass:**  3128.2003

(9.366e-07)

**Charge States:** 3

**Start Position**: 483

**End Position**: 493

| Profile Score | Tandem Score | TDA q-value | Volume | Spectrum Matches |
| --- | --- | --- | --- | --- |
| -19.6239 | 112.5784 | 0.0000 | 5.2749e+06 | 1 |

**Adduct Signal Fractions**

- **Unmodified**: 5.2749e+06

### N(N)GTYDHDVYRDEALNNR Fuc 1 Hex 7 HexNAc 3

**Mass:**  3940.5844

(2.390e-06)

**Charge States:** 4

**Start Position**: 483

**End Position**: 500

| Profile Score | Tandem Score | TDA q-value | Volume | Spectrum Matches |
| --- | --- | --- | --- | --- |
| -19.0164 | 112.2024 | 0.0000 | 7.1295e+07 | 1 |

**Adduct Signal Fractions**

- **Unmodified**: 7.1295e+07

### N(N)GTYDHDVYRDEALN(D)NR Hex 6 HexNAc 3

**Mass:**  3633.4543

(2.726e-04)

**Charge States:** 4

**Start Position**: 483

**End Position**: 500

| Profile Score | Tandem Score | TDA q-value | Volume | Spectrum Matches |
| --- | --- | --- | --- | --- |
| -5.9395 | 112.1918 | 0.0000 | 5.8359e+07 | 1 |

**Adduct Signal Fractions**

- **Unmodified**: 5.8359e+07

### N(N)GTYDHDVYR Fuc 1 Hex 6 HexNAc 5

**Mass:**  3372.3063

(-2.717e-07)

**Charge States:** 3

**Start Position**: 483

**End Position**: 493

| Profile Score | Tandem Score | TDA q-value | Volume | Spectrum Matches |
| --- | --- | --- | --- | --- |
| -4.3249 | 112.1908 | 0.0000 | 6.0898e+07 | 4 |

**Adduct Signal Fractions**

- **Unmodified**: 6.0898e+07

### N(N)GTYDHDVYR Fuc 2 Hex 4 HexNAc 3

**Mass:**  2788.0980

(4.573e-07)

**Charge States:** 3

**Start Position**: 483

**End Position**: 493

| Profile Score | Tandem Score | TDA q-value | Volume | Spectrum Matches |
| --- | --- | --- | --- | --- |
| -18.6836 | 111.7255 | 0.0000 | 5.1851e+06 | 1 |

**Adduct Signal Fractions**

- **Unmodified**: 5.1851e+06

### N(N)GTYDHDVYRDEALNNR Fuc 1 Hex 9 HexNAc 4

**Mass:**  4467.7224

(-8.471e-06)

**Charge States:** 4

**Start Position**: 483

**End Position**: 500

| Profile Score | Tandem Score | TDA q-value | Volume | Spectrum Matches |
| --- | --- | --- | --- | --- |
| 8.8188 | 111.5234 | 0.0000 | 5.1206e+07 | 1 |

**Adduct Signal Fractions**

- **Unmodified**: 5.1206e+07

### RGSN(N)SSFFSR Fuc 1 Hex 5 HexNAc 6

**Mass:**  3318.3407

(1.194e-07)

**Charge States:** 3

**Start Position**: 141

**End Position**: 151

| Profile Score | Tandem Score | TDA q-value | Volume | Spectrum Matches |
| --- | --- | --- | --- | --- |
| -5.0763 | 110.8920 | 0.0000 | 4.4132e+07 | 1 |

**Adduct Signal Fractions**

- **Unmodified**: 4.4132e+07

### N(N)GTYDHDVYRDEALNNR Hex 7 HexNAc 3

**Mass:**  3794.5222

(1.313e-06)

**Charge States:** 4

**Start Position**: 483

**End Position**: 500

| Profile Score | Tandem Score | TDA q-value | Volume | Spectrum Matches |
| --- | --- | --- | --- | --- |
| -19.6617 | 109.6974 | 0.0000 | 4.6999e+07 | 2 |

**Adduct Signal Fractions**

- **Unmodified**: 4.6999e+07

### N(N)GTYDHDVYRDEALNNR Fuc 3 Hex 5 HexNAc 5

**Mass:**  4314.7497

(1.043e-06)

**Charge States:** 4

**Start Position**: 483

**End Position**: 500

| Profile Score | Tandem Score | TDA q-value | Volume | Spectrum Matches |
| --- | --- | --- | --- | --- |
| -18.4942 | 108.3648 | 0.0000 | 7.4151e+07 | 2 |

**Adduct Signal Fractions**

- **Unmodified**: 7.4151e+07

### RGSN(N)SSFFSR Fuc 3 Hex 7 HexNAc 7

**Mass:**  4137.6804

(9.487e-06)

**Charge States:** 4

**Start Position**: 141

**End Position**: 151

| Profile Score | Tandem Score | TDA q-value | Volume | Spectrum Matches |
| --- | --- | --- | --- | --- |
| -20.4763 | 107.7763 | 0.0000 | 3.1674e+07 | 1 |

**Adduct Signal Fractions**

- **Unmodified**: 3.1674e+07

### YPALN(N)VTMPNNEQFDK Hex 6 HexNAc 2

**Mass:**  3258.3576

(1.408e-06)

**Charge States:** 3

**Start Position**: 161

**End Position**: 177

| Profile Score | Tandem Score | TDA q-value | Volume | Spectrum Matches |
| --- | --- | --- | --- | --- |
| -20.3472 | 107.2581 | 0.0037 | 2.1440e+06 | 1 |

**Adduct Signal Fractions**

- **Unmodified**: 2.1440e+06

### N(N)GTYDHDVYR Fuc 1 Hex 6 HexNAc 6

**Mass:**  3575.3869

(3.359e-07)

**Charge States:** 4

**Start Position**: 483

**End Position**: 493

| Profile Score | Tandem Score | TDA q-value | Volume | Spectrum Matches |
| --- | --- | --- | --- | --- |
| -19.5773 | 106.4511 | 0.0067 | 1.3882e+07 | 2 |

**Adduct Signal Fractions**

- **Unmodified**: 1.3882e+07

### N(N)GTYDHDVYRDEALNNR Fuc 2 Hex 6 HexNAc 6

**Mass:**  4533.8244

(1.352e-06)

**Charge States:** 4

**Start Position**: 483

**End Position**: 500

| Profile Score | Tandem Score | TDA q-value | Volume | Spectrum Matches |
| --- | --- | --- | --- | --- |
| -19.6463 | 106.0255 | 0.0067 | 4.7954e+07 | 1 |

**Adduct Signal Fractions**

- **Unmodified**: 4.7954e+07

### YPALN(N)VTMPNNEQFDK Fuc 2 Hex 3 HexNAc 4 Neu5Ac 1

**Mass:**  3761.5413

(-6.186e-06)

**Charge States:** 3

**Start Position**: 161

**End Position**: 177

| Profile Score | Tandem Score | TDA q-value | Volume | Spectrum Matches |
| --- | --- | --- | --- | --- |
| -19.7738 | 103.9700 | 0.0067 | 2.6612e+06 | 1 |

**Adduct Signal Fractions**

- **Unmodified**: 2.6612e+06

### RGSN(N)SSFFSR Fuc 2 Hex 5 HexNAc 6 Neu5Ac 2

**Mass:**  4046.5541

(-7.898e-06)

**Charge States:** 3, 4

**Start Position**: 141

**End Position**: 151

| Profile Score | Tandem Score | TDA q-value | Volume | Spectrum Matches |
| --- | --- | --- | --- | --- |
| 10.3815 | 103.5330 | 0.0067 | 1.0615e+08 | 1 |

**Adduct Signal Fractions**

- **Unmodified**: 1.0615e+08

### N(N)GTYDHDVYRDEALN(D)NR Fuc 1 Hex 5 HexNAc 4

**Mass:**  3820.5371

(2.600e-04)

**Charge States:** 3, 4, 5

**Start Position**: 483

**End Position**: 500

| Profile Score | Tandem Score | TDA q-value | Volume | Spectrum Matches |
| --- | --- | --- | --- | --- |
| -5.6632 | 102.7645 | 0.0067 | 1.0457e+09 | 19 |

**Adduct Signal Fractions**

- **Unmodified**: 1.0457e+09

### N(N)GTYDHDVYRDEALNNR Fuc 4 Hex 6 HexNAc 5

**Mass:**  4622.8581

(7.093e-07)

**Charge States:** 4

**Start Position**: 483

**End Position**: 500

| Profile Score | Tandem Score | TDA q-value | Volume | Spectrum Matches |
| --- | --- | --- | --- | --- |
| -18.7675 | 102.5008 | 0.0067 | 3.8923e+07 | 1 |

**Adduct Signal Fractions**

- **Unmodified**: 3.8923e+07

### ISIYWTIVKPGDILLIN(N)STGNLIAPR Fuc 2 Hex 4 HexNAc 4 Neu5Ac 1

**Mass:**  4910.3527

(-3.976e-06)

**Charge States:** 4

**Start Position**: 230

**End Position**: 256

| Profile Score | Tandem Score | TDA q-value | Volume | Spectrum Matches |
| --- | --- | --- | --- | --- |
| -19.6342 | 102.2116 | 0.0067 | 8.5612e+06 | 1 |

**Adduct Signal Fractions**

- **Unmodified**: 8.5612e+06

### N(N)GTYDHDVYRDEALNNR Fuc 1 Hex 7 HexNAc 6

**Mass:**  4549.8137

(2.406e-06)

**Charge States:** 4

**Start Position**: 483

**End Position**: 500

| Profile Score | Tandem Score | TDA q-value | Volume | Spectrum Matches |
| --- | --- | --- | --- | --- |
| -20.9346 | 101.7853 | 0.0067 | 3.4935e+07 | 1 |

**Adduct Signal Fractions**

- **Unmodified**: 3.4935e+07

### RGSN(N)SSFFSR Fuc 2 Hex 7 HexNAc 7

**Mass:**  3991.6257

(8.190e-06)

**Charge States:** 3, 4

**Start Position**: 141

**End Position**: 151

| Profile Score | Tandem Score | TDA q-value | Volume | Spectrum Matches |
| --- | --- | --- | --- | --- |
| -19.6413 | 100.5839 | 0.0096 | 7.5778e+07 | 1 |

**Adduct Signal Fractions**

- **Unmodified**: 7.5778e+07

### SLVASSGTLEFNNESFNWAGVTHN(N)GTSSSC(C)R Hex 6 HexNAc 4

**Mass:**  5100.1315

(2.631e-06)

**Charge States:** 4

**Start Position**: 110

**End Position**: 141

| Profile Score | Tandem Score | TDA q-value | Volume | Spectrum Matches |
| --- | --- | --- | --- | --- |
| -18.8788 | 100.3284 | 0.0096 | 2.5509e+07 | 2 |

**Adduct Signal Fractions**

- **Unmodified**: 2.5509e+07

### RGSN(N)SSFFSR Fuc 1 Hex 7 HexNAc 3 Neu5Ac 1

**Mass:**  3324.2986

(-1.496e-06)

**Charge States:** 3

**Start Position**: 141

**End Position**: 151

| Profile Score | Tandem Score | TDA q-value | Volume | Spectrum Matches |
| --- | --- | --- | --- | --- |
| -5.4601 | 98.4073 | 0.0096 | 1.5986e+07 | 2 |

**Adduct Signal Fractions**

- **Unmodified**: 1.5986e+07

### LNWLTHLNSKYPALN(N)VTM(O)PNNEQFDK Hex 8 HexNAc 2

**Mass:**  4805.1184

(2.836e-06)

**Charge States:** 4

**Start Position**: 151

**End Position**: 177

| Profile Score | Tandem Score | TDA q-value | Volume | Spectrum Matches |
| --- | --- | --- | --- | --- |
| -17.7626 | 98.0540 | 0.0096 | 3.8715e+07 | 2 |

**Adduct Signal Fractions**

- **Unmodified**: 3.8715e+07

### RGSN(N)SSFFSR Hex 5 HexNAc 6 Neu5Ac 3

**Mass:**  4045.5688

(-8.101e-07)

**Charge States:** 3, 4

**Start Position**: 141

**End Position**: 151

| Profile Score | Tandem Score | TDA q-value | Volume | Spectrum Matches |
| --- | --- | --- | --- | --- |
| 14.3883 | 97.5174 | 0.0096 | 5.8428e+08 | 3 |

**Adduct Signal Fractions**

- **Unmodified**: 5.8428e+08

### C(C)KSEC(C)ITPN(N)GSIPN(D)DKPFQNVNR Hex 9 HexNAc 2

**Mass:**  4539.8791

(2.172e-04)

**Charge States:** 4

**Start Position**: 277

**End Position**: 300

| Profile Score | Tandem Score | TDA q-value | Volume | Spectrum Matches |
| --- | --- | --- | --- | --- |
| -6.3842 | 96.7203 | 0.0096 | 4.4373e+07 | 1 |

**Adduct Signal Fractions**

- **Unmodified**: 4.4373e+07

### ISIYWTIVKPGDILLIN(N)STGNLIAPR Hex 9 HexNAc 2

**Mass:**  4733.2711

(4.238e-04)

**Start Position**: 230

**End Position**: 256

| Profile Score | Tandem Score | TDA q-value | Volume | Spectrum Matches |
| --- | --- | --- | --- | --- |
| 0.0000 | 96.3208 | 0.0096 | 0.0000e+00 | 1 |

Adduct:

No Chromatogram Found

### N(N)GTYDHDVYR Fuc 3 Hex 6 HexNAc 5

**Mass:**  3664.4196

(1.241e-07)

**Charge States:** 3

**Start Position**: 483

**End Position**: 493

| Profile Score | Tandem Score | TDA q-value | Volume | Spectrum Matches |
| --- | --- | --- | --- | --- |
| -21.3651 | 95.9716 | 0.0096 | 6.2640e+06 | 1 |

**Adduct Signal Fractions**

- **Unmodified**: 6.2640e+06

### N(N)GTYDHDVYR Hex 7 HexNAc 3

**Mass:**  2982.1406

(3.429e-07)

**Charge States:** 3

**Start Position**: 483

**End Position**: 493

| Profile Score | Tandem Score | TDA q-value | Volume | Spectrum Matches |
| --- | --- | --- | --- | --- |
| -20.3221 | 95.7100 | 0.0096 | 6.9689e+06 | 1 |

**Adduct Signal Fractions**

- **Unmodified**: 6.9689e+06

### SEC(C)ITPN(N)GSIPN(D)DKPFQNVNR Hex 8 HexNAc 2

**Mass:**  4089.7143

(2.449e-04)

**Charge States:** 3, 4

**Start Position**: 279

**End Position**: 300

| Profile Score | Tandem Score | TDA q-value | Volume | Spectrum Matches |
| --- | --- | --- | --- | --- |
| 9.5316 | 95.6561 | 0.0096 | 1.8147e+08 | 3 |

**Adduct Signal Fractions**

- **Unmodified**: 1.8147e+08

### N(N)GTYDHDVYR Fuc 1 Hex 4 HexNAc 3

**Mass:**  2642.0390

(3.745e-08)

**Charge States:** 3

**Start Position**: 483

**End Position**: 493

| Profile Score | Tandem Score | TDA q-value | Volume | Spectrum Matches |
| --- | --- | --- | --- | --- |
| -19.5062 | 92.9929 | 0.0158 | 3.8240e+06 | 1 |

**Adduct Signal Fractions**

- **Unmodified**: 3.8240e+06

### SEC(C)ITPN(N)GSIPN(D)DKPFQNVNR Hex 7 HexNAc 2

**Mass:**  3927.6662

(2.530e-04)

**Charge States:** 3, 4

**Start Position**: 279

**End Position**: 300

| Profile Score | Tandem Score | TDA q-value | Volume | Spectrum Matches |
| --- | --- | --- | --- | --- |
| 11.6929 | 92.9584 | 0.0158 | 3.3462e+08 | 4 |

**Adduct Signal Fractions**

- **Unmodified**: 3.3462e+08

### N(N)GTYDHDVYR Hex 5 HexNAc 2

**Mass:**  2454.9580

(1.399e-06)

**Charge States:** 3

**Start Position**: 483

**End Position**: 493

| Profile Score | Tandem Score | TDA q-value | Volume | Spectrum Matches |
| --- | --- | --- | --- | --- |
| -20.4348 | 91.5182 | 0.0210 | 4.7521e+06 | 1 |

**Adduct Signal Fractions**

- **Unmodified**: 4.7521e+06

### SEC(C)ITPN(N)GSIPN(D)DKPFQNVNR Hex 9 HexNAc 2

**Mass:**  4251.7663

(2.327e-04)

**Charge States:** 3, 4

**Start Position**: 279

**End Position**: 300

| Profile Score | Tandem Score | TDA q-value | Volume | Spectrum Matches |
| --- | --- | --- | --- | --- |
| 6.4609 | 91.4064 | 0.0210 | 2.4373e+08 | 4 |

**Adduct Signal Fractions**

- **Unmodified**: 2.4373e+08

### N(N)GTYDHDVYR Hex 5 HexNAc 3

**Mass:**  2658.0419

(3.026e-06)

**Charge States:** 3

**Start Position**: 483

**End Position**: 493

| Profile Score | Tandem Score | TDA q-value | Volume | Spectrum Matches |
| --- | --- | --- | --- | --- |
| -20.0013 | 90.9597 | 0.0210 | 3.6410e+06 | 1 |

**Adduct Signal Fractions**

- **Unmodified**: 3.6410e+06

### IEVTN(N)ATELVQN Hex 5 HexNAc 2

**Mass:**  2546.1057

(2.066e-06)

**Charge States:** 3

**Start Position**: 34

**End Position**: 46

| Profile Score | Tandem Score | TDA q-value | Volume | Spectrum Matches |
| --- | --- | --- | --- | --- |
| -19.9213 | 89.0360 | 0.0210 | 1.4555e+06 | 1 |

**Adduct Signal Fractions**

- **Unmodified**: 1.4555e+06

### SLVASSGTLEFNNESFNWAGVTHN(N)GTSSSC(C)R Fuc 1 Hex 5 HexNAc 6 Neu5Ac 2

**Mass:**  6072.4479

(-4.356e-06)

**Charge States:** 4, 5

**Start Position**: 110

**End Position**: 141

| Profile Score | Tandem Score | TDA q-value | Volume | Spectrum Matches |
| --- | --- | --- | --- | --- |
| -18.8330 | 88.0994 | 0.0238 | 1.7056e+07 | 2 |

**Adduct Signal Fractions**

- **Unmodified**: 1.7056e+07

### RGSN(N)SSFFSR Hex 7 HexNAc 3

**Mass:**  2887.1522

(7.629e-07)

**Charge States:** 3

**Start Position**: 141

**End Position**: 151

| Profile Score | Tandem Score | TDA q-value | Volume | Spectrum Matches |
| --- | --- | --- | --- | --- |
| -18.6694 | 85.9996 | 0.0265 | 1.0895e+07 | 1 |

**Adduct Signal Fractions**

- **Unmodified**: 1.0895e+07

### SLVASSGTLEFNNESFNWAGVTHN(N)GTSSSC(C)R Fuc 1 Hex 7 HexNAc 6 Neu5Ac 2

**Mass:**  6396.5456

(-3.135e-06)

**Charge States:** 4

**Start Position**: 110

**End Position**: 141

| Profile Score | Tandem Score | TDA q-value | Volume | Spectrum Matches |
| --- | --- | --- | --- | --- |
| -3.7808 | 84.4177 | 0.0353 | 4.6829e+07 | 2 |

**Adduct Signal Fractions**

- **Unmodified**: 4.6829e+07

### N(N)GTYDHDVYR Fuc 2 Hex 4 HexNAc 9

**Mass:**  4008.5869

(5.026e-04)

**Start Position**: 483

**End Position**: 493

| Profile Score | Tandem Score | TDA q-value | Volume | Spectrum Matches |
| --- | --- | --- | --- | --- |
| 0.0000 | 83.4283 | 0.0407 | 0.0000e+00 | 1 |

Adduct:

No Chromatogram Found

### N(N)GTYDHDVYRDEALN(D)NR Fuc 3 Hex 5 HexNAc 4

**Mass:**  4112.6550

(2.411e-04)

**Charge States:** 4

**Start Position**: 483

**End Position**: 500

| Profile Score | Tandem Score | TDA q-value | Volume | Spectrum Matches |
| --- | --- | --- | --- | --- |
| -5.5675 | 83.2462 | 0.0407 | 2.3460e+08 | 2 |

**Adduct Signal Fractions**

- **Unmodified**: 2.3460e+08

### RGSN(N)SSFFSR Fuc 2 Hex 10 HexNAc 6

**Mass:**  4274.6618

(-1.153e-07)

**Charge States:** 4

**Start Position**: 141

**End Position**: 151

| Profile Score | Tandem Score | TDA q-value | Volume | Spectrum Matches |
| --- | --- | --- | --- | --- |
| -19.2960 | 81.8039 | 0.0431 | 2.9735e+06 | 1 |

**Adduct Signal Fractions**

- **Unmodified**: 2.9735e+06

### cus|SWZHA|A/Switzerland/9715293/2013

**>cus|SWZHA|A/Switzerland/9715293/2013**  
 `QKLPGNDNSTATLCLGHHAVPNGTIVKTITNDRIEVTNATELVQNSSIGEICDSPHQILDGENCTLIDALLGDPQCDGFQ  
NKKWDLFVERSKAYSNCYPYDVPDYASLRSLVASSGTLEFNNESFNWAGVTQNGTSSSCRRGSNSSFFSRLNWLTHLNSK  
YPALNVTMPNNEQFDKLYIWGVHHPVTDKDQIFLYAQSSGRITVSTKRSQQAVIPNIGYRPRIRDIPSRISIYWTIVKPG  
DILLINSTGNLIAPRGYFKIRSGKSSIMRSDAPIGKCKSECITPNGSIPNDKPFQNVNRITYGACPRYVKQSTLKLATGM  
RNVPERQTRGIFGAIAGFIENGWEGMVDGWYGFRHQNSEGRGQAADLKSTQAAIDQINGKLNRLIGKTNEKFHQIEKEFS  
EVEGRIQDLEKYVEDTKIDLWSYNAELLVALENQHTIDLTDSEMNKLFEKTKKQLRENAEDMGNGCFKIYHKCDNACIGS  
IRNGTYDHDVYRDEALNNRFQIKGVELKSGYKDWILWISFAISCFLLCVALLGFIMWACQKGNIRCNICI`

| Site | Type | Count |
| --- | --- | --- |
| 483 | N-linked | 55 |
| 165 | N-linked | 14 |
| 38 | N-linked | 1 |
| 144 | N-linked | 48 |
| 246 | N-linked | 13 |
| 285 | N-linked | 14 |
| 22 | N-linked | 1 |

| Mass | Sequence | MS_2_ Score | `q`-value | MS_1_ Score | Signal | Protein Coordinates | Tandem Scans | Start Time | Apex Time | End Time |
| --- | --- | --- | --- | --- | --- | --- | --- | --- | --- | --- |
| 2547.0504 | RGSN(N)SSFFSR Fuc 1 Hex 4 HexNAc 3 | 192.551 | 0.0 | -4.996 | 5.388e+07 | 140 - 150 | 2 | 19.55 | 20.03 | 20.03 |
| 2522.0186 | RGSN(N)SSFFSR Hex 6 HexNAc 2 | 187.745 | 0.0 | -2.516 | 3.694e+08 | 140 - 150 | 3 | 19.06 | 19.39 | 19.55 |
| 2684.0732 | RGSN(N)SSFFSR Hex 7 HexNAc 2 | 187.187 | 0.0 | 12.351 | 1.951e+08 | 140 - 150 | 4 | 18.39 | 19.23 | 19.39 |
| 2871.1576 | RGSN(N)SSFFSR Fuc 1 Hex 6 HexNAc 3 | 182.895 | 0.0 | 14.803 | 2.509e+08 | 140 - 150 | 4 | 18.22 | 19.06 | 19.39 |
| 2912.1825 | RGSN(N)SSFFSR Fuc 1 Hex 5 HexNAc 4 | 181.650 | 0.0 | 14.114 | 6.666e+08 | 140 - 150 | 7 | 18.56 | 19.39 | 20.20 |
| 3115.2626 | RGSN(N)SSFFSR Fuc 1 Hex 5 HexNAc 5 | 180.569 | 0.0 | 14.508 | 1.563e+09 | 140 - 150 | 8 | 18.56 | 19.70 | 20.20 |
| 2359.9658 | RGSN(N)SSFFSR Hex 5 HexNAc 2 | 178.787 | 0.0 | 13.212 | 4.030e+08 | 140 - 150 | 4 | 18.90 | 19.70 | 20.03 |
| 2725.0980 | RGSN(N)SSFFSR Hex 6 HexNAc 3 | 178.109 | 0.0 | 14.634 | 4.578e+08 | 140 - 150 | 4 | 18.56 | 19.39 | 19.55 |
| 3436.4109 | YPALN(N)VTM(O)PNNEQFDK Hex 7 HexNAc 2 | 177.098 | 0.0 | 15.501 | 5.389e+08 | 160 - 176 | 5 | 35.14 | 35.47 | 35.81 |
| 3598.4682 | YPALN(N)VTM(O)PNNEQFDK Hex 8 HexNAc 2 | 176.212 | 0.0 | 3.140 | 2.675e+09 | 160 - 176 | 7 | 34.80 | 35.31 | 112.83 |
| 2563.0462 | RGSN(N)SSFFSR Hex 5 HexNAc 3 | 173.704 | 0.0 | -3.801 | 5.759e+07 | 140 - 150 | 1 | 19.55 | 19.70 | 19.86 |
| 3760.5158 | YPALN(N)VTM(O)PNNEQFDK Hex 9 HexNAc 2 | 172.800 | 0.0 | 14.596 | 2.315e+08 | 160 - 176 | 4 | 34.97 | 35.31 | 35.64 |
| 2709.1028 | RGSN(N)SSFFSR Fuc 1 Hex 5 HexNAc 3 | 170.682 | 0.0 | -3.753 | 7.876e+07 | 140 - 150 | 2 | 19.39 | 19.39 | 19.86 |
| 3058.2427 | RGSN(N)SSFFSR Fuc 2 Hex 5 HexNAc 4 | 170.114 | 0.0 | 14.584 | 2.337e+08 | 140 - 150 | 4 | 18.39 | 19.06 | 19.23 |
| 2766.1252 | RGSN(N)SSFFSR Hex 5 HexNAc 4 | 167.709 | 0.0 | -3.932 | 9.122e+07 | 140 - 150 | 2 | 19.39 | 19.55 | 20.03 |
| 2400.9928 | RGSN(N)SSFFSR Hex 4 HexNAc 3 | 166.588 | 0.0 | -19.581 | 1.368e+07 | 140 - 150 | 1 | 20.03 | 20.03 | 20.03 |
| 3582.5528 | YPALN(N)VTMPNNEQFDK Hex 8 HexNAc 2 | 166.026 | 0.0 | 12.696 | 3.046e+08 | 160 - 176 | 8 | 42.69 | 43.03 | 44.21 |
| 4085.9555 | TIVKPGDILLIN(N)STGNLIAPR Fuc 2 Hex 3 HexNAc 4 Neu5Ac 1 | 164.824 | 0.0 | -17.883 | 4.489e+07 | 234 - 255 | 1 | 53.73 | 53.73 | 53.90 |
| 4068.9306 | TIVKPGDILLIN(N)STGNLIAPR Hex 9 HexNAc 2 | 164.431 | 0.0 | 19.101 | 4.872e+08 | 234 - 255 | 5 | 53.21 | 53.73 | 54.41 |
| 3204.3002 | RGSN(N)SSFFSR Fuc 3 Hex 5 HexNAc 4 | 162.992 | 0.0 | -2.872 | 8.324e+07 | 140 - 150 | 2 | 18.73 | 18.90 | 19.06 |
| 3153.2315 | N(N)GTYDHDVYR Fuc 2 Hex 5 HexNAc 4 | 160.895 | 0.0 | 15.565 | 2.631e+08 | 482 - 492 | 5 | 14.50 | 14.50 | 15.14 |
| 3423.3728 | RGSN(N)SSFFSR Fuc 2 Hex 6 HexNAc 5 | 159.764 | 0.0 | -20.097 | 1.162e+07 | 140 - 150 | 1 | 18.56 | 18.73 | 18.73 |
| 3278.3221 | RGSN(N)SSFFSR Fuc 1 Hex 6 HexNAc 5 | 159.653 | 0.0 | 0.000 | 0.000e+00 | 140 - 150 | 1 | - | - | - |
| 3906.8782 | TIVKPGDILLIN(N)STGNLIAPR Hex 8 HexNAc 2 | 159.423 | 0.0 | -3.316 | 5.211e+07 | 234 - 255 | 3 | 53.73 | 53.90 | 54.07 |
| 2953.2100 | RGSN(N)SSFFSR Fuc 1 Hex 4 HexNAc 5 | 158.502 | 0.0 | -4.283 | 5.371e+07 | 140 - 150 | 2 | 19.39 | 19.55 | 19.86 |
| 3261.3219 | RGSN(N)SSFFSR Fuc 2 Hex 5 HexNAc 5 | 158.360 | 0.0 | 13.968 | 3.148e+08 | 140 - 150 | 5 | 18.39 | 19.23 | 19.39 |
| 2527.9656 | GSN(N)SSFFSR Hex 7 HexNAc 2 | 158.330 | 0.0 | -5.120 | 3.999e+07 | 141 - 150 | 1 | 22.78 | 22.94 | 23.11 |
| 3420.4105 | YPALN(N)VTMPNNEQFDK Hex 7 HexNAc 2 | 155.533 | 0.0 | -3.237 | 4.252e+07 | 160 - 176 | 3 | 42.69 | 43.03 | 43.20 |
| 4376.8446 | C(C)KSEC(C)ITPN(N)GSIPNDKPFQNVNR Hex 8 HexNAc 2 | 155.184 | 0.0 | -3.881 | 1.376e+08 | 276 - 299 | 1 | 29.66 | 29.66 | 29.99 |
| 2756.0817 | GSN(N)SSFFSR Fuc 1 Hex 5 HexNAc 4 | 155.043 | 0.0 | 11.847 | 2.522e+08 | 141 - 150 | 2 | 22.94 | 22.94 | 23.90 |
| 4538.8973 | C(C)KSEC(C)ITPN(N)GSIPNDKPFQNVNR Hex 9 HexNAc 2 | 154.476 | 0.0 | -3.717 | 2.292e+08 | 276 - 299 | 3 | 29.50 | 29.66 | 29.83 |
| 3407.3813 | RGSN(N)SSFFSR Fuc 3 Hex 5 HexNAc 5 | 154.260 | 0.0 | -18.509 | 3.239e+07 | 140 - 150 | 3 | 18.73 | 18.90 | 18.90 |
| 2969.2045 | RGSN(N)SSFFSR Hex 5 HexNAc 5 | 154.224 | 0.0 | 12.946 | 2.980e+08 | 140 - 150 | 3 | 18.73 | 19.70 | 19.86 |
| 3778.5267 | N(N)GTYDHDVYRDEALNNR Fuc 1 Hex 6 HexNAc 3 | 153.311 | 0.0 | -3.624 | 2.971e+08 | 482 - 499 | 2 | 20.65 | 20.65 | 21.11 |
| 2928.1777 | RGSN(N)SSFFSR Hex 6 HexNAc 4 | 152.623 | 0.0 | 9.607 | 1.161e+08 | 140 - 150 | 2 | 19.23 | 19.39 | 20.03 |
| 3112.3065 | YPALN(N)VTM(O)PNNEQFDK Hex 5 HexNAc 2 | 151.800 | 0.0 | 12.942 | 3.132e+07 | 160 - 176 | 2 | 35.47 | 35.98 | 36.15 |
| 3007.1745 | N(N)GTYDHDVYR Fuc 1 Hex 5 HexNAc 4 | 151.798 | 0.0 | 16.144 | 4.201e+08 | 482 - 492 | 6 | 14.50 | 14.82 | 15.31 |
| 4214.7922 | C(C)KSEC(C)ITPN(N)GSIPNDKPFQNVNR Hex 7 HexNAc 2 | 151.758 | 0.0 | -17.484 | 2.394e+08 | 276 - 299 | 1 | 29.66 | 29.83 | 29.83 |
| 4731.2739 | ISIYWTIVKPGDILLIN(N)STGNLIAPR Hex 9 HexNAc 2 | 151.247 | 0.0 | 19.938 | 8.766e+08 | 229 - 255 | 10 | 68.58 | 69.36 | 69.85 |
| 3274.3575 | YPALN(N)VTM(O)PNNEQFDK Hex 6 HexNAc 2 | 150.623 | 0.0 | 12.604 | 4.201e+07 | 160 - 176 | 3 | 35.31 | 35.64 | 35.98 |
| 2365.9177 | GSN(N)SSFFSR Hex 6 HexNAc 2 | 150.460 | 0.0 | -3.698 | 1.255e+08 | 141 - 150 | 2 | 22.94 | 23.26 | 23.26 |
| 2846.1269 | RGSN(N)SSFFSR Hex 8 HexNAc 2 | 149.909 | 0.0 | -3.088 | 7.197e+07 | 140 - 150 | 1 | 18.90 | 19.06 | 19.23 |
| 2203.8645 | GSN(N)SSFFSR Hex 5 HexNAc 2 | 148.743 | 0.0 | -2.280 | 2.013e+08 | 141 - 150 | 3 | 23.26 | 23.59 | 23.75 |
| 3017.2163 | RGSN(N)SSFFSR Fuc 2 Hex 6 HexNAc 3 | 147.861 | 0.0 | -17.894 | 1.807e+07 | 140 - 150 | 1 | 19.06 | 19.06 | 19.06 |
| 3074.2355 | RGSN(N)SSFFSR Fuc 1 Hex 6 HexNAc 4 | 147.736 | 0.0 | -5.259 | 3.861e+07 | 140 - 150 | 2 | 19.06 | 19.70 | 19.86 |
| 4893.3257 | ISIYWTIVKPGDILLIN(N)STGNLIAPR Hex 10 HexNAc 2 | 147.623 | 0.0 | -4.106 | 4.252e+07 | 229 - 255 | 4 | 69.02 | 69.19 | 69.53 |
| 3172.2854 | RGSN(N)SSFFSR Hex 5 HexNAc 6 | 147.236 | 0.0 | -2.092 | 2.472e+08 | 140 - 150 | 2 | 19.70 | 19.86 | 20.03 |
| 4569.2192 | ISIYWTIVKPGDILLIN(N)STGNLIAPR Hex 8 HexNAc 2 | 146.263 | 0.0 | 15.516 | 9.151e+07 | 229 - 255 | 5 | 69.02 | 69.36 | 69.70 |
| 2959.1623 | GSN(N)SSFFSR Fuc 1 Hex 5 HexNAc 5 | 144.682 | 0.0 | 13.094 | 6.069e+08 | 141 - 150 | 5 | 22.61 | 23.26 | 23.75 |
| 2855.1602 | RGSN(N)SSFFSR Fuc 2 Hex 5 HexNAc 3 | 143.885 | 0.0 | -17.295 | 2.300e+07 | 140 - 150 | 1 | 19.39 | 19.39 | 19.39 |
| 4748.2969 | ISIYWTIVKPGDILLIN(N)STGNLIAPR Fuc 2 Hex 3 HexNAc 4 Neu5Ac 1 | 143.225 | 0.0 | 19.502 | 1.505e+08 | 229 - 255 | 3 | 69.19 | 69.36 | 69.85 |
| 3899.5114 | N(N)GTYDHDVYRDEALNNR Fuc 1 Hex 8 HexNAc 2 | 142.507 | 0.0 | 13.853 | 6.512e+08 | 482 - 499 | 3 | 22.27 | 22.61 | 22.94 |
| 3965.6101 | N(N)GTYDHDVYRDEALNNR Fuc 2 Hex 5 HexNAc 4 | 141.997 | 0.0 | 17.083 | 2.403e+09 | 482 - 499 | 4 | 20.35 | 20.65 | 20.96 |
| 4230.9816 | TIVKPGDILLIN(N)STGNLIAPR Hex 10 HexNAc 2 | 141.435 | 0.0 | -19.163 | 1.652e+07 | 234 - 255 | 2 | 53.56 | 53.73 | 53.73 |
| 3627.6336 | PGDILLIN(N)STGNLIAPR Hex 9 HexNAc 2 | 141.327 | 0.0 | -18.503 | 2.537e+07 | 238 - 255 | 3 | 52.02 | 52.02 | 52.19 |
| 3267.3355 | N(N)GTYDHDVYRDEALNNR Hex 5 HexNAc 2 | 141.244 | 0.0 | -4.851 | 9.602e+07 | 482 - 499 | 1 | 20.96 | 21.11 | 21.28 |
| 3356.3131 | N(N)GTYDHDVYR Fuc 2 Hex 5 HexNAc 5 | 140.452 | 0.0 | -19.137 | 5.374e+07 | 482 - 492 | 4 | 14.66 | 14.66 | 14.82 |
| 3277.3155 | RGSN(N)SSFFSR Fuc 1 Hex 6 HexNAc 5 | 140.002 | 0.0 | -3.774 | 8.049e+07 | 140 - 150 | 3 | 18.73 | 18.90 | 19.23 |
| 3967.8799 | IVKPGDILLIN(N)STGNLIAPR Hex 9 HexNAc 2 | 138.589 | 0.0 | -18.347 | 9.414e+06 | 235 - 255 | 2 | 53.04 | 53.04 | 53.21 |
| 3008.3236 | HAVPN(N)GTIVK Fuc 1 Hex 5 HexNAc 5 | 138.124 | 0.0 | 0.000 | 0.000e+00 | 17 - 27 | 1 | - | - | - |
| 3454.4201 | N(N)GTYDHDVYRDEALNNR Fuc 1 Hex 4 HexNAc 3 | 137.699 | 0.0 | -3.000 | 8.758e+07 | 482 - 499 | 1 | 20.96 | 21.28 | 21.28 |
| 3777.5420 | YPALN(N)VTM(O)PNNEQFDK Fuc 2 Hex 3 HexNAc 4 Neu5Ac 1 | 137.455 | 0.0 | -18.197 | 1.653e+07 | 160 - 176 | 1 | 35.14 | 35.31 | 35.31 |
| 3299.2877 | N(N)GTYDHDVYR Fuc 3 Hex 5 HexNAc 4 | 136.394 | 0.0 | -19.073 | 8.878e+07 | 482 - 492 | 2 | 14.35 | 14.35 | 14.50 |
| 3599.4518 | YPALN(N)VTM(O)PN(D)NEQFDK Hex 8 HexNAc 2 | 135.779 | 0.0 | 9.640 | 3.209e+08 | 160 - 176 | 27 | 35.81 | 36.15 | 37.34 |
| 4248.6497 | N(N)GTYDHDVYRDEALNNR Fuc 2 Hex 8 HexNAc 3 | 135.368 | 0.0 | 11.575 | 1.855e+08 | 482 - 499 | 2 | 21.77 | 22.44 | 22.78 |
| 3632.4656 | N(N)GTYDHDVYRDEALNNR Hex 6 HexNAc 3 | 135.262 | 0.0 | -3.901 | 2.274e+08 | 482 - 499 | 3 | 20.65 | 20.96 | 21.11 |
| 3819.5501 | N(N)GTYDHDVYRDEALNNR Fuc 1 Hex 5 HexNAc 4 | 133.678 | 0.0 | 16.117 | 3.378e+09 | 482 - 499 | 6 | 20.50 | 20.80 | 21.28 |
| 3706.4150 | RGSN(N)SSFFSR Fuc 2 Hex 9 HexNAc 4 | 132.856 | 0.0 | 9.312 | 3.226e+07 | 140 - 150 | 2 | 21.11 | 21.28 | 21.77 |
| 3673.4923 | N(N)GTYDHDVYRDEALNNR Hex 5 HexNAc 4 | 132.477 | 0.0 | 13.013 | 1.752e+08 | 482 - 499 | 3 | 20.65 | 20.96 | 21.28 |
| 4088.7191 | SEC(C)ITPN(N)GSIPNDKPFQNVNR Hex 8 HexNAc 2 | 132.310 | 0.0 | 16.325 | 9.453e+08 | 278 - 299 | 4 | 32.45 | 32.61 | 33.11 |
| 3090.2327 | RGSN(N)SSFFSR Hex 7 HexNAc 4 | 132.124 | 0.0 | -19.496 | 1.752e+07 | 140 - 150 | 1 | 19.23 | 19.39 | 19.39 |
| 3518.3639 | N(N)GTYDHDVYR Fuc 2 Hex 6 HexNAc 5 | 131.894 | 0.0 | -19.755 | 2.006e+07 | 482 - 492 | 2 | 14.50 | 14.50 | 14.50 |
| 3926.6684 | SEC(C)ITPN(N)GSIPNDKPFQNVNR Hex 7 HexNAc 2 | 131.470 | 0.0 | 16.149 | 1.580e+09 | 278 - 299 | 6 | 32.45 | 32.61 | 33.11 |
| 2966.1475 | N(N)GTYDHDVYR Fuc 1 Hex 6 HexNAc 3 | 131.091 | 0.0 | -18.003 | 3.579e+07 | 482 - 492 | 1 | 14.50 | 14.50 | 14.66 |
| 4185.6732 | RGSN(N)SSFFSR Fuc 1 Hex 3 HexNAc 9 Neu5Ac 2 | 130.506 | 0.0 | -17.685 | 4.991e+07 | 140 - 150 | 1 | 21.77 | 21.77 | 21.94 |
| 4264.6437 | N(N)GTYDHDVYRDEALNNR Fuc 1 Hex 9 HexNAc 3 | 130.103 | 0.0 | -3.379 | 1.513e+08 | 482 - 499 | 2 | 22.27 | 22.27 | 22.78 |
| 2820.0893 | N(N)GTYDHDVYR Hex 6 HexNAc 3 | 129.951 | 0.0 | -18.914 | 1.997e+07 | 482 - 492 | 1 | 14.66 | 14.66 | 14.66 |
| 4022.6301 | N(N)GTYDHDVYRDEALNNR Fuc 1 Hex 5 HexNAc 5 | 129.623 | 0.0 | 10.621 | 2.208e+09 | 482 - 499 | 7 | 19.86 | 20.96 | 21.28 |
| 4250.7727 | SEC(C)ITPN(N)GSIPNDKPFQNVNR Hex 9 HexNAc 2 | 129.278 | 0.0 | -1.638 | 1.438e+09 | 278 - 299 | 3 | 32.45 | 32.45 | 32.94 |
| 3764.6174 | SEC(C)ITPN(N)GSIPNDKPFQNVNR Hex 6 HexNAc 2 | 129.272 | 0.0 | -2.891 | 2.213e+08 | 278 - 299 | 3 | 32.61 | 32.77 | 33.11 |
| 4330.7424 | N(N)GTYDHDVYRDEALNNR Fuc 2 Hex 6 HexNAc 5 | 128.209 | 0.0 | 13.543 | 2.475e+08 | 482 - 499 | 1 | 20.35 | 20.50 | 20.96 |
| 3600.4777 | N(N)GTYDHDVYRDEALNNR Fuc 2 Hex 4 HexNAc 3 | 128.165 | 0.0 | -16.840 | 7.534e+07 | 482 - 499 | 2 | 20.96 | 20.96 | 21.11 |
| 2693.1077 | RGSN(N)SSFFSR Fuc 2 Hex 4 HexNAc 3 | 127.462 | 0.0 | -18.359 | 2.218e+07 | 140 - 150 | 1 | 19.70 | 19.70 | 19.70 |
| 3480.3978 | RGSN(N)SSFFSR Fuc 1 Hex 6 HexNAc 6 | 126.299 | 0.0 | -4.716 | 4.814e+07 | 140 - 150 | 3 | 18.73 | 19.23 | 19.23 |
| 4476.8006 | N(N)GTYDHDVYRDEALNNR Fuc 3 Hex 6 HexNAc 5 | 126.139 | 0.0 | -4.353 | 1.245e+08 | 482 - 499 | 1 | 20.35 | 20.50 | 20.65 |
| 4048.6397 | RGSN(N)SSFFSR Fuc 1 Hex 7 HexNAc 8 | 125.486 | 0.0 | -18.144 | 5.510e+07 | 140 - 150 | 1 | 23.42 | 23.42 | 23.59 |
| 4184.6844 | N(N)GTYDHDVYRDEALNNR Fuc 1 Hex 6 HexNAc 5 | 124.693 | 0.0 | 11.420 | 6.097e+08 | 482 - 499 | 3 | 20.50 | 20.65 | 21.11 |
| 3616.4721 | N(N)GTYDHDVYRDEALNNR Fuc 1 Hex 5 HexNAc 3 | 124.205 | 0.0 | 12.382 | 1.122e+08 | 482 - 499 | 2 | 20.80 | 21.11 | 21.44 |
| 3211.2617 | N(N)GTYDHDVYR Fuc 1 Hex 5 HexNAc 5 | 123.157 | 0.0 | 0.000 | 0.000e+00 | 482 - 492 | 1 | - | - | - |
| 3602.5666 | SEC(C)ITPN(N)GSIPNDKPFQNVNR Hex 5 HexNAc 2 | 122.741 | 0.0 | -4.235 | 6.902e+07 | 278 - 299 | 2 | 32.77 | 32.94 | 33.11 |
| 4102.5902 | N(N)GTYDHDVYRDEALNNR Fuc 1 Hex 8 HexNAc 3 | 121.156 | 0.0 | 11.022 | 3.417e+08 | 482 - 499 | 5 | 21.60 | 22.61 | 23.11 |
| 3762.5291 | N(N)GTYDHDVYRDEALNNR Fuc 2 Hex 5 HexNAc 3 | 120.998 | 0.0 | -4.264 | 5.741e+07 | 482 - 499 | 1 | 20.80 | 20.80 | 21.11 |
| 4111.6681 | N(N)GTYDHDVYRDEALNNR Fuc 3 Hex 5 HexNAc 4 | 120.915 | 0.0 | 15.727 | 9.365e+08 | 482 - 499 | 3 | 20.35 | 20.50 | 20.96 |
| 3744.5159 | YPALN(N)VTMPNNEQFDK Hex 9 HexNAc 2 | 120.572 | 0.0 | -4.523 | 2.403e+07 | 160 - 176 | 3 | 42.69 | 42.86 | 43.03 |
| 3414.3042 | RGSN(N)SSFFSR Hex 9 HexNAc 4 | 120.212 | 0.0 | -4.653 | 1.582e+07 | 140 - 150 | 1 | 21.44 | 21.60 | 21.77 |
| 3210.2535 | N(N)GTYDHDVYR Fuc 1 Hex 5 HexNAc 5 | 119.999 | 0.0 | 10.250 | 1.991e+08 | 482 - 492 | 6 | 14.82 | 14.98 | 15.81 |
| 4912.0910 | SEC(C)ITPN(N)GSIPNDKPFQNVNR Fuc 2 Hex 5 HexNAc 7 | 119.740 | 0.0 | -6.382 | 1.334e+06 | 278 - 299 | 1 | 72.45 | 72.66 | 72.72 |
| 4387.7631 | N(N)GTYDHDVYRDEALNNR Fuc 1 Hex 6 HexNAc 6 | 119.599 | 0.0 | 7.062 | 1.596e+08 | 482 - 499 | 2 | 20.03 | 20.80 | 20.96 |
| 4267.7988 | SEC(C)ITPN(N)GSIPNDKPFQNVNR Fuc 2 Hex 3 HexNAc 4 Neu5Ac 1 | 118.874 | 0.0 | -21.113 | 4.557e+07 | 278 - 299 | 1 | 32.45 | 32.45 | 32.61 |
| 3099.2658 | RGSN(N)SSFFSR Fuc 2 Hex 4 HexNAc 5 | 118.395 | 0.0 | -18.371 | 1.592e+07 | 140 - 150 | 1 | 19.06 | 19.23 | 19.23 |
| 3267.2739 | N(N)GTYDHDVYR Hex 5 HexNAc 6 | 117.749 | 0.0 | -5.679 | 2.985e+07 | 482 - 492 | 3 | 14.35 | 14.66 | 14.82 |
| 4168.6873 | N(N)GTYDHDVYRDEALNNR Fuc 2 Hex 5 HexNAc 5 | 117.118 | 0.0 | -3.208 | 4.334e+08 | 482 - 499 | 2 | 20.65 | 20.80 | 21.11 |
| 4743.2696 | TIVKPGDILLIN(N)STGNLIAPR Fuc 4 Hex 4 HexNAc 5 Neu5Ac 1 | 114.465 | 0.0 | 13.979 | 5.224e+07 | 234 - 255 | 6 | 72.66 | 73.30 | 73.75 |
| 3966.5985 | N(N)GTYDHDVYRDEALN(D)NR Fuc 2 Hex 5 HexNAc 4 | 114.372 | 0.0 | 6.385 | 6.913e+08 | 482 - 499 | 4 | 21.11 | 21.28 | 21.94 |
| 3779.5152 | N(N)GTYDHDVYRDEALN(D)NR Fuc 1 Hex 6 HexNAc 3 | 114.197 | 0.0 | -5.772 | 7.151e+07 | 482 - 499 | 2 | 21.28 | 21.28 | 21.94 |
| 4410.7024 | N(N)GTYDHDVYRDEALNNR Fuc 2 Hex 9 HexNAc 3 | 113.309 | 0.0 | -4.357 | 1.058e+08 | 482 - 499 | 2 | 22.11 | 22.27 | 22.61 |
| 2861.1179 | N(N)GTYDHDVYR Hex 5 HexNAc 4 | 112.854 | 0.0 | -19.169 | 1.546e+07 | 482 - 492 | 1 | 14.66 | 14.82 | 14.82 |
| 3437.3979 | YPALN(N)VTM(O)PN(D)NEQFDK Hex 7 HexNAc 2 | 112.643 | 0.0 | 10.605 | 5.022e+07 | 160 - 176 | 3 | 35.81 | 36.15 | 37.00 |
| 3128.2003 | N(N)GTYDHDVYR Fuc 1 Hex 7 HexNAc 3 | 112.578 | 0.0 | -19.624 | 5.275e+06 | 482 - 492 | 1 | 14.35 | 14.35 | 14.35 |
| 3940.5839 | N(N)GTYDHDVYRDEALNNR Fuc 1 Hex 7 HexNAc 3 | 112.202 | 0.0 | -19.016 | 7.130e+07 | 482 - 499 | 1 | 20.50 | 20.65 | 20.65 |
| 3633.4552 | N(N)GTYDHDVYRDEALN(D)NR Hex 6 HexNAc 3 | 112.192 | 0.0 | -5.940 | 5.836e+07 | 482 - 499 | 1 | 21.44 | 21.44 | 22.11 |
| 3372.3056 | N(N)GTYDHDVYR Fuc 1 Hex 6 HexNAc 5 | 112.191 | 0.0 | -4.325 | 6.090e+07 | 482 - 492 | 4 | 14.50 | 14.66 | 14.98 |
| 2788.0980 | N(N)GTYDHDVYR Fuc 2 Hex 4 HexNAc 3 | 111.725 | 0.0 | -18.684 | 5.185e+06 | 482 - 492 | 1 | 14.82 | 14.82 | 14.82 |
| 4467.7224 | N(N)GTYDHDVYRDEALNNR Fuc 1 Hex 9 HexNAc 4 | 111.523 | 0.0 | 8.819 | 5.121e+07 | 482 - 499 | 1 | 21.94 | 22.44 | 22.61 |
| 3318.3409 | RGSN(N)SSFFSR Fuc 1 Hex 5 HexNAc 6 | 110.892 | 0.0 | -5.076 | 4.413e+07 | 140 - 150 | 1 | 19.23 | 19.39 | 19.55 |
| 3794.5221 | N(N)GTYDHDVYRDEALNNR Hex 7 HexNAc 3 | 109.697 | 0.0 | -19.662 | 4.700e+07 | 482 - 499 | 2 | 20.65 | 20.80 | 20.80 |
| 4314.7491 | N(N)GTYDHDVYRDEALNNR Fuc 3 Hex 5 HexNAc 5 | 108.365 | 0.0 | -18.494 | 7.415e+07 | 482 - 499 | 2 | 20.50 | 20.65 | 20.65 |
| 4137.6801 | RGSN(N)SSFFSR Fuc 3 Hex 7 HexNAc 7 | 107.776 | 0.0 | -20.476 | 3.167e+07 | 140 - 150 | 1 | 22.94 | 23.11 | 23.11 |
| 3258.3573 | YPALN(N)VTMPNNEQFDK Hex 6 HexNAc 2 | 107.258 | 0.004 | -20.347 | 2.144e+06 | 160 - 176 | 1 | 43.20 | 43.20 | 43.37 |
| 3575.3869 | N(N)GTYDHDVYR Fuc 1 Hex 6 HexNAc 6 | 106.451 | 0.007 | -19.577 | 1.388e+07 | 482 - 492 | 2 | 14.82 | 14.98 | 14.98 |
| 4533.8258 | N(N)GTYDHDVYRDEALNNR Fuc 2 Hex 6 HexNAc 6 | 106.025 | 0.007 | -19.646 | 4.795e+07 | 482 - 499 | 1 | 20.65 | 20.65 | 20.80 |
| 3761.5413 | YPALN(N)VTMPNNEQFDK Fuc 2 Hex 3 HexNAc 4 Neu5Ac 1 | 103.970 | 0.007 | -19.774 | 2.661e+06 | 160 - 176 | 1 | 42.86 | 42.86 | 42.86 |
| 4046.5557 | RGSN(N)SSFFSR Fuc 2 Hex 5 HexNAc 6 Neu5Ac 2 | 103.533 | 0.007 | 10.381 | 1.062e+08 | 140 - 150 | 1 | 22.94 | 22.94 | 23.75 |
| 3820.5389 | N(N)GTYDHDVYRDEALN(D)NR Fuc 1 Hex 5 HexNAc 4 | 102.764 | 0.007 | -5.663 | 1.046e+09 | 482 - 499 | 19 | 21.44 | 21.44 | 22.11 |
| 4622.8581 | N(N)GTYDHDVYRDEALNNR Fuc 4 Hex 6 HexNAc 5 | 102.501 | 0.007 | -18.767 | 3.892e+07 | 482 - 499 | 1 | 20.35 | 20.35 | 20.35 |
| 4910.3514 | ISIYWTIVKPGDILLIN(N)STGNLIAPR Fuc 2 Hex 4 HexNAc 4 Neu5Ac 1 | 102.212 | 0.007 | -19.634 | 8.561e+06 | 229 - 255 | 1 | 69.19 | 69.19 | 69.36 |
| 4549.8187 | N(N)GTYDHDVYRDEALNNR Fuc 1 Hex 7 HexNAc 6 | 101.785 | 0.007 | -20.935 | 3.493e+07 | 482 - 499 | 1 | 20.35 | 20.50 | 20.50 |
| 3991.6214 | RGSN(N)SSFFSR Fuc 2 Hex 7 HexNAc 7 | 100.584 | 0.010 | -19.641 | 7.578e+07 | 140 - 150 | 1 | 23.11 | 23.26 | 23.26 |
| 3324.2985 | RGSN(N)SSFFSR Fuc 1 Hex 7 HexNAc 3 Neu5Ac 1 | 98.407 | 0.010 | -5.460 | 1.599e+07 | 140 - 150 | 2 | 22.27 | 22.61 | 22.61 |
| 4805.1179 | LNWLTHLNSKYPALN(N)VTM(O)PNNEQFDK Hex 8 HexNAc 2 | 98.054 | 0.010 | -17.763 | 3.871e+07 | 150 - 176 | 2 | 48.44 | 48.44 | 48.61 |
| 4045.5690 | RGSN(N)SSFFSR Hex 5 HexNAc 6 Neu5Ac 3 | 97.517 | 0.010 | 14.388 | 5.843e+08 | 140 - 150 | 3 | 22.11 | 22.44 | 22.78 |
| 4539.8795 | C(C)KSEC(C)ITPN(N)GSIPN(D)DKPFQNVNR Hex 9 HexNAc 2 | 96.720 | 0.010 | -6.384 | 4.437e+07 | 276 - 299 | 1 | 30.96 | 31.12 | 31.29 |
| 4733.2711 | ISIYWTIVKPGDILLIN(N)STGNLIAPR Hex 9 HexNAc 2 | 96.321 | 0.010 | 0.000 | 0.000e+00 | 229 - 255 | 1 | - | - | - |
| 3664.4196 | N(N)GTYDHDVYR Fuc 3 Hex 6 HexNAc 5 | 95.972 | 0.010 | -21.365 | 6.264e+06 | 482 - 492 | 1 | 14.35 | 14.35 | 14.35 |
| 2982.1401 | N(N)GTYDHDVYR Hex 7 HexNAc 3 | 95.710 | 0.010 | -20.322 | 6.969e+06 | 482 - 492 | 1 | 14.50 | 14.50 | 14.66 |
| 4089.7124 | SEC(C)ITPN(N)GSIPN(D)DKPFQNVNR Hex 8 HexNAc 2 | 95.656 | 0.010 | 9.532 | 1.815e+08 | 278 - 299 | 3 | 33.11 | 34.13 | 34.13 |
| 2642.0390 | N(N)GTYDHDVYR Fuc 1 Hex 4 HexNAc 3 | 92.993 | 0.016 | -19.506 | 3.824e+06 | 482 - 492 | 1 | 14.98 | 14.98 | 14.98 |
| 3927.6598 | SEC(C)ITPN(N)GSIPN(D)DKPFQNVNR Hex 7 HexNAc 2 | 92.958 | 0.016 | 11.693 | 3.346e+08 | 278 - 299 | 4 | 33.28 | 34.13 | 34.30 |
| 2454.9580 | N(N)GTYDHDVYR Hex 5 HexNAc 2 | 91.518 | 0.021 | -20.435 | 4.752e+06 | 482 - 492 | 1 | 14.82 | 14.82 | 14.82 |
| 4251.7638 | SEC(C)ITPN(N)GSIPN(D)DKPFQNVNR Hex 9 HexNAc 2 | 91.406 | 0.021 | 6.461 | 2.437e+08 | 278 - 299 | 4 | 33.11 | 33.96 | 34.13 |
| 2658.0419 | N(N)GTYDHDVYR Hex 5 HexNAc 3 | 90.960 | 0.021 | -20.001 | 3.641e+06 | 482 - 492 | 1 | 14.82 | 14.82 | 14.82 |
| 2546.1057 | IEVTN(N)ATELVQN Hex 5 HexNAc 2 | 89.036 | 0.021 | -19.921 | 1.456e+06 | 33 - 45 | 1 | 36.15 | 36.15 | 36.15 |
| 2887.1522 | RGSN(N)SSFFSR Hex 7 HexNAc 3 | 86.000 | 0.027 | -18.669 | 1.089e+07 | 140 - 150 | 1 | 19.23 | 19.23 | 19.23 |
| 4008.5869 | N(N)GTYDHDVYR Fuc 2 Hex 4 HexNAc 9 | 83.428 | 0.041 | 0.000 | 0.000e+00 | 482 - 492 | 1 | - | - | - |
| 4112.6562 | N(N)GTYDHDVYRDEALN(D)NR Fuc 3 Hex 5 HexNAc 4 | 83.246 | 0.041 | -5.567 | 2.346e+08 | 482 - 499 | 2 | 21.11 | 21.11 | 21.77 |
| 4274.6618 | RGSN(N)SSFFSR Fuc 2 Hex 10 HexNAc 6 | 81.804 | 0.043 | -19.296 | 2.973e+06 | 140 - 150 | 1 | 24.87 | 24.87 | 24.87 |

### RGSN(N)SSFFSR Fuc 1 Hex 4 HexNAc 3

**Mass:**  2547.0510

(6.508e-07)

**Charge States:** 3

**Start Position**: 141

**End Position**: 151

| Profile Score | Tandem Score | TDA q-value | Volume | Spectrum Matches |
| --- | --- | --- | --- | --- |
| -4.9956 | 192.5506 | 0.0000 | 5.3877e+07 | 2 |

**Adduct Signal Fractions**

- **Unmodified**: 5.3877e+07

### RGSN(N)SSFFSR Hex 6 HexNAc 2

**Mass:**  2522.0185

(1.115e-06)

**Charge States:** 3

**Start Position**: 141

**End Position**: 151

| Profile Score | Tandem Score | TDA q-value | Volume | Spectrum Matches |
| --- | --- | --- | --- | --- |
| -2.5158 | 187.7451 | 0.0000 | 3.6936e+08 | 3 |

**Adduct Signal Fractions**

- **Unmodified**: 3.6936e+08

### RGSN(N)SSFFSR Hex 7 HexNAc 2

**Mass:**  2684.0733

(9.619e-07)

**Charge States:** 3

**Start Position**: 141

**End Position**: 151

| Profile Score | Tandem Score | TDA q-value | Volume | Spectrum Matches |
| --- | --- | --- | --- | --- |
| 12.3506 | 187.1874 | 0.0000 | 1.9512e+08 | 4 |

**Adduct Signal Fractions**

- **Unmodified**: 1.9512e+08

### RGSN(N)SSFFSR Fuc 1 Hex 6 HexNAc 3

**Mass:**  2871.1582

(1.072e-06)

**Charge States:** 3

**Start Position**: 141

**End Position**: 151

| Profile Score | Tandem Score | TDA q-value | Volume | Spectrum Matches |
| --- | --- | --- | --- | --- |
| 14.8032 | 182.8952 | 0.0000 | 2.5088e+08 | 4 |

**Adduct Signal Fractions**

- **Unmodified**: 2.5088e+08

### RGSN(N)SSFFSR Fuc 1 Hex 5 HexNAc 4

**Mass:**  2912.1817

(9.044e-07)

**Charge States:** 3

**Start Position**: 141

**End Position**: 151

| Profile Score | Tandem Score | TDA q-value | Volume | Spectrum Matches |
| --- | --- | --- | --- | --- |
| 14.1140 | 181.6497 | 0.0000 | 6.6662e+08 | 7 |

**Adduct Signal Fractions**

- **Unmodified**: 6.6662e+08

### RGSN(N)SSFFSR Fuc 1 Hex 5 HexNAc 5

**Mass:**  3115.2621

(5.407e-07)

**Charge States:** 3, 4

**Start Position**: 141

**End Position**: 151

| Profile Score | Tandem Score | TDA q-value | Volume | Spectrum Matches |
| --- | --- | --- | --- | --- |
| 14.5079 | 180.5694 | 0.0000 | 1.5630e+09 | 8 |

**Adduct Signal Fractions**

- **Unmodified**: 1.5630e+09

### RGSN(N)SSFFSR Hex 5 HexNAc 2

**Mass:**  2359.9654

(5.595e-07)

**Charge States:** 3

**Start Position**: 141

**End Position**: 151

| Profile Score | Tandem Score | TDA q-value | Volume | Spectrum Matches |
| --- | --- | --- | --- | --- |
| 13.2119 | 178.7873 | 0.0000 | 4.0297e+08 | 4 |

**Adduct Signal Fractions**

- **Unmodified**: 4.0297e+08

### RGSN(N)SSFFSR Hex 6 HexNAc 3

**Mass:**  2725.0977

(-1.898e-07)

**Charge States:** 3

**Start Position**: 141

**End Position**: 151

| Profile Score | Tandem Score | TDA q-value | Volume | Spectrum Matches |
| --- | --- | --- | --- | --- |
| 14.6345 | 178.1088 | 0.0000 | 4.5779e+08 | 4 |

**Adduct Signal Fractions**

- **Unmodified**: 4.5779e+08

### YPALN(N)VTM(O)PNNEQFDK Hex 7 HexNAc 2

**Mass:**  3436.4126

(3.463e-06)

**Charge States:** 2, 3, 4

**Start Position**: 161

**End Position**: 177

| Profile Score | Tandem Score | TDA q-value | Volume | Spectrum Matches |
| --- | --- | --- | --- | --- |
| 15.5006 | 177.0981 | 0.0000 | 5.3888e+08 | 5 |

**Adduct Signal Fractions**

- **Unmodified**: 5.3888e+08

### YPALN(N)VTM(O)PNNEQFDK Hex 8 HexNAc 2

**Mass:**  3598.4752

(2.875e-06)

**Charge States:** 2, 3, 4

**Start Position**: 161

**End Position**: 177

| Profile Score | Tandem Score | TDA q-value | Volume | Spectrum Matches |
| --- | --- | --- | --- | --- |
| 3.1404 | 176.2119 | 0.0000 | 2.6753e+09 | 7 |

**Adduct Signal Fractions**

- **Unmodified**: 2.6753e+09

### RGSN(N)SSFFSR Hex 5 HexNAc 3

**Mass:**  2563.0460

(6.692e-07)

**Charge States:** 3

**Start Position**: 141

**End Position**: 151

| Profile Score | Tandem Score | TDA q-value | Volume | Spectrum Matches |
| --- | --- | --- | --- | --- |
| -3.8011 | 173.7038 | 0.0000 | 5.7586e+07 | 1 |

**Adduct Signal Fractions**

- **Unmodified**: 5.7586e+07

### YPALN(N)VTM(O)PNNEQFDK Hex 9 HexNAc 2

**Mass:**  3760.5159

(2.292e-06)

**Charge States:** 3, 4

**Start Position**: 161

**End Position**: 177

| Profile Score | Tandem Score | TDA q-value | Volume | Spectrum Matches |
| --- | --- | --- | --- | --- |
| 14.5962 | 172.8004 | 0.0000 | 2.3152e+08 | 4 |

**Adduct Signal Fractions**

- **Unmodified**: 2.3152e+08

### RGSN(N)SSFFSR Fuc 1 Hex 5 HexNAc 3

**Mass:**  2709.1034

(3.935e-07)

**Charge States:** 3

**Start Position**: 141

**End Position**: 151

| Profile Score | Tandem Score | TDA q-value | Volume | Spectrum Matches |
| --- | --- | --- | --- | --- |
| -3.7535 | 170.6815 | 0.0000 | 7.8760e+07 | 2 |

**Adduct Signal Fractions**

- **Unmodified**: 7.8760e+07

### RGSN(N)SSFFSR Fuc 2 Hex 5 HexNAc 4

**Mass:**  3058.2427

(1.256e-06)

**Charge States:** 3

**Start Position**: 141

**End Position**: 151

| Profile Score | Tandem Score | TDA q-value | Volume | Spectrum Matches |
| --- | --- | --- | --- | --- |
| 14.5839 | 170.1136 | 0.0000 | 2.3374e+08 | 4 |

**Adduct Signal Fractions**

- **Unmodified**: 2.3374e+08

### RGSN(N)SSFFSR Hex 5 HexNAc 4

**Mass:**  2766.1254

(3.885e-07)

**Charge States:** 3

**Start Position**: 141

**End Position**: 151

| Profile Score | Tandem Score | TDA q-value | Volume | Spectrum Matches |
| --- | --- | --- | --- | --- |
| -3.9319 | 167.7085 | 0.0000 | 9.1216e+07 | 2 |

**Adduct Signal Fractions**

- **Unmodified**: 9.1216e+07

### RGSN(N)SSFFSR Hex 4 HexNAc 3

**Mass:**  2400.9928

(4.905e-07)

**Charge States:** 3

**Start Position**: 141

**End Position**: 151

| Profile Score | Tandem Score | TDA q-value | Volume | Spectrum Matches |
| --- | --- | --- | --- | --- |
| -19.5807 | 166.5880 | 0.0000 | 1.3682e+07 | 1 |

**Adduct Signal Fractions**

- **Unmodified**: 1.3682e+07

### YPALN(N)VTMPNNEQFDK Hex 8 HexNAc 2

**Mass:**  3582.4641

(1.475e-06)

**Charge States:** 3, 4

**Start Position**: 161

**End Position**: 177

| Profile Score | Tandem Score | TDA q-value | Volume | Spectrum Matches |
| --- | --- | --- | --- | --- |
| 12.6961 | 166.0260 | 0.0000 | 3.0460e+08 | 8 |

**Adduct Signal Fractions**

- **Unmodified**: 3.0460e+08

### TIVKPGDILLIN(N)STGNLIAPR Fuc 2 Hex 3 HexNAc 4 Neu5Ac 1

**Mass:**  4085.9555

(-5.101e-06)

**Charge States:** 4

**Start Position**: 235

**End Position**: 256

| Profile Score | Tandem Score | TDA q-value | Volume | Spectrum Matches |
| --- | --- | --- | --- | --- |
| -17.8830 | 164.8242 | 0.0000 | 4.4893e+07 | 1 |

**Adduct Signal Fractions**

- **Unmodified**: 4.4893e+07

### TIVKPGDILLIN(N)STGNLIAPR Hex 9 HexNAc 2

**Mass:**  4068.9309

(1.612e-06)

**Charge States:** 3, 4

**Start Position**: 235

**End Position**: 256

| Profile Score | Tandem Score | TDA q-value | Volume | Spectrum Matches |
| --- | --- | --- | --- | --- |
| 19.1007 | 164.4311 | 0.0000 | 4.8723e+08 | 5 |

**Adduct Signal Fractions**

- **Unmodified**: 4.8723e+08

### RGSN(N)SSFFSR Fuc 3 Hex 5 HexNAc 4

**Mass:**  3204.3001

(1.079e-06)

**Charge States:** 3

**Start Position**: 141

**End Position**: 151

| Profile Score | Tandem Score | TDA q-value | Volume | Spectrum Matches |
| --- | --- | --- | --- | --- |
| -2.8720 | 162.9923 | 0.0000 | 8.3240e+07 | 2 |

**Adduct Signal Fractions**

- **Unmodified**: 8.3240e+07

### N(N)GTYDHDVYR Fuc 2 Hex 5 HexNAc 4

**Mass:**  3153.2320

(4.713e-07)

**Charge States:** 3

**Start Position**: 483

**End Position**: 493

| Profile Score | Tandem Score | TDA q-value | Volume | Spectrum Matches |
| --- | --- | --- | --- | --- |
| 15.5653 | 160.8949 | 0.0000 | 2.6313e+08 | 5 |

**Adduct Signal Fractions**

- **Unmodified**: 2.6313e+08

### RGSN(N)SSFFSR Fuc 2 Hex 6 HexNAc 5

**Mass:**  3423.3748

(1.828e-07)

**Charge States:** 3, 4

**Start Position**: 141

**End Position**: 151

| Profile Score | Tandem Score | TDA q-value | Volume | Spectrum Matches |
| --- | --- | --- | --- | --- |
| -20.0967 | 159.7642 | 0.0000 | 1.1616e+07 | 1 |

**Adduct Signal Fractions**

- **Unmodified**: 1.1616e+07

### RGSN(N)SSFFSR Fuc 1 Hex 6 HexNAc 5

**Mass:**  3278.3221

(3.076e-04)

**Start Position**: 141

**End Position**: 151

| Profile Score | Tandem Score | TDA q-value | Volume | Spectrum Matches |
| --- | --- | --- | --- | --- |
| 0.0000 | 159.6527 | 0.0000 | 0.0000e+00 | 1 |

Adduct:

No Chromatogram Found

### TIVKPGDILLIN(N)STGNLIAPR Hex 8 HexNAc 2

**Mass:**  3906.8791

(1.733e-06)

**Charge States:** 4

**Start Position**: 235

**End Position**: 256

| Profile Score | Tandem Score | TDA q-value | Volume | Spectrum Matches |
| --- | --- | --- | --- | --- |
| -3.3159 | 159.4225 | 0.0000 | 5.2110e+07 | 3 |

**Adduct Signal Fractions**

- **Unmodified**: 5.2110e+07

### RGSN(N)SSFFSR Fuc 1 Hex 4 HexNAc 5

**Mass:**  2953.2092

(1.195e-06)

**Charge States:** 3

**Start Position**: 141

**End Position**: 151

| Profile Score | Tandem Score | TDA q-value | Volume | Spectrum Matches |
| --- | --- | --- | --- | --- |
| -4.2827 | 158.5016 | 0.0000 | 5.3706e+07 | 2 |

**Adduct Signal Fractions**

- **Unmodified**: 5.3706e+07

### RGSN(N)SSFFSR Fuc 2 Hex 5 HexNAc 5

**Mass:**  3261.3217

(8.735e-07)

**Charge States:** 3, 4

**Start Position**: 141

**End Position**: 151

| Profile Score | Tandem Score | TDA q-value | Volume | Spectrum Matches |
| --- | --- | --- | --- | --- |
| 13.9685 | 158.3598 | 0.0000 | 3.1477e+08 | 5 |

**Adduct Signal Fractions**

- **Unmodified**: 3.1477e+08

### GSN(N)SSFFSR Hex 7 HexNAc 2

**Mass:**  2527.9703

(2.971e-07)

**Charge States:** 2

**Start Position**: 142

**End Position**: 151

| Profile Score | Tandem Score | TDA q-value | Volume | Spectrum Matches |
| --- | --- | --- | --- | --- |
| -5.1203 | 158.3305 | 0.0000 | 3.9991e+07 | 1 |

**Adduct Signal Fractions**

- **Unmodified**: 3.9991e+07

### YPALN(N)VTMPNNEQFDK Hex 7 HexNAc 2

**Mass:**  3420.4099

(1.609e-06)

**Charge States:** 3

**Start Position**: 161

**End Position**: 177

| Profile Score | Tandem Score | TDA q-value | Volume | Spectrum Matches |
| --- | --- | --- | --- | --- |
| -3.2375 | 155.5333 | 0.0000 | 4.2517e+07 | 3 |

**Adduct Signal Fractions**

- **Unmodified**: 4.2517e+07

### C(C)KSEC(C)ITPN(N)GSIPNDKPFQNVNR Hex 8 HexNAc 2

**Mass:**  4376.8444

(8.521e-07)

**Charge States:** 4

**Start Position**: 277

**End Position**: 300

| Profile Score | Tandem Score | TDA q-value | Volume | Spectrum Matches |
| --- | --- | --- | --- | --- |
| -3.8808 | 155.1839 | 0.0000 | 1.3761e+08 | 1 |

**Adduct Signal Fractions**

- **Unmodified**: 1.3761e+08

### GSN(N)SSFFSR Fuc 1 Hex 5 HexNAc 4

**Mass:**  2756.0824

(7.186e-07)

**Charge States:** 3

**Start Position**: 142

**End Position**: 151

| Profile Score | Tandem Score | TDA q-value | Volume | Spectrum Matches |
| --- | --- | --- | --- | --- |
| 11.8469 | 155.0426 | 0.0000 | 2.5216e+08 | 2 |

**Adduct Signal Fractions**

- **Unmodified**: 2.5216e+08

### C(C)KSEC(C)ITPN(N)GSIPNDKPFQNVNR Hex 9 HexNAc 2

**Mass:**  4538.8977

(-8.052e-07)

**Charge States:** 4

**Start Position**: 277

**End Position**: 300

| Profile Score | Tandem Score | TDA q-value | Volume | Spectrum Matches |
| --- | --- | --- | --- | --- |
| -3.7168 | 154.4760 | 0.0000 | 2.2917e+08 | 3 |

**Adduct Signal Fractions**

- **Unmodified**: 2.2917e+08

### RGSN(N)SSFFSR Fuc 3 Hex 5 HexNAc 5

**Mass:**  3407.3798

(3.486e-06)

**Charge States:** 3, 4

**Start Position**: 141

**End Position**: 151

| Profile Score | Tandem Score | TDA q-value | Volume | Spectrum Matches |
| --- | --- | --- | --- | --- |
| -18.5087 | 154.2605 | 0.0000 | 3.2394e+07 | 3 |

**Adduct Signal Fractions**

- **Unmodified**: 3.2394e+07

### RGSN(N)SSFFSR Hex 5 HexNAc 5

**Mass:**  2969.2036

(5.628e-07)

**Charge States:** 3, 4

**Start Position**: 141

**End Position**: 151

| Profile Score | Tandem Score | TDA q-value | Volume | Spectrum Matches |
| --- | --- | --- | --- | --- |
| 12.9465 | 154.2240 | 0.0000 | 2.9795e+08 | 3 |

**Adduct Signal Fractions**

- **Unmodified**: 2.9795e+08

### N(N)GTYDHDVYRDEALNNR Fuc 1 Hex 6 HexNAc 3

**Mass:**  3778.5278

(1.442e-06)

**Charge States:** 4

**Start Position**: 483

**End Position**: 500

| Profile Score | Tandem Score | TDA q-value | Volume | Spectrum Matches |
| --- | --- | --- | --- | --- |
| -3.6244 | 153.3110 | 0.0000 | 2.9715e+08 | 2 |

**Adduct Signal Fractions**

- **Unmodified**: 2.9715e+08

### RGSN(N)SSFFSR Hex 6 HexNAc 4

**Mass:**  2928.1756

(-6.265e-07)

**Charge States:** 3

**Start Position**: 141

**End Position**: 151

| Profile Score | Tandem Score | TDA q-value | Volume | Spectrum Matches |
| --- | --- | --- | --- | --- |
| 9.6071 | 152.6227 | 0.0000 | 1.1608e+08 | 2 |

**Adduct Signal Fractions**

- **Unmodified**: 1.1608e+08

### YPALN(N)VTM(O)PNNEQFDK Hex 5 HexNAc 2

**Mass:**  3112.3075

(3.926e-06)

**Charge States:** 3

**Start Position**: 161

**End Position**: 177

| Profile Score | Tandem Score | TDA q-value | Volume | Spectrum Matches |
| --- | --- | --- | --- | --- |
| 12.9425 | 151.7996 | 0.0000 | 3.1322e+07 | 2 |

**Adduct Signal Fractions**

- **Unmodified**: 3.1322e+07

### N(N)GTYDHDVYR Fuc 1 Hex 5 HexNAc 4

**Mass:**  3007.1744

(-1.024e-06)

**Charge States:** 3, 4

**Start Position**: 483

**End Position**: 493

| Profile Score | Tandem Score | TDA q-value | Volume | Spectrum Matches |
| --- | --- | --- | --- | --- |
| 16.1445 | 151.7980 | 0.0000 | 4.2008e+08 | 6 |

**Adduct Signal Fractions**

- **Unmodified**: 4.2008e+08

### C(C)KSEC(C)ITPN(N)GSIPNDKPFQNVNR Hex 7 HexNAc 2

**Mass:**  4214.7932

(5.565e-07)

**Charge States:** 3, 4, 5

**Start Position**: 277

**End Position**: 300

| Profile Score | Tandem Score | TDA q-value | Volume | Spectrum Matches |
| --- | --- | --- | --- | --- |
| -17.4842 | 151.7581 | 0.0000 | 2.3944e+08 | 1 |

**Adduct Signal Fractions**

- **Unmodified**: 2.3944e+08

### ISIYWTIVKPGDILLIN(N)STGNLIAPR Hex 9 HexNAc 2

**Mass:**  4731.2751

(7.743e-07)

**Charge States:** 3, 4, 5

**Start Position**: 230

**End Position**: 256

| Profile Score | Tandem Score | TDA q-value | Volume | Spectrum Matches |
| --- | --- | --- | --- | --- |
| 19.9380 | 151.2474 | 0.0000 | 8.7664e+08 | 10 |

**Adduct Signal Fractions**

- **Unmodified**: 8.7664e+08

### YPALN(N)VTM(O)PNNEQFDK Hex 6 HexNAc 2

**Mass:**  3274.3581

(3.017e-06)

**Charge States:** 3

**Start Position**: 161

**End Position**: 177

| Profile Score | Tandem Score | TDA q-value | Volume | Spectrum Matches |
| --- | --- | --- | --- | --- |
| 12.6040 | 150.6227 | 0.0000 | 4.2009e+07 | 3 |

**Adduct Signal Fractions**

- **Unmodified**: 4.2009e+07

### GSN(N)SSFFSR Hex 6 HexNAc 2

**Mass:**  2365.9186

(-6.407e-07)

**Charge States:** 2

**Start Position**: 142

**End Position**: 151

| Profile Score | Tandem Score | TDA q-value | Volume | Spectrum Matches |
| --- | --- | --- | --- | --- |
| -3.6984 | 150.4600 | 0.0000 | 1.2551e+08 | 2 |

**Adduct Signal Fractions**

- **Unmodified**: 1.2551e+08

### RGSN(N)SSFFSR Hex 8 HexNAc 2

**Mass:**  2846.1271

(1.304e-06)

**Charge States:** 3

**Start Position**: 141

**End Position**: 151

| Profile Score | Tandem Score | TDA q-value | Volume | Spectrum Matches |
| --- | --- | --- | --- | --- |
| -3.0880 | 149.9087 | 0.0000 | 7.1970e+07 | 1 |

**Adduct Signal Fractions**

- **Unmodified**: 7.1970e+07

### GSN(N)SSFFSR Hex 5 HexNAc 2

**Mass:**  2203.8646

(1.877e-07)

**Charge States:** 2, 3

**Start Position**: 142

**End Position**: 151

| Profile Score | Tandem Score | TDA q-value | Volume | Spectrum Matches |
| --- | --- | --- | --- | --- |
| -2.2803 | 148.7433 | 0.0000 | 2.0125e+08 | 3 |

**Adduct Signal Fractions**

- **Unmodified**: 2.0125e+08

### RGSN(N)SSFFSR Fuc 2 Hex 6 HexNAc 3

**Mass:**  3017.2163

(1.087e-06)

**Charge States:** 3

**Start Position**: 141

**End Position**: 151

| Profile Score | Tandem Score | TDA q-value | Volume | Spectrum Matches |
| --- | --- | --- | --- | --- |
| -17.8940 | 147.8611 | 0.0000 | 1.8067e+07 | 1 |

**Adduct Signal Fractions**

- **Unmodified**: 1.8067e+07

### RGSN(N)SSFFSR Fuc 1 Hex 6 HexNAc 4

**Mass:**  3074.2326

(-6.253e-07)

**Charge States:** 3

**Start Position**: 141

**End Position**: 151

| Profile Score | Tandem Score | TDA q-value | Volume | Spectrum Matches |
| --- | --- | --- | --- | --- |
| -5.2591 | 147.7356 | 0.0000 | 3.8606e+07 | 2 |

**Adduct Signal Fractions**

- **Unmodified**: 3.8606e+07

### ISIYWTIVKPGDILLIN(N)STGNLIAPR Hex 10 HexNAc 2

**Mass:**  4893.3254

(1.306e-06)

**Charge States:** 4

**Start Position**: 230

**End Position**: 256

| Profile Score | Tandem Score | TDA q-value | Volume | Spectrum Matches |
| --- | --- | --- | --- | --- |
| -4.1061 | 147.6235 | 0.0000 | 4.2517e+07 | 4 |

**Adduct Signal Fractions**

- **Unmodified**: 4.2517e+07

### RGSN(N)SSFFSR Hex 5 HexNAc 6

**Mass:**  3172.2852

(8.048e-07)

**Charge States:** 3

**Start Position**: 141

**End Position**: 151

| Profile Score | Tandem Score | TDA q-value | Volume | Spectrum Matches |
| --- | --- | --- | --- | --- |
| -2.0918 | 147.2361 | 0.0000 | 2.4724e+08 | 2 |

**Adduct Signal Fractions**

- **Unmodified**: 2.4724e+08

### ISIYWTIVKPGDILLIN(N)STGNLIAPR Hex 8 HexNAc 2

**Mass:**  4569.2194

(1.374e-06)

**Charge States:** 4, 5

**Start Position**: 230

**End Position**: 256

| Profile Score | Tandem Score | TDA q-value | Volume | Spectrum Matches |
| --- | --- | --- | --- | --- |
| 15.5162 | 146.2627 | 0.0000 | 9.1510e+07 | 5 |

**Adduct Signal Fractions**

- **Unmodified**: 9.1510e+07

### GSN(N)SSFFSR Fuc 1 Hex 5 HexNAc 5

**Mass:**  2959.1629

(6.772e-07)

**Charge States:** 3

**Start Position**: 142

**End Position**: 151

| Profile Score | Tandem Score | TDA q-value | Volume | Spectrum Matches |
| --- | --- | --- | --- | --- |
| 13.0938 | 144.6823 | 0.0000 | 6.0685e+08 | 5 |

**Adduct Signal Fractions**

- **Unmodified**: 6.0685e+08

### RGSN(N)SSFFSR Fuc 2 Hex 5 HexNAc 3

**Mass:**  2855.1602

(2.574e-08)

**Charge States:** 3

**Start Position**: 141

**End Position**: 151

| Profile Score | Tandem Score | TDA q-value | Volume | Spectrum Matches |
| --- | --- | --- | --- | --- |
| -17.2945 | 143.8853 | 0.0000 | 2.3003e+07 | 1 |

**Adduct Signal Fractions**

- **Unmodified**: 2.3003e+07

### ISIYWTIVKPGDILLIN(N)STGNLIAPR Fuc 2 Hex 3 HexNAc 4 Neu5Ac 1

**Mass:**  4748.2956

(-4.944e-06)

**Charge States:** 4

**Start Position**: 230

**End Position**: 256

| Profile Score | Tandem Score | TDA q-value | Volume | Spectrum Matches |
| --- | --- | --- | --- | --- |
| 19.5023 | 143.2254 | 0.0000 | 1.5050e+08 | 3 |

**Adduct Signal Fractions**

- **Unmodified**: 1.5050e+08

### N(N)GTYDHDVYRDEALNNR Fuc 1 Hex 8 HexNAc 2

**Mass:**  3899.5141

(-9.234e-06)

**Charge States:** 3, 4

**Start Position**: 483

**End Position**: 500

| Profile Score | Tandem Score | TDA q-value | Volume | Spectrum Matches |
| --- | --- | --- | --- | --- |
| 13.8532 | 142.5070 | 0.0000 | 6.5117e+08 | 3 |

**Adduct Signal Fractions**

- **Unmodified**: 6.5117e+08

### N(N)GTYDHDVYRDEALNNR Fuc 2 Hex 5 HexNAc 4

**Mass:**  3965.6105

(5.995e-07)

**Charge States:** 4

**Start Position**: 483

**End Position**: 500

| Profile Score | Tandem Score | TDA q-value | Volume | Spectrum Matches |
| --- | --- | --- | --- | --- |
| 17.0830 | 141.9970 | 0.0000 | 2.4031e+09 | 4 |

**Adduct Signal Fractions**

- **Unmodified**: 2.4031e+09

### TIVKPGDILLIN(N)STGNLIAPR Hex 10 HexNAc 2

**Mass:**  4230.9831

(4.531e-06)

**Charge States:** 4

**Start Position**: 235

**End Position**: 256

| Profile Score | Tandem Score | TDA q-value | Volume | Spectrum Matches |
| --- | --- | --- | --- | --- |
| -19.1627 | 141.4347 | 0.0000 | 1.6522e+07 | 2 |

**Adduct Signal Fractions**

- **Unmodified**: 1.6522e+07

### PGDILLIN(N)STGNLIAPR Hex 9 HexNAc 2

**Mass:**  3627.6337

(1.507e-06)

**Charge States:** 3

**Start Position**: 239

**End Position**: 256

| Profile Score | Tandem Score | TDA q-value | Volume | Spectrum Matches |
| --- | --- | --- | --- | --- |
| -18.5031 | 141.3272 | 0.0000 | 2.5371e+07 | 3 |

**Adduct Signal Fractions**

- **Unmodified**: 2.5371e+07

### N(N)GTYDHDVYRDEALNNR Hex 5 HexNAc 2

**Mass:**  3267.3359

(1.108e-06)

**Charge States:** 4

**Start Position**: 483

**End Position**: 500

| Profile Score | Tandem Score | TDA q-value | Volume | Spectrum Matches |
| --- | --- | --- | --- | --- |
| -4.8506 | 141.2443 | 0.0000 | 9.6018e+07 | 1 |

**Adduct Signal Fractions**

- **Unmodified**: 9.6018e+07

### N(N)GTYDHDVYR Fuc 2 Hex 5 HexNAc 5

**Mass:**  3356.3128

(1.349e-06)

**Charge States:** 3, 4

**Start Position**: 483

**End Position**: 493

| Profile Score | Tandem Score | TDA q-value | Volume | Spectrum Matches |
| --- | --- | --- | --- | --- |
| -19.1373 | 140.4520 | 0.0000 | 5.3740e+07 | 4 |

**Adduct Signal Fractions**

- **Unmodified**: 5.3740e+07

### RGSN(N)SSFFSR Fuc 1 Hex 6 HexNAc 5

**Mass:**  3277.3163

(6.881e-07)

**Charge States:** 3

**Start Position**: 141

**End Position**: 151

| Profile Score | Tandem Score | TDA q-value | Volume | Spectrum Matches |
| --- | --- | --- | --- | --- |
| -3.7741 | 140.0018 | 0.0000 | 8.0495e+07 | 3 |

**Adduct Signal Fractions**

- **Unmodified**: 8.0495e+07

### IVKPGDILLIN(N)STGNLIAPR Hex 9 HexNAc 2

**Mass:**  3967.8808

(1.256e-06)

**Charge States:** 4

**Start Position**: 236

**End Position**: 256

| Profile Score | Tandem Score | TDA q-value | Volume | Spectrum Matches |
| --- | --- | --- | --- | --- |
| -18.3474 | 138.5892 | 0.0000 | 9.4137e+06 | 2 |

**Adduct Signal Fractions**

- **Unmodified**: 9.4137e+06

### HAVPN(N)GTIVK Fuc 1 Hex 5 HexNAc 5

**Mass:**  3008.3236

(6.711e-04)

**Start Position**: 18

**End Position**: 28

| Profile Score | Tandem Score | TDA q-value | Volume | Spectrum Matches |
| --- | --- | --- | --- | --- |
| 0.0000 | 138.1240 | 0.0000 | 0.0000e+00 | 1 |

Adduct:

No Chromatogram Found

### N(N)GTYDHDVYRDEALNNR Fuc 1 Hex 4 HexNAc 3

**Mass:**  3454.4199

(1.262e-06)

**Charge States:** 4

**Start Position**: 483

**End Position**: 500

| Profile Score | Tandem Score | TDA q-value | Volume | Spectrum Matches |
| --- | --- | --- | --- | --- |
| -3.0003 | 137.6993 | 0.0000 | 8.7585e+07 | 1 |

**Adduct Signal Fractions**

- **Unmodified**: 8.7585e+07

### YPALN(N)VTM(O)PNNEQFDK Fuc 2 Hex 3 HexNAc 4 Neu5Ac 1

**Mass:**  3777.5403

(-5.067e-06)

**Charge States:** 3

**Start Position**: 161

**End Position**: 177

| Profile Score | Tandem Score | TDA q-value | Volume | Spectrum Matches |
| --- | --- | --- | --- | --- |
| -18.1974 | 137.4550 | 0.0000 | 1.6530e+07 | 1 |

**Adduct Signal Fractions**

- **Unmodified**: 1.6530e+07

### N(N)GTYDHDVYR Fuc 3 Hex 5 HexNAc 4

**Mass:**  3299.2868

(-8.528e-08)

**Charge States:** 3, 4

**Start Position**: 483

**End Position**: 493

| Profile Score | Tandem Score | TDA q-value | Volume | Spectrum Matches |
| --- | --- | --- | --- | --- |
| -19.0732 | 136.3943 | 0.0000 | 8.8780e+07 | 2 |

**Adduct Signal Fractions**

- **Unmodified**: 8.8780e+07

### YPALN(N)VTM(O)PN(D)NEQFDK Hex 8 HexNAc 2

**Mass:**  3599.4523

(2.770e-04)

**Charge States:** 2, 3, 4

**Start Position**: 161

**End Position**: 177

| Profile Score | Tandem Score | TDA q-value | Volume | Spectrum Matches |
| --- | --- | --- | --- | --- |
| 9.6400 | 135.7791 | 0.0000 | 3.2087e+08 | 27 |

**Adduct Signal Fractions**

- **Unmodified**: 3.2087e+08

### N(N)GTYDHDVYRDEALNNR Fuc 2 Hex 8 HexNAc 3

**Mass:**  4248.6501

(-8.395e-06)

**Charge States:** 3, 4

**Start Position**: 483

**End Position**: 500

| Profile Score | Tandem Score | TDA q-value | Volume | Spectrum Matches |
| --- | --- | --- | --- | --- |
| 11.5747 | 135.3678 | 0.0000 | 1.8553e+08 | 2 |

**Adduct Signal Fractions**

- **Unmodified**: 1.8553e+08

### N(N)GTYDHDVYRDEALNNR Hex 6 HexNAc 3

**Mass:**  3632.4658

(3.700e-07)

**Charge States:** 4

**Start Position**: 483

**End Position**: 500

| Profile Score | Tandem Score | TDA q-value | Volume | Spectrum Matches |
| --- | --- | --- | --- | --- |
| -3.9010 | 135.2617 | 0.0000 | 2.2741e+08 | 3 |

**Adduct Signal Fractions**

- **Unmodified**: 2.2741e+08

### N(N)GTYDHDVYRDEALNNR Fuc 1 Hex 5 HexNAc 4

**Mass:**  3819.5498

(5.985e-07)

**Charge States:** 3, 4, 5

**Start Position**: 483

**End Position**: 500

| Profile Score | Tandem Score | TDA q-value | Volume | Spectrum Matches |
| --- | --- | --- | --- | --- |
| 16.1167 | 133.6776 | 0.0000 | 3.3785e+09 | 6 |

**Adduct Signal Fractions**

- **Unmodified**: 3.3785e+09

### RGSN(N)SSFFSR Fuc 2 Hex 9 HexNAc 4

**Mass:**  3706.4090

(-8.421e-06)

**Charge States:** 3

**Start Position**: 141

**End Position**: 151

| Profile Score | Tandem Score | TDA q-value | Volume | Spectrum Matches |
| --- | --- | --- | --- | --- |
| 9.3122 | 132.8555 | 0.0000 | 3.2256e+07 | 2 |

**Adduct Signal Fractions**

- **Unmodified**: 3.2256e+07

### N(N)GTYDHDVYRDEALNNR Hex 5 HexNAc 4

**Mass:**  3673.4924

(-1.171e-07)

**Charge States:** 4

**Start Position**: 483

**End Position**: 500

| Profile Score | Tandem Score | TDA q-value | Volume | Spectrum Matches |
| --- | --- | --- | --- | --- |
| 13.0127 | 132.4771 | 0.0000 | 1.7520e+08 | 3 |

**Adduct Signal Fractions**

- **Unmodified**: 1.7520e+08

### SEC(C)ITPN(N)GSIPNDKPFQNVNR Hex 8 HexNAc 2

**Mass:**  4088.7170

(1.306e-06)

**Charge States:** 3, 4

**Start Position**: 279

**End Position**: 300

| Profile Score | Tandem Score | TDA q-value | Volume | Spectrum Matches |
| --- | --- | --- | --- | --- |
| 16.3255 | 132.3095 | 0.0000 | 9.4531e+08 | 4 |

**Adduct Signal Fractions**

- **Unmodified**: 9.4531e+08

### RGSN(N)SSFFSR Hex 7 HexNAc 4

**Mass:**  3090.2323

(9.053e-07)

**Charge States:** 3

**Start Position**: 141

**End Position**: 151

| Profile Score | Tandem Score | TDA q-value | Volume | Spectrum Matches |
| --- | --- | --- | --- | --- |
| -19.4958 | 132.1240 | 0.0000 | 1.7522e+07 | 1 |

**Adduct Signal Fractions**

- **Unmodified**: 1.7522e+07

### N(N)GTYDHDVYR Fuc 2 Hex 6 HexNAc 5

**Mass:**  3518.3637

(6.556e-07)

**Charge States:** 3, 4

**Start Position**: 483

**End Position**: 493

| Profile Score | Tandem Score | TDA q-value | Volume | Spectrum Matches |
| --- | --- | --- | --- | --- |
| -19.7549 | 131.8935 | 0.0000 | 2.0062e+07 | 2 |

**Adduct Signal Fractions**

- **Unmodified**: 2.0062e+07

### SEC(C)ITPN(N)GSIPNDKPFQNVNR Hex 7 HexNAc 2

**Mass:**  3926.6682

(1.794e-06)

**Charge States:** 3, 4

**Start Position**: 279

**End Position**: 300

| Profile Score | Tandem Score | TDA q-value | Volume | Spectrum Matches |
| --- | --- | --- | --- | --- |
| 16.1490 | 131.4705 | 0.0000 | 1.5801e+09 | 6 |

**Adduct Signal Fractions**

- **Unmodified**: 1.5801e+09

### N(N)GTYDHDVYR Fuc 1 Hex 6 HexNAc 3

**Mass:**  2966.1476

(1.028e-06)

**Charge States:** 3

**Start Position**: 483

**End Position**: 493

| Profile Score | Tandem Score | TDA q-value | Volume | Spectrum Matches |
| --- | --- | --- | --- | --- |
| -18.0030 | 131.0909 | 0.0000 | 3.5788e+07 | 1 |

**Adduct Signal Fractions**

- **Unmodified**: 3.5788e+07

### RGSN(N)SSFFSR Fuc 1 Hex 3 HexNAc 9 Neu5Ac 2

**Mass:**  4185.6726

(2.154e-06)

**Charge States:** 4

**Start Position**: 141

**End Position**: 151

| Profile Score | Tandem Score | TDA q-value | Volume | Spectrum Matches |
| --- | --- | --- | --- | --- |
| -17.6853 | 130.5065 | 0.0000 | 4.9908e+07 | 1 |

**Adduct Signal Fractions**

- **Unmodified**: 4.9908e+07

### N(N)GTYDHDVYRDEALNNR Fuc 1 Hex 9 HexNAc 3

**Mass:**  4264.6452

(-8.339e-06)

**Charge States:** 4

**Start Position**: 483

**End Position**: 500

| Profile Score | Tandem Score | TDA q-value | Volume | Spectrum Matches |
| --- | --- | --- | --- | --- |
| -3.3790 | 130.1030 | 0.0000 | 1.5132e+08 | 2 |

**Adduct Signal Fractions**

- **Unmodified**: 1.5132e+08

### N(N)GTYDHDVYR Hex 6 HexNAc 3

**Mass:**  2820.0893

(9.621e-07)

**Charge States:** 3

**Start Position**: 483

**End Position**: 493

| Profile Score | Tandem Score | TDA q-value | Volume | Spectrum Matches |
| --- | --- | --- | --- | --- |
| -18.9141 | 129.9509 | 0.0000 | 1.9966e+07 | 1 |

**Adduct Signal Fractions**

- **Unmodified**: 1.9966e+07

### N(N)GTYDHDVYRDEALNNR Fuc 1 Hex 5 HexNAc 5

**Mass:**  4022.6284

(2.793e-08)

**Charge States:** 3, 4, 5

**Start Position**: 483

**End Position**: 500

| Profile Score | Tandem Score | TDA q-value | Volume | Spectrum Matches |
| --- | --- | --- | --- | --- |
| 10.6215 | 129.6230 | 0.0000 | 2.2084e+09 | 7 |

**Adduct Signal Fractions**

- **Unmodified**: 2.2084e+09

### SEC(C)ITPN(N)GSIPNDKPFQNVNR Hex 9 HexNAc 2

**Mass:**  4250.7761

(1.961e-06)

**Charge States:** 3, 4

**Start Position**: 279

**End Position**: 300

| Profile Score | Tandem Score | TDA q-value | Volume | Spectrum Matches |
| --- | --- | --- | --- | --- |
| -1.6379 | 129.2779 | 0.0000 | 1.4383e+09 | 3 |

**Adduct Signal Fractions**

- **Unmodified**: 1.4383e+09

### SEC(C)ITPN(N)GSIPNDKPFQNVNR Hex 6 HexNAc 2

**Mass:**  3764.6201

(1.825e-06)

**Charge States:** 3, 4

**Start Position**: 279

**End Position**: 300

| Profile Score | Tandem Score | TDA q-value | Volume | Spectrum Matches |
| --- | --- | --- | --- | --- |
| -2.8912 | 129.2717 | 0.0000 | 2.2129e+08 | 3 |

**Adduct Signal Fractions**

- **Unmodified**: 2.2129e+08

### N(N)GTYDHDVYRDEALNNR Fuc 2 Hex 6 HexNAc 5

**Mass:**  4330.7419

(7.426e-07)

**Charge States:** 4

**Start Position**: 483

**End Position**: 500

| Profile Score | Tandem Score | TDA q-value | Volume | Spectrum Matches |
| --- | --- | --- | --- | --- |
| 13.5430 | 128.2094 | 0.0000 | 2.4748e+08 | 1 |

**Adduct Signal Fractions**

- **Unmodified**: 2.4748e+08

### N(N)GTYDHDVYRDEALNNR Fuc 2 Hex 4 HexNAc 3

**Mass:**  3600.4768

(5.918e-07)

**Charge States:** 4

**Start Position**: 483

**End Position**: 500

| Profile Score | Tandem Score | TDA q-value | Volume | Spectrum Matches |
| --- | --- | --- | --- | --- |
| -16.8400 | 128.1653 | 0.0000 | 7.5336e+07 | 2 |

**Adduct Signal Fractions**

- **Unmodified**: 7.5336e+07

### RGSN(N)SSFFSR Fuc 2 Hex 4 HexNAc 3

**Mass:**  2693.1077

(1.058e-07)

**Charge States:** 3

**Start Position**: 141

**End Position**: 151

| Profile Score | Tandem Score | TDA q-value | Volume | Spectrum Matches |
| --- | --- | --- | --- | --- |
| -18.3592 | 127.4619 | 0.0000 | 2.2176e+07 | 1 |

**Adduct Signal Fractions**

- **Unmodified**: 2.2176e+07

### RGSN(N)SSFFSR Fuc 1 Hex 6 HexNAc 6

**Mass:**  3480.3945

(9.978e-07)

**Charge States:** 3, 4

**Start Position**: 141

**End Position**: 151

| Profile Score | Tandem Score | TDA q-value | Volume | Spectrum Matches |
| --- | --- | --- | --- | --- |
| -4.7156 | 126.2987 | 0.0000 | 4.8137e+07 | 3 |

**Adduct Signal Fractions**

- **Unmodified**: 4.8137e+07

### N(N)GTYDHDVYRDEALNNR Fuc 3 Hex 6 HexNAc 5

**Mass:**  4476.7995

(1.694e-06)

**Charge States:** 4

**Start Position**: 483

**End Position**: 500

| Profile Score | Tandem Score | TDA q-value | Volume | Spectrum Matches |
| --- | --- | --- | --- | --- |
| -4.3527 | 126.1391 | 0.0000 | 1.2450e+08 | 1 |

**Adduct Signal Fractions**

- **Unmodified**: 1.2450e+08

### RGSN(N)SSFFSR Fuc 1 Hex 7 HexNAc 8

**Mass:**  4048.6357

(7.609e-06)

**Charge States:** 4

**Start Position**: 141

**End Position**: 151

| Profile Score | Tandem Score | TDA q-value | Volume | Spectrum Matches |
| --- | --- | --- | --- | --- |
| -18.1438 | 125.4858 | 0.0000 | 5.5097e+07 | 1 |

**Adduct Signal Fractions**

- **Unmodified**: 5.5097e+07

### N(N)GTYDHDVYRDEALNNR Fuc 1 Hex 6 HexNAc 5

**Mass:**  4184.6852

(6.421e-07)

**Charge States:** 4

**Start Position**: 483

**End Position**: 500

| Profile Score | Tandem Score | TDA q-value | Volume | Spectrum Matches |
| --- | --- | --- | --- | --- |
| 11.4200 | 124.6928 | 0.0000 | 6.0972e+08 | 3 |

**Adduct Signal Fractions**

- **Unmodified**: 6.0972e+08

### N(N)GTYDHDVYRDEALNNR Fuc 1 Hex 5 HexNAc 3

**Mass:**  3616.4746

(1.387e-06)

**Charge States:** 4

**Start Position**: 483

**End Position**: 500

| Profile Score | Tandem Score | TDA q-value | Volume | Spectrum Matches |
| --- | --- | --- | --- | --- |
| 12.3823 | 124.2055 | 0.0000 | 1.1224e+08 | 2 |

**Adduct Signal Fractions**

- **Unmodified**: 1.1224e+08

### N(N)GTYDHDVYR Fuc 1 Hex 5 HexNAc 5

**Mass:**  3211.2617

(3.150e-04)

**Start Position**: 483

**End Position**: 493

| Profile Score | Tandem Score | TDA q-value | Volume | Spectrum Matches |
| --- | --- | --- | --- | --- |
| 0.0000 | 123.1572 | 0.0000 | 0.0000e+00 | 1 |

Adduct:

No Chromatogram Found

### SEC(C)ITPN(N)GSIPNDKPFQNVNR Hex 5 HexNAc 2

**Mass:**  3602.5651

(3.057e-06)

**Charge States:** 4

**Start Position**: 279

**End Position**: 300

| Profile Score | Tandem Score | TDA q-value | Volume | Spectrum Matches |
| --- | --- | --- | --- | --- |
| -4.2348 | 122.7406 | 0.0000 | 6.9018e+07 | 2 |

**Adduct Signal Fractions**

- **Unmodified**: 6.9018e+07

### N(N)GTYDHDVYRDEALNNR Fuc 1 Hex 8 HexNAc 3

**Mass:**  4102.5915

(-9.120e-06)

**Charge States:** 3, 4

**Start Position**: 483

**End Position**: 500

| Profile Score | Tandem Score | TDA q-value | Volume | Spectrum Matches |
| --- | --- | --- | --- | --- |
| 11.0217 | 121.1555 | 0.0000 | 3.4167e+08 | 5 |

**Adduct Signal Fractions**

- **Unmodified**: 3.4167e+08

### N(N)GTYDHDVYRDEALNNR Fuc 2 Hex 5 HexNAc 3

**Mass:**  3762.5300

(7.107e-07)

**Charge States:** 4

**Start Position**: 483

**End Position**: 500

| Profile Score | Tandem Score | TDA q-value | Volume | Spectrum Matches |
| --- | --- | --- | --- | --- |
| -4.2644 | 120.9983 | 0.0000 | 5.7408e+07 | 1 |

**Adduct Signal Fractions**

- **Unmodified**: 5.7408e+07

### N(N)GTYDHDVYRDEALNNR Fuc 3 Hex 5 HexNAc 4

**Mass:**  4111.6677

(7.547e-07)

**Charge States:** 4

**Start Position**: 483

**End Position**: 500

| Profile Score | Tandem Score | TDA q-value | Volume | Spectrum Matches |
| --- | --- | --- | --- | --- |
| 15.7272 | 120.9145 | 0.0000 | 9.3651e+08 | 3 |

**Adduct Signal Fractions**

- **Unmodified**: 9.3651e+08

### YPALN(N)VTMPNNEQFDK Hex 9 HexNAc 2

**Mass:**  3744.5165

(1.593e-06)

**Charge States:** 3

**Start Position**: 161

**End Position**: 177

| Profile Score | Tandem Score | TDA q-value | Volume | Spectrum Matches |
| --- | --- | --- | --- | --- |
| -4.5228 | 120.5715 | 0.0000 | 2.4029e+07 | 3 |

**Adduct Signal Fractions**

- **Unmodified**: 2.4029e+07

### RGSN(N)SSFFSR Hex 9 HexNAc 4

**Mass:**  3414.3092

(-7.509e-06)

**Charge States:** 3

**Start Position**: 141

**End Position**: 151

| Profile Score | Tandem Score | TDA q-value | Volume | Spectrum Matches |
| --- | --- | --- | --- | --- |
| -4.6533 | 120.2115 | 0.0000 | 1.5816e+07 | 1 |

**Adduct Signal Fractions**

- **Unmodified**: 1.5816e+07

### N(N)GTYDHDVYR Fuc 1 Hex 5 HexNAc 5

**Mass:**  3210.2528

(2.071e-06)

**Charge States:** 3, 4

**Start Position**: 483

**End Position**: 493

| Profile Score | Tandem Score | TDA q-value | Volume | Spectrum Matches |
| --- | --- | --- | --- | --- |
| 10.2504 | 119.9988 | 0.0000 | 1.9905e+08 | 6 |

**Adduct Signal Fractions**

- **Unmodified**: 1.9905e+08

### SEC(C)ITPN(N)GSIPNDKPFQNVNR Fuc 2 Hex 5 HexNAc 7

**Mass:**  4912.0931

(4.157e-04)

**Charge States:** 4

**Start Position**: 279

**End Position**: 300

| Profile Score | Tandem Score | TDA q-value | Volume | Spectrum Matches |
| --- | --- | --- | --- | --- |
| -6.3819 | 119.7401 | 0.0000 | 1.3342e+06 | 1 |

**Adduct Signal Fractions**

- **Unmodified**: 1.3342e+06

### N(N)GTYDHDVYRDEALNNR Fuc 1 Hex 6 HexNAc 6

**Mass:**  4387.7653

(1.176e-06)

**Charge States:** 4

**Start Position**: 483

**End Position**: 500

| Profile Score | Tandem Score | TDA q-value | Volume | Spectrum Matches |
| --- | --- | --- | --- | --- |
| 7.0621 | 119.5992 | 0.0000 | 1.5964e+08 | 2 |

**Adduct Signal Fractions**

- **Unmodified**: 1.5964e+08

### SEC(C)ITPN(N)GSIPNDKPFQNVNR Fuc 2 Hex 3 HexNAc 4 Neu5Ac 1

**Mass:**  4267.7985

(-5.084e-06)

**Charge States:** 4

**Start Position**: 279

**End Position**: 300

| Profile Score | Tandem Score | TDA q-value | Volume | Spectrum Matches |
| --- | --- | --- | --- | --- |
| -21.1132 | 118.8737 | 0.0000 | 4.5567e+07 | 1 |

**Adduct Signal Fractions**

- **Unmodified**: 4.5567e+07

### RGSN(N)SSFFSR Fuc 2 Hex 4 HexNAc 5

**Mass:**  3099.2671

(2.865e-07)

**Charge States:** 3

**Start Position**: 141

**End Position**: 151

| Profile Score | Tandem Score | TDA q-value | Volume | Spectrum Matches |
| --- | --- | --- | --- | --- |
| -18.3711 | 118.3946 | 0.0000 | 1.5922e+07 | 1 |

**Adduct Signal Fractions**

- **Unmodified**: 1.5922e+07

### N(N)GTYDHDVYR Hex 5 HexNAc 6

**Mass:**  3267.2744

(4.575e-07)

**Charge States:** 3

**Start Position**: 483

**End Position**: 493

| Profile Score | Tandem Score | TDA q-value | Volume | Spectrum Matches |
| --- | --- | --- | --- | --- |
| -5.6793 | 117.7487 | 0.0000 | 2.9854e+07 | 3 |

**Adduct Signal Fractions**

- **Unmodified**: 2.9854e+07

### N(N)GTYDHDVYRDEALNNR Fuc 2 Hex 5 HexNAc 5

**Mass:**  4168.6857

(1.035e-06)

**Charge States:** 4

**Start Position**: 483

**End Position**: 500

| Profile Score | Tandem Score | TDA q-value | Volume | Spectrum Matches |
| --- | --- | --- | --- | --- |
| -3.2080 | 117.1181 | 0.0000 | 4.3341e+08 | 2 |

**Adduct Signal Fractions**

- **Unmodified**: 4.3341e+08

### TIVKPGDILLIN(N)STGNLIAPR Fuc 4 Hex 4 HexNAc 5 Neu5Ac 1

**Mass:**  4743.2702

(9.607e-06)

**Charge States:** 3, 4

**Start Position**: 235

**End Position**: 256

| Profile Score | Tandem Score | TDA q-value | Volume | Spectrum Matches |
| --- | --- | --- | --- | --- |
| 13.9786 | 114.4653 | 0.0000 | 5.2235e+07 | 6 |

**Adduct Signal Fractions**

- **Unmodified**: 5.2235e+07

### N(N)GTYDHDVYRDEALN(D)NR Fuc 2 Hex 5 HexNAc 4

**Mass:**  3966.5974

(2.503e-04)

**Charge States:** 3, 4

**Start Position**: 483

**End Position**: 500

| Profile Score | Tandem Score | TDA q-value | Volume | Spectrum Matches |
| --- | --- | --- | --- | --- |
| 6.3855 | 114.3721 | 0.0000 | 6.9127e+08 | 4 |

**Adduct Signal Fractions**

- **Unmodified**: 6.9127e+08

### N(N)GTYDHDVYRDEALN(D)NR Fuc 1 Hex 6 HexNAc 3

**Mass:**  3779.5144

(2.626e-04)

**Charge States:** 4

**Start Position**: 483

**End Position**: 500

| Profile Score | Tandem Score | TDA q-value | Volume | Spectrum Matches |
| --- | --- | --- | --- | --- |
| -5.7720 | 114.1971 | 0.0000 | 7.1511e+07 | 2 |

**Adduct Signal Fractions**

- **Unmodified**: 7.1511e+07

### N(N)GTYDHDVYRDEALNNR Fuc 2 Hex 9 HexNAc 3

**Mass:**  4410.7038

(-8.407e-06)

**Charge States:** 3, 4

**Start Position**: 483

**End Position**: 500

| Profile Score | Tandem Score | TDA q-value | Volume | Spectrum Matches |
| --- | --- | --- | --- | --- |
| -4.3575 | 113.3093 | 0.0000 | 1.0581e+08 | 2 |

**Adduct Signal Fractions**

- **Unmodified**: 1.0581e+08

### N(N)GTYDHDVYR Hex 5 HexNAc 4

**Mass:**  2861.1189

(8.125e-07)

**Charge States:** 3

**Start Position**: 483

**End Position**: 493

| Profile Score | Tandem Score | TDA q-value | Volume | Spectrum Matches |
| --- | --- | --- | --- | --- |
| -19.1687 | 112.8543 | 0.0000 | 1.5462e+07 | 1 |

**Adduct Signal Fractions**

- **Unmodified**: 1.5462e+07

### YPALN(N)VTM(O)PN(D)NEQFDK Hex 7 HexNAc 2

**Mass:**  3437.3966

(2.897e-04)

**Charge States:** 3, 4

**Start Position**: 161

**End Position**: 177

| Profile Score | Tandem Score | TDA q-value | Volume | Spectrum Matches |
| --- | --- | --- | --- | --- |
| 10.6049 | 112.6425 | 0.0000 | 5.0218e+07 | 3 |

**Adduct Signal Fractions**

- **Unmodified**: 5.0218e+07

### N(N)GTYDHDVYR Fuc 1 Hex 7 HexNAc 3

**Mass:**  3128.2003

(9.366e-07)

**Charge States:** 3

**Start Position**: 483

**End Position**: 493

| Profile Score | Tandem Score | TDA q-value | Volume | Spectrum Matches |
| --- | --- | --- | --- | --- |
| -19.6239 | 112.5784 | 0.0000 | 5.2749e+06 | 1 |

**Adduct Signal Fractions**

- **Unmodified**: 5.2749e+06

### N(N)GTYDHDVYRDEALNNR Fuc 1 Hex 7 HexNAc 3

**Mass:**  3940.5844

(2.390e-06)

**Charge States:** 4

**Start Position**: 483

**End Position**: 500

| Profile Score | Tandem Score | TDA q-value | Volume | Spectrum Matches |
| --- | --- | --- | --- | --- |
| -19.0164 | 112.2024 | 0.0000 | 7.1295e+07 | 1 |

**Adduct Signal Fractions**

- **Unmodified**: 7.1295e+07

### N(N)GTYDHDVYRDEALN(D)NR Hex 6 HexNAc 3

**Mass:**  3633.4543

(2.726e-04)

**Charge States:** 4

**Start Position**: 483

**End Position**: 500

| Profile Score | Tandem Score | TDA q-value | Volume | Spectrum Matches |
| --- | --- | --- | --- | --- |
| -5.9395 | 112.1918 | 0.0000 | 5.8359e+07 | 1 |

**Adduct Signal Fractions**

- **Unmodified**: 5.8359e+07

### N(N)GTYDHDVYR Fuc 1 Hex 6 HexNAc 5

**Mass:**  3372.3063

(-2.717e-07)

**Charge States:** 3

**Start Position**: 483

**End Position**: 493

| Profile Score | Tandem Score | TDA q-value | Volume | Spectrum Matches |
| --- | --- | --- | --- | --- |
| -4.3249 | 112.1908 | 0.0000 | 6.0898e+07 | 4 |

**Adduct Signal Fractions**

- **Unmodified**: 6.0898e+07

### N(N)GTYDHDVYR Fuc 2 Hex 4 HexNAc 3

**Mass:**  2788.0980

(4.573e-07)

**Charge States:** 3

**Start Position**: 483

**End Position**: 493

| Profile Score | Tandem Score | TDA q-value | Volume | Spectrum Matches |
| --- | --- | --- | --- | --- |
| -18.6836 | 111.7255 | 0.0000 | 5.1851e+06 | 1 |

**Adduct Signal Fractions**

- **Unmodified**: 5.1851e+06

### N(N)GTYDHDVYRDEALNNR Fuc 1 Hex 9 HexNAc 4

**Mass:**  4467.7224

(-8.471e-06)

**Charge States:** 4

**Start Position**: 483

**End Position**: 500

| Profile Score | Tandem Score | TDA q-value | Volume | Spectrum Matches |
| --- | --- | --- | --- | --- |
| 8.8188 | 111.5234 | 0.0000 | 5.1206e+07 | 1 |

**Adduct Signal Fractions**

- **Unmodified**: 5.1206e+07

### RGSN(N)SSFFSR Fuc 1 Hex 5 HexNAc 6

**Mass:**  3318.3407

(1.194e-07)

**Charge States:** 3

**Start Position**: 141

**End Position**: 151

| Profile Score | Tandem Score | TDA q-value | Volume | Spectrum Matches |
| --- | --- | --- | --- | --- |
| -5.0763 | 110.8920 | 0.0000 | 4.4132e+07 | 1 |

**Adduct Signal Fractions**

- **Unmodified**: 4.4132e+07

### N(N)GTYDHDVYRDEALNNR Hex 7 HexNAc 3

**Mass:**  3794.5222

(1.313e-06)

**Charge States:** 4

**Start Position**: 483

**End Position**: 500

| Profile Score | Tandem Score | TDA q-value | Volume | Spectrum Matches |
| --- | --- | --- | --- | --- |
| -19.6617 | 109.6974 | 0.0000 | 4.6999e+07 | 2 |

**Adduct Signal Fractions**

- **Unmodified**: 4.6999e+07

### N(N)GTYDHDVYRDEALNNR Fuc 3 Hex 5 HexNAc 5

**Mass:**  4314.7497

(1.043e-06)

**Charge States:** 4

**Start Position**: 483

**End Position**: 500

| Profile Score | Tandem Score | TDA q-value | Volume | Spectrum Matches |
| --- | --- | --- | --- | --- |
| -18.4942 | 108.3648 | 0.0000 | 7.4151e+07 | 2 |

**Adduct Signal Fractions**

- **Unmodified**: 7.4151e+07

### RGSN(N)SSFFSR Fuc 3 Hex 7 HexNAc 7

**Mass:**  4137.6804

(9.487e-06)

**Charge States:** 4

**Start Position**: 141

**End Position**: 151

| Profile Score | Tandem Score | TDA q-value | Volume | Spectrum Matches |
| --- | --- | --- | --- | --- |
| -20.4763 | 107.7763 | 0.0000 | 3.1674e+07 | 1 |

**Adduct Signal Fractions**

- **Unmodified**: 3.1674e+07

### YPALN(N)VTMPNNEQFDK Hex 6 HexNAc 2

**Mass:**  3258.3576

(1.408e-06)

**Charge States:** 3

**Start Position**: 161

**End Position**: 177

| Profile Score | Tandem Score | TDA q-value | Volume | Spectrum Matches |
| --- | --- | --- | --- | --- |
| -20.3472 | 107.2581 | 0.0037 | 2.1440e+06 | 1 |

**Adduct Signal Fractions**

- **Unmodified**: 2.1440e+06

### N(N)GTYDHDVYR Fuc 1 Hex 6 HexNAc 6

**Mass:**  3575.3869

(3.359e-07)

**Charge States:** 4

**Start Position**: 483

**End Position**: 493

| Profile Score | Tandem Score | TDA q-value | Volume | Spectrum Matches |
| --- | --- | --- | --- | --- |
| -19.5773 | 106.4511 | 0.0067 | 1.3882e+07 | 2 |

**Adduct Signal Fractions**

- **Unmodified**: 1.3882e+07

### N(N)GTYDHDVYRDEALNNR Fuc 2 Hex 6 HexNAc 6

**Mass:**  4533.8244

(1.352e-06)

**Charge States:** 4

**Start Position**: 483

**End Position**: 500

| Profile Score | Tandem Score | TDA q-value | Volume | Spectrum Matches |
| --- | --- | --- | --- | --- |
| -19.6463 | 106.0255 | 0.0067 | 4.7954e+07 | 1 |

**Adduct Signal Fractions**

- **Unmodified**: 4.7954e+07

### YPALN(N)VTMPNNEQFDK Fuc 2 Hex 3 HexNAc 4 Neu5Ac 1

**Mass:**  3761.5413

(-6.186e-06)

**Charge States:** 3

**Start Position**: 161

**End Position**: 177

| Profile Score | Tandem Score | TDA q-value | Volume | Spectrum Matches |
| --- | --- | --- | --- | --- |
| -19.7738 | 103.9700 | 0.0067 | 2.6612e+06 | 1 |

**Adduct Signal Fractions**

- **Unmodified**: 2.6612e+06

### RGSN(N)SSFFSR Fuc 2 Hex 5 HexNAc 6 Neu5Ac 2

**Mass:**  4046.5541

(-7.898e-06)

**Charge States:** 3, 4

**Start Position**: 141

**End Position**: 151

| Profile Score | Tandem Score | TDA q-value | Volume | Spectrum Matches |
| --- | --- | --- | --- | --- |
| 10.3815 | 103.5330 | 0.0067 | 1.0615e+08 | 1 |

**Adduct Signal Fractions**

- **Unmodified**: 1.0615e+08

### N(N)GTYDHDVYRDEALN(D)NR Fuc 1 Hex 5 HexNAc 4

**Mass:**  3820.5371

(2.600e-04)

**Charge States:** 3, 4, 5

**Start Position**: 483

**End Position**: 500

| Profile Score | Tandem Score | TDA q-value | Volume | Spectrum Matches |
| --- | --- | --- | --- | --- |
| -5.6632 | 102.7645 | 0.0067 | 1.0457e+09 | 19 |

**Adduct Signal Fractions**

- **Unmodified**: 1.0457e+09

### N(N)GTYDHDVYRDEALNNR Fuc 4 Hex 6 HexNAc 5

**Mass:**  4622.8581

(7.093e-07)

**Charge States:** 4

**Start Position**: 483

**End Position**: 500

| Profile Score | Tandem Score | TDA q-value | Volume | Spectrum Matches |
| --- | --- | --- | --- | --- |
| -18.7675 | 102.5008 | 0.0067 | 3.8923e+07 | 1 |

**Adduct Signal Fractions**

- **Unmodified**: 3.8923e+07

### ISIYWTIVKPGDILLIN(N)STGNLIAPR Fuc 2 Hex 4 HexNAc 4 Neu5Ac 1

**Mass:**  4910.3527

(-3.976e-06)

**Charge States:** 4

**Start Position**: 230

**End Position**: 256

| Profile Score | Tandem Score | TDA q-value | Volume | Spectrum Matches |
| --- | --- | --- | --- | --- |
| -19.6342 | 102.2116 | 0.0067 | 8.5612e+06 | 1 |

**Adduct Signal Fractions**

- **Unmodified**: 8.5612e+06

### N(N)GTYDHDVYRDEALNNR Fuc 1 Hex 7 HexNAc 6

**Mass:**  4549.8137

(2.406e-06)

**Charge States:** 4

**Start Position**: 483

**End Position**: 500

| Profile Score | Tandem Score | TDA q-value | Volume | Spectrum Matches |
| --- | --- | --- | --- | --- |
| -20.9346 | 101.7853 | 0.0067 | 3.4935e+07 | 1 |

**Adduct Signal Fractions**

- **Unmodified**: 3.4935e+07

### RGSN(N)SSFFSR Fuc 2 Hex 7 HexNAc 7

**Mass:**  3991.6257

(8.190e-06)

**Charge States:** 3, 4

**Start Position**: 141

**End Position**: 151

| Profile Score | Tandem Score | TDA q-value | Volume | Spectrum Matches |
| --- | --- | --- | --- | --- |
| -19.6413 | 100.5839 | 0.0096 | 7.5778e+07 | 1 |

**Adduct Signal Fractions**

- **Unmodified**: 7.5778e+07

### RGSN(N)SSFFSR Fuc 1 Hex 7 HexNAc 3 Neu5Ac 1

**Mass:**  3324.2986

(-1.496e-06)

**Charge States:** 3

**Start Position**: 141

**End Position**: 151

| Profile Score | Tandem Score | TDA q-value | Volume | Spectrum Matches |
| --- | --- | --- | --- | --- |
| -5.4601 | 98.4073 | 0.0096 | 1.5986e+07 | 2 |

**Adduct Signal Fractions**

- **Unmodified**: 1.5986e+07

### LNWLTHLNSKYPALN(N)VTM(O)PNNEQFDK Hex 8 HexNAc 2

**Mass:**  4805.1184

(2.836e-06)

**Charge States:** 4

**Start Position**: 151

**End Position**: 177

| Profile Score | Tandem Score | TDA q-value | Volume | Spectrum Matches |
| --- | --- | --- | --- | --- |
| -17.7626 | 98.0540 | 0.0096 | 3.8715e+07 | 2 |

**Adduct Signal Fractions**

- **Unmodified**: 3.8715e+07

### RGSN(N)SSFFSR Hex 5 HexNAc 6 Neu5Ac 3

**Mass:**  4045.5688

(-8.101e-07)

**Charge States:** 3, 4

**Start Position**: 141

**End Position**: 151

| Profile Score | Tandem Score | TDA q-value | Volume | Spectrum Matches |
| --- | --- | --- | --- | --- |
| 14.3883 | 97.5174 | 0.0096 | 5.8428e+08 | 3 |

**Adduct Signal Fractions**

- **Unmodified**: 5.8428e+08

### C(C)KSEC(C)ITPN(N)GSIPN(D)DKPFQNVNR Hex 9 HexNAc 2

**Mass:**  4539.8791

(2.172e-04)

**Charge States:** 4

**Start Position**: 277

**End Position**: 300

| Profile Score | Tandem Score | TDA q-value | Volume | Spectrum Matches |
| --- | --- | --- | --- | --- |
| -6.3842 | 96.7203 | 0.0096 | 4.4373e+07 | 1 |

**Adduct Signal Fractions**

- **Unmodified**: 4.4373e+07

### ISIYWTIVKPGDILLIN(N)STGNLIAPR Hex 9 HexNAc 2

**Mass:**  4733.2711

(4.238e-04)

**Start Position**: 230

**End Position**: 256

| Profile Score | Tandem Score | TDA q-value | Volume | Spectrum Matches |
| --- | --- | --- | --- | --- |
| 0.0000 | 96.3208 | 0.0096 | 0.0000e+00 | 1 |

Adduct:

No Chromatogram Found

### N(N)GTYDHDVYR Fuc 3 Hex 6 HexNAc 5

**Mass:**  3664.4196

(1.241e-07)

**Charge States:** 3

**Start Position**: 483

**End Position**: 493

| Profile Score | Tandem Score | TDA q-value | Volume | Spectrum Matches |
| --- | --- | --- | --- | --- |
| -21.3651 | 95.9716 | 0.0096 | 6.2640e+06 | 1 |

**Adduct Signal Fractions**

- **Unmodified**: 6.2640e+06

### N(N)GTYDHDVYR Hex 7 HexNAc 3

**Mass:**  2982.1406

(3.429e-07)

**Charge States:** 3

**Start Position**: 483

**End Position**: 493

| Profile Score | Tandem Score | TDA q-value | Volume | Spectrum Matches |
| --- | --- | --- | --- | --- |
| -20.3221 | 95.7100 | 0.0096 | 6.9689e+06 | 1 |

**Adduct Signal Fractions**

- **Unmodified**: 6.9689e+06

### SEC(C)ITPN(N)GSIPN(D)DKPFQNVNR Hex 8 HexNAc 2

**Mass:**  4089.7143

(2.449e-04)

**Charge States:** 3, 4

**Start Position**: 279

**End Position**: 300

| Profile Score | Tandem Score | TDA q-value | Volume | Spectrum Matches |
| --- | --- | --- | --- | --- |
| 9.5316 | 95.6561 | 0.0096 | 1.8147e+08 | 3 |

**Adduct Signal Fractions**

- **Unmodified**: 1.8147e+08

### N(N)GTYDHDVYR Fuc 1 Hex 4 HexNAc 3

**Mass:**  2642.0390

(3.745e-08)

**Charge States:** 3

**Start Position**: 483

**End Position**: 493

| Profile Score | Tandem Score | TDA q-value | Volume | Spectrum Matches |
| --- | --- | --- | --- | --- |
| -19.5062 | 92.9929 | 0.0158 | 3.8240e+06 | 1 |

**Adduct Signal Fractions**

- **Unmodified**: 3.8240e+06

### SEC(C)ITPN(N)GSIPN(D)DKPFQNVNR Hex 7 HexNAc 2

**Mass:**  3927.6662

(2.530e-04)

**Charge States:** 3, 4

**Start Position**: 279

**End Position**: 300

| Profile Score | Tandem Score | TDA q-value | Volume | Spectrum Matches |
| --- | --- | --- | --- | --- |
| 11.6929 | 92.9584 | 0.0158 | 3.3462e+08 | 4 |

**Adduct Signal Fractions**

- **Unmodified**: 3.3462e+08

### N(N)GTYDHDVYR Hex 5 HexNAc 2

**Mass:**  2454.9580

(1.399e-06)

**Charge States:** 3

**Start Position**: 483

**End Position**: 493

| Profile Score | Tandem Score | TDA q-value | Volume | Spectrum Matches |
| --- | --- | --- | --- | --- |
| -20.4348 | 91.5182 | 0.0210 | 4.7521e+06 | 1 |

**Adduct Signal Fractions**

- **Unmodified**: 4.7521e+06

### SEC(C)ITPN(N)GSIPN(D)DKPFQNVNR Hex 9 HexNAc 2

**Mass:**  4251.7663

(2.327e-04)

**Charge States:** 3, 4

**Start Position**: 279

**End Position**: 300

| Profile Score | Tandem Score | TDA q-value | Volume | Spectrum Matches |
| --- | --- | --- | --- | --- |
| 6.4609 | 91.4064 | 0.0210 | 2.4373e+08 | 4 |

**Adduct Signal Fractions**

- **Unmodified**: 2.4373e+08

### N(N)GTYDHDVYR Hex 5 HexNAc 3

**Mass:**  2658.0419

(3.026e-06)

**Charge States:** 3

**Start Position**: 483

**End Position**: 493

| Profile Score | Tandem Score | TDA q-value | Volume | Spectrum Matches |
| --- | --- | --- | --- | --- |
| -20.0013 | 90.9597 | 0.0210 | 3.6410e+06 | 1 |

**Adduct Signal Fractions**

- **Unmodified**: 3.6410e+06

### IEVTN(N)ATELVQN Hex 5 HexNAc 2

**Mass:**  2546.1057

(2.066e-06)

**Charge States:** 3

**Start Position**: 34

**End Position**: 46

| Profile Score | Tandem Score | TDA q-value | Volume | Spectrum Matches |
| --- | --- | --- | --- | --- |
| -19.9213 | 89.0360 | 0.0210 | 1.4555e+06 | 1 |

**Adduct Signal Fractions**

- **Unmodified**: 1.4555e+06

### RGSN(N)SSFFSR Hex 7 HexNAc 3

**Mass:**  2887.1522

(7.629e-07)

**Charge States:** 3

**Start Position**: 141

**End Position**: 151

| Profile Score | Tandem Score | TDA q-value | Volume | Spectrum Matches |
| --- | --- | --- | --- | --- |
| -18.6694 | 85.9996 | 0.0265 | 1.0895e+07 | 1 |

**Adduct Signal Fractions**

- **Unmodified**: 1.0895e+07

### N(N)GTYDHDVYR Fuc 2 Hex 4 HexNAc 9

**Mass:**  4008.5869

(5.026e-04)

**Start Position**: 483

**End Position**: 493

| Profile Score | Tandem Score | TDA q-value | Volume | Spectrum Matches |
| --- | --- | --- | --- | --- |
| 0.0000 | 83.4283 | 0.0407 | 0.0000e+00 | 1 |

Adduct:

No Chromatogram Found

### N(N)GTYDHDVYRDEALN(D)NR Fuc 3 Hex 5 HexNAc 4

**Mass:**  4112.6550

(2.411e-04)

**Charge States:** 4

**Start Position**: 483

**End Position**: 500

| Profile Score | Tandem Score | TDA q-value | Volume | Spectrum Matches |
| --- | --- | --- | --- | --- |
| -5.5675 | 83.2462 | 0.0407 | 2.3460e+08 | 2 |

**Adduct Signal Fractions**

- **Unmodified**: 2.3460e+08

### RGSN(N)SSFFSR Fuc 2 Hex 10 HexNAc 6

**Mass:**  4274.6618

(-1.153e-07)

**Charge States:** 4

**Start Position**: 141

**End Position**: 151

| Profile Score | Tandem Score | TDA q-value | Volume | Spectrum Matches |
| --- | --- | --- | --- | --- |
| -19.2960 | 81.8039 | 0.0431 | 2.9735e+06 | 1 |

**Adduct Signal Fractions**

- **Unmodified**: 2.9735e+06

### cus|SWZNA|A/Switzerland/9715293/2013

**>cus|SWZNA|A/Switzerland/9715293/2013**  
 `MNPNQKIITIGSVSLTISTICFFMQIAILITTVTLHFKQYEFNSPPNNQVMLCEPTIIERNITEIVYLTNTTIEKEICPK  
PAEYRNWSKPQCGITGFAPFSKDNSIRLSAGGDIWVTREPYVSCDPDKCYQFALGQGTTLNNVHSNNTVRDRTPYRTLLM  
NELGVPFHLGTKQVCIAWSSSSCHDGKAWLHVCITGDDKNATASFIYNGRLVDSVVSWSKDILRTQESECVCINGTCTVV  
MTDGSASGKADTKILFIEEGKIVHTSTLSGSAQHVEECSCYPRYPGVRCVCRDNWKGSNRPIVDINIKDHSIVSSYVCSG  
LVGDTPRKNDSSSSSHCLDPNNEEGGHGVKGWAFDDGNDVWMGRTINETSRLGYETFKVIEGWSNPKSKLQTNRQVIVDR  
GDRSGYSGIFSVEGKSCINRCFYVELIRGRKEETEVLWTSNSIVVFCGTSGTYGTGSWPDGADLNLMPI`

| Site | Type | Count |
| --- | --- | --- |
| 329 | N-linked | 7 |
| 86 | N-linked | 7 |
| 367 | N-linked | 3 |
| 200 | N-linked | 2 |

| Mass | Sequence | MS_2_ Score | `q`-value | MS_1_ Score | Signal | Protein Coordinates | Tandem Scans | Start Time | Apex Time | End Time |
| --- | --- | --- | --- | --- | --- | --- | --- | --- | --- | --- |
| 2521.9926 | TIN(N)ETSR Hex 8 HexNAc 2 | 170.959 | 0.0 | -20.451 | 1.225e+07 | 364 - 371 | 1 | 11.37 | 11.37 | 11.54 |
| 3626.5148 | N(N)WSKPQC(C)GITGFAPFSK Hex 8 HexNAc 2 | 161.559 | 0.0 | -1.460 | 5.415e+08 | 85 - 102 | 4 | 43.54 | 43.71 | 43.88 |
| 3573.4439 | N(N)WSKPQC(C)GITGFAPF Hex 9 HexNAc 2 | 154.551 | 0.0 | -16.788 | 1.548e+08 | 85 - 100 | 3 | 52.02 | 52.02 | 52.19 |
| 3670.4714 | KN(N)DSSSSSHC(C)LDPNNEEGGHGVK Hex 5 HexNAc 2 | 152.529 | 0.0 | -17.630 | 5.706e+07 | 327 - 350 | 2 | 13.36 | 13.36 | 13.36 |
| 2359.9426 | TIN(N)ETSR Hex 7 HexNAc 2 | 149.811 | 0.0 | -20.451 | 6.710e+06 | 364 - 371 | 1 | 11.37 | 11.37 | 11.54 |
| 3464.4599 | N(N)WSKPQC(C)GITGFAPFSK Hex 7 HexNAc 2 | 149.459 | 0.0 | -3.826 | 2.893e+07 | 85 - 102 | 1 | 43.54 | 43.71 | 43.88 |
| 3411.3900 | N(N)WSKPQC(C)GITGFAPF Hex 8 HexNAc 2 | 148.166 | 0.0 | -3.326 | 1.439e+08 | 85 - 100 | 4 | 52.02 | 52.19 | 52.53 |
| 3788.5688 | N(N)WSKPQC(C)GITGFAPFSK Hex 9 HexNAc 2 | 147.696 | 0.0 | -1.089 | 6.115e+08 | 85 - 102 | 4 | 43.37 | 43.54 | 43.88 |
| 2791.1317 | TIN(N)ETSR Fuc 1 Hex 5 HexNAc 5 | 146.384 | 0.0 | -20.546 | 1.040e+07 | 364 - 371 | 1 | 11.70 | 11.70 | 11.87 |
| 4279.7100 | KN(N)DSSSSSHC(C)LDPNNEEGGHGVK Hex 5 HexNAc 5 | 142.180 | 0.0 | -17.767 | 7.175e+07 | 327 - 350 | 2 | 13.36 | 13.36 | 13.53 |
| 3834.5245 | KN(N)DSSSSSHC(C)LDPNNEEGGHGVK Hex 6 HexNAc 2 | 132.988 | 0.0 | 0.000 | 0.000e+00 | 327 - 350 | 1 | - | - | - |
| 3590.4705 | N(N)WSKPQC(C)GITGFAPF Fuc 2 Hex 3 HexNAc 4 Neu5Ac 1 | 132.549 | 0.0 | -18.286 | 2.455e+07 | 85 - 100 | 1 | 52.02 | 52.02 | 52.19 |
| 3249.3353 | N(N)WSKPQC(C)GITGFAPF Hex 7 HexNAc 2 | 130.181 | 0.0 | -20.009 | 6.168e+06 | 85 - 100 | 1 | 52.36 | 52.36 | 52.53 |
| 4428.7651 | KN(N)DSSSSSHC(C)LDPNNEEGGHGVK Fuc 1 Hex 5 HexNAc 5 | 115.508 | 0.0 | 0.000 | 0.000e+00 | 327 - 350 | 1 | - | - | - |
| 4359.6721 | KN(N)DSSSSSHC(C)LDPNNEEGGHGVK Hex 8 HexNAc 3 | 109.135 | 0.0 | -20.641 | 7.033e+06 | 327 - 350 | 1 | 14.19 | 14.19 | 14.19 |
| 3995.5831 | KN(N)DSSSSSHC(C)LDPNNEEGGHGVK Hex 7 HexNAc 2 | 105.076 | 0.007 | 0.000 | 0.000e+00 | 327 - 350 | 1 | - | - | - |
| 4472.8980 | AWLHVC(C)ITGDDKN(N)ATASFIYNGR Hex 9 HexNAc 2 | 90.869 | 0.021 | -19.141 | 6.041e+06 | 187 - 210 | 1 | 46.91 | 47.08 | 47.08 |
| 3835.5465 | KN(N)DSSSSSHC(C)LDPNNEEGGHGVK Hex 6 HexNAc 2 | 86.505 | 0.024 | 6.820 | 6.271e+07 | 327 - 350 | 3 | 20.03 | 20.50 | 20.65 |
| 3184.2856 | N(N)ATASFIYNGR Fuc 1 Hex 5 HexNAc 5 | 81.686 | 0.043 | -19.009 | 1.175e+07 | 199 - 210 | 1 | 24.71 | 24.87 | 24.87 |

### TIN(N)ETSR Hex 8 HexNAc 2

**Mass:**  2521.9925

(9.840e-07)

**Charge States:** 2

**Start Position**: 365

**End Position**: 372

| Profile Score | Tandem Score | TDA q-value | Volume | Spectrum Matches |
| --- | --- | --- | --- | --- |
| -20.4508 | 170.9593 | 0.0000 | 1.2251e+07 | 1 |

**Adduct Signal Fractions**

- **Unmodified**: 1.2251e+07

### N(N)WSKPQC(C)GITGFAPFSK Hex 8 HexNAc 2

**Mass:**  3626.5138

(1.530e-06)

**Charge States:** 3, 4

**Start Position**: 86

**End Position**: 103

| Profile Score | Tandem Score | TDA q-value | Volume | Spectrum Matches |
| --- | --- | --- | --- | --- |
| -1.4599 | 161.5588 | 0.0000 | 5.4155e+08 | 4 |

**Adduct Signal Fractions**

- **Unmodified**: 5.4155e+08

### N(N)WSKPQC(C)GITGFAPF Hex 9 HexNAc 2

**Mass:**  3573.4440

(1.872e-06)

**Charge States:** 3, 4

**Start Position**: 86

**End Position**: 101

| Profile Score | Tandem Score | TDA q-value | Volume | Spectrum Matches |
| --- | --- | --- | --- | --- |
| -16.7883 | 154.5507 | 0.0000 | 1.5483e+08 | 3 |

**Adduct Signal Fractions**

- **Unmodified**: 1.5483e+08

### KN(N)DSSSSSHC(C)LDPNNEEGGHGVK Hex 5 HexNAc 2

**Mass:**  3670.4720

(7.090e-07)

**Charge States:** 4, 5

**Start Position**: 328

**End Position**: 351

| Profile Score | Tandem Score | TDA q-value | Volume | Spectrum Matches |
| --- | --- | --- | --- | --- |
| -17.6303 | 152.5288 | 0.0000 | 5.7064e+07 | 2 |

**Adduct Signal Fractions**

- **Unmodified**: 5.7064e+07

### TIN(N)ETSR Hex 7 HexNAc 2

**Mass:**  2359.9410

(1.589e-06)

**Charge States:** 2

**Start Position**: 365

**End Position**: 372

| Profile Score | Tandem Score | TDA q-value | Volume | Spectrum Matches |
| --- | --- | --- | --- | --- |
| -20.4513 | 149.8115 | 0.0000 | 6.7096e+06 | 1 |

**Adduct Signal Fractions**

- **Unmodified**: 6.7096e+06

### N(N)WSKPQC(C)GITGFAPFSK Hex 7 HexNAc 2

**Mass:**  3464.4600

(6.262e-07)

**Charge States:** 3, 4

**Start Position**: 86

**End Position**: 103

| Profile Score | Tandem Score | TDA q-value | Volume | Spectrum Matches |
| --- | --- | --- | --- | --- |
| -3.8256 | 149.4593 | 0.0000 | 2.8925e+07 | 1 |

**Adduct Signal Fractions**

- **Unmodified**: 2.8925e+07

### N(N)WSKPQC(C)GITGFAPF Hex 8 HexNAc 2

**Mass:**  3411.3902

(1.784e-06)

**Charge States:** 3

**Start Position**: 86

**End Position**: 101

| Profile Score | Tandem Score | TDA q-value | Volume | Spectrum Matches |
| --- | --- | --- | --- | --- |
| -3.3260 | 148.1661 | 0.0000 | 1.4387e+08 | 4 |

**Adduct Signal Fractions**

- **Unmodified**: 1.4387e+08

### N(N)WSKPQC(C)GITGFAPFSK Hex 9 HexNAc 2

**Mass:**  3788.5684

(1.888e-06)

**Charge States:** 3, 4

**Start Position**: 86

**End Position**: 103

| Profile Score | Tandem Score | TDA q-value | Volume | Spectrum Matches |
| --- | --- | --- | --- | --- |
| -1.0894 | 147.6965 | 0.0000 | 6.1148e+08 | 4 |

**Adduct Signal Fractions**

- **Unmodified**: 6.1148e+08

### TIN(N)ETSR Fuc 1 Hex 5 HexNAc 5

**Mass:**  2791.1305

(1.078e-06)

**Charge States:** 3

**Start Position**: 365

**End Position**: 372

| Profile Score | Tandem Score | TDA q-value | Volume | Spectrum Matches |
| --- | --- | --- | --- | --- |
| -20.5464 | 146.3836 | 0.0000 | 1.0396e+07 | 1 |

**Adduct Signal Fractions**

- **Unmodified**: 1.0396e+07

### KN(N)DSSSSSHC(C)LDPNNEEGGHGVK Hex 5 HexNAc 5

**Mass:**  4279.7109

(8.157e-07)

**Charge States:** 4, 5

**Start Position**: 328

**End Position**: 351

| Profile Score | Tandem Score | TDA q-value | Volume | Spectrum Matches |
| --- | --- | --- | --- | --- |
| -17.7671 | 142.1800 | 0.0000 | 7.1748e+07 | 2 |

**Adduct Signal Fractions**

- **Unmodified**: 7.1748e+07

### KN(N)DSSSSSHC(C)LDPNNEEGGHGVK Hex 6 HexNAc 2

**Mass:**  3834.5245

(5.224e-04)

**Start Position**: 328

**End Position**: 351

| Profile Score | Tandem Score | TDA q-value | Volume | Spectrum Matches |
| --- | --- | --- | --- | --- |
| 0.0000 | 132.9876 | 0.0000 | 0.0000e+00 | 1 |

Adduct:

No Chromatogram Found

### N(N)WSKPQC(C)GITGFAPF Fuc 2 Hex 3 HexNAc 4 Neu5Ac 1

**Mass:**  3590.4702

(-5.614e-06)

**Charge States:** 3

**Start Position**: 86

**End Position**: 101

| Profile Score | Tandem Score | TDA q-value | Volume | Spectrum Matches |
| --- | --- | --- | --- | --- |
| -18.2861 | 132.5492 | 0.0000 | 2.4548e+07 | 1 |

**Adduct Signal Fractions**

- **Unmodified**: 2.4548e+07

### N(N)WSKPQC(C)GITGFAPF Hex 7 HexNAc 2

**Mass:**  3249.3352

(1.107e-06)

**Charge States:** 3

**Start Position**: 86

**End Position**: 101

| Profile Score | Tandem Score | TDA q-value | Volume | Spectrum Matches |
| --- | --- | --- | --- | --- |
| -20.0093 | 130.1806 | 0.0000 | 6.1678e+06 | 1 |

**Adduct Signal Fractions**

- **Unmodified**: 6.1678e+06

### KN(N)DSSSSSHC(C)LDPNNEEGGHGVK Fuc 1 Hex 5 HexNAc 5

**Mass:**  4428.7651

(6.778e-04)

**Start Position**: 328

**End Position**: 351

| Profile Score | Tandem Score | TDA q-value | Volume | Spectrum Matches |
| --- | --- | --- | --- | --- |
| 0.0000 | 115.5078 | 0.0000 | 0.0000e+00 | 1 |

Adduct:

No Chromatogram Found

### KN(N)DSSSSSHC(C)LDPNNEEGGHGVK Hex 8 HexNAc 3

**Mass:**  4359.6721

(-8.108e-06)

**Charge States:** 4

**Start Position**: 328

**End Position**: 351

| Profile Score | Tandem Score | TDA q-value | Volume | Spectrum Matches |
| --- | --- | --- | --- | --- |
| -20.6412 | 109.1345 | 0.0000 | 7.0327e+06 | 1 |

**Adduct Signal Fractions**

- **Unmodified**: 7.0327e+06

### KN(N)DSSSSSHC(C)LDPNNEEGGHGVK Hex 7 HexNAc 2

**Mass:**  3995.5831

(2.523e-04)

**Start Position**: 328

**End Position**: 351

| Profile Score | Tandem Score | TDA q-value | Volume | Spectrum Matches |
| --- | --- | --- | --- | --- |
| 0.0000 | 105.0758 | 0.0067 | 0.0000e+00 | 1 |

Adduct:

No Chromatogram Found

### AWLHVC(C)ITGDDKN(N)ATASFIYNGR Hex 9 HexNAc 2

**Mass:**  4472.8977

(3.377e-06)

**Charge States:** 4

**Start Position**: 188

**End Position**: 211

| Profile Score | Tandem Score | TDA q-value | Volume | Spectrum Matches |
| --- | --- | --- | --- | --- |
| -19.1412 | 90.8695 | 0.0210 | 6.0411e+06 | 1 |

**Adduct Signal Fractions**

- **Unmodified**: 6.0411e+06

### KN(N)DSSSSSHC(C)LDPNNEEGGHGVK Hex 6 HexNAc 2

**Mass:**  3835.5456

(7.861e-04)

**Charge States:** 4

**Start Position**: 328

**End Position**: 351

| Profile Score | Tandem Score | TDA q-value | Volume | Spectrum Matches |
| --- | --- | --- | --- | --- |
| 6.8203 | 86.5052 | 0.0238 | 6.2713e+07 | 3 |

**Adduct Signal Fractions**

- **Unmodified**: 6.2713e+07

### N(N)ATASFIYNGR Fuc 1 Hex 5 HexNAc 5

**Mass:**  3184.2841

(-7.344e-06)

**Charge States:** 3

**Start Position**: 200

**End Position**: 211

| Profile Score | Tandem Score | TDA q-value | Volume | Spectrum Matches |
| --- | --- | --- | --- | --- |
| -19.0094 | 81.6863 | 0.0431 | 1.1749e+07 | 1 |

**Adduct Signal Fractions**

- **Unmodified**: 1.1749e+07

### tr|E1C041|E1C041\_CHICK

**>tr|E1C041|E1C041\_CHICK**  
 `MRTGAGEAVGVLCLVSLVAALDYKYHHSEELEAYLKEVHAAYPALTHLHSIGRSVEGRDLWVLVLGRFPTQHKIGIPEFK  
YVANMHGDETVGREILLHLIDHLVTNYGRDPVITRLLNNTRIHIMPTMNPDGFEATVVPDCYYSRGRYNKNGEDLNRNFP  
DAFENNNNLIQPETQAVINWIKNETFVLSANLHGGALVASYTFDNGNSVTGTSNGYSRSPDDDVFIHLAKTYSFNHASMY  
KGTGCDSKQTFPDGITNGYSWYQLEGGMQDYNYVWGQCFEITLELSCCKYPPAEQLEKFWRDNKVALVEYIKQVHLGVKG  
QVTDKNGNPIPNAIVEAKGRPHICPYRTNEHGEYFLLLLPGKYVINATVPGFKSMLKTVDIPDNTANFSALKQDFSFPEV  
SVKPRPASCPRTPLYQELERSSAAVKPTLHILVLVIVTISILK`

| Site | Type | Count |
| --- | --- | --- |
| 366 | N-linked | 4 |

| Mass | Sequence | MS_2_ Score | `q`-value | MS_1_ Score | Signal | Protein Coordinates | Tandem Scans | Start Time | Apex Time | End Time |
| --- | --- | --- | --- | --- | --- | --- | --- | --- | --- | --- |
| 2910.2465 | YVIN(N)ATVPGFK Hex 8 HexNAc 2 | 148.049 | 0.0 | -3.240 | 1.506e+07 | 362 - 373 | 2 | 41.34 | 41.51 | 41.68 |
| 3072.2992 | YVIN(N)ATVPGFK Hex 9 HexNAc 2 | 146.735 | 0.0 | -1.424 | 4.676e+07 | 362 - 373 | 3 | 41.34 | 41.51 | 41.68 |
| 3234.3540 | YVIN(N)ATVPGFK Hex 10 HexNAc 2 | 143.349 | 0.0 | -18.430 | 5.249e+06 | 362 - 373 | 2 | 41.34 | 41.34 | 41.51 |
| 3089.3261 | YVIN(N)ATVPGFK Fuc 2 Hex 3 HexNAc 4 Neu5Ac 1 | 142.013 | 0.0 | -18.090 | 6.788e+06 | 362 - 373 | 1 | 41.34 | 41.51 | 41.51 |

### YVIN(N)ATVPGFK Hex 8 HexNAc 2

**Mass:**  2910.2462

(1.651e-06)

**Charge States:** 3

**Start Position**: 363

**End Position**: 374

| Profile Score | Tandem Score | TDA q-value | Volume | Spectrum Matches |
| --- | --- | --- | --- | --- |
| -3.2402 | 148.0489 | 0.0000 | 1.5057e+07 | 2 |

**Adduct Signal Fractions**

- **Unmodified**: 1.5057e+07

### YVIN(N)ATVPGFK Hex 9 HexNAc 2

**Mass:**  3072.2990

(2.175e-06)

**Charge States:** 3

**Start Position**: 363

**End Position**: 374

| Profile Score | Tandem Score | TDA q-value | Volume | Spectrum Matches |
| --- | --- | --- | --- | --- |
| -1.4237 | 146.7354 | 0.0000 | 4.6759e+07 | 3 |

**Adduct Signal Fractions**

- **Unmodified**: 4.6759e+07

### YVIN(N)ATVPGFK Hex 10 HexNAc 2

**Mass:**  3234.3539

(2.063e-06)

**Charge States:** 3

**Start Position**: 363

**End Position**: 374

| Profile Score | Tandem Score | TDA q-value | Volume | Spectrum Matches |
| --- | --- | --- | --- | --- |
| -18.4297 | 143.3494 | 0.0000 | 5.2494e+06 | 2 |

**Adduct Signal Fractions**

- **Unmodified**: 5.2494e+06

### YVIN(N)ATVPGFK Fuc 2 Hex 3 HexNAc 4 Neu5Ac 1

**Mass:**  3089.3252

(-5.722e-06)

**Charge States:** 3

**Start Position**: 363

**End Position**: 374

| Profile Score | Tandem Score | TDA q-value | Volume | Spectrum Matches |
| --- | --- | --- | --- | --- |
| -18.0896 | 142.0128 | 0.0000 | 6.7884e+06 | 1 |

**Adduct Signal Fractions**

- **Unmodified**: 6.7884e+06

### tr|A0A1D5PGT3|A0A1D5PGT3\_CHICK

**>tr|A0A1D5PGT3|A0A1D5PGT3\_CHICK**  
 `MVLPGPPGMARSEEVHRLTENVYKTIMEQFNPSLRNFISMGKTYEKALASMTYAAKGYFDALVKMGELASESQGSKELGD  
VLFQMAEVHRQIQNQLEEMLKSFHNELLTQLEQKVELDSRYLSAALKKYQTEQRNKGDSLDKCQAELKKLRKKSQGSKNP  
QKYSDKELQYIEAISNKQGELENYVSDGYKTALTEERRRFCFLVEKQCAVAKSAITYHAKGKEMLTQKLPLWQQSCSDPN  
KIPDRAIQLMQQMAASSNGTIMPSPLSTSKSNLIISDPIPGAKPLPVPPELAPFVGRMQEAMPAVNGVSGPDSDDYNHWS  
EVKPAQPKSLSPPQPQKQLSDSYSNTLPVRKNVTPKNSYASAENKTLPRSSSMAAGLERNGRTRVQAIFSHAAGDNSTLL  
SFKEGDLITLLVPEARDGWHYGESEKTKLRGWFPFSYTRVLDNDGSERVHASLQQGKSSSTGNLLDKDDIAIPPPDYGMS  
SQAFPSQNVNTFKQRPYSVAVPAFSQDYLMPYGCAIKDYSSGLCQPPPAHYKPYARSTAGLDDYGTRSVSSPDVEVARF`

| Site | Type | Count |
| --- | --- | --- |
| 352 | N-linked | 1 |

| Mass | Sequence | MS_2_ Score | `q`-value | MS_1_ Score | Signal | Protein Coordinates | Tandem Scans | Start Time | Apex Time | End Time |
| --- | --- | --- | --- | --- | --- | --- | --- | --- | --- | --- |
| 4425.7693 | KN(N)VTPK Fuc 2 Hex 10 HexNAc 9 | 153.813 | 0.0 | -19.751 | 2.625e+07 | 350 - 356 | 2 | 13.36 | 13.36 | 13.53 |

### KN(N)VTPK Fuc 2 Hex 10 HexNAc 9

**Mass:**  4425.7699

(-1.107e-06)

**Charge States:** 5

**Start Position**: 351

**End Position**: 357

| Profile Score | Tandem Score | TDA q-value | Volume | Spectrum Matches |
| --- | --- | --- | --- | --- |
| -19.7508 | 153.8129 | 0.0000 | 2.6253e+07 | 2 |

**Adduct Signal Fractions**

- **Unmodified**: 2.6253e+07

### tr|A0A1D5P0L1|A0A1D5P0L1\_CHICK

**>tr|A0A1D5P0L1|A0A1D5P0L1\_CHICK**  
 `MVLPGPPGMARSEEVHRLTENVYKTIMEQFNPSLRNFISMGKTYEKALASMTYAAKGYFDALVKMGELASESQGSKELGD  
VLFQMAEVHRQIQNQLEEMLKSFHNELLTQLEQKVELDSRYLSAALKKYQTEQRNKGDSLDKCQAELKKLRKKSQGSKNP  
QKYSDKELQYIEAISNKQGELENYVSDGYKTALTEERRRFCFLVEKQCAVAKSAITYHAKGKEMLTQKLPLWQQSCSDPN  
KIPDRAIQLMQQMAASSNGTIMPSPLSTSKSNLIISDPIPGAKPLPVPPELAPFVGRMQEAMPAVNGVSGPDSDDYNHWS  
EVKPAQPKSLSPPQPQKQLSDSYSNTLPVRKNVTPKNSYASAENKTLPRSSSMAAGLERNGRTRVQAIFSHAAGDNSTLL  
SFKEGDLITLLVPEARDGWHYGESEKTKLRGWFPFSYTRVLDNDGSERVHASLQQGKSSSTGNLLDKDDIAIPPPDYGMS  
SQAFPSQNVNTFKQRPYSVAVPAFSQDYLMPYGCAIKDYSSGLCQPPPAHYKPYARSTAGLDDYGTRSVSRNPFANVQLK  
PTVTNDRSAPLIS`

| Site | Type | Count |
| --- | --- | --- |
| 352 | N-linked | 1 |

| Mass | Sequence | MS_2_ Score | `q`-value | MS_1_ Score | Signal | Protein Coordinates | Tandem Scans | Start Time | Apex Time | End Time |
| --- | --- | --- | --- | --- | --- | --- | --- | --- | --- | --- |
| 4425.7693 | KN(N)VTPK Fuc 2 Hex 10 HexNAc 9 | 153.813 | 0.0 | -19.751 | 2.625e+07 | 350 - 356 | 2 | 13.36 | 13.36 | 13.53 |

### KN(N)VTPK Fuc 2 Hex 10 HexNAc 9

**Mass:**  4425.7699

(-1.107e-06)

**Charge States:** 5

**Start Position**: 351

**End Position**: 357

| Profile Score | Tandem Score | TDA q-value | Volume | Spectrum Matches |
| --- | --- | --- | --- | --- |
| -19.7508 | 153.8129 | 0.0000 | 2.6253e+07 | 2 |

**Adduct Signal Fractions**

- **Unmodified**: 2.6253e+07

### tr|A0A1D5PBT7|A0A1D5PBT7\_CHICK

**>tr|A0A1D5PBT7|A0A1D5PBT7\_CHICK**  
 `MVLPGPPGMARSEEVHRLTENVYKTIMEQFNPSLRNFISMGKTYEKALASMTYAAKGYFDALVKMGELASESQGSKELGD  
VLFQMAEVHRQIQNQLEEMLKSFHNELLTQLEQKVELDSRYLSAALKKYQTEQRNKGDSLDKCQAELKKLRKKSQGSKNP  
QKYSDKELQYIEAISNKQGELENYVSDGYKTALTEERRRFCFLVEKQCAVAKSAITYHAKGKEMLTQKLPLWQQSCSDPN  
KIPDRAIQLMQQMAASSNGTIMPSPLSTSKSNLIISDPIPGAKPLPVPPELAPFVGRMQEAMPAVNGVSGPDSDDYNHWS  
EVKPAQPKSLSPPQPQKQLSDSYSNTLPVRKNVTPKNSYASAENKTLPRSSSMAAGLERNGRTRVQAIFSHAAGDNSTLL  
SFKEGDLITLLVPEARDGWHYGESEKTKLRGWFPFSYTRVLDNDGSERVHASLQQGKSSSTGNLLDKDDIAIPPPDYGMS  
SQAFPSQNVNTFKQRPYSVAVPAFSQGLDDYGTRSVSSGSSSLVSTV`

| Site | Type | Count |
| --- | --- | --- |
| 352 | N-linked | 1 |

| Mass | Sequence | MS_2_ Score | `q`-value | MS_1_ Score | Signal | Protein Coordinates | Tandem Scans | Start Time | Apex Time | End Time |
| --- | --- | --- | --- | --- | --- | --- | --- | --- | --- | --- |
| 4425.7693 | KN(N)VTPK Fuc 2 Hex 10 HexNAc 9 | 153.813 | 0.0 | -19.751 | 2.625e+07 | 350 - 356 | 2 | 13.36 | 13.36 | 13.53 |

### KN(N)VTPK Fuc 2 Hex 10 HexNAc 9

**Mass:**  4425.7699

(-1.107e-06)

**Charge States:** 5

**Start Position**: 351

**End Position**: 357

| Profile Score | Tandem Score | TDA q-value | Volume | Spectrum Matches |
| --- | --- | --- | --- | --- |
| -19.7508 | 153.8129 | 0.0000 | 2.6253e+07 | 2 |

**Adduct Signal Fractions**

- **Unmodified**: 2.6253e+07

### tr|A0A1D5PRN6|A0A1D5PRN6\_CHICK

**>tr|A0A1D5PRN6|A0A1D5PRN6\_CHICK**  
 `MVLPGPPGMARSEEVHRLTENVYKTIMEQFNPSLRNFISMGKTYEKALASMTYAAKGYFDALVKMGELASESQGSKELGD  
VLFQMAEVHRQIQNQLEEMLKSFHNELLTQLEQKVELDSRYLSAALKKYQTEQRNKGDSLDKCQAELKKLRKKSQGSKNP  
QKYSDKELQYIEAISNKQGELENYVSDGYKTALTEERRRFCFLVEKQCAVAKSAITYHAKGKEMLTQKLPLWQQSCSDPN  
KIPDRAIQLMQQMAASSNGTIMPSPLSTSKSNLIISDPIPGAKPLPVPPELAPFVGRMQEAMPAVNGVSGPDSDDYNHWS  
EVKPAQPKSLSPPQPQKQLSDSYSNTLPVRKNVTPKNSYASAENKTLPRSSSMAAGLERNGRTRVQAIFSHAAGDNSTLL  
SFKEGDLITLLVPEARDGWHYGESEKTKLRGWFPFSYTRVLDNDGSERVHASLQQGKSSSTGNLLDKDDIAIPPPDYGMS  
SQAFPSQNVNTFKQRPYSVAVPAFSQDYLMPYGCAIKDYSSGLCQPPPAHYKPYARSTAGLDDYGTRSVSSGSSSLVSTV`

| Site | Type | Count |
| --- | --- | --- |
| 352 | N-linked | 1 |

| Mass | Sequence | MS_2_ Score | `q`-value | MS_1_ Score | Signal | Protein Coordinates | Tandem Scans | Start Time | Apex Time | End Time |
| --- | --- | --- | --- | --- | --- | --- | --- | --- | --- | --- |
| 4425.7693 | KN(N)VTPK Fuc 2 Hex 10 HexNAc 9 | 153.813 | 0.0 | -19.751 | 2.625e+07 | 350 - 356 | 2 | 13.36 | 13.36 | 13.53 |

### KN(N)VTPK Fuc 2 Hex 10 HexNAc 9

**Mass:**  4425.7699

(-1.107e-06)

**Charge States:** 5

**Start Position**: 351

**End Position**: 357

| Profile Score | Tandem Score | TDA q-value | Volume | Spectrum Matches |
| --- | --- | --- | --- | --- |
| -19.7508 | 153.8129 | 0.0000 | 2.6253e+07 | 2 |

**Adduct Signal Fractions**

- **Unmodified**: 2.6253e+07

### tr|A0A1D5P780|A0A1D5P780\_CHICK

**>tr|A0A1D5P780|A0A1D5P780\_CHICK**  
 `MRGIILALVLTLVDPGFNSRRSYLYNYEGSMLNGLQDRSLGKAGVRLSSKLEISGLPENAYLLKVRSPQVEEYNGVWPRD  
PFTRSSKITQVISSCFTRLFKFEYSSGRIGNIYAPEDCPDLCVNIVRGILNMFQMTIKKSQNVYELQEAGIGGICHARYV  
IQEDRKNSRIYVTRTVDLNNCQEKVQKSIGMAYIYPCPVDVMKERLTKGTTAFSYKLKQSDSGTLITDVSSRQVYQISPF  
NEPTGVAVMEARQQLTLVEVRSERGSAPDVPMQNYGSLRYRFPAVLPQMPLQLIKTKNPEQRIIETLQHIVLNNQQDFHD  
DVSYRFLEVVQLCRIANADNLESIWRQVSDKPRYRRWLLSAVSASGTTETLKFLKNRIRNDDLNYIQTLLTVSLTLHLLQ  
ADEHTLPIAADLMTSSRIQKNPVLQQVACLGYSSVVNRYCSQTSACPKEALQPIHDLADEAISRGREDKMKLALKCIGNM  
GEPASLKRILKFLPISSSSAADIPVHIQIDAITALKKIAWKDPKTVQGYLIQILADQSLPPEVRMMACAVIFETRPALAL  
ITTIANVAMKESNMQVASFVYSHMKSLSKSRLPFMYNISSACNIALKLLSPKLDSMSYRYSKVIRADTYFDNYRVGATGE  
IFVVNSPRTMFPSAIISKLMANSAGSVADLVEVGIRVEGLADVIMKRNIPFAEYPTYKQIKELGKALQGWKELPTETPLV  
SAYLKILGQEVAFININKELLQQVMKTVVEPADRNAAIKRIANQIRNSIAGQWTQPVWMGELRYVVPSCLGLPLEYGSYT  
TALARAAVSVEGKMTPPLTGDFRLSQLLESTMQIRSDLKPSLYVHTVATMGVNTEYFQHAVEIQGEVQTRMPMKFDAKID  
VKLKNLKIETNPCREETEIVVGRHKAFAVSRNIGELGVEKRTSILPEDAPLDVTEEPFQTSERASREHFAMQGPDSMPRK  
QSHSSREDLRRSTGKRAHKRDICLKMHHIGCQLCFSRRSRDASFIQNTYLHKLIGEHEAKIVLMPVHTDADIDKIQLEIQ  
AGSRAAARIITEVNPESEEEDESSPYEDIQAKLKRILGIDSMFKVANKTRHPKNRPSKKGNTVLAEFGTEPDAKTSSSSS  
SASSTATSSSSSSASSPNRKKPMDEEENDQVKQARNKDASSSSRSSKSSNSSKRSSSKSSNSSKRSSSSSSSSSSSSSSS  
SRSSSSSSSSSSNSKSSSSSSKSSSSSSRSRSSSKSSSSSSSSSSSSSSKSSSSRSSSSSSKSSSHHSHSHHSGHLNGSS  
SSSSSSRSVSHHSHEHHSGHLEDDSSSSSSSSVLSKIWGRHEIYQYRFRSAHRQEFPKRKLPGDRATSRYSSTRSSHDTS  
RAASWPKFLGDIKTPVLAAFLHGISNNKKTGGLQLVVYADTDSVRPRVQVFVTNLTDSSKWKLCADASVRNAHKAVAYVK  
WGWDCRDYKVSTELVTGRFAGHPAAQVKLEWPKVPSNVRSVVEWFYEFVPGAAFMLGFSERMDKNPSRQARMVVALTSPR  
TCDVVVKLPDMILYQKAVRLPLSLPVGPRIPASELQSPIWNVFAEAPSAVLENLKARCSVSYNKIKTFNEVKFNYSMPAN  
CYHILVQDCSSELKFLVMMKSAGEATNLKAINIKIGSHEIDMHPVNGQVKLLVDGAESPTANISLISAGASLWIHNENQG  
FALAAPGHGIDKLYFDGKTITIQVPLWMAGKTCGICGKYDAECEQEYRMPNGYLAKNAVSFGHSWILEEAPCRGACKLHR  
SFVKLEKTVQLAGVDSKCYSTEPVLRCAKGCSATKTTPVTVGFHCLPADSANSLTDKQMKYDQKSEDMQDTVDAHTACSC  
ENEECST`

| Site | Type | Count |
| --- | --- | --- |
| 1414 | N-linked | 1 |

| Mass | Sequence | MS_2_ Score | `q`-value | MS_1_ Score | Signal | Protein Coordinates | Tandem Scans | Start Time | Apex Time | End Time |
| --- | --- | --- | --- | --- | --- | --- | --- | --- | --- | --- |
| 2815.2350 | VQVFVTN(N)LTDSSK Hex 6 HexNAc 2 | 118.099 | 0.0 | -17.662 | 2.609e+06 | 1407 - 1420 | 1 | 37.51 | 37.51 | 37.51 |

### VQVFVTN(N)LTDSSK Hex 6 HexNAc 2

**Mass:**  2815.2350

(2.904e-06)

**Charge States:** 3

**Start Position**: 1408

**End Position**: 1421

| Profile Score | Tandem Score | TDA q-value | Volume | Spectrum Matches |
| --- | --- | --- | --- | --- |
| -17.6619 | 118.0993 | 0.0000 | 2.6090e+06 | 1 |

**Adduct Signal Fractions**

- **Unmodified**: 2.6090e+06

### sp|P02845|VIT2\_CHICK

**>sp|P02845|VIT2\_CHICK**  
 `MRGIILALVLTLVGSQKFDIDPGFNSRRSYLYNYEGSMLNGLQDRSLGKAGVRLSSKLEISGLPENAYLLKVRSPQVEEY  
NGVWPRDPFTRSSKITQVISSCFTRLFKFEYSSGRIGNIYAPEDCPDLCVNIVRGILNMFQMTIKKSQNVYELQEAGIGG  
ICHARYVIQEDRKNSRIYVTRTVDLNNCQEKVQKSIGMAYIYPCPVDVMKERLTKGTTAFSYKLKQSDSGTLITDVSSRQ  
VYQISPFNEPTGVAVMEARQQLTLVEVRSERGSAPDVPMQNYGSLRYRFPAVLPQMPLQLIKTKNPEQRIVETLQHIVLN  
NQQDFHDDVSYRFLEVVQLCRIANADNLESIWRQVSDKPRYRRWLLSAVSASGTTETLKFLKNRIRNDDLNYIQTLLTVS  
LTLHLLQADEHTLPIAADLMTSSRIQKNPVLQQVACLGYSSVVNRYCSQTSACPKEALQPIHDLADEAISRGREDKMKLA  
LKCIGNMGEPASLKRILKFLPISSSSAADIPVHIQIDAITALKKIAWKDPKTVQGYLIQILADQSLPPEVRMMACAVIFE  
TRPALALITTIANVAMKESNMQVASFVYSHMKSLSKSRLPFMYNISSACNIALKLLSPKLDSMSYRYSKVIRADTYFDNY  
RVGATGEIFVVNSPRTMFPSAIISKLMANSAGSVADLVEVGIRVEGLADVIMKRNIPFAEYPTYKQIKELGKALQGWKEL  
PTETPLVSAYLKILGQEVAFININKELLQQVMKTVVEPADRNAAIKRIANQIRNSIAGQWTQPVWMGELRYVVPSCLGLP  
LEYGSYTTALARAAVSVEGKMTPPLTGDFRLSQLLESTMQIRSDLKPSLYVHTVATMGVNTEYFQHAVEIQGEVQTRMPM  
KFDAKIDVKLKNLKIETNPCREETEIVVGRHKAFAVSRNIGELGVEKRTSILPEDAPLDVTEEPFQTSERASREHFAMQG  
PDSMPRKQSHSSREDLRRSTGKRAHKRDICLKMHHIGCQLCFSRRSRDASFIQNTYLHKLIGEHEAKIVLMPVHTDADID  
KIQLEIQAGSRAAARIITEVNPESEEEDESSPYEDIQAKLKRILGIDSMFKVANKTRHPKNRPSKKGNTVLAEFGTEPDA  
KTSSSSSSASSTATSSSSSSASSPNRKKPMDEEENDQVKQARNKDASSSSRSSKSSNSSKRSSSKSSNSSKRSSSSSSSS  
SSSSRSSSSSSSSSSNSKSSSSSSKSSSSSSRSRSSSKSSSSSSSSSSSSSSKSSSSRSSSSSSKSSSHHSHSHHSGHLN  
GSSSSSSSSRSVSHHSHEHHSGHLEDDSSSSSSSSVLSKIWGRHEIYQYRFRSAHRQEFPKRKLPGDRATSRYSSTRSSH  
DTSRAASWPKFLGDIKTPVLAAFLHGISNNKKTGGLQLVVYADTDSVRPRVQVFVTNLTDSSKWKLCADASVRNAHKAVA  
YVKWGWDCRDYKVSTELVTGRFAGHPAAQVKLEWPKVPSNVRSVVEWFYEFVPGAAFMLGFSERMDKNPSRQARMVVALT  
SPRTCDVVVKLPDIILYQKAVRLPLSLPVGPRIPASELQPPIWNVFAEAPSAVLENLKARCSVSYNKIKTFNEVKFNYSM  
PANCYHILVQDCSSELKFLVMMKSAGEATNLKAINIKIGSHEIDMHPVNGQVKLLVDGAESPTANISLISAGASLWIHNE  
NQGFALAAPGHGIDKLYFDGKTITIQVPLWMAGKTCGICGKYDAECEQEYRMPNGYLAKNAVSFGHSWILEEAPCRGACK  
LHRSFVKLEKTVQLAGVDSKCYSTEPVLRCAKGCSATKTTPVTVGFHCLPADSANSLTDKQMKYDQKSEDMQDTVDAHTT  
CSCENEECST`

| Site | Type | Count |
| --- | --- | --- |
| 1417 | N-linked | 1 |

| Mass | Sequence | MS_2_ Score | `q`-value | MS_1_ Score | Signal | Protein Coordinates | Tandem Scans | Start Time | Apex Time | End Time |
| --- | --- | --- | --- | --- | --- | --- | --- | --- | --- | --- |
| 2815.2350 | VQVFVTN(N)LTDSSK Hex 6 HexNAc 2 | 118.099 | 0.0 | -17.662 | 2.609e+06 | 1410 - 1423 | 1 | 37.51 | 37.51 | 37.51 |

### VQVFVTN(N)LTDSSK Hex 6 HexNAc 2

**Mass:**  2815.2350

(2.904e-06)

**Charge States:** 3

**Start Position**: 1411

**End Position**: 1424

| Profile Score | Tandem Score | TDA q-value | Volume | Spectrum Matches |
| --- | --- | --- | --- | --- |
| -17.6619 | 118.0993 | 0.0000 | 2.6090e+06 | 1 |

**Adduct Signal Fractions**

- **Unmodified**: 2.6090e+06

### tr|F1NFL6|F1NFL6\_CHICK

**>tr|F1NFL6|F1NFL6\_CHICK**  
 `MRGIILALVLTLVGSQKFDIDPGFNSRRSYLYNYEGSMLNGLQDRSLGKAGVRLSSKLEISGLPENAYLLKVRSPQVEEY  
NGVWPRDPFTRSSKITQVISSCFTRLFKFEYSSGRIGNIYAPEDCPDLCVNIVRGILNMFQMTIKKSQNVYELQEAGIGG  
ICHARYVIQEDRKNSRIYVTRTVDLNNCQEKVQKSIGMAYIYPCPVDVMKERLTKGTTAFSYKLKQSDSGTLITDVSSRQ  
VYQISPFNEPTGVAVMEARQQLTLVEVRSERGSAPDVPMQNYGSLRYRFPAVLPQMPLQLIKTKNPEQRIIETLQHIVLN  
NQQDFHDDVSYRFLEVVQLCRIANADNLESIWRQVSDKPRYRRWLLSAVSASGTTETLKFLKNRIRNDDLNYIQTLLTVS  
LTLHLLQADEHTLPIAADLMTSSRIQKNPVLQQVACLGYSSVVNRYCSQTSACPKEALQPIHDLADEAISRGREDKMKLA  
LKCIGNMGEPASLKRILKFLPISSSSAADIPVHIQIDAITALKKIAWKDPKTVQGYLIQILADQSLPPEVRMMACAVIFE  
TRPALALITTIANVAMKESNMQVASFVYSHMKSLSKSRLPFMYNISSACNIALKLLSPKLDSMSYRYSKVIRADTYFDNY  
RVGATGEIFVVNSPRTMFPSAIISKLMANSAGSVADLVEVGIRVEGLADVIMKRNIPFAEYPTYKQIKELGKALQGWKEL  
PTETPLVSAYLKILGQEVAFININKELLQQVMKTVVEPADRNAAIKRIANQIRNSIAGQWTQPVWMGELRYVVPSCLGLP  
LEYGSYTTALARAAVSVEGKMTPPLTGDFRLSQLLESTMQIRSDLKPSLYVHTVATMGVNTEYFQHAVEIQGEVQTRMPM  
KFDAKIDVKLKNLKIETNPCREETEIVVGRHKAFAVSRNIGELGVEKRTSILPEDAPLDVTEEPFQTSERASREHFAMQG  
PDSMPRKQSHSSREDLRRSTGKRAHKRDICLKMHHIGCQLCFSRRSRDASFIQNTYLHKLIGEHEAKIVLMPVHTDADID  
KIQLEIQAGSRAAARIITEVNPESEEEDESSPYEDIQAKLKRILGIDSMFKVANKTRHPKNRPSKKGNTVLAEFGTEPDA  
KTSSSSSSASSTATSSSSSSASSPNRKKPMDEEENDQVKQARNKDASSSSRSSKSSNSSKRSSSKSSNSSKRSSSSSSSS  
SSSSSSSSRSSSSSSSSSSNSKSSSSSSKSSSSSSRSRSSSKSSSSSSSSSSSSSSKSSSSRSSSSSSKSSSHHSHSHHS  
GHLNGSSSSSSSSRSVSHHSHEHHSGHLEDDSSSSSSSSVLSKIWGRHEIYQYRFRSAHRQEFPKRKLPGDRATSRYSST  
RSSHDTSRAASWPKFLGDIKTPVLAAFLHGISNNKKTGGLQLVVYADTDSVRPRVQVFVTNLTDSSKWKLCADASVRNAH  
KAVAYVKWGWDCRDYKVSTELVTGRFAGHPAAQVKLEWPKVPSNVRSVVEWFYEFVPGAAFMLGFSERMDKNPSRQARMV  
VALTSPRTCDVVVKLPDMILYQKAVRLPLSLPVGPRIPASELQSPIWNVFAEAPSAVLENLKARCSVSYNKIKTFNEVKF  
NYSMPANCYHILVQDCSSELKFLVMMKSAGEATNLKAINIKIGSHEIDMHPVNGQVKLLVDGAESPTANISLISAGASLW  
IHNENQGFALAAPGHGIDKLYFDGKTITIQVPLWMAGKTCGICGKYDAECEQEYRMPNGYLAKNAVSFGHSWILEEAPCR  
GACKLHRSFVKLEKTVQLAGVDSKCYSTEPVLRCAKGCSATKTTPVTVGFHCLPADSANSLTDKQMKYDQKSEDMQDTVD  
AHTACSCENEECST`

| Site | Type | Count |
| --- | --- | --- |
| 1421 | N-linked | 1 |

| Mass | Sequence | MS_2_ Score | `q`-value | MS_1_ Score | Signal | Protein Coordinates | Tandem Scans | Start Time | Apex Time | End Time |
| --- | --- | --- | --- | --- | --- | --- | --- | --- | --- | --- |
| 2815.2350 | VQVFVTN(N)LTDSSK Hex 6 HexNAc 2 | 118.099 | 0.0 | -17.662 | 2.609e+06 | 1414 - 1427 | 1 | 37.51 | 37.51 | 37.51 |

### VQVFVTN(N)LTDSSK Hex 6 HexNAc 2

**Mass:**  2815.2350

(2.904e-06)

**Charge States:** 3

**Start Position**: 1415

**End Position**: 1428

| Profile Score | Tandem Score | TDA q-value | Volume | Spectrum Matches |
| --- | --- | --- | --- | --- |
| -17.6619 | 118.0993 | 0.0000 | 2.6090e+06 | 1 |

**Adduct Signal Fractions**

- **Unmodified**: 2.6090e+06
