# Supplementary material for: Measuring Site-specific Glycosylation Similarity between Influenza a Virus Variants with Statistical Certainty
Source: Mol Cell Proteomics. 2020 Nov 25;19(9):1533–45. doi: 10.1074/mcp.RA120.002031 (PMC8143645; doi:10.1074/mcp.RA120.002031)
Supplement: Supplementary file 2 [file mmc2.zip › mmc2/159250_2_supp_555293_qcjklw.zip!/20190301_SWZeggL2_021119_tryp_HILIC_DDA_1ug_02_glycopeptides.html]

## 20190301\_SWZeggL2\_021119\_tryp\_HILIC\_DDA\_1ug\_02 @ GlycopeptideHypothesis-5e596fc5c5d64f22bbe904acf5d589db

**MS^1^ Mass Accuracy:** 10.0 PPM  
**Grouping Tolerance:** 15.0 PPM  
**MS^n^ Mass Accuracy:** 20.0 PPM  
**Minimum `q`-Value For Clustering:** 0.05

**Glycopeptide Hypothesis:**
GlycopeptideHypothesis-5e596fc5c5d64f22bbe904acf5d589db  
**Sample Searched:**
20190301\_SWZeggL2\_021119\_tryp\_HILIC\_DDA\_1ug\_02

| Protein | Matched Glycopeptides |
| --- | --- |
| cus\|SWZHA\|A/Switzerland/9715293/2013 | 152 |
| cus\|SWZNA\|A/Switzerland/9715293/2013 | 12 |
| tr\|E1C041\|E1C041\_CHICK | 2 |
| tr\|A0A1D5PBT7\|A0A1D5PBT7\_CHICK | 1 |
| tr\|A0A1D5PGT3\|A0A1D5PGT3\_CHICK | 1 |
| tr\|A0A1D5PRN6\|A0A1D5PRN6\_CHICK | 1 |
| tr\|A0A1D5P0L1\|A0A1D5P0L1\_CHICK | 1 |
| tr\|A0A1D5PNI3\|A0A1D5PNI3\_CHICK | 1 |
| tr\|A0A1D5PUR1\|A0A1D5PUR1\_CHICK | 1 |

### cus|SWZHA|A/Switzerland/9715293/2013

**>cus|SWZHA|A/Switzerland/9715293/2013**  
 `QKLPGNDNSTATLCLGHHAVPNGTIVKTITNDRIEVTNATELVQNSSIGEICDSPHQILDGENCTLIDALLGDPQCDGFQ  
NKKWDLFVERSKAYSNCYPYDVPDYASLRSLVASSGTLEFNNESFNWAGVTQNGTSSSCRRGSNSSFFSRLNWLTHLNSK  
YPALNVTMPNNEQFDKLYIWGVHHPVTDKDQIFLYAQSSGRITVSTKRSQQAVIPNIGYRPRIRDIPSRISIYWTIVKPG  
DILLINSTGNLIAPRGYFKIRSGKSSIMRSDAPIGKCKSECITPNGSIPNDKPFQNVNRITYGACPRYVKQSTLKLATGM  
RNVPERQTRGIFGAIAGFIENGWEGMVDGWYGFRHQNSEGRGQAADLKSTQAAIDQINGKLNRLIGKTNEKFHQIEKEFS  
EVEGRIQDLEKYVEDTKIDLWSYNAELLVALENQHTIDLTDSEMNKLFEKTKKQLRENAEDMGNGCFKIYHKCDNACIGS  
IRNGTYDHDVYRDEALNNRFQIKGVELKSGYKDWILWISFAISCFLLCVALLGFIMWACQKGNIRCNICI`

| Site | Type | Count |
| --- | --- | --- |
| 483 | N-linked | 53 |
| 133 | N-linked | 2 |
| 38 | N-linked | 1 |
| 144 | N-linked | 48 |
| 246 | N-linked | 17 |
| 165 | N-linked | 19 |
| 285 | N-linked | 9 |
| 22 | N-linked | 3 |

| Mass | Sequence | MS_2_ Score | `q`-value | MS_1_ Score | Signal | Protein Coordinates | Tandem Scans | Start Time | Apex Time | End Time |
| --- | --- | --- | --- | --- | --- | --- | --- | --- | --- | --- |
| 2684.0718 | RGSN(N)SSFFSR Hex 7 HexNAc 2 | 188.079 | 0.0 | 12.299 | 1.506e+08 | 140 - 150 | 4 | 18.76 | 19.60 | 19.76 |
| 2359.9701 | RGSN(N)SSFFSR Hex 5 HexNAc 2 | 186.185 | 0.0 | -1.163 | 4.851e+08 | 140 - 150 | 6 | 19.27 | 19.92 | 81.96 |
| 3760.5077 | YPALN(N)VTM(O)PNNEQFDK Hex 9 HexNAc 2 | 184.720 | 0.0 | 16.180 | 1.813e+09 | 160 - 176 | 12 | 34.66 | 35.15 | 35.31 |
| 3115.2639 | RGSN(N)SSFFSR Fuc 1 Hex 5 HexNAc 5 | 182.720 | 0.0 | 13.217 | 1.107e+09 | 140 - 150 | 8 | 18.93 | 19.92 | 20.24 |
| 3598.4550 | YPALN(N)VTM(O)PNNEQFDK Hex 8 HexNAc 2 | 179.713 | 0.0 | 14.953 | 1.981e+09 | 160 - 176 | 8 | 34.66 | 35.15 | 35.47 |
| 2928.1771 | RGSN(N)SSFFSR Hex 6 HexNAc 4 | 176.832 | 0.0 | -18.552 | 4.733e+07 | 140 - 150 | 1 | 19.60 | 19.60 | 19.76 |
| 2846.1241 | RGSN(N)SSFFSR Hex 8 HexNAc 2 | 173.994 | 0.0 | -2.890 | 5.509e+07 | 140 - 150 | 4 | 18.93 | 19.43 | 19.43 |
| 2871.1555 | RGSN(N)SSFFSR Fuc 1 Hex 6 HexNAc 3 | 173.586 | 0.0 | -2.921 | 9.993e+07 | 140 - 150 | 2 | 19.10 | 19.43 | 19.60 |
| 3117.2726 | RGSN(N)SSFFSR Fuc 1 Hex 5 HexNAc 5 | 173.430 | 0.0 | 0.000 | 0.000e+00 | 140 - 150 | 1 | - | - | - |
| 2766.1263 | RGSN(N)SSFFSR Hex 5 HexNAc 4 | 171.403 | 0.0 | 8.114 | 9.685e+07 | 140 - 150 | 1 | 19.76 | 19.76 | 20.40 |
| 2547.0523 | RGSN(N)SSFFSR Fuc 1 Hex 4 HexNAc 3 | 171.259 | 0.0 | -5.246 | 4.509e+07 | 140 - 150 | 2 | 19.92 | 20.24 | 20.40 |
| 2725.0989 | RGSN(N)SSFFSR Hex 6 HexNAc 3 | 170.413 | 0.0 | 13.475 | 3.192e+08 | 140 - 150 | 5 | 18.93 | 19.76 | 20.07 |
| 4068.9364 | TIVKPGDILLIN(N)STGNLIAPR Hex 9 HexNAc 2 | 169.741 | 0.0 | 16.991 | 4.667e+08 | 234 - 255 | 5 | 53.04 | 53.55 | 54.24 |
| 3261.3205 | RGSN(N)SSFFSR Fuc 2 Hex 5 HexNAc 5 | 169.426 | 0.0 | -4.389 | 7.465e+07 | 140 - 150 | 3 | 19.10 | 19.60 | 19.60 |
| 2522.0195 | RGSN(N)SSFFSR Hex 6 HexNAc 2 | 169.312 | 0.0 | -17.131 | 2.266e+08 | 140 - 150 | 4 | 19.60 | 19.76 | 80.18 |
| 3436.4033 | YPALN(N)VTM(O)PNNEQFDK Hex 7 HexNAc 2 | 168.181 | 0.0 | 12.599 | 1.906e+08 | 160 - 176 | 4 | 34.83 | 35.15 | 35.64 |
| 2709.1052 | RGSN(N)SSFFSR Fuc 1 Hex 5 HexNAc 3 | 165.810 | 0.0 | -5.058 | 5.279e+07 | 140 - 150 | 2 | 19.60 | 19.92 | 20.07 |
| 2969.2054 | RGSN(N)SSFFSR Hex 5 HexNAc 5 | 165.505 | 0.0 | 10.336 | 2.522e+08 | 140 - 150 | 7 | 19.10 | 19.92 | 20.07 |
| 3582.4659 | YPALN(N)VTMPNNEQFDK Hex 8 HexNAc 2 | 165.358 | 0.0 | 16.731 | 1.438e+08 | 160 - 176 | 5 | 42.38 | 42.89 | 43.06 |
| 3058.2398 | RGSN(N)SSFFSR Fuc 2 Hex 5 HexNAc 4 | 164.120 | 0.0 | -3.402 | 8.267e+07 | 140 - 150 | 2 | 19.27 | 19.43 | 19.60 |
| 3744.5173 | YPALN(N)VTMPNNEQFDK Hex 9 HexNAc 2 | 161.497 | 0.0 | 15.617 | 1.491e+08 | 160 - 176 | 4 | 42.38 | 42.72 | 43.06 |
| 2912.1836 | RGSN(N)SSFFSR Fuc 1 Hex 5 HexNAc 4 | 161.393 | 0.0 | 14.875 | 6.175e+08 | 140 - 150 | 6 | 18.93 | 19.76 | 20.07 |
| 2953.2105 | RGSN(N)SSFFSR Fuc 1 Hex 4 HexNAc 5 | 160.720 | 0.0 | -17.780 | 1.630e+07 | 140 - 150 | 1 | 19.76 | 19.76 | 19.76 |
| 3153.2302 | N(N)GTYDHDVYR Fuc 2 Hex 5 HexNAc 4 | 160.417 | 0.0 | -3.634 | 1.198e+08 | 482 - 492 | 3 | 15.22 | 15.22 | 15.55 |
| 3277.3174 | RGSN(N)SSFFSR Fuc 1 Hex 6 HexNAc 5 | 159.873 | 0.0 | -4.356 | 7.008e+07 | 140 - 150 | 3 | 19.10 | 19.27 | 19.60 |
| 3112.3064 | YPALN(N)VTM(O)PNNEQFDK Hex 5 HexNAc 2 | 159.815 | 0.0 | 15.725 | 1.025e+08 | 160 - 176 | 3 | 35.47 | 35.81 | 36.15 |
| 3007.1715 | N(N)GTYDHDVYR Fuc 1 Hex 5 HexNAc 4 | 159.689 | 0.0 | 15.136 | 3.687e+08 | 482 - 492 | 5 | 15.22 | 15.38 | 16.04 |
| 2365.9186 | GSN(N)SSFFSR Hex 6 HexNAc 2 | 159.217 | 0.0 | -4.618 | 1.043e+08 | 141 - 150 | 2 | 23.31 | 23.48 | 23.64 |
| 2563.0462 | RGSN(N)SSFFSR Hex 5 HexNAc 3 | 158.615 | 0.0 | -4.360 | 6.266e+07 | 140 - 150 | 1 | 19.60 | 20.07 | 20.07 |
| 2693.1089 | RGSN(N)SSFFSR Fuc 2 Hex 4 HexNAc 3 | 157.094 | 0.0 | -20.885 | 1.377e+07 | 140 - 150 | 1 | 19.92 | 19.92 | 20.07 |
| 2959.1602 | GSN(N)SSFFSR Fuc 1 Hex 5 HexNAc 5 | 154.722 | 0.0 | -2.851 | 4.148e+08 | 141 - 150 | 4 | 23.31 | 23.48 | 23.80 |
| 3777.5336 | YPALN(N)VTM(O)PNNEQFDK Fuc 2 Hex 3 HexNAc 4 Neu5Ac 1 | 153.262 | 0.0 | -4.380 | 1.048e+08 | 160 - 176 | 2 | 34.83 | 35.15 | 35.31 |
| 4230.9853 | TIVKPGDILLIN(N)STGNLIAPR Hex 10 HexNAc 2 | 151.120 | 0.0 | -17.917 | 3.495e+07 | 234 - 255 | 1 | 53.38 | 53.38 | 53.55 |
| 4538.8991 | C(C)KSEC(C)ITPN(N)GSIPNDKPFQNVNR Hex 9 HexNAc 2 | 150.933 | 0.0 | -4.201 | 8.669e+07 | 276 - 299 | 1 | 29.35 | 29.69 | 29.86 |
| 2929.1836 | RGSN(N)SSFFSR Hex 6 HexNAc 4 | 150.266 | 0.0 | 0.000 | 0.000e+00 | 140 - 150 | 1 | - | - | - |
| 2756.0802 | GSN(N)SSFFSR Fuc 1 Hex 5 HexNAc 4 | 149.989 | 0.0 | 12.542 | 1.801e+08 | 141 - 150 | 3 | 23.16 | 23.16 | 23.80 |
| 3172.2860 | RGSN(N)SSFFSR Hex 5 HexNAc 6 | 149.444 | 0.0 | 5.331 | 1.640e+08 | 140 - 150 | 5 | 19.27 | 20.24 | 20.24 |
| 4569.2190 | ISIYWTIVKPGDILLIN(N)STGNLIAPR Hex 8 HexNAc 2 | 148.809 | 0.0 | 14.807 | 1.005e+08 | 229 - 255 | 5 | 68.75 | 69.09 | 69.42 |
| 2950.2532 | YPALN(N)VTM(O)PNNEQFDK Hex 4 HexNAc 2 | 148.413 | 0.0 | -2.350 | 1.741e+07 | 160 - 176 | 1 | 35.81 | 35.98 | 36.15 |
| 2203.8637 | GSN(N)SSFFSR Hex 5 HexNAc 2 | 147.997 | 0.0 | -1.459 | 1.496e+08 | 141 - 150 | 3 | 23.64 | 23.80 | 23.96 |
| 4376.8470 | C(C)KSEC(C)ITPN(N)GSIPNDKPFQNVNR Hex 8 HexNAc 2 | 147.524 | 0.0 | -18.974 | 4.092e+07 | 276 - 299 | 1 | 29.69 | 29.69 | 29.86 |
| 4731.2733 | ISIYWTIVKPGDILLIN(N)STGNLIAPR Hex 9 HexNAc 2 | 146.613 | 0.0 | 16.662 | 1.455e+09 | 229 - 255 | 10 | 67.21 | 69.09 | 69.59 |
| 3204.3002 | RGSN(N)SSFFSR Fuc 3 Hex 5 HexNAc 4 | 146.296 | 0.0 | -17.503 | 1.868e+07 | 140 - 150 | 1 | 19.10 | 19.27 | 19.27 |
| 3922.5649 | YPALN(N)VTM(O)PNNEQFDK Hex 10 HexNAc 2 | 145.852 | 0.0 | -5.717 | 4.215e+07 | 160 - 176 | 1 | 34.83 | 34.99 | 35.31 |
| 3090.2335 | RGSN(N)SSFFSR Hex 7 HexNAc 4 | 145.702 | 0.0 | -19.532 | 1.698e+07 | 140 - 150 | 1 | 19.60 | 19.60 | 19.76 |
| 3074.2363 | RGSN(N)SSFFSR Fuc 1 Hex 6 HexNAc 4 | 143.759 | 0.0 | -7.454 | 2.373e+07 | 140 - 150 | 2 | 19.43 | 20.07 | 20.07 |
| 3274.3542 | YPALN(N)VTM(O)PNNEQFDK Hex 6 HexNAc 2 | 142.341 | 0.0 | -3.711 | 2.518e+07 | 160 - 176 | 2 | 35.47 | 35.47 | 35.81 |
| 3420.4118 | YPALN(N)VTMPNNEQFDK Hex 7 HexNAc 2 | 142.159 | 0.0 | -4.269 | 1.249e+07 | 160 - 176 | 1 | 42.72 | 42.89 | 43.06 |
| 4893.3246 | ISIYWTIVKPGDILLIN(N)STGNLIAPR Hex 10 HexNAc 2 | 140.967 | 0.0 | -3.728 | 1.418e+08 | 229 - 255 | 4 | 68.75 | 68.92 | 69.25 |
| 3761.5417 | YPALN(N)VTMPNNEQFDK Fuc 2 Hex 3 HexNAc 4 Neu5Ac 1 | 140.666 | 0.0 | -3.237 | 1.050e+07 | 160 - 176 | 2 | 42.55 | 42.72 | 42.89 |
| 4748.2948 | ISIYWTIVKPGDILLIN(N)STGNLIAPR Fuc 2 Hex 3 HexNAc 4 Neu5Ac 1 | 140.533 | 0.0 | 12.988 | 1.653e+08 | 229 - 255 | 6 | 68.57 | 69.09 | 69.59 |
| 3315.4713 | LGHHAVPN(N)GTIVK Fuc 1 Hex 5 HexNAc 5 | 140.333 | 0.0 | 0.000 | 0.000e+00 | 14 - 27 | 1 | - | - | - |
| 3761.5057 | YPALN(N)VTM(O)PN(D)NEQFDK Hex 9 HexNAc 2 | 139.840 | 0.0 | 10.176 | 5.263e+08 | 160 - 176 | 7 | 35.31 | 35.98 | 36.83 |
| 4910.3514 | ISIYWTIVKPGDILLIN(N)STGNLIAPR Fuc 2 Hex 4 HexNAc 4 Neu5Ac 1 | 139.819 | 0.0 | -19.388 | 1.460e+07 | 229 - 255 | 2 | 68.92 | 68.92 | 69.09 |
| 3267.3338 | N(N)GTYDHDVYRDEALNNR Hex 5 HexNAc 2 | 139.646 | 0.0 | -19.674 | 7.695e+07 | 482 - 499 | 1 | 21.18 | 21.34 | 21.34 |
| 3632.4649 | N(N)GTYDHDVYRDEALNNR Hex 6 HexNAc 3 | 139.067 | 0.0 | -4.352 | 1.772e+08 | 482 - 499 | 2 | 20.85 | 21.02 | 21.34 |
| 3050.1961 | WAGVTQN(N)GTSSSC(C)R Hex 7 HexNAc 2 | 138.746 | 0.0 | -18.874 | 3.571e+06 | 126 - 140 | 1 | 18.60 | 18.76 | 18.76 |
| 2568.9958 | GSN(N)SSFFSR Hex 6 HexNAc 3 | 138.124 | 0.0 | -4.078 | 1.469e+08 | 141 - 150 | 3 | 23.31 | 23.48 | 23.64 |
| 3131.2557 | RGSN(N)SSFFSR Hex 6 HexNAc 5 | 137.748 | 0.0 | -4.795 | 1.216e+07 | 140 - 150 | 1 | 19.27 | 19.43 | 19.60 |
| 2690.0255 | GSN(N)SSFFSR Hex 8 HexNAc 2 | 137.235 | 0.0 | -18.072 | 1.793e+07 | 141 - 150 | 2 | 23.00 | 23.00 | 23.00 |
| 5568.7555 | IRDIPSRISIYWTIVKPGDILLIN(N)STGNLIAPR Hex 9 HexNAc 2 | 136.688 | 0.0 | 15.880 | 2.261e+08 | 222 - 255 | 5 | 64.79 | 65.19 | 65.54 |
| 3599.4521 | YPALN(N)VTM(O)PN(D)NEQFDK Hex 8 HexNAc 2 | 136.448 | 0.0 | 9.631 | 4.306e+08 | 160 - 176 | 6 | 35.64 | 35.98 | 37.00 |
| 4185.6686 | RGSN(N)SSFFSR Fuc 1 Hex 3 HexNAc 9 Neu5Ac 2 | 134.305 | 0.0 | 9.373 | 1.856e+08 | 140 - 150 | 2 | 21.34 | 21.34 | 22.16 |
| 4088.7209 | SEC(C)ITPN(N)GSIPNDKPFQNVNR Hex 8 HexNAc 2 | 134.256 | 0.0 | 16.804 | 6.040e+08 | 278 - 299 | 3 | 32.30 | 32.47 | 32.96 |
| 3906.8840 | TIVKPGDILLIN(N)STGNLIAPR Hex 8 HexNAc 2 | 133.813 | 0.0 | -19.481 | 2.285e+07 | 234 - 255 | 2 | 53.55 | 53.55 | 53.72 |
| 3926.6710 | SEC(C)ITPN(N)GSIPNDKPFQNVNR Hex 7 HexNAc 2 | 133.687 | 0.0 | -2.720 | 7.238e+08 | 278 - 299 | 4 | 32.47 | 32.63 | 32.96 |
| 4022.6294 | N(N)GTYDHDVYRDEALNNR Fuc 1 Hex 5 HexNAc 5 | 132.482 | 0.0 | 12.418 | 1.424e+09 | 482 - 499 | 7 | 20.07 | 21.18 | 21.50 |
| 3778.5253 | N(N)GTYDHDVYRDEALNNR Fuc 1 Hex 6 HexNAc 3 | 132.317 | 0.0 | -4.719 | 1.532e+08 | 482 - 499 | 1 | 20.70 | 20.85 | 21.18 |
| 4250.7739 | SEC(C)ITPN(N)GSIPNDKPFQNVNR Hex 9 HexNAc 2 | 132.157 | 0.0 | -1.970 | 1.104e+09 | 278 - 299 | 3 | 32.30 | 32.47 | 32.79 |
| 4085.9593 | TIVKPGDILLIN(N)STGNLIAPR Fuc 2 Hex 3 HexNAc 4 Neu5Ac 1 | 131.793 | 0.0 | -3.863 | 3.128e+07 | 234 - 255 | 1 | 53.38 | 53.55 | 53.72 |
| 3211.2581 | N(N)GTYDHDVYR Fuc 1 Hex 5 HexNAc 5 | 131.677 | 0.0 | 0.000 | 0.000e+00 | 482 - 492 | 1 | - | - | - |
| 3006.2949 | HAVPN(N)GTIVK Fuc 1 Hex 5 HexNAc 5 | 131.324 | 0.0 | -20.558 | 5.223e+06 | 17 - 27 | 1 | 16.52 | 16.52 | 16.52 |
| 4184.6831 | N(N)GTYDHDVYRDEALNNR Fuc 1 Hex 6 HexNAc 5 | 130.638 | 0.0 | 14.116 | 5.247e+08 | 482 - 499 | 3 | 20.56 | 20.85 | 21.18 |
| 3819.5482 | N(N)GTYDHDVYRDEALNNR Fuc 1 Hex 5 HexNAc 4 | 130.451 | 0.0 | 16.578 | 3.098e+09 | 482 - 499 | 6 | 20.70 | 21.02 | 21.34 |
| 4114.6665 | N(N)GTYDHDVYRDEALNNR Fuc 3 Hex 5 HexNAc 4 | 129.840 | 0.0 | 0.000 | 0.000e+00 | 482 - 499 | 1 | - | - | - |
| 3480.3951 | RGSN(N)SSFFSR Fuc 1 Hex 6 HexNAc 6 | 129.597 | 0.0 | -4.879 | 3.285e+07 | 140 - 150 | 4 | 19.10 | 19.10 | 19.60 |
| 3673.4909 | N(N)GTYDHDVYRDEALNNR Hex 5 HexNAc 4 | 128.045 | 0.0 | -3.977 | 1.841e+08 | 482 - 499 | 2 | 20.85 | 21.02 | 21.34 |
| 3454.4170 | N(N)GTYDHDVYRDEALNNR Fuc 1 Hex 4 HexNAc 3 | 127.797 | 0.0 | -4.409 | 1.027e+08 | 482 - 499 | 3 | 21.18 | 21.34 | 21.50 |
| 2804.2189 | HAVPN(N)GTIVK Fuc 1 Hex 5 HexNAc 4 | 126.593 | 0.0 | 0.000 | 0.000e+00 | 17 - 27 | 1 | - | - | - |
| 3616.4713 | N(N)GTYDHDVYRDEALNNR Fuc 1 Hex 5 HexNAc 3 | 126.275 | 0.0 | -4.211 | 7.086e+07 | 482 - 499 | 1 | 21.02 | 21.18 | 21.34 |
| 3591.4407 | N(N)GTYDHDVYRDEALNNR Hex 7 HexNAc 2 | 125.857 | 0.0 | -19.737 | 3.932e+07 | 482 - 499 | 1 | 20.85 | 20.85 | 21.02 |
| 2872.1590 | RGSN(N)SSFFSR Fuc 1 Hex 6 HexNAc 3 | 125.626 | 0.0 | 0.000 | 0.000e+00 | 140 - 150 | 1 | - | - | - |
| 4159.6130 | GSN(N)SSFFSR Fuc 2 Hex 9 HexNAc 7 | 125.294 | 0.0 | -18.130 | 2.534e+07 | 141 - 150 | 1 | 22.49 | 22.49 | 22.49 |
| 3334.3365 | RGSN(N)SSFFSR Hex 6 HexNAc 6 | 124.740 | 0.0 | -5.623 | 1.519e+07 | 140 - 150 | 1 | 19.27 | 19.43 | 19.60 |
| 2610.0231 | GSN(N)SSFFSR Hex 5 HexNAc 4 | 124.298 | 0.0 | -5.168 | 4.896e+07 | 141 - 150 | 1 | 23.31 | 23.48 | 23.64 |
| 3764.6205 | SEC(C)ITPN(N)GSIPNDKPFQNVNR Hex 6 HexNAc 2 | 123.902 | 0.0 | -3.498 | 9.211e+07 | 278 - 299 | 3 | 32.63 | 32.79 | 32.96 |
| 2400.9941 | RGSN(N)SSFFSR Hex 4 HexNAc 3 | 123.356 | 0.0 | -20.343 | 1.620e+07 | 140 - 150 | 1 | 20.24 | 20.24 | 20.40 |
| 3470.4119 | N(N)GTYDHDVYRDEALNNR Hex 5 HexNAc 3 | 122.564 | 0.0 | -4.565 | 4.798e+07 | 482 - 499 | 1 | 21.18 | 21.18 | 21.50 |
| 3966.5943 | N(N)GTYDHDVYRDEALN(D)NR Fuc 2 Hex 5 HexNAc 4 | 122.519 | 0.0 | 4.290 | 4.039e+08 | 482 - 499 | 4 | 21.34 | 21.50 | 22.33 |
| 3299.2892 | N(N)GTYDHDVYR Fuc 3 Hex 5 HexNAc 4 | 122.476 | 0.0 | -20.133 | 1.951e+07 | 482 - 492 | 3 | 15.05 | 15.05 | 15.22 |
| 3372.3058 | N(N)GTYDHDVYR Fuc 1 Hex 6 HexNAc 5 | 121.865 | 0.0 | -4.869 | 3.644e+07 | 482 - 492 | 3 | 15.22 | 15.22 | 15.55 |
| 2406.9419 | GSN(N)SSFFSR Hex 5 HexNAc 3 | 121.652 | 0.0 | -18.582 | 1.075e+07 | 141 - 150 | 1 | 23.80 | 23.80 | 23.80 |
| 3899.5085 | N(N)GTYDHDVYRDEALNNR Fuc 1 Hex 8 HexNAc 2 | 121.566 | 0.0 | 14.418 | 6.578e+08 | 482 - 499 | 4 | 22.49 | 22.66 | 23.16 |
| 2861.1136 | N(N)GTYDHDVYR Hex 5 HexNAc 4 | 121.505 | 0.0 | -18.713 | 1.598e+07 | 482 - 492 | 2 | 15.38 | 15.38 | 15.38 |
| 2820.0869 | N(N)GTYDHDVYR Hex 6 HexNAc 3 | 121.291 | 0.0 | -18.227 | 1.420e+07 | 482 - 492 | 1 | 15.38 | 15.38 | 15.38 |
| 4168.6870 | N(N)GTYDHDVYRDEALNNR Fuc 2 Hex 5 HexNAc 5 | 121.282 | 0.0 | -3.713 | 1.803e+08 | 482 - 499 | 1 | 20.70 | 20.85 | 21.18 |
| 3965.6081 | N(N)GTYDHDVYRDEALNNR Fuc 2 Hex 5 HexNAc 4 | 120.728 | 0.0 | -2.246 | 1.114e+09 | 482 - 499 | 4 | 20.70 | 20.85 | 21.18 |
| 3210.2525 | N(N)GTYDHDVYR Fuc 1 Hex 5 HexNAc 5 | 120.610 | 0.0 | -3.368 | 1.309e+08 | 482 - 492 | 3 | 15.38 | 15.55 | 15.71 |
| 3105.2198 | GSN(N)SSFFSR Fuc 2 Hex 5 HexNAc 5 | 119.568 | 0.048 | -18.409 | 1.580e+07 | 141 - 150 | 1 | 23.00 | 23.00 | 23.00 |
| 3794.5211 | N(N)GTYDHDVYRDEALNNR Hex 7 HexNAc 3 | 119.246 | 0.0 | -18.843 | 3.924e+07 | 482 - 499 | 1 | 20.85 | 20.85 | 21.02 |
| 2772.0765 | GSN(N)SSFFSR Hex 6 HexNAc 4 | 119.005 | 0.048 | -5.206 | 2.379e+07 | 141 - 150 | 1 | 23.31 | 23.31 | 23.80 |
| 4102.5891 | N(N)GTYDHDVYRDEALNNR Fuc 1 Hex 8 HexNAc 3 | 118.810 | 0.0 | 11.818 | 2.819e+08 | 482 - 499 | 3 | 22.33 | 22.83 | 23.00 |
| 4387.7610 | N(N)GTYDHDVYRDEALNNR Fuc 1 Hex 6 HexNAc 6 | 118.659 | 0.0 | -4.015 | 6.801e+07 | 482 - 499 | 1 | 20.70 | 20.85 | 21.02 |
| 3602.5684 | SEC(C)ITPN(N)GSIPNDKPFQNVNR Hex 5 HexNAc 2 | 116.849 | 0.0 | -18.515 | 3.849e+07 | 278 - 299 | 1 | 32.79 | 32.79 | 32.96 |
| 3627.6354 | PGDILLIN(N)STGNLIAPR Hex 9 HexNAc 2 | 116.773 | 0.0 | -19.275 | 2.272e+07 | 238 - 255 | 1 | 51.85 | 51.85 | 52.02 |
| 3820.5364 | N(N)GTYDHDVYRDEALN(D)NR Fuc 1 Hex 5 HexNAc 4 | 116.152 | 0.0 | 5.846 | 1.378e+09 | 482 - 499 | 9 | 21.50 | 21.67 | 22.33 |
| 3096.3027 | YPALN(N)VTMPNNEQFDK Hex 5 HexNAc 2 | 113.835 | 0.0 | -4.150 | 6.149e+06 | 160 - 176 | 1 | 43.22 | 43.39 | 43.56 |
| 3429.3850 | N(N)GTYDHDVYRDEALNNR Hex 6 HexNAc 2 | 113.775 | 0.0 | -19.968 | 1.855e+07 | 482 - 499 | 1 | 21.02 | 21.02 | 21.18 |
| 3633.4530 | N(N)GTYDHDVYRDEALN(D)NR Hex 6 HexNAc 3 | 113.727 | 0.0 | -5.796 | 7.438e+07 | 482 - 499 | 2 | 21.50 | 21.67 | 22.16 |
| 3600.4761 | N(N)GTYDHDVYRDEALNNR Fuc 2 Hex 4 HexNAc 3 | 113.201 | 0.0 | -18.834 | 2.711e+07 | 482 - 499 | 1 | 21.02 | 21.18 | 21.18 |
| 3583.4505 | YPALN(N)VTMPN(D)NEQFDK Hex 8 HexNAc 2 | 112.374 | 0.0 | -6.303 | 2.457e+07 | 160 - 176 | 1 | 43.56 | 43.90 | 43.90 |
| 3745.5041 | YPALN(N)VTMPN(D)NEQFDK Hex 9 HexNAc 2 | 112.322 | 0.0 | -5.531 | 2.501e+07 | 160 - 176 | 1 | 43.56 | 43.73 | 43.90 |
| 3437.3992 | YPALN(N)VTM(O)PN(D)NEQFDK Hex 7 HexNAc 2 | 112.005 | 0.0 | 10.440 | 3.110e+07 | 160 - 176 | 2 | 35.64 | 36.15 | 36.66 |
| 2642.0440 | N(N)GTYDHDVYR Fuc 1 Hex 4 HexNAc 3 | 111.826 | 0.0 | -21.772 | 2.490e+06 | 482 - 492 | 1 | 15.55 | 15.55 | 15.55 |
| 4264.6430 | N(N)GTYDHDVYRDEALNNR Fuc 1 Hex 9 HexNAc 3 | 110.168 | 0.0 | -3.557 | 1.560e+08 | 482 - 499 | 2 | 22.33 | 22.49 | 22.83 |
| 3644.6541 | PGDILLIN(N)STGNLIAPR Fuc 2 Hex 3 HexNAc 4 Neu5Ac 1 | 110.148 | 0.0 | -20.148 | 2.392e+06 | 238 - 255 | 1 | 51.85 | 51.85 | 51.85 |
| 4410.6994 | N(N)GTYDHDVYRDEALNNR Fuc 2 Hex 9 HexNAc 3 | 109.839 | 0.0 | -4.878 | 4.569e+07 | 482 - 499 | 1 | 22.33 | 22.49 | 22.66 |
| 5406.7035 | IRDIPSRISIYWTIVKPGDILLIN(N)STGNLIAPR Hex 8 HexNAc 2 | 109.140 | 0.0 | -18.932 | 1.021e+07 | 222 - 255 | 1 | 65.19 | 65.37 | 65.37 |
| 3423.3762 | RGSN(N)SSFFSR Fuc 2 Hex 6 HexNAc 5 | 108.575 | 0.0 | -20.112 | 4.265e+06 | 140 - 150 | 1 | 18.93 | 19.10 | 19.10 |
| 4476.8010 | N(N)GTYDHDVYRDEALNNR Fuc 3 Hex 6 HexNAc 5 | 108.420 | 0.0 | -20.441 | 2.147e+07 | 482 - 499 | 1 | 20.56 | 20.56 | 20.70 |
| 2806.1149 | N(N)GTYDHDVYR Fuc 1 Hex 5 HexNAc 3 | 108.011 | 0.0 | 0.000 | 0.000e+00 | 482 - 492 | 1 | - | - | - |
| 4039.6225 | RGSN(N)SSFFSR Fuc 4 Hex 8 HexNAc 5 | 106.929 | 0.0 | -20.975 | 5.314e+06 | 140 - 150 | 1 | 21.99 | 21.99 | 21.99 |
| 4330.7401 | N(N)GTYDHDVYRDEALNNR Fuc 2 Hex 6 HexNAc 5 | 105.073 | 0.004 | -4.681 | 1.043e+08 | 482 - 499 | 1 | 20.56 | 20.70 | 20.85 |
| 2966.1496 | N(N)GTYDHDVYR Fuc 1 Hex 6 HexNAc 3 | 103.846 | 0.004 | -20.959 | 7.834e+06 | 482 - 492 | 1 | 15.22 | 15.22 | 15.22 |
| 4251.7662 | SEC(C)ITPN(N)GSIPN(D)DKPFQNVNR Hex 9 HexNAc 2 | 101.926 | 0.012 | 8.923 | 2.675e+08 | 278 - 299 | 3 | 32.96 | 33.81 | 34.15 |
| 3154.1955 | RGSN(N)SSFFSR Fuc 1 Hex 9 HexNAc 2 | 101.496 | 0.012 | -20.407 | 2.134e+06 | 140 - 150 | 3 | 21.82 | 21.82 | 21.82 |
| 3298.2562 | N(N)GTYDHDVYR Fuc 1 Hex 5 HexNAc 4 Neu5Ac 1 | 99.666 | 0.012 | -21.210 | 3.346e+06 | 482 - 492 | 1 | 16.52 | 16.52 | 16.52 |
| 4629.7767 | N(N)GTYDHDVYRDEALNNR Fuc 1 Hex 10 HexNAc 4 | 99.088 | 0.012 | -5.443 | 2.720e+07 | 482 - 499 | 1 | 22.16 | 22.33 | 22.66 |
| 2546.1108 | IEVTN(N)ATELVQN Hex 5 HexNAc 2 | 97.472 | 0.015 | -19.769 | 1.883e+06 | 33 - 45 | 1 | 35.98 | 36.15 | 36.15 |
| 3981.6027 | N(N)GTYDHDVYRDEALNNR Fuc 1 Hex 6 HexNAc 4 | 96.312 | 0.015 | -19.718 | 1.907e+07 | 482 - 499 | 1 | 20.85 | 20.85 | 20.85 |
| 5299.5717 | DIPSRISIYWTIVKPGDILLIN(N)STGNLIAPR Hex 9 HexNAc 2 | 94.992 | 0.023 | -4.428 | 1.935e+07 | 224 - 255 | 3 | 69.59 | 69.77 | 69.93 |
| 4314.7502 | N(N)GTYDHDVYRDEALNNR Fuc 3 Hex 5 HexNAc 5 | 94.835 | 0.023 | -19.206 | 3.393e+07 | 482 - 499 | 1 | 20.70 | 20.70 | 20.85 |
| 4112.6551 | N(N)GTYDHDVYRDEALN(D)NR Fuc 3 Hex 5 HexNAc 4 | 92.919 | 0.026 | 8.513 | 9.792e+07 | 482 - 499 | 2 | 21.02 | 21.34 | 21.99 |
| 4732.2677 | ISIYWTIVKPGDILLIN(N)STGN(D)LIAPR Hex 9 HexNAc 2 | 92.815 | 0.026 | -5.464 | 1.754e+07 | 229 - 255 | 2 | 69.77 | 69.77 | 70.11 |
| 4401.7463 | N(N)GTYDHDVYRDEALNNR Fuc 1 Hex 5 HexNAc 4 Neu5Ac 2 | 92.726 | 0.026 | -19.968 | 1.618e+07 | 482 - 499 | 2 | 24.27 | 24.27 | 24.44 |
| 3455.4044 | N(N)GTYDHDVYRDEALN(D)NR Fuc 1 Hex 4 HexNAc 3 | 92.640 | 0.026 | -20.939 | 1.749e+07 | 482 - 499 | 1 | 21.82 | 21.82 | 21.99 |
| 4023.6179 | N(N)GTYDHDVYRDEALN(D)NR Fuc 1 Hex 5 HexNAc 5 | 91.068 | 0.026 | 8.910 | 6.057e+08 | 482 - 499 | 6 | 21.50 | 21.67 | 23.00 |
| 4336.8532 | N(N)GTYDHDVYRDEALN(D)NRFQIK Fuc 1 Hex 5 HexNAc 4 | 89.736 | 0.029 | -20.692 | 5.291e+07 | 482 - 503 | 1 | 34.15 | 34.15 | 34.32 |
| 3446.3236 | N(N)GTYDHDVYR Fuc 2 Hex 5 HexNAc 4 Neu5Ac 1 | 89.347 | 0.029 | 0.000 | 0.000e+00 | 482 - 492 | 1 | - | - | - |
| 4248.6487 | N(N)GTYDHDVYRDEALNNR Fuc 2 Hex 8 HexNAc 3 | 88.482 | 0.029 | -4.452 | 7.248e+07 | 482 - 499 | 1 | 22.33 | 22.66 | 22.83 |
| 3657.4986 | N(N)GTYDHDVYRDEALNNR Fuc 1 Hex 4 HexNAc 4 | 88.120 | 0.029 | -5.507 | 1.950e+07 | 482 - 499 | 2 | 20.85 | 21.18 | 21.18 |
| 4111.6691 | N(N)GTYDHDVYRDEALNNR Fuc 3 Hex 5 HexNAc 4 | 88.097 | 0.031 | -16.096 | 3.265e+08 | 482 - 499 | 2 | 20.70 | 20.70 | 20.85 |
| 4046.5589 | RGSN(N)SSFFSR Fuc 2 Hex 5 HexNAc 6 Neu5Ac 2 | 88.042 | 0.031 | -18.496 | 3.729e+07 | 140 - 150 | 1 | 23.00 | 23.00 | 23.00 |
| 3356.3117 | N(N)GTYDHDVYR Fuc 2 Hex 5 HexNAc 5 | 87.876 | 0.031 | -19.621 | 5.117e+06 | 482 - 492 | 2 | 15.38 | 15.38 | 15.38 |
| 5003.1280 | SLVASSGTLEFNNESFNWAGVTQN(N)GTSSSC(C)RR Hex 7 HexNAc 2 | 86.736 | 0.031 | -19.700 | 2.715e+06 | 109 - 141 | 1 | 50.49 | 50.66 | 50.66 |
| 3008.1769 | N(N)GTYDHDVYR Fuc 1 Hex 5 HexNAc 4 | 85.420 | 0.037 | 0.000 | 0.000e+00 | 482 - 492 | 1 | - | - | - |
| 5144.2762 | YPALN(N)VTM(O)PNNEQFDKLYIWGVHHPVTDK Hex 8 HexNAc 2 | 85.319 | 0.037 | -18.052 | 9.204e+06 | 160 - 189 | 1 | 49.98 | 49.98 | 50.15 |
| 3017.2093 | RGSN(N)SSFFSR Fuc 2 Hex 6 HexNAc 3 | 84.965 | 0.037 | -18.098 | 6.740e+06 | 140 - 150 | 1 | 19.43 | 19.43 | 19.43 |
| 3407.3838 | RGSN(N)SSFFSR Fuc 3 Hex 5 HexNAc 5 | 84.849 | 0.037 | -19.323 | 5.428e+06 | 140 - 150 | 2 | 19.27 | 19.27 | 19.27 |
| 4089.7145 | SEC(C)ITPN(N)GSIPN(D)DKPFQNVNR Hex 8 HexNAc 2 | 82.581 | 0.037 | 10.685 | 1.298e+08 | 278 - 299 | 4 | 32.96 | 33.98 | 34.15 |
| 4255.0056 | WTIVKPGDILLIN(N)STGNLIAPR Hex 9 HexNAc 2 | 82.444 | 0.041 | -19.083 | 2.044e+06 | 233 - 255 | 1 | 60.64 | 60.64 | 60.64 |
| 4760.2575 | TIVKPGDILLIN(N)STGNLIAPR Hex 7 HexNAc 7 | 81.671 | 0.047 | -19.206 | 6.959e+06 | 234 - 255 | 1 | 65.88 | 65.88 | 65.88 |

### RGSN(N)SSFFSR Hex 7 HexNAc 2

**Mass:**  2684.0722

(-3.127e-08)

**Charge States:** 3

**Start Position**: 141

**End Position**: 151

| Profile Score | Tandem Score | TDA q-value | Volume | Spectrum Matches |
| --- | --- | --- | --- | --- |
| 12.2987 | 188.0787 | 0.0000 | 1.5057e+08 | 4 |

**Adduct Signal Fractions**

- **Unmodified**: 1.5057e+08

### RGSN(N)SSFFSR Hex 5 HexNAc 2

**Mass:**  2359.9674

(1.011e-06)

**Charge States:** 2, 3

**Start Position**: 141

**End Position**: 151

| Profile Score | Tandem Score | TDA q-value | Volume | Spectrum Matches |
| --- | --- | --- | --- | --- |
| -1.1630 | 186.1845 | 0.0000 | 4.8507e+08 | 6 |

**Adduct Signal Fractions**

- **Unmodified**: 4.8507e+08

### YPALN(N)VTM(O)PNNEQFDK Hex 9 HexNAc 2

**Mass:**  3760.5060

(-3.748e-07)

**Charge States:** 2, 3, 4

**Start Position**: 161

**End Position**: 177

| Profile Score | Tandem Score | TDA q-value | Volume | Spectrum Matches |
| --- | --- | --- | --- | --- |
| 16.1797 | 184.7199 | 0.0000 | 1.8132e+09 | 12 |

**Adduct Signal Fractions**

- **Unmodified**: 1.8132e+09

### RGSN(N)SSFFSR Fuc 1 Hex 5 HexNAc 5

**Mass:**  3115.2636

(1.648e-06)

**Charge States:** 3, 4

**Start Position**: 141

**End Position**: 151

| Profile Score | Tandem Score | TDA q-value | Volume | Spectrum Matches |
| --- | --- | --- | --- | --- |
| 13.2173 | 182.7197 | 0.0000 | 1.1066e+09 | 8 |

**Adduct Signal Fractions**

- **Unmodified**: 1.1066e+09

### YPALN(N)VTM(O)PNNEQFDK Hex 8 HexNAc 2

**Mass:**  3598.4536

(-1.189e-07)

**Charge States:** 2, 3, 4

**Start Position**: 161

**End Position**: 177

| Profile Score | Tandem Score | TDA q-value | Volume | Spectrum Matches |
| --- | --- | --- | --- | --- |
| 14.9532 | 179.7132 | 0.0000 | 1.9808e+09 | 8 |

**Adduct Signal Fractions**

- **Unmodified**: 1.9808e+09

### RGSN(N)SSFFSR Hex 6 HexNAc 4

**Mass:**  2928.1765

(-4.598e-08)

**Charge States:** 3

**Start Position**: 141

**End Position**: 151

| Profile Score | Tandem Score | TDA q-value | Volume | Spectrum Matches |
| --- | --- | --- | --- | --- |
| -18.5519 | 176.8316 | 0.0000 | 4.7328e+07 | 1 |

**Adduct Signal Fractions**

- **Unmodified**: 4.7328e+07

### RGSN(N)SSFFSR Hex 8 HexNAc 2

**Mass:**  2846.1238

(2.393e-06)

**Charge States:** 3

**Start Position**: 141

**End Position**: 151

| Profile Score | Tandem Score | TDA q-value | Volume | Spectrum Matches |
| --- | --- | --- | --- | --- |
| -2.8902 | 173.9935 | 0.0000 | 5.5089e+07 | 4 |

**Adduct Signal Fractions**

- **Unmodified**: 5.5089e+07

### RGSN(N)SSFFSR Fuc 1 Hex 6 HexNAc 3

**Mass:**  2871.1563

(-1.613e-07)

**Charge States:** 3

**Start Position**: 141

**End Position**: 151

| Profile Score | Tandem Score | TDA q-value | Volume | Spectrum Matches |
| --- | --- | --- | --- | --- |
| -2.9214 | 173.5862 | 0.0000 | 9.9927e+07 | 2 |

**Adduct Signal Fractions**

- **Unmodified**: 9.9927e+07

### RGSN(N)SSFFSR Fuc 1 Hex 5 HexNAc 5

**Mass:**  3117.2726

(6.457e-04)

**Start Position**: 141

**End Position**: 151

| Profile Score | Tandem Score | TDA q-value | Volume | Spectrum Matches |
| --- | --- | --- | --- | --- |
| 0.0000 | 173.4297 | 0.0000 | 0.0000e+00 | 1 |

Adduct:

No Chromatogram Found

### RGSN(N)SSFFSR Hex 5 HexNAc 4

**Mass:**  2766.1250

(4.979e-07)

**Charge States:** 3

**Start Position**: 141

**End Position**: 151

| Profile Score | Tandem Score | TDA q-value | Volume | Spectrum Matches |
| --- | --- | --- | --- | --- |
| 8.1138 | 171.4033 | 0.0000 | 9.6846e+07 | 1 |

**Adduct Signal Fractions**

- **Unmodified**: 9.6846e+07

### RGSN(N)SSFFSR Fuc 1 Hex 4 HexNAc 3

**Mass:**  2547.0529

(1.378e-06)

**Charge States:** 3

**Start Position**: 141

**End Position**: 151

| Profile Score | Tandem Score | TDA q-value | Volume | Spectrum Matches |
| --- | --- | --- | --- | --- |
| -5.2465 | 171.2589 | 0.0000 | 4.5087e+07 | 2 |

**Adduct Signal Fractions**

- **Unmodified**: 4.5087e+07

### RGSN(N)SSFFSR Hex 6 HexNAc 3

**Mass:**  2725.0991

(5.880e-07)

**Charge States:** 3

**Start Position**: 141

**End Position**: 151

| Profile Score | Tandem Score | TDA q-value | Volume | Spectrum Matches |
| --- | --- | --- | --- | --- |
| 13.4751 | 170.4128 | 0.0000 | 3.1922e+08 | 5 |

**Adduct Signal Fractions**

- **Unmodified**: 3.1922e+08

### TIVKPGDILLIN(N)STGNLIAPR Hex 9 HexNAc 2

**Mass:**  4068.9387

(2.920e-06)

**Charge States:** 3, 4, 5

**Start Position**: 235

**End Position**: 256

| Profile Score | Tandem Score | TDA q-value | Volume | Spectrum Matches |
| --- | --- | --- | --- | --- |
| 16.9909 | 169.7409 | 0.0000 | 4.6667e+08 | 5 |

**Adduct Signal Fractions**

- **Unmodified**: 4.6667e+08

### RGSN(N)SSFFSR Fuc 2 Hex 5 HexNAc 5

**Mass:**  3261.3195

(1.222e-07)

**Charge States:** 3

**Start Position**: 141

**End Position**: 151

| Profile Score | Tandem Score | TDA q-value | Volume | Spectrum Matches |
| --- | --- | --- | --- | --- |
| -4.3892 | 169.4259 | 0.0000 | 7.4647e+07 | 3 |

**Adduct Signal Fractions**

- **Unmodified**: 7.4647e+07

### RGSN(N)SSFFSR Hex 6 HexNAc 2

**Mass:**  2522.0194

(6.597e-07)

**Charge States:** 2

**Start Position**: 141

**End Position**: 151

| Profile Score | Tandem Score | TDA q-value | Volume | Spectrum Matches |
| --- | --- | --- | --- | --- |
| -17.1306 | 169.3120 | 0.0000 | 2.2663e+08 | 4 |

**Adduct Signal Fractions**

- **Unmodified**: 2.2663e+08

### YPALN(N)VTM(O)PNNEQFDK Hex 7 HexNAc 2

**Mass:**  3436.4020

(1.221e-06)

**Charge States:** 3, 4

**Start Position**: 161

**End Position**: 177

| Profile Score | Tandem Score | TDA q-value | Volume | Spectrum Matches |
| --- | --- | --- | --- | --- |
| 12.5994 | 168.1808 | 0.0000 | 1.9058e+08 | 4 |

**Adduct Signal Fractions**

- **Unmodified**: 1.9058e+08

### RGSN(N)SSFFSR Fuc 1 Hex 5 HexNAc 3

**Mass:**  2709.1052

(1.373e-06)

**Charge States:** 3

**Start Position**: 141

**End Position**: 151

| Profile Score | Tandem Score | TDA q-value | Volume | Spectrum Matches |
| --- | --- | --- | --- | --- |
| -5.0579 | 165.8097 | 0.0000 | 5.2789e+07 | 2 |

**Adduct Signal Fractions**

- **Unmodified**: 5.2789e+07

### RGSN(N)SSFFSR Hex 5 HexNAc 5

**Mass:**  2969.2053

(8.597e-07)

**Charge States:** 3

**Start Position**: 141

**End Position**: 151

| Profile Score | Tandem Score | TDA q-value | Volume | Spectrum Matches |
| --- | --- | --- | --- | --- |
| 10.3361 | 165.5046 | 0.0000 | 2.5220e+08 | 7 |

**Adduct Signal Fractions**

- **Unmodified**: 2.5220e+08

### YPALN(N)VTMPNNEQFDK Hex 8 HexNAc 2

**Mass:**  3582.4663

(2.114e-06)

**Charge States:** 3, 4

**Start Position**: 161

**End Position**: 177

| Profile Score | Tandem Score | TDA q-value | Volume | Spectrum Matches |
| --- | --- | --- | --- | --- |
| 16.7311 | 165.3583 | 0.0000 | 1.4384e+08 | 5 |

**Adduct Signal Fractions**

- **Unmodified**: 1.4384e+08

### RGSN(N)SSFFSR Fuc 2 Hex 5 HexNAc 4

**Mass:**  3058.2392

(-9.053e-08)

**Charge States:** 3

**Start Position**: 141

**End Position**: 151

| Profile Score | Tandem Score | TDA q-value | Volume | Spectrum Matches |
| --- | --- | --- | --- | --- |
| -3.4023 | 164.1197 | 0.0000 | 8.2667e+07 | 2 |

**Adduct Signal Fractions**

- **Unmodified**: 8.2667e+07

### YPALN(N)VTMPNNEQFDK Hex 9 HexNAc 2

**Mass:**  3744.5158

(2.509e-06)

**Charge States:** 3, 4

**Start Position**: 161

**End Position**: 177

| Profile Score | Tandem Score | TDA q-value | Volume | Spectrum Matches |
| --- | --- | --- | --- | --- |
| 15.6174 | 161.4970 | 0.0000 | 1.4906e+08 | 4 |

**Adduct Signal Fractions**

- **Unmodified**: 1.4906e+08

### RGSN(N)SSFFSR Fuc 1 Hex 5 HexNAc 4

**Mass:**  2912.1833

(1.550e-06)

**Charge States:** 3, 4

**Start Position**: 141

**End Position**: 151

| Profile Score | Tandem Score | TDA q-value | Volume | Spectrum Matches |
| --- | --- | --- | --- | --- |
| 14.8748 | 161.3931 | 0.0000 | 6.1746e+08 | 6 |

**Adduct Signal Fractions**

- **Unmodified**: 6.1746e+08

### RGSN(N)SSFFSR Fuc 1 Hex 4 HexNAc 5

**Mass:**  2953.2105

(7.746e-07)

**Charge States:** 3

**Start Position**: 141

**End Position**: 151

| Profile Score | Tandem Score | TDA q-value | Volume | Spectrum Matches |
| --- | --- | --- | --- | --- |
| -17.7797 | 160.7201 | 0.0000 | 1.6298e+07 | 1 |

**Adduct Signal Fractions**

- **Unmodified**: 1.6298e+07

### N(N)GTYDHDVYR Fuc 2 Hex 5 HexNAc 4

**Mass:**  3153.2298

(2.841e-07)

**Charge States:** 3

**Start Position**: 483

**End Position**: 493

| Profile Score | Tandem Score | TDA q-value | Volume | Spectrum Matches |
| --- | --- | --- | --- | --- |
| -3.6336 | 160.4168 | 0.0000 | 1.1980e+08 | 3 |

**Adduct Signal Fractions**

- **Unmodified**: 1.1980e+08

### RGSN(N)SSFFSR Fuc 1 Hex 6 HexNAc 5

**Mass:**  3277.3181

(1.852e-06)

**Charge States:** 3, 4

**Start Position**: 141

**End Position**: 151

| Profile Score | Tandem Score | TDA q-value | Volume | Spectrum Matches |
| --- | --- | --- | --- | --- |
| -4.3558 | 159.8730 | 0.0000 | 7.0080e+07 | 3 |

**Adduct Signal Fractions**

- **Unmodified**: 7.0080e+07

### YPALN(N)VTM(O)PNNEQFDK Hex 5 HexNAc 2

**Mass:**  3112.3068

(3.969e-06)

**Charge States:** 3, 4

**Start Position**: 161

**End Position**: 177

| Profile Score | Tandem Score | TDA q-value | Volume | Spectrum Matches |
| --- | --- | --- | --- | --- |
| 15.7255 | 159.8152 | 0.0000 | 1.0255e+08 | 3 |

**Adduct Signal Fractions**

- **Unmodified**: 1.0255e+08

### N(N)GTYDHDVYR Fuc 1 Hex 5 HexNAc 4

**Mass:**  3007.1709

(-1.277e-06)

**Charge States:** 3

**Start Position**: 483

**End Position**: 493

| Profile Score | Tandem Score | TDA q-value | Volume | Spectrum Matches |
| --- | --- | --- | --- | --- |
| 15.1363 | 159.6889 | 0.0000 | 3.6875e+08 | 5 |

**Adduct Signal Fractions**

- **Unmodified**: 3.6875e+08

### GSN(N)SSFFSR Hex 6 HexNAc 2

**Mass:**  2365.9171

(5.700e-07)

**Charge States:** 2, 3

**Start Position**: 142

**End Position**: 151

| Profile Score | Tandem Score | TDA q-value | Volume | Spectrum Matches |
| --- | --- | --- | --- | --- |
| -4.6180 | 159.2168 | 0.0000 | 1.0433e+08 | 2 |

**Adduct Signal Fractions**

- **Unmodified**: 1.0433e+08

### RGSN(N)SSFFSR Hex 5 HexNAc 3

**Mass:**  2563.0462

(8.423e-07)

**Charge States:** 3

**Start Position**: 141

**End Position**: 151

| Profile Score | Tandem Score | TDA q-value | Volume | Spectrum Matches |
| --- | --- | --- | --- | --- |
| -4.3605 | 158.6154 | 0.0000 | 6.2662e+07 | 1 |

**Adduct Signal Fractions**

- **Unmodified**: 6.2662e+07

### RGSN(N)SSFFSR Fuc 2 Hex 4 HexNAc 3

**Mass:**  2693.1092

(6.874e-07)

**Charge States:** 3

**Start Position**: 141

**End Position**: 151

| Profile Score | Tandem Score | TDA q-value | Volume | Spectrum Matches |
| --- | --- | --- | --- | --- |
| -20.8850 | 157.0938 | 0.0000 | 1.3771e+07 | 1 |

**Adduct Signal Fractions**

- **Unmodified**: 1.3771e+07

### GSN(N)SSFFSR Fuc 1 Hex 5 HexNAc 5

**Mass:**  2959.1601

(-4.119e-07)

**Charge States:** 3

**Start Position**: 142

**End Position**: 151

| Profile Score | Tandem Score | TDA q-value | Volume | Spectrum Matches |
| --- | --- | --- | --- | --- |
| -2.8510 | 154.7222 | 0.0000 | 4.1483e+08 | 4 |

**Adduct Signal Fractions**

- **Unmodified**: 4.1483e+08

### YPALN(N)VTM(O)PNNEQFDK Fuc 2 Hex 3 HexNAc 4 Neu5Ac 1

**Mass:**  3777.5326

(-7.170e-06)

**Charge States:** 3

**Start Position**: 161

**End Position**: 177

| Profile Score | Tandem Score | TDA q-value | Volume | Spectrum Matches |
| --- | --- | --- | --- | --- |
| -4.3797 | 153.2622 | 0.0000 | 1.0484e+08 | 2 |

**Adduct Signal Fractions**

- **Unmodified**: 1.0484e+08

### TIVKPGDILLIN(N)STGNLIAPR Hex 10 HexNAc 2

**Mass:**  4230.9851

(2.062e-06)

**Charge States:** 3, 4

**Start Position**: 235

**End Position**: 256

| Profile Score | Tandem Score | TDA q-value | Volume | Spectrum Matches |
| --- | --- | --- | --- | --- |
| -17.9169 | 151.1200 | 0.0000 | 3.4948e+07 | 1 |

**Adduct Signal Fractions**

- **Unmodified**: 3.4948e+07

### C(C)KSEC(C)ITPN(N)GSIPNDKPFQNVNR Hex 9 HexNAc 2

**Mass:**  4538.9006

(1.644e-06)

**Charge States:** 4

**Start Position**: 277

**End Position**: 300

| Profile Score | Tandem Score | TDA q-value | Volume | Spectrum Matches |
| --- | --- | --- | --- | --- |
| -4.2012 | 150.9335 | 0.0000 | 8.6691e+07 | 1 |

**Adduct Signal Fractions**

- **Unmodified**: 8.6691e+07

### RGSN(N)SSFFSR Hex 6 HexNAc 4

**Mass:**  2929.1836

(3.439e-04)

**Start Position**: 141

**End Position**: 151

| Profile Score | Tandem Score | TDA q-value | Volume | Spectrum Matches |
| --- | --- | --- | --- | --- |
| 0.0000 | 150.2660 | 0.0000 | 0.0000e+00 | 1 |

Adduct:

No Chromatogram Found

### GSN(N)SSFFSR Fuc 1 Hex 5 HexNAc 4

**Mass:**  2756.0793

(-3.091e-07)

**Charge States:** 3

**Start Position**: 142

**End Position**: 151

| Profile Score | Tandem Score | TDA q-value | Volume | Spectrum Matches |
| --- | --- | --- | --- | --- |
| 12.5423 | 149.9894 | 0.0000 | 1.8011e+08 | 3 |

**Adduct Signal Fractions**

- **Unmodified**: 1.8011e+08

### RGSN(N)SSFFSR Hex 5 HexNAc 6

**Mass:**  3172.2863

(8.911e-07)

**Charge States:** 3, 4

**Start Position**: 141

**End Position**: 151

| Profile Score | Tandem Score | TDA q-value | Volume | Spectrum Matches |
| --- | --- | --- | --- | --- |
| 5.3310 | 149.4439 | 0.0000 | 1.6405e+08 | 5 |

**Adduct Signal Fractions**

- **Unmodified**: 1.6405e+08

### ISIYWTIVKPGDILLIN(N)STGNLIAPR Hex 8 HexNAc 2

**Mass:**  4569.2199

(1.444e-06)

**Charge States:** 4

**Start Position**: 230

**End Position**: 256

| Profile Score | Tandem Score | TDA q-value | Volume | Spectrum Matches |
| --- | --- | --- | --- | --- |
| 14.8068 | 148.8093 | 0.0000 | 1.0052e+08 | 5 |

**Adduct Signal Fractions**

- **Unmodified**: 1.0052e+08

### YPALN(N)VTM(O)PNNEQFDK Hex 4 HexNAc 2

**Mass:**  2950.2537

(3.802e-06)

**Charge States:** 3

**Start Position**: 161

**End Position**: 177

| Profile Score | Tandem Score | TDA q-value | Volume | Spectrum Matches |
| --- | --- | --- | --- | --- |
| -2.3496 | 148.4127 | 0.0000 | 1.7413e+07 | 1 |

**Adduct Signal Fractions**

- **Unmodified**: 1.7413e+07

### GSN(N)SSFFSR Hex 5 HexNAc 2

**Mass:**  2203.8639

(-4.769e-07)

**Charge States:** 2

**Start Position**: 142

**End Position**: 151

| Profile Score | Tandem Score | TDA q-value | Volume | Spectrum Matches |
| --- | --- | --- | --- | --- |
| -1.4588 | 147.9972 | 0.0000 | 1.4956e+08 | 3 |

**Adduct Signal Fractions**

- **Unmodified**: 1.4956e+08

### C(C)KSEC(C)ITPN(N)GSIPNDKPFQNVNR Hex 8 HexNAc 2

**Mass:**  4376.8469

(1.437e-06)

**Charge States:** 4

**Start Position**: 277

**End Position**: 300

| Profile Score | Tandem Score | TDA q-value | Volume | Spectrum Matches |
| --- | --- | --- | --- | --- |
| -18.9743 | 147.5241 | 0.0000 | 4.0921e+07 | 1 |

**Adduct Signal Fractions**

- **Unmodified**: 4.0921e+07

### ISIYWTIVKPGDILLIN(N)STGNLIAPR Hex 9 HexNAc 2

**Mass:**  4731.2746

(1.800e-06)

**Charge States:** 3, 4, 5

**Start Position**: 230

**End Position**: 256

| Profile Score | Tandem Score | TDA q-value | Volume | Spectrum Matches |
| --- | --- | --- | --- | --- |
| 16.6623 | 146.6126 | 0.0000 | 1.4551e+09 | 10 |

**Adduct Signal Fractions**

- **Unmodified**: 1.4551e+09

### RGSN(N)SSFFSR Fuc 3 Hex 5 HexNAc 4

**Mass:**  3204.3019

(1.886e-07)

**Charge States:** 3

**Start Position**: 141

**End Position**: 151

| Profile Score | Tandem Score | TDA q-value | Volume | Spectrum Matches |
| --- | --- | --- | --- | --- |
| -17.5026 | 146.2962 | 0.0000 | 1.8684e+07 | 1 |

**Adduct Signal Fractions**

- **Unmodified**: 1.8684e+07

### YPALN(N)VTM(O)PNNEQFDK Hex 10 HexNAc 2

**Mass:**  3922.5628

(1.769e-06)

**Charge States:** 3

**Start Position**: 161

**End Position**: 177

| Profile Score | Tandem Score | TDA q-value | Volume | Spectrum Matches |
| --- | --- | --- | --- | --- |
| -5.7169 | 145.8524 | 0.0000 | 4.2154e+07 | 1 |

**Adduct Signal Fractions**

- **Unmodified**: 4.2154e+07

### RGSN(N)SSFFSR Hex 7 HexNAc 4

**Mass:**  3090.2316

(7.354e-07)

**Charge States:** 3

**Start Position**: 141

**End Position**: 151

| Profile Score | Tandem Score | TDA q-value | Volume | Spectrum Matches |
| --- | --- | --- | --- | --- |
| -19.5323 | 145.7017 | 0.0000 | 1.6976e+07 | 1 |

**Adduct Signal Fractions**

- **Unmodified**: 1.6976e+07

### RGSN(N)SSFFSR Fuc 1 Hex 6 HexNAc 4

**Mass:**  3074.2384

(4.608e-07)

**Charge States:** 3

**Start Position**: 141

**End Position**: 151

| Profile Score | Tandem Score | TDA q-value | Volume | Spectrum Matches |
| --- | --- | --- | --- | --- |
| -7.4545 | 143.7586 | 0.0000 | 2.3725e+07 | 2 |

**Adduct Signal Fractions**

- **Unmodified**: 2.3725e+07

### YPALN(N)VTM(O)PNNEQFDK Hex 6 HexNAc 2

**Mass:**  3274.3474

(-2.093e-07)

**Charge States:** 3

**Start Position**: 161

**End Position**: 177

| Profile Score | Tandem Score | TDA q-value | Volume | Spectrum Matches |
| --- | --- | --- | --- | --- |
| -3.7108 | 142.3414 | 0.0000 | 2.5184e+07 | 2 |

**Adduct Signal Fractions**

- **Unmodified**: 2.5184e+07

### YPALN(N)VTMPNNEQFDK Hex 7 HexNAc 2

**Mass:**  3420.4085

(7.499e-07)

**Charge States:** 3

**Start Position**: 161

**End Position**: 177

| Profile Score | Tandem Score | TDA q-value | Volume | Spectrum Matches |
| --- | --- | --- | --- | --- |
| -4.2690 | 142.1592 | 0.0000 | 1.2491e+07 | 1 |

**Adduct Signal Fractions**

- **Unmodified**: 1.2491e+07

### ISIYWTIVKPGDILLIN(N)STGNLIAPR Hex 10 HexNAc 2

**Mass:**  4893.3249

(1.212e-06)

**Charge States:** 4, 5

**Start Position**: 230

**End Position**: 256

| Profile Score | Tandem Score | TDA q-value | Volume | Spectrum Matches |
| --- | --- | --- | --- | --- |
| -3.7275 | 140.9673 | 0.0000 | 1.4179e+08 | 4 |

**Adduct Signal Fractions**

- **Unmodified**: 1.4179e+08

### YPALN(N)VTMPNNEQFDK Fuc 2 Hex 3 HexNAc 4 Neu5Ac 1

**Mass:**  3761.5416

(-6.807e-06)

**Charge States:** 3

**Start Position**: 161

**End Position**: 177

| Profile Score | Tandem Score | TDA q-value | Volume | Spectrum Matches |
| --- | --- | --- | --- | --- |
| -3.2365 | 140.6660 | 0.0000 | 1.0499e+07 | 2 |

**Adduct Signal Fractions**

- **Unmodified**: 1.0499e+07

### ISIYWTIVKPGDILLIN(N)STGNLIAPR Fuc 2 Hex 3 HexNAc 4 Neu5Ac 1

**Mass:**  4748.2946

(-4.678e-06)

**Charge States:** 4

**Start Position**: 230

**End Position**: 256

| Profile Score | Tandem Score | TDA q-value | Volume | Spectrum Matches |
| --- | --- | --- | --- | --- |
| 12.9884 | 140.5333 | 0.0000 | 1.6535e+08 | 6 |

**Adduct Signal Fractions**

- **Unmodified**: 1.6535e+08

### LGHHAVPN(N)GTIVK Fuc 1 Hex 5 HexNAc 5

**Mass:**  3315.4713

(6.038e-04)

**Start Position**: 15

**End Position**: 28

| Profile Score | Tandem Score | TDA q-value | Volume | Spectrum Matches |
| --- | --- | --- | --- | --- |
| 0.0000 | 140.3330 | 0.0000 | 0.0000e+00 | 1 |

Adduct:

No Chromatogram Found

### YPALN(N)VTM(O)PN(D)NEQFDK Hex 9 HexNAc 2

**Mass:**  3761.5065

(2.668e-04)

**Charge States:** 2, 3, 4

**Start Position**: 161

**End Position**: 177

| Profile Score | Tandem Score | TDA q-value | Volume | Spectrum Matches |
| --- | --- | --- | --- | --- |
| 10.1760 | 139.8404 | 0.0000 | 5.2629e+08 | 7 |

**Adduct Signal Fractions**

- **Unmodified**: 5.2629e+08

### ISIYWTIVKPGDILLIN(N)STGNLIAPR Fuc 2 Hex 4 HexNAc 4 Neu5Ac 1

**Mass:**  4910.3527

(-3.934e-06)

**Charge States:** 4

**Start Position**: 230

**End Position**: 256

| Profile Score | Tandem Score | TDA q-value | Volume | Spectrum Matches |
| --- | --- | --- | --- | --- |
| -19.3876 | 139.8193 | 0.0000 | 1.4601e+07 | 2 |

**Adduct Signal Fractions**

- **Unmodified**: 1.4601e+07

### N(N)GTYDHDVYRDEALNNR Hex 5 HexNAc 2

**Mass:**  3267.3337

(3.712e-07)

**Charge States:** 3, 4

**Start Position**: 483

**End Position**: 500

| Profile Score | Tandem Score | TDA q-value | Volume | Spectrum Matches |
| --- | --- | --- | --- | --- |
| -19.6742 | 139.6457 | 0.0000 | 7.6949e+07 | 1 |

**Adduct Signal Fractions**

- **Unmodified**: 7.6949e+07

### N(N)GTYDHDVYRDEALNNR Hex 6 HexNAc 3

**Mass:**  3632.4641

(-1.064e-07)

**Charge States:** 4

**Start Position**: 483

**End Position**: 500

| Profile Score | Tandem Score | TDA q-value | Volume | Spectrum Matches |
| --- | --- | --- | --- | --- |
| -4.3515 | 139.0674 | 0.0000 | 1.7720e+08 | 2 |

**Adduct Signal Fractions**

- **Unmodified**: 1.7720e+08

### WAGVTQN(N)GTSSSC(C)R Hex 7 HexNAc 2

**Mass:**  3050.1974

(-4.600e-07)

**Charge States:** 3

**Start Position**: 127

**End Position**: 141

| Profile Score | Tandem Score | TDA q-value | Volume | Spectrum Matches |
| --- | --- | --- | --- | --- |
| -18.8742 | 138.7458 | 0.0000 | 3.5708e+06 | 1 |

**Adduct Signal Fractions**

- **Unmodified**: 3.5708e+06

### GSN(N)SSFFSR Hex 6 HexNAc 3

**Mass:**  2568.9964

(-1.853e-07)

**Charge States:** 3

**Start Position**: 142

**End Position**: 151

| Profile Score | Tandem Score | TDA q-value | Volume | Spectrum Matches |
| --- | --- | --- | --- | --- |
| -4.0775 | 138.1236 | 0.0000 | 1.4690e+08 | 3 |

**Adduct Signal Fractions**

- **Unmodified**: 1.4690e+08

### RGSN(N)SSFFSR Hex 6 HexNAc 5

**Mass:**  3131.2545

(-4.324e-07)

**Charge States:** 3

**Start Position**: 141

**End Position**: 151

| Profile Score | Tandem Score | TDA q-value | Volume | Spectrum Matches |
| --- | --- | --- | --- | --- |
| -4.7953 | 137.7480 | 0.0000 | 1.2164e+07 | 1 |

**Adduct Signal Fractions**

- **Unmodified**: 1.2164e+07

### GSN(N)SSFFSR Hex 8 HexNAc 2

**Mass:**  2690.0264

(6.200e-07)

**Charge States:** 2, 3

**Start Position**: 142

**End Position**: 151

| Profile Score | Tandem Score | TDA q-value | Volume | Spectrum Matches |
| --- | --- | --- | --- | --- |
| -18.0715 | 137.2346 | 0.0000 | 1.7928e+07 | 2 |

**Adduct Signal Fractions**

- **Unmodified**: 1.7928e+07

### IRDIPSRISIYWTIVKPGDILLIN(N)STGNLIAPR Hex 9 HexNAc 2

**Mass:**  5568.7563

(1.401e-06)

**Charge States:** 5

**Start Position**: 223

**End Position**: 256

| Profile Score | Tandem Score | TDA q-value | Volume | Spectrum Matches |
| --- | --- | --- | --- | --- |
| 15.8799 | 136.6875 | 0.0000 | 2.2607e+08 | 5 |

**Adduct Signal Fractions**

- **Unmodified**: 2.2607e+08

### YPALN(N)VTM(O)PN(D)NEQFDK Hex 8 HexNAc 2

**Mass:**  3599.4505

(2.779e-04)

**Charge States:** 2, 3, 4

**Start Position**: 161

**End Position**: 177

| Profile Score | Tandem Score | TDA q-value | Volume | Spectrum Matches |
| --- | --- | --- | --- | --- |
| 9.6307 | 136.4482 | 0.0000 | 4.3059e+08 | 6 |

**Adduct Signal Fractions**

- **Unmodified**: 4.3059e+08

### RGSN(N)SSFFSR Fuc 1 Hex 3 HexNAc 9 Neu5Ac 2

**Mass:**  4185.6696

(1.060e-06)

**Charge States:** 4

**Start Position**: 141

**End Position**: 151

| Profile Score | Tandem Score | TDA q-value | Volume | Spectrum Matches |
| --- | --- | --- | --- | --- |
| 9.3726 | 134.3050 | 0.0000 | 1.8556e+08 | 2 |

**Adduct Signal Fractions**

- **Unmodified**: 1.8556e+08

### SEC(C)ITPN(N)GSIPNDKPFQNVNR Hex 8 HexNAc 2

**Mass:**  4088.7194

(1.630e-06)

**Charge States:** 3, 4

**Start Position**: 279

**End Position**: 300

| Profile Score | Tandem Score | TDA q-value | Volume | Spectrum Matches |
| --- | --- | --- | --- | --- |
| 16.8041 | 134.2563 | 0.0000 | 6.0404e+08 | 3 |

**Adduct Signal Fractions**

- **Unmodified**: 6.0404e+08

### TIVKPGDILLIN(N)STGNLIAPR Hex 8 HexNAc 2

**Mass:**  3906.8872

(1.985e-06)

**Charge States:** 3, 4

**Start Position**: 235

**End Position**: 256

| Profile Score | Tandem Score | TDA q-value | Volume | Spectrum Matches |
| --- | --- | --- | --- | --- |
| -19.4814 | 133.8134 | 0.0000 | 2.2848e+07 | 2 |

**Adduct Signal Fractions**

- **Unmodified**: 2.2848e+07

### SEC(C)ITPN(N)GSIPNDKPFQNVNR Hex 7 HexNAc 2

**Mass:**  3926.6755

(-1.808e-07)

**Charge States:** 3, 5

**Start Position**: 279

**End Position**: 300

| Profile Score | Tandem Score | TDA q-value | Volume | Spectrum Matches |
| --- | --- | --- | --- | --- |
| -2.7199 | 133.6870 | 0.0000 | 7.2380e+08 | 4 |

**Adduct Signal Fractions**

- **Unmodified**: 7.2380e+08

### N(N)GTYDHDVYRDEALNNR Fuc 1 Hex 5 HexNAc 5

**Mass:**  4022.6301

(4.821e-07)

**Charge States:** 3, 4, 5

**Start Position**: 483

**End Position**: 500

| Profile Score | Tandem Score | TDA q-value | Volume | Spectrum Matches |
| --- | --- | --- | --- | --- |
| 12.4177 | 132.4815 | 0.0000 | 1.4235e+09 | 7 |

**Adduct Signal Fractions**

- **Unmodified**: 1.4235e+09

### N(N)GTYDHDVYRDEALNNR Fuc 1 Hex 6 HexNAc 3

**Mass:**  3778.5270

(1.264e-06)

**Charge States:** 4

**Start Position**: 483

**End Position**: 500

| Profile Score | Tandem Score | TDA q-value | Volume | Spectrum Matches |
| --- | --- | --- | --- | --- |
| -4.7194 | 132.3172 | 0.0000 | 1.5321e+08 | 1 |

**Adduct Signal Fractions**

- **Unmodified**: 1.5321e+08

### SEC(C)ITPN(N)GSIPNDKPFQNVNR Hex 9 HexNAc 2

**Mass:**  4250.7746

(8.342e-07)

**Charge States:** 3, 4

**Start Position**: 279

**End Position**: 300

| Profile Score | Tandem Score | TDA q-value | Volume | Spectrum Matches |
| --- | --- | --- | --- | --- |
| -1.9703 | 132.1566 | 0.0000 | 1.1037e+09 | 3 |

**Adduct Signal Fractions**

- **Unmodified**: 1.1037e+09

### TIVKPGDILLIN(N)STGNLIAPR Fuc 2 Hex 3 HexNAc 4 Neu5Ac 1

**Mass:**  4085.9589

(-3.561e-06)

**Charge States:** 4

**Start Position**: 235

**End Position**: 256

| Profile Score | Tandem Score | TDA q-value | Volume | Spectrum Matches |
| --- | --- | --- | --- | --- |
| -3.8627 | 131.7935 | 0.0000 | 3.1280e+07 | 1 |

**Adduct Signal Fractions**

- **Unmodified**: 3.1280e+07

### N(N)GTYDHDVYR Fuc 1 Hex 5 HexNAc 5

**Mass:**  3211.2581

(3.139e-04)

**Start Position**: 483

**End Position**: 493

| Profile Score | Tandem Score | TDA q-value | Volume | Spectrum Matches |
| --- | --- | --- | --- | --- |
| 0.0000 | 131.6775 | 0.0000 | 0.0000e+00 | 1 |

Adduct:

No Chromatogram Found

### HAVPN(N)GTIVK Fuc 1 Hex 5 HexNAc 5

**Mass:**  3006.2949

(-3.708e-06)

**Charge States:** 3

**Start Position**: 18

**End Position**: 28

| Profile Score | Tandem Score | TDA q-value | Volume | Spectrum Matches |
| --- | --- | --- | --- | --- |
| -20.5577 | 131.3241 | 0.0000 | 5.2225e+06 | 1 |

**Adduct Signal Fractions**

- **Unmodified**: 5.2225e+06

### N(N)GTYDHDVYRDEALNNR Fuc 1 Hex 6 HexNAc 5

**Mass:**  4184.6843

(7.438e-07)

**Charge States:** 4

**Start Position**: 483

**End Position**: 500

| Profile Score | Tandem Score | TDA q-value | Volume | Spectrum Matches |
| --- | --- | --- | --- | --- |
| 14.1158 | 130.6380 | 0.0000 | 5.2466e+08 | 3 |

**Adduct Signal Fractions**

- **Unmodified**: 5.2466e+08

### N(N)GTYDHDVYRDEALNNR Fuc 1 Hex 5 HexNAc 4

**Mass:**  3819.5463

(3.679e-07)

**Charge States:** 3, 4, 5

**Start Position**: 483

**End Position**: 500

| Profile Score | Tandem Score | TDA q-value | Volume | Spectrum Matches |
| --- | --- | --- | --- | --- |
| 16.5783 | 130.4509 | 0.0000 | 3.0982e+09 | 6 |

**Adduct Signal Fractions**

- **Unmodified**: 3.0982e+09

### N(N)GTYDHDVYRDEALNNR Fuc 3 Hex 5 HexNAc 4

**Mass:**  4114.6665

(7.301e-04)

**Start Position**: 483

**End Position**: 500

| Profile Score | Tandem Score | TDA q-value | Volume | Spectrum Matches |
| --- | --- | --- | --- | --- |
| 0.0000 | 129.8398 | 0.0000 | 0.0000e+00 | 1 |

Adduct:

No Chromatogram Found

### RGSN(N)SSFFSR Fuc 1 Hex 6 HexNAc 6

**Mass:**  3480.3957

(6.837e-07)

**Charge States:** 3, 4

**Start Position**: 141

**End Position**: 151

| Profile Score | Tandem Score | TDA q-value | Volume | Spectrum Matches |
| --- | --- | --- | --- | --- |
| -4.8786 | 129.5971 | 0.0000 | 3.2851e+07 | 4 |

**Adduct Signal Fractions**

- **Unmodified**: 3.2851e+07

### N(N)GTYDHDVYRDEALNNR Hex 5 HexNAc 4

**Mass:**  3673.4897

(-3.044e-07)

**Charge States:** 4

**Start Position**: 483

**End Position**: 500

| Profile Score | Tandem Score | TDA q-value | Volume | Spectrum Matches |
| --- | --- | --- | --- | --- |
| -3.9765 | 128.0450 | 0.0000 | 1.8409e+08 | 2 |

**Adduct Signal Fractions**

- **Unmodified**: 1.8409e+08

### N(N)GTYDHDVYRDEALNNR Fuc 1 Hex 4 HexNAc 3

**Mass:**  3454.4184

(1.203e-06)

**Charge States:** 4

**Start Position**: 483

**End Position**: 500

| Profile Score | Tandem Score | TDA q-value | Volume | Spectrum Matches |
| --- | --- | --- | --- | --- |
| -4.4093 | 127.7970 | 0.0000 | 1.0274e+08 | 3 |

**Adduct Signal Fractions**

- **Unmodified**: 1.0274e+08

### HAVPN(N)GTIVK Fuc 1 Hex 5 HexNAc 4

**Mass:**  2804.2189

(3.539e-04)

**Start Position**: 18

**End Position**: 28

| Profile Score | Tandem Score | TDA q-value | Volume | Spectrum Matches |
| --- | --- | --- | --- | --- |
| 0.0000 | 126.5931 | 0.0000 | 0.0000e+00 | 1 |

Adduct:

No Chromatogram Found

### N(N)GTYDHDVYRDEALNNR Fuc 1 Hex 5 HexNAc 3

**Mass:**  3616.4733

(1.045e-06)

**Charge States:** 4

**Start Position**: 483

**End Position**: 500

| Profile Score | Tandem Score | TDA q-value | Volume | Spectrum Matches |
| --- | --- | --- | --- | --- |
| -4.2112 | 126.2747 | 0.0000 | 7.0856e+07 | 1 |

**Adduct Signal Fractions**

- **Unmodified**: 7.0856e+07

### N(N)GTYDHDVYRDEALNNR Hex 7 HexNAc 2

**Mass:**  3591.4409

(8.387e-07)

**Charge States:** 4

**Start Position**: 483

**End Position**: 500

| Profile Score | Tandem Score | TDA q-value | Volume | Spectrum Matches |
| --- | --- | --- | --- | --- |
| -19.7372 | 125.8573 | 0.0000 | 3.9323e+07 | 1 |

**Adduct Signal Fractions**

- **Unmodified**: 3.9323e+07

### RGSN(N)SSFFSR Fuc 1 Hex 6 HexNAc 3

**Mass:**  2872.1590

(3.496e-04)

**Start Position**: 141

**End Position**: 151

| Profile Score | Tandem Score | TDA q-value | Volume | Spectrum Matches |
| --- | --- | --- | --- | --- |
| 0.0000 | 125.6258 | 0.0000 | 0.0000e+00 | 1 |

Adduct:

No Chromatogram Found

### GSN(N)SSFFSR Fuc 2 Hex 9 HexNAc 7

**Mass:**  4159.6130

(6.016e-06)

**Charge States:** 3, 4

**Start Position**: 142

**End Position**: 151

| Profile Score | Tandem Score | TDA q-value | Volume | Spectrum Matches |
| --- | --- | --- | --- | --- |
| -18.1296 | 125.2939 | 0.0000 | 2.5340e+07 | 1 |

**Adduct Signal Fractions**

- **Unmodified**: 2.5340e+07

### RGSN(N)SSFFSR Hex 6 HexNAc 6

**Mass:**  3334.3368

(4.868e-07)

**Charge States:** 3

**Start Position**: 141

**End Position**: 151

| Profile Score | Tandem Score | TDA q-value | Volume | Spectrum Matches |
| --- | --- | --- | --- | --- |
| -5.6229 | 124.7401 | 0.0000 | 1.5188e+07 | 1 |

**Adduct Signal Fractions**

- **Unmodified**: 1.5188e+07

### GSN(N)SSFFSR Hex 5 HexNAc 4

**Mass:**  2610.0243

(6.209e-07)

**Charge States:** 3

**Start Position**: 142

**End Position**: 151

| Profile Score | Tandem Score | TDA q-value | Volume | Spectrum Matches |
| --- | --- | --- | --- | --- |
| -5.1678 | 124.2981 | 0.0000 | 4.8964e+07 | 1 |

**Adduct Signal Fractions**

- **Unmodified**: 4.8964e+07

### SEC(C)ITPN(N)GSIPNDKPFQNVNR Hex 6 HexNAc 2

**Mass:**  3764.6267

(2.065e-06)

**Charge States:** 3, 4

**Start Position**: 279

**End Position**: 300

| Profile Score | Tandem Score | TDA q-value | Volume | Spectrum Matches |
| --- | --- | --- | --- | --- |
| -3.4983 | 123.9023 | 0.0000 | 9.2111e+07 | 3 |

**Adduct Signal Fractions**

- **Unmodified**: 9.2111e+07

### RGSN(N)SSFFSR Hex 4 HexNAc 3

**Mass:**  2400.9931

(1.480e-06)

**Charge States:** 3

**Start Position**: 141

**End Position**: 151

| Profile Score | Tandem Score | TDA q-value | Volume | Spectrum Matches |
| --- | --- | --- | --- | --- |
| -20.3426 | 123.3564 | 0.0000 | 1.6198e+07 | 1 |

**Adduct Signal Fractions**

- **Unmodified**: 1.6198e+07

### N(N)GTYDHDVYRDEALNNR Hex 5 HexNAc 3

**Mass:**  3470.4138

(3.939e-07)

**Charge States:** 4

**Start Position**: 483

**End Position**: 500

| Profile Score | Tandem Score | TDA q-value | Volume | Spectrum Matches |
| --- | --- | --- | --- | --- |
| -4.5646 | 122.5636 | 0.0000 | 4.7977e+07 | 1 |

**Adduct Signal Fractions**

- **Unmodified**: 4.7977e+07

### N(N)GTYDHDVYRDEALN(D)NR Fuc 2 Hex 5 HexNAc 4

**Mass:**  3966.5898

(2.496e-04)

**Charge States:** 3, 4

**Start Position**: 483

**End Position**: 500

| Profile Score | Tandem Score | TDA q-value | Volume | Spectrum Matches |
| --- | --- | --- | --- | --- |
| 4.2898 | 122.5185 | 0.0000 | 4.0391e+08 | 4 |

**Adduct Signal Fractions**

- **Unmodified**: 4.0391e+08

### N(N)GTYDHDVYR Fuc 3 Hex 5 HexNAc 4

**Mass:**  3299.2904

(1.073e-06)

**Charge States:** 3

**Start Position**: 483

**End Position**: 493

| Profile Score | Tandem Score | TDA q-value | Volume | Spectrum Matches |
| --- | --- | --- | --- | --- |
| -20.1331 | 122.4764 | 0.0000 | 1.9514e+07 | 3 |

**Adduct Signal Fractions**

- **Unmodified**: 1.9514e+07

### N(N)GTYDHDVYR Fuc 1 Hex 6 HexNAc 5

**Mass:**  3372.3056

(7.513e-07)

**Charge States:** 3, 4

**Start Position**: 483

**End Position**: 493

| Profile Score | Tandem Score | TDA q-value | Volume | Spectrum Matches |
| --- | --- | --- | --- | --- |
| -4.8687 | 121.8653 | 0.0000 | 3.6440e+07 | 3 |

**Adduct Signal Fractions**

- **Unmodified**: 3.6440e+07

### GSN(N)SSFFSR Hex 5 HexNAc 3

**Mass:**  2406.9419

(-5.518e-07)

**Charge States:** 3

**Start Position**: 142

**End Position**: 151

| Profile Score | Tandem Score | TDA q-value | Volume | Spectrum Matches |
| --- | --- | --- | --- | --- |
| -18.5818 | 121.6521 | 0.0000 | 1.0750e+07 | 1 |

**Adduct Signal Fractions**

- **Unmodified**: 1.0750e+07

### N(N)GTYDHDVYRDEALNNR Fuc 1 Hex 8 HexNAc 2

**Mass:**  3899.5102

(-9.840e-06)

**Charge States:** 3, 4

**Start Position**: 483

**End Position**: 500

| Profile Score | Tandem Score | TDA q-value | Volume | Spectrum Matches |
| --- | --- | --- | --- | --- |
| 14.4176 | 121.5657 | 0.0000 | 6.5779e+08 | 4 |

**Adduct Signal Fractions**

- **Unmodified**: 6.5779e+08

### N(N)GTYDHDVYR Hex 5 HexNAc 4

**Mass:**  2861.1141

(3.243e-07)

**Charge States:** 3, 4

**Start Position**: 483

**End Position**: 493

| Profile Score | Tandem Score | TDA q-value | Volume | Spectrum Matches |
| --- | --- | --- | --- | --- |
| -18.7133 | 121.5047 | 0.0000 | 1.5980e+07 | 2 |

**Adduct Signal Fractions**

- **Unmodified**: 1.5980e+07

### N(N)GTYDHDVYR Hex 6 HexNAc 3

**Mass:**  2820.0869

(1.031e-07)

**Charge States:** 3

**Start Position**: 483

**End Position**: 493

| Profile Score | Tandem Score | TDA q-value | Volume | Spectrum Matches |
| --- | --- | --- | --- | --- |
| -18.2274 | 121.2914 | 0.0000 | 1.4201e+07 | 1 |

**Adduct Signal Fractions**

- **Unmodified**: 1.4201e+07

### N(N)GTYDHDVYRDEALNNR Fuc 2 Hex 5 HexNAc 5

**Mass:**  4168.6882

(5.387e-07)

**Charge States:** 4

**Start Position**: 483

**End Position**: 500

| Profile Score | Tandem Score | TDA q-value | Volume | Spectrum Matches |
| --- | --- | --- | --- | --- |
| -3.7132 | 121.2825 | 0.0000 | 1.8035e+08 | 1 |

**Adduct Signal Fractions**

- **Unmodified**: 1.8035e+08

### N(N)GTYDHDVYRDEALNNR Fuc 2 Hex 5 HexNAc 4

**Mass:**  3965.6079

(8.775e-07)

**Charge States:** 3, 4

**Start Position**: 483

**End Position**: 500

| Profile Score | Tandem Score | TDA q-value | Volume | Spectrum Matches |
| --- | --- | --- | --- | --- |
| -2.2457 | 120.7285 | 0.0000 | 1.1141e+09 | 4 |

**Adduct Signal Fractions**

- **Unmodified**: 1.1141e+09

### N(N)GTYDHDVYR Fuc 1 Hex 5 HexNAc 5

**Mass:**  3210.2525

(-7.393e-07)

**Charge States:** 3

**Start Position**: 483

**End Position**: 493

| Profile Score | Tandem Score | TDA q-value | Volume | Spectrum Matches |
| --- | --- | --- | --- | --- |
| -3.3680 | 120.6102 | 0.0000 | 1.3091e+08 | 3 |

**Adduct Signal Fractions**

- **Unmodified**: 1.3091e+08

### GSN(N)SSFFSR Fuc 2 Hex 5 HexNAc 5

**Mass:**  3105.2195

(5.977e-07)

**Charge States:** 3, 4

**Start Position**: 142

**End Position**: 151

| Profile Score | Tandem Score | TDA q-value | Volume | Spectrum Matches |
| --- | --- | --- | --- | --- |
| -18.4094 | 119.5682 | 0.0476 | 1.5800e+07 | 1 |

**Adduct Signal Fractions**

- **Unmodified**: 1.5800e+07

### N(N)GTYDHDVYRDEALNNR Hex 7 HexNAc 3

**Mass:**  3794.5214

(1.120e-06)

**Charge States:** 4

**Start Position**: 483

**End Position**: 500

| Profile Score | Tandem Score | TDA q-value | Volume | Spectrum Matches |
| --- | --- | --- | --- | --- |
| -18.8429 | 119.2461 | 0.0000 | 3.9240e+07 | 1 |

**Adduct Signal Fractions**

- **Unmodified**: 3.9240e+07

### GSN(N)SSFFSR Hex 6 HexNAc 4

**Mass:**  2772.0824

(2.470e-06)

**Charge States:** 3

**Start Position**: 142

**End Position**: 151

| Profile Score | Tandem Score | TDA q-value | Volume | Spectrum Matches |
| --- | --- | --- | --- | --- |
| -5.2061 | 119.0053 | 0.0476 | 2.3787e+07 | 1 |

**Adduct Signal Fractions**

- **Unmodified**: 2.3787e+07

### N(N)GTYDHDVYRDEALNNR Fuc 1 Hex 8 HexNAc 3

**Mass:**  4102.5891

(-8.927e-06)

**Charge States:** 3, 4

**Start Position**: 483

**End Position**: 500

| Profile Score | Tandem Score | TDA q-value | Volume | Spectrum Matches |
| --- | --- | --- | --- | --- |
| 11.8180 | 118.8104 | 0.0000 | 2.8190e+08 | 3 |

**Adduct Signal Fractions**

- **Unmodified**: 2.8190e+08

### N(N)GTYDHDVYRDEALNNR Fuc 1 Hex 6 HexNAc 6

**Mass:**  4387.7624

(4.821e-07)

**Charge States:** 4

**Start Position**: 483

**End Position**: 500

| Profile Score | Tandem Score | TDA q-value | Volume | Spectrum Matches |
| --- | --- | --- | --- | --- |
| -4.0148 | 118.6591 | 0.0000 | 6.8012e+07 | 1 |

**Adduct Signal Fractions**

- **Unmodified**: 6.8012e+07

### SEC(C)ITPN(N)GSIPNDKPFQNVNR Hex 5 HexNAc 2

**Mass:**  3602.5678

(4.263e-06)

**Charge States:** 4

**Start Position**: 279

**End Position**: 300

| Profile Score | Tandem Score | TDA q-value | Volume | Spectrum Matches |
| --- | --- | --- | --- | --- |
| -18.5146 | 116.8489 | 0.0000 | 3.8487e+07 | 1 |

**Adduct Signal Fractions**

- **Unmodified**: 3.8487e+07

### PGDILLIN(N)STGNLIAPR Hex 9 HexNAc 2

**Mass:**  3627.6341

(1.559e-06)

**Charge States:** 3

**Start Position**: 239

**End Position**: 256

| Profile Score | Tandem Score | TDA q-value | Volume | Spectrum Matches |
| --- | --- | --- | --- | --- |
| -19.2750 | 116.7734 | 0.0000 | 2.2720e+07 | 1 |

**Adduct Signal Fractions**

- **Unmodified**: 2.2720e+07

### N(N)GTYDHDVYRDEALN(D)NR Fuc 1 Hex 5 HexNAc 4

**Mass:**  3820.5368

(2.571e-04)

**Charge States:** 3, 4, 5

**Start Position**: 483

**End Position**: 500

| Profile Score | Tandem Score | TDA q-value | Volume | Spectrum Matches |
| --- | --- | --- | --- | --- |
| 5.8459 | 116.1523 | 0.0000 | 1.3778e+09 | 9 |

**Adduct Signal Fractions**

- **Unmodified**: 1.3778e+09

### YPALN(N)VTMPNNEQFDK Hex 5 HexNAc 2

**Mass:**  3096.3067

(2.087e-06)

**Charge States:** 3

**Start Position**: 161

**End Position**: 177

| Profile Score | Tandem Score | TDA q-value | Volume | Spectrum Matches |
| --- | --- | --- | --- | --- |
| -4.1499 | 113.8355 | 0.0000 | 6.1486e+06 | 1 |

**Adduct Signal Fractions**

- **Unmodified**: 6.1486e+06

### N(N)GTYDHDVYRDEALNNR Hex 6 HexNAc 2

**Mass:**  3429.3840

(-3.017e-07)

**Charge States:** 4

**Start Position**: 483

**End Position**: 500

| Profile Score | Tandem Score | TDA q-value | Volume | Spectrum Matches |
| --- | --- | --- | --- | --- |
| -19.9684 | 113.7747 | 0.0000 | 1.8554e+07 | 1 |

**Adduct Signal Fractions**

- **Unmodified**: 1.8554e+07

### N(N)GTYDHDVYRDEALN(D)NR Hex 6 HexNAc 3

**Mass:**  3633.4519

(2.720e-04)

**Charge States:** 4

**Start Position**: 483

**End Position**: 500

| Profile Score | Tandem Score | TDA q-value | Volume | Spectrum Matches |
| --- | --- | --- | --- | --- |
| -5.7961 | 113.7270 | 0.0000 | 7.4384e+07 | 2 |

**Adduct Signal Fractions**

- **Unmodified**: 7.4384e+07

### N(N)GTYDHDVYRDEALNNR Fuc 2 Hex 4 HexNAc 3

**Mass:**  3600.4777

(8.872e-07)

**Charge States:** 4

**Start Position**: 483

**End Position**: 500

| Profile Score | Tandem Score | TDA q-value | Volume | Spectrum Matches |
| --- | --- | --- | --- | --- |
| -18.8336 | 113.2011 | 0.0000 | 2.7114e+07 | 1 |

**Adduct Signal Fractions**

- **Unmodified**: 2.7114e+07

### YPALN(N)VTMPN(D)NEQFDK Hex 8 HexNAc 2

**Mass:**  3583.4525

(2.756e-04)

**Charge States:** 3, 4

**Start Position**: 161

**End Position**: 177

| Profile Score | Tandem Score | TDA q-value | Volume | Spectrum Matches |
| --- | --- | --- | --- | --- |
| -6.3031 | 112.3741 | 0.0000 | 2.4568e+07 | 1 |

**Adduct Signal Fractions**

- **Unmodified**: 2.4568e+07

### YPALN(N)VTMPN(D)NEQFDK Hex 9 HexNAc 2

**Mass:**  3745.5012

(2.641e-04)

**Charge States:** 3

**Start Position**: 161

**End Position**: 177

| Profile Score | Tandem Score | TDA q-value | Volume | Spectrum Matches |
| --- | --- | --- | --- | --- |
| -5.5309 | 112.3223 | 0.0000 | 2.5012e+07 | 1 |

**Adduct Signal Fractions**

- **Unmodified**: 2.5012e+07

### YPALN(N)VTM(O)PN(D)NEQFDK Hex 7 HexNAc 2

**Mass:**  3437.3981

(2.902e-04)

**Charge States:** 2, 3

**Start Position**: 161

**End Position**: 177

| Profile Score | Tandem Score | TDA q-value | Volume | Spectrum Matches |
| --- | --- | --- | --- | --- |
| 10.4398 | 112.0052 | 0.0000 | 3.1099e+07 | 2 |

**Adduct Signal Fractions**

- **Unmodified**: 3.1099e+07

### N(N)GTYDHDVYR Fuc 1 Hex 4 HexNAc 3

**Mass:**  2642.0440

(1.880e-06)

**Charge States:** 3

**Start Position**: 483

**End Position**: 493

| Profile Score | Tandem Score | TDA q-value | Volume | Spectrum Matches |
| --- | --- | --- | --- | --- |
| -21.7721 | 111.8258 | 0.0000 | 2.4901e+06 | 1 |

**Adduct Signal Fractions**

- **Unmodified**: 2.4901e+06

### N(N)GTYDHDVYRDEALNNR Fuc 1 Hex 9 HexNAc 3

**Mass:**  4264.6433

(-8.559e-06)

**Charge States:** 4

**Start Position**: 483

**End Position**: 500

| Profile Score | Tandem Score | TDA q-value | Volume | Spectrum Matches |
| --- | --- | --- | --- | --- |
| -3.5566 | 110.1684 | 0.0000 | 1.5603e+08 | 2 |

**Adduct Signal Fractions**

- **Unmodified**: 1.5603e+08

### PGDILLIN(N)STGNLIAPR Fuc 2 Hex 3 HexNAc 4 Neu5Ac 1

**Mass:**  3644.6541

(-7.499e-06)

**Charge States:** 3

**Start Position**: 239

**End Position**: 256

| Profile Score | Tandem Score | TDA q-value | Volume | Spectrum Matches |
| --- | --- | --- | --- | --- |
| -20.1475 | 110.1481 | 0.0000 | 2.3915e+06 | 1 |

**Adduct Signal Fractions**

- **Unmodified**: 2.3915e+06

### N(N)GTYDHDVYRDEALNNR Fuc 2 Hex 9 HexNAc 3

**Mass:**  4410.7033

(-9.396e-06)

**Charge States:** 4

**Start Position**: 483

**End Position**: 500

| Profile Score | Tandem Score | TDA q-value | Volume | Spectrum Matches |
| --- | --- | --- | --- | --- |
| -4.8777 | 109.8390 | 0.0000 | 4.5692e+07 | 1 |

**Adduct Signal Fractions**

- **Unmodified**: 4.5692e+07

### IRDIPSRISIYWTIVKPGDILLIN(N)STGNLIAPR Hex 8 HexNAc 2

**Mass:**  5406.7019

(2.122e-06)

**Charge States:** 5

**Start Position**: 223

**End Position**: 256

| Profile Score | Tandem Score | TDA q-value | Volume | Spectrum Matches |
| --- | --- | --- | --- | --- |
| -18.9319 | 109.1400 | 0.0000 | 1.0210e+07 | 1 |

**Adduct Signal Fractions**

- **Unmodified**: 1.0210e+07

### RGSN(N)SSFFSR Fuc 2 Hex 6 HexNAc 5

**Mass:**  3423.3777

(6.645e-08)

**Charge States:** 3

**Start Position**: 141

**End Position**: 151

| Profile Score | Tandem Score | TDA q-value | Volume | Spectrum Matches |
| --- | --- | --- | --- | --- |
| -20.1124 | 108.5750 | 0.0000 | 4.2652e+06 | 1 |

**Adduct Signal Fractions**

- **Unmodified**: 4.2652e+06

### N(N)GTYDHDVYRDEALNNR Fuc 3 Hex 6 HexNAc 5

**Mass:**  4476.7971

(8.298e-08)

**Charge States:** 4

**Start Position**: 483

**End Position**: 500

| Profile Score | Tandem Score | TDA q-value | Volume | Spectrum Matches |
| --- | --- | --- | --- | --- |
| -20.4411 | 108.4195 | 0.0000 | 2.1470e+07 | 1 |

**Adduct Signal Fractions**

- **Unmodified**: 2.1470e+07

### N(N)GTYDHDVYR Fuc 1 Hex 5 HexNAc 3

**Mass:**  2806.1149

(7.215e-04)

**Start Position**: 483

**End Position**: 493

| Profile Score | Tandem Score | TDA q-value | Volume | Spectrum Matches |
| --- | --- | --- | --- | --- |
| 0.0000 | 108.0107 | 0.0000 | 0.0000e+00 | 1 |

Adduct:

No Chromatogram Found

### RGSN(N)SSFFSR Fuc 4 Hex 8 HexNAc 5

**Mass:**  4039.6225

(7.258e-06)

**Charge States:** 4

**Start Position**: 141

**End Position**: 151

| Profile Score | Tandem Score | TDA q-value | Volume | Spectrum Matches |
| --- | --- | --- | --- | --- |
| -20.9748 | 106.9294 | 0.0000 | 5.3142e+06 | 1 |

**Adduct Signal Fractions**

- **Unmodified**: 5.3142e+06

### N(N)GTYDHDVYRDEALNNR Fuc 2 Hex 6 HexNAc 5

**Mass:**  4330.7414

(-2.249e-07)

**Charge States:** 4

**Start Position**: 483

**End Position**: 500

| Profile Score | Tandem Score | TDA q-value | Volume | Spectrum Matches |
| --- | --- | --- | --- | --- |
| -4.6815 | 105.0727 | 0.0041 | 1.0425e+08 | 1 |

**Adduct Signal Fractions**

- **Unmodified**: 1.0425e+08

### N(N)GTYDHDVYR Fuc 1 Hex 6 HexNAc 3

**Mass:**  2966.1496

(1.709e-06)

**Charge States:** 3

**Start Position**: 483

**End Position**: 493

| Profile Score | Tandem Score | TDA q-value | Volume | Spectrum Matches |
| --- | --- | --- | --- | --- |
| -20.9585 | 103.8456 | 0.0041 | 7.8343e+06 | 1 |

**Adduct Signal Fractions**

- **Unmodified**: 7.8343e+06

### SEC(C)ITPN(N)GSIPN(D)DKPFQNVNR Hex 9 HexNAc 2

**Mass:**  4251.7678

(2.352e-04)

**Charge States:** 3, 4

**Start Position**: 279

**End Position**: 300

| Profile Score | Tandem Score | TDA q-value | Volume | Spectrum Matches |
| --- | --- | --- | --- | --- |
| 8.9235 | 101.9260 | 0.0117 | 2.6749e+08 | 3 |

**Adduct Signal Fractions**

- **Unmodified**: 2.6749e+08

### RGSN(N)SSFFSR Fuc 1 Hex 9 HexNAc 2

**Mass:**  3154.1955

(-9.215e-06)

**Charge States:** 3

**Start Position**: 141

**End Position**: 151

| Profile Score | Tandem Score | TDA q-value | Volume | Spectrum Matches |
| --- | --- | --- | --- | --- |
| -20.4071 | 101.4956 | 0.0117 | 2.1343e+06 | 3 |

**Adduct Signal Fractions**

- **Unmodified**: 2.1343e+06

### N(N)GTYDHDVYR Fuc 1 Hex 5 HexNAc 4 Neu5Ac 1

**Mass:**  3298.2562

(-3.070e-06)

**Charge States:** 3

**Start Position**: 483

**End Position**: 493

| Profile Score | Tandem Score | TDA q-value | Volume | Spectrum Matches |
| --- | --- | --- | --- | --- |
| -21.2097 | 99.6660 | 0.0117 | 3.3456e+06 | 1 |

**Adduct Signal Fractions**

- **Unmodified**: 3.3456e+06

### N(N)GTYDHDVYRDEALNNR Fuc 1 Hex 10 HexNAc 4

**Mass:**  4629.7785

(-7.028e-06)

**Charge States:** 4

**Start Position**: 483

**End Position**: 500

| Profile Score | Tandem Score | TDA q-value | Volume | Spectrum Matches |
| --- | --- | --- | --- | --- |
| -5.4425 | 99.0882 | 0.0117 | 2.7204e+07 | 1 |

**Adduct Signal Fractions**

- **Unmodified**: 2.7204e+07

### IEVTN(N)ATELVQN Hex 5 HexNAc 2

**Mass:**  2546.1110

(4.016e-06)

**Charge States:** 3

**Start Position**: 34

**End Position**: 46

| Profile Score | Tandem Score | TDA q-value | Volume | Spectrum Matches |
| --- | --- | --- | --- | --- |
| -19.7693 | 97.4719 | 0.0154 | 1.8835e+06 | 1 |

**Adduct Signal Fractions**

- **Unmodified**: 1.8835e+06

### N(N)GTYDHDVYRDEALNNR Fuc 1 Hex 6 HexNAc 4

**Mass:**  3981.6027

(2.539e-07)

**Charge States:** 4

**Start Position**: 483

**End Position**: 500

| Profile Score | Tandem Score | TDA q-value | Volume | Spectrum Matches |
| --- | --- | --- | --- | --- |
| -19.7175 | 96.3124 | 0.0154 | 1.9073e+07 | 1 |

**Adduct Signal Fractions**

- **Unmodified**: 1.9073e+07

### DIPSRISIYWTIVKPGDILLIN(N)STGNLIAPR Hex 9 HexNAc 2

**Mass:**  5299.5749

(2.641e-06)

**Charge States:** 4, 5

**Start Position**: 225

**End Position**: 256

| Profile Score | Tandem Score | TDA q-value | Volume | Spectrum Matches |
| --- | --- | --- | --- | --- |
| -4.4281 | 94.9920 | 0.0226 | 1.9348e+07 | 3 |

**Adduct Signal Fractions**

- **Unmodified**: 1.9348e+07

### N(N)GTYDHDVYRDEALNNR Fuc 3 Hex 5 HexNAc 5

**Mass:**  4314.7492

(1.225e-06)

**Charge States:** 4

**Start Position**: 483

**End Position**: 500

| Profile Score | Tandem Score | TDA q-value | Volume | Spectrum Matches |
| --- | --- | --- | --- | --- |
| -19.2057 | 94.8355 | 0.0226 | 3.3930e+07 | 1 |

**Adduct Signal Fractions**

- **Unmodified**: 3.3930e+07

### N(N)GTYDHDVYRDEALN(D)NR Fuc 3 Hex 5 HexNAc 4

**Mass:**  4112.6520

(2.423e-04)

**Charge States:** 4

**Start Position**: 483

**End Position**: 500

| Profile Score | Tandem Score | TDA q-value | Volume | Spectrum Matches |
| --- | --- | --- | --- | --- |
| 8.5127 | 92.9186 | 0.0255 | 9.7917e+07 | 2 |

**Adduct Signal Fractions**

- **Unmodified**: 9.7917e+07

### ISIYWTIVKPGDILLIN(N)STGN(D)LIAPR Hex 9 HexNAc 2

**Mass:**  4732.2648

(2.111e-04)

**Charge States:** 4

**Start Position**: 230

**End Position**: 256

| Profile Score | Tandem Score | TDA q-value | Volume | Spectrum Matches |
| --- | --- | --- | --- | --- |
| -5.4644 | 92.8150 | 0.0255 | 1.7537e+07 | 2 |

**Adduct Signal Fractions**

- **Unmodified**: 1.7537e+07

### N(N)GTYDHDVYRDEALNNR Fuc 1 Hex 5 HexNAc 4 Neu5Ac 2

**Mass:**  4401.7463

(1.561e-06)

**Charge States:** 4

**Start Position**: 483

**End Position**: 500

| Profile Score | Tandem Score | TDA q-value | Volume | Spectrum Matches |
| --- | --- | --- | --- | --- |
| -19.9679 | 92.7261 | 0.0255 | 1.6181e+07 | 2 |

**Adduct Signal Fractions**

- **Unmodified**: 1.6181e+07

### N(N)GTYDHDVYRDEALN(D)NR Fuc 1 Hex 4 HexNAc 3

**Mass:**  3455.4047

(2.861e-04)

**Charge States:** 4

**Start Position**: 483

**End Position**: 500

| Profile Score | Tandem Score | TDA q-value | Volume | Spectrum Matches |
| --- | --- | --- | --- | --- |
| -20.9389 | 92.6401 | 0.0255 | 1.7495e+07 | 1 |

**Adduct Signal Fractions**

- **Unmodified**: 1.7495e+07

### N(N)GTYDHDVYRDEALN(D)NR Fuc 1 Hex 5 HexNAc 5

**Mass:**  4023.6157

(2.460e-04)

**Charge States:** 3, 4, 5

**Start Position**: 483

**End Position**: 500

| Profile Score | Tandem Score | TDA q-value | Volume | Spectrum Matches |
| --- | --- | --- | --- | --- |
| 8.9099 | 91.0682 | 0.0255 | 6.0566e+08 | 6 |

**Adduct Signal Fractions**

- **Unmodified**: 6.0566e+08

### N(N)GTYDHDVYRDEALN(D)NRFQIK Fuc 1 Hex 5 HexNAc 4

**Mass:**  4336.8527

(2.301e-04)

**Charge States:** 5

**Start Position**: 483

**End Position**: 504

| Profile Score | Tandem Score | TDA q-value | Volume | Spectrum Matches |
| --- | --- | --- | --- | --- |
| -20.6920 | 89.7358 | 0.0288 | 5.2914e+07 | 1 |

**Adduct Signal Fractions**

- **Unmodified**: 5.2914e+07

### N(N)GTYDHDVYR Fuc 2 Hex 5 HexNAc 4 Neu5Ac 1

**Mass:**  3446.3236

(5.804e-04)

**Start Position**: 483

**End Position**: 493

| Profile Score | Tandem Score | TDA q-value | Volume | Spectrum Matches |
| --- | --- | --- | --- | --- |
| 0.0000 | 89.3468 | 0.0288 | 0.0000e+00 | 1 |

Adduct:

No Chromatogram Found

### N(N)GTYDHDVYRDEALNNR Fuc 2 Hex 8 HexNAc 3

**Mass:**  4248.6491

(-8.605e-06)

**Charge States:** 4

**Start Position**: 483

**End Position**: 500

| Profile Score | Tandem Score | TDA q-value | Volume | Spectrum Matches |
| --- | --- | --- | --- | --- |
| -4.4517 | 88.4824 | 0.0288 | 7.2484e+07 | 1 |

**Adduct Signal Fractions**

- **Unmodified**: 7.2484e+07

### N(N)GTYDHDVYRDEALNNR Fuc 1 Hex 4 HexNAc 4

**Mass:**  3657.5039

(2.171e-06)

**Charge States:** 4

**Start Position**: 483

**End Position**: 500

| Profile Score | Tandem Score | TDA q-value | Volume | Spectrum Matches |
| --- | --- | --- | --- | --- |
| -5.5073 | 88.1202 | 0.0288 | 1.9504e+07 | 2 |

**Adduct Signal Fractions**

- **Unmodified**: 1.9504e+07

### N(N)GTYDHDVYRDEALNNR Fuc 3 Hex 5 HexNAc 4

**Mass:**  4111.6686

(1.217e-06)

**Charge States:** 4

**Start Position**: 483

**End Position**: 500

| Profile Score | Tandem Score | TDA q-value | Volume | Spectrum Matches |
| --- | --- | --- | --- | --- |
| -16.0965 | 88.0970 | 0.0315 | 3.2651e+08 | 2 |

**Adduct Signal Fractions**

- **Unmodified**: 3.2651e+08

### RGSN(N)SSFFSR Fuc 2 Hex 5 HexNAc 6 Neu5Ac 2

**Mass:**  4046.5588

(-7.504e-06)

**Charge States:** 3, 4

**Start Position**: 141

**End Position**: 151

| Profile Score | Tandem Score | TDA q-value | Volume | Spectrum Matches |
| --- | --- | --- | --- | --- |
| -18.4962 | 88.0421 | 0.0315 | 3.7288e+07 | 1 |

**Adduct Signal Fractions**

- **Unmodified**: 3.7288e+07

### N(N)GTYDHDVYR Fuc 2 Hex 5 HexNAc 5

**Mass:**  3356.3117

(1.002e-06)

**Charge States:** 3

**Start Position**: 483

**End Position**: 493

| Profile Score | Tandem Score | TDA q-value | Volume | Spectrum Matches |
| --- | --- | --- | --- | --- |
| -19.6206 | 87.8755 | 0.0315 | 5.1166e+06 | 2 |

**Adduct Signal Fractions**

- **Unmodified**: 5.1166e+06

### SLVASSGTLEFNNESFNWAGVTQN(N)GTSSSC(C)RR Hex 7 HexNAc 2

**Mass:**  5003.1286

(2.251e-06)

**Charge States:** 4

**Start Position**: 110

**End Position**: 142

| Profile Score | Tandem Score | TDA q-value | Volume | Spectrum Matches |
| --- | --- | --- | --- | --- |
| -19.7003 | 86.7362 | 0.0315 | 2.7151e+06 | 1 |

**Adduct Signal Fractions**

- **Unmodified**: 2.7151e+06

### N(N)GTYDHDVYR Fuc 1 Hex 5 HexNAc 4

**Mass:**  3008.1769

(3.344e-04)

**Start Position**: 483

**End Position**: 493

| Profile Score | Tandem Score | TDA q-value | Volume | Spectrum Matches |
| --- | --- | --- | --- | --- |
| 0.0000 | 85.4204 | 0.0374 | 0.0000e+00 | 1 |

Adduct:

No Chromatogram Found

### YPALN(N)VTM(O)PNNEQFDKLYIWGVHHPVTDK Hex 8 HexNAc 2

**Mass:**  5144.2751

(2.327e-06)

**Charge States:** 5

**Start Position**: 161

**End Position**: 190

| Profile Score | Tandem Score | TDA q-value | Volume | Spectrum Matches |
| --- | --- | --- | --- | --- |
| -18.0520 | 85.3190 | 0.0374 | 9.2041e+06 | 1 |

**Adduct Signal Fractions**

- **Unmodified**: 9.2041e+06

### RGSN(N)SSFFSR Fuc 2 Hex 6 HexNAc 3

**Mass:**  3017.2093

(-1.201e-06)

**Charge States:** 3

**Start Position**: 141

**End Position**: 151

| Profile Score | Tandem Score | TDA q-value | Volume | Spectrum Matches |
| --- | --- | --- | --- | --- |
| -18.0980 | 84.9652 | 0.0374 | 6.7402e+06 | 1 |

**Adduct Signal Fractions**

- **Unmodified**: 6.7402e+06

### RGSN(N)SSFFSR Fuc 3 Hex 5 HexNAc 5

**Mass:**  3407.3816

(1.411e-06)

**Charge States:** 3, 4

**Start Position**: 141

**End Position**: 151

| Profile Score | Tandem Score | TDA q-value | Volume | Spectrum Matches |
| --- | --- | --- | --- | --- |
| -19.3229 | 84.8486 | 0.0374 | 5.4282e+06 | 2 |

**Adduct Signal Fractions**

- **Unmodified**: 5.4282e+06

### SEC(C)ITPN(N)GSIPN(D)DKPFQNVNR Hex 8 HexNAc 2

**Mass:**  4089.7202

(2.436e-04)

**Charge States:** 3, 4

**Start Position**: 279

**End Position**: 300

| Profile Score | Tandem Score | TDA q-value | Volume | Spectrum Matches |
| --- | --- | --- | --- | --- |
| 10.6849 | 82.5810 | 0.0374 | 1.2978e+08 | 4 |

**Adduct Signal Fractions**

- **Unmodified**: 1.2978e+08

### WTIVKPGDILLIN(N)STGNLIAPR Hex 9 HexNAc 2

**Mass:**  4255.0056

(6.501e-07)

**Charge States:** 4

**Start Position**: 234

**End Position**: 256

| Profile Score | Tandem Score | TDA q-value | Volume | Spectrum Matches |
| --- | --- | --- | --- | --- |
| -19.0834 | 82.4442 | 0.0405 | 2.0439e+06 | 1 |

**Adduct Signal Fractions**

- **Unmodified**: 2.0439e+06

### TIVKPGDILLIN(N)STGNLIAPR Hex 7 HexNAc 7

**Mass:**  4760.2575

(9.059e-06)

**Charge States:** 4

**Start Position**: 235

**End Position**: 256

| Profile Score | Tandem Score | TDA q-value | Volume | Spectrum Matches |
| --- | --- | --- | --- | --- |
| -19.2064 | 81.6708 | 0.0470 | 6.9594e+06 | 1 |

**Adduct Signal Fractions**

- **Unmodified**: 6.9594e+06

### cus|SWZNA|A/Switzerland/9715293/2013

**>cus|SWZNA|A/Switzerland/9715293/2013**  
 `MNPNQKIITIGSVSLTISTICFFMQIAILITTVTLHFKQYEFNSPPNNQVMLCEPTIIERNITEIVYLTNTTIEKEICPK  
PAEYRNWSKPQCGITGFAPFSKDNSIRLSAGGDIWVTREPYVSCDPDKCYQFALGQGTTLNNVHSNNTVRDRTPYRTLLM  
NELGVPFHLGTKQVCIAWSSSSCHDGKAWLHVCITGDDKNATASFIYNGRLVDSVVSWSKDILRTQESECVCINGTCTVV  
MTDGSASGKADTKILFIEEGKIVHTSTLSGSAQHVEECSCYPRYPGVRCVCRDNWKGSNRPIVDINIKDHSIVSSYVCSG  
LVGDTPRKNDSSSSSHCLDPNNEEGGHGVKGWAFDDGNDVWMGRTINETSRLGYETFKVIEGWSNPKSKLQTNRQVIVDR  
GDRSGYSGIFSVEGKSCINRCFYVELIRGRKEETEVLWTSNSIVVFCGTSGTYGTGSWPDGADLNLMPI`

| Site | Type | Count |
| --- | --- | --- |
| 329 | N-linked | 3 |
| 86 | N-linked | 8 |
| 200 | N-linked | 1 |

| Mass | Sequence | MS_2_ Score | `q`-value | MS_1_ Score | Signal | Protein Coordinates | Tandem Scans | Start Time | Apex Time | End Time |
| --- | --- | --- | --- | --- | --- | --- | --- | --- | --- | --- |
| 3411.3898 | N(N)WSKPQC(C)GITGFAPF Hex 8 HexNAc 2 | 158.649 | 0.0 | -3.323 | 9.764e+07 | 85 - 100 | 4 | 51.85 | 52.02 | 52.19 |
| 3626.5173 | N(N)WSKPQC(C)GITGFAPFSK Hex 8 HexNAc 2 | 156.958 | 0.0 | 17.111 | 4.504e+08 | 85 - 102 | 4 | 43.39 | 43.56 | 44.07 |
| 3788.5697 | N(N)WSKPQC(C)GITGFAPFSK Hex 9 HexNAc 2 | 155.085 | 0.0 | 18.613 | 4.575e+08 | 85 - 102 | 4 | 43.22 | 43.39 | 43.90 |
| 3573.4453 | N(N)WSKPQC(C)GITGFAPF Hex 9 HexNAc 2 | 144.672 | 0.0 | -3.005 | 1.105e+08 | 85 - 100 | 4 | 51.68 | 51.85 | 52.19 |
| 3464.4614 | N(N)WSKPQC(C)GITGFAPFSK Hex 7 HexNAc 2 | 141.312 | 0.0 | -4.629 | 3.587e+07 | 85 - 102 | 2 | 43.39 | 43.56 | 43.73 |
| 3670.4730 | KN(N)DSSSSSHC(C)LDPNNEEGGHGVK Hex 5 HexNAc 2 | 129.007 | 0.0 | -18.854 | 5.624e+07 | 327 - 350 | 1 | 14.21 | 14.21 | 14.21 |
| 4472.8937 | AWLHVC(C)ITGDDKN(N)ATASFIYNGR Hex 9 HexNAc 2 | 124.825 | 0.0 | -18.754 | 5.703e+06 | 187 - 210 | 1 | 46.76 | 46.93 | 46.93 |
| 3249.3409 | N(N)WSKPQC(C)GITGFAPF Hex 7 HexNAc 2 | 121.371 | 0.0 | -19.412 | 5.956e+06 | 85 - 100 | 1 | 52.19 | 52.19 | 52.36 |
| 3590.4719 | N(N)WSKPQC(C)GITGFAPF Fuc 2 Hex 3 HexNAc 4 Neu5Ac 1 | 118.159 | 0.0 | -19.411 | 9.706e+06 | 85 - 100 | 1 | 51.85 | 51.85 | 52.02 |
| 3140.3607 | N(N)WSKPQC(C)GITGFAPFSK Hex 5 HexNAc 2 | 94.624 | 0.023 | -17.637 | 2.881e+06 | 85 - 102 | 1 | 44.07 | 44.07 | 44.07 |
| 4076.6284 | KN(N)DSSSSSHC(C)LDPNNEEGGHGVK Hex 5 HexNAc 4 | 84.344 | 0.037 | -20.311 | 1.404e+07 | 327 - 350 | 1 | 14.21 | 14.21 | 14.21 |
| 4359.6691 | KN(N)DSSSSSHC(C)LDPNNEEGGHGVK Hex 8 HexNAc 3 | 81.903 | 0.044 | -20.785 | 9.199e+06 | 327 - 350 | 1 | 14.84 | 14.84 | 14.84 |

### N(N)WSKPQC(C)GITGFAPF Hex 8 HexNAc 2

**Mass:**  3411.3898

(1.601e-06)

**Charge States:** 3

**Start Position**: 86

**End Position**: 101

| Profile Score | Tandem Score | TDA q-value | Volume | Spectrum Matches |
| --- | --- | --- | --- | --- |
| -3.3233 | 158.6491 | 0.0000 | 9.7643e+07 | 4 |

**Adduct Signal Fractions**

- **Unmodified**: 9.7643e+07

### N(N)WSKPQC(C)GITGFAPFSK Hex 8 HexNAc 2

**Mass:**  3626.5164

(1.818e-06)

**Charge States:** 3, 4

**Start Position**: 86

**End Position**: 103

| Profile Score | Tandem Score | TDA q-value | Volume | Spectrum Matches |
| --- | --- | --- | --- | --- |
| 17.1110 | 156.9584 | 0.0000 | 4.5041e+08 | 4 |

**Adduct Signal Fractions**

- **Unmodified**: 4.5041e+08

### N(N)WSKPQC(C)GITGFAPFSK Hex 9 HexNAc 2

**Mass:**  3788.5687

(2.148e-06)

**Charge States:** 3, 4

**Start Position**: 86

**End Position**: 103

| Profile Score | Tandem Score | TDA q-value | Volume | Spectrum Matches |
| --- | --- | --- | --- | --- |
| 18.6127 | 155.0847 | 0.0000 | 4.5748e+08 | 4 |

**Adduct Signal Fractions**

- **Unmodified**: 4.5748e+08

### N(N)WSKPQC(C)GITGFAPF Hex 9 HexNAc 2

**Mass:**  3573.4451

(2.218e-06)

**Charge States:** 3

**Start Position**: 86

**End Position**: 101

| Profile Score | Tandem Score | TDA q-value | Volume | Spectrum Matches |
| --- | --- | --- | --- | --- |
| -3.0050 | 144.6720 | 0.0000 | 1.1054e+08 | 4 |

**Adduct Signal Fractions**

- **Unmodified**: 1.1054e+08

### N(N)WSKPQC(C)GITGFAPFSK Hex 7 HexNAc 2

**Mass:**  3464.4644

(1.636e-06)

**Charge States:** 3, 4

**Start Position**: 86

**End Position**: 103

| Profile Score | Tandem Score | TDA q-value | Volume | Spectrum Matches |
| --- | --- | --- | --- | --- |
| -4.6287 | 141.3122 | 0.0000 | 3.5866e+07 | 2 |

**Adduct Signal Fractions**

- **Unmodified**: 3.5866e+07

### KN(N)DSSSSSHC(C)LDPNNEEGGHGVK Hex 5 HexNAc 2

**Mass:**  3670.4720

(1.411e-06)

**Charge States:** 4, 5

**Start Position**: 328

**End Position**: 351

| Profile Score | Tandem Score | TDA q-value | Volume | Spectrum Matches |
| --- | --- | --- | --- | --- |
| -18.8541 | 129.0070 | 0.0000 | 5.6240e+07 | 1 |

**Adduct Signal Fractions**

- **Unmodified**: 5.6240e+07

### AWLHVC(C)ITGDDKN(N)ATASFIYNGR Hex 9 HexNAc 2

**Mass:**  4472.8937

(2.264e-06)

**Charge States:** 4

**Start Position**: 188

**End Position**: 211

| Profile Score | Tandem Score | TDA q-value | Volume | Spectrum Matches |
| --- | --- | --- | --- | --- |
| -18.7544 | 124.8250 | 0.0000 | 5.7029e+06 | 1 |

**Adduct Signal Fractions**

- **Unmodified**: 5.7029e+06

### N(N)WSKPQC(C)GITGFAPF Hex 7 HexNAc 2

**Mass:**  3249.3418

(3.071e-06)

**Charge States:** 3

**Start Position**: 86

**End Position**: 101

| Profile Score | Tandem Score | TDA q-value | Volume | Spectrum Matches |
| --- | --- | --- | --- | --- |
| -19.4118 | 121.3715 | 0.0000 | 5.9564e+06 | 1 |

**Adduct Signal Fractions**

- **Unmodified**: 5.9564e+06

### N(N)WSKPQC(C)GITGFAPF Fuc 2 Hex 3 HexNAc 4 Neu5Ac 1

**Mass:**  3590.4728

(-4.882e-06)

**Charge States:** 3

**Start Position**: 86

**End Position**: 101

| Profile Score | Tandem Score | TDA q-value | Volume | Spectrum Matches |
| --- | --- | --- | --- | --- |
| -19.4109 | 118.1588 | 0.0000 | 9.7061e+06 | 1 |

**Adduct Signal Fractions**

- **Unmodified**: 9.7061e+06

### N(N)WSKPQC(C)GITGFAPFSK Hex 5 HexNAc 2

**Mass:**  3140.3604

(2.308e-06)

**Charge States:** 3, 4

**Start Position**: 86

**End Position**: 103

| Profile Score | Tandem Score | TDA q-value | Volume | Spectrum Matches |
| --- | --- | --- | --- | --- |
| -17.6368 | 94.6244 | 0.0226 | 2.8807e+06 | 1 |

**Adduct Signal Fractions**

- **Unmodified**: 2.8807e+06

### KN(N)DSSSSSHC(C)LDPNNEEGGHGVK Hex 5 HexNAc 4

**Mass:**  4076.6284

(6.709e-08)

**Charge States:** 5

**Start Position**: 328

**End Position**: 351

| Profile Score | Tandem Score | TDA q-value | Volume | Spectrum Matches |
| --- | --- | --- | --- | --- |
| -20.3111 | 84.3441 | 0.0374 | 1.4037e+07 | 1 |

**Adduct Signal Fractions**

- **Unmodified**: 1.4037e+07

### KN(N)DSSSSSHC(C)LDPNNEEGGHGVK Hex 8 HexNAc 3

**Mass:**  4359.6691

(-8.712e-06)

**Charge States:** 4

**Start Position**: 328

**End Position**: 351

| Profile Score | Tandem Score | TDA q-value | Volume | Spectrum Matches |
| --- | --- | --- | --- | --- |
| -20.7852 | 81.9031 | 0.0438 | 9.1985e+06 | 1 |

**Adduct Signal Fractions**

- **Unmodified**: 9.1985e+06

### tr|E1C041|E1C041\_CHICK

**>tr|E1C041|E1C041\_CHICK**  
 `MRTGAGEAVGVLCLVSLVAALDYKYHHSEELEAYLKEVHAAYPALTHLHSIGRSVEGRDLWVLVLGRFPTQHKIGIPEFK  
YVANMHGDETVGREILLHLIDHLVTNYGRDPVITRLLNNTRIHIMPTMNPDGFEATVVPDCYYSRGRYNKNGEDLNRNFP  
DAFENNNNLIQPETQAVINWIKNETFVLSANLHGGALVASYTFDNGNSVTGTSNGYSRSPDDDVFIHLAKTYSFNHASMY  
KGTGCDSKQTFPDGITNGYSWYQLEGGMQDYNYVWGQCFEITLELSCCKYPPAEQLEKFWRDNKVALVEYIKQVHLGVKG  
QVTDKNGNPIPNAIVEAKGRPHICPYRTNEHGEYFLLLLPGKYVINATVPGFKSMLKTVDIPDNTANFSALKQDFSFPEV  
SVKPRPASCPRTPLYQELERSSAAVKPTLHILVLVIVTISILK`

| Site | Type | Count |
| --- | --- | --- |
| 366 | N-linked | 2 |

| Mass | Sequence | MS_2_ Score | `q`-value | MS_1_ Score | Signal | Protein Coordinates | Tandem Scans | Start Time | Apex Time | End Time |
| --- | --- | --- | --- | --- | --- | --- | --- | --- | --- | --- |
| 3072.3004 | YVIN(N)ATVPGFK Hex 9 HexNAc 2 | 153.617 | 0.0 | -3.165 | 3.750e+07 | 362 - 373 | 2 | 41.20 | 41.20 | 41.54 |
| 2910.2461 | YVIN(N)ATVPGFK Hex 8 HexNAc 2 | 144.170 | 0.0 | -3.702 | 1.185e+07 | 362 - 373 | 1 | 41.20 | 41.37 | 41.54 |

### YVIN(N)ATVPGFK Hex 9 HexNAc 2

**Mass:**  3072.3019

(2.431e-06)

**Charge States:** 3

**Start Position**: 363

**End Position**: 374

| Profile Score | Tandem Score | TDA q-value | Volume | Spectrum Matches |
| --- | --- | --- | --- | --- |
| -3.1645 | 153.6173 | 0.0000 | 3.7496e+07 | 2 |

**Adduct Signal Fractions**

- **Unmodified**: 3.7496e+07

### YVIN(N)ATVPGFK Hex 8 HexNAc 2

**Mass:**  2910.2461

(1.614e-06)

**Charge States:** 3

**Start Position**: 363

**End Position**: 374

| Profile Score | Tandem Score | TDA q-value | Volume | Spectrum Matches |
| --- | --- | --- | --- | --- |
| -3.7022 | 144.1696 | 0.0000 | 1.1852e+07 | 1 |

**Adduct Signal Fractions**

- **Unmodified**: 1.1852e+07

### tr|A0A1D5PBT7|A0A1D5PBT7\_CHICK

**>tr|A0A1D5PBT7|A0A1D5PBT7\_CHICK**  
 `MVLPGPPGMARSEEVHRLTENVYKTIMEQFNPSLRNFISMGKTYEKALASMTYAAKGYFDALVKMGELASESQGSKELGD  
VLFQMAEVHRQIQNQLEEMLKSFHNELLTQLEQKVELDSRYLSAALKKYQTEQRNKGDSLDKCQAELKKLRKKSQGSKNP  
QKYSDKELQYIEAISNKQGELENYVSDGYKTALTEERRRFCFLVEKQCAVAKSAITYHAKGKEMLTQKLPLWQQSCSDPN  
KIPDRAIQLMQQMAASSNGTIMPSPLSTSKSNLIISDPIPGAKPLPVPPELAPFVGRMQEAMPAVNGVSGPDSDDYNHWS  
EVKPAQPKSLSPPQPQKQLSDSYSNTLPVRKNVTPKNSYASAENKTLPRSSSMAAGLERNGRTRVQAIFSHAAGDNSTLL  
SFKEGDLITLLVPEARDGWHYGESEKTKLRGWFPFSYTRVLDNDGSERVHASLQQGKSSSTGNLLDKDDIAIPPPDYGMS  
SQAFPSQNVNTFKQRPYSVAVPAFSQGLDDYGTRSVSSGSSSLVSTV`

| Site | Type | Count |
| --- | --- | --- |
| 352 | N-linked | 1 |

| Mass | Sequence | MS_2_ Score | `q`-value | MS_1_ Score | Signal | Protein Coordinates | Tandem Scans | Start Time | Apex Time | End Time |
| --- | --- | --- | --- | --- | --- | --- | --- | --- | --- | --- |
| 4279.7108 | KN(N)VTPK Fuc 1 Hex 10 HexNAc 9 | 124.702 | 0.0 | -19.175 | 5.521e+07 | 350 - 356 | 2 | 14.10 | 14.21 | 14.21 |

### KN(N)VTPK Fuc 1 Hex 10 HexNAc 9

**Mass:**  4279.7131

(-1.793e-06)

**Charge States:** 5

**Start Position**: 351

**End Position**: 357

| Profile Score | Tandem Score | TDA q-value | Volume | Spectrum Matches |
| --- | --- | --- | --- | --- |
| -19.1747 | 124.7024 | 0.0000 | 5.5208e+07 | 2 |

**Adduct Signal Fractions**

- **Unmodified**: 5.5208e+07

### tr|A0A1D5PGT3|A0A1D5PGT3\_CHICK

**>tr|A0A1D5PGT3|A0A1D5PGT3\_CHICK**  
 `MVLPGPPGMARSEEVHRLTENVYKTIMEQFNPSLRNFISMGKTYEKALASMTYAAKGYFDALVKMGELASESQGSKELGD  
VLFQMAEVHRQIQNQLEEMLKSFHNELLTQLEQKVELDSRYLSAALKKYQTEQRNKGDSLDKCQAELKKLRKKSQGSKNP  
QKYSDKELQYIEAISNKQGELENYVSDGYKTALTEERRRFCFLVEKQCAVAKSAITYHAKGKEMLTQKLPLWQQSCSDPN  
KIPDRAIQLMQQMAASSNGTIMPSPLSTSKSNLIISDPIPGAKPLPVPPELAPFVGRMQEAMPAVNGVSGPDSDDYNHWS  
EVKPAQPKSLSPPQPQKQLSDSYSNTLPVRKNVTPKNSYASAENKTLPRSSSMAAGLERNGRTRVQAIFSHAAGDNSTLL  
SFKEGDLITLLVPEARDGWHYGESEKTKLRGWFPFSYTRVLDNDGSERVHASLQQGKSSSTGNLLDKDDIAIPPPDYGMS  
SQAFPSQNVNTFKQRPYSVAVPAFSQDYLMPYGCAIKDYSSGLCQPPPAHYKPYARSTAGLDDYGTRSVSSPDVEVARF`

| Site | Type | Count |
| --- | --- | --- |
| 352 | N-linked | 1 |

| Mass | Sequence | MS_2_ Score | `q`-value | MS_1_ Score | Signal | Protein Coordinates | Tandem Scans | Start Time | Apex Time | End Time |
| --- | --- | --- | --- | --- | --- | --- | --- | --- | --- | --- |
| 4279.7108 | KN(N)VTPK Fuc 1 Hex 10 HexNAc 9 | 124.702 | 0.0 | -19.175 | 5.521e+07 | 350 - 356 | 2 | 14.10 | 14.21 | 14.21 |

### KN(N)VTPK Fuc 1 Hex 10 HexNAc 9

**Mass:**  4279.7131

(-1.793e-06)

**Charge States:** 5

**Start Position**: 351

**End Position**: 357

| Profile Score | Tandem Score | TDA q-value | Volume | Spectrum Matches |
| --- | --- | --- | --- | --- |
| -19.1747 | 124.7024 | 0.0000 | 5.5208e+07 | 2 |

**Adduct Signal Fractions**

- **Unmodified**: 5.5208e+07

### tr|A0A1D5PRN6|A0A1D5PRN6\_CHICK

**>tr|A0A1D5PRN6|A0A1D5PRN6\_CHICK**  
 `MVLPGPPGMARSEEVHRLTENVYKTIMEQFNPSLRNFISMGKTYEKALASMTYAAKGYFDALVKMGELASESQGSKELGD  
VLFQMAEVHRQIQNQLEEMLKSFHNELLTQLEQKVELDSRYLSAALKKYQTEQRNKGDSLDKCQAELKKLRKKSQGSKNP  
QKYSDKELQYIEAISNKQGELENYVSDGYKTALTEERRRFCFLVEKQCAVAKSAITYHAKGKEMLTQKLPLWQQSCSDPN  
KIPDRAIQLMQQMAASSNGTIMPSPLSTSKSNLIISDPIPGAKPLPVPPELAPFVGRMQEAMPAVNGVSGPDSDDYNHWS  
EVKPAQPKSLSPPQPQKQLSDSYSNTLPVRKNVTPKNSYASAENKTLPRSSSMAAGLERNGRTRVQAIFSHAAGDNSTLL  
SFKEGDLITLLVPEARDGWHYGESEKTKLRGWFPFSYTRVLDNDGSERVHASLQQGKSSSTGNLLDKDDIAIPPPDYGMS  
SQAFPSQNVNTFKQRPYSVAVPAFSQDYLMPYGCAIKDYSSGLCQPPPAHYKPYARSTAGLDDYGTRSVSSGSSSLVSTV`

| Site | Type | Count |
| --- | --- | --- |
| 352 | N-linked | 1 |

| Mass | Sequence | MS_2_ Score | `q`-value | MS_1_ Score | Signal | Protein Coordinates | Tandem Scans | Start Time | Apex Time | End Time |
| --- | --- | --- | --- | --- | --- | --- | --- | --- | --- | --- |
| 4279.7108 | KN(N)VTPK Fuc 1 Hex 10 HexNAc 9 | 124.702 | 0.0 | -19.175 | 5.521e+07 | 350 - 356 | 2 | 14.10 | 14.21 | 14.21 |

### KN(N)VTPK Fuc 1 Hex 10 HexNAc 9

**Mass:**  4279.7131

(-1.793e-06)

**Charge States:** 5

**Start Position**: 351

**End Position**: 357

| Profile Score | Tandem Score | TDA q-value | Volume | Spectrum Matches |
| --- | --- | --- | --- | --- |
| -19.1747 | 124.7024 | 0.0000 | 5.5208e+07 | 2 |

**Adduct Signal Fractions**

- **Unmodified**: 5.5208e+07

### tr|A0A1D5P0L1|A0A1D5P0L1\_CHICK

**>tr|A0A1D5P0L1|A0A1D5P0L1\_CHICK**  
 `MVLPGPPGMARSEEVHRLTENVYKTIMEQFNPSLRNFISMGKTYEKALASMTYAAKGYFDALVKMGELASESQGSKELGD  
VLFQMAEVHRQIQNQLEEMLKSFHNELLTQLEQKVELDSRYLSAALKKYQTEQRNKGDSLDKCQAELKKLRKKSQGSKNP  
QKYSDKELQYIEAISNKQGELENYVSDGYKTALTEERRRFCFLVEKQCAVAKSAITYHAKGKEMLTQKLPLWQQSCSDPN  
KIPDRAIQLMQQMAASSNGTIMPSPLSTSKSNLIISDPIPGAKPLPVPPELAPFVGRMQEAMPAVNGVSGPDSDDYNHWS  
EVKPAQPKSLSPPQPQKQLSDSYSNTLPVRKNVTPKNSYASAENKTLPRSSSMAAGLERNGRTRVQAIFSHAAGDNSTLL  
SFKEGDLITLLVPEARDGWHYGESEKTKLRGWFPFSYTRVLDNDGSERVHASLQQGKSSSTGNLLDKDDIAIPPPDYGMS  
SQAFPSQNVNTFKQRPYSVAVPAFSQDYLMPYGCAIKDYSSGLCQPPPAHYKPYARSTAGLDDYGTRSVSRNPFANVQLK  
PTVTNDRSAPLIS`

| Site | Type | Count |
| --- | --- | --- |
| 352 | N-linked | 1 |

| Mass | Sequence | MS_2_ Score | `q`-value | MS_1_ Score | Signal | Protein Coordinates | Tandem Scans | Start Time | Apex Time | End Time |
| --- | --- | --- | --- | --- | --- | --- | --- | --- | --- | --- |
| 4279.7108 | KN(N)VTPK Fuc 1 Hex 10 HexNAc 9 | 124.702 | 0.0 | -19.175 | 5.521e+07 | 350 - 356 | 2 | 14.10 | 14.21 | 14.21 |

### KN(N)VTPK Fuc 1 Hex 10 HexNAc 9

**Mass:**  4279.7131

(-1.793e-06)

**Charge States:** 5

**Start Position**: 351

**End Position**: 357

| Profile Score | Tandem Score | TDA q-value | Volume | Spectrum Matches |
| --- | --- | --- | --- | --- |
| -19.1747 | 124.7024 | 0.0000 | 5.5208e+07 | 2 |

**Adduct Signal Fractions**

- **Unmodified**: 5.5208e+07

### tr|A0A1D5PNI3|A0A1D5PNI3\_CHICK

**>tr|A0A1D5PNI3|A0A1D5PNI3\_CHICK**  
 `MTTSTLAQTTSLPGTSTPTPASSTLMTTSTPAQTSLMPGHFKAQLLHLLHFTTTSTPAQTTSVPASSSPTPASSALPTAT  
TPAQTPGTSSPTPASSALPTTTTPAETTTFPATSSTTPASSAPPTTSTPAQTTSLPRTSTPTPASSALTTTTTPEQTTTV  
PGTSTPTPASSTVLTTSTPVQTTSMPGISSPTPASSALLTKSTPAQTTTVPATSSPTPASSALPTTTTPEQTTTESGTST  
PTPASSGPPTTSTPAETTSFPATSSPTPASSTLMTTSTPAQTTTVPGTSSPTPVSSALLTTRTLAQTKASLQLNSSPTSV  
SSALSTTSTPAETTTVPGTSIPTAASSTLLTTSTLAQTTSFPATSTPTPSSSTLMTTSTRAETKTSLQLNSSPTPASLAL  
PTTSTPAQTTSVPTSSSPTPASSALSTTSTPALTTALRSTSQIMSPSSPATSPSPTATSAGTVTTAQETTSPAGTTNALT  
STIRTTSTQTLSSSSPPYTTAALSYTAIPTSTPATPTTSAPQTTIASSSTPTESTALLTTTVPSTTQDISSQTAQTSNAA  
TSSTTNSATSARLTRTHAETTSSTTTFTSHSRPSASQTSTPGTTISPLPATTAQQTTAPAGTTPPTTASTTLFSTRETSS  
KEEETSTVVTSSQTPASSVLPSTSTPAQTTSMPSTSSPTPASSTLMTTSTLAQTTSLPGTSVPTPASSAPRTTTTPAQTT  
SVPGNSSPIPPYSTLLTTSTLAQTTSLPGTSTPTPASSTLMTTSTPAETKTSLLLNSSPNICIFCSSDYNHSCTDNISAR  
HFKPNSCTAIKHSCRKQTFITTFTSRARTSASWTTTPGTTTSALTATTARETTTPAGTTHPHNSNIETTSTSTIKAPTRP  
PTTPAGHERFTVNFTITNLPYDSDLQIPHSAKLNATQRVMTTLLNRLLKDTSIGPSFLGCATTAFR`

| Site | Type | Count |
| --- | --- | --- |
| 893 | N-linked | 1 |

| Mass | Sequence | MS_2_ Score | `q`-value | MS_1_ Score | Signal | Protein Coordinates | Tandem Scans | Start Time | Apex Time | End Time |
| --- | --- | --- | --- | --- | --- | --- | --- | --- | --- | --- |
| 4160.8529 | FTVN(N)FTITNLPYDSDLQIPHSAK Hex 7 HexNAc 2 | 103.042 | 0.008 | -3.741 | 2.173e+07 | 889 - 912 | 3 | 58.33 | 58.50 | 58.67 |

### FTVN(N)FTITNLPYDSDLQIPHSAK Hex 7 HexNAc 2

**Mass:**  4160.8537

(1.750e-06)

**Charge States:** 4

**Start Position**: 890

**End Position**: 913

| Profile Score | Tandem Score | TDA q-value | Volume | Spectrum Matches |
| --- | --- | --- | --- | --- |
| -3.7413 | 103.0416 | 0.0082 | 2.1729e+07 | 3 |

**Adduct Signal Fractions**

- **Unmodified**: 2.1729e+07

### tr|A0A1D5PUR1|A0A1D5PUR1\_CHICK

**>tr|A0A1D5PUR1|A0A1D5PUR1\_CHICK**  
 `MTTSTLAQTTSLPGTSTPTPASSTLMTTSTPAQTSLMPGHFKAQLLHLLHFTTTSTPAQTTSVPASSSPTPASSALPTAT  
TPAQTPGTSSPTPASSALPTTTTPAETTTFPATSSTTPASSAPPTTSTPAQTTSLPRTSTPTPASSALTTTTTPEQTTTV  
PGTSTPTPASSTVLTTSTPVQTTSMPGISSPTPASSALLTKSTPAQTTTVPATSSPTPASSALPTTTTPEQTTTESGTST  
PTPASSGPPTTSTPAETTSVPGTSYPNPASSSLMTTSTLAQTTSLPATSTPSLASSTLMTKSTFAPTTALPGTSTPTPAS  
SAPPTTSTPAQTTSVPGTSTATSASSTLLTTSTPAQTTSFPATSSPTPASSTLMTTSTPAQTTTVPGTSSPTPVSSALLT  
TRTLAQTKASLQLNSSPTSVSSALSTTSTPAETTTVPGTSIPTAASSTLLTTSTLAQTTSFPATSTPTPSSSTLMTTSTR  
AETKTSLQLNSSPTPASLALPTTSTPAQTTSVPTSSSPTPASSALSTTSTPALTTALRSTSQIMSPSSPATSPSPTATSA  
GTVTTAQETTSPAGTTNALTSTIRTTSTQTLSSSSPPYTTAALSYTAIPTSTPATPTTSAPQTTIASSSTPTESTALLTT  
TVPSTTQDISSQTAQTSNAATSSTTNSATSARLTRTHAETTSSTTTFTSHSRPSASQTSTPGTTISPLPATTAQQTTAPA  
GTTPPTTASTTLFSTRETSSKEEETSTVVTSSQTPASSVLPSTSTPAQTTSMPSTSSPTPASSTLMTTSTLAQTTSLPGT  
SVPTPASSAPRTTTTPAQTTSVPGNSSPIPPYSTLLTTSTLAQTTSLPGTSTPTPASSTLMTTSTPAETKTSLLLNSSPN  
ICIFCSSDYNHSCTDNISARHFKPNSCTAIKHSCRKQTFITTFTSRARTSASWTTTPGTTTSALTATTARETTTPAGTTH  
PHNSNIETTSTSTIKAPTRPPTTPAGHERFTVNFTITNLPYDSDLQIPHSAKLNATQRVMTTLLNRLLKDTSIGPSFLGC  
ATTAFR`

| Site | Type | Count |
| --- | --- | --- |
| 993 | N-linked | 1 |

| Mass | Sequence | MS_2_ Score | `q`-value | MS_1_ Score | Signal | Protein Coordinates | Tandem Scans | Start Time | Apex Time | End Time |
| --- | --- | --- | --- | --- | --- | --- | --- | --- | --- | --- |
| 4160.8529 | FTVN(N)FTITNLPYDSDLQIPHSAK Hex 7 HexNAc 2 | 103.042 | 0.008 | -3.741 | 2.173e+07 | 989 - 1012 | 3 | 58.33 | 58.50 | 58.67 |

### FTVN(N)FTITNLPYDSDLQIPHSAK Hex 7 HexNAc 2

**Mass:**  4160.8537

(1.750e-06)

**Charge States:** 4

**Start Position**: 990

**End Position**: 1013

| Profile Score | Tandem Score | TDA q-value | Volume | Spectrum Matches |
| --- | --- | --- | --- | --- |
| -3.7413 | 103.0416 | 0.0082 | 2.1729e+07 | 3 |

**Adduct Signal Fractions**

- **Unmodified**: 2.1729e+07
