# Supplementary material for: Measuring Site-specific Glycosylation Similarity between Influenza a Virus Variants with Statistical Certainty
Source: Mol Cell Proteomics. 2020 Nov 25;19(9):1533–45. doi: 10.1074/mcp.RA120.002031 (PMC8143645; doi:10.1074/mcp.RA120.002031)
Supplement: Supplementary file 2 [file mmc2.zip › mmc2/159250_2_supp_555293_qcjklw.zip!/20190301_SWZeggL2_021119_tryp_HILIC_DDA_1ug_03_glycopeptides.html]

## 20190301\_SWZeggL2\_021119\_tryp\_HILIC\_DDA\_1ug\_03 @ GlycopeptideHypothesis-fcde6e19be4f437b97ec7256f0ecaee8

**MS^1^ Mass Accuracy:** 10.0 PPM  
**Grouping Tolerance:** 15.0 PPM  
**MS^n^ Mass Accuracy:** 20.0 PPM  
**Minimum `q`-Value For Clustering:** 0.05

**Glycopeptide Hypothesis:**
GlycopeptideHypothesis-fcde6e19be4f437b97ec7256f0ecaee8  
**Sample Searched:**
20190301\_SWZeggL2\_021119\_tryp\_HILIC\_DDA\_1ug\_03

| Protein | Matched Glycopeptides |
| --- | --- |
| cus\|SWZHA\|A/Switzerland/9715293/2013 | 154 |
| cus\|SWZNA\|A/Switzerland/9715293/2013 | 13 |
| tr\|E1C041\|E1C041\_CHICK | 3 |
| tr\|A0A1D5PNI3\|A0A1D5PNI3\_CHICK | 2 |
| tr\|A0A1D5PUR1\|A0A1D5PUR1\_CHICK | 2 |
| tr\|F1NUU6\|F1NUU6\_CHICK | 1 |

### cus|SWZHA|A/Switzerland/9715293/2013

**>cus|SWZHA|A/Switzerland/9715293/2013**  
 `QKLPGNDNSTATLCLGHHAVPNGTIVKTITNDRIEVTNATELVQNSSIGEICDSPHQILDGENCTLIDALLGDPQCDGFQ  
NKKWDLFVERSKAYSNCYPYDVPDYASLRSLVASSGTLEFNNESFNWAGVTQNGTSSSCRRGSNSSFFSRLNWLTHLNSK  
YPALNVTMPNNEQFDKLYIWGVHHPVTDKDQIFLYAQSSGRITVSTKRSQQAVIPNIGYRPRIRDIPSRISIYWTIVKPG  
DILLINSTGNLIAPRGYFKIRSGKSSIMRSDAPIGKCKSECITPNGSIPNDKPFQNVNRITYGACPRYVKQSTLKLATGM  
RNVPERQTRGIFGAIAGFIENGWEGMVDGWYGFRHQNSEGRGQAADLKSTQAAIDQINGKLNRLIGKTNEKFHQIEKEFS  
EVEGRIQDLEKYVEDTKIDLWSYNAELLVALENQHTIDLTDSEMNKLFEKTKKQLRENAEDMGNGCFKIYHKCDNACIGS  
IRNGTYDHDVYRDEALNNRFQIKGVELKSGYKDWILWISFAISCFLLCVALLGFIMWACQKGNIRCNICI`

| Site | Type | Count |
| --- | --- | --- |
| 483 | N-linked | 59 |
| 165 | N-linked | 23 |
| 38 | N-linked | 1 |
| 144 | N-linked | 35 |
| 246 | N-linked | 21 |
| 133 | N-linked | 2 |
| 285 | N-linked | 10 |
| 22 | N-linked | 3 |

| Mass | Sequence | MS_2_ Score | `q`-value | MS_1_ Score | Signal | Protein Coordinates | Tandem Scans | Start Time | Apex Time | End Time |
| --- | --- | --- | --- | --- | --- | --- | --- | --- | --- | --- |
| 2522.0214 | RGSN(N)SSFFSR Hex 6 HexNAc 2 | 194.026 | 0.0 | -3.452 | 4.730e+08 | 140 - 150 | 5 | 18.94 | 19.43 | 19.58 |
| 2725.1027 | RGSN(N)SSFFSR Hex 6 HexNAc 3 | 191.512 | 0.0 | 12.492 | 6.028e+08 | 140 - 150 | 4 | 18.94 | 19.58 | 19.58 |
| 2359.9701 | RGSN(N)SSFFSR Hex 5 HexNAc 2 | 189.848 | 0.0 | 11.587 | 5.121e+08 | 140 - 150 | 5 | 19.11 | 19.73 | 20.46 |
| 2684.0731 | RGSN(N)SSFFSR Hex 7 HexNAc 2 | 187.540 | 0.0 | 13.742 | 3.011e+08 | 140 - 150 | 2 | 18.61 | 19.43 | 19.43 |
| 3115.2674 | RGSN(N)SSFFSR Fuc 1 Hex 5 HexNAc 5 | 186.481 | 0.0 | 12.347 | 1.407e+09 | 140 - 150 | 10 | 18.94 | 19.58 | 19.88 |
| 3204.3009 | RGSN(N)SSFFSR Fuc 3 Hex 5 HexNAc 4 | 181.431 | 0.0 | -18.539 | 4.862e+07 | 140 - 150 | 1 | 18.94 | 19.11 | 19.11 |
| 3598.4681 | YPALN(N)VTM(O)PNNEQFDK Hex 8 HexNAc 2 | 179.333 | 0.0 | 2.454 | 1.720e+09 | 160 - 176 | 8 | 34.11 | 34.61 | 112.84 |
| 3760.5184 | YPALN(N)VTM(O)PNNEQFDK Hex 9 HexNAc 2 | 177.682 | 0.0 | 17.908 | 1.724e+09 | 160 - 176 | 7 | 34.11 | 34.44 | 112.84 |
| 3006.3114 | HAVPN(N)GTIVK Fuc 1 Hex 5 HexNAc 5 | 176.497 | 0.0 | -21.011 | 1.263e+07 | 17 - 27 | 1 | 16.27 | 16.43 | 16.43 |
| 3277.3175 | RGSN(N)SSFFSR Fuc 1 Hex 6 HexNAc 5 | 173.178 | 0.0 | 11.311 | 1.586e+08 | 140 - 150 | 3 | 18.61 | 19.11 | 19.43 |
| 2547.0551 | RGSN(N)SSFFSR Fuc 1 Hex 4 HexNAc 3 | 172.981 | 0.0 | -5.043 | 7.139e+07 | 140 - 150 | 1 | 19.88 | 20.03 | 20.16 |
| 2912.1857 | RGSN(N)SSFFSR Fuc 1 Hex 5 HexNAc 4 | 172.546 | 0.0 | 14.719 | 1.151e+09 | 140 - 150 | 5 | 18.77 | 19.43 | 19.88 |
| 2766.1289 | RGSN(N)SSFFSR Hex 5 HexNAc 4 | 171.821 | 0.0 | 11.333 | 1.426e+08 | 140 - 150 | 1 | 18.94 | 19.58 | 19.58 |
| 3744.5289 | YPALN(N)VTMPNNEQFDK Hex 9 HexNAc 2 | 170.772 | 0.0 | 18.571 | 1.776e+08 | 160 - 176 | 5 | 42.03 | 42.20 | 42.71 |
| 3261.3223 | RGSN(N)SSFFSR Fuc 2 Hex 5 HexNAc 5 | 170.458 | 0.0 | -4.522 | 1.472e+08 | 140 - 150 | 1 | 18.94 | 19.27 | 19.43 |
| 3582.4778 | YPALN(N)VTMPNNEQFDK Hex 8 HexNAc 2 | 170.438 | 0.0 | -1.736 | 1.638e+08 | 160 - 176 | 6 | 42.03 | 42.37 | 42.54 |
| 4068.9388 | TIVKPGDILLIN(N)STGNLIAPR Hex 9 HexNAc 2 | 169.202 | 0.0 | 17.084 | 4.710e+08 | 234 - 255 | 6 | 52.95 | 53.12 | 53.63 |
| 3436.4134 | YPALN(N)VTM(O)PNNEQFDK Hex 7 HexNAc 2 | 168.661 | 0.0 | 14.828 | 1.369e+08 | 160 - 176 | 6 | 34.28 | 34.61 | 35.11 |
| 3777.5421 | YPALN(N)VTM(O)PNNEQFDK Fuc 2 Hex 3 HexNAc 4 Neu5Ac 1 | 166.047 | 0.0 | -4.296 | 8.882e+07 | 160 - 176 | 1 | 34.28 | 34.44 | 34.78 |
| 3274.3593 | YPALN(N)VTM(O)PNNEQFDK Hex 6 HexNAc 2 | 164.271 | 0.0 | 6.575 | 1.743e+07 | 160 - 176 | 4 | 34.44 | 34.44 | 35.28 |
| 2871.1574 | RGSN(N)SSFFSR Fuc 1 Hex 6 HexNAc 3 | 163.747 | 0.0 | -3.192 | 1.956e+08 | 140 - 150 | 1 | 19.27 | 19.27 | 19.58 |
| 2969.2099 | RGSN(N)SSFFSR Hex 5 HexNAc 5 | 163.563 | 0.0 | 11.188 | 3.820e+08 | 140 - 150 | 3 | 18.94 | 19.58 | 19.88 |
| 3153.2298 | N(N)GTYDHDVYR Fuc 2 Hex 5 HexNAc 4 | 162.772 | 0.0 | -17.796 | 1.691e+08 | 482 - 492 | 2 | 15.29 | 15.29 | 15.44 |
| 3112.3142 | YPALN(N)VTM(O)PNNEQFDK Hex 5 HexNAc 2 | 161.845 | 0.0 | -3.646 | 2.756e+07 | 160 - 176 | 2 | 34.94 | 35.45 | 35.45 |
| 2709.1056 | RGSN(N)SSFFSR Fuc 1 Hex 5 HexNAc 3 | 160.718 | 0.0 | 10.346 | 6.190e+07 | 140 - 150 | 1 | 19.27 | 19.58 | 19.88 |
| 3172.2902 | RGSN(N)SSFFSR Hex 5 HexNAc 6 | 160.271 | 0.0 | -6.154 | 1.873e+08 | 140 - 150 | 2 | 19.11 | 19.88 | 19.88 |
| 2568.9945 | GSN(N)SSFFSR Hex 6 HexNAc 3 | 158.034 | 0.0 | -17.242 | 2.531e+08 | 141 - 150 | 3 | 23.02 | 23.02 | 23.16 |
| 2203.8653 | GSN(N)SSFFSR Hex 5 HexNAc 2 | 157.455 | 0.0 | 14.654 | 3.930e+08 | 141 - 150 | 4 | 23.16 | 23.46 | 23.77 |
| 2928.1806 | RGSN(N)SSFFSR Hex 6 HexNAc 4 | 157.088 | 0.0 | 10.571 | 1.001e+08 | 140 - 150 | 1 | 18.94 | 19.43 | 19.58 |
| 4569.2281 | ISIYWTIVKPGDILLIN(N)STGNLIAPR Hex 8 HexNAc 2 | 155.355 | 0.0 | 15.034 | 1.674e+08 | 229 - 255 | 5 | 68.47 | 68.81 | 69.15 |
| 3334.3365 | RGSN(N)SSFFSR Hex 6 HexNAc 6 | 154.850 | 0.0 | -18.609 | 3.014e+07 | 140 - 150 | 1 | 19.11 | 19.11 | 19.27 |
| 2563.0506 | RGSN(N)SSFFSR Hex 5 HexNAc 3 | 154.545 | 0.0 | -19.757 | 4.417e+07 | 140 - 150 | 1 | 19.73 | 19.88 | 19.88 |
| 4731.2906 | ISIYWTIVKPGDILLIN(N)STGNLIAPR Hex 9 HexNAc 2 | 154.540 | 0.0 | 17.508 | 2.227e+09 | 229 - 255 | 10 | 66.76 | 68.81 | 69.32 |
| 3420.4249 | YPALN(N)VTMPNNEQFDK Hex 7 HexNAc 2 | 152.398 | 0.0 | -3.855 | 1.085e+07 | 160 - 176 | 1 | 42.20 | 42.37 | 42.54 |
| 3058.2423 | RGSN(N)SSFFSR Fuc 2 Hex 5 HexNAc 4 | 151.669 | 0.0 | -3.628 | 2.019e+08 | 140 - 150 | 1 | 18.77 | 19.27 | 19.27 |
| 3922.5686 | YPALN(N)VTM(O)PNNEQFDK Hex 10 HexNAc 2 | 149.639 | 0.0 | 14.075 | 5.023e+07 | 160 - 176 | 1 | 34.11 | 34.44 | 34.78 |
| 4376.8520 | C(C)KSEC(C)ITPN(N)GSIPNDKPFQNVNR Hex 8 HexNAc 2 | 149.192 | 0.0 | -17.257 | 3.623e+07 | 276 - 299 | 1 | 29.16 | 29.16 | 29.32 |
| 3210.2527 | N(N)GTYDHDVYR Fuc 1 Hex 5 HexNAc 5 | 149.143 | 0.0 | -3.176 | 2.102e+08 | 482 - 492 | 5 | 15.44 | 15.44 | 15.93 |
| 3627.6411 | PGDILLIN(N)STGNLIAPR Hex 9 HexNAc 2 | 149.100 | 0.0 | -18.983 | 3.860e+07 | 238 - 255 | 2 | 51.42 | 51.42 | 51.59 |
| 4230.9876 | TIVKPGDILLIN(N)STGNLIAPR Hex 10 HexNAc 2 | 148.919 | 0.0 | -3.518 | 3.646e+07 | 234 - 255 | 3 | 52.95 | 53.12 | 53.29 |
| 4748.2969 | ISIYWTIVKPGDILLIN(N)STGNLIAPR Fuc 2 Hex 3 HexNAc 4 Neu5Ac 1 | 148.639 | 0.0 | -4.896 | 2.891e+08 | 229 - 255 | 4 | 68.64 | 68.81 | 69.15 |
| 3007.1729 | N(N)GTYDHDVYR Fuc 1 Hex 5 HexNAc 4 | 148.167 | 0.0 | -2.686 | 5.676e+08 | 482 - 492 | 5 | 15.29 | 15.44 | 15.77 |
| 2950.2603 | YPALN(N)VTM(O)PNNEQFDK Hex 4 HexNAc 2 | 148.061 | 0.0 | -4.234 | 4.228e+06 | 160 - 176 | 1 | 35.28 | 35.45 | 35.62 |
| 4893.3295 | ISIYWTIVKPGDILLIN(N)STGNLIAPR Hex 10 HexNAc 2 | 147.346 | 0.0 | 16.642 | 2.224e+08 | 229 - 255 | 5 | 68.30 | 68.64 | 69.15 |
| 3761.5502 | YPALN(N)VTMPNNEQFDK Fuc 2 Hex 3 HexNAc 4 Neu5Ac 1 | 147.301 | 0.0 | -4.374 | 1.364e+07 | 160 - 176 | 2 | 42.03 | 42.20 | 42.37 |
| 3480.3980 | RGSN(N)SSFFSR Fuc 1 Hex 6 HexNAc 6 | 146.565 | 0.0 | -5.050 | 6.606e+07 | 140 - 150 | 3 | 18.94 | 19.27 | 19.27 |
| 4085.9540 | TIVKPGDILLIN(N)STGNLIAPR Fuc 2 Hex 3 HexNAc 4 Neu5Ac 1 | 145.786 | 0.0 | -18.221 | 2.999e+07 | 234 - 255 | 1 | 53.12 | 53.12 | 53.29 |
| 3131.2553 | RGSN(N)SSFFSR Hex 6 HexNAc 5 | 144.697 | 0.0 | -20.172 | 1.719e+07 | 140 - 150 | 2 | 19.11 | 19.27 | 19.27 |
| 2846.1257 | RGSN(N)SSFFSR Hex 8 HexNAc 2 | 143.286 | 0.0 | -18.158 | 1.425e+08 | 140 - 150 | 3 | 19.27 | 19.27 | 19.43 |
| 3779.5143 | N(N)GTYDHDVYRDEALN(D)NR Fuc 1 Hex 6 HexNAc 3 | 142.582 | 0.0 | -6.381 | 7.254e+07 | 482 - 499 | 1 | 20.76 | 20.76 | 21.41 |
| 3632.4688 | N(N)GTYDHDVYRDEALNNR Hex 6 HexNAc 3 | 142.291 | 0.0 | -3.516 | 3.730e+08 | 482 - 499 | 2 | 20.16 | 20.30 | 20.61 |
| 4910.3571 | ISIYWTIVKPGDILLIN(N)STGNLIAPR Fuc 2 Hex 4 HexNAc 4 Neu5Ac 1 | 141.297 | 0.0 | -4.684 | 3.154e+07 | 229 - 255 | 3 | 68.30 | 68.64 | 68.81 |
| 3616.4729 | N(N)GTYDHDVYRDEALNNR Fuc 1 Hex 5 HexNAc 3 | 140.384 | 0.0 | -3.307 | 1.368e+08 | 482 - 499 | 2 | 20.16 | 20.46 | 20.61 |
| 3599.4635 | YPALN(N)VTM(O)PN(D)NEQFDK Hex 8 HexNAc 2 | 137.210 | 0.0 | 9.650 | 2.286e+08 | 160 - 176 | 7 | 35.11 | 35.45 | 36.47 |
| 3778.5296 | N(N)GTYDHDVYRDEALNNR Fuc 1 Hex 6 HexNAc 3 | 136.933 | 0.0 | 10.244 | 1.977e+08 | 482 - 499 | 1 | 20.03 | 20.16 | 20.61 |
| 3965.6151 | N(N)GTYDHDVYRDEALNNR Fuc 2 Hex 5 HexNAc 4 | 136.483 | 0.0 | 13.826 | 1.157e+09 | 482 - 499 | 3 | 19.88 | 20.03 | 20.46 |
| 3761.5196 | YPALN(N)VTM(O)PN(D)NEQFDK Hex 9 HexNAc 2 | 134.762 | 0.0 | 8.089 | 2.723e+08 | 160 - 176 | 8 | 34.94 | 35.45 | 36.30 |
| 4250.7761 | SEC(C)ITPN(N)GSIPNDKPFQNVNR Hex 9 HexNAc 2 | 134.681 | 0.0 | -0.862 | 4.465e+08 | 278 - 299 | 4 | 31.90 | 32.07 | 32.24 |
| 4185.6716 | RGSN(N)SSFFSR Fuc 1 Hex 3 HexNAc 9 Neu5Ac 2 | 134.071 | 0.0 | 10.018 | 3.263e+08 | 140 - 150 | 3 | 20.46 | 20.61 | 21.41 |
| 3819.5542 | N(N)GTYDHDVYRDEALNNR Fuc 1 Hex 5 HexNAc 4 | 134.053 | 0.0 | 17.216 | 6.338e+09 | 482 - 499 | 7 | 19.88 | 20.30 | 20.61 |
| 3454.4170 | N(N)GTYDHDVYRDEALNNR Fuc 1 Hex 4 HexNAc 3 | 133.411 | 0.0 | -4.504 | 2.006e+08 | 482 - 499 | 2 | 20.46 | 20.61 | 20.92 |
| 4184.6894 | N(N)GTYDHDVYRDEALNNR Fuc 1 Hex 6 HexNAc 5 | 132.827 | 0.0 | 6.490 | 5.073e+08 | 482 - 499 | 2 | 19.88 | 20.03 | 20.46 |
| 2820.0867 | N(N)GTYDHDVYR Hex 6 HexNAc 3 | 132.443 | 0.0 | -20.019 | 1.667e+07 | 482 - 492 | 1 | 15.29 | 15.29 | 15.44 |
| 4088.7239 | SEC(C)ITPN(N)GSIPNDKPFQNVNR Hex 8 HexNAc 2 | 132.429 | 0.0 | -1.952 | 2.594e+08 | 278 - 299 | 4 | 31.90 | 32.07 | 32.41 |
| 3934.5881 | RGSN(N)SSFFSR Fuc 3 Hex 7 HexNAc 6 | 130.960 | 0.0 | -19.715 | 4.183e+07 | 140 - 150 | 1 | 21.57 | 21.57 | 21.73 |
| 3778.5471 | YPALN(N)VTM(O)PN(D)NEQFDK Fuc 2 Hex 3 HexNAc 4 Neu5Ac 1 | 130.504 | 0.0 | -5.635 | 1.291e+07 | 160 - 176 | 2 | 34.94 | 35.45 | 35.45 |
| 4168.6916 | N(N)GTYDHDVYRDEALNNR Fuc 2 Hex 5 HexNAc 5 | 130.032 | 0.0 | -4.246 | 1.896e+08 | 482 - 499 | 1 | 20.03 | 20.16 | 20.46 |
| 5568.7633 | IRDIPSRISIYWTIVKPGDILLIN(N)STGNLIAPR Hex 9 HexNAc 2 | 129.386 | 0.0 | 17.347 | 3.526e+08 | 222 - 255 | 7 | 64.37 | 65.05 | 65.56 |
| 4387.7619 | N(N)GTYDHDVYRDEALNNR Fuc 1 Hex 6 HexNAc 6 | 129.094 | 0.0 | -4.385 | 8.842e+07 | 482 - 499 | 2 | 20.16 | 20.16 | 20.46 |
| 3470.4119 | N(N)GTYDHDVYRDEALNNR Hex 5 HexNAc 3 | 128.292 | 0.0 | -4.157 | 9.222e+07 | 482 - 499 | 1 | 20.46 | 20.61 | 20.76 |
| 3926.6718 | SEC(C)ITPN(N)GSIPNDKPFQNVNR Hex 7 HexNAc 2 | 128.242 | 0.0 | -3.444 | 3.742e+08 | 278 - 299 | 3 | 31.90 | 32.07 | 32.41 |
| 3090.2295 | RGSN(N)SSFFSR Hex 7 HexNAc 4 | 128.097 | 0.0 | -19.198 | 2.591e+07 | 140 - 150 | 2 | 19.43 | 19.43 | 19.43 |
| 4102.5927 | N(N)GTYDHDVYRDEALNNR Fuc 1 Hex 8 HexNAc 3 | 127.720 | 0.0 | 11.035 | 4.941e+08 | 482 - 499 | 4 | 21.25 | 22.06 | 22.23 |
| 4255.0174 | WTIVKPGDILLIN(N)STGNLIAPR Hex 9 HexNAc 2 | 127.409 | 0.0 | -18.516 | 6.575e+06 | 233 - 255 | 2 | 60.27 | 60.27 | 60.44 |
| 3313.4663 | LGHHAVPN(N)GTIVK Fuc 1 Hex 5 HexNAc 5 | 127.057 | 0.0 | -18.375 | 8.594e+06 | 14 - 27 | 1 | 17.16 | 17.16 | 17.16 |
| 3764.6196 | SEC(C)ITPN(N)GSIPNDKPFQNVNR Hex 6 HexNAc 2 | 127.054 | 0.0 | -3.499 | 4.380e+07 | 278 - 299 | 2 | 32.07 | 32.24 | 32.41 |
| 5128.2062 | SEC(C)ITPN(N)GSIPNDKPFQNVNR Fuc 4 Hex 4 HexNAc 6 Neu5Ac 1 | 127.041 | 0.0 | 7.975 | 3.570e+08 | 278 - 299 | 3 | 31.74 | 32.24 | 32.41 |
| 3267.3338 | N(N)GTYDHDVYRDEALNNR Hex 5 HexNAc 2 | 125.802 | 0.0 | -5.022 | 1.545e+08 | 482 - 499 | 2 | 20.46 | 20.61 | 20.92 |
| 3634.4806 | N(N)GTYDHDVYRDEALNNR Hex 6 HexNAc 3 | 125.640 | 0.0 | 0.000 | 0.000e+00 | 482 - 499 | 1 | - | - | - |
| 4111.6746 | N(N)GTYDHDVYRDEALNNR Fuc 3 Hex 5 HexNAc 4 | 125.260 | 0.0 | -4.309 | 2.138e+08 | 482 - 499 | 1 | 19.88 | 19.88 | 20.16 |
| 3673.4945 | N(N)GTYDHDVYRDEALNNR Hex 5 HexNAc 4 | 124.686 | 0.0 | 13.642 | 3.743e+08 | 482 - 499 | 4 | 20.16 | 20.30 | 20.76 |
| 4022.6318 | N(N)GTYDHDVYRDEALNNR Fuc 1 Hex 5 HexNAc 5 | 124.337 | 0.0 | -2.229 | 2.393e+09 | 482 - 499 | 5 | 20.16 | 20.46 | 20.61 |
| 3299.2888 | N(N)GTYDHDVYR Fuc 3 Hex 5 HexNAc 4 | 123.538 | 0.0 | -20.579 | 2.143e+07 | 482 - 492 | 1 | 15.12 | 15.12 | 15.29 |
| 3899.5104 | N(N)GTYDHDVYRDEALNNR Fuc 1 Hex 8 HexNAc 2 | 123.453 | 0.0 | 11.500 | 1.233e+09 | 482 - 499 | 4 | 21.73 | 22.06 | 22.40 |
| 5299.5746 | DIPSRISIYWTIVKPGDILLIN(N)STGNLIAPR Hex 9 HexNAc 2 | 123.450 | 0.0 | -4.039 | 2.457e+07 | 224 - 255 | 2 | 69.15 | 69.32 | 69.49 |
| 4264.6419 | N(N)GTYDHDVYRDEALNNR Fuc 1 Hex 9 HexNAc 3 | 122.657 | 0.0 | -3.936 | 2.852e+08 | 482 - 499 | 2 | 21.57 | 21.73 | 22.06 |
| 3967.8934 | IVKPGDILLIN(N)STGNLIAPR Hex 9 HexNAc 2 | 120.856 | 0.0 | -18.254 | 1.580e+07 | 235 - 255 | 2 | 52.27 | 52.44 | 52.44 |
| 4048.6379 | RGSN(N)SSFFSR Fuc 1 Hex 7 HexNAc 8 | 120.521 | 0.0 | -20.306 | 3.772e+07 | 140 - 150 | 1 | 22.72 | 22.72 | 22.72 |
| 3309.3653 | N(N)GTYDHDVYRDEALNNR Hex 4 HexNAc 3 | 120.471 | 0.0 | 0.000 | 0.000e+00 | 482 - 499 | 1 | - | - | - |
| 4467.7205 | N(N)GTYDHDVYRDEALNNR Fuc 1 Hex 9 HexNAc 4 | 119.560 | 0.0 | 10.137 | 6.085e+07 | 482 - 499 | 1 | 21.41 | 21.90 | 22.06 |
| 4335.8648 | N(N)GTYDHDVYRDEALNNRFQIK Fuc 1 Hex 5 HexNAc 4 | 119.281 | 0.0 | -17.397 | 2.059e+07 | 482 - 503 | 1 | 31.90 | 31.90 | 31.90 |
| 3602.5715 | SEC(C)ITPN(N)GSIPNDKPFQNVNR Hex 5 HexNAc 2 | 119.181 | 0.0 | -5.174 | 2.601e+07 | 278 - 299 | 1 | 32.24 | 32.41 | 32.58 |
| 4248.6478 | N(N)GTYDHDVYRDEALNNR Fuc 2 Hex 8 HexNAc 3 | 118.932 | 0.0 | -3.985 | 1.248e+08 | 482 - 499 | 1 | 21.73 | 21.90 | 22.06 |
| 2803.2304 | HAVPN(N)GTIVK Fuc 1 Hex 5 HexNAc 4 | 118.588 | 0.0 | -21.689 | 4.522e+06 | 17 - 27 | 1 | 16.27 | 16.27 | 16.27 |
| 3820.5390 | N(N)GTYDHDVYRDEALN(D)NR Fuc 1 Hex 5 HexNAc 4 | 117.656 | 0.0 | 5.292 | 2.869e+09 | 482 - 499 | 19 | 20.76 | 20.92 | 21.57 |
| 4336.8610 | N(N)GTYDHDVYRDEALN(D)NRFQIK Fuc 1 Hex 5 HexNAc 4 | 117.261 | 0.0 | -20.445 | 2.248e+07 | 482 - 503 | 1 | 33.43 | 33.43 | 33.60 |
| 4023.6203 | N(N)GTYDHDVYRDEALN(D)NR Fuc 1 Hex 5 HexNAc 5 | 117.204 | 0.0 | 8.763 | 1.169e+09 | 482 - 499 | 7 | 20.76 | 20.92 | 22.56 |
| 4410.7004 | N(N)GTYDHDVYRDEALNNR Fuc 2 Hex 9 HexNAc 3 | 116.967 | 0.0 | -4.642 | 7.919e+07 | 482 - 499 | 1 | 21.57 | 21.57 | 21.90 |
| 2546.1181 | IEVTN(N)ATELVQN Hex 5 HexNAc 2 | 115.676 | 0.0 | -17.901 | 7.032e+05 | 33 - 45 | 1 | 35.45 | 35.45 | 35.45 |
| 3591.4463 | N(N)GTYDHDVYRDEALNNR Hex 7 HexNAc 2 | 115.125 | 0.0 | -19.547 | 4.174e+07 | 482 - 499 | 1 | 20.16 | 20.16 | 20.30 |
| 4083.0635 | ISIYWTIVKPGDILLIN(N)STGNLIAPR Hex 5 HexNAc 2 | 114.994 | 0.0 | -18.680 | 1.401e+07 | 229 - 255 | 1 | 69.32 | 69.49 | 69.49 |
| 3762.5302 | N(N)GTYDHDVYRDEALNNR Fuc 2 Hex 5 HexNAc 3 | 114.754 | 0.0 | -5.243 | 4.708e+07 | 482 - 499 | 7 | 20.16 | 20.16 | 20.46 |
| 3600.4799 | N(N)GTYDHDVYRDEALNNR Fuc 2 Hex 4 HexNAc 3 | 114.224 | 0.0 | -5.139 | 7.033e+07 | 482 - 499 | 1 | 20.30 | 20.46 | 20.61 |
| 3372.3049 | N(N)GTYDHDVYR Fuc 1 Hex 6 HexNAc 5 | 113.882 | 0.0 | -19.010 | 7.267e+07 | 482 - 492 | 2 | 15.29 | 15.29 | 15.44 |
| 3414.3029 | RGSN(N)SSFFSR Hex 9 HexNAc 4 | 113.881 | 0.0 | -19.360 | 2.490e+07 | 140 - 150 | 1 | 21.08 | 21.25 | 21.25 |
| 4776.2632 | TIVKPGDILLIN(N)STGNLIAPR Fuc 4 Hex 6 HexNAc 5 | 112.774 | 0.0 | -18.450 | 3.650e+07 | 234 - 255 | 2 | 66.93 | 66.93 | 67.10 |
| 3753.4581 | N(N)GTYDHDVYRDEALNNR Hex 8 HexNAc 2 | 112.269 | 0.0 | -17.244 | 3.568e+07 | 482 - 499 | 1 | 22.06 | 22.06 | 22.06 |
| 3374.3143 | N(N)GTYDHDVYR Fuc 1 Hex 6 HexNAc 5 | 111.450 | 0.0 | 0.000 | 0.000e+00 | 482 - 492 | 1 | - | - | - |
| 3745.5092 | YPALN(N)VTMPN(D)NEQFDK Hex 9 HexNAc 2 | 111.415 | 0.0 | -6.028 | 2.810e+07 | 160 - 176 | 3 | 43.04 | 43.21 | 43.38 |
| 3598.3273 | N(N)GTYDHDVYR Fuc 2 Hex 9 HexNAc 3 | 111.254 | 0.0 | 0.000 | 0.000e+00 | 482 - 492 | 1 | - | - | - |
| 3050.1958 | WAGVTQN(N)GTSSSC(C)R Hex 7 HexNAc 2 | 110.895 | 0.0 | -16.587 | 9.322e+06 | 126 - 140 | 1 | 18.44 | 18.44 | 18.44 |
| 2642.0412 | N(N)GTYDHDVYR Fuc 1 Hex 4 HexNAc 3 | 109.670 | 0.0 | -20.284 | 1.660e+07 | 482 - 492 | 1 | 15.44 | 15.60 | 15.60 |
| 2454.9554 | N(N)GTYDHDVYR Hex 5 HexNAc 2 | 109.112 | 0.0 | -20.284 | 1.130e+07 | 482 - 492 | 1 | 15.44 | 15.44 | 15.44 |
| 3966.5973 | N(N)GTYDHDVYRDEALN(D)NR Fuc 2 Hex 5 HexNAc 4 | 109.103 | 0.0 | 8.190 | 7.276e+08 | 482 - 499 | 6 | 20.61 | 20.76 | 21.57 |
| 3455.4108 | N(N)GTYDHDVYRDEALN(D)NR Fuc 1 Hex 4 HexNAc 3 | 109.081 | 0.0 | -6.448 | 4.743e+07 | 482 - 499 | 1 | 21.08 | 21.08 | 21.73 |
| 4733.2810 | ISIYWTIVKPGDILLIN(N)STGNLIAPR Hex 4 HexNAc 6 | 108.894 | 0.0 | -20.113 | 9.405e+06 | 229 - 255 | 32 | 67.61 | 67.61 | 67.61 |
| 2861.1156 | N(N)GTYDHDVYR Hex 5 HexNAc 4 | 108.257 | 0.0 | -18.817 | 2.007e+07 | 482 - 492 | 1 | 15.44 | 15.44 | 15.44 |
| 4112.6541 | N(N)GTYDHDVYRDEALN(D)NR Fuc 3 Hex 5 HexNAc 4 | 108.095 | 0.0 | -5.665 | 1.130e+08 | 482 - 499 | 2 | 20.30 | 20.61 | 20.76 |
| 3858.4835 | N(N)GTYDHDVYR Fuc 1 Hex 9 HexNAc 5 | 107.716 | 0.0 | -19.014 | 5.291e+07 | 482 - 492 | 2 | 20.76 | 20.92 | 20.92 |
| 4089.7190 | SEC(C)ITPN(N)GSIPN(D)DKPFQNVNR Hex 8 HexNAc 2 | 106.840 | 0.0 | 8.981 | 7.799e+07 | 278 - 299 | 3 | 32.58 | 33.43 | 33.43 |
| 3583.4592 | YPALN(N)VTMPN(D)NEQFDK Hex 8 HexNAc 2 | 106.209 | 0.0 | -5.786 | 2.658e+07 | 160 - 176 | 2 | 43.21 | 43.21 | 43.55 |
| 3794.5273 | N(N)GTYDHDVYRDEALNNR Hex 7 HexNAc 3 | 105.510 | 0.0 | -20.203 | 3.107e+07 | 482 - 499 | 1 | 20.16 | 20.16 | 20.16 |
| 3298.2680 | N(N)GTYDHDVYR Fuc 1 Hex 5 HexNAc 4 Neu5Ac 1 | 104.545 | 0.0 | -20.454 | 7.575e+06 | 482 - 492 | 1 | 16.27 | 16.27 | 16.27 |
| 2966.1445 | N(N)GTYDHDVYR Fuc 1 Hex 6 HexNAc 3 | 104.199 | 0.0 | -18.253 | 1.715e+07 | 482 - 492 | 1 | 15.29 | 15.29 | 15.29 |
| 3633.4557 | N(N)GTYDHDVYRDEALN(D)NR Hex 6 HexNAc 3 | 103.982 | 0.0 | 3.505 | 1.522e+08 | 482 - 499 | 2 | 20.76 | 20.92 | 21.57 |
| 3956.5331 | RGSN(N)SSFFSR Fuc 2 Hex 10 HexNAc 3 Neu5Ac 1 | 103.470 | 0.0 | 10.797 | 2.692e+08 | 140 - 150 | 4 | 21.41 | 21.73 | 22.06 |
| 3437.4086 | YPALN(N)VTM(O)PN(D)NEQFDK Hex 7 HexNAc 2 | 103.304 | 0.0 | 7.489 | 1.262e+07 | 160 - 176 | 1 | 35.28 | 35.45 | 36.13 |
| 3300.2965 | N(N)GTYDHDVYR Fuc 3 Hex 5 HexNAc 4 | 103.074 | 0.0 | 0.000 | 0.000e+00 | 482 - 492 | 1 | - | - | - |
| 2658.0333 | N(N)GTYDHDVYR Hex 5 HexNAc 3 | 100.539 | 0.0 | -20.096 | 7.486e+06 | 482 - 492 | 1 | 15.44 | 15.44 | 15.44 |
| 3356.3087 | N(N)GTYDHDVYR Fuc 2 Hex 5 HexNAc 5 | 100.269 | 0.0 | -20.959 | 1.079e+07 | 482 - 492 | 1 | 15.29 | 15.29 | 15.29 |
| 3979.5914 | YPALN(N)VTM(O)PNNEQFDK Hex 3 HexNAc 5 Neu5Ac 2 | 99.663 | 0.0 | 11.603 | 2.342e+07 | 160 - 176 | 1 | 33.60 | 34.28 | 34.94 |
| 5306.3397 | YPALN(N)VTM(O)PNNEQFDKLYIWGVHHPVTDK Hex 9 HexNAc 2 | 99.172 | 0.0 | -17.897 | 1.364e+07 | 160 - 189 | 1 | 49.55 | 49.55 | 49.72 |
| 5730.8179 | IRDIPSRISIYWTIVKPGDILLIN(N)STGNLIAPR Hex 10 HexNAc 2 | 99.003 | 0.0 | -19.234 | 2.332e+07 | 222 - 255 | 1 | 64.87 | 64.87 | 65.05 |
| 3845.5655 | RGSN(N)SSFFSR Fuc 1 Hex 7 HexNAc 7 | 98.502 | 0.0 | -18.355 | 1.721e+08 | 140 - 150 | 3 | 22.56 | 22.72 | 22.72 |
| 3876.5765 | N(N)GTYDHDVYRDEALNNR Hex 5 HexNAc 5 | 98.423 | 0.0 | 8.700 | 9.985e+08 | 482 - 499 | 4 | 19.58 | 19.88 | 20.46 |
| 4732.2749 | ISIYWTIVKPGDILLIN(N)STGN(D)LIAPR Hex 9 HexNAc 2 | 97.461 | 0.003 | -5.425 | 2.332e+07 | 229 - 255 | 7 | 69.32 | 69.49 | 69.83 |
| 5144.2874 | YPALN(N)VTM(O)PNNEQFDKLYIWGVHHPVTDK Hex 8 HexNAc 2 | 96.978 | 0.003 | -16.805 | 1.043e+07 | 160 - 189 | 1 | 49.55 | 49.55 | 49.72 |
| 4747.2802 | ISIYWTIVKPGDILLIN(N)STGNLIAPR Hex 3 HexNAc 4 Neu5Ac 2 | 95.031 | 0.007 | -18.705 | 2.933e+07 | 229 - 255 | 2 | 68.47 | 68.47 | 68.47 |
| 4128.6031 | RGSN(N)SSFFSR Fuc 1 Hex 10 HexNAc 6 | 93.478 | 0.010 | -20.119 | 1.855e+07 | 140 - 150 | 1 | 24.21 | 24.21 | 24.37 |
| 4805.1160 | LNWLTHLNSKYPALN(N)VTM(O)PNNEQFDK Hex 8 HexNAc 2 | 92.704 | 0.010 | -18.876 | 2.368e+07 | 150 - 176 | 2 | 47.86 | 48.03 | 48.03 |
| 3927.6635 | SEC(C)ITPN(N)GSIPN(D)DKPFQNVNR Hex 7 HexNAc 2 | 92.646 | 0.013 | 10.256 | 9.171e+07 | 278 - 299 | 3 | 32.58 | 33.43 | 33.60 |
| 3657.4997 | N(N)GTYDHDVYRDEALNNR Fuc 1 Hex 4 HexNAc 4 | 92.171 | 0.013 | -5.787 | 3.282e+07 | 482 - 499 | 3 | 20.16 | 20.46 | 20.46 |
| 3900.4934 | N(N)GTYDHDVYRDEALN(D)NR Fuc 1 Hex 8 HexNAc 2 | 91.454 | 0.016 | -20.671 | 1.383e+08 | 482 - 499 | 1 | 22.56 | 22.56 | 22.72 |
| 3267.2747 | N(N)GTYDHDVYR Hex 5 HexNAc 6 | 89.792 | 0.019 | -19.542 | 2.523e+07 | 482 - 492 | 2 | 16.10 | 16.10 | 16.10 |
| 4265.6330 | RGSN(N)SSFFSR Fuc 1 Hex 6 HexNAc 7 Neu5Ac 2 | 89.047 | 0.023 | -4.837 | 9.160e+07 | 140 - 150 | 1 | 22.23 | 22.23 | 22.72 |
| 4251.7695 | SEC(C)ITPN(N)GSIPN(D)DKPFQNVNR Hex 9 HexNAc 2 | 88.729 | 0.026 | 8.055 | 1.689e+08 | 278 - 299 | 5 | 32.41 | 33.26 | 33.43 |
| 5003.1390 | SLVASSGTLEFNNESFNWAGVTQN(N)GTSSSC(C)RR Hex 7 HexNAc 2 | 85.922 | 0.038 | -19.831 | 4.011e+06 | 109 - 141 | 3 | 50.23 | 50.23 | 50.41 |
| 5406.7069 | IRDIPSRISIYWTIVKPGDILLIN(N)STGNLIAPR Hex 8 HexNAc 2 | 85.547 | 0.038 | -4.381 | 2.240e+07 | 222 - 255 | 3 | 64.87 | 65.05 | 65.22 |
| 3407.3827 | RGSN(N)SSFFSR Fuc 3 Hex 5 HexNAc 5 | 84.694 | 0.041 | -18.333 | 1.197e+07 | 140 - 150 | 2 | 19.11 | 19.11 | 19.11 |
| 3300.2761 | N(N)GTYDHDVYR Fuc 1 Hex 5 HexNAc 4 Neu5Ac 1 | 84.487 | 0.044 | 0.000 | 0.000e+00 | 482 - 492 | 1 | - | - | - |
| 3643.4503 | RGSN(N)SSFFSR Fuc 1 Hex 7 HexNAc 6 | 83.694 | 0.047 | 0.000 | 0.000e+00 | 140 - 150 | 1 | - | - | - |
| 3575.3915 | N(N)GTYDHDVYR Fuc 1 Hex 6 HexNAc 6 | 82.871 | 0.049 | -20.385 | 1.288e+07 | 482 - 492 | 1 | 15.44 | 15.44 | 15.44 |
| 3906.8844 | TIVKPGDILLIN(N)STGNLIAPR Hex 8 HexNAc 2 | 82.403 | 0.049 | -19.414 | 2.499e+07 | 234 - 255 | 2 | 53.12 | 53.29 | 53.29 |
| 3834.5894 | YPALN(N)VTM(O)PNNEQFDK Fuc 1 Hex 3 HexNAc 5 Neu5Ac 1 | 82.064 | 0.049 | -4.806 | 1.604e+07 | 160 - 176 | 2 | 33.60 | 33.94 | 33.94 |

### RGSN(N)SSFFSR Hex 6 HexNAc 2

**Mass:**  2522.0200

(1.331e-06)

**Charge States:** 3

**Start Position**: 141

**End Position**: 151

| Profile Score | Tandem Score | TDA q-value | Volume | Spectrum Matches |
| --- | --- | --- | --- | --- |
| -3.4521 | 194.0258 | 0.0000 | 4.7298e+08 | 5 |

**Adduct Signal Fractions**

- **Unmodified**: 4.7298e+08

### RGSN(N)SSFFSR Hex 6 HexNAc 3

**Mass:**  2725.1039

(1.296e-06)

**Charge States:** 3

**Start Position**: 141

**End Position**: 151

| Profile Score | Tandem Score | TDA q-value | Volume | Spectrum Matches |
| --- | --- | --- | --- | --- |
| 12.4923 | 191.5120 | 0.0000 | 6.0278e+08 | 4 |

**Adduct Signal Fractions**

- **Unmodified**: 6.0278e+08

### RGSN(N)SSFFSR Hex 5 HexNAc 2

**Mass:**  2359.9705

(2.339e-06)

**Charge States:** 3

**Start Position**: 141

**End Position**: 151

| Profile Score | Tandem Score | TDA q-value | Volume | Spectrum Matches |
| --- | --- | --- | --- | --- |
| 11.5874 | 189.8478 | 0.0000 | 5.1213e+08 | 5 |

**Adduct Signal Fractions**

- **Unmodified**: 5.1213e+08

### RGSN(N)SSFFSR Hex 7 HexNAc 2

**Mass:**  2684.0729

(1.058e-06)

**Charge States:** 3

**Start Position**: 141

**End Position**: 151

| Profile Score | Tandem Score | TDA q-value | Volume | Spectrum Matches |
| --- | --- | --- | --- | --- |
| 13.7419 | 187.5398 | 0.0000 | 3.0111e+08 | 2 |

**Adduct Signal Fractions**

- **Unmodified**: 3.0111e+08

### RGSN(N)SSFFSR Fuc 1 Hex 5 HexNAc 5

**Mass:**  3115.2672

(2.339e-06)

**Charge States:** 3, 4

**Start Position**: 141

**End Position**: 151

| Profile Score | Tandem Score | TDA q-value | Volume | Spectrum Matches |
| --- | --- | --- | --- | --- |
| 12.3470 | 186.4814 | 0.0000 | 1.4067e+09 | 10 |

**Adduct Signal Fractions**

- **Unmodified**: 1.4067e+09

### RGSN(N)SSFFSR Fuc 3 Hex 5 HexNAc 4

**Mass:**  3204.3008

(1.545e-06)

**Charge States:** 3

**Start Position**: 141

**End Position**: 151

| Profile Score | Tandem Score | TDA q-value | Volume | Spectrum Matches |
| --- | --- | --- | --- | --- |
| -18.5386 | 181.4306 | 0.0000 | 4.8615e+07 | 1 |

**Adduct Signal Fractions**

- **Unmodified**: 4.8615e+07

### YPALN(N)VTM(O)PNNEQFDK Hex 8 HexNAc 2

**Mass:**  3598.4719

(3.502e-06)

**Charge States:** 2, 3, 4

**Start Position**: 161

**End Position**: 177

| Profile Score | Tandem Score | TDA q-value | Volume | Spectrum Matches |
| --- | --- | --- | --- | --- |
| 2.4538 | 179.3333 | 0.0000 | 1.7203e+09 | 8 |

**Adduct Signal Fractions**

- **Unmodified**: 1.7203e+09

### YPALN(N)VTM(O)PNNEQFDK Hex 9 HexNAc 2

**Mass:**  3760.5192

(3.616e-06)

**Charge States:** 2, 3, 4

**Start Position**: 161

**End Position**: 177

| Profile Score | Tandem Score | TDA q-value | Volume | Spectrum Matches |
| --- | --- | --- | --- | --- |
| 17.9083 | 177.6818 | 0.0000 | 1.7240e+09 | 7 |

**Adduct Signal Fractions**

- **Unmodified**: 1.7240e+09

### HAVPN(N)GTIVK Fuc 1 Hex 5 HexNAc 5

**Mass:**  3006.3070

(3.693e-06)

**Charge States:** 3

**Start Position**: 18

**End Position**: 28

| Profile Score | Tandem Score | TDA q-value | Volume | Spectrum Matches |
| --- | --- | --- | --- | --- |
| -21.0109 | 176.4970 | 0.0000 | 1.2632e+07 | 1 |

**Adduct Signal Fractions**

- **Unmodified**: 1.2632e+07

### RGSN(N)SSFFSR Fuc 1 Hex 6 HexNAc 5

**Mass:**  3277.3178

(1.199e-06)

**Charge States:** 3

**Start Position**: 141

**End Position**: 151

| Profile Score | Tandem Score | TDA q-value | Volume | Spectrum Matches |
| --- | --- | --- | --- | --- |
| 11.3105 | 173.1781 | 0.0000 | 1.5859e+08 | 3 |

**Adduct Signal Fractions**

- **Unmodified**: 1.5859e+08

### RGSN(N)SSFFSR Fuc 1 Hex 4 HexNAc 3

**Mass:**  2547.0554

(2.383e-06)

**Charge States:** 3

**Start Position**: 141

**End Position**: 151

| Profile Score | Tandem Score | TDA q-value | Volume | Spectrum Matches |
| --- | --- | --- | --- | --- |
| -5.0431 | 172.9815 | 0.0000 | 7.1394e+07 | 1 |

**Adduct Signal Fractions**

- **Unmodified**: 7.1394e+07

### RGSN(N)SSFFSR Fuc 1 Hex 5 HexNAc 4

**Mass:**  2912.1844

(9.817e-07)

**Charge States:** 3

**Start Position**: 141

**End Position**: 151

| Profile Score | Tandem Score | TDA q-value | Volume | Spectrum Matches |
| --- | --- | --- | --- | --- |
| 14.7188 | 172.5459 | 0.0000 | 1.1514e+09 | 5 |

**Adduct Signal Fractions**

- **Unmodified**: 1.1514e+09

### RGSN(N)SSFFSR Hex 5 HexNAc 4

**Mass:**  2766.1305

(2.464e-06)

**Charge States:** 3

**Start Position**: 141

**End Position**: 151

| Profile Score | Tandem Score | TDA q-value | Volume | Spectrum Matches |
| --- | --- | --- | --- | --- |
| 11.3326 | 171.8209 | 0.0000 | 1.4262e+08 | 1 |

**Adduct Signal Fractions**

- **Unmodified**: 1.4262e+08

### YPALN(N)VTMPNNEQFDK Hex 9 HexNAc 2

**Mass:**  3744.5260

(5.955e-06)

**Charge States:** 3, 4

**Start Position**: 161

**End Position**: 177

| Profile Score | Tandem Score | TDA q-value | Volume | Spectrum Matches |
| --- | --- | --- | --- | --- |
| 18.5715 | 170.7719 | 0.0000 | 1.7762e+08 | 5 |

**Adduct Signal Fractions**

- **Unmodified**: 1.7762e+08

### RGSN(N)SSFFSR Fuc 2 Hex 5 HexNAc 5

**Mass:**  3261.3209

(1.653e-06)

**Charge States:** 3

**Start Position**: 141

**End Position**: 151

| Profile Score | Tandem Score | TDA q-value | Volume | Spectrum Matches |
| --- | --- | --- | --- | --- |
| -4.5223 | 170.4578 | 0.0000 | 1.4722e+08 | 1 |

**Adduct Signal Fractions**

- **Unmodified**: 1.4722e+08

### YPALN(N)VTMPNNEQFDK Hex 8 HexNAc 2

**Mass:**  3582.4788

(5.576e-06)

**Charge States:** 2, 3, 4

**Start Position**: 161

**End Position**: 177

| Profile Score | Tandem Score | TDA q-value | Volume | Spectrum Matches |
| --- | --- | --- | --- | --- |
| -1.7358 | 170.4385 | 0.0000 | 1.6379e+08 | 6 |

**Adduct Signal Fractions**

- **Unmodified**: 1.6379e+08

### TIVKPGDILLIN(N)STGNLIAPR Hex 9 HexNAc 2

**Mass:**  4068.9421

(4.221e-06)

**Charge States:** 3, 4, 5

**Start Position**: 235

**End Position**: 256

| Profile Score | Tandem Score | TDA q-value | Volume | Spectrum Matches |
| --- | --- | --- | --- | --- |
| 17.0840 | 169.2024 | 0.0000 | 4.7103e+08 | 6 |

**Adduct Signal Fractions**

- **Unmodified**: 4.7103e+08

### YPALN(N)VTM(O)PNNEQFDK Hex 7 HexNAc 2

**Mass:**  3436.4115

(2.709e-06)

**Charge States:** 2, 3, 4

**Start Position**: 161

**End Position**: 177

| Profile Score | Tandem Score | TDA q-value | Volume | Spectrum Matches |
| --- | --- | --- | --- | --- |
| 14.8280 | 168.6614 | 0.0000 | 1.3690e+08 | 6 |

**Adduct Signal Fractions**

- **Unmodified**: 1.3690e+08

### YPALN(N)VTM(O)PNNEQFDK Fuc 2 Hex 3 HexNAc 4 Neu5Ac 1

**Mass:**  3777.5396

(-4.870e-06)

**Charge States:** 3

**Start Position**: 161

**End Position**: 177

| Profile Score | Tandem Score | TDA q-value | Volume | Spectrum Matches |
| --- | --- | --- | --- | --- |
| -4.2956 | 166.0474 | 0.0000 | 8.8823e+07 | 1 |

**Adduct Signal Fractions**

- **Unmodified**: 8.8823e+07

### YPALN(N)VTM(O)PNNEQFDK Hex 6 HexNAc 2

**Mass:**  3274.3548

(6.291e-06)

**Charge States:** 3

**Start Position**: 161

**End Position**: 177

| Profile Score | Tandem Score | TDA q-value | Volume | Spectrum Matches |
| --- | --- | --- | --- | --- |
| 6.5746 | 164.2713 | 0.0000 | 1.7433e+07 | 4 |

**Adduct Signal Fractions**

- **Unmodified**: 1.7433e+07

### RGSN(N)SSFFSR Fuc 1 Hex 6 HexNAc 3

**Mass:**  2871.1574

(7.923e-07)

**Charge States:** 3

**Start Position**: 141

**End Position**: 151

| Profile Score | Tandem Score | TDA q-value | Volume | Spectrum Matches |
| --- | --- | --- | --- | --- |
| -3.1916 | 163.7470 | 0.0000 | 1.9562e+08 | 1 |

**Adduct Signal Fractions**

- **Unmodified**: 1.9562e+08

### RGSN(N)SSFFSR Hex 5 HexNAc 5

**Mass:**  2969.2100

(5.268e-06)

**Charge States:** 3

**Start Position**: 141

**End Position**: 151

| Profile Score | Tandem Score | TDA q-value | Volume | Spectrum Matches |
| --- | --- | --- | --- | --- |
| 11.1883 | 163.5626 | 0.0000 | 3.8198e+08 | 3 |

**Adduct Signal Fractions**

- **Unmodified**: 3.8198e+08

### N(N)GTYDHDVYR Fuc 2 Hex 5 HexNAc 4

**Mass:**  3153.2298

(2.527e-07)

**Charge States:** 3

**Start Position**: 483

**End Position**: 493

| Profile Score | Tandem Score | TDA q-value | Volume | Spectrum Matches |
| --- | --- | --- | --- | --- |
| -17.7961 | 162.7723 | 0.0000 | 1.6911e+08 | 2 |

**Adduct Signal Fractions**

- **Unmodified**: 1.6911e+08

### YPALN(N)VTM(O)PNNEQFDK Hex 5 HexNAc 2

**Mass:**  3112.3148

(6.262e-06)

**Charge States:** 3, 4

**Start Position**: 161

**End Position**: 177

| Profile Score | Tandem Score | TDA q-value | Volume | Spectrum Matches |
| --- | --- | --- | --- | --- |
| -3.6459 | 161.8451 | 0.0000 | 2.7556e+07 | 2 |

**Adduct Signal Fractions**

- **Unmodified**: 2.7556e+07

### RGSN(N)SSFFSR Fuc 1 Hex 5 HexNAc 3

**Mass:**  2709.1078

(1.085e-06)

**Charge States:** 3

**Start Position**: 141

**End Position**: 151

| Profile Score | Tandem Score | TDA q-value | Volume | Spectrum Matches |
| --- | --- | --- | --- | --- |
| 10.3460 | 160.7176 | 0.0000 | 6.1896e+07 | 1 |

**Adduct Signal Fractions**

- **Unmodified**: 6.1896e+07

### RGSN(N)SSFFSR Hex 5 HexNAc 6

**Mass:**  3172.2899

(1.778e-06)

**Charge States:** 3

**Start Position**: 141

**End Position**: 151

| Profile Score | Tandem Score | TDA q-value | Volume | Spectrum Matches |
| --- | --- | --- | --- | --- |
| -6.1544 | 160.2707 | 0.0000 | 1.8733e+08 | 2 |

**Adduct Signal Fractions**

- **Unmodified**: 1.8733e+08

### GSN(N)SSFFSR Hex 6 HexNAc 3

**Mass:**  2568.9950

(9.775e-07)

**Charge States:** 2, 3

**Start Position**: 142

**End Position**: 151

| Profile Score | Tandem Score | TDA q-value | Volume | Spectrum Matches |
| --- | --- | --- | --- | --- |
| -17.2425 | 158.0336 | 0.0000 | 2.5309e+08 | 3 |

**Adduct Signal Fractions**

- **Unmodified**: 2.5309e+08

### GSN(N)SSFFSR Hex 5 HexNAc 2

**Mass:**  2203.8666

(-2.014e-07)

**Charge States:** 2, 3

**Start Position**: 142

**End Position**: 151

| Profile Score | Tandem Score | TDA q-value | Volume | Spectrum Matches |
| --- | --- | --- | --- | --- |
| 14.6544 | 157.4554 | 0.0000 | 3.9302e+08 | 4 |

**Adduct Signal Fractions**

- **Unmodified**: 3.9302e+08

### RGSN(N)SSFFSR Hex 6 HexNAc 4

**Mass:**  2928.1807

(1.404e-06)

**Charge States:** 3

**Start Position**: 141

**End Position**: 151

| Profile Score | Tandem Score | TDA q-value | Volume | Spectrum Matches |
| --- | --- | --- | --- | --- |
| 10.5712 | 157.0876 | 0.0000 | 1.0014e+08 | 1 |

**Adduct Signal Fractions**

- **Unmodified**: 1.0014e+08

### ISIYWTIVKPGDILLIN(N)STGNLIAPR Hex 8 HexNAc 2

**Mass:**  4569.2307

(3.755e-06)

**Charge States:** 3, 4, 5

**Start Position**: 230

**End Position**: 256

| Profile Score | Tandem Score | TDA q-value | Volume | Spectrum Matches |
| --- | --- | --- | --- | --- |
| 15.0342 | 155.3545 | 0.0000 | 1.6744e+08 | 5 |

**Adduct Signal Fractions**

- **Unmodified**: 1.6744e+08

### RGSN(N)SSFFSR Hex 6 HexNAc 6

**Mass:**  3334.3361

(2.742e-07)

**Charge States:** 3

**Start Position**: 141

**End Position**: 151

| Profile Score | Tandem Score | TDA q-value | Volume | Spectrum Matches |
| --- | --- | --- | --- | --- |
| -18.6091 | 154.8495 | 0.0000 | 3.0136e+07 | 1 |

**Adduct Signal Fractions**

- **Unmodified**: 3.0136e+07

### RGSN(N)SSFFSR Hex 5 HexNAc 3

**Mass:**  2563.0499

(3.153e-06)

**Charge States:** 3

**Start Position**: 141

**End Position**: 151

| Profile Score | Tandem Score | TDA q-value | Volume | Spectrum Matches |
| --- | --- | --- | --- | --- |
| -19.7568 | 154.5447 | 0.0000 | 4.4173e+07 | 1 |

**Adduct Signal Fractions**

- **Unmodified**: 4.4173e+07

### ISIYWTIVKPGDILLIN(N)STGNLIAPR Hex 9 HexNAc 2

**Mass:**  4731.2946

(5.982e-06)

**Charge States:** 3, 4, 5

**Start Position**: 230

**End Position**: 256

| Profile Score | Tandem Score | TDA q-value | Volume | Spectrum Matches |
| --- | --- | --- | --- | --- |
| 17.5083 | 154.5401 | 0.0000 | 2.2272e+09 | 10 |

**Adduct Signal Fractions**

- **Unmodified**: 2.2272e+09

### YPALN(N)VTMPNNEQFDK Hex 7 HexNAc 2

**Mass:**  3420.4235

(5.158e-06)

**Charge States:** 3

**Start Position**: 161

**End Position**: 177

| Profile Score | Tandem Score | TDA q-value | Volume | Spectrum Matches |
| --- | --- | --- | --- | --- |
| -3.8550 | 152.3985 | 0.0000 | 1.0847e+07 | 1 |

**Adduct Signal Fractions**

- **Unmodified**: 1.0847e+07

### RGSN(N)SSFFSR Fuc 2 Hex 5 HexNAc 4

**Mass:**  3058.2421

(8.629e-07)

**Charge States:** 3

**Start Position**: 141

**End Position**: 151

| Profile Score | Tandem Score | TDA q-value | Volume | Spectrum Matches |
| --- | --- | --- | --- | --- |
| -3.6283 | 151.6691 | 0.0000 | 2.0191e+08 | 1 |

**Adduct Signal Fractions**

- **Unmodified**: 2.0191e+08

### YPALN(N)VTM(O)PNNEQFDK Hex 10 HexNAc 2

**Mass:**  3922.5694

(9.613e-07)

**Charge States:** 3, 4

**Start Position**: 161

**End Position**: 177

| Profile Score | Tandem Score | TDA q-value | Volume | Spectrum Matches |
| --- | --- | --- | --- | --- |
| 14.0751 | 149.6392 | 0.0000 | 5.0227e+07 | 1 |

**Adduct Signal Fractions**

- **Unmodified**: 5.0227e+07

### C(C)KSEC(C)ITPN(N)GSIPNDKPFQNVNR Hex 8 HexNAc 2

**Mass:**  4376.8493

(1.971e-06)

**Charge States:** 4

**Start Position**: 277

**End Position**: 300

| Profile Score | Tandem Score | TDA q-value | Volume | Spectrum Matches |
| --- | --- | --- | --- | --- |
| -17.2571 | 149.1917 | 0.0000 | 3.6229e+07 | 1 |

**Adduct Signal Fractions**

- **Unmodified**: 3.6229e+07

### N(N)GTYDHDVYR Fuc 1 Hex 5 HexNAc 5

**Mass:**  3210.2525

(5.920e-07)

**Charge States:** 3

**Start Position**: 483

**End Position**: 493

| Profile Score | Tandem Score | TDA q-value | Volume | Spectrum Matches |
| --- | --- | --- | --- | --- |
| -3.1763 | 149.1428 | 0.0000 | 2.1017e+08 | 5 |

**Adduct Signal Fractions**

- **Unmodified**: 2.1017e+08

### PGDILLIN(N)STGNLIAPR Hex 9 HexNAc 2

**Mass:**  3627.6417

(3.938e-06)

**Charge States:** 3, 4

**Start Position**: 239

**End Position**: 256

| Profile Score | Tandem Score | TDA q-value | Volume | Spectrum Matches |
| --- | --- | --- | --- | --- |
| -18.9835 | 149.0995 | 0.0000 | 3.8596e+07 | 2 |

**Adduct Signal Fractions**

- **Unmodified**: 3.8596e+07

### TIVKPGDILLIN(N)STGNLIAPR Hex 10 HexNAc 2

**Mass:**  4230.9914

(2.887e-06)

**Charge States:** 4

**Start Position**: 235

**End Position**: 256

| Profile Score | Tandem Score | TDA q-value | Volume | Spectrum Matches |
| --- | --- | --- | --- | --- |
| -3.5178 | 148.9192 | 0.0000 | 3.6464e+07 | 3 |

**Adduct Signal Fractions**

- **Unmodified**: 3.6464e+07

### ISIYWTIVKPGDILLIN(N)STGNLIAPR Fuc 2 Hex 3 HexNAc 4 Neu5Ac 1

**Mass:**  4748.2917

(-5.770e-06)

**Charge States:** 4, 5

**Start Position**: 230

**End Position**: 256

| Profile Score | Tandem Score | TDA q-value | Volume | Spectrum Matches |
| --- | --- | --- | --- | --- |
| -4.8957 | 148.6394 | 0.0000 | 2.8915e+08 | 4 |

**Adduct Signal Fractions**

- **Unmodified**: 2.8915e+08

### N(N)GTYDHDVYR Fuc 1 Hex 5 HexNAc 4

**Mass:**  3007.1735

(-8.609e-08)

**Charge States:** 3, 4

**Start Position**: 483

**End Position**: 493

| Profile Score | Tandem Score | TDA q-value | Volume | Spectrum Matches |
| --- | --- | --- | --- | --- |
| -2.6865 | 148.1668 | 0.0000 | 5.6758e+08 | 5 |

**Adduct Signal Fractions**

- **Unmodified**: 5.6758e+08

### YPALN(N)VTM(O)PNNEQFDK Hex 4 HexNAc 2

**Mass:**  2950.2586

(5.489e-06)

**Charge States:** 3

**Start Position**: 161

**End Position**: 177

| Profile Score | Tandem Score | TDA q-value | Volume | Spectrum Matches |
| --- | --- | --- | --- | --- |
| -4.2337 | 148.0605 | 0.0000 | 4.2281e+06 | 1 |

**Adduct Signal Fractions**

- **Unmodified**: 4.2281e+06

### ISIYWTIVKPGDILLIN(N)STGNLIAPR Hex 10 HexNAc 2

**Mass:**  4893.3288

(1.525e-06)

**Charge States:** 4, 5

**Start Position**: 230

**End Position**: 256

| Profile Score | Tandem Score | TDA q-value | Volume | Spectrum Matches |
| --- | --- | --- | --- | --- |
| 16.6416 | 147.3461 | 0.0000 | 2.2238e+08 | 5 |

**Adduct Signal Fractions**

- **Unmodified**: 2.2238e+08

### YPALN(N)VTMPNNEQFDK Fuc 2 Hex 3 HexNAc 4 Neu5Ac 1

**Mass:**  3761.5497

(-3.951e-06)

**Charge States:** 3

**Start Position**: 161

**End Position**: 177

| Profile Score | Tandem Score | TDA q-value | Volume | Spectrum Matches |
| --- | --- | --- | --- | --- |
| -4.3741 | 147.3006 | 0.0000 | 1.3644e+07 | 2 |

**Adduct Signal Fractions**

- **Unmodified**: 1.3644e+07

### RGSN(N)SSFFSR Fuc 1 Hex 6 HexNAc 6

**Mass:**  3480.3989

(1.344e-07)

**Charge States:** 3, 4

**Start Position**: 141

**End Position**: 151

| Profile Score | Tandem Score | TDA q-value | Volume | Spectrum Matches |
| --- | --- | --- | --- | --- |
| -5.0504 | 146.5646 | 0.0000 | 6.6057e+07 | 3 |

**Adduct Signal Fractions**

- **Unmodified**: 6.6057e+07

### TIVKPGDILLIN(N)STGNLIAPR Fuc 2 Hex 3 HexNAc 4 Neu5Ac 1

**Mass:**  4085.9514

(-6.151e-06)

**Charge States:** 4

**Start Position**: 235

**End Position**: 256

| Profile Score | Tandem Score | TDA q-value | Volume | Spectrum Matches |
| --- | --- | --- | --- | --- |
| -18.2207 | 145.7857 | 0.0000 | 2.9993e+07 | 1 |

**Adduct Signal Fractions**

- **Unmodified**: 2.9993e+07

### RGSN(N)SSFFSR Hex 6 HexNAc 5

**Mass:**  3131.2516

(-1.410e-06)

**Charge States:** 3

**Start Position**: 141

**End Position**: 151

| Profile Score | Tandem Score | TDA q-value | Volume | Spectrum Matches |
| --- | --- | --- | --- | --- |
| -20.1723 | 144.6970 | 0.0000 | 1.7191e+07 | 2 |

**Adduct Signal Fractions**

- **Unmodified**: 1.7191e+07

### RGSN(N)SSFFSR Hex 8 HexNAc 2

**Mass:**  2846.1260

(8.752e-07)

**Charge States:** 3

**Start Position**: 141

**End Position**: 151

| Profile Score | Tandem Score | TDA q-value | Volume | Spectrum Matches |
| --- | --- | --- | --- | --- |
| -18.1585 | 143.2856 | 0.0000 | 1.4249e+08 | 3 |

**Adduct Signal Fractions**

- **Unmodified**: 1.4249e+08

### N(N)GTYDHDVYRDEALN(D)NR Fuc 1 Hex 6 HexNAc 3

**Mass:**  3779.5156

(2.629e-04)

**Charge States:** 4

**Start Position**: 483

**End Position**: 500

| Profile Score | Tandem Score | TDA q-value | Volume | Spectrum Matches |
| --- | --- | --- | --- | --- |
| -6.3812 | 142.5821 | 0.0000 | 7.2543e+07 | 1 |

**Adduct Signal Fractions**

- **Unmodified**: 7.2543e+07

### N(N)GTYDHDVYRDEALNNR Hex 6 HexNAc 3

**Mass:**  3632.4680

(2.584e-06)

**Charge States:** 4

**Start Position**: 483

**End Position**: 500

| Profile Score | Tandem Score | TDA q-value | Volume | Spectrum Matches |
| --- | --- | --- | --- | --- |
| -3.5160 | 142.2905 | 0.0000 | 3.7301e+08 | 2 |

**Adduct Signal Fractions**

- **Unmodified**: 3.7301e+08

### ISIYWTIVKPGDILLIN(N)STGNLIAPR Fuc 2 Hex 4 HexNAc 4 Neu5Ac 1

**Mass:**  4910.3571

(-3.032e-06)

**Charge States:** 4

**Start Position**: 230

**End Position**: 256

| Profile Score | Tandem Score | TDA q-value | Volume | Spectrum Matches |
| --- | --- | --- | --- | --- |
| -4.6839 | 141.2972 | 0.0000 | 3.1536e+07 | 3 |

**Adduct Signal Fractions**

- **Unmodified**: 3.1536e+07

### N(N)GTYDHDVYRDEALNNR Fuc 1 Hex 5 HexNAc 3

**Mass:**  3616.4768

(7.558e-07)

**Charge States:** 4

**Start Position**: 483

**End Position**: 500

| Profile Score | Tandem Score | TDA q-value | Volume | Spectrum Matches |
| --- | --- | --- | --- | --- |
| -3.3070 | 140.3837 | 0.0000 | 1.3675e+08 | 2 |

**Adduct Signal Fractions**

- **Unmodified**: 1.3675e+08

### YPALN(N)VTM(O)PN(D)NEQFDK Hex 8 HexNAc 2

**Mass:**  3599.4622

(2.802e-04)

**Charge States:** 2, 3, 4

**Start Position**: 161

**End Position**: 177

| Profile Score | Tandem Score | TDA q-value | Volume | Spectrum Matches |
| --- | --- | --- | --- | --- |
| 9.6498 | 137.2097 | 0.0000 | 2.2862e+08 | 7 |

**Adduct Signal Fractions**

- **Unmodified**: 2.2862e+08

### N(N)GTYDHDVYRDEALNNR Fuc 1 Hex 6 HexNAc 3

**Mass:**  3778.5305

(3.914e-06)

**Charge States:** 4

**Start Position**: 483

**End Position**: 500

| Profile Score | Tandem Score | TDA q-value | Volume | Spectrum Matches |
| --- | --- | --- | --- | --- |
| 10.2439 | 136.9331 | 0.0000 | 1.9772e+08 | 1 |

**Adduct Signal Fractions**

- **Unmodified**: 1.9772e+08

### N(N)GTYDHDVYRDEALNNR Fuc 2 Hex 5 HexNAc 4

**Mass:**  3965.6164

(2.253e-06)

**Charge States:** 3, 4

**Start Position**: 483

**End Position**: 500

| Profile Score | Tandem Score | TDA q-value | Volume | Spectrum Matches |
| --- | --- | --- | --- | --- |
| 13.8262 | 136.4834 | 0.0000 | 1.1572e+09 | 3 |

**Adduct Signal Fractions**

- **Unmodified**: 1.1572e+09

### YPALN(N)VTM(O)PN(D)NEQFDK Hex 9 HexNAc 2

**Mass:**  3761.5189

(2.691e-04)

**Charge States:** 3, 4

**Start Position**: 161

**End Position**: 177

| Profile Score | Tandem Score | TDA q-value | Volume | Spectrum Matches |
| --- | --- | --- | --- | --- |
| 8.0886 | 134.7620 | 0.0000 | 2.7232e+08 | 8 |

**Adduct Signal Fractions**

- **Unmodified**: 2.7232e+08

### SEC(C)ITPN(N)GSIPNDKPFQNVNR Hex 9 HexNAc 2

**Mass:**  4250.7805

(2.259e-06)

**Charge States:** 3, 4

**Start Position**: 279

**End Position**: 300

| Profile Score | Tandem Score | TDA q-value | Volume | Spectrum Matches |
| --- | --- | --- | --- | --- |
| -0.8624 | 134.6807 | 0.0000 | 4.4646e+08 | 4 |

**Adduct Signal Fractions**

- **Unmodified**: 4.4646e+08

### RGSN(N)SSFFSR Fuc 1 Hex 3 HexNAc 9 Neu5Ac 2

**Mass:**  4185.6672

(2.962e-06)

**Charge States:** 4

**Start Position**: 141

**End Position**: 151

| Profile Score | Tandem Score | TDA q-value | Volume | Spectrum Matches |
| --- | --- | --- | --- | --- |
| 10.0180 | 134.0706 | 0.0000 | 3.2634e+08 | 3 |

**Adduct Signal Fractions**

- **Unmodified**: 3.2634e+08

### N(N)GTYDHDVYRDEALNNR Fuc 1 Hex 5 HexNAc 4

**Mass:**  3819.5512

(2.143e-06)

**Charge States:** 3, 4, 5

**Start Position**: 483

**End Position**: 500

| Profile Score | Tandem Score | TDA q-value | Volume | Spectrum Matches |
| --- | --- | --- | --- | --- |
| 17.2158 | 134.0530 | 0.0000 | 6.3379e+09 | 7 |

**Adduct Signal Fractions**

- **Unmodified**: 6.3379e+09

### N(N)GTYDHDVYRDEALNNR Fuc 1 Hex 4 HexNAc 3

**Mass:**  3454.4155

(1.561e-06)

**Charge States:** 4

**Start Position**: 483

**End Position**: 500

| Profile Score | Tandem Score | TDA q-value | Volume | Spectrum Matches |
| --- | --- | --- | --- | --- |
| -4.5041 | 133.4107 | 0.0000 | 2.0061e+08 | 2 |

**Adduct Signal Fractions**

- **Unmodified**: 2.0061e+08

### N(N)GTYDHDVYRDEALNNR Fuc 1 Hex 6 HexNAc 5

**Mass:**  4184.6921

(2.614e-06)

**Charge States:** 3, 4

**Start Position**: 483

**End Position**: 500

| Profile Score | Tandem Score | TDA q-value | Volume | Spectrum Matches |
| --- | --- | --- | --- | --- |
| 6.4896 | 132.8270 | 0.0000 | 5.0734e+08 | 2 |

**Adduct Signal Fractions**

- **Unmodified**: 5.0734e+08

### N(N)GTYDHDVYR Hex 6 HexNAc 3

**Mass:**  2820.0866

(-7.889e-09)

**Charge States:** 3

**Start Position**: 483

**End Position**: 493

| Profile Score | Tandem Score | TDA q-value | Volume | Spectrum Matches |
| --- | --- | --- | --- | --- |
| -20.0185 | 132.4434 | 0.0000 | 1.6666e+07 | 1 |

**Adduct Signal Fractions**

- **Unmodified**: 1.6666e+07

### SEC(C)ITPN(N)GSIPNDKPFQNVNR Hex 8 HexNAc 2

**Mass:**  4088.7258

(6.703e-07)

**Charge States:** 3, 4

**Start Position**: 279

**End Position**: 300

| Profile Score | Tandem Score | TDA q-value | Volume | Spectrum Matches |
| --- | --- | --- | --- | --- |
| -1.9519 | 132.4288 | 0.0000 | 2.5936e+08 | 4 |

**Adduct Signal Fractions**

- **Unmodified**: 2.5936e+08

### RGSN(N)SSFFSR Fuc 3 Hex 7 HexNAc 6

**Mass:**  3934.5869

(6.357e-06)

**Charge States:** 4

**Start Position**: 141

**End Position**: 151

| Profile Score | Tandem Score | TDA q-value | Volume | Spectrum Matches |
| --- | --- | --- | --- | --- |
| -19.7145 | 130.9597 | 0.0000 | 4.1830e+07 | 1 |

**Adduct Signal Fractions**

- **Unmodified**: 4.1830e+07

### YPALN(N)VTM(O)PN(D)NEQFDK Fuc 2 Hex 3 HexNAc 4 Neu5Ac 1

**Mass:**  3778.5437

(2.625e-04)

**Charge States:** 3

**Start Position**: 161

**End Position**: 177

| Profile Score | Tandem Score | TDA q-value | Volume | Spectrum Matches |
| --- | --- | --- | --- | --- |
| -5.6345 | 130.5037 | 0.0000 | 1.2914e+07 | 2 |

**Adduct Signal Fractions**

- **Unmodified**: 1.2914e+07

### N(N)GTYDHDVYRDEALNNR Fuc 2 Hex 5 HexNAc 5

**Mass:**  4168.6931

(3.059e-06)

**Charge States:** 4

**Start Position**: 483

**End Position**: 500

| Profile Score | Tandem Score | TDA q-value | Volume | Spectrum Matches |
| --- | --- | --- | --- | --- |
| -4.2460 | 130.0322 | 0.0000 | 1.8958e+08 | 1 |

**Adduct Signal Fractions**

- **Unmodified**: 1.8958e+08

### IRDIPSRISIYWTIVKPGDILLIN(N)STGNLIAPR Hex 9 HexNAc 2

**Mass:**  5568.7637

(2.981e-06)

**Charge States:** 5, 6, 7

**Start Position**: 223

**End Position**: 256

| Profile Score | Tandem Score | TDA q-value | Volume | Spectrum Matches |
| --- | --- | --- | --- | --- |
| 17.3474 | 129.3857 | 0.0000 | 3.5263e+08 | 7 |

**Adduct Signal Fractions**

- **Unmodified**: 3.5263e+08

### N(N)GTYDHDVYRDEALNNR Fuc 1 Hex 6 HexNAc 6

**Mass:**  4387.7614

(2.541e-07)

**Charge States:** 4

**Start Position**: 483

**End Position**: 500

| Profile Score | Tandem Score | TDA q-value | Volume | Spectrum Matches |
| --- | --- | --- | --- | --- |
| -4.3850 | 129.0944 | 0.0000 | 8.8418e+07 | 2 |

**Adduct Signal Fractions**

- **Unmodified**: 8.8418e+07

### N(N)GTYDHDVYRDEALNNR Hex 5 HexNAc 3

**Mass:**  3470.4081

(9.133e-07)

**Charge States:** 4

**Start Position**: 483

**End Position**: 500

| Profile Score | Tandem Score | TDA q-value | Volume | Spectrum Matches |
| --- | --- | --- | --- | --- |
| -4.1571 | 128.2922 | 0.0000 | 9.2218e+07 | 1 |

**Adduct Signal Fractions**

- **Unmodified**: 9.2218e+07

### SEC(C)ITPN(N)GSIPNDKPFQNVNR Hex 7 HexNAc 2

**Mass:**  3926.6748

(1.436e-06)

**Charge States:** 3, 4

**Start Position**: 279

**End Position**: 300

| Profile Score | Tandem Score | TDA q-value | Volume | Spectrum Matches |
| --- | --- | --- | --- | --- |
| -3.4440 | 128.2416 | 0.0000 | 3.7420e+08 | 3 |

**Adduct Signal Fractions**

- **Unmodified**: 3.7420e+08

### RGSN(N)SSFFSR Hex 7 HexNAc 4

**Mass:**  3090.2327

(1.029e-06)

**Charge States:** 3, 4

**Start Position**: 141

**End Position**: 151

| Profile Score | Tandem Score | TDA q-value | Volume | Spectrum Matches |
| --- | --- | --- | --- | --- |
| -19.1978 | 128.0966 | 0.0000 | 2.5910e+07 | 2 |

**Adduct Signal Fractions**

- **Unmodified**: 2.5910e+07

### N(N)GTYDHDVYRDEALNNR Fuc 1 Hex 8 HexNAc 3

**Mass:**  4102.5949

(-8.793e-06)

**Charge States:** 3, 4

**Start Position**: 483

**End Position**: 500

| Profile Score | Tandem Score | TDA q-value | Volume | Spectrum Matches |
| --- | --- | --- | --- | --- |
| 11.0349 | 127.7203 | 0.0000 | 4.9407e+08 | 4 |

**Adduct Signal Fractions**

- **Unmodified**: 4.9407e+08

### WTIVKPGDILLIN(N)STGNLIAPR Hex 9 HexNAc 2

**Mass:**  4255.0168

(3.264e-06)

**Charge States:** 4

**Start Position**: 234

**End Position**: 256

| Profile Score | Tandem Score | TDA q-value | Volume | Spectrum Matches |
| --- | --- | --- | --- | --- |
| -18.5162 | 127.4094 | 0.0000 | 6.5748e+06 | 2 |

**Adduct Signal Fractions**

- **Unmodified**: 6.5748e+06

### LGHHAVPN(N)GTIVK Fuc 1 Hex 5 HexNAc 5

**Mass:**  3313.4663

(-1.308e-06)

**Charge States:** 4

**Start Position**: 15

**End Position**: 28

| Profile Score | Tandem Score | TDA q-value | Volume | Spectrum Matches |
| --- | --- | --- | --- | --- |
| -18.3747 | 127.0572 | 0.0000 | 8.5942e+06 | 1 |

**Adduct Signal Fractions**

- **Unmodified**: 8.5942e+06

### SEC(C)ITPN(N)GSIPNDKPFQNVNR Hex 6 HexNAc 2

**Mass:**  3764.6138

(3.480e-06)

**Charge States:** 3, 4, 5

**Start Position**: 279

**End Position**: 300

| Profile Score | Tandem Score | TDA q-value | Volume | Spectrum Matches |
| --- | --- | --- | --- | --- |
| -3.4995 | 127.0543 | 0.0000 | 4.3795e+07 | 2 |

**Adduct Signal Fractions**

- **Unmodified**: 4.3795e+07

### SEC(C)ITPN(N)GSIPNDKPFQNVNR Fuc 4 Hex 4 HexNAc 6 Neu5Ac 1

**Mass:**  5128.2119

(6.889e-06)

**Charge States:** 4, 5

**Start Position**: 279

**End Position**: 300

| Profile Score | Tandem Score | TDA q-value | Volume | Spectrum Matches |
| --- | --- | --- | --- | --- |
| 7.9752 | 127.0405 | 0.0000 | 3.5703e+08 | 3 |

**Adduct Signal Fractions**

- **Unmodified**: 3.5703e+08

### N(N)GTYDHDVYRDEALNNR Hex 5 HexNAc 2

**Mass:**  3267.3312

(-2.426e-07)

**Charge States:** 4

**Start Position**: 483

**End Position**: 500

| Profile Score | Tandem Score | TDA q-value | Volume | Spectrum Matches |
| --- | --- | --- | --- | --- |
| -5.0218 | 125.8024 | 0.0000 | 1.5445e+08 | 2 |

**Adduct Signal Fractions**

- **Unmodified**: 1.5445e+08

### N(N)GTYDHDVYRDEALNNR Hex 6 HexNAc 3

**Mass:**  3634.4806

(5.551e-04)

**Start Position**: 483

**End Position**: 500

| Profile Score | Tandem Score | TDA q-value | Volume | Spectrum Matches |
| --- | --- | --- | --- | --- |
| 0.0000 | 125.6402 | 0.0000 | 0.0000e+00 | 1 |

Adduct:

No Chromatogram Found

### N(N)GTYDHDVYRDEALNNR Fuc 3 Hex 5 HexNAc 4

**Mass:**  4111.6726

(1.957e-06)

**Charge States:** 4

**Start Position**: 483

**End Position**: 500

| Profile Score | Tandem Score | TDA q-value | Volume | Spectrum Matches |
| --- | --- | --- | --- | --- |
| -4.3091 | 125.2600 | 0.0000 | 2.1377e+08 | 1 |

**Adduct Signal Fractions**

- **Unmodified**: 2.1377e+08

### N(N)GTYDHDVYRDEALNNR Hex 5 HexNAc 4

**Mass:**  3673.4951

(1.600e-06)

**Charge States:** 4

**Start Position**: 483

**End Position**: 500

| Profile Score | Tandem Score | TDA q-value | Volume | Spectrum Matches |
| --- | --- | --- | --- | --- |
| 13.6419 | 124.6865 | 0.0000 | 3.7431e+08 | 4 |

**Adduct Signal Fractions**

- **Unmodified**: 3.7431e+08

### N(N)GTYDHDVYRDEALNNR Fuc 1 Hex 5 HexNAc 5

**Mass:**  4022.6332

(1.274e-06)

**Charge States:** 3, 4, 5

**Start Position**: 483

**End Position**: 500

| Profile Score | Tandem Score | TDA q-value | Volume | Spectrum Matches |
| --- | --- | --- | --- | --- |
| -2.2288 | 124.3366 | 0.0000 | 2.3926e+09 | 5 |

**Adduct Signal Fractions**

- **Unmodified**: 2.3926e+09

### N(N)GTYDHDVYR Fuc 3 Hex 5 HexNAc 4

**Mass:**  3299.2893

(7.692e-07)

**Charge States:** 3

**Start Position**: 483

**End Position**: 493

| Profile Score | Tandem Score | TDA q-value | Volume | Spectrum Matches |
| --- | --- | --- | --- | --- |
| -20.5787 | 123.5381 | 0.0000 | 2.1433e+07 | 1 |

**Adduct Signal Fractions**

- **Unmodified**: 2.1433e+07

### N(N)GTYDHDVYRDEALNNR Fuc 1 Hex 8 HexNAc 2

**Mass:**  3899.5156

(-9.969e-06)

**Charge States:** 3, 4

**Start Position**: 483

**End Position**: 500

| Profile Score | Tandem Score | TDA q-value | Volume | Spectrum Matches |
| --- | --- | --- | --- | --- |
| 11.5001 | 123.4528 | 0.0000 | 1.2329e+09 | 4 |

**Adduct Signal Fractions**

- **Unmodified**: 1.2329e+09

### DIPSRISIYWTIVKPGDILLIN(N)STGNLIAPR Hex 9 HexNAc 2

**Mass:**  5299.5749

(2.218e-06)

**Charge States:** 4

**Start Position**: 225

**End Position**: 256

| Profile Score | Tandem Score | TDA q-value | Volume | Spectrum Matches |
| --- | --- | --- | --- | --- |
| -4.0391 | 123.4499 | 0.0000 | 2.4566e+07 | 2 |

**Adduct Signal Fractions**

- **Unmodified**: 2.4566e+07

### N(N)GTYDHDVYRDEALNNR Fuc 1 Hex 9 HexNAc 3

**Mass:**  4264.6413

(-9.424e-06)

**Charge States:** 3, 4

**Start Position**: 483

**End Position**: 500

| Profile Score | Tandem Score | TDA q-value | Volume | Spectrum Matches |
| --- | --- | --- | --- | --- |
| -3.9360 | 122.6572 | 0.0000 | 2.8520e+08 | 2 |

**Adduct Signal Fractions**

- **Unmodified**: 2.8520e+08

### IVKPGDILLIN(N)STGNLIAPR Hex 9 HexNAc 2

**Mass:**  3967.8967

(5.283e-06)

**Charge States:** 4

**Start Position**: 236

**End Position**: 256

| Profile Score | Tandem Score | TDA q-value | Volume | Spectrum Matches |
| --- | --- | --- | --- | --- |
| -18.2538 | 120.8564 | 0.0000 | 1.5802e+07 | 2 |

**Adduct Signal Fractions**

- **Unmodified**: 1.5802e+07

### RGSN(N)SSFFSR Fuc 1 Hex 7 HexNAc 8

**Mass:**  4048.6379

(8.173e-06)

**Charge States:** 4

**Start Position**: 141

**End Position**: 151

| Profile Score | Tandem Score | TDA q-value | Volume | Spectrum Matches |
| --- | --- | --- | --- | --- |
| -20.3059 | 120.5211 | 0.0000 | 3.7723e+07 | 1 |

**Adduct Signal Fractions**

- **Unmodified**: 3.7723e+07

### N(N)GTYDHDVYRDEALNNR Hex 4 HexNAc 3

**Mass:**  3309.3653

(3.043e-04)

**Start Position**: 483

**End Position**: 500

| Profile Score | Tandem Score | TDA q-value | Volume | Spectrum Matches |
| --- | --- | --- | --- | --- |
| 0.0000 | 120.4709 | 0.0000 | 0.0000e+00 | 1 |

Adduct:

No Chromatogram Found

### N(N)GTYDHDVYRDEALNNR Fuc 1 Hex 9 HexNAc 4

**Mass:**  4467.7228

(-9.290e-06)

**Charge States:** 4

**Start Position**: 483

**End Position**: 500

| Profile Score | Tandem Score | TDA q-value | Volume | Spectrum Matches |
| --- | --- | --- | --- | --- |
| 10.1368 | 119.5601 | 0.0000 | 6.0853e+07 | 1 |

**Adduct Signal Fractions**

- **Unmodified**: 6.0853e+07

### N(N)GTYDHDVYRDEALNNRFQIK Fuc 1 Hex 5 HexNAc 4

**Mass:**  4335.8654

(2.440e-06)

**Charge States:** 5, 6

**Start Position**: 483

**End Position**: 504

| Profile Score | Tandem Score | TDA q-value | Volume | Spectrum Matches |
| --- | --- | --- | --- | --- |
| -17.3971 | 119.2805 | 0.0000 | 2.0587e+07 | 1 |

**Adduct Signal Fractions**

- **Unmodified**: 2.0587e+07

### SEC(C)ITPN(N)GSIPNDKPFQNVNR Hex 5 HexNAc 2

**Mass:**  3602.5684

(5.454e-06)

**Charge States:** 4

**Start Position**: 279

**End Position**: 300

| Profile Score | Tandem Score | TDA q-value | Volume | Spectrum Matches |
| --- | --- | --- | --- | --- |
| -5.1735 | 119.1807 | 0.0000 | 2.6005e+07 | 1 |

**Adduct Signal Fractions**

- **Unmodified**: 2.6005e+07

### N(N)GTYDHDVYRDEALNNR Fuc 2 Hex 8 HexNAc 3

**Mass:**  4248.6477

(-8.997e-06)

**Charge States:** 4

**Start Position**: 483

**End Position**: 500

| Profile Score | Tandem Score | TDA q-value | Volume | Spectrum Matches |
| --- | --- | --- | --- | --- |
| -3.9854 | 118.9319 | 0.0000 | 1.2478e+08 | 1 |

**Adduct Signal Fractions**

- **Unmodified**: 1.2478e+08

### HAVPN(N)GTIVK Fuc 1 Hex 5 HexNAc 4

**Mass:**  2803.2304

(1.293e-06)

**Charge States:** 3

**Start Position**: 18

**End Position**: 28

| Profile Score | Tandem Score | TDA q-value | Volume | Spectrum Matches |
| --- | --- | --- | --- | --- |
| -21.6894 | 118.5878 | 0.0000 | 4.5223e+06 | 1 |

**Adduct Signal Fractions**

- **Unmodified**: 4.5223e+06

### N(N)GTYDHDVYRDEALN(D)NR Fuc 1 Hex 5 HexNAc 4

**Mass:**  3820.5366

(2.591e-04)

**Charge States:** 3, 4, 5

**Start Position**: 483

**End Position**: 500

| Profile Score | Tandem Score | TDA q-value | Volume | Spectrum Matches |
| --- | --- | --- | --- | --- |
| 5.2922 | 117.6561 | 0.0000 | 2.8691e+09 | 19 |

**Adduct Signal Fractions**

- **Unmodified**: 2.8691e+09

### N(N)GTYDHDVYRDEALN(D)NRFQIK Fuc 1 Hex 5 HexNAc 4

**Mass:**  4336.8600

(2.318e-04)

**Charge States:** 5

**Start Position**: 483

**End Position**: 504

| Profile Score | Tandem Score | TDA q-value | Volume | Spectrum Matches |
| --- | --- | --- | --- | --- |
| -20.4452 | 117.2606 | 0.0000 | 2.2482e+07 | 1 |

**Adduct Signal Fractions**

- **Unmodified**: 2.2482e+07

### N(N)GTYDHDVYRDEALN(D)NR Fuc 1 Hex 5 HexNAc 5

**Mass:**  4023.6176

(2.468e-04)

**Charge States:** 3, 4, 5

**Start Position**: 483

**End Position**: 500

| Profile Score | Tandem Score | TDA q-value | Volume | Spectrum Matches |
| --- | --- | --- | --- | --- |
| 8.7628 | 117.2036 | 0.0000 | 1.1692e+09 | 7 |

**Adduct Signal Fractions**

- **Unmodified**: 1.1692e+09

### N(N)GTYDHDVYRDEALNNR Fuc 2 Hex 9 HexNAc 3

**Mass:**  4410.7009

(-8.587e-06)

**Charge States:** 4

**Start Position**: 483

**End Position**: 500

| Profile Score | Tandem Score | TDA q-value | Volume | Spectrum Matches |
| --- | --- | --- | --- | --- |
| -4.6419 | 116.9666 | 0.0000 | 7.9195e+07 | 1 |

**Adduct Signal Fractions**

- **Unmodified**: 7.9195e+07

### IEVTN(N)ATELVQN Hex 5 HexNAc 2

**Mass:**  2546.1181

(6.934e-06)

**Charge States:** 3

**Start Position**: 34

**End Position**: 46

| Profile Score | Tandem Score | TDA q-value | Volume | Spectrum Matches |
| --- | --- | --- | --- | --- |
| -17.9014 | 115.6758 | 0.0000 | 7.0322e+05 | 1 |

**Adduct Signal Fractions**

- **Unmodified**: 7.0322e+05

### N(N)GTYDHDVYRDEALNNR Hex 7 HexNAc 2

**Mass:**  3591.4465

(2.404e-06)

**Charge States:** 4

**Start Position**: 483

**End Position**: 500

| Profile Score | Tandem Score | TDA q-value | Volume | Spectrum Matches |
| --- | --- | --- | --- | --- |
| -19.5466 | 115.1248 | 0.0000 | 4.1739e+07 | 1 |

**Adduct Signal Fractions**

- **Unmodified**: 4.1739e+07

### ISIYWTIVKPGDILLIN(N)STGNLIAPR Hex 5 HexNAc 2

**Mass:**  4083.0649

(2.984e-06)

**Charge States:** 3, 4

**Start Position**: 230

**End Position**: 256

| Profile Score | Tandem Score | TDA q-value | Volume | Spectrum Matches |
| --- | --- | --- | --- | --- |
| -18.6804 | 114.9944 | 0.0000 | 1.4009e+07 | 1 |

**Adduct Signal Fractions**

- **Unmodified**: 1.4009e+07

### N(N)GTYDHDVYRDEALNNR Fuc 2 Hex 5 HexNAc 3

**Mass:**  3762.5324

(1.364e-06)

**Charge States:** 4

**Start Position**: 483

**End Position**: 500

| Profile Score | Tandem Score | TDA q-value | Volume | Spectrum Matches |
| --- | --- | --- | --- | --- |
| -5.2429 | 114.7537 | 0.0000 | 4.7084e+07 | 7 |

**Adduct Signal Fractions**

- **Unmodified**: 4.7084e+07

### N(N)GTYDHDVYRDEALNNR Fuc 2 Hex 4 HexNAc 3

**Mass:**  3600.4814

(1.892e-06)

**Charge States:** 4

**Start Position**: 483

**End Position**: 500

| Profile Score | Tandem Score | TDA q-value | Volume | Spectrum Matches |
| --- | --- | --- | --- | --- |
| -5.1393 | 114.2235 | 0.0000 | 7.0327e+07 | 1 |

**Adduct Signal Fractions**

- **Unmodified**: 7.0327e+07

### N(N)GTYDHDVYR Fuc 1 Hex 6 HexNAc 5

**Mass:**  3372.3041

(1.459e-06)

**Charge States:** 3, 4

**Start Position**: 483

**End Position**: 493

| Profile Score | Tandem Score | TDA q-value | Volume | Spectrum Matches |
| --- | --- | --- | --- | --- |
| -19.0100 | 113.8825 | 0.0000 | 7.2671e+07 | 2 |

**Adduct Signal Fractions**

- **Unmodified**: 7.2671e+07

### RGSN(N)SSFFSR Hex 9 HexNAc 4

**Mass:**  3414.3019

(-9.707e-06)

**Charge States:** 3

**Start Position**: 141

**End Position**: 151

| Profile Score | Tandem Score | TDA q-value | Volume | Spectrum Matches |
| --- | --- | --- | --- | --- |
| -19.3598 | 113.8807 | 0.0000 | 2.4896e+07 | 1 |

**Adduct Signal Fractions**

- **Unmodified**: 2.4896e+07

### TIVKPGDILLIN(N)STGNLIAPR Fuc 4 Hex 6 HexNAc 5

**Mass:**  4776.2648

(6.270e-06)

**Charge States:** 3, 4

**Start Position**: 235

**End Position**: 256

| Profile Score | Tandem Score | TDA q-value | Volume | Spectrum Matches |
| --- | --- | --- | --- | --- |
| -18.4501 | 112.7739 | 0.0000 | 3.6504e+07 | 2 |

**Adduct Signal Fractions**

- **Unmodified**: 3.6504e+07

### N(N)GTYDHDVYRDEALNNR Hex 8 HexNAc 2

**Mass:**  3753.4582

(-8.666e-06)

**Charge States:** 3, 4

**Start Position**: 483

**End Position**: 500

| Profile Score | Tandem Score | TDA q-value | Volume | Spectrum Matches |
| --- | --- | --- | --- | --- |
| -17.2444 | 112.2691 | 0.0000 | 3.5677e+07 | 1 |

**Adduct Signal Fractions**

- **Unmodified**: 3.5677e+07

### N(N)GTYDHDVYR Fuc 1 Hex 6 HexNAc 5

**Mass:**  3374.3143

(5.963e-04)

**Start Position**: 483

**End Position**: 493

| Profile Score | Tandem Score | TDA q-value | Volume | Spectrum Matches |
| --- | --- | --- | --- | --- |
| 0.0000 | 111.4503 | 0.0000 | 0.0000e+00 | 1 |

Adduct:

No Chromatogram Found

### YPALN(N)VTMPN(D)NEQFDK Hex 9 HexNAc 2

**Mass:**  3745.5038

(2.650e-04)

**Charge States:** 3

**Start Position**: 161

**End Position**: 177

| Profile Score | Tandem Score | TDA q-value | Volume | Spectrum Matches |
| --- | --- | --- | --- | --- |
| -6.0279 | 111.4152 | 0.0000 | 2.8096e+07 | 3 |

**Adduct Signal Fractions**

- **Unmodified**: 2.8096e+07

### N(N)GTYDHDVYR Fuc 2 Hex 9 HexNAc 3

**Mass:**  3598.3273

(-9.362e-06)

**Start Position**: 483

**End Position**: 493

| Profile Score | Tandem Score | TDA q-value | Volume | Spectrum Matches |
| --- | --- | --- | --- | --- |
| 0.0000 | 111.2537 | 0.0000 | 0.0000e+00 | 1 |

Adduct:

No Chromatogram Found

### WAGVTQN(N)GTSSSC(C)R Hex 7 HexNAc 2

**Mass:**  3050.1958

(1.406e-06)

**Charge States:** 3

**Start Position**: 127

**End Position**: 141

| Profile Score | Tandem Score | TDA q-value | Volume | Spectrum Matches |
| --- | --- | --- | --- | --- |
| -16.5874 | 110.8946 | 0.0000 | 9.3219e+06 | 1 |

**Adduct Signal Fractions**

- **Unmodified**: 9.3219e+06

### N(N)GTYDHDVYR Fuc 1 Hex 4 HexNAc 3

**Mass:**  2642.0410

(1.010e-06)

**Charge States:** 3

**Start Position**: 483

**End Position**: 493

| Profile Score | Tandem Score | TDA q-value | Volume | Spectrum Matches |
| --- | --- | --- | --- | --- |
| -20.2838 | 109.6702 | 0.0000 | 1.6604e+07 | 1 |

**Adduct Signal Fractions**

- **Unmodified**: 1.6604e+07

### N(N)GTYDHDVYR Hex 5 HexNAc 2

**Mass:**  2454.9554

(3.773e-07)

**Charge States:** 3

**Start Position**: 483

**End Position**: 493

| Profile Score | Tandem Score | TDA q-value | Volume | Spectrum Matches |
| --- | --- | --- | --- | --- |
| -20.2836 | 109.1118 | 0.0000 | 1.1299e+07 | 1 |

**Adduct Signal Fractions**

- **Unmodified**: 1.1299e+07

### N(N)GTYDHDVYRDEALN(D)NR Fuc 2 Hex 5 HexNAc 4

**Mass:**  3966.5969

(2.502e-04)

**Charge States:** 3, 4

**Start Position**: 483

**End Position**: 500

| Profile Score | Tandem Score | TDA q-value | Volume | Spectrum Matches |
| --- | --- | --- | --- | --- |
| 8.1900 | 109.1029 | 0.0000 | 7.2759e+08 | 6 |

**Adduct Signal Fractions**

- **Unmodified**: 7.2759e+08

### N(N)GTYDHDVYRDEALN(D)NR Fuc 1 Hex 4 HexNAc 3

**Mass:**  3455.4079

(2.870e-04)

**Charge States:** 4

**Start Position**: 483

**End Position**: 500

| Profile Score | Tandem Score | TDA q-value | Volume | Spectrum Matches |
| --- | --- | --- | --- | --- |
| -6.4480 | 109.0808 | 0.0000 | 4.7427e+07 | 1 |

**Adduct Signal Fractions**

- **Unmodified**: 4.7427e+07

### ISIYWTIVKPGDILLIN(N)STGNLIAPR Hex 4 HexNAc 6

**Mass:**  4733.2810

(-8.136e-06)

**Charge States:** 4

**Start Position**: 230

**End Position**: 256

| Profile Score | Tandem Score | TDA q-value | Volume | Spectrum Matches |
| --- | --- | --- | --- | --- |
| -20.1126 | 108.8940 | 0.0000 | 9.4052e+06 | 32 |

**Adduct Signal Fractions**

- **Unmodified**: 9.4052e+06

### N(N)GTYDHDVYR Hex 5 HexNAc 4

**Mass:**  2861.1156

(8.384e-07)

**Charge States:** 3

**Start Position**: 483

**End Position**: 493

| Profile Score | Tandem Score | TDA q-value | Volume | Spectrum Matches |
| --- | --- | --- | --- | --- |
| -18.8172 | 108.2574 | 0.0000 | 2.0067e+07 | 1 |

**Adduct Signal Fractions**

- **Unmodified**: 2.0067e+07

### N(N)GTYDHDVYRDEALN(D)NR Fuc 3 Hex 5 HexNAc 4

**Mass:**  4112.6491

(2.415e-04)

**Charge States:** 4

**Start Position**: 483

**End Position**: 500

| Profile Score | Tandem Score | TDA q-value | Volume | Spectrum Matches |
| --- | --- | --- | --- | --- |
| -5.6654 | 108.0952 | 0.0000 | 1.1300e+08 | 2 |

**Adduct Signal Fractions**

- **Unmodified**: 1.1300e+08

### N(N)GTYDHDVYR Fuc 1 Hex 9 HexNAc 5

**Mass:**  3858.4829

(5.961e-06)

**Charge States:** 5

**Start Position**: 483

**End Position**: 493

| Profile Score | Tandem Score | TDA q-value | Volume | Spectrum Matches |
| --- | --- | --- | --- | --- |
| -19.0138 | 107.7163 | 0.0000 | 5.2914e+07 | 2 |

**Adduct Signal Fractions**

- **Unmodified**: 5.2914e+07

### SEC(C)ITPN(N)GSIPN(D)DKPFQNVNR Hex 8 HexNAc 2

**Mass:**  4089.7294

(2.443e-04)

**Charge States:** 3, 4

**Start Position**: 279

**End Position**: 300

| Profile Score | Tandem Score | TDA q-value | Volume | Spectrum Matches |
| --- | --- | --- | --- | --- |
| 8.9812 | 106.8403 | 0.0000 | 7.7987e+07 | 3 |

**Adduct Signal Fractions**

- **Unmodified**: 7.7987e+07

### YPALN(N)VTMPN(D)NEQFDK Hex 8 HexNAc 2

**Mass:**  3583.4536

(2.777e-04)

**Charge States:** 3

**Start Position**: 161

**End Position**: 177

| Profile Score | Tandem Score | TDA q-value | Volume | Spectrum Matches |
| --- | --- | --- | --- | --- |
| -5.7862 | 106.2093 | 0.0000 | 2.6578e+07 | 2 |

**Adduct Signal Fractions**

- **Unmodified**: 2.6578e+07

### N(N)GTYDHDVYRDEALNNR Hex 7 HexNAc 3

**Mass:**  3794.5273

(2.646e-06)

**Charge States:** 4

**Start Position**: 483

**End Position**: 500

| Profile Score | Tandem Score | TDA q-value | Volume | Spectrum Matches |
| --- | --- | --- | --- | --- |
| -20.2030 | 105.5101 | 0.0000 | 3.1067e+07 | 1 |

**Adduct Signal Fractions**

- **Unmodified**: 3.1067e+07

### N(N)GTYDHDVYR Fuc 1 Hex 5 HexNAc 4 Neu5Ac 1

**Mass:**  3298.2680

(4.846e-07)

**Charge States:** 3

**Start Position**: 483

**End Position**: 493

| Profile Score | Tandem Score | TDA q-value | Volume | Spectrum Matches |
| --- | --- | --- | --- | --- |
| -20.4537 | 104.5453 | 0.0000 | 7.5754e+06 | 1 |

**Adduct Signal Fractions**

- **Unmodified**: 7.5754e+06

### N(N)GTYDHDVYR Fuc 1 Hex 6 HexNAc 3

**Mass:**  2966.1445

(-1.720e-08)

**Charge States:** 3

**Start Position**: 483

**End Position**: 493

| Profile Score | Tandem Score | TDA q-value | Volume | Spectrum Matches |
| --- | --- | --- | --- | --- |
| -18.2529 | 104.1991 | 0.0000 | 1.7155e+07 | 1 |

**Adduct Signal Fractions**

- **Unmodified**: 1.7155e+07

### N(N)GTYDHDVYRDEALN(D)NR Hex 6 HexNAc 3

**Mass:**  3633.4540

(2.724e-04)

**Charge States:** 4

**Start Position**: 483

**End Position**: 500

| Profile Score | Tandem Score | TDA q-value | Volume | Spectrum Matches |
| --- | --- | --- | --- | --- |
| 3.5050 | 103.9819 | 0.0000 | 1.5222e+08 | 2 |

**Adduct Signal Fractions**

- **Unmodified**: 1.5222e+08

### RGSN(N)SSFFSR Fuc 2 Hex 10 HexNAc 3 Neu5Ac 1

**Mass:**  3956.5317

(3.006e-06)

**Charge States:** 3, 4

**Start Position**: 141

**End Position**: 151

| Profile Score | Tandem Score | TDA q-value | Volume | Spectrum Matches |
| --- | --- | --- | --- | --- |
| 10.7965 | 103.4704 | 0.0000 | 2.6925e+08 | 4 |

**Adduct Signal Fractions**

- **Unmodified**: 2.6925e+08

### YPALN(N)VTM(O)PN(D)NEQFDK Hex 7 HexNAc 2

**Mass:**  3437.4094

(2.935e-04)

**Charge States:** 3, 4

**Start Position**: 161

**End Position**: 177

| Profile Score | Tandem Score | TDA q-value | Volume | Spectrum Matches |
| --- | --- | --- | --- | --- |
| 7.4892 | 103.3039 | 0.0000 | 1.2624e+07 | 1 |

**Adduct Signal Fractions**

- **Unmodified**: 1.2624e+07

### N(N)GTYDHDVYR Fuc 3 Hex 5 HexNAc 4

**Mass:**  3300.2965

(3.060e-04)

**Start Position**: 483

**End Position**: 493

| Profile Score | Tandem Score | TDA q-value | Volume | Spectrum Matches |
| --- | --- | --- | --- | --- |
| 0.0000 | 103.0744 | 0.0000 | 0.0000e+00 | 1 |

Adduct:

No Chromatogram Found

### N(N)GTYDHDVYR Hex 5 HexNAc 3

**Mass:**  2658.0333

(-2.426e-07)

**Charge States:** 3

**Start Position**: 483

**End Position**: 493

| Profile Score | Tandem Score | TDA q-value | Volume | Spectrum Matches |
| --- | --- | --- | --- | --- |
| -20.0964 | 100.5385 | 0.0000 | 7.4858e+06 | 1 |

**Adduct Signal Fractions**

- **Unmodified**: 7.4858e+06

### N(N)GTYDHDVYR Fuc 2 Hex 5 HexNAc 5

**Mass:**  3356.3087

(1.398e-07)

**Charge States:** 3

**Start Position**: 483

**End Position**: 493

| Profile Score | Tandem Score | TDA q-value | Volume | Spectrum Matches |
| --- | --- | --- | --- | --- |
| -20.9593 | 100.2689 | 0.0000 | 1.0792e+07 | 1 |

**Adduct Signal Fractions**

- **Unmodified**: 1.0792e+07

### YPALN(N)VTM(O)PNNEQFDK Hex 3 HexNAc 5 Neu5Ac 2

**Mass:**  3979.5847

(-8.482e-06)

**Charge States:** 3

**Start Position**: 161

**End Position**: 177

| Profile Score | Tandem Score | TDA q-value | Volume | Spectrum Matches |
| --- | --- | --- | --- | --- |
| 11.6033 | 99.6626 | 0.0000 | 2.3421e+07 | 1 |

**Adduct Signal Fractions**

- **Unmodified**: 2.3421e+07

### YPALN(N)VTM(O)PNNEQFDKLYIWGVHHPVTDK Hex 9 HexNAc 2

**Mass:**  5306.3394

(4.466e-06)

**Charge States:** 5

**Start Position**: 161

**End Position**: 190

| Profile Score | Tandem Score | TDA q-value | Volume | Spectrum Matches |
| --- | --- | --- | --- | --- |
| -17.8968 | 99.1724 | 0.0000 | 1.3640e+07 | 1 |

**Adduct Signal Fractions**

- **Unmodified**: 1.3640e+07

### IRDIPSRISIYWTIVKPGDILLIN(N)STGNLIAPR Hex 10 HexNAc 2

**Mass:**  5730.8157

(2.519e-06)

**Charge States:** 5

**Start Position**: 223

**End Position**: 256

| Profile Score | Tandem Score | TDA q-value | Volume | Spectrum Matches |
| --- | --- | --- | --- | --- |
| -19.2343 | 99.0035 | 0.0000 | 2.3318e+07 | 1 |

**Adduct Signal Fractions**

- **Unmodified**: 2.3318e+07

### RGSN(N)SSFFSR Fuc 1 Hex 7 HexNAc 7

**Mass:**  3845.5649

(9.552e-06)

**Charge States:** 3, 4

**Start Position**: 141

**End Position**: 151

| Profile Score | Tandem Score | TDA q-value | Volume | Spectrum Matches |
| --- | --- | --- | --- | --- |
| -18.3555 | 98.5018 | 0.0000 | 1.7213e+08 | 3 |

**Adduct Signal Fractions**

- **Unmodified**: 1.7213e+08

### N(N)GTYDHDVYRDEALNNR Hex 5 HexNAc 5

**Mass:**  3876.5781

(1.831e-06)

**Charge States:** 3, 4

**Start Position**: 483

**End Position**: 500

| Profile Score | Tandem Score | TDA q-value | Volume | Spectrum Matches |
| --- | --- | --- | --- | --- |
| 8.7000 | 98.4228 | 0.0000 | 9.9849e+08 | 4 |

**Adduct Signal Fractions**

- **Unmodified**: 9.9849e+08

### ISIYWTIVKPGDILLIN(N)STGN(D)LIAPR Hex 9 HexNAc 2

**Mass:**  4732.2756

(2.134e-04)

**Charge States:** 4

**Start Position**: 230

**End Position**: 256

| Profile Score | Tandem Score | TDA q-value | Volume | Spectrum Matches |
| --- | --- | --- | --- | --- |
| -5.4249 | 97.4610 | 0.0033 | 2.3319e+07 | 7 |

**Adduct Signal Fractions**

- **Unmodified**: 2.3319e+07

### YPALN(N)VTM(O)PNNEQFDKLYIWGVHHPVTDK Hex 8 HexNAc 2

**Mass:**  5144.2867

(4.609e-06)

**Charge States:** 5

**Start Position**: 161

**End Position**: 190

| Profile Score | Tandem Score | TDA q-value | Volume | Spectrum Matches |
| --- | --- | --- | --- | --- |
| -16.8051 | 96.9779 | 0.0033 | 1.0430e+07 | 1 |

**Adduct Signal Fractions**

- **Unmodified**: 1.0430e+07

### ISIYWTIVKPGDILLIN(N)STGNLIAPR Hex 3 HexNAc 4 Neu5Ac 2

**Mass:**  4747.2795

(-4.065e-06)

**Charge States:** 3, 4, 5

**Start Position**: 230

**End Position**: 256

| Profile Score | Tandem Score | TDA q-value | Volume | Spectrum Matches |
| --- | --- | --- | --- | --- |
| -18.7047 | 95.0313 | 0.0066 | 2.9332e+07 | 2 |

**Adduct Signal Fractions**

- **Unmodified**: 2.9332e+07

### RGSN(N)SSFFSR Fuc 1 Hex 10 HexNAc 6

**Mass:**  4128.6125

(1.899e-06)

**Charge States:** 4

**Start Position**: 141

**End Position**: 151

| Profile Score | Tandem Score | TDA q-value | Volume | Spectrum Matches |
| --- | --- | --- | --- | --- |
| -20.1187 | 93.4779 | 0.0098 | 1.8546e+07 | 1 |

**Adduct Signal Fractions**

- **Unmodified**: 1.8546e+07

### LNWLTHLNSKYPALN(N)VTM(O)PNNEQFDK Hex 8 HexNAc 2

**Mass:**  4805.1159

(4.803e-06)

**Charge States:** 4, 5

**Start Position**: 151

**End Position**: 177

| Profile Score | Tandem Score | TDA q-value | Volume | Spectrum Matches |
| --- | --- | --- | --- | --- |
| -18.8757 | 92.7037 | 0.0098 | 2.3675e+07 | 2 |

**Adduct Signal Fractions**

- **Unmodified**: 2.3675e+07

### SEC(C)ITPN(N)GSIPN(D)DKPFQNVNR Hex 7 HexNAc 2

**Mass:**  3927.6674

(2.544e-04)

**Charge States:** 3, 4

**Start Position**: 279

**End Position**: 300

| Profile Score | Tandem Score | TDA q-value | Volume | Spectrum Matches |
| --- | --- | --- | --- | --- |
| 10.2565 | 92.6455 | 0.0129 | 9.1709e+07 | 3 |

**Adduct Signal Fractions**

- **Unmodified**: 9.1709e+07

### N(N)GTYDHDVYRDEALNNR Fuc 1 Hex 4 HexNAc 4

**Mass:**  3657.4977

(4.782e-07)

**Charge States:** 4

**Start Position**: 483

**End Position**: 500

| Profile Score | Tandem Score | TDA q-value | Volume | Spectrum Matches |
| --- | --- | --- | --- | --- |
| -5.7871 | 92.1714 | 0.0129 | 3.2824e+07 | 3 |

**Adduct Signal Fractions**

- **Unmodified**: 3.2824e+07

### N(N)GTYDHDVYRDEALN(D)NR Fuc 1 Hex 8 HexNAc 2

**Mass:**  3900.4963

(2.430e-04)

**Charge States:** 3, 4

**Start Position**: 483

**End Position**: 500

| Profile Score | Tandem Score | TDA q-value | Volume | Spectrum Matches |
| --- | --- | --- | --- | --- |
| -20.6708 | 91.4541 | 0.0162 | 1.3830e+08 | 1 |

**Adduct Signal Fractions**

- **Unmodified**: 1.3830e+08

### N(N)GTYDHDVYR Hex 5 HexNAc 6

**Mass:**  3267.2730

(1.648e-06)

**Charge States:** 3, 4

**Start Position**: 483

**End Position**: 493

| Profile Score | Tandem Score | TDA q-value | Volume | Spectrum Matches |
| --- | --- | --- | --- | --- |
| -19.5424 | 89.7919 | 0.0194 | 2.5232e+07 | 2 |

**Adduct Signal Fractions**

- **Unmodified**: 2.5232e+07

### RGSN(N)SSFFSR Fuc 1 Hex 6 HexNAc 7 Neu5Ac 2

**Mass:**  4265.6345

(-6.728e-06)

**Charge States:** 4

**Start Position**: 141

**End Position**: 151

| Profile Score | Tandem Score | TDA q-value | Volume | Spectrum Matches |
| --- | --- | --- | --- | --- |
| -4.8370 | 89.0467 | 0.0225 | 9.1598e+07 | 1 |

**Adduct Signal Fractions**

- **Unmodified**: 9.1598e+07

### SEC(C)ITPN(N)GSIPN(D)DKPFQNVNR Hex 9 HexNAc 2

**Mass:**  4251.7717

(2.359e-04)

**Charge States:** 3, 4

**Start Position**: 279

**End Position**: 300

| Profile Score | Tandem Score | TDA q-value | Volume | Spectrum Matches |
| --- | --- | --- | --- | --- |
| 8.0545 | 88.7288 | 0.0256 | 1.6889e+08 | 5 |

**Adduct Signal Fractions**

- **Unmodified**: 1.6889e+08

### SLVASSGTLEFNNESFNWAGVTQN(N)GTSSSC(C)RR Hex 7 HexNAc 2

**Mass:**  5003.1389

(4.943e-06)

**Charge States:** 4

**Start Position**: 110

**End Position**: 142

| Profile Score | Tandem Score | TDA q-value | Volume | Spectrum Matches |
| --- | --- | --- | --- | --- |
| -19.8309 | 85.9221 | 0.0376 | 4.0109e+06 | 3 |

**Adduct Signal Fractions**

- **Unmodified**: 4.0109e+06

### IRDIPSRISIYWTIVKPGDILLIN(N)STGNLIAPR Hex 8 HexNAc 2

**Mass:**  5406.7062

(8.472e-07)

**Charge States:** 5

**Start Position**: 223

**End Position**: 256

| Profile Score | Tandem Score | TDA q-value | Volume | Spectrum Matches |
| --- | --- | --- | --- | --- |
| -4.3808 | 85.5472 | 0.0376 | 2.2396e+07 | 3 |

**Adduct Signal Fractions**

- **Unmodified**: 2.2396e+07

### RGSN(N)SSFFSR Fuc 3 Hex 5 HexNAc 5

**Mass:**  3407.3827

(1.891e-06)

**Charge States:** 3

**Start Position**: 141

**End Position**: 151

| Profile Score | Tandem Score | TDA q-value | Volume | Spectrum Matches |
| --- | --- | --- | --- | --- |
| -18.3327 | 84.6941 | 0.0408 | 1.1973e+07 | 2 |

**Adduct Signal Fractions**

- **Unmodified**: 1.1973e+07

### N(N)GTYDHDVYR Fuc 1 Hex 5 HexNAc 4 Neu5Ac 1

**Mass:**  3300.2761

(6.093e-04)

**Start Position**: 483

**End Position**: 493

| Profile Score | Tandem Score | TDA q-value | Volume | Spectrum Matches |
| --- | --- | --- | --- | --- |
| 0.0000 | 84.4869 | 0.0437 | 0.0000e+00 | 1 |

Adduct:

No Chromatogram Found

### RGSN(N)SSFFSR Fuc 1 Hex 7 HexNAc 6

**Mass:**  3643.4503

(2.757e-04)

**Start Position**: 141

**End Position**: 151

| Profile Score | Tandem Score | TDA q-value | Volume | Spectrum Matches |
| --- | --- | --- | --- | --- |
| 0.0000 | 83.6938 | 0.0467 | 0.0000e+00 | 1 |

Adduct:

No Chromatogram Found

### N(N)GTYDHDVYR Fuc 1 Hex 6 HexNAc 6

**Mass:**  3575.3915

(2.450e-06)

**Charge States:** 4

**Start Position**: 483

**End Position**: 493

| Profile Score | Tandem Score | TDA q-value | Volume | Spectrum Matches |
| --- | --- | --- | --- | --- |
| -20.3846 | 82.8713 | 0.0494 | 1.2877e+07 | 1 |

**Adduct Signal Fractions**

- **Unmodified**: 1.2877e+07

### TIVKPGDILLIN(N)STGNLIAPR Hex 8 HexNAc 2

**Mass:**  3906.8884

(4.538e-06)

**Charge States:** 3, 4

**Start Position**: 235

**End Position**: 256

| Profile Score | Tandem Score | TDA q-value | Volume | Spectrum Matches |
| --- | --- | --- | --- | --- |
| -19.4135 | 82.4034 | 0.0494 | 2.4989e+07 | 2 |

**Adduct Signal Fractions**

- **Unmodified**: 2.4989e+07

### YPALN(N)VTM(O)PNNEQFDK Fuc 1 Hex 3 HexNAc 5 Neu5Ac 1

**Mass:**  3834.6000

(-1.979e-06)

**Charge States:** 3

**Start Position**: 161

**End Position**: 177

| Profile Score | Tandem Score | TDA q-value | Volume | Spectrum Matches |
| --- | --- | --- | --- | --- |
| -4.8062 | 82.0642 | 0.0494 | 1.6040e+07 | 2 |

**Adduct Signal Fractions**

- **Unmodified**: 1.6040e+07

### cus|SWZNA|A/Switzerland/9715293/2013

**>cus|SWZNA|A/Switzerland/9715293/2013**  
 `MNPNQKIITIGSVSLTISTICFFMQIAILITTVTLHFKQYEFNSPPNNQVMLCEPTIIERNITEIVYLTNTTIEKEICPK  
PAEYRNWSKPQCGITGFAPFSKDNSIRLSAGGDIWVTREPYVSCDPDKCYQFALGQGTTLNNVHSNNTVRDRTPYRTLLM  
NELGVPFHLGTKQVCIAWSSSSCHDGKAWLHVCITGDDKNATASFIYNGRLVDSVVSWSKDILRTQESECVCINGTCTVV  
MTDGSASGKADTKILFIEEGKIVHTSTLSGSAQHVEECSCYPRYPGVRCVCRDNWKGSNRPIVDINIKDHSIVSSYVCSG  
LVGDTPRKNDSSSSSHCLDPNNEEGGHGVKGWAFDDGNDVWMGRTINETSRLGYETFKVIEGWSNPKSKLQTNRQVIVDR  
GDRSGYSGIFSVEGKSCINRCFYVELIRGRKEETEVLWTSNSIVVFCGTSGTYGTGSWPDGADLNLMPI`

| Site | Type | Count |
| --- | --- | --- |
| 329 | N-linked | 3 |
| 86 | N-linked | 8 |
| 200 | N-linked | 2 |

| Mass | Sequence | MS_2_ Score | `q`-value | MS_1_ Score | Signal | Protein Coordinates | Tandem Scans | Start Time | Apex Time | End Time |
| --- | --- | --- | --- | --- | --- | --- | --- | --- | --- | --- |
| 3788.5757 | N(N)WSKPQC(C)GITGFAPFSK Hex 9 HexNAc 2 | 158.620 | 0.0 | -1.327 | 5.802e+08 | 85 - 102 | 4 | 42.88 | 43.04 | 43.21 |
| 3626.5229 | N(N)WSKPQC(C)GITGFAPFSK Hex 8 HexNAc 2 | 157.978 | 0.0 | -1.166 | 5.769e+08 | 85 - 102 | 4 | 43.04 | 43.21 | 43.55 |
| 3411.3961 | N(N)WSKPQC(C)GITGFAPF Hex 8 HexNAc 2 | 157.296 | 0.0 | -2.783 | 1.049e+08 | 85 - 100 | 4 | 51.59 | 51.76 | 51.93 |
| 3573.4481 | N(N)WSKPQC(C)GITGFAPF Hex 9 HexNAc 2 | 145.324 | 0.0 | -16.840 | 1.268e+08 | 85 - 100 | 4 | 51.59 | 51.59 | 51.76 |
| 4472.9081 | AWLHVC(C)ITGDDKN(N)ATASFIYNGR Hex 9 HexNAc 2 | 144.817 | 0.0 | -20.047 | 3.833e+06 | 187 - 210 | 1 | 46.33 | 46.33 | 46.50 |
| 3670.4705 | KN(N)DSSSSSHC(C)LDPNNEEGGHGVK Hex 5 HexNAc 2 | 136.506 | 0.0 | -20.434 | 3.847e+07 | 327 - 350 | 2 | 14.32 | 14.48 | 14.48 |
| 3590.4699 | N(N)WSKPQC(C)GITGFAPF Fuc 2 Hex 3 HexNAc 4 Neu5Ac 1 | 133.961 | 0.0 | -18.081 | 1.130e+07 | 85 - 100 | 1 | 51.59 | 51.59 | 51.76 |
| 3464.4710 | N(N)WSKPQC(C)GITGFAPFSK Hex 7 HexNAc 2 | 130.819 | 0.0 | -4.119 | 4.017e+07 | 85 - 102 | 2 | 43.04 | 43.21 | 43.38 |
| 4281.7133 | KN(N)DSSSSSHC(C)LDPNNEEGGHGVK Hex 5 HexNAc 5 | 128.169 | 0.0 | 0.000 | 0.000e+00 | 327 - 350 | 2 | - | - | - |
| 3249.3427 | N(N)WSKPQC(C)GITGFAPF Hex 7 HexNAc 2 | 127.115 | 0.0 | -18.990 | 6.464e+06 | 85 - 100 | 1 | 51.93 | 51.93 | 52.10 |
| 4310.8546 | AWLHVC(C)ITGDDKN(N)ATASFIYNGR Hex 8 HexNAc 2 | 124.673 | 0.0 | -5.400 | 3.431e+06 | 187 - 210 | 1 | 46.33 | 46.50 | 46.67 |
| 3140.3769 | N(N)WSKPQC(C)GITGFAPFSK Hex 5 HexNAc 2 | 115.680 | 0.0 | -17.372 | 1.561e+06 | 85 - 102 | 1 | 43.72 | 43.72 | 43.72 |
| 4359.6643 | KN(N)DSSSSSHC(C)LDPNNEEGGHGVK Hex 8 HexNAc 3 | 111.559 | 0.0 | -19.804 | 9.391e+06 | 327 - 350 | 1 | 14.81 | 14.81 | 14.81 |

### N(N)WSKPQC(C)GITGFAPFSK Hex 9 HexNAc 2

**Mass:**  3788.5724

(4.816e-06)

**Charge States:** 3, 4

**Start Position**: 86

**End Position**: 103

| Profile Score | Tandem Score | TDA q-value | Volume | Spectrum Matches |
| --- | --- | --- | --- | --- |
| -1.3269 | 158.6205 | 0.0000 | 5.8025e+08 | 4 |

**Adduct Signal Fractions**

- **Unmodified**: 5.8025e+08

### N(N)WSKPQC(C)GITGFAPFSK Hex 8 HexNAc 2

**Mass:**  3626.5189

(3.262e-06)

**Charge States:** 3, 4

**Start Position**: 86

**End Position**: 103

| Profile Score | Tandem Score | TDA q-value | Volume | Spectrum Matches |
| --- | --- | --- | --- | --- |
| -1.1659 | 157.9780 | 0.0000 | 5.7692e+08 | 4 |

**Adduct Signal Fractions**

- **Unmodified**: 5.7692e+08

### N(N)WSKPQC(C)GITGFAPF Hex 8 HexNAc 2

**Mass:**  3411.3971

(3.732e-06)

**Charge States:** 4

**Start Position**: 86

**End Position**: 101

| Profile Score | Tandem Score | TDA q-value | Volume | Spectrum Matches |
| --- | --- | --- | --- | --- |
| -2.7833 | 157.2956 | 0.0000 | 1.0490e+08 | 4 |

**Adduct Signal Fractions**

- **Unmodified**: 1.0490e+08

### N(N)WSKPQC(C)GITGFAPF Hex 9 HexNAc 2

**Mass:**  3573.4488

(3.177e-06)

**Charge States:** 2, 3, 4

**Start Position**: 86

**End Position**: 101

| Profile Score | Tandem Score | TDA q-value | Volume | Spectrum Matches |
| --- | --- | --- | --- | --- |
| -16.8396 | 145.3240 | 0.0000 | 1.2682e+08 | 4 |

**Adduct Signal Fractions**

- **Unmodified**: 1.2682e+08

### AWLHVC(C)ITGDDKN(N)ATASFIYNGR Hex 9 HexNAc 2

**Mass:**  4472.9060

(5.018e-06)

**Charge States:** 4

**Start Position**: 188

**End Position**: 211

| Profile Score | Tandem Score | TDA q-value | Volume | Spectrum Matches |
| --- | --- | --- | --- | --- |
| -20.0470 | 144.8172 | 0.0000 | 3.8335e+06 | 1 |

**Adduct Signal Fractions**

- **Unmodified**: 3.8335e+06

### KN(N)DSSSSSHC(C)LDPNNEEGGHGVK Hex 5 HexNAc 2

**Mass:**  3670.4739

(-9.941e-07)

**Charge States:** 5

**Start Position**: 328

**End Position**: 351

| Profile Score | Tandem Score | TDA q-value | Volume | Spectrum Matches |
| --- | --- | --- | --- | --- |
| -20.4343 | 136.5057 | 0.0000 | 3.8472e+07 | 2 |

**Adduct Signal Fractions**

- **Unmodified**: 3.8472e+07

### N(N)WSKPQC(C)GITGFAPF Fuc 2 Hex 3 HexNAc 4 Neu5Ac 1

**Mass:**  3590.4688

(-6.011e-06)

**Charge States:** 3

**Start Position**: 86

**End Position**: 101

| Profile Score | Tandem Score | TDA q-value | Volume | Spectrum Matches |
| --- | --- | --- | --- | --- |
| -18.0808 | 133.9612 | 0.0000 | 1.1299e+07 | 1 |

**Adduct Signal Fractions**

- **Unmodified**: 1.1299e+07

### N(N)WSKPQC(C)GITGFAPFSK Hex 7 HexNAc 2

**Mass:**  3464.4688

(2.965e-06)

**Charge States:** 4

**Start Position**: 86

**End Position**: 103

| Profile Score | Tandem Score | TDA q-value | Volume | Spectrum Matches |
| --- | --- | --- | --- | --- |
| -4.1191 | 130.8186 | 0.0000 | 4.0171e+07 | 2 |

**Adduct Signal Fractions**

- **Unmodified**: 4.0171e+07

### KN(N)DSSSSSHC(C)LDPNNEEGGHGVK Hex 5 HexNAc 5

**Mass:**  4281.7133

(4.687e-04)

**Start Position**: 328

**End Position**: 351

| Profile Score | Tandem Score | TDA q-value | Volume | Spectrum Matches |
| --- | --- | --- | --- | --- |
| 0.0000 | 128.1688 | 0.0000 | 0.0000e+00 | 2 |

Adduct:

No Chromatogram Found

### N(N)WSKPQC(C)GITGFAPF Hex 7 HexNAc 2

**Mass:**  3249.3426

(3.352e-06)

**Charge States:** 3

**Start Position**: 86

**End Position**: 101

| Profile Score | Tandem Score | TDA q-value | Volume | Spectrum Matches |
| --- | --- | --- | --- | --- |
| -18.9905 | 127.1152 | 0.0000 | 6.4644e+06 | 1 |

**Adduct Signal Fractions**

- **Unmodified**: 6.4644e+06

### AWLHVC(C)ITGDDKN(N)ATASFIYNGR Hex 8 HexNAc 2

**Mass:**  4310.8566

(7.343e-06)

**Charge States:** 4

**Start Position**: 188

**End Position**: 211

| Profile Score | Tandem Score | TDA q-value | Volume | Spectrum Matches |
| --- | --- | --- | --- | --- |
| -5.4001 | 124.6728 | 0.0000 | 3.4306e+06 | 1 |

**Adduct Signal Fractions**

- **Unmodified**: 3.4306e+06

### N(N)WSKPQC(C)GITGFAPFSK Hex 5 HexNAc 2

**Mass:**  3140.3769

(7.533e-06)

**Charge States:** 3

**Start Position**: 86

**End Position**: 103

| Profile Score | Tandem Score | TDA q-value | Volume | Spectrum Matches |
| --- | --- | --- | --- | --- |
| -17.3716 | 115.6799 | 0.0000 | 1.5613e+06 | 1 |

**Adduct Signal Fractions**

- **Unmodified**: 1.5613e+06

### KN(N)DSSSSSHC(C)LDPNNEEGGHGVK Hex 8 HexNAc 3

**Mass:**  4359.6643

(-9.875e-06)

**Charge States:** 4

**Start Position**: 328

**End Position**: 351

| Profile Score | Tandem Score | TDA q-value | Volume | Spectrum Matches |
| --- | --- | --- | --- | --- |
| -19.8042 | 111.5588 | 0.0000 | 9.3906e+06 | 1 |

**Adduct Signal Fractions**

- **Unmodified**: 9.3906e+06

### tr|E1C041|E1C041\_CHICK

**>tr|E1C041|E1C041\_CHICK**  
 `MRTGAGEAVGVLCLVSLVAALDYKYHHSEELEAYLKEVHAAYPALTHLHSIGRSVEGRDLWVLVLGRFPTQHKIGIPEFK  
YVANMHGDETVGREILLHLIDHLVTNYGRDPVITRLLNNTRIHIMPTMNPDGFEATVVPDCYYSRGRYNKNGEDLNRNFP  
DAFENNNNLIQPETQAVINWIKNETFVLSANLHGGALVASYTFDNGNSVTGTSNGYSRSPDDDVFIHLAKTYSFNHASMY  
KGTGCDSKQTFPDGITNGYSWYQLEGGMQDYNYVWGQCFEITLELSCCKYPPAEQLEKFWRDNKVALVEYIKQVHLGVKG  
QVTDKNGNPIPNAIVEAKGRPHICPYRTNEHGEYFLLLLPGKYVINATVPGFKSMLKTVDIPDNTANFSALKQDFSFPEV  
SVKPRPASCPRTPLYQELERSSAAVKPTLHILVLVIVTISILK`

| Site | Type | Count |
| --- | --- | --- |
| 366 | N-linked | 3 |

| Mass | Sequence | MS_2_ Score | `q`-value | MS_1_ Score | Signal | Protein Coordinates | Tandem Scans | Start Time | Apex Time | End Time |
| --- | --- | --- | --- | --- | --- | --- | --- | --- | --- | --- |
| 2910.2560 | YVIN(N)ATVPGFK Hex 8 HexNAc 2 | 171.986 | 0.0 | -17.744 | 1.209e+07 | 362 - 373 | 1 | 40.86 | 40.86 | 41.03 |
| 3072.3099 | YVIN(N)ATVPGFK Hex 9 HexNAc 2 | 155.392 | 0.0 | -1.736 | 3.899e+07 | 362 - 373 | 3 | 40.70 | 40.86 | 41.03 |
| 3089.3369 | YVIN(N)ATVPGFK Fuc 2 Hex 3 HexNAc 4 Neu5Ac 1 | 125.933 | 0.0 | -17.487 | 2.805e+06 | 362 - 373 | 1 | 40.86 | 40.86 | 40.86 |

### YVIN(N)ATVPGFK Hex 8 HexNAc 2

**Mass:**  2910.2589

(5.997e-06)

**Charge States:** 3

**Start Position**: 363

**End Position**: 374

| Profile Score | Tandem Score | TDA q-value | Volume | Spectrum Matches |
| --- | --- | --- | --- | --- |
| -17.7440 | 171.9859 | 0.0000 | 1.2088e+07 | 1 |

**Adduct Signal Fractions**

- **Unmodified**: 1.2088e+07

### YVIN(N)ATVPGFK Hex 9 HexNAc 2

**Mass:**  3072.3107

(5.760e-06)

**Charge States:** 3

**Start Position**: 363

**End Position**: 374

| Profile Score | Tandem Score | TDA q-value | Volume | Spectrum Matches |
| --- | --- | --- | --- | --- |
| -1.7361 | 155.3924 | 0.0000 | 3.8987e+07 | 3 |

**Adduct Signal Fractions**

- **Unmodified**: 3.8987e+07

### YVIN(N)ATVPGFK Fuc 2 Hex 3 HexNAc 4 Neu5Ac 1

**Mass:**  3089.3369

(-3.344e-06)

**Charge States:** 3

**Start Position**: 363

**End Position**: 374

| Profile Score | Tandem Score | TDA q-value | Volume | Spectrum Matches |
| --- | --- | --- | --- | --- |
| -17.4865 | 125.9331 | 0.0000 | 2.8045e+06 | 1 |

**Adduct Signal Fractions**

- **Unmodified**: 2.8045e+06

### tr|A0A1D5PNI3|A0A1D5PNI3\_CHICK

**>tr|A0A1D5PNI3|A0A1D5PNI3\_CHICK**  
 `MTTSTLAQTTSLPGTSTPTPASSTLMTTSTPAQTSLMPGHFKAQLLHLLHFTTTSTPAQTTSVPASSSPTPASSALPTAT  
TPAQTPGTSSPTPASSALPTTTTPAETTTFPATSSTTPASSAPPTTSTPAQTTSLPRTSTPTPASSALTTTTTPEQTTTV  
PGTSTPTPASSTVLTTSTPVQTTSMPGISSPTPASSALLTKSTPAQTTTVPATSSPTPASSALPTTTTPEQTTTESGTST  
PTPASSGPPTTSTPAETTSFPATSSPTPASSTLMTTSTPAQTTTVPGTSSPTPVSSALLTTRTLAQTKASLQLNSSPTSV  
SSALSTTSTPAETTTVPGTSIPTAASSTLLTTSTLAQTTSFPATSTPTPSSSTLMTTSTRAETKTSLQLNSSPTPASLAL  
PTTSTPAQTTSVPTSSSPTPASSALSTTSTPALTTALRSTSQIMSPSSPATSPSPTATSAGTVTTAQETTSPAGTTNALT  
STIRTTSTQTLSSSSPPYTTAALSYTAIPTSTPATPTTSAPQTTIASSSTPTESTALLTTTVPSTTQDISSQTAQTSNAA  
TSSTTNSATSARLTRTHAETTSSTTTFTSHSRPSASQTSTPGTTISPLPATTAQQTTAPAGTTPPTTASTTLFSTRETSS  
KEEETSTVVTSSQTPASSVLPSTSTPAQTTSMPSTSSPTPASSTLMTTSTLAQTTSLPGTSVPTPASSAPRTTTTPAQTT  
SVPGNSSPIPPYSTLLTTSTLAQTTSLPGTSTPTPASSTLMTTSTPAETKTSLLLNSSPNICIFCSSDYNHSCTDNISAR  
HFKPNSCTAIKHSCRKQTFITTFTSRARTSASWTTTPGTTTSALTATTARETTTPAGTTHPHNSNIETTSTSTIKAPTRP  
PTTPAGHERFTVNFTITNLPYDSDLQIPHSAKLNATQRVMTTLLNRLLKDTSIGPSFLGCATTAFR`

| Site | Type | Count |
| --- | --- | --- |
| 893 | N-linked | 2 |

| Mass | Sequence | MS_2_ Score | `q`-value | MS_1_ Score | Signal | Protein Coordinates | Tandem Scans | Start Time | Apex Time | End Time |
| --- | --- | --- | --- | --- | --- | --- | --- | --- | --- | --- |
| 4160.8580 | FTVN(N)FTITNLPYDSDLQIPHSAK Hex 7 HexNAc 2 | 123.220 | 0.0 | -18.347 | 3.646e+07 | 889 - 912 | 2 | 58.05 | 58.05 | 58.22 |
| 3998.8013 | FTVN(N)FTITNLPYDSDLQIPHSAK Hex 6 HexNAc 2 | 107.263 | 0.0 | -17.726 | 1.317e+07 | 889 - 912 | 2 | 58.22 | 58.22 | 58.39 |

### FTVN(N)FTITNLPYDSDLQIPHSAK Hex 7 HexNAc 2

**Mass:**  4160.8601

(3.384e-06)

**Charge States:** 3, 4

**Start Position**: 890

**End Position**: 913

| Profile Score | Tandem Score | TDA q-value | Volume | Spectrum Matches |
| --- | --- | --- | --- | --- |
| -18.3471 | 123.2201 | 0.0000 | 3.6464e+07 | 2 |

**Adduct Signal Fractions**

- **Unmodified**: 3.6464e+07

### FTVN(N)FTITNLPYDSDLQIPHSAK Hex 6 HexNAc 2

**Mass:**  3998.8015

(2.116e-06)

**Charge States:** 4

**Start Position**: 890

**End Position**: 913

| Profile Score | Tandem Score | TDA q-value | Volume | Spectrum Matches |
| --- | --- | --- | --- | --- |
| -17.7256 | 107.2631 | 0.0000 | 1.3170e+07 | 2 |

**Adduct Signal Fractions**

- **Unmodified**: 1.3170e+07

### tr|A0A1D5PUR1|A0A1D5PUR1\_CHICK

**>tr|A0A1D5PUR1|A0A1D5PUR1\_CHICK**  
 `MTTSTLAQTTSLPGTSTPTPASSTLMTTSTPAQTSLMPGHFKAQLLHLLHFTTTSTPAQTTSVPASSSPTPASSALPTAT  
TPAQTPGTSSPTPASSALPTTTTPAETTTFPATSSTTPASSAPPTTSTPAQTTSLPRTSTPTPASSALTTTTTPEQTTTV  
PGTSTPTPASSTVLTTSTPVQTTSMPGISSPTPASSALLTKSTPAQTTTVPATSSPTPASSALPTTTTPEQTTTESGTST  
PTPASSGPPTTSTPAETTSVPGTSYPNPASSSLMTTSTLAQTTSLPATSTPSLASSTLMTKSTFAPTTALPGTSTPTPAS  
SAPPTTSTPAQTTSVPGTSTATSASSTLLTTSTPAQTTSFPATSSPTPASSTLMTTSTPAQTTTVPGTSSPTPVSSALLT  
TRTLAQTKASLQLNSSPTSVSSALSTTSTPAETTTVPGTSIPTAASSTLLTTSTLAQTTSFPATSTPTPSSSTLMTTSTR  
AETKTSLQLNSSPTPASLALPTTSTPAQTTSVPTSSSPTPASSALSTTSTPALTTALRSTSQIMSPSSPATSPSPTATSA  
GTVTTAQETTSPAGTTNALTSTIRTTSTQTLSSSSPPYTTAALSYTAIPTSTPATPTTSAPQTTIASSSTPTESTALLTT  
TVPSTTQDISSQTAQTSNAATSSTTNSATSARLTRTHAETTSSTTTFTSHSRPSASQTSTPGTTISPLPATTAQQTTAPA  
GTTPPTTASTTLFSTRETSSKEEETSTVVTSSQTPASSVLPSTSTPAQTTSMPSTSSPTPASSTLMTTSTLAQTTSLPGT  
SVPTPASSAPRTTTTPAQTTSVPGNSSPIPPYSTLLTTSTLAQTTSLPGTSTPTPASSTLMTTSTPAETKTSLLLNSSPN  
ICIFCSSDYNHSCTDNISARHFKPNSCTAIKHSCRKQTFITTFTSRARTSASWTTTPGTTTSALTATTARETTTPAGTTH  
PHNSNIETTSTSTIKAPTRPPTTPAGHERFTVNFTITNLPYDSDLQIPHSAKLNATQRVMTTLLNRLLKDTSIGPSFLGC  
ATTAFR`

| Site | Type | Count |
| --- | --- | --- |
| 993 | N-linked | 2 |

| Mass | Sequence | MS_2_ Score | `q`-value | MS_1_ Score | Signal | Protein Coordinates | Tandem Scans | Start Time | Apex Time | End Time |
| --- | --- | --- | --- | --- | --- | --- | --- | --- | --- | --- |
| 4160.8580 | FTVN(N)FTITNLPYDSDLQIPHSAK Hex 7 HexNAc 2 | 123.220 | 0.0 | -18.347 | 3.646e+07 | 989 - 1012 | 2 | 58.05 | 58.05 | 58.22 |
| 3998.8013 | FTVN(N)FTITNLPYDSDLQIPHSAK Hex 6 HexNAc 2 | 107.263 | 0.0 | -17.726 | 1.317e+07 | 989 - 1012 | 2 | 58.22 | 58.22 | 58.39 |

### FTVN(N)FTITNLPYDSDLQIPHSAK Hex 7 HexNAc 2

**Mass:**  4160.8601

(3.384e-06)

**Charge States:** 3, 4

**Start Position**: 990

**End Position**: 1013

| Profile Score | Tandem Score | TDA q-value | Volume | Spectrum Matches |
| --- | --- | --- | --- | --- |
| -18.3471 | 123.2201 | 0.0000 | 3.6464e+07 | 2 |

**Adduct Signal Fractions**

- **Unmodified**: 3.6464e+07

### FTVN(N)FTITNLPYDSDLQIPHSAK Hex 6 HexNAc 2

**Mass:**  3998.8015

(2.116e-06)

**Charge States:** 4

**Start Position**: 990

**End Position**: 1013

| Profile Score | Tandem Score | TDA q-value | Volume | Spectrum Matches |
| --- | --- | --- | --- | --- |
| -17.7256 | 107.2631 | 0.0000 | 1.3170e+07 | 2 |

**Adduct Signal Fractions**

- **Unmodified**: 1.3170e+07

### tr|F1NUU6|F1NUU6\_CHICK

**>tr|F1NUU6|F1NUU6\_CHICK**  
 `MGCSTILVFLTLLSFASAHRKLLLFLLDGFRFDYIDDAELESLPGFRDIIGMGVKVDYMTPDFPSLSYPNYYTLMTGRHC  
EVHQMIGNYMWDPKTNVSFDIGVNKESLLPLWWNGSEPLWVTMTKEKKNVFMYYWPGCEVEILGVRPSYCREYFSVPTDQ  
NFADAISDALEALRNGSAEMAAVYYERIDVEGHHYGPWSEQRKSALKELDKAMSNMITQIKNKGLQHDVNVLLFSDHGMT  
DISWTDKVIELKNYINMSDTIQMKDRGPVVSLWPVPEKHTEIYQKLKAVEHMNVYNIQDIPERFFYKKGKFVSPLTLVAE  
EGWFIVESRDKLPFWENGTGKREAWQNGWHGYDNELMEMRGIFLAYGPDFRSNYRAPPIRSVDVYNVMCSLAGVQPQPNN  
GSWSRVECMLKNTAVRVPLCTRSGCVLALTLLLLFWLL`

| Site | Type | Count |
| --- | --- | --- |
| 337 | N-linked | 1 |

| Mass | Sequence | MS_2_ Score | `q`-value | MS_1_ Score | Signal | Protein Coordinates | Tandem Scans | Start Time | Apex Time | End Time |
| --- | --- | --- | --- | --- | --- | --- | --- | --- | --- | --- |
| 3607.5578 | DKLPFWEN(N)GTGKR Fuc 3 Hex 5 HexNAc 4 | 137.450 | 0.0 | -18.681 | 6.560e+06 | 329 - 342 | 2 | 28.17 | 28.17 | 28.17 |

### DKLPFWEN(N)GTGKR Fuc 3 Hex 5 HexNAc 4

**Mass:**  3607.5578

(3.704e-06)

**Charge States:** 4

**Start Position**: 330

**End Position**: 343

| Profile Score | Tandem Score | TDA q-value | Volume | Spectrum Matches |
| --- | --- | --- | --- | --- |
| -18.6815 | 137.4505 | 0.0000 | 6.5599e+06 | 2 |

**Adduct Signal Fractions**

- **Unmodified**: 6.5599e+06
