# Supplementary material for: Measuring Site-specific Glycosylation Similarity between Influenza a Virus Variants with Statistical Certainty
Source: Mol Cell Proteomics. 2020 Nov 25;19(9):1533–45. doi: 10.1074/mcp.RA120.002031 (PMC8143645; doi:10.1074/mcp.RA120.002031)
Supplement: Supplementary file 2 [file mmc2.zip › mmc2/159250_2_supp_555293_qcjklw.zip!/20190301_SWZeggL2_021119_tryp_HILIC_DDA_1ug_04_glycopeptides.html]

## 20190301\_SWZeggL2\_021119\_tryp\_HILIC\_DDA\_1ug\_04 @ GlycopeptideHypothesis-7d0279944fe140abae76d511bf0bf893

**MS^1^ Mass Accuracy:** 10.0 PPM  
**Grouping Tolerance:** 15.0 PPM  
**MS^n^ Mass Accuracy:** 20.0 PPM  
**Minimum `q`-Value For Clustering:** 0.05

**Glycopeptide Hypothesis:**
GlycopeptideHypothesis-7d0279944fe140abae76d511bf0bf893  
**Sample Searched:**
20190301\_SWZeggL2\_021119\_tryp\_HILIC\_DDA\_1ug\_04

| Protein | Matched Glycopeptides |
| --- | --- |
| cus\|SWZHA\|A/Switzerland/9715293/2013 | 125 |
| cus\|SWZNA\|A/Switzerland/9715293/2013 | 13 |
| tr\|E1C041\|E1C041\_CHICK | 3 |
| tr\|A0A1D5PNI3\|A0A1D5PNI3\_CHICK | 2 |
| tr\|A0A1D5PUR1\|A0A1D5PUR1\_CHICK | 2 |
| tr\|F1NUU6\|F1NUU6\_CHICK | 1 |
| tr\|A0A1D5P780\|A0A1D5P780\_CHICK | 1 |
| sp\|P02845\|VIT2\_CHICK | 1 |
| tr\|F1NFL6\|F1NFL6\_CHICK | 1 |

### cus|SWZHA|A/Switzerland/9715293/2013

**>cus|SWZHA|A/Switzerland/9715293/2013**  
 `QKLPGNDNSTATLCLGHHAVPNGTIVKTITNDRIEVTNATELVQNSSIGEICDSPHQILDGENCTLIDALLGDPQCDGFQ  
NKKWDLFVERSKAYSNCYPYDVPDYASLRSLVASSGTLEFNNESFNWAGVTQNGTSSSCRRGSNSSFFSRLNWLTHLNSK  
YPALNVTMPNNEQFDKLYIWGVHHPVTDKDQIFLYAQSSGRITVSTKRSQQAVIPNIGYRPRIRDIPSRISIYWTIVKPG  
DILLINSTGNLIAPRGYFKIRSGKSSIMRSDAPIGKCKSECITPNGSIPNDKPFQNVNRITYGACPRYVKQSTLKLATGM  
RNVPERQTRGIFGAIAGFIENGWEGMVDGWYGFRHQNSEGRGQAADLKSTQAAIDQINGKLNRLIGKTNEKFHQIEKEFS  
EVEGRIQDLEKYVEDTKIDLWSYNAELLVALENQHTIDLTDSEMNKLFEKTKKQLRENAEDMGNGCFKIYHKCDNACIGS  
IRNGTYDHDVYRDEALNNRFQIKGVELKSGYKDWILWISFAISCFLLCVALLGFIMWACQKGNIRCNICI`

| Site | Type | Count |
| --- | --- | --- |
| 483 | N-linked | 42 |
| 165 | N-linked | 20 |
| 144 | N-linked | 30 |
| 22 | N-linked | 2 |
| 133 | N-linked | 1 |
| 285 | N-linked | 11 |
| 246 | N-linked | 19 |

| Mass | Sequence | MS_2_ Score | `q`-value | MS_1_ Score | Signal | Protein Coordinates | Tandem Scans | Start Time | Apex Time | End Time |
| --- | --- | --- | --- | --- | --- | --- | --- | --- | --- | --- |
| 2725.0978 | RGSN(N)SSFFSR Hex 6 HexNAc 3 | 195.197 | 0.0 | -3.017 | 4.661e+08 | 140 - 150 | 2 | 19.68 | 19.68 | 19.97 |
| 3115.2613 | RGSN(N)SSFFSR Fuc 1 Hex 5 HexNAc 5 | 189.741 | 0.0 | 12.972 | 1.322e+09 | 140 - 150 | 7 | 19.20 | 19.82 | 20.12 |
| 2684.0718 | RGSN(N)SSFFSR Hex 7 HexNAc 2 | 187.663 | 0.0 | -3.556 | 2.820e+08 | 140 - 150 | 3 | 19.20 | 19.68 | 19.68 |
| 2522.0184 | RGSN(N)SSFFSR Hex 6 HexNAc 2 | 187.218 | 0.0 | -4.458 | 4.187e+08 | 140 - 150 | 3 | 19.20 | 19.68 | 19.82 |
| 2359.9664 | RGSN(N)SSFFSR Hex 5 HexNAc 2 | 185.574 | 0.0 | 10.876 | 4.715e+08 | 140 - 150 | 4 | 19.36 | 19.97 | 20.25 |
| 3760.5174 | YPALN(N)VTM(O)PNNEQFDK Hex 9 HexNAc 2 | 182.390 | 0.0 | 2.496 | 1.694e+09 | 160 - 176 | 7 | 33.88 | 34.22 | 112.83 |
| 3598.4661 | YPALN(N)VTM(O)PNNEQFDK Hex 8 HexNAc 2 | 177.414 | 0.0 | 4.366 | 1.626e+09 | 160 - 176 | 7 | 33.88 | 34.38 | 112.83 |
| 2912.1828 | RGSN(N)SSFFSR Fuc 1 Hex 5 HexNAc 4 | 177.234 | 0.0 | 13.834 | 1.090e+09 | 140 - 150 | 5 | 19.03 | 19.68 | 20.40 |
| 3744.5242 | YPALN(N)VTMPNNEQFDK Hex 9 HexNAc 2 | 176.534 | 0.0 | 16.629 | 1.933e+08 | 160 - 176 | 5 | 41.77 | 42.10 | 42.61 |
| 2547.0503 | RGSN(N)SSFFSR Fuc 1 Hex 4 HexNAc 3 | 176.332 | 0.0 | -19.522 | 7.747e+07 | 140 - 150 | 2 | 20.12 | 20.25 | 20.25 |
| 2871.1571 | RGSN(N)SSFFSR Fuc 1 Hex 6 HexNAc 3 | 175.380 | 0.0 | -4.274 | 2.224e+08 | 140 - 150 | 2 | 19.20 | 19.53 | 19.68 |
| 3582.4712 | YPALN(N)VTMPNNEQFDK Hex 8 HexNAc 2 | 173.870 | 0.0 | 16.047 | 1.782e+08 | 160 - 176 | 3 | 41.77 | 42.10 | 42.44 |
| 2726.0908 | RGSN(N)SSFFSR Hex 6 HexNAc 3 | 171.512 | 0.0 | 0.000 | 0.000e+00 | 140 - 150 | 1 | - | - | - |
| 2400.9946 | RGSN(N)SSFFSR Hex 4 HexNAc 3 | 170.264 | 0.0 | -19.337 | 3.635e+07 | 140 - 150 | 1 | 20.12 | 20.25 | 20.25 |
| 3172.2852 | RGSN(N)SSFFSR Hex 5 HexNAc 6 | 168.929 | 0.0 | -4.344 | 1.317e+08 | 140 - 150 | 2 | 19.82 | 19.97 | 20.12 |
| 3131.2588 | RGSN(N)SSFFSR Hex 6 HexNAc 5 | 168.769 | 0.0 | -19.552 | 2.136e+07 | 140 - 150 | 1 | 19.36 | 19.53 | 19.53 |
| 3761.5451 | YPALN(N)VTMPNNEQFDK Fuc 2 Hex 3 HexNAc 4 Neu5Ac 1 | 167.013 | 0.0 | -4.398 | 2.111e+07 | 160 - 176 | 3 | 41.77 | 42.10 | 42.27 |
| 3277.3162 | RGSN(N)SSFFSR Fuc 1 Hex 6 HexNAc 5 | 165.333 | 0.0 | -4.035 | 1.634e+08 | 140 - 150 | 2 | 19.20 | 19.36 | 19.53 |
| 4068.9399 | TIVKPGDILLIN(N)STGNLIAPR Hex 9 HexNAc 2 | 164.542 | 0.0 | 16.990 | 4.681e+08 | 234 - 255 | 5 | 52.73 | 52.89 | 53.74 |
| 3777.5415 | YPALN(N)VTM(O)PNNEQFDK Fuc 2 Hex 3 HexNAc 4 Neu5Ac 1 | 163.979 | 0.0 | 13.439 | 1.116e+08 | 160 - 176 | 1 | 34.05 | 34.22 | 34.71 |
| 3274.3574 | YPALN(N)VTM(O)PNNEQFDK Hex 6 HexNAc 2 | 163.861 | 0.0 | 9.298 | 1.147e+07 | 160 - 176 | 2 | 34.38 | 34.71 | 35.22 |
| 2969.2043 | RGSN(N)SSFFSR Hex 5 HexNAc 5 | 163.646 | 0.0 | 11.034 | 3.017e+08 | 140 - 150 | 3 | 19.20 | 19.82 | 20.12 |
| 2846.1265 | RGSN(N)SSFFSR Hex 8 HexNAc 2 | 163.643 | 0.0 | -17.439 | 1.178e+08 | 140 - 150 | 2 | 18.87 | 19.53 | 19.53 |
| 3204.3011 | RGSN(N)SSFFSR Fuc 3 Hex 5 HexNAc 4 | 162.781 | 0.0 | -17.361 | 4.302e+07 | 140 - 150 | 1 | 19.20 | 19.36 | 19.36 |
| 3436.4101 | YPALN(N)VTM(O)PNNEQFDK Hex 7 HexNAc 2 | 161.571 | 0.0 | 15.018 | 1.273e+08 | 160 - 176 | 4 | 34.05 | 34.55 | 35.05 |
| 3112.3073 | YPALN(N)VTM(O)PNNEQFDK Hex 5 HexNAc 2 | 161.468 | 0.0 | 13.989 | 3.511e+07 | 160 - 176 | 3 | 34.71 | 35.22 | 35.39 |
| 4085.9575 | TIVKPGDILLIN(N)STGNLIAPR Fuc 2 Hex 3 HexNAc 4 Neu5Ac 1 | 161.296 | 0.0 | -17.530 | 3.921e+07 | 234 - 255 | 1 | 52.89 | 52.89 | 53.06 |
| 2766.1230 | RGSN(N)SSFFSR Hex 5 HexNAc 4 | 161.245 | 0.0 | -4.812 | 1.406e+08 | 140 - 150 | 2 | 19.20 | 19.82 | 19.97 |
| 2756.0827 | GSN(N)SSFFSR Fuc 1 Hex 5 HexNAc 4 | 160.200 | 0.0 | 13.755 | 4.093e+08 | 141 - 150 | 2 | 22.82 | 22.97 | 23.40 |
| 2928.1777 | RGSN(N)SSFFSR Hex 6 HexNAc 4 | 160.046 | 0.0 | -4.556 | 9.737e+07 | 140 - 150 | 1 | 19.36 | 19.68 | 19.82 |
| 3922.5695 | YPALN(N)VTM(O)PNNEQFDK Hex 10 HexNAc 2 | 159.578 | 0.0 | 14.642 | 4.429e+07 | 160 - 176 | 1 | 34.05 | 34.22 | 34.71 |
| 2365.9171 | GSN(N)SSFFSR Hex 6 HexNAc 2 | 159.348 | 0.0 | -3.024 | 2.404e+08 | 141 - 150 | 2 | 23.11 | 23.11 | 23.40 |
| 4230.9912 | TIVKPGDILLIN(N)STGNLIAPR Hex 10 HexNAc 2 | 159.325 | 0.0 | -3.282 | 3.872e+07 | 234 - 255 | 1 | 52.73 | 52.89 | 53.06 |
| 3153.2308 | N(N)GTYDHDVYR Fuc 2 Hex 5 HexNAc 4 | 159.111 | 0.0 | -17.830 | 1.655e+08 | 482 - 492 | 2 | 15.73 | 15.73 | 15.89 |
| 3006.3120 | HAVPN(N)GTIVK Fuc 1 Hex 5 HexNAc 5 | 158.817 | 0.0 | -18.798 | 1.449e+07 | 17 - 27 | 1 | 16.71 | 16.71 | 16.71 |
| 3058.2421 | RGSN(N)SSFFSR Fuc 2 Hex 5 HexNAc 4 | 158.744 | 0.0 | -3.867 | 1.738e+08 | 140 - 150 | 2 | 18.87 | 19.53 | 19.53 |
| 3210.2545 | N(N)GTYDHDVYR Fuc 1 Hex 5 HexNAc 5 | 158.576 | 0.0 | 12.324 | 2.554e+08 | 482 - 492 | 5 | 15.57 | 15.89 | 16.38 |
| 2203.8664 | GSN(N)SSFFSR Hex 5 HexNAc 2 | 158.421 | 0.0 | 14.293 | 3.861e+08 | 141 - 150 | 5 | 23.25 | 23.55 | 23.86 |
| 3632.4685 | N(N)GTYDHDVYRDEALNNR Hex 6 HexNAc 3 | 156.538 | 0.0 | -2.939 | 3.502e+08 | 482 - 499 | 3 | 20.25 | 20.40 | 20.71 |
| 3420.4196 | YPALN(N)VTMPNNEQFDK Hex 7 HexNAc 2 | 156.502 | 0.0 | -5.091 | 1.065e+07 | 160 - 176 | 1 | 41.94 | 42.10 | 42.44 |
| 4376.8531 | C(C)KSEC(C)ITPN(N)GSIPNDKPFQNVNR Hex 8 HexNAc 2 | 156.468 | 0.0 | -18.359 | 2.249e+07 | 276 - 299 | 1 | 29.09 | 29.09 | 29.26 |
| 3261.3217 | RGSN(N)SSFFSR Fuc 2 Hex 5 HexNAc 5 | 156.462 | 0.0 | -4.580 | 1.542e+08 | 140 - 150 | 2 | 19.36 | 19.53 | 19.68 |
| 3906.8857 | TIVKPGDILLIN(N)STGNLIAPR Hex 8 HexNAc 2 | 155.566 | 0.0 | -19.406 | 2.518e+07 | 234 - 255 | 3 | 52.89 | 53.06 | 53.06 |
| 4748.2989 | ISIYWTIVKPGDILLIN(N)STGNLIAPR Fuc 2 Hex 3 HexNAc 4 Neu5Ac 1 | 153.581 | 0.0 | -4.670 | 2.781e+08 | 229 - 255 | 4 | 68.47 | 68.47 | 68.81 |
| 3627.6395 | PGDILLIN(N)STGNLIAPR Hex 9 HexNAc 2 | 152.331 | 0.0 | -19.025 | 3.716e+07 | 238 - 255 | 1 | 51.20 | 51.20 | 51.37 |
| 2685.0829 | RGSN(N)SSFFSR Hex 7 HexNAc 2 | 151.433 | 0.0 | 0.000 | 0.000e+00 | 140 - 150 | 1 | - | - | - |
| 3934.5893 | RGSN(N)SSFFSR Fuc 3 Hex 7 HexNAc 6 | 151.425 | 0.0 | -5.850 | 6.151e+07 | 140 - 150 | 2 | 21.51 | 21.51 | 21.84 |
| 4538.9061 | C(C)KSEC(C)ITPN(N)GSIPNDKPFQNVNR Hex 9 HexNAc 2 | 150.628 | 0.0 | -17.752 | 4.884e+07 | 276 - 299 | 2 | 29.09 | 29.09 | 29.26 |
| 4893.3319 | ISIYWTIVKPGDILLIN(N)STGNLIAPR Hex 10 HexNAc 2 | 150.524 | 0.0 | 15.855 | 1.919e+08 | 229 - 255 | 5 | 67.95 | 68.47 | 68.81 |
| 3050.1945 | WAGVTQN(N)GTSSSC(C)R Hex 7 HexNAc 2 | 150.379 | 0.0 | -15.415 | 9.786e+06 | 126 - 140 | 1 | 18.70 | 18.70 | 18.70 |
| 3599.4576 | YPALN(N)VTM(O)PN(D)NEQFDK Hex 8 HexNAc 2 | 150.181 | 0.0 | 9.236 | 2.601e+08 | 160 - 176 | 9 | 34.88 | 35.22 | 36.23 |
| 4731.2894 | ISIYWTIVKPGDILLIN(N)STGNLIAPR Hex 9 HexNAc 2 | 149.639 | 0.0 | 17.885 | 1.938e+09 | 229 - 255 | 8 | 68.13 | 68.47 | 69.15 |
| 3007.1753 | N(N)GTYDHDVYR Fuc 1 Hex 5 HexNAc 4 | 148.306 | 0.0 | 14.818 | 5.772e+08 | 482 - 492 | 6 | 15.73 | 15.89 | 16.38 |
| 3965.6090 | N(N)GTYDHDVYRDEALNNR Fuc 2 Hex 5 HexNAc 4 | 148.126 | 0.0 | 13.412 | 1.087e+09 | 482 - 499 | 3 | 19.97 | 20.12 | 20.55 |
| 3480.3964 | RGSN(N)SSFFSR Fuc 1 Hex 6 HexNAc 6 | 147.801 | 0.0 | -5.040 | 5.462e+07 | 140 - 150 | 2 | 19.20 | 19.20 | 19.53 |
| 4569.2272 | ISIYWTIVKPGDILLIN(N)STGNLIAPR Hex 8 HexNAc 2 | 146.700 | 0.0 | 14.490 | 1.430e+08 | 229 - 255 | 6 | 68.30 | 68.64 | 68.98 |
| 3267.3366 | N(N)GTYDHDVYRDEALNNR Hex 5 HexNAc 2 | 143.425 | 0.0 | -5.026 | 1.777e+08 | 482 - 499 | 1 | 20.55 | 20.71 | 20.86 |
| 4910.3555 | ISIYWTIVKPGDILLIN(N)STGNLIAPR Fuc 2 Hex 4 HexNAc 4 Neu5Ac 1 | 141.165 | 0.0 | -4.401 | 2.683e+07 | 229 - 255 | 1 | 68.30 | 68.47 | 68.64 |
| 2950.2548 | YPALN(N)VTM(O)PNNEQFDK Hex 4 HexNAc 2 | 140.454 | 0.0 | -16.806 | 4.992e+06 | 160 - 176 | 1 | 35.22 | 35.22 | 35.39 |
| 4214.7999 | C(C)KSEC(C)ITPN(N)GSIPNDKPFQNVNR Hex 7 HexNAc 2 | 140.384 | 0.0 | -18.213 | 2.875e+07 | 276 - 299 | 1 | 29.09 | 29.26 | 29.26 |
| 4185.6718 | RGSN(N)SSFFSR Fuc 1 Hex 3 HexNAc 9 Neu5Ac 2 | 138.543 | 0.0 | 9.649 | 3.437e+08 | 140 - 150 | 3 | 20.55 | 20.71 | 21.35 |
| 3967.8891 | IVKPGDILLIN(N)STGNLIAPR Hex 9 HexNAc 2 | 138.338 | 0.0 | -4.598 | 1.650e+07 | 235 - 255 | 2 | 52.05 | 52.22 | 52.39 |
| 3560.3558 | RGSN(N)SSFFSR Fuc 1 Hex 9 HexNAc 4 | 137.926 | 0.0 | 10.912 | 1.121e+08 | 140 - 150 | 3 | 21.19 | 21.84 | 22.00 |
| 3673.4962 | N(N)GTYDHDVYRDEALNNR Hex 5 HexNAc 4 | 137.846 | 0.0 | -3.590 | 3.539e+08 | 482 - 499 | 4 | 20.25 | 20.40 | 20.71 |
| 3819.5525 | N(N)GTYDHDVYRDEALNNR Fuc 1 Hex 5 HexNAc 4 | 137.768 | 0.0 | 17.200 | 5.535e+09 | 482 - 499 | 6 | 20.12 | 20.40 | 20.71 |
| 3313.4682 | LGHHAVPN(N)GTIVK Fuc 1 Hex 5 HexNAc 5 | 137.572 | 0.0 | -19.870 | 6.712e+06 | 14 - 27 | 1 | 17.43 | 17.43 | 17.43 |
| 3454.4208 | N(N)GTYDHDVYRDEALNNR Fuc 1 Hex 4 HexNAc 3 | 137.513 | 0.0 | -3.662 | 2.250e+08 | 482 - 499 | 2 | 20.55 | 20.71 | 20.86 |
| 3470.4155 | N(N)GTYDHDVYRDEALNNR Hex 5 HexNAc 3 | 137.347 | 0.0 | -17.648 | 9.443e+07 | 482 - 499 | 1 | 20.55 | 20.55 | 20.71 |
| 4022.6323 | N(N)GTYDHDVYRDEALNNR Fuc 1 Hex 5 HexNAc 5 | 136.904 | 0.0 | -2.461 | 2.336e+09 | 482 - 499 | 4 | 20.25 | 20.55 | 20.71 |
| 4896.3259 | ISIYWTIVKPGDILLIN(N)STGNLIAPR Hex 10 HexNAc 2 | 136.168 | 0.0 | 0.000 | 0.000e+00 | 229 - 255 | 1 | - | - | - |
| 4184.6836 | N(N)GTYDHDVYRDEALNNR Fuc 1 Hex 6 HexNAc 5 | 135.605 | 0.0 | 9.384 | 4.277e+08 | 482 - 499 | 2 | 19.97 | 20.12 | 20.55 |
| 3334.3383 | RGSN(N)SSFFSR Hex 6 HexNAc 6 | 134.259 | 0.0 | -19.238 | 2.511e+07 | 140 - 150 | 1 | 19.36 | 19.36 | 19.36 |
| 3761.5110 | YPALN(N)VTM(O)PN(D)NEQFDK Hex 9 HexNAc 2 | 134.015 | 0.0 | 9.157 | 2.544e+08 | 160 - 176 | 10 | 34.88 | 35.22 | 36.07 |
| 4088.7235 | SEC(C)ITPN(N)GSIPNDKPFQNVNR Hex 8 HexNAc 2 | 133.863 | 0.0 | 15.711 | 4.796e+08 | 278 - 299 | 4 | 31.68 | 32.02 | 32.35 |
| 4111.6672 | N(N)GTYDHDVYRDEALNNR Fuc 3 Hex 5 HexNAc 4 | 131.146 | 0.0 | -19.732 | 1.382e+08 | 482 - 499 | 1 | 19.97 | 19.97 | 20.12 |
| 4387.7646 | N(N)GTYDHDVYRDEALNNR Fuc 1 Hex 6 HexNAc 6 | 131.053 | 0.0 | -5.188 | 9.115e+07 | 482 - 499 | 1 | 20.12 | 20.25 | 20.55 |
| 4102.5914 | N(N)GTYDHDVYRDEALNNR Fuc 1 Hex 8 HexNAc 3 | 130.497 | 0.0 | 11.639 | 5.385e+08 | 482 - 499 | 4 | 21.35 | 22.00 | 22.33 |
| 3778.5253 | N(N)GTYDHDVYRDEALNNR Fuc 1 Hex 6 HexNAc 3 | 129.753 | 0.0 | -4.937 | 1.557e+08 | 482 - 499 | 3 | 20.12 | 20.25 | 20.55 |
| 3778.5378 | YPALN(N)VTM(O)PN(D)NEQFDK Fuc 2 Hex 3 HexNAc 4 Neu5Ac 1 | 129.170 | 0.0 | -5.810 | 1.458e+07 | 160 - 176 | 2 | 34.88 | 35.22 | 35.39 |
| 2820.0901 | N(N)GTYDHDVYR Hex 6 HexNAc 3 | 128.885 | 0.0 | -20.154 | 1.541e+07 | 482 - 492 | 1 | 15.73 | 15.89 | 15.89 |
| 4250.7765 | SEC(C)ITPN(N)GSIPNDKPFQNVNR Hex 9 HexNAc 2 | 128.563 | 0.0 | -1.970 | 6.769e+08 | 278 - 299 | 4 | 31.68 | 31.85 | 32.19 |
| 3899.5115 | N(N)GTYDHDVYRDEALNNR Fuc 1 Hex 8 HexNAc 2 | 128.202 | 0.0 | 14.577 | 1.224e+09 | 482 - 499 | 4 | 21.67 | 22.00 | 22.33 |
| 5568.7640 | IRDIPSRISIYWTIVKPGDILLIN(N)STGNLIAPR Hex 9 HexNAc 2 | 127.161 | 0.0 | 17.014 | 3.558e+08 | 222 - 255 | 5 | 64.19 | 64.87 | 65.04 |
| 3820.5379 | N(N)GTYDHDVYRDEALN(D)NR Fuc 1 Hex 5 HexNAc 4 | 125.885 | 0.0 | 6.632 | 2.813e+09 | 482 - 499 | 10 | 20.86 | 20.86 | 21.67 |
| 5144.2849 | YPALN(N)VTM(O)PNNEQFDKLYIWGVHHPVTDK Hex 8 HexNAc 2 | 123.992 | 0.0 | -3.918 | 1.131e+07 | 160 - 189 | 1 | 49.33 | 49.33 | 49.67 |
| 3764.6190 | SEC(C)ITPN(N)GSIPNDKPFQNVNR Hex 6 HexNAc 2 | 123.975 | 0.0 | -3.458 | 1.026e+08 | 278 - 299 | 3 | 31.85 | 32.02 | 32.35 |
| 5299.5754 | DIPSRISIYWTIVKPGDILLIN(N)STGNLIAPR Hex 9 HexNAc 2 | 123.943 | 0.0 | -4.488 | 2.241e+07 | 224 - 255 | 2 | 68.98 | 69.15 | 69.32 |
| 3926.6720 | SEC(C)ITPN(N)GSIPNDKPFQNVNR Hex 7 HexNAc 2 | 123.712 | 0.0 | -1.302 | 8.282e+08 | 278 - 299 | 3 | 31.85 | 32.02 | 32.19 |
| 4248.6488 | N(N)GTYDHDVYRDEALNNR Fuc 2 Hex 8 HexNAc 3 | 122.743 | 0.0 | -17.796 | 1.219e+08 | 482 - 499 | 2 | 21.84 | 21.84 | 22.00 |
| 4264.6440 | N(N)GTYDHDVYRDEALNNR Fuc 1 Hex 9 HexNAc 3 | 122.387 | 0.0 | -3.313 | 2.418e+08 | 482 - 499 | 2 | 21.51 | 21.84 | 21.84 |
| 3372.3066 | N(N)GTYDHDVYR Fuc 1 Hex 6 HexNAc 5 | 122.077 | 0.0 | -19.282 | 7.785e+07 | 482 - 492 | 3 | 15.73 | 15.73 | 15.89 |
| 3616.4743 | N(N)GTYDHDVYRDEALNNR Fuc 1 Hex 5 HexNAc 3 | 121.374 | 0.0 | -3.757 | 1.499e+08 | 482 - 499 | 2 | 20.40 | 20.55 | 20.86 |
| 3437.4027 | YPALN(N)VTM(O)PN(D)NEQFDK Hex 7 HexNAc 2 | 120.704 | 0.0 | 6.263 | 1.397e+07 | 160 - 176 | 3 | 35.22 | 35.22 | 35.90 |
| 4335.8671 | N(N)GTYDHDVYRDEALNNRFQIK Fuc 1 Hex 5 HexNAc 4 | 119.953 | 0.0 | -18.282 | 4.672e+07 | 482 - 503 | 1 | 31.68 | 31.68 | 31.85 |
| 5306.3394 | YPALN(N)VTM(O)PNNEQFDKLYIWGVHHPVTDK Hex 9 HexNAc 2 | 119.659 | 0.0 | -18.187 | 1.350e+07 | 160 - 189 | 1 | 49.33 | 49.33 | 49.50 |
| 3299.2872 | N(N)GTYDHDVYR Fuc 3 Hex 5 HexNAc 4 | 119.577 | 0.0 | -19.797 | 2.202e+07 | 482 - 492 | 1 | 15.57 | 15.57 | 15.73 |
| 4168.6881 | N(N)GTYDHDVYRDEALNNR Fuc 2 Hex 5 HexNAc 5 | 119.200 | 0.005 | -4.040 | 1.520e+08 | 482 - 499 | 1 | 20.12 | 20.25 | 20.40 |
| 3602.5670 | SEC(C)ITPN(N)GSIPNDKPFQNVNR Hex 5 HexNAc 2 | 118.215 | 0.005 | -18.784 | 4.718e+07 | 278 - 299 | 2 | 32.19 | 32.35 | 32.35 |
| 3600.4788 | N(N)GTYDHDVYRDEALNNR Fuc 2 Hex 4 HexNAc 3 | 117.705 | 0.008 | -4.746 | 8.188e+07 | 482 - 499 | 11 | 20.40 | 20.55 | 20.71 |
| 4467.7196 | N(N)GTYDHDVYRDEALNNR Fuc 1 Hex 9 HexNAc 4 | 117.227 | 0.008 | -18.855 | 4.523e+07 | 482 - 499 | 1 | 21.84 | 21.84 | 22.00 |
| 4083.0682 | ISIYWTIVKPGDILLIN(N)STGNLIAPR Hex 5 HexNAc 2 | 116.844 | 0.008 | -18.437 | 1.216e+07 | 229 - 255 | 2 | 69.15 | 69.32 | 69.32 |
| 4410.7023 | N(N)GTYDHDVYRDEALNNR Fuc 2 Hex 9 HexNAc 3 | 116.833 | 0.008 | -4.734 | 8.772e+07 | 482 - 499 | 1 | 21.51 | 21.67 | 21.84 |
| 3583.4571 | YPALN(N)VTMPN(D)NEQFDK Hex 8 HexNAc 2 | 115.238 | 0.008 | -6.071 | 2.705e+07 | 160 - 176 | 2 | 42.95 | 43.12 | 43.28 |
| 3794.5217 | N(N)GTYDHDVYRDEALNNR Hex 7 HexNAc 3 | 115.179 | 0.008 | -19.705 | 3.821e+07 | 482 - 499 | 1 | 20.25 | 20.25 | 20.25 |
| 5406.7103 | IRDIPSRISIYWTIVKPGDILLIN(N)STGNLIAPR Hex 8 HexNAc 2 | 112.476 | 0.008 | -4.685 | 2.091e+07 | 222 - 255 | 3 | 64.70 | 64.87 | 65.04 |
| 3298.2692 | N(N)GTYDHDVYR Fuc 1 Hex 5 HexNAc 4 Neu5Ac 1 | 112.376 | 0.008 | -20.931 | 8.346e+06 | 482 - 492 | 1 | 16.55 | 16.71 | 16.71 |
| 2861.1183 | N(N)GTYDHDVYR Hex 5 HexNAc 4 | 110.862 | 0.008 | -18.214 | 2.904e+07 | 482 - 492 | 1 | 15.73 | 15.89 | 15.89 |
| 4255.0188 | WTIVKPGDILLIN(N)STGNLIAPR Hex 9 HexNAc 2 | 109.194 | 0.008 | -19.507 | 6.452e+06 | 233 - 255 | 2 | 60.03 | 60.20 | 60.20 |
| 3591.4479 | N(N)GTYDHDVYRDEALNNR Hex 7 HexNAc 2 | 108.568 | 0.008 | -19.546 | 2.145e+07 | 482 - 499 | 1 | 20.25 | 20.25 | 20.25 |
| 3745.5111 | YPALN(N)VTMPN(D)NEQFDK Hex 9 HexNAc 2 | 107.422 | 0.012 | -5.834 | 3.331e+07 | 160 - 176 | 2 | 42.95 | 43.12 | 43.28 |
| 3657.5049 | N(N)GTYDHDVYRDEALNNR Fuc 1 Hex 4 HexNAc 4 | 107.326 | 0.012 | -5.669 | 3.316e+07 | 482 - 499 | 1 | 20.40 | 20.55 | 20.71 |
| 4089.7143 | SEC(C)ITPN(N)GSIPN(D)DKPFQNVNR Hex 8 HexNAc 2 | 105.665 | 0.012 | 9.574 | 9.188e+07 | 278 - 299 | 3 | 32.35 | 33.20 | 33.20 |
| 3927.6599 | SEC(C)ITPN(N)GSIPN(D)DKPFQNVNR Hex 7 HexNAc 2 | 105.334 | 0.012 | 8.250 | 9.883e+07 | 278 - 299 | 3 | 32.35 | 33.20 | 33.37 |
| 3633.4551 | N(N)GTYDHDVYRDEALN(D)NR Hex 6 HexNAc 3 | 105.174 | 0.012 | -5.988 | 1.492e+08 | 482 - 499 | 2 | 20.86 | 21.03 | 21.51 |
| 2966.1483 | N(N)GTYDHDVYR Fuc 1 Hex 6 HexNAc 3 | 104.689 | 0.012 | -18.031 | 2.038e+07 | 482 - 492 | 1 | 15.73 | 15.73 | 15.73 |
| 3356.3106 | N(N)GTYDHDVYR Fuc 2 Hex 5 HexNAc 5 | 103.211 | 0.015 | -19.574 | 1.065e+07 | 482 - 492 | 1 | 15.73 | 15.73 | 15.73 |
| 4048.6414 | RGSN(N)SSFFSR Fuc 1 Hex 7 HexNAc 8 | 103.184 | 0.015 | -18.562 | 6.208e+07 | 140 - 150 | 1 | 22.66 | 22.66 | 22.82 |
| 5730.8071 | IRDIPSRISIYWTIVKPGDILLIN(N)STGNLIAPR Hex 10 HexNAc 2 | 103.101 | 0.015 | -19.721 | 1.882e+07 | 222 - 255 | 1 | 64.70 | 64.70 | 64.70 |
| 3674.4843 | N(N)GTYDHDVYRDEALN(D)NR Hex 5 HexNAc 4 | 103.044 | 0.015 | 7.816 | 1.746e+08 | 482 - 499 | 2 | 20.71 | 21.03 | 21.67 |
| 4112.6578 | N(N)GTYDHDVYRDEALN(D)NR Fuc 3 Hex 5 HexNAc 4 | 98.760 | 0.022 | 6.426 | 1.819e+08 | 482 - 499 | 2 | 20.55 | 20.71 | 21.35 |
| 4251.7647 | SEC(C)ITPN(N)GSIPN(D)DKPFQNVNR Hex 9 HexNAc 2 | 97.948 | 0.022 | 6.908 | 1.894e+08 | 278 - 299 | 6 | 32.35 | 33.20 | 33.20 |
| 3900.4982 | N(N)GTYDHDVYRDEALN(D)NR Fuc 1 Hex 8 HexNAc 2 | 96.156 | 0.022 | 8.396 | 4.079e+08 | 482 - 499 | 3 | 22.50 | 22.50 | 23.40 |
| 4747.2808 | ISIYWTIVKPGDILLIN(N)STGNLIAPR Hex 3 HexNAc 4 Neu5Ac 2 | 91.372 | 0.040 | -18.851 | 2.317e+07 | 229 - 255 | 3 | 68.13 | 68.30 | 68.30 |
| 4305.6706 | N(N)GTYDHDVYRDEALNNR Fuc 1 Hex 8 HexNAc 4 | 90.533 | 0.040 | -19.763 | 2.092e+07 | 482 - 499 | 1 | 21.51 | 21.67 | 21.67 |
| 3520.3793 | N(N)GTYDHDVYR Fuc 2 Hex 6 HexNAc 5 | 90.097 | 0.043 | -22.106 | 1.334e+06 | 482 - 492 | 2 | 52.39 | 52.39 | 52.39 |

### RGSN(N)SSFFSR Hex 6 HexNAc 3

**Mass:**  2725.0984

(-2.667e-07)

**Charge States:** 3

**Start Position**: 141

**End Position**: 151

| Profile Score | Tandem Score | TDA q-value | Volume | Spectrum Matches |
| --- | --- | --- | --- | --- |
| -3.0167 | 195.1966 | 0.0000 | 4.6614e+08 | 2 |

**Adduct Signal Fractions**

- **Unmodified**: 4.6614e+08

### RGSN(N)SSFFSR Fuc 1 Hex 5 HexNAc 5

**Mass:**  3115.2603

(4.581e-07)

**Charge States:** 3, 4

**Start Position**: 141

**End Position**: 151

| Profile Score | Tandem Score | TDA q-value | Volume | Spectrum Matches |
| --- | --- | --- | --- | --- |
| 12.9720 | 189.7406 | 0.0000 | 1.3222e+09 | 7 |

**Adduct Signal Fractions**

- **Unmodified**: 1.3222e+09

### RGSN(N)SSFFSR Hex 7 HexNAc 2

**Mass:**  2684.0711

(1.320e-07)

**Charge States:** 3

**Start Position**: 141

**End Position**: 151

| Profile Score | Tandem Score | TDA q-value | Volume | Spectrum Matches |
| --- | --- | --- | --- | --- |
| -3.5562 | 187.6629 | 0.0000 | 2.8201e+08 | 3 |

**Adduct Signal Fractions**

- **Unmodified**: 2.8201e+08

### RGSN(N)SSFFSR Hex 6 HexNAc 2

**Mass:**  2522.0183

(2.294e-07)

**Charge States:** 3

**Start Position**: 141

**End Position**: 151

| Profile Score | Tandem Score | TDA q-value | Volume | Spectrum Matches |
| --- | --- | --- | --- | --- |
| -4.4582 | 187.2178 | 0.0000 | 4.1869e+08 | 3 |

**Adduct Signal Fractions**

- **Unmodified**: 4.1869e+08

### RGSN(N)SSFFSR Hex 5 HexNAc 2

**Mass:**  2359.9663

(4.037e-07)

**Charge States:** 3

**Start Position**: 141

**End Position**: 151

| Profile Score | Tandem Score | TDA q-value | Volume | Spectrum Matches |
| --- | --- | --- | --- | --- |
| 10.8757 | 185.5737 | 0.0000 | 4.7146e+08 | 4 |

**Adduct Signal Fractions**

- **Unmodified**: 4.7146e+08

### YPALN(N)VTM(O)PNNEQFDK Hex 9 HexNAc 2

**Mass:**  3760.5174

(3.607e-06)

**Charge States:** 2, 3, 4

**Start Position**: 161

**End Position**: 177

| Profile Score | Tandem Score | TDA q-value | Volume | Spectrum Matches |
| --- | --- | --- | --- | --- |
| 2.4956 | 182.3897 | 0.0000 | 1.6937e+09 | 7 |

**Adduct Signal Fractions**

- **Unmodified**: 1.6937e+09

### YPALN(N)VTM(O)PNNEQFDK Hex 8 HexNAc 2

**Mass:**  3598.4650

(3.073e-06)

**Charge States:** 2, 3, 4

**Start Position**: 161

**End Position**: 177

| Profile Score | Tandem Score | TDA q-value | Volume | Spectrum Matches |
| --- | --- | --- | --- | --- |
| 4.3656 | 177.4144 | 0.0000 | 1.6256e+09 | 7 |

**Adduct Signal Fractions**

- **Unmodified**: 1.6256e+09

### RGSN(N)SSFFSR Fuc 1 Hex 5 HexNAc 4

**Mass:**  2912.1824

(2.254e-07)

**Charge States:** 3

**Start Position**: 141

**End Position**: 151

| Profile Score | Tandem Score | TDA q-value | Volume | Spectrum Matches |
| --- | --- | --- | --- | --- |
| 13.8343 | 177.2338 | 0.0000 | 1.0895e+09 | 5 |

**Adduct Signal Fractions**

- **Unmodified**: 1.0895e+09

### YPALN(N)VTMPNNEQFDK Hex 9 HexNAc 2

**Mass:**  3744.5246

(2.783e-06)

**Charge States:** 3, 4

**Start Position**: 161

**End Position**: 177

| Profile Score | Tandem Score | TDA q-value | Volume | Spectrum Matches |
| --- | --- | --- | --- | --- |
| 16.6286 | 176.5337 | 0.0000 | 1.9330e+08 | 5 |

**Adduct Signal Fractions**

- **Unmodified**: 1.9330e+08

### RGSN(N)SSFFSR Fuc 1 Hex 4 HexNAc 3

**Mass:**  2547.0516

(-4.498e-07)

**Charge States:** 3

**Start Position**: 141

**End Position**: 151

| Profile Score | Tandem Score | TDA q-value | Volume | Spectrum Matches |
| --- | --- | --- | --- | --- |
| -19.5217 | 176.3320 | 0.0000 | 7.7466e+07 | 2 |

**Adduct Signal Fractions**

- **Unmodified**: 7.7466e+07

### RGSN(N)SSFFSR Fuc 1 Hex 6 HexNAc 3

**Mass:**  2871.1582

(1.101e-06)

**Charge States:** 3

**Start Position**: 141

**End Position**: 151

| Profile Score | Tandem Score | TDA q-value | Volume | Spectrum Matches |
| --- | --- | --- | --- | --- |
| -4.2738 | 175.3796 | 0.0000 | 2.2236e+08 | 2 |

**Adduct Signal Fractions**

- **Unmodified**: 2.2236e+08

### YPALN(N)VTMPNNEQFDK Hex 8 HexNAc 2

**Mass:**  3582.4689

(4.797e-06)

**Charge States:** 3, 4

**Start Position**: 161

**End Position**: 177

| Profile Score | Tandem Score | TDA q-value | Volume | Spectrum Matches |
| --- | --- | --- | --- | --- |
| 16.0474 | 173.8696 | 0.0000 | 1.7817e+08 | 3 |

**Adduct Signal Fractions**

- **Unmodified**: 1.7817e+08

### RGSN(N)SSFFSR Hex 6 HexNAc 3

**Mass:**  2726.0908

(3.646e-04)

**Start Position**: 141

**End Position**: 151

| Profile Score | Tandem Score | TDA q-value | Volume | Spectrum Matches |
| --- | --- | --- | --- | --- |
| 0.0000 | 171.5124 | 0.0000 | 0.0000e+00 | 1 |

Adduct:

No Chromatogram Found

### RGSN(N)SSFFSR Hex 4 HexNAc 3

**Mass:**  2400.9939

(9.875e-07)

**Charge States:** 3

**Start Position**: 141

**End Position**: 151

| Profile Score | Tandem Score | TDA q-value | Volume | Spectrum Matches |
| --- | --- | --- | --- | --- |
| -19.3370 | 170.2640 | 0.0000 | 3.6346e+07 | 1 |

**Adduct Signal Fractions**

- **Unmodified**: 3.6346e+07

### RGSN(N)SSFFSR Hex 5 HexNAc 6

**Mass:**  3172.2863

(1.151e-06)

**Charge States:** 3

**Start Position**: 141

**End Position**: 151

| Profile Score | Tandem Score | TDA q-value | Volume | Spectrum Matches |
| --- | --- | --- | --- | --- |
| -4.3438 | 168.9290 | 0.0000 | 1.3174e+08 | 2 |

**Adduct Signal Fractions**

- **Unmodified**: 1.3174e+08

### RGSN(N)SSFFSR Hex 6 HexNAc 5

**Mass:**  3131.2604

(4.021e-07)

**Charge States:** 3

**Start Position**: 141

**End Position**: 151

| Profile Score | Tandem Score | TDA q-value | Volume | Spectrum Matches |
| --- | --- | --- | --- | --- |
| -19.5522 | 168.7686 | 0.0000 | 2.1357e+07 | 1 |

**Adduct Signal Fractions**

- **Unmodified**: 2.1357e+07

### YPALN(N)VTMPNNEQFDK Fuc 2 Hex 3 HexNAc 4 Neu5Ac 1

**Mass:**  3761.5468

(-4.036e-06)

**Charge States:** 3

**Start Position**: 161

**End Position**: 177

| Profile Score | Tandem Score | TDA q-value | Volume | Spectrum Matches |
| --- | --- | --- | --- | --- |
| -4.3984 | 167.0126 | 0.0000 | 2.1113e+07 | 3 |

**Adduct Signal Fractions**

- **Unmodified**: 2.1113e+07

### RGSN(N)SSFFSR Fuc 1 Hex 6 HexNAc 5

**Mass:**  3277.3159

(6.683e-07)

**Charge States:** 3

**Start Position**: 141

**End Position**: 151

| Profile Score | Tandem Score | TDA q-value | Volume | Spectrum Matches |
| --- | --- | --- | --- | --- |
| -4.0346 | 165.3326 | 0.0000 | 1.6342e+08 | 2 |

**Adduct Signal Fractions**

- **Unmodified**: 1.6342e+08

### TIVKPGDILLIN(N)STGNLIAPR Hex 9 HexNAc 2

**Mass:**  4068.9436

(4.202e-06)

**Charge States:** 3, 4

**Start Position**: 235

**End Position**: 256

| Profile Score | Tandem Score | TDA q-value | Volume | Spectrum Matches |
| --- | --- | --- | --- | --- |
| 16.9898 | 164.5422 | 0.0000 | 4.6814e+08 | 5 |

**Adduct Signal Fractions**

- **Unmodified**: 4.6814e+08

### YPALN(N)VTM(O)PNNEQFDK Fuc 2 Hex 3 HexNAc 4 Neu5Ac 1

**Mass:**  3777.5429

(-4.670e-06)

**Charge States:** 3

**Start Position**: 161

**End Position**: 177

| Profile Score | Tandem Score | TDA q-value | Volume | Spectrum Matches |
| --- | --- | --- | --- | --- |
| 13.4385 | 163.9787 | 0.0000 | 1.1163e+08 | 1 |

**Adduct Signal Fractions**

- **Unmodified**: 1.1163e+08

### YPALN(N)VTM(O)PNNEQFDK Hex 6 HexNAc 2

**Mass:**  3274.3540

(1.771e-06)

**Charge States:** 3

**Start Position**: 161

**End Position**: 177

| Profile Score | Tandem Score | TDA q-value | Volume | Spectrum Matches |
| --- | --- | --- | --- | --- |
| 9.2978 | 163.8613 | 0.0000 | 1.1473e+07 | 2 |

**Adduct Signal Fractions**

- **Unmodified**: 1.1473e+07

### RGSN(N)SSFFSR Hex 5 HexNAc 5

**Mass:**  2969.2025

(9.939e-07)

**Charge States:** 3

**Start Position**: 141

**End Position**: 151

| Profile Score | Tandem Score | TDA q-value | Volume | Spectrum Matches |
| --- | --- | --- | --- | --- |
| 11.0341 | 163.6457 | 0.0000 | 3.0168e+08 | 3 |

**Adduct Signal Fractions**

- **Unmodified**: 3.0168e+08

### RGSN(N)SSFFSR Hex 8 HexNAc 2

**Mass:**  2846.1265

(1.085e-06)

**Charge States:** 3

**Start Position**: 141

**End Position**: 151

| Profile Score | Tandem Score | TDA q-value | Volume | Spectrum Matches |
| --- | --- | --- | --- | --- |
| -17.4390 | 163.6432 | 0.0000 | 1.1778e+08 | 2 |

**Adduct Signal Fractions**

- **Unmodified**: 1.1778e+08

### RGSN(N)SSFFSR Fuc 3 Hex 5 HexNAc 4

**Mass:**  3204.3015

(8.435e-07)

**Charge States:** 3

**Start Position**: 141

**End Position**: 151

| Profile Score | Tandem Score | TDA q-value | Volume | Spectrum Matches |
| --- | --- | --- | --- | --- |
| -17.3608 | 162.7805 | 0.0000 | 4.3022e+07 | 1 |

**Adduct Signal Fractions**

- **Unmodified**: 4.3022e+07

### YPALN(N)VTM(O)PNNEQFDK Hex 7 HexNAc 2

**Mass:**  3436.4090

(2.315e-06)

**Charge States:** 2, 3, 4

**Start Position**: 161

**End Position**: 177

| Profile Score | Tandem Score | TDA q-value | Volume | Spectrum Matches |
| --- | --- | --- | --- | --- |
| 15.0178 | 161.5705 | 0.0000 | 1.2726e+08 | 4 |

**Adduct Signal Fractions**

- **Unmodified**: 1.2726e+08

### YPALN(N)VTM(O)PNNEQFDK Hex 5 HexNAc 2

**Mass:**  3112.3075

(5.275e-06)

**Charge States:** 3

**Start Position**: 161

**End Position**: 177

| Profile Score | Tandem Score | TDA q-value | Volume | Spectrum Matches |
| --- | --- | --- | --- | --- |
| 13.9889 | 161.4675 | 0.0000 | 3.5108e+07 | 3 |

**Adduct Signal Fractions**

- **Unmodified**: 3.5108e+07

### TIVKPGDILLIN(N)STGNLIAPR Fuc 2 Hex 3 HexNAc 4 Neu5Ac 1

**Mass:**  4085.9558

(-5.088e-06)

**Charge States:** 4

**Start Position**: 235

**End Position**: 256

| Profile Score | Tandem Score | TDA q-value | Volume | Spectrum Matches |
| --- | --- | --- | --- | --- |
| -17.5298 | 161.2956 | 0.0000 | 3.9210e+07 | 1 |

**Adduct Signal Fractions**

- **Unmodified**: 3.9210e+07

### RGSN(N)SSFFSR Hex 5 HexNAc 4

**Mass:**  2766.1219

(1.167e-06)

**Charge States:** 3

**Start Position**: 141

**End Position**: 151

| Profile Score | Tandem Score | TDA q-value | Volume | Spectrum Matches |
| --- | --- | --- | --- | --- |
| -4.8116 | 161.2449 | 0.0000 | 1.4059e+08 | 2 |

**Adduct Signal Fractions**

- **Unmodified**: 1.4059e+08

### GSN(N)SSFFSR Fuc 1 Hex 5 HexNAc 4

**Mass:**  2756.0835

(3.460e-07)

**Charge States:** 3

**Start Position**: 142

**End Position**: 151

| Profile Score | Tandem Score | TDA q-value | Volume | Spectrum Matches |
| --- | --- | --- | --- | --- |
| 13.7554 | 160.2000 | 0.0000 | 4.0932e+08 | 2 |

**Adduct Signal Fractions**

- **Unmodified**: 4.0932e+08

### RGSN(N)SSFFSR Hex 6 HexNAc 4

**Mass:**  2928.1779

(4.838e-07)

**Charge States:** 3

**Start Position**: 141

**End Position**: 151

| Profile Score | Tandem Score | TDA q-value | Volume | Spectrum Matches |
| --- | --- | --- | --- | --- |
| -4.5563 | 160.0464 | 0.0000 | 9.7370e+07 | 1 |

**Adduct Signal Fractions**

- **Unmodified**: 9.7370e+07

### YPALN(N)VTM(O)PNNEQFDK Hex 10 HexNAc 2

**Mass:**  3922.5697

(2.641e-06)

**Charge States:** 3, 4

**Start Position**: 161

**End Position**: 177

| Profile Score | Tandem Score | TDA q-value | Volume | Spectrum Matches |
| --- | --- | --- | --- | --- |
| 14.6416 | 159.5779 | 0.0000 | 4.4293e+07 | 1 |

**Adduct Signal Fractions**

- **Unmodified**: 4.4293e+07

### GSN(N)SSFFSR Hex 6 HexNAc 2

**Mass:**  2365.9171

(1.738e-07)

**Charge States:** 2

**Start Position**: 142

**End Position**: 151

| Profile Score | Tandem Score | TDA q-value | Volume | Spectrum Matches |
| --- | --- | --- | --- | --- |
| -3.0245 | 159.3483 | 0.0000 | 2.4036e+08 | 2 |

**Adduct Signal Fractions**

- **Unmodified**: 2.4036e+08

### TIVKPGDILLIN(N)STGNLIAPR Hex 10 HexNAc 2

**Mass:**  4230.9938

(3.237e-06)

**Charge States:** 4

**Start Position**: 235

**End Position**: 256

| Profile Score | Tandem Score | TDA q-value | Volume | Spectrum Matches |
| --- | --- | --- | --- | --- |
| -3.2816 | 159.3253 | 0.0000 | 3.8723e+07 | 1 |

**Adduct Signal Fractions**

- **Unmodified**: 3.8723e+07

### N(N)GTYDHDVYR Fuc 2 Hex 5 HexNAc 4

**Mass:**  3153.2305

(4.796e-07)

**Charge States:** 3

**Start Position**: 483

**End Position**: 493

| Profile Score | Tandem Score | TDA q-value | Volume | Spectrum Matches |
| --- | --- | --- | --- | --- |
| -17.8297 | 159.1108 | 0.0000 | 1.6550e+08 | 2 |

**Adduct Signal Fractions**

- **Unmodified**: 1.6550e+08

### HAVPN(N)GTIVK Fuc 1 Hex 5 HexNAc 5

**Mass:**  3006.3120

(1.917e-06)

**Charge States:** 3

**Start Position**: 18

**End Position**: 28

| Profile Score | Tandem Score | TDA q-value | Volume | Spectrum Matches |
| --- | --- | --- | --- | --- |
| -18.7978 | 158.8169 | 0.0000 | 1.4489e+07 | 1 |

**Adduct Signal Fractions**

- **Unmodified**: 1.4489e+07

### RGSN(N)SSFFSR Fuc 2 Hex 5 HexNAc 4

**Mass:**  3058.2423

(8.895e-07)

**Charge States:** 3

**Start Position**: 141

**End Position**: 151

| Profile Score | Tandem Score | TDA q-value | Volume | Spectrum Matches |
| --- | --- | --- | --- | --- |
| -3.8674 | 158.7439 | 0.0000 | 1.7381e+08 | 2 |

**Adduct Signal Fractions**

- **Unmodified**: 1.7381e+08

### N(N)GTYDHDVYR Fuc 1 Hex 5 HexNAc 5

**Mass:**  3210.2547

(1.265e-06)

**Charge States:** 3, 4

**Start Position**: 483

**End Position**: 493

| Profile Score | Tandem Score | TDA q-value | Volume | Spectrum Matches |
| --- | --- | --- | --- | --- |
| 12.3241 | 158.5761 | 0.0000 | 2.5535e+08 | 5 |

**Adduct Signal Fractions**

- **Unmodified**: 2.5535e+08

### GSN(N)SSFFSR Hex 5 HexNAc 2

**Mass:**  2203.8666

(7.634e-07)

**Charge States:** 2, 3

**Start Position**: 142

**End Position**: 151

| Profile Score | Tandem Score | TDA q-value | Volume | Spectrum Matches |
| --- | --- | --- | --- | --- |
| 14.2934 | 158.4208 | 0.0000 | 3.8606e+08 | 5 |

**Adduct Signal Fractions**

- **Unmodified**: 3.8606e+08

### N(N)GTYDHDVYRDEALNNR Hex 6 HexNAc 3

**Mass:**  3632.4687

(5.963e-07)

**Charge States:** 4

**Start Position**: 483

**End Position**: 500

| Profile Score | Tandem Score | TDA q-value | Volume | Spectrum Matches |
| --- | --- | --- | --- | --- |
| -2.9394 | 156.5382 | 0.0000 | 3.5015e+08 | 3 |

**Adduct Signal Fractions**

- **Unmodified**: 3.5015e+08

### YPALN(N)VTMPNNEQFDK Hex 7 HexNAc 2

**Mass:**  3420.4173

(4.873e-06)

**Charge States:** 3

**Start Position**: 161

**End Position**: 177

| Profile Score | Tandem Score | TDA q-value | Volume | Spectrum Matches |
| --- | --- | --- | --- | --- |
| -5.0914 | 156.5018 | 0.0000 | 1.0653e+07 | 1 |

**Adduct Signal Fractions**

- **Unmodified**: 1.0653e+07

### C(C)KSEC(C)ITPN(N)GSIPNDKPFQNVNR Hex 8 HexNAc 2

**Mass:**  4376.8547

(3.240e-06)

**Charge States:** 4, 5

**Start Position**: 277

**End Position**: 300

| Profile Score | Tandem Score | TDA q-value | Volume | Spectrum Matches |
| --- | --- | --- | --- | --- |
| -18.3593 | 156.4680 | 0.0000 | 2.2486e+07 | 1 |

**Adduct Signal Fractions**

- **Unmodified**: 2.2486e+07

### RGSN(N)SSFFSR Fuc 2 Hex 5 HexNAc 5

**Mass:**  3261.3224

(1.350e-06)

**Charge States:** 3

**Start Position**: 141

**End Position**: 151

| Profile Score | Tandem Score | TDA q-value | Volume | Spectrum Matches |
| --- | --- | --- | --- | --- |
| -4.5805 | 156.4620 | 0.0000 | 1.5420e+08 | 2 |

**Adduct Signal Fractions**

- **Unmodified**: 1.5420e+08

### TIVKPGDILLIN(N)STGNLIAPR Hex 8 HexNAc 2

**Mass:**  3906.8889

(3.497e-06)

**Charge States:** 3, 4

**Start Position**: 235

**End Position**: 256

| Profile Score | Tandem Score | TDA q-value | Volume | Spectrum Matches |
| --- | --- | --- | --- | --- |
| -19.4065 | 155.5662 | 0.0000 | 2.5180e+07 | 3 |

**Adduct Signal Fractions**

- **Unmodified**: 2.5180e+07

### ISIYWTIVKPGDILLIN(N)STGNLIAPR Fuc 2 Hex 3 HexNAc 4 Neu5Ac 1

**Mass:**  4748.2980

(-4.992e-06)

**Charge States:** 4, 5

**Start Position**: 230

**End Position**: 256

| Profile Score | Tandem Score | TDA q-value | Volume | Spectrum Matches |
| --- | --- | --- | --- | --- |
| -4.6699 | 153.5814 | 0.0000 | 2.7809e+08 | 4 |

**Adduct Signal Fractions**

- **Unmodified**: 2.7809e+08

### PGDILLIN(N)STGNLIAPR Hex 9 HexNAc 2

**Mass:**  3627.6395

(3.117e-06)

**Charge States:** 3, 4

**Start Position**: 239

**End Position**: 256

| Profile Score | Tandem Score | TDA q-value | Volume | Spectrum Matches |
| --- | --- | --- | --- | --- |
| -19.0247 | 152.3306 | 0.0000 | 3.7155e+07 | 1 |

**Adduct Signal Fractions**

- **Unmodified**: 3.7155e+07

### RGSN(N)SSFFSR Hex 7 HexNAc 2

**Mass:**  2685.0829

(3.771e-04)

**Start Position**: 141

**End Position**: 151

| Profile Score | Tandem Score | TDA q-value | Volume | Spectrum Matches |
| --- | --- | --- | --- | --- |
| 0.0000 | 151.4326 | 0.0000 | 0.0000e+00 | 1 |

Adduct:

No Chromatogram Found

### RGSN(N)SSFFSR Fuc 3 Hex 7 HexNAc 6

**Mass:**  3934.5881

(6.641e-06)

**Charge States:** 4

**Start Position**: 141

**End Position**: 151

| Profile Score | Tandem Score | TDA q-value | Volume | Spectrum Matches |
| --- | --- | --- | --- | --- |
| -5.8501 | 151.4248 | 0.0000 | 6.1505e+07 | 2 |

**Adduct Signal Fractions**

- **Unmodified**: 6.1505e+07

### C(C)KSEC(C)ITPN(N)GSIPNDKPFQNVNR Hex 9 HexNAc 2

**Mass:**  4538.9064

(2.923e-06)

**Charge States:** 4

**Start Position**: 277

**End Position**: 300

| Profile Score | Tandem Score | TDA q-value | Volume | Spectrum Matches |
| --- | --- | --- | --- | --- |
| -17.7516 | 150.6278 | 0.0000 | 4.8842e+07 | 2 |

**Adduct Signal Fractions**

- **Unmodified**: 4.8842e+07

### ISIYWTIVKPGDILLIN(N)STGNLIAPR Hex 10 HexNAc 2

**Mass:**  4893.3327

(2.748e-06)

**Charge States:** 4, 5

**Start Position**: 230

**End Position**: 256

| Profile Score | Tandem Score | TDA q-value | Volume | Spectrum Matches |
| --- | --- | --- | --- | --- |
| 15.8552 | 150.5245 | 0.0000 | 1.9186e+08 | 5 |

**Adduct Signal Fractions**

- **Unmodified**: 1.9186e+08

### WAGVTQN(N)GTSSSC(C)R Hex 7 HexNAc 2

**Mass:**  3050.1945

(9.760e-07)

**Charge States:** 3

**Start Position**: 127

**End Position**: 141

| Profile Score | Tandem Score | TDA q-value | Volume | Spectrum Matches |
| --- | --- | --- | --- | --- |
| -15.4155 | 150.3786 | 0.0000 | 9.7858e+06 | 1 |

**Adduct Signal Fractions**

- **Unmodified**: 9.7858e+06

### YPALN(N)VTM(O)PN(D)NEQFDK Hex 8 HexNAc 2

**Mass:**  3599.4567

(2.822e-04)

**Charge States:** 2, 3, 4

**Start Position**: 161

**End Position**: 177

| Profile Score | Tandem Score | TDA q-value | Volume | Spectrum Matches |
| --- | --- | --- | --- | --- |
| 9.2361 | 150.1815 | 0.0000 | 2.6005e+08 | 9 |

**Adduct Signal Fractions**

- **Unmodified**: 2.6005e+08

### ISIYWTIVKPGDILLIN(N)STGNLIAPR Hex 9 HexNAc 2

**Mass:**  4731.2951

(1.662e-06)

**Charge States:** 3, 4, 5

**Start Position**: 230

**End Position**: 256

| Profile Score | Tandem Score | TDA q-value | Volume | Spectrum Matches |
| --- | --- | --- | --- | --- |
| 17.8849 | 149.6387 | 0.0000 | 1.9384e+09 | 8 |

**Adduct Signal Fractions**

- **Unmodified**: 1.9384e+09

### N(N)GTYDHDVYR Fuc 1 Hex 5 HexNAc 4

**Mass:**  3007.1755

(8.879e-07)

**Charge States:** 3

**Start Position**: 483

**End Position**: 493

| Profile Score | Tandem Score | TDA q-value | Volume | Spectrum Matches |
| --- | --- | --- | --- | --- |
| 14.8181 | 148.3058 | 0.0000 | 5.7720e+08 | 6 |

**Adduct Signal Fractions**

- **Unmodified**: 5.7720e+08

### N(N)GTYDHDVYRDEALNNR Fuc 2 Hex 5 HexNAc 4

**Mass:**  3965.6076

(4.102e-08)

**Charge States:** 3, 4

**Start Position**: 483

**End Position**: 500

| Profile Score | Tandem Score | TDA q-value | Volume | Spectrum Matches |
| --- | --- | --- | --- | --- |
| 13.4122 | 148.1258 | 0.0000 | 1.0870e+09 | 3 |

**Adduct Signal Fractions**

- **Unmodified**: 1.0870e+09

### RGSN(N)SSFFSR Fuc 1 Hex 6 HexNAc 6

**Mass:**  3480.3964

(9.624e-07)

**Charge States:** 3

**Start Position**: 141

**End Position**: 151

| Profile Score | Tandem Score | TDA q-value | Volume | Spectrum Matches |
| --- | --- | --- | --- | --- |
| -5.0399 | 147.8007 | 0.0000 | 5.4621e+07 | 2 |

**Adduct Signal Fractions**

- **Unmodified**: 5.4621e+07

### ISIYWTIVKPGDILLIN(N)STGNLIAPR Hex 8 HexNAc 2

**Mass:**  4569.2292

(3.434e-06)

**Charge States:** 3, 4, 5

**Start Position**: 230

**End Position**: 256

| Profile Score | Tandem Score | TDA q-value | Volume | Spectrum Matches |
| --- | --- | --- | --- | --- |
| 14.4895 | 146.6995 | 0.0000 | 1.4299e+08 | 6 |

**Adduct Signal Fractions**

- **Unmodified**: 1.4299e+08

### N(N)GTYDHDVYRDEALNNR Hex 5 HexNAc 2

**Mass:**  3267.3364

(1.402e-06)

**Charge States:** 4

**Start Position**: 483

**End Position**: 500

| Profile Score | Tandem Score | TDA q-value | Volume | Spectrum Matches |
| --- | --- | --- | --- | --- |
| -5.0259 | 143.4251 | 0.0000 | 1.7768e+08 | 1 |

**Adduct Signal Fractions**

- **Unmodified**: 1.7768e+08

### ISIYWTIVKPGDILLIN(N)STGNLIAPR Fuc 2 Hex 4 HexNAc 4 Neu5Ac 1

**Mass:**  4910.3561

(-3.258e-06)

**Charge States:** 4

**Start Position**: 230

**End Position**: 256

| Profile Score | Tandem Score | TDA q-value | Volume | Spectrum Matches |
| --- | --- | --- | --- | --- |
| -4.4014 | 141.1653 | 0.0000 | 2.6831e+07 | 1 |

**Adduct Signal Fractions**

- **Unmodified**: 2.6831e+07

### YPALN(N)VTM(O)PNNEQFDK Hex 4 HexNAc 2

**Mass:**  2950.2540

(3.968e-06)

**Charge States:** 3

**Start Position**: 161

**End Position**: 177

| Profile Score | Tandem Score | TDA q-value | Volume | Spectrum Matches |
| --- | --- | --- | --- | --- |
| -16.8060 | 140.4535 | 0.0000 | 4.9915e+06 | 1 |

**Adduct Signal Fractions**

- **Unmodified**: 4.9915e+06

### C(C)KSEC(C)ITPN(N)GSIPNDKPFQNVNR Hex 7 HexNAc 2

**Mass:**  4214.8000

(3.502e-06)

**Charge States:** 4, 5

**Start Position**: 277

**End Position**: 300

| Profile Score | Tandem Score | TDA q-value | Volume | Spectrum Matches |
| --- | --- | --- | --- | --- |
| -18.2131 | 140.3837 | 0.0000 | 2.8751e+07 | 1 |

**Adduct Signal Fractions**

- **Unmodified**: 2.8751e+07

### RGSN(N)SSFFSR Fuc 1 Hex 3 HexNAc 9 Neu5Ac 2

**Mass:**  4185.6721

(1.566e-06)

**Charge States:** 4

**Start Position**: 141

**End Position**: 151

| Profile Score | Tandem Score | TDA q-value | Volume | Spectrum Matches |
| --- | --- | --- | --- | --- |
| 9.6491 | 138.5433 | 0.0000 | 3.4369e+08 | 3 |

**Adduct Signal Fractions**

- **Unmodified**: 3.4369e+08

### IVKPGDILLIN(N)STGNLIAPR Hex 9 HexNAc 2

**Mass:**  3967.8915

(3.982e-06)

**Charge States:** 4

**Start Position**: 236

**End Position**: 256

| Profile Score | Tandem Score | TDA q-value | Volume | Spectrum Matches |
| --- | --- | --- | --- | --- |
| -4.5982 | 138.3378 | 0.0000 | 1.6498e+07 | 2 |

**Adduct Signal Fractions**

- **Unmodified**: 1.6498e+07

### RGSN(N)SSFFSR Fuc 1 Hex 9 HexNAc 4

**Mass:**  3560.3560

(-9.709e-06)

**Charge States:** 3, 4

**Start Position**: 141

**End Position**: 151

| Profile Score | Tandem Score | TDA q-value | Volume | Spectrum Matches |
| --- | --- | --- | --- | --- |
| 10.9122 | 137.9259 | 0.0000 | 1.1205e+08 | 3 |

**Adduct Signal Fractions**

- **Unmodified**: 1.1205e+08

### N(N)GTYDHDVYRDEALNNR Hex 5 HexNAc 4

**Mass:**  3673.4960

(5.169e-07)

**Charge States:** 4

**Start Position**: 483

**End Position**: 500

| Profile Score | Tandem Score | TDA q-value | Volume | Spectrum Matches |
| --- | --- | --- | --- | --- |
| -3.5901 | 137.8464 | 0.0000 | 3.5387e+08 | 4 |

**Adduct Signal Fractions**

- **Unmodified**: 3.5387e+08

### N(N)GTYDHDVYRDEALNNR Fuc 1 Hex 5 HexNAc 4

**Mass:**  3819.5519

(1.199e-06)

**Charge States:** 3, 4, 5

**Start Position**: 483

**End Position**: 500

| Profile Score | Tandem Score | TDA q-value | Volume | Spectrum Matches |
| --- | --- | --- | --- | --- |
| 17.2001 | 137.7675 | 0.0000 | 5.5350e+09 | 6 |

**Adduct Signal Fractions**

- **Unmodified**: 5.5350e+09

### LGHHAVPN(N)GTIVK Fuc 1 Hex 5 HexNAc 5

**Mass:**  3313.4682

(-6.918e-07)

**Charge States:** 4

**Start Position**: 15

**End Position**: 28

| Profile Score | Tandem Score | TDA q-value | Volume | Spectrum Matches |
| --- | --- | --- | --- | --- |
| -19.8696 | 137.5724 | 0.0000 | 6.7116e+06 | 1 |

**Adduct Signal Fractions**

- **Unmodified**: 6.7116e+06

### N(N)GTYDHDVYRDEALNNR Fuc 1 Hex 4 HexNAc 3

**Mass:**  3454.4218

(7.883e-07)

**Charge States:** 4

**Start Position**: 483

**End Position**: 500

| Profile Score | Tandem Score | TDA q-value | Volume | Spectrum Matches |
| --- | --- | --- | --- | --- |
| -3.6615 | 137.5131 | 0.0000 | 2.2503e+08 | 2 |

**Adduct Signal Fractions**

- **Unmodified**: 2.2503e+08

### N(N)GTYDHDVYRDEALNNR Hex 5 HexNAc 3

**Mass:**  3470.4157

(1.233e-06)

**Charge States:** 4

**Start Position**: 483

**End Position**: 500

| Profile Score | Tandem Score | TDA q-value | Volume | Spectrum Matches |
| --- | --- | --- | --- | --- |
| -17.6480 | 137.3472 | 0.0000 | 9.4429e+07 | 1 |

**Adduct Signal Fractions**

- **Unmodified**: 9.4429e+07

### N(N)GTYDHDVYRDEALNNR Fuc 1 Hex 5 HexNAc 5

**Mass:**  4022.6330

(1.045e-06)

**Charge States:** 3, 4, 5

**Start Position**: 483

**End Position**: 500

| Profile Score | Tandem Score | TDA q-value | Volume | Spectrum Matches |
| --- | --- | --- | --- | --- |
| -2.4612 | 136.9041 | 0.0000 | 2.3356e+09 | 4 |

**Adduct Signal Fractions**

- **Unmodified**: 2.3356e+09

### ISIYWTIVKPGDILLIN(N)STGNLIAPR Hex 10 HexNAc 2

**Mass:**  4896.3259

(6.145e-04)

**Start Position**: 230

**End Position**: 256

| Profile Score | Tandem Score | TDA q-value | Volume | Spectrum Matches |
| --- | --- | --- | --- | --- |
| 0.0000 | 136.1677 | 0.0000 | 0.0000e+00 | 1 |

Adduct:

No Chromatogram Found

### N(N)GTYDHDVYRDEALNNR Fuc 1 Hex 6 HexNAc 5

**Mass:**  4184.6818

(1.598e-07)

**Charge States:** 4

**Start Position**: 483

**End Position**: 500

| Profile Score | Tandem Score | TDA q-value | Volume | Spectrum Matches |
| --- | --- | --- | --- | --- |
| 9.3836 | 135.6054 | 0.0000 | 4.2774e+08 | 2 |

**Adduct Signal Fractions**

- **Unmodified**: 4.2774e+08

### RGSN(N)SSFFSR Hex 6 HexNAc 6

**Mass:**  3334.3353

(-6.280e-10)

**Charge States:** 3, 4

**Start Position**: 141

**End Position**: 151

| Profile Score | Tandem Score | TDA q-value | Volume | Spectrum Matches |
| --- | --- | --- | --- | --- |
| -19.2378 | 134.2595 | 0.0000 | 2.5109e+07 | 1 |

**Adduct Signal Fractions**

- **Unmodified**: 2.5109e+07

### YPALN(N)VTM(O)PN(D)NEQFDK Hex 9 HexNAc 2

**Mass:**  3761.5091

(2.658e-04)

**Charge States:** 2, 3, 4

**Start Position**: 161

**End Position**: 177

| Profile Score | Tandem Score | TDA q-value | Volume | Spectrum Matches |
| --- | --- | --- | --- | --- |
| 9.1566 | 134.0153 | 0.0000 | 2.5438e+08 | 10 |

**Adduct Signal Fractions**

- **Unmodified**: 2.5438e+08

### SEC(C)ITPN(N)GSIPNDKPFQNVNR Hex 8 HexNAc 2

**Mass:**  4088.7260

(1.785e-06)

**Charge States:** 3, 4

**Start Position**: 279

**End Position**: 300

| Profile Score | Tandem Score | TDA q-value | Volume | Spectrum Matches |
| --- | --- | --- | --- | --- |
| 15.7111 | 133.8627 | 0.0000 | 4.7963e+08 | 4 |

**Adduct Signal Fractions**

- **Unmodified**: 4.7963e+08

### N(N)GTYDHDVYRDEALNNR Fuc 3 Hex 5 HexNAc 4

**Mass:**  4111.6686

(9.721e-07)

**Charge States:** 4

**Start Position**: 483

**End Position**: 500

| Profile Score | Tandem Score | TDA q-value | Volume | Spectrum Matches |
| --- | --- | --- | --- | --- |
| -19.7321 | 131.1463 | 0.0000 | 1.3825e+08 | 1 |

**Adduct Signal Fractions**

- **Unmodified**: 1.3825e+08

### N(N)GTYDHDVYRDEALNNR Fuc 1 Hex 6 HexNAc 6

**Mass:**  4387.7727

(2.787e-06)

**Charge States:** 4

**Start Position**: 483

**End Position**: 500

| Profile Score | Tandem Score | TDA q-value | Volume | Spectrum Matches |
| --- | --- | --- | --- | --- |
| -5.1884 | 131.0526 | 0.0000 | 9.1152e+07 | 1 |

**Adduct Signal Fractions**

- **Unmodified**: 9.1152e+07

### N(N)GTYDHDVYRDEALNNR Fuc 1 Hex 8 HexNAc 3

**Mass:**  4102.5895

(-8.702e-06)

**Charge States:** 3, 4

**Start Position**: 483

**End Position**: 500

| Profile Score | Tandem Score | TDA q-value | Volume | Spectrum Matches |
| --- | --- | --- | --- | --- |
| 11.6386 | 130.4967 | 0.0000 | 5.3851e+08 | 4 |

**Adduct Signal Fractions**

- **Unmodified**: 5.3851e+08

### N(N)GTYDHDVYRDEALNNR Fuc 1 Hex 6 HexNAc 3

**Mass:**  3778.5253

(7.920e-07)

**Charge States:** 4

**Start Position**: 483

**End Position**: 500

| Profile Score | Tandem Score | TDA q-value | Volume | Spectrum Matches |
| --- | --- | --- | --- | --- |
| -4.9375 | 129.7530 | 0.0000 | 1.5570e+08 | 3 |

**Adduct Signal Fractions**

- **Unmodified**: 1.5570e+08

### YPALN(N)VTM(O)PN(D)NEQFDK Fuc 2 Hex 3 HexNAc 4 Neu5Ac 1

**Mass:**  3778.5316

(2.611e-04)

**Charge States:** 3

**Start Position**: 161

**End Position**: 177

| Profile Score | Tandem Score | TDA q-value | Volume | Spectrum Matches |
| --- | --- | --- | --- | --- |
| -5.8099 | 129.1703 | 0.0000 | 1.4585e+07 | 2 |

**Adduct Signal Fractions**

- **Unmodified**: 1.4585e+07

### N(N)GTYDHDVYR Hex 6 HexNAc 3

**Mass:**  2820.0910

(5.788e-07)

**Charge States:** 3

**Start Position**: 483

**End Position**: 493

| Profile Score | Tandem Score | TDA q-value | Volume | Spectrum Matches |
| --- | --- | --- | --- | --- |
| -20.1539 | 128.8847 | 0.0000 | 1.5412e+07 | 1 |

**Adduct Signal Fractions**

- **Unmodified**: 1.5412e+07

### SEC(C)ITPN(N)GSIPNDKPFQNVNR Hex 9 HexNAc 2

**Mass:**  4250.7824

(-1.618e-07)

**Charge States:** 3, 4

**Start Position**: 279

**End Position**: 300

| Profile Score | Tandem Score | TDA q-value | Volume | Spectrum Matches |
| --- | --- | --- | --- | --- |
| -1.9696 | 128.5628 | 0.0000 | 6.7688e+08 | 4 |

**Adduct Signal Fractions**

- **Unmodified**: 6.7688e+08

### N(N)GTYDHDVYRDEALNNR Fuc 1 Hex 8 HexNAc 2

**Mass:**  3899.5117

(-9.704e-06)

**Charge States:** 3, 4

**Start Position**: 483

**End Position**: 500

| Profile Score | Tandem Score | TDA q-value | Volume | Spectrum Matches |
| --- | --- | --- | --- | --- |
| 14.5770 | 128.2018 | 0.0000 | 1.2239e+09 | 4 |

**Adduct Signal Fractions**

- **Unmodified**: 1.2239e+09

### IRDIPSRISIYWTIVKPGDILLIN(N)STGNLIAPR Hex 9 HexNAc 2

**Mass:**  5568.7637

(3.112e-06)

**Charge States:** 5

**Start Position**: 223

**End Position**: 256

| Profile Score | Tandem Score | TDA q-value | Volume | Spectrum Matches |
| --- | --- | --- | --- | --- |
| 17.0143 | 127.1610 | 0.0000 | 3.5581e+08 | 5 |

**Adduct Signal Fractions**

- **Unmodified**: 3.5581e+08

### N(N)GTYDHDVYRDEALN(D)NR Fuc 1 Hex 5 HexNAc 4

**Mass:**  3820.5373

(2.597e-04)

**Charge States:** 3, 4, 5

**Start Position**: 483

**End Position**: 500

| Profile Score | Tandem Score | TDA q-value | Volume | Spectrum Matches |
| --- | --- | --- | --- | --- |
| 6.6323 | 125.8854 | 0.0000 | 2.8126e+09 | 10 |

**Adduct Signal Fractions**

- **Unmodified**: 2.8126e+09

### YPALN(N)VTM(O)PNNEQFDKLYIWGVHHPVTDK Hex 8 HexNAc 2

**Mass:**  5144.2855

(4.384e-06)

**Charge States:** 5

**Start Position**: 161

**End Position**: 190

| Profile Score | Tandem Score | TDA q-value | Volume | Spectrum Matches |
| --- | --- | --- | --- | --- |
| -3.9184 | 123.9916 | 0.0000 | 1.1309e+07 | 1 |

**Adduct Signal Fractions**

- **Unmodified**: 1.1309e+07

### SEC(C)ITPN(N)GSIPNDKPFQNVNR Hex 6 HexNAc 2

**Mass:**  3764.6188

(2.548e-06)

**Charge States:** 4

**Start Position**: 279

**End Position**: 300

| Profile Score | Tandem Score | TDA q-value | Volume | Spectrum Matches |
| --- | --- | --- | --- | --- |
| -3.4575 | 123.9747 | 0.0000 | 1.0264e+08 | 3 |

**Adduct Signal Fractions**

- **Unmodified**: 1.0264e+08

### DIPSRISIYWTIVKPGDILLIN(N)STGNLIAPR Hex 9 HexNAc 2

**Mass:**  5299.5739

(1.997e-06)

**Charge States:** 4, 5

**Start Position**: 225

**End Position**: 256

| Profile Score | Tandem Score | TDA q-value | Volume | Spectrum Matches |
| --- | --- | --- | --- | --- |
| -4.4876 | 123.9430 | 0.0000 | 2.2411e+07 | 2 |

**Adduct Signal Fractions**

- **Unmodified**: 2.2411e+07

### SEC(C)ITPN(N)GSIPNDKPFQNVNR Hex 7 HexNAc 2

**Mass:**  3926.6740

(8.427e-07)

**Charge States:** 3

**Start Position**: 279

**End Position**: 300

| Profile Score | Tandem Score | TDA q-value | Volume | Spectrum Matches |
| --- | --- | --- | --- | --- |
| -1.3019 | 123.7120 | 0.0000 | 8.2820e+08 | 3 |

**Adduct Signal Fractions**

- **Unmodified**: 8.2820e+08

### N(N)GTYDHDVYRDEALNNR Fuc 2 Hex 8 HexNAc 3

**Mass:**  4248.6491

(-8.589e-06)

**Charge States:** 4

**Start Position**: 483

**End Position**: 500

| Profile Score | Tandem Score | TDA q-value | Volume | Spectrum Matches |
| --- | --- | --- | --- | --- |
| -17.7958 | 122.7434 | 0.0000 | 1.2188e+08 | 2 |

**Adduct Signal Fractions**

- **Unmodified**: 1.2188e+08

### N(N)GTYDHDVYRDEALNNR Fuc 1 Hex 9 HexNAc 3

**Mass:**  4264.6437

(-8.261e-06)

**Charge States:** 4

**Start Position**: 483

**End Position**: 500

| Profile Score | Tandem Score | TDA q-value | Volume | Spectrum Matches |
| --- | --- | --- | --- | --- |
| -3.3132 | 122.3872 | 0.0000 | 2.4183e+08 | 2 |

**Adduct Signal Fractions**

- **Unmodified**: 2.4183e+08

### N(N)GTYDHDVYR Fuc 1 Hex 6 HexNAc 5

**Mass:**  3372.3059

(7.530e-07)

**Charge States:** 3, 4

**Start Position**: 483

**End Position**: 493

| Profile Score | Tandem Score | TDA q-value | Volume | Spectrum Matches |
| --- | --- | --- | --- | --- |
| -19.2821 | 122.0768 | 0.0000 | 7.7852e+07 | 3 |

**Adduct Signal Fractions**

- **Unmodified**: 7.7852e+07

### N(N)GTYDHDVYRDEALNNR Fuc 1 Hex 5 HexNAc 3

**Mass:**  3616.4750

(1.243e-06)

**Charge States:** 4

**Start Position**: 483

**End Position**: 500

| Profile Score | Tandem Score | TDA q-value | Volume | Spectrum Matches |
| --- | --- | --- | --- | --- |
| -3.7566 | 121.3739 | 0.0000 | 1.4990e+08 | 2 |

**Adduct Signal Fractions**

- **Unmodified**: 1.4990e+08

### YPALN(N)VTM(O)PN(D)NEQFDK Hex 7 HexNAc 2

**Mass:**  3437.4007

(2.909e-04)

**Charge States:** 3

**Start Position**: 161

**End Position**: 177

| Profile Score | Tandem Score | TDA q-value | Volume | Spectrum Matches |
| --- | --- | --- | --- | --- |
| 6.2627 | 120.7043 | 0.0000 | 1.3969e+07 | 3 |

**Adduct Signal Fractions**

- **Unmodified**: 1.3969e+07

### N(N)GTYDHDVYRDEALNNRFQIK Fuc 1 Hex 5 HexNAc 4

**Mass:**  4335.8675

(2.946e-06)

**Charge States:** 5

**Start Position**: 483

**End Position**: 504

| Profile Score | Tandem Score | TDA q-value | Volume | Spectrum Matches |
| --- | --- | --- | --- | --- |
| -18.2820 | 119.9528 | 0.0000 | 4.6722e+07 | 1 |

**Adduct Signal Fractions**

- **Unmodified**: 4.6722e+07

### YPALN(N)VTM(O)PNNEQFDKLYIWGVHHPVTDK Hex 9 HexNAc 2

**Mass:**  5306.3406

(4.667e-06)

**Charge States:** 5

**Start Position**: 161

**End Position**: 190

| Profile Score | Tandem Score | TDA q-value | Volume | Spectrum Matches |
| --- | --- | --- | --- | --- |
| -18.1868 | 119.6587 | 0.0000 | 1.3504e+07 | 1 |

**Adduct Signal Fractions**

- **Unmodified**: 1.3504e+07

### N(N)GTYDHDVYR Fuc 3 Hex 5 HexNAc 4

**Mass:**  3299.2879

(2.949e-07)

**Charge States:** 3

**Start Position**: 483

**End Position**: 493

| Profile Score | Tandem Score | TDA q-value | Volume | Spectrum Matches |
| --- | --- | --- | --- | --- |
| -19.7966 | 119.5766 | 0.0000 | 2.2022e+07 | 1 |

**Adduct Signal Fractions**

- **Unmodified**: 2.2022e+07

### N(N)GTYDHDVYRDEALNNR Fuc 2 Hex 5 HexNAc 5

**Mass:**  4168.6882

(4.557e-07)

**Charge States:** 4

**Start Position**: 483

**End Position**: 500

| Profile Score | Tandem Score | TDA q-value | Volume | Spectrum Matches |
| --- | --- | --- | --- | --- |
| -4.0396 | 119.1999 | 0.0048 | 1.5204e+08 | 1 |

**Adduct Signal Fractions**

- **Unmodified**: 1.5204e+08

### SEC(C)ITPN(N)GSIPNDKPFQNVNR Hex 5 HexNAc 2

**Mass:**  3602.5636

(1.981e-06)

**Charge States:** 4

**Start Position**: 279

**End Position**: 300

| Profile Score | Tandem Score | TDA q-value | Volume | Spectrum Matches |
| --- | --- | --- | --- | --- |
| -18.7843 | 118.2153 | 0.0048 | 4.7180e+07 | 2 |

**Adduct Signal Fractions**

- **Unmodified**: 4.7180e+07

### N(N)GTYDHDVYRDEALNNR Fuc 2 Hex 4 HexNAc 3

**Mass:**  3600.4792

(1.325e-06)

**Charge States:** 4

**Start Position**: 483

**End Position**: 500

| Profile Score | Tandem Score | TDA q-value | Volume | Spectrum Matches |
| --- | --- | --- | --- | --- |
| -4.7459 | 117.7052 | 0.0084 | 8.1879e+07 | 11 |

**Adduct Signal Fractions**

- **Unmodified**: 8.1879e+07

### N(N)GTYDHDVYRDEALNNR Fuc 1 Hex 9 HexNAc 4

**Mass:**  4467.7194

(-9.105e-06)

**Charge States:** 4

**Start Position**: 483

**End Position**: 500

| Profile Score | Tandem Score | TDA q-value | Volume | Spectrum Matches |
| --- | --- | --- | --- | --- |
| -18.8547 | 117.2269 | 0.0084 | 4.5230e+07 | 1 |

**Adduct Signal Fractions**

- **Unmodified**: 4.5230e+07

### ISIYWTIVKPGDILLIN(N)STGNLIAPR Hex 5 HexNAc 2

**Mass:**  4083.0688

(2.874e-06)

**Charge States:** 3, 4

**Start Position**: 230

**End Position**: 256

| Profile Score | Tandem Score | TDA q-value | Volume | Spectrum Matches |
| --- | --- | --- | --- | --- |
| -18.4365 | 116.8444 | 0.0084 | 1.2159e+07 | 2 |

**Adduct Signal Fractions**

- **Unmodified**: 1.2159e+07

### N(N)GTYDHDVYRDEALNNR Fuc 2 Hex 9 HexNAc 3

**Mass:**  4410.7019

(-8.311e-06)

**Charge States:** 4

**Start Position**: 483

**End Position**: 500

| Profile Score | Tandem Score | TDA q-value | Volume | Spectrum Matches |
| --- | --- | --- | --- | --- |
| -4.7336 | 116.8328 | 0.0084 | 8.7718e+07 | 1 |

**Adduct Signal Fractions**

- **Unmodified**: 8.7718e+07

### YPALN(N)VTMPN(D)NEQFDK Hex 8 HexNAc 2

**Mass:**  3583.4584

(2.790e-04)

**Charge States:** 3

**Start Position**: 161

**End Position**: 177

| Profile Score | Tandem Score | TDA q-value | Volume | Spectrum Matches |
| --- | --- | --- | --- | --- |
| -6.0713 | 115.2378 | 0.0084 | 2.7051e+07 | 2 |

**Adduct Signal Fractions**

- **Unmodified**: 2.7051e+07

### N(N)GTYDHDVYRDEALNNR Hex 7 HexNAc 3

**Mass:**  3794.5217

(1.201e-06)

**Charge States:** 4

**Start Position**: 483

**End Position**: 500

| Profile Score | Tandem Score | TDA q-value | Volume | Spectrum Matches |
| --- | --- | --- | --- | --- |
| -19.7046 | 115.1792 | 0.0084 | 3.8212e+07 | 1 |

**Adduct Signal Fractions**

- **Unmodified**: 3.8212e+07

### IRDIPSRISIYWTIVKPGDILLIN(N)STGNLIAPR Hex 8 HexNAc 2

**Mass:**  5406.7111

(3.137e-06)

**Charge States:** 5

**Start Position**: 223

**End Position**: 256

| Profile Score | Tandem Score | TDA q-value | Volume | Spectrum Matches |
| --- | --- | --- | --- | --- |
| -4.6849 | 112.4763 | 0.0084 | 2.0910e+07 | 3 |

**Adduct Signal Fractions**

- **Unmodified**: 2.0910e+07

### N(N)GTYDHDVYR Fuc 1 Hex 5 HexNAc 4 Neu5Ac 1

**Mass:**  3298.2702

(1.102e-06)

**Charge States:** 3

**Start Position**: 483

**End Position**: 493

| Profile Score | Tandem Score | TDA q-value | Volume | Spectrum Matches |
| --- | --- | --- | --- | --- |
| -20.9306 | 112.3763 | 0.0084 | 8.3460e+06 | 1 |

**Adduct Signal Fractions**

- **Unmodified**: 8.3460e+06

### N(N)GTYDHDVYR Hex 5 HexNAc 4

**Mass:**  2861.1183

(1.769e-06)

**Charge States:** 3

**Start Position**: 483

**End Position**: 493

| Profile Score | Tandem Score | TDA q-value | Volume | Spectrum Matches |
| --- | --- | --- | --- | --- |
| -18.2141 | 110.8616 | 0.0084 | 2.9041e+07 | 1 |

**Adduct Signal Fractions**

- **Unmodified**: 2.9041e+07

### WTIVKPGDILLIN(N)STGNLIAPR Hex 9 HexNAc 2

**Mass:**  4255.0158

(2.721e-06)

**Charge States:** 4

**Start Position**: 234

**End Position**: 256

| Profile Score | Tandem Score | TDA q-value | Volume | Spectrum Matches |
| --- | --- | --- | --- | --- |
| -19.5071 | 109.1939 | 0.0084 | 6.4515e+06 | 2 |

**Adduct Signal Fractions**

- **Unmodified**: 6.4515e+06

### N(N)GTYDHDVYRDEALNNR Hex 7 HexNAc 2

**Mass:**  3591.4479

(2.855e-06)

**Charge States:** 4

**Start Position**: 483

**End Position**: 500

| Profile Score | Tandem Score | TDA q-value | Volume | Spectrum Matches |
| --- | --- | --- | --- | --- |
| -19.5457 | 108.5680 | 0.0084 | 2.1454e+07 | 1 |

**Adduct Signal Fractions**

- **Unmodified**: 2.1454e+07

### YPALN(N)VTMPN(D)NEQFDK Hex 9 HexNAc 2

**Mass:**  3745.5133

(2.654e-04)

**Charge States:** 3

**Start Position**: 161

**End Position**: 177

| Profile Score | Tandem Score | TDA q-value | Volume | Spectrum Matches |
| --- | --- | --- | --- | --- |
| -5.8337 | 107.4223 | 0.0120 | 3.3311e+07 | 2 |

**Adduct Signal Fractions**

- **Unmodified**: 3.3311e+07

### N(N)GTYDHDVYRDEALNNR Fuc 1 Hex 4 HexNAc 4

**Mass:**  3657.5102

(3.873e-06)

**Charge States:** 4

**Start Position**: 483

**End Position**: 500

| Profile Score | Tandem Score | TDA q-value | Volume | Spectrum Matches |
| --- | --- | --- | --- | --- |
| -5.6689 | 107.3257 | 0.0120 | 3.3165e+07 | 1 |

**Adduct Signal Fractions**

- **Unmodified**: 3.3165e+07

### SEC(C)ITPN(N)GSIPN(D)DKPFQNVNR Hex 8 HexNAc 2

**Mass:**  4089.7197

(2.459e-04)

**Charge States:** 3, 4

**Start Position**: 279

**End Position**: 300

| Profile Score | Tandem Score | TDA q-value | Volume | Spectrum Matches |
| --- | --- | --- | --- | --- |
| 9.5743 | 105.6654 | 0.0120 | 9.1876e+07 | 3 |

**Adduct Signal Fractions**

- **Unmodified**: 9.1876e+07

### SEC(C)ITPN(N)GSIPN(D)DKPFQNVNR Hex 7 HexNAc 2

**Mass:**  3927.6650

(2.554e-04)

**Charge States:** 3

**Start Position**: 279

**End Position**: 300

| Profile Score | Tandem Score | TDA q-value | Volume | Spectrum Matches |
| --- | --- | --- | --- | --- |
| 8.2496 | 105.3339 | 0.0120 | 9.8829e+07 | 3 |

**Adduct Signal Fractions**

- **Unmodified**: 9.8829e+07

### N(N)GTYDHDVYRDEALN(D)NR Hex 6 HexNAc 3

**Mass:**  3633.4545

(2.743e-04)

**Charge States:** 4

**Start Position**: 483

**End Position**: 500

| Profile Score | Tandem Score | TDA q-value | Volume | Spectrum Matches |
| --- | --- | --- | --- | --- |
| -5.9883 | 105.1741 | 0.0120 | 1.4917e+08 | 2 |

**Adduct Signal Fractions**

- **Unmodified**: 1.4917e+08

### N(N)GTYDHDVYR Fuc 1 Hex 6 HexNAc 3

**Mass:**  2966.1483

(1.248e-06)

**Charge States:** 3

**Start Position**: 483

**End Position**: 493

| Profile Score | Tandem Score | TDA q-value | Volume | Spectrum Matches |
| --- | --- | --- | --- | --- |
| -18.0313 | 104.6894 | 0.0120 | 2.0380e+07 | 1 |

**Adduct Signal Fractions**

- **Unmodified**: 2.0380e+07

### N(N)GTYDHDVYR Fuc 2 Hex 5 HexNAc 5

**Mass:**  3356.3106

(6.377e-07)

**Charge States:** 3

**Start Position**: 483

**End Position**: 493

| Profile Score | Tandem Score | TDA q-value | Volume | Spectrum Matches |
| --- | --- | --- | --- | --- |
| -19.5740 | 103.2114 | 0.0154 | 1.0652e+07 | 1 |

**Adduct Signal Fractions**

- **Unmodified**: 1.0652e+07

### RGSN(N)SSFFSR Fuc 1 Hex 7 HexNAc 8

**Mass:**  4048.6447

(9.848e-06)

**Charge States:** 4

**Start Position**: 141

**End Position**: 151

| Profile Score | Tandem Score | TDA q-value | Volume | Spectrum Matches |
| --- | --- | --- | --- | --- |
| -18.5622 | 103.1843 | 0.0154 | 6.2083e+07 | 1 |

**Adduct Signal Fractions**

- **Unmodified**: 6.2083e+07

### IRDIPSRISIYWTIVKPGDILLIN(N)STGNLIAPR Hex 10 HexNAc 2

**Mass:**  5730.8071

(1.022e-06)

**Charge States:** 4, 5

**Start Position**: 223

**End Position**: 256

| Profile Score | Tandem Score | TDA q-value | Volume | Spectrum Matches |
| --- | --- | --- | --- | --- |
| -19.7213 | 103.1011 | 0.0154 | 1.8818e+07 | 1 |

**Adduct Signal Fractions**

- **Unmodified**: 1.8818e+07

### N(N)GTYDHDVYRDEALN(D)NR Hex 5 HexNAc 4

**Mass:**  3674.4804

(2.714e-04)

**Charge States:** 3, 4

**Start Position**: 483

**End Position**: 500

| Profile Score | Tandem Score | TDA q-value | Volume | Spectrum Matches |
| --- | --- | --- | --- | --- |
| 7.8155 | 103.0444 | 0.0154 | 1.7459e+08 | 2 |

**Adduct Signal Fractions**

- **Unmodified**: 1.7459e+08

### N(N)GTYDHDVYRDEALN(D)NR Fuc 3 Hex 5 HexNAc 4

**Mass:**  4112.6574

(2.418e-04)

**Charge States:** 4

**Start Position**: 483

**End Position**: 500

| Profile Score | Tandem Score | TDA q-value | Volume | Spectrum Matches |
| --- | --- | --- | --- | --- |
| 6.4264 | 98.7598 | 0.0225 | 1.8185e+08 | 2 |

**Adduct Signal Fractions**

- **Unmodified**: 1.8185e+08

### SEC(C)ITPN(N)GSIPN(D)DKPFQNVNR Hex 9 HexNAc 2

**Mass:**  4251.7687

(2.353e-04)

**Charge States:** 3, 4

**Start Position**: 279

**End Position**: 300

| Profile Score | Tandem Score | TDA q-value | Volume | Spectrum Matches |
| --- | --- | --- | --- | --- |
| 6.9078 | 97.9483 | 0.0225 | 1.8945e+08 | 6 |

**Adduct Signal Fractions**

- **Unmodified**: 1.8945e+08

### N(N)GTYDHDVYRDEALN(D)NR Fuc 1 Hex 8 HexNAc 2

**Mass:**  3900.5036

(2.439e-04)

**Charge States:** 3, 4

**Start Position**: 483

**End Position**: 500

| Profile Score | Tandem Score | TDA q-value | Volume | Spectrum Matches |
| --- | --- | --- | --- | --- |
| 8.3959 | 96.1559 | 0.0225 | 4.0794e+08 | 3 |

**Adduct Signal Fractions**

- **Unmodified**: 4.0794e+08

### ISIYWTIVKPGDILLIN(N)STGNLIAPR Hex 3 HexNAc 4 Neu5Ac 2

**Mass:**  4747.2805

(-3.830e-06)

**Charge States:** 5

**Start Position**: 230

**End Position**: 256

| Profile Score | Tandem Score | TDA q-value | Volume | Spectrum Matches |
| --- | --- | --- | --- | --- |
| -18.8506 | 91.3718 | 0.0403 | 2.3171e+07 | 3 |

**Adduct Signal Fractions**

- **Unmodified**: 2.3171e+07

### N(N)GTYDHDVYRDEALNNR Fuc 1 Hex 8 HexNAc 4

**Mass:**  4305.6726

(-8.093e-06)

**Charge States:** 4

**Start Position**: 483

**End Position**: 500

| Profile Score | Tandem Score | TDA q-value | Volume | Spectrum Matches |
| --- | --- | --- | --- | --- |
| -19.7629 | 90.5329 | 0.0403 | 2.0922e+07 | 1 |

**Adduct Signal Fractions**

- **Unmodified**: 2.0922e+07

### N(N)GTYDHDVYR Fuc 2 Hex 6 HexNAc 5

**Mass:**  3520.3793

(5.701e-04)

**Charge States:** 4

**Start Position**: 483

**End Position**: 493

| Profile Score | Tandem Score | TDA q-value | Volume | Spectrum Matches |
| --- | --- | --- | --- | --- |
| -22.1065 | 90.0969 | 0.0435 | 1.3342e+06 | 2 |

**Adduct Signal Fractions**

- **Unmodified**: 1.3342e+06

### cus|SWZNA|A/Switzerland/9715293/2013

**>cus|SWZNA|A/Switzerland/9715293/2013**  
 `MNPNQKIITIGSVSLTISTICFFMQIAILITTVTLHFKQYEFNSPPNNQVMLCEPTIIERNITEIVYLTNTTIEKEICPK  
PAEYRNWSKPQCGITGFAPFSKDNSIRLSAGGDIWVTREPYVSCDPDKCYQFALGQGTTLNNVHSNNTVRDRTPYRTLLM  
NELGVPFHLGTKQVCIAWSSSSCHDGKAWLHVCITGDDKNATASFIYNGRLVDSVVSWSKDILRTQESECVCINGTCTVV  
MTDGSASGKADTKILFIEEGKIVHTSTLSGSAQHVEECSCYPRYPGVRCVCRDNWKGSNRPIVDINIKDHSIVSSYVCSG  
LVGDTPRKNDSSSSSHCLDPNNEEGGHGVKGWAFDDGNDVWMGRTINETSRLGYETFKVIEGWSNPKSKLQTNRQVIVDR  
GDRSGYSGIFSVEGKSCINRCFYVELIRGRKEETEVLWTSNSIVVFCGTSGTYGTGSWPDGADLNLMPI`

| Site | Type | Count |
| --- | --- | --- |
| 329 | N-linked | 2 |
| 86 | N-linked | 9 |
| 200 | N-linked | 2 |

| Mass | Sequence | MS_2_ Score | `q`-value | MS_1_ Score | Signal | Protein Coordinates | Tandem Scans | Start Time | Apex Time | End Time |
| --- | --- | --- | --- | --- | --- | --- | --- | --- | --- | --- |
| 3788.5787 | N(N)WSKPQC(C)GITGFAPFSK Hex 9 HexNAc 2 | 161.257 | 0.0 | -1.075 | 6.359e+08 | 85 - 102 | 3 | 42.61 | 42.78 | 43.12 |
| 3626.5224 | N(N)WSKPQC(C)GITGFAPFSK Hex 8 HexNAc 2 | 158.165 | 0.0 | -1.088 | 6.037e+08 | 85 - 102 | 4 | 42.78 | 42.95 | 43.28 |
| 3411.3972 | N(N)WSKPQC(C)GITGFAPF Hex 8 HexNAc 2 | 158.011 | 0.0 | -16.863 | 1.048e+08 | 85 - 100 | 3 | 51.54 | 51.54 | 51.71 |
| 3573.4490 | N(N)WSKPQC(C)GITGFAPF Hex 9 HexNAc 2 | 152.898 | 0.0 | -16.575 | 1.291e+08 | 85 - 100 | 3 | 51.37 | 51.37 | 51.54 |
| 3464.4693 | N(N)WSKPQC(C)GITGFAPFSK Hex 7 HexNAc 2 | 148.336 | 0.0 | -4.022 | 4.228e+07 | 85 - 102 | 2 | 42.95 | 43.12 | 43.28 |
| 3590.4726 | N(N)WSKPQC(C)GITGFAPF Fuc 2 Hex 3 HexNAc 4 Neu5Ac 1 | 132.094 | 0.0 | -18.770 | 1.344e+07 | 85 - 100 | 1 | 51.37 | 51.37 | 51.54 |
| 4279.7105 | KN(N)DSSSSSHC(C)LDPNNEEGGHGVK Hex 5 HexNAc 5 | 129.103 | 0.0 | -18.814 | 6.634e+07 | 327 - 350 | 3 | 14.92 | 14.92 | 14.92 |
| 3249.3423 | N(N)WSKPQC(C)GITGFAPF Hex 7 HexNAc 2 | 127.682 | 0.0 | -18.462 | 6.743e+06 | 85 - 100 | 2 | 51.71 | 51.71 | 51.88 |
| 3302.4147 | N(N)WSKPQC(C)GITGFAPFSK Hex 6 HexNAc 2 | 125.320 | 0.0 | -18.993 | 5.060e+06 | 85 - 102 | 1 | 43.28 | 43.28 | 43.28 |
| 3670.4712 | KN(N)DSSSSSHC(C)LDPNNEEGGHGVK Hex 5 HexNAc 2 | 125.143 | 0.0 | -19.431 | 5.365e+07 | 327 - 350 | 2 | 14.92 | 14.92 | 14.92 |
| 2978.3179 | N(N)WSKPQC(C)GITGFAPFSK Hex 4 HexNAc 2 | 104.020 | 0.012 | -20.572 | 6.118e+05 | 85 - 102 | 1 | 43.62 | 43.62 | 43.62 |
| 4472.8974 | AWLHVC(C)ITGDDKN(N)ATASFIYNGR Hex 9 HexNAc 2 | 98.389 | 0.022 | -19.203 | 4.236e+06 | 187 - 210 | 1 | 46.11 | 46.28 | 46.28 |
| 4310.8461 | AWLHVC(C)ITGDDKN(N)ATASFIYNGR Hex 8 HexNAc 2 | 89.316 | 0.043 | -18.672 | 2.037e+06 | 187 - 210 | 1 | 46.28 | 46.28 | 46.28 |

### N(N)WSKPQC(C)GITGFAPFSK Hex 9 HexNAc 2

**Mass:**  3788.5761

(5.808e-06)

**Charge States:** 3, 4

**Start Position**: 86

**End Position**: 103

| Profile Score | Tandem Score | TDA q-value | Volume | Spectrum Matches |
| --- | --- | --- | --- | --- |
| -1.0747 | 161.2572 | 0.0000 | 6.3595e+08 | 3 |

**Adduct Signal Fractions**

- **Unmodified**: 6.3595e+08

### N(N)WSKPQC(C)GITGFAPFSK Hex 8 HexNAc 2

**Mass:**  3626.5197

(2.719e-06)

**Charge States:** 3, 4

**Start Position**: 86

**End Position**: 103

| Profile Score | Tandem Score | TDA q-value | Volume | Spectrum Matches |
| --- | --- | --- | --- | --- |
| -1.0881 | 158.1646 | 0.0000 | 6.0369e+08 | 4 |

**Adduct Signal Fractions**

- **Unmodified**: 6.0369e+08

### N(N)WSKPQC(C)GITGFAPF Hex 8 HexNAc 2

**Mass:**  3411.3975

(3.797e-06)

**Charge States:** 2, 3, 4

**Start Position**: 86

**End Position**: 101

| Profile Score | Tandem Score | TDA q-value | Volume | Spectrum Matches |
| --- | --- | --- | --- | --- |
| -16.8629 | 158.0110 | 0.0000 | 1.0476e+08 | 3 |

**Adduct Signal Fractions**

- **Unmodified**: 1.0476e+08

### N(N)WSKPQC(C)GITGFAPF Hex 9 HexNAc 2

**Mass:**  3573.4488

(3.209e-06)

**Charge States:** 2, 3, 4

**Start Position**: 86

**End Position**: 101

| Profile Score | Tandem Score | TDA q-value | Volume | Spectrum Matches |
| --- | --- | --- | --- | --- |
| -16.5753 | 152.8975 | 0.0000 | 1.2908e+08 | 3 |

**Adduct Signal Fractions**

- **Unmodified**: 1.2908e+08

### N(N)WSKPQC(C)GITGFAPFSK Hex 7 HexNAc 2

**Mass:**  3464.4728

(4.096e-06)

**Charge States:** 3

**Start Position**: 86

**End Position**: 103

| Profile Score | Tandem Score | TDA q-value | Volume | Spectrum Matches |
| --- | --- | --- | --- | --- |
| -4.0223 | 148.3357 | 0.0000 | 4.2275e+07 | 2 |

**Adduct Signal Fractions**

- **Unmodified**: 4.2275e+07

### N(N)WSKPQC(C)GITGFAPF Fuc 2 Hex 3 HexNAc 4 Neu5Ac 1

**Mass:**  3590.4739

(-4.625e-06)

**Charge States:** 3

**Start Position**: 86

**End Position**: 101

| Profile Score | Tandem Score | TDA q-value | Volume | Spectrum Matches |
| --- | --- | --- | --- | --- |
| -18.7695 | 132.0942 | 0.0000 | 1.3442e+07 | 1 |

**Adduct Signal Fractions**

- **Unmodified**: 1.3442e+07

### KN(N)DSSSSSHC(C)LDPNNEEGGHGVK Hex 5 HexNAc 5

**Mass:**  4279.7112

(8.872e-07)

**Charge States:** 4, 5

**Start Position**: 328

**End Position**: 351

| Profile Score | Tandem Score | TDA q-value | Volume | Spectrum Matches |
| --- | --- | --- | --- | --- |
| -18.8141 | 129.1034 | 0.0000 | 6.6339e+07 | 3 |

**Adduct Signal Fractions**

- **Unmodified**: 6.6339e+07

### N(N)WSKPQC(C)GITGFAPF Hex 7 HexNAc 2

**Mass:**  3249.3422

(3.285e-06)

**Charge States:** 3

**Start Position**: 86

**End Position**: 101

| Profile Score | Tandem Score | TDA q-value | Volume | Spectrum Matches |
| --- | --- | --- | --- | --- |
| -18.4622 | 127.6818 | 0.0000 | 6.7433e+06 | 2 |

**Adduct Signal Fractions**

- **Unmodified**: 6.7433e+06

### N(N)WSKPQC(C)GITGFAPFSK Hex 6 HexNAc 2

**Mass:**  3302.4138

(2.444e-06)

**Charge States:** 3, 4

**Start Position**: 86

**End Position**: 103

| Profile Score | Tandem Score | TDA q-value | Volume | Spectrum Matches |
| --- | --- | --- | --- | --- |
| -18.9934 | 125.3200 | 0.0000 | 5.0598e+06 | 1 |

**Adduct Signal Fractions**

- **Unmodified**: 5.0598e+06

### KN(N)DSSSSSHC(C)LDPNNEEGGHGVK Hex 5 HexNAc 2

**Mass:**  3670.4705

(7.723e-07)

**Charge States:** 4, 5

**Start Position**: 328

**End Position**: 351

| Profile Score | Tandem Score | TDA q-value | Volume | Spectrum Matches |
| --- | --- | --- | --- | --- |
| -19.4306 | 125.1429 | 0.0000 | 5.3651e+07 | 2 |

**Adduct Signal Fractions**

- **Unmodified**: 5.3651e+07

### N(N)WSKPQC(C)GITGFAPFSK Hex 4 HexNAc 2

**Mass:**  2978.3179

(5.953e-06)

**Charge States:** 3

**Start Position**: 86

**End Position**: 103

| Profile Score | Tandem Score | TDA q-value | Volume | Spectrum Matches |
| --- | --- | --- | --- | --- |
| -20.5717 | 104.0199 | 0.0120 | 6.1182e+05 | 1 |

**Adduct Signal Fractions**

- **Unmodified**: 6.1182e+05

### AWLHVC(C)ITGDDKN(N)ATASFIYNGR Hex 9 HexNAc 2

**Mass:**  4472.8977

(3.034e-06)

**Charge States:** 4

**Start Position**: 188

**End Position**: 211

| Profile Score | Tandem Score | TDA q-value | Volume | Spectrum Matches |
| --- | --- | --- | --- | --- |
| -19.2026 | 98.3892 | 0.0225 | 4.2356e+06 | 1 |

**Adduct Signal Fractions**

- **Unmodified**: 4.2356e+06

### AWLHVC(C)ITGDDKN(N)ATASFIYNGR Hex 8 HexNAc 2

**Mass:**  4310.8469

(3.753e-06)

**Charge States:** 4, 5

**Start Position**: 188

**End Position**: 211

| Profile Score | Tandem Score | TDA q-value | Volume | Spectrum Matches |
| --- | --- | --- | --- | --- |
| -18.6721 | 89.3160 | 0.0435 | 2.0373e+06 | 1 |

**Adduct Signal Fractions**

- **Unmodified**: 2.0373e+06

### tr|E1C041|E1C041\_CHICK

**>tr|E1C041|E1C041\_CHICK**  
 `MRTGAGEAVGVLCLVSLVAALDYKYHHSEELEAYLKEVHAAYPALTHLHSIGRSVEGRDLWVLVLGRFPTQHKIGIPEFK  
YVANMHGDETVGREILLHLIDHLVTNYGRDPVITRLLNNTRIHIMPTMNPDGFEATVVPDCYYSRGRYNKNGEDLNRNFP  
DAFENNNNLIQPETQAVINWIKNETFVLSANLHGGALVASYTFDNGNSVTGTSNGYSRSPDDDVFIHLAKTYSFNHASMY  
KGTGCDSKQTFPDGITNGYSWYQLEGGMQDYNYVWGQCFEITLELSCCKYPPAEQLEKFWRDNKVALVEYIKQVHLGVKG  
QVTDKNGNPIPNAIVEAKGRPHICPYRTNEHGEYFLLLLPGKYVINATVPGFKSMLKTVDIPDNTANFSALKQDFSFPEV  
SVKPRPASCPRTPLYQELERSSAAVKPTLHILVLVIVTISILK`

| Site | Type | Count |
| --- | --- | --- |
| 366 | N-linked | 3 |

| Mass | Sequence | MS_2_ Score | `q`-value | MS_1_ Score | Signal | Protein Coordinates | Tandem Scans | Start Time | Apex Time | End Time |
| --- | --- | --- | --- | --- | --- | --- | --- | --- | --- | --- |
| 3072.3043 | YVIN(N)ATVPGFK Hex 9 HexNAc 2 | 164.047 | 0.0 | -2.630 | 4.768e+07 | 362 - 373 | 2 | 40.44 | 40.61 | 40.78 |
| 2910.2526 | YVIN(N)ATVPGFK Hex 8 HexNAc 2 | 151.168 | 0.0 | -3.026 | 1.338e+07 | 362 - 373 | 1 | 40.61 | 40.78 | 40.95 |
| 3089.3290 | YVIN(N)ATVPGFK Fuc 2 Hex 3 HexNAc 4 Neu5Ac 1 | 143.529 | 0.0 | -15.602 | 5.268e+06 | 362 - 373 | 1 | 40.61 | 40.61 | 40.78 |

### YVIN(N)ATVPGFK Hex 9 HexNAc 2

**Mass:**  3072.3041

(3.230e-06)

**Charge States:** 3

**Start Position**: 363

**End Position**: 374

| Profile Score | Tandem Score | TDA q-value | Volume | Spectrum Matches |
| --- | --- | --- | --- | --- |
| -2.6301 | 164.0466 | 0.0000 | 4.7679e+07 | 2 |

**Adduct Signal Fractions**

- **Unmodified**: 4.7679e+07

### YVIN(N)ATVPGFK Hex 8 HexNAc 2

**Mass:**  2910.2550

(4.639e-06)

**Charge States:** 3

**Start Position**: 363

**End Position**: 374

| Profile Score | Tandem Score | TDA q-value | Volume | Spectrum Matches |
| --- | --- | --- | --- | --- |
| -3.0258 | 151.1675 | 0.0000 | 1.3380e+07 | 1 |

**Adduct Signal Fractions**

- **Unmodified**: 1.3380e+07

### YVIN(N)ATVPGFK Fuc 2 Hex 3 HexNAc 4 Neu5Ac 1

**Mass:**  3089.3292

(-5.890e-06)

**Charge States:** 3

**Start Position**: 363

**End Position**: 374

| Profile Score | Tandem Score | TDA q-value | Volume | Spectrum Matches |
| --- | --- | --- | --- | --- |
| -15.6025 | 143.5285 | 0.0000 | 5.2679e+06 | 1 |

**Adduct Signal Fractions**

- **Unmodified**: 5.2679e+06

### tr|A0A1D5PNI3|A0A1D5PNI3\_CHICK

**>tr|A0A1D5PNI3|A0A1D5PNI3\_CHICK**  
 `MTTSTLAQTTSLPGTSTPTPASSTLMTTSTPAQTSLMPGHFKAQLLHLLHFTTTSTPAQTTSVPASSSPTPASSALPTAT  
TPAQTPGTSSPTPASSALPTTTTPAETTTFPATSSTTPASSAPPTTSTPAQTTSLPRTSTPTPASSALTTTTTPEQTTTV  
PGTSTPTPASSTVLTTSTPVQTTSMPGISSPTPASSALLTKSTPAQTTTVPATSSPTPASSALPTTTTPEQTTTESGTST  
PTPASSGPPTTSTPAETTSFPATSSPTPASSTLMTTSTPAQTTTVPGTSSPTPVSSALLTTRTLAQTKASLQLNSSPTSV  
SSALSTTSTPAETTTVPGTSIPTAASSTLLTTSTLAQTTSFPATSTPTPSSSTLMTTSTRAETKTSLQLNSSPTPASLAL  
PTTSTPAQTTSVPTSSSPTPASSALSTTSTPALTTALRSTSQIMSPSSPATSPSPTATSAGTVTTAQETTSPAGTTNALT  
STIRTTSTQTLSSSSPPYTTAALSYTAIPTSTPATPTTSAPQTTIASSSTPTESTALLTTTVPSTTQDISSQTAQTSNAA  
TSSTTNSATSARLTRTHAETTSSTTTFTSHSRPSASQTSTPGTTISPLPATTAQQTTAPAGTTPPTTASTTLFSTRETSS  
KEEETSTVVTSSQTPASSVLPSTSTPAQTTSMPSTSSPTPASSTLMTTSTLAQTTSLPGTSVPTPASSAPRTTTTPAQTT  
SVPGNSSPIPPYSTLLTTSTLAQTTSLPGTSTPTPASSTLMTTSTPAETKTSLLLNSSPNICIFCSSDYNHSCTDNISAR  
HFKPNSCTAIKHSCRKQTFITTFTSRARTSASWTTTPGTTTSALTATTARETTTPAGTTHPHNSNIETTSTSTIKAPTRP  
PTTPAGHERFTVNFTITNLPYDSDLQIPHSAKLNATQRVMTTLLNRLLKDTSIGPSFLGCATTAFR`

| Site | Type | Count |
| --- | --- | --- |
| 893 | N-linked | 2 |

| Mass | Sequence | MS_2_ Score | `q`-value | MS_1_ Score | Signal | Protein Coordinates | Tandem Scans | Start Time | Apex Time | End Time |
| --- | --- | --- | --- | --- | --- | --- | --- | --- | --- | --- |
| 4160.8578 | FTVN(N)FTITNLPYDSDLQIPHSAK Hex 7 HexNAc 2 | 117.172 | 0.008 | -18.167 | 3.736e+07 | 889 - 912 | 3 | 57.82 | 57.99 | 57.99 |
| 3998.8037 | FTVN(N)FTITNLPYDSDLQIPHSAK Hex 6 HexNAc 2 | 90.094 | 0.043 | -18.475 | 1.469e+07 | 889 - 912 | 2 | 57.99 | 57.99 | 58.16 |

### FTVN(N)FTITNLPYDSDLQIPHSAK Hex 7 HexNAc 2

**Mass:**  4160.8571

(3.332e-06)

**Charge States:** 3, 4

**Start Position**: 890

**End Position**: 913

| Profile Score | Tandem Score | TDA q-value | Volume | Spectrum Matches |
| --- | --- | --- | --- | --- |
| -18.1671 | 117.1720 | 0.0084 | 3.7364e+07 | 3 |

**Adduct Signal Fractions**

- **Unmodified**: 3.7364e+07

### FTVN(N)FTITNLPYDSDLQIPHSAK Hex 6 HexNAc 2

**Mass:**  3998.8051

(3.038e-06)

**Charge States:** 4

**Start Position**: 890

**End Position**: 913

| Profile Score | Tandem Score | TDA q-value | Volume | Spectrum Matches |
| --- | --- | --- | --- | --- |
| -18.4752 | 90.0943 | 0.0435 | 1.4693e+07 | 2 |

**Adduct Signal Fractions**

- **Unmodified**: 1.4693e+07

### tr|A0A1D5PUR1|A0A1D5PUR1\_CHICK

**>tr|A0A1D5PUR1|A0A1D5PUR1\_CHICK**  
 `MTTSTLAQTTSLPGTSTPTPASSTLMTTSTPAQTSLMPGHFKAQLLHLLHFTTTSTPAQTTSVPASSSPTPASSALPTAT  
TPAQTPGTSSPTPASSALPTTTTPAETTTFPATSSTTPASSAPPTTSTPAQTTSLPRTSTPTPASSALTTTTTPEQTTTV  
PGTSTPTPASSTVLTTSTPVQTTSMPGISSPTPASSALLTKSTPAQTTTVPATSSPTPASSALPTTTTPEQTTTESGTST  
PTPASSGPPTTSTPAETTSVPGTSYPNPASSSLMTTSTLAQTTSLPATSTPSLASSTLMTKSTFAPTTALPGTSTPTPAS  
SAPPTTSTPAQTTSVPGTSTATSASSTLLTTSTPAQTTSFPATSSPTPASSTLMTTSTPAQTTTVPGTSSPTPVSSALLT  
TRTLAQTKASLQLNSSPTSVSSALSTTSTPAETTTVPGTSIPTAASSTLLTTSTLAQTTSFPATSTPTPSSSTLMTTSTR  
AETKTSLQLNSSPTPASLALPTTSTPAQTTSVPTSSSPTPASSALSTTSTPALTTALRSTSQIMSPSSPATSPSPTATSA  
GTVTTAQETTSPAGTTNALTSTIRTTSTQTLSSSSPPYTTAALSYTAIPTSTPATPTTSAPQTTIASSSTPTESTALLTT  
TVPSTTQDISSQTAQTSNAATSSTTNSATSARLTRTHAETTSSTTTFTSHSRPSASQTSTPGTTISPLPATTAQQTTAPA  
GTTPPTTASTTLFSTRETSSKEEETSTVVTSSQTPASSVLPSTSTPAQTTSMPSTSSPTPASSTLMTTSTLAQTTSLPGT  
SVPTPASSAPRTTTTPAQTTSVPGNSSPIPPYSTLLTTSTLAQTTSLPGTSTPTPASSTLMTTSTPAETKTSLLLNSSPN  
ICIFCSSDYNHSCTDNISARHFKPNSCTAIKHSCRKQTFITTFTSRARTSASWTTTPGTTTSALTATTARETTTPAGTTH  
PHNSNIETTSTSTIKAPTRPPTTPAGHERFTVNFTITNLPYDSDLQIPHSAKLNATQRVMTTLLNRLLKDTSIGPSFLGC  
ATTAFR`

| Site | Type | Count |
| --- | --- | --- |
| 993 | N-linked | 2 |

| Mass | Sequence | MS_2_ Score | `q`-value | MS_1_ Score | Signal | Protein Coordinates | Tandem Scans | Start Time | Apex Time | End Time |
| --- | --- | --- | --- | --- | --- | --- | --- | --- | --- | --- |
| 4160.8578 | FTVN(N)FTITNLPYDSDLQIPHSAK Hex 7 HexNAc 2 | 117.172 | 0.008 | -18.167 | 3.736e+07 | 989 - 1012 | 3 | 57.82 | 57.99 | 57.99 |
| 3998.8037 | FTVN(N)FTITNLPYDSDLQIPHSAK Hex 6 HexNAc 2 | 90.094 | 0.043 | -18.475 | 1.469e+07 | 989 - 1012 | 2 | 57.99 | 57.99 | 58.16 |

### FTVN(N)FTITNLPYDSDLQIPHSAK Hex 7 HexNAc 2

**Mass:**  4160.8571

(3.332e-06)

**Charge States:** 3, 4

**Start Position**: 990

**End Position**: 1013

| Profile Score | Tandem Score | TDA q-value | Volume | Spectrum Matches |
| --- | --- | --- | --- | --- |
| -18.1671 | 117.1720 | 0.0084 | 3.7364e+07 | 3 |

**Adduct Signal Fractions**

- **Unmodified**: 3.7364e+07

### FTVN(N)FTITNLPYDSDLQIPHSAK Hex 6 HexNAc 2

**Mass:**  3998.8051

(3.038e-06)

**Charge States:** 4

**Start Position**: 990

**End Position**: 1013

| Profile Score | Tandem Score | TDA q-value | Volume | Spectrum Matches |
| --- | --- | --- | --- | --- |
| -18.4752 | 90.0943 | 0.0435 | 1.4693e+07 | 2 |

**Adduct Signal Fractions**

- **Unmodified**: 1.4693e+07

### tr|F1NUU6|F1NUU6\_CHICK

**>tr|F1NUU6|F1NUU6\_CHICK**  
 `MGCSTILVFLTLLSFASAHRKLLLFLLDGFRFDYIDDAELESLPGFRDIIGMGVKVDYMTPDFPSLSYPNYYTLMTGRHC  
EVHQMIGNYMWDPKTNVSFDIGVNKESLLPLWWNGSEPLWVTMTKEKKNVFMYYWPGCEVEILGVRPSYCREYFSVPTDQ  
NFADAISDALEALRNGSAEMAAVYYERIDVEGHHYGPWSEQRKSALKELDKAMSNMITQIKNKGLQHDVNVLLFSDHGMT  
DISWTDKVIELKNYINMSDTIQMKDRGPVVSLWPVPEKHTEIYQKLKAVEHMNVYNIQDIPERFFYKKGKFVSPLTLVAE  
EGWFIVESRDKLPFWENGTGKREAWQNGWHGYDNELMEMRGIFLAYGPDFRSNYRAPPIRSVDVYNVMCSLAGVQPQPNN  
GSWSRVECMLKNTAVRVPLCTRSGCVLALTLLLLFWLL`

| Site | Type | Count |
| --- | --- | --- |
| 337 | N-linked | 1 |

| Mass | Sequence | MS_2_ Score | `q`-value | MS_1_ Score | Signal | Protein Coordinates | Tandem Scans | Start Time | Apex Time | End Time |
| --- | --- | --- | --- | --- | --- | --- | --- | --- | --- | --- |
| 3607.5510 | DKLPFWEN(N)GTGKR Fuc 3 Hex 5 HexNAc 4 | 141.559 | 0.0 | -19.968 | 6.553e+06 | 329 - 342 | 1 | 28.10 | 28.10 | 28.10 |

### DKLPFWEN(N)GTGKR Fuc 3 Hex 5 HexNAc 4

**Mass:**  3607.5510

(1.756e-06)

**Charge States:** 4

**Start Position**: 330

**End Position**: 343

| Profile Score | Tandem Score | TDA q-value | Volume | Spectrum Matches |
| --- | --- | --- | --- | --- |
| -19.9677 | 141.5589 | 0.0000 | 6.5533e+06 | 1 |

**Adduct Signal Fractions**

- **Unmodified**: 6.5533e+06

### tr|A0A1D5P780|A0A1D5P780\_CHICK

**>tr|A0A1D5P780|A0A1D5P780\_CHICK**  
 `MRGIILALVLTLVDPGFNSRRSYLYNYEGSMLNGLQDRSLGKAGVRLSSKLEISGLPENAYLLKVRSPQVEEYNGVWPRD  
PFTRSSKITQVISSCFTRLFKFEYSSGRIGNIYAPEDCPDLCVNIVRGILNMFQMTIKKSQNVYELQEAGIGGICHARYV  
IQEDRKNSRIYVTRTVDLNNCQEKVQKSIGMAYIYPCPVDVMKERLTKGTTAFSYKLKQSDSGTLITDVSSRQVYQISPF  
NEPTGVAVMEARQQLTLVEVRSERGSAPDVPMQNYGSLRYRFPAVLPQMPLQLIKTKNPEQRIIETLQHIVLNNQQDFHD  
DVSYRFLEVVQLCRIANADNLESIWRQVSDKPRYRRWLLSAVSASGTTETLKFLKNRIRNDDLNYIQTLLTVSLTLHLLQ  
ADEHTLPIAADLMTSSRIQKNPVLQQVACLGYSSVVNRYCSQTSACPKEALQPIHDLADEAISRGREDKMKLALKCIGNM  
GEPASLKRILKFLPISSSSAADIPVHIQIDAITALKKIAWKDPKTVQGYLIQILADQSLPPEVRMMACAVIFETRPALAL  
ITTIANVAMKESNMQVASFVYSHMKSLSKSRLPFMYNISSACNIALKLLSPKLDSMSYRYSKVIRADTYFDNYRVGATGE  
IFVVNSPRTMFPSAIISKLMANSAGSVADLVEVGIRVEGLADVIMKRNIPFAEYPTYKQIKELGKALQGWKELPTETPLV  
SAYLKILGQEVAFININKELLQQVMKTVVEPADRNAAIKRIANQIRNSIAGQWTQPVWMGELRYVVPSCLGLPLEYGSYT  
TALARAAVSVEGKMTPPLTGDFRLSQLLESTMQIRSDLKPSLYVHTVATMGVNTEYFQHAVEIQGEVQTRMPMKFDAKID  
VKLKNLKIETNPCREETEIVVGRHKAFAVSRNIGELGVEKRTSILPEDAPLDVTEEPFQTSERASREHFAMQGPDSMPRK  
QSHSSREDLRRSTGKRAHKRDICLKMHHIGCQLCFSRRSRDASFIQNTYLHKLIGEHEAKIVLMPVHTDADIDKIQLEIQ  
AGSRAAARIITEVNPESEEEDESSPYEDIQAKLKRILGIDSMFKVANKTRHPKNRPSKKGNTVLAEFGTEPDAKTSSSSS  
SASSTATSSSSSSASSPNRKKPMDEEENDQVKQARNKDASSSSRSSKSSNSSKRSSSKSSNSSKRSSSSSSSSSSSSSSS  
SRSSSSSSSSSSNSKSSSSSSKSSSSSSRSRSSSKSSSSSSSSSSSSSSKSSSSRSSSSSSKSSSHHSHSHHSGHLNGSS  
SSSSSSRSVSHHSHEHHSGHLEDDSSSSSSSSVLSKIWGRHEIYQYRFRSAHRQEFPKRKLPGDRATSRYSSTRSSHDTS  
RAASWPKFLGDIKTPVLAAFLHGISNNKKTGGLQLVVYADTDSVRPRVQVFVTNLTDSSKWKLCADASVRNAHKAVAYVK  
WGWDCRDYKVSTELVTGRFAGHPAAQVKLEWPKVPSNVRSVVEWFYEFVPGAAFMLGFSERMDKNPSRQARMVVALTSPR  
TCDVVVKLPDMILYQKAVRLPLSLPVGPRIPASELQSPIWNVFAEAPSAVLENLKARCSVSYNKIKTFNEVKFNYSMPAN  
CYHILVQDCSSELKFLVMMKSAGEATNLKAINIKIGSHEIDMHPVNGQVKLLVDGAESPTANISLISAGASLWIHNENQG  
FALAAPGHGIDKLYFDGKTITIQVPLWMAGKTCGICGKYDAECEQEYRMPNGYLAKNAVSFGHSWILEEAPCRGACKLHR  
SFVKLEKTVQLAGVDSKCYSTEPVLRCAKGCSATKTTPVTVGFHCLPADSANSLTDKQMKYDQKSEDMQDTVDAHTACSC  
ENEECST`

| Site | Type | Count |
| --- | --- | --- |
| 1414 | N-linked | 1 |

| Mass | Sequence | MS_2_ Score | `q`-value | MS_1_ Score | Signal | Protein Coordinates | Tandem Scans | Start Time | Apex Time | End Time |
| --- | --- | --- | --- | --- | --- | --- | --- | --- | --- | --- |
| 2815.2447 | VQVFVTN(N)LTDSSK Hex 6 HexNAc 2 | 121.005 | 0.0 | -19.391 | 2.038e+06 | 1407 - 1420 | 1 | 36.57 | 36.57 | 36.57 |

### VQVFVTN(N)LTDSSK Hex 6 HexNAc 2

**Mass:**  2815.2447

(6.352e-06)

**Charge States:** 3

**Start Position**: 1408

**End Position**: 1421

| Profile Score | Tandem Score | TDA q-value | Volume | Spectrum Matches |
| --- | --- | --- | --- | --- |
| -19.3907 | 121.0051 | 0.0000 | 2.0379e+06 | 1 |

**Adduct Signal Fractions**

- **Unmodified**: 2.0379e+06

### sp|P02845|VIT2\_CHICK

**>sp|P02845|VIT2\_CHICK**  
 `MRGIILALVLTLVGSQKFDIDPGFNSRRSYLYNYEGSMLNGLQDRSLGKAGVRLSSKLEISGLPENAYLLKVRSPQVEEY  
NGVWPRDPFTRSSKITQVISSCFTRLFKFEYSSGRIGNIYAPEDCPDLCVNIVRGILNMFQMTIKKSQNVYELQEAGIGG  
ICHARYVIQEDRKNSRIYVTRTVDLNNCQEKVQKSIGMAYIYPCPVDVMKERLTKGTTAFSYKLKQSDSGTLITDVSSRQ  
VYQISPFNEPTGVAVMEARQQLTLVEVRSERGSAPDVPMQNYGSLRYRFPAVLPQMPLQLIKTKNPEQRIVETLQHIVLN  
NQQDFHDDVSYRFLEVVQLCRIANADNLESIWRQVSDKPRYRRWLLSAVSASGTTETLKFLKNRIRNDDLNYIQTLLTVS  
LTLHLLQADEHTLPIAADLMTSSRIQKNPVLQQVACLGYSSVVNRYCSQTSACPKEALQPIHDLADEAISRGREDKMKLA  
LKCIGNMGEPASLKRILKFLPISSSSAADIPVHIQIDAITALKKIAWKDPKTVQGYLIQILADQSLPPEVRMMACAVIFE  
TRPALALITTIANVAMKESNMQVASFVYSHMKSLSKSRLPFMYNISSACNIALKLLSPKLDSMSYRYSKVIRADTYFDNY  
RVGATGEIFVVNSPRTMFPSAIISKLMANSAGSVADLVEVGIRVEGLADVIMKRNIPFAEYPTYKQIKELGKALQGWKEL  
PTETPLVSAYLKILGQEVAFININKELLQQVMKTVVEPADRNAAIKRIANQIRNSIAGQWTQPVWMGELRYVVPSCLGLP  
LEYGSYTTALARAAVSVEGKMTPPLTGDFRLSQLLESTMQIRSDLKPSLYVHTVATMGVNTEYFQHAVEIQGEVQTRMPM  
KFDAKIDVKLKNLKIETNPCREETEIVVGRHKAFAVSRNIGELGVEKRTSILPEDAPLDVTEEPFQTSERASREHFAMQG  
PDSMPRKQSHSSREDLRRSTGKRAHKRDICLKMHHIGCQLCFSRRSRDASFIQNTYLHKLIGEHEAKIVLMPVHTDADID  
KIQLEIQAGSRAAARIITEVNPESEEEDESSPYEDIQAKLKRILGIDSMFKVANKTRHPKNRPSKKGNTVLAEFGTEPDA  
KTSSSSSSASSTATSSSSSSASSPNRKKPMDEEENDQVKQARNKDASSSSRSSKSSNSSKRSSSKSSNSSKRSSSSSSSS  
SSSSRSSSSSSSSSSNSKSSSSSSKSSSSSSRSRSSSKSSSSSSSSSSSSSSKSSSSRSSSSSSKSSSHHSHSHHSGHLN  
GSSSSSSSSRSVSHHSHEHHSGHLEDDSSSSSSSSVLSKIWGRHEIYQYRFRSAHRQEFPKRKLPGDRATSRYSSTRSSH  
DTSRAASWPKFLGDIKTPVLAAFLHGISNNKKTGGLQLVVYADTDSVRPRVQVFVTNLTDSSKWKLCADASVRNAHKAVA  
YVKWGWDCRDYKVSTELVTGRFAGHPAAQVKLEWPKVPSNVRSVVEWFYEFVPGAAFMLGFSERMDKNPSRQARMVVALT  
SPRTCDVVVKLPDIILYQKAVRLPLSLPVGPRIPASELQPPIWNVFAEAPSAVLENLKARCSVSYNKIKTFNEVKFNYSM  
PANCYHILVQDCSSELKFLVMMKSAGEATNLKAINIKIGSHEIDMHPVNGQVKLLVDGAESPTANISLISAGASLWIHNE  
NQGFALAAPGHGIDKLYFDGKTITIQVPLWMAGKTCGICGKYDAECEQEYRMPNGYLAKNAVSFGHSWILEEAPCRGACK  
LHRSFVKLEKTVQLAGVDSKCYSTEPVLRCAKGCSATKTTPVTVGFHCLPADSANSLTDKQMKYDQKSEDMQDTVDAHTT  
CSCENEECST`

| Site | Type | Count |
| --- | --- | --- |
| 1417 | N-linked | 1 |

| Mass | Sequence | MS_2_ Score | `q`-value | MS_1_ Score | Signal | Protein Coordinates | Tandem Scans | Start Time | Apex Time | End Time |
| --- | --- | --- | --- | --- | --- | --- | --- | --- | --- | --- |
| 2815.2447 | VQVFVTN(N)LTDSSK Hex 6 HexNAc 2 | 121.005 | 0.0 | -19.391 | 2.038e+06 | 1410 - 1423 | 1 | 36.57 | 36.57 | 36.57 |

### VQVFVTN(N)LTDSSK Hex 6 HexNAc 2

**Mass:**  2815.2447

(6.352e-06)

**Charge States:** 3

**Start Position**: 1411

**End Position**: 1424

| Profile Score | Tandem Score | TDA q-value | Volume | Spectrum Matches |
| --- | --- | --- | --- | --- |
| -19.3907 | 121.0051 | 0.0000 | 2.0379e+06 | 1 |

**Adduct Signal Fractions**

- **Unmodified**: 2.0379e+06

### tr|F1NFL6|F1NFL6\_CHICK

**>tr|F1NFL6|F1NFL6\_CHICK**  
 `MRGIILALVLTLVGSQKFDIDPGFNSRRSYLYNYEGSMLNGLQDRSLGKAGVRLSSKLEISGLPENAYLLKVRSPQVEEY  
NGVWPRDPFTRSSKITQVISSCFTRLFKFEYSSGRIGNIYAPEDCPDLCVNIVRGILNMFQMTIKKSQNVYELQEAGIGG  
ICHARYVIQEDRKNSRIYVTRTVDLNNCQEKVQKSIGMAYIYPCPVDVMKERLTKGTTAFSYKLKQSDSGTLITDVSSRQ  
VYQISPFNEPTGVAVMEARQQLTLVEVRSERGSAPDVPMQNYGSLRYRFPAVLPQMPLQLIKTKNPEQRIIETLQHIVLN  
NQQDFHDDVSYRFLEVVQLCRIANADNLESIWRQVSDKPRYRRWLLSAVSASGTTETLKFLKNRIRNDDLNYIQTLLTVS  
LTLHLLQADEHTLPIAADLMTSSRIQKNPVLQQVACLGYSSVVNRYCSQTSACPKEALQPIHDLADEAISRGREDKMKLA  
LKCIGNMGEPASLKRILKFLPISSSSAADIPVHIQIDAITALKKIAWKDPKTVQGYLIQILADQSLPPEVRMMACAVIFE  
TRPALALITTIANVAMKESNMQVASFVYSHMKSLSKSRLPFMYNISSACNIALKLLSPKLDSMSYRYSKVIRADTYFDNY  
RVGATGEIFVVNSPRTMFPSAIISKLMANSAGSVADLVEVGIRVEGLADVIMKRNIPFAEYPTYKQIKELGKALQGWKEL  
PTETPLVSAYLKILGQEVAFININKELLQQVMKTVVEPADRNAAIKRIANQIRNSIAGQWTQPVWMGELRYVVPSCLGLP  
LEYGSYTTALARAAVSVEGKMTPPLTGDFRLSQLLESTMQIRSDLKPSLYVHTVATMGVNTEYFQHAVEIQGEVQTRMPM  
KFDAKIDVKLKNLKIETNPCREETEIVVGRHKAFAVSRNIGELGVEKRTSILPEDAPLDVTEEPFQTSERASREHFAMQG  
PDSMPRKQSHSSREDLRRSTGKRAHKRDICLKMHHIGCQLCFSRRSRDASFIQNTYLHKLIGEHEAKIVLMPVHTDADID  
KIQLEIQAGSRAAARIITEVNPESEEEDESSPYEDIQAKLKRILGIDSMFKVANKTRHPKNRPSKKGNTVLAEFGTEPDA  
KTSSSSSSASSTATSSSSSSASSPNRKKPMDEEENDQVKQARNKDASSSSRSSKSSNSSKRSSSKSSNSSKRSSSSSSSS  
SSSSSSSSRSSSSSSSSSSNSKSSSSSSKSSSSSSRSRSSSKSSSSSSSSSSSSSSKSSSSRSSSSSSKSSSHHSHSHHS  
GHLNGSSSSSSSSRSVSHHSHEHHSGHLEDDSSSSSSSSVLSKIWGRHEIYQYRFRSAHRQEFPKRKLPGDRATSRYSST  
RSSHDTSRAASWPKFLGDIKTPVLAAFLHGISNNKKTGGLQLVVYADTDSVRPRVQVFVTNLTDSSKWKLCADASVRNAH  
KAVAYVKWGWDCRDYKVSTELVTGRFAGHPAAQVKLEWPKVPSNVRSVVEWFYEFVPGAAFMLGFSERMDKNPSRQARMV  
VALTSPRTCDVVVKLPDMILYQKAVRLPLSLPVGPRIPASELQSPIWNVFAEAPSAVLENLKARCSVSYNKIKTFNEVKF  
NYSMPANCYHILVQDCSSELKFLVMMKSAGEATNLKAINIKIGSHEIDMHPVNGQVKLLVDGAESPTANISLISAGASLW  
IHNENQGFALAAPGHGIDKLYFDGKTITIQVPLWMAGKTCGICGKYDAECEQEYRMPNGYLAKNAVSFGHSWILEEAPCR  
GACKLHRSFVKLEKTVQLAGVDSKCYSTEPVLRCAKGCSATKTTPVTVGFHCLPADSANSLTDKQMKYDQKSEDMQDTVD  
AHTACSCENEECST`

| Site | Type | Count |
| --- | --- | --- |
| 1421 | N-linked | 1 |

| Mass | Sequence | MS_2_ Score | `q`-value | MS_1_ Score | Signal | Protein Coordinates | Tandem Scans | Start Time | Apex Time | End Time |
| --- | --- | --- | --- | --- | --- | --- | --- | --- | --- | --- |
| 2815.2447 | VQVFVTN(N)LTDSSK Hex 6 HexNAc 2 | 121.005 | 0.0 | -19.391 | 2.038e+06 | 1414 - 1427 | 1 | 36.57 | 36.57 | 36.57 |

### VQVFVTN(N)LTDSSK Hex 6 HexNAc 2

**Mass:**  2815.2447

(6.352e-06)

**Charge States:** 3

**Start Position**: 1415

**End Position**: 1428

| Profile Score | Tandem Score | TDA q-value | Volume | Spectrum Matches |
| --- | --- | --- | --- | --- |
| -19.3907 | 121.0051 | 0.0000 | 2.0379e+06 | 1 |

**Adduct Signal Fractions**

- **Unmodified**: 2.0379e+06
