# Supplementary material for: Measuring Site-specific Glycosylation Similarity between Influenza a Virus Variants with Statistical Certainty
Source: Mol Cell Proteomics. 2020 Nov 25;19(9):1533–45. doi: 10.1074/mcp.RA120.002031 (PMC8143645; doi:10.1074/mcp.RA120.002031)
Supplement: Supplementary file 2 [file mmc2.zip › mmc2/159250_2_supp_555293_qcjklw.zip!/20190301_SWZeggL2_021119_tryp_HILIC_DDA_1ug_01_glycopeptides.html]

## 20190301\_SWZeggL2\_021119\_tryp\_HILIC\_DDA\_1ug\_01 @ GlycopeptideHypothesis-7f0ee1579ffe4b55a6981093529de994

**MS^1^ Mass Accuracy:** 10.0 PPM  
**Grouping Tolerance:** 15.0 PPM  
**MS^n^ Mass Accuracy:** 20.0 PPM  
**Minimum `q`-Value For Clustering:** 0.05

**Glycopeptide Hypothesis:**
GlycopeptideHypothesis-7f0ee1579ffe4b55a6981093529de994  
**Sample Searched:**
20190301\_SWZeggL2\_021119\_tryp\_HILIC\_DDA\_1ug\_01

| Protein | Matched Glycopeptides |
| --- | --- |
| cus\|SWZHA\|A/Switzerland/9715293/2013 | 140 |
| cus\|SWZNA\|A/Switzerland/9715293/2013 | 18 |
| tr\|F1NUU6\|F1NUU6\_CHICK | 2 |
| tr\|E1C041\|E1C041\_CHICK | 2 |
| tr\|A0A1D5PNI3\|A0A1D5PNI3\_CHICK | 1 |
| tr\|A0A1D5PUR1\|A0A1D5PUR1\_CHICK | 1 |

### cus|SWZHA|A/Switzerland/9715293/2013

**>cus|SWZHA|A/Switzerland/9715293/2013**  
 `QKLPGNDNSTATLCLGHHAVPNGTIVKTITNDRIEVTNATELVQNSSIGEICDSPHQILDGENCTLIDALLGDPQCDGFQ  
NKKWDLFVERSKAYSNCYPYDVPDYASLRSLVASSGTLEFNNESFNWAGVTQNGTSSSCRRGSNSSFFSRLNWLTHLNSK  
YPALNVTMPNNEQFDKLYIWGVHHPVTDKDQIFLYAQSSGRITVSTKRSQQAVIPNIGYRPRIRDIPSRISIYWTIVKPG  
DILLINSTGNLIAPRGYFKIRSGKSSIMRSDAPIGKCKSECITPNGSIPNDKPFQNVNRITYGACPRYVKQSTLKLATGM  
RNVPERQTRGIFGAIAGFIENGWEGMVDGWYGFRHQNSEGRGQAADLKSTQAAIDQINGKLNRLIGKTNEKFHQIEKEFS  
EVEGRIQDLEKYVEDTKIDLWSYNAELLVALENQHTIDLTDSEMNKLFEKTKKQLRENAEDMGNGCFKIYHKCDNACIGS  
IRNGTYDHDVYRDEALNNRFQIKGVELKSGYKDWILWISFAISCFLLCVALLGFIMWACQKGNIRCNICI`

| Site | Type | Count |
| --- | --- | --- |
| 483 | N-linked | 48 |
| 165 | N-linked | 20 |
| 38 | N-linked | 1 |
| 144 | N-linked | 42 |
| 246 | N-linked | 15 |
| 285 | N-linked | 12 |
| 22 | N-linked | 2 |

| Mass | Sequence | MS_2_ Score | `q`-value | MS_1_ Score | Signal | Protein Coordinates | Tandem Scans | Start Time | Apex Time | End Time |
| --- | --- | --- | --- | --- | --- | --- | --- | --- | --- | --- |
| 2846.1260 | RGSN(N)SSFFSR Hex 8 HexNAc 2 | 187.724 | 0.0 | -17.215 | 6.115e+07 | 140 - 150 | 2 | 18.96 | 19.12 | 19.12 |
| 2684.0729 | RGSN(N)SSFFSR Hex 7 HexNAc 2 | 187.135 | 0.0 | 13.704 | 1.769e+08 | 140 - 150 | 4 | 18.62 | 19.29 | 19.45 |
| 2359.9656 | RGSN(N)SSFFSR Hex 5 HexNAc 2 | 187.094 | 0.0 | 13.149 | 5.813e+08 | 140 - 150 | 6 | 18.96 | 19.61 | 20.08 |
| 2522.0202 | RGSN(N)SSFFSR Hex 6 HexNAc 2 | 186.956 | 0.0 | 12.969 | 2.790e+08 | 140 - 150 | 3 | 18.96 | 19.45 | 19.92 |
| 3115.2623 | RGSN(N)SSFFSR Fuc 1 Hex 5 HexNAc 5 | 183.607 | 0.0 | 13.971 | 1.410e+09 | 140 - 150 | 9 | 18.79 | 19.61 | 20.08 |
| 3598.4608 | YPALN(N)VTM(O)PNNEQFDK Hex 8 HexNAc 2 | 178.409 | 0.0 | 15.678 | 2.306e+09 | 160 - 176 | 4 | 34.63 | 35.12 | 35.45 |
| 2928.1791 | RGSN(N)SSFFSR Hex 6 HexNAc 4 | 178.241 | 0.0 | 13.667 | 8.025e+07 | 140 - 150 | 2 | 18.96 | 19.45 | 19.92 |
| 2547.0498 | RGSN(N)SSFFSR Fuc 1 Hex 4 HexNAc 3 | 175.885 | 0.0 | -4.858 | 7.873e+07 | 140 - 150 | 2 | 19.61 | 20.08 | 20.08 |
| 2969.2046 | RGSN(N)SSFFSR Hex 5 HexNAc 5 | 174.782 | 0.0 | 12.912 | 3.878e+08 | 140 - 150 | 5 | 18.79 | 19.61 | 19.92 |
| 2725.0999 | RGSN(N)SSFFSR Hex 6 HexNAc 3 | 174.241 | 0.0 | 13.222 | 4.033e+08 | 140 - 150 | 5 | 18.62 | 19.45 | 19.61 |
| 2912.1845 | RGSN(N)SSFFSR Fuc 1 Hex 5 HexNAc 4 | 173.430 | 0.0 | 13.779 | 6.795e+08 | 140 - 150 | 7 | 18.62 | 19.45 | 20.08 |
| 3760.5150 | YPALN(N)VTM(O)PNNEQFDK Hex 9 HexNAc 2 | 173.219 | 0.0 | 19.016 | 2.174e+09 | 160 - 176 | 5 | 34.47 | 34.97 | 35.29 |
| 3058.2425 | RGSN(N)SSFFSR Fuc 2 Hex 5 HexNAc 4 | 173.104 | 0.0 | -3.162 | 1.075e+08 | 140 - 150 | 2 | 18.96 | 19.12 | 19.29 |
| 3261.3235 | RGSN(N)SSFFSR Fuc 2 Hex 5 HexNAc 5 | 172.323 | 0.0 | 12.727 | 9.162e+07 | 140 - 150 | 3 | 18.79 | 19.29 | 19.45 |
| 2871.1577 | RGSN(N)SSFFSR Fuc 1 Hex 6 HexNAc 3 | 171.560 | 0.0 | 12.948 | 1.311e+08 | 140 - 150 | 3 | 18.62 | 19.12 | 19.45 |
| 2766.1253 | RGSN(N)SSFFSR Hex 5 HexNAc 4 | 171.487 | 0.0 | 12.395 | 1.392e+08 | 140 - 150 | 3 | 18.96 | 19.45 | 20.08 |
| 4068.9328 | TIVKPGDILLIN(N)STGNLIAPR Hex 9 HexNAc 2 | 170.767 | 0.0 | 16.865 | 3.486e+08 | 234 - 255 | 6 | 53.33 | 53.50 | 54.02 |
| 3436.4040 | YPALN(N)VTM(O)PNNEQFDK Hex 7 HexNAc 2 | 168.369 | 0.0 | 13.150 | 1.935e+08 | 160 - 176 | 2 | 34.80 | 35.29 | 35.45 |
| 2568.9975 | GSN(N)SSFFSR Hex 6 HexNAc 3 | 167.200 | 0.0 | -17.435 | 1.177e+08 | 141 - 150 | 2 | 23.12 | 23.28 | 23.28 |
| 2400.9933 | RGSN(N)SSFFSR Hex 4 HexNAc 3 | 166.587 | 0.0 | -18.575 | 3.267e+07 | 140 - 150 | 1 | 19.92 | 20.08 | 20.08 |
| 3582.4659 | YPALN(N)VTMPNNEQFDK Hex 8 HexNAc 2 | 165.398 | 0.0 | -2.259 | 1.511e+08 | 160 - 176 | 4 | 42.50 | 42.84 | 43.01 |
| 3744.6392 | YPALN(N)VTMPNNEQFDK Hex 9 HexNAc 2 | 163.810 | 0.0 | 10.102 | 1.834e+08 | 160 - 176 | 7 | 42.50 | 42.67 | 43.86 |
| 2709.1037 | RGSN(N)SSFFSR Fuc 1 Hex 5 HexNAc 3 | 162.672 | 0.0 | 9.247 | 5.928e+07 | 140 - 150 | 2 | 19.12 | 19.75 | 19.75 |
| 2953.2060 | RGSN(N)SSFFSR Fuc 1 Hex 4 HexNAc 5 | 160.890 | 0.0 | -5.894 | 3.660e+07 | 140 - 150 | 2 | 19.45 | 19.61 | 19.92 |
| 3007.1738 | N(N)GTYDHDVYR Fuc 1 Hex 5 HexNAc 4 | 160.868 | 0.0 | 16.104 | 5.436e+08 | 482 - 492 | 5 | 14.69 | 14.85 | 15.50 |
| 3112.3048 | YPALN(N)VTM(O)PNNEQFDK Hex 5 HexNAc 2 | 160.420 | 0.0 | -3.223 | 1.021e+08 | 160 - 176 | 3 | 35.45 | 35.78 | 35.95 |
| 3277.3167 | RGSN(N)SSFFSR Fuc 1 Hex 6 HexNAc 5 | 160.358 | 0.0 | 12.590 | 9.488e+07 | 140 - 150 | 5 | 18.29 | 18.96 | 19.29 |
| 3090.2350 | RGSN(N)SSFFSR Hex 7 HexNAc 4 | 159.898 | 0.0 | -18.961 | 1.409e+07 | 140 - 150 | 4 | 19.29 | 19.45 | 19.45 |
| 3210.2538 | N(N)GTYDHDVYR Fuc 1 Hex 5 HexNAc 5 | 158.802 | 0.0 | -3.493 | 1.618e+08 | 482 - 492 | 4 | 14.85 | 15.01 | 15.34 |
| 3627.6338 | PGDILLIN(N)STGNLIAPR Hex 9 HexNAc 2 | 156.803 | 0.0 | -18.765 | 2.108e+07 | 238 - 255 | 1 | 51.80 | 51.97 | 51.97 |
| 2756.0835 | GSN(N)SSFFSR Fuc 1 Hex 5 HexNAc 4 | 155.592 | 0.0 | 13.682 | 2.224e+08 | 141 - 150 | 2 | 22.81 | 22.98 | 23.60 |
| 3172.2842 | RGSN(N)SSFFSR Hex 5 HexNAc 6 | 155.113 | 0.0 | -4.103 | 2.601e+08 | 140 - 150 | 3 | 19.29 | 19.92 | 20.08 |
| 2563.0450 | RGSN(N)SSFFSR Hex 5 HexNAc 3 | 154.875 | 0.0 | -2.956 | 7.254e+07 | 140 - 150 | 1 | 19.61 | 19.75 | 19.92 |
| 3008.3203 | HAVPN(N)GTIVK Fuc 1 Hex 5 HexNAc 5 | 154.202 | 0.0 | 0.000 | 0.000e+00 | 17 - 27 | 1 | - | - | - |
| 3204.3006 | RGSN(N)SSFFSR Fuc 3 Hex 5 HexNAc 4 | 153.207 | 0.0 | -17.798 | 2.331e+07 | 140 - 150 | 1 | 18.79 | 18.79 | 18.96 |
| 3761.5376 | YPALN(N)VTMPNNEQFDK Fuc 2 Hex 3 HexNAc 4 Neu5Ac 1 | 152.098 | 0.0 | 9.265 | 2.220e+07 | 160 - 176 | 2 | 42.00 | 42.67 | 42.84 |
| 4085.9564 | TIVKPGDILLIN(N)STGNLIAPR Fuc 2 Hex 3 HexNAc 4 Neu5Ac 1 | 151.871 | 0.0 | -17.698 | 2.742e+07 | 234 - 255 | 1 | 53.50 | 53.50 | 53.67 |
| 3777.5404 | YPALN(N)VTM(O)PNNEQFDK Fuc 2 Hex 3 HexNAc 4 Neu5Ac 1 | 151.766 | 0.0 | 14.797 | 1.946e+08 | 160 - 176 | 1 | 34.63 | 34.97 | 35.29 |
| 4569.2226 | ISIYWTIVKPGDILLIN(N)STGNLIAPR Hex 8 HexNAc 2 | 151.356 | 0.0 | 15.254 | 1.147e+08 | 229 - 255 | 6 | 68.71 | 69.23 | 69.39 |
| 3274.3570 | YPALN(N)VTM(O)PNNEQFDK Hex 6 HexNAc 2 | 151.227 | 0.0 | 11.144 | 3.685e+07 | 160 - 176 | 1 | 35.12 | 35.45 | 35.78 |
| 3074.2368 | RGSN(N)SSFFSR Fuc 1 Hex 6 HexNAc 4 | 150.881 | 0.0 | -5.568 | 3.435e+07 | 140 - 150 | 2 | 19.12 | 19.75 | 19.92 |
| 3922.5694 | YPALN(N)VTM(O)PNNEQFDK Hex 10 HexNAc 2 | 149.523 | 0.0 | -3.166 | 6.233e+07 | 160 - 176 | 1 | 34.63 | 34.97 | 35.12 |
| 3420.4139 | YPALN(N)VTMPNNEQFDK Hex 7 HexNAc 2 | 149.474 | 0.0 | -4.377 | 1.068e+07 | 160 - 176 | 1 | 42.67 | 42.84 | 43.01 |
| 2365.9191 | GSN(N)SSFFSR Hex 6 HexNAc 2 | 149.227 | 0.0 | -18.652 | 9.129e+07 | 141 - 150 | 3 | 23.12 | 23.12 | 23.28 |
| 3632.4680 | N(N)GTYDHDVYRDEALNNR Hex 6 HexNAc 3 | 147.557 | 0.0 | -3.912 | 2.256e+08 | 482 - 499 | 3 | 20.69 | 20.85 | 21.17 |
| 4731.2814 | ISIYWTIVKPGDILLIN(N)STGNLIAPR Hex 9 HexNAc 2 | 147.518 | 0.0 | 18.029 | 1.615e+09 | 229 - 255 | 9 | 67.85 | 69.05 | 69.57 |
| 4376.8462 | C(C)KSEC(C)ITPN(N)GSIPNDKPFQNVNR Hex 8 HexNAc 2 | 146.954 | 0.0 | -4.151 | 4.493e+07 | 276 - 299 | 1 | 29.48 | 29.65 | 29.82 |
| 4893.3277 | ISIYWTIVKPGDILLIN(N)STGNLIAPR Hex 10 HexNAc 2 | 145.260 | 0.0 | 16.971 | 1.583e+08 | 229 - 255 | 5 | 68.54 | 69.05 | 69.23 |
| 2959.1616 | GSN(N)SSFFSR Fuc 1 Hex 5 HexNAc 5 | 144.929 | 0.0 | -3.233 | 3.630e+08 | 141 - 150 | 4 | 23.12 | 23.28 | 23.60 |
| 2813.1047 | GSN(N)SSFFSR Hex 5 HexNAc 5 | 143.700 | 0.0 | 8.690 | 1.435e+08 | 141 - 150 | 3 | 22.81 | 23.44 | 23.60 |
| 2610.0238 | GSN(N)SSFFSR Hex 5 HexNAc 4 | 143.385 | 0.0 | -5.248 | 4.088e+07 | 141 - 150 | 1 | 23.12 | 23.28 | 23.44 |
| 3778.5283 | N(N)GTYDHDVYRDEALNNR Fuc 1 Hex 6 HexNAc 3 | 142.895 | 0.0 | -4.277 | 1.452e+08 | 482 - 499 | 1 | 20.69 | 20.69 | 21.01 |
| 2803.2284 | HAVPN(N)GTIVK Fuc 1 Hex 5 HexNAc 4 | 142.278 | 0.0 | -20.926 | 2.992e+06 | 17 - 27 | 1 | 15.84 | 16.00 | 16.00 |
| 4748.2976 | ISIYWTIVKPGDILLIN(N)STGNLIAPR Fuc 2 Hex 3 HexNAc 4 Neu5Ac 1 | 142.105 | 0.0 | 15.172 | 2.577e+08 | 229 - 255 | 6 | 68.37 | 69.05 | 69.57 |
| 3153.2311 | N(N)GTYDHDVYR Fuc 2 Hex 5 HexNAc 4 | 141.473 | 0.0 | -3.676 | 1.115e+08 | 482 - 492 | 3 | 14.52 | 14.69 | 14.85 |
| 3267.3343 | N(N)GTYDHDVYRDEALNNR Hex 5 HexNAc 2 | 141.108 | 0.0 | -4.943 | 1.068e+08 | 482 - 499 | 1 | 21.01 | 21.17 | 21.32 |
| 4910.3549 | ISIYWTIVKPGDILLIN(N)STGNLIAPR Fuc 2 Hex 4 HexNAc 4 Neu5Ac 1 | 140.832 | 0.0 | -18.305 | 2.426e+07 | 229 - 255 | 2 | 68.88 | 69.05 | 69.05 |
| 4230.9839 | TIVKPGDILLIN(N)STGNLIAPR Hex 10 HexNAc 2 | 140.494 | 0.0 | -4.498 | 3.146e+07 | 234 - 255 | 2 | 53.33 | 53.50 | 53.84 |
| 2693.1085 | RGSN(N)SSFFSR Fuc 2 Hex 4 HexNAc 3 | 140.126 | 0.0 | -20.444 | 1.754e+07 | 140 - 150 | 2 | 19.61 | 19.61 | 19.75 |
| 2203.8655 | GSN(N)SSFFSR Hex 5 HexNAc 2 | 139.372 | 0.0 | -1.916 | 2.024e+08 | 141 - 150 | 3 | 23.28 | 23.60 | 23.76 |
| 2690.0252 | GSN(N)SSFFSR Hex 8 HexNAc 2 | 137.919 | 0.0 | -18.560 | 2.231e+07 | 141 - 150 | 1 | 22.64 | 22.81 | 22.81 |
| 3926.6725 | SEC(C)ITPN(N)GSIPNDKPFQNVNR Hex 7 HexNAc 2 | 137.560 | 0.0 | 15.230 | 6.471e+08 | 278 - 299 | 3 | 32.28 | 32.60 | 32.94 |
| 4022.6307 | N(N)GTYDHDVYRDEALNNR Fuc 1 Hex 5 HexNAc 5 | 137.387 | 0.0 | 11.531 | 1.641e+09 | 482 - 499 | 6 | 20.08 | 21.01 | 21.17 |
| 3965.6108 | N(N)GTYDHDVYRDEALNNR Fuc 2 Hex 5 HexNAc 4 | 137.072 | 0.0 | -1.946 | 1.181e+09 | 482 - 499 | 4 | 20.55 | 20.69 | 21.01 |
| 4102.5900 | N(N)GTYDHDVYRDEALNNR Fuc 1 Hex 8 HexNAc 3 | 137.034 | 0.0 | 11.400 | 3.161e+08 | 482 - 499 | 3 | 21.82 | 22.64 | 22.81 |
| 3454.4189 | N(N)GTYDHDVYRDEALNNR Fuc 1 Hex 4 HexNAc 3 | 136.248 | 0.0 | -4.451 | 1.328e+08 | 482 - 499 | 1 | 21.01 | 21.17 | 21.32 |
| 3131.2594 | RGSN(N)SSFFSR Hex 6 HexNAc 5 | 135.214 | 0.0 | -5.133 | 1.437e+07 | 140 - 150 | 1 | 18.96 | 18.96 | 19.29 |
| 3819.5517 | N(N)GTYDHDVYRDEALNNR Fuc 1 Hex 5 HexNAc 4 | 134.171 | 0.0 | 17.196 | 3.862e+09 | 482 - 499 | 7 | 19.92 | 20.85 | 21.17 |
| 4250.7734 | SEC(C)ITPN(N)GSIPNDKPFQNVNR Hex 9 HexNAc 2 | 133.065 | 0.0 | -1.994 | 1.042e+09 | 278 - 299 | 3 | 32.28 | 32.45 | 32.77 |
| 3967.8831 | IVKPGDILLIN(N)STGNLIAPR Hex 9 HexNAc 2 | 132.722 | 0.0 | -19.494 | 1.168e+07 | 235 - 255 | 2 | 52.82 | 52.82 | 52.99 |
| 3480.3971 | RGSN(N)SSFFSR Fuc 1 Hex 6 HexNAc 6 | 132.102 | 0.0 | -5.196 | 3.613e+07 | 140 - 150 | 3 | 18.79 | 18.79 | 19.29 |
| 4088.7223 | SEC(C)ITPN(N)GSIPNDKPFQNVNR Hex 8 HexNAc 2 | 131.844 | 0.0 | 16.688 | 5.727e+08 | 278 - 299 | 3 | 32.28 | 32.45 | 32.94 |
| 3794.5236 | N(N)GTYDHDVYRDEALNNR Hex 7 HexNAc 3 | 131.491 | 0.0 | -19.794 | 2.367e+07 | 482 - 499 | 1 | 20.69 | 20.69 | 20.69 |
| 3673.4939 | N(N)GTYDHDVYRDEALNNR Hex 5 HexNAc 4 | 131.456 | 0.0 | -3.535 | 2.301e+08 | 482 - 499 | 3 | 20.69 | 20.85 | 21.17 |
| 3017.2161 | RGSN(N)SSFFSR Fuc 2 Hex 6 HexNAc 3 | 130.873 | 0.0 | -18.345 | 9.704e+06 | 140 - 150 | 1 | 19.12 | 19.12 | 19.12 |
| 5568.7558 | IRDIPSRISIYWTIVKPGDILLIN(N)STGNLIAPR Hex 9 HexNAc 2 | 130.407 | 0.0 | -2.945 | 1.717e+08 | 222 - 255 | 5 | 65.11 | 65.28 | 65.62 |
| 2820.0904 | N(N)GTYDHDVYR Hex 6 HexNAc 3 | 130.379 | 0.0 | -20.060 | 1.868e+07 | 482 - 492 | 1 | 14.69 | 14.69 | 14.85 |
| 4168.6921 | N(N)GTYDHDVYRDEALNNR Fuc 2 Hex 5 HexNAc 5 | 129.729 | 0.0 | -17.751 | 1.508e+08 | 482 - 499 | 1 | 20.69 | 20.69 | 20.85 |
| 3599.4513 | YPALN(N)VTM(O)PN(D)NEQFDK Hex 8 HexNAc 2 | 129.502 | 0.0 | 9.696 | 4.160e+08 | 160 - 176 | 6 | 35.61 | 35.95 | 36.80 |
| 3899.5098 | N(N)GTYDHDVYRDEALNNR Fuc 1 Hex 8 HexNAc 2 | 128.065 | 0.0 | -2.423 | 6.442e+08 | 482 - 499 | 4 | 22.31 | 22.48 | 22.81 |
| 3470.4138 | N(N)GTYDHDVYRDEALNNR Hex 5 HexNAc 3 | 127.818 | 0.0 | -17.808 | 4.963e+07 | 482 - 499 | 1 | 21.01 | 21.17 | 21.17 |
| 4111.6670 | N(N)GTYDHDVYRDEALNNR Fuc 3 Hex 5 HexNAc 4 | 127.151 | 0.0 | -17.857 | 2.746e+08 | 482 - 499 | 1 | 20.55 | 20.55 | 20.69 |
| 3616.4736 | N(N)GTYDHDVYRDEALNNR Fuc 1 Hex 5 HexNAc 3 | 126.967 | 0.0 | -3.813 | 8.241e+07 | 482 - 499 | 1 | 20.85 | 21.01 | 21.17 |
| 3761.5050 | YPALN(N)VTM(O)PN(D)NEQFDK Hex 9 HexNAc 2 | 126.542 | 0.0 | 8.465 | 4.844e+08 | 160 - 176 | 5 | 35.45 | 35.95 | 36.97 |
| 3778.5295 | YPALN(N)VTM(O)PN(D)NEQFDK Fuc 2 Hex 3 HexNAc 4 Neu5Ac 1 | 126.060 | 0.0 | 7.792 | 3.572e+07 | 160 - 176 | 1 | 35.45 | 35.95 | 36.12 |
| 2950.2525 | YPALN(N)VTM(O)PNNEQFDK Hex 4 HexNAc 2 | 125.381 | 0.0 | -3.156 | 1.833e+07 | 160 - 176 | 1 | 35.78 | 35.95 | 36.12 |
| 4214.7945 | C(C)KSEC(C)ITPN(N)GSIPNDKPFQNVNR Hex 7 HexNAc 2 | 124.949 | 0.0 | -3.824 | 5.923e+07 | 276 - 299 | 1 | 29.48 | 29.65 | 29.82 |
| 3764.6199 | SEC(C)ITPN(N)GSIPNDKPFQNVNR Hex 6 HexNAc 2 | 123.233 | 0.0 | -2.642 | 8.320e+07 | 278 - 299 | 2 | 32.60 | 32.60 | 32.94 |
| 3820.5390 | N(N)GTYDHDVYRDEALN(D)NR Fuc 1 Hex 5 HexNAc 4 | 123.010 | 0.0 | -7.279 | 1.687e+09 | 482 - 499 | 6 | 21.32 | 21.49 | 22.31 |
| 2861.1173 | N(N)GTYDHDVYR Hex 5 HexNAc 4 | 121.965 | 0.0 | -19.225 | 2.300e+07 | 482 - 492 | 2 | 14.85 | 14.85 | 14.85 |
| 4184.6839 | N(N)GTYDHDVYRDEALNNR Fuc 1 Hex 6 HexNAc 5 | 120.995 | 0.0 | -3.269 | 5.745e+08 | 482 - 499 | 4 | 20.55 | 20.69 | 21.01 |
| 3602.5655 | SEC(C)ITPN(N)GSIPNDKPFQNVNR Hex 5 HexNAc 2 | 120.840 | 0.0 | -19.114 | 3.504e+07 | 278 - 299 | 1 | 32.77 | 32.77 | 32.94 |
| 4387.7651 | N(N)GTYDHDVYRDEALNNR Fuc 1 Hex 6 HexNAc 6 | 120.676 | 0.0 | -4.647 | 8.732e+07 | 482 - 499 | 1 | 20.55 | 20.85 | 21.01 |
| 3356.3072 | N(N)GTYDHDVYR Fuc 2 Hex 5 HexNAc 5 | 120.176 | 0.0 | -20.015 | 1.692e+07 | 482 - 492 | 2 | 14.69 | 14.69 | 14.85 |
| 4267.7995 | SEC(C)ITPN(N)GSIPNDKPFQNVNR Fuc 2 Hex 3 HexNAc 4 Neu5Ac 1 | 119.560 | 0.0 | -18.818 | 3.901e+07 | 278 - 299 | 1 | 32.45 | 32.45 | 32.60 |
| 4330.7415 | N(N)GTYDHDVYRDEALNNR Fuc 2 Hex 6 HexNAc 5 | 119.484 | 0.0 | -4.355 | 1.048e+08 | 482 - 499 | 1 | 20.39 | 20.55 | 20.69 |
| 4264.6445 | N(N)GTYDHDVYRDEALNNR Fuc 1 Hex 9 HexNAc 3 | 119.168 | 0.0 | -2.978 | 1.778e+08 | 482 - 499 | 2 | 22.14 | 22.31 | 22.64 |
| 3966.5970 | N(N)GTYDHDVYRDEALN(D)NR Fuc 2 Hex 5 HexNAc 4 | 118.124 | 0.0 | 4.310 | 5.101e+08 | 482 - 499 | 3 | 21.17 | 21.32 | 21.98 |
| 4083.0603 | ISIYWTIVKPGDILLIN(N)STGNLIAPR Hex 5 HexNAc 2 | 117.888 | 0.0 | -4.438 | 7.668e+06 | 229 - 255 | 1 | 69.57 | 69.74 | 69.91 |
| 4549.8123 | N(N)GTYDHDVYRDEALNNR Fuc 1 Hex 7 HexNAc 6 | 116.711 | 0.0 | -20.586 | 2.742e+07 | 482 - 499 | 1 | 20.39 | 20.39 | 20.55 |
| 3591.4442 | N(N)GTYDHDVYRDEALNNR Hex 7 HexNAc 2 | 114.478 | 0.0 | -18.946 | 3.273e+07 | 482 - 499 | 1 | 20.69 | 20.69 | 20.85 |
| 3299.2882 | N(N)GTYDHDVYR Fuc 3 Hex 5 HexNAc 4 | 113.644 | 0.0 | -19.485 | 2.671e+07 | 482 - 492 | 3 | 14.36 | 14.52 | 14.52 |
| 2914.2127 | RGSN(N)SSFFSR Fuc 1 Hex 5 HexNAc 4 | 113.153 | 0.0 | 0.000 | 0.000e+00 | 140 - 150 | 1 | - | - | - |
| 3981.6022 | N(N)GTYDHDVYRDEALNNR Fuc 1 Hex 6 HexNAc 4 | 113.040 | 0.0 | -19.636 | 2.624e+07 | 482 - 499 | 1 | 20.55 | 20.69 | 20.69 |
| 2642.0402 | N(N)GTYDHDVYR Fuc 1 Hex 4 HexNAc 3 | 112.849 | 0.0 | -20.034 | 8.080e+06 | 482 - 492 | 1 | 14.85 | 15.01 | 15.01 |
| 3372.3071 | N(N)GTYDHDVYR Fuc 1 Hex 6 HexNAc 5 | 112.513 | 0.0 | -5.035 | 6.301e+07 | 482 - 492 | 4 | 14.52 | 14.69 | 15.01 |
| 3437.4007 | YPALN(N)VTM(O)PN(D)NEQFDK Hex 7 HexNAc 2 | 112.457 | 0.0 | 7.988 | 3.257e+07 | 160 - 176 | 3 | 35.61 | 35.95 | 36.80 |
| 2966.1513 | N(N)GTYDHDVYR Fuc 1 Hex 6 HexNAc 3 | 112.416 | 0.0 | -18.373 | 1.038e+07 | 482 - 492 | 1 | 14.52 | 14.52 | 14.52 |
| 3600.4761 | N(N)GTYDHDVYRDEALNNR Fuc 2 Hex 4 HexNAc 3 | 111.722 | 0.0 | -4.817 | 4.101e+07 | 482 - 499 | 1 | 20.85 | 21.01 | 21.17 |
| 3779.5134 | N(N)GTYDHDVYRDEALN(D)NR Fuc 1 Hex 6 HexNAc 3 | 111.304 | 0.0 | -5.837 | 5.751e+07 | 482 - 499 | 1 | 21.32 | 21.32 | 21.82 |
| 2658.0353 | N(N)GTYDHDVYR Hex 5 HexNAc 3 | 111.264 | 0.0 | -21.122 | 4.657e+06 | 482 - 492 | 1 | 14.85 | 14.85 | 14.85 |
| 2546.1071 | IEVTN(N)ATELVQN Hex 5 HexNAc 2 | 110.971 | 0.0 | -21.168 | 1.189e+06 | 33 - 45 | 1 | 35.95 | 35.95 | 35.95 |
| 3178.2379 | RGSN(N)SSFFSR Hex 7 HexNAc 3 Neu5Ac 1 | 109.466 | 0.004 | -21.286 | 3.536e+06 | 140 - 150 | 1 | 22.48 | 22.48 | 22.48 |
| 4047.5609 | RGSN(N)SSFFSR Fuc 2 Hex 5 HexNAc 6 Neu5Ac 2 | 109.366 | 0.004 | 0.000 | 0.000e+00 | 140 - 150 | 1 | - | - | - |
| 4476.7918 | N(N)GTYDHDVYRDEALNNR Fuc 3 Hex 6 HexNAc 5 | 107.943 | 0.004 | -19.896 | 2.588e+07 | 482 - 499 | 1 | 20.39 | 20.39 | 20.55 |
| 3583.4495 | YPALN(N)VTMPN(D)NEQFDK Hex 8 HexNAc 2 | 106.653 | 0.004 | -6.319 | 2.178e+07 | 160 - 176 | 2 | 43.52 | 43.86 | 44.03 |
| 4410.6999 | N(N)GTYDHDVYRDEALNNR Fuc 2 Hex 9 HexNAc 3 | 105.910 | 0.004 | -4.580 | 7.026e+07 | 482 - 499 | 1 | 22.14 | 22.14 | 22.64 |
| 4023.6200 | N(N)GTYDHDVYRDEALN(D)NR Fuc 1 Hex 5 HexNAc 5 | 104.133 | 0.004 | 10.310 | 7.295e+08 | 482 - 499 | 7 | 21.17 | 21.49 | 22.81 |
| 3318.3441 | RGSN(N)SSFFSR Fuc 1 Hex 5 HexNAc 6 | 103.738 | 0.004 | -21.095 | 1.053e+07 | 140 - 150 | 1 | 19.29 | 19.45 | 19.45 |
| 2779.0678 | N(N)GTYDHDVYR Hex 7 HexNAc 2 | 103.627 | 0.004 | -20.109 | 3.606e+06 | 482 - 492 | 1 | 14.52 | 14.52 | 14.52 |
| 4401.7526 | N(N)GTYDHDVYRDEALNNR Fuc 1 Hex 5 HexNAc 4 Neu5Ac 2 | 102.963 | 0.004 | -19.134 | 1.609e+07 | 482 - 499 | 1 | 24.08 | 24.08 | 24.08 |
| 3876.5726 | N(N)GTYDHDVYRDEALNNR Hex 5 HexNAc 5 | 102.714 | 0.004 | 12.026 | 1.037e+09 | 482 - 499 | 6 | 20.08 | 20.55 | 21.01 |
| 4089.7132 | SEC(C)ITPN(N)GSIPN(D)DKPFQNVNR Hex 8 HexNAc 2 | 102.253 | 0.004 | 8.138 | 1.347e+08 | 278 - 299 | 2 | 33.11 | 33.96 | 33.96 |
| 3334.3365 | RGSN(N)SSFFSR Hex 6 HexNAc 6 | 100.652 | 0.004 | -5.423 | 1.753e+07 | 140 - 150 | 1 | 18.96 | 19.12 | 19.29 |
| 3423.3700 | RGSN(N)SSFFSR Fuc 2 Hex 6 HexNAc 5 | 97.792 | 0.008 | -19.503 | 4.214e+06 | 140 - 150 | 1 | 18.62 | 18.79 | 18.79 |
| 2454.9552 | N(N)GTYDHDVYR Hex 5 HexNAc 2 | 97.496 | 0.008 | -18.694 | 9.048e+06 | 482 - 492 | 1 | 14.85 | 14.85 | 14.85 |
| 4045.5692 | RGSN(N)SSFFSR Hex 5 HexNAc 6 Neu5Ac 3 | 94.190 | 0.015 | 13.808 | 3.516e+08 | 140 - 150 | 3 | 22.14 | 22.48 | 22.81 |
| 5306.3296 | YPALN(N)VTM(O)PNNEQFDKLYIWGVHHPVTDK Hex 9 HexNAc 2 | 93.467 | 0.018 | -18.457 | 1.303e+07 | 160 - 189 | 1 | 49.92 | 49.92 | 50.10 |
| 2805.1060 | N(N)GTYDHDVYR Fuc 1 Hex 5 HexNAc 3 | 93.223 | 0.018 | 0.000 | 0.000e+00 | 482 - 492 | 1 | - | - | - |
| 5406.7031 | IRDIPSRISIYWTIVKPGDILLIN(N)STGNLIAPR Hex 8 HexNAc 2 | 93.047 | 0.018 | -4.456 | 1.147e+07 | 222 - 255 | 2 | 65.28 | 65.28 | 65.62 |
| 5299.5722 | DIPSRISIYWTIVKPGDILLIN(N)STGNLIAPR Hex 9 HexNAc 2 | 92.964 | 0.018 | -4.686 | 1.489e+07 | 224 - 255 | 2 | 69.57 | 69.74 | 69.91 |
| 3927.6580 | SEC(C)ITPN(N)GSIPN(D)DKPFQNVNR Hex 7 HexNAc 2 | 92.752 | 0.018 | 8.984 | 1.447e+08 | 278 - 299 | 3 | 33.11 | 33.96 | 34.13 |
| 4251.7634 | SEC(C)ITPN(N)GSIPN(D)DKPFQNVNR Hex 9 HexNAc 2 | 92.191 | 0.021 | 8.929 | 2.947e+08 | 278 - 299 | 4 | 32.94 | 33.79 | 33.96 |
| 3657.4990 | N(N)GTYDHDVYRDEALNNR Fuc 1 Hex 4 HexNAc 4 | 91.429 | 0.021 | 10.634 | 4.257e+07 | 482 - 499 | 1 | 20.69 | 21.01 | 21.32 |
| 4336.8508 | N(N)GTYDHDVYRDEALN(D)NRFQIK Fuc 1 Hex 5 HexNAc 4 | 90.653 | 0.021 | -20.689 | 5.439e+07 | 482 - 503 | 2 | 34.13 | 34.13 | 34.30 |
| 4112.6543 | N(N)GTYDHDVYRDEALN(D)NR Fuc 3 Hex 5 HexNAc 4 | 90.092 | 0.021 | 8.600 | 1.178e+08 | 482 - 499 | 1 | 20.85 | 21.17 | 21.82 |
| 4538.8990 | C(C)KSEC(C)ITPN(N)GSIPNDKPFQNVNR Hex 9 HexNAc 2 | 88.226 | 0.032 | -4.072 | 7.910e+07 | 276 - 299 | 1 | 29.32 | 29.65 | 29.65 |
| 3834.5570 | YPALN(N)VTM(O)PNNEQFDK Fuc 1 Hex 3 HexNAc 5 Neu5Ac 1 | 87.517 | 0.039 | -4.449 | 4.817e+07 | 160 - 176 | 1 | 34.97 | 35.29 | 35.29 |
| 3684.6566 | DILLIN(N)STGNLIAPR Hex 6 HexNAc 4 Neu5Ac 1 | 86.146 | 0.042 | -17.297 | 3.254e+07 | 240 - 255 | 3 | 52.48 | 52.65 | 52.65 |
| 3837.5605 | YPALN(N)VTM(O)PN(D)NEQFDK Fuc 3 Hex 3 HexNAc 5 | 85.648 | 0.049 | 0.000 | 0.000e+00 | 160 - 176 | 1 | - | - | - |

### RGSN(N)SSFFSR Hex 8 HexNAc 2

**Mass:**  2846.1258

(1.297e-06)

**Charge States:** 3

**Start Position**: 141

**End Position**: 151

| Profile Score | Tandem Score | TDA q-value | Volume | Spectrum Matches |
| --- | --- | --- | --- | --- |
| -17.2145 | 187.7241 | 0.0000 | 6.1147e+07 | 2 |

**Adduct Signal Fractions**

- **Unmodified**: 6.1147e+07

### RGSN(N)SSFFSR Hex 7 HexNAc 2

**Mass:**  2684.0729

(6.068e-07)

**Charge States:** 3

**Start Position**: 141

**End Position**: 151

| Profile Score | Tandem Score | TDA q-value | Volume | Spectrum Matches |
| --- | --- | --- | --- | --- |
| 13.7040 | 187.1352 | 0.0000 | 1.7692e+08 | 4 |

**Adduct Signal Fractions**

- **Unmodified**: 1.7692e+08

### RGSN(N)SSFFSR Hex 5 HexNAc 2

**Mass:**  2359.9660

(4.009e-07)

**Charge States:** 3

**Start Position**: 141

**End Position**: 151

| Profile Score | Tandem Score | TDA q-value | Volume | Spectrum Matches |
| --- | --- | --- | --- | --- |
| 13.1493 | 187.0944 | 0.0000 | 5.8129e+08 | 6 |

**Adduct Signal Fractions**

- **Unmodified**: 5.8129e+08

### RGSN(N)SSFFSR Hex 6 HexNAc 2

**Mass:**  2522.0202

(7.310e-07)

**Charge States:** 3

**Start Position**: 141

**End Position**: 151

| Profile Score | Tandem Score | TDA q-value | Volume | Spectrum Matches |
| --- | --- | --- | --- | --- |
| 12.9694 | 186.9557 | 0.0000 | 2.7900e+08 | 3 |

**Adduct Signal Fractions**

- **Unmodified**: 2.7900e+08

### RGSN(N)SSFFSR Fuc 1 Hex 5 HexNAc 5

**Mass:**  3115.2625

(1.579e-06)

**Charge States:** 3, 4

**Start Position**: 141

**End Position**: 151

| Profile Score | Tandem Score | TDA q-value | Volume | Spectrum Matches |
| --- | --- | --- | --- | --- |
| 13.9705 | 183.6074 | 0.0000 | 1.4097e+09 | 9 |

**Adduct Signal Fractions**

- **Unmodified**: 1.4097e+09

### YPALN(N)VTM(O)PNNEQFDK Hex 8 HexNAc 2

**Mass:**  3598.4628

(2.554e-06)

**Charge States:** 2, 3, 4

**Start Position**: 161

**End Position**: 177

| Profile Score | Tandem Score | TDA q-value | Volume | Spectrum Matches |
| --- | --- | --- | --- | --- |
| 15.6778 | 178.4092 | 0.0000 | 2.3056e+09 | 4 |

**Adduct Signal Fractions**

- **Unmodified**: 2.3056e+09

### RGSN(N)SSFFSR Hex 6 HexNAc 4

**Mass:**  2928.1789

(9.790e-07)

**Charge States:** 3

**Start Position**: 141

**End Position**: 151

| Profile Score | Tandem Score | TDA q-value | Volume | Spectrum Matches |
| --- | --- | --- | --- | --- |
| 13.6672 | 178.2411 | 0.0000 | 8.0251e+07 | 2 |

**Adduct Signal Fractions**

- **Unmodified**: 8.0251e+07

### RGSN(N)SSFFSR Fuc 1 Hex 4 HexNAc 3

**Mass:**  2547.0505

(4.182e-07)

**Charge States:** 3

**Start Position**: 141

**End Position**: 151

| Profile Score | Tandem Score | TDA q-value | Volume | Spectrum Matches |
| --- | --- | --- | --- | --- |
| -4.8584 | 175.8848 | 0.0000 | 7.8728e+07 | 2 |

**Adduct Signal Fractions**

- **Unmodified**: 7.8728e+07

### RGSN(N)SSFFSR Hex 5 HexNAc 5

**Mass:**  2969.2040

(3.133e-07)

**Charge States:** 3, 4

**Start Position**: 141

**End Position**: 151

| Profile Score | Tandem Score | TDA q-value | Volume | Spectrum Matches |
| --- | --- | --- | --- | --- |
| 12.9121 | 174.7820 | 0.0000 | 3.8779e+08 | 5 |

**Adduct Signal Fractions**

- **Unmodified**: 3.8779e+08

### RGSN(N)SSFFSR Hex 6 HexNAc 3

**Mass:**  2725.0999

(9.607e-07)

**Charge States:** 3

**Start Position**: 141

**End Position**: 151

| Profile Score | Tandem Score | TDA q-value | Volume | Spectrum Matches |
| --- | --- | --- | --- | --- |
| 13.2217 | 174.2408 | 0.0000 | 4.0334e+08 | 5 |

**Adduct Signal Fractions**

- **Unmodified**: 4.0334e+08

### RGSN(N)SSFFSR Fuc 1 Hex 5 HexNAc 4

**Mass:**  2912.1848

(1.064e-06)

**Charge States:** 3

**Start Position**: 141

**End Position**: 151

| Profile Score | Tandem Score | TDA q-value | Volume | Spectrum Matches |
| --- | --- | --- | --- | --- |
| 13.7794 | 173.4296 | 0.0000 | 6.7950e+08 | 7 |

**Adduct Signal Fractions**

- **Unmodified**: 6.7950e+08

### YPALN(N)VTM(O)PNNEQFDK Hex 9 HexNAc 2

**Mass:**  3760.5188

(2.758e-06)

**Charge States:** 2, 3, 4

**Start Position**: 161

**End Position**: 177

| Profile Score | Tandem Score | TDA q-value | Volume | Spectrum Matches |
| --- | --- | --- | --- | --- |
| 19.0160 | 173.2190 | 0.0000 | 2.1736e+09 | 5 |

**Adduct Signal Fractions**

- **Unmodified**: 2.1736e+09

### RGSN(N)SSFFSR Fuc 2 Hex 5 HexNAc 4

**Mass:**  3058.2421

(8.402e-07)

**Charge States:** 3

**Start Position**: 141

**End Position**: 151

| Profile Score | Tandem Score | TDA q-value | Volume | Spectrum Matches |
| --- | --- | --- | --- | --- |
| -3.1615 | 173.1044 | 0.0000 | 1.0747e+08 | 2 |

**Adduct Signal Fractions**

- **Unmodified**: 1.0747e+08

### RGSN(N)SSFFSR Fuc 2 Hex 5 HexNAc 5

**Mass:**  3261.3224

(1.049e-06)

**Charge States:** 3

**Start Position**: 141

**End Position**: 151

| Profile Score | Tandem Score | TDA q-value | Volume | Spectrum Matches |
| --- | --- | --- | --- | --- |
| 12.7269 | 172.3225 | 0.0000 | 9.1625e+07 | 3 |

**Adduct Signal Fractions**

- **Unmodified**: 9.1625e+07

### RGSN(N)SSFFSR Fuc 1 Hex 6 HexNAc 3

**Mass:**  2871.1572

(7.289e-07)

**Charge States:** 3

**Start Position**: 141

**End Position**: 151

| Profile Score | Tandem Score | TDA q-value | Volume | Spectrum Matches |
| --- | --- | --- | --- | --- |
| 12.9476 | 171.5597 | 0.0000 | 1.3107e+08 | 3 |

**Adduct Signal Fractions**

- **Unmodified**: 1.3107e+08

### RGSN(N)SSFFSR Hex 5 HexNAc 4

**Mass:**  2766.1263

(9.233e-07)

**Charge States:** 3

**Start Position**: 141

**End Position**: 151

| Profile Score | Tandem Score | TDA q-value | Volume | Spectrum Matches |
| --- | --- | --- | --- | --- |
| 12.3950 | 171.4871 | 0.0000 | 1.3922e+08 | 3 |

**Adduct Signal Fractions**

- **Unmodified**: 1.3922e+08

### TIVKPGDILLIN(N)STGNLIAPR Hex 9 HexNAc 2

**Mass:**  4068.9328

(2.766e-06)

**Charge States:** 3, 4

**Start Position**: 235

**End Position**: 256

| Profile Score | Tandem Score | TDA q-value | Volume | Spectrum Matches |
| --- | --- | --- | --- | --- |
| 16.8653 | 170.7668 | 0.0000 | 3.4859e+08 | 6 |

**Adduct Signal Fractions**

- **Unmodified**: 3.4859e+08

### YPALN(N)VTM(O)PNNEQFDK Hex 7 HexNAc 2

**Mass:**  3436.4016

(2.582e-07)

**Charge States:** 3, 4

**Start Position**: 161

**End Position**: 177

| Profile Score | Tandem Score | TDA q-value | Volume | Spectrum Matches |
| --- | --- | --- | --- | --- |
| 13.1498 | 168.3687 | 0.0000 | 1.9355e+08 | 2 |

**Adduct Signal Fractions**

- **Unmodified**: 1.9355e+08

### GSN(N)SSFFSR Hex 6 HexNAc 3

**Mass:**  2568.9966

(3.013e-07)

**Charge States:** 2, 3

**Start Position**: 142

**End Position**: 151

| Profile Score | Tandem Score | TDA q-value | Volume | Spectrum Matches |
| --- | --- | --- | --- | --- |
| -17.4348 | 167.2004 | 0.0000 | 1.1768e+08 | 2 |

**Adduct Signal Fractions**

- **Unmodified**: 1.1768e+08

### RGSN(N)SSFFSR Hex 4 HexNAc 3

**Mass:**  2400.9931

(9.907e-07)

**Charge States:** 3

**Start Position**: 141

**End Position**: 151

| Profile Score | Tandem Score | TDA q-value | Volume | Spectrum Matches |
| --- | --- | --- | --- | --- |
| -18.5750 | 166.5870 | 0.0000 | 3.2673e+07 | 1 |

**Adduct Signal Fractions**

- **Unmodified**: 3.2673e+07

### YPALN(N)VTMPNNEQFDK Hex 8 HexNAc 2

**Mass:**  3582.4667

(2.205e-06)

**Charge States:** 3, 4

**Start Position**: 161

**End Position**: 177

| Profile Score | Tandem Score | TDA q-value | Volume | Spectrum Matches |
| --- | --- | --- | --- | --- |
| -2.2592 | 165.3975 | 0.0000 | 1.5111e+08 | 4 |

**Adduct Signal Fractions**

- **Unmodified**: 1.5111e+08

### YPALN(N)VTMPNNEQFDK Hex 9 HexNAc 2

**Mass:**  3744.5194

(2.066e-06)

**Charge States:** 3, 4

**Start Position**: 161

**End Position**: 177

| Profile Score | Tandem Score | TDA q-value | Volume | Spectrum Matches |
| --- | --- | --- | --- | --- |
| 10.1019 | 163.8097 | 0.0000 | 1.8340e+08 | 7 |

**Adduct Signal Fractions**

- **Unmodified**: 1.8340e+08

### RGSN(N)SSFFSR Fuc 1 Hex 5 HexNAc 3

**Mass:**  2709.1040

(6.154e-07)

**Charge States:** 3

**Start Position**: 141

**End Position**: 151

| Profile Score | Tandem Score | TDA q-value | Volume | Spectrum Matches |
| --- | --- | --- | --- | --- |
| 9.2469 | 162.6720 | 0.0000 | 5.9278e+07 | 2 |

**Adduct Signal Fractions**

- **Unmodified**: 5.9278e+07

### RGSN(N)SSFFSR Fuc 1 Hex 4 HexNAc 5

**Mass:**  2953.2052

(-1.502e-06)

**Charge States:** 3

**Start Position**: 141

**End Position**: 151

| Profile Score | Tandem Score | TDA q-value | Volume | Spectrum Matches |
| --- | --- | --- | --- | --- |
| -5.8941 | 160.8897 | 0.0000 | 3.6602e+07 | 2 |

**Adduct Signal Fractions**

- **Unmodified**: 3.6602e+07

### N(N)GTYDHDVYR Fuc 1 Hex 5 HexNAc 4

**Mass:**  3007.1735

(1.442e-06)

**Charge States:** 3

**Start Position**: 483

**End Position**: 493

| Profile Score | Tandem Score | TDA q-value | Volume | Spectrum Matches |
| --- | --- | --- | --- | --- |
| 16.1041 | 160.8681 | 0.0000 | 5.4360e+08 | 5 |

**Adduct Signal Fractions**

- **Unmodified**: 5.4360e+08

### YPALN(N)VTM(O)PNNEQFDK Hex 5 HexNAc 2

**Mass:**  3112.3061

(3.473e-06)

**Charge States:** 3, 4

**Start Position**: 161

**End Position**: 177

| Profile Score | Tandem Score | TDA q-value | Volume | Spectrum Matches |
| --- | --- | --- | --- | --- |
| -3.2232 | 160.4200 | 0.0000 | 1.0205e+08 | 3 |

**Adduct Signal Fractions**

- **Unmodified**: 1.0205e+08

### RGSN(N)SSFFSR Fuc 1 Hex 6 HexNAc 5

**Mass:**  3277.3170

(-2.958e-07)

**Charge States:** 3

**Start Position**: 141

**End Position**: 151

| Profile Score | Tandem Score | TDA q-value | Volume | Spectrum Matches |
| --- | --- | --- | --- | --- |
| 12.5898 | 160.3582 | 0.0000 | 9.4879e+07 | 5 |

**Adduct Signal Fractions**

- **Unmodified**: 9.4879e+07

### RGSN(N)SSFFSR Hex 7 HexNAc 4

**Mass:**  3090.2371

(1.095e-06)

**Charge States:** 3

**Start Position**: 141

**End Position**: 151

| Profile Score | Tandem Score | TDA q-value | Volume | Spectrum Matches |
| --- | --- | --- | --- | --- |
| -18.9614 | 159.8980 | 0.0000 | 1.4089e+07 | 4 |

**Adduct Signal Fractions**

- **Unmodified**: 1.4089e+07

### N(N)GTYDHDVYR Fuc 1 Hex 5 HexNAc 5

**Mass:**  3210.2536

(1.768e-06)

**Charge States:** 3, 4

**Start Position**: 483

**End Position**: 493

| Profile Score | Tandem Score | TDA q-value | Volume | Spectrum Matches |
| --- | --- | --- | --- | --- |
| -3.4934 | 158.8021 | 0.0000 | 1.6185e+08 | 4 |

**Adduct Signal Fractions**

- **Unmodified**: 1.6185e+08

### PGDILLIN(N)STGNLIAPR Hex 9 HexNAc 2

**Mass:**  3627.6326

(2.202e-06)

**Charge States:** 3, 4

**Start Position**: 239

**End Position**: 256

| Profile Score | Tandem Score | TDA q-value | Volume | Spectrum Matches |
| --- | --- | --- | --- | --- |
| -18.7655 | 156.8032 | 0.0000 | 2.1081e+07 | 1 |

**Adduct Signal Fractions**

- **Unmodified**: 2.1081e+07

### GSN(N)SSFFSR Fuc 1 Hex 5 HexNAc 4

**Mass:**  2756.0833

(1.002e-06)

**Charge States:** 3

**Start Position**: 142

**End Position**: 151

| Profile Score | Tandem Score | TDA q-value | Volume | Spectrum Matches |
| --- | --- | --- | --- | --- |
| 13.6822 | 155.5921 | 0.0000 | 2.2236e+08 | 2 |

**Adduct Signal Fractions**

- **Unmodified**: 2.2236e+08

### RGSN(N)SSFFSR Hex 5 HexNAc 6

**Mass:**  3172.2837

(2.041e-06)

**Charge States:** 3

**Start Position**: 141

**End Position**: 151

| Profile Score | Tandem Score | TDA q-value | Volume | Spectrum Matches |
| --- | --- | --- | --- | --- |
| -4.1028 | 155.1126 | 0.0000 | 2.6007e+08 | 3 |

**Adduct Signal Fractions**

- **Unmodified**: 2.6007e+08

### RGSN(N)SSFFSR Hex 5 HexNAc 3

**Mass:**  2563.0451

(2.950e-07)

**Charge States:** 3

**Start Position**: 141

**End Position**: 151

| Profile Score | Tandem Score | TDA q-value | Volume | Spectrum Matches |
| --- | --- | --- | --- | --- |
| -2.9557 | 154.8748 | 0.0000 | 7.2538e+07 | 1 |

**Adduct Signal Fractions**

- **Unmodified**: 7.2538e+07

### HAVPN(N)GTIVK Fuc 1 Hex 5 HexNAc 5

**Mass:**  3008.3203

(6.700e-04)

**Start Position**: 18

**End Position**: 28

| Profile Score | Tandem Score | TDA q-value | Volume | Spectrum Matches |
| --- | --- | --- | --- | --- |
| 0.0000 | 154.2025 | 0.0000 | 0.0000e+00 | 1 |

Adduct:

No Chromatogram Found

### RGSN(N)SSFFSR Fuc 3 Hex 5 HexNAc 4

**Mass:**  3204.2997

(7.262e-07)

**Charge States:** 3

**Start Position**: 141

**End Position**: 151

| Profile Score | Tandem Score | TDA q-value | Volume | Spectrum Matches |
| --- | --- | --- | --- | --- |
| -17.7978 | 153.2071 | 0.0000 | 2.3312e+07 | 1 |

**Adduct Signal Fractions**

- **Unmodified**: 2.3312e+07

### YPALN(N)VTMPNNEQFDK Fuc 2 Hex 3 HexNAc 4 Neu5Ac 1

**Mass:**  3761.5446

(-5.374e-06)

**Charge States:** 3

**Start Position**: 161

**End Position**: 177

| Profile Score | Tandem Score | TDA q-value | Volume | Spectrum Matches |
| --- | --- | --- | --- | --- |
| 9.2645 | 152.0980 | 0.0000 | 2.2199e+07 | 2 |

**Adduct Signal Fractions**

- **Unmodified**: 2.2199e+07

### TIVKPGDILLIN(N)STGNLIAPR Fuc 2 Hex 3 HexNAc 4 Neu5Ac 1

**Mass:**  4085.9553

(-5.164e-06)

**Charge States:** 4

**Start Position**: 235

**End Position**: 256

| Profile Score | Tandem Score | TDA q-value | Volume | Spectrum Matches |
| --- | --- | --- | --- | --- |
| -17.6977 | 151.8714 | 0.0000 | 2.7418e+07 | 1 |

**Adduct Signal Fractions**

- **Unmodified**: 2.7418e+07

### YPALN(N)VTM(O)PNNEQFDK Fuc 2 Hex 3 HexNAc 4 Neu5Ac 1

**Mass:**  3777.5407

(-5.376e-06)

**Charge States:** 3

**Start Position**: 161

**End Position**: 177

| Profile Score | Tandem Score | TDA q-value | Volume | Spectrum Matches |
| --- | --- | --- | --- | --- |
| 14.7966 | 151.7660 | 0.0000 | 1.9456e+08 | 1 |

**Adduct Signal Fractions**

- **Unmodified**: 1.9456e+08

### ISIYWTIVKPGDILLIN(N)STGNLIAPR Hex 8 HexNAc 2

**Mass:**  4569.2199

(2.618e-06)

**Charge States:** 3, 4

**Start Position**: 230

**End Position**: 256

| Profile Score | Tandem Score | TDA q-value | Volume | Spectrum Matches |
| --- | --- | --- | --- | --- |
| 15.2541 | 151.3557 | 0.0000 | 1.1468e+08 | 6 |

**Adduct Signal Fractions**

- **Unmodified**: 1.1468e+08

### YPALN(N)VTM(O)PNNEQFDK Hex 6 HexNAc 2

**Mass:**  3274.3515

(1.029e-06)

**Charge States:** 3

**Start Position**: 161

**End Position**: 177

| Profile Score | Tandem Score | TDA q-value | Volume | Spectrum Matches |
| --- | --- | --- | --- | --- |
| 11.1443 | 151.2274 | 0.0000 | 3.6855e+07 | 1 |

**Adduct Signal Fractions**

- **Unmodified**: 3.6855e+07

### RGSN(N)SSFFSR Fuc 1 Hex 6 HexNAc 4

**Mass:**  3074.2366

(6.939e-07)

**Charge States:** 3

**Start Position**: 141

**End Position**: 151

| Profile Score | Tandem Score | TDA q-value | Volume | Spectrum Matches |
| --- | --- | --- | --- | --- |
| -5.5678 | 150.8807 | 0.0000 | 3.4350e+07 | 2 |

**Adduct Signal Fractions**

- **Unmodified**: 3.4350e+07

### YPALN(N)VTM(O)PNNEQFDK Hex 10 HexNAc 2

**Mass:**  3922.5694

(2.053e-06)

**Charge States:** 3

**Start Position**: 161

**End Position**: 177

| Profile Score | Tandem Score | TDA q-value | Volume | Spectrum Matches |
| --- | --- | --- | --- | --- |
| -3.1656 | 149.5233 | 0.0000 | 6.2334e+07 | 1 |

**Adduct Signal Fractions**

- **Unmodified**: 6.2334e+07

### YPALN(N)VTMPNNEQFDK Hex 7 HexNAc 2

**Mass:**  3420.4140

(2.337e-06)

**Charge States:** 3

**Start Position**: 161

**End Position**: 177

| Profile Score | Tandem Score | TDA q-value | Volume | Spectrum Matches |
| --- | --- | --- | --- | --- |
| -4.3772 | 149.4743 | 0.0000 | 1.0682e+07 | 1 |

**Adduct Signal Fractions**

- **Unmodified**: 1.0682e+07

### GSN(N)SSFFSR Hex 6 HexNAc 2

**Mass:**  2365.9205

(1.599e-06)

**Charge States:** 2, 3

**Start Position**: 142

**End Position**: 151

| Profile Score | Tandem Score | TDA q-value | Volume | Spectrum Matches |
| --- | --- | --- | --- | --- |
| -18.6524 | 149.2274 | 0.0000 | 9.1295e+07 | 3 |

**Adduct Signal Fractions**

- **Unmodified**: 9.1295e+07

### N(N)GTYDHDVYRDEALNNR Hex 6 HexNAc 3

**Mass:**  3632.4685

(1.139e-06)

**Charge States:** 4

**Start Position**: 483

**End Position**: 500

| Profile Score | Tandem Score | TDA q-value | Volume | Spectrum Matches |
| --- | --- | --- | --- | --- |
| -3.9125 | 147.5574 | 0.0000 | 2.2561e+08 | 3 |

**Adduct Signal Fractions**

- **Unmodified**: 2.2561e+08

### ISIYWTIVKPGDILLIN(N)STGNLIAPR Hex 9 HexNAc 2

**Mass:**  4731.2844

(2.527e-06)

**Charge States:** 3, 4, 5

**Start Position**: 230

**End Position**: 256

| Profile Score | Tandem Score | TDA q-value | Volume | Spectrum Matches |
| --- | --- | --- | --- | --- |
| 18.0289 | 147.5180 | 0.0000 | 1.6148e+09 | 9 |

**Adduct Signal Fractions**

- **Unmodified**: 1.6148e+09

### C(C)KSEC(C)ITPN(N)GSIPNDKPFQNVNR Hex 8 HexNAc 2

**Mass:**  4376.8459

(1.605e-06)

**Charge States:** 4

**Start Position**: 277

**End Position**: 300

| Profile Score | Tandem Score | TDA q-value | Volume | Spectrum Matches |
| --- | --- | --- | --- | --- |
| -4.1509 | 146.9544 | 0.0000 | 4.4929e+07 | 1 |

**Adduct Signal Fractions**

- **Unmodified**: 4.4929e+07

### ISIYWTIVKPGDILLIN(N)STGNLIAPR Hex 10 HexNAc 2

**Mass:**  4893.3278

(1.647e-06)

**Charge States:** 4

**Start Position**: 230

**End Position**: 256

| Profile Score | Tandem Score | TDA q-value | Volume | Spectrum Matches |
| --- | --- | --- | --- | --- |
| 16.9708 | 145.2598 | 0.0000 | 1.5831e+08 | 5 |

**Adduct Signal Fractions**

- **Unmodified**: 1.5831e+08

### GSN(N)SSFFSR Fuc 1 Hex 5 HexNAc 5

**Mass:**  2959.1618

(4.981e-07)

**Charge States:** 3

**Start Position**: 142

**End Position**: 151

| Profile Score | Tandem Score | TDA q-value | Volume | Spectrum Matches |
| --- | --- | --- | --- | --- |
| -3.2335 | 144.9285 | 0.0000 | 3.6305e+08 | 4 |

**Adduct Signal Fractions**

- **Unmodified**: 3.6305e+08

### GSN(N)SSFFSR Hex 5 HexNAc 5

**Mass:**  2813.1042

(7.743e-07)

**Charge States:** 3

**Start Position**: 142

**End Position**: 151

| Profile Score | Tandem Score | TDA q-value | Volume | Spectrum Matches |
| --- | --- | --- | --- | --- |
| 8.6903 | 143.7001 | 0.0000 | 1.4350e+08 | 3 |

**Adduct Signal Fractions**

- **Unmodified**: 1.4350e+08

### GSN(N)SSFFSR Hex 5 HexNAc 4

**Mass:**  2610.0228

(6.668e-08)

**Charge States:** 3

**Start Position**: 142

**End Position**: 151

| Profile Score | Tandem Score | TDA q-value | Volume | Spectrum Matches |
| --- | --- | --- | --- | --- |
| -5.2481 | 143.3846 | 0.0000 | 4.0880e+07 | 1 |

**Adduct Signal Fractions**

- **Unmodified**: 4.0880e+07

### N(N)GTYDHDVYRDEALNNR Fuc 1 Hex 6 HexNAc 3

**Mass:**  3778.5285

(1.639e-06)

**Charge States:** 4

**Start Position**: 483

**End Position**: 500

| Profile Score | Tandem Score | TDA q-value | Volume | Spectrum Matches |
| --- | --- | --- | --- | --- |
| -4.2769 | 142.8952 | 0.0000 | 1.4523e+08 | 1 |

**Adduct Signal Fractions**

- **Unmodified**: 1.4523e+08

### HAVPN(N)GTIVK Fuc 1 Hex 5 HexNAc 4

**Mass:**  2803.2297

(-7.473e-08)

**Charge States:** 3

**Start Position**: 18

**End Position**: 28

| Profile Score | Tandem Score | TDA q-value | Volume | Spectrum Matches |
| --- | --- | --- | --- | --- |
| -20.9257 | 142.2777 | 0.0000 | 2.9921e+06 | 1 |

**Adduct Signal Fractions**

- **Unmodified**: 2.9921e+06

### ISIYWTIVKPGDILLIN(N)STGNLIAPR Fuc 2 Hex 3 HexNAc 4 Neu5Ac 1

**Mass:**  4748.2966

(-4.570e-06)

**Charge States:** 4, 5

**Start Position**: 230

**End Position**: 256

| Profile Score | Tandem Score | TDA q-value | Volume | Spectrum Matches |
| --- | --- | --- | --- | --- |
| 15.1717 | 142.1051 | 0.0000 | 2.5771e+08 | 6 |

**Adduct Signal Fractions**

- **Unmodified**: 2.5771e+08

### N(N)GTYDHDVYR Fuc 2 Hex 5 HexNAc 4

**Mass:**  3153.2316

(9.812e-09)

**Charge States:** 3

**Start Position**: 483

**End Position**: 493

| Profile Score | Tandem Score | TDA q-value | Volume | Spectrum Matches |
| --- | --- | --- | --- | --- |
| -3.6758 | 141.4729 | 0.0000 | 1.1151e+08 | 3 |

**Adduct Signal Fractions**

- **Unmodified**: 1.1151e+08

### N(N)GTYDHDVYRDEALNNR Hex 5 HexNAc 2

**Mass:**  3267.3334

(1.545e-07)

**Charge States:** 4

**Start Position**: 483

**End Position**: 500

| Profile Score | Tandem Score | TDA q-value | Volume | Spectrum Matches |
| --- | --- | --- | --- | --- |
| -4.9430 | 141.1077 | 0.0000 | 1.0679e+08 | 1 |

**Adduct Signal Fractions**

- **Unmodified**: 1.0679e+08

### ISIYWTIVKPGDILLIN(N)STGNLIAPR Fuc 2 Hex 4 HexNAc 4 Neu5Ac 1

**Mass:**  4910.3557

(-3.833e-06)

**Charge States:** 4

**Start Position**: 230

**End Position**: 256

| Profile Score | Tandem Score | TDA q-value | Volume | Spectrum Matches |
| --- | --- | --- | --- | --- |
| -18.3051 | 140.8319 | 0.0000 | 2.4260e+07 | 2 |

**Adduct Signal Fractions**

- **Unmodified**: 2.4260e+07

### TIVKPGDILLIN(N)STGNLIAPR Hex 10 HexNAc 2

**Mass:**  4230.9846

(2.008e-06)

**Charge States:** 4

**Start Position**: 235

**End Position**: 256

| Profile Score | Tandem Score | TDA q-value | Volume | Spectrum Matches |
| --- | --- | --- | --- | --- |
| -4.4981 | 140.4942 | 0.0000 | 3.1464e+07 | 2 |

**Adduct Signal Fractions**

- **Unmodified**: 3.1464e+07

### RGSN(N)SSFFSR Fuc 2 Hex 4 HexNAc 3

**Mass:**  2693.1062

(-3.907e-07)

**Charge States:** 3

**Start Position**: 141

**End Position**: 151

| Profile Score | Tandem Score | TDA q-value | Volume | Spectrum Matches |
| --- | --- | --- | --- | --- |
| -20.4439 | 140.1263 | 0.0000 | 1.7538e+07 | 2 |

**Adduct Signal Fractions**

- **Unmodified**: 1.7538e+07

### GSN(N)SSFFSR Hex 5 HexNAc 2

**Mass:**  2203.8658

(8.786e-07)

**Charge States:** 2, 3

**Start Position**: 142

**End Position**: 151

| Profile Score | Tandem Score | TDA q-value | Volume | Spectrum Matches |
| --- | --- | --- | --- | --- |
| -1.9164 | 139.3724 | 0.0000 | 2.0242e+08 | 3 |

**Adduct Signal Fractions**

- **Unmodified**: 2.0242e+08

### GSN(N)SSFFSR Hex 8 HexNAc 2

**Mass:**  2690.0257

(5.766e-07)

**Charge States:** 2, 3

**Start Position**: 142

**End Position**: 151

| Profile Score | Tandem Score | TDA q-value | Volume | Spectrum Matches |
| --- | --- | --- | --- | --- |
| -18.5602 | 137.9190 | 0.0000 | 2.2306e+07 | 1 |

**Adduct Signal Fractions**

- **Unmodified**: 2.2306e+07

### SEC(C)ITPN(N)GSIPNDKPFQNVNR Hex 7 HexNAc 2

**Mass:**  3926.6777

(3.432e-07)

**Charge States:** 3

**Start Position**: 279

**End Position**: 300

| Profile Score | Tandem Score | TDA q-value | Volume | Spectrum Matches |
| --- | --- | --- | --- | --- |
| 15.2302 | 137.5598 | 0.0000 | 6.4707e+08 | 3 |

**Adduct Signal Fractions**

- **Unmodified**: 6.4707e+08

### N(N)GTYDHDVYRDEALNNR Fuc 1 Hex 5 HexNAc 5

**Mass:**  4022.6315

(5.474e-08)

**Charge States:** 3, 4

**Start Position**: 483

**End Position**: 500

| Profile Score | Tandem Score | TDA q-value | Volume | Spectrum Matches |
| --- | --- | --- | --- | --- |
| 11.5314 | 137.3872 | 0.0000 | 1.6410e+09 | 6 |

**Adduct Signal Fractions**

- **Unmodified**: 1.6410e+09

### N(N)GTYDHDVYRDEALNNR Fuc 2 Hex 5 HexNAc 4

**Mass:**  3965.6110

(1.113e-06)

**Charge States:** 4

**Start Position**: 483

**End Position**: 500

| Profile Score | Tandem Score | TDA q-value | Volume | Spectrum Matches |
| --- | --- | --- | --- | --- |
| -1.9457 | 137.0721 | 0.0000 | 1.1810e+09 | 4 |

**Adduct Signal Fractions**

- **Unmodified**: 1.1810e+09

### N(N)GTYDHDVYRDEALNNR Fuc 1 Hex 8 HexNAc 3

**Mass:**  4102.5895

(-9.018e-06)

**Charge States:** 3, 4

**Start Position**: 483

**End Position**: 500

| Profile Score | Tandem Score | TDA q-value | Volume | Spectrum Matches |
| --- | --- | --- | --- | --- |
| 11.3998 | 137.0340 | 0.0000 | 3.1608e+08 | 3 |

**Adduct Signal Fractions**

- **Unmodified**: 3.1608e+08

### N(N)GTYDHDVYRDEALNNR Fuc 1 Hex 4 HexNAc 3

**Mass:**  3454.4194

(7.958e-07)

**Charge States:** 4

**Start Position**: 483

**End Position**: 500

| Profile Score | Tandem Score | TDA q-value | Volume | Spectrum Matches |
| --- | --- | --- | --- | --- |
| -4.4512 | 136.2479 | 0.0000 | 1.3276e+08 | 1 |

**Adduct Signal Fractions**

- **Unmodified**: 1.3276e+08

### RGSN(N)SSFFSR Hex 6 HexNAc 5

**Mass:**  3131.2608

(1.563e-06)

**Charge States:** 3

**Start Position**: 141

**End Position**: 151

| Profile Score | Tandem Score | TDA q-value | Volume | Spectrum Matches |
| --- | --- | --- | --- | --- |
| -5.1325 | 135.2137 | 0.0000 | 1.4368e+07 | 1 |

**Adduct Signal Fractions**

- **Unmodified**: 1.4368e+07

### N(N)GTYDHDVYRDEALNNR Fuc 1 Hex 5 HexNAc 4

**Mass:**  3819.5512

(5.716e-07)

**Charge States:** 3, 4, 5

**Start Position**: 483

**End Position**: 500

| Profile Score | Tandem Score | TDA q-value | Volume | Spectrum Matches |
| --- | --- | --- | --- | --- |
| 17.1961 | 134.1706 | 0.0000 | 3.8622e+09 | 7 |

**Adduct Signal Fractions**

- **Unmodified**: 3.8622e+09

### SEC(C)ITPN(N)GSIPNDKPFQNVNR Hex 9 HexNAc 2

**Mass:**  4250.7727

(1.212e-06)

**Charge States:** 3, 4

**Start Position**: 279

**End Position**: 300

| Profile Score | Tandem Score | TDA q-value | Volume | Spectrum Matches |
| --- | --- | --- | --- | --- |
| -1.9940 | 133.0654 | 0.0000 | 1.0423e+09 | 3 |

**Adduct Signal Fractions**

- **Unmodified**: 1.0423e+09

### IVKPGDILLIN(N)STGNLIAPR Hex 9 HexNAc 2

**Mass:**  3967.8823

(1.622e-06)

**Charge States:** 3, 4

**Start Position**: 236

**End Position**: 256

| Profile Score | Tandem Score | TDA q-value | Volume | Spectrum Matches |
| --- | --- | --- | --- | --- |
| -19.4940 | 132.7219 | 0.0000 | 1.1682e+07 | 2 |

**Adduct Signal Fractions**

- **Unmodified**: 1.1682e+07

### RGSN(N)SSFFSR Fuc 1 Hex 6 HexNAc 6

**Mass:**  3480.3949

(1.204e-06)

**Charge States:** 3, 4

**Start Position**: 141

**End Position**: 151

| Profile Score | Tandem Score | TDA q-value | Volume | Spectrum Matches |
| --- | --- | --- | --- | --- |
| -5.1957 | 132.1024 | 0.0000 | 3.6134e+07 | 3 |

**Adduct Signal Fractions**

- **Unmodified**: 3.6134e+07

### SEC(C)ITPN(N)GSIPNDKPFQNVNR Hex 8 HexNAc 2

**Mass:**  4088.7197

(2.285e-06)

**Charge States:** 3, 4

**Start Position**: 279

**End Position**: 300

| Profile Score | Tandem Score | TDA q-value | Volume | Spectrum Matches |
| --- | --- | --- | --- | --- |
| 16.6878 | 131.8444 | 0.0000 | 5.7269e+08 | 3 |

**Adduct Signal Fractions**

- **Unmodified**: 5.7269e+08

### N(N)GTYDHDVYRDEALNNR Hex 7 HexNAc 3

**Mass:**  3794.5236

(1.704e-06)

**Charge States:** 4

**Start Position**: 483

**End Position**: 500

| Profile Score | Tandem Score | TDA q-value | Volume | Spectrum Matches |
| --- | --- | --- | --- | --- |
| -19.7939 | 131.4908 | 0.0000 | 2.3665e+07 | 1 |

**Adduct Signal Fractions**

- **Unmodified**: 2.3665e+07

### N(N)GTYDHDVYRDEALNNR Hex 5 HexNAc 4

**Mass:**  3673.4943

(9.594e-07)

**Charge States:** 4

**Start Position**: 483

**End Position**: 500

| Profile Score | Tandem Score | TDA q-value | Volume | Spectrum Matches |
| --- | --- | --- | --- | --- |
| -3.5347 | 131.4560 | 0.0000 | 2.3008e+08 | 3 |

**Adduct Signal Fractions**

- **Unmodified**: 2.3008e+08

### RGSN(N)SSFFSR Fuc 2 Hex 6 HexNAc 3

**Mass:**  3017.2161

(1.039e-06)

**Charge States:** 3

**Start Position**: 141

**End Position**: 151

| Profile Score | Tandem Score | TDA q-value | Volume | Spectrum Matches |
| --- | --- | --- | --- | --- |
| -18.3449 | 130.8726 | 0.0000 | 9.7039e+06 | 1 |

**Adduct Signal Fractions**

- **Unmodified**: 9.7039e+06

### IRDIPSRISIYWTIVKPGDILLIN(N)STGNLIAPR Hex 9 HexNAc 2

**Mass:**  5568.7551

(1.209e-06)

**Charge States:** 4, 5, 7

**Start Position**: 223

**End Position**: 256

| Profile Score | Tandem Score | TDA q-value | Volume | Spectrum Matches |
| --- | --- | --- | --- | --- |
| -2.9446 | 130.4066 | 0.0000 | 1.7173e+08 | 5 |

**Adduct Signal Fractions**

- **Unmodified**: 1.7173e+08

### N(N)GTYDHDVYR Hex 6 HexNAc 3

**Mass:**  2820.0889

(8.110e-07)

**Charge States:** 3

**Start Position**: 483

**End Position**: 493

| Profile Score | Tandem Score | TDA q-value | Volume | Spectrum Matches |
| --- | --- | --- | --- | --- |
| -20.0598 | 130.3786 | 0.0000 | 1.8680e+07 | 1 |

**Adduct Signal Fractions**

- **Unmodified**: 1.8680e+07

### N(N)GTYDHDVYRDEALNNR Fuc 2 Hex 5 HexNAc 5

**Mass:**  4168.6936

(1.751e-06)

**Charge States:** 4

**Start Position**: 483

**End Position**: 500

| Profile Score | Tandem Score | TDA q-value | Volume | Spectrum Matches |
| --- | --- | --- | --- | --- |
| -17.7513 | 129.7286 | 0.0000 | 1.5076e+08 | 1 |

**Adduct Signal Fractions**

- **Unmodified**: 1.5076e+08

### YPALN(N)VTM(O)PN(D)NEQFDK Hex 8 HexNAc 2

**Mass:**  3599.4494

(2.774e-04)

**Charge States:** 2, 3, 4

**Start Position**: 161

**End Position**: 177

| Profile Score | Tandem Score | TDA q-value | Volume | Spectrum Matches |
| --- | --- | --- | --- | --- |
| 9.6957 | 129.5018 | 0.0000 | 4.1601e+08 | 6 |

**Adduct Signal Fractions**

- **Unmodified**: 4.1601e+08

### N(N)GTYDHDVYRDEALNNR Fuc 1 Hex 8 HexNAc 2

**Mass:**  3899.5097

(-9.170e-06)

**Charge States:** 3, 4

**Start Position**: 483

**End Position**: 500

| Profile Score | Tandem Score | TDA q-value | Volume | Spectrum Matches |
| --- | --- | --- | --- | --- |
| -2.4233 | 128.0651 | 0.0000 | 6.4415e+08 | 4 |

**Adduct Signal Fractions**

- **Unmodified**: 6.4415e+08

### N(N)GTYDHDVYRDEALNNR Hex 5 HexNAc 3

**Mass:**  3470.4133

(8.789e-07)

**Charge States:** 4

**Start Position**: 483

**End Position**: 500

| Profile Score | Tandem Score | TDA q-value | Volume | Spectrum Matches |
| --- | --- | --- | --- | --- |
| -17.8076 | 127.8182 | 0.0000 | 4.9631e+07 | 1 |

**Adduct Signal Fractions**

- **Unmodified**: 4.9631e+07

### N(N)GTYDHDVYRDEALNNR Fuc 3 Hex 5 HexNAc 4

**Mass:**  4111.6667

(4.575e-07)

**Charge States:** 4

**Start Position**: 483

**End Position**: 500

| Profile Score | Tandem Score | TDA q-value | Volume | Spectrum Matches |
| --- | --- | --- | --- | --- |
| -17.8574 | 127.1512 | 0.0000 | 2.7459e+08 | 1 |

**Adduct Signal Fractions**

- **Unmodified**: 2.7459e+08

### N(N)GTYDHDVYRDEALNNR Fuc 1 Hex 5 HexNAc 3

**Mass:**  3616.4731

(1.294e-06)

**Charge States:** 4

**Start Position**: 483

**End Position**: 500

| Profile Score | Tandem Score | TDA q-value | Volume | Spectrum Matches |
| --- | --- | --- | --- | --- |
| -3.8128 | 126.9666 | 0.0000 | 8.2411e+07 | 1 |

**Adduct Signal Fractions**

- **Unmodified**: 8.2411e+07

### YPALN(N)VTM(O)PN(D)NEQFDK Hex 9 HexNAc 2

**Mass:**  3761.5054

(2.665e-04)

**Charge States:** 3, 4

**Start Position**: 161

**End Position**: 177

| Profile Score | Tandem Score | TDA q-value | Volume | Spectrum Matches |
| --- | --- | --- | --- | --- |
| 8.4645 | 126.5418 | 0.0000 | 4.8439e+08 | 5 |

**Adduct Signal Fractions**

- **Unmodified**: 4.8439e+08

### YPALN(N)VTM(O)PN(D)NEQFDK Fuc 2 Hex 3 HexNAc 4 Neu5Ac 1

**Mass:**  3778.5280

(2.563e-04)

**Charge States:** 3

**Start Position**: 161

**End Position**: 177

| Profile Score | Tandem Score | TDA q-value | Volume | Spectrum Matches |
| --- | --- | --- | --- | --- |
| 7.7925 | 126.0602 | 0.0000 | 3.5717e+07 | 1 |

**Adduct Signal Fractions**

- **Unmodified**: 3.5717e+07

### YPALN(N)VTM(O)PNNEQFDK Hex 4 HexNAc 2

**Mass:**  2950.2524

(4.177e-06)

**Charge States:** 3

**Start Position**: 161

**End Position**: 177

| Profile Score | Tandem Score | TDA q-value | Volume | Spectrum Matches |
| --- | --- | --- | --- | --- |
| -3.1565 | 125.3805 | 0.0000 | 1.8335e+07 | 1 |

**Adduct Signal Fractions**

- **Unmodified**: 1.8335e+07

### C(C)KSEC(C)ITPN(N)GSIPNDKPFQNVNR Hex 7 HexNAc 2

**Mass:**  4214.7946

(1.652e-06)

**Charge States:** 4

**Start Position**: 277

**End Position**: 300

| Profile Score | Tandem Score | TDA q-value | Volume | Spectrum Matches |
| --- | --- | --- | --- | --- |
| -3.8239 | 124.9492 | 0.0000 | 5.9230e+07 | 1 |

**Adduct Signal Fractions**

- **Unmodified**: 5.9230e+07

### SEC(C)ITPN(N)GSIPNDKPFQNVNR Hex 6 HexNAc 2

**Mass:**  3764.6182

(2.422e-06)

**Charge States:** 3

**Start Position**: 279

**End Position**: 300

| Profile Score | Tandem Score | TDA q-value | Volume | Spectrum Matches |
| --- | --- | --- | --- | --- |
| -2.6416 | 123.2335 | 0.0000 | 8.3198e+07 | 2 |

**Adduct Signal Fractions**

- **Unmodified**: 8.3198e+07

### N(N)GTYDHDVYRDEALN(D)NR Fuc 1 Hex 5 HexNAc 4

**Mass:**  3820.5371

(2.587e-04)

**Charge States:** 3, 4, 5

**Start Position**: 483

**End Position**: 500

| Profile Score | Tandem Score | TDA q-value | Volume | Spectrum Matches |
| --- | --- | --- | --- | --- |
| -7.2794 | 123.0098 | 0.0000 | 1.6869e+09 | 6 |

**Adduct Signal Fractions**

- **Unmodified**: 1.6869e+09

### N(N)GTYDHDVYR Hex 5 HexNAc 4

**Mass:**  2861.1178

(1.592e-06)

**Charge States:** 3, 4

**Start Position**: 483

**End Position**: 493

| Profile Score | Tandem Score | TDA q-value | Volume | Spectrum Matches |
| --- | --- | --- | --- | --- |
| -19.2252 | 121.9653 | 0.0000 | 2.2999e+07 | 2 |

**Adduct Signal Fractions**

- **Unmodified**: 2.2999e+07

### N(N)GTYDHDVYRDEALNNR Fuc 1 Hex 6 HexNAc 5

**Mass:**  4184.6848

(3.582e-07)

**Charge States:** 3, 4

**Start Position**: 483

**End Position**: 500

| Profile Score | Tandem Score | TDA q-value | Volume | Spectrum Matches |
| --- | --- | --- | --- | --- |
| -3.2690 | 120.9950 | 0.0000 | 5.7447e+08 | 4 |

**Adduct Signal Fractions**

- **Unmodified**: 5.7447e+08

### SEC(C)ITPN(N)GSIPNDKPFQNVNR Hex 5 HexNAc 2

**Mass:**  3602.5636

(3.092e-06)

**Charge States:** 4

**Start Position**: 279

**End Position**: 300

| Profile Score | Tandem Score | TDA q-value | Volume | Spectrum Matches |
| --- | --- | --- | --- | --- |
| -19.1135 | 120.8405 | 0.0000 | 3.5040e+07 | 1 |

**Adduct Signal Fractions**

- **Unmodified**: 3.5040e+07

### N(N)GTYDHDVYRDEALNNR Fuc 1 Hex 6 HexNAc 6

**Mass:**  4387.7658

(1.287e-06)

**Charge States:** 4

**Start Position**: 483

**End Position**: 500

| Profile Score | Tandem Score | TDA q-value | Volume | Spectrum Matches |
| --- | --- | --- | --- | --- |
| -4.6472 | 120.6758 | 0.0000 | 8.7324e+07 | 1 |

**Adduct Signal Fractions**

- **Unmodified**: 8.7324e+07

### N(N)GTYDHDVYR Fuc 2 Hex 5 HexNAc 5

**Mass:**  3356.3069

(-4.462e-07)

**Charge States:** 3

**Start Position**: 483

**End Position**: 493

| Profile Score | Tandem Score | TDA q-value | Volume | Spectrum Matches |
| --- | --- | --- | --- | --- |
| -20.0152 | 120.1765 | 0.0000 | 1.6920e+07 | 2 |

**Adduct Signal Fractions**

- **Unmodified**: 1.6920e+07

### SEC(C)ITPN(N)GSIPNDKPFQNVNR Fuc 2 Hex 3 HexNAc 4 Neu5Ac 1

**Mass:**  4267.7990

(-5.044e-06)

**Charge States:** 4

**Start Position**: 279

**End Position**: 300

| Profile Score | Tandem Score | TDA q-value | Volume | Spectrum Matches |
| --- | --- | --- | --- | --- |
| -18.8183 | 119.5598 | 0.0000 | 3.9013e+07 | 1 |

**Adduct Signal Fractions**

- **Unmodified**: 3.9013e+07

### N(N)GTYDHDVYRDEALNNR Fuc 2 Hex 6 HexNAc 5

**Mass:**  4330.7409

(5.000e-07)

**Charge States:** 4

**Start Position**: 483

**End Position**: 500

| Profile Score | Tandem Score | TDA q-value | Volume | Spectrum Matches |
| --- | --- | --- | --- | --- |
| -4.3549 | 119.4838 | 0.0000 | 1.0482e+08 | 1 |

**Adduct Signal Fractions**

- **Unmodified**: 1.0482e+08

### N(N)GTYDHDVYRDEALNNR Fuc 1 Hex 9 HexNAc 3

**Mass:**  4264.6452

(-8.341e-06)

**Charge States:** 4

**Start Position**: 483

**End Position**: 500

| Profile Score | Tandem Score | TDA q-value | Volume | Spectrum Matches |
| --- | --- | --- | --- | --- |
| -2.9776 | 119.1677 | 0.0000 | 1.7783e+08 | 2 |

**Adduct Signal Fractions**

- **Unmodified**: 1.7783e+08

### N(N)GTYDHDVYRDEALN(D)NR Fuc 2 Hex 5 HexNAc 4

**Mass:**  3966.5959

(2.495e-04)

**Charge States:** 3, 4

**Start Position**: 483

**End Position**: 500

| Profile Score | Tandem Score | TDA q-value | Volume | Spectrum Matches |
| --- | --- | --- | --- | --- |
| 4.3104 | 118.1236 | 0.0000 | 5.1007e+08 | 3 |

**Adduct Signal Fractions**

- **Unmodified**: 5.1007e+08

### ISIYWTIVKPGDILLIN(N)STGNLIAPR Hex 5 HexNAc 2

**Mass:**  4083.0622

(1.773e-06)

**Charge States:** 3, 4

**Start Position**: 230

**End Position**: 256

| Profile Score | Tandem Score | TDA q-value | Volume | Spectrum Matches |
| --- | --- | --- | --- | --- |
| -4.4376 | 117.8880 | 0.0000 | 7.6675e+06 | 1 |

**Adduct Signal Fractions**

- **Unmodified**: 7.6675e+06

### N(N)GTYDHDVYRDEALNNR Fuc 1 Hex 7 HexNAc 6

**Mass:**  4549.8063

(-1.456e-06)

**Charge States:** 4

**Start Position**: 483

**End Position**: 500

| Profile Score | Tandem Score | TDA q-value | Volume | Spectrum Matches |
| --- | --- | --- | --- | --- |
| -20.5859 | 116.7107 | 0.0000 | 2.7420e+07 | 1 |

**Adduct Signal Fractions**

- **Unmodified**: 2.7420e+07

### N(N)GTYDHDVYRDEALNNR Hex 7 HexNAc 2

**Mass:**  3591.4436

(1.633e-06)

**Charge States:** 4

**Start Position**: 483

**End Position**: 500

| Profile Score | Tandem Score | TDA q-value | Volume | Spectrum Matches |
| --- | --- | --- | --- | --- |
| -18.9459 | 114.4776 | 0.0000 | 3.2728e+07 | 1 |

**Adduct Signal Fractions**

- **Unmodified**: 3.2728e+07

### N(N)GTYDHDVYR Fuc 3 Hex 5 HexNAc 4

**Mass:**  3299.2879

(1.041e-06)

**Charge States:** 3

**Start Position**: 483

**End Position**: 493

| Profile Score | Tandem Score | TDA q-value | Volume | Spectrum Matches |
| --- | --- | --- | --- | --- |
| -19.4846 | 113.6438 | 0.0000 | 2.6706e+07 | 3 |

**Adduct Signal Fractions**

- **Unmodified**: 2.6706e+07

### RGSN(N)SSFFSR Fuc 1 Hex 5 HexNAc 4

**Mass:**  2914.2127

(6.974e-04)

**Start Position**: 141

**End Position**: 151

| Profile Score | Tandem Score | TDA q-value | Volume | Spectrum Matches |
| --- | --- | --- | --- | --- |
| 0.0000 | 113.1534 | 0.0000 | 0.0000e+00 | 1 |

Adduct:

No Chromatogram Found

### N(N)GTYDHDVYRDEALNNR Fuc 1 Hex 6 HexNAc 4

**Mass:**  3981.6044

(7.174e-07)

**Charge States:** 4

**Start Position**: 483

**End Position**: 500

| Profile Score | Tandem Score | TDA q-value | Volume | Spectrum Matches |
| --- | --- | --- | --- | --- |
| -19.6364 | 113.0397 | 0.0000 | 2.6238e+07 | 1 |

**Adduct Signal Fractions**

- **Unmodified**: 2.6238e+07

### N(N)GTYDHDVYR Fuc 1 Hex 4 HexNAc 3

**Mass:**  2642.0390

(4.305e-08)

**Charge States:** 3

**Start Position**: 483

**End Position**: 493

| Profile Score | Tandem Score | TDA q-value | Volume | Spectrum Matches |
| --- | --- | --- | --- | --- |
| -20.0342 | 112.8488 | 0.0000 | 8.0796e+06 | 1 |

**Adduct Signal Fractions**

- **Unmodified**: 8.0796e+06

### N(N)GTYDHDVYR Fuc 1 Hex 6 HexNAc 5

**Mass:**  3372.3059

(2.929e-06)

**Charge States:** 3

**Start Position**: 483

**End Position**: 493

| Profile Score | Tandem Score | TDA q-value | Volume | Spectrum Matches |
| --- | --- | --- | --- | --- |
| -5.0349 | 112.5128 | 0.0000 | 6.3007e+07 | 4 |

**Adduct Signal Fractions**

- **Unmodified**: 6.3007e+07

### YPALN(N)VTM(O)PN(D)NEQFDK Hex 7 HexNAc 2

**Mass:**  3437.4021

(2.932e-04)

**Charge States:** 3, 4

**Start Position**: 161

**End Position**: 177

| Profile Score | Tandem Score | TDA q-value | Volume | Spectrum Matches |
| --- | --- | --- | --- | --- |
| 7.9878 | 112.4569 | 0.0000 | 3.2572e+07 | 3 |

**Adduct Signal Fractions**

- **Unmodified**: 3.2572e+07

### N(N)GTYDHDVYR Fuc 1 Hex 6 HexNAc 3

**Mass:**  2966.1513

(2.263e-06)

**Charge States:** 3

**Start Position**: 483

**End Position**: 493

| Profile Score | Tandem Score | TDA q-value | Volume | Spectrum Matches |
| --- | --- | --- | --- | --- |
| -18.3725 | 112.4160 | 0.0000 | 1.0384e+07 | 1 |

**Adduct Signal Fractions**

- **Unmodified**: 1.0384e+07

### N(N)GTYDHDVYRDEALNNR Fuc 2 Hex 4 HexNAc 3

**Mass:**  3600.4775

(8.099e-07)

**Charge States:** 4

**Start Position**: 483

**End Position**: 500

| Profile Score | Tandem Score | TDA q-value | Volume | Spectrum Matches |
| --- | --- | --- | --- | --- |
| -4.8171 | 111.7222 | 0.0000 | 4.1011e+07 | 1 |

**Adduct Signal Fractions**

- **Unmodified**: 4.1011e+07

### N(N)GTYDHDVYRDEALN(D)NR Fuc 1 Hex 6 HexNAc 3

**Mass:**  3779.5144

(2.626e-04)

**Charge States:** 4

**Start Position**: 483

**End Position**: 500

| Profile Score | Tandem Score | TDA q-value | Volume | Spectrum Matches |
| --- | --- | --- | --- | --- |
| -5.8367 | 111.3041 | 0.0000 | 5.7507e+07 | 1 |

**Adduct Signal Fractions**

- **Unmodified**: 5.7507e+07

### N(N)GTYDHDVYR Hex 5 HexNAc 3

**Mass:**  2658.0353

(5.154e-07)

**Charge States:** 3

**Start Position**: 483

**End Position**: 493

| Profile Score | Tandem Score | TDA q-value | Volume | Spectrum Matches |
| --- | --- | --- | --- | --- |
| -21.1215 | 111.2644 | 0.0000 | 4.6573e+06 | 1 |

**Adduct Signal Fractions**

- **Unmodified**: 4.6573e+06

### IEVTN(N)ATELVQN Hex 5 HexNAc 2

**Mass:**  2546.1071

(2.653e-06)

**Charge States:** 3

**Start Position**: 34

**End Position**: 46

| Profile Score | Tandem Score | TDA q-value | Volume | Spectrum Matches |
| --- | --- | --- | --- | --- |
| -21.1678 | 110.9711 | 0.0000 | 1.1886e+06 | 1 |

**Adduct Signal Fractions**

- **Unmodified**: 1.1886e+06

### RGSN(N)SSFFSR Hex 7 HexNAc 3 Neu5Ac 1

**Mass:**  3178.2379

(-2.377e-06)

**Charge States:** 3

**Start Position**: 141

**End Position**: 151

| Profile Score | Tandem Score | TDA q-value | Volume | Spectrum Matches |
| --- | --- | --- | --- | --- |
| -21.2859 | 109.4660 | 0.0038 | 3.5359e+06 | 1 |

**Adduct Signal Fractions**

- **Unmodified**: 3.5359e+06

### RGSN(N)SSFFSR Fuc 2 Hex 5 HexNAc 6 Neu5Ac 2

**Mass:**  4047.5609

(2.401e-04)

**Start Position**: 141

**End Position**: 151

| Profile Score | Tandem Score | TDA q-value | Volume | Spectrum Matches |
| --- | --- | --- | --- | --- |
| 0.0000 | 109.3661 | 0.0038 | 0.0000e+00 | 1 |

Adduct:

No Chromatogram Found

### N(N)GTYDHDVYRDEALNNR Fuc 3 Hex 6 HexNAc 5

**Mass:**  4476.7927

(-9.648e-07)

**Charge States:** 4

**Start Position**: 483

**End Position**: 500

| Profile Score | Tandem Score | TDA q-value | Volume | Spectrum Matches |
| --- | --- | --- | --- | --- |
| -19.8957 | 107.9426 | 0.0038 | 2.5879e+07 | 1 |

**Adduct Signal Fractions**

- **Unmodified**: 2.5879e+07

### YPALN(N)VTMPN(D)NEQFDK Hex 8 HexNAc 2

**Mass:**  3583.4489

(2.764e-04)

**Charge States:** 3

**Start Position**: 161

**End Position**: 177

| Profile Score | Tandem Score | TDA q-value | Volume | Spectrum Matches |
| --- | --- | --- | --- | --- |
| -6.3191 | 106.6530 | 0.0038 | 2.1775e+07 | 2 |

**Adduct Signal Fractions**

- **Unmodified**: 2.1775e+07

### N(N)GTYDHDVYRDEALNNR Fuc 2 Hex 9 HexNAc 3

**Mass:**  4410.6999

(-8.824e-06)

**Charge States:** 4

**Start Position**: 483

**End Position**: 500

| Profile Score | Tandem Score | TDA q-value | Volume | Spectrum Matches |
| --- | --- | --- | --- | --- |
| -4.5805 | 105.9102 | 0.0038 | 7.0261e+07 | 1 |

**Adduct Signal Fractions**

- **Unmodified**: 7.0261e+07

### N(N)GTYDHDVYRDEALN(D)NR Fuc 1 Hex 5 HexNAc 5

**Mass:**  4023.6174

(2.462e-04)

**Charge States:** 3, 4, 5

**Start Position**: 483

**End Position**: 500

| Profile Score | Tandem Score | TDA q-value | Volume | Spectrum Matches |
| --- | --- | --- | --- | --- |
| 10.3095 | 104.1325 | 0.0038 | 7.2946e+08 | 7 |

**Adduct Signal Fractions**

- **Unmodified**: 7.2946e+08

### RGSN(N)SSFFSR Fuc 1 Hex 5 HexNAc 6

**Mass:**  3318.3444

(1.210e-06)

**Charge States:** 3

**Start Position**: 141

**End Position**: 151

| Profile Score | Tandem Score | TDA q-value | Volume | Spectrum Matches |
| --- | --- | --- | --- | --- |
| -21.0953 | 103.7377 | 0.0038 | 1.0526e+07 | 1 |

**Adduct Signal Fractions**

- **Unmodified**: 1.0526e+07

### N(N)GTYDHDVYR Hex 7 HexNAc 2

**Mass:**  2779.0678

(2.749e-06)

**Charge States:** 3

**Start Position**: 483

**End Position**: 493

| Profile Score | Tandem Score | TDA q-value | Volume | Spectrum Matches |
| --- | --- | --- | --- | --- |
| -20.1090 | 103.6269 | 0.0038 | 3.6064e+06 | 1 |

**Adduct Signal Fractions**

- **Unmodified**: 3.6064e+06

### N(N)GTYDHDVYRDEALNNR Fuc 1 Hex 5 HexNAc 4 Neu5Ac 2

**Mass:**  4401.7526

(2.975e-06)

**Charge States:** 4

**Start Position**: 483

**End Position**: 500

| Profile Score | Tandem Score | TDA q-value | Volume | Spectrum Matches |
| --- | --- | --- | --- | --- |
| -19.1337 | 102.9632 | 0.0038 | 1.6086e+07 | 1 |

**Adduct Signal Fractions**

- **Unmodified**: 1.6086e+07

### N(N)GTYDHDVYRDEALNNR Hex 5 HexNAc 5

**Mass:**  3876.5712

(4.764e-07)

**Charge States:** 3, 4

**Start Position**: 483

**End Position**: 500

| Profile Score | Tandem Score | TDA q-value | Volume | Spectrum Matches |
| --- | --- | --- | --- | --- |
| 12.0264 | 102.7135 | 0.0038 | 1.0372e+09 | 6 |

**Adduct Signal Fractions**

- **Unmodified**: 1.0372e+09

### SEC(C)ITPN(N)GSIPN(D)DKPFQNVNR Hex 8 HexNAc 2

**Mass:**  4089.7206

(2.458e-04)

**Charge States:** 3, 4

**Start Position**: 279

**End Position**: 300

| Profile Score | Tandem Score | TDA q-value | Volume | Spectrum Matches |
| --- | --- | --- | --- | --- |
| 8.1383 | 102.2533 | 0.0038 | 1.3473e+08 | 2 |

**Adduct Signal Fractions**

- **Unmodified**: 1.3473e+08

### RGSN(N)SSFFSR Hex 6 HexNAc 6

**Mass:**  3334.3339

(1.129e-06)

**Charge States:** 3

**Start Position**: 141

**End Position**: 151

| Profile Score | Tandem Score | TDA q-value | Volume | Spectrum Matches |
| --- | --- | --- | --- | --- |
| -5.4234 | 100.6516 | 0.0038 | 1.7533e+07 | 1 |

**Adduct Signal Fractions**

- **Unmodified**: 1.7533e+07

### RGSN(N)SSFFSR Fuc 2 Hex 6 HexNAc 5

**Mass:**  3423.3718

(-1.236e-06)

**Charge States:** 3

**Start Position**: 141

**End Position**: 151

| Profile Score | Tandem Score | TDA q-value | Volume | Spectrum Matches |
| --- | --- | --- | --- | --- |
| -19.5035 | 97.7922 | 0.0075 | 4.2144e+06 | 1 |

**Adduct Signal Fractions**

- **Unmodified**: 4.2144e+06

### N(N)GTYDHDVYR Hex 5 HexNAc 2

**Mass:**  2454.9552

(2.711e-07)

**Charge States:** 3

**Start Position**: 483

**End Position**: 493

| Profile Score | Tandem Score | TDA q-value | Volume | Spectrum Matches |
| --- | --- | --- | --- | --- |
| -18.6945 | 97.4957 | 0.0075 | 9.0476e+06 | 1 |

**Adduct Signal Fractions**

- **Unmodified**: 9.0476e+06

### RGSN(N)SSFFSR Hex 5 HexNAc 6 Neu5Ac 3

**Mass:**  4045.5693

(-9.052e-07)

**Charge States:** 3, 4

**Start Position**: 141

**End Position**: 151

| Profile Score | Tandem Score | TDA q-value | Volume | Spectrum Matches |
| --- | --- | --- | --- | --- |
| 13.8077 | 94.1901 | 0.0149 | 3.5158e+08 | 3 |

**Adduct Signal Fractions**

- **Unmodified**: 3.5158e+08

### YPALN(N)VTM(O)PNNEQFDKLYIWGVHHPVTDK Hex 9 HexNAc 2

**Mass:**  5306.3296

(2.640e-06)

**Charge States:** 5

**Start Position**: 161

**End Position**: 190

| Profile Score | Tandem Score | TDA q-value | Volume | Spectrum Matches |
| --- | --- | --- | --- | --- |
| -18.4574 | 93.4674 | 0.0182 | 1.3026e+07 | 1 |

**Adduct Signal Fractions**

- **Unmodified**: 1.3026e+07

### N(N)GTYDHDVYR Fuc 1 Hex 5 HexNAc 3

**Mass:**  2805.1060

(3.617e-04)

**Start Position**: 483

**End Position**: 493

| Profile Score | Tandem Score | TDA q-value | Volume | Spectrum Matches |
| --- | --- | --- | --- | --- |
| 0.0000 | 93.2229 | 0.0182 | 0.0000e+00 | 1 |

Adduct:

No Chromatogram Found

### IRDIPSRISIYWTIVKPGDILLIN(N)STGNLIAPR Hex 8 HexNAc 2

**Mass:**  5406.7025

(1.327e-06)

**Charge States:** 5

**Start Position**: 223

**End Position**: 256

| Profile Score | Tandem Score | TDA q-value | Volume | Spectrum Matches |
| --- | --- | --- | --- | --- |
| -4.4564 | 93.0472 | 0.0182 | 1.1469e+07 | 2 |

**Adduct Signal Fractions**

- **Unmodified**: 1.1469e+07

### DIPSRISIYWTIVKPGDILLIN(N)STGNLIAPR Hex 9 HexNAc 2

**Mass:**  5299.5744

(2.124e-06)

**Charge States:** 4

**Start Position**: 225

**End Position**: 256

| Profile Score | Tandem Score | TDA q-value | Volume | Spectrum Matches |
| --- | --- | --- | --- | --- |
| -4.6864 | 92.9640 | 0.0182 | 1.4885e+07 | 2 |

**Adduct Signal Fractions**

- **Unmodified**: 1.4885e+07

### SEC(C)ITPN(N)GSIPN(D)DKPFQNVNR Hex 7 HexNAc 2

**Mass:**  3927.6625

(2.548e-04)

**Charge States:** 3

**Start Position**: 279

**End Position**: 300

| Profile Score | Tandem Score | TDA q-value | Volume | Spectrum Matches |
| --- | --- | --- | --- | --- |
| 8.9842 | 92.7525 | 0.0182 | 1.4471e+08 | 3 |

**Adduct Signal Fractions**

- **Unmodified**: 1.4471e+08

### SEC(C)ITPN(N)GSIPN(D)DKPFQNVNR Hex 9 HexNAc 2

**Mass:**  4251.7653

(2.347e-04)

**Charge States:** 3, 4

**Start Position**: 279

**End Position**: 300

| Profile Score | Tandem Score | TDA q-value | Volume | Spectrum Matches |
| --- | --- | --- | --- | --- |
| 8.9285 | 92.1913 | 0.0214 | 2.9470e+08 | 4 |

**Adduct Signal Fractions**

- **Unmodified**: 2.9470e+08

### N(N)GTYDHDVYRDEALNNR Fuc 1 Hex 4 HexNAc 4

**Mass:**  3657.5007

(1.297e-06)

**Charge States:** 4

**Start Position**: 483

**End Position**: 500

| Profile Score | Tandem Score | TDA q-value | Volume | Spectrum Matches |
| --- | --- | --- | --- | --- |
| 10.6339 | 91.4293 | 0.0214 | 4.2574e+07 | 1 |

**Adduct Signal Fractions**

- **Unmodified**: 4.2574e+07

### N(N)GTYDHDVYRDEALN(D)NRFQIK Fuc 1 Hex 5 HexNAc 4

**Mass:**  4336.8496

(2.294e-04)

**Charge States:** 5

**Start Position**: 483

**End Position**: 504

| Profile Score | Tandem Score | TDA q-value | Volume | Spectrum Matches |
| --- | --- | --- | --- | --- |
| -20.6895 | 90.6529 | 0.0214 | 5.4395e+07 | 2 |

**Adduct Signal Fractions**

- **Unmodified**: 5.4395e+07

### N(N)GTYDHDVYRDEALN(D)NR Fuc 3 Hex 5 HexNAc 4

**Mass:**  4112.6516

(2.398e-04)

**Charge States:** 4

**Start Position**: 483

**End Position**: 500

| Profile Score | Tandem Score | TDA q-value | Volume | Spectrum Matches |
| --- | --- | --- | --- | --- |
| 8.6003 | 90.0919 | 0.0214 | 1.1782e+08 | 1 |

**Adduct Signal Fractions**

- **Unmodified**: 1.1782e+08

### C(C)KSEC(C)ITPN(N)GSIPNDKPFQNVNR Hex 9 HexNAc 2

**Mass:**  4538.8986

(1.207e-06)

**Charge States:** 4

**Start Position**: 277

**End Position**: 300

| Profile Score | Tandem Score | TDA q-value | Volume | Spectrum Matches |
| --- | --- | --- | --- | --- |
| -4.0721 | 88.2256 | 0.0319 | 7.9102e+07 | 1 |

**Adduct Signal Fractions**

- **Unmodified**: 7.9102e+07

### YPALN(N)VTM(O)PNNEQFDK Fuc 1 Hex 3 HexNAc 5 Neu5Ac 1

**Mass:**  3834.5535

(-5.977e-06)

**Charge States:** 3

**Start Position**: 161

**End Position**: 177

| Profile Score | Tandem Score | TDA q-value | Volume | Spectrum Matches |
| --- | --- | --- | --- | --- |
| -4.4488 | 87.5171 | 0.0389 | 4.8167e+07 | 1 |

**Adduct Signal Fractions**

- **Unmodified**: 4.8167e+07

### DILLIN(N)STGNLIAPR Hex 6 HexNAc 4 Neu5Ac 1

**Mass:**  3684.6575

(1.290e-06)

**Charge States:** 3, 4

**Start Position**: 241

**End Position**: 256

| Profile Score | Tandem Score | TDA q-value | Volume | Spectrum Matches |
| --- | --- | --- | --- | --- |
| -17.2969 | 86.1458 | 0.0420 | 3.2544e+07 | 3 |

**Adduct Signal Fractions**

- **Unmodified**: 3.2544e+07

### YPALN(N)VTM(O)PN(D)NEQFDK Fuc 3 Hex 3 HexNAc 5

**Mass:**  3837.5605

(5.108e-04)

**Start Position**: 161

**End Position**: 177

| Profile Score | Tandem Score | TDA q-value | Volume | Spectrum Matches |
| --- | --- | --- | --- | --- |
| 0.0000 | 85.6476 | 0.0488 | 0.0000e+00 | 1 |

Adduct:

No Chromatogram Found

### cus|SWZNA|A/Switzerland/9715293/2013

**>cus|SWZNA|A/Switzerland/9715293/2013**  
 `MNPNQKIITIGSVSLTISTICFFMQIAILITTVTLHFKQYEFNSPPNNQVMLCEPTIIERNITEIVYLTNTTIEKEICPK  
PAEYRNWSKPQCGITGFAPFSKDNSIRLSAGGDIWVTREPYVSCDPDKCYQFALGQGTTLNNVHSNNTVRDRTPYRTLLM  
NELGVPFHLGTKQVCIAWSSSSCHDGKAWLHVCITGDDKNATASFIYNGRLVDSVVSWSKDILRTQESECVCINGTCTVV  
MTDGSASGKADTKILFIEEGKIVHTSTLSGSAQHVEECSCYPRYPGVRCVCRDNWKGSNRPIVDINIKDHSIVSSYVCSG  
LVGDTPRKNDSSSSSHCLDPNNEEGGHGVKGWAFDDGNDVWMGRTINETSRLGYETFKVIEGWSNPKSKLQTNRQVIVDR  
GDRSGYSGIFSVEGKSCINRCFYVELIRGRKEETEVLWTSNSIVVFCGTSGTYGTGSWPDGADLNLMPI`

| Site | Type | Count |
| --- | --- | --- |
| 329 | N-linked | 6 |
| 86 | N-linked | 8 |
| 367 | N-linked | 2 |
| 200 | N-linked | 2 |

| Mass | Sequence | MS_2_ Score | `q`-value | MS_1_ Score | Signal | Protein Coordinates | Tandem Scans | Start Time | Apex Time | End Time |
| --- | --- | --- | --- | --- | --- | --- | --- | --- | --- | --- |
| 2521.9918 | TIN(N)ETSR Hex 8 HexNAc 2 | 158.419 | 0.0 | -17.785 | 6.207e+06 | 364 - 371 | 3 | 11.55 | 11.55 | 11.55 |
| 3788.5713 | N(N)WSKPQC(C)GITGFAPFSK Hex 9 HexNAc 2 | 157.176 | 0.0 | -1.041 | 4.201e+08 | 85 - 102 | 3 | 43.18 | 43.35 | 43.52 |
| 3573.4440 | N(N)WSKPQC(C)GITGFAPF Hex 9 HexNAc 2 | 153.558 | 0.0 | -2.904 | 9.669e+07 | 85 - 100 | 2 | 51.80 | 51.97 | 52.14 |
| 3670.4720 | KN(N)DSSSSSHC(C)LDPNNEEGGHGVK Hex 5 HexNAc 2 | 151.703 | 0.0 | -18.644 | 5.560e+07 | 327 - 350 | 2 | 13.37 | 13.37 | 13.54 |
| 2791.1342 | TIN(N)ETSR Fuc 1 Hex 5 HexNAc 5 | 148.016 | 0.0 | -19.715 | 2.091e+07 | 364 - 371 | 1 | 11.88 | 11.88 | 11.88 |
| 3626.5167 | N(N)WSKPQC(C)GITGFAPFSK Hex 8 HexNAc 2 | 147.779 | 0.0 | -1.175 | 4.208e+08 | 85 - 102 | 4 | 43.35 | 43.52 | 43.69 |
| 3590.4702 | N(N)WSKPQC(C)GITGFAPF Fuc 2 Hex 3 HexNAc 4 Neu5Ac 1 | 146.747 | 0.0 | -17.893 | 1.450e+07 | 85 - 100 | 1 | 51.80 | 51.97 | 51.97 |
| 3411.3889 | N(N)WSKPQC(C)GITGFAPF Hex 8 HexNAc 2 | 143.892 | 0.0 | -3.225 | 8.613e+07 | 85 - 100 | 4 | 51.97 | 52.14 | 52.31 |
| 4279.7084 | KN(N)DSSSSSHC(C)LDPNNEEGGHGVK Hex 5 HexNAc 5 | 135.411 | 0.0 | -18.902 | 6.900e+07 | 327 - 350 | 3 | 13.37 | 13.37 | 13.54 |
| 3805.5956 | N(N)WSKPQC(C)GITGFAPFSK Fuc 2 Hex 3 HexNAc 4 Neu5Ac 1 | 126.056 | 0.0 | -19.958 | 7.845e+06 | 85 - 102 | 1 | 43.35 | 43.35 | 43.35 |
| 4472.8898 | AWLHVC(C)ITGDDKN(N)ATASFIYNGR Hex 9 HexNAc 2 | 118.425 | 0.0 | -17.599 | 4.685e+06 | 187 - 210 | 1 | 46.87 | 46.87 | 46.87 |
| 4156.5909 | KN(N)DSSSSSHC(C)LDPNNEEGGHGVK Hex 8 HexNAc 2 | 115.745 | 0.0 | -20.488 | 5.301e+06 | 327 - 350 | 1 | 14.19 | 14.19 | 14.36 |
| 4361.7073 | KN(N)DSSSSSHC(C)LDPNNEEGGHGVK Hex 8 HexNAc 3 | 115.620 | 0.0 | 0.000 | 0.000e+00 | 327 - 350 | 1 | - | - | - |
| 4310.8415 | AWLHVC(C)ITGDDKN(N)ATASFIYNGR Hex 8 HexNAc 2 | 115.384 | 0.0 | -17.281 | 4.721e+06 | 187 - 210 | 1 | 46.87 | 46.87 | 47.04 |
| 3464.4623 | N(N)WSKPQC(C)GITGFAPFSK Hex 7 HexNAc 2 | 111.943 | 0.0 | -4.115 | 3.557e+07 | 85 - 102 | 2 | 43.35 | 43.69 | 43.69 |
| 3249.3392 | N(N)WSKPQC(C)GITGFAPF Hex 7 HexNAc 2 | 111.847 | 0.0 | -19.807 | 4.651e+06 | 85 - 100 | 1 | 52.14 | 52.14 | 52.31 |
| 4076.6239 | KN(N)DSSSSSHC(C)LDPNNEEGGHGVK Hex 5 HexNAc 4 | 111.415 | 0.0 | -18.248 | 2.066e+07 | 327 - 350 | 2 | 13.37 | 13.37 | 13.37 |
| 4425.7690 | KN(N)DSSSSSHC(C)LDPNNEEGGHGVK Fuc 1 Hex 5 HexNAc 5 | 111.169 | 0.0 | -20.691 | 1.238e+07 | 327 - 350 | 2 | 13.37 | 13.54 | 13.54 |

### TIN(N)ETSR Hex 8 HexNAc 2

**Mass:**  2521.9918

(7.297e-07)

**Charge States:** 2

**Start Position**: 365

**End Position**: 372

| Profile Score | Tandem Score | TDA q-value | Volume | Spectrum Matches |
| --- | --- | --- | --- | --- |
| -17.7854 | 158.4190 | 0.0000 | 6.2072e+06 | 3 |

**Adduct Signal Fractions**

- **Unmodified**: 6.2072e+06

### N(N)WSKPQC(C)GITGFAPFSK Hex 9 HexNAc 2

**Mass:**  3788.5702

(3.672e-06)

**Charge States:** 3, 4

**Start Position**: 86

**End Position**: 103

| Profile Score | Tandem Score | TDA q-value | Volume | Spectrum Matches |
| --- | --- | --- | --- | --- |
| -1.0412 | 157.1765 | 0.0000 | 4.2014e+08 | 3 |

**Adduct Signal Fractions**

- **Unmodified**: 4.2014e+08

### N(N)WSKPQC(C)GITGFAPF Hex 9 HexNAc 2

**Mass:**  3573.4436

(1.865e-06)

**Charge States:** 3

**Start Position**: 86

**End Position**: 101

| Profile Score | Tandem Score | TDA q-value | Volume | Spectrum Matches |
| --- | --- | --- | --- | --- |
| -2.9037 | 153.5579 | 0.0000 | 9.6690e+07 | 2 |

**Adduct Signal Fractions**

- **Unmodified**: 9.6690e+07

### KN(N)DSSSSSHC(C)LDPNNEEGGHGVK Hex 5 HexNAc 2

**Mass:**  3670.4723

(7.151e-07)

**Charge States:** 4

**Start Position**: 328

**End Position**: 351

| Profile Score | Tandem Score | TDA q-value | Volume | Spectrum Matches |
| --- | --- | --- | --- | --- |
| -18.6445 | 151.7027 | 0.0000 | 5.5595e+07 | 2 |

**Adduct Signal Fractions**

- **Unmodified**: 5.5595e+07

### TIN(N)ETSR Fuc 1 Hex 5 HexNAc 5

**Mass:**  2791.1345

(2.499e-06)

**Charge States:** 2, 3

**Start Position**: 365

**End Position**: 372

| Profile Score | Tandem Score | TDA q-value | Volume | Spectrum Matches |
| --- | --- | --- | --- | --- |
| -19.7151 | 148.0165 | 0.0000 | 2.0907e+07 | 1 |

**Adduct Signal Fractions**

- **Unmodified**: 2.0907e+07

### N(N)WSKPQC(C)GITGFAPFSK Hex 8 HexNAc 2

**Mass:**  3626.5164

(1.515e-06)

**Charge States:** 3, 4

**Start Position**: 86

**End Position**: 103

| Profile Score | Tandem Score | TDA q-value | Volume | Spectrum Matches |
| --- | --- | --- | --- | --- |
| -1.1746 | 147.7787 | 0.0000 | 4.2083e+08 | 4 |

**Adduct Signal Fractions**

- **Unmodified**: 4.2083e+08

### N(N)WSKPQC(C)GITGFAPF Fuc 2 Hex 3 HexNAc 4 Neu5Ac 1

**Mass:**  3590.4699

(-5.314e-06)

**Charge States:** 3

**Start Position**: 86

**End Position**: 101

| Profile Score | Tandem Score | TDA q-value | Volume | Spectrum Matches |
| --- | --- | --- | --- | --- |
| -17.8929 | 146.7467 | 0.0000 | 1.4504e+07 | 1 |

**Adduct Signal Fractions**

- **Unmodified**: 1.4504e+07

### N(N)WSKPQC(C)GITGFAPF Hex 8 HexNAc 2

**Mass:**  3411.3887

(1.332e-06)

**Charge States:** 3

**Start Position**: 86

**End Position**: 101

| Profile Score | Tandem Score | TDA q-value | Volume | Spectrum Matches |
| --- | --- | --- | --- | --- |
| -3.2254 | 143.8916 | 0.0000 | 8.6129e+07 | 4 |

**Adduct Signal Fractions**

- **Unmodified**: 8.6129e+07

### KN(N)DSSSSSHC(C)LDPNNEEGGHGVK Hex 5 HexNAc 5

**Mass:**  4279.7094

(4.399e-07)

**Charge States:** 4, 5

**Start Position**: 328

**End Position**: 351

| Profile Score | Tandem Score | TDA q-value | Volume | Spectrum Matches |
| --- | --- | --- | --- | --- |
| -18.9019 | 135.4105 | 0.0000 | 6.8996e+07 | 3 |

**Adduct Signal Fractions**

- **Unmodified**: 6.8996e+07

### N(N)WSKPQC(C)GITGFAPFSK Fuc 2 Hex 3 HexNAc 4 Neu5Ac 1

**Mass:**  3805.5956

(-5.708e-06)

**Charge States:** 4

**Start Position**: 86

**End Position**: 103

| Profile Score | Tandem Score | TDA q-value | Volume | Spectrum Matches |
| --- | --- | --- | --- | --- |
| -19.9584 | 126.0556 | 0.0000 | 7.8452e+06 | 1 |

**Adduct Signal Fractions**

- **Unmodified**: 7.8452e+06

### AWLHVC(C)ITGDDKN(N)ATASFIYNGR Hex 9 HexNAc 2

**Mass:**  4472.8898

(1.388e-06)

**Charge States:** 4

**Start Position**: 188

**End Position**: 211

| Profile Score | Tandem Score | TDA q-value | Volume | Spectrum Matches |
| --- | --- | --- | --- | --- |
| -17.5994 | 118.4254 | 0.0000 | 4.6853e+06 | 1 |

**Adduct Signal Fractions**

- **Unmodified**: 4.6853e+06

### KN(N)DSSSSSHC(C)LDPNNEEGGHGVK Hex 8 HexNAc 2

**Mass:**  4156.5939

(-8.138e-06)

**Charge States:** 4

**Start Position**: 328

**End Position**: 351

| Profile Score | Tandem Score | TDA q-value | Volume | Spectrum Matches |
| --- | --- | --- | --- | --- |
| -20.4885 | 115.7451 | 0.0000 | 5.3008e+06 | 1 |

**Adduct Signal Fractions**

- **Unmodified**: 5.3008e+06

### KN(N)DSSSSSHC(C)LDPNNEEGGHGVK Hex 8 HexNAc 3

**Mass:**  4361.7073

(4.587e-04)

**Start Position**: 328

**End Position**: 351

| Profile Score | Tandem Score | TDA q-value | Volume | Spectrum Matches |
| --- | --- | --- | --- | --- |
| 0.0000 | 115.6195 | 0.0000 | 0.0000e+00 | 1 |

Adduct:

No Chromatogram Found

### AWLHVC(C)ITGDDKN(N)ATASFIYNGR Hex 8 HexNAc 2

**Mass:**  4310.8410

(2.381e-06)

**Charge States:** 4

**Start Position**: 188

**End Position**: 211

| Profile Score | Tandem Score | TDA q-value | Volume | Spectrum Matches |
| --- | --- | --- | --- | --- |
| -17.2812 | 115.3841 | 0.0000 | 4.7211e+06 | 1 |

**Adduct Signal Fractions**

- **Unmodified**: 4.7211e+06

### N(N)WSKPQC(C)GITGFAPFSK Hex 7 HexNAc 2

**Mass:**  3464.4636

(1.053e-06)

**Charge States:** 3, 4

**Start Position**: 86

**End Position**: 103

| Profile Score | Tandem Score | TDA q-value | Volume | Spectrum Matches |
| --- | --- | --- | --- | --- |
| -4.1155 | 111.9433 | 0.0000 | 3.5572e+07 | 2 |

**Adduct Signal Fractions**

- **Unmodified**: 3.5572e+07

### N(N)WSKPQC(C)GITGFAPF Hex 7 HexNAc 2

**Mass:**  3249.3374

(1.727e-06)

**Charge States:** 3

**Start Position**: 86

**End Position**: 101

| Profile Score | Tandem Score | TDA q-value | Volume | Spectrum Matches |
| --- | --- | --- | --- | --- |
| -19.8072 | 111.8469 | 0.0000 | 4.6506e+06 | 1 |

**Adduct Signal Fractions**

- **Unmodified**: 4.6506e+06

### KN(N)DSSSSSHC(C)LDPNNEEGGHGVK Hex 5 HexNAc 4

**Mass:**  4076.6305

(5.899e-07)

**Charge States:** 4, 5

**Start Position**: 328

**End Position**: 351

| Profile Score | Tandem Score | TDA q-value | Volume | Spectrum Matches |
| --- | --- | --- | --- | --- |
| -18.2484 | 111.4151 | 0.0000 | 2.0657e+07 | 2 |

**Adduct Signal Fractions**

- **Unmodified**: 2.0657e+07

### KN(N)DSSSSSHC(C)LDPNNEEGGHGVK Fuc 1 Hex 5 HexNAc 5

**Mass:**  4425.7681

(1.154e-06)

**Charge States:** 5

**Start Position**: 328

**End Position**: 351

| Profile Score | Tandem Score | TDA q-value | Volume | Spectrum Matches |
| --- | --- | --- | --- | --- |
| -20.6913 | 111.1686 | 0.0000 | 1.2380e+07 | 2 |

**Adduct Signal Fractions**

- **Unmodified**: 1.2380e+07

### tr|F1NUU6|F1NUU6\_CHICK

**>tr|F1NUU6|F1NUU6\_CHICK**  
 `MGCSTILVFLTLLSFASAHRKLLLFLLDGFRFDYIDDAELESLPGFRDIIGMGVKVDYMTPDFPSLSYPNYYTLMTGRHC  
EVHQMIGNYMWDPKTNVSFDIGVNKESLLPLWWNGSEPLWVTMTKEKKNVFMYYWPGCEVEILGVRPSYCREYFSVPTDQ  
NFADAISDALEALRNGSAEMAAVYYERIDVEGHHYGPWSEQRKSALKELDKAMSNMITQIKNKGLQHDVNVLLFSDHGMT  
DISWTDKVIELKNYINMSDTIQMKDRGPVVSLWPVPEKHTEIYQKLKAVEHMNVYNIQDIPERFFYKKGKFVSPLTLVAE  
EGWFIVESRDKLPFWENGTGKREAWQNGWHGYDNELMEMRGIFLAYGPDFRSNYRAPPIRSVDVYNVMCSLAGVQPQPNN  
GSWSRVECMLKNTAVRVPLCTRSGCVLALTLLLLFWLL`

| Site | Type | Count |
| --- | --- | --- |
| 337 | N-linked | 2 |

| Mass | Sequence | MS_2_ Score | `q`-value | MS_1_ Score | Signal | Protein Coordinates | Tandem Scans | Start Time | Apex Time | End Time |
| --- | --- | --- | --- | --- | --- | --- | --- | --- | --- | --- |
| 3315.4335 | DKLPFWEN(N)GTGKR Fuc 1 Hex 5 HexNAc 4 | 142.869 | 0.0 | -18.546 | 1.082e+07 | 329 - 342 | 1 | 29.15 | 29.15 | 29.15 |
| 3451.4474 | DKLPFWEN(N)GTGK Fuc 3 Hex 5 HexNAc 4 | 98.321 | 0.008 | -19.783 | 2.956e+06 | 329 - 341 | 1 | 32.60 | 32.60 | 32.60 |

### DKLPFWEN(N)GTGKR Fuc 1 Hex 5 HexNAc 4

**Mass:**  3315.4335

(1.472e-06)

**Charge States:** 4

**Start Position**: 330

**End Position**: 343

| Profile Score | Tandem Score | TDA q-value | Volume | Spectrum Matches |
| --- | --- | --- | --- | --- |
| -18.5459 | 142.8695 | 0.0000 | 1.0816e+07 | 1 |

**Adduct Signal Fractions**

- **Unmodified**: 1.0816e+07

### DKLPFWEN(N)GTGK Fuc 3 Hex 5 HexNAc 4

**Mass:**  3451.4474

(1.181e-06)

**Charge States:** 3

**Start Position**: 330

**End Position**: 342

| Profile Score | Tandem Score | TDA q-value | Volume | Spectrum Matches |
| --- | --- | --- | --- | --- |
| -19.7826 | 98.3208 | 0.0075 | 2.9559e+06 | 1 |

**Adduct Signal Fractions**

- **Unmodified**: 2.9559e+06

### tr|E1C041|E1C041\_CHICK

**>tr|E1C041|E1C041\_CHICK**  
 `MRTGAGEAVGVLCLVSLVAALDYKYHHSEELEAYLKEVHAAYPALTHLHSIGRSVEGRDLWVLVLGRFPTQHKIGIPEFK  
YVANMHGDETVGREILLHLIDHLVTNYGRDPVITRLLNNTRIHIMPTMNPDGFEATVVPDCYYSRGRYNKNGEDLNRNFP  
DAFENNNNLIQPETQAVINWIKNETFVLSANLHGGALVASYTFDNGNSVTGTSNGYSRSPDDDVFIHLAKTYSFNHASMY  
KGTGCDSKQTFPDGITNGYSWYQLEGGMQDYNYVWGQCFEITLELSCCKYPPAEQLEKFWRDNKVALVEYIKQVHLGVKG  
QVTDKNGNPIPNAIVEAKGRPHICPYRTNEHGEYFLLLLPGKYVINATVPGFKSMLKTVDIPDNTANFSALKQDFSFPEV  
SVKPRPASCPRTPLYQELERSSAAVKPTLHILVLVIVTISILK`

| Site | Type | Count |
| --- | --- | --- |
| 366 | N-linked | 2 |

| Mass | Sequence | MS_2_ Score | `q`-value | MS_1_ Score | Signal | Protein Coordinates | Tandem Scans | Start Time | Apex Time | End Time |
| --- | --- | --- | --- | --- | --- | --- | --- | --- | --- | --- |
| 3072.3007 | YVIN(N)ATVPGFK Hex 9 HexNAc 2 | 161.390 | 0.0 | -17.944 | 2.909e+07 | 362 - 373 | 1 | 41.18 | 41.18 | 41.35 |
| 2910.2472 | YVIN(N)ATVPGFK Hex 8 HexNAc 2 | 155.762 | 0.0 | -18.391 | 7.737e+06 | 362 - 373 | 1 | 41.18 | 41.35 | 41.35 |

### YVIN(N)ATVPGFK Hex 9 HexNAc 2

**Mass:**  3072.3015

(2.418e-06)

**Charge States:** 2, 3

**Start Position**: 363

**End Position**: 374

| Profile Score | Tandem Score | TDA q-value | Volume | Spectrum Matches |
| --- | --- | --- | --- | --- |
| -17.9437 | 161.3905 | 0.0000 | 2.9095e+07 | 1 |

**Adduct Signal Fractions**

- **Unmodified**: 2.9095e+07

### YVIN(N)ATVPGFK Hex 8 HexNAc 2

**Mass:**  2910.2473

(1.998e-06)

**Charge States:** 3

**Start Position**: 363

**End Position**: 374

| Profile Score | Tandem Score | TDA q-value | Volume | Spectrum Matches |
| --- | --- | --- | --- | --- |
| -18.3913 | 155.7619 | 0.0000 | 7.7367e+06 | 1 |

**Adduct Signal Fractions**

- **Unmodified**: 7.7367e+06

### tr|A0A1D5PNI3|A0A1D5PNI3\_CHICK

**>tr|A0A1D5PNI3|A0A1D5PNI3\_CHICK**  
 `MTTSTLAQTTSLPGTSTPTPASSTLMTTSTPAQTSLMPGHFKAQLLHLLHFTTTSTPAQTTSVPASSSPTPASSALPTAT  
TPAQTPGTSSPTPASSALPTTTTPAETTTFPATSSTTPASSAPPTTSTPAQTTSLPRTSTPTPASSALTTTTTPEQTTTV  
PGTSTPTPASSTVLTTSTPVQTTSMPGISSPTPASSALLTKSTPAQTTTVPATSSPTPASSALPTTTTPEQTTTESGTST  
PTPASSGPPTTSTPAETTSFPATSSPTPASSTLMTTSTPAQTTTVPGTSSPTPVSSALLTTRTLAQTKASLQLNSSPTSV  
SSALSTTSTPAETTTVPGTSIPTAASSTLLTTSTLAQTTSFPATSTPTPSSSTLMTTSTRAETKTSLQLNSSPTPASLAL  
PTTSTPAQTTSVPTSSSPTPASSALSTTSTPALTTALRSTSQIMSPSSPATSPSPTATSAGTVTTAQETTSPAGTTNALT  
STIRTTSTQTLSSSSPPYTTAALSYTAIPTSTPATPTTSAPQTTIASSSTPTESTALLTTTVPSTTQDISSQTAQTSNAA  
TSSTTNSATSARLTRTHAETTSSTTTFTSHSRPSASQTSTPGTTISPLPATTAQQTTAPAGTTPPTTASTTLFSTRETSS  
KEEETSTVVTSSQTPASSVLPSTSTPAQTTSMPSTSSPTPASSTLMTTSTLAQTTSLPGTSVPTPASSAPRTTTTPAQTT  
SVPGNSSPIPPYSTLLTTSTLAQTTSLPGTSTPTPASSTLMTTSTPAETKTSLLLNSSPNICIFCSSDYNHSCTDNISAR  
HFKPNSCTAIKHSCRKQTFITTFTSRARTSASWTTTPGTTTSALTATTARETTTPAGTTHPHNSNIETTSTSTIKAPTRP  
PTTPAGHERFTVNFTITNLPYDSDLQIPHSAKLNATQRVMTTLLNRLLKDTSIGPSFLGCATTAFR`

| Site | Type | Count |
| --- | --- | --- |
| 893 | N-linked | 1 |

| Mass | Sequence | MS_2_ Score | `q`-value | MS_1_ Score | Signal | Protein Coordinates | Tandem Scans | Start Time | Apex Time | End Time |
| --- | --- | --- | --- | --- | --- | --- | --- | --- | --- | --- |
| 4160.8534 | FTVN(N)FTITNLPYDSDLQIPHSAK Hex 7 HexNAc 2 | 113.686 | 0.0 | -3.244 | 2.282e+07 | 889 - 912 | 3 | 58.28 | 58.45 | 58.62 |

### FTVN(N)FTITNLPYDSDLQIPHSAK Hex 7 HexNAc 2

**Mass:**  4160.8527

(1.437e-06)

**Charge States:** 4

**Start Position**: 890

**End Position**: 913

| Profile Score | Tandem Score | TDA q-value | Volume | Spectrum Matches |
| --- | --- | --- | --- | --- |
| -3.2445 | 113.6864 | 0.0000 | 2.2819e+07 | 3 |

**Adduct Signal Fractions**

- **Unmodified**: 2.2819e+07

### tr|A0A1D5PUR1|A0A1D5PUR1\_CHICK

**>tr|A0A1D5PUR1|A0A1D5PUR1\_CHICK**  
 `MTTSTLAQTTSLPGTSTPTPASSTLMTTSTPAQTSLMPGHFKAQLLHLLHFTTTSTPAQTTSVPASSSPTPASSALPTAT  
TPAQTPGTSSPTPASSALPTTTTPAETTTFPATSSTTPASSAPPTTSTPAQTTSLPRTSTPTPASSALTTTTTPEQTTTV  
PGTSTPTPASSTVLTTSTPVQTTSMPGISSPTPASSALLTKSTPAQTTTVPATSSPTPASSALPTTTTPEQTTTESGTST  
PTPASSGPPTTSTPAETTSVPGTSYPNPASSSLMTTSTLAQTTSLPATSTPSLASSTLMTKSTFAPTTALPGTSTPTPAS  
SAPPTTSTPAQTTSVPGTSTATSASSTLLTTSTPAQTTSFPATSSPTPASSTLMTTSTPAQTTTVPGTSSPTPVSSALLT  
TRTLAQTKASLQLNSSPTSVSSALSTTSTPAETTTVPGTSIPTAASSTLLTTSTLAQTTSFPATSTPTPSSSTLMTTSTR  
AETKTSLQLNSSPTPASLALPTTSTPAQTTSVPTSSSPTPASSALSTTSTPALTTALRSTSQIMSPSSPATSPSPTATSA  
GTVTTAQETTSPAGTTNALTSTIRTTSTQTLSSSSPPYTTAALSYTAIPTSTPATPTTSAPQTTIASSSTPTESTALLTT  
TVPSTTQDISSQTAQTSNAATSSTTNSATSARLTRTHAETTSSTTTFTSHSRPSASQTSTPGTTISPLPATTAQQTTAPA  
GTTPPTTASTTLFSTRETSSKEEETSTVVTSSQTPASSVLPSTSTPAQTTSMPSTSSPTPASSTLMTTSTLAQTTSLPGT  
SVPTPASSAPRTTTTPAQTTSVPGNSSPIPPYSTLLTTSTLAQTTSLPGTSTPTPASSTLMTTSTPAETKTSLLLNSSPN  
ICIFCSSDYNHSCTDNISARHFKPNSCTAIKHSCRKQTFITTFTSRARTSASWTTTPGTTTSALTATTARETTTPAGTTH  
PHNSNIETTSTSTIKAPTRPPTTPAGHERFTVNFTITNLPYDSDLQIPHSAKLNATQRVMTTLLNRLLKDTSIGPSFLGC  
ATTAFR`

| Site | Type | Count |
| --- | --- | --- |
| 993 | N-linked | 1 |

| Mass | Sequence | MS_2_ Score | `q`-value | MS_1_ Score | Signal | Protein Coordinates | Tandem Scans | Start Time | Apex Time | End Time |
| --- | --- | --- | --- | --- | --- | --- | --- | --- | --- | --- |
| 4160.8534 | FTVN(N)FTITNLPYDSDLQIPHSAK Hex 7 HexNAc 2 | 113.686 | 0.0 | -3.244 | 2.282e+07 | 989 - 1012 | 3 | 58.28 | 58.45 | 58.62 |

### FTVN(N)FTITNLPYDSDLQIPHSAK Hex 7 HexNAc 2

**Mass:**  4160.8527

(1.437e-06)

**Charge States:** 4

**Start Position**: 990

**End Position**: 1013

| Profile Score | Tandem Score | TDA q-value | Volume | Spectrum Matches |
| --- | --- | --- | --- | --- |
| -3.2445 | 113.6864 | 0.0000 | 2.2819e+07 | 3 |

**Adduct Signal Fractions**

- **Unmodified**: 2.2819e+07
